# Supplementary material for: A single-cell pan-cancer analysis to show the variability of tumor-infiltrating myeloid cells in immune checkpoint blockade
Source: Nat Commun. 2024 Jul 21;15:6142. doi: 10.1038/s41467-024-50478-8 (PMC11271490; doi:10.1038/s41467-024-50478-8)
Supplement: Supplementary file 1 — Supplementary Information [file 41467_2024_50478_MOESM1_ESM.pdf]

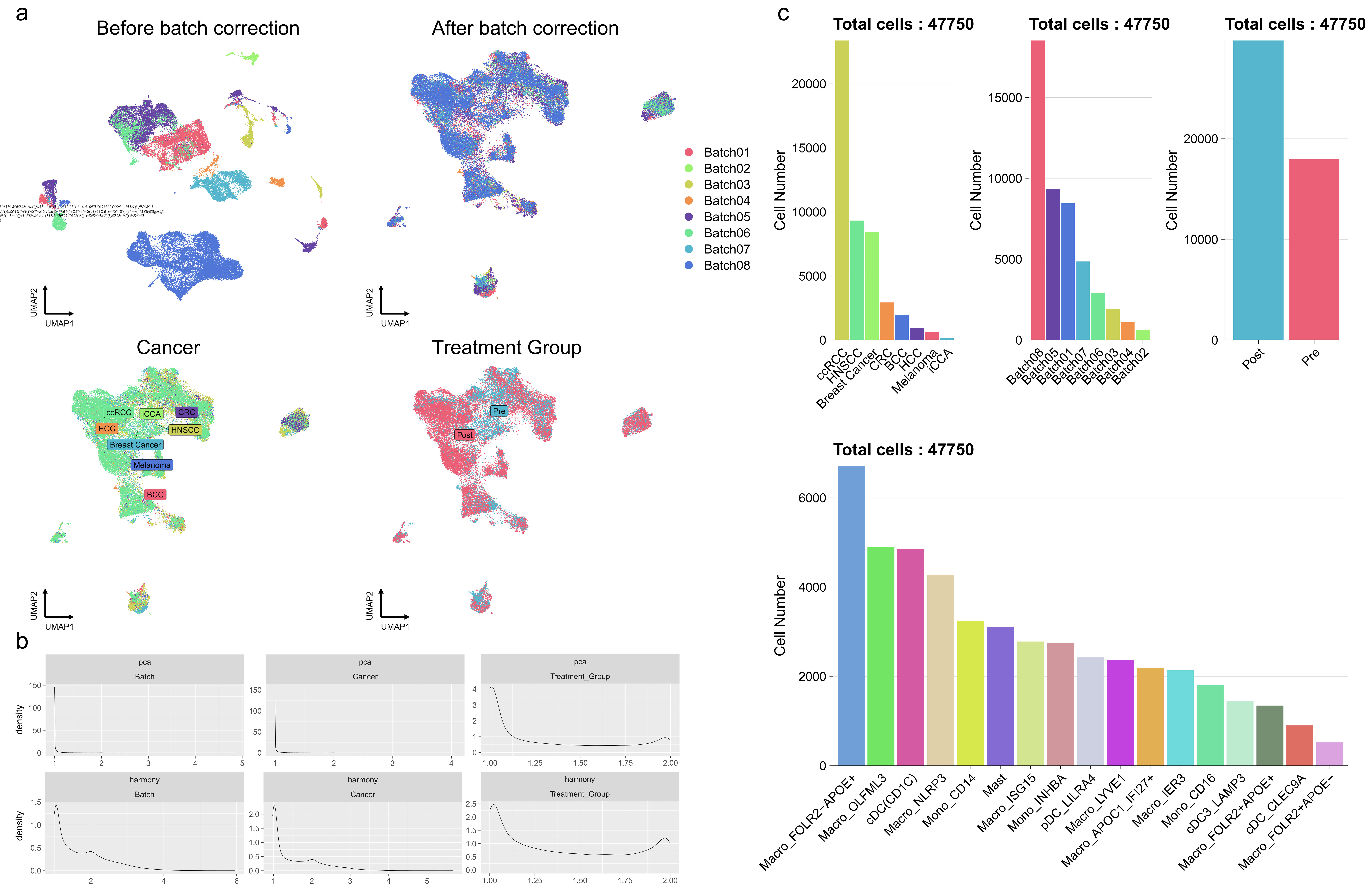

Supplementary Fig. 1. Batch correction based on UMAP embeddings. (a) UMAP projections of the batches before batch correction. (b) The line graph illustrates the density performance of the LISI index across different batch algorithms. (c) UMAP representation after batch correction.

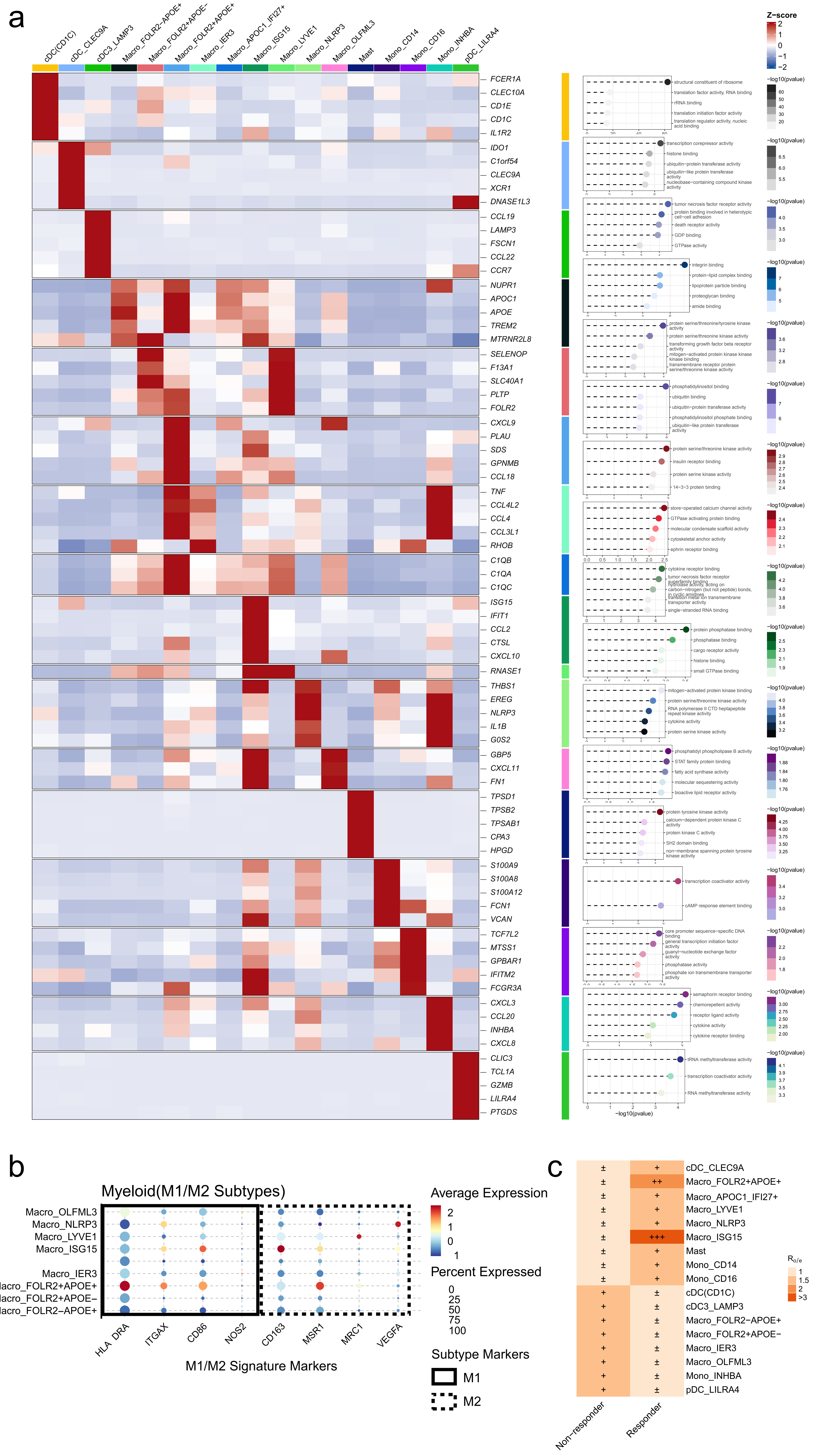

Supplementary Fig. 2. (a) Expressions of signature genes in M1 and M2 macrophages. (b) Dot size represents percentage of cells expressing the genes, and color gradient indicates average expression of the genes. (c) Rectangular outlines represent M1/M2 markers.

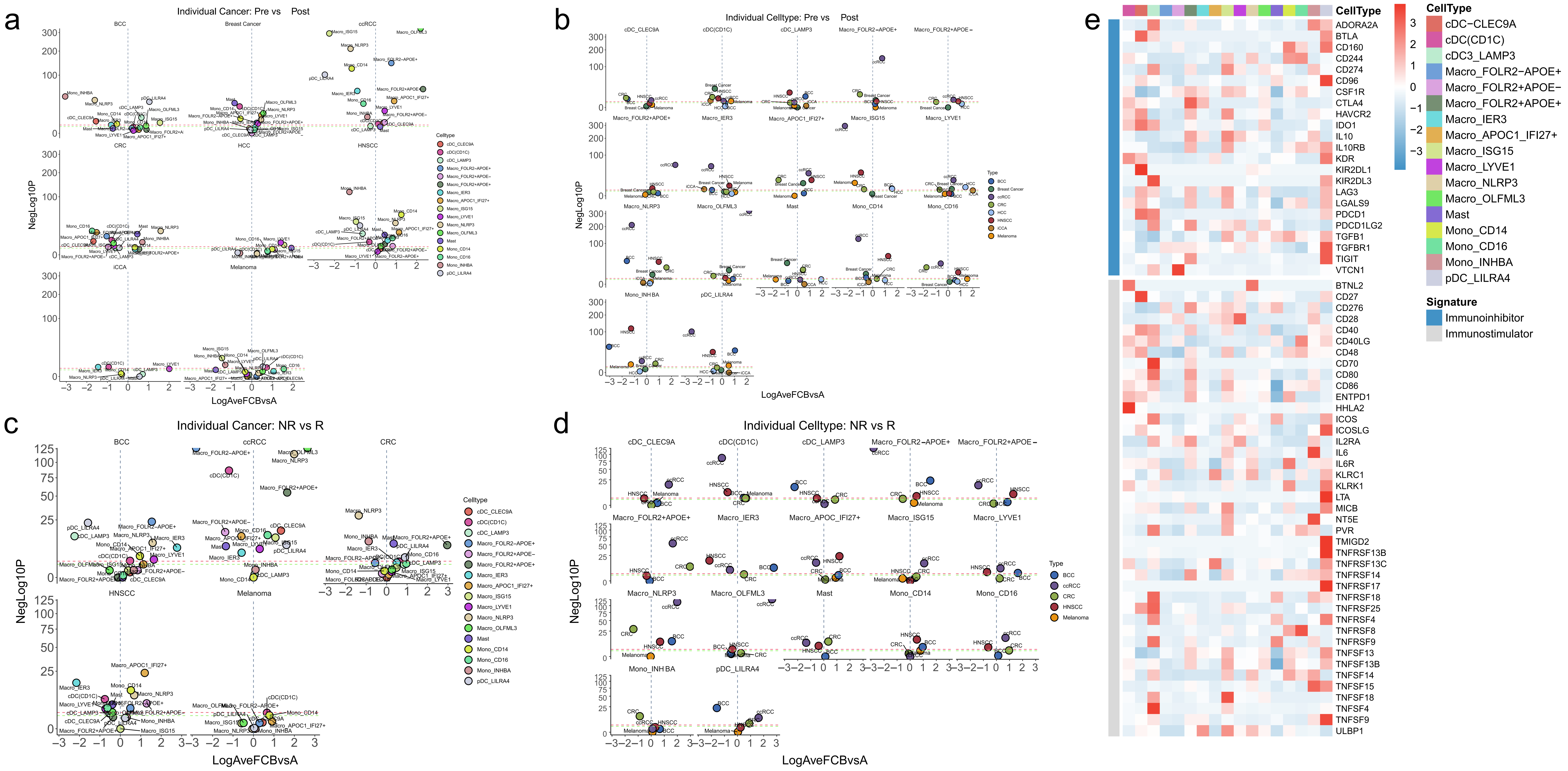

Supplementary Fig. 3. Comparison of the proportions between the subgroups across TIMs. Comparing between (a-b) the Pre and Post groups, and (c-d) the R and NR groups. (e) Expression of immunoinhibitors and immunostimulators across various cell types.

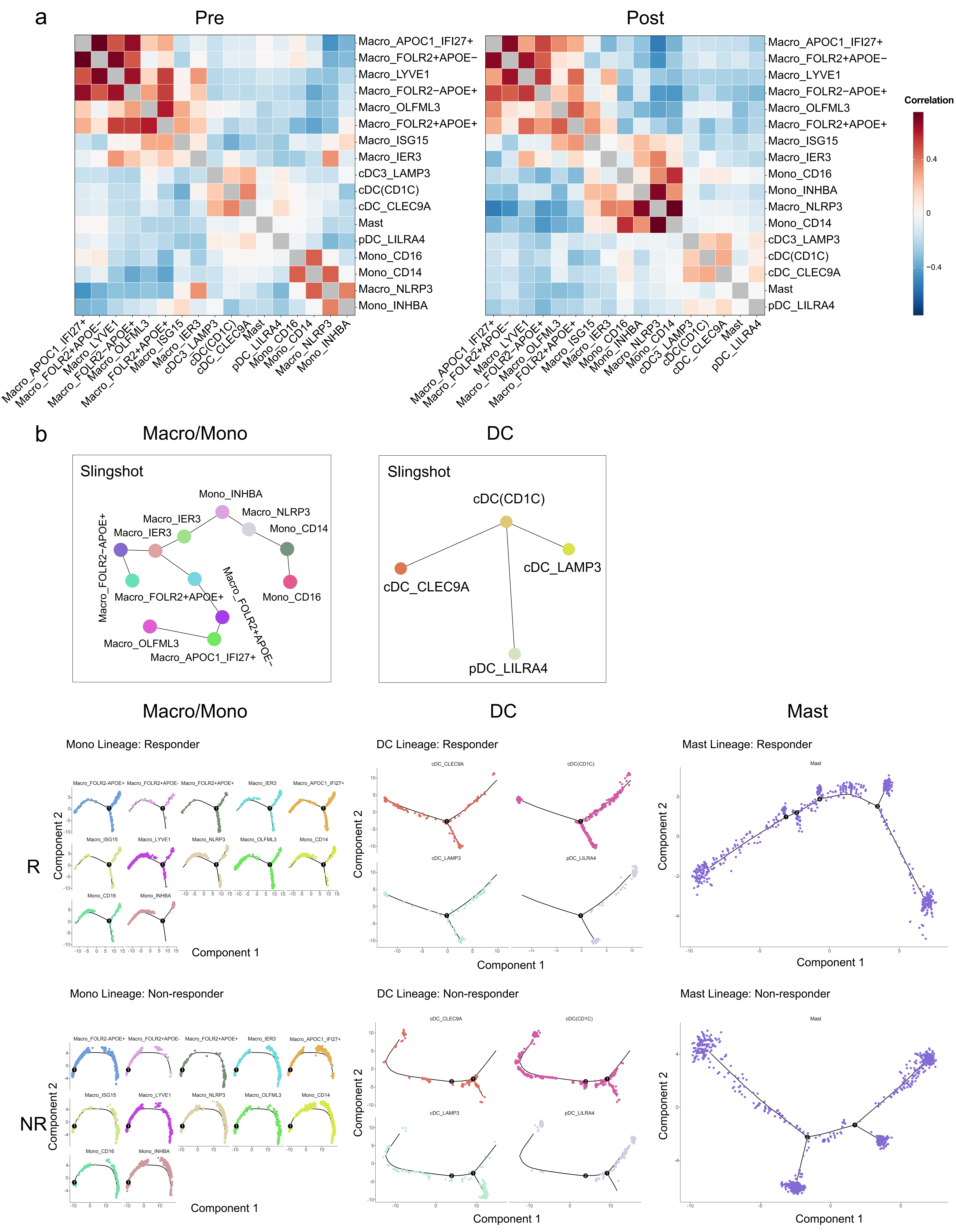

Supplementary Fig. 4. (a) Correlation analysis revealed that the TME underwent significant remodeling following immunotherapy. (b) Pseudotime trajectories of all cell types in the response groups. Colored by cell types.

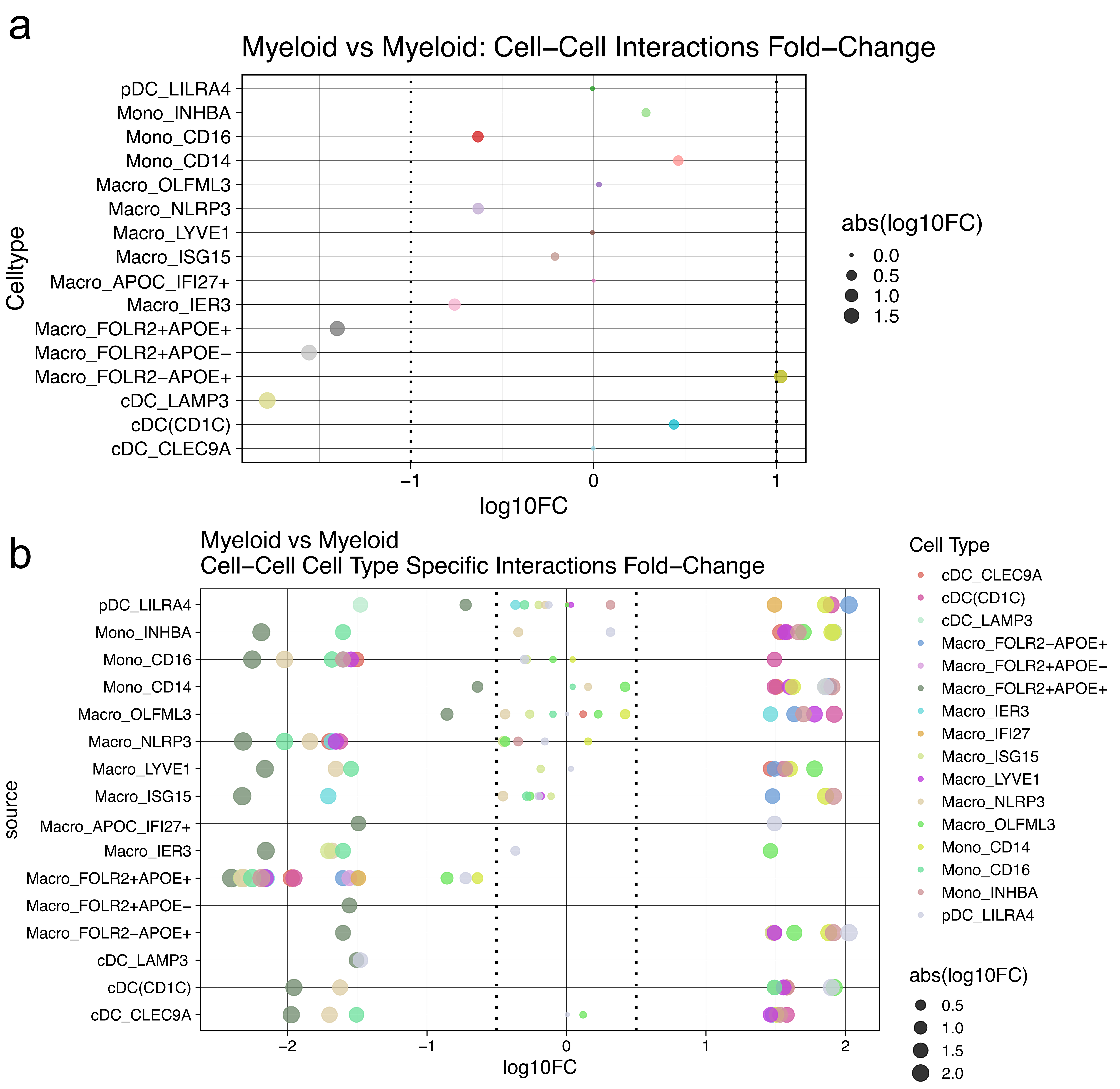

Supplementary Fig. 5. Cell-cell interaction patterns across TIM cell types in response groups. (a) Fold change of combined ligand and receptor interaction frequencies of each TIM between the R (numerator) and NR (denominator) groups. (b) Fold change of combined ligand and receptor interaction frequencies of each TIM with other TIMs between the R (numerator) and NR (denominator) groups.

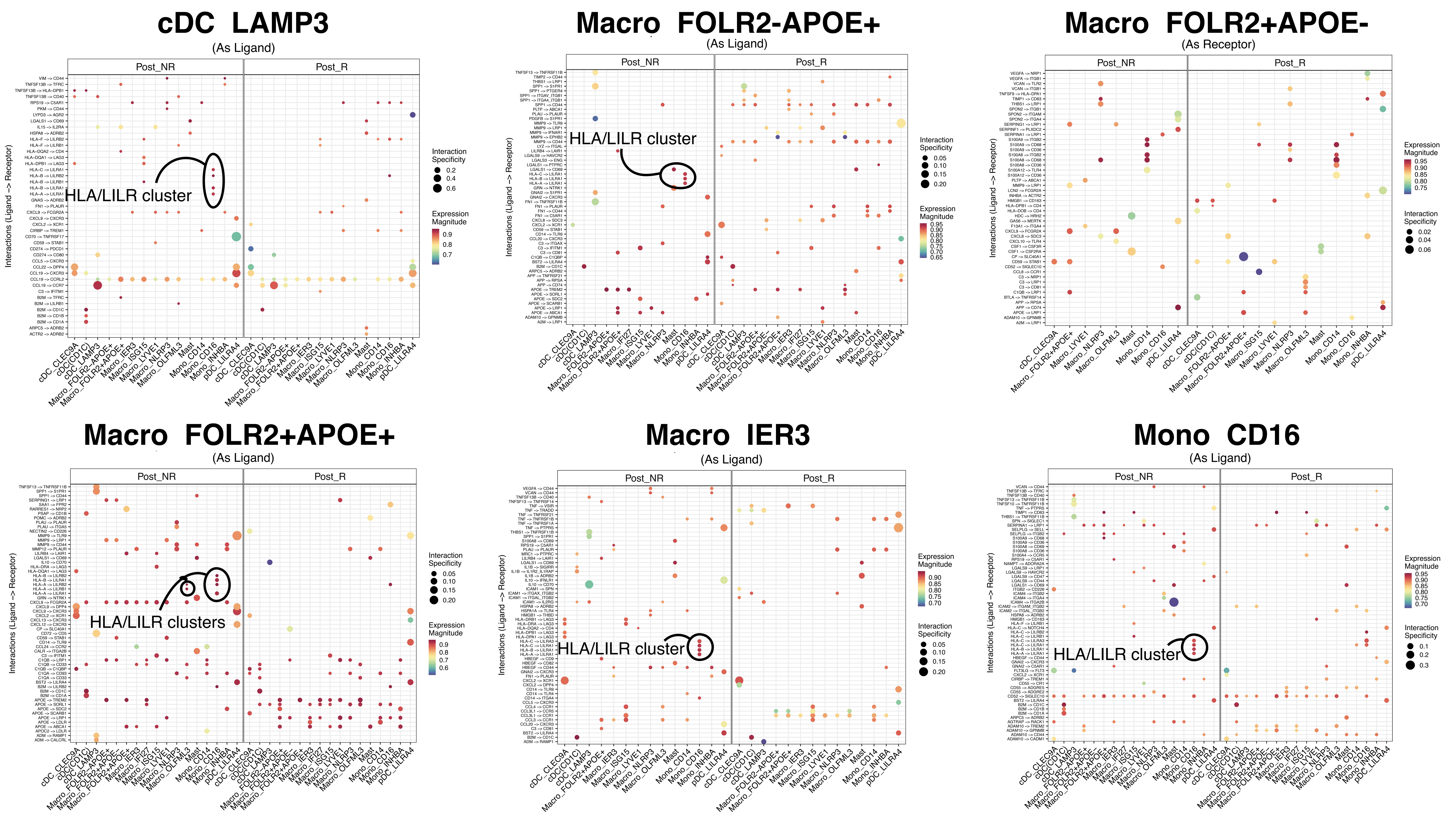

Supplementary Fig. 6. Top ligand-receptor binding interactions in the TIMs. Dot size represented interaction specificity and color gradient indicates the expression of the interaction pairs.

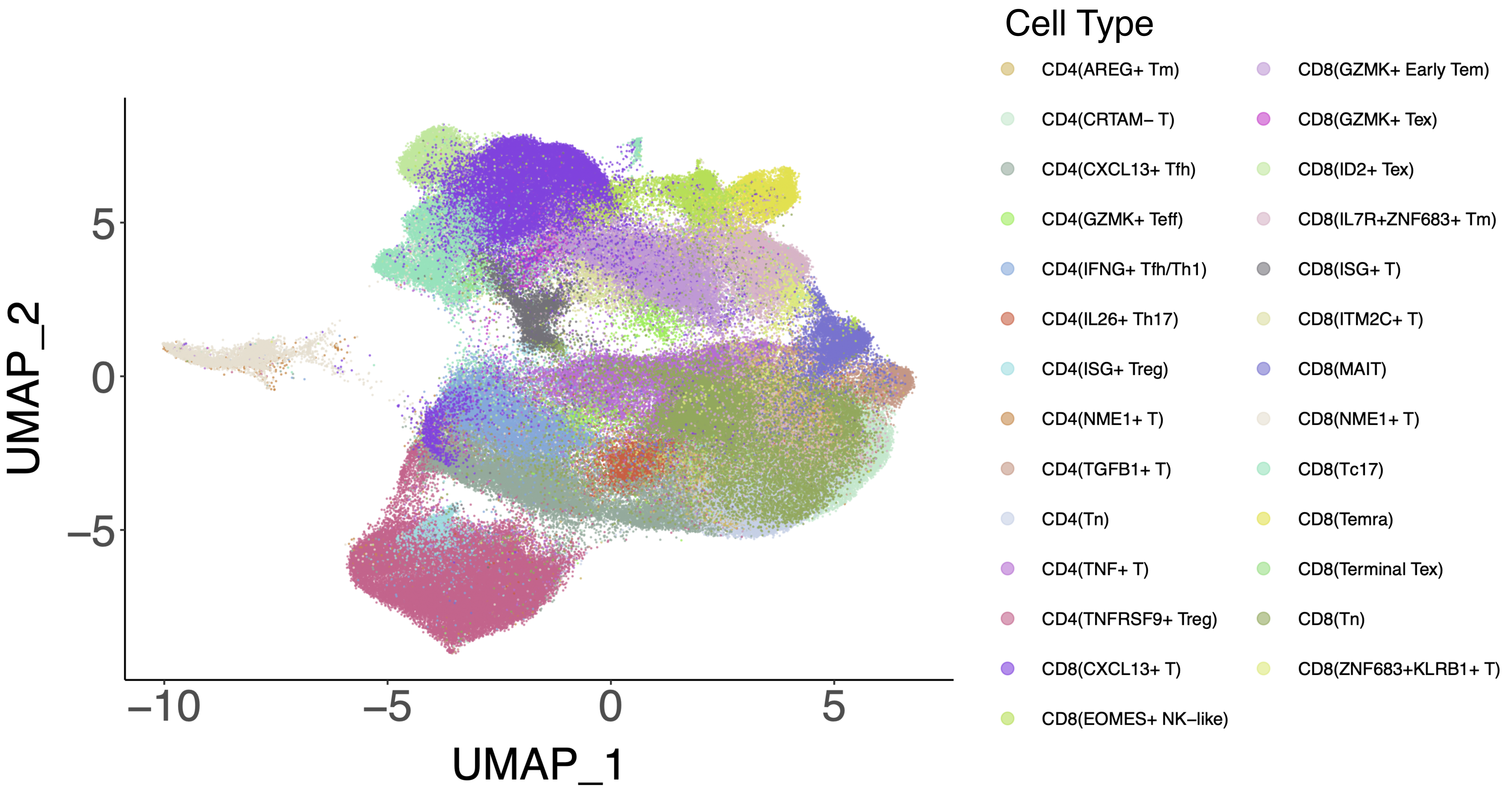

Supplementary Fig. 7. UMAP displaying T cell types of post-treatment samples. UMAP projections showing CD4 and CD8 T cell types represented in the same cancer samples.

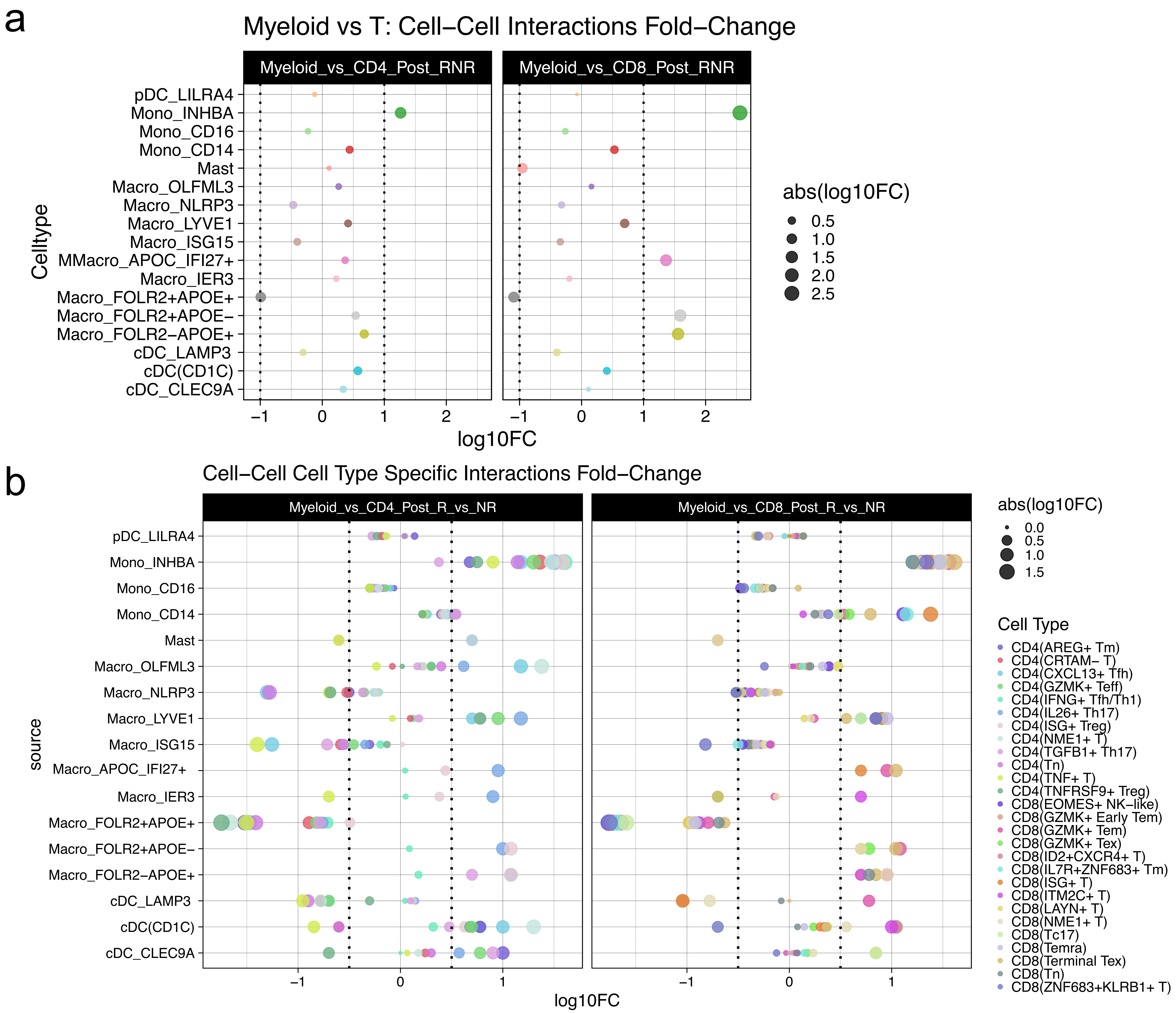

Supplementary Fig. 8. Cell-cell interaction patterns between TIMs and CD4 and CD8 T-cells in response groups. (a) Fold change of combined ligand and receptor interaction frequencies of TIM versus CD4 or CD8 T-cells between the R (numerator) and NR (denominator) groups. (b) Fold change of combined ligand and receptor interaction frequencies of each TIM with CD4 or CD8 T-cells between the R (numerator) and NR (denominator) groups.

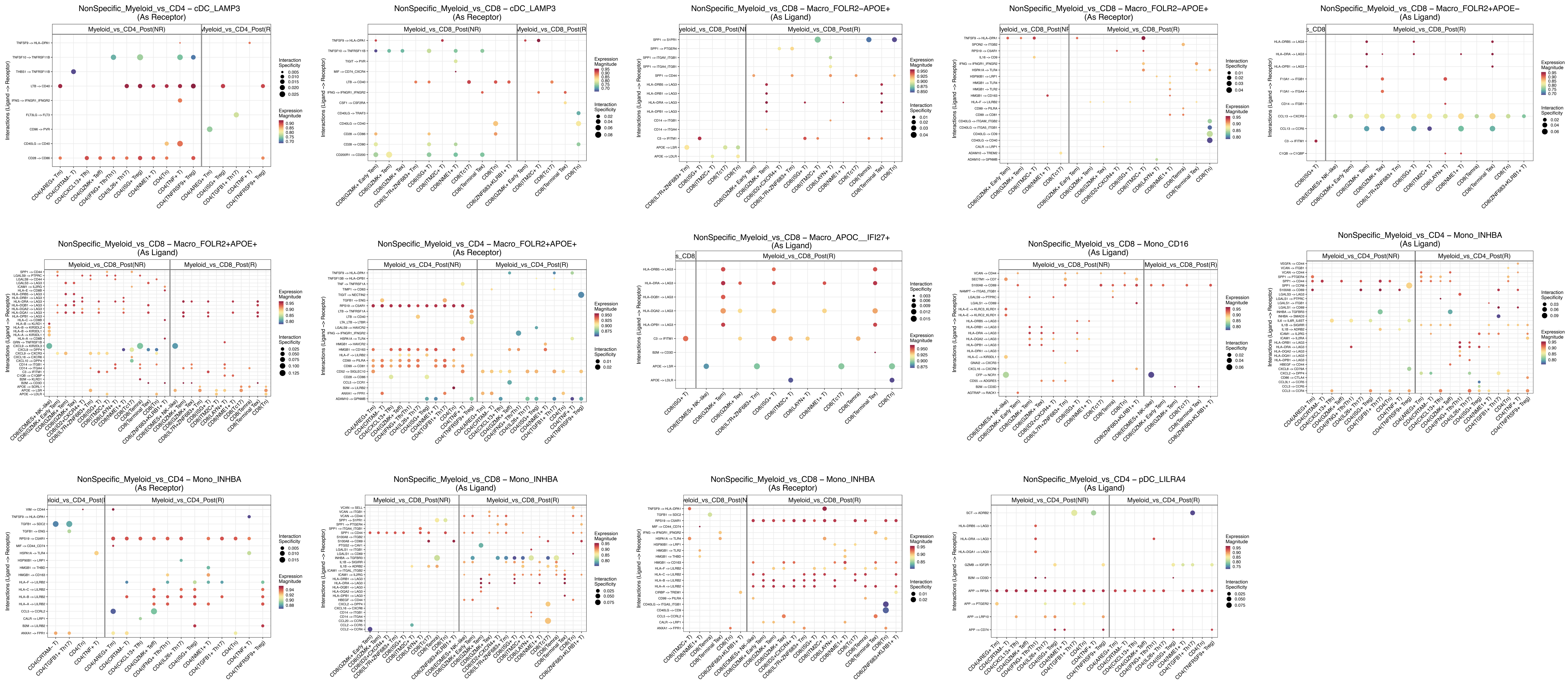

Supplementary Fig. 9. Top ligand-receptor binding interactions of the TIMs with CD4 and CD8 T-cells. Dot size represents interaction specificity and color gradient indicate the expression of the interaction pairs.

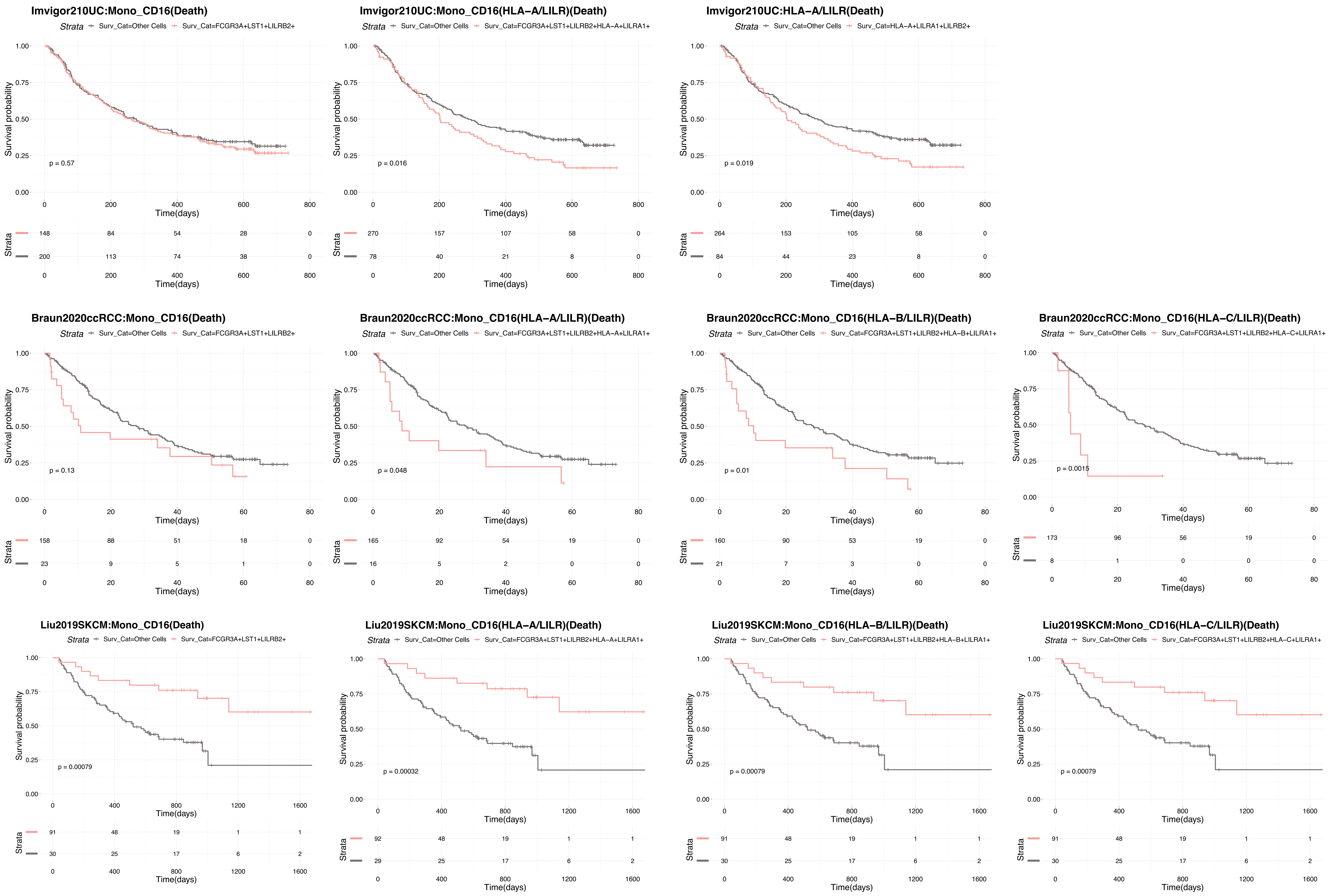

Supplementary Fig. 10. Survival analysis based on the expression of the signature genes of Mono_CD16.

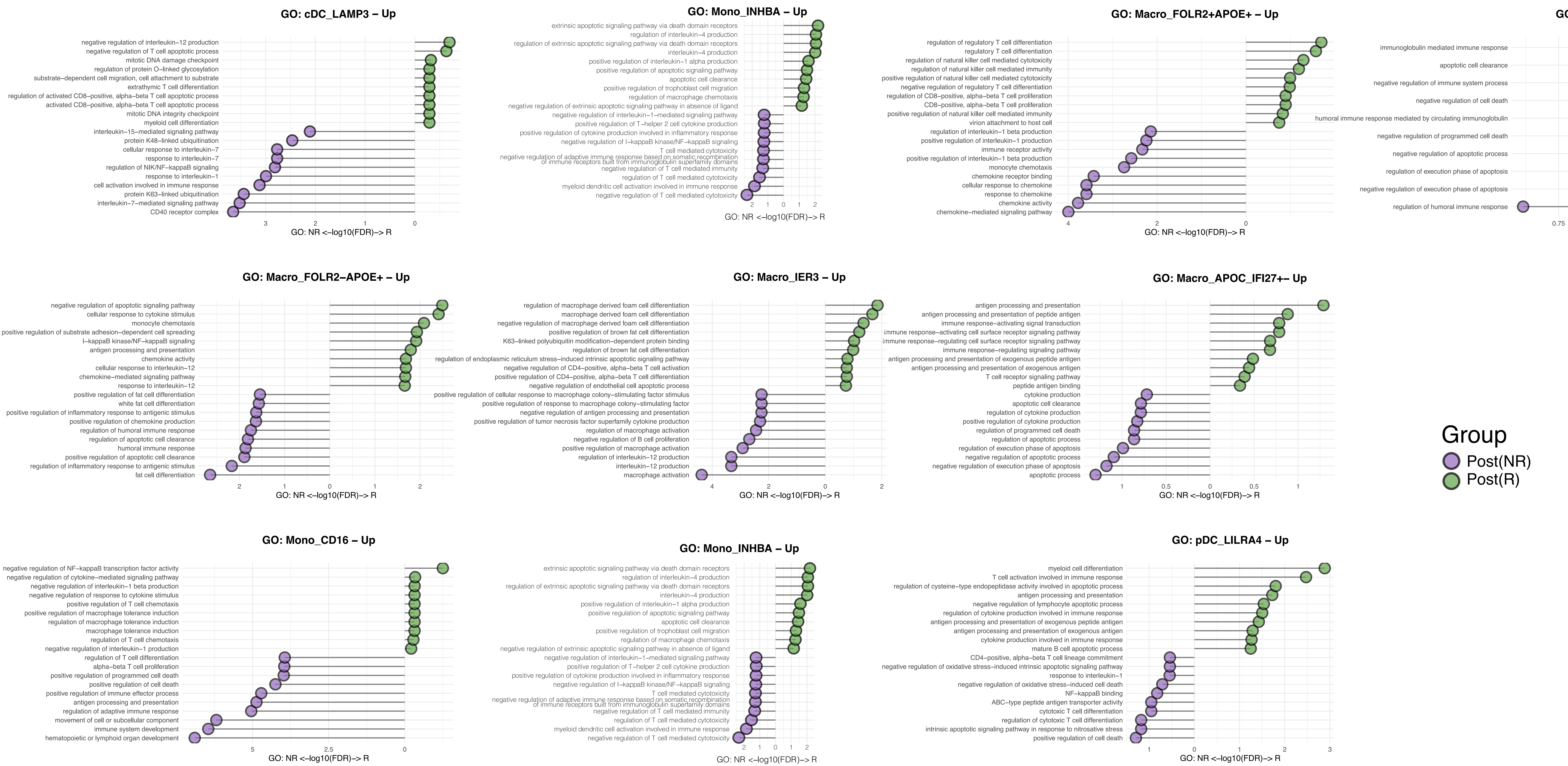

Supplementary Fig. 11. Top GO immune processes of the highly interacting TIMs in both response groups.

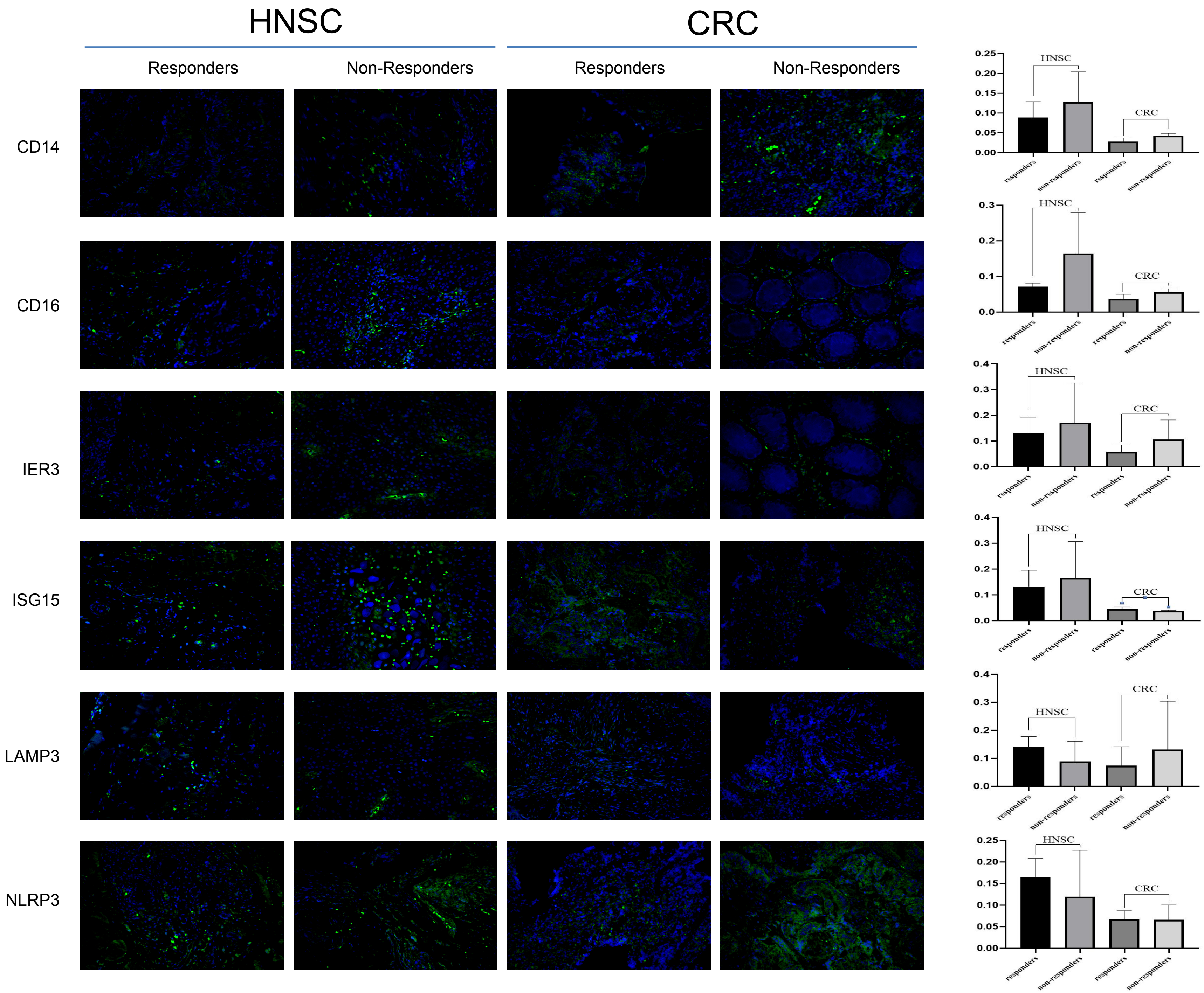

Supplementary Fig. 12. Representative markers showed increased expression levels among responsive patients.
