# Supplementary material for: A single-cell pan-cancer analysis to show the variability of tumor-infiltrating myeloid cells in immune checkpoint blockade
Source: Nat Commun. 2024 Jul 21;15:6142. doi: 10.1038/s41467-024-50478-8 (PMC11271490; doi:10.1038/s41467-024-50478-8)
Supplement: Supplementary file 3 — Description of Additional Supplementary Files [file 41467_2024_50478_MOESM3_ESM.pdf]

Description of Additional Supplementary Files

Supplementary Data 1. Sample information across cancer types in the atlas.

Supplementary Data 2. DEGs of all cell types. Non-parametric two-sided Wilcoxon rank sum test was used for the differential expression testing. Multiple testing correction was made using Bonferroni. Only DEGs with p-adjusted values < 0.05 were retained.

Supplementary Data 3. Enrichment analysis results with reference to hallmark pathway signatures from MSigDB in each pseudotime branch of the two cell type categories. Kolmogorov–Smirnov (KS) test was used for in the enrichment analysis. Multiple testing correction was done using FDR.

Supplementary Data 4. List of top TFs (AUC > 0.2) in each cancer type across all TIMs.

Supplementary Data 5. List of top cell-cell interactions (aggregated rank < 0.01) between the TIMs. The P values shown are derived from the CellPhoneDB empirical shuffling method, which calculates the likelihood of cell-type specificity for a given receptor-ligand complex. P-values derived from the proportion of shuffled means that are as high as or higher than the observed mean. Aggregated rank values are obtained from Liana R package using the RRA method, to provide a quantitative value to assess interactions which are consistently ranked higher than random.

Supplementary Data 6. List of top cell-cell interactions (aggregated rank < 0.01) between the TIMs and CD4 or CD8 T-cells. The P values shown are derived from the CellPhoneDB empirical shuffling method, which calculates the likelihood of cell-type specificity for a given receptor-ligand complex. P-values derived from the proportion of shuffled means that are as high as or higher than the observed mean. Aggregated rank values are obtained from Liana R package using the RRA method, to provide a quantitative value to assess interactions which are consistently ranked higher than random.
