# Supplementary material for: A single-cell pan-cancer analysis to show the variability of tumor-infiltrating myeloid cells in immune checkpoint blockade
Source: Nat Commun. 2024 Jul 21;15:6142. doi: 10.1038/s41467-024-50478-8 (PMC11271490; doi:10.1038/s41467-024-50478-8)
Supplement: Supplementary file 4 — Supplementary Data 1 [file 41467_2024_50478_MOESM4_ESM.pdf]

Dataset_ID	Cancer	Sample_ID	Patient_ID	Treatment_Group	Treatment	Drug_Name	Response
GSE123813	BCC	bcc.su002.pre	bcc.su002	Pre	None	None	Responder
GSE123813	BCC	bcc.su002.post.tumor.cd45	bcc.su002	Post	Anti-PD1	Pembrolizumab	Responder
GSE123813	BCC	bcc.su003.pre	bcc.su003	Pre	None	None	Responder
GSE123813	BCC	bcc.su003.post.tcell.cd45	bcc.su003	Post	Anti-PD1	Pembrolizumab	Responder
GSE123813	BCC	bcc.su003.post.all	bcc.su003	Post	Anti-PD1	Pembrolizumab	Responder
GSE123813	BCC	bcc.su004.pre.tumor.cd45	bcc.su004	Pre	None	None	Responder
GSE123813	BCC	bcc.su004.post.tcell	bcc.su004	Post	Anti-PD1	Cemiplimab	Responder
GSE123813	BCC	bcc.su004.post.tumor.cd45	bcc.su004	Post	Anti-PD1	Cemiplimab	Responder
GSE123813	BCC	bcc.su005.pre.tumor	bcc.su005	Pre	None	None	Non-responder
GSE123813	BCC	bcc.su005.pre.cd45	bcc.su005	Pre	None	None	Non-responder
GSE123813	BCC	bcc.su005.post.tcell	bcc.su005	Post	Anti-PD1	Pembrolizumab	Non-responder
GSE123813	BCC	bcc.su005.post.tumor.cd45	bcc.su005	Post	Anti-PD1	Pembrolizumab	Non-responder
GSE123813	BCC	bcc.su007.pre.tumor.cd45	bcc.su007	Pre	None	None	Non-responder
GSE123813	BCC	bcc.su007.post	bcc.su007	Post	Anti-PD1	Pembrolizumab	Non-responder
GSE123813	BCC	bcc.su009.post.tcell	bcc.su009	Post	Anti-PD1	Cemiplimab	Responder
GSE123813	BCC	bcc.su012.pre.tcell	bcc.su012	Pre	None	None	Responder
EGAD00001006608	Breast Cancer	BIOKEY_13_Pre	BIOKEY_13	Pre	None	None	NotAvailable
EGAD00001006608	Breast Cancer	BIOKEY_13_On	BIOKEY_13	Post	Anti-PD1	Pembrolizumab	NotAvailable
EGAD00001006608	Breast Cancer	BIOKEY_10_Pre	BIOKEY_10	Pre	None	None	NotAvailable
EGAD00001006608	Breast Cancer	BIOKEY_10_On	BIOKEY_10	Post	Anti-PD1	Pembrolizumab	NotAvailable
EGAD00001006608	Breast Cancer	BIOKEY_16_Pre	BIOKEY_16	Pre	None	None	NotAvailable
EGAD00001006608	Breast Cancer	BIOKEY_16_On	BIOKEY_16	Post	Anti-PD1	Pembrolizumab	NotAvailable
EGAD00001006608	Breast Cancer	BIOKEY_14_Pre	BIOKEY_14	Pre	None	None	NotAvailable
EGAD00001006608	Breast Cancer	BIOKEY_14_On	BIOKEY_14	Post	Anti-PD1	Pembrolizumab	NotAvailable
EGAD00001006608	Breast Cancer	BIOKEY_19_Pre	BIOKEY_19	Pre	None	None	NotAvailable
EGAD00001006608	Breast Cancer	BIOKEY_19_On	BIOKEY_19	Post	Anti-PD1	Pembrolizumab	NotAvailable
EGAD00001006608	Breast Cancer	BIOKEY_23_Pre	BIOKEY_23	Pre	None	None	NotAvailable
EGAD00001006608	Breast Cancer	BIOKEY_23_On	BIOKEY_23	Post	Anti-PD1	Pembrolizumab	NotAvailable
EGAD00001006608	Breast Cancer	BIOKEY_26_Pre	BIOKEY_26	Pre	None	None	NotAvailable
EGAD00001006608	Breast Cancer	BIOKEY_26_On	BIOKEY_26	Post	Anti-PD1	Pembrolizumab	NotAvailable
EGAD00001006608	Breast Cancer	BIOKEY_28_Pre	BIOKEY_28	Pre	None	None	NotAvailable
EGAD00001006608	Breast Cancer	BIOKEY_28_On	BIOKEY_28	Post	Anti-PD1	Pembrolizumab	NotAvailable
EGAD00001006608	Breast Cancer	BIOKEY_3_Pre	BIOKEY_3	Pre	None	None	NotAvailable
EGAD00001006608	Breast Cancer	BIOKEY_3_On	BIOKEY_3	Post	Anti-PD1	Pembrolizumab	NotAvailable

EGAD00001006608	Breast Cancer	BIOKEY_15_Pre	BIOKEY_15	Pre	None	None	NotAvailable
EGAD00001006608	Breast Cancer	BIOKEY_15_On	BIOKEY_15	Post	Anti-PD1	Pembrolizumab	NotAvailable
EGAD00001006608	Breast Cancer	BIOKEY_8_Pre	BIOKEY_8	Pre	None	None	NotAvailable
EGAD00001006608	Breast Cancer	BIOKEY_8_On	BIOKEY_8	Post	Anti-PD1	Pembrolizumab	NotAvailable
EGAD00001006608	Breast Cancer	BIOKEY_5_Pre	BIOKEY_5	Pre	None	None	NotAvailable
EGAD00001006608	Breast Cancer	BIOKEY_5_On	BIOKEY_5	Post	Anti-PD1	Pembrolizumab	NotAvailable
EGAD00001006608	Breast Cancer	BIOKEY_30_Pre	BIOKEY_30	Pre	None	None	NotAvailable
EGAD00001006608	Breast Cancer	BIOKEY_30_On	BIOKEY_30	Post	Anti-PD1	Pembrolizumab	NotAvailable
EGAD00001006608	Breast Cancer	BIOKEY_12_Pre	BIOKEY_12	Pre	None	None	NotAvailable
EGAD00001006608	Breast Cancer	BIOKEY_12_On	BIOKEY_12	Post	Anti-PD1	Pembrolizumab	NotAvailable
EGAD00001006608	Breast Cancer	BIOKEY_1_Pre	BIOKEY_1	Pre	None	None	NotAvailable
EGAD00001006608	Breast Cancer	BIOKEY_1_On	BIOKEY_1	Post	Anti-PD1	Pembrolizumab	NotAvailable
EGAD00001006608	Breast Cancer	BIOKEY_31_Pre	BIOKEY_31	Pre	None	None	NotAvailable
EGAD00001006608	Breast Cancer	BIOKEY_31_On	BIOKEY_31	Post	Anti-PD1	Pembrolizumab	NotAvailable
EGAD00001006608	Breast Cancer	BIOKEY_20_Pre	BIOKEY_20	Pre	None	None	NotAvailable
EGAD00001006608	Breast Cancer	BIOKEY_20_On	BIOKEY_20	Post	Anti-PD1	Pembrolizumab	NotAvailable
EGAD00001006608	Breast Cancer	BIOKEY_22_Pre	BIOKEY_22	Pre	None	None	NotAvailable
EGAD00001006608	Breast Cancer	BIOKEY_22_On	BIOKEY_22	Post	Anti-PD1	Pembrolizumab	NotAvailable
EGAD00001006608	Breast Cancer	BIOKEY_25_Pre	BIOKEY_25	Pre	None	None	NotAvailable
EGAD00001006608	Breast Cancer	BIOKEY_25_On	BIOKEY_25	Post	Anti-PD1	Pembrolizumab	NotAvailable
EGAD00001006608	Breast Cancer	BIOKEY_21_Pre	BIOKEY_21	Pre	None	None	NotAvailable
EGAD00001006608	Breast Cancer	BIOKEY_21_On	BIOKEY_21	Post	Anti-PD1	Pembrolizumab	NotAvailable
EGAD00001006608	Breast Cancer	BIOKEY_29_Pre	BIOKEY_29	Pre	None	None	NotAvailable
EGAD00001006608	Breast Cancer	BIOKEY_29_On	BIOKEY_29	Post	Anti-PD1	Pembrolizumab	NotAvailable
EGAD00001006608	Breast Cancer	BIOKEY_4_Pre	BIOKEY_4	Pre	None	None	NotAvailable
EGAD00001006608	Breast Cancer	BIOKEY_4_On	BIOKEY_4	Post	Anti-PD1	Pembrolizumab	NotAvailable
EGAD00001006608	Breast Cancer	BIOKEY_9_Pre	BIOKEY_9	Pre	None	None	NotAvailable
EGAD00001006608	Breast Cancer	BIOKEY_9_On	BIOKEY_9	Post	Anti-PD1	Pembrolizumab	NotAvailable
EGAD00001006608	Breast Cancer	BIOKEY_18_Pre	BIOKEY_18	Pre	None	None	NotAvailable
EGAD00001006608	Breast Cancer	BIOKEY_18_On	BIOKEY_18	Post	Anti-PD1	Pembrolizumab	NotAvailable
EGAD00001006608	Breast Cancer	BIOKEY_11_Pre	BIOKEY_11	Pre	None	None	NotAvailable
EGAD00001006608	Breast Cancer	BIOKEY_11_On	BIOKEY_11	Post	Anti-PD1	Pembrolizumab	NotAvailable
EGAD00001006608	Breast Cancer	BIOKEY_7_Pre	BIOKEY_7	Pre	None	None	NotAvailable
EGAD00001006608	Breast Cancer	BIOKEY_7_On	BIOKEY_7	Post	Anti-PD1	Pembrolizumab	NotAvailable
EGAD00001006608	Breast Cancer	BIOKEY_2_Pre	BIOKEY_2	Pre	None	None	NotAvailable

EGAD00001006608	Breast Cancer	BIOKEY_2_On	BIOKEY_2	Post	Anti-PD1	Pembrolizumab	NotAvailable
EGAD00001006608	Breast Cancer	BIOKEY_6_Pre	BIOKEY_6	Pre	None	None	NotAvailable
EGAD00001006608	Breast Cancer	BIOKEY_6_On	BIOKEY_6	Post	Anti-PD1	Pembrolizumab	NotAvailable
EGAD00001006608	Breast Cancer	BIOKEY_17_Pre	BIOKEY_17	Pre	None	None	NotAvailable
EGAD00001006608	Breast Cancer	BIOKEY_17_On	BIOKEY_17	Post	Anti-PD1	Pembrolizumab	NotAvailable
EGAD00001006608	Breast Cancer	BIOKEY_27_Pre	BIOKEY_27	Pre	None	None	NotAvailable
EGAD00001006608	Breast Cancer	BIOKEY_27_On	BIOKEY_27	Post	Anti-PD1	Pembrolizumab	NotAvailable
EGAD00001006608	Breast Cancer	BIOKEY_24_Pre	BIOKEY_24	Pre	None	None	NotAvailable
EGAD00001006608	Breast Cancer	BIOKEY_24_On	BIOKEY_24	Post	Anti-PD1	Pembrolizumab	NotAvailable
SCP1288	ccRCC	P76_scRNA	P76_scRN	Pre	None	None	None
SCP1288	ccRCC	P90_scRNA	P90_scRN	Pre	None	None	None
SCP1288	ccRCC	P915_scRNA	P915_scRN	Post	Anti-PD1+CTLA4	Not Provided	Responder
SRZ190804	ccRCC	UT1_Center	UT1	Pre	None	None	None
SRZ190804	ccRCC	UT1_Upper	UT1	Pre	None	None	None
SRZ190804	ccRCC	UT1_Lower	UT1	Pre	None	None	None
SRZ190804	ccRCC	UT2_Center	UT2	Pre	None	None	None
SRZ190804	ccRCC	UT2_Far	UT2	Pre	None	None	None
SRZ190804	ccRCC	UT2_Near	UT2	Pre	None	None	None
SRZ190804	ccRCC	t1_SupraLateral	t1	Post	Anti-PD1	Nivolumab	None
SRZ190804	ccRCC	t1_LowerLateral	t1	Post	Anti-PD1	Nivolumab	None
SRZ190804	ccRCC	t1_LowerMedial	t1	Post	Anti-PD1	Nivolumab	None
SRZ190804	ccRCC	t2_Center	t2	Post	Anti-PD1+CTLA4	Nivolumab/Ipilimumab	Non-responder
SRZ190804	ccRCC	t2_Far	t2	Post	Anti-PD1+CTLA4	Nivolumab/Ipilimumab	Non-responder
SRZ190804	ccRCC	t2_Near	t2	Post	Anti-PD1+CTLA4	Nivolumab/Ipilimumab	Non-responder
SRZ190804	ccRCC	t3_Center	t3	Post	Anti-PD1+CTLA4	Nivolumab/Ipilimumab	Responder
SRZ190804	ccRCC	t3_Far	t3	Post	Anti-PD1+CTLA4	Nivolumab/Ipilimumab	Responder
SRZ190804	ccRCC	t3_Near	t3	Post	Anti-PD1+CTLA4	Nivolumab/Ipilimumab	Responder
SRZ190804	ccRCC	t4_Center	t4	Post	Anti-PD1+CTLA4	Nivolumab/Ipilimumab	Responder
SRZ190804	ccRCC	t4_Lateral	t4	Post	Anti-PD1+CTLA4	Nivolumab/Ipilimumab	Responder
SRZ190804	ccRCC	t4_Medial	t4	Post	Anti-PD1+CTLA4	Nivolumab/Ipilimumab	Responder
InHouseData	CRC	CK	CK	Post	Anti-PD1	Nivolumab	Responder
InHouseData	CRC	CSY	CSY	Pre	None	None	Responder
InHouseData	CRC	CSY-T	CSY	Post	Anti-PD1	Nivolumab	Responder
InHouseData	CRC	KYSY-6874	GXP001	Pre	None	None	Responder
InHouseData	CRC	KYSY-6926	SC002	Pre	None	None	None

InHouseData	CRC	KYSY-7086	LLM003	Pre	None	None	Non-responder
InHouseData	CRC	KYSY-7380	GXP001	Post	Anti-PD1	Nivolumab	Responder
InHouseData	CRC	KYSY-7612	LLM003	Post	Anti-PD1	Nivolumab	Non-responder
InHouseData	CRC	LJW-1	LJW	Pre	None	None	None
InHouseData	CRC	LJW-2	LJW	Pre	None	None	None
InHouseData	CRC	LLH	LLH	Pre	None	None	None
InHouseData	CRC	LYL	LYL	Post	Anti-PD1	Nivolumab	Non-responder
InHouseData	CRC	WYH	WYH	Post	Anti-PD1	Nivolumab	Non-responder
InHouseData	CRC	WYX	WYX	Pre	None	None	None
InHouseData	CRC	XKL-BS	XKL-BS	Post	Anti-PD1	Nivolumab	Responder
InHouseData	CRC	YT	YT	Pre	None	None	Responder
InHouseData	CRC	YT06	YT	Post	Anti-PD1	Nivolumab	Responder
InHouseData	CRC	ZJL	ZJL	Pre	None	None	None
InHouseData	CRC	ZXF	ZXF	Post	Anti-PD1	Nivolumab	Responder
InHouseData	CRC	ZYL	ZYL	Post	Anti-PD1	Nivolumab	Non-responder
GSE151530	HCC	H70	H70	Pre	None	None	NotAvailable
GSE151530	HCC	H72	H72	Pre	None	None	NotAvailable
GSE151530	HCC	H62	H62	Pre	None	None	NotAvailable
GSE151530	HCC	H41	H41	Pre	None	None	NotAvailable
GSE151530	HCC	H43	H43	Post	Anti-PD-L1/Anti-CTL	Durvalumab/Tremelimu	NotAvailable
GSE151530	HCC	H65	H65	Post	Anti-PD-L1/Anti-CTL	Durvalumab/Tremelimu	NotAvailable
GSE151530	HCC	H21	H21	Pre	None	None	NotAvailable
GSE151530	HCC	H38	H38	Pre	None	None	NotAvailable
GSE151530	HCC	H37	H37	Post	Anti-PD-L1/Anti-CTL	Durvalumab/Tremelimu	NotAvailable
GSE200996	HNSCC	P18_pre-Tx	P18	Pre	None	None	NotAvailable
GSE200996	HNSCC	P23_pre-Tx	P23	Pre	None	None	Non-responder
GSE200996	HNSCC	P24_pre-Tx	P24	Pre	None	None	Non-responder
GSE200996	HNSCC	P27_pre-Tx	P27	Pre	None	None	Non-responder
GSE200996	HNSCC	P29_pre-Tx	P29	Pre	None	None	Non-responder
GSE200996	HNSCC	P32_pre-Tx	P32	Pre	None	None	Non-responder
GSE200996	HNSCC	P13_post-Tx	P13	Post	Anti-PD1+CTLA4	Nivolumab+Ipilimumab	Responder
GSE200996	HNSCC	P14_post-Tx	P14	Post	Anti-PD1	Nivolumab	Non-responder
GSE200996	HNSCC	P15_post-Tx	P15	Post	Anti-PD1	Nivolumab	Non-responder
GSE200996	HNSCC	P16_post-Tx	P16	Post	Anti-PD1+CTLA4	Nivolumab+Ipilimumab	NotAvailable
GSE200996	HNSCC	P18_post-Tx	P18	Post	Anti-PD1+CTLA4	Nivolumab+Ipilimumab	NotAvailable

GSE200996	HNSCC	P19_post-Tx	P19	Post	Anti-PD1	Nivolumab	NotAvailable
GSE200996	HNSCC	P20_post-Tx	P20	Post	Anti-PD1	Nivolumab	NotAvailable
GSE200996	HNSCC	P21_post-Tx	P21	Post	Anti-PD1+CTLA4	Nivolumab+Ipilimumab	Non-responder
GSE200996	HNSCC	P22_post-Tx	P22	Post	Anti-PD1	Nivolumab	NotAvailable
GSE200996	HNSCC	P23_post-Tx	P23	Post	Anti-PD1	Nivolumab	Non-responder
GSE200996	HNSCC	P24_post-Tx	P24	Post	Anti-PD1+CTLA4	Nivolumab+Ipilimumab	Non-responder
GSE200996	HNSCC	P25_post-Tx	P25	Post	Anti-PD1+CTLA4	Nivolumab+Ipilimumab	Non-responder
GSE200996	HNSCC	P26_post-Tx	P26	Post	Anti-PD1+CTLA4	Nivolumab+Ipilimumab	Responder
GSE200996	HNSCC	P27_post-Tx	P27	Post	Anti-PD1	Nivolumab	Non-responder
GSE200996	HNSCC	P28_post-Tx	P28	Post	Anti-PD1	Nivolumab	Responder
GSE200996	HNSCC	P29_post-Tx	P29	Post	Anti-PD1	Nivolumab	Non-responder
GSE200996	HNSCC	P30_post-Tx	P30	Post	Anti-PD1+CTLA4	Nivolumab+Ipilimumab	NotAvailable
GSE200996	HNSCC	P31_post-Tx	P31	Post	Anti-PD1	Nivolumab	Non-responder
GSE200996	HNSCC	P32_post-Tx	P32	Post	Anti-PD1+CTLA4	Nivolumab+Ipilimumab	Non-responder
GSE151530	iCCA	C60	C60	Pre	None	None	NotAvailable
GSE151530	iCCA	C42	C42	Post	Anti-PD1	Pembrolizumab	NotAvailable
GSE151530	iCCA	C66	C66	Post	Anti-PD1	Pembrolizumab	NotAvailable
GSE151530	iCCA	C25	C25	Post	Anti-PD-L1/Anti-CTL	Durvalumab/Tremelimu	NotAvailable
GSE151530	iCCA	C29	C29	Post	Anti-PD-L1/Anti-CTL	Durvalumab/Tremelimu	NotAvailable
GSE151530	iCCA	C39	C39	Post	Anti-PD1	Pembrolizumab	NotAvailable
GSE151530	iCCA	C35	C35	Post	Anti-PD1	Pembrolizumab	NotAvailable
GSE120575	Melanoma	Pre_P2_Non-responder_anti-PD1	Pre_P2	Pre	None	None	Non-responder
GSE120575	Melanoma	Post_P2_Non-responder_anti-PD1	Post_P2	Post	Anti-PD1	Pembrolizumab	Non-responder
GSE120575	Melanoma	Pre_P3_Non-responder_anti-PD1	Pre_P3	Pre	None	None	Non-responder
GSE120575	Melanoma	Post_P3_Non-responder_anti-PD1	Post_P3	Post	Anti-PD1	Pembrolizumab	Non-responder
GSE120575	Melanoma	Post_P3_2_Non-responder_anti-PD	Post_P3_2	Post	Anti-PD1	Pembrolizumab	Non-responder
GSE120575	Melanoma	Post_P7_Responder_anti-CTLA4+PD	Post_P7	Post	Anti-PD1+CTLA4	Not Provided	Responder
GSE120575	Melanoma	Pre_P8_Responder_anti-CTLA4+PD1	Pre_P8	Pre	None	None	Responder
GSE120575	Melanoma	Post_P8_Responder_anti-CTLA4+PD	Post_P8	Post	Anti-PD1+CTLA4	Not Provided	Responder
GSE120575	Melanoma	Post_P10_Non-responder_anti-PD1	Post_P10	Post	Anti-PD1	Pembrolizumab	Non-responder
GSE120575	Melanoma	Post_P11_Non-responder_anti-PD1	Post_P11	Post	Anti-PD1	Pembrolizumab	Non-responder
GSE120575	Melanoma	Pre_P12_Non-responder_anti-PD1	Pre_P12	Pre	None	None	Non-responder
GSE120575	Melanoma	Post_P12_Non-responder_anti-PD1	Post_P12	Post	Anti-PD1	Pembrolizumab	Non-responder
GSE120575	Melanoma	Post_P13_Non-responder_anti-CTL	Post_P13	Post	Anti-PD1+CTLA4	Not Provided	Non-responder
GSE120575	Melanoma	Post_P14_Non-responder_anti-PD1	Post_P14	Post	Anti-PD1	Pembrolizumab	Non-responder

GSE120575	Melanoma	Pre_P15_Non-responder_anti-PD1	Pre_P15	Pre	None	None	Non-responder
GSE120575	Melanoma	Post_P15_Non-responder_anti-PD1	Post_P15	Post	Anti-PD1	Pembrolizumab	Non-responder
GSE120575	Melanoma	Post_P16_Non-responder_anti-PD1	Post_P16	Post	Anti-PD1	Pembrolizumab	Non-responder
GSE120575	Melanoma	Post_P18_Non-responder_anti-PD1	Post_P18	Post	Anti-PD1	Pembrolizumab	Non-responder
GSE120575	Melanoma	Post_P19_Responder_anti-PD1	Post_P19	Post	Anti-PD1	Pembrolizumab	Responder
GSE120575	Melanoma	Pre_P20_Non-responder_anti-PD1	Pre_P20	Pre	None	None	Non-responder
GSE120575	Melanoma	Post_P20_Non-responder_anti-PD1	Post_P20	Post	Anti-PD1	Pembrolizumab	Non-responder
GSE120575	Melanoma	Post_P21_Responder_anti-PD1	Post_P21	Post	Anti-PD1	Pembrolizumab	Responder
GSE120575	Melanoma	Post_P22_Non-responder_anti-PD1	Post_P22	Post	Anti-PD1	Pembrolizumab	Non-responder
GSE120575	Melanoma	Post_P23_Non-responder_anti-PD1	Post_P23	Post	Anti-PD1	Pembrolizumab	Non-responder
GSE120575	Melanoma	Post_P23_2_Non-responder_anti-P	Post_P23_2	Post	Anti-PD1	Pembrolizumab	Non-responder
GSE120575	Melanoma	Pre_P24_Responder_anti-PD1	Pre_P24	Pre	None	None	Responder
GSE120575	Melanoma	Pre_P26_Responder_anti-CTLA4+PI	Pre_P26	Pre	None	None	Responder
GSE120575	Melanoma	Pre_P27_Non-responder_anti-PD1	Pre_P27	Pre	None	None	Non-responder
GSE120575	Melanoma	Pre_P29_Responder_anti-PD1	Pre_P29	Pre	None	None	Responder
GSE120575	Melanoma	Post_P30_Non-responder_anti-PD1	Post_P30	Post	Anti-PD1	Pembrolizumab	Non-responder
GSE120575	Melanoma	Pre_P31_Non-responder_anti-PD1	Pre_P31	Pre	None	None	Non-responder
GSE120575	Melanoma	Post_P17_Responder_anti-PD1	Post_P17	Post	Anti-PD1	Pembrolizumab	Responder
