# Supplementary material for: A single-cell pan-cancer analysis to show the variability of tumor-infiltrating myeloid cells in immune checkpoint blockade
Source: Nat Commun. 2024 Jul 21;15:6142. doi: 10.1038/s41467-024-50478-8 (PMC11271490; doi:10.1038/s41467-024-50478-8)
Supplement: Supplementary file 5 — Supplementary Data 2 [file 41467_2024_50478_MOESM5_ESM.pdf]

cDC_CLEC9A

cluster	gene	p_val	avg_log2FC	pct.1	pct.2	p_val_adj
cDC_CLEC9A	C1orf54	0,00000E+00	3,90182E+00	0,893	0,271	0,00000E+00
cDC_CLEC9A	IDO1	0,00000E+00	3,88486E+00	0,796	0,097	0,00000E+00
cDC_CLEC9A	CLEC9A	0,00000E+00	3,70914E+00	0,813	0,029	0,00000E+00
cDC_CLEC9A	DNASE1L3	0,00000E+00	3,47489E+00	0,74	0,054	0,00000E+00
cDC_CLEC9A	XCR1	0,00000E+00	3,43715E+00	0,573	0,001	0,00000E+00
cDC_CLEC9A	S100B	0,00000E+00	3,39614E+00	0,526	0,093	0,00000E+00
cDC_CLEC9A	CPVL	0,00000E+00	3,32299E+00	0,917	0,557	0,00000E+00
cDC_CLEC9A	WDFY4	0,00000E+00	3,28789E+00	0,817	0,15	0,00000E+00
cDC_CLEC9A	CPNE3	0,00000E+00	2,97972E+00	0,879	0,231	0,00000E+00
cDC_CLEC9A	CADM1	0,00000E+00	2,93786E+00	0,621	0,107	0,00000E+00
cDC_CLEC9A	ASB2	0,00000E+00	2,93445E+00	0,486	0,021	0,00000E+00
cDC_CLEC9A	CCND1	0,00000E+00	2,90696E+00	0,738	0,154	0,00000E+00
cDC_CLEC9A	HLA-DOB	0,00000E+00	2,87068E+00	0,612	0,061	0,00000E+00
cDC_CLEC9A	LGALS2	0,00000E+00	2,80855E+00	0,878	0,245	0,00000E+00
cDC_CLEC9A	CLNK	0,00000E+00	2,70916E+00	0,688	0,015	0,00000E+00
cDC_CLEC9A	SNX3	0,00000E+00	2,69664E+00	0,95	0,685	0,00000E+00
cDC_CLEC9A	NAAA	1,66442E-295	2,65785E+00	0,811	0,359	3,06536E-291
cDC_CLEC9A	RGCC	0,00000E+00	2,51532E+00	0,83	0,215	0,00000E+00
cDC_CLEC9A	NET1	0,00000E+00	2,46964E+00	0,687	0,086	0,00000E+00
cDC_CLEC9A	FLT3	0,00000E+00	2,40039E+00	0,647	0,06	0,00000E+00
cDC_CLEC9A	IRF8	0,00000E+00	2,37404E+00	0,922	0,368	0,00000E+00
cDC_CLEC9A	VAC14	0,00000E+00	2,35567E+00	0,68	0,148	0,00000E+00
cDC_CLEC9A	CST3	2,92045E-255	2,35245E+00	0,986	0,949	5,37859E-251
cDC_CLEC9A	NDRG2	2,57972E-203	2,34110E+00	0,514	0,138	4,75107E-199
cDC_CLEC9A	CST7	1,84934E-165	2,31145E+00	0,466	0,138	3,40593E-161
cDC_CLEC9A	PPT1	1,68543E-119	2,26748E+00	0,851	0,647	3,10405E-115
cDC_CLEC9A	DBN1	0,00000E+00	2,24074E+00	0,432	0,01	0,00000E+00
cDC_CLEC9A	BTLA	0,00000E+00	2,20388E+00	0,459	0,013	0,00000E+00
cDC_CLEC9A	TACSTD2	0,00000E+00	2,16906E+00	0,442	0,018	0,00000E+00
cDC_CLEC9A	TMEM14A	0,00000E+00	2,14830E+00	0,622	0,058	0,00000E+00
cDC_CLEC9A	CSRP1	2,18302E-203	2,14564E+00	0,654	0,25	4,02047E-199
cDC_CLEC9A	GSTP1	1,90282E-259	2,13820E+00	0,951	0,821	3,50442E-255
cDC_CLEC9A	BATF3	0,00000E+00	2,13564E+00	0,588	0,064	0,00000E+00
cDC_CLEC9A	TAP1	1,00062E-226	2,12977E+00	0,814	0,415	1,84284E-222
cDC_CLEC9A	CYB5R3	2,70326E-215	2,10203E+00	0,779	0,403	4,97859E-211
cDC_CLEC9A	CNN2	1,72437E-289	2,07956E+00	0,834	0,394	3,17578E-285
cDC_CLEC9A	AP1G2	1,93264E-135	2,07666E+00	0,437	0,137	3,55935E-131
cDC_CLEC9A	ID2	9,73257E-147	2,07501E+00	0,854	0,551	1,79245E-142
cDC_CLEC9A	LSP1	0,00000E+00	2,07053E+00	0,963	0,665	0,00000E+00
cDC_CLEC9A	SLAMF7	7,81674E-308	2,02004E+00	0,699	0,2	1,43961E-303

cDC_CLEC9A	CCSER1	0,00000E+00	2,01265E+00	0,482	0,026	0,00000E+00
cDC_CLEC9A	ANPEP	2,09148E-186	2,00706E+00	0,622	0,201	3,85187E-182
cDC_CLEC9A	UCP2	5,37180E-178	1,99304E+00	0,894	0,629	9,89324E-174
cDC_CLEC9A	SLAMF8	6,49050E-73	1,92516E+00	0,612	0,32	1,19536E-68
cDC_CLEC9A	PLCD1	0,00000E+00	1,92333E+00	0,436	0,037	0,00000E+00
cDC_CLEC9A	TMSB4X	5,67488E-253	1,91117E+00	0,998	0,983	1,04514E-248
cDC_CLEC9A	ACTG1	2,81732E-273	1,88790E+00	0,982	0,921	5,18867E-269
cDC_CLEC9A	HLA-DPA1	9,42257E-126	1,88548E+00	0,996	0,896	1,73536E-121
cDC_CLEC9A	CAMK2D	0,00000E+00	1,86883E+00	0,666	0,159	0,00000E+00
cDC_CLEC9A	NAPSA	0,00000E+00	1,86676E+00	0,694	0,17	0,00000E+00
cDC_CLEC9A	SUSD3	4,73134E-210	1,86286E+00	0,407	0,094	8,71371E-206
cDC_CLEC9A	BCL6	2,13902E-135	1,83894E+00	0,42	0,127	3,93943E-131
cDC_CLEC9A	HLA-DPB1	1,28587E-153	1,81608E+00	0,993	0,881	2,36819E-149
cDC_CLEC9A	SUB1	8,45249E-194	1,81149E+00	0,903	0,72	1,55670E-189
cDC_CLEC9A	NLRC5	4,12894E-154	1,80054E+00	0,409	0,115	7,60426E-150
cDC_CLEC9A	ICAM3	0,00000E+00	1,79880E+00	0,708	0,187	0,00000E+00
cDC_CLEC9A	PPA1	4,07519E-193	1,79159E+00	0,824	0,454	7,50528E-189
cDC_CLEC9A	CYP2S1	5,43914E-169	1,78777E+00	0,432	0,119	1,00173E-164
cDC_CLEC9A	FUCA1	1,34478E-30	1,78420E+00	0,436	0,247	2,47668E-26
cDC_CLEC9A	PNMA1	0,00000E+00	1,77454E+00	0,39	0,054	0,00000E+00
cDC_CLEC9A	ACTB	1,15807E-265	1,77390E+00	0,999	0,991	2,13281E-261
cDC_CLEC9A	RGS10	1,53888E-159	1,75986E+00	0,922	0,737	2,83416E-155
cDC_CLEC9A	CD40	4,52363E-28	1,75576E+00	0,473	0,289	8,33117E-24
cDC_CLEC9A	SRP19	2,95758E-96	1,75511E+00	0,439	0,177	5,44698E-92
cDC_CLEC9A	C1QBP	7,89464E-149	1,74041E+00	0,76	0,423	1,45396E-144
cDC_CLEC9A	CCDC126	0,00000E+00	1,74003E+00	0,493	0,05	0,00000E+00
cDC_CLEC9A	APOL3	2,21354E-113	1,73821E+00	0,48	0,176	4,07667E-109
cDC_CLEC9A	MPEG1	1,23344E-126	1,73273E+00	0,728	0,367	2,27163E-122
cDC_CLEC9A	C1orf21	0,00000E+00	1,72720E+00	0,53	0,051	0,00000E+00
cDC_CLEC9A	CDK2AP2	1,13690E-212	1,71561E+00	0,794	0,39	2,09384E-208
cDC_CLEC9A	HLA-DQB1	2,13336E-152	1,70983E+00	0,977	0,813	3,92902E-148
cDC_CLEC9A	CD74	6,70841E-142	1,70962E+00	0,999	0,962	1,23549E-137
cDC_CLEC9A	DCTPP1	6,96608E-148	1,69524E+00	0,53	0,19	1,28294E-143
cDC_CLEC9A	PRDX2	1,17766E-116	1,68754E+00	0,507	0,191	2,16890E-112
cDC_CLEC9A	ATG3	1,34296E-83	1,67601E+00	0,753	0,518	2,47332E-79
cDC_CLEC9A	PON2	5,84254E-135	1,66834E+00	0,418	0,125	1,07602E-130
cDC_CLEC9A	LSM6	9,24424E-143	1,66309E+00	0,7	0,344	1,70251E-138
cDC_CLEC9A	ASAP1	1,91648E-304	1,66124E+00	0,696	0,213	3,52959E-300
cDC_CLEC9A	GSTM2	3,83695E-226	1,65977E+00	0,304	0,052	7,06651E-222
cDC_CLEC9A	NAP1L1	8,84331E-128	1,65188E+00	0,854	0,615	1,62867E-123
cDC_CLEC9A	HDAC9	1,34112E-282	1,65042E+00	0,541	0,126	2,46994E-278

cDC_CLEC9A	LIMD2	6,33067E-241	1,64923E+00	0,878	0,474	1,16592E-236
cDC_CLEC9A	ACSS2	1,43936E-93	1,64106E+00	0,301	0,091	2,65087E-89
cDC_CLEC9A	RHOF	2,17293E-306	1,64059E+00	0,612	0,149	4,00188E-302
cDC_CLEC9A	NDUFC2	1,92845E-41	1,63890E+00	0,498	0,302	3,55163E-37
cDC_CLEC9A	ISG20	1,18435E-26	1,63807E+00	0,493	0,307	2,18121E-22
cDC_CLEC9A	PIK3CB	2,15176E-202	1,63635E+00	0,45	0,114	3,96290E-198
cDC_CLEC9A	CD226	0,00000E+00	1,62875E+00	0,402	0,046	0,00000E+00
cDC_CLEC9A	PRCP	2,64521E-43	1,62255E+00	0,627	0,385	4,87169E-39
cDC_CLEC9A	GCSAM	0,00000E+00	1,62128E+00	0,453	0,017	0,00000E+00
cDC_CLEC9A	SERPINB9	1,91360E-108	1,62067E+00	0,706	0,369	3,52427E-104
cDC_CLEC9A	DCXR	5,67725E-73	1,60756E+00	0,651	0,362	1,04558E-68
cDC_CLEC9A	ZNF366	0,00000E+00	1,60202E+00	0,501	0,031	0,00000E+00
cDC_CLEC9A	NDUFS4	1,50338E-77	1,57502E+00	0,573	0,281	2,76878E-73
cDC_CLEC9A	RPL4	7,49184E-70	1,57287E+00	0,627	0,474	1,37977E-65
cDC_CLEC9A	HLA-DQA1	9,94178E-119	1,57049E+00	0,967	0,78	1,83098E-114
cDC_CLEC9A	PPM1J	0,00000E+00	1,56403E+00	0,434	0,021	0,00000E+00
cDC_CLEC9A	SNRPA	7,11836E-63	1,55849E+00	0,557	0,291	1,31099E-58
cDC_CLEC9A	FARP2	7,68118E-119	1,55165E+00	0,321	0,089	1,41464E-114
cDC_CLEC9A	RPL8	8,04865E-58	1,54807E+00	0,656	0,571	1,48232E-53
cDC_CLEC9A	PDLIM7	4,98408E-77	1,54222E+00	0,453	0,192	9,17919E-73
cDC_CLEC9A	TRPV2	3,24888E-73	1,54159E+00	0,467	0,204	5,98346E-69
cDC_CLEC9A	ANXA6	2,49328E-220	1,52727E+00	0,648	0,217	4,59188E-216
cDC_CLEC9A	LMNA	7,19549E-121	1,52254E+00	0,726	0,362	1,32519E-116
cDC_CLEC9A	PSMB9	5,39392E-132	1,52199E+00	0,881	0,653	9,93397E-128
cDC_CLEC9A	BID	4,75919E-101	1,51286E+00	0,721	0,408	8,76500E-97
cDC_CLEC9A	LCP1	1,02749E-109	1,51029E+00	0,884	0,7	1,89233E-105
cDC_CLEC9A	RPL18A	1,80401E-62	1,49403E+00	0,656	0,561	3,32245E-58
cDC_CLEC9A	PTP4A2	2,89348E-44	1,49252E+00	0,627	0,4	5,32892E-40
cDC_CLEC9A	DUSP4	3,81672E-212	1,49104E+00	0,44	0,11	7,02926E-208
cDC_CLEC9A	HLA-DRA	2,17494E-41	1,49075E+00	0,992	0,924	4,00558E-37
cDC_CLEC9A	FLOT2	1,64082E-127	1,48997E+00	0,578	0,236	3,02189E-123
cDC_CLEC9A	COX5A	2,25976E-63	1,48585E+00	0,846	0,691	4,16179E-59
cDC_CLEC9A	MAP3K13	4,52468E-136	1,48557E+00	0,55	0,206	8,33311E-132
cDC_CLEC9A	CKLF	1,79065E-81	1,48357E+00	0,754	0,501	3,29783E-77
cDC_CLEC9A	TMEM50B	5,27085E-171	1,48269E+00	0,569	0,194	9,70732E-167
cDC_CLEC9A	CD48	6,55552E-59	1,46453E+00	0,736	0,474	1,20733E-54
cDC_CLEC9A	ENSA	1,56551E-197	1,46327E+00	0,814	0,466	2,88321E-193
cDC_CLEC9A	SNX22	0,00000E+00	1,45822E+00	0,449	0,018	0,00000E+00
cDC_CLEC9A	RNH1	6,52043E-64	1,45524E+00	0,812	0,614	1,20087E-59
cDC_CLEC9A	RPS23	7,20988E-64	1,45453E+00	0,656	0,566	1,32784E-59
cDC_CLEC9A	PSMB4	6,51000E-23	1,45037E+00	0,562	0,369	1,19895E-18

cDC_CLEC9A	KCNK6	2,45330E-293	1,44704E+00	0,621	0,166	4,51825E-289
cDC_CLEC9A	RPL27A	1,14515E-34	1,44541E+00	0,617	0,461	2,10902E-30
cDC_CLEC9A	BIRC3	3,77519E-82	1,44334E+00	0,511	0,217	6,95277E-78
cDC_CLEC9A	AIM2	5,23094E-296	1,44100E+00	0,427	0,079	9,63383E-292
cDC_CLEC9A	PTPN7	9,19969E-74	1,43213E+00	0,363	0,137	1,69431E-69
cDC_CLEC9A	EEF1B2	1,59496E-142	1,42775E+00	0,927	0,84	2,93743E-138
cDC_CLEC9A	DCAF13	5,20883E-48	1,42575E+00	0,302	0,128	9,59311E-44
cDC_CLEC9A	CIITA	5,71365E-83	1,42473E+00	0,626	0,319	1,05228E-78
cDC_CLEC9A	CKS2	1,72923E-106	1,42298E+00	0,558	0,237	3,18471E-102
cDC_CLEC9A	PTDSS1	2,33223E-44	1,42243E+00	0,497	0,265	4,29527E-40
cDC_CLEC9A	PSTPIP1	3,82960E-177	1,41259E+00	0,534	0,166	7,05297E-173
cDC_CLEC9A	AGPAT1	1,01204E-182	1,41200E+00	0,517	0,155	1,86387E-178
cDC_CLEC9A	VDAC3	3,83306E-36	1,41041E+00	0,528	0,308	7,05934E-32
cDC_CLEC9A	NEDD9	1,29009E-71	1,40924E+00	0,312	0,11	2,37596E-67
cDC_CLEC9A	MCM5	6,06958E-141	1,40287E+00	0,568	0,217	1,11783E-136
cDC_CLEC9A	PSMA6	1,62171E-48	1,40278E+00	0,682	0,462	2,98670E-44
cDC_CLEC9A	MSRB1	6,00919E-52	1,40079E+00	0,402	0,187	1,10671E-47
cDC_CLEC9A	EEF1G	1,28757E-42	1,39894E+00	0,492	0,292	2,37131E-38
cDC_CLEC9A	RPS11	6,64099E-33	1,38864E+00	0,63	0,501	1,22307E-28
cDC_CLEC9A	ELOVL5	3,34403E-162	1,38428E+00	0,678	0,28	6,15870E-158
cDC_CLEC9A	SERPINF2	0,00000E+00	1,38388E+00	0,608	0,114	0,00000E+00
cDC_CLEC9A	TTF2	5,36845E-81	1,38373E+00	0,251	0,075	9,88707E-77
cDC_CLEC9A	P2RY6	5,61590E-204	1,38023E+00	0,588	0,187	1,03428E-199
cDC_CLEC9A	PTPRE	2,72260E-176	1,37803E+00	0,829	0,432	5,01421E-172
cDC_CLEC9A	MAP4K1	0,00000E+00	1,37795E+00	0,506	0,095	0,00000E+00
cDC_CLEC9A	RPS18	1,03358E-41	1,37594E+00	0,66	0,558	1,90354E-37
cDC_CLEC9A	DENND1B	3,43674E-227	1,37130E+00	0,607	0,188	6,32945E-223
cDC_CLEC9A	NUBPL	1,26230E-246	1,37064E+00	0,289	0,044	2,32478E-242
cDC_CLEC9A	TXN	1,32669E-99	1,36994E+00	0,872	0,663	2,44337E-95
cDC_CLEC9A	PSME2	1,21322E-83	1,36782E+00	0,908	0,712	2,23438E-79
cDC_CLEC9A	UBE2V2	1,56058E-56	1,36754E+00	0,508	0,254	2,87412E-52
cDC_CLEC9A	AIP	5,22464E-40	1,36730E+00	0,57	0,335	9,62221E-36
cDC_CLEC9A	SDHA	6,46992E-58	1,36710E+00	0,494	0,247	1,19156E-53
cDC_CLEC9A	SERPINB1	4,03290E-58	1,36640E+00	0,816	0,66	7,42740E-54
cDC_CLEC9A	RPS16	8,84912E-40	1,36379E+00	0,634	0,543	1,62974E-35
cDC_CLEC9A	LYRM4	2,19614E-231	1,36016E+00	0,514	0,134	4,04462E-227
cDC_CLEC9A	TPM3	6,93547E-79	1,35885E+00	0,919	0,801	1,27730E-74
cDC_CLEC9A	MYADM	7,93663E-64	1,35874E+00	0,606	0,329	1,46169E-59
cDC_CLEC9A	HMGN1	5,87018E-80	1,35522E+00	0,832	0,621	1,08111E-75
cDC_CLEC9A	CFL1	2,93452E-139	1,35306E+00	0,97	0,921	5,40451E-135
cDC_CLEC9A	SLC1A5	8,30978E-77	1,35291E+00	0,512	0,234	1,53041E-72

cDC_CLEC9A	NSMAF	7,69846E-54	1,34557E+00	0,37	0,162	1,41783E-49
cDC_CLEC9A	ITGB7	7,62927E-296	1,33861E+00	0,444	0,085	1,40508E-291
cDC_CLEC9A	FNBP1	1,19686E-199	1,33805E+00	0,812	0,426	2,20426E-195
cDC_CLEC9A	WDR41	6,24867E-79	1,33666E+00	0,404	0,159	1,15082E-74
cDC_CLEC9A	EIF6	2,15140E-81	1,33428E+00	0,688	0,4	3,96224E-77
cDC_CLEC9A	PAK1	1,61548E-77	1,33262E+00	0,736	0,431	2,97524E-73
cDC_CLEC9A	RPSA	1,14305E-54	1,33217E+00	0,626	0,493	2,10516E-50
cDC_CLEC9A	GABARAPL2	1,32257E-15	1,33056E+00	0,726	0,575	2,43577E-11
cDC_CLEC9A	ARPC2	2,88476E-83	1,32625E+00	0,914	0,858	5,31286E-79
cDC_CLEC9A	SCNM1	3,68704E-56	1,32494E+00	0,599	0,33	6,79043E-52
cDC_CLEC9A	ERAP2	3,93785E-104	1,32166E+00	0,44	0,166	7,25234E-100
cDC_CLEC9A	G3BP2	1,21486E-102	1,32129E+00	0,584	0,271	2,23740E-98
cDC_CLEC9A	TNFRSF10B	9,89373E-29	1,31893E+00	0,26	0,126	1,82213E-24
cDC_CLEC9A	GDI2	1,55838E-65	1,31471E+00	0,839	0,647	2,87007E-61
cDC_CLEC9A	ETFA	1,54383E-41	1,31425E+00	0,568	0,331	2,84327E-37
cDC_CLEC9A	LIMA1	0,00000E+00	1,31024E+00	0,431	0,076	0,00000E+00
cDC_CLEC9A	TPD52	1,26593E-143	1,30961E+00	0,346	0,089	2,33145E-139
cDC_CLEC9A	S100A10	5,05306E-163	1,30748E+00	0,959	0,835	9,30623E-159
cDC_CLEC9A	PRMT2	3,46546E-52	1,30679E+00	0,689	0,423	6,38234E-48
cDC_CLEC9A	ADAM19	9,85800E-153	1,30440E+00	0,392	0,104	1,81555E-148
cDC_CLEC9A	IDH2	6,52234E-62	1,30288E+00	0,666	0,406	1,20122E-57
cDC_CLEC9A	INPP5D	1,04961E-70	1,29853E+00	0,597	0,306	1,93306E-66
cDC_CLEC9A	RPS5	8,28870E-50	1,29833E+00	0,64	0,515	1,52653E-45
cDC_CLEC9A	VAV3	0,00000E+00	1,29107E+00	0,443	0,07	0,00000E+00
cDC_CLEC9A	HAVCR2	8,59498E-24	1,29073E+00	0,643	0,431	1,58294E-19
cDC_CLEC9A	UNC119	2,21486E-117	1,28736E+00	0,601	0,261	4,07910E-113
cDC_CLEC9A	SNRNP25	1,77199E-109	1,28732E+00	0,467	0,177	3,26346E-105
cDC_CLEC9A	RPL23A	2,85164E-48	1,28313E+00	0,643	0,523	5,25186E-44
cDC_CLEC9A	TAGLN2	2,62041E-175	1,28306E+00	0,919	0,751	4,82600E-171
cDC_CLEC9A	RAN	7,88644E-44	1,27953E+00	0,792	0,655	1,45245E-39
cDC_CLEC9A	FGD2	3,81917E-58	1,27877E+00	0,616	0,339	7,03376E-54
cDC_CLEC9A	DUSP18	1,00452E-135	1,27720E+00	0,316	0,08	1,85002E-131
cDC_CLEC9A	RPS21	2,51858E-53	1,27513E+00	0,647	0,541	4,63847E-49
cDC_CLEC9A	DBNL	3,32963E-62	1,27344E+00	0,733	0,487	6,13217E-58
cDC_CLEC9A	C1orf162	2,87635E-54	1,27251E+00	0,896	0,704	5,29737E-50
cDC_CLEC9A	SNAP23	1,52092E-29	1,27036E+00	0,603	0,396	2,80108E-25
cDC_CLEC9A	TUBA1A	3,81901E-75	1,26968E+00	0,711	0,457	7,03346E-71
cDC_CLEC9A	NME1-NME2	2,24627E-07	1,26850E+00	0,284	0,238	4,13695E-03
cDC_CLEC9A	NDUFV2	1,25517E-80	1,26405E+00	0,777	0,53	2,31164E-76
cDC_CLEC9A	PPM1M	1,57922E-131	1,26051E+00	0,487	0,176	2,90845E-127
cDC_CLEC9A	PSME1	5,86673E-86	1,25177E+00	0,884	0,752	1,08048E-81

cDC_CLEC9A	UBL7	3,63451E-126	1,25149E+00	0,594	0,247	6,69368E-122
cDC_CLEC9A	LPP	8,73460E-78	1,24728E+00	0,556	0,262	1,60865E-73
cDC_CLEC9A	TOMM34	4,02346E-181	1,24660E+00	0,402	0,099	7,41000E-177
cDC_CLEC9A	C20orf27	1,75006E-155	1,24465E+00	0,71	0,338	3,22308E-151
cDC_CLEC9A	TRMT112	5,98068E-100	1,24344E+00	0,83	0,614	1,10146E-95
cDC_CLEC9A	THEM4	4,31651E-261	1,24231E+00	0,394	0,075	7,94972E-257
cDC_CLEC9A	MYCBP	1,01508E-43	1,24206E+00	0,251	0,1	1,86946E-39
cDC_CLEC9A	CD59	9,10568E-68	1,24121E+00	0,634	0,335	1,67699E-63
cDC_CLEC9A	OSBPL9	2,65957E-88	1,23833E+00	0,429	0,164	4,89813E-84
cDC_CLEC9A	IFT20	2,76916E-118	1,23620E+00	0,549	0,225	5,09996E-114
cDC_CLEC9A	RPS29	1,26275E-24	1,23532E+00	0,624	0,5	2,32560E-20
cDC_CLEC9A	GYPC	1,14744E-141	1,22814E+00	0,688	0,293	2,11324E-137
cDC_CLEC9A	EIF3M	4,40165E-44	1,22588E+00	0,729	0,495	8,10652E-40
cDC_CLEC9A	RAB7B	0,00000E+00	1,22268E+00	0,398	0,034	0,00000E+00
cDC_CLEC9A	B3GNTL1	1,32782E-156	1,21889E+00	0,308	0,069	2,44545E-152
cDC_CLEC9A	STX12	2,18497E-35	1,21566E+00	0,53	0,308	4,02406E-31
cDC_CLEC9A	TAPBP	5,12138E-42	1,21415E+00	0,761	0,576	9,43204E-38
cDC_CLEC9A	MYL6	1,31759E-56	1,21362E+00	0,96	0,941	2,42660E-52
cDC_CLEC9A	MYD88	1,79980E-73	1,21051E+00	0,64	0,352	3,31469E-69
cDC_CLEC9A	HSPA8	1,00003E-55	1,21042E+00	0,914	0,818	1,84175E-51
cDC_CLEC9A	TMSB10	9,09109E-139	1,20799E+00	0,998	0,984	1,67431E-134
cDC_CLEC9A	RPL10	2,21984E-30	1,20796E+00	0,659	0,593	4,08828E-26
cDC_CLEC9A	ZFAND2A	8,05993E-26	1,20556E+00	0,307	0,168	1,48440E-21
cDC_CLEC9A	RPL19	2,10496E-38	1,20446E+00	0,657	0,579	3,87671E-34
cDC_CLEC9A	RPS24	2,57076E-37	1,19951E+00	0,65	0,576	4,73457E-33
cDC_CLEC9A	RPS3	1,77013E-25	1,19814E+00	0,643	0,566	3,26005E-21
cDC_CLEC9A	VMO1	7,60646E-14	1,19508E+00	0,333	0,225	1,40088E-09
cDC_CLEC9A	RPL14	8,60450E-51	1,19417E+00	0,644	0,541	1,58469E-46
cDC_CLEC9A	ACTR3	9,63262E-86	1,19160E+00	0,843	0,66	1,77404E-81
cDC_CLEC9A	TUBA1B	8,53150E-20	1,19029E+00	0,866	0,754	1,57125E-15
cDC_CLEC9A	RPL21	9,28643E-37	1,18549E+00	0,644	0,539	1,71028E-32
cDC_CLEC9A	ANP32E	1,47512E-34	1,18300E+00	0,344	0,176	2,71672E-30
cDC_CLEC9A	RPL13A	3,68544E-16	1,17753E+00	0,623	0,519	6,78747E-12
cDC_CLEC9A	GTF3C6	2,66347E-20	1,17595E+00	0,551	0,368	4,90532E-16
cDC_CLEC9A	SELPLG	2,69617E-121	1,17467E+00	0,686	0,337	4,96553E-117
cDC_CLEC9A	CNOT2	2,77360E-18	1,17403E+00	0,43	0,268	5,10814E-14
cDC_CLEC9A	SOD1	3,52154E-24	1,17353E+00	0,747	0,56	6,48561E-20
cDC_CLEC9A	MZT2A	4,86112E-292	1,17280E+00	0,694	0,221	8,95273E-288
cDC_CLEC9A	RPL7	1,10328E-20	1,17265E+00	0,617	0,495	2,03191E-16
cDC_CLEC9A	HPS5	1,49063E-117	1,17248E+00	0,51	0,197	2,74529E-113
cDC_CLEC9A	RPS6	3,39699E-48	1,17111E+00	0,656	0,542	6,25623E-44

cDC_CLEC9A	PPIA	1,22991E-79	1,17081E+00	0,949	0,894	2,26512E-75
cDC_CLEC9A	PTTG1	2,98790E-74	1,16972E+00	0,254	0,082	5,50282E-70
cDC_CLEC9A	PDLIM1	0,00000E+00	1,16938E+00	0,373	0,042	0,00000E+00
cDC_CLEC9A	RPL5	3,61248E-41	1,16869E+00	0,636	0,515	6,65310E-37
cDC_CLEC9A	EGLN3	0,00000E+00	1,16816E+00	0,442	0,046	0,00000E+00
cDC_CLEC9A	RAB7A	3,62847E-43	1,16766E+00	0,806	0,63	6,68256E-39
cDC_CLEC9A	SMCO4	2,62866E-61	1,16742E+00	0,697	0,413	4,84120E-57
cDC_CLEC9A	CCDC12	3,41551E-89	1,16655E+00	0,592	0,281	6,29034E-85
cDC_CLEC9A	NAALADL1	2,19777E-265	1,16088E+00	0,293	0,042	4,04763E-261
cDC_CLEC9A	ARF5	1,66863E-47	1,15988E+00	0,791	0,61	3,07312E-43
cDC_CLEC9A	FOXN2	2,66743E-44	1,15933E+00	0,48	0,258	4,91261E-40
cDC_CLEC9A	OSBPL3	2,58563E-77	1,15902E+00	0,357	0,134	4,76196E-73
cDC_CLEC9A	ATP1A1	1,26869E-80	1,15777E+00	0,7	0,425	2,33655E-76
cDC_CLEC9A	FYN	2,67102E-90	1,15612E+00	0,273	0,078	4,91922E-86
cDC_CLEC9A	CS	1,77739E-44	1,15580E+00	0,441	0,227	3,27341E-40
cDC_CLEC9A	GSTM4	8,94469E-114	1,15339E+00	0,331	0,097	1,64734E-109
cDC_CLEC9A	PTPN22	2,28705E-196	1,15269E+00	0,418	0,101	4,21207E-192
cDC_CLEC9A	RPL31	2,10181E-25	1,15198E+00	0,622	0,494	3,87090E-21
cDC_CLEC9A	TUBB	5,79467E-11	1,14800E+00	0,773	0,659	1,06721E-06
cDC_CLEC9A	GMPS	1,73022E-26	1,14579E+00	0,268	0,134	3,18655E-22
cDC_CLEC9A	EIF5A	8,21401E-61	1,14442E+00	0,759	0,553	1,51277E-56
cDC_CLEC9A	BCL2L11	7,54842E-78	1,14250E+00	0,353	0,132	1,39019E-73
cDC_CLEC9A	RPS3A	6,36456E-43	1,14211E+00	0,652	0,565	1,17216E-38
cDC_CLEC9A	PFDN2	1,43553E-79	1,14199E+00	0,728	0,437	2,64381E-75
cDC_CLEC9A	TWF2	1,92458E-50	1,14187E+00	0,544	0,33	3,54450E-46
cDC_CLEC9A	NAGA	8,89859E-31	1,14155E+00	0,57	0,357	1,63885E-26
cDC_CLEC9A	CORO1A	6,28746E-57	1,14034E+00	0,842	0,642	1,15796E-52
cDC_CLEC9A	CLNS1A	5,58652E-14	1,13811E+00	0,589	0,418	1,02887E-09
cDC_CLEC9A	PSMA3	9,05566E-40	1,13538E+00	0,599	0,367	1,66778E-35
cDC_CLEC9A	RPS15	3,74082E-30	1,13520E+00	0,65	0,572	6,88946E-26
cDC_CLEC9A	RPL10A	5,80253E-50	1,13401E+00	0,638	0,494	1,06865E-45
cDC_CLEC9A	SNRPD1	6,80568E-48	1,12721E+00	0,629	0,387	1,25340E-43
cDC_CLEC9A	FAM118A	4,35402E-64	1,12459E+00	0,383	0,164	8,01879E-60
cDC_CLEC9A	NDUFA13	2,35402E-11	1,12131E+00	0,552	0,43	4,33541E-07
cDC_CLEC9A	CLIC2	1,33946E-180	1,11901E+00	0,51	0,155	2,46688E-176
cDC_CLEC9A	OTUB1	3,79790E-47	1,11866E+00	0,658	0,426	6,99459E-43
cDC_CLEC9A	MTMR14	7,16354E-39	1,11621E+00	0,531	0,306	1,31931E-34
cDC_CLEC9A	SERTAD3	1,84853E-119	1,11414E+00	0,472	0,168	3,40444E-115
cDC_CLEC9A	RPS25	2,62010E-36	1,11132E+00	0,642	0,546	4,82545E-32
cDC_CLEC9A	HLA-DQB2	9,80104E-12	1,11093E+00	0,457	0,314	1,80506E-07
cDC_CLEC9A	PSMB6	5,67741E-18	1,11051E+00	0,723	0,573	1,04561E-13

cDC_CLEC9A	HLA-DRB1	6,79342E-61	1,11028E+00	0,986	0,916	1,25114E-56
cDC_CLEC9A	PARVG	5,34960E-57	1,10714E+00	0,711	0,445	9,85236E-53
cDC_CLEC9A	LACC1	6,29022E-86	1,10426E+00	0,319	0,105	1,15847E-81
cDC_CLEC9A	HLA-DMA	1,32710E-07	1,10174E+00	0,914	0,779	2,44411E-03
cDC_CLEC9A	CPNE1	1,41183E-50	1,09933E+00	0,459	0,229	2,60016E-46
cDC_CLEC9A	MCOLN2	2,53266E-248	1,09678E+00	0,478	0,11	4,66441E-244
cDC_CLEC9A	SAP18	3,74661E-55	1,09635E+00	0,824	0,645	6,90013E-51
cDC_CLEC9A	RPL26	1,37818E-21	1,09517E+00	0,637	0,541	2,53819E-17
cDC_CLEC9A	PTK2	0,00000E+00	1,09348E+00	0,378	0,053	0,00000E+00
cDC_CLEC9A	TLR10	0,00000E+00	1,09266E+00	0,374	0,039	0,00000E+00
cDC_CLEC9A	ENO1	4,99068E-34	1,08859E+00	0,854	0,759	9,19133E-30
cDC_CLEC9A	IL16	1,85071E-133	1,08549E+00	0,553	0,215	3,40845E-129
cDC_CLEC9A	DAPP1	5,38857E-154	1,08472E+00	0,589	0,211	9,92414E-150
cDC_CLEC9A	ADSL	8,02408E-70	1,08405E+00	0,469	0,211	1,47779E-65
cDC_CLEC9A	ACTN1	1,04964E-85	1,08300E+00	0,623	0,313	1,93313E-81
cDC_CLEC9A	HCK	6,30045E-23	1,08222E+00	0,699	0,517	1,16035E-18
cDC_CLEC9A	SAMHD1	5,66022E-46	1,08216E+00	0,837	0,642	1,04244E-41
cDC_CLEC9A	RPL3	8,53570E-35	1,07910E+00	0,646	0,531	1,57202E-30
cDC_CLEC9A	SLC25A3	4,29516E-33	1,07641E+00	0,814	0,712	7,91039E-29
cDC_CLEC9A	YDJC	7,06447E-51	1,07383E+00	0,339	0,148	1,30106E-46
cDC_CLEC9A	PABPC1	2,29464E-88	1,07274E+00	0,96	0,891	4,22604E-84
cDC_CLEC9A	UBA52	2,87080E-26	1,07160E+00	0,948	0,92	5,28716E-22
cDC_CLEC9A	NELFCD	3,25179E-28	1,07088E+00	0,279	0,138	5,98882E-24
cDC_CLEC9A	DPYSL2	8,80388E-28	1,07016E+00	0,531	0,32	1,62141E-23
cDC_CLEC9A	HMGA1	2,53933E-144	1,07015E+00	0,741	0,398	4,67668E-140
cDC_CLEC9A	RPL11	1,92588E-23	1,07003E+00	0,66	0,579	3,54690E-19
cDC_CLEC9A	OAZ2	1,46358E-25	1,06859E+00	0,463	0,28	2,69547E-21
cDC_CLEC9A	ARHGDIB	6,50158E-15	1,06838E+00	0,9	0,821	1,19740E-10
cDC_CLEC9A	MCCC2	2,77984E-60	1,06573E+00	0,271	0,098	5,11962E-56
cDC_CLEC9A	BIN1	4,12478E-136	1,06273E+00	0,523	0,183	7,59661E-132
cDC_CLEC9A	RMDN1	2,58749E-28	1,06242E+00	0,299	0,151	4,76539E-24
cDC_CLEC9A	RPS8	7,79673E-42	1,06115E+00	0,654	0,577	1,43592E-37
cDC_CLEC9A	MYCL	2,61251E-189	1,06114E+00	0,368	0,083	4,81147E-185
cDC_CLEC9A	ARPC1B	1,43105E-31	1,06054E+00	0,874	0,795	2,63557E-27
cDC_CLEC9A	DAXX	1,14498E-76	1,06032E+00	0,392	0,154	2,10871E-72
cDC_CLEC9A	GNG10	3,02570E-10	1,05788E+00	0,472	0,335	5,57243E-06
cDC_CLEC9A	HSPA1B	1,05714E-26	1,05757E+00	0,661	0,502	1,94693E-22
cDC_CLEC9A	RPS14	1,68188E-28	1,05708E+00	0,653	0,574	3,09752E-24
cDC_CLEC9A	APOL2	2,49632E-50	1,05654E+00	0,299	0,124	4,59747E-46
cDC_CLEC9A	EIF3D	8,03521E-23	1,05318E+00	0,672	0,47	1,47984E-18
cDC_CLEC9A	TSTD1	3,52388E-83	1,05215E+00	0,529	0,234	6,48993E-79

cDC_CLEC9A	NAV1	3,41604E-200	1,05157E+00	0,431	0,107	6,29132E-196
cDC_CLEC9A	RPL36A	3,60129E-25	1,05097E+00	0,58	0,451	6,63250E-21
cDC_CLEC9A	RPL23	3,83578E-22	1,05075E+00	0,597	0,45	7,06435E-18
cDC_CLEC9A	UTP6	1,31245E-16	1,05026E+00	0,298	0,174	2,41713E-12
cDC_CLEC9A	PTRHD1	4,94157E-91	1,05007E+00	0,668	0,343	9,10088E-87
cDC_CLEC9A	SLC24A4	0,00000E+00	1,05000E+00	0,261	0,027	0,00000E+00
cDC_CLEC9A	MIF	1,58613E-59	1,04992E+00	0,862	0,721	2,92118E-55
cDC_CLEC9A	PARM1	0,00000E+00	1,04838E+00	0,341	0,031	0,00000E+00
cDC_CLEC9A	PSME3	3,46286E-35	1,04828E+00	0,384	0,199	6,37754E-31
cDC_CLEC9A	EVL	1,17777E-96	1,04723E+00	0,681	0,351	2,16910E-92
cDC_CLEC9A	IDH3G	2,87803E-13	1,04630E+00	0,528	0,356	5,30046E-09
cDC_CLEC9A	COX7C	6,88973E-103	1,04198E+00	0,924	0,827	1,26888E-98
cDC_CLEC9A	KIF16B	6,56556E-275	1,04194E+00	0,447	0,09	1,20918E-270
cDC_CLEC9A	RAB30	3,51386E-292	1,03856E+00	0,254	0,03	6,47148E-288
cDC_CLEC9A	RPLP0	8,33180E-50	1,03765E+00	0,643	0,541	1,53447E-45
cDC_CLEC9A	RPLP2	4,93935E-18	1,03574E+00	0,647	0,569	9,09679E-14
cDC_CLEC9A	BIK	0,00000E+00	1,03548E+00	0,279	0,017	0,00000E+00
cDC_CLEC9A	HSH2D	1,19494E-287	1,03520E+00	0,429	0,079	2,20072E-283
cDC_CLEC9A	PDIA3	1,80693E-31	1,03504E+00	0,82	0,673	3,32783E-27
cDC_CLEC9A	RPS4X	1,72096E-22	1,03477E+00	0,649	0,564	3,16948E-18
cDC_CLEC9A	RPL9	9,57067E-20	1,03301E+00	0,639	0,542	1,76263E-15
cDC_CLEC9A	RPL12	5,04217E-16	1,03272E+00	0,652	0,574	9,28617E-12
cDC_CLEC9A	NRBF2	7,62466E-55	1,03258E+00	0,484	0,239	1,40423E-50
cDC_CLEC9A	HNRNPA1	9,99372E-51	1,03166E+00	0,909	0,799	1,84054E-46
cDC_CLEC9A	TPMT	9,68304E-143	1,03037E+00	0,564	0,213	1,78333E-138
cDC_CLEC9A	RPL17	1,11378E-18	1,03000E+00	0,547	0,431	2,05126E-14
cDC_CLEC9A	NDUFS8	9,81187E-14	1,02824E+00	0,66	0,492	1,80705E-09
cDC_CLEC9A	EHD4	1,27949E-61	1,02806E+00	0,466	0,215	2,35643E-57
cDC_CLEC9A	RPL28	4,84100E-32	1,02805E+00	0,662	0,593	8,91568E-28
cDC_CLEC9A	CKAP2	1,63255E-66	1,02790E+00	0,279	0,099	3,00666E-62
cDC_CLEC9A	PLCG2	9,64398E-81	1,02631E+00	0,402	0,155	1,77613E-76
cDC_CLEC9A	RNF4	7,26938E-40	1,02591E+00	0,363	0,178	1,33880E-35
cDC_CLEC9A	MYL12A	2,07269E-43	1,02269E+00	0,9	0,823	3,81728E-39
cDC_CLEC9A	EIF3H	1,47743E-45	1,02230E+00	0,821	0,654	2,72098E-41
cDC_CLEC9A	P2RY14	1,58396E-287	1,02061E+00	0,403	0,071	2,91717E-283
cDC_CLEC9A	FGL2	1,55755E-44	1,01990E+00	0,822	0,61	2,86853E-40
cDC_CLEC9A	PTGES2	2,49090E-96	1,01655E+00	0,504	0,215	4,58749E-92
cDC_CLEC9A	ABRACL	7,31528E-45	1,01644E+00	0,727	0,493	1,34726E-40
cDC_CLEC9A	SRI	1,62483E-72	1,01639E+00	0,703	0,429	2,99245E-68
cDC_CLEC9A	SYMPK	1,96364E-51	1,01520E+00	0,359	0,159	3,61644E-47
cDC_CLEC9A	CCT4	4,68954E-20	1,01518E+00	0,609	0,429	8,63673E-16

cDC_CLEC9A	RPS17	1,89629E-20	1,01498E+00	0,554	0,404	3,49240E-16
cDC_CLEC9A	EDEM1	2,15316E-65	1,01329E+00	0,43	0,188	3,96548E-61
cDC_CLEC9A	EIF3L	4,67002E-66	1,01259E+00	0,787	0,596	8,60078E-62
cDC_CLEC9A	RAB8B	5,34297E-41	1,01222E+00	0,471	0,246	9,84016E-37
cDC_CLEC9A	TMEM243	1,00236E-120	1,01129E+00	0,609	0,263	1,84604E-116
cDC_CLEC9A	SEMA4A	5,17816E-78	1,01051E+00	0,492	0,218	9,53661E-74
cDC_CLEC9A	RPL37A	1,21071E-18	1,00759E+00	0,639	0,536	2,22977E-14
cDC_CLEC9A	SFT2D2	5,38126E-77	1,00564E+00	0,574	0,288	9,91066E-73
cDC_CLEC9A	SRSF7	2,97282E-85	1,00451E+00	0,797	0,547	5,47504E-81
cDC_CLEC9A	RPL41	1,05076E-19	1,00200E+00	0,65	0,573	1,93518E-15
cDC_CLEC9A	PSMB1	1,23266E-18	1,00192E+00	0,772	0,626	2,27019E-14
cDC_CLEC9A	MRPL18	1,87431E-101	1,00171E+00	0,664	0,343	3,45191E-97
cDC_CLEC9A	CACYBP	6,77736E-79	1,00128E+00	0,686	0,396	1,24819E-74
cDC_CLEC9A	F11R	1,73259E-54	1,00113E+00	0,292	0,116	3,19090E-50
cDC_CLEC9A	ARL2	3,57150E-21	9,99904E-01	0,338	0,194	6,57762E-17
cDC_CLEC9A	RPLP1	8,40495E-35	9,99652E-01	0,661	0,597	1,54794E-30
cDC_CLEC9A	ACER3	3,67244E-41	9,99141E-01	0,453	0,234	6,76352E-37
cDC_CLEC9A	CD52	2,29280E-46	9,98185E-01	0,656	0,385	4,22266E-42
cDC_CLEC9A	NABP1	1,65505E-15	9,98130E-01	0,488	0,337	3,04811E-11
cDC_CLEC9A	GLRX3	3,18542E-24	9,97957E-01	0,419	0,245	5,86658E-20
cDC_CLEC9A	LGALS9	8,16976E-12	9,97153E-01	0,71	0,545	1,50462E-07
cDC_CLEC9A	NPM1	3,04510E-30	9,96179E-01	0,862	0,764	5,60815E-26
cDC_CLEC9A	HLA-DQA2	1,45902E-34	9,94350E-01	0,67	0,509	2,68707E-30
cDC_CLEC9A	SHTN1	2,32007E-239	9,94103E-01	0,482	0,13	4,27288E-235
cDC_CLEC9A	ITGB1BP1	1,01948E-24	9,89951E-01	0,418	0,247	1,87757E-20
cDC_CLEC9A	NDEL1	7,83787E-35	9,88181E-01	0,362	0,186	1,44350E-30
cDC_CLEC9A	PSMA2	3,12246E-28	9,87817E-01	0,373	0,222	5,75064E-24
cDC_CLEC9A	NIPSNAP3A	3,79944E-36	9,86386E-01	0,377	0,192	6,99743E-32
cDC_CLEC9A	RPS20	4,12987E-12	9,85825E-01	0,572	0,449	7,60597E-08
cDC_CLEC9A	PSMB8	6,10827E-52	9,83541E-01	0,789	0,573	1,12496E-47
cDC_CLEC9A	EGLN2	7,55256E-48	9,83378E-01	0,304	0,13	1,39095E-43
cDC_CLEC9A	PSMG2	1,68838E-08	9,83023E-01	0,572	0,421	3,10949E-04
cDC_CLEC9A	TAP2	4,64231E-113	9,82068E-01	0,462	0,183	8,54974E-109
cDC_CLEC9A	RPL7A	9,60387E-32	9,81394E-01	0,656	0,557	1,76875E-27
cDC_CLEC9A	COTL1	2,54039E-79	9,80749E-01	0,86	0,709	4,67864E-75
cDC_CLEC9A	PSMD14	9,49496E-24	9,79819E-01	0,379	0,215	1,74869E-19
cDC_CLEC9A	TIMM10	1,21897E-24	9,77512E-01	0,421	0,25	2,24497E-20
cDC_CLEC9A	LMF2	2,33667E-19	9,76945E-01	0,343	0,202	4,30344E-15
cDC_CLEC9A	TOMM6	3,50882E-33	9,76673E-01	0,27	0,132	6,46220E-29
cDC_CLEC9A	STARD7	1,00531E-38	9,76007E-01	0,528	0,301	1,85149E-34
cDC_CLEC9A	ACAA1	2,25295E-21	9,75726E-01	0,562	0,374	4,14926E-17

cDC_CLEC9A	RPL13	6,44509E-18	9,75542E-01	0,658	0,58	1,18699E-13
cDC_CLEC9A	CNPPD1	2,89465E-73	9,75422E-01	0,544	0,266	5,33107E-69
cDC_CLEC9A	CCDC6	1,74297E-161	9,74682E-01	0,497	0,157	3,21003E-157
cDC_CLEC9A	MRPL3	3,40552E-57	9,73411E-01	0,534	0,278	6,27194E-53
cDC_CLEC9A	VCP	9,98006E-45	9,73289E-01	0,69	0,462	1,83803E-40
cDC_CLEC9A	UBE2N	1,23951E-68	9,73202E-01	0,651	0,369	2,28280E-64
cDC_CLEC9A	PAFAH1B3	3,13489E-100	9,73021E-01	0,367	0,121	5,77353E-96
cDC_CLEC9A	CAP1	1,53384E-31	9,72673E-01	0,834	0,732	2,82487E-27
cDC_CLEC9A	DBNDD2	4,38042E-34	9,72336E-01	0,382	0,202	8,06743E-30
cDC_CLEC9A	ELOVL1	1,10035E-15	9,70742E-01	0,517	0,357	2,02652E-11
cDC_CLEC9A	DNM1L	8,42714E-22	9,69933E-01	0,311	0,172	1,55203E-17
cDC_CLEC9A	SRP14	6,79285E-20	9,69495E-01	0,902	0,835	1,25104E-15
cDC_CLEC9A	CNOT8	1,07997E-31	9,68546E-01	0,393	0,21	1,98898E-27
cDC_CLEC9A	FPGS	1,39363E-10	9,67708E-01	0,316	0,204	2,56666E-06
cDC_CLEC9A	NOB1	1,86382E-17	9,67442E-01	0,272	0,154	3,43259E-13
cDC_CLEC9A	PMM1	3,67835E-109	9,67206E-01	0,332	0,099	6,77441E-105
cDC_CLEC9A	CHN2	7,11243E-107	9,66656E-01	0,419	0,144	1,30990E-102
cDC_CLEC9A	HLA-DRB5	2,46287E-38	9,66649E-01	0,948	0,845	4,53587E-34
cDC_CLEC9A	GYG1	1,47430E-18	9,66038E-01	0,434	0,27	2,71522E-14
cDC_CLEC9A	DECR1	3,94641E-40	9,63226E-01	0,579	0,343	7,26811E-36
cDC_CLEC9A	HSPA1A	1,72811E-16	9,63207E-01	0,743	0,662	3,18266E-12
cDC_CLEC9A	IGFLR1	9,83946E-10	9,62901E-01	0,501	0,357	1,81213E-05
cDC_CLEC9A	RPS27	4,22160E-22	9,60319E-01	0,649	0,565	7,77493E-18
cDC_CLEC9A	DGUOK	4,53660E-10	9,59823E-01	0,518	0,368	8,35505E-06
cDC_CLEC9A	PPM1G	5,06992E-39	9,58686E-01	0,591	0,364	9,33728E-35
cDC_CLEC9A	TRERF1	7,94297E-280	9,57547E-01	0,342	0,054	1,46286E-275
cDC_CLEC9A	PTPN2	6,45700E-55	9,57074E-01	0,649	0,381	1,18919E-50
cDC_CLEC9A	B4GALT4	1,52856E-110	9,56980E-01	0,297	0,082	2,81514E-106
cDC_CLEC9A	GM2A	3,09993E-12	9,56232E-01	0,544	0,372	5,70915E-08
cDC_CLEC9A	RPL35	1,90363E-39	9,55855E-01	0,649	0,529	3,50592E-35
cDC_CLEC9A	LDHB	2,48025E-28	9,54828E-01	0,698	0,508	4,56788E-24
cDC_CLEC9A	C18orf32	7,73893E-33	9,53277E-01	0,477	0,276	1,42528E-28
cDC_CLEC9A	MPST	2,60386E-50	9,53059E-01	0,43	0,206	4,79553E-46
cDC_CLEC9A	XRCC6	8,88154E-29	9,51439E-01	0,681	0,491	1,63571E-24
cDC_CLEC9A	CCNB1IP1	2,16528E-84	9,50398E-01	0,319	0,105	3,98780E-80
cDC_CLEC9A	MSL3	2,14025E-93	9,46955E-01	0,499	0,203	3,94171E-89
cDC_CLEC9A	OAT	6,85637E-45	9,46121E-01	0,343	0,157	1,26274E-40
cDC_CLEC9A	ITSN2	7,86525E-55	9,44404E-01	0,657	0,381	1,44854E-50
cDC_CLEC9A	MAP4	1,66532E-58	9,43253E-01	0,507	0,253	3,06702E-54
cDC_CLEC9A	PPP6R2	1,02383E-25	9,42205E-01	0,271	0,138	1,88558E-21
cDC_CLEC9A	HMGN4	6,08545E-46	9,41730E-01	0,479	0,244	1,12076E-41

cDC_CLEC9A	EVI2B	2,15631E-22	9,41722E-01	0,554	0,373	3,97128E-18
cDC_CLEC9A	HSPD1	1,41774E-38	9,41287E-01	0,728	0,532	2,61104E-34
cDC_CLEC9A	SNRPE	2,46003E-37	9,39333E-01	0,564	0,338	4,53063E-33
cDC_CLEC9A	PLEK	1,39080E-53	9,35826E-01	0,796	0,557	2,56143E-49
cDC_CLEC9A	BASP1	4,70808E-179	9,34259E-01	0,757	0,339	8,67086E-175
cDC_CLEC9A	DGAT2	0,00000E+00	9,33556E-01	0,348	0,028	0,00000E+00
cDC_CLEC9A	APRT	4,70629E-38	9,32881E-01	0,798	0,62	8,66757E-34
cDC_CLEC9A	CRIP1	2,17447E-67	9,32504E-01	0,557	0,288	4,00472E-63
cDC_CLEC9A	UBE2F	6,60057E-24	9,31850E-01	0,427	0,256	1,21563E-19
cDC_CLEC9A	RPL6	2,51528E-28	9,31403E-01	0,646	0,552	4,63239E-24
cDC_CLEC9A	PTMS	2,41178E-96	9,30170E-01	0,659	0,357	4,44177E-92
cDC_CLEC9A	HNRNPM	4,47225E-13	9,28387E-01	0,621	0,455	8,23654E-09
cDC_CLEC9A	UQCRH	4,74356E-22	9,28292E-01	0,827	0,699	8,73621E-18
cDC_CLEC9A	COX6A1	3,06520E-31	9,28186E-01	0,798	0,678	5,64518E-27
cDC_CLEC9A	ARF6	1,84489E-104	9,27597E-01	0,709	0,413	3,39774E-100
cDC_CLEC9A	NME4	4,15791E-161	9,27026E-01	0,596	0,224	7,65763E-157
cDC_CLEC9A	SLC46A3	2,90370E-92	9,26419E-01	0,39	0,142	5,34774E-88
cDC_CLEC9A	CFLAR	2,81728E-09	9,25435E-01	0,669	0,509	5,18859E-05
cDC_CLEC9A	ATF7IP	1,64895E-47	9,24960E-01	0,389	0,184	3,03686E-43
cDC_CLEC9A	PSMD8	6,37749E-14	9,24495E-01	0,67	0,522	1,17454E-09
cDC_CLEC9A	SRSF2	8,23709E-34	9,23768E-01	0,74	0,554	1,51702E-29
cDC_CLEC9A	RPL29	2,01103E-25	9,22647E-01	0,646	0,556	3,70371E-21
cDC_CLEC9A	SCARF1	7,28822E-137	9,21264E-01	0,306	0,075	1,34227E-132
cDC_CLEC9A	PFN1	6,93564E-94	9,21138E-01	0,972	0,947	1,27734E-89
cDC_CLEC9A	YWHAZ	8,19291E-26	9,19864E-01	0,823	0,71	1,50889E-21
cDC_CLEC9A	INPP5F	4,14075E-137	9,19113E-01	0,279	0,064	7,62602E-133
cDC_CLEC9A	OXA1L	2,89253E-20	9,19113E-01	0,63	0,452	5,32717E-16
cDC_CLEC9A	RPL32	2,74187E-28	9,18564E-01	0,659	0,577	5,04971E-24
cDC_CLEC9A	LRRK2	3,18787E-68	9,17490E-01	0,463	0,206	5,87109E-64
cDC_CLEC9A	ACSL5	2,84994E-129	9,17337E-01	0,449	0,145	5,24873E-125
cDC_CLEC9A	STT3A	2,41358E-32	9,16958E-01	0,317	0,156	4,44508E-28
cDC_CLEC9A	CNDP2	5,13850E-13	9,16672E-01	0,617	0,451	9,46357E-09
cDC_CLEC9A	LDHA	3,07413E-17	9,16593E-01	0,799	0,712	5,66163E-13
cDC_CLEC9A	NDUFB5	3,05046E-16	9,15686E-01	0,629	0,45	5,61803E-12
cDC_CLEC9A	IDH3A	1,84475E-152	9,15077E-01	0,491	0,156	3,39748E-148
cDC_CLEC9A	PPIB	2,82610E-35	9,14761E-01	0,878	0,791	5,20483E-31
cDC_CLEC9A	POLD2	1,30412E-66	9,13806E-01	0,319	0,119	2,40180E-62
cDC_CLEC9A	NDUFA9	8,14142E-38	9,13502E-01	0,308	0,147	1,49940E-33
cDC_CLEC9A	ITPA	3,01492E-15	9,11389E-01	0,42	0,272	5,55258E-11
cDC_CLEC9A	GPR157	3,18197E-180	9,10132E-01	0,293	0,058	5,86023E-176
cDC_CLEC9A	SULF2	4,15526E-126	9,08943E-01	0,466	0,153	7,65275E-122

cDC_CLEC9A	PRMT1	4,84185E-57	9,08471E-01	0,564	0,302	8,91724E-53
cDC_CLEC9A	YIF1B	1,16435E-38	9,08328E-01	0,584	0,346	2,14438E-34
cDC_CLEC9A	PIM2	1,09228E-186	9,08115E-01	0,393	0,095	2,01165E-182
cDC_CLEC9A	RPS15A	4,67947E-26	9,07366E-01	0,652	0,567	8,61819E-22
cDC_CLEC9A	SNX1	1,02150E-16	9,06866E-01	0,463	0,304	1,88129E-12
cDC_CLEC9A	SUCLG1	2,98384E-23	9,06587E-01	0,493	0,307	5,49534E-19
cDC_CLEC9A	RGS18	1,17804E-26	9,06483E-01	0,358	0,194	2,16959E-22
cDC_CLEC9A	DSTN	4,97579E-92	9,06105E-01	0,66	0,341	9,16391E-88
cDC_CLEC9A	M6PR	9,35011E-10	9,06027E-01	0,7	0,561	1,72201E-05
cDC_CLEC9A	COMMD10	5,11148E-20	9,05585E-01	0,311	0,178	9,41382E-16
cDC_CLEC9A	TRMT6	9,72046E-70	9,04301E-01	0,298	0,104	1,79022E-65
cDC_CLEC9A	RPS12	9,68503E-33	9,03245E-01	0,659	0,581	1,78369E-28
cDC_CLEC9A	CALM2	6,70085E-11	9,01841E-01	0,863	0,78	1,23410E-06
cDC_CLEC9A	MRPS27	2,12939E-59	9,00567E-01	0,281	0,103	3,92169E-55
cDC_CLEC9A	ARHGAP17	2,28327E-52	9,00103E-01	0,397	0,185	4,20510E-48
cDC_CLEC9A	OFD1	5,34122E-78	8,99590E-01	0,433	0,177	9,83693E-74
cDC_CLEC9A	KATNA1	8,49297E-75	8,99442E-01	0,287	0,095	1,56415E-70
cDC_CLEC9A	PDCD5	8,24062E-18	8,99142E-01	0,522	0,348	1,51767E-13
cDC_CLEC9A	DST	1,13610E-57	8,98406E-01	0,453	0,209	2,09236E-53
cDC_CLEC9A	MPC2	3,23838E-21	8,98331E-01	0,554	0,356	5,96413E-17
cDC_CLEC9A	RAB32	3,64198E-107	8,97882E-01	0,671	0,369	6,70743E-103
cDC_CLEC9A	PSMA1	4,61694E-14	8,97609E-01	0,507	0,358	8,50303E-10
cDC_CLEC9A	SMIM14	7,67455E-79	8,97427E-01	0,554	0,264	1,41342E-74
cDC_CLEC9A	CCT8	2,23366E-44	8,95324E-01	0,647	0,408	4,11373E-40
cDC_CLEC9A	RPL36	1,05509E-20	8,95084E-01	0,642	0,551	1,94316E-16
cDC_CLEC9A	SIGLEC10	2,52032E-26	8,94997E-01	0,522	0,317	4,64168E-22
cDC_CLEC9A	ZYX	5,80800E-55	8,94814E-01	0,811	0,642	1,06966E-50
cDC_CLEC9A	RUSC1	7,02784E-120	8,93760E-01	0,329	0,091	1,29432E-115
cDC_CLEC9A	MFNG	4,55125E-52	8,91952E-01	0,396	0,183	8,38203E-48
cDC_CLEC9A	GTF3A	8,62760E-28	8,90834E-01	0,679	0,476	1,58894E-23
cDC_CLEC9A	GNAO1	0,00000E+00	8,90803E-01	0,34	0,011	0,00000E+00
cDC_CLEC9A	HINT1	2,48457E-92	8,90250E-01	0,862	0,72	4,57582E-88
cDC_CLEC9A	SNX20	1,57021E-71	8,89431E-01	0,47	0,214	2,89185E-67
cDC_CLEC9A	SRP9	1,41101E-18	8,89064E-01	0,634	0,462	2,59865E-14
cDC_CLEC9A	CDK2AP1	1,38481E-57	8,88662E-01	0,331	0,139	2,55040E-53
cDC_CLEC9A	TPM4	3,33097E-37	8,88295E-01	0,799	0,615	6,13465E-33
cDC_CLEC9A	COPS6	1,33296E-18	8,88162E-01	0,513	0,339	2,45490E-14
cDC_CLEC9A	CLN3	1,67925E-38	8,87429E-01	0,334	0,159	3,09268E-34
cDC_CLEC9A	RAB5A	5,03632E-31	8,87338E-01	0,507	0,306	9,27540E-27
cDC_CLEC9A	RAB11A	2,72013E-77	8,85413E-01	0,693	0,396	5,00967E-73
cDC_CLEC9A	CCNDBP1	8,66734E-18	8,85244E-01	0,431	0,27	1,59626E-13

cDC_CLEC9A	DYSF	9,29002E-194	8,84160E-01	0,344	0,073	1,71094E-189
cDC_CLEC9A	SNX8	1,94247E-18	8,82515E-01	0,36	0,216	3,57744E-14
cDC_CLEC9A	CEP164	5,97918E-62	8,81391E-01	0,272	0,098	1,10119E-57
cDC_CLEC9A	TBC1D22A	1,01547E-09	8,80742E-01	0,337	0,226	1,87019E-05
cDC_CLEC9A	TIMM17B	2,45108E-66	8,79404E-01	0,609	0,319	4,51415E-62
cDC_CLEC9A	RPL30	3,50959E-24	8,79010E-01	0,661	0,579	6,46360E-20
cDC_CLEC9A	FDFT1	3,34184E-71	8,77245E-01	0,53	0,255	6,15467E-67
cDC_CLEC9A	COMMD5	4,81518E-14	8,77206E-01	0,313	0,196	8,86811E-10
cDC_CLEC9A	CCT2	2,47240E-89	8,76970E-01	0,648	0,334	4,55342E-85
cDC_CLEC9A	HTT	1,32450E-56	8,75558E-01	0,359	0,152	2,43934E-52
cDC_CLEC9A	SNRPD2	1,24128E-31	8,73345E-01	0,813	0,675	2,28606E-27
cDC_CLEC9A	PPA2	1,80527E-29	8,72184E-01	0,408	0,228	3,32477E-25
cDC_CLEC9A	CYFIP2	5,61820E-193	8,71931E-01	0,336	0,067	1,03470E-188
cDC_CLEC9A	RPS2	7,95433E-34	8,71858E-01	0,647	0,561	1,46495E-29
cDC_CLEC9A	LASP1	1,53188E-49	8,70591E-01	0,563	0,305	2,82126E-45
cDC_CLEC9A	TIMM50	1,54953E-11	8,69432E-01	0,279	0,174	2,85377E-07
cDC_CLEC9A	ARMCX6	3,35861E-08	8,69049E-01	0,287	0,191	6,18555E-04
cDC_CLEC9A	CHD6	2,88729E-34	8,68962E-01	0,293	0,138	5,31752E-30
cDC_CLEC9A	PARP1	6,06594E-39	8,68828E-01	0,554	0,33	1,11716E-34
cDC_CLEC9A	RAB11FIP1	2,14487E-139	8,68301E-01	0,567	0,215	3,95021E-135
cDC_CLEC9A	PSMC6	1,89552E-41	8,67730E-01	0,469	0,25	3,49098E-37
cDC_CLEC9A	STK32C	7,24889E-131	8,67541E-01	0,324	0,085	1,33503E-126
cDC_CLEC9A	HSD17B10	6,79206E-31	8,65654E-01	0,512	0,302	1,25089E-26
cDC_CLEC9A	RPS7	5,66238E-33	8,65250E-01	0,646	0,559	1,04284E-28
cDC_CLEC9A	PLEKHA5	2,69302E-205	8,64134E-01	0,274	0,046	4,95973E-201
cDC_CLEC9A	SPINT2	2,41325E-40	8,61653E-01	0,712	0,494	4,44448E-36
cDC_CLEC9A	C17orf49	5,59108E-12	8,61622E-01	0,354	0,227	1,02971E-07
cDC_CLEC9A	SET	6,72970E-20	8,60359E-01	0,703	0,524	1,23941E-15
cDC_CLEC9A	PGAM1	2,41544E-11	8,59405E-01	0,699	0,563	4,44851E-07
cDC_CLEC9A	PMAIP1	6,68568E-24	8,59331E-01	0,324	0,183	1,23130E-19
cDC_CLEC9A	AKR7A2	2,83560E-62	8,58542E-01	0,5	0,245	5,22232E-58
cDC_CLEC9A	DEK	3,62273E-33	8,57883E-01	0,754	0,571	6,67198E-29
cDC_CLEC9A	TMED3	2,99307E-55	8,56910E-01	0,516	0,27	5,51234E-51
cDC_CLEC9A	UROD	6,16574E-09	8,56482E-01	0,342	0,232	1,13554E-04
cDC_CLEC9A	PTMA	1,43485E-97	8,54962E-01	0,986	0,962	2,64257E-93
cDC_CLEC9A	R3HDM4	4,82071E-106	8,53041E-01	0,53	0,215	8,87830E-102
cDC_CLEC9A	PTPMT1	4,13380E-13	8,52712E-01	0,379	0,246	7,61322E-09
cDC_CLEC9A	GNG5	1,58840E-24	8,52627E-01	0,871	0,792	2,92536E-20
cDC_CLEC9A	HNRNPC	1,44112E-17	8,51918E-01	0,759	0,645	2,65411E-13
cDC_CLEC9A	CPQ	3,04661E-10	8,51295E-01	0,332	0,22	5,61094E-06
cDC_CLEC9A	NME1	2,80907E-52	8,51255E-01	0,346	0,152	5,17346E-48

cDC_CLEC9A	APOBEC3G	4,03954E-15	8,49242E-01	0,314	0,189	7,43962E-11
cDC_CLEC9A	TOX	0,00000E+00	8,49056E-01	0,3	0,012	0,00000E+00
cDC_CLEC9A	C19orf53	1,87570E-21	8,48892E-01	0,767	0,581	3,45448E-17
cDC_CLEC9A	UXT	3,12852E-16	8,48374E-01	0,708	0,538	5,76179E-12
cDC_CLEC9A	HSPB11	2,82799E-97	8,48205E-01	0,569	0,261	5,20831E-93
cDC_CLEC9A	MTIF3	5,86440E-08	8,47368E-01	0,408	0,293	1,08005E-03
cDC_CLEC9A	NME2	4,51227E-49	8,47142E-01	0,531	0,356	8,31025E-45
cDC_CLEC9A	RPS13	9,82491E-24	8,46921E-01	0,649	0,569	1,80945E-19
cDC_CLEC9A	RPS28	2,83210E-14	8,46277E-01	0,651	0,581	5,21588E-10
cDC_CLEC9A	PGLS	8,31484E-39	8,45709E-01	0,726	0,539	1,53134E-34
cDC_CLEC9A	VOPP1	3,25539E-43	8,43996E-01	0,589	0,342	5,99546E-39
cDC_CLEC9A	EIF3F	5,21047E-40	8,43942E-01	0,82	0,663	9,59612E-36
cDC_CLEC9A	MRPL51	7,04120E-21	8,43731E-01	0,628	0,436	1,29678E-16
cDC_CLEC9A	TBCB	4,61109E-35	8,42120E-01	0,672	0,464	8,49225E-31
cDC_CLEC9A	TRAPPC4	1,32316E-06	8,41224E-01	0,293	0,206	2,43687E-02
cDC_CLEC9A	HNRNPA2B1	1,31181E-23	8,41160E-01	0,903	0,83	2,41596E-19
cDC_CLEC9A	CCT5	1,34113E-27	8,40924E-01	0,577	0,377	2,46996E-23
cDC_CLEC9A	MAGEF1	1,65112E-238	8,40187E-01	0,407	0,085	3,04088E-234
cDC_CLEC9A	UBE2A	1,27434E-30	8,38986E-01	0,616	0,398	2,34696E-26
cDC_CLEC9A	NDUFAB1	1,69345E-10	8,36754E-01	0,61	0,473	3,11883E-06
cDC_CLEC9A	DLGAP4	2,32986E-96	8,36095E-01	0,479	0,191	4,29090E-92
cDC_CLEC9A	EIF4EBP1	7,55492E-12	8,35917E-01	0,559	0,407	1,39139E-07
cDC_CLEC9A	RHOC	6,80403E-42	8,34600E-01	0,526	0,305	1,25310E-37
cDC_CLEC9A	FBL	2,12269E-48	8,32817E-01	0,599	0,343	3,90935E-44
cDC_CLEC9A	TYK2	1,33619E-23	8,30275E-01	0,348	0,196	2,46086E-19
cDC_CLEC9A	SARNP	1,36451E-44	8,30103E-01	0,293	0,124	2,51303E-40
cDC_CLEC9A	CDKN2C	1,08560E-225	8,29349E-01	0,287	0,047	1,99936E-221
cDC_CLEC9A	APOBR	7,07099E-115	8,28469E-01	0,506	0,188	1,30226E-110
cDC_CLEC9A	NUTF2	2,41641E-22	8,28409E-01	0,568	0,381	4,45029E-18
cDC_CLEC9A	TSPAN3	1,34013E-15	8,27789E-01	0,479	0,315	2,46811E-11
cDC_CLEC9A	UBE2L3	2,08489E-27	8,27543E-01	0,703	0,498	3,83975E-23
cDC_CLEC9A	MRPS23	1,35526E-16	8,26761E-01	0,346	0,209	2,49599E-12
cDC_CLEC9A	ARPC5L	3,30229E-53	8,25925E-01	0,512	0,266	6,08182E-49
cDC_CLEC9A	PDIA6	5,22577E-18	8,23618E-01	0,672	0,517	9,62430E-14
cDC_CLEC9A	SLC25A5	1,46769E-36	8,23366E-01	0,844	0,742	2,70304E-32
cDC_CLEC9A	TMEM97	6,53120E-170	8,21682E-01	0,254	0,047	1,20285E-165
cDC_CLEC9A	NASP	2,69668E-22	8,21113E-01	0,43	0,273	4,96648E-18
cDC_CLEC9A	CDK4	1,38683E-07	8,20660E-01	0,328	0,23	2,55413E-03
cDC_CLEC9A	SPATS2	1,31636E-80	8,20246E-01	0,299	0,099	2,42434E-76
cDC_CLEC9A	PDHB	3,22612E-21	8,19482E-01	0,346	0,196	5,94154E-17
cDC_CLEC9A	ZNF296	7,57074E-203	8,19160E-01	0,258	0,041	1,39430E-198

cDC_CLEC9A	SNRPA1	2,10361E-42	8,18984E-01	0,4	0,202	3,87422E-38
cDC_CLEC9A	IGFBP7	1,03428E-155	8,17774E-01	0,486	0,153	1,90484E-151
cDC_CLEC9A	PPP6C	1,15651E-09	8,17556E-01	0,388	0,264	2,12995E-05
cDC_CLEC9A	TRA2A	2,78660E-33	8,15566E-01	0,457	0,252	5,13208E-29
cDC_CLEC9A	NSFL1C	1,33532E-10	8,15356E-01	0,399	0,272	2,45926E-06
cDC_CLEC9A	FUT8	1,92982E-195	8,14808E-01	0,286	0,052	3,55415E-191
cDC_CLEC9A	HNRNPR	6,30459E-34	8,14269E-01	0,569	0,357	1,16112E-29
cDC_CLEC9A	CD38	6,54021E-49	8,13646E-01	0,392	0,182	1,20451E-44
cDC_CLEC9A	HNRNPLL	7,46784E-106	8,13311E-01	0,419	0,147	1,37535E-101
cDC_CLEC9A	ETV6	7,95252E-72	8,12215E-01	0,556	0,285	1,46462E-67
cDC_CLEC9A	PEX16	1,67910E-10	8,10916E-01	0,259	0,165	3,09239E-06
cDC_CLEC9A	PMPCB	9,67485E-23	8,10063E-01	0,406	0,239	1,78182E-18
cDC_CLEC9A	BTF3	2,06161E-58	8,09346E-01	0,909	0,845	3,79686E-54
cDC_CLEC9A	INF2	1,75417E-76	8,08110E-01	0,348	0,126	3,23066E-72
cDC_CLEC9A	TUBB6	2,79809E-45	8,07787E-01	0,363	0,17	5,15323E-41
cDC_CLEC9A	GRN	1,60287E-30	8,06404E-01	0,849	0,822	2,95200E-26
cDC_CLEC9A	ATP6V0A2	5,02769E-113	8,06292E-01	0,377	0,118	9,25950E-109
cDC_CLEC9A	STK25	4,50584E-12	8,06031E-01	0,282	0,173	8,29841E-08
cDC_CLEC9A	DCTN3	3,03244E-15	8,03884E-01	0,526	0,357	5,58485E-11
cDC_CLEC9A	LDLRAD4	4,72591E-103	8,03517E-01	0,513	0,207	8,70371E-99
cDC_CLEC9A	UBAP2L	5,89059E-08	8,02850E-01	0,288	0,2	1,08487E-03
cDC_CLEC9A	ANAPC5	3,15784E-14	8,01773E-01	0,436	0,285	5,81580E-10
cDC_CLEC9A	RPL35A	2,25991E-17	8,01507E-01	0,646	0,56	4,16208E-13
cDC_CLEC9A	RNPS1	1,05823E-16	8,00082E-01	0,569	0,402	1,94894E-12
cDC_CLEC9A	CBX6	2,74190E-81	8,00023E-01	0,357	0,126	5,04976E-77
cDC_CLEC9A	MIF4GD	4,11469E-31	7,99544E-01	0,522	0,308	7,57803E-27
cDC_CLEC9A	SPIDR	1,35999E-23	7,99076E-01	0,276	0,144	2,50469E-19
cDC_CLEC9A	ZSWIM7	2,52179E-08	7,97990E-01	0,359	0,247	4,64439E-04
cDC_CLEC9A	STMN1	8,21966E-11	7,97772E-01	0,377	0,285	1,51382E-06
cDC_CLEC9A	DOCK2	1,90169E-09	7,96309E-01	0,48	0,346	3,50233E-05
cDC_CLEC9A	RUVBL2	1,89066E-30	7,96249E-01	0,307	0,154	3,48203E-26
cDC_CLEC9A	EID1	1,39340E-13	7,95930E-01	0,733	0,577	2,56622E-09
cDC_CLEC9A	TRABD	4,83089E-64	7,94762E-01	0,633	0,352	8,89706E-60
cDC_CLEC9A	REEP5	7,75239E-11	7,94495E-01	0,72	0,56	1,42776E-06
cDC_CLEC9A	RPL22	8,72462E-41	7,93825E-01	0,639	0,528	1,60681E-36
cDC_CLEC9A	TTC1	2,06134E-12	7,93682E-01	0,377	0,248	3,79636E-08
cDC_CLEC9A	VRK2	6,43063E-30	7,93408E-01	0,349	0,186	1,18433E-25
cDC_CLEC9A	RPL27	5,15453E-22	7,93025E-01	0,621	0,503	9,49310E-18
cDC_CLEC9A	DDX39A	1,06182E-06	7,92561E-01	0,392	0,29	1,95556E-02
cDC_CLEC9A	PPP1CA	2,03906E-06	7,91753E-01	0,729	0,62	3,75534E-02
cDC_CLEC9A	ARPC4	1,73270E-11	7,90769E-01	0,532	0,407	3,19111E-07

cDC_CLEC9A	CSK	2,95812E-64	7,89571E-01	0,614	0,342	5,44798E-60
cDC_CLEC9A	DOCK10	2,31482E-26	7,87767E-01	0,513	0,319	4,26321E-22
cDC_CLEC9A	VDAC2	1,33048E-09	7,86961E-01	0,694	0,55	2,45035E-05
cDC_CLEC9A	ECHS1	1,32838E-06	7,86602E-01	0,501	0,367	2,44648E-02
cDC_CLEC9A	MED8	6,18023E-20	7,86374E-01	0,373	0,219	1,13821E-15
cDC_CLEC9A	GTF2A2	8,90705E-29	7,86255E-01	0,619	0,403	1,64041E-24
cDC_CLEC9A	RPS19	3,32417E-07	7,85941E-01	0,65	0,574	6,12212E-03
cDC_CLEC9A	SNX4	4,39058E-13	7,85429E-01	0,268	0,16	8,08613E-09
cDC_CLEC9A	TBCA	1,18890E-31	7,84125E-01	0,77	0,585	2,18960E-27
cDC_CLEC9A	SEC13	3,57858E-40	7,83443E-01	0,622	0,398	6,59067E-36
cDC_CLEC9A	STK17B	3,80621E-08	7,83241E-01	0,593	0,441	7,00990E-04
cDC_CLEC9A	MBD4	6,39393E-19	7,82670E-01	0,399	0,247	1,17757E-14
cDC_CLEC9A	LY75	4,81622E-115	7,81016E-01	0,323	0,092	8,87004E-111
cDC_CLEC9A	LAP3	2,64387E-08	7,80888E-01	0,707	0,583	4,86921E-04
cDC_CLEC9A	EIF3E	4,56795E-19	7,80485E-01	0,768	0,602	8,41279E-15
cDC_CLEC9A	RPL38	1,05896E-06	7,80091E-01	0,602	0,49	1,95029E-02
cDC_CLEC9A	FARSA	7,60885E-24	7,79126E-01	0,372	0,211	1,40132E-19
cDC_CLEC9A	PSMB3	1,05691E-18	7,78395E-01	0,787	0,641	1,94651E-14
cDC_CLEC9A	RPL18	2,31388E-22	7,78373E-01	0,644	0,561	4,26147E-18
cDC_CLEC9A	RANGRF	1,22442E-20	7,78285E-01	0,28	0,153	2,25501E-16
cDC_CLEC9A	RRP7A	1,26472E-30	7,75460E-01	0,481	0,291	2,32924E-26
cDC_CLEC9A	B2M	9,21850E-38	7,74682E-01	0,994	0,995	1,69777E-33
cDC_CLEC9A	PDE6D	3,54726E-45	7,73245E-01	0,34	0,155	6,53298E-41
cDC_CLEC9A	MDH1	1,16730E-08	7,72987E-01	0,524	0,383	2,14982E-04
cDC_CLEC9A	SMARCE1	3,19547E-19	7,72904E-01	0,379	0,226	5,88509E-15
cDC_CLEC9A	ACAP1	1,11963E-108	7,72620E-01	0,403	0,131	2,06202E-104
cDC_CLEC9A	UQCRFS1	7,92045E-07	7,71697E-01	0,612	0,49	1,45871E-02
cDC_CLEC9A	NCKAP1L	7,19560E-13	7,71267E-01	0,598	0,426	1,32521E-08
cDC_CLEC9A	VIM	1,98366E-71	7,71143E-01	0,97	0,943	3,65331E-67
cDC_CLEC9A	EIF3I	1,70691E-17	7,70205E-01	0,641	0,462	3,14361E-13
cDC_CLEC9A	RPL15	1,60020E-19	7,69954E-01	0,646	0,551	2,94708E-15
cDC_CLEC9A	CPSF3	4,12476E-31	7,69850E-01	0,259	0,121	7,59657E-27
cDC_CLEC9A	AP2B1	1,42547E-08	7,69747E-01	0,273	0,184	2,62529E-04
cDC_CLEC9A	IMP4	5,16442E-10	7,69064E-01	0,388	0,264	9,51131E-06
cDC_CLEC9A	HLA-B	8,56930E-14	7,68925E-01	0,974	0,968	1,57821E-09
cDC_CLEC9A	SDAD1	1,50525E-07	7,68734E-01	0,293	0,2	2,77222E-03
cDC_CLEC9A	SAE1	7,46920E-24	7,68219E-01	0,288	0,152	1,37560E-19
cDC_CLEC9A	MDH2	4,78513E-14	7,66648E-01	0,673	0,519	8,81278E-10
cDC_CLEC9A	POMP	2,08612E-34	7,66360E-01	0,854	0,718	3,84202E-30
cDC_CLEC9A	ILF3	8,68978E-37	7,66338E-01	0,606	0,362	1,60040E-32
cDC_CLEC9A	IL18BP	2,42096E-20	7,65965E-01	0,329	0,194	4,45868E-16

cDC_CLEC9A	APH1A	3,15456E-39	7,65885E-01	0,688	0,495	5,80975E-35
cDC_CLEC9A	SNRPD3	1,59435E-10	7,65657E-01	0,479	0,368	2,93631E-06
cDC_CLEC9A	PEBP1	3,47896E-10	7,65521E-01	0,733	0,561	6,40721E-06
cDC_CLEC9A	EDF1	1,32704E-10	7,65342E-01	0,85	0,748	2,44401E-06
cDC_CLEC9A	EXOSC5	1,04890E-26	7,64997E-01	0,319	0,167	1,93177E-22
cDC_CLEC9A	INSIG1	2,32664E-28	7,63103E-01	0,491	0,291	4,28497E-24
cDC_CLEC9A	VDR	3,56319E-75	7,63004E-01	0,291	0,099	6,56232E-71
cDC_CLEC9A	HPRT1	4,28875E-15	7,61571E-01	0,409	0,261	7,89859E-11
cDC_CLEC9A	CCT6A	1,00674E-33	7,61017E-01	0,661	0,444	1,85411E-29
cDC_CLEC9A	CBFA2T3	0,00000E+00	7,60472E-01	0,392	0,055	0,00000E+00
cDC_CLEC9A	BRK1	2,04299E-28	7,60215E-01	0,841	0,724	3,76258E-24
cDC_CLEC9A	STK4	1,22385E-20	7,60171E-01	0,618	0,425	2,25396E-16
cDC_CLEC9A	ATG12	1,83498E-06	7,59383E-01	0,363	0,261	3,37949E-02
cDC_CLEC9A	ACP1	1,47216E-09	7,57728E-01	0,47	0,332	2,71128E-05
cDC_CLEC9A	TCP1	1,70584E-26	7,57107E-01	0,528	0,333	3,14164E-22
cDC_CLEC9A	HSPE1	2,20817E-42	7,57096E-01	0,794	0,599	4,06678E-38
cDC_CLEC9A	TRIM69	3,36938E-30	7,56707E-01	0,388	0,213	6,20539E-26
cDC_CLEC9A	DCP2	1,70189E-18	7,56573E-01	0,419	0,263	3,13438E-14
cDC_CLEC9A	HNRNPH2	1,35030E-11	7,55746E-01	0,333	0,217	2,48685E-07
cDC_CLEC9A	CENPW	1,18679E-51	7,55735E-01	0,253	0,097	2,18572E-47
cDC_CLEC9A	ACOT7	2,95342E-90	7,55729E-01	0,286	0,084	5,43932E-86
cDC_CLEC9A	DPP4	0,00000E+00	7,55367E-01	0,271	0,017	0,00000E+00
cDC_CLEC9A	C5orf15	5,25672E-19	7,54812E-01	0,377	0,225	9,68130E-15
cDC_CLEC9A	TXN2	5,95436E-19	7,54146E-01	0,579	0,393	1,09661E-14
cDC_CLEC9A	POP7	6,70292E-28	7,53682E-01	0,3	0,151	1,23448E-23
cDC_CLEC9A	NUP62	1,10970E-14	7,53655E-01	0,367	0,234	2,04373E-10
cDC_CLEC9A	HMOX2	1,81563E-60	7,53277E-01	0,421	0,19	3,34384E-56
cDC_CLEC9A	WDR77	3,70860E-59	7,53268E-01	0,349	0,144	6,83013E-55
cDC_CLEC9A	FIBP	2,88445E-18	7,52905E-01	0,54	0,364	5,31230E-14
cDC_CLEC9A	HPS1	6,33222E-21	7,52792E-01	0,532	0,348	1,16620E-16
cDC_CLEC9A	YWHAQ	2,45392E-11	7,52633E-01	0,571	0,427	4,51939E-07
cDC_CLEC9A	FBXO7	1,73968E-26	7,52359E-01	0,514	0,318	3,20396E-22
cDC_CLEC9A	HNRNPK	3,45885E-14	7,52278E-01	0,814	0,707	6,37016E-10
cDC_CLEC9A	KPNA2	9,58099E-19	7,52191E-01	0,342	0,207	1,76453E-14
cDC_CLEC9A	HNRNPH3	6,35585E-20	7,51042E-01	0,522	0,349	1,17056E-15
cDC_CLEC9A	NDUFA2	5,93322E-12	7,50990E-01	0,708	0,549	1,09272E-07
cDC_CLEC9A	NFAT5	1,00472E-41	7,50777E-01	0,344	0,161	1,85040E-37
cDC_CLEC9A	BLOC1S2	1,03582E-20	7,48849E-01	0,527	0,327	1,90768E-16
cDC_CLEC9A	LRBA	3,76911E-178	7,48049E-01	0,34	0,075	6,94157E-174
cDC_CLEC9A	PCNP	4,71540E-10	7,47939E-01	0,458	0,32	8,68436E-06
cDC_CLEC9A	COPB1	7,43317E-16	7,47538E-01	0,369	0,229	1,36897E-11

cDC_CLEC9A	MRPS11	1,09083E-10	7,47026E-01	0,317	0,204	2,00898E-06
cDC_CLEC9A	MMADHC	5,88729E-35	7,46361E-01	0,569	0,344	1,08426E-30
cDC_CLEC9A	FABP5	6,47631E-41	7,45760E-01	0,681	0,442	1,19274E-36
cDC_CLEC9A	SLC25A6	5,43569E-56	7,45696E-01	0,903	0,815	1,00109E-51
cDC_CLEC9A	LCMT1	2,04553E-24	7,43349E-01	0,302	0,162	3,76726E-20
cDC_CLEC9A	VPS72	1,28476E-26	7,43104E-01	0,312	0,161	2,36614E-22
cDC_CLEC9A	EIF4B	3,94123E-15	7,42709E-01	0,668	0,514	7,25856E-11
cDC_CLEC9A	STX18	7,50894E-55	7,42519E-01	0,342	0,144	1,38292E-50
cDC_CLEC9A	THYN1	5,96346E-44	7,41920E-01	0,442	0,222	1,09829E-39
cDC_CLEC9A	FDPS	9,79996E-22	7,41869E-01	0,391	0,232	1,80486E-17
cDC_CLEC9A	MPG	1,00397E-37	7,41268E-01	0,541	0,318	1,84901E-33
cDC_CLEC9A	TMEM14B	6,60253E-09	7,41192E-01	0,567	0,42	1,21599E-04
cDC_CLEC9A	HMGB2	1,43476E-06	7,40623E-01	0,324	0,4	2,64240E-02
cDC_CLEC9A	P2RY10	4,56604E-298	7,40475E-01	0,256	0,029	8,40927E-294
cDC_CLEC9A	DYNC1LI1	3,61523E-55	7,39248E-01	0,459	0,22	6,65816E-51
cDC_CLEC9A	ATPAF2	1,92036E-41	7,39023E-01	0,319	0,145	3,53673E-37
cDC_CLEC9A	DYNLL1	3,02922E-25	7,38252E-01	0,846	0,705	5,57892E-21
cDC_CLEC9A	NDUFB3	2,89258E-09	7,36658E-01	0,612	0,465	5,32727E-05
cDC_CLEC9A	NACA	9,89849E-30	7,35997E-01	0,961	0,918	1,82301E-25
cDC_CLEC9A	RPL37	3,40739E-27	7,35620E-01	0,65	0,567	6,27540E-23
cDC_CLEC9A	SERPINB6	4,88012E-35	7,35069E-01	0,636	0,418	8,98772E-31
cDC_CLEC9A	COPS3	2,83699E-13	7,35023E-01	0,387	0,248	5,22488E-09
cDC_CLEC9A	UPF2	3,14636E-82	7,34523E-01	0,499	0,217	5,79466E-78
cDC_CLEC9A	PRKAR1A	1,06965E-41	7,33588E-01	0,72	0,476	1,96998E-37
cDC_CLEC9A	ZCCHC10	3,36567E-34	7,33566E-01	0,292	0,137	6,19855E-30
cDC_CLEC9A	CUL1	2,02196E-24	7,33300E-01	0,382	0,215	3,72384E-20
cDC_CLEC9A	TP53RK	8,69687E-66	7,31800E-01	0,278	0,098	1,60170E-61
cDC_CLEC9A	SPECC1	8,18995E-26	7,31411E-01	0,324	0,173	1,50834E-21
cDC_CLEC9A	ADD3	2,03379E-36	7,30514E-01	0,317	0,151	3,74562E-32
cDC_CLEC9A	RBMX	5,01497E-25	7,30191E-01	0,619	0,421	9,23606E-21
cDC_CLEC9A	TAF15	6,03873E-54	7,29127E-01	0,563	0,305	1,11215E-49
cDC_CLEC9A	DUSP2	2,45811E-58	7,28836E-01	0,552	0,296	4,52710E-54
cDC_CLEC9A	AMD1	1,97879E-25	7,28833E-01	0,47	0,282	3,64434E-21
cDC_CLEC9A	CLASRP	1,67553E-38	7,28819E-01	0,364	0,18	3,08582E-34
cDC_CLEC9A	REPIN1	1,19653E-190	7,28765E-01	0,486	0,138	2,20366E-186
cDC_CLEC9A	MGST1	9,42211E-54	7,28414E-01	0,283	0,109	1,73527E-49
cDC_CLEC9A	CCPG1	1,38794E-18	7,27985E-01	0,382	0,229	2,55616E-14
cDC_CLEC9A	SSB	2,17742E-21	7,27862E-01	0,633	0,449	4,01015E-17
cDC_CLEC9A	THOC6	9,33956E-25	7,27638E-01	0,304	0,161	1,72007E-20
cDC_CLEC9A	APIP	1,25296E-16	7,26901E-01	0,372	0,231	2,30757E-12
cDC_CLEC9A	DESI1	4,58722E-47	7,26458E-01	0,442	0,223	8,44829E-43

cDC_CLEC9A	ST3GAL5	7,08582E-75	7,24428E-01	0,293	0,102	1,30499E-70
cDC_CLEC9A	COX6C	1,94077E-16	7,24016E-01	0,841	0,717	3,57432E-12
cDC_CLEC9A	HAX1	1,89610E-06	7,23721E-01	0,47	0,344	3,49204E-02
cDC_CLEC9A	BAG3	1,87373E-25	7,20125E-01	0,263	0,141	3,45085E-21
cDC_CLEC9A	RPL34	3,80887E-08	7,19900E-01	0,657	0,574	7,01480E-04
cDC_CLEC9A	ARHGAP31	1,51511E-55	7,19287E-01	0,439	0,204	2,79038E-51
cDC_CLEC9A	TUBA1C	4,86865E-35	7,19173E-01	0,61	0,375	8,96660E-31
cDC_CLEC9A	CALR	8,65373E-25	7,17674E-01	0,839	0,721	1,59376E-20
cDC_CLEC9A	DNAJC4	5,68906E-69	7,17468E-01	0,65	0,357	1,04775E-64
cDC_CLEC9A	TCTN3	1,57562E-97	7,17440E-01	0,317	0,099	2,90182E-93
cDC_CLEC9A	PPP1R2	1,82855E-17	7,16844E-01	0,569	0,39	3,36765E-13
cDC_CLEC9A	SERBP1	1,68990E-19	7,16842E-01	0,741	0,592	3,11229E-15
cDC_CLEC9A	SNRPF	1,00795E-84	7,16678E-01	0,693	0,387	1,85634E-80
cDC_CLEC9A	YY1AP1	9,27192E-15	7,15280E-01	0,303	0,184	1,70761E-10
cDC_CLEC9A	CHD9	4,49472E-10	7,15144E-01	0,481	0,34	8,27792E-06
cDC_CLEC9A	CFDP1	1,59145E-22	7,15123E-01	0,39	0,225	2,93098E-18
cDC_CLEC9A	DNAJC15	4,65980E-55	7,14734E-01	0,728	0,495	8,58196E-51
cDC_CLEC9A	CLEC7A	5,62965E-11	7,14356E-01	0,703	0,521	1,03681E-06
cDC_CLEC9A	AK3	9,07932E-28	7,14031E-01	0,288	0,145	1,67214E-23
cDC_CLEC9A	AIG1	5,05956E-23	7,13414E-01	0,326	0,179	9,31819E-19
cDC_CLEC9A	ZEB1	0,00000E+00	7,12849E-01	0,29	0,03	0,00000E+00
cDC_CLEC9A	RALB	1,14708E-26	7,12647E-01	0,404	0,225	2,11257E-22
cDC_CLEC9A	WDR46	2,53173E-15	7,10567E-01	0,261	0,153	4,66269E-11
cDC_CLEC9A	RPL39	3,74819E-08	7,09276E-01	0,65	0,58	6,90305E-04
cDC_CLEC9A	IWS1	1,95694E-29	7,09266E-01	0,329	0,169	3,60410E-25
cDC_CLEC9A	ROGDI	2,79302E-73	7,08475E-01	0,401	0,164	5,14390E-69
cDC_CLEC9A	E2F4	1,39685E-39	7,08452E-01	0,377	0,184	2,57257E-35
cDC_CLEC9A	NOP16	2,14914E-80	7,08339E-01	0,35	0,126	3,95808E-76
cDC_CLEC9A	MYO9A	9,16741E-135	7,07898E-01	0,326	0,084	1,68836E-130
cDC_CLEC9A	TECR	1,47016E-42	7,07091E-01	0,618	0,375	2,70760E-38
cDC_CLEC9A	ECE1	9,75453E-162	7,07071E-01	0,322	0,071	1,79649E-157
cDC_CLEC9A	MCRS1	2,17907E-15	7,04128E-01	0,287	0,171	4,01320E-11
cDC_CLEC9A	AUP1	6,97211E-14	7,03946E-01	0,678	0,522	1,28405E-09
cDC_CLEC9A	CD83	1,75013E-18	7,03382E-01	0,653	0,485	3,22322E-14
cDC_CLEC9A	USP10	4,95713E-28	7,02810E-01	0,386	0,209	9,12955E-24
cDC_CLEC9A	PTBP1	1,27444E-19	7,02482E-01	0,474	0,304	2,34714E-15
cDC_CLEC9A	EIF3G	7,23737E-10	7,02145E-01	0,744	0,611	1,33291E-05
cDC_CLEC9A	MRPS34	2,96834E-08	7,01132E-01	0,527	0,392	5,46680E-04
cDC_CLEC9A	LSM7	3,28531E-57	7,00557E-01	0,747	0,51	6,05056E-53
cDC_CLEC9A	NFATC2	6,33553E-179	6,98797E-01	0,45	0,122	1,16681E-174
cDC_CLEC9A	ANKRD44	6,82857E-24	6,97808E-01	0,476	0,288	1,25762E-19

cDC_CLEC9A	FARS2	5,00852E-147	6,97621E-01	0,326	0,079	9,22420E-143
cDC_CLEC9A	HMGN3	2,49924E-42	6,97612E-01	0,766	0,518	4,60284E-38
cDC_CLEC9A	NDUFS3	2,36103E-12	6,97419E-01	0,487	0,338	4,34830E-08
cDC_CLEC9A	MIIP	3,11099E-36	6,97257E-01	0,448	0,241	5,72952E-32
cDC_CLEC9A	SLC9A9	1,35121E-73	6,97082E-01	0,318	0,113	2,48853E-69
cDC_CLEC9A	MOB4	2,00168E-29	6,96149E-01	0,277	0,136	3,68649E-25
cDC_CLEC9A	DHX36	2,90636E-29	6,95886E-01	0,481	0,282	5,35265E-25
cDC_CLEC9A	EXOSC8	1,67112E-35	6,95248E-01	0,299	0,142	3,07769E-31
cDC_CLEC9A	LSM12	5,71143E-19	6,94202E-01	0,371	0,221	1,05187E-14
cDC_CLEC9A	CORO7	7,48226E-32	6,93811E-01	0,302	0,149	1,37801E-27
cDC_CLEC9A	LSM2	3,62239E-33	6,93751E-01	0,573	0,347	6,67135E-29
cDC_CLEC9A	BROX	6,01730E-10	6,92937E-01	0,302	0,202	1,10821E-05
cDC_CLEC9A	NAP1L4	6,12031E-22	6,92452E-01	0,366	0,211	1,12718E-17
cDC_CLEC9A	FNIP2	6,75870E-70	6,92430E-01	0,48	0,216	1,24475E-65
cDC_CLEC9A	TRPC4AP	1,15780E-09	6,92063E-01	0,327	0,218	2,13232E-05
cDC_CLEC9A	HMGB1	4,08370E-34	6,92059E-01	0,903	0,791	7,52095E-30
cDC_CLEC9A	SSU72	2,22497E-12	6,91750E-01	0,541	0,38	4,09773E-08
cDC_CLEC9A	TRAPPC2L	3,66198E-08	6,91369E-01	0,381	0,267	6,74426E-04
cDC_CLEC9A	EIF4H	4,38536E-23	6,90965E-01	0,628	0,449	8,07651E-19
cDC_CLEC9A	MRPL9	2,70197E-34	6,90470E-01	0,376	0,191	4,97622E-30
cDC_CLEC9A	SMDT1	4,72787E-59	6,90415E-01	0,723	0,478	8,70731E-55
cDC_CLEC9A	TFG	5,94636E-10	6,90296E-01	0,382	0,259	1,09514E-05
cDC_CLEC9A	CCDC167	1,19308E-33	6,89869E-01	0,27	0,125	2,19729E-29
cDC_CLEC9A	KIF2A	2,47163E-108	6,88573E-01	0,466	0,169	4,55200E-104
cDC_CLEC9A	MRPS24	2,63745E-35	6,88274E-01	0,348	0,174	4,85739E-31
cDC_CLEC9A	SF3A3	5,75718E-09	6,87785E-01	0,323	0,217	1,06030E-04
cDC_CLEC9A	ROMO1	6,15480E-38	6,86492E-01	0,726	0,527	1,13353E-33
cDC_CLEC9A	BAZ1A	5,32837E-32	6,86388E-01	0,636	0,414	9,81325E-28
cDC_CLEC9A	MLF2	1,19422E-16	6,86210E-01	0,62	0,449	2,19940E-12
cDC_CLEC9A	MCM3AP	3,91541E-13	6,86107E-01	0,279	0,172	7,21101E-09
cDC_CLEC9A	THOC7	3,15516E-21	6,86015E-01	0,514	0,335	5,81085E-17
cDC_CLEC9A	PPP1CC	1,22465E-38	6,84729E-01	0,573	0,348	2,25544E-34
cDC_CLEC9A	TUBB4B	1,02093E-14	6,84356E-01	0,538	0,377	1,88025E-10
cDC_CLEC9A	PSMC2	4,30606E-14	6,83754E-01	0,432	0,284	7,93046E-10
cDC_CLEC9A	TRIAP1	1,25290E-17	6,83004E-01	0,259	0,144	2,30746E-13
cDC_CLEC9A	EWSR1	1,20668E-13	6,82799E-01	0,621	0,46	2,22235E-09
cDC_CLEC9A	WDR61	9,45012E-22	6,82635E-01	0,378	0,218	1,74043E-17
cDC_CLEC9A	BLOC1S6	7,25991E-60	6,82597E-01	0,542	0,279	1,33706E-55
cDC_CLEC9A	HIGD2A	6,07790E-11	6,80760E-01	0,817	0,713	1,11937E-06
cDC_CLEC9A	RBBP4	1,24672E-11	6,80613E-01	0,52	0,368	2,29608E-07
cDC_CLEC9A	SHMT2	1,31654E-30	6,80066E-01	0,384	0,206	2,42467E-26

cDC_CLEC9A	EIF1AX	1,04558E-11	6,79681E-01	0,553	0,397	1,92564E-07
cDC_CLEC9A	DAP3	1,44053E-09	6,79651E-01	0,451	0,315	2,65303E-05
cDC_CLEC9A	OSTF1	9,30077E-07	6,78957E-01	0,69	0,547	1,71292E-02
cDC_CLEC9A	DEGS1	1,21651E-23	6,78868E-01	0,511	0,323	2,24045E-19
cDC_CLEC9A	UBE2I	1,05081E-28	6,78760E-01	0,711	0,523	1,93527E-24
cDC_CLEC9A	LRRC41	1,98875E-21	6,77837E-01	0,261	0,137	3,66268E-17
cDC_CLEC9A	SAMM50	2,52932E-59	6,77829E-01	0,363	0,153	4,65825E-55
cDC_CLEC9A	RPN2	1,80919E-14	6,77514E-01	0,424	0,282	3,33199E-10
cDC_CLEC9A	COMMD8	5,20796E-11	6,76172E-01	0,343	0,224	9,59150E-07
cDC_CLEC9A	RAB8A	3,52094E-13	6,75818E-01	0,6	0,422	6,48452E-09
cDC_CLEC9A	COPZ1	1,01983E-13	6,75163E-01	0,543	0,379	1,87823E-09
cDC_CLEC9A	LBR	3,22219E-45	6,74437E-01	0,359	0,166	5,93431E-41
cDC_CLEC9A	RANBP1	6,22132E-38	6,72000E-01	0,582	0,363	1,14578E-33
cDC_CLEC9A	MTCH2	2,97226E-18	6,70754E-01	0,496	0,319	5,47401E-14
cDC_CLEC9A	LAMTOR1	1,38777E-06	6,69894E-01	0,753	0,646	2,55585E-02
cDC_CLEC9A	TERF2IP	3,12106E-24	6,69781E-01	0,532	0,334	5,74805E-20
cDC_CLEC9A	C3orf38	2,50601E-21	6,68864E-01	0,267	0,143	4,61531E-17
cDC_CLEC9A	DNTTIP1	4,09199E-19	6,67179E-01	0,37	0,221	7,53621E-15
cDC_CLEC9A	MCUR1	8,88702E-118	6,64847E-01	0,5	0,191	1,63672E-113
cDC_CLEC9A	IFITM2	2,16769E-65	6,64779E-01	0,421	0,651	3,99224E-61
cDC_CLEC9A	USP1	1,64829E-80	6,64691E-01	0,393	0,152	3,03566E-76
cDC_CLEC9A	RICTOR	4,05720E-19	6,63878E-01	0,263	0,147	7,47215E-15
cDC_CLEC9A	NDUFAF4	3,37471E-22	6,63778E-01	0,256	0,134	6,21519E-18
cDC_CLEC9A	GART	2,41241E-22	6,63030E-01	0,259	0,135	4,44293E-18
cDC_CLEC9A	HMG20B	3,50514E-07	6,62815E-01	0,338	0,237	6,45542E-03
cDC_CLEC9A	SCFD1	2,19426E-25	6,62503E-01	0,294	0,154	4,04117E-21
cDC_CLEC9A	EIF2A	2,85067E-12	6,62248E-01	0,451	0,305	5,25008E-08
cDC_CLEC9A	UBL5	2,18849E-12	6,60642E-01	0,86	0,753	4,03055E-08
cDC_CLEC9A	RAB11FIP4	0,00000E+00	6,60603E-01	0,27	0,032	0,00000E+00
cDC_CLEC9A	LRRFIP2	5,23142E-14	6,60411E-01	0,387	0,249	9,63471E-10
cDC_CLEC9A	SF1	1,17864E-24	6,59552E-01	0,694	0,5	2,17070E-20
cDC_CLEC9A	PSMC3	8,95272E-13	6,58082E-01	0,457	0,316	1,64882E-08
cDC_CLEC9A	COX7A2L	2,51456E-10	6,57549E-01	0,641	0,487	4,63106E-06
cDC_CLEC9A	CCDC88A	9,57307E-40	6,57035E-01	0,718	0,498	1,76307E-35
cDC_CLEC9A	NMI	1,66205E-25	6,56995E-01	0,486	0,296	3,06100E-21
cDC_CLEC9A	TIMM17A	1,17123E-40	6,56943E-01	0,491	0,267	2,15705E-36
cDC_CLEC9A	PLOD3	1,87628E-21	6,56873E-01	0,3	0,163	3,45554E-17
cDC_CLEC9A	QPCT	1,73900E-69	6,56046E-01	0,356	0,139	3,20272E-65
cDC_CLEC9A	LYZ	4,28878E-29	6,55836E-01	0,931	0,806	7,89864E-25
cDC_CLEC9A	BZW2	4,28824E-92	6,55398E-01	0,426	0,16	7,89765E-88
cDC_CLEC9A	IGBP1	1,91105E-08	6,55016E-01	0,479	0,342	3,51958E-04

cDC_CLEC9A	DDT	2,05018E-41	6,54613E-01	0,658	0,436	3,77581E-37
cDC_CLEC9A	NMT1	1,63189E-42	6,54514E-01	0,361	0,171	3,00546E-38
cDC_CLEC9A	CBX3	1,41818E-26	6,54482E-01	0,609	0,403	2,61186E-22
cDC_CLEC9A	PSMD2	2,28652E-08	6,54270E-01	0,442	0,318	4,21108E-04
cDC_CLEC9A	ARL2BP	7,08067E-08	6,54092E-01	0,321	0,217	1,30405E-03
cDC_CLEC9A	RSL24D1	8,31085E-22	6,53481E-01	0,626	0,43	1,53061E-17
cDC_CLEC9A	HCCS	1,54778E-26	6,52811E-01	0,283	0,144	2,85054E-22
cDC_CLEC9A	YTHDF2	1,61232E-16	6,52653E-01	0,436	0,278	2,96942E-12
cDC_CLEC9A	MALSU1	1,31553E-06	6,52563E-01	0,253	0,173	2,42281E-02
cDC_CLEC9A	TRIM22	6,49471E-34	6,51902E-01	0,648	0,412	1,19613E-29
cDC_CLEC9A	NRBP1	1,07566E-09	6,51318E-01	0,44	0,305	1,98104E-05
cDC_CLEC9A	PLCB2	4,85406E-28	6,50504E-01	0,423	0,238	8,93973E-24
cDC_CLEC9A	UFC1	2,70570E-06	6,50410E-01	0,627	0,5	4,98309E-02
cDC_CLEC9A	ARGLU1	6,91851E-25	6,50172E-01	0,648	0,429	1,27418E-20
cDC_CLEC9A	RPL24	1,51969E-20	6,50073E-01	0,631	0,537	2,79882E-16
cDC_CLEC9A	PSMD7	2,01915E-06	6,49048E-01	0,521	0,403	3,71867E-02
cDC_CLEC9A	SNX17	1,10737E-09	6,48788E-01	0,568	0,43	2,03944E-05
cDC_CLEC9A	FXR1	1,68074E-06	6,48773E-01	0,396	0,285	3,09541E-02
cDC_CLEC9A	HSDL2	6,76279E-101	6,46331E-01	0,426	0,153	1,24550E-96
cDC_CLEC9A	FAM162A	4,25044E-46	6,45899E-01	0,589	0,341	7,82803E-42
cDC_CLEC9A	HNRNPD	4,69822E-30	6,45666E-01	0,556	0,339	8,65271E-26
cDC_CLEC9A	RCN2	4,47482E-99	6,45509E-01	0,41	0,146	8,24128E-95
cDC_CLEC9A	FAM136A	8,32302E-29	6,44815E-01	0,363	0,196	1,53285E-24
cDC_CLEC9A	CSNK2B	7,03501E-09	6,44290E-01	0,447	0,335	1,29564E-04
cDC_CLEC9A	ATP6V1H	2,22506E-17	6,44281E-01	0,343	0,209	4,09789E-13
cDC_CLEC9A	SFPQ	2,13577E-20	6,44043E-01	0,684	0,504	3,93345E-16
cDC_CLEC9A	SDHC	1,01676E-06	6,44029E-01	0,58	0,439	1,87256E-02
cDC_CLEC9A	PSMB7	4,11332E-15	6,43073E-01	0,534	0,36	7,57550E-11
cDC_CLEC9A	TRA2B	3,33950E-07	6,42675E-01	0,616	0,468	6,15037E-03
cDC_CLEC9A	OLA1	4,71891E-26	6,41747E-01	0,5	0,305	8,69081E-22
cDC_CLEC9A	VPS29	3,15189E-14	6,41472E-01	0,701	0,532	5,80483E-10
cDC_CLEC9A	SLTM	2,25671E-16	6,40903E-01	0,522	0,352	4,15618E-12
cDC_CLEC9A	ACTR10	8,59165E-08	6,40868E-01	0,292	0,198	1,58232E-03
cDC_CLEC9A	COPS8	3,78905E-16	6,38451E-01	0,271	0,157	6,97829E-12
cDC_CLEC9A	STK17A	8,64408E-150	6,37035E-01	0,544	0,186	1,59198E-145
cDC_CLEC9A	TBC1D10C	2,96259E-98	6,36939E-01	0,314	0,095	5,45621E-94
cDC_CLEC9A	RPS26	8,12266E-14	6,36525E-01	0,623	0,522	1,49595E-09
cDC_CLEC9A	ATP13A1	1,20180E-21	6,35196E-01	0,29	0,156	2,21336E-17
cDC_CLEC9A	LRRK1	5,89082E-29	6,35072E-01	0,308	0,16	1,08491E-24
cDC_CLEC9A	ABHD14B	3,45117E-22	6,34903E-01	0,431	0,259	6,35602E-18
cDC_CLEC9A	CLK1	1,38637E-23	6,33531E-01	0,578	0,379	2,55327E-19

cDC_CLEC9A	MT-ATP8	7,32744E-13	6,32466E-01	0,518	0,381	1,34949E-08
cDC_CLEC9A	FLNB	6,95564E-134	6,32221E-01	0,318	0,08	1,28102E-129
cDC_CLEC9A	SEH1L	1,13046E-36	6,31524E-01	0,252	0,108	2,08197E-32
cDC_CLEC9A	TRAP1	1,34377E-53	6,30826E-01	0,323	0,133	2,47482E-49
cDC_CLEC9A	ABCF2	1,74304E-59	6,30745E-01	0,293	0,111	3,21015E-55
cDC_CLEC9A	MRPS33	3,40542E-15	6,30207E-01	0,362	0,228	6,27177E-11
cDC_CLEC9A	NFKBID	9,99716E-15	6,29868E-01	0,338	0,211	1,84118E-10
cDC_CLEC9A	PLEKHO1	6,79468E-45	6,29328E-01	0,613	0,356	1,25138E-40
cDC_CLEC9A	VDAC1	7,61673E-18	6,29236E-01	0,698	0,531	1,40277E-13
cDC_CLEC9A	ACAA2	7,91830E-15	6,27030E-01	0,331	0,202	1,45831E-10
cDC_CLEC9A	SLC38A1	2,50734E-175	6,25812E-01	0,342	0,075	4,61777E-171
cDC_CLEC9A	FKBP1B	0,00000E+00	6,25616E-01	0,313	0,024	0,00000E+00
cDC_CLEC9A	EVI2A	1,17620E-32	6,25012E-01	0,476	0,27	2,16620E-28
cDC_CLEC9A	ACADM	2,35342E-21	6,24992E-01	0,302	0,165	4,33429E-17
cDC_CLEC9A	RAB29	4,06837E-164	6,24675E-01	0,399	0,11	7,49272E-160
cDC_CLEC9A	NCOA7	1,04145E-154	6,24390E-01	0,496	0,158	1,91804E-150
cDC_CLEC9A	FAM135A	2,09075E-95	6,24145E-01	0,322	0,1	3,85054E-91
cDC_CLEC9A	RNF5	2,47400E-23	6,23594E-01	0,459	0,28	4,55637E-19
cDC_CLEC9A	MIA3	9,69172E-18	6,23457E-01	0,364	0,219	1,78492E-13
cDC_CLEC9A	GRPEL1	2,34707E-30	6,23416E-01	0,389	0,211	4,32260E-26
cDC_CLEC9A	MT-ND4L	2,03488E-12	6,23350E-01	0,59	0,513	3,74763E-08
cDC_CLEC9A	HSPBP1	4,70704E-95	6,21561E-01	0,306	0,092	8,66896E-91
cDC_CLEC9A	BCLAF1	2,74564E-10	6,21533E-01	0,521	0,378	5,05665E-06
cDC_CLEC9A	DCLRE1C	5,50552E-46	6,21329E-01	0,264	0,108	1,01395E-41
cDC_CLEC9A	PSMD13	4,21327E-18	6,21081E-01	0,492	0,32	7,75958E-14
cDC_CLEC9A	BAD	1,01073E-07	6,21049E-01	0,264	0,177	1,86146E-03
cDC_CLEC9A	ABHD2	1,36076E-07	6,20733E-01	0,342	0,239	2,50612E-03
cDC_CLEC9A	ITGAL	1,14716E-61	6,20046E-01	0,376	0,156	2,11272E-57
cDC_CLEC9A	DIMT1	6,51391E-59	6,19296E-01	0,299	0,114	1,19967E-54
cDC_CLEC9A	DDX39B	8,16731E-17	6,18943E-01	0,331	0,205	1,50417E-12
cDC_CLEC9A	TSPAN33	1,08844E-70	6,18223E-01	0,292	0,103	2,00457E-66
cDC_CLEC9A	CINP	2,11685E-10	6,17954E-01	0,273	0,175	3,89860E-06
cDC_CLEC9A	SPG21	2,09431E-09	6,17715E-01	0,584	0,435	3,85709E-05
cDC_CLEC9A	GINM1	7,82016E-45	6,17695E-01	0,477	0,251	1,44024E-40
cDC_CLEC9A	FAM110A	6,58358E-40	6,17614E-01	0,346	0,164	1,21250E-35
cDC_CLEC9A	COMMD3	3,76097E-18	6,16697E-01	0,27	0,152	6,92659E-14
cDC_CLEC9A	PLEKHJ1	3,83466E-20	6,15182E-01	0,406	0,247	7,06229E-16
cDC_CLEC9A	DNAJB1	2,88026E-09	6,13974E-01	0,659	0,556	5,30458E-05
cDC_CLEC9A	CDV3	9,22448E-33	6,13877E-01	0,477	0,271	1,69887E-28
cDC_CLEC9A	RPS27A	5,24698E-16	6,13173E-01	0,658	0,574	9,66337E-12
cDC_CLEC9A	SEPHS2	1,21000E-84	6,12720E-01	0,501	0,222	2,22846E-80

cDC_CLEC9A	MTRNR2L12	1,28475E-26	6,12207E-01	0,864	0,764	2,36613E-22
cDC_CLEC9A	CBL	1,40461E-93	6,12201E-01	0,449	0,176	2,58688E-89
cDC_CLEC9A	PHB2	2,99750E-20	6,12183E-01	0,658	0,49	5,52050E-16
cDC_CLEC9A	LSM5	3,99835E-41	6,11223E-01	0,574	0,339	7,36377E-37
cDC_CLEC9A	FKBP3	7,54048E-13	6,10938E-01	0,362	0,233	1,38873E-08
cDC_CLEC9A	ECHDC1	1,13521E-33	6,09150E-01	0,473	0,266	2,09072E-29
cDC_CLEC9A	PLK3	5,17234E-10	6,08029E-01	0,372	0,25	9,52591E-06
cDC_CLEC9A	MRPS6	3,55369E-21	6,07288E-01	0,502	0,321	6,54483E-17
cDC_CLEC9A	SNRPG	5,95484E-24	6,05732E-01	0,728	0,554	1,09670E-19
cDC_CLEC9A	PAICS	1,43509E-58	6,05251E-01	0,271	0,1	2,64301E-54
cDC_CLEC9A	ENOPH1	3,33639E-15	6,05078E-01	0,278	0,162	6,14462E-11
cDC_CLEC9A	FAR1	2,81251E-26	6,04972E-01	0,344	0,188	5,17979E-22
cDC_CLEC9A	RBM3	5,30552E-35	6,04968E-01	0,813	0,672	9,77117E-31
cDC_CLEC9A	TOMM22	8,47537E-23	6,04578E-01	0,609	0,42	1,56091E-18
cDC_CLEC9A	MAP4K5	7,11408E-34	6,04502E-01	0,288	0,136	1,31020E-29
cDC_CLEC9A	ZNF672	2,06546E-24	6,02459E-01	0,299	0,159	3,80396E-20
cDC_CLEC9A	PDPK1	9,41824E-25	6,02294E-01	0,326	0,177	1,73456E-20
cDC_CLEC9A	PCSK7	7,58655E-09	6,02140E-01	0,257	0,168	1,39722E-04
cDC_CLEC9A	POGZ	1,30104E-19	6,02133E-01	0,331	0,19	2,39613E-15
cDC_CLEC9A	EIF2B1	4,45153E-20	6,01138E-01	0,298	0,165	8,19839E-16
cDC_CLEC9A	MRPL15	1,02361E-07	6,00596E-01	0,302	0,208	1,88519E-03
cDC_CLEC9A	BUB3	5,32178E-17	6,00198E-01	0,403	0,255	9,80113E-13
cDC_CLEC9A	PKM	3,36542E-09	5,98770E-01	0,859	0,782	6,19809E-05
cDC_CLEC9A	ATP5F1B	8,05850E-35	5,97886E-01	0,473	0,31	1,48413E-30
cDC_CLEC9A	RBPJ	2,50837E-16	5,96994E-01	0,658	0,463	4,61966E-12
cDC_CLEC9A	ADCY7	7,85002E-32	5,96650E-01	0,422	0,229	1,44574E-27
cDC_CLEC9A	PSMA5	8,19695E-31	5,95801E-01	0,64	0,431	1,50963E-26
cDC_CLEC9A	IL2RG	1,21215E-14	5,95652E-01	0,49	0,321	2,23242E-10
cDC_CLEC9A	SPOP	1,63181E-10	5,93701E-01	0,392	0,265	3,00530E-06
cDC_CLEC9A	EIF2S1	2,75365E-30	5,92065E-01	0,416	0,226	5,07140E-26
cDC_CLEC9A	AIMP2	4,73063E-13	5,91713E-01	0,291	0,177	8,71240E-09
cDC_CLEC9A	CLTB	2,13319E-46	5,90657E-01	0,603	0,356	3,92869E-42
cDC_CLEC9A	DCTD	1,34598E-19	5,90442E-01	0,349	0,204	2,47890E-15
cDC_CLEC9A	SUPT16H	4,64654E-17	5,89284E-01	0,329	0,2	8,55753E-13
cDC_CLEC9A	TMA7	2,91099E-38	5,88875E-01	0,904	0,791	5,36116E-34
cDC_CLEC9A	ANP32B	3,40145E-13	5,87889E-01	0,708	0,569	6,26445E-09
cDC_CLEC9A	DCAF7	3,33549E-21	5,87513E-01	0,489	0,309	6,14296E-17
cDC_CLEC9A	SIGIRR	3,62455E-07	5,87017E-01	0,362	0,255	6,67533E-03
cDC_CLEC9A	SCML1	8,32005E-110	5,86399E-01	0,37	0,117	1,53230E-105
cDC_CLEC9A	JMJD1C	5,06871E-23	5,86378E-01	0,627	0,424	9,33504E-19
cDC_CLEC9A	C7orf50	4,35412E-16	5,85995E-01	0,506	0,334	8,01898E-12

cDC_CLEC9A	RSU1	1,75777E-17	5,85525E-01	0,5	0,333	3,23728E-13
cDC_CLEC9A	FLII	6,50140E-10	5,85418E-01	0,462	0,322	1,19736E-05
cDC_CLEC9A	RPS27L	3,06090E-14	5,85373E-01	0,519	0,359	5,63726E-10
cDC_CLEC9A	SPINT1	6,70803E-31	5,84840E-01	0,299	0,146	1,23542E-26
cDC_CLEC9A	ESYT1	6,40347E-15	5,84253E-01	0,27	0,159	1,17933E-10
cDC_CLEC9A	LUC7L	1,92304E-08	5,84109E-01	0,273	0,182	3,54167E-04
cDC_CLEC9A	ASPSCR1	2,63155E-19	5,83489E-01	0,258	0,14	4,84653E-15
cDC_CLEC9A	PLEKHA2	2,42006E-24	5,82814E-01	0,394	0,226	4,45703E-20
cDC_CLEC9A	PSMA4	7,87769E-07	5,82095E-01	0,603	0,466	1,45083E-02
cDC_CLEC9A	SSNA1	2,93201E-07	5,81596E-01	0,539	0,402	5,39988E-03
cDC_CLEC9A	ITGA4	1,24713E-07	5,80837E-01	0,498	0,366	2,29684E-03
cDC_CLEC9A	ADAM15	3,55296E-70	5,79684E-01	0,372	0,147	6,54348E-66
cDC_CLEC9A	MRPS36	1,25691E-28	5,78307E-01	0,534	0,324	2,31485E-24
cDC_CLEC9A	MRPL40	2,32430E-20	5,78232E-01	0,434	0,269	4,28067E-16
cDC_CLEC9A	MRPL14	1,97470E-12	5,78091E-01	0,496	0,341	3,63681E-08
cDC_CLEC9A	MRPS25	4,45264E-22	5,77186E-01	0,37	0,213	8,20043E-18
cDC_CLEC9A	MGST2	4,33741E-07	5,77053E-01	0,498	0,356	7,98821E-03
cDC_CLEC9A	PNRC2	7,66449E-35	5,74788E-01	0,417	0,226	1,41157E-30
cDC_CLEC9A	PPP6R1	4,29959E-55	5,73713E-01	0,344	0,143	7,91856E-51
cDC_CLEC9A	CNOT7	4,20823E-12	5,73465E-01	0,452	0,305	7,75030E-08
cDC_CLEC9A	ATL3	1,93909E-12	5,72716E-01	0,308	0,195	3,57122E-08
cDC_CLEC9A	CAT	5,99091E-15	5,72140E-01	0,578	0,406	1,10335E-10
cDC_CLEC9A	DHPS	1,35459E-24	5,71143E-01	0,386	0,214	2,49474E-20
cDC_CLEC9A	DEF6	6,78623E-25	5,71106E-01	0,412	0,238	1,24982E-20
cDC_CLEC9A	DEF8	1,63127E-30	5,70585E-01	0,326	0,166	3,00432E-26
cDC_CLEC9A	FUBP1	3,94872E-26	5,70493E-01	0,33	0,177	7,27236E-22
cDC_CLEC9A	CXCR3	0,00000E+00	5,69920E-01	0,4	0,046	0,00000E+00
cDC_CLEC9A	EEF1A1	4,25117E-08	5,69336E-01	0,992	0,984	7,82938E-04
cDC_CLEC9A	MPDU1	8,49736E-08	5,68607E-01	0,327	0,225	1,56496E-03
cDC_CLEC9A	TRADD	2,48571E-24	5,68134E-01	0,416	0,243	4,57793E-20
cDC_CLEC9A	ATP6V1A	1,23981E-06	5,67833E-01	0,354	0,253	2,28337E-02
cDC_CLEC9A	DNAJC8	1,96432E-06	5,67516E-01	0,541	0,411	3,61769E-02
cDC_CLEC9A	RAB34	2,09975E-12	5,66124E-01	0,407	0,27	3,86711E-08
cDC_CLEC9A	SRSF3	1,08382E-28	5,66017E-01	0,751	0,567	1,99607E-24
cDC_CLEC9A	ADAM28	7,42615E-29	5,65676E-01	0,418	0,235	1,36767E-24
cDC_CLEC9A	REC8	5,86148E-219	5,64913E-01	0,282	0,046	1,07951E-214
cDC_CLEC9A	ENY2	3,43837E-09	5,64579E-01	0,727	0,593	6,33245E-05
cDC_CLEC9A	RAB10	1,89812E-06	5,63837E-01	0,616	0,487	3,49577E-02
cDC_CLEC9A	TAX1BP3	1,51666E-25	5,63166E-01	0,327	0,171	2,79324E-21
cDC_CLEC9A	FBXO6	1,31110E-09	5,63138E-01	0,26	0,168	2,41466E-05
cDC_CLEC9A	DTD1	4,35910E-18	5,63110E-01	0,271	0,152	8,02816E-14

cDC_CLEC9A	NUDC	6,59447E-08	5,62702E-01	0,5	0,378	1,21450E-03
cDC_CLEC9A	ELP5	1,47164E-16	5,62534E-01	0,316	0,187	2,71031E-12
cDC_CLEC9A	GNG7	1,50655E-96	5,62399E-01	0,311	0,095	2,77461E-92
cDC_CLEC9A	NOL7	4,85052E-16	5,62122E-01	0,587	0,411	8,93321E-12
cDC_CLEC9A	CSNK1A1	5,33417E-09	5,61350E-01	0,602	0,46	9,82395E-05
cDC_CLEC9A	PXN	8,40566E-117	5,61125E-01	0,429	0,14	1,54807E-112
cDC_CLEC9A	SLC25A24	2,69065E-07	5,59917E-01	0,32	0,224	4,95538E-03
cDC_CLEC9A	NSMCE1	1,25955E-55	5,59883E-01	0,52	0,266	2,31971E-51
cDC_CLEC9A	SMARCA5	5,21760E-63	5,59771E-01	0,576	0,306	9,60925E-59
cDC_CLEC9A	BCCIP	2,08115E-30	5,58964E-01	0,384	0,207	3,83285E-26
cDC_CLEC9A	STARD3	1,87473E-07	5,58774E-01	0,344	0,24	3,45268E-03
cDC_CLEC9A	MRPS15	4,64508E-24	5,57758E-01	0,503	0,314	8,55485E-20
cDC_CLEC9A	UBE2K	4,04206E-35	5,55603E-01	0,499	0,281	7,44425E-31
cDC_CLEC9A	TOR3A	6,68012E-27	5,55544E-01	0,411	0,234	1,23028E-22
cDC_CLEC9A	ATXN10	1,07050E-20	5,55195E-01	0,447	0,273	1,97154E-16
cDC_CLEC9A	FAU	1,95153E-13	5,54677E-01	0,96	0,957	3,59414E-09
cDC_CLEC9A	TRAM1	1,06232E-08	5,54335E-01	0,59	0,448	1,95648E-04
cDC_CLEC9A	U2SURP	1,43549E-14	5,53869E-01	0,48	0,324	2,64374E-10
cDC_CLEC9A	RTCA	6,52554E-10	5,53035E-01	0,284	0,185	1,20181E-05
cDC_CLEC9A	TUBA4A	3,86119E-104	5,52255E-01	0,269	0,072	7,11115E-100
cDC_CLEC9A	IER5	3,44178E-19	5,50602E-01	0,482	0,323	6,33872E-15
cDC_CLEC9A	DCUN1D5	1,60285E-21	5,50139E-01	0,278	0,148	2,95196E-17
cDC_CLEC9A	UTP18	5,14647E-09	5,50071E-01	0,298	0,196	9,47825E-05
cDC_CLEC9A	PARK7	1,98732E-16	5,49937E-01	0,791	0,656	3,66005E-12
cDC_CLEC9A	TXNL1	2,60965E-22	5,49883E-01	0,571	0,377	4,80620E-18
cDC_CLEC9A	CISD1	2,74146E-45	5,48830E-01	0,34	0,152	5,04894E-41
cDC_CLEC9A	KDM1B	3,65438E-61	5,47678E-01	0,278	0,101	6,73026E-57
cDC_CLEC9A	RPS9	6,48857E-07	5,46785E-01	0,634	0,553	1,19500E-02
cDC_CLEC9A	ADRM1	6,07155E-18	5,46369E-01	0,602	0,425	1,11820E-13
cDC_CLEC9A	TRAPPC1	1,07645E-38	5,46328E-01	0,793	0,606	1,98249E-34
cDC_CLEC9A	SEC31A	2,18897E-16	5,45923E-01	0,46	0,296	4,03142E-12
cDC_CLEC9A	ZNF207	4,12351E-15	5,45499E-01	0,574	0,402	7,59426E-11
cDC_CLEC9A	HNRNPF	3,91439E-09	5,45039E-01	0,686	0,563	7,20913E-05
cDC_CLEC9A	DNAJC1	1,66181E-06	5,44686E-01	0,397	0,288	3,06056E-02
cDC_CLEC9A	THRAP3	8,59070E-08	5,44451E-01	0,557	0,418	1,58215E-03
cDC_CLEC9A	NBEAL2	2,46829E-156	5,44145E-01	0,331	0,078	4,54586E-152
cDC_CLEC9A	IRF3	1,50595E-13	5,43926E-01	0,294	0,179	2,77352E-09
cDC_CLEC9A	RAB4B	1,27293E-33	5,43497E-01	0,266	0,122	2,34436E-29
cDC_CLEC9A	GGNBP2	1,35950E-08	5,42993E-01	0,463	0,338	2,50379E-04
cDC_CLEC9A	HLA-F	1,04667E-14	5,42978E-01	0,687	0,512	1,92766E-10
cDC_CLEC9A	CLEC2B	5,71110E-09	5,42836E-01	0,616	0,467	1,05181E-04

cDC_CLEC9A	WDR1	1,11477E-45	5,42250E-01	0,732	0,537	2,05306E-41
cDC_CLEC9A	SS18L2	1,89777E-47	5,41581E-01	0,464	0,235	3,49513E-43
cDC_CLEC9A	SMC3	4,07967E-18	5,40857E-01	0,323	0,19	7,51352E-14
cDC_CLEC9A	GLTP	3,42772E-52	5,40814E-01	0,423	0,195	6,31283E-48
cDC_CLEC9A	GMDS	5,44426E-43	5,40421E-01	0,254	0,104	1,00267E-38
cDC_CLEC9A	PSMB10	1,55626E-06	5,40039E-01	0,698	0,564	2,86616E-02
cDC_CLEC9A	AP3D1	2,03812E-54	5,39721E-01	0,46	0,223	3,75361E-50
cDC_CLEC9A	TES	1,89938E-26	5,39516E-01	0,472	0,276	3,49808E-22
cDC_CLEC9A	PTOV1	6,26025E-47	5,39236E-01	0,342	0,153	1,15295E-42
cDC_CLEC9A	SNRNP40	6,84851E-36	5,39094E-01	0,389	0,197	1,26129E-31
cDC_CLEC9A	MRPL20	5,72511E-08	5,37162E-01	0,598	0,465	1,05439E-03
cDC_CLEC9A	PDE4B	1,46720E-09	5,37152E-01	0,494	0,365	2,70214E-05
cDC_CLEC9A	POLR2I	1,21615E-06	5,36916E-01	0,4	0,292	2,23978E-02
cDC_CLEC9A	NOSIP	6,65782E-13	5,36087E-01	0,483	0,325	1,22617E-08
cDC_CLEC9A	PDZD11	1,64792E-06	5,35353E-01	0,26	0,181	3,03498E-02
cDC_CLEC9A	LRRCC1	1,01658E-212	5,34436E-01	0,334	0,065	1,87223E-208
cDC_CLEC9A	PSMB5	1,30037E-17	5,34271E-01	0,483	0,312	2,39489E-13
cDC_CLEC9A	GID8	4,50197E-17	5,34056E-01	0,38	0,231	8,29128E-13
cDC_CLEC9A	SUPT5H	8,03609E-26	5,32393E-01	0,421	0,239	1,48001E-21
cDC_CLEC9A	THBD	2,01571E-48	5,32080E-01	0,424	0,203	3,71233E-44
cDC_CLEC9A	MRPL13	6,61190E-26	5,32015E-01	0,411	0,234	1,21771E-21
cDC_CLEC9A	SNRPB	1,18377E-23	5,31648E-01	0,682	0,505	2,18016E-19
cDC_CLEC9A	GNB2	8,85676E-23	5,31545E-01	0,68	0,554	1,63115E-18
cDC_CLEC9A	NCL	1,40621E-21	5,31326E-01	0,783	0,623	2,58982E-17
cDC_CLEC9A	GSTK1	4,97290E-19	5,30011E-01	0,797	0,641	9,15860E-15
cDC_CLEC9A	RUBCNL	3,88519E-196	5,29696E-01	0,32	0,064	7,15536E-192
cDC_CLEC9A	CCSER2	9,59843E-29	5,28622E-01	0,281	0,139	1,76774E-24
cDC_CLEC9A	OXSR1	5,73065E-91	5,27909E-01	0,458	0,179	1,05541E-86
cDC_CLEC9A	ARPP19	5,00174E-09	5,27408E-01	0,387	0,266	9,21171E-05
cDC_CLEC9A	IFITM1	4,38896E-13	5,26497E-01	0,157	0,256	8,08314E-09
cDC_CLEC9A	VAMP8	1,64472E-08	5,26185E-01	0,834	0,78	3,02907E-04
cDC_CLEC9A	PPHLN1	5,02763E-35	5,25583E-01	0,412	0,215	9,25938E-31
cDC_CLEC9A	ORAI2	5,21176E-13	5,24454E-01	0,273	0,167	9,59849E-09
cDC_CLEC9A	POP4	7,58321E-10	5,24382E-01	0,313	0,208	1,39660E-05
cDC_CLEC9A	NOLC1	9,80198E-42	5,23868E-01	0,348	0,165	1,80523E-37
cDC_CLEC9A	CYTIP	1,09166E-22	5,23659E-01	0,597	0,387	2,01052E-18
cDC_CLEC9A	VBP1	1,81468E-48	5,22331E-01	0,417	0,201	3,34210E-44
cDC_CLEC9A	CLPP	7,78175E-19	5,21828E-01	0,426	0,262	1,43316E-14
cDC_CLEC9A	ARL6IP5	1,65184E-18	5,21470E-01	0,622	0,655	3,04218E-14
cDC_CLEC9A	LMNB1	1,46411E-109	5,20654E-01	0,431	0,15	2,69644E-105
cDC_CLEC9A	FBXO27	0,00000E+00	5,20205E-01	0,273	0,006	0,00000E+00

cDC_CLEC9A	MVP	2,10555E-24	5,19762E-01	0,57	0,37	3,87779E-20
cDC_CLEC9A	UBE2G1	2,70125E-13	5,19555E-01	0,28	0,17	4,97490E-09
cDC_CLEC9A	IFI16	4,64057E-08	5,17799E-01	0,633	0,497	8,54653E-04
cDC_CLEC9A	INIP	9,61620E-32	5,17566E-01	0,274	0,13	1,77102E-27
cDC_CLEC9A	PPP1R7	5,44540E-19	5,16978E-01	0,442	0,276	1,00288E-14
cDC_CLEC9A	COPS7A	2,56416E-13	5,16935E-01	0,337	0,213	4,72240E-09
cDC_CLEC9A	RGS12	1,23836E-25	5,16755E-01	0,258	0,131	2,28069E-21
cDC_CLEC9A	DLD	1,19254E-12	5,16502E-01	0,303	0,189	2,19630E-08
cDC_CLEC9A	AKIRIN1	1,79545E-12	5,16180E-01	0,356	0,229	3,30667E-08
cDC_CLEC9A	BOLA3	1,60322E-30	5,16087E-01	0,346	0,181	2,95266E-26
cDC_CLEC9A	ERH	8,18455E-22	5,15702E-01	0,707	0,525	1,50735E-17
cDC_CLEC9A	ARRDC1	1,97944E-14	5,15649E-01	0,446	0,291	3,64553E-10
cDC_CLEC9A	DR1	6,67093E-16	5,15538E-01	0,381	0,233	1,22858E-11
cDC_CLEC9A	CCAR1	1,06721E-22	5,15079E-01	0,439	0,264	1,96549E-18
cDC_CLEC9A	PSMD3	8,57186E-44	5,14850E-01	0,507	0,276	1,57868E-39
cDC_CLEC9A	FAM104A	8,61095E-26	5,14303E-01	0,261	0,131	1,58588E-21
cDC_CLEC9A	RALY	1,33545E-25	5,13792E-01	0,672	0,489	2,45949E-21
cDC_CLEC9A	GNA15	8,60998E-60	5,13699E-01	0,573	0,301	1,58570E-55
cDC_CLEC9A	OGDH	1,52060E-13	5,13684E-01	0,313	0,195	2,80049E-09
cDC_CLEC9A	NFYC	1,54379E-07	5,13436E-01	0,362	0,256	2,84320E-03
cDC_CLEC9A	CSRNP1	2,69752E-09	5,12576E-01	0,413	0,289	4,96802E-05
cDC_CLEC9A	CDC42SE2	5,19649E-35	5,12418E-01	0,488	0,27	9,57038E-31
cDC_CLEC9A	PSMD9	1,76950E-10	5,12358E-01	0,374	0,252	3,25888E-06
cDC_CLEC9A	REEP3	1,57185E-48	5,12182E-01	0,422	0,203	2,89488E-44
cDC_CLEC9A	EMC6	2,09236E-13	5,11982E-01	0,47	0,31	3,85350E-09
cDC_CLEC9A	RUFY3	6,11751E-36	5,10917E-01	0,28	0,128	1,12666E-31
cDC_CLEC9A	UBXN1	4,51450E-11	5,09398E-01	0,766	0,626	8,31435E-07
cDC_CLEC9A	COPE	1,97549E-07	5,09264E-01	0,734	0,604	3,63826E-03
cDC_CLEC9A	PPP3CC	2,29902E-48	5,09143E-01	0,319	0,135	4,23410E-44
cDC_CLEC9A	NUBP1	7,19611E-10	5,09092E-01	0,343	0,232	1,32531E-05
cDC_CLEC9A	MAPRE2	1,68133E-20	5,08944E-01	0,361	0,212	3,09650E-16
cDC_CLEC9A	STIP1	7,10014E-20	5,08577E-01	0,443	0,278	1,30763E-15
cDC_CLEC9A	DCAF11	1,50593E-16	5,08522E-01	0,29	0,167	2,77347E-12
cDC_CLEC9A	GTF2E2	5,67395E-47	5,08343E-01	0,381	0,18	1,04497E-42
cDC_CLEC9A	PQBP1	5,63582E-34	5,08305E-01	0,533	0,31	1,03795E-29
cDC_CLEC9A	SUGT1	3,88650E-08	5,08305E-01	0,403	0,282	7,15776E-04
cDC_CLEC9A	TTC9C	8,67553E-25	5,07692E-01	0,282	0,148	1,59777E-20
cDC_CLEC9A	UCK2	1,72333E-83	5,07509E-01	0,292	0,093	3,17385E-79
cDC_CLEC9A	EIF3J	1,25186E-20	5,07397E-01	0,399	0,241	2,30555E-16
cDC_CLEC9A	CEP63	1,28144E-15	5,07321E-01	0,26	0,15	2,36002E-11
cDC_CLEC9A	DDOST	1,02161E-14	5,06954E-01	0,614	0,46	1,88149E-10

cDC_CLEC9A	SBNO1	2,44583E-41	5,06378E-01	0,349	0,166	4,50448E-37
cDC_CLEC9A	ANXA4	1,11840E-12	5,05656E-01	0,46	0,306	2,05976E-08
cDC_CLEC9A	ILF2	5,68894E-18	5,04369E-01	0,562	0,388	1,04773E-13
cDC_CLEC9A	MICAL1	1,45973E-28	5,04264E-01	0,484	0,29	2,68839E-24
cDC_CLEC9A	PDCD10	4,15671E-07	5,04260E-01	0,43	0,312	7,65542E-03
cDC_CLEC9A	SKA2	3,50361E-23	5,04057E-01	0,251	0,129	6,45260E-19
cDC_CLEC9A	TRNAU1AP	3,35511E-14	5,04004E-01	0,28	0,168	6,17911E-10
cDC_CLEC9A	RAB4A	3,30204E-25	5,03898E-01	0,439	0,259	6,08137E-21
cDC_CLEC9A	CHTF8	1,07525E-16	5,03599E-01	0,298	0,174	1,98029E-12
cDC_CLEC9A	BCL2A1	3,36516E-11	5,02900E-01	0,673	0,466	6,19761E-07
cDC_CLEC9A	AHNAK	4,76787E-16	5,02716E-01	0,748	0,6	8,78099E-12
cDC_CLEC9A	COX17	9,07005E-34	5,02338E-01	0,703	0,486	1,67043E-29
cDC_CLEC9A	HLA-A	2,22926E-06	5,01829E-01	0,949	0,95	4,10564E-02
cDC_CLEC9A	DENR	2,72476E-08	5,01747E-01	0,354	0,246	5,01820E-04
cDC_CLEC9A	PRPSAP2	1,32105E-40	5,01662E-01	0,251	0,103	2,43298E-36
cDC_CLEC9A	PDCD6	3,50538E-12	5,01065E-01	0,58	0,422	6,45587E-08
cDC_CLEC9A	NOP2	3,31529E-61	5,00307E-01	0,284	0,104	6,10577E-57
cDC_CLEC9A	EIF4E	5,02933E-11	4,99971E-01	0,433	0,295	9,26251E-07
cDC_CLEC9A	CAMTA1	3,34593E-44	4,99517E-01	0,61	0,359	6,16221E-40
cDC_CLEC9A	HSPA9	5,25276E-09	4,99447E-01	0,502	0,368	9,67401E-05
cDC_CLEC9A	POLR2K	2,78780E-10	4,99294E-01	0,468	0,323	5,13429E-06
cDC_CLEC9A	MFF	9,67610E-35	4,99236E-01	0,54	0,315	1,78205E-30
cDC_CLEC9A	MRPL47	9,53424E-23	4,98919E-01	0,368	0,212	1,75592E-18
cDC_CLEC9A	MRPL22	3,51588E-37	4,97754E-01	0,371	0,186	6,47520E-33
cDC_CLEC9A	MBP	1,11075E-68	4,97688E-01	0,602	0,313	2,04568E-64
cDC_CLEC9A	HDAC2	5,13913E-33	4,97636E-01	0,461	0,264	9,46473E-29
cDC_CLEC9A	VCPIP1	2,12508E-30	4,97551E-01	0,277	0,134	3,91377E-26
cDC_CLEC9A	PSMB2	1,07848E-15	4,97307E-01	0,611	0,438	1,98624E-11
cDC_CLEC9A	ACTR1B	2,79453E-24	4,96271E-01	0,259	0,131	5,14669E-20
cDC_CLEC9A	ATP6V1D	9,62781E-24	4,96130E-01	0,419	0,244	1,77315E-19
cDC_CLEC9A	RAP1GDS1	2,38606E-25	4,95273E-01	0,341	0,184	4,39440E-21
cDC_CLEC9A	GPR137B	3,55720E-16	4,95051E-01	0,441	0,295	6,55129E-12
cDC_CLEC9A	NADSYN1	3,09722E-10	4,94581E-01	0,288	0,187	5,70416E-06
cDC_CLEC9A	SSR1	1,41888E-06	4,93071E-01	0,601	0,461	2,61315E-02
cDC_CLEC9A	MRPL36	8,36046E-13	4,92661E-01	0,36	0,228	1,53975E-08
cDC_CLEC9A	RFTN1	1,75629E-43	4,92600E-01	0,408	0,196	3,23455E-39
cDC_CLEC9A	MEA1	6,62880E-08	4,91523E-01	0,514	0,388	1,22083E-03
cDC_CLEC9A	NDUFA10	1,13802E-07	4,89888E-01	0,457	0,325	2,09590E-03
cDC_CLEC9A	SLC12A7	1,53943E-18	4,88168E-01	0,288	0,165	2,83517E-14
cDC_CLEC9A	ARHGAP1	3,35437E-31	4,88166E-01	0,272	0,129	6,17775E-27
cDC_CLEC9A	ARFGAP2	1,07546E-08	4,86658E-01	0,298	0,196	1,98067E-04

cDC_CLEC9A	MED28	7,61057E-16	4,86037E-01	0,457	0,292	1,40164E-11
cDC_CLEC9A	NOP56	4,56929E-11	4,84408E-01	0,318	0,207	8,41526E-07
cDC_CLEC9A	EI24	1,54347E-18	4,83213E-01	0,291	0,168	2,84261E-14
cDC_CLEC9A	TNFAIP8	4,62199E-12	4,83206E-01	0,486	0,333	8,51233E-08
cDC_CLEC9A	HINT2	1,63324E-12	4,82773E-01	0,35	0,228	3,00794E-08
cDC_CLEC9A	CCT7	6,57328E-08	4,81418E-01	0,474	0,347	1,21060E-03
cDC_CLEC9A	SP140	5,79294E-116	4,79888E-01	0,339	0,099	1,06688E-111
cDC_CLEC9A	PSMG4	1,77489E-24	4,79825E-01	0,267	0,137	3,26882E-20
cDC_CLEC9A	ANAPC15	1,31417E-14	4,79596E-01	0,326	0,201	2,42031E-10
cDC_CLEC9A	TSC22D4	1,47807E-16	4,79397E-01	0,419	0,267	2,72217E-12
cDC_CLEC9A	NR4A1	2,09646E-07	4,78780E-01	0,547	0,417	3,86105E-03
cDC_CLEC9A	CLASP2	5,71801E-17	4,78419E-01	0,287	0,167	1,05309E-12
cDC_CLEC9A	PPP1R10	1,06498E-15	4,76590E-01	0,462	0,305	1,96137E-11
cDC_CLEC9A	CCDC91	3,46193E-34	4,75501E-01	0,354	0,18	6,37583E-30
cDC_CLEC9A	HNRNPL	1,73474E-19	4,74463E-01	0,452	0,286	3,19488E-15
cDC_CLEC9A	MRPL11	7,81356E-17	4,73990E-01	0,453	0,291	1,43902E-12
cDC_CLEC9A	POLDIP3	1,26330E-30	4,73763E-01	0,301	0,147	2,32662E-26
cDC_CLEC9A	ZFAND6	2,03824E-18	4,72334E-01	0,488	0,308	3,75383E-14
cDC_CLEC9A	ST8SIA4	1,25188E-34	4,72081E-01	0,513	0,293	2,30559E-30
cDC_CLEC9A	GTF2B	6,49325E-38	4,71973E-01	0,491	0,275	1,19586E-33
cDC_CLEC9A	AATF	1,08485E-06	4,70518E-01	0,326	0,232	1,99796E-02
cDC_CLEC9A	VPS4A	9,21770E-21	4,69345E-01	0,326	0,183	1,69762E-16
cDC_CLEC9A	HSPA4	5,22588E-08	4,69090E-01	0,418	0,299	9,62451E-04
cDC_CLEC9A	TAPBPL	1,32300E-13	4,67456E-01	0,316	0,194	2,43656E-09
cDC_CLEC9A	VPS51	2,05955E-19	4,67335E-01	0,359	0,211	3,79307E-15
cDC_CLEC9A	SRRT	3,78170E-32	4,66728E-01	0,358	0,187	6,96476E-28
cDC_CLEC9A	URM1	1,21088E-14	4,66498E-01	0,383	0,244	2,23008E-10
cDC_CLEC9A	KIAA0040	4,82681E-47	4,65668E-01	0,271	0,109	8,88954E-43
cDC_CLEC9A	TXNDC15	3,55680E-09	4,65624E-01	0,282	0,186	6,55057E-05
cDC_CLEC9A	ELMO1	4,57454E-14	4,65272E-01	0,502	0,336	8,42493E-10
cDC_CLEC9A	AASDHPPT	7,67415E-10	4,65245E-01	0,277	0,179	1,41335E-05
cDC_CLEC9A	PRKRA	2,62282E-46	4,63621E-01	0,356	0,159	4,83045E-42
cDC_CLEC9A	MARCKSL1	1,37652E-149	4,63281E-01	0,65	0,258	2,53513E-145
cDC_CLEC9A	API5	3,11169E-17	4,60989E-01	0,31	0,181	5,73080E-13
cDC_CLEC9A	CMC1	1,06216E-13	4,60507E-01	0,354	0,225	1,95618E-09
cDC_CLEC9A	UVRAG	1,28125E-40	4,60119E-01	0,517	0,287	2,35968E-36
cDC_CLEC9A	CCDC25	2,26944E-11	4,59788E-01	0,27	0,169	4,17963E-07
cDC_CLEC9A	NIT2	1,85941E-27	4,59693E-01	0,326	0,169	3,42448E-23
cDC_CLEC9A	TOMM7	1,43975E-07	4,59369E-01	0,867	0,789	2,65158E-03
cDC_CLEC9A	ADAM8	8,81389E-15	4,58480E-01	0,364	0,224	1,62325E-10
cDC_CLEC9A	EBPL	1,13853E-76	4,58338E-01	0,301	0,101	2,09683E-72

cDC_CLEC9A	DNAJB9	2,97296E-18	4,57803E-01	0,343	0,204	5,47530E-14
cDC_CLEC9A	MTX2	3,80404E-36	4,57758E-01	0,267	0,118	7,00591E-32
cDC_CLEC9A	COMTD1	2,35090E-60	4,57396E-01	0,298	0,113	4,32965E-56
cDC_CLEC9A	FCHO1	3,45426E-128	4,57362E-01	0,308	0,078	6,36171E-124
cDC_CLEC9A	CRELD2	5,52602E-43	4,57320E-01	0,409	0,203	1,01773E-38
cDC_CLEC9A	MRPL19	8,07317E-21	4,56160E-01	0,278	0,152	1,48684E-16
cDC_CLEC9A	EHBP1L1	8,25505E-13	4,55629E-01	0,358	0,23	1,52033E-08
cDC_CLEC9A	CAND1	9,04889E-74	4,55615E-01	0,404	0,162	1,66653E-69
cDC_CLEC9A	GSPT1	2,91909E-22	4,55598E-01	0,413	0,243	5,37609E-18
cDC_CLEC9A	RBM4	1,03489E-20	4,55435E-01	0,284	0,153	1,90597E-16
cDC_CLEC9A	SLC25A19	1,83200E-10	4,54743E-01	0,252	0,163	3,37400E-06
cDC_CLEC9A	GAPDH	1,79012E-06	4,54658E-01	0,978	0,96	3,29686E-02
cDC_CLEC9A	TANK	2,82567E-14	4,54542E-01	0,447	0,291	5,20404E-10
cDC_CLEC9A	IFNGR1	4,78594E-12	4,54336E-01	0,58	0,581	8,81426E-08
cDC_CLEC9A	HDDC2	1,50683E-08	4,53789E-01	0,252	0,165	2,77513E-04
cDC_CLEC9A	GNB4	3,22401E-09	4,53484E-01	0,471	0,334	5,93766E-05
cDC_CLEC9A	PPP2R1A	1,65102E-18	4,53464E-01	0,567	0,393	3,04068E-14
cDC_CLEC9A	RPL7L1	2,70254E-12	4,52634E-01	0,253	0,155	4,97727E-08
cDC_CLEC9A	NOTCH2	8,23672E-10	4,52304E-01	0,451	0,315	1,51696E-05
cDC_CLEC9A	SND1	1,08348E-07	4,51618E-01	0,462	0,343	1,99544E-03
cDC_CLEC9A	RSL1D1	9,13640E-13	4,48919E-01	0,647	0,466	1,68265E-08
cDC_CLEC9A	GGCT	2,13289E-13	4,48817E-01	0,338	0,218	3,92815E-09
cDC_CLEC9A	GCA	7,87483E-08	4,48740E-01	0,533	0,399	1,45031E-03
cDC_CLEC9A	STRAP	1,36905E-25	4,48720E-01	0,544	0,352	2,52137E-21
cDC_CLEC9A	FBXW5	1,03105E-10	4,47591E-01	0,454	0,312	1,89889E-06
cDC_CLEC9A	NEMF	2,47521E-10	4,47497E-01	0,412	0,286	4,55860E-06
cDC_CLEC9A	PFKFB3	2,09494E-12	4,47493E-01	0,484	0,331	3,85825E-08
cDC_CLEC9A	ARRB1	1,79656E-12	4,47238E-01	0,269	0,163	3,30873E-08
cDC_CLEC9A	RBM25	7,23278E-19	4,47010E-01	0,681	0,491	1,33206E-14
cDC_CLEC9A	ACLY	2,18464E-14	4,46653E-01	0,264	0,159	4,02344E-10
cDC_CLEC9A	SRSF11	1,82697E-13	4,43808E-01	0,68	0,512	3,36473E-09
cDC_CLEC9A	YIPF4	2,00084E-11	4,43667E-01	0,393	0,262	3,68494E-07
cDC_CLEC9A	SNRPB2	9,86892E-16	4,43565E-01	0,576	0,403	1,81756E-11
cDC_CLEC9A	SPN	7,74441E-99	4,42564E-01	0,446	0,162	1,42629E-94
cDC_CLEC9A	SLC12A9	1,26702E-59	4,42354E-01	0,411	0,181	2,33348E-55
cDC_CLEC9A	RBX1	6,16405E-07	4,41814E-01	0,741	0,617	1,13523E-02
cDC_CLEC9A	MTDH	4,81976E-26	4,41620E-01	0,806	0,692	8,87656E-22
cDC_CLEC9A	CLDND1	1,65547E-12	4,41365E-01	0,289	0,181	3,04887E-08
cDC_CLEC9A	SLC35A4	2,09761E-09	4,40912E-01	0,257	0,166	3,86317E-05
cDC_CLEC9A	SAFB	2,81720E-12	4,40144E-01	0,377	0,244	5,18843E-08
cDC_CLEC9A	RABGGTB	3,13478E-15	4,39932E-01	0,398	0,254	5,77332E-11

cDC_CLEC9A	PDLIM2	2,67805E-20	4,38179E-01	0,267	0,144	4,93217E-16
cDC_CLEC9A	DCUN1D1	5,63379E-38	4,37743E-01	0,297	0,136	1,03758E-33
cDC_CLEC9A	MRPL55	1,85194E-12	4,36064E-01	0,412	0,275	3,41071E-08
cDC_CLEC9A	AP1M1	1,64656E-12	4,35469E-01	0,376	0,245	3,03247E-08
cDC_CLEC9A	LARP4B	7,70869E-19	4,35320E-01	0,267	0,147	1,41971E-14
cDC_CLEC9A	MLEC	2,56539E-15	4,35120E-01	0,502	0,344	4,72468E-11
cDC_CLEC9A	PDCL3	1,15683E-12	4,34825E-01	0,347	0,218	2,13053E-08
cDC_CLEC9A	EIF5B	6,96759E-57	4,33141E-01	0,632	0,354	1,28322E-52
cDC_CLEC9A	ATF6B	2,08438E-06	4,33077E-01	0,391	0,285	3,83880E-02
cDC_CLEC9A	G3BP1	2,69439E-12	4,32695E-01	0,449	0,305	4,96226E-08
cDC_CLEC9A	CYC1	3,93529E-08	4,32591E-01	0,572	0,432	7,24762E-04
cDC_CLEC9A	AHSA1	1,94218E-15	4,31403E-01	0,453	0,296	3,57691E-11
cDC_CLEC9A	MRPL21	2,82736E-13	4,30623E-01	0,406	0,263	5,20716E-09
cDC_CLEC9A	YPEL5	1,49589E-09	4,30423E-01	0,58	0,431	2,75498E-05
cDC_CLEC9A	ENTPD1	5,58772E-07	4,30287E-01	0,418	0,307	1,02909E-02
cDC_CLEC9A	POLR3K	5,80601E-17	4,30154E-01	0,271	0,157	1,06929E-12
cDC_CLEC9A	NUDCD2	8,88669E-23	4,29931E-01	0,378	0,219	1,63666E-18
cDC_CLEC9A	CTBP1	1,67029E-14	4,29611E-01	0,29	0,176	3,07618E-10
cDC_CLEC9A	LYPLA2	1,03645E-22	4,28861E-01	0,388	0,221	1,90882E-18
cDC_CLEC9A	CYLD	5,45307E-38	4,28541E-01	0,452	0,243	1,00429E-33
cDC_CLEC9A	PPP2CA	3,97239E-15	4,27654E-01	0,494	0,331	7,31595E-11
cDC_CLEC9A	NDUFA7	6,93589E-34	4,26569E-01	0,306	0,15	1,27738E-29
cDC_CLEC9A	TCERG1	5,14497E-34	4,25629E-01	0,393	0,207	9,47549E-30
cDC_CLEC9A	U2AF1L4	2,03247E-19	4,25500E-01	0,272	0,152	3,74319E-15
cDC_CLEC9A	SMIM12	6,67186E-13	4,24709E-01	0,404	0,265	1,22876E-08
cDC_CLEC9A	PCBP2	2,32875E-22	4,24339E-01	0,856	0,797	4,28886E-18
cDC_CLEC9A	AMZ2	1,27103E-36	4,24288E-01	0,428	0,223	2,34086E-32
cDC_CLEC9A	TTC17	7,09827E-08	4,23918E-01	0,312	0,218	1,30729E-03
cDC_CLEC9A	RBM42	1,54459E-17	4,21488E-01	0,458	0,293	2,84467E-13
cDC_CLEC9A	LSM10	3,86725E-07	4,21456E-01	0,51	0,373	7,12232E-03
cDC_CLEC9A	GORASP2	6,78581E-28	4,21331E-01	0,388	0,211	1,24974E-23
cDC_CLEC9A	CTTNBP2NL	1,66788E-32	4,20137E-01	0,321	0,162	3,07174E-28
cDC_CLEC9A	ATP5F1A	5,18681E-35	4,18731E-01	0,426	0,251	9,55255E-31
cDC_CLEC9A	PIH1D1	1,19205E-07	4,18690E-01	0,358	0,252	2,19539E-03
cDC_CLEC9A	CASC3	1,59112E-14	4,18591E-01	0,299	0,18	2,93037E-10
cDC_CLEC9A	HDAC1	1,34152E-28	4,18397E-01	0,318	0,164	2,47067E-24
cDC_CLEC9A	RPL36AL	5,91255E-07	4,18141E-01	0,59	0,491	1,08891E-02
cDC_CLEC9A	PCBP1	1,33741E-08	4,17664E-01	0,702	0,609	2,46311E-04
cDC_CLEC9A	LSM14A	1,34198E-32	4,17339E-01	0,523	0,313	2,47153E-28
cDC_CLEC9A	AGGF1	3,17702E-32	4,16214E-01	0,389	0,206	5,85112E-28
cDC_CLEC9A	SRSF10	4,99507E-16	4,16038E-01	0,423	0,276	9,19942E-12

cDC_CLEC9A	RPS6KB2	8,11303E-39	4,15791E-01	0,296	0,132	1,49418E-34
cDC_CLEC9A	LONP2	1,82161E-09	4,14157E-01	0,332	0,226	3,35486E-05
cDC_CLEC9A	SNRNP200	1,31064E-09	4,13665E-01	0,372	0,257	2,41380E-05
cDC_CLEC9A	PIKFYVE	1,08524E-28	4,13509E-01	0,267	0,131	1,99869E-24
cDC_CLEC9A	SHKBP1	1,04856E-07	4,12103E-01	0,503	0,367	1,93114E-03
cDC_CLEC9A	ATP5MF	1,19999E-23	4,11749E-01	0,49	0,353	2,21003E-19
cDC_CLEC9A	RBBP6	1,40175E-16	4,10963E-01	0,44	0,286	2,58160E-12
cDC_CLEC9A	GNL1	7,64363E-23	4,10035E-01	0,25	0,128	1,40773E-18
cDC_CLEC9A	KLHL6	3,01664E-10	4,08811E-01	0,293	0,195	5,55575E-06
cDC_CLEC9A	ELAVL1	9,09493E-32	4,08699E-01	0,374	0,199	1,67501E-27
cDC_CLEC9A	C1orf35	1,68335E-22	4,07630E-01	0,301	0,164	3,10022E-18
cDC_CLEC9A	N4BP2	3,71189E-51	4,07353E-01	0,253	0,094	6,83618E-47
cDC_CLEC9A	SEPTIN6	3,61208E-70	4,07183E-01	0,253	0,086	6,65236E-66
cDC_CLEC9A	RRM2B	8,94445E-34	4,06508E-01	0,272	0,125	1,64730E-29
cDC_CLEC9A	TNPO1	2,61170E-06	4,06026E-01	0,346	0,248	4,80998E-02
cDC_CLEC9A	IMPA2	2,15267E-69	4,05962E-01	0,279	0,095	3,96457E-65
cDC_CLEC9A	TNIP2	1,27677E-33	4,05748E-01	0,357	0,182	2,35143E-29
cDC_CLEC9A	SPNS1	7,42073E-60	4,05133E-01	0,502	0,244	1,36668E-55
cDC_CLEC9A	ATP5MC3	3,69175E-21	4,04491E-01	0,477	0,351	6,79909E-17
cDC_CLEC9A	CCNC	3,63403E-29	4,03307E-01	0,314	0,16	6,69279E-25
cDC_CLEC9A	ATP5PB	2,78174E-31	4,03271E-01	0,44	0,28	5,12314E-27
cDC_CLEC9A	CAMK2G	1,25629E-59	4,02639E-01	0,281	0,103	2,31370E-55
cDC_CLEC9A	SMC1A	1,84883E-09	4,01573E-01	0,362	0,251	3,40500E-05
cDC_CLEC9A	CDC42EP3	4,75426E-22	4,01433E-01	0,32	0,172	8,75592E-18
cDC_CLEC9A	BUD23	4,75436E-103	4,00299E-01	0,369	0,125	8,75610E-99
cDC_CLEC9A	COA4	1,45227E-15	4,00269E-01	0,437	0,283	2,67464E-11
cDC_CLEC9A	SRSF1	6,79694E-28	3,99958E-01	0,454	0,265	1,25179E-23
cDC_CLEC9A	CYCS	1,33206E-07	3,99641E-01	0,656	0,521	2,45325E-03
cDC_CLEC9A	RACK1	3,25912E-13	3,99539E-01	0,54	0,466	6,00233E-09
cDC_CLEC9A	ANXA5	9,45884E-24	3,98558E-01	0,779	0,773	1,74203E-19
cDC_CLEC9A	TAF12	1,16984E-07	3,97165E-01	0,323	0,222	2,15450E-03
cDC_CLEC9A	OSTC	1,70303E-11	3,96097E-01	0,643	0,496	3,13646E-07
cDC_CLEC9A	GOLIM4	1,01492E-13	3,95566E-01	0,347	0,219	1,86917E-09
cDC_CLEC9A	GPBP1	5,47264E-16	3,95421E-01	0,507	0,341	1,00790E-11
cDC_CLEC9A	PDIA4	1,20934E-18	3,95105E-01	0,487	0,318	2,22723E-14
cDC_CLEC9A	FASTK	1,38056E-06	3,95018E-01	0,254	0,176	2,54258E-02
cDC_CLEC9A	STYX	1,74062E-38	3,94948E-01	0,252	0,107	3,20569E-34
cDC_CLEC9A	NBEAL1	4,59577E-08	3,94600E-01	0,326	0,231	8,46403E-04
cDC_CLEC9A	BAK1	1,88144E-13	3,94296E-01	0,25	0,149	3,46505E-09
cDC_CLEC9A	SLBP	1,53349E-22	3,93842E-01	0,384	0,23	2,82423E-18
cDC_CLEC9A	HSPH1	3,37339E-30	3,93323E-01	0,622	0,411	6,21277E-26

cDC_CLEC9A	ATP2B1	2,48299E-15	3,91493E-01	0,511	0,345	4,57292E-11
cDC_CLEC9A	PSENEN	5,88966E-23	3,91299E-01	0,472	0,291	1,08470E-18
cDC_CLEC9A	PSMD11	5,70486E-18	3,90928E-01	0,477	0,305	1,05066E-13
cDC_CLEC9A	RAB1B	2,25410E-13	3,90252E-01	0,513	0,353	4,15137E-09
cDC_CLEC9A	TXNDC17	5,39490E-08	3,90200E-01	0,571	0,431	9,93579E-04
cDC_CLEC9A	LAG3	6,66524E-116	3,89236E-01	0,261	0,065	1,22754E-111
cDC_CLEC9A	CAPRIN1	2,07573E-12	3,89077E-01	0,482	0,333	3,82287E-08
cDC_CLEC9A	AHCY	1,82846E-06	3,88765E-01	0,358	0,26	3,36748E-02
cDC_CLEC9A	IRF5	7,86969E-15	3,88314E-01	0,403	0,26	1,44936E-10
cDC_CLEC9A	UCHL3	7,94810E-56	3,88058E-01	0,45	0,214	1,46380E-51
cDC_CLEC9A	ZDHHC24	3,16761E-15	3,87871E-01	0,37	0,228	5,83378E-11
cDC_CLEC9A	ARCN1	3,10089E-07	3,87676E-01	0,307	0,214	5,71091E-03
cDC_CLEC9A	RBBP7	1,17606E-23	3,87159E-01	0,359	0,202	2,16594E-19
cDC_CLEC9A	GCC2	1,00252E-23	3,86681E-01	0,459	0,277	1,84635E-19
cDC_CLEC9A	HNRNPA3	3,30016E-22	3,86270E-01	0,771	0,627	6,07790E-18
cDC_CLEC9A	CRLF3	3,39783E-07	3,85543E-01	0,267	0,182	6,25778E-03
cDC_CLEC9A	TMX1	2,69610E-17	3,84669E-01	0,504	0,333	4,96541E-13
cDC_CLEC9A	OVCA2	3,77807E-15	3,83231E-01	0,262	0,151	6,95807E-11
cDC_CLEC9A	SZRD1	2,70613E-08	3,83230E-01	0,409	0,292	4,98389E-04
cDC_CLEC9A	PSMD6	6,31332E-12	3,83083E-01	0,414	0,276	1,16272E-07
cDC_CLEC9A	KDM2A	1,49293E-19	3,82988E-01	0,34	0,198	2,74953E-15
cDC_CLEC9A	NOP58	5,36472E-59	3,82634E-01	0,48	0,233	9,88020E-55
cDC_CLEC9A	HSD17B12	9,96709E-32	3,82399E-01	0,436	0,243	1,83564E-27
cDC_CLEC9A	GTF2H1	2,23265E-18	3,82257E-01	0,254	0,141	4,11188E-14
cDC_CLEC9A	ZCCHC7	1,21900E-28	3,82173E-01	0,26	0,124	2,24503E-24
cDC_CLEC9A	THOC3	5,38148E-30	3,81194E-01	0,252	0,119	9,91108E-26
cDC_CLEC9A	CTNNBL1	2,12251E-25	3,80972E-01	0,398	0,227	3,90903E-21
cDC_CLEC9A	FRG1	1,08410E-10	3,80669E-01	0,357	0,239	1,99658E-06
cDC_CLEC9A	PAF1	3,45507E-19	3,80618E-01	0,316	0,181	6,36321E-15
cDC_CLEC9A	ILKAP	1,83269E-12	3,80298E-01	0,277	0,169	3,37526E-08
cDC_CLEC9A	BAG6	2,64155E-13	3,80290E-01	0,416	0,27	4,86494E-09
cDC_CLEC9A	AAMP	1,00460E-06	3,79726E-01	0,409	0,291	1,85017E-02
cDC_CLEC9A	MZT2B	5,91605E-75	3,77612E-01	0,642	0,351	1,08956E-70
cDC_CLEC9A	COX7B	1,20614E-06	3,77296E-01	0,792	0,664	2,22134E-02
cDC_CLEC9A	NUBP2	3,14388E-11	3,77070E-01	0,306	0,197	5,79009E-07
cDC_CLEC9A	RAB3GAP1	1,96128E-11	3,76962E-01	0,272	0,17	3,61208E-07
cDC_CLEC9A	HDAC3	1,08992E-15	3,76009E-01	0,348	0,21	2,00730E-11
cDC_CLEC9A	DOCK5	1,02982E-19	3,75250E-01	0,284	0,157	1,89662E-15
cDC_CLEC9A	UBA1	9,59984E-08	3,75228E-01	0,424	0,301	1,76800E-03
cDC_CLEC9A	PA2G4	4,91768E-18	3,74654E-01	0,602	0,43	9,05689E-14
cDC_CLEC9A	DCTN2	2,32701E-08	3,74491E-01	0,461	0,327	4,28566E-04

cDC_CLEC9A	EIF4G1	7,95397E-07	3,74355E-01	0,441	0,33	1,46488E-02
cDC_CLEC9A	SSRP1	2,95719E-84	3,74087E-01	0,47	0,199	5,44626E-80
cDC_CLEC9A	PFDN5	2,27070E-06	3,74006E-01	0,923	0,902	4,18195E-02
cDC_CLEC9A	CAPN2	6,15514E-29	3,73656E-01	0,412	0,23	1,13359E-24
cDC_CLEC9A	ZFP36L1	1,92029E-08	3,73057E-01	0,759	0,725	3,53660E-04
cDC_CLEC9A	TXNDC9	2,10847E-30	3,72661E-01	0,31	0,154	3,88317E-26
cDC_CLEC9A	MAD2L2	4,17440E-10	3,70984E-01	0,338	0,222	7,68800E-06
cDC_CLEC9A	MRPL43	1,86299E-06	3,69444E-01	0,476	0,348	3,43107E-02
cDC_CLEC9A	CCNG2	1,96906E-68	3,69344E-01	0,314	0,116	3,62642E-64
cDC_CLEC9A	CYB5B	1,57940E-16	3,68890E-01	0,316	0,188	2,90878E-12
cDC_CLEC9A	RASSF2	8,42714E-47	3,68798E-01	0,5	0,26	1,55203E-42
cDC_CLEC9A	EHMT1	5,56338E-09	3,67613E-01	0,253	0,166	1,02461E-04
cDC_CLEC9A	DRAM2	9,51060E-24	3,67411E-01	0,27	0,392	1,75157E-19
cDC_CLEC9A	MIS18BP1	1,84460E-21	3,67267E-01	0,549	0,357	3,39721E-17
cDC_CLEC9A	ECH1	8,94194E-10	3,66944E-01	0,408	0,286	1,64684E-05
cDC_CLEC9A	NHP2	6,27097E-36	3,66756E-01	0,636	0,408	1,15492E-31
cDC_CLEC9A	SERTAD2	3,91476E-35	3,66569E-01	0,358	0,182	7,20981E-31
cDC_CLEC9A	CIAO1	1,99812E-14	3,65496E-01	0,383	0,244	3,67993E-10
cDC_CLEC9A	PRR14L	1,31732E-31	3,65331E-01	0,266	0,125	2,42611E-27
cDC_CLEC9A	GSS	7,39120E-11	3,65193E-01	0,264	0,166	1,36124E-06
cDC_CLEC9A	DSE	5,07187E-22	3,64595E-01	0,614	0,435	9,34087E-18
cDC_CLEC9A	LSM4	4,10175E-29	3,62609E-01	0,634	0,428	7,55419E-25
cDC_CLEC9A	ATAD2B	8,70509E-44	3,61812E-01	0,314	0,139	1,60322E-39
cDC_CLEC9A	PIK3CG	2,19741E-27	3,61070E-01	0,252	0,121	4,04697E-23
cDC_CLEC9A	NDUFC1	1,76560E-06	3,61002E-01	0,59	0,455	3,25170E-02
cDC_CLEC9A	NUP210	9,07276E-155	3,59010E-01	0,373	0,095	1,67093E-150
cDC_CLEC9A	KEAP1	1,88896E-16	3,58287E-01	0,266	0,151	3,47890E-12
cDC_CLEC9A	DUSP1	3,22602E-10	3,58112E-01	0,789	0,851	5,94136E-06
cDC_CLEC9A	EXOSC1	4,72713E-07	3,58091E-01	0,269	0,185	8,70596E-03
cDC_CLEC9A	MYH9	6,75067E-11	3,57789E-01	0,713	0,576	1,24327E-06
cDC_CLEC9A	ATP5MG	1,28739E-15	3,57672E-01	0,517	0,417	2,37098E-11
cDC_CLEC9A	RGL2	2,12230E-26	3,56968E-01	0,256	0,125	3,90863E-22
cDC_CLEC9A	TSC22D3	2,38263E-17	3,55988E-01	0,659	0,695	4,38808E-13
cDC_CLEC9A	MRPL37	2,08880E-15	3,55516E-01	0,362	0,228	3,84694E-11
cDC_CLEC9A	MZT1	7,15270E-34	3,55456E-01	0,324	0,16	1,31731E-29
cDC_CLEC9A	ATF4	8,49812E-12	3,55262E-01	0,571	0,402	1,56510E-07
cDC_CLEC9A	CMC2	7,65391E-08	3,55128E-01	0,386	0,273	1,40962E-03
cDC_CLEC9A	PDAP1	4,31883E-13	3,55059E-01	0,487	0,331	7,95399E-09
cDC_CLEC9A	RAD21	4,48192E-15	3,54667E-01	0,506	0,344	8,25436E-11
cDC_CLEC9A	DDX27	2,80633E-22	3,53085E-01	0,403	0,234	5,16842E-18
cDC_CLEC9A	ZCRB1	5,46226E-23	3,52036E-01	0,449	0,269	1,00599E-18

cDC_CLEC9A	CHCHD3	3,43313E-11	3,50649E-01	0,373	0,247	6,32279E-07
cDC_CLEC9A	PNN	5,64900E-28	3,49551E-01	0,51	0,311	1,04038E-23
cDC_CLEC9A	SPI1	3,38820E-26	3,48882E-01	0,88	0,765	6,24004E-22
cDC_CLEC9A	TTC19	1,56226E-42	3,48626E-01	0,338	0,153	2,87721E-38
cDC_CLEC9A	RAD50	2,73231E-20	3,48504E-01	0,29	0,161	5,03210E-16
cDC_CLEC9A	AFF3	2,43905E-181	3,46492E-01	0,256	0,044	4,49200E-177
cDC_CLEC9A	BRD2	5,37928E-42	3,45999E-01	0,716	0,483	9,90703E-38
cDC_CLEC9A	CPSF6	2,62392E-50	3,45858E-01	0,399	0,184	4,83248E-46
cDC_CLEC9A	MARK3	1,76496E-08	3,45721E-01	0,291	0,195	3,25054E-04
cDC_CLEC9A	UBE2V1	3,37726E-33	3,45601E-01	0,347	0,178	6,21991E-29
cDC_CLEC9A	ABI1	1,15249E-06	3,45340E-01	0,454	0,338	2,12254E-02
cDC_CLEC9A	GRSF1	1,97942E-13	3,44681E-01	0,493	0,337	3,64550E-09
cDC_CLEC9A	NDUFV1	8,51355E-10	3,44437E-01	0,548	0,405	1,56794E-05
cDC_CLEC9A	RNF10	1,81379E-09	3,43539E-01	0,394	0,272	3,34046E-05
cDC_CLEC9A	DNAJC19	2,03822E-11	3,43244E-01	0,351	0,231	3,75380E-07
cDC_CLEC9A	DDB1	3,00300E-10	3,42599E-01	0,336	0,222	5,53062E-06
cDC_CLEC9A	LPGAT1	3,63184E-39	3,42206E-01	0,369	0,183	6,68875E-35
cDC_CLEC9A	GTF2F2	1,94722E-51	3,41920E-01	0,374	0,168	3,58619E-47
cDC_CLEC9A	DAZAP2	1,61335E-12	3,41647E-01	0,764	0,742	2,97130E-08
cDC_CLEC9A	MRPL32	4,44563E-16	3,41644E-01	0,299	0,174	8,18752E-12
cDC_CLEC9A	SAT1	2,10411E-82	3,41603E-01	0,933	0,948	3,87514E-78
cDC_CLEC9A	NDUFA6	3,35673E-14	3,41599E-01	0,677	0,525	6,18208E-10
cDC_CLEC9A	ARF3	5,73346E-15	3,41416E-01	0,49	0,334	1,05593E-10
cDC_CLEC9A	KLF6	1,30901E-06	3,40977E-01	0,777	0,798	2,41081E-02
cDC_CLEC9A	CBX5	1,54493E-27	3,40807E-01	0,253	0,124	2,84530E-23
cDC_CLEC9A	PACS1	3,32726E-50	3,39820E-01	0,272	0,107	6,12781E-46
cDC_CLEC9A	MAPK1IP1L	8,18756E-24	3,39605E-01	0,542	0,34	1,50790E-19
cDC_CLEC9A	AFF1	2,80733E-11	3,38751E-01	0,34	0,225	5,17026E-07
cDC_CLEC9A	SMCHD1	1,43327E-09	3,37817E-01	0,453	0,322	2,63966E-05
cDC_CLEC9A	NDUFA4	9,35798E-10	3,37355E-01	0,844	0,743	1,72346E-05
cDC_CLEC9A	FAM177A1	7,56024E-14	3,36916E-01	0,469	0,313	1,39237E-09
cDC_CLEC9A	SERF2	3,50192E-07	3,36855E-01	0,976	0,953	6,44949E-03
cDC_CLEC9A	SGK1	4,58951E-15	3,35927E-01	0,533	0,606	8,45251E-11
cDC_CLEC9A	NKTR	2,38573E-11	3,35832E-01	0,416	0,281	4,39380E-07
cDC_CLEC9A	ABHD16A	2,51832E-17	3,35098E-01	0,257	0,142	4,63798E-13
cDC_CLEC9A	DDX49	7,39026E-25	3,33780E-01	0,262	0,131	1,36106E-20
cDC_CLEC9A	AKNA	8,88364E-31	3,32300E-01	0,453	0,252	1,63610E-26
cDC_CLEC9A	KDM2B	1,50547E-18	3,31975E-01	0,268	0,146	2,77263E-14
cDC_CLEC9A	BLCAP	2,51210E-14	3,29488E-01	0,304	0,184	4,62654E-10
cDC_CLEC9A	VASH1	2,58182E-16	3,29237E-01	0,333	0,201	4,75494E-12
cDC_CLEC9A	SUDS3	2,34337E-20	3,28802E-01	0,276	0,152	4,31578E-16

cDC_CLEC9A	MEMO1	2,51143E-37	3,28725E-01	0,254	0,109	4,62530E-33
cDC_CLEC9A	MAP3K11	1,17640E-12	3,28493E-01	0,373	0,242	2,16657E-08
cDC_CLEC9A	NXT1	1,09939E-18	3,28335E-01	0,353	0,206	2,02475E-14
cDC_CLEC9A	METTL26	4,25500E-37	3,27397E-01	0,363	0,189	7,83644E-33
cDC_CLEC9A	PHF5A	2,58477E-12	3,27043E-01	0,388	0,256	4,76038E-08
cDC_CLEC9A	WDR18	1,06728E-26	3,26474E-01	0,291	0,148	1,96561E-22
cDC_CLEC9A	TUFM	9,55006E-10	3,26297E-01	0,653	0,507	1,75883E-05
cDC_CLEC9A	PPIE	3,94579E-08	3,26176E-01	0,259	0,175	7,26696E-04
cDC_CLEC9A	PRKX	1,05604E-12	3,25899E-01	0,262	0,159	1,94491E-08
cDC_CLEC9A	TICAM1	1,00963E-34	3,25705E-01	0,307	0,143	1,85943E-30
cDC_CLEC9A	HTATSF1	9,04522E-20	3,25694E-01	0,286	0,158	1,66586E-15
cDC_CLEC9A	RFX5	1,21351E-17	3,25575E-01	0,298	0,171	2,23493E-13
cDC_CLEC9A	SRP72	2,85883E-11	3,24737E-01	0,492	0,344	5,26511E-07
cDC_CLEC9A	RTRAF	9,76906E-27	3,24345E-01	0,424	0,262	1,79917E-22
cDC_CLEC9A	MRPS10	1,61901E-08	3,23764E-01	0,27	0,179	2,98173E-04
cDC_CLEC9A	ANKRD12	4,24117E-11	3,22903E-01	0,637	0,467	7,81096E-07
cDC_CLEC9A	DUT	9,55557E-15	3,22645E-01	0,558	0,399	1,75985E-10
cDC_CLEC9A	SMARCC1	4,65825E-37	3,22581E-01	0,407	0,208	8,57910E-33
cDC_CLEC9A	TBC1D5	4,09484E-09	3,22420E-01	0,298	0,198	7,54148E-05
cDC_CLEC9A	ZNF622	9,61118E-12	3,21910E-01	0,272	0,168	1,77009E-07
cDC_CLEC9A	MRPS5	2,80377E-08	3,21863E-01	0,361	0,253	5,16371E-04
cDC_CLEC9A	RABL6	4,71025E-21	3,19918E-01	0,28	0,15	8,67488E-17
cDC_CLEC9A	HNRNPA0	1,74894E-32	3,19870E-01	0,707	0,506	3,22102E-28
cDC_CLEC9A	DNAJA2	1,60923E-09	3,18135E-01	0,454	0,314	2,96372E-05
cDC_CLEC9A	FCHSD2	5,89695E-17	3,18097E-01	0,363	0,222	1,08604E-12
cDC_CLEC9A	FBXO9	2,65243E-11	3,17112E-01	0,274	0,173	4,88498E-07
cDC_CLEC9A	SMARCA4	9,87964E-16	3,16943E-01	0,296	0,177	1,81953E-11
cDC_CLEC9A	EIF2S2	7,22071E-10	3,16187E-01	0,612	0,459	1,32984E-05
cDC_CLEC9A	MRPS2	2,95833E-24	3,15849E-01	0,263	0,133	5,44836E-20
cDC_CLEC9A	ZNF721	7,27393E-34	3,15242E-01	0,251	0,111	1,33964E-29
cDC_CLEC9A	BICD2	7,66009E-36	3,14046E-01	0,331	0,158	1,41076E-31
cDC_CLEC9A	MED29	2,51067E-10	3,13582E-01	0,342	0,233	4,62390E-06
cDC_CLEC9A	VHL	5,61439E-69	3,12432E-01	0,41	0,17	1,03400E-64
cDC_CLEC9A	MAGOH	4,08791E-18	3,12118E-01	0,516	0,346	7,52870E-14
cDC_CLEC9A	OXLD1	2,31478E-08	3,10854E-01	0,261	0,173	4,26313E-04
cDC_CLEC9A	ISOC2	2,72817E-20	3,10720E-01	0,367	0,215	5,02446E-16
cDC_CLEC9A	CHMP1B	1,39844E-09	3,10643E-01	0,533	0,404	2,57551E-05
cDC_CLEC9A	UBA2	2,43532E-23	3,08453E-01	0,39	0,227	4,48513E-19
cDC_CLEC9A	TMEM256	3,05465E-12	3,06802E-01	0,577	0,408	5,62574E-08
cDC_CLEC9A	PACSIN2	1,25930E-30	3,06768E-01	0,463	0,266	2,31925E-26
cDC_CLEC9A	DNAJA1	1,79506E-08	3,06689E-01	0,721	0,602	3,30595E-04

cDC_CLEC9A	NSMCE4A	5,43859E-31	3,06591E-01	0,309	0,155	1,00163E-26
cDC_CLEC9A	JPT1	1,34368E-27	3,06152E-01	0,448	0,28	2,47466E-23
cDC_CLEC9A	FUS	3,51577E-09	3,06014E-01	0,72	0,585	6,47500E-05
cDC_CLEC9A	BCL10	1,11312E-12	3,05548E-01	0,266	0,164	2,05003E-08
cDC_CLEC9A	EAPP	3,22955E-08	3,05415E-01	0,336	0,23	5,94785E-04
cDC_CLEC9A	SH3BP1	2,14070E-43	3,04607E-01	0,462	0,246	3,94254E-39
cDC_CLEC9A	TPT1	6,37102E-17	3,04568E-01	0,981	0,974	1,17335E-12
cDC_CLEC9A	MRPS14	2,70897E-21	3,04562E-01	0,324	0,181	4,98911E-17
cDC_CLEC9A	AXL	7,01498E-08	3,04490E-01	0,213	0,281	1,29195E-03
cDC_CLEC9A	DTNBP1	2,74036E-07	3,04257E-01	0,259	0,177	5,04692E-03
cDC_CLEC9A	SDR39U1	4,86013E-18	3,03426E-01	0,288	0,164	8,95089E-14
cDC_CLEC9A	RAD23A	1,94104E-06	3,02255E-01	0,628	0,502	3,57481E-02
cDC_CLEC9A	DDX54	1,08934E-20	3,01786E-01	0,324	0,182	2,00624E-16
cDC_CLEC9A	PIN4	1,25240E-07	3,01533E-01	0,373	0,261	2,30655E-03
cDC_CLEC9A	STAT6	2,57876E-12	2,99205E-01	0,447	0,302	4,74930E-08
cDC_CLEC9A	ZNF330	1,73370E-06	2,99075E-01	0,303	0,209	3,19296E-02
cDC_CLEC9A	TAF7	5,99649E-07	2,98405E-01	0,413	0,3	1,10437E-02
cDC_CLEC9A	IAH1	1,10878E-26	2,98151E-01	0,498	0,306	2,04204E-22
cDC_CLEC9A	CRYBG3	5,07846E-53	2,97768E-01	0,28	0,109	9,35300E-49
cDC_CLEC9A	SDF2L1	6,23790E-14	2,97322E-01	0,507	0,351	1,14883E-09
cDC_CLEC9A	GPAA1	3,96873E-11	2,97161E-01	0,384	0,254	7,30922E-07
cDC_CLEC9A	RPA3	1,50894E-23	2,97151E-01	0,268	0,141	2,77902E-19
cDC_CLEC9A	MRPS21	6,53470E-10	2,96665E-01	0,683	0,524	1,20350E-05
cDC_CLEC9A	CAMK1D	5,55651E-13	2,95887E-01	0,337	0,218	1,02334E-08
cDC_CLEC9A	UROS	1,72892E-10	2,95071E-01	0,306	0,202	3,18416E-06
cDC_CLEC9A	SYNCRIP	2,59486E-15	2,94726E-01	0,482	0,319	4,77896E-11
cDC_CLEC9A	NCBP2	6,31673E-08	2,94663E-01	0,297	0,203	1,16335E-03
cDC_CLEC9A	SEC23B	3,91787E-08	2,94609E-01	0,26	0,174	7,21553E-04
cDC_CLEC9A	ZC3H15	4,38617E-10	2,94562E-01	0,598	0,435	8,07800E-06
cDC_CLEC9A	PPM1K	2,43519E-21	2,93887E-01	0,287	0,153	4,48490E-17
cDC_CLEC9A	KTN1	7,24347E-13	2,93780E-01	0,657	0,49	1,33403E-08
cDC_CLEC9A	DPM2	4,89021E-16	2,93265E-01	0,349	0,213	9,00631E-12
cDC_CLEC9A	SRSF8	2,45139E-79	2,93212E-01	0,263	0,08	4,51473E-75
cDC_CLEC9A	MRPL17	2,24520E-19	2,91530E-01	0,37	0,227	4,13498E-15
cDC_CLEC9A	FAM204A	1,35421E-09	2,90843E-01	0,413	0,285	2,49404E-05
cDC_CLEC9A	FNBP4	3,22484E-17	2,90777E-01	0,458	0,297	5,93918E-13
cDC_CLEC9A	PSMD1	5,18292E-07	2,89825E-01	0,382	0,275	9,54539E-03
cDC_CLEC9A	MT-CYB	1,51548E-06	2,89663E-01	0,643	0,577	2,79106E-02
cDC_CLEC9A	ALDOA	1,02998E-10	2,88842E-01	0,783	0,759	1,89691E-06
cDC_CLEC9A	DNAJB14	2,32147E-09	2,88316E-01	0,301	0,201	4,27545E-05
cDC_CLEC9A	RNASEH1	1,82573E-19	2,87936E-01	0,269	0,145	3,36245E-15

cDC_CLEC9A	ARHGAP30	7,53468E-19	2,87773E-01	0,502	0,329	1,38766E-14
cDC_CLEC9A	CMPK1	4,07368E-07	2,86671E-01	0,388	0,275	7,50250E-03
cDC_CLEC9A	MT-ND6	1,34001E-19	2,86223E-01	0,453	0,299	2,46789E-15
cDC_CLEC9A	SRSF4	3,66170E-08	2,85997E-01	0,482	0,35	6,74375E-04
cDC_CLEC9A	LRRC59	2,58385E-19	2,85984E-01	0,444	0,28	4,75868E-15
cDC_CLEC9A	SRPK1	1,47249E-71	2,85668E-01	0,309	0,109	2,71189E-67
cDC_CLEC9A	ATP5F1C	1,01530E-23	2,85562E-01	0,437	0,285	1,86988E-19
cDC_CLEC9A	AIDA	1,34241E-41	2,84855E-01	0,4	0,201	2,47232E-37
cDC_CLEC9A	USP34	4,29497E-14	2,84680E-01	0,339	0,209	7,91005E-10
cDC_CLEC9A	SPPL2A	1,41696E-07	2,83214E-01	0,306	0,341	2,60962E-03
cDC_CLEC9A	SMIM20	6,37233E-09	2,83208E-01	0,339	0,236	1,17359E-04
cDC_CLEC9A	PRRC2A	4,31026E-13	2,83183E-01	0,376	0,246	7,93821E-09
cDC_CLEC9A	MED25	3,31101E-45	2,82979E-01	0,3	0,127	6,09788E-41
cDC_CLEC9A	RC3H1	7,55915E-15	2,81078E-01	0,312	0,193	1,39217E-10
cDC_CLEC9A	ABCE1	1,80456E-37	2,81001E-01	0,339	0,162	3,32345E-33
cDC_CLEC9A	CPPED1	3,27772E-11	2,80692E-01	0,401	0,268	6,03658E-07
cDC_CLEC9A	MANF	1,57327E-18	2,80212E-01	0,459	0,292	2,89749E-14
cDC_CLEC9A	RHOG	1,53178E-10	2,78162E-01	0,756	0,726	2,82108E-06
cDC_CLEC9A	PDHA1	4,28360E-08	2,78148E-01	0,28	0,189	7,88911E-04
cDC_CLEC9A	TGOLN2	5,96861E-07	2,77091E-01	0,516	0,514	1,09924E-02
cDC_CLEC9A	UBE2J1	1,72005E-45	2,76252E-01	0,553	0,297	3,16783E-41
cDC_CLEC9A	NUCKS1	2,83639E-13	2,75963E-01	0,65	0,498	5,22377E-09
cDC_CLEC9A	C6orf62	6,77765E-10	2,75686E-01	0,307	0,365	1,24824E-05
cDC_CLEC9A	NARF	6,20989E-27	2,75365E-01	0,366	0,203	1,14368E-22
cDC_CLEC9A	NDUFB11	9,40145E-10	2,74212E-01	0,777	0,66	1,73147E-05
cDC_CLEC9A	CUL5	7,11537E-10	2,74022E-01	0,267	0,17	1,31044E-05
cDC_CLEC9A	TRIM28	4,68488E-11	2,71962E-01	0,294	0,19	8,62815E-07
cDC_CLEC9A	AGPS	9,19207E-36	2,71904E-01	0,291	0,133	1,69290E-31
cDC_CLEC9A	SLC25A33	1,30453E-108	2,71569E-01	0,33	0,097	2,40255E-104
cDC_CLEC9A	C5orf24	1,20912E-06	2,70683E-01	0,254	0,176	2,22684E-02
cDC_CLEC9A	PHB	2,05503E-06	2,70269E-01	0,514	0,386	3,78474E-02
cDC_CLEC9A	EFHD2	9,81416E-35	2,69817E-01	0,662	0,426	1,80747E-30
cDC_CLEC9A	PHLDA2	6,28172E-28	2,69603E-01	0,318	0,164	1,15690E-23
cDC_CLEC9A	LSM1	2,20129E-10	2,69466E-01	0,356	0,235	4,05412E-06
cDC_CLEC9A	OAZ1	3,50136E-95	2,69463E-01	0,924	0,937	6,44846E-91
cDC_CLEC9A	TXNRD1	4,19403E-10	2,69401E-01	0,289	0,188	7,72414E-06
cDC_CLEC9A	SSBP4	3,13341E-50	2,69221E-01	0,326	0,138	5,77081E-46
cDC_CLEC9A	AFTPH	4,18055E-19	2,68876E-01	0,343	0,203	7,69932E-15
cDC_CLEC9A	MAN1A2	1,67532E-19	2,68483E-01	0,306	0,171	3,08544E-15
cDC_CLEC9A	CLIP1	3,93636E-17	2,67846E-01	0,361	0,217	7,24959E-13
cDC_CLEC9A	BTG2	2,70862E-20	2,67749E-01	0,664	0,495	4,98847E-16

cDC_CLEC9A	COMMD2	2,39224E-12	2,67175E-01	0,304	0,189	4,40578E-08
cDC_CLEC9A	TMUB1	9,12406E-20	2,66578E-01	0,427	0,259	1,68038E-15
cDC_CLEC9A	GAA	2,51233E-13	2,66153E-01	0,324	0,388	4,62696E-09
cDC_CLEC9A	METAP2	9,98911E-08	2,65419E-01	0,403	0,291	1,83969E-03
cDC_CLEC9A	PRPF8	7,71951E-10	2,65076E-01	0,438	0,305	1,42170E-05
cDC_CLEC9A	TMPO	4,97524E-15	2,64649E-01	0,339	0,213	9,16291E-11
cDC_CLEC9A	NBPF10	1,40987E-10	2,63939E-01	0,303	0,2	2,59655E-06
cDC_CLEC9A	C19orf38	3,46520E-15	2,63885E-01	0,376	0,231	6,38186E-11
cDC_CLEC9A	EEF2	6,68613E-07	2,63033E-01	0,903	0,827	1,23138E-02
cDC_CLEC9A	PHYKPL	2,05008E-12	2,62876E-01	0,389	0,256	3,77564E-08
cDC_CLEC9A	SMC5	1,38601E-36	2,62811E-01	0,33	0,158	2,55261E-32
cDC_CLEC9A	BOD1L1	3,60159E-37	2,62779E-01	0,462	0,25	6,63305E-33
cDC_CLEC9A	RPAIN	6,39519E-21	2,61429E-01	0,271	0,145	1,17780E-16
cDC_CLEC9A	MPPE1	1,35733E-14	2,60538E-01	0,274	0,165	2,49979E-10
cDC_CLEC9A	RPA2	2,70845E-28	2,59814E-01	0,261	0,126	4,98816E-24
cDC_CLEC9A	CTNNBIP1	1,29503E-90	2,59181E-01	0,378	0,134	2,38505E-86
cDC_CLEC9A	STX3	1,49948E-52	2,57792E-01	0,31	0,123	2,76159E-48
cDC_CLEC9A	PRKAG1	1,44736E-08	2,57302E-01	0,289	0,193	2,66560E-04
cDC_CLEC9A	WDR43	4,36836E-38	2,56692E-01	0,287	0,127	8,04521E-34
cDC_CLEC9A	CBFB	3,80711E-38	2,55606E-01	0,327	0,154	7,01156E-34
cDC_CLEC9A	POLR2J3	6,14398E-07	2,55005E-01	0,28	0,194	1,13154E-02
cDC_CLEC9A	GNA13	2,29725E-06	2,54897E-01	0,44	0,332	4,23085E-02
cDC_CLEC9A	ZC3HAV1	2,99614E-10	2,54316E-01	0,387	0,269	5,51799E-06
cDC_CLEC9A	TRIM26	1,51122E-41	2,53846E-01	0,303	0,134	2,78322E-37
cDC_CLEC9A	TPGS2	1,31563E-20	2,53418E-01	0,319	0,179	2,42299E-16
cDC_CLEC9A	RNF138	2,27994E-13	2,52164E-01	0,26	0,155	4,19896E-09
cDC_CLEC9A	HYOU1	3,80952E-07	-2,50258E-01	0,251	0,17	7,01599E-03
cDC_CLEC9A	SELL	2,41892E-59	-2,52562E-01	0,43	0,197	4,45492E-55
cDC_CLEC9A	RNF149	6,75828E-20	-2,63448E-01	0,61	0,627	1,24467E-15
cDC_CLEC9A	NUMB	7,27301E-09	-2,64685E-01	0,282	0,334	1,33947E-04
cDC_CLEC9A	MGAT1	2,91741E-33	-2,77361E-01	0,569	0,625	5,37300E-29
cDC_CLEC9A	CDKN1A	2,35440E-13	-2,77468E-01	0,434	0,527	4,33610E-09
cDC_CLEC9A	WIPF1	3,88462E-09	-2,78433E-01	0,458	0,466	7,15431E-05
cDC_CLEC9A	RTCB	1,20671E-09	-2,78682E-01	0,239	0,296	2,22239E-05
cDC_CLEC9A	MANBA	8,33607E-08	-2,85217E-01	0,292	0,337	1,53525E-03
cDC_CLEC9A	ATP6AP2	6,50502E-59	-2,86804E-01	0,456	0,594	1,19803E-54
cDC_CLEC9A	QKI	2,70033E-18	-2,87264E-01	0,271	0,37	4,97320E-14
cDC_CLEC9A	C4orf48	1,99757E-10	-2,88874E-01	0,533	0,528	3,67893E-06
cDC_CLEC9A	LRPAP1	5,79999E-27	-2,89330E-01	0,416	0,497	1,06818E-22
cDC_CLEC9A	OCIAD1	1,94097E-07	-2,93418E-01	0,423	0,434	3,57468E-03
cDC_CLEC9A	QSOX1	3,89738E-11	-3,00434E-01	0,218	0,29	7,17780E-07

cDC_CLEC9A	SOAT1	1,05327E-13	-3,00909E-01	0,201	0,287	1,93980E-09
cDC_CLEC9A	CXCR4	1,90538E-11	-3,03512E-01	0,539	0,581	3,50913E-07
cDC_CLEC9A	SLC8A1	1,99896E-06	-3,05993E-01	0,343	0,366	3,68149E-02
cDC_CLEC9A	PPP1R14B	3,38628E-38	-3,06920E-01	0,31	0,141	6,23651E-34
cDC_CLEC9A	STX6	4,36848E-20	-3,10524E-01	0,132	0,253	8,04543E-16
cDC_CLEC9A	NOP10	5,50234E-28	-3,17349E-01	0,607	0,632	1,01337E-23
cDC_CLEC9A	STX4	1,15468E-06	-3,19027E-01	0,274	0,313	2,12658E-02
cDC_CLEC9A	RAB24	2,89278E-09	-3,20285E-01	0,212	0,272	5,32763E-05
cDC_CLEC9A	RAB20	1,99752E-17	-3,33044E-01	0,247	0,349	3,67883E-13
cDC_CLEC9A	PNPLA2	1,99038E-06	-3,33666E-01	0,271	0,302	3,66568E-02
cDC_CLEC9A	SDCBP	1,39369E-105	-3,40605E-01	0,553	0,727	2,56675E-101
cDC_CLEC9A	ATOX1	1,72225E-08	-3,41679E-01	0,621	0,575	3,17187E-04
cDC_CLEC9A	TKT	3,88979E-20	-3,45361E-01	0,581	0,608	7,16382E-16
cDC_CLEC9A	FKBP5	6,39263E-28	-3,46022E-01	0,423	0,52	1,17733E-23
cDC_CLEC9A	NFKBIZ	2,97599E-13	-3,46702E-01	0,409	0,455	5,48088E-09
cDC_CLEC9A	SCAMP2	7,30612E-20	-3,53287E-01	0,462	0,504	1,34557E-15
cDC_CLEC9A	TGIF1	1,13789E-17	-3,56492E-01	0,214	0,326	2,09565E-13
cDC_CLEC9A	SLC43A2	1,62395E-15	-3,57889E-01	0,291	0,37	2,99083E-11
cDC_CLEC9A	IFI27L2	3,51820E-15	-3,60295E-01	0,344	0,41	6,47947E-11
cDC_CLEC9A	TYMP	7,05262E-20	-3,61206E-01	0,8	0,793	1,29888E-15
cDC_CLEC9A	REL	8,49184E-07	-3,63796E-01	0,61	0,598	1,56394E-02
cDC_CLEC9A	RBM47	1,74491E-11	-3,64771E-01	0,302	0,36	3,21360E-07
cDC_CLEC9A	TSPAN13	1,75717E-189	-3,65945E-01	0,348	0,071	3,23618E-185
cDC_CLEC9A	TCIRG1	9,73116E-28	-3,71039E-01	0,371	0,469	1,79219E-23
cDC_CLEC9A	GK	2,13449E-12	-3,73797E-01	0,286	0,366	3,93110E-08
cDC_CLEC9A	MEF2C	1,00001E-08	-3,77906E-01	0,379	0,418	1,84171E-04
cDC_CLEC9A	BIN2	1,54404E-15	-3,86109E-01	0,224	0,315	2,84365E-11
cDC_CLEC9A	KCTD12	1,39910E-15	-3,87105E-01	0,452	0,492	2,57672E-11
cDC_CLEC9A	HVCN1	2,46535E-10	-3,87967E-01	0,182	0,255	4,54043E-06
cDC_CLEC9A	NFIC	9,28516E-08	-3,94189E-01	0,244	0,296	1,71005E-03
cDC_CLEC9A	LIMS1	5,90031E-28	-3,95215E-01	0,496	0,556	1,08666E-23
cDC_CLEC9A	SRGN	6,62068E-111	-4,00472E-01	0,874	0,944	1,21933E-106
cDC_CLEC9A	SH3BP2	3,23045E-07	-4,00868E-01	0,246	0,295	5,94952E-03
cDC_CLEC9A	GBP4	1,09587E-47	-4,03478E-01	0,067	0,281	2,01827E-43
cDC_CLEC9A	CREBL2	1,28260E-17	-4,06250E-01	0,191	0,293	2,36217E-13
cDC_CLEC9A	RASSF4	1,23087E-47	-4,06766E-01	0,343	0,51	2,26689E-43
cDC_CLEC9A	NCOA4	1,96396E-44	-4,09315E-01	0,394	0,533	3,61702E-40
cDC_CLEC9A	PARVB	2,01360E-12	-4,11204E-01	0,219	0,297	3,70845E-08
cDC_CLEC9A	RAB13	1,86604E-29	-4,27220E-01	0,154	0,308	3,43669E-25
cDC_CLEC9A	HTATIP2	7,85904E-20	-4,31011E-01	0,177	0,293	1,44740E-15
cDC_CLEC9A	LEPROT	3,48907E-17	-4,31045E-01	0,268	0,357	6,42581E-13

cDC_CLEC9A	CD151	1,12824E-07	-4,31918E-01	0,302	0,338	2,07788E-03
cDC_CLEC9A	TNFSF13B	7,47132E-64	-4,33931E-01	0,419	0,582	1,37599E-59
cDC_CLEC9A	LACTB	1,85011E-13	-4,36875E-01	0,249	0,325	3,40735E-09
cDC_CLEC9A	CD84	1,65461E-22	-4,39375E-01	0,29	0,394	3,04730E-18
cDC_CLEC9A	ELF1	1,09244E-07	-4,44440E-01	0,484	0,488	2,01195E-03
cDC_CLEC9A	N4BP2L2	1,48050E-13	-4,48004E-01	0,41	0,46	2,72663E-09
cDC_CLEC9A	PRNP	7,75927E-35	-4,51594E-01	0,244	0,404	1,42903E-30
cDC_CLEC9A	TM6SF1	6,31168E-18	-4,53339E-01	0,168	0,274	1,16242E-13
cDC_CLEC9A	CD81	7,11870E-26	-4,56747E-01	0,364	0,463	1,31105E-21
cDC_CLEC9A	AOAH	1,32237E-12	-4,59444E-01	0,302	0,376	2,43542E-08
cDC_CLEC9A	S100A11	1,14517E-165	-4,65981E-01	0,923	0,938	2,10906E-161
cDC_CLEC9A	ACP5	4,00428E-14	-4,66219E-01	0,354	0,401	7,37469E-10
cDC_CLEC9A	LY96	3,97429E-21	-4,70890E-01	0,382	0,459	7,31946E-17
cDC_CLEC9A	OGFRL1	2,73158E-13	-4,75417E-01	0,23	0,311	5,03075E-09
cDC_CLEC9A	PRKCD	7,26641E-18	-4,88785E-01	0,15	0,259	1,33825E-13
cDC_CLEC9A	LST1	1,28322E-127	-4,91268E-01	0,496	0,719	2,36330E-123
cDC_CLEC9A	PSAP	5,07045E-124	-4,92083E-01	0,887	0,917	9,33825E-120
cDC_CLEC9A	GNAQ	2,07507E-14	-4,98935E-01	0,259	0,342	3,82165E-10
cDC_CLEC9A	PTGS1	1,32308E-31	-5,07095E-01	0,101	0,26	2,43671E-27
cDC_CLEC9A	TNFSF10	1,79927E-13	-5,11350E-01	0,293	0,362	3,31371E-09
cDC_CLEC9A	PIK3AP1	9,79139E-23	-5,16052E-01	0,181	0,307	1,80328E-18
cDC_CLEC9A	FOSL2	2,10846E-12	-5,20844E-01	0,216	0,297	3,88314E-08
cDC_CLEC9A	USF2	7,08643E-21	-5,21557E-01	0,239	0,348	1,30511E-16
cDC_CLEC9A	TGFBI	7,28201E-14	-5,22509E-01	0,627	0,598	1,34113E-09
cDC_CLEC9A	ABHD12	1,78925E-28	-5,23576E-01	0,257	0,382	3,29526E-24
cDC_CLEC9A	LYN	1,79678E-11	-5,24132E-01	0,421	0,453	3,30913E-07
cDC_CLEC9A	CD302	1,32391E-32	-5,24836E-01	0,194	0,352	2,43825E-28
cDC_CLEC9A	RASGEF1B	1,40205E-21	-5,25235E-01	0,137	0,271	2,58216E-17
cDC_CLEC9A	HEXB	2,33603E-09	-5,26678E-01	0,517	0,514	4,30228E-05
cDC_CLEC9A	SWAP70	1,37001E-39	-5,27389E-01	0,151	0,338	2,52316E-35
cDC_CLEC9A	CITED2	1,56136E-09	-5,35071E-01	0,276	0,336	2,87556E-05
cDC_CLEC9A	CACUL1	8,28839E-18	-5,37341E-01	0,199	0,305	1,52647E-13
cDC_CLEC9A	SSH2	2,76411E-19	-5,40535E-01	0,206	0,313	5,09066E-15
cDC_CLEC9A	TMBIM1	2,56257E-08	-5,41689E-01	0,328	0,362	4,71949E-04
cDC_CLEC9A	FEZ2	3,11125E-12	-5,43349E-01	0,291	0,351	5,72999E-08
cDC_CLEC9A	BAZ2B	2,29002E-13	-5,57451E-01	0,173	0,262	4,21753E-09
cDC_CLEC9A	RAC2	4,13888E-20	-5,59381E-01	0,396	0,498	7,62258E-16
cDC_CLEC9A	UBE2D1	6,06704E-09	-5,60824E-01	0,328	0,363	1,11737E-04
cDC_CLEC9A	ITPR2	2,18528E-30	-5,61047E-01	0,117	0,281	4,02463E-26
cDC_CLEC9A	CYBA	1,22381E-56	-5,61589E-01	0,881	0,898	2,25389E-52
cDC_CLEC9A	GPX4	2,75743E-133	-5,74176E-01	0,754	0,846	5,07835E-129

cDC_CLEC9A	CRTAP	2,08834E-26	-5,80022E-01	0,448	0,522	3,84610E-22
cDC_CLEC9A	HEBP1	2,56211E-08	-5,82296E-01	0,263	0,31	4,71863E-04
cDC_CLEC9A	SKIL	7,45573E-15	-5,89388E-01	0,233	0,333	1,37312E-10
cDC_CLEC9A	BCL11A	1,07522E-71	-5,98813E-01	0,257	0,08	1,98023E-67
cDC_CLEC9A	SH3KBP1	2,03193E-28	-5,99925E-01	0,281	0,417	3,74220E-24
cDC_CLEC9A	GPX1	8,79080E-29	-6,00200E-01	0,478	0,55	1,61900E-24
cDC_CLEC9A	LAMP2	1,02602E-34	-6,06892E-01	0,244	0,404	1,88963E-30
cDC_CLEC9A	RCSD1	9,56920E-29	-6,14210E-01	0,233	0,379	1,76236E-24
cDC_CLEC9A	RB1	1,14477E-25	-6,15325E-01	0,286	0,404	2,10833E-21
cDC_CLEC9A	AKR1B1	8,74263E-37	-6,16645E-01	0,348	0,47	1,61013E-32
cDC_CLEC9A	BTK	2,71913E-07	-6,18196E-01	0,271	0,311	5,00782E-03
cDC_CLEC9A	ZFAND5	3,39042E-25	-6,20612E-01	0,544	0,586	6,24413E-21
cDC_CLEC9A	PDXK	3,59130E-17	-6,22968E-01	0,207	0,303	6,61410E-13
cDC_CLEC9A	EMP3	2,44115E-20	-6,29587E-01	0,702	0,729	4,49587E-16
cDC_CLEC9A	ATF3	4,24199E-10	-6,35662E-01	0,378	0,434	7,81248E-06
cDC_CLEC9A	NUP214	1,52635E-27	-6,35919E-01	0,176	0,317	2,81109E-23
cDC_CLEC9A	SLC2A3	1,87753E-08	-6,36409E-01	0,362	0,435	3,45784E-04
cDC_CLEC9A	FOSB	1,46594E-06	-6,38520E-01	0,603	0,579	2,69981E-02
cDC_CLEC9A	NCF1	6,76255E-14	-6,44929E-01	0,464	0,501	1,24546E-09
cDC_CLEC9A	PSTPIP2	2,19952E-58	-6,48292E-01	0,018	0,255	4,05086E-54
cDC_CLEC9A	SLC3A2	1,38012E-32	-6,60177E-01	0,329	0,466	2,54177E-28
cDC_CLEC9A	BMP2K	5,45044E-31	-6,61468E-01	0,137	0,295	1,00381E-26
cDC_CLEC9A	IL18	1,11910E-52	-6,62311E-01	0,244	0,446	2,06104E-48
cDC_CLEC9A	YWHAH	4,47790E-17	-6,64806E-01	0,521	0,532	8,24694E-13
cDC_CLEC9A	ACP2	2,20264E-20	-6,68748E-01	0,14	0,26	4,05661E-16
cDC_CLEC9A	FXYD5	1,60505E-101	-6,75263E-01	0,758	0,826	2,95601E-97
cDC_CLEC9A	LIPA	3,49761E-67	-6,79164E-01	0,287	0,505	6,44154E-63
cDC_CLEC9A	BRI3	3,96800E-22	-6,79925E-01	0,317	0,404	7,30786E-18
cDC_CLEC9A	TACC1	2,21369E-15	-6,80863E-01	0,358	0,422	4,07695E-11
cDC_CLEC9A	IL17RA	4,34141E-23	-6,81857E-01	0,179	0,304	7,99557E-19
cDC_CLEC9A	CREG1	1,46192E-42	-6,88570E-01	0,391	0,515	2,69243E-38
cDC_CLEC9A	FOXO3	9,63547E-50	-6,89376E-01	0,038	0,252	1,77456E-45
cDC_CLEC9A	PNRC1	5,55656E-30	-6,92965E-01	0,752	0,779	1,02335E-25
cDC_CLEC9A	IQGAP2	3,46492E-11	-6,94200E-01	0,241	0,305	6,38134E-07
cDC_CLEC9A	CMTM7	1,63325E-21	-6,95360E-01	0,317	0,413	3,00796E-17
cDC_CLEC9A	FRMD4B	8,52209E-65	-6,95850E-01	0,029	0,29	1,56951E-60
cDC_CLEC9A	UTRN	2,44944E-32	-6,96189E-01	0,122	0,292	4,51113E-28
cDC_CLEC9A	EMILIN2	8,75828E-20	-6,99635E-01	0,196	0,303	1,61301E-15
cDC_CLEC9A	CHST11	4,30336E-42	-6,99707E-01	0,089	0,281	7,92549E-38
cDC_CLEC9A	JUN	3,89987E-27	-7,01380E-01	0,597	0,712	7,18238E-23
cDC_CLEC9A	GABARAPL1	8,84266E-36	-7,03905E-01	0,112	0,295	1,62855E-31

cDC_CLEC9A	CD4	6,68238E-45	-7,04473E-01	0,401	0,541	1,23069E-40
cDC_CLEC9A	APOL6	1,15458E-53	-7,05133E-01	0,038	0,265	2,12639E-49
cDC_CLEC9A	CTSH	6,03539E-106	-7,07485E-01	0,577	0,713	1,11154E-101
cDC_CLEC9A	HIF1A	9,00659E-33	-7,08051E-01	0,457	0,551	1,65874E-28
cDC_CLEC9A	TSPAN14	4,57009E-43	-7,08303E-01	0,137	0,337	8,41674E-39
cDC_CLEC9A	LYSMD2	1,39740E-56	-7,12792E-01	0,059	0,294	2,57360E-52
cDC_CLEC9A	CTSS	8,85398E-133	-7,26490E-01	0,731	0,818	1,63064E-128
cDC_CLEC9A	GPCPD1	4,91951E-15	-7,27727E-01	0,19	0,282	9,06026E-11
cDC_CLEC9A	SLA	3,72750E-38	-7,28700E-01	0,251	0,42	6,86494E-34
cDC_CLEC9A	ABCA1	4,66990E-40	-7,32898E-01	0,119	0,319	8,60056E-36
cDC_CLEC9A	MS4A6A	9,04029E-137	-7,43606E-01	0,432	0,72	1,66495E-132
cDC_CLEC9A	PIK3R1	2,04472E-30	-7,49767E-01	0,173	0,324	3,76575E-26
cDC_CLEC9A	FBXL5	5,60714E-08	-7,49941E-01	0,222	0,275	1,03267E-03
cDC_CLEC9A	TIMP2	2,18389E-41	-7,56851E-01	0,18	0,364	4,02207E-37
cDC_CLEC9A	LY6E	3,07217E-21	-7,58231E-01	0,493	0,548	5,65802E-17
cDC_CLEC9A	LTA4H	1,71157E-24	-7,58570E-01	0,356	0,449	3,15219E-20
cDC_CLEC9A	CD63	3,56480E-139	-7,63092E-01	0,718	0,82	6,56529E-135
cDC_CLEC9A	ACSL1	3,31679E-44	-7,66806E-01	0,197	0,392	6,10853E-40
cDC_CLEC9A	PEA15	1,64891E-34	-7,72460E-01	0,408	0,519	3,03679E-30
cDC_CLEC9A	LAPTM4A	5,50218E-55	-7,78570E-01	0,517	0,63	1,01334E-50
cDC_CLEC9A	LCP2	6,45627E-55	-7,81140E-01	0,408	0,58	1,18905E-50
cDC_CLEC9A	TMEM51	1,82084E-65	-7,84742E-01	0,01	0,266	3,35344E-61
cDC_CLEC9A	CAMK1	2,32347E-51	-7,86623E-01	0,041	0,26	4,27913E-47
cDC_CLEC9A	CMTM3	3,00571E-35	-7,87411E-01	0,191	0,365	5,53562E-31
cDC_CLEC9A	GIMAP1	1,32769E-60	-7,89391E-01	0,026	0,271	2,44520E-56
cDC_CLEC9A	PARP14	1,53046E-26	-7,92770E-01	0,242	0,38	2,81865E-22
cDC_CLEC9A	PLBD1	3,55745E-13	-7,98199E-01	0,336	0,394	6,55175E-09
cDC_CLEC9A	TGFBR2	1,67730E-37	-8,01138E-01	0,159	0,339	3,08907E-33
cDC_CLEC9A	RBMS1	2,12917E-09	-8,07827E-01	0,267	0,311	3,92129E-05
cDC_CLEC9A	RENBP	4,63885E-61	-8,09596E-01	0,052	0,302	8,54338E-57
cDC_CLEC9A	HSD17B4	5,54883E-09	-8,11030E-01	0,201	0,26	1,02193E-04
cDC_CLEC9A	BNIP3L	7,44780E-67	-8,12730E-01	0,368	0,567	1,37166E-62
cDC_CLEC9A	SLC15A3	3,13482E-66	-8,18246E-01	0,024	0,288	5,77339E-62
cDC_CLEC9A	CPM	7,65966E-74	-8,24596E-01	0,089	0,375	1,41068E-69
cDC_CLEC9A	IFI44L	3,13866E-41	-8,27739E-01	0,063	0,257	5,78048E-37
cDC_CLEC9A	ANXA1	1,82241E-62	-8,28675E-01	0,464	0,692	3,35634E-58
cDC_CLEC9A	SLC16A3	3,20962E-45	-8,29151E-01	0,372	0,515	5,91116E-41
cDC_CLEC9A	PAG1	2,77955E-36	-8,29347E-01	0,099	0,274	5,11909E-32
cDC_CLEC9A	ADAM10	3,53142E-16	-8,35781E-01	0,189	0,289	6,50381E-12
cDC_CLEC9A	PTK2B	1,12125E-38	-8,40206E-01	0,072	0,257	2,06500E-34
cDC_CLEC9A	BLVRB	1,53514E-14	-8,41563E-01	0,468	0,491	2,82726E-10

cDC_CLEC9A	CCDC50	5,63496E-33	-8,45342E-01	0,111	0,277	1,03779E-28
cDC_CLEC9A	EPB41L3	1,70310E-33	-8,48207E-01	0,141	0,307	3,13660E-29
cDC_CLEC9A	CD44	1,76574E-79	-8,59678E-01	0,514	0,68	3,25197E-75
cDC_CLEC9A	ERCC1	1,79577E-07	-8,61062E-01	0,357	0,382	3,30726E-03
cDC_CLEC9A	GNS	2,92649E-60	-8,62447E-01	0,191	0,435	5,38972E-56
cDC_CLEC9A	IFITM3	3,10828E-201	-8,66407E-01	0,282	0,774	5,72451E-197
cDC_CLEC9A	MCOLN1	8,55040E-26	-8,78295E-01	0,14	0,277	1,57473E-21
cDC_CLEC9A	CTNND1	1,29810E-28	-8,91084E-01	0,119	0,275	2,39072E-24
cDC_CLEC9A	SMAP2	6,72769E-60	-8,98940E-01	0,233	0,475	1,23904E-55
cDC_CLEC9A	LAPTM5	6,97889E-173	-9,01717E-01	0,822	0,9	1,28530E-168
cDC_CLEC9A	ETS2	2,26348E-55	-9,07299E-01	0,2	0,43	4,16866E-51
cDC_CLEC9A	GLRX	8,36661E-48	-9,09846E-01	0,407	0,55	1,54088E-43
cDC_CLEC9A	ADORA3	2,37400E-36	-9,16752E-01	0,089	0,266	4,37219E-32
cDC_CLEC9A	SOCS3	3,07567E-07	-9,17732E-01	0,326	0,358	5,66446E-03
cDC_CLEC9A	SLC1A3	1,41135E-72	-9,24133E-01	0,086	0,368	2,59928E-68
cDC_CLEC9A	STXBP2	6,46049E-22	-9,24426E-01	0,332	0,421	1,18983E-17
cDC_CLEC9A	TBXAS1	1,46323E-59	-9,30718E-01	0,312	0,522	2,69483E-55
cDC_CLEC9A	SCPEP1	5,42480E-89	-9,37915E-01	0,089	0,414	9,99085E-85
cDC_CLEC9A	AZI2	6,02540E-43	-9,42776E-01	0,107	0,306	1,10970E-38
cDC_CLEC9A	RNF13	9,47703E-69	-9,45337E-01	0,3	0,522	1,74539E-64
cDC_CLEC9A	KCNMA1	5,82160E-66	-9,51377E-01	0,009	0,265	1,07216E-61
cDC_CLEC9A	CD99	2,69374E-50	-9,51829E-01	0,51	0,622	4,96107E-46
cDC_CLEC9A	GPR183	3,45957E-09	-9,56259E-01	0,407	0,458	6,37148E-05
cDC_CLEC9A	PEPD	1,34135E-30	-9,57299E-01	0,227	0,366	2,47037E-26
cDC_CLEC9A	TNFRSF1A	8,87455E-23	-9,64950E-01	0,366	0,45	1,63443E-18
cDC_CLEC9A	DNASE2	2,42224E-71	-9,71089E-01	0,092	0,372	4,46104E-67
cDC_CLEC9A	PHACTR1	7,50433E-10	-9,81041E-01	0,241	0,303	1,38207E-05
cDC_CLEC9A	IRAK3	6,59345E-64	-9,82190E-01	0,008	0,259	1,21432E-59
cDC_CLEC9A	LPXN	2,43298E-53	-9,85618E-01	0,118	0,352	4,48083E-49
cDC_CLEC9A	CLEC4A	3,07275E-53	-9,88999E-01	0,072	0,302	5,65909E-49
cDC_CLEC9A	TSPAN4	7,40108E-46	-9,90062E-01	0,092	0,297	1,36306E-41
cDC_CLEC9A	TMEM205	4,11284E-16	-9,93829E-01	0,192	0,286	7,57461E-12
cDC_CLEC9A	LRRC25	1,38298E-58	-9,97349E-01	0,092	0,338	2,54704E-54
cDC_CLEC9A	LHFPL2	1,47241E-67	-9,99383E-01	0,026	0,288	2,71175E-63
cDC_CLEC9A	IL13RA1	4,76281E-75	-1,00050E+00	0,104	0,394	8,77166E-71
cDC_CLEC9A	NAMPT	8,63723E-30	-1,00343E+00	0,5	0,575	1,59072E-25
cDC_CLEC9A	RHBDF2	1,68373E-62	-1,01099E+00	0,072	0,329	3,10093E-58
cDC_CLEC9A	DOCK4	3,21969E-70	-1,01297E+00	0,018	0,291	5,92971E-66
cDC_CLEC9A	CEBPD	8,24337E-43	-1,01683E+00	0,426	0,571	1,51818E-38
cDC_CLEC9A	SERPING1	4,10937E-52	-1,02615E+00	0,19	0,401	7,56822E-48
cDC_CLEC9A	GRINA	2,76254E-75	-1,04489E+00	0,539	0,686	5,08777E-71

cDC_CLEC9A	MGAT4A	2,09872E-51	-1,04546E+00	0,141	0,361	3,86521E-47
cDC_CLEC9A	APLP2	1,36036E-94	-1,05122E+00	0,349	0,6	2,50537E-90
cDC_CLEC9A	KYNU	3,15803E-37	-1,05696E+00	0,137	0,319	5,81615E-33
cDC_CLEC9A	ATP6V0C	5,79628E-75	-1,06656E+00	0,621	0,7	1,06750E-70
cDC_CLEC9A	ENG	1,33019E-51	-1,06848E+00	0,119	0,352	2,44981E-47
cDC_CLEC9A	GIMAP7	5,01770E-55	-1,07174E+00	0,029	0,26	9,24109E-51
cDC_CLEC9A	NAIP	3,70409E-57	-1,07228E+00	0,141	0,375	6,82182E-53
cDC_CLEC9A	SLC25A37	3,59309E-19	-1,07577E+00	0,181	0,293	6,61739E-15
cDC_CLEC9A	DPYD	1,58103E-70	-1,08481E+00	0,09	0,371	2,91179E-66
cDC_CLEC9A	TFEC	2,98091E-78	-1,08511E+00	0,008	0,301	5,48994E-74
cDC_CLEC9A	LGALS3BP	9,22461E-68	-1,08550E+00	0,054	0,325	1,69890E-63
cDC_CLEC9A	ELL2	5,78189E-50	-1,08744E+00	0,066	0,288	1,06485E-45
cDC_CLEC9A	FNDC3B	1,03781E-55	-1,09917E+00	0,051	0,283	1,91134E-51
cDC_CLEC9A	LPAR6	1,72049E-50	-1,10247E+00	0,1	0,326	3,16863E-46
cDC_CLEC9A	ZNF331	4,68357E-14	-1,12288E+00	0,24	0,346	8,62573E-10
cDC_CLEC9A	SIRPA	3,85998E-99	-1,12365E+00	0,009	0,357	7,10892E-95
cDC_CLEC9A	S100A6	4,45743E-93	-1,12526E+00	0,779	0,876	8,20924E-89
cDC_CLEC9A	TCF4	2,86331E-18	-1,12858E+00	0,164	0,275	5,27337E-14
cDC_CLEC9A	TOM1	6,96997E-40	-1,13893E+00	0,101	0,287	1,28366E-35
cDC_CLEC9A	GBP1	1,14212E-51	-1,14171E+00	0,123	0,35	2,10345E-47
cDC_CLEC9A	KLF2	5,53419E-43	-1,16986E+00	0,084	0,281	1,01923E-38
cDC_CLEC9A	FBP1	6,23694E-19	-1,17131E+00	0,259	0,351	1,14866E-14
cDC_CLEC9A	FTH1	2,08063E-234	-1,17757E+00	0,982	0,998	3,83189E-230
cDC_CLEC9A	LAMP1	1,49407E-110	-1,19355E+00	0,029	0,404	2,75162E-106
cDC_CLEC9A	MPP1	4,50349E-82	-1,19438E+00	0,113	0,418	8,29408E-78
cDC_CLEC9A	CAPG	1,21998E-189	-1,19867E+00	0,406	0,747	2,24684E-185
cDC_CLEC9A	IFI6	2,32746E-96	-1,20824E+00	0,161	0,495	4,28648E-92
cDC_CLEC9A	CTSA	7,10136E-77	-1,22064E+00	0,266	0,51	1,30786E-72
cDC_CLEC9A	ATP6V1B2	4,35632E-117	-1,22604E+00	0,187	0,541	8,02304E-113
cDC_CLEC9A	ARHGAP18	1,65203E-82	-1,23578E+00	0,204	0,481	3,04255E-78
cDC_CLEC9A	GAPT	2,11577E-62	-1,24237E+00	0,012	0,259	3,89660E-58
cDC_CLEC9A	GPR34	1,68326E-75	-1,28248E+00	0,024	0,311	3,10005E-71
cDC_CLEC9A	TNFRSF1B	4,73401E-112	-1,28409E+00	0,191	0,547	8,71862E-108
cDC_CLEC9A	PLAUR	2,68365E-65	-1,28775E+00	0,428	0,645	4,94248E-61
cDC_CLEC9A	ARRB2	2,11247E-115	-1,28869E+00	0,434	0,671	3,89053E-111
cDC_CLEC9A	CARD16	3,53114E-135	-1,29081E+00	0,164	0,559	6,50330E-131
cDC_CLEC9A	SORL1	4,06368E-94	-1,30788E+00	0,031	0,37	7,48407E-90
cDC_CLEC9A	BTG1	3,94981E-112	-1,31162E+00	0,638	0,813	7,27437E-108
cDC_CLEC9A	LTC4S	1,01937E-41	-1,32966E+00	0,097	0,287	1,87738E-37
cDC_CLEC9A	LGALS3	6,63720E-106	-1,34124E+00	0,529	0,728	1,22237E-101
cDC_CLEC9A	NPC2	2,98664E-213	-1,34867E+00	0,81	0,885	5,50050E-209

cDC_CLEC9A	UPP1	4,93068E-52	-1,35739E+00	0,221	0,435	9,08083E-48
cDC_CLEC9A	SCARB2	1,71070E-96	-1,36052E+00	0,032	0,378	3,15059E-92
cDC_CLEC9A	TAGAP	1,78318E-33	-1,36326E+00	0,107	0,275	3,28408E-29
cDC_CLEC9A	TXNIP	5,37020E-90	-1,36530E+00	0,521	0,702	9,89029E-86
cDC_CLEC9A	NPL	2,26764E-65	-1,37454E+00	0,009	0,263	4,17631E-61
cDC_CLEC9A	CASP4	1,46164E-79	-1,37891E+00	0,157	0,449	2,69190E-75
cDC_CLEC9A	RGS2	3,10094E-40	-1,38003E+00	0,492	0,628	5,71101E-36
cDC_CLEC9A	FYB1	2,32554E-55	-1,38636E+00	0,096	0,322	4,28294E-51
cDC_CLEC9A	TSPO	3,61053E-122	-1,38824E+00	0,632	0,768	6,64952E-118
cDC_CLEC9A	XAF1	3,25816E-54	-1,39238E+00	0,093	0,332	6,00055E-50
cDC_CLEC9A	TLR2	3,52475E-95	-1,39333E+00	0,051	0,393	6,49154E-91
cDC_CLEC9A	NLRP3	1,92838E-15	-1,39343E+00	0,164	0,265	3,55150E-11
cDC_CLEC9A	PTAFR	9,42890E-107	-1,39867E+00	0,013	0,383	1,73652E-102
cDC_CLEC9A	DMXL2	4,99698E-69	-1,40427E+00	0,008	0,272	9,20293E-65
cDC_CLEC9A	HNMT	3,20048E-98	-1,41571E+00	0,039	0,387	5,89433E-94
cDC_CLEC9A	TIMP1	7,16238E-18	-1,41764E+00	0,598	0,651	1,31910E-13
cDC_CLEC9A	PLXDC2	5,31987E-126	-1,42689E+00	0,163	0,551	9,79760E-122
cDC_CLEC9A	LILRB1	2,70752E-104	-1,42698E+00	0,017	0,384	4,98643E-100
cDC_CLEC9A	GBP5	1,79544E-47	-1,44789E+00	0,044	0,25	3,30667E-43
cDC_CLEC9A	GBP2	1,00738E-75	-1,48633E+00	0,089	0,378	1,85529E-71
cDC_CLEC9A	CEBPB	1,83257E-72	-1,49765E+00	0,24	0,476	3,37504E-68
cDC_CLEC9A	FPR3	3,12019E-92	-1,50331E+00	0,014	0,345	5,74645E-88
cDC_CLEC9A	PMP22	5,07250E-70	-1,50627E+00	0,012	0,282	9,34202E-66
cDC_CLEC9A	MS4A4A	5,02315E-113	-1,51039E+00	0,047	0,427	9,25113E-109
cDC_CLEC9A	PLD4	1,04072E-66	-1,51179E+00	0,067	0,337	1,91670E-62
cDC_CLEC9A	CLEC4E	1,59993E-78	-1,51655E+00	0,006	0,298	2,94660E-74
cDC_CLEC9A	ITM2B	1,29245E-265	-1,52475E+00	0,742	0,909	2,38031E-261
cDC_CLEC9A	LAIR1	9,71714E-170	-1,52530E+00	0,024	0,529	1,78961E-165
cDC_CLEC9A	ALOX5	8,52644E-137	-1,53566E+00	0,049	0,482	1,57031E-132
cDC_CLEC9A	MARCKS	1,74711E-116	-1,55058E+00	0,177	0,539	3,21765E-112
cDC_CLEC9A	NCF2	4,08958E-112	-1,55813E+00	0,101	0,472	7,53178E-108
cDC_CLEC9A	ZFP36L2	1,64344E-109	-1,56384E+00	0,449	0,699	3,02672E-105
cDC_CLEC9A	HCST	2,68213E-122	-1,61074E+00	0,356	0,659	4,93967E-118
cDC_CLEC9A	CSF3R	1,75611E-51	-1,61250E+00	0,174	0,398	3,23423E-47
cDC_CLEC9A	LRP1	6,75301E-130	-1,61572E+00	0,008	0,43	1,24370E-125
cDC_CLEC9A	A2M	1,53750E-75	-1,62596E+00	0,104	0,395	2,83161E-71
cDC_CLEC9A	CSTA	7,06142E-112	-1,62663E+00	0,062	0,437	1,30050E-107
cDC_CLEC9A	SOD2	7,56878E-59	-1,63483E+00	0,549	0,653	1,39394E-54
cDC_CLEC9A	RNF213	6,31686E-123	-1,65123E+00	0,117	0,512	1,16338E-118
cDC_CLEC9A	SLC7A7	1,18561E-131	-1,65153E+00	0,053	0,474	2,18355E-127
cDC_CLEC9A	ASAH1	2,56192E-161	-1,66058E+00	0,371	0,695	4,71829E-157

cDC_CLEC9A	SLCO2B1	5,40828E-106	-1,72850E+00	0,011	0,376	9,96043E-102
cDC_CLEC9A	IGSF6	2,23939E-175	-1,73039E+00	0,056	0,567	4,12428E-171
cDC_CLEC9A	OAS1	7,24941E-49	-1,73473E+00	0,118	0,331	1,33512E-44
cDC_CLEC9A	AGTRAP	1,20393E-85	-1,78788E+00	0,05	0,367	2,21727E-81
cDC_CLEC9A	HBEGF	9,23965E-52	-1,78872E+00	0,056	0,28	1,70167E-47
cDC_CLEC9A	OLR1	1,18248E-94	-1,79081E+00	0,016	0,354	2,17778E-90
cDC_CLEC9A	DOK2	1,92938E-82	-1,80360E+00	0,038	0,351	3,55334E-78
cDC_CLEC9A	DUSP6	1,05653E-59	-1,80814E+00	0,046	0,293	1,94580E-55
cDC_CLEC9A	GIMAP4	2,93165E-112	-1,81058E+00	0,049	0,435	5,39921E-108
cDC_CLEC9A	DAB2	1,35076E-120	-1,81853E+00	0,042	0,441	2,48769E-116
cDC_CLEC9A	RHOB	7,24896E-93	-1,82032E+00	0,238	0,548	1,33504E-88
cDC_CLEC9A	CTSC	2,74394E-124	-1,88159E+00	0,443	0,682	5,05352E-120
cDC_CLEC9A	LILRB4	1,82291E-172	-1,89341E+00	0,064	0,559	3,35725E-168
cDC_CLEC9A	FPR1	1,08844E-118	-1,89421E+00	0,06	0,455	2,00457E-114
cDC_CLEC9A	MS4A7	3,40042E-187	-1,94046E+00	0,231	0,671	6,26255E-183
cDC_CLEC9A	FCER1G	0,00000E+00	-1,94564E+00	0,484	0,921	0,00000E+00
cDC_CLEC9A	RNF130	2,56037E-275	-1,95790E+00	0,08	0,721	4,71544E-271
cDC_CLEC9A	PDK4	3,43652E-66	-1,95881E+00	0,049	0,316	6,32903E-62
cDC_CLEC9A	CD300A	1,27989E-119	-1,96436E+00	0,076	0,468	2,35717E-115
cDC_CLEC9A	MSR1	3,33316E-108	-1,96488E+00	0,017	0,387	6,13868E-104
cDC_CLEC9A	LILRB2	1,53990E-140	-1,97116E+00	0,021	0,465	2,83604E-136
cDC_CLEC9A	SAMSN1	2,74795E-128	-1,99274E+00	0,053	0,472	5,06090E-124
cDC_CLEC9A	FTL	0,00000E+00	-2,01828E+00	0,984	0,992	0,00000E+00
cDC_CLEC9A	C3AR1	6,77199E-124	-2,04202E+00	0,014	0,423	1,24720E-119
cDC_CLEC9A	TNFSF13	1,05262E-108	-2,05839E+00	0,086	0,45	1,93861E-104
cDC_CLEC9A	CASP1	2,90604E-129	-2,06812E+00	0,05	0,471	5,35205E-125
cDC_CLEC9A	STAB1	1,25413E-91	-2,08011E+00	0,02	0,351	2,30974E-87
cDC_CLEC9A	PILRA	1,23892E-156	-2,08058E+00	0,028	0,502	2,28171E-152
cDC_CLEC9A	IFI30	8,67772E-17	-2,10467E+00	0,457	0,571	1,59818E-12
cDC_CLEC9A	PLTP	6,12744E-82	-2,18598E+00	0,03	0,334	1,12849E-77
cDC_CLEC9A	NINJ1	1,51083E-125	-2,19383E+00	0,189	0,567	2,78249E-121
cDC_CLEC9A	TMEM176A	2,61128E-129	-2,20486E+00	0,037	0,456	4,80920E-125
cDC_CLEC9A	C5AR1	1,96698E-121	-2,27443E+00	0,013	0,415	3,62258E-117
cDC_CLEC9A	FCGR1A	2,21675E-119	-2,27576E+00	0,02	0,418	4,08259E-115
cDC_CLEC9A	CD68	1,45617E-225	-2,30671E+00	0,464	0,787	2,68182E-221
cDC_CLEC9A	FCGR2B	1,56464E-124	-2,31705E+00	0,019	0,433	2,88159E-120
cDC_CLEC9A	TREM2	1,50137E-117	-2,35602E+00	0,02	0,414	2,76507E-113
cDC_CLEC9A	VSIG4	1,06343E-83	-2,38182E+00	0,042	0,354	1,95851E-79
cDC_CLEC9A	ZEB2	6,59060E-218	-2,38611E+00	0,03	0,616	1,21379E-213
cDC_CLEC9A	IER3	6,24802E-124	-2,41509E+00	0,132	0,54	1,15070E-119
cDC_CLEC9A	C3	8,19549E-102	-2,42367E+00	0,033	0,391	1,50936E-97

cDC_CLEC9A	CFD	5,59096E-162	-2,47794E+00	0,11	0,571	1,02969E-157
cDC_CLEC9A	CD9	2,68822E-104	-2,50826E+00	0,154	0,491	4,95089E-100
cDC_CLEC9A	GPNMB	1,60435E-87	-2,50861E+00	0,02	0,341	2,95474E-83
cDC_CLEC9A	MAFB	1,36265E-205	-2,55071E+00	0,046	0,604	2,50960E-201
cDC_CLEC9A	PLIN2	1,00743E-85	-2,56228E+00	0,214	0,503	1,85538E-81
cDC_CLEC9A	CYBB	2,54239E-229	-2,56248E+00	0,031	0,635	4,68232E-225
cDC_CLEC9A	SERPINA1	5,40656E-209	-2,56709E+00	0,318	0,729	9,95726E-205
cDC_CLEC9A	TYROBP	0,00000E+00	-2,57992E+00	0,442	0,957	0,00000E+00
cDC_CLEC9A	CSF1R	2,35522E-204	-2,65070E+00	0,02	0,582	4,33761E-200
cDC_CLEC9A	TMEM176B	7,76750E-172	-2,78107E+00	0,041	0,544	1,43054E-167
cDC_CLEC9A	SLC11A1	5,91729E-147	-2,80530E+00	0,02	0,476	1,08979E-142
cDC_CLEC9A	S100A4	1,40044E-247	-2,83003E+00	0,422	0,834	2,57919E-243
cDC_CLEC9A	MT2A	1,31050E-139	-2,88862E+00	0,28	0,635	2,41355E-135
cDC_CLEC9A	CTSB	0,00000E+00	-2,97892E+00	0,497	0,879	0,00000E+00
cDC_CLEC9A	GLUL	0,00000E+00	-2,98684E+00	0,163	0,816	0,00000E+00
cDC_CLEC9A	CD163	5,73650E-139	-3,07018E+00	0,012	0,455	1,05649E-134
cDC_CLEC9A	FCGR2A	1,06953E-233	-3,11928E+00	0,037	0,642	1,96975E-229
cDC_CLEC9A	CTSL	9,26939E-97	-3,32624E+00	0,207	0,525	1,70714E-92
cDC_CLEC9A	APOC1	1,02527E-93	-3,46588E+00	0,228	0,513	1,88824E-89
cDC_CLEC9A	ALOX5AP	3,12915E-153	-3,50521E+00	0,049	0,514	5,76295E-149
cDC_CLEC9A	CTSD	7,67043E-260	-3,66895E+00	0,317	0,778	1,41266E-255
cDC_CLEC9A	CCL4	1,60015E-38	-3,74640E+00	0,13	0,313	2,94699E-34
cDC_CLEC9A	C1QB	1,41427E-134	-3,76357E+00	0,174	0,553	2,60466E-130
cDC_CLEC9A	CCL3	1,18804E-83	-3,76775E+00	0,069	0,382	2,18801E-79
cDC_CLEC9A	FCGR3A	1,55384E-203	-3,85023E+00	0,029	0,585	2,86170E-199
cDC_CLEC9A	FCN1	3,89617E-63	-3,93477E+00	0,009	0,255	7,17558E-59
cDC_CLEC9A	CD14	9,47927E-228	-4,01735E+00	0,038	0,635	1,74580E-223
cDC_CLEC9A	RNASE1	3,01221E-64	-4,18204E+00	0,024	0,277	5,54759E-60
cDC_CLEC9A	IL1B	1,98955E-61	-4,18786E+00	0,086	0,34	3,66415E-57
cDC_CLEC9A	C1QA	6,78340E-170	-4,35728E+00	0,113	0,575	1,24930E-165
cDC_CLEC9A	C1QC	7,49059E-161	-4,38434E+00	0,102	0,554	1,37954E-156
cDC_CLEC9A	S100A9	7,86672E-108	-4,56313E+00	0,114	0,463	1,44881E-103
cDC_CLEC9A	S100A8	1,14361E-91	-4,62951E+00	0,029	0,358	2,10619E-87
cDC_CLEC9A	APOE	3,24850E-134	-4,68125E+00	0,107	0,514	5,98277E-130

cDC(CD1C)

cluster	gene	p_val	avg_log2FC	pct.1	pct.2	p_val_adj
cDC(CD1C)	CD1C	0,00000E+00	3,56187E+00	0,58	0,036	0,00000E+00
cDC(CD1C)	FCER1A	0,00000E+00	3,47187E+00	0,671	0,078	0,00000E+00
cDC(CD1C)	CD1E	0,00000E+00	2,87761E+00	0,53	0,029	0,00000E+00
cDC(CD1C)	CLEC10A	0,00000E+00	2,78717E+00	0,714	0,168	0,00000E+00
cDC(CD1C)	IL1R2	0,00000E+00	1,99904E+00	0,323	0,073	0,00000E+00
cDC(CD1C)	NDRG2	0,00000E+00	1,79536E+00	0,545	0,1	0,00000E+00
cDC(CD1C)	HLA-DQB2	0,00000E+00	1,70638E+00	0,602	0,285	0,00000E+00
cDC(CD1C)	INSIG1	8,78174E-83	1,67854E+00	0,416	0,281	1,61733E-78
cDC(CD1C)	S100B	0,00000E+00	1,60751E+00	0,291	0,08	0,00000E+00
cDC(CD1C)	CST7	0,00000E+00	1,58062E+00	0,456	0,109	0,00000E+00
cDC(CD1C)	PPA1	0,00000E+00	1,55613E+00	0,804	0,422	0,00000E+00
cDC(CD1C)	PKIB	0,00000E+00	1,48949E+00	0,628	0,202	0,00000E+00
cDC(CD1C)	HLA-DQA1	0,00000E+00	1,40908E+00	0,986	0,76	0,00000E+00
cDC(CD1C)	HLA-DPB1	0,00000E+00	1,40054E+00	0,995	0,871	0,00000E+00
cDC(CD1C)	HLA-DQB1	0,00000E+00	1,33191E+00	0,987	0,797	0,00000E+00
cDC(CD1C)	CFP	7,32778E-226	1,32308E+00	0,395	0,186	1,34956E-221
cDC(CD1C)	HLA-DPA1	0,00000E+00	1,30364E+00	0,994	0,887	0,00000E+00
cDC(CD1C)	HLA-DRA	0,00000E+00	1,28582E+00	0,998	0,917	0,00000E+00
cDC(CD1C)	FCGR2B	0,00000E+00	1,27694E+00	0,74	0,389	0,00000E+00
cDC(CD1C)	HLA-DQA2	0,00000E+00	1,19721E+00	0,764	0,484	0,00000E+00
cDC(CD1C)	LGALS2	0,00000E+00	1,17660E+00	0,485	0,231	0,00000E+00
cDC(CD1C)	CYP2S1	0,00000E+00	1,17021E+00	0,354	0,1	0,00000E+00
cDC(CD1C)	MAT2A	3,11087E-48	1,15188E+00	0,563	0,434	5,72928E-44
cDC(CD1C)	CD1D	0,00000E+00	1,13887E+00	0,388	0,139	0,00000E+00
cDC(CD1C)	GSN	0,00000E+00	1,12767E+00	0,898	0,604	0,00000E+00
cDC(CD1C)	TUBA1A	3,05645E-193	1,12383E+00	0,651	0,441	5,62906E-189
cDC(CD1C)	CST3	0,00000E+00	1,08607E+00	0,99	0,945	0,00000E+00
cDC(CD1C)	COTL1	3,17912E-154	1,07713E+00	0,843	0,697	5,85498E-150
cDC(CD1C)	CNN2	0,00000E+00	1,04002E+00	0,696	0,369	0,00000E+00
cDC(CD1C)	HLA-DMB	3,98555E-228	1,02918E+00	0,861	0,614	7,34019E-224
cDC(CD1C)	LYZ	7,13277E-99	1,01106E+00	0,927	0,795	1,31364E-94
cDC(CD1C)	CLEC4A	1,56915E-276	9,77984E-01	0,52	0,272	2,88990E-272
cDC(CD1C)	CKLF	0,00000E+00	9,77585E-01	0,73	0,48	0,00000E+00
cDC(CD1C)	ACAA1	4,44119E-150	9,68152E-01	0,547	0,359	8,17934E-146
cDC(CD1C)	PRCP	5,75564E-297	9,64394E-01	0,624	0,363	1,06002E-292
cDC(CD1C)	AXL	1,11229E-267	9,61820E-01	0,487	0,257	2,04850E-263
cDC(CD1C)	HLA-DRB1	0,00000E+00	9,47737E-01	0,991	0,909	0,00000E+00
cDC(CD1C)	CIITA	1,60156E-188	9,37674E-01	0,508	0,304	2,94959E-184
cDC(CD1C)	HLA-DOA	1,05921E-242	9,27061E-01	0,625	0,367	1,95074E-238

cDC(CD1C)	MAD1L1	1,38552E-66	9,23434E-01	0,28	0,173	2,55172E-62
cDC(CD1C)	HLA-DRB5	0,00000E+00	9,08399E-01	0,964	0,834	0,00000E+00
cDC(CD1C)	CD74	0,00000E+00	9,06823E-01	0,999	0,959	0,00000E+00
cDC(CD1C)	HLA-DMA	0,00000E+00	9,01670E-01	0,957	0,762	0,00000E+00
cDC(CD1C)	EIF3L	0,00000E+00	8,96965E-01	0,854	0,571	0,00000E+00
cDC(CD1C)	RPSA	5,23826E-72	8,94802E-01	0,543	0,49	9,64730E-68
cDC(CD1C)	RPS18	3,73946E-59	8,89496E-01	0,564	0,559	6,88696E-55
cDC(CD1C)	ETHE1	3,21738E-192	8,88774E-01	0,532	0,32	5,92545E-188
cDC(CD1C)	RPL8	5,10857E-27	8,77071E-01	0,561	0,574	9,40846E-23
cDC(CD1C)	TUBA1B	1,15325E-266	8,72900E-01	0,923	0,737	2,12393E-262
cDC(CD1C)	YIF1B	4,39871E-164	8,68831E-01	0,525	0,331	8,10111E-160
cDC(CD1C)	RPL4	1,26277E-88	8,57248E-01	0,54	0,47	2,32564E-84
cDC(CD1C)	GSTP1	1,93917E-74	8,48507E-01	0,926	0,812	3,57138E-70
cDC(CD1C)	CSF2RA	1,96256E-307	8,45230E-01	0,661	0,39	3,61445E-303
cDC(CD1C)	SGK1	5,73360E-186	8,37142E-01	0,771	0,586	1,05596E-181
cDC(CD1C)	RPS11	2,38358E-31	8,36404E-01	0,539	0,5	4,38984E-27
cDC(CD1C)	GDI2	0,00000E+00	8,23716E-01	0,87	0,626	0,00000E+00
cDC(CD1C)	ACTB	3,02778E-244	8,19058E-01	0,998	0,99	5,57626E-240
cDC(CD1C)	CPVL	7,11726E-217	8,14872E-01	0,773	0,54	1,31079E-212
cDC(CD1C)	PAK1	0,00000E+00	8,11463E-01	0,684	0,409	0,00000E+00
cDC(CD1C)	IL13RA1	0,00000E+00	8,11367E-01	0,636	0,36	0,00000E+00
cDC(CD1C)	RPL18A	2,92288E-47	8,09191E-01	0,561	0,563	5,38307E-43
cDC(CD1C)	PON2	5,72433E-288	8,08006E-01	0,297	0,112	1,05425E-283
cDC(CD1C)	LSP1	0,00000E+00	8,06661E-01	0,912	0,644	0,00000E+00
cDC(CD1C)	RPS24	1,05291E-37	8,02749E-01	0,563	0,579	1,93915E-33
cDC(CD1C)	EIF3K	1,44743E-221	7,99286E-01	0,924	0,771	2,66573E-217
cDC(CD1C)	RPL10A	3,34612E-80	7,95206E-01	0,551	0,491	6,16256E-76
cDC(CD1C)	ELMO1	3,82941E-213	7,86496E-01	0,546	0,316	7,05263E-209
cDC(CD1C)	IMPDH2	8,54539E-217	7,83420E-01	0,461	0,252	1,57380E-212
cDC(CD1C)	RPS29	2,54676E-33	7,78030E-01	0,54	0,498	4,69037E-29
cDC(CD1C)	JAML	0,00000E+00	7,77701E-01	0,39	0,178	0,00000E+00
cDC(CD1C)	SLC38A1	0,00000E+00	7,77195E-01	0,296	0,056	0,00000E+00
cDC(CD1C)	COPS3	1,81875E-221	7,76091E-01	0,437	0,23	3,34960E-217
cDC(CD1C)	LY86	1,52255E-136	7,76045E-01	0,689	0,468	2,80408E-132
cDC(CD1C)	EEF1G	9,22908E-24	7,72164E-01	0,362	0,288	1,69972E-19
cDC(CD1C)	DEF6	3,42859E-269	7,71119E-01	0,445	0,218	6,31444E-265
cDC(CD1C)	IL2RG	2,00793E-227	7,70601E-01	0,529	0,301	3,69801E-223
cDC(CD1C)	ACTG1	0,00000E+00	7,67390E-01	0,989	0,915	0,00000E+00
cDC(CD1C)	SPINT2	0,00000E+00	7,66072E-01	0,751	0,469	0,00000E+00
cDC(CD1C)	CTSH	5,84152E-289	7,63897E-01	0,911	0,687	1,07583E-284

cDC(CD1C)	CLNS1A	1,23868E-268	7,62519E-01	0,648	0,396	2,28128E-264
cDC(CD1C)	LPXN	3,27857E-203	7,59784E-01	0,549	0,325	6,03814E-199
cDC(CD1C)	RPL38	1,44391E-23	7,59622E-01	0,53	0,488	2,65925E-19
cDC(CD1C)	CORO1A	3,24421E-235	7,59619E-01	0,847	0,623	5,97487E-231
cDC(CD1C)	C1orf162	5,65200E-190	7,56263E-01	0,871	0,689	1,04093E-185
cDC(CD1C)	YWHAH	0,00000E+00	7,54743E-01	0,745	0,508	0,00000E+00
cDC(CD1C)	RPL26	2,21915E-18	7,51066E-01	0,552	0,541	4,08701E-14
cDC(CD1C)	RPL5	1,32775E-41	7,49331E-01	0,549	0,514	2,44531E-37
cDC(CD1C)	RPS5	6,91231E-54	7,39496E-01	0,549	0,514	1,27304E-49
cDC(CD1C)	RPS10	4,96816E-28	7,39334E-01	0,355	0,275	9,14986E-24
cDC(CD1C)	GLIPR1	0,00000E+00	7,36551E-01	0,816	0,557	0,00000E+00
cDC(CD1C)	RPS6	3,43325E-60	7,31518E-01	0,56	0,542	6,32301E-56
cDC(CD1C)	EEF1B2	0,00000E+00	7,31168E-01	0,964	0,828	0,00000E+00
cDC(CD1C)	APEX1	6,65183E-242	7,29442E-01	0,665	0,416	1,22507E-237
cDC(CD1C)	STK17B	0,00000E+00	7,27834E-01	0,699	0,416	0,00000E+00
cDC(CD1C)	RPL7	1,69009E-23	7,23982E-01	0,536	0,493	3,11263E-19
cDC(CD1C)	NME1-NME2	1,64292E-35	7,23084E-01	0,309	0,231	3,02576E-31
cDC(CD1C)	RPS15	5,31542E-32	7,17995E-01	0,564	0,575	9,78940E-28
cDC(CD1C)	RPL15	2,57241E-52	7,16753E-01	0,56	0,552	4,73761E-48
cDC(CD1C)	FBL	8,41258E-237	7,15447E-01	0,559	0,324	1,54935E-232
cDC(CD1C)	FES	5,80331E-45	7,15431E-01	0,348	0,244	1,06880E-40
cDC(CD1C)	ENTPD1	5,49679E-199	7,14031E-01	0,5	0,288	1,01234E-194
cDC(CD1C)	CDC37	4,26370E-108	7,11742E-01	0,781	0,622	7,85245E-104
cDC(CD1C)	BTF3	0,00000E+00	7,09850E-01	0,955	0,834	0,00000E+00
cDC(CD1C)	RPL27A	2,36910E-40	7,08215E-01	0,527	0,457	4,36317E-36
cDC(CD1C)	AREG	3,14302E-108	7,07830E-01	0,423	0,259	5,78850E-104
cDC(CD1C)	RPS17	1,04667E-30	7,06900E-01	0,476	0,399	1,92766E-26
cDC(CD1C)	ALDH2	4,39299E-229	7,06270E-01	0,755	0,527	8,09057E-225
cDC(CD1C)	TMSB4X	1,71406E-71	7,06102E-01	0,998	0,982	3,15678E-67
cDC(CD1C)	RPL3	7,87040E-59	7,04929E-01	0,559	0,53	1,44949E-54
cDC(CD1C)	SLC25A5	1,28835E-281	7,03082E-01	0,911	0,725	2,37275E-277
cDC(CD1C)	RBM3	2,42218E-208	7,01602E-01	0,842	0,656	4,46092E-204
cDC(CD1C)	MYD88	4,05955E-75	7,00238E-01	0,488	0,342	7,47647E-71
cDC(CD1C)	HCLS1	3,08197E-224	6,99686E-01	0,891	0,705	5,67607E-220
cDC(CD1C)	GLA	5,88490E-15	6,98819E-01	0,372	0,304	1,08382E-10
cDC(CD1C)	GTF2A2	2,90124E-52	6,94118E-01	0,523	0,394	5,34322E-48
cDC(CD1C)	PABPC1	3,05798E-285	6,94005E-01	0,974	0,883	5,63189E-281
cDC(CD1C)	RPS3	5,54527E-33	6,93681E-01	0,562	0,568	1,02127E-28
cDC(CD1C)	DOK1	3,11403E-96	6,90126E-01	0,362	0,224	5,73511E-92
cDC(CD1C)	MS4A6A	2,39142E-122	6,84604E-01	0,844	0,7	4,40428E-118

cDC(CD1C)	PEA15	0,00000E+00	6,83247E-01	0,752	0,491	0,00000E+00
cDC(CD1C)	PPA2	9,72645E-162	6,81633E-01	0,387	0,213	1,79132E-157
cDC(CD1C)	IL18	1,15369E-264	6,78513E-01	0,677	0,415	2,12476E-260
cDC(CD1C)	RPS14	2,86242E-34	6,75488E-01	0,564	0,577	5,27173E-30
cDC(CD1C)	PPP1CB	4,25126E-23	6,73885E-01	0,484	0,387	7,82954E-19
cDC(CD1C)	RPS19	9,49535E-59	6,73519E-01	0,567	0,576	1,74876E-54
cDC(CD1C)	RPL31	2,96215E-21	6,70502E-01	0,531	0,493	5,45540E-17
cDC(CD1C)	TMEM14C	0,00000E+00	6,65195E-01	0,755	0,504	0,00000E+00
cDC(CD1C)	ADAM28	1,24188E-183	6,63373E-01	0,407	0,22	2,28718E-179
cDC(CD1C)	ARF5	1,04423E-227	6,62991E-01	0,793	0,593	1,92315E-223
cDC(CD1C)	LAMTOR1	9,89557E-156	6,60252E-01	0,816	0,629	1,82247E-151
cDC(CD1C)	RPL19	7,82930E-24	6,54412E-01	0,564	0,582	1,44192E-19
cDC(CD1C)	ARHGDIB	1,79348E-167	6,53685E-01	0,95	0,808	3,30306E-163
cDC(CD1C)	CIB1	2,32905E-154	6,53634E-01	0,773	0,594	4,28941E-150
cDC(CD1C)	RPL9	5,31183E-35	6,52662E-01	0,556	0,542	9,78280E-31
cDC(CD1C)	LDHA	2,01219E-111	6,51576E-01	0,854	0,698	3,70585E-107
cDC(CD1C)	RPL23	4,17879E-22	6,48438E-01	0,512	0,446	7,69607E-18
cDC(CD1C)	RPS23	1,19030E-50	6,46460E-01	0,563	0,569	2,19217E-46
cDC(CD1C)	TRAPPC6A	4,92623E-98	6,43409E-01	0,366	0,227	9,07264E-94
cDC(CD1C)	PDHB	7,39928E-30	6,42593E-01	0,264	0,192	1,36272E-25
cDC(CD1C)	OLA1	2,35814E-133	6,42240E-01	0,465	0,291	4,34299E-129
cDC(CD1C)	APMAP	3,92731E-81	6,42029E-01	0,424	0,287	7,23293E-77
cDC(CD1C)	DECR1	1,84990E-76	6,41947E-01	0,477	0,332	3,40696E-72
cDC(CD1C)	RPL21	1,42101E-22	6,41123E-01	0,553	0,54	2,61707E-18
cDC(CD1C)	ACTR3	1,45074E-87	6,40965E-01	0,787	0,65	2,67182E-83
cDC(CD1C)	RPL27	2,61739E-43	6,40238E-01	0,542	0,501	4,82045E-39
cDC(CD1C)	RPL17	3,47060E-16	6,39314E-01	0,483	0,428	6,39180E-12
cDC(CD1C)	SAMHD1	4,45222E-159	6,37197E-01	0,825	0,626	8,19965E-155
cDC(CD1C)	CLIC2	1,46559E-172	6,36462E-01	0,302	0,145	2,69917E-168
cDC(CD1C)	AOAH	8,16435E-55	6,35594E-01	0,489	0,362	1,50363E-50
cDC(CD1C)	HMGN1	4,00486E-303	6,35199E-01	0,843	0,601	7,37576E-299
cDC(CD1C)	PTRHD1	5,14530E-261	6,34894E-01	0,566	0,324	9,47610E-257
cDC(CD1C)	PLBD1	2,47642E-99	6,33311E-01	0,545	0,376	4,56082E-95
cDC(CD1C)	HNRNPA1	0,00000E+00	6,33192E-01	0,944	0,785	0,00000E+00
cDC(CD1C)	PRMT2	7,63310E-151	6,31471E-01	0,604	0,408	1,40579E-146
cDC(CD1C)	PHACTR1	9,38818E-123	6,31335E-01	0,459	0,284	1,72902E-118
cDC(CD1C)	SLC25A3	1,27862E-144	6,31289E-01	0,868	0,696	2,35483E-140
cDC(CD1C)	STK38L	1,25233E-104	6,30784E-01	0,319	0,183	2,30642E-100
cDC(CD1C)	MIF4GD	1,52901E-241	6,30602E-01	0,514	0,289	2,81598E-237
cDC(CD1C)	MTHFD2	2,75796E-96	6,27978E-01	0,554	0,388	5,07933E-92

cDC(CD1C)	RPL13A	5,13249E-18	6,27793E-01	0,545	0,518	9,45250E-14
cDC(CD1C)	UQCRC1	5,00660E-26	6,26771E-01	0,612	0,493	9,22066E-22
cDC(CD1C)	HPRT1	3,01691E-128	6,23987E-01	0,408	0,247	5,55625E-124
cDC(CD1C)	IFNGR1	2,38296E-138	6,23894E-01	0,749	0,562	4,38870E-134
cDC(CD1C)	RPS9	5,65143E-32	6,22490E-01	0,558	0,554	1,04082E-27
cDC(CD1C)	RPL41	2,27824E-16	6,22194E-01	0,559	0,576	4,19583E-12
cDC(CD1C)	CCND3	5,95415E-219	6,21850E-01	0,508	0,29	1,09658E-214
cDC(CD1C)	LITAF	2,05277E-139	6,20525E-01	0,884	0,726	3,78059E-135
cDC(CD1C)	TMSB10	3,28811E-73	6,19534E-01	0,998	0,983	6,05570E-69
cDC(CD1C)	XRCC6	2,15724E-117	6,19324E-01	0,662	0,476	3,97300E-113
cDC(CD1C)	TSC22D3	1,22821E-93	6,17905E-01	0,811	0,681	2,26200E-89
cDC(CD1C)	ABI3	2,73945E-126	6,17769E-01	0,564	0,374	5,04525E-122
cDC(CD1C)	EIF3E	2,37539E-231	6,17312E-01	0,804	0,583	4,37476E-227
cDC(CD1C)	TMEM123	1,46954E-62	6,16149E-01	0,584	0,44	2,70645E-58
cDC(CD1C)	PTGER4	6,54548E-76	6,16109E-01	0,38	0,257	1,20548E-71
cDC(CD1C)	RPS13	1,89512E-20	6,12835E-01	0,562	0,572	3,49023E-16
cDC(CD1C)	VDAC1	2,63505E-96	6,12465E-01	0,694	0,516	4,85297E-92
cDC(CD1C)	RPL36A	3,66146E-26	6,11696E-01	0,505	0,448	6,74331E-22
cDC(CD1C)	GABARAPL2	7,99967E-114	6,08830E-01	0,729	0,561	1,47330E-109
cDC(CD1C)	UBE2E2	1,08500E-123	6,08805E-01	0,449	0,283	1,99824E-119
cDC(CD1C)	SRSF2	3,68443E-135	6,06677E-01	0,72	0,539	6,78561E-131
cDC(CD1C)	RPS27	1,07497E-33	6,05737E-01	0,563	0,567	1,97977E-29
cDC(CD1C)	RNPS1	1,56406E-99	6,05697E-01	0,559	0,387	2,88053E-95
cDC(CD1C)	HDAC9	3,77597E-167	6,03936E-01	0,264	0,119	6,95421E-163
cDC(CD1C)	RPL23A	2,68297E-46	6,03419E-01	0,551	0,523	4,94122E-42
cDC(CD1C)	CYTIP	1,02466E-219	6,01406E-01	0,605	0,367	1,88711E-215
cDC(CD1C)	NDUFS8	3,36139E-98	6,00689E-01	0,65	0,478	6,19067E-94
cDC(CD1C)	RPL35	1,01038E-37	5,99948E-01	0,552	0,529	1,86081E-33
cDC(CD1C)	PLSCR1	4,53275E-93	5,99932E-01	0,702	0,524	8,34796E-89
cDC(CD1C)	ZFP36L2	3,60941E-193	5,99857E-01	0,838	0,678	6,64744E-189
cDC(CD1C)	EXOSC5	1,55003E-108	5,98512E-01	0,285	0,156	2,85469E-104
cDC(CD1C)	RPS21	6,53807E-31	5,98042E-01	0,551	0,542	1,20412E-26
cDC(CD1C)	NDEL1	6,45580E-31	5,95070E-01	0,257	0,182	1,18896E-26
cDC(CD1C)	EEF1A1	0,00000E+00	5,94881E-01	0,998	0,983	0,00000E+00
cDC(CD1C)	MBOAT7	1,32487E-109	5,94711E-01	0,388	0,234	2,44001E-105
cDC(CD1C)	DNTTIP2	4,30883E-23	5,92692E-01	0,345	0,267	7,93558E-19
cDC(CD1C)	RNH1	2,80466E-65	5,91232E-01	0,757	0,602	5,16535E-61
cDC(CD1C)	PSME2	8,97036E-13	5,90408E-01	0,825	0,703	1,65207E-08
cDC(CD1C)	EIF3G	1,48158E-228	5,90258E-01	0,818	0,591	2,72863E-224
cDC(CD1C)	C20orf27	1,75878E-205	5,90212E-01	0,545	0,322	3,23914E-201

cDC(CD1C)	TXN2	1,73233E-71	5,89768E-01	0,531	0,382	3,19043E-67
cDC(CD1C)	RPL11	1,41899E-15	5,89338E-01	0,564	0,582	2,61335E-11
cDC(CD1C)	MXD1	6,04626E-08	5,88793E-01	0,353	0,296	1,11354E-03
cDC(CD1C)	OXA1L	2,40694E-193	5,88259E-01	0,656	0,433	4,43286E-189
cDC(CD1C)	UFC1	4,26600E-108	5,86976E-01	0,665	0,484	7,85669E-104
cDC(CD1C)	SCNM1	5,40066E-134	5,86549E-01	0,497	0,316	9,94640E-130
cDC(CD1C)	EIF4A1	3,58168E-69	5,86138E-01	0,777	0,663	6,59637E-65
cDC(CD1C)	NIPSNAP3A	1,62020E-79	5,85672E-01	0,301	0,183	2,98391E-75
cDC(CD1C)	RPS25	6,67765E-35	5,85552E-01	0,558	0,546	1,22982E-30
cDC(CD1C)	RBPJ	2,29402E-168	5,85343E-01	0,656	0,445	4,22489E-164
cDC(CD1C)	CAT	2,86415E-215	5,84914E-01	0,613	0,386	5,27490E-211
cDC(CD1C)	NR4A3	8,61074E-115	5,84531E-01	0,438	0,281	1,58584E-110
cDC(CD1C)	UBA52	7,45992E-225	5,84103E-01	0,979	0,914	1,37389E-220
cDC(CD1C)	RPL10	7,56665E-18	5,83837E-01	0,567	0,597	1,39355E-13
cDC(CD1C)	MPST	2,89021E-152	5,83386E-01	0,359	0,194	5,32290E-148
cDC(CD1C)	MNDA	4,80486E-128	5,82302E-01	0,703	0,514	8,84910E-124
cDC(CD1C)	PGLS	7,22186E-118	5,81824E-01	0,706	0,524	1,33005E-113
cDC(CD1C)	RPS16	4,79818E-30	5,81749E-01	0,553	0,544	8,83681E-26
cDC(CD1C)	RPL18	1,71589E-25	5,80496E-01	0,559	0,563	3,16015E-21
cDC(CD1C)	AKIRIN1	2,40683E-73	5,80033E-01	0,341	0,219	4,43266E-69
cDC(CD1C)	IFITM2	2,55088E-48	5,79937E-01	0,767	0,633	4,69795E-44
cDC(CD1C)	SLC25A6	2,36916E-249	5,79222E-01	0,948	0,801	4,36328E-245
cDC(CD1C)	RPS3A	9,41865E-40	5,79210E-01	0,561	0,567	1,73463E-35
cDC(CD1C)	PSTPIP1	2,13210E-83	5,78798E-01	0,279	0,161	3,92669E-79
cDC(CD1C)	SLAMF8	2,45925E-67	5,75885E-01	0,449	0,311	4,52921E-63
cDC(CD1C)	EIF3F	2,01314E-305	5,75449E-01	0,864	0,644	3,70759E-301
cDC(CD1C)	DUSP23	3,12085E-112	5,75086E-01	0,543	0,365	5,74767E-108
cDC(CD1C)	DCXR	1,67545E-133	5,74762E-01	0,532	0,349	3,08569E-129
cDC(CD1C)	C7orf50	1,11971E-84	5,74339E-01	0,472	0,322	2,06217E-80
cDC(CD1C)	NPM1	2,54667E-306	5,73749E-01	0,932	0,747	4,69020E-302
cDC(CD1C)	PRELID1	2,55160E-93	5,73654E-01	0,815	0,67	4,69929E-89
cDC(CD1C)	SPINT1	2,03269E-254	5,73103E-01	0,313	0,13	3,74360E-250
cDC(CD1C)	TMEM109	5,03142E-250	5,71188E-01	0,585	0,344	9,26637E-246
cDC(CD1C)	RPL29	1,27662E-26	5,70830E-01	0,557	0,558	2,35114E-22
cDC(CD1C)	EIF3H	9,95983E-196	5,69499E-01	0,842	0,637	1,83430E-191
cDC(CD1C)	CEACAM4	0,00000E+00	5,69065E-01	0,26	0,083	0,00000E+00
cDC(CD1C)	TES	1,29693E-156	5,68635E-01	0,449	0,261	2,38856E-152
cDC(CD1C)	SMDT1	4,56087E-114	5,68364E-01	0,639	0,465	8,39976E-110
cDC(CD1C)	EIF3M	7,56420E-166	5,68306E-01	0,689	0,478	1,39310E-161
cDC(CD1C)	HDAC3	1,40347E-101	5,65568E-01	0,334	0,199	2,58477E-97

cDC(CD1C)	RPL37A	8,40301E-32	5,63840E-01	0,555	0,536	1,54758E-27
cDC(CD1C)	AP1S2	4,86919E-70	5,63091E-01	0,715	0,563	8,96758E-66
cDC(CD1C)	SPECC1	7,29563E-89	5,59939E-01	0,282	0,164	1,34364E-84
cDC(CD1C)	RIPK2	5,41156E-37	5,59896E-01	0,361	0,264	9,96647E-33
cDC(CD1C)	RPS2	9,10976E-32	5,58975E-01	0,56	0,563	1,67774E-27
cDC(CD1C)	RPL13	4,27073E-31	5,58361E-01	0,566	0,583	7,86540E-27
cDC(CD1C)	TNFAIP8L2	1,44746E-158	5,56813E-01	0,423	0,242	2,66578E-154
cDC(CD1C)	RPS15A	4,53612E-20	5,55763E-01	0,559	0,57	8,35417E-16
cDC(CD1C)	RASSF5	3,61054E-111	5,54105E-01	0,413	0,255	6,64953E-107
cDC(CD1C)	RPL35A	8,55355E-19	5,53792E-01	0,557	0,563	1,57531E-14
cDC(CD1C)	MAP3K13	4,59327E-63	5,53539E-01	0,313	0,201	8,45942E-59
cDC(CD1C)	MAP3K8	2,45714E-76	5,51946E-01	0,613	0,467	4,52531E-72
cDC(CD1C)	CAPN2	1,68349E-93	5,51319E-01	0,358	0,22	3,10048E-89
cDC(CD1C)	ARPC1B	2,56404E-73	5,51281E-01	0,9	0,785	4,72218E-69
cDC(CD1C)	SEC11A	6,52389E-188	5,51025E-01	0,843	0,647	1,20150E-183
cDC(CD1C)	RPL36	7,48001E-22	5,49687E-01	0,557	0,552	1,37759E-17
cDC(CD1C)	RGS10	8,54920E-251	5,49685E-01	0,911	0,721	1,57451E-246
cDC(CD1C)	RPS7	4,22147E-28	5,49445E-01	0,559	0,561	7,77469E-24
cDC(CD1C)	RPS8	8,13549E-16	5,45969E-01	0,565	0,58	1,49831E-11
cDC(CD1C)	RPL34	2,50780E-14	5,45953E-01	0,56	0,577	4,61862E-10
cDC(CD1C)	CFL1	2,20809E-162	5,45379E-01	0,982	0,915	4,06663E-158
cDC(CD1C)	SNRPE	2,56112E-48	5,44453E-01	0,448	0,33	4,71681E-44
cDC(CD1C)	NAP1L1	3,34311E-178	5,43685E-01	0,805	0,598	6,15700E-174
cDC(CD1C)	RPL6	3,52638E-27	5,43666E-01	0,557	0,553	6,49453E-23
cDC(CD1C)	ARF6	1,10767E-182	5,42486E-01	0,602	0,398	2,04000E-178
cDC(CD1C)	LST1	2,24374E-172	5,40254E-01	0,899	0,694	4,13229E-168
cDC(CD1C)	RALA	2,39812E-98	5,40047E-01	0,516	0,358	4,41662E-94
cDC(CD1C)	CYCS	6,30280E-61	5,39679E-01	0,66	0,509	1,16079E-56
cDC(CD1C)	LAT2	3,36849E-101	5,39485E-01	0,611	0,436	6,20374E-97
cDC(CD1C)	FILIP1L	2,42250E-253	5,38829E-01	0,305	0,126	4,46152E-249
cDC(CD1C)	RPL32	1,80312E-26	5,38166E-01	0,564	0,581	3,32080E-22
cDC(CD1C)	PA2G4	3,66816E-81	5,36440E-01	0,576	0,418	6,75564E-77
cDC(CD1C)	NDUFA10	8,32339E-94	5,36117E-01	0,467	0,312	1,53292E-89
cDC(CD1C)	PHB2	1,58531E-154	5,36062E-01	0,672	0,473	2,91966E-150
cDC(CD1C)	LMBR1L	4,98671E-88	5,35980E-01	0,25	0,14	9,18403E-84
cDC(CD1C)	BRK1	9,37394E-159	5,35177E-01	0,867	0,71	1,72640E-154
cDC(CD1C)	MRPL3	2,49796E-217	5,33749E-01	0,478	0,261	4,60050E-213
cDC(CD1C)	RPL14	5,54705E-24	5,32751E-01	0,556	0,541	1,02160E-19
cDC(CD1C)	PDCL3	2,01989E-98	5,31985E-01	0,344	0,207	3,72003E-94
cDC(CD1C)	RPLP0	4,44690E-28	5,30505E-01	0,556	0,541	8,18986E-24

cDC(CD1C)	SNX20	8,04229E-137	5,28408E-01	0,36	0,203	1,48115E-132
cDC(CD1C)	IGSF6	9,52474E-153	5,27831E-01	0,753	0,535	1,75417E-148
cDC(CD1C)	PPT1	5,73672E-138	5,26396E-01	0,843	0,629	1,05653E-133
cDC(CD1C)	DUSP2	5,16167E-14	5,25403E-01	0,36	0,294	9,50626E-10
cDC(CD1C)	RPL7A	5,34226E-32	5,24751E-01	0,562	0,558	9,83884E-28
cDC(CD1C)	CASP1	2,33895E-71	5,23025E-01	0,617	0,446	4,30765E-67
cDC(CD1C)	ITGB2	9,83059E-80	5,22964E-01	0,909	0,747	1,81050E-75
cDC(CD1C)	DCTPP1	3,22017E-83	5,22461E-01	0,301	0,185	5,93058E-79
cDC(CD1C)	SCP2	5,62248E-63	5,21502E-01	0,601	0,445	1,03549E-58
cDC(CD1C)	PDCD6	1,57667E-71	5,21465E-01	0,559	0,41	2,90376E-67
cDC(CD1C)	CAP1	1,65048E-144	5,21205E-01	0,866	0,718	3,03969E-140
cDC(CD1C)	DAPP1	2,14196E-111	5,18705E-01	0,348	0,204	3,94485E-107
cDC(CD1C)	NFKBID	3,62067E-37	5,18427E-01	0,292	0,205	6,66819E-33
cDC(CD1C)	CCT2	2,22401E-71	5,18200E-01	0,466	0,326	4,09596E-67
cDC(CD1C)	GLIPR2	1,94344E-37	5,15812E-01	0,482	0,365	3,57923E-33
cDC(CD1C)	PFN1	2,11419E-125	5,15787E-01	0,987	0,943	3,89371E-121
cDC(CD1C)	MTCH2	2,03343E-39	5,13397E-01	0,419	0,312	3,74496E-35
cDC(CD1C)	RPS4X	6,02077E-25	5,13341E-01	0,562	0,566	1,10884E-20
cDC(CD1C)	SNX8	2,64760E-67	5,12930E-01	0,319	0,207	4,87608E-63
cDC(CD1C)	RPLP2	9,09738E-13	5,12194E-01	0,559	0,572	1,67547E-08
cDC(CD1C)	DNMT1	8,64510E-95	5,11540E-01	0,45	0,302	1,59217E-90
cDC(CD1C)	DOK2	3,25650E-138	5,10817E-01	0,521	0,325	5,99749E-134
cDC(CD1C)	CDC42	5,97826E-33	5,10772E-01	0,804	0,685	1,10102E-28
cDC(CD1C)	PGK1	1,16131E-30	5,10234E-01	0,821	0,693	2,13879E-26
cDC(CD1C)	PPIA	1,19691E-183	5,09311E-01	0,975	0,885	2,20435E-179
cDC(CD1C)	PRKAG1	2,32059E-81	5,04865E-01	0,299	0,183	4,27383E-77
cDC(CD1C)	CD33	3,86920E-89	5,04835E-01	0,438	0,293	7,12590E-85
cDC(CD1C)	CCNH	2,18627E-126	5,04353E-01	0,54	0,352	4,02646E-122
cDC(CD1C)	DEGS1	9,84787E-96	5,04211E-01	0,468	0,31	1,81368E-91
cDC(CD1C)	HIGD2A	3,34227E-97	5,04027E-01	0,854	0,699	6,15546E-93
cDC(CD1C)	EZR	5,05083E-64	5,03649E-01	0,519	0,386	9,30211E-60
cDC(CD1C)	LCP1	1,70964E-50	5,02825E-01	0,805	0,692	3,14864E-46
cDC(CD1C)	PID1	7,48254E-111	5,01986E-01	0,265	0,135	1,37806E-106
cDC(CD1C)	NDUFS7	2,35146E-38	5,01352E-01	0,667	0,527	4,33068E-34
cDC(CD1C)	FKBP1A	1,61968E-82	5,00536E-01	0,821	0,68	2,98296E-78
cDC(CD1C)	ANXA2	4,40894E-10	5,00138E-01	0,839	0,732	8,11994E-06
cDC(CD1C)	USP10	3,29421E-56	5,00109E-01	0,305	0,201	6,06694E-52
cDC(CD1C)	DDOST	6,54389E-141	4,99079E-01	0,641	0,443	1,20519E-136
cDC(CD1C)	CD86	5,60318E-114	4,97842E-01	0,669	0,475	1,03194E-109
cDC(CD1C)	PSME1	4,86404E-52	4,97097E-01	0,88	0,74	8,95811E-48

cDC(CD1C)

cDC(CD1C)	BZW2	4,41400E-157	4,96959E-01	0,302	0,149	8,12927E-153
cDC(CD1C)	HSD17B10	9,00720E-86	4,96391E-01	0,438	0,291	1,65886E-81
cDC(CD1C)	PRPF31	7,71934E-32	4,95686E-01	0,286	0,206	1,42167E-27
cDC(CD1C)	MICAL1	2,46287E-111	4,95092E-01	0,439	0,277	4,53587E-107
cDC(CD1C)	SF3A3	6,59555E-62	4,94519E-01	0,317	0,208	1,21470E-57
cDC(CD1C)	SRP9	7,88399E-54	4,94434E-01	0,584	0,452	1,45199E-49
cDC(CD1C)	SUSD3	0,00000E+00	4,94276E-01	0,259	0,082	0,00000E+00
cDC(CD1C)	PSMA5	3,49722E-55	4,94076E-01	0,562	0,42	6,44083E-51
cDC(CD1C)	RIN3	1,58045E-121	4,94062E-01	0,577	0,385	2,91072E-117
cDC(CD1C)	ASAP1	3,51773E-130	4,93843E-01	0,364	0,206	6,47860E-126
cDC(CD1C)	CDC123	1,57908E-83	4,93795E-01	0,351	0,222	2,90819E-79
cDC(CD1C)	RTN1	4,92085E-232	4,91829E-01	0,254	0,097	9,06272E-228
cDC(CD1C)	TIMM17A	3,00253E-58	4,91581E-01	0,378	0,259	5,52975E-54
cDC(CD1C)	EIF3I	1,71149E-127	4,91524E-01	0,635	0,446	3,15205E-123
cDC(CD1C)	VASP	1,78464E-124	4,91235E-01	0,626	0,452	3,28677E-120
cDC(CD1C)	RPL37	3,42138E-31	4,91178E-01	0,56	0,569	6,30116E-27
cDC(CD1C)	TUFM	8,74208E-131	4,90596E-01	0,691	0,489	1,61003E-126
cDC(CD1C)	CAPZA1	7,58825E-28	4,88895E-01	0,594	0,483	1,39753E-23
cDC(CD1C)	ENO1	3,51133E-49	4,88491E-01	0,872	0,749	6,46682E-45
cDC(CD1C)	MFNG	1,44519E-64	4,88388E-01	0,282	0,176	2,66161E-60
cDC(CD1C)	TPI1	6,46663E-78	4,86012E-01	0,933	0,814	1,19096E-73
cDC(CD1C)	NDUFV1	3,82595E-88	4,85548E-01	0,551	0,392	7,04625E-84
cDC(CD1C)	RPL28	4,86280E-12	4,85478E-01	0,568	0,597	8,95581E-08
cDC(CD1C)	RALY	2,71931E-115	4,83752E-01	0,656	0,474	5,00815E-111
cDC(CD1C)	GGCT	5,33735E-88	4,82966E-01	0,335	0,207	9,82979E-84
cDC(CD1C)	ARPC2	4,58993E-64	4,82117E-01	0,945	0,849	8,45327E-60
cDC(CD1C)	LAMTOR3	3,59887E-19	4,81916E-01	0,321	0,251	6,62804E-15
cDC(CD1C)	ZNF385A	4,49523E-196	4,81224E-01	0,618	0,389	8,27887E-192
cDC(CD1C)	HGSNAT	6,46154E-124	4,80113E-01	0,318	0,173	1,19002E-119
cDC(CD1C)	FGR	1,29397E-148	4,79310E-01	0,655	0,431	2,38311E-144
cDC(CD1C)	ALKBH7	2,35834E-111	4,79139E-01	0,659	0,473	4,34335E-107
cDC(CD1C)	FAM162A	1,33858E-109	4,78757E-01	0,499	0,328	2,46527E-105
cDC(CD1C)	ADAM8	5,67074E-271	4,78472E-01	0,437	0,203	1,04438E-266
cDC(CD1C)	NTMT1	2,15721E-40	4,78387E-01	0,282	0,196	3,97294E-36
cDC(CD1C)	ACAP1	5,66415E-306	4,78382E-01	0,313	0,116	1,04317E-301
cDC(CD1C)	EEF2	9,01146E-283	4,78041E-01	0,946	0,815	1,65964E-278
cDC(CD1C)	C1QBP	1,16840E-158	4,77457E-01	0,618	0,408	2,15184E-154
cDC(CD1C)	RPL30	4,55598E-11	4,76912E-01	0,564	0,582	8,39075E-07
cDC(CD1C)	RNASE6	0,00000E+00	4,76402E-01	0,784	0,512	0,00000E+00
cDC(CD1C)	PLCB2	1,25364E-68	4,75740E-01	0,351	0,229	2,30883E-64

cDC(CD1C)	GPR108	6,40531E-70	4,75720E-01	0,37	0,245	1,17967E-65
cDC(CD1C)	TMEM230	2,23405E-64	4,75326E-01	0,588	0,436	4,11444E-60
cDC(CD1C)	SMCO4	5,35635E-51	4,73412E-01	0,533	0,405	9,86480E-47
cDC(CD1C)	ETS2	8,94691E-112	4,72462E-01	0,595	0,407	1,64775E-107
cDC(CD1C)	MYADM	2,41149E-53	4,71397E-01	0,45	0,321	4,44124E-49
cDC(CD1C)	SSNA1	6,46656E-44	4,71366E-01	0,517	0,392	1,19095E-39
cDC(CD1C)	CCT6A	1,95308E-130	4,67378E-01	0,62	0,429	3,59698E-126
cDC(CD1C)	SEMA4A	4,66201E-26	4,67074E-01	0,293	0,215	8,58603E-22
cDC(CD1C)	MRPL55	2,39071E-49	4,66828E-01	0,373	0,267	4,40297E-45
cDC(CD1C)	PTP4A1	2,33748E-32	4,65792E-01	0,3	0,224	4,30494E-28
cDC(CD1C)	RANBP1	1,09885E-73	4,64613E-01	0,499	0,352	2,02375E-69
cDC(CD1C)	RAB7A	1,37004E-34	4,63461E-01	0,758	0,619	2,52319E-30
cDC(CD1C)	TIMM10	8,74698E-73	4,63367E-01	0,365	0,241	1,61093E-68
cDC(CD1C)	PPHLN1	1,09481E-99	4,62367E-01	0,341	0,205	2,01632E-95
cDC(CD1C)	EID1	2,47810E-107	4,62196E-01	0,741	0,561	4,56392E-103
cDC(CD1C)	RBM39	3,18979E-44	4,62008E-01	0,742	0,609	5,87464E-40
cDC(CD1C)	SLC8A1	4,96547E-142	4,61726E-01	0,543	0,345	9,14491E-138
cDC(CD1C)	COMMD8	2,37824E-121	4,61253E-01	0,362	0,211	4,38001E-117
cDC(CD1C)	HINT1	4,54467E-263	4,60775E-01	0,898	0,703	8,36992E-259
cDC(CD1C)	POP4	1,18686E-27	4,60319E-01	0,276	0,202	2,18584E-23
cDC(CD1C)	RBMX	1,56604E-119	4,58847E-01	0,594	0,406	2,88417E-115
cDC(CD1C)	PUF60	1,25809E-53	4,57859E-01	0,426	0,308	2,31702E-49
cDC(CD1C)	RGS18	2,18269E-167	4,57818E-01	0,352	0,18	4,01985E-163
cDC(CD1C)	GLRX3	2,14172E-69	4,56440E-01	0,357	0,235	3,94440E-65
cDC(CD1C)	RHOC	6,25160E-61	4,55378E-01	0,421	0,297	1,15136E-56
cDC(CD1C)	SET	4,65713E-117	4,55005E-01	0,696	0,509	8,57704E-113
cDC(CD1C)	NDUFA13	4,47910E-08	4,54991E-01	0,481	0,426	8,24917E-04
cDC(CD1C)	NACA	1,62867E-201	4,53707E-01	0,979	0,912	2,99952E-197
cDC(CD1C)	CYC1	2,44033E-60	4,53486E-01	0,561	0,42	4,49435E-56
cDC(CD1C)	TPT1	6,77273E-275	4,52428E-01	0,994	0,972	1,24733E-270
cDC(CD1C)	HMGN2	2,12305E-43	4,52260E-01	0,861	0,72	3,91002E-39
cDC(CD1C)	TPM3	1,40129E-30	4,51878E-01	0,889	0,794	2,58076E-26
cDC(CD1C)	EIF6	7,40958E-90	4,50863E-01	0,551	0,389	1,36462E-85
cDC(CD1C)	CD48	5,81494E-31	4,50081E-01	0,599	0,466	1,07094E-26
cDC(CD1C)	STK4	1,28578E-121	4,49628E-01	0,596	0,41	2,36802E-117
cDC(CD1C)	SERPINB9	1,27358E-30	4,49397E-01	0,463	0,365	2,34556E-26
cDC(CD1C)	TM6SF1	2,86791E-134	4,49057E-01	0,422	0,255	5,28182E-130
cDC(CD1C)	PSMA4	1,91075E-16	4,48270E-01	0,559	0,458	3,51902E-12
cDC(CD1C)	CD52	5,45503E-36	4,47876E-01	0,495	0,378	1,00465E-31
cDC(CD1C)	C17orf49	1,78754E-27	4,47197E-01	0,298	0,222	3,29211E-23

cDC(CD1C)	MYL12A	1,00007E-108	4,46872E-01	0,922	0,814	1,84182E-104
cDC(CD1C)	ICAM3	0,00000E+00	4,46746E-01	0,43	0,17	0,00000E+00
cDC(CD1C)	RGS19	2,07084E-154	4,46013E-01	0,67	0,476	3,81387E-150
cDC(CD1C)	STX11	6,97039E-23	4,44471E-01	0,402	0,32	1,28374E-18
cDC(CD1C)	ELOVL1	3,61783E-128	4,44337E-01	0,523	0,342	6,66296E-124
cDC(CD1C)	VPS35	1,20381E-127	4,44194E-01	0,606	0,421	2,21706E-123
cDC(CD1C)	NARF	3,65796E-38	4,44091E-01	0,284	0,197	6,73687E-34
cDC(CD1C)	CLK1	1,04414E-15	4,42967E-01	0,455	0,374	1,92299E-11
cDC(CD1C)	COX8A	1,41202E-14	4,42512E-01	0,866	0,768	2,60051E-10
cDC(CD1C)	RPL39	1,35078E-07	4,42165E-01	0,56	0,583	2,48772E-03
cDC(CD1C)	PSMB10	3,69057E-22	4,41483E-01	0,673	0,555	6,79692E-18
cDC(CD1C)	AHR	1,04722E-111	4,41462E-01	0,507	0,338	1,92866E-107
cDC(CD1C)	ANXA1	4,03898E-77	4,41187E-01	0,837	0,671	7,43858E-73
cDC(CD1C)	ATF4	2,30199E-25	4,40297E-01	0,5	0,395	4,23958E-21
cDC(CD1C)	LMNA	9,27209E-12	4,40121E-01	0,408	0,364	1,70764E-07
cDC(CD1C)	PLXDC2	1,08952E-83	4,39684E-01	0,714	0,525	2,00656E-79
cDC(CD1C)	UQCRH	1,41743E-68	4,39245E-01	0,835	0,687	2,61048E-64
cDC(CD1C)	MRPL33	1,67594E-41	4,39171E-01	0,476	0,36	3,08658E-37
cDC(CD1C)	PARVG	2,92647E-82	4,38390E-01	0,592	0,434	5,38967E-78
cDC(CD1C)	NDUFV2	1,45522E-35	4,38079E-01	0,645	0,522	2,68007E-31
cDC(CD1C)	FGL2	2,13801E-126	4,37737E-01	0,816	0,591	3,93757E-122
cDC(CD1C)	ILF2	2,04430E-102	4,37341E-01	0,545	0,374	3,76498E-98
cDC(CD1C)	CCT3	6,59181E-105	4,36735E-01	0,566	0,393	1,21401E-100
cDC(CD1C)	MBP	9,25854E-106	4,36723E-01	0,466	0,302	1,70515E-101
cDC(CD1C)	ITGB1BP1	1,18063E-31	4,36010E-01	0,328	0,241	2,17437E-27
cDC(CD1C)	UNC119	4,97533E-119	4,34649E-01	0,415	0,251	9,16306E-115
cDC(CD1C)	COX4I1	2,24165E-53	4,34114E-01	0,959	0,89	4,12845E-49
cDC(CD1C)	JMJD1C	8,97940E-51	4,34056E-01	0,549	0,414	1,65374E-46
cDC(CD1C)	FAU	7,98689E-143	4,33833E-01	0,987	0,953	1,47094E-138
cDC(CD1C)	NFE2L2	2,42822E-11	4,33367E-01	0,595	0,511	4,47206E-07
cDC(CD1C)	SERPINF2	0,00000E+00	4,33120E-01	0,307	0,102	0,00000E+00
cDC(CD1C)	CX3CR1	6,69558E-151	4,32931E-01	0,273	0,126	1,23313E-146
cDC(CD1C)	IDH2	3,10557E-157	4,32352E-01	0,59	0,391	5,71953E-153
cDC(CD1C)	BLOC1S6	4,48430E-95	4,32285E-01	0,419	0,268	8,25873E-91
cDC(CD1C)	SCIMP	1,43807E-26	4,31831E-01	0,353	0,269	2,64849E-22
cDC(CD1C)	CLIC1	3,51051E-44	4,31756E-01	0,944	0,866	6,46530E-40
cDC(CD1C)	ARPC5	5,19753E-25	4,31145E-01	0,858	0,752	9,57230E-21
cDC(CD1C)	CNPPD1	7,21292E-162	4,30466E-01	0,436	0,252	1,32840E-157
cDC(CD1C)	HMGA1	1,24048E-239	4,30254E-01	0,616	0,38	2,28459E-235
cDC(CD1C)	HAVCR2	1,57721E-82	4,30149E-01	0,588	0,418	2,90475E-78

cDC(CD1C)	MRPL14	4,77113E-09	4,29763E-01	0,4	0,338	8,78698E-05
cDC(CD1C)	IL18BP	1,31400E-29	4,29197E-01	0,263	0,189	2,41999E-25
cDC(CD1C)	ELOVL5	4,52175E-134	4,28254E-01	0,447	0,27	8,32770E-130
cDC(CD1C)	SLC2A3	1,47926E-09	4,27318E-01	0,516	0,424	2,72436E-05
cDC(CD1C)	TNFRSF1A	2,51737E-33	4,25331E-01	0,552	0,437	4,63624E-29
cDC(CD1C)	NHP2	9,07747E-105	4,25085E-01	0,566	0,395	1,67180E-100
cDC(CD1C)	TCP1	2,65347E-36	4,24951E-01	0,427	0,327	4,88689E-32
cDC(CD1C)	NAPSA	0,00000E+00	4,23354E-01	0,425	0,153	0,00000E+00
cDC(CD1C)	CCT7	7,77492E-81	4,23231E-01	0,482	0,334	1,43191E-76
cDC(CD1C)	MSN	7,94879E-74	4,23147E-01	0,78	0,622	1,46393E-69
cDC(CD1C)	CLPP	2,40473E-27	4,22663E-01	0,338	0,257	4,42879E-23
cDC(CD1C)	HNRNPK	1,10140E-81	4,21521E-01	0,845	0,694	2,02845E-77
cDC(CD1C)	PSMB3	6,96845E-28	4,21205E-01	0,765	0,63	1,28338E-23
cDC(CD1C)	RPS12	2,28444E-13	4,20573E-01	0,563	0,584	4,20725E-09
cDC(CD1C)	FUNDC2	1,92309E-21	4,20347E-01	0,438	0,351	3,54176E-17
cDC(CD1C)	RSL1D1	9,14461E-123	4,20261E-01	0,644	0,45	1,68416E-118
cDC(CD1C)	PTDSS1	1,68366E-125	4,20162E-01	0,418	0,253	3,10079E-121
cDC(CD1C)	MRPS16	8,39634E-12	4,19314E-01	0,355	0,295	1,54635E-07
cDC(CD1C)	UVRAG	1,29592E-164	4,19089E-01	0,462	0,272	2,38670E-160
cDC(CD1C)	PDIA3	4,78378E-39	4,18940E-01	0,796	0,662	8,81029E-35
cDC(CD1C)	SNX5	2,59159E-37	4,18677E-01	0,501	0,387	4,77293E-33
cDC(CD1C)	SERPINB1	2,67677E-67	4,18571E-01	0,79	0,649	4,92981E-63
cDC(CD1C)	PNRC2	9,42130E-12	4,18361E-01	0,278	0,225	1,73512E-07
cDC(CD1C)	SUPT4H1	5,93082E-133	4,17986E-01	0,632	0,442	1,09228E-128
cDC(CD1C)	POLD4	4,01549E-94	4,17589E-01	0,72	0,552	7,39532E-90
cDC(CD1C)	CHP1	9,66142E-16	4,17582E-01	0,292	0,231	1,77934E-11
cDC(CD1C)	ATIC	9,03143E-65	4,17531E-01	0,265	0,165	1,66332E-60
cDC(CD1C)	ATP6AP1	1,44824E-45	4,16865E-01	0,678	0,529	2,66723E-41
cDC(CD1C)	CNBP	2,83985E-76	4,16858E-01	0,847	0,702	5,23014E-72
cDC(CD1C)	KCNK6	6,37262E-289	4,16854E-01	0,36	0,153	1,17364E-284
cDC(CD1C)	MEF2C	1,02362E-136	4,15919E-01	0,6	0,397	1,88520E-132
cDC(CD1C)	EIF4H	3,29594E-86	4,15832E-01	0,592	0,437	6,07013E-82
cDC(CD1C)	ARRDC1	3,07324E-69	4,15185E-01	0,409	0,281	5,65999E-65
cDC(CD1C)	PAPOLA	1,30756E-35	4,14837E-01	0,564	0,443	2,40814E-31
cDC(CD1C)	TIMM9	1,07901E-58	4,14813E-01	0,258	0,163	1,98721E-54
cDC(CD1C)	FOXN3	1,05177E-101	4,14741E-01	0,512	0,343	1,93705E-97
cDC(CD1C)	EIF5A	7,39745E-58	4,14596E-01	0,679	0,543	1,36239E-53
cDC(CD1C)	ARL4C	3,31358E-96	4,14525E-01	0,474	0,316	6,10263E-92
cDC(CD1C)	MANBA	3,57067E-11	4,14221E-01	0,395	0,33	6,57611E-07
cDC(CD1C)	LIMD2	6,16175E-133	4,12137E-01	0,663	0,461	1,13481E-128

cDC(CD1C)	ACOT9	1,14868E-17	4,12073E-01	0,342	0,272	2,11553E-13
cDC(CD1C)	FLOT2	4,68363E-138	4,11937E-01	0,393	0,225	8,62584E-134
cDC(CD1C)	UQCRC2	1,24184E-76	4,11649E-01	0,555	0,399	2,28710E-72
cDC(CD1C)	B3GNT5	2,08463E-87	4,10849E-01	0,26	0,148	3,83927E-83
cDC(CD1C)	ETFA	8,72058E-69	4,10153E-01	0,458	0,322	1,60607E-64
cDC(CD1C)	PRDX3	1,21080E-28	4,09652E-01	0,585	0,468	2,22993E-24
cDC(CD1C)	CCNDBP1	1,12487E-36	4,09347E-01	0,358	0,264	2,07167E-32
cDC(CD1C)	VDAC2	1,10640E-69	4,08190E-01	0,701	0,536	2,03767E-65
cDC(CD1C)	SNRPB	7,87062E-63	4,07310E-01	0,642	0,493	1,44953E-58
cDC(CD1C)	TYK2	1,65603E-34	4,07079E-01	0,273	0,19	3,04990E-30
cDC(CD1C)	CHMP1B	1,10562E-33	4,07053E-01	0,494	0,397	2,03621E-29
cDC(CD1C)	PCBP2	3,05020E-231	4,07013E-01	0,914	0,785	5,61756E-227
cDC(CD1C)	HNRNPA3	8,13180E-42	4,06488E-01	0,752	0,615	1,49763E-37
cDC(CD1C)	CCNI	7,09096E-181	4,04757E-01	0,916	0,771	1,30594E-176
cDC(CD1C)	RAB11A	9,81860E-26	4,04423E-01	0,492	0,391	1,80829E-21
cDC(CD1C)	HMOX2	9,78539E-51	4,03763E-01	0,278	0,185	1,80218E-46
cDC(CD1C)	MOB3A	4,86817E-29	4,02871E-01	0,3	0,218	8,96571E-25
cDC(CD1C)	MRPL11	3,62011E-77	4,02700E-01	0,417	0,28	6,66715E-73
cDC(CD1C)	VIM	2,28650E-84	4,02473E-01	0,983	0,939	4,21104E-80
cDC(CD1C)	PSMC2	2,82461E-20	4,01934E-01	0,355	0,279	5,20208E-16
cDC(CD1C)	GRPEL1	1,51643E-37	4,01282E-01	0,293	0,206	2,79281E-33
cDC(CD1C)	CNDP2	6,61900E-60	4,01077E-01	0,581	0,44	1,21902E-55
cDC(CD1C)	NDUFB5	1,19993E-80	4,00122E-01	0,598	0,437	2,20991E-76
cDC(CD1C)	CFLAR	2,33255E-11	3,99972E-01	0,595	0,503	4,29586E-07
cDC(CD1C)	RPS20	3,47871E-11	3,98598E-01	0,498	0,446	6,40674E-07
cDC(CD1C)	EIF2S3	9,35949E-95	3,98131E-01	0,608	0,434	1,72374E-90
cDC(CD1C)	COX7C	1,69915E-21	3,98072E-01	0,918	0,819	3,12933E-17
cDC(CD1C)	PSMB1	1,44304E-60	3,97470E-01	0,776	0,613	2,65765E-56
cDC(CD1C)	IGBP1	6,14404E-99	3,97448E-01	0,488	0,328	1,13155E-94
cDC(CD1C)	VPS29	8,36145E-58	3,96161E-01	0,669	0,52	1,53993E-53
cDC(CD1C)	AK2	3,53410E-39	3,96008E-01	0,441	0,332	6,50875E-35
cDC(CD1C)	RAB24	1,35048E-77	3,95239E-01	0,394	0,256	2,48718E-73
cDC(CD1C)	PSMB6	7,85840E-37	3,94664E-01	0,697	0,562	1,44728E-32
cDC(CD1C)	YWHAB	7,59832E-42	3,94308E-01	0,891	0,776	1,39938E-37
cDC(CD1C)	HADHA	5,14185E-94	3,93585E-01	0,674	0,503	9,46975E-90
cDC(CD1C)	MRPL9	1,60533E-51	3,92362E-01	0,279	0,185	2,95654E-47
cDC(CD1C)	DDX54	2,58366E-49	3,91857E-01	0,266	0,175	4,75833E-45
cDC(CD1C)	PFDN5	3,79448E-55	3,91491E-01	0,965	0,895	6,98830E-51
cDC(CD1C)	RPL22	2,07403E-23	3,91077E-01	0,548	0,528	3,81973E-19
cDC(CD1C)	PMPCB	1,87722E-58	3,90960E-01	0,341	0,231	3,45728E-54

cDC(CD1C)	MRPS15	6,60507E-67	3,90904E-01	0,435	0,305	1,21646E-62
cDC(CD1C)	KLF4	2,73917E-66	3,90597E-01	0,574	0,433	5,04473E-62
cDC(CD1C)	STK17A	5,93004E-233	3,90565E-01	0,365	0,173	1,09214E-228
cDC(CD1C)	RAB31	4,06869E-117	3,89629E-01	0,8	0,625	7,49330E-113
cDC(CD1C)	SH3BP5	1,24143E-61	3,88196E-01	0,448	0,325	2,28634E-57
cDC(CD1C)	PSMC3	3,27735E-27	3,87707E-01	0,4	0,31	6,03589E-23
cDC(CD1C)	GTF3A	6,91067E-82	3,87648E-01	0,633	0,463	1,27274E-77
cDC(CD1C)	CMTM6	1,91514E-91	3,87578E-01	0,757	0,601	3,52711E-87
cDC(CD1C)	CPSF6	4,58252E-64	3,87472E-01	0,282	0,177	8,43963E-60
cDC(CD1C)	DYNC1LI1	2,00219E-74	3,87247E-01	0,333	0,212	3,68743E-70
cDC(CD1C)	CNOT8	1,21550E-85	3,85472E-01	0,329	0,201	2,23859E-81
cDC(CD1C)	COPZ1	1,49111E-45	3,85267E-01	0,492	0,37	2,74619E-41
cDC(CD1C)	YWHAE	5,10895E-55	3,84843E-01	0,71	0,566	9,40916E-51
cDC(CD1C)	SEC13	7,93703E-110	3,84080E-01	0,558	0,384	1,46176E-105
cDC(CD1C)	ERCC1	8,39570E-122	3,83751E-01	0,543	0,363	1,54624E-117
cDC(CD1C)	FKBP5	1,03729E-81	3,83647E-01	0,658	0,502	1,91038E-77
cDC(CD1C)	CUL1	1,27790E-38	3,82886E-01	0,294	0,21	2,35351E-34
cDC(CD1C)	RUNX3	1,26931E-121	3,82275E-01	0,296	0,163	2,33768E-117
cDC(CD1C)	FLII	1,73777E-23	3,82224E-01	0,403	0,315	3,20046E-19
cDC(CD1C)	CELF2	3,39073E-101	3,81860E-01	0,703	0,521	6,24471E-97
cDC(CD1C)	IL27RA	1,73730E-175	3,81438E-01	0,288	0,134	3,19959E-171
cDC(CD1C)	AKAP13	3,44018E-66	3,81318E-01	0,759	0,618	6,33578E-62
cDC(CD1C)	ARL6IP4	5,32126E-54	3,81298E-01	0,763	0,619	9,80017E-50
cDC(CD1C)	ARHGAP15	7,70577E-81	3,80857E-01	0,375	0,241	1,41917E-76
cDC(CD1C)	RAB34	1,79099E-33	3,80445E-01	0,355	0,263	3,29846E-29
cDC(CD1C)	CXCL16	1,76672E-72	3,80402E-01	0,795	0,635	3,25377E-68
cDC(CD1C)	ANXA5	3,39989E-09	3,79705E-01	0,88	0,761	6,26157E-05
cDC(CD1C)	PGAM1	3,35496E-36	3,79567E-01	0,681	0,552	6,17884E-32
cDC(CD1C)	RPL24	3,08941E-16	3,79455E-01	0,546	0,537	5,68976E-12
cDC(CD1C)	SLC31A2	3,85075E-19	3,79260E-01	0,535	0,432	7,09193E-15
cDC(CD1C)	DDT	7,63267E-47	3,78637E-01	0,555	0,427	1,40571E-42
cDC(CD1C)	TET2	2,70077E-49	3,78469E-01	0,338	0,229	4,97402E-45
cDC(CD1C)	SDHC	2,61245E-45	3,77815E-01	0,56	0,428	4,81134E-41
cDC(CD1C)	GPRIN3	5,98265E-216	3,77036E-01	0,42	0,216	1,10182E-211
cDC(CD1C)	RAN	3,33494E-82	3,76231E-01	0,808	0,64	6,14195E-78
cDC(CD1C)	PSMD4	2,35776E-44	3,76229E-01	0,524	0,399	4,34229E-40
cDC(CD1C)	MTMR14	2,46533E-88	3,75926E-01	0,444	0,295	4,54039E-84
cDC(CD1C)	OGFRL1	1,36430E-192	3,75890E-01	0,498	0,288	2,51264E-188
cDC(CD1C)	ZSWIM7	3,21571E-91	3,75778E-01	0,374	0,235	5,92238E-87
cDC(CD1C)	PSTPIP2	7,88027E-125	3,75720E-01	0,391	0,235	1,45131E-120

cDC(CD1C)	ATG3	1,06395E-70	3,75214E-01	0,664	0,507	1,95947E-66
cDC(CD1C)	CAMLG	6,59261E-80	3,74719E-01	0,418	0,278	1,21416E-75
cDC(CD1C)	LSM7	6,85060E-101	3,74241E-01	0,67	0,497	1,26168E-96
cDC(CD1C)	TIPARP	5,59317E-61	3,74190E-01	0,275	0,175	1,03009E-56
cDC(CD1C)	MFF	3,63047E-39	3,73677E-01	0,416	0,309	6,68623E-35
cDC(CD1C)	STRAP	2,21075E-91	3,72925E-01	0,499	0,339	4,07153E-87
cDC(CD1C)	ARID5A	2,05655E-43	3,72910E-01	0,383	0,277	3,78755E-39
cDC(CD1C)	LAMTOR2	4,66242E-62	3,72840E-01	0,673	0,528	8,58678E-58
cDC(CD1C)	SRP72	7,80983E-89	3,72245E-01	0,483	0,331	1,43834E-84
cDC(CD1C)	POLR2E	8,72297E-72	3,70434E-01	0,672	0,514	1,60651E-67
cDC(CD1C)	HPS1	2,43099E-73	3,69875E-01	0,478	0,337	4,47716E-69
cDC(CD1C)	ALCAM	3,47461E-102	3,68678E-01	0,402	0,252	6,39918E-98
cDC(CD1C)	AGPAT1	8,91864E-190	3,68612E-01	0,309	0,146	1,64255E-185
cDC(CD1C)	CARD9	6,07083E-143	3,68227E-01	0,321	0,17	1,11806E-138
cDC(CD1C)	GABARAPL1	4,24293E-08	3,67835E-01	0,333	0,287	7,81421E-04
cDC(CD1C)	P2RY13	5,28561E-204	3,66431E-01	0,364	0,177	9,73451E-200
cDC(CD1C)	GADD45GIP1	1,00172E-26	3,66293E-01	0,536	0,427	1,84488E-22
cDC(CD1C)	PPIF	7,50856E-07	3,66077E-01	0,41	0,352	1,38285E-02
cDC(CD1C)	SMARCE1	1,80996E-46	3,66038E-01	0,319	0,219	3,33340E-42
cDC(CD1C)	SNRPD1	3,84983E-64	3,65907E-01	0,518	0,377	7,09023E-60
cDC(CD1C)	TWF2	1,78504E-29	3,65705E-01	0,41	0,326	3,28751E-25
cDC(CD1C)	PKM	1,05501E-60	3,64951E-01	0,893	0,771	1,94301E-56
cDC(CD1C)	IFT20	8,54006E-124	3,64574E-01	0,37	0,215	1,57282E-119
cDC(CD1C)	FIBP	5,62439E-89	3,63385E-01	0,508	0,352	1,03584E-84
cDC(CD1C)	TPGS2	2,15658E-37	3,63011E-01	0,252	0,174	3,97177E-33
cDC(CD1C)	DBI	3,49056E-12	3,62480E-01	0,809	0,714	6,42857E-08
cDC(CD1C)	PSMA1	8,53580E-42	3,62429E-01	0,46	0,35	1,57204E-37
cDC(CD1C)	MRPL4	8,72854E-70	3,61927E-01	0,391	0,265	1,60754E-65
cDC(CD1C)	WDR61	1,37161E-76	3,61383E-01	0,33	0,209	2,52610E-72
cDC(CD1C)	TMEM256	4,85714E-84	3,61377E-01	0,553	0,395	8,94539E-80
cDC(CD1C)	CALM2	2,91156E-08	3,61267E-01	0,881	0,771	5,36221E-04
cDC(CD1C)	NDUFA8	7,83896E-33	3,61189E-01	0,377	0,281	1,44370E-28
cDC(CD1C)	CTNND1	3,17538E-125	3,60958E-01	0,417	0,256	5,84810E-121
cDC(CD1C)	POLR2G	2,28763E-112	3,60312E-01	0,524	0,355	4,21313E-108
cDC(CD1C)	GMFG	1,18391E-33	3,59616E-01	0,792	0,66	2,18041E-29
cDC(CD1C)	CCDC47	3,23642E-68	3,59280E-01	0,421	0,291	5,96052E-64
cDC(CD1C)	CYB5R3	4,13316E-88	3,59052E-01	0,555	0,394	7,61205E-84
cDC(CD1C)	SPOP	2,08419E-137	3,58932E-01	0,42	0,25	3,83845E-133
cDC(CD1C)	RFX5	1,59141E-50	3,58714E-01	0,254	0,165	2,93089E-46
cDC(CD1C)	DDX27	1,47551E-57	3,58590E-01	0,335	0,226	2,71746E-53

cDC(CD1C)	HNRNPH2	7,14221E-11	3,57532E-01	0,263	0,214	1,31538E-06
cDC(CD1C)	LSM2	5,79214E-87	3,57509E-01	0,488	0,335	1,06674E-82
cDC(CD1C)	FOXN2	2,77046E-98	3,54685E-01	0,393	0,247	5,10237E-94
cDC(CD1C)	NDUFB3	1,37604E-08	3,54579E-01	0,537	0,459	2,53425E-04
cDC(CD1C)	PRKAR1A	2,06373E-26	3,53491E-01	0,582	0,469	3,80078E-22
cDC(CD1C)	FDPS	2,38075E-43	3,53197E-01	0,32	0,225	4,38462E-39
cDC(CD1C)	RAP1B	5,09009E-28	3,51453E-01	0,684	0,567	9,37442E-24
cDC(CD1C)	YWHAQ	3,70247E-47	3,51331E-01	0,551	0,416	6,81884E-43
cDC(CD1C)	CD302	2,40873E-24	3,51113E-01	0,435	0,339	4,43616E-20
cDC(CD1C)	DNAJC4	7,41787E-156	3,51067E-01	0,542	0,342	1,36615E-151
cDC(CD1C)	MLX	2,56804E-56	3,50581E-01	0,476	0,345	4,72956E-52
cDC(CD1C)	NXT1	1,21765E-58	3,50503E-01	0,302	0,198	2,24255E-54
cDC(CD1C)	SLC25A39	4,76847E-63	3,49059E-01	0,536	0,394	8,78208E-59
cDC(CD1C)	CHD1	6,04970E-37	3,48867E-01	0,391	0,287	1,11417E-32
cDC(CD1C)	UQCRFS1	7,77309E-60	3,48742E-01	0,622	0,478	1,43157E-55
cDC(CD1C)	PTPN6	3,32631E-101	3,48457E-01	0,659	0,493	6,12606E-97
cDC(CD1C)	SRGAP1	4,62111E-92	3,48288E-01	0,271	0,159	8,51069E-88
cDC(CD1C)	IDH3G	1,47780E-50	3,48218E-01	0,471	0,347	2,72166E-46
cDC(CD1C)	FDFT1	6,58594E-143	3,47297E-01	0,414	0,243	1,21293E-138
cDC(CD1C)	SUB1	8,31737E-81	3,47082E-01	0,849	0,709	1,53181E-76
cDC(CD1C)	CSRNP1	1,44997E-38	3,46606E-01	0,384	0,281	2,67041E-34
cDC(CD1C)	ESD	1,50415E-53	3,46527E-01	0,479	0,351	2,77019E-49
cDC(CD1C)	SLBP	3,21048E-30	3,46362E-01	0,306	0,225	5,91274E-26
cDC(CD1C)	RHOF	1,72212E-234	3,46318E-01	0,326	0,139	3,17163E-230
cDC(CD1C)	TOMM22	7,71413E-115	3,46267E-01	0,589	0,405	1,42071E-110
cDC(CD1C)	CSNK1A1	2,50425E-34	3,46178E-01	0,571	0,45	4,61208E-30
cDC(CD1C)	ARPC3	2,49104E-104	3,45887E-01	0,964	0,884	4,58775E-100
cDC(CD1C)	SSR1	9,56803E-127	3,44697E-01	0,632	0,444	1,76214E-122
cDC(CD1C)	REL	7,45624E-104	3,43804E-01	0,756	0,581	1,37322E-99
cDC(CD1C)	SUCLG1	2,60082E-75	3,43607E-01	0,434	0,297	4,78992E-71
cDC(CD1C)	SNRPA	5,67887E-129	3,43425E-01	0,45	0,279	1,04588E-124
cDC(CD1C)	ATP1B1	6,22614E-60	3,43345E-01	0,317	0,21	1,14667E-55
cDC(CD1C)	NCOA4	4,90177E-37	3,42865E-01	0,651	0,517	9,02758E-33
cDC(CD1C)	UBE2N	3,64963E-67	3,42618E-01	0,499	0,36	6,72153E-63
cDC(CD1C)	DENND1B	8,39222E-153	3,42009E-01	0,341	0,179	1,54560E-148
cDC(CD1C)	LTB	6,09871E-211	3,41885E-01	0,295	0,129	1,12320E-206
cDC(CD1C)	R3HDM4	4,82382E-104	3,41816E-01	0,35	0,207	8,88404E-100
cDC(CD1C)	VPS51	7,13525E-61	3,41295E-01	0,31	0,202	1,31410E-56
cDC(CD1C)	PAIP2	4,26506E-25	3,40214E-01	0,614	0,5	7,85496E-21
cDC(CD1C)	CRIP1	1,19245E-34	3,39956E-01	0,381	0,283	2,19613E-30

cDC(CD1C)

cDC(CD1C)	FUOM	2,92763E-21	3,39896E-01	0,451	0,368	5,39182E-17
cDC(CD1C)	PSMD8	2,93560E-46	3,39660E-01	0,649	0,511	5,40649E-42
cDC(CD1C)	ARL14EP	1,18075E-97	3,39606E-01	0,274	0,154	2,17458E-93
cDC(CD1C)	RPS27A	1,78553E-12	3,38951E-01	0,561	0,577	3,28841E-08
cDC(CD1C)	ISOC2	2,61434E-38	3,37371E-01	0,295	0,209	4,81482E-34
cDC(CD1C)	RABGGTB	1,40227E-33	3,37204E-01	0,338	0,248	2,58256E-29
cDC(CD1C)	GSTK1	6,36193E-33	3,37174E-01	0,776	0,63	1,17168E-28
cDC(CD1C)	IDH3B	1,01346E-71	3,36356E-01	0,377	0,248	1,86649E-67
cDC(CD1C)	EIF2A	1,48279E-97	3,35513E-01	0,445	0,292	2,73085E-93
cDC(CD1C)	C19orf53	3,49737E-33	3,35448E-01	0,703	0,571	6,44111E-29
cDC(CD1C)	COX7A2L	3,08652E-114	3,35081E-01	0,66	0,471	5,68444E-110
cDC(CD1C)	ZC3H15	1,22561E-50	3,34939E-01	0,561	0,424	2,25721E-46
cDC(CD1C)	NDUFAB1	3,18416E-23	3,34907E-01	0,57	0,464	5,86426E-19
cDC(CD1C)	MRPL13	7,05875E-35	3,34618E-01	0,314	0,229	1,30001E-30
cDC(CD1C)	ACER3	6,90692E-32	3,34486E-01	0,313	0,229	1,27205E-27
cDC(CD1C)	DBNDD2	4,45799E-76	3,33559E-01	0,309	0,194	8,21029E-72
cDC(CD1C)	COA4	2,58260E-51	3,33550E-01	0,386	0,275	4,75638E-47
cDC(CD1C)	PPP1CA	1,40266E-64	3,32934E-01	0,751	0,607	2,58328E-60
cDC(CD1C)	SRGAP2	1,32315E-10	3,32927E-01	0,271	0,223	2,43684E-06
cDC(CD1C)	ADCY7	1,71320E-29	3,32526E-01	0,305	0,224	3,15521E-25
cDC(CD1C)	CREB5	8,99236E-82	3,32341E-01	0,254	0,144	1,65612E-77
cDC(CD1C)	PRMT1	7,84559E-105	3,32132E-01	0,451	0,29	1,44492E-100
cDC(CD1C)	LRRFIP1	2,77462E-45	3,31841E-01	0,792	0,671	5,11002E-41
cDC(CD1C)	AHSA1	5,03086E-48	3,31822E-01	0,397	0,288	9,26534E-44
cDC(CD1C)	RHOG	1,71813E-88	3,31481E-01	0,855	0,712	3,16428E-84
cDC(CD1C)	AKR1B1	1,23116E-53	3,31423E-01	0,612	0,452	2,26743E-49
cDC(CD1C)	PSMB4	1,76900E-14	3,31135E-01	0,443	0,365	3,25797E-10
cDC(CD1C)	MRPS24	1,00343E-43	3,30437E-01	0,256	0,168	1,84801E-39
cDC(CD1C)	UBE2K	1,83671E-42	3,30201E-01	0,378	0,275	3,38267E-38
cDC(CD1C)	TAOK3	2,01397E-48	3,30053E-01	0,572	0,438	3,70913E-44
cDC(CD1C)	FPR1	2,64004E-80	3,30017E-01	0,592	0,432	4,86216E-76
cDC(CD1C)	CPPED1	2,74633E-31	3,29707E-01	0,353	0,261	5,05791E-27
cDC(CD1C)	C1orf43	4,08841E-45	3,28300E-01	0,672	0,532	7,52963E-41
cDC(CD1C)	ILF3	2,72851E-56	3,28119E-01	0,488	0,352	5,02510E-52
cDC(CD1C)	GLUD1	1,65142E-65	3,28044E-01	0,447	0,314	3,04143E-61
cDC(CD1C)	NDUFB2	1,15627E-07	3,26035E-01	0,812	0,704	2,12951E-03
cDC(CD1C)	SERBP1	1,05303E-109	3,25626E-01	0,758	0,576	1,93936E-105
cDC(CD1C)	TBCA	3,08459E-21	3,25225E-01	0,692	0,577	5,68090E-17
cDC(CD1C)	ELOF1	3,44503E-20	3,24926E-01	0,274	0,21	6,34471E-16
cDC(CD1C)	PQBP1	8,15225E-114	3,24316E-01	0,465	0,297	1,50140E-109

cDC(CD1C)	PTMA	2,62197E-124	3,23785E-01	0,991	0,959	4,82888E-120
cDC(CD1C)	ZNF655	7,86156E-12	3,23117E-01	0,262	0,212	1,44786E-07
cDC(CD1C)	PHB	1,21377E-60	3,22921E-01	0,517	0,374	2,23540E-56
cDC(CD1C)	CD53	1,56279E-27	3,22639E-01	0,809	0,664	2,87820E-23
cDC(CD1C)	SMIM14	3,25920E-73	3,22265E-01	0,383	0,256	6,00247E-69
cDC(CD1C)	PTPRC	3,05654E-95	3,21470E-01	0,828	0,674	5,62923E-91
cDC(CD1C)	TEX264	7,85139E-24	3,21235E-01	0,382	0,299	1,44599E-19
cDC(CD1C)	SNRPD3	3,56342E-47	3,20973E-01	0,474	0,358	6,56276E-43
cDC(CD1C)	MT-CO2	3,64048E-10	3,20502E-01	0,562	0,6	6,70468E-06
cDC(CD1C)	PDE4A	2,44382E-63	3,20317E-01	0,268	0,164	4,50079E-59
cDC(CD1C)	PYURF	3,91997E-91	3,20142E-01	0,557	0,394	7,21940E-87
cDC(CD1C)	RASSF4	4,44946E-18	3,20129E-01	0,611	0,495	8,19457E-14
cDC(CD1C)	GCA	2,25324E-91	3,19844E-01	0,552	0,385	4,14980E-87
cDC(CD1C)	WDR83OS	4,04778E-34	3,19794E-01	0,735	0,608	7,45480E-30
cDC(CD1C)	SNRPF	6,44829E-118	3,19569E-01	0,552	0,375	1,18758E-113
cDC(CD1C)	BIN1	9,60591E-172	3,18638E-01	0,338	0,173	1,76912E-167
cDC(CD1C)	SMAP2	9,50289E-83	3,18584E-01	0,62	0,454	1,75015E-78
cDC(CD1C)	HMGB1	2,49025E-102	3,18428E-01	0,91	0,78	4,58630E-98
cDC(CD1C)	UCHL3	2,38776E-61	3,18014E-01	0,316	0,208	4,39753E-57
cDC(CD1C)	SLC12A9	3,97233E-155	3,17166E-01	0,328	0,169	7,31585E-151
cDC(CD1C)	SNRPD2	1,99435E-63	3,17106E-01	0,805	0,663	3,67300E-59
cDC(CD1C)	SSBP1	7,45983E-60	3,16447E-01	0,624	0,471	1,37388E-55
cDC(CD1C)	HAX1	4,46345E-46	3,16442E-01	0,452	0,334	8,22033E-42
cDC(CD1C)	LGALS9	1,47415E-08	3,16352E-01	0,632	0,538	2,71494E-04
cDC(CD1C)	CCDC88A	1,16530E-86	3,16304E-01	0,653	0,485	2,14614E-82
cDC(CD1C)	SLC50A1	3,29548E-34	3,16241E-01	0,291	0,209	6,06928E-30
cDC(CD1C)	HPS5	2,65704E-96	3,16060E-01	0,32	0,19	4,89347E-92
cDC(CD1C)	GHITM	1,26016E-60	3,15768E-01	0,64	0,489	2,32084E-56
cDC(CD1C)	COPS7A	1,53214E-28	3,15620E-01	0,282	0,207	2,82175E-24
cDC(CD1C)	APRT	1,28735E-161	3,14228E-01	0,796	0,604	2,37091E-157
cDC(CD1C)	LYPLA1	7,24643E-73	3,14115E-01	0,452	0,313	1,33457E-68
cDC(CD1C)	ANP32B	3,31306E-145	3,13809E-01	0,75	0,552	6,10167E-141
cDC(CD1C)	HSPA9	6,01471E-29	3,13534E-01	0,458	0,36	1,10773E-24
cDC(CD1C)	GPX1	7,15773E-27	3,13085E-01	0,616	0,542	1,31824E-22
cDC(CD1C)	FIS1	2,50635E-37	3,12943E-01	0,649	0,512	4,61595E-33
cDC(CD1C)	MRPL24	3,17218E-22	3,12770E-01	0,283	0,217	5,84221E-18
cDC(CD1C)	TOR2A	3,09557E-66	3,12083E-01	0,3	0,191	5,70112E-62
cDC(CD1C)	GK	1,21319E-07	3,11520E-01	0,425	0,358	2,23433E-03
cDC(CD1C)	GAPDH	4,07594E-123	3,11461E-01	0,992	0,956	7,50666E-119
cDC(CD1C)	DAZAP2	1,16032E-50	3,11385E-01	0,85	0,73	2,13696E-46

cDC(CD1C)	ECHS1	1,02109E-80	3,10832E-01	0,508	0,354	1,88054E-76
cDC(CD1C)	AP2S1	4,42845E-38	3,10570E-01	0,837	0,706	8,15587E-34
cDC(CD1C)	CEBPD	1,75866E-90	3,10524E-01	0,718	0,551	3,23893E-86
cDC(CD1C)	PFDN2	4,89112E-37	3,10403E-01	0,551	0,43	9,00797E-33
cDC(CD1C)	SIPA1	2,57907E-40	3,10379E-01	0,289	0,199	4,74988E-36
cDC(CD1C)	HNRNPDL	1,19530E-59	3,10229E-01	0,805	0,66	2,20139E-55
cDC(CD1C)	HNRNPC	1,33863E-64	3,09827E-01	0,776	0,633	2,46536E-60
cDC(CD1C)	MBNL1	1,88172E-45	3,09451E-01	0,636	0,512	3,46556E-41
cDC(CD1C)	UXT	1,04203E-87	3,08871E-01	0,687	0,525	1,91911E-83
cDC(CD1C)	EWSR1	2,21923E-75	3,07649E-01	0,603	0,447	4,08716E-71
cDC(CD1C)	MPV17	9,06400E-43	3,06776E-01	0,404	0,299	1,66932E-38
cDC(CD1C)	MPC1	5,19402E-24	3,06396E-01	0,394	0,308	9,56583E-20
cDC(CD1C)	HADHB	5,11750E-38	3,05662E-01	0,462	0,349	9,42489E-34
cDC(CD1C)	EIF3D	4,78475E-98	3,05660E-01	0,629	0,456	8,81207E-94
cDC(CD1C)	ANXA6	2,69913E-32	3,05580E-01	0,304	0,216	4,97098E-28
cDC(CD1C)	MRPL21	2,41915E-26	3,05094E-01	0,337	0,258	4,45536E-22
cDC(CD1C)	AIG1	7,69518E-67	3,04485E-01	0,275	0,172	1,41722E-62
cDC(CD1C)	CLDND1	2,94514E-58	3,04470E-01	0,272	0,173	5,42407E-54
cDC(CD1C)	CKS2	2,16117E-12	3,03633E-01	0,291	0,238	3,98023E-08
cDC(CD1C)	MYL6	7,84231E-24	3,03136E-01	0,971	0,938	1,44432E-19
cDC(CD1C)	CSRP1	4,71649E-67	3,03117E-01	0,37	0,245	8,68637E-63
cDC(CD1C)	SH3BGRL	2,75277E-88	3,03008E-01	0,866	0,703	5,06977E-84
cDC(CD1C)	ZFAND1	9,51881E-92	3,02962E-01	0,262	0,148	1,75308E-87
cDC(CD1C)	SRA1	7,75766E-24	3,02798E-01	0,474	0,376	1,42873E-19
cDC(CD1C)	CAMTA1	2,18182E-41	3,02633E-01	0,466	0,353	4,01826E-37
cDC(CD1C)	PFDN4	7,26546E-64	3,02268E-01	0,312	0,202	1,33808E-59
cDC(CD1C)	CLTB	3,55293E-142	3,02175E-01	0,524	0,342	6,54343E-138
cDC(CD1C)	APPL1	1,10665E-78	3,02107E-01	0,494	0,347	2,03811E-74
cDC(CD1C)	ARPC4	3,63218E-07	3,02029E-01	0,451	0,405	6,68939E-03
cDC(CD1C)	SDHB	3,87054E-11	3,01697E-01	0,471	0,391	7,12837E-07
cDC(CD1C)	PARL	1,31012E-68	3,01167E-01	0,404	0,277	2,41285E-64
cDC(CD1C)	PRR13	3,60967E-09	3,01051E-01	0,639	0,574	6,64793E-05
cDC(CD1C)	PSMA7	1,11470E-12	3,01025E-01	0,849	0,734	2,05294E-08
cDC(CD1C)	MCM5	1,04812E-114	3,00832E-01	0,356	0,208	1,93032E-110
cDC(CD1C)	ANKRD44	6,03972E-132	3,00219E-01	0,449	0,274	1,11233E-127
cDC(CD1C)	HNRNPA0	4,44195E-177	3,00155E-01	0,705	0,488	8,18074E-173
cDC(CD1C)	CCT8	7,35017E-92	3,00045E-01	0,566	0,395	1,35368E-87
cDC(CD1C)	TBC1D22A	1,16148E-109	2,99620E-01	0,359	0,214	2,13909E-105
cDC(CD1C)	NUBP1	1,87673E-44	2,98666E-01	0,321	0,224	3,45638E-40
cDC(CD1C)	C14orf119	1,46339E-22	2,98648E-01	0,361	0,284	2,69512E-18

cDC(CD1C)	CCDC124	1,38579E-33	2,98577E-01	0,448	0,342	2,55221E-29
cDC(CD1C)	NABP1	2,82371E-07	2,98550E-01	0,393	0,333	5,20042E-03
cDC(CD1C)	DNAJC8	6,58983E-19	2,98477E-01	0,494	0,404	1,21365E-14
cDC(CD1C)	PRDX5	2,39197E-15	2,97786E-01	0,713	0,588	4,40530E-11
cDC(CD1C)	VRK2	2,51925E-28	2,97783E-01	0,251	0,182	4,63970E-24
cDC(CD1C)	FKBP3	4,02569E-24	2,97694E-01	0,3	0,228	7,41411E-20
cDC(CD1C)	COX5A	8,06155E-115	2,97654E-01	0,854	0,676	1,48470E-110
cDC(CD1C)	CNOT7	1,10936E-67	2,97559E-01	0,425	0,295	2,04310E-63
cDC(CD1C)	ARMCX6	6,22259E-43	2,97249E-01	0,273	0,184	1,14601E-38
cDC(CD1C)	HNRNPA2B1	1,69430E-66	2,97061E-01	0,922	0,822	3,12039E-62
cDC(CD1C)	NQO2	2,93604E-80	2,96869E-01	0,378	0,248	5,40731E-76
cDC(CD1C)	NCOR1	1,03639E-53	2,96680E-01	0,519	0,385	1,90871E-49
cDC(CD1C)	CMTM3	2,87267E-63	2,96055E-01	0,487	0,348	5,29059E-59
cDC(CD1C)	CAMK1D	4,28566E-194	2,95148E-01	0,389	0,201	7,89289E-190
cDC(CD1C)	MRPS26	1,43612E-43	2,94867E-01	0,292	0,201	2,64491E-39
cDC(CD1C)	LIMS1	2,71849E-39	2,94380E-01	0,68	0,541	5,00665E-35
cDC(CD1C)	HNRNPH1	3,32458E-08	2,94184E-01	0,464	0,398	6,12288E-04
cDC(CD1C)	DAP3	1,40160E-56	2,93642E-01	0,427	0,305	2,58133E-52
cDC(CD1C)	SF3A1	1,28320E-56	2,93231E-01	0,397	0,277	2,36327E-52
cDC(CD1C)	NDUFA6	3,30089E-24	2,93142E-01	0,632	0,516	6,07924E-20
cDC(CD1C)	DBNL	6,49579E-81	2,92757E-01	0,637	0,476	1,19633E-76
cDC(CD1C)	DUSP22	6,01743E-86	2,92714E-01	0,34	0,209	1,10823E-81
cDC(CD1C)	APAF1	4,66012E-145	2,92631E-01	0,281	0,137	8,58254E-141
cDC(CD1C)	MMADHC	2,39307E-47	2,91799E-01	0,455	0,336	4,40731E-43
cDC(CD1C)	DERA	4,65078E-39	2,91681E-01	0,27	0,188	8,56534E-35
cDC(CD1C)	TMA7	1,10241E-25	2,91059E-01	0,875	0,784	2,03032E-21
cDC(CD1C)	RNF166	1,33355E-97	2,90995E-01	0,372	0,229	2,45599E-93
cDC(CD1C)	NEDD8	1,43489E-11	2,90967E-01	0,678	0,576	2,64264E-07
cDC(CD1C)	RARA	2,07529E-80	2,90799E-01	0,31	0,186	3,82207E-76
cDC(CD1C)	PTEN	1,30226E-37	2,89847E-01	0,348	0,252	2,39837E-33
cDC(CD1C)	S100A10	9,84532E-26	2,89828E-01	0,917	0,829	1,81321E-21
cDC(CD1C)	GUSB	4,60329E-13	2,89310E-01	0,408	0,339	8,47788E-09
cDC(CD1C)	SHMT2	4,19686E-73	2,89255E-01	0,313	0,198	7,72935E-69
cDC(CD1C)	EIF4E2	1,36229E-63	2,88857E-01	0,496	0,357	2,50892E-59
cDC(CD1C)	KCNMB1	1,98877E-154	2,88819E-01	0,303	0,152	3,66272E-150
cDC(CD1C)	APEH	3,87289E-27	2,88604E-01	0,261	0,191	7,13270E-23
cDC(CD1C)	NAGA	5,55442E-55	2,88076E-01	0,476	0,348	1,02296E-50
cDC(CD1C)	SNRPA1	1,78456E-44	2,85313E-01	0,288	0,197	3,28663E-40
cDC(CD1C)	SNX17	5,95816E-87	2,85295E-01	0,577	0,416	1,09731E-82
cDC(CD1C)	BANF1	1,86326E-87	2,85264E-01	0,706	0,53	3,43157E-83

cDC(CD1C)	GPSM3	3,27606E-133	2,85185E-01	0,838	0,666	6,03351E-129
cDC(CD1C)	WAS	6,28043E-152	2,85157E-01	0,718	0,508	1,15667E-147
cDC(CD1C)	PCMT1	1,56210E-25	2,84871E-01	0,421	0,329	2,87692E-21
cDC(CD1C)	MPC2	2,03241E-138	2,84694E-01	0,532	0,34	3,74308E-134
cDC(CD1C)	TRPV2	3,92217E-23	2,84386E-01	0,269	0,203	7,22346E-19
cDC(CD1C)	BAIAP2	5,20976E-172	2,84275E-01	0,271	0,124	9,59482E-168
cDC(CD1C)	ASCC2	4,36556E-44	2,84078E-01	0,295	0,202	8,04005E-40
cDC(CD1C)	IFITM3	6,78779E-11	2,83665E-01	0,848	0,755	1,25011E-06
cDC(CD1C)	SDHD	3,88963E-20	2,83377E-01	0,415	0,336	7,16352E-16
cDC(CD1C)	HAUS4	5,64806E-126	2,83073E-01	0,358	0,205	1,04020E-121
cDC(CD1C)	FHL3	2,60868E-222	2,82886E-01	0,383	0,185	4,80441E-218
cDC(CD1C)	GSDMD	2,10688E-08	2,82545E-01	0,349	0,295	3,88025E-04
cDC(CD1C)	JAGN1	1,66642E-34	2,82502E-01	0,282	0,203	3,06905E-30
cDC(CD1C)	DOCK10	1,25454E-44	2,81710E-01	0,421	0,311	2,31049E-40
cDC(CD1C)	ACO2	3,58291E-41	2,81371E-01	0,36	0,26	6,59864E-37
cDC(CD1C)	VBP1	2,60603E-78	2,81318E-01	0,312	0,193	4,79953E-74
cDC(CD1C)	GLS	2,13523E-14	2,81281E-01	0,253	0,199	3,93246E-10
cDC(CD1C)	MTDH	4,96500E-117	2,81212E-01	0,838	0,678	9,14404E-113
cDC(CD1C)	HNRNPH3	6,65185E-38	2,81141E-01	0,448	0,341	1,22507E-33
cDC(CD1C)	MAF1	1,12411E-81	2,81084E-01	0,529	0,373	2,07028E-77
cDC(CD1C)	CSK	6,70349E-73	2,80668E-01	0,472	0,333	1,23458E-68
cDC(CD1C)	CLEC5A	3,64642E-108	2,80196E-01	0,316	0,176	6,71562E-104
cDC(CD1C)	COMMD6	1,00736E-195	2,80191E-01	0,88	0,719	1,85526E-191
cDC(CD1C)	ATXN10	1,67511E-132	2,80090E-01	0,432	0,259	3,08506E-128
cDC(CD1C)	MDH2	7,69969E-111	2,79614E-01	0,687	0,503	1,41805E-106
cDC(CD1C)	FBP1	1,96613E-32	2,79529E-01	0,449	0,338	3,62102E-28
cDC(CD1C)	SNAPIN	7,79187E-23	2,79364E-01	0,358	0,28	1,43503E-18
cDC(CD1C)	TRIM69	7,58223E-08	2,79141E-01	0,25	0,213	1,39642E-03
cDC(CD1C)	CHCHD2	3,17019E-24	2,78992E-01	0,907	0,805	5,83853E-20
cDC(CD1C)	CLEC7A	8,50792E-59	2,78536E-01	0,674	0,507	1,56690E-54
cDC(CD1C)	HNRNPF	2,37382E-112	2,78378E-01	0,727	0,547	4,37186E-108
cDC(CD1C)	ZCRB1	1,62088E-56	2,77532E-01	0,375	0,261	2,98517E-52
cDC(CD1C)	AFF4	6,18363E-18	2,77329E-01	0,456	0,368	1,13884E-13
cDC(CD1C)	RBM42	1,45692E-52	2,77322E-01	0,402	0,285	2,68322E-48
cDC(CD1C)	UBXN1	2,46770E-96	2,77271E-01	0,789	0,61	4,54476E-92
cDC(CD1C)	TRAPPC1	3,16303E-58	2,77264E-01	0,737	0,595	5,82535E-54
cDC(CD1C)	GTF2B	1,84928E-45	2,76623E-01	0,374	0,268	3,40583E-41
cDC(CD1C)	BCCIP	1,17630E-52	2,76618E-01	0,3	0,2	2,16640E-48
cDC(CD1C)	MRPL52	8,52058E-39	2,76390E-01	0,569	0,447	1,56923E-34
cDC(CD1C)	PRDX2	9,52620E-44	2,75600E-01	0,278	0,188	1,75444E-39

cDC(CD1C)	PSMD6	2,48510E-29	2,75533E-01	0,357	0,27	4,57681E-25
cDC(CD1C)	NONO	2,94712E-62	2,75462E-01	0,562	0,416	5,42772E-58
cDC(CD1C)	TOR1AIP1	8,27428E-21	2,75204E-01	0,261	0,197	1,52387E-16
cDC(CD1C)	CTNNA1	3,10915E-13	2,74846E-01	0,427	0,356	5,72612E-09
cDC(CD1C)	PRKDC	8,35028E-58	2,74495E-01	0,357	0,242	1,53787E-53
cDC(CD1C)	EIF4EBP1	2,15864E-33	2,74446E-01	0,513	0,398	3,97557E-29
cDC(CD1C)	BCKDK	1,07227E-20	2,74418E-01	0,393	0,311	1,97480E-16
cDC(CD1C)	NSA2	1,20663E-89	2,74143E-01	0,641	0,469	2,22225E-85
cDC(CD1C)	BEX4	1,10626E-37	2,73488E-01	0,425	0,318	2,03740E-33
cDC(CD1C)	MZT2A	1,15611E-151	2,73415E-01	0,382	0,213	2,12920E-147
cDC(CD1C)	DNAJA2	6,36756E-59	2,73371E-01	0,427	0,304	1,17271E-54
cDC(CD1C)	PRPF38B	1,29497E-88	2,73348E-01	0,586	0,419	2,38496E-84
cDC(CD1C)	LAP3	1,21631E-21	2,73226E-01	0,708	0,571	2,24007E-17
cDC(CD1C)	OTUB1	1,67860E-35	2,73009E-01	0,535	0,418	3,09147E-31
cDC(CD1C)	BLOC1S2	1,09572E-09	2,72800E-01	0,386	0,324	2,01798E-05
cDC(CD1C)	NANS	3,81901E-24	2,72685E-01	0,476	0,38	7,03348E-20
cDC(CD1C)	AAMP	1,13778E-45	2,72513E-01	0,39	0,282	2,09546E-41
cDC(CD1C)	IL16	1,62446E-114	2,72512E-01	0,353	0,206	2,99176E-110
cDC(CD1C)	PSMD14	1,76584E-24	2,72409E-01	0,281	0,211	3,25214E-20
cDC(CD1C)	GPI	1,15227E-42	2,72403E-01	0,527	0,403	2,12213E-38
cDC(CD1C)	GNA13	3,06781E-08	2,72138E-01	0,382	0,329	5,64998E-04
cDC(CD1C)	DNAJC15	4,61548E-52	2,71685E-01	0,627	0,485	8,50032E-48
cDC(CD1C)	OSTC	4,53253E-153	2,71269E-01	0,688	0,478	8,34756E-149
cDC(CD1C)	KPNA2	7,32014E-44	2,71213E-01	0,288	0,2	1,34815E-39
cDC(CD1C)	STIP1	1,18606E-31	2,70857E-01	0,362	0,272	2,18437E-27
cDC(CD1C)	HERPUD1	3,83373E-277	2,70672E-01	0,854	0,662	7,06058E-273
cDC(CD1C)	CALM1	1,71656E-07	2,70661E-01	0,877	0,786	3,16138E-03
cDC(CD1C)	DCTN3	6,81771E-30	2,70331E-01	0,452	0,35	1,25562E-25
cDC(CD1C)	HMGN3	1,96744E-43	2,70103E-01	0,65	0,509	3,62343E-39
cDC(CD1C)	OAZ1	7,80352E-14	2,70019E-01	0,977	0,932	1,43717E-09
cDC(CD1C)	UBE2D3	4,55531E-21	2,69957E-01	0,831	0,726	8,38952E-17
cDC(CD1C)	RAB32	4,29094E-101	2,69941E-01	0,523	0,358	7,90262E-97
cDC(CD1C)	SIL1	2,19259E-06	2,69873E-01	0,271	0,234	4,03809E-02
cDC(CD1C)	SNRNP40	1,17026E-71	2,69863E-01	0,303	0,189	2,15526E-67
cDC(CD1C)	ALOX5	2,84533E-162	2,69639E-01	0,672	0,452	5,24025E-158
cDC(CD1C)	SNAP23	5,40312E-68	2,69527E-01	0,532	0,385	9,95093E-64
cDC(CD1C)	CTNNBL1	7,50263E-12	2,69463E-01	0,277	0,225	1,38176E-07
cDC(CD1C)	TMEM14B	2,60694E-67	2,68883E-01	0,552	0,408	4,80120E-63
cDC(CD1C)	PHF5A	9,42088E-63	2,68499E-01	0,365	0,246	1,73504E-58
cDC(CD1C)	ARHGAP30	7,47729E-43	2,68339E-01	0,435	0,32	1,37709E-38

cDC(CD1C)	IGFLR1	4,46552E-80	2,67600E-01	0,49	0,345	8,22414E-76
cDC(CD1C)	PRPF38A	2,69540E-54	2,67167E-01	0,275	0,178	4,96412E-50
cDC(CD1C)	RBM8A	6,96570E-37	2,67149E-01	0,647	0,521	1,28287E-32
cDC(CD1C)	CS	2,32544E-27	2,66408E-01	0,3	0,223	4,28277E-23
cDC(CD1C)	SPAG9	1,05655E-19	2,65735E-01	0,375	0,296	1,94584E-15
cDC(CD1C)	COX14	6,05535E-12	2,65688E-01	0,544	0,453	1,11521E-07
cDC(CD1C)	EIF4B	2,38556E-115	2,65485E-01	0,686	0,498	4,39348E-111
cDC(CD1C)	ERGIC2	1,65914E-26	2,65375E-01	0,281	0,209	3,05563E-22
cDC(CD1C)	SNRPG	8,48494E-16	2,64953E-01	0,642	0,547	1,56267E-11
cDC(CD1C)	SRSF10	4,66286E-22	2,63979E-01	0,352	0,271	8,58759E-18
cDC(CD1C)	ARL6IP5	2,32786E-84	2,63603E-01	0,807	0,638	4,28722E-80
cDC(CD1C)	CASP4	7,06159E-19	2,63557E-01	0,542	0,432	1,30053E-14
cDC(CD1C)	SKIL	1,46611E-56	2,63499E-01	0,442	0,319	2,70014E-52
cDC(CD1C)	ATXN2L	6,35352E-23	2,62801E-01	0,299	0,223	1,17013E-18
cDC(CD1C)	AIP	7,67104E-93	2,62633E-01	0,482	0,324	1,41278E-88
cDC(CD1C)	IAH1	9,61403E-56	2,62429E-01	0,419	0,297	1,77062E-51
cDC(CD1C)	FGD4	3,13423E-41	2,62256E-01	0,312	0,22	5,77230E-37
cDC(CD1C)	BHLHE40	1,15876E-53	2,62052E-01	0,45	0,328	2,13408E-49
cDC(CD1C)	GLO1	5,45534E-150	2,61967E-01	0,394	0,226	1,00471E-145
cDC(CD1C)	EDF1	1,23314E-49	2,61947E-01	0,868	0,736	2,27108E-45
cDC(CD1C)	OTULINL	2,68160E-113	2,61377E-01	0,277	0,152	4,93870E-109
cDC(CD1C)	SRSF1	8,45480E-53	2,61265E-01	0,371	0,257	1,55712E-48
cDC(CD1C)	ANAPC15	3,34694E-34	2,60808E-01	0,277	0,195	6,16407E-30
cDC(CD1C)	SUMF1	8,45130E-39	2,60800E-01	0,257	0,178	1,55648E-34
cDC(CD1C)	RACK1	2,12334E-14	2,60649E-01	0,47	0,467	3,91055E-10
cDC(CD1C)	UBL3	1,67517E-105	2,60405E-01	0,376	0,23	3,08516E-101
cDC(CD1C)	FXYD5	4,31214E-67	2,60385E-01	0,922	0,814	7,94168E-63
cDC(CD1C)	URM1	1,49374E-59	2,60044E-01	0,347	0,236	2,75103E-55
cDC(CD1C)	LEPROTL1	6,96657E-30	2,59904E-01	0,494	0,388	1,28303E-25
cDC(CD1C)	MDH1	1,90064E-24	2,59528E-01	0,477	0,375	3,50041E-20
cDC(CD1C)	THOC7	6,83221E-73	2,59440E-01	0,468	0,324	1,25829E-68
cDC(CD1C)	VASH1	8,26685E-85	2,59297E-01	0,313	0,191	1,52251E-80
cDC(CD1C)	COMMD5	1,32604E-18	2,59178E-01	0,25	0,192	2,44217E-14
cDC(CD1C)	ARIH2	4,59802E-19	2,58751E-01	0,321	0,251	8,46817E-15
cDC(CD1C)	PSMC1	8,71645E-26	2,58678E-01	0,411	0,321	1,60531E-21
cDC(CD1C)	RILPL2	1,16097E-16	2,58332E-01	0,537	0,447	2,13815E-12
cDC(CD1C)	BID	1,50741E-16	2,58163E-01	0,486	0,406	2,77621E-12
cDC(CD1C)	NUP98	9,76164E-29	2,58042E-01	0,278	0,202	1,79780E-24
cDC(CD1C)	COX6B1	1,18369E-06	2,57821E-01	0,892	0,802	2,17999E-02
cDC(CD1C)	UBE2V2	5,07828E-59	2,57305E-01	0,361	0,247	9,35266E-55

cDC(CD1C)	CCDC6	1,90005E-95	2,57213E-01	0,269	0,151	3,49933E-91
cDC(CD1C)	DRAM2	4,34838E-16	2,56438E-01	0,468	0,381	8,00842E-12
cDC(CD1C)	PACSIN2	4,00779E-79	2,56391E-01	0,389	0,256	7,38115E-75
cDC(CD1C)	SPI1	2,05642E-97	2,56191E-01	0,892	0,753	3,78730E-93
cDC(CD1C)	ATRAID	2,09753E-24	2,55948E-01	0,564	0,455	3,86303E-20
cDC(CD1C)	COMMD9	4,24935E-32	2,55923E-01	0,402	0,304	7,82602E-28
cDC(CD1C)	EMC4	6,46406E-19	2,55369E-01	0,44	0,352	1,19049E-14
cDC(CD1C)	UQCRB	1,44225E-20	2,55030E-01	0,869	0,762	2,65620E-16
cDC(CD1C)	PPP2R1A	2,39374E-43	2,54698E-01	0,499	0,385	4,40855E-39
cDC(CD1C)	PHPT1	3,40606E-32	2,53876E-01	0,549	0,435	6,27293E-28
cDC(CD1C)	GNG5	3,41871E-07	2,53793E-01	0,865	0,785	6,29623E-03
cDC(CD1C)	SUMO3	1,46022E-69	2,53730E-01	0,617	0,466	2,68929E-65
cDC(CD1C)	VOPP1	5,58519E-49	2,53645E-01	0,46	0,334	1,02862E-44
cDC(CD1C)	ROCK1	9,36627E-102	2,52736E-01	0,67	0,493	1,72499E-97
cDC(CD1C)	UBE2L3	2,77219E-27	2,52520E-01	0,608	0,49	5,10554E-23
cDC(CD1C)	MAP2K1	3,39276E-128	2,52519E-01	0,48	0,304	6,24845E-124
cDC(CD1C)	MRPL18	6,03201E-44	2,52493E-01	0,454	0,337	1,11092E-39
cDC(CD1C)	CIAO1	2,51114E-23	2,52273E-01	0,313	0,239	4,62476E-19
cDC(CD1C)	PICALM	4,78422E-90	2,52271E-01	0,575	0,412	8,81110E-86
cDC(CD1C)	SDCCAG8	3,23942E-20	2,51766E-01	0,411	0,329	5,96605E-16
cDC(CD1C)	ARHGEF2	3,28074E-09	2,51416E-01	0,295	0,244	6,04213E-05
cDC(CD1C)	POLR1D	1,00580E-96	2,51263E-01	0,629	0,45	1,85238E-92
cDC(CD1C)	TBCB	1,45659E-53	2,51229E-01	0,593	0,454	2,68259E-49
cDC(CD1C)	TKT	3,25862E-43	2,51102E-01	0,745	0,592	6,00141E-39
cDC(CD1C)	ARHGAP31	1,02654E-89	2,50741E-01	0,322	0,196	1,89058E-85
cDC(CD1C)	SLC25A11	4,84883E-30	2,50605E-01	0,387	0,295	8,93010E-26
cDC(CD1C)	PSMA6	1,06268E-15	2,50316E-01	0,544	0,457	1,95715E-11
cDC(CD1C)	IMP4	7,67069E-71	2,50281E-01	0,379	0,253	1,41271E-66
cDC(CD1C)	HSPD1	1,77425E-103	2,50271E-01	0,701	0,516	3,26763E-99
cDC(CD1C)	UBE2J2	1,35096E-45	2,50071E-01	0,286	0,191	2,48807E-41
cDC(CD1C)	FCHSD2	3,68899E-23	-2,50912E-01	0,294	0,217	6,79401E-19
cDC(CD1C)	ZEB2	4,88129E-49	-2,53859E-01	0,575	0,609	8,98988E-45
cDC(CD1C)	BLVRB	1,37650E-41	-2,54728E-01	0,461	0,494	2,53511E-37
cDC(CD1C)	SEC62	2,97707E-17	-2,55412E-01	0,56	0,554	5,48286E-13
cDC(CD1C)	ICAM1	6,93004E-37	-2,63874E-01	0,3	0,369	1,27631E-32
cDC(CD1C)	ISCU	5,23477E-15	-2,64150E-01	0,556	0,535	9,64087E-11
cDC(CD1C)	PLD4	0,00000E+00	-2,67532E-01	0,673	0,293	0,00000E+00
cDC(CD1C)	CPM	3,14996E-13	-2,71975E-01	0,355	0,371	5,80128E-09
cDC(CD1C)	GM2A	2,86011E-23	-2,75176E-01	0,345	0,378	5,26746E-19
cDC(CD1C)	LAMP2	2,88143E-34	-2,78526E-01	0,356	0,407	5,30673E-30

cDC(CD1C)	LYN	2,87883E-08	-2,80097E-01	0,455	0,452	5,30194E-04
cDC(CD1C)	MORF4L2	1,75144E-06	-2,83174E-01	0,314	0,27	3,22562E-02
cDC(CD1C)	DICER1	5,89442E-17	-2,84786E-01	0,232	0,274	1,08557E-12
cDC(CD1C)	TOM1	5,72786E-17	-2,86056E-01	0,248	0,288	1,05490E-12
cDC(CD1C)	HNMT	1,15331E-40	-2,88630E-01	0,323	0,387	2,12406E-36
cDC(CD1C)	SNX6	2,63215E-17	-2,90667E-01	0,522	0,515	4,84763E-13
cDC(CD1C)	RAC2	4,70105E-14	-2,91331E-01	0,595	0,485	8,65793E-10
cDC(CD1C)	HPCAL1	9,82422E-15	-2,91730E-01	0,213	0,25	1,80933E-10
cDC(CD1C)	TSPAN3	1,00480E-36	-2,95800E-01	0,408	0,308	1,85053E-32
cDC(CD1C)	GIMAP1	5,43130E-29	-2,96011E-01	0,206	0,273	1,00028E-24
cDC(CD1C)	OLR1	3,33458E-27	-2,98875E-01	0,298	0,353	6,14130E-23
cDC(CD1C)	CD69	5,78856E-24	-2,99905E-01	0,286	0,209	1,06608E-19
cDC(CD1C)	LTC4S	7,14978E-39	-3,03051E-01	0,385	0,272	1,31678E-34
cDC(CD1C)	HLA-A	3,01271E-58	-3,05204E-01	0,975	0,947	5,54850E-54
cDC(CD1C)	ORMDL1	1,03457E-19	-3,09772E-01	0,388	0,307	1,90536E-15
cDC(CD1C)	LMO4	2,33393E-07	-3,13138E-01	0,279	0,232	4,29840E-03
cDC(CD1C)	ADAP2	8,96412E-09	-3,14148E-01	0,455	0,444	1,65092E-04
cDC(CD1C)	CALM3	1,15683E-38	-3,17835E-01	0,482	0,511	2,13053E-34
cDC(CD1C)	GNS	1,28161E-50	-3,18563E-01	0,36	0,438	2,36035E-46
cDC(CD1C)	CTSS	8,58023E-83	-3,25798E-01	0,881	0,809	1,58022E-78
cDC(CD1C)	RENBP	2,06903E-57	-3,27466E-01	0,212	0,307	3,81053E-53
cDC(CD1C)	TSPAN4	4,56761E-60	-3,27761E-01	0,21	0,303	8,41216E-56
cDC(CD1C)	BMP2K	1,85337E-42	-3,30356E-01	0,223	0,299	3,41335E-38
cDC(CD1C)	CCDC50	2,02568E-93	-3,34390E-01	0,411	0,258	3,73069E-89
cDC(CD1C)	SPCS1	5,73138E-45	-3,36720E-01	0,751	0,591	1,05555E-40
cDC(CD1C)	A2M	1,69607E-06	-3,38260E-01	0,388	0,39	3,12365E-02
cDC(CD1C)	HLA-E	9,85365E-177	-3,38422E-01	0,934	0,901	1,81475E-172
cDC(CD1C)	PARP14	8,54234E-16	-3,40937E-01	0,351	0,38	1,57324E-11
cDC(CD1C)	IRF1	2,50863E-12	-3,41015E-01	0,421	0,441	4,62015E-08
cDC(CD1C)	IFI35	2,59584E-07	-3,41766E-01	0,339	0,347	4,78076E-03
cDC(CD1C)	ACADVL	8,79047E-08	-3,46388E-01	0,538	0,461	1,61894E-03
cDC(CD1C)	ATOX1	4,89657E-44	-3,50295E-01	0,57	0,576	9,01800E-40
cDC(CD1C)	CPNE3	4,79577E-17	-3,50469E-01	0,304	0,236	8,83237E-13
cDC(CD1C)	ELOB	2,34377E-06	-3,60925E-01	0,404	0,398	4,31652E-02
cDC(CD1C)	GPR34	2,06220E-11	-3,62384E-01	0,274	0,31	3,79795E-07
cDC(CD1C)	PLP2	2,80357E-100	-3,62663E-01	0,576	0,385	5,16333E-96
cDC(CD1C)	GIMAP7	2,11047E-07	-3,64249E-01	0,235	0,258	3,88685E-03
cDC(CD1C)	SCPEP1	6,94812E-07	-3,69104E-01	0,411	0,407	1,27964E-02
cDC(CD1C)	PDXK	2,35788E-10	-3,70001E-01	0,281	0,304	4,34251E-06
cDC(CD1C)	IER2	1,92009E-13	-3,72911E-01	0,727	0,621	3,53624E-09

cDC(CD1C)	PRDX1	1,08517E-106	-3,74590E-01	0,75	0,747	1,99855E-102
cDC(CD1C)	SEC61B	2,19854E-16	-3,77275E-01	0,833	0,73	4,04906E-12
cDC(CD1C)	LGALS3	1,79933E-101	-3,77523E-01	0,712	0,726	3,31382E-97
cDC(CD1C)	TNFRSF1B	6,16556E-13	-3,82352E-01	0,553	0,538	1,13551E-08
cDC(CD1C)	SAMSN1	1,58420E-79	-3,88988E-01	0,375	0,474	2,91761E-75
cDC(CD1C)	GNPDA1	2,03545E-07	-3,89694E-01	0,24	0,261	3,74868E-03
cDC(CD1C)	RHOB	6,32190E-07	-3,90340E-01	0,555	0,54	1,16430E-02
cDC(CD1C)	SLC43A3	1,22328E-42	-3,96260E-01	0,172	0,25	2,25291E-38
cDC(CD1C)	PFKFB3	1,11781E-22	-3,96687E-01	0,295	0,339	2,05868E-18
cDC(CD1C)	SLC1A3	1,70810E-59	-3,96724E-01	0,27	0,373	3,14580E-55
cDC(CD1C)	DAPK1	3,40118E-10	-4,00424E-01	0,315	0,337	6,26396E-06
cDC(CD1C)	RAB13	4,29608E-28	-4,05754E-01	0,252	0,311	7,91209E-24
cDC(CD1C)	SLC25A37	2,84401E-16	-4,21486E-01	0,259	0,295	5,23782E-12
cDC(CD1C)	ACP5	4,16237E-15	-4,22344E-01	0,383	0,402	7,66583E-11
cDC(CD1C)	CD84	6,84254E-20	-4,27939E-01	0,356	0,396	1,26019E-15
cDC(CD1C)	RBMS1	2,83283E-11	-4,29416E-01	0,29	0,313	5,21722E-07
cDC(CD1C)	CTSA	7,01213E-46	-4,33673E-01	0,481	0,509	1,29142E-41
cDC(CD1C)	LILRB1	2,83724E-10	-4,37077E-01	0,364	0,378	5,22534E-06
cDC(CD1C)	CTSZ	6,76547E-21	-4,37493E-01	0,698	0,647	1,24600E-16
cDC(CD1C)	MCOLN1	4,00854E-35	-4,38220E-01	0,215	0,281	7,38253E-31
cDC(CD1C)	CYSTM1	8,60075E-21	-4,40729E-01	0,315	0,357	1,58400E-16
cDC(CD1C)	SLC7A7	5,17437E-55	-4,40891E-01	0,404	0,473	9,52963E-51
cDC(CD1C)	EML4	4,18749E-15	-4,47860E-01	0,33	0,359	7,71210E-11
cDC(CD1C)	AZI2	6,24025E-31	-4,48402E-01	0,239	0,309	1,14927E-26
cDC(CD1C)	GIMAP4	3,77450E-16	-4,51305E-01	0,403	0,43	6,95149E-12
cDC(CD1C)	CYBB	1,93431E-28	-4,51743E-01	0,648	0,621	3,56242E-24
cDC(CD1C)	NFKBIA	1,75587E-44	-4,56223E-01	0,83	0,806	3,23379E-40
cDC(CD1C)	ALOX5AP	9,28769E-181	-4,56724E-01	0,698	0,483	1,71051E-176
cDC(CD1C)	BCL2A1	1,02455E-46	-4,59710E-01	0,421	0,476	1,88692E-42
cDC(CD1C)	MYO9B	1,37031E-46	-4,60762E-01	0,321	0,396	2,52371E-42
cDC(CD1C)	MT2A	2,34562E-78	-4,62067E-01	0,577	0,634	4,31993E-74
cDC(CD1C)	CD99	2,98223E-23	-4,64584E-01	0,627	0,62	5,49236E-19
cDC(CD1C)	MGAT4A	2,94229E-07	-4,64959E-01	0,344	0,359	5,41882E-03
cDC(CD1C)	FTH1	0,00000E+00	-4,70523E-01	0,999	0,997	0,00000E+00
cDC(CD1C)	PAG1	3,31171E-35	-4,70783E-01	0,209	0,278	6,09918E-31
cDC(CD1C)	ATP6V0C	3,04816E-56	-4,79819E-01	0,724	0,695	5,61379E-52
cDC(CD1C)	APLP2	1,59976E-87	-4,80018E-01	0,553	0,6	2,94628E-83
cDC(CD1C)	CLEC4E	7,23395E-12	-4,86737E-01	0,257	0,296	1,33228E-07
cDC(CD1C)	RPS27L	8,43525E-16	-4,86978E-01	0,351	0,363	1,55352E-11
cDC(CD1C)	BRI3	1,47372E-75	-4,87584E-01	0,317	0,412	2,71415E-71

cDC(CD1C)

cDC(CD1C)	TIMP2	2,50278E-87	-4,89990E-01	0,259	0,372	4,60936E-83
cDC(CD1C)	LY6E	4,92349E-08	-5,06197E-01	0,579	0,544	9,06758E-04
cDC(CD1C)	FCN1	2,19999E-17	-5,13320E-01	0,217	0,254	4,05172E-13
cDC(CD1C)	PDE4DIP	1,31782E-42	-5,15560E-01	0,251	0,33	2,42702E-38
cDC(CD1C)	NINJ1	4,08745E-153	-5,15616E-01	0,433	0,574	7,52786E-149
cDC(CD1C)	C3	2,15303E-06	-5,19641E-01	0,369	0,386	3,96523E-02
cDC(CD1C)	CD81	2,10105E-129	-5,27820E-01	0,35	0,474	3,86951E-125
cDC(CD1C)	C3AR1	2,96146E-44	-5,30313E-01	0,344	0,424	5,45411E-40
cDC(CD1C)	PILRA	1,15175E-14	-5,33718E-01	0,496	0,493	2,12118E-10
cDC(CD1C)	JUND	1,55255E-23	-5,46452E-01	0,436	0,457	2,85933E-19
cDC(CD1C)	LGALS3BP	3,02786E-47	-5,55623E-01	0,251	0,328	5,57641E-43
cDC(CD1C)	KCNMA1	1,93476E-120	-5,61954E-01	0,127	0,275	3,56324E-116
cDC(CD1C)	CREG1	2,13865E-105	-5,62497E-01	0,443	0,521	3,93875E-101
cDC(CD1C)	LIPA	6,15855E-85	-5,62552E-01	0,444	0,507	1,13422E-80
cDC(CD1C)	RNF213	4,55403E-38	-5,66175E-01	0,484	0,507	8,38715E-34
cDC(CD1C)	PIK3AP1	3,13290E-61	-5,66618E-01	0,221	0,314	5,76986E-57
cDC(CD1C)	IL1B	4,71024E-17	-5,74595E-01	0,427	0,325	8,67486E-13
cDC(CD1C)	NFKBIZ	5,45065E-09	-5,75710E-01	0,464	0,453	1,00385E-04
cDC(CD1C)	HIGD1A	2,53360E-11	-5,90173E-01	0,256	0,211	4,66614E-07
cDC(CD1C)	ACP2	3,07381E-75	-5,91585E-01	0,161	0,268	5,66103E-71
cDC(CD1C)	TNFSF10	2,28702E-10	-5,97441E-01	0,351	0,361	4,21201E-06
cDC(CD1C)	LAIR1	4,10216E-56	-6,04784E-01	0,455	0,526	7,55494E-52
cDC(CD1C)	CD63	1,74872E-186	-6,15364E-01	0,844	0,815	3,22061E-182
cDC(CD1C)	ELL2	3,24357E-138	-6,15615E-01	0,136	0,301	5,97368E-134
cDC(CD1C)	STXBP2	1,96148E-53	-6,27928E-01	0,357	0,427	3,61245E-49
cDC(CD1C)	LILRB4	9,49886E-42	-6,28698E-01	0,538	0,551	1,74941E-37
cDC(CD1C)	LHFPL2	3,14888E-158	-6,46479E-01	0,124	0,302	5,79930E-154
cDC(CD1C)	GBP5	2,44815E-40	-6,52387E-01	0,178	0,254	4,50875E-36
cDC(CD1C)	LRP1	1,28128E-134	-6,53507E-01	0,287	0,437	2,35973E-130
cDC(CD1C)	MPP1	2,14326E-102	-6,57435E-01	0,305	0,424	3,94724E-98
cDC(CD1C)	FCGR1A	9,16794E-53	-6,59146E-01	0,341	0,419	1,68846E-48
cDC(CD1C)	LAMP1	6,43710E-140	-6,61765E-01	0,26	0,412	1,18552E-135
cDC(CD1C)	PMP22	3,39250E-36	-6,69998E-01	0,221	0,284	6,24797E-32
cDC(CD1C)	PLIN2	3,91516E-65	-6,79951E-01	0,434	0,505	7,21056E-61
cDC(CD1C)	CSTB	5,05709E-65	-6,81371E-01	0,84	0,784	9,31364E-61
cDC(CD1C)	TREM2	3,50288E-47	-6,82983E-01	0,333	0,415	6,45126E-43
cDC(CD1C)	LILRB2	1,24572E-25	-6,88075E-01	0,431	0,46	2,29425E-21
cDC(CD1C)	MAFB	3,40342E-123	-6,94074E-01	0,489	0,605	6,26808E-119
cDC(CD1C)	GLUL	7,55154E-238	-7,20010E-01	0,803	0,804	1,39077E-233
cDC(CD1C)	PSAP	0,00000E+00	-7,31884E-01	0,935	0,914	0,00000E+00

cDC(CD1C)	CEBPB	4,20592E-50	-7,41627E-01	0,432	0,476	7,74604E-46
cDC(CD1C)	NCF1	5,36916E-07	-7,48834E-01	0,513	0,499	9,88839E-03
cDC(CD1C)	TMEM176A	4,27801E-127	-7,50954E-01	0,315	0,464	7,87881E-123
cDC(CD1C)	KLF2	1,79575E-72	-7,54516E-01	0,181	0,288	3,30724E-68
cDC(CD1C)	VAT1	6,16275E-132	-7,57911E-01	0,102	0,251	1,13499E-127
cDC(CD1C)	FNDC3B	2,95146E-80	-7,65705E-01	0,175	0,291	5,43570E-76
cDC(CD1C)	PLAC8	4,54771E-265	-7,67188E-01	0,404	0,182	8,37551E-261
cDC(CD1C)	ASAH1	6,73520E-232	-7,69786E-01	0,619	0,697	1,24042E-227
cDC(CD1C)	SCARB2	2,90570E-135	-7,72653E-01	0,23	0,388	5,35143E-131
cDC(CD1C)	IER3	2,42924E-27	-7,80193E-01	0,524	0,534	4,47392E-23
cDC(CD1C)	ISG15	2,60916E-49	-7,81455E-01	0,369	0,434	4,80528E-45
cDC(CD1C)	MSR1	2,94356E-72	-7,83446E-01	0,282	0,392	5,42116E-68
cDC(CD1C)	ABCA1	9,67757E-141	-7,86465E-01	0,17	0,331	1,78232E-136
cDC(CD1C)	CD68	9,33287E-150	-7,89509E-01	0,798	0,779	1,71883E-145
cDC(CD1C)	FUCA1	5,78620E-57	-7,93252E-01	0,169	0,259	1,06564E-52
cDC(CD1C)	IFI6	1,34472E-61	-8,14172E-01	0,426	0,496	2,47657E-57
cDC(CD1C)	MS4A4A	1,56110E-129	-8,26377E-01	0,3	0,434	2,87507E-125
cDC(CD1C)	DNASE2	1,34014E-64	-8,29700E-01	0,281	0,377	2,46813E-60
cDC(CD1C)	NR3C1	3,05087E-15	-8,35573E-01	0,467	0,379	5,61878E-11
cDC(CD1C)	MS4A7	1,97614E-65	-8,44116E-01	0,706	0,658	3,63946E-61
cDC(CD1C)	HMOX1	2,02040E-44	-8,57386E-01	0,522	0,547	3,72097E-40
cDC(CD1C)	MX1	2,19925E-15	-8,62373E-01	0,306	0,336	4,05035E-11
cDC(CD1C)	MPEG1	9,51288E-50	-8,81641E-01	0,3	0,382	1,75199E-45
cDC(CD1C)	IFI44L	9,73967E-76	-9,08760E-01	0,152	0,265	1,79375E-71
cDC(CD1C)	TXN	1,12228E-43	-9,17988E-01	0,669	0,667	2,06691E-39
cDC(CD1C)	OAS1	6,63270E-69	-9,23777E-01	0,231	0,338	1,22154E-64
cDC(CD1C)	CTSC	9,98579E-45	-9,27648E-01	0,734	0,671	1,83908E-40
cDC(CD1C)	SERPING1	8,20400E-117	-9,42945E-01	0,282	0,41	1,51093E-112
cDC(CD1C)	TMEM176B	7,55157E-156	-9,48155E-01	0,396	0,55	1,39077E-151
cDC(CD1C)	SLCO2B1	8,28975E-85	-9,70764E-01	0,262	0,381	1,52672E-80
cDC(CD1C)	STAB1	1,09264E-152	-9,90952E-01	0,189	0,363	2,01231E-148
cDC(CD1C)	DUSP6	4,52287E-42	-1,00279E+00	0,224	0,296	8,32977E-38
cDC(CD1C)	MARCKS	8,86277E-230	-1,00353E+00	0,38	0,55	1,63226E-225
cDC(CD1C)	DMXL2	7,42534E-228	-1,02242E+00	0,077	0,289	1,36752E-223
cDC(CD1C)	PLD3	2,87285E-181	-1,02961E+00	0,395	0,524	5,29092E-177
cDC(CD1C)	FTL	0,00000E+00	-1,03858E+00	0,994	0,991	0,00000E+00
cDC(CD1C)	C1QC	1,64174E-52	-1,05755E+00	0,553	0,545	3,02360E-48
cDC(CD1C)	CTSB	0,00000E+00	-1,05802E+00	0,905	0,868	0,00000E+00
cDC(CD1C)	CD163	9,73855E-70	-1,07789E+00	0,354	0,457	1,79355E-65
cDC(CD1C)	C1QB	9,70594E-46	-1,08651E+00	0,577	0,542	1,78754E-41

cDC(CD1C)	C1QA	1,19455E-66	-1,10495E+00	0,578	0,565	2,20000E-62
cDC(CD1C)	CD82	1,38673E-153	-1,12764E+00	0,092	0,257	2,55395E-149
cDC(CD1C)	SOD2	8,59849E-183	-1,16337E+00	0,575	0,66	1,58358E-178
cDC(CD1C)	DAB2	6,66334E-131	-1,20068E+00	0,315	0,447	1,22719E-126
cDC(CD1C)	EGR1	6,81396E-25	-1,20173E+00	0,241	0,291	1,25493E-20
cDC(CD1C)	CD14	6,13268E-256	-1,24192E+00	0,473	0,641	1,12946E-251
cDC(CD1C)	NPL	4,52070E-256	-1,28092E+00	0,058	0,281	8,32577E-252
cDC(CD1C)	LGMN	8,06497E-115	-1,31356E+00	0,308	0,421	1,48533E-110
cDC(CD1C)	SERPINF1	6,08776E-142	-1,37868E+00	0,588	0,384	1,12118E-137
cDC(CD1C)	SLC11A1	0,00000E+00	-1,49879E+00	0,215	0,496	0,00000E+00
cDC(CD1C)	C5AR1	0,00000E+00	-1,52795E+00	0,144	0,437	0,00000E+00
cDC(CD1C)	PLTP	1,04117E-212	-1,71804E+00	0,14	0,35	1,91752E-208
cDC(CD1C)	CFD	0,00000E+00	-1,75003E+00	0,342	0,587	0,00000E+00
cDC(CD1C)	FCGR3A	2,83801E-273	-1,84385E+00	0,4	0,595	5,22676E-269
cDC(CD1C)	S100A9	2,43900E-272	-1,96812E+00	0,241	0,48	4,49190E-268
cDC(CD1C)	APOC1	3,76523E-155	-1,97267E+00	0,414	0,518	6,93443E-151
cDC(CD1C)	CTSD	0,00000E+00	-2,04057E+00	0,624	0,786	0,00000E+00
cDC(CD1C)	GPNMB	0,00000E+00	-2,06226E+00	0,093	0,362	0,00000E+00
cDC(CD1C)	CCL3	6,08115E-102	-2,08246E+00	0,268	0,388	1,11997E-97
cDC(CD1C)	APOE	7,88763E-280	-2,30710E+00	0,317	0,528	1,45267E-275
cDC(CD1C)	CCL4	1,34251E-105	-2,47182E+00	0,192	0,323	2,47250E-101
cDC(CD1C)	CTSL	0,00000E+00	-2,85670E+00	0,254	0,549	0,00000E+00
cDC(CD1C)	S100A8	3,43629E-264	-2,88167E+00	0,143	0,376	6,32861E-260
cDC(CD1C)	RNASE1	1,34192E-228	-3,74936E+00	0,089	0,293	2,47141E-224

cDC_LAMP3

cluster	gene	p_val	avg_log2FC	pct.1	pct.2	p_val_adj
cDC_LAMP3	CCL22	0,00000E+00	4,50884E+00	0,496	0,02	0,00000E+00
cDC_LAMP3	FSCN1	0,00000E+00	4,39031E+00	0,804	0,093	0,00000E+00
cDC_LAMP3	CCL19	0,00000E+00	4,04089E+00	0,574	0,017	0,00000E+00
cDC_LAMP3	LAMP3	0,00000E+00	3,68888E+00	0,789	0,042	0,00000E+00
cDC_LAMP3	CCR7	0,00000E+00	3,68542E+00	0,698	0,033	0,00000E+00
cDC_LAMP3	MARCKSl	0,00000E+00	3,58057E+00	0,828	0,248	0,00000E+00
cDC_LAMP3	TXN	0,00000E+00	3,44289E+00	0,894	0,66	0,00000E+00
cDC_LAMP3	CCL17	0,00000E+00	3,29998E+00	0,382	0,017	0,00000E+00
cDC_LAMP3	BIRC3	0,00000E+00	3,10003E+00	0,789	0,205	0,00000E+00
cDC_LAMP3	CST7	0,00000E+00	2,75656E+00	0,675	0,128	0,00000E+00
cDC_LAMP3	EBI3	0,00000E+00	2,58869E+00	0,606	0,146	0,00000E+00
cDC_LAMP3	RAMP1	0,00000E+00	2,16620E+00	0,351	0,021	0,00000E+00
cDC_LAMP3	IDO1	0,00000E+00	2,08646E+00	0,655	0,093	0,00000E+00
cDC_LAMP3	IL7R	0,00000E+00	2,07507E+00	0,589	0,118	0,00000E+00
cDC_LAMP3	NCCRP1	0,00000E+00	2,00310E+00	0,329	0	0,00000E+00
cDC_LAMP3	IL32	0,00000E+00	1,99576E+00	0,574	0,118	0,00000E+00
cDC_LAMP3	LAD1	0,00000E+00	1,91473E+00	0,529	0,005	0,00000E+00
cDC_LAMP3	TRAF1	0,00000E+00	1,90295E+00	0,648	0,117	0,00000E+00
cDC_LAMP3	DAPP1	0,00000E+00	1,86703E+00	0,701	0,203	0,00000E+00
cDC_LAMP3	CRIP1	9,35013E-236	1,83254E+00	0,633	0,282	1,72201E-231
cDC_LAMP3	KIF2A	0,00000E+00	1,82768E+00	0,59	0,162	0,00000E+00
cDC_LAMP3	GPR157	0,00000E+00	1,82316E+00	0,587	0,046	0,00000E+00
cDC_LAMP3	TBC1D4	0,00000E+00	1,73988E+00	0,55	0,09	0,00000E+00
cDC_LAMP3	PPA1	0,00000E+00	1,71387E+00	0,846	0,449	0,00000E+00
cDC_LAMP3	AOC1	0,00000E+00	1,71383E+00	0,25	0,004	0,00000E+00
cDC_LAMP3	CLIC2	0,00000E+00	1,67052E+00	0,616	0,147	0,00000E+00
cDC_LAMP3	RAB9A	0,00000E+00	1,63569E+00	0,633	0,229	0,00000E+00
cDC_LAMP3	GRSF1	0,00000E+00	1,62622E+00	0,713	0,328	0,00000E+00
cDC_LAMP3	HSPB1	1,02481E-40	1,60025E+00	0,764	0,655	1,88740E-36
cDC_LAMP3	RPS27L	6,10976E-274	1,54245E+00	0,693	0,352	1,12523E-269
cDC_LAMP3	NUB1	0,00000E+00	1,52242E+00	0,736	0,3	0,00000E+00
cDC_LAMP3	DUSP4	0,00000E+00	1,51998E+00	0,53	0,103	0,00000E+00
cDC_LAMP3	DUSP5	0,00000E+00	1,50132E+00	0,539	0,122	0,00000E+00
cDC_LAMP3	ANXA6	0,00000E+00	1,49225E+00	0,594	0,213	0,00000E+00
cDC_LAMP3	CDKN1A	0,00000E+00	1,46852E+00	0,838	0,515	0,00000E+00
cDC_LAMP3	KDM2B	0,00000E+00	1,45539E+00	0,568	0,135	0,00000E+00
cDC_LAMP3	CD40	0,00000E+00	1,43987E+00	0,665	0,28	0,00000E+00
cDC_LAMP3	GADD45/	0,00000E+00	1,42854E+00	0,408	0,064	0,00000E+00
cDC_LAMP3	CD80	0,00000E+00	1,40528E+00	0,444	0,083	0,00000E+00
cDC_LAMP3	CD83	0,00000E+00	1,38427E+00	0,809	0,479	0,00000E+00

cDC_LAMP3	TUBA1C	3,05596E-211	1,37885E+00	0,667	0,37	5,62816E-207
cDC_LAMP3	RELB	0,00000E+00	1,37661E+00	0,657	0,232	0,00000E+00
cDC_LAMP3	RHOF	0,00000E+00	1,36977E+00	0,613	0,144	0,00000E+00
cDC_LAMP3	HSPD1	1,54342E-21	1,36828E+00	0,64	0,532	2,84251E-17
cDC_LAMP3	MGLL	0,00000E+00	1,35386E+00	0,572	0,145	0,00000E+00
cDC_LAMP3	CD200	0,00000E+00	1,35266E+00	0,45	0,004	0,00000E+00
cDC_LAMP3	NFKB1	9,42486E-282	1,34311E+00	0,651	0,277	1,73578E-277
cDC_LAMP3	FKBP4	3,39540E-18	1,34123E+00	0,309	0,225	6,25331E-14
cDC_LAMP3	LY75	0,00000E+00	1,28849E+00	0,497	0,083	0,00000E+00
cDC_LAMP3	S1PR1	0,00000E+00	1,26830E+00	0,251	0,015	0,00000E+00
cDC_LAMP3	CACYBP	3,85756E-12	1,26714E+00	0,481	0,399	7,10447E-08
cDC_LAMP3	CALCRL	0,00000E+00	1,25770E+00	0,333	0,048	0,00000E+00
cDC_LAMP3	POGLUT1	0,00000E+00	1,25536E+00	0,52	0,09	0,00000E+00
cDC_LAMP3	BASP1	1,04117E-258	1,25115E+00	0,685	0,336	1,91752E-254
cDC_LAMP3	ACHE	0,00000E+00	1,24964E+00	0,354	0,007	0,00000E+00
cDC_LAMP3	LSP1	0,00000E+00	1,24853E+00	0,912	0,663	0,00000E+00
cDC_LAMP3	GPR137E	1,09598E-225	1,22435E+00	0,62	0,288	2,01847E-221
cDC_LAMP3	TVP23A	0,00000E+00	1,21433E+00	0,439	0,065	0,00000E+00
cDC_LAMP3	ADORA2/	0,00000E+00	1,21143E+00	0,336	0,021	0,00000E+00
cDC_LAMP3	GABARAI	1,03228E-180	1,19599E+00	0,78	0,571	1,90114E-176
cDC_LAMP3	STK4	3,30880E-250	1,18824E+00	0,72	0,42	6,09381E-246
cDC_LAMP3	GSN	1,10204E-159	1,17834E+00	0,83	0,628	2,02962E-155
cDC_LAMP3	RAB8B	0,00000E+00	1,17189E+00	0,622	0,239	0,00000E+00
cDC_LAMP3	PNRC1	5,30667E-191	1,16799E+00	0,871	0,776	9,77330E-187
cDC_LAMP3	MALT1	6,54118E-284	1,15452E+00	0,514	0,169	1,20469E-279
cDC_LAMP3	RFTN1	0,00000E+00	1,15077E+00	0,615	0,187	0,00000E+00
cDC_LAMP3	SINHCAF	0,00000E+00	1,12750E+00	0,36	0,079	0,00000E+00
cDC_LAMP3	CSF2RA	5,79103E-211	1,12440E+00	0,702	0,408	1,06653E-206
cDC_LAMP3	UBD	0,00000E+00	1,12437E+00	0,274	0,038	0,00000E+00
cDC_LAMP3	TRIP10	0,00000E+00	1,12245E+00	0,499	0,081	0,00000E+00
cDC_LAMP3	BCL2L14	0,00000E+00	1,12021E+00	0,316	0,015	0,00000E+00
cDC_LAMP3	NDE1	0,00000E+00	1,10517E+00	0,508	0,148	0,00000E+00
cDC_LAMP3	HSPE1	1,89552E-07	1,09346E+00	0,646	0,602	3,49099E-03
cDC_LAMP3	PTPN1	5,02947E-228	1,08094E+00	0,702	0,392	9,26277E-224
cDC_LAMP3	REL	2,19838E-142	1,08001E+00	0,77	0,593	4,04876E-138
cDC_LAMP3	SYNGR2	1,98486E-248	1,07350E+00	0,856	0,724	3,65551E-244
cDC_LAMP3	NRP2	1,52639E-275	1,06783E+00	0,548	0,199	2,81115E-271
cDC_LAMP3	NET1	0,00000E+00	1,05433E+00	0,461	0,086	0,00000E+00
cDC_LAMP3	IL4I1	1,72451E-243	1,04711E+00	0,584	0,224	3,17604E-239
cDC_LAMP3	FNBP1	3,44300E-235	1,04069E+00	0,723	0,424	6,34098E-231
cDC_LAMP3	CXCL9	7,17948E-108	1,03622E+00	0,366	0,152	1,32224E-103

cDC_LAMP3	CRLF2	0,00000E+00	1,03406E+00	0,344	0,021	0,00000E+00
cDC_LAMP3	GCSAM	0,00000E+00	1,02197E+00	0,303	0,016	0,00000E+00
cDC_LAMP3	SLCO5A1	0,00000E+00	1,02108E+00	0,398	0,005	0,00000E+00
cDC_LAMP3	HSPH1	1,11143E-11	1,00802E+00	0,497	0,412	2,04692E-07
cDC_LAMP3	TSPAN15	5,34447E-123	9,97211E-01	0,277	0,09	9,84290E-119
cDC_LAMP3	MREG	0,00000E+00	9,88848E-01	0,418	0,039	0,00000E+00
cDC_LAMP3	TMEM17	2,42576E-90	9,85634E-01	0,591	0,444	4,46753E-86
cDC_LAMP3	ARHGAP:	0,00000E+00	9,73646E-01	0,464	0,121	0,00000E+00
cDC_LAMP3	CASP7	1,62816E-185	9,68634E-01	0,341	0,102	2,99858E-181
cDC_LAMP3	TBC1D8	7,66912E-274	9,44596E-01	0,513	0,17	1,41242E-269
cDC_LAMP3	TNIP2	3,29597E-193	9,41074E-01	0,471	0,176	6,07019E-189
cDC_LAMP3	USP12	0,00000E+00	9,40929E-01	0,455	0,09	0,00000E+00
cDC_LAMP3	TUBB6	4,13509E-291	9,38844E-01	0,508	0,163	7,61559E-287
cDC_LAMP3	CFLAR	1,91175E-255	9,36928E-01	0,794	0,503	3,52087E-251
cDC_LAMP3	SIAH2	0,00000E+00	9,34886E-01	0,495	0,13	0,00000E+00
cDC_LAMP3	IL15	0,00000E+00	9,27193E-01	0,463	0,118	0,00000E+00
cDC_LAMP3	SOCS2	0,00000E+00	9,25917E-01	0,364	0,026	0,00000E+00
cDC_LAMP3	NFKB2	6,24712E-251	9,00737E-01	0,572	0,216	1,15053E-246
cDC_LAMP3	LIMCH1	0,00000E+00	8,99346E-01	0,3	0,008	0,00000E+00
cDC_LAMP3	CD274	0,00000E+00	8,98012E-01	0,442	0,065	0,00000E+00
cDC_LAMP3	HMGN3	1,21086E-94	8,94815E-01	0,693	0,518	2,23004E-90
cDC_LAMP3	GSTP1	8,04775E-168	8,93634E-01	0,902	0,821	1,48215E-163
cDC_LAMP3	VOPP1	9,77215E-244	8,91313E-01	0,671	0,337	1,79974E-239
cDC_LAMP3	MTHFD2	1,32404E-132	8,90347E-01	0,637	0,397	2,43849E-128
cDC_LAMP3	HSPA8	1,43701E-41	8,86803E-01	0,877	0,818	2,64654E-37
cDC_LAMP3	RALA	3,85419E-102	8,79819E-01	0,604	0,367	7,09826E-98
cDC_LAMP3	MAP3K1	0,00000E+00	8,73923E-01	0,407	0,057	0,00000E+00
cDC_LAMP3	ID2	2,38871E-156	8,62049E-01	0,766	0,551	4,39929E-152
cDC_LAMP3	TOR3A	4,36071E-53	8,59436E-01	0,411	0,232	8,03112E-49
cDC_LAMP3	ALDH2	5,68887E-145	8,59427E-01	0,768	0,544	1,04772E-140
cDC_LAMP3	NABP1	2,08415E-156	8,49982E-01	0,614	0,331	3,83838E-152
cDC_LAMP3	CEP350	6,57857E-181	8,44080E-01	0,581	0,281	1,21158E-176
cDC_LAMP3	CERS6	0,00000E+00	8,38873E-01	0,493	0,132	0,00000E+00
cDC_LAMP3	BTG1	2,68296E-158	8,30705E-01	0,896	0,807	4,94121E-154
cDC_LAMP3	SERPINB!	1,17681E-46	8,24582E-01	0,535	0,37	2,16734E-42
cDC_LAMP3	PTMS	1,76182E-146	8,24426E-01	0,633	0,354	3,24474E-142
cDC_LAMP3	ANKRD3:	0,00000E+00	7,98875E-01	0,349	0,009	0,00000E+00
cDC_LAMP3	PIM3	4,18048E-121	7,95036E-01	0,536	0,278	7,69919E-117
cDC_LAMP3	FAM118/	1,26489E-178	7,91386E-01	0,442	0,16	2,32955E-174
cDC_LAMP3	RAB5B	2,82516E-224	7,91080E-01	0,503	0,186	5,20310E-220
cDC_LAMP3	MAP3K1:	1,88157E-229	7,85970E-01	0,524	0,203	3,46528E-225

cDC_LAMP3	CD86	1,14447E-102	7,85193E-01	0,678	0,489	2,10777E-98
cDC_LAMP3	RAB29	2,27992E-293	7,83281E-01	0,409	0,107	4,19893E-289
cDC_LAMP3	TTYH2	0,00000E+00	7,83191E-01	0,388	0,088	0,00000E+00
cDC_LAMP3	ETV3	0,00000E+00	7,80058E-01	0,508	0,135	0,00000E+00
cDC_LAMP3	RASSF4	1,79562E-101	7,75304E-01	0,698	0,501	3,30700E-97
cDC_LAMP3	YWHAQ	9,65379E-119	7,73191E-01	0,65	0,423	1,77794E-114
cDC_LAMP3	TRAFD1	0,00000E+00	7,71564E-01	0,538	0,168	0,00000E+00
cDC_LAMP3	FLT3	0,00000E+00	7,68585E-01	0,404	0,061	0,00000E+00
cDC_LAMP3	GPX4	8,60162E-164	7,67546E-01	0,903	0,843	1,58416E-159
cDC_LAMP3	P2RY10	0,00000E+00	7,67530E-01	0,329	0,024	0,00000E+00
cDC_LAMP3	GABPB1	2,12126E-146	7,60956E-01	0,327	0,106	3,90673E-142
cDC_LAMP3	SNX11	5,00677E-224	7,57255E-01	0,379	0,111	9,22097E-220
cDC_LAMP3	CHST7	0,00000E+00	7,56728E-01	0,314	0,039	0,00000E+00
cDC_LAMP3	HMSD	0,00000E+00	7,54119E-01	0,331	0,003	0,00000E+00
cDC_LAMP3	AHSA1	9,52425E-17	7,50848E-01	0,398	0,296	1,75408E-12
cDC_LAMP3	CYB5A	4,95133E-158	7,41821E-01	0,514	0,232	9,11887E-154
cDC_LAMP3	MCOLN2	3,37904E-241	7,41116E-01	0,387	0,109	6,22318E-237
cDC_LAMP3	SOCS1	2,24294E-178	7,39201E-01	0,491	0,195	4,13081E-174
cDC_LAMP3	SLC7A11	1,53461E-217	7,37242E-01	0,261	0,056	2,82629E-213
cDC_LAMP3	NBEAL1	2,54286E-35	7,36970E-01	0,372	0,228	4,68319E-31
cDC_LAMP3	CMTM6	5,22659E-112	7,32732E-01	0,748	0,612	9,62582E-108
cDC_LAMP3	NECTIN2	2,36894E-80	7,24045E-01	0,348	0,173	4,36288E-76
cDC_LAMP3	PFN1	3,78594E-291	7,18655E-01	0,975	0,946	6,97256E-287
cDC_LAMP3	ASMTL	3,33843E-179	7,15962E-01	0,38	0,127	6,14838E-175
cDC_LAMP3	ARAP2	0,00000E+00	7,15775E-01	0,402	0,094	0,00000E+00
cDC_LAMP3	GRINA	6,34881E-92	7,15336E-01	0,777	0,68	1,16926E-87
cDC_LAMP3	ACTB	6,30340E-247	7,10895E-01	0,998	0,991	1,16090E-242
cDC_LAMP3	GRAMD1	2,88561E-182	7,10005E-01	0,453	0,174	5,31443E-178
cDC_LAMP3	LRRFIP2	4,60037E-239	7,06776E-01	0,589	0,242	8,47249E-235
cDC_LAMP3	PDCL3	9,88238E-77	7,01491E-01	0,404	0,215	1,82004E-72
cDC_LAMP3	IL2RG	1,44764E-254	7,00788E-01	0,688	0,313	2,66612E-250
cDC_LAMP3	ZBTB10	1,87209E-245	7,00247E-01	0,332	0,08	3,44782E-241
cDC_LAMP3	ATF5	1,21306E-110	6,97326E-01	0,614	0,353	2,23410E-106
cDC_LAMP3	MYO1G	4,38269E-191	6,95579E-01	0,598	0,274	8,07159E-187
cDC_LAMP3	CCNG2	4,52138E-160	6,93968E-01	0,344	0,113	8,32703E-156
cDC_LAMP3	GLS	1,42957E-155	6,93419E-01	0,466	0,196	2,63283E-151
cDC_LAMP3	NUAK2	0,00000E+00	6,91369E-01	0,365	0,055	0,00000E+00
cDC_LAMP3	MMD	2,91121E-259	6,90210E-01	0,409	0,114	5,36157E-255
cDC_LAMP3	TNFRSF9	0,00000E+00	6,88158E-01	0,321	0,026	0,00000E+00
cDC_LAMP3	TOMM34	0,00000E+00	6,86821E-01	0,481	0,093	0,00000E+00
cDC_LAMP3	CNN2	1,19897E-95	6,86282E-01	0,62	0,395	2,20815E-91

cDC_LAMP3	ACTG1	9,41184E-167	6,85168E-01	0,963	0,921	1,73338E-162
cDC_LAMP3	REPIN1	2,05944E-256	6,79545E-01	0,447	0,136	3,79288E-252
cDC_LAMP3	RNF145	4,27413E-123	6,77151E-01	0,54	0,267	7,87166E-119
cDC_LAMP3	PDCD1LG	6,35500E-306	6,70825E-01	0,36	0,081	1,17040E-301
cDC_LAMP3	HLA-DOE	0,00000E+00	6,53631E-01	0,33	0,063	0,00000E+00
cDC_LAMP3	TIFAB	7,29168E-276	6,47865E-01	0,288	0,058	1,34291E-271
cDC_LAMP3	RPL35A	2,98970E-167	6,46256E-01	0,796	0,555	5,50613E-163
cDC_LAMP3	TNFRSF1	0,00000E+00	6,45376E-01	0,31	0,048	0,00000E+00
cDC_LAMP3	ANXA7	9,19859E-125	6,38846E-01	0,627	0,365	1,69410E-120
cDC_LAMP3	SNN	3,07799E-241	6,38353E-01	0,44	0,137	5,66873E-237
cDC_LAMP3	NMRK1	4,92088E-297	6,34017E-01	0,425	0,114	9,06279E-293
cDC_LAMP3	STARD7	1,26303E-129	6,30682E-01	0,554	0,298	2,32612E-125
cDC_LAMP3	GPR132	4,91212E-235	6,28878E-01	0,426	0,127	9,04665E-231
cDC_LAMP3	CYTIP	9,45943E-200	6,28106E-01	0,69	0,382	1,74214E-195
cDC_LAMP3	TUBB2A	2,81694E-149	6,27174E-01	0,293	0,09	5,18796E-145
cDC_LAMP3	MAP4K4	2,68623E-151	6,23989E-01	0,378	0,134	4,94722E-147
cDC_LAMP3	RUBCN	6,16216E-281	6,23775E-01	0,355	0,081	1,13488E-276
cDC_LAMP3	H6PD	2,23157E-214	6,16177E-01	0,344	0,094	4,10988E-210
cDC_LAMP3	RPS26	2,12302E-110	6,14572E-01	0,763	0,517	3,90997E-106
cDC_LAMP3	PPDPF	1,49484E-63	6,12728E-01	0,841	0,765	2,75305E-59
cDC_LAMP3	TMSB10	1,57344E-233	6,09732E-01	0,992	0,984	2,89780E-229
cDC_LAMP3	CCDC28E	0,00000E+00	6,08705E-01	0,315	0,039	0,00000E+00
cDC_LAMP3	TRADD	2,16355E-170	6,05608E-01	0,534	0,237	3,98461E-166
cDC_LAMP3	UBB	1,25964E-26	6,03299E-01	0,848	0,809	2,31989E-22
cDC_LAMP3	BCL2A1	1,96550E-99	5,98867E-01	0,702	0,463	3,61987E-95
cDC_LAMP3	NAV1	5,21250E-233	5,98837E-01	0,381	0,105	9,59986E-229
cDC_LAMP3	TUBB	3,48092E-146	5,98613E-01	0,823	0,656	6,41081E-142
cDC_LAMP3	SAMSN1	2,35755E-93	5,98055E-01	0,638	0,459	4,34190E-89
cDC_LAMP3	EEF1A1	5,02398E-260	5,95792E-01	0,994	0,984	9,25266E-256
cDC_LAMP3	SWAP70	2,90565E-72	5,95355E-01	0,532	0,328	5,35133E-68
cDC_LAMP3	ARPP19	5,32204E-131	5,92900E-01	0,526	0,261	9,80160E-127
cDC_LAMP3	TSPAN33	7,41061E-218	5,92436E-01	0,355	0,099	1,36481E-213
cDC_LAMP3	RAB30	0,00000E+00	5,90990E-01	0,374	0,023	0,00000E+00
cDC_LAMP3	TNFAIP8	1,79896E-116	5,90453E-01	0,583	0,328	3,31314E-112
cDC_LAMP3	MARCKS	7,86319E-59	5,89597E-01	0,688	0,528	1,44816E-54
cDC_LAMP3	DENND4.	3,30270E-89	5,87709E-01	0,282	0,108	6,08258E-85
cDC_LAMP3	NAA25	2,13562E-302	5,86475E-01	0,409	0,104	3,93317E-298
cDC_LAMP3	BID	1,83094E-14	5,86297E-01	0,492	0,412	3,37204E-10
cDC_LAMP3	HAPLN3	6,53279E-201	5,81907E-01	0,359	0,107	1,20314E-196
cDC_LAMP3	SERPINB:	2,91977E-19	5,80071E-01	0,731	0,661	5,37734E-15
cDC_LAMP3	BCL3	1,49085E-123	5,71591E-01	0,466	0,21	2,74570E-119

cDC_LAMP3	RACK1	1,57233E-26	5,69923E-01	0,561	0,464	2,89576E-22
cDC_LAMP3	LMNB1	3,71465E-158	5,67952E-01	0,403	0,148	6,84127E-154
cDC_LAMP3	KYNU	8,87460E-112	5,65457E-01	0,565	0,308	1,63444E-107
cDC_LAMP3	PTGES2	2,89660E-144	5,64293E-01	0,478	0,213	5,33468E-140
cDC_LAMP3	STAT5A	1,13070E-53	5,62056E-01	0,385	0,216	2,08240E-49
cDC_LAMP3	NME2	9,13994E-87	5,61615E-01	0,614	0,351	1,68330E-82
cDC_LAMP3	PEA15	4,38869E-83	5,60113E-01	0,702	0,511	8,08266E-79
cDC_LAMP3	BZW1	8,38948E-128	5,56869E-01	0,675	0,435	1,54509E-123
cDC_LAMP3	GPBP1	4,60075E-143	5,55977E-01	0,615	0,336	8,47321E-139
cDC_LAMP3	SPRED2	0,00000E+00	5,55350E-01	0,341	0,049	0,00000E+00
cDC_LAMP3	SQSTM1	1,54410E-63	5,53667E-01	0,779	0,671	2,84378E-59
cDC_LAMP3	DCAF6	1,61406E-203	5,50678E-01	0,438	0,147	2,97261E-199
cDC_LAMP3	LRRK1	1,10891E-133	5,45992E-01	0,392	0,156	2,04227E-129
cDC_LAMP3	RAB11A	2,04809E-65	5,45290E-01	0,599	0,396	3,77197E-61
cDC_LAMP3	C17orf49	3,72766E-284	5,45227E-01	0,584	0,219	6,86523E-280
cDC_LAMP3	SQLE	1,63192E-243	5,44285E-01	0,311	0,071	3,00551E-239
cDC_LAMP3	CYTH1	1,42751E-157	5,42713E-01	0,613	0,318	2,62904E-153
cDC_LAMP3	TNFAIP2	7,04149E-90	5,42631E-01	0,62	0,399	1,29683E-85
cDC_LAMP3	ERICH1	7,55838E-99	5,41993E-01	0,533	0,315	1,39203E-94
cDC_LAMP3	RHOC	4,33283E-33	5,38434E-01	0,436	0,305	7,97977E-29
cDC_LAMP3	RPL37	9,08041E-112	5,35411E-01	0,804	0,561	1,67234E-107
cDC_LAMP3	HIVEP1	0,00000E+00	5,34490E-01	0,4	0,084	0,00000E+00
cDC_LAMP3	ATOX1	2,66290E-79	5,34390E-01	0,749	0,57	4,90426E-75
cDC_LAMP3	BIRC2	1,85633E-158	5,34099E-01	0,49	0,206	3,41881E-154
cDC_LAMP3	PPP1R16	0,00000E+00	5,33133E-01	0,307	0,032	0,00000E+00
cDC_LAMP3	PPP1R18	9,84939E-65	5,32755E-01	0,668	0,496	1,81396E-60
cDC_LAMP3	CBLB	1,72644E-166	5,27796E-01	0,325	0,099	3,17959E-162
cDC_LAMP3	ARNTL2	0,00000E+00	5,26657E-01	0,302	0,024	0,00000E+00
cDC_LAMP3	LGALS2	7,86888E-61	5,24890E-01	0,45	0,251	1,44921E-56
cDC_LAMP3	ZNF296	0,00000E+00	5,21133E-01	0,261	0,039	0,00000E+00
cDC_LAMP3	G3BP2	9,15081E-66	5,18764E-01	0,479	0,271	1,68531E-61
cDC_LAMP3	JAK1	4,73703E-101	5,17497E-01	0,724	0,516	8,72419E-97
cDC_LAMP3	CLPTM1	4,82768E-117	5,15849E-01	0,498	0,252	8,89114E-113
cDC_LAMP3	CHD3	2,34009E-202	5,15652E-01	0,417	0,135	4,30974E-198
cDC_LAMP3	IL13RA1	2,67318E-76	5,15478E-01	0,608	0,382	4,92320E-72
cDC_LAMP3	UVRAG	2,22672E-105	5,14849E-01	0,527	0,284	4,10095E-101
cDC_LAMP3	RPS27A	1,19463E-165	5,12252E-01	0,816	0,568	2,20015E-161
cDC_LAMP3	ST8SIA4	1,61824E-59	5,09762E-01	0,467	0,292	2,98031E-55
cDC_LAMP3	SLC41A2	3,53888E-192	5,08747E-01	0,282	0,069	6,51756E-188
cDC_LAMP3	RPL36AL	5,75584E-128	5,04546E-01	0,729	0,486	1,06005E-123
cDC_LAMP3	DNAJB6	1,61333E-63	5,03433E-01	0,669	0,518	2,97128E-59

cDC_LAMP3	FYTTD1	1,94833E-180	5,02739E-01	0,539	0,239	3,58825E-176
cDC_LAMP3	ISCU	3,79939E-95	5,01781E-01	0,716	0,532	6,99733E-91
cDC_LAMP3	PRRG4	8,06093E-102	5,00523E-01	0,297	0,113	1,48458E-97
cDC_LAMP3	PTGIR	0,00000E+00	4,94971E-01	0,273	0,028	0,00000E+00
cDC_LAMP3	HLA-DQA	2,06680E-22	4,86943E-01	0,615	0,509	3,80642E-18
cDC_LAMP3	GLIPR2	6,11462E-72	4,86935E-01	0,586	0,37	1,12613E-67
cDC_LAMP3	RAC1	1,84070E-35	4,84623E-01	0,766	0,686	3,39001E-31
cDC_LAMP3	ANKLE2	8,30860E-91	4,84342E-01	0,355	0,157	1,53019E-86
cDC_LAMP3	BET1	4,12006E-137	4,84223E-01	0,363	0,134	7,58792E-133
cDC_LAMP3	SUB1	9,22147E-158	4,82427E-01	0,851	0,719	1,69832E-153
cDC_LAMP3	TMUB1	2,93389E-32	4,79305E-01	0,409	0,257	5,40335E-28
cDC_LAMP3	TES	9,37501E-50	4,78964E-01	0,453	0,274	1,72660E-45
cDC_LAMP3	ARL6IP5	3,23400E-71	4,77610E-01	0,766	0,651	5,95605E-67
cDC_LAMP3	HLA-DQA	1,15467E-22	4,72655E-01	0,904	0,78	2,12656E-18
cDC_LAMP3	HMGCS1	6,49120E-157	4,69084E-01	0,302	0,091	1,19549E-152
cDC_LAMP3	LYST	3,21956E-177	4,66128E-01	0,529	0,221	5,92946E-173
cDC_LAMP3	GNG5	1,32734E-141	4,57599E-01	0,867	0,791	2,44456E-137
cDC_LAMP3	VAC14	3,98847E-104	4,54742E-01	0,358	0,152	7,34556E-100
cDC_LAMP3	CCSER2	6,85471E-80	4,52619E-01	0,314	0,136	1,26243E-75
cDC_LAMP3	OSTF1	1,40138E-128	4,44980E-01	0,734	0,544	2,58092E-124
cDC_LAMP3	ZDHHC18	1,10049E-257	4,43479E-01	0,268	0,052	2,02677E-253
cDC_LAMP3	RAP1B	1,08634E-77	4,42932E-01	0,713	0,575	2,00072E-73
cDC_LAMP3	RIC1	4,79112E-148	4,42752E-01	0,305	0,096	8,82380E-144
cDC_LAMP3	JPT1	4,33003E-18	4,42743E-01	0,379	0,28	7,97461E-14
cDC_LAMP3	RUFY3	9,85838E-181	4,36536E-01	0,38	0,123	1,81562E-176
cDC_LAMP3	AXL	9,98220E-34	4,28999E-01	0,137	0,285	1,83842E-29
cDC_LAMP3	SEMA7A	0,00000E+00	4,27699E-01	0,308	0,048	0,00000E+00
cDC_LAMP3	HINT1	2,84734E-181	4,23678E-01	0,861	0,718	5,24394E-177
cDC_LAMP3	LMBR1L	5,86995E-70	4,23357E-01	0,321	0,146	1,08107E-65
cDC_LAMP3	EDEM1	2,07732E-89	4,19576E-01	0,399	0,186	3,82580E-85
cDC_LAMP3	CDK2	8,02096E-292	4,19038E-01	0,256	0,044	1,47722E-287
cDC_LAMP3	SNAP23	1,02527E-17	4,18990E-01	0,516	0,396	1,88824E-13
cDC_LAMP3	MAPK8	5,60773E-176	4,17185E-01	0,282	0,074	1,03278E-171
cDC_LAMP3	RPL36	8,28208E-113	4,16799E-01	0,787	0,545	1,52531E-108
cDC_LAMP3	GRK3	8,51830E-109	4,16547E-01	0,262	0,089	1,56882E-104
cDC_LAMP3	TPRA1	4,41977E-42	4,15841E-01	0,293	0,16	8,13989E-38
cDC_LAMP3	MOB3A	3,03870E-72	4,15006E-01	0,425	0,22	5,59638E-68
cDC_LAMP3	MRPS6	4,80588E-48	4,13863E-01	0,484	0,32	8,85099E-44
cDC_LAMP3	LRCH3	5,27505E-94	4,09372E-01	0,264	0,094	9,71506E-90
cDC_LAMP3	CASP3	2,32990E-121	4,08589E-01	0,325	0,118	4,29097E-117
cDC_LAMP3	TSTD1	8,27134E-98	4,06814E-01	0,468	0,232	1,52333E-93

cDC_LAMP3	ETV6	1,16708E-96	4,05349E-01	0,524	0,283	2,14940E-92
cDC_LAMP3	TFG	6,01052E-89	4,04953E-01	0,487	0,255	1,10696E-84
cDC_LAMP3	MKNK2	7,07760E-14	3,97388E-01	0,33	0,23	1,30348E-09
cDC_LAMP3	CSRP1	6,37393E-41	3,97157E-01	0,418	0,253	1,17389E-36
cDC_LAMP3	RPS7	2,04638E-141	3,97035E-01	0,802	0,553	3,76882E-137
cDC_LAMP3	TOR1AIP	6,91834E-84	3,96347E-01	0,318	0,135	1,27415E-79
cDC_LAMP3	ZFAND2A	1,80001E-14	3,95562E-01	0,252	0,168	3,31508E-10
cDC_LAMP3	PNMA1	2,45272E-208	3,87109E-01	0,25	0,055	4,51717E-204
cDC_LAMP3	RNF115	1,62426E-92	3,85321E-01	0,44	0,214	2,99140E-88
cDC_LAMP3	RPL14	8,84122E-142	3,84604E-01	0,794	0,535	1,62829E-137
cDC_LAMP3	CDC42SE	7,14540E-66	3,84578E-01	0,589	0,379	1,31597E-61
cDC_LAMP3	TRIR	3,43997E-20	3,81499E-01	0,437	0,333	6,33540E-16
cDC_LAMP3	TUBA1A	1,47050E-46	3,79728E-01	0,618	0,457	2,70821E-42
cDC_LAMP3	DLGAP4	5,07691E-76	3,77877E-01	0,388	0,19	9,35014E-72
cDC_LAMP3	MOB1B	4,27158E-133	3,77451E-01	0,385	0,149	7,86697E-129
cDC_LAMP3	NDUFV2	1,42489E-37	3,73751E-01	0,687	0,529	2,62422E-33
cDC_LAMP3	PCBP1	1,11707E-53	3,71252E-01	0,732	0,607	2,05731E-49
cDC_LAMP3	HSPA4	2,20425E-11	3,70945E-01	0,394	0,298	4,05956E-07
cDC_LAMP3	UBE2F	5,21044E-83	3,70917E-01	0,458	0,253	9,59606E-79
cDC_LAMP3	ATP6V0A	1,37251E-82	3,69356E-01	0,293	0,117	2,52775E-78
cDC_LAMP3	DOK2	5,37337E-32	3,67711E-01	0,21	0,349	9,89613E-28
cDC_LAMP3	CUL1	1,18111E-72	3,67291E-01	0,41	0,212	2,17525E-68
cDC_LAMP3	ST3GAL5	2,05966E-119	3,66911E-01	0,286	0,1	3,79328E-115
cDC_LAMP3	SLC44A1	1,48681E-94	3,66811E-01	0,394	0,179	2,73825E-90
cDC_LAMP3	RPL22	3,04405E-75	3,64345E-01	0,765	0,523	5,60623E-71
cDC_LAMP3	RSRP1	1,18587E-61	3,63197E-01	0,481	0,275	2,18401E-57
cDC_LAMP3	MYO1C	3,28677E-67	3,60318E-01	0,257	0,109	6,05324E-63
cDC_LAMP3	REST	3,92097E-57	3,58194E-01	0,41	0,227	7,22125E-53
cDC_LAMP3	ANTXR2	2,24706E-204	3,50372E-01	0,442	0,148	4,13842E-200
cDC_LAMP3	MINK1	3,77167E-77	3,48844E-01	0,403	0,201	6,94628E-73
cDC_LAMP3	APOBEC3	1,59186E-41	3,48205E-01	0,332	0,187	2,93173E-37
cDC_LAMP3	TAP2	3,38494E-54	3,47643E-01	0,34	0,184	6,23404E-50
cDC_LAMP3	IRF4	0,00000E+00	3,47147E-01	0,451	0,081	0,00000E+00
cDC_LAMP3	HMG20B	3,06733E-10	3,47107E-01	0,314	0,236	5,64911E-06
cDC_LAMP3	ZBTB38	3,22386E-80	3,45276E-01	0,383	0,188	5,93739E-76
cDC_LAMP3	SESN3	4,81448E-51	3,42486E-01	0,273	0,132	8,86683E-47
cDC_LAMP3	RAP2B	2,43434E-45	3,41713E-01	0,483	0,31	4,48332E-41
cDC_LAMP3	ACOT9	4,62029E-90	3,40968E-01	0,501	0,272	8,50918E-86
cDC_LAMP3	RPL10	7,34661E-192	3,40141E-01	0,832	0,587	1,35303E-187
cDC_LAMP3	NFAT5	1,21950E-43	3,39316E-01	0,307	0,16	2,24596E-39
cDC_LAMP3	CYP2S1	9,04616E-66	3,38930E-01	0,274	0,121	1,66603E-61

cDC_LAMP3	ARHGAP:	2,01615E-68	3,38744E-01	0,291	0,131	3,71314E-64
cDC_LAMP3	SUZ12	1,99282E-68	3,38604E-01	0,313	0,145	3,67018E-64
cDC_LAMP3	MRPL18	4,70869E-09	3,36968E-01	0,446	0,346	8,67200E-05
cDC_LAMP3	IRF1	6,44412E-69	3,36503E-01	0,623	0,433	1,18681E-64
cDC_LAMP3	HLX	2,78434E-102	3,35006E-01	0,25	0,084	5,12793E-98
cDC_LAMP3	DNAJC15	8,69363E-24	3,34933E-01	0,62	0,496	1,60111E-19
cDC_LAMP3	NOP53	3,34097E-22	3,34072E-01	0,396	0,281	6,15306E-18
cDC_LAMP3	MYL6	2,01254E-119	3,34049E-01	0,949	0,941	3,70649E-115
cDC_LAMP3	RUNX3	7,12361E-63	3,32444E-01	0,348	0,172	1,31195E-58
cDC_LAMP3	CEP135	2,37869E-133	3,30332E-01	0,272	0,083	4,38083E-129
cDC_LAMP3	TMEM13	1,67019E-103	3,29912E-01	0,352	0,143	3,07599E-99
cDC_LAMP3	PELI1	1,88669E-92	3,26364E-01	0,498	0,255	3,47473E-88
cDC_LAMP3	ZC3H12A	1,48893E-35	3,23529E-01	0,285	0,154	2,74215E-31
cDC_LAMP3	PITPNB	1,87898E-69	3,21887E-01	0,339	0,159	3,46051E-65
cDC_LAMP3	SPPL2A	3,10999E-86	3,21190E-01	0,563	0,334	5,72767E-82
cDC_LAMP3	TNIP1	8,51354E-107	3,20283E-01	0,601	0,338	1,56794E-102
cDC_LAMP3	BAZ1A	5,87939E-33	3,18851E-01	0,571	0,414	1,08281E-28
cDC_LAMP3	POLR2E	1,09446E-46	3,18124E-01	0,681	0,525	2,01567E-42
cDC_LAMP3	HSPA6	3,47999E-29	3,18037E-01	0,141	0,291	6,40909E-25
cDC_LAMP3	STIP1	7,75370E-14	3,17682E-01	0,379	0,279	1,42800E-09
cDC_LAMP3	SUCO	1,50291E-78	3,17223E-01	0,291	0,12	2,76791E-74
cDC_LAMP3	VDAC2	1,08258E-82	3,16208E-01	0,725	0,547	1,99379E-78
cDC_LAMP3	TBC1D10	1,41149E-195	3,14875E-01	0,373	0,113	2,59954E-191
cDC_LAMP3	MAFF	2,34930E-74	3,12987E-01	0,328	0,151	4,32671E-70
cDC_LAMP3	SMNDC1	1,56812E-57	3,12966E-01	0,398	0,218	2,88801E-53
cDC_LAMP3	GTF2E2	9,09044E-102	3,12577E-01	0,399	0,177	1,67419E-97
cDC_LAMP3	FOXP1	3,40347E-55	3,11595E-01	0,533	0,339	6,26816E-51
cDC_LAMP3	PDLIM5	8,22129E-64	3,08968E-01	0,429	0,229	1,51411E-59
cDC_LAMP3	RCN1	3,10180E-33	3,08954E-01	0,296	0,173	5,71259E-29
cDC_LAMP3	ATF4	2,43724E-63	3,08120E-01	0,6	0,399	4,48866E-59
cDC_LAMP3	ARHGAP	3,65806E-53	3,05894E-01	0,287	0,142	6,73705E-49
cDC_LAMP3	PMAIP1	3,19564E-125	3,00963E-01	0,425	0,178	5,88541E-121
cDC_LAMP3	CDK17	1,67565E-129	3,00768E-01	0,316	0,107	3,08604E-125
cDC_LAMP3	DNAJA1	1,29663E-29	2,99043E-01	0,704	0,601	2,38800E-25
cDC_LAMP3	ZNF267	4,33985E-69	2,98871E-01	0,435	0,228	7,99271E-65
cDC_LAMP3	LAMTOR	7,09892E-54	2,96529E-01	0,436	0,253	1,30741E-49
cDC_LAMP3	CDK6	7,97619E-73	2,92385E-01	0,291	0,126	1,46897E-68
cDC_LAMP3	ENSA	3,33453E-28	2,91604E-01	0,607	0,468	6,14120E-24
cDC_LAMP3	C15orf48	1,21959E-40	2,88086E-01	0,507	0,34	2,24613E-36
cDC_LAMP3	MBNL2	9,07764E-64	2,87810E-01	0,303	0,143	1,67183E-59
cDC_LAMP3	LCP1	8,09202E-47	2,87679E-01	0,794	0,701	1,49031E-42

cDC_LAMP3	DPP9	8,46149E-58	2,87622E-01	0,325	0,16	1,55835E-53
cDC_LAMP3	SF3B6	4,67668E-46	2,86558E-01	0,473	0,284	8,61303E-42
cDC_LAMP3	RALY	1,85787E-39	2,86294E-01	0,645	0,488	3,42163E-35
cDC_LAMP3	PSIP1	1,02777E-74	2,86234E-01	0,326	0,149	1,89285E-70
cDC_LAMP3	NARF	1,24604E-09	2,86193E-01	0,285	0,204	2,29483E-05
cDC_LAMP3	PPM1K	4,31997E-215	2,86186E-01	0,446	0,147	7,95608E-211
cDC_LAMP3	DAZAP1	6,95179E-44	2,85749E-01	0,333	0,181	1,28031E-39
cDC_LAMP3	PPP2CA	2,31974E-29	2,85334E-01	0,48	0,33	4,27227E-25
cDC_LAMP3	TMSB4X	5,35830E-63	2,83382E-01	0,99	0,984	9,86838E-59
cDC_LAMP3	ADAM19	1,35294E-235	2,79742E-01	0,378	0,101	2,49171E-231
cDC_LAMP3	FAAP20	2,45924E-53	2,77610E-01	0,412	0,228	4,52919E-49
cDC_LAMP3	PLGRKT	2,20021E-94	2,76686E-01	0,425	0,205	4,05213E-90
cDC_LAMP3	GFPT1	3,45784E-99	2,75353E-01	0,259	0,091	6,36831E-95
cDC_LAMP3	ATP5MG	1,40506E-08	2,74882E-01	0,497	0,416	2,58770E-04
cDC_LAMP3	ICAM1	7,37604E-42	2,74718E-01	0,543	0,356	1,35844E-37
cDC_LAMP3	SMIM14	1,42639E-29	2,74222E-01	0,401	0,265	2,62698E-25
cDC_LAMP3	SELENOV	5,91869E-18	2,73832E-01	0,393	0,288	1,09005E-13
cDC_LAMP3	NIT2	3,92206E-60	2,71531E-01	0,335	0,167	7,22325E-56
cDC_LAMP3	RPS19	3,34988E-140	2,70034E-01	0,821	0,568	6,16947E-136
cDC_LAMP3	NFKBIB	1,15405E-27	2,68863E-01	0,271	0,156	2,12541E-23
cDC_LAMP3	SRD5A1	4,06147E-70	2,68621E-01	0,252	0,102	7,48001E-66
cDC_LAMP3	RPL28	5,15545E-110	2,67840E-01	0,826	0,587	9,49480E-106
cDC_LAMP3	SERTAD2	1,37563E-77	2,67753E-01	0,381	0,179	2,53349E-73
cDC_LAMP3	TRIB1	5,24823E-74	2,67251E-01	0,446	0,235	9,66567E-70
cDC_LAMP3	RELA	4,33465E-51	2,60177E-01	0,397	0,22	7,98312E-47
cDC_LAMP3	CXCL16	1,83736E-06	2,57854E-01	0,678	0,65	3,38387E-02
cDC_LAMP3	BZW2	6,20516E-86	2,57795E-01	0,356	0,159	1,14280E-81
cDC_LAMP3	IKZF1	3,61742E-53	2,57102E-01	0,46	0,277	6,66220E-49
cDC_LAMP3	WTAP	2,81057E-40	2,56910E-01	0,54	0,36	5,17622E-36
cDC_LAMP3	NR4A3	7,21140E-78	2,54968E-01	0,514	0,29	1,32812E-73
cDC_LAMP3	RAD21	3,29002E-34	2,53694E-01	0,499	0,342	6,05923E-30
cDC_LAMP3	EED	7,80097E-117	2,53180E-01	0,264	0,085	1,43670E-112
cDC_LAMP3	MCM5	7,76585E-59	2,53148E-01	0,395	0,218	1,43024E-54
cDC_LAMP3	UBE2E1	1,27700E-18	2,51469E-01	0,42	0,306	2,35185E-14
cDC_LAMP3	CCNI	3,66162E-32	2,51414E-01	0,846	0,784	6,74360E-28
cDC_LAMP3	PRELID3E	3,08853E-66	2,51038E-01	0,286	0,127	5,68815E-62
cDC_LAMP3	SYMPK	2,61824E-32	-2,50757E-01	0,284	0,159	4,82202E-28
cDC_LAMP3	PSMC4	7,39478E-09	-2,52813E-01	0,426	0,333	1,36190E-04
cDC_LAMP3	ST3GAL1	5,11682E-10	-2,52951E-01	0,305	0,227	9,42365E-06
cDC_LAMP3	RNF10	2,26983E-08	-2,56372E-01	0,36	0,272	4,18035E-04
cDC_LAMP3	USP48	3,07524E-07	-2,56534E-01	0,255	0,19	5,66367E-03

cDC_LAMP3	G6PD	2,68478E-06	-2,57955E-01	0,221	0,262	4,94456E-02
cDC_LAMP3	SRSF11	1,39229E-06	-2,59335E-01	0,606	0,513	2,56418E-02
cDC_LAMP3	MT-CO1	1,83052E-66	-2,59914E-01	0,813	0,592	3,37127E-62
cDC_LAMP3	FAM32A	3,73037E-08	-2,60001E-01	0,405	0,314	6,87021E-04
cDC_LAMP3	SLAMF7	5,76580E-56	-2,61150E-01	0,38	0,204	1,06189E-51
cDC_LAMP3	MAT2B	1,29438E-10	-2,61468E-01	0,433	0,332	2,38386E-06
cDC_LAMP3	HNRNPC	3,58931E-11	-2,61722E-01	0,727	0,645	6,61043E-07
cDC_LAMP3	IFITM3	2,39605E-45	-2,63914E-01	0,622	0,769	4,41281E-41
cDC_LAMP3	CNOT1	7,92624E-10	-2,66784E-01	0,299	0,216	1,45978E-05
cDC_LAMP3	STX6	1,37708E-09	-2,68610E-01	0,196	0,253	2,53616E-05
cDC_LAMP3	TDG	2,85328E-07	-2,70664E-01	0,29	0,217	5,25489E-03
cDC_LAMP3	FKBP8	1,36132E-08	-2,70785E-01	0,515	0,538	2,50715E-04
cDC_LAMP3	SRP54	3,27088E-07	-2,71777E-01	0,272	0,204	6,02397E-03
cDC_LAMP3	PPHLN1	2,68660E-10	-2,72447E-01	0,301	0,216	4,94791E-06
cDC_LAMP3	CD2BP2	6,26329E-08	-2,73218E-01	0,33	0,248	1,15351E-03
cDC_LAMP3	TMEM13	8,19647E-15	-2,74961E-01	0,201	0,275	1,50954E-10
cDC_LAMP3	HMGN4	8,64578E-20	-2,75606E-01	0,363	0,245	1,59229E-15
cDC_LAMP3	OGFR	2,00523E-09	-2,76015E-01	0,29	0,336	3,69303E-05
cDC_LAMP3	RPLP2	5,57946E-41	-2,77370E-01	0,8	0,563	1,02757E-36
cDC_LAMP3	PTBP1	1,96669E-07	-2,79461E-01	0,389	0,304	3,62205E-03
cDC_LAMP3	TSEN34	4,11280E-08	-2,79858E-01	0,244	0,292	7,57454E-04
cDC_LAMP3	EFTUD2	5,53797E-09	-2,80272E-01	0,267	0,193	1,01993E-04
cDC_LAMP3	RPS3A	9,23951E-84	-2,80414E-01	0,809	0,559	1,70164E-79
cDC_LAMP3	CAP1	3,27689E-14	-2,82709E-01	0,714	0,734	6,03505E-10
cDC_LAMP3	PMPCB	1,50007E-19	-2,82955E-01	0,358	0,238	2,76267E-15
cDC_LAMP3	MSRB1	2,46652E-09	-2,85218E-01	0,26	0,189	4,54259E-05
cDC_LAMP3	ANP32A	4,73330E-07	-2,86468E-01	0,445	0,471	8,71732E-03
cDC_LAMP3	EPN1	9,06883E-23	-2,88504E-01	0,272	0,362	1,67021E-18
cDC_LAMP3	ATP5IF1	2,58558E-07	-2,88612E-01	0,226	0,266	4,76186E-03
cDC_LAMP3	DCTN2	1,79414E-15	-2,95448E-01	0,446	0,326	3,30427E-11
cDC_LAMP3	NDUFB1[illegible]	2,28771E-11	-2,96501E-01	0,65	0,662	4,21328E-07
cDC_LAMP3	MCTS1	2,97556E-10	-2,97498E-01	0,242	0,297	5,48009E-06
cDC_LAMP3	LMNA	3,11845E-12	-2,98854E-01	0,298	0,371	5,74325E-08
cDC_LAMP3	RNF5	9,45456E-10	-3,00536E-01	0,37	0,28	1,74125E-05
cDC_LAMP3	ENO1	5,09628E-16	-3,01662E-01	0,8	0,76	9,38582E-12
cDC_LAMP3	MT-CO2	9,99680E-59	-3,02706E-01	0,81	0,59	1,84111E-54
cDC_LAMP3	PSMD4	1,29002E-08	-3,03102E-01	0,516	0,409	2,37583E-04
cDC_LAMP3	BBX	1,25968E-14	-3,03620E-01	0,219	0,3	2,31994E-10
cDC_LAMP3	PSTPIP2	1,68267E-21	-3,04290E-01	0,142	0,254	3,09897E-17
cDC_LAMP3	OSBPL8	2,91945E-08	-3,04446E-01	0,393	0,43	5,37675E-04
cDC_LAMP3	FOXN3	2,66229E-08	-3,06438E-01	0,309	0,362	4,90313E-04

cDC_LAMP3	DHPS	1,12554E-17	-3,07332E-01	0,324	0,214	2,07291E-13
cDC_LAMP3	RBM4	5,31915E-48	-3,07436E-01	0,302	0,151	9,79628E-44
cDC_LAMP3	TMA7	7,80521E-08	-3,07588E-01	0,788	0,794	1,43748E-03
cDC_LAMP3	MRPS11	1,52164E-06	-3,11049E-01	0,265	0,205	2,80240E-02
cDC_LAMP3	SRSF10	3,89415E-29	-3,12040E-01	0,426	0,274	7,17185E-25
cDC_LAMP3	PSMD14	2,34576E-07	-3,12215E-01	0,283	0,216	4,32019E-03
cDC_LAMP3	RPL36A	1,96581E-37	-3,12310E-01	0,654	0,447	3,62044E-33
cDC_LAMP3	HK1	2,41370E-18	-3,12437E-01	0,178	0,265	4,44531E-14
cDC_LAMP3	PSMB3	1,18137E-08	-3,13614E-01	0,655	0,643	2,17572E-04
cDC_LAMP3	MRPS7	2,13212E-07	-3,14145E-01	0,273	0,311	3,92673E-03
cDC_LAMP3	EIF6	2,39380E-07	-3,14271E-01	0,498	0,403	4,40866E-03
cDC_LAMP3	EIF4A3	7,39715E-07	-3,15120E-01	0,378	0,403	1,36233E-02
cDC_LAMP3	RPLP0	9,32433E-97	-3,17327E-01	0,789	0,535	1,71726E-92
cDC_LAMP3	ZDHHC12	6,40554E-09	-3,17925E-01	0,344	0,259	1,17971E-04
cDC_LAMP3	ETFB	9,61514E-12	-3,18050E-01	0,243	0,309	1,77082E-07
cDC_LAMP3	MAPK1IP	9,29687E-19	-3,19281E-01	0,471	0,34	1,71221E-14
cDC_LAMP3	RPL5	2,35332E-78	-3,20318E-01	0,756	0,51	4,33410E-74
cDC_LAMP3	PSMB5	9,55486E-09	-3,20522E-01	0,407	0,313	1,75972E-04
cDC_LAMP3	KLF6	1,08241E-10	-3,20831E-01	0,817	0,797	1,99348E-06
cDC_LAMP3	CHD2	1,22552E-11	-3,21506E-01	0,396	0,296	2,25704E-07
cDC_LAMP3	ELOVL5	4,63127E-08	-3,21555E-01	0,372	0,285	8,52941E-04
cDC_LAMP3	CDC123	1,54002E-10	-3,22280E-01	0,318	0,232	2,83625E-06
cDC_LAMP3	NDUFS8	8,56240E-13	-3,25009E-01	0,465	0,496	1,57694E-08
cDC_LAMP3	POMP	2,52527E-17	-3,26554E-01	0,691	0,721	4,65079E-13
cDC_LAMP3	YBX1	8,48685E-26	-3,26910E-01	0,857	0,862	1,56302E-21
cDC_LAMP3	DPYSL2	1,91541E-07	-3,28584E-01	0,396	0,321	3,52761E-03
cDC_LAMP3	N4BP2L2	2,80258E-13	-3,29443E-01	0,56	0,456	5,16151E-09
cDC_LAMP3	BOD1L1	1,30836E-07	-3,30573E-01	0,209	0,255	2,40962E-03
cDC_LAMP3	DCK	6,78569E-11	-3,30643E-01	0,372	0,277	1,24972E-06
cDC_LAMP3	HYOU1	4,18542E-14	-3,31110E-01	0,256	0,169	7,70829E-10
cDC_LAMP3	ZNF207	7,21148E-13	-3,31493E-01	0,518	0,401	1,32814E-08
cDC_LAMP3	CYB5R3	2,89155E-07	-3,32021E-01	0,497	0,407	5,32537E-03
cDC_LAMP3	ANAPC11	1,52421E-14	-3,32682E-01	0,592	0,608	2,80714E-10
cDC_LAMP3	ARAF	3,24365E-13	-3,33653E-01	0,252	0,168	5,97382E-09
cDC_LAMP3	RPL37A	1,29010E-31	-3,33763E-01	0,757	0,531	2,37599E-27
cDC_LAMP3	NAP1L4	4,46365E-10	-3,36150E-01	0,29	0,211	8,22070E-06
cDC_LAMP3	LPCAT2	1,32227E-46	-3,36184E-01	0,145	0,315	2,43523E-42
cDC_LAMP3	UBE2J2	1,09492E-07	-3,37091E-01	0,269	0,199	2,01651E-03
cDC_LAMP3	XRN2	5,08787E-12	-3,38506E-01	0,344	0,398	9,37034E-08
cDC_LAMP3	TOMM6	3,45949E-39	-3,40027E-01	0,259	0,131	6,37134E-35
cDC_LAMP3	DNAJB9	8,71972E-07	-3,40501E-01	0,266	0,205	1,60591E-02

cDC_LAMP3	LPXN	5,42979E-23	-3,41284E-01	0,227	0,352	1,00000E-18
cDC_LAMP3	QKI	6,73000E-21	-3,43851E-01	0,279	0,371	1,23946E-16
cDC_LAMP3	PARVB	2,77728E-12	-3,44697E-01	0,225	0,297	5,11492E-08
cDC_LAMP3	RPL10A	3,83287E-63	-3,45049E-01	0,734	0,489	7,05900E-59
cDC_LAMP3	HNRNPR	1,23931E-08	-3,45502E-01	0,456	0,358	2,28243E-04
cDC_LAMP3	RHBDF2	4,54053E-07	-3,45580E-01	0,284	0,326	8,36229E-03
cDC_LAMP3	SERP1	1,84906E-15	-3,47182E-01	0,797	0,82	3,40541E-11
cDC_LAMP3	RPL7	4,27693E-26	-3,47950E-01	0,709	0,491	7,87682E-22
cDC_LAMP3	BCKDK	1,34592E-08	-3,48342E-01	0,273	0,321	2,47878E-04
cDC_LAMP3	ZFR	6,96297E-09	-3,48726E-01	0,33	0,246	1,28237E-04
cDC_LAMP3	ZFP36L1	8,52777E-56	-3,49284E-01	0,812	0,723	1,57056E-51
cDC_LAMP3	GNAQ	2,25695E-13	-3,49627E-01	0,274	0,343	4,15663E-09
cDC_LAMP3	TCF25	3,71171E-08	-3,50622E-01	0,549	0,562	6,83585E-04
cDC_LAMP3	EIF4H	2,43588E-06	-3,50939E-01	0,54	0,45	4,48617E-02
cDC_LAMP3	SYAP1	4,73732E-09	-3,51499E-01	0,298	0,346	8,72472E-05
cDC_LAMP3	COX7A2L	1,47241E-07	-3,52537E-01	0,579	0,487	2,71174E-03
cDC_LAMP3	CAPZB	3,95193E-09	-3,53454E-01	0,766	0,76	7,27826E-05
cDC_LAMP3	LRRFIP1	7,57783E-08	-3,53593E-01	0,654	0,685	1,39561E-03
cDC_LAMP3	OST4	1,35901E-16	-3,54751E-01	0,83	0,776	2,50288E-12
cDC_LAMP3	MRPL43	1,74638E-09	-3,56638E-01	0,304	0,351	3,21631E-05
cDC_LAMP3	RRBP1	2,34713E-09	-3,58947E-01	0,341	0,386	4,32271E-05
cDC_LAMP3	UBE2V1	1,13084E-25	-3,59483E-01	0,293	0,178	2,08267E-21
cDC_LAMP3	NUDT3	1,73943E-19	-3,59826E-01	0,241	0,333	3,20350E-15
cDC_LAMP3	SUPT16H	6,52005E-07	-3,60044E-01	0,267	0,201	1,20080E-02
cDC_LAMP3	VPS35	8,71159E-13	-3,60742E-01	0,389	0,441	1,60441E-08
cDC_LAMP3	UQCRB	2,87782E-30	-3,63583E-01	0,75	0,774	5,30009E-26
cDC_LAMP3	SUCLG1	4,71258E-07	-3,63701E-01	0,391	0,308	8,67916E-03
cDC_LAMP3	CIB1	4,54345E-41	-3,63823E-01	0,52	0,615	8,36767E-37
cDC_LAMP3	DNPH1	8,79613E-15	-3,64927E-01	0,272	0,349	1,61998E-10
cDC_LAMP3	SDHC	3,06140E-11	-3,65633E-01	0,411	0,442	5,63818E-07
cDC_LAMP3	UQCC2	4,82267E-31	-3,65915E-01	0,189	0,314	8,88191E-27
cDC_LAMP3	ZMIZ1	1,79026E-18	-3,66495E-01	0,173	0,268	3,29711E-14
cDC_LAMP3	UBE2W	6,57431E-26	-3,67059E-01	0,264	0,155	1,21079E-21
cDC_LAMP3	TIMM8B	2,65153E-18	-3,67927E-01	0,349	0,419	4,88333E-14
cDC_LAMP3	HGS	1,62915E-11	-3,69079E-01	0,293	0,205	3,00040E-07
cDC_LAMP3	JTB	4,22208E-26	-3,70819E-01	0,494	0,563	7,77580E-22
cDC_LAMP3	RPL4	1,64451E-82	-3,71518E-01	0,721	0,47	3,02870E-78
cDC_LAMP3	SELENOH	5,48499E-08	-3,72751E-01	0,3	0,332	1,01017E-03
cDC_LAMP3	NHP2	6,36461E-12	-3,73643E-01	0,366	0,414	1,17217E-07
cDC_LAMP3	MRPS5	6,68467E-07	-3,74471E-01	0,217	0,256	1,23112E-02
cDC_LAMP3	ME2	4,12677E-23	-3,77170E-01	0,205	0,308	7,60026E-19

cDC_LAMP3	MRPL52	1,58358E-06	-3,78294E-01	0,45	0,46	2,91648E-02
cDC_LAMP3	PCBD1	1,00334E-09	-3,78819E-01	0,316	0,362	1,84785E-05
cDC_LAMP3	YTHDF2	6,87979E-08	-3,78987E-01	0,364	0,278	1,26705E-03
cDC_LAMP3	NDUFA1	3,21552E-09	-3,79086E-01	0,701	0,687	5,92202E-05
cDC_LAMP3	TMEM9E	9,38843E-15	-3,80035E-01	0,312	0,375	1,72907E-10
cDC_LAMP3	MRPL11	9,70266E-08	-3,82770E-01	0,247	0,295	1,78694E-03
cDC_LAMP3	XRCC6	1,45375E-06	-3,83105E-01	0,588	0,492	2,67737E-02
cDC_LAMP3	DBNL	4,80967E-13	-3,83221E-01	0,596	0,489	8,85797E-09
cDC_LAMP3	CD84	2,26356E-07	-3,83915E-01	0,355	0,393	4,16880E-03
cDC_LAMP3	LUC7L	9,64235E-32	-3,85322E-01	0,31	0,18	1,77583E-27
cDC_LAMP3	PPIG	1,47267E-07	-3,85474E-01	0,435	0,453	2,71222E-03
cDC_LAMP3	HLA-C	2,71046E-18	-3,86443E-01	0,945	0,951	4,99186E-14
cDC_LAMP3	RABGGTI	7,18245E-23	-3,88002E-01	0,384	0,253	1,32279E-18
cDC_LAMP3	CACUL1	9,84291E-11	-3,88354E-01	0,242	0,305	1,81277E-06
cDC_LAMP3	RWDD1	3,89750E-10	-3,89644E-01	0,425	0,458	7,17803E-06
cDC_LAMP3	TUFM	2,15714E-06	-3,90755E-01	0,507	0,51	3,97280E-02
cDC_LAMP3	EIF2S3	1,64186E-08	-3,93058E-01	0,423	0,452	3,02381E-04
cDC_LAMP3	RNASEH2	2,32086E-10	-3,95884E-01	0,296	0,348	4,27433E-06
cDC_LAMP3	UBE2K	2,50783E-09	-3,96072E-01	0,373	0,283	4,61867E-05
cDC_LAMP3	CHCHD7	4,24425E-08	-3,96965E-01	0,211	0,259	7,81664E-04
cDC_LAMP3	TFEC	9,50256E-40	-3,97064E-01	0,135	0,3	1,75009E-35
cDC_LAMP3	FEZ2	1,26436E-26	-3,98644E-01	0,245	0,354	2,32858E-22
cDC_LAMP3	ACSL3	9,64041E-10	-4,00026E-01	0,196	0,255	1,77547E-05
cDC_LAMP3	RGS10	2,78583E-27	-4,02430E-01	0,705	0,741	5,13067E-23
cDC_LAMP3	PPP1CC	2,36296E-08	-4,02564E-01	0,309	0,353	4,35186E-04
cDC_LAMP3	BLOC1S1	1,86956E-20	-4,03376E-01	0,398	0,474	3,44318E-16
cDC_LAMP3	RER1	2,95921E-07	-4,05130E-01	0,452	0,461	5,44998E-03
cDC_LAMP3	SERF2	9,55524E-63	-4,05620E-01	0,928	0,955	1,75979E-58
cDC_LAMP3	VPS36	1,00490E-10	-4,05981E-01	0,226	0,289	1,85072E-06
cDC_LAMP3	SPG7	5,22221E-11	-4,07136E-01	0,309	0,218	9,61774E-07
cDC_LAMP3	NEDD8	5,24396E-13	-4,07320E-01	0,573	0,587	9,65780E-09
cDC_LAMP3	MGMT	9,27041E-21	-4,10155E-01	0,162	0,259	1,70733E-16
cDC_LAMP3	TIMM13	9,63527E-17	-4,10335E-01	0,323	0,397	1,77453E-12
cDC_LAMP3	CHTOP	5,37695E-08	-4,10624E-01	0,282	0,21	9,90274E-04
cDC_LAMP3	RPL13A	4,66248E-30	-4,11384E-01	0,74	0,514	8,58690E-26
cDC_LAMP3	YPEL5	8,40317E-55	-4,11411E-01	0,611	0,429	1,54761E-50
cDC_LAMP3	ATP6V1B	5,05386E-66	-4,13035E-01	0,368	0,54	9,30769E-62
cDC_LAMP3	HNRNPA	5,17067E-07	-4,13044E-01	0,801	0,833	9,52283E-03
cDC_LAMP3	PICALM	3,97176E-15	-4,13179E-01	0,36	0,431	7,31479E-11
cDC_LAMP3	CISD3	1,68333E-32	-4,13642E-01	0,181	0,309	3,10020E-28
cDC_LAMP3	PRKCSH	3,35872E-11	-4,14064E-01	0,264	0,321	6,18575E-07

cDC_LAMP3	MRPL34	1,82913E-07	-4,14507E-01	0,34	0,374	3,36870E-03
cDC_LAMP3	RPS4Y1	1,57636E-26	-4,16044E-01	0,298	0,172	2,90319E-22
cDC_LAMP3	PARL	1,00650E-10	-4,16185E-01	0,234	0,292	1,85367E-06
cDC_LAMP3	RPL23A	2,26785E-52	-4,16935E-01	0,761	0,518	4,17670E-48
cDC_LAMP3	CAMK1	1,46230E-75	-4,19252E-01	0,047	0,262	2,69311E-71
cDC_LAMP3	SOCS3	2,43499E-09	-4,19836E-01	0,459	0,354	4,48451E-05
cDC_LAMP3	CLTC	2,58824E-06	-4,20293E-01	0,409	0,42	4,76677E-02
cDC_LAMP3	ARHGAP:	7,92628E-07	-4,20464E-01	0,291	0,333	1,45978E-02
cDC_LAMP3	KRTCAP2	1,72891E-13	-4,20475E-01	0,57	0,578	3,18413E-09
cDC_LAMP3	YWHAZ	4,33862E-53	-4,22076E-01	0,8	0,71	7,99045E-49
cDC_LAMP3	IDH2	8,55259E-21	-4,22675E-01	0,316	0,414	1,57513E-16
cDC_LAMP3	MCTP1	2,64361E-24	-4,22756E-01	0,134	0,251	4,86873E-20
cDC_LAMP3	TAF9	1,92811E-07	-4,22788E-01	0,264	0,303	3,55100E-03
cDC_LAMP3	CHMP1A	1,80911E-09	-4,24881E-01	0,261	0,186	3,33183E-05
cDC_LAMP3	MT-ND5	8,82627E-48	-4,25907E-01	0,736	0,531	1,62553E-43
cDC_LAMP3	HBP1	1,17414E-09	-4,26785E-01	0,32	0,237	2,16241E-05
cDC_LAMP3	GUSB	7,36214E-27	-4,27880E-01	0,239	0,349	1,35589E-22
cDC_LAMP3	TPD52L2	3,90804E-08	-4,28438E-01	0,224	0,27	7,19745E-04
cDC_LAMP3	TGIF1	7,18087E-13	-4,28600E-01	0,422	0,321	1,32250E-08
cDC_LAMP3	PPP1R7	5,18067E-08	-4,29855E-01	0,234	0,281	9,54124E-04
cDC_LAMP3	TCEAL4	5,18680E-29	-4,30287E-01	0,159	0,284	9,55254E-25
cDC_LAMP3	CORO1B	9,20611E-09	-4,30731E-01	0,444	0,473	1,69549E-04
cDC_LAMP3	POLR2F	6,92286E-12	-4,32090E-01	0,251	0,315	1,27498E-07
cDC_LAMP3	PSMB4	2,18492E-23	-4,32611E-01	0,512	0,368	4,02396E-19
cDC_LAMP3	NDUFA5	7,71880E-12	-4,33950E-01	0,228	0,295	1,42157E-07
cDC_LAMP3	MPG	2,58024E-07	-4,34048E-01	0,283	0,324	4,75202E-03
cDC_LAMP3	NDUFS6	8,67122E-23	-4,34405E-01	0,439	0,509	1,59698E-18
cDC_LAMP3	NDUFA3	5,73018E-14	-4,35583E-01	0,52	0,547	1,05533E-09
cDC_LAMP3	AIMP1	3,76209E-07	-4,36442E-01	0,252	0,293	6,92863E-03
cDC_LAMP3	GUK1	1,53699E-22	-4,37390E-01	0,689	0,711	2,83067E-18
cDC_LAMP3	PJA2	2,41639E-07	-4,40009E-01	0,234	0,277	4,45026E-03
cDC_LAMP3	SERPINB(	7,02450E-17	-4,40642E-01	0,36	0,424	1,29370E-12
cDC_LAMP3	GNG10	2,77136E-16	-4,41190E-01	0,266	0,34	5,10401E-12
cDC_LAMP3	BRK1	4,05602E-20	-4,41255E-01	0,692	0,727	7,46997E-16
cDC_LAMP3	SNX3	5,33339E-08	-4,41677E-01	0,679	0,69	9,82251E-04
cDC_LAMP3	IFNGR2	4,53320E-11	-4,42295E-01	0,652	0,562	8,34880E-07
cDC_LAMP3	SH3BP5	2,63016E-48	-4,43059E-01	0,161	0,343	4,84396E-44
cDC_LAMP3	HSD17B1	2,13694E-07	-4,45766E-01	0,266	0,307	3,93560E-03
cDC_LAMP3	HSPA5	1,66177E-36	-4,48539E-01	0,441	0,565	3,06049E-32
cDC_LAMP3	ELOVL1	6,75378E-11	-4,49559E-01	0,461	0,357	1,24384E-06
cDC_LAMP3	SETX	7,00571E-21	-4,50906E-01	0,184	0,283	1,29024E-16

cDC_LAMP3	LMAN1	8,53956E-19	-4,55776E-01	0,202	0,292	1,57273E-14
cDC_LAMP3	FAM120/	1,81672E-23	-4,56118E-01	0,191	0,297	3,34586E-19
cDC_LAMP3	ECHDC1	1,87597E-31	-4,56806E-01	0,14	0,274	3,45497E-27
cDC_LAMP3	MIDN	3,97812E-16	-4,57487E-01	0,206	0,284	7,32650E-12
cDC_LAMP3	MBP	1,39535E-36	-4,57924E-01	0,174	0,323	2,56982E-32
cDC_LAMP3	NUCB1	2,20652E-33	-4,58292E-01	0,454	0,546	4,06375E-29
cDC_LAMP3	NDUFV3	2,08411E-22	-4,60161E-01	0,178	0,284	3,83830E-18
cDC_LAMP3	GRB2	2,36349E-32	-4,60423E-01	0,635	0,681	4,35284E-28
cDC_LAMP3	CHMP2A	6,58459E-09	-4,63369E-01	0,476	0,492	1,21268E-04
cDC_LAMP3	FTH1	1,95243E-10	-4,63870E-01	0,993	0,997	3,59579E-06
cDC_LAMP3	SPI1	1,28424E-35	-4,65104E-01	0,713	0,769	2,36518E-31
cDC_LAMP3	PCM1	1,13946E-18	-4,66770E-01	0,227	0,318	2,09853E-14
cDC_LAMP3	CD151	3,99888E-16	-4,66809E-01	0,269	0,34	7,36474E-12
cDC_LAMP3	EMC7	1,51442E-15	-4,67370E-01	0,252	0,323	2,78910E-11
cDC_LAMP3	B2M	4,84977E-17	-4,67435E-01	0,992	0,996	8,93182E-13
cDC_LAMP3	SLIRP	4,45094E-14	-4,67800E-01	0,376	0,428	8,19729E-10
cDC_LAMP3	RNPEP	1,72713E-11	-4,68131E-01	0,273	0,333	3,18085E-07
cDC_LAMP3	SLC8A1	6,41192E-51	-4,68143E-01	0,175	0,371	1,18088E-46
cDC_LAMP3	PEBP1	1,80113E-08	-4,68643E-01	0,559	0,565	3,31714E-04
cDC_LAMP3	IGFLR1	9,02145E-11	-4,69113E-01	0,465	0,356	1,66148E-06
cDC_LAMP3	NCOA4	2,06999E-19	-4,70025E-01	0,464	0,533	3,81231E-15
cDC_LAMP3	TMBIM1	2,05041E-16	-4,71692E-01	0,283	0,364	3,77625E-12
cDC_LAMP3	TACC1	1,34183E-13	-4,72021E-01	0,36	0,423	2,47125E-09
cDC_LAMP3	POLE4	1,49683E-30	-4,72184E-01	0,207	0,335	2,75670E-26
cDC_LAMP3	COX14	3,52255E-12	-4,73536E-01	0,411	0,464	6,48747E-08
cDC_LAMP3	OAZ2	3,83898E-16	-4,75751E-01	0,2	0,286	7,07024E-12
cDC_LAMP3	NDUFB5	1,61034E-14	-4,75973E-01	0,403	0,455	2,96577E-10
cDC_LAMP3	ERP44	2,79803E-12	-4,77691E-01	0,392	0,43	5,15313E-08
cDC_LAMP3	TWF2	1,34950E-16	-4,77833E-01	0,458	0,33	2,48537E-12
cDC_LAMP3	C7orf50	2,51079E-08	-4,77934E-01	0,286	0,339	4,62412E-04
cDC_LAMP3	PFDN5	3,77850E-62	-4,80046E-01	0,867	0,903	6,95886E-58
cDC_LAMP3	PLIN3	1,03452E-07	-4,80301E-01	0,337	0,373	1,90527E-03
cDC_LAMP3	PRPF38B	7,29855E-08	-4,80754E-01	0,412	0,437	1,34417E-03
cDC_LAMP3	RBMX	2,20426E-07	-4,80971E-01	0,517	0,422	4,05958E-03
cDC_LAMP3	PABPC4	9,81766E-07	-4,81344E-01	0,404	0,424	1,80812E-02
cDC_LAMP3	FDFT1	1,57556E-12	-4,82005E-01	0,349	0,257	2,90172E-08
cDC_LAMP3	DDX18	4,57880E-14	-4,82446E-01	0,307	0,372	8,43278E-10
cDC_LAMP3	DNM2	5,94235E-08	-4,82711E-01	0,255	0,298	1,09440E-03
cDC_LAMP3	DNMT1	1,20408E-16	-4,83179E-01	0,235	0,32	2,21756E-12
cDC_LAMP3	DECR1	2,14168E-22	-4,84990E-01	0,248	0,35	3,94433E-18
cDC_LAMP3	ZNF385A	7,47812E-42	-4,85463E-01	0,255	0,417	1,37725E-37

cDC_LAMP3	SLC31A2	7,70983E-09	-4,85579E-01	0,416	0,443	1,41992E-04
cDC_LAMP3	TMEM51	1,66046E-33	-4,86142E-01	0,131	0,266	3,05806E-29
cDC_LAMP3	CMTM7	1,52408E-33	-4,86417E-01	0,284	0,415	2,80689E-29
cDC_LAMP3	ATP6V1C	4,80999E-10	-4,86451E-01	0,218	0,275	8,85855E-06
cDC_LAMP3	SNAP29	2,60232E-12	-4,89027E-01	0,223	0,287	4,79269E-08
cDC_LAMP3	NDUFA8	5,75779E-09	-4,89063E-01	0,241	0,292	1,06041E-04
cDC_LAMP3	KDELR1	2,66086E-17	-4,90505E-01	0,508	0,54	4,90051E-13
cDC_LAMP3	CCDC85E	1,13915E-32	-4,90667E-01	0,272	0,401	2,09798E-28
cDC_LAMP3	RAB31	3,47675E-49	-4,90693E-01	0,56	0,645	6,40313E-45
cDC_LAMP3	OS9	2,83906E-13	-4,91363E-01	0,505	0,538	5,22869E-09
cDC_LAMP3	RPS17	1,78569E-34	-4,92121E-01	0,611	0,401	3,28871E-30
cDC_LAMP3	AHNAK	2,89013E-24	-4,93286E-01	0,507	0,606	5,32275E-20
cDC_LAMP3	CHMP2B	8,18849E-08	-4,94913E-01	0,253	0,3	1,50807E-03
cDC_LAMP3	TMED5	1,08743E-06	-4,95474E-01	0,35	0,374	2,00272E-02
cDC_LAMP3	CREB1	6,47379E-14	-4,95518E-01	0,284	0,192	1,19228E-09
cDC_LAMP3	ARMCX3	1,79659E-14	-4,98703E-01	0,181	0,261	3,30878E-10
cDC_LAMP3	TOMM7	5,50299E-28	-4,99106E-01	0,758	0,792	1,01349E-23
cDC_LAMP3	DNAJC3	2,57259E-16	-5,01259E-01	0,286	0,366	4,73793E-12
cDC_LAMP3	SKP1	6,12435E-07	-5,01664E-01	0,744	0,687	1,12792E-02
cDC_LAMP3	COX6B1	4,34843E-27	-5,04351E-01	0,801	0,811	8,00851E-23
cDC_LAMP3	COX5B	1,55963E-50	-5,04902E-01	0,725	0,774	2,87237E-46
cDC_LAMP3	DDX21	2,64766E-17	-5,04964E-01	0,366	0,439	4,87619E-13
cDC_LAMP3	PLEKHB2	4,95084E-08	-5,06693E-01	0,414	0,441	9,11797E-04
cDC_LAMP3	CHCHD1(	8,66888E-31	-5,07996E-01	0,43	0,519	1,59655E-26
cDC_LAMP3	POLD4	5,25391E-17	-5,08532E-01	0,535	0,57	9,67613E-13
cDC_LAMP3	COX7B	5,22279E-29	-5,10484E-01	0,64	0,667	9,61882E-25
cDC_LAMP3	BAG1	2,21996E-33	-5,11796E-01	0,244	0,379	4,08851E-29
cDC_LAMP3	RUNX1	1,40475E-12	-5,12438E-01	0,21	0,28	2,58713E-08
cDC_LAMP3	GNB4	9,57610E-22	-5,12438E-01	0,241	0,339	1,76363E-17
cDC_LAMP3	RCSD1	4,19247E-14	-5,15572E-01	0,302	0,378	7,72127E-10
cDC_LAMP3	LAMP1	9,81037E-26	-5,15655E-01	0,297	0,4	1,80678E-21
cDC_LAMP3	MAX	1,32067E-20	-5,16743E-01	0,312	0,399	2,43228E-16
cDC_LAMP3	PIK3R1	5,26876E-51	-5,18229E-01	0,143	0,327	9,70347E-47
cDC_LAMP3	DICER1	3,91393E-27	-5,18595E-01	0,154	0,273	7,20828E-23
cDC_LAMP3	COX7A2	3,33805E-10	-5,19007E-01	0,706	0,721	6,14768E-06
cDC_LAMP3	SSR2	2,36364E-06	-5,19017E-01	0,667	0,652	4,35312E-02
cDC_LAMP3	ATM	2,52037E-30	-5,19076E-01	0,133	0,263	4,64177E-26
cDC_LAMP3	NMT1	1,14242E-13	-5,20076E-01	0,256	0,172	2,10399E-09
cDC_LAMP3	MT-ND3	4,27331E-28	-5,21515E-01	0,776	0,563	7,87016E-24
cDC_LAMP3	RPL31	4,24397E-30	-5,22175E-01	0,706	0,49	7,81611E-26
cDC_LAMP3	PAG1	1,00819E-33	-5,22337E-01	0,138	0,275	1,85678E-29

cDC_LAMP3	PTK2B	2,63925E-14	-5,23162E-01	0,346	0,251	4,86071E-10
cDC_LAMP3	CBR1	5,73370E-23	-5,23558E-01	0,216	0,32	1,05598E-18
cDC_LAMP3	MIF4GD	4,56419E-20	-5,23597E-01	0,218	0,315	8,40587E-16
cDC_LAMP3	RABGAP1	1,03421E-12	-5,24319E-01	0,344	0,25	1,90471E-08
cDC_LAMP3	MTPN	5,45946E-12	-5,25043E-01	0,494	0,528	1,00547E-07
cDC_LAMP3	CPNE1	7,43260E-20	-5,25609E-01	0,347	0,23	1,36886E-15
cDC_LAMP3	NDUFA4	2,57669E-44	-5,26145E-01	0,706	0,746	4,74548E-40
cDC_LAMP3	TGFB1	1,18879E-50	-5,27674E-01	0,293	0,458	2,18939E-46
cDC_LAMP3	TMX1	3,71450E-08	-5,28040E-01	0,294	0,337	6,84100E-04
cDC_LAMP3	QSOX1	5,19752E-18	-5,29545E-01	0,206	0,291	9,57228E-14
cDC_LAMP3	TMEM10	7,17256E-16	-5,30061E-01	0,287	0,371	1,32097E-11
cDC_LAMP3	PHF20	9,34253E-22	-5,30909E-01	0,174	0,278	1,72061E-17
cDC_LAMP3	SLC43A2	2,10994E-31	-5,32961E-01	0,242	0,372	3,88588E-27
cDC_LAMP3	CHMP4A	1,11109E-17	-5,33904E-01	0,271	0,176	2,04629E-13
cDC_LAMP3	MRPS15	4,98197E-12	-5,35401E-01	0,258	0,32	9,17529E-08
cDC_LAMP3	DRAP1	7,07052E-60	-5,36238E-01	0,544	0,661	1,30218E-55
cDC_LAMP3	RPL17	7,10959E-59	-5,37161E-01	0,634	0,427	1,30937E-54
cDC_LAMP3	KPNA2	1,70706E-11	-5,38313E-01	0,291	0,207	3,14390E-07
cDC_LAMP3	EZR	6,02426E-19	-5,38592E-01	0,535	0,396	1,10949E-14
cDC_LAMP3	SCAND1	2,38777E-14	-5,38605E-01	0,499	0,536	4,39756E-10
cDC_LAMP3	GPSM3	7,59899E-43	-5,38670E-01	0,597	0,686	1,39951E-38
cDC_LAMP3	FLI1	3,13116E-38	-5,39230E-01	0,118	0,266	5,76666E-34
cDC_LAMP3	UQCRH	5,96485E-34	-5,40537E-01	0,662	0,703	1,09855E-29
cDC_LAMP3	FAM204A	1,60691E-19	-5,41098E-01	0,197	0,29	2,95945E-15
cDC_LAMP3	FUCA2	8,34263E-44	-5,41426E-01	0,152	0,313	1,53646E-39
cDC_LAMP3	ABHD14B	1,65202E-14	-5,43109E-01	0,186	0,265	3,04252E-10
cDC_LAMP3	ITGB1	2,41083E-16	-5,43168E-01	0,414	0,477	4,44002E-12
cDC_LAMP3	PDXK	1,29553E-58	-5,44969E-01	0,115	0,307	2,38597E-54
cDC_LAMP3	RPS20	1,34067E-15	-5,45606E-01	0,627	0,445	2,46911E-11
cDC_LAMP3	USP4	2,27433E-06	-5,45773E-01	0,262	0,297	4,18863E-02
cDC_LAMP3	EEF1G	3,90099E-48	-5,47007E-01	0,462	0,29	7,18446E-44
cDC_LAMP3	COX8A	2,54586E-51	-5,47290E-01	0,732	0,779	4,68871E-47
cDC_LAMP3	PPP1CA	1,70814E-12	-5,50411E-01	0,606	0,622	3,14588E-08
cDC_LAMP3	TTC3	6,41857E-07	-5,52143E-01	0,288	0,33	1,18211E-02
cDC_LAMP3	RHOA	1,06825E-09	-5,53422E-01	0,878	0,878	1,96739E-05
cDC_LAMP3	CSK	1,08289E-07	-5,53483E-01	0,305	0,348	1,99435E-03
cDC_LAMP3	FIS1	3,10466E-12	-5,53719E-01	0,492	0,527	5,71785E-08
cDC_LAMP3	APOL6	1,09873E-46	-5,53942E-01	0,1	0,265	2,02353E-42
cDC_LAMP3	RAB32	3,52949E-34	-5,55295E-01	0,246	0,379	6,50026E-30
cDC_LAMP3	GPR65	1,55613E-15	-5,56046E-01	0,238	0,33	2,86592E-11
cDC_LAMP3	SAP30BP	8,54188E-07	-5,56531E-01	0,352	0,268	1,57316E-02

cDC_LAMP3	SCAF11	1,61071E-08	-5,57304E-01	0,479	0,49	2,96645E-04
cDC_LAMP3	SH3BP2	2,75000E-24	-5,59229E-01	0,185	0,298	5,06467E-20
cDC_LAMP3	SEC11C	1,35732E-06	-5,59366E-01	0,287	0,327	2,49978E-02
cDC_LAMP3	STX7	3,64164E-07	-5,59652E-01	0,389	0,415	6,70681E-03
cDC_LAMP3	RHBDD2	2,50580E-14	-5,59737E-01	0,188	0,26	4,61493E-10
cDC_LAMP3	PRDX4	9,88650E-21	-5,61537E-01	0,189	0,29	1,82080E-16
cDC_LAMP3	RPS11	3,37376E-22	-5,62444E-01	0,705	0,498	6,21346E-18
cDC_LAMP3	AP2M1	1,06655E-15	-5,64235E-01	0,564	0,584	1,96426E-11
cDC_LAMP3	SLC25A5	3,13535E-54	-5,64823E-01	0,696	0,745	5,77438E-50
cDC_LAMP3	DDX3X	1,27892E-17	-5,64921E-01	0,417	0,48	2,35540E-13
cDC_LAMP3	EIF4A1	6,08341E-31	-5,67128E-01	0,595	0,677	1,12038E-26
cDC_LAMP3	CLTA	4,45080E-18	-5,67331E-01	0,64	0,649	8,19704E-14
cDC_LAMP3	NDUFS2	1,06855E-07	-5,68617E-01	0,32	0,362	1,96794E-03
cDC_LAMP3	VPS28	3,11889E-15	-5,69013E-01	0,592	0,613	5,74406E-11
cDC_LAMP3	CD81	2,44534E-80	-5,69927E-01	0,255	0,468	4,50359E-76
cDC_LAMP3	GGA2	1,12162E-18	-5,70165E-01	0,175	0,273	2,06570E-14
cDC_LAMP3	NDUFA2	9,73102E-20	-5,70744E-01	0,507	0,553	1,79216E-15
cDC_LAMP3	EML4	6,50179E-36	-5,71590E-01	0,214	0,36	1,19743E-31
cDC_LAMP3	MBD4	1,09872E-12	-5,71639E-01	0,18	0,252	2,02351E-08
cDC_LAMP3	MRPL3	2,25062E-12	-5,72238E-01	0,216	0,285	4,14496E-08
cDC_LAMP3	C4orf48	1,48214E-26	-5,72736E-01	0,444	0,53	2,72966E-22
cDC_LAMP3	PHYKPL	1,87717E-09	-5,73125E-01	0,346	0,256	3,45718E-05
cDC_LAMP3	XBP1	1,78015E-18	-5,74029E-01	0,34	0,428	3,27851E-14
cDC_LAMP3	VASP	9,66636E-26	-5,74965E-01	0,373	0,473	1,78025E-21
cDC_LAMP3	MRPL40	9,99935E-13	-5,75033E-01	0,205	0,274	1,84158E-08
cDC_LAMP3	MCL1	1,18052E-25	-5,75973E-01	0,746	0,77	2,17416E-21
cDC_LAMP3	KLF4	4,56722E-09	-5,76468E-01	0,403	0,448	8,41145E-05
cDC_LAMP3	CREG1	4,62683E-08	-5,78271E-01	0,472	0,514	8,52124E-04
cDC_LAMP3	SMIM19	8,02092E-08	-5,79284E-01	0,211	0,261	1,47721E-03
cDC_LAMP3	TMEM17	5,98357E-24	-5,81562E-01	0,415	0,484	1,10199E-19
cDC_LAMP3	FOSL2	5,12329E-21	-5,81762E-01	0,198	0,298	9,43556E-17
cDC_LAMP3	BNIP2	1,66532E-06	-5,83980E-01	0,383	0,401	3,06703E-02
cDC_LAMP3	FIBP	1,52228E-09	-5,85051E-01	0,326	0,369	2,80358E-05
cDC_LAMP3	RAB1B	2,53737E-07	-5,85531E-01	0,323	0,357	4,67307E-03
cDC_LAMP3	AKR7A2	4,23263E-09	-5,86818E-01	0,195	0,251	7,79523E-05
cDC_LAMP3	POLR3GL	1,59851E-27	-5,87186E-01	0,178	0,301	2,94398E-23
cDC_LAMP3	TRAPPC2	1,72733E-08	-5,87536E-01	0,218	0,271	3,18123E-04
cDC_LAMP3	UQCR10	5,44698E-47	-5,89322E-01	0,679	0,728	1,00317E-42
cDC_LAMP3	RNASEK	1,01320E-19	-5,92794E-01	0,602	0,461	1,86601E-15
cDC_LAMP3	UGCG	2,24749E-30	-5,93057E-01	0,316	0,184	4,13921E-26
cDC_LAMP3	SNX29	2,84834E-22	-5,94346E-01	0,161	0,263	5,24579E-18

cDC_LAMP3	MLEC	1,51738E-36	-5,96443E-01	0,209	0,351	2,79455E-32
cDC_LAMP3	CCDC107	2,86624E-19	-5,96496E-01	0,217	0,31	5,27875E-15
cDC_LAMP3	NR4A2	9,88834E-35	-5,97050E-01	0,317	0,455	1,82114E-30
cDC_LAMP3	AK2	7,53572E-07	-5,99233E-01	0,301	0,344	1,38785E-02
cDC_LAMP3	TALDO1	8,79025E-07	-6,00690E-01	0,656	0,662	1,61890E-02
cDC_LAMP3	ELL2	6,44609E-12	-6,01160E-01	0,225	0,286	1,18718E-07
cDC_LAMP3	IL17RA	5,01986E-53	-6,01218E-01	0,128	0,307	9,24508E-49
cDC_LAMP3	COA6	3,79828E-11	-6,02587E-01	0,193	0,254	6,99529E-07
cDC_LAMP3	CSTB	3,24548E-10	-6,04131E-01	0,794	0,789	5,97720E-06
cDC_LAMP3	TNFAIP3	6,16363E-10	-6,04625E-01	0,679	0,539	1,13516E-05
cDC_LAMP3	CCNH	5,84284E-36	-6,05700E-01	0,219	0,376	1,07608E-31
cDC_LAMP3	TPT1	7,76159E-18	-6,07704E-01	0,974	0,974	1,42945E-13
cDC_LAMP3	CTBS	1,72683E-06	-6,08661E-01	0,236	0,27	3,18030E-02
cDC_LAMP3	NAA38	9,28438E-21	-6,09071E-01	0,329	0,419	1,70990E-16
cDC_LAMP3	RPS10	4,03998E-12	-6,09433E-01	0,404	0,28	7,44042E-08
cDC_LAMP3	PYURF	1,54505E-14	-6,10425E-01	0,347	0,412	2,84553E-10
cDC_LAMP3	MSN	6,13063E-25	-6,10618E-01	0,578	0,64	1,12908E-20
cDC_LAMP3	LAMP2	1,49290E-16	-6,13703E-01	0,342	0,403	2,74948E-12
cDC_LAMP3	TGFBR2	2,54695E-39	-6,13775E-01	0,18	0,34	4,69071E-35
cDC_LAMP3	SAT1	6,91332E-23	-6,14107E-01	0,898	0,949	1,27323E-18
cDC_LAMP3	RIN3	5,25933E-40	-6,14124E-01	0,252	0,409	9,68610E-36
cDC_LAMP3	UPP1	9,98993E-23	-6,14932E-01	0,343	0,434	1,83985E-18
cDC_LAMP3	COPS6	1,25522E-13	-6,15654E-01	0,283	0,345	2,31173E-09
cDC_LAMP3	NDUFAF3	2,48606E-11	-6,16249E-01	0,443	0,472	4,57857E-07
cDC_LAMP3	MT-ND4L	6,04315E-124	-6,16765E-01	0,767	0,506	1,11297E-119
cDC_LAMP3	BSG	5,79002E-11	-6,17933E-01	0,546	0,564	1,06635E-06
cDC_LAMP3	ENY2	3,26823E-14	-6,18219E-01	0,569	0,596	6,01910E-10
cDC_LAMP3	DAZAP2	2,60140E-14	-6,18960E-01	0,725	0,743	4,79099E-10
cDC_LAMP3	MRPL16	2,31507E-11	-6,20385E-01	0,2	0,261	4,26367E-07
cDC_LAMP3	SNX5	3,22007E-19	-6,20401E-01	0,323	0,4	5,93040E-15
cDC_LAMP3	RNF7	2,51132E-16	-6,21395E-01	0,51	0,546	4,62511E-12
cDC_LAMP3	EEF1B2	1,96056E-11	-6,22872E-01	0,848	0,841	3,61076E-07
cDC_LAMP3	SERTAD1	1,33523E-06	-6,24767E-01	0,292	0,327	2,45910E-02
cDC_LAMP3	TXN2	5,40741E-12	-6,25661E-01	0,348	0,398	9,95882E-08
cDC_LAMP3	SRA1	5,19045E-11	-6,26250E-01	0,343	0,387	9,55924E-07
cDC_LAMP3	ATP6AP1	6,75355E-70	-6,27288E-01	0,371	0,549	1,24380E-65
cDC_LAMP3	IRAK3	3,51506E-48	-6,27920E-01	0,092	0,259	6,47369E-44
cDC_LAMP3	GPR108	4,88959E-07	-6,28050E-01	0,217	0,259	9,00515E-03
cDC_LAMP3	HMGB2	2,23753E-31	-6,28968E-01	0,283	0,402	4,12086E-27
cDC_LAMP3	C20orf27	3,57953E-68	-6,28985E-01	0,129	0,351	6,59242E-64
cDC_LAMP3	WDR83O	1,61618E-15	-6,29540E-01	0,627	0,62	2,97653E-11

cDC_LAMP3	UQCRQ	8,44251E-53	-6,31078E-01	0,599	0,668	1,55486E-48
cDC_LAMP3	PTTG1IP	4,43704E-33	-6,32532E-01	0,4	0,505	8,17169E-29
cDC_LAMP3	LIMS1	5,19554E-71	-6,34074E-01	0,378	0,561	9,56863E-67
cDC_LAMP3	HSBP1	8,05159E-36	-6,35184E-01	0,526	0,603	1,48286E-31
cDC_LAMP3	DHRS4	8,84391E-24	-6,35283E-01	0,169	0,274	1,62878E-19
cDC_LAMP3	ARL6IP4	3,04760E-13	-6,35473E-01	0,623	0,634	5,61276E-09
cDC_LAMP3	RPL27A	2,62098E-26	-6,35779E-01	0,674	0,457	4,82706E-22
cDC_LAMP3	TRMT1	1,72514E-28	-6,36620E-01	0,159	0,278	3,17719E-24
cDC_LAMP3	EFCAB14	4,05618E-14	-6,36917E-01	0,196	0,27	7,47026E-10
cDC_LAMP3	PA2G4	9,44893E-24	-6,37400E-01	0,348	0,436	1,74021E-19
cDC_LAMP3	COMMD'	1,25052E-22	-6,37523E-01	0,213	0,317	2,30309E-18
cDC_LAMP3	PHC2	2,06605E-57	-6,40926E-01	0,068	0,25	3,80504E-53
cDC_LAMP3	MRPS18I	2,78635E-08	-6,42071E-01	0,229	0,279	5,13162E-04
cDC_LAMP3	NUMB	9,98652E-19	-6,42824E-01	0,243	0,336	1,83922E-14
cDC_LAMP3	LEPROTL	4,64250E-39	-6,43864E-01	0,259	0,403	8,55009E-35
cDC_LAMP3	GPCPD1	7,84099E-21	-6,43933E-01	0,185	0,283	1,44407E-16
cDC_LAMP3	WASF2	2,68335E-22	-6,44064E-01	0,52	0,57	4,94192E-18
cDC_LAMP3	METTL7A	2,82906E-38	-6,44885E-01	0,1	0,252	5,21029E-34
cDC_LAMP3	ARL4C	2,00007E-14	-6,45596E-01	0,257	0,335	3,68353E-10
cDC_LAMP3	UNC93B1	1,45658E-55	-6,45635E-01	0,16	0,342	2,68259E-51
cDC_LAMP3	TMEM23	2,01510E-08	-6,47132E-01	0,425	0,452	3,71122E-04
cDC_LAMP3	JUND	3,18669E-23	-6,49640E-01	0,382	0,457	5,86892E-19
cDC_LAMP3	MGST3	1,21534E-12	-6,49945E-01	0,497	0,543	2,23829E-08
cDC_LAMP3	TMEM70	7,24637E-07	-6,50075E-01	0,254	0,291	1,33456E-02
cDC_LAMP3	ATRAID	7,55102E-21	-6,51321E-01	0,396	0,468	1,39067E-16
cDC_LAMP3	C6orf62	1,44415E-08	-6,51748E-01	0,461	0,361	2,65970E-04
cDC_LAMP3	RASGEF1	7,66814E-19	-6,52022E-01	0,174	0,272	1,41224E-14
cDC_LAMP3	PEPD	1,23151E-37	-6,52071E-01	0,231	0,367	2,26807E-33
cDC_LAMP3	RB1	5,07449E-12	-6,52156E-01	0,353	0,404	9,34568E-08
cDC_LAMP3	KCNMA1	2,79681E-54	-6,53538E-01	0,083	0,266	5,15088E-50
cDC_LAMP3	EIF2AK2	1,63898E-26	-6,53677E-01	0,15	0,265	3,01851E-22
cDC_LAMP3	FKBP5	6,45674E-20	-6,55069E-01	0,451	0,52	1,18914E-15
cDC_LAMP3	NAAA	1,38362E-10	-6,55508E-01	0,308	0,369	2,54821E-06
cDC_LAMP3	NACA	1,76033E-16	-6,55717E-01	0,912	0,919	3,24200E-12
cDC_LAMP3	TMCO1	2,12762E-22	-6,55832E-01	0,266	0,361	3,91844E-18
cDC_LAMP3	CALHM6	1,37941E-18	-6,56515E-01	0,176	0,264	2,54046E-14
cDC_LAMP3	YIF1A	4,58531E-12	-6,57686E-01	0,218	0,287	8,44476E-08
cDC_LAMP3	MLX	2,16437E-08	-6,60383E-01	0,32	0,36	3,98611E-04
cDC_LAMP3	PIGT	2,67711E-19	-6,60625E-01	0,201	0,294	4,93043E-15
cDC_LAMP3	CUTA	1,45247E-36	-6,61652E-01	0,364	0,489	2,67502E-32
cDC_LAMP3	DPP7	5,62437E-17	-6,62681E-01	0,523	0,558	1,03584E-12

cDC_LAMP3	MRPL23	7,02606E-43	-6,63005E-01	0,204	0,361	1,29399E-38
cDC_LAMP3	ACP2	1,69265E-21	-6,63761E-01	0,166	0,26	3,11736E-17
cDC_LAMP3	HEBP1	1,11012E-46	-6,64501E-01	0,147	0,314	2,04451E-42
cDC_LAMP3	AGPAT2	9,36520E-23	-6,64876E-01	0,191	0,295	1,72479E-18
cDC_LAMP3	CASP4	1,81826E-29	-6,64956E-01	0,336	0,446	3,34869E-25
cDC_LAMP3	CAT	1,06290E-09	-6,67004E-01	0,37	0,41	1,95755E-05
cDC_LAMP3	RILPL2	4,57530E-31	-6,68138E-01	0,33	0,46	8,42633E-27
cDC_LAMP3	COMMD	2,00859E-32	-6,69231E-01	0,7	0,737	3,69923E-28
cDC_LAMP3	RAP1A	1,32754E-52	-6,71214E-01	0,444	0,569	2,44493E-48
cDC_LAMP3	TM6SF1	4,61959E-61	-6,72921E-01	0,08	0,278	8,50790E-57
cDC_LAMP3	RBM47	6,92986E-08	-6,73335E-01	0,316	0,36	1,27627E-03
cDC_LAMP3	WAS	1,82399E-63	-6,75011E-01	0,354	0,535	3,35924E-59
cDC_LAMP3	PRDX1	3,21002E-62	-6,76078E-01	0,67	0,749	5,91190E-58
cDC_LAMP3	RENBP	7,44500E-60	-6,77043E-01	0,111	0,303	1,37115E-55
cDC_LAMP3	RNF213	1,91840E-12	-6,78719E-01	0,468	0,506	3,53311E-08
cDC_LAMP3	SERINC3	1,13820E-11	-6,79077E-01	0,287	0,342	2,09623E-07
cDC_LAMP3	MRPS34	3,34622E-16	-6,80148E-01	0,328	0,397	6,16273E-12
cDC_LAMP3	BEX4	1,44806E-07	-6,81507E-01	0,287	0,33	2,66689E-03
cDC_LAMP3	TGOLN2	3,14122E-51	-6,82646E-01	0,367	0,518	5,78519E-47
cDC_LAMP3	ETS2	3,23295E-32	-6,83345E-01	0,293	0,43	5,95413E-28
cDC_LAMP3	MEF2A	3,02849E-36	-6,83460E-01	0,287	0,426	5,57756E-32
cDC_LAMP3	OCIAD1	2,58702E-11	-6,83532E-01	0,399	0,435	4,76452E-07
cDC_LAMP3	STX12	9,21910E-13	-6,83558E-01	0,243	0,314	1,69788E-08
cDC_LAMP3	MTDH	7,02900E-77	-6,85179E-01	0,579	0,698	1,29453E-72
cDC_LAMP3	MPV17	2,16967E-11	-6,87849E-01	0,25	0,311	3,99588E-07
cDC_LAMP3	SSBP1	8,49766E-12	-6,88837E-01	0,455	0,488	1,56501E-07
cDC_LAMP3	USF2	7,95485E-36	-6,88910E-01	0,218	0,349	1,46504E-31
cDC_LAMP3	POU2F2	1,08231E-49	-6,90095E-01	0,099	0,275	1,99329E-45
cDC_LAMP3	PGD	2,46901E-08	-6,90889E-01	0,442	0,466	4,54717E-04
cDC_LAMP3	DOCK4	2,90720E-54	-6,92919E-01	0,105	0,291	5,35418E-50
cDC_LAMP3	U2AF1	4,73146E-31	-6,97448E-01	0,142	0,273	8,71393E-27
cDC_LAMP3	ITPR2	1,18647E-32	-6,98103E-01	0,145	0,282	2,18512E-28
cDC_LAMP3	NDUFB2	5,11948E-40	-6,98408E-01	0,653	0,716	9,42855E-36
cDC_LAMP3	NUDT22	4,37452E-07	-6,99152E-01	0,229	0,274	8,05655E-03
cDC_LAMP3	IL10RB	1,23278E-07	-7,01156E-01	0,213	0,256	2,27040E-03
cDC_LAMP3	BTF3L4	2,06910E-23	-7,02033E-01	0,281	0,381	3,81066E-19
cDC_LAMP3	SH3BGRL	2,62514E-36	-7,04815E-01	0,672	0,721	4,83472E-32
cDC_LAMP3	IFNGR1	3,20035E-23	-7,05940E-01	0,513	0,583	5,89409E-19
cDC_LAMP3	TNFAIP8I	9,68012E-37	-7,06650E-01	0,12	0,265	1,78279E-32
cDC_LAMP3	PPCS	8,07349E-10	-7,06691E-01	0,344	0,392	1,48689E-05
cDC_LAMP3	NANS	2,51382E-28	-7,07941E-01	0,269	0,393	4,62970E-24

cDC_LAMP3	MCOLN1	7,78460E-63	-7,08866E-01	0,083	0,28	1,43369E-58
cDC_LAMP3	SYPL1	1,17199E-59	-7,10083E-01	0,113	0,309	2,15845E-55
cDC_LAMP3	ADIPOR1	1,83902E-25	-7,10758E-01	0,383	0,464	3,38693E-21
cDC_LAMP3	TECR	4,54523E-31	-7,11176E-01	0,262	0,383	8,37095E-27
cDC_LAMP3	ST14	4,29942E-72	-7,11208E-01	0,059	0,273	7,91823E-68
cDC_LAMP3	MYL12A	1,61704E-10	-7,12859E-01	0,771	0,826	2,97809E-06
cDC_LAMP3	SDCCAG8	4,87208E-24	-7,13531E-01	0,231	0,34	8,97291E-20
cDC_LAMP3	ZSWIM7	9,63551E-17	-7,14769E-01	0,166	0,252	1,77457E-12
cDC_LAMP3	TXNDC17	1,45957E-06	-7,15630E-01	0,423	0,434	2,68808E-02
cDC_LAMP3	YWHAH	9,13426E-23	-7,17257E-01	0,485	0,533	1,68226E-18
cDC_LAMP3	AHCY	7,16610E-23	-7,17490E-01	0,161	0,265	1,31978E-18
cDC_LAMP3	EMC4	4,20685E-08	-7,18618E-01	0,329	0,362	7,74776E-04
cDC_LAMP3	KLF10	4,69703E-12	-7,19539E-01	0,225	0,292	8,65052E-08
cDC_LAMP3	TRAM1	5,18804E-10	-7,21296E-01	0,417	0,451	9,55482E-06
cDC_LAMP3	RPL38	5,77517E-26	-7,21770E-01	0,695	0,486	1,06361E-21
cDC_LAMP3	NUDT16	1,34065E-76	-7,28583E-01	0,184	0,415	2,46908E-72
cDC_LAMP3	ATP6V0C	5,68662E-59	-7,28872E-01	0,672	0,699	1,04731E-54
cDC_LAMP3	MTMR14	8,13544E-17	-7,30124E-01	0,222	0,313	1,49830E-12
cDC_LAMP3	IFI27L2	9,07885E-74	-7,33029E-01	0,193	0,416	1,67205E-69
cDC_LAMP3	NDUFB10	1,86839E-19	-7,34555E-01	0,519	0,56	3,44101E-15
cDC_LAMP3	IRF9	1,28670E-12	-7,35568E-01	0,262	0,178	2,36972E-08
cDC_LAMP3	YWHAB	1,89561E-41	-7,35859E-01	0,743	0,789	3,49115E-37
cDC_LAMP3	VAMP5	5,72180E-16	-7,35936E-01	0,442	0,481	1,05378E-11
cDC_LAMP3	LAMTOR	4,26743E-34	-7,37105E-01	0,581	0,65	7,85933E-30
cDC_LAMP3	ARHGAP1	1,73193E-23	-7,37949E-01	0,147	0,258	3,18970E-19
cDC_LAMP3	FKBP1A	9,52734E-26	-7,38768E-01	0,659	0,695	1,75465E-21
cDC_LAMP3	MGAT1	3,04414E-29	-7,39453E-01	0,545	0,627	5,60640E-25
cDC_LAMP3	DOCK8	2,25742E-16	-7,41122E-01	0,375	0,45	4,15748E-12
cDC_LAMP3	LAMTOR	2,24832E-24	-7,41215E-01	0,479	0,545	4,14073E-20
cDC_LAMP3	GIMAP1	3,05147E-86	-7,43346E-01	0,04	0,273	5,61990E-82
cDC_LAMP3	GNAI2	1,18337E-64	-7,45088E-01	0,574	0,677	2,17941E-60
cDC_LAMP3	FKBP2	8,46529E-25	-7,47760E-01	0,309	0,412	1,55905E-20
cDC_LAMP3	NQO2	4,20800E-08	-7,47947E-01	0,212	0,263	7,74987E-04
cDC_LAMP3	GNPTG	1,16974E-27	-7,48504E-01	0,332	0,432	2,15432E-23
cDC_LAMP3	MAN2B1	8,73145E-10	-7,48529E-01	0,449	0,475	1,60807E-05
cDC_LAMP3	ARF1	5,70094E-09	-7,48751E-01	0,682	0,699	1,04994E-04
cDC_LAMP3	STAT1	7,30903E-11	-7,50436E-01	0,481	0,519	1,34610E-06
cDC_LAMP3	GPI	1,18108E-19	-7,50519E-01	0,338	0,418	2,17520E-15
cDC_LAMP3	SOAT1	3,08122E-32	-7,50620E-01	0,157	0,29	5,67468E-28
cDC_LAMP3	DGCR6L	9,64671E-29	-7,50950E-01	0,144	0,265	1,77663E-24
cDC_LAMP3	PHACTR1	1,02767E-37	-7,53182E-01	0,148	0,306	1,89265E-33

cDC_LAMP3	VAMP3	2,10802E-13	-7,53702E-01	0,288	0,353	3,88234E-09
cDC_LAMP3	CALM1	4,46984E-32	-7,55169E-01	0,735	0,797	8,23210E-28
cDC_LAMP3	PLK3	1,00166E-45	-7,56061E-01	0,097	0,257	1,84475E-41
cDC_LAMP3	IFI35	3,24455E-20	-7,56934E-01	0,256	0,349	5,97548E-16
cDC_LAMP3	AP3S1	3,38123E-07	-7,57764E-01	0,315	0,347	6,22721E-03
cDC_LAMP3	CD44	2,42543E-15	-7,58847E-01	0,576	0,68	4,46691E-11
cDC_LAMP3	MDH2	1,98948E-17	-7,60426E-01	0,476	0,523	3,66402E-13
cDC_LAMP3	DNAJB11	2,85480E-12	-7,61170E-01	0,244	0,313	5,25768E-08
cDC_LAMP3	ARL5A	1,65637E-57	-7,61985E-01	0,252	0,435	3,05054E-53
cDC_LAMP3	FERMT3	2,32147E-48	-7,62566E-01	0,34	0,487	4,27545E-44
cDC_LAMP3	ABCA1	7,60763E-63	-7,63497E-01	0,116	0,321	1,40110E-58
cDC_LAMP3	PGK1	1,23875E-18	-7,65614E-01	0,67	0,707	2,28141E-14
cDC_LAMP3	EMP3	4,68147E-107	-7,65742E-01	0,513	0,735	8,62187E-103
cDC_LAMP3	TMEM20	1,05731E-30	-7,65914E-01	0,161	0,288	1,94725E-26
cDC_LAMP3	GLA	1,18962E-08	-7,66266E-01	0,259	0,313	2,19093E-04
cDC_LAMP3	USP15	5,04579E-19	-7,66637E-01	0,345	0,427	9,29284E-15
cDC_LAMP3	PRDX5	4,37761E-28	-7,67854E-01	0,538	0,603	8,06225E-24
cDC_LAMP3	IL18	6,47892E-56	-7,68492E-01	0,25	0,448	1,19322E-51
cDC_LAMP3	ERGIC3	8,11164E-24	-7,70161E-01	0,435	0,5	1,49392E-19
cDC_LAMP3	LSM10	3,11706E-43	-7,72229E-01	0,229	0,38	5,74069E-39
cDC_LAMP3	DYNLRB1	2,51692E-14	-7,73971E-01	0,402	0,465	4,63540E-10
cDC_LAMP3	LSM7	6,33932E-31	-7,74956E-01	0,432	0,517	1,16751E-26
cDC_LAMP3	HMGB1	1,83316E-86	-7,76256E-01	0,701	0,796	3,37614E-82
cDC_LAMP3	PLSCR1	4,16391E-38	-7,76946E-01	0,392	0,547	7,66868E-34
cDC_LAMP3	NCF4	8,60602E-51	-7,80057E-01	0,259	0,428	1,58497E-46
cDC_LAMP3	PTPN18	1,28080E-69	-7,81512E-01	0,142	0,358	2,35885E-65
cDC_LAMP3	TEX264	1,43981E-14	-7,81542E-01	0,237	0,31	2,65170E-10
cDC_LAMP3	TLN1	1,24051E-55	-7,81730E-01	0,419	0,559	2,28464E-51
cDC_LAMP3	CTSA	1,56765E-96	-7,83130E-01	0,285	0,513	2,88714E-92
cDC_LAMP3	SUMF2	5,34454E-07	-7,83401E-01	0,257	0,296	9,84304E-03
cDC_LAMP3	FRMD4B	2,60923E-64	-7,84096E-01	0,086	0,291	4,80542E-60
cDC_LAMP3	IRF5	7,72923E-13	-7,85852E-01	0,191	0,264	1,42349E-08
cDC_LAMP3	RABAC1	6,56686E-18	-7,86592E-01	0,465	0,527	1,20942E-13
cDC_LAMP3	PPIB	1,02371E-35	-7,86957E-01	0,754	0,794	1,88538E-31
cDC_LAMP3	CYFIP1	1,98076E-32	-7,89777E-01	0,201	0,327	3,64797E-28
cDC_LAMP3	PLXNB2	6,07630E-16	-7,91177E-01	0,188	0,267	1,11907E-11
cDC_LAMP3	EIF3K	1,12749E-75	-7,91674E-01	0,71	0,789	2,07650E-71
cDC_LAMP3	PTPRC	7,04301E-67	-7,92166E-01	0,524	0,695	1,29711E-62
cDC_LAMP3	RPL23	8,58906E-17	-7,92321E-01	0,638	0,447	1,58185E-12
cDC_LAMP3	CANX	1,04983E-25	-7,93171E-01	0,589	0,641	1,93347E-21
cDC_LAMP3	SDF2L1	1,60506E-32	-7,95338E-01	0,227	0,358	2,95603E-28

cDC_LAMP3	SERINC1	4,10870E-07	-7,96351E-01	0,377	0,405	7,56699E-03
cDC_LAMP3	UCP2	1,13643E-24	-7,96539E-01	0,547	0,637	2,09296E-20
cDC_LAMP3	FBXL5	1,75096E-17	-7,96705E-01	0,192	0,277	3,22474E-13
cDC_LAMP3	PER1	1,19538E-24	-7,97438E-01	0,21	0,323	2,20154E-20
cDC_LAMP3	MAT2A	3,39600E-24	-8,00545E-01	0,357	0,449	6,25441E-20
cDC_LAMP3	PIK3AP1	4,91501E-70	-8,02312E-01	0,094	0,311	9,05198E-66
cDC_LAMP3	P4HB	6,96492E-14	-8,02787E-01	0,613	0,629	1,28273E-09
cDC_LAMP3	ALOX5	1,02901E-62	-8,02800E-01	0,274	0,48	1,89512E-58
cDC_LAMP3	MT-ND1	6,55196E-16	-8,03264E-01	0,764	0,563	1,20668E-11
cDC_LAMP3	BRI3	3,99161E-42	-8,04579E-01	0,278	0,406	7,35134E-38
cDC_LAMP3	NCF1	2,27086E-59	-8,07149E-01	0,313	0,506	4,18225E-55
cDC_LAMP3	LEPROT	8,45814E-37	-8,08150E-01	0,22	0,359	1,55773E-32
cDC_LAMP3	OAZ1	1,55553E-84	-8,08787E-01	0,893	0,938	2,86482E-80
cDC_LAMP3	TSPAN14	9,95016E-25	-8,09692E-01	0,229	0,336	1,83252E-20
cDC_LAMP3	MRPS18(	4,49091E-24	-8,10415E-01	0,197	0,305	8,27090E-20
cDC_LAMP3	CITED2	1,20029E-54	-8,10804E-01	0,158	0,34	2,21057E-50
cDC_LAMP3	PDIA6	4,37202E-27	-8,11827E-01	0,425	0,523	8,05196E-23
cDC_LAMP3	GTF3A	4,60974E-38	-8,11924E-01	0,349	0,484	8,48975E-34
cDC_LAMP3	NEU1	5,58132E-24	-8,12638E-01	0,203	0,308	1,02791E-19
cDC_LAMP3	SYK	1,43397E-51	-8,15655E-01	0,276	0,448	2,64094E-47
cDC_LAMP3	HADHB	1,11531E-09	-8,16284E-01	0,316	0,361	2,05406E-05
cDC_LAMP3	MT2A	1,60381E-14	-8,18266E-01	0,556	0,63	2,95373E-10
cDC_LAMP3	SEC62	2,22559E-46	-8,18709E-01	0,436	0,558	4,09887E-42
cDC_LAMP3	GIT2	7,17511E-15	-8,18825E-01	0,22	0,297	1,32144E-10
cDC_LAMP3	TSPAN4	4,39532E-45	-8,21396E-01	0,142	0,298	8,09486E-41
cDC_LAMP3	RTN3	1,77064E-24	-8,26932E-01	0,329	0,421	3,26100E-20
cDC_LAMP3	CNIH4	9,23108E-21	-8,27658E-01	0,2	0,289	1,70009E-16
cDC_LAMP3	ABI3	3,04737E-96	-8,27851E-01	0,129	0,401	5,61235E-92
cDC_LAMP3	ARPC1B	1,28452E-88	-8,28836E-01	0,684	0,8	2,36570E-84
cDC_LAMP3	YIF1B	2,21708E-32	-8,29125E-01	0,225	0,354	4,08319E-28
cDC_LAMP3	WSB1	2,55942E-42	-8,29911E-01	0,559	0,643	4,71369E-38
cDC_LAMP3	DCTN3	1,15209E-14	-8,30399E-01	0,295	0,362	2,12181E-10
cDC_LAMP3	CAPZA2	6,07817E-33	-8,32687E-01	0,464	0,552	1,11942E-28
cDC_LAMP3	AP1B1	5,87880E-73	-8,33150E-01	0,183	0,4	1,08270E-68
cDC_LAMP3	DDIT4	1,95805E-53	-8,33663E-01	0,309	0,502	3,60614E-49
cDC_LAMP3	CLEC2B	6,01443E-139	-8,37024E-01	0,136	0,48	1,10768E-134
cDC_LAMP3	IRF2BP2	1,10596E-21	-8,37839E-01	0,227	0,325	2,03685E-17
cDC_LAMP3	SPCS3	5,97295E-18	-8,38789E-01	0,384	0,447	1,10004E-13
cDC_LAMP3	SDCBP	9,16453E-81	-8,38804E-01	0,614	0,727	1,68783E-76
cDC_LAMP3	CASP1	8,54896E-103	-8,39638E-01	0,188	0,472	1,57446E-98
cDC_LAMP3	KLF2	3,55935E-52	-8,40107E-01	0,104	0,282	6,55526E-48

cDC_LAMP3	ZNF106	7,60158E-38	-8,41532E-01	0,199	0,348	1,39998E-33
cDC_LAMP3	BTK	2,75867E-43	-8,42235E-01	0,154	0,316	5,08065E-39
cDC_LAMP3	CALR	7,40054E-37	-8,42306E-01	0,671	0,725	1,36296E-32
cDC_LAMP3	CMTM3	5,02661E-81	-8,42826E-01	0,127	0,369	9,25751E-77
cDC_LAMP3	NDUFS3	6,39896E-12	-8,42888E-01	0,284	0,342	1,17850E-07
cDC_LAMP3	SH3BGRL	5,39387E-192	-8,43001E-01	0,839	0,936	9,93388E-188
cDC_LAMP3	CD302	4,27066E-93	-8,45288E-01	0,092	0,357	7,86527E-89
cDC_LAMP3	DAD1	1,12546E-39	-8,45292E-01	0,523	0,603	2,07276E-35
cDC_LAMP3	PPP1R15	3,37713E-14	-8,47349E-01	0,618	0,659	6,21966E-10
cDC_LAMP3	ARPC3	4,19976E-186	-8,49399E-01	0,796	0,895	7,73469E-182
cDC_LAMP3	CD93	1,03147E-79	-8,50383E-01	0,061	0,286	1,89966E-75
cDC_LAMP3	SPCS2	1,31793E-11	-8,50745E-01	0,458	0,494	2,42724E-07
cDC_LAMP3	DPYD	2,61372E-42	-8,51248E-01	0,205	0,371	4,81369E-38
cDC_LAMP3	REEP5	1,86609E-55	-8,51756E-01	0,436	0,567	3,43679E-51
cDC_LAMP3	GCA	2,19958E-10	-8,53276E-01	0,357	0,403	4,05097E-06
cDC_LAMP3	UQCRC1	6,27226E-26	-8,53703E-01	0,437	0,508	1,15516E-21
cDC_LAMP3	JUN	7,42298E-25	-8,54799E-01	0,637	0,712	1,36709E-20
cDC_LAMP3	GABARAI	4,92198E-29	-8,54807E-01	0,166	0,296	9,06482E-25
cDC_LAMP3	STX10	3,53284E-49	-8,57769E-01	0,145	0,315	6,50643E-45
cDC_LAMP3	KDELR2	3,77105E-18	-8,58048E-01	0,456	0,508	6,94515E-14
cDC_LAMP3	SLC1A3	2,56352E-63	-8,59351E-01	0,16	0,369	4,72123E-59
cDC_LAMP3	LHFPL2	3,07602E-77	-8,59993E-01	0,067	0,29	5,66511E-73
cDC_LAMP3	IQGAP2	8,77977E-71	-8,60173E-01	0,096	0,31	1,61697E-66
cDC_LAMP3	DRAM2	2,61943E-29	-8,60880E-01	0,273	0,394	4,82421E-25
cDC_LAMP3	NDUFS5	2,28237E-75	-8,61768E-01	0,527	0,658	4,20343E-71
cDC_LAMP3	PDIA4	1,40131E-18	-8,63223E-01	0,234	0,324	2,58079E-14
cDC_LAMP3	NAA20	1,11387E-14	-8,64234E-01	0,236	0,309	2,05142E-10
cDC_LAMP3	LY6E	2,10627E-28	-8,64448E-01	0,428	0,551	3,87911E-24
cDC_LAMP3	FKBP15	3,08385E-58	-8,64588E-01	0,088	0,278	5,67952E-54
cDC_LAMP3	CMC2	5,22449E-09	-8,70612E-01	0,224	0,277	9,62195E-05
cDC_LAMP3	AUP1	1,76537E-11	-8,70621E-01	0,51	0,526	3,25128E-07
cDC_LAMP3	GM2A	2,49854E-59	-8,71181E-01	0,183	0,381	4,60157E-55
cDC_LAMP3	SORL1	4,75220E-68	-8,71425E-01	0,145	0,37	8,75212E-64
cDC_LAMP3	HAVCR2	1,65294E-53	-8,73349E-01	0,252	0,441	3,04423E-49
cDC_LAMP3	JUNB	7,03995E-20	-8,77125E-01	0,791	0,827	1,29655E-15
cDC_LAMP3	TMBIM6	6,50128E-30	-8,77409E-01	0,737	0,779	1,19734E-25
cDC_LAMP3	HNMT	2,30953E-37	-8,77707E-01	0,242	0,385	4,25346E-33
cDC_LAMP3	VMP1	6,06537E-59	-8,78552E-01	0,399	0,555	1,11706E-54
cDC_LAMP3	FPR3	1,39398E-57	-8,82467E-01	0,147	0,345	2,56730E-53
cDC_LAMP3	TXNDC12	8,71677E-17	-8,82795E-01	0,268	0,343	1,60537E-12
cDC_LAMP3	NUP214	5,43375E-55	-8,85526E-01	0,14	0,32	1,00073E-50

cDC_LAMP3	TMED10	3,84489E-61	-8,85633E-01	0,416	0,572	7,08114E-57
cDC_LAMP3	LAPTM4/	1,06667E-53	-8,85707E-01	0,492	0,632	1,96448E-49
cDC_LAMP3	NOP10	3,08715E-95	-8,87532E-01	0,453	0,637	5,68561E-91
cDC_LAMP3	B4GALT1	1,01287E-29	-8,89385E-01	0,202	0,329	1,86540E-25
cDC_LAMP3	SSB	1,11816E-23	-8,89628E-01	0,376	0,455	2,05932E-19
cDC_LAMP3	PDCD4	3,82038E-08	-8,89864E-01	0,209	0,26	7,03599E-04
cDC_LAMP3	THEMIS2	1,09655E-08	-8,91961E-01	0,456	0,496	2,01952E-04
cDC_LAMP3	TSPAN3	5,76477E-15	-8,96059E-01	0,237	0,321	1,06170E-10
cDC_LAMP3	CALCOC(	7,13067E-49	-8,98957E-01	0,19	0,364	1,31326E-44
cDC_LAMP3	SRSF5	6,71407E-10	-8,99340E-01	0,567	0,579	1,23653E-05
cDC_LAMP3	GNPDA1	6,63617E-31	-8,99464E-01	0,138	0,263	1,22218E-26
cDC_LAMP3	FES	1,00448E-59	-8,99541E-01	0,072	0,26	1,84995E-55
cDC_LAMP3	HLA-E	6,40335E-101	-9,00762E-01	0,837	0,906	1,17930E-96
cDC_LAMP3	STAT2	4,73913E-25	-9,00769E-01	0,186	0,298	8,72806E-21
cDC_LAMP3	ATP6V1F	7,47572E-136	-9,02939E-01	0,617	0,769	1,37680E-131
cDC_LAMP3	ADORA3	1,63782E-73	-9,05998E-01	0,053	0,27	3,01638E-69
cDC_LAMP3	SPG21	2,13039E-60	-9,07207E-01	0,257	0,443	3,92354E-56
cDC_LAMP3	DHRS7	8,98876E-15	-9,07321E-01	0,433	0,487	1,65546E-10
cDC_LAMP3	PTAFR	2,60137E-89	-9,12015E-01	0,117	0,384	4,79094E-85
cDC_LAMP3	TOM1	2,37344E-46	-9,13707E-01	0,124	0,289	4,37117E-42
cDC_LAMP3	SON	1,64442E-09	-9,14316E-01	0,67	0,674	3,02853E-05
cDC_LAMP3	CAPG	2,64784E-52	-9,14400E-01	0,647	0,744	4,87654E-48
cDC_LAMP3	SLC16A3	1,06274E-57	-9,14652E-01	0,353	0,517	1,95725E-53
cDC_LAMP3	HVCN1	5,89109E-43	-9,14970E-01	0,103	0,259	1,08496E-38
cDC_LAMP3	EVL	6,88753E-67	-9,17327E-01	0,142	0,364	1,26848E-62
cDC_LAMP3	RAB1A	2,23949E-11	-9,19531E-01	0,354	0,397	4,12446E-07
cDC_LAMP3	AKR1A1	1,52387E-74	-9,20182E-01	0,408	0,582	2,80651E-70
cDC_LAMP3	SLAMF8	8,13127E-78	-9,20258E-01	0,088	0,333	1,49754E-73
cDC_LAMP3	UBAC2	8,83167E-15	-9,22730E-01	0,225	0,312	1,62653E-10
cDC_LAMP3	ACAP2	1,87111E-28	-9,23507E-01	0,329	0,434	3,44603E-24
cDC_LAMP3	SIGLEC1(	1,36953E-80	-9,25794E-01	0,086	0,328	2,52226E-76
cDC_LAMP3	SCAMP2	1,38636E-50	-9,27308E-01	0,365	0,507	2,55325E-46
cDC_LAMP3	APBB1IP	1,00739E-104	-9,32819E-01	0,202	0,487	1,85531E-100
cDC_LAMP3	JMJD1C	5,82828E-07	-9,34291E-01	0,403	0,428	1,07339E-02
cDC_LAMP3	PPIF	3,00452E-07	-9,36380E-01	0,331	0,359	5,53342E-03
cDC_LAMP3	TM9SF2	1,54380E-22	-9,36419E-01	0,344	0,43	2,84321E-18
cDC_LAMP3	TIMP2	4,82149E-118	-9,37360E-01	0,073	0,37	8,87974E-114
cDC_LAMP3	AZI2	9,66686E-64	-9,38186E-01	0,108	0,308	1,78035E-59
cDC_LAMP3	CST3	1,92953E-177	-9,40201E-01	0,865	0,952	3,55362E-173
cDC_LAMP3	GIMAP7	4,76528E-70	-9,40207E-01	0,054	0,262	8,77622E-66
cDC_LAMP3	PTGS1	1,76314E-71	-9,40723E-01	0,051	0,263	3,24717E-67

cDC_LAMP3	TMEM21	3,41853E-49	-9,45261E-01	0,545	0,64	6,29590E-45
cDC_LAMP3	SDHB	6,25362E-16	-9,46861E-01	0,338	0,401	1,15173E-11
cDC_LAMP3	NDUFB7	1,21109E-22	-9,48097E-01	0,468	0,531	2,23046E-18
cDC_LAMP3	CDK5RAF	8,02954E-12	-9,49410E-01	0,244	0,309	1,47880E-07
cDC_LAMP3	AP2S1	1,65399E-61	-9,50627E-01	0,64	0,722	3,04616E-57
cDC_LAMP3	ADAM10	8,60784E-37	-9,50971E-01	0,151	0,292	1,58531E-32
cDC_LAMP3	BNIP3L	1,38876E-50	-9,53878E-01	0,439	0,567	2,55768E-46
cDC_LAMP3	MPP1	3,76127E-88	-9,53895E-01	0,169	0,42	6,92713E-84
cDC_LAMP3	SCIMP	1,13890E-56	-9,54002E-01	0,092	0,284	2,09751E-52
cDC_LAMP3	SCARB2	8,73120E-90	-9,56216E-01	0,127	0,379	1,60802E-85
cDC_LAMP3	PTPMT1	1,48491E-11	-9,59260E-01	0,186	0,25	2,73477E-07
cDC_LAMP3	LMAN2	2,97092E-26	-9,59408E-01	0,444	0,513	5,47154E-22
cDC_LAMP3	SCP2	9,53876E-31	-9,61544E-01	0,362	0,464	1,75675E-26
cDC_LAMP3	GAA	1,06072E-40	-9,62546E-01	0,25	0,391	1,95352E-36
cDC_LAMP3	COMT	3,31108E-46	-9,63275E-01	0,451	0,561	6,09801E-42
cDC_LAMP3	ENG	1,07938E-56	-9,63565E-01	0,166	0,353	1,98789E-52
cDC_LAMP3	ALDOA	7,40478E-52	-9,64594E-01	0,728	0,761	1,36374E-47
cDC_LAMP3	CHD9	1,22305E-29	-9,65452E-01	0,22	0,347	2,25248E-25
cDC_LAMP3	CCNL1	2,61551E-15	-9,65665E-01	0,499	0,526	4,81698E-11
cDC_LAMP3	MYD88	7,62149E-30	-9,69642E-01	0,239	0,361	1,40365E-25
cDC_LAMP3	FYB1	5,95211E-62	-9,69673E-01	0,127	0,324	1,09620E-57
cDC_LAMP3	ARHGEF1	1,00486E-09	-9,69704E-01	0,216	0,273	1,85065E-05
cDC_LAMP3	MGST2	4,37249E-75	-9,72179E-01	0,131	0,366	8,05281E-71
cDC_LAMP3	NDUFB9	1,92955E-25	-9,74476E-01	0,495	0,552	3,55365E-21
cDC_LAMP3	LY96	3,87402E-61	-9,77983E-01	0,273	0,463	7,13478E-57
cDC_LAMP3	MAPKAP	5,99168E-23	-9,79835E-01	0,187	0,292	1,10349E-18
cDC_LAMP3	LIMD2	7,08837E-31	-9,89277E-01	0,375	0,485	1,30547E-26
cDC_LAMP3	PLBD1	1,31766E-69	-9,91691E-01	0,184	0,399	2,42674E-65
cDC_LAMP3	TMED9	8,93660E-26	-9,91864E-01	0,464	0,526	1,64585E-21
cDC_LAMP3	HCLS1	2,52974E-85	-9,93346E-01	0,579	0,728	4,65902E-81
cDC_LAMP3	AKAP9	3,13937E-18	-9,94016E-01	0,301	0,388	5,78178E-14
cDC_LAMP3	TMEM14	1,29660E-67	-9,96613E-01	0,243	0,44	2,38794E-63
cDC_LAMP3	METRNL	3,19190E-54	-1,00100E+00	0,084	0,259	5,87851E-50
cDC_LAMP3	MNDA	1,01605E-39	-1,00171E+00	0,393	0,538	1,87126E-35
cDC_LAMP3	SLC3A2	1,96722E-11	-1,00397E+00	0,408	0,465	3,62304E-07
cDC_LAMP3	LILRA2	9,58772E-64	-1,00447E+00	0,058	0,253	1,76577E-59
cDC_LAMP3	ADAP2	1,49958E-52	-1,00486E+00	0,292	0,45	2,76178E-48
cDC_LAMP3	DNASE2	4,38341E-89	-1,00531E+00	0,125	0,374	8,07292E-85
cDC_LAMP3	ANXA1	3,47965E-168	-1,00978E+00	0,358	0,698	6,40847E-164
cDC_LAMP3	AKR1B1	9,08881E-105	-1,01185E+00	0,209	0,476	1,67389E-100
cDC_LAMP3	NAGA	2,76376E-44	-1,01213E+00	0,206	0,365	5,09002E-40

cDC_LAMP3	NLRP3	1,30115E-49	-1,01516E+00	0,093	0,268	2,39632E-45
cDC_LAMP3	CARD16	1,44556E-51	-1,01630E+00	0,392	0,557	2,66229E-47
cDC_LAMP3	ITGAX	1,67667E-72	-1,01976E+00	0,168	0,407	3,08793E-68
cDC_LAMP3	UTRN	1,85034E-49	-1,02485E+00	0,117	0,294	3,40776E-45
cDC_LAMP3	IFITM2	1,16218E-50	-1,02584E+00	0,488	0,651	2,14039E-46
cDC_LAMP3	ACSL1	2,28574E-17	-1,02639E+00	0,318	0,391	4,20966E-13
cDC_LAMP3	CD63	1,31202E-187	-1,02698E+00	0,67	0,822	2,41635E-183
cDC_LAMP3	EIF4EBP1	2,11018E-92	-1,02863E+00	0,161	0,417	3,88632E-88
cDC_LAMP3	ATP6V0B	4,99369E-76	-1,02947E+00	0,713	0,793	9,19689E-72
cDC_LAMP3	NDUFS7	5,88404E-38	-1,03303E+00	0,441	0,544	1,08366E-33
cDC_LAMP3	S100A11	6,93711E-166	-1,03392E+00	0,882	0,939	1,27761E-161
cDC_LAMP3	CLIC1	4,44083E-184	-1,03430E+00	0,704	0,88	8,17868E-180
cDC_LAMP3	TMEM59	3,64136E-82	-1,03501E+00	0,465	0,622	6,70629E-78
cDC_LAMP3	HLA-DM/	1,32417E-111	-1,03817E+00	0,643	0,786	2,43872E-107
cDC_LAMP3	SP100	2,97809E-39	-1,03913E+00	0,33	0,471	5,48475E-35
cDC_LAMP3	SPTLC2	7,11443E-39	-1,03967E+00	0,164	0,314	1,31027E-34
cDC_LAMP3	SSR1	4,96919E-46	-1,04446E+00	0,323	0,468	9,15177E-42
cDC_LAMP3	CTNND1	6,13689E-21	-1,05059E+00	0,171	0,275	1,13023E-16
cDC_LAMP3	LGALS3	7,58428E-56	-1,05208E+00	0,651	0,727	1,39680E-51
cDC_LAMP3	CD164	7,75539E-41	-1,05338E+00	0,474	0,56	1,42831E-36
cDC_LAMP3	GNS	6,48205E-58	-1,05628E+00	0,255	0,436	1,19380E-53
cDC_LAMP3	AREG	1,52674E-31	-1,05848E+00	0,148	0,279	2,81180E-27
cDC_LAMP3	DUSP23	3,91382E-78	-1,06211E+00	0,155	0,39	7,20808E-74
cDC_LAMP3	HERPUD1	5,34765E-65	-1,06652E+00	0,543	0,685	9,84877E-61
cDC_LAMP3	GSTO1	1,92367E-80	-1,06686E+00	0,458	0,624	3,54282E-76
cDC_LAMP3	CALM2	7,47083E-18	-1,06814E+00	0,757	0,783	1,37590E-13
cDC_LAMP3	SNX10	1,38869E-77	-1,06816E+00	0,273	0,493	2,55756E-73
cDC_LAMP3	PPT1	7,08916E-104	-1,06973E+00	0,43	0,658	1,30561E-99
cDC_LAMP3	IFNAR2	6,40029E-09	-1,07020E+00	0,218	0,269	1,17874E-04
cDC_LAMP3	S100A6	6,78038E-74	-1,07099E+00	0,792	0,877	1,24874E-69
cDC_LAMP3	CYBA	1,52067E-230	-1,07621E+00	0,791	0,901	2,80063E-226
cDC_LAMP3	SKAP2	1,48650E-87	-1,07654E+00	0,161	0,413	2,73769E-83
cDC_LAMP3	KCNAB2	4,93497E-65	-1,07915E+00	0,083	0,287	9,08873E-61
cDC_LAMP3	NAIP	6,24807E-103	-1,07976E+00	0,096	0,379	1,15071E-98
cDC_LAMP3	BTG2	4,51886E-48	-1,08155E+00	0,353	0,503	8,32238E-44
cDC_LAMP3	LRRC25	1,19354E-114	-1,08299E+00	0,049	0,342	2,19815E-110
cDC_LAMP3	SHISA5	6,24297E-44	-1,08358E+00	0,225	0,375	1,14977E-39
cDC_LAMP3	MEF2C	1,80183E-91	-1,08518E+00	0,157	0,426	3,31843E-87
cDC_LAMP3	PNP	8,80917E-42	-1,08669E+00	0,108	0,264	1,62238E-37
cDC_LAMP3	MYO1F	1,10046E-88	-1,08701E+00	0,212	0,467	2,02672E-84
cDC_LAMP3	IER2	1,45565E-25	-1,08744E+00	0,556	0,634	2,68087E-21

cDC_LAMP3	SASH3	6,19628E-29	-1,09093E+00	0,163	0,288	1,14117E-24
cDC_LAMP3	SH3TC1	8,00726E-37	-1,09275E+00	0,17	0,318	1,47470E-32
cDC_LAMP3	CPM	1,61176E-109	-1,09302E+00	0,088	0,378	2,96838E-105
cDC_LAMP3	FGR	1,33534E-85	-1,09649E+00	0,203	0,462	2,45930E-81
cDC_LAMP3	ITGB2	1,21719E-220	-1,09670E+00	0,437	0,773	2,24170E-216
cDC_LAMP3	PMP22	1,58607E-84	-1,09848E+00	0,052	0,284	2,92106E-80
cDC_LAMP3	IVNS1AB	6,21880E-26	-1,09920E+00	0,259	0,37	1,14532E-21
cDC_LAMP3	ERCC1	9,69294E-53	-1,09991E+00	0,204	0,387	1,78515E-48
cDC_LAMP3	TMBIM4	1,99404E-24	-1,10458E+00	0,236	0,334	3,67242E-20
cDC_LAMP3	LPAR6	5,84594E-83	-1,10742E+00	0,088	0,329	1,07665E-78
cDC_LAMP3	BST2	1,51629E-58	-1,10846E+00	0,635	0,728	2,79256E-54
cDC_LAMP3	FUCA1	6,63972E-68	-1,10958E+00	0,057	0,256	1,22284E-63
cDC_LAMP3	FBP1	2,20256E-87	-1,11249E+00	0,102	0,357	4,05646E-83
cDC_LAMP3	ANXA5	7,94411E-102	-1,11394E+00	0,658	0,777	1,46307E-97
cDC_LAMP3	AP1S2	1,56909E-51	-1,11548E+00	0,467	0,582	2,88980E-47
cDC_LAMP3	EVI2A	3,17028E-59	-1,11990E+00	0,088	0,279	5,83870E-55
cDC_LAMP3	TNFRSF1	3,44417E-31	-1,12129E+00	0,337	0,452	6,34312E-27
cDC_LAMP3	KCTD12	3,65509E-169	-1,12227E+00	0,113	0,503	6,73159E-165
cDC_LAMP3	CD99	2,35520E-82	-1,12595E+00	0,456	0,625	4,33757E-78
cDC_LAMP3	PTPN6	3,63257E-93	-1,12888E+00	0,25	0,518	6,69011E-89
cDC_LAMP3	SCPEP1	1,61988E-72	-1,12893E+00	0,192	0,415	2,98333E-68
cDC_LAMP3	CEBPD	1,47421E-98	-1,13208E+00	0,322	0,576	2,71504E-94
cDC_LAMP3	HSD17B1	1,45103E-62	-1,14084E+00	0,211	0,41	2,67237E-58
cDC_LAMP3	HSP90B1	2,16499E-54	-1,14462E+00	0,599	0,703	3,98726E-50
cDC_LAMP3	MGAT4A	8,68645E-113	-1,14650E+00	0,069	0,366	1,59978E-108
cDC_LAMP3	GSTK1	7,18307E-32	-1,14832E+00	0,59	0,646	1,32291E-27
cDC_LAMP3	ARAP1	2,60590E-45	-1,15086E+00	0,134	0,297	4,79929E-41
cDC_LAMP3	RGS19	1,49849E-91	-1,15116E+00	0,252	0,503	2,75976E-87
cDC_LAMP3	INPP5D	6,19878E-40	-1,15361E+00	0,159	0,316	1,14163E-35
cDC_LAMP3	LTBR	1,73091E-36	-1,16449E+00	0,213	0,353	3,18781E-32
cDC_LAMP3	GPR34	8,98945E-109	-1,17367E+00	0,036	0,314	1,65559E-104
cDC_LAMP3	RGS1	5,84127E-16	-1,18078E+00	0,784	0,638	1,07579E-11
cDC_LAMP3	RAB20	2,50525E-100	-1,18084E+00	0,079	0,355	4,61392E-96
cDC_LAMP3	DMXL2	2,15900E-48	-1,18168E+00	0,108	0,272	3,97623E-44
cDC_LAMP3	TFRC	2,46670E-15	-1,18649E+00	0,344	0,234	4,54293E-11
cDC_LAMP3	SAMHD1	5,29320E-169	-1,19216E+00	0,278	0,657	9,74849E-165
cDC_LAMP3	XAF1	2,68631E-48	-1,19565E+00	0,162	0,333	4,94737E-44
cDC_LAMP3	ZFP36	4,52439E-47	-1,19941E+00	0,716	0,848	8,33257E-43
cDC_LAMP3	DOCK2	1,38401E-24	-1,20208E+00	0,236	0,352	2,54893E-20
cDC_LAMP3	ZEB2	3,97132E-40	-1,20251E+00	0,512	0,608	7,31398E-36
cDC_LAMP3	CCND3	2,26053E-25	-1,20440E+00	0,2	0,316	4,16322E-21

cDC_LAMP3	UBE2L6	4,93474E-44	-1,20760E+00	0,439	0,575	9,08831E-40
cDC_LAMP3	ITM2B	2,42057E-181	-1,20819E+00	0,813	0,909	4,45796E-177
cDC_LAMP3	FGL2	7,96747E-179	-1,20922E+00	0,202	0,627	1,46737E-174
cDC_LAMP3	HLA-DMI	1,31298E-123	-1,21222E+00	0,389	0,647	2,41812E-119
cDC_LAMP3	PRNP	9,56730E-76	-1,21452E+00	0,179	0,408	1,76201E-71
cDC_LAMP3	CD33	1,82091E-93	-1,22035E+00	0,059	0,315	3,35357E-89
cDC_LAMP3	LAT2	2,13855E-57	-1,22747E+00	0,264	0,46	3,93857E-53
cDC_LAMP3	TRIM22	2,61797E-15	-1,22940E+00	0,34	0,419	4,82151E-11
cDC_LAMP3	AOAH	1,86225E-67	-1,23383E+00	0,154	0,381	3,42970E-63
cDC_LAMP3	BCAP31	1,02963E-62	-1,23779E+00	0,465	0,587	1,89627E-58
cDC_LAMP3	LILRB2	4,65385E-62	-1,23903E+00	0,255	0,463	8,57100E-58
cDC_LAMP3	TAGAP	9,11169E-54	-1,24430E+00	0,092	0,278	1,67810E-49
cDC_LAMP3	LILRB1	4,58848E-72	-1,24575E+00	0,15	0,384	8,45060E-68
cDC_LAMP3	APEX1	2,40536E-31	-1,25266E+00	0,328	0,445	4,42996E-27
cDC_LAMP3	SERPING	4,63649E-89	-1,25357E+00	0,152	0,405	8,53902E-85
cDC_LAMP3	PARVG	1,35152E-93	-1,25390E+00	0,19	0,458	2,48909E-89
cDC_LAMP3	LAMTOR	8,24670E-107	-1,25491E+00	0,574	0,718	1,51879E-102
cDC_LAMP3	LTC4S	8,02959E-79	-1,25907E+00	0,063	0,291	1,47881E-74
cDC_LAMP3	NPL	4,71705E-72	-1,26365E+00	0,058	0,265	8,68740E-68
cDC_LAMP3	PRELID1	7,61217E-114	-1,26416E+00	0,491	0,691	1,40193E-109
cDC_LAMP3	RBPJ	1,52012E-29	-1,26623E+00	0,369	0,47	2,79960E-25
cDC_LAMP3	BIN2	1,86645E-79	-1,26713E+00	0,089	0,321	3,43744E-75
cDC_LAMP3	CLEC7A	1,13052E-106	-1,26738E+00	0,256	0,533	2,08208E-102
cDC_LAMP3	SLC25A3	3,02810E-84	-1,27169E+00	0,063	0,298	5,57684E-80
cDC_LAMP3	SELPLG	4,24931E-10	-1,27625E+00	0,434	0,341	7,82596E-06
cDC_LAMP3	ARHGAP	3,42607E-160	-1,27692E+00	0,117	0,487	6,30979E-156
cDC_LAMP3	CLEC4E	1,39117E-86	-1,27708E+00	0,054	0,299	2,56211E-82
cDC_LAMP3	HIF1A	3,93818E-26	-1,27956E+00	0,468	0,552	7,25294E-22
cDC_LAMP3	SP110	7,75142E-43	-1,28026E+00	0,233	0,395	1,42758E-38
cDC_LAMP3	ABRACL	1,32620E-94	-1,28215E+00	0,259	0,505	2,44247E-90
cDC_LAMP3	TLR2	2,95239E-126	-1,28563E+00	0,076	0,396	5,43741E-122
cDC_LAMP3	CRTAP	1,22771E-63	-1,28599E+00	0,351	0,526	2,26107E-59
cDC_LAMP3	TCIRG1	6,28887E-48	-1,28875E+00	0,325	0,472	1,15822E-43
cDC_LAMP3	SGK1	4,24554E-98	-1,28888E+00	0,319	0,614	7,81901E-94
cDC_LAMP3	LGMN	9,92522E-34	-1,28968E+00	0,304	0,413	1,82793E-29
cDC_LAMP3	LRPAP1	8,79341E-73	-1,29010E+00	0,313	0,501	1,61948E-68
cDC_LAMP3	LCP2	1,29209E-172	-1,29360E+00	0,182	0,589	2,37965E-168
cDC_LAMP3	RAB24	8,18958E-47	-1,29890E+00	0,107	0,275	1,50828E-42
cDC_LAMP3	HEXB	3,75883E-118	-1,29964E+00	0,236	0,523	6,92264E-114
cDC_LAMP3	ERP29	1,64404E-84	-1,30307E+00	0,45	0,629	3,02784E-80
cDC_LAMP3	LGALS3B	4,46084E-80	-1,30414E+00	0,096	0,327	8,21553E-76

cDC_LAMP3	IFI16	1,01422E-92	-1,31063E+00	0,256	0,507	1,86789E-88
cDC_LAMP3	A2M	5,35364E-79	-1,31198E+00	0,154	0,397	9,85979E-75
cDC_LAMP3	RHOB	2,51872E-88	-1,31201E+00	0,314	0,549	4,63872E-84
cDC_LAMP3	LILRB4	2,63560E-118	-1,31358E+00	0,277	0,558	4,85399E-114
cDC_LAMP3	SSR4	1,30097E-51	-1,31734E+00	0,695	0,755	2,39600E-47
cDC_LAMP3	GAPT	5,24527E-59	-1,32056E+00	0,068	0,26	9,66022E-55
cDC_LAMP3	HMOX1	4,37441E-54	-1,32656E+00	0,394	0,55	8,05635E-50
cDC_LAMP3	STXBP2	1,58528E-25	-1,32983E+00	0,337	0,422	2,91961E-21
cDC_LAMP3	CD9	5,53914E-127	-1,33133E+00	0,191	0,494	1,02014E-122
cDC_LAMP3	NAGK	1,86245E-84	-1,33434E+00	0,261	0,484	3,43008E-80
cDC_LAMP3	GLRX	4,67754E-138	-1,33473E+00	0,221	0,558	8,61462E-134
cDC_LAMP3	CYTH4	2,11749E-84	-1,34018E+00	0,079	0,322	3,89979E-80
cDC_LAMP3	LST1	9,86151E-191	-1,34120E+00	0,422	0,724	1,81619E-186
cDC_LAMP3	RNF13	1,90985E-126	-1,34374E+00	0,232	0,526	3,51737E-122
cDC_LAMP3	CD4	1,72991E-96	-1,34596E+00	0,303	0,546	3,18597E-92
cDC_LAMP3	ARHGDIE	1,02111E-287	-1,34703E+00	0,397	0,836	1,88058E-283
cDC_LAMP3	AGTRAP	2,36934E-117	-1,34859E+00	0,072	0,37	4,36361E-113
cDC_LAMP3	TPP1	1,23655E-83	-1,35311E+00	0,398	0,601	2,27736E-79
cDC_LAMP3	PLD4	3,94505E-56	-1,36182E+00	0,136	0,338	7,26561E-52
cDC_LAMP3	DUSP1	1,48117E-26	-1,36402E+00	0,792	0,852	2,72788E-22
cDC_LAMP3	ARHGAP	4,19391E-92	-1,36516E+00	0,134	0,393	7,72392E-88
cDC_LAMP3	GPX1	3,74433E-73	-1,36814E+00	0,392	0,554	6,89594E-69
cDC_LAMP3	FCGR2B	2,20194E-108	-1,37079E+00	0,122	0,435	4,05531E-104
cDC_LAMP3	GLIPR1	8,60741E-128	-1,37174E+00	0,259	0,593	1,58523E-123
cDC_LAMP3	TYROBP	5,17112E-276	-1,37476E+00	0,869	0,949	9,52366E-272
cDC_LAMP3	CD53	3,41369E-161	-1,37550E+00	0,363	0,688	6,28699E-157
cDC_LAMP3	CEBPB	3,43086E-126	-1,37621E+00	0,195	0,48	6,31862E-122
cDC_LAMP3	CD48	1,24396E-99	-1,37770E+00	0,214	0,487	2,29100E-95
cDC_LAMP3	ATP6AP2	1,30063E-113	-1,38020E+00	0,364	0,598	2,39537E-109
cDC_LAMP3	PLXDC2	7,59231E-149	-1,39186E+00	0,198	0,555	1,39828E-144
cDC_LAMP3	SLC7A7	2,93132E-147	-1,40119E+00	0,131	0,476	5,39861E-143
cDC_LAMP3	NAMPT	5,61192E-33	-1,40572E+00	0,496	0,576	1,03355E-28
cDC_LAMP3	CD37	3,72161E-156	-1,41810E+00	0,501	0,74	6,85409E-152
cDC_LAMP3	PLP2	3,40667E-39	-1,42381E+00	0,275	0,408	6,27407E-35
cDC_LAMP3	LRP1	3,51407E-166	-1,42569E+00	0,055	0,433	6,47186E-162
cDC_LAMP3	ARHGAP	1,09337E-34	-1,43382E+00	0,111	0,25	2,01365E-30
cDC_LAMP3	PTPRE	2,66163E-110	-1,43404E+00	0,15	0,449	4,90192E-106
cDC_LAMP3	SLA	9,14781E-125	-1,44354E+00	0,105	0,427	1,68475E-120
cDC_LAMP3	MX1	5,17687E-20	-1,45078E+00	0,238	0,336	9,53425E-16
cDC_LAMP3	VAMP8	4,97677E-190	-1,45264E+00	0,608	0,786	9,16572E-186
cDC_LAMP3	CNPY3	1,05949E-110	-1,45278E+00	0,405	0,635	1,95127E-106

cDC_LAMP3	EVI2B	9,41283E-105	-1,45335E+00	0,108	0,385	1,73356E-100
cDC_LAMP3	CLEC12A	5,29981E-84	-1,45513E+00	0,036	0,267	9,76065E-80
cDC_LAMP3	CSF3R	3,31911E-109	-1,46217E+00	0,108	0,402	6,11281E-105
cDC_LAMP3	FXYD5	1,41997E-246	-1,46821E+00	0,56	0,833	2,61516E-242
cDC_LAMP3	MX2	3,71495E-36	-1,46980E+00	0,136	0,274	6,84182E-32
cDC_LAMP3	CORO1C	9,77981E-24	-1,47371E+00	0,244	0,347	1,80115E-19
cDC_LAMP3	ZFP36L2	1,38167E-201	-1,48140E+00	0,333	0,706	2,54463E-197
cDC_LAMP3	TSPO	2,63316E-204	-1,48751E+00	0,539	0,773	4,84949E-200
cDC_LAMP3	HM13	1,10707E-79	-1,48917E+00	0,346	0,545	2,03889E-75
cDC_LAMP3	HCST	1,42337E-142	-1,49295E+00	0,404	0,661	2,62142E-138
cDC_LAMP3	HEXA	1,49297E-121	-1,49459E+00	0,167	0,479	2,74960E-117
cDC_LAMP3	CTSC	2,64130E-64	-1,49587E+00	0,577	0,681	4,86447E-60
cDC_LAMP3	CKLF	7,54060E-143	-1,49927E+00	0,159	0,516	1,38875E-138
cDC_LAMP3	SLCO2B1	3,53090E-123	-1,49958E+00	0,069	0,379	6,50286E-119
cDC_LAMP3	LY86	6,57297E-130	-1,50112E+00	0,169	0,5	1,21054E-125
cDC_LAMP3	TKT	2,74385E-101	-1,50658E+00	0,416	0,613	5,05334E-97
cDC_LAMP3	RNF130	5,10778E-210	-1,51245E+00	0,394	0,719	9,40700E-206
cDC_LAMP3	GMFG	4,30901E-189	-1,51381E+00	0,343	0,683	7,93590E-185
cDC_LAMP3	GIMAP4	4,25235E-134	-1,54985E+00	0,106	0,438	7,83155E-130
cDC_LAMP3	C3	1,65087E-105	-1,56577E+00	0,104	0,393	3,04041E-101
cDC_LAMP3	MFSD1	4,08726E-157	-1,56798E+00	0,271	0,601	7,52751E-153
cDC_LAMP3	NCF2	2,18573E-148	-1,57595E+00	0,131	0,475	4,02546E-144
cDC_LAMP3	CD52	1,00167E-37	-1,59582E+00	0,257	0,394	1,84478E-33
cDC_LAMP3	ACP5	7,26510E-102	-1,60496E+00	0,131	0,408	1,33801E-97
cDC_LAMP3	BLVRB	5,39759E-123	-1,61083E+00	0,216	0,499	9,94074E-119
cDC_LAMP3	TXNIP	1,24473E-162	-1,61158E+00	0,387	0,709	2,29241E-158
cDC_LAMP3	PDK4	4,11111E-84	-1,61247E+00	0,076	0,318	7,57143E-80
cDC_LAMP3	LTA4H	7,57236E-53	-1,61330E+00	0,297	0,452	1,39460E-48
cDC_LAMP3	GRN	2,08282E-142	-1,61812E+00	0,744	0,825	3,83594E-138
cDC_LAMP3	APLP2	3,62597E-133	-1,62208E+00	0,353	0,602	6,67795E-129
cDC_LAMP3	TNFSF10	8,19870E-99	-1,62249E+00	0,097	0,368	1,50995E-94
cDC_LAMP3	LYZ	3,73149E-94	-1,63067E+00	0,668	0,813	6,87228E-90
cDC_LAMP3	RNF149	2,85517E-132	-1,64033E+00	0,37	0,635	5,25836E-128
cDC_LAMP3	TSC22D3	1,45655E-106	-1,64501E+00	0,445	0,702	2,68253E-102
cDC_LAMP3	CPVL	2,81754E-155	-1,64544E+00	0,193	0,575	5,18906E-151
cDC_LAMP3	IGSF6	1,76197E-139	-1,64917E+00	0,222	0,567	3,24502E-135
cDC_LAMP3	TNFSF13	1,67330E-102	-1,65031E+00	0,181	0,452	3,08172E-98
cDC_LAMP3	ZNF331	1,08858E-76	-1,65591E+00	0,115	0,352	2,00484E-72
cDC_LAMP3	PYCARD	4,56505E-295	-1,68445E+00	0,217	0,721	8,40745E-291
cDC_LAMP3	CD300A	2,56448E-144	-1,68982E+00	0,113	0,471	4,72300E-140
cDC_LAMP3	C1orf162	6,26548E-280	-1,70264E+00	0,191	0,724	1,15391E-275

cDC_LAMP3	FPR1	6,33341E-106	-1,70673E+00	0,162	0,457	1,16642E-101
cDC_LAMP3	TBXAS1	7,55127E-174	-1,71009E+00	0,134	0,53	1,39072E-169
cDC_LAMP3	FOSB	7,46041E-123	-1,71632E+00	0,305	0,588	1,37398E-118
cDC_LAMP3	C3AR1	8,80692E-152	-1,73770E+00	0,067	0,427	1,62197E-147
cDC_LAMP3	LIPA	6,91238E-162	-1,74203E+00	0,146	0,512	1,27305E-157
cDC_LAMP3	OAS1	4,37696E-71	-1,74259E+00	0,118	0,334	8,06104E-67
cDC_LAMP3	MSR1	1,28429E-137	-1,74601E+00	0,058	0,391	2,36528E-133
cDC_LAMP3	PLD3	3,95149E-136	-1,74639E+00	0,231	0,519	7,27746E-132
cDC_LAMP3	HBEGF	4,41797E-95	-1,76759E+00	0,033	0,283	8,13657E-91
cDC_LAMP3	DAB2	3,18304E-176	-1,76995E+00	0,054	0,445	5,86221E-172
cDC_LAMP3	CORO1A	8,70795E-122	-1,78792E+00	0,412	0,653	1,60374E-117
cDC_LAMP3	C5AR1	4,56494E-120	-1,81194E+00	0,111	0,417	8,40725E-116
cDC_LAMP3	MAFB	2,08326E-241	-1,81226E+00	0,138	0,608	3,83673E-237
cDC_LAMP3	RNASE6	2,73548E-163	-1,81537E+00	0,152	0,551	5,03794E-159
cDC_LAMP3	ISG15	6,32167E-18	-1,84396E+00	0,346	0,43	1,16426E-13
cDC_LAMP3	OLR1	5,48090E-102	-1,87246E+00	0,078	0,356	1,00942E-97
cDC_LAMP3	FCGR1A	2,20166E-145	-1,89217E+00	0,073	0,421	4,05480E-141
cDC_LAMP3	RGS2	2,30205E-70	-1,89564E+00	0,455	0,631	4,23969E-66
cDC_LAMP3	MS4A7	3,71159E-213	-1,91116E+00	0,296	0,674	6,83564E-209
cDC_LAMP3	SLC2A3	7,47857E-96	-1,91721E+00	0,172	0,442	1,37733E-91
cDC_LAMP3	TREM2	3,90065E-132	-1,91984E+00	0,097	0,416	7,18382E-128
cDC_LAMP3	NPC2	1,27265E-272	-1,94536E+00	0,745	0,888	2,34384E-268
cDC_LAMP3	IFI44L	5,03711E-40	-1,95365E+00	0,111	0,258	9,27684E-36
cDC_LAMP3	CSF1R	7,40469E-207	-1,96195E+00	0,138	0,585	1,36372E-202
cDC_LAMP3	MS4A4A	1,45877E-176	-1,97422E+00	0,047	0,432	2,68662E-172
cDC_LAMP3	PILRA	8,53167E-165	-1,99064E+00	0,129	0,504	1,57128E-160
cDC_LAMP3	LAIR1	8,69630E-197	-1,99122E+00	0,099	0,532	1,60160E-192
cDC_LAMP3	CYBB	1,75542E-244	-1,99507E+00	0,159	0,639	3,23296E-240
cDC_LAMP3	PLTP	6,55229E-118	-2,03169E+00	0,045	0,337	1,20674E-113
cDC_LAMP3	CTSS	1,10655E-254	-2,03585E+00	0,586	0,824	2,03794E-250
cDC_LAMP3	STAB1	4,18040E-144	-2,05470E+00	0,021	0,355	7,69904E-140
cDC_LAMP3	TIMP1	9,85973E-62	-2,09279E+00	0,479	0,655	1,81587E-57
cDC_LAMP3	DUSP6	1,19111E-101	-2,13139E+00	0,035	0,296	2,19367E-97
cDC_LAMP3	SERPINA	4,52989E-228	-2,14251E+00	0,368	0,732	8,34270E-224
cDC_LAMP3	FCGR2A	8,04690E-242	-2,14927E+00	0,168	0,645	1,48200E-237
cDC_LAMP3	ALOX5AP	1,31530E-126	-2,16230E+00	0,21	0,514	2,42238E-122
cDC_LAMP3	RAC2	3,31390E-145	-2,16447E+00	0,155	0,507	6,10320E-141
cDC_LAMP3	RNASET2	2,82112E-293	-2,16565E+00	0,306	0,754	5,19565E-289
cDC_LAMP3	MPEG1	7,17387E-125	-2,18362E+00	0,069	0,383	1,32121E-120
cDC_LAMP3	EGR1	1,29940E-80	-2,18536E+00	0,064	0,293	2,39311E-76
cDC_LAMP3	LAPTM5	0,00000E+00	-2,20278E+00	0,532	0,909	0,00000E+00

cDC_LAMP3	IFI6	1,34136E-86	-2,22247E+00	0,263	0,495	2,47039E-82
cDC_LAMP3	FOS	1,35632E-213	-2,23878E+00	0,53	0,853	2,49793E-209
cDC_LAMP3	FTL	0,00000E+00	-2,24874E+00	0,949	0,993	0,00000E+00
cDC_LAMP3	GLUL	0,00000E+00	-2,26395E+00	0,358	0,818	0,00000E+00
cDC_LAMP3	CD163	4,20577E-187	-2,29319E+00	0,054	0,459	7,74576E-183
cDC_LAMP3	ASAH1	1,53476E-261	-2,30489E+00	0,294	0,701	2,82657E-257
cDC_LAMP3	FCGRT	0,00000E+00	-2,31891E+00	0,23	0,754	0,00000E+00
cDC_LAMP3	S100A4	5,23212E-297	-2,32863E+00	0,471	0,837	9,63599E-293
cDC_LAMP3	IL1B	8,94371E-66	-2,36018E+00	0,136	0,341	1,64716E-61
cDC_LAMP3	AIF1	0,00000E+00	-2,37007E+00	0,37	0,836	0,00000E+00
cDC_LAMP3	VSIG4	1,16111E-128	-2,37694E+00	0,043	0,357	2,13842E-124
cDC_LAMP3	CD68	0,00000E+00	-2,39140E+00	0,335	0,794	0,00000E+00
cDC_LAMP3	MS4A6A	0,00000E+00	-2,40089E+00	0,197	0,731	0,00000E+00
cDC_LAMP3	GPNMB	6,28416E-124	-2,43297E+00	0,042	0,344	1,15735E-119
cDC_LAMP3	PSAP	0,00000E+00	-2,50382E+00	0,695	0,923	0,00000E+00
cDC_LAMP3	CFD	6,75236E-233	-2,55773E+00	0,142	0,576	1,24358E-228
cDC_LAMP3	FCER1G	0,00000E+00	-2,62509E+00	0,389	0,929	0,00000E+00
cDC_LAMP3	SLC11A1	1,41968E-217	-2,63070E+00	0,037	0,481	2,61463E-213
cDC_LAMP3	APOC1	2,81859E-88	-2,63406E+00	0,337	0,513	5,19099E-84
cDC_LAMP3	C1QB	1,56777E-171	-2,75131E+00	0,217	0,556	2,88737E-167
cDC_LAMP3	PLAUR	1,28994E-223	-2,75281E+00	0,213	0,654	2,37568E-219
cDC_LAMP3	CTSB	0,00000E+00	-2,83031E+00	0,637	0,879	0,00000E+00
cDC_LAMP3	C1QA	1,97420E-214	-2,93508E+00	0,169	0,579	3,63589E-210
cDC_LAMP3	C1QC	1,81218E-193	-2,96869E+00	0,182	0,557	3,33750E-189
cDC_LAMP3	PLIN2	6,30344E-144	-3,06528E+00	0,175	0,508	1,16091E-139
cDC_LAMP3	TGFBI	3,36469E-241	-3,06642E+00	0,14	0,613	6,19675E-237
cDC_LAMP3	FCGR3A	4,39245E-256	-3,22226E+00	0,107	0,589	8,08958E-252
cDC_LAMP3	CCL4	6,75656E-67	-3,25706E+00	0,109	0,316	1,24435E-62
cDC_LAMP3	CTSD	0,00000E+00	-3,38482E+00	0,337	0,783	0,00000E+00
cDC_LAMP3	CTSL	2,27048E-224	-3,65948E+00	0,096	0,532	4,18154E-220
cDC_LAMP3	APOE	1,59504E-161	-3,72010E+00	0,184	0,517	2,93759E-157
cDC_LAMP3	FCN1	9,52389E-87	-3,76909E+00	0,029	0,258	1,75402E-82
cDC_LAMP3	CCL3	3,27546E-111	-3,78263E+00	0,096	0,384	6,03242E-107
cDC_LAMP3	S100A8	1,43198E-122	-3,79406E+00	0,063	0,361	2,63728E-118
cDC_LAMP3	RNASE1	4,13801E-92	-3,84843E+00	0,039	0,28	7,62097E-88
cDC_LAMP3	CD14	0,00000E+00	-3,93192E+00	0,062	0,641	0,00000E+00
cDC_LAMP3	S100A9	9,07121E-177	-3,97228E+00	0,086	0,468	1,67065E-172

Macro_FOLR2-APOE+

cluster	gene	p_val	avg_log2FC	pct.1	pct.2	p_val_adj
Macro_FOLR2-APOE+	APOE	0,00000E+00	2,02419E+00	0,934	0,437	0,00000E+00
Macro_FOLR2-APOE+	APOC1	0,00000E+00	1,83649E+00	0,917	0,44	0,00000E+00
Macro_FOLR2-APOE+	TREM2	0,00000E+00	1,36386E+00	0,869	0,331	0,00000E+00
Macro_FOLR2-APOE+	NUPR1	0,00000E+00	1,23292E+00	0,613	0,151	0,00000E+00
Macro_FOLR2-APOE+	MTRNR2L8	0,00000E+00	1,23209E+00	0,699	0,398	0,00000E+00
Macro_FOLR2-APOE+	RNASE1	0,00000E+00	1,20621E+00	0,668	0,208	0,00000E+00
Macro_FOLR2-APOE+	GPNMB	0,00000E+00	9,56336E-01	0,802	0,258	0,00000E+00
Macro_FOLR2-APOE+	PDK4	0,00000E+00	8,49671E-01	0,633	0,258	0,00000E+00
Macro_FOLR2-APOE+	KCNMA1	0,00000E+00	8,48780E-01	0,593	0,206	0,00000E+00
Macro_FOLR2-APOE+	LPL	0,00000E+00	8,38240E-01	0,308	0,032	0,00000E+00
Macro_FOLR2-APOE+	GCHFR	0,00000E+00	8,29954E-01	0,398	0,149	0,00000E+00
Macro_FOLR2-APOE+	CFD	0,00000E+00	8,23814E-01	0,817	0,521	0,00000E+00
Macro_FOLR2-APOE+	RHOB	0,00000E+00	8,15223E-01	0,79	0,501	0,00000E+00
Macro_FOLR2-APOE+	A2M	0,00000E+00	8,09641E-01	0,809	0,321	0,00000E+00
Macro_FOLR2-APOE+	C1QC	0,00000E+00	7,95708E-01	0,959	0,478	0,00000E+00
Macro_FOLR2-APOE+	DAB2	0,00000E+00	7,22958E-01	0,829	0,369	0,00000E+00
Macro_FOLR2-APOE+	GPX1	0,00000E+00	6,43685E-01	0,8	0,508	0,00000E+00
Macro_FOLR2-APOE+	MAFB	0,00000E+00	6,43064E-01	0,893	0,545	0,00000E+00
Macro_FOLR2-APOE+	ALOX15B	0,00000E+00	6,16647E-01	0,335	0,107	0,00000E+00
Macro_FOLR2-APOE+	CD81	0,00000E+00	6,14659E-01	0,777	0,41	0,00000E+00
Macro_FOLR2-APOE+	ADORA3	0,00000E+00	6,06755E-01	0,544	0,217	0,00000E+00
Macro_FOLR2-APOE+	GPR34	0,00000E+00	6,05843E-01	0,608	0,257	0,00000E+00
Macro_FOLR2-APOE+	C3	0,00000E+00	5,93908E-01	0,767	0,321	0,00000E+00
Macro_FOLR2-APOE+	SCD	0,00000E+00	5,90089E-01	0,387	0,11	0,00000E+00
Macro_FOLR2-APOE+	LIPA	0,00000E+00	5,66767E-01	0,844	0,445	0,00000E+00
Macro_FOLR2-APOE+	MTRNR2L12	9,08655E-85	5,28241E-01	0,82	0,757	1,67347E-80
Macro_FOLR2-APOE+	CTSD	0,00000E+00	5,17188E-01	0,975	0,735	0,00000E+00
Macro_FOLR2-APOE+	CPM	0,00000E+00	5,12728E-01	0,661	0,322	0,00000E+00
Macro_FOLR2-APOE+	CEBPA	0,00000E+00	4,92307E-01	0,322	0,129	0,00000E+00
Macro_FOLR2-APOE+	PLTP	0,00000E+00	4,91810E-01	0,732	0,263	0,00000E+00
Macro_FOLR2-APOE+	AKR1B1	0,00000E+00	4,83477E-01	0,768	0,419	0,00000E+00
Macro_FOLR2-APOE+	CTSZ	0,00000E+00	4,81474E-01	0,856	0,619	0,00000E+00
Macro_FOLR2-APOE+	OLFML3	0,00000E+00	4,65544E-01	0,429	0,193	0,00000E+00
Macro_FOLR2-APOE+	C1QA	0,00000E+00	4,52045E-01	0,958	0,502	0,00000E+00
Macro_FOLR2-APOE+	MMP2	0,00000E+00	4,42168E-01	0,264	0,067	0,00000E+00
Macro_FOLR2-APOE+	SLC1A3	0,00000E+00	4,38568E-01	0,66	0,314	0,00000E+00
Macro_FOLR2-APOE+	ABHD12	0,00000E+00	4,24827E-01	0,638	0,337	0,00000E+00
Macro_FOLR2-APOE+	FABP3	0,00000E+00	4,11553E-01	0,281	0,045	0,00000E+00
Macro_FOLR2-APOE+	HMOX1	0,00000E+00	4,06093E-01	0,797	0,504	0,00000E+00
Macro_FOLR2-APOE+	TMEM37	0,00000E+00	3,96351E-01	0,274	0,075	0,00000E+00
Macro_FOLR2-APOE+	CD9	0,00000E+00	3,92656E-01	0,834	0,428	0,00000E+00
Macro_FOLR2-APOE+	MSR1	0,00000E+00	3,88645E-01	0,723	0,325	0,00000E+00
Macro_FOLR2-APOE+	VAT1	0,00000E+00	3,83917E-01	0,474	0,197	0,00000E+00
Macro_FOLR2-APOE+	GAL3ST4	0,00000E+00	3,80061E-01	0,37	0,135	0,00000E+00
Macro_FOLR2-APOE+	ALDH1A1	0,00000E+00	3,76121E-01	0,403	0,166	0,00000E+00

Macro_FOLR2-APOE+	DNASE2	0,00000E+00	3,73263E-01	0,634	0,323	0,00000E+00
Macro_FOLR2-APOE+	MMP14	0,00000E+00	3,72860E-01	0,405	0,195	0,00000E+00
Macro_FOLR2-APOE+	GYPC	0,00000E+00	3,69822E-01	0,522	0,265	0,00000E+00
Macro_FOLR2-APOE+	TIMP2	0,00000E+00	3,67099E-01	0,582	0,325	0,00000E+00
Macro_FOLR2-APOE+	SLCO2B1	0,00000E+00	3,57387E-01	0,716	0,313	0,00000E+00
Macro_FOLR2-APOE+	FMNL2	0,00000E+00	3,44347E-01	0,413	0,197	0,00000E+00
Macro_FOLR2-APOE+	FRMD4A	0,00000E+00	3,32883E-01	0,368	0,156	0,00000E+00
Macro_FOLR2-APOE+	CD151	7,85001E-223	3,31530E-01	0,496	0,312	1,44574E-218
Macro_FOLR2-APOE+	YBX1	0,00000E+00	3,27550E-01	0,956	0,846	0,00000E+00
Macro_FOLR2-APOE+	FTL	0,00000E+00	3,25185E-01	1	0,99	0,00000E+00
Macro_FOLR2-APOE+	PLEC	3,71914E-212	3,24555E-01	0,524	0,338	6,84954E-208
Macro_FOLR2-APOE+	C1QB	0,00000E+00	3,22576E-01	0,951	0,48	0,00000E+00
Macro_FOLR2-APOE+	LHFPL2	0,00000E+00	3,15949E-01	0,5	0,248	0,00000E+00
Macro_FOLR2-APOE+	MERTK	0,00000E+00	3,08700E-01	0,428	0,153	0,00000E+00
Macro_FOLR2-APOE+	EPHX1	4,97922E-259	3,05543E-01	0,269	0,115	9,17023E-255
Macro_FOLR2-APOE+	FCHO2	0,00000E+00	2,99335E-01	0,399	0,185	0,00000E+00
Macro_FOLR2-APOE+	TSPAN4	0,00000E+00	2,98140E-01	0,557	0,25	0,00000E+00
Macro_FOLR2-APOE+	JUN	1,11652E-213	2,95867E-01	0,863	0,684	2,05630E-209
Macro_FOLR2-APOE+	AP1B1	0,00000E+00	2,95201E-01	0,645	0,353	0,00000E+00
Macro_FOLR2-APOE+	LRP1	0,00000E+00	2,84675E-01	0,679	0,38	0,00000E+00
Macro_FOLR2-APOE+	SRGAP1	1,68404E-223	2,80126E-01	0,307	0,148	3,10150E-219
Macro_FOLR2-APOE+	FN1	3,59684E-88	2,69637E-01	0,322	0,204	6,62429E-84
Macro_FOLR2-APOE+	DST	0,00000E+00	2,66489E-01	0,385	0,186	0,00000E+00
Macro_FOLR2-APOE+	SDC3	5,41664E-192	2,64019E-01	0,272	0,132	9,97583E-188
Macro_FOLR2-APOE+	FUCA1	0,00000E+00	2,61930E-01	0,513	0,207	0,00000E+00
Macro_FOLR2-APOE+	PLXDC2	0,00000E+00	2,54991E-01	0,811	0,5	0,00000E+00
Macro_FOLR2-APOE+	PIK3IP1	0,00000E+00	2,52212E-01	0,394	0,159	0,00000E+00
Macro_FOLR2-APOE+	NME3	1,59695E-42	-2,51075E-01	0,435	0,347	2,94110E-38
Macro_FOLR2-APOE+	CALU	1,27533E-84	-2,51973E-01	0,315	0,211	2,34878E-80
Macro_FOLR2-APOE+	SERPINB6	1,36460E-133	-2,54018E-01	0,559	0,4	2,51318E-129
Macro_FOLR2-APOE+	SLC43A2	6,13872E-18	-2,54657E-01	0,426	0,359	1,13057E-13
Macro_FOLR2-APOE+	NCF1	1,65754E-92	-2,55496E-01	0,613	0,482	3,05268E-88
Macro_FOLR2-APOE+	PRKDC	8,49450E-15	-2,56033E-01	0,293	0,248	1,56443E-10
Macro_FOLR2-APOE+	GLUD1	1,63610E-09	-2,56584E-01	0,366	0,322	3,01320E-05
Macro_FOLR2-APOE+	COA3	1,46152E-68	-2,57638E-01	0,411	0,305	2,69169E-64
Macro_FOLR2-APOE+	KLHL24	7,34032E-43	-2,59457E-01	0,254	0,181	1,35187E-38
Macro_FOLR2-APOE+	MRPS21	1,48423E-36	-2,59464E-01	0,611	0,514	2,73351E-32
Macro_FOLR2-APOE+	PABPC4	5,83251E-149	-2,61473E-01	0,564	0,401	1,07417E-144
Macro_FOLR2-APOE+	RNF135	2,01928E-55	-2,61715E-01	0,312	0,226	3,71890E-51
Macro_FOLR2-APOE+	FNDC3A	5,61302E-44	-2,61735E-01	0,31	0,229	1,03375E-39
Macro_FOLR2-APOE+	ZNF24	1,34024E-06	-2,63778E-01	0,26	0,235	2,46831E-02
Macro_FOLR2-APOE+	ADAM17	1,84117E-14	-2,63896E-01	0,266	0,22	3,39088E-10
Macro_FOLR2-APOE+	TUBA1C	4,83969E-57	-2,65061E-01	0,472	0,364	8,91327E-53
Macro_FOLR2-APOE+	USP9X	2,94618E-45	-2,65402E-01	0,343	0,263	5,42597E-41
Macro_FOLR2-APOE+	SLC40A1	2,60163E-168	-2,66026E-01	0,372	0,208	4,79143E-164
Macro_FOLR2-APOE+	POLDIP2	2,28737E-24	-2,66587E-01	0,263	0,21	4,21266E-20

Macro_FOLR2-APOE+	TNPO1	2,18253E-28	-2,67221E-01	0,303	0,241	4,01957E-24
Macro_FOLR2-APOE+	ARHGAP24	3,30497E-84	-2,68018E-01	0,29	0,183	6,08676E-80
Macro_FOLR2-APOE+	ERP44	3,19869E-39	-2,68679E-01	0,503	0,417	5,89102E-35
Macro_FOLR2-APOE+	HTATIP2	5,16209E-64	-2,68786E-01	0,376	0,277	9,50702E-60
Macro_FOLR2-APOE+	TMEM14C	4,65729E-141	-2,70994E-01	0,676	0,505	8,57734E-137
Macro_FOLR2-APOE+	AXL	0,00000E+00	-2,71022E-01	0,485	0,247	0,00000E+00
Macro_FOLR2-APOE+	CHID1	2,61375E-150	-2,71095E-01	0,321	0,189	4,81375E-146
Macro_FOLR2-APOE+	KDSR	6,16888E-49	-2,71378E-01	0,266	0,191	1,13612E-44
Macro_FOLR2-APOE+	MGAT4A	1,10875E-133	-2,72980E-01	0,494	0,335	2,04199E-129
Macro_FOLR2-APOE+	CLEC5A	5,42911E-134	-2,73442E-01	0,304	0,172	9,99880E-130
Macro_FOLR2-APOE+	TUSC2	9,92674E-16	-2,73895E-01	0,251	0,208	1,82821E-11
Macro_FOLR2-APOE+	ATP5IF1	1,41074E-24	-2,76056E-01	0,216	0,273	2,59816E-20
Macro_FOLR2-APOE+	DNAJB12	7,79131E-17	-2,76970E-01	0,284	0,237	1,43493E-12
Macro_FOLR2-APOE+	FCGRT	0,00000E+00	-2,77698E-01	0,937	0,706	0,00000E+00
Macro_FOLR2-APOE+	RB1	1,95234E-46	-2,78984E-01	0,487	0,388	3,59562E-42
Macro_FOLR2-APOE+	GNPDA1	2,25842E-127	-2,79660E-01	0,373	0,24	4,15934E-123
Macro_FOLR2-APOE+	PCBP2	1,54619E-21	-2,80119E-01	0,891	0,783	2,84762E-17
Macro_FOLR2-APOE+	WIPI1	2,19120E-112	-2,80849E-01	0,25	0,142	4,03552E-108
Macro_FOLR2-APOE+	OSTM1	1,77507E-39	-2,81173E-01	0,288	0,215	3,26915E-35
Macro_FOLR2-APOE+	FOXN2	1,73433E-13	-2,82590E-01	0,298	0,256	3,19412E-09
Macro_FOLR2-APOE+	SLC35F6	2,26641E-210	-2,83431E-01	0,27	0,127	4,17404E-206
Macro_FOLR2-APOE+	CTBS	5,08468E-34	-2,83883E-01	0,33	0,259	9,36446E-30
Macro_FOLR2-APOE+	DYNC1H1	2,78447E-17	-2,85532E-01	0,43	0,371	5,12817E-13
Macro_FOLR2-APOE+	MRPS18B	9,96922E-61	-2,85904E-01	0,358	0,264	1,83603E-56
Macro_FOLR2-APOE+	SNX27	3,44818E-36	-2,86213E-01	0,307	0,235	6,35051E-32
Macro_FOLR2-APOE+	COA6	4,40461E-07	-2,86366E-01	0,278	0,248	8,11197E-03
Macro_FOLR2-APOE+	LONP2	3,72600E-16	-2,87002E-01	0,267	0,221	6,86217E-12
Macro_FOLR2-APOE+	SIRT2	1,00661E-37	-2,87099E-01	0,285	0,216	1,85387E-33
Macro_FOLR2-APOE+	MZT2B	3,56432E-10	-2,87248E-01	0,396	0,351	6,56441E-06
Macro_FOLR2-APOE+	LSM8	5,55988E-42	-2,88002E-01	0,181	0,252	1,02396E-37
Macro_FOLR2-APOE+	NUCB1	9,40350E-126	-2,88710E-01	0,676	0,521	1,73184E-121
Macro_FOLR2-APOE+	NSL1	1,41048E-12	-2,89637E-01	0,281	0,241	2,59768E-08
Macro_FOLR2-APOE+	FCGR3A	0,00000E+00	-2,90127E-01	0,897	0,522	0,00000E+00
Macro_FOLR2-APOE+	MACF1	2,98129E-25	-2,90280E-01	0,379	0,311	5,49064E-21
Macro_FOLR2-APOE+	CD63	0,00000E+00	-2,91009E-01	0,96	0,795	0,00000E+00
Macro_FOLR2-APOE+	PRKCSH	2,64285E-29	-2,91665E-01	0,381	0,31	4,86734E-25
Macro_FOLR2-APOE+	UQCR10	2,77334E-52	-2,92330E-01	0,836	0,709	5,10766E-48
Macro_FOLR2-APOE+	RAD23A	8,24594E-50	-2,92505E-01	0,598	0,489	1,51865E-45
Macro_FOLR2-APOE+	EIF2AK4	5,07400E-23	-2,92931E-01	0,29	0,233	9,34479E-19
Macro_FOLR2-APOE+	IDH1	6,67506E-131	-2,93015E-01	0,364	0,227	1,22935E-126
Macro_FOLR2-APOE+	NOP53	5,17422E-42	-2,93467E-01	0,214	0,296	9,52936E-38
Macro_FOLR2-APOE+	CXCL16	0,00000E+00	-2,94871E-01	0,858	0,617	0,00000E+00
Macro_FOLR2-APOE+	STAU1	5,53266E-13	-2,94973E-01	0,328	0,284	1,01895E-08
Macro_FOLR2-APOE+	SLC7A7	4,36571E-250	-2,95650E-01	0,665	0,433	8,04033E-246
Macro_FOLR2-APOE+	ELOC	1,12119E-46	-2,95968E-01	0,187	0,266	2,06489E-42
Macro_FOLR2-APOE+	TFEC	1,59288E-34	-2,96882E-01	0,367	0,283	2,93361E-30

Macro_FOLR2-APOE+	DUT	2,55066E-42	-2,96942E-01	0,482	0,389	4,69755E-38
Macro_FOLR2-APOE+	NSA2	1,60627E-22	-2,97297E-01	0,553	0,475	2,95826E-18
Macro_FOLR2-APOE+	TPRKB	5,42296E-07	-2,97336E-01	0,266	0,237	9,98747E-03
Macro_FOLR2-APOE+	LMBRD1	1,23578E-52	-2,98227E-01	0,347	0,258	2,27594E-48
Macro_FOLR2-APOE+	CYB5A	7,00151E-68	-2,98438E-01	0,326	0,226	1,28947E-63
Macro_FOLR2-APOE+	VPS36	1,73020E-27	-2,98656E-01	0,342	0,278	3,18650E-23
Macro_FOLR2-APOE+	SLC15A3	1,11185E-255	-2,99034E-01	0,451	0,255	2,04770E-251
Macro_FOLR2-APOE+	PRDX1	0,00000E+00	-2,99067E-01	0,926	0,718	0,00000E+00
Macro_FOLR2-APOE+	RNF167	1,59195E-15	-2,99206E-01	0,335	0,287	2,93190E-11
Macro_FOLR2-APOE+	MIDN	3,15457E-15	-3,00032E-01	0,249	0,287	5,80977E-11
Macro_FOLR2-APOE+	MEA1	7,02010E-38	-3,00319E-01	0,464	0,378	1,29289E-33
Macro_FOLR2-APOE+	MED8	1,10492E-18	-3,00837E-01	0,262	0,216	2,03494E-14
Macro_FOLR2-APOE+	SELENOW	5,42981E-36	-3,01194E-01	0,229	0,302	1,00001E-31
Macro_FOLR2-APOE+	TRAPPC1	8,84871E-14	-3,01318E-01	0,694	0,596	1,62967E-09
Macro_FOLR2-APOE+	NUMA1	4,24733E-21	-3,01869E-01	0,279	0,226	7,82231E-17
Macro_FOLR2-APOE+	PRKAG2	8,13636E-22	-3,01905E-01	0,268	0,215	1,49847E-17
Macro_FOLR2-APOE+	LY86	2,76582E-271	-3,02097E-01	0,691	0,457	5,09381E-267
Macro_FOLR2-APOE+	SMIM20	7,96499E-70	-3,02390E-01	0,32	0,224	1,46691E-65
Macro_FOLR2-APOE+	HNRNPUL1	2,55671E-09	-3,02452E-01	0,456	0,404	4,70870E-05
Macro_FOLR2-APOE+	S100A4	2,59237E-21	-3,03258E-01	0,909	0,813	4,77438E-17
Macro_FOLR2-APOE+	SOAT1	3,73124E-107	-3,04506E-01	0,397	0,268	6,87183E-103
Macro_FOLR2-APOE+	POLR2I	3,52493E-24	-3,05807E-01	0,348	0,286	6,49186E-20
Macro_FOLR2-APOE+	EIF4EBP1	1,03907E-139	-3,06734E-01	0,547	0,387	1,91365E-135
Macro_FOLR2-APOE+	MRPL27	1,75338E-35	-3,07048E-01	0,347	0,278	3,22920E-31
Macro_FOLR2-APOE+	SYNCRIP	7,45985E-12	-3,07075E-01	0,359	0,316	1,37388E-07
Macro_FOLR2-APOE+	PBDC1	9,56120E-46	-3,07119E-01	0,268	0,195	1,76089E-41
Macro_FOLR2-APOE+	SMC1A	5,33465E-08	-3,07343E-01	0,28	0,249	9,82483E-04
Macro_FOLR2-APOE+	CD74	0,00000E+00	-3,09064E-01	1	0,957	0,00000E+00
Macro_FOLR2-APOE+	TMEM126B	1,97214E-49	-3,10059E-01	0,277	0,198	3,63209E-45
Macro_FOLR2-APOE+	B3GAT3	1,82561E-20	-3,11000E-01	0,285	0,234	3,36222E-16
Macro_FOLR2-APOE+	SCAMP2	5,02313E-175	-3,11271E-01	0,652	0,479	9,25110E-171
Macro_FOLR2-APOE+	FKBP15	5,10075E-37	-3,11420E-01	0,336	0,262	9,39406E-33
Macro_FOLR2-APOE+	AGPAT2	1,18441E-63	-3,11811E-01	0,377	0,277	2,18132E-59
Macro_FOLR2-APOE+	SHARPIN	4,50143E-39	-3,11989E-01	0,314	0,242	8,29029E-35
Macro_FOLR2-APOE+	KPNB1	2,43013E-11	-3,12320E-01	0,49	0,432	4,47557E-07
Macro_FOLR2-APOE+	PAPSS1	5,37936E-23	-3,12607E-01	0,283	0,228	9,90716E-19
Macro_FOLR2-APOE+	MTX1	8,77599E-31	-3,13644E-01	0,252	0,193	1,61627E-26
Macro_FOLR2-APOE+	NCF4	2,61809E-73	-3,13665E-01	0,531	0,405	4,82174E-69
Macro_FOLR2-APOE+	SNAP29	1,60738E-32	-3,14066E-01	0,344	0,275	2,96032E-28
Macro_FOLR2-APOE+	ERLEC1	4,57323E-19	-3,14567E-01	0,321	0,268	8,42251E-15
Macro_FOLR2-APOE+	DYNLRB1	8,79855E-187	-3,16177E-01	0,63	0,435	1,62043E-182
Macro_FOLR2-APOE+	S100A11	0,00000E+00	-3,16330E-01	0,992	0,928	0,00000E+00
Macro_FOLR2-APOE+	PTGS1	6,71924E-60	-3,16362E-01	0,344	0,243	1,23748E-55
Macro_FOLR2-APOE+	COPS2	2,12432E-06	-3,17405E-01	0,254	0,228	3,91235E-02
Macro_FOLR2-APOE+	NDUFB11	2,55777E-10	-3,17934E-01	0,744	0,648	4,71064E-06
Macro_FOLR2-APOE+	MRPL43	1,18421E-70	-3,18187E-01	0,44	0,335	2,18096E-66

Macro_FOLR2-APOE+	MKKS	8,45503E-20	-3,20130E-01	0,253	0,202	1,55716E-15
Macro_FOLR2-APOE+	CHCHD1	1,30608E-15	-3,20602E-01	0,311	0,263	2,40541E-11
Macro_FOLR2-APOE+	GTF2I	1,09093E-17	-3,21370E-01	0,358	0,304	2,00917E-13
Macro_FOLR2-APOE+	NT5C	1,09430E-43	-3,22033E-01	0,427	0,339	2,01537E-39
Macro_FOLR2-APOE+	APIP	7,45881E-44	-3,22196E-01	0,298	0,223	1,37369E-39
Macro_FOLR2-APOE+	HSF1	8,67417E-10	-3,22860E-01	0,305	0,268	1,59752E-05
Macro_FOLR2-APOE+	CCDC88A	7,66795E-09	-3,22879E-01	0,557	0,493	1,41221E-04
Macro_FOLR2-APOE+	REXO2	2,84406E-61	-3,23595E-01	0,252	0,169	5,23791E-57
Macro_FOLR2-APOE+	AIMP1	2,54819E-11	-3,23623E-01	0,327	0,286	4,69301E-07
Macro_FOLR2-APOE+	SNU13	7,44073E-43	-3,23730E-01	0,237	0,314	1,37036E-38
Macro_FOLR2-APOE+	CCDC47	1,01459E-23	-3,24542E-01	0,355	0,296	1,86858E-19
Macro_FOLR2-APOE+	TMEM141	4,84522E-07	-3,24577E-01	0,286	0,257	8,92344E-03
Macro_FOLR2-APOE+	TMEM70	2,60439E-24	-3,24623E-01	0,345	0,281	4,79651E-20
Macro_FOLR2-APOE+	PJA2	1,52911E-07	-3,25098E-01	0,306	0,271	2,81616E-03
Macro_FOLR2-APOE+	BTF3L4	9,03347E-46	-3,25223E-01	0,455	0,365	1,66369E-41
Macro_FOLR2-APOE+	VMA21	2,49096E-11	-3,25226E-01	0,415	0,368	4,58761E-07
Macro_FOLR2-APOE+	HAGH	1,79938E-47	-3,25434E-01	0,313	0,235	3,31391E-43
Macro_FOLR2-APOE+	RRAGA	1,56438E-22	-3,26252E-01	0,278	0,225	2,88111E-18
Macro_FOLR2-APOE+	ATP6V0A1	1,60864E-70	-3,27362E-01	0,267	0,175	2,96263E-66
Macro_FOLR2-APOE+	LEPROT	5,50693E-66	-3,27422E-01	0,448	0,34	1,01421E-61
Macro_FOLR2-APOE+	GPX4	3,36067E-175	-3,27501E-01	0,951	0,827	6,18934E-171
Macro_FOLR2-APOE+	NDUFA8	1,52569E-43	-3,27958E-01	0,359	0,279	2,80986E-39
Macro_FOLR2-APOE+	DCTD	1,07292E-33	-3,28299E-01	0,26	0,198	1,97600E-29
Macro_FOLR2-APOE+	GUSB	2,07106E-173	-3,28332E-01	0,486	0,323	3,81427E-169
Macro_FOLR2-APOE+	NOL7	2,69146E-19	-3,29099E-01	0,47	0,405	4,95685E-15
Macro_FOLR2-APOE+	DHRS4	1,18420E-18	-3,29869E-01	0,313	0,264	2,18094E-14
Macro_FOLR2-APOE+	RBM42	4,42840E-11	-3,30331E-01	0,33	0,291	8,15578E-07
Macro_FOLR2-APOE+	ABHD14B	4,74252E-45	-3,30376E-01	0,333	0,251	8,73429E-41
Macro_FOLR2-APOE+	NAIP	1,95676E-12	-3,31477E-01	0,425	0,362	3,60377E-08
Macro_FOLR2-APOE+	RNF130	2,13358E-159	-3,31612E-01	0,867	0,683	3,92941E-155
Macro_FOLR2-APOE+	CSRP1	9,15395E-70	-3,31624E-01	0,342	0,244	1,68588E-65
Macro_FOLR2-APOE+	PHF5A	4,59238E-07	-3,31668E-01	0,282	0,254	8,45778E-03
Macro_FOLR2-APOE+	FNDC3B	4,66565E-19	-3,31856E-01	0,333	0,27	8,59273E-15
Macro_FOLR2-APOE+	RRBP1	6,50083E-17	-3,32208E-01	0,44	0,376	1,19726E-12
Macro_FOLR2-APOE+	GNB1	1,29028E-13	-3,32712E-01	0,436	0,381	2,37630E-09
Macro_FOLR2-APOE+	RAPGEF1	2,96544E-08	-3,32860E-01	0,29	0,254	5,46145E-04
Macro_FOLR2-APOE+	ATP5PD	1,59175E-50	-3,32879E-01	0,243	0,332	2,93152E-46
Macro_FOLR2-APOE+	MTCH1	9,25149E-36	-3,33043E-01	0,534	0,443	1,70385E-31
Macro_FOLR2-APOE+	HLA-DQA2	3,60858E-115	-3,34268E-01	0,721	0,478	6,64593E-111
Macro_FOLR2-APOE+	WASF2	8,25490E-83	-3,34507E-01	0,687	0,549	1,52031E-78
Macro_FOLR2-APOE+	CYBB	5,44361E-132	-3,36165E-01	0,795	0,596	1,00255E-127
Macro_FOLR2-APOE+	TRAPPC6A	1,11002E-16	-3,36380E-01	0,282	0,235	2,04432E-12
Macro_FOLR2-APOE+	SPG21	8,73693E-100	-3,37755E-01	0,554	0,419	1,60908E-95
Macro_FOLR2-APOE+	SF3B6	3,75005E-48	-3,38052E-01	0,222	0,301	6,90647E-44
Macro_FOLR2-APOE+	DPM3	2,16779E-09	-3,38483E-01	0,319	0,28	3,99242E-05
Macro_FOLR2-APOE+	MSN	4,39192E-65	-3,38876E-01	0,758	0,618	8,08860E-61

Macro_FOLR2-APOE+	STOM	4,95696E-106	-3,40758E-01	0,466	0,326	9,12923E-102
Macro_FOLR2-APOE+	LMF2	2,54893E-28	-3,41305E-01	0,252	0,197	4,69437E-24
Macro_FOLR2-APOE+	DUSP23	2,48257E-129	-3,41636E-01	0,515	0,362	4,57215E-125
Macro_FOLR2-APOE+	HMG20B	2,24058E-17	-3,41859E-01	0,279	0,232	4,12648E-13
Macro_FOLR2-APOE+	C4orf48	1,66807E-42	-3,41932E-01	0,618	0,513	3,07209E-38
Macro_FOLR2-APOE+	ZCRB1	1,05640E-28	-3,42826E-01	0,327	0,263	1,94558E-24
Macro_FOLR2-APOE+	RGS10	1,03618E-49	-3,43093E-01	0,864	0,72	1,90834E-45
Macro_FOLR2-APOE+	BCAP31	1,56042E-292	-3,44675E-01	0,76	0,555	2,87383E-288
Macro_FOLR2-APOE+	SRPK2	7,95236E-24	-3,46680E-01	0,261	0,205	1,46459E-19
Macro_FOLR2-APOE+	VAMP3	2,92355E-82	-3,47314E-01	0,453	0,334	5,38431E-78
Macro_FOLR2-APOE+	MRPL4	6,15283E-16	-3,48829E-01	0,317	0,271	1,13317E-11
Macro_FOLR2-APOE+	RHOA	2,63660E-249	-3,49199E-01	0,962	0,865	4,85583E-245
Macro_FOLR2-APOE+	SLBP	4,89604E-11	-3,49242E-01	0,265	0,228	9,01704E-07
Macro_FOLR2-APOE+	RTRAF	4,80154E-53	-3,50229E-01	0,193	0,277	8,84300E-49
Macro_FOLR2-APOE+	RSF1	1,98825E-16	-3,50309E-01	0,388	0,336	3,66177E-12
Macro_FOLR2-APOE+	COMMD4	1,47373E-53	-3,50922E-01	0,308	0,225	2,71416E-49
Macro_FOLR2-APOE+	IMP3	1,05716E-28	-3,51264E-01	0,374	0,305	1,94697E-24
Macro_FOLR2-APOE+	HPCAL1	9,16374E-19	-3,51589E-01	0,293	0,239	1,68769E-14
Macro_FOLR2-APOE+	MRPS15	1,11402E-47	-3,52481E-01	0,391	0,306	2,05170E-43
Macro_FOLR2-APOE+	TM2D3	1,03040E-17	-3,54726E-01	0,254	0,207	1,89769E-13
Macro_FOLR2-APOE+	CCPG1	1,46434E-25	-3,54969E-01	0,283	0,223	2,69687E-21
Macro_FOLR2-APOE+	CNPPD1	1,50589E-11	-3,55181E-01	0,305	0,265	2,77339E-07
Macro_FOLR2-APOE+	LAPTM5	0,00000E+00	-3,55579E-01	0,988	0,883	0,00000E+00
Macro_FOLR2-APOE+	MRPL54	4,37756E-16	-3,55650E-01	0,453	0,394	8,06214E-12
Macro_FOLR2-APOE+	SLAMF8	6,39082E-91	-3,56019E-01	0,444	0,306	1,17700E-86
Macro_FOLR2-APOE+	SMIM19	8,39071E-44	-3,56261E-01	0,327	0,249	1,54532E-39
Macro_FOLR2-APOE+	ADAM28	7,76262E-66	-3,56443E-01	0,321	0,225	1,42964E-61
Macro_FOLR2-APOE+	CNTRL	3,95435E-10	-3,56644E-01	0,273	0,236	7,28273E-06
Macro_FOLR2-APOE+	LRPAP1	9,30857E-204	-3,56739E-01	0,654	0,47	1,71436E-199
Macro_FOLR2-APOE+	SUMO3	1,44112E-35	-3,58696E-01	0,567	0,467	2,65411E-31
Macro_FOLR2-APOE+	CHCHD5	3,61467E-14	-3,58998E-01	0,342	0,292	6,65714E-10
Macro_FOLR2-APOE+	RAB5IF	5,11147E-66	-3,59845E-01	0,161	0,253	9,41380E-62
Macro_FOLR2-APOE+	TCF12	2,11246E-54	-3,60679E-01	0,294	0,211	3,89052E-50
Macro_FOLR2-APOE+	ADI1	8,28690E-15	-3,60718E-01	0,349	0,296	1,52620E-10
Macro_FOLR2-APOE+	BFAR	2,41346E-14	-3,60899E-01	0,252	0,212	4,44487E-10
Macro_FOLR2-APOE+	BCAP29	1,32470E-38	-3,61048E-01	0,27	0,2	2,43970E-34
Macro_FOLR2-APOE+	ZNHIT1	1,46611E-29	-3,62330E-01	0,549	0,467	2,70013E-25
Macro_FOLR2-APOE+	REEP4	6,28257E-27	-3,62665E-01	0,293	0,232	1,15706E-22
Macro_FOLR2-APOE+	FOLR2	1,49533E-223	-3,62973E-01	0,326	0,157	2,75395E-219
Macro_FOLR2-APOE+	ADH5	9,31950E-50	-3,63121E-01	0,31	0,23	1,71637E-45
Macro_FOLR2-APOE+	HLA-DOA	9,52805E-215	-3,63438E-01	0,567	0,364	1,75478E-210
Macro_FOLR2-APOE+	ECHS1	2,01009E-61	-3,63957E-01	0,46	0,354	3,70197E-57
Macro_FOLR2-APOE+	POLR2F	2,58355E-37	-3,64225E-01	0,381	0,302	4,75812E-33
Macro_FOLR2-APOE+	CHMP2B	1,77802E-34	-3,64855E-01	0,362	0,288	3,27459E-30
Macro_FOLR2-APOE+	PUM1	8,13057E-28	-3,64880E-01	0,296	0,234	1,49741E-23
Macro_FOLR2-APOE+	RWDD1	1,46776E-27	-3,65146E-01	0,527	0,446	2,70317E-23

Macro_FOLR2-APOE+	P2RX4	1,15854E-138	-3,65326E-01	0,35	0,214	2,13368E-134
Macro_FOLR2-APOE+	TMEM33	1,18837E-33	-3,65449E-01	0,276	0,209	2,18862E-29
Macro_FOLR2-APOE+	ITGB1	4,17362E-58	-3,65462E-01	0,574	0,458	7,68655E-54
Macro_FOLR2-APOE+	GOLGA4	1,78576E-10	-3,65884E-01	0,374	0,329	3,28884E-06
Macro_FOLR2-APOE+	BEX4	2,91778E-114	-3,66984E-01	0,446	0,309	5,37367E-110
Macro_FOLR2-APOE+	ACER3	8,47996E-29	-3,67180E-01	0,292	0,229	1,56175E-24
Macro_FOLR2-APOE+	ZNF385A	7,22065E-24	-3,67237E-01	0,485	0,4	1,32983E-19
Macro_FOLR2-APOE+	DOK1	5,67410E-64	-3,67476E-01	0,318	0,225	1,04500E-59
Macro_FOLR2-APOE+	EPS15	6,20212E-10	-3,68681E-01	0,28	0,243	1,14225E-05
Macro_FOLR2-APOE+	STX4	2,10756E-26	-3,68692E-01	0,371	0,303	3,88149E-22
Macro_FOLR2-APOE+	OLR1	0,00000E+00	-3,69028E-01	0,586	0,309	0,00000E+00
Macro_FOLR2-APOE+	TBXAS1	1,79268E-198	-3,69587E-01	0,695	0,49	3,30159E-194
Macro_FOLR2-APOE+	MFSD10	1,64694E-25	-3,69646E-01	0,321	0,262	3,03317E-21
Macro_FOLR2-APOE+	STMP1	1,32652E-60	-3,69748E-01	0,18	0,269	2,44305E-56
Macro_FOLR2-APOE+	ZNF581	4,56256E-28	-3,70911E-01	0,25	0,192	8,40287E-24
Macro_FOLR2-APOE+	PKM	2,19814E-18	-3,71319E-01	0,879	0,768	4,04832E-14
Macro_FOLR2-APOE+	ANP32B	3,71235E-21	-3,71887E-01	0,65	0,559	6,83703E-17
Macro_FOLR2-APOE+	RNASEH2C	3,40570E-17	-3,72160E-01	0,395	0,338	6,27227E-13
Macro_FOLR2-APOE+	ATP6AP2	9,23529E-236	-3,73089E-01	0,766	0,563	1,70086E-231
Macro_FOLR2-APOE+	TTC1	9,95500E-17	-3,73481E-01	0,291	0,244	1,83341E-12
Macro_FOLR2-APOE+	HECTD1	1,79813E-07	-3,73710E-01	0,26	0,227	3,31162E-03
Macro_FOLR2-APOE+	LSM2	1,80529E-13	-3,74727E-01	0,394	0,344	3,32480E-09
Macro_FOLR2-APOE+	CD302	2,15758E-27	-3,76184E-01	0,42	0,337	3,97362E-23
Macro_FOLR2-APOE+	SMARCC2	1,54154E-19	-3,76704E-01	0,267	0,218	2,83905E-15
Macro_FOLR2-APOE+	BASP1	6,02548E-07	-3,76848E-01	0,337	0,348	1,10971E-02
Macro_FOLR2-APOE+	PRKAG1	2,55707E-39	-3,77172E-01	0,252	0,185	4,70935E-35
Macro_FOLR2-APOE+	SMAP2	1,11500E-29	-3,77709E-01	0,553	0,457	2,05350E-25
Macro_FOLR2-APOE+	CCDC115	3,08348E-16	-3,78426E-01	0,306	0,259	5,67884E-12
Macro_FOLR2-APOE+	TPR	4,39046E-10	-3,78458E-01	0,431	0,383	8,08590E-06
Macro_FOLR2-APOE+	CLPP	2,51633E-30	-3,78928E-01	0,32	0,256	4,63433E-26
Macro_FOLR2-APOE+	MRPL24	6,67591E-47	-3,79242E-01	0,289	0,213	1,22950E-42
Macro_FOLR2-APOE+	MFSD1	0,00000E+00	-3,79352E-01	0,798	0,558	0,00000E+00
Macro_FOLR2-APOE+	NQO2	1,31494E-34	-3,80468E-01	0,323	0,251	2,42172E-30
Macro_FOLR2-APOE+	TSPAN14	6,14718E-137	-3,80666E-01	0,468	0,311	1,13213E-132
Macro_FOLR2-APOE+	SEC63	1,39914E-22	-3,80688E-01	0,289	0,233	2,57680E-18
Macro_FOLR2-APOE+	ADAR	2,46152E-10	-3,82123E-01	0,477	0,42	4,53338E-06
Macro_FOLR2-APOE+	TMEM165	1,47292E-20	-3,82249E-01	0,451	0,383	2,71268E-16
Macro_FOLR2-APOE+	MRPL13	1,63209E-16	-3,82513E-01	0,276	0,231	3,00581E-12
Macro_FOLR2-APOE+	NEK6	3,42652E-100	-3,82578E-01	0,338	0,223	6,31062E-96
Macro_FOLR2-APOE+	KLHDC3	4,35583E-23	-3,82677E-01	0,289	0,234	8,02213E-19
Macro_FOLR2-APOE+	MAP4	1,32785E-23	-3,82999E-01	0,307	0,25	2,44549E-19
Macro_FOLR2-APOE+	ARMCX3	6,42160E-24	-3,83898E-01	0,312	0,249	1,18267E-19
Macro_FOLR2-APOE+	KDELR2	2,23939E-96	-3,84316E-01	0,626	0,487	4,12428E-92
Macro_FOLR2-APOE+	ACO2	4,04227E-18	-3,84767E-01	0,31	0,263	7,44466E-14
Macro_FOLR2-APOE+	PHF20L1	1,90449E-06	-3,84783E-01	0,229	0,251	3,50749E-02
Macro_FOLR2-APOE+	ARSA	7,23472E-16	-3,85222E-01	0,273	0,228	1,33242E-11

Macro_FOLR2-APOE+	ATP5PF	4,08793E-61	-3,85604E-01	0,239	0,337	7,52875E-57
Macro_FOLR2-APOE+	ITPR2	3,94456E-08	-3,87459E-01	0,31	0,273	7,26469E-04
Macro_FOLR2-APOE+	GLUL	0,00000E+00	-3,88194E-01	0,964	0,777	0,00000E+00
Macro_FOLR2-APOE+	MRPL11	1,13876E-15	-3,89186E-01	0,335	0,287	2,09725E-11
Macro_FOLR2-APOE+	RNASEH2B	6,03539E-22	-3,89555E-01	0,355	0,294	1,11154E-17
Macro_FOLR2-APOE+	VMO1	2,79770E-214	-3,89888E-01	0,379	0,203	5,15252E-210
Macro_FOLR2-APOE+	RHBDF2	1,86566E-74	-3,91275E-01	0,422	0,308	3,43598E-70
Macro_FOLR2-APOE+	TBC1D1	1,43096E-18	-3,91340E-01	0,264	0,216	2,63540E-14
Macro_FOLR2-APOE+	FAM50A	7,91869E-13	-3,91454E-01	0,344	0,298	1,45838E-08
Macro_FOLR2-APOE+	ACTN1	9,72560E-20	-3,91651E-01	0,377	0,309	1,79116E-15
Macro_FOLR2-APOE+	ATP5F1A	3,18849E-58	-3,91752E-01	0,184	0,266	5,87225E-54
Macro_FOLR2-APOE+	NUDCD2	9,72528E-20	-3,92124E-01	0,263	0,215	1,79111E-15
Macro_FOLR2-APOE+	DNPH1	3,59652E-131	-3,92376E-01	0,476	0,326	6,62371E-127
Macro_FOLR2-APOE+	PPT1	0,00000E+00	-3,92643E-01	0,852	0,618	0,00000E+00
Macro_FOLR2-APOE+	SREK1	1,40387E-09	-3,93676E-01	0,267	0,231	2,58551E-05
Macro_FOLR2-APOE+	TACC1	5,00792E-19	-3,93738E-01	0,482	0,411	9,22309E-15
Macro_FOLR2-APOE+	TEX264	1,91463E-61	-3,93955E-01	0,389	0,295	3,52618E-57
Macro_FOLR2-APOE+	PITHD1	6,70639E-26	-3,94747E-01	0,278	0,221	1,23512E-21
Macro_FOLR2-APOE+	STMN1	7,32535E-164	-3,95274E-01	0,438	0,262	1,34911E-159
Macro_FOLR2-APOE+	ZNF511	1,39363E-09	-3,95666E-01	0,251	0,217	2,56664E-05
Macro_FOLR2-APOE+	ITFG1	5,12546E-25	-3,95987E-01	0,261	0,207	9,43956E-21
Macro_FOLR2-APOE+	ANXA4	1,23711E-91	-3,96376E-01	0,411	0,292	2,27838E-87
Macro_FOLR2-APOE+	SYPL1	1,94535E-68	-3,96588E-01	0,393	0,288	3,58276E-64
Macro_FOLR2-APOE+	CNIH4	1,20337E-36	-3,96656E-01	0,353	0,275	2,21624E-32
Macro_FOLR2-APOE+	TMED9	2,63748E-217	-3,97206E-01	0,692	0,496	4,85745E-213
Macro_FOLR2-APOE+	ASPH	7,06874E-51	-3,97882E-01	0,294	0,211	1,30185E-46
Macro_FOLR2-APOE+	COX20	2,92725E-11	-3,98226E-01	0,293	0,253	5,39112E-07
Macro_FOLR2-APOE+	AP2S1	9,05847E-153	-3,99103E-01	0,855	0,697	1,66830E-148
Macro_FOLR2-APOE+	UBE2G2	3,22379E-13	-4,00248E-01	0,257	0,217	5,93725E-09
Macro_FOLR2-APOE+	SELENOH	3,45568E-55	-4,01464E-01	0,252	0,344	6,36433E-51
Macro_FOLR2-APOE+	LARP7	2,15044E-17	-4,03204E-01	0,32	0,269	3,96047E-13
Macro_FOLR2-APOE+	ZC3H13	1,22586E-09	-4,03253E-01	0,286	0,25	2,25767E-05
Macro_FOLR2-APOE+	TRMT1	6,46847E-07	-4,05375E-01	0,303	0,269	1,19130E-02
Macro_FOLR2-APOE+	SNX29	8,26999E-64	-4,06081E-01	0,346	0,246	1,52308E-59
Macro_FOLR2-APOE+	SEM1	8,16646E-69	-4,06375E-01	0,235	0,337	1,50402E-64
Macro_FOLR2-APOE+	PRDX4	4,06198E-82	-4,06637E-01	0,381	0,271	7,48095E-78
Macro_FOLR2-APOE+	RCBTB2	1,53121E-45	-4,06895E-01	0,25	0,175	2,82002E-41
Macro_FOLR2-APOE+	GBA	3,49716E-128	-4,07473E-01	0,307	0,183	6,44072E-124
Macro_FOLR2-APOE+	SNX6	4,83484E-46	-4,07477E-01	0,617	0,499	8,90433E-42
Macro_FOLR2-APOE+	HVCN1	6,97064E-45	-4,07503E-01	0,322	0,243	1,28378E-40
Macro_FOLR2-APOE+	APMAP	3,61274E-78	-4,07968E-01	0,395	0,285	6,65358E-74
Macro_FOLR2-APOE+	FGFR1OP2	7,70934E-29	-4,08959E-01	0,365	0,296	1,41983E-24
Macro_FOLR2-APOE+	CBR1	2,70403E-40	-4,09428E-01	0,391	0,304	4,98000E-36
Macro_FOLR2-APOE+	UBR4	1,20123E-17	-4,09602E-01	0,289	0,239	2,21231E-13
Macro_FOLR2-APOE+	UROD	4,17310E-69	-4,10327E-01	0,314	0,222	7,68559E-65
Macro_FOLR2-APOE+	TXNDC12	1,03663E-59	-4,10477E-01	0,422	0,328	1,90916E-55

Macro_FOLR2-APOE+	IMMT	3,89598E-14	-4,10575E-01	0,269	0,229	7,17524E-10
Macro_FOLR2-APOE+	TMEM147	2,69475E-188	-4,10640E-01	0,589	0,408	4,96291E-184
Macro_FOLR2-APOE+	STX12	8,14720E-34	-4,12771E-01	0,377	0,302	1,50047E-29
Macro_FOLR2-APOE+	PICALM	1,88159E-44	-4,13481E-01	0,516	0,415	3,46532E-40
Macro_FOLR2-APOE+	DCAF7	1,86242E-09	-4,13686E-01	0,347	0,306	3,43001E-05
Macro_FOLR2-APOE+	GPSM3	2,62675E-09	-4,13697E-01	0,729	0,676	4,83768E-05
Macro_FOLR2-APOE+	EAPP	4,88911E-16	-4,14146E-01	0,271	0,225	9,00427E-12
Macro_FOLR2-APOE+	STARD3NL	2,76993E-07	-4,14618E-01	0,298	0,266	5,10137E-03
Macro_FOLR2-APOE+	PNKD	5,27762E-23	-4,14787E-01	0,377	0,313	9,71980E-19
Macro_FOLR2-APOE+	LTA4H	1,66935E-201	-4,14894E-01	0,603	0,422	3,07444E-197
Macro_FOLR2-APOE+	ALKBH7	2,52196E-60	-4,15544E-01	0,589	0,476	4,64470E-56
Macro_FOLR2-APOE+	BLOC1S2	1,22137E-73	-4,15857E-01	0,423	0,316	2,24939E-69
Macro_FOLR2-APOE+	RBX1	4,20578E-11	-4,16468E-01	0,7	0,606	7,74579E-07
Macro_FOLR2-APOE+	ENTPD1	5,31127E-48	-4,18685E-01	0,389	0,296	9,78177E-44
Macro_FOLR2-APOE+	UQCRB	1,55146E-11	-4,19490E-01	0,845	0,762	2,85732E-07
Macro_FOLR2-APOE+	RNF13	1,97142E-276	-4,20066E-01	0,704	0,487	3,63076E-272
Macro_FOLR2-APOE+	MRPS12	2,04543E-13	-4,21391E-01	0,321	0,277	3,76707E-09
Macro_FOLR2-APOE+	NAA38	3,80124E-16	-4,21663E-01	0,474	0,407	7,00074E-12
Macro_FOLR2-APOE+	DOK3	1,84962E-23	-4,22364E-01	0,279	0,22	3,40645E-19
Macro_FOLR2-APOE+	S100A6	4,26416E-07	-4,22758E-01	0,921	0,867	7,85329E-03
Macro_FOLR2-APOE+	RBM47	6,47396E-22	-4,23604E-01	0,417	0,349	1,19231E-17
Macro_FOLR2-APOE+	SF3B2	8,51259E-08	-4,24025E-01	0,564	0,508	1,56776E-03
Macro_FOLR2-APOE+	PTAFR	2,14389E-44	-4,24224E-01	0,469	0,361	3,94839E-40
Macro_FOLR2-APOE+	SMIM12	1,62962E-08	-4,24356E-01	0,298	0,263	3,00128E-04
Macro_FOLR2-APOE+	MEAF6	1,95898E-27	-4,24548E-01	0,374	0,306	3,60785E-23
Macro_FOLR2-APOE+	MRPL37	4,25450E-33	-4,24584E-01	0,285	0,221	7,83551E-29
Macro_FOLR2-APOE+	WNK1	1,87904E-49	-4,24828E-01	0,44	0,343	3,46062E-45
Macro_FOLR2-APOE+	NUTF2	8,80110E-40	-4,25397E-01	0,455	0,373	1,62090E-35
Macro_FOLR2-APOE+	BSG	1,48019E-74	-4,25444E-01	0,671	0,545	2,72607E-70
Macro_FOLR2-APOE+	BTG1	4,06968E-07	-4,25520E-01	0,875	0,799	7,49513E-03
Macro_FOLR2-APOE+	RASSF4	1,36469E-141	-4,25896E-01	0,669	0,48	2,51335E-137
Macro_FOLR2-APOE+	GNA13	2,06045E-08	-4,26790E-01	0,369	0,328	3,79473E-04
Macro_FOLR2-APOE+	MIEN1	2,22515E-27	-4,27163E-01	0,462	0,386	4,09806E-23
Macro_FOLR2-APOE+	DENND3	1,29428E-35	-4,27578E-01	0,296	0,222	2,38367E-31
Macro_FOLR2-APOE+	TMEM176A	1,20520E-55	-4,28230E-01	0,587	0,426	2,21962E-51
Macro_FOLR2-APOE+	STARD3	1,40401E-48	-4,28342E-01	0,311	0,231	2,58576E-44
Macro_FOLR2-APOE+	TYROBP	0,00000E+00	-4,28822E-01	0,995	0,939	0,00000E+00
Macro_FOLR2-APOE+	COPS7A	1,98142E-16	-4,30592E-01	0,252	0,209	3,64919E-12
Macro_FOLR2-APOE+	CPQ	3,56973E-43	-4,31190E-01	0,288	0,212	6,57437E-39
Macro_FOLR2-APOE+	CELF2	1,43716E-30	-4,31234E-01	0,625	0,525	2,64683E-26
Macro_FOLR2-APOE+	TTC3	2,10584E-52	-4,31548E-01	0,414	0,314	3,87832E-48
Macro_FOLR2-APOE+	ATP6V1F	2,86581E-213	-4,31598E-01	0,894	0,743	5,27797E-209
Macro_FOLR2-APOE+	ATP5PB	8,12707E-68	-4,31659E-01	0,202	0,296	1,49676E-63
Macro_FOLR2-APOE+	C1orf54	1,44462E-116	-4,31689E-01	0,409	0,262	2,66055E-112
Macro_FOLR2-APOE+	CMTM7	6,17981E-122	-4,31746E-01	0,54	0,39	1,13814E-117
Macro_FOLR2-APOE+	CMC1	1,86793E-19	-4,32444E-01	0,269	0,22	3,44016E-15

Macro_FOLR2-APOE+	SLC25A11	8,03194E-32	-4,32989E-01	0,365	0,294	1,47924E-27
Macro_FOLR2-APOE+	MAF1	1,35945E-53	-4,34628E-01	0,475	0,374	2,50371E-49
Macro_FOLR2-APOE+	FAM162A	1,98850E-29	-4,34814E-01	0,409	0,335	3,66222E-25
Macro_FOLR2-APOE+	ZSWIM7	7,14571E-36	-4,34855E-01	0,312	0,239	1,31603E-31
Macro_FOLR2-APOE+	DPYD	8,12048E-27	-4,34945E-01	0,438	0,354	1,49555E-22
Macro_FOLR2-APOE+	THRAP3	2,02252E-09	-4,35550E-01	0,461	0,413	3,72487E-05
Macro_FOLR2-APOE+	ATP6AP1	6,44819E-258	-4,35827E-01	0,722	0,515	1,18756E-253
Macro_FOLR2-APOE+	C5AR1	1,34397E-178	-4,35884E-01	0,612	0,374	2,47520E-174
Macro_FOLR2-APOE+	CAMLG	2,39212E-09	-4,36555E-01	0,327	0,287	4,40557E-05
Macro_FOLR2-APOE+	RDH11	7,50901E-64	-4,36687E-01	0,255	0,17	1,38294E-59
Macro_FOLR2-APOE+	PCMTD1	2,25311E-14	-4,37159E-01	0,276	0,232	4,14955E-10
Macro_FOLR2-APOE+	PPP1R7	7,80627E-24	-4,37708E-01	0,33	0,271	1,43768E-19
Macro_FOLR2-APOE+	FGD4	1,79835E-16	-4,37710E-01	0,274	0,222	3,31202E-12
Macro_FOLR2-APOE+	SF3A1	3,23183E-13	-4,38484E-01	0,327	0,283	5,95206E-09
Macro_FOLR2-APOE+	GRHPR	6,31991E-42	-4,39224E-01	0,359	0,28	1,16394E-37
Macro_FOLR2-APOE+	MCTS1	5,39123E-25	-4,39533E-01	0,349	0,287	9,92904E-21
Macro_FOLR2-APOE+	HLA-A	2,83919E-110	-4,39693E-01	0,993	0,943	5,22894E-106
Macro_FOLR2-APOE+	CCDC90B	2,69374E-20	-4,39735E-01	0,287	0,236	4,96106E-16
Macro_FOLR2-APOE+	CSF2RA	1,63205E-84	-4,39903E-01	0,533	0,398	3,00576E-80
Macro_FOLR2-APOE+	JTB	1,84089E-23	-4,40118E-01	0,639	0,548	3,39037E-19
Macro_FOLR2-APOE+	MTSS1	1,78513E-47	-4,40907E-01	0,274	0,187	3,28767E-43
Macro_FOLR2-APOE+	RAB9A	1,23209E-28	-4,41056E-01	0,297	0,232	2,26914E-24
Macro_FOLR2-APOE+	FCGR1A	2,07511E-287	-4,41416E-01	0,644	0,373	3,82173E-283
Macro_FOLR2-APOE+	FUOM	2,20855E-70	-4,41671E-01	0,475	0,36	4,06748E-66
Macro_FOLR2-APOE+	RSRP1	3,64672E-66	-4,42521E-01	0,201	0,294	6,71616E-62
Macro_FOLR2-APOE+	SIGLEC10	1,17774E-17	-4,42535E-01	0,377	0,311	2,16905E-13
Macro_FOLR2-APOE+	RTN3	7,37625E-76	-4,43136E-01	0,524	0,401	1,35848E-71
Macro_FOLR2-APOE+	TOLLIP	2,52063E-13	-4,43160E-01	0,274	0,232	4,64224E-09
Macro_FOLR2-APOE+	CLTC	2,05743E-28	-4,44027E-01	0,489	0,409	3,78917E-24
Macro_FOLR2-APOE+	LY96	1,84885E-99	-4,44192E-01	0,582	0,437	3,40502E-95
Macro_FOLR2-APOE+	SRSF4	5,93473E-14	-4,44298E-01	0,397	0,346	1,09300E-09
Macro_FOLR2-APOE+	SCAND1	1,39330E-30	-4,44879E-01	0,615	0,521	2,56604E-26
Macro_FOLR2-APOE+	CRTAP	2,04495E-159	-4,45162E-01	0,675	0,496	3,76619E-155
Macro_FOLR2-APOE+	PIGT	6,93944E-76	-4,45270E-01	0,381	0,276	1,27804E-71
Macro_FOLR2-APOE+	TOMM20	4,90883E-35	-4,45601E-01	0,617	0,521	9,04060E-31
Macro_FOLR2-APOE+	THYN1	2,33025E-20	-4,45747E-01	0,269	0,219	4,29162E-16
Macro_FOLR2-APOE+	HLA-DPB1	5,70373E-202	-4,46102E-01	0,99	0,866	1,05046E-197
Macro_FOLR2-APOE+	MRPS25	3,64267E-28	-4,46345E-01	0,266	0,208	6,70870E-24
Macro_FOLR2-APOE+	TPST2	8,24270E-81	-4,46362E-01	0,299	0,198	1,51806E-76
Macro_FOLR2-APOE+	DPY30	4,24749E-30	-4,46678E-01	0,3	0,235	7,82260E-26
Macro_FOLR2-APOE+	TAF12	2,21835E-08	-4,47413E-01	0,251	0,219	4,08553E-04
Macro_FOLR2-APOE+	TMEM243	1,32743E-07	-4,47455E-01	0,298	0,264	2,44472E-03
Macro_FOLR2-APOE+	DPM2	8,74759E-30	-4,47657E-01	0,266	0,208	1,61104E-25
Macro_FOLR2-APOE+	BBX	2,45725E-07	-4,48033E-01	0,327	0,293	4,52552E-03
Macro_FOLR2-APOE+	SLC39A1	1,56777E-66	-4,48453E-01	0,358	0,264	2,88736E-62
Macro_FOLR2-APOE+	METTL5	1,26506E-34	-4,48605E-01	0,293	0,227	2,32986E-30

Macro_FOLR2-APOE+	FYB1	1,89152E-40	-4,48671E-01	0,258	0,327	3,48361E-36
Macro_FOLR2-APOE+	YIF1A	1,16349E-37	-4,48724E-01	0,346	0,274	2,14279E-33
Macro_FOLR2-APOE+	WBP2	1,96156E-53	-4,49614E-01	0,471	0,369	3,61261E-49
Macro_FOLR2-APOE+	SPAG7	3,41928E-14	-4,50461E-01	0,367	0,319	6,29728E-10
Macro_FOLR2-APOE+	C14orf119	2,41023E-49	-4,50560E-01	0,363	0,28	4,43893E-45
Macro_FOLR2-APOE+	NDUFA2	1,61555E-13	-4,50575E-01	0,611	0,542	2,97537E-09
Macro_FOLR2-APOE+	TSPO	2,87033E-201	-4,50881E-01	0,91	0,742	5,28628E-197
Macro_FOLR2-APOE+	GNS	1,27013E-57	-4,51246E-01	0,535	0,413	2,33920E-53
Macro_FOLR2-APOE+	OLA1	1,60935E-17	-4,51687E-01	0,354	0,302	2,96394E-13
Macro_FOLR2-APOE+	QSOX1	5,40509E-10	-4,51933E-01	0,323	0,283	9,95456E-06
Macro_FOLR2-APOE+	ATP5F1C	3,61440E-78	-4,51995E-01	0,2	0,302	6,65665E-74
Macro_FOLR2-APOE+	SLA	5,47781E-187	-4,52096E-01	0,586	0,389	1,00885E-182
Macro_FOLR2-APOE+	TMED5	7,08496E-13	-4,52097E-01	0,417	0,366	1,30484E-08
Macro_FOLR2-APOE+	ATP5MF	3,10076E-78	-4,53586E-01	0,259	0,372	5,71067E-74
Macro_FOLR2-APOE+	ATRX	1,59480E-08	-4,53666E-01	0,434	0,382	2,93715E-04
Macro_FOLR2-APOE+	AKNA	3,34824E-18	-4,54408E-01	0,217	0,262	6,16646E-14
Macro_FOLR2-APOE+	IRF5	1,15854E-38	-4,56286E-01	0,326	0,252	2,13368E-34
Macro_FOLR2-APOE+	TNFAIP8L2	9,85010E-18	-4,56977E-01	0,304	0,253	1,81409E-13
Macro_FOLR2-APOE+	CDK4	1,05295E-48	-4,57705E-01	0,299	0,22	1,93922E-44
Macro_FOLR2-APOE+	MGST2	1,92266E-106	-4,58082E-01	0,48	0,339	3,54096E-102
Macro_FOLR2-APOE+	POLR2K	1,69212E-17	-4,58207E-01	0,372	0,318	3,11638E-13
Macro_FOLR2-APOE+	TMED3	1,49868E-45	-4,58272E-01	0,342	0,263	2,76013E-41
Macro_FOLR2-APOE+	STX7	1,65844E-36	-4,58562E-01	0,488	0,402	3,05435E-32
Macro_FOLR2-APOE+	ABHD2	7,72120E-24	-4,59545E-01	0,292	0,233	1,42201E-19
Macro_FOLR2-APOE+	MAX	7,29704E-14	-4,59786E-01	0,446	0,388	1,34390E-09
Macro_FOLR2-APOE+	TMEM9B	7,99943E-42	-4,60450E-01	0,446	0,361	1,47325E-37
Macro_FOLR2-APOE+	CAPZB	1,21173E-69	-4,60454E-01	0,877	0,741	2,23165E-65
Macro_FOLR2-APOE+	AZIN1	1,46468E-51	-4,62211E-01	0,398	0,307	2,69750E-47
Macro_FOLR2-APOE+	NDUFA5	1,98064E-27	-4,62227E-01	0,35	0,284	3,64775E-23
Macro_FOLR2-APOE+	DDAH2	1,69514E-59	-4,62411E-01	0,517	0,403	3,12194E-55
Macro_FOLR2-APOE+	GLRX	3,42055E-56	-4,62974E-01	0,665	0,528	6,29963E-52
Macro_FOLR2-APOE+	DPP7	5,07887E-236	-4,63194E-01	0,722	0,529	9,35376E-232
Macro_FOLR2-APOE+	FIS1	1,31975E-70	-4,63350E-01	0,635	0,508	2,43059E-66
Macro_FOLR2-APOE+	GYG1	5,57488E-16	-4,64739E-01	0,314	0,266	1,02673E-11
Macro_FOLR2-APOE+	PRPF31	4,53737E-20	-4,64858E-01	0,254	0,208	8,35648E-16
Macro_FOLR2-APOE+	PSMD1	1,30395E-10	-4,65405E-01	0,311	0,272	2,40148E-06
Macro_FOLR2-APOE+	SUGT1	4,49005E-13	-4,65717E-01	0,322	0,278	8,26932E-09
Macro_FOLR2-APOE+	CEP170	2,92201E-87	-4,65901E-01	0,45	0,327	5,38146E-83
Macro_FOLR2-APOE+	DDB1	2,64048E-14	-4,66455E-01	0,261	0,219	4,86297E-10
Macro_FOLR2-APOE+	JUND	6,68106E-13	-4,66942E-01	0,442	0,457	1,23045E-08
Macro_FOLR2-APOE+	SLC1A5	3,05512E-17	-4,68372E-01	0,278	0,233	5,62661E-13
Macro_FOLR2-APOE+	RBM25	1,77026E-07	-4,68582E-01	0,545	0,486	3,26029E-03
Macro_FOLR2-APOE+	SLIRP	1,32682E-18	-4,68737E-01	0,481	0,418	2,44360E-14
Macro_FOLR2-APOE+	RSU1	2,19875E-49	-4,68857E-01	0,412	0,324	4,04944E-45
Macro_FOLR2-APOE+	GTF2F1	7,64028E-08	-4,70526E-01	0,281	0,25	1,40711E-03
Macro_FOLR2-APOE+	FKBP4	3,36476E-12	-4,71156E-01	0,264	0,222	6,19687E-08

Macro_FOLR2-APOE+	CYB5R3	1,15227E-66	-4,71178E-01	0,508	0,394	2,12213E-62
Macro_FOLR2-APOE+	TMEM134	4,42648E-77	-4,71263E-01	0,366	0,258	8,15224E-73
Macro_FOLR2-APOE+	KTN1	2,03648E-14	-4,72581E-01	0,554	0,483	3,75058E-10
Macro_FOLR2-APOE+	ATP5MC3	5,99325E-80	-4,74051E-01	0,258	0,369	1,10378E-75
Macro_FOLR2-APOE+	MRPL15	1,05570E-25	-4,75537E-01	0,257	0,202	1,94429E-21
Macro_FOLR2-APOE+	EFHD2	3,93139E-21	-4,75823E-01	0,403	0,435	7,24045E-17
Macro_FOLR2-APOE+	COMMD7	6,36558E-25	-4,75895E-01	0,366	0,303	1,17235E-20
Macro_FOLR2-APOE+	SIGLEC9	1,62307E-94	-4,76401E-01	0,31	0,199	2,98920E-90
Macro_FOLR2-APOE+	SSNA1	8,65091E-26	-4,76810E-01	0,466	0,394	1,59324E-21
Macro_FOLR2-APOE+	CCDC107	9,19841E-77	-4,77345E-01	0,402	0,291	1,69407E-72
Macro_FOLR2-APOE+	ISCU	5,63251E-145	-4,77750E-01	0,683	0,513	1,03734E-140
Macro_FOLR2-APOE+	HSBP1	1,49489E-06	-4,77791E-01	0,661	0,591	2,75314E-02
Macro_FOLR2-APOE+	APLP2	3,87297E-202	-4,78747E-01	0,776	0,565	7,13284E-198
Macro_FOLR2-APOE+	BROX	2,08412E-25	-4,78889E-01	0,253	0,196	3,83832E-21
Macro_FOLR2-APOE+	PSMC1	9,95665E-26	-4,79219E-01	0,386	0,321	1,83372E-21
Macro_FOLR2-APOE+	DDT	2,20558E-22	-4,79908E-01	0,504	0,43	4,06202E-18
Macro_FOLR2-APOE+	SNRNP200	1,18208E-22	-4,80329E-01	0,307	0,251	2,17704E-18
Macro_FOLR2-APOE+	CTSA	0,00000E+00	-4,80350E-01	0,715	0,472	0,00000E+00
Macro_FOLR2-APOE+	MANBA	8,73457E-15	-4,81003E-01	0,384	0,329	1,60865E-10
Macro_FOLR2-APOE+	SIL1	7,53626E-25	-4,81114E-01	0,288	0,23	1,38795E-20
Macro_FOLR2-APOE+	SCAMP3	7,00052E-33	-4,81177E-01	0,305	0,242	1,28929E-28
Macro_FOLR2-APOE+	SDF2L1	2,67126E-16	-4,81874E-01	0,406	0,346	4,91967E-12
Macro_FOLR2-APOE+	AP2M1	1,44270E-65	-4,81905E-01	0,693	0,565	2,65701E-61
Macro_FOLR2-APOE+	DYNC1I2	1,22358E-18	-4,81945E-01	0,296	0,245	2,25347E-14
Macro_FOLR2-APOE+	ELOB	2,51434E-96	-4,81961E-01	0,281	0,418	4,63066E-92
Macro_FOLR2-APOE+	TXNL1	1,60304E-20	-4,82246E-01	0,434	0,373	2,95231E-16
Macro_FOLR2-APOE+	SPNS1	6,53658E-10	-4,82375E-01	0,28	0,244	1,20384E-05
Macro_FOLR2-APOE+	ERH	1,21114E-14	-4,82404E-01	0,593	0,518	2,23055E-10
Macro_FOLR2-APOE+	VAMP8	1,27662E-192	-4,82407E-01	0,927	0,757	2,35115E-188
Macro_FOLR2-APOE+	MRPS16	1,55756E-37	-4,84384E-01	0,367	0,29	2,86856E-33
Macro_FOLR2-APOE+	IRF2	7,77692E-10	-4,84395E-01	0,362	0,322	1,43227E-05
Macro_FOLR2-APOE+	SLC38A10	5,98761E-20	-4,85046E-01	0,27	0,221	1,10274E-15
Macro_FOLR2-APOE+	GIMAP4	1,68812E-99	-4,85374E-01	0,578	0,403	3,10900E-95
Macro_FOLR2-APOE+	CAST	3,53074E-18	-4,85598E-01	0,604	0,519	6,50257E-14
Macro_FOLR2-APOE+	FNBP1	2,22620E-14	-4,85755E-01	0,414	0,436	4,09998E-10
Macro_FOLR2-APOE+	DDX21	1,77556E-14	-4,87988E-01	0,417	0,44	3,27005E-10
Macro_FOLR2-APOE+	ST13	9,73944E-56	-4,88611E-01	0,705	0,589	1,79371E-51
Macro_FOLR2-APOE+	CSF1R	1,64306E-163	-4,89295E-01	0,759	0,541	3,02603E-159
Macro_FOLR2-APOE+	EML4	1,62300E-06	-4,89585E-01	0,395	0,35	2,98909E-02
Macro_FOLR2-APOE+	NKTR	7,29820E-09	-4,90213E-01	0,316	0,279	1,34411E-04
Macro_FOLR2-APOE+	LGMN	3,17723E-251	-4,90332E-01	0,598	0,378	5,85151E-247
Macro_FOLR2-APOE+	NOSIP	9,96052E-14	-4,91591E-01	0,369	0,321	1,83443E-09
Macro_FOLR2-APOE+	SEC14L1	4,41022E-44	-4,91771E-01	0,478	0,382	8,12230E-40
Macro_FOLR2-APOE+	RHEB	2,39233E-33	-4,92363E-01	0,54	0,45	4,40595E-29
Macro_FOLR2-APOE+	ELL2	2,44712E-09	-4,93823E-01	0,331	0,276	4,50686E-05
Macro_FOLR2-APOE+	KCNAB2	9,12470E-29	-4,93859E-01	0,34	0,271	1,68050E-24

Macro_FOLR2-APOE+	GGA2	3,79229E-63	-4,93946E-01	0,354	0,257	6,98427E-59
Macro_FOLR2-APOE+	SLU7	3,26498E-16	-4,94132E-01	0,281	0,235	6,01311E-12
Macro_FOLR2-APOE+	RBBP6	5,11881E-23	-4,94301E-01	0,244	0,297	9,42731E-19
Macro_FOLR2-APOE+	SUMF2	2,00605E-126	-4,94591E-01	0,413	0,275	3,69455E-122
Macro_FOLR2-APOE+	SLC25A39	9,46232E-128	-4,94603E-01	0,538	0,387	1,74267E-123
Macro_FOLR2-APOE+	TMEM219	3,20516E-92	-4,95857E-01	0,766	0,616	5,90295E-88
Macro_FOLR2-APOE+	CANX	2,63482E-92	-4,97096E-01	0,762	0,619	4,85255E-88
Macro_FOLR2-APOE+	EMD	2,08224E-06	-4,98552E-01	0,407	0,418	3,83487E-02
Macro_FOLR2-APOE+	COMT	5,25990E-175	-4,98629E-01	0,711	0,533	9,68716E-171
Macro_FOLR2-APOE+	BNIP3L	7,61692E-60	-5,00346E-01	0,682	0,544	1,40281E-55
Macro_FOLR2-APOE+	COMMD9	2,19306E-68	-5,00675E-01	0,405	0,299	4,03895E-64
Macro_FOLR2-APOE+	CIRBP	1,86605E-22	-5,00848E-01	0,797	0,687	3,43670E-18
Macro_FOLR2-APOE+	SHOC2	1,16406E-23	-5,02104E-01	0,219	0,272	2,14386E-19
Macro_FOLR2-APOE+	COMMD1	3,52248E-10	-5,02335E-01	0,359	0,316	6,48735E-06
Macro_FOLR2-APOE+	LTC4S	1,58661E-52	-5,02895E-01	0,381	0,268	2,92206E-48
Macro_FOLR2-APOE+	ARPP19	2,04718E-26	-5,04371E-01	0,324	0,26	3,77029E-22
Macro_FOLR2-APOE+	DCTN4	6,06108E-20	-5,04533E-01	0,272	0,219	1,11627E-15
Macro_FOLR2-APOE+	USP4	3,94133E-46	-5,04614E-01	0,369	0,284	7,25875E-42
Macro_FOLR2-APOE+	IKZF1	6,78193E-15	-5,04843E-01	0,25	0,288	1,24903E-10
Macro_FOLR2-APOE+	ZDHHC12	2,47066E-11	-5,05154E-01	0,294	0,257	4,55022E-07
Macro_FOLR2-APOE+	GLOD4	1,45050E-24	-5,05906E-01	0,268	0,214	2,67138E-20
Macro_FOLR2-APOE+	NDUFB2	1,34288E-38	-5,06496E-01	0,81	0,699	2,47319E-34
Macro_FOLR2-APOE+	AHCY	1,24858E-31	-5,07039E-01	0,32	0,253	2,29952E-27
Macro_FOLR2-APOE+	TUBB	1,36069E-70	-5,07130E-01	0,787	0,64	2,50598E-66
Macro_FOLR2-APOE+	IGBP1	5,70936E-14	-5,08873E-01	0,388	0,337	1,05149E-09
Macro_FOLR2-APOE+	RALY	9,47435E-20	-5,09045E-01	0,561	0,482	1,74489E-15
Macro_FOLR2-APOE+	MRPS33	2,50431E-10	-5,09060E-01	0,259	0,225	4,61219E-06
Macro_FOLR2-APOE+	VASP	4,05445E-08	-5,09351E-01	0,466	0,47	7,46707E-04
Macro_FOLR2-APOE+	MRPS7	2,32723E-43	-5,09419E-01	0,38	0,299	4,28605E-39
Macro_FOLR2-APOE+	CAPNS1	9,74585E-28	-5,10302E-01	0,432	0,36	1,79489E-23
Macro_FOLR2-APOE+	DGCR6L	3,53501E-25	-5,11606E-01	0,313	0,253	6,51043E-21
Macro_FOLR2-APOE+	PIK3R5	6,59306E-79	-5,11616E-01	0,35	0,242	1,21424E-74
Macro_FOLR2-APOE+	MRPS34	1,44992E-51	-5,11713E-01	0,479	0,381	2,67032E-47
Macro_FOLR2-APOE+	ATP5F1D	2,02042E-92	-5,13176E-01	0,239	0,356	3,72101E-88
Macro_FOLR2-APOE+	SNAPIN	9,82156E-36	-5,13272E-01	0,35	0,278	1,80884E-31
Macro_FOLR2-APOE+	HMGA1	1,06761E-20	-5,13694E-01	0,374	0,409	1,96621E-16
Macro_FOLR2-APOE+	HSD17B11	8,92194E-28	-5,14222E-01	0,475	0,393	1,64315E-23
Macro_FOLR2-APOE+	GMPR2	5,80891E-29	-5,14438E-01	0,271	0,211	1,06983E-24
Macro_FOLR2-APOE+	JAK2	2,00904E-42	-5,14572E-01	0,176	0,25	3,70004E-38
Macro_FOLR2-APOE+	RUNX1	6,10056E-36	-5,14793E-01	0,223	0,287	1,12354E-31
Macro_FOLR2-APOE+	SNRPD3	1,14305E-37	-5,15103E-01	0,453	0,357	2,10516E-33
Macro_FOLR2-APOE+	SH3GLB1	3,41453E-33	-5,15238E-01	0,499	0,415	6,28855E-29
Macro_FOLR2-APOE+	MRPL3	4,22416E-25	-5,15558E-01	0,333	0,275	7,77964E-21
Macro_FOLR2-APOE+	METAP2	2,92694E-17	-5,15596E-01	0,34	0,285	5,39055E-13
Macro_FOLR2-APOE+	LTBR	2,91462E-117	-5,15722E-01	0,469	0,33	5,36786E-113
Macro_FOLR2-APOE+	SERBP1	2,68059E-16	-5,15946E-01	0,672	0,582	4,93684E-12

Macro_FOLR2-APOE+	KXD1	1,97855E-26	-5,16264E-01	0,411	0,341	3,64390E-22
Macro_FOLR2-APOE+	PA2G4	1,42758E-11	-5,16344E-01	0,484	0,425	2,62918E-07
Macro_FOLR2-APOE+	SDCCAG8	9,44719E-126	-5,16624E-01	0,463	0,316	1,73989E-121
Macro_FOLR2-APOE+	CSNK1D	6,03937E-09	-5,16694E-01	0,378	0,337	1,11227E-04
Macro_FOLR2-APOE+	ATOX1	5,08929E-09	-5,16792E-01	0,642	0,565	9,37295E-05
Macro_FOLR2-APOE+	PDCD10	2,22788E-15	-5,17715E-01	0,357	0,307	4,10309E-11
Macro_FOLR2-APOE+	OCIAD1	2,30429E-40	-5,17743E-01	0,515	0,421	4,24381E-36
Macro_FOLR2-APOE+	INTS10	8,97729E-40	-5,17746E-01	0,306	0,232	1,65335E-35
Macro_FOLR2-APOE+	COA4	6,72334E-08	-5,18614E-01	0,314	0,281	1,23824E-03
Macro_FOLR2-APOE+	MRPL36	1,52918E-07	-5,18961E-01	0,257	0,227	2,81629E-03
Macro_FOLR2-APOE+	SERPING1	9,87626E-88	-5,19121E-01	0,546	0,373	1,81891E-83
Macro_FOLR2-APOE+	TSTD1	4,64097E-09	-5,19345E-01	0,272	0,234	8,54727E-05
Macro_FOLR2-APOE+	TUFM	3,59162E-65	-5,19501E-01	0,61	0,493	6,61469E-61
Macro_FOLR2-APOE+	OSGEP	5,72326E-12	-5,19546E-01	0,267	0,229	1,05405E-07
Macro_FOLR2-APOE+	ETHE1	5,39193E-19	-5,20128E-01	0,395	0,333	9,93031E-15
Macro_FOLR2-APOE+	CYB561D2	3,83481E-20	-5,20660E-01	0,275	0,225	7,06257E-16
Macro_FOLR2-APOE+	CAP1	4,79614E-55	-5,20730E-01	0,85	0,714	8,83305E-51
Macro_FOLR2-APOE+	GSN	7,82161E-173	-5,21674E-01	0,824	0,603	1,44051E-168
Macro_FOLR2-APOE+	HLA-DMA	0,00000E+00	-5,22011E-01	0,955	0,753	0,00000E+00
Macro_FOLR2-APOE+	ACSL3	3,41384E-16	-5,22762E-01	0,299	0,246	6,28726E-12
Macro_FOLR2-APOE+	MKNK1	3,91216E-63	-5,22850E-01	0,288	0,197	7,20502E-59
Macro_FOLR2-APOE+	NDUFB9	3,93671E-44	-5,23629E-01	0,637	0,536	7,25025E-40
Macro_FOLR2-APOE+	JAK1	5,01643E-08	-5,24461E-01	0,523	0,522	9,23876E-04
Macro_FOLR2-APOE+	DRAM2	1,18521E-136	-5,24825E-01	0,522	0,369	2,18280E-132
Macro_FOLR2-APOE+	ANAPC13	5,32630E-37	-5,25466E-01	0,291	0,22	9,80945E-33
Macro_FOLR2-APOE+	HLA-DQB1	4,37382E-112	-5,25609E-01	0,965	0,791	8,05527E-108
Macro_FOLR2-APOE+	FARSA	4,00365E-24	-5,25691E-01	0,259	0,207	7,37352E-20
Macro_FOLR2-APOE+	CTNNA1	7,17466E-45	-5,26084E-01	0,437	0,351	1,32136E-40
Macro_FOLR2-APOE+	PHPT1	5,99399E-10	-5,27025E-01	0,494	0,439	1,10391E-05
Macro_FOLR2-APOE+	EFCAB14	1,04256E-23	-5,27074E-01	0,32	0,259	1,92009E-19
Macro_FOLR2-APOE+	ITPA	1,57214E-34	-5,27623E-01	0,335	0,265	2,89541E-30
Macro_FOLR2-APOE+	ADAM10	2,56559E-15	-5,28806E-01	0,333	0,28	4,72505E-11
Macro_FOLR2-APOE+	MRPL16	1,44464E-12	-5,29686E-01	0,292	0,253	2,66060E-08
Macro_FOLR2-APOE+	PNRC1	2,19691E-12	-5,30130E-01	0,841	0,769	4,04604E-08
Macro_FOLR2-APOE+	PSTPIP2	5,38548E-21	-5,30651E-01	0,211	0,257	9,91845E-17
Macro_FOLR2-APOE+	ACSL4	1,22154E-18	-5,30673E-01	0,24	0,281	2,24971E-14
Macro_FOLR2-APOE+	COX14	7,37231E-18	-5,30698E-01	0,524	0,452	1,35776E-13
Macro_FOLR2-APOE+	NCKAP1L	4,72232E-22	-5,30885E-01	0,491	0,419	8,69709E-18
Macro_FOLR2-APOE+	SUCLG1	3,16086E-12	-5,32258E-01	0,35	0,304	5,82135E-08
Macro_FOLR2-APOE+	SNX3	2,24259E-13	-5,32684E-01	0,784	0,674	4,13018E-09
Macro_FOLR2-APOE+	PCM1	2,50866E-17	-5,32847E-01	0,365	0,308	4,62020E-13
Macro_FOLR2-APOE+	SDHC	7,09484E-61	-5,33201E-01	0,54	0,425	1,30666E-56
Macro_FOLR2-APOE+	PRNP	2,05909E-75	-5,33339E-01	0,514	0,383	3,79222E-71
Macro_FOLR2-APOE+	METTL23	6,38238E-11	-5,33486E-01	0,252	0,216	1,17544E-06
Macro_FOLR2-APOE+	TRAPPC2L	1,47157E-29	-5,34178E-01	0,327	0,26	2,71018E-25
Macro_FOLR2-APOE+	FXYD5	6,12531E-80	-5,34484E-01	0,922	0,809	1,12810E-75

Macro_FOLR2-APOE+	SNW1	5,82947E-14	-5,34570E-01	0,329	0,283	1,07361E-09
Macro_FOLR2-APOE+	SARAF	1,09600E-93	-5,34838E-01	0,324	0,445	2,01850E-89
Macro_FOLR2-APOE+	NSFL1C	1,32629E-11	-5,35709E-01	0,308	0,269	2,44263E-07
Macro_FOLR2-APOE+	CELF1	1,19517E-06	-5,35888E-01	0,314	0,28	2,20115E-02
Macro_FOLR2-APOE+	PTTG1IP	7,65512E-54	-5,35962E-01	0,602	0,486	1,40984E-49
Macro_FOLR2-APOE+	HINT2	4,44740E-21	-5,36115E-01	0,277	0,222	8,19078E-17
Macro_FOLR2-APOE+	STX10	2,79580E-10	-5,37778E-01	0,349	0,303	5,14902E-06
Macro_FOLR2-APOE+	LAMTOR1	1,42964E-50	-5,38760E-01	0,756	0,63	2,63297E-46
Macro_FOLR2-APOE+	HADHA	1,53315E-38	-5,39901E-01	0,605	0,507	2,82361E-34
Macro_FOLR2-APOE+	HLA-DPA1	0,00000E+00	-5,40112E-01	0,994	0,882	0,00000E+00
Macro_FOLR2-APOE+	SERINC1	3,07322E-81	-5,40245E-01	0,511	0,387	5,65995E-77
Macro_FOLR2-APOE+	MRPL21	2,18810E-11	-5,40989E-01	0,3	0,261	4,02982E-07
Macro_FOLR2-APOE+	IFNAR1	1,07640E-12	-5,42180E-01	0,354	0,307	1,98240E-08
Macro_FOLR2-APOE+	LILRB4	0,00000E+00	-5,43448E-01	0,782	0,511	0,00000E+00
Macro_FOLR2-APOE+	ADIPOR1	3,79200E-104	-5,43843E-01	0,582	0,442	6,98373E-100
Macro_FOLR2-APOE+	RAB20	6,87445E-105	-5,44583E-01	0,472	0,326	1,26607E-100
Macro_FOLR2-APOE+	PLEKHO1	6,75363E-10	-5,44946E-01	0,336	0,365	1,24382E-05
Macro_FOLR2-APOE+	BLOC1S6	8,10755E-17	-5,45430E-01	0,248	0,289	1,49317E-12
Macro_FOLR2-APOE+	HK1	6,91894E-13	-5,45699E-01	0,3	0,256	1,27426E-08
Macro_FOLR2-APOE+	CIB1	8,71692E-33	-5,45853E-01	0,708	0,597	1,60540E-28
Macro_FOLR2-APOE+	NDUFB6	2,48517E-20	-5,46130E-01	0,421	0,36	4,57694E-16
Macro_FOLR2-APOE+	CAPRIN1	3,28095E-09	-5,46237E-01	0,371	0,33	6,04253E-05
Macro_FOLR2-APOE+	RNF166	2,13460E-09	-5,46408E-01	0,273	0,239	3,93130E-05
Macro_FOLR2-APOE+	S100A10	4,23835E-31	-5,46485E-01	0,933	0,822	7,80576E-27
Macro_FOLR2-APOE+	UGP2	3,70225E-14	-5,46929E-01	0,344	0,293	6,81843E-10
Macro_FOLR2-APOE+	EMC6	3,91354E-08	-5,46950E-01	0,342	0,308	7,20756E-04
Macro_FOLR2-APOE+	NR1H2	2,76027E-14	-5,47728E-01	0,374	0,324	5,08360E-10
Macro_FOLR2-APOE+	SLC31A2	2,25815E-83	-5,48697E-01	0,568	0,422	4,15883E-79
Macro_FOLR2-APOE+	ATRAID	1,74681E-147	-5,49441E-01	0,606	0,443	3,21711E-143
Macro_FOLR2-APOE+	CAPG	0,00000E+00	-5,50753E-01	0,914	0,713	0,00000E+00
Macro_FOLR2-APOE+	NAA20	2,20866E-78	-5,51244E-01	0,4	0,292	4,06768E-74
Macro_FOLR2-APOE+	TMED4	1,43268E-58	-5,51371E-01	0,375	0,28	2,63857E-54
Macro_FOLR2-APOE+	SLC11A1	0,00000E+00	-5,52802E-01	0,716	0,426	0,00000E+00
Macro_FOLR2-APOE+	TPD52L2	1,71610E-35	-5,53113E-01	0,332	0,259	3,16053E-31
Macro_FOLR2-APOE+	SPSB3	2,36706E-10	-5,53329E-01	0,323	0,285	4,35941E-06
Macro_FOLR2-APOE+	NEU1	4,42414E-34	-5,53337E-01	0,372	0,293	8,14794E-30
Macro_FOLR2-APOE+	GGNBP2	1,42678E-07	-5,53872E-01	0,372	0,335	2,62771E-03
Macro_FOLR2-APOE+	TRIR	1,13258E-107	-5,54041E-01	0,23	0,353	2,08587E-103
Macro_FOLR2-APOE+	NDUFAF3	7,87265E-97	-5,54121E-01	0,59	0,452	1,44991E-92
Macro_FOLR2-APOE+	DDX24	5,65691E-39	-5,54353E-01	0,564	0,463	1,04183E-34
Macro_FOLR2-APOE+	TMEM176B	3,30861E-72	-5,54398E-01	0,711	0,506	6,09346E-68
Macro_FOLR2-APOE+	NDUFB7	1,16236E-46	-5,55997E-01	0,619	0,515	2,14071E-42
Macro_FOLR2-APOE+	MLX	2,15930E-51	-5,56616E-01	0,439	0,345	3,97678E-47
Macro_FOLR2-APOE+	ATP5F1B	1,39562E-100	-5,56812E-01	0,216	0,329	2,57032E-96
Macro_FOLR2-APOE+	FUNDC2	1,58580E-43	-5,57120E-01	0,435	0,347	2,92057E-39
Macro_FOLR2-APOE+	AATF	3,07714E-10	-5,57753E-01	0,261	0,229	5,66717E-06

Macro_FOLR2-APOE+	IMP4	3,43874E-50	-5,59297E-01	0,338	0,254	6,33312E-46
Macro_FOLR2-APOE+	YIPF3	1,78975E-43	-5,59423E-01	0,395	0,313	3,29619E-39
Macro_FOLR2-APOE+	RAB6A	3,53053E-25	-5,59512E-01	0,355	0,291	6,50218E-21
Macro_FOLR2-APOE+	VDAC1	5,16231E-81	-5,59864E-01	0,655	0,514	9,50743E-77
Macro_FOLR2-APOE+	PNISR	1,03793E-15	-5,62132E-01	0,561	0,483	1,91155E-11
Macro_FOLR2-APOE+	VPS35	1,99823E-09	-5,62306E-01	0,488	0,432	3,68014E-05
Macro_FOLR2-APOE+	VPS26A	4,77676E-15	-5,63010E-01	0,268	0,224	8,79735E-11
Macro_FOLR2-APOE+	NDUFA6	1,52106E-22	-5,64082E-01	0,599	0,516	2,80133E-18
Macro_FOLR2-APOE+	SMCHD1	9,27479E-15	-5,64696E-01	0,293	0,329	1,70814E-10
Macro_FOLR2-APOE+	AHR	4,98521E-21	-5,66101E-01	0,322	0,36	9,18126E-17
Macro_FOLR2-APOE+	TIMM10	1,24168E-09	-5,66441E-01	0,285	0,248	2,28680E-05
Macro_FOLR2-APOE+	PIN1	1,13749E-37	-5,66450E-01	0,417	0,335	2,09492E-33
Macro_FOLR2-APOE+	C4orf3	1,85432E-21	-5,67050E-01	0,686	0,668	3,41511E-17
Macro_FOLR2-APOE+	SEC11A	4,72622E-38	-5,67139E-01	0,767	0,651	8,70429E-34
Macro_FOLR2-APOE+	RSRC2	1,14457E-12	-5,67356E-01	0,438	0,383	2,10795E-08
Macro_FOLR2-APOE+	C9orf78	1,59712E-15	-5,67358E-01	0,38	0,329	2,94142E-11
Macro_FOLR2-APOE+	DPYSL2	2,00689E-29	-5,67443E-01	0,385	0,314	3,69609E-25
Macro_FOLR2-APOE+	ATP6V1D	6,41751E-20	-5,67547E-01	0,293	0,24	1,18191E-15
Macro_FOLR2-APOE+	RANBP1	9,66439E-19	-5,67927E-01	0,419	0,359	1,77989E-14
Macro_FOLR2-APOE+	NCOA4	1,11607E-44	-5,69051E-01	0,623	0,516	2,05546E-40
Macro_FOLR2-APOE+	AURKAIP1	1,44776E-21	-5,69946E-01	0,589	0,507	2,66634E-17
Macro_FOLR2-APOE+	HMGB2	2,12464E-29	-5,70074E-01	0,477	0,386	3,91294E-25
Macro_FOLR2-APOE+	BCKDK	2,94599E-23	-5,72426E-01	0,372	0,31	5,42564E-19
Macro_FOLR2-APOE+	SAT2	4,43892E-11	-5,72908E-01	0,485	0,425	8,17516E-07
Macro_FOLR2-APOE+	PLEKHB2	2,17403E-28	-5,74060E-01	0,51	0,429	4,00390E-24
Macro_FOLR2-APOE+	IQGAP1	1,13172E-09	-5,74583E-01	0,625	0,552	2,08430E-05
Macro_FOLR2-APOE+	FBXW5	1,45624E-14	-5,75432E-01	0,355	0,308	2,68196E-10
Macro_FOLR2-APOE+	MPP1	6,26631E-249	-5,75457E-01	0,599	0,382	1,15407E-244
Macro_FOLR2-APOE+	VPS25	5,23427E-20	-5,75677E-01	0,253	0,205	9,63995E-16
Macro_FOLR2-APOE+	ARID4B	6,06246E-10	-5,75835E-01	0,351	0,377	1,11652E-05
Macro_FOLR2-APOE+	ITGB2	4,73526E-227	-5,76165E-01	0,937	0,735	8,72093E-223
Macro_FOLR2-APOE+	GOLGA7	1,52076E-20	-5,76218E-01	0,34	0,285	2,80078E-16
Macro_FOLR2-APOE+	CNOT7	1,33156E-07	-5,76366E-01	0,341	0,303	2,45233E-03
Macro_FOLR2-APOE+	GLIPR1	1,22781E-90	-5,77293E-01	0,725	0,56	2,26126E-86
Macro_FOLR2-APOE+	SH3BGRL	1,53595E-120	-5,78891E-01	0,845	0,7	2,82877E-116
Macro_FOLR2-APOE+	MPC1	9,99323E-20	-5,79145E-01	0,369	0,308	1,84045E-15
Macro_FOLR2-APOE+	ATF6B	1,34282E-25	-5,79895E-01	0,34	0,279	2,47307E-21
Macro_FOLR2-APOE+	GLB1	5,77295E-89	-5,80868E-01	0,387	0,276	1,06320E-84
Macro_FOLR2-APOE+	NFYC	1,94052E-08	-5,81086E-01	0,286	0,254	3,57386E-04
Macro_FOLR2-APOE+	ACTR1A	7,29011E-16	-5,81190E-01	0,328	0,281	1,34262E-11
Macro_FOLR2-APOE+	CIAO1	1,62166E-18	-5,82419E-01	0,289	0,239	2,98661E-14
Macro_FOLR2-APOE+	TGIF1	5,23065E-64	-5,83123E-01	0,423	0,307	9,63328E-60
Macro_FOLR2-APOE+	EIF6	2,49983E-19	-5,83783E-01	0,461	0,396	4,60394E-15
Macro_FOLR2-APOE+	EIF4G1	3,24006E-16	-5,83952E-01	0,376	0,325	5,96722E-12
Macro_FOLR2-APOE+	DEGS1	1,34838E-24	-5,84393E-01	0,382	0,317	2,48332E-20
Macro_FOLR2-APOE+	LAMP2	1,90206E-160	-5,84654E-01	0,547	0,378	3,50302E-156

Macro_FOLR2-APOE+	ZNF106	1,95036E-07	-5,84937E-01	0,379	0,338	3,59198E-03
Macro_FOLR2-APOE+	LSM10	5,67509E-14	-5,85657E-01	0,422	0,368	1,04518E-09
Macro_FOLR2-APOE+	VIM	2,28209E-07	-5,86327E-01	0,977	0,938	4,20293E-03
Macro_FOLR2-APOE+	TIMM13	1,04434E-26	-5,86488E-01	0,461	0,384	1,92336E-22
Macro_FOLR2-APOE+	CUTA	3,63873E-33	-5,86874E-01	0,562	0,473	6,70145E-29
Macro_FOLR2-APOE+	RAB2A	2,61894E-14	-5,87993E-01	0,536	0,478	4,82331E-10
Macro_FOLR2-APOE+	TNFSF13	1,29953E-201	-5,89906E-01	0,604	0,417	2,39335E-197
Macro_FOLR2-APOE+	ORMDL2	1,40704E-31	-5,90138E-01	0,357	0,29	2,59135E-27
Macro_FOLR2-APOE+	TNFRSF1A	1,36324E-91	-5,90267E-01	0,569	0,429	2,51067E-87
Macro_FOLR2-APOE+	EMC3	1,26335E-65	-5,90452E-01	0,408	0,305	2,32672E-61
Macro_FOLR2-APOE+	ALDH2	2,28054E-111	-5,90659E-01	0,698	0,526	4,20007E-107
Macro_FOLR2-APOE+	TRIM38	4,96035E-07	-5,90955E-01	0,265	0,288	9,13548E-03
Macro_FOLR2-APOE+	ANXA7	2,55934E-19	-5,90991E-01	0,429	0,363	4,71354E-15
Macro_FOLR2-APOE+	NUBP1	2,06225E-19	-5,91187E-01	0,276	0,227	3,79804E-15
Macro_FOLR2-APOE+	LEPROTL1	7,21094E-25	-5,91761E-01	0,462	0,389	1,32804E-20
Macro_FOLR2-APOE+	HLA-E	5,21171E-94	-5,92537E-01	0,973	0,893	9,59841E-90
Macro_FOLR2-APOE+	PYURF	4,35203E-08	-5,92656E-01	0,451	0,404	8,01514E-04
Macro_FOLR2-APOE+	NDUFC1	1,36983E-13	-5,92711E-01	0,509	0,449	2,52281E-09
Macro_FOLR2-APOE+	FCGR2A	1,64966E-183	-5,93100E-01	0,825	0,599	3,03817E-179
Macro_FOLR2-APOE+	DNMT1	8,03058E-15	-5,94293E-01	0,361	0,31	1,47899E-10
Macro_FOLR2-APOE+	DHRS7	1,19523E-163	-5,95093E-01	0,63	0,461	2,20126E-159
Macro_FOLR2-APOE+	SCPEP1	1,82621E-117	-5,95880E-01	0,552	0,384	3,36333E-113
Macro_FOLR2-APOE+	VPS28	3,67544E-121	-5,96142E-01	0,741	0,591	6,76905E-117
Macro_FOLR2-APOE+	RNF5	1,38256E-10	-5,96523E-01	0,319	0,277	2,54626E-06
Macro_FOLR2-APOE+	TRAPPC3	2,16258E-20	-5,96927E-01	0,403	0,345	3,98282E-16
Macro_FOLR2-APOE+	LAIR1	8,30983E-208	-5,98483E-01	0,715	0,487	1,53042E-203
Macro_FOLR2-APOE+	TAOK3	5,17953E-09	-5,99781E-01	0,495	0,444	9,53915E-05
Macro_FOLR2-APOE+	REEP5	2,57634E-96	-5,99961E-01	0,682	0,543	4,74484E-92
Macro_FOLR2-APOE+	PTGER4	1,53035E-15	-6,01161E-01	0,316	0,262	2,81845E-11
Macro_FOLR2-APOE+	LAMTOR2	3,03085E-41	-6,01179E-01	0,637	0,527	5,58191E-37
Macro_FOLR2-APOE+	SKAP2	2,18678E-34	-6,02496E-01	0,483	0,393	4,02740E-30
Macro_FOLR2-APOE+	CALM3	8,00766E-30	-6,03592E-01	0,584	0,495	1,47477E-25
Macro_FOLR2-APOE+	AKIRIN2	2,97171E-08	-6,04185E-01	0,467	0,414	5,47300E-04
Macro_FOLR2-APOE+	VSIR	4,06188E-100	-6,04893E-01	0,177	0,289	7,48077E-96
Macro_FOLR2-APOE+	PSMC2	8,60171E-22	-6,04977E-01	0,336	0,278	1,58418E-17
Macro_FOLR2-APOE+	NECAP2	5,01959E-60	-6,05315E-01	0,443	0,338	9,24459E-56
Macro_FOLR2-APOE+	PSMC4	2,44749E-15	-6,06234E-01	0,379	0,329	4,50753E-11
Macro_FOLR2-APOE+	LPP	8,93839E-41	-6,06295E-01	0,335	0,256	1,64618E-36
Macro_FOLR2-APOE+	NDUFS5	1,69851E-85	-6,06433E-01	0,77	0,635	3,12814E-81
Macro_FOLR2-APOE+	PRKCD	2,69861E-08	-6,07229E-01	0,232	0,26	4,97003E-04
Macro_FOLR2-APOE+	PDCD5	4,04140E-10	-6,07414E-01	0,39	0,345	7,44304E-06
Macro_FOLR2-APOE+	ITSN2	1,01382E-15	-6,07987E-01	0,434	0,379	1,86715E-11
Macro_FOLR2-APOE+	DYNLL1	3,33561E-12	-6,08349E-01	0,792	0,694	6,14319E-08
Macro_FOLR2-APOE+	NRP1	2,96256E-104	-6,09326E-01	0,255	0,149	5,45615E-100
Macro_FOLR2-APOE+	PTPMT1	4,43639E-54	-6,09394E-01	0,322	0,236	8,17051E-50
Macro_FOLR2-APOE+	PSMB5	4,03056E-30	-6,09860E-01	0,374	0,306	7,42309E-26

Macro_FOLR2-APOE+	COPS5	7,36850E-08	-6,10059E-01	0,306	0,275	1,35706E-03
Macro_FOLR2-APOE+	MS4A6A	1,04154E-296	-6,10811E-01	0,933	0,679	1,91820E-292
Macro_FOLR2-APOE+	NDUFS3	2,36807E-56	-6,11029E-01	0,422	0,327	4,36127E-52
Macro_FOLR2-APOE+	SDHD	8,95151E-20	-6,11101E-01	0,397	0,335	1,64860E-15
Macro_FOLR2-APOE+	MRPS5	1,72830E-15	-6,11164E-01	0,295	0,249	3,18301E-11
Macro_FOLR2-APOE+	TBC1D9B	4,02312E-15	-6,11483E-01	0,271	0,227	7,40938E-11
Macro_FOLR2-APOE+	MIS18BP1	1,01754E-15	-6,11486E-01	0,408	0,353	1,87400E-11
Macro_FOLR2-APOE+	CHD4	5,37922E-08	-6,12225E-01	0,306	0,272	9,90690E-04
Macro_FOLR2-APOE+	TUBGCP2	3,99994E-20	-6,13905E-01	0,353	0,295	7,36668E-16
Macro_FOLR2-APOE+	SBDS	1,61894E-09	-6,14096E-01	0,318	0,278	2,98160E-05
Macro_FOLR2-APOE+	ATF5	3,58316E-19	-6,15000E-01	0,331	0,366	6,59910E-15
Macro_FOLR2-APOE+	FCGR1B	8,77111E-55	-6,15760E-01	0,278	0,192	1,61538E-50
Macro_FOLR2-APOE+	TMEM205	9,96869E-30	-6,16199E-01	0,346	0,275	1,83593E-25
Macro_FOLR2-APOE+	MTIF3	4,94880E-21	-6,17220E-01	0,344	0,287	9,11420E-17
Macro_FOLR2-APOE+	HAVCR2	3,68678E-130	-6,17654E-01	0,584	0,411	6,78994E-126
Macro_FOLR2-APOE+	NDUFS2	6,13911E-26	-6,20188E-01	0,418	0,351	1,13064E-21
Macro_FOLR2-APOE+	MRPL20	6,42015E-09	-6,20266E-01	0,518	0,46	1,18240E-04
Macro_FOLR2-APOE+	PDIA6	5,39961E-22	-6,20640E-01	0,597	0,508	9,94447E-18
Macro_FOLR2-APOE+	PSMC3	7,50745E-21	-6,21223E-01	0,369	0,311	1,38265E-16
Macro_FOLR2-APOE+	MMADHC	1,54386E-26	-6,21787E-01	0,407	0,339	2,84333E-22
Macro_FOLR2-APOE+	TMEM230	5,66193E-88	-6,21865E-01	0,562	0,434	1,04276E-83
Macro_FOLR2-APOE+	RAB1B	3,19976E-07	-6,22936E-01	0,386	0,351	5,89300E-03
Macro_FOLR2-APOE+	SAR1B	1,61148E-18	-6,23264E-01	0,283	0,234	2,96785E-14
Macro_FOLR2-APOE+	STOML2	8,33277E-12	-6,23392E-01	0,311	0,27	1,53465E-07
Macro_FOLR2-APOE+	NDUFB5	7,93626E-39	-6,23518E-01	0,536	0,44	1,46162E-34
Macro_FOLR2-APOE+	PPIG	6,04478E-13	-6,24721E-01	0,506	0,444	1,11327E-08
Macro_FOLR2-APOE+	MDH2	6,52942E-28	-6,24740E-01	0,601	0,509	1,20252E-23
Macro_FOLR2-APOE+	PSMG2	2,33369E-20	-6,26985E-01	0,482	0,414	4,29795E-16
Macro_FOLR2-APOE+	SDCBP	4,07889E-212	-6,28567E-01	0,874	0,699	7,51208E-208
Macro_FOLR2-APOE+	SRP72	4,05722E-20	-6,28573E-01	0,397	0,338	7,47218E-16
Macro_FOLR2-APOE+	YIF1B	2,21766E-11	-6,28760E-01	0,388	0,344	4,08427E-07
Macro_FOLR2-APOE+	FOXP1	4,26211E-09	-6,30709E-01	0,329	0,348	7,84953E-05
Macro_FOLR2-APOE+	MFF	3,60607E-10	-6,30774E-01	0,354	0,314	6,64130E-06
Macro_FOLR2-APOE+	HADHB	2,63120E-41	-6,30997E-01	0,435	0,348	4,84588E-37
Macro_FOLR2-APOE+	EMC7	7,61701E-35	-6,31569E-01	0,384	0,31	1,40283E-30
Macro_FOLR2-APOE+	SYAP1	1,32382E-28	-6,31705E-01	0,299	0,352	2,43809E-24
Macro_FOLR2-APOE+	CYLD	1,08003E-26	-6,31943E-01	0,199	0,255	1,98908E-22
Macro_FOLR2-APOE+	PRCP	1,50019E-50	-6,32140E-01	0,484	0,374	2,76290E-46
Macro_FOLR2-APOE+	AKR1A1	1,20837E-183	-6,34757E-01	0,747	0,549	2,22546E-179
Macro_FOLR2-APOE+	TMC6	2,19790E-44	-6,35197E-01	0,292	0,216	4,04787E-40
Macro_FOLR2-APOE+	NDUFB4	3,66677E-07	-6,35535E-01	0,654	0,583	6,75309E-03
Macro_FOLR2-APOE+	FTH1	4,64255E-67	-6,37216E-01	1	0,997	8,55018E-63
Macro_FOLR2-APOE+	FKBP2	1,39134E-120	-6,38469E-01	0,547	0,387	2,56243E-116
Macro_FOLR2-APOE+	CAMTA1	1,60813E-06	-6,38660E-01	0,396	0,359	2,96170E-02
Macro_FOLR2-APOE+	LYPLA2	8,52489E-10	-6,38945E-01	0,252	0,22	1,57003E-05
Macro_FOLR2-APOE+	TECR	2,37870E-101	-6,39184E-01	0,493	0,36	4,38086E-97

Macro_FOLR2-APOE+	SOD1	8,47390E-87	-6,39457E-01	0,686	0,544	1,56064E-82
Macro_FOLR2-APOE+	NUP214	1,06528E-28	-6,40318E-01	0,383	0,303	1,96193E-24
Macro_FOLR2-APOE+	OS9	9,28123E-105	-6,41523E-01	0,657	0,517	1,70932E-100
Macro_FOLR2-APOE+	HEXB	5,34600E-214	-6,42404E-01	0,685	0,486	9,84573E-210
Macro_FOLR2-APOE+	RAB18	8,14265E-25	-6,42549E-01	0,334	0,272	1,49963E-20
Macro_FOLR2-APOE+	C7orf50	3,52068E-12	-6,43836E-01	0,381	0,33	6,48403E-08
Macro_FOLR2-APOE+	NDUFB10	5,45570E-38	-6,44395E-01	0,646	0,544	1,00478E-33
Macro_FOLR2-APOE+	NDUFS8	5,69957E-63	-6,44710E-01	0,597	0,478	1,04969E-58
Macro_FOLR2-APOE+	TMEM50A	1,18393E-33	-6,44963E-01	0,697	0,587	2,18045E-29
Macro_FOLR2-APOE+	PRPF8	1,05249E-09	-6,46022E-01	0,342	0,302	1,93837E-05
Macro_FOLR2-APOE+	IDH2	5,68981E-20	-6,46560E-01	0,473	0,401	1,04789E-15
Macro_FOLR2-APOE+	NME2	2,15962E-77	-6,47193E-01	0,282	0,372	3,97737E-73
Macro_FOLR2-APOE+	MIF4GD	6,63429E-09	-6,47344E-01	0,345	0,307	1,22184E-04
Macro_FOLR2-APOE+	NDUFS7	5,30960E-64	-6,49296E-01	0,647	0,524	9,77869E-60
Macro_FOLR2-APOE+	CLTA	6,96141E-79	-6,50633E-01	0,765	0,63	1,28208E-74
Macro_FOLR2-APOE+	IMPDH2	8,40770E-08	-6,51951E-01	0,302	0,268	1,54845E-03
Macro_FOLR2-APOE+	CD58	1,59358E-73	-6,52099E-01	0,407	0,298	2,93490E-69
Macro_FOLR2-APOE+	BLVRB	9,22940E-100	-6,52458E-01	0,631	0,467	1,69978E-95
Macro_FOLR2-APOE+	NANS	1,10396E-47	-6,52895E-01	0,474	0,376	2,03316E-43
Macro_FOLR2-APOE+	STX8	4,75996E-37	-6,53305E-01	0,299	0,228	8,76641E-33
Macro_FOLR2-APOE+	TOM1	2,30387E-24	-6,53988E-01	0,343	0,274	4,24304E-20
Macro_FOLR2-APOE+	CTNND1	1,36264E-35	-6,54194E-01	0,336	0,262	2,50957E-31
Macro_FOLR2-APOE+	HDLBP	2,49685E-41	-6,54353E-01	0,488	0,397	4,59845E-37
Macro_FOLR2-APOE+	SSU72	1,10499E-11	-6,54520E-01	0,361	0,387	2,03507E-07
Macro_FOLR2-APOE+	CD14	0,00000E+00	-6,56521E-01	0,889	0,58	0,00000E+00
Macro_FOLR2-APOE+	PHB	1,73458E-73	-6,57643E-01	0,487	0,373	3,19457E-69
Macro_FOLR2-APOE+	RTCB	3,38449E-13	-6,57758E-01	0,334	0,289	6,23322E-09
Macro_FOLR2-APOE+	JKAMP	9,78414E-13	-6,59105E-01	0,25	0,214	1,80194E-08
Macro_FOLR2-APOE+	GPS1	5,34444E-50	-6,59480E-01	0,335	0,254	9,84286E-46
Macro_FOLR2-APOE+	SYF2	5,04993E-09	-6,60637E-01	0,468	0,417	9,30045E-05
Macro_FOLR2-APOE+	PFN1	4,26637E-11	-6,60723E-01	0,989	0,94	7,85738E-07
Macro_FOLR2-APOE+	TYMP	6,12134E-15	-6,61452E-01	0,882	0,779	1,12737E-10
Macro_FOLR2-APOE+	DHRS3	1,99716E-241	-6,61875E-01	0,286	0,131	3,67817E-237
Macro_FOLR2-APOE+	ALOX5	5,18682E-42	-6,62093E-01	0,572	0,458	9,55256E-38
Macro_FOLR2-APOE+	NDUFAB1	1,89954E-12	-6,62109E-01	0,529	0,466	3,49838E-08
Macro_FOLR2-APOE+	TMEM14B	9,27693E-36	-6,62212E-01	0,496	0,41	1,70853E-31
Macro_FOLR2-APOE+	NDUFS4	1,04786E-17	-6,62376E-01	0,332	0,28	1,92984E-13
Macro_FOLR2-APOE+	SNRPC	1,37340E-27	-6,62506E-01	0,484	0,408	2,52939E-23
Macro_FOLR2-APOE+	ACSL1	3,62583E-81	-6,62513E-01	0,512	0,369	6,67769E-77
Macro_FOLR2-APOE+	DOK2	2,40011E-37	-6,63601E-01	0,425	0,332	4,42029E-33
Macro_FOLR2-APOE+	SNX2	6,16086E-60	-6,64015E-01	0,537	0,425	1,13465E-55
Macro_FOLR2-APOE+	ANXA5	9,73476E-183	-6,64608E-01	0,918	0,749	1,79285E-178
Macro_FOLR2-APOE+	PPP2R1A	1,08369E-12	-6,66129E-01	0,441	0,389	1,99583E-08
Macro_FOLR2-APOE+	YWHAB	2,90340E-17	-6,66853E-01	0,882	0,772	5,34719E-13
Macro_FOLR2-APOE+	MPDU1	1,70794E-09	-6,67340E-01	0,254	0,223	3,14552E-05
Macro_FOLR2-APOE+	POLR2E	9,80682E-57	-6,69472E-01	0,629	0,514	1,80612E-52

Macro_FOLR2-APOE+	SPPL2A	1,08191E-12	-6,69833E-01	0,384	0,334	1,99255E-08
Macro_FOLR2-APOE+	FIBP	5,82969E-11	-6,69901E-01	0,408	0,361	1,07365E-06
Macro_FOLR2-APOE+	NUDC	1,07435E-17	-6,70732E-01	0,431	0,372	1,97864E-13
Macro_FOLR2-APOE+	AKR7A2	8,98771E-12	-6,71307E-01	0,284	0,244	1,65527E-07
Macro_FOLR2-APOE+	SELENOK	6,24278E-101	-6,71536E-01	0,18	0,294	1,14973E-96
Macro_FOLR2-APOE+	PLXNB2	1,02237E-27	-6,71986E-01	0,321	0,256	1,88290E-23
Macro_FOLR2-APOE+	C19orf53	2,53088E-16	-6,73036E-01	0,657	0,573	4,66112E-12
Macro_FOLR2-APOE+	PARL	8,53961E-14	-6,73092E-01	0,33	0,283	1,57274E-09
Macro_FOLR2-APOE+	TMEM179B	2,01835E-43	-6,73617E-01	0,566	0,468	3,71720E-39
Macro_FOLR2-APOE+	PSMD7	8,51575E-16	-6,73628E-01	0,457	0,397	1,56835E-11
Macro_FOLR2-APOE+	ARFGAP3	2,27075E-13	-6,73633E-01	0,237	0,273	4,18203E-09
Macro_FOLR2-APOE+	ATP5MG	1,11917E-148	-6,73826E-01	0,286	0,44	2,06118E-144
Macro_FOLR2-APOE+	SNX1	1,99577E-21	-6,73948E-01	0,355	0,299	3,67562E-17
Macro_FOLR2-APOE+	ATP6V1E1	1,09849E-39	-6,76007E-01	0,403	0,32	2,02309E-35
Macro_FOLR2-APOE+	MRPL28	5,97269E-07	-6,78814E-01	0,292	0,263	1,09999E-02
Macro_FOLR2-APOE+	BLOC1S1	6,33173E-16	-6,79013E-01	0,545	0,46	1,16611E-11
Macro_FOLR2-APOE+	IK	2,12394E-07	-6,79564E-01	0,368	0,334	3,91165E-03
Macro_FOLR2-APOE+	TSC22D3	1,30455E-140	-6,80124E-01	0,831	0,672	2,40258E-136
Macro_FOLR2-APOE+	DNAJB6	1,07576E-12	-6,80977E-01	0,581	0,512	1,98123E-08
Macro_FOLR2-APOE+	PFKL	3,78226E-52	-6,83042E-01	0,477	0,38	6,96579E-48
Macro_FOLR2-APOE+	PLIN3	2,08433E-25	-6,83205E-01	0,429	0,362	3,83872E-21
Macro_FOLR2-APOE+	UFC1	7,49832E-34	-6,83302E-01	0,586	0,489	1,38097E-29
Macro_FOLR2-APOE+	RABAC1	4,83124E-58	-6,83742E-01	0,622	0,509	8,89770E-54
Macro_FOLR2-APOE+	RPS27L	6,09570E-21	-6,83898E-01	0,309	0,371	1,12265E-16
Macro_FOLR2-APOE+	HSD17B10	2,17822E-08	-6,84140E-01	0,337	0,301	4,01162E-04
Macro_FOLR2-APOE+	ATP5F1E	3,16712E-176	-6,87015E-01	0,311	0,504	5,83289E-172
Macro_FOLR2-APOE+	BANF1	7,91699E-12	-6,87064E-01	0,614	0,538	1,45807E-07
Macro_FOLR2-APOE+	PIH1D1	9,72128E-08	-6,87457E-01	0,28	0,249	1,79037E-03
Macro_FOLR2-APOE+	LY6E	2,51701E-19	-6,87702E-01	0,648	0,531	4,63557E-15
Macro_FOLR2-APOE+	ANXA2	0,00000E+00	-6,88103E-01	0,91	0,715	0,00000E+00
Macro_FOLR2-APOE+	SRGAP2	1,30964E-43	-6,89104E-01	0,295	0,217	2,41197E-39
Macro_FOLR2-APOE+	DCTN2	6,00603E-38	-6,89483E-01	0,395	0,319	1,10613E-33
Macro_FOLR2-APOE+	PRMT2	6,30131E-21	-6,89611E-01	0,492	0,417	1,16051E-16
Macro_FOLR2-APOE+	ACP1	7,64271E-26	-6,89802E-01	0,391	0,325	1,40756E-21
Macro_FOLR2-APOE+	SMIM7	5,86891E-11	-6,89807E-01	0,271	0,233	1,08088E-06
Macro_FOLR2-APOE+	MYO1G	7,87834E-23	-6,89820E-01	0,243	0,29	1,45095E-18
Macro_FOLR2-APOE+	IFNGR2	8,80963E-62	-6,90017E-01	0,681	0,545	1,62247E-57
Macro_FOLR2-APOE+	IDH3G	1,83723E-14	-6,90086E-01	0,403	0,352	3,38363E-10
Macro_FOLR2-APOE+	PPP1R10	2,03607E-12	-6,90614E-01	0,279	0,313	3,74982E-08
Macro_FOLR2-APOE+	WDR61	1,76994E-12	-6,91931E-01	0,253	0,216	3,25970E-08
Macro_FOLR2-APOE+	SH3TC1	1,24411E-47	-6,91996E-01	0,393	0,3	2,29128E-43
Macro_FOLR2-APOE+	COLGALT1	4,30980E-96	-6,92512E-01	0,329	0,214	7,93736E-92
Macro_FOLR2-APOE+	WDR1	1,07791E-12	-6,95655E-01	0,61	0,53	1,98520E-08
Macro_FOLR2-APOE+	HPS1	7,50006E-45	-6,96042E-01	0,429	0,339	1,38129E-40
Macro_FOLR2-APOE+	PSMD11	1,09163E-13	-6,96078E-01	0,347	0,302	2,01045E-09
Macro_FOLR2-APOE+	HLA-DQA1	2,98106E-51	-6,98398E-01	0,947	0,757	5,49021E-47

Macro_FOLR2-APOE+	PPCS	2,58683E-34	-6,98931E-01	0,46	0,379	4,76416E-30
Macro_FOLR2-APOE+	CPNE3	1,69374E-06	-6,99147E-01	0,271	0,238	3,11936E-02
Macro_FOLR2-APOE+	TMED10	1,32198E-169	-7,01488E-01	0,718	0,543	2,43469E-165
Macro_FOLR2-APOE+	WDR83OS	2,04495E-28	-7,01855E-01	0,697	0,608	3,76618E-24
Macro_FOLR2-APOE+	ZFAND5	6,82887E-16	-7,02906E-01	0,661	0,573	1,25767E-11
Macro_FOLR2-APOE+	LAPTM4A	1,09036E-57	-7,02996E-01	0,742	0,61	2,00811E-53
Macro_FOLR2-APOE+	RAB8B	8,02826E-07	-7,03529E-01	0,232	0,253	1,47856E-02
Macro_FOLR2-APOE+	ATP6V0B	1,06771E-151	-7,03546E-01	0,909	0,772	1,96640E-147
Macro_FOLR2-APOE+	LYN	2,31085E-40	-7,04024E-01	0,408	0,459	4,25590E-36
Macro_FOLR2-APOE+	CNIH1	1,46909E-06	-7,04046E-01	0,423	0,38	2,70562E-02
Macro_FOLR2-APOE+	C1orf43	1,02196E-58	-7,04967E-01	0,644	0,53	1,88214E-54
Macro_FOLR2-APOE+	RNF181	6,59192E-34	-7,05165E-01	0,648	0,548	1,21403E-29
Macro_FOLR2-APOE+	IDS	3,55954E-13	-7,05336E-01	0,379	0,401	6,55561E-09
Macro_FOLR2-APOE+	NCL	9,62667E-10	-7,07930E-01	0,696	0,614	1,77294E-05
Macro_FOLR2-APOE+	GIT2	4,43667E-22	-7,08585E-01	0,344	0,286	8,17101E-18
Macro_FOLR2-APOE+	ELMO1	1,01654E-25	-7,08759E-01	0,397	0,33	1,87216E-21
Macro_FOLR2-APOE+	RHOG	3,49787E-08	-7,09107E-01	0,822	0,711	6,44202E-04
Macro_FOLR2-APOE+	ANAPC16	2,36987E-06	-7,09123E-01	0,526	0,473	4,36459E-02
Macro_FOLR2-APOE+	AUP1	9,58919E-90	-7,09441E-01	0,64	0,507	1,76604E-85
Macro_FOLR2-APOE+	YWHAE	1,78232E-12	-7,12331E-01	0,647	0,57	3,28250E-08
Macro_FOLR2-APOE+	RNASET2	3,21942E-52	-7,12368E-01	0,863	0,72	5,92920E-48
Macro_FOLR2-APOE+	GSTO1	3,76647E-125	-7,12788E-01	0,755	0,597	6,93671E-121
Macro_FOLR2-APOE+	TMEM123	2,71628E-20	-7,13973E-01	0,522	0,444	5,00258E-16
Macro_FOLR2-APOE+	PILRA	8,42974E-110	-7,14244E-01	0,652	0,467	1,55251E-105
Macro_FOLR2-APOE+	UNC119	1,37429E-09	-7,14553E-01	0,241	0,272	2,53103E-05
Macro_FOLR2-APOE+	CAPN1	3,75137E-21	-7,15687E-01	0,313	0,259	6,90889E-17
Macro_FOLR2-APOE+	PSMD9	5,72620E-08	-7,15849E-01	0,283	0,25	1,05459E-03
Macro_FOLR2-APOE+	LSM4	1,10005E-22	-7,16587E-01	0,495	0,421	2,02595E-18
Macro_FOLR2-APOE+	SAMD9L	2,82423E-08	-7,16886E-01	0,259	0,283	5,20138E-04
Macro_FOLR2-APOE+	RSL1D1	2,42068E-26	-7,16976E-01	0,542	0,458	4,45817E-22
Macro_FOLR2-APOE+	ANAPC11	2,22867E-64	-7,18620E-01	0,717	0,59	4,10454E-60
Macro_FOLR2-APOE+	AIP	1,54790E-11	-7,19102E-01	0,379	0,333	2,85077E-07
Macro_FOLR2-APOE+	GABARAPL1	7,24436E-24	-7,19225E-01	0,357	0,281	1,33419E-19
Macro_FOLR2-APOE+	LMNA	6,85285E-53	-7,19671E-01	0,485	0,35	1,26209E-48
Macro_FOLR2-APOE+	ATP6V1B2	1,12328E-65	-7,19783E-01	0,656	0,515	2,06874E-61
Macro_FOLR2-APOE+	TIMMDC1	2,38558E-18	-7,20682E-01	0,269	0,221	4,39353E-14
Macro_FOLR2-APOE+	BST2	1,65803E-43	-7,21192E-01	0,851	0,704	3,05359E-39
Macro_FOLR2-APOE+	TP53I13	7,40616E-18	-7,22692E-01	0,271	0,225	1,36399E-13
Macro_FOLR2-APOE+	BECN1	7,72882E-08	-7,23419E-01	0,288	0,256	1,42342E-03
Macro_FOLR2-APOE+	RPS17	5,42244E-63	-7,24094E-01	0,292	0,426	9,98651E-59
Macro_FOLR2-APOE+	TXNDC17	1,92438E-19	-7,25290E-01	0,489	0,425	3,54413E-15
Macro_FOLR2-APOE+	GNAS	5,85409E-79	-7,25429E-01	0,727	0,59	1,07815E-74
Macro_FOLR2-APOE+	LRRC25	7,92494E-60	-7,25794E-01	0,428	0,318	1,45954E-55
Macro_FOLR2-APOE+	AIF1	4,43399E-07	-7,26976E-01	0,963	0,799	8,16608E-03
Macro_FOLR2-APOE+	TNIP1	5,90293E-25	-7,28927E-01	0,304	0,353	1,08714E-20
Macro_FOLR2-APOE+	MTCH2	2,16230E-25	-7,28934E-01	0,377	0,314	3,98231E-21

Macro_FOLR2-APOE+	EID1	1,37208E-18	-7,29310E-01	0,658	0,567	2,52697E-14
Macro_FOLR2-APOE+	TMCO1	7,88999E-32	-7,29573E-01	0,425	0,348	1,45310E-27
Macro_FOLR2-APOE+	SNX5	4,93894E-57	-7,31422E-01	0,489	0,383	9,09604E-53
Macro_FOLR2-APOE+	MAT2B	3,85953E-14	-7,31440E-01	0,377	0,329	7,10809E-10
Macro_FOLR2-APOE+	ZC3H15	4,34910E-07	-7,32184E-01	0,476	0,432	8,00974E-03
Macro_FOLR2-APOE+	PTGES3	1,44782E-19	-7,32328E-01	0,734	0,629	2,66645E-15
Macro_FOLR2-APOE+	COPA	2,70208E-10	-7,32455E-01	0,375	0,332	4,97642E-06
Macro_FOLR2-APOE+	ILK	2,23864E-10	-7,34208E-01	0,457	0,408	4,12290E-06
Macro_FOLR2-APOE+	AFF4	2,80464E-09	-7,36251E-01	0,423	0,369	5,16530E-05
Macro_FOLR2-APOE+	CTSB	0,00000E+00	-7,36865E-01	0,996	0,852	0,00000E+00
Macro_FOLR2-APOE+	CALR	2,55255E-45	-7,36985E-01	0,837	0,705	4,70102E-41
Macro_FOLR2-APOE+	EMC4	4,40202E-53	-7,36986E-01	0,442	0,347	8,10720E-49
Macro_FOLR2-APOE+	PTPN1	8,10277E-65	-7,37405E-01	0,33	0,413	1,49229E-60
Macro_FOLR2-APOE+	AP3S1	9,56161E-09	-7,37651E-01	0,383	0,34	1,76096E-04
Macro_FOLR2-APOE+	ATP6V0E1	3,85154E-84	-7,37746E-01	0,91	0,777	7,09339E-80
Macro_FOLR2-APOE+	PRDX3	8,05232E-44	-7,38970E-01	0,567	0,466	1,48300E-39
Macro_FOLR2-APOE+	TXN2	2,29881E-20	-7,39447E-01	0,453	0,387	4,23372E-16
Macro_FOLR2-APOE+	ARL6IP4	4,38672E-64	-7,39694E-01	0,751	0,615	8,07903E-60
Macro_FOLR2-APOE+	PDIA4	5,51467E-07	-7,39925E-01	0,351	0,316	1,01564E-02
Macro_FOLR2-APOE+	CAT	5,76919E-34	-7,39930E-01	0,484	0,397	1,06251E-29
Macro_FOLR2-APOE+	SGK1	1,05193E-104	-7,39974E-01	0,773	0,577	1,93735E-100
Macro_FOLR2-APOE+	CACYBP	7,41725E-12	-7,41367E-01	0,451	0,393	1,36604E-07
Macro_FOLR2-APOE+	SCP2	9,54126E-92	-7,42279E-01	0,578	0,442	1,75721E-87
Macro_FOLR2-APOE+	MAPRE1	3,69771E-09	-7,42546E-01	0,429	0,383	6,81007E-05
Macro_FOLR2-APOE+	SERINC3	3,78163E-12	-7,45077E-01	0,383	0,333	6,96463E-08
Macro_FOLR2-APOE+	MYO1F	7,50295E-21	-7,46623E-01	0,534	0,447	1,38182E-16
Macro_FOLR2-APOE+	RASSF2	1,64966E-26	-7,47806E-01	0,218	0,272	3,03818E-22
Macro_FOLR2-APOE+	SF3B5	2,43377E-12	-7,47874E-01	0,616	0,541	4,48227E-08
Macro_FOLR2-APOE+	SRSF11	2,08809E-09	-7,49043E-01	0,572	0,506	3,84564E-05
Macro_FOLR2-APOE+	VTI1B	2,55613E-59	-7,49825E-01	0,465	0,363	4,70762E-55
Macro_FOLR2-APOE+	KLF6	1,09822E-06	-7,51516E-01	0,862	0,787	2,02259E-02
Macro_FOLR2-APOE+	POLD4	1,37177E-18	-7,51591E-01	0,642	0,558	2,52639E-14
Macro_FOLR2-APOE+	ARAP1	3,41425E-11	-7,52910E-01	0,332	0,285	6,28802E-07
Macro_FOLR2-APOE+	UBE2I	8,73618E-07	-7,55190E-01	0,582	0,518	1,60894E-02
Macro_FOLR2-APOE+	PWP1	5,17972E-37	-7,55336E-01	0,268	0,202	9,53948E-33
Macro_FOLR2-APOE+	ERP29	2,62087E-26	-7,55552E-01	0,722	0,607	4,82685E-22
Macro_FOLR2-APOE+	TAF1D	4,67503E-18	-7,55939E-01	0,441	0,376	8,61001E-14
Macro_FOLR2-APOE+	LAMTOR3	5,23631E-08	-7,56490E-01	0,287	0,254	9,64371E-04
Macro_FOLR2-APOE+	MBOAT7	1,06739E-54	-7,57436E-01	0,18	0,26	1,96580E-50
Macro_FOLR2-APOE+	RNPS1	1,33251E-06	-7,57663E-01	0,442	0,399	2,45409E-02
Macro_FOLR2-APOE+	PCMT1	3,00869E-11	-7,58192E-01	0,377	0,332	5,54110E-07
Macro_FOLR2-APOE+	STK4	4,80544E-12	-7,60409E-01	0,414	0,431	8,85018E-08
Macro_FOLR2-APOE+	UVRAG	3,66443E-23	-7,61318E-01	0,246	0,298	6,74878E-19
Macro_FOLR2-APOE+	IAH1	4,69898E-12	-7,63349E-01	0,347	0,303	8,65410E-08
Macro_FOLR2-APOE+	ERGIC3	1,23263E-60	-7,63618E-01	0,594	0,482	2,27014E-56
Macro_FOLR2-APOE+	PCBP1	5,77793E-25	-7,63885E-01	0,631	0,607	1,06412E-20

Macro_FOLR2-APOE+	ESD	4,70358E-26	-7,64195E-01	0,423	0,354	8,66259E-22
Macro_FOLR2-APOE+	TMBIM6	1,24006E-177	-7,64615E-01	0,912	0,756	2,28382E-173
Macro_FOLR2-APOE+	AP1M1	3,86421E-09	-7,64631E-01	0,276	0,243	7,11672E-05
Macro_FOLR2-APOE+	DAD1	6,37618E-55	-7,64978E-01	0,703	0,584	1,17430E-50
Macro_FOLR2-APOE+	PFDN1	1,06585E-09	-7,66439E-01	0,321	0,281	1,96298E-05
Macro_FOLR2-APOE+	DNAJC8	7,44668E-22	-7,66828E-01	0,472	0,404	1,37146E-17
Macro_FOLR2-APOE+	MDM2	1,15999E-30	-7,68851E-01	0,253	0,189	2,13635E-26
Macro_FOLR2-APOE+	HLA-DRA	5,02864E-265	-7,72275E-01	0,999	0,913	9,26124E-261
Macro_FOLR2-APOE+	PTK2B	3,84908E-29	-7,73075E-01	0,312	0,244	7,08886E-25
Macro_FOLR2-APOE+	EVI2A	1,67301E-11	-7,73570E-01	0,311	0,268	3,08117E-07
Macro_FOLR2-APOE+	NELFE	1,97705E-10	-7,74362E-01	0,323	0,285	3,64114E-06
Macro_FOLR2-APOE+	NDUFV1	8,51297E-37	-7,74833E-01	0,48	0,396	1,56783E-32
Macro_FOLR2-APOE+	NDUFA12	1,19425E-07	-7,74969E-01	0,547	0,485	2,19946E-03
Macro_FOLR2-APOE+	TMED2	1,16536E-29	-7,75952E-01	0,575	0,487	2,14625E-25
Macro_FOLR2-APOE+	TMSB4X	7,62420E-104	-7,75957E-01	0,999	0,981	1,40415E-99
Macro_FOLR2-APOE+	REL	2,51865E-46	-7,76341E-01	0,578	0,602	4,63860E-42
Macro_FOLR2-APOE+	CPPED1	2,63464E-11	-7,76404E-01	0,246	0,274	4,85222E-07
Macro_FOLR2-APOE+	UBE2J1	5,15027E-84	-7,76522E-01	0,213	0,316	9,48526E-80
Macro_FOLR2-APOE+	TCF25	3,33883E-10	-7,76636E-01	0,62	0,552	6,14913E-06
Macro_FOLR2-APOE+	LILRA2	1,98948E-20	-7,78662E-01	0,297	0,239	3,66402E-16
Macro_FOLR2-APOE+	UQCRH	1,31178E-10	-7,79104E-01	0,78	0,689	2,41591E-06
Macro_FOLR2-APOE+	UQCRC2	3,13784E-33	-7,79851E-01	0,487	0,403	5,77895E-29
Macro_FOLR2-APOE+	PHB2	7,00452E-10	-7,80242E-01	0,548	0,484	1,29002E-05
Macro_FOLR2-APOE+	PDCD6IP	2,12756E-19	-7,81865E-01	0,422	0,36	3,91833E-15
Macro_FOLR2-APOE+	NAGA	2,70292E-65	-7,83257E-01	0,452	0,346	4,97797E-61
Macro_FOLR2-APOE+	VDAC2	9,91050E-20	-7,83430E-01	0,622	0,542	1,82522E-15
Macro_FOLR2-APOE+	CCT3	1,33955E-24	-7,84077E-01	0,472	0,4	2,46704E-20
Macro_FOLR2-APOE+	BAZ1A	1,35993E-58	-7,85716E-01	0,347	0,43	2,50459E-54
Macro_FOLR2-APOE+	XBP1	1,53308E-12	-7,85909E-01	0,412	0,427	2,82348E-08
Macro_FOLR2-APOE+	ELOVL1	8,40335E-12	-7,86788E-01	0,397	0,355	1,54764E-07
Macro_FOLR2-APOE+	SASH3	2,83907E-09	-7,87876E-01	0,318	0,278	5,22871E-05
Macro_FOLR2-APOE+	RGS19	2,31831E-19	-7,88235E-01	0,573	0,483	4,26963E-15
Macro_FOLR2-APOE+	EIF2A	2,54078E-07	-7,88285E-01	0,337	0,303	4,67935E-03
Macro_FOLR2-APOE+	YME1L1	1,16869E-11	-7,88635E-01	0,357	0,382	2,15238E-07
Macro_FOLR2-APOE+	CD163	0,00000E+00	-7,89322E-01	0,722	0,402	0,00000E+00
Macro_FOLR2-APOE+	GPR108	6,82315E-14	-7,90291E-01	0,295	0,252	1,25662E-09
Macro_FOLR2-APOE+	DAP	1,11732E-10	-7,90795E-01	0,261	0,224	2,05777E-06
Macro_FOLR2-APOE+	NOTCH2	5,40602E-18	-7,90976E-01	0,282	0,323	9,95626E-14
Macro_FOLR2-APOE+	NOP10	2,43491E-16	-7,91905E-01	0,703	0,62	4,48437E-12
Macro_FOLR2-APOE+	DNTTIP2	9,04873E-09	-7,92909E-01	0,251	0,279	1,66650E-04
Macro_FOLR2-APOE+	XRCC5	9,03032E-11	-7,93195E-01	0,525	0,467	1,66311E-06
Macro_FOLR2-APOE+	PSMD2	1,04269E-17	-7,93311E-01	0,365	0,313	1,92032E-13
Macro_FOLR2-APOE+	HSD17B4	3,92874E-19	-7,93350E-01	0,304	0,251	7,23557E-15
Macro_FOLR2-APOE+	CALHM6	3,37225E-101	-7,93468E-01	0,168	0,276	6,21067E-97
Macro_FOLR2-APOE+	CSDE1	5,53529E-07	-7,93822E-01	0,666	0,595	1,01943E-02
Macro_FOLR2-APOE+	DBI	9,14634E-13	-7,95011E-01	0,805	0,711	1,68448E-08

Macro_FOLR2-APOE+	GRB2	1,25355E-14	-7,95185E-01	0,764	0,666	2,30866E-10
Macro_FOLR2-APOE+	BIN2	7,17380E-11	-7,97915E-01	0,353	0,307	1,32120E-06
Macro_FOLR2-APOE+	DSTN	4,54082E-19	-7,99880E-01	0,401	0,338	8,36284E-15
Macro_FOLR2-APOE+	SEC31A	1,37863E-06	-8,00450E-01	0,325	0,294	2,53903E-02
Macro_FOLR2-APOE+	HM13	1,58728E-115	-8,01132E-01	0,655	0,52	2,92329E-111
Macro_FOLR2-APOE+	ETV6	5,96936E-83	-8,01708E-01	0,204	0,305	1,09938E-78
Macro_FOLR2-APOE+	PRDX5	2,17148E-40	-8,02329E-01	0,701	0,584	3,99921E-36
Macro_FOLR2-APOE+	DERL2	1,25752E-08	-8,02536E-01	0,262	0,231	2,31597E-04
Macro_FOLR2-APOE+	SPCS2	3,89372E-43	-8,02873E-01	0,582	0,478	7,17106E-39
Macro_FOLR2-APOE+	TUBA1B	3,86151E-70	-8,03076E-01	0,878	0,736	7,11174E-66
Macro_FOLR2-APOE+	BACH1	4,14280E-11	-8,03219E-01	0,288	0,316	7,62980E-07
Macro_FOLR2-APOE+	RAB10	2,42615E-11	-8,04960E-01	0,545	0,481	4,46825E-07
Macro_FOLR2-APOE+	TBCB	1,63862E-12	-8,06174E-01	0,522	0,459	3,01785E-08
Macro_FOLR2-APOE+	AMD1	2,00562E-09	-8,06192E-01	0,263	0,289	3,69376E-05
Macro_FOLR2-APOE+	ATP6V0D1	3,64202E-35	-8,07644E-01	0,654	0,558	6,70751E-31
Macro_FOLR2-APOE+	IGSF6	3,89622E-60	-8,07810E-01	0,685	0,536	7,17567E-56
Macro_FOLR2-APOE+	SF1	9,51274E-07	-8,09004E-01	0,509	0,503	1,75196E-02
Macro_FOLR2-APOE+	RNH1	1,03326E-135	-8,10909E-01	0,761	0,595	1,90296E-131
Macro_FOLR2-APOE+	MGAT1	1,08187E-148	-8,12109E-01	0,773	0,6	1,99249E-144
Macro_FOLR2-APOE+	ADRM1	1,28290E-09	-8,12835E-01	0,471	0,422	2,36272E-05
Macro_FOLR2-APOE+	UBL5	7,00973E-08	-8,12931E-01	0,799	0,748	1,29098E-03
Macro_FOLR2-APOE+	HIGD2A	1,48352E-12	-8,13760E-01	0,808	0,699	2,73219E-08
Macro_FOLR2-APOE+	BUB3	5,49721E-07	-8,15112E-01	0,283	0,254	1,01242E-02
Macro_FOLR2-APOE+	HCLS1	4,57315E-21	-8,15389E-01	0,824	0,707	8,42238E-17
Macro_FOLR2-APOE+	CUEDC2	6,73975E-07	-8,16798E-01	0,322	0,29	1,24126E-02
Macro_FOLR2-APOE+	TMEM59	2,93984E-53	-8,17167E-01	0,716	0,601	5,41431E-49
Macro_FOLR2-APOE+	CNPY3	2,92233E-27	-8,17253E-01	0,726	0,612	5,38206E-23
Macro_FOLR2-APOE+	MRPL33	7,36853E-14	-8,18446E-01	0,344	0,377	1,35706E-09
Macro_FOLR2-APOE+	CD86	5,32962E-17	-8,18547E-01	0,574	0,482	9,81557E-13
Macro_FOLR2-APOE+	CD33	2,99546E-22	-8,18838E-01	0,366	0,298	5,51674E-18
Macro_FOLR2-APOE+	SERP1	3,25894E-59	-8,18938E-01	0,864	0,812	6,00199E-55
Macro_FOLR2-APOE+	SNX17	6,18774E-18	-8,19269E-01	0,487	0,424	1,13960E-13
Macro_FOLR2-APOE+	P4HB	1,74910E-59	-8,19852E-01	0,741	0,611	3,22133E-55
Macro_FOLR2-APOE+	SLC43A3	1,84597E-68	-8,20096E-01	0,329	0,228	3,39972E-64
Macro_FOLR2-APOE+	GBP4	3,15240E-45	-8,22149E-01	0,22	0,287	5,80577E-41
Macro_FOLR2-APOE+	NDUFA10	3,33613E-42	-8,22157E-01	0,4	0,316	6,14416E-38
Macro_FOLR2-APOE+	PUF60	8,69974E-15	-8,22870E-01	0,36	0,313	1,60223E-10
Macro_FOLR2-APOE+	UQCRQ	4,25404E-12	-8,24703E-01	0,73	0,655	7,83466E-08
Macro_FOLR2-APOE+	PSMC5	1,93587E-25	-8,25015E-01	0,471	0,401	3,56529E-21
Macro_FOLR2-APOE+	TRABD	1,77373E-07	-8,25539E-01	0,341	0,36	3,26668E-03
Macro_FOLR2-APOE+	PYCARD	6,68195E-15	-8,26830E-01	0,816	0,688	1,23061E-10
Macro_FOLR2-APOE+	CTSL	0,00000E+00	-8,27438E-01	0,826	0,469	0,00000E+00
Macro_FOLR2-APOE+	F13A1	3,26199E-19	-8,27757E-01	0,28	0,225	6,00760E-15
Macro_FOLR2-APOE+	HLA-B	1,47381E-06	-8,28005E-01	0,997	0,963	2,71431E-02
Macro_FOLR2-APOE+	GNG10	3,75540E-19	-8,28253E-01	0,302	0,344	6,91632E-15
Macro_FOLR2-APOE+	WTAP	1,44257E-15	-8,28677E-01	0,335	0,37	2,65678E-11

Macro_FOLR2-APOE+	LAT2	1,37611E-32	-8,28749E-01	0,535	0,441	2,53438E-28
Macro_FOLR2-APOE+	NMI	8,55900E-22	-8,30162E-01	0,257	0,307	1,57631E-17
Macro_FOLR2-APOE+	DCTN3	1,37106E-10	-8,30362E-01	0,4	0,354	2,52509E-06
Macro_FOLR2-APOE+	TRAM1	2,48244E-07	-8,30516E-01	0,497	0,443	4,57191E-03
Macro_FOLR2-APOE+	DAP3	1,95733E-09	-8,31908E-01	0,351	0,312	3,60481E-05
Macro_FOLR2-APOE+	RER1	8,07975E-32	-8,34217E-01	0,531	0,449	1,48805E-27
Macro_FOLR2-APOE+	CCT5	1,27332E-15	-8,34280E-01	0,43	0,373	2,34508E-11
Macro_FOLR2-APOE+	SNRPG	2,55370E-07	-8,34311E-01	0,567	0,555	4,70315E-03
Macro_FOLR2-APOE+	AAMP	9,25250E-08	-8,34338E-01	0,319	0,288	1,70403E-03
Macro_FOLR2-APOE+	RNASE6	1,93979E-289	-8,34605E-01	0,752	0,505	3,57251E-285
Macro_FOLR2-APOE+	SND1	4,05920E-13	-8,36137E-01	0,388	0,338	7,47582E-09
Macro_FOLR2-APOE+	UBXN4	4,21720E-09	-8,36137E-01	0,573	0,512	7,76682E-05
Macro_FOLR2-APOE+	TMEM208	2,92642E-25	-8,36639E-01	0,385	0,322	5,38959E-21
Macro_FOLR2-APOE+	PSMB6	9,43478E-42	-8,37247E-01	0,677	0,56	1,73760E-37
Macro_FOLR2-APOE+	CCT6A	4,13716E-08	-8,38746E-01	0,492	0,441	7,61941E-04
Macro_FOLR2-APOE+	PSMD8	3,96538E-39	-8,38801E-01	0,611	0,511	7,30304E-35
Macro_FOLR2-APOE+	IFI16	5,45586E-11	-8,39685E-01	0,566	0,489	1,00481E-06
Macro_FOLR2-APOE+	LMAN2	1,16418E-42	-8,40913E-01	0,596	0,497	2,14407E-38
Macro_FOLR2-APOE+	RNF7	1,96951E-08	-8,41446E-01	0,602	0,535	3,62725E-04
Macro_FOLR2-APOE+	RBPJ	2,84682E-12	-8,41932E-01	0,529	0,456	5,24298E-08
Macro_FOLR2-APOE+	NEMF	1,02851E-07	-8,42945E-01	0,319	0,283	1,89422E-03
Macro_FOLR2-APOE+	PFKFB3	1,47698E-07	-8,43392E-01	0,32	0,336	2,72015E-03
Macro_FOLR2-APOE+	PRDX6	1,03007E-30	-8,44865E-01	0,643	0,545	1,89707E-26
Macro_FOLR2-APOE+	ATP5MC2	3,89862E-181	-8,45517E-01	0,288	0,457	7,18009E-177
Macro_FOLR2-APOE+	RNF149	6,48126E-35	-8,45576E-01	0,722	0,611	1,19365E-30
Macro_FOLR2-APOE+	CHD9	1,75486E-09	-8,46039E-01	0,382	0,337	3,23193E-05
Macro_FOLR2-APOE+	G3BP2	2,13474E-13	-8,47161E-01	0,248	0,282	3,93155E-09
Macro_FOLR2-APOE+	PSEN1	1,15417E-12	-8,47348E-01	0,256	0,216	2,12564E-08
Macro_FOLR2-APOE+	EIF3H	7,36090E-15	-8,47370E-01	0,737	0,645	1,35566E-10
Macro_FOLR2-APOE+	SDHA	1,59231E-12	-8,47762E-01	0,284	0,246	2,93255E-08
Macro_FOLR2-APOE+	GRN	0,00000E+00	-8,48151E-01	0,973	0,798	0,00000E+00
Macro_FOLR2-APOE+	CYC1	7,04413E-24	-8,49005E-01	0,499	0,424	1,29732E-19
Macro_FOLR2-APOE+	LSP1	3,64443E-10	-8,49607E-01	0,769	0,655	6,71194E-06
Macro_FOLR2-APOE+	ATG12	2,23070E-09	-8,49651E-01	0,295	0,258	4,10827E-05
Macro_FOLR2-APOE+	OXA1L	1,17421E-23	-8,49729E-01	0,525	0,444	2,16255E-19
Macro_FOLR2-APOE+	MPV17	5,40000E-67	-8,51846E-01	0,395	0,295	9,94518E-63
Macro_FOLR2-APOE+	SSBP1	8,07383E-15	-8,53546E-01	0,547	0,477	1,48696E-10
Macro_FOLR2-APOE+	NCF2	2,70732E-29	-8,55148E-01	0,554	0,45	4,98606E-25
Macro_FOLR2-APOE+	SEC62	5,65485E-13	-8,57638E-01	0,618	0,544	1,04145E-08
Macro_FOLR2-APOE+	RELB	9,88626E-106	-8,58977E-01	0,146	0,261	1,82075E-101
Macro_FOLR2-APOE+	STAT3	9,00015E-16	-8,59175E-01	0,492	0,508	1,65756E-11
Macro_FOLR2-APOE+	C1orf162	1,44259E-37	-8,61642E-01	0,849	0,684	2,65681E-33
Macro_FOLR2-APOE+	HNRNPU	2,64810E-09	-8,61794E-01	0,648	0,619	4,87701E-05
Macro_FOLR2-APOE+	LITAF	1,23031E-17	-8,63989E-01	0,847	0,725	2,26587E-13
Macro_FOLR2-APOE+	ARHGAP4	2,22366E-14	-8,66361E-01	0,433	0,377	4,09531E-10
Macro_FOLR2-APOE+	TAF9	1,72041E-25	-8,67113E-01	0,358	0,293	3,16847E-21

Macro_FOLR2-APOE+	GHITM	4,29807E-15	-8,67978E-01	0,566	0,494	7,91575E-11
Macro_FOLR2-APOE+	UBXN1	2,28893E-28	-8,68515E-01	0,716	0,614	4,21553E-24
Macro_FOLR2-APOE+	NHP2	5,84307E-14	-8,68894E-01	0,466	0,404	1,07612E-09
Macro_FOLR2-APOE+	ETFA	5,63035E-16	-8,69841E-01	0,379	0,328	1,03694E-11
Macro_FOLR2-APOE+	SERTAD1	2,00999E-23	-8,70914E-01	0,286	0,332	3,70180E-19
Macro_FOLR2-APOE+	MRFAP1	1,12492E-26	-8,71038E-01	0,466	0,39	2,07176E-22
Macro_FOLR2-APOE+	SDHB	6,85767E-27	-8,74781E-01	0,464	0,388	1,26298E-22
Macro_FOLR2-APOE+	IFNGR1	4,19600E-59	-8,77098E-01	0,696	0,563	7,72777E-55
Macro_FOLR2-APOE+	BRK1	5,43658E-09	-8,78170E-01	0,808	0,712	1,00125E-04
Macro_FOLR2-APOE+	CLN8	4,60694E-103	-8,80690E-01	0,34	0,218	8,48460E-99
Macro_FOLR2-APOE+	PAPOLA	9,20907E-07	-8,80742E-01	0,497	0,449	1,69603E-02
Macro_FOLR2-APOE+	CBX3	2,40909E-09	-8,81952E-01	0,391	0,41	4,43682E-05
Macro_FOLR2-APOE+	SP100	1,71518E-13	-8,82611E-01	0,517	0,458	3,15884E-09
Macro_FOLR2-APOE+	EIF3I	6,27992E-12	-8,85841E-01	0,52	0,456	1,15657E-07
Macro_FOLR2-APOE+	NFKB1	5,32301E-117	-8,86378E-01	0,181	0,306	9,80338E-113
Macro_FOLR2-APOE+	PIM3	4,64427E-62	-8,86503E-01	0,215	0,298	8,55335E-58
Macro_FOLR2-APOE+	RBBP4	5,89041E-08	-8,87209E-01	0,409	0,364	1,08484E-03
Macro_FOLR2-APOE+	MDH1	3,96035E-67	-8,88273E-01	0,48	0,37	7,29377E-63
Macro_FOLR2-APOE+	GTF3A	1,06369E-63	-8,89699E-01	0,583	0,463	1,95900E-59
Macro_FOLR2-APOE+	ARL6IP1	2,62954E-41	-8,89725E-01	0,493	0,402	4,84283E-37
Macro_FOLR2-APOE+	POLR2G	1,00318E-07	-8,89992E-01	0,409	0,366	1,84756E-03
Macro_FOLR2-APOE+	SHISA5	3,63481E-12	-8,90242E-01	0,413	0,364	6,69423E-08
Macro_FOLR2-APOE+	CLEC12A	2,35771E-10	-8,91245E-01	0,297	0,254	4,34219E-06
Macro_FOLR2-APOE+	PSMD6	7,10073E-07	-8,92502E-01	0,304	0,274	1,30774E-02
Macro_FOLR2-APOE+	CHMP2A	2,79611E-24	-8,95562E-01	0,56	0,48	5,14959E-20
Macro_FOLR2-APOE+	ATP2B1	6,14526E-12	-8,95951E-01	0,33	0,352	1,13177E-07
Macro_FOLR2-APOE+	PSMA7	2,11211E-25	-8,96131E-01	0,84	0,73	3,88987E-21
Macro_FOLR2-APOE+	TM9SF2	1,70067E-09	-8,97693E-01	0,469	0,421	3,13213E-05
Macro_FOLR2-APOE+	HSP90AA1	3,84660E-19	-8,98314E-01	0,957	0,867	7,08429E-15
Macro_FOLR2-APOE+	TMSB10	2,66013E-106	-8,99020E-01	0,998	0,982	4,89916E-102
Macro_FOLR2-APOE+	CMTM6	2,14681E-17	-8,99820E-01	0,636	0,613	3,95378E-13
Macro_FOLR2-APOE+	TALDO1	2,17987E-27	-9,00226E-01	0,745	0,648	4,01467E-23
Macro_FOLR2-APOE+	RHOC	4,09934E-25	-9,00976E-01	0,37	0,299	7,54975E-21
Macro_FOLR2-APOE+	MT-CO3	2,57280E-140	-9,01285E-01	0,382	0,624	4,73832E-136
Macro_FOLR2-APOE+	TRMT112	4,37315E-39	-9,02314E-01	0,715	0,602	8,05403E-35
Macro_FOLR2-APOE+	SIVA1	3,04527E-23	-9,02970E-01	0,522	0,444	5,60847E-19
Macro_FOLR2-APOE+	SEC13	3,82284E-10	-9,05193E-01	0,442	0,395	7,04053E-06
Macro_FOLR2-APOE+	SSR3	4,67752E-101	-9,05539E-01	0,723	0,575	8,61460E-97
Macro_FOLR2-APOE+	CD300A	2,00938E-17	-9,05642E-01	0,531	0,449	3,70067E-13
Macro_FOLR2-APOE+	CDK5RAP3	9,01647E-24	-9,05710E-01	0,359	0,298	1,66056E-19
Macro_FOLR2-APOE+	CSF3R	1,16666E-39	-9,10793E-01	0,488	0,378	2,14864E-35
Macro_FOLR2-APOE+	TSPAN3	2,31913E-44	-9,10950E-01	0,391	0,306	4,27114E-40
Macro_FOLR2-APOE+	MRPL55	5,45069E-09	-9,13288E-01	0,309	0,273	1,00385E-04
Macro_FOLR2-APOE+	NEDD8	1,40731E-17	-9,14058E-01	0,662	0,574	2,59185E-13
Macro_FOLR2-APOE+	LAMTOR5	1,23348E-25	-9,14365E-01	0,578	0,495	2,27169E-21
Macro_FOLR2-APOE+	SSB	7,50904E-10	-9,16367E-01	0,506	0,443	1,38294E-05

Macro_FOLR2-APOE+	C12orf57	5,91324E-17	-9,16615E-01	0,305	0,338	1,08904E-12
Macro_FOLR2-APOE+	LGALS9	3,64191E-49	-9,16813E-01	0,658	0,53	6,70731E-45
Macro_FOLR2-APOE+	RPS19BP1	7,79102E-28	-9,16878E-01	0,208	0,268	1,43487E-23
Macro_FOLR2-APOE+	EIF4H	6,22732E-13	-9,18575E-01	0,505	0,444	1,14688E-08
Macro_FOLR2-APOE+	COMMD6	9,01996E-47	-9,19715E-01	0,761	0,731	1,66121E-42
Macro_FOLR2-APOE+	PARP9	1,98980E-07	-9,24056E-01	0,233	0,258	3,66461E-03
Macro_FOLR2-APOE+	C1QBP	1,14230E-12	-9,25094E-01	0,487	0,42	2,10377E-08
Macro_FOLR2-APOE+	M6PR	2,97698E-28	-9,26627E-01	0,647	0,55	5,48270E-24
Macro_FOLR2-APOE+	NDUFA11	1,39836E-19	-9,27667E-01	0,53	0,442	2,57536E-15
Macro_FOLR2-APOE+	RACK1	2,53180E-230	-9,27693E-01	0,299	0,495	4,66282E-226
Macro_FOLR2-APOE+	PPP1CA	1,94522E-13	-9,28871E-01	0,703	0,609	3,58251E-09
Macro_FOLR2-APOE+	DDX3X	8,91095E-24	-9,30368E-01	0,451	0,482	1,64113E-19
Macro_FOLR2-APOE+	LAMTOR4	3,01224E-15	-9,30672E-01	0,8	0,7	5,54763E-11
Macro_FOLR2-APOE+	RAB7A	8,50212E-35	-9,31218E-01	0,738	0,616	1,56584E-30
Macro_FOLR2-APOE+	CAPZA2	4,32524E-39	-9,32760E-01	0,639	0,534	7,96580E-35
Macro_FOLR2-APOE+	VAPA	2,84563E-50	-9,33047E-01	0,698	0,588	5,24079E-46
Macro_FOLR2-APOE+	SEC11C	5,49404E-29	-9,35633E-01	0,386	0,316	1,01184E-24
Macro_FOLR2-APOE+	CNDP2	3,27086E-47	-9,35930E-01	0,549	0,439	6,02395E-43
Macro_FOLR2-APOE+	SFT2D1	4,84029E-16	-9,36966E-01	0,516	0,451	8,91436E-12
Macro_FOLR2-APOE+	ORMDL1	1,82716E-19	-9,37901E-01	0,367	0,307	3,36509E-15
Macro_FOLR2-APOE+	GPI	5,84543E-58	-9,38560E-01	0,508	0,401	1,07655E-53
Macro_FOLR2-APOE+	PGD	5,27920E-19	-9,42678E-01	0,526	0,456	9,72271E-15
Macro_FOLR2-APOE+	MCL1	2,04028E-103	-9,44194E-01	0,773	0,769	3,75758E-99
Macro_FOLR2-APOE+	TSG101	8,74260E-10	-9,45107E-01	0,263	0,231	1,61012E-05
Macro_FOLR2-APOE+	CHD1	2,38843E-60	-9,45749E-01	0,223	0,309	4,39877E-56
Macro_FOLR2-APOE+	NASP	1,40830E-06	-9,47035E-01	0,253	0,279	2,59366E-02
Macro_FOLR2-APOE+	SFPQ	8,51755E-25	-9,48536E-01	0,488	0,51	1,56868E-20
Macro_FOLR2-APOE+	SAP18	8,04272E-09	-9,49972E-01	0,726	0,635	1,48123E-04
Macro_FOLR2-APOE+	CTSC	4,71528E-67	-9,50326E-01	0,835	0,652	8,68413E-63
Macro_FOLR2-APOE+	RAB34	8,29570E-07	-9,51217E-01	0,298	0,268	1,52782E-02
Macro_FOLR2-APOE+	PPIB	2,63017E-24	-9,53609E-01	0,891	0,776	4,84398E-20
Macro_FOLR2-APOE+	ARID5A	5,76397E-08	-9,55418E-01	0,266	0,291	1,06155E-03
Macro_FOLR2-APOE+	DDX39A	1,10734E-07	-9,59502E-01	0,32	0,287	2,03938E-03
Macro_FOLR2-APOE+	GPBP1	2,25490E-30	-9,60782E-01	0,295	0,352	4,15285E-26
Macro_FOLR2-APOE+	OSTC	3,85523E-08	-9,63520E-01	0,557	0,49	7,10017E-04
Macro_FOLR2-APOE+	KLF10	6,07401E-07	-9,63551E-01	0,272	0,293	1,11865E-02
Macro_FOLR2-APOE+	EEF1A1	3,94157E-08	-9,64484E-01	0,997	0,982	7,25920E-04
Macro_FOLR2-APOE+	TKT	1,09114E-29	-9,65021E-01	0,701	0,592	2,00956E-25
Macro_FOLR2-APOE+	CD82	3,30316E-07	-9,65343E-01	0,274	0,234	6,08344E-03
Macro_FOLR2-APOE+	COPS6	1,20068E-27	-9,69427E-01	0,402	0,333	2,21130E-23
Macro_FOLR2-APOE+	NCSTN	3,37348E-09	-9,70198E-01	0,26	0,229	6,21294E-05
Macro_FOLR2-APOE+	SLC3A2	2,30512E-42	-9,70939E-01	0,555	0,448	4,24534E-38
Macro_FOLR2-APOE+	ITGAX	1,88470E-13	-9,72195E-01	0,464	0,389	3,47106E-09
Macro_FOLR2-APOE+	HSPA1A	3,33974E-127	-9,74552E-01	0,822	0,638	6,15079E-123
Macro_FOLR2-APOE+	ELOVL5	1,23293E-30	-9,74666E-01	0,237	0,296	2,27069E-26
Macro_FOLR2-APOE+	PSMF1	1,64561E-33	-9,75093E-01	0,493	0,409	3,03072E-29

Macro_FOLR2-APOE+	SRP14	1,47819E-08	-9,75117E-01	0,926	0,822	2,72238E-04
Macro_FOLR2-APOE+	CHURC1	4,93252E-12	-9,75186E-01	0,289	0,323	9,08423E-08
Macro_FOLR2-APOE+	IDH3B	5,93511E-11	-9,77262E-01	0,293	0,256	1,09307E-06
Macro_FOLR2-APOE+	PARVG	1,84105E-20	-9,77718E-01	0,518	0,439	3,39067E-16
Macro_FOLR2-APOE+	SLC25A5	1,62173E-27	-9,78149E-01	0,853	0,726	2,98673E-23
Macro_FOLR2-APOE+	CD164	6,30929E-51	-9,79584E-01	0,657	0,541	1,16198E-46
Macro_FOLR2-APOE+	APRT	3,04199E-08	-9,82879E-01	0,698	0,611	5,60244E-04
Macro_FOLR2-APOE+	RABGAP1L	6,91308E-17	-9,85684E-01	0,218	0,258	1,27318E-12
Macro_FOLR2-APOE+	SRSF10	1,37846E-20	-9,86521E-01	0,237	0,286	2,53871E-16
Macro_FOLR2-APOE+	BTF3	6,33355E-08	-9,86627E-01	0,918	0,834	1,16645E-03
Macro_FOLR2-APOE+	RASSF5	6,36462E-89	-9,86940E-01	0,182	0,286	1,17217E-84
Macro_FOLR2-APOE+	SMIM14	9,07529E-08	-9,87093E-01	0,248	0,272	1,67140E-03
Macro_FOLR2-APOE+	SLC38A2	1,51045E-22	-9,88115E-01	0,269	0,315	2,78179E-18
Macro_FOLR2-APOE+	MATR3	5,22587E-19	-9,88849E-01	0,277	0,22	9,62449E-15
Macro_FOLR2-APOE+	MT2A	2,14669E-39	-9,90252E-01	0,613	0,631	3,95355E-35
Macro_FOLR2-APOE+	TNFAIP8	9,05545E-82	-9,94600E-01	0,244	0,351	1,66774E-77
Macro_FOLR2-APOE+	PTMA	5,89985E-254	-9,94639E-01	0,989	0,958	1,08658E-249
Macro_FOLR2-APOE+	CTSH	8,98819E-90	-9,95200E-01	0,877	0,683	1,65535E-85
Macro_FOLR2-APOE+	PHACTR1	3,45559E-39	-9,95502E-01	0,249	0,31	6,36415E-35
Macro_FOLR2-APOE+	TMEM109	8,20928E-12	-9,98123E-01	0,417	0,361	1,51190E-07
Macro_FOLR2-APOE+	APEX1	1,87441E-10	-9,99494E-01	0,496	0,433	3,45210E-06
Macro_FOLR2-APOE+	SH3BGRL3	1,09372E-34	-1,00095E+00	0,972	0,926	2,01431E-30
Macro_FOLR2-APOE+	CSRNP1	1,13193E-147	-1,00432E+00	0,169	0,312	2,08468E-143
Macro_FOLR2-APOE+	CLEC4A	4,20288E-10	-1,00529E+00	0,279	0,3	7,74045E-06
Macro_FOLR2-APOE+	DOCK2	1,02757E-19	-1,00688E+00	0,401	0,34	1,89248E-15
Macro_FOLR2-APOE+	GNA15	2,51408E-34	-1,00950E+00	0,253	0,315	4,63018E-30
Macro_FOLR2-APOE+	DECR1	9,18561E-08	-1,01170E+00	0,382	0,341	1,69171E-03
Macro_FOLR2-APOE+	RPL22L1	1,36279E-53	-1,01211E+00	0,204	0,286	2,50985E-49
Macro_FOLR2-APOE+	INPP5D	1,21036E-11	-1,01449E+00	0,35	0,305	2,22913E-07
Macro_FOLR2-APOE+	PSMD4	9,46582E-27	-1,01946E+00	0,475	0,402	1,74332E-22
Macro_FOLR2-APOE+	LILRB2	2,65677E-15	-1,02232E+00	0,456	0,457	4,89297E-11
Macro_FOLR2-APOE+	FYTTD1	1,42141E-10	-1,02342E+00	0,223	0,253	2,61781E-06
Macro_FOLR2-APOE+	EIF3G	3,22620E-07	-1,02370E+00	0,681	0,603	5,94170E-03
Macro_FOLR2-APOE+	SF3B1	9,54634E-11	-1,02619E+00	0,546	0,485	1,75815E-06
Macro_FOLR2-APOE+	FCER1G	5,48107E-68	-1,02812E+00	0,991	0,9	1,00945E-63
Macro_FOLR2-APOE+	ADPGK	8,15725E-24	-1,03222E+00	0,461	0,391	1,50232E-19
Macro_FOLR2-APOE+	TAGAP	2,19774E-13	-1,03433E+00	0,244	0,277	4,04758E-09
Macro_FOLR2-APOE+	U2AF1	7,88167E-58	-1,03712E+00	0,362	0,254	1,45157E-53
Macro_FOLR2-APOE+	VDAC3	4,35166E-07	-1,04031E+00	0,34	0,308	8,01445E-03
Macro_FOLR2-APOE+	CD4	8,23477E-115	-1,04148E+00	0,69	0,514	1,51660E-110
Macro_FOLR2-APOE+	COX5A	1,39985E-18	-1,04388E+00	0,791	0,678	2,57811E-14
Macro_FOLR2-APOE+	ERCC1	7,63998E-10	-1,04459E+00	0,426	0,374	1,40706E-05
Macro_FOLR2-APOE+	HNRNPA2B1	3,76731E-58	-1,04679E+00	0,881	0,824	6,93825E-54
Macro_FOLR2-APOE+	CCT7	1,29918E-09	-1,04853E+00	0,384	0,344	2,39269E-05
Macro_FOLR2-APOE+	ALOX5AP	0,00000E+00	-1,04879E+00	0,749	0,465	0,00000E+00
Macro_FOLR2-APOE+	STX11	2,89810E-54	-1,04912E+00	0,261	0,339	5,33743E-50

Macro_FOLR2-APOE+	ANKRD44	1,78596E-28	-1,05112E+00	0,239	0,3	3,28919E-24
Macro_FOLR2-APOE+	MAP3K8	3,20519E-22	-1,05324E+00	0,463	0,485	5,90299E-18
Macro_FOLR2-APOE+	VPS29	7,10384E-24	-1,05475E+00	0,613	0,522	1,30832E-19
Macro_FOLR2-APOE+	OAS1	3,41012E-13	-1,05548E+00	0,381	0,318	6,28042E-09
Macro_FOLR2-APOE+	SERPINB1	9,92287E-45	-1,05843E+00	0,679	0,66	1,82750E-40
Macro_FOLR2-APOE+	TNFRSF1B	3,67442E-07	-1,05945E+00	0,57	0,535	6,76719E-03
Macro_FOLR2-APOE+	FGR	1,07629E-41	-1,05952E+00	0,549	0,438	1,98221E-37
Macro_FOLR2-APOE+	FPR1	7,82180E-16	-1,06031E+00	0,529	0,435	1,44054E-11
Macro_FOLR2-APOE+	CORO1C	5,89099E-109	-1,06051E+00	0,461	0,325	1,08494E-104
Macro_FOLR2-APOE+	FGD2	2,14450E-12	-1,06097E+00	0,389	0,337	3,94953E-08
Macro_FOLR2-APOE+	SMCO4	1,65950E-15	-1,06113E+00	0,403	0,42	3,05630E-11
Macro_FOLR2-APOE+	EIF4G2	6,34909E-13	-1,06262E+00	0,637	0,616	1,16931E-08
Macro_FOLR2-APOE+	MIF	5,75934E-30	-1,06417E+00	0,741	0,721	1,06070E-25
Macro_FOLR2-APOE+	DDOST	1,70047E-30	-1,06438E+00	0,533	0,452	3,13176E-26
Macro_FOLR2-APOE+	PSMB7	5,51776E-11	-1,06978E+00	0,406	0,356	1,01620E-06
Macro_FOLR2-APOE+	RAB1A	2,35286E-15	-1,06996E+00	0,448	0,388	4,33326E-11
Macro_FOLR2-APOE+	MLF2	1,96931E-20	-1,07135E+00	0,518	0,442	3,62688E-16
Macro_FOLR2-APOE+	TRA2B	5,85850E-07	-1,07306E+00	0,465	0,472	1,07896E-02
Macro_FOLR2-APOE+	NONO	2,99982E-16	-1,07333E+00	0,485	0,422	5,52477E-12
Macro_FOLR2-APOE+	PAIP2	2,52822E-06	-1,07409E+00	0,557	0,504	4,65622E-02
Macro_FOLR2-APOE+	MCTP1	1,38382E-12	-1,07487E+00	0,219	0,253	2,54858E-08
Macro_FOLR2-APOE+	GCA	7,82803E-50	-1,07736E+00	0,343	0,411	1,44169E-45
Macro_FOLR2-APOE+	GTF2B	3,70323E-20	-1,07784E+00	0,237	0,286	6,82024E-16
Macro_FOLR2-APOE+	ZNF706	1,14197E-21	-1,07800E+00	0,678	0,577	2,10317E-17
Macro_FOLR2-APOE+	NAP1L1	2,40084E-15	-1,07813E+00	0,696	0,607	4,42163E-11
Macro_FOLR2-APOE+	CDK2AP2	4,60644E-08	-1,08110E+00	0,385	0,399	8,48367E-04
Macro_FOLR2-APOE+	NABP1	2,02711E-25	-1,08275E+00	0,298	0,346	3,73334E-21
Macro_FOLR2-APOE+	RBM8A	7,58656E-18	-1,08439E+00	0,521	0,536	1,39722E-13
Macro_FOLR2-APOE+	FAU	2,67908E-260	-1,08616E+00	0,982	0,953	4,93406E-256
Macro_FOLR2-APOE+	KYNU	6,34421E-11	-1,08809E+00	0,289	0,32	1,16841E-06
Macro_FOLR2-APOE+	SYNGR2	1,31073E-92	-1,08876E+00	0,87	0,705	2,41398E-88
Macro_FOLR2-APOE+	MAN2B1	4,55337E-27	-1,09254E+00	0,55	0,462	8,38594E-23
Macro_FOLR2-APOE+	RIPK2	5,19549E-38	-1,09270E+00	0,22	0,282	9,56854E-34
Macro_FOLR2-APOE+	BZW1	3,02609E-37	-1,09302E+00	0,399	0,45	5,57315E-33
Macro_FOLR2-APOE+	SEC61G	1,39995E-38	-1,09310E+00	0,58	0,603	2,57828E-34
Macro_FOLR2-APOE+	NINJ1	6,95550E-54	-1,09355E+00	0,694	0,538	1,28099E-49
Macro_FOLR2-APOE+	TWF2	1,87028E-11	-1,09448E+00	0,312	0,338	3,44450E-07
Macro_FOLR2-APOE+	COPZ1	9,12482E-22	-1,09459E+00	0,436	0,373	1,68052E-17
Macro_FOLR2-APOE+	RILPL2	2,05266E-33	-1,09529E+00	0,424	0,461	3,78038E-29
Macro_FOLR2-APOE+	TET2	1,46538E-42	-1,09646E+00	0,18	0,25	2,69880E-38
Macro_FOLR2-APOE+	PNP	1,12929E-21	-1,09915E+00	0,219	0,265	2,07982E-17
Macro_FOLR2-APOE+	TUBB4B	2,61005E-11	-1,10155E+00	0,357	0,383	4,80694E-07
Macro_FOLR2-APOE+	EIF3D	9,72228E-08	-1,10209E+00	0,521	0,466	1,79055E-03
Macro_FOLR2-APOE+	TMA7	1,87874E-120	-1,10239E+00	0,791	0,794	3,46007E-116
Macro_FOLR2-APOE+	OST4	2,87255E-07	-1,10335E+00	0,822	0,77	5,29037E-03
Macro_FOLR2-APOE+	IFI35	3,83113E-21	-1,10423E+00	0,31	0,352	7,05579E-17

Macro_FOLR2-APOE+	ACTB	1,55072E-16	-1,10455E+00	0,999	0,99	2,85597E-12
Macro_FOLR2-APOE+	ZEB2	2,82979E-13	-1,10509E+00	0,624	0,602	5,21163E-09
Macro_FOLR2-APOE+	PAK1	9,28922E-39	-1,10874E+00	0,395	0,444	1,71080E-34
Macro_FOLR2-APOE+	ARPC1B	1,54797E-12	-1,10937E+00	0,9	0,78	2,85089E-08
Macro_FOLR2-APOE+	USP15	1,02244E-15	-1,11231E+00	0,399	0,429	1,88303E-11
Macro_FOLR2-APOE+	CDC42SE2	1,59943E-27	-1,11370E+00	0,226	0,282	2,94567E-23
Macro_FOLR2-APOE+	HBEGF	5,80726E-10	-1,11412E+00	0,261	0,278	1,06952E-05
Macro_FOLR2-APOE+	TNFSF13B	7,83402E-13	-1,11661E+00	0,615	0,573	1,44279E-08
Macro_FOLR2-APOE+	NAAA	2,26131E-25	-1,12277E+00	0,333	0,373	4,16466E-21
Macro_FOLR2-APOE+	PPIF	4,03930E-17	-1,12333E+00	0,334	0,362	7,43919E-13
Macro_FOLR2-APOE+	PRKAR1A	1,18217E-10	-1,12639E+00	0,468	0,482	2,17720E-06
Macro_FOLR2-APOE+	COX6A1	1,03435E-34	-1,12826E+00	0,692	0,678	1,90496E-30
Macro_FOLR2-APOE+	SNRPD2	1,81000E-08	-1,12848E+00	0,713	0,672	3,33348E-04
Macro_FOLR2-APOE+	PTPN2	2,00665E-32	-1,12877E+00	0,335	0,394	3,69564E-28
Macro_FOLR2-APOE+	WSB1	1,22253E-16	-1,12956E+00	0,655	0,638	2,25153E-12
Macro_FOLR2-APOE+	GAPT	7,33693E-11	-1,13000E+00	0,234	0,258	1,35124E-06
Macro_FOLR2-APOE+	IST1	3,67007E-14	-1,13074E+00	0,304	0,257	6,75916E-10
Macro_FOLR2-APOE+	EIF5A	6,80304E-07	-1,13078E+00	0,616	0,547	1,25292E-02
Macro_FOLR2-APOE+	GBP2	2,82960E-18	-1,13192E+00	0,355	0,376	5,21128E-14
Macro_FOLR2-APOE+	UBA52	2,04747E-56	-1,13279E+00	0,97	0,912	3,77082E-52
Macro_FOLR2-APOE+	FUS	2,96652E-20	-1,13356E+00	0,579	0,589	5,46344E-16
Macro_FOLR2-APOE+	MT-ATP6	1,77643E-150	-1,13550E+00	0,379	0,61	3,27165E-146
Macro_FOLR2-APOE+	HSP90B1	1,42871E-07	-1,13626E+00	0,785	0,686	2,63126E-03
Macro_FOLR2-APOE+	FLOT1	2,46868E-12	-1,13879E+00	0,521	0,525	4,54657E-08
Macro_FOLR2-APOE+	FOSL2	5,31470E-71	-1,14002E+00	0,218	0,308	9,78809E-67
Macro_FOLR2-APOE+	C18orf32	8,50114E-11	-1,14372E+00	0,254	0,284	1,56565E-06
Macro_FOLR2-APOE+	PTP4A1	1,14284E-15	-1,14764E+00	0,278	0,224	2,10478E-11
Macro_FOLR2-APOE+	ARPC2	6,17476E-33	-1,14798E+00	0,925	0,848	1,13721E-28
Macro_FOLR2-APOE+	TANK	4,00290E-12	-1,15071E+00	0,262	0,299	7,37214E-08
Macro_FOLR2-APOE+	MT-ND4	2,18977E-108	-1,15113E+00	0,38	0,606	4,03289E-104
Macro_FOLR2-APOE+	UBE2D3	6,90902E-07	-1,15229E+00	0,788	0,728	1,27243E-02
Macro_FOLR2-APOE+	CHP1	2,60220E-07	-1,15311E+00	0,267	0,233	4,79247E-03
Macro_FOLR2-APOE+	IVNS1ABP	1,17234E-65	-1,15602E+00	0,291	0,379	2,15909E-61
Macro_FOLR2-APOE+	UBC	9,11666E-155	-1,15829E+00	0,975	0,886	1,67901E-150
Macro_FOLR2-APOE+	TNFAIP2	3,18911E-44	-1,15962E+00	0,508	0,389	5,87338E-40
Macro_FOLR2-APOE+	DBNL	1,39977E-07	-1,16331E+00	0,543	0,484	2,57795E-03
Macro_FOLR2-APOE+	MT-ND3	6,81129E-110	-1,16490E+00	0,378	0,6	1,25443E-105
Macro_FOLR2-APOE+	OAZ1	1,18754E-63	-1,16674E+00	0,988	0,928	2,18709E-59
Macro_FOLR2-APOE+	MT-ND6	5,44412E-81	-1,16833E+00	0,212	0,317	1,00264E-76
Macro_FOLR2-APOE+	MBNL1	1,59935E-17	-1,17155E+00	0,517	0,526	2,94553E-13
Macro_FOLR2-APOE+	GK	2,59212E-71	-1,17229E+00	0,285	0,377	4,77390E-67
Macro_FOLR2-APOE+	CALM1	1,13421E-46	-1,17428E+00	0,889	0,78	2,08888E-42
Macro_FOLR2-APOE+	ZC3HAV1	2,71926E-27	-1,17474E+00	0,224	0,278	5,00805E-23
Macro_FOLR2-APOE+	HCST	1,13950E-125	-1,17477E+00	0,599	0,662	2,09862E-121
Macro_FOLR2-APOE+	PSMA5	4,37456E-10	-1,17976E+00	0,424	0,436	8,05662E-06
Macro_FOLR2-APOE+	SSR2	4,14597E-16	-1,18512E+00	0,734	0,639	7,63564E-12

Macro_FOLR2-APOE+	TPP1	2,18609E-196	-1,19274E+00	0,752	0,57	4,02612E-192
Macro_FOLR2-APOE+	MAPK1IP1L	4,15118E-08	-1,19453E+00	0,323	0,347	7,64523E-04
Macro_FOLR2-APOE+	EEF1D	1,99396E-09	-1,19740E+00	0,929	0,862	3,67228E-05
Macro_FOLR2-APOE+	ZFP36L1	3,04169E-37	-1,20133E+00	0,833	0,708	5,60188E-33
Macro_FOLR2-APOE+	SLC25A37	1,64142E-21	-1,20327E+00	0,262	0,296	3,02300E-17
Macro_FOLR2-APOE+	LDHB	3,89013E-11	-1,21275E+00	0,575	0,501	7,16446E-07
Macro_FOLR2-APOE+	EIF1	9,17918E-55	-1,21462E+00	0,979	0,938	1,69053E-50
Macro_FOLR2-APOE+	RAN	8,21867E-14	-1,21544E+00	0,748	0,643	1,51363E-09
Macro_FOLR2-APOE+	MYL6	9,36087E-10	-1,21560E+00	0,98	0,935	1,72399E-05
Macro_FOLR2-APOE+	GBP5	1,17458E-87	-1,21731E+00	0,161	0,26	2,16322E-83
Macro_FOLR2-APOE+	HLA-DMB	4,30080E-119	-1,22241E+00	0,812	0,611	7,92078E-115
Macro_FOLR2-APOE+	IFI6	1,89192E-107	-1,22250E+00	0,646	0,463	3,48434E-103
Macro_FOLR2-APOE+	MAP2K3	9,56394E-43	-1,22261E+00	0,209	0,28	1,76139E-38
Macro_FOLR2-APOE+	FDFT1	1,61469E-08	-1,22492E+00	0,237	0,264	2,97378E-04
Macro_FOLR2-APOE+	EIF4A2	5,05759E-15	-1,23163E+00	0,627	0,551	9,31456E-11
Macro_FOLR2-APOE+	FERMT3	3,72215E-10	-1,24150E+00	0,537	0,474	6,85509E-06
Macro_FOLR2-APOE+	SNAP23	3,24194E-13	-1,24461E+00	0,379	0,403	5,97068E-09
Macro_FOLR2-APOE+	EIF5	1,31721E-09	-1,24467E+00	0,66	0,581	2,42590E-05
Macro_FOLR2-APOE+	RPL22	4,21511E-250	-1,24491E+00	0,359	0,558	7,76297E-246
Macro_FOLR2-APOE+	TPM3	3,39597E-07	-1,25024E+00	0,87	0,792	6,25437E-03
Macro_FOLR2-APOE+	EIF4A1	3,99400E-83	-1,25160E+00	0,794	0,655	7,35575E-79
Macro_FOLR2-APOE+	PTPRC	6,28654E-49	-1,25287E+00	0,71	0,687	1,15779E-44
Macro_FOLR2-APOE+	NPM1	1,96549E-77	-1,25383E+00	0,889	0,746	3,61984E-73
Macro_FOLR2-APOE+	CD53	1,02504E-33	-1,25524E+00	0,792	0,66	1,88782E-29
Macro_FOLR2-APOE+	CPVL	3,66369E-34	-1,25623E+00	0,702	0,541	6,74743E-30
Macro_FOLR2-APOE+	MRPS6	1,29545E-25	-1,25707E+00	0,282	0,332	2,38583E-21
Macro_FOLR2-APOE+	CALM2	5,05061E-78	-1,25977E+00	0,904	0,762	9,30171E-74
Macro_FOLR2-APOE+	CHMP1B	9,67986E-44	-1,26174E+00	0,359	0,414	1,78274E-39
Macro_FOLR2-APOE+	ATP1B3	9,14987E-13	-1,26703E+00	0,614	0,533	1,68513E-08
Macro_FOLR2-APOE+	RPL37	1,14789E-287	-1,26978E+00	0,37	0,6	2,11407E-283
Macro_FOLR2-APOE+	KDM6B	2,31581E-178	-1,27170E+00	0,204	0,363	4,26504E-174
Macro_FOLR2-APOE+	MNDA	4,50663E-24	-1,27215E+00	0,548	0,531	8,29986E-20
Macro_FOLR2-APOE+	PRR13	6,44133E-29	-1,27311E+00	0,578	0,581	1,18630E-24
Macro_FOLR2-APOE+	FOSB	2,85066E-59	-1,27680E+00	0,569	0,581	5,25006E-55
Macro_FOLR2-APOE+	PTP4A2	4,85110E-16	-1,27701E+00	0,379	0,409	8,93428E-12
Macro_FOLR2-APOE+	ATG3	1,19796E-22	-1,28104E+00	0,516	0,524	2,20628E-18
Macro_FOLR2-APOE+	FKBP1A	2,35996E-28	-1,28663E+00	0,723	0,69	4,34633E-24
Macro_FOLR2-APOE+	UQCRC1	2,07729E-43	-1,28950E+00	0,585	0,493	3,82574E-39
Macro_FOLR2-APOE+	RPL36	7,88776E-196	-1,29384E+00	0,371	0,582	1,45269E-191
Macro_FOLR2-APOE+	NDUFA13	1,60335E-06	-1,30137E+00	0,43	0,432	2,95289E-02
Macro_FOLR2-APOE+	JMJD1C	9,55644E-07	-1,30215E+00	0,421	0,429	1,76001E-02
Macro_FOLR2-APOE+	ID2	2,36681E-06	-1,30577E+00	0,637	0,544	4,35895E-02
Macro_FOLR2-APOE+	DNAJB1	3,74060E-97	-1,30709E+00	0,683	0,537	6,88906E-93
Macro_FOLR2-APOE+	ARF1	1,90616E-16	-1,30795E+00	0,788	0,684	3,51058E-12
Macro_FOLR2-APOE+	CARD16	2,00797E-47	-1,31691E+00	0,536	0,554	3,69809E-43
Macro_FOLR2-APOE+	LIMD2	7,82483E-211	-1,31963E+00	0,348	0,504	1,44110E-206

Macro_FOLR2-APOE+	CD47	4,56999E-17	-1,32107E+00	0,399	0,425	8,41655E-13
Macro_FOLR2-APOE+	TRAPPC5	1,97182E-72	-1,32435E+00	0,159	0,251	3,63151E-68
Macro_FOLR2-APOE+	PET100	3,29024E-08	-1,32556E+00	0,387	0,408	6,05964E-04
Macro_FOLR2-APOE+	SRSF3	3,35120E-39	-1,32571E+00	0,549	0,574	6,17190E-35
Macro_FOLR2-APOE+	NACA	1,75727E-36	-1,33218E+00	0,97	0,911	3,23637E-32
Macro_FOLR2-APOE+	CCND3	4,85317E-14	-1,33380E+00	0,284	0,317	8,93809E-10
Macro_FOLR2-APOE+	DUSP1	2,26658E-16	-1,33540E+00	0,931	0,837	4,17437E-12
Macro_FOLR2-APOE+	HERPUD1	2,42407E-109	-1,34013E+00	0,824	0,658	4,46441E-105
Macro_FOLR2-APOE+	TPI1	2,53072E-18	-1,34073E+00	0,889	0,816	4,66082E-14
Macro_FOLR2-APOE+	CTSS	2,67687E-36	-1,34458E+00	0,942	0,796	4,93000E-32
Macro_FOLR2-APOE+	CFLAR	3,28049E-32	-1,34464E+00	0,489	0,516	6,04167E-28
Macro_FOLR2-APOE+	CCNL1	2,52558E-23	-1,34686E+00	0,508	0,528	4,65135E-19
Macro_FOLR2-APOE+	ANKRD10	6,51835E-47	-1,35080E+00	0,41	0,319	1,20048E-42
Macro_FOLR2-APOE+	PGAM1	7,21016E-17	-1,35527E+00	0,573	0,564	1,32790E-12
Macro_FOLR2-APOE+	TREM1	9,52334E-36	-1,36254E+00	0,309	0,226	1,75391E-31
Macro_FOLR2-APOE+	IER3	1,79683E-51	-1,36592E+00	0,503	0,537	3,30923E-47
Macro_FOLR2-APOE+	VAMP5	4,25729E-65	-1,36730E+00	0,432	0,488	7,84065E-61
Macro_FOLR2-APOE+	TRA2A	1,79463E-20	-1,36735E+00	0,216	0,262	3,30516E-16
Macro_FOLR2-APOE+	PPIA	9,61425E-14	-1,36819E+00	0,972	0,882	1,77066E-09
Macro_FOLR2-APOE+	MARCKSL1	2,21230E-200	-1,36872E+00	0,123	0,288	4,07439E-196
Macro_FOLR2-APOE+	JUNB	3,27938E-50	-1,37031E+00	0,871	0,819	6,03964E-46
Macro_FOLR2-APOE+	RPN1	4,33049E-75	-1,37312E+00	0,205	0,309	7,97547E-71
Macro_FOLR2-APOE+	STAT1	1,92802E-44	-1,37807E+00	0,494	0,522	3,55084E-40
Macro_FOLR2-APOE+	ATF3	1,31161E-24	-1,37811E+00	0,408	0,437	2,41560E-20
Macro_FOLR2-APOE+	PSMA4	1,92609E-18	-1,37826E+00	0,452	0,471	3,54729E-14
Macro_FOLR2-APOE+	GLIPR2	1,53770E-126	-1,37833E+00	0,266	0,395	2,83199E-122
Macro_FOLR2-APOE+	NAGK	1,71551E-15	-1,38190E+00	0,545	0,466	3,15945E-11
Macro_FOLR2-APOE+	MT-ND2	1,40976E-109	-1,38501E+00	0,377	0,592	2,59635E-105
Macro_FOLR2-APOE+	MX2	1,78427E-64	-1,39044E+00	0,195	0,282	3,28609E-60
Macro_FOLR2-APOE+	PRELID1	2,09690E-53	-1,39511E+00	0,705	0,681	3,86186E-49
Macro_FOLR2-APOE+	FLII	6,26066E-23	-1,40326E+00	0,378	0,315	1,15303E-18
Macro_FOLR2-APOE+	ACADVL	1,55062E-20	-1,40607E+00	0,529	0,459	2,85577E-16
Macro_FOLR2-APOE+	SOCS3	5,12762E-221	-1,40701E+00	0,201	0,383	9,44355E-217
Macro_FOLR2-APOE+	IER2	2,84263E-47	-1,40832E+00	0,624	0,633	5,23528E-43
Macro_FOLR2-APOE+	RGS1	1,15061E-235	-1,41337E+00	0,849	0,609	2,11908E-231
Macro_FOLR2-APOE+	PSMB10	2,52406E-11	-1,41874E+00	0,594	0,562	4,64857E-07
Macro_FOLR2-APOE+	CASP4	7,95585E-17	-1,41989E+00	0,426	0,446	1,46523E-12
Macro_FOLR2-APOE+	MT-CYB	5,54444E-174	-1,43142E+00	0,379	0,611	1,02112E-169
Macro_FOLR2-APOE+	HLA-F	1,08387E-19	-1,44410E+00	0,504	0,517	1,99617E-15
Macro_FOLR2-APOE+	HSPA1B	2,37703E-83	-1,44503E+00	0,65	0,481	4,37778E-79
Macro_FOLR2-APOE+	PSMB9	6,96101E-143	-1,44659E+00	0,636	0,661	1,28201E-138
Macro_FOLR2-APOE+	TGFBI	1,43074E-225	-1,44662E+00	0,787	0,568	2,63500E-221
Macro_FOLR2-APOE+	PPP1CB	3,19592E-08	-1,44871E+00	0,381	0,4	5,88593E-04
Macro_FOLR2-APOE+	B2M	6,41926E-44	-1,44895E+00	0,999	0,995	1,18224E-39
Macro_FOLR2-APOE+	MXD1	7,32337E-92	-1,45749E+00	0,209	0,317	1,34874E-87
Macro_FOLR2-APOE+	DUSP6	1,61071E-28	-1,46053E+00	0,247	0,295	2,96644E-24

Macro_FOLR2-APOE+	RAB24	9,85403E-96	-1,46446E+00	0,176	0,286	1,81482E-91
Macro_FOLR2-APOE+	RPL36AL	3,17805E-210	-1,46696E+00	0,34	0,518	5,85301E-206
Macro_FOLR2-APOE+	CSNK2B	2,62940E-52	-1,46908E+00	0,273	0,348	4,84257E-48
Macro_FOLR2-APOE+	BHLHE40	1,46440E-50	-1,46980E+00	0,284	0,349	2,69699E-46
Macro_FOLR2-APOE+	MT-CO1	8,01455E-169	-1,47489E+00	0,383	0,634	1,47604E-164
Macro_FOLR2-APOE+	SOD2	4,31644E-23	-1,47621E+00	0,683	0,646	7,94958E-19
Macro_FOLR2-APOE+	PLD4	1,50443E-53	-1,48104E+00	0,425	0,317	2,77071E-49
Macro_FOLR2-APOE+	BID	9,48349E-20	-1,48179E+00	0,388	0,419	1,74657E-15
Macro_FOLR2-APOE+	PDE4B	1,86782E-57	-1,48961E+00	0,3	0,378	3,43997E-53
Macro_FOLR2-APOE+	TRIM22	4,18992E-09	-1,49065E+00	0,406	0,419	7,71658E-05
Macro_FOLR2-APOE+	NME1-NME2	2,25049E-48	-1,50238E+00	0,328	0,225	4,14473E-44
Macro_FOLR2-APOE+	RPS2	2,25498E-246	-1,50293E+00	0,373	0,593	4,15300E-242
Macro_FOLR2-APOE+	LDHA	3,22975E-11	-1,50428E+00	0,806	0,699	5,94823E-07
Macro_FOLR2-APOE+	IRF1	2,87003E-177	-1,50436E+00	0,31	0,46	5,28573E-173
Macro_FOLR2-APOE+	SERPINF1	0,00000E+00	-1,50748E+00	0,652	0,364	0,00000E+00
Macro_FOLR2-APOE+	PLSCR1	8,30229E-28	-1,50760E+00	0,532	0,544	1,52903E-23
Macro_FOLR2-APOE+	SELPLG	1,30288E-13	-1,51187E+00	0,393	0,336	2,39951E-09
Macro_FOLR2-APOE+	RPN2	1,37981E-32	-1,51200E+00	0,228	0,294	2,54119E-28
Macro_FOLR2-APOE+	NLRP3	1,31193E-87	-1,51992E+00	0,176	0,277	2,41618E-83
Macro_FOLR2-APOE+	PTPRE	1,12154E-170	-1,52033E+00	0,314	0,46	2,06554E-166
Macro_FOLR2-APOE+	CDKN1A	1,58960E-67	-1,52633E+00	0,491	0,531	2,92756E-63
Macro_FOLR2-APOE+	RPS21	2,18734E-247	-1,53074E+00	0,366	0,572	4,02842E-243
Macro_FOLR2-APOE+	ALDOA	4,29694E-18	-1,53112E+00	0,831	0,748	7,91368E-14
Macro_FOLR2-APOE+	STK17B	2,66050E-190	-1,53527E+00	0,305	0,467	4,89985E-186
Macro_FOLR2-APOE+	LYZ	4,03298E-11	-1,53741E+00	0,958	0,784	7,42754E-07
Macro_FOLR2-APOE+	CKS2	1,17333E-59	-1,54026E+00	0,173	0,255	2,16093E-55
Macro_FOLR2-APOE+	INSIG1	5,90460E-47	-1,54512E+00	0,232	0,305	1,08745E-42
Macro_FOLR2-APOE+	CNN2	4,80527E-134	-1,54752E+00	0,291	0,42	8,84987E-130
Macro_FOLR2-APOE+	CD44	1,31335E-35	-1,55318E+00	0,697	0,673	2,41880E-31
Macro_FOLR2-APOE+	CRIP1	1,47214E-233	-1,55385E+00	0,132	0,319	2,71124E-229
Macro_FOLR2-APOE+	HIGD1A	1,54988E-28	-1,56721E+00	0,27	0,207	2,85441E-24
Macro_FOLR2-APOE+	CD48	1,49205E-119	-1,57103E+00	0,393	0,493	2,74791E-115
Macro_FOLR2-APOE+	SLC25A3	6,66516E-15	-1,57370E+00	0,76	0,706	1,22752E-10
Macro_FOLR2-APOE+	TCF4	8,31099E-16	-1,57521E+00	0,326	0,265	1,53064E-11
Macro_FOLR2-APOE+	SRGN	2,41183E-138	-1,57943E+00	0,979	0,937	4,44188E-134
Macro_FOLR2-APOE+	RPS26	9,29242E-276	-1,58218E+00	0,35	0,553	1,71139E-271
Macro_FOLR2-APOE+	RPL35	5,30439E-157	-1,58613E+00	0,366	0,558	9,76909E-153
Macro_FOLR2-APOE+	IGFLR1	3,95792E-25	-1,58661E+00	0,317	0,366	7,28929E-21
Macro_FOLR2-APOE+	STXBP2	3,02037E-124	-1,58707E+00	0,319	0,436	5,56262E-120
Macro_FOLR2-APOE+	LAP3	2,30972E-49	-1,59831E+00	0,588	0,585	4,25381E-45
Macro_FOLR2-APOE+	SEC61B	1,65648E-39	-1,60051E+00	0,78	0,734	3,05074E-35
Macro_FOLR2-APOE+	SPCS1	2,46991E-11	-1,60217E+00	0,691	0,594	4,54883E-07
Macro_FOLR2-APOE+	C15orf48	4,57618E-89	-1,60799E+00	0,254	0,359	8,42796E-85
Macro_FOLR2-APOE+	ICAM1	3,61157E-114	-1,61012E+00	0,258	0,379	6,65142E-110
Macro_FOLR2-APOE+	MPEG1	1,98046E-14	-1,61032E+00	0,362	0,375	3,64742E-10
Macro_FOLR2-APOE+	TAP1	2,95734E-143	-1,61810E+00	0,315	0,44	5,44654E-139

Macro_FOLR2-APOE+	CIITA	4,60393E-19	-1,62428E+00	0,289	0,33	8,47905E-15
Macro_FOLR2-APOE+	MYADM	7,78488E-113	-1,64014E+00	0,229	0,351	1,43374E-108
Macro_FOLR2-APOE+	RPL27A	1,51624E-65	-1,64153E+00	0,348	0,483	2,79245E-61
Macro_FOLR2-APOE+	HNRNPA1	1,05167E-26	-1,65250E+00	0,868	0,791	1,93685E-22
Macro_FOLR2-APOE+	MX1	3,71263E-09	-1,66278E+00	0,324	0,334	6,83755E-05
Macro_FOLR2-APOE+	TNFSF10	1,06026E-214	-1,66330E+00	0,212	0,384	1,95269E-210
Macro_FOLR2-APOE+	ZFP36	1,98174E-134	-1,67101E+00	0,875	0,839	3,64977E-130
Macro_FOLR2-APOE+	RPL12	3,02643E-259	-1,67263E+00	0,377	0,608	5,57378E-255
Macro_FOLR2-APOE+	PLP2	9,72791E-64	-1,68026E+00	0,341	0,415	1,79159E-59
Macro_FOLR2-APOE+	ARPC4	1,22843E-10	-1,68203E+00	0,401	0,411	2,26240E-06
Macro_FOLR2-APOE+	NR4A2	1,58506E-112	-1,68322E+00	0,37	0,464	2,91921E-108
Macro_FOLR2-APOE+	RPS29	2,39277E-107	-1,68546E+00	0,354	0,526	4,40676E-103
Macro_FOLR2-APOE+	LCP1	2,71208E-186	-1,68979E+00	0,657	0,711	4,99483E-182
Macro_FOLR2-APOE+	HSPA8	7,50882E-20	-1,69386E+00	0,887	0,809	1,38290E-15
Macro_FOLR2-APOE+	NR4A3	2,15150E-172	-1,69868E+00	0,166	0,318	3,96241E-168
Macro_FOLR2-APOE+	MT-ND1	2,13473E-132	-1,70747E+00	0,377	0,6	3,93153E-128
Macro_FOLR2-APOE+	RPLP1	0,00000E+00	-1,70946E+00	0,382	0,633	0,00000E+00
Macro_FOLR2-APOE+	RPS27A	0,00000E+00	-1,70956E+00	0,372	0,609	0,00000E+00
Macro_FOLR2-APOE+	RPL24	9,33476E-294	-1,71400E+00	0,361	0,567	1,71918E-289
Macro_FOLR2-APOE+	SUB1	4,81546E-227	-1,71551E+00	0,691	0,729	8,86863E-223
Macro_FOLR2-APOE+	RPL32	0,00000E+00	-1,71781E+00	0,376	0,612	0,00000E+00
Macro_FOLR2-APOE+	RPL39	1,47689E-302	-1,72683E+00	0,376	0,614	2,71999E-298
Macro_FOLR2-APOE+	HNRNPC	2,05601E-16	-1,72724E+00	0,668	0,644	3,78655E-12
Macro_FOLR2-APOE+	YPEL5	5,17035E-16	-1,73171E+00	0,419	0,437	9,52223E-12
Macro_FOLR2-APOE+	PSMB8	5,14919E-52	-1,74385E+00	0,567	0,578	9,48326E-48
Macro_FOLR2-APOE+	MYL12A	1,61039E-41	-1,76368E+00	0,883	0,815	2,96586E-37
Macro_FOLR2-APOE+	MT-ND5	3,92873E-171	-1,76457E+00	0,37	0,564	7,23554E-167
Macro_FOLR2-APOE+	CD52	2,65780E-48	-1,76538E+00	0,336	0,399	4,89487E-44
Macro_FOLR2-APOE+	RPL23	1,40641E-85	-1,76558E+00	0,329	0,473	2,59018E-81
Macro_FOLR2-APOE+	TAGLN2	4,06620E-75	-1,76610E+00	0,778	0,751	7,48872E-71
Macro_FOLR2-APOE+	MYD88	1,89722E-47	-1,76736E+00	0,294	0,368	3,49411E-43
Macro_FOLR2-APOE+	PLIN2	2,21248E-272	-1,77044E+00	0,717	0,462	4,07473E-268
Macro_FOLR2-APOE+	GBP1	1,09381E-113	-1,77151E+00	0,251	0,361	2,01446E-109
Macro_FOLR2-APOE+	RPL37A	4,02028E-150	-1,77372E+00	0,367	0,566	7,40415E-146
Macro_FOLR2-APOE+	RPL34	0,00000E+00	-1,78492E+00	0,372	0,609	0,00000E+00
Macro_FOLR2-APOE+	MT-ATP8	1,29118E-149	-1,78733E+00	0,235	0,408	2,37797E-145
Macro_FOLR2-APOE+	RPL14	4,49128E-283	-1,79935E+00	0,362	0,572	8,27159E-279
Macro_FOLR2-APOE+	BTG2	1,29139E-75	-1,80072E+00	0,452	0,506	2,37835E-71
Macro_FOLR2-APOE+	MT-CO2	5,73430E-257	-1,80216E+00	0,382	0,631	1,05609E-252
Macro_FOLR2-APOE+	RPS19	2,79058E-239	-1,80269E+00	0,379	0,607	5,13942E-235
Macro_FOLR2-APOE+	IL2RG	8,18129E-88	-1,80363E+00	0,235	0,339	1,50675E-83
Macro_FOLR2-APOE+	EGR1	2,10481E-97	-1,80809E+00	0,196	0,301	3,87643E-93
Macro_FOLR2-APOE+	RPS20	5,12845E-65	-1,81011E+00	0,336	0,47	9,44506E-61
Macro_FOLR2-APOE+	RPLP2	8,57144E-236	-1,81858E+00	0,376	0,602	1,57860E-231
Macro_FOLR2-APOE+	RPL28	0,00000E+00	-1,82001E+00	0,381	0,629	0,00000E+00
Macro_FOLR2-APOE+	PSMA6	1,92635E-86	-1,82409E+00	0,391	0,478	3,54776E-82

Macro_FOLR2-APOE+	RPL38	1,96854E-89	-1,82650E+00	0,354	0,515	3,62546E-85
Macro_FOLR2-APOE+	DNAJA1	1,60867E-63	-1,82871E+00	0,577	0,609	2,96269E-59
Macro_FOLR2-APOE+	CORO1A	1,13255E-54	-1,83050E+00	0,644	0,646	2,08582E-50
Macro_FOLR2-APOE+	YWHAZ	1,54674E-56	-1,83184E+00	0,729	0,71	2,84864E-52
Macro_FOLR2-APOE+	PPA1	2,33967E-208	-1,83955E+00	0,344	0,48	4,30897E-204
Macro_FOLR2-APOE+	RPS28	5,86040E-290	-1,86624E+00	0,378	0,615	1,07931E-285
Macro_FOLR2-APOE+	SRSF5	1,76536E-32	-1,86780E+00	0,563	0,582	3,25126E-28
Macro_FOLR2-APOE+	RPS7	0,00000E+00	-1,86854E+00	0,367	0,592	0,00000E+00
Macro_FOLR2-APOE+	RPS15	1,01740E-283	-1,87134E+00	0,376	0,606	1,87375E-279
Macro_FOLR2-APOE+	RPS12	0,00000E+00	-1,87204E+00	0,376	0,616	0,00000E+00
Macro_FOLR2-APOE+	CCL3	3,18942E-19	-1,87914E+00	0,464	0,361	5,87395E-15
Macro_FOLR2-APOE+	RAC2	1,87645E-129	-1,88115E+00	0,415	0,51	3,45586E-125
Macro_FOLR2-APOE+	NFKBIZ	8,75753E-211	-1,88225E+00	0,319	0,477	1,61288E-206
Macro_FOLR2-APOE+	NFKBIA	2,46902E-101	-1,88402E+00	0,845	0,802	4,54719E-97
Macro_FOLR2-APOE+	ISG20	3,22937E-195	-1,88736E+00	0,168	0,334	5,94754E-191
Macro_FOLR2-APOE+	PLEK	1,47528E-125	-1,88743E+00	0,492	0,573	2,71703E-121
Macro_FOLR2-APOE+	RBM39	1,99898E-41	-1,89058E+00	0,607	0,625	3,68152E-37
Macro_FOLR2-APOE+	RPL35A	0,00000E+00	-1,89227E+00	0,366	0,594	0,00000E+00
Macro_FOLR2-APOE+	PSME2	1,01922E-63	-1,89417E+00	0,76	0,708	1,87709E-59
Macro_FOLR2-APOE+	TUBA1A	1,96067E-46	-1,89925E+00	0,42	0,469	3,61096E-42
Macro_FOLR2-APOE+	GABARAP	3,12744E-283	-1,90189E+00	0,843	0,827	5,75980E-279
Macro_FOLR2-APOE+	RPS25	1,01710E-226	-1,90599E+00	0,366	0,577	1,87319E-222
Macro_FOLR2-APOE+	RPL21	9,27532E-185	-1,90841E+00	0,364	0,57	1,70824E-180
Macro_FOLR2-APOE+	RPL27	6,15845E-142	-1,91295E+00	0,358	0,529	1,13420E-137
Macro_FOLR2-APOE+	CLK1	1,03375E-07	-1,91921E+00	0,368	0,385	1,90386E-03
Macro_FOLR2-APOE+	RPS23	9,25223E-296	-1,93049E+00	0,372	0,6	1,70398E-291
Macro_FOLR2-APOE+	RPS15A	0,00000E+00	-1,93929E+00	0,373	0,601	0,00000E+00
Macro_FOLR2-APOE+	RPL29	0,00000E+00	-1,94089E+00	0,368	0,589	0,00000E+00
Macro_FOLR2-APOE+	CREM	2,24975E-15	-1,95096E+00	0,35	0,382	4,14337E-11
Macro_FOLR2-APOE+	RPL30	0,00000E+00	-1,96259E+00	0,373	0,614	0,00000E+00
Macro_FOLR2-APOE+	RPS4X	2,37385E-292	-1,96642E+00	0,37	0,597	4,37192E-288
Macro_FOLR2-APOE+	RPL18	0,00000E+00	-1,97553E+00	0,37	0,594	0,00000E+00
Macro_FOLR2-APOE+	CD55	2,56999E-139	-1,98499E+00	0,321	0,447	4,73315E-135
Macro_FOLR2-APOE+	PSME1	8,26103E-93	-1,99369E+00	0,789	0,748	1,52143E-88
Macro_FOLR2-APOE+	NAMPT	8,48165E-85	-2,00237E+00	0,536	0,58	1,56206E-80
Macro_FOLR2-APOE+	RPL41	2,30676E-268	-2,00929E+00	0,374	0,607	4,24836E-264
Macro_FOLR2-APOE+	RPL13A	5,96527E-129	-2,01989E+00	0,363	0,546	1,09862E-124
Macro_FOLR2-APOE+	RPS24	0,00000E+00	-2,02990E+00	0,374	0,611	0,00000E+00
Macro_FOLR2-APOE+	RPS14	0,00000E+00	-2,03724E+00	0,377	0,608	0,00000E+00
Macro_FOLR2-APOE+	TNFAIP3	6,70720E-114	-2,04022E+00	0,478	0,553	1,23526E-109
Macro_FOLR2-APOE+	RPL7A	0,00000E+00	-2,04023E+00	0,368	0,59	0,00000E+00
Macro_FOLR2-APOE+	PLAUR	2,48992E-17	-2,04938E+00	0,69	0,633	4,58568E-13
Macro_FOLR2-APOE+	RPS18	8,48750E-210	-2,05026E+00	0,375	0,59	1,56314E-205
Macro_FOLR2-APOE+	SERPINB9	8,39060E-150	-2,06490E+00	0,253	0,395	1,54530E-145
Macro_FOLR2-APOE+	IFITM3	1,44846E-159	-2,07119E+00	0,789	0,76	2,66762E-155
Macro_FOLR2-APOE+	RPL15	4,33074E-206	-2,07192E+00	0,37	0,583	7,97592E-202

Macro_FOLR2-APOE+	CD83	2,16932E-131	-2,09505E+00	0,404	0,502	3,99524E-127
Macro_FOLR2-APOE+	RPL13	3,42303E-290	-2,10548E+00	0,379	0,615	6,30419E-286
Macro_FOLR2-APOE+	RNASEK	2,74353E-133	-2,11666E+00	0,375	0,48	5,05275E-129
Macro_FOLR2-APOE+	RPL11	0,00000E+00	-2,12631E+00	0,374	0,614	0,00000E+00
Macro_FOLR2-APOE+	PPP1R15A	1,20351E-65	-2,12970E+00	0,65	0,659	2,21650E-61
Macro_FOLR2-APOE+	NR4A1	4,94208E-286	-2,13018E+00	0,243	0,449	9,10182E-282
Macro_FOLR2-APOE+	PABPC1	3,02066E-110	-2,14020E+00	0,94	0,885	5,56316E-106
Macro_FOLR2-APOE+	RPS9	4,58165E-260	-2,14264E+00	0,371	0,584	8,43803E-256
Macro_FOLR2-APOE+	RPS27	1,55713E-221	-2,16041E+00	0,372	0,598	2,86776E-217
Macro_FOLR2-APOE+	RPS6	1,67436E-206	-2,18174E+00	0,369	0,573	3,08367E-202
Macro_FOLR2-APOE+	RPL18A	0,00000E+00	-2,20018E+00	0,37	0,594	0,00000E+00
Macro_FOLR2-APOE+	RPS10	1,42132E-16	-2,22060E+00	0,236	0,291	2,61765E-12
Macro_FOLR2-APOE+	RPS16	5,44092E-221	-2,22644E+00	0,369	0,573	1,00205E-216
Macro_FOLR2-APOE+	DDIT4	1,59971E-12	-2,23737E+00	0,501	0,495	2,94618E-08
Macro_FOLR2-APOE+	RPSA	7,22558E-188	-2,24697E+00	0,348	0,519	1,33074E-183
Macro_FOLR2-APOE+	LGALS2	0,00000E+00	-2,25562E+00	0,062	0,289	0,00000E+00
Macro_FOLR2-APOE+	RPL8	0,00000E+00	-2,26106E+00	0,371	0,606	0,00000E+00
Macro_FOLR2-APOE+	RPL10	0,00000E+00	-2,26466E+00	0,38	0,629	0,00000E+00
Macro_FOLR2-APOE+	RPL9	9,36107E-199	-2,26547E+00	0,367	0,572	1,72403E-194
Macro_FOLR2-APOE+	RPS8	0,00000E+00	-2,28531E+00	0,374	0,611	0,00000E+00
Macro_FOLR2-APOE+	RPL19	0,00000E+00	-2,30803E+00	0,376	0,614	0,00000E+00
Macro_FOLR2-APOE+	RPL31	1,05462E-127	-2,33238E+00	0,349	0,521	1,94229E-123
Macro_FOLR2-APOE+	RPL26	4,30638E-260	-2,33942E+00	0,358	0,572	7,93106E-256
Macro_FOLR2-APOE+	IFI30	5,45298E-68	-2,35212E+00	0,553	0,571	1,00427E-63
Macro_FOLR2-APOE+	RPS13	0,00000E+00	-2,36410E+00	0,371	0,603	0,00000E+00
Macro_FOLR2-APOE+	RPL23A	6,22760E-199	-2,38193E+00	0,359	0,553	1,14694E-194
Macro_FOLR2-APOE+	RPS5	5,00187E-224	-2,38917E+00	0,355	0,544	9,21194E-220
Macro_FOLR2-APOE+	SLC2A3	5,60696E-228	-2,39430E+00	0,285	0,458	1,03263E-223
Macro_FOLR2-APOE+	RPS3	0,00000E+00	-2,40510E+00	0,37	0,6	0,00000E+00
Macro_FOLR2-APOE+	RPL5	4,18724E-251	-2,42538E+00	0,35	0,545	7,71163E-247
Macro_FOLR2-APOE+	RPL36A	1,90127E-187	-2,42935E+00	0,314	0,476	3,50158E-183
Macro_FOLR2-APOE+	IFITM1	6,52011E-291	-2,44638E+00	0,082	0,282	1,20081E-286
Macro_FOLR2-APOE+	RPS11	3,20794E-153	-2,45763E+00	0,354	0,528	5,90807E-149
Macro_FOLR2-APOE+	RPLP0	1,65198E-294	-2,46160E+00	0,365	0,572	3,04245E-290
Macro_FOLR2-APOE+	RPL6	0,00000E+00	-2,47449E+00	0,365	0,584	0,00000E+00
Macro_FOLR2-APOE+	RPL3	4,95839E-183	-2,47716E+00	0,365	0,56	9,13187E-179
Macro_FOLR2-APOE+	RPL10A	1,94114E-181	-2,52517E+00	0,347	0,521	3,57500E-177
Macro_FOLR2-APOE+	RPS3A	0,00000E+00	-2,53297E+00	0,369	0,599	0,00000E+00
Macro_FOLR2-APOE+	CXCR4	5,08358E-16	-2,54582E+00	0,682	0,564	9,36243E-12
Macro_FOLR2-APOE+	BCL2A1	0,00000E+00	-2,56492E+00	0,297	0,499	0,00000E+00
Macro_FOLR2-APOE+	ISG15	1,57992E-38	-2,60636E+00	0,393	0,433	2,90973E-34
Macro_FOLR2-APOE+	DUSP2	6,38494E-196	-2,61718E+00	0,161	0,324	1,17591E-191
Macro_FOLR2-APOE+	RPL7	1,35262E-147	-2,65382E+00	0,341	0,523	2,49113E-143
Macro_FOLR2-APOE+	S100A8	1,73276E-13	-2,73060E+00	0,354	0,352	3,19122E-09
Macro_FOLR2-APOE+	RGS2	2,42543E-80	-2,80936E+00	0,592	0,631	4,46692E-76
Macro_FOLR2-APOE+	IRF7	7,40178E-94	-2,82125E+00	0,32	0,412	1,36319E-89

Macro_FOLR2-APOE+	MT-ND4L	4,16729E-246	-2,94776E+00	0,338	0,543	7,67489E-242
Macro_FOLR2-APOE+	IL1B	2,19218E-156	-3,04870E+00	0,217	0,354	4,03734E-152
Macro_FOLR2-APOE+	IFITM2	0,00000E+00	-3,05171E+00	0,523	0,667	0,00000E+00
Macro_FOLR2-APOE+	TIMP1	9,91182E-224	-3,06741E+00	0,579	0,662	1,82546E-219
Macro_FOLR2-APOE+	RPL4	2,79135E-180	-3,10546E+00	0,338	0,5	5,14083E-176
Macro_FOLR2-APOE+	AREG	5,63980E-297	-3,20950E+00	0,099	0,304	1,03868E-292
Macro_FOLR2-APOE+	GPR183	2,67581E-09	-3,32418E+00	0,468	0,455	4,92803E-05
Macro_FOLR2-APOE+	RPL17	1,28035E-213	-3,37086E+00	0,285	0,457	2,35802E-209
Macro_FOLR2-APOE+	EEF1G	5,88469E-207	-3,42459E+00	0,153	0,319	1,08378E-202
Macro_FOLR2-APOE+	FCN1	1,30417E-302	-3,70120E+00	0,078	0,279	2,40189E-298

Macro_FOLR2+APOE-

cluster	gene	p_val	avg_log2FC	pct.1	pct.2	p_val_adj
Macro_FOLR2+APOE-	SELENOP	3,94969E-218	3,31749E+00	0,46	0,088	7,27414E-214
Macro_FOLR2+APOE-	SLC40A1	6,25818E-106	2,18039E+00	0,548	0,227	1,15257E-101
Macro_FOLR2+APOE-	FOLR2	3,29429E-98	1,50015E+00	0,476	0,177	6,06709E-94
Macro_FOLR2+APOE-	F13A1	1,23807E-68	1,40619E+00	0,484	0,229	2,28016E-64
Macro_FOLR2+APOE-	PLTP	5,79580E-71	1,37689E+00	0,586	0,326	1,06741E-66
Macro_FOLR2+APOE-	RNASE1	8,08054E-102	1,26443E+00	0,631	0,268	1,48819E-97
Macro_FOLR2+APOE-	PDK4	1,92514E-17	1,21692E+00	0,414	0,309	3,54553E-13
Macro_FOLR2+APOE-	DAB2	4,46052E-25	1,07313E+00	0,524	0,433	8,21495E-21
Macro_FOLR2+APOE-	C1QC	2,60084E-118	1,06204E+00	0,951	0,541	4,78996E-114
Macro_FOLR2+APOE-	C1QA	1,28277E-116	1,00720E+00	0,953	0,562	2,36248E-112
Macro_FOLR2+APOE-	GPR34	2,86889E-13	9,07567E-01	0,38	0,305	5,28364E-09
Macro_FOLR2+APOE-	A2M	2,18030E-16	8,80506E-01	0,475	0,388	4,01546E-12
Macro_FOLR2+APOE-	STAB1	2,45092E-24	8,54804E-01	0,463	0,344	4,51386E-20
Macro_FOLR2+APOE-	C1QB	1,58682E-99	7,68041E-01	0,953	0,541	2,92244E-95
Macro_FOLR2+APOE-	MS4A4A	6,63925E-27	7,60491E-01	0,524	0,419	1,22275E-22
Macro_FOLR2+APOE-	SLCO2B1	2,24307E-10	7,56353E-01	0,409	0,369	4,13107E-06
Macro_FOLR2+APOE-	TMEM176A	8,51062E-13	5,91255E-01	0,503	0,448	1,56740E-08
Macro_FOLR2+APOE-	ITM2B	6,43971E-47	4,94794E-01	0,874	0,907	1,18600E-42
Macro_FOLR2+APOE-	MS4A7	3,82346E-27	3,70099E-01	0,691	0,662	7,04167E-23
Macro_FOLR2+APOE-	TMEM176B	4,19602E-13	3,34973E-01	0,574	0,534	7,72781E-09
Macro_FOLR2+APOE-	LAMP1	2,89002E-10	3,24303E-01	0,203	0,399	5,32254E-06
Macro_FOLR2+APOE-	SELENOT	5,53232E-13	3,12513E-01	0,094	0,253	1,01889E-08
Macro_FOLR2+APOE-	ATP5ME	4,43622E-14	3,12391E-01	0,124	0,311	8,17019E-10
Macro_FOLR2+APOE-	SPOP	1,07400E-13	2,92631E-01	0,102	0,269	1,97799E-09
Macro_FOLR2+APOE-	GNPDA1	6,40657E-07	2,86676E-01	0,136	0,26	1,17990E-02
Macro_FOLR2+APOE-	RAB4A	1,29245E-09	2,66220E-01	0,121	0,264	2,38030E-05
Macro_FOLR2+APOE-	EPN1	5,93317E-11	2,51087E-01	0,171	0,361	1,09271E-06
Macro_FOLR2+APOE-	HLA-DRA	6,55075E-29	-2,51864E-01	0,974	0,924	1,20645E-24
Macro_FOLR2+APOE-	SCARB2	1,79822E-10	-2,54500E-01	0,186	0,374	3,31178E-06
Macro_FOLR2+APOE-	PPP6C	4,78633E-20	-2,54794E-01	0,073	0,268	8,81498E-16
Macro_FOLR2+APOE-	PEPD	3,79490E-12	-2,56332E-01	0,169	0,365	6,98906E-08
Macro_FOLR2+APOE-	HNRNPA0	6,94217E-29	-2,57864E-01	0,19	0,513	1,27854E-24
Macro_FOLR2+APOE-	GNPTAB	1,43556E-14	-2,57945E-01	0,107	0,281	2,64386E-10
Macro_FOLR2+APOE-	LY86	3,54229E-08	-2,58308E-01	0,282	0,492	6,52383E-04
Macro_FOLR2+APOE-	CD163	2,73605E-11	-2,59040E-01	0,48	0,447	5,03898E-07
Macro_FOLR2+APOE-	BAG1	3,10144E-20	-2,60062E-01	0,141	0,377	5,71192E-16
Macro_FOLR2+APOE-	LHFPL2	2,86989E-18	-2,61707E-01	0,092	0,286	5,28547E-14
Macro_FOLR2+APOE-	VPS13C	1,96702E-16	-2,62140E-01	0,154	0,373	3,62266E-12
Macro_FOLR2+APOE-	STX12	2,51958E-12	-2,62291E-01	0,137	0,314	4,64031E-08
Macro_FOLR2+APOE-	TXNL4A	2,54981E-20	-2,62736E-01	0,079	0,282	4,69599E-16
Macro_FOLR2+APOE-	TIMM10	2,55574E-21	-2,62881E-01	0,06	0,255	4,70691E-17
Macro_FOLR2+APOE-	BAZ2B	1,28149E-13	-2,63137E-01	0,1	0,263	2,36013E-09
Macro_FOLR2+APOE-	PYURF	1,55604E-21	-2,63525E-01	0,154	0,413	2,86576E-17
Macro_FOLR2+APOE-	CCNI	2,04193E-09	-2,63566E-01	0,488	0,789	3,76062E-05
Macro_FOLR2+APOE-	ACIN1	2,50714E-17	-2,64173E-01	0,092	0,279	4,61740E-13
Macro_FOLR2+APOE-	SNX17	2,67140E-20	-2,64516E-01	0,173	0,435	4,91991E-16

Macro_FOLR2+APOE-	COA3	1,10683E-16	-2,65183E-01	0,119	0,322	2,03846E-12
Macro_FOLR2+APOE-	SPI1	8,64315E-21	-2,65788E-01	0,465	0,77	1,59181E-16
Macro_FOLR2+APOE-	BLOC1S6	4,84162E-16	-2,66437E-01	0,102	0,286	8,91682E-12
Macro_FOLR2+APOE-	TMEM243	1,27695E-09	-2,67491E-01	0,126	0,271	2,35177E-05
Macro_FOLR2+APOE-	C4orf3	2,16636E-28	-2,68117E-01	0,294	0,675	3,98978E-24
Macro_FOLR2+APOE-	PTPN2	6,17312E-28	-2,68383E-01	0,117	0,389	1,13690E-23
Macro_FOLR2+APOE-	DICER1	6,40607E-15	-2,68906E-01	0,1	0,272	1,17981E-10
Macro_FOLR2+APOE-	TLN1	1,44253E-21	-2,68938E-01	0,252	0,559	2,65671E-17
Macro_FOLR2+APOE-	TNRC6B	2,74294E-12	-2,69825E-01	0,113	0,274	5,05166E-08
Macro_FOLR2+APOE-	ATF6B	1,87969E-13	-2,70082E-01	0,117	0,289	3,46183E-09
Macro_FOLR2+APOE-	ANXA7	1,84224E-24	-2,70242E-01	0,119	0,375	3,39285E-20
Macro_FOLR2+APOE-	PPP4R2	3,42353E-19	-2,71241E-01	0,079	0,27	6,30512E-15
Macro_FOLR2+APOE-	RUNX1	5,10838E-15	-2,71864E-01	0,104	0,28	9,40811E-11
Macro_FOLR2+APOE-	SNRPG	1,44000E-31	-2,73860E-01	0,211	0,561	2,65205E-27
Macro_FOLR2+APOE-	MRPS18B	4,94844E-11	-2,73978E-01	0,122	0,279	9,11354E-07
Macro_FOLR2+APOE-	RSF1	8,88300E-15	-2,74008E-01	0,145	0,345	1,63598E-10
Macro_FOLR2+APOE-	ANKRD12	2,13890E-18	-2,74547E-01	0,209	0,473	3,93921E-14
Macro_FOLR2+APOE-	MRPS16	6,27946E-18	-2,75027E-01	0,102	0,303	1,15649E-13
Macro_FOLR2+APOE-	BCAP31	4,56388E-11	-2,76423E-01	0,318	0,587	8,40530E-07
Macro_FOLR2+APOE-	DPM3	5,39574E-19	-2,80206E-01	0,089	0,287	9,93734E-15
Macro_FOLR2+APOE-	NAA38	7,99756E-18	-2,80366E-01	0,175	0,419	1,47291E-13
Macro_FOLR2+APOE-	LASP1	9,37915E-20	-2,80958E-01	0,1	0,312	1,72736E-15
Macro_FOLR2+APOE-	SF3B2	1,21387E-18	-2,81000E-01	0,232	0,519	2,23558E-14
Macro_FOLR2+APOE-	SPG21	1,63566E-25	-2,81327E-01	0,156	0,441	3,01240E-21
Macro_FOLR2+APOE-	TMEM141	8,22459E-17	-2,82412E-01	0,083	0,263	1,51472E-12
Macro_FOLR2+APOE-	UBE2D2	6,51724E-25	-2,83030E-01	0,235	0,569	1,20028E-20
Macro_FOLR2+APOE-	CELF1	1,92942E-14	-2,83412E-01	0,111	0,287	3,55341E-10
Macro_FOLR2+APOE-	PCBP2	2,87885E-08	-2,86435E-01	0,499	0,801	5,30198E-04
Macro_FOLR2+APOE-	PLXDC2	3,41627E-10	-2,86588E-01	0,315	0,547	6,29175E-06
Macro_FOLR2+APOE-	SH3BP1	1,57461E-22	-2,87622E-01	0,055	0,252	2,89996E-18
Macro_FOLR2+APOE-	MRPS21	1,29344E-21	-2,88033E-01	0,226	0,531	2,38212E-17
Macro_FOLR2+APOE-	HSD17B11	5,58575E-12	-2,88789E-01	0,198	0,406	1,02873E-07
Macro_FOLR2+APOE-	DDX18	2,93333E-21	-2,90293E-01	0,132	0,373	5,40231E-17
Macro_FOLR2+APOE-	ARMCX3	1,66675E-12	-2,90708E-01	0,104	0,26	3,06965E-08
Macro_FOLR2+APOE-	CSRP1	7,24465E-12	-2,91219E-01	0,105	0,26	1,33425E-07
Macro_FOLR2+APOE-	YWHAE	9,20400E-16	-2,91419E-01	0,282	0,584	1,69510E-11
Macro_FOLR2+APOE-	NDUFB7	2,03133E-16	-2,91488E-01	0,25	0,533	3,74110E-12
Macro_FOLR2+APOE-	TMEM9B	1,27873E-11	-2,91520E-01	0,177	0,375	2,35504E-07
Macro_FOLR2+APOE-	CLTB	1,36351E-21	-2,91609E-01	0,124	0,363	2,51117E-17
Macro_FOLR2+APOE-	ARF6	5,33131E-26	-2,91735E-01	0,143	0,422	9,81867E-22
Macro_FOLR2+APOE-	PHIP	9,43170E-21	-2,92114E-01	0,111	0,331	1,73704E-16
Macro_FOLR2+APOE-	HSF1	4,84078E-17	-2,92930E-01	0,09	0,275	8,91526E-13
Macro_FOLR2+APOE-	NDUFB9	6,11389E-22	-2,93312E-01	0,241	0,554	1,12599E-17
Macro_FOLR2+APOE-	GLUD1	1,45421E-16	-2,93629E-01	0,128	0,33	2,67821E-12
Macro_FOLR2+APOE-	SMDT1	2,10147E-27	-2,93805E-01	0,175	0,486	3,87027E-23
Macro_FOLR2+APOE-	ARID1A	1,25804E-17	-2,94259E-01	0,087	0,27	2,31693E-13
Macro_FOLR2+APOE-	SEC31A	3,90322E-17	-2,94304E-01	0,105	0,301	7,18855E-13

Macro_FOLR2+APOE-	DDRGK1	7,13501E-21	-2,94512E-01	0,102	0,324	1,31405E-16
Macro_FOLR2+APOE-	FIBP	8,92378E-18	-2,94832E-01	0,145	0,37	1,64349E-13
Macro_FOLR2+APOE-	UQCC2	3,78612E-18	-2,94848E-01	0,107	0,313	6,97291E-14
Macro_FOLR2+APOE-	DHX36	1,40029E-21	-2,95708E-01	0,079	0,288	2,57892E-17
Macro_FOLR2+APOE-	MAP3K2	1,74832E-21	-2,96375E-01	0,096	0,316	3,21988E-17
Macro_FOLR2+APOE-	ARPC5L	2,31218E-18	-2,96616E-01	0,083	0,273	4,25834E-14
Macro_FOLR2+APOE-	COMMD7	2,19025E-21	-2,97227E-01	0,096	0,314	4,03377E-17
Macro_FOLR2+APOE-	PLIN3	2,48619E-28	-2,97319E-01	0,105	0,375	4,57882E-24
Macro_FOLR2+APOE-	TECR	2,23791E-11	-2,98700E-01	0,185	0,381	4,12157E-07
Macro_FOLR2+APOE-	MRPS34	1,97284E-17	-2,99184E-01	0,164	0,397	3,63337E-13
Macro_FOLR2+APOE-	APBB1IP	3,13144E-24	-2,99450E-01	0,192	0,482	5,76717E-20
Macro_FOLR2+APOE-	LENG8	2,94208E-14	-3,03471E-01	0,113	0,286	5,41843E-10
Macro_FOLR2+APOE-	RSL24D1	1,56399E-25	-3,03767E-01	0,153	0,437	2,88040E-21
Macro_FOLR2+APOE-	DPYD	2,99413E-18	-3,04144E-01	0,143	0,368	5,51428E-14
Macro_FOLR2+APOE-	NDUFS3	4,18746E-10	-3,04674E-01	0,166	0,342	7,71204E-06
Macro_FOLR2+APOE-	TPR	2,42473E-20	-3,05670E-01	0,147	0,392	4,46563E-16
Macro_FOLR2+APOE-	MRPL20	8,95541E-18	-3,06413E-01	0,205	0,471	1,64932E-13
Macro_FOLR2+APOE-	MYCBP2	2,48688E-17	-3,06552E-01	0,128	0,339	4,58009E-13
Macro_FOLR2+APOE-	PABPN1	4,63190E-19	-3,06622E-01	0,09	0,289	8,53057E-15
Macro_FOLR2+APOE-	TADA3	4,58098E-21	-3,06888E-01	0,09	0,304	8,43680E-17
Macro_FOLR2+APOE-	TTC1	3,52751E-11	-3,07071E-01	0,105	0,252	6,49662E-07
Macro_FOLR2+APOE-	NUCB1	1,25185E-12	-3,07220E-01	0,275	0,546	2,30554E-08
Macro_FOLR2+APOE-	PHF20L1	6,76010E-20	-3,08324E-01	0,064	0,25	1,24501E-15
Macro_FOLR2+APOE-	PPCS	2,82612E-17	-3,08535E-01	0,162	0,393	5,20487E-13
Macro_FOLR2+APOE-	DSE	6,18172E-26	-3,09728E-01	0,16	0,442	1,13849E-21
Macro_FOLR2+APOE-	DDT	1,30421E-22	-3,10312E-01	0,169	0,443	2,40197E-18
Macro_FOLR2+APOE-	AP2M1	2,51040E-11	-3,10484E-01	0,307	0,586	4,62341E-07
Macro_FOLR2+APOE-	RNMT	1,83934E-17	-3,10945E-01	0,073	0,25	3,38752E-13
Macro_FOLR2+APOE-	G3BP1	1,54262E-18	-3,12383E-01	0,105	0,31	2,84104E-14
Macro_FOLR2+APOE-	ELF2	4,66318E-15	-3,12835E-01	0,085	0,25	8,58818E-11
Macro_FOLR2+APOE-	RNASEH2B	8,78393E-14	-3,12908E-01	0,124	0,304	1,61774E-09
Macro_FOLR2+APOE-	PSMC6	8,64706E-15	-3,13558E-01	0,089	0,256	1,59253E-10
Macro_FOLR2+APOE-	RAB2A	2,76211E-15	-3,15900E-01	0,228	0,489	5,08698E-11
Macro_FOLR2+APOE-	MIDN	2,24111E-24	-3,17093E-01	0,068	0,284	4,12745E-20
Macro_FOLR2+APOE-	PAFAH1B1	7,77192E-19	-3,17131E-01	0,124	0,344	1,43135E-14
Macro_FOLR2+APOE-	DUSP3	2,30806E-17	-3,17160E-01	0,083	0,265	4,25076E-13
Macro_FOLR2+APOE-	PTAFR	5,65540E-20	-3,17257E-01	0,145	0,378	1,04156E-15
Macro_FOLR2+APOE-	TFG	4,40572E-18	-3,17528E-01	0,079	0,264	8,11402E-14
Macro_FOLR2+APOE-	AHNAK	3,05678E-13	-3,17955E-01	0,339	0,606	5,62967E-09
Macro_FOLR2+APOE-	TCEA1	1,86928E-24	-3,18540E-01	0,185	0,477	3,44265E-20
Macro_FOLR2+APOE-	SERINC3	4,75877E-22	-3,18677E-01	0,111	0,343	8,76423E-18
Macro_FOLR2+APOE-	JTB	6,01583E-21	-3,18845E-01	0,25	0,564	1,10794E-16
Macro_FOLR2+APOE-	CLEC2B	9,16991E-19	-3,19511E-01	0,209	0,473	1,68882E-14
Macro_FOLR2+APOE-	SUMO3	4,53526E-23	-3,20455E-01	0,196	0,484	8,35259E-19
Macro_FOLR2+APOE-	LARP7	2,95503E-13	-3,20866E-01	0,109	0,278	5,44228E-09
Macro_FOLR2+APOE-	GRINA	1,69034E-23	-3,21315E-01	0,341	0,687	3,11310E-19
Macro_FOLR2+APOE-	TMEM123	1,24722E-12	-3,22321E-01	0,224	0,457	2,29701E-08

Macro_FOLR2+APOE-	YWHAG	2,13676E-25	-3,22448E-01	0,085	0,32	3,93527E-21
Macro_FOLR2+APOE-	PHF5A	1,19899E-15	-3,23904E-01	0,087	0,26	2,20818E-11
Macro_FOLR2+APOE-	ST13	1,91831E-13	-3,24683E-01	0,315	0,609	3,53296E-09
Macro_FOLR2+APOE-	DEGS1	1,42421E-15	-3,25528E-01	0,132	0,328	2,62297E-11
Macro_FOLR2+APOE-	DCAF7	8,15216E-24	-3,25659E-01	0,087	0,315	1,50138E-19
Macro_FOLR2+APOE-	FGD2	3,62950E-07	-3,25850E-01	0,194	0,346	6,68446E-03
Macro_FOLR2+APOE-	MTDH	2,13062E-21	-3,25997E-01	0,352	0,698	3,92396E-17
Macro_FOLR2+APOE-	SZRD1	7,53008E-23	-3,26115E-01	0,079	0,296	1,38682E-18
Macro_FOLR2+APOE-	CIR1	1,04902E-15	-3,26220E-01	0,1	0,279	1,93199E-11
Macro_FOLR2+APOE-	SCAMP2	1,04444E-07	-3,26422E-01	0,282	0,506	1,92355E-03
Macro_FOLR2+APOE-	UQCRQ	5,69637E-15	-3,27198E-01	0,345	0,669	1,04910E-10
Macro_FOLR2+APOE-	MRPS18C	6,03794E-17	-3,28233E-01	0,105	0,304	1,11201E-12
Macro_FOLR2+APOE-	TOMM22	6,93279E-21	-3,28376E-01	0,168	0,427	1,27681E-16
Macro_FOLR2+APOE-	ENG	3,12308E-07	-3,28581E-01	0,198	0,349	5,75178E-03
Macro_FOLR2+APOE-	MEA1	1,59361E-22	-3,28803E-01	0,137	0,393	2,93495E-18
Macro_FOLR2+APOE-	G6PD	8,49239E-22	-3,29730E-01	0,064	0,263	1,56404E-17
Macro_FOLR2+APOE-	KMT2C	1,43213E-20	-3,30277E-01	0,111	0,333	2,63755E-16
Macro_FOLR2+APOE-	TNFAIP8L2	3,18289E-12	-3,30384E-01	0,105	0,262	5,86194E-08
Macro_FOLR2+APOE-	CRTAP	5,86241E-09	-3,31949E-01	0,296	0,524	1,07968E-04
Macro_FOLR2+APOE-	SMCHD1	4,63918E-21	-3,33175E-01	0,105	0,327	8,54398E-17
Macro_FOLR2+APOE-	FUCA2	9,99645E-15	-3,33497E-01	0,124	0,31	1,84105E-10
Macro_FOLR2+APOE-	KRAS	1,18485E-24	-3,33692E-01	0,089	0,323	2,18213E-20
Macro_FOLR2+APOE-	LSM6	1,02289E-16	-3,34139E-01	0,139	0,353	1,88385E-12
Macro_FOLR2+APOE-	ZCRB1	1,01540E-20	-3,34270E-01	0,075	0,274	1,87007E-16
Macro_FOLR2+APOE-	TTC3	7,51251E-13	-3,35117E-01	0,145	0,33	1,38358E-08
Macro_FOLR2+APOE-	GADD45GIP1	1,03082E-20	-3,35390E-01	0,175	0,441	1,89847E-16
Macro_FOLR2+APOE-	MRPL40	1,28323E-18	-3,35718E-01	0,083	0,274	2,36333E-14
Macro_FOLR2+APOE-	U2SURP	1,42934E-15	-3,36538E-01	0,132	0,329	2,63242E-11
Macro_FOLR2+APOE-	BLOC1S1	2,15117E-17	-3,37366E-01	0,217	0,475	3,96182E-13
Macro_FOLR2+APOE-	NUDT1	1,44868E-13	-3,38334E-01	0,1	0,264	2,66804E-09
Macro_FOLR2+APOE-	PPDPF	3,90810E-28	-3,38469E-01	0,405	0,772	7,19754E-24
Macro_FOLR2+APOE-	PIN1	2,29096E-18	-3,39180E-01	0,128	0,349	4,21927E-14
Macro_FOLR2+APOE-	SMCO4	3,98371E-38	-3,41314E-01	0,102	0,422	7,33680E-34
Macro_FOLR2+APOE-	MAX	1,57764E-16	-3,41792E-01	0,168	0,399	2,90554E-12
Macro_FOLR2+APOE-	PACSIN2	2,56811E-19	-3,42492E-01	0,079	0,272	4,72970E-15
Macro_FOLR2+APOE-	TNPO1	8,51620E-15	-3,42980E-01	0,087	0,252	1,56843E-10
Macro_FOLR2+APOE-	PSTPIP2	1,09742E-16	-3,43256E-01	0,081	0,253	2,02112E-12
Macro_FOLR2+APOE-	APLP2	1,35484E-08	-3,43329E-01	0,354	0,598	2,49522E-04
Macro_FOLR2+APOE-	DEK	6,98341E-24	-3,43884E-01	0,252	0,578	1,28613E-19
Macro_FOLR2+APOE-	TGFB1	8,45145E-25	-3,44137E-01	0,177	0,456	1,55650E-20
Macro_FOLR2+APOE-	IMP3	8,31632E-15	-3,44280E-01	0,126	0,317	1,53162E-10
Macro_FOLR2+APOE-	COX16	1,17685E-25	-3,44442E-01	0,1	0,349	2,16741E-21
Macro_FOLR2+APOE-	NCOR2	8,15040E-17	-3,45647E-01	0,09	0,272	1,50106E-12
Macro_FOLR2+APOE-	APMAP	2,01038E-07	-3,46100E-01	0,16	0,302	3,70252E-03
Macro_FOLR2+APOE-	SNRPA	6,00984E-14	-3,46780E-01	0,119	0,298	1,10683E-09
Macro_FOLR2+APOE-	SNRNP200	4,12593E-13	-3,46950E-01	0,102	0,26	7,59873E-09
Macro_FOLR2+APOE-	NCOR1	3,52263E-22	-3,47173E-01	0,147	0,402	6,48763E-18

Macro_FOLR2+APOE-	ATRX	2,02139E-18	-3,47639E-01	0,156	0,392	3,72279E-14
Macro_FOLR2+APOE-	ZNHIT1	6,61654E-27	-3,48966E-01	0,171	0,482	1,21857E-22
Macro_FOLR2+APOE-	SUMO1	4,87829E-24	-3,48994E-01	0,177	0,472	8,98434E-20
Macro_FOLR2+APOE-	ARHGDIA	2,00423E-31	-3,49183E-01	0,239	0,605	3,69118E-27
Macro_FOLR2+APOE-	PLEKHO1	1,08892E-16	-3,49420E-01	0,149	0,363	2,00546E-12
Macro_FOLR2+APOE-	NOSIP	5,11479E-15	-3,49523E-01	0,132	0,33	9,41990E-11
Macro_FOLR2+APOE-	AP2S1	9,42584E-07	-3,50041E-01	0,439	0,723	1,73596E-02
Macro_FOLR2+APOE-	TMSB4X	4,37866E-15	-3,50075E-01	0,994	0,984	8,06418E-11
Macro_FOLR2+APOE-	SNX3	5,27303E-11	-3,50126E-01	0,394	0,693	9,71133E-07
Macro_FOLR2+APOE-	RNF167	1,34405E-16	-3,50223E-01	0,105	0,296	2,47533E-12
Macro_FOLR2+APOE-	MGAT4A	1,29783E-11	-3,50751E-01	0,169	0,359	2,39021E-07
Macro_FOLR2+APOE-	DPYSL2	5,03697E-11	-3,51114E-01	0,153	0,325	9,27659E-07
Macro_FOLR2+APOE-	UBQLN1	3,37422E-17	-3,51238E-01	0,083	0,265	6,21431E-13
Macro_FOLR2+APOE-	TMEM230	2,67481E-15	-3,51493E-01	0,207	0,454	4,92620E-11
Macro_FOLR2+APOE-	ITGA4	6,02227E-13	-3,51989E-01	0,171	0,371	1,10912E-08
Macro_FOLR2+APOE-	DYNLL1	1,08805E-10	-3,52072E-01	0,414	0,711	2,00386E-06
Macro_FOLR2+APOE-	STOM	8,54361E-18	-3,52317E-01	0,132	0,348	1,57348E-13
Macro_FOLR2+APOE-	WDR33	4,38307E-23	-3,52630E-01	0,056	0,258	8,07230E-19
Macro_FOLR2+APOE-	DDX17	6,18563E-19	-3,52708E-01	0,247	0,535	1,13921E-14
Macro_FOLR2+APOE-	OXA1L	1,37955E-16	-3,53012E-01	0,203	0,458	2,54072E-12
Macro_FOLR2+APOE-	PRKDC	5,99772E-15	-3,54536E-01	0,089	0,256	1,10460E-10
Macro_FOLR2+APOE-	CHCHD5	5,68543E-22	-3,54867E-01	0,087	0,301	1,04709E-17
Macro_FOLR2+APOE-	CELF2	9,33811E-25	-3,54931E-01	0,23	0,543	1,71980E-20
Macro_FOLR2+APOE-	LACTB	2,06076E-33	-3,55039E-01	0,06	0,326	3,79530E-29
Macro_FOLR2+APOE-	OSTF1	3,07243E-18	-3,55318E-01	0,25	0,553	5,65849E-14
Macro_FOLR2+APOE-	STARD7	5,34378E-23	-3,56697E-01	0,087	0,308	9,84164E-19
Macro_FOLR2+APOE-	NIPBL	8,54251E-17	-3,57559E-01	0,139	0,353	1,57327E-12
Macro_FOLR2+APOE-	HTATIP2	2,81264E-14	-3,58270E-01	0,115	0,293	5,18004E-10
Macro_FOLR2+APOE-	ACTR2	2,21303E-24	-3,59160E-01	0,279	0,626	4,07573E-20
Macro_FOLR2+APOE-	GOLGB1	2,44965E-15	-3,59305E-01	0,105	0,284	4,51152E-11
Macro_FOLR2+APOE-	MRPL4	2,34836E-14	-3,59642E-01	0,105	0,279	4,32497E-10
Macro_FOLR2+APOE-	SNRPF	1,19530E-29	-3,59996E-01	0,113	0,396	2,20138E-25
Macro_FOLR2+APOE-	CD14	1,44127E-19	-3,60588E-01	0,687	0,623	2,65438E-15
Macro_FOLR2+APOE-	USP4	2,17782E-15	-3,60703E-01	0,113	0,298	4,01089E-11
Macro_FOLR2+APOE-	MANF	1,85454E-21	-3,62522E-01	0,087	0,297	3,41551E-17
Macro_FOLR2+APOE-	COX7B	3,15305E-27	-3,62972E-01	0,303	0,671	5,80698E-23
Macro_FOLR2+APOE-	JOSD2	6,56047E-21	-3,63732E-01	0,105	0,331	1,20824E-16
Macro_FOLR2+APOE-	LSM7	2,52709E-26	-3,64066E-01	0,2	0,518	4,65414E-22
Macro_FOLR2+APOE-	ADAR	1,31211E-23	-3,64542E-01	0,158	0,431	2,41652E-19
Macro_FOLR2+APOE-	PRCP	1,64800E-16	-3,64737E-01	0,168	0,392	3,03513E-12
Macro_FOLR2+APOE-	ZC3H15	2,55896E-21	-3,65341E-01	0,171	0,441	4,71283E-17
Macro_FOLR2+APOE-	NT5C	5,45268E-14	-3,66178E-01	0,153	0,353	1,00422E-09
Macro_FOLR2+APOE-	CNIH4	2,97350E-27	-3,66184E-01	0,06	0,289	5,47630E-23
Macro_FOLR2+APOE-	SRSF9	2,85688E-24	-3,66397E-01	0,128	0,382	5,26151E-20
Macro_FOLR2+APOE-	TOP1	2,80238E-28	-3,67115E-01	0,202	0,524	5,16114E-24
Macro_FOLR2+APOE-	RBM42	4,81606E-20	-3,67250E-01	0,092	0,299	8,86974E-16
Macro_FOLR2+APOE-	CHMP4B	6,30757E-38	-3,67288E-01	0,098	0,412	1,16166E-33

Macro_FOLR2+APOE-	JPT1	3,11864E-21	-3,67586E-01	0,081	0,285	5,74359E-17
Macro_FOLR2+APOE-	FAM162A	4,09939E-17	-3,67958E-01	0,134	0,348	7,54985E-13
Macro_FOLR2+APOE-	VASP	6,98132E-48	-3,68051E-01	0,105	0,474	1,28575E-43
Macro_FOLR2+APOE-	UBE2D1	5,50934E-23	-3,68658E-01	0,121	0,365	1,01465E-18
Macro_FOLR2+APOE-	CCS	5,77343E-12	-3,69073E-01	0,107	0,263	1,06329E-07
Macro_FOLR2+APOE-	CITED2	1,14581E-20	-3,69267E-01	0,119	0,337	2,11024E-16
Macro_FOLR2+APOE-	NEU1	7,23381E-21	-3,69452E-01	0,096	0,307	1,33225E-16
Macro_FOLR2+APOE-	TRAPPC2L	1,25475E-11	-3,70235E-01	0,115	0,271	2,31087E-07
Macro_FOLR2+APOE-	ABHD14B	3,89284E-12	-3,71453E-01	0,107	0,264	7,16945E-08
Macro_FOLR2+APOE-	DNAJC1	5,26901E-14	-3,72331E-01	0,117	0,292	9,70394E-10
Macro_FOLR2+APOE-	SLA	6,78800E-11	-3,72438E-01	0,218	0,419	1,25015E-06
Macro_FOLR2+APOE-	ENTPD1	2,78492E-12	-3,73557E-01	0,137	0,311	5,12899E-08
Macro_FOLR2+APOE-	RAB5C	2,14131E-20	-3,73832E-01	0,188	0,463	3,94365E-16
Macro_FOLR2+APOE-	GMFG	8,41553E-11	-3,75713E-01	0,382	0,676	1,54989E-06
Macro_FOLR2+APOE-	COX7A2L	4,46363E-25	-3,75746E-01	0,188	0,493	8,22066E-21
Macro_FOLR2+APOE-	SSU72	4,92223E-20	-3,76997E-01	0,147	0,386	9,06527E-16
Macro_FOLR2+APOE-	MRPL52	8,95127E-27	-3,77303E-01	0,164	0,463	1,64856E-22
Macro_FOLR2+APOE-	EIF3H	2,37767E-12	-3,78159E-01	0,36	0,661	4,37895E-08
Macro_FOLR2+APOE-	SLC25A6	1,42358E-08	-3,81457E-01	0,546	0,819	2,62181E-04
Macro_FOLR2+APOE-	MRPS15	4,99254E-18	-3,82124E-01	0,113	0,32	9,19475E-14
Macro_FOLR2+APOE-	LPCAT2	4,29265E-16	-3,82125E-01	0,121	0,312	7,90577E-12
Macro_FOLR2+APOE-	UGP2	4,20413E-15	-3,82135E-01	0,115	0,303	7,74275E-11
Macro_FOLR2+APOE-	UBE2R2	3,43428E-24	-3,82461E-01	0,058	0,267	6,32491E-20
Macro_FOLR2+APOE-	EMILIN2	2,89495E-19	-3,82666E-01	0,1	0,304	5,33164E-15
Macro_FOLR2+APOE-	SERINC1	4,78438E-14	-3,82905E-01	0,185	0,406	8,81139E-10
Macro_FOLR2+APOE-	BTF3L4	3,75827E-17	-3,83054E-01	0,154	0,381	6,92161E-13
Macro_FOLR2+APOE-	FUOM	2,05104E-16	-3,83396E-01	0,154	0,379	3,77740E-12
Macro_FOLR2+APOE-	RANBP1	1,15751E-15	-3,83918E-01	0,153	0,37	2,13179E-11
Macro_FOLR2+APOE-	C7orf50	2,18808E-21	-3,84320E-01	0,111	0,34	4,02979E-17
Macro_FOLR2+APOE-	GOLGA7	7,47462E-16	-3,85302E-01	0,107	0,295	1,37660E-11
Macro_FOLR2+APOE-	RSRP1	3,66118E-29	-3,85901E-01	0,049	0,284	6,74280E-25
Macro_FOLR2+APOE-	MMADHC	6,54647E-17	-3,87393E-01	0,136	0,351	1,20566E-12
Macro_FOLR2+APOE-	NDUFA3	1,86240E-15	-3,87847E-01	0,267	0,549	3,42998E-11
Macro_FOLR2+APOE-	TSPAN3	1,16815E-12	-3,87860E-01	0,139	0,32	2,15137E-08
Macro_FOLR2+APOE-	SUMF2	6,82288E-10	-3,88166E-01	0,139	0,296	1,25657E-05
Macro_FOLR2+APOE-	SF3B4	6,00268E-30	-3,88487E-01	0,056	0,302	1,10551E-25
Macro_FOLR2+APOE-	MIER1	8,09486E-16	-3,88833E-01	0,085	0,255	1,49083E-11
Macro_FOLR2+APOE-	MAP2K1	3,09919E-21	-3,89124E-01	0,104	0,324	5,70777E-17
Macro_FOLR2+APOE-	RTN4	1,91156E-24	-3,89901E-01	0,303	0,653	3,52051E-20
Macro_FOLR2+APOE-	CCDC115	3,53555E-17	-3,90203E-01	0,085	0,268	6,51142E-13
Macro_FOLR2+APOE-	TBRG1	4,64774E-16	-3,90555E-01	0,145	0,354	8,55974E-12
Macro_FOLR2+APOE-	EEF2	1,29816E-07	-3,92448E-01	0,573	0,831	2,39082E-03
Macro_FOLR2+APOE-	EIF4EBP1	2,49945E-16	-3,92477E-01	0,177	0,412	4,60324E-12
Macro_FOLR2+APOE-	PCM1	5,35475E-14	-3,92548E-01	0,132	0,318	9,86184E-10
Macro_FOLR2+APOE-	NDUFA8	2,02565E-20	-3,92631E-01	0,087	0,293	3,73065E-16
Macro_FOLR2+APOE-	DGCR6L	5,19948E-12	-3,93215E-01	0,107	0,263	9,57588E-08
Macro_FOLR2+APOE-	ELF1	1,45921E-37	-3,93835E-01	0,149	0,491	2,68743E-33

Macro_FOLR2+APOE-	CLEC4E	7,44001E-07	-3,94282E-01	0,168	0,293	1,37023E-02
Macro_FOLR2+APOE-	FOXN2	3,80141E-15	-3,95504E-01	0,094	0,264	7,00106E-11
Macro_FOLR2+APOE-	HNRNPUL1	1,88451E-25	-3,97177E-01	0,141	0,414	3,47070E-21
Macro_FOLR2+APOE-	FAM133B	4,31837E-19	-3,97475E-01	0,096	0,298	7,95314E-15
Macro_FOLR2+APOE-	BAX	4,09678E-34	-3,97886E-01	0,192	0,542	7,54505E-30
Macro_FOLR2+APOE-	RNASEH2C	2,22428E-22	-3,98857E-01	0,111	0,349	4,09645E-18
Macro_FOLR2+APOE-	EIF2AK2	1,32192E-13	-3,99024E-01	0,1	0,263	2,43458E-09
Macro_FOLR2+APOE-	CTNNA1	5,90559E-19	-3,99292E-01	0,136	0,365	1,08763E-14
Macro_FOLR2+APOE-	CLNS1A	6,85250E-20	-4,00283E-01	0,168	0,424	1,26202E-15
Macro_FOLR2+APOE-	TMBIM1	4,44295E-14	-4,00501E-01	0,16	0,364	8,18259E-10
Macro_FOLR2+APOE-	ARPC1A	1,48297E-11	-4,00708E-01	0,115	0,274	2,73118E-07
Macro_FOLR2+APOE-	COMT	2,52563E-10	-4,00921E-01	0,301	0,56	4,65144E-06
Macro_FOLR2+APOE-	NAGK	4,32225E-21	-4,01769E-01	0,2	0,48	7,96028E-17
Macro_FOLR2+APOE-	METAP2	2,52480E-17	-4,02238E-01	0,1	0,295	4,64993E-13
Macro_FOLR2+APOE-	TACC1	1,03351E-18	-4,02738E-01	0,177	0,424	1,90341E-14
Macro_FOLR2+APOE-	PHF11	7,50163E-17	-4,02744E-01	0,1	0,29	1,38158E-12
Macro_FOLR2+APOE-	RAPGEF1	3,77052E-19	-4,02775E-01	0,073	0,261	6,94417E-15
Macro_FOLR2+APOE-	MRPL11	2,33471E-20	-4,02873E-01	0,089	0,296	4,29983E-16
Macro_FOLR2+APOE-	ODF3B	7,74845E-20	-4,03185E-01	0,124	0,348	1,42703E-15
Macro_FOLR2+APOE-	SH3BP2	5,08627E-19	-4,03897E-01	0,096	0,297	9,36739E-15
Macro_FOLR2+APOE-	DRAM2	2,74599E-07	-4,03899E-01	0,218	0,392	5,05729E-03
Macro_FOLR2+APOE-	CIB1	3,10119E-31	-4,04255E-01	0,256	0,617	5,71147E-27
Macro_FOLR2+APOE-	HSPA6	6,42142E-08	-4,05545E-01	0,158	0,288	1,18263E-03
Macro_FOLR2+APOE-	ABI1	1,91277E-26	-4,06046E-01	0,092	0,343	3,52274E-22
Macro_FOLR2+APOE-	RAB31	1,57159E-16	-4,06811E-01	0,341	0,646	2,89440E-12
Macro_FOLR2+APOE-	PRDX5	3,74352E-21	-4,07046E-01	0,273	0,604	6,89443E-17
Macro_FOLR2+APOE-	PSMG2	1,00631E-12	-4,07895E-01	0,202	0,427	1,85332E-08
Macro_FOLR2+APOE-	DNAJC8	5,91460E-23	-4,08086E-01	0,149	0,416	1,08929E-18
Macro_FOLR2+APOE-	C1orf54	9,25767E-08	-4,08257E-01	0,151	0,284	1,70498E-03
Macro_FOLR2+APOE-	SIVA1	5,40893E-27	-4,08760E-01	0,16	0,458	9,96162E-23
Macro_FOLR2+APOE-	PLEC	3,14045E-22	-4,08869E-01	0,13	0,367	5,78377E-18
Macro_FOLR2+APOE-	UQCRH	5,04880E-22	-4,09410E-01	0,363	0,705	9,29837E-18
Macro_FOLR2+APOE-	SF3B5	8,38834E-19	-4,09518E-01	0,256	0,555	1,54488E-14
Macro_FOLR2+APOE-	LYPLA1	2,86997E-17	-4,10143E-01	0,122	0,33	5,28562E-13
Macro_FOLR2+APOE-	MPC2	2,69585E-16	-4,10377E-01	0,149	0,362	4,96495E-12
Macro_FOLR2+APOE-	JUND	2,01182E-26	-4,10464E-01	0,173	0,458	3,70516E-22
Macro_FOLR2+APOE-	TSPAN14	7,26230E-18	-4,10680E-01	0,124	0,335	1,33750E-13
Macro_FOLR2+APOE-	LAMTOR1	8,64147E-18	-4,10791E-01	0,335	0,651	1,59150E-13
Macro_FOLR2+APOE-	INPP5D	2,54862E-09	-4,11093E-01	0,158	0,313	4,69379E-05
Macro_FOLR2+APOE-	SAT2	6,77595E-14	-4,11567E-01	0,205	0,436	1,24793E-09
Macro_FOLR2+APOE-	SCAF11	2,29817E-27	-4,11780E-01	0,179	0,493	4,23254E-23
Macro_FOLR2+APOE-	RIN3	2,85952E-20	-4,11920E-01	0,164	0,407	5,26638E-16
Macro_FOLR2+APOE-	SRP72	1,02619E-15	-4,12685E-01	0,141	0,349	1,88993E-11
Macro_FOLR2+APOE-	PPP1R7	1,45058E-14	-4,12953E-01	0,105	0,281	2,67154E-10
Macro_FOLR2+APOE-	PCMT1	1,34276E-19	-4,14180E-01	0,119	0,341	2,47296E-15
Macro_FOLR2+APOE-	PPP1CC	1,28424E-21	-4,14196E-01	0,119	0,354	2,36519E-17
Macro_FOLR2+APOE-	ZNFX1	1,57573E-20	-4,14283E-01	0,066	0,259	2,90202E-16

Macro_FOLR2+APOE-	MRPL21	2,04811E-14	-4,14538E-01	0,098	0,268	3,77201E-10
Macro_FOLR2+APOE-	KCNAB2	1,92553E-12	-4,14538E-01	0,119	0,282	3,54625E-08
Macro_FOLR2+APOE-	CUTA	2,30093E-18	-4,15055E-01	0,213	0,488	4,23763E-14
Macro_FOLR2+APOE-	PAK2	1,40880E-35	-4,15525E-01	0,177	0,533	2,59460E-31
Macro_FOLR2+APOE-	MRPL3	1,26048E-09	-4,15643E-01	0,136	0,284	2,32142E-05
Macro_FOLR2+APOE-	NAAA	7,90508E-20	-4,15665E-01	0,139	0,37	1,45588E-15
Macro_FOLR2+APOE-	RTN3	5,48124E-20	-4,16397E-01	0,168	0,421	1,00948E-15
Macro_FOLR2+APOE-	FCGR1A	1,31551E-07	-4,16435E-01	0,245	0,413	2,42277E-03
Macro_FOLR2+APOE-	GLRX3	5,80278E-18	-4,16800E-01	0,072	0,25	1,06870E-13
Macro_FOLR2+APOE-	OSBPL8	8,84667E-29	-4,17232E-01	0,141	0,432	1,62929E-24
Macro_FOLR2+APOE-	NDUFB6	1,29306E-25	-4,17286E-01	0,113	0,371	2,38143E-21
Macro_FOLR2+APOE-	SOAT1	3,52390E-20	-4,17990E-01	0,085	0,288	6,48996E-16
Macro_FOLR2+APOE-	CCDC124	3,81294E-17	-4,18351E-01	0,139	0,355	7,02229E-13
Macro_FOLR2+APOE-	AOAH	2,32585E-06	-4,18843E-01	0,226	0,376	4,28353E-02
Macro_FOLR2+APOE-	BBX	8,90169E-15	-4,19348E-01	0,117	0,3	1,63943E-10
Macro_FOLR2+APOE-	PSMB2	2,82597E-33	-4,21694E-01	0,132	0,445	5,20459E-29
Macro_FOLR2+APOE-	GDI2	4,99377E-11	-4,22004E-01	0,365	0,654	9,19703E-07
Macro_FOLR2+APOE-	CNPPD1	1,68006E-19	-4,22444E-01	0,079	0,273	3,09417E-15
Macro_FOLR2+APOE-	WNK1	1,71530E-17	-4,22450E-01	0,141	0,359	3,15907E-13
Macro_FOLR2+APOE-	PPP1R12A	5,71364E-23	-4,23406E-01	0,13	0,38	1,05228E-18
Macro_FOLR2+APOE-	PRNP	3,88475E-09	-4,24233E-01	0,218	0,403	7,15455E-05
Macro_FOLR2+APOE-	CD2BP2	3,77240E-20	-4,24650E-01	0,064	0,253	6,94763E-16
Macro_FOLR2+APOE-	MTCH2	3,49066E-25	-4,24673E-01	0,087	0,325	6,42874E-21
Macro_FOLR2+APOE-	CIITA	5,74396E-22	-4,24736E-01	0,104	0,327	1,05786E-17
Macro_FOLR2+APOE-	HSPA4	3,61099E-20	-4,24774E-01	0,094	0,303	6,65035E-16
Macro_FOLR2+APOE-	NDUFS7	1,39215E-18	-4,24844E-01	0,25	0,544	2,56393E-14
Macro_FOLR2+APOE-	GTF3C6	9,15081E-15	-4,25357E-01	0,158	0,374	1,68530E-10
Macro_FOLR2+APOE-	GLG1	1,09024E-17	-4,25442E-01	0,1	0,293	2,00790E-13
Macro_FOLR2+APOE-	EIF3D	1,15825E-14	-4,25746E-01	0,226	0,477	2,13314E-10
Macro_FOLR2+APOE-	METTL9	9,21128E-20	-4,26516E-01	0,134	0,361	1,69644E-15
Macro_FOLR2+APOE-	COX17	1,77180E-36	-4,27021E-01	0,149	0,494	3,26313E-32
Macro_FOLR2+APOE-	JAK1	4,33110E-25	-4,27491E-01	0,215	0,525	7,97658E-21
Macro_FOLR2+APOE-	LSM10	1,10641E-24	-4,27577E-01	0,121	0,378	2,03767E-20
Macro_FOLR2+APOE-	MTPN	2,28133E-29	-4,27712E-01	0,2	0,53	4,20152E-25
Macro_FOLR2+APOE-	RBBP6	1,86552E-23	-4,27863E-01	0,075	0,292	3,43572E-19
Macro_FOLR2+APOE-	CEP170	3,14170E-13	-4,28055E-01	0,154	0,346	5,78606E-09
Macro_FOLR2+APOE-	COX6B1	5,87224E-11	-4,28820E-01	0,507	0,814	1,08149E-06
Macro_FOLR2+APOE-	NANS	2,48154E-22	-4,29866E-01	0,139	0,392	4,57026E-18
Macro_FOLR2+APOE-	VOPP1	7,15498E-19	-4,30049E-01	0,13	0,35	1,31773E-14
Macro_FOLR2+APOE-	PIK3AP1	2,78376E-22	-4,30143E-01	0,089	0,307	5,12684E-18
Macro_FOLR2+APOE-	EFCAB14	6,93656E-13	-4,30790E-01	0,107	0,27	1,27751E-08
Macro_FOLR2+APOE-	CHMP5	4,01898E-17	-4,31092E-01	0,171	0,41	7,40176E-13
Macro_FOLR2+APOE-	TUFM	6,08878E-19	-4,31818E-01	0,23	0,513	1,12137E-14
Macro_FOLR2+APOE-	NELFE	2,21667E-16	-4,31975E-01	0,104	0,292	4,08245E-12
Macro_FOLR2+APOE-	MIS18BP1	8,30231E-17	-4,32276E-01	0,147	0,363	1,52904E-12
Macro_FOLR2+APOE-	GCC2	1,71780E-18	-4,33854E-01	0,089	0,283	3,16367E-14
Macro_FOLR2+APOE-	TSEN34	9,54201E-20	-4,34324E-01	0,09	0,293	1,75735E-15

Macro_FOLR2+APOE-	GTF2I	8,77774E-20	-4,34730E-01	0,104	0,314	1,61660E-15
Macro_FOLR2+APOE-	IFNAR1	2,31144E-14	-4,35407E-01	0,128	0,315	4,25697E-10
Macro_FOLR2+APOE-	SNAP29	2,45028E-19	-4,35790E-01	0,089	0,287	4,51268E-15
Macro_FOLR2+APOE-	RBX1	8,58628E-25	-4,36100E-01	0,273	0,623	1,58133E-20
Macro_FOLR2+APOE-	PHF20	9,31058E-15	-4,36595E-01	0,104	0,277	1,71473E-10
Macro_FOLR2+APOE-	MRPL54	1,39340E-22	-4,36609E-01	0,143	0,405	2,56623E-18
Macro_FOLR2+APOE-	JUN	1,42503E-20	-4,37000E-01	0,399	0,713	2,62448E-16
Macro_FOLR2+APOE-	TMED5	1,14325E-19	-4,37174E-01	0,139	0,375	2,10552E-15
Macro_FOLR2+APOE-	CAMTA1	9,96871E-18	-4,37224E-01	0,141	0,367	1,83594E-13
Macro_FOLR2+APOE-	PRMT2	2,12829E-14	-4,37243E-01	0,2	0,43	3,91966E-10
Macro_FOLR2+APOE-	LMBRD1	3,21315E-11	-4,37348E-01	0,121	0,272	5,91766E-07
Macro_FOLR2+APOE-	DDX24	1,40175E-17	-4,37838E-01	0,217	0,48	2,58160E-13
Macro_FOLR2+APOE-	CHCHD7	3,58919E-18	-4,38189E-01	0,075	0,259	6,61022E-14
Macro_FOLR2+APOE-	AIP	1,13401E-24	-4,39695E-01	0,1	0,343	2,08850E-20
Macro_FOLR2+APOE-	TPD52L2	3,58839E-18	-4,40112E-01	0,083	0,271	6,60873E-14
Macro_FOLR2+APOE-	COX7C	1,76379E-07	-4,41729E-01	0,569	0,832	3,24837E-03
Macro_FOLR2+APOE-	GPS1	1,32422E-17	-4,41872E-01	0,083	0,267	2,43881E-13
Macro_FOLR2+APOE-	TRIP12	1,18829E-15	-4,42335E-01	0,1	0,278	2,18848E-11
Macro_FOLR2+APOE-	XRCC5	1,54771E-23	-4,42409E-01	0,186	0,479	2,85042E-19
Macro_FOLR2+APOE-	FNBP4	3,71492E-22	-4,43239E-01	0,089	0,303	6,84177E-18
Macro_FOLR2+APOE-	SYK	4,16133E-19	-4,43607E-01	0,186	0,446	7,66392E-15
Macro_FOLR2+APOE-	MRPL43	5,15256E-20	-4,43626E-01	0,124	0,352	9,48947E-16
Macro_FOLR2+APOE-	ATP6V1C1	2,56884E-17	-4,44120E-01	0,09	0,275	4,73104E-13
Macro_FOLR2+APOE-	PSMF1	2,29540E-25	-4,44512E-01	0,147	0,424	4,22745E-21
Macro_FOLR2+APOE-	GNB2	1,74677E-35	-4,45676E-01	0,205	0,561	3,21702E-31
Macro_FOLR2+APOE-	NOL7	2,57031E-26	-4,45803E-01	0,137	0,418	4,73373E-22
Macro_FOLR2+APOE-	FCGR3A	4,04689E-07	-4,46807E-01	0,612	0,575	7,45316E-03
Macro_FOLR2+APOE-	KTN1	5,15683E-25	-4,47131E-01	0,19	0,497	9,49733E-21
Macro_FOLR2+APOE-	FAM204A	4,44002E-16	-4,47324E-01	0,104	0,289	8,17719E-12
Macro_FOLR2+APOE-	TRAPPC1	1,21429E-34	-4,48441E-01	0,232	0,614	2,23635E-30
Macro_FOLR2+APOE-	ERP29	2,85580E-16	-4,48880E-01	0,326	0,627	5,25953E-12
Macro_FOLR2+APOE-	ACP5	1,93332E-12	-4,49432E-01	0,209	0,402	3,56060E-08
Macro_FOLR2+APOE-	GRHPR	3,30175E-15	-4,49485E-01	0,109	0,293	6,08083E-11
Macro_FOLR2+APOE-	PRMT1	1,35011E-20	-4,50127E-01	0,094	0,309	2,48650E-16
Macro_FOLR2+APOE-	COX6C	1,80350E-13	-4,51970E-01	0,405	0,723	3,32151E-09
Macro_FOLR2+APOE-	RBBP4	1,52717E-15	-4,52015E-01	0,158	0,373	2,81259E-11
Macro_FOLR2+APOE-	CXCL16	5,23601E-12	-4,52197E-01	0,384	0,654	9,64315E-08
Macro_FOLR2+APOE-	PCGF5	1,86040E-21	-4,53203E-01	0,072	0,275	3,42630E-17
Macro_FOLR2+APOE-	GNAI3	1,29284E-29	-4,53687E-01	0,085	0,349	2,38102E-25
Macro_FOLR2+APOE-	CCDC12	1,64587E-20	-4,53960E-01	0,085	0,289	3,03120E-16
Macro_FOLR2+APOE-	ETFA	1,03830E-18	-4,54103E-01	0,121	0,338	1,91223E-14
Macro_FOLR2+APOE-	FXR1	1,95752E-15	-4,54182E-01	0,107	0,29	3,60517E-11
Macro_FOLR2+APOE-	RSU1	4,73309E-16	-4,54365E-01	0,132	0,338	8,71693E-12
Macro_FOLR2+APOE-	NQO2	3,96937E-15	-4,54738E-01	0,092	0,263	7,31039E-11
Macro_FOLR2+APOE-	ACSL3	2,32822E-19	-4,54875E-01	0,07	0,256	4,28788E-15
Macro_FOLR2+APOE-	NDUFC1	2,24104E-23	-4,55098E-01	0,173	0,461	4,12733E-19
Macro_FOLR2+APOE-	COX20	1,40162E-19	-4,55230E-01	0,072	0,261	2,58137E-15

Macro_FOLR2+APOE-	NDUFA11	2,77274E-17	-4,55479E-01	0,205	0,457	5,10655E-13
Macro_FOLR2+APOE-	POLR2L	1,57435E-35	-4,55740E-01	0,243	0,631	2,89948E-31
Macro_FOLR2+APOE-	ERP44	3,96544E-23	-4,55924E-01	0,16	0,432	7,30315E-19
Macro_FOLR2+APOE-	DOCK10	1,88910E-17	-4,56556E-01	0,121	0,325	3,47916E-13
Macro_FOLR2+APOE-	PCNP	5,38745E-16	-4,57133E-01	0,126	0,325	9,92208E-12
Macro_FOLR2+APOE-	DNMT1	1,20171E-12	-4,58177E-01	0,141	0,319	2,21319E-08
Macro_FOLR2+APOE-	RER1	2,28679E-23	-4,58905E-01	0,175	0,464	4,21158E-19
Macro_FOLR2+APOE-	YTHDF2	3,02132E-17	-4,59274E-01	0,094	0,283	5,56437E-13
Macro_FOLR2+APOE-	POLE4	2,91673E-27	-4,59369E-01	0,085	0,334	5,37175E-23
Macro_FOLR2+APOE-	ATP6V1A	6,28873E-18	-4,60213E-01	0,075	0,257	1,15820E-13
Macro_FOLR2+APOE-	TRAM1	4,39164E-22	-4,61175E-01	0,179	0,453	8,08809E-18
Macro_FOLR2+APOE-	CAST	2,79221E-21	-4,61374E-01	0,235	0,534	5,14241E-17
Macro_FOLR2+APOE-	HK1	1,11189E-18	-4,61891E-01	0,077	0,264	2,04776E-14
Macro_FOLR2+APOE-	TMEM258	2,59541E-16	-4,61903E-01	0,341	0,671	4,77997E-12
Macro_FOLR2+APOE-	SURF4	5,80086E-20	-4,62031E-01	0,089	0,293	1,06834E-15
Macro_FOLR2+APOE-	EMD	1,60013E-31	-4,62245E-01	0,122	0,42	2,94696E-27
Macro_FOLR2+APOE-	CLTA	3,26942E-09	-4,62566E-01	0,367	0,652	6,02130E-05
Macro_FOLR2+APOE-	USP8	2,01052E-15	-4,63843E-01	0,098	0,274	3,70278E-11
Macro_FOLR2+APOE-	CLPP	1,13079E-16	-4,65096E-01	0,087	0,267	2,08257E-12
Macro_FOLR2+APOE-	RBMS1	5,57353E-20	-4,65293E-01	0,102	0,313	1,02648E-15
Macro_FOLR2+APOE-	SH3TC1	1,69925E-11	-4,65755E-01	0,145	0,315	3,12951E-07
Macro_FOLR2+APOE-	ACTR1A	1,72217E-20	-4,67923E-01	0,085	0,29	3,17172E-16
Macro_FOLR2+APOE-	DENR	3,94048E-17	-4,68042E-01	0,075	0,25	7,25718E-13
Macro_FOLR2+APOE-	PRDX6	3,33635E-22	-4,68565E-01	0,247	0,562	6,14456E-18
Macro_FOLR2+APOE-	PTTG1IP	1,40687E-23	-4,69553E-01	0,205	0,506	2,59104E-19
Macro_FOLR2+APOE-	PAK1	1,14893E-39	-4,71106E-01	0,111	0,441	2,11598E-35
Macro_FOLR2+APOE-	HMGN3	2,46038E-19	-4,72481E-01	0,235	0,526	4,53128E-15
Macro_FOLR2+APOE-	DDAH2	1,40725E-20	-4,72842E-01	0,166	0,422	2,59173E-16
Macro_FOLR2+APOE-	MGST3	2,95907E-16	-4,72891E-01	0,271	0,545	5,44972E-12
Macro_FOLR2+APOE-	SLC25A39	1,72591E-13	-4,73539E-01	0,19	0,411	3,17861E-09
Macro_FOLR2+APOE-	C12orf57	3,14550E-17	-4,74039E-01	0,132	0,336	5,79307E-13
Macro_FOLR2+APOE-	NUB1	2,01109E-19	-4,74418E-01	0,107	0,316	3,70383E-15
Macro_FOLR2+APOE-	ACO2	1,00496E-17	-4,74999E-01	0,085	0,272	1,85083E-13
Macro_FOLR2+APOE-	BIN2	1,81008E-16	-4,75657E-01	0,119	0,316	3,33363E-12
Macro_FOLR2+APOE-	FBXL5	1,65454E-22	-4,75725E-01	0,07	0,276	3,04716E-18
Macro_FOLR2+APOE-	COMMD9	1,53677E-13	-4,76761E-01	0,132	0,316	2,83026E-09
Macro_FOLR2+APOE-	MTCH1	2,86799E-27	-4,76761E-01	0,16	0,459	5,28197E-23
Macro_FOLR2+APOE-	SEC62	6,78211E-20	-4,76791E-01	0,254	0,558	1,24906E-15
Macro_FOLR2+APOE-	TMEM50A	7,31553E-24	-4,76915E-01	0,266	0,607	1,34730E-19
Macro_FOLR2+APOE-	GTF2F1	5,68464E-15	-4,77216E-01	0,089	0,257	1,04694E-10
Macro_FOLR2+APOE-	MYO1F	4,59591E-18	-4,77285E-01	0,207	0,462	8,46428E-14
Macro_FOLR2+APOE-	PNISR	6,13076E-14	-4,77432E-01	0,245	0,496	1,12910E-09
Macro_FOLR2+APOE-	CD164	7,80207E-12	-4,77828E-01	0,286	0,56	1,43691E-07
Macro_FOLR2+APOE-	CDV3	1,58146E-21	-4,77866E-01	0,073	0,277	2,91257E-17
Macro_FOLR2+APOE-	POLR2I	1,85456E-18	-4,79136E-01	0,096	0,297	3,41555E-14
Macro_FOLR2+APOE-	PEA15	3,91248E-23	-4,79307E-01	0,23	0,52	7,20561E-19
Macro_FOLR2+APOE-	TRAPPC3	7,54224E-16	-4,79731E-01	0,143	0,355	1,38905E-11

Macro_FOLR2+APOE-	COMMD6	4,61028E-36	-4,82245E-01	0,337	0,74	8,49075E-32
Macro_FOLR2+APOE-	CCAR1	1,81342E-14	-4,82718E-01	0,1	0,269	3,33978E-10
Macro_FOLR2+APOE-	HM13	8,37011E-28	-4,82948E-01	0,217	0,543	1,54152E-23
Macro_FOLR2+APOE-	HMGB1	2,30771E-09	-4,83156E-01	0,492	0,797	4,25011E-05
Macro_FOLR2+APOE-	SDCCAG8	3,91943E-12	-4,84854E-01	0,156	0,339	7,21841E-08
Macro_FOLR2+APOE-	ZFAND6	2,73984E-19	-4,84883E-01	0,104	0,314	5,04596E-15
Macro_FOLR2+APOE-	PDCD4	6,28671E-15	-4,85330E-01	0,094	0,261	1,15782E-10
Macro_FOLR2+APOE-	PTGS1	1,02358E-14	-4,85887E-01	0,094	0,259	1,88513E-10
Macro_FOLR2+APOE-	UBXN1	2,36952E-12	-4,86429E-01	0,339	0,632	4,36394E-08
Macro_FOLR2+APOE-	CHCHD3	3,98327E-13	-4,86494E-01	0,094	0,251	7,33599E-09
Macro_FOLR2+APOE-	PARP14	4,91254E-23	-4,86801E-01	0,132	0,38	9,04743E-19
Macro_FOLR2+APOE-	IRAK3	7,20955E-16	-4,87052E-01	0,089	0,256	1,32778E-11
Macro_FOLR2+APOE-	PPIG	6,74176E-28	-4,87132E-01	0,154	0,456	1,24163E-23
Macro_FOLR2+APOE-	HP1BP3	1,33595E-25	-4,87539E-01	0,137	0,41	2,46043E-21
Macro_FOLR2+APOE-	PNN	1,83434E-21	-4,87686E-01	0,098	0,317	3,37831E-17
Macro_FOLR2+APOE-	TAX1BP1	6,18444E-25	-4,87803E-01	0,2	0,502	1,13899E-20
Macro_FOLR2+APOE-	ATP6V0B	1,93874E-10	-4,88043E-01	0,484	0,794	3,57058E-06
Macro_FOLR2+APOE-	GM2A	1,10964E-27	-4,88367E-01	0,113	0,378	2,04362E-23
Macro_FOLR2+APOE-	ATXN10	7,46070E-17	-4,88746E-01	0,094	0,278	1,37404E-12
Macro_FOLR2+APOE-	PAG1	1,13511E-20	-4,88826E-01	0,077	0,273	2,09054E-16
Macro_FOLR2+APOE-	MYH9	1,41390E-27	-4,89293E-01	0,245	0,582	2,60399E-23
Macro_FOLR2+APOE-	TMEM134	2,23130E-13	-4,89299E-01	0,107	0,275	4,10939E-09
Macro_FOLR2+APOE-	TXNL1	1,18372E-19	-4,89550E-01	0,145	0,384	2,18005E-15
Macro_FOLR2+APOE-	BIRC6	5,12201E-15	-4,90303E-01	0,087	0,251	9,43320E-11
Macro_FOLR2+APOE-	CSDE1	1,58564E-17	-4,90349E-01	0,294	0,608	2,92028E-13
Macro_FOLR2+APOE-	EPC1	3,70280E-25	-4,91053E-01	0,107	0,353	6,81944E-21
Macro_FOLR2+APOE-	CYB5R3	9,83616E-36	-4,91105E-01	0,105	0,414	1,81153E-31
Macro_FOLR2+APOE-	THRAP3	3,85859E-25	-4,91170E-01	0,147	0,423	7,10637E-21
Macro_FOLR2+APOE-	ETNK1	1,61945E-15	-4,91913E-01	0,083	0,25	2,98255E-11
Macro_FOLR2+APOE-	LRRC59	2,20150E-30	-4,93451E-01	0,047	0,286	4,05450E-26
Macro_FOLR2+APOE-	KDM5A	3,57260E-17	-4,94047E-01	0,115	0,314	6,57965E-13
Macro_FOLR2+APOE-	COA4	1,58829E-19	-4,94177E-01	0,087	0,288	2,92515E-15
Macro_FOLR2+APOE-	PIK3R5	6,50939E-13	-4,94208E-01	0,102	0,259	1,19883E-08
Macro_FOLR2+APOE-	NDUFB8	7,53431E-13	-4,95617E-01	0,122	0,289	1,38759E-08
Macro_FOLR2+APOE-	ARID4B	1,66564E-20	-4,95640E-01	0,139	0,376	3,06761E-16
Macro_FOLR2+APOE-	EPSTI1	2,53760E-16	-4,96508E-01	0,185	0,412	4,67349E-12
Macro_FOLR2+APOE-	PPP2R1A	5,30598E-26	-4,96557E-01	0,13	0,399	9,77202E-22
Macro_FOLR2+APOE-	ALOX5	4,87506E-19	-4,96946E-01	0,215	0,477	8,97839E-15
Macro_FOLR2+APOE-	MPG	1,47360E-18	-4,97294E-01	0,113	0,325	2,71394E-14
Macro_FOLR2+APOE-	TMEM59	3,40834E-08	-4,98160E-01	0,348	0,62	6,27714E-04
Macro_FOLR2+APOE-	RHBDF2	3,74786E-12	-4,98361E-01	0,149	0,326	6,90243E-08
Macro_FOLR2+APOE-	SERBP1	4,11161E-27	-4,99073E-01	0,254	0,598	7,57235E-23
Macro_FOLR2+APOE-	IRF2	2,99533E-14	-4,99231E-01	0,137	0,33	5,51651E-10
Macro_FOLR2+APOE-	LRPAP1	1,32422E-17	-4,99278E-01	0,23	0,499	2,43882E-13
Macro_FOLR2+APOE-	LEPROTL1	1,64654E-18	-4,99541E-01	0,162	0,402	3,03244E-14
Macro_FOLR2+APOE-	EIF4E2	4,49103E-38	-5,00688E-01	0,075	0,375	8,27113E-34
Macro_FOLR2+APOE-	FXYD5	6,17873E-08	-5,01154E-01	0,54	0,828	1,13794E-03

Macro_FOLR2+APOE-	ERGIC3	2,31159E-21	-5,01530E-01	0,207	0,501	4,25726E-17
Macro_FOLR2+APOE-	DHRS4	7,32441E-17	-5,01761E-01	0,09	0,273	1,34894E-12
Macro_FOLR2+APOE-	DYNC1H1	1,63421E-21	-5,02057E-01	0,139	0,382	3,00972E-17
Macro_FOLR2+APOE-	SPSB3	2,48963E-13	-5,02125E-01	0,119	0,292	4,58515E-09
Macro_FOLR2+APOE-	TMOD3	6,70944E-25	-5,02653E-01	0,104	0,347	1,23568E-20
Macro_FOLR2+APOE-	SMG1	2,67804E-18	-5,02787E-01	0,115	0,32	4,93214E-14
Macro_FOLR2+APOE-	EFHD2	3,36075E-38	-5,04358E-01	0,113	0,434	6,18949E-34
Macro_FOLR2+APOE-	EIF1AX	9,28054E-33	-5,05021E-01	0,107	0,403	1,70920E-28
Macro_FOLR2+APOE-	RSRC2	1,71041E-16	-5,05058E-01	0,166	0,393	3,15006E-12
Macro_FOLR2+APOE-	SCPEP1	1,53945E-15	-5,06308E-01	0,183	0,41	2,83521E-11
Macro_FOLR2+APOE-	MANBA	7,27682E-25	-5,06459E-01	0,098	0,339	1,34017E-20
Macro_FOLR2+APOE-	TEX264	1,01661E-16	-5,06762E-01	0,113	0,31	1,87229E-12
Macro_FOLR2+APOE-	FOXP1	5,04966E-20	-5,07413E-01	0,124	0,347	9,29996E-16
Macro_FOLR2+APOE-	UBE2I	9,21899E-28	-5,07513E-01	0,203	0,531	1,69786E-23
Macro_FOLR2+APOE-	ARL6IP5	1,74652E-21	-5,07529E-01	0,318	0,659	3,21657E-17
Macro_FOLR2+APOE-	SEC14L1	1,95705E-11	-5,07641E-01	0,196	0,397	3,60430E-07
Macro_FOLR2+APOE-	SUGT1	6,75667E-20	-5,07963E-01	0,085	0,286	1,24438E-15
Macro_FOLR2+APOE-	SYNCRIP	1,81996E-25	-5,08214E-01	0,087	0,325	3,35183E-21
Macro_FOLR2+APOE-	NDUFS8	1,05120E-22	-5,08226E-01	0,202	0,498	1,93599E-18
Macro_FOLR2+APOE-	LYSMD2	2,43035E-26	-5,08249E-01	0,064	0,293	4,47598E-22
Macro_FOLR2+APOE-	NDUFS2	2,70511E-19	-5,08820E-01	0,134	0,363	4,98200E-15
Macro_FOLR2+APOE-	IRF5	4,70334E-18	-5,09742E-01	0,081	0,264	8,66214E-14
Macro_FOLR2+APOE-	ARHGEF2	4,14698E-18	-5,09955E-01	0,072	0,251	7,63750E-14
Macro_FOLR2+APOE-	USP16	3,93603E-22	-5,10480E-01	0,072	0,277	7,24899E-18
Macro_FOLR2+APOE-	RNF213	3,41729E-24	-5,11839E-01	0,222	0,508	6,29363E-20
Macro_FOLR2+APOE-	FBL	1,25682E-22	-5,12401E-01	0,113	0,35	2,31469E-18
Macro_FOLR2+APOE-	DYNLRB1	4,45078E-10	-5,12464E-01	0,25	0,465	8,19700E-06
Macro_FOLR2+APOE-	RPS17	1,63523E-10	-5,13065E-01	0,217	0,409	3,01161E-06
Macro_FOLR2+APOE-	RNF114	3,54235E-22	-5,14279E-01	0,13	0,373	6,52395E-18
Macro_FOLR2+APOE-	CTNND1	1,90248E-10	-5,14382E-01	0,122	0,274	3,50380E-06
Macro_FOLR2+APOE-	KLF2	1,76893E-15	-5,14650E-01	0,107	0,279	3,25784E-11
Macro_FOLR2+APOE-	RBPJ	5,43822E-07	-5,15059E-01	0,269	0,469	1,00156E-02
Macro_FOLR2+APOE-	MRPL51	7,57157E-18	-5,15188E-01	0,186	0,443	1,39446E-13
Macro_FOLR2+APOE-	DDX21	1,95184E-36	-5,15656E-01	0,124	0,44	3,59470E-32
Macro_FOLR2+APOE-	MTIF3	4,74019E-18	-5,15761E-01	0,098	0,297	8,73001E-14
Macro_FOLR2+APOE-	CASP4	3,99277E-22	-5,16254E-01	0,175	0,446	7,35349E-18
Macro_FOLR2+APOE-	PPP1R10	1,38579E-18	-5,16880E-01	0,109	0,311	2,55220E-14
Macro_FOLR2+APOE-	ESD	8,04628E-18	-5,17659E-01	0,143	0,366	1,48188E-13
Macro_FOLR2+APOE-	PDE4DIP	3,25628E-14	-5,19165E-01	0,141	0,324	5,99708E-10
Macro_FOLR2+APOE-	NUP214	4,38516E-12	-5,19279E-01	0,145	0,316	8,07615E-08
Macro_FOLR2+APOE-	GNPTG	4,41992E-14	-5,19585E-01	0,202	0,431	8,14016E-10
Macro_FOLR2+APOE-	YIPF3	1,52774E-15	-5,19612E-01	0,128	0,326	2,81363E-11
Macro_FOLR2+APOE-	BAG6	3,03618E-20	-5,20562E-01	0,077	0,275	5,59173E-16
Macro_FOLR2+APOE-	DNAJA2	1,44622E-23	-5,21434E-01	0,09	0,319	2,66350E-19
Macro_FOLR2+APOE-	KDELR2	5,99171E-26	-5,21472E-01	0,196	0,51	1,10349E-21
Macro_FOLR2+APOE-	SBDS	2,74110E-18	-5,21600E-01	0,092	0,286	5,04828E-14
Macro_FOLR2+APOE-	NDUFB4	4,87797E-20	-5,22019E-01	0,273	0,597	8,98376E-16

Macro_FOLR2+APOE-	UXT	8,00016E-26	-5,22263E-01	0,22	0,545	1,47339E-21
Macro_FOLR2+APOE-	LAMTOR2	2,13343E-17	-5,22459E-01	0,26	0,546	3,92914E-13
Macro_FOLR2+APOE-	SLTM	1,46236E-23	-5,22723E-01	0,115	0,358	2,69323E-19
Macro_FOLR2+APOE-	RAB11A	2,79435E-25	-5,22983E-01	0,134	0,405	5,14636E-21
Macro_FOLR2+APOE-	COPS5	9,03377E-16	-5,23113E-01	0,1	0,281	1,66375E-11
Macro_FOLR2+APOE-	SNW1	8,84198E-17	-5,23188E-01	0,102	0,292	1,62843E-12
Macro_FOLR2+APOE-	RAB6A	2,09862E-14	-5,24847E-01	0,119	0,302	3,86502E-10
Macro_FOLR2+APOE-	SRSF11	2,36428E-21	-5,25947E-01	0,224	0,519	4,35429E-17
Macro_FOLR2+APOE-	IRF2BP2	1,59629E-19	-5,26871E-01	0,111	0,325	2,93989E-15
Macro_FOLR2+APOE-	HNRNPA3	1,01272E-27	-5,26913E-01	0,275	0,633	1,86512E-23
Macro_FOLR2+APOE-	ITGB2	6,91079E-09	-5,27226E-01	0,524	0,766	1,27276E-04
Macro_FOLR2+APOE-	SLC15A3	2,06176E-12	-5,29038E-01	0,119	0,285	3,79715E-08
Macro_FOLR2+APOE-	MAN2B1	1,17390E-09	-5,29146E-01	0,26	0,477	2,16197E-05
Macro_FOLR2+APOE-	MFF	8,19903E-15	-5,29483E-01	0,128	0,322	1,51002E-10
Macro_FOLR2+APOE-	PPT1	1,44841E-08	-5,29728E-01	0,392	0,654	2,66754E-04
Macro_FOLR2+APOE-	SAMHD1	7,76820E-18	-5,30304E-01	0,352	0,649	1,43067E-13
Macro_FOLR2+APOE-	SELENOK	4,88895E-30	-5,30308E-01	0,045	0,28	9,00397E-26
Macro_FOLR2+APOE-	ARF3	2,42327E-21	-5,30411E-01	0,111	0,34	4,46294E-17
Macro_FOLR2+APOE-	NDUFA5	3,52977E-20	-5,30601E-01	0,089	0,295	6,50078E-16
Macro_FOLR2+APOE-	HPRT1	5,49276E-17	-5,30847E-01	0,085	0,265	1,01160E-12
Macro_FOLR2+APOE-	MIEN1	1,95359E-24	-5,31062E-01	0,136	0,399	3,59793E-20
Macro_FOLR2+APOE-	YWHAB	1,46057E-11	-5,31239E-01	0,471	0,791	2,68994E-07
Macro_FOLR2+APOE-	MLX	1,09331E-18	-5,31482E-01	0,136	0,361	2,01354E-14
Macro_FOLR2+APOE-	TMEM165	4,43940E-25	-5,32210E-01	0,13	0,395	8,17605E-21
Macro_FOLR2+APOE-	PPP4C	1,61568E-33	-5,32635E-01	0,173	0,517	2,97560E-29
Macro_FOLR2+APOE-	HVCN1	4,83567E-17	-5,33659E-01	0,079	0,256	8,90586E-13
Macro_FOLR2+APOE-	HNRNPF	1,05525E-31	-5,33796E-01	0,215	0,569	1,94346E-27
Macro_FOLR2+APOE-	SPAG7	1,00738E-17	-5,33973E-01	0,119	0,328	1,85529E-13
Macro_FOLR2+APOE-	RRBP1	3,41784E-21	-5,34028E-01	0,145	0,388	6,29464E-17
Macro_FOLR2+APOE-	PARVG	1,73747E-29	-5,34251E-01	0,153	0,453	3,19991E-25
Macro_FOLR2+APOE-	ROMO1	4,21933E-33	-5,35590E-01	0,185	0,534	7,77075E-29
Macro_FOLR2+APOE-	EIF3I	1,59702E-25	-5,35642E-01	0,173	0,469	2,94123E-21
Macro_FOLR2+APOE-	TRIM38	1,45322E-19	-5,36257E-01	0,089	0,287	2,67640E-15
Macro_FOLR2+APOE-	IGFLR1	2,25655E-29	-5,36641E-01	0,098	0,362	4,15589E-25
Macro_FOLR2+APOE-	POMP	2,02480E-32	-5,37471E-01	0,337	0,725	3,72908E-28
Macro_FOLR2+APOE-	GGA2	4,15066E-17	-5,37483E-01	0,089	0,272	7,64427E-13
Macro_FOLR2+APOE-	PQBP1	5,03355E-19	-5,37485E-01	0,107	0,317	9,27029E-15
Macro_FOLR2+APOE-	POLD4	1,20634E-22	-5,40111E-01	0,262	0,573	2,22172E-18
Macro_FOLR2+APOE-	MPC1	3,08816E-14	-5,40372E-01	0,13	0,319	5,68746E-10
Macro_FOLR2+APOE-	PLEKHB2	5,35386E-23	-5,40546E-01	0,164	0,443	9,86021E-19
Macro_FOLR2+APOE-	TOMM20	6,42982E-26	-5,40712E-01	0,217	0,538	1,18418E-21
Macro_FOLR2+APOE-	SSB	5,88249E-23	-5,40779E-01	0,177	0,455	1,08338E-18
Macro_FOLR2+APOE-	RNF181	2,52512E-18	-5,40874E-01	0,258	0,565	4,65051E-14
Macro_FOLR2+APOE-	DYNC1I2	2,51842E-14	-5,41081E-01	0,09	0,254	4,63818E-10
Macro_FOLR2+APOE-	TMED3	1,57046E-17	-5,41821E-01	0,089	0,276	2,89231E-13
Macro_FOLR2+APOE-	PRPF8	2,64372E-13	-5,42407E-01	0,13	0,309	4,86894E-09
Macro_FOLR2+APOE-	ITGB1	1,91590E-30	-5,42501E-01	0,162	0,478	3,52852E-26

Macro_FOLR2+APOE-	C11orf58	8,27091E-24	-5,42896E-01	0,235	0,563	1,52325E-19
Macro_FOLR2+APOE-	CNN2	7,55225E-34	-5,43007E-01	0,111	0,406	1,39090E-29
Macro_FOLR2+APOE-	GLB1	1,02088E-13	-5,44032E-01	0,119	0,294	1,88016E-09
Macro_FOLR2+APOE-	SHOC2	4,98837E-27	-5,45362E-01	0,047	0,267	9,18709E-23
Macro_FOLR2+APOE-	TMEM147	2,74798E-12	-5,46405E-01	0,211	0,436	5,06095E-08
Macro_FOLR2+APOE-	RPS27L	2,31657E-22	-5,46874E-01	0,124	0,365	4,26643E-18
Macro_FOLR2+APOE-	NEDD8	6,08750E-27	-5,47802E-01	0,249	0,591	1,12114E-22
Macro_FOLR2+APOE-	CAT	8,55281E-11	-5,48106E-01	0,207	0,411	1,57517E-06
Macro_FOLR2+APOE-	SF3A1	1,55189E-18	-5,48540E-01	0,094	0,291	2,85812E-14
Macro_FOLR2+APOE-	GNAS	5,77712E-09	-5,49673E-01	0,348	0,613	1,06397E-04
Macro_FOLR2+APOE-	SPAG9	1,82808E-25	-5,50411E-01	0,077	0,306	3,36677E-21
Macro_FOLR2+APOE-	OCIAD1	4,46045E-21	-5,50995E-01	0,171	0,437	8,21481E-17
Macro_FOLR2+APOE-	RHOG	6,10891E-17	-5,51953E-01	0,399	0,731	1,12508E-12
Macro_FOLR2+APOE-	ELOVL5	1,27492E-24	-5,52195E-01	0,07	0,29	2,34801E-20
Macro_FOLR2+APOE-	HIGD2A	2,34802E-18	-5,52346E-01	0,379	0,718	4,32435E-14
Macro_FOLR2+APOE-	C1orf43	3,60585E-20	-5,53174E-01	0,247	0,55	6,64090E-16
Macro_FOLR2+APOE-	ZC3H13	1,52300E-16	-5,53626E-01	0,083	0,257	2,80490E-12
Macro_FOLR2+APOE-	CHMP2B	5,72174E-19	-5,54379E-01	0,098	0,301	1,05377E-14
Macro_FOLR2+APOE-	ZMIZ1	7,02939E-26	-5,54663E-01	0,053	0,268	1,29460E-21
Macro_FOLR2+APOE-	CAPRIN1	3,62953E-21	-5,54821E-01	0,111	0,338	6,68450E-17
Macro_FOLR2+APOE-	ZYX	1,73305E-28	-5,54848E-01	0,298	0,649	3,19176E-24
Macro_FOLR2+APOE-	RTCB	7,20024E-15	-5,54887E-01	0,113	0,297	1,32607E-10
Macro_FOLR2+APOE-	TMEM14B	5,41151E-22	-5,55130E-01	0,158	0,425	9,96638E-18
Macro_FOLR2+APOE-	RHEB	5,46246E-27	-5,55289E-01	0,164	0,466	1,00602E-22
Macro_FOLR2+APOE-	OSTC	1,64894E-33	-5,55545E-01	0,166	0,503	3,03685E-29
Macro_FOLR2+APOE-	UBE2N	1,61029E-32	-5,57180E-01	0,092	0,377	2,96567E-28
Macro_FOLR2+APOE-	PDIA4	5,16183E-20	-5,57425E-01	0,107	0,323	9,50655E-16
Macro_FOLR2+APOE-	PCBP1	1,18307E-33	-5,57805E-01	0,252	0,615	2,17886E-29
Macro_FOLR2+APOE-	PGLS	5,98600E-16	-5,57825E-01	0,267	0,546	1,10244E-11
Macro_FOLR2+APOE-	COX14	1,53878E-28	-5,58708E-01	0,158	0,465	2,83397E-24
Macro_FOLR2+APOE-	REEP5	2,23252E-21	-5,59442E-01	0,254	0,566	4,11164E-17
Macro_FOLR2+APOE-	AGPAT2	5,87389E-20	-5,60029E-01	0,09	0,294	1,08179E-15
Macro_FOLR2+APOE-	ERGIC1	3,68800E-29	-5,60586E-01	0,056	0,294	6,79219E-25
Macro_FOLR2+APOE-	PIGT	6,75629E-14	-5,61292E-01	0,117	0,293	1,24431E-09
Macro_FOLR2+APOE-	DCP2	5,47553E-16	-5,61462E-01	0,092	0,268	1,00843E-11
Macro_FOLR2+APOE-	ZFR	2,32730E-17	-5,61934E-01	0,075	0,251	4,28618E-13
Macro_FOLR2+APOE-	CD9	1,15357E-19	-5,61999E-01	0,25	0,488	2,12452E-15
Macro_FOLR2+APOE-	MFSD10	3,36386E-17	-5,62156E-01	0,089	0,272	6,19523E-13
Macro_FOLR2+APOE-	SLC16A3	9,14693E-34	-5,62276E-01	0,183	0,516	1,68459E-29
Macro_FOLR2+APOE-	SYF2	3,16214E-22	-5,63317E-01	0,16	0,428	5,82372E-18
Macro_FOLR2+APOE-	SCNM1	4,27208E-16	-5,64647E-01	0,132	0,337	7,86790E-12
Macro_FOLR2+APOE-	SNRPC	1,56173E-20	-5,65230E-01	0,162	0,422	2,87623E-16
Macro_FOLR2+APOE-	SEC61A1	9,39499E-18	-5,65316E-01	0,109	0,313	1,73028E-13
Macro_FOLR2+APOE-	MAGOH	1,68960E-24	-5,65894E-01	0,105	0,352	3,11175E-20
Macro_FOLR2+APOE-	HNRNPD	2,94787E-21	-5,66437E-01	0,115	0,346	5,42909E-17
Macro_FOLR2+APOE-	SRSF4	3,93800E-24	-5,67208E-01	0,111	0,356	7,25262E-20
Macro_FOLR2+APOE-	SDHD	9,81352E-16	-5,67398E-01	0,141	0,346	1,80736E-11

Macro_FOLR2+APOE-	ATP6V1E1	1,81966E-23	-5,67953E-01	0,1	0,334	3,35127E-19
Macro_FOLR2+APOE-	PDCD10	3,00771E-20	-5,68235E-01	0,102	0,317	5,53930E-16
Macro_FOLR2+APOE-	LAPTM4A	6,91077E-08	-5,68238E-01	0,371	0,631	1,27276E-03
Macro_FOLR2+APOE-	SMARCB1	3,94610E-23	-5,69295E-01	0,079	0,297	7,26753E-19
Macro_FOLR2+APOE-	DSTN	3,60395E-17	-5,69533E-01	0,136	0,349	6,63739E-13
Macro_FOLR2+APOE-	NOTCH2	4,78048E-16	-5,69795E-01	0,126	0,319	8,80420E-12
Macro_FOLR2+APOE-	NSFL1C	2,74690E-16	-5,70317E-01	0,094	0,276	5,05896E-12
Macro_FOLR2+APOE-	EID1	4,26070E-20	-5,71250E-01	0,271	0,583	7,84693E-16
Macro_FOLR2+APOE-	UFC1	1,15883E-20	-5,71308E-01	0,215	0,506	2,13423E-16
Macro_FOLR2+APOE-	CARD16	1,23051E-13	-5,72658E-01	0,286	0,555	2,26624E-09
Macro_FOLR2+APOE-	IDH2	4,40483E-23	-5,73172E-01	0,151	0,414	8,11238E-19
Macro_FOLR2+APOE-	NUDT21	1,42537E-16	-5,73812E-01	0,089	0,269	2,62510E-12
Macro_FOLR2+APOE-	ADRM1	1,32001E-29	-5,73827E-01	0,136	0,432	2,43107E-25
Macro_FOLR2+APOE-	RSL1D1	3,31895E-25	-5,74149E-01	0,177	0,473	6,11251E-21
Macro_FOLR2+APOE-	AKAP9	1,62817E-12	-5,74557E-01	0,185	0,388	2,99859E-08
Macro_FOLR2+APOE-	CTSA	9,22810E-11	-5,74573E-01	0,277	0,508	1,69954E-06
Macro_FOLR2+APOE-	SMIM14	2,18016E-17	-5,74591E-01	0,089	0,271	4,01520E-13
Macro_FOLR2+APOE-	CDC42	3,04724E-14	-5,74990E-01	0,371	0,7	5,61210E-10
Macro_FOLR2+APOE-	DCK	3,10473E-20	-5,75411E-01	0,081	0,282	5,71797E-16
Macro_FOLR2+APOE-	CAPN1	1,89522E-15	-5,78486E-01	0,094	0,269	3,49042E-11
Macro_FOLR2+APOE-	SUCLG1	9,22328E-19	-5,78567E-01	0,105	0,313	1,69865E-14
Macro_FOLR2+APOE-	LST1	3,70138E-12	-5,78824E-01	0,461	0,718	6,81684E-08
Macro_FOLR2+APOE-	NDUFA1	8,97385E-26	-5,79325E-01	0,324	0,691	1,65271E-21
Macro_FOLR2+APOE-	COPA	3,57649E-27	-5,79447E-01	0,09	0,34	6,58682E-23
Macro_FOLR2+APOE-	CUX1	1,16595E-19	-5,79492E-01	0,13	0,353	2,14733E-15
Macro_FOLR2+APOE-	NDUFB2	2,59502E-16	-5,79523E-01	0,405	0,718	4,77926E-12
Macro_FOLR2+APOE-	STARD3NL	1,62533E-22	-5,79641E-01	0,066	0,273	2,99337E-18
Macro_FOLR2+APOE-	CYC1	7,81804E-23	-5,80784E-01	0,164	0,437	1,43985E-18
Macro_FOLR2+APOE-	PLEKHJ1	1,93223E-13	-5,80813E-01	0,092	0,251	3,55859E-09
Macro_FOLR2+APOE-	MAF1	2,12619E-21	-5,80917E-01	0,143	0,391	3,91580E-17
Macro_FOLR2+APOE-	COX5B	3,17953E-17	-5,80938E-01	0,435	0,776	5,85574E-13
Macro_FOLR2+APOE-	HMGA1	4,38408E-38	-5,81078E-01	0,096	0,408	8,07415E-34
Macro_FOLR2+APOE-	PARL	3,03328E-13	-5,81966E-01	0,119	0,292	5,58638E-09
Macro_FOLR2+APOE-	YIPF4	5,42053E-18	-5,82127E-01	0,081	0,266	9,98298E-14
Macro_FOLR2+APOE-	DOCK8	4,00704E-25	-5,82347E-01	0,169	0,451	7,37976E-21
Macro_FOLR2+APOE-	ARF4	8,61078E-31	-5,82491E-01	0,143	0,449	1,58585E-26
Macro_FOLR2+APOE-	PSMD2	6,96467E-20	-5,82960E-01	0,107	0,322	1,28268E-15
Macro_FOLR2+APOE-	NECAP2	8,91212E-17	-5,83220E-01	0,139	0,355	1,64134E-12
Macro_FOLR2+APOE-	TAOK3	8,33292E-25	-5,84072E-01	0,169	0,454	1,53467E-20
Macro_FOLR2+APOE-	SUMO2	1,51632E-11	-5,84155E-01	0,461	0,787	2,79261E-07
Macro_FOLR2+APOE-	IER5	5,53405E-25	-5,84920E-01	0,096	0,329	1,01921E-20
Macro_FOLR2+APOE-	ZSWIM7	3,98899E-14	-5,86787E-01	0,09	0,251	7,34652E-10
Macro_FOLR2+APOE-	CSNK1D	7,09648E-23	-5,86813E-01	0,109	0,346	1,30696E-18
Macro_FOLR2+APOE-	PRRC2C	3,08742E-17	-5,88422E-01	0,296	0,597	5,68611E-13
Macro_FOLR2+APOE-	CDC26	8,27345E-24	-5,89099E-01	0,06	0,271	1,52372E-19
Macro_FOLR2+APOE-	ADAM10	2,69643E-16	-5,89134E-01	0,104	0,289	4,96602E-12
Macro_FOLR2+APOE-	HDAC2	5,37173E-14	-5,90788E-01	0,102	0,269	9,89311E-10

Macro_FOLR2+APOE-	SEC11A	9,62779E-13	-5,90974E-01	0,375	0,67	1,77315E-08
Macro_FOLR2+APOE-	RNH1	4,24468E-23	-5,91776E-01	0,286	0,622	7,81742E-19
Macro_FOLR2+APOE-	RAB18	5,09488E-17	-5,92157E-01	0,094	0,283	9,38324E-13
Macro_FOLR2+APOE-	TMED10	6,39785E-24	-5,92400E-01	0,243	0,571	1,17829E-19
Macro_FOLR2+APOE-	TBCA	6,76139E-26	-5,93350E-01	0,245	0,593	1,24524E-21
Macro_FOLR2+APOE-	HCST	1,38540E-13	-5,94232E-01	0,36	0,657	2,55148E-09
Macro_FOLR2+APOE-	AKR7A2	5,86631E-14	-5,94242E-01	0,09	0,251	1,08040E-09
Macro_FOLR2+APOE-	FAM50A	8,19487E-24	-5,94594E-01	0,083	0,307	1,50925E-19
Macro_FOLR2+APOE-	TMED9	3,23052E-12	-5,94830E-01	0,273	0,527	5,94965E-08
Macro_FOLR2+APOE-	ABRACL	1,03535E-20	-5,95721E-01	0,218	0,501	1,90681E-16
Macro_FOLR2+APOE-	C1orf162	3,72531E-08	-5,95944E-01	0,463	0,71	6,86090E-04
Macro_FOLR2+APOE-	POLR1D	1,01508E-21	-5,96296E-01	0,19	0,471	1,86947E-17
Macro_FOLR2+APOE-	GNA13	8,72676E-28	-5,96812E-01	0,087	0,337	1,60721E-23
Macro_FOLR2+APOE-	ZNF385A	5,33770E-25	-5,97672E-01	0,147	0,415	9,83044E-21
Macro_FOLR2+APOE-	ACSL4	4,46851E-27	-5,98497E-01	0,055	0,278	8,22966E-23
Macro_FOLR2+APOE-	CCDC59	1,32952E-18	-5,98559E-01	0,07	0,251	2,44858E-14
Macro_FOLR2+APOE-	FMNL1	1,53896E-28	-5,98708E-01	0,126	0,406	2,83430E-24
Macro_FOLR2+APOE-	BSG	4,11668E-21	-5,98919E-01	0,254	0,566	7,58170E-17
Macro_FOLR2+APOE-	ARGLU1	9,96758E-25	-5,98963E-01	0,158	0,436	1,83573E-20
Macro_FOLR2+APOE-	STK4	5,69442E-24	-5,99134E-01	0,162	0,432	1,04874E-19
Macro_FOLR2+APOE-	CEBPB	3,90107E-33	-5,99190E-01	0,166	0,475	7,18460E-29
Macro_FOLR2+APOE-	GNS	1,29101E-23	-5,99732E-01	0,162	0,433	2,37766E-19
Macro_FOLR2+APOE-	PTBP3	5,05042E-18	-5,99930E-01	0,121	0,328	9,30136E-14
Macro_FOLR2+APOE-	ARL6IP4	6,98918E-23	-6,01256E-01	0,292	0,638	1,28720E-18
Macro_FOLR2+APOE-	ANKRD11	1,89299E-23	-6,01319E-01	0,09	0,315	3,48632E-19
Macro_FOLR2+APOE-	DNAJC3	6,29131E-25	-6,01726E-01	0,115	0,367	1,15867E-20
Macro_FOLR2+APOE-	CD40	1,24134E-28	-6,02413E-01	0,058	0,295	2,28617E-24
Macro_FOLR2+APOE-	PTRHD1	2,65313E-25	-6,02535E-01	0,104	0,351	4,88627E-21
Macro_FOLR2+APOE-	SERP1	1,51335E-23	-6,02794E-01	0,467	0,823	2,78713E-19
Macro_FOLR2+APOE-	TMX1	9,11796E-21	-6,03796E-01	0,113	0,339	1,67925E-16
Macro_FOLR2+APOE-	EVI2B	2,21384E-12	-6,03898E-01	0,181	0,378	4,07723E-08
Macro_FOLR2+APOE-	GLIPR1	5,69073E-08	-6,03977E-01	0,345	0,586	1,04806E-03
Macro_FOLR2+APOE-	MIF4GD	6,48933E-14	-6,05453E-01	0,13	0,314	1,19514E-09
Macro_FOLR2+APOE-	SRRM1	8,79696E-30	-6,05552E-01	0,218	0,568	1,62014E-25
Macro_FOLR2+APOE-	CALM3	7,39371E-23	-6,06196E-01	0,211	0,511	1,36170E-18
Macro_FOLR2+APOE-	CMTM7	8,71462E-16	-6,06486E-01	0,183	0,414	1,60497E-11
Macro_FOLR2+APOE-	MRPS12	5,28526E-22	-6,06818E-01	0,075	0,285	9,73387E-18
Macro_FOLR2+APOE-	CDC42SE1	1,13146E-29	-6,07171E-01	0,107	0,388	2,08381E-25
Macro_FOLR2+APOE-	CANX	4,61577E-18	-6,07664E-01	0,32	0,643	8,50086E-14
Macro_FOLR2+APOE-	ENSA	2,37315E-43	-6,07916E-01	0,119	0,476	4,37064E-39
Macro_FOLR2+APOE-	SRRM2	7,50921E-29	-6,08965E-01	0,232	0,573	1,38297E-24
Macro_FOLR2+APOE-	ACAP2	1,79172E-19	-6,09678E-01	0,177	0,434	3,29981E-15
Macro_FOLR2+APOE-	PTPN1	3,29539E-45	-6,10018E-01	0,072	0,405	6,06912E-41
Macro_FOLR2+APOE-	LAMTOR4	1,20000E-09	-6,10917E-01	0,412	0,718	2,21003E-05
Macro_FOLR2+APOE-	ECHDC1	3,02172E-20	-6,11026E-01	0,075	0,272	5,56511E-16
Macro_FOLR2+APOE-	BCKDK	3,10266E-19	-6,11253E-01	0,109	0,321	5,71416E-15
Macro_FOLR2+APOE-	BECN1	1,80883E-16	-6,11414E-01	0,085	0,262	3,33132E-12

Macro_FOLR2+APOE-	CYTH4	1,28266E-11	-6,11611E-01	0,145	0,317	2,36228E-07
Macro_FOLR2+APOE-	LSM14A	1,24573E-18	-6,12316E-01	0,111	0,319	2,29426E-14
Macro_FOLR2+APOE-	PARP9	9,79056E-20	-6,12705E-01	0,068	0,256	1,80313E-15
Macro_FOLR2+APOE-	PFDN2	1,10421E-25	-6,13716E-01	0,158	0,445	2,03362E-21
Macro_FOLR2+APOE-	ETFB	3,38937E-19	-6,13804E-01	0,1	0,309	6,24219E-15
Macro_FOLR2+APOE-	UBE2J1	4,07324E-32	-6,14152E-01	0,053	0,304	7,50169E-28
Macro_FOLR2+APOE-	UVRAG	2,72570E-19	-6,14536E-01	0,092	0,293	5,01992E-15
Macro_FOLR2+APOE-	ARPC5	5,11207E-12	-6,15119E-01	0,439	0,766	9,41489E-08
Macro_FOLR2+APOE-	QSOX1	4,39869E-21	-6,15333E-01	0,085	0,291	8,10107E-17
Macro_FOLR2+APOE-	SET	3,21596E-26	-6,15808E-01	0,213	0,531	5,92284E-22
Macro_FOLR2+APOE-	NCF1	1,92111E-08	-6,16520E-01	0,301	0,502	3,53811E-04
Macro_FOLR2+APOE-	DOK2	1,47145E-08	-6,16999E-01	0,186	0,347	2,70997E-04
Macro_FOLR2+APOE-	TMSB10	2,36092E-07	-6,17395E-01	0,976	0,984	4,34811E-03
Macro_FOLR2+APOE-	CD86	1,01961E-23	-6,17605E-01	0,209	0,498	1,87781E-19
Macro_FOLR2+APOE-	BANF1	8,65145E-26	-6,18328E-01	0,224	0,552	1,59334E-21
Macro_FOLR2+APOE-	CNOT7	1,06328E-12	-6,19388E-01	0,134	0,31	1,95824E-08
Macro_FOLR2+APOE-	KXD1	5,02757E-21	-6,19389E-01	0,122	0,354	9,25928E-17
Macro_FOLR2+APOE-	MBP	6,25044E-32	-6,19851E-01	0,064	0,322	1,15114E-27
Macro_FOLR2+APOE-	LSM5	1,82773E-25	-6,20642E-01	0,1	0,347	3,36612E-21
Macro_FOLR2+APOE-	YBX3	4,92687E-27	-6,22141E-01	0,1	0,351	9,07381E-23
Macro_FOLR2+APOE-	CAPNS1	9,88625E-28	-6,22159E-01	0,107	0,373	1,82075E-23
Macro_FOLR2+APOE-	PPP1R11	6,01170E-28	-6,22205E-01	0,072	0,314	1,10717E-23
Macro_FOLR2+APOE-	IMPDH2	5,54519E-13	-6,22549E-01	0,109	0,275	1,02126E-08
Macro_FOLR2+APOE-	HNRNPL	3,37981E-19	-6,23207E-01	0,092	0,291	6,22460E-15
Macro_FOLR2+APOE-	EMC6	3,45056E-24	-6,23327E-01	0,085	0,316	6,35489E-20
Macro_FOLR2+APOE-	AHCY	2,37256E-17	-6,23806E-01	0,083	0,264	4,36954E-13
Macro_FOLR2+APOE-	CAPZA2	3,85716E-09	-6,24189E-01	0,301	0,552	7,10373E-05
Macro_FOLR2+APOE-	ATP2B1	2,74025E-19	-6,26174E-01	0,13	0,351	5,04672E-15
Macro_FOLR2+APOE-	RAB5A	2,79946E-21	-6,26843E-01	0,094	0,312	5,15577E-17
Macro_FOLR2+APOE-	AZIN1	2,43102E-25	-6,26993E-01	0,085	0,322	4,47722E-21
Macro_FOLR2+APOE-	LCP2	2,87438E-19	-6,27913E-01	0,284	0,58	5,29375E-15
Macro_FOLR2+APOE-	DAP3	2,59661E-12	-6,28516E-01	0,139	0,32	4,78218E-08
Macro_FOLR2+APOE-	PAPOLA	1,93238E-19	-6,29484E-01	0,188	0,458	3,55887E-15
Macro_FOLR2+APOE-	LRRFIP2	7,85287E-24	-6,29846E-01	0,053	0,254	1,44626E-19
Macro_FOLR2+APOE-	STOML2	3,87901E-21	-6,30349E-01	0,075	0,278	7,14398E-17
Macro_FOLR2+APOE-	ANAPC5	3,67535E-18	-6,31093E-01	0,094	0,29	6,76889E-14
Macro_FOLR2+APOE-	TOMM5	4,47742E-26	-6,31142E-01	0,085	0,329	8,24606E-22
Macro_FOLR2+APOE-	CASP1	3,89014E-18	-6,31429E-01	0,211	0,466	7,16448E-14
Macro_FOLR2+APOE-	CSK	7,94039E-30	-6,31760E-01	0,087	0,35	1,46238E-25
Macro_FOLR2+APOE-	DOCK2	4,01532E-18	-6,31769E-01	0,132	0,351	7,39502E-14
Macro_FOLR2+APOE-	PSMB5	5,69765E-20	-6,32888E-01	0,102	0,318	1,04934E-15
Macro_FOLR2+APOE-	CCDC107	2,69182E-18	-6,33205E-01	0,105	0,309	4,95753E-14
Macro_FOLR2+APOE-	TMEM109	1,92258E-24	-6,33400E-01	0,122	0,372	3,54082E-20
Macro_FOLR2+APOE-	ARIH2	1,23272E-15	-6,33451E-01	0,089	0,26	2,27030E-11
Macro_FOLR2+APOE-	EML4	1,01695E-25	-6,33517E-01	0,109	0,359	1,87291E-21
Macro_FOLR2+APOE-	GPAA1	1,54523E-16	-6,34742E-01	0,083	0,258	2,84584E-12
Macro_FOLR2+APOE-	TAPBP	3,34577E-39	-6,36095E-01	0,202	0,583	6,16190E-35

Macro_FOLR2+APOE-	TMEM30A	3,29710E-18	-6,36131E-01	0,105	0,307	6,07227E-14
Macro_FOLR2+APOE-	MPP1	3,40816E-11	-6,36377E-01	0,207	0,415	6,27681E-07
Macro_FOLR2+APOE-	UFM1	7,70464E-26	-6,36389E-01	0,087	0,329	1,41896E-21
Macro_FOLR2+APOE-	LRRC25	2,82701E-16	-6,36829E-01	0,134	0,336	5,20651E-12
Macro_FOLR2+APOE-	SLIRP	1,57931E-33	-6,37158E-01	0,119	0,43	2,90861E-29
Macro_FOLR2+APOE-	SDHC	8,67788E-22	-6,37597E-01	0,171	0,444	1,59821E-17
Macro_FOLR2+APOE-	LAT2	1,97643E-19	-6,37864E-01	0,198	0,457	3,63999E-15
Macro_FOLR2+APOE-	GPCPD1	1,01270E-27	-6,38910E-01	0,056	0,283	1,86509E-23
Macro_FOLR2+APOE-	COPB2	1,02093E-19	-6,39399E-01	0,068	0,256	1,88024E-15
Macro_FOLR2+APOE-	SNX1	1,04916E-17	-6,39431E-01	0,107	0,309	1,93224E-13
Macro_FOLR2+APOE-	HINT1	2,68515E-11	-6,41024E-01	0,431	0,726	4,94524E-07
Macro_FOLR2+APOE-	MICAL1	2,89633E-24	-6,41471E-01	0,073	0,296	5,33417E-20
Macro_FOLR2+APOE-	COX8A	7,18091E-25	-6,41517E-01	0,411	0,782	1,32251E-20
Macro_FOLR2+APOE-	PSMD1	6,53126E-20	-6,42328E-01	0,083	0,28	1,20286E-15
Macro_FOLR2+APOE-	ACTN1	3,80074E-26	-6,43061E-01	0,083	0,321	6,99982E-22
Macro_FOLR2+APOE-	EIF4H	1,39364E-29	-6,43070E-01	0,151	0,456	2,56667E-25
Macro_FOLR2+APOE-	WBP2	4,21740E-22	-6,43184E-01	0,139	0,386	7,76718E-18
Macro_FOLR2+APOE-	VCP	2,30997E-28	-6,43281E-01	0,166	0,469	4,25427E-24
Macro_FOLR2+APOE-	C14orf119	1,09135E-19	-6,43394E-01	0,09	0,294	2,00995E-15
Macro_FOLR2+APOE-	AZI2	1,59555E-15	-6,44050E-01	0,119	0,304	2,93852E-11
Macro_FOLR2+APOE-	PRDX4	4,21665E-16	-6,44291E-01	0,104	0,289	7,76581E-12
Macro_FOLR2+APOE-	SP110	3,62994E-16	-6,44979E-01	0,173	0,392	6,68527E-12
Macro_FOLR2+APOE-	MRPS5	4,11573E-17	-6,45065E-01	0,079	0,257	7,57994E-13
Macro_FOLR2+APOE-	HADHB	3,92236E-21	-6,45490E-01	0,126	0,363	7,22382E-17
Macro_FOLR2+APOE-	TUBB	1,36549E-18	-6,46255E-01	0,352	0,664	2,51482E-14
Macro_FOLR2+APOE-	ARAP1	2,19241E-13	-6,46397E-01	0,121	0,294	4,03776E-09
Macro_FOLR2+APOE-	SRSF1	2,37255E-16	-6,47118E-01	0,092	0,27	4,36953E-12
Macro_FOLR2+APOE-	ATF5	9,33739E-33	-6,47404E-01	0,087	0,364	1,71967E-28
Macro_FOLR2+APOE-	DNASE2	4,24704E-18	-6,48389E-01	0,153	0,369	7,82178E-14
Macro_FOLR2+APOE-	LIPA	1,50567E-08	-6,48448E-01	0,315	0,503	2,77299E-04
Macro_FOLR2+APOE-	MAP4	5,64196E-17	-6,48791E-01	0,083	0,26	1,03908E-12
Macro_FOLR2+APOE-	UBE2V2	3,65290E-18	-6,49446E-01	0,077	0,261	6,72754E-14
Macro_FOLR2+APOE-	LYN	9,71237E-32	-6,50066E-01	0,145	0,455	1,78873E-27
Macro_FOLR2+APOE-	IK	1,86383E-24	-6,50091E-01	0,1	0,341	3,43262E-20
Macro_FOLR2+APOE-	ITGB1BP1	8,88000E-17	-6,50735E-01	0,077	0,252	1,63543E-12
Macro_FOLR2+APOE-	PTGES3	5,39592E-20	-6,51868E-01	0,32	0,647	9,93767E-16
Macro_FOLR2+APOE-	ANXA4	6,50661E-21	-6,52371E-01	0,096	0,311	1,19832E-16
Macro_FOLR2+APOE-	RNASET2	1,10423E-06	-6,54280E-01	0,497	0,743	2,03366E-02
Macro_FOLR2+APOE-	THOC7	2,62450E-21	-6,54301E-01	0,111	0,341	4,83355E-17
Macro_FOLR2+APOE-	SCAND1	5,59634E-33	-6,54864E-01	0,19	0,539	1,03068E-28
Macro_FOLR2+APOE-	SKAP2	1,37762E-17	-6,54928E-01	0,171	0,408	2,53717E-13
Macro_FOLR2+APOE-	HSD17B10	2,79769E-21	-6,54983E-01	0,092	0,309	5,15251E-17
Macro_FOLR2+APOE-	YTHDC1	1,00754E-20	-6,54998E-01	0,096	0,308	1,85559E-16
Macro_FOLR2+APOE-	CNIH1	2,13511E-22	-6,55383E-01	0,139	0,388	3,93224E-18
Macro_FOLR2+APOE-	MORF4L1	3,54081E-21	-6,55436E-01	0,303	0,655	6,52111E-17
Macro_FOLR2+APOE-	EIF4E	4,98808E-21	-6,56030E-01	0,09	0,3	9,18655E-17
Macro_FOLR2+APOE-	RHBDD2	6,87060E-19	-6,56327E-01	0,075	0,26	1,26536E-14

Macro_FOLR2+APOE-	GRSF1	3,18554E-15	-6,56473E-01	0,143	0,342	5,86680E-11
Macro_FOLR2+APOE-	PER1	1,14128E-15	-6,57918E-01	0,132	0,322	2,10189E-11
Macro_FOLR2+APOE-	SLC7A7	7,93857E-16	-6,58852E-01	0,224	0,468	1,46205E-11
Macro_FOLR2+APOE-	DAD1	3,94714E-14	-6,58884E-01	0,305	0,604	7,26944E-10
Macro_FOLR2+APOE-	STX10	4,93891E-22	-6,59324E-01	0,092	0,312	9,09599E-18
Macro_FOLR2+APOE-	DNAJC7	2,13010E-23	-6,61104E-01	0,119	0,368	3,92300E-19
Macro_FOLR2+APOE-	TNFSF10	7,80252E-29	-6,61251E-01	0,105	0,363	1,43699E-24
Macro_FOLR2+APOE-	ATF4	1,82845E-26	-6,61563E-01	0,134	0,409	3,36746E-22
Macro_FOLR2+APOE-	EMC3	6,70589E-18	-6,61926E-01	0,115	0,322	1,23502E-13
Macro_FOLR2+APOE-	RALY	1,46523E-30	-6,62288E-01	0,171	0,496	2,69852E-26
Macro_FOLR2+APOE-	RRP7A	4,91855E-24	-6,64351E-01	0,075	0,297	9,05849E-20
Macro_FOLR2+APOE-	SERF2	3,49749E-07	-6,64521E-01	0,823	0,955	6,44134E-03
Macro_FOLR2+APOE-	BNIP3L	1,69673E-22	-6,65342E-01	0,254	0,567	3,12486E-18
Macro_FOLR2+APOE-	ERICH1	2,09375E-26	-6,65789E-01	0,085	0,324	3,85605E-22
Macro_FOLR2+APOE-	SNX2	3,54106E-15	-6,66454E-01	0,203	0,444	6,52157E-11
Macro_FOLR2+APOE-	PTDSS1	3,29544E-19	-6,66700E-01	0,079	0,272	6,06922E-15
Macro_FOLR2+APOE-	LAMTOR3	1,20494E-22	-6,66875E-01	0,058	0,261	2,21914E-18
Macro_FOLR2+APOE-	TRABD	4,28806E-24	-6,68007E-01	0,115	0,36	7,89732E-20
Macro_FOLR2+APOE-	PTGER4	7,90268E-12	-6,71669E-01	0,117	0,271	1,45544E-07
Macro_FOLR2+APOE-	C9orf78	3,88919E-25	-6,71822E-01	0,096	0,339	7,16272E-21
Macro_FOLR2+APOE-	EEF1B2	2,63530E-08	-6,72034E-01	0,599	0,844	4,85342E-04
Macro_FOLR2+APOE-	TIMM17A	2,04792E-24	-6,72142E-01	0,06	0,274	3,77166E-20
Macro_FOLR2+APOE-	ARHGAP15	1,61851E-14	-6,72299E-01	0,092	0,256	2,98080E-10
Macro_FOLR2+APOE-	LAMP2	3,75886E-13	-6,73594E-01	0,19	0,404	6,92269E-09
Macro_FOLR2+APOE-	EIF2A	2,34781E-16	-6,74182E-01	0,115	0,31	4,32395E-12
Macro_FOLR2+APOE-	TWF2	1,61603E-25	-6,74390E-01	0,09	0,337	2,97623E-21
Macro_FOLR2+APOE-	YWHAQ	1,84060E-28	-6,74475E-01	0,143	0,433	3,38983E-24
Macro_FOLR2+APOE-	CLEC4A	4,52754E-11	-6,74739E-01	0,139	0,299	8,33838E-07
Macro_FOLR2+APOE-	SRP9	1,42298E-20	-6,75545E-01	0,186	0,468	2,62071E-16
Macro_FOLR2+APOE-	TM9SF2	1,14033E-21	-6,76013E-01	0,166	0,43	2,10014E-17
Macro_FOLR2+APOE-	RNF5	9,37828E-20	-6,76816E-01	0,085	0,285	1,72720E-15
Macro_FOLR2+APOE-	KRTCAP2	4,42742E-24	-6,77217E-01	0,252	0,581	8,15398E-20
Macro_FOLR2+APOE-	RPL22L1	1,27583E-23	-6,78477E-01	0,064	0,276	2,34970E-19
Macro_FOLR2+APOE-	ATP6V0D1	4,81383E-38	-6,79948E-01	0,2	0,575	8,86563E-34
Macro_FOLR2+APOE-	POLR2A	1,78755E-21	-6,80491E-01	0,096	0,311	3,29213E-17
Macro_FOLR2+APOE-	PSMA1	1,30283E-15	-6,80994E-01	0,153	0,363	2,39943E-11
Macro_FOLR2+APOE-	UBE2L3	1,98745E-32	-6,82282E-01	0,169	0,505	3,66028E-28
Macro_FOLR2+APOE-	RPN1	6,30115E-23	-6,82384E-01	0,079	0,297	1,16048E-18
Macro_FOLR2+APOE-	DMXL2	3,42340E-18	-6,82396E-01	0,085	0,269	6,30488E-14
Macro_FOLR2+APOE-	SF1	2,81876E-31	-6,83248E-01	0,175	0,507	5,19131E-27
Macro_FOLR2+APOE-	BLOC1S2	6,76408E-29	-6,83373E-01	0,079	0,334	1,24574E-24
Macro_FOLR2+APOE-	SNRPD3	3,79471E-21	-6,83445E-01	0,136	0,373	6,98871E-17
Macro_FOLR2+APOE-	YIF1A	4,80112E-21	-6,84492E-01	0,081	0,287	8,84223E-17
Macro_FOLR2+APOE-	PTPMT1	1,78938E-14	-6,85048E-01	0,087	0,25	3,29550E-10
Macro_FOLR2+APOE-	PRDX3	4,06519E-23	-6,85641E-01	0,188	0,483	7,48685E-19
Macro_FOLR2+APOE-	FBXW5	1,58961E-24	-6,85702E-01	0,085	0,317	2,92758E-20
Macro_FOLR2+APOE-	MRPL28	2,88508E-19	-6,85709E-01	0,077	0,269	5,31344E-15

Macro_FOLR2+APOE-	NFYC	6,24075E-19	-6,85771E-01	0,073	0,26	1,14936E-14
Macro_FOLR2+APOE-	PSMD7	1,17975E-28	-6,85883E-01	0,126	0,409	2,17275E-24
Macro_FOLR2+APOE-	HADHA	1,23920E-27	-6,86306E-01	0,202	0,524	2,28224E-23
Macro_FOLR2+APOE-	SPNS1	2,83401E-20	-6,86348E-01	0,064	0,251	5,21939E-16
Macro_FOLR2+APOE-	PHPT1	5,51297E-31	-6,87103E-01	0,139	0,45	1,01532E-26
Macro_FOLR2+APOE-	PDCD5	2,77440E-23	-6,87146E-01	0,113	0,354	5,10961E-19
Macro_FOLR2+APOE-	HPS1	2,01547E-16	-6,87178E-01	0,141	0,354	3,71189E-12
Macro_FOLR2+APOE-	ATP6AP1	4,26894E-21	-6,87638E-01	0,245	0,547	7,86210E-17
Macro_FOLR2+APOE-	SSNA1	1,68261E-27	-6,88966E-01	0,128	0,407	3,09887E-23
Macro_FOLR2+APOE-	RPL24	2,21828E-06	-6,89439E-01	0,362	0,54	4,08541E-02
Macro_FOLR2+APOE-	SND1	2,19257E-19	-6,90188E-01	0,124	0,347	4,03806E-15
Macro_FOLR2+APOE-	CHD4	1,48111E-19	-6,90839E-01	0,083	0,279	2,72777E-15
Macro_FOLR2+APOE-	PYCARD	2,00310E-10	-6,91746E-01	0,431	0,709	3,68911E-06
Macro_FOLR2+APOE-	PIAS1	1,07644E-17	-6,92384E-01	0,119	0,324	1,98248E-13
Macro_FOLR2+APOE-	ASAH1	2,34420E-06	-6,93604E-01	0,452	0,691	4,31732E-02
Macro_FOLR2+APOE-	NDUFB10	1,53540E-26	-6,94349E-01	0,224	0,562	2,82774E-22
Macro_FOLR2+APOE-	HAVCR2	4,33392E-14	-6,95320E-01	0,209	0,438	7,98179E-10
Macro_FOLR2+APOE-	OS9	5,45340E-20	-6,95797E-01	0,239	0,54	1,00435E-15
Macro_FOLR2+APOE-	VAPA	6,58918E-20	-6,95965E-01	0,286	0,607	1,21353E-15
Macro_FOLR2+APOE-	IFNGR1	1,12597E-16	-6,96365E-01	0,296	0,585	2,07370E-12
Macro_FOLR2+APOE-	TXNDC12	1,13447E-19	-6,96479E-01	0,122	0,344	2,08935E-15
Macro_FOLR2+APOE-	MRPS7	5,88426E-22	-6,97290E-01	0,092	0,313	1,08370E-17
Macro_FOLR2+APOE-	RAB8A	9,74166E-27	-6,97420E-01	0,145	0,429	1,79412E-22
Macro_FOLR2+APOE-	FAU	2,16222E-15	-6,97981E-01	0,842	0,958	3,98215E-11
Macro_FOLR2+APOE-	CACYBP	4,39940E-22	-6,99779E-01	0,149	0,404	8,10238E-18
Macro_FOLR2+APOE-	RASSF2	2,24010E-21	-7,00016E-01	0,07	0,267	4,12559E-17
Macro_FOLR2+APOE-	MRPL16	5,62066E-24	-7,00074E-01	0,055	0,261	1,03516E-19
Macro_FOLR2+APOE-	TIMM17B	1,05252E-24	-7,00372E-01	0,09	0,327	1,93842E-20
Macro_FOLR2+APOE-	FNDC3B	2,26925E-23	-7,00462E-01	0,072	0,281	4,17928E-19
Macro_FOLR2+APOE-	RBM17	1,77987E-21	-7,01173E-01	0,154	0,414	3,27799E-17
Macro_FOLR2+APOE-	UBA1	1,07541E-22	-7,01819E-01	0,087	0,305	1,98059E-18
Macro_FOLR2+APOE-	BTF3	1,75845E-08	-7,01880E-01	0,573	0,849	3,23854E-04
Macro_FOLR2+APOE-	TIMM13	3,53225E-25	-7,02672E-01	0,13	0,397	6,50534E-21
Macro_FOLR2+APOE-	SPCS3	1,61495E-31	-7,03193E-01	0,137	0,448	2,97425E-27
Macro_FOLR2+APOE-	PSMD8	7,62314E-27	-7,03327E-01	0,207	0,529	1,40395E-22
Macro_FOLR2+APOE-	AKIRIN2	8,05693E-25	-7,04283E-01	0,153	0,425	1,48385E-20
Macro_FOLR2+APOE-	CLINT1	2,01727E-24	-7,05048E-01	0,068	0,285	3,71521E-20
Macro_FOLR2+APOE-	SH3GLB1	3,88812E-33	-7,05562E-01	0,121	0,43	7,16075E-29
Macro_FOLR2+APOE-	SPPL2A	1,85218E-25	-7,06732E-01	0,096	0,343	3,41116E-21
Macro_FOLR2+APOE-	SNRPD1	5,42584E-28	-7,06950E-01	0,119	0,395	9,99278E-24
Macro_FOLR2+APOE-	MAT2B	2,63901E-17	-7,07211E-01	0,128	0,338	4,86027E-13
Macro_FOLR2+APOE-	ARRDC1	2,24937E-16	-7,07329E-01	0,107	0,296	4,14267E-12
Macro_FOLR2+APOE-	SCAMP3	4,74857E-17	-7,07690E-01	0,077	0,253	8,74543E-13
Macro_FOLR2+APOE-	N4BP2L2	1,01672E-22	-7,07870E-01	0,179	0,462	1,87249E-18
Macro_FOLR2+APOE-	PRKCD	8,13577E-23	-7,08300E-01	0,058	0,259	1,49836E-18
Macro_FOLR2+APOE-	TXNDC17	2,15711E-28	-7,08669E-01	0,139	0,437	3,97274E-24
Macro_FOLR2+APOE-	C19orf53	6,12829E-26	-7,08800E-01	0,249	0,588	1,12865E-21

Macro_FOLR2+APOE-	KLF10	2,74306E-19	-7,08879E-01	0,096	0,292	5,05189E-15
Macro_FOLR2+APOE-	BTK	2,91178E-20	-7,09814E-01	0,102	0,313	5,36263E-16
Macro_FOLR2+APOE-	OST4	4,17712E-12	-7,10912E-01	0,45	0,781	7,69300E-08
Macro_FOLR2+APOE-	DERL1	1,06591E-22	-7,11127E-01	0,068	0,276	1,96309E-18
Macro_FOLR2+APOE-	LMAN2	6,78230E-24	-7,11634E-01	0,209	0,514	1,24910E-19
Macro_FOLR2+APOE-	YIF1B	6,66829E-22	-7,11650E-01	0,117	0,353	1,22810E-17
Macro_FOLR2+APOE-	DPP7	4,70669E-14	-7,11774E-01	0,282	0,56	8,66831E-10
Macro_FOLR2+APOE-	WDR83OS	5,11562E-18	-7,11955E-01	0,309	0,624	9,42143E-14
Macro_FOLR2+APOE-	NDUFAB1	7,26086E-32	-7,12220E-01	0,153	0,479	1,33723E-27
Macro_FOLR2+APOE-	HDLBP	1,40708E-23	-7,13063E-01	0,145	0,413	2,59142E-19
Macro_FOLR2+APOE-	CCDC88A	6,18289E-37	-7,13554E-01	0,16	0,506	1,13870E-32
Macro_FOLR2+APOE-	ECH1	4,83619E-15	-7,14698E-01	0,109	0,29	8,90681E-11
Macro_FOLR2+APOE-	GSTK1	1,42531E-17	-7,19355E-01	0,33	0,648	2,62499E-13
Macro_FOLR2+APOE-	SFT2D1	9,81363E-15	-7,19946E-01	0,215	0,463	1,80738E-10
Macro_FOLR2+APOE-	GBP4	1,87341E-23	-7,21011E-01	0,072	0,28	3,45026E-19
Macro_FOLR2+APOE-	SERTAD1	2,20842E-28	-7,21811E-01	0,083	0,328	4,06725E-24
Macro_FOLR2+APOE-	ERCC1	3,35854E-24	-7,22687E-01	0,126	0,384	6,18543E-20
Macro_FOLR2+APOE-	ARHGEF1	9,89412E-17	-7,22900E-01	0,09	0,273	1,82220E-12
Macro_FOLR2+APOE-	NAA20	2,91548E-18	-7,23472E-01	0,105	0,309	5,36944E-14
Macro_FOLR2+APOE-	NAGA	6,01107E-14	-7,23648E-01	0,158	0,363	1,10706E-09
Macro_FOLR2+APOE-	PNRC1	2,44613E-31	-7,23964E-01	0,42	0,783	4,50505E-27
Macro_FOLR2+APOE-	TUBGCP2	1,73727E-22	-7,24013E-01	0,087	0,306	3,19952E-18
Macro_FOLR2+APOE-	ELL2	5,16401E-27	-7,25148E-01	0,062	0,287	9,51056E-23
Macro_FOLR2+APOE-	G3BP2	1,39882E-22	-7,25913E-01	0,072	0,279	2,57620E-18
Macro_FOLR2+APOE-	PFKL	4,11412E-21	-7,26784E-01	0,147	0,396	7,57698E-17
Macro_FOLR2+APOE-	LAP3	8,01609E-26	-7,26909E-01	0,275	0,589	1,47632E-21
Macro_FOLR2+APOE-	BACH1	2,48593E-23	-7,27076E-01	0,09	0,315	4,57833E-19
Macro_FOLR2+APOE-	RAB1B	9,65107E-29	-7,27336E-01	0,096	0,359	1,77744E-24
Macro_FOLR2+APOE-	SMIM12	8,22707E-24	-7,27734E-01	0,06	0,27	1,51518E-19
Macro_FOLR2+APOE-	EIF3M	1,63986E-33	-7,29960E-01	0,168	0,503	3,02014E-29
Macro_FOLR2+APOE-	ORMDL1	1,73506E-16	-7,30291E-01	0,119	0,318	3,19546E-12
Macro_FOLR2+APOE-	UBE2K	5,73400E-26	-7,31297E-01	0,062	0,288	1,05603E-21
Macro_FOLR2+APOE-	LTBR	1,21138E-14	-7,32814E-01	0,151	0,351	2,23099E-10
Macro_FOLR2+APOE-	SCP2	1,06349E-20	-7,33580E-01	0,194	0,464	1,95864E-16
Macro_FOLR2+APOE-	SOD1	2,23549E-25	-7,33906E-01	0,239	0,567	4,11710E-21
Macro_FOLR2+APOE-	NCL	8,67295E-17	-7,34790E-01	0,32	0,629	1,59730E-12
Macro_FOLR2+APOE-	AFF4	7,80187E-24	-7,36084E-01	0,126	0,38	1,43687E-19
Macro_FOLR2+APOE-	NAP1L1	3,85622E-30	-7,36195E-01	0,271	0,623	7,10201E-26
Macro_FOLR2+APOE-	WAC	3,75861E-21	-7,36662E-01	0,085	0,292	6,92224E-17
Macro_FOLR2+APOE-	ARPC1B	1,96277E-14	-7,39050E-01	0,507	0,8	3,61483E-10
Macro_FOLR2+APOE-	CAP1	1,67480E-25	-7,39884E-01	0,367	0,738	3,08447E-21
Macro_FOLR2+APOE-	PLXNB2	1,56418E-14	-7,40694E-01	0,098	0,267	2,88074E-10
Macro_FOLR2+APOE-	ILK	3,88067E-20	-7,41248E-01	0,162	0,418	7,14704E-16
Macro_FOLR2+APOE-	MDH2	3,00798E-28	-7,45760E-01	0,2	0,526	5,53979E-24
Macro_FOLR2+APOE-	PRKAR1A	6,29446E-33	-7,46219E-01	0,158	0,484	1,15925E-28
Macro_FOLR2+APOE-	MYD88	5,74683E-38	-7,46867E-01	0,07	0,361	1,05839E-33
Macro_FOLR2+APOE-	EVI2A	3,23330E-13	-7,47661E-01	0,109	0,275	5,95477E-09

Macro_FOLR2+APOE-	CUEDC2	1,90203E-18	-7,49218E-01	0,098	0,297	3,50297E-14
Macro_FOLR2+APOE-	EIF4A2	4,92285E-10	-7,50027E-01	0,316	0,565	9,06642E-06
Macro_FOLR2+APOE-	PSMC3	3,42135E-19	-7,50958E-01	0,109	0,321	6,30110E-15
Macro_FOLR2+APOE-	POLR2J	9,24008E-18	-7,51245E-01	0,107	0,308	1,70175E-13
Macro_FOLR2+APOE-	SRP14	2,19831E-12	-7,51752E-01	0,535	0,84	4,04862E-08
Macro_FOLR2+APOE-	SH3KBP1	3,32885E-21	-7,51997E-01	0,164	0,417	6,13075E-17
Macro_FOLR2+APOE-	TYMP	2,22877E-35	-7,53088E-01	0,465	0,797	4,10472E-31
Macro_FOLR2+APOE-	ANXA11	5,46641E-33	-7,53567E-01	0,198	0,547	1,00675E-28
Macro_FOLR2+APOE-	NDUFS5	6,54578E-20	-7,53736E-01	0,328	0,658	1,20554E-15
Macro_FOLR2+APOE-	TMCO1	3,25731E-14	-7,54688E-01	0,154	0,361	5,99898E-10
Macro_FOLR2+APOE-	MAPRE1	1,89471E-26	-7,54741E-01	0,122	0,392	3,48948E-22
Macro_FOLR2+APOE-	VDAC1	4,80558E-23	-7,55036E-01	0,23	0,537	8,85043E-19
Macro_FOLR2+APOE-	MDH1	6,46408E-17	-7,55806E-01	0,158	0,388	1,19049E-12
Macro_FOLR2+APOE-	GBP2	3,44979E-31	-7,57717E-01	0,1	0,376	6,35349E-27
Macro_FOLR2+APOE-	STAT3	6,46845E-32	-7,58054E-01	0,181	0,51	1,19129E-27
Macro_FOLR2+APOE-	HNRNPR	3,57588E-25	-7,58151E-01	0,113	0,364	6,58570E-21
Macro_FOLR2+APOE-	TNFSF13B	1,43107E-11	-7,58271E-01	0,326	0,582	2,63560E-07
Macro_FOLR2+APOE-	EIF3F	3,58092E-15	-7,58925E-01	0,354	0,67	6,59497E-11
Macro_FOLR2+APOE-	DGUOK	3,98291E-18	-7,59023E-01	0,145	0,373	7,33532E-14
Macro_FOLR2+APOE-	PHYKPL	9,31687E-16	-7,60044E-01	0,089	0,261	1,71589E-11
Macro_FOLR2+APOE-	ANAPC16	1,98685E-28	-7,60350E-01	0,173	0,484	3,65919E-24
Macro_FOLR2+APOE-	VPS29	2,36057E-24	-7,60704E-01	0,222	0,539	4,34746E-20
Macro_FOLR2+APOE-	CD46	2,11941E-16	-7,61658E-01	0,121	0,319	3,90331E-12
Macro_FOLR2+APOE-	PHACTR1	4,24862E-23	-7,62881E-01	0,087	0,304	7,82469E-19
Macro_FOLR2+APOE-	CSTA	3,36666E-33	-7,63553E-01	0,134	0,433	6,20037E-29
Macro_FOLR2+APOE-	PSMB6	8,12234E-26	-7,64352E-01	0,239	0,58	1,49589E-21
Macro_FOLR2+APOE-	CMC2	1,25927E-19	-7,65137E-01	0,081	0,278	2,31920E-15
Macro_FOLR2+APOE-	GNG5	1,67592E-38	-7,66246E-01	0,405	0,798	3,08653E-34
Macro_FOLR2+APOE-	SDHB	8,92175E-30	-7,67206E-01	0,119	0,402	1,64312E-25
Macro_FOLR2+APOE-	RAB8B	2,33981E-22	-7,68327E-01	0,056	0,252	4,30923E-18
Macro_FOLR2+APOE-	RPL36AL	3,33926E-22	-7,69033E-01	0,222	0,496	6,14991E-18
Macro_FOLR2+APOE-	FBXO7	9,56827E-22	-7,69162E-01	0,1	0,325	1,76219E-17
Macro_FOLR2+APOE-	DNTTIP2	3,43253E-24	-7,69256E-01	0,064	0,278	6,32170E-20
Macro_FOLR2+APOE-	VPS28	6,35640E-17	-7,69555E-01	0,303	0,616	1,17066E-12
Macro_FOLR2+APOE-	IDH3G	1,36757E-20	-7,69907E-01	0,128	0,362	2,51866E-16
Macro_FOLR2+APOE-	PSMA5	2,89033E-32	-7,70947E-01	0,132	0,438	5,32311E-28
Macro_FOLR2+APOE-	TXN2	1,68728E-25	-7,71550E-01	0,13	0,4	3,10746E-21
Macro_FOLR2+APOE-	XBP1	1,71130E-33	-7,71571E-01	0,122	0,428	3,15171E-29
Macro_FOLR2+APOE-	SNRPE	1,09819E-33	-7,72321E-01	0,07	0,345	2,02254E-29
Macro_FOLR2+APOE-	PIH1D1	1,68009E-17	-7,72664E-01	0,077	0,256	3,09423E-13
Macro_FOLR2+APOE-	CTSH	2,99899E-16	-7,72980E-01	0,431	0,713	5,52325E-12
Macro_FOLR2+APOE-	APP	1,72394E-24	-7,73221E-01	0,087	0,311	3,17497E-20
Macro_FOLR2+APOE-	FKBP2	5,63273E-14	-7,74020E-01	0,194	0,412	1,03738E-09
Macro_FOLR2+APOE-	NUTF2	2,02035E-31	-7,75015E-01	0,104	0,388	3,72088E-27
Macro_FOLR2+APOE-	UNC119	1,68900E-22	-7,75025E-01	0,066	0,27	3,11063E-18
Macro_FOLR2+APOE-	GIT2	1,02668E-16	-7,75034E-01	0,105	0,297	1,89084E-12
Macro_FOLR2+APOE-	MRPL33	1,74101E-28	-7,75619E-01	0,105	0,375	3,20641E-24

Macro_FOLR2+APOE-	SNAP23	7,01024E-22	-7,75887E-01	0,147	0,402	1,29108E-17
Macro_FOLR2+APOE-	RPS19BP1	4,76877E-23	-7,75999E-01	0,058	0,262	8,78265E-19
Macro_FOLR2+APOE-	HAX1	5,07720E-28	-7,77447E-01	0,09	0,349	9,35068E-24
Macro_FOLR2+APOE-	TMBIM4	9,31822E-15	-7,77487E-01	0,137	0,333	1,71614E-10
Macro_FOLR2+APOE-	HMGB2	8,31140E-24	-7,78837E-01	0,149	0,402	1,53071E-19
Macro_FOLR2+APOE-	ANAPC11	2,87143E-22	-7,79306E-01	0,273	0,612	5,28831E-18
Macro_FOLR2+APOE-	PSENEN	3,15373E-24	-7,79438E-01	0,073	0,297	5,80822E-20
Macro_FOLR2+APOE-	GGNBP2	2,28878E-23	-7,80857E-01	0,107	0,343	4,21525E-19
Macro_FOLR2+APOE-	TNFAIP2	2,46631E-18	-7,81069E-01	0,177	0,408	4,54220E-14
Macro_FOLR2+APOE-	TAF7	4,21813E-21	-7,81299E-01	0,092	0,304	7,76852E-17
Macro_FOLR2+APOE-	TMA7	1,01839E-26	-7,81622E-01	0,429	0,797	1,87557E-22
Macro_FOLR2+APOE-	CCT2	1,35646E-20	-7,81759E-01	0,117	0,343	2,49819E-16
Macro_FOLR2+APOE-	COPS3	1,29562E-15	-7,81851E-01	0,085	0,253	2,38613E-11
Macro_FOLR2+APOE-	IFNAR2	5,41511E-17	-7,82586E-01	0,089	0,27	9,97301E-13
Macro_FOLR2+APOE-	POLR2G	6,35263E-24	-7,83288E-01	0,122	0,375	1,16996E-19
Macro_FOLR2+APOE-	SHISA5	7,46801E-23	-7,83294E-01	0,128	0,374	1,37538E-18
Macro_FOLR2+APOE-	CTNNB1	4,71636E-18	-7,84068E-01	0,153	0,382	8,68613E-14
Macro_FOLR2+APOE-	SLC25A5	7,10486E-22	-7,85021E-01	0,414	0,748	1,30850E-17
Macro_FOLR2+APOE-	SSR1	4,47204E-16	-7,85025E-01	0,215	0,466	8,23617E-12
Macro_FOLR2+APOE-	UQCRFS1	2,75218E-36	-7,86263E-01	0,153	0,497	5,06868E-32
Macro_FOLR2+APOE-	PELI1	1,16101E-22	-7,86290E-01	0,064	0,264	2,13823E-18
Macro_FOLR2+APOE-	PILRA	1,28830E-18	-7,86867E-01	0,234	0,496	2,37267E-14
Macro_FOLR2+APOE-	ATP6V0E1	8,93722E-14	-7,87546E-01	0,482	0,799	1,64597E-09
Macro_FOLR2+APOE-	VTI1B	1,75054E-18	-7,87995E-01	0,147	0,38	3,22396E-14
Macro_FOLR2+APOE-	EIF1B	1,08364E-22	-7,88730E-01	0,181	0,463	1,99575E-18
Macro_FOLR2+APOE-	CCT4	5,99532E-28	-7,89751E-01	0,147	0,436	1,10416E-23
Macro_FOLR2+APOE-	RAB10	2,81514E-22	-7,89921E-01	0,203	0,493	5,18464E-18
Macro_FOLR2+APOE-	RBMX	1,19159E-25	-7,90131E-01	0,149	0,428	2,19456E-21
Macro_FOLR2+APOE-	ARPC3	7,42624E-07	-7,90295E-01	0,665	0,895	1,36769E-02
Macro_FOLR2+APOE-	SP100	7,77917E-20	-7,92327E-01	0,198	0,469	1,43269E-15
Macro_FOLR2+APOE-	AMD1	1,76362E-21	-7,92397E-01	0,081	0,288	3,24805E-17
Macro_FOLR2+APOE-	USP15	5,12724E-23	-7,92398E-01	0,16	0,427	9,44284E-19
Macro_FOLR2+APOE-	SDF2L1	1,36904E-24	-7,92914E-01	0,111	0,357	2,52135E-20
Macro_FOLR2+APOE-	PLBD1	3,02370E-21	-7,94607E-01	0,153	0,396	5,56874E-17
Macro_FOLR2+APOE-	TCF25	3,20395E-25	-7,94891E-01	0,239	0,565	5,90071E-21
Macro_FOLR2+APOE-	AP1S2	8,38384E-20	-7,95871E-01	0,282	0,582	1,54405E-15
Macro_FOLR2+APOE-	SDHA	7,71729E-14	-7,96092E-01	0,092	0,253	1,42129E-09
Macro_FOLR2+APOE-	DCTN3	1,39515E-20	-7,96918E-01	0,128	0,363	2,56945E-16
Macro_FOLR2+APOE-	FLOT1	4,24079E-30	-7,97700E-01	0,202	0,528	7,81027E-26
Macro_FOLR2+APOE-	IQGAP1	4,16952E-33	-7,97926E-01	0,211	0,566	7,67901E-29
Macro_FOLR2+APOE-	CCDC50	1,25579E-16	-7,97973E-01	0,096	0,276	2,31278E-12
Macro_FOLR2+APOE-	ALDH2	5,56069E-22	-8,00504E-01	0,256	0,554	1,02411E-17
Macro_FOLR2+APOE-	SF3B1	3,92629E-18	-8,00647E-01	0,226	0,496	7,23104E-14
Macro_FOLR2+APOE-	RPL27	2,78746E-07	-8,01871E-01	0,326	0,507	5,13366E-03
Macro_FOLR2+APOE-	CPPED1	1,97502E-25	-8,02191E-01	0,056	0,273	3,63739E-21
Macro_FOLR2+APOE-	SSBP1	8,34824E-30	-8,02694E-01	0,169	0,491	1,53749E-25
Macro_FOLR2+APOE-	TUBA1C	2,22971E-36	-8,02769E-01	0,087	0,383	4,10646E-32

Macro_FOLR2+APOE-	ITPA	1,83947E-21	-8,03470E-01	0,073	0,277	3,38775E-17
Macro_FOLR2+APOE-	IL10RA	7,17971E-18	-8,03592E-01	0,205	0,467	1,32229E-13
Macro_FOLR2+APOE-	PRR13	4,42022E-28	-8,03933E-01	0,241	0,584	8,14071E-24
Macro_FOLR2+APOE-	NUDT22	2,30316E-19	-8,04057E-01	0,081	0,275	4,24173E-15
Macro_FOLR2+APOE-	RAB20	1,90823E-16	-8,05835E-01	0,143	0,349	3,51439E-12
Macro_FOLR2+APOE-	RBM25	3,86762E-33	-8,06540E-01	0,169	0,498	7,12299E-29
Macro_FOLR2+APOE-	CHD9	4,20012E-16	-8,08594E-01	0,139	0,345	7,73535E-12
Macro_FOLR2+APOE-	HMGN1	8,22460E-26	-8,08695E-01	0,288	0,629	1,51473E-21
Macro_FOLR2+APOE-	TRMT112	1,59827E-13	-8,09049E-01	0,322	0,622	2,94353E-09
Macro_FOLR2+APOE-	PSMC2	4,94238E-22	-8,09921E-01	0,077	0,289	9,10238E-18
Macro_FOLR2+APOE-	MTMR14	2,99452E-21	-8,09964E-01	0,096	0,312	5,51502E-17
Macro_FOLR2+APOE-	PFKFB3	1,16983E-25	-8,10602E-01	0,098	0,337	2,15447E-21
Macro_FOLR2+APOE-	RABAC1	1,20039E-17	-8,14291E-01	0,247	0,528	2,21076E-13
Macro_FOLR2+APOE-	GSDMD	1,09453E-16	-8,14826E-01	0,109	0,303	2,01580E-12
Macro_FOLR2+APOE-	SEC13	2,19968E-21	-8,15430E-01	0,151	0,405	4,05115E-17
Macro_FOLR2+APOE-	GRB2	3,10369E-32	-8,15782E-01	0,298	0,684	5,71606E-28
Macro_FOLR2+APOE-	CLEC7A	2,21482E-23	-8,15885E-01	0,23	0,528	4,07903E-19
Macro_FOLR2+APOE-	SAMD9L	6,11976E-26	-8,16702E-01	0,06	0,282	1,12708E-21
Macro_FOLR2+APOE-	STAT1	2,90627E-24	-8,16737E-01	0,232	0,521	5,35248E-20
Macro_FOLR2+APOE-	STX4	7,17359E-32	-8,17837E-01	0,058	0,315	1,32116E-27
Macro_FOLR2+APOE-	UBE2E2	3,07708E-20	-8,18110E-01	0,094	0,302	5,66707E-16
Macro_FOLR2+APOE-	MYO1G	1,69860E-26	-8,18711E-01	0,062	0,286	3,12832E-22
Macro_FOLR2+APOE-	PPM1G	3,46894E-33	-8,18894E-01	0,087	0,371	6,38875E-29
Macro_FOLR2+APOE-	CBX3	1,52454E-31	-8,19164E-01	0,115	0,41	2,80775E-27
Macro_FOLR2+APOE-	CORO1C	4,92413E-25	-8,19353E-01	0,102	0,347	9,06877E-21
Macro_FOLR2+APOE-	RABGAP1L	1,54834E-16	-8,19941E-01	0,081	0,254	2,85158E-12
Macro_FOLR2+APOE-	M6PR	1,63176E-26	-8,22641E-01	0,232	0,567	3,00521E-22
Macro_FOLR2+APOE-	CSTB	2,30383E-22	-8,22821E-01	0,49	0,793	4,24297E-18
Macro_FOLR2+APOE-	GTF3A	1,33219E-16	-8,24402E-01	0,222	0,483	2,45349E-12
Macro_FOLR2+APOE-	ARFGAP3	2,31564E-27	-8,25121E-01	0,049	0,27	4,26472E-23
Macro_FOLR2+APOE-	HCK	5,36582E-30	-8,25810E-01	0,203	0,524	9,88223E-26
Macro_FOLR2+APOE-	ZEB2	4,17579E-24	-8,28139E-01	0,279	0,609	7,69054E-20
Macro_FOLR2+APOE-	IAH1	7,84983E-20	-8,28280E-01	0,102	0,312	1,44570E-15
Macro_FOLR2+APOE-	NDUFA6	2,68573E-38	-8,28384E-01	0,168	0,532	4,94630E-34
Macro_FOLR2+APOE-	AK2	1,32267E-28	-8,28425E-01	0,087	0,346	2,43595E-24
Macro_FOLR2+APOE-	MBOAT7	2,74189E-26	-8,30234E-01	0,041	0,252	5,04974E-22
Macro_FOLR2+APOE-	EIF2S3	1,48593E-24	-8,31160E-01	0,168	0,454	2,73664E-20
Macro_FOLR2+APOE-	RNPS1	8,25258E-29	-8,31427E-01	0,124	0,408	1,51988E-24
Macro_FOLR2+APOE-	UBXN4	8,18889E-25	-8,31917E-01	0,211	0,524	1,50815E-20
Macro_FOLR2+APOE-	ETV6	2,97024E-32	-8,32180E-01	0,047	0,293	5,47029E-28
Macro_FOLR2+APOE-	RIOK3	1,57462E-24	-8,33214E-01	0,06	0,273	2,89997E-20
Macro_FOLR2+APOE-	IDS	1,92381E-33	-8,33885E-01	0,107	0,401	3,54309E-29
Macro_FOLR2+APOE-	PSMD6	3,96054E-16	-8,33902E-01	0,098	0,281	7,29412E-12
Macro_FOLR2+APOE-	GLRX	2,08846E-27	-8,34209E-01	0,228	0,551	3,84632E-23
Macro_FOLR2+APOE-	ATP6V1F	1,06110E-23	-8,34228E-01	0,414	0,768	1,95422E-19
Macro_FOLR2+APOE-	ZNF706	5,53271E-20	-8,34233E-01	0,284	0,595	1,01896E-15
Macro_FOLR2+APOE-	TMEM179B	3,93733E-24	-8,34267E-01	0,192	0,485	7,25139E-20

Macro_FOLR2+APOE-	IVNS1ABP	9,36361E-36	-8,34596E-01	0,083	0,37	1,72450E-31
Macro_FOLR2+APOE-	CHCHD2	1,61275E-26	-8,34830E-01	0,463	0,819	2,97021E-22
Macro_FOLR2+APOE-	COPS6	6,58199E-16	-8,35429E-01	0,137	0,345	1,21220E-11
Macro_FOLR2+APOE-	BLVRA	1,09401E-22	-8,36001E-01	0,136	0,386	2,01484E-18
Macro_FOLR2+APOE-	EWSR1	1,40117E-31	-8,36218E-01	0,151	0,466	2,58053E-27
Macro_FOLR2+APOE-	ACOT9	7,01978E-25	-8,36918E-01	0,064	0,281	1,29283E-20
Macro_FOLR2+APOE-	TMEM205	2,68278E-20	-8,37806E-01	0,085	0,287	4,94088E-16
Macro_FOLR2+APOE-	C6orf62	4,48605E-14	-8,38130E-01	0,16	0,367	8,26196E-10
Macro_FOLR2+APOE-	MRFAP1	8,39678E-19	-8,38858E-01	0,162	0,403	1,54643E-14
Macro_FOLR2+APOE-	HMGN2	2,01470E-13	-8,39272E-01	0,443	0,737	3,71046E-09
Macro_FOLR2+APOE-	IL10RB	2,05367E-20	-8,39597E-01	0,066	0,257	3,78224E-16
Macro_FOLR2+APOE-	TNFAIP8	8,46231E-23	-8,40211E-01	0,107	0,339	1,55850E-18
Macro_FOLR2+APOE-	PRPF40A	4,35634E-31	-8,40278E-01	0,147	0,459	8,02308E-27
Macro_FOLR2+APOE-	ATP6V1B2	4,28540E-22	-8,40593E-01	0,239	0,538	7,89242E-18
Macro_FOLR2+APOE-	AP3S1	2,16010E-21	-8,40889E-01	0,117	0,349	3,97825E-17
Macro_FOLR2+APOE-	TMEM208	5,19567E-17	-8,41327E-01	0,126	0,333	9,56886E-13
Macro_FOLR2+APOE-	RAB7A	7,09674E-29	-8,42564E-01	0,275	0,637	1,30701E-24
Macro_FOLR2+APOE-	NEMF	7,11671E-19	-8,46147E-01	0,092	0,291	1,31068E-14
Macro_FOLR2+APOE-	MRPL55	1,63787E-13	-8,46215E-01	0,109	0,28	3,01646E-09
Macro_FOLR2+APOE-	PTK2B	7,18264E-13	-8,47419E-01	0,1	0,255	1,32283E-08
Macro_FOLR2+APOE-	UQCRC2	4,09678E-30	-8,47783E-01	0,124	0,418	7,54504E-26
Macro_FOLR2+APOE-	POLR2E	2,41799E-21	-8,47830E-01	0,234	0,533	4,45320E-17
Macro_FOLR2+APOE-	MCTP1	2,49212E-16	-8,48635E-01	0,079	0,25	4,58974E-12
Macro_FOLR2+APOE-	FAM32A	2,42486E-20	-8,49441E-01	0,102	0,319	4,46587E-16
Macro_FOLR2+APOE-	CCT6A	5,14044E-27	-8,50038E-01	0,162	0,451	9,46714E-23
Macro_FOLR2+APOE-	ARPC2	2,03859E-20	-8,50378E-01	0,565	0,862	3,75447E-16
Macro_FOLR2+APOE-	YME1L1	2,68089E-37	-8,50570E-01	0,081	0,382	4,93739E-33
Macro_FOLR2+APOE-	RAB24	1,18456E-28	-8,51005E-01	0,047	0,273	2,18161E-24
Macro_FOLR2+APOE-	SLC31A2	1,42538E-21	-8,51609E-01	0,179	0,445	2,62512E-17
Macro_FOLR2+APOE-	TNFSF13	3,98947E-08	-8,52018E-01	0,258	0,446	7,34740E-04
Macro_FOLR2+APOE-	SQSTM1	1,30636E-31	-8,52140E-01	0,316	0,678	2,40593E-27
Macro_FOLR2+APOE-	ATG12	1,39054E-17	-8,53146E-01	0,083	0,265	2,56096E-13
Macro_FOLR2+APOE-	PFDN1	9,35546E-20	-8,53424E-01	0,087	0,289	1,72299E-15
Macro_FOLR2+APOE-	FAM177A1	2,37943E-33	-8,53527E-01	0,056	0,319	4,38220E-29
Macro_FOLR2+APOE-	TNIP1	4,69664E-33	-8,54066E-01	0,077	0,349	8,64980E-29
Macro_FOLR2+APOE-	ACP1	1,87490E-22	-8,54796E-01	0,105	0,337	3,45300E-18
Macro_FOLR2+APOE-	WDR1	2,56127E-39	-8,54926E-01	0,179	0,545	4,71709E-35
Macro_FOLR2+APOE-	SKIL	1,01267E-30	-8,55147E-01	0,075	0,334	1,86504E-26
Macro_FOLR2+APOE-	RAP1B	3,95636E-34	-8,55528E-01	0,217	0,583	7,28643E-30
Macro_FOLR2+APOE-	BRK1	8,21004E-20	-8,56158E-01	0,373	0,73	1,51204E-15
Macro_FOLR2+APOE-	PUF60	3,09998E-19	-8,59651E-01	0,107	0,322	5,70923E-15
Macro_FOLR2+APOE-	GTF2A2	1,86413E-24	-8,61144E-01	0,139	0,41	3,43317E-20
Macro_FOLR2+APOE-	TSC22D3	7,74094E-10	-8,62004E-01	0,414	0,697	1,42565E-05
Macro_FOLR2+APOE-	PRELID1	5,11886E-33	-8,62722E-01	0,315	0,689	9,42741E-29
Macro_FOLR2+APOE-	UBAC2	1,73094E-19	-8,62940E-01	0,102	0,311	3,18787E-15
Macro_FOLR2+APOE-	DNAJB6	3,44088E-31	-8,63063E-01	0,192	0,526	6,33707E-27
Macro_FOLR2+APOE-	FKBP1A	1,35713E-25	-8,63310E-01	0,343	0,698	2,49943E-21

Macro_FOLR2+APOE-	LIMS1	5,96132E-36	-8,63647E-01	0,207	0,559	1,09790E-31
Macro_FOLR2+APOE-	GPR65	2,51470E-18	-8,64725E-01	0,121	0,329	4,63133E-14
Macro_FOLR2+APOE-	CYTH1	1,14356E-22	-8,64870E-01	0,102	0,33	2,10609E-18
Macro_FOLR2+APOE-	DCXR	5,12221E-24	-8,65343E-01	0,119	0,37	9,43357E-20
Macro_FOLR2+APOE-	MSN	5,91156E-39	-8,65425E-01	0,25	0,642	1,08873E-34
Macro_FOLR2+APOE-	TOM1	3,93721E-21	-8,65872E-01	0,081	0,286	7,25117E-17
Macro_FOLR2+APOE-	RASSF5	9,56027E-28	-8,66069E-01	0,049	0,274	1,76071E-23
Macro_FOLR2+APOE-	NDUFV2	1,19879E-23	-8,66308E-01	0,222	0,538	2,20781E-19
Macro_FOLR2+APOE-	LGALS9	2,34302E-26	-8,66493E-01	0,232	0,551	4,31515E-22
Macro_FOLR2+APOE-	SASH3	1,14323E-24	-8,66577E-01	0,068	0,286	2,10549E-20
Macro_FOLR2+APOE-	SYAP1	1,89021E-38	-8,66943E-01	0,06	0,348	3,48121E-34
Macro_FOLR2+APOE-	MT-ND6	5,97891E-19	-8,67292E-01	0,102	0,304	1,10114E-14
Macro_FOLR2+APOE-	STAT2	7,65668E-27	-8,68322E-01	0,068	0,297	1,41013E-22
Macro_FOLR2+APOE-	TMED2	2,24414E-26	-8,69483E-01	0,196	0,503	4,13303E-22
Macro_FOLR2+APOE-	CDC42SE2	4,48030E-19	-8,70226E-01	0,083	0,276	8,25137E-15
Macro_FOLR2+APOE-	PAIP2	1,41430E-21	-8,70686E-01	0,217	0,515	2,60472E-17
Macro_FOLR2+APOE-	DDOST	6,70104E-19	-8,73077E-01	0,2	0,466	1,23413E-14
Macro_FOLR2+APOE-	EIF3E	1,36953E-10	-8,73253E-01	0,337	0,609	2,52226E-06
Macro_FOLR2+APOE-	PKM	1,38218E-37	-8,73287E-01	0,392	0,788	2,54555E-33
Macro_FOLR2+APOE-	SFT2D2	1,73730E-23	-8,75380E-01	0,077	0,296	3,19958E-19
Macro_FOLR2+APOE-	CCNDBP1	2,10431E-16	-8,75560E-01	0,094	0,275	3,87551E-12
Macro_FOLR2+APOE-	CHURC1	7,69434E-27	-8,75702E-01	0,079	0,321	1,41707E-22
Macro_FOLR2+APOE-	XRCC6	1,36288E-30	-8,75721E-01	0,177	0,499	2,51002E-26
Macro_FOLR2+APOE-	CDK5RAP3	1,92974E-16	-8,76088E-01	0,115	0,309	3,55400E-12
Macro_FOLR2+APOE-	CDK2AP2	1,61717E-31	-8,76193E-01	0,113	0,401	2,97834E-27
Macro_FOLR2+APOE-	BZW1	2,71857E-35	-8,76316E-01	0,128	0,446	5,00679E-31
Macro_FOLR2+APOE-	GPBP1	8,43630E-25	-8,77544E-01	0,104	0,347	1,55371E-20
Macro_FOLR2+APOE-	GCA	1,96567E-24	-8,79501E-01	0,141	0,405	3,62017E-20
Macro_FOLR2+APOE-	PHB	6,75443E-23	-8,80232E-01	0,137	0,392	1,24396E-18
Macro_FOLR2+APOE-	RNF144B	5,51167E-26	-8,81021E-01	0,111	0,361	1,01508E-21
Macro_FOLR2+APOE-	MAPKAPK3	1,03107E-28	-8,83131E-01	0,056	0,292	1,89891E-24
Macro_FOLR2+APOE-	UBE2F	1,02874E-19	-8,83369E-01	0,072	0,261	1,89463E-15
Macro_FOLR2+APOE-	BAZ1A	1,59443E-43	-8,83376E-01	0,089	0,422	2,93647E-39
Macro_FOLR2+APOE-	UBA52	1,18006E-10	-8,83547E-01	0,729	0,923	2,17331E-06
Macro_FOLR2+APOE-	DCTN2	2,53032E-23	-8,83550E-01	0,098	0,332	4,66010E-19
Macro_FOLR2+APOE-	ELOVL1	2,10859E-27	-8,84582E-01	0,104	0,363	3,88339E-23
Macro_FOLR2+APOE-	PFN1	5,47213E-28	-8,85053E-01	0,81	0,949	1,00780E-23
Macro_FOLR2+APOE-	ACTN4	1,01573E-35	-8,85361E-01	0,068	0,351	1,87066E-31
Macro_FOLR2+APOE-	TNFRSF14	4,76596E-23	-8,86844E-01	0,171	0,452	8,77748E-19
Macro_FOLR2+APOE-	ZNF207	2,41570E-27	-8,88026E-01	0,13	0,408	4,44900E-23
Macro_FOLR2+APOE-	NUMB	2,22904E-23	-8,89059E-01	0,104	0,336	4,10522E-19
Macro_FOLR2+APOE-	UCP2	1,29357E-18	-8,90330E-01	0,331	0,638	2,38236E-14
Macro_FOLR2+APOE-	RABGGTB	6,25089E-16	-8,90963E-01	0,085	0,259	1,15123E-11
Macro_FOLR2+APOE-	NOP10	3,26599E-35	-8,90997E-01	0,245	0,636	6,01497E-31
Macro_FOLR2+APOE-	MGAT1	1,72265E-17	-8,92080E-01	0,32	0,628	3,17261E-13
Macro_FOLR2+APOE-	NDUFA12	2,19992E-23	-8,92956E-01	0,198	0,497	4,05159E-19
Macro_FOLR2+APOE-	GLUL	3,97314E-08	-8,93053E-01	0,601	0,806	7,31733E-04

Macro_FOLR2+APOE-	C18orf32	3,43467E-22	-8,93788E-01	0,073	0,283	6,32564E-18
Macro_FOLR2+APOE-	BASP1	3,63289E-43	-8,94028E-01	0,047	0,35	6,69069E-39
Macro_FOLR2+APOE-	SEC11C	6,98165E-21	-8,94486E-01	0,105	0,328	1,28581E-16
Macro_FOLR2+APOE-	MNDA	1,69155E-24	-8,95849E-01	0,243	0,537	3,11534E-20
Macro_FOLR2+APOE-	VDAC2	1,32328E-23	-8,96136E-01	0,241	0,556	2,43708E-19
Macro_FOLR2+APOE-	PDCD6IP	3,44133E-20	-8,97021E-01	0,136	0,371	6,33791E-16
Macro_FOLR2+APOE-	SNX10	1,14295E-39	-8,97147E-01	0,149	0,49	2,10498E-35
Macro_FOLR2+APOE-	NINJ1	4,18154E-10	-8,98004E-01	0,328	0,562	7,70114E-06
Macro_FOLR2+APOE-	ILF3	3,35965E-26	-8,98543E-01	0,113	0,369	6,18746E-22
Macro_FOLR2+APOE-	TES	4,70173E-25	-8,98751E-01	0,066	0,282	8,65918E-21
Macro_FOLR2+APOE-	MAPK1IP1L	9,31521E-31	-9,01530E-01	0,081	0,347	1,71558E-26
Macro_FOLR2+APOE-	MRPS6	1,96879E-24	-9,01901E-01	0,094	0,327	3,62592E-20
Macro_FOLR2+APOE-	ETS2	9,58504E-27	-9,04230E-01	0,158	0,429	1,76528E-22
Macro_FOLR2+APOE-	NMI	1,96559E-28	-9,04731E-01	0,064	0,302	3,62003E-24
Macro_FOLR2+APOE-	SRSF10	1,04665E-22	-9,04848E-01	0,072	0,281	1,92761E-18
Macro_FOLR2+APOE-	RBM6	1,41789E-13	-9,05239E-01	0,102	0,265	2,61132E-09
Macro_FOLR2+APOE-	OAS1	1,27410E-12	-9,05906E-01	0,151	0,329	2,34650E-08
Macro_FOLR2+APOE-	SUPT4H1	1,09098E-34	-9,08880E-01	0,143	0,465	2,00926E-30
Macro_FOLR2+APOE-	ARL6IP1	1,92746E-14	-9,08987E-01	0,192	0,417	3,54981E-10
Macro_FOLR2+APOE-	OAZ2	1,95698E-17	-9,09247E-01	0,094	0,285	3,60417E-13
Macro_FOLR2+APOE-	IMP4	1,45977E-17	-9,11378E-01	0,085	0,268	2,68845E-13
Macro_FOLR2+APOE-	PDIA6	1,18649E-30	-9,12678E-01	0,192	0,524	2,18515E-26
Macro_FOLR2+APOE-	NRBP1	3,67475E-25	-9,13088E-01	0,079	0,31	6,76779E-21
Macro_FOLR2+APOE-	CALR	3,92926E-22	-9,13405E-01	0,411	0,727	7,23653E-18
Macro_FOLR2+APOE-	GPR108	1,61265E-18	-9,14977E-01	0,075	0,26	2,97001E-14
Macro_FOLR2+APOE-	UBL5	9,47020E-34	-9,15458E-01	0,348	0,76	1,74413E-29
Macro_FOLR2+APOE-	OTUB1	4,89852E-29	-9,15778E-01	0,139	0,434	9,02160E-25
Macro_FOLR2+APOE-	IL2RG	2,89265E-27	-9,17845E-01	0,087	0,327	5,32739E-23
Macro_FOLR2+APOE-	TGIF1	2,29378E-21	-9,18623E-01	0,105	0,326	4,22445E-17
Macro_FOLR2+APOE-	ATP1A1	2,19694E-36	-9,18730E-01	0,117	0,433	4,04610E-32
Macro_FOLR2+APOE-	PLSCR1	4,15633E-21	-9,18971E-01	0,247	0,545	7,65472E-17
Macro_FOLR2+APOE-	PDCD6	1,00422E-27	-9,19482E-01	0,143	0,428	1,84947E-23
Macro_FOLR2+APOE-	DDX3X	3,18488E-44	-9,20819E-01	0,124	0,482	5,86560E-40
Macro_FOLR2+APOE-	NASP	1,62007E-21	-9,21144E-01	0,073	0,278	2,98368E-17
Macro_FOLR2+APOE-	EIF3K	2,85617E-20	-9,21790E-01	0,444	0,791	5,26021E-16
Macro_FOLR2+APOE-	CLEC12A	3,49217E-15	-9,21848E-01	0,094	0,262	6,43152E-11
Macro_FOLR2+APOE-	PSMD11	6,32021E-24	-9,23181E-01	0,085	0,311	1,16399E-19
Macro_FOLR2+APOE-	CFL1	1,08235E-11	-9,24578E-01	0,759	0,924	1,99337E-07
Macro_FOLR2+APOE-	CSNK1A1	2,01192E-36	-9,26091E-01	0,136	0,466	3,70534E-32
Macro_FOLR2+APOE-	NR1H2	3,94678E-31	-9,26985E-01	0,072	0,334	7,26879E-27
Macro_FOLR2+APOE-	NDUFC2	5,67163E-17	-9,27001E-01	0,111	0,308	1,04454E-12
Macro_FOLR2+APOE-	MAP1LC3B	9,77388E-37	-9,28788E-01	0,2	0,562	1,80006E-32
Macro_FOLR2+APOE-	RNF7	8,64577E-35	-9,31048E-01	0,19	0,548	1,59229E-30
Macro_FOLR2+APOE-	CHMP2A	3,24730E-23	-9,31469E-01	0,196	0,495	5,98056E-19
Macro_FOLR2+APOE-	EMC4	4,17400E-17	-9,32755E-01	0,143	0,363	7,68725E-13
Macro_FOLR2+APOE-	NSMCE1	5,10893E-24	-9,33493E-01	0,064	0,273	9,40912E-20
Macro_FOLR2+APOE-	ACSL1	4,14218E-25	-9,36013E-01	0,137	0,392	7,62865E-21

Macro_FOLR2+APOE-	DHRS7	5,54727E-19	-9,37826E-01	0,218	0,488	1,02164E-14
Macro_FOLR2+APOE-	BRD2	2,73778E-35	-9,37863E-01	0,158	0,491	5,04216E-31
Macro_FOLR2+APOE-	SEC61G	3,39240E-42	-9,38924E-01	0,205	0,604	6,24778E-38
Macro_FOLR2+APOE-	CNPY3	3,76458E-20	-9,39281E-01	0,313	0,631	6,93323E-16
Macro_FOLR2+APOE-	CALCOCO2	2,85961E-16	-9,40641E-01	0,151	0,361	5,26655E-12
Macro_FOLR2+APOE-	RAB34	1,93923E-17	-9,42238E-01	0,089	0,274	3,57148E-13
Macro_FOLR2+APOE-	SRA1	1,02440E-33	-9,43998E-01	0,098	0,389	1,88663E-29
Macro_FOLR2+APOE-	MPV17	4,53129E-17	-9,45650E-01	0,113	0,312	8,34527E-13
Macro_FOLR2+APOE-	VDAC3	3,50464E-19	-9,46172E-01	0,105	0,315	6,45450E-15
Macro_FOLR2+APOE-	LRRFIP1	5,86729E-52	-9,48052E-01	0,25	0,688	1,08058E-47
Macro_FOLR2+APOE-	PPP1CA	2,33702E-29	-9,49517E-01	0,269	0,626	4,30409E-25
Macro_FOLR2+APOE-	IFNGR2	6,40118E-24	-9,50555E-01	0,252	0,568	1,17891E-19
Macro_FOLR2+APOE-	SSR3	2,96887E-15	-9,51468E-01	0,303	0,6	5,46776E-11
Macro_FOLR2+APOE-	PET100	1,01951E-25	-9,52630E-01	0,132	0,408	1,87763E-21
Macro_FOLR2+APOE-	METRNL	6,00398E-21	-9,52749E-01	0,064	0,256	1,10575E-16
Macro_FOLR2+APOE-	EIF4G2	4,69001E-38	-9,53376E-01	0,234	0,623	8,63759E-34
Macro_FOLR2+APOE-	CCT3	1,11623E-26	-9,54608E-01	0,134	0,414	2,05576E-22
Macro_FOLR2+APOE-	FUS	2,36351E-33	-9,54635E-01	0,232	0,591	4,35288E-29
Macro_FOLR2+APOE-	HNRNPU	1,10865E-39	-9,55934E-01	0,232	0,628	2,04180E-35
Macro_FOLR2+APOE-	CYSTM1	1,57228E-37	-9,56211E-01	0,064	0,356	2,89567E-33
Macro_FOLR2+APOE-	IST1	6,79625E-19	-9,57002E-01	0,077	0,266	1,25167E-14
Macro_FOLR2+APOE-	MPEG1	2,20047E-09	-9,57748E-01	0,202	0,376	4,05261E-05
Macro_FOLR2+APOE-	CSF3R	2,03614E-24	-9,58869E-01	0,139	0,396	3,74996E-20
Macro_FOLR2+APOE-	SAP18	4,48433E-24	-9,58984E-01	0,299	0,652	8,25878E-20
Macro_FOLR2+APOE-	PTBP1	4,35939E-27	-9,60030E-01	0,072	0,309	8,02869E-23
Macro_FOLR2+APOE-	ATP6V1G1	6,55118E-38	-9,60152E-01	0,313	0,724	1,20653E-33
Macro_FOLR2+APOE-	PTPRC	6,26965E-40	-9,63525E-01	0,303	0,694	1,15468E-35
Macro_FOLR2+APOE-	HSD17B4	8,85060E-12	-9,64000E-01	0,107	0,26	1,63001E-07
Macro_FOLR2+APOE-	XAF1	1,92189E-19	-9,64113E-01	0,117	0,33	3,53954E-15
Macro_FOLR2+APOE-	LILRB4	3,37088E-09	-9,65696E-01	0,331	0,552	6,20814E-05
Macro_FOLR2+APOE-	DNAJC4	8,73765E-20	-9,66066E-01	0,134	0,365	1,60921E-15
Macro_FOLR2+APOE-	FDFT1	3,12955E-18	-9,68316E-01	0,079	0,262	5,76368E-14
Macro_FOLR2+APOE-	AAMP	8,85293E-21	-9,69421E-01	0,087	0,295	1,63044E-16
Macro_FOLR2+APOE-	CHD2	4,91592E-25	-9,70975E-01	0,077	0,301	9,05365E-21
Macro_FOLR2+APOE-	ORMDL2	3,14913E-25	-9,71065E-01	0,073	0,302	5,79976E-21
Macro_FOLR2+APOE-	PPP2CA	8,34908E-28	-9,71565E-01	0,085	0,337	1,53765E-23
Macro_FOLR2+APOE-	EIF1	3,14820E-14	-9,71971E-01	0,755	0,946	5,79804E-10
Macro_FOLR2+APOE-	B4GALT1	2,11366E-20	-9,73563E-01	0,111	0,327	3,89273E-16
Macro_FOLR2+APOE-	RANBP2	2,78587E-24	-9,75351E-01	0,087	0,314	5,13073E-20
Macro_FOLR2+APOE-	MRPL18	2,11151E-28	-9,75362E-01	0,094	0,352	3,88877E-24
Macro_FOLR2+APOE-	MOB1A	8,17372E-34	-9,75829E-01	0,218	0,587	1,50535E-29
Macro_FOLR2+APOE-	AGTRAP	2,88115E-26	-9,76264E-01	0,111	0,364	5,30621E-22
Macro_FOLR2+APOE-	BNIP2	4,95634E-20	-9,77482E-01	0,156	0,404	9,12809E-16
Macro_FOLR2+APOE-	HMGN4	2,02958E-25	-9,77810E-01	0,043	0,25	3,73787E-21
Macro_FOLR2+APOE-	ITGAX	2,21419E-38	-9,80671E-01	0,094	0,403	4,07787E-34
Macro_FOLR2+APOE-	DUSP6	2,94799E-18	-9,80906E-01	0,102	0,29	5,42932E-14
Macro_FOLR2+APOE-	PSMB1	6,09964E-31	-9,81112E-01	0,262	0,633	1,12337E-26

Macro_FOLR2+APOE-	EIF3G	9,69625E-19	-9,81727E-01	0,294	0,617	1,78576E-14
Macro_FOLR2+APOE-	STAT6	1,02153E-20	-9,84111E-01	0,096	0,307	1,88134E-16
Macro_FOLR2+APOE-	CNBP	7,61579E-23	-9,85110E-01	0,373	0,721	1,40260E-18
Macro_FOLR2+APOE-	GHITM	5,09394E-33	-9,85970E-01	0,171	0,508	9,38152E-29
Macro_FOLR2+APOE-	SYNGR2	2,32538E-11	-9,87186E-01	0,454	0,731	4,28266E-07
Macro_FOLR2+APOE-	PSMB4	8,18877E-25	-9,88043E-01	0,121	0,376	1,50813E-20
Macro_FOLR2+APOE-	EIF6	5,64449E-26	-9,88973E-01	0,136	0,408	1,03955E-21
Macro_FOLR2+APOE-	ARF5	2,35706E-31	-9,89785E-01	0,256	0,618	4,34099E-27
Macro_FOLR2+APOE-	NHP2	2,38826E-30	-9,92583E-01	0,124	0,416	4,39846E-26
Macro_FOLR2+APOE-	EEF1D	2,15311E-11	-9,93925E-01	0,578	0,875	3,96539E-07
Macro_FOLR2+APOE-	CMTM6	3,46241E-42	-9,94157E-01	0,224	0,621	6,37673E-38
Macro_FOLR2+APOE-	APEX1	2,31902E-15	-9,95292E-01	0,209	0,444	4,27095E-11
Macro_FOLR2+APOE-	DNAJB11	5,80937E-25	-9,96520E-01	0,081	0,313	1,06991E-20
Macro_FOLR2+APOE-	AKAP13	1,31546E-27	-9,98426E-01	0,286	0,637	2,42267E-23
Macro_FOLR2+APOE-	CHD1	2,91636E-29	-9,98730E-01	0,06	0,3	5,37106E-25
Macro_FOLR2+APOE-	LITAF	1,16439E-19	-9,99817E-01	0,416	0,745	2,14446E-15
Macro_FOLR2+APOE-	PIM3	8,98146E-35	-1,00035E+00	0,036	0,289	1,65411E-30
Macro_FOLR2+APOE-	SLC25A3	1,24956E-26	-1,00114E+00	0,352	0,718	2,30132E-22
Macro_FOLR2+APOE-	NAPA	4,84947E-41	-1,00307E+00	0,147	0,499	8,93127E-37
Macro_FOLR2+APOE-	HSPD1	3,68776E-35	-1,00329E+00	0,19	0,539	6,79175E-31
Macro_FOLR2+APOE-	EDF1	9,70396E-23	-1,00371E+00	0,388	0,754	1,78718E-18
Macro_FOLR2+APOE-	TNFRSF1B	3,67922E-41	-1,00412E+00	0,185	0,544	6,77602E-37
Macro_FOLR2+APOE-	MVP	1,42942E-34	-1,00465E+00	0,087	0,377	2,63257E-30
Macro_FOLR2+APOE-	CCT5	9,04606E-31	-1,00503E+00	0,102	0,384	1,66601E-26
Macro_FOLR2+APOE-	PPP1CB	1,31835E-24	-1,00724E+00	0,136	0,4	2,42801E-20
Macro_FOLR2+APOE-	KMT2E	2,81575E-25	-1,00815E+00	0,192	0,488	5,18576E-21
Macro_FOLR2+APOE-	LILRB2	2,17404E-28	-1,01046E+00	0,171	0,46	4,00393E-24
Macro_FOLR2+APOE-	CKLF	5,26996E-32	-1,01134E+00	0,186	0,509	9,70568E-28
Macro_FOLR2+APOE-	BTG1	1,06145E-49	-1,01249E+00	0,422	0,814	1,95486E-45
Macro_FOLR2+APOE-	MX2	1,04135E-20	-1,01260E+00	0,075	0,272	1,91785E-16
Macro_FOLR2+APOE-	BUB3	7,79602E-23	-1,01280E+00	0,058	0,26	1,43579E-18
Macro_FOLR2+APOE-	DDX39A	9,74931E-16	-1,01479E+00	0,107	0,294	1,79553E-11
Macro_FOLR2+APOE-	UBE2B	4,71908E-27	-1,01532E+00	0,185	0,492	8,69113E-23
Macro_FOLR2+APOE-	LGALS2	6,86073E-25	-1,01900E+00	0,055	0,259	1,26354E-20
Macro_FOLR2+APOE-	COPE	6,32618E-31	-1,01910E+00	0,247	0,611	1,16509E-26
Macro_FOLR2+APOE-	PSMA3	3,90220E-28	-1,01962E+00	0,105	0,375	7,18669E-24
Macro_FOLR2+APOE-	MT-ATP8	1,51144E-07	-1,02005E+00	0,228	0,386	2,78362E-03
Macro_FOLR2+APOE-	LAMTOR5	3,28017E-30	-1,02015E+00	0,177	0,511	6,04108E-26
Macro_FOLR2+APOE-	TCP1	9,69783E-19	-1,02051E+00	0,122	0,339	1,78605E-14
Macro_FOLR2+APOE-	MYL12B	1,33601E-26	-1,02165E+00	0,405	0,781	2,46052E-22
Macro_FOLR2+APOE-	ENY2	2,09639E-33	-1,02439E+00	0,228	0,6	3,86093E-29
Macro_FOLR2+APOE-	EMC7	1,47979E-26	-1,02603E+00	0,083	0,323	2,72533E-22
Macro_FOLR2+APOE-	COX6A1	3,12727E-28	-1,02608E+00	0,316	0,684	5,75950E-24
Macro_FOLR2+APOE-	PLK3	1,95724E-24	-1,02874E+00	0,051	0,255	3,60464E-20
Macro_FOLR2+APOE-	DECR1	2,25965E-24	-1,02886E+00	0,105	0,35	4,16160E-20
Macro_FOLR2+APOE-	HNRNPH3	3,09214E-24	-1,02926E+00	0,109	0,355	5,69479E-20
Macro_FOLR2+APOE-	C1QBP	2,12922E-27	-1,02942E+00	0,145	0,433	3,92139E-23

Macro_FOLR2+APOE-	VAMP5	1,11577E-24	-1,02988E+00	0,205	0,483	2,05492E-20
Macro_FOLR2+APOE-	LY6E	1,13693E-37	-1,03269E+00	0,213	0,551	2,09388E-33
Macro_FOLR2+APOE-	ACTG1	3,04515E-24	-1,03503E+00	0,736	0,924	5,60825E-20
Macro_FOLR2+APOE-	CSNK2B	5,53980E-19	-1,03556E+00	0,124	0,34	1,02026E-14
Macro_FOLR2+APOE-	CD53	1,41900E-19	-1,03808E+00	0,348	0,682	2,61338E-15
Macro_FOLR2+APOE-	C5AR1	2,01250E-20	-1,03808E+00	0,185	0,41	3,70641E-16
Macro_FOLR2+APOE-	WSB1	4,30729E-36	-1,04067E+00	0,26	0,645	7,93275E-32
Macro_FOLR2+APOE-	REL	1,54287E-54	-1,04162E+00	0,183	0,603	2,84151E-50
Macro_FOLR2+APOE-	SFPQ	5,51694E-39	-1,04294E+00	0,16	0,511	1,01605E-34
Macro_FOLR2+APOE-	ARID5A	7,62050E-26	-1,04413E+00	0,066	0,29	1,40347E-21
Macro_FOLR2+APOE-	NDUFA10	7,27212E-20	-1,04513E+00	0,113	0,33	1,33931E-15
Macro_FOLR2+APOE-	BCAS2	6,63965E-30	-1,05011E+00	0,038	0,266	1,22282E-25
Macro_FOLR2+APOE-	CD58	9,45033E-24	-1,05347E+00	0,089	0,316	1,74047E-19
Macro_FOLR2+APOE-	MORF4L2	8,29210E-20	-1,05373E+00	0,079	0,277	1,52716E-15
Macro_FOLR2+APOE-	RNF149	5,66295E-28	-1,05553E+00	0,282	0,631	1,04295E-23
Macro_FOLR2+APOE-	PSMB10	1,93204E-25	-1,05665E+00	0,249	0,57	3,55824E-21
Macro_FOLR2+APOE-	SPCS2	3,68451E-23	-1,05781E+00	0,203	0,496	6,78577E-19
Macro_FOLR2+APOE-	CCND3	3,97226E-17	-1,06426E+00	0,121	0,314	7,31571E-13
Macro_FOLR2+APOE-	NFKB1	5,13152E-35	-1,06440E+00	0,038	0,291	9,45072E-31
Macro_FOLR2+APOE-	SSR2	8,42468E-23	-1,06448E+00	0,303	0,656	1,55157E-18
Macro_FOLR2+APOE-	DBI	1,74322E-37	-1,06510E+00	0,326	0,728	3,21049E-33
Macro_FOLR2+APOE-	COX7A2	1,01189E-24	-1,06679E+00	0,367	0,725	1,86361E-20
Macro_FOLR2+APOE-	HNRNPA2B1	3,52920E-32	-1,06717E+00	0,456	0,836	6,49972E-28
Macro_FOLR2+APOE-	CD37	1,52191E-28	-1,06806E+00	0,388	0,736	2,80290E-24
Macro_FOLR2+APOE-	RILPL2	9,48640E-37	-1,06994E+00	0,134	0,459	1,74711E-32
Macro_FOLR2+APOE-	CNDP2	1,11767E-29	-1,07091E+00	0,154	0,458	2,05841E-25
Macro_FOLR2+APOE-	RPN2	1,04045E-19	-1,07248E+00	0,087	0,287	1,91619E-15
Macro_FOLR2+APOE-	BCLAF1	3,11196E-28	-1,07250E+00	0,115	0,384	5,73129E-24
Macro_FOLR2+APOE-	CNOT2	5,22365E-14	-1,07281E+00	0,104	0,273	9,62039E-10
Macro_FOLR2+APOE-	HNRNPDL	5,25956E-28	-1,07329E+00	0,311	0,679	9,68653E-24
Macro_FOLR2+APOE-	ATG3	2,98269E-37	-1,07442E+00	0,173	0,527	5,49322E-33
Macro_FOLR2+APOE-	SERPINA1	8,58518E-23	-1,07551E+00	0,458	0,724	1,58113E-18
Macro_FOLR2+APOE-	TMEM167A	3,95941E-35	-1,07711E+00	0,098	0,404	7,29205E-31
Macro_FOLR2+APOE-	GNA15	7,06139E-31	-1,07787E+00	0,06	0,309	1,30050E-26
Macro_FOLR2+APOE-	WTAP	1,12336E-31	-1,07820E+00	0,096	0,368	2,06889E-27
Macro_FOLR2+APOE-	NCF2	9,67393E-25	-1,07887E+00	0,188	0,468	1,78165E-20
Macro_FOLR2+APOE-	RALA	3,94190E-43	-1,08038E+00	0,064	0,378	7,25979E-39
Macro_FOLR2+APOE-	SHKBP1	7,98647E-34	-1,08251E+00	0,087	0,373	1,47087E-29
Macro_FOLR2+APOE-	NDUFB3	1,28561E-37	-1,08530E+00	0,134	0,471	2,36771E-33
Macro_FOLR2+APOE-	LUC7L3	9,41644E-14	-1,08564E+00	0,16	0,361	1,73423E-09
Macro_FOLR2+APOE-	SON	1,02253E-29	-1,08637E+00	0,301	0,678	1,88319E-25
Macro_FOLR2+APOE-	HEXB	5,06103E-17	-1,08644E+00	0,241	0,517	9,32090E-13
Macro_FOLR2+APOE-	TBCB	1,23362E-24	-1,08806E+00	0,179	0,471	2,27197E-20
Macro_FOLR2+APOE-	NFE2L2	2,54706E-29	-1,08920E+00	0,202	0,523	4,69092E-25
Macro_FOLR2+APOE-	COX5A	1,16868E-33	-1,08927E+00	0,311	0,699	2,15235E-29
Macro_FOLR2+APOE-	PSMB8	1,40376E-38	-1,08981E+00	0,211	0,581	2,58530E-34
Macro_FOLR2+APOE-	ZC3HAV1	2,23428E-21	-1,09032E+00	0,073	0,273	4,11488E-17

Macro_FOLR2+APOE-	GADD45B	2,33190E-34	-1,09113E+00	0,311	0,646	4,29466E-30
Macro_FOLR2+APOE-	STIP1	2,59178E-21	-1,09131E+00	0,079	0,284	4,77329E-17
Macro_FOLR2+APOE-	SRI	1,74652E-25	-1,09205E+00	0,156	0,437	3,21656E-21
Macro_FOLR2+APOE-	GUK1	2,13883E-29	-1,09244E+00	0,331	0,714	3,93908E-25
Macro_FOLR2+APOE-	PSMD9	7,27510E-22	-1,09245E+00	0,06	0,256	1,33985E-17
Macro_FOLR2+APOE-	DBNL	4,47428E-34	-1,09540E+00	0,164	0,496	8,24028E-30
Macro_FOLR2+APOE-	PSMC5	2,46506E-20	-1,09877E+00	0,162	0,414	4,53991E-16
Macro_FOLR2+APOE-	PSMD4	1,70145E-21	-1,10161E+00	0,156	0,415	3,13356E-17
Macro_FOLR2+APOE-	PPP1R2	6,96959E-28	-1,10194E+00	0,121	0,396	1,28359E-23
Macro_FOLR2+APOE-	CD300A	6,03613E-20	-1,10839E+00	0,198	0,463	1,11167E-15
Macro_FOLR2+APOE-	UBE2L6	5,41810E-28	-1,10973E+00	0,243	0,574	9,97852E-24
Macro_FOLR2+APOE-	TALDO1	9,72492E-29	-1,11383E+00	0,296	0,666	1,79104E-24
Macro_FOLR2+APOE-	FERMT3	1,58755E-30	-1,11468E+00	0,166	0,486	2,92379E-26
Macro_FOLR2+APOE-	P4HB	5,04383E-27	-1,11514E+00	0,277	0,633	9,28922E-23
Macro_FOLR2+APOE-	IFI35	7,26096E-33	-1,11556E+00	0,077	0,349	1,33725E-28
Macro_FOLR2+APOE-	RNF10	1,84745E-20	-1,11697E+00	0,077	0,277	3,40246E-16
Macro_FOLR2+APOE-	RHOC	4,49627E-21	-1,11707E+00	0,096	0,312	8,28078E-17
Macro_FOLR2+APOE-	S100A11	2,15708E-23	-1,11814E+00	0,804	0,939	3,97270E-19
Macro_FOLR2+APOE-	ANXA5	1,64740E-15	-1,11824E+00	0,471	0,777	3,03402E-11
Macro_FOLR2+APOE-	RPL23	3,67657E-10	-1,12635E+00	0,25	0,455	6,77114E-06
Macro_FOLR2+APOE-	BUD31	8,00084E-24	-1,12676E+00	0,115	0,363	1,47351E-19
Macro_FOLR2+APOE-	CCT7	5,67395E-23	-1,12737E+00	0,113	0,352	1,04497E-18
Macro_FOLR2+APOE-	ZFP36L1	1,91550E-10	-1,12980E+00	0,482	0,728	3,52778E-06
Macro_FOLR2+APOE-	AHSA1	4,10510E-21	-1,13305E+00	0,09	0,301	7,56036E-17
Macro_FOLR2+APOE-	DYNLT1	1,81926E-29	-1,13530E+00	0,186	0,513	3,35052E-25
Macro_FOLR2+APOE-	UBE2D3	3,92854E-30	-1,13548E+00	0,362	0,741	7,23520E-26
Macro_FOLR2+APOE-	SAP30BP	1,69872E-16	-1,13661E+00	0,092	0,273	3,12853E-12
Macro_FOLR2+APOE-	DAZAP2	1,21297E-22	-1,13886E+00	0,384	0,746	2,23392E-18
Macro_FOLR2+APOE-	FPR1	3,84128E-29	-1,13932E+00	0,166	0,451	7,07448E-25
Macro_FOLR2+APOE-	ETF1	2,13211E-31	-1,14153E+00	0,066	0,322	3,92670E-27
Macro_FOLR2+APOE-	SLC38A2	6,03537E-29	-1,14692E+00	0,068	0,311	1,11153E-24
Macro_FOLR2+APOE-	CLIC1	1,38682E-23	-1,15096E+00	0,565	0,878	2,55410E-19
Macro_FOLR2+APOE-	RAB1A	1,10059E-23	-1,15152E+00	0,137	0,399	2,02696E-19
Macro_FOLR2+APOE-	IFI44L	3,98892E-08	-1,15202E+00	0,13	0,255	7,34640E-04
Macro_FOLR2+APOE-	NABP1	1,32933E-24	-1,15326E+00	0,102	0,342	2,44824E-20
Macro_FOLR2+APOE-	PGD	2,73930E-33	-1,15480E+00	0,153	0,469	5,04497E-29
Macro_FOLR2+APOE-	TAF9	1,23266E-21	-1,15627E+00	0,09	0,304	2,27019E-17
Macro_FOLR2+APOE-	GTF2B	3,62679E-29	-1,15736E+00	0,049	0,282	6,67946E-25
Macro_FOLR2+APOE-	HSP90AB1	1,32723E-15	-1,15739E+00	0,535	0,829	2,44437E-11
Macro_FOLR2+APOE-	HSPA9	3,85126E-25	-1,15783E+00	0,117	0,373	7,09287E-21
Macro_FOLR2+APOE-	LILRB1	9,06604E-36	-1,16137E+00	0,087	0,38	1,66969E-31
Macro_FOLR2+APOE-	HSPA1A	4,45339E-11	-1,16204E+00	0,437	0,666	8,20181E-07
Macro_FOLR2+APOE-	HNRNPM	7,39229E-35	-1,16361E+00	0,137	0,461	1,36144E-30
Macro_FOLR2+APOE-	HNRNPK	1,77149E-29	-1,16409E+00	0,335	0,713	3,26255E-25
Macro_FOLR2+APOE-	KLF4	3,36906E-36	-1,16660E+00	0,139	0,45	6,20481E-32
Macro_FOLR2+APOE-	CYTIP	1,07457E-40	-1,16799E+00	0,085	0,394	1,97903E-36
Macro_FOLR2+APOE-	JMJD1C	8,92042E-26	-1,16820E+00	0,154	0,431	1,64287E-21

Macro_FOLR2+APOE-	GAPT	2,15479E-20	-1,17008E+00	0,07	0,257	3,96848E-16
Macro_FOLR2+APOE-	ZFAND5	9,96928E-42	-1,17112E+00	0,22	0,589	1,83604E-37
Macro_FOLR2+APOE-	SKP1	6,34506E-13	-1,17663E+00	0,375	0,692	1,16857E-08
Macro_FOLR2+APOE-	SNRPD2	4,88633E-30	-1,17727E+00	0,301	0,682	8,99916E-26
Macro_FOLR2+APOE-	UBL7	4,80950E-17	-1,17795E+00	0,079	0,256	8,85766E-13
Macro_FOLR2+APOE-	GABARAPL2	5,76583E-32	-1,17966E+00	0,226	0,582	1,06189E-27
Macro_FOLR2+APOE-	NONO	2,54794E-28	-1,17969E+00	0,141	0,434	4,69255E-24
Macro_FOLR2+APOE-	PSMB3	4,82169E-24	-1,18115E+00	0,294	0,647	8,88010E-20
Macro_FOLR2+APOE-	MLF2	1,53942E-28	-1,18130E+00	0,153	0,456	2,83515E-24
Macro_FOLR2+APOE-	RIPK2	3,94437E-25	-1,18145E+00	0,062	0,276	7,26434E-21
Macro_FOLR2+APOE-	LIMD2	7,75272E-55	-1,18334E+00	0,102	0,486	1,42782E-50
Macro_FOLR2+APOE-	U2AF1	1,64928E-15	-1,18590E+00	0,104	0,271	3,03748E-11
Macro_FOLR2+APOE-	SSR4	2,40156E-19	-1,18632E+00	0,414	0,757	4,42295E-15
Macro_FOLR2+APOE-	PSMA4	2,10931E-33	-1,18650E+00	0,149	0,472	3,88471E-29
Macro_FOLR2+APOE-	DDX5	5,79522E-20	-1,18701E+00	0,492	0,837	1,06731E-15
Macro_FOLR2+APOE-	ARL8B	1,93589E-33	-1,18739E+00	0,111	0,407	3,56532E-29
Macro_FOLR2+APOE-	EIF5A	3,75679E-27	-1,18780E+00	0,234	0,56	6,91888E-23
Macro_FOLR2+APOE-	SRSF7	5,55058E-28	-1,18975E+00	0,23	0,555	1,02225E-23
Macro_FOLR2+APOE-	RAN	9,14912E-41	-1,18996E+00	0,26	0,662	1,68499E-36
Macro_FOLR2+APOE-	CDC37	4,96433E-36	-1,19185E+00	0,26	0,642	9,14281E-32
Macro_FOLR2+APOE-	TANK	7,97923E-28	-1,19240E+00	0,062	0,296	1,46953E-23
Macro_FOLR2+APOE-	ACTR3	9,29531E-41	-1,19281E+00	0,262	0,668	1,71192E-36
Macro_FOLR2+APOE-	IDH3B	4,18457E-20	-1,19393E+00	0,072	0,264	7,70672E-16
Macro_FOLR2+APOE-	RBM8A	6,62268E-41	-1,19547E+00	0,168	0,538	1,21970E-36
Macro_FOLR2+APOE-	FYTTD1	2,70717E-26	-1,19547E+00	0,041	0,251	4,98580E-22
Macro_FOLR2+APOE-	COPZ1	2,13734E-30	-1,19606E+00	0,105	0,385	3,93634E-26
Macro_FOLR2+APOE-	CCT8	2,68879E-25	-1,19643E+00	0,145	0,416	4,95195E-21
Macro_FOLR2+APOE-	GSTP1	1,28116E-20	-1,19842E+00	0,522	0,827	2,35951E-16
Macro_FOLR2+APOE-	OAZ1	2,19278E-09	-1,20262E+00	0,774	0,938	4,03844E-05
Macro_FOLR2+APOE-	MBNL1	1,96342E-33	-1,20304E+00	0,192	0,529	3,61603E-29
Macro_FOLR2+APOE-	IRF1	1,64476E-46	-1,20384E+00	0,102	0,442	3,02915E-42
Macro_FOLR2+APOE-	MT-ND5	8,58853E-07	-1,20468E+00	0,348	0,539	1,58175E-02
Macro_FOLR2+APOE-	ADPGK	8,06614E-25	-1,20784E+00	0,136	0,404	1,48554E-20
Macro_FOLR2+APOE-	EMP3	1,60771E-45	-1,20910E+00	0,322	0,733	2,96092E-41
Macro_FOLR2+APOE-	HNRNPC	1,44901E-23	-1,20951E+00	0,298	0,651	2,66865E-19
Macro_FOLR2+APOE-	PTPRE	3,81516E-36	-1,21570E+00	0,126	0,443	7,02638E-32
Macro_FOLR2+APOE-	CYCS	1,26366E-41	-1,21813E+00	0,16	0,528	2,32728E-37
Macro_FOLR2+APOE-	ACADVL	1,35990E-29	-1,21961E+00	0,16	0,473	2,50453E-25
Macro_FOLR2+APOE-	PSMD13	4,38529E-23	-1,22235E+00	0,096	0,326	8,07638E-19
Macro_FOLR2+APOE-	GABARAPL1	1,00762E-32	-1,22266E+00	0,047	0,294	1,85574E-28
Macro_FOLR2+APOE-	THEMIS2	1,40164E-29	-1,22475E+00	0,183	0,498	2,58139E-25
Macro_FOLR2+APOE-	STRAP	6,69282E-35	-1,22483E+00	0,073	0,359	1,23262E-30
Macro_FOLR2+APOE-	PTP4A2	1,93436E-33	-1,22541E+00	0,109	0,408	3,56251E-29
Macro_FOLR2+APOE-	HLA-F	2,47493E-24	-1,23015E+00	0,209	0,519	4,55807E-20
Macro_FOLR2+APOE-	TPM4	1,84222E-42	-1,23195E+00	0,235	0,622	3,39282E-38
Macro_FOLR2+APOE-	ACTB	1,66589E-30	-1,23357E+00	0,97	0,991	3,06807E-26
Macro_FOLR2+APOE-	HSPA1B	5,45334E-14	-1,23401E+00	0,298	0,507	1,00434E-09

Macro_FOLR2+APOE-	FLII	9,62826E-22	-1,23411E+00	0,104	0,327	1,77324E-17
Macro_FOLR2+APOE-	PARK7	3,50550E-20	-1,23541E+00	0,333	0,662	6,45608E-16
Macro_FOLR2+APOE-	GPI	6,19341E-21	-1,23584E+00	0,162	0,419	1,14064E-16
Macro_FOLR2+APOE-	CAPZA1	9,53065E-27	-1,23632E+00	0,185	0,498	1,75526E-22
Macro_FOLR2+APOE-	ILF2	1,15925E-34	-1,23843E+00	0,098	0,395	2,13499E-30
Macro_FOLR2+APOE-	CRIP1	9,29761E-28	-1,23989E+00	0,064	0,295	1,71234E-23
Macro_FOLR2+APOE-	HSPH1	3,20672E-30	-1,24057E+00	0,136	0,418	5,90581E-26
Macro_FOLR2+APOE-	NR3C1	8,53711E-26	-1,24224E+00	0,13	0,391	1,57228E-21
Macro_FOLR2+APOE-	CSRNP1	5,46649E-42	-1,24475E+00	0,017	0,295	1,00676E-37
Macro_FOLR2+APOE-	LGALS1	6,33194E-40	-1,24662E+00	0,531	0,839	1,16615E-35
Macro_FOLR2+APOE-	EIF3L	2,61404E-19	-1,24987E+00	0,299	0,603	4,81428E-15
Macro_FOLR2+APOE-	ARF1	4,70128E-25	-1,25486E+00	0,335	0,703	8,65835E-21
Macro_FOLR2+APOE-	PSMB7	1,09952E-28	-1,25486E+00	0,1	0,366	2,02499E-24
Macro_FOLR2+APOE-	GABARAP	2,46028E-32	-1,25730E+00	0,492	0,833	4,53110E-28
Macro_FOLR2+APOE-	LTA4H	8,05179E-29	-1,26127E+00	0,164	0,451	1,48290E-24
Macro_FOLR2+APOE-	TCIRG1	1,68484E-35	-1,26375E+00	0,149	0,471	3,10297E-31
Macro_FOLR2+APOE-	HSP90AA1	1,35788E-14	-1,26508E+00	0,635	0,882	2,50081E-10
Macro_FOLR2+APOE-	LILRA2	3,06441E-26	-1,26762E+00	0,041	0,25	5,64373E-22
Macro_FOLR2+APOE-	GSTO1	4,46469E-37	-1,26884E+00	0,25	0,624	8,22261E-33
Macro_FOLR2+APOE-	PSMA7	6,63998E-23	-1,26951E+00	0,401	0,749	1,22288E-18
Macro_FOLR2+APOE-	TPP1	1,32412E-09	-1,26960E+00	0,324	0,598	2,43863E-05
Macro_FOLR2+APOE-	TKT	3,32255E-30	-1,27084E+00	0,267	0,611	6,11915E-26
Macro_FOLR2+APOE-	TSPO	2,37148E-26	-1,27545E+00	0,433	0,769	4,36755E-22
Macro_FOLR2+APOE-	VMP1	1,77799E-23	-1,28147E+00	0,245	0,554	3,27453E-19
Macro_FOLR2+APOE-	TCF4	2,47178E-12	-1,28229E+00	0,117	0,275	4,55228E-08
Macro_FOLR2+APOE-	TPM3	2,60812E-31	-1,28366E+00	0,424	0,808	4,80337E-27
Macro_FOLR2+APOE-	SPCS1	3,86714E-23	-1,28467E+00	0,286	0,611	7,12211E-19
Macro_FOLR2+APOE-	LDHB	4,69465E-26	-1,28902E+00	0,205	0,515	8,64613E-22
Macro_FOLR2+APOE-	SRSF5	2,41218E-35	-1,29040E+00	0,215	0,583	4,44252E-31
Macro_FOLR2+APOE-	NDUFA13	2,57040E-23	-1,29088E+00	0,168	0,435	4,73391E-19
Macro_FOLR2+APOE-	APRT	1,18359E-43	-1,29233E+00	0,226	0,628	2,17982E-39
Macro_FOLR2+APOE-	CD47	3,21941E-33	-1,29695E+00	0,121	0,425	5,92919E-29
Macro_FOLR2+APOE-	ACAA1	1,28165E-25	-1,29881E+00	0,121	0,381	2,36041E-21
Macro_FOLR2+APOE-	PPIB	3,06024E-25	-1,30910E+00	0,444	0,796	5,63604E-21
Macro_FOLR2+APOE-	RBM3	4,76808E-30	-1,30978E+00	0,313	0,679	8,78137E-26
Macro_FOLR2+APOE-	STX11	3,55643E-40	-1,30993E+00	0,045	0,331	6,54988E-36
Macro_FOLR2+APOE-	TRA2B	2,33010E-45	-1,31030E+00	0,117	0,475	4,29134E-41
Macro_FOLR2+APOE-	MYL12A	1,24109E-17	-1,31227E+00	0,497	0,828	2,28571E-13
Macro_FOLR2+APOE-	GLIPR2	4,13187E-59	-1,31472E+00	0,021	0,381	7,60966E-55
Macro_FOLR2+APOE-	FTH1	4,94921E-25	-1,31472E+00	0,989	0,997	9,11497E-21
Macro_FOLR2+APOE-	HERPUD1	9,57263E-07	-1,31610E+00	0,437	0,684	1,76299E-02
Macro_FOLR2+APOE-	TRA2A	3,89644E-25	-1,31852E+00	0,049	0,258	7,17607E-21
Macro_FOLR2+APOE-	SRSF3	1,85472E-44	-1,32010E+00	0,186	0,575	3,41583E-40
Macro_FOLR2+APOE-	CAPG	4,45234E-34	-1,32231E+00	0,38	0,745	8,19987E-30
Macro_FOLR2+APOE-	PPIF	2,93704E-42	-1,33121E+00	0,06	0,361	5,40915E-38
Macro_FOLR2+APOE-	SUB1	2,22653E-41	-1,33176E+00	0,318	0,728	4,10060E-37
Macro_FOLR2+APOE-	LPXN	1,34711E-30	-1,33236E+00	0,087	0,351	2,48097E-26

Macro_FOLR2+APOE-	SAMSN1	1,06014E-42	-1,33281E+00	0,126	0,468	1,95246E-38
Macro_FOLR2+APOE-	SRSF2	1,10818E-41	-1,34480E+00	0,181	0,562	2,04094E-37
Macro_FOLR2+APOE-	TAGAP	1,17268E-31	-1,34642E+00	0,038	0,275	2,15973E-27
Macro_FOLR2+APOE-	MAP2K3	2,46461E-32	-1,34859E+00	0,034	0,273	4,53908E-28
Macro_FOLR2+APOE-	MAP3K8	7,79100E-41	-1,35560E+00	0,143	0,486	1,43487E-36
Macro_FOLR2+APOE-	KDM6B	3,54685E-44	-1,35781E+00	0,041	0,344	6,53224E-40
Macro_FOLR2+APOE-	SERPINB1	2,07246E-56	-1,35899E+00	0,235	0,668	3,81684E-52
Macro_FOLR2+APOE-	MARCKSL1	1,36293E-31	-1,36008E+00	0,036	0,268	2,51011E-27
Macro_FOLR2+APOE-	TPI1	8,05206E-34	-1,36169E+00	0,458	0,83	1,48295E-29
Macro_FOLR2+APOE-	HSP90B1	1,35466E-26	-1,36780E+00	0,365	0,704	2,49488E-22
Macro_FOLR2+APOE-	PPA1	1,12751E-55	-1,37149E+00	0,089	0,465	2,07654E-51
Macro_FOLR2+APOE-	HNRNPA1	1,19952E-17	-1,37362E+00	0,497	0,805	2,20915E-13
Macro_FOLR2+APOE-	KYNU	2,90882E-31	-1,38275E+00	0,066	0,318	5,35717E-27
Macro_FOLR2+APOE-	IFI6	7,46809E-20	-1,38293E+00	0,237	0,491	1,37540E-15
Macro_FOLR2+APOE-	FABP5	3,34094E-42	-1,38413E+00	0,113	0,45	6,15301E-38
Macro_FOLR2+APOE-	RPSA	3,83726E-07	-1,38673E+00	0,324	0,497	7,06708E-03
Macro_FOLR2+APOE-	NLRP3	8,12921E-29	-1,38932E+00	0,043	0,265	1,49716E-24
Macro_FOLR2+APOE-	ARPC4	1,13460E-28	-1,39195E+00	0,13	0,413	2,08960E-24
Macro_FOLR2+APOE-	MIF	1,07654E-52	-1,39250E+00	0,299	0,729	1,98266E-48
Macro_FOLR2+APOE-	SNRPB	1,34982E-40	-1,39574E+00	0,153	0,512	2,48596E-36
Macro_FOLR2+APOE-	PNP	1,47687E-34	-1,39697E+00	0,021	0,262	2,71995E-30
Macro_FOLR2+APOE-	UPP1	6,00517E-39	-1,39795E+00	0,115	0,435	1,10597E-34
Macro_FOLR2+APOE-	SLC25A37	1,29759E-28	-1,40503E+00	0,062	0,294	2,38978E-24
Macro_FOLR2+APOE-	MTHFD2	3,16164E-33	-1,41081E+00	0,113	0,408	5,82280E-29
Macro_FOLR2+APOE-	RBM39	6,71591E-41	-1,42261E+00	0,23	0,627	1,23687E-36
Macro_FOLR2+APOE-	CFLAR	3,99033E-42	-1,43288E+00	0,156	0,516	7,34899E-38
Macro_FOLR2+APOE-	CCNL1	7,07149E-44	-1,43397E+00	0,16	0,53	1,30236E-39
Macro_FOLR2+APOE-	PDIA3	9,07136E-41	-1,43498E+00	0,284	0,68	1,67067E-36
Macro_FOLR2+APOE-	SLC3A2	1,02542E-31	-1,43655E+00	0,156	0,467	1,88851E-27
Macro_FOLR2+APOE-	MYL6	1,33018E-24	-1,43825E+00	0,761	0,944	2,44979E-20
Macro_FOLR2+APOE-	GK	1,51606E-46	-1,43944E+00	0,049	0,368	2,79213E-42
Macro_FOLR2+APOE-	GAPDH	7,25781E-42	-1,44052E+00	0,8	0,962	1,33667E-37
Macro_FOLR2+APOE-	PGAM1	1,16826E-47	-1,44242E+00	0,169	0,57	2,15159E-43
Macro_FOLR2+APOE-	KLF6	1,07316E-53	-1,44569E+00	0,38	0,802	1,97644E-49
Macro_FOLR2+APOE-	PSMB9	5,43075E-48	-1,45213E+00	0,25	0,662	1,00018E-43
Macro_FOLR2+APOE-	MYADM	1,96121E-39	-1,45560E+00	0,051	0,337	3,61196E-35
Macro_FOLR2+APOE-	TUBB4B	9,19881E-47	-1,46346E+00	0,055	0,383	1,69415E-42
Macro_FOLR2+APOE-	UBB	2,34008E-10	-1,46582E+00	0,516	0,814	4,30972E-06
Macro_FOLR2+APOE-	PTPN6	8,41083E-39	-1,46751E+00	0,166	0,514	1,54902E-34
Macro_FOLR2+APOE-	PSME1	9,71090E-34	-1,47113E+00	0,365	0,759	1,78846E-29
Macro_FOLR2+APOE-	FOSL2	1,68947E-41	-1,47275E+00	0,021	0,299	3,11149E-37
Macro_FOLR2+APOE-	HSPA8	1,69606E-20	-1,47316E+00	0,518	0,823	3,12364E-16
Macro_FOLR2+APOE-	STK17B	1,58942E-44	-1,47950E+00	0,105	0,448	2,92724E-40
Macro_FOLR2+APOE-	S100A4	4,16517E-48	-1,48717E+00	0,503	0,83	7,67099E-44
Macro_FOLR2+APOE-	MCL1	2,78864E-82	-1,48964E+00	0,267	0,775	5,13584E-78
Macro_FOLR2+APOE-	CALM1	2,77109E-25	-1,49020E+00	0,456	0,799	5,10351E-21
Macro_FOLR2+APOE-	EIF5	1,24319E-32	-1,49253E+00	0,239	0,596	2,28958E-28

Macro_FOLR2+APOE-	SH3BGRL3	2,97779E-65	-1,49816E+00	0,674	0,936	5,48420E-61
Macro_FOLR2+APOE-	STXBP2	1,45923E-44	-1,51365E+00	0,09	0,423	2,68746E-40
Macro_FOLR2+APOE-	LCP1	6,70063E-57	-1,51627E+00	0,258	0,709	1,23405E-52
Macro_FOLR2+APOE-	HSPE1	2,07013E-37	-1,52081E+00	0,241	0,607	3,81257E-33
Macro_FOLR2+APOE-	LYZ	4,86915E-09	-1,52444E+00	0,684	0,81	8,96750E-05
Macro_FOLR2+APOE-	EIF4A1	1,75978E-20	-1,53181E+00	0,384	0,677	3,24098E-16
Macro_FOLR2+APOE-	HIF1A	3,88649E-43	-1,53604E+00	0,179	0,553	7,15774E-39
Macro_FOLR2+APOE-	CCNH	1,00574E-31	-1,53693E+00	0,102	0,374	1,85228E-27
Macro_FOLR2+APOE-	RPL36A	7,65348E-13	-1,53837E+00	0,239	0,456	1,40954E-08
Macro_FOLR2+APOE-	PSMA6	1,19679E-50	-1,54241E+00	0,098	0,47	2,20412E-46
Macro_FOLR2+APOE-	ALOX5AP	3,03436E-10	-1,54708E+00	0,326	0,507	5,58839E-06
Macro_FOLR2+APOE-	TRIM22	3,17550E-26	-1,55833E+00	0,145	0,42	5,84833E-22
Macro_FOLR2+APOE-	MX1	1,03476E-27	-1,55968E+00	0,089	0,336	1,90573E-23
Macro_FOLR2+APOE-	FOSB	2,40882E-54	-1,56470E+00	0,192	0,584	4,43632E-50
Macro_FOLR2+APOE-	SELPLG	3,71402E-15	-1,56822E+00	0,147	0,346	6,84012E-11
Macro_FOLR2+APOE-	HSPA5	9,89217E-47	-1,57224E+00	0,181	0,566	1,82184E-42
Macro_FOLR2+APOE-	CHMP1B	2,21830E-43	-1,57982E+00	0,089	0,41	4,08544E-39
Macro_FOLR2+APOE-	SEC61B	6,24134E-31	-1,58646E+00	0,377	0,744	1,14947E-26
Macro_FOLR2+APOE-	UQCRC1	6,17474E-34	-1,59240E+00	0,171	0,509	1,13720E-29
Macro_FOLR2+APOE-	MAT2A	7,62738E-31	-1,59384E+00	0,149	0,45	1,40473E-26
Macro_FOLR2+APOE-	IER3	1,10634E-29	-1,59443E+00	0,247	0,536	2,03755E-25
Macro_FOLR2+APOE-	TAGLN2	3,63940E-25	-1,59655E+00	0,414	0,758	6,70269E-21
Macro_FOLR2+APOE-	COTL1	2,66596E-43	-1,60774E+00	0,339	0,716	4,90990E-39
Macro_FOLR2+APOE-	LMNA	1,46663E-32	-1,62329E+00	0,1	0,372	2,70109E-28
Macro_FOLR2+APOE-	YPEL5	2,24255E-26	-1,63320E+00	0,158	0,437	4,13010E-22
Macro_FOLR2+APOE-	LGALS3	8,72737E-38	-1,63625E+00	0,379	0,728	1,60732E-33
Macro_FOLR2+APOE-	S100A6	7,96237E-74	-1,63646E+00	0,518	0,879	1,46643E-69
Macro_FOLR2+APOE-	ANKRD10	1,98839E-19	-1,63841E+00	0,119	0,334	3,66202E-15
Macro_FOLR2+APOE-	MT2A	1,36837E-52	-1,63858E+00	0,245	0,632	2,52013E-48
Macro_FOLR2+APOE-	INSIG1	2,65731E-24	-1,64736E+00	0,081	0,298	4,89397E-20
Macro_FOLR2+APOE-	IFRD1	3,00689E-30	-1,66208E+00	0,055	0,294	5,53779E-26
Macro_FOLR2+APOE-	LSP1	6,74949E-65	-1,66253E+00	0,247	0,676	1,24305E-60
Macro_FOLR2+APOE-	HBEGF	7,47810E-31	-1,66293E+00	0,045	0,278	1,37724E-26
Macro_FOLR2+APOE-	RPS11	5,97185E-07	-1,67064E+00	0,318	0,506	1,09984E-02
Macro_FOLR2+APOE-	RPL4	1,49112E-09	-1,67080E+00	0,288	0,479	2,74620E-05
Macro_FOLR2+APOE-	SLC11A1	8,39941E-37	-1,67261E+00	0,158	0,471	1,54692E-32
Macro_FOLR2+APOE-	FBP1	1,68610E-33	-1,68574E+00	0,081	0,352	3,10529E-29
Macro_FOLR2+APOE-	ID2	1,25605E-42	-1,68799E+00	0,2	0,561	2,31327E-38
Macro_FOLR2+APOE-	CTSL	8,68882E-12	-1,69499E+00	0,32	0,521	1,60022E-07
Macro_FOLR2+APOE-	TAP1	8,51066E-47	-1,70261E+00	0,087	0,426	1,56741E-42
Macro_FOLR2+APOE-	SGK1	1,31113E-39	-1,70514E+00	0,26	0,609	2,41470E-35
Macro_FOLR2+APOE-	MXD1	2,14866E-35	-1,70527E+00	0,045	0,305	3,95719E-31
Macro_FOLR2+APOE-	PDE4B	3,61178E-40	-1,70788E+00	0,07	0,371	6,65182E-36
Macro_FOLR2+APOE-	PSME2	5,01925E-28	-1,71756E+00	0,388	0,719	9,24396E-24
Macro_FOLR2+APOE-	BHLHE40	1,96947E-43	-1,71967E+00	0,043	0,343	3,62718E-39
Macro_FOLR2+APOE-	S100A10	9,51355E-46	-1,72362E+00	0,507	0,841	1,75211E-41
Macro_FOLR2+APOE-	BID	2,44201E-44	-1,72458E+00	0,083	0,418	4,49746E-40

Macro_FOLR2+APOE-	TXN	9,43369E-35	-1,72737E+00	0,305	0,671	1,73740E-30
Macro_FOLR2+APOE-	SOCS3	2,87912E-53	-1,73366E+00	0,026	0,361	5,30248E-49
Macro_FOLR2+APOE-	ANXA1	8,47069E-41	-1,75985E+00	0,311	0,692	1,56005E-36
Macro_FOLR2+APOE-	GBP1	1,62510E-36	-1,76175E+00	0,072	0,349	2,99295E-32
Macro_FOLR2+APOE-	PABPC1	2,22564E-26	-1,77290E+00	0,618	0,896	4,09897E-22
Macro_FOLR2+APOE-	TGFBI	4,16093E-09	-1,78908E+00	0,358	0,601	7,66318E-05
Macro_FOLR2+APOE-	RPL17	1,54676E-18	-1,79347E+00	0,2	0,436	2,84867E-14
Macro_FOLR2+APOE-	ALDOA	2,97973E-38	-1,79561E+00	0,403	0,764	5,48777E-34
Macro_FOLR2+APOE-	OLR1	1,46126E-44	-1,80065E+00	0,045	0,351	2,69121E-40
Macro_FOLR2+APOE-	IFITM2	2,62612E-10	-1,81871E+00	0,407	0,649	4,83653E-06
Macro_FOLR2+APOE-	RPS10	2,03502E-08	-1,84070E+00	0,151	0,285	3,74790E-04
Macro_FOLR2+APOE-	RAC2	4,27004E-49	-1,84125E+00	0,132	0,501	7,86413E-45
Macro_FOLR2+APOE-	VIM	5,30695E-88	-1,86053E+00	0,637	0,947	9,77381E-84
Macro_FOLR2+APOE-	RNASEK	1,71058E-38	-1,86312E+00	0,143	0,469	3,15037E-34
Macro_FOLR2+APOE-	PLEK	3,08849E-56	-1,87909E+00	0,153	0,566	5,68808E-52
Macro_FOLR2+APOE-	FGR	2,69378E-46	-1,88479E+00	0,111	0,458	4,96114E-42
Macro_FOLR2+APOE-	DNAJB1	9,80123E-29	-1,90386E+00	0,26	0,561	1,80509E-24
Macro_FOLR2+APOE-	IER2	3,97309E-61	-1,92122E+00	0,217	0,637	7,31724E-57
Macro_FOLR2+APOE-	CORO1A	9,50232E-54	-1,92413E+00	0,234	0,651	1,75004E-49
Macro_FOLR2+APOE-	JUNB	7,89985E-70	-1,93146E+00	0,399	0,831	1,45492E-65
Macro_FOLR2+APOE-	HNRNPH1	6,14170E-28	-1,93542E+00	0,128	0,408	1,13112E-23
Macro_FOLR2+APOE-	ISG20	1,38255E-38	-1,93719E+00	0,041	0,314	2,54623E-34
Macro_FOLR2+APOE-	CD48	3,69039E-65	-1,94200E+00	0,073	0,484	6,79659E-61
Macro_FOLR2+APOE-	EZR	1,33793E-33	-1,94418E+00	0,113	0,403	2,46406E-29
Macro_FOLR2+APOE-	ENO1	1,06151E-48	-1,95458E+00	0,343	0,766	1,95499E-44
Macro_FOLR2+APOE-	DNAJA1	3,95666E-45	-1,96289E+00	0,215	0,609	7,28697E-41
Macro_FOLR2+APOE-	IFITM1	1,03496E-28	-1,96528E+00	0,038	0,257	1,90608E-24
Macro_FOLR2+APOE-	ATP1B3	6,06833E-53	-1,96779E+00	0,153	0,549	1,11760E-48
Macro_FOLR2+APOE-	FLNA	3,28973E-58	-1,97030E+00	0,075	0,456	6,05869E-54
Macro_FOLR2+APOE-	ANXA2	3,97332E-35	-1,97357E+00	0,399	0,747	7,31766E-31
Macro_FOLR2+APOE-	CLK1	1,75108E-30	-1,98096E+00	0,107	0,386	3,22496E-26
Macro_FOLR2+APOE-	EGR1	6,93573E-32	-1,98290E+00	0,049	0,289	1,27735E-27
Macro_FOLR2+APOE-	CD44	4,39294E-75	-1,99644E+00	0,205	0,682	8,09047E-71
Macro_FOLR2+APOE-	FOS	6,51230E-43	-1,99818E+00	0,522	0,847	1,19937E-38
Macro_FOLR2+APOE-	CREM	1,08013E-24	-2,00119E+00	0,132	0,38	1,98928E-20
Macro_FOLR2+APOE-	PLP2	5,34972E-44	-2,01003E+00	0,087	0,408	9,85258E-40
Macro_FOLR2+APOE-	SAT1	1,48384E-11	-2,01362E+00	0,847	0,949	2,73278E-07
Macro_FOLR2+APOE-	RASGEF1B	3,55985E-34	-2,01693E+00	0,028	0,272	6,55617E-30
Macro_FOLR2+APOE-	NR4A3	5,27607E-45	-2,02998E+00	0,013	0,3	9,71694E-41
Macro_FOLR2+APOE-	EEF1G	6,90386E-23	-2,04364E+00	0,087	0,298	1,27148E-18
Macro_FOLR2+APOE-	YWHAZ	1,25898E-57	-2,04451E+00	0,269	0,717	2,31866E-53
Macro_FOLR2+APOE-	TUBA1A	1,02043E-39	-2,04496E+00	0,136	0,466	1,87933E-35
Macro_FOLR2+APOE-	ICAM1	5,41826E-49	-2,07176E+00	0,04	0,366	9,97881E-45
Macro_FOLR2+APOE-	RGS1	1,81639E-08	-2,07400E+00	0,439	0,645	3,34525E-04
Macro_FOLR2+APOE-	GLA	1,22177E-32	-2,08105E+00	0,058	0,314	2,25014E-28
Macro_FOLR2+APOE-	PGK1	1,96004E-38	-2,10615E+00	0,309	0,711	3,60980E-34
Macro_FOLR2+APOE-	ATF3	1,74141E-48	-2,10981E+00	0,094	0,437	3,20716E-44

Macro_FOLR2+APOE-	LDHA	4,08940E-48	-2,11957E+00	0,301	0,719	7,53144E-44
Macro_FOLR2+APOE-	SRGN	1,06712E-70	-2,13378E+00	0,678	0,946	1,96532E-66
Macro_FOLR2+APOE-	BTG2	4,67518E-57	-2,13607E+00	0,113	0,503	8,61029E-53
Macro_FOLR2+APOE-	EIF4A3	8,28068E-35	-2,18320E+00	0,107	0,406	1,52505E-30
Macro_FOLR2+APOE-	DUSP1	9,28320E-45	-2,22940E+00	0,503	0,854	1,70969E-40
Macro_FOLR2+APOE-	SERPINB9	1,69146E-52	-2,23936E+00	0,04	0,379	3,11517E-48
Macro_FOLR2+APOE-	NR4A2	1,95802E-62	-2,33921E+00	0,068	0,455	3,60609E-58
Macro_FOLR2+APOE-	ZFP36	1,25645E-86	-2,37259E+00	0,407	0,849	2,31400E-82
Macro_FOLR2+APOE-	SLC2A3	2,75807E-55	-2,37440E+00	0,075	0,438	5,07955E-51
Macro_FOLR2+APOE-	CD55	2,41747E-53	-2,37946E+00	0,073	0,433	4,45225E-49
Macro_FOLR2+APOE-	ZNF331	5,44440E-36	-2,38067E+00	0,073	0,347	1,00269E-31
Macro_FOLR2+APOE-	NAMPT	3,46296E-63	-2,39092E+00	0,156	0,578	6,37774E-59
Macro_FOLR2+APOE-	CDKN1A	9,77742E-72	-2,39895E+00	0,098	0,53	1,80071E-67
Macro_FOLR2+APOE-	NR4A1	8,17649E-68	-2,42796E+00	0,03	0,424	1,50586E-63
Macro_FOLR2+APOE-	NFKBIZ	6,58945E-59	-2,43827E+00	0,079	0,459	1,21358E-54
Macro_FOLR2+APOE-	ISG15	1,42078E-28	-2,46305E+00	0,154	0,431	2,61665E-24
Macro_FOLR2+APOE-	PPP1R15A	7,69401E-74	-2,47047E+00	0,194	0,663	1,41701E-69
Macro_FOLR2+APOE-	CCL4	3,62052E-26	-2,58143E+00	0,092	0,312	6,66791E-22
Macro_FOLR2+APOE-	PLIN2	1,02050E-28	-2,62550E+00	0,213	0,501	1,87946E-24
Macro_FOLR2+APOE-	NFKBIA	1,09397E-73	-2,69404E+00	0,373	0,813	2,01476E-69
Macro_FOLR2+APOE-	DDIT4	2,20689E-46	-2,71129E+00	0,147	0,5	4,06443E-42
Macro_FOLR2+APOE-	SOD2	7,75187E-68	-2,78169E+00	0,222	0,656	1,42766E-63
Macro_FOLR2+APOE-	TNFAIP3	1,65455E-71	-2,78678E+00	0,113	0,548	3,04719E-67
Macro_FOLR2+APOE-	IRF7	5,34157E-40	-2,81587E+00	0,094	0,403	9,83758E-36
Macro_FOLR2+APOE-	CD52	1,20357E-54	-2,82476E+00	0,045	0,394	2,21661E-50
Macro_FOLR2+APOE-	CD83	6,60964E-77	-2,88752E+00	0,051	0,493	1,21730E-72
Macro_FOLR2+APOE-	CCL3	6,27164E-28	-2,89002E+00	0,136	0,378	1,15505E-23
Macro_FOLR2+APOE-	RGS2	1,25103E-62	-2,89138E+00	0,209	0,63	2,30403E-58
Macro_FOLR2+APOE-	S100A9	4,33002E-28	-2,93219E+00	0,205	0,459	7,97459E-24
Macro_FOLR2+APOE-	C15orf48	3,57191E-47	-2,93387E+00	0,04	0,348	6,57839E-43
Macro_FOLR2+APOE-	DUSP2	2,54930E-45	-3,12855E+00	0,015	0,304	4,69505E-41
Macro_FOLR2+APOE-	IRF8	2,26195E-32	-3,13148E+00	0,104	0,381	4,16584E-28
Macro_FOLR2+APOE-	CXCR4	4,56318E-33	-3,19147E+00	0,264	0,584	8,40401E-29
Macro_FOLR2+APOE-	IFI30	1,10690E-48	-3,27577E+00	0,19	0,573	2,03858E-44
Macro_FOLR2+APOE-	PLAUR	3,14880E-94	-3,31491E+00	0,132	0,646	5,79915E-90
Macro_FOLR2+APOE-	BCL2A1	1,12222E-81	-3,34530E+00	0,028	0,475	2,06679E-77
Macro_FOLR2+APOE-	AREG	2,00931E-39	-3,48397E+00	0,019	0,278	3,70054E-35
Macro_FOLR2+APOE-	FCN1	1,29410E-31	-3,50426E+00	0,028	0,253	2,38334E-27
Macro_FOLR2+APOE-	TIMP1	6,69041E-63	-3,70454E+00	0,232	0,655	1,23217E-58
Macro_FOLR2+APOE-	S100A8	2,35002E-37	-3,79972E+00	0,085	0,355	4,32804E-33
Macro_FOLR2+APOE-	GPR183	4,19762E-38	-3,81094E+00	0,154	0,46	7,73075E-34
Macro_FOLR2+APOE-	IL1B	1,22271E-44	-4,28592E+00	0,047	0,338	2,25187E-40

cluster	gene	p_val	avg_log2FC	pct.1	pct.2	p_val_adj
Macro_FOLR2+APOE+	GPNMB	0,00000E+00	2,79766E+00	0,929	0,318	0,00000E+00
Macro_FOLR2+APOE+	CCL18	0,00000E+00	2,77510E+00	0,346	0,045	0,00000E+00
Macro_FOLR2+APOE+	CCL3	2,13967E-57	2,75279E+00	0,618	0,369	3,94062E-53
Macro_FOLR2+APOE+	UBD	0,00000E+00	2,70747E+00	0,288	0,038	0,00000E+00
Macro_FOLR2+APOE+	SDS	2,32382E-97	2,69373E+00	0,487	0,211	4,27977E-93
Macro_FOLR2+APOE+	PLAU	3,94500E-271	2,58767E+00	0,587	0,183	7,26550E-267
Macro_FOLR2+APOE+	CXCL9	0,00000E+00	2,45619E+00	0,714	0,142	0,00000E+00
Macro_FOLR2+APOE+	NR1H3	0,00000E+00	2,43995E+00	0,743	0,133	0,00000E+00
Macro_FOLR2+APOE+	LGMN	0,00000E+00	2,43445E+00	0,93	0,394	0,00000E+00
Macro_FOLR2+APOE+	CCL3L1	1,86049E-33	2,43039E+00	0,291	0,158	3,42647E-29
Macro_FOLR2+APOE+	CCL4	8,52731E-82	2,42922E+00	0,571	0,302	1,57047E-77
Macro_FOLR2+APOE+	ACP5	0,00000E+00	2,39401E+00	0,876	0,386	0,00000E+00
Macro_FOLR2+APOE+	CTSD	0,00000E+00	2,34969E+00	0,997	0,763	0,00000E+00
Macro_FOLR2+APOE+	C1QB	0,00000E+00	2,33503E+00	0,986	0,533	0,00000E+00
Macro_FOLR2+APOE+	RGS1	1,23480E-37	2,30782E+00	0,911	0,635	2,27413E-33
Macro_FOLR2+APOE+	CTSB	0,00000E+00	2,22015E+00	0,999	0,868	0,00000E+00
Macro_FOLR2+APOE+	C1QA	0,00000E+00	2,21635E+00	0,987	0,554	0,00000E+00
Macro_FOLR2+APOE+	A2M	2,32966E-184	2,20409E+00	0,836	0,376	4,29054E-180
Macro_FOLR2+APOE+	CYP27A1	0,00000E+00	2,18857E+00	0,446	0,085	0,00000E+00
Macro_FOLR2+APOE+	C3	7,39527E-08	2,09682E+00	0,532	0,38	1,36199E-03
Macro_FOLR2+APOE+	GLA	9,51718E-160	2,09625E+00	0,691	0,3	1,75278E-155
Macro_FOLR2+APOE+	LIPA	3,36320E-227	2,07016E+00	0,932	0,489	6,19401E-223
Macro_FOLR2+APOE+	TMEM176B	0,00000E+00	2,04732E+00	0,98	0,522	0,00000E+00
Macro_FOLR2+APOE+	EGR1	9,61840E-41	2,04609E+00	0,493	0,28	1,77142E-36
Macro_FOLR2+APOE+	APOC1	0,00000E+00	2,03441E+00	0,962	0,494	0,00000E+00
Macro_FOLR2+APOE+	FOLR2	7,12675E-188	1,99491E+00	0,505	0,171	1,31253E-183
Macro_FOLR2+APOE+	CXCL2	1,37653E-26	1,99106E+00	0,371	0,223	2,53516E-22
Macro_FOLR2+APOE+	SGK1	1,70966E-12	1,98854E+00	0,856	0,597	3,14867E-08
Macro_FOLR2+APOE+	PRDM1	8,55151E-39	1,97794E+00	0,359	0,193	1,57493E-34
Macro_FOLR2+APOE+	CD63	0,00000E+00	1,97759E+00	0,996	0,813	0,00000E+00
Macro_FOLR2+APOE+	MMP19	3,00216E-82	1,97060E+00	0,316	0,122	5,52907E-78
Macro_FOLR2+APOE+	TREM2	1,35369E-112	1,93713E+00	0,781	0,396	2,49309E-108
Macro_FOLR2+APOE+	PLD3	0,00000E+00	1,91446E+00	0,975	0,497	0,00000E+00
Macro_FOLR2+APOE+	SOD2	1,62767E-65	1,90680E+00	0,916	0,644	2,99768E-61
Macro_FOLR2+APOE+	PSAP	5,51374E-284	1,90387E+00	1	0,914	1,01546E-279
Macro_FOLR2+APOE+	DHRS3	5,66574E-156	1,86175E+00	0,44	0,144	1,04346E-151
Macro_FOLR2+APOE+	PLA2G7	0,00000E+00	1,83758E+00	0,661	0,182	0,00000E+00
Macro_FOLR2+APOE+	CCL4L2	2,48120E-46	1,83666E+00	0,356	0,19	4,56962E-42
Macro_FOLR2+APOE+	C1QC	0,00000E+00	1,83565E+00	0,988	0,533	0,00000E+00
Macro_FOLR2+APOE+	CD9	7,12030E-18	1,83259E+00	0,701	0,479	1,31135E-13
Macro_FOLR2+APOE+	MSR1	5,87608E-100	1,82229E+00	0,758	0,37	1,08220E-95
Macro_FOLR2+APOE+	CTSL	0,00000E+00	1,82059E+00	0,969	0,506	0,00000E+00
Macro_FOLR2+APOE+	HLA-DMB	2,57250E-232	1,81915E+00	0,975	0,629	4,73778E-228
Macro_FOLR2+APOE+	IFI30	2,62973E-80	1,81606E+00	0,853	0,56	4,84318E-76
Macro_FOLR2+APOE+	ATF3	4,15409E-10	1,80701E+00	0,574	0,429	7,65058E-06
Macro_FOLR2+APOE+	ADAMDEC1	0,00000E+00	1,79221E+00	0,641	0,075	0,00000E+00

Macro_FOLR2+APOE+	PRDX1	0,00000E+00	1,78652E+00	0,989	0,74	0,00000E+00
Macro_FOLR2+APOE+	RASGEF1B	1,84726E-11	1,77018E+00	0,395	0,265	3,40209E-07
Macro_FOLR2+APOE+	MGLL	2,09870E-230	1,75653E+00	0,495	0,148	3,86518E-226
Macro_FOLR2+APOE+	PLTP	5,01694E-273	1,75599E+00	0,817	0,314	9,23969E-269
Macro_FOLR2+APOE+	LGALS3	1,38309E-153	1,74849E+00	0,967	0,717	2,54723E-149
Macro_FOLR2+APOE+	CTSC	0,00000E+00	1,73271E+00	0,991	0,669	0,00000E+00
Macro_FOLR2+APOE+	APOE	0,00000E+00	1,73037E+00	0,97	0,493	0,00000E+00
Macro_FOLR2+APOE+	ACP2	0,00000E+00	1,72872E+00	0,833	0,241	0,00000E+00
Macro_FOLR2+APOE+	CTSA	1,25371E-295	1,72424E+00	0,939	0,493	2,30896E-291
Macro_FOLR2+APOE+	C2	0,00000E+00	1,71213E+00	0,738	0,146	0,00000E+00
Macro_FOLR2+APOE+	LGALS3BP	0,00000E+00	1,70262E+00	0,817	0,305	0,00000E+00
Macro_FOLR2+APOE+	DNASE2	0,00000E+00	1,68990E+00	0,864	0,353	0,00000E+00
Macro_FOLR2+APOE+	CD72	7,66052E-269	1,68567E+00	0,615	0,195	1,41084E-264
Macro_FOLR2+APOE+	DUSP1	5,22449E-09	1,67385E+00	0,942	0,848	9,62194E-05
Macro_FOLR2+APOE+	DUSP2	9,03333E-07	1,67337E+00	0,399	0,298	1,66367E-02
Macro_FOLR2+APOE+	FABP5	1,76812E-55	1,63765E+00	0,728	0,438	3,25635E-51
Macro_FOLR2+APOE+	MMP9	0,00000E+00	1,63496E+00	0,468	0,097	0,00000E+00
Macro_FOLR2+APOE+	SLCO2B1	9,89939E-271	1,62396E+00	0,876	0,355	1,82317E-266
Macro_FOLR2+APOE+	RAB20	9,22104E-178	1,62180E+00	0,781	0,334	1,69824E-173
Macro_FOLR2+APOE+	DDIT3	1,76136E-20	1,61497E+00	0,309	0,181	3,24391E-16
Macro_FOLR2+APOE+	ASAH1	1,24692E-187	1,60955E+00	0,98	0,68	2,29645E-183
Macro_FOLR2+APOE+	SERPING1	0,00000E+00	1,59980E+00	0,938	0,381	0,00000E+00
Macro_FOLR2+APOE+	MGAT1	8,15267E-53	1,59882E+00	0,906	0,616	1,50148E-48
Macro_FOLR2+APOE+	TMEM176A	0,00000E+00	1,59667E+00	0,964	0,434	0,00000E+00
Macro_FOLR2+APOE+	TGFBI	1,71079E-42	1,58791E+00	0,893	0,59	3,15076E-38
Macro_FOLR2+APOE+	CREG1	0,00000E+00	1,58531E+00	0,956	0,5	0,00000E+00
Macro_FOLR2+APOE+	SCPEP1	0,00000E+00	1,58000E+00	0,889	0,394	0,00000E+00
Macro_FOLR2+APOE+	IL18BP	4,13510E-302	1,57537E+00	0,606	0,185	7,61562E-298
Macro_FOLR2+APOE+	TNF	1,79644E-11	1,57399E+00	0,251	0,17	3,30851E-07
Macro_FOLR2+APOE+	PPP1R15A	1,16574E-08	1,57304E+00	0,76	0,655	2,14695E-04
Macro_FOLR2+APOE+	TCF12	1,42778E-18	1,56160E+00	0,354	0,219	2,62954E-14
Macro_FOLR2+APOE+	HSPA1B	3,63531E-24	1,55684E+00	0,73	0,498	6,69515E-20
Macro_FOLR2+APOE+	CAPG	3,22483E-174	1,54811E+00	0,968	0,734	5,93917E-170
Macro_FOLR2+APOE+	NPL	0,00000E+00	1,54067E+00	0,78	0,243	0,00000E+00
Macro_FOLR2+APOE+	CTSH	1,76160E-145	1,53733E+00	0,97	0,702	3,24434E-141
Macro_FOLR2+APOE+	MPP1	1,84299E-173	1,52783E+00	0,839	0,4	3,39424E-169
Macro_FOLR2+APOE+	SLC38A6	3,94504E-259	1,52628E+00	0,496	0,136	7,26559E-255
Macro_FOLR2+APOE+	IGSF6	7,04799E-167	1,52189E+00	0,915	0,547	1,29803E-162
Macro_FOLR2+APOE+	C3AR1	1,47325E-127	1,51834E+00	0,821	0,404	2,71328E-123
Macro_FOLR2+APOE+	LAIR1	4,27345E-153	1,51047E+00	0,911	0,508	7,87042E-149
Macro_FOLR2+APOE+	GRN	2,83816E-243	1,50842E+00	0,999	0,818	5,22704E-239
Macro_FOLR2+APOE+	IL4I1	2,06791E-302	1,50690E+00	0,68	0,222	3,80847E-298
Macro_FOLR2+APOE+	CKS2	1,11606E-23	1,50672E+00	0,397	0,239	2,05544E-19
Macro_FOLR2+APOE+	C1orf54	1,41686E-292	1,50309E+00	0,752	0,269	2,60943E-288
Macro_FOLR2+APOE+	HEXA	4,25822E-132	1,50145E+00	0,872	0,458	7,84236E-128
Macro_FOLR2+APOE+	LMNA	1,73117E-27	1,50049E+00	0,606	0,362	3,18830E-23
Macro_FOLR2+APOE+	FPR3	5,70292E-169	1,49302E+00	0,743	0,327	1,05031E-164

Macro_FOLR2+APOE+	TSC22D1	3,59454E-154	1,48958E+00	0,444	0,154	6,62006E-150
Macro_FOLR2+APOE+	GNPDA1	1,03759E-176	1,48716E+00	0,647	0,248	1,91093E-172
Macro_FOLR2+APOE+	TSPAN4	0,00000E+00	1,48436E+00	0,814	0,278	0,00000E+00
Macro_FOLR2+APOE+	AXL	5,43811E-98	1,48379E+00	0,603	0,271	1,00154E-93
Macro_FOLR2+APOE+	ADM	1,70078E-38	1,48340E+00	0,282	0,14	3,13232E-34
Macro_FOLR2+APOE+	FTL	6,27614E-283	1,47923E+00	1	0,991	1,15588E-278
Macro_FOLR2+APOE+	MFSD1	1,38096E-110	1,45606E+00	0,932	0,581	2,54332E-106
Macro_FOLR2+APOE+	GLUL	2,08402E-155	1,45590E+00	0,99	0,798	3,83814E-151
Macro_FOLR2+APOE+	FCGR3A	4,10653E-77	1,45148E+00	0,926	0,565	7,56300E-73
Macro_FOLR2+APOE+	HSPA1A	1,58840E-15	1,44143E+00	0,875	0,657	2,92536E-11
Macro_FOLR2+APOE+	LAMP2	3,83560E-214	1,43652E+00	0,838	0,389	7,06403E-210
Macro_FOLR2+APOE+	CD68	2,00103E-93	1,42928E+00	0,922	0,777	3,68530E-89
Macro_FOLR2+APOE+	DNPH1	7,07233E-282	1,42843E+00	0,828	0,333	1,30251E-277
Macro_FOLR2+APOE+	CCL5	9,88756E-208	1,41933E+00	0,4	0,11	1,82099E-203
Macro_FOLR2+APOE+	CPM	1,45402E-185	1,40761E+00	0,785	0,357	2,67786E-181
Macro_FOLR2+APOE+	GM2A	0,00000E+00	1,40155E+00	0,885	0,36	0,00000E+00
Macro_FOLR2+APOE+	PILRA	9,55910E-69	1,38660E+00	0,812	0,484	1,76050E-64
Macro_FOLR2+APOE+	HLA-DRA	8,48811E-48	1,38482E+00	1	0,923	1,56325E-43
Macro_FOLR2+APOE+	IFI27	1,47506E-246	1,38409E+00	0,514	0,162	2,71661E-242
Macro_FOLR2+APOE+	FUCA1	0,00000E+00	1,38357E+00	0,736	0,236	0,00000E+00
Macro_FOLR2+APOE+	SMPDL3A	8,82037E-269	1,38087E+00	0,499	0,135	1,62445E-264
Macro_FOLR2+APOE+	IER3	3,98093E-12	1,37767E+00	0,716	0,527	7,33167E-08
Macro_FOLR2+APOE+	AKR1B1	4,05163E-174	1,37748E+00	0,871	0,456	7,46188E-170
Macro_FOLR2+APOE+	C6orf62	1,71482E-23	1,37722E+00	0,578	0,358	3,15818E-19
Macro_FOLR2+APOE+	GAA	2,54900E-195	1,37452E+00	0,829	0,374	4,69449E-191
Macro_FOLR2+APOE+	TFRC	9,52341E-162	1,37323E+00	0,571	0,228	1,75393E-157
Macro_FOLR2+APOE+	PLEKHO2	5,98317E-186	1,37321E+00	0,562	0,198	1,10192E-181
Macro_FOLR2+APOE+	PIK3IP1	5,30977E-151	1,36837E+00	0,504	0,183	9,77900E-147
Macro_FOLR2+APOE+	P2RX7	2,30918E-97	1,36205E+00	0,391	0,152	4,25282E-93
Macro_FOLR2+APOE+	GALM	2,19529E-265	1,36174E+00	0,603	0,185	4,04306E-261
Macro_FOLR2+APOE+	TAGAP	9,90282E-23	1,35687E+00	0,436	0,267	1,82380E-18
Macro_FOLR2+APOE+	CD81	7,07395E-282	1,35462E+00	0,929	0,448	1,30281E-277
Macro_FOLR2+APOE+	ATOX1	0,00000E+00	1,35357E+00	0,947	0,565	0,00000E+00
Macro_FOLR2+APOE+	ATP6V1F	6,30901E-126	1,35033E+00	0,969	0,758	1,16193E-121
Macro_FOLR2+APOE+	HLA-DOA	1,78822E-128	1,34855E+00	0,795	0,381	3,29337E-124
Macro_FOLR2+APOE+	HEXB	3,12562E-152	1,34331E+00	0,882	0,503	5,75646E-148
Macro_FOLR2+APOE+	SLC43A3	3,83754E-91	1,33474E+00	0,522	0,234	7,06760E-87
Macro_FOLR2+APOE+	SLC15A3	1,85274E-191	1,33456E+00	0,686	0,271	3,41219E-187
Macro_FOLR2+APOE+	AKR1A1	3,23214E-253	1,32949E+00	0,953	0,566	5,95263E-249
Macro_FOLR2+APOE+	SDCBP	3,55536E-61	1,32709E+00	0,969	0,716	6,54790E-57
Macro_FOLR2+APOE+	ANXA2	4,24926E-47	1,32563E+00	0,961	0,736	7,82586E-43
Macro_FOLR2+APOE+	NRP2	4,11050E-291	1,32261E+00	0,646	0,197	7,57031E-287
Macro_FOLR2+APOE+	SLAMF8	3,43521E-292	1,32134E+00	0,808	0,311	6,32662E-288
Macro_FOLR2+APOE+	PLIN2	4,24509E-41	1,32051E+00	0,799	0,489	7,81819E-37
Macro_FOLR2+APOE+	NAGK	1,15354E-102	1,30541E+00	0,842	0,467	2,12448E-98
Macro_FOLR2+APOE+	ANKRD10	4,93572E-08	1,30014E+00	0,478	0,328	9,09011E-04
Macro_FOLR2+APOE+	TANK	4,09088E-21	1,29171E+00	0,478	0,288	7,53418E-17

Macro_FOLR2+APOE+	SEMA4A	2,51419E-143	1,29004E+00	0,542	0,214	4,63038E-139
Macro_FOLR2+APOE+	SDHB	4,08341E-141	1,28802E+00	0,781	0,388	7,52041E-137
Macro_FOLR2+APOE+	NPC2	6,13124E-79	1,28761E+00	0,994	0,881	1,12919E-74
Macro_FOLR2+APOE+	PPT1	1,57072E-26	1,28431E+00	0,919	0,643	2,89280E-22
Macro_FOLR2+APOE+	HLA-DPA1	1,85536E-25	1,28207E+00	1	0,895	3,41701E-21
Macro_FOLR2+APOE+	DNAJB1	4,56404E-15	1,27665E+00	0,778	0,552	8,40559E-11
Macro_FOLR2+APOE+	SGPL1	7,90843E-272	1,27481E+00	0,539	0,158	1,45650E-267
Macro_FOLR2+APOE+	CTSZ	0,00000E+00	1,26447E+00	0,965	0,643	0,00000E+00
Macro_FOLR2+APOE+	VAT1	2,33901E-208	1,26430E+00	0,627	0,225	4,30776E-204
Macro_FOLR2+APOE+	FLII	9,61985E-29	1,26244E+00	0,544	0,318	1,77169E-24
Macro_FOLR2+APOE+	KLF6	1,34407E-06	1,26238E+00	0,888	0,795	2,47537E-02
Macro_FOLR2+APOE+	TNFRSF14	7,70353E-160	1,25954E+00	0,855	0,437	1,41876E-155
Macro_FOLR2+APOE+	SDSL	3,18243E-161	1,25494E+00	0,6	0,229	5,86108E-157
Macro_FOLR2+APOE+	TNFAIP2	1,15104E-64	1,24996E+00	0,724	0,397	2,11987E-60
Macro_FOLR2+APOE+	RABGGTA	1,93684E-63	1,24917E+00	0,36	0,159	3,56707E-59
Macro_FOLR2+APOE+	HSD17B4	1,50113E-117	1,24624E+00	0,585	0,249	2,76463E-113
Macro_FOLR2+APOE+	MS4A4A	2,33478E-228	1,24602E+00	0,838	0,408	4,29996E-224
Macro_FOLR2+APOE+	ADAM28	7,54901E-73	1,24452E+00	0,49	0,231	1,39030E-68
Macro_FOLR2+APOE+	CYFIP1	2,37689E-156	1,23477E+00	0,719	0,312	4,37752E-152
Macro_FOLR2+APOE+	RARRES1	0,00000E+00	1,23473E+00	0,449	0,043	0,00000E+00
Macro_FOLR2+APOE+	PLXND1	2,01006E-177	1,23181E+00	0,603	0,222	3,70193E-173
Macro_FOLR2+APOE+	NUPR1	4,92808E-209	1,22406E+00	0,581	0,205	9,07605E-205
Macro_FOLR2+APOE+	TCN2	0,00000E+00	1,22196E+00	0,786	0,203	0,00000E+00
Macro_FOLR2+APOE+	MGAT4A	2,86520E-116	1,21938E+00	0,725	0,346	5,27683E-112
Macro_FOLR2+APOE+	CTNNB1	6,10737E-10	1,21843E+00	0,553	0,374	1,12479E-05
Macro_FOLR2+APOE+	ALDH1A1	0,00000E+00	1,21648E+00	0,624	0,187	0,00000E+00
Macro_FOLR2+APOE+	PLA2G15	1,50372E-183	1,21327E+00	0,464	0,146	2,76939E-179
Macro_FOLR2+APOE+	KLHDC8B	1,62541E-291	1,20881E+00	0,369	0,077	2,99351E-287
Macro_FOLR2+APOE+	MAN2B1	2,25014E-35	1,20455E+00	0,678	0,468	4,14407E-31
Macro_FOLR2+APOE+	FCER1G	2,86076E-81	1,19993E+00	1	0,91	5,26866E-77
Macro_FOLR2+APOE+	HLA-DMA	4,89544E-61	1,19845E+00	0,991	0,775	9,01593E-57
Macro_FOLR2+APOE+	IFNGR1	1,41736E-23	1,19647E+00	0,856	0,573	2,61034E-19
Macro_FOLR2+APOE+	HSD17B14	0,00000E+00	1,19361E+00	0,426	0,089	0,00000E+00
Macro_FOLR2+APOE+	HSPA8	2,22346E-15	1,18252E+00	0,979	0,815	4,09494E-11
Macro_FOLR2+APOE+	PSMC2	5,20057E-120	1,18183E+00	0,625	0,277	9,57789E-116
Macro_FOLR2+APOE+	HLA-A	3,69721E-215	1,18018E+00	0,999	0,948	6,80916E-211
Macro_FOLR2+APOE+	SLC3A2	5,20719E-58	1,17828E+00	0,778	0,454	9,59008E-54
Macro_FOLR2+APOE+	CMKLR1	0,00000E+00	1,17572E+00	0,623	0,135	0,00000E+00
Macro_FOLR2+APOE+	CXCL3	3,05353E-22	1,16915E+00	0,271	0,159	5,62369E-18
Macro_FOLR2+APOE+	SAT1	6,03549E-28	1,16876E+00	0,999	0,946	1,11156E-23
Macro_FOLR2+APOE+	ATP2C1	8,62093E-82	1,16864E+00	0,414	0,177	1,58772E-77
Macro_FOLR2+APOE+	SLC40A1	4,48469E-225	1,16862E+00	0,603	0,22	8,25946E-221
Macro_FOLR2+APOE+	GBA	5,67118E-124	1,16832E+00	0,487	0,192	1,04446E-119
Macro_FOLR2+APOE+	SLC7A7	1,90303E-143	1,16725E+00	0,862	0,454	3,50482E-139
Macro_FOLR2+APOE+	DMXL2	7,88778E-164	1,16341E+00	0,636	0,257	1,45269E-159
Macro_FOLR2+APOE+	CTSS	7,57273E-163	1,16194E+00	0,996	0,811	1,39467E-158
Macro_FOLR2+APOE+	CBR1	5,84500E-170	1,16101E+00	0,718	0,305	1,07647E-165

Macro_FOLR2+APOE+	GCHFR	4,21417E-135	1,16078E+00	0,469	0,176	7,76124E-131
Macro_FOLR2+APOE+	ATP6AP1	1,84703E-114	1,15800E+00	0,897	0,534	3,40167E-110
Macro_FOLR2+APOE+	RAB7A	3,36411E-34	1,15759E+00	0,898	0,625	6,19568E-30
Macro_FOLR2+APOE+	GBP2	4,48564E-115	1,15728E+00	0,755	0,362	8,26121E-111
Macro_FOLR2+APOE+	RASSF4	1,00323E-126	1,15609E+00	0,87	0,496	1,84765E-122
Macro_FOLR2+APOE+	B2M	1,85923E-141	1,15542E+00	1	0,995	3,42415E-137
Macro_FOLR2+APOE+	RIPK2	7,16406E-10	1,15509E+00	0,402	0,27	1,31941E-05
Macro_FOLR2+APOE+	DENND2D	2,78763E-169	1,15477E+00	0,361	0,104	5,13398E-165
Macro_FOLR2+APOE+	VMO1	1,01394E-51	1,15347E+00	0,443	0,221	1,86737E-47
Macro_FOLR2+APOE+	TRPV2	1,21991E-134	1,15211E+00	0,517	0,201	2,24671E-130
Macro_FOLR2+APOE+	TUBA1B	1,35298E-25	1,15149E+00	0,945	0,751	2,49178E-21
Macro_FOLR2+APOE+	MRPL15	1,52264E-65	1,14591E+00	0,436	0,203	2,80424E-61
Macro_FOLR2+APOE+	COMMD4	4,68605E-103	1,14369E+00	0,533	0,228	8,63030E-99
Macro_FOLR2+APOE+	CDK5RAP3	9,63958E-43	1,14150E+00	0,542	0,3	1,77532E-38
Macro_FOLR2+APOE+	CSF1R	1,82440E-68	1,14056E+00	0,923	0,561	3,36000E-64
Macro_FOLR2+APOE+	VAMP5	4,22980E-230	1,13865E+00	0,885	0,469	7,79002E-226
Macro_FOLR2+APOE+	SLC31A1	1,52038E-300	1,13777E+00	0,656	0,202	2,80008E-296
Macro_FOLR2+APOE+	LYZ	1,39366E-54	1,13493E+00	0,986	0,803	2,56670E-50
Macro_FOLR2+APOE+	CD84	1,35090E-174	1,13450E+00	0,824	0,38	2,48795E-170
Macro_FOLR2+APOE+	SLAMF7	6,17472E-247	1,12768E+00	0,594	0,199	1,13720E-242
Macro_FOLR2+APOE+	MS4A7	4,79500E-89	1,12291E+00	0,948	0,654	8,83095E-85
Macro_FOLR2+APOE+	SLC35F6	8,66872E-130	1,12030E+00	0,402	0,14	1,59652E-125
Macro_FOLR2+APOE+	EIF4A3	4,91749E-50	1,11736E+00	0,69	0,394	9,05655E-46
Macro_FOLR2+APOE+	BLVRA	5,12697E-132	1,11707E+00	0,775	0,372	9,44233E-128
Macro_FOLR2+APOE+	ICAM1	1,87236E-74	1,11102E+00	0,666	0,353	3,44833E-70
Macro_FOLR2+APOE+	HLA-DQA1	2,16781E-160	1,10774E+00	0,999	0,777	3,99245E-156
Macro_FOLR2+APOE+	PTAFR	4,96972E-174	1,10680E+00	0,811	0,363	9,15274E-170
Macro_FOLR2+APOE+	ATP6AP2	4,31734E-110	1,10325E+00	0,909	0,582	7,95125E-106
Macro_FOLR2+APOE+	BLVRB	3,03672E-128	1,10031E+00	0,862	0,479	5,59272E-124
Macro_FOLR2+APOE+	UBB	3,77902E-48	1,09957E+00	0,971	0,806	6,95982E-44
Macro_FOLR2+APOE+	HMOX1	2,38524E-116	1,09411E+00	0,903	0,534	4,39289E-112
Macro_FOLR2+APOE+	RDX	2,35417E-138	1,09228E+00	0,558	0,222	4,33568E-134
Macro_FOLR2+APOE+	TUBB4B	7,91935E-12	1,09110E+00	0,562	0,374	1,45851E-07
Macro_FOLR2+APOE+	MERTK	6,43544E-173	1,08910E+00	0,524	0,182	1,18522E-168
Macro_FOLR2+APOE+	PEPD	1,91827E-109	1,08688E+00	0,731	0,352	3,53287E-105
Macro_FOLR2+APOE+	CD38	0,00000E+00	1,08553E+00	0,62	0,173	0,00000E+00
Macro_FOLR2+APOE+	OAZ2	2,39762E-162	1,08299E+00	0,667	0,272	4,41570E-158
Macro_FOLR2+APOE+	ACAA2	4,93133E-124	1,08093E+00	0,499	0,196	9,08203E-120
Macro_FOLR2+APOE+	SLC7A8	0,00000E+00	1,08035E+00	0,346	0,061	0,00000E+00
Macro_FOLR2+APOE+	ABHD12	4,29066E-239	1,07137E+00	0,841	0,366	7,90211E-235
Macro_FOLR2+APOE+	GUSB	1,45773E-96	1,07039E+00	0,69	0,336	2,68470E-92
Macro_FOLR2+APOE+	CLEC7A	4,83738E-25	1,07007E+00	0,815	0,516	8,90901E-21
Macro_FOLR2+APOE+	ADAP2	9,03019E-91	1,06947E+00	0,81	0,434	1,66309E-86
Macro_FOLR2+APOE+	LILRB4	3,43309E-210	1,06918E+00	0,922	0,539	6,32271E-206
Macro_FOLR2+APOE+	HAMP	7,62917E-136	1,06597E+00	0,257	0,07	1,40506E-131
Macro_FOLR2+APOE+	SCAMP2	2,07356E-40	1,06533E+00	0,805	0,494	3,81888E-36
Macro_FOLR2+APOE+	PTGER4	1,76321E-41	1,06522E+00	0,488	0,263	3,24731E-37

Macro_FOLR2+APOE+	ZNF267	6,08314E-20	1,06516E+00	0,382	0,23	1,12033E-15
Macro_FOLR2+APOE+	CCRL2	5,64567E-108	1,06496E+00	0,452	0,177	1,03976E-103
Macro_FOLR2+APOE+	ARRDC3	7,28441E-13	1,06468E+00	0,283	0,183	1,34157E-08
Macro_FOLR2+APOE+	MRPL55	2,44283E-62	1,06181E+00	0,542	0,27	4,49897E-58
Macro_FOLR2+APOE+	P2RX4	2,48227E-138	1,05928E+00	0,574	0,223	4,57160E-134
Macro_FOLR2+APOE+	TGIF1	9,32500E-26	1,05867E+00	0,541	0,317	1,71738E-21
Macro_FOLR2+APOE+	LY86	3,95510E-20	1,05859E+00	0,73	0,483	7,28412E-16
Macro_FOLR2+APOE+	HM13	4,01083E-49	1,05806E+00	0,833	0,531	7,38675E-45
Macro_FOLR2+APOE+	STOM	2,23508E-162	1,05693E+00	0,749	0,334	4,11634E-158
Macro_FOLR2+APOE+	IL18	4,64301E-112	1,05519E+00	0,805	0,432	8,55103E-108
Macro_FOLR2+APOE+	KMO	1,23003E-32	1,05350E+00	0,257	0,128	2,26535E-28
Macro_FOLR2+APOE+	TSG101	3,38657E-47	1,05305E+00	0,452	0,229	6,23705E-43
Macro_FOLR2+APOE+	ATP6V0E1	3,99360E-74	1,05101E+00	0,967	0,791	7,35501E-70
Macro_FOLR2+APOE+	TPP1	2,93100E-58	1,05080E+00	0,911	0,586	5,39803E-54
Macro_FOLR2+APOE+	CXCL16	7,88638E-38	1,05073E+00	0,923	0,643	1,45243E-33
Macro_FOLR2+APOE+	ARL6IP1	1,90663E-110	1,04699E+00	0,786	0,404	3,51144E-106
Macro_FOLR2+APOE+	TMED1	4,66394E-103	1,04675E+00	0,354	0,129	8,58957E-99
Macro_FOLR2+APOE+	LY96	3,97568E-160	1,04545E+00	0,861	0,445	7,32201E-156
Macro_FOLR2+APOE+	ATP6V0A1	4,86017E-136	1,04526E+00	0,485	0,179	8,95097E-132
Macro_FOLR2+APOE+	IST1	2,36181E-53	1,04499E+00	0,506	0,257	4,34975E-49
Macro_FOLR2+APOE+	PDE4DIP	3,11880E-102	1,04462E+00	0,655	0,312	5,74389E-98
Macro_FOLR2+APOE+	RNF130	3,56840E-75	1,04410E+00	0,957	0,702	6,57193E-71
Macro_FOLR2+APOE+	PDHA1	9,03706E-65	1,04362E+00	0,404	0,184	1,66435E-60
Macro_FOLR2+APOE+	ZFAND2A	1,08911E-21	1,04242E+00	0,286	0,167	2,00582E-17
Macro_FOLR2+APOE+	HNMT	8,25506E-169	1,04102E+00	0,811	0,368	1,52033E-164
Macro_FOLR2+APOE+	ALAS1	6,22150E-145	1,04073E+00	0,4	0,133	1,14581E-140
Macro_FOLR2+APOE+	SLC11A2	6,20183E-96	1,04059E+00	0,377	0,145	1,14219E-91
Macro_FOLR2+APOE+	ST3GAL6	2,32948E-23	1,03986E+00	0,276	0,153	4,29021E-19
Macro_FOLR2+APOE+	CNDP2	1,54421E-70	1,03688E+00	0,801	0,445	2,84396E-66
Macro_FOLR2+APOE+	ARAP1	6,17418E-64	1,03353E+00	0,559	0,284	1,13710E-59
Macro_FOLR2+APOE+	UTP18	9,23156E-53	1,03326E+00	0,395	0,192	1,70018E-48
Macro_FOLR2+APOE+	FUOM	1,45220E-121	1,03208E+00	0,757	0,365	2,67451E-117
Macro_FOLR2+APOE+	PSMD4	3,65048E-35	1,03197E+00	0,685	0,404	6,72310E-31
Macro_FOLR2+APOE+	BCAP31	6,86971E-161	1,03098E+00	0,938	0,574	1,26519E-156
Macro_FOLR2+APOE+	RNASET2	1,67417E-55	1,02977E+00	0,959	0,734	3,08331E-51
Macro_FOLR2+APOE+	FOS	5,44196E-25	1,02914E+00	0,918	0,841	1,00225E-20
Macro_FOLR2+APOE+	SLC1A3	8,84979E-88	1,02891E+00	0,717	0,352	1,62987E-83
Macro_FOLR2+APOE+	MT-ND4L	1,87550E-28	1,02538E+00	0,742	0,507	3,45411E-24
Macro_FOLR2+APOE+	CNPY2	2,39115E-132	1,02503E+00	0,469	0,179	4,40377E-128
Macro_FOLR2+APOE+	LMAN2	3,21088E-68	1,02395E+00	0,836	0,502	5,91349E-64
Macro_FOLR2+APOE+	MT1X	1,32571E-48	1,02336E+00	0,437	0,229	2,44156E-44
Macro_FOLR2+APOE+	TUBB	1,50339E-24	1,02302E+00	0,903	0,654	2,76879E-20
Macro_FOLR2+APOE+	TBC1D2	4,78953E-153	1,01752E+00	0,315	0,089	8,82088E-149
Macro_FOLR2+APOE+	TM6SF1	8,74177E-32	1,01550E+00	0,481	0,266	1,60997E-27
Macro_FOLR2+APOE+	GPX3	1,96467E-137	1,01500E+00	0,478	0,183	3,61833E-133
Macro_FOLR2+APOE+	OAT	4,70418E-52	1,01327E+00	0,334	0,155	8,66369E-48
Macro_FOLR2+APOE+	NT5C2	9,85443E-52	1,01228E+00	0,346	0,163	1,81489E-47

Macro_FOLR2+APOE+	RNH1	1,00435E-36	1,00976E+00	0,891	0,61	1,84971E-32
Macro_FOLR2+APOE+	CLEC4E	5,31593E-56	1,00783E+00	0,552	0,284	9,79034E-52
Macro_FOLR2+APOE+	RNF13	1,61742E-64	1,00637E+00	0,837	0,508	2,97880E-60
Macro_FOLR2+APOE+	TYROBP	4,96221E-36	1,00569E+00	0,999	0,945	9,13889E-32
Macro_FOLR2+APOE+	COMT	3,31380E-70	1,00474E+00	0,873	0,548	6,10302E-66
Macro_FOLR2+APOE+	NINJ1	8,57309E-72	1,00398E+00	0,895	0,55	1,57891E-67
Macro_FOLR2+APOE+	DPP7	2,35022E-87	1,00321E+00	0,888	0,547	4,32841E-83
Macro_FOLR2+APOE+	FCGR2A	8,96471E-37	1,00202E+00	0,912	0,623	1,65103E-32
Macro_FOLR2+APOE+	RNASEK	2,48710E-18	9,99269E-01	0,621	0,461	4,58050E-14
Macro_FOLR2+APOE+	CRTAP	2,93038E-07	9,96812E-01	0,746	0,515	5,39689E-03
Macro_FOLR2+APOE+	STAT1	1,72722E-258	9,94788E-01	0,938	0,505	3,18101E-254
Macro_FOLR2+APOE+	SLC29A3	1,96102E-183	9,94464E-01	0,42	0,127	3,61161E-179
Macro_FOLR2+APOE+	TALDO1	3,98913E-51	9,92786E-01	0,908	0,655	7,34679E-47
Macro_FOLR2+APOE+	GPR84	1,22127E-57	9,90714E-01	0,261	0,109	2,24921E-53
Macro_FOLR2+APOE+	SDC3	0,00000E+00	9,89552E-01	0,61	0,139	0,00000E+00
Macro_FOLR2+APOE+	ATRAID	4,42288E-58	9,89364E-01	0,79	0,456	8,14561E-54
Macro_FOLR2+APOE+	LITAF	2,06920E-14	9,88946E-01	0,933	0,736	3,81085E-10
Macro_FOLR2+APOE+	HADHB	1,03647E-65	9,88509E-01	0,67	0,351	1,90886E-61
Macro_FOLR2+APOE+	METTL17	2,50125E-23	9,86251E-01	0,256	0,141	4,60655E-19
Macro_FOLR2+APOE+	NQO2	5,66571E-35	9,85227E-01	0,461	0,256	1,04345E-30
Macro_FOLR2+APOE+	IFNGR2	4,34730E-63	9,84798E-01	0,875	0,555	8,00643E-59
Macro_FOLR2+APOE+	CD163	3,76407E-162	9,80929E-01	0,845	0,435	6,93229E-158
Macro_FOLR2+APOE+	UCP2	2,18644E-53	9,79982E-01	0,92	0,626	4,02678E-49
Macro_FOLR2+APOE+	FCGR1A	2,81140E-158	9,78438E-01	0,807	0,399	5,17775E-154
Macro_FOLR2+APOE+	LPP	2,49244E-25	9,76993E-01	0,449	0,262	4,59033E-21
Macro_FOLR2+APOE+	GPR34	1,60134E-72	9,75891E-01	0,591	0,298	2,94919E-68
Macro_FOLR2+APOE+	CDK4	1,94768E-89	9,75530E-01	0,51	0,223	3,58704E-85
Macro_FOLR2+APOE+	NDUFA12	5,12719E-46	9,75469E-01	0,798	0,485	9,44275E-42
Macro_FOLR2+APOE+	LHFPL2	6,72760E-205	9,72531E-01	0,71	0,271	1,23902E-200
Macro_FOLR2+APOE+	SQSTM1	1,68750E-41	9,72397E-01	0,923	0,667	3,10787E-37
Macro_FOLR2+APOE+	SLC37A2	3,67432E-76	9,72252E-01	0,375	0,159	6,76700E-72
Macro_FOLR2+APOE+	CD82	1,22047E-42	9,70630E-01	0,452	0,234	2,24774E-38
Macro_FOLR2+APOE+	MAGED2	2,63189E-66	9,70231E-01	0,386	0,172	4,84715E-62
Macro_FOLR2+APOE+	HLA-DPB1	1,11608E-30	9,69188E-01	0,999	0,88	2,05549E-26
Macro_FOLR2+APOE+	MGST3	8,26174E-96	9,68601E-01	0,874	0,532	1,52156E-91
Macro_FOLR2+APOE+	SLC25A39	5,84534E-47	9,67699E-01	0,708	0,4	1,07654E-42
Macro_FOLR2+APOE+	GPS2	2,59028E-28	9,66656E-01	0,264	0,141	4,77052E-24
Macro_FOLR2+APOE+	ACSL1	3,47797E-10	9,65211E-01	0,581	0,383	6,40537E-06
Macro_FOLR2+APOE+	ALCAM	3,56737E-33	9,64461E-01	0,461	0,262	6,57002E-29
Macro_FOLR2+APOE+	CD74	7,83728E-29	9,64118E-01	1	0,962	1,44339E-24
Macro_FOLR2+APOE+	DRAM1	6,63987E-227	9,64103E-01	0,61	0,207	1,22286E-222
Macro_FOLR2+APOE+	SELENOP	4,06680E-239	9,63875E-01	0,355	0,084	7,48982E-235
Macro_FOLR2+APOE+	APMAP	5,35039E-98	9,61342E-01	0,627	0,291	9,85381E-94
Macro_FOLR2+APOE+	NEK6	1,63397E-92	9,59922E-01	0,519	0,231	3,00929E-88
Macro_FOLR2+APOE+	LTA4H	4,39381E-29	9,59875E-01	0,68	0,441	8,09209E-25
Macro_FOLR2+APOE+	CD40	2,95760E-206	9,57793E-01	0,703	0,28	5,44701E-202
Macro_FOLR2+APOE+	PPA2	8,70768E-54	9,55765E-01	0,455	0,225	1,60369E-49

Macro_FOLR2+APOE+	BLOC1S1	2,24944E-67	9,54221E-01	0,752	0,464	4,14280E-63
Macro_FOLR2+APOE+	ATP6V0B	1,25844E-53	9,53591E-01	0,97	0,786	2,31767E-49
Macro_FOLR2+APOE+	ITGAX	3,35979E-29	9,50355E-01	0,636	0,393	6,18772E-25
Macro_FOLR2+APOE+	GLB1	4,93890E-107	9,49966E-01	0,626	0,282	9,09597E-103
Macro_FOLR2+APOE+	LACTB2	1,64127E-76	9,49866E-01	0,3	0,115	3,02273E-72
Macro_FOLR2+APOE+	SOAT1	3,96255E-136	9,49083E-01	0,65	0,275	7,29782E-132
Macro_FOLR2+APOE+	IRAK1	1,57432E-60	9,48432E-01	0,361	0,163	2,89942E-56
Macro_FOLR2+APOE+	TTYH3	1,19084E-293	9,45231E-01	0,661	0,211	2,19317E-289
Macro_FOLR2+APOE+	HGS	1,76460E-21	9,45208E-01	0,347	0,203	3,24987E-17
Macro_FOLR2+APOE+	FABP3	6,85291E-143	9,44319E-01	0,27	0,073	1,26210E-138
Macro_FOLR2+APOE+	GPX4	5,18825E-48	9,43955E-01	0,984	0,84	9,55520E-44
Macro_FOLR2+APOE+	RAB1A	3,49462E-86	9,43679E-01	0,743	0,386	6,43604E-82
Macro_FOLR2+APOE+	KCNMA1	0,00000E+00	9,43669E-01	0,745	0,246	0,00000E+00
Macro_FOLR2+APOE+	ABCD4	4,12574E-71	9,39347E-01	0,298	0,118	7,59837E-67
Macro_FOLR2+APOE+	ZFP36	3,53472E-37	9,37908E-01	0,917	0,842	6,50989E-33
Macro_FOLR2+APOE+	CD14	5,87313E-144	9,37522E-01	0,955	0,614	1,08165E-139
Macro_FOLR2+APOE+	GAL3ST4	3,52245E-183	9,37315E-01	0,482	0,159	6,48729E-179
Macro_FOLR2+APOE+	CD53	1,01753E-54	9,35462E-01	0,944	0,671	1,87399E-50
Macro_FOLR2+APOE+	AMDHD2	4,86941E-176	9,35461E-01	0,447	0,141	8,96798E-172
Macro_FOLR2+APOE+	DUSP10	1,50427E-36	9,34750E-01	0,27	0,132	2,77041E-32
Macro_FOLR2+APOE+	PSMB1	5,22613E-19	9,33205E-01	0,882	0,622	9,62496E-15
Macro_FOLR2+APOE+	LRPAP1	9,31892E-126	9,32775E-01	0,865	0,485	1,71627E-121
Macro_FOLR2+APOE+	EBI3	3,85334E-85	9,32070E-01	0,377	0,153	7,09670E-81
Macro_FOLR2+APOE+	PRDX4	5,10586E-144	9,31338E-01	0,651	0,276	9,40346E-140
Macro_FOLR2+APOE+	SMIM20	6,39526E-75	9,30105E-01	0,499	0,23	1,17781E-70
Macro_FOLR2+APOE+	DBI	4,03949E-117	9,29327E-01	0,958	0,717	7,43953E-113
Macro_FOLR2+APOE+	SLC39A1	4,22124E-51	9,28883E-01	0,509	0,27	7,77425E-47
Macro_FOLR2+APOE+	CALR	9,39494E-116	9,28416E-01	0,964	0,716	1,73027E-111
Macro_FOLR2+APOE+	IDH1	1,52714E-298	9,27799E-01	0,7	0,233	2,81254E-294
Macro_FOLR2+APOE+	COLGALT1	1,09550E-169	9,27471E-01	0,592	0,22	2,01759E-165
Macro_FOLR2+APOE+	ITM2B	1,15898E-46	9,25936E-01	0,997	0,904	2,13450E-42
Macro_FOLR2+APOE+	PSME2	8,21483E-105	9,25221E-01	0,965	0,708	1,51293E-100
Macro_FOLR2+APOE+	DAB2	1,49479E-163	9,24952E-01	0,879	0,421	2,75295E-159
Macro_FOLR2+APOE+	LAPTM5	1,35940E-13	9,24726E-01	0,996	0,895	2,50361E-09
Macro_FOLR2+APOE+	TSPAN14	3,16533E-54	9,24377E-01	0,612	0,325	5,82958E-50
Macro_FOLR2+APOE+	NUBP1	1,13146E-47	9,24353E-01	0,449	0,228	2,08381E-43
Macro_FOLR2+APOE+	ZFAND5	6,26498E-27	9,23236E-01	0,827	0,578	1,15382E-22
Macro_FOLR2+APOE+	DAPK1	5,60751E-62	9,21938E-01	0,616	0,327	1,03274E-57
Macro_FOLR2+APOE+	ABCA1	4,64545E-122	9,21611E-01	0,661	0,305	8,55553E-118
Macro_FOLR2+APOE+	AOAH	8,79023E-53	9,21179E-01	0,667	0,366	1,61890E-48
Macro_FOLR2+APOE+	LGALS9	2,78069E-109	9,20644E-01	0,895	0,538	5,12121E-105
Macro_FOLR2+APOE+	NME1	8,41963E-64	9,19870E-01	0,336	0,15	1,55064E-59
Macro_FOLR2+APOE+	COX7A2	3,52145E-12	9,19452E-01	0,921	0,715	6,48545E-08
Macro_FOLR2+APOE+	RBM47	6,55347E-85	9,18898E-01	0,683	0,349	1,20695E-80
Macro_FOLR2+APOE+	OLFML3	4,07875E-78	9,17352E-01	0,472	0,219	7,51183E-74
Macro_FOLR2+APOE+	RPN2	2,85687E-236	9,16654E-01	0,711	0,272	5,26150E-232
Macro_FOLR2+APOE+	INTS10	7,26318E-24	9,15521E-01	0,413	0,237	1,33766E-19

Macro_FOLR2+APOE+	ATP6V0D1	8,34027E-35	9,14793E-01	0,836	0,564	1,53603E-30
Macro_FOLR2+APOE+	CD58	4,10255E-23	9,14775E-01	0,514	0,308	7,55566E-19
Macro_FOLR2+APOE+	FCGR1B	5,26207E-83	9,14655E-01	0,46	0,197	9,69116E-79
Macro_FOLR2+APOE+	APIP	6,15730E-77	9,14397E-01	0,491	0,226	1,13399E-72
Macro_FOLR2+APOE+	UBC	5,77656E-07	9,14221E-01	0,993	0,896	1,06387E-02
Macro_FOLR2+APOE+	PTGS1	2,17546E-12	9,13115E-01	0,393	0,253	4,00654E-08
Macro_FOLR2+APOE+	NCEH1	7,50388E-199	9,12988E-01	0,429	0,125	1,38199E-194
Macro_FOLR2+APOE+	HMGCL	3,65046E-105	9,12340E-01	0,392	0,147	6,72305E-101
Macro_FOLR2+APOE+	TMEM147	3,18136E-92	9,10566E-01	0,792	0,423	5,85911E-88
Macro_FOLR2+APOE+	ARHGEF10L	3,94273E-147	9,09097E-01	0,369	0,117	7,26133E-143
Macro_FOLR2+APOE+	EIF4A1	1,18161E-11	9,08888E-01	0,712	0,673	2,17616E-07
Macro_FOLR2+APOE+	CIITA	1,39228E-42	9,07674E-01	0,566	0,318	2,56416E-38
Macro_FOLR2+APOE+	GSN	1,76940E-86	9,07481E-01	0,939	0,625	3,25871E-82
Macro_FOLR2+APOE+	ATP6V1H	5,26563E-94	9,05949E-01	0,478	0,204	9,69771E-90
Macro_FOLR2+APOE+	AVPI1	1,84398E-66	9,04928E-01	0,357	0,156	3,39606E-62
Macro_FOLR2+APOE+	RNASE1	3,25822E-86	9,03562E-01	0,548	0,264	6,00066E-82
Macro_FOLR2+APOE+	DDOST	4,12142E-36	9,03013E-01	0,737	0,455	7,59042E-32
Macro_FOLR2+APOE+	GSTO1	5,28378E-83	9,01806E-01	0,911	0,611	9,73114E-79
Macro_FOLR2+APOE+	ARL8B	2,87348E-18	9,00385E-01	0,623	0,397	5,29208E-14
Macro_FOLR2+APOE+	ABHD2	9,41506E-87	8,98915E-01	0,515	0,233	1,73397E-82
Macro_FOLR2+APOE+	DEGS1	8,93591E-51	8,98592E-01	0,598	0,318	1,64573E-46
Macro_FOLR2+APOE+	ID3	4,14561E-73	8,98314E-01	0,33	0,139	7,63498E-69
Macro_FOLR2+APOE+	NABP1	4,84982E-44	8,97345E-01	0,592	0,332	8,93191E-40
Macro_FOLR2+APOE+	TCEAL4	5,42442E-87	8,96629E-01	0,585	0,271	9,99015E-83
Macro_FOLR2+APOE+	PPFIA1	2,07074E-56	8,95213E-01	0,325	0,144	3,81368E-52
Macro_FOLR2+APOE+	PDCD5	4,94693E-39	8,94707E-01	0,616	0,343	9,11077E-35
Macro_FOLR2+APOE+	CORO1B	1,89913E-104	8,94543E-01	0,838	0,462	3,49763E-100
Macro_FOLR2+APOE+	CCT5	1,23615E-29	8,94211E-01	0,631	0,374	2,27661E-25
Macro_FOLR2+APOE+	LAMP1	0,00000E+00	8,92465E-01	0,885	0,383	0,00000E+00
Macro_FOLR2+APOE+	TWF2	3,71797E-160	8,92095E-01	0,726	0,323	6,84738E-156
Macro_FOLR2+APOE+	MT-ATP6	1,76129E-73	8,91352E-01	0,834	0,571	3,24377E-69
Macro_FOLR2+APOE+	FLVCR2	1,41900E-190	8,89834E-01	0,413	0,12	2,61337E-186
Macro_FOLR2+APOE+	ATP6V1B2	2,99981E-73	8,88618E-01	0,87	0,525	5,52474E-69
Macro_FOLR2+APOE+	STAB1	5,44632E-116	8,88040E-01	0,692	0,335	1,00305E-111
Macro_FOLR2+APOE+	PCBD1	2,12614E-173	8,86742E-01	0,772	0,349	3,91571E-169
Macro_FOLR2+APOE+	SMS	8,01272E-47	8,86468E-01	0,722	0,418	1,47570E-42
Macro_FOLR2+APOE+	GIMAP4	3,47735E-108	8,86111E-01	0,824	0,416	6,40423E-104
Macro_FOLR2+APOE+	TPCN1	4,53519E-117	8,85855E-01	0,391	0,139	8,35246E-113
Macro_FOLR2+APOE+	JKAMP	5,67649E-58	8,83462E-01	0,441	0,212	1,04544E-53
Macro_FOLR2+APOE+	TM2D2	8,80563E-87	8,83172E-01	0,451	0,194	1,62173E-82
Macro_FOLR2+APOE+	ITGB2	1,71900E-58	8,82332E-01	0,983	0,757	3,16589E-54
Macro_FOLR2+APOE+	TCP1	5,90967E-24	8,82058E-01	0,551	0,331	1,08838E-19
Macro_FOLR2+APOE+	CYB561D2	8,92007E-61	8,81845E-01	0,471	0,225	1,64281E-56
Macro_FOLR2+APOE+	VOPP1	1,60883E-195	8,81115E-01	0,771	0,335	2,96298E-191
Macro_FOLR2+APOE+	TMEM59	9,88803E-45	8,80715E-01	0,881	0,61	1,82108E-40
Macro_FOLR2+APOE+	FAM20C	7,89117E-152	8,80414E-01	0,295	0,081	1,45332E-147
Macro_FOLR2+APOE+	HLA-DQB2	8,67326E-31	8,79440E-01	0,519	0,311	1,59735E-26

Macro_FOLR2+APOE+	MS4A6A	4,94555E-81	8,79136E-01	0,964	0,708	9,10822E-77
Macro_FOLR2+APOE+	TMEM37	0,00000E+00	8,77975E-01	0,446	0,093	0,00000E+00
Macro_FOLR2+APOE+	GNS	7,32222E-98	8,74341E-01	0,785	0,42	1,34853E-93
Macro_FOLR2+APOE+	DYNC1I2	2,05397E-56	8,74216E-01	0,49	0,246	3,78281E-52
Macro_FOLR2+APOE+	UROD	2,33494E-74	8,70055E-01	0,489	0,227	4,30026E-70
Macro_FOLR2+APOE+	ADH5	1,05825E-89	8,69981E-01	0,521	0,233	1,94898E-85
Macro_FOLR2+APOE+	PLCB2	3,64726E-21	8,69603E-01	0,397	0,237	6,71716E-17
Macro_FOLR2+APOE+	VSIG4	5,03904E-95	8,68880E-01	0,618	0,34	9,28041E-91
Macro_FOLR2+APOE+	EMC4	8,43737E-39	8,68783E-01	0,62	0,353	1,55391E-34
Macro_FOLR2+APOE+	ADPGK	5,96712E-54	8,67329E-01	0,707	0,392	1,09897E-49
Macro_FOLR2+APOE+	CALU	4,06180E-118	8,67238E-01	0,525	0,217	7,48062E-114
Macro_FOLR2+APOE+	CD59	4,67871E-161	8,66603E-01	0,729	0,329	8,61678E-157
Macro_FOLR2+APOE+	RAB5C	1,68524E-107	8,66296E-01	0,815	0,45	3,10371E-103
Macro_FOLR2+APOE+	DDX39B	1,22579E-14	8,66070E-01	0,318	0,204	2,25755E-10
Macro_FOLR2+APOE+	WIPI1	1,12049E-125	8,65489E-01	0,42	0,15	2,06361E-121
Macro_FOLR2+APOE+	ASPSCR1	1,10061E-49	8,65212E-01	0,303	0,137	2,02699E-45
Macro_FOLR2+APOE+	RIT1	2,51144E-65	8,63945E-01	0,397	0,179	4,62531E-61
Macro_FOLR2+APOE+	JUNB	1,99208E-06	8,62796E-01	0,945	0,823	3,66881E-02
Macro_FOLR2+APOE+	ABL2	5,04904E-44	8,62130E-01	0,364	0,182	9,29881E-40
Macro_FOLR2+APOE+	ORMDL2	1,84369E-50	8,61792E-01	0,548	0,292	3,39552E-46
Macro_FOLR2+APOE+	ELOVL1	3,11117E-24	8,61407E-01	0,592	0,354	5,72983E-20
Macro_FOLR2+APOE+	MPDU1	2,74803E-73	8,61238E-01	0,475	0,22	5,06104E-69
Macro_FOLR2+APOE+	MT-CO1	2,26869E-52	8,60824E-01	0,833	0,592	4,17825E-48
Macro_FOLR2+APOE+	SIGLEC10	1,62724E-156	8,59854E-01	0,693	0,31	2,99689E-152
Macro_FOLR2+APOE+	MARCKS	1,47770E-140	8,59760E-01	0,927	0,521	2,72147E-136
Macro_FOLR2+APOE+	SLC8B1	4,69596E-93	8,59498E-01	0,395	0,158	8,64855E-89
Macro_FOLR2+APOE+	CD86	1,46832E-65	8,59442E-01	0,836	0,485	2,70421E-61
Macro_FOLR2+APOE+	CCDC47	9,84099E-62	8,59125E-01	0,572	0,296	1,81242E-57
Macro_FOLR2+APOE+	CXCL10	1,67691E-185	8,58992E-01	0,452	0,144	3,08837E-181
Macro_FOLR2+APOE+	SLC31A2	1,39316E-113	8,57791E-01	0,818	0,431	2,56579E-109
Macro_FOLR2+APOE+	SLC36A1	2,26619E-191	8,56151E-01	0,333	0,084	4,17364E-187
Macro_FOLR2+APOE+	TNS3	1,09602E-271	8,55386E-01	0,589	0,182	2,01855E-267
Macro_FOLR2+APOE+	ADIPOR1	9,12001E-54	8,54384E-01	0,769	0,453	1,67963E-49
Macro_FOLR2+APOE+	PSMF1	4,23228E-45	8,54211E-01	0,728	0,412	7,79458E-41
Macro_FOLR2+APOE+	KYNU	5,09582E-20	8,54018E-01	0,51	0,31	9,38497E-16
Macro_FOLR2+APOE+	IVNS1ABP	6,11685E-24	8,53023E-01	0,597	0,36	1,12654E-19
Macro_FOLR2+APOE+	CCR1	1,17332E-150	8,52998E-01	0,565	0,218	2,16090E-146
Macro_FOLR2+APOE+	ATP6V1E1	1,61287E-91	8,51641E-01	0,661	0,322	2,97042E-87
Macro_FOLR2+APOE+	MT-CO3	4,21823E-64	8,51563E-01	0,831	0,583	7,76871E-60
Macro_FOLR2+APOE+	MT-ATP8	1,21541E-39	8,50704E-01	0,581	0,378	2,23842E-35
Macro_FOLR2+APOE+	WDR61	8,19533E-42	8,49283E-01	0,415	0,215	1,50933E-37
Macro_FOLR2+APOE+	MCTP1	1,60711E-32	8,49206E-01	0,433	0,242	2,95981E-28
Macro_FOLR2+APOE+	RNF111	8,63873E-25	8,48298E-01	0,26	0,141	1,59099E-20
Macro_FOLR2+APOE+	SOD1	7,08861E-54	8,46193E-01	0,875	0,555	1,30551E-49
Macro_FOLR2+APOE+	HLA-DRB1	1,50905E-54	8,45888E-01	1	0,915	2,77922E-50
Macro_FOLR2+APOE+	CYBB	1,40138E-71	8,45144E-01	0,93	0,615	2,58093E-67
Macro_FOLR2+APOE+	SH3GLB1	4,79335E-37	8,44079E-01	0,715	0,419	8,82791E-33

Macro_FOLR2+APOE+	GADD45G	1,29416E-293	8,43335E-01	0,485	0,124	2,38345E-289
Macro_FOLR2+APOE+	ARL14EP	1,14637E-10	8,42209E-01	0,253	0,164	2,11127E-06
Macro_FOLR2+APOE+	VPS29	8,65611E-33	8,42022E-01	0,829	0,527	1,59420E-28
Macro_FOLR2+APOE+	SCARB2	6,65293E-246	8,41646E-01	0,841	0,358	1,22527E-241
Macro_FOLR2+APOE+	CDS2	3,54840E-136	8,41557E-01	0,473	0,176	6,53509E-132
Macro_FOLR2+APOE+	ALDH2	3,39194E-74	8,40381E-01	0,86	0,542	6,24693E-70
Macro_FOLR2+APOE+	SNX8	2,40747E-102	8,40218E-01	0,503	0,21	4,43384E-98
Macro_FOLR2+APOE+	SNX2	5,38090E-77	8,39927E-01	0,775	0,431	9,91001E-73
Macro_FOLR2+APOE+	RNF14	7,91458E-46	8,39914E-01	0,26	0,117	1,45763E-41
Macro_FOLR2+APOE+	CCDC107	4,40402E-76	8,39135E-01	0,615	0,298	8,11089E-72
Macro_FOLR2+APOE+	EFTUD2	3,69078E-41	8,36880E-01	0,372	0,19	6,79730E-37
Macro_FOLR2+APOE+	CAPZB	3,48385E-44	8,36452E-01	0,968	0,754	6,41622E-40
Macro_FOLR2+APOE+	SNX29	2,39999E-38	8,36411E-01	0,465	0,254	4,42007E-34
Macro_FOLR2+APOE+	SYNGR2	2,24618E-43	8,33459E-01	0,958	0,721	4,13679E-39
Macro_FOLR2+APOE+	PCK2	5,01095E-148	8,33400E-01	0,399	0,13	9,22867E-144
Macro_FOLR2+APOE+	VKORC1	9,17167E-137	8,32938E-01	0,567	0,234	1,68915E-132
Macro_FOLR2+APOE+	FYB1	2,96070E-252	8,31730E-01	0,748	0,305	5,45272E-248
Macro_FOLR2+APOE+	MMP14	0,00000E+00	8,29980E-01	0,693	0,211	0,00000E+00
Macro_FOLR2+APOE+	EPB41L2	6,21143E-72	8,29931E-01	0,423	0,192	1,14396E-67
Macro_FOLR2+APOE+	TAPBPL	1,70761E-108	8,29882E-01	0,472	0,188	3,14490E-104
Macro_FOLR2+APOE+	SPIN1	6,65947E-55	8,29735E-01	0,265	0,112	1,22647E-50
Macro_FOLR2+APOE+	PTRH2	3,11477E-50	8,29278E-01	0,288	0,128	5,73647E-46
Macro_FOLR2+APOE+	UBAC2	2,90701E-24	8,28855E-01	0,516	0,303	5,35385E-20
Macro_FOLR2+APOE+	GIT2	1,82895E-30	8,28666E-01	0,507	0,288	3,36837E-26
Macro_FOLR2+APOE+	GATM	4,39635E-46	8,28620E-01	0,322	0,155	8,09676E-42
Macro_FOLR2+APOE+	SMCO4	3,46375E-120	8,28359E-01	0,782	0,407	6,37920E-116
Macro_FOLR2+APOE+	ANAPC13	4,51481E-68	8,26691E-01	0,481	0,223	8,31493E-64
Macro_FOLR2+APOE+	ARPC4	1,70113E-73	8,26170E-01	0,727	0,401	3,13298E-69
Macro_FOLR2+APOE+	MPC2	7,54831E-18	8,25453E-01	0,566	0,354	1,39017E-13
Macro_FOLR2+APOE+	MPC1	6,03670E-84	8,25221E-01	0,635	0,308	1,11178E-79
Macro_FOLR2+APOE+	C5AR1	8,69052E-36	8,25042E-01	0,679	0,4	1,60053E-31
Macro_FOLR2+APOE+	FCGR2B	1,40147E-10	8,23526E-01	0,624	0,419	2,58109E-06
Macro_FOLR2+APOE+	IL10RB	2,40151E-63	8,20869E-01	0,497	0,248	4,42285E-59
Macro_FOLR2+APOE+	CYB5R1	1,59861E-123	8,20382E-01	0,525	0,208	2,94415E-119
Macro_FOLR2+APOE+	IRF5	3,63884E-34	8,19900E-01	0,464	0,256	6,70165E-30
Macro_FOLR2+APOE+	CRELD2	7,74532E-64	8,18593E-01	0,431	0,2	1,42646E-59
Macro_FOLR2+APOE+	ANXA7	1,40646E-46	8,17710E-01	0,655	0,364	2,59027E-42
Macro_FOLR2+APOE+	CALM3	2,93343E-57	8,17532E-01	0,83	0,498	5,40249E-53
Macro_FOLR2+APOE+	CYCS	4,02099E-28	8,17394E-01	0,793	0,516	7,40545E-24
Macro_FOLR2+APOE+	PSMD6	4,17357E-29	8,16811E-01	0,481	0,273	7,68647E-25
Macro_FOLR2+APOE+	RAPGEF1	4,68599E-50	8,16596E-01	0,484	0,253	8,63018E-46
Macro_FOLR2+APOE+	NELFCD	1,26020E-25	8,15709E-01	0,256	0,137	2,32092E-21
Macro_FOLR2+APOE+	GBP1	1,67302E-170	8,14669E-01	0,766	0,334	3,08119E-166
Macro_FOLR2+APOE+	IL2RA	1,19738E-286	8,13762E-01	0,324	0,062	2,20522E-282
Macro_FOLR2+APOE+	NANS	1,97039E-67	8,13131E-01	0,708	0,38	3,62886E-63
Macro_FOLR2+APOE+	BRI3	3,88998E-251	8,12622E-01	0,851	0,389	7,16418E-247
Macro_FOLR2+APOE+	SYPL1	9,15334E-31	8,10223E-01	0,523	0,297	1,68577E-26

Macro_FOLR2+APOE+	RPL22L1	8,70843E-67	8,09781E-01	0,545	0,266	1,60383E-62
Macro_FOLR2+APOE+	PGD	4,85378E-114	8,08595E-01	0,827	0,455	8,93921E-110
Macro_FOLR2+APOE+	PABPC4	4,47284E-26	8,07597E-01	0,68	0,416	8,23763E-22
Macro_FOLR2+APOE+	PDIA6	2,54974E-63	8,07397E-01	0,842	0,511	4,69586E-59
Macro_FOLR2+APOE+	EIF4E	7,73604E-16	8,05683E-01	0,468	0,293	1,42475E-11
Macro_FOLR2+APOE+	RAB18	8,01836E-80	8,04709E-01	0,577	0,272	1,47674E-75
Macro_FOLR2+APOE+	MTHFD1	5,57298E-89	8,04462E-01	0,288	0,102	1,02638E-84
Macro_FOLR2+APOE+	LRRC25	1,83772E-72	8,04369E-01	0,645	0,324	3,38452E-68
Macro_FOLR2+APOE+	ENG	5,35345E-73	8,04226E-01	0,656	0,338	9,85946E-69
Macro_FOLR2+APOE+	PSMD11	1,19421E-53	8,03730E-01	0,571	0,301	2,19938E-49
Macro_FOLR2+APOE+	TTC19	5,36265E-18	8,03041E-01	0,26	0,154	9,87639E-14
Macro_FOLR2+APOE+	PSMB4	2,54843E-45	8,02796E-01	0,658	0,365	4,69345E-41
Macro_FOLR2+APOE+	CALHM6	2,53566E-287	8,01875E-01	0,711	0,248	4,66993E-283
Macro_FOLR2+APOE+	FMNL2	2,43232E-217	8,01857E-01	0,625	0,215	4,47961E-213
Macro_FOLR2+APOE+	PIK3R5	1,05015E-08	8,01477E-01	0,371	0,254	1,93407E-04
Macro_FOLR2+APOE+	DENND3	7,29643E-27	8,00671E-01	0,401	0,228	1,34378E-22
Macro_FOLR2+APOE+	DCTN2	1,24551E-34	8,00185E-01	0,565	0,322	2,29386E-30
Macro_FOLR2+APOE+	ISCU	5,06129E-86	7,99712E-01	0,863	0,528	9,32138E-82
Macro_FOLR2+APOE+	HLA-B	6,56721E-152	7,98794E-01	1	0,967	1,20948E-147
Macro_FOLR2+APOE+	FRMD4A	1,01412E-197	7,97402E-01	0,538	0,175	1,86771E-193
Macro_FOLR2+APOE+	MT-ND1	2,26304E-68	7,96833E-01	0,83	0,561	4,16785E-64
Macro_FOLR2+APOE+	SLC2A5	3,56648E-166	7,96288E-01	0,254	0,06	6,56839E-162
Macro_FOLR2+APOE+	SNX10	5,53649E-134	7,95978E-01	0,842	0,476	1,01966E-129
Macro_FOLR2+APOE+	PLEKHO1	4,42503E-163	7,95754E-01	0,768	0,349	8,14959E-159
Macro_FOLR2+APOE+	ITGB1	1,97565E-23	7,95705E-01	0,732	0,467	3,63856E-19
Macro_FOLR2+APOE+	ARPC1B	1,82092E-18	7,95674E-01	0,969	0,792	3,35359E-14
Macro_FOLR2+APOE+	MDM2	6,24621E-28	7,95390E-01	0,35	0,194	1,15036E-23
Macro_FOLR2+APOE+	REXO2	1,38320E-58	7,94743E-01	0,381	0,175	2,54743E-54
Macro_FOLR2+APOE+	SERINC1	1,53195E-43	7,94735E-01	0,685	0,396	2,82139E-39
Macro_FOLR2+APOE+	MAP1LC3B	8,61583E-16	7,94620E-01	0,813	0,55	1,58678E-11
Macro_FOLR2+APOE+	DCP1A	1,64237E-24	7,93983E-01	0,269	0,146	3,02475E-20
Macro_FOLR2+APOE+	MLX	5,95920E-37	7,93975E-01	0,618	0,351	1,09751E-32
Macro_FOLR2+APOE+	GOSR1	1,16276E-45	7,93648E-01	0,32	0,152	2,14146E-41
Macro_FOLR2+APOE+	CTNNA1	1,29674E-25	7,93511E-01	0,592	0,356	2,38821E-21
Macro_FOLR2+APOE+	MT-ND4	1,74190E-76	7,92536E-01	0,824	0,567	3,20805E-72
Macro_FOLR2+APOE+	YIF1B	2,06903E-72	7,92004E-01	0,657	0,342	3,81052E-68
Macro_FOLR2+APOE+	CEP170	5,18923E-10	7,90705E-01	0,501	0,34	9,55700E-06
Macro_FOLR2+APOE+	IL15	3,49234E-31	7,90266E-01	0,251	0,125	6,43184E-27
Macro_FOLR2+APOE+	LAP3	2,08190E-117	7,89792E-01	0,931	0,575	3,83424E-113
Macro_FOLR2+APOE+	ABI3	4,18081E-85	7,89660E-01	0,741	0,383	7,69979E-81
Macro_FOLR2+APOE+	PDCD10	2,17608E-37	7,89006E-01	0,55	0,307	4,00768E-33
Macro_FOLR2+APOE+	FCGRT	2,05609E-116	7,88884E-01	0,98	0,731	3,78670E-112
Macro_FOLR2+APOE+	MT-CO2	7,30010E-36	7,88120E-01	0,833	0,589	1,34446E-31
Macro_FOLR2+APOE+	ME1	1,80058E-289	7,86966E-01	0,403	0,088	3,31613E-285
Macro_FOLR2+APOE+	EDEM2	6,05917E-100	7,86579E-01	0,482	0,2	1,11592E-95
Macro_FOLR2+APOE+	ABI1	4,30058E-75	7,85207E-01	0,649	0,331	7,92038E-71
Macro_FOLR2+APOE+	ST6GAL1	1,26480E-40	7,85124E-01	0,333	0,167	2,32938E-36

Macro_FOLR2+APOE+	NDUFA13	1,81463E-58	7,84855E-01	0,725	0,423	3,34201E-54
Macro_FOLR2+APOE+	TPD52L2	1,09058E-65	7,84837E-01	0,533	0,261	2,00853E-61
Macro_FOLR2+APOE+	CASP4	8,33076E-53	7,84422E-01	0,757	0,434	1,53428E-48
Macro_FOLR2+APOE+	HAVCR2	1,15358E-66	7,84162E-01	0,764	0,426	2,12454E-62
Macro_FOLR2+APOE+	PLEK	1,58063E-22	7,82396E-01	0,823	0,554	2,91105E-18
Macro_FOLR2+APOE+	EIF4A2	3,73927E-11	7,81179E-01	0,805	0,555	6,88662E-07
Macro_FOLR2+APOE+	PTPMT1	8,30015E-75	7,81098E-01	0,513	0,241	1,52864E-70
Macro_FOLR2+APOE+	PLBD1	1,85613E-67	7,79949E-01	0,718	0,383	3,41843E-63
Macro_FOLR2+APOE+	CORO1C	1,48232E-63	7,78725E-01	0,647	0,335	2,73000E-59
Macro_FOLR2+APOE+	NDUFB9	1,48466E-39	7,78646E-01	0,836	0,542	2,73429E-35
Macro_FOLR2+APOE+	HSD3B7	3,90630E-277	7,78044E-01	0,373	0,081	7,19423E-273
Macro_FOLR2+APOE+	SCARB1	2,55815E-204	7,76742E-01	0,487	0,149	4,71135E-200
Macro_FOLR2+APOE+	MFSD11	9,03816E-49	7,76523E-01	0,325	0,151	1,66456E-44
Macro_FOLR2+APOE+	CYC1	7,52584E-59	7,76379E-01	0,75	0,425	1,38603E-54
Macro_FOLR2+APOE+	MRPL40	7,86829E-101	7,74555E-01	0,592	0,263	1,44910E-96
Macro_FOLR2+APOE+	NDUFC2	1,20367E-96	7,74307E-01	0,586	0,297	2,21680E-92
Macro_FOLR2+APOE+	WDR45B	7,99040E-25	7,74267E-01	0,369	0,209	1,47159E-20
Macro_FOLR2+APOE+	SDHD	3,28569E-120	7,71816E-01	0,707	0,334	6,05126E-116
Macro_FOLR2+APOE+	ENO1	7,18998E-13	7,71249E-01	0,953	0,755	1,32418E-08
Macro_FOLR2+APOE+	SLC23A2	2,15350E-87	7,68271E-01	0,274	0,097	3,96610E-83
Macro_FOLR2+APOE+	SORBS3	1,90083E-175	7,67545E-01	0,464	0,151	3,50075E-171
Macro_FOLR2+APOE+	SRP54	3,25975E-35	7,67520E-01	0,379	0,201	6,00349E-31
Macro_FOLR2+APOE+	CD300A	3,42512E-34	7,67174E-01	0,738	0,452	6,30805E-30
Macro_FOLR2+APOE+	CHMP1A	2,80855E-39	7,67075E-01	0,359	0,183	5,17251E-35
Macro_FOLR2+APOE+	GRINA	1,05531E-127	7,66470E-01	0,957	0,675	1,94356E-123
Macro_FOLR2+APOE+	GGA1	1,99753E-93	7,65958E-01	0,525	0,231	3,67886E-89
Macro_FOLR2+APOE+	TMSB4X	1,40790E-58	7,65517E-01	1	0,983	2,59292E-54
Macro_FOLR2+APOE+	DOCK4	1,06073E-35	7,65336E-01	0,501	0,279	1,95355E-31
Macro_FOLR2+APOE+	SUCLG2	2,12742E-66	7,65227E-01	0,407	0,184	3,91808E-62
Macro_FOLR2+APOE+	IL32	0,00000E+00	7,64575E-01	0,533	0,12	0,00000E+00
Macro_FOLR2+APOE+	EML4	2,72306E-61	7,62423E-01	0,623	0,348	5,01506E-57
Macro_FOLR2+APOE+	MGST2	5,22426E-99	7,61897E-01	0,7	0,349	9,62152E-95
Macro_FOLR2+APOE+	LIMS1	1,28504E-23	7,61099E-01	0,82	0,547	2,36666E-19
Macro_FOLR2+APOE+	COPG1	5,12530E-55	7,60516E-01	0,386	0,184	9,43926E-51
Macro_FOLR2+APOE+	ABCC5	5,15691E-146	7,60205E-01	0,373	0,118	9,49748E-142
Macro_FOLR2+APOE+	SLC35B1	6,35542E-73	7,59384E-01	0,442	0,199	1,17048E-68
Macro_FOLR2+APOE+	PRDX5	7,57809E-26	7,58695E-01	0,869	0,593	1,39566E-21
Macro_FOLR2+APOE+	LAMTOR2	4,55605E-50	7,58187E-01	0,845	0,534	8,39088E-46
Macro_FOLR2+APOE+	ETV5	9,29428E-54	7,55672E-01	0,403	0,193	1,71173E-49
Macro_FOLR2+APOE+	THUMPD3	1,96202E-55	7,55645E-01	0,262	0,109	3,61344E-51
Macro_FOLR2+APOE+	MT-ND5	3,68520E-45	7,55412E-01	0,819	0,529	6,78703E-41
Macro_FOLR2+APOE+	AMPD3	1,22239E-74	7,54477E-01	0,318	0,128	2,25128E-70
Macro_FOLR2+APOE+	SLC25A5	7,19204E-08	7,54012E-01	0,941	0,738	1,32456E-03
Macro_FOLR2+APOE+	CALM2	2,48751E-08	7,53310E-01	0,968	0,777	4,58124E-04
Macro_FOLR2+APOE+	TRPM2	7,01769E-120	7,51818E-01	0,466	0,177	1,29245E-115
Macro_FOLR2+APOE+	IL1B	2,43626E-15	7,51458E-01	0,266	0,337	4,48687E-11
Macro_FOLR2+APOE+	FTH1	1,19569E-06	7,50964E-01	1	0,997	2,20211E-02

Macro_FOLR2+APOE+	COX6C	5,79833E-41	7,49992E-01	0,935	0,713	1,06788E-36
Macro_FOLR2+APOE+	NAGA	4,93089E-74	7,49970E-01	0,687	0,351	9,08122E-70
Macro_FOLR2+APOE+	NDUFA11	1,30028E-28	7,49836E-01	0,695	0,448	2,39473E-24
Macro_FOLR2+APOE+	COMMD1	1,26166E-60	7,49560E-01	0,617	0,314	2,32359E-56
Macro_FOLR2+APOE+	ZNF331	3,39101E-66	7,48363E-01	0,65	0,336	6,24523E-62
Macro_FOLR2+APOE+	TMBIM4	1,18242E-101	7,47821E-01	0,644	0,322	2,17766E-97
Macro_FOLR2+APOE+	BAG6	1,91049E-40	7,47679E-01	0,495	0,266	3,51855E-36
Macro_FOLR2+APOE+	RCBTB2	2,00548E-16	7,47167E-01	0,302	0,182	3,69349E-12
Macro_FOLR2+APOE+	PPIH	2,64808E-41	7,46561E-01	0,392	0,198	4,87698E-37
Macro_FOLR2+APOE+	MT-ND2	3,16189E-81	7,46190E-01	0,825	0,554	5,82325E-77
Macro_FOLR2+APOE+	TMBIM6	4,19456E-35	7,43957E-01	0,975	0,772	7,72512E-31
Macro_FOLR2+APOE+	MDH1	1,67523E-101	7,42643E-01	0,74	0,375	3,08527E-97
Macro_FOLR2+APOE+	PCMT1	3,05615E-40	7,42555E-01	0,593	0,331	5,62852E-36
Macro_FOLR2+APOE+	PTTG1IP	2,68462E-33	7,42399E-01	0,799	0,494	4,94426E-29
Macro_FOLR2+APOE+	CANX	7,58631E-31	7,41798E-01	0,913	0,631	1,39717E-26
Macro_FOLR2+APOE+	TPGS2	2,43171E-30	7,40596E-01	0,332	0,177	4,47848E-26
Macro_FOLR2+APOE+	NECAP2	1,22586E-120	7,39929E-01	0,725	0,342	2,25767E-116
Macro_FOLR2+APOE+	COPA	2,25486E-35	7,39776E-01	0,578	0,331	4,15278E-31
Macro_FOLR2+APOE+	GIMAP5	1,58440E-166	7,39318E-01	0,446	0,149	2,91799E-162
Macro_FOLR2+APOE+	TMED3	7,51162E-78	7,38788E-01	0,559	0,266	1,38341E-73
Macro_FOLR2+APOE+	IRF3	2,41866E-50	7,38300E-01	0,368	0,176	4,45445E-46
Macro_FOLR2+APOE+	M6PR	8,47671E-69	7,38067E-01	0,876	0,555	1,56116E-64
Macro_FOLR2+APOE+	FNIP2	2,59463E-82	7,38040E-01	0,473	0,214	4,77854E-78
Macro_FOLR2+APOE+	NEU1	3,74758E-137	7,37424E-01	0,681	0,294	6,90191E-133
Macro_FOLR2+APOE+	GBP5	7,41025E-235	7,37101E-01	0,669	0,234	1,36475E-230
Macro_FOLR2+APOE+	TMEM70	1,23057E-117	7,36930E-01	0,623	0,28	2,26634E-113
Macro_FOLR2+APOE+	MT-ND3	1,33832E-26	7,36035E-01	0,824	0,562	2,46478E-22
Macro_FOLR2+APOE+	JPT1	1,49103E-266	7,35671E-01	0,719	0,27	2,74603E-262
Macro_FOLR2+APOE+	DAP3	1,04357E-47	7,35422E-01	0,58	0,31	1,92195E-43
Macro_FOLR2+APOE+	HLA-DQB1	2,56371E-51	7,34722E-01	0,997	0,811	4,72158E-47
Macro_FOLR2+APOE+	CPVL	1,13153E-47	7,34423E-01	0,868	0,555	2,08394E-43
Macro_FOLR2+APOE+	CD4	3,15922E-85	7,34092E-01	0,879	0,529	5,81834E-81
Macro_FOLR2+APOE+	SIL1	8,66039E-137	7,33490E-01	0,577	0,228	1,59498E-132
Macro_FOLR2+APOE+	ATG7	4,10703E-126	7,32113E-01	0,479	0,182	7,56391E-122
Macro_FOLR2+APOE+	FAM20A	0,00000E+00	7,32059E-01	0,422	0,09	0,00000E+00
Macro_FOLR2+APOE+	VAC14	4,49637E-127	7,31266E-01	0,42	0,151	8,28096E-123
Macro_FOLR2+APOE+	SGK3	3,43293E-95	7,31222E-01	0,409	0,163	6,32243E-91
Macro_FOLR2+APOE+	GALC	1,52767E-83	7,30899E-01	0,399	0,163	2,81350E-79
Macro_FOLR2+APOE+	MANBA	6,95522E-50	7,30605E-01	0,61	0,328	1,28094E-45
Macro_FOLR2+APOE+	DOK3	4,40277E-96	7,30329E-01	0,521	0,22	8,10858E-92
Macro_FOLR2+APOE+	DNAJB11	4,02019E-48	7,30139E-01	0,563	0,303	7,40399E-44
Macro_FOLR2+APOE+	SERPINH1	4,39860E-89	7,29332E-01	0,266	0,091	8,10090E-85
Macro_FOLR2+APOE+	HSPA9	2,44517E-29	7,29284E-01	0,609	0,364	4,50327E-25
Macro_FOLR2+APOE+	CIAO1	1,02197E-31	7,28834E-01	0,433	0,241	1,88216E-27
Macro_FOLR2+APOE+	PPP1R15B	1,64512E-12	7,26028E-01	0,311	0,203	3,02982E-08
Macro_FOLR2+APOE+	TLR1	1,27624E-50	7,25711E-01	0,406	0,199	2,35046E-46
Macro_FOLR2+APOE+	HLA-E	6,70315E-53	7,25361E-01	0,993	0,902	1,23452E-48

Macro_FOLR2+APOE+	UBE2L6	2,18323E-59	7,25006E-01	0,886	0,561	4,02085E-55
Macro_FOLR2+APOE+	CFDP1	3,37511E-35	7,24457E-01	0,413	0,222	6,21593E-31
Macro_FOLR2+APOE+	ETFB	2,70908E-103	7,24178E-01	0,631	0,298	4,98932E-99
Macro_FOLR2+APOE+	PSMB7	2,23061E-53	7,23437E-01	0,647	0,355	4,10811E-49
Macro_FOLR2+APOE+	ZNF622	6,95529E-43	7,23042E-01	0,336	0,165	1,28096E-38
Macro_FOLR2+APOE+	ITSN1	1,53201E-50	7,22579E-01	0,252	0,107	2,82151E-46
Macro_FOLR2+APOE+	MAPKAP1	1,81116E-75	7,20699E-01	0,457	0,205	3,33562E-71
Macro_FOLR2+APOE+	ALG3	2,33794E-62	7,20487E-01	0,42	0,195	4,30579E-58
Macro_FOLR2+APOE+	GRB2	6,49945E-37	7,19898E-01	0,929	0,672	1,19700E-32
Macro_FOLR2+APOE+	INPPL1	4,01364E-75	7,18915E-01	0,327	0,131	7,39192E-71
Macro_FOLR2+APOE+	PMP22	5,29667E-157	7,18885E-01	0,656	0,266	9,75489E-153
Macro_FOLR2+APOE+	GBGT1	1,69940E-124	7,18269E-01	0,334	0,108	3,12979E-120
Macro_FOLR2+APOE+	HCK	1,23275E-24	7,17548E-01	0,804	0,512	2,27036E-20
Macro_FOLR2+APOE+	S100A11	2,55939E-09	7,16247E-01	0,994	0,936	4,71363E-05
Macro_FOLR2+APOE+	ITGAV	2,68530E-48	7,16157E-01	0,44	0,223	4,94551E-44
Macro_FOLR2+APOE+	ALPK1	3,34679E-63	7,15656E-01	0,277	0,113	6,16378E-59
Macro_FOLR2+APOE+	AHSA1	5,88588E-29	7,15171E-01	0,511	0,293	1,08400E-24
Macro_FOLR2+APOE+	PLXDC2	6,70381E-14	7,14544E-01	0,832	0,536	1,23464E-09
Macro_FOLR2+APOE+	ILK	7,01653E-34	7,14255E-01	0,699	0,407	1,29223E-29
Macro_FOLR2+APOE+	ALDH3A2	4,16181E-81	7,12713E-01	0,312	0,119	7,66480E-77
Macro_FOLR2+APOE+	TMEM51	5,76738E-126	7,12241E-01	0,597	0,252	1,06218E-121
Macro_FOLR2+APOE+	PHPT1	1,14783E-33	7,12017E-01	0,734	0,438	2,11397E-29
Macro_FOLR2+APOE+	KCNAB2	2,26219E-60	7,12001E-01	0,539	0,273	4,16628E-56
Macro_FOLR2+APOE+	SNAP23	9,56230E-44	7,11126E-01	0,688	0,391	1,76109E-39
Macro_FOLR2+APOE+	NAAA	2,43168E-79	7,09420E-01	0,698	0,358	4,47843E-75
Macro_FOLR2+APOE+	BTF3L4	4,78922E-41	7,09103E-01	0,66	0,37	8,82031E-37
Macro_FOLR2+APOE+	PSMA5	2,37792E-40	7,08494E-01	0,725	0,426	4,37942E-36
Macro_FOLR2+APOE+	ENOSF1	8,30529E-116	7,08215E-01	0,321	0,105	1,52959E-111
Macro_FOLR2+APOE+	EMC7	9,92930E-110	7,08062E-01	0,675	0,31	1,82868E-105
Macro_FOLR2+APOE+	ANP32E	2,21655E-22	7,07469E-01	0,307	0,176	4,08222E-18
Macro_FOLR2+APOE+	DRAM2	8,17475E-95	7,06878E-01	0,746	0,38	1,50554E-90
Macro_FOLR2+APOE+	PEX16	6,20837E-21	7,06315E-01	0,285	0,163	1,14339E-16
Macro_FOLR2+APOE+	LILRB3	8,49806E-65	7,04882E-01	0,464	0,223	1,56509E-60
Macro_FOLR2+APOE+	MILR1	1,17924E-130	7,04337E-01	0,373	0,125	2,17181E-126
Macro_FOLR2+APOE+	GNG10	3,50373E-68	7,04008E-01	0,571	0,331	6,45282E-64
Macro_FOLR2+APOE+	PSMD10	1,50113E-19	7,03991E-01	0,252	0,144	2,76462E-15
Macro_FOLR2+APOE+	FUCA2	8,07413E-139	7,03425E-01	0,682	0,298	1,48701E-134
Macro_FOLR2+APOE+	NSMAF	1,37690E-18	7,03299E-01	0,277	0,163	2,53584E-14
Macro_FOLR2+APOE+	GNPTG	1,62968E-57	7,02789E-01	0,721	0,42	3,00137E-53
Macro_FOLR2+APOE+	BAZ2B	9,04980E-18	7,02382E-01	0,415	0,256	1,66670E-13
Macro_FOLR2+APOE+	PSMA7	1,85120E-21	7,02297E-01	0,946	0,74	3,40936E-17
Macro_FOLR2+APOE+	NDUFB7	3,25073E-45	7,01091E-01	0,836	0,521	5,98686E-41
Macro_FOLR2+APOE+	SEC22B	2,39521E-115	7,00975E-01	0,368	0,131	4,41126E-111
Macro_FOLR2+APOE+	MT2A	1,19775E-42	7,00008E-01	0,879	0,621	2,20590E-38
Macro_FOLR2+APOE+	PSEN1	3,24389E-15	6,96890E-01	0,346	0,218	5,97427E-11
Macro_FOLR2+APOE+	NUP93	1,20877E-37	6,96494E-01	0,257	0,122	2,22620E-33
Macro_FOLR2+APOE+	TYMP	5,83878E-111	6,96185E-01	0,983	0,788	1,07533E-106

Macro_FOLR2+APOE+	PSMB10	1,01704E-11	6,94884E-01	0,766	0,561	1,87309E-07
Macro_FOLR2+APOE+	ESYT1	7,49272E-36	6,94847E-01	0,309	0,157	1,37993E-31
Macro_FOLR2+APOE+	TFEC	3,11568E-150	6,94086E-01	0,673	0,284	5,73816E-146
Macro_FOLR2+APOE+	DYNLRB1	5,64757E-29	6,93841E-01	0,708	0,456	1,04011E-24
Macro_FOLR2+APOE+	PSMC3	2,29964E-60	6,92907E-01	0,6	0,311	4,23525E-56
Macro_FOLR2+APOE+	ADAM17	4,65635E-14	6,92570E-01	0,352	0,223	8,57561E-10
Macro_FOLR2+APOE+	PDE4B	1,47055E-10	6,92232E-01	0,539	0,362	2,70832E-06
Macro_FOLR2+APOE+	TBK1	7,16752E-30	6,91937E-01	0,258	0,133	1,32004E-25
Macro_FOLR2+APOE+	NXF1	1,60389E-20	6,91624E-01	0,347	0,207	2,95388E-16
Macro_FOLR2+APOE+	COMMD2	2,83782E-20	6,90817E-01	0,324	0,188	5,22641E-16
Macro_FOLR2+APOE+	MITF	3,38855E-88	6,90729E-01	0,403	0,163	6,24069E-84
Macro_FOLR2+APOE+	MT-ND6	7,10602E-70	6,90384E-01	0,572	0,294	1,30872E-65
Macro_FOLR2+APOE+	MRPL37	8,97751E-41	6,89556E-01	0,432	0,224	1,65339E-36
Macro_FOLR2+APOE+	DOCK10	3,49827E-16	6,88744E-01	0,507	0,317	6,44276E-12
Macro_FOLR2+APOE+	EIF2S1	1,07635E-56	6,87339E-01	0,457	0,223	1,98232E-52
Macro_FOLR2+APOE+	MRRF	9,41498E-37	6,87180E-01	0,26	0,125	1,73396E-32
Macro_FOLR2+APOE+	OLFML2B	2,75583E-178	6,86465E-01	0,445	0,143	5,07541E-174
Macro_FOLR2+APOE+	NFIC	1,08452E-83	6,86327E-01	0,603	0,286	1,99737E-79
Macro_FOLR2+APOE+	STX8	5,92708E-45	6,85985E-01	0,449	0,232	1,09159E-40
Macro_FOLR2+APOE+	PSMC1	2,26952E-62	6,85978E-01	0,621	0,321	4,17978E-58
Macro_FOLR2+APOE+	NT5C	7,58095E-25	6,85682E-01	0,58	0,345	1,39618E-20
Macro_FOLR2+APOE+	ABHD3	2,33799E-83	6,85021E-01	0,34	0,133	4,30587E-79
Macro_FOLR2+APOE+	PIGS	1,33601E-41	6,84635E-01	0,314	0,151	2,46052E-37
Macro_FOLR2+APOE+	COX20	1,73578E-26	6,84318E-01	0,442	0,254	3,19678E-22
Macro_FOLR2+APOE+	RAC1	5,42231E-85	6,84100E-01	0,933	0,681	9,98626E-81
Macro_FOLR2+APOE+	TAF9	6,51874E-15	6,83885E-01	0,468	0,297	1,20056E-10
Macro_FOLR2+APOE+	MAEA	3,56192E-52	6,83436E-01	0,334	0,153	6,55999E-48
Macro_FOLR2+APOE+	PDXDC1	2,32620E-54	6,83359E-01	0,395	0,19	4,28416E-50
Macro_FOLR2+APOE+	KDSR	3,81808E-70	6,83149E-01	0,432	0,195	7,03176E-66
Macro_FOLR2+APOE+	IQGAP2	1,26088E-95	6,83012E-01	0,629	0,294	2,32216E-91
Macro_FOLR2+APOE+	SURF1	1,16261E-59	6,82888E-01	0,611	0,319	2,14118E-55
Macro_FOLR2+APOE+	BLOC1S2	2,66690E-76	6,81396E-01	0,635	0,322	4,91164E-72
Macro_FOLR2+APOE+	TMED10	1,56811E-46	6,81309E-01	0,879	0,558	2,88799E-42
Macro_FOLR2+APOE+	RPS27L	4,15794E-161	6,79672E-01	0,753	0,351	7,65768E-157
Macro_FOLR2+APOE+	FCHO2	6,83148E-243	6,78970E-01	0,629	0,203	1,25815E-238
Macro_FOLR2+APOE+	FKBP2	2,19233E-07	6,78053E-01	0,536	0,405	4,03761E-03
Macro_FOLR2+APOE+	CCPG1	4,18142E-59	6,77623E-01	0,457	0,225	7,70092E-55
Macro_FOLR2+APOE+	RAP2B	8,51684E-147	6,77231E-01	0,695	0,304	1,56855E-142
Macro_FOLR2+APOE+	ECHS1	4,96031E-83	6,76538E-01	0,699	0,36	9,13540E-79
Macro_FOLR2+APOE+	PDCD1LG2	3,01232E-253	6,75854E-01	0,362	0,081	5,54778E-249
Macro_FOLR2+APOE+	COPE	1,24863E-19	6,75800E-01	0,856	0,599	2,29960E-15
Macro_FOLR2+APOE+	ARHGAP17	5,08691E-79	6,75368E-01	0,414	0,183	9,36857E-75
Macro_FOLR2+APOE+	CHMP2A	9,78979E-42	6,74240E-01	0,797	0,483	1,80298E-37
Macro_FOLR2+APOE+	CLTA	4,01506E-85	6,74087E-01	0,929	0,641	7,39453E-81
Macro_FOLR2+APOE+	HSPE1	1,64703E-17	6,73562E-01	0,863	0,595	3,03334E-13
Macro_FOLR2+APOE+	ILVBL	1,04304E-93	6,73538E-01	0,304	0,107	1,92096E-89
Macro_FOLR2+APOE+	SERPINB6	7,77689E-78	6,73436E-01	0,772	0,412	1,43227E-73

Macro_FOLR2+APOE+	BMP2K	2,77504E-112	6,73186E-01	0,627	0,282	5,11079E-108
Macro_FOLR2+APOE+	ARL1	4,96763E-94	6,72957E-01	0,43	0,175	9,14889E-90
Macro_FOLR2+APOE+	SHISA5	2,65979E-22	6,72735E-01	0,603	0,364	4,89853E-18
Macro_FOLR2+APOE+	PWP1	4,93893E-28	6,72716E-01	0,372	0,207	9,09602E-24
Macro_FOLR2+APOE+	ZBTB8OS	2,62627E-45	6,71476E-01	0,428	0,217	4,83681E-41
Macro_FOLR2+APOE+	NDUFB3	1,03433E-62	6,70850E-01	0,799	0,458	1,90493E-58
Macro_FOLR2+APOE+	FNDC3B	1,09582E-49	6,69923E-01	0,513	0,272	2,01817E-45
Macro_FOLR2+APOE+	COPS8	2,46196E-26	6,69784E-01	0,287	0,156	4,53420E-22
Macro_FOLR2+APOE+	TOM1	1,10187E-32	6,69112E-01	0,499	0,278	2,02932E-28
Macro_FOLR2+APOE+	WSB2	4,04141E-139	6,68820E-01	0,313	0,093	7,44307E-135
Macro_FOLR2+APOE+	OSER1	1,16931E-20	6,68351E-01	0,363	0,213	2,15351E-16
Macro_FOLR2+APOE+	METTL5	2,91956E-51	6,67041E-01	0,464	0,229	5,37695E-47
Macro_FOLR2+APOE+	FNDC3A	6,86474E-46	6,66599E-01	0,46	0,234	1,26428E-41
Macro_FOLR2+APOE+	MAPK6	4,34244E-49	6,66328E-01	0,273	0,121	7,99746E-45
Macro_FOLR2+APOE+	NCF2	4,39288E-24	6,66116E-01	0,734	0,457	8,09037E-20
Macro_FOLR2+APOE+	GNAS	3,82951E-18	6,65443E-01	0,875	0,602	7,05280E-14
Macro_FOLR2+APOE+	FGL2	5,02843E-37	6,65407E-01	0,909	0,605	9,26086E-33
Macro_FOLR2+APOE+	DPM2	5,47575E-51	6,65005E-01	0,426	0,21	1,00847E-46
Macro_FOLR2+APOE+	BNIP2	2,33487E-15	6,64628E-01	0,614	0,395	4,30013E-11
Macro_FOLR2+APOE+	YIF1A	3,12950E-81	6,64318E-01	0,589	0,276	5,76361E-77
Macro_FOLR2+APOE+	PSMC5	9,99803E-35	6,64232E-01	0,684	0,403	1,84134E-30
Macro_FOLR2+APOE+	MYDGF	8,05800E-234	6,62746E-01	0,716	0,299	1,48404E-229
Macro_FOLR2+APOE+	SRI	1,70246E-46	6,62031E-01	0,741	0,425	3,13543E-42
Macro_FOLR2+APOE+	ZBTB38	1,09248E-33	6,61564E-01	0,356	0,189	2,01203E-29
Macro_FOLR2+APOE+	TATDN1	1,53645E-26	6,60638E-01	0,303	0,163	2,82968E-22
Macro_FOLR2+APOE+	SAE1	1,76668E-31	6,60455E-01	0,289	0,151	3,25369E-27
Macro_FOLR2+APOE+	PSMD2	3,29291E-35	6,60107E-01	0,554	0,313	6,06455E-31
Macro_FOLR2+APOE+	EXOSC4	1,55243E-55	6,59989E-01	0,41	0,194	2,85912E-51
Macro_FOLR2+APOE+	ENTPD1	4,05014E-60	6,59389E-01	0,586	0,301	7,45915E-56
Macro_FOLR2+APOE+	TMEM30A	3,48205E-63	6,59116E-01	0,578	0,297	6,41289E-59
Macro_FOLR2+APOE+	DYNLL1	2,59896E-15	6,59107E-01	0,935	0,701	4,78651E-11
Macro_FOLR2+APOE+	MMADHC	2,58529E-45	6,59064E-01	0,622	0,34	4,76133E-41
Macro_FOLR2+APOE+	APOL3	2,03475E-153	6,58527E-01	0,487	0,173	3,74739E-149
Macro_FOLR2+APOE+	PDIA3	4,04702E-33	6,58523E-01	0,927	0,668	7,45339E-29
Macro_FOLR2+APOE+	STK25	2,81960E-51	6,58188E-01	0,359	0,17	5,19286E-47
Macro_FOLR2+APOE+	ASL	7,19755E-28	6,57231E-01	0,258	0,135	1,32557E-23
Macro_FOLR2+APOE+	TLR4	1,71466E-119	6,57223E-01	0,533	0,215	3,15788E-115
Macro_FOLR2+APOE+	ANXA1	6,88674E-08	6,56437E-01	0,864	0,683	1,26833E-03
Macro_FOLR2+APOE+	CYTH1	2,20298E-21	6,56341E-01	0,52	0,322	4,05723E-17
Macro_FOLR2+APOE+	WBP2	3,59942E-63	6,56067E-01	0,691	0,375	6,62905E-59
Macro_FOLR2+APOE+	PEBP1	2,59114E-66	6,54670E-01	0,887	0,555	4,77211E-62
Macro_FOLR2+APOE+	SYNCRIP	4,13340E-24	6,54527E-01	0,533	0,316	7,61248E-20
Macro_FOLR2+APOE+	PSMG1	2,94379E-50	6,53313E-01	0,274	0,12	5,42158E-46
Macro_FOLR2+APOE+	SDCCAG8	2,36155E-30	6,53292E-01	0,571	0,33	4,34927E-26
Macro_FOLR2+APOE+	RNF135	7,59091E-82	6,53244E-01	0,516	0,23	1,39802E-77
Macro_FOLR2+APOE+	SPPL2A	3,87173E-131	6,52992E-01	0,714	0,33	7,13056E-127
Macro_FOLR2+APOE+	HTATIP2	2,52929E-99	6,51684E-01	0,621	0,282	4,65820E-95

Macro_FOLR2+APOE+	NASP	1,31695E-26	6,51473E-01	0,469	0,27	2,42542E-22
Macro_FOLR2+APOE+	ADA2	0,00000E+00	6,50515E-01	0,687	0,216	0,00000E+00
Macro_FOLR2+APOE+	VAMP3	2,81337E-47	6,50472E-01	0,627	0,343	5,18138E-43
Macro_FOLR2+APOE+	SCD	5,83604E-224	6,50360E-01	0,48	0,139	1,07482E-219
Macro_FOLR2+APOE+	RGS12	5,62115E-74	6,49435E-01	0,319	0,128	1,03525E-69
Macro_FOLR2+APOE+	RNASE6	9,26994E-31	6,49404E-01	0,843	0,531	1,70725E-26
Macro_FOLR2+APOE+	RPS6KA1	2,99839E-98	6,48690E-01	0,372	0,144	5,52213E-94
Macro_FOLR2+APOE+	PPP1CC	4,71896E-27	6,47985E-01	0,585	0,345	8,69091E-23
Macro_FOLR2+APOE+	NDUFS7	2,04978E-27	6,47817E-01	0,821	0,533	3,77509E-23
Macro_FOLR2+APOE+	COQ2	1,53248E-118	6,47785E-01	0,431	0,16	2,82236E-114
Macro_FOLR2+APOE+	RFX5	2,23507E-43	6,46995E-01	0,343	0,169	4,11632E-39
Macro_FOLR2+APOE+	BSDC1	2,12813E-20	6,46116E-01	0,267	0,153	3,91937E-16
Macro_FOLR2+APOE+	ATP1B3	1,27608E-08	6,45678E-01	0,774	0,538	2,35016E-04
Macro_FOLR2+APOE+	DYRK4	1,61542E-67	6,45636E-01	0,331	0,138	2,97511E-63
Macro_FOLR2+APOE+	PEF1	1,34534E-25	6,44945E-01	0,422	0,242	2,47772E-21
Macro_FOLR2+APOE+	WDFY1	7,24479E-56	6,44679E-01	0,335	0,151	1,33427E-51
Macro_FOLR2+APOE+	ALDOA	2,33691E-07	6,44504E-01	0,787	0,759	4,30389E-03
Macro_FOLR2+APOE+	TXNDC15	3,08889E-41	6,44203E-01	0,363	0,182	5,68880E-37
Macro_FOLR2+APOE+	PLOD1	2,27730E-98	6,43964E-01	0,395	0,153	4,19410E-94
Macro_FOLR2+APOE+	SERTAD1	2,32308E-06	6,43525E-01	0,455	0,322	4,27842E-02
Macro_FOLR2+APOE+	CLN8	1,58598E-85	6,43519E-01	0,511	0,228	2,92090E-81
Macro_FOLR2+APOE+	HSPD1	2,34404E-08	6,43415E-01	0,787	0,528	4,31702E-04
Macro_FOLR2+APOE+	NDUFA3	3,78093E-28	6,43371E-01	0,818	0,538	6,96334E-24
Macro_FOLR2+APOE+	AZIN1	1,00656E-09	6,43219E-01	0,469	0,315	1,85379E-05
Macro_FOLR2+APOE+	BSG	6,68886E-55	6,41883E-01	0,884	0,554	1,23189E-50
Macro_FOLR2+APOE+	DDRGK1	5,41506E-115	6,41845E-01	0,683	0,311	9,97292E-111
Macro_FOLR2+APOE+	GTF2B	8,58194E-14	6,41834E-01	0,43	0,275	1,58054E-09
Macro_FOLR2+APOE+	PPIL3	2,96102E-46	6,40097E-01	0,266	0,119	5,45330E-42
Macro_FOLR2+APOE+	UQCRC2	1,27259E-57	6,39314E-01	0,724	0,406	2,34372E-53
Macro_FOLR2+APOE+	PDXK	4,14022E-160	6,39255E-01	0,688	0,29	7,62504E-156
Macro_FOLR2+APOE+	MRPS22	9,50384E-45	6,38595E-01	0,328	0,157	1,75032E-40
Macro_FOLR2+APOE+	RFXANK	1,53978E-35	6,37982E-01	0,368	0,193	2,83581E-31
Macro_FOLR2+APOE+	ACP1	1,02928E-33	6,37214E-01	0,578	0,327	1,89562E-29
Macro_FOLR2+APOE+	NELFE	2,31067E-28	6,36111E-01	0,498	0,284	4,25555E-24
Macro_FOLR2+APOE+	PRCP	6,48726E-50	6,36073E-01	0,689	0,381	1,19476E-45
Macro_FOLR2+APOE+	TUBGCP2	2,08344E-37	6,35941E-01	0,536	0,297	3,83706E-33
Macro_FOLR2+APOE+	SCAMP3	1,70367E-59	6,35641E-01	0,493	0,244	3,13766E-55
Macro_FOLR2+APOE+	AP2M1	1,56545E-34	6,34681E-01	0,859	0,575	2,88308E-30
Macro_FOLR2+APOE+	C1orf43	1,63036E-16	6,34500E-01	0,812	0,539	3,00264E-12
Macro_FOLR2+APOE+	RPN1	6,78689E-157	6,34460E-01	0,664	0,284	1,24994E-152
Macro_FOLR2+APOE+	APH1B	1,51118E-140	6,33986E-01	0,483	0,174	2,78314E-136
Macro_FOLR2+APOE+	ARHGEF2	1,25919E-47	6,33606E-01	0,473	0,243	2,31905E-43
Macro_FOLR2+APOE+	TRIM14	3,95513E-137	6,33187E-01	0,461	0,166	7,28416E-133
Macro_FOLR2+APOE+	TMED5	6,49280E-40	6,32366E-01	0,634	0,365	1,19578E-35
Macro_FOLR2+APOE+	PNRC2	9,19107E-47	6,32106E-01	0,438	0,224	1,69272E-42
Macro_FOLR2+APOE+	REEP5	9,22229E-48	6,31488E-01	0,873	0,554	1,69847E-43
Macro_FOLR2+APOE+	YIPF1	2,78706E-52	6,31256E-01	0,299	0,134	5,13292E-48

Macro_FOLR2+APOE+	DBNDD2	3,06795E-77	6,29837E-01	0,435	0,199	5,65024E-73
Macro_FOLR2+APOE+	RIOK3	8,95098E-13	6,29813E-01	0,415	0,267	1,64850E-08
Macro_FOLR2+APOE+	OSTM1	9,11906E-92	6,29298E-01	0,504	0,217	1,67946E-87
Macro_FOLR2+APOE+	AGAP3	1,23284E-77	6,29053E-01	0,314	0,124	2,27052E-73
Macro_FOLR2+APOE+	TANGO2	4,82586E-55	6,29051E-01	0,335	0,15	8,88779E-51
Macro_FOLR2+APOE+	APLP2	4,82583E-18	6,29048E-01	0,869	0,587	8,88773E-14
Macro_FOLR2+APOE+	SLC29A1	9,08928E-117	6,28945E-01	0,433	0,164	1,67397E-112
Macro_FOLR2+APOE+	GNB4	2,07974E-149	6,28448E-01	0,725	0,325	3,83025E-145
Macro_FOLR2+APOE+	RDH11	8,43785E-108	6,27631E-01	0,448	0,174	1,55400E-103
Macro_FOLR2+APOE+	ARHGAP18	3,83917E-91	6,27344E-01	0,84	0,466	7,07059E-87
Macro_FOLR2+APOE+	CPQ	3,34257E-61	6,26338E-01	0,452	0,216	6,15602E-57
Macro_FOLR2+APOE+	VDAC2	2,50750E-06	6,26187E-01	0,776	0,546	4,61806E-02
Macro_FOLR2+APOE+	ARHGAP22	3,43927E-38	6,25821E-01	0,274	0,132	6,33409E-34
Macro_FOLR2+APOE+	CYBA	1,02705E-149	6,25612E-01	0,997	0,895	1,89152E-145
Macro_FOLR2+APOE+	NRBF2	3,05950E-40	6,25547E-01	0,447	0,238	5,63467E-36
Macro_FOLR2+APOE+	VTI1B	4,95860E-77	6,25477E-01	0,715	0,368	9,13225E-73
Macro_FOLR2+APOE+	ZC3H7A	9,36510E-23	6,25378E-01	0,283	0,159	1,72477E-18
Macro_FOLR2+APOE+	C17orf49	7,83978E-16	6,24845E-01	0,357	0,226	1,44385E-11
Macro_FOLR2+APOE+	TMBIM1	2,39615E-72	6,24835E-01	0,679	0,352	4,41300E-68
Macro_FOLR2+APOE+	CUTA	7,71372E-32	6,24727E-01	0,778	0,477	1,42064E-27
Macro_FOLR2+APOE+	SLC35A4	4,33120E-38	6,22828E-01	0,324	0,163	7,97677E-34
Macro_FOLR2+APOE+	HELZ	8,72483E-21	6,21899E-01	0,35	0,207	1,60685E-16
Macro_FOLR2+APOE+	AP2A2	1,20861E-176	6,21514E-01	0,588	0,217	2,22590E-172
Macro_FOLR2+APOE+	IRF2	1,22952E-24	6,21056E-01	0,538	0,322	2,26440E-20
Macro_FOLR2+APOE+	PSMD14	6,77898E-83	6,19935E-01	0,477	0,211	1,24849E-78
Macro_FOLR2+APOE+	ECH1	2,92979E-99	6,19861E-01	0,6	0,279	5,39580E-95
Macro_FOLR2+APOE+	RAB5A	2,56899E-22	6,19582E-01	0,509	0,304	4,73131E-18
Macro_FOLR2+APOE+	DTNBP1	1,40571E-98	6,18725E-01	0,429	0,171	2,58890E-94
Macro_FOLR2+APOE+	ALDH3B1	2,10080E-55	6,18685E-01	0,368	0,17	3,86905E-51
Macro_FOLR2+APOE+	C14orf119	1,85837E-55	6,18532E-01	0,552	0,284	3,42256E-51
Macro_FOLR2+APOE+	RALGDS	1,25887E-178	6,18374E-01	0,521	0,18	2,31846E-174
Macro_FOLR2+APOE+	HLA-C	1,01007E-31	6,17887E-01	0,997	0,949	1,86024E-27
Macro_FOLR2+APOE+	AATF	2,06120E-24	6,17487E-01	0,398	0,229	3,79611E-20
Macro_FOLR2+APOE+	BCAP29	1,91728E-42	6,17237E-01	0,402	0,204	3,53105E-38
Macro_FOLR2+APOE+	DCTN6	6,66607E-43	6,16883E-01	0,301	0,144	1,22769E-38
Macro_FOLR2+APOE+	RSU1	7,98490E-47	6,15743E-01	0,599	0,329	1,47058E-42
Macro_FOLR2+APOE+	VPS26A	6,71303E-51	6,15605E-01	0,446	0,224	1,23634E-46
Macro_FOLR2+APOE+	HEBP1	1,30663E-98	6,15249E-01	0,643	0,3	2,40643E-94
Macro_FOLR2+APOE+	SNX6	2,43619E-89	6,14886E-01	0,866	0,506	4,48672E-85
Macro_FOLR2+APOE+	SBDS	5,10324E-34	6,14245E-01	0,5	0,277	9,39864E-30
Macro_FOLR2+APOE+	PLBD2	4,45449E-248	6,12617E-01	0,552	0,168	8,20384E-244
Macro_FOLR2+APOE+	TBXAS1	3,21367E-20	6,12479E-01	0,792	0,51	5,91862E-16
Macro_FOLR2+APOE+	UBE2E2	5,76217E-72	6,12345E-01	0,586	0,291	1,06122E-67
Macro_FOLR2+APOE+	NOP10	1,38782E-31	6,11753E-01	0,885	0,624	2,55594E-27
Macro_FOLR2+APOE+	SLC30A9	4,80977E-25	6,11453E-01	0,256	0,137	8,85816E-21
Macro_FOLR2+APOE+	SRPRB	4,05393E-48	6,11119E-01	0,311	0,144	7,46612E-44
Macro_FOLR2+APOE+	NTAN1	1,42229E-174	6,10716E-01	0,516	0,176	2,61943E-170

Macro_FOLR2+APOE+	FRMD4B	2,10423E-86	6,09784E-01	0,593	0,276	3,87537E-82
Macro_FOLR2+APOE+	POLR2J3	2,06022E-34	6,09249E-01	0,356	0,191	3,79431E-30
Macro_FOLR2+APOE+	SAP30BP	7,95346E-15	6,09110E-01	0,426	0,266	1,46479E-10
Macro_FOLR2+APOE+	ANXA4	1,44046E-73	6,08230E-01	0,61	0,3	2,65290E-69
Macro_FOLR2+APOE+	ZFYVE21	9,55261E-53	6,07436E-01	0,278	0,121	1,75930E-48
Macro_FOLR2+APOE+	MRPL18	2,23813E-16	6,06695E-01	0,545	0,343	4,12197E-12
Macro_FOLR2+APOE+	HNRNPH3	1,41988E-07	6,06672E-01	0,512	0,348	2,61499E-03
Macro_FOLR2+APOE+	EIF4G1	3,46127E-24	6,06495E-01	0,532	0,327	6,37462E-20
Macro_FOLR2+APOE+	DDB2	1,68015E-54	6,06236E-01	0,275	0,118	3,09433E-50
Macro_FOLR2+APOE+	ATP6V1A	3,99188E-106	6,05868E-01	0,557	0,246	7,35184E-102
Macro_FOLR2+APOE+	ABR	2,91008E-129	6,05787E-01	0,51	0,201	5,35950E-125
Macro_FOLR2+APOE+	CHP1	6,99763E-13	6,04894E-01	0,362	0,234	1,28875E-08
Macro_FOLR2+APOE+	NDUFAB1	1,01849E-46	6,04865E-01	0,786	0,466	1,87576E-42
Macro_FOLR2+APOE+	SLC25A11	4,83894E-73	6,04826E-01	0,591	0,296	8,91188E-69
Macro_FOLR2+APOE+	MRC1	1,16935E-186	6,04624E-01	0,392	0,116	2,15359E-182
Macro_FOLR2+APOE+	PSMA3	2,53092E-66	6,04542E-01	0,668	0,363	4,66120E-62
Macro_FOLR2+APOE+	TSR2	2,11897E-29	6,04434E-01	0,325	0,174	3,90251E-25
Macro_FOLR2+APOE+	DOK1	1,68770E-49	6,04384E-01	0,457	0,232	3,10824E-45
Macro_FOLR2+APOE+	NBN	1,89367E-47	6,04261E-01	0,4	0,202	3,48757E-43
Macro_FOLR2+APOE+	TTC1	6,83987E-49	6,04101E-01	0,478	0,244	1,25970E-44
Macro_FOLR2+APOE+	TMEM179B	6,20508E-48	6,03902E-01	0,783	0,473	1,14279E-43
Macro_FOLR2+APOE+	GPR137B	3,85694E-143	6,03858E-01	0,679	0,287	7,10333E-139
Macro_FOLR2+APOE+	ARRDC1	2,89723E-30	6,03155E-01	0,508	0,288	5,33583E-26
Macro_FOLR2+APOE+	COQ10B	1,34583E-13	6,03051E-01	0,273	0,17	2,47862E-09
Macro_FOLR2+APOE+	GSDMD	5,16043E-92	6,02953E-01	0,62	0,292	9,50397E-88
Macro_FOLR2+APOE+	OSTF1	9,32592E-13	6,02114E-01	0,799	0,542	1,71755E-08
Macro_FOLR2+APOE+	CCT4	1,35127E-14	6,01792E-01	0,659	0,426	2,48863E-10
Macro_FOLR2+APOE+	ACADVL	7,45006E-20	6,01740E-01	0,73	0,461	1,37208E-15
Macro_FOLR2+APOE+	UQCRC1	5,50022E-25	6,01334E-01	0,774	0,498	1,01298E-20
Macro_FOLR2+APOE+	MYO1E	2,71504E-68	6,01219E-01	0,324	0,137	5,00029E-64
Macro_FOLR2+APOE+	CMTM7	1,00922E-38	6,01098E-01	0,704	0,403	1,85868E-34
Macro_FOLR2+APOE+	TMEM50A	8,83475E-28	6,00973E-01	0,874	0,595	1,62710E-23
Macro_FOLR2+APOE+	PRMT2	3,16881E-15	6,00535E-01	0,667	0,421	5,83600E-11
Macro_FOLR2+APOE+	VSIR	8,24158E-200	6,00468E-01	0,666	0,262	1,51785E-195
Macro_FOLR2+APOE+	TMEM218	2,07460E-24	6,00299E-01	0,252	0,135	3,82079E-20
Macro_FOLR2+APOE+	LMF2	4,10158E-72	5,99771E-01	0,442	0,198	7,55388E-68
Macro_FOLR2+APOE+	PHF11	7,73483E-28	5,99026E-01	0,492	0,281	1,42452E-23
Macro_FOLR2+APOE+	TNFSF13B	9,13021E-134	5,98850E-01	0,935	0,569	1,68151E-129
Macro_FOLR2+APOE+	IL15RA	1,82306E-205	5,98762E-01	0,403	0,11	3,35754E-201
Macro_FOLR2+APOE+	CHFR	6,56461E-39	5,98514E-01	0,28	0,135	1,20900E-34
Macro_FOLR2+APOE+	PSMC4	1,94583E-36	5,98244E-01	0,577	0,329	3,58363E-32
Macro_FOLR2+APOE+	PSMG2	8,29571E-25	5,97671E-01	0,685	0,416	1,52782E-20
Macro_FOLR2+APOE+	MYADM	1,21108E-10	5,97267E-01	0,31	0,335	2,23045E-06
Macro_FOLR2+APOE+	SSBP1	1,62989E-21	5,97257E-01	0,746	0,479	3,00177E-17
Macro_FOLR2+APOE+	ETHE1	3,48766E-59	5,96562E-01	0,641	0,333	6,42323E-55
Macro_FOLR2+APOE+	SLU7	7,99629E-46	5,95118E-01	0,458	0,235	1,47268E-41
Macro_FOLR2+APOE+	COPS7A	8,19566E-59	5,95092E-01	0,436	0,208	1,50939E-54

Macro_FOLR2+APOE+	ARHGAP4	2,84462E-34	5,95090E-01	0,643	0,377	5,23894E-30
Macro_FOLR2+APOE+	LTBR	4,98233E-55	5,94950E-01	0,64	0,341	9,17596E-51
Macro_FOLR2+APOE+	STIP1	3,56589E-25	5,94890E-01	0,474	0,276	6,56729E-21
Macro_FOLR2+APOE+	DAD1	9,47046E-14	5,94283E-01	0,848	0,593	1,74417E-09
Macro_FOLR2+APOE+	TMEM9B	1,49469E-38	5,93589E-01	0,638	0,365	2,75276E-34
Macro_FOLR2+APOE+	DTYMK	9,55542E-61	5,93013E-01	0,277	0,113	1,75982E-56
Macro_FOLR2+APOE+	RENBP	6,99327E-187	5,92993E-01	0,716	0,285	1,28795E-182
Macro_FOLR2+APOE+	TUFM	1,20742E-18	5,92347E-01	0,766	0,502	2,22370E-14
Macro_FOLR2+APOE+	LAPTM4A	6,47303E-32	5,91769E-01	0,882	0,621	1,19214E-27
Macro_FOLR2+APOE+	TRAPPC6A	3,17302E-25	5,91657E-01	0,407	0,236	5,84375E-21
Macro_FOLR2+APOE+	SIRPA	6,67581E-141	5,90898E-01	0,736	0,339	1,22948E-136
Macro_FOLR2+APOE+	NOSIP	3,76804E-26	5,90684E-01	0,554	0,321	6,93959E-22
Macro_FOLR2+APOE+	SDHC	3,77547E-55	5,90565E-01	0,745	0,432	6,95329E-51
Macro_FOLR2+APOE+	TIMMDC1	6,22647E-36	5,90476E-01	0,417	0,222	1,14673E-31
Macro_FOLR2+APOE+	RHOC	1,89871E-15	5,90041E-01	0,48	0,304	3,49686E-11
Macro_FOLR2+APOE+	LUZP1	1,75949E-41	5,89939E-01	0,269	0,126	3,24045E-37
Macro_FOLR2+APOE+	GLMP	0,00000E+00	5,89755E-01	0,592	0,135	0,00000E+00
Macro_FOLR2+APOE+	AMD1	3,80630E-33	5,89615E-01	0,498	0,279	7,01006E-29
Macro_FOLR2+APOE+	EBP	3,71880E-44	5,89520E-01	0,263	0,118	6,84892E-40
Macro_FOLR2+APOE+	CSRP1	1,38894E-20	5,88742E-01	0,423	0,253	2,55800E-16
Macro_FOLR2+APOE+	VAMP8	1,97501E-66	5,88411E-01	0,972	0,775	3,63738E-62
Macro_FOLR2+APOE+	HSPA5	5,02503E-12	5,88266E-01	0,787	0,555	9,25460E-08
Macro_FOLR2+APOE+	EPHX1	0,00000E+00	5,88255E-01	0,536	0,125	0,00000E+00
Macro_FOLR2+APOE+	DLD	9,66304E-47	5,87999E-01	0,376	0,186	1,77964E-42
Macro_FOLR2+APOE+	RHEB	8,54264E-42	5,87857E-01	0,769	0,453	1,57330E-37
Macro_FOLR2+APOE+	DNAJB6	1,75940E-14	5,87568E-01	0,788	0,514	3,24028E-10
Macro_FOLR2+APOE+	SDF2L1	1,42872E-57	5,87206E-01	0,641	0,346	2,63128E-53
Macro_FOLR2+APOE+	ZNF330	1,75005E-56	5,86954E-01	0,427	0,205	3,22307E-52
Macro_FOLR2+APOE+	DHRS7	7,32773E-12	5,86499E-01	0,715	0,478	1,34955E-07
Macro_FOLR2+APOE+	CIAPIN1	1,27584E-38	5,86492E-01	0,263	0,124	2,34972E-34
Macro_FOLR2+APOE+	CALHM2	1,32287E-21	5,86456E-01	0,276	0,155	2,43633E-17
Macro_FOLR2+APOE+	NFE2L2	5,17215E-10	5,86020E-01	0,746	0,513	9,52554E-06
Macro_FOLR2+APOE+	DEDD2	3,02227E-45	5,85691E-01	0,364	0,178	5,56611E-41
Macro_FOLR2+APOE+	CD47	2,30520E-93	5,84945E-01	0,761	0,412	4,24548E-89
Macro_FOLR2+APOE+	MAFB	6,78584E-101	5,84733E-01	0,935	0,584	1,24975E-96
Macro_FOLR2+APOE+	COMMD3	1,05463E-73	5,84119E-01	0,353	0,149	1,94230E-69
Macro_FOLR2+APOE+	AMPD2	1,51921E-06	5,83830E-01	0,258	0,182	2,79793E-02
Macro_FOLR2+APOE+	SLC50A1	1,81360E-72	5,83808E-01	0,461	0,21	3,34012E-68
Macro_FOLR2+APOE+	P4HA1	8,09895E-36	5,83678E-01	0,398	0,214	1,49158E-31
Macro_FOLR2+APOE+	ATP5MC3	4,60145E-162	5,83325E-01	0,731	0,342	8,47450E-158
Macro_FOLR2+APOE+	ATP6V0C	4,19883E-54	5,82514E-01	0,726	0,697	7,73299E-50
Macro_FOLR2+APOE+	ANAPC11	4,91613E-17	5,82143E-01	0,868	0,6	9,05403E-13
Macro_FOLR2+APOE+	CHORDC1	2,08159E-07	5,82114E-01	0,331	0,231	3,83367E-03
Macro_FOLR2+APOE+	PSMD13	2,66191E-34	5,82000E-01	0,562	0,317	4,90244E-30
Macro_FOLR2+APOE+	CYB5A	2,55644E-214	5,81593E-01	0,641	0,228	4,70819E-210
Macro_FOLR2+APOE+	TNFSF13	8,88258E-21	5,81359E-01	0,597	0,439	1,63590E-16
Macro_FOLR2+APOE+	APEH	4,42503E-50	5,81275E-01	0,395	0,193	8,14958E-46

Macro_FOLR2+APOE+	CHCHD10	2,30959E-169	5,81074E-01	0,888	0,505	4,25357E-165
Macro_FOLR2+APOE+	CLTC	1,92476E-45	5,80738E-01	0,705	0,412	3,54483E-41
Macro_FOLR2+APOE+	GAPDH	2,06169E-12	5,80379E-01	0,996	0,959	3,79702E-08
Macro_FOLR2+APOE+	WTAP	7,97349E-19	5,80345E-01	0,579	0,359	1,46848E-14
Macro_FOLR2+APOE+	STXBP2	8,03829E-15	5,79575E-01	0,655	0,413	1,48041E-10
Macro_FOLR2+APOE+	TUBA1C	5,44410E-45	5,79361E-01	0,652	0,372	1,00264E-40
Macro_FOLR2+APOE+	ME2	2,86441E-61	5,78725E-01	0,577	0,298	5,27538E-57
Macro_FOLR2+APOE+	ARL6IP5	5,89617E-24	5,77613E-01	0,905	0,648	1,08590E-19
Macro_FOLR2+APOE+	ARPP19	3,21708E-29	5,76874E-01	0,461	0,263	5,92490E-25
Macro_FOLR2+APOE+	HLA-F	2,83631E-88	5,76178E-01	0,857	0,505	5,22363E-84
Macro_FOLR2+APOE+	ATP1A1	5,53553E-19	5,76022E-01	0,668	0,423	1,01948E-14
Macro_FOLR2+APOE+	OSTC	1,64495E-15	5,75197E-01	0,755	0,492	3,02951E-11
Macro_FOLR2+APOE+	TRAP1	3,63054E-39	5,74747E-01	0,277	0,133	6,68637E-35
Macro_FOLR2+APOE+	RAB13	1,69391E-126	5,74655E-01	0,654	0,295	3,11967E-122
Macro_FOLR2+APOE+	CXCL12	2,69989E-138	5,73881E-01	0,281	0,081	4,97239E-134
Macro_FOLR2+APOE+	CDC27	2,52177E-40	5,73515E-01	0,305	0,149	4,64434E-36
Macro_FOLR2+APOE+	SERPINA1	2,44111E-15	5,73280E-01	0,935	0,715	4,49579E-11
Macro_FOLR2+APOE+	LCP2	3,34209E-11	5,72819E-01	0,818	0,57	6,15512E-07
Macro_FOLR2+APOE+	ALG5	5,18028E-91	5,72193E-01	0,456	0,192	9,54051E-87
Macro_FOLR2+APOE+	TMED9	5,38676E-53	5,71923E-01	0,827	0,515	9,92079E-49
Macro_FOLR2+APOE+	CLIC2	1,75889E-98	5,71896E-01	0,4	0,154	3,23935E-94
Macro_FOLR2+APOE+	LASP1	4,74844E-36	5,71675E-01	0,535	0,303	8,74520E-32
Macro_FOLR2+APOE+	EGLN2	2,33370E-29	5,71299E-01	0,251	0,13	4,29797E-25
Macro_FOLR2+APOE+	ASAP1	2,57118E-56	5,70887E-01	0,44	0,216	4,73534E-52
Macro_FOLR2+APOE+	TIMM23	2,07443E-74	5,70722E-01	0,301	0,119	3,82048E-70
Macro_FOLR2+APOE+	ARFIP1	6,10739E-30	5,70408E-01	0,275	0,143	1,12480E-25
Macro_FOLR2+APOE+	IWS1	8,47065E-19	5,70342E-01	0,286	0,169	1,56004E-14
Macro_FOLR2+APOE+	NAA20	1,84431E-115	5,70009E-01	0,658	0,297	3,39667E-111
Macro_FOLR2+APOE+	AKT1	3,31334E-44	5,69973E-01	0,293	0,138	6,10217E-40
Macro_FOLR2+APOE+	FBP1	1,02994E-60	5,69169E-01	0,618	0,342	1,89684E-56
Macro_FOLR2+APOE+	RAB6A	4,60287E-19	5,69101E-01	0,479	0,295	8,47710E-15
Macro_FOLR2+APOE+	CNOT2	5,27529E-15	5,68967E-01	0,423	0,266	9,71550E-11
Macro_FOLR2+APOE+	MGME1	2,42349E-95	5,67997E-01	0,287	0,098	4,46334E-91
Macro_FOLR2+APOE+	METTL7A	2,19771E-30	5,67987E-01	0,429	0,242	4,04752E-26
Macro_FOLR2+APOE+	ADIPOR2	2,99817E-37	5,66821E-01	0,263	0,127	5,52173E-33
Macro_FOLR2+APOE+	GNL2	2,45202E-28	5,66220E-01	0,3	0,16	4,51589E-24
Macro_FOLR2+APOE+	DDX39A	3,26534E-13	5,65980E-01	0,446	0,287	6,01377E-09
Macro_FOLR2+APOE+	MDK	9,35398E-242	5,64858E-01	0,364	0,087	1,72272E-237
Macro_FOLR2+APOE+	GPI	2,17070E-42	5,64701E-01	0,702	0,408	3,99778E-38
Macro_FOLR2+APOE+	MYL6	2,36245E-18	5,64461E-01	0,992	0,94	4,35093E-14
Macro_FOLR2+APOE+	JOSD2	4,72566E-63	5,64406E-01	0,623	0,32	8,70325E-59
Macro_FOLR2+APOE+	COX6A1	1,72489E-34	5,64373E-01	0,911	0,673	3,17674E-30
Macro_FOLR2+APOE+	REEP4	6,10535E-124	5,64281E-01	0,561	0,231	1,12442E-119
Macro_FOLR2+APOE+	CDCP1	2,30996E-89	5,64083E-01	0,327	0,123	4,25426E-85
Macro_FOLR2+APOE+	RPS20	4,67128E-97	5,63572E-01	0,792	0,441	8,60310E-93
Macro_FOLR2+APOE+	ACTR1B	2,31167E-29	5,63311E-01	0,255	0,13	4,25739E-25
Macro_FOLR2+APOE+	DGUOK	1,97762E-17	5,62777E-01	0,586	0,365	3,64218E-13

Macro_FOLR2+APOE+	OTUB1	8,42787E-17	5,62330E-01	0,671	0,423	1,55216E-12
Macro_FOLR2+APOE+	IFI27L2	3,32938E-61	5,62245E-01	0,73	0,4	6,13172E-57
Macro_FOLR2+APOE+	RPS19BP1	3,72405E-153	5,61833E-01	0,611	0,249	6,85859E-149
Macro_FOLR2+APOE+	NGDN	4,02629E-11	5,61710E-01	0,252	0,161	7,41522E-07
Macro_FOLR2+APOE+	LAMTOR5	1,58448E-33	5,60717E-01	0,802	0,499	2,91813E-29
Macro_FOLR2+APOE+	PLGRKT	2,12834E-108	5,60688E-01	0,501	0,203	3,91977E-104
Macro_FOLR2+APOE+	TMUB2	1,25742E-39	5,60208E-01	0,374	0,191	2,31578E-35
Macro_FOLR2+APOE+	NCOA4	7,01847E-38	5,59650E-01	0,827	0,522	1,29259E-33
Macro_FOLR2+APOE+	GSTK1	3,55804E-29	5,58865E-01	0,9	0,637	6,55285E-25
Macro_FOLR2+APOE+	GPCPD1	1,52355E-20	5,58113E-01	0,449	0,275	2,80593E-16
Macro_FOLR2+APOE+	MAN1B1	4,54990E-72	5,57146E-01	0,342	0,141	8,37956E-68
Macro_FOLR2+APOE+	ENOPH1	4,93017E-65	5,57003E-01	0,363	0,159	9,07989E-61
Macro_FOLR2+APOE+	PSMD7	3,98381E-49	5,56413E-01	0,697	0,397	7,33698E-45
Macro_FOLR2+APOE+	GPATCH2L	8,28492E-24	5,56397E-01	0,301	0,172	1,52583E-19
Macro_FOLR2+APOE+	ACTR10	4,48461E-46	5,56046E-01	0,391	0,194	8,25930E-42
Macro_FOLR2+APOE+	PRNP	9,76819E-58	5,56023E-01	0,721	0,392	1,79901E-53
Macro_FOLR2+APOE+	VPS25	4,24092E-44	5,55818E-01	0,406	0,206	7,81050E-40
Macro_FOLR2+APOE+	EIF1B	7,65447E-07	5,55769E-01	0,658	0,454	1,40972E-02
Macro_FOLR2+APOE+	SLC46A3	2,70547E-68	5,55744E-01	0,337	0,141	4,98267E-64
Macro_FOLR2+APOE+	CNOT1	2,06251E-24	5,55723E-01	0,371	0,214	3,79853E-20
Macro_FOLR2+APOE+	TAPBP	1,75459E-46	5,54925E-01	0,878	0,57	3,23144E-42
Macro_FOLR2+APOE+	ADAM10	1,55858E-46	5,54818E-01	0,527	0,28	2,87043E-42
Macro_FOLR2+APOE+	WASF2	5,02203E-25	5,54240E-01	0,853	0,56	9,24907E-21
Macro_FOLR2+APOE+	SRA1	2,73257E-13	5,53900E-01	0,6	0,38	5,03258E-09
Macro_FOLR2+APOE+	TMEM199	3,66593E-75	5,53636E-01	0,358	0,147	6,75155E-71
Macro_FOLR2+APOE+	RNF10	1,28249E-27	5,53306E-01	0,469	0,269	2,36197E-23
Macro_FOLR2+APOE+	NDUFA9	2,96159E-114	5,53164E-01	0,388	0,143	5,45436E-110
Macro_FOLR2+APOE+	GPAA1	3,06231E-56	5,52893E-01	0,498	0,249	5,63985E-52
Macro_FOLR2+APOE+	BLNK	2,58080E-46	5,52839E-01	0,349	0,165	4,75307E-42
Macro_FOLR2+APOE+	C6orf89	9,90357E-72	5,52018E-01	0,406	0,177	1,82394E-67
Macro_FOLR2+APOE+	ENTPD6	4,30646E-58	5,51290E-01	0,291	0,124	7,93121E-54
Macro_FOLR2+APOE+	MRPS14	8,62933E-65	5,51025E-01	0,394	0,177	1,58926E-60
Macro_FOLR2+APOE+	ACE	0,00000E+00	5,50612E-01	0,329	0,031	0,00000E+00
Macro_FOLR2+APOE+	P4HB	4,06199E-69	5,50561E-01	0,914	0,621	7,48097E-65
Macro_FOLR2+APOE+	ZCRB1	1,98589E-78	5,50190E-01	0,564	0,264	3,65741E-74
Macro_FOLR2+APOE+	PLK3	1,21800E-13	5,49865E-01	0,388	0,248	2,24319E-09
Macro_FOLR2+APOE+	RANBP1	8,24450E-22	5,49740E-01	0,589	0,361	1,51839E-17
Macro_FOLR2+APOE+	SLC16A3	3,85324E-45	5,49706E-01	0,81	0,503	7,09651E-41
Macro_FOLR2+APOE+	HSPB1	5,27257E-51	5,49654E-01	0,938	0,65	9,71050E-47
Macro_FOLR2+APOE+	LPAR6	4,84206E-69	5,49580E-01	0,619	0,313	8,91763E-65
Macro_FOLR2+APOE+	INSIG1	1,45283E-17	5,49373E-01	0,457	0,29	2,67568E-13
Macro_FOLR2+APOE+	HSPH1	2,36221E-13	5,49237E-01	0,625	0,409	4,35049E-09
Macro_FOLR2+APOE+	GABARAP	1,37883E-06	5,49013E-01	0,861	0,828	2,53939E-02
Macro_FOLR2+APOE+	EPB41L3	2,94489E-56	5,48897E-01	0,57	0,296	5,42360E-52
Macro_FOLR2+APOE+	METRNL	5,88932E-10	5,48731E-01	0,369	0,251	1,08464E-05
Macro_FOLR2+APOE+	POR	1,88531E-58	5,48651E-01	0,386	0,177	3,47218E-54
Macro_FOLR2+APOE+	MAN2C1	8,28918E-31	5,48575E-01	0,304	0,161	1,52662E-26

Macro_FOLR2+APOE+	MOB1A	1,03971E-22	5,48137E-01	0,847	0,576	1,91483E-18
Macro_FOLR2+APOE+	VPS8	3,16750E-50	5,46850E-01	0,281	0,125	5,83359E-46
Macro_FOLR2+APOE+	SF3A3	5,30670E-34	5,46721E-01	0,399	0,213	9,77335E-30
Macro_FOLR2+APOE+	DPP3	6,70367E-59	5,46306E-01	0,272	0,113	1,23461E-54
Macro_FOLR2+APOE+	IMP4	1,13327E-27	5,46135E-01	0,458	0,261	2,08715E-23
Macro_FOLR2+APOE+	BEX4	1,77181E-49	5,46135E-01	0,591	0,321	3,26314E-45
Macro_FOLR2+APOE+	COPZ1	4,18384E-43	5,45787E-01	0,658	0,374	7,70538E-39
Macro_FOLR2+APOE+	RIN2	4,16001E-33	5,45776E-01	0,273	0,137	7,66148E-29
Macro_FOLR2+APOE+	SPAG7	1,95868E-54	5,45577E-01	0,608	0,318	3,60731E-50
Macro_FOLR2+APOE+	RCN1	2,24482E-103	5,44988E-01	0,433	0,169	4,13428E-99
Macro_FOLR2+APOE+	TEX264	5,63983E-104	5,44746E-01	0,65	0,298	1,03869E-99
Macro_FOLR2+APOE+	CARD9	2,41753E-46	5,44718E-01	0,371	0,18	4,45236E-42
Macro_FOLR2+APOE+	PAPSS1	1,50336E-40	5,44609E-01	0,434	0,23	2,76874E-36
Macro_FOLR2+APOE+	NDUFA6	1,28711E-32	5,44009E-01	0,818	0,519	2,37047E-28
Macro_FOLR2+APOE+	SWAP70	1,72170E-46	5,43984E-01	0,602	0,327	3,17085E-42
Macro_FOLR2+APOE+	TXNDC17	1,07479E-42	5,43718E-01	0,72	0,426	1,97944E-38
Macro_FOLR2+APOE+	KIFC3	1,33011E-103	5,43379E-01	0,297	0,099	2,44967E-99
Macro_FOLR2+APOE+	OS9	2,04265E-25	5,42984E-01	0,814	0,529	3,76195E-21
Macro_FOLR2+APOE+	SSH1	1,51310E-51	5,42810E-01	0,279	0,123	2,78667E-47
Macro_FOLR2+APOE+	TBC1D22A	5,74664E-33	5,42646E-01	0,41	0,223	1,05836E-28
Macro_FOLR2+APOE+	LARP4	8,77421E-48	5,42624E-01	0,313	0,147	1,61595E-43
Macro_FOLR2+APOE+	CACYBP	4,52138E-39	5,42551E-01	0,684	0,393	8,32702E-35
Macro_FOLR2+APOE+	TIMM9	2,57915E-29	5,42137E-01	0,317	0,169	4,75001E-25
Macro_FOLR2+APOE+	SLC2A8	1,21258E-179	5,41968E-01	0,395	0,115	2,23321E-175
Macro_FOLR2+APOE+	NADK	3,27723E-147	5,41831E-01	0,576	0,225	6,03568E-143
Macro_FOLR2+APOE+	WDR11	4,30876E-25	5,41806E-01	0,269	0,147	7,93544E-21
Macro_FOLR2+APOE+	CHURC1	2,71942E-35	5,41639E-01	0,555	0,311	5,00835E-31
Macro_FOLR2+APOE+	RABGGTB	1,19294E-28	5,41618E-01	0,441	0,251	2,19704E-24
Macro_FOLR2+APOE+	IL7R	1,77996E-66	5,41449E-01	0,298	0,127	3,27816E-62
Macro_FOLR2+APOE+	NAGPA	3,67260E-85	5,41272E-01	0,374	0,15	6,76384E-81
Macro_FOLR2+APOE+	BIRC2	3,27805E-07	5,40637E-01	0,307	0,212	6,03719E-03
Macro_FOLR2+APOE+	AIF1	1,53655E-29	5,40433E-01	0,984	0,818	2,82986E-25
Macro_FOLR2+APOE+	CEP63	5,31099E-24	5,39935E-01	0,272	0,148	9,78124E-20
Macro_FOLR2+APOE+	ADORA3	9,86738E-44	5,39849E-01	0,469	0,257	1,81727E-39
Macro_FOLR2+APOE+	GIMAP7	9,25216E-151	5,39540E-01	0,617	0,245	1,70397E-146
Macro_FOLR2+APOE+	RGL1	1,94923E-205	5,39315E-01	0,452	0,136	3,58989E-201
Macro_FOLR2+APOE+	MIF4GD	7,91020E-48	5,39140E-01	0,576	0,304	1,45682E-43
Macro_FOLR2+APOE+	HDAC2	1,00805E-28	5,38878E-01	0,457	0,262	1,85652E-24
Macro_FOLR2+APOE+	RAB42	0,00000E+00	5,38876E-01	0,346	0,051	0,00000E+00
Macro_FOLR2+APOE+	ACAT1	5,29148E-73	5,38693E-01	0,373	0,158	9,74532E-69
Macro_FOLR2+APOE+	CHMP3	1,68316E-95	5,38682E-01	0,516	0,225	3,09987E-91
Macro_FOLR2+APOE+	CLIC4	8,83906E-54	5,38439E-01	0,451	0,223	1,62789E-49
Macro_FOLR2+APOE+	MTMR14	3,65190E-81	5,38362E-01	0,621	0,301	6,72571E-77
Macro_FOLR2+APOE+	TMEM115	5,95806E-43	5,38302E-01	0,283	0,133	1,09730E-38
Macro_FOLR2+APOE+	GPR108	1,03018E-49	5,37950E-01	0,488	0,251	1,89729E-45
Macro_FOLR2+APOE+	NUCB1	1,97012E-73	5,37863E-01	0,865	0,534	3,62837E-69
Macro_FOLR2+APOE+	TAX1BP1	3,86825E-28	5,37672E-01	0,782	0,49	7,12416E-24

Macro_FOLR2+APOE+	OGFOD3	1,19889E-95	5,37346E-01	0,362	0,136	2,20799E-91
Macro_FOLR2+APOE+	FDPS	4,11426E-38	5,37324E-01	0,429	0,229	7,57723E-34
Macro_FOLR2+APOE+	CUL9	2,93417E-222	5,37294E-01	0,275	0,057	5,40385E-218
Macro_FOLR2+APOE+	MED11	7,63727E-50	5,36713E-01	0,317	0,145	1,40656E-45
Macro_FOLR2+APOE+	NRBP1	1,35183E-33	5,36697E-01	0,534	0,301	2,48967E-29
Macro_FOLR2+APOE+	PFKL	1,67560E-54	5,36029E-01	0,696	0,384	3,08594E-50
Macro_FOLR2+APOE+	ZBTB1	2,19693E-28	5,35992E-01	0,281	0,149	4,04608E-24
Macro_FOLR2+APOE+	FERMT3	4,63432E-78	5,35762E-01	0,827	0,473	8,53503E-74
Macro_FOLR2+APOE+	GLRX3	1,95711E-42	5,35457E-01	0,464	0,242	3,60441E-38
Macro_FOLR2+APOE+	SMIM15	4,18935E-73	5,35173E-01	0,461	0,21	7,71553E-69
Macro_FOLR2+APOE+	PPP2R1A	4,18998E-18	5,34768E-01	0,626	0,39	7,71669E-14
Macro_FOLR2+APOE+	SNX14	1,05557E-43	5,34303E-01	0,339	0,167	1,94404E-39
Macro_FOLR2+APOE+	GSTZ1	6,74655E-72	5,33569E-01	0,295	0,115	1,24251E-67
Macro_FOLR2+APOE+	MRPL11	9,99246E-39	5,33436E-01	0,529	0,287	1,84031E-34
Macro_FOLR2+APOE+	SIGLEC1	2,44844E-229	5,33203E-01	0,557	0,174	4,50928E-225
Macro_FOLR2+APOE+	MORF4L2	5,18154E-32	5,33164E-01	0,479	0,268	9,54285E-28
Macro_FOLR2+APOE+	RMDN3	3,32124E-97	5,32608E-01	0,414	0,166	6,11673E-93
Macro_FOLR2+APOE+	LSM2	2,99350E-37	5,32581E-01	0,615	0,343	5,51313E-33
Macro_FOLR2+APOE+	DUSP23	2,38077E-30	5,32304E-01	0,643	0,376	4,38466E-26
Macro_FOLR2+APOE+	RHBDF2	3,76763E-62	5,32279E-01	0,618	0,316	6,93884E-58
Macro_FOLR2+APOE+	AP1B1	4,65232E-87	5,31942E-01	0,747	0,383	8,56818E-83
Macro_FOLR2+APOE+	TMEM205	3,86648E-74	5,31738E-01	0,579	0,276	7,12090E-70
Macro_FOLR2+APOE+	WAC	5,51419E-12	5,30863E-01	0,44	0,286	1,01555E-07
Macro_FOLR2+APOE+	ERP44	5,09740E-72	5,30230E-01	0,754	0,419	9,38788E-68
Macro_FOLR2+APOE+	AGTRAP	3,09736E-63	5,30184E-01	0,676	0,352	5,70442E-59
Macro_FOLR2+APOE+	SNRPA1	2,93618E-33	5,30040E-01	0,376	0,201	5,40756E-29
Macro_FOLR2+APOE+	GLOD4	1,66926E-47	5,28920E-01	0,433	0,216	3,07428E-43
Macro_FOLR2+APOE+	SPG21	3,93686E-73	5,28534E-01	0,772	0,428	7,25051E-69
Macro_FOLR2+APOE+	ACOT13	8,28781E-181	5,28342E-01	0,542	0,188	1,52637E-176
Macro_FOLR2+APOE+	TECR	7,94548E-74	5,28216E-01	0,711	0,369	1,46332E-69
Macro_FOLR2+APOE+	MT-CYB	5,00129E-45	5,27835E-01	0,829	0,571	9,21087E-41
Macro_FOLR2+APOE+	MRPL24	6,02995E-55	5,27778E-01	0,443	0,217	1,11054E-50
Macro_FOLR2+APOE+	CCT7	8,14594E-32	5,27555E-01	0,594	0,342	1,50024E-27
Macro_FOLR2+APOE+	SPCS3	2,80319E-54	5,27456E-01	0,766	0,435	5,16264E-50
Macro_FOLR2+APOE+	GK	8,51794E-22	5,26466E-01	0,571	0,358	1,56875E-17
Macro_FOLR2+APOE+	COA3	2,31952E-49	5,26316E-01	0,589	0,312	4,27186E-45
Macro_FOLR2+APOE+	SDAD1	1,22601E-35	5,26069E-01	0,376	0,197	2,25794E-31
Macro_FOLR2+APOE+	CHCHD6	8,81181E-232	5,25708E-01	0,332	0,077	1,62287E-227
Macro_FOLR2+APOE+	ADRM1	1,26336E-26	5,25076E-01	0,702	0,421	2,32673E-22
Macro_FOLR2+APOE+	RBM39	3,51149E-08	5,24997E-01	0,778	0,618	6,46710E-04
Macro_FOLR2+APOE+	CSTB	2,34957E-116	5,24937E-01	0,977	0,784	4,32721E-112
Macro_FOLR2+APOE+	TSPO	4,07294E-15	5,24931E-01	0,944	0,761	7,50114E-11
Macro_FOLR2+APOE+	PPP2CA	2,71270E-22	5,24882E-01	0,548	0,328	4,99598E-18
Macro_FOLR2+APOE+	POMP	6,25992E-39	5,24569E-01	0,937	0,714	1,15289E-34
Macro_FOLR2+APOE+	CAPZA1	3,98998E-12	5,24195E-01	0,734	0,487	7,34835E-08
Macro_FOLR2+APOE+	LPCAT2	1,05264E-24	5,23339E-01	0,522	0,303	1,93865E-20
Macro_FOLR2+APOE+	BCKDK	7,27014E-62	5,23278E-01	0,606	0,311	1,33894E-57

Macro_FOLR2+APOE+	ATP5F1B	2,85302E-176	5,22637E-01	0,705	0,302	5,25441E-172
Macro_FOLR2+APOE+	ABHD5	1,62374E-48	5,22574E-01	0,357	0,172	2,99044E-44
Macro_FOLR2+APOE+	POLR2G	1,45560E-20	5,22332E-01	0,592	0,366	2,68079E-16
Macro_FOLR2+APOE+	GPR183	1,70787E-07	5,21870E-01	0,643	0,451	3,14539E-03
Macro_FOLR2+APOE+	DTD1	3,20532E-33	5,21800E-01	0,296	0,151	5,90324E-29
Macro_FOLR2+APOE+	PARL	7,56764E-60	5,21272E-01	0,554	0,282	1,39373E-55
Macro_FOLR2+APOE+	ELAC2	9,18672E-39	5,20995E-01	0,255	0,12	1,69192E-34
Macro_FOLR2+APOE+	TMEM123	1,17632E-32	5,20961E-01	0,743	0,446	2,16642E-28
Macro_FOLR2+APOE+	TMC6	4,97171E-83	5,20930E-01	0,489	0,219	9,15639E-79
Macro_FOLR2+APOE+	PPM1M	1,61390E-86	5,20183E-01	0,417	0,175	2,97232E-82
Macro_FOLR2+APOE+	EEA1	2,50534E-49	5,19570E-01	0,447	0,227	4,61409E-45
Macro_FOLR2+APOE+	TXNDC12	1,05910E-20	5,19106E-01	0,527	0,336	1,95054E-16
Macro_FOLR2+APOE+	SSR3	2,19399E-15	5,18878E-01	0,862	0,589	4,04068E-11
Macro_FOLR2+APOE+	IL10RA	1,98491E-38	5,18866E-01	0,756	0,456	3,65561E-34
Macro_FOLR2+APOE+	SLA	3,00367E-30	5,18640E-01	0,674	0,409	5,53186E-26
Macro_FOLR2+APOE+	ACTR1A	3,22503E-53	5,17841E-01	0,545	0,281	5,93955E-49
Macro_FOLR2+APOE+	GAB2	8,03496E-25	5,17601E-01	0,291	0,161	1,47980E-20
Macro_FOLR2+APOE+	PSMC6	6,75459E-35	5,17381E-01	0,456	0,249	1,24399E-30
Macro_FOLR2+APOE+	STARD7	1,53549E-46	5,17366E-01	0,556	0,298	2,82791E-42
Macro_FOLR2+APOE+	ISOC2	1,07511E-106	5,17285E-01	0,504	0,209	1,98004E-102
Macro_FOLR2+APOE+	UBE2E3	1,48359E-12	5,17046E-01	0,372	0,238	2,73232E-08
Macro_FOLR2+APOE+	NIT2	1,29855E-60	5,17009E-01	0,372	0,166	2,39155E-56
Macro_FOLR2+APOE+	SIGLEC9	2,76316E-50	5,16354E-01	0,421	0,209	5,08891E-46
Macro_FOLR2+APOE+	TMEM251	1,76842E-134	5,16266E-01	0,38	0,126	3,25689E-130
Macro_FOLR2+APOE+	TMEM86A	4,76256E-224	5,16065E-01	0,407	0,106	8,77121E-220
Macro_FOLR2+APOE+	SEC62	8,84488E-12	5,15732E-01	0,812	0,547	1,62896E-07
Macro_FOLR2+APOE+	MANF	2,13014E-50	5,15567E-01	0,545	0,288	3,92307E-46
Macro_FOLR2+APOE+	LEPROTL1	3,15967E-51	5,15247E-01	0,704	0,39	5,81916E-47
Macro_FOLR2+APOE+	PFKFB3	3,96848E-31	5,15134E-01	0,545	0,328	7,30875E-27
Macro_FOLR2+APOE+	SLC25A19	6,26828E-58	5,14828E-01	0,35	0,159	1,15443E-53
Macro_FOLR2+APOE+	HDAC1	3,09593E-16	5,14393E-01	0,274	0,164	5,70177E-12
Macro_FOLR2+APOE+	SPRED1	9,65694E-179	5,13268E-01	0,421	0,132	1,77852E-174
Macro_FOLR2+APOE+	KEAP1	9,07347E-76	5,13238E-01	0,356	0,147	1,67106E-71
Macro_FOLR2+APOE+	CD33	5,55236E-27	5,12670E-01	0,526	0,301	1,02258E-22
Macro_FOLR2+APOE+	GUCD1	2,26304E-44	5,12580E-01	0,264	0,119	4,16784E-40
Macro_FOLR2+APOE+	TIMP2	8,08054E-185	5,12144E-01	0,804	0,348	1,48819E-180
Macro_FOLR2+APOE+	BCL2L1	3,40410E-86	5,12125E-01	0,484	0,211	6,26933E-82
Macro_FOLR2+APOE+	CLEC2B	3,74349E-56	5,11696E-01	0,799	0,46	6,89439E-52
Macro_FOLR2+APOE+	SQOR	2,35257E-303	5,11545E-01	0,607	0,18	4,33272E-299
Macro_FOLR2+APOE+	NTPCR	6,49940E-64	5,11175E-01	0,337	0,144	1,19699E-59
Macro_FOLR2+APOE+	UBE2K	9,82864E-33	5,10864E-01	0,499	0,279	1,81014E-28
Macro_FOLR2+APOE+	RAB10	2,72824E-43	5,10792E-01	0,797	0,481	5,02460E-39
Macro_FOLR2+APOE+	SUGT1	1,48493E-27	5,09977E-01	0,481	0,278	2,73480E-23
Macro_FOLR2+APOE+	SHKBP1	1,43746E-13	5,09957E-01	0,561	0,364	2,64737E-09
Macro_FOLR2+APOE+	UBE2N	6,51462E-13	5,09819E-01	0,574	0,368	1,19980E-08
Macro_FOLR2+APOE+	TMEM14C	4,57034E-09	5,09718E-01	0,774	0,522	8,41719E-05
Macro_FOLR2+APOE+	EVI5	8,63251E-30	5,09430E-01	0,315	0,168	1,58985E-25

Macro_FOLR2+APOE+	MR1	1,50256E-119	5,09418E-01	0,438	0,162	2,76726E-115
Macro_FOLR2+APOE+	ST3GAL1	6,86473E-71	5,08983E-01	0,475	0,223	1,26428E-66
Macro_FOLR2+APOE+	HIBADH	5,84140E-83	5,08920E-01	0,29	0,106	1,07581E-78
Macro_FOLR2+APOE+	STX11	1,00236E-14	5,08686E-01	0,509	0,323	1,84605E-10
Macro_FOLR2+APOE+	HAUS4	8,08278E-23	5,08371E-01	0,374	0,217	1,48861E-18
Macro_FOLR2+APOE+	FUBP1	4,61035E-08	5,07901E-01	0,26	0,177	8,49088E-04
Macro_FOLR2+APOE+	GTF3A	9,32496E-15	5,07812E-01	0,722	0,473	1,71738E-10
Macro_FOLR2+APOE+	SAR1B	8,64760E-45	5,06871E-01	0,456	0,234	1,59263E-40
Macro_FOLR2+APOE+	SIDT2	1,84038E-68	5,06583E-01	0,385	0,169	3,38942E-64
Macro_FOLR2+APOE+	GNA13	7,70121E-29	5,06409E-01	0,554	0,328	1,41833E-24
Macro_FOLR2+APOE+	SGMS1	6,41977E-42	5,06178E-01	0,259	0,118	1,18233E-37
Macro_FOLR2+APOE+	TMEM140	1,39993E-133	5,05013E-01	0,355	0,113	2,57825E-129
Macro_FOLR2+APOE+	MAPRE1	3,78544E-26	5,05000E-01	0,632	0,382	6,97164E-22
Macro_FOLR2+APOE+	MPV17	2,78233E-70	5,04532E-01	0,611	0,301	5,12421E-66
Macro_FOLR2+APOE+	GBP4	8,95427E-171	5,04033E-01	0,685	0,265	1,64911E-166
Macro_FOLR2+APOE+	POLR2A	4,82733E-10	5,03496E-01	0,457	0,304	8,89049E-06
Macro_FOLR2+APOE+	SPTLC1	6,44673E-45	5,03152E-01	0,322	0,154	1,18729E-40
Macro_FOLR2+APOE+	SNRPD1	1,69838E-18	5,02843E-01	0,622	0,385	3,12791E-14
Macro_FOLR2+APOE+	SCAND1	2,60170E-20	5,02825E-01	0,804	0,527	4,79155E-16
Macro_FOLR2+APOE+	IRF9	2,17125E-74	5,02718E-01	0,395	0,175	3,99879E-70
Macro_FOLR2+APOE+	NRP1	2,04717E-186	5,02586E-01	0,476	0,155	3,77027E-182
Macro_FOLR2+APOE+	MRPL9	1,99318E-35	5,02494E-01	0,363	0,19	3,67084E-31
Macro_FOLR2+APOE+	FBXW2	3,45483E-38	5,02205E-01	0,286	0,139	6,36277E-34
Macro_FOLR2+APOE+	GHITM	2,02500E-31	5,01865E-01	0,78	0,496	3,72945E-27
Macro_FOLR2+APOE+	AHR	8,33375E-30	5,01858E-01	0,568	0,349	1,53483E-25
Macro_FOLR2+APOE+	TBC1D2B	9,02491E-143	5,01159E-01	0,391	0,127	1,66212E-138
Macro_FOLR2+APOE+	LSM5	2,55470E-24	5,00853E-01	0,565	0,337	4,70499E-20
Macro_FOLR2+APOE+	LILRB5	7,39657E-273	5,00536E-01	0,331	0,067	1,36223E-268
Macro_FOLR2+APOE+	FAR1	1,43219E-17	4,99696E-01	0,311	0,188	2,63766E-13
Macro_FOLR2+APOE+	GGCX	2,58169E-49	4,99695E-01	0,264	0,115	4,75469E-45
Macro_FOLR2+APOE+	OSBPL3	1,65470E-32	4,99676E-01	0,266	0,134	3,04747E-28
Macro_FOLR2+APOE+	SSNA1	6,65541E-13	4,99623E-01	0,615	0,398	1,22573E-08
Macro_FOLR2+APOE+	PSMA1	6,23679E-44	4,99462E-01	0,613	0,354	1,14863E-39
Macro_FOLR2+APOE+	MRPS18C	9,13400E-66	4,99361E-01	0,59	0,293	1,68221E-61
Macro_FOLR2+APOE+	TXNRD1	1,43751E-66	4,99324E-01	0,406	0,184	2,64746E-62
Macro_FOLR2+APOE+	NDUFA5	5,04803E-40	4,99179E-01	0,528	0,286	9,29696E-36
Macro_FOLR2+APOE+	SUMO3	3,82115E-32	4,99126E-01	0,771	0,473	7,03742E-28
Macro_FOLR2+APOE+	ATP6V1G1	2,86175E-09	4,98967E-01	0,94	0,713	5,27048E-05
Macro_FOLR2+APOE+	TMEM33	1,17787E-59	4,98924E-01	0,445	0,212	2,16929E-55
Macro_FOLR2+APOE+	PTPN18	5,11631E-33	4,98455E-01	0,599	0,344	9,42271E-29
Macro_FOLR2+APOE+	SCIMP	2,06849E-79	4,97667E-01	0,572	0,269	3,80953E-75
Macro_FOLR2+APOE+	BTK	3,62965E-16	4,97583E-01	0,487	0,306	6,68473E-12
Macro_FOLR2+APOE+	CHID1	5,58816E-86	4,97369E-01	0,466	0,2	1,02917E-81
Macro_FOLR2+APOE+	ITFG1	6,48673E-75	4,96739E-01	0,464	0,207	1,19466E-70
Macro_FOLR2+APOE+	TRNAU1AP	1,26253E-47	4,96227E-01	0,345	0,165	2,32520E-43
Macro_FOLR2+APOE+	SELENOS	8,77634E-259	4,95778E-01	0,639	0,215	1,61634E-254
Macro_FOLR2+APOE+	PTPN6	3,31765E-14	4,94868E-01	0,769	0,503	6,11011E-10

Macro_FOLR2+APOE+	RPL12	4,51731E-16	4,94862E-01	0,829	0,568	8,31953E-12
Macro_FOLR2+APOE+	CISD2	2,23389E-112	4,94750E-01	0,737	0,359	4,11416E-108
Macro_FOLR2+APOE+	LAMTOR3	8,31376E-37	4,94597E-01	0,463	0,252	1,53115E-32
Macro_FOLR2+APOE+	RABAC1	2,23738E-17	4,94506E-01	0,786	0,517	4,12058E-13
Macro_FOLR2+APOE+	AKAP13	3,27530E-14	4,94374E-01	0,759	0,629	6,03212E-10
Macro_FOLR2+APOE+	ASPH	1,16442E-54	4,94349E-01	0,44	0,217	2,14451E-50
Macro_FOLR2+APOE+	ADAM9	7,67733E-109	4,94060E-01	0,526	0,22	1,41393E-104
Macro_FOLR2+APOE+	RPL38	4,46478E-42	4,93572E-01	0,799	0,483	8,22279E-38
Macro_FOLR2+APOE+	CHTOP	1,73757E-21	4,93518E-01	0,356	0,208	3,20007E-17
Macro_FOLR2+APOE+	GSS	1,19664E-57	4,93466E-01	0,36	0,162	2,20384E-53
Macro_FOLR2+APOE+	RTN3	3,62222E-40	4,93329E-01	0,711	0,41	6,67105E-36
Macro_FOLR2+APOE+	SOCS3	8,23329E-19	4,93329E-01	0,549	0,352	1,51633E-14
Macro_FOLR2+APOE+	VIM	1,49872E-50	4,92823E-01	0,974	0,942	2,76019E-46
Macro_FOLR2+APOE+	SUCLG1	1,10573E-39	4,92687E-01	0,551	0,304	2,03642E-35
Macro_FOLR2+APOE+	NFKBIE	2,48395E-102	4,92127E-01	0,446	0,184	4,57470E-98
Macro_FOLR2+APOE+	COPS6	3,60256E-43	4,91769E-01	0,611	0,335	6,63484E-39
Macro_FOLR2+APOE+	CCDC28A	3,88155E-67	4,91470E-01	0,451	0,207	7,14866E-63
Macro_FOLR2+APOE+	HPS3	4,17143E-44	4,91447E-01	0,355	0,178	7,68252E-40
Macro_FOLR2+APOE+	RPAIN	6,82049E-89	4,89960E-01	0,361	0,141	1,25613E-84
Macro_FOLR2+APOE+	SPCS2	8,89622E-34	4,89683E-01	0,783	0,484	1,63842E-29
Macro_FOLR2+APOE+	PDZD11	1,19071E-89	4,89559E-01	0,426	0,176	2,19292E-85
Macro_FOLR2+APOE+	PLEKHB2	1,35158E-39	4,89366E-01	0,731	0,432	2,48920E-35
Macro_FOLR2+APOE+	KIN	2,39190E-32	4,89167E-01	0,272	0,137	4,40516E-28
Macro_FOLR2+APOE+	GMPPA	2,52777E-68	4,88686E-01	0,318	0,131	4,65539E-64
Macro_FOLR2+APOE+	EDEM1	6,64406E-17	4,88611E-01	0,309	0,189	1,22364E-12
Macro_FOLR2+APOE+	NCF4	4,56035E-57	4,88566E-01	0,741	0,413	8,39879E-53
Macro_FOLR2+APOE+	IAH1	3,48618E-25	4,88416E-01	0,516	0,303	6,42049E-21
Macro_FOLR2+APOE+	FBXO7	3,27598E-45	4,88370E-01	0,582	0,315	6,03337E-41
Macro_FOLR2+APOE+	NSMCE4A	4,02896E-28	4,88336E-01	0,288	0,154	7,42013E-24
Macro_FOLR2+APOE+	GANAB	3,74115E-78	4,88293E-01	0,441	0,194	6,89008E-74
Macro_FOLR2+APOE+	PHKG2	8,08679E-55	4,88084E-01	0,335	0,152	1,48934E-50
Macro_FOLR2+APOE+	MFSD12	1,05995E-141	4,87104E-01	0,492	0,181	1,95212E-137
Macro_FOLR2+APOE+	VPS4A	9,21023E-64	4,86974E-01	0,395	0,18	1,69625E-59
Macro_FOLR2+APOE+	MRPL32	9,58554E-32	4,86352E-01	0,328	0,172	1,76537E-27
Macro_FOLR2+APOE+	CCNK	1,17440E-14	4,86072E-01	0,305	0,192	2,16289E-10
Macro_FOLR2+APOE+	DNMT1	3,74358E-62	4,85963E-01	0,601	0,309	6,89455E-58
Macro_FOLR2+APOE+	PPIF	1,36753E-23	4,85552E-01	0,588	0,351	2,51858E-19
Macro_FOLR2+APOE+	TLR2	2,54894E-17	4,85392E-01	0,606	0,38	4,69438E-13
Macro_FOLR2+APOE+	CAPNS1	5,79290E-32	4,84372E-01	0,625	0,362	1,06688E-27
Macro_FOLR2+APOE+	PSMB3	6,72746E-21	4,84188E-01	0,885	0,636	1,23900E-16
Macro_FOLR2+APOE+	RPL27A	4,93100E-88	4,84141E-01	0,796	0,454	9,08141E-84
Macro_FOLR2+APOE+	NDUFA8	1,20142E-60	4,84133E-01	0,56	0,283	2,21266E-56
Macro_FOLR2+APOE+	INF2	1,24184E-38	4,84025E-01	0,263	0,127	2,28710E-34
Macro_FOLR2+APOE+	RAB32	2,73806E-79	4,83933E-01	0,715	0,365	5,04269E-75
Macro_FOLR2+APOE+	RPS4Y1	1,45871E-94	4,83355E-01	0,404	0,169	2,68650E-90
Macro_FOLR2+APOE+	NSFL1C	7,75992E-62	4,82839E-01	0,545	0,267	1,42914E-57
Macro_FOLR2+APOE+	ADPRH	7,72275E-33	4,82300E-01	0,26	0,131	1,42230E-28

Macro_FOLR2+APOE+	STARD3	9,06104E-95	4,82134E-01	0,53	0,234	1,66877E-90
Macro_FOLR2+APOE+	MCFD2	2,31041E-103	4,80870E-01	0,489	0,198	4,25508E-99
Macro_FOLR2+APOE+	TMEM9	2,79339E-47	4,80626E-01	0,257	0,113	5,14458E-43
Macro_FOLR2+APOE+	TM9SF2	2,92828E-36	4,80489E-01	0,703	0,419	5,39301E-32
Macro_FOLR2+APOE+	UQCR10	1,83362E-32	4,78587E-01	0,932	0,721	3,37699E-28
Macro_FOLR2+APOE+	ZFR	4,41935E-18	4,78586E-01	0,401	0,245	8,13913E-14
Macro_FOLR2+APOE+	MRPS16	5,26451E-44	4,78243E-01	0,545	0,294	9,69564E-40
Macro_FOLR2+APOE+	LPXN	1,00177E-26	4,78069E-01	0,577	0,341	1,84495E-22
Macro_FOLR2+APOE+	FIBP	9,01171E-23	4,77455E-01	0,606	0,361	1,65969E-18
Macro_FOLR2+APOE+	CHKA	1,25665E-45	4,77204E-01	0,259	0,116	2,31437E-41
Macro_FOLR2+APOE+	SEC22C	7,60461E-58	4,77056E-01	0,284	0,12	1,40054E-53
Macro_FOLR2+APOE+	LILRA6	3,53057E-57	4,76419E-01	0,336	0,155	6,50226E-53
Macro_FOLR2+APOE+	ADD1	8,41219E-20	4,76108E-01	0,373	0,223	1,54927E-15
Macro_FOLR2+APOE+	GNPAT	3,56788E-29	4,75694E-01	0,292	0,154	6,57097E-25
Macro_FOLR2+APOE+	PPIE	1,27443E-52	4,75433E-01	0,365	0,171	2,34711E-48
Macro_FOLR2+APOE+	RAB11A	1,13486E-18	4,75346E-01	0,633	0,395	2,09008E-14
Macro_FOLR2+APOE+	CMTM3	3,65342E-43	4,75304E-01	0,634	0,354	6,72850E-39
Macro_FOLR2+APOE+	ZDHHC24	9,12964E-116	4,74789E-01	0,539	0,222	1,68141E-111
Macro_FOLR2+APOE+	UXS1	9,27767E-64	4,74767E-01	0,266	0,105	1,70867E-59
Macro_FOLR2+APOE+	MTHFD2	1,13487E-20	4,74270E-01	0,631	0,398	2,09009E-16
Macro_FOLR2+APOE+	STX12	5,54331E-62	4,73774E-01	0,602	0,304	1,02091E-57
Macro_FOLR2+APOE+	TSPAN31	8,66657E-57	4,73616E-01	0,32	0,14	1,59612E-52
Macro_FOLR2+APOE+	MAD1L1	1,45354E-07	4,73444E-01	0,263	0,182	2,67699E-03
Macro_FOLR2+APOE+	VCP	2,17011E-17	4,73151E-01	0,716	0,459	3,99669E-13
Macro_FOLR2+APOE+	MRPS33	1,10864E-55	4,72744E-01	0,458	0,224	2,04179E-51
Macro_FOLR2+APOE+	SLC25A24	1,59823E-82	4,72376E-01	0,481	0,218	2,94347E-78
Macro_FOLR2+APOE+	OVCA2	1,79252E-19	4,72362E-01	0,259	0,15	3,30128E-15
Macro_FOLR2+APOE+	COMMD8	1,37862E-35	4,71131E-01	0,416	0,221	2,53901E-31
Macro_FOLR2+APOE+	BTG3	8,93767E-14	4,70924E-01	0,264	0,163	1,64605E-09
Macro_FOLR2+APOE+	RGS10	9,08902E-15	4,70465E-01	0,953	0,734	1,67392E-10
Macro_FOLR2+APOE+	PRKACB	5,90427E-41	4,70414E-01	0,339	0,168	1,08739E-36
Macro_FOLR2+APOE+	GAPVD1	3,81125E-34	4,70357E-01	0,329	0,17	7,01919E-30
Macro_FOLR2+APOE+	MACF1	5,15229E-41	4,70238E-01	0,557	0,314	9,48897E-37
Macro_FOLR2+APOE+	SLC39A3	1,69198E-101	4,69272E-01	0,318	0,112	3,11611E-97
Macro_FOLR2+APOE+	HNRNPR	1,07702E-11	4,68906E-01	0,551	0,355	1,98355E-07
Macro_FOLR2+APOE+	NEDD8	2,69080E-15	4,68782E-01	0,82	0,58	4,95565E-11
Macro_FOLR2+APOE+	SNX5	1,38674E-62	4,67736E-01	0,718	0,389	2,55395E-58
Macro_FOLR2+APOE+	TSPAN3	1,56295E-59	4,67706E-01	0,597	0,31	2,87848E-55
Macro_FOLR2+APOE+	BST2	2,65986E-42	4,67140E-01	0,949	0,719	4,89867E-38
Macro_FOLR2+APOE+	SRP9	1,84252E-16	4,66485E-01	0,716	0,458	3,39337E-12
Macro_FOLR2+APOE+	SLAIN2	6,69823E-28	4,66108E-01	0,264	0,138	1,23361E-23
Macro_FOLR2+APOE+	IFFO1	1,08113E-112	4,66011E-01	0,408	0,151	1,99111E-108
Macro_FOLR2+APOE+	ATP5PD	3,10384E-171	4,65824E-01	0,703	0,308	5,71634E-167
Macro_FOLR2+APOE+	TFEB	2,47685E-95	4,65443E-01	0,369	0,143	4,56161E-91
Macro_FOLR2+APOE+	PITPNA	1,53149E-48	4,64966E-01	0,315	0,146	2,82055E-44
Macro_FOLR2+APOE+	GDE1	2,48291E-129	4,64890E-01	0,545	0,213	4,57277E-125
Macro_FOLR2+APOE+	PLXNB2	2,37574E-47	4,64467E-01	0,493	0,258	4,37540E-43

Macro_FOLR2+APOE+	TSNAX	1,23015E-79	4,64329E-01	0,333	0,131	2,26557E-75
Macro_FOLR2+APOE+	DEDD	2,80864E-16	4,63832E-01	0,274	0,164	5,17266E-12
Macro_FOLR2+APOE+	MKNK1	5,20193E-131	4,63690E-01	0,513	0,201	9,58040E-127
Macro_FOLR2+APOE+	NDUFB8	3,64548E-109	4,63598E-01	0,578	0,279	6,71388E-105
Macro_FOLR2+APOE+	UROS	1,37500E-100	4,63592E-01	0,48	0,196	2,53235E-96
Macro_FOLR2+APOE+	XIAP	1,27273E-27	4,63487E-01	0,316	0,172	2,34399E-23
Macro_FOLR2+APOE+	TMSB10	3,34145E-21	4,63423E-01	0,999	0,984	6,15395E-17
Macro_FOLR2+APOE+	SRSF6	3,57437E-10	4,63318E-01	0,307	0,206	6,58292E-06
Macro_FOLR2+APOE+	CUL1	6,83099E-24	4,63127E-01	0,371	0,214	1,25806E-19
Macro_FOLR2+APOE+	ETNK1	3,37640E-39	4,62757E-01	0,454	0,242	6,21832E-35
Macro_FOLR2+APOE+	CREBL2	2,12989E-151	4,62355E-01	0,673	0,28	3,92261E-147
Macro_FOLR2+APOE+	HDLBP	2,24258E-50	4,62256E-01	0,712	0,401	4,13015E-46
Macro_FOLR2+APOE+	SLC25A36	1,08561E-20	4,62157E-01	0,319	0,186	1,99936E-16
Macro_FOLR2+APOE+	EPS15	3,94326E-19	4,62041E-01	0,397	0,244	7,26230E-15
Macro_FOLR2+APOE+	CD276	1,96837E-101	4,61254E-01	0,296	0,101	3,62515E-97
Macro_FOLR2+APOE+	ERGIC3	2,71999E-20	4,61188E-01	0,769	0,49	5,00941E-16
Macro_FOLR2+APOE+	WIPF1	2,69013E-36	4,60889E-01	0,744	0,457	4,95441E-32
Macro_FOLR2+APOE+	STYXL1	1,97407E-31	4,60750E-01	0,303	0,157	3,63565E-27
Macro_FOLR2+APOE+	N4BP1	9,90434E-53	4,60663E-01	0,268	0,116	1,82408E-48
Macro_FOLR2+APOE+	TBC1D9B	5,59588E-42	4,60611E-01	0,433	0,227	1,03059E-37
Macro_FOLR2+APOE+	FMN1	3,82402E-90	4,60494E-01	0,371	0,146	7,04270E-86
Macro_FOLR2+APOE+	RBM3	4,16060E-09	4,60254E-01	0,836	0,67	7,66258E-05
Macro_FOLR2+APOE+	RNF4	4,04629E-11	4,60004E-01	0,275	0,179	7,45205E-07
Macro_FOLR2+APOE+	IDH2	5,85977E-44	4,59797E-01	0,709	0,403	1,07919E-39
Macro_FOLR2+APOE+	IL12RB1	1,71284E-92	4,59654E-01	0,27	0,091	3,15453E-88
Macro_FOLR2+APOE+	HVCN1	8,43146E-125	4,59504E-01	0,589	0,244	1,55282E-120
Macro_FOLR2+APOE+	MLST8	4,58507E-53	4,59396E-01	0,3	0,133	8,44432E-49
Macro_FOLR2+APOE+	OLA1	4,68406E-18	4,58760E-01	0,495	0,304	8,62663E-14
Macro_FOLR2+APOE+	MVP	1,77823E-81	4,58138E-01	0,72	0,364	3,27496E-77
Macro_FOLR2+APOE+	SNRPA	4,41630E-08	4,58058E-01	0,432	0,292	8,13350E-04
Macro_FOLR2+APOE+	JAGN1	1,73834E-34	4,57922E-01	0,387	0,206	3,20151E-30
Macro_FOLR2+APOE+	SAR1A	1,90684E-76	4,57596E-01	0,51	0,236	3,51182E-72
Macro_FOLR2+APOE+	HNRNPLL	1,41943E-31	4,57250E-01	0,287	0,148	2,61416E-27
Macro_FOLR2+APOE+	SCFD1	5,75953E-35	4,57146E-01	0,301	0,152	1,06073E-30
Macro_FOLR2+APOE+	COX8A	1,39294E-06	4,57097E-01	0,943	0,773	2,56538E-02
Macro_FOLR2+APOE+	VMP1	1,52766E-14	4,56943E-01	0,802	0,543	2,81350E-10
Macro_FOLR2+APOE+	ARHGAP10	2,23380E-86	4,56730E-01	0,328	0,126	4,11399E-82
Macro_FOLR2+APOE+	MRPS7	1,44409E-82	4,56357E-01	0,621	0,301	2,65959E-78
Macro_FOLR2+APOE+	TCIRG1	4,17257E-45	4,56340E-01	0,749	0,459	7,68463E-41
Macro_FOLR2+APOE+	EIF6	5,10701E-15	4,56023E-01	0,632	0,399	9,40558E-11
Macro_FOLR2+APOE+	SUMF2	2,18200E-52	4,55793E-01	0,554	0,287	4,01858E-48
Macro_FOLR2+APOE+	HPS1	1,41635E-42	4,55692E-01	0,613	0,344	2,60848E-38
Macro_FOLR2+APOE+	DPAGT1	9,14240E-64	4,55513E-01	0,296	0,122	1,68376E-59
Macro_FOLR2+APOE+	MCOLN1	3,67441E-136	4,55155E-01	0,634	0,264	6,76717E-132
Macro_FOLR2+APOE+	CCDC90B	4,96450E-37	4,54982E-01	0,446	0,237	9,14312E-33
Macro_FOLR2+APOE+	IDH3B	3,41240E-26	4,54526E-01	0,446	0,256	6,28462E-22
Macro_FOLR2+APOE+	GOLT1B	3,61684E-39	4,54400E-01	0,335	0,169	6,66113E-35

Macro_FOLR2+APOE+	KCNE3	5,03870E-50	4,54099E-01	0,279	0,122	9,27978E-46
Macro_FOLR2+APOE+	CAPZA2	7,42986E-26	4,54015E-01	0,822	0,541	1,36836E-21
Macro_FOLR2+APOE+	SNX1	1,23244E-57	4,53974E-01	0,575	0,299	2,26978E-53
Macro_FOLR2+APOE+	GMPR2	2,68644E-49	4,53523E-01	0,43	0,214	4,94762E-45
Macro_FOLR2+APOE+	GMIP	5,11971E-24	4,53083E-01	0,371	0,215	9,42898E-20
Macro_FOLR2+APOE+	SEC23B	2,11293E-42	4,52900E-01	0,342	0,171	3,89139E-38
Macro_FOLR2+APOE+	IGFLR1	5,26379E-70	4,52607E-01	0,641	0,351	9,69432E-66
Macro_FOLR2+APOE+	CISD3	2,51937E-93	4,52422E-01	0,631	0,296	4,63992E-89
Macro_FOLR2+APOE+	PDK3	6,10891E-67	4,52129E-01	0,288	0,116	1,12508E-62
Macro_FOLR2+APOE+	GSAP	2,59741E-106	4,51465E-01	0,461	0,184	4,78366E-102
Macro_FOLR2+APOE+	TYK2	1,21714E-09	4,51117E-01	0,293	0,196	2,24161E-05
Macro_FOLR2+APOE+	TMEM167A	2,86945E-19	4,51047E-01	0,635	0,393	5,28468E-15
Macro_FOLR2+APOE+	RTCB	9,30672E-62	4,50408E-01	0,564	0,287	1,71402E-57
Macro_FOLR2+APOE+	C18orf32	5,23700E-45	4,50368E-01	0,501	0,274	9,64499E-41
Macro_FOLR2+APOE+	MRPL52	4,06681E-15	4,50192E-01	0,71	0,452	7,48985E-11
Macro_FOLR2+APOE+	SEPHS2	6,05902E-119	4,49748E-01	0,531	0,219	1,11589E-114
Macro_FOLR2+APOE+	MFSD5	2,30122E-62	4,49088E-01	0,293	0,122	4,23815E-58
Macro_FOLR2+APOE+	PPP2R3C	6,71486E-43	4,48758E-01	0,42	0,212	1,23668E-38
Macro_FOLR2+APOE+	SMIM19	9,46486E-41	4,48386E-01	0,484	0,253	1,74314E-36
Macro_FOLR2+APOE+	ACAA1	2,71508E-23	4,47805E-01	0,618	0,371	5,00037E-19
Macro_FOLR2+APOE+	DDX6	3,34448E-07	4,47306E-01	0,329	0,232	6,15953E-03
Macro_FOLR2+APOE+	COA6	3,30944E-122	4,46642E-01	0,582	0,243	6,09500E-118
Macro_FOLR2+APOE+	HPRT1	4,75355E-22	4,45999E-01	0,438	0,258	8,75461E-18
Macro_FOLR2+APOE+	PLSCR1	1,12820E-11	4,45863E-01	0,782	0,535	2,07781E-07
Macro_FOLR2+APOE+	DOK2	2,83301E-48	4,45622E-01	0,615	0,337	5,21755E-44
Macro_FOLR2+APOE+	GSTM4	4,83509E-162	4,45609E-01	0,336	0,095	8,90478E-158
Macro_FOLR2+APOE+	CTBP1	1,48796E-44	4,45596E-01	0,35	0,173	2,74037E-40
Macro_FOLR2+APOE+	PSMB5	7,83843E-89	4,45232E-01	0,64	0,306	1,44360E-84
Macro_FOLR2+APOE+	NCSTN	7,68587E-76	4,45217E-01	0,489	0,226	1,41551E-71
Macro_FOLR2+APOE+	ARL2BP	8,99092E-33	4,45033E-01	0,398	0,214	1,65586E-28
Macro_FOLR2+APOE+	PPP1R12A	3,42189E-13	4,44834E-01	0,581	0,371	6,30210E-09
Macro_FOLR2+APOE+	ACOX1	4,07114E-51	4,44711E-01	0,27	0,117	7,49782E-47
Macro_FOLR2+APOE+	RBM23	1,03261E-31	4,44350E-01	0,358	0,192	1,90177E-27
Macro_FOLR2+APOE+	PNKD	1,00436E-74	4,43814E-01	0,625	0,313	1,84973E-70
Macro_FOLR2+APOE+	CAPN1	2,82321E-56	4,43809E-01	0,517	0,26	5,19951E-52
Macro_FOLR2+APOE+	SDC4	8,82792E-113	4,42929E-01	0,282	0,089	1,62584E-108
Macro_FOLR2+APOE+	DGCR6L	1,51117E-77	4,42817E-01	0,549	0,253	2,78313E-73
Macro_FOLR2+APOE+	SNRPG	1,20334E-08	4,42399E-01	0,802	0,55	2,21619E-04
Macro_FOLR2+APOE+	NAA15	8,60982E-34	4,42238E-01	0,26	0,129	1,58567E-29
Macro_FOLR2+APOE+	DHRS4L2	2,29761E-87	4,41634E-01	0,387	0,156	4,23150E-83
Macro_FOLR2+APOE+	DDX5	9,09183E-38	4,41185E-01	0,953	0,829	1,67444E-33
Macro_FOLR2+APOE+	SNRNP200	1,13429E-24	4,40552E-01	0,429	0,254	2,08903E-20
Macro_FOLR2+APOE+	ARL2	8,46619E-127	4,40544E-01	0,486	0,188	1,55922E-122
Macro_FOLR2+APOE+	UNC50	3,40326E-50	4,40498E-01	0,362	0,172	6,26779E-46
Macro_FOLR2+APOE+	ATP5IF1	5,34164E-219	4,40176E-01	0,673	0,253	9,83769E-215
Macro_FOLR2+APOE+	DST	3,23570E-39	4,39961E-01	0,396	0,209	5,95919E-35
Macro_FOLR2+APOE+	MTG1	4,23806E-47	4,39758E-01	0,295	0,136	7,80523E-43

Macro_FOLR2+APOE+	ASCC2	7,61505E-22	4,39604E-01	0,357	0,207	1,40246E-17
Macro_FOLR2+APOE+	CBFB	2,21416E-38	4,39478E-01	0,309	0,153	4,07781E-34
Macro_FOLR2+APOE+	CD209	3,71153E-220	4,39145E-01	0,258	0,051	6,83553E-216
Macro_FOLR2+APOE+	RPL36AL	1,20106E-34	4,39091E-01	0,792	0,484	2,21199E-30
Macro_FOLR2+APOE+	MRPS5	6,92794E-34	4,38953E-01	0,458	0,249	1,27592E-29
Macro_FOLR2+APOE+	SRC	3,76499E-46	4,38729E-01	0,26	0,116	6,93398E-42
Macro_FOLR2+APOE+	RBM5	2,67813E-13	4,38137E-01	0,313	0,199	4,93231E-09
Macro_FOLR2+APOE+	DTX2	6,51345E-90	4,38031E-01	0,394	0,158	1,19958E-85
Macro_FOLR2+APOE+	SH3BP2	1,24210E-46	4,37935E-01	0,536	0,288	2,28757E-42
Macro_FOLR2+APOE+	YY1AP1	1,30887E-18	4,37925E-01	0,309	0,182	2,41054E-14
Macro_FOLR2+APOE+	SIRT2	5,21878E-63	4,37579E-01	0,46	0,219	9,61142E-59
Macro_FOLR2+APOE+	ZNF655	2,66187E-20	4,37563E-01	0,361	0,213	4,90236E-16
Macro_FOLR2+APOE+	EMC2	1,16112E-48	4,37538E-01	0,309	0,142	2,13843E-44
Macro_FOLR2+APOE+	HAPLN3	1,55807E-172	4,37482E-01	0,369	0,107	2,86951E-168
Macro_FOLR2+APOE+	SLC35B2	2,95341E-69	4,37195E-01	0,298	0,12	5,43929E-65
Macro_FOLR2+APOE+	NDUFV1	4,96476E-40	4,37056E-01	0,694	0,4	9,14360E-36
Macro_FOLR2+APOE+	NKIRAS2	3,74543E-15	4,36888E-01	0,324	0,202	6,89796E-11
Macro_FOLR2+APOE+	CHMP2B	1,49729E-51	4,36264E-01	0,562	0,291	2,75756E-47
Macro_FOLR2+APOE+	MICAL1	1,95593E-44	4,36004E-01	0,525	0,287	3,60223E-40
Macro_FOLR2+APOE+	ATP5PO	3,32929E-145	4,35759E-01	0,549	0,225	6,13156E-141
Macro_FOLR2+APOE+	RFC2	7,61715E-40	4,35615E-01	0,295	0,144	1,40285E-35
Macro_FOLR2+APOE+	ARRDC2	4,82283E-70	4,35524E-01	0,434	0,199	8,88221E-66
Macro_FOLR2+APOE+	DYNC1H1	9,49850E-18	4,35308E-01	0,582	0,374	1,74934E-13
Macro_FOLR2+APOE+	TBC1D5	8,52349E-35	4,34962E-01	0,368	0,195	1,56977E-30
Macro_FOLR2+APOE+	ZCCHC10	1,62970E-25	4,34344E-01	0,257	0,136	3,00142E-21
Macro_FOLR2+APOE+	GUK1	1,98869E-13	4,33754E-01	0,924	0,704	3,66256E-09
Macro_FOLR2+APOE+	SMURF2	7,98162E-84	4,33592E-01	0,254	0,089	1,46998E-79
Macro_FOLR2+APOE+	ITGAM	1,18019E-16	4,33513E-01	0,383	0,238	2,17355E-12
Macro_FOLR2+APOE+	SMAD2	4,41110E-23	4,33099E-01	0,342	0,194	8,12393E-19
Macro_FOLR2+APOE+	MBD4	9,53987E-54	4,32746E-01	0,484	0,243	1,75696E-49
Macro_FOLR2+APOE+	IGF2R	4,81081E-193	4,32621E-01	0,577	0,205	8,86007E-189
Macro_FOLR2+APOE+	TBRG4	1,59425E-41	4,32548E-01	0,272	0,127	2,93614E-37
Macro_FOLR2+APOE+	ZMYM2	3,52410E-13	4,32419E-01	0,314	0,2	6,49034E-09
Macro_FOLR2+APOE+	MYO1C	2,76358E-50	4,32338E-01	0,254	0,109	5,08969E-46
Macro_FOLR2+APOE+	LDHB	4,60344E-19	4,32023E-01	0,758	0,504	8,47816E-15
Macro_FOLR2+APOE+	TMEM109	1,44694E-34	4,31944E-01	0,632	0,361	2,66483E-30
Macro_FOLR2+APOE+	PDCD6IP	2,18007E-23	4,31885E-01	0,601	0,362	4,01503E-19
Macro_FOLR2+APOE+	PLEKHM2	1,62918E-137	4,31743E-01	0,415	0,142	3,00046E-133
Macro_FOLR2+APOE+	CLCN7	6,30344E-141	4,31579E-01	0,47	0,171	1,16090E-136
Macro_FOLR2+APOE+	STX10	1,31624E-30	4,31190E-01	0,532	0,303	2,42411E-26
Macro_FOLR2+APOE+	USO1	2,17886E-48	4,31065E-01	0,444	0,226	4,01281E-44
Macro_FOLR2+APOE+	CAMK2D	3,80006E-31	4,30750E-01	0,312	0,165	6,99857E-27
Macro_FOLR2+APOE+	PPP1R7	7,31544E-57	4,30717E-01	0,54	0,272	1,34728E-52
Macro_FOLR2+APOE+	SLC46A1	1,67300E-202	4,30607E-01	0,251	0,051	3,08117E-198
Macro_FOLR2+APOE+	RELA	5,74901E-17	4,30518E-01	0,362	0,221	1,05880E-12
Macro_FOLR2+APOE+	BST1	1,36569E-73	4,30412E-01	0,324	0,129	2,51519E-69
Macro_FOLR2+APOE+	UBE2D1	4,33790E-78	4,30045E-01	0,673	0,354	7,98911E-74

Macro_FOLR2+APOE+	BABAM1	1,19489E-63	4,29528E-01	0,424	0,198	2,20062E-59
Macro_FOLR2+APOE+	C1orf122	3,19371E-97	4,29434E-01	0,499	0,212	5,88185E-93
Macro_FOLR2+APOE+	CMC2	7,82050E-32	4,29122E-01	0,484	0,27	1,44030E-27
Macro_FOLR2+APOE+	MRPL27	1,56118E-70	4,28427E-01	0,575	0,28	2,87522E-66
Macro_FOLR2+APOE+	NPTN	2,99196E-41	4,28304E-01	0,418	0,223	5,51030E-37
Macro_FOLR2+APOE+	MTX1	2,64400E-67	4,28155E-01	0,426	0,195	4,86945E-63
Macro_FOLR2+APOE+	SPHK1	4,53325E-48	4,27126E-01	0,362	0,175	8,34888E-44
Macro_FOLR2+APOE+	STK10	2,25988E-07	4,26858E-01	0,349	0,241	4,16201E-03
Macro_FOLR2+APOE+	LILRB1	1,57456E-47	4,26719E-01	0,664	0,368	2,89988E-43
Macro_FOLR2+APOE+	C19orf25	9,78750E-19	4,26379E-01	0,284	0,166	1,80256E-14
Macro_FOLR2+APOE+	YIPF3	1,29182E-55	4,25373E-01	0,609	0,316	2,37915E-51
Macro_FOLR2+APOE+	GPR155	6,14836E-119	4,25354E-01	0,387	0,137	1,13234E-114
Macro_FOLR2+APOE+	YWHAH	1,70102E-55	4,25291E-01	0,833	0,523	3,13276E-51
Macro_FOLR2+APOE+	NMRK1	7,53621E-94	4,24714E-01	0,322	0,118	1,38794E-89
Macro_FOLR2+APOE+	NSF	1,74444E-24	4,24071E-01	0,279	0,154	3,21274E-20
Macro_FOLR2+APOE+	TNPO1	4,27725E-37	4,23920E-01	0,455	0,244	7,87742E-33
Macro_FOLR2+APOE+	CLDND1	1,99015E-11	4,23635E-01	0,28	0,18	3,66526E-07
Macro_FOLR2+APOE+	LRP10	1,60793E-90	4,23597E-01	0,6	0,286	2,96132E-86
Macro_FOLR2+APOE+	AP2S1	7,78598E-35	4,23250E-01	0,942	0,713	1,43394E-30
Macro_FOLR2+APOE+	ARF4	2,28737E-16	4,23073E-01	0,688	0,439	4,21266E-12
Macro_FOLR2+APOE+	ACOT9	5,12085E-29	4,22511E-01	0,481	0,273	9,43107E-25
Macro_FOLR2+APOE+	PRR13	1,44876E-47	4,22375E-01	0,874	0,572	2,66818E-43
Macro_FOLR2+APOE+	FN1	2,26328E-12	4,21900E-01	0,326	0,217	4,16828E-08
Macro_FOLR2+APOE+	TRAPPC3	2,41971E-49	4,21741E-01	0,645	0,345	4,45638E-45
Macro_FOLR2+APOE+	SMIM7	4,09934E-55	4,21730E-01	0,466	0,231	7,54976E-51
Macro_FOLR2+APOE+	CTBS	5,41875E-18	4,21109E-01	0,427	0,265	9,97971E-14
Macro_FOLR2+APOE+	SDHA	2,71302E-50	4,20945E-01	0,478	0,245	4,99656E-46
Macro_FOLR2+APOE+	SFT2D1	1,74150E-48	4,20763E-01	0,777	0,451	3,20731E-44
Macro_FOLR2+APOE+	CHD4	4,09045E-23	4,20729E-01	0,451	0,272	7,53339E-19
Macro_FOLR2+APOE+	RPL15	7,93859E-19	4,20400E-01	0,818	0,545	1,46205E-14
Macro_FOLR2+APOE+	AGA	6,08126E-51	4,20204E-01	0,251	0,106	1,11999E-46
Macro_FOLR2+APOE+	NDUFC1	1,47819E-44	4,19970E-01	0,764	0,449	2,72238E-40
Macro_FOLR2+APOE+	RFTN1	1,69114E-16	4,19938E-01	0,322	0,197	3,11457E-12
Macro_FOLR2+APOE+	DDB1	3,68828E-28	4,19893E-01	0,388	0,22	6,79270E-24
Macro_FOLR2+APOE+	APOL4	5,37866E-122	4,19603E-01	0,278	0,084	9,90589E-118
Macro_FOLR2+APOE+	EFHD2	3,59439E-59	4,19510E-01	0,741	0,421	6,61978E-55
Macro_FOLR2+APOE+	MTX2	2,07125E-51	4,19388E-01	0,269	0,116	3,81462E-47
Macro_FOLR2+APOE+	AKIRIN2	2,93688E-23	4,19354E-01	0,67	0,414	5,40884E-19
Macro_FOLR2+APOE+	MYO5A	1,82627E-97	4,18945E-01	0,463	0,192	3,36344E-93
Macro_FOLR2+APOE+	IFI6	6,26945E-87	4,18887E-01	0,836	0,478	1,15465E-82
Macro_FOLR2+APOE+	SLC22A18	1,10845E-187	4,18356E-01	0,504	0,164	2,04143E-183
Macro_FOLR2+APOE+	PLEKHA2	3,67645E-48	4,18328E-01	0,438	0,223	6,77092E-44
Macro_FOLR2+APOE+	RNF114	3,16805E-94	4,18141E-01	0,727	0,36	5,83460E-90
Macro_FOLR2+APOE+	RILPL2	2,16970E-06	4,18126E-01	0,659	0,45	3,99594E-02
Macro_FOLR2+APOE+	SELENOW	3,11677E-186	4,17934E-01	0,679	0,28	5,74016E-182
Macro_FOLR2+APOE+	PSMA6	1,20317E-06	4,17668E-01	0,631	0,461	2,21587E-02
Macro_FOLR2+APOE+	IL21R	1,45691E-178	4,17444E-01	0,278	0,067	2,68319E-174

Macro_FOLR2+APOE+	PUM1	8,49414E-21	4,17214E-01	0,397	0,238	1,56437E-16
Macro_FOLR2+APOE+	RPS9	5,97900E-29	4,16322E-01	0,826	0,546	1,10115E-24
Macro_FOLR2+APOE+	ENPP2	2,02798E-208	4,16254E-01	0,33	0,083	3,73494E-204
Macro_FOLR2+APOE+	PLIN3	2,03448E-31	4,16201E-01	0,624	0,364	3,74690E-27
Macro_FOLR2+APOE+	LARP7	3,43778E-47	4,16174E-01	0,514	0,269	6,33136E-43
Macro_FOLR2+APOE+	COMMD9	4,34185E-67	4,16063E-01	0,603	0,306	7,99638E-63
Macro_FOLR2+APOE+	PSMD8	5,83988E-23	4,15888E-01	0,791	0,518	1,07553E-18
Macro_FOLR2+APOE+	NDUFS8	1,57427E-41	4,15188E-01	0,804	0,486	2,89934E-37
Macro_FOLR2+APOE+	ESD	1,21760E-29	4,14989E-01	0,608	0,357	2,24245E-25
Macro_FOLR2+APOE+	CHMP1B	1,81991E-20	4,14934E-01	0,607	0,401	3,35173E-16
Macro_FOLR2+APOE+	MTMR6	2,80971E-51	4,14193E-01	0,294	0,131	5,17465E-47
Macro_FOLR2+APOE+	WDR46	1,25184E-25	4,14138E-01	0,28	0,151	2,30551E-21
Macro_FOLR2+APOE+	MRPS18B	1,46960E-59	4,14082E-01	0,545	0,269	2,70657E-55
Macro_FOLR2+APOE+	PLOD3	2,14542E-97	4,14045E-01	0,401	0,159	3,95123E-93
Macro_FOLR2+APOE+	EVI2B	4,74670E-38	4,13883E-01	0,643	0,369	8,74200E-34
Macro_FOLR2+APOE+	TRMT112	6,47130E-07	4,13765E-01	0,867	0,611	1,19182E-02
Macro_FOLR2+APOE+	C7orf50	9,82983E-48	4,12915E-01	0,609	0,329	1,81036E-43
Macro_FOLR2+APOE+	GPN3	6,03098E-64	4,12097E-01	0,381	0,17	1,11073E-59
Macro_FOLR2+APOE+	ATPAF2	6,87783E-40	4,11844E-01	0,296	0,144	1,26669E-35
Macro_FOLR2+APOE+	DUSP3	2,83593E-76	4,11784E-01	0,538	0,255	5,22293E-72
Macro_FOLR2+APOE+	CCM2	9,29736E-74	4,11769E-01	0,442	0,196	1,71230E-69
Macro_FOLR2+APOE+	ATP5F1A	3,97133E-188	4,11705E-01	0,634	0,244	7,31400E-184
Macro_FOLR2+APOE+	TRAPPC4	9,15559E-52	4,11325E-01	0,413	0,202	1,68619E-47
Macro_FOLR2+APOE+	YWHAZ	9,24381E-21	4,10749E-01	0,847	0,709	1,70243E-16
Macro_FOLR2+APOE+	MFSD10	5,56368E-35	4,10670E-01	0,477	0,264	1,02466E-30
Macro_FOLR2+APOE+	XRCC6	2,54872E-10	4,10144E-01	0,734	0,488	4,69397E-06
Macro_FOLR2+APOE+	ZMPSTE24	1,29093E-51	4,10143E-01	0,31	0,14	2,37750E-47
Macro_FOLR2+APOE+	TFPT	1,92915E-141	4,09922E-01	0,581	0,234	3,55291E-137
Macro_FOLR2+APOE+	TRAPPC12	1,31942E-41	4,08901E-01	0,285	0,135	2,42997E-37
Macro_FOLR2+APOE+	MRPS24	8,60402E-10	4,08849E-01	0,263	0,175	1,58460E-05
Macro_FOLR2+APOE+	MRPS12	1,66873E-34	4,08774E-01	0,502	0,276	3,07330E-30
Macro_FOLR2+APOE+	EIF4H	1,68870E-07	4,08443E-01	0,67	0,446	3,11009E-03
Macro_FOLR2+APOE+	STAMBP	1,57239E-45	4,08369E-01	0,341	0,165	2,89588E-41
Macro_FOLR2+APOE+	BTN3A2	8,24639E-48	4,08161E-01	0,429	0,214	1,51874E-43
Macro_FOLR2+APOE+	GLO1	2,36477E-52	4,07280E-01	0,461	0,237	4,35520E-48
Macro_FOLR2+APOE+	DUSP6	1,33554E-08	4,07253E-01	0,403	0,285	2,45966E-04
Macro_FOLR2+APOE+	CCT3	1,30163E-24	4,07166E-01	0,66	0,403	2,39721E-20
Macro_FOLR2+APOE+	MDH2	1,74317E-13	4,07162E-01	0,777	0,515	3,21040E-09
Macro_FOLR2+APOE+	UBA7	1,66110E-41	4,06822E-01	0,335	0,164	3,05926E-37
Macro_FOLR2+APOE+	SAP18	3,98438E-08	4,06581E-01	0,881	0,641	7,33803E-04
Macro_FOLR2+APOE+	POLR2F	2,37776E-49	4,06511E-01	0,579	0,305	4,37911E-45
Macro_FOLR2+APOE+	MTF2	1,08440E-19	4,06371E-01	0,301	0,175	1,99714E-15
Macro_FOLR2+APOE+	RELB	1,58279E-21	4,06125E-01	0,407	0,24	2,91503E-17
Macro_FOLR2+APOE+	PRKAG1	2,15935E-24	4,05922E-01	0,335	0,19	3,97687E-20
Macro_FOLR2+APOE+	RHOT2	2,73521E-34	4,05274E-01	0,328	0,169	5,03744E-30
Macro_FOLR2+APOE+	ROGDI	1,79372E-36	4,05124E-01	0,323	0,164	3,30349E-32
Macro_FOLR2+APOE+	GGA2	2,38487E-60	4,04508E-01	0,528	0,263	4,39221E-56

Macro_FOLR2+APOE+	SYS1	1,05223E-114	4,04425E-01	0,426	0,161	1,93789E-110
Macro_FOLR2+APOE+	AUP1	4,01284E-32	4,04219E-01	0,806	0,517	7,39044E-28
Macro_FOLR2+APOE+	ACO2	2,60492E-59	4,04178E-01	0,523	0,263	4,79748E-55
Macro_FOLR2+APOE+	GAB3	1,84889E-34	4,03461E-01	0,254	0,124	3,40511E-30
Macro_FOLR2+APOE+	CACUL1	8,13893E-43	4,03268E-01	0,542	0,296	1,49895E-38
Macro_FOLR2+APOE+	AFG3L2	6,22571E-66	4,02755E-01	0,395	0,178	1,14659E-61
Macro_FOLR2+APOE+	TAF12	3,55512E-34	4,02635E-01	0,405	0,218	6,54747E-30
Macro_FOLR2+APOE+	STX18	6,36616E-62	4,02297E-01	0,331	0,142	1,17246E-57
Macro_FOLR2+APOE+	BUD31	1,01360E-23	4,02029E-01	0,589	0,353	1,86674E-19
Macro_FOLR2+APOE+	SIGLEC7	5,92099E-109	4,01850E-01	0,445	0,175	1,09047E-104
Macro_FOLR2+APOE+	FBXL5	1,32496E-44	4,01359E-01	0,507	0,267	2,44018E-40
Macro_FOLR2+APOE+	WDR81	7,93088E-95	4,00930E-01	0,338	0,127	1,46063E-90
Macro_FOLR2+APOE+	PPIG	2,03890E-27	4,00898E-01	0,727	0,445	3,75505E-23
Macro_FOLR2+APOE+	RPS5	2,27265E-16	4,00859E-01	0,802	0,509	4,18554E-12
Macro_FOLR2+APOE+	LIMK1	8,78979E-87	4,00796E-01	0,373	0,148	1,61882E-82
Macro_FOLR2+APOE+	PSME3	1,80852E-25	4,00454E-01	0,351	0,198	3,33075E-21
Macro_FOLR2+APOE+	CD300C	1,58035E-08	3,99864E-01	0,352	0,234	2,91053E-04
Macro_FOLR2+APOE+	MKLN1	4,53149E-16	3,99216E-01	0,269	0,161	8,34565E-12
Macro_FOLR2+APOE+	STX4	1,37337E-79	3,98885E-01	0,612	0,304	2,52934E-75
Macro_FOLR2+APOE+	POLR3C	4,62195E-39	3,98074E-01	0,266	0,126	8,51224E-35
Macro_FOLR2+APOE+	SNX24	6,99331E-223	3,98064E-01	0,344	0,081	1,28796E-218
Macro_FOLR2+APOE+	DDX56	2,03601E-24	3,98016E-01	0,26	0,14	3,74972E-20
Macro_FOLR2+APOE+	NUTF2	1,25353E-18	3,97092E-01	0,611	0,378	2,30862E-14
Macro_FOLR2+APOE+	MAP2K2	1,92153E-54	3,97054E-01	0,345	0,159	3,53889E-50
Macro_FOLR2+APOE+	MAP3K11	9,08268E-28	3,96676E-01	0,42	0,24	1,67276E-23
Macro_FOLR2+APOE+	CD302	2,14785E-47	3,96494E-01	0,624	0,341	3,95569E-43
Macro_FOLR2+APOE+	COPS4	5,38012E-37	3,96357E-01	0,283	0,139	9,90856E-33
Macro_FOLR2+APOE+	ZDHHC12	5,51329E-100	3,96287E-01	0,574	0,253	1,01538E-95
Macro_FOLR2+APOE+	REX1BD	1,48240E-208	3,96267E-01	0,661	0,249	2,73014E-204
Macro_FOLR2+APOE+	CDK5	1,61361E-39	3,95996E-01	0,275	0,13	2,97178E-35
Macro_FOLR2+APOE+	NUDT21	3,51043E-37	3,95802E-01	0,483	0,26	6,46516E-33
Macro_FOLR2+APOE+	TFG	6,24462E-56	3,95666E-01	0,51	0,254	1,15007E-51
Macro_FOLR2+APOE+	CLK3	1,62785E-12	3,95298E-01	0,262	0,165	2,99801E-08
Macro_FOLR2+APOE+	ZNF706	9,57781E-15	3,95289E-01	0,844	0,584	1,76394E-10
Macro_FOLR2+APOE+	LPCAT1	6,72397E-31	3,95115E-01	0,278	0,144	1,23835E-26
Macro_FOLR2+APOE+	ARFGAP2	4,66426E-23	3,95063E-01	0,337	0,194	8,59018E-19
Macro_FOLR2+APOE+	AIMP2	3,74095E-35	3,95058E-01	0,338	0,174	6,88971E-31
Macro_FOLR2+APOE+	ATXN1	1,65064E-19	3,94978E-01	0,291	0,17	3,03999E-15
Macro_FOLR2+APOE+	KDELR2	4,52366E-37	3,94407E-01	0,789	0,498	8,33122E-33
Macro_FOLR2+APOE+	SLC8A1	4,68573E-36	3,94395E-01	0,629	0,358	8,62972E-32
Macro_FOLR2+APOE+	PTK2B	3,87707E-15	3,94212E-01	0,399	0,25	7,14040E-11
Macro_FOLR2+APOE+	ZDHHC7	3,50337E-60	3,93968E-01	0,422	0,2	6,45215E-56
Macro_FOLR2+APOE+	SNX27	4,83541E-69	3,93798E-01	0,504	0,237	8,90538E-65
Macro_FOLR2+APOE+	PSMD1	1,59993E-68	3,93602E-01	0,55	0,27	2,94660E-64
Macro_FOLR2+APOE+	ACLY	5,04919E-26	3,93472E-01	0,287	0,157	9,29909E-22
Macro_FOLR2+APOE+	HSF1	2,95467E-22	3,93277E-01	0,452	0,268	5,44162E-18
Macro_FOLR2+APOE+	PARP10	1,19205E-73	3,92853E-01	0,461	0,207	2,19540E-69

Macro_FOLR2+APOE+	ARL4C	2,58018E-96	3,92475E-01	0,65	0,323	4,75192E-92
Macro_FOLR2+APOE+	NSMCE2	3,52289E-61	3,92147E-01	0,307	0,13	6,48811E-57
Macro_FOLR2+APOE+	NXT1	3,47987E-11	3,91928E-01	0,315	0,206	6,40887E-07
Macro_FOLR2+APOE+	NDUFB5	3,88423E-43	3,91852E-01	0,753	0,445	7,15359E-39
Macro_FOLR2+APOE+	AAMP	1,75010E-40	3,91838E-01	0,53	0,286	3,22317E-36
Macro_FOLR2+APOE+	CTNNBL1	1,69286E-29	3,91644E-01	0,406	0,225	3,11774E-25
Macro_FOLR2+APOE+	PPID	1,12858E-38	3,91476E-01	0,346	0,174	2,07851E-34
Macro_FOLR2+APOE+	CFL1	9,89891E-09	3,91395E-01	0,993	0,92	1,82308E-04
Macro_FOLR2+APOE+	NENF	1,35212E-213	3,91341E-01	0,664	0,246	2,49020E-209
Macro_FOLR2+APOE+	MYLIP	5,41562E-16	3,90441E-01	0,274	0,164	9,97394E-12
Macro_FOLR2+APOE+	ERAP2	2,74341E-41	3,90337E-01	0,336	0,166	5,05253E-37
Macro_FOLR2+APOE+	UQCR11	1,53866E-11	3,89897E-01	0,944	0,749	2,83376E-07
Macro_FOLR2+APOE+	ATP5MF	1,32293E-132	3,89707E-01	0,731	0,345	2,43643E-128
Macro_FOLR2+APOE+	ILKAP	2,34816E-26	3,89624E-01	0,303	0,167	4,32461E-22
Macro_FOLR2+APOE+	ARMC10	2,48879E-67	3,89465E-01	0,346	0,147	4,58360E-63
Macro_FOLR2+APOE+	PPIP5K2	9,16344E-26	3,89450E-01	0,31	0,172	1,68763E-21
Macro_FOLR2+APOE+	RALB	2,87606E-34	3,89448E-01	0,416	0,223	5,29684E-30
Macro_FOLR2+APOE+	MRPL51	1,20303E-48	3,89116E-01	0,749	0,431	2,21562E-44
Macro_FOLR2+APOE+	KXD1	9,74177E-26	3,88687E-01	0,574	0,345	1,79414E-21
Macro_FOLR2+APOE+	MAPK1IP1L	9,55724E-13	3,88637E-01	0,529	0,338	1,76016E-08
Macro_FOLR2+APOE+	LACC1	3,15937E-143	3,88543E-01	0,336	0,103	5,81861E-139
Macro_FOLR2+APOE+	HLA-DRB5	3,72082E-11	3,88364E-01	0,961	0,844	6,85264E-07
Macro_FOLR2+APOE+	MAF	1,57217E-192	3,87172E-01	0,496	0,157	2,89546E-188
Macro_FOLR2+APOE+	TOR1AIP1	1,87652E-26	3,87089E-01	0,356	0,199	3,45598E-22
Macro_FOLR2+APOE+	TMEM50B	6,94851E-17	3,86850E-01	0,327	0,197	1,27971E-12
Macro_FOLR2+APOE+	CNIH1	2,61267E-12	3,86603E-01	0,584	0,38	4,81176E-08
Macro_FOLR2+APOE+	MAP1S	8,07757E-39	3,86491E-01	0,316	0,158	1,48765E-34
Macro_FOLR2+APOE+	PTMS	5,70911E-167	3,86215E-01	0,778	0,351	1,05145E-162
Macro_FOLR2+APOE+	PHF20L1	8,31983E-09	3,85957E-01	0,367	0,245	1,53226E-04
Macro_FOLR2+APOE+	UBXN4	9,92889E-15	3,85847E-01	0,772	0,514	1,82860E-10
Macro_FOLR2+APOE+	APOL1	2,03212E-196	3,85736E-01	0,343	0,088	3,74255E-192
Macro_FOLR2+APOE+	SUMF1	2,51978E-42	3,85372E-01	0,36	0,181	4,64068E-38
Macro_FOLR2+APOE+	THOC2	3,22516E-22	3,85200E-01	0,427	0,251	5,93977E-18
Macro_FOLR2+APOE+	SF3A1	4,78462E-10	3,84888E-01	0,434	0,285	8,81183E-06
Macro_FOLR2+APOE+	FOXN3	3,47063E-22	3,84736E-01	0,588	0,354	6,39187E-18
Macro_FOLR2+APOE+	BIN2	3,22378E-36	3,84236E-01	0,555	0,307	5,93723E-32
Macro_FOLR2+APOE+	STARD3NL	7,45854E-56	3,84228E-01	0,525	0,263	1,37364E-51
Macro_FOLR2+APOE+	CTTNBP2NL	7,53169E-32	3,84191E-01	0,307	0,161	1,38711E-27
Macro_FOLR2+APOE+	ERLEC1	1,23764E-64	3,83274E-01	0,545	0,268	2,27936E-60
Macro_FOLR2+APOE+	ROMO1	3,03466E-08	3,83228E-01	0,748	0,524	5,58894E-04
Macro_FOLR2+APOE+	GPX1	3,35699E-08	3,83219E-01	0,46	0,552	6,18257E-04
Macro_FOLR2+APOE+	SLC37A4	1,86337E-153	3,82831E-01	0,262	0,066	3,43177E-149
Macro_FOLR2+APOE+	LYPLAL1	4,81069E-74	3,82525E-01	0,396	0,171	8,85984E-70
Macro_FOLR2+APOE+	SELENOT	2,18912E-210	3,82296E-01	0,65	0,24	4,03170E-206
Macro_FOLR2+APOE+	CIAO2A	5,98652E-143	3,82199E-01	0,419	0,15	1,10254E-138
Macro_FOLR2+APOE+	HCST	1,10932E-12	3,81968E-01	0,885	0,647	2,04304E-08
Macro_FOLR2+APOE+	RER1	1,30214E-73	3,81797E-01	0,801	0,451	2,39815E-69

Macro_FOLR2+APOE+	EIF4E2	1,45108E-10	3,81583E-01	0,563	0,366	2,67246E-06
Macro_FOLR2+APOE+	UQCC2	6,84651E-91	3,81343E-01	0,643	0,301	1,26092E-86
Macro_FOLR2+APOE+	FAM50A	4,26718E-105	3,81242E-01	0,638	0,295	7,85886E-101
Macro_FOLR2+APOE+	SREBF1	8,76911E-136	3,80719E-01	0,347	0,11	1,61501E-131
Macro_FOLR2+APOE+	TNIP2	1,68480E-45	3,79920E-01	0,365	0,18	3,10289E-41
Macro_FOLR2+APOE+	DDX1	1,22088E-41	3,79833E-01	0,269	0,124	2,24849E-37
Macro_FOLR2+APOE+	NECTIN2	1,11852E-230	3,79806E-01	0,54	0,167	2,05999E-226
Macro_FOLR2+APOE+	OCIAD1	1,46627E-30	3,79722E-01	0,716	0,426	2,70043E-26
Macro_FOLR2+APOE+	NUB1	4,05073E-50	3,79517E-01	0,577	0,306	7,46022E-46
Macro_FOLR2+APOE+	IL6ST	5,48173E-36	3,79405E-01	0,387	0,207	1,00957E-31
Macro_FOLR2+APOE+	WIPI2	8,17629E-73	3,79167E-01	0,511	0,24	1,50583E-68
Macro_FOLR2+APOE+	FAM136A	9,15305E-36	3,78983E-01	0,371	0,194	1,68572E-31
Macro_FOLR2+APOE+	NDFIP1	2,67152E-62	3,78211E-01	0,832	0,494	4,92013E-58
Macro_FOLR2+APOE+	COPB1	5,31897E-32	3,77942E-01	0,406	0,226	9,79594E-28
Macro_FOLR2+APOE+	ITPA	2,46729E-78	3,77694E-01	0,568	0,266	4,54402E-74
Macro_FOLR2+APOE+	PSMD12	2,08285E-33	3,77477E-01	0,377	0,203	3,83599E-29
Macro_FOLR2+APOE+	TMUB1	9,98007E-38	3,77474E-01	0,475	0,256	1,83803E-33
Macro_FOLR2+APOE+	GOLIM4	1,51292E-64	3,77400E-01	0,455	0,215	2,78635E-60
Macro_FOLR2+APOE+	YWHAB	3,48303E-09	3,77202E-01	0,942	0,783	6,41469E-05
Macro_FOLR2+APOE+	GLUD1	2,20199E-26	3,76567E-01	0,542	0,322	4,05541E-22
Macro_FOLR2+APOE+	YTHDF3	1,94314E-24	3,76531E-01	0,313	0,177	3,57869E-20
Macro_FOLR2+APOE+	PTDSS1	2,92428E-14	3,76416E-01	0,42	0,265	5,38564E-10
Macro_FOLR2+APOE+	EMC3	1,27280E-76	3,76362E-01	0,626	0,311	2,34411E-72
Macro_FOLR2+APOE+	ARIH2	1,85318E-17	3,76325E-01	0,414	0,254	3,41301E-13
Macro_FOLR2+APOE+	POLR2E	6,53398E-08	3,76309E-01	0,766	0,523	1,20336E-03
Macro_FOLR2+APOE+	DYNC1LI1	8,68911E-28	3,75875E-01	0,394	0,219	1,60027E-23
Macro_FOLR2+APOE+	GABARAPL1	4,02308E-22	3,75473E-01	0,488	0,286	7,40930E-18
Macro_FOLR2+APOE+	TSPAN33	2,83753E-76	3,75411E-01	0,273	0,102	5,22587E-72
Macro_FOLR2+APOE+	MYO9B	5,60630E-64	3,75063E-01	0,699	0,379	1,03251E-59
Macro_FOLR2+APOE+	TK2	6,99807E-54	3,74729E-01	0,326	0,147	1,28883E-49
Macro_FOLR2+APOE+	PBRM1	2,36614E-51	3,74181E-01	0,451	0,228	4,35772E-47
Macro_FOLR2+APOE+	PIGT	1,45478E-94	3,74069E-01	0,612	0,282	2,67926E-90
Macro_FOLR2+APOE+	ARF3	9,98540E-66	3,74025E-01	0,647	0,328	1,83901E-61
Macro_FOLR2+APOE+	SURF4	1,57066E-44	3,73959E-01	0,526	0,284	2,89268E-40
Macro_FOLR2+APOE+	WDR45	8,35935E-47	3,73938E-01	0,397	0,196	1,53954E-42
Macro_FOLR2+APOE+	GIMAP2	7,36981E-50	3,73753E-01	0,393	0,189	1,35730E-45
Macro_FOLR2+APOE+	ANKRD22	3,42098E-111	3,73696E-01	0,528	0,217	6,30043E-107
Macro_FOLR2+APOE+	SSB	3,38884E-37	3,73464E-01	0,741	0,444	6,24122E-33
Macro_FOLR2+APOE+	GIMAP8	5,26681E-108	3,72685E-01	0,38	0,138	9,69988E-104
Macro_FOLR2+APOE+	CD48	3,63880E-37	3,72576E-01	0,771	0,471	6,70157E-33
Macro_FOLR2+APOE+	SLC9A9	8,35008E-48	3,71999E-01	0,256	0,113	1,53783E-43
Macro_FOLR2+APOE+	HAUS1	4,56567E-44	3,71912E-01	0,283	0,13	8,40860E-40
Macro_FOLR2+APOE+	ALDH9A1	8,80689E-114	3,71668E-01	0,486	0,192	1,62197E-109
Macro_FOLR2+APOE+	NGRN	7,53483E-33	3,71533E-01	0,343	0,184	1,38769E-28
Macro_FOLR2+APOE+	RREB1	4,72804E-44	3,71494E-01	0,353	0,177	8,70764E-40
Macro_FOLR2+APOE+	PEX2	7,74994E-37	3,71130E-01	0,307	0,152	1,42731E-32
Macro_FOLR2+APOE+	CDV3	2,61824E-71	3,70848E-01	0,55	0,267	4,82200E-67

Macro_FOLR2+APOE+	RAB2A	5,68386E-31	3,70679E-01	0,772	0,478	1,04680E-26
Macro_FOLR2+APOE+	ATP5PF	1,23199E-151	3,70096E-01	0,705	0,312	2,26895E-147
Macro_FOLR2+APOE+	DUSP22	6,00937E-07	3,70096E-01	0,318	0,22	1,10675E-02
Macro_FOLR2+APOE+	KLHDC3	2,15629E-70	3,69368E-01	0,499	0,234	3,97124E-66
Macro_FOLR2+APOE+	RAB1B	1,09182E-17	3,69192E-01	0,557	0,35	2,01080E-13
Macro_FOLR2+APOE+	NOL8	2,96907E-45	3,68443E-01	0,312	0,148	5,46813E-41
Macro_FOLR2+APOE+	CUL4A	4,22046E-34	3,68319E-01	0,31	0,159	7,77283E-30
Macro_FOLR2+APOE+	STRAP	2,60347E-12	3,68258E-01	0,545	0,35	4,79481E-08
Macro_FOLR2+APOE+	DHX29	1,97844E-57	3,68047E-01	0,443	0,212	3,64370E-53
Macro_FOLR2+APOE+	FEM1B	6,29213E-81	3,67777E-01	0,312	0,119	1,15882E-76
Macro_FOLR2+APOE+	DYNLT1	3,04239E-49	3,67738E-01	0,817	0,5	5,60318E-45
Macro_FOLR2+APOE+	COX5A	1,42637E-25	3,67675E-01	0,929	0,687	2,62694E-21
Macro_FOLR2+APOE+	SERTAD3	2,28423E-21	3,67668E-01	0,3	0,171	4,20686E-17
Macro_FOLR2+APOE+	CORO7	4,98624E-76	3,67352E-01	0,353	0,147	9,18316E-72
Macro_FOLR2+APOE+	RTCA	3,42854E-55	3,67294E-01	0,388	0,181	6,31434E-51
Macro_FOLR2+APOE+	CSF2RA	3,03251E-20	3,66889E-01	0,658	0,41	5,58497E-16
Macro_FOLR2+APOE+	DAZAP2	2,13822E-15	3,66828E-01	0,895	0,738	3,93796E-11
Macro_FOLR2+APOE+	PNPLA6	1,17460E-67	3,66784E-01	0,43	0,2	2,16326E-63
Macro_FOLR2+APOE+	TRAPPC2L	1,03704E-90	3,66566E-01	0,572	0,261	1,90991E-86
Macro_FOLR2+APOE+	PCM1	4,32670E-22	3,66478E-01	0,515	0,31	7,96849E-18
Macro_FOLR2+APOE+	FBXO9	1,22923E-28	3,66001E-01	0,317	0,171	2,26387E-24
Macro_FOLR2+APOE+	LRP1	1,78970E-66	3,65486E-01	0,772	0,412	3,29609E-62
Macro_FOLR2+APOE+	PMF1	7,05059E-69	3,65264E-01	0,262	0,102	1,29851E-64
Macro_FOLR2+APOE+	APEX2	6,34383E-73	3,65144E-01	0,289	0,113	1,16834E-68
Macro_FOLR2+APOE+	NDUFB6	8,64678E-45	3,65010E-01	0,648	0,36	1,59248E-40
Macro_FOLR2+APOE+	TXNL1	1,52158E-33	3,64726E-01	0,639	0,374	2,80229E-29
Macro_FOLR2+APOE+	IFNAR1	4,78088E-86	3,64452E-01	0,631	0,304	8,80494E-82
Macro_FOLR2+APOE+	POP7	3,41044E-54	3,64370E-01	0,33	0,149	6,28100E-50
Macro_FOLR2+APOE+	FKBP15	5,16264E-96	3,64131E-01	0,584	0,263	9,50804E-92
Macro_FOLR2+APOE+	TPM4	6,41737E-07	3,63902E-01	0,832	0,612	1,18189E-02
Macro_FOLR2+APOE+	BCAT1	7,07608E-39	3,63896E-01	0,44	0,232	1,30320E-34
Macro_FOLR2+APOE+	CREB3	9,28583E-64	3,63538E-01	0,342	0,147	1,71017E-59
Macro_FOLR2+APOE+	KLHL6	7,92291E-77	3,63371E-01	0,429	0,191	1,45916E-72
Macro_FOLR2+APOE+	UBAP2L	1,41651E-33	3,62788E-01	0,368	0,196	2,60879E-29
Macro_FOLR2+APOE+	FXYD5	3,76187E-55	3,62756E-01	0,932	0,822	6,92824E-51
Macro_FOLR2+APOE+	YKT6	6,56518E-37	3,62631E-01	0,301	0,149	1,20911E-32
Macro_FOLR2+APOE+	WDR41	1,97692E-20	3,62165E-01	0,28	0,16	3,64090E-16
Macro_FOLR2+APOE+	RPL17	2,32369E-22	3,61975E-01	0,697	0,426	4,27953E-18
Macro_FOLR2+APOE+	ABHD16A	5,37727E-64	3,61324E-01	0,32	0,139	9,90332E-60
Macro_FOLR2+APOE+	MAT2A	4,96149E-13	3,61150E-01	0,661	0,44	9,13758E-09
Macro_FOLR2+APOE+	BET1L	3,06275E-21	3,60789E-01	0,27	0,152	5,64067E-17
Macro_FOLR2+APOE+	RB1	1,85533E-15	3,60644E-01	0,594	0,397	3,41696E-11
Macro_FOLR2+APOE+	SMIM12	3,75039E-33	3,60639E-01	0,475	0,262	6,90710E-29
Macro_FOLR2+APOE+	SLC25A1	6,17132E-51	3,60414E-01	0,333	0,155	1,13657E-46
Macro_FOLR2+APOE+	SAMSN1	6,36675E-13	3,60029E-01	0,712	0,457	1,17257E-08
Macro_FOLR2+APOE+	DERL1	9,15731E-86	3,59896E-01	0,571	0,265	1,68650E-81
Macro_FOLR2+APOE+	MRPL21	8,93276E-50	3,59877E-01	0,506	0,259	1,64515E-45

Macro_FOLR2+APOE+	E2F4	3,18498E-22	3,59583E-01	0,321	0,184	5,86577E-18
Macro_FOLR2+APOE+	SMIM30	0,00000E+00	3,59461E-01	0,487	0,113	0,00000E+00
Macro_FOLR2+APOE+	FAM204A	1,77463E-18	3,59409E-01	0,466	0,282	3,26833E-14
Macro_FOLR2+APOE+	APOBEC3G	1,85897E-57	3,59109E-01	0,396	0,186	3,42367E-53
Macro_FOLR2+APOE+	PPIB	5,89752E-09	3,58868E-01	0,969	0,787	1,08615E-04
Macro_FOLR2+APOE+	NCLN	2,05986E-61	3,58812E-01	0,292	0,122	3,79364E-57
Macro_FOLR2+APOE+	CD300LF	4,55491E-182	3,58707E-01	0,507	0,166	8,38877E-178
Macro_FOLR2+APOE+	GYG1	1,00231E-51	3,58646E-01	0,525	0,266	1,84596E-47
Macro_FOLR2+APOE+	HMGXB3	1,03936E-50	3,58313E-01	0,292	0,131	1,91420E-46
Macro_FOLR2+APOE+	SDR39U1	1,29658E-17	3,58012E-01	0,277	0,163	2,38791E-13
Macro_FOLR2+APOE+	RAB5IF	6,35323E-176	3,57890E-01	0,597	0,23	1,17007E-171
Macro_FOLR2+APOE+	CHCHD5	2,67194E-29	3,57849E-01	0,511	0,293	4,92092E-25
Macro_FOLR2+APOE+	SEC13	3,69378E-16	3,57580E-01	0,629	0,395	6,80284E-12
Macro_FOLR2+APOE+	PHACTR2	3,85443E-131	3,57526E-01	0,458	0,168	7,09871E-127
Macro_FOLR2+APOE+	PDIA4	3,47074E-102	3,56126E-01	0,663	0,311	6,39207E-98
Macro_FOLR2+APOE+	TKT	3,04996E-17	3,56117E-01	0,86	0,6	5,61711E-13
Macro_FOLR2+APOE+	CEPT1	1,90626E-51	3,56040E-01	0,292	0,13	3,51075E-47
Macro_FOLR2+APOE+	LAG3	5,09201E-275	3,55878E-01	0,314	0,062	9,37796E-271
Macro_FOLR2+APOE+	XRN1	2,41283E-28	3,55811E-01	0,376	0,211	4,44370E-24
Macro_FOLR2+APOE+	NDUFAF3	5,00669E-32	3,55585E-01	0,756	0,463	9,22082E-28
Macro_FOLR2+APOE+	SFXN3	3,52320E-53	3,55554E-01	0,338	0,155	6,48868E-49
Macro_FOLR2+APOE+	CRYL1	3,16572E-54	3,55071E-01	0,45	0,22	5,83030E-50
Macro_FOLR2+APOE+	ARID5A	1,98756E-40	3,54904E-01	0,515	0,281	3,66049E-36
Macro_FOLR2+APOE+	PML	3,79751E-45	3,54313E-01	0,433	0,224	6,99387E-41
Macro_FOLR2+APOE+	CMC1	1,93820E-57	3,53574E-01	0,458	0,221	3,56958E-53
Macro_FOLR2+APOE+	PACSIN2	4,49223E-52	3,52747E-01	0,51	0,263	8,27335E-48
Macro_FOLR2+APOE+	APOO	3,95147E-132	3,52393E-01	0,296	0,087	7,27743E-128
Macro_FOLR2+APOE+	ACTB	3,22715E-44	3,52179E-01	1	0,991	5,94344E-40
Macro_FOLR2+APOE+	ERP29	8,39301E-24	3,51610E-01	0,888	0,616	1,54574E-19
Macro_FOLR2+APOE+	CEBPA	6,15757E-226	3,51410E-01	0,496	0,147	1,13404E-221
Macro_FOLR2+APOE+	LRCH4	1,41182E-16	3,51397E-01	0,276	0,167	2,60015E-12
Macro_FOLR2+APOE+	UQCRQ	3,62806E-11	3,51293E-01	0,9	0,659	6,68180E-07
Macro_FOLR2+APOE+	HMOX2	5,84474E-65	3,51262E-01	0,414	0,188	1,07643E-60
Macro_FOLR2+APOE+	STAT6	3,00473E-14	3,51192E-01	0,467	0,3	5,53381E-10
Macro_FOLR2+APOE+	SPG7	5,92955E-29	3,51089E-01	0,387	0,216	1,09204E-24
Macro_FOLR2+APOE+	TMEM223	4,27317E-30	3,51058E-01	0,276	0,143	7,86989E-26
Macro_FOLR2+APOE+	IKBKE	8,24876E-183	3,50907E-01	0,354	0,095	1,51917E-178
Macro_FOLR2+APOE+	ADSL	5,77335E-25	3,50786E-01	0,37	0,212	1,06328E-20
Macro_FOLR2+APOE+	ATP1B1	2,51857E-124	3,50734E-01	0,528	0,212	4,63845E-120
Macro_FOLR2+APOE+	UCHL3	1,42992E-18	3,50037E-01	0,359	0,214	2,63349E-14
Macro_FOLR2+APOE+	RPSA	3,02322E-29	3,49676E-01	0,804	0,486	5,56786E-25
Macro_FOLR2+APOE+	PARP1	1,17959E-45	3,49314E-01	0,6	0,327	2,17245E-41
Macro_FOLR2+APOE+	TPM3	7,17293E-11	3,49038E-01	0,943	0,799	1,32104E-06
Macro_FOLR2+APOE+	ACTG1	3,88167E-30	3,48909E-01	0,991	0,92	7,14888E-26
Macro_FOLR2+APOE+	TMEM219	1,79941E-12	3,48859E-01	0,889	0,629	3,31397E-08
Macro_FOLR2+APOE+	UBE2Z	4,75149E-70	3,48197E-01	0,391	0,172	8,75082E-66
Macro_FOLR2+APOE+	IER3IP1	6,86699E-80	3,48090E-01	0,457	0,204	1,26469E-75

Macro_FOLR2+APOE+	POLR3GL	4,10889E-57	3,47835E-01	0,574	0,289	7,56734E-53
Macro_FOLR2+APOE+	CAMK1D	1,76279E-36	3,47569E-01	0,408	0,215	3,24654E-32
Macro_FOLR2+APOE+	EIF1	3,75820E-144	3,47288E-01	0,987	0,943	6,92148E-140
Macro_FOLR2+APOE+	MICU1	2,29669E-59	3,47246E-01	0,359	0,162	4,22981E-55
Macro_FOLR2+APOE+	ADCK2	8,30967E-88	3,47181E-01	0,274	0,096	1,53039E-83
Macro_FOLR2+APOE+	ACBD5	4,71275E-66	3,47050E-01	0,291	0,118	8,67947E-62
Macro_FOLR2+APOE+	MRPS15	1,79357E-69	3,47022E-01	0,622	0,309	3,30321E-65
Macro_FOLR2+APOE+	RAP1B	1,03781E-07	3,46859E-01	0,818	0,572	1,91133E-03
Macro_FOLR2+APOE+	SLC6A6	1,80603E-21	3,46829E-01	0,311	0,181	3,32617E-17
Macro_FOLR2+APOE+	NAIP	4,84769E-53	3,46792E-01	0,642	0,363	8,92800E-49
Macro_FOLR2+APOE+	RAB34	2,57975E-36	3,46598E-01	0,487	0,266	4,75112E-32
Macro_FOLR2+APOE+	CUEDC2	5,67252E-31	3,46436E-01	0,506	0,289	1,04471E-26
Macro_FOLR2+APOE+	SCO2	5,70866E-15	3,46376E-01	0,331	0,215	1,05136E-10
Macro_FOLR2+APOE+	TMED7	1,52041E-80	3,46115E-01	0,38	0,157	2,80014E-76
Macro_FOLR2+APOE+	RFK	7,35027E-34	3,46065E-01	0,252	0,125	1,35370E-29
Macro_FOLR2+APOE+	ST8SIA4	1,24641E-45	3,45926E-01	0,531	0,29	2,29551E-41
Macro_FOLR2+APOE+	GLIPR2	1,91458E-19	3,45688E-01	0,58	0,371	3,52608E-15
Macro_FOLR2+APOE+	GALNT6	2,22523E-44	3,45662E-01	0,269	0,122	4,09820E-40
Macro_FOLR2+APOE+	ETFA	1,44996E-35	3,45565E-01	0,584	0,328	2,67040E-31
Macro_FOLR2+APOE+	GMFB	4,17891E-47	3,45548E-01	0,342	0,164	7,69630E-43
Macro_FOLR2+APOE+	ERAL1	2,05945E-30	3,45045E-01	0,258	0,131	3,79288E-26
Macro_FOLR2+APOE+	MEAF6	2,92579E-10	3,45040E-01	0,471	0,311	5,38843E-06
Macro_FOLR2+APOE+	DCUN1D5	1,80989E-36	3,44904E-01	0,295	0,146	3,33328E-32
Macro_FOLR2+APOE+	DENND4B	2,00098E-26	3,44887E-01	0,27	0,145	3,68520E-22
Macro_FOLR2+APOE+	NR1H2	8,60823E-25	3,44416E-01	0,545	0,324	1,58538E-20
Macro_FOLR2+APOE+	DNAJC3	1,80948E-45	3,44193E-01	0,641	0,356	3,33252E-41
Macro_FOLR2+APOE+	GNAI3	1,51453E-38	3,44012E-01	0,61	0,338	2,78931E-34
Macro_FOLR2+APOE+	RAB31	2,52251E-09	3,43611E-01	0,883	0,636	4,64571E-05
Macro_FOLR2+APOE+	SDF4	3,86164E-69	3,43424E-01	0,755	0,422	7,11198E-65
Macro_FOLR2+APOE+	ATF6B	2,98633E-28	3,43420E-01	0,491	0,281	5,49993E-24
Macro_FOLR2+APOE+	ZYX	2,60014E-08	3,42976E-01	0,857	0,639	4,78867E-04
Macro_FOLR2+APOE+	CCNDBP1	7,28010E-24	3,42943E-01	0,458	0,268	1,34078E-19
Macro_FOLR2+APOE+	PTPRJ	4,77245E-124	3,42727E-01	0,415	0,15	8,78942E-120
Macro_FOLR2+APOE+	ERAP1	1,23643E-39	3,42602E-01	0,261	0,123	2,27713E-35
Macro_FOLR2+APOE+	TNFSF12	2,25718E-186	3,42349E-01	0,565	0,199	4,15706E-182
Macro_FOLR2+APOE+	ZNF638	5,90395E-26	3,42131E-01	0,422	0,244	1,08733E-21
Macro_FOLR2+APOE+	SPTAN1	1,47340E-50	3,42093E-01	0,359	0,175	2,71356E-46
Macro_FOLR2+APOE+	AP3B1	4,65435E-47	3,41925E-01	0,457	0,234	8,57192E-43
Macro_FOLR2+APOE+	PRPF8	3,29481E-12	3,41906E-01	0,461	0,303	6,06806E-08
Macro_FOLR2+APOE+	MESD	7,48205E-222	3,41841E-01	0,528	0,169	1,37797E-217
Macro_FOLR2+APOE+	FKBP4	1,19030E-29	3,41690E-01	0,403	0,223	2,19217E-25
Macro_FOLR2+APOE+	OSCAR	4,00641E-32	3,41358E-01	0,406	0,221	7,37860E-28
Macro_FOLR2+APOE+	TMX2	4,77018E-56	3,41187E-01	0,376	0,173	8,78525E-52
Macro_FOLR2+APOE+	CRBN	2,08590E-22	3,40843E-01	0,315	0,178	3,84159E-18
Macro_FOLR2+APOE+	SCCPDH	8,75138E-171	3,40766E-01	0,452	0,145	1,61174E-166
Macro_FOLR2+APOE+	TIPRL	1,42018E-37	3,40722E-01	0,398	0,208	2,61555E-33
Macro_FOLR2+APOE+	NDUFS1	1,06084E-43	3,40693E-01	0,282	0,131	1,95374E-39

Macro_FOLR2+APOE+	PMPCB	1,03064E-24	3,40671E-01	0,413	0,237	1,89812E-20
Macro_FOLR2+APOE+	OARD1	2,09506E-30	3,40634E-01	0,272	0,139	3,85847E-26
Macro_FOLR2+APOE+	NEMF	1,39260E-28	3,40628E-01	0,492	0,283	2,56475E-24
Macro_FOLR2+APOE+	OSBPL1A	8,80310E-143	3,40368E-01	0,433	0,148	1,62127E-138
Macro_FOLR2+APOE+	ZFYVE16	3,84565E-53	3,40338E-01	0,416	0,202	7,08253E-49
Macro_FOLR2+APOE+	MRPL14	7,81705E-47	3,40133E-01	0,624	0,336	1,43967E-42
Macro_FOLR2+APOE+	NUDT5	1,14635E-42	3,39204E-01	0,432	0,225	2,11124E-38
Macro_FOLR2+APOE+	STAT5A	1,30451E-59	3,38840E-01	0,447	0,215	2,40252E-55
Macro_FOLR2+APOE+	NAA60	1,30624E-77	3,38735E-01	0,283	0,107	2,40570E-73
Macro_FOLR2+APOE+	AIM2	1,15884E-214	3,38573E-01	0,334	0,079	2,13423E-210
Macro_FOLR2+APOE+	BNIP3L	1,19614E-07	3,38227E-01	0,808	0,556	2,20293E-03
Macro_FOLR2+APOE+	PPARD	3,46726E-61	3,37911E-01	0,269	0,109	6,38565E-57
Macro_FOLR2+APOE+	HDAC3	1,87811E-36	3,37823E-01	0,392	0,208	3,45891E-32
Macro_FOLR2+APOE+	AIG1	6,32599E-35	3,37644E-01	0,342	0,178	1,16506E-30
Macro_FOLR2+APOE+	TSPYL1	4,61842E-12	3,37466E-01	0,275	0,176	8,50575E-08
Macro_FOLR2+APOE+	ATP5F1C	1,85463E-167	3,37321E-01	0,673	0,277	3,41568E-163
Macro_FOLR2+APOE+	BUB3	2,37658E-18	3,37159E-01	0,421	0,253	4,37695E-14
Macro_FOLR2+APOE+	CARD8	2,08699E-21	3,37037E-01	0,376	0,221	3,84361E-17
Macro_FOLR2+APOE+	RWDD1	7,38369E-26	3,36904E-01	0,719	0,45	1,35985E-21
Macro_FOLR2+APOE+	C19orf53	1,50068E-09	3,36804E-01	0,839	0,577	2,76380E-05
Macro_FOLR2+APOE+	CCDC115	3,23320E-39	3,36625E-01	0,479	0,26	5,95459E-35
Macro_FOLR2+APOE+	MRPS26	5,57102E-45	3,36230E-01	0,41	0,204	1,02601E-40
Macro_FOLR2+APOE+	SLC4A7	9,15407E-44	3,35952E-01	0,435	0,223	1,68591E-39
Macro_FOLR2+APOE+	CLNS1A	1,95839E-12	3,35923E-01	0,638	0,415	3,60677E-08
Macro_FOLR2+APOE+	ARFRP1	3,05831E-26	3,35835E-01	0,292	0,157	5,63248E-22
Macro_FOLR2+APOE+	SBF2	1,71757E-43	3,35611E-01	0,304	0,144	3,16325E-39
Macro_FOLR2+APOE+	OPTN	2,27720E-144	3,35462E-01	0,358	0,111	4,19391E-140
Macro_FOLR2+APOE+	EXOC4	6,41086E-37	3,35116E-01	0,3	0,15	1,18069E-32
Macro_FOLR2+APOE+	NDUFB2	2,23413E-24	3,34583E-01	0,923	0,709	4,11461E-20
Macro_FOLR2+APOE+	SRGN	1,63223E-51	3,34474E-01	0,991	0,941	3,00608E-47
Macro_FOLR2+APOE+	GLRX	9,33986E-36	3,34283E-01	0,839	0,539	1,72012E-31
Macro_FOLR2+APOE+	HAGH	6,13545E-80	3,34072E-01	0,518	0,238	1,12997E-75
Macro_FOLR2+APOE+	NMRAL1	4,27552E-84	3,33843E-01	0,395	0,162	7,87423E-80
Macro_FOLR2+APOE+	ZFAND6	4,18705E-25	3,33120E-01	0,522	0,305	7,71129E-21
Macro_FOLR2+APOE+	TLN1	5,35690E-15	3,33065E-01	0,815	0,548	9,86580E-11
Macro_FOLR2+APOE+	ARID5B	3,06344E-94	3,32949E-01	0,374	0,146	5,64194E-90
Macro_FOLR2+APOE+	PRKAG2	8,44243E-23	3,32910E-01	0,376	0,218	1,55484E-18
Macro_FOLR2+APOE+	WDR18	3,91843E-44	3,32635E-01	0,308	0,147	7,21658E-40
Macro_FOLR2+APOE+	HDDC2	5,41850E-79	3,32621E-01	0,387	0,161	9,97925E-75
Macro_FOLR2+APOE+	DDAH2	7,76195E-17	3,32334E-01	0,65	0,412	1,42952E-12
Macro_FOLR2+APOE+	SPINT2	1,30080E-41	3,32264E-01	0,8	0,489	2,39568E-37
Macro_FOLR2+APOE+	RNF141	2,04733E-28	3,32084E-01	0,303	0,161	3,77057E-24
Macro_FOLR2+APOE+	FIS1	5,08709E-17	3,31969E-01	0,791	0,518	9,36889E-13
Macro_FOLR2+APOE+	NDUFV2	2,37442E-06	3,31863E-01	0,733	0,528	4,37298E-02
Macro_FOLR2+APOE+	PTBP1	2,23701E-19	3,31746E-01	0,492	0,301	4,11991E-15
Macro_FOLR2+APOE+	OSBPL11	1,24070E-58	3,31628E-01	0,301	0,129	2,28499E-54
Macro_FOLR2+APOE+	BAK1	2,12043E-53	3,31523E-01	0,32	0,146	3,90520E-49

Macro_FOLR2+APOE+	SH3PXD2B	2,33747E-135	3,31192E-01	0,333	0,105	4,30491E-131
Macro_FOLR2+APOE+	MRPS11	7,96421E-29	3,31153E-01	0,365	0,202	1,46677E-24
Macro_FOLR2+APOE+	TMEM14B	1,68303E-18	3,31082E-01	0,661	0,415	3,09964E-14
Macro_FOLR2+APOE+	SRP19	2,67207E-63	3,31068E-01	0,389	0,176	4,92116E-59
Macro_FOLR2+APOE+	TM9SF4	4,75328E-33	3,31059E-01	0,26	0,13	8,75412E-29
Macro_FOLR2+APOE+	SLC38A7	1,85305E-150	3,31004E-01	0,28	0,074	3,41277E-146
Macro_FOLR2+APOE+	GLTP	3,56284E-12	3,30956E-01	0,307	0,196	6,56167E-08
Macro_FOLR2+APOE+	TMEM150A	1,05119E-62	3,30874E-01	0,304	0,127	1,93597E-58
Macro_FOLR2+APOE+	PRDX3	5,96942E-34	3,30819E-01	0,758	0,472	1,09939E-29
Macro_FOLR2+APOE+	NOL7	6,37244E-14	3,30530E-01	0,642	0,408	1,17361E-09
Macro_FOLR2+APOE+	AAK1	1,32512E-67	3,30323E-01	0,438	0,201	2,44047E-63
Macro_FOLR2+APOE+	DRG1	2,35453E-33	3,30230E-01	0,341	0,177	4,33633E-29
Macro_FOLR2+APOE+	DRG2	1,33927E-25	3,30051E-01	0,254	0,135	2,46653E-21
Macro_FOLR2+APOE+	WBP1L	1,96272E-46	3,29895E-01	0,321	0,153	3,61474E-42
Macro_FOLR2+APOE+	LARP1	9,77926E-86	3,29846E-01	0,469	0,205	1,80105E-81
Macro_FOLR2+APOE+	XRN2	3,43339E-18	3,29516E-01	0,616	0,39	6,32327E-14
Macro_FOLR2+APOE+	MAX	3,49375E-72	3,29426E-01	0,721	0,387	6,43444E-68
Macro_FOLR2+APOE+	BANF1	2,68323E-09	3,28692E-01	0,787	0,541	4,94170E-05
Macro_FOLR2+APOE+	AKR7A2	5,07738E-40	3,28649E-01	0,458	0,244	9,35100E-36
Macro_FOLR2+APOE+	RPS10	6,77463E-74	3,28193E-01	0,528	0,276	1,24768E-69
Macro_FOLR2+APOE+	NHP2	3,09815E-10	3,27683E-01	0,638	0,406	5,70586E-06
Macro_FOLR2+APOE+	PSMA2	2,27490E-79	3,27388E-01	0,477	0,217	4,18968E-75
Macro_FOLR2+APOE+	ETF1	2,31335E-18	3,27282E-01	0,51	0,313	4,26049E-14
Macro_FOLR2+APOE+	EIF4EBP1	6,29385E-43	3,26467E-01	0,712	0,401	1,15914E-38
Macro_FOLR2+APOE+	HAX1	2,76434E-29	3,26411E-01	0,583	0,339	5,09109E-25
Macro_FOLR2+APOE+	XPO6	2,33656E-71	3,26248E-01	0,336	0,138	4,30325E-67
Macro_FOLR2+APOE+	FLYWCH2	9,47400E-33	3,26051E-01	0,296	0,151	1,74483E-28
Macro_FOLR2+APOE+	LILRB2	2,68024E-44	3,25992E-01	0,754	0,448	4,93620E-40
Macro_FOLR2+APOE+	IL2RG	6,14884E-50	3,25865E-01	0,573	0,317	1,13243E-45
Macro_FOLR2+APOE+	G6PC3	4,10777E-107	3,25781E-01	0,496	0,202	7,56528E-103
Macro_FOLR2+APOE+	ABCC3	4,52178E-90	3,25305E-01	0,327	0,121	8,32776E-86
Macro_FOLR2+APOE+	TCEAL3	3,53301E-76	3,25146E-01	0,381	0,162	6,50675E-72
Macro_FOLR2+APOE+	AMZ2	1,09359E-24	3,25142E-01	0,39	0,222	2,01407E-20
Macro_FOLR2+APOE+	TOR1A	2,78414E-62	3,24339E-01	0,422	0,193	5,12755E-58
Macro_FOLR2+APOE+	CDIPT	7,63095E-69	3,24305E-01	0,481	0,223	1,40539E-64
Macro_FOLR2+APOE+	PDCL	2,59750E-68	3,23834E-01	0,281	0,111	4,78381E-64
Macro_FOLR2+APOE+	TOR3A	2,11802E-113	3,23751E-01	0,534	0,229	3,90076E-109
Macro_FOLR2+APOE+	AGPAT1	4,67108E-14	3,23717E-01	0,26	0,159	8,60273E-10
Macro_FOLR2+APOE+	MCM5	1,41281E-18	3,23551E-01	0,368	0,219	2,60198E-14
Macro_FOLR2+APOE+	ATP6V1C1	2,83472E-77	3,23380E-01	0,56	0,265	5,22071E-73
Macro_FOLR2+APOE+	MYOF	5,31548E-28	3,22700E-01	0,376	0,213	9,78953E-24
Macro_FOLR2+APOE+	SCOC	1,37261E-60	3,22613E-01	0,368	0,164	2,52794E-56
Macro_FOLR2+APOE+	CHTF8	1,15373E-07	3,22157E-01	0,252	0,174	2,12483E-03
Macro_FOLR2+APOE+	ELOB	1,26314E-101	3,21803E-01	0,746	0,388	2,32632E-97
Macro_FOLR2+APOE+	UPF3A	8,11127E-34	3,21484E-01	0,324	0,168	1,49385E-29
Macro_FOLR2+APOE+	TFAM	3,76048E-33	3,21425E-01	0,356	0,188	6,92568E-29
Macro_FOLR2+APOE+	RPL28	3,63389E-07	3,21138E-01	0,833	0,588	6,69253E-03

Macro_FOLR2+APOE+	EPSTI1	5,34814E-104	3,20650E-01	0,797	0,398	9,84967E-100
Macro_FOLR2+APOE+	PMVK	2,25075E-63	3,20623E-01	0,487	0,234	4,14520E-59
Macro_FOLR2+APOE+	SIKE1	7,82440E-35	3,20611E-01	0,263	0,128	1,44102E-30
Macro_FOLR2+APOE+	HPS5	2,03520E-46	3,19989E-01	0,393	0,198	3,74824E-42
Macro_FOLR2+APOE+	RPL31	3,91794E-46	3,19842E-01	0,806	0,488	7,21568E-42
Macro_FOLR2+APOE+	VPS35	7,19719E-12	3,19669E-01	0,663	0,433	1,32551E-07
Macro_FOLR2+APOE+	FUNDC2	1,59963E-24	3,19453E-01	0,592	0,353	2,94604E-20
Macro_FOLR2+APOE+	ELL2	4,66271E-17	3,19013E-01	0,458	0,279	8,58730E-13
Macro_FOLR2+APOE+	OTOA	6,73279E-270	3,18989E-01	0,349	0,073	1,23998E-265
Macro_FOLR2+APOE+	PHACTR4	1,92639E-51	3,18930E-01	0,35	0,164	3,54783E-47
Macro_FOLR2+APOE+	CNIH4	3,57876E-97	3,18898E-01	0,598	0,277	6,59100E-93
Macro_FOLR2+APOE+	CHMP5	8,62842E-75	3,18831E-01	0,737	0,397	1,58910E-70
Macro_FOLR2+APOE+	SELENOF	3,97670E-182	3,18711E-01	0,631	0,24	7,32388E-178
Macro_FOLR2+APOE+	THRAP3	1,54645E-17	3,18493E-01	0,65	0,413	2,84809E-13
Macro_FOLR2+APOE+	THAP7	6,16672E-49	3,18454E-01	0,286	0,127	1,13572E-44
Macro_FOLR2+APOE+	IMPACT	1,49371E-51	3,17875E-01	0,286	0,125	2,75097E-47
Macro_FOLR2+APOE+	RAD23A	5,52763E-20	3,17382E-01	0,762	0,497	1,01802E-15
Macro_FOLR2+APOE+	FARSA	4,09894E-41	3,17235E-01	0,406	0,209	7,54902E-37
Macro_FOLR2+APOE+	BROX	9,70629E-22	3,17160E-01	0,346	0,2	1,78761E-17
Macro_FOLR2+APOE+	PRDX6	1,23182E-09	3,16844E-01	0,799	0,551	2,26865E-05
Macro_FOLR2+APOE+	ARMCX3	1,53008E-17	3,16529E-01	0,415	0,254	2,81795E-13
Macro_FOLR2+APOE+	GNPTAB	5,52713E-148	3,16200E-01	0,644	0,268	1,01793E-143
Macro_FOLR2+APOE+	DDHD1	6,63386E-57	3,16070E-01	0,285	0,122	1,22176E-52
Macro_FOLR2+APOE+	SETDB2	1,65452E-53	3,15980E-01	0,283	0,124	3,04714E-49
Macro_FOLR2+APOE+	SLC20A1	1,21012E-74	3,15912E-01	0,481	0,222	2,22867E-70
Macro_FOLR2+APOE+	COX16	7,59006E-12	3,15615E-01	0,508	0,341	1,39786E-07
Macro_FOLR2+APOE+	PES1	2,34629E-31	3,15481E-01	0,301	0,158	4,32117E-27
Macro_FOLR2+APOE+	LSM12	4,39573E-38	3,15318E-01	0,416	0,219	8,09562E-34
Macro_FOLR2+APOE+	ZNF524	5,70937E-28	3,15106E-01	0,403	0,227	1,05150E-23
Macro_FOLR2+APOE+	CSF3R	5,73954E-12	3,15106E-01	0,6	0,387	1,05705E-07
Macro_FOLR2+APOE+	POLE3	1,17661E-15	3,15049E-01	0,349	0,217	2,16696E-11
Macro_FOLR2+APOE+	DCXR	2,77614E-23	3,14940E-01	0,601	0,361	5,11282E-19
Macro_FOLR2+APOE+	SAMD9L	3,37526E-76	3,14833E-01	0,567	0,271	6,21622E-72
Macro_FOLR2+APOE+	LYSMD2	8,21947E-92	3,14671E-01	0,613	0,281	1,51378E-87
Macro_FOLR2+APOE+	RPL13A	3,64477E-56	3,14653E-01	0,818	0,512	6,71257E-52
Macro_FOLR2+APOE+	MTCH1	9,85069E-34	3,13943E-01	0,736	0,447	1,81420E-29
Macro_FOLR2+APOE+	COX14	8,93342E-53	3,13895E-01	0,769	0,453	1,64527E-48
Macro_FOLR2+APOE+	U2SURP	9,50216E-21	3,13867E-01	0,53	0,321	1,75001E-16
Macro_FOLR2+APOE+	NDUFS3	2,02523E-67	3,13846E-01	0,646	0,332	3,72986E-63
Macro_FOLR2+APOE+	RANBP3	1,91816E-56	3,13467E-01	0,312	0,137	3,53267E-52
Macro_FOLR2+APOE+	CKS1B	8,47823E-52	3,12302E-01	0,262	0,112	1,56144E-47
Macro_FOLR2+APOE+	ALOX15B	1,06079E-133	3,11997E-01	0,391	0,132	1,95365E-129
Macro_FOLR2+APOE+	PTPN12	9,41210E-39	3,11773E-01	0,421	0,221	1,73343E-34
Macro_FOLR2+APOE+	TMEM19	6,55226E-50	3,11496E-01	0,298	0,134	1,20673E-45
Macro_FOLR2+APOE+	SAFB2	2,88621E-07	3,11478E-01	0,324	0,224	5,31553E-03
Macro_FOLR2+APOE+	LACTB	2,34484E-113	3,11366E-01	0,681	0,313	4,31849E-109
Macro_FOLR2+APOE+	ATP2A2	7,94641E-100	3,11203E-01	0,522	0,225	1,46349E-95

Macro_FOLR2+APOE+	IFI35	2,10103E-94	3,11138E-01	0,696	0,336	3,86948E-90
Macro_FOLR2+APOE+	ERGIC2	1,09616E-32	3,10754E-01	0,396	0,212	2,01880E-28
Macro_FOLR2+APOE+	COMMD7	4,84399E-31	3,10322E-01	0,528	0,305	8,92118E-27
Macro_FOLR2+APOE+	DERA	7,96889E-61	3,09575E-01	0,412	0,19	1,46763E-56
Macro_FOLR2+APOE+	RRAS	2,26308E-149	3,09317E-01	0,287	0,077	4,16791E-145
Macro_FOLR2+APOE+	TGM2	2,89837E-243	3,09238E-01	0,385	0,094	5,33794E-239
Macro_FOLR2+APOE+	ACBD6	6,52747E-32	3,08128E-01	0,33	0,173	1,20216E-27
Macro_FOLR2+APOE+	TDP2	1,82753E-90	3,08085E-01	0,359	0,137	3,36576E-86
Macro_FOLR2+APOE+	C1QBP	1,02000E-21	3,08074E-01	0,686	0,422	1,87854E-17
Macro_FOLR2+APOE+	SMC3	5,63289E-26	3,07812E-01	0,338	0,189	1,03741E-21
Macro_FOLR2+APOE+	FAM78A	3,68893E-81	3,06955E-01	0,267	0,095	6,79391E-77
Macro_FOLR2+APOE+	GYPC	4,57922E-57	3,06534E-01	0,566	0,293	8,43355E-53
Macro_FOLR2+APOE+	GPS1	7,74460E-21	3,06409E-01	0,432	0,261	1,42632E-16
Macro_FOLR2+APOE+	AP1M1	1,64967E-12	3,06258E-01	0,376	0,243	3,03820E-08
Macro_FOLR2+APOE+	ITPR2	2,96930E-91	3,05924E-01	0,575	0,269	5,46856E-87
Macro_FOLR2+APOE+	RPS19	1,48678E-21	3,05522E-01	0,83	0,568	2,73821E-17
Macro_FOLR2+APOE+	ATP5MG	7,62248E-73	3,05513E-01	0,75	0,409	1,40383E-68
Macro_FOLR2+APOE+	C1orf52	4,32273E-12	3,04748E-01	0,262	0,164	7,96116E-08
Macro_FOLR2+APOE+	MANBAL	4,41898E-83	3,04666E-01	0,266	0,093	8,13843E-79
Macro_FOLR2+APOE+	ATP5F1E	6,78004E-49	3,04598E-01	0,767	0,469	1,24868E-44
Macro_FOLR2+APOE+	PTRH1	3,55200E-90	3,04414E-01	0,296	0,105	6,54172E-86
Macro_FOLR2+APOE+	MICOS10	8,05466E-100	3,04261E-01	0,452	0,196	1,48343E-95
Macro_FOLR2+APOE+	CAMLG	1,33293E-12	3,04147E-01	0,449	0,288	2,45485E-08
Macro_FOLR2+APOE+	NBR1	2,33387E-44	3,04115E-01	0,361	0,179	4,29829E-40
Macro_FOLR2+APOE+	PTP4A2	2,54957E-15	3,03307E-01	0,632	0,398	4,69555E-11
Macro_FOLR2+APOE+	ZNF32	1,25514E-68	3,03036E-01	0,329	0,135	2,31160E-64
Macro_FOLR2+APOE+	VAV1	1,82797E-55	3,03013E-01	0,399	0,187	3,36657E-51
Macro_FOLR2+APOE+	NFE2L1	3,87879E-115	3,02930E-01	0,385	0,139	7,14357E-111
Macro_FOLR2+APOE+	METTL23	2,30427E-33	3,02903E-01	0,399	0,216	4,24377E-29
Macro_FOLR2+APOE+	MYL12B	1,95378E-07	3,02251E-01	0,933	0,772	3,59828E-03
Macro_FOLR2+APOE+	PFN1	1,06445E-08	3,02248E-01	0,996	0,946	1,96039E-04
Macro_FOLR2+APOE+	TMEM165	1,16453E-52	3,02165E-01	0,685	0,384	2,14472E-48
Macro_FOLR2+APOE+	PRPF40A	6,79566E-10	3,01649E-01	0,667	0,449	1,25156E-05
Macro_FOLR2+APOE+	MAN2B2	2,37845E-83	3,01541E-01	0,321	0,123	4,38040E-79
Macro_FOLR2+APOE+	TMEM160	6,25830E-49	3,01016E-01	0,678	0,374	1,15259E-44
Macro_FOLR2+APOE+	RHOQ	1,16243E-92	3,00984E-01	0,501	0,214	2,14084E-88
Macro_FOLR2+APOE+	MGMT	1,55802E-57	3,00941E-01	0,501	0,249	2,86941E-53
Macro_FOLR2+APOE+	NOB1	2,83969E-27	3,00836E-01	0,286	0,153	5,22986E-23
Macro_FOLR2+APOE+	ARHGAP21	7,93602E-44	3,00566E-01	0,258	0,116	1,46158E-39
Macro_FOLR2+APOE+	USB1	5,30082E-50	3,00549E-01	0,373	0,179	9,76252E-46
Macro_FOLR2+APOE+	PSMD9	1,96203E-16	3,00367E-01	0,405	0,25	3,61346E-12
Macro_FOLR2+APOE+	RPL27	6,99515E-35	2,99643E-01	0,801	0,496	1,28830E-30
Macro_FOLR2+APOE+	MRPL54	1,40405E-34	2,99354E-01	0,677	0,395	2,58583E-30
Macro_FOLR2+APOE+	SULT1A1	1,88248E-39	2,99231E-01	0,317	0,155	3,46696E-35
Macro_FOLR2+APOE+	MPPE1	7,91214E-31	2,99227E-01	0,313	0,163	1,45718E-26
Macro_FOLR2+APOE+	NTMT1	2,36380E-56	2,99069E-01	0,42	0,199	4,35341E-52
Macro_FOLR2+APOE+	ZNF385A	6,55493E-26	2,99002E-01	0,66	0,405	1,20722E-21

Macro_FOLR2+APOE+	DYSF	6,58735E-142	2,98696E-01	0,27	0,073	1,21319E-137
Macro_FOLR2+APOE+	ATP5F1D	2,26297E-121	2,98570E-01	0,708	0,329	4,16770E-117
Macro_FOLR2+APOE+	RPL3	1,21798E-26	2,98095E-01	0,816	0,525	2,24315E-22
Macro_FOLR2+APOE+	UFM1	6,33195E-21	2,98093E-01	0,529	0,32	1,16616E-16
Macro_FOLR2+APOE+	OAS3	1,29499E-35	2,98036E-01	0,286	0,143	2,38499E-31
Macro_FOLR2+APOE+	GSPT1	8,13882E-32	2,97891E-01	0,439	0,24	1,49893E-27
Macro_FOLR2+APOE+	RAB35	1,75714E-122	2,97022E-01	0,43	0,157	3,23612E-118
Macro_FOLR2+APOE+	DNAJC8	9,54436E-27	2,96849E-01	0,672	0,406	1,75779E-22
Macro_FOLR2+APOE+	SMIM4	4,59669E-139	2,96715E-01	0,482	0,177	8,46572E-135
Macro_FOLR2+APOE+	DDX27	2,78655E-10	2,96414E-01	0,353	0,234	5,13200E-06
Macro_FOLR2+APOE+	KDELR1	4,30185E-25	2,96144E-01	0,809	0,531	7,92272E-21
Macro_FOLR2+APOE+	STT3A	2,34205E-39	2,96085E-01	0,309	0,154	4,31335E-35
Macro_FOLR2+APOE+	CDC123	1,09071E-32	2,95708E-01	0,417	0,23	2,00876E-28
Macro_FOLR2+APOE+	NAA10	2,90071E-82	2,95270E-01	0,42	0,18	5,34225E-78
Macro_FOLR2+APOE+	PSMB8	3,34123E-23	2,95137E-01	0,849	0,569	6,15354E-19
Macro_FOLR2+APOE+	APPL2	1,41424E-107	2,95064E-01	0,269	0,084	2,60461E-103
Macro_FOLR2+APOE+	EHD4	6,24759E-57	2,94978E-01	0,437	0,213	1,15062E-52
Macro_FOLR2+APOE+	DHRS7B	3,66768E-65	2,94943E-01	0,258	0,1	6,75476E-61
Macro_FOLR2+APOE+	SASH1	8,92297E-226	2,94800E-01	0,295	0,062	1,64334E-221
Macro_FOLR2+APOE+	SNRPB2	7,14219E-20	2,94799E-01	0,638	0,4	1,31538E-15
Macro_FOLR2+APOE+	PLXNC1	9,03443E-90	2,94537E-01	0,526	0,238	1,66387E-85
Macro_FOLR2+APOE+	ECI2	2,90075E-51	2,93999E-01	0,297	0,132	5,34231E-47
Macro_FOLR2+APOE+	CHD9	4,29919E-19	2,93879E-01	0,548	0,337	7,91782E-15
Macro_FOLR2+APOE+	HINT2	3,23714E-15	2,92972E-01	0,358	0,226	5,96184E-11
Macro_FOLR2+APOE+	PSMB6	5,54007E-28	2,92935E-01	0,842	0,568	1,02031E-23
Macro_FOLR2+APOE+	SF3B5	1,46854E-09	2,92815E-01	0,792	0,545	2,70462E-05
Macro_FOLR2+APOE+	NUBP2	1,80291E-28	2,92619E-01	0,356	0,195	3,32042E-24
Macro_FOLR2+APOE+	CISD1	8,63527E-70	2,92432E-01	0,353	0,15	1,59036E-65
Macro_FOLR2+APOE+	HSBP1L1	2,70446E-126	2,92157E-01	0,306	0,093	4,98080E-122
Macro_FOLR2+APOE+	CSNK2B	8,82614E-70	2,92147E-01	0,62	0,329	1,62551E-65
Macro_FOLR2+APOE+	TMEM60	4,54558E-79	2,91719E-01	0,438	0,189	8,37159E-75
Macro_FOLR2+APOE+	QKI	1,09438E-52	2,91690E-01	0,654	0,36	2,01553E-48
Macro_FOLR2+APOE+	MRPL57	1,82752E-161	2,91538E-01	0,636	0,258	3,36574E-157
Macro_FOLR2+APOE+	FAH	4,39407E-69	2,91534E-01	0,333	0,138	8,09256E-65
Macro_FOLR2+APOE+	LAMTOR1	1,09340E-16	2,91417E-01	0,891	0,64	2,01371E-12
Macro_FOLR2+APOE+	SGTA	1,15971E-49	2,91025E-01	0,335	0,157	2,13583E-45
Macro_FOLR2+APOE+	TMEM230	5,76150E-26	2,90977E-01	0,716	0,444	1,06110E-21
Macro_FOLR2+APOE+	TMEM11	1,35534E-53	2,90532E-01	0,353	0,161	2,49614E-49
Macro_FOLR2+APOE+	CCDC112	1,38433E-34	2,90327E-01	0,344	0,18	2,54953E-30
Macro_FOLR2+APOE+	TIMM10	4,47120E-36	2,90292E-01	0,454	0,247	8,23461E-32
Macro_FOLR2+APOE+	EXOSC5	2,03181E-11	2,90212E-01	0,263	0,167	3,74199E-07
Macro_FOLR2+APOE+	SLC39A11	3,53126E-109	2,90052E-01	0,322	0,109	6,50351E-105
Macro_FOLR2+APOE+	GART	8,07920E-41	2,90022E-01	0,281	0,133	1,48795E-36
Macro_FOLR2+APOE+	SSR4	1,71432E-07	2,89929E-01	0,938	0,748	3,15726E-03
Macro_FOLR2+APOE+	SMARCA2	1,48863E-21	2,89574E-01	0,371	0,218	2,74161E-17
Macro_FOLR2+APOE+	UFC1	1,07285E-19	2,89430E-01	0,766	0,495	1,97587E-15
Macro_FOLR2+APOE+	ELP5	2,59638E-28	2,89425E-01	0,34	0,185	4,78175E-24

Macro_FOLR2+APOE+	ARSB	2,86314E-55	2,89346E-01	0,25	0,103	5,27304E-51
Macro_FOLR2+APOE+	RAP1GDS1	7,29986E-11	2,89168E-01	0,283	0,184	1,34442E-06
Macro_FOLR2+APOE+	C15orf48	5,05511E-66	2,89045E-01	0,637	0,336	9,30999E-62
Macro_FOLR2+APOE+	BRWD1	2,67166E-10	2,88893E-01	0,283	0,187	4,92040E-06
Macro_FOLR2+APOE+	MED4	8,58206E-26	2,88881E-01	0,342	0,192	1,58056E-21
Macro_FOLR2+APOE+	CNTLN	1,46468E-53	2,88175E-01	0,317	0,144	2,69749E-49
Macro_FOLR2+APOE+	MED10	2,65519E-28	2,87761E-01	0,428	0,241	4,89006E-24
Macro_FOLR2+APOE+	DFFA	5,02996E-32	2,87718E-01	0,25	0,124	9,26368E-28
Macro_FOLR2+APOE+	SECTM1	1,13600E-122	2,87685E-01	0,431	0,158	2,09218E-118
Macro_FOLR2+APOE+	APOBEC3C	1,70466E-43	2,87248E-01	0,391	0,197	3,13947E-39
Macro_FOLR2+APOE+	TLE3	2,66160E-18	2,87171E-01	0,304	0,182	4,90186E-14
Macro_FOLR2+APOE+	USP4	2,67798E-21	2,87154E-01	0,48	0,29	4,93204E-17
Macro_FOLR2+APOE+	MRPL20	4,14250E-25	2,86962E-01	0,74	0,46	7,62924E-21
Macro_FOLR2+APOE+	UBE2F	6,06196E-73	2,86960E-01	0,522	0,252	1,11643E-68
Macro_FOLR2+APOE+	FAM120A	3,50666E-34	2,86904E-01	0,516	0,287	6,45822E-30
Macro_FOLR2+APOE+	RPS3	5,66983E-12	2,86784E-01	0,824	0,56	1,04421E-07
Macro_FOLR2+APOE+	B4GALT1	7,48769E-34	2,86739E-01	0,542	0,319	1,37901E-29
Macro_FOLR2+APOE+	PRXL2A	0,00000E+00	2,86213E-01	0,262	0,038	0,00000E+00
Macro_FOLR2+APOE+	GCC2	4,73639E-52	2,86047E-01	0,528	0,273	8,72301E-48
Macro_FOLR2+APOE+	SUPT16H	4,40516E-27	2,86042E-01	0,355	0,198	8,11298E-23
Macro_FOLR2+APOE+	SEC11C	4,49218E-72	2,86003E-01	0,631	0,317	8,27325E-68
Macro_FOLR2+APOE+	CYBRD1	1,42771E-47	2,85416E-01	0,257	0,112	2,62941E-43
Macro_FOLR2+APOE+	TMEM87A	1,51047E-43	2,85377E-01	0,379	0,189	2,78183E-39
Macro_FOLR2+APOE+	PAQR8	4,23930E-111	2,85326E-01	0,303	0,099	7,80752E-107
Macro_FOLR2+APOE+	ST13	1,99947E-08	2,84792E-01	0,847	0,598	3,68243E-04
Macro_FOLR2+APOE+	AIP	2,11435E-12	2,84770E-01	0,523	0,335	3,89400E-08
Macro_FOLR2+APOE+	LBR	1,43528E-27	2,84424E-01	0,307	0,166	2,64336E-23
Macro_FOLR2+APOE+	ADAMTSL4	2,44038E-55	2,84038E-01	0,262	0,111	4,49445E-51
Macro_FOLR2+APOE+	SPINT1	1,41006E-19	2,83856E-01	0,254	0,146	2,59690E-15
Macro_FOLR2+APOE+	FGFR1OP2	3,71816E-55	2,83806E-01	0,578	0,298	6,84774E-51
Macro_FOLR2+APOE+	GON4L	5,71925E-19	2,83673E-01	0,333	0,197	1,05331E-14
Macro_FOLR2+APOE+	GNGT2	2,93455E-89	2,83628E-01	0,295	0,104	5,40456E-85
Macro_FOLR2+APOE+	IK	3,95267E-23	2,83107E-01	0,559	0,332	7,27963E-19
Macro_FOLR2+APOE+	RWDD4	1,91746E-38	2,82809E-01	0,311	0,154	3,53139E-34
Macro_FOLR2+APOE+	EIF2A	2,46416E-07	2,82799E-01	0,446	0,304	4,53824E-03
Macro_FOLR2+APOE+	CYBC1	2,45627E-211	2,82578E-01	0,569	0,189	4,52371E-207
Macro_FOLR2+APOE+	USP22	2,15485E-27	2,82575E-01	0,368	0,208	3,96858E-23
Macro_FOLR2+APOE+	RBBP7	1,11782E-29	2,82509E-01	0,368	0,2	2,05870E-25
Macro_FOLR2+APOE+	PCSK7	2,84698E-45	2,82347E-01	0,343	0,165	5,24328E-41
Macro_FOLR2+APOE+	ODF3B	5,18449E-30	2,82174E-01	0,582	0,339	9,54827E-26
Macro_FOLR2+APOE+	NRAS	2,20146E-26	2,81340E-01	0,263	0,14	4,05443E-22
Macro_FOLR2+APOE+	DDX41	1,35677E-45	2,81247E-01	0,343	0,166	2,49876E-41
Macro_FOLR2+APOE+	TRAPPC5	5,06628E-40	2,80805E-01	0,414	0,233	9,33057E-36
Macro_FOLR2+APOE+	PSMB2	3,71379E-45	2,80449E-01	0,725	0,433	6,83968E-41
Macro_FOLR2+APOE+	GLS	4,44737E-15	2,80238E-01	0,323	0,201	8,19073E-11
Macro_FOLR2+APOE+	DKC1	9,36621E-25	2,80205E-01	0,252	0,136	1,72497E-20
Macro_FOLR2+APOE+	SEC14L1	1,28000E-19	2,80119E-01	0,615	0,389	2,35738E-15

Macro_FOLR2+APOE+	TOR2A	8,09592E-51	2,79988E-01	0,404	0,197	1,49102E-46
Macro_FOLR2+APOE+	TXLNA	8,31396E-48	2,79380E-01	0,259	0,115	1,53118E-43
Macro_FOLR2+APOE+	SESN1	3,28125E-11	2,79344E-01	0,317	0,207	6,04309E-07
Macro_FOLR2+APOE+	UBQLN1	1,39734E-22	2,78911E-01	0,44	0,257	2,57349E-18
Macro_FOLR2+APOE+	SRPK2	3,98146E-18	2,78705E-01	0,348	0,209	7,33265E-14
Macro_FOLR2+APOE+	TRPC4AP	2,36104E-37	2,78638E-01	0,406	0,214	4,34832E-33
Macro_FOLR2+APOE+	RHBDD2	6,43929E-26	2,78595E-01	0,436	0,252	1,18592E-21
Macro_FOLR2+APOE+	KPNB1	1,13877E-31	2,78502E-01	0,724	0,432	2,09727E-27
Macro_FOLR2+APOE+	VEZT	8,87371E-34	2,78395E-01	0,271	0,135	1,63427E-29
Macro_FOLR2+APOE+	ISCA2	8,69133E-87	2,78173E-01	0,475	0,204	1,60068E-82
Macro_FOLR2+APOE+	GLG1	3,41486E-34	2,78164E-01	0,508	0,285	6,28915E-30
Macro_FOLR2+APOE+	NDUFV3	1,65451E-96	2,78021E-01	0,604	0,271	3,04711E-92
Macro_FOLR2+APOE+	RAB8B	8,01583E-13	2,77994E-01	0,385	0,246	1,47627E-08
Macro_FOLR2+APOE+	SARAF	1,27346E-87	2,77935E-01	0,766	0,418	2,34533E-83
Macro_FOLR2+APOE+	FKBP1A	1,55406E-06	2,77797E-01	0,903	0,688	2,86212E-02
Macro_FOLR2+APOE+	CHCHD7	3,04688E-34	2,77797E-01	0,46	0,251	5,61144E-30
Macro_FOLR2+APOE+	TAF1D	2,43817E-06	2,77584E-01	0,55	0,38	4,49038E-02
Macro_FOLR2+APOE+	SNRPC	1,28431E-49	2,77461E-01	0,732	0,41	2,36532E-45
Macro_FOLR2+APOE+	DAGLB	6,50499E-13	2,77392E-01	0,267	0,168	1,19802E-08
Macro_FOLR2+APOE+	CREBRF	2,09895E-37	2,77137E-01	0,397	0,211	3,86563E-33
Macro_FOLR2+APOE+	PPP6R3	1,10464E-12	2,76796E-01	0,254	0,161	2,03442E-08
Macro_FOLR2+APOE+	PXDC1	1,28191E-115	2,76771E-01	0,256	0,075	2,36090E-111
Macro_FOLR2+APOE+	USP9X	2,62115E-48	2,76491E-01	0,51	0,267	4,82737E-44
Macro_FOLR2+APOE+	DGKZ	5,74060E-19	2,76443E-01	0,335	0,199	1,05725E-14
Macro_FOLR2+APOE+	TM7SF3	3,24159E-98	2,75823E-01	0,383	0,145	5,97004E-94
Macro_FOLR2+APOE+	MRPL2	1,31997E-39	2,75717E-01	0,31	0,151	2,43099E-35
Macro_FOLR2+APOE+	NDUFS4	2,83281E-37	2,75630E-01	0,506	0,281	5,21719E-33
Macro_FOLR2+APOE+	EXOC7	4,45384E-52	2,75420E-01	0,372	0,175	8,20264E-48
Macro_FOLR2+APOE+	THEMIS2	1,75438E-36	2,75160E-01	0,801	0,486	3,23104E-32
Macro_FOLR2+APOE+	S100A13	3,26759E-112	2,74660E-01	0,292	0,094	6,01792E-108
Macro_FOLR2+APOE+	ORAI2	1,54298E-25	2,74506E-01	0,301	0,165	2,84170E-21
Macro_FOLR2+APOE+	ANKRD13D	7,37389E-41	2,74477E-01	0,32	0,157	1,35805E-36
Macro_FOLR2+APOE+	TMEM127	7,28745E-93	2,74166E-01	0,441	0,182	1,34213E-88
Macro_FOLR2+APOE+	CLIC1	2,38750E-27	2,73792E-01	0,966	0,872	4,39706E-23
Macro_FOLR2+APOE+	MVB12A	1,34635E-74	2,73640E-01	0,45	0,2	2,47958E-70
Macro_FOLR2+APOE+	SEC61A1	3,20190E-40	2,73499E-01	0,551	0,304	5,89693E-36
Macro_FOLR2+APOE+	C5orf15	4,54349E-56	2,73180E-01	0,452	0,222	8,36774E-52
Macro_FOLR2+APOE+	BCAS2	2,91470E-25	2,72718E-01	0,449	0,258	5,36800E-21
Macro_FOLR2+APOE+	ATP5PB	9,56668E-153	2,72656E-01	0,656	0,272	1,76190E-148
Macro_FOLR2+APOE+	UBE2G2	1,61574E-18	2,72249E-01	0,362	0,219	2,97570E-14
Macro_FOLR2+APOE+	FAM104A	8,80535E-37	2,72097E-01	0,269	0,13	1,62168E-32
Macro_FOLR2+APOE+	CES2	1,96006E-51	2,72033E-01	0,257	0,109	3,60985E-47
Macro_FOLR2+APOE+	ILF3	8,07447E-07	2,71531E-01	0,53	0,362	1,48708E-02
Macro_FOLR2+APOE+	FBXO6	7,52340E-124	2,70882E-01	0,44	0,161	1,38558E-119
Macro_FOLR2+APOE+	EFCAB14	1,22318E-25	2,70658E-01	0,455	0,262	2,25272E-21
Macro_FOLR2+APOE+	TPRKB	8,73650E-21	2,70614E-01	0,402	0,237	1,60900E-16
Macro_FOLR2+APOE+	C1GALT1	1,36486E-41	2,70586E-01	0,291	0,14	2,51366E-37

Macro_FOLR2+APOE+	PI4KB	3,42439E-26	2,70581E-01	0,283	0,154	6,30669E-22
Macro_FOLR2+APOE+	PANK2	8,34933E-48	2,70010E-01	0,387	0,19	1,53770E-43
Macro_FOLR2+APOE+	YIPF2	1,39603E-65	2,69705E-01	0,308	0,127	2,57106E-61
Macro_FOLR2+APOE+	COX5B	6,33937E-19	2,69687E-01	0,948	0,767	1,16752E-14
Macro_FOLR2+APOE+	UNC13D	3,73382E-43	2,69125E-01	0,275	0,127	6,87658E-39
Macro_FOLR2+APOE+	TSEN34	1,22147E-55	2,68932E-01	0,551	0,283	2,24958E-51
Macro_FOLR2+APOE+	DDX54	5,77620E-29	2,68809E-01	0,334	0,18	1,06380E-24
Macro_FOLR2+APOE+	VBP1	4,40147E-22	2,68789E-01	0,347	0,201	8,10619E-18
Macro_FOLR2+APOE+	LEPROT	3,84655E-49	2,68781E-01	0,637	0,347	7,08420E-45
Macro_FOLR2+APOE+	SLC49A3	0,00000E+00	2,68572E-01	0,324	0,043	0,00000E+00
Macro_FOLR2+APOE+	RIC8A	1,86559E-36	2,68354E-01	0,354	0,181	3,43585E-32
Macro_FOLR2+APOE+	LRRFIP2	8,09218E-18	2,68265E-01	0,406	0,248	1,49034E-13
Macro_FOLR2+APOE+	BID	1,39117E-13	2,68221E-01	0,635	0,408	2,56211E-09
Macro_FOLR2+APOE+	POP4	6,53516E-47	2,68147E-01	0,41	0,204	1,20358E-42
Macro_FOLR2+APOE+	HSDL2	9,99569E-18	2,68044E-01	0,266	0,155	1,84091E-13
Macro_FOLR2+APOE+	COPRS	1,00475E-161	2,67902E-01	0,338	0,096	1,85045E-157
Macro_FOLR2+APOE+	AURKAIP1	8,07329E-25	2,67758E-01	0,793	0,51	1,48686E-20
Macro_FOLR2+APOE+	SNRPN	2,10534E-114	2,67627E-01	0,34	0,117	3,87741E-110
Macro_FOLR2+APOE+	FMNL3	1,03767E-38	2,67280E-01	0,379	0,197	1,91109E-34
Macro_FOLR2+APOE+	NFU1	5,29795E-67	2,67200E-01	0,354	0,151	9,75723E-63
Macro_FOLR2+APOE+	GPR137	2,12675E-102	2,67101E-01	0,304	0,104	3,91684E-98
Macro_FOLR2+APOE+	LYAR	6,56889E-20	2,66536E-01	0,266	0,151	1,20979E-15
Macro_FOLR2+APOE+	NOP56	9,54377E-51	2,66519E-01	0,416	0,203	1,75768E-46
Macro_FOLR2+APOE+	FIG4	6,96412E-69	2,65818E-01	0,253	0,095	1,28258E-64
Macro_FOLR2+APOE+	WBP11	9,06610E-48	2,65321E-01	0,432	0,218	1,66970E-43
Macro_FOLR2+APOE+	GTF3C6	6,50296E-82	2,65257E-01	0,701	0,362	1,19765E-77
Macro_FOLR2+APOE+	DHRS4	6,43586E-34	2,65033E-01	0,48	0,265	1,18529E-29
Macro_FOLR2+APOE+	COX7B	4,42447E-17	2,64063E-01	0,895	0,66	8,14854E-13
Macro_FOLR2+APOE+	RANGRF	1,92819E-25	2,63392E-01	0,28	0,151	3,55115E-21
Macro_FOLR2+APOE+	STOML2	2,98117E-55	2,63369E-01	0,533	0,268	5,49042E-51
Macro_FOLR2+APOE+	BIRC6	7,41230E-17	2,63197E-01	0,396	0,245	1,36512E-12
Macro_FOLR2+APOE+	UGP2	3,12279E-76	2,62845E-01	0,597	0,292	5,75124E-72
Macro_FOLR2+APOE+	MPZL1	2,92486E-78	2,62828E-01	0,404	0,171	5,38672E-74
Macro_FOLR2+APOE+	NPC1	1,35984E-120	2,62688E-01	0,356	0,121	2,50442E-116
Macro_FOLR2+APOE+	FDFT1	2,86727E-14	2,62685E-01	0,403	0,256	5,28064E-10
Macro_FOLR2+APOE+	SAMHD1	2,09644E-10	2,62365E-01	0,912	0,638	3,86102E-06
Macro_FOLR2+APOE+	TMEM134	7,88930E-44	2,62229E-01	0,509	0,266	1,45297E-39
Macro_FOLR2+APOE+	HK1	8,16584E-31	2,62071E-01	0,461	0,257	1,50390E-26
Macro_FOLR2+APOE+	DERL2	3,81029E-63	2,61712E-01	0,476	0,228	7,01741E-59
Macro_FOLR2+APOE+	MRPL35	4,34215E-85	2,61541E-01	0,339	0,13	7,99694E-81
Macro_FOLR2+APOE+	PET100	5,23414E-12	2,61413E-01	0,62	0,399	9,63972E-08
Macro_FOLR2+APOE+	MRPS18A	2,25184E-57	2,60946E-01	0,358	0,162	4,14722E-53
Macro_FOLR2+APOE+	ZRANB2	9,73891E-12	2,60907E-01	0,363	0,235	1,79362E-07
Macro_FOLR2+APOE+	SELENOH	1,15589E-119	2,60702E-01	0,697	0,32	2,12881E-115
Macro_FOLR2+APOE+	BRD7	2,75229E-28	2,60648E-01	0,262	0,137	5,06889E-24
Macro_FOLR2+APOE+	TMED2	9,57533E-18	2,60455E-01	0,756	0,492	1,76349E-13
Macro_FOLR2+APOE+	EIF3I	8,82943E-13	2,60156E-01	0,703	0,459	1,62612E-08

Macro_FOLR2+APOE+	MRPS25	2,49126E-69	2,59992E-01	0,458	0,209	4,58815E-65
Macro_FOLR2+APOE+	TGOLN2	2,23101E-29	2,59898E-01	0,8	0,505	4,10885E-25
Macro_FOLR2+APOE+	CCDC91	2,53710E-36	2,59799E-01	0,349	0,178	4,67258E-32
Macro_FOLR2+APOE+	MAT2B	2,14251E-31	2,59733E-01	0,569	0,329	3,94586E-27
Macro_FOLR2+APOE+	SNX17	1,07107E-28	2,59354E-01	0,705	0,425	1,97260E-24
Macro_FOLR2+APOE+	NDUFB10	1,31376E-19	2,59150E-01	0,815	0,551	2,41955E-15
Macro_FOLR2+APOE+	NDUFA1	9,89432E-12	2,58918E-01	0,9	0,681	1,82224E-07
Macro_FOLR2+APOE+	TMEM101	1,20016E-39	2,58850E-01	0,326	0,16	2,21034E-35
Macro_FOLR2+APOE+	C20orf27	1,68730E-32	2,58835E-01	0,594	0,337	3,10750E-28
Macro_FOLR2+APOE+	NDUFA4	5,00961E-26	2,58692E-01	0,942	0,739	9,22621E-22
Macro_FOLR2+APOE+	MPHOSPH6	4,34791E-18	2,58446E-01	0,292	0,172	8,00755E-14
Macro_FOLR2+APOE+	STX6	4,73414E-37	2,58355E-01	0,461	0,245	8,71886E-33
Macro_FOLR2+APOE+	RPL26L1	1,94352E-138	2,57805E-01	0,414	0,141	3,57939E-134
Macro_FOLR2+APOE+	OXA1L	2,32747E-07	2,57662E-01	0,665	0,449	4,28651E-03
Macro_FOLR2+APOE+	GIMAP6	1,78564E-103	2,57626E-01	0,362	0,132	3,28860E-99
Macro_FOLR2+APOE+	MRAS	5,13238E-134	2,57550E-01	0,301	0,088	9,45231E-130
Macro_FOLR2+APOE+	TSTD1	6,55456E-23	2,57458E-01	0,402	0,234	1,20715E-18
Macro_FOLR2+APOE+	LPIN2	5,15406E-65	2,57339E-01	0,464	0,217	9,49224E-61
Macro_FOLR2+APOE+	VPS13C	4,69391E-15	2,57303E-01	0,562	0,365	8,64478E-11
Macro_FOLR2+APOE+	PRPS2	5,74388E-51	2,57141E-01	0,312	0,142	1,05785E-46
Macro_FOLR2+APOE+	SLC38A10	3,61759E-53	2,56997E-01	0,45	0,221	6,66252E-49
Macro_FOLR2+APOE+	UBE2I	4,06463E-08	2,56621E-01	0,768	0,52	7,48582E-04
Macro_FOLR2+APOE+	FBXL15	7,49536E-63	2,56011E-01	0,439	0,206	1,38042E-58
Macro_FOLR2+APOE+	ATG12	4,73073E-24	2,55788E-01	0,446	0,258	8,71258E-20
Macro_FOLR2+APOE+	UBE2V1	5,06150E-18	2,55445E-01	0,289	0,178	9,32177E-14
Macro_FOLR2+APOE+	ATG3	1,21311E-12	2,55355E-01	0,789	0,515	2,23419E-08
Macro_FOLR2+APOE+	NME6	5,02018E-52	2,55208E-01	0,265	0,114	9,24567E-48
Macro_FOLR2+APOE+	HSBP1	1,28788E-21	2,55157E-01	0,87	0,593	2,37189E-17
Macro_FOLR2+APOE+	SEM1	9,62692E-123	2,54776E-01	0,686	0,312	1,77299E-118
Macro_FOLR2+APOE+	RNF187	1,56667E-88	2,54599E-01	0,361	0,142	2,88533E-84
Macro_FOLR2+APOE+	GCLC	2,46431E-114	2,54516E-01	0,34	0,117	4,53851E-110
Macro_FOLR2+APOE+	TMEM126A	4,82480E-47	2,54280E-01	0,425	0,213	8,88583E-43
Macro_FOLR2+APOE+	CASP1	5,63821E-16	2,54005E-01	0,719	0,456	1,03839E-11
Macro_FOLR2+APOE+	SLC35E3	7,84957E-47	2,53961E-01	0,278	0,124	1,44566E-42
Macro_FOLR2+APOE+	RPS16	4,94912E-13	2,53821E-01	0,822	0,537	9,11480E-09
Macro_FOLR2+APOE+	TNFRSF1A	5,30741E-56	2,52746E-01	0,771	0,439	9,77466E-52
Macro_FOLR2+APOE+	ABCG1	3,76264E-105	2,52125E-01	0,446	0,178	6,92965E-101
Macro_FOLR2+APOE+	SCYL1	4,67702E-53	2,51944E-01	0,375	0,178	8,61367E-49
Macro_FOLR2+APOE+	PPP1CA	2,36947E-09	2,51874E-01	0,873	0,615	4,36386E-05
Macro_FOLR2+APOE+	UBA52	9,49440E-48	2,51711E-01	0,983	0,919	1,74858E-43
Macro_FOLR2+APOE+	QSOX1	6,40474E-39	2,51680E-01	0,513	0,282	1,17956E-34
Macro_FOLR2+APOE+	CCNC	5,60465E-28	2,50815E-01	0,298	0,159	1,03221E-23
Macro_FOLR2+APOE+	CYTH2	8,55220E-24	2,50678E-01	0,257	0,14	1,57506E-19
Macro_FOLR2+APOE+	ZBED1	8,06697E-78	2,50641E-01	0,334	0,135	1,48569E-73
Macro_FOLR2+APOE+	SLC52A2	1,62594E-64	2,50619E-01	0,388	0,174	2,99449E-60
Macro_FOLR2+APOE+	VMA21	1,32106E-41	2,50536E-01	0,653	0,367	2,43300E-37
Macro_FOLR2+APOE+	TMEM18	2,11752E-23	-2,50148E-01	0,334	0,19	3,89983E-19

Macro_FOLR2+APOE+	CRYZL1	1,30686E-26	-2,50482E-01	0,251	0,132	2,40684E-22
Macro_FOLR2+APOE+	PRPF38A	9,58813E-09	-2,50914E-01	0,279	0,185	1,76585E-04
Macro_FOLR2+APOE+	FAM53C	3,48309E-13	-2,53695E-01	0,272	0,171	6,41481E-09
Macro_FOLR2+APOE+	MRPL36	7,47830E-35	-2,54092E-01	0,425	0,225	1,37728E-30
Macro_FOLR2+APOE+	MZT2A	1,68089E-18	-2,54632E-01	0,379	0,226	3,09570E-14
Macro_FOLR2+APOE+	NDRG1	5,68110E-22	-2,55741E-01	0,334	0,194	1,04629E-17
Macro_FOLR2+APOE+	PNKP	5,96160E-25	-2,58377E-01	0,315	0,174	1,09795E-20
Macro_FOLR2+APOE+	HECTD1	2,42381E-12	-2,58522E-01	0,353	0,228	4,46394E-08
Macro_FOLR2+APOE+	RXRA	1,15098E-17	-2,59855E-01	0,274	0,159	2,11975E-13
Macro_FOLR2+APOE+	CD37	1,08664E-09	-2,61407E-01	0,891	0,728	2,00127E-05
Macro_FOLR2+APOE+	CD55	5,10879E-35	-2,62297E-01	0,362	0,431	9,40886E-31
Macro_FOLR2+APOE+	PCBP2	2,94039E-37	-2,67452E-01	0,911	0,795	5,41532E-33
Macro_FOLR2+APOE+	SPAG9	6,45060E-07	-2,69768E-01	0,432	0,3	1,18801E-02
Macro_FOLR2+APOE+	IFIT3	2,58577E-105	-2,80428E-01	0,498	0,203	4,76221E-101
Macro_FOLR2+APOE+	FAM111A	3,15777E-29	-2,85426E-01	0,334	0,178	5,81567E-25
Macro_FOLR2+APOE+	MICU2	4,50543E-17	-2,89517E-01	0,257	0,149	8,29766E-13
Macro_FOLR2+APOE+	SLC2A3	7,34923E-07	-2,89656E-01	0,461	0,433	1,35351E-02
Macro_FOLR2+APOE+	RSRP1	1,81309E-18	-2,95713E-01	0,454	0,276	3,33916E-14
Macro_FOLR2+APOE+	IKBKB	9,16868E-16	-2,96833E-01	0,257	0,154	1,68860E-11
Macro_FOLR2+APOE+	SNRNP40	6,98099E-16	-2,98697E-01	0,324	0,197	1,28569E-11
Macro_FOLR2+APOE+	CYB5B	1,00722E-24	-2,99665E-01	0,335	0,186	1,85499E-20
Macro_FOLR2+APOE+	PTRHD1	5,88538E-09	-3,01132E-01	0,528	0,344	1,08391E-04
Macro_FOLR2+APOE+	AFF1	2,37914E-15	-3,02022E-01	0,352	0,224	4,38166E-11
Macro_FOLR2+APOE+	GPATCH8	3,06961E-14	-3,02698E-01	0,281	0,173	5,65329E-10
Macro_FOLR2+APOE+	SNRNP27	1,26636E-24	-3,04199E-01	0,32	0,178	2,33225E-20
Macro_FOLR2+APOE+	SP3	5,25653E-07	-3,04716E-01	0,267	0,184	9,68095E-03
Macro_FOLR2+APOE+	UVRAG	1,88291E-11	-3,05973E-01	0,438	0,287	3,46776E-07
Macro_FOLR2+APOE+	LRRFIP1	8,39264E-11	-3,11779E-01	0,846	0,679	1,54567E-06
Macro_FOLR2+APOE+	DHPS	1,12469E-09	-3,12534E-01	0,323	0,215	2,07134E-05
Macro_FOLR2+APOE+	CASP8	3,19513E-24	-3,15789E-01	0,282	0,154	5,88448E-20
Macro_FOLR2+APOE+	CHST12	2,07983E-36	-3,16526E-01	0,266	0,127	3,83042E-32
Macro_FOLR2+APOE+	COPS5	9,14711E-20	-3,19133E-01	0,458	0,274	1,68462E-15
Macro_FOLR2+APOE+	NUCB2	4,19845E-73	-3,19710E-01	0,44	0,195	7,73228E-69
Macro_FOLR2+APOE+	PRKCB	1,81943E-06	-3,19742E-01	0,318	0,224	3,35084E-02
Macro_FOLR2+APOE+	RNF5	1,71745E-21	-3,19833E-01	0,464	0,278	3,16303E-17
Macro_FOLR2+APOE+	WDFY4	3,60060E-13	-3,23365E-01	0,257	0,16	6,63123E-09
Macro_FOLR2+APOE+	STK17B	1,22631E-33	-3,26058E-01	0,374	0,446	2,25849E-29
Macro_FOLR2+APOE+	POGZ	1,36212E-08	-3,26309E-01	0,282	0,19	2,50862E-04
Macro_FOLR2+APOE+	LSP1	3,97289E-67	-3,27965E-01	0,592	0,673	7,31687E-63
Macro_FOLR2+APOE+	TNRC6B	1,02330E-06	-3,31442E-01	0,387	0,269	1,88461E-02
Macro_FOLR2+APOE+	TNFRSF21	5,32141E-10	-3,31918E-01	0,25	0,166	9,80044E-06
Macro_FOLR2+APOE+	MX1	5,95362E-54	-3,36438E-01	0,617	0,325	1,09648E-49
Macro_FOLR2+APOE+	SP110	1,29916E-10	-3,38946E-01	0,59	0,384	2,39266E-06
Macro_FOLR2+APOE+	KATNBL1	5,76850E-18	-3,40398E-01	0,301	0,178	1,06239E-13
Macro_FOLR2+APOE+	TNIP1	3,44427E-15	-3,42460E-01	0,544	0,34	6,34331E-11
Macro_FOLR2+APOE+	TRABD	1,91215E-06	-3,44212E-01	0,53	0,352	3,52160E-02
Macro_FOLR2+APOE+	LGALS2	2,35371E-10	-3,46614E-01	0,373	0,254	4,33483E-06

Macro_FOLR2+APOE+	PHYKPL	5,08304E-13	-3,59154E-01	0,401	0,255	9,36143E-09
Macro_FOLR2+APOE+	PPP1R14B	1,14599E-35	-3,61496E-01	0,284	0,14	2,11058E-31
Macro_FOLR2+APOE+	GNL3	1,55424E-15	-3,61905E-01	0,278	0,17	2,86245E-11
Macro_FOLR2+APOE+	NACA	2,31991E-88	-3,64308E-01	0,982	0,917	4,27257E-84
Macro_FOLR2+APOE+	CALCOCO2	1,42896E-15	-3,65830E-01	0,554	0,353	2,63171E-11
Macro_FOLR2+APOE+	CCNI	3,34942E-50	-3,68110E-01	0,893	0,782	6,16862E-46
Macro_FOLR2+APOE+	PRPF31	2,08818E-46	-3,81818E-01	0,418	0,208	3,84581E-42
Macro_FOLR2+APOE+	RNF126	3,75027E-16	-3,83728E-01	0,309	0,185	6,90687E-12
Macro_FOLR2+APOE+	BCL3	2,77746E-09	-3,87791E-01	0,322	0,215	5,11525E-05
Macro_FOLR2+APOE+	FYTTD1	1,93836E-08	-3,93637E-01	0,365	0,245	3,56988E-04
Macro_FOLR2+APOE+	GNA15	8,96831E-07	-3,94646E-01	0,443	0,302	1,65169E-02
Macro_FOLR2+APOE+	TMA7	7,17997E-51	-3,95803E-01	0,902	0,79	1,32234E-46
Macro_FOLR2+APOE+	CERS2	3,94922E-26	-3,96429E-01	0,326	0,179	7,27327E-22
Macro_FOLR2+APOE+	KDM5A	1,11829E-06	-4,02797E-01	0,442	0,308	2,05956E-02
Macro_FOLR2+APOE+	C22orf39	4,98926E-27	-4,07939E-01	0,342	0,188	9,18872E-23
Macro_FOLR2+APOE+	CSF2RB	7,37563E-49	-4,08156E-01	0,397	0,196	1,35837E-44
Macro_FOLR2+APOE+	USP48	1,12297E-08	-4,28476E-01	0,283	0,189	2,06817E-04
Macro_FOLR2+APOE+	NKG7	1,25207E-45	-4,45810E-01	0,268	0,123	2,30594E-41
Macro_FOLR2+APOE+	COMMD6	6,55889E-42	-4,46883E-01	0,853	0,732	1,20795E-37
Macro_FOLR2+APOE+	BTG1	3,72004E-39	-4,49513E-01	0,883	0,808	6,85119E-35
Macro_FOLR2+APOE+	CCDC50	5,56608E-24	-4,56830E-01	0,464	0,268	1,02511E-19
Macro_FOLR2+APOE+	RPS17	1,69374E-27	-4,67524E-01	0,683	0,399	3,11936E-23
Macro_FOLR2+APOE+	MSL3	1,49527E-06	-4,87901E-01	0,297	0,206	2,75385E-02
Macro_FOLR2+APOE+	S100A6	2,63330E-93	-4,99502E-01	0,885	0,874	4,84975E-89
Macro_FOLR2+APOE+	IGKC	1,76778E-64	-5,06428E-01	0,352	0,152	3,25572E-60
Macro_FOLR2+APOE+	SOCS1	4,12380E-33	-5,27247E-01	0,376	0,199	7,59480E-29
Macro_FOLR2+APOE+	MYO1G	1,35402E-06	-5,27725E-01	0,266	0,284	2,49371E-02
Macro_FOLR2+APOE+	UBL7	5,27555E-21	-5,50356E-01	0,423	0,249	9,71597E-17
Macro_FOLR2+APOE+	IFIT2	6,12097E-48	-5,58221E-01	0,385	0,187	1,12730E-43
Macro_FOLR2+APOE+	TCF4	4,91589E-63	-5,96569E-01	0,539	0,265	9,05360E-59
Macro_FOLR2+APOE+	IFI44L	4,76851E-64	-6,14862E-01	0,515	0,246	8,78216E-60
Macro_FOLR2+APOE+	PLD4	1,06591E-23	-6,16686E-01	0,245	0,335	1,96310E-19
Macro_FOLR2+APOE+	ISG15	6,54619E-70	-6,25500E-01	0,76	0,418	1,20561E-65
Macro_FOLR2+APOE+	ERN1	2,58014E-13	-6,30368E-01	0,271	0,17	4,75184E-09
Macro_FOLR2+APOE+	MTRNR2L12	3,48523E-27	-6,71374E-01	0,695	0,768	6,41874E-23
Macro_FOLR2+APOE+	CYB561A3	2,21760E-103	-7,13528E-01	0,494	0,209	4,08416E-99
Macro_FOLR2+APOE+	IRF8	1,71262E-42	-7,45885E-01	0,661	0,37	3,15413E-38
Macro_FOLR2+APOE+	RAC2	2,26344E-24	-7,57500E-01	0,485	0,497	4,16857E-20
Macro_FOLR2+APOE+	S100A4	4,11181E-148	-8,39303E-01	0,789	0,827	7,57272E-144
Macro_FOLR2+APOE+	PLP2	9,76246E-11	-8,42729E-01	0,418	0,404	1,79795E-06
Macro_FOLR2+APOE+	SERPINF1	2,36977E-49	-8,85641E-01	0,67	0,397	4,36441E-45
Macro_FOLR2+APOE+	GAPT	7,87225E-36	-9,68655E-01	0,128	0,258	1,44983E-31
Macro_FOLR2+APOE+	CD52	6,95859E-07	-1,00523E+00	0,406	0,389	1,28156E-02
Macro_FOLR2+APOE+	DDIT4	2,10790E-09	-1,08298E+00	0,501	0,496	3,88213E-05
Macro_FOLR2+APOE+	TIMP1	3,85918E-20	-1,11266E+00	0,745	0,647	7,10745E-16
Macro_FOLR2+APOE+	MARCKSL1	4,00435E-09	-1,21986E+00	0,234	0,266	7,37480E-05
Macro_FOLR2+APOE+	IFITM2	1,68611E-23	-1,29729E+00	0,747	0,644	3,10530E-19

Macro_FOLR2+APOE+	CXCL8	5,07961E-11	-1,48701E+00	0,289	0,191	9,35511E-07
Macro_FOLR2+APOE+	S100A9	2,46938E-19	-1,56786E+00	0,707	0,449	4,54785E-15
Macro_FOLR2+APOE+	AREG	3,14509E-39	-1,64074E+00	0,132	0,28	5,79231E-35
Macro_FOLR2+APOE+	FCN1	2,78994E-41	-1,99704E+00	0,11	0,255	5,13823E-37

cluster	gene	p_val	avg_log2FC	pct.1	pct.2	p_val_adj
Macro_IER3	CCL4L2	0,00000E+00	1,56191E+00	0,558	0,177	0,00000E+00
Macro_IER3	RHOB	1,55682E-263	1,25872E+00	0,827	0,528	2,86719E-259
Macro_IER3	CCL4	0,00000E+00	1,14426E+00	0,696	0,292	0,00000E+00
Macro_IER3	CCL3L1	0,00000E+00	1,07988E+00	0,495	0,147	0,00000E+00
Macro_IER3	TNF	8,66918E-222	1,01527E+00	0,419	0,161	1,59660E-217
Macro_IER3	GADD45B	0,00000E+00	9,83105E-01	0,906	0,63	0,00000E+00
Macro_IER3	IER3	0,00000E+00	9,79968E-01	0,881	0,516	0,00000E+00
Macro_IER3	SGK1	0,00000E+00	9,79695E-01	0,902	0,591	0,00000E+00
Macro_IER3	JUN	6,70496E-203	9,01307E-01	0,915	0,7	1,23485E-198
Macro_IER3	C5AR1	3,58351E-244	8,90786E-01	0,722	0,393	6,59975E-240
Macro_IER3	BAG3	9,08354E-107	8,87712E-01	0,298	0,136	1,67292E-102
Macro_IER3	NR4A2	0,00000E+00	8,68140E-01	0,851	0,432	0,00000E+00
Macro_IER3	EGR1	3,52559E-227	8,42925E-01	0,574	0,273	6,49308E-223
Macro_IER3	BCAS2	7,50375E-73	8,18384E-01	0,416	0,256	1,38197E-68
Macro_IER3	CCL3	0,00000E+00	8,13948E-01	0,782	0,357	0,00000E+00
Macro_IER3	KLF2	1,64567E-161	7,88913E-01	0,524	0,265	3,03083E-157
Macro_IER3	EGR2	3,86175E-276	7,77699E-01	0,318	0,089	7,11219E-272
Macro_IER3	CEBPD	1,56309E-135	7,28837E-01	0,805	0,557	2,87874E-131
Macro_IER3	ATF3	0,00000E+00	7,28276E-01	0,813	0,415	0,00000E+00
Macro_IER3	ZFP36L2	1,85124E-153	7,00585E-01	0,888	0,685	3,40943E-149
Macro_IER3	KLF4	1,48160E-245	6,71379E-01	0,75	0,433	2,72867E-241
Macro_IER3	C3	7,90714E-174	6,54817E-01	0,666	0,371	1,45626E-169
Macro_IER3	IER2	5,05645E-263	6,46786E-01	0,896	0,62	9,31247E-259
Macro_IER3	DUSP2	4,05074E-191	6,23690E-01	0,572	0,288	7,46024E-187
Macro_IER3	PRDM1	2,06794E-169	6,17038E-01	0,424	0,187	3,80853E-165
Macro_IER3	PIK3R1	4,71904E-90	6,08665E-01	0,51	0,312	8,69105E-86
Macro_IER3	RBKS	3,83025E-170	5,96007E-01	0,361	0,143	7,05416E-166
Macro_IER3	CITED2	4,56544E-79	5,92842E-01	0,507	0,327	8,40818E-75
Macro_IER3	PHLDA1	5,31827E-97	5,89983E-01	0,299	0,138	9,79466E-93
Macro_IER3	NR4A1	0,00000E+00	5,82120E-01	0,776	0,403	0,00000E+00
Macro_IER3	OSM	2,00259E-88	5,59523E-01	0,293	0,137	3,68818E-84
Macro_IER3	ZFAND5	4,55410E-172	5,53271E-01	0,812	0,575	8,38729E-168
Macro_IER3	TNFAIP3	5,78384E-193	5,41572E-01	0,81	0,53	1,06521E-188
Macro_IER3	CHMP1B	1,04151E-143	5,37945E-01	0,634	0,396	1,91814E-139
Macro_IER3	FOSB	1,16324E-218	5,28450E-01	0,868	0,566	2,14234E-214
Macro_IER3	PDK4	1,57298E-71	5,22762E-01	0,484	0,303	2,89696E-67
Macro_IER3	MAFB	1,86601E-120	5,17574E-01	0,852	0,581	3,43662E-116
Macro_IER3	CX3CR1	5,05654E-123	5,15649E-01	0,315	0,133	9,31263E-119
Macro_IER3	KLF6	2,44259E-196	5,13409E-01	0,938	0,791	4,49852E-192

Macro_IER3	PNRC1	1,16114E-153	5,00869E-01	0,936	0,772	2,13848E-149
Macro_IER3	FOS	8,10361E-275	4,85899E-01	0,969	0,837	1,49244E-270
Macro_IER3	TFRC	1,07271E-14	4,77910E-01	0,308	0,234	1,97561E-10
Macro_IER3	HSPA6	7,99396E-52	4,71283E-01	0,431	0,28	1,47225E-47
Macro_IER3	MAFF	3,05178E-120	4,68752E-01	0,332	0,148	5,62047E-116
Macro_IER3	GPR34	8,62166E-128	4,58847E-01	0,542	0,295	1,58785E-123
Macro_IER3	PHACTR1	1,09656E-155	4,57115E-01	0,549	0,29	2,01953E-151
Macro_IER3	CXCL2	2,73038E-194	4,30649E-01	0,485	0,215	5,02854E-190
Macro_IER3	CXCL3	1,37835E-136	4,16329E-01	0,353	0,153	2,53850E-132
Macro_IER3	ZFP36	3,83649E-291	4,14251E-01	0,978	0,838	7,06566E-287
Macro_IER3	FILIP1L	1,80852E-123	4,05150E-01	0,316	0,136	3,33075E-119
Macro_IER3	HBEGF	3,51036E-140	4,04529E-01	0,514	0,264	6,46504E-136
Macro_IER3	CSF1R	1,29274E-173	4,03503E-01	0,849	0,558	2,38084E-169
Macro_IER3	DUSP1	2,79171E-307	3,98191E-01	0,98	0,844	5,14149E-303
Macro_IER3	NFKBIA	6,93750E-206	3,95938E-01	0,947	0,802	1,27768E-201
Macro_IER3	TLR5	2,16458E-76	3,90293E-01	0,25	0,115	3,98650E-72
Macro_IER3	DNAJB1	8,57575E-152	3,88705E-01	0,771	0,548	1,57940E-147
Macro_IER3	AKR1B1	1,70229E-80	3,86716E-01	0,677	0,458	3,13510E-76
Macro_IER3	CKS2	1,59980E-68	3,85768E-01	0,394	0,236	2,94634E-64
Macro_IER3	PELI1	5,57593E-91	3,78414E-01	0,447	0,253	1,02692E-86
Macro_IER3	BTG1	1,47193E-59	3,77724E-01	0,911	0,805	2,71085E-55
Macro_IER3	ARL5A	1,74716E-62	3,72211E-01	0,597	0,421	3,21774E-58
Macro_IER3	TAGAP	3,59636E-117	3,69193E-01	0,477	0,262	6,62342E-113
Macro_IER3	AOAH	9,53681E-99	3,68345E-01	0,596	0,364	1,75640E-94
Macro_IER3	HSPA1A	1,20644E-84	3,65649E-01	0,789	0,658	2,22190E-80
Macro_IER3	TRIB1	1,29404E-73	3,65169E-01	0,402	0,233	2,38324E-69
Macro_IER3	SLC1A3	4,67015E-78	3,62141E-01	0,55	0,354	8,60101E-74
Macro_IER3	MEF2A	1,23358E-97	3,61167E-01	0,625	0,412	2,27188E-93
Macro_IER3	ARRDC2	2,80760E-38	3,57546E-01	0,316	0,2	5,17076E-34
Macro_IER3	NLRP3	3,86046E-161	3,52389E-01	0,515	0,251	7,10981E-157
Macro_IER3	CLEC7A	2,32355E-130	3,50754E-01	0,773	0,513	4,27927E-126
Macro_IER3	FYB1	3,45452E-39	3,48014E-01	0,441	0,312	6,36220E-35
Macro_IER3	TREM2	1,70524E-80	3,47852E-01	0,626	0,396	3,14054E-76
Macro_IER3	IER5	5,38048E-70	3,47368E-01	0,497	0,318	9,90923E-66
Macro_IER3	IL1B	4,43544E-229	3,46256E-01	0,651	0,32	8,16875E-225
Macro_IER3	ETV5	3,48758E-41	3,40436E-01	0,312	0,194	6,42307E-37
Macro_IER3	VSIR	2,54168E-39	3,39539E-01	0,391	0,268	4,68101E-35
Macro_IER3	HLA-DQB1	1,02676E-163	3,29311E-01	0,971	0,809	1,89098E-159
Macro_IER3	FCGR1A	1,86338E-60	3,29190E-01	0,608	0,402	3,43179E-56
Macro_IER3	PLXDC2	1,18393E-129	3,28095E-01	0,791	0,532	2,18045E-125

Macro_IER3	FRMD4A	1,78098E-45	3,23252E-01	0,299	0,18	3,28003E-41
Macro_IER3	TGIF1	5,01988E-86	3,12014E-01	0,506	0,315	9,24511E-82
Macro_IER3	SRGAP1	1,78841E-71	3,11700E-01	0,315	0,164	3,29372E-67
Macro_IER3	YBX3	1,58070E-50	3,11374E-01	0,5	0,341	2,91117E-46
Macro_IER3	REL	4,48258E-97	3,05485E-01	0,799	0,589	8,25556E-93
Macro_IER3	NFKBIZ	1,47735E-145	3,01188E-01	0,704	0,443	2,72084E-141
Macro_IER3	OTUD1	8,29391E-67	2,97472E-01	0,277	0,141	1,52749E-62
Macro_IER3	HLA-DRB1	1,19671E-106	2,95141E-01	0,987	0,914	2,20398E-102
Macro_IER3	CSRNP1	2,65694E-134	2,95035E-01	0,524	0,281	4,89329E-130
Macro_IER3	HLA-DQA1	4,24596E-115	2,90359E-01	0,949	0,776	7,81979E-111
Macro_IER3	A2M	4,63440E-105	2,87066E-01	0,624	0,378	8,53517E-101
Macro_IER3	ZFHX3	2,73241E-75	2,81352E-01	0,397	0,224	5,03227E-71
Macro_IER3	KLF7	4,98609E-70	2,76755E-01	0,265	0,13	9,18289E-66
Macro_IER3	NEU1	6,03290E-46	2,73167E-01	0,438	0,298	1,11108E-41
Macro_IER3	FAM111A	5,83234E-31	2,72775E-01	0,281	0,178	1,07414E-26
Macro_IER3	C1QC	7,53591E-110	2,71760E-01	0,853	0,532	1,38789E-105
Macro_IER3	HSPH1	3,22619E-82	2,71689E-01	0,59	0,407	5,94168E-78
Macro_IER3	HSPA1B	6,75808E-96	2,70222E-01	0,657	0,498	1,24464E-91
Macro_IER3	LPAR6	1,10244E-104	2,68169E-01	0,542	0,312	2,03037E-100
Macro_IER3	MYLIP	1,78430E-63	2,67046E-01	0,299	0,161	3,28615E-59
Macro_IER3	OGFRL1	7,35517E-52	2,66355E-01	0,453	0,303	1,35460E-47
Macro_IER3	JUNB	4,71585E-272	2,63808E-01	0,97	0,819	8,68517E-268
Macro_IER3	DOCK4	7,42342E-78	2,58456E-01	0,465	0,277	1,36717E-73
Macro_IER3	IFNGR2	6,66787E-19	-2,50006E-01	0,685	0,559	1,22802E-14
Macro_IER3	ARSA	3,30471E-08	-2,50880E-01	0,29	0,231	6,08628E-04
Macro_IER3	ARF4	9,63395E-16	-2,50910E-01	0,547	0,441	1,77429E-11
Macro_IER3	STX7	9,45707E-11	-2,51505E-01	0,497	0,41	1,74171E-06
Macro_IER3	PTGS2	6,08576E-70	-2,51742E-01	0,333	0,172	1,12081E-65
Macro_IER3	ILK	3,02163E-07	-2,51752E-01	0,489	0,412	5,56493E-03
Macro_IER3	FAM53C	1,05203E-23	-2,52909E-01	0,256	0,17	1,93753E-19
Macro_IER3	ADCY7	3,36195E-10	-2,53363E-01	0,295	0,23	6,19171E-06
Macro_IER3	PLD3	8,57604E-14	-2,54663E-01	0,647	0,504	1,57945E-09
Macro_IER3	PCM1	1,31351E-15	-2,54750E-01	0,4	0,312	2,41908E-11
Macro_IER3	ATRX	2,06696E-13	-2,54755E-01	0,474	0,385	3,80673E-09
Macro_IER3	MAP1LC3B	1,01042E-12	-2,55110E-01	0,65	0,553	1,86089E-08
Macro_IER3	HLA-E	1,20729E-14	-2,56320E-01	0,955	0,902	2,22347E-10
Macro_IER3	FUS	8,65937E-13	-2,56341E-01	0,687	0,583	1,59480E-08
Macro_IER3	PTDSS1	8,98631E-08	-2,56508E-01	0,325	0,267	1,65501E-03
Macro_IER3	RRAGA	9,49060E-11	-2,56731E-01	0,292	0,23	1,74788E-06
Macro_IER3	ZFR	8,98898E-07	-2,57154E-01	0,298	0,247	1,65550E-02

Macro_IER3	NSL1	4,95901E-08	-2,57405E-01	0,303	0,244	9,13301E-04
Macro_IER3	PPP1CC	1,28124E-06	-2,58235E-01	0,414	0,349	2,35966E-02
Macro_IER3	SYPL1	1,01278E-18	-2,59287E-01	0,396	0,299	1,86523E-14
Macro_IER3	RAP2B	5,82338E-07	-2,60134E-01	0,376	0,312	1,07249E-02
Macro_IER3	SAMHD1	3,04261E-16	-2,60584E-01	0,774	0,64	5,60358E-12
Macro_IER3	PRNP	8,01169E-31	-2,60886E-01	0,534	0,395	1,47551E-26
Macro_IER3	YPEL5	2,86128E-23	-2,62589E-01	0,545	0,429	5,26963E-19
Macro_IER3	PTPRC	6,56199E-26	-2,62704E-01	0,811	0,684	1,20852E-21
Macro_IER3	NAGA	1,31463E-15	-2,62710E-01	0,457	0,356	2,42116E-11
Macro_IER3	UBE2D3	6,06866E-13	-2,63178E-01	0,833	0,732	1,11767E-08
Macro_IER3	VPS35	1,12483E-09	-2,65231E-01	0,521	0,436	2,07159E-05
Macro_IER3	CHD2	1,36918E-22	-2,66253E-01	0,396	0,294	2,52161E-18
Macro_IER3	LITAF	5,80516E-37	-2,66881E-01	0,864	0,736	1,06914E-32
Macro_IER3	HNRNPDL	9,21086E-21	-2,67129E-01	0,792	0,669	1,69636E-16
Macro_IER3	PARVG	1,22556E-15	-2,67165E-01	0,554	0,445	2,25712E-11
Macro_IER3	SAT1	1,12593E-65	-2,67816E-01	0,995	0,946	2,07363E-61
Macro_IER3	PTTG1IP	1,10704E-11	-2,67844E-01	0,595	0,498	2,03884E-07
Macro_IER3	EIF3H	9,16270E-08	-2,69376E-01	0,75	0,653	1,68750E-03
Macro_IER3	CCDC107	3,48775E-12	-2,69731E-01	0,38	0,303	6,42339E-08
Macro_IER3	ITGB2	2,04385E-14	-2,71202E-01	0,908	0,757	3,76417E-10
Macro_IER3	PNPLA8	3,70601E-11	-2,71736E-01	0,312	0,239	6,82536E-07
Macro_IER3	CAPNS1	3,35904E-08	-2,72343E-01	0,334	0,372	6,18634E-04
Macro_IER3	FCHSD2	2,54088E-12	-2,72463E-01	0,293	0,221	4,67954E-08
Macro_IER3	LTC4S	1,28748E-55	-2,72993E-01	0,457	0,276	2,37116E-51
Macro_IER3	COMT	5,48926E-08	-2,72998E-01	0,652	0,553	1,01096E-03
Macro_IER3	SRRM1	1,97483E-06	-2,73091E-01	0,639	0,561	3,63704E-02
Macro_IER3	EIF3L	1,07980E-21	-2,73171E-01	0,718	0,595	1,98866E-17
Macro_IER3	POLR3GL	1,07103E-06	-2,75144E-01	0,356	0,295	1,97252E-02
Macro_IER3	NR1H2	1,48935E-08	-2,75156E-01	0,396	0,328	2,74293E-04
Macro_IER3	CCS	1,89236E-07	-2,75488E-01	0,318	0,259	3,48516E-03
Macro_IER3	WNK1	1,82750E-08	-2,76940E-01	0,428	0,353	3,36571E-04
Macro_IER3	MYO1F	2,67309E-15	-2,77268E-01	0,568	0,455	4,92303E-11
Macro_IER3	ESD	1,45801E-06	-2,78411E-01	0,427	0,361	2,68522E-02
Macro_IER3	WASF2	4,16535E-21	-2,78919E-01	0,688	0,563	7,67132E-17
Macro_IER3	DPYSL2	3,26005E-23	-2,79113E-01	0,429	0,319	6,00404E-19
Macro_IER3	COPS2	3,36198E-08	-2,79464E-01	0,286	0,229	6,19176E-04
Macro_IER3	OSBPL8	4,62648E-10	-2,79826E-01	0,516	0,425	8,52058E-06
Macro_IER3	FOLR2	2,96277E-55	-2,80546E-01	0,312	0,174	5,45653E-51
Macro_IER3	CTNNB1	4,41156E-11	-2,80618E-01	0,459	0,376	8,12477E-07
Macro_IER3	PEPD	1,18861E-13	-2,81220E-01	0,456	0,359	2,18907E-09

Macro_IER3	LRRC25	3,44717E-16	-2,81920E-01	0,43	0,329	6,34865E-12
Macro_IER3	C12orf57	3,34033E-18	-2,82098E-01	0,429	0,329	6,15188E-14
Macro_IER3	ANP32B	9,73357E-08	-2,82272E-01	0,659	0,568	1,79263E-03
Macro_IER3	DDX24	6,92545E-08	-2,83139E-01	0,551	0,474	1,27546E-03
Macro_IER3	SRSF7	5,51383E-64	-2,83178E-01	0,715	0,544	1,01548E-59
Macro_IER3	USP36	7,56560E-55	-2,83499E-01	0,303	0,171	1,39336E-50
Macro_IER3	TBRG1	2,33997E-13	-2,83947E-01	0,437	0,348	4,30952E-09
Macro_IER3	AMPD2	4,30775E-14	-2,83972E-01	0,251	0,181	7,93359E-10
Macro_IER3	ARID4A	8,22354E-07	-2,83976E-01	0,282	0,233	1,51453E-02
Macro_IER3	RABGGTB	1,36682E-06	-2,86796E-01	0,308	0,254	2,51727E-02
Macro_IER3	ZNF330	2,08514E-07	-2,87025E-01	0,258	0,209	3,84020E-03
Macro_IER3	DNAJB9	6,39487E-08	-2,89941E-01	0,256	0,204	1,17774E-03
Macro_IER3	MTMR14	9,42017E-07	-2,91787E-01	0,369	0,307	1,73491E-02
Macro_IER3	IVNS1ABP	9,28468E-36	-2,92264E-01	0,505	0,36	1,70996E-31
Macro_IER3	SRGAP2	5,43072E-09	-2,92805E-01	0,287	0,225	1,00018E-04
Macro_IER3	CANX	4,93585E-08	-2,93172E-01	0,741	0,634	9,09035E-04
Macro_IER3	MTCH1	2,48335E-06	-2,93256E-01	0,533	0,452	4,57358E-02
Macro_IER3	ISCU	7,98319E-09	-2,93536E-01	0,638	0,532	1,47026E-04
Macro_IER3	ATRAID	2,63712E-15	-2,95144E-01	0,568	0,461	4,85678E-11
Macro_IER3	GIMAP2	3,33246E-13	-2,95513E-01	0,263	0,192	6,13740E-09
Macro_IER3	ACER3	1,36967E-09	-2,95655E-01	0,293	0,235	2,52252E-05
Macro_IER3	HEXB	4,30024E-29	-2,95737E-01	0,664	0,507	7,91976E-25
Macro_IER3	COX6B1	6,81357E-13	-2,95785E-01	0,856	0,809	1,25485E-08
Macro_IER3	LAPTM5	2,59711E-11	-2,97418E-01	0,961	0,895	4,78310E-07
Macro_IER3	IQGAP1	6,06073E-08	-2,97892E-01	0,646	0,558	1,11620E-03
Macro_IER3	SNX6	1,76181E-10	-2,99265E-01	0,611	0,511	3,24472E-06
Macro_IER3	BNIP3L	2,76079E-27	-2,99342E-01	0,702	0,557	5,08455E-23
Macro_IER3	ITSN2	2,20324E-20	-3,00070E-01	0,498	0,381	4,05770E-16
Macro_IER3	TNRC6B	6,60580E-09	-3,00446E-01	0,333	0,269	1,21659E-04
Macro_IER3	PSAP	9,85291E-09	-3,00822E-01	0,98	0,913	1,81461E-04
Macro_IER3	TPT1	4,95463E-18	-3,01351E-01	0,985	0,974	9,12495E-14
Macro_IER3	ARRDC3	2,02364E-25	-3,02424E-01	0,273	0,182	3,72693E-21
Macro_IER3	GPR108	1,56736E-07	-3,03634E-01	0,312	0,255	2,88661E-03
Macro_IER3	TRAPPC1	2,02697E-08	-3,04213E-01	0,614	0,609	3,73308E-04
Macro_IER3	NDUFS2	3,69265E-11	-3,04474E-01	0,444	0,357	6,80074E-07
Macro_IER3	CTSH	1,70780E-15	-3,04653E-01	0,848	0,704	3,14525E-11
Macro_IER3	SIGIRR	7,27168E-07	-3,05585E-01	0,313	0,254	1,33923E-02
Macro_IER3	NXT1	2,78971E-15	-3,05840E-01	0,276	0,205	5,13781E-11
Macro_IER3	EIF2A	3,47075E-09	-3,06351E-01	0,374	0,305	6,39207E-05
Macro_IER3	GRHPR	2,16891E-07	-3,06636E-01	0,347	0,289	3,99449E-03

Macro_IER3	TMEM176B	1,72666E-12	-3,06640E-01	0,681	0,528	3,17998E-08
Macro_IER3	EEF1A1	2,54944E-09	-3,07007E-01	0,992	0,984	4,69530E-05
Macro_IER3	APMAP	8,50327E-08	-3,08900E-01	0,359	0,298	1,56605E-03
Macro_IER3	HNRNPF	2,00645E-07	-3,09469E-01	0,654	0,561	3,69528E-03
Macro_IER3	CEP170	2,74982E-30	-3,09496E-01	0,463	0,339	5,06434E-26
Macro_IER3	CD300C	3,65941E-14	-3,10739E-01	0,312	0,234	6,73953E-10
Macro_IER3	DNMT1	5,07177E-10	-3,11125E-01	0,386	0,314	9,34067E-06
Macro_IER3	DAP3	3,92671E-08	-3,11699E-01	0,376	0,315	7,23182E-04
Macro_IER3	ABHD14B	7,99093E-13	-3,12655E-01	0,335	0,259	1,47169E-08
Macro_IER3	TMX1	2,27974E-17	-3,13158E-01	0,429	0,332	4,19861E-13
Macro_IER3	EIF4A2	5,42969E-51	-3,15084E-01	0,717	0,555	9,99986E-47
Macro_IER3	SUMF2	1,57759E-12	-3,15154E-01	0,373	0,291	2,90546E-08
Macro_IER3	TMEM243	4,85208E-10	-3,15325E-01	0,335	0,266	8,93608E-06
Macro_IER3	SNX5	4,66774E-15	-3,15435E-01	0,494	0,394	8,59657E-11
Macro_IER3	LMNA	1,65544E-21	-3,18190E-01	0,469	0,364	3,04882E-17
Macro_IER3	SBDS	6,04697E-16	-3,20124E-01	0,364	0,28	1,11367E-11
Macro_IER3	SERINC1	1,69030E-20	-3,20453E-01	0,508	0,399	3,11303E-16
Macro_IER3	G6PC3	4,95823E-07	-3,20654E-01	0,256	0,208	9,13158E-03
Macro_IER3	TUBA1B	1,28686E-07	-3,21077E-01	0,862	0,751	2,37001E-03
Macro_IER3	CD14	3,38407E-101	-3,21172E-01	0,867	0,612	6,23245E-97
Macro_IER3	RNASE6	8,68196E-24	-3,22032E-01	0,695	0,532	1,59896E-19
Macro_IER3	CD86	8,65055E-32	-3,22227E-01	0,647	0,488	1,59317E-27
Macro_IER3	METTL23	2,19901E-07	-3,22803E-01	0,27	0,219	4,04992E-03
Macro_IER3	GAS6	2,10388E-09	-3,22959E-01	0,263	0,205	3,87471E-05
Macro_IER3	VSIG4	1,05212E-47	-3,25288E-01	0,524	0,34	1,93768E-43
Macro_IER3	PIK3R5	4,61609E-11	-3,25632E-01	0,328	0,254	8,50145E-07
Macro_IER3	DCTN2	6,16402E-07	-3,27097E-01	0,39	0,326	1,13523E-02
Macro_IER3	POLD4	2,71456E-06	-3,27829E-01	0,662	0,565	4,99941E-02
Macro_IER3	OCIAD1	1,29145E-07	-3,28547E-01	0,506	0,431	2,37847E-03
Macro_IER3	FCGR1B	1,04375E-35	-3,28917E-01	0,315	0,199	1,92228E-31
Macro_IER3	SCAMP2	5,90220E-33	-3,28938E-01	0,646	0,496	1,08701E-28
Macro_IER3	IFI16	6,49835E-08	-3,29727E-01	0,585	0,496	1,19680E-03
Macro_IER3	BCL2A1	7,34445E-44	-3,30170E-01	0,621	0,463	1,35263E-39
Macro_IER3	EVI2A	2,39759E-08	-3,30406E-01	0,332	0,271	4,41564E-04
Macro_IER3	GK	3,28475E-36	-3,30712E-01	0,507	0,358	6,04953E-32
Macro_IER3	CMTM6	5,02149E-10	-3,30830E-01	0,72	0,612	9,24809E-06
Macro_IER3	AKIRIN2	2,72090E-13	-3,32469E-01	0,518	0,417	5,01108E-09
Macro_IER3	EIF3E	8,38918E-20	-3,32863E-01	0,723	0,6	1,54504E-15
Macro_IER3	RGS19	3,42026E-09	-3,33508E-01	0,587	0,491	6,29909E-05
Macro_IER3	HAVCR2	1,66846E-08	-3,36009E-01	0,515	0,432	3,07280E-04

Macro_IER3	SLC1A5	4,25694E-10	-3,37950E-01	0,301	0,236	7,84001E-06
Macro_IER3	EIF2S3	5,10704E-16	-3,38600E-01	0,551	0,447	9,40564E-12
Macro_IER3	CD300A	2,29639E-35	-3,38748E-01	0,609	0,453	4,22927E-31
Macro_IER3	NDUFA10	6,94985E-07	-3,38941E-01	0,383	0,325	1,27995E-02
Macro_IER3	CYCS	2,72991E-15	-3,39143E-01	0,62	0,519	5,02767E-11
Macro_IER3	ARAP1	3,16835E-07	-3,40085E-01	0,352	0,289	5,83515E-03
Macro_IER3	BAZ2B	1,61592E-30	-3,41042E-01	0,37	0,256	2,97604E-26
Macro_IER3	GMPR2	7,23495E-11	-3,41535E-01	0,281	0,217	1,33246E-06
Macro_IER3	TXNDC12	1,16826E-08	-3,42495E-01	0,409	0,338	2,15158E-04
Macro_IER3	ARMCX6	4,71403E-13	-3,42560E-01	0,254	0,19	8,68183E-09
Macro_IER3	SEC11A	1,15907E-15	-3,42578E-01	0,775	0,662	2,13466E-11
Macro_IER3	PRRC2C	2,19670E-12	-3,42929E-01	0,693	0,589	4,04565E-08
Macro_IER3	PPP1CB	1,26072E-13	-3,43791E-01	0,494	0,393	2,32187E-09
Macro_IER3	UBAC2	3,15571E-28	-3,44118E-01	0,421	0,304	5,81188E-24
Macro_IER3	EIF4A3	6,46585E-100	-3,45527E-01	0,596	0,393	1,19082E-95
Macro_IER3	MS4A4A	4,61717E-30	-3,45728E-01	0,568	0,413	8,50345E-26
Macro_IER3	BCAP29	6,06065E-10	-3,47078E-01	0,268	0,207	1,11619E-05
Macro_IER3	VAPA	2,11320E-07	-3,47101E-01	0,696	0,599	3,89189E-03
Macro_IER3	IL16	1,45804E-12	-3,47756E-01	0,288	0,218	2,68527E-08
Macro_IER3	ATP6V1C1	2,38818E-07	-3,50308E-01	0,328	0,271	4,39831E-03
Macro_IER3	TGOLN2	2,58651E-08	-3,51872E-01	0,606	0,509	4,76357E-04
Macro_IER3	MGAT1	3,85914E-32	-3,51881E-01	0,766	0,618	7,10738E-28
Macro_IER3	UQCR11	8,39213E-11	-3,52368E-01	0,785	0,753	1,54558E-06
Macro_IER3	THBD	2,03224E-26	-3,52524E-01	0,302	0,203	3,74277E-22
Macro_IER3	ATP1A1	5,44504E-13	-3,56113E-01	0,522	0,425	1,00281E-08
Macro_IER3	STAT3	6,85974E-07	-3,56234E-01	0,58	0,502	1,26336E-02
Macro_IER3	SH3KBP1	2,97815E-16	-3,56266E-01	0,519	0,41	5,48487E-12
Macro_IER3	ZC3HAV1	9,22617E-14	-3,56570E-01	0,344	0,267	1,69918E-09
Macro_IER3	RNF166	8,99225E-22	-3,57266E-01	0,336	0,24	1,65610E-17
Macro_IER3	HSPE1	7,44355E-24	-3,59277E-01	0,706	0,598	1,37088E-19
Macro_IER3	FCN1	3,71122E-17	-3,60222E-01	0,18	0,254	6,83495E-13
Macro_IER3	SNAP23	1,46017E-09	-3,60788E-01	0,48	0,396	2,68920E-05
Macro_IER3	VAMP3	8,97840E-17	-3,61247E-01	0,447	0,346	1,65355E-12
Macro_IER3	RNF149	2,29114E-49	-3,62856E-01	0,788	0,619	4,21959E-45
Macro_IER3	POLR2L	2,18035E-08	-3,64953E-01	0,632	0,627	4,01555E-04
Macro_IER3	ACSL1	3,72350E-27	-3,65163E-01	0,511	0,383	6,85758E-23
Macro_IER3	STARD3	5,10088E-11	-3,66479E-01	0,306	0,239	9,39429E-07
Macro_IER3	BROX	1,02628E-08	-3,67381E-01	0,256	0,202	1,89010E-04
Macro_IER3	ASAH1	3,96410E-12	-3,67482E-01	0,819	0,683	7,30069E-08
Macro_IER3	AXL	1,48492E-29	-3,67520E-01	0,391	0,275	2,73478E-25

Macro_IER3	EIF3D	1,81396E-12	-3,67578E-01	0,57	0,469	3,34077E-08
Macro_IER3	TMEM176A	4,73988E-11	-3,69041E-01	0,576	0,443	8,72943E-07
Macro_IER3	HINT2	2,03932E-06	-3,70769E-01	0,28	0,228	3,75581E-02
Macro_IER3	HNRNPH3	3,49778E-12	-3,70917E-01	0,432	0,348	6,44186E-08
Macro_IER3	STX12	3,68941E-18	-3,71321E-01	0,403	0,308	6,79479E-14
Macro_IER3	CD68	4,31488E-18	-3,73337E-01	0,909	0,775	7,94672E-14
Macro_IER3	COX5B	5,74449E-08	-3,73409E-01	0,82	0,77	1,05796E-03
Macro_IER3	TMEM123	5,35543E-17	-3,74548E-01	0,563	0,45	9,86310E-13
Macro_IER3	EIF1	2,27922E-18	-3,74615E-01	0,977	0,943	4,19765E-14
Macro_IER3	DDX5	3,95060E-48	-3,75058E-01	0,94	0,828	7,27582E-44
Macro_IER3	CSNK1D	3,89926E-12	-3,75913E-01	0,422	0,339	7,18126E-08
Macro_IER3	EIF5	2,17554E-19	-3,77285E-01	0,698	0,587	4,00669E-15
Macro_IER3	UTRN	1,59021E-08	-3,79207E-01	0,352	0,285	2,92870E-04
Macro_IER3	UBE2B	5,29136E-10	-3,80184E-01	0,581	0,484	9,74509E-06
Macro_IER3	RNASET2	1,57029E-52	-3,81129E-01	0,89	0,733	2,89200E-48
Macro_IER3	SRGN	2,37819E-55	-3,82037E-01	0,982	0,941	4,37991E-51
Macro_IER3	SRSF5	1,55045E-29	-3,82967E-01	0,705	0,573	2,85545E-25
Macro_IER3	GIT2	8,59815E-21	-3,83895E-01	0,385	0,29	1,58352E-16
Macro_IER3	MATR3	2,51432E-12	-3,84596E-01	0,293	0,225	4,63062E-08
Macro_IER3	GLUL	1,17739E-14	-3,84785E-01	0,93	0,798	2,16839E-10
Macro_IER3	MT2A	1,40922E-06	-3,84911E-01	0,634	0,628	2,59535E-02
Macro_IER3	GPS1	2,42616E-07	-3,85757E-01	0,319	0,263	4,46826E-03
Macro_IER3	ARL8B	1,72591E-08	-3,88883E-01	0,479	0,4	3,17861E-04
Macro_IER3	DPP7	1,54975E-14	-3,89122E-01	0,667	0,551	2,85418E-10
Macro_IER3	DOK2	5,06063E-08	-3,89181E-01	0,414	0,342	9,32016E-04
Macro_IER3	TMEM219	3,38251E-09	-3,89328E-01	0,745	0,632	6,22957E-05
Macro_IER3	SRSF1	1,28698E-08	-3,90313E-01	0,329	0,266	2,37024E-04
Macro_IER3	SKAP2	1,47015E-10	-3,90703E-01	0,486	0,402	2,70757E-06
Macro_IER3	ENG	4,82233E-21	-3,91903E-01	0,46	0,342	8,88129E-17
Macro_IER3	FES	6,11247E-09	-3,92661E-01	0,316	0,251	1,12573E-04
Macro_IER3	CD46	4,52115E-09	-3,93490E-01	0,383	0,314	8,32660E-05
Macro_IER3	ATG7	8,26033E-17	-3,93683E-01	0,263	0,187	1,52131E-12
Macro_IER3	DEF6	2,73034E-08	-3,93776E-01	0,297	0,239	5,02847E-04
Macro_IER3	NABP1	8,43236E-22	-3,98405E-01	0,447	0,334	1,55299E-17
Macro_IER3	MIF4GD	8,72080E-12	-4,00044E-01	0,388	0,308	1,60611E-07
Macro_IER3	NDUFA4	1,34330E-10	-4,01662E-01	0,792	0,742	2,47395E-06
Macro_IER3	LTBR	2,34871E-21	-4,02662E-01	0,457	0,344	4,32561E-17
Macro_IER3	FBXL5	7,32819E-07	-4,05608E-01	0,335	0,271	1,34963E-02
Macro_IER3	RGS2	2,50059E-105	-4,05702E-01	0,804	0,617	4,60533E-101
Macro_IER3	SPINT2	1,09041E-10	-4,06622E-01	0,604	0,493	2,00821E-06

Macro_IER3	SRSF3	6,33177E-15	-4,07505E-01	0,688	0,565	1,16612E-10
Macro_IER3	LY96	7,85179E-14	-4,09693E-01	0,562	0,452	1,44606E-09
Macro_IER3	C9orf72	1,60136E-16	-4,12180E-01	0,306	0,222	2,94922E-12
Macro_IER3	IGSF6	4,68429E-14	-4,12998E-01	0,695	0,551	8,62706E-10
Macro_IER3	GNG10	2,40468E-15	-4,14175E-01	0,438	0,333	4,42871E-11
Macro_IER3	SF3A1	1,00069E-06	-4,14468E-01	0,345	0,286	1,84297E-02
Macro_IER3	NUP214	1,40938E-14	-4,16161E-01	0,404	0,31	2,59565E-10
Macro_IER3	ASPH	2,25817E-07	-4,17609E-01	0,275	0,22	4,15886E-03
Macro_IER3	LAPTM4A	5,22537E-14	-4,18412E-01	0,751	0,622	9,62355E-10
Macro_IER3	NASP	4,88081E-08	-4,19506E-01	0,334	0,273	8,98899E-04
Macro_IER3	TCP1	2,41666E-10	-4,21444E-01	0,406	0,334	4,45076E-06
Macro_IER3	FKBP1A	4,18839E-12	-4,22483E-01	0,715	0,693	7,71376E-08
Macro_IER3	METRNL	2,25744E-07	-4,23676E-01	0,31	0,252	4,15752E-03
Macro_IER3	PTMA	1,38397E-07	-4,23785E-01	0,982	0,961	2,54885E-03
Macro_IER3	TNFRSF1A	2,74209E-07	-4,24084E-01	0,528	0,445	5,05011E-03
Macro_IER3	THEMIS2	2,64610E-23	-4,24412E-01	0,624	0,489	4,87332E-19
Macro_IER3	APLP2	6,82032E-11	-4,24741E-01	0,72	0,589	1,25610E-06
Macro_IER3	TYMP	3,64927E-07	-4,25717E-01	0,836	0,791	6,72086E-03
Macro_IER3	TOR3A	2,72293E-10	-4,26189E-01	0,295	0,235	5,01481E-06
Macro_IER3	ARHGEF1	4,06458E-16	-4,27195E-01	0,359	0,267	7,48574E-12
Macro_IER3	CTSA	3,34930E-13	-4,29155E-01	0,637	0,5	6,16841E-09
Macro_IER3	CMTM7	4,09150E-12	-4,29195E-01	0,505	0,407	7,53531E-08
Macro_IER3	HELZ	1,26760E-08	-4,31075E-01	0,265	0,208	2,33453E-04
Macro_IER3	NCF1	5,18838E-08	-4,31361E-01	0,601	0,495	9,55544E-04
Macro_IER3	ATP2B1	6,93273E-26	-4,32611E-01	0,471	0,343	1,27680E-21
Macro_IER3	RNF13	1,41054E-29	-4,33366E-01	0,658	0,511	2,59779E-25
Macro_IER3	CKLF	8,73859E-11	-4,34285E-01	0,614	0,501	1,60939E-06
Macro_IER3	CFLAR	4,02896E-17	-4,35516E-01	0,626	0,506	7,42013E-13
Macro_IER3	PNISR	1,72343E-07	-4,35923E-01	0,578	0,49	3,17404E-03
Macro_IER3	CLK1	1,45055E-64	-4,36416E-01	0,55	0,375	2,67148E-60
Macro_IER3	NAAA	1,88858E-06	-4,39400E-01	0,435	0,364	3,47820E-02
Macro_IER3	GPCPD1	4,00084E-08	-4,40855E-01	0,344	0,277	7,36836E-04
Macro_IER3	APEX1	1,96892E-13	-4,41259E-01	0,548	0,437	3,62616E-09
Macro_IER3	CLEC4A	2,63510E-22	-4,43731E-01	0,41	0,292	4,85307E-18
Macro_IER3	MFF	1,80609E-08	-4,44624E-01	0,384	0,316	3,32628E-04
Macro_IER3	MBNL1	2,10939E-12	-4,46298E-01	0,624	0,52	3,88487E-08
Macro_IER3	PHB2	6,22112E-09	-4,46380E-01	0,584	0,489	1,14574E-04
Macro_IER3	APOC1	2,64388E-14	-4,46401E-01	0,654	0,5	4,86924E-10
Macro_IER3	ZEB2	1,93967E-10	-4,46662E-01	0,704	0,601	3,57229E-06
Macro_IER3	DOCK10	6,81218E-13	-4,47614E-01	0,4	0,319	1,25460E-08

Macro_IER3	NCOR1	2,77812E-09	-4,49723E-01	0,478	0,395	5,11646E-05
Macro_IER3	SLC38A2	8,40454E-14	-4,49750E-01	0,39	0,305	1,54786E-09
Macro_IER3	TAF7	1,24939E-09	-4,51252E-01	0,365	0,299	2,30101E-05
Macro_IER3	RALA	1,84387E-06	-4,51839E-01	0,349	0,375	3,39586E-02
Macro_IER3	MAT2A	7,97451E-69	-4,53467E-01	0,625	0,438	1,46866E-64
Macro_IER3	ATF4	1,80025E-06	-4,53496E-01	0,474	0,402	3,31553E-02
Macro_IER3	FPR1	1,02393E-19	-4,55039E-01	0,578	0,442	1,88577E-15
Macro_IER3	NDUFA1	8,30418E-08	-4,55172E-01	0,708	0,686	1,52938E-03
Macro_IER3	TNFRSF1B	5,25846E-14	-4,55704E-01	0,669	0,534	9,68451E-10
Macro_IER3	SNX2	1,20737E-08	-4,58127E-01	0,526	0,437	2,22362E-04
Macro_IER3	TAX1BP1	9,74288E-09	-4,58172E-01	0,581	0,494	1,79435E-04
Macro_IER3	NEK6	2,64076E-13	-4,58558E-01	0,312	0,235	4,86350E-09
Macro_IER3	DNAJA1	1,50889E-40	-4,61625E-01	0,74	0,598	2,77892E-36
Macro_IER3	FNBP4	1,09000E-10	-4,61879E-01	0,37	0,297	2,00744E-06
Macro_IER3	SF3B1	5,64868E-21	-4,62083E-01	0,603	0,488	1,04032E-16
Macro_IER3	MICAL1	1,04764E-12	-4,64897E-01	0,368	0,29	1,92944E-08
Macro_IER3	ARRDC1	1,68549E-08	-4,64982E-01	0,359	0,291	3,10417E-04
Macro_IER3	HNRNPA2B1	1,56270E-06	-4,67333E-01	0,919	0,828	2,87802E-02
Macro_IER3	NFKBID	2,31541E-93	-4,68013E-01	0,394	0,205	4,26429E-89
Macro_IER3	CCT6A	9,36513E-10	-4,68455E-01	0,54	0,444	1,72478E-05
Macro_IER3	CCR1	1,23118E-06	-4,68863E-01	0,278	0,225	2,26746E-02
Macro_IER3	EMB	8,51630E-21	-4,69791E-01	0,297	0,207	1,56845E-16
Macro_IER3	AKR7A2	2,21043E-08	-4,71756E-01	0,305	0,247	4,07095E-04
Macro_IER3	MYO1G	3,63134E-10	-4,73009E-01	0,239	0,285	6,68784E-06
Macro_IER3	ATP1B3	3,80253E-20	-4,73900E-01	0,662	0,539	7,00311E-16
Macro_IER3	PTGES3	5,87201E-12	-4,76637E-01	0,758	0,639	1,08145E-07
Macro_IER3	CIITA	3,38028E-26	-4,76777E-01	0,438	0,319	6,22546E-22
Macro_IER3	TMEM59	3,88603E-08	-4,77622E-01	0,72	0,613	7,15690E-04
Macro_IER3	C4orf3	3,60215E-11	-4,78698E-01	0,679	0,67	6,63408E-07
Macro_IER3	SDCCAG8	2,99780E-27	-4,79405E-01	0,451	0,332	5,52105E-23
Macro_IER3	COX7B	1,37418E-09	-4,80226E-01	0,693	0,665	2,53083E-05
Macro_IER3	POMP	3,20859E-16	-4,80643E-01	0,763	0,718	5,90926E-12
Macro_IER3	HSPA9	9,12396E-22	-4,81033E-01	0,474	0,366	1,68036E-17
Macro_IER3	GTF2B	5,54731E-11	-4,81119E-01	0,346	0,276	1,02165E-06
Macro_IER3	PPIF	2,11017E-06	-4,82864E-01	0,419	0,355	3,88630E-02
Macro_IER3	PLEK	1,69452E-06	-4,83645E-01	0,642	0,558	3,12081E-02
Macro_IER3	RPLP1	1,51728E-09	-4,85778E-01	0,555	0,6	2,79437E-05
Macro_IER3	DOCK2	2,19508E-09	-4,86843E-01	0,421	0,345	4,04267E-05
Macro_IER3	ARPC1B	4,66196E-21	-4,88060E-01	0,815	0,796	8,58593E-17
Macro_IER3	ATOX1	4,84079E-18	-4,88564E-01	0,558	0,576	8,91528E-14

Macro_IER3	ST3GAL1	6,16429E-09	-4,89425E-01	0,288	0,227	1,13528E-04
Macro_IER3	BLNK	4,09387E-43	-4,90147E-01	0,283	0,165	7,53967E-39
Macro_IER3	NDUFS5	7,21995E-09	-4,91267E-01	0,677	0,653	1,32970E-04
Macro_IER3	NAGK	6,94179E-10	-4,91447E-01	0,571	0,473	1,27847E-05
Macro_IER3	OSGEP	1,28342E-11	-4,92524E-01	0,297	0,231	2,36367E-07
Macro_IER3	SON	1,88689E-07	-4,93237E-01	0,763	0,67	3,47508E-03
Macro_IER3	GLIPR2	1,21920E-22	-4,95525E-01	0,303	0,38	2,24540E-18
Macro_IER3	PFDN5	1,05776E-09	-4,95871E-01	0,932	0,901	1,94809E-05
Macro_IER3	EIF3F	1,93729E-10	-4,96710E-01	0,769	0,661	3,56790E-06
Macro_IER3	FAU	1,48292E-15	-4,97701E-01	0,964	0,956	2,73109E-11
Macro_IER3	PRELID1	3,51079E-15	-4,98499E-01	0,689	0,684	6,46583E-11
Macro_IER3	LAMTOR5	9,54655E-08	-5,00253E-01	0,499	0,507	1,75819E-03
Macro_IER3	RSL1D1	2,49624E-07	-5,01976E-01	0,542	0,466	4,59732E-03
Macro_IER3	TMSB4X	1,40218E-11	-5,02719E-01	0,994	0,983	2,58240E-07
Macro_IER3	PGLS	1,24912E-08	-5,03410E-01	0,639	0,538	2,30050E-04
Macro_IER3	PLIN2	1,72974E-57	-5,05431E-01	0,668	0,49	3,18566E-53
Macro_IER3	CLNS1A	5,14737E-12	-5,05771E-01	0,511	0,417	9,47991E-08
Macro_IER3	STRAP	7,03188E-08	-5,06477E-01	0,43	0,352	1,29506E-03
Macro_IER3	TSPYL2	6,92234E-22	-5,08997E-01	0,252	0,167	1,27489E-17
Macro_IER3	NDUFV1	8,73626E-14	-5,11921E-01	0,503	0,403	1,60896E-09
Macro_IER3	TPP1	1,18738E-18	-5,13027E-01	0,715	0,59	2,18679E-14
Macro_IER3	PLAUR	1,45259E-72	-5,15046E-01	0,821	0,632	2,67523E-68
Macro_IER3	VMP1	9,18833E-08	-5,16723E-01	0,641	0,546	1,69221E-03
Macro_IER3	ARL6IP1	8,54847E-08	-5,19068E-01	0,485	0,412	1,57437E-03
Macro_IER3	UBA52	1,98106E-11	-5,20428E-01	0,941	0,92	3,64852E-07
Macro_IER3	SNRPG	1,69381E-06	-5,21341E-01	0,567	0,556	3,11950E-02
Macro_IER3	PSMB3	2,63287E-06	-5,25878E-01	0,677	0,642	4,84896E-02
Macro_IER3	TBCA	4,60221E-07	-5,27270E-01	0,602	0,588	8,47588E-03
Macro_IER3	PKM	2,28663E-24	-5,27766E-01	0,816	0,782	4,21128E-20
Macro_IER3	LCP1	1,67319E-15	-5,27821E-01	0,715	0,703	3,08151E-11
Macro_IER3	PSMG2	4,71968E-09	-5,33313E-01	0,504	0,42	8,69224E-05
Macro_IER3	COX6C	2,68809E-11	-5,34781E-01	0,754	0,718	4,95065E-07
Macro_IER3	ERN1	7,83393E-22	-5,34937E-01	0,252	0,169	1,44278E-17
Macro_IER3	B4GALT1	1,49678E-48	-5,35485E-01	0,477	0,318	2,75661E-44
Macro_IER3	CHD9	1,02322E-09	-5,35675E-01	0,414	0,34	1,88446E-05
Macro_IER3	CACYBP	1,02707E-06	-5,39614E-01	0,463	0,399	1,89155E-02
Macro_IER3	JKAMP	3,95696E-12	-5,39720E-01	0,285	0,216	7,28753E-08
Macro_IER3	HSPA8	2,44107E-32	-5,42801E-01	0,922	0,815	4,49572E-28
Macro_IER3	RPL34	3,91242E-11	-5,44720E-01	0,54	0,577	7,20551E-07
Macro_IER3	COX7C	1,68142E-10	-5,44905E-01	0,88	0,826	3,09668E-06

Macro_IER3	GRN	6,39677E-10	-5,46055E-01	0,925	0,818	1,17809E-05
Macro_IER3	DHRS7	4,32992E-20	-5,46364E-01	0,6	0,48	7,97441E-16
Macro_IER3	TUBGCP2	2,40288E-08	-5,48901E-01	0,364	0,3	4,42538E-04
Macro_IER3	BEX4	1,25145E-09	-5,50584E-01	0,401	0,325	2,30480E-05
Macro_IER3	TECR	9,15959E-12	-5,50834E-01	0,466	0,375	1,68692E-07
Macro_IER3	COX6A1	1,26134E-09	-5,52870E-01	0,709	0,678	2,32302E-05
Macro_IER3	SERINC3	4,29526E-09	-5,53513E-01	0,408	0,337	7,91059E-05
Macro_IER3	ATP6V1F	2,00898E-08	-5,54410E-01	0,809	0,762	3,69994E-04
Macro_IER3	TMSB10	1,72747E-75	-5,54645E-01	0,988	0,984	3,18147E-71
Macro_IER3	LTA4H	1,48002E-07	-5,56925E-01	0,535	0,444	2,72574E-03
Macro_IER3	ATP6V1G1	1,04891E-12	-5,57074E-01	0,753	0,718	1,93178E-08
Macro_IER3	NPM1	8,00108E-13	-5,57462E-01	0,875	0,761	1,47356E-08
Macro_IER3	FTH1	1,89464E-06	-5,58307E-01	0,999	0,997	3,48935E-02
Macro_IER3	CAPZA2	3,90132E-09	-5,60972E-01	0,641	0,545	7,18507E-05
Macro_IER3	GBP1	2,89456E-07	-5,62652E-01	0,323	0,347	5,33091E-03
Macro_IER3	RBM6	9,07017E-09	-5,67155E-01	0,322	0,26	1,67045E-04
Macro_IER3	GSTO1	6,32029E-14	-5,67216E-01	0,63	0,619	1,16401E-09
Macro_IER3	RPL8	2,05078E-10	-5,69368E-01	0,544	0,574	3,77692E-06
Macro_IER3	PDCD6IP	4,84682E-09	-5,72093E-01	0,442	0,365	8,92638E-05
Macro_IER3	TMEM109	7,53704E-13	-5,72251E-01	0,459	0,365	1,38810E-08
Macro_IER3	LAMP2	2,53495E-09	-5,72373E-01	0,488	0,397	4,66862E-05
Macro_IER3	COTL1	3,36162E-07	-5,73858E-01	0,726	0,711	6,19109E-03
Macro_IER3	TALDO1	7,13605E-18	-5,76168E-01	0,67	0,662	1,31425E-13
Macro_IER3	PFN1	2,35636E-78	-5,76550E-01	0,96	0,946	4,33971E-74
Macro_IER3	TINF2	2,50133E-06	-5,82650E-01	0,273	0,225	4,60669E-02
Macro_IER3	NXF1	3,28703E-14	-5,90054E-01	0,279	0,208	6,05372E-10
Macro_IER3	MYADM	4,64620E-08	-5,90872E-01	0,405	0,331	8,55690E-04
Macro_IER3	DUSP6	8,10107E-12	-5,95476E-01	0,364	0,285	1,49197E-07
Macro_IER3	SRSF2	7,17568E-15	-5,95626E-01	0,671	0,552	1,32155E-10
Macro_IER3	ANKRD44	4,62397E-12	-5,95953E-01	0,365	0,288	8,51596E-08
Macro_IER3	PPDPF	1,11698E-50	-5,96222E-01	0,752	0,768	2,05714E-46
Macro_IER3	EIF4H	7,66741E-07	-5,97051E-01	0,53	0,449	1,41211E-02
Macro_IER3	NDUFB9	1,29443E-06	-6,01677E-01	0,552	0,55	2,38395E-02
Macro_IER3	RPS27A	9,89177E-10	-6,04411E-01	0,536	0,577	1,82177E-05
Macro_IER3	PTPRE	1,09174E-19	-6,05439E-01	0,559	0,434	2,01066E-15
Macro_IER3	COPS3	3,45568E-08	-6,07643E-01	0,306	0,249	6,36432E-04
Macro_IER3	STMN1	8,33609E-07	-6,09564E-01	0,349	0,284	1,53526E-02
Macro_IER3	UBC	4,07654E-27	-6,14520E-01	0,965	0,896	7,50775E-23
Macro_IER3	CTSC	4,59488E-17	-6,15368E-01	0,836	0,67	8,46239E-13
Macro_IER3	RPS7	6,50091E-08	-6,16225E-01	0,533	0,562	1,19727E-03

Macro_IER3	COX8A	4,10622E-24	-6,16906E-01	0,796	0,777	7,56243E-20
Macro_IER3	SERPINB1	1,64053E-06	-6,19535E-01	0,685	0,662	3,02136E-02
Macro_IER3	ATP6V0E1	4,68602E-13	-6,21494E-01	0,838	0,794	8,63024E-09
Macro_IER3	PRR13	6,02759E-09	-6,21807E-01	0,559	0,581	1,11010E-04
Macro_IER3	KRTCAP2	7,94865E-07	-6,23392E-01	0,67	0,573	1,46390E-02
Macro_IER3	RPL28	2,08151E-10	-6,28426E-01	0,553	0,596	3,83353E-06
Macro_IER3	UQCRQ	1,97438E-06	-6,30076E-01	0,693	0,665	3,63622E-02
Macro_IER3	ACTN4	1,01693E-07	-6,30620E-01	0,314	0,35	1,87289E-03
Macro_IER3	AMZ2	3,27240E-11	-6,32948E-01	0,289	0,224	6,02677E-07
Macro_IER3	ABRACL	1,16208E-08	-6,33645E-01	0,486	0,498	2,14020E-04
Macro_IER3	RPL11	2,75996E-09	-6,33732E-01	0,544	0,582	5,08301E-05
Macro_IER3	LGALS3	1,72736E-17	-6,34344E-01	0,755	0,723	3,18128E-13
Macro_IER3	RBMX	1,45950E-07	-6,37742E-01	0,502	0,422	2,68797E-03
Macro_IER3	CD164	3,29801E-11	-6,38275E-01	0,658	0,553	6,07394E-07
Macro_IER3	ANKRD10	1,62068E-38	-6,38471E-01	0,465	0,326	2,98481E-34
Macro_IER3	AUP1	4,13254E-10	-6,40290E-01	0,619	0,521	7,61090E-06
Macro_IER3	SLC25A3	2,47476E-07	-6,44261E-01	0,797	0,71	4,55776E-03
Macro_IER3	COX4I1	5,47364E-24	-6,46638E-01	0,931	0,895	1,00808E-19
Macro_IER3	S100A11	1,41575E-30	-6,47188E-01	0,96	0,936	2,60738E-26
Macro_IER3	VMO1	1,01946E-14	-6,52933E-01	0,303	0,224	1,87753E-10
Macro_IER3	CD4	3,02717E-20	-6,54013E-01	0,682	0,532	5,57513E-16
Macro_IER3	EIF4A1	6,82984E-10	-6,54166E-01	0,77	0,67	1,25785E-05
Macro_IER3	GAPT	1,09977E-19	-6,54433E-01	0,354	0,25	2,02545E-15
Macro_IER3	ANAPC5	5,21272E-08	-6,55650E-01	0,346	0,285	9,60027E-04
Macro_IER3	FCER1G	1,92495E-22	-6,56081E-01	0,97	0,91	3,54519E-18
Macro_IER3	CRIP1	3,26936E-09	-6,56568E-01	0,236	0,295	6,02118E-05
Macro_IER3	EMP3	3,97758E-23	-6,60443E-01	0,724	0,729	7,32550E-19
Macro_IER3	EIF3G	3,32678E-15	-6,61575E-01	0,719	0,609	6,12694E-11
Macro_IER3	SERF2	6,20666E-98	-6,68639E-01	0,961	0,953	1,14308E-93
Macro_IER3	NR3C1	6,80864E-07	-6,72796E-01	0,455	0,385	1,25395E-02
Macro_IER3	RPL30	1,62221E-06	-6,73004E-01	0,544	0,582	2,98762E-02
Macro_IER3	RPL24	1,14084E-06	-6,75035E-01	0,527	0,539	2,10108E-02
Macro_IER3	S100A4	1,31549E-16	-6,80274E-01	0,842	0,826	2,42274E-12
Macro_IER3	PDCD4	2,66209E-17	-6,81861E-01	0,346	0,255	4,90277E-13
Macro_IER3	RPL19	1,01370E-06	-6,85205E-01	0,546	0,582	1,86694E-02
Macro_IER3	OST4	8,45839E-22	-6,86990E-01	0,81	0,776	1,55778E-17
Macro_IER3	COX5A	9,75585E-12	-6,87226E-01	0,732	0,692	1,79674E-07
Macro_IER3	RPL39	1,15100E-11	-6,88915E-01	0,547	0,582	2,11980E-07
Macro_IER3	ACTB	5,20299E-41	-6,92975E-01	0,99	0,991	9,58235E-37
Macro_IER3	NACA	5,80000E-09	-7,01159E-01	0,948	0,918	1,06819E-04

Macro_IER3	GPI	1,44530E-06	-7,03007E-01	0,49	0,413	2,66181E-02
Macro_IER3	CNIH4	8,01801E-13	-7,05736E-01	0,229	0,289	1,47668E-08
Macro_IER3	STX8	1,52689E-09	-7,07505E-01	0,298	0,235	2,81207E-05
Macro_IER3	PSME1	2,11407E-18	-7,07525E-01	0,783	0,753	3,89349E-14
Macro_IER3	CLN8	1,80974E-07	-7,11292E-01	0,286	0,233	3,33299E-03
Macro_IER3	MYL12B	1,93853E-21	-7,12176E-01	0,811	0,775	3,57019E-17
Macro_IER3	DBI	7,00692E-08	-7,13006E-01	0,767	0,722	1,29046E-03
Macro_IER3	TMA7	1,10069E-17	-7,13277E-01	0,819	0,792	2,02715E-13
Macro_IER3	RPS12	1,66778E-07	-7,16329E-01	0,548	0,584	3,07155E-03
Macro_IER3	RPLP2	4,86672E-11	-7,18040E-01	0,543	0,572	8,96303E-07
Macro_IER3	CFL1	2,03861E-38	-7,20286E-01	0,951	0,921	3,75451E-34
Macro_IER3	NOP10	1,84474E-16	-7,21988E-01	0,627	0,632	3,39745E-12
Macro_IER3	RPL35A	5,86589E-10	-7,22123E-01	0,531	0,564	1,08032E-05
Macro_IER3	EDF1	2,03948E-06	-7,22987E-01	0,795	0,748	3,75612E-02
Macro_IER3	ARPC2	1,30433E-24	-7,24260E-01	0,901	0,857	2,40219E-20
Macro_IER3	SLC3A2	5,12570E-13	-7,25906E-01	0,562	0,459	9,44000E-09
Macro_IER3	PRDX1	2,50330E-08	-7,26641E-01	0,805	0,744	4,61033E-04
Macro_IER3	RAB1A	4,48139E-11	-7,30768E-01	0,477	0,392	8,25337E-07
Macro_IER3	B2M	2,93415E-18	-7,32527E-01	0,991	0,996	5,40383E-14
Macro_IER3	FLOT1	2,12236E-11	-7,34186E-01	0,616	0,52	3,90876E-07
Macro_IER3	CCDC50	9,38565E-07	-7,42735E-01	0,335	0,271	1,72855E-02
Macro_IER3	PLSCR1	2,81475E-18	-7,46534E-01	0,671	0,536	5,18393E-14
Macro_IER3	FLNA	3,29141E-23	-7,49503E-01	0,374	0,455	6,06180E-19
Macro_IER3	RBM39	4,68531E-19	-7,50362E-01	0,742	0,617	8,62894E-15
Macro_IER3	RPS28	7,14008E-11	-7,53170E-01	0,544	0,584	1,31499E-06
Macro_IER3	TNFSF10	4,06577E-08	-7,58439E-01	0,34	0,361	7,48793E-04
Macro_IER3	CHCHD2	2,60058E-26	-7,59490E-01	0,855	0,814	4,78949E-22
Macro_IER3	HINT1	4,71023E-12	-7,63083E-01	0,775	0,72	8,67483E-08
Macro_IER3	PPIA	2,14147E-14	-7,66296E-01	0,942	0,892	3,94394E-10
Macro_IER3	TPM3	4,70060E-15	-7,67354E-01	0,853	0,801	8,65709E-11
Macro_IER3	OAZ1	1,15478E-38	-7,77264E-01	0,956	0,936	2,12676E-34
Macro_IER3	SNRPD2	4,27253E-09	-7,81463E-01	0,707	0,676	7,86872E-05
Macro_IER3	CD163	1,07342E-49	-7,82848E-01	0,638	0,438	1,97692E-45
Macro_IER3	SEC61G	8,59190E-23	-7,85909E-01	0,581	0,601	1,58237E-18
Macro_IER3	SH3BGRL3	1,02111E-98	-7,89892E-01	0,929	0,933	1,88058E-94
Macro_IER3	CLIC1	1,03148E-41	-8,00038E-01	0,895	0,873	1,89968E-37
Macro_IER3	COX7A2	2,14209E-22	-8,03831E-01	0,739	0,72	3,94510E-18
Macro_IER3	RPS8	3,03169E-08	-8,04314E-01	0,543	0,58	5,58347E-04
Macro_IER3	PPIB	1,72339E-07	-8,04456E-01	0,851	0,79	3,17397E-03
Macro_IER3	PABPC1	5,98268E-20	-8,06419E-01	0,955	0,89	1,10183E-15

Macro_IER3	DDOST	1,15324E-06	-8,10231E-01	0,539	0,46	2,12392E-02
Macro_IER3	RPL36AL	2,32839E-09	-8,10383E-01	0,486	0,493	4,28820E-05
Macro_IER3	UBL5	6,53901E-27	-8,21935E-01	0,761	0,755	1,20429E-22
Macro_IER3	SSR4	1,96428E-10	-8,25183E-01	0,805	0,751	3,61762E-06
Macro_IER3	CALM3	1,41777E-11	-8,25963E-01	0,492	0,508	2,61110E-07
Macro_IER3	PSMB9	6,72776E-16	-8,31656E-01	0,688	0,656	1,23905E-11
Macro_IER3	GUK1	5,57928E-16	-8,33206E-01	0,728	0,709	1,02754E-11
Macro_IER3	PSMA4	2,61967E-08	-8,37149E-01	0,451	0,469	4,82464E-04
Macro_IER3	LGALS1	1,14627E-39	-8,38517E-01	0,85	0,835	2,11108E-35
Macro_IER3	GNG5	2,04281E-30	-8,41495E-01	0,824	0,792	3,76224E-26
Macro_IER3	MARCKSL1	5,70496E-11	-8,42692E-01	0,216	0,267	1,05068E-06
Macro_IER3	MIF	7,81496E-08	-8,44424E-01	0,755	0,723	1,43928E-03
Macro_IER3	SERPINB9	2,60467E-22	-8,45266E-01	0,501	0,369	4,79701E-18
Macro_IER3	SRP14	1,07459E-26	-8,46164E-01	0,868	0,835	1,97907E-22
Macro_IER3	VIM	5,04595E-38	-8,49424E-01	0,947	0,943	9,29314E-34
Macro_IER3	PARK7	5,69654E-09	-8,54663E-01	0,693	0,657	1,04913E-04
Macro_IER3	CTSB	1,64998E-09	-8,69718E-01	0,969	0,867	3,03877E-05
Macro_IER3	GSTP1	4,84114E-19	-8,75280E-01	0,879	0,821	8,91592E-15
Macro_IER3	GPNMB	6,56723E-07	-8,86868E-01	0,42	0,331	1,20949E-02
Macro_IER3	LSP1	1,62436E-08	-8,87219E-01	0,719	0,669	2,99158E-04
Macro_IER3	CXCR4	3,55661E-110	-8,87330E-01	0,776	0,571	6,55021E-106
Macro_IER3	CALM1	5,78506E-08	-8,94917E-01	0,844	0,793	1,06543E-03
Macro_IER3	MYL6	3,49242E-120	-8,95214E-01	0,942	0,941	6,43199E-116
Macro_IER3	TXNDC17	1,82807E-07	-9,02827E-01	0,409	0,435	3,36675E-03
Macro_IER3	EZR	7,10579E-10	-9,06570E-01	0,479	0,396	1,30867E-05
Macro_IER3	PLD4	3,73072E-78	-9,08399E-01	0,535	0,323	6,87087E-74
Macro_IER3	LDLRAD4	6,10846E-08	-9,15227E-01	0,267	0,21	1,12500E-03
Macro_IER3	PSMA7	6,57351E-27	-9,41389E-01	0,769	0,744	1,21064E-22
Macro_IER3	PGAM1	2,78996E-12	-9,42668E-01	0,561	0,565	5,13826E-08
Macro_IER3	CSTB	7,04756E-41	-9,52120E-01	0,814	0,788	1,29795E-36
Macro_IER3	ANXA2	4,65918E-27	-9,54804E-01	0,77	0,742	8,58081E-23
Macro_IER3	GAPDH	2,66515E-79	-9,58200E-01	0,962	0,96	4,90840E-75
Macro_IER3	TGFBI	6,61624E-38	-9,62761E-01	0,761	0,591	1,21851E-33
Macro_IER3	IL2RG	1,93642E-34	-9,73592E-01	0,215	0,329	3,56631E-30
Macro_IER3	CYSTM1	4,16964E-30	-9,88318E-01	0,258	0,357	7,67923E-26
Macro_IER3	TREM1	5,47866E-08	-9,99598E-01	0,307	0,234	1,00900E-03
Macro_IER3	PSMB8	1,88367E-07	-1,00731E+00	0,591	0,576	3,46916E-03
Macro_IER3	S100A6	7,84823E-105	-1,01115E+00	0,814	0,877	1,44541E-100
Macro_IER3	TPI1	9,08152E-18	-1,01123E+00	0,868	0,824	1,67254E-13
Macro_IER3	TAP1	2,72754E-11	-1,01497E+00	0,394	0,424	5,02331E-07

Macro_IER3	GPR183	9,88709E-190	-1,01679E+00	0,744	0,443	1,82091E-185
Macro_IER3	PSME2	1,85823E-29	-1,02493E+00	0,726	0,715	3,42230E-25
Macro_IER3	SUB1	4,19204E-38	-1,02885E+00	0,729	0,723	7,72048E-34
Macro_IER3	HNRNPH1	2,48816E-07	-1,03808E+00	0,478	0,401	4,58244E-03
Macro_IER3	DDIT4	7,57422E-20	-1,04225E+00	0,617	0,49	1,39494E-15
Macro_IER3	STXBP2	2,78349E-17	-1,06322E+00	0,364	0,422	5,12635E-13
Macro_IER3	LY6E	1,99886E-35	-1,09412E+00	0,48	0,551	3,68130E-31
Macro_IER3	CREM	8,04230E-57	-1,14121E+00	0,55	0,37	1,48115E-52
Macro_IER3	ENO1	9,39031E-34	-1,14155E+00	0,77	0,761	1,72941E-29
Macro_IER3	LDHA	7,09970E-08	-1,16299E+00	0,747	0,712	1,30755E-03
Macro_IER3	PGK1	1,56732E-07	-1,19990E+00	0,752	0,704	2,88653E-03
Macro_IER3	S100A10	4,27231E-63	-1,24271E+00	0,823	0,838	7,86831E-59
Macro_IER3	MYL12A	2,06761E-64	-1,29959E+00	0,834	0,824	3,80792E-60
Macro_IER3	SEC61B	3,56066E-27	-1,37083E+00	0,764	0,739	6,55767E-23
Macro_IER3	TCF4	1,68796E-08	-1,41216E+00	0,338	0,27	3,10871E-04
Macro_IER3	PLP2	2,41169E-23	-1,42403E+00	0,336	0,407	4,44161E-19
Macro_IER3	RAC2	1,94272E-24	-1,43041E+00	0,454	0,498	3,57790E-20
Macro_IER3	ALDOA	9,14907E-43	-1,45194E+00	0,776	0,759	1,68499E-38
Macro_IER3	CD52	1,11167E-73	-1,46573E+00	0,222	0,398	2,04736E-69
Macro_IER3	IFI6	3,77603E-14	-1,46860E+00	0,467	0,489	6,95431E-10
Macro_IER3	TAGLN2	1,93144E-31	-1,47555E+00	0,757	0,754	3,55713E-27
Macro_IER3	MX1	6,70521E-10	-1,52526E+00	0,296	0,335	1,23490E-05
Macro_IER3	ISG20	6,70802E-21	-1,64912E+00	0,241	0,314	1,23542E-16
Macro_IER3	IFITM2	2,82489E-12	-1,65446E+00	0,668	0,646	5,20260E-08
Macro_IER3	TXN	6,14441E-61	-1,74307E+00	0,615	0,67	1,13162E-56
Macro_IER3	IFITM1	2,80735E-31	-1,91088E+00	0,161	0,259	5,17029E-27
Macro_IER3	TIMP1	1,03455E-34	-2,05039E+00	0,593	0,653	1,90532E-30
Macro_IER3	S100A9	1,12378E-31	-2,05087E+00	0,365	0,46	2,06967E-27
Macro_IER3	IRF8	5,36824E-21	-2,06329E+00	0,504	0,372	9,88670E-17
Macro_IER3	ISG15	2,84593E-22	-2,28511E+00	0,363	0,431	5,24135E-18
Macro_IER3	S100A8	8,60746E-31	-2,39036E+00	0,258	0,357	1,58524E-26
Macro_IER3	IRF7	3,63772E-12	-2,51381E+00	0,367	0,401	6,69959E-08

cluster	gene	p_val	avg_log2FC	pct.1	pct.2	p_val_adj
Macro_IFI27	APOC1	6,81258E-286	1,23778E+00	0,797	0,493	1,25467E-281
Macro_IFI27	IFI27	3,46133E-57	1,07767E+00	0,278	0,167	6,37473E-53
Macro_IFI27	APOE	4,28386E-287	1,06197E+00	0,841	0,491	7,88959E-283
Macro_IFI27	NUPR1	4,59311E-33	9,36146E-01	0,299	0,212	8,45914E-29
Macro_IFI27	A2M	8,13962E-15	9,18042E-01	0,41	0,388	1,49907E-10
Macro_IFI27	C1QC	2,42680E-306	9,04803E-01	0,896	0,529	4,46944E-302
Macro_IFI27	C1QA	1,62379E-266	6,92314E-01	0,893	0,551	2,99054E-262
Macro_IFI27	C1QB	1,19668E-260	6,27787E-01	0,881	0,53	2,20392E-256
Macro_IFI27	FTL	2,54152E-222	5,57294E-01	0,999	0,991	4,68071E-218
Macro_IFI27	GPNMB	1,07571E-33	5,35205E-01	0,417	0,331	1,98114E-29
Macro_IFI27	ATP5F1E	1,69653E-12	5,24366E-01	0,482	0,477	3,12450E-08
Macro_IFI27	ATP5MC3	1,91484E-08	5,24066E-01	0,244	0,359	3,52656E-04
Macro_IFI27	TREM2	7,95080E-40	4,93189E-01	0,486	0,403	1,46430E-35
Macro_IFI27	PTMS	2,84361E-11	4,75490E-01	0,246	0,368	5,23708E-07
Macro_IFI27	ATP5MF	1,70603E-10	4,71435E-01	0,238	0,362	3,14199E-06
Macro_IFI27	ATP5PB	1,50852E-21	4,35224E-01	0,161	0,288	2,77825E-17
Macro_IFI27	CHCHD10	4,94114E-07	4,34267E-01	0,369	0,523	9,10010E-03
Macro_IFI27	ATP5PD	1,29549E-21	4,30465E-01	0,187	0,326	2,38590E-17
Macro_IFI27	ATP5IF1	1,75131E-18	4,29761E-01	0,156	0,27	3,22539E-14
Macro_IFI27	SELENOW	1,91867E-17	4,20175E-01	0,176	0,297	3,53361E-13
Macro_IFI27	LAMP1	1,33980E-24	3,94114E-01	0,237	0,405	2,46751E-20
Macro_IFI27	PRDX1	1,69085E-63	3,93177E-01	0,734	0,748	3,11405E-59
Macro_IFI27	CTSD	5,49019E-82	3,83751E-01	0,802	0,768	1,01113E-77
Macro_IFI27	ATP5F1B	1,41293E-21	3,76401E-01	0,184	0,32	2,60220E-17
Macro_IFI27	JPT1	2,07525E-18	3,70330E-01	0,169	0,288	3,82199E-14
Macro_IFI27	PLD3	7,57481E-14	3,51770E-01	0,495	0,512	1,39505E-09
Macro_IFI27	ATP5F1C	7,55791E-32	3,49109E-01	0,147	0,295	1,39194E-27
Macro_IFI27	FYB1	7,26998E-13	3,47672E-01	0,213	0,323	1,33891E-08
Macro_IFI27	LIPA	2,64745E-15	3,45805E-01	0,489	0,502	4,87581E-11
Macro_IFI27	SEM1	1,33028E-31	3,40903E-01	0,172	0,33	2,44998E-27
Macro_IFI27	ATP5PF	2,20628E-27	3,33629E-01	0,178	0,33	4,06330E-23
Macro_IFI27	NME2	7,91207E-15	3,21004E-01	0,234	0,365	1,45717E-10
Macro_IFI27	ATP5ME	7,61900E-29	3,02809E-01	0,167	0,316	1,40319E-24
Macro_IFI27	ELOB	7,96643E-15	2,93126E-01	0,259	0,405	1,46718E-10
Macro_IFI27	CREBL2	3,36974E-09	2,88970E-01	0,199	0,295	6,20605E-05
Macro_IFI27	REX1BD	1,71703E-32	2,87767E-01	0,128	0,267	3,16225E-28
Macro_IFI27	TMEM176A	5,10200E-09	2,77290E-01	0,442	0,449	9,39635E-05
Macro_IFI27	MRPL57	7,83239E-40	2,76947E-01	0,122	0,276	1,44249E-35
Macro_IFI27	RAB13	1,47274E-10	2,74594E-01	0,207	0,309	2,71235E-06
Macro_IFI27	SELENOF	2,83324E-40	2,54046E-01	0,11	0,257	5,21798E-36
Macro_IFI27	HNRNPAB	1,31848E-75	-2,50103E-01	0,115	0,328	2,42825E-71
Macro_IFI27	TMEM230	2,86597E-32	-2,50809E-01	0,262	0,461	5,27826E-28

Macro_IFI27	MRPS21	2,72827E-58	-2,50834E-01	0,28	0,539	5,02466E-54
Macro_IFI27	MEF2A	3,22116E-30	-2,51157E-01	0,253	0,429	5,93242E-26
Macro_IFI27	PNPLA2	3,50899E-66	-2,52351E-01	0,118	0,31	6,46251E-62
Macro_IFI27	PPP1R11	5,44286E-51	-2,52577E-01	0,14	0,319	1,00241E-46
Macro_IFI27	PDAP1	5,99814E-59	-2,52806E-01	0,145	0,343	1,10468E-54
Macro_IFI27	MAP3K11	2,74288E-61	-2,52986E-01	0,083	0,252	5,05157E-57
Macro_IFI27	KRAS	9,35967E-75	-2,53367E-01	0,12	0,33	1,72377E-70
Macro_IFI27	PYURF	3,40160E-65	-2,57251E-01	0,19	0,421	6,26472E-61
Macro_IFI27	NPC2	9,41747E-48	-2,57273E-01	0,828	0,887	1,73442E-43
Macro_IFI27	PDCD10	2,73846E-57	-2,57884E-01	0,131	0,323	5,04342E-53
Macro_IFI27	SZRD1	4,61499E-62	-2,58725E-01	0,114	0,302	8,49943E-58
Macro_IFI27	MAP7D1	2,07393E-67	-2,59011E-01	0,101	0,289	3,81956E-63
Macro_IFI27	NDUFS8	3,58489E-34	-2,59346E-01	0,29	0,505	6,60229E-30
Macro_IFI27	QKI	1,18893E-76	-2,59360E-01	0,149	0,378	2,18966E-72
Macro_IFI27	GNPDA1	2,12310E-20	-2,59430E-01	0,151	0,264	3,91011E-16
Macro_IFI27	WASF2	2,73643E-36	-2,59656E-01	0,341	0,579	5,03969E-32
Macro_IFI27	RCSD1	5,01241E-31	-2,60053E-01	0,218	0,384	9,23135E-27
Macro_IFI27	HLA-A	1,28131E-12	-2,60265E-01	0,882	0,953	2,35980E-08
Macro_IFI27	MTCH2	5,86083E-43	-2,61053E-01	0,156	0,331	1,07939E-38
Macro_IFI27	GSPT1	1,27536E-58	-2,61725E-01	0,087	0,254	2,34884E-54
Macro_IFI27	C14orf119	9,24573E-44	-2,63793E-01	0,135	0,3	1,70279E-39
Macro_IFI27	SF3B4	1,67073E-58	-2,64966E-01	0,123	0,308	3,07697E-54
Macro_IFI27	DICER1	2,37540E-44	-2,64991E-01	0,122	0,277	4,37477E-40
Macro_IFI27	CLPTM1	5,37959E-39	-2,66039E-01	0,12	0,266	9,90758E-35
Macro_IFI27	RTN4	3,04014E-96	-2,66055E-01	0,342	0,664	5,59903E-92
Macro_IFI27	WIPF1	3,48607E-64	-2,66120E-01	0,232	0,477	6,42030E-60
Macro_IFI27	ERGIC1	5,74586E-46	-2,66372E-01	0,136	0,299	1,05821E-41
Macro_IFI27	PLXDC2	1,88984E-25	-2,66413E-01	0,355	0,553	3,48052E-21
Macro_IFI27	PRPF4B	1,11467E-53	-2,67556E-01	0,12	0,294	2,05289E-49
Macro_IFI27	PIK3AP1	1,45685E-73	-2,67709E-01	0,111	0,314	2,68308E-69
Macro_IFI27	FIBP	3,96897E-56	-2,67718E-01	0,17	0,377	7,30966E-52
Macro_IFI27	COX6C	2,83665E-11	-2,68771E-01	0,51	0,729	5,22425E-07
Macro_IFI27	DDX18	1,54969E-58	-2,68960E-01	0,17	0,38	2,85407E-54
Macro_IFI27	TGOLN2	1,11321E-54	-2,69492E-01	0,276	0,525	2,05020E-50
Macro_IFI27	ANXA4	2,16419E-51	-2,69557E-01	0,136	0,317	3,98579E-47
Macro_IFI27	PABPC4	8,92795E-42	-2,69585E-01	0,233	0,433	1,64426E-37
Macro_IFI27	EPS15	5,43300E-37	-2,69683E-01	0,117	0,254	1,00060E-32
Macro_IFI27	NDUFC1	4,54110E-54	-2,69691E-01	0,235	0,468	8,36335E-50
Macro_IFI27	COMMD9	3,41318E-25	-2,70517E-01	0,179	0,32	6,28606E-21
Macro_IFI27	CHCHD3	7,68766E-51	-2,70976E-01	0,099	0,257	1,41584E-46
Macro_IFI27	MRPS36	1,58779E-69	-2,72601E-01	0,127	0,338	2,92423E-65
Macro_IFI27	CMIP	4,51409E-66	-2,73389E-01	0,078	0,25	8,31359E-62
Macro_IFI27	TMEM9B	2,08541E-44	-2,75300E-01	0,19	0,381	3,84070E-40

Macro_IFI27	TRAPPC1	2,18917E-73	-2,75581E-01	0,329	0,623	4,03179E-69
Macro_IFI27	TRIM44	4,30510E-65	-2,77632E-01	0,106	0,293	7,92869E-61
Macro_IFI27	MRPL27	3,45021E-50	-2,77953E-01	0,123	0,296	6,35424E-46
Macro_IFI27	IMP3	1,40076E-40	-2,78095E-01	0,156	0,323	2,57979E-36
Macro_IFI27	SNF8	9,58846E-62	-2,78509E-01	0,115	0,303	1,76591E-57
Macro_IFI27	UBE2R2	4,10562E-66	-2,78903E-01	0,092	0,273	7,56132E-62
Macro_IFI27	SMIM19	3,15502E-29	-2,79186E-01	0,136	0,265	5,81060E-25
Macro_IFI27	AIF1	1,78750E-07	-2,79572E-01	0,781	0,824	3,29203E-03
Macro_IFI27	GNB2	1,25818E-90	-2,80365E-01	0,272	0,57	2,31720E-86
Macro_IFI27	NDUFAB1	3,05934E-52	-2,80560E-01	0,246	0,486	5,63438E-48
Macro_IFI27	MFSD10	9,59001E-58	-2,81462E-01	0,104	0,278	1,76619E-53
Macro_IFI27	S100A11	7,62327E-16	-2,81784E-01	0,916	0,938	1,40398E-11
Macro_IFI27	DPY30	8,77279E-39	-2,82928E-01	0,111	0,251	1,61568E-34
Macro_IFI27	CHMP4B	1,32061E-112	-2,83146E-01	0,141	0,421	2,43217E-108
Macro_IFI27	DDRGK1	6,19426E-41	-2,83611E-01	0,157	0,329	1,14080E-36
Macro_IFI27	SNAP29	2,58924E-43	-2,83870E-01	0,132	0,292	4,76860E-39
Macro_IFI27	POLR2L	2,09103E-82	-2,84185E-01	0,33	0,641	3,85104E-78
Macro_IFI27	PPP4R2	2,13091E-73	-2,85806E-01	0,085	0,276	3,92449E-69
Macro_IFI27	PHF5A	2,04502E-36	-2,85986E-01	0,123	0,265	3,76632E-32
Macro_IFI27	SNRPF	4,77769E-87	-2,86027E-01	0,156	0,404	8,79907E-83
Macro_IFI27	CTSA	1,27869E-14	-2,86209E-01	0,347	0,513	2,35496E-10
Macro_IFI27	DUSP23	1,13600E-35	-2,86377E-01	0,212	0,392	2,09218E-31
Macro_IFI27	ERLEC1	2,62760E-48	-2,87756E-01	0,12	0,283	4,83925E-44
Macro_IFI27	POMP	6,70669E-39	-2,87846E-01	0,465	0,733	1,23517E-34
Macro_IFI27	GBP4	3,19010E-44	-2,88031E-01	0,132	0,284	5,87521E-40
Macro_IFI27	TXNL4A	1,08273E-64	-2,88667E-01	0,102	0,289	1,99406E-60
Macro_IFI27	BSG	9,79009E-47	-2,89659E-01	0,322	0,575	1,80304E-42
Macro_IFI27	IGSF6	1,23506E-06	-2,89736E-01	0,417	0,564	2,27460E-02
Macro_IFI27	TUBA1C	6,66939E-61	-2,90343E-01	0,174	0,389	1,22830E-56
Macro_IFI27	ABI3	8,15896E-21	-2,91487E-01	0,253	0,4	1,50264E-16
Macro_IFI27	EIF4EBP1	5,02341E-30	-2,91741E-01	0,242	0,418	9,25162E-26
Macro_IFI27	KRCC1	1,35074E-43	-2,92774E-01	0,12	0,274	2,48767E-39
Macro_IFI27	PRRC2A	6,33167E-51	-2,93016E-01	0,099	0,256	1,16610E-46
Macro_IFI27	BRD4	1,03966E-82	-2,93616E-01	0,119	0,342	1,91475E-78
Macro_IFI27	CHMP2B	4,91183E-56	-2,94270E-01	0,125	0,307	9,04612E-52
Macro_IFI27	NAA38	2,84356E-49	-2,95082E-01	0,212	0,426	5,23698E-45
Macro_IFI27	CSRP1	5,11678E-30	-2,95308E-01	0,134	0,264	9,42358E-26
Macro_IFI27	PPP1CC	3,89214E-56	-2,96027E-01	0,161	0,361	7,16816E-52
Macro_IFI27	STAU1	6,03900E-55	-2,96123E-01	0,121	0,299	1,11220E-50
Macro_IFI27	TAF15	7,40334E-58	-2,96899E-01	0,133	0,318	1,36347E-53
Macro_IFI27	PRPF6	1,64213E-53	-2,97356E-01	0,113	0,282	3,02432E-49
Macro_IFI27	FRMD4B	5,90375E-28	-2,98411E-01	0,158	0,291	1,08729E-23
Macro_IFI27	ATRAID	2,94352E-31	-2,98559E-01	0,272	0,475	5,42107E-27

Macro_IFI27	AKR7A2	3,18655E-37	-2,99228E-01	0,116	0,256	5,86867E-33
Macro_IFI27	GRHPR	1,44009E-39	-3,00281E-01	0,142	0,298	2,65221E-35
Macro_IFI27	PDE4DIP	6,52691E-38	-3,00983E-01	0,173	0,329	1,20206E-33
Macro_IFI27	HTATIP2	2,70291E-30	-3,01625E-01	0,156	0,298	4,97794E-26
Macro_IFI27	ARL6IP5	3,83122E-47	-3,01666E-01	0,398	0,667	7,05597E-43
Macro_IFI27	SNX29	5,31519E-22	-3,02001E-01	0,151	0,265	9,78898E-18
Macro_IFI27	CUEDC2	3,90705E-60	-3,02012E-01	0,117	0,303	7,19562E-56
Macro_IFI27	AXL	1,34810E-11	-3,02109E-01	0,191	0,284	2,48280E-07
Macro_IFI27	ZFP36L2	6,00935E-42	-3,02408E-01	0,467	0,705	1,10674E-37
Macro_IFI27	MRPL21	5,93459E-41	-3,02528E-01	0,122	0,273	1,09297E-36
Macro_IFI27	CTDNEP1	1,45197E-53	-3,02643E-01	0,104	0,269	2,67409E-49
Macro_IFI27	PTGES3	7,03958E-43	-3,02852E-01	0,387	0,656	1,29648E-38
Macro_IFI27	MEA1	6,94861E-46	-3,03287E-01	0,198	0,399	1,27972E-41
Macro_IFI27	KXD1	4,89471E-51	-3,03534E-01	0,165	0,36	9,01459E-47
Macro_IFI27	AIMP1	8,22879E-49	-3,05184E-01	0,129	0,3	1,51550E-44
Macro_IFI27	NSL1	1,38223E-38	-3,05362E-01	0,113	0,253	2,54566E-34
Macro_IFI27	TNFAIP8L2	4,67958E-28	-3,05837E-01	0,139	0,266	8,61838E-24
Macro_IFI27	PSMC1	3,95425E-49	-3,06323E-01	0,154	0,338	7,28254E-45
Macro_IFI27	ARPC1A	1,57677E-35	-3,06542E-01	0,132	0,279	2,90393E-31
Macro_IFI27	ITGB1	6,78438E-93	-3,06574E-01	0,209	0,487	1,24948E-88
Macro_IFI27	RHEB	1,59320E-67	-3,06968E-01	0,222	0,474	2,93419E-63
Macro_IFI27	RNASEH2B	1,02667E-34	-3,07632E-01	0,158	0,309	1,89082E-30
Macro_IFI27	TMEM141	4,33805E-50	-3,08884E-01	0,107	0,269	7,98939E-46
Macro_IFI27	TMEM256	9,47487E-62	-3,09282E-01	0,197	0,422	1,74499E-57
Macro_IFI27	SNX6	1,41516E-29	-3,09332E-01	0,316	0,525	2,60629E-25
Macro_IFI27	ARHGDIA	1,71677E-96	-3,11580E-01	0,295	0,616	3,16178E-92
Macro_IFI27	RBMS1	2,78044E-69	-3,12533E-01	0,119	0,319	5,12073E-65
Macro_IFI27	TRAPPC2L	1,40085E-40	-3,12685E-01	0,125	0,277	2,57995E-36
Macro_IFI27	MT-ND6	5,27763E-39	-3,13302E-01	0,15	0,309	9,71982E-35
Macro_IFI27	HNRNPA0	5,21822E-112	-3,14139E-01	0,215	0,524	9,61040E-108
Macro_IFI27	GADD45GIP1	5,26095E-53	-3,14754E-01	0,226	0,448	9,68910E-49
Macro_IFI27	NDUFB4	1,90988E-58	-3,15123E-01	0,324	0,606	3,51743E-54
Macro_IFI27	FOXO3	6,77521E-48	-3,15448E-01	0,104	0,254	1,24779E-43
Macro_IFI27	GTF3C6	1,13374E-58	-3,15553E-01	0,167	0,381	2,08801E-54
Macro_IFI27	CHCHD5	8,22840E-47	-3,16918E-01	0,138	0,307	1,51542E-42
Macro_IFI27	TMBIM1	2,65966E-43	-3,17455E-01	0,186	0,37	4,89830E-39
Macro_IFI27	SNRPA	2,11011E-47	-3,18037E-01	0,137	0,304	3,88620E-43
Macro_IFI27	DUT	7,57681E-35	-3,18190E-01	0,228	0,41	1,39542E-30
Macro_IFI27	ZYX	1,06022E-39	-3,18321E-01	0,406	0,656	1,95261E-35
Macro_IFI27	MRPS18B	8,45051E-29	-3,19132E-01	0,148	0,283	1,55633E-24
Macro_IFI27	FUOM	3,44494E-25	-3,19426E-01	0,222	0,384	6,34455E-21
Macro_IFI27	RSU1	4,82228E-44	-3,19752E-01	0,166	0,344	8,88120E-40
Macro_IFI27	JTB	5,35769E-47	-3,20117E-01	0,318	0,572	9,86726E-43

Macro_IFI27	MRPL11	1,72368E-43	-3,20165E-01	0,136	0,301	3,17450E-39
Macro_IFI27	MKRN1	9,74711E-60	-3,20518E-01	0,115	0,299	1,79512E-55
Macro_IFI27	GNAQ	1,33196E-73	-3,21681E-01	0,136	0,351	2,45308E-69
Macro_IFI27	HLA-DPA1	2,42034E-70	-3,22640E-01	0,945	0,895	4,45754E-66
Macro_IFI27	ARPC5L	9,62840E-60	-3,23148E-01	0,103	0,279	1,77326E-55
Macro_IFI27	DPM3	1,08068E-56	-3,23200E-01	0,115	0,293	1,99029E-52
Macro_IFI27	SDHC	2,59294E-30	-3,23665E-01	0,256	0,45	4,77542E-26
Macro_IFI27	CMC2	7,99122E-61	-3,24034E-01	0,104	0,284	1,47174E-56
Macro_IFI27	PCGF5	1,64948E-61	-3,25872E-01	0,101	0,281	3,03786E-57
Macro_IFI27	NAIP	5,44498E-36	-3,26070E-01	0,209	0,379	1,00280E-31
Macro_IFI27	MPHOSPH8	6,87997E-53	-3,26277E-01	0,181	0,379	1,26708E-48
Macro_IFI27	TERF2IP	4,04380E-63	-3,26772E-01	0,143	0,347	7,44746E-59
Macro_IFI27	STMN1	1,50914E-16	-3,27143E-01	0,187	0,291	2,77939E-12
Macro_IFI27	MRPL4	2,68859E-49	-3,27467E-01	0,117	0,285	4,95158E-45
Macro_IFI27	PARP1	2,27492E-41	-3,27974E-01	0,171	0,342	4,18971E-37
Macro_IFI27	PRPF38B	5,15220E-45	-3,29592E-01	0,238	0,446	9,48881E-41
Macro_IFI27	RNF167	3,70084E-46	-3,30367E-01	0,135	0,301	6,81583E-42
Macro_IFI27	UBE2D2	3,28586E-85	-3,31583E-01	0,277	0,579	6,05158E-81
Macro_IFI27	LY86	1,04563E-19	-3,32594E-01	0,323	0,498	1,92574E-15
Macro_IFI27	YWHAQ	3,50666E-79	-3,33384E-01	0,189	0,442	6,45821E-75
Macro_IFI27	TOMM20	7,50748E-61	-3,34006E-01	0,282	0,547	1,38265E-56
Macro_IFI27	COX20	1,87008E-40	-3,34100E-01	0,119	0,266	3,44413E-36
Macro_IFI27	PKIB	3,42486E-28	-3,35001E-01	0,134	0,25	6,30756E-24
Macro_IFI27	NDUFB7	5,25715E-46	-3,35259E-01	0,295	0,541	9,68210E-42
Macro_IFI27	CD99	1,21573E-41	-3,35887E-01	0,384	0,632	2,23901E-37
Macro_IFI27	DYNLL1	1,76532E-23	-3,36227E-01	0,479	0,719	3,25118E-19
Macro_IFI27	DNAJA2	9,26666E-57	-3,36889E-01	0,137	0,325	1,70664E-52
Macro_IFI27	TXNDC12	1,75295E-38	-3,36994E-01	0,178	0,349	3,22841E-34
Macro_IFI27	KHDRBS1	3,24234E-95	-3,37250E-01	0,192	0,468	5,97142E-91
Macro_IFI27	EIF2S2	1,32519E-79	-3,37582E-01	0,209	0,474	2,44061E-75
Macro_IFI27	SNX3	1,34391E-44	-3,37952E-01	0,433	0,702	2,47507E-40
Macro_IFI27	PHF3	3,93227E-58	-3,38240E-01	0,125	0,307	7,24207E-54
Macro_IFI27	EPB41L3	2,89741E-52	-3,38381E-01	0,137	0,312	5,33616E-48
Macro_IFI27	TYMP	2,41319E-26	-3,39085E-01	0,61	0,802	4,44437E-22
Macro_IFI27	ZNHIT1	1,83346E-67	-3,39232E-01	0,234	0,491	3,37669E-63
Macro_IFI27	AZI2	2,71996E-27	-3,39500E-01	0,174	0,308	5,00935E-23
Macro_IFI27	MAP4	2,75654E-49	-3,40333E-01	0,106	0,265	5,07672E-45
Macro_IFI27	PACSIN2	1,82227E-66	-3,41127E-01	0,093	0,278	3,35608E-62
Macro_IFI27	C18orf32	6,55395E-46	-3,41424E-01	0,125	0,288	1,20704E-41
Macro_IFI27	SRSF9	2,11050E-97	-3,41548E-01	0,136	0,391	3,88692E-93
Macro_IFI27	SCAND1	1,19604E-57	-3,41923E-01	0,284	0,547	2,20274E-53
Macro_IFI27	PRDX3	6,72738E-30	-3,42565E-01	0,286	0,489	1,23898E-25
Macro_IFI27	LY6E	1,85328E-24	-3,42908E-01	0,375	0,556	3,41318E-20

Macro_IFI27	STARD7	2,77906E-58	-3,43231E-01	0,126	0,314	5,11819E-54
Macro_IFI27	YIF1A	1,46434E-43	-3,43581E-01	0,131	0,292	2,69687E-39
Macro_IFI27	SCP2	5,13053E-26	-3,43878E-01	0,28	0,47	9,44889E-22
Macro_IFI27	IDS	2,29719E-92	-3,43973E-01	0,156	0,409	4,23073E-88
Macro_IFI27	TOP1	1,26167E-89	-3,45216E-01	0,246	0,534	2,32362E-85
Macro_IFI27	RBM42	1,46410E-47	-3,45585E-01	0,134	0,304	2,69643E-43
Macro_IFI27	BTF3L4	8,01334E-39	-3,45766E-01	0,202	0,386	1,47582E-34
Macro_IFI27	MGAT4A	1,07620E-23	-3,45836E-01	0,218	0,364	1,98204E-19
Macro_IFI27	SOAT1	2,10316E-45	-3,45996E-01	0,131	0,293	3,87339E-41
Macro_IFI27	NSA2	2,72252E-56	-3,46141E-01	0,255	0,497	5,01407E-52
Macro_IFI27	MRPL23	1,80215E-32	-3,47564E-01	0,206	0,364	3,31902E-28
Macro_IFI27	CTBS	8,83330E-38	-3,48396E-01	0,131	0,276	1,62683E-33
Macro_IFI27	SETX	4,58473E-65	-3,48462E-01	0,104	0,288	8,44371E-61
Macro_IFI27	RAD23A	4,88474E-66	-3,48489E-01	0,254	0,517	8,99622E-62
Macro_IFI27	PHPT1	3,69860E-67	-3,49036E-01	0,212	0,458	6,81172E-63
Macro_IFI27	RWDD1	1,34019E-52	-3,49792E-01	0,237	0,468	2,46822E-48
Macro_IFI27	SLC8A1	1,77371E-53	-3,50120E-01	0,177	0,374	3,26663E-49
Macro_IFI27	SUMO2	7,54657E-23	-3,50800E-01	0,544	0,795	1,38985E-18
Macro_IFI27	ZSWIM7	6,49792E-35	-3,50859E-01	0,121	0,256	1,19672E-30
Macro_IFI27	LAPTM5	1,97953E-06	-3,51743E-01	0,789	0,903	3,64571E-02
Macro_IFI27	PHIP	2,00231E-74	-3,52916E-01	0,127	0,338	3,68765E-70
Macro_IFI27	UFM1	2,32950E-69	-3,53456E-01	0,128	0,336	4,29024E-65
Macro_IFI27	ADIPOR1	3,41978E-34	-3,53725E-01	0,266	0,471	6,29822E-30
Macro_IFI27	HMGA1	1,04148E-84	-3,53770E-01	0,167	0,415	1,91810E-80
Macro_IFI27	CCNI	8,63746E-60	-3,53821E-01	0,497	0,799	1,59076E-55
Macro_IFI27	LPCAT2	1,93764E-49	-3,54028E-01	0,143	0,318	3,56856E-45
Macro_IFI27	ENSA	6,72453E-84	-3,55622E-01	0,212	0,485	1,23846E-79
Macro_IFI27	UGP2	1,38172E-33	-3,55720E-01	0,156	0,307	2,54472E-29
Macro_IFI27	CD2BP2	1,16262E-58	-3,55908E-01	0,09	0,258	2,14119E-54
Macro_IFI27	AP2M1	2,54398E-46	-3,56445E-01	0,335	0,595	4,68525E-42
Macro_IFI27	NAA20	8,17271E-26	-3,56473E-01	0,171	0,314	1,50517E-21
Macro_IFI27	U2SURP	1,82092E-57	-3,56841E-01	0,144	0,336	3,35358E-53
Macro_IFI27	EFHD2	4,92877E-110	-3,56930E-01	0,16	0,443	9,07732E-106
Macro_IFI27	NUFIP2	4,92951E-68	-3,57032E-01	0,082	0,258	9,07867E-64
Macro_IFI27	GDI1	1,29398E-57	-3,57875E-01	0,131	0,318	2,38312E-53
Macro_IFI27	CNPPD1	6,29201E-46	-3,57955E-01	0,12	0,278	1,15880E-41
Macro_IFI27	UBE3A	4,30484E-73	-3,58056E-01	0,099	0,293	7,92822E-69
Macro_IFI27	MEAF6	6,66505E-50	-3,58444E-01	0,144	0,324	1,22750E-45
Macro_IFI27	HAX1	1,48832E-71	-3,58720E-01	0,141	0,356	2,74104E-67
Macro_IFI27	BOD1L1	4,12900E-67	-3,58748E-01	0,085	0,262	7,60438E-63
Macro_IFI27	MED28	1,33264E-50	-3,58991E-01	0,13	0,303	2,45432E-46
Macro_IFI27	TMEM219	8,57296E-17	-3,59342E-01	0,422	0,647	1,57888E-12
Macro_IFI27	FAM133B	2,71898E-78	-3,59597E-01	0,099	0,305	5,00755E-74

Macro_IFI27	SH3BP5	2,65457E-48	-3,59628E-01	0,167	0,345	4,88893E-44
Macro_IFI27	PHC2	1,06652E-48	-3,59785E-01	0,101	0,251	1,96420E-44
Macro_IFI27	ATP6V1C1	1,48551E-41	-3,59957E-01	0,127	0,28	2,73586E-37
Macro_IFI27	YIPF3	2,49820E-42	-3,61854E-01	0,16	0,332	4,60093E-38
Macro_IFI27	NDUFS3	8,01801E-34	-3,62373E-01	0,182	0,348	1,47668E-29
Macro_IFI27	XRN2	5,73834E-72	-3,62395E-01	0,173	0,407	1,05683E-67
Macro_IFI27	RAB5C	1,13461E-59	-3,64232E-01	0,23	0,472	2,08961E-55
Macro_IFI27	GPAA1	1,06869E-46	-3,64557E-01	0,108	0,263	1,96820E-42
Macro_IFI27	FOXN3	2,10223E-57	-3,64798E-01	0,167	0,37	3,87168E-53
Macro_IFI27	MRPS18C	3,96947E-40	-3,64857E-01	0,147	0,309	7,31057E-36
Macro_IFI27	COMMD1	1,08604E-52	-3,65023E-01	0,144	0,331	2,00016E-48
Macro_IFI27	GLB1	4,90491E-37	-3,65124E-01	0,146	0,299	9,03337E-33
Macro_IFI27	TGFB1	2,84841E-102	-3,65505E-01	0,187	0,466	5,24593E-98
Macro_IFI27	WDR83OS	4,36734E-43	-3,65519E-01	0,376	0,632	8,04334E-39
Macro_IFI27	STAT1	4,18179E-11	-3,66192E-01	0,369	0,525	7,70161E-07
Macro_IFI27	SNRPD1	5,20122E-65	-3,66480E-01	0,176	0,402	9,57909E-61
Macro_IFI27	NT5C	1,11149E-40	-3,66782E-01	0,18	0,359	2,04703E-36
Macro_IFI27	SLC25A39	6,13523E-31	-3,67183E-01	0,234	0,417	1,12993E-26
Macro_IFI27	MANF	4,74531E-69	-3,67225E-01	0,109	0,304	8,73944E-65
Macro_IFI27	DNAJC15	7,21524E-95	-3,68140E-01	0,223	0,513	1,32883E-90
Macro_IFI27	HSPA4	2,73428E-60	-3,68195E-01	0,123	0,31	5,03573E-56
Macro_IFI27	WNK1	8,10884E-59	-3,69088E-01	0,163	0,366	1,49341E-54
Macro_IFI27	TMED3	1,10780E-47	-3,69244E-01	0,12	0,282	2,04023E-43
Macro_IFI27	DNAJB12	2,26834E-38	-3,69925E-01	0,112	0,25	4,17761E-34
Macro_IFI27	ALDH2	7,47681E-18	-3,69940E-01	0,38	0,559	1,37700E-13
Macro_IFI27	MRPL54	1,68905E-65	-3,69959E-01	0,182	0,413	3,11072E-61
Macro_IFI27	VAMP2	1,13178E-66	-3,70008E-01	0,111	0,298	2,08440E-62
Macro_IFI27	CD84	6,82196E-22	-3,70050E-01	0,252	0,399	1,25640E-17
Macro_IFI27	TOMM5	1,45276E-54	-3,70532E-01	0,145	0,335	2,67555E-50
Macro_IFI27	DDT	2,25536E-48	-3,70765E-01	0,235	0,45	4,15370E-44
Macro_IFI27	LRP1	9,61861E-41	-3,71933E-01	0,244	0,431	1,77146E-36
Macro_IFI27	EIF1AX	9,04037E-100	-3,72039E-01	0,147	0,412	1,66497E-95
Macro_IFI27	USP9X	2,99966E-38	-3,72071E-01	0,133	0,281	5,52448E-34
Macro_IFI27	HAGH	3,96318E-44	-3,72094E-01	0,105	0,252	7,29898E-40
Macro_IFI27	SSU72	2,04066E-80	-3,73014E-01	0,155	0,394	3,75829E-76
Macro_IFI27	OXA1L	2,33203E-44	-3,73171E-01	0,25	0,465	4,29489E-40
Macro_IFI27	SEC11A	7,85422E-27	-3,73430E-01	0,424	0,679	1,44651E-22
Macro_IFI27	NDUFB2	5,99876E-23	-3,73448E-01	0,481	0,726	1,10479E-18
Macro_IFI27	SRP9	4,74244E-45	-3,74257E-01	0,253	0,475	8,73416E-41
Macro_IFI27	CAPN1	1,39281E-46	-3,74444E-01	0,115	0,274	2,56514E-42
Macro_IFI27	MTCH1	1,96618E-68	-3,76053E-01	0,22	0,467	3,62111E-64
Macro_IFI27	METTL7A	1,12344E-29	-3,77964E-01	0,131	0,253	2,06903E-25
Macro_IFI27	PAK2	3,14084E-120	-3,78214E-01	0,218	0,544	5,78448E-116

Macro_IFI27	COMMD7	6,56952E-40	-3,78320E-01	0,154	0,319	1,20991E-35
Macro_IFI27	UQCRH	3,98758E-44	-3,79230E-01	0,435	0,714	7,34392E-40
Macro_IFI27	GUSB	1,32060E-34	-3,80052E-01	0,188	0,354	2,43214E-30
Macro_IFI27	RPL22	2,85192E-15	-3,80243E-01	0,393	0,537	5,25239E-11
Macro_IFI27	HNRNPUL1	1,65435E-87	-3,80389E-01	0,165	0,423	3,04682E-83
Macro_IFI27	SMC1A	4,82797E-58	-3,80767E-01	0,094	0,261	8,89166E-54
Macro_IFI27	ERH	1,27495E-63	-3,81007E-01	0,272	0,541	2,34808E-59
Macro_IFI27	GLUD1	6,49482E-69	-3,81145E-01	0,131	0,337	1,19615E-64
Macro_IFI27	ROCK1	1,75264E-107	-3,81453E-01	0,218	0,525	3,22783E-103
Macro_IFI27	COX6B1	2,13954E-23	-3,81484E-01	0,576	0,822	3,94039E-19
Macro_IFI27	NDUFA6	3,47993E-57	-3,81930E-01	0,28	0,54	6,40899E-53
Macro_IFI27	FXYD5	6,38072E-21	-3,83068E-01	0,572	0,837	1,17514E-16
Macro_IFI27	SELENOK	8,08856E-92	-3,84083E-01	0,076	0,287	1,48967E-87
Macro_IFI27	NDUFA5	2,72615E-49	-3,84472E-01	0,128	0,301	5,02074E-45
Macro_IFI27	SNX27	9,87417E-44	-3,84790E-01	0,106	0,252	1,81853E-39
Macro_IFI27	NUCB1	7,06033E-52	-3,85246E-01	0,293	0,555	1,30030E-47
Macro_IFI27	SENP6	2,21932E-61	-3,86101E-01	0,086	0,254	4,08732E-57
Macro_IFI27	STX10	3,86509E-52	-3,86319E-01	0,141	0,318	7,11833E-48
Macro_IFI27	TRAPPC3	1,89735E-62	-3,86353E-01	0,151	0,363	3,49434E-58
Macro_IFI27	RNF114	1,94376E-53	-3,87672E-01	0,175	0,38	3,57982E-49
Macro_IFI27	EPSTI1	1,11904E-43	-3,87816E-01	0,224	0,418	2,06095E-39
Macro_IFI27	FUNDC2	2,95800E-45	-3,88115E-01	0,18	0,368	5,44774E-41
Macro_IFI27	KPNB1	8,29300E-72	-3,88421E-01	0,204	0,451	1,52732E-67
Macro_IFI27	C9orf78	6,77858E-48	-3,90281E-01	0,161	0,344	1,24841E-43
Macro_IFI27	TMEM147	1,78046E-31	-3,91135E-01	0,251	0,442	3,27908E-27
Macro_IFI27	HLA-DMA	8,70954E-22	-3,91451E-01	0,703	0,785	1,60404E-17
Macro_IFI27	STX12	2,56696E-47	-3,92703E-01	0,145	0,32	4,72757E-43
Macro_IFI27	STARD3NL	3,69937E-46	-3,93088E-01	0,119	0,278	6,81313E-42
Macro_IFI27	DBI	2,57860E-15	-3,93337E-01	0,509	0,734	4,74900E-11
Macro_IFI27	SLC39A1	1,30649E-39	-3,94478E-01	0,131	0,284	2,40617E-35
Macro_IFI27	VAMP5	9,96759E-14	-3,94814E-01	0,34	0,487	1,83573E-09
Macro_IFI27	ATP6V0E1	1,13137E-12	-3,94822E-01	0,573	0,806	2,08365E-08
Macro_IFI27	CUTA	1,04268E-47	-3,95156E-01	0,264	0,496	1,92030E-43
Macro_IFI27	EIF2AK2	1,11032E-43	-3,95608E-01	0,116	0,268	2,04488E-39
Macro_IFI27	STOML2	3,28778E-57	-3,96162E-01	0,108	0,284	6,05510E-53
Macro_IFI27	NCF4	1,17998E-38	-3,96228E-01	0,236	0,432	2,17318E-34
Macro_IFI27	SNRPC	4,04298E-43	-3,96469E-01	0,221	0,428	7,44595E-39
Macro_IFI27	FAM50A	5,85567E-55	-3,96568E-01	0,13	0,313	1,07844E-50
Macro_IFI27	ITPR2	1,57168E-42	-3,96847E-01	0,13	0,285	2,89457E-38
Macro_IFI27	GPS1	2,42166E-35	-3,96860E-01	0,13	0,272	4,45998E-31
Macro_IFI27	ETHE1	3,33443E-40	-3,96935E-01	0,176	0,349	6,14102E-36
Macro_IFI27	VPS4B	1,32129E-62	-3,98116E-01	0,103	0,284	2,43341E-58
Macro_IFI27	ACTR2	1,83248E-106	-3,98351E-01	0,304	0,637	3,37489E-102

Macro_IFI27	MPP1	4,88309E-26	-3,99872E-01	0,254	0,42	8,99319E-22
Macro_IFI27	CDKN1B	1,58390E-60	-4,00131E-01	0,089	0,254	2,91706E-56
Macro_IFI27	EFCAB14	1,46798E-45	-4,00376E-01	0,118	0,275	2,70359E-41
Macro_IFI27	COX14	6,72910E-49	-4,01511E-01	0,246	0,472	1,23930E-44
Macro_IFI27	MDH1	4,20010E-23	-4,02244E-01	0,231	0,393	7,73533E-19
Macro_IFI27	NDUFAF3	4,91021E-42	-4,03889E-01	0,261	0,481	9,04313E-38
Macro_IFI27	STAG2	4,26082E-59	-4,03996E-01	0,086	0,252	7,84716E-55
Macro_IFI27	TOMM7	3,04635E-32	-4,04634E-01	0,535	0,803	5,61046E-28
Macro_IFI27	NDUFB6	4,61279E-49	-4,04717E-01	0,18	0,377	8,49538E-45
Macro_IFI27	OLA1	1,00178E-50	-4,04834E-01	0,139	0,317	1,84498E-46
Macro_IFI27	YIPF4	1,33925E-43	-4,05025E-01	0,118	0,271	2,46649E-39
Macro_IFI27	IL17RA	4,76160E-73	-4,05321E-01	0,11	0,311	8,76943E-69
Macro_IFI27	PTPN18	2,25397E-44	-4,05402E-01	0,179	0,359	4,15113E-40
Macro_IFI27	SUMF2	7,66884E-25	-4,05542E-01	0,169	0,301	1,41237E-20
Macro_IFI27	MTDH	1,27462E-95	-4,05937E-01	0,373	0,71	2,34747E-91
Macro_IFI27	CRTAP	9,52140E-25	-4,07756E-01	0,333	0,53	1,75356E-20
Macro_IFI27	CCDC115	3,74555E-43	-4,08104E-01	0,12	0,273	6,89818E-39
Macro_IFI27	C7orf50	7,78768E-60	-4,08230E-01	0,146	0,346	1,43426E-55
Macro_IFI27	VPS36	3,07171E-46	-4,09668E-01	0,132	0,295	5,65716E-42
Macro_IFI27	SOD1	1,03466E-30	-4,10029E-01	0,349	0,574	1,90554E-26
Macro_IFI27	RNASEH2C	6,32318E-56	-4,10455E-01	0,157	0,355	1,16454E-51
Macro_IFI27	FKBP15	2,92209E-46	-4,11480E-01	0,12	0,28	5,38160E-42
Macro_IFI27	RAB5A	3,60986E-55	-4,11605E-01	0,133	0,318	6,64828E-51
Macro_IFI27	UBE2V2	8,31976E-45	-4,12038E-01	0,111	0,266	1,53225E-40
Macro_IFI27	HOOK3	1,32995E-76	-4,12231E-01	0,113	0,321	2,44937E-72
Macro_IFI27	GOLGB1	1,62411E-62	-4,12507E-01	0,108	0,29	2,99112E-58
Macro_IFI27	PIGT	2,46075E-35	-4,13145E-01	0,146	0,298	4,53197E-31
Macro_IFI27	MRPS12	2,12505E-46	-4,13374E-01	0,125	0,29	3,91370E-42
Macro_IFI27	RALY	1,54184E-57	-4,13910E-01	0,257	0,504	2,83961E-53
Macro_IFI27	RASSF4	1,73892E-08	-4,13982E-01	0,371	0,513	3,20257E-04
Macro_IFI27	CPNE3	1,28270E-43	-4,14097E-01	0,104	0,25	2,36234E-39
Macro_IFI27	LSM4	2,02132E-40	-4,14778E-01	0,236	0,441	3,72266E-36
Macro_IFI27	PPT1	4,21224E-13	-4,14867E-01	0,469	0,66	7,75767E-09
Macro_IFI27	GLRX	2,10564E-31	-4,15444E-01	0,344	0,557	3,87796E-27
Macro_IFI27	SYNCRIP	1,82441E-70	-4,15491E-01	0,125	0,332	3,36001E-66
Macro_IFI27	MRPL28	6,88472E-46	-4,15782E-01	0,116	0,274	1,26796E-41
Macro_IFI27	FXR1	8,67600E-53	-4,18097E-01	0,123	0,295	1,59786E-48
Macro_IFI27	COMT	1,66870E-33	-4,18112E-01	0,335	0,568	3,07325E-29
Macro_IFI27	SLC25A6	2,26776E-26	-4,18630E-01	0,577	0,828	4,17654E-22
Macro_IFI27	NOL7	2,05326E-75	-4,18956E-01	0,182	0,426	3,78149E-71
Macro_IFI27	PPP1R7	8,85576E-36	-4,18962E-01	0,139	0,286	1,63097E-31
Macro_IFI27	GNAI2	1,72906E-112	-4,19157E-01	0,349	0,689	3,18442E-108
Macro_IFI27	PLEKHO1	1,38121E-67	-4,19445E-01	0,155	0,371	2,54377E-63

Macro_IFI27	ROMO1	2,55344E-72	-4,19447E-01	0,263	0,543	4,70267E-68
Macro_IFI27	MS4A6A	1,77055E-30	-4,19526E-01	0,693	0,716	3,26083E-26
Macro_IFI27	NCOA4	5,83074E-33	-4,19861E-01	0,319	0,541	1,07385E-28
Macro_IFI27	COPB2	1,85849E-60	-4,20428E-01	0,089	0,262	3,42278E-56
Macro_IFI27	PKM	2,11506E-28	-4,21305E-01	0,564	0,794	3,89531E-24
Macro_IFI27	ATP6AP1	9,61069E-24	-4,21594E-01	0,341	0,554	1,77000E-19
Macro_IFI27	PFDN2	5,01948E-73	-4,22034E-01	0,203	0,454	9,24438E-69
Macro_IFI27	TSEN34	2,34987E-60	-4,22210E-01	0,115	0,299	4,32775E-56
Macro_IFI27	HSF1	2,14757E-59	-4,22427E-01	0,104	0,281	3,95518E-55
Macro_IFI27	ODF3B	4,44904E-65	-4,22698E-01	0,148	0,355	8,19379E-61
Macro_IFI27	DCAF7	3,77704E-58	-4,22766E-01	0,131	0,321	6,95617E-54
Macro_IFI27	PHF20L1	2,81256E-68	-4,22989E-01	0,079	0,256	5,17988E-64
Macro_IFI27	NCKAP1L	4,66818E-59	-4,23977E-01	0,209	0,44	8,59739E-55
Macro_IFI27	TIMM17A	1,73402E-58	-4,24793E-01	0,103	0,279	3,19355E-54
Macro_IFI27	NUB1	1,22545E-45	-4,25495E-01	0,15	0,321	2,25691E-41
Macro_IFI27	TXNDC17	3,81339E-59	-4,25520E-01	0,209	0,445	7,02313E-55
Macro_IFI27	CIRBP	8,14480E-53	-4,25908E-01	0,418	0,716	1,50003E-48
Macro_IFI27	POLR2F	3,19863E-59	-4,26448E-01	0,13	0,322	5,89092E-55
Macro_IFI27	METTL9	6,34919E-68	-4,27219E-01	0,155	0,369	1,16933E-63
Macro_IFI27	TRMT1	9,43166E-50	-4,27403E-01	0,118	0,282	1,73703E-45
Macro_IFI27	GNB1	8,87641E-74	-4,27677E-01	0,166	0,399	1,63477E-69
Macro_IFI27	COPS6	1,05451E-44	-4,27728E-01	0,169	0,351	1,94210E-40
Macro_IFI27	RNF145	1,73572E-86	-4,28538E-01	0,079	0,285	3,19667E-82
Macro_IFI27	GOLGA4	5,71546E-76	-4,28684E-01	0,129	0,345	1,05262E-71
Macro_IFI27	ANXA11	2,20853E-68	-4,28857E-01	0,282	0,555	4,06745E-64
Macro_IFI27	RRBP1	1,09456E-95	-4,29733E-01	0,143	0,397	2,01585E-91
Macro_IFI27	LEPROTL1	1,30614E-45	-4,29866E-01	0,207	0,408	2,40552E-41
Macro_IFI27	TCEA1	8,08034E-68	-4,30351E-01	0,23	0,486	1,48816E-63
Macro_IFI27	ACIN1	7,48712E-67	-4,31124E-01	0,099	0,285	1,37890E-62
Macro_IFI27	TMEM165	8,37015E-92	-4,31177E-01	0,149	0,404	1,54153E-87
Macro_IFI27	SERPINB6	1,44897E-41	-4,32434E-01	0,229	0,432	2,66857E-37
Macro_IFI27	TRADD	2,68060E-60	-4,32691E-01	0,085	0,254	4,93686E-56
Macro_IFI27	SIGLEC10	2,75859E-37	-4,32999E-01	0,17	0,328	5,08049E-33
Macro_IFI27	CACUL1	6,74471E-41	-4,33754E-01	0,149	0,31	1,24217E-36
Macro_IFI27	HNRNPA3	5,06448E-86	-4,33964E-01	0,329	0,644	9,32726E-82
Macro_IFI27	ETFB	1,98704E-48	-4,33969E-01	0,14	0,315	3,65953E-44
Macro_IFI27	NRBF2	3,67217E-61	-4,34517E-01	0,082	0,252	6,76304E-57
Macro_IFI27	SNRPG	1,39683E-86	-4,35486E-01	0,271	0,571	2,57254E-82
Macro_IFI27	COPZ1	1,19106E-49	-4,35496E-01	0,188	0,391	2,19357E-45
Macro_IFI27	THRAP3	6,83236E-83	-4,35501E-01	0,179	0,432	1,25832E-78
Macro_IFI27	DERL1	1,75403E-45	-4,35701E-01	0,12	0,281	3,23040E-41
Macro_IFI27	MRPL52	8,24230E-87	-4,35984E-01	0,199	0,472	1,51798E-82
Macro_IFI27	TMEM243	6,03727E-39	-4,36006E-01	0,128	0,276	1,11188E-34

Macro_IFI27	CAPRIN1	1,28629E-49	-4,36508E-01	0,159	0,344	2,36896E-45
Macro_IFI27	DENR	4,96346E-57	-4,36714E-01	0,09	0,256	9,14121E-53
Macro_IFI27	KTN1	5,44223E-84	-4,36782E-01	0,231	0,506	1,00230E-79
Macro_IFI27	CNOT7	1,05941E-52	-4,36854E-01	0,135	0,316	1,95111E-48
Macro_IFI27	WDR33	1,78213E-58	-4,37084E-01	0,095	0,264	3,28216E-54
Macro_IFI27	TYROBP	1,05114E-24	-4,38071E-01	0,932	0,948	1,93588E-20
Macro_IFI27	PCBP1	1,33472E-98	-4,40197E-01	0,319	0,625	2,45815E-94
Macro_IFI27	METAP2	3,33645E-54	-4,40785E-01	0,124	0,301	6,14474E-50
Macro_IFI27	USP8	1,05459E-61	-4,41263E-01	0,102	0,28	1,94223E-57
Macro_IFI27	ALKBH7	9,25525E-38	-4,41385E-01	0,287	0,502	1,70454E-33
Macro_IFI27	TOMM22	4,25168E-56	-4,41585E-01	0,208	0,434	7,83033E-52
Macro_IFI27	DYNC1I2	1,11131E-51	-4,41679E-01	0,101	0,26	2,04670E-47
Macro_IFI27	PPP4C	1,38240E-93	-4,42382E-01	0,23	0,527	2,54597E-89
Macro_IFI27	MYCBP2	9,73311E-70	-4,42745E-01	0,139	0,346	1,79255E-65
Macro_IFI27	TXNL1	3,66412E-51	-4,43298E-01	0,187	0,39	6,74821E-47
Macro_IFI27	ENG	7,19439E-22	-4,43874E-01	0,216	0,354	1,32499E-17
Macro_IFI27	RTN3	6,93613E-45	-4,44078E-01	0,223	0,428	1,27743E-40
Macro_IFI27	ATP6V1E1	4,39134E-42	-4,44078E-01	0,164	0,339	8,08753E-38
Macro_IFI27	RNF166	2,99174E-40	-4,44500E-01	0,111	0,25	5,50989E-36
Macro_IFI27	PRKCSH	1,47270E-56	-4,44535E-01	0,141	0,328	2,71228E-52
Macro_IFI27	SF3B2	2,87310E-81	-4,44930E-01	0,246	0,529	5,29139E-77
Macro_IFI27	POLE4	2,57869E-69	-4,45257E-01	0,132	0,341	4,74917E-65
Macro_IFI27	LSM10	1,94733E-54	-4,45661E-01	0,178	0,385	3,58639E-50
Macro_IFI27	TMSB4X	1,02618E-35	-4,46886E-01	0,994	0,983	1,88991E-31
Macro_IFI27	SF3A1	1,16699E-52	-4,46985E-01	0,125	0,297	2,14924E-48
Macro_IFI27	EEF2	1,29203E-22	-4,47710E-01	0,597	0,839	2,37953E-18
Macro_IFI27	ENTPD1	1,62321E-39	-4,48431E-01	0,158	0,317	2,98947E-35
Macro_IFI27	PRMT1	1,12839E-55	-4,48582E-01	0,131	0,315	2,07816E-51
Macro_IFI27	SNRPD3	1,76331E-53	-4,48980E-01	0,181	0,379	3,24749E-49
Macro_IFI27	TMOD3	1,52665E-82	-4,49227E-01	0,128	0,355	2,81163E-78
Macro_IFI27	CHCHD7	7,52260E-51	-4,49787E-01	0,103	0,265	1,38544E-46
Macro_IFI27	PRKDC	1,12299E-57	-4,49902E-01	0,094	0,262	2,06822E-53
Macro_IFI27	NIPBL	1,47665E-72	-4,50979E-01	0,142	0,361	2,71954E-68
Macro_IFI27	LARP7	6,68244E-43	-4,51290E-01	0,128	0,283	1,23071E-38
Macro_IFI27	FCGR3A	1,77207E-28	-4,51315E-01	0,622	0,573	3,26363E-24
Macro_IFI27	RSF1	1,98644E-59	-4,51544E-01	0,152	0,352	3,65843E-55
Macro_IFI27	HVCN1	6,92776E-30	-4,51775E-01	0,132	0,26	1,27589E-25
Macro_IFI27	CNIH4	3,14452E-55	-4,51789E-01	0,118	0,294	5,79126E-51
Macro_IFI27	LASP1	9,53479E-59	-4,51869E-01	0,13	0,318	1,75602E-54
Macro_IFI27	MGST2	1,08303E-26	-4,52721E-01	0,211	0,366	1,99461E-22
Macro_IFI27	MED10	6,96999E-41	-4,53024E-01	0,109	0,253	1,28366E-36
Macro_IFI27	MDH2	8,36714E-51	-4,54241E-01	0,286	0,533	1,54098E-46
Macro_IFI27	ARMCX3	4,31935E-48	-4,54522E-01	0,109	0,265	7,95495E-44

Macro_IFI27	ABCA1	1,86107E-64	-4,55649E-01	0,135	0,324	3,42753E-60
Macro_IFI27	BAG6	8,32529E-57	-4,55654E-01	0,105	0,281	1,53327E-52
Macro_IFI27	NUDT1	6,74166E-43	-4,55889E-01	0,119	0,269	1,24161E-38
Macro_IFI27	TMEM259	6,34193E-74	-4,55968E-01	0,073	0,255	1,16799E-69
Macro_IFI27	PJA2	3,26095E-60	-4,56061E-01	0,107	0,284	6,00569E-56
Macro_IFI27	SMARCB1	9,63169E-68	-4,56221E-01	0,109	0,304	1,77387E-63
Macro_IFI27	TMED4	2,07037E-47	-4,56520E-01	0,134	0,301	3,81300E-43
Macro_IFI27	THOC7	6,32951E-54	-4,57056E-01	0,155	0,347	1,16571E-49
Macro_IFI27	COX5B	4,28614E-23	-4,57748E-01	0,535	0,784	7,89378E-19
Macro_IFI27	IGBP1	3,66672E-47	-4,57825E-01	0,167	0,353	6,75299E-43
Macro_IFI27	CAMLG	1,49058E-47	-4,58039E-01	0,135	0,3	2,74520E-43
Macro_IFI27	TLN1	1,78437E-98	-4,58064E-01	0,26	0,569	3,28628E-94
Macro_IFI27	LSM14A	7,18375E-60	-4,58464E-01	0,133	0,325	1,32303E-55
Macro_IFI27	MAP3K2	3,98536E-91	-4,59525E-01	0,1	0,324	7,33984E-87
Macro_IFI27	SUCLG1	1,24431E-60	-4,59593E-01	0,129	0,319	2,29165E-56
Macro_IFI27	COX17	2,69714E-104	-4,59697E-01	0,205	0,503	4,96732E-100
Macro_IFI27	RABAC1	3,60898E-41	-4,59868E-01	0,305	0,535	6,64666E-37
Macro_IFI27	GTF2I	5,32110E-65	-4,59919E-01	0,125	0,321	9,79986E-61
Macro_IFI27	RSL24D1	1,77232E-77	-4,60207E-01	0,196	0,445	3,26407E-73
Macro_IFI27	ZNFX1	3,03322E-69	-4,60329E-01	0,083	0,265	5,58628E-65
Macro_IFI27	GHITM	2,58425E-51	-4,60888E-01	0,271	0,516	4,75941E-47
Macro_IFI27	HLA-DRA	8,13086E-60	-4,61002E-01	0,963	0,923	1,49746E-55
Macro_IFI27	EIF3A	1,07147E-94	-4,61163E-01	0,215	0,503	1,97332E-90
Macro_IFI27	SERINC1	1,55742E-56	-4,61343E-01	0,198	0,414	2,86830E-52
Macro_IFI27	CD40	1,37388E-44	-4,61429E-01	0,137	0,299	2,53028E-40
Macro_IFI27	TMEM50A	1,70416E-62	-4,61810E-01	0,328	0,616	3,13856E-58
Macro_IFI27	SH3GLB1	2,62913E-56	-4,62463E-01	0,212	0,437	4,84206E-52
Macro_IFI27	ADAR	3,10289E-83	-4,62598E-01	0,182	0,44	5,71459E-79
Macro_IFI27	SMIM12	1,85083E-55	-4,63290E-01	0,104	0,276	3,40868E-51
Macro_IFI27	ZMIZ1	5,80268E-81	-4,63346E-01	0,078	0,274	1,06868E-76
Macro_IFI27	YWHAE	2,82751E-73	-4,63533E-01	0,301	0,594	5,20743E-69
Macro_IFI27	FOXN2	4,83451E-46	-4,65144E-01	0,115	0,269	8,90372E-42
Macro_IFI27	CHMP5	1,11708E-49	-4,66759E-01	0,205	0,417	2,05733E-45
Macro_IFI27	OCIAD1	1,65882E-54	-4,67257E-01	0,219	0,445	3,05505E-50
Macro_IFI27	CCDC47	4,48539E-52	-4,67318E-01	0,135	0,312	8,26075E-48
Macro_IFI27	HK1	1,63021E-57	-4,67595E-01	0,1	0,27	3,00235E-53
Macro_IFI27	EMD	2,67790E-94	-4,67917E-01	0,165	0,429	4,93188E-90
Macro_IFI27	PUM1	7,13120E-50	-4,67956E-01	0,097	0,25	1,31335E-45
Macro_IFI27	LEPROT	1,34487E-57	-4,68046E-01	0,163	0,364	2,47684E-53
Macro_IFI27	HPCAL1	6,39840E-50	-4,69036E-01	0,099	0,254	1,17839E-45
Macro_IFI27	BCAP31	2,64802E-17	-4,69358E-01	0,384	0,593	4,87685E-13
Macro_IFI27	IQGAP2	6,20682E-64	-4,70137E-01	0,122	0,312	1,14311E-59
Macro_IFI27	EMC6	5,21074E-47	-4,70182E-01	0,145	0,321	9,59662E-43

Macro_IFI27	TMEM59	1,02904E-35	-4,70364E-01	0,374	0,629	1,89518E-31
Macro_IFI27	TPD52L2	1,14119E-45	-4,71840E-01	0,119	0,276	2,10172E-41
Macro_IFI27	CTNNA1	1,22378E-68	-4,72795E-01	0,153	0,373	2,25383E-64
Macro_IFI27	RAPGEF1	2,49643E-73	-4,72902E-01	0,081	0,268	4,59767E-69
Macro_IFI27	PRKCD	1,37783E-61	-4,73276E-01	0,091	0,264	2,53755E-57
Macro_IFI27	NCOR2	8,83753E-74	-4,74044E-01	0,088	0,278	1,62761E-69
Macro_IFI27	MAP2K1	1,67925E-86	-4,74277E-01	0,109	0,332	3,09267E-82
Macro_IFI27	MORF4L1	1,71777E-63	-4,74815E-01	0,365	0,665	3,16361E-59
Macro_IFI27	HMGB1	1,23505E-24	-4,75531E-01	0,553	0,805	2,27459E-20
Macro_IFI27	UBE2L3	9,56382E-70	-4,75616E-01	0,246	0,514	1,76137E-65
Macro_IFI27	SPNS1	3,11885E-49	-4,75778E-01	0,101	0,256	5,74398E-45
Macro_IFI27	MRPS34	6,27436E-37	-4,76097E-01	0,214	0,403	1,15555E-32
Macro_IFI27	TBCA	1,14384E-61	-4,76437E-01	0,325	0,602	2,10662E-57
Macro_IFI27	WBP2	1,52584E-48	-4,76998E-01	0,192	0,393	2,81014E-44
Macro_IFI27	MRPL16	1,18687E-41	-4,78047E-01	0,116	0,266	2,18586E-37
Macro_IFI27	RNMT	1,60658E-56	-4,78706E-01	0,093	0,255	2,95883E-52
Macro_IFI27	BLOC1S2	9,34384E-52	-4,78723E-01	0,15	0,339	1,72086E-47
Macro_IFI27	HINT1	9,46361E-28	-4,78816E-01	0,501	0,733	1,74291E-23
Macro_IFI27	COA4	1,29179E-47	-4,78863E-01	0,126	0,294	2,37909E-43
Macro_IFI27	SRSF4	3,01961E-66	-4,78913E-01	0,151	0,363	5,56121E-62
Macro_IFI27	NOSIP	1,62403E-45	-4,79188E-01	0,158	0,336	2,99097E-41
Macro_IFI27	UBQLN1	2,40676E-71	-4,79613E-01	0,084	0,271	4,43254E-67
Macro_IFI27	TMEM134	2,16194E-28	-4,79819E-01	0,146	0,279	3,98165E-24
Macro_IFI27	BIN2	2,35726E-44	-4,79910E-01	0,154	0,321	4,34136E-40
Macro_IFI27	SMIM14	3,58638E-64	-4,80673E-01	0,098	0,277	6,60504E-60
Macro_IFI27	MIER1	1,48490E-54	-4,81142E-01	0,098	0,261	2,73473E-50
Macro_IFI27	USP16	3,08603E-60	-4,81522E-01	0,106	0,282	5,68355E-56
Macro_IFI27	CBX3	3,74706E-71	-4,81648E-01	0,181	0,418	6,90095E-67
Macro_IFI27	SAMD9L	6,54769E-63	-4,81963E-01	0,107	0,288	1,20589E-58
Macro_IFI27	SNRNP200	4,51063E-53	-4,82905E-01	0,104	0,266	8,30722E-49
Macro_IFI27	IMPDH2	2,57247E-38	-4,83426E-01	0,131	0,28	4,73772E-34
Macro_IFI27	TEX264	1,37304E-37	-4,83549E-01	0,157	0,315	2,52873E-33
Macro_IFI27	PGD	5,15386E-41	-4,83804E-01	0,26	0,476	9,49187E-37
Macro_IFI27	ST8SIA4	3,85053E-68	-4,83835E-01	0,112	0,306	7,09153E-64
Macro_IFI27	STX7	2,27623E-52	-4,83872E-01	0,208	0,424	4,19213E-48
Macro_IFI27	FBXW5	1,33021E-49	-4,84114E-01	0,143	0,323	2,44985E-45
Macro_IFI27	BLVRB	4,61197E-20	-4,84441E-01	0,326	0,498	8,49387E-16
Macro_IFI27	PSMD1	3,06030E-53	-4,84716E-01	0,114	0,285	5,63615E-49
Macro_IFI27	IDH2	1,51551E-48	-4,85115E-01	0,214	0,421	2,79111E-44
Macro_IFI27	DHRS7	4,40496E-31	-4,85542E-01	0,29	0,494	8,11261E-27
Macro_IFI27	PSTPIP2	4,57986E-53	-4,87096E-01	0,101	0,258	8,43473E-49
Macro_IFI27	ELF2	7,33083E-58	-4,87426E-01	0,092	0,256	1,35012E-53
Macro_IFI27	AP2S1	7,42794E-18	-4,87545E-01	0,495	0,73	1,36800E-13

Macro_IFI27	PMP22	1,89322E-26	-4,88005E-01	0,159	0,283	3,48674E-22
Macro_IFI27	PABPN1	1,34965E-84	-4,88014E-01	0,089	0,297	2,48565E-80
Macro_IFI27	VPS35	1,78806E-77	-4,88970E-01	0,198	0,451	3,29307E-73
Macro_IFI27	CITED2	3,38862E-92	-4,89518E-01	0,116	0,345	6,24083E-88
Macro_IFI27	NQO2	1,21494E-50	-4,90513E-01	0,107	0,269	2,23756E-46
Macro_IFI27	PA2G4	7,13570E-61	-4,90575E-01	0,21	0,444	1,31418E-56
Macro_IFI27	ACO2	2,46608E-48	-4,91472E-01	0,115	0,277	4,54178E-44
Macro_IFI27	POLR2I	2,02111E-46	-4,91836E-01	0,135	0,302	3,72228E-42
Macro_IFI27	BLOC1S6	1,85539E-81	-4,93291E-01	0,088	0,293	3,41707E-77
Macro_IFI27	BST2	7,57257E-14	-4,94093E-01	0,522	0,735	1,39464E-09
Macro_IFI27	SSNA1	2,98637E-64	-4,94711E-01	0,186	0,415	5,49999E-60
Macro_IFI27	CLTC	1,43749E-80	-4,94840E-01	0,181	0,431	2,64742E-76
Macro_IFI27	MIEN1	2,40401E-61	-4,95312E-01	0,185	0,407	4,42746E-57
Macro_IFI27	DPYD	6,39784E-53	-4,95586E-01	0,18	0,375	1,17829E-48
Macro_IFI27	CDC26	1,84920E-50	-4,97054E-01	0,112	0,276	3,40568E-46
Macro_IFI27	COX7C	6,19199E-22	-4,97790E-01	0,601	0,84	1,14038E-17
Macro_IFI27	MCOLN1	1,63402E-47	-4,98055E-01	0,12	0,282	3,00938E-43
Macro_IFI27	AGPAT2	5,76031E-51	-4,98321E-01	0,128	0,299	1,06088E-46
Macro_IFI27	TMX1	1,95685E-64	-4,98683E-01	0,141	0,345	3,60393E-60
Macro_IFI27	RPS17	1,97220E-40	-4,98852E-01	0,219	0,416	3,63219E-36
Macro_IFI27	LAMTOR1	7,11026E-32	-4,99023E-01	0,408	0,659	1,30950E-27
Macro_IFI27	EPC1	3,68841E-82	-4,99768E-01	0,135	0,361	6,79295E-78
Macro_IFI27	DHX36	4,12738E-75	-5,00101E-01	0,096	0,295	7,60139E-71
Macro_IFI27	SHOC2	1,49158E-74	-5,00112E-01	0,083	0,273	2,74704E-70
Macro_IFI27	NDUFA3	2,20758E-32	-5,00205E-01	0,326	0,557	4,06570E-28
Macro_IFI27	CDV3	4,85268E-71	-5,00447E-01	0,093	0,284	8,93718E-67
Macro_IFI27	ATP6V1A	2,53239E-60	-5,00941E-01	0,091	0,262	4,66390E-56
Macro_IFI27	MRPL43	5,02278E-53	-5,01210E-01	0,163	0,359	9,25045E-49
Macro_IFI27	PARL	1,40060E-33	-5,01544E-01	0,15	0,297	2,57949E-29
Macro_IFI27	GLG1	1,10933E-67	-5,02066E-01	0,106	0,3	2,04305E-63
Macro_IFI27	SBDS	2,52563E-75	-5,02145E-01	0,094	0,292	4,65146E-71
Macro_IFI27	CD93	1,64273E-65	-5,02430E-01	0,108	0,287	3,02542E-61
Macro_IFI27	FAM204A	2,63651E-38	-5,02438E-01	0,141	0,294	4,85566E-34
Macro_IFI27	SSBP1	4,74925E-57	-5,03677E-01	0,254	0,498	8,74669E-53
Macro_IFI27	DPP7	1,66352E-48	-5,04008E-01	0,317	0,568	3,06370E-44
Macro_IFI27	UBR4	8,34714E-66	-5,04295E-01	0,081	0,254	1,53729E-61
Macro_IFI27	DAP3	9,73707E-45	-5,05047E-01	0,151	0,326	1,79328E-40
Macro_IFI27	TIMM10	5,71317E-43	-5,05215E-01	0,112	0,26	1,05219E-38
Macro_IFI27	SUGT1	2,35442E-48	-5,05410E-01	0,125	0,292	4,33613E-44
Macro_IFI27	QSOX1	2,37308E-56	-5,05591E-01	0,12	0,297	4,37050E-52
Macro_IFI27	BPTF	4,53754E-83	-5,05932E-01	0,092	0,299	8,35678E-79
Macro_IFI27	NUDT21	6,87218E-50	-5,06344E-01	0,11	0,274	1,26565E-45
Macro_IFI27	DAPK1	1,53732E-36	-5,06921E-01	0,181	0,343	2,83129E-32

Macro_IFI27	HPRT1	2,50128E-43	-5,08158E-01	0,118	0,27	4,60661E-39
Macro_IFI27	ARF3	5,70383E-81	-5,08176E-01	0,121	0,347	1,05047E-76
Macro_IFI27	PPM1G	8,09422E-72	-5,08354E-01	0,156	0,378	1,49071E-67
Macro_IFI27	NDUFA12	3,75383E-48	-5,08545E-01	0,269	0,504	6,91343E-44
Macro_IFI27	MPG	8,30167E-45	-5,08606E-01	0,156	0,33	1,52892E-40
Macro_IFI27	GTF2F1	7,77995E-47	-5,08849E-01	0,108	0,262	1,43283E-42
Macro_IFI27	DNAJC1	4,05494E-57	-5,09105E-01	0,121	0,298	7,46799E-53
Macro_IFI27	REEP5	3,90407E-64	-5,09369E-01	0,296	0,576	7,19012E-60
Macro_IFI27	LRPAP1	1,80684E-36	-5,09526E-01	0,292	0,505	3,32765E-32
Macro_IFI27	SF3B5	5,33394E-68	-5,09635E-01	0,286	0,565	9,82352E-64
Macro_IFI27	EIF3K	3,47674E-22	-5,10434E-01	0,536	0,799	6,40311E-18
Macro_IFI27	MRPL20	1,06992E-73	-5,10455E-01	0,218	0,48	1,97047E-69
Macro_IFI27	KDM5A	4,19696E-70	-5,10763E-01	0,119	0,321	7,72954E-66
Macro_IFI27	PFKL	6,77804E-55	-5,12082E-01	0,189	0,403	1,24831E-50
Macro_IFI27	PET100	7,93597E-88	-5,12926E-01	0,162	0,417	1,46157E-83
Macro_IFI27	OSTF1	1,69528E-56	-5,13014E-01	0,298	0,561	3,12220E-52
Macro_IFI27	HP1BP3	3,61006E-75	-5,14015E-01	0,181	0,418	6,64864E-71
Macro_IFI27	EID1	1,78717E-75	-5,14370E-01	0,297	0,593	3,29144E-71
Macro_IFI27	VASP	4,07104E-113	-5,14399E-01	0,189	0,483	7,49764E-109
Macro_IFI27	MAF1	9,70666E-54	-5,14736E-01	0,188	0,398	1,78768E-49
Macro_IFI27	ARPC5	1,44720E-27	-5,15910E-01	0,52	0,774	2,66532E-23
Macro_IFI27	SPOP	6,22347E-52	-5,17084E-01	0,111	0,275	1,14618E-47
Macro_IFI27	FGL2	3,73185E-22	-5,17371E-01	0,439	0,622	6,87295E-18
Macro_IFI27	EIF2A	3,61377E-44	-5,17663E-01	0,149	0,315	6,65547E-40
Macro_IFI27	BNIP3L	4,47242E-51	-5,18413E-01	0,327	0,575	8,23686E-47
Macro_IFI27	FGFR1OP2	3,20354E-68	-5,19180E-01	0,115	0,315	5,89996E-64
Macro_IFI27	C1orf43	5,14561E-40	-5,19749E-01	0,31	0,558	9,47667E-36
Macro_IFI27	DSE	3,59619E-117	-5,21367E-01	0,163	0,452	6,62309E-113
Macro_IFI27	IRF5	1,19505E-32	-5,22583E-01	0,134	0,268	2,20093E-28
Macro_IFI27	NDUFS7	1,57336E-42	-5,23185E-01	0,312	0,552	2,89766E-38
Macro_IFI27	UNC119	2,82217E-63	-5,24361E-01	0,098	0,276	5,19759E-59
Macro_IFI27	SCAMP2	4,42401E-31	-5,24424E-01	0,303	0,513	8,14769E-27
Macro_IFI27	PFN1	7,23100E-08	-5,24763E-01	0,884	0,95	1,33173E-03
Macro_IFI27	PRDX5	1,70359E-37	-5,24873E-01	0,355	0,613	3,13750E-33
Macro_IFI27	SUMO1	2,28257E-76	-5,24992E-01	0,215	0,481	4,20381E-72
Macro_IFI27	RANBP1	6,88281E-60	-5,25621E-01	0,166	0,377	1,26761E-55
Macro_IFI27	CMTM7	1,28798E-52	-5,25948E-01	0,208	0,421	2,37207E-48
Macro_IFI27	CXCL16	5,86947E-12	-5,25955E-01	0,474	0,66	1,08098E-07
Macro_IFI27	ANAPC11	3,48832E-47	-5,26738E-01	0,352	0,62	6,42444E-43
Macro_IFI27	SIGIRR	9,53552E-42	-5,28633E-01	0,117	0,263	1,75616E-37
Macro_IFI27	KMT2C	3,15291E-67	-5,29252E-01	0,137	0,339	5,80672E-63
Macro_IFI27	PHB	1,70630E-46	-5,29869E-01	0,196	0,398	3,14249E-42
Macro_IFI27	BANF1	9,55185E-51	-5,30270E-01	0,305	0,56	1,75916E-46

Macro_IFI27	APMAP	7,89882E-32	-5,30513E-01	0,162	0,307	1,45473E-27
Macro_IFI27	SERBP1	3,59799E-83	-5,30598E-01	0,306	0,608	6,62643E-79
Macro_IFI27	XPO1	2,75684E-70	-5,30746E-01	0,081	0,263	5,07727E-66
Macro_IFI27	FAM177A1	5,77028E-72	-5,30994E-01	0,119	0,326	1,06271E-67
Macro_IFI27	SDCCAG8	2,13798E-34	-5,32389E-01	0,182	0,344	3,93752E-30
Macro_IFI27	SAR1A	3,81038E-56	-5,32565E-01	0,089	0,251	7,01757E-52
Macro_IFI27	SLC43A2	6,02869E-88	-5,32991E-01	0,143	0,379	1,11030E-83
Macro_IFI27	SLC25A11	2,19350E-46	-5,33017E-01	0,14	0,312	4,03977E-42
Macro_IFI27	RNF13	5,77364E-39	-5,33050E-01	0,305	0,528	1,06333E-34
Macro_IFI27	ATP6V1D	3,08682E-46	-5,34428E-01	0,103	0,254	5,68499E-42
Macro_IFI27	ARID1A	1,98854E-72	-5,35040E-01	0,089	0,277	3,66229E-68
Macro_IFI27	TPR	9,21338E-72	-5,35225E-01	0,171	0,4	1,69683E-67
Macro_IFI27	UBE2N	1,00220E-70	-5,35524E-01	0,158	0,384	1,84575E-66
Macro_IFI27	SMAP2	2,56589E-80	-5,35534E-01	0,224	0,483	4,72559E-76
Macro_IFI27	DIAPH1	1,09025E-74	-5,35980E-01	0,068	0,251	2,00792E-70
Macro_IFI27	TBXAS1	9,62853E-18	-5,36216E-01	0,344	0,527	1,77329E-13
Macro_IFI27	HDLBP	9,11461E-45	-5,37123E-01	0,217	0,419	1,67864E-40
Macro_IFI27	LSM6	2,01827E-70	-5,37270E-01	0,145	0,36	3,71706E-66
Macro_IFI27	RAB8A	1,47795E-45	-5,37378E-01	0,223	0,436	2,72193E-41
Macro_IFI27	DNM2	6,51430E-77	-5,38507E-01	0,102	0,306	1,19974E-72
Macro_IFI27	CYB5R4	5,12628E-52	-5,38726E-01	0,105	0,269	9,44106E-48
Macro_IFI27	PCM1	1,74164E-63	-5,38786E-01	0,13	0,325	3,20759E-59
Macro_IFI27	CUX1	1,69712E-71	-5,38871E-01	0,144	0,36	3,12558E-67
Macro_IFI27	GNAI3	6,24200E-74	-5,40101E-01	0,137	0,356	1,14959E-69
Macro_IFI27	CTSB	6,91537E-70	-5,40437E-01	0,879	0,872	1,27360E-65
Macro_IFI27	TNFRSF1A	2,33512E-50	-5,40467E-01	0,235	0,459	4,30059E-46
Macro_IFI27	LAMP2	8,12137E-27	-5,40516E-01	0,24	0,409	1,49571E-22
Macro_IFI27	LTBR	4,63933E-34	-5,40793E-01	0,191	0,357	8,54425E-30
Macro_IFI27	PSMC2	2,16656E-40	-5,40874E-01	0,135	0,294	3,99015E-36
Macro_IFI27	FKBP2	6,25522E-43	-5,41201E-01	0,225	0,418	1,15202E-38
Macro_IFI27	RSRP1	3,82753E-121	-5,41504E-01	0,052	0,292	7,04916E-117
Macro_IFI27	RNF5	3,32135E-44	-5,41672E-01	0,13	0,29	6,11693E-40
Macro_IFI27	TMEM14B	3,14878E-53	-5,42306E-01	0,212	0,432	5,79910E-49
Macro_IFI27	RAB1B	1,64364E-59	-5,42895E-01	0,16	0,366	3,02708E-55
Macro_IFI27	DEK	1,50558E-83	-5,43114E-01	0,288	0,588	2,77282E-79
Macro_IFI27	IRF2	3,81526E-42	-5,43309E-01	0,166	0,336	7,02656E-38
Macro_IFI27	MIDN	3,92842E-115	-5,43848E-01	0,058	0,292	7,23497E-111
Macro_IFI27	SNAPIN	2,13147E-38	-5,44917E-01	0,141	0,295	3,92553E-34
Macro_IFI27	SAT2	2,05073E-89	-5,45422E-01	0,183	0,446	3,77682E-85
Macro_IFI27	NEU1	1,21659E-61	-5,45441E-01	0,125	0,313	2,24059E-57
Macro_IFI27	CYLD	3,62264E-70	-5,46155E-01	0,076	0,255	6,67182E-66
Macro_IFI27	MRPL14	1,02504E-72	-5,46440E-01	0,135	0,354	1,88782E-68
Macro_IFI27	RALA	4,43262E-88	-5,47837E-01	0,144	0,385	8,16355E-84

Macro_IFI27	NDUFB9	1,16679E-57	-5,48406E-01	0,299	0,563	2,14888E-53
Macro_IFI27	SFT2D1	2,39011E-88	-5,48847E-01	0,198	0,472	4,40187E-84
Macro_IFI27	TMEM205	2,58686E-37	-5,49066E-01	0,142	0,291	4,76421E-33
Macro_IFI27	CEP350	1,15348E-90	-5,49217E-01	0,085	0,3	2,12436E-86
Macro_IFI27	RAP1B	7,02036E-73	-5,50120E-01	0,302	0,593	1,29294E-68
Macro_IFI27	PTK2B	3,44342E-66	-5,50191E-01	0,088	0,262	6,34174E-62
Macro_IFI27	SMDT1	5,61591E-80	-5,51115E-01	0,226	0,495	1,03428E-75
Macro_IFI27	IKZF1	5,15684E-83	-5,51669E-01	0,088	0,292	9,49735E-79
Macro_IFI27	STX4	1,20242E-66	-5,52727E-01	0,123	0,322	2,21450E-62
Macro_IFI27	NSFL1C	7,76005E-50	-5,53008E-01	0,116	0,282	1,42917E-45
Macro_IFI27	SNRPB2	3,77715E-73	-5,53062E-01	0,177	0,417	6,95638E-69
Macro_IFI27	HEXB	6,26738E-16	-5,53302E-01	0,343	0,522	1,15426E-11
Macro_IFI27	FCGR2A	1,64102E-10	-5,54312E-01	0,469	0,639	3,02227E-06
Macro_IFI27	MACF1	1,28339E-77	-5,54844E-01	0,118	0,33	2,36363E-73
Macro_IFI27	GPR108	1,14382E-50	-5,55779E-01	0,106	0,265	2,10658E-46
Macro_IFI27	NANS	4,86475E-60	-5,56044E-01	0,184	0,399	8,95941E-56
Macro_IFI27	STAB1	2,66099E-11	-5,56655E-01	0,253	0,349	4,90074E-07
Macro_IFI27	NDUFC2	5,07563E-50	-5,57795E-01	0,142	0,313	9,34780E-46
Macro_IFI27	MSN	1,05124E-86	-5,58766E-01	0,336	0,652	1,93606E-82
Macro_IFI27	CIB1	1,84991E-54	-5,58799E-01	0,355	0,625	3,40697E-50
Macro_IFI27	RIN3	1,24656E-66	-5,58878E-01	0,193	0,414	2,29579E-62
Macro_IFI27	MRPL3	4,01472E-37	-5,59379E-01	0,141	0,29	7,39390E-33
Macro_IFI27	FNBP1	3,78942E-121	-5,60761E-01	0,153	0,447	6,97897E-117
Macro_IFI27	PPP2R1A	1,29699E-53	-5,61851E-01	0,192	0,406	2,38867E-49
Macro_IFI27	SPPL2A	7,30822E-66	-5,61974E-01	0,142	0,35	1,34596E-61
Macro_IFI27	ACSL4	2,10770E-94	-5,62191E-01	0,072	0,285	3,88176E-90
Macro_IFI27	MAGOH	5,40599E-76	-5,62358E-01	0,136	0,359	9,95621E-72
Macro_IFI27	EVI2A	6,20127E-47	-5,62452E-01	0,122	0,281	1,14209E-42
Macro_IFI27	FNDC3B	1,34731E-72	-5,62580E-01	0,098	0,288	2,48134E-68
Macro_IFI27	BLOC1S1	2,61353E-43	-5,62724E-01	0,266	0,482	4,81334E-39
Macro_IFI27	CALR	2,08225E-13	-5,62912E-01	0,532	0,733	3,83488E-09
Macro_IFI27	YTHDF2	5,83790E-69	-5,62916E-01	0,099	0,29	1,07517E-64
Macro_IFI27	MLX	8,87843E-52	-5,63110E-01	0,17	0,368	1,63514E-47
Macro_IFI27	ACOT9	1,75671E-87	-5,63956E-01	0,08	0,289	3,23533E-83
Macro_IFI27	YWHAB	5,72764E-31	-5,64026E-01	0,524	0,801	1,05486E-26
Macro_IFI27	AHR	1,36801E-89	-5,64061E-01	0,134	0,365	2,51947E-85
Macro_IFI27	ACTR1A	2,50775E-51	-5,65007E-01	0,124	0,296	4,61853E-47
Macro_IFI27	RAB18	6,74694E-49	-5,65654E-01	0,121	0,288	1,24258E-44
Macro_IFI27	SERP1	4,55617E-61	-5,66436E-01	0,532	0,833	8,39110E-57
Macro_IFI27	PLEKHB2	9,24395E-86	-5,66525E-01	0,189	0,452	1,70246E-81
Macro_IFI27	TACC1	3,88937E-96	-5,67475E-01	0,169	0,433	7,16306E-92
Macro_IFI27	TFG	3,80873E-48	-5,68259E-01	0,11	0,269	7,01454E-44
Macro_IFI27	UFC1	1,73153E-47	-5,68417E-01	0,274	0,513	3,18895E-43

Macro_IFI27	CCT2	2,12436E-62	-5,68439E-01	0,146	0,349	3,91243E-58
Macro_IFI27	TMEM208	8,36994E-52	-5,69322E-01	0,151	0,339	1,54149E-47
Macro_IFI27	TIMM17B	2,25264E-66	-5,69632E-01	0,132	0,334	4,14869E-62
Macro_IFI27	HAVCR2	6,64909E-48	-5,69636E-01	0,236	0,445	1,22456E-43
Macro_IFI27	IRAK3	1,00209E-68	-5,69896E-01	0,085	0,262	1,84554E-64
Macro_IFI27	PTAFR	3,36217E-39	-5,70085E-01	0,209	0,384	6,19211E-35
Macro_IFI27	XRCC5	7,83808E-66	-5,70807E-01	0,236	0,487	1,44354E-61
Macro_IFI27	SNW1	2,46522E-45	-5,71980E-01	0,133	0,297	4,54019E-41
Macro_IFI27	SSR1	5,50661E-68	-5,72697E-01	0,224	0,475	1,01415E-63
Macro_IFI27	FKBP5	1,53858E-60	-5,73081E-01	0,286	0,529	2,83360E-56
Macro_IFI27	ECHDC1	5,73379E-56	-5,73291E-01	0,106	0,277	1,05599E-51
Macro_IFI27	GNS	3,34319E-65	-5,73320E-01	0,21	0,441	6,15716E-61
Macro_IFI27	RNF181	2,62609E-44	-5,73374E-01	0,318	0,573	4,83647E-40
Macro_IFI27	NDUFA11	1,35629E-39	-5,73418E-01	0,26	0,464	2,49788E-35
Macro_IFI27	SH3BP2	8,96420E-72	-5,73650E-01	0,107	0,304	1,65094E-67
Macro_IFI27	CCT4	1,36470E-65	-5,73746E-01	0,205	0,444	2,51337E-61
Macro_IFI27	SPAG7	1,95373E-50	-5,74172E-01	0,151	0,335	3,59819E-46
Macro_IFI27	HIGD2A	1,29358E-27	-5,74358E-01	0,468	0,727	2,38239E-23
Macro_IFI27	USP4	6,66885E-48	-5,74364E-01	0,135	0,303	1,22820E-43
Macro_IFI27	ALOX5	6,01336E-51	-5,75581E-01	0,266	0,484	1,10748E-46
Macro_IFI27	TMEM258	7,92962E-67	-5,75801E-01	0,376	0,681	1,46040E-62
Macro_IFI27	BCKDK	1,30122E-52	-5,75934E-01	0,142	0,328	2,39646E-48
Macro_IFI27	TNRC6B	1,27858E-56	-5,75943E-01	0,109	0,28	2,35476E-52
Macro_IFI27	LAIR1	1,51047E-19	-5,77118E-01	0,349	0,527	2,78183E-15
Macro_IFI27	NELFE	1,59463E-54	-5,77158E-01	0,121	0,298	2,93683E-50
Macro_IFI27	PSMC4	2,18743E-52	-5,77714E-01	0,155	0,345	4,02859E-48
Macro_IFI27	CAMTA1	8,30031E-70	-5,78712E-01	0,15	0,374	1,52867E-65
Macro_IFI27	MTPN	1,90205E-121	-5,79597E-01	0,219	0,541	3,50300E-117
Macro_IFI27	LSM7	1,22697E-94	-5,79677E-01	0,235	0,528	2,25971E-90
Macro_IFI27	CAPNS1	5,18651E-86	-5,80573E-01	0,14	0,381	9,55199E-82
Macro_IFI27	DNAJC3	3,25112E-84	-5,81820E-01	0,14	0,375	5,98758E-80
Macro_IFI27	LITAF	1,21455E-23	-5,81855E-01	0,537	0,752	2,23683E-19
Macro_IFI27	EMC7	4,25378E-51	-5,82149E-01	0,146	0,329	7,83419E-47
Macro_IFI27	ACTN1	8,69374E-74	-5,82165E-01	0,123	0,328	1,60113E-69
Macro_IFI27	SNX1	9,18461E-45	-5,82280E-01	0,146	0,314	1,69153E-40
Macro_IFI27	VPS13C	5,12877E-68	-5,82330E-01	0,164	0,381	9,44566E-64
Macro_IFI27	ATP6V1B2	3,87781E-36	-5,82672E-01	0,325	0,545	7,14176E-32
Macro_IFI27	HMGN1	1,85067E-45	-5,83484E-01	0,379	0,637	3,40838E-41
Macro_IFI27	CCDC59	2,11484E-54	-5,83599E-01	0,095	0,257	3,89490E-50
Macro_IFI27	PTEN	4,94782E-57	-5,83640E-01	0,103	0,27	9,11241E-53
Macro_IFI27	UBE2E2	6,93466E-40	-5,84352E-01	0,147	0,307	1,27716E-35
Macro_IFI27	PPCS	1,44371E-35	-5,84594E-01	0,216	0,399	2,65889E-31
Macro_IFI27	DYNC1H1	2,39509E-82	-5,85085E-01	0,153	0,39	4,41104E-78

Macro_IFI27	C19orf53	1,14946E-54	-5,85284E-01	0,325	0,597	2,11695E-50
Macro_IFI27	EMILIN2	1,53603E-86	-5,85463E-01	0,098	0,311	2,82891E-82
Macro_IFI27	ADAM10	3,20579E-68	-5,85791E-01	0,106	0,296	5,90411E-64
Macro_IFI27	WDR1	2,48630E-79	-5,85853E-01	0,267	0,554	4,57901E-75
Macro_IFI27	ASH1L	1,90191E-71	-5,86023E-01	0,077	0,256	3,50275E-67
Macro_IFI27	PSMC3	3,34361E-43	-5,86691E-01	0,154	0,327	6,15793E-39
Macro_IFI27	DCP2	1,58313E-47	-5,86840E-01	0,116	0,273	2,91564E-43
Macro_IFI27	IL6R	1,48569E-83	-5,87749E-01	0,06	0,25	2,73620E-79
Macro_IFI27	PDCD6	5,30806E-61	-5,88166E-01	0,206	0,436	9,77586E-57
Macro_IFI27	FBXO7	3,21524E-47	-5,88960E-01	0,152	0,33	5,92151E-43
Macro_IFI27	NDUFB10	9,20993E-49	-5,89869E-01	0,314	0,57	1,69619E-44
Macro_IFI27	MMADHC	4,67860E-62	-5,90566E-01	0,15	0,358	8,61658E-58
Macro_IFI27	UBXN1	1,58777E-49	-5,93755E-01	0,365	0,641	2,92419E-45
Macro_IFI27	DCTN3	3,71267E-63	-5,93893E-01	0,158	0,37	6,83763E-59
Macro_IFI27	PAG1	2,65463E-91	-5,94004E-01	0,073	0,281	4,88903E-87
Macro_IFI27	UQCRFS1	2,44206E-80	-5,94874E-01	0,228	0,505	4,49755E-76
Macro_IFI27	ARF6	1,34989E-133	-5,95059E-01	0,132	0,432	2,48609E-129
Macro_IFI27	AKNA	1,04312E-81	-5,95825E-01	0,072	0,264	1,92112E-77
Macro_IFI27	TBRG1	1,75530E-62	-5,96051E-01	0,155	0,361	3,23274E-58
Macro_IFI27	NDUFB3	4,27044E-58	-5,96672E-01	0,233	0,479	7,86488E-54
Macro_IFI27	AZIN1	2,82245E-58	-5,96829E-01	0,136	0,328	5,19810E-54
Macro_IFI27	CAPZA2	6,14556E-40	-5,97084E-01	0,318	0,56	1,13183E-35
Macro_IFI27	ATRX	5,61980E-82	-5,97805E-01	0,159	0,4	1,03500E-77
Macro_IFI27	RNASE6	2,37681E-07	-5,97824E-01	0,398	0,546	4,37737E-03
Macro_IFI27	SNX17	1,94167E-56	-5,97893E-01	0,216	0,443	3,57597E-52
Macro_IFI27	M6PR	1,04918E-51	-5,98125E-01	0,315	0,576	1,93228E-47
Macro_IFI27	HADHB	1,96738E-42	-5,98223E-01	0,185	0,368	3,62333E-38
Macro_IFI27	ARHGEF2	3,56862E-61	-5,98684E-01	0,087	0,257	6,57233E-57
Macro_IFI27	PAFAH1B1	7,19045E-95	-5,98981E-01	0,113	0,353	1,32426E-90
Macro_IFI27	GCC2	6,00447E-61	-5,99024E-01	0,109	0,289	1,10584E-56
Macro_IFI27	EML4	5,25632E-72	-5,99166E-01	0,149	0,366	9,68057E-68
Macro_IFI27	PICALM	4,99195E-68	-5,99217E-01	0,205	0,44	9,19367E-64
Macro_IFI27	PHF11	3,92949E-56	-5,99269E-01	0,118	0,296	7,23694E-52
Macro_IFI27	BBX	9,50516E-65	-5,99640E-01	0,118	0,306	1,75057E-60
Macro_IFI27	UBE2K	6,38622E-66	-6,00004E-01	0,104	0,294	1,17615E-61
Macro_IFI27	PRNP	4,97874E-52	-6,00254E-01	0,206	0,41	9,16935E-48
Macro_IFI27	TSPO	3,95715E-14	-6,00635E-01	0,576	0,775	7,28789E-10
Macro_IFI27	GGA2	5,75006E-46	-6,01386E-01	0,119	0,277	1,05899E-41
Macro_IFI27	PHF20	3,22014E-66	-6,01671E-01	0,1	0,283	5,93054E-62
Macro_IFI27	LIMS1	1,07837E-90	-6,01769E-01	0,273	0,569	1,98603E-86
Macro_IFI27	SUPT4H1	1,14058E-59	-6,03664E-01	0,236	0,472	2,10061E-55
Macro_IFI27	NDUFS4	4,49528E-45	-6,03948E-01	0,13	0,295	8,27895E-41
Macro_IFI27	ILK	2,05070E-65	-6,04599E-01	0,194	0,426	3,77677E-61

Macro_IFI27	ARL6IP4	3,44432E-45	-6,04933E-01	0,378	0,646	6,34341E-41
Macro_IFI27	ANXA7	3,01601E-48	-6,05038E-01	0,182	0,382	5,55459E-44
Macro_IFI27	RRP7A	3,44875E-61	-6,05241E-01	0,117	0,303	6,35156E-57
Macro_IFI27	FLOT2	2,02131E-59	-6,05373E-01	0,084	0,25	3,72265E-55
Macro_IFI27	DDAH2	2,44192E-59	-6,05635E-01	0,204	0,429	4,49728E-55
Macro_IFI27	HADHA	1,66338E-80	-6,05903E-01	0,252	0,534	3,06345E-76
Macro_IFI27	TSPAN14	1,17513E-50	-6,06920E-01	0,157	0,342	2,16423E-46
Macro_IFI27	LTC4S	1,70261E-21	-6,07203E-01	0,182	0,289	3,13569E-17
Macro_IFI27	COX6A1	3,53091E-46	-6,07670E-01	0,423	0,692	6,50288E-42
Macro_IFI27	NMI	1,69076E-57	-6,08013E-01	0,124	0,308	3,11386E-53
Macro_IFI27	NAPA	5,33909E-72	-6,08176E-01	0,246	0,507	9,83299E-68
Macro_IFI27	PSMD11	5,86055E-57	-6,08274E-01	0,13	0,317	1,07934E-52
Macro_IFI27	CEBPD	1,63020E-101	-6,08908E-01	0,297	0,581	3,00234E-97
Macro_IFI27	UQCRC2	1,63512E-40	-6,09561E-01	0,226	0,424	3,01140E-36
Macro_IFI27	SYF2	1,28955E-59	-6,10100E-01	0,208	0,435	2,37497E-55
Macro_IFI27	DNAJC8	1,53006E-50	-6,10198E-01	0,209	0,423	2,81791E-46
Macro_IFI27	MBP	1,23495E-98	-6,10334E-01	0,097	0,329	2,27440E-94
Macro_IFI27	RBM47	3,34352E-73	-6,10520E-01	0,149	0,369	6,15776E-69
Macro_IFI27	UVRAG	1,63057E-60	-6,11818E-01	0,116	0,299	3,00301E-56
Macro_IFI27	SPCS3	1,43873E-90	-6,11977E-01	0,188	0,457	2,64971E-86
Macro_IFI27	UBE2J1	6,08242E-100	-6,12218E-01	0,083	0,312	1,12020E-95
Macro_IFI27	G3BP2	2,90108E-72	-6,12282E-01	0,094	0,286	5,34291E-68
Macro_IFI27	CHURC1	1,48403E-65	-6,12659E-01	0,127	0,327	2,73314E-61
Macro_IFI27	NOP10	7,25132E-66	-6,12748E-01	0,35	0,645	1,33548E-61
Macro_IFI27	DCK	1,46702E-48	-6,13343E-01	0,123	0,287	2,70180E-44
Macro_IFI27	CSK	2,62100E-89	-6,13879E-01	0,123	0,358	4,82710E-85
Macro_IFI27	NR1H2	2,63836E-73	-6,13943E-01	0,128	0,34	4,85907E-69
Macro_IFI27	ATF6B	4,01939E-48	-6,14370E-01	0,129	0,295	7,40251E-44
Macro_IFI27	TRAM1	2,81992E-57	-6,14531E-01	0,227	0,461	5,19345E-53
Macro_IFI27	PTTG1IP	4,35101E-77	-6,16164E-01	0,242	0,515	8,01325E-73
Macro_IFI27	HMGN3	4,86044E-65	-6,16409E-01	0,274	0,535	8,95147E-61
Macro_IFI27	TECR	6,90398E-36	-6,16425E-01	0,209	0,387	1,27151E-31
Macro_IFI27	JAK1	2,32422E-122	-6,16664E-01	0,215	0,537	4,28052E-118
Macro_IFI27	PCNP	2,51696E-58	-6,16673E-01	0,139	0,332	4,63549E-54
Macro_IFI27	CNIH1	2,97426E-61	-6,16768E-01	0,179	0,395	5,47769E-57
Macro_IFI27	RPS26	4,93579E-20	-6,17498E-01	0,379	0,531	9,09024E-16
Macro_IFI27	LTA4H	6,13790E-23	-6,17579E-01	0,292	0,455	1,13042E-18
Macro_IFI27	SNX5	2,34851E-42	-6,17747E-01	0,211	0,407	4,32526E-38
Macro_IFI27	ZC3H13	5,35996E-66	-6,18504E-01	0,086	0,264	9,87143E-62
Macro_IFI27	RHOG	6,96203E-33	-6,19081E-01	0,473	0,739	1,28220E-28
Macro_IFI27	PTRHD1	2,57476E-70	-6,19171E-01	0,145	0,359	4,74193E-66
Macro_IFI27	SNX2	1,95471E-35	-6,19203E-01	0,249	0,45	3,60000E-31
Macro_IFI27	KYNU	6,41497E-81	-6,19253E-01	0,113	0,325	1,18144E-76

Macro_IFI27	NDUFB8	1,09956E-48	-6,19736E-01	0,13	0,295	2,02507E-44
Macro_IFI27	DNMT1	5,31061E-41	-6,19920E-01	0,16	0,325	9,78055E-37
Macro_IFI27	PCMT1	5,06443E-55	-6,20183E-01	0,153	0,347	9,32715E-51
Macro_IFI27	UBE2I	3,37837E-70	-6,20557E-01	0,264	0,54	6,22195E-66
Macro_IFI27	SCNM1	1,15555E-41	-6,20570E-01	0,17	0,343	2,12818E-37
Macro_IFI27	DEGS1	1,77023E-48	-6,20630E-01	0,153	0,335	3,26023E-44
Macro_IFI27	HNRNPL	2,79943E-59	-6,20717E-01	0,116	0,297	5,15571E-55
Macro_IFI27	CAST	1,95403E-75	-6,21057E-01	0,272	0,544	3,59874E-71
Macro_IFI27	PRMT2	6,51217E-48	-6,21298E-01	0,224	0,437	1,19935E-43
Macro_IFI27	PIN1	1,78633E-53	-6,21473E-01	0,159	0,355	3,28989E-49
Macro_IFI27	ARPP19	1,44687E-59	-6,21767E-01	0,101	0,277	2,66470E-55
Macro_IFI27	SDF2L1	4,59818E-63	-6,22286E-01	0,157	0,364	8,46846E-59
Macro_IFI27	HMGN4	7,23758E-44	-6,22325E-01	0,107	0,255	1,33295E-39
Macro_IFI27	PLXNB2	8,63326E-53	-6,22513E-01	0,109	0,272	1,58999E-48
Macro_IFI27	MIF4GD	1,48170E-39	-6,23579E-01	0,156	0,32	2,72885E-35
Macro_IFI27	NDUFS5	1,73166E-46	-6,25230E-01	0,391	0,667	3,18920E-42
Macro_IFI27	C1QBP	1,28680E-60	-6,25415E-01	0,209	0,44	2,36989E-56
Macro_IFI27	AKIRIN2	2,65676E-85	-6,25957E-01	0,177	0,433	4,89296E-81
Macro_IFI27	SYAP1	3,02529E-114	-6,26466E-01	0,1	0,356	5,57168E-110
Macro_IFI27	RAB6A	1,35886E-48	-6,27157E-01	0,136	0,308	2,50262E-44
Macro_IFI27	POU2F2	8,07975E-63	-6,27803E-01	0,104	0,278	1,48805E-58
Macro_IFI27	TLR2	1,30536E-52	-6,28402E-01	0,2	0,395	2,40408E-48
Macro_IFI27	PRCP	7,17552E-43	-6,29335E-01	0,206	0,398	1,32152E-38
Macro_IFI27	POLR1D	5,94899E-67	-6,29731E-01	0,228	0,48	1,09563E-62
Macro_IFI27	ETFA	5,22066E-41	-6,29988E-01	0,17	0,343	9,61490E-37
Macro_IFI27	LYN	1,26493E-123	-6,30033E-01	0,167	0,466	2,32962E-119
Macro_IFI27	RPL38	1,11272E-14	-6,30078E-01	0,342	0,499	2,04929E-10
Macro_IFI27	TMED5	3,18366E-84	-6,30116E-01	0,146	0,384	5,86335E-80
Macro_IFI27	TUBGCP2	2,07134E-65	-6,30559E-01	0,119	0,312	3,81479E-61
Macro_IFI27	RTCB	1,34432E-45	-6,30852E-01	0,135	0,303	2,47584E-41
Macro_IFI27	COX5A	3,03147E-19	-6,31791E-01	0,472	0,705	5,58306E-15
Macro_IFI27	PDIA4	1,51226E-58	-6,31822E-01	0,137	0,33	2,78512E-54
Macro_IFI27	MTIF3	3,04876E-49	-6,32688E-01	0,131	0,303	5,61491E-45
Macro_IFI27	TTC3	7,20920E-49	-6,33156E-01	0,158	0,337	1,32772E-44
Macro_IFI27	SCAMP3	9,97661E-42	-6,33556E-01	0,111	0,258	1,83739E-37
Macro_IFI27	ACP1	3,64876E-41	-6,34289E-01	0,169	0,342	6,71993E-37
Macro_IFI27	HSD17B4	4,77960E-47	-6,34681E-01	0,109	0,266	8,80258E-43
Macro_IFI27	MICAL1	7,66228E-66	-6,36262E-01	0,113	0,302	1,41116E-61
Macro_IFI27	PSENEN	9,91541E-47	-6,37011E-01	0,135	0,302	1,82612E-42
Macro_IFI27	NUDT22	2,11204E-53	-6,37418E-01	0,11	0,28	3,88974E-49
Macro_IFI27	DCXR	4,58407E-51	-6,37595E-01	0,179	0,376	8,44248E-47
Macro_IFI27	SRI	1,61942E-65	-6,37686E-01	0,207	0,445	2,98249E-61
Macro_IFI27	NKTR	8,08227E-66	-6,37769E-01	0,106	0,292	1,48851E-61

Macro_IFI27	MPC1	9,73395E-55	-6,37874E-01	0,138	0,325	1,79270E-50
Macro_IFI27	SLC16A3	7,66764E-105	-6,39232E-01	0,234	0,525	1,41215E-100
Macro_IFI27	PDIA6	3,05929E-66	-6,39533E-01	0,269	0,532	5,63430E-62
Macro_IFI27	MYO9B	1,78594E-93	-6,40212E-01	0,148	0,4	3,28917E-89
Macro_IFI27	OTUB1	2,79068E-75	-6,40473E-01	0,19	0,442	5,13960E-71
Macro_IFI27	SPAG9	2,80971E-91	-6,40943E-01	0,094	0,314	5,17464E-87
Macro_IFI27	LRRFIP2	4,81349E-72	-6,41007E-01	0,079	0,26	8,86501E-68
Macro_IFI27	UBA1	2,63321E-80	-6,41601E-01	0,102	0,313	4,84958E-76
Macro_IFI27	TMEM30A	8,91922E-66	-6,41843E-01	0,118	0,314	1,64265E-61
Macro_IFI27	DECR1	1,04776E-52	-6,42203E-01	0,161	0,356	1,92966E-48
Macro_IFI27	MAPRE1	1,51496E-77	-6,44297E-01	0,16	0,4	2,79010E-73
Macro_IFI27	TMBIM6	4,60951E-20	-6,44578E-01	0,528	0,79	8,48933E-16
Macro_IFI27	TRIP12	1,42353E-78	-6,45173E-01	0,087	0,285	2,62172E-74
Macro_IFI27	CCT6A	1,44741E-72	-6,45231E-01	0,21	0,46	2,66569E-68
Macro_IFI27	TAX1BP1	2,00822E-78	-6,45445E-01	0,238	0,511	3,69853E-74
Macro_IFI27	TMED9	1,02855E-43	-6,46187E-01	0,3	0,535	1,89428E-39
Macro_IFI27	CCAR1	3,42378E-68	-6,46487E-01	0,091	0,276	6,30557E-64
Macro_IFI27	CCDC88A	1,29217E-122	-6,46533E-01	0,199	0,517	2,37979E-118
Macro_IFI27	CAP1	4,81061E-78	-6,46753E-01	0,427	0,748	8,85970E-74
Macro_IFI27	ZNF106	1,01350E-54	-6,47623E-01	0,161	0,352	1,86657E-50
Macro_IFI27	COPS3	1,00682E-38	-6,49318E-01	0,116	0,258	1,85427E-34
Macro_IFI27	BASP1	9,81947E-118	-6,49443E-01	0,1	0,359	1,80845E-113
Macro_IFI27	PGLS	1,62577E-51	-6,49518E-01	0,312	0,554	2,99419E-47
Macro_IFI27	CSTA	7,19331E-73	-6,49605E-01	0,209	0,44	1,32479E-68
Macro_IFI27	SRP72	2,96003E-57	-6,49829E-01	0,158	0,356	5,45149E-53
Macro_IFI27	POLR2E	5,06404E-58	-6,50157E-01	0,283	0,542	9,32645E-54
Macro_IFI27	GRSF1	1,57568E-64	-6,50198E-01	0,145	0,349	2,90193E-60
Macro_IFI27	MAT2B	3,19824E-41	-6,50422E-01	0,168	0,343	5,89021E-37
Macro_IFI27	CLINT1	1,85276E-61	-6,50705E-01	0,11	0,291	3,41224E-57
Macro_IFI27	TRIM38	1,10438E-79	-6,53549E-01	0,091	0,294	2,03393E-75
Macro_IFI27	RPL36AL	2,50249E-45	-6,55300E-01	0,286	0,503	4,60884E-41
Macro_IFI27	RNASET2	9,64124E-20	-6,55651E-01	0,527	0,75	1,77563E-15
Macro_IFI27	MFF	2,87322E-50	-6,55822E-01	0,147	0,328	5,29162E-46
Macro_IFI27	CANX	7,32528E-58	-6,56025E-01	0,37	0,652	1,34910E-53
Macro_IFI27	ARHGAP30	4,50474E-75	-6,56100E-01	0,128	0,342	8,29638E-71
Macro_IFI27	PILRA	1,41367E-15	-6,56232E-01	0,345	0,5	2,60356E-11
Macro_IFI27	CCDC107	9,78820E-56	-6,57673E-01	0,132	0,315	1,80269E-51
Macro_IFI27	RNF7	2,07067E-66	-6,58747E-01	0,284	0,557	3,81355E-62
Macro_IFI27	RBM25	8,59209E-114	-6,58754E-01	0,205	0,508	1,58241E-109
Macro_IFI27	MYH9	1,16510E-121	-6,59616E-01	0,264	0,594	2,14576E-117
Macro_IFI27	CPPED1	1,04010E-69	-6,59764E-01	0,092	0,279	1,91555E-65
Macro_IFI27	GNA13	1,17111E-103	-6,60409E-01	0,101	0,345	2,15684E-99
Macro_IFI27	RAB34	2,15375E-47	-6,60750E-01	0,119	0,28	3,96656E-43

Macro_IFI27	ZC3H15	4,37507E-82	-6,60852E-01	0,191	0,45	8,05756E-78
Macro_IFI27	RPL32	8,83930E-10	-6,60929E-01	0,532	0,581	1,62793E-05
Macro_IFI27	MAN2B1	1,25496E-29	-6,61020E-01	0,286	0,483	2,31126E-25
Macro_IFI27	NUDC	1,42432E-58	-6,61611E-01	0,178	0,39	2,62316E-54
Macro_IFI27	IL10RB	1,24402E-64	-6,61750E-01	0,086	0,263	2,29112E-60
Macro_IFI27	ELL2	5,34890E-85	-6,62211E-01	0,088	0,294	9,85106E-81
Macro_IFI27	PDCD5	3,63938E-58	-6,62771E-01	0,158	0,36	6,70265E-54
Macro_IFI27	CYCS	6,08721E-86	-6,63566E-01	0,254	0,537	1,12108E-81
Macro_IFI27	ATP6V1G1	3,87081E-63	-6,63568E-01	0,427	0,733	7,12888E-59
Macro_IFI27	SPSB3	2,35838E-52	-6,63803E-01	0,125	0,298	4,34343E-48
Macro_IFI27	CSF2RA	8,40336E-47	-6,64062E-01	0,228	0,426	1,54765E-42
Macro_IFI27	TNIP1	8,83174E-96	-6,64160E-01	0,117	0,357	1,62654E-91
Macro_IFI27	CSDE1	6,04770E-62	-6,64190E-01	0,336	0,617	1,11381E-57
Macro_IFI27	RHOB	1,36684E-82	-6,64251E-01	0,3	0,553	2,51731E-78
Macro_IFI27	RBBP6	1,44630E-111	-6,64257E-01	0,067	0,3	2,66365E-107
Macro_IFI27	PARP14	8,52716E-96	-6,64271E-01	0,14	0,388	1,57045E-91
Macro_IFI27	FBP1	4,75630E-16	-6,64815E-01	0,233	0,355	8,75968E-12
Macro_IFI27	TRIB1	1,78854E-95	-6,65071E-01	0,049	0,25	3,29396E-91
Macro_IFI27	ARL4C	1,16534E-87	-6,65761E-01	0,119	0,343	2,14621E-83
Macro_IFI27	TIMM13	2,36578E-68	-6,67014E-01	0,177	0,405	4,35705E-64
Macro_IFI27	CNDP2	2,73829E-44	-6,68371E-01	0,249	0,465	5,04310E-40
Macro_IFI27	PSMG2	1,17777E-49	-6,68395E-01	0,217	0,434	2,16910E-45
Macro_IFI27	CELF2	1,06755E-89	-6,69032E-01	0,26	0,553	1,96610E-85
Macro_IFI27	TRMT112	1,11444E-60	-6,69091E-01	0,339	0,632	2,05246E-56
Macro_IFI27	TUFM	5,46086E-57	-6,69114E-01	0,27	0,521	1,00573E-52
Macro_IFI27	EIF3D	7,75391E-52	-6,69630E-01	0,248	0,485	1,42804E-47
Macro_IFI27	PLIN3	3,89958E-66	-6,69797E-01	0,165	0,382	7,18186E-62
Macro_IFI27	CSNK1D	5,06891E-85	-6,69802E-01	0,126	0,354	9,33542E-81
Macro_IFI27	LAMTOR3	2,64731E-56	-6,70212E-01	0,097	0,266	4,87556E-52
Macro_IFI27	THOC2	2,23424E-59	-6,70503E-01	0,094	0,263	4,11479E-55
Macro_IFI27	TMEM123	2,41112E-58	-6,70746E-01	0,23	0,465	4,44055E-54
Macro_IFI27	HDAC2	1,13109E-51	-6,71806E-01	0,111	0,275	2,08313E-47
Macro_IFI27	SCIMP	2,10994E-56	-6,71924E-01	0,113	0,286	3,88587E-52
Macro_IFI27	MAPKAPK3	1,10377E-63	-6,72297E-01	0,111	0,298	2,03281E-59
Macro_IFI27	SSB	2,43939E-58	-6,73210E-01	0,228	0,463	4,49262E-54
Macro_IFI27	CYB5R3	5,43883E-80	-6,73317E-01	0,173	0,422	1,00167E-75
Macro_IFI27	LGALS9	4,31423E-40	-6,73592E-01	0,323	0,559	7,94552E-36
Macro_IFI27	SSH2	6,69309E-65	-6,73673E-01	0,13	0,32	1,23267E-60
Macro_IFI27	PUF60	1,12597E-50	-6,73724E-01	0,145	0,328	2,07370E-46
Macro_IFI27	PLEKHJ1	2,48644E-48	-6,73926E-01	0,102	0,257	4,57928E-44
Macro_IFI27	SRA1	2,43099E-83	-6,74012E-01	0,154	0,397	4,47715E-79
Macro_IFI27	UXT	5,58378E-79	-6,74024E-01	0,267	0,555	1,02836E-74
Macro_IFI27	PSMD9	3,07211E-57	-6,74169E-01	0,095	0,262	5,65790E-53

Macro_IFI27	TMED10	1,05604E-30	-6,74367E-01	0,352	0,578	1,94491E-26
Macro_IFI27	UBL5	2,36328E-63	-6,75086E-01	0,465	0,769	4,35246E-59
Macro_IFI27	SEC13	2,22841E-58	-6,75336E-01	0,191	0,412	4,10407E-54
Macro_IFI27	RAB10	7,44093E-87	-6,75429E-01	0,22	0,503	1,37040E-82
Macro_IFI27	NRBP1	1,73486E-64	-6,75975E-01	0,121	0,316	3,19510E-60
Macro_IFI27	CALCOCO2	2,11772E-62	-6,76007E-01	0,16	0,368	3,90020E-58
Macro_IFI27	SEC62	7,83953E-81	-6,76489E-01	0,275	0,568	1,44381E-76
Macro_IFI27	ABRACL	2,65235E-77	-6,77142E-01	0,239	0,51	4,88483E-73
Macro_IFI27	EIF4E	1,18042E-77	-6,77226E-01	0,102	0,307	2,17398E-73
Macro_IFI27	RPS21	2,02172E-16	-6,77699E-01	0,422	0,548	3,72341E-12
Macro_IFI27	SCAF11	2,36468E-106	-6,79037E-01	0,205	0,503	4,35504E-102
Macro_IFI27	CEBPB	2,06593E-139	-6,79348E-01	0,173	0,486	3,80482E-135
Macro_IFI27	AURKAIP1	1,50981E-81	-6,79471E-01	0,247	0,531	2,78063E-77
Macro_IFI27	MIF	6,29830E-47	-6,79680E-01	0,469	0,736	1,15996E-42
Macro_IFI27	SEC61G	9,35665E-130	-6,82564E-01	0,267	0,616	1,72321E-125
Macro_IFI27	LAPTM4A	1,55111E-37	-6,82668E-01	0,391	0,64	2,85669E-33
Macro_IFI27	SLTM	1,00757E-83	-6,83006E-01	0,133	0,366	1,85564E-79
Macro_IFI27	NDUFS2	4,68462E-46	-6,83499E-01	0,18	0,369	8,62766E-42
Macro_IFI27	RABGGTB	3,95300E-47	-6,84091E-01	0,108	0,264	7,28024E-43
Macro_IFI27	GLIPR2	4,49265E-92	-6,84289E-01	0,137	0,388	8,27411E-88
Macro_IFI27	TPM4	1,56673E-89	-6,85180E-01	0,323	0,632	2,88545E-85
Macro_IFI27	GRB2	1,91113E-71	-6,85865E-01	0,384	0,694	3,51973E-67
Macro_IFI27	ARFGAP3	2,84052E-77	-6,85981E-01	0,083	0,277	5,23138E-73
Macro_IFI27	COX8A	9,23445E-31	-6,86188E-01	0,527	0,79	1,70071E-26
Macro_IFI27	SPCS2	3,40119E-50	-6,86423E-01	0,266	0,504	6,26398E-46
Macro_IFI27	LAMTOR5	9,35600E-70	-6,86883E-01	0,251	0,519	1,72309E-65
Macro_IFI27	ZDHHC12	6,43158E-61	-6,87139E-01	0,095	0,27	1,18450E-56
Macro_IFI27	ACSL3	6,94309E-58	-6,87151E-01	0,095	0,261	1,27871E-53
Macro_IFI27	PTDSS1	3,24836E-45	-6,87624E-01	0,121	0,277	5,98251E-41
Macro_IFI27	PNPLA8	1,69923E-69	-6,87698E-01	0,076	0,251	3,12948E-65
Macro_IFI27	ERICH1	3,91994E-62	-6,87730E-01	0,136	0,33	7,21936E-58
Macro_IFI27	IDH3G	1,56276E-65	-6,89895E-01	0,156	0,369	2,87814E-61
Macro_IFI27	RBBP4	4,54024E-59	-6,90412E-01	0,171	0,38	8,36176E-55
Macro_IFI27	EIF4E2	7,60096E-86	-6,90706E-01	0,14	0,383	1,39987E-81
Macro_IFI27	TXN2	1,03501E-43	-6,91381E-01	0,208	0,406	1,90618E-39
Macro_IFI27	NCF2	3,74902E-48	-6,91805E-01	0,263	0,474	6,90458E-44
Macro_IFI27	CLNS1A	1,70301E-49	-6,92064E-01	0,218	0,431	3,13643E-45
Macro_IFI27	PIH1D1	3,46972E-37	-6,93006E-01	0,12	0,26	6,39019E-33
Macro_IFI27	ARF5	1,14773E-62	-6,94054E-01	0,348	0,626	2,11378E-58
Macro_IFI27	SYK	1,04370E-72	-6,94338E-01	0,208	0,454	1,92219E-68
Macro_IFI27	PYCARD	2,03897E-23	-6,95060E-01	0,499	0,716	3,75517E-19
Macro_IFI27	DCTN2	9,51981E-40	-6,95427E-01	0,167	0,337	1,75326E-35
Macro_IFI27	ELF1	4,20601E-134	-6,96719E-01	0,182	0,502	7,74620E-130

Macro_IFI27	SMCHD1	1,23423E-95	-6,98061E-01	0,105	0,335	2,27309E-91
Macro_IFI27	C3AR1	1,27227E-19	-6,98248E-01	0,275	0,422	2,34315E-15
Macro_IFI27	RPS29	1,84757E-09	-6,99831E-01	0,375	0,508	3,40267E-05
Macro_IFI27	SAMHD1	1,77890E-60	-7,00314E-01	0,397	0,658	3,27621E-56
Macro_IFI27	COMMD6	2,43114E-84	-7,00546E-01	0,434	0,75	4,47743E-80
Macro_IFI27	NECAP2	1,06764E-62	-7,00733E-01	0,153	0,362	1,96628E-58
Macro_IFI27	TKT	1,28728E-38	-7,01304E-01	0,377	0,618	2,37079E-34
Macro_IFI27	COX7A2L	9,47797E-58	-7,01465E-01	0,256	0,501	1,74556E-53
Macro_IFI27	PSMB6	3,93216E-46	-7,02059E-01	0,33	0,588	7,24187E-42
Macro_IFI27	CSF1R	3,30803E-21	-7,02106E-01	0,388	0,58	6,09239E-17
Macro_IFI27	GGNBP2	3,64365E-72	-7,03098E-01	0,137	0,35	6,71051E-68
Macro_IFI27	ATXN10	1,99336E-53	-7,03272E-01	0,115	0,284	3,67118E-49
Macro_IFI27	IFI6	2,11863E-07	-7,03304E-01	0,37	0,494	3,90188E-03
Macro_IFI27	COX7A2	2,80090E-59	-7,03453E-01	0,437	0,734	5,15841E-55
Macro_IFI27	MRPS5	6,82124E-58	-7,04120E-01	0,094	0,263	1,25627E-53
Macro_IFI27	MT-ND4	2,70457E-18	-7,04738E-01	0,455	0,58	4,98101E-14
Macro_IFI27	NCF1	7,63882E-19	-7,04765E-01	0,345	0,508	1,40684E-14
Macro_IFI27	DAD1	2,83167E-67	-7,05591E-01	0,326	0,614	5,21508E-63
Macro_IFI27	RHBDD2	9,75535E-53	-7,05928E-01	0,105	0,265	1,79664E-48
Macro_IFI27	BIRC6	2,44463E-66	-7,06225E-01	0,083	0,257	4,50228E-62
Macro_IFI27	EIF4B	9,87592E-65	-7,06542E-01	0,275	0,529	1,81885E-60
Macro_IFI27	MRPL55	9,12633E-49	-7,06803E-01	0,12	0,285	1,68080E-44
Macro_IFI27	CIAO1	4,34122E-54	-7,06810E-01	0,093	0,254	7,99523E-50
Macro_IFI27	EMC3	8,81813E-45	-7,08297E-01	0,153	0,328	1,62404E-40
Macro_IFI27	NFYC	6,83303E-59	-7,09730E-01	0,094	0,266	1,25844E-54
Macro_IFI27	PPP2CA	7,58086E-84	-7,10353E-01	0,12	0,345	1,39617E-79
Macro_IFI27	GNG5	6,86960E-53	-7,11069E-01	0,547	0,805	1,26517E-48
Macro_IFI27	HSD17B10	7,56371E-59	-7,11254E-01	0,125	0,315	1,39301E-54
Macro_IFI27	MRFAP1	1,59027E-52	-7,11561E-01	0,201	0,41	2,92879E-48
Macro_IFI27	PIK3R5	1,64697E-48	-7,12181E-01	0,109	0,265	3,03322E-44
Macro_IFI27	IFNGR2	1,12401E-54	-7,12342E-01	0,32	0,576	2,07009E-50
Macro_IFI27	AP3S1	1,43467E-73	-7,12445E-01	0,136	0,357	2,64223E-69
Macro_IFI27	DPYSL2	3,23617E-48	-7,12548E-01	0,154	0,332	5,96005E-44
Macro_IFI27	LAMTOR4	4,68468E-51	-7,12632E-01	0,434	0,728	8,62778E-47
Macro_IFI27	CDC42SE1	7,13878E-88	-7,13049E-01	0,15	0,396	1,31475E-83
Macro_IFI27	KCNAB2	6,66674E-61	-7,13683E-01	0,11	0,289	1,22781E-56
Macro_IFI27	SSR3	5,39345E-32	-7,13853E-01	0,365	0,607	9,93312E-28
Macro_IFI27	CHMP2A	7,36028E-75	-7,14619E-01	0,239	0,504	1,35554E-70
Macro_IFI27	ERP29	1,65429E-54	-7,14951E-01	0,37	0,635	3,04670E-50
Macro_IFI27	SHISA5	9,41515E-56	-7,15321E-01	0,177	0,38	1,73399E-51
Macro_IFI27	TMBIM4	2,64265E-54	-7,16607E-01	0,156	0,339	4,86697E-50
Macro_IFI27	NHP2	3,69115E-66	-7,16887E-01	0,191	0,423	6,79799E-62
Macro_IFI27	SP100	3,98976E-66	-7,17026E-01	0,229	0,478	7,34794E-62

Macro_IFI27	RGS19	1,91641E-56	-7,18266E-01	0,267	0,507	3,52945E-52
Macro_IFI27	ATG12	3,35950E-63	-7,18269E-01	0,094	0,271	6,18719E-59
Macro_IFI27	RASSF2	6,84314E-78	-7,18416E-01	0,081	0,273	1,26030E-73
Macro_IFI27	ARF4	3,12547E-92	-7,19088E-01	0,186	0,458	5,75619E-88
Macro_IFI27	PPIG	6,07989E-76	-7,19094E-01	0,21	0,464	1,11973E-71
Macro_IFI27	VDAC2	7,72380E-53	-7,19926E-01	0,305	0,565	1,42249E-48
Macro_IFI27	SEC11C	3,09272E-51	-7,20002E-01	0,149	0,334	5,69587E-47
Macro_IFI27	MANBA	2,76698E-73	-7,21241E-01	0,135	0,346	5,09595E-69
Macro_IFI27	SH3KBP1	1,48152E-84	-7,21765E-01	0,176	0,426	2,72851E-80
Macro_IFI27	PFDN1	1,20738E-52	-7,22573E-01	0,12	0,295	2,22362E-48
Macro_IFI27	RIOK3	2,63474E-76	-7,22677E-01	0,086	0,28	4,85240E-72
Macro_IFI27	TMA7	4,11427E-100	-7,22858E-01	0,475	0,809	7,57725E-96
Macro_IFI27	PIAS1	3,13793E-63	-7,23579E-01	0,134	0,331	5,77912E-59
Macro_IFI27	YIF1B	6,93900E-57	-7,23765E-01	0,161	0,36	1,27796E-52
Macro_IFI27	PDCD6IP	5,64549E-70	-7,24788E-01	0,159	0,379	1,03973E-65
Macro_IFI27	ILF2	1,20778E-67	-7,24798E-01	0,175	0,402	2,22436E-63
Macro_IFI27	DDX21	9,41121E-140	-7,24821E-01	0,143	0,451	1,73326E-135
Macro_IFI27	ZFR	2,57368E-61	-7,24875E-01	0,088	0,257	4,73995E-57
Macro_IFI27	CCT7	8,06403E-46	-7,25154E-01	0,171	0,358	1,48515E-41
Macro_IFI27	PSMA7	4,29105E-57	-7,25264E-01	0,464	0,759	7,90283E-53
Macro_IFI27	FKBP1A	8,04969E-45	-7,25416E-01	0,431	0,707	1,48251E-40
Macro_IFI27	ELOVL1	6,84187E-52	-7,25541E-01	0,172	0,37	1,26007E-47
Macro_IFI27	PNN	1,50985E-66	-7,26121E-01	0,128	0,324	2,78069E-62
Macro_IFI27	CHD4	1,69024E-57	-7,26191E-01	0,111	0,285	3,11292E-53
Macro_IFI27	SLC31A2	4,69090E-48	-7,26374E-01	0,24	0,452	8,63922E-44
Macro_IFI27	TMEM179B	6,88509E-64	-7,26978E-01	0,242	0,493	1,26803E-59
Macro_IFI27	PRR13	8,62370E-47	-7,27519E-01	0,341	0,592	1,58823E-42
Macro_IFI27	ANAPC16	5,88724E-73	-7,27941E-01	0,231	0,493	1,08425E-68
Macro_IFI27	PSMC6	5,27319E-42	-7,27977E-01	0,113	0,261	9,71163E-38
Macro_IFI27	C12orf57	3,99081E-81	-7,28727E-01	0,126	0,343	7,34987E-77
Macro_IFI27	ATP6V0B	2,48747E-25	-7,29986E-01	0,535	0,803	4,58118E-21
Macro_IFI27	TMCO1	8,79489E-63	-7,30385E-01	0,156	0,368	1,61975E-58
Macro_IFI27	HNRNPD	3,35807E-108	-7,31299E-01	0,103	0,355	6,18456E-104
Macro_IFI27	PSMD2	4,44522E-51	-7,31872E-01	0,144	0,328	8,18676E-47
Macro_IFI27	ZNF638	5,45478E-61	-7,32217E-01	0,088	0,256	1,00461E-56
Macro_IFI27	IFI16	7,50900E-52	-7,32520E-01	0,277	0,511	1,38293E-47
Macro_IFI27	OSTC	2,57039E-66	-7,32836E-01	0,253	0,511	4,73388E-62
Macro_IFI27	ACAP2	7,03024E-84	-7,32978E-01	0,187	0,443	1,29476E-79
Macro_IFI27	DNAJB6	9,45010E-90	-7,33044E-01	0,245	0,535	1,74042E-85
Macro_IFI27	VCP	6,55101E-100	-7,33319E-01	0,193	0,479	1,20650E-95
Macro_IFI27	BTK	3,28637E-46	-7,33754E-01	0,149	0,318	6,05250E-42
Macro_IFI27	CDC42	6,43795E-82	-7,34629E-01	0,382	0,712	1,18568E-77
Macro_IFI27	PTPN1	1,00580E-132	-7,34996E-01	0,122	0,414	1,85237E-128

Macro_IFI27	SAP18	8,87481E-62	-7,35453E-01	0,372	0,661	1,63447E-57
Macro_IFI27	ELMO1	6,29541E-36	-7,35786E-01	0,185	0,347	1,15943E-31
Macro_IFI27	RER1	7,05644E-63	-7,36335E-01	0,224	0,472	1,29959E-58
Macro_IFI27	RBM17	8,48308E-78	-7,37397E-01	0,177	0,422	1,56233E-73
Macro_IFI27	RUNX1	2,68325E-103	-7,37653E-01	0,066	0,288	4,94174E-99
Macro_IFI27	STK4	3,42989E-114	-7,38496E-01	0,158	0,442	6,31682E-110
Macro_IFI27	ANKRD11	5,30140E-100	-7,38561E-01	0,091	0,324	9,76360E-96
Macro_IFI27	MTMR14	1,89167E-63	-7,38566E-01	0,123	0,319	3,48388E-59
Macro_IFI27	AK2	6,06121E-66	-7,38990E-01	0,143	0,352	1,11629E-61
Macro_IFI27	TOX4	6,44994E-57	-7,39032E-01	0,115	0,291	1,18789E-52
Macro_IFI27	AFF4	6,92498E-116	-7,39664E-01	0,122	0,389	1,27537E-111
Macro_IFI27	ITGA4	3,44213E-77	-7,40627E-01	0,154	0,379	6,33936E-73
Macro_IFI27	RHBDF2	4,09209E-59	-7,40635E-01	0,143	0,333	7,53640E-55
Macro_IFI27	SIVA1	1,35854E-62	-7,41383E-01	0,226	0,466	2,50201E-58
Macro_IFI27	AIP	6,68470E-54	-7,42334E-01	0,156	0,349	1,23112E-49
Macro_IFI27	MRPL18	4,97802E-71	-7,42477E-01	0,144	0,359	9,16802E-67
Macro_IFI27	BRK1	2,03606E-47	-7,42883E-01	0,449	0,739	3,74981E-43
Macro_IFI27	EIF1B	8,10860E-66	-7,43111E-01	0,225	0,472	1,49336E-61
Macro_IFI27	SKP1	1,04209E-50	-7,43707E-01	0,416	0,702	1,91921E-46
Macro_IFI27	SMCO4	8,72924E-65	-7,43736E-01	0,194	0,429	1,60766E-60
Macro_IFI27	ACSL1	1,90604E-78	-7,43776E-01	0,171	0,399	3,51035E-74
Macro_IFI27	SH2B3	2,61814E-81	-7,43837E-01	0,077	0,274	4,82182E-77
Macro_IFI27	DNTTIP2	7,14194E-76	-7,44556E-01	0,09	0,284	1,31533E-71
Macro_IFI27	GLRX3	3,00853E-54	-7,45272E-01	0,093	0,255	5,54082E-50
Macro_IFI27	HNRNPR	2,35026E-81	-7,45542E-01	0,14	0,371	4,32847E-77
Macro_IFI27	NCL	3,02229E-62	-7,46854E-01	0,358	0,639	5,56615E-58
Macro_IFI27	UBXN4	6,78679E-88	-7,48029E-01	0,244	0,534	1,24992E-83
Macro_IFI27	PSMD7	2,24340E-68	-7,48159E-01	0,182	0,416	4,13167E-64
Macro_IFI27	SRSF10	4,01631E-74	-7,49032E-01	0,093	0,288	7,39685E-70
Macro_IFI27	PTBP3	2,04598E-84	-7,49813E-01	0,114	0,336	3,76807E-80
Macro_IFI27	SRP14	3,07131E-12	-7,50481E-01	0,624	0,846	5,65643E-08
Macro_IFI27	CHCHD2	7,02229E-43	-7,50979E-01	0,566	0,828	1,29329E-38
Macro_IFI27	CACYBP	1,78291E-62	-7,51224E-01	0,19	0,412	3,28359E-58
Macro_IFI27	IAH1	1,84190E-45	-7,51975E-01	0,146	0,317	3,39223E-41
Macro_IFI27	IER5	5,49845E-115	-7,52074E-01	0,089	0,338	1,01265E-110
Macro_IFI27	PRPF40A	1,54784E-76	-7,52427E-01	0,209	0,467	2,85065E-72
Macro_IFI27	BECN1	2,95459E-49	-7,52990E-01	0,107	0,268	5,44147E-45
Macro_IFI27	HNRNPH3	9,68163E-59	-7,53347E-01	0,157	0,362	1,78307E-54
Macro_IFI27	PPP1R10	4,36568E-91	-7,54178E-01	0,096	0,319	8,04027E-87
Macro_IFI27	MT-CO1	1,39431E-09	-7,54921E-01	0,586	0,6	2,56790E-05
Macro_IFI27	MRPL33	1,96795E-88	-7,54926E-01	0,14	0,383	3,62436E-84
Macro_IFI27	POLR2J	1,63093E-55	-7,55333E-01	0,13	0,314	3,00368E-51
Macro_IFI27	CCS	2,29012E-56	-7,55826E-01	0,101	0,269	4,21772E-52

Macro_IFI27	VKORC1	3,81928E-45	-7,56038E-01	0,103	0,251	7,03396E-41
Macro_IFI27	TWF2	2,24091E-52	-7,57315E-01	0,151	0,343	4,12709E-48
Macro_IFI27	GLIPR1	3,66477E-49	-7,57736E-01	0,345	0,595	6,74940E-45
Macro_IFI27	DNAJB11	2,02582E-69	-7,60127E-01	0,118	0,32	3,73095E-65
Macro_IFI27	RAB1A	2,01032E-81	-7,60391E-01	0,161	0,407	3,70241E-77
Macro_IFI27	SKAP2	4,28068E-78	-7,60561E-01	0,174	0,416	7,88373E-74
Macro_IFI27	LAP3	8,49712E-17	-7,60672E-01	0,406	0,594	1,56491E-12
Macro_IFI27	LCP2	5,48773E-73	-7,60941E-01	0,318	0,589	1,01068E-68
Macro_IFI27	COPA	4,39208E-78	-7,62505E-01	0,127	0,348	8,08889E-74
Macro_IFI27	PSMC5	3,97361E-51	-7,63080E-01	0,207	0,421	7,31820E-47
Macro_IFI27	VTI1B	1,68041E-41	-7,63338E-01	0,198	0,386	3,09481E-37
Macro_IFI27	NDUFA10	5,04404E-48	-7,66091E-01	0,155	0,336	9,28960E-44
Macro_IFI27	EIF4G2	2,85203E-107	-7,66628E-01	0,301	0,634	5,25259E-103
Macro_IFI27	SAFB	2,13833E-55	-7,66867E-01	0,093	0,254	3,93817E-51
Macro_IFI27	MPV17	7,56207E-38	-7,67213E-01	0,155	0,317	1,39271E-33
Macro_IFI27	PARP9	4,71197E-62	-7,67969E-01	0,089	0,262	8,67804E-58
Macro_IFI27	RPL30	4,01936E-07	-7,68339E-01	0,533	0,583	7,40245E-03
Macro_IFI27	GSTP1	8,69875E-19	-7,68627E-01	0,622	0,833	1,60205E-14
Macro_IFI27	ARIH2	1,84427E-53	-7,68711E-01	0,101	0,266	3,39660E-49
Macro_IFI27	ACTG1	2,39688E-12	-7,69473E-01	0,813	0,928	4,41433E-08
Macro_IFI27	RSRC2	2,41043E-77	-7,69684E-01	0,164	0,401	4,43929E-73
Macro_IFI27	YBX3	5,31276E-110	-7,69922E-01	0,109	0,359	9,78451E-106
Macro_IFI27	SEC14L1	3,23511E-61	-7,70756E-01	0,188	0,405	5,95811E-57
Macro_IFI27	VIM	1,94807E-45	-7,71083E-01	0,841	0,948	3,58776E-41
Macro_IFI27	PHB2	8,98190E-60	-7,71563E-01	0,255	0,505	1,65420E-55
Macro_IFI27	BUB3	1,49845E-49	-7,72663E-01	0,105	0,265	2,75970E-45
Macro_IFI27	SRRM1	3,89554E-116	-7,72903E-01	0,251	0,579	7,17441E-112
Macro_IFI27	IK	4,44399E-57	-7,74506E-01	0,151	0,347	8,18450E-53
Macro_IFI27	OSBPL8	2,99834E-119	-7,74632E-01	0,156	0,442	5,52204E-115
Macro_IFI27	ETNK1	1,14277E-66	-7,74699E-01	0,081	0,256	2,10463E-62
Macro_IFI27	BLVRA	3,60500E-51	-7,75559E-01	0,19	0,392	6,63932E-47
Macro_IFI27	HSPB1	6,70285E-18	-7,75569E-01	0,496	0,666	1,23446E-13
Macro_IFI27	MIIP	2,15834E-57	-7,75640E-01	0,088	0,252	3,97502E-53
Macro_IFI27	NEDD8	1,54154E-70	-7,76144E-01	0,313	0,6	2,83906E-66
Macro_IFI27	GTF3A	2,70465E-47	-7,77952E-01	0,261	0,491	4,98116E-43
Macro_IFI27	CLEC4A	7,55034E-38	-7,79494E-01	0,154	0,304	1,39055E-33
Macro_IFI27	FOXP1	3,45250E-96	-7,80067E-01	0,12	0,356	6,35847E-92
Macro_IFI27	TNFSF13B	2,38632E-25	-7,80476E-01	0,394	0,588	4,39488E-21
Macro_IFI27	CMTM6	2,54014E-127	-7,81522E-01	0,287	0,632	4,67818E-123
Macro_IFI27	GMFG	7,51081E-56	-7,82868E-01	0,412	0,686	1,38327E-51
Macro_IFI27	CCNDBP1	4,12336E-49	-7,82986E-01	0,119	0,281	7,59399E-45
Macro_IFI27	MIS18BP1	9,82015E-64	-7,83879E-01	0,166	0,37	1,80858E-59
Macro_IFI27	ADRM1	4,21447E-67	-7,83960E-01	0,199	0,44	7,76179E-63

Macro_IFI27	RELB	3,42593E-103	-7,84061E-01	0,044	0,255	6,30953E-99
Macro_IFI27	ERGIC3	8,98549E-62	-7,84109E-01	0,255	0,51	1,65486E-57
Macro_IFI27	DMXL2	1,52632E-67	-7,85142E-01	0,094	0,276	2,81102E-63
Macro_IFI27	PPP1R12A	2,78451E-98	-7,86047E-01	0,135	0,389	5,12823E-94
Macro_IFI27	PSMD13	6,45415E-74	-7,86719E-01	0,12	0,333	1,18866E-69
Macro_IFI27	ESD	6,49503E-44	-7,89496E-01	0,184	0,372	1,19619E-39
Macro_IFI27	SUPT5H	5,38322E-68	-7,90434E-01	0,076	0,25	9,91427E-64
Macro_IFI27	RPL14	1,17461E-11	-7,91149E-01	0,434	0,548	2,16328E-07
Macro_IFI27	APLP2	2,41302E-76	-7,91401E-01	0,331	0,608	4,44407E-72
Macro_IFI27	PELI1	1,17494E-73	-7,91645E-01	0,087	0,27	2,16389E-69
Macro_IFI27	ATP6V0D1	1,48088E-72	-7,91945E-01	0,297	0,584	2,72734E-68
Macro_IFI27	OST4	1,12250E-67	-7,92418E-01	0,488	0,791	2,06732E-63
Macro_IFI27	FES	5,20372E-55	-7,92574E-01	0,099	0,262	9,58369E-51
Macro_IFI27	ITGB2	6,19564E-09	-7,92922E-01	0,59	0,772	1,14105E-04
Macro_IFI27	LDHB	1,80854E-42	-7,93054E-01	0,298	0,521	3,33079E-38
Macro_IFI27	TNFAIP8	1,92166E-83	-7,93935E-01	0,124	0,346	3,53913E-79
Macro_IFI27	UQCRC1	9,83529E-62	-7,94654E-01	0,259	0,517	1,81136E-57
Macro_IFI27	RAB7A	4,62234E-51	-7,94804E-01	0,37	0,646	8,51297E-47
Macro_IFI27	PEF1	2,04974E-52	-7,96135E-01	0,096	0,255	3,77501E-48
Macro_IFI27	PQBP1	1,09761E-56	-7,96363E-01	0,135	0,323	2,02147E-52
Macro_IFI27	OS9	1,21974E-64	-7,97238E-01	0,281	0,549	2,24639E-60
Macro_IFI27	VAPA	3,50437E-79	-7,97371E-01	0,315	0,617	6,45400E-75
Macro_IFI27	SASH3	2,19915E-51	-7,98014E-01	0,123	0,292	4,05017E-47
Macro_IFI27	PSMD6	1,74143E-42	-7,99340E-01	0,127	0,286	3,20720E-38
Macro_IFI27	YTHDC1	1,81079E-83	-7,99694E-01	0,104	0,315	3,33493E-79
Macro_IFI27	LPP	1,09359E-59	-8,00775E-01	0,102	0,275	2,01407E-55
Macro_IFI27	NAGA	8,67972E-52	-8,01076E-01	0,175	0,369	1,59854E-47
Macro_IFI27	POLD4	5,93949E-43	-8,01114E-01	0,343	0,58	1,09388E-38
Macro_IFI27	TALDO1	4,62521E-38	-8,02500E-01	0,416	0,674	8,51825E-34
Macro_IFI27	PTBP1	2,57580E-79	-8,03176E-01	0,104	0,316	4,74386E-75
Macro_IFI27	RAB20	5,00674E-52	-8,03185E-01	0,168	0,355	9,22092E-48
Macro_IFI27	EDF1	9,99133E-44	-8,03401E-01	0,469	0,763	1,84010E-39
Macro_IFI27	GDI2	3,55843E-58	-8,03474E-01	0,38	0,664	6,55355E-54
Macro_IFI27	HM13	1,21188E-45	-8,03994E-01	0,31	0,55	2,23192E-41
Macro_IFI27	MT-CYB	6,20740E-08	-8,04910E-01	0,489	0,583	1,14322E-03
Macro_IFI27	TMED2	4,43727E-62	-8,05902E-01	0,256	0,511	8,17212E-58
Macro_IFI27	RPS27A	3,21355E-10	-8,06276E-01	0,513	0,579	5,91839E-06
Macro_IFI27	ORMDL1	2,19953E-47	-8,07191E-01	0,15	0,323	4,05087E-43
Macro_IFI27	FBXL5	9,79635E-57	-8,09243E-01	0,11	0,282	1,80419E-52
Macro_IFI27	RNF213	4,84166E-85	-8,09506E-01	0,254	0,517	8,91688E-81
Macro_IFI27	PNRC1	5,18201E-133	-8,09819E-01	0,454	0,795	9,54371E-129
Macro_IFI27	HNRNPF	2,13968E-69	-8,10560E-01	0,295	0,578	3,94065E-65
Macro_IFI27	HCLS1	1,35960E-30	-8,10760E-01	0,466	0,736	2,50398E-26

Macro_IFI27	EIF3H	8,24298E-38	-8,11047E-01	0,406	0,67	1,51811E-33
Macro_IFI27	PPP1CA	1,03678E-69	-8,12396E-01	0,341	0,635	1,90944E-65
Macro_IFI27	GBP1	2,67071E-43	-8,12759E-01	0,182	0,354	4,91864E-39
Macro_IFI27	MTHFD2	6,69389E-60	-8,12824E-01	0,196	0,415	1,23281E-55
Macro_IFI27	SF1	1,56133E-119	-8,12847E-01	0,206	0,518	2,87549E-115
Macro_IFI27	EIF3I	6,46784E-70	-8,12877E-01	0,224	0,477	1,19118E-65
Macro_IFI27	IFI35	2,44443E-67	-8,13162E-01	0,146	0,356	4,50191E-63
Macro_IFI27	ANAPC5	9,19898E-57	-8,13238E-01	0,118	0,296	1,69418E-52
Macro_IFI27	RSL1D1	2,60533E-54	-8,14332E-01	0,247	0,48	4,79824E-50
Macro_IFI27	AP1M1	2,91028E-47	-8,14345E-01	0,104	0,254	5,35986E-43
Macro_IFI27	SPTLC2	7,08608E-56	-8,14405E-01	0,136	0,318	1,30504E-51
Macro_IFI27	PSMD8	9,96365E-61	-8,14639E-01	0,276	0,537	1,83501E-56
Macro_IFI27	KRTCAP2	3,02432E-67	-8,14914E-01	0,309	0,59	5,56989E-63
Macro_IFI27	ETV6	1,00330E-109	-8,16088E-01	0,068	0,301	1,84777E-105
Macro_IFI27	SDHB	2,70816E-37	-8,16739E-01	0,218	0,408	4,98761E-33
Macro_IFI27	RBCK1	9,34437E-72	-8,16891E-01	0,16	0,384	1,72095E-67
Macro_IFI27	FAM32A	5,73777E-59	-8,19106E-01	0,133	0,326	1,05673E-54
Macro_IFI27	ARHGAP15	1,75297E-33	-8,19505E-01	0,127	0,26	3,22845E-29
Macro_IFI27	AMD1	2,04900E-82	-8,20838E-01	0,088	0,295	3,77364E-78
Macro_IFI27	CD86	2,17440E-57	-8,21855E-01	0,27	0,506	4,00460E-53
Macro_IFI27	NEMF	3,87082E-53	-8,22077E-01	0,124	0,296	7,12890E-49
Macro_IFI27	PRDX6	3,77614E-53	-8,23018E-01	0,313	0,57	6,95452E-49
Macro_IFI27	ARPC2	9,96332E-32	-8,23157E-01	0,651	0,869	1,83494E-27
Macro_IFI27	FMNL1	6,14420E-129	-8,24521E-01	0,126	0,416	1,13158E-124
Macro_IFI27	SURF4	1,46788E-75	-8,25723E-01	0,099	0,3	2,70339E-71
Macro_IFI27	CPVL	5,93776E-23	-8,25906E-01	0,388	0,572	1,09356E-18
Macro_IFI27	RPL37A	5,53336E-07	-8,25979E-01	0,437	0,543	1,01908E-02
Macro_IFI27	RAB11A	6,70646E-67	-8,26002E-01	0,182	0,412	1,23513E-62
Macro_IFI27	DDX24	8,08938E-93	-8,26147E-01	0,211	0,49	1,48982E-88
Macro_IFI27	MEF2C	2,28163E-79	-8,26256E-01	0,189	0,429	4,20208E-75
Macro_IFI27	RPS15A	3,14918E-14	-8,26489E-01	0,487	0,573	5,79984E-10
Macro_IFI27	IRF2BP2	1,12177E-97	-8,27339E-01	0,1	0,333	2,06597E-93
Macro_IFI27	HMGB2	1,46349E-74	-8,27411E-01	0,182	0,409	2,69530E-70
Macro_IFI27	TOM1	5,74111E-83	-8,27580E-01	0,088	0,293	1,05734E-78
Macro_IFI27	GPCPD1	1,74514E-83	-8,27827E-01	0,085	0,29	3,21403E-79
Macro_IFI27	ANXA5	5,56877E-11	-8,28228E-01	0,584	0,782	1,02560E-06
Macro_IFI27	MAP1LC3B	2,52978E-117	-8,28651E-01	0,247	0,573	4,65910E-113
Macro_IFI27	CNPY3	3,69769E-52	-8,28991E-01	0,364	0,64	6,81004E-48
Macro_IFI27	IMP4	8,47256E-40	-8,29535E-01	0,124	0,273	1,56039E-35
Macro_IFI27	NAGK	3,75724E-39	-8,29717E-01	0,274	0,487	6,91970E-35
Macro_IFI27	GCA	2,82951E-81	-8,31319E-01	0,169	0,413	5,21110E-77
Macro_IFI27	RAB8B	4,81874E-80	-8,32530E-01	0,07	0,259	8,87468E-76
Macro_IFI27	PPP1R2	1,88321E-91	-8,32640E-01	0,153	0,405	3,46831E-87

Macro_IFI27	TMEM109	1,04708E-46	-8,34027E-01	0,189	0,378	1,92842E-42
Macro_IFI27	COX4I1	1,07044E-08	-8,34053E-01	0,699	0,906	1,97143E-04
Macro_IFI27	PNISR	4,98032E-82	-8,35070E-01	0,236	0,506	9,17226E-78
Macro_IFI27	LAT2	3,30609E-58	-8,35203E-01	0,237	0,465	6,08883E-54
Macro_IFI27	PTMA	3,36861E-59	-8,35502E-01	0,851	0,967	6,20396E-55
Macro_IFI27	COPE	7,56216E-73	-8,36053E-01	0,327	0,62	1,39272E-68
Macro_IFI27	CELF1	1,02039E-84	-8,36220E-01	0,087	0,295	1,87925E-80
Macro_IFI27	BAZ1A	1,20811E-141	-8,38074E-01	0,128	0,433	2,22498E-137
Macro_IFI27	APRT	1,09361E-68	-8,38115E-01	0,342	0,637	2,01410E-64
Macro_IFI27	RPS7	1,49791E-11	-8,38233E-01	0,468	0,565	2,75871E-07
Macro_IFI27	ARHGEF1	2,80502E-76	-8,41527E-01	0,088	0,28	5,16601E-72
Macro_IFI27	PSMB7	3,92799E-62	-8,41646E-01	0,161	0,373	7,23418E-58
Macro_IFI27	OAS1	1,06380E-28	-8,42506E-01	0,189	0,334	1,95920E-24
Macro_IFI27	NOTCH2	1,06442E-80	-8,44038E-01	0,116	0,327	1,96034E-76
Macro_IFI27	TGFBI	5,39397E-40	-8,44237E-01	0,379	0,609	9,93407E-36
Macro_IFI27	HMGN2	9,00253E-18	-8,44635E-01	0,505	0,745	1,65800E-13
Macro_IFI27	WAC	1,47165E-85	-8,45737E-01	0,089	0,3	2,71033E-81
Macro_IFI27	CD58	1,23770E-55	-8,47632E-01	0,138	0,322	2,27946E-51
Macro_IFI27	SRSF1	6,65926E-65	-8,48350E-01	0,095	0,277	1,22644E-60
Macro_IFI27	NAAA	3,84592E-64	-8,48619E-01	0,167	0,377	7,08302E-60
Macro_IFI27	CD46	9,67425E-52	-8,48681E-01	0,143	0,325	1,78171E-47
Macro_IFI27	GABARAPL1	2,79400E-81	-8,48715E-01	0,099	0,301	5,14572E-77
Macro_IFI27	CLEC12A	3,75207E-24	-8,48719E-01	0,153	0,265	6,91018E-20
Macro_IFI27	POLR2G	9,44081E-58	-8,48909E-01	0,173	0,382	1,73871E-53
Macro_IFI27	NSMCE1	5,69793E-64	-8,49772E-01	0,1	0,279	1,04939E-59
Macro_IFI27	VPS28	7,55653E-48	-8,50456E-01	0,353	0,625	1,39169E-43
Macro_IFI27	ACTN4	4,14539E-109	-8,50465E-01	0,104	0,36	7,63457E-105
Macro_IFI27	LILRA2	4,87199E-54	-8,50809E-01	0,097	0,255	8,97275E-50
Macro_IFI27	RPS25	8,47838E-07	-8,50824E-01	0,444	0,552	1,56146E-02
Macro_IFI27	FAU	6,44709E-69	-8,50863E-01	0,805	0,964	1,18736E-64
Macro_IFI27	SHKBP1	2,69555E-86	-8,51185E-01	0,14	0,381	4,96439E-82
Macro_IFI27	CCT3	2,21348E-56	-8,52248E-01	0,199	0,421	4,07656E-52
Macro_IFI27	TAF7	7,39119E-81	-8,52539E-01	0,103	0,311	1,36124E-76
Macro_IFI27	DGUOK	2,09752E-77	-8,55428E-01	0,15	0,382	3,86301E-73
Macro_IFI27	AAMP	1,36074E-52	-8,55807E-01	0,125	0,301	2,50607E-48
Macro_IFI27	GIT2	5,16871E-51	-8,56268E-01	0,129	0,302	9,51921E-47
Macro_IFI27	CFL1	6,84716E-11	-8,56313E-01	0,81	0,927	1,26104E-06
Macro_IFI27	XBP1	1,49402E-129	-8,56732E-01	0,141	0,439	2,75154E-125
Macro_IFI27	NDUFV2	4,94157E-52	-8,57015E-01	0,3	0,545	9,10088E-48
Macro_IFI27	P4HB	5,23969E-50	-8,58091E-01	0,375	0,641	9,64993E-46
Macro_IFI27	FNBP4	8,58567E-73	-8,58133E-01	0,111	0,309	1,58122E-68
Macro_IFI27	IDH3B	1,07164E-40	-8,58171E-01	0,121	0,268	1,97363E-36
Macro_IFI27	CCDC50	4,04840E-54	-8,58319E-01	0,115	0,282	7,45594E-50

Macro_IFI27	CHD2	3,56209E-82	-8,58610E-01	0,098	0,308	6,56030E-78
Macro_IFI27	AHNAK	2,43856E-119	-8,58757E-01	0,302	0,618	4,49109E-115
Macro_IFI27	RPS12	8,93021E-07	-8,59543E-01	0,555	0,584	1,64468E-02
Macro_IFI27	SLA	6,07692E-43	-8,59664E-01	0,236	0,426	1,11919E-38
Macro_IFI27	CTNND1	1,51894E-43	-8,59863E-01	0,126	0,279	2,79742E-39
Macro_IFI27	APEX1	2,64173E-45	-8,60130E-01	0,243	0,451	4,86528E-41
Macro_IFI27	LMAN2	1,67844E-55	-8,61161E-01	0,27	0,523	3,09119E-51
Macro_IFI27	PTGER4	5,53736E-72	-8,61359E-01	0,089	0,278	1,01982E-67
Macro_IFI27	ATP1A1	2,68452E-113	-8,61900E-01	0,157	0,443	4,94407E-109
Macro_IFI27	PAK1	4,49548E-87	-8,62277E-01	0,193	0,449	8,27933E-83
Macro_IFI27	LRRC25	3,92229E-47	-8,64054E-01	0,165	0,341	7,22368E-43
Macro_IFI27	TES	6,71569E-90	-8,65835E-01	0,078	0,29	1,23683E-85
Macro_IFI27	HPS1	7,45734E-70	-8,65898E-01	0,144	0,362	1,37342E-65
Macro_IFI27	GSTK1	1,06439E-42	-8,66618E-01	0,395	0,656	1,96029E-38
Macro_IFI27	RNPS1	1,19985E-84	-8,66685E-01	0,166	0,416	2,20977E-80
Macro_IFI27	CD163	1,15178E-13	-8,66760E-01	0,328	0,453	2,12122E-09
Macro_IFI27	SH3TC1	3,46334E-53	-8,66876E-01	0,143	0,321	6,37844E-49
Macro_IFI27	MNDA	2,47532E-52	-8,67415E-01	0,312	0,544	4,55880E-48
Macro_IFI27	ARGLU1	7,42064E-125	-8,68781E-01	0,152	0,447	1,36666E-120
Macro_IFI27	PSMB1	8,19496E-47	-8,69168E-01	0,372	0,642	1,50927E-42
Macro_IFI27	EIF2S3	7,57352E-44	-8,70158E-01	0,248	0,461	1,39481E-39
Macro_IFI27	SERINC3	1,77545E-71	-8,70193E-01	0,137	0,35	3,26985E-67
Macro_IFI27	PSMF1	3,31924E-45	-8,71547E-01	0,221	0,431	6,11304E-41
Macro_IFI27	BZW1	9,71849E-114	-8,71919E-01	0,165	0,456	1,78985E-109
Macro_IFI27	RABGAP1L	7,20240E-49	-8,72207E-01	0,105	0,26	1,32647E-44
Macro_IFI27	XRCC6	1,25599E-62	-8,72241E-01	0,251	0,507	2,31316E-58
Macro_IFI27	STRAP	7,34241E-81	-8,72884E-01	0,135	0,366	1,35225E-76
Macro_IFI27	DDX17	6,94449E-118	-8,73673E-01	0,226	0,546	1,27897E-113
Macro_IFI27	GUK1	4,34970E-48	-8,74803E-01	0,44	0,723	8,01084E-44
Macro_IFI27	EMP3	1,35166E-110	-8,75888E-01	0,41	0,744	2,48934E-106
Macro_IFI27	SPINT2	3,83080E-54	-8,76442E-01	0,274	0,509	7,05519E-50
Macro_IFI27	SND1	2,70101E-68	-8,76501E-01	0,143	0,355	4,97445E-64
Macro_IFI27	PHYKPL	1,63179E-53	-8,76933E-01	0,103	0,266	3,00527E-49
Macro_IFI27	BACH1	4,76748E-89	-8,77130E-01	0,103	0,323	8,78026E-85
Macro_IFI27	EIF4G1	2,57412E-80	-8,77857E-01	0,123	0,343	4,74075E-76
Macro_IFI27	SNRPE	1,39405E-66	-8,78284E-01	0,142	0,352	2,56742E-62
Macro_IFI27	RPL35A	4,20356E-23	-8,79602E-01	0,448	0,568	7,74170E-19
Macro_IFI27	PGAM1	5,91795E-88	-8,82357E-01	0,276	0,579	1,08991E-83
Macro_IFI27	BCAS2	9,93103E-89	-8,84101E-01	0,068	0,273	1,82900E-84
Macro_IFI27	OAZ2	4,10694E-50	-8,85987E-01	0,122	0,291	7,56374E-46
Macro_IFI27	MOB1A	1,10836E-93	-8,87239E-01	0,282	0,598	2,04126E-89
Macro_IFI27	BAZ2B	2,04604E-60	-8,87432E-01	0,097	0,269	3,76818E-56
Macro_IFI27	RAN	1,20663E-46	-8,87516E-01	0,398	0,67	2,22225E-42

Macro_IFI27	LRRFIP1	1,80355E-179	-8,88349E-01	0,302	0,702	3,32161E-175
Macro_IFI27	ARL8B	6,89171E-112	-8,89363E-01	0,141	0,416	1,26925E-107
Macro_IFI27	SEC31A	1,98985E-67	-8,89556E-01	0,114	0,308	3,66471E-63
Macro_IFI27	NUP214	1,36386E-47	-8,90644E-01	0,153	0,322	2,51181E-43
Macro_IFI27	RPL18	7,60661E-13	-8,90743E-01	0,471	0,567	1,40091E-08
Macro_IFI27	CSF3R	1,44537E-55	-8,92199E-01	0,2	0,403	2,66194E-51
Macro_IFI27	ARPC1B	5,42274E-12	-8,94222E-01	0,594	0,806	9,98706E-08
Macro_IFI27	PAPOLA	2,42745E-97	-8,94271E-01	0,188	0,468	4,47064E-93
Macro_IFI27	APP	7,04032E-77	-8,96291E-01	0,113	0,318	1,29662E-72
Macro_IFI27	UBE2L6	2,77505E-16	-8,97393E-01	0,388	0,579	5,11081E-12
Macro_IFI27	NDUFV1	2,86709E-44	-8,97702E-01	0,214	0,417	5,28032E-40
Macro_IFI27	TAF9	6,24434E-54	-8,97710E-01	0,132	0,31	1,15002E-49
Macro_IFI27	MBOAT7	6,10563E-104	-8,97840E-01	0,047	0,259	1,12447E-99
Macro_IFI27	TAOK3	5,79276E-94	-8,97926E-01	0,191	0,464	1,06685E-89
Macro_IFI27	SKIL	2,10608E-94	-8,98642E-01	0,11	0,342	3,87877E-90
Macro_IFI27	CDC42SE2	1,01322E-72	-8,98773E-01	0,091	0,283	1,86605E-68
Macro_IFI27	TRABD	4,31458E-101	-8,98908E-01	0,119	0,369	7,94616E-97
Macro_IFI27	EIF6	4,93000E-72	-8,99126E-01	0,18	0,416	9,07958E-68
Macro_IFI27	EIF3E	8,32236E-63	-8,99191E-01	0,339	0,618	1,53273E-58
Macro_IFI27	SPCS1	4,61406E-63	-8,99985E-01	0,341	0,62	8,49771E-59
Macro_IFI27	DOK2	7,45019E-43	-9,00764E-01	0,179	0,353	1,37210E-38
Macro_IFI27	UBE2F	2,38555E-41	-9,00919E-01	0,117	0,266	4,39347E-37
Macro_IFI27	RNH1	6,73895E-49	-9,01204E-01	0,359	0,63	1,24111E-44
Macro_IFI27	SQSTM1	9,94296E-80	-9,02389E-01	0,386	0,688	1,83120E-75
Macro_IFI27	DOCK10	1,13834E-66	-9,02830E-01	0,131	0,332	2,09649E-62
Macro_IFI27	XAF1	4,00439E-69	-9,03393E-01	0,133	0,337	7,37489E-65
Macro_IFI27	ARID4B	5,54595E-107	-9,03420E-01	0,125	0,385	1,02140E-102
Macro_IFI27	TSPAN3	3,59844E-35	-9,05348E-01	0,166	0,326	6,62724E-31
Macro_IFI27	FYTTD1	2,18179E-66	-9,07059E-01	0,081	0,257	4,01820E-62
Macro_IFI27	CAT	3,20217E-54	-9,07421E-01	0,203	0,419	5,89744E-50
Macro_IFI27	BUD31	3,00474E-69	-9,09156E-01	0,151	0,37	5,53383E-65
Macro_IFI27	CTNNB1	9,25543E-93	-9,09494E-01	0,142	0,391	1,70457E-88
Macro_IFI27	DOCK8	1,21904E-104	-9,11154E-01	0,179	0,461	2,24511E-100
Macro_IFI27	SFT2D2	1,05243E-81	-9,11232E-01	0,095	0,303	1,93826E-77
Macro_IFI27	HCST	3,59481E-83	-9,11434E-01	0,37	0,667	6,62057E-79
Macro_IFI27	ITSN2	2,22283E-87	-9,12250E-01	0,155	0,398	4,09378E-83
Macro_IFI27	EMC4	2,36260E-48	-9,12466E-01	0,176	0,37	4,35120E-44
Macro_IFI27	NAP1L1	9,25426E-87	-9,13934E-01	0,332	0,633	1,70436E-82
Macro_IFI27	PSMD4	1,85751E-57	-9,14182E-01	0,2	0,422	3,42098E-53
Macro_IFI27	NUMB	1,38699E-68	-9,15041E-01	0,137	0,343	2,55441E-64
Macro_IFI27	TAF1D	9,57237E-70	-9,15352E-01	0,172	0,395	1,76294E-65
Macro_IFI27	RPL24	2,53608E-30	-9,15856E-01	0,377	0,546	4,67071E-26
Macro_IFI27	PAIP2	1,21776E-66	-9,16328E-01	0,26	0,524	2,24275E-62

Macro_IFI27	GSDMD	1,29183E-56	-9,16392E-01	0,126	0,309	2,37917E-52
Macro_IFI27	CHD9	1,20399E-48	-9,18061E-01	0,168	0,352	2,21738E-44
Macro_IFI27	EIF4H	8,67493E-68	-9,18265E-01	0,216	0,464	1,59766E-63
Macro_IFI27	IFNGR1	2,68362E-37	-9,18322E-01	0,354	0,592	4,94242E-33
Macro_IFI27	CEP170	1,50704E-71	-9,19250E-01	0,142	0,354	2,77551E-67
Macro_IFI27	HSPA9	2,29707E-89	-9,19798E-01	0,138	0,382	4,23052E-85
Macro_IFI27	GSTO1	3,41157E-46	-9,20236E-01	0,375	0,631	6,28310E-42
Macro_IFI27	MT2A	9,31565E-36	-9,23154E-01	0,444	0,637	1,71566E-31
Macro_IFI27	SET	3,65790E-72	-9,23921E-01	0,266	0,54	6,73675E-68
Macro_IFI27	PSMB10	1,07660E-39	-9,24930E-01	0,342	0,578	1,98278E-35
Macro_IFI27	ETF1	6,53431E-94	-9,25125E-01	0,099	0,33	1,20342E-89
Macro_IFI27	YME1L1	1,38564E-122	-9,25391E-01	0,116	0,391	2,55193E-118
Macro_IFI27	ARAP1	1,99874E-61	-9,25397E-01	0,116	0,3	3,68108E-57
Macro_IFI27	DDOST	4,82579E-63	-9,26137E-01	0,233	0,474	8,88765E-59
Macro_IFI27	KLF2	2,52080E-99	-9,26692E-01	0,073	0,287	4,64256E-95
Macro_IFI27	PRPF8	1,82062E-65	-9,27102E-01	0,123	0,316	3,35304E-61
Macro_IFI27	SNAP23	2,03413E-55	-9,27256E-01	0,193	0,409	3,74625E-51
Macro_IFI27	SDHA	2,87837E-49	-9,28751E-01	0,102	0,258	5,30110E-45
Macro_IFI27	CKLF	3,33018E-83	-9,29530E-01	0,254	0,518	6,13319E-79
Macro_IFI27	AOAH	5,34913E-34	-9,29907E-01	0,216	0,382	9,85149E-30
Macro_IFI27	CD4	4,93459E-37	-9,30259E-01	0,328	0,549	9,08804E-33
Macro_IFI27	HNRNPM	2,18745E-107	-9,31428E-01	0,178	0,471	4,02862E-103
Macro_IFI27	JUND	4,31060E-210	-9,31552E-01	0,101	0,472	7,93883E-206
Macro_IFI27	LILRB1	1,15420E-85	-9,31849E-01	0,15	0,387	2,12570E-81
Macro_IFI27	NASP	2,10780E-57	-9,31918E-01	0,11	0,283	3,88194E-53
Macro_IFI27	CCDC12	1,33316E-59	-9,34170E-01	0,116	0,295	2,45528E-55
Macro_IFI27	RPL34	3,08865E-17	-9,34190E-01	0,496	0,579	5,68837E-13
Macro_IFI27	MT-ND1	2,28614E-08	-9,34314E-01	0,464	0,574	4,21038E-04
Macro_IFI27	UTRN	1,51870E-62	-9,34750E-01	0,115	0,297	2,79700E-58
Macro_IFI27	ARID5A	6,38085E-87	-9,34879E-01	0,086	0,297	1,17516E-82
Macro_IFI27	CYC1	4,77921E-64	-9,35422E-01	0,207	0,445	8,80186E-60
Macro_IFI27	TCF25	8,81039E-89	-9,35959E-01	0,272	0,576	1,62261E-84
Macro_IFI27	TM9SF2	6,18414E-75	-9,36186E-01	0,193	0,439	1,13893E-70
Macro_IFI27	MYO1G	3,84502E-106	-9,37806E-01	0,068	0,294	7,08136E-102
Macro_IFI27	HNRNPA2B1	7,08172E-94	-9,38867E-01	0,531	0,846	1,30424E-89
Macro_IFI27	CRIP1	6,56795E-62	-9,39867E-01	0,119	0,301	1,20962E-57
Macro_IFI27	PRKAR1A	1,10522E-94	-9,39935E-01	0,209	0,493	2,03549E-90
Macro_IFI27	AGTRAP	1,33443E-63	-9,40536E-01	0,167	0,37	2,45762E-59
Macro_IFI27	SERPINB1	4,59626E-119	-9,40537E-01	0,344	0,678	8,46493E-115
Macro_IFI27	BTF3	2,30831E-31	-9,41390E-01	0,604	0,858	4,25121E-27
Macro_IFI27	PPIB	1,02639E-22	-9,42497E-01	0,573	0,803	1,89029E-18
Macro_IFI27	PLSCR1	1,59937E-87	-9,43129E-01	0,267	0,555	2,94557E-83
Macro_IFI27	PRRC2C	3,42280E-148	-9,43947E-01	0,245	0,61	6,30378E-144

Macro_IFI27	UBA52	3,02666E-26	-9,44611E-01	0,757	0,928	5,57420E-22
Macro_IFI27	CYTH1	1,07163E-86	-9,45355E-01	0,113	0,338	1,97361E-82
Macro_IFI27	MYO1F	1,47548E-94	-9,45630E-01	0,199	0,472	2,71740E-90
Macro_IFI27	ARHGAP4	1,21772E-65	-9,45716E-01	0,175	0,395	2,24268E-61
Macro_IFI27	GTF2A2	1,28019E-74	-9,47447E-01	0,176	0,418	2,35773E-70
Macro_IFI27	SERPINA1	6,66729E-14	-9,49947E-01	0,572	0,728	1,22791E-09
Macro_IFI27	ARL6IP1	4,02821E-86	-9,51243E-01	0,173	0,426	7,41876E-82
Macro_IFI27	CASP1	2,26528E-58	-9,53461E-01	0,243	0,474	4,17196E-54
Macro_IFI27	GABARAPL2	7,16169E-93	-9,53814E-01	0,287	0,592	1,31897E-88
Macro_IFI27	TNFRSF14	6,43813E-58	-9,54208E-01	0,227	0,46	1,18571E-53
Macro_IFI27	SSR4	6,74954E-53	-9,54629E-01	0,472	0,767	1,24306E-48
Macro_IFI27	VSIG4	4,28180E-08	-9,55048E-01	0,27	0,352	7,88578E-04
Macro_IFI27	SNRPB	3,84226E-120	-9,55487E-01	0,206	0,523	7,07628E-116
Macro_IFI27	TAPBP	3,98459E-97	-9,56016E-01	0,276	0,594	7,33842E-93
Macro_IFI27	MARCKSL1	5,40291E-94	-9,56361E-01	0,066	0,275	9,95054E-90
Macro_IFI27	GPBP1	2,53432E-104	-9,57449E-01	0,109	0,355	4,66745E-100
Macro_IFI27	OLR1	4,81775E-41	-9,58020E-01	0,198	0,355	8,87286E-37
Macro_IFI27	C1orf162	1,66666E-35	-9,60138E-01	0,491	0,718	3,06948E-31
Macro_IFI27	ERCC1	1,89017E-70	-9,60637E-01	0,167	0,392	3,48112E-66
Macro_IFI27	RPL27	5,92485E-13	-9,61972E-01	0,375	0,511	1,09118E-08
Macro_IFI27	CCT5	1,90607E-67	-9,62189E-01	0,166	0,392	3,51041E-63
Macro_IFI27	ELOVL5	4,84214E-79	-9,62617E-01	0,096	0,297	8,91776E-75
Macro_IFI27	CARD16	4,32376E-83	-9,63085E-01	0,287	0,564	7,96307E-79
Macro_IFI27	ZNF706	5,34792E-65	-9,63880E-01	0,327	0,604	9,84926E-61
Macro_IFI27	TXN	1,02700E-33	-9,64346E-01	0,448	0,678	1,89143E-29
Macro_IFI27	CYTH4	2,81805E-75	-9,64859E-01	0,116	0,324	5,19000E-71
Macro_IFI27	SSR2	2,15959E-55	-9,66788E-01	0,371	0,666	3,97732E-51
Macro_IFI27	C11orf58	4,21656E-70	-9,68378E-01	0,287	0,573	7,76564E-66
Macro_IFI27	NABP1	1,32532E-105	-9,70091E-01	0,105	0,351	2,44083E-101
Macro_IFI27	NINJ1	5,20465E-37	-9,70196E-01	0,359	0,569	9,58541E-33
Macro_IFI27	PARVG	3,58614E-60	-9,71307E-01	0,228	0,461	6,60459E-56
Macro_IFI27	IQGAP1	3,36827E-159	-9,71508E-01	0,219	0,578	6,20335E-155
Macro_IFI27	AP1S2	1,45148E-95	-9,72124E-01	0,295	0,592	2,67319E-91
Macro_IFI27	RIPK2	4,14754E-91	-9,73027E-01	0,073	0,283	7,63852E-87
Macro_IFI27	IFITM1	1,19086E-43	-9,74094E-01	0,114	0,261	2,19320E-39
Macro_IFI27	S100A6	5,05720E-113	-9,75872E-01	0,655	0,885	9,31385E-109
Macro_IFI27	ZFAND6	1,91108E-71	-9,76776E-01	0,116	0,321	3,51964E-67
Macro_IFI27	RPLP2	3,46311E-12	-9,76792E-01	0,482	0,575	6,37801E-08
Macro_IFI27	PNP	2,00307E-101	-9,79859E-01	0,055	0,269	3,68906E-97
Macro_IFI27	NFKB1	2,97290E-127	-9,80072E-01	0,053	0,299	5,47519E-123
Macro_IFI27	SMG1	1,31599E-87	-9,80219E-01	0,108	0,328	2,42366E-83
Macro_IFI27	CD37	4,00544E-74	-9,80747E-01	0,442	0,747	7,37682E-70
Macro_IFI27	LENG8	6,98154E-91	-9,81747E-01	0,081	0,294	1,28579E-86

Macro_IFI27	SRSF11	2,20914E-102	-9,83080E-01	0,229	0,529	4,06857E-98
Macro_IFI27	UBE2D3	2,93805E-76	-9,83125E-01	0,437	0,751	5,41101E-72
Macro_IFI27	ENY2	3,12293E-94	-9,83457E-01	0,295	0,61	5,75149E-90
Macro_IFI27	EIF3G	2,81872E-54	-9,83629E-01	0,349	0,627	5,19124E-50
Macro_IFI27	IFNAR2	5,30698E-65	-9,84440E-01	0,095	0,276	9,77386E-61
Macro_IFI27	SEC61A1	3,43505E-70	-9,86680E-01	0,118	0,32	6,32633E-66
Macro_IFI27	MCTP1	3,75127E-64	-9,89171E-01	0,084	0,256	6,90871E-60
Macro_IFI27	FBL	4,06137E-78	-9,90656E-01	0,134	0,358	7,47982E-74
Macro_IFI27	VPS29	2,56564E-62	-9,91222E-01	0,279	0,547	4,72514E-58
Macro_IFI27	PSMA5	7,64173E-70	-9,91723E-01	0,2	0,446	1,40738E-65
Macro_IFI27	GTF2B	1,47261E-74	-9,92336E-01	0,093	0,288	2,71211E-70
Macro_IFI27	AUP1	3,52657E-77	-9,94389E-01	0,259	0,538	6,49488E-73
Macro_IFI27	BNIP2	9,68806E-80	-9,94594E-01	0,169	0,412	1,78425E-75
Macro_IFI27	B2M	1,00431E-40	-9,96582E-01	0,994	0,995	1,84964E-36
Macro_IFI27	EIF5A	7,44405E-54	-9,96946E-01	0,32	0,568	1,37097E-49
Macro_IFI27	EWSR1	6,75880E-88	-9,97540E-01	0,205	0,475	1,24477E-83
Macro_IFI27	RPS23	1,49668E-08	-9,98083E-01	0,511	0,571	2,75643E-04
Macro_IFI27	DYNLT1	3,18471E-74	-9,98247E-01	0,249	0,522	5,86528E-70
Macro_IFI27	PER1	6,81700E-96	-9,99941E-01	0,103	0,33	1,25549E-91
Macro_IFI27	RANBP2	3,04039E-98	-1,00118E+00	0,093	0,322	5,59948E-94
Macro_IFI27	PTPN2	3,82673E-81	-1,00118E+00	0,156	0,397	7,04769E-77
Macro_IFI27	BCLAF1	5,07415E-96	-1,00153E+00	0,141	0,393	9,34506E-92
Macro_IFI27	CD33	1,34744E-40	-1,00168E+00	0,154	0,315	2,48159E-36
Macro_IFI27	SNRPD2	1,88513E-77	-1,00211E+00	0,377	0,692	3,47185E-73
Macro_IFI27	RPN1	1,85379E-78	-1,00263E+00	0,097	0,304	3,41413E-74
Macro_IFI27	CHD1	2,16561E-111	-1,00439E+00	0,071	0,308	3,98841E-107
Macro_IFI27	EIF3F	1,92701E-46	-1,00505E+00	0,405	0,679	3,54897E-42
Macro_IFI27	STAT3	1,27835E-149	-1,00627E+00	0,183	0,521	2,35434E-145
Macro_IFI27	GBP2	1,07920E-60	-1,00639E+00	0,179	0,382	1,98757E-56
Macro_IFI27	INPP5D	1,84526E-43	-1,00699E+00	0,153	0,319	3,39842E-39
Macro_IFI27	MRPS6	2,06665E-74	-1,00700E+00	0,125	0,335	3,80615E-70
Macro_IFI27	CLEC7A	6,68416E-61	-1,00812E+00	0,287	0,536	1,23102E-56
Macro_IFI27	STK10	6,74310E-76	-1,00900E+00	0,07	0,252	1,24188E-71
Macro_IFI27	AKAP9	2,92743E-80	-1,01047E+00	0,162	0,397	5,39146E-76
Macro_IFI27	CD164	3,16272E-88	-1,01205E+00	0,275	0,571	5,82477E-84
Macro_IFI27	ECH1	9,48332E-71	-1,01341E+00	0,103	0,297	1,74654E-66
Macro_IFI27	PIM3	7,36726E-128	-1,01529E+00	0,05	0,297	1,35683E-123
Macro_IFI27	PFDN5	1,50732E-21	-1,01616E+00	0,695	0,912	2,77604E-17
Macro_IFI27	ARRDC1	1,52566E-63	-1,01726E+00	0,115	0,303	2,80981E-59
Macro_IFI27	S100A10	1,02380E-43	-1,01817E+00	0,658	0,846	1,88554E-39
Macro_IFI27	GNAS	1,88462E-88	-1,01908E+00	0,308	0,624	3,47090E-84
Macro_IFI27	LSP1	1,55650E-82	-1,02070E+00	0,417	0,683	2,86660E-78
Macro_IFI27	PSMB9	6,64426E-65	-1,02113E+00	0,396	0,67	1,22367E-60

Macro_IFI27	ANKRD44	1,82238E-76	-1,02196E+00	0,1	0,301	3,35627E-72
Macro_IFI27	POLR2A	1,01209E-93	-1,02396E+00	0,096	0,319	1,86397E-89
Macro_IFI27	NACA	2,19715E-11	-1,02407E+00	0,755	0,927	4,04650E-07
Macro_IFI27	WTAP	1,81049E-112	-1,02501E+00	0,116	0,377	3,33438E-108
Macro_IFI27	CCT8	2,94495E-64	-1,02515E+00	0,194	0,423	5,42371E-60
Macro_IFI27	TNFAIP2	1,35183E-64	-1,02659E+00	0,2	0,416	2,48966E-60
Macro_IFI27	PSMA6	2,24770E-66	-1,02701E+00	0,229	0,477	4,13959E-62
Macro_IFI27	DNAJC4	1,51297E-53	-1,02718E+00	0,175	0,371	2,78643E-49
Macro_IFI27	ALOX5AP	8,18706E-28	-1,02738E+00	0,35	0,512	1,50781E-23
Macro_IFI27	VDAC3	1,76162E-57	-1,02755E+00	0,133	0,321	3,24438E-53
Macro_IFI27	KMT2E	5,92940E-119	-1,02782E+00	0,194	0,499	1,09202E-114
Macro_IFI27	UBAC2	1,57061E-64	-1,02809E+00	0,125	0,318	2,89260E-60
Macro_IFI27	MT-ND2	1,11780E-32	-1,02839E+00	0,395	0,57	2,05864E-28
Macro_IFI27	SLC3A2	1,07063E-97	-1,02852E+00	0,199	0,476	1,97179E-93
Macro_IFI27	TBCB	7,80286E-75	-1,02874E+00	0,22	0,48	1,43705E-70
Macro_IFI27	CDK2AP2	5,74620E-84	-1,02926E+00	0,166	0,409	1,05828E-79
Macro_IFI27	RPS24	3,92838E-09	-1,03376E+00	0,528	0,58	7,23490E-05
Macro_IFI27	PSMA1	1,06298E-74	-1,03483E+00	0,148	0,371	1,95770E-70
Macro_IFI27	PLK3	5,85323E-89	-1,03572E+00	0,063	0,261	1,07799E-84
Macro_IFI27	TNFSF10	4,97717E-77	-1,03653E+00	0,151	0,37	9,16645E-73
Macro_IFI27	HSP90AB1	1,02135E-47	-1,03655E+00	0,601	0,837	1,88101E-43
Macro_IFI27	RPS20	1,71270E-33	-1,03841E+00	0,272	0,46	3,15428E-29
Macro_IFI27	LIMD2	6,66613E-128	-1,03973E+00	0,197	0,496	1,22770E-123
Macro_IFI27	SH3BGRL3	3,26121E-53	-1,04130E+00	0,79	0,94	6,00617E-49
Macro_IFI27	RBM8A	8,05243E-119	-1,04193E+00	0,224	0,549	1,48302E-114
Macro_IFI27	TGIF1	1,08730E-70	-1,04301E+00	0,131	0,333	2,00248E-66
Macro_IFI27	PSME2	9,57534E-09	-1,04708E+00	0,543	0,724	1,76349E-04
Macro_IFI27	EEF1B2	7,83770E-26	-1,04720E+00	0,63	0,852	1,44347E-21
Macro_IFI27	ATG3	1,37461E-77	-1,04810E+00	0,257	0,536	2,53162E-73
Macro_IFI27	EIF3L	5,70958E-49	-1,04975E+00	0,355	0,612	1,05153E-44
Macro_IFI27	MLF2	1,45051E-77	-1,05147E+00	0,203	0,464	2,67141E-73
Macro_IFI27	MT-ND5	1,43296E-19	-1,05521E+00	0,38	0,545	2,63909E-15
Macro_IFI27	HNRNPU	2,81519E-162	-1,05566E+00	0,266	0,641	5,18474E-158
Macro_IFI27	GPR65	3,77245E-52	-1,05695E+00	0,152	0,335	6,94772E-48
Macro_IFI27	MYL12B	1,83404E-53	-1,05748E+00	0,503	0,79	3,37776E-49
Macro_IFI27	SYNGR2	1,90646E-10	-1,06127E+00	0,534	0,737	3,51112E-06
Macro_IFI27	MYL6	2,30045E-43	-1,06142E+00	0,801	0,948	4,23674E-39
Macro_IFI27	NDUFA13	2,12183E-62	-1,06288E+00	0,216	0,442	3,90778E-58
Macro_IFI27	TCP1	6,71013E-79	-1,06332E+00	0,126	0,347	1,23581E-74
Macro_IFI27	SERTAD1	1,29345E-121	-1,06368E+00	0,083	0,337	2,38214E-117
Macro_IFI27	MT-CO2	1,21414E-07	-1,06464E+00	0,567	0,598	2,23607E-03
Macro_IFI27	RBPJ	2,05173E-79	-1,06498E+00	0,219	0,479	3,77867E-75
Macro_IFI27	SFPQ	3,23786E-177	-1,06519E+00	0,166	0,524	5,96317E-173

Macro_IFI27	JUN	7,53313E-126	-1,06593E+00	0,417	0,724	1,38738E-121
Macro_IFI27	NFE2L2	1,67710E-126	-1,06694E+00	0,214	0,534	3,08872E-122
Macro_IFI27	RASSF5	2,50577E-112	-1,06790E+00	0,054	0,282	4,61488E-108
Macro_IFI27	AHSA1	1,08724E-56	-1,06877E+00	0,125	0,307	2,00237E-52
Macro_IFI27	KLF10	3,09686E-71	-1,06879E+00	0,109	0,299	5,70348E-67
Macro_IFI27	ATP2B1	4,62750E-92	-1,06920E+00	0,128	0,359	8,52248E-88
Macro_IFI27	NCOR1	6,19643E-84	-1,06961E+00	0,164	0,41	1,14120E-79
Macro_IFI27	STX11	1,12952E-124	-1,07389E+00	0,079	0,34	2,08024E-120
Macro_IFI27	CLIC1	4,52084E-48	-1,07434E+00	0,654	0,885	8,32603E-44
Macro_IFI27	RPL29	5,31895E-12	-1,07539E+00	0,471	0,562	9,79591E-08
Macro_IFI27	LPXN	2,50439E-77	-1,07663E+00	0,137	0,358	4,61234E-73
Macro_IFI27	CNBP	1,02412E-35	-1,07702E+00	0,458	0,729	1,88613E-31
Macro_IFI27	UBL7	1,13809E-38	-1,07800E+00	0,118	0,26	2,09602E-34
Macro_IFI27	GNA15	6,63455E-118	-1,08133E+00	0,071	0,318	1,22188E-113
Macro_IFI27	DNAJC7	2,42620E-73	-1,08277E+00	0,151	0,376	4,46833E-69
Macro_IFI27	PDIA3	2,30315E-69	-1,08298E+00	0,401	0,689	4,24171E-65
Macro_IFI27	CD300A	1,07229E-68	-1,08324E+00	0,228	0,471	1,97484E-64
Macro_IFI27	TPP1	1,49606E-51	-1,08423E+00	0,342	0,607	2,75529E-47
Macro_IFI27	GAPT	1,64130E-46	-1,08782E+00	0,115	0,261	3,02279E-42
Macro_IFI27	RNF144B	1,30253E-97	-1,08850E+00	0,129	0,369	2,39887E-93
Macro_IFI27	S100A4	1,53310E-101	-1,08877E+00	0,602	0,837	2,82350E-97
Macro_IFI27	FLOT1	1,21614E-107	-1,09165E+00	0,234	0,539	2,23977E-103
Macro_IFI27	RPL11	2,92081E-08	-1,09213E+00	0,539	0,583	5,37926E-04
Macro_IFI27	PDCD4	3,05538E-46	-1,09427E+00	0,114	0,266	5,62709E-42
Macro_IFI27	DBNL	2,67205E-71	-1,09660E+00	0,242	0,504	4,92112E-67
Macro_IFI27	UBE2B	3,65671E-93	-1,09881E+00	0,218	0,502	6,73457E-89
Macro_IFI27	RILPL2	1,80376E-144	-1,10124E+00	0,154	0,47	3,32199E-140
Macro_IFI27	SRRM2	4,52665E-148	-1,10158E+00	0,231	0,585	8,33674E-144
Macro_IFI27	RNF10	1,07204E-69	-1,10164E+00	0,094	0,283	1,97438E-65
Macro_IFI27	ANKRD12	3,82002E-127	-1,10230E+00	0,176	0,484	7,03534E-123
Macro_IFI27	N4BP2L2	2,90174E-77	-1,10267E+00	0,215	0,471	5,34414E-73
Macro_IFI27	DOCK2	2,28794E-73	-1,10337E+00	0,142	0,358	4,21371E-69
Macro_IFI27	DDX3X	3,56363E-192	-1,10347E+00	0,129	0,494	6,56314E-188
Macro_IFI27	PPA1	2,11164E-72	-1,10382E+00	0,232	0,472	3,88900E-68
Macro_IFI27	ARF1	2,52716E-42	-1,10458E+00	0,435	0,712	4,65427E-38
Macro_IFI27	CSNK1A1	4,46277E-124	-1,10476E+00	0,169	0,477	8,21909E-120
Macro_IFI27	CD48	1,16456E-94	-1,10781E+00	0,216	0,492	2,14477E-90
Macro_IFI27	TUBB4B	5,84444E-119	-1,10781E+00	0,118	0,392	1,07637E-114
Macro_IFI27	CDK5RAP3	1,33091E-50	-1,10934E+00	0,141	0,315	2,45113E-46
Macro_IFI27	FGD2	9,47268E-44	-1,11193E+00	0,179	0,353	1,74458E-39
Macro_IFI27	EEF1D	6,60272E-58	-1,11361E+00	0,583	0,885	1,21602E-53
Macro_IFI27	IFITM3	2,09401E-15	-1,11446E+00	0,616	0,772	3,85654E-11
Macro_IFI27	IRF1	4,20217E-118	-1,11447E+00	0,169	0,452	7,73913E-114

Macro_IFI27	MORF4L2	9,80259E-66	-1,11808E+00	0,098	0,283	1,80534E-61
Macro_IFI27	HLA-F	5,26138E-69	-1,11855E+00	0,26	0,528	9,68988E-65
Macro_IFI27	STAT2	2,94885E-79	-1,12008E+00	0,099	0,304	5,43090E-75
Macro_IFI27	CASP4	1,83685E-62	-1,12111E+00	0,219	0,454	3,38293E-58
Macro_IFI27	PFKFB3	6,40457E-123	-1,12234E+00	0,089	0,346	1,17953E-118
Macro_IFI27	RNASEK	1,25673E-54	-1,12403E+00	0,251	0,476	2,31453E-50
Macro_IFI27	LILRB2	2,26327E-105	-1,12437E+00	0,198	0,47	4,16827E-101
Macro_IFI27	RPL27A	1,52278E-30	-1,12455E+00	0,295	0,472	2,80450E-26
Macro_IFI27	PSMB3	9,24263E-65	-1,12491E+00	0,361	0,657	1,70221E-60
Macro_IFI27	FERMT3	2,10096E-74	-1,12545E+00	0,23	0,495	3,86935E-70
Macro_IFI27	HSP90B1	1,54240E-68	-1,12689E+00	0,427	0,713	2,84064E-64
Macro_IFI27	CORO1C	3,84755E-56	-1,12734E+00	0,156	0,353	7,08604E-52
Macro_IFI27	ACTR3	5,39372E-104	-1,12828E+00	0,344	0,679	9,93362E-100
Macro_IFI27	USP15	2,07426E-103	-1,12905E+00	0,164	0,437	3,82017E-99
Macro_IFI27	SP110	1,21650E-78	-1,13012E+00	0,171	0,401	2,24042E-74
Macro_IFI27	SLC25A3	2,70591E-62	-1,13533E+00	0,435	0,727	4,98347E-58
Macro_IFI27	MX1	5,26948E-55	-1,13592E+00	0,155	0,341	9,70480E-51
Macro_IFI27	FDFT1	1,07620E-60	-1,13641E+00	0,096	0,268	1,98203E-56
Macro_IFI27	RPL7A	3,18654E-11	-1,13646E+00	0,468	0,563	5,86865E-07
Macro_IFI27	RHOC	7,80963E-61	-1,14860E+00	0,13	0,318	1,43830E-56
Macro_IFI27	GPI	8,77118E-48	-1,14906E+00	0,216	0,426	1,61539E-43
Macro_IFI27	ARPC4	1,57686E-82	-1,14945E+00	0,176	0,421	2,90411E-78
Macro_IFI27	CD53	3,01769E-50	-1,15578E+00	0,416	0,691	5,55768E-46
Macro_IFI27	DDX39A	8,36146E-69	-1,15888E+00	0,106	0,3	1,53993E-64
Macro_IFI27	HCK	2,27059E-99	-1,16188E+00	0,24	0,534	4,18175E-95
Macro_IFI27	MVP	5,68837E-85	-1,16367E+00	0,145	0,385	1,04763E-80
Macro_IFI27	PARK7	1,02481E-52	-1,16410E+00	0,399	0,671	1,88740E-48
Macro_IFI27	METRNL	3,44036E-96	-1,16456E+00	0,057	0,264	6,33611E-92
Macro_IFI27	CALM1	2,16421E-49	-1,16752E+00	0,537	0,807	3,98583E-45
Macro_IFI27	C5AR1	8,38791E-89	-1,16755E+00	0,186	0,418	1,54480E-84
Macro_IFI27	EVI2B	2,94359E-63	-1,16775E+00	0,176	0,386	5,42122E-59
Macro_IFI27	RPS14	8,22622E-08	-1,17031E+00	0,525	0,578	1,51502E-03
Macro_IFI27	PRELID1	6,44548E-77	-1,17098E+00	0,401	0,698	1,18706E-72
Macro_IFI27	STIP1	7,03139E-76	-1,17485E+00	0,093	0,291	1,29497E-71
Macro_IFI27	HSPD1	4,55712E-113	-1,17843E+00	0,24	0,549	8,39284E-109
Macro_IFI27	ZFAND5	8,21529E-138	-1,17905E+00	0,265	0,601	1,51301E-133
Macro_IFI27	STAT6	5,66991E-92	-1,17955E+00	0,094	0,315	1,04423E-87
Macro_IFI27	RPL21	1,57063E-14	-1,18328E+00	0,427	0,547	2,89263E-10
Macro_IFI27	TUBA1A	2,97353E-114	-1,18447E+00	0,188	0,475	5,47635E-110
Macro_IFI27	TANK	2,04350E-86	-1,18542E+00	0,091	0,303	3,76352E-82
Macro_IFI27	RPN2	8,18411E-64	-1,18973E+00	0,105	0,293	1,50727E-59
Macro_IFI27	PPP1CB	1,62133E-112	-1,19224E+00	0,136	0,41	2,98599E-108
Macro_IFI27	SF3B1	8,16501E-96	-1,19258E+00	0,215	0,507	1,50375E-91

Macro_IFI27	GABARAP	5,49282E-08	-1,19695E+00	0,677	0,837	1,01161E-03
Macro_IFI27	TMEM167A	1,57207E-105	-1,19821E+00	0,141	0,413	2,89528E-101
Macro_IFI27	BTG1	3,03113E-232	-1,20085E+00	0,422	0,829	5,58243E-228
Macro_IFI27	TAGAP	6,69882E-101	-1,20125E+00	0,065	0,282	1,23372E-96
Macro_IFI27	RPS13	2,63239E-07	-1,20128E+00	0,515	0,574	4,84807E-03
Macro_IFI27	PSMA3	1,37053E-56	-1,20175E+00	0,175	0,381	2,52410E-52
Macro_IFI27	ILF3	2,37306E-85	-1,20225E+00	0,141	0,377	4,37046E-81
Macro_IFI27	LST1	2,31628E-42	-1,20350E+00	0,495	0,725	4,26589E-38
Macro_IFI27	SAP30BP	7,09025E-61	-1,20719E+00	0,102	0,279	1,30581E-56
Macro_IFI27	REL	6,01136E-211	-1,21063E+00	0,225	0,616	1,10711E-206
Macro_IFI27	RPL9	1,79941E-08	-1,21368E+00	0,443	0,548	3,31398E-04
Macro_IFI27	CD47	1,35610E-90	-1,21481E+00	0,173	0,433	2,49753E-86
Macro_IFI27	BRD2	3,78272E-160	-1,21678E+00	0,165	0,503	6,96664E-156
Macro_IFI27	CCND3	3,26504E-60	-1,22035E+00	0,135	0,321	6,01322E-56
Macro_IFI27	RPS8	2,01521E-08	-1,22117E+00	0,531	0,58	3,71141E-04
Macro_IFI27	COTL1	9,46811E-50	-1,22416E+00	0,488	0,723	1,74374E-45
Macro_IFI27	MYL12A	1,31906E-45	-1,22479E+00	0,581	0,836	2,42931E-41
Macro_IFI27	IL2RG	1,11578E-58	-1,22714E+00	0,142	0,333	2,05494E-54
Macro_IFI27	CSRNP1	2,04282E-166	-1,22743E+00	0,024	0,304	3,76226E-162
Macro_IFI27	HNRNPA1	7,87943E-43	-1,23074E+00	0,549	0,814	1,45116E-38
Macro_IFI27	PTPRC	9,38242E-147	-1,23213E+00	0,344	0,707	1,72796E-142
Macro_IFI27	CDC37	1,35963E-110	-1,23654E+00	0,319	0,653	2,50404E-106
Macro_IFI27	RNF149	4,90579E-114	-1,23664E+00	0,308	0,642	9,03499E-110
Macro_IFI27	PSMA4	1,87374E-69	-1,23807E+00	0,227	0,48	3,45087E-65
Macro_IFI27	ETS2	8,41494E-130	-1,23882E+00	0,151	0,439	1,54978E-125
Macro_IFI27	FUS	1,11316E-159	-1,23893E+00	0,236	0,604	2,05011E-155
Macro_IFI27	ADPGK	1,02001E-80	-1,24132E+00	0,169	0,412	1,87855E-76
Macro_IFI27	HNRNPC	1,04476E-92	-1,24832E+00	0,337	0,662	1,92413E-88
Macro_IFI27	MAPK1IP1L	2,55593E-96	-1,24865E+00	0,114	0,355	4,70725E-92
Macro_IFI27	RBMX	2,24767E-87	-1,25482E+00	0,178	0,437	4,13954E-83
Macro_IFI27	ENO1	6,84212E-49	-1,25622E+00	0,495	0,774	1,26011E-44
Macro_IFI27	PSMB8	9,65491E-74	-1,25838E+00	0,312	0,589	1,77815E-69
Macro_IFI27	NONO	7,78170E-79	-1,25931E+00	0,191	0,442	1,43316E-74
Macro_IFI27	MAP2K3	7,92736E-98	-1,26197E+00	0,066	0,28	1,45998E-93
Macro_IFI27	RPL18A	8,25610E-10	-1,26231E+00	0,492	0,566	1,52053E-05
Macro_IFI27	MGAT1	1,27894E-71	-1,26417E+00	0,341	0,638	2,35543E-67
Macro_IFI27	PHACTR1	2,09857E-128	-1,26426E+00	0,063	0,313	3,86493E-124
Macro_IFI27	IL10RA	8,33800E-103	-1,26930E+00	0,196	0,477	1,53561E-98
Macro_IFI27	TSC22D3	8,16999E-69	-1,26994E+00	0,416	0,708	1,50467E-64
Macro_IFI27	PTP4A2	2,64730E-113	-1,27136E+00	0,141	0,417	4,87553E-109
Macro_IFI27	IST1	1,06868E-52	-1,27257E+00	0,107	0,271	1,96819E-48
Macro_IFI27	SUB1	4,37344E-115	-1,27308E+00	0,396	0,739	8,05456E-111
Macro_IFI27	RPL5	4,83462E-23	-1,27493E+00	0,374	0,524	8,90393E-19

Macro_IFI27	ARHGAP9	7,78926E-57	-1,27587E+00	0,09	0,253	1,43455E-52
Macro_IFI27	HNRNPK	1,43716E-92	-1,27705E+00	0,388	0,725	2,64681E-88
Macro_IFI27	RBM3	2,26065E-84	-1,27843E+00	0,38	0,689	4,16344E-80
Macro_IFI27	EIF3M	2,82898E-76	-1,28004E+00	0,244	0,512	5,21013E-72
Macro_IFI27	LUC7L3	4,82471E-65	-1,28067E+00	0,159	0,369	8,88566E-61
Macro_IFI27	FPR1	8,45611E-93	-1,28234E+00	0,206	0,46	1,55736E-88
Macro_IFI27	ANXA1	4,08798E-103	-1,28301E+00	0,405	0,702	7,52883E-99
Macro_IFI27	TPM3	4,02076E-79	-1,28323E+00	0,505	0,818	7,40503E-75
Macro_IFI27	RPL26	6,04966E-21	-1,28485E+00	0,413	0,549	1,11417E-16
Macro_IFI27	GK	5,23659E-133	-1,28553E+00	0,101	0,377	9,64423E-129
Macro_IFI27	B4GALT1	7,55526E-102	-1,28711E+00	0,099	0,336	1,39145E-97
Macro_IFI27	DAZAP2	3,78120E-54	-1,28827E+00	0,45	0,756	6,96384E-50
Macro_IFI27	U2AF1	5,23803E-41	-1,28897E+00	0,135	0,276	9,64689E-37
Macro_IFI27	RPL23	2,99644E-56	-1,29022E+00	0,239	0,463	5,51854E-52
Macro_IFI27	TRIM22	6,07628E-87	-1,29528E+00	0,177	0,428	1,11907E-82
Macro_IFI27	ATF4	2,14812E-100	-1,29813E+00	0,153	0,418	3,95619E-96
Macro_IFI27	CSNK2B	1,95824E-76	-1,29961E+00	0,132	0,347	3,60648E-72
Macro_IFI27	LMNA	1,41370E-108	-1,30984E+00	0,125	0,38	2,60361E-104
Macro_IFI27	CFLAR	4,10937E-161	-1,31531E+00	0,187	0,527	7,56822E-157
Macro_IFI27	RPSA	1,18346E-09	-1,31628E+00	0,373	0,501	2,17957E-05
Macro_IFI27	MX2	2,45271E-87	-1,31753E+00	0,078	0,279	4,51715E-83
Macro_IFI27	CYTIP	1,35635E-120	-1,31837E+00	0,134	0,403	2,49798E-116
Macro_IFI27	PPIF	7,00465E-120	-1,32184E+00	0,106	0,37	1,29005E-115
Macro_IFI27	IGFLR1	1,13468E-63	-1,32554E+00	0,16	0,369	2,08975E-59
Macro_IFI27	SLC25A37	2,77530E-93	-1,32647E+00	0,089	0,301	5,11128E-89
Macro_IFI27	EIF1	3,79990E-118	-1,32700E+00	0,735	0,954	6,99828E-114
Macro_IFI27	RPL31	1,23367E-30	-1,32727E+00	0,319	0,505	2,27206E-26
Macro_IFI27	ZC3HAV1	8,61307E-101	-1,33072E+00	0,064	0,281	1,58627E-96
Macro_IFI27	MBNL1	2,58937E-136	-1,33373E+00	0,208	0,54	4,76884E-132
Macro_IFI27	UPP1	7,29558E-122	-1,33403E+00	0,158	0,444	1,34363E-117
Macro_IFI27	HSPA8	1,21247E-28	-1,33596E+00	0,634	0,829	2,23300E-24
Macro_IFI27	STXBP2	5,65537E-164	-1,33716E+00	0,115	0,434	1,04155E-159
Macro_IFI27	CCNH	1,56105E-98	-1,34629E+00	0,138	0,382	2,87499E-94
Macro_IFI27	SRSF3	1,51380E-155	-1,34857E+00	0,225	0,587	2,78796E-151
Macro_IFI27	IVNS1ABP	5,04173E-125	-1,35062E+00	0,107	0,379	9,28535E-121
Macro_IFI27	RPS11	5,85483E-30	-1,35135E+00	0,33	0,512	1,07828E-25
Macro_IFI27	TPI1	4,45999E-51	-1,35319E+00	0,573	0,838	8,21397E-47
Macro_IFI27	SAMSN1	1,50133E-160	-1,35681E+00	0,153	0,479	2,76499E-156
Macro_IFI27	ACAA1	5,84936E-89	-1,35796E+00	0,143	0,389	1,07728E-84
Macro_IFI27	RAB24	2,91013E-84	-1,35930E+00	0,081	0,28	5,35959E-80
Macro_IFI27	FOSL2	2,10835E-144	-1,35976E+00	0,045	0,307	3,88294E-140
Macro_IFI27	HSPA6	7,93572E-53	-1,36028E+00	0,131	0,294	1,46152E-48
Macro_IFI27	SLC38A2	3,81601E-133	-1,36166E+00	0,06	0,32	7,02794E-129

Macro_IFI27	TAP1	1,59019E-124	-1,36174E+00	0,146	0,436	2,92866E-120
Macro_IFI27	IFI44L	5,83083E-43	-1,36208E+00	0,116	0,26	1,07386E-38
Macro_IFI27	RPL6	1,97076E-14	-1,36387E+00	0,443	0,559	3,62955E-10
Macro_IFI27	FLII	2,69753E-62	-1,36494E+00	0,137	0,333	4,96805E-58
Macro_IFI27	AKAP13	1,09026E-165	-1,36661E+00	0,281	0,65	2,00794E-161
Macro_IFI27	ZNF207	1,83869E-99	-1,36734E+00	0,153	0,417	3,38631E-95
Macro_IFI27	SRSF7	7,35682E-128	-1,36976E+00	0,241	0,567	1,35491E-123
Macro_IFI27	UBB	4,54457E-53	-1,37363E+00	0,549	0,823	8,36973E-49
Macro_IFI27	TRA2B	8,53602E-173	-1,37383E+00	0,139	0,487	1,57208E-168
Macro_IFI27	ATP1B3	2,69679E-143	-1,38107E+00	0,229	0,56	4,96668E-139
Macro_IFI27	EIF4A1	5,72055E-61	-1,38903E+00	0,428	0,686	1,05355E-56
Macro_IFI27	HSPA5	3,72720E-144	-1,39276E+00	0,24	0,577	6,86438E-140
Macro_IFI27	BID	2,50448E-119	-1,39368E+00	0,143	0,427	4,61250E-115
Macro_IFI27	ITGAX	4,35109E-100	-1,39774E+00	0,156	0,411	8,01340E-96
Macro_IFI27	HNRNPDL	8,35838E-118	-1,39848E+00	0,344	0,691	1,53936E-113
Macro_IFI27	CNOT2	3,38022E-73	-1,39969E+00	0,089	0,28	6,22536E-69
Macro_IFI27	ISG20	8,34557E-100	-1,40531E+00	0,091	0,322	1,53700E-95
Macro_IFI27	RPL8	4,72894E-14	-1,40813E+00	0,497	0,576	8,70928E-10
Macro_IFI27	NR3C1	5,87947E-98	-1,40872E+00	0,146	0,399	1,08282E-93
Macro_IFI27	CAPZA1	1,75251E-106	-1,40960E+00	0,207	0,508	3,22760E-102
Macro_IFI27	CNN2	5,88783E-124	-1,41274E+00	0,133	0,415	1,08436E-119
Macro_IFI27	HSPE1	7,68242E-104	-1,41435E+00	0,305	0,617	1,41487E-99
Macro_IFI27	CKS2	1,75770E-94	-1,41569E+00	0,052	0,252	3,23715E-90
Macro_IFI27	RPS5	1,21575E-17	-1,41572E+00	0,385	0,524	2,23906E-13
Macro_IFI27	IFI30	1,94370E-40	-1,42075E+00	0,356	0,579	3,57972E-36
Macro_IFI27	PTPN6	7,44684E-109	-1,42583E+00	0,225	0,524	1,37148E-104
Macro_IFI27	PSME1	9,40421E-38	-1,42912E+00	0,507	0,766	1,73197E-33
Macro_IFI27	PLD4	6,59892E-09	-1,43984E+00	0,254	0,336	1,21532E-04
Macro_IFI27	PTPRE	1,56196E-179	-1,43985E+00	0,116	0,455	2,87665E-175
Macro_IFI27	C15orf48	4,40969E-48	-1,44019E+00	0,186	0,352	8,12133E-44
Macro_IFI27	TNFRSF1B	4,83527E-172	-1,44182E+00	0,208	0,556	8,90513E-168
Macro_IFI27	WSB1	1,92760E-191	-1,44199E+00	0,257	0,659	3,55007E-187
Macro_IFI27	HSP90AA1	5,01012E-124	-1,44232E+00	0,632	0,891	9,22715E-120
Macro_IFI27	ZEB2	8,56847E-178	-1,44370E+00	0,243	0,623	1,57806E-173
Macro_IFI27	CD44	6,73011E-141	-1,44489E+00	0,348	0,692	1,23948E-136
Macro_IFI27	RPL13A	8,91906E-14	-1,44799E+00	0,393	0,527	1,64262E-09
Macro_IFI27	SEC61B	1,51235E-112	-1,45459E+00	0,419	0,755	2,78530E-108
Macro_IFI27	LGALS2	2,71250E-64	-1,45796E+00	0,094	0,265	4,99562E-60
Macro_IFI27	SRSF2	1,60002E-134	-1,46467E+00	0,241	0,573	2,94675E-130
Macro_IFI27	EIF4A2	5,80305E-84	-1,46571E+00	0,282	0,575	1,06875E-79
Macro_IFI27	PLEK	2,46908E-139	-1,46701E+00	0,234	0,578	4,54731E-135
Macro_IFI27	THEMIS2	1,30393E-97	-1,47232E+00	0,223	0,508	2,40144E-93
Macro_IFI27	MAP3K8	4,45471E-162	-1,47297E+00	0,165	0,498	8,20424E-158

Macro_IFI27	KLF4	1,59969E-188	-1,48076E+00	0,12	0,463	2,94616E-184
Macro_IFI27	TRA2A	2,11376E-80	-1,48119E+00	0,073	0,264	3,89292E-76
Macro_IFI27	FLNA	3,32382E-157	-1,48687E+00	0,146	0,466	6,12147E-153
Macro_IFI27	LDHA	9,70442E-78	-1,48992E+00	0,433	0,728	1,78726E-73
Macro_IFI27	RPL10A	6,83929E-17	-1,48994E+00	0,363	0,503	1,25959E-12
Macro_IFI27	PABPC1	1,27568E-65	-1,49040E+00	0,666	0,904	2,34943E-61
Macro_IFI27	KDM6B	5,10876E-190	-1,49146E+00	0,039	0,355	9,40880E-186
Macro_IFI27	RPS16	4,23202E-13	-1,50328E+00	0,451	0,549	7,79411E-09
Macro_IFI27	SGK1	1,37040E-96	-1,50668E+00	0,342	0,617	2,52387E-92
Macro_IFI27	RBM6	6,61008E-62	-1,50820E+00	0,098	0,271	1,21738E-57
Macro_IFI27	UCP2	3,82583E-23	-1,51588E+00	0,424	0,644	7,04603E-19
Macro_IFI27	RPS3A	8,84767E-08	-1,51842E+00	0,499	0,57	1,62947E-03
Macro_IFI27	SON	7,71617E-179	-1,51977E+00	0,293	0,693	1,42109E-174
Macro_IFI27	RPL23A	1,81098E-08	-1,51990E+00	0,425	0,53	3,33528E-04
Macro_IFI27	SOCS3	5,04107E-192	-1,52209E+00	0,047	0,372	9,28414E-188
Macro_IFI27	MCL1	0,00000E+00	-1,52778E+00	0,323	0,791	0,00000E+00
Macro_IFI27	MYD88	2,35677E-120	-1,52987E+00	0,102	0,37	4,34046E-116
Macro_IFI27	JMJD1C	1,23907E-131	-1,53755E+00	0,144	0,441	2,28200E-127
Macro_IFI27	PSMB4	2,47706E-64	-1,53845E+00	0,167	0,383	4,56201E-60
Macro_IFI27	EIF5	3,10545E-108	-1,54189E+00	0,286	0,607	5,71931E-104
Macro_IFI27	SLC11A1	5,14942E-98	-1,54312E+00	0,227	0,479	9,48368E-94
Macro_IFI27	RPL36A	4,73270E-62	-1,54947E+00	0,238	0,464	8,71621E-58
Macro_IFI27	ALDOA	1,20769E-15	-1,55244E+00	0,579	0,768	2,22421E-11
Macro_IFI27	C6orf62	3,03269E-90	-1,55255E+00	0,136	0,375	5,58531E-86
Macro_IFI27	SOD2	2,54096E-90	-1,56325E+00	0,386	0,664	4,67969E-86
Macro_IFI27	MPEG1	2,10924E-100	-1,57344E+00	0,137	0,385	3,88459E-96
Macro_IFI27	TCF4	1,12665E-53	-1,58269E+00	0,118	0,281	2,07495E-49
Macro_IFI27	DUSP6	1,30819E-90	-1,59474E+00	0,088	0,298	2,40929E-86
Macro_IFI27	YWHAZ	4,91294E-143	-1,60536E+00	0,361	0,729	9,04816E-139
Macro_IFI27	HIF1A	2,88384E-160	-1,61806E+00	0,214	0,565	5,31117E-156
Macro_IFI27	CHMP1B	6,87407E-171	-1,62695E+00	0,103	0,421	1,26600E-166
Macro_IFI27	FGR	1,61257E-101	-1,62977E+00	0,198	0,466	2,96988E-97
Macro_IFI27	GADD45B	7,75518E-225	-1,63071E+00	0,265	0,661	1,42827E-220
Macro_IFI27	PDE4B	2,83452E-144	-1,63398E+00	0,094	0,38	5,22033E-140
Macro_IFI27	UBC	1,34491E-21	-1,63403E+00	0,726	0,907	2,47692E-17
Macro_IFI27	NLRP3	9,02711E-112	-1,63672E+00	0,052	0,273	1,66252E-107
Macro_IFI27	CIITA	1,68229E-69	-1,64321E+00	0,131	0,334	3,09828E-65
Macro_IFI27	YPEL5	1,29779E-122	-1,64529E+00	0,159	0,447	2,39015E-118
Macro_IFI27	HSPH1	2,00121E-164	-1,64757E+00	0,111	0,43	3,68563E-160
Macro_IFI27	PGK1	8,76160E-58	-1,64886E+00	0,43	0,72	1,61362E-53
Macro_IFI27	RPL4	3,27742E-21	-1,65154E+00	0,318	0,485	6,03602E-17
Macro_IFI27	PLP2	9,44170E-134	-1,65262E+00	0,131	0,417	1,73888E-129
Macro_IFI27	CDKN1A	2,70848E-218	-1,65937E+00	0,155	0,543	4,98821E-214

Macro_IFI27	ACADVL	9,00344E-84	-1,65955E+00	0,212	0,481	1,65816E-79
Macro_IFI27	TCIRG1	2,06177E-109	-1,66863E+00	0,189	0,481	3,79717E-105
Macro_IFI27	TAGLN2	5,69041E-85	-1,68033E+00	0,468	0,768	1,04800E-80
Macro_IFI27	IFRD1	1,02152E-103	-1,71049E+00	0,076	0,302	1,88133E-99
Macro_IFI27	ANKRD10	2,03160E-56	-1,71755E+00	0,15	0,341	3,74160E-52
Macro_IFI27	RPL17	3,30650E-51	-1,72136E+00	0,235	0,443	6,08958E-47
Macro_IFI27	MXD1	2,69931E-142	-1,73612E+00	0,05	0,314	4,97131E-138
Macro_IFI27	HERPUD1	4,07592E-57	-1,74835E+00	0,421	0,694	7,50663E-53
Macro_IFI27	HBEGF	1,24479E-121	-1,75580E+00	0,053	0,286	2,29253E-117
Macro_IFI27	ICAM1	1,28392E-161	-1,75839E+00	0,073	0,376	2,36459E-157
Macro_IFI27	MAT2A	8,82096E-129	-1,76027E+00	0,16	0,46	1,62456E-124
Macro_IFI27	SELPLG	6,26733E-61	-1,76193E+00	0,157	0,353	1,15425E-56
Macro_IFI27	BHLHE40	1,94031E-168	-1,76267E+00	0,056	0,354	3,57346E-164
Macro_IFI27	CCNL1	4,23465E-222	-1,76856E+00	0,148	0,544	7,79896E-218
Macro_IFI27	MYADM	1,18110E-157	-1,77180E+00	0,058	0,347	2,17522E-153
Macro_IFI27	RAC2	8,97914E-153	-1,78700E+00	0,185	0,511	1,65369E-148
Macro_IFI27	STK17B	1,05442E-203	-1,78979E+00	0,105	0,461	1,94193E-199
Macro_IFI27	LCP1	5,75097E-198	-1,80253E+00	0,332	0,722	1,05916E-193
Macro_IFI27	CLK1	7,59487E-154	-1,81452E+00	0,094	0,396	1,39875E-149
Macro_IFI27	RPL7	2,58566E-27	-1,82083E+00	0,322	0,506	4,76201E-23
Macro_IFI27	SRGN	6,32912E-218	-1,82898E+00	0,733	0,953	1,16563E-213
Macro_IFI27	INSIG1	1,35195E-116	-1,83367E+00	0,068	0,306	2,48989E-112
Macro_IFI27	CORO1A	9,51563E-128	-1,84055E+00	0,337	0,661	1,75249E-123
Macro_IFI27	RASGEF1B	2,37620E-120	-1,84215E+00	0,045	0,28	4,37625E-116
Macro_IFI27	GLA	1,63268E-89	-1,84884E+00	0,101	0,321	3,00691E-85
Macro_IFI27	VMP1	2,14064E-130	-1,86902E+00	0,24	0,566	3,94241E-126
Macro_IFI27	EZR	1,13995E-141	-1,87778E+00	0,116	0,413	2,09944E-137
Macro_IFI27	CD52	3,74241E-131	-1,88036E+00	0,125	0,403	6,89240E-127
Macro_IFI27	ISG15	6,46346E-60	-1,88747E+00	0,22	0,438	1,19038E-55
Macro_IFI27	ID2	9,78776E-154	-1,90031E+00	0,244	0,572	1,80261E-149
Macro_IFI27	EIF4A3	1,60431E-122	-1,91413E+00	0,135	0,415	2,95465E-118
Macro_IFI27	RPS10	1,34796E-51	-1,92391E+00	0,123	0,291	2,48253E-47
Macro_IFI27	KLF6	3,12928E-283	-1,93979E+00	0,385	0,817	5,76320E-279
Macro_IFI27	DDX5	2,98329E-172	-1,94223E+00	0,469	0,85	5,49432E-168
Macro_IFI27	DNAJA1	7,04142E-189	-1,95453E+00	0,238	0,622	1,29682E-184
Macro_IFI27	SAT1	2,05398E-56	-1,96358E+00	0,876	0,951	3,78282E-52
Macro_IFI27	NR4A3	1,10082E-177	-1,99669E+00	0,02	0,31	2,02738E-173
Macro_IFI27	ZFP36L1	3,79921E-120	-2,00046E+00	0,429	0,74	6,99700E-116
Macro_IFI27	HNRNPH1	2,27564E-128	-2,00968E+00	0,13	0,418	4,19104E-124
Macro_IFI27	SRSF5	2,10887E-173	-2,03819E+00	0,222	0,596	3,88391E-169
Macro_IFI27	CCL4	2,81813E-69	-2,06718E+00	0,135	0,318	5,19014E-65
Macro_IFI27	RGS1	3,50220E-32	-2,06784E+00	0,483	0,65	6,45001E-28
Macro_IFI27	IER3	2,75052E-221	-2,08608E+00	0,175	0,55	5,06563E-217

Macro_IFI27	CREM	1,60777E-131	-2,09333E+00	0,11	0,391	2,96103E-127
Macro_IFI27	BTG2	3,18919E-223	-2,19017E+00	0,134	0,516	5,87354E-219
Macro_IFI27	EGR1	2,99459E-145	-2,19140E+00	0,037	0,298	5,51513E-141
Macro_IFI27	HSPA1A	6,09826E-150	-2,19853E+00	0,368	0,678	1,12312E-145
Macro_IFI27	RBM39	2,62124E-201	-2,19968E+00	0,238	0,641	4,82753E-197
Macro_IFI27	ATF3	4,67579E-223	-2,21027E+00	0,083	0,45	8,61140E-219
Macro_IFI27	SERPINB9	1,16495E-161	-2,21371E+00	0,087	0,389	2,14549E-157
Macro_IFI27	EEF1G	5,44147E-51	-2,21436E+00	0,14	0,303	1,00216E-46
Macro_IFI27	JUNB	0,00000E+00	-2,28623E+00	0,431	0,845	0,00000E+00
Macro_IFI27	FOSB	0,00000E+00	-2,34896E+00	0,126	0,602	0,00000E+00
Macro_IFI27	NFKBIZ	1,06823E-273	-2,35854E+00	0,063	0,473	1,96735E-269
Macro_IFI27	IER2	0,00000E+00	-2,37877E+00	0,194	0,653	0,00000E+00
Macro_IFI27	IRF7	3,81169E-134	-2,39145E+00	0,125	0,412	7,01999E-130
Macro_IFI27	CD55	3,94237E-191	-2,41991E+00	0,108	0,445	7,26066E-187
Macro_IFI27	NR4A2	1,57039E-281	-2,44578E+00	0,057	0,47	2,89219E-277
Macro_IFI27	PLIN2	8,55036E-82	-2,45016E+00	0,26	0,509	1,57472E-77
Macro_IFI27	FOS	1,23068E-253	-2,46826E+00	0,517	0,859	2,26654E-249
Macro_IFI27	DUSP1	4,18100E-229	-2,46907E+00	0,509	0,867	7,70015E-225
Macro_IFI27	TNFAIP3	1,01899E-260	-2,49427E+00	0,152	0,562	1,87667E-256
Macro_IFI27	ZNF331	6,28045E-119	-2,53109E+00	0,106	0,356	1,15667E-114
Macro_IFI27	BCL2A1	2,31475E-213	-2,57233E+00	0,115	0,487	4,26307E-209
Macro_IFI27	NFKBIA	2,70543E-277	-2,57766E+00	0,438	0,826	4,98260E-273
Macro_IFI27	ZFP36	0,00000E+00	-2,58780E+00	0,415	0,865	0,00000E+00
Macro_IFI27	CCL3	1,18081E-92	-2,59448E+00	0,164	0,386	2,17471E-88
Macro_IFI27	HSPA1B	5,31053E-136	-2,59810E+00	0,231	0,518	9,78040E-132
Macro_IFI27	NAMPT	3,89956E-283	-2,61144E+00	0,156	0,593	7,18182E-279
Macro_IFI27	IFITM2	1,47141E-154	-2,65287E+00	0,32	0,662	2,70989E-150
Macro_IFI27	S100A9	6,93537E-80	-2,67583E+00	0,25	0,466	1,27729E-75
Macro_IFI27	DNAJB1	2,13057E-172	-2,67658E+00	0,234	0,573	3,92387E-168
Macro_IFI27	DDIT4	4,41246E-139	-2,69215E+00	0,205	0,51	8,12642E-135
Macro_IFI27	PLAUR	6,61390E-262	-2,77682E+00	0,241	0,66	1,21808E-257
Macro_IFI27	CD83	3,36185E-290	-2,84678E+00	0,079	0,508	6,19152E-286
Macro_IFI27	NR4A1	1,66104E-281	-2,84951E+00	0,032	0,438	3,05913E-277
Macro_IFI27	DUSP2	1,07492E-167	-2,88590E+00	0,029	0,314	1,97969E-163
Macro_IFI27	SLC2A3	3,63349E-258	-3,00440E+00	0,06	0,452	6,69180E-254
Macro_IFI27	IRF8	2,54174E-85	-3,06485E+00	0,156	0,389	4,68112E-81
Macro_IFI27	S100A8	7,64587E-99	-3,08447E+00	0,141	0,362	1,40814E-94
Macro_IFI27	CXCR4	1,11246E-121	-3,08532E+00	0,296	0,594	2,04882E-117
Macro_IFI27	PPP1R15A	0,00000E+00	-3,16829E+00	0,176	0,681	0,00000E+00
Macro_IFI27	AREG	5,04317E-151	-3,25124E+00	0,028	0,287	9,28800E-147
Macro_IFI27	TIMP1	1,08083E-254	-3,37729E+00	0,26	0,669	1,99056E-250
Macro_IFI27	RGS2	9,58692E-262	-3,51593E+00	0,233	0,645	1,76562E-257
Macro_IFI27	FCN1	1,09320E-126	-3,68368E+00	0,034	0,261	2,01334E-122

Macro_IFI27	GPR183	9,05174E-164	-3,83090E+00	0,156	0,471	1,66706E-159
Macro_IFI27	IL1B	1,08660E-167	-3,89127E+00	0,059	0,349	2,00119E-163

cluster	gene	p_val	avg_log2FC	pct.1	pct.2	p_val_adj
Macro_ISG15	CCL2	0,00000E+00	4,77126E+00	0,409	0,1	0,00000E+00
Macro_ISG15	CTSL	0,00000E+00	3,80394E+00	0,781	0,503	0,00000E+00
Macro_ISG15	SPP1	7,50051E-181	3,73355E+00	0,428	0,214	1,38137E-176
Macro_ISG15	ISG15	0,00000E+00	3,64848E+00	0,963	0,395	0,00000E+00
Macro_ISG15	IFIT1	0,00000E+00	3,53239E+00	0,686	0,076	0,00000E+00
Macro_ISG15	CXCL10	0,00000E+00	3,52093E+00	0,42	0,136	0,00000E+00
Macro_ISG15	APOBEC3A	0,00000E+00	3,49779E+00	0,51	0,114	0,00000E+00
Macro_ISG15	IFI6	0,00000E+00	3,48509E+00	0,938	0,461	0,00000E+00
Macro_ISG15	IFIT3	0,00000E+00	3,37550E+00	0,799	0,175	0,00000E+00
Macro_ISG15	RSAD2	0,00000E+00	3,28598E+00	0,616	0,082	0,00000E+00
Macro_ISG15	CD163	9,88243E-133	3,09843E+00	0,673	0,433	1,82005E-128
Macro_ISG15	CCL8	0,00000E+00	2,77104E+00	0,264	0,029	0,00000E+00
Macro_ISG15	IFITM3	0,00000E+00	2,68333E+00	0,988	0,751	0,00000E+00
Macro_ISG15	OASL	0,00000E+00	2,66499E+00	0,598	0,081	0,00000E+00
Macro_ISG15	LY6E	0,00000E+00	2,63727E+00	0,942	0,523	0,00000E+00
Macro_ISG15	IFIT2	0,00000E+00	2,57873E+00	0,66	0,164	0,00000E+00
Macro_ISG15	IL1RN	0,00000E+00	2,53446E+00	0,509	0,201	0,00000E+00
Macro_ISG15	IFITM1	0,00000E+00	2,51975E+00	0,804	0,22	0,00000E+00
Macro_ISG15	CD14	4,13689E-130	2,51194E+00	0,825	0,611	7,61891E-126
Macro_ISG15	MX1	0,00000E+00	2,49675E+00	0,896	0,298	0,00000E+00
Macro_ISG15	PLIN2	1,11254E-31	2,48336E+00	0,576	0,493	2,04896E-27
Macro_ISG15	TGFBI	8,28640E-108	2,44710E+00	0,745	0,59	1,52611E-103
Macro_ISG15	VSIG4	9,11361E-44	2,35093E+00	0,462	0,341	1,67845E-39
Macro_ISG15	HSPA6	1,51100E-159	2,25511E+00	0,487	0,274	2,78281E-155
Macro_ISG15	RNASE1	6,22724E-84	2,24586E+00	0,435	0,262	1,14687E-79
Macro_ISG15	MX2	0,00000E+00	2,15468E+00	0,737	0,241	0,00000E+00
Macro_ISG15	VMO1	7,68192E-13	2,14109E+00	0,278	0,224	1,41478E-08
Macro_ISG15	OAS3	0,00000E+00	2,13916E+00	0,595	0,119	0,00000E+00
Macro_ISG15	SLC11A1	0,00000E+00	2,13307E+00	0,795	0,447	0,00000E+00
Macro_ISG15	CTSB	3,12104E-174	2,11323E+00	0,946	0,867	5,74802E-170
Macro_ISG15	TNFSF10	0,00000E+00	2,11239E+00	0,693	0,34	0,00000E+00
Macro_ISG15	TIMP1	5,93610E-112	2,09409E+00	0,819	0,64	1,09325E-107
Macro_ISG15	IL10	1,62423E-205	2,08350E+00	0,253	0,082	2,99134E-201
Macro_ISG15	IFI30	2,39472E-264	2,06292E+00	0,822	0,553	4,41035E-260
Macro_ISG15	IFI44	0,00000E+00	2,06220E+00	0,594	0,189	0,00000E+00
Macro_ISG15	FCGR2A	2,32314E-88	2,05865E+00	0,79	0,621	4,27854E-84
Macro_ISG15	OAS1	0,00000E+00	2,05422E+00	0,732	0,302	0,00000E+00
Macro_ISG15	CD300E	1,24251E-114	2,03221E+00	0,316	0,157	2,28833E-110
Macro_ISG15	ADM	1,85810E-99	2,01249E+00	0,279	0,135	3,42206E-95
Macro_ISG15	CCR1	9,83614E-255	2,00248E+00	0,474	0,212	1,81152E-250
Macro_ISG15	IFI44L	0,00000E+00	2,00150E+00	0,767	0,222	0,00000E+00
Macro_ISG15	VCAN	2,61879E-67	1,99605E+00	0,371	0,216	4,82303E-63

Macro_ISG15	GLUL	3,31532E-216	1,98913E+00	0,934	0,796	6,10583E-212
Macro_ISG15	SDS	0,00000E+00	1,96718E+00	0,503	0,202	0,00000E+00
Macro_ISG15	TREM1	1,21958E-87	1,95955E+00	0,396	0,228	2,24610E-83
Macro_ISG15	IFITM2	0,00000E+00	1,95370E+00	0,906	0,63	0,00000E+00
Macro_ISG15	HIF1A	1,96376E-110	1,94661E+00	0,702	0,54	3,61666E-106
Macro_ISG15	DDIT3	1,21378E-58	1,94384E+00	0,297	0,177	2,23543E-54
Macro_ISG15	FCGR3A	5,43473E-184	1,93845E+00	0,823	0,56	1,00091E-179
Macro_ISG15	FCGR1A	3,57051E-90	1,92593E+00	0,592	0,4	6,57580E-86
Macro_ISG15	GBP1	3,57734E-298	1,92415E+00	0,648	0,327	6,58838E-294
Macro_ISG15	HERC5	0,00000E+00	1,92207E+00	0,576	0,095	0,00000E+00
Macro_ISG15	LILRA6	7,26706E-108	1,91187E+00	0,306	0,151	1,33837E-103
Macro_ISG15	ZFP36L1	8,67534E-13	1,90859E+00	0,801	0,721	1,59774E-08
Macro_ISG15	PLSCR1	0,00000E+00	1,87883E+00	0,795	0,526	0,00000E+00
Macro_ISG15	P2RX4	1,00294E-64	1,87243E+00	0,361	0,225	1,84711E-60
Macro_ISG15	SIGLEC1	0,00000E+00	1,86260E+00	0,628	0,158	0,00000E+00
Macro_ISG15	XAF1	0,00000E+00	1,85307E+00	0,749	0,302	0,00000E+00
Macro_ISG15	DHRS3	4,06032E-72	1,83929E+00	0,268	0,145	7,47789E-68
Macro_ISG15	FCGR1B	1,75585E-34	1,83520E+00	0,296	0,199	3,23375E-30
Macro_ISG15	ALDOA	2,64944E-73	1,82559E+00	0,853	0,754	4,87948E-69
Macro_ISG15	CTSD	3,85932E-266	1,82269E+00	0,898	0,761	7,10772E-262
Macro_ISG15	LILRA5	6,28171E-101	1,81868E+00	0,337	0,176	1,15690E-96
Macro_ISG15	LGALS3BP	1,84948E-300	1,81429E+00	0,586	0,303	3,40618E-296
Macro_ISG15	FPR1	3,66493E-52	1,79647E+00	0,575	0,44	6,74970E-48
Macro_ISG15	EPSTI1	0,00000E+00	1,78989E+00	0,793	0,386	0,00000E+00
Macro_ISG15	IL4I1	2,79569E-285	1,78197E+00	0,499	0,218	5,14883E-281
Macro_ISG15	THBS1	1,05139E-107	1,78147E+00	0,339	0,169	1,93634E-103
Macro_ISG15	IFI27	0,00000E+00	1,77148E+00	0,417	0,157	0,00000E+00
Macro_ISG15	DDX60L	0,00000E+00	1,76621E+00	0,46	0,152	0,00000E+00
Macro_ISG15	FLOT1	2,83030E-86	1,74772E+00	0,672	0,515	5,21256E-82
Macro_ISG15	ENG	2,62302E-60	1,74556E+00	0,487	0,339	4,83082E-56
Macro_ISG15	MMP19	1,68679E-125	1,74196E+00	0,274	0,118	3,10657E-121
Macro_ISG15	OAS2	0,00000E+00	1,74041E+00	0,61	0,182	0,00000E+00
Macro_ISG15	HPSE	0,00000E+00	1,73579E+00	0,358	0,107	0,00000E+00
Macro_ISG15	MXD1	2,82842E-98	1,71165E+00	0,47	0,291	5,20910E-94
Macro_ISG15	IFI35	0,00000E+00	1,70225E+00	0,708	0,324	0,00000E+00
Macro_ISG15	PMP22	4,42301E-28	1,70184E+00	0,36	0,272	8,14585E-24
Macro_ISG15	APLP2	6,51535E-121	1,69982E+00	0,762	0,584	1,19993E-116
Macro_ISG15	MPP1	6,17835E-74	1,68999E+00	0,563	0,403	1,13787E-69
Macro_ISG15	STAB1	2,27592E-137	1,66026E+00	0,566	0,331	4,19155E-133
Macro_ISG15	LDHA	1,94927E-28	1,65545E+00	0,789	0,709	3,58996E-24
Macro_ISG15	IRF9	8,35113E-46	1,63841E+00	0,275	0,175	1,53803E-41
Macro_ISG15	C3AR1	5,19551E-42	1,63547E+00	0,555	0,407	9,56858E-38
Macro_ISG15	C15orf48	3,22004E-171	1,63296E+00	0,583	0,33	5,93034E-167

Macro_ISG15	HK3	1,33695E-108	1,62857E+00	0,265	0,12	2,46227E-104
Macro_ISG15	CLEC5A	2,78258E-27	1,61552E+00	0,268	0,186	5,12468E-23
Macro_ISG15	SHISA5	1,99871E-278	1,61314E+00	0,637	0,354	3,68102E-274
Macro_ISG15	USP18	0,00000E+00	1,60916E+00	0,37	0,051	0,00000E+00
Macro_ISG15	DDX58	0,00000E+00	1,60881E+00	0,369	0,082	0,00000E+00
Macro_ISG15	SLCO2B1	8,14440E-30	1,60365E+00	0,48	0,362	1,49995E-25
Macro_ISG15	FN1	1,04204E-50	1,59476E+00	0,333	0,214	1,91913E-46
Macro_ISG15	FPR3	5,75127E-271	1,59440E+00	0,605	0,322	1,05921E-266
Macro_ISG15	GBP5	5,79789E-174	1,58712E+00	0,458	0,233	1,06780E-169
Macro_ISG15	ANPEP	1,12937E-76	1,55740E+00	0,348	0,2	2,07996E-72
Macro_ISG15	PLAUR	1,18736E-186	1,55231E+00	0,836	0,629	2,18676E-182
Macro_ISG15	FABP5	9,51469E-98	1,54887E+00	0,597	0,437	1,75232E-93
Macro_ISG15	GBP2	9,83880E-24	1,54614E+00	0,472	0,366	1,81201E-19
Macro_ISG15	S100A9	8,49207E-112	1,54445E+00	0,672	0,443	1,56398E-107
Macro_ISG15	DYNLT1	2,35320E-286	1,54050E+00	0,747	0,494	4,33390E-282
Macro_ISG15	UPP1	2,39645E-108	1,54050E+00	0,613	0,42	4,41354E-104
Macro_ISG15	CD36	5,60954E-62	1,53216E+00	0,325	0,193	1,03311E-57
Macro_ISG15	HEXB	1,08478E-68	1,53187E+00	0,628	0,507	1,99785E-64
Macro_ISG15	FCER1G	1,12653E-151	1,52297E+00	0,966	0,91	2,07473E-147
Macro_ISG15	RNF213	0,00000E+00	1,51969E+00	0,802	0,486	0,00000E+00
Macro_ISG15	S100A11	0,00000E+00	1,50724E+00	0,99	0,934	0,00000E+00
Macro_ISG15	SERPING1	0,00000E+00	1,50226E+00	0,709	0,378	0,00000E+00
Macro_ISG15	PARP9	0,00000E+00	1,49529E+00	0,534	0,237	0,00000E+00
Macro_ISG15	SERPINA1	5,11668E-44	1,49420E+00	0,853	0,713	9,42340E-40
Macro_ISG15	MS4A4A	1,46860E-134	1,49149E+00	0,629	0,407	2,70471E-130
Macro_ISG15	TPI1	1,69050E-15	1,48938E+00	0,876	0,823	3,11339E-11
Macro_ISG15	DDIT4	1,97135E-34	1,48713E+00	0,589	0,49	3,63063E-30
Macro_ISG15	MT2A	0,00000E+00	1,47179E+00	0,88	0,613	0,00000E+00
Macro_ISG15	CD82	1,92775E-69	1,47103E+00	0,381	0,231	3,55034E-65
Macro_ISG15	GAPDH	1,96158E-21	1,47067E+00	0,975	0,959	3,61265E-17
Macro_ISG15	GSTO1	3,34596E-57	1,47014E+00	0,729	0,613	6,16225E-53
Macro_ISG15	MERTK	7,46733E-128	1,46760E+00	0,364	0,181	1,37526E-123
Macro_ISG15	TYMP	0,00000E+00	1,45231E+00	0,956	0,783	0,00000E+00
Macro_ISG15	OSM	1,35873E-89	1,45097E+00	0,273	0,136	2,50238E-85
Macro_ISG15	OLR1	1,78071E-84	1,44570E+00	0,513	0,337	3,27952E-80
Macro_ISG15	MSR1	7,99331E-88	1,44120E+00	0,559	0,369	1,47213E-83
Macro_ISG15	LAIR1	1,20836E-99	1,44023E+00	0,689	0,509	2,22544E-95
Macro_ISG15	ANXA2	2,38278E-38	1,41136E+00	0,841	0,737	4,38836E-34
Macro_ISG15	PARP14	0,00000E+00	1,41032E+00	0,709	0,356	0,00000E+00
Macro_ISG15	RIN2	2,11979E-136	1,40376E+00	0,298	0,131	3,90402E-132
Macro_ISG15	CASP4	2,36620E-29	1,40271E+00	0,545	0,437	4,35783E-25
Macro_ISG15	CTSA	1,34975E-143	1,39291E+00	0,679	0,495	2,48584E-139
Macro_ISG15	HAVCR2	8,25682E-104	1,39209E+00	0,612	0,424	1,52066E-99

Macro_ISG15	CALM1	2,04675E-66	1,38592E+00	0,858	0,791	3,76950E-62
Macro_ISG15	MGAT1	1,36745E-76	1,38360E+00	0,74	0,617	2,51843E-72
Macro_ISG15	CCRL2	5,71048E-45	1,38113E+00	0,284	0,179	1,05170E-40
Macro_ISG15	TCN2	2,72284E-224	1,37769E+00	0,446	0,205	5,01466E-220
Macro_ISG15	PLAU	2,11764E-56	1,36838E+00	0,305	0,187	3,90006E-52
Macro_ISG15	WIPI1	4,36067E-56	1,36572E+00	0,26	0,151	8,03105E-52
Macro_ISG15	NINJ1	4,45781E-171	1,36412E+00	0,765	0,547	8,20996E-167
Macro_ISG15	GIMAP4	3,58812E-108	1,36244E+00	0,621	0,416	6,60823E-104
Macro_ISG15	CCL3	4,39258E-14	1,35312E+00	0,446	0,371	8,08982E-10
Macro_ISG15	HCK	8,41137E-22	1,35309E+00	0,612	0,515	1,54912E-17
Macro_ISG15	ISG20	0,00000E+00	1,34840E+00	0,688	0,288	0,00000E+00
Macro_ISG15	CMPK2	0,00000E+00	1,34678E+00	0,417	0,087	0,00000E+00
Macro_ISG15	HELZ2	0,00000E+00	1,34147E+00	0,33	0,064	0,00000E+00
Macro_ISG15	POR	7,65116E-93	1,34132E+00	0,323	0,174	1,40911E-88
Macro_ISG15	ADAM9	5,67916E-27	1,34083E+00	0,314	0,223	1,04593E-22
Macro_ISG15	LILRB2	3,03144E-124	1,33937E+00	0,64	0,446	5,58301E-120
Macro_ISG15	BLVRA	5,21283E-128	1,33789E+00	0,571	0,371	9,60047E-124
Macro_ISG15	VAMP5	3,68979E-200	1,33634E+00	0,721	0,465	6,79549E-196
Macro_ISG15	SAT1	2,75876E-198	1,33548E+00	0,994	0,945	5,08082E-194
Macro_ISG15	CD63	2,21484E-55	1,33462E+00	0,887	0,814	4,07906E-51
Macro_ISG15	GPI	1,30364E-19	1,33188E+00	0,496	0,411	2,40092E-15
Macro_ISG15	PLA2G7	3,69773E-102	1,33128E+00	0,341	0,187	6,81011E-98
Macro_ISG15	HSPB1	1,36313E-88	1,32711E+00	0,777	0,651	2,51047E-84
Macro_ISG15	ENO1	1,25284E-14	1,32105E+00	0,821	0,757	2,30736E-10
Macro_ISG15	S100A10	1,03985E-47	1,32093E+00	0,928	0,832	1,91509E-43
Macro_ISG15	PLD3	2,77775E-112	1,32073E+00	0,667	0,501	5,11578E-108
Macro_ISG15	IDH1	3,72235E-44	1,32045E+00	0,352	0,24	6,85545E-40
Macro_ISG15	CD68	3,68224E-167	1,31906E+00	0,9	0,773	6,78157E-163
Macro_ISG15	SNX2	2,36537E-54	1,31693E+00	0,559	0,434	4,35631E-50
Macro_ISG15	CD300A	1,05216E-85	1,31667E+00	0,625	0,45	1,93776E-81
Macro_ISG15	MT1X	1,97491E-76	1,30870E+00	0,375	0,227	3,63718E-72
Macro_ISG15	FCGRT	2,72701E-47	1,30827E+00	0,823	0,733	5,02233E-43
Macro_ISG15	PTAFR	1,31101E-53	1,30589E+00	0,511	0,367	2,41449E-49
Macro_ISG15	NPL	3,80521E-87	1,30566E+00	0,42	0,249	7,00805E-83
Macro_ISG15	GRB2	9,65219E-174	1,30260E+00	0,83	0,67	1,77764E-169
Macro_ISG15	TFRC	1,26854E-47	1,30187E+00	0,347	0,23	2,33627E-43
Macro_ISG15	LILRB1	2,77541E-173	1,30056E+00	0,599	0,363	5,11147E-169
Macro_ISG15	VKORC1	1,12546E-08	1,28976E+00	0,288	0,241	2,07276E-04
Macro_ISG15	TNFSF13B	0,00000E+00	1,28607E+00	0,827	0,564	0,00000E+00
Macro_ISG15	UBE2L6	0,00000E+00	1,28566E+00	0,81	0,556	0,00000E+00
Macro_ISG15	IFIH1	0,00000E+00	1,28547E+00	0,441	0,145	0,00000E+00
Macro_ISG15	SLC43A3	1,09016E-63	1,28496E+00	0,372	0,234	2,00775E-59
Macro_ISG15	TMEM167A	5,36754E-30	1,28294E+00	0,494	0,394	9,88541E-26

Macro_ISG15	LAMP2	1,74159E-41	1,28181E+00	0,514	0,395	3,20748E-37
Macro_ISG15	COLGALT1	1,25421E-58	1,27533E+00	0,352	0,223	2,30988E-54
Macro_ISG15	RCBTB2	5,47366E-51	1,27136E+00	0,291	0,179	1,00808E-46
Macro_ISG15	AZI2	1,48846E-65	1,27098E+00	0,443	0,293	2,74129E-61
Macro_ISG15	SIGLEC9	1,76484E-49	1,27062E+00	0,324	0,208	3,25030E-45
Macro_ISG15	NT5C3A	0,00000E+00	1,27025E+00	0,425	0,152	0,00000E+00
Macro_ISG15	TMEM123	1,97576E-111	1,27004E+00	0,622	0,444	3,63876E-107
Macro_ISG15	SLC2A3	2,73846E-36	1,26620E+00	0,569	0,425	5,04342E-32
Macro_ISG15	LGALS9	2,81898E-212	1,26565E+00	0,753	0,535	5,19172E-208
Macro_ISG15	LAT2	1,65023E-23	1,26555E+00	0,553	0,448	3,03922E-19
Macro_ISG15	LAPTM5	7,61793E-119	1,26276E+00	0,955	0,895	1,40299E-114
Macro_ISG15	IER3	6,02924E-59	1,25754E+00	0,67	0,524	1,11040E-54
Macro_ISG15	SLA	7,37056E-32	1,25739E+00	0,527	0,41	1,35744E-27
Macro_ISG15	GLIPR2	8,63055E-157	1,25723E+00	0,589	0,364	1,58949E-152
Macro_ISG15	SSB	7,16277E-179	1,25458E+00	0,647	0,44	1,31917E-174
Macro_ISG15	TYROBP	0,00000E+00	1,25448E+00	0,992	0,944	0,00000E+00
Macro_ISG15	AQP9	2,36363E-232	1,24648E+00	0,311	0,104	4,35310E-228
Macro_ISG15	DOK2	1,25464E-25	1,24540E+00	0,437	0,339	2,31068E-21
Macro_ISG15	SP110	4,75786E-205	1,24415E+00	0,624	0,376	8,76254E-201
Macro_ISG15	LRPAP1	7,85776E-92	1,24357E+00	0,64	0,487	1,44716E-87
Macro_ISG15	LGMN	5,15815E-139	1,24191E+00	0,594	0,398	9,49976E-135
Macro_ISG15	FTL	2,78759E-109	1,24048E+00	0,999	0,991	5,13391E-105
Macro_ISG15	THBD	1,30184E-76	1,24038E+00	0,341	0,199	2,39760E-72
Macro_ISG15	CHMP5	2,38801E-117	1,23999E+00	0,579	0,396	4,39800E-113
Macro_ISG15	LAP3	1,85569E-225	1,23860E+00	0,805	0,572	3,41762E-221
Macro_ISG15	SRGN	1,33057E-82	1,23764E+00	0,963	0,941	2,45051E-78
Macro_ISG15	EIF2AK2	0,00000E+00	1,23499E+00	0,592	0,241	0,00000E+00
Macro_ISG15	BUD31	3,28523E-23	1,23237E+00	0,44	0,355	6,05041E-19
Macro_ISG15	RAB20	5,51808E-91	1,22897E+00	0,513	0,337	1,01627E-86
Macro_ISG15	SLC31A2	7,65077E-17	1,22778E+00	0,521	0,437	1,40904E-12
Macro_ISG15	CSF1R	4,42197E-55	1,22738E+00	0,709	0,563	8,14394E-51
Macro_ISG15	IFNGR2	3,56409E-11	1,22220E+00	0,623	0,561	6,56399E-07
Macro_ISG15	MTRNR2L8	1,30366E-72	1,22070E+00	0,557	0,433	2,40095E-68
Macro_ISG15	DOK3	5,03487E-81	1,21953E+00	0,373	0,219	9,27272E-77
Macro_ISG15	CD53	5,49442E-20	1,21901E+00	0,759	0,673	1,01191E-15
Macro_ISG15	LILRA2	1,30764E-62	1,21798E+00	0,376	0,239	2,40828E-58
Macro_ISG15	RGL1	1,04801E-153	1,21786E+00	0,311	0,134	1,93012E-149
Macro_ISG15	NAMPT	5,98817E-32	1,21771E+00	0,682	0,567	1,10284E-27
Macro_ISG15	SLC25A37	1,08975E-94	1,21614E+00	0,451	0,281	2,00699E-90
Macro_ISG15	FTH1	8,97248E-30	1,21561E+00	1	0,997	1,65246E-25
Macro_ISG15	SDCBP	1,96759E-17	1,21539E+00	0,786	0,719	3,62372E-13
Macro_ISG15	ZFAND2A	4,01057E-36	1,21494E+00	0,25	0,166	7,38627E-32
Macro_ISG15	CYSTM1	1,96791E-164	1,21249E+00	0,566	0,34	3,62430E-160

Macro_ISG15	MS4A7	1,30156E-65	1,21080E+00	0,811	0,653	2,39708E-61
Macro_ISG15	SCO2	2,49658E-289	1,20805E+00	0,476	0,202	4,59795E-285
Macro_ISG15	ACSL1	3,40469E-55	1,20671E+00	0,528	0,38	6,27042E-51
Macro_ISG15	C9orf72	2,18857E-37	1,20594E+00	0,323	0,219	4,03069E-33
Macro_ISG15	DUSP6	3,55213E-16	1,20407E+00	0,357	0,284	6,54196E-12
Macro_ISG15	PILRA	9,53768E-109	1,19849E+00	0,673	0,482	1,75655E-104
Macro_ISG15	ACTN1	1,26729E-28	1,19604E+00	0,414	0,313	2,33397E-24
Macro_ISG15	BLVRB	9,39702E-16	1,19512E+00	0,572	0,485	1,73065E-11
Macro_ISG15	STAT2	1,03281E-298	1,19332E+00	0,576	0,277	1,90213E-294
Macro_ISG15	TMBIM1	1,35511E-36	1,19137E+00	0,47	0,355	2,49571E-32
Macro_ISG15	MKNK1	2,69225E-21	1,18694E+00	0,279	0,206	4,95831E-17
Macro_ISG15	TPP1	1,65961E-20	1,18648E+00	0,665	0,591	3,05651E-16
Macro_ISG15	GBA	3,11407E-78	1,18455E+00	0,336	0,192	5,73517E-74
Macro_ISG15	STXBP2	3,42939E-106	1,18333E+00	0,595	0,409	6,31590E-102
Macro_ISG15	C2	3,19583E-61	1,18205E+00	0,274	0,156	5,88576E-57
Macro_ISG15	LILRB3	1,80409E-82	1,18189E+00	0,371	0,221	3,32259E-78
Macro_ISG15	ACP5	1,30371E-48	1,18175E+00	0,516	0,393	2,40104E-44
Macro_ISG15	TCIRG1	2,77317E-137	1,17987E+00	0,661	0,455	5,10735E-133
Macro_ISG15	FCN1	1,53550E-34	1,17874E+00	0,359	0,244	2,82794E-30
Macro_ISG15	LRRC25	8,65736E-22	1,17844E+00	0,421	0,328	1,59443E-17
Macro_ISG15	CARD16	1,32219E-73	1,17608E+00	0,702	0,542	2,43507E-69
Macro_ISG15	BCL2A1	1,51425E-36	1,17433E+00	0,585	0,463	2,78880E-32
Macro_ISG15	CALM3	8,69332E-113	1,17098E+00	0,68	0,497	1,60105E-108
Macro_ISG15	TNFRSF1A	2,37496E-60	1,16791E+00	0,579	0,44	4,37396E-56
Macro_ISG15	BAG3	1,97362E-93	1,16790E+00	0,266	0,136	3,63481E-89
Macro_ISG15	NOP10	3,76055E-74	1,16137E+00	0,75	0,624	6,92580E-70
Macro_ISG15	MCOLN1	2,49065E-46	1,16067E+00	0,391	0,267	4,58704E-42
Macro_ISG15	TNFSF13	6,01095E-12	1,15927E+00	0,513	0,439	1,10704E-07
Macro_ISG15	PGAM1	1,67625E-30	1,15628E+00	0,648	0,56	3,08714E-26
Macro_ISG15	SHKBP1	2,31420E-32	1,15596E+00	0,47	0,364	4,26205E-28
Macro_ISG15	RAB1A	2,27759E-40	1,15580E+00	0,504	0,389	4,19464E-36
Macro_ISG15	FNDC3B	3,43433E-148	1,15531E+00	0,485	0,266	6,32501E-144
Macro_ISG15	CBR1	9,38410E-124	1,15498E+00	0,492	0,306	1,72827E-119
Macro_ISG15	C5AR1	6,18076E-129	1,15221E+00	0,616	0,395	1,13831E-124
Macro_ISG15	C1orf162	1,58745E-37	1,15188E+00	0,818	0,701	2,92361E-33
Macro_ISG15	SCARB2	3,41400E-179	1,15174E+00	0,586	0,359	6,28756E-175
Macro_ISG15	SIGLEC7	3,99424E-58	1,14896E+00	0,296	0,176	7,35618E-54
Macro_ISG15	QSOX1	1,62968E-75	1,14487E+00	0,437	0,279	3,00139E-71
Macro_ISG15	TOR1B	1,29248E-307	1,14315E+00	0,33	0,102	2,38036E-303
Macro_ISG15	NMI	8,10556E-147	1,14117E+00	0,493	0,288	1,49280E-142
Macro_ISG15	BNIP3L	1,52613E-14	1,13696E+00	0,634	0,559	2,81068E-10
Macro_ISG15	IFI16	1,97493E-134	1,13615E+00	0,694	0,488	3,63723E-130
Macro_ISG15	B2M	1,54951E-91	1,13459E+00	0,999	0,995	2,85373E-87

Macro_ISG15	SP100	1,42632E-72	1,13311E+00	0,608	0,458	2,62685E-68
Macro_ISG15	ARRDC3	1,40319E-26	1,12965E+00	0,262	0,181	2,58426E-22
Macro_ISG15	ANXA5	1,38919E-09	1,12657E+00	0,837	0,769	2,55847E-05
Macro_ISG15	GPNMB	1,86575E-82	1,12468E+00	0,513	0,324	3,43614E-78
Macro_ISG15	BST2	7,01692E-241	1,12337E+00	0,871	0,716	1,29231E-236
Macro_ISG15	ACP2	1,45270E-50	1,12229E+00	0,374	0,25	2,67544E-46
Macro_ISG15	CXCL16	4,81566E-10	1,12060E+00	0,716	0,647	8,86899E-06
Macro_ISG15	HSP90AA1	7,69902E-41	1,11570E+00	0,919	0,877	1,41793E-36
Macro_ISG15	LGALS3	1,48731E-64	1,11307E+00	0,823	0,718	2,73917E-60
Macro_ISG15	TRIM25	8,66652E-81	1,11243E+00	0,251	0,126	1,59611E-76
Macro_ISG15	CREG1	3,72932E-64	1,10945E+00	0,634	0,505	6,86830E-60
Macro_ISG15	GRN	3,05189E-179	1,10865E+00	0,907	0,818	5,62066E-175
Macro_ISG15	SNX8	3,82584E-11	1,10589E+00	0,268	0,215	7,04605E-07
Macro_ISG15	ATP6V1B2	6,55220E-52	1,10560E+00	0,665	0,527	1,20672E-47
Macro_ISG15	CD48	2,47323E-25	1,10545E+00	0,584	0,473	4,55494E-21
Macro_ISG15	ATP6V1F	2,62431E-132	1,10400E+00	0,853	0,758	4,83318E-128
Macro_ISG15	HGS	4,10915E-11	1,10383E+00	0,255	0,205	7,56782E-07
Macro_ISG15	ORMDL2	5,76212E-26	1,09976E+00	0,383	0,294	1,06121E-21
Macro_ISG15	CNIH4	1,84711E-47	1,09892E+00	0,4	0,279	3,40183E-43
Macro_ISG15	ITGA5	5,66790E-98	1,09791E+00	0,349	0,188	1,04386E-93
Macro_ISG15	SH3BGRL3	1,77268E-109	1,09652E+00	0,967	0,931	3,26475E-105
Macro_ISG15	NCF2	4,64606E-56	1,09554E+00	0,605	0,456	8,55665E-52
Macro_ISG15	PTP4A1	1,14299E-58	1,09381E+00	0,352	0,224	2,10505E-54
Macro_ISG15	TRIM22	3,76606E-190	1,09349E+00	0,647	0,403	6,93594E-186
Macro_ISG15	DBNDD2	2,52265E-15	1,08887E+00	0,268	0,202	4,64597E-11
Macro_ISG15	DNAJB1	1,99932E-23	1,08601E+00	0,627	0,554	3,68215E-19
Macro_ISG15	LTBR	2,91657E-45	1,08442E+00	0,47	0,342	5,37144E-41
Macro_ISG15	ACADVL	3,12516E-41	1,08281E+00	0,571	0,463	5,75560E-37
Macro_ISG15	NADK	7,80401E-68	1,08147E+00	0,361	0,227	1,43726E-63
Macro_ISG15	REEP5	9,86356E-29	1,07968E+00	0,653	0,557	1,81657E-24
Macro_ISG15	HBEGF	1,70671E-50	1,07913E+00	0,401	0,268	3,14325E-46
Macro_ISG15	CLIC1	2,21237E-60	1,07641E+00	0,917	0,872	4,07452E-56
Macro_ISG15	HEXA	6,42481E-25	1,07505E+00	0,557	0,464	1,18326E-20
Macro_ISG15	NCSTN	1,18910E-32	1,07473E+00	0,323	0,227	2,18997E-28
Macro_ISG15	SPTLC2	4,97784E-67	1,07272E+00	0,451	0,301	9,16769E-63
Macro_ISG15	PSMA5	1,30401E-12	1,07206E+00	0,495	0,431	2,40159E-08
Macro_ISG15	SAMD9L	0,00000E+00	1,07205E+00	0,593	0,26	0,00000E+00
Macro_ISG15	RNF181	5,20004E-50	1,07169E+00	0,675	0,555	9,57691E-46
Macro_ISG15	RIPK2	2,71246E-22	1,06851E+00	0,351	0,269	4,99555E-18
Macro_ISG15	GK	5,93735E-18	1,06831E+00	0,44	0,36	1,09348E-13
Macro_ISG15	ASAH1	8,26941E-34	1,06681E+00	0,781	0,683	1,52298E-29
Macro_ISG15	HSPA1A	2,12463E-67	1,06182E+00	0,759	0,658	3,91293E-63
Macro_ISG15	RALB	2,04516E-10	1,06042E+00	0,278	0,226	3,76657E-06

Macro_ISG15	PSMA6	1,30751E-55	1,05647E+00	0,599	0,458	2,40804E-51
Macro_ISG15	CD86	3,43009E-49	1,05570E+00	0,611	0,488	6,31720E-45
Macro_ISG15	ATP6V1C1	2,37388E-19	1,05529E+00	0,346	0,269	4,37197E-15
Macro_ISG15	DHX58	4,94800E-223	1,04874E+00	0,25	0,078	9,11274E-219
Macro_ISG15	SAR1A	7,75457E-20	1,04827E+00	0,315	0,239	1,42816E-15
Macro_ISG15	EPB41L3	3,95820E-28	1,04672E+00	0,398	0,298	7,28982E-24
Macro_ISG15	SLC7A7	6,36891E-31	1,04342E+00	0,59	0,458	1,17296E-26
Macro_ISG15	MRPS18C	2,01011E-90	1,04133E+00	0,456	0,292	3,70201E-86
Macro_ISG15	FKBP4	1,45305E-32	1,03968E+00	0,314	0,223	2,67609E-28
Macro_ISG15	SOCS3	4,25540E-45	1,03737E+00	0,481	0,35	7,83717E-41
Macro_ISG15	PSMA4	3,17899E-55	1,03609E+00	0,589	0,461	5,85474E-51
Macro_ISG15	LY96	2,61808E-35	1,03588E+00	0,571	0,45	4,82171E-31
Macro_ISG15	GNG5	2,15469E-152	1,03326E+00	0,901	0,787	3,96829E-148
Macro_ISG15	CASP1	1,06580E-44	1,03276E+00	0,581	0,456	1,96288E-40
Macro_ISG15	SLC39A1	7,91709E-12	1,02954E+00	0,333	0,274	1,45809E-07
Macro_ISG15	TPM3	6,47668E-30	1,02921E+00	0,859	0,8	1,19281E-25
Macro_ISG15	ANKRD22	2,93744E-08	1,02853E+00	0,266	0,223	5,40988E-04
Macro_ISG15	SLC16A3	4,18398E-179	1,02837E+00	0,717	0,499	7,70564E-175
Macro_ISG15	RB1	1,68348E-46	1,02741E+00	0,516	0,395	3,10046E-42
Macro_ISG15	ADAP2	8,64488E-115	1,02621E+00	0,623	0,434	1,59213E-110
Macro_ISG15	IL10RB	3,50027E-60	1,02240E+00	0,382	0,247	6,44645E-56
Macro_ISG15	CAPG	3,01097E-124	1,02235E+00	0,85	0,734	5,54530E-120
Macro_ISG15	NXF1	1,20960E-24	1,02072E+00	0,288	0,207	2,22772E-20
Macro_ISG15	TLR2	7,44006E-19	1,01803E+00	0,476	0,381	1,37024E-14
Macro_ISG15	STIP1	1,43974E-12	1,01725E+00	0,337	0,278	2,65156E-08
Macro_ISG15	MYO1G	1,80967E-49	1,01521E+00	0,409	0,276	3,33286E-45
Macro_ISG15	HCST	2,79505E-203	1,01517E+00	0,82	0,643	5,14764E-199
Macro_ISG15	PSMB9	2,87882E-146	1,01473E+00	0,829	0,647	5,30192E-142
Macro_ISG15	MYD88	7,42579E-49	1,01465E+00	0,481	0,35	1,36761E-44
Macro_ISG15	HNMT	1,91294E-49	1,01415E+00	0,507	0,373	3,52306E-45
Macro_ISG15	ATG3	2,43929E-23	1,01329E+00	0,607	0,518	4,49244E-19
Macro_ISG15	ACOT9	4,96307E-63	1,01329E+00	0,41	0,271	9,14049E-59
Macro_ISG15	TNS3	6,39184E-59	1,01314E+00	0,311	0,187	1,17718E-54
Macro_ISG15	AGTRAP	1,05758E-30	1,01262E+00	0,472	0,354	1,94774E-26
Macro_ISG15	RAB8A	4,69799E-125	1,01093E+00	0,606	0,415	8,65229E-121
Macro_ISG15	STOM	2,51343E-41	1,00998E+00	0,457	0,339	4,62898E-37
Macro_ISG15	DMXL2	1,71039E-110	1,00800E+00	0,448	0,256	3,15002E-106
Macro_ISG15	CD300C	2,21902E-23	1,00782E+00	0,317	0,232	4,08678E-19
Macro_ISG15	PTPN6	1,71646E-17	1,00572E+00	0,594	0,505	3,16120E-13
Macro_ISG15	LHFPL2	1,43491E-157	1,00555E+00	0,49	0,271	2,64268E-153
Macro_ISG15	SRGAP2	3,95286E-44	1,00332E+00	0,333	0,221	7,27998E-40
Macro_ISG15	PSEN1	2,57870E-07	1,00201E+00	0,263	0,219	4,74919E-03
Macro_ISG15	LGALS1	1,80793E-106	1,00117E+00	0,924	0,83	3,32967E-102

Macro_ISG15	MLKL	7,48408E-114	1,00009E+00	0,262	0,116	1,37834E-109
Macro_ISG15	GPR108	2,93667E-13	9,97571E-01	0,318	0,254	5,40847E-09
Macro_ISG15	SLC3A2	1,21220E-63	9,96971E-01	0,581	0,456	2,23250E-59
Macro_ISG15	ALOX5AP	2,43293E-17	9,96779E-01	0,462	0,508	4,48072E-13
Macro_ISG15	SAMD9	0,00000E+00	9,96740E-01	0,446	0,157	0,00000E+00
Macro_ISG15	RASGEF1B	1,76683E-31	9,91206E-01	0,366	0,263	3,25397E-27
Macro_ISG15	PARP12	4,75539E-246	9,91049E-01	0,391	0,158	8,75800E-242
Macro_ISG15	RTCB	1,44123E-170	9,90274E-01	0,501	0,282	2,65432E-166
Macro_ISG15	CHMP2A	6,12971E-13	9,85481E-01	0,554	0,488	1,12891E-08
Macro_ISG15	HLA-F	1,75484E-47	9,83901E-01	0,641	0,508	3,23189E-43
Macro_ISG15	ADAR	4,05756E-239	9,82475E-01	0,674	0,413	7,47281E-235
Macro_ISG15	HMOX1	1,85637E-67	9,81474E-01	0,7	0,535	3,41887E-63
Macro_ISG15	RAB8B	5,39407E-39	9,81197E-01	0,356	0,244	9,93426E-35
Macro_ISG15	UBA7	1,45860E-111	9,81148E-01	0,32	0,16	2,68629E-107
Macro_ISG15	MIF	1,40695E-75	9,80986E-01	0,838	0,717	2,59118E-71
Macro_ISG15	CSF3R	6,89038E-81	9,79646E-01	0,554	0,384	1,26900E-76
Macro_ISG15	NAGK	5,00073E-63	9,77560E-01	0,606	0,469	9,20985E-59
Macro_ISG15	PML	3,93490E-278	9,76180E-01	0,482	0,214	7,24690E-274
Macro_ISG15	PSAP	2,89418E-64	9,76012E-01	0,957	0,914	5,33020E-60
Macro_ISG15	ARF1	3,45770E-14	9,74197E-01	0,757	0,695	6,36804E-10
Macro_ISG15	OAZ1	5,83521E-50	9,72224E-01	0,964	0,935	1,07467E-45
Macro_ISG15	MGST3	2,19295E-26	9,71824E-01	0,624	0,537	4,03876E-22
Macro_ISG15	TMEM176A	2,80802E-07	9,71171E-01	0,5	0,445	5,17154E-03
Macro_ISG15	BST1	3,75842E-75	9,70815E-01	0,25	0,127	6,92188E-71
Macro_ISG15	CXCL2	2,09990E-06	9,70461E-01	0,27	0,224	3,86739E-02
Macro_ISG15	CALR	9,07982E-81	9,69065E-01	0,817	0,718	1,67223E-76
Macro_ISG15	ARPC5	4,25408E-27	9,68678E-01	0,826	0,759	7,83473E-23
Macro_ISG15	TM2D2	1,49636E-14	9,68645E-01	0,256	0,198	2,75584E-10
Macro_ISG15	TOM1	8,97653E-74	9,66450E-01	0,43	0,275	1,65321E-69
Macro_ISG15	PHF11	1,71000E-103	9,65598E-01	0,453	0,277	3,14931E-99
Macro_ISG15	BACH1	7,13443E-13	9,62331E-01	0,376	0,309	1,31395E-08
Macro_ISG15	GBP4	6,49188E-95	9,61537E-01	0,446	0,267	1,19561E-90
Macro_ISG15	ID3	2,40868E-94	9,58509E-01	0,273	0,137	4,43607E-90
Macro_ISG15	TPD52L2	4,65748E-62	9,58377E-01	0,4	0,261	8,57768E-58
Macro_ISG15	ATP6V0B	1,42315E-22	9,56873E-01	0,824	0,789	2,62102E-18
Macro_ISG15	SNX1	1,75056E-27	9,53606E-01	0,397	0,301	3,22400E-23
Macro_ISG15	SPPL2A	2,33591E-40	9,53409E-01	0,45	0,334	4,30205E-36
Macro_ISG15	PSMA7	4,11840E-20	9,52974E-01	0,807	0,742	7,58487E-16
Macro_ISG15	SECTM1	2,16175E-79	9,52763E-01	0,293	0,157	3,98130E-75
Macro_ISG15	ZBTB8OS	1,90809E-11	9,51256E-01	0,274	0,22	3,51412E-07
Macro_ISG15	HSPA1B	6,06889E-56	9,48242E-01	0,616	0,498	1,11771E-51
Macro_ISG15	FLII	2,80302E-15	9,46505E-01	0,39	0,32	5,16233E-11
Macro_ISG15	MFSD1	3,14082E-11	9,44287E-01	0,645	0,588	5,78445E-07

Macro_ISG15	ADPGK	3,87898E-41	9,43766E-01	0,51	0,394	7,14392E-37
Macro_ISG15	SLC25A19	1,63393E-96	9,43294E-01	0,3	0,156	3,00920E-92
Macro_ISG15	EMP3	1,59814E-51	9,42780E-01	0,836	0,722	2,94330E-47
Macro_ISG15	RAB5C	6,22095E-59	9,40664E-01	0,592	0,452	1,14571E-54
Macro_ISG15	HLA-A	5,03354E-179	9,40577E-01	0,983	0,948	9,27026E-175
Macro_ISG15	COMT	1,06590E-86	9,40352E-01	0,683	0,55	1,96307E-82
Macro_ISG15	ITGAM	2,30095E-24	9,40216E-01	0,322	0,237	4,23765E-20
Macro_ISG15	TREM2	8,12520E-68	9,39911E-01	0,556	0,397	1,49642E-63
Macro_ISG15	CTNND1	9,74009E-10	9,39447E-01	0,326	0,269	1,79383E-05
Macro_ISG15	VMP1	3,75168E-25	9,38762E-01	0,645	0,545	6,90946E-21
Macro_ISG15	RHBDD2	5,77898E-08	9,37620E-01	0,303	0,255	1,06431E-03
Macro_ISG15	TAP1	6,22364E-120	9,36203E-01	0,623	0,41	1,14621E-115
Macro_ISG15	IRAK1	1,60709E-86	9,35559E-01	0,3	0,16	2,95978E-82
Macro_ISG15	ARPC4	2,03575E-08	9,35190E-01	0,465	0,406	3,74924E-04
Macro_ISG15	TSPAN14	1,86533E-16	9,35051E-01	0,411	0,328	3,43539E-12
Macro_ISG15	XRN1	5,60671E-62	9,33580E-01	0,335	0,208	1,03259E-57
Macro_ISG15	EHD4	6,70565E-72	9,26604E-01	0,349	0,211	1,23498E-67
Macro_ISG15	CNDP2	2,41023E-42	9,25655E-01	0,572	0,447	4,43892E-38
Macro_ISG15	KYNU	1,03008E-07	9,23379E-01	0,364	0,312	1,89711E-03
Macro_ISG15	TRAPPC5	4,41164E-50	9,22661E-01	0,352	0,231	8,12492E-46
Macro_ISG15	HM13	1,20052E-46	9,21777E-01	0,646	0,533	2,21100E-42
Macro_ISG15	RAB10	2,61805E-33	9,15171E-01	0,591	0,484	4,82166E-29
Macro_ISG15	CAPZA1	9,75504E-14	9,14075E-01	0,567	0,49	1,79658E-09
Macro_ISG15	GNS	4,10527E-74	9,13815E-01	0,582	0,421	7,56067E-70
Macro_ISG15	CTSC	6,37670E-73	9,12706E-01	0,773	0,672	1,17440E-68
Macro_ISG15	AUP1	1,55956E-12	9,10027E-01	0,586	0,522	2,87224E-08
Macro_ISG15	LRP1	9,23832E-112	9,09684E-01	0,62	0,41	1,70142E-107
Macro_ISG15	ICAM2	5,33216E-36	9,08701E-01	0,253	0,16	9,82024E-32
Macro_ISG15	CLEC7A	5,86351E-07	9,08575E-01	0,584	0,521	1,07988E-02
Macro_ISG15	GCA	9,91047E-07	9,03337E-01	0,454	0,399	1,82521E-02
Macro_ISG15	LILRB4	1,53668E-123	9,02530E-01	0,736	0,538	2,83010E-119
Macro_ISG15	GABARAP	4,07383E-26	9,02309E-01	0,881	0,826	7,50277E-22
Macro_ISG15	PLIN3	6,25444E-21	9,01810E-01	0,454	0,367	1,15188E-16
Macro_ISG15	TMEM205	1,38981E-26	9,01557E-01	0,371	0,279	2,55961E-22
Macro_ISG15	FKBP15	2,32449E-43	9,00049E-01	0,378	0,266	4,28101E-39
Macro_ISG15	PTK2B	1,12318E-54	9,00032E-01	0,378	0,246	2,06856E-50
Macro_ISG15	TPM4	1,30656E-121	8,97753E-01	0,771	0,609	2,40630E-117
Macro_ISG15	RBMS1	1,32561E-42	8,97637E-01	0,42	0,303	2,44138E-38
Macro_ISG15	CTNNB1	1,37787E-13	8,95133E-01	0,447	0,375	2,53763E-09
Macro_ISG15	RAB24	2,54399E-30	8,93840E-01	0,363	0,265	4,68526E-26
Macro_ISG15	PIK3R5	1,03191E-14	8,93333E-01	0,318	0,254	1,90047E-10
Macro_ISG15	NAPA	2,32852E-222	8,93201E-01	0,708	0,482	4,28844E-218
Macro_ISG15	ARAP1	2,09078E-38	8,92292E-01	0,396	0,285	3,85059E-34

Macro_ISG15	RPS27L	1,61090E-18	8,92286E-01	0,412	0,359	2,96680E-14
Macro_ISG15	EGR1	1,30563E-17	8,90636E-01	0,37	0,281	2,40458E-13
Macro_ISG15	ARID5A	1,35770E-25	8,88695E-01	0,371	0,283	2,50047E-21
Macro_ISG15	SCIMP	4,52100E-34	8,88609E-01	0,376	0,272	8,32633E-30
Macro_ISG15	CTSH	7,67168E-09	8,88297E-01	0,793	0,705	1,41289E-04
Macro_ISG15	IFNAR2	2,27445E-19	8,87291E-01	0,342	0,263	4,18885E-15
Macro_ISG15	CYB5R4	2,08168E-09	8,85273E-01	0,311	0,259	3,83383E-05
Macro_ISG15	HLA-B	1,04929E-177	8,84254E-01	0,991	0,967	1,93249E-173
Macro_ISG15	GABARAPL1	4,41103E-32	8,83437E-01	0,382	0,286	8,12380E-28
Macro_ISG15	GAA	1,13162E-53	8,83116E-01	0,512	0,379	2,08411E-49
Macro_ISG15	SOD2	5,85360E-30	8,81888E-01	0,733	0,646	1,07806E-25
Macro_ISG15	BLOC1S1	2,75183E-25	8,81341E-01	0,567	0,466	5,06804E-21
Macro_ISG15	OSCAR	5,35719E-157	8,81093E-01	0,42	0,214	9,86634E-153
Macro_ISG15	TMEM50A	4,26470E-40	8,80508E-01	0,687	0,598	7,85429E-36
Macro_ISG15	LMAN2	2,75667E-22	8,80390E-01	0,591	0,506	5,07696E-18
Macro_ISG15	TMBIM6	4,54437E-22	8,79927E-01	0,827	0,775	8,36937E-18
Macro_ISG15	PLA2G15	9,67406E-54	8,79250E-01	0,257	0,149	1,78167E-49
Macro_ISG15	MYL6	2,83803E-74	8,79240E-01	0,971	0,94	5,22680E-70
Macro_ISG15	SIRPA	9,47294E-113	8,79057E-01	0,53	0,339	1,74463E-108
Macro_ISG15	DRAP1	5,00780E-211	8,77243E-01	0,819	0,647	9,22286E-207
Macro_ISG15	IRF2	3,02579E-15	8,76632E-01	0,391	0,324	5,57260E-11
Macro_ISG15	SASH3	3,63164E-51	8,76198E-01	0,403	0,276	6,68839E-47
Macro_ISG15	IL10RA	2,54961E-36	8,75589E-01	0,572	0,458	4,69563E-32
Macro_ISG15	PSMD11	2,02588E-08	8,75416E-01	0,355	0,305	3,73107E-04
Macro_ISG15	PSMD7	9,41940E-07	8,75412E-01	0,45	0,403	1,73477E-02
Macro_ISG15	C1QC	1,03382E-07	8,73432E-01	0,576	0,544	1,90399E-03
Macro_ISG15	NUPR1	6,95027E-57	8,71558E-01	0,336	0,208	1,28003E-52
Macro_ISG15	LGALS8	1,29479E-35	8,71266E-01	0,286	0,19	2,38462E-31
Macro_ISG15	GADD45B	6,82833E-30	8,71200E-01	0,727	0,637	1,25757E-25
Macro_ISG15	ANXA4	4,68706E-45	8,69847E-01	0,42	0,302	8,63215E-41
Macro_ISG15	RBCK1	5,55682E-66	8,69340E-01	0,513	0,365	1,02340E-61
Macro_ISG15	SLC15A3	6,43779E-44	8,69285E-01	0,395	0,276	1,18565E-39
Macro_ISG15	SDSL	8,12909E-92	8,68292E-01	0,39	0,23	1,49713E-87
Macro_ISG15	STAT1	8,83816E-216	8,66351E-01	0,771	0,502	1,62772E-211
Macro_ISG15	GAL3ST4	2,19532E-107	8,65083E-01	0,318	0,159	4,04312E-103
Macro_ISG15	NPTN	4,20471E-28	8,64733E-01	0,312	0,223	7,74381E-24
Macro_ISG15	S100A8	4,99562E-134	8,63966E-01	0,577	0,338	9,20044E-130
Macro_ISG15	PRNP	1,47006E-07	8,59622E-01	0,455	0,398	2,70740E-03
Macro_ISG15	EML4	5,07015E-53	8,59594E-01	0,481	0,348	9,33770E-49
Macro_ISG15	SDHB	1,40172E-22	8,59389E-01	0,477	0,394	2,58155E-18
Macro_ISG15	DTX3L	1,20269E-213	8,59226E-01	0,41	0,181	2,21499E-209
Macro_ISG15	RHOG	2,91816E-10	8,58924E-01	0,792	0,723	5,37438E-06
Macro_ISG15	APOE	1,66446E-55	8,58832E-01	0,634	0,499	3,06543E-51

Macro_ISG15	NEU1	2,28086E-30	8,58166E-01	0,403	0,298	4,20066E-26
Macro_ISG15	PSME2	5,81230E-121	8,57778E-01	0,862	0,706	1,07045E-116
Macro_ISG15	LIMS1	2,14456E-30	8,57174E-01	0,66	0,549	3,94964E-26
Macro_ISG15	ATF5	2,18306E-138	8,56598E-01	0,552	0,349	4,02055E-134
Macro_ISG15	GALM	1,83159E-99	8,55539E-01	0,344	0,187	3,37325E-95
Macro_ISG15	PTTG1IP	1,51113E-14	8,54139E-01	0,57	0,498	2,78306E-10
Macro_ISG15	HSPE1	6,08612E-31	8,52541E-01	0,679	0,598	1,12088E-26
Macro_ISG15	MVP	1,84004E-26	8,52032E-01	0,463	0,368	3,38881E-22
Macro_ISG15	SNX10	5,08632E-36	8,50139E-01	0,587	0,48	9,36748E-32
Macro_ISG15	ZEB2	5,53782E-12	8,49323E-01	0,686	0,6	1,01990E-07
Macro_ISG15	LPAR6	5,18043E-75	8,48293E-01	0,476	0,313	9,54079E-71
Macro_ISG15	ADRM1	3,31473E-07	8,48008E-01	0,48	0,425	6,10473E-03
Macro_ISG15	PSMD14	6,41858E-07	8,47615E-01	0,255	0,216	1,18211E-02
Macro_ISG15	RAB7A	4,73675E-09	8,46728E-01	0,685	0,63	8,72367E-05
Macro_ISG15	CTNNA1	5,02627E-11	8,46354E-01	0,423	0,359	9,25688E-07
Macro_ISG15	TSPO	5,87217E-24	8,46199E-01	0,844	0,761	1,08148E-19
Macro_ISG15	CD81	4,80968E-114	8,44542E-01	0,64	0,451	8,85798E-110
Macro_ISG15	ATP6V0D1	2,16461E-34	8,39394E-01	0,659	0,566	3,98656E-30
Macro_ISG15	P4HA1	1,07519E-25	8,37821E-01	0,297	0,214	1,98017E-21
Macro_ISG15	ATP6AP1	1,99469E-30	8,37667E-01	0,628	0,539	3,67362E-26
Macro_ISG15	EWSR1	1,14932E-08	8,36495E-01	0,519	0,459	2,11670E-04
Macro_ISG15	CTSS	1,22800E-07	8,36074E-01	0,884	0,812	2,26160E-03
Macro_ISG15	VTI1B	1,16000E-06	8,35779E-01	0,421	0,375	2,13637E-02
Macro_ISG15	CFL1	1,58149E-18	8,35705E-01	0,954	0,92	2,91263E-14
Macro_ISG15	PSMA3	4,82293E-14	8,35608E-01	0,438	0,368	8,88239E-10
Macro_ISG15	SH3GLB1	5,19065E-18	8,33975E-01	0,499	0,422	9,55961E-14
Macro_ISG15	IGSF6	3,42500E-08	8,33509E-01	0,621	0,553	6,30783E-04
Macro_ISG15	FKBP1A	5,07181E-11	8,33449E-01	0,75	0,691	9,34075E-07
Macro_ISG15	MCFD2	4,27607E-09	8,31148E-01	0,25	0,204	7,87524E-05
Macro_ISG15	EDEM2	1,99657E-22	8,28896E-01	0,283	0,203	3,67709E-18
Macro_ISG15	SPATS2L	1,43745E-196	8,28209E-01	0,449	0,219	2,64735E-192
Macro_ISG15	SLC43A2	1,17736E-87	8,27050E-01	0,528	0,358	2,16835E-83
Macro_ISG15	ADAM8	1,19950E-70	8,26919E-01	0,358	0,219	2,20912E-66
Macro_ISG15	KRTCAP2	8,26461E-36	8,25498E-01	0,672	0,572	1,52209E-31
Macro_ISG15	MARCKS	1,09119E-160	8,21857E-01	0,745	0,519	2,00965E-156
Macro_ISG15	CD47	1,59027E-42	8,21808E-01	0,531	0,415	2,92880E-38
Macro_ISG15	HLA-E	1,30885E-70	8,20807E-01	0,956	0,901	2,41052E-66
Macro_ISG15	ST14	7,15904E-116	8,18973E-01	0,438	0,256	1,31848E-111
Macro_ISG15	CLIC4	9,64117E-18	8,16783E-01	0,296	0,225	1,77561E-13
Macro_ISG15	SIL1	1,40901E-28	8,15909E-01	0,325	0,233	2,59497E-24
Macro_ISG15	SIDT2	3,03893E-31	8,14776E-01	0,258	0,17	5,59680E-27
Macro_ISG15	G0S2	2,96345E-36	8,14185E-01	0,278	0,18	5,45778E-32
Macro_ISG15	SDF2L1	2,93310E-36	8,13229E-01	0,462	0,348	5,40189E-32

Macro_ISG15	TINF2	1,02338E-12	8,12437E-01	0,282	0,224	1,88477E-08
Macro_ISG15	MOB1A	8,52105E-23	8,10155E-01	0,666	0,578	1,56932E-18
Macro_ISG15	CTNNBL1	7,38074E-12	8,07798E-01	0,284	0,227	1,35931E-07
Macro_ISG15	NABP1	4,17568E-16	8,04454E-01	0,414	0,335	7,69035E-12
Macro_ISG15	ATP6V1G1	5,60574E-31	8,01304E-01	0,781	0,715	1,03241E-26
Macro_ISG15	CMC2	1,29598E-33	8,01199E-01	0,371	0,27	2,38680E-29
Macro_ISG15	NASP	4,45262E-10	7,97544E-01	0,326	0,272	8,20039E-06
Macro_ISG15	GLRX	4,10495E-108	7,96456E-01	0,708	0,538	7,56009E-104
Macro_ISG15	VPS29	4,59257E-21	7,95575E-01	0,616	0,53	8,45813E-17
Macro_ISG15	SAT2	7,41967E-63	7,95355E-01	0,565	0,425	1,36648E-58
Macro_ISG15	MRPL28	1,51696E-11	7,94383E-01	0,32	0,264	2,79379E-07
Macro_ISG15	MYOF	3,45835E-44	7,93492E-01	0,32	0,212	6,36925E-40
Macro_ISG15	PSMB6	1,88072E-08	7,89245E-01	0,625	0,573	3,46371E-04
Macro_ISG15	CCT5	1,38723E-06	7,88230E-01	0,423	0,379	2,55485E-02
Macro_ISG15	M6PR	1,74273E-09	7,86821E-01	0,612	0,561	3,20958E-05
Macro_ISG15	ST8SIA4	1,94564E-80	7,86516E-01	0,447	0,288	3,58328E-76
Macro_ISG15	NUCB1	4,79737E-27	7,85003E-01	0,629	0,538	8,83532E-23
Macro_ISG15	PDLIM7	7,68914E-51	7,84695E-01	0,307	0,19	1,41611E-46
Macro_ISG15	PNKD	5,83007E-62	7,82340E-01	0,453	0,314	1,07372E-57
Macro_ISG15	CLTA	3,72804E-31	7,80869E-01	0,728	0,644	6,86593E-27
Macro_ISG15	CMC1	8,83481E-15	7,80624E-01	0,289	0,223	1,62711E-10
Macro_ISG15	JOSD2	1,38468E-57	7,80501E-01	0,459	0,32	2,55017E-53
Macro_ISG15	SAP30BP	8,92007E-09	7,80287E-01	0,319	0,268	1,64281E-04
Macro_ISG15	TMEM106A	7,18831E-81	7,79847E-01	0,282	0,15	1,32387E-76
Macro_ISG15	TAGAP	1,27248E-50	7,79411E-01	0,389	0,265	2,34352E-46
Macro_ISG15	MNDA	1,58018E-53	7,78930E-01	0,686	0,524	2,91022E-49
Macro_ISG15	RNF149	7,65052E-10	7,78283E-01	0,675	0,624	1,40900E-05
Macro_ISG15	GPCPD1	4,88007E-50	7,76382E-01	0,4	0,273	8,98762E-46
Macro_ISG15	BCKDK	3,42265E-22	7,76314E-01	0,401	0,314	6,30350E-18
Macro_ISG15	EMC7	5,58944E-11	7,75798E-01	0,378	0,317	1,02941E-06
Macro_ISG15	MAT2A	2,31493E-41	7,75157E-01	0,558	0,44	4,26341E-37
Macro_ISG15	ME2	7,28519E-25	7,74459E-01	0,383	0,301	1,34171E-20
Macro_ISG15	DNASE2	8,61881E-70	7,74192E-01	0,519	0,358	1,58733E-65
Macro_ISG15	IFNAR1	2,32201E-06	7,73653E-01	0,356	0,311	4,27644E-02
Macro_ISG15	SH3BP2	4,21956E-67	7,73125E-01	0,433	0,286	7,77117E-63
Macro_ISG15	PCBP1	1,92273E-35	7,72611E-01	0,704	0,605	3,54109E-31
Macro_ISG15	PLXND1	1,03390E-55	7,71244E-01	0,352	0,226	1,90414E-51
Macro_ISG15	SLC22A18	2,67582E-48	7,68520E-01	0,276	0,167	4,92806E-44
Macro_ISG15	GNPTG	7,13506E-27	7,68428E-01	0,52	0,423	1,31406E-22
Macro_ISG15	DDX60	8,75223E-176	7,68300E-01	0,251	0,088	1,61190E-171
Macro_ISG15	CDKN1A	5,09103E-46	7,67569E-01	0,659	0,517	9,37615E-42
Macro_ISG15	CSTB	8,35136E-44	7,67165E-01	0,873	0,784	1,53807E-39
Macro_ISG15	PLEKHO2	2,09725E-84	7,66978E-01	0,348	0,2	3,86251E-80

Macro_ISG15	PUF60	7,97499E-10	7,66224E-01	0,374	0,317	1,46875E-05
Macro_ISG15	APOL6	2,05195E-185	7,65720E-01	0,475	0,247	3,77908E-181
Macro_ISG15	GUSB	7,91075E-16	7,65662E-01	0,416	0,342	1,45692E-11
Macro_ISG15	PKM	1,18239E-42	7,65254E-01	0,869	0,778	2,17760E-38
Macro_ISG15	PSMB8	1,74487E-34	7,62465E-01	0,682	0,57	3,21352E-30
Macro_ISG15	CFD	6,40158E-87	7,61259E-01	0,762	0,55	1,17898E-82
Macro_ISG15	PNP	2,62949E-13	7,61074E-01	0,318	0,255	4,84272E-09
Macro_ISG15	ITGB1	1,33255E-08	7,60774E-01	0,526	0,472	2,45415E-04
Macro_ISG15	METRNL	5,42175E-105	7,60565E-01	0,425	0,244	9,98524E-101
Macro_ISG15	UBE2F	5,68574E-23	7,59442E-01	0,337	0,254	1,04714E-18
Macro_ISG15	CIB1	1,68623E-15	7,58626E-01	0,691	0,608	3,10553E-11
Macro_ISG15	PSMB3	4,10445E-36	7,56755E-01	0,732	0,638	7,55917E-32
Macro_ISG15	ECH1	2,22028E-19	7,55208E-01	0,364	0,284	4,08909E-15
Macro_ISG15	TMC6	1,29127E-64	7,54215E-01	0,351	0,219	2,37813E-60
Macro_ISG15	RNF135	1,35560E-15	7,52651E-01	0,299	0,234	2,49660E-11
Macro_ISG15	ATP6V1H	2,60900E-12	7,50537E-01	0,264	0,209	4,80499E-08
Macro_ISG15	XRCC5	4,85844E-07	7,50237E-01	0,451	0,477	8,94778E-03
Macro_ISG15	ATP6V0E1	5,68329E-17	7,50232E-01	0,828	0,794	1,04669E-12
Macro_ISG15	ZNFX1	2,36836E-170	7,49470E-01	0,465	0,244	4,36181E-166
Macro_ISG15	APOBEC3G	5,79703E-69	7,48628E-01	0,314	0,184	1,06764E-64
Macro_ISG15	OS9	3,14514E-07	7,47815E-01	0,59	0,533	5,79241E-03
Macro_ISG15	BLOC1S2	2,80232E-14	7,45388E-01	0,39	0,327	5,16104E-10
Macro_ISG15	PSMB7	7,26968E-09	7,45222E-01	0,415	0,36	1,33886E-04
Macro_ISG15	ARPC2	1,18548E-06	7,44834E-01	0,905	0,856	2,18331E-02
Macro_ISG15	ADAM10	4,28918E-17	7,42800E-01	0,359	0,283	7,89938E-13
Macro_ISG15	TFEC	1,54881E-93	7,40616E-01	0,46	0,285	2,85244E-89
Macro_ISG15	SRA1	2,20527E-30	7,40044E-01	0,482	0,38	4,06145E-26
Macro_ISG15	AIF1	1,30364E-38	7,39438E-01	0,94	0,815	2,40091E-34
Macro_ISG15	HSBP1	3,46464E-74	7,37286E-01	0,728	0,593	6,38082E-70
Macro_ISG15	DOCK4	4,52594E-26	7,36956E-01	0,378	0,28	8,33542E-22
Macro_ISG15	TRIM14	1,21110E-95	7,36415E-01	0,314	0,166	2,23049E-91
Macro_ISG15	WSB1	1,78201E-35	7,35069E-01	0,736	0,635	3,28192E-31
Macro_ISG15	NT5C2	4,74380E-55	7,34511E-01	0,274	0,161	8,73665E-51
Macro_ISG15	DYNLL1	5,64009E-25	7,33288E-01	0,78	0,703	1,03874E-20
Macro_ISG15	BCAP31	1,67093E-12	7,32994E-01	0,636	0,581	3,07735E-08
Macro_ISG15	PRELID1	6,74376E-15	7,31514E-01	0,754	0,68	1,24200E-10
Macro_ISG15	CD164	2,05445E-22	7,30992E-01	0,635	0,553	3,78368E-18
Macro_ISG15	TTYH3	1,77438E-129	7,29749E-01	0,398	0,213	3,26787E-125
Macro_ISG15	CAPNS1	1,56889E-14	7,29534E-01	0,439	0,366	2,88943E-10
Macro_ISG15	CMTM7	4,56235E-19	7,29463E-01	0,491	0,406	8,40248E-15
Macro_ISG15	RER1	6,57946E-10	7,28980E-01	0,521	0,457	1,21174E-05
Macro_ISG15	FUCA2	1,68142E-30	7,28833E-01	0,399	0,303	3,09667E-26
Macro_ISG15	TMED5	4,88305E-18	7,28616E-01	0,451	0,368	8,99311E-14

Macro_ISG15	MAFB	4,50684E-87	7,26907E-01	0,774	0,582	8,30024E-83
Macro_ISG15	TANK	4,57984E-08	7,25662E-01	0,341	0,291	8,43470E-04
Macro_ISG15	FCGR2B	2,86480E-09	7,24830E-01	0,399	0,427	5,27610E-05
Macro_ISG15	BSG	6,61351E-53	7,22961E-01	0,668	0,557	1,21801E-48
Macro_ISG15	TLE3	6,60550E-48	7,21248E-01	0,286	0,18	1,21653E-43
Macro_ISG15	LTA4H	1,70891E-34	7,19805E-01	0,36	0,453	3,14729E-30
Macro_ISG15	MFSD10	1,01115E-11	7,19716E-01	0,327	0,267	1,86223E-07
Macro_ISG15	P4HB	5,43809E-20	7,19690E-01	0,704	0,624	1,00153E-15
Macro_ISG15	FAM200B	1,18354E-06	7,19465E-01	0,251	0,21	2,17972E-02
Macro_ISG15	C6orf62	1,42098E-65	7,16909E-01	0,503	0,356	2,61702E-61
Macro_ISG15	RNH1	1,44987E-12	7,12097E-01	0,684	0,614	2,67023E-08
Macro_ISG15	ASGR1	1,86068E-62	7,09738E-01	0,312	0,186	3,42681E-58
Macro_ISG15	ANAPC11	3,46072E-16	7,08682E-01	0,676	0,604	6,37361E-12
Macro_ISG15	PPP4C	3,46396E-28	7,06313E-01	0,607	0,507	6,37957E-24
Macro_ISG15	PIK3AP1	6,38141E-70	7,05813E-01	0,447	0,296	1,17526E-65
Macro_ISG15	DNAJA1	2,35617E-39	7,03521E-01	0,696	0,599	4,33935E-35
Macro_ISG15	RHBDF2	3,47234E-60	7,01892E-01	0,459	0,316	6,39502E-56
Macro_ISG15	NECAP2	1,67653E-09	7,01699E-01	0,406	0,349	3,08767E-05
Macro_ISG15	ITGAX	1,27712E-29	6,99829E-01	0,504	0,393	2,35206E-25
Macro_ISG15	SLC37A2	2,40305E-45	6,99730E-01	0,261	0,159	4,42570E-41
Macro_ISG15	DNMT1	6,67052E-15	6,99229E-01	0,385	0,313	1,22851E-10
Macro_ISG15	NANS	7,07173E-08	6,98820E-01	0,435	0,387	1,30240E-03
Macro_ISG15	VPS26A	1,40794E-15	6,98055E-01	0,292	0,226	2,59301E-11
Macro_ISG15	ACSL3	1,52194E-17	6,97555E-01	0,321	0,249	2,80295E-13
Macro_ISG15	CEP170	9,93251E-15	6,95938E-01	0,414	0,34	1,82927E-10
Macro_ISG15	OAZ2	3,10273E-11	6,95574E-01	0,341	0,28	5,71429E-07
Macro_ISG15	DEK	5,46403E-31	6,94672E-01	0,665	0,569	1,00631E-26
Macro_ISG15	PRR13	2,84369E-31	6,94183E-01	0,67	0,575	5,23722E-27
Macro_ISG15	SLC31A1	8,14340E-30	6,92551E-01	0,296	0,21	1,49977E-25
Macro_ISG15	CYTH1	8,74222E-46	6,90747E-01	0,445	0,32	1,61005E-41
Macro_ISG15	SNRPB	6,08923E-09	6,89682E-01	0,566	0,505	1,12145E-04
Macro_ISG15	STARD3NL	7,65164E-07	6,89284E-01	0,311	0,268	1,40920E-02
Macro_ISG15	NSFL1C	2,41184E-12	6,89244E-01	0,331	0,271	4,44189E-08
Macro_ISG15	TNFRSF1B	2,67764E-54	6,89115E-01	0,669	0,532	4,93140E-50
Macro_ISG15	UBXN11	3,41561E-27	6,88143E-01	0,289	0,201	6,29053E-23
Macro_ISG15	SURF4	3,92523E-09	6,87200E-01	0,342	0,288	7,22910E-05
Macro_ISG15	PDCD6IP	2,67150E-14	6,86530E-01	0,438	0,364	4,92009E-10
Macro_ISG15	STK10	2,20602E-08	6,85935E-01	0,289	0,241	4,06284E-04
Macro_ISG15	ZDHHC12	2,21856E-19	6,85880E-01	0,333	0,257	4,08592E-15
Macro_ISG15	SFT2D1	1,99407E-09	6,81961E-01	0,514	0,456	3,67247E-05
Macro_ISG15	GALK1	1,93713E-13	6,81009E-01	0,282	0,222	3,56761E-09
Macro_ISG15	B3GAT3	7,95276E-20	6,79710E-01	0,311	0,237	1,46466E-15
Macro_ISG15	HK1	7,40801E-42	6,78935E-01	0,372	0,256	1,36433E-37

Macro_ISG15	UBC	3,22159E-11	6,78898E-01	0,933	0,897	5,93320E-07
Macro_ISG15	C4orf3	6,03885E-84	6,76805E-01	0,796	0,663	1,11218E-79
Macro_ISG15	SPHK1	1,55095E-232	6,76651E-01	0,406	0,166	2,85639E-228
Macro_ISG15	UQCRC1	3,28424E-25	6,76425E-01	0,597	0,5	6,04858E-21
Macro_ISG15	NKIRAS2	1,01663E-12	6,75705E-01	0,255	0,202	1,87233E-08
Macro_ISG15	SCPEP1	8,31534E-16	6,74896E-01	0,487	0,403	1,53144E-11
Macro_ISG15	NUP62	2,45025E-47	6,72488E-01	0,343	0,23	4,51263E-43
Macro_ISG15	HPCAL1	3,05483E-33	6,72250E-01	0,342	0,241	5,62607E-29
Macro_ISG15	ENY2	2,21784E-25	6,71774E-01	0,681	0,59	4,08460E-21
Macro_ISG15	GGA1	1,51072E-28	6,71198E-01	0,327	0,234	2,78229E-24
Macro_ISG15	PTPN12	4,04365E-09	6,70838E-01	0,272	0,224	7,44719E-05
Macro_ISG15	NDUFB7	5,44117E-29	6,70832E-01	0,622	0,524	1,00210E-24
Macro_ISG15	TMSB10	1,26669E-153	6,69550E-01	0,991	0,984	2,33286E-149
Macro_ISG15	FBXL15	7,28924E-39	6,69423E-01	0,305	0,207	1,34246E-34
Macro_ISG15	ARL8B	1,91492E-23	6,69160E-01	0,494	0,398	3,52670E-19
Macro_ISG15	ABCA1	5,44214E-63	6,66750E-01	0,458	0,306	1,00228E-58
Macro_ISG15	LYPLA2	2,83619E-15	6,66550E-01	0,282	0,221	5,22341E-11
Macro_ISG15	AP2S1	6,46105E-15	6,66242E-01	0,778	0,716	1,18993E-10
Macro_ISG15	LYN	8,37623E-59	6,65986E-01	0,572	0,445	1,54265E-54
Macro_ISG15	GCH1	1,13575E-99	6,64740E-01	0,298	0,15	2,09171E-95
Macro_ISG15	MYL12A	8,14925E-63	6,64623E-01	0,886	0,821	1,50085E-58
Macro_ISG15	SNX6	2,51370E-33	6,64359E-01	0,608	0,51	4,62948E-29
Macro_ISG15	BIN2	2,58156E-06	6,64202E-01	0,363	0,311	4,75446E-02
Macro_ISG15	RRBP1	5,21332E-74	6,63273E-01	0,531	0,376	9,60136E-70
Macro_ISG15	S100A6	2,99429E-46	6,62772E-01	0,944	0,87	5,51459E-42
Macro_ISG15	TMED9	2,49810E-08	6,62750E-01	0,58	0,52	4,60075E-04
Macro_ISG15	HSH2D	1,31936E-240	6,62729E-01	0,254	0,075	2,42987E-236
Macro_ISG15	ODF3B	1,10867E-86	6,61156E-01	0,506	0,336	2,04185E-82
Macro_ISG15	TOR1A	8,23041E-24	6,60442E-01	0,273	0,195	1,51580E-19
Macro_ISG15	NRBP1	1,24039E-10	6,58426E-01	0,363	0,304	2,28442E-06
Macro_ISG15	DDAH2	5,63169E-22	6,58395E-01	0,505	0,413	1,03719E-17
Macro_ISG15	HSPA5	1,00891E-10	6,57445E-01	0,619	0,558	1,85812E-06
Macro_ISG15	HMGN2	2,96341E-25	6,56874E-01	0,796	0,73	5,45771E-21
Macro_ISG15	GIMAP7	1,48062E-29	6,55178E-01	0,346	0,25	2,72687E-25
Macro_ISG15	PSMC2	1,87625E-08	6,53786E-01	0,331	0,284	3,45549E-04
Macro_ISG15	TGIF1	1,02448E-31	6,53336E-01	0,42	0,318	1,88679E-27
Macro_ISG15	IVNS1ABP	5,16321E-20	6,53239E-01	0,449	0,361	9,50908E-16
Macro_ISG15	TBC1D9B	2,24288E-44	6,52885E-01	0,337	0,227	4,13072E-40
Macro_ISG15	SERF2	5,12459E-107	6,52826E-01	0,971	0,953	9,43795E-103
Macro_ISG15	WDFY1	2,47195E-50	6,45589E-01	0,256	0,15	4,55259E-46
Macro_ISG15	CORO1C	3,43580E-32	6,45168E-01	0,445	0,338	6,32771E-28
Macro_ISG15	RABAC1	1,18586E-25	6,43004E-01	0,607	0,52	2,18399E-21
Macro_ISG15	PFKFB3	2,57035E-38	6,40328E-01	0,444	0,327	4,73382E-34

Macro_ISG15	COX5B	9,59920E-65	6,39847E-01	0,856	0,767	1,76788E-60
Macro_ISG15	LCP2	7,05314E-68	6,38403E-01	0,728	0,567	1,29898E-63
Macro_ISG15	TWF2	2,96429E-09	6,36998E-01	0,384	0,331	5,45934E-05
Macro_ISG15	IRF5	1,03180E-14	6,35658E-01	0,326	0,258	1,90026E-10
Macro_ISG15	WIPF1	1,66890E-70	6,35349E-01	0,603	0,457	3,07362E-66
Macro_ISG15	CFLAR	8,55042E-14	6,33644E-01	0,588	0,507	1,57473E-09
Macro_ISG15	ISCA2	1,82335E-11	6,32759E-01	0,26	0,209	3,35806E-07
Macro_ISG15	COX6A1	3,04322E-14	6,30527E-01	0,757	0,675	5,60469E-10
Macro_ISG15	ATOX1	4,67326E-86	6,28461E-01	0,711	0,567	8,60674E-82
Macro_ISG15	GNA13	1,55587E-41	6,27754E-01	0,446	0,327	2,86545E-37
Macro_ISG15	STAT3	3,42804E-27	6,27614E-01	0,601	0,5	6,31343E-23
Macro_ISG15	FMNL1	1,37791E-41	6,26562E-01	0,525	0,395	2,53769E-37
Macro_ISG15	GSDMD	3,24218E-35	6,24890E-01	0,402	0,294	5,97111E-31
Macro_ISG15	GUK1	6,43251E-23	6,23665E-01	0,781	0,706	1,18468E-18
Macro_ISG15	DPYD	6,44043E-07	6,23254E-01	0,42	0,363	1,18613E-02
Macro_ISG15	ATF3	4,89881E-24	6,22417E-01	0,527	0,427	9,02214E-20
Macro_ISG15	MIIP	2,09275E-41	6,22182E-01	0,35	0,238	3,85422E-37
Macro_ISG15	PRDX5	2,57091E-09	6,21897E-01	0,655	0,597	4,73485E-05
Macro_ISG15	DRAM1	6,63045E-28	6,21857E-01	0,298	0,213	1,22113E-23
Macro_ISG15	ADAMTSL4	7,56597E-122	6,19998E-01	0,251	0,107	1,39342E-117
Macro_ISG15	NAIP	1,11960E-37	6,19805E-01	0,485	0,364	2,06196E-33
Macro_ISG15	PCGF5	2,75759E-11	6,18856E-01	0,326	0,269	5,07865E-07
Macro_ISG15	SEC61A1	1,09836E-30	6,18280E-01	0,407	0,305	2,02285E-26
Macro_ISG15	RGS19	9,37887E-13	6,18125E-01	0,57	0,491	1,72731E-08
Macro_ISG15	C18orf32	5,41831E-18	6,16751E-01	0,349	0,276	9,97891E-14
Macro_ISG15	PHC2	8,92639E-66	6,16749E-01	0,376	0,236	1,64397E-61
Macro_ISG15	SERTAD1	2,41422E-34	6,16698E-01	0,425	0,319	4,44627E-30
Macro_ISG15	EIF4G1	6,87366E-11	6,16631E-01	0,389	0,329	1,26592E-06
Macro_ISG15	MT-ND4L	1,69580E-06	6,14697E-01	0,506	0,515	3,12315E-02
Macro_ISG15	RNF114	4,86151E-64	6,13687E-01	0,502	0,362	8,95344E-60
Macro_ISG15	CYTH4	9,30787E-43	6,13597E-01	0,428	0,308	1,71423E-38
Macro_ISG15	TMEM179B	9,95615E-20	6,12274E-01	0,55	0,478	1,83362E-15
Macro_ISG15	ARL6IP1	9,08734E-09	6,11351E-01	0,465	0,412	1,67362E-04
Macro_ISG15	HLA-C	1,03579E-10	6,10994E-01	0,98	0,949	1,90761E-06
Macro_ISG15	ATP6V0C	1,47671E-140	6,10820E-01	0,811	0,691	2,71966E-136
Macro_ISG15	NUDC	5,40473E-08	6,09588E-01	0,427	0,377	9,95389E-04
Macro_ISG15	RBM47	1,36745E-36	6,09563E-01	0,468	0,352	2,51843E-32
Macro_ISG15	GNAI3	2,98616E-10	6,09236E-01	0,399	0,343	5,49961E-06
Macro_ISG15	U2AF1	5,87280E-09	6,06565E-01	0,319	0,266	1,08159E-04
Macro_ISG15	PDIA6	3,13270E-11	6,06337E-01	0,579	0,516	5,76949E-07
Macro_ISG15	FOSL2	1,90229E-32	6,06253E-01	0,4	0,289	3,50345E-28
Macro_ISG15	NR1H2	9,90202E-14	6,05565E-01	0,393	0,327	1,82365E-09
Macro_ISG15	RNASET2	2,05015E-36	6,05514E-01	0,83	0,735	3,77576E-32

Macro_ISG15	BCAT1	9,67415E-16	6,04722E-01	0,305	0,234	1,78169E-11
Macro_ISG15	DNAJB6	1,01040E-07	6,04558E-01	0,567	0,519	1,86085E-03
Macro_ISG15	YIF1B	6,34550E-19	6,03562E-01	0,428	0,346	1,16865E-14
Macro_ISG15	ZYX	5,84919E-14	6,01723E-01	0,73	0,64	1,07725E-09
Macro_ISG15	VAT1	5,45313E-20	6,01022E-01	0,314	0,231	1,00430E-15
Macro_ISG15	FERMT3	4,18051E-24	6,00288E-01	0,571	0,477	7,69924E-20
Macro_ISG15	UNC93B1	1,61024E-97	5,98239E-01	0,5	0,326	2,96558E-93
Macro_ISG15	CALM2	4,90626E-32	5,98032E-01	0,767	0,783	9,03586E-28
Macro_ISG15	NCF1	9,74965E-19	5,97082E-01	0,601	0,494	1,79559E-14
Macro_ISG15	FGD2	3,56675E-20	5,96776E-01	0,424	0,34	6,56889E-16
Macro_ISG15	TMEM30A	9,83479E-33	5,96007E-01	0,4	0,299	1,81127E-28
Macro_ISG15	TNFRSF14	3,45730E-22	5,95596E-01	0,537	0,444	6,36730E-18
Macro_ISG15	AP2M1	7,11854E-11	5,95296E-01	0,64	0,58	1,31102E-06
Macro_ISG15	GMFG	6,20435E-07	5,95284E-01	0,73	0,67	1,14266E-02
Macro_ISG15	PTPRE	1,01491E-22	5,93986E-01	0,537	0,434	1,86916E-18
Macro_ISG15	XPO1	3,15008E-28	5,93864E-01	0,337	0,25	5,80151E-24
Macro_ISG15	DNAJC8	1,06324E-06	5,93641E-01	0,461	0,411	1,95817E-02
Macro_ISG15	PAPSS1	7,14272E-20	5,92889E-01	0,308	0,231	1,31548E-15
Macro_ISG15	PLEKHB2	5,51682E-12	5,92872E-01	0,508	0,436	1,01603E-07
Macro_ISG15	RAB31	5,39652E-19	5,91738E-01	0,743	0,637	9,93876E-15
Macro_ISG15	TIMP2	1,46284E-103	5,90800E-01	0,535	0,35	2,69411E-99
Macro_ISG15	POMP	1,70082E-40	5,90781E-01	0,816	0,714	3,13240E-36
Macro_ISG15	RNF7	5,84557E-12	5,90675E-01	0,601	0,541	1,07658E-07
Macro_ISG15	CACYBP	1,42023E-09	5,89801E-01	0,451	0,398	2,61564E-05
Macro_ISG15	PSMB10	5,09327E-32	5,89705E-01	0,663	0,561	9,38028E-28
Macro_ISG15	USP15	6,28003E-32	5,89273E-01	0,523	0,418	1,15659E-27
Macro_ISG15	SPCS3	7,77925E-41	5,88183E-01	0,557	0,438	1,43270E-36
Macro_ISG15	HMGB2	9,81936E-13	5,88025E-01	0,455	0,395	1,80843E-08
Macro_ISG15	TUBA1C	2,09728E-42	5,87934E-01	0,488	0,373	3,86255E-38
Macro_ISG15	GTF2F1	2,67145E-12	5,87642E-01	0,311	0,251	4,92002E-08
Macro_ISG15	SERPINB9	7,63555E-14	5,86885E-01	0,45	0,37	1,40624E-09
Macro_ISG15	COPE	1,14510E-41	5,84891E-01	0,706	0,601	2,10893E-37
Macro_ISG15	ZNF706	6,28373E-07	5,84887E-01	0,641	0,588	1,15727E-02
Macro_ISG15	DNAJC7	1,02385E-06	5,83494E-01	0,411	0,363	1,88563E-02
Macro_ISG15	LAMTOR2	6,50271E-21	5,82037E-01	0,623	0,538	1,19760E-16
Macro_ISG15	MBD2	1,41044E-17	5,81748E-01	0,265	0,199	2,59761E-13
Macro_ISG15	ERP44	9,03535E-28	5,81413E-01	0,515	0,423	1,66404E-23
Macro_ISG15	SNRPC	7,79054E-18	5,78691E-01	0,492	0,414	1,43478E-13
Macro_ISG15	FOS	3,24954E-13	5,78679E-01	0,88	0,841	5,98468E-09
Macro_ISG15	PLK3	1,13759E-11	5,76191E-01	0,31	0,249	2,09511E-07
Macro_ISG15	DERL1	6,04494E-09	5,75553E-01	0,32	0,271	1,11330E-04
Macro_ISG15	TLR1	1,29941E-18	5,75199E-01	0,27	0,2	2,39313E-14
Macro_ISG15	MIA3	6,69736E-08	5,73944E-01	0,265	0,219	1,23345E-03

Macro_ISG15	TXNDC17	5,00388E-50	5,73423E-01	0,554	0,426	9,21564E-46
Macro_ISG15	TPMT	2,28447E-06	5,73154E-01	0,255	0,218	4,20731E-02
Macro_ISG15	MSN	9,63598E-09	5,69881E-01	0,703	0,634	1,77466E-04
Macro_ISG15	PPDPF	5,57678E-114	5,68317E-01	0,86	0,762	1,02708E-109
Macro_ISG15	NDUFS5	2,23519E-33	5,68030E-01	0,737	0,649	4,11655E-29
Macro_ISG15	PNPLA6	1,71584E-38	5,66922E-01	0,299	0,2	3,16006E-34
Macro_ISG15	CSNK1D	2,58998E-15	5,66158E-01	0,411	0,339	4,76996E-11
Macro_ISG15	NDRG1	8,15054E-24	5,66065E-01	0,27	0,193	1,50109E-19
Macro_ISG15	RALGDS	1,72049E-48	5,65853E-01	0,294	0,183	3,16862E-44
Macro_ISG15	LEPROTL1	4,30460E-11	5,64691E-01	0,461	0,395	7,92778E-07
Macro_ISG15	MEA1	7,95098E-07	5,64621E-01	0,436	0,387	1,46433E-02
Macro_ISG15	C7orf50	2,15517E-37	5,64016E-01	0,438	0,331	3,96918E-33
Macro_ISG15	COA3	7,20333E-15	5,63417E-01	0,387	0,316	1,32664E-10
Macro_ISG15	IL4R	2,77506E-38	5,63257E-01	0,302	0,2	5,11083E-34
Macro_ISG15	GNB1	1,03081E-20	5,63117E-01	0,471	0,383	1,89844E-16
Macro_ISG15	ROMO1	9,44034E-46	5,62678E-01	0,65	0,523	1,73863E-41
Macro_ISG15	ATP6V1E1	1,66362E-09	5,62152E-01	0,385	0,328	3,06389E-05
Macro_ISG15	MYO9B	2,01667E-70	5,61681E-01	0,539	0,379	3,71409E-66
Macro_ISG15	POLR2F	1,14494E-09	5,60777E-01	0,361	0,31	2,10864E-05
Macro_ISG15	ABR	7,09942E-41	5,60023E-01	0,307	0,204	1,30750E-36
Macro_ISG15	TLR4	2,16003E-15	5,57649E-01	0,286	0,22	3,97813E-11
Macro_ISG15	DOCK8	2,32114E-54	5,56906E-01	0,576	0,44	4,27484E-50
Macro_ISG15	CMKLR1	1,14448E-73	5,56601E-01	0,265	0,141	2,10780E-69
Macro_ISG15	FBXO6	5,12331E-43	5,56168E-01	0,26	0,164	9,43559E-39
Macro_ISG15	DYNLRB1	1,65752E-35	5,55904E-01	0,562	0,457	3,05265E-31
Macro_ISG15	UBE2D1	1,29805E-06	5,55514E-01	0,407	0,36	2,39061E-02
Macro_ISG15	ARHGDIB	3,18648E-08	5,54695E-01	0,831	0,822	5,86854E-04
Macro_ISG15	LEPROT	1,04804E-07	5,54443E-01	0,405	0,352	1,93018E-03
Macro_ISG15	GTF3C6	1,59384E-20	5,52926E-01	0,454	0,366	2,93537E-16
Macro_ISG15	ARSA	2,39831E-70	5,51725E-01	0,367	0,226	4,41697E-66
Macro_ISG15	BRI3	6,68142E-80	5,49084E-01	0,557	0,393	1,23052E-75
Macro_ISG15	TMEM51	1,20344E-55	5,48546E-01	0,384	0,254	2,21638E-51
Macro_ISG15	RRP7A	1,27618E-15	5,48365E-01	0,361	0,29	2,35035E-11
Macro_ISG15	PRDX1	6,67351E-09	5,47683E-01	0,789	0,744	1,22906E-04
Macro_ISG15	ZNF385A	4,68502E-53	5,46647E-01	0,548	0,404	8,62840E-49
Macro_ISG15	PLBD1	3,22499E-10	5,45403E-01	0,359	0,395	5,93946E-06
Macro_ISG15	SCAMP3	4,34116E-09	5,44567E-01	0,3	0,248	7,99511E-05
Macro_ISG15	DSE	1,61630E-55	5,42227E-01	0,576	0,43	2,97674E-51
Macro_ISG15	IRF1	2,63020E-08	5,41503E-01	0,508	0,434	4,84403E-04
Macro_ISG15	PYCARD	4,01510E-32	5,41332E-01	0,794	0,701	7,39460E-28
Macro_ISG15	OSTM1	3,58601E-10	5,40651E-01	0,275	0,222	6,60436E-06
Macro_ISG15	DNM2	2,13829E-42	5,37833E-01	0,405	0,29	3,93809E-38
Macro_ISG15	PLXDC2	4,06971E-13	5,37360E-01	0,632	0,539	7,49518E-09

Macro_ISG15	PFDN1	4,97123E-10	5,37031E-01	0,339	0,283	9,15551E-06
Macro_ISG15	EIF3I	7,97885E-08	5,36899E-01	0,437	0,467	1,46947E-03
Macro_ISG15	TRMT112	3,63276E-09	5,36885E-01	0,673	0,615	6,69045E-05
Macro_ISG15	NTMT1	1,27711E-10	5,36624E-01	0,253	0,202	2,35206E-06
Macro_ISG15	ICAM1	2,83814E-10	5,36588E-01	0,42	0,358	5,22701E-06
Macro_ISG15	IMPDH1	7,37156E-08	5,34911E-01	0,261	0,215	1,35762E-03
Macro_ISG15	MRPL18	3,75539E-13	5,34265E-01	0,405	0,345	6,91629E-09
Macro_ISG15	ACTG1	2,49561E-24	5,34196E-01	0,935	0,922	4,59617E-20
Macro_ISG15	AP1B1	2,37410E-15	5,31567E-01	0,462	0,389	4,37239E-11
Macro_ISG15	TMEM259	9,98186E-34	5,30657E-01	0,339	0,241	1,83836E-29
Macro_ISG15	KCNAB2	2,24753E-27	5,29148E-01	0,369	0,275	4,13927E-23
Macro_ISG15	TMEM219	5,45946E-23	5,28917E-01	0,711	0,632	1,00547E-18
Macro_ISG15	SMIM15	2,12323E-11	5,28285E-01	0,268	0,214	3,91035E-07
Macro_ISG15	POLD4	3,67888E-12	5,28145E-01	0,642	0,565	6,77539E-08
Macro_ISG15	IDH2	1,48789E-36	5,27807E-01	0,517	0,405	2,74025E-32
Macro_ISG15	AHSA1	4,10389E-12	5,26444E-01	0,353	0,296	7,55813E-08
Macro_ISG15	C14orf119	8,39211E-11	5,24453E-01	0,347	0,289	1,54557E-06
Macro_ISG15	THEMIS2	3,65954E-75	5,24349E-01	0,635	0,486	6,73978E-71
Macro_ISG15	MRPL27	8,52915E-48	5,23208E-01	0,405	0,281	1,57081E-43
Macro_ISG15	NOSIP	1,77980E-12	5,23002E-01	0,387	0,324	3,27786E-08
Macro_ISG15	CUEDC2	2,45258E-09	5,22898E-01	0,343	0,292	4,51692E-05
Macro_ISG15	ETNK1	3,13165E-08	5,21405E-01	0,294	0,245	5,76755E-04
Macro_ISG15	FYTTD1	2,94294E-09	5,20562E-01	0,3	0,245	5,42001E-05
Macro_ISG15	SZRD1	6,65575E-37	5,18701E-01	0,394	0,288	1,22579E-32
Macro_ISG15	NEK6	2,30606E-19	5,18328E-01	0,31	0,234	4,24707E-15
Macro_ISG15	IER5	2,87216E-34	5,18277E-01	0,421	0,32	5,28966E-30
Macro_ISG15	PFDN5	7,22860E-19	5,16265E-01	0,919	0,901	1,33129E-14
Macro_ISG15	RAC1	4,71571E-53	5,15651E-01	0,78	0,682	8,68492E-49
Macro_ISG15	UQCR10	1,08742E-65	5,15498E-01	0,816	0,721	2,00271E-61
Macro_ISG15	CHD1	2,04915E-15	5,15490E-01	0,362	0,293	3,77393E-11
Macro_ISG15	MAP1LC3B	2,84510E-12	5,14802E-01	0,617	0,554	5,23982E-08
Macro_ISG15	SAP18	9,43276E-12	5,14724E-01	0,705	0,644	1,73723E-07
Macro_ISG15	CHORDC1	1,04179E-11	5,13574E-01	0,286	0,231	1,91866E-07
Macro_ISG15	HSPH1	4,41275E-13	5,11886E-01	0,461	0,412	8,12696E-09
Macro_ISG15	DUSP23	1,03376E-21	5,11311E-01	0,472	0,378	1,90388E-17
Macro_ISG15	MED15	1,07112E-24	5,10604E-01	0,296	0,215	1,97268E-20
Macro_ISG15	DESI1	1,54037E-15	5,09930E-01	0,287	0,224	2,83690E-11
Macro_ISG15	ARF3	3,53108E-17	5,08801E-01	0,409	0,333	6,50320E-13
Macro_ISG15	RNASEH2C	1,24048E-21	5,08788E-01	0,428	0,341	2,28458E-17
Macro_ISG15	CD300LF	2,34847E-43	5,08441E-01	0,272	0,17	4,32518E-39
Macro_ISG15	OTUB1	4,35527E-15	5,07399E-01	0,499	0,426	8,02110E-11
Macro_ISG15	COA6	4,21723E-62	5,06546E-01	0,376	0,245	7,76687E-58
Macro_ISG15	PFN1	6,17994E-51	5,04791E-01	0,974	0,945	1,13816E-46

Macro_ISG15	PDXK	2,25245E-30	5,04651E-01	0,398	0,296	4,14833E-26
Macro_ISG15	DOCK10	9,97000E-22	5,04313E-01	0,405	0,318	1,83617E-17
Macro_ISG15	RBX1	7,28335E-75	5,04288E-01	0,738	0,612	1,34137E-70
Macro_ISG15	JMJD6	3,26378E-11	5,01918E-01	0,26	0,208	6,01090E-07
Macro_ISG15	TAGLN2	5,70873E-24	5,01541E-01	0,739	0,755	1,05138E-19
Macro_ISG15	MRPL14	4,84862E-12	5,00804E-01	0,403	0,34	8,92971E-08
Macro_ISG15	ZNHIT1	1,92408E-07	5,00479E-01	0,53	0,476	3,54357E-03
Macro_ISG15	BAZ1A	5,38939E-41	4,99341E-01	0,532	0,412	9,92564E-37
Macro_ISG15	NAGA	6,84139E-10	4,98726E-01	0,421	0,357	1,25998E-05
Macro_ISG15	SSR3	4,46780E-07	4,98529E-01	0,581	0,597	8,22836E-03
Macro_ISG15	MBD4	2,05440E-15	4,97341E-01	0,31	0,246	3,78359E-11
Macro_ISG15	CEBPB	1,01925E-95	4,96281E-01	0,652	0,461	1,87716E-91
Macro_ISG15	ATP13A1	1,72358E-55	4,96162E-01	0,26	0,152	3,17431E-51
Macro_ISG15	AK2	1,69907E-23	4,95392E-01	0,428	0,337	3,12918E-19
Macro_ISG15	TMOD3	1,05669E-13	4,95198E-01	0,411	0,34	1,94610E-09
Macro_ISG15	MGAT4A	7,50316E-38	4,94702E-01	0,468	0,35	1,38186E-33
Macro_ISG15	UBL5	1,24531E-18	4,94162E-01	0,832	0,75	2,29348E-14
Macro_ISG15	PRKCD	1,25958E-17	4,93795E-01	0,321	0,252	2,31977E-13
Macro_ISG15	CALHM6	1,70530E-45	4,93246E-01	0,365	0,255	3,14065E-41
Macro_ISG15	TRPV2	2,48084E-13	4,91601E-01	0,263	0,206	4,56895E-09
Macro_ISG15	BAX	9,11489E-49	4,90984E-01	0,636	0,532	1,67869E-44
Macro_ISG15	ACIN1	6,51596E-11	4,90185E-01	0,331	0,273	1,20004E-06
Macro_ISG15	FNIP2	4,25580E-36	4,88233E-01	0,316	0,215	7,83791E-32
Macro_ISG15	ARHGAP18	2,62888E-29	4,87825E-01	0,575	0,47	4,84161E-25
Macro_ISG15	KPNB1	2,79540E-08	4,87743E-01	0,491	0,437	5,14829E-04
Macro_ISG15	TRPC4AP	4,58879E-29	4,86727E-01	0,302	0,215	8,45118E-25
Macro_ISG15	MMP14	8,40842E-51	4,84790E-01	0,333	0,218	1,54858E-46
Macro_ISG15	SLC38A10	1,01433E-33	4,83783E-01	0,317	0,222	1,86809E-29
Macro_ISG15	POLE4	4,27192E-08	4,83744E-01	0,378	0,328	7,86759E-04
Macro_ISG15	CTDSP1	1,21731E-11	4,83743E-01	0,267	0,211	2,24191E-07
Macro_ISG15	SLC8A1	6,71593E-18	4,83607E-01	0,449	0,36	1,23687E-13
Macro_ISG15	ARHGAP30	5,89586E-09	4,83461E-01	0,387	0,328	1,08584E-04
Macro_ISG15	TUBGCP2	3,34445E-10	4,83034E-01	0,355	0,3	6,15947E-06
Macro_ISG15	NFKBIZ	2,42051E-08	4,83017E-01	0,41	0,457	4,45785E-04
Macro_ISG15	PUM1	8,23412E-13	4,82927E-01	0,298	0,239	1,51648E-08
Macro_ISG15	NRP2	2,89448E-09	4,80756E-01	0,259	0,207	5,33077E-05
Macro_ISG15	AOAH	3,58707E-09	4,79556E-01	0,437	0,371	6,60630E-05
Macro_ISG15	PARP10	1,75596E-157	4,78320E-01	0,407	0,203	3,23394E-153
Macro_ISG15	CORO1A	1,64346E-39	4,77286E-01	0,755	0,639	3,02676E-35
Macro_ISG15	PYGL	4,37042E-22	4,77267E-01	0,278	0,198	8,04901E-18
Macro_ISG15	RHEB	6,84820E-14	4,77133E-01	0,526	0,458	1,26123E-09
Macro_ISG15	RABGAP1L	1,25995E-106	4,77060E-01	0,416	0,242	2,32045E-102
Macro_ISG15	CAPN2	6,17347E-27	4,75229E-01	0,318	0,229	1,13697E-22

Macro_ISG15	FKBP3	6,87118E-07	4,75164E-01	0,274	0,233	1,26546E-02
Macro_ISG15	CORO1B	8,02227E-11	4,74122E-01	0,535	0,469	1,47746E-06
Macro_ISG15	GCHFR	1,38942E-70	4,74085E-01	0,309	0,177	2,55890E-66
Macro_ISG15	PLEKHO1	2,31532E-95	4,73979E-01	0,522	0,351	4,26413E-91
Macro_ISG15	FNDC3A	2,17789E-17	4,73091E-01	0,306	0,236	4,01103E-13
Macro_ISG15	STARD3	1,72859E-13	4,72748E-01	0,299	0,238	3,18354E-09
Macro_ISG15	PLBD2	8,08418E-26	4,72508E-01	0,252	0,175	1,48886E-21
Macro_ISG15	RNPEPL1	2,22860E-13	4,72059E-01	0,253	0,196	4,10441E-09
Macro_ISG15	FAM50A	1,51759E-15	4,71294E-01	0,369	0,3	2,79494E-11
Macro_ISG15	GNB2	2,20423E-20	4,67919E-01	0,635	0,552	4,05953E-16
Macro_ISG15	FGFR1OP2	5,34071E-13	4,66815E-01	0,367	0,302	9,83599E-09
Macro_ISG15	GRINA	2,00662E-36	4,66779E-01	0,777	0,677	3,69560E-32
Macro_ISG15	UBR4	3,20380E-08	4,65891E-01	0,291	0,243	5,90044E-04
Macro_ISG15	RALA	4,24257E-33	4,65650E-01	0,47	0,368	7,81353E-29
Macro_ISG15	DCTN2	7,33791E-07	4,64695E-01	0,297	0,331	1,35142E-02
Macro_ISG15	NDUFB2	6,02811E-46	4,64409E-01	0,799	0,709	1,11020E-41
Macro_ISG15	FYB1	6,25119E-50	4,64391E-01	0,431	0,311	1,15128E-45
Macro_ISG15	ERGIC1	4,94542E-08	4,64096E-01	0,343	0,288	9,10798E-04
Macro_ISG15	SLC6A6	1,11065E-26	4,63986E-01	0,261	0,18	2,04549E-22
Macro_ISG15	FGL2	2,48920E-15	4,63699E-01	0,714	0,608	4,58436E-11
Macro_ISG15	ST3GAL1	3,45814E-12	4,62893E-01	0,287	0,226	6,36886E-08
Macro_ISG15	AP1S2	1,52491E-32	4,61320E-01	0,493	0,584	2,80844E-28
Macro_ISG15	NDUFS7	6,14663E-08	4,60983E-01	0,592	0,538	1,13203E-03
Macro_ISG15	TMEM127	7,83774E-24	4,60475E-01	0,261	0,185	1,44348E-19
Macro_ISG15	CHCHD7	8,13391E-11	4,59994E-01	0,308	0,254	1,49802E-06
Macro_ISG15	COX8A	6,20997E-09	4,59483E-01	0,818	0,775	1,14369E-04
Macro_ISG15	PFDN2	2,51514E-11	4,58488E-01	0,502	0,438	4,63214E-07
Macro_ISG15	UBE2L3	1,31668E-14	4,58459E-01	0,569	0,498	2,42493E-10
Macro_ISG15	IST1	2,40180E-06	4,58221E-01	0,301	0,262	4,42340E-02
Macro_ISG15	TSC22D1	2,90382E-47	4,57485E-01	0,26	0,156	5,34796E-43
Macro_ISG15	SH3BGRL	4,23100E-13	4,57420E-01	0,705	0,721	7,79223E-09
Macro_ISG15	COX6B1	8,32564E-25	4,56805E-01	0,875	0,807	1,53333E-20
Macro_ISG15	REEP4	2,73102E-18	4,52917E-01	0,31	0,236	5,02972E-14
Macro_ISG15	SNRPG	1,11946E-13	4,52461E-01	0,622	0,553	2,06170E-09
Macro_ISG15	RNF19B	1,11795E-70	4,51498E-01	0,272	0,149	2,05893E-66
Macro_ISG15	FKBP2	1,61051E-12	4,51384E-01	0,479	0,405	2,96608E-08
Macro_ISG15	KLF6	1,99894E-08	4,50678E-01	0,8	0,797	3,68145E-04
Macro_ISG15	LARP1	8,35703E-23	4,49996E-01	0,285	0,208	1,53911E-18
Macro_ISG15	UCP2	7,37496E-24	4,49343E-01	0,71	0,629	1,35825E-19
Macro_ISG15	GPX4	8,49971E-11	4,49285E-01	0,883	0,842	1,56539E-06
Macro_ISG15	COPA	2,33053E-13	4,49274E-01	0,403	0,334	4,29213E-09
Macro_ISG15	PFKL	6,65741E-11	4,48319E-01	0,457	0,389	1,22610E-06
Macro_ISG15	COX20	2,17486E-12	4,47073E-01	0,314	0,256	4,00545E-08

Macro_ISG15	ARHGAP15	8,07487E-08	4,47025E-01	0,216	0,257	1,48715E-03
Macro_ISG15	CIR1	3,95588E-15	4,46643E-01	0,341	0,274	7,28554E-11
Macro_ISG15	NCF4	1,83291E-19	4,46574E-01	0,51	0,417	3,37567E-15
Macro_ISG15	TCF12	7,37196E-11	4,46427E-01	0,272	0,22	1,35769E-06
Macro_ISG15	IRF7	0,00000E+00	4,46312E-01	0,823	0,373	0,00000E+00
Macro_ISG15	CDK5RAP3	3,14262E-10	4,46063E-01	0,366	0,303	5,78775E-06
Macro_ISG15	CNP	1,84111E-96	4,45837E-01	0,302	0,156	3,39078E-92
Macro_ISG15	RAP1B	3,11925E-08	4,45233E-01	0,559	0,581	5,74472E-04
Macro_ISG15	GNB4	7,38478E-56	4,44958E-01	0,458	0,329	1,36005E-51
Macro_ISG15	STX11	9,39686E-19	4,44901E-01	0,409	0,323	1,73062E-14
Macro_ISG15	LSM10	3,58555E-14	4,44347E-01	0,444	0,371	6,60351E-10
Macro_ISG15	TRIP12	2,14527E-11	4,44266E-01	0,332	0,273	3,95095E-07
Macro_ISG15	WBP2	1,33064E-10	4,42541E-01	0,439	0,38	2,45064E-06
Macro_ISG15	PLEK	9,81640E-13	4,41892E-01	0,643	0,557	1,80789E-08
Macro_ISG15	SSR2	3,55798E-24	4,41795E-01	0,609	0,655	6,55273E-20
Macro_ISG15	TXNIP	7,93375E-17	4,41585E-01	0,812	0,692	1,46116E-12
Macro_ISG15	C19orf38	1,44396E-24	4,40819E-01	0,318	0,229	2,65935E-20
Macro_ISG15	SEMA4A	7,64115E-42	4,40201E-01	0,32	0,217	1,40727E-37
Macro_ISG15	STX4	2,42919E-45	4,40053E-01	0,427	0,305	4,47385E-41
Macro_ISG15	PATL1	3,59500E-50	4,37661E-01	0,253	0,151	6,62091E-46
Macro_ISG15	UBA1	1,08480E-35	4,36341E-01	0,406	0,297	1,99788E-31
Macro_ISG15	PTBP1	3,53082E-13	4,35539E-01	0,374	0,303	6,50272E-09
Macro_ISG15	TFPT	7,94358E-29	4,33675E-01	0,327	0,239	1,46297E-24
Macro_ISG15	OGFR	2,41872E-84	4,33557E-01	0,488	0,325	4,45456E-80
Macro_ISG15	LACTB	1,02934E-11	4,33426E-01	0,383	0,32	1,89573E-07
Macro_ISG15	PPP1R18	8,22386E-25	4,31940E-01	0,597	0,495	1,51459E-20
Macro_ISG15	ITGAV	1,21158E-10	4,29586E-01	0,282	0,225	2,23136E-06
Macro_ISG15	LYSMD2	4,05764E-30	4,28765E-01	0,378	0,285	7,47296E-26
Macro_ISG15	SH3TC1	3,28638E-26	4,27456E-01	0,397	0,308	6,05252E-22
Macro_ISG15	CYTIP	5,03095E-23	4,27098E-01	0,318	0,396	9,26550E-19
Macro_ISG15	VAMP8	7,37128E-21	4,26282E-01	0,837	0,777	1,35757E-16
Macro_ISG15	TRIM38	1,32204E-53	4,23753E-01	0,407	0,277	2,43480E-49
Macro_ISG15	CHCHD1	3,46836E-10	4,23393E-01	0,321	0,267	6,38768E-06
Macro_ISG15	PPIB	1,86725E-26	4,20944E-01	0,847	0,789	3,43891E-22
Macro_ISG15	STAU1	3,02270E-11	4,20504E-01	0,349	0,287	5,56691E-07
Macro_ISG15	DPP7	5,48891E-20	4,16059E-01	0,625	0,552	1,01089E-15
Macro_ISG15	COX7A2	3,46449E-24	4,15455E-01	0,795	0,716	6,38056E-20
Macro_ISG15	ATP13A3	2,06219E-31	4,14681E-01	0,304	0,205	3,79794E-27
Macro_ISG15	DPH3	2,40923E-15	4,14386E-01	0,318	0,25	4,43708E-11
Macro_ISG15	ARHGAP27	2,26247E-41	4,14245E-01	0,293	0,19	4,16679E-37
Macro_ISG15	MS4A6A	1,05808E-06	4,14136E-01	0,819	0,708	1,94866E-02
Macro_ISG15	TMEM165	6,59731E-07	4,13934E-01	0,436	0,39	1,21503E-02
Macro_ISG15	NDUFB3	1,00992E-20	4,12177E-01	0,548	0,462	1,85998E-16

Macro_ISG15	CCS	4,23110E-14	4,11563E-01	0,322	0,258	7,79242E-10
Macro_ISG15	ELL2	9,00169E-54	4,11274E-01	0,415	0,276	1,65784E-49
Macro_ISG15	PDCD5	6,87751E-12	4,09525E-01	0,409	0,347	1,26663E-07
Macro_ISG15	KCTD12	9,88811E-36	4,09221E-01	0,611	0,483	1,82109E-31
Macro_ISG15	RAPGEF1	1,98119E-25	4,08535E-01	0,339	0,254	3,64875E-21
Macro_ISG15	CFDP1	5,08933E-09	4,08085E-01	0,272	0,225	9,37302E-05
Macro_ISG15	ABCD1	2,33684E-106	4,07415E-01	0,25	0,113	4,30375E-102
Macro_ISG15	COMMD4	2,76768E-25	4,06157E-01	0,318	0,232	5,09724E-21
Macro_ISG15	APOBR	5,84184E-68	4,06011E-01	0,319	0,186	1,07589E-63
Macro_ISG15	CYFIP1	4,98893E-13	4,05932E-01	0,394	0,319	9,18812E-09
Macro_ISG15	BEST1	7,49117E-11	4,05366E-01	0,282	0,226	1,37965E-06
Macro_ISG15	PPT1	3,41226E-08	4,05273E-01	0,648	0,651	6,28436E-04
Macro_ISG15	SUMO3	1,55812E-18	4,05251E-01	0,562	0,476	2,86958E-14
Macro_ISG15	UBXN1	1,91192E-26	4,04812E-01	0,577	0,632	3,52118E-22
Macro_ISG15	MED29	1,53817E-15	4,04604E-01	0,296	0,231	2,83286E-11
Macro_ISG15	GPBP1	4,12731E-09	4,03799E-01	0,403	0,34	7,60127E-05
Macro_ISG15	MACF1	4,06596E-22	4,02995E-01	0,403	0,315	7,48829E-18
Macro_ISG15	GNPDA1	4,74459E-15	4,00051E-01	0,324	0,255	8,73811E-11
Macro_ISG15	SDF2	1,99770E-08	3,99186E-01	0,281	0,234	3,67917E-04
Macro_ISG15	CD151	1,42263E-09	3,97802E-01	0,392	0,334	2,62005E-05
Macro_ISG15	BCL2L1	9,25764E-14	3,97362E-01	0,272	0,215	1,70498E-09
Macro_ISG15	NDUFB5	2,34802E-07	3,97118E-01	0,429	0,455	4,32435E-03
Macro_ISG15	SLC25A3	3,11464E-25	3,96226E-01	0,695	0,715	5,73624E-21
Macro_ISG15	MFSD12	1,23631E-97	3,95427E-01	0,335	0,18	2,27692E-93
Macro_ISG15	VEGFA	1,05012E-80	3,95347E-01	0,364	0,208	1,93400E-76
Macro_ISG15	NDUFA1	3,72274E-11	3,94432E-01	0,757	0,683	6,85617E-07
Macro_ISG15	ACOT13	1,23048E-34	3,94112E-01	0,285	0,193	2,26618E-30
Macro_ISG15	PQBP1	1,45098E-07	3,93570E-01	0,276	0,317	2,67228E-03
Macro_ISG15	CCT2	2,10452E-06	3,90903E-01	0,309	0,342	3,87590E-02
Macro_ISG15	MED8	5,88855E-07	3,89820E-01	0,264	0,22	1,08449E-02
Macro_ISG15	ANO6	5,97361E-17	3,89798E-01	0,286	0,218	1,10016E-12
Macro_ISG15	GNAI2	4,05467E-50	3,89318E-01	0,78	0,667	7,46748E-46
Macro_ISG15	NDUFA3	4,87345E-18	3,89268E-01	0,626	0,541	8,97543E-14
Macro_ISG15	ADAM17	6,47434E-08	3,89057E-01	0,271	0,223	1,19238E-03
Macro_ISG15	CISD2	2,74748E-21	3,88515E-01	0,449	0,365	5,06004E-17
Macro_ISG15	EMILIN2	1,81407E-58	3,87883E-01	0,438	0,293	3,34097E-54
Macro_ISG15	PRDM1	1,65344E-21	3,87833E-01	0,27	0,193	3,04513E-17
Macro_ISG15	COX16	3,01338E-26	3,87023E-01	0,438	0,34	5,54975E-22
Macro_ISG15	PPIF	2,82778E-33	3,85810E-01	0,46	0,352	5,20792E-29
Macro_ISG15	ZNF331	1,07601E-51	3,84910E-01	0,471	0,337	1,98169E-47
Macro_ISG15	DUSP3	3,77933E-11	3,84822E-01	0,32	0,259	6,96039E-07
Macro_ISG15	ARRDC1	1,22300E-07	3,84414E-01	0,341	0,291	2,25240E-03
Macro_ISG15	DEF6	3,25182E-08	3,84304E-01	0,286	0,238	5,98887E-04

Macro_ISG15	SMCHD1	6,15499E-76	3,83611E-01	0,475	0,315	1,13356E-71
Macro_ISG15	ARHGAP25	2,25594E-24	3,83134E-01	0,264	0,186	4,15477E-20
Macro_ISG15	RNPEP	7,40221E-20	3,81772E-01	0,41	0,326	1,36326E-15
Macro_ISG15	TMEM230	3,68129E-07	3,79886E-01	0,423	0,453	6,77983E-03
Macro_ISG15	SOAT1	4,57292E-29	3,78722E-01	0,375	0,28	8,42196E-25
Macro_ISG15	SEC61G	5,92087E-12	3,78187E-01	0,682	0,595	1,09045E-07
Macro_ISG15	MEF2A	1,49593E-08	3,78083E-01	0,481	0,418	2,75506E-04
Macro_ISG15	ARHGAP4	8,97056E-34	3,77469E-01	0,493	0,378	1,65211E-29
Macro_ISG15	BANF1	7,80865E-08	3,77444E-01	0,599	0,545	1,43812E-03
Macro_ISG15	MRPL40	1,37623E-28	3,76792E-01	0,358	0,267	2,53461E-24
Macro_ISG15	GOLGA4	1,56548E-07	3,76027E-01	0,378	0,332	2,88314E-03
Macro_ISG15	MBOAT7	1,49404E-13	3,75734E-01	0,306	0,246	2,75157E-09
Macro_ISG15	COX4I1	5,53989E-63	3,75172E-01	0,878	0,898	1,02028E-58
Macro_ISG15	SPI1	7,76441E-89	3,74762E-01	0,881	0,76	1,42997E-84
Macro_ISG15	SQOR	8,75961E-64	3,74326E-01	0,305	0,185	1,61326E-59
Macro_ISG15	GLA	8,42331E-11	3,74097E-01	0,363	0,308	1,55132E-06
Macro_ISG15	LAMP1	2,38059E-55	3,73691E-01	0,529	0,389	4,38433E-51
Macro_ISG15	LRP10	8,23143E-20	3,72904E-01	0,369	0,29	1,51598E-15
Macro_ISG15	LSP1	9,35244E-09	3,72241E-01	0,781	0,664	1,72244E-04
Macro_ISG15	CDV3	7,01636E-09	3,72118E-01	0,32	0,272	1,29220E-04
Macro_ISG15	NECTIN2	1,36032E-73	3,71730E-01	0,298	0,17	2,50531E-69
Macro_ISG15	COX14	8,78758E-13	3,70441E-01	0,532	0,458	1,61841E-08
Macro_ISG15	TOLLIP	2,90812E-16	3,70200E-01	0,301	0,234	5,35588E-12
Macro_ISG15	TET2	5,36437E-10	3,69376E-01	0,293	0,237	9,87956E-06
Macro_ISG15	ZMIZ1	3,14348E-46	3,68692E-01	0,377	0,258	5,78934E-42
Macro_ISG15	ACAP2	1,31358E-08	3,68347E-01	0,489	0,427	2,41922E-04
Macro_ISG15	SLC25A24	4,03009E-27	3,67717E-01	0,306	0,221	7,42221E-23
Macro_ISG15	CD93	5,80623E-24	3,67444E-01	0,366	0,274	1,06933E-19
Macro_ISG15	PDAP1	1,08317E-07	3,67069E-01	0,381	0,331	1,99488E-03
Macro_ISG15	EVL	2,00882E-09	3,67026E-01	0,419	0,353	3,69965E-05
Macro_ISG15	UBE2A	1,46498E-10	3,66752E-01	0,46	0,398	2,69806E-06
Macro_ISG15	TAPBP	5,03015E-11	3,65182E-01	0,645	0,575	9,26403E-07
Macro_ISG15	GM2A	1,13616E-42	3,64441E-01	0,488	0,368	2,09246E-38
Macro_ISG15	UQCRQ	3,99283E-15	3,63676E-01	0,737	0,661	7,35359E-11
Macro_ISG15	DENND3	1,92658E-18	3,62772E-01	0,3	0,228	3,54818E-14
Macro_ISG15	COX7B	4,08027E-24	3,62756E-01	0,749	0,662	7,51464E-20
Macro_ISG15	POLR1D	1,18474E-06	3,62713E-01	0,44	0,47	2,18193E-02
Macro_ISG15	UBALD2	1,92724E-56	3,62595E-01	0,283	0,169	3,54940E-52
Macro_ISG15	RAP2B	7,65832E-21	3,61139E-01	0,391	0,31	1,41043E-16
Macro_ISG15	FBXO7	3,70302E-07	3,60504E-01	0,367	0,319	6,81986E-03
Macro_ISG15	CLPTM1	1,29170E-06	3,60335E-01	0,301	0,257	2,37892E-02
Macro_ISG15	CAPN1	2,10759E-07	3,60318E-01	0,309	0,264	3,88155E-03
Macro_ISG15	ETFB	4,08869E-09	3,59532E-01	0,359	0,304	7,53015E-05

Macro_ISG15	JAK2	1,06299E-15	3,57984E-01	0,302	0,236	1,95770E-11
Macro_ISG15	HCLS1	2,18994E-14	3,57796E-01	0,727	0,723	4,03322E-10
Macro_ISG15	CREM	2,97705E-60	3,57738E-01	0,518	0,369	5,48282E-56
Macro_ISG15	CD38	4,17498E-64	3,54840E-01	0,305	0,178	7,68907E-60
Macro_ISG15	RAB13	2,26994E-24	3,54511E-01	0,39	0,299	4,18055E-20
Macro_ISG15	RAD23A	2,46678E-11	3,53298E-01	0,564	0,501	4,54306E-07
Macro_ISG15	EIF4A2	1,94215E-12	3,52078E-01	0,529	0,564	3,57687E-08
Macro_ISG15	CCDC85B	7,40644E-32	3,51189E-01	0,492	0,392	1,36404E-27
Macro_ISG15	EIF3G	2,31524E-06	3,51161E-01	0,604	0,614	4,26397E-02
Macro_ISG15	DECR1	1,20485E-07	3,51110E-01	0,395	0,344	2,21897E-03
Macro_ISG15	SAMHD1	1,12471E-35	3,50818E-01	0,768	0,638	2,07138E-31
Macro_ISG15	EMD	2,81857E-07	3,50146E-01	0,467	0,413	5,19096E-03
Macro_ISG15	APOC1	7,85179E-36	3,48134E-01	0,61	0,501	1,44606E-31
Macro_ISG15	ST13	1,99093E-07	3,48121E-01	0,582	0,607	3,66670E-03
Macro_ISG15	LENG8	4,09566E-15	3,47022E-01	0,349	0,28	7,54299E-11
Macro_ISG15	ELF1	1,74045E-07	3,46826E-01	0,536	0,485	3,20539E-03
Macro_ISG15	NDUFB6	1,40643E-07	3,45408E-01	0,414	0,365	2,59022E-03
Macro_ISG15	TGFB1	7,79122E-66	3,44289E-01	0,606	0,444	1,43491E-61
Macro_ISG15	CAMK1	6,50368E-21	3,43361E-01	0,335	0,251	1,19778E-16
Macro_ISG15	FKBP8	2,62937E-39	3,41646E-01	0,643	0,531	4,84251E-35
Macro_ISG15	SCAND1	2,44900E-11	3,41403E-01	0,6	0,531	4,51033E-07
Macro_ISG15	RAB5IF	1,54534E-32	3,41292E-01	0,321	0,235	2,84605E-28
Macro_ISG15	NDUFC1	2,34303E-11	3,40370E-01	0,516	0,454	4,31515E-07
Macro_ISG15	SEMA4D	4,52134E-39	3,40105E-01	0,272	0,175	8,32695E-35
Macro_ISG15	MAP3K8	3,29112E-19	3,39812E-01	0,542	0,479	6,06125E-15
Macro_ISG15	YWHAH	8,74610E-10	3,38811E-01	0,588	0,528	1,61077E-05
Macro_ISG15	VDAC2	3,23004E-09	3,38556E-01	0,53	0,554	5,94877E-05
Macro_ISG15	DDX3X	9,60840E-23	3,38296E-01	0,569	0,472	1,76958E-18
Macro_ISG15	STRAP	2,44775E-12	3,38085E-01	0,303	0,359	4,50801E-08
Macro_ISG15	PER1	1,82596E-10	3,37208E-01	0,379	0,316	3,36286E-06
Macro_ISG15	CUL1	5,55436E-21	3,36659E-01	0,289	0,214	1,02295E-16
Macro_ISG15	CD2BP2	3,68236E-08	3,36185E-01	0,296	0,248	6,78180E-04
Macro_ISG15	MCUB	4,06035E-48	3,36172E-01	0,261	0,162	7,47794E-44
Macro_ISG15	ATP1A1	1,82328E-07	3,35089E-01	0,491	0,426	3,35794E-03
Macro_ISG15	TUBB4B	7,52929E-10	3,34881E-01	0,436	0,376	1,38667E-05
Macro_ISG15	DDX5	7,62235E-09	3,34668E-01	0,841	0,832	1,40381E-04
Macro_ISG15	TAP2	9,54673E-40	3,33661E-01	0,279	0,183	1,75822E-35
Macro_ISG15	EFR3A	4,12245E-10	3,32964E-01	0,291	0,239	7,59231E-06
Macro_ISG15	ATP6V0A1	5,55700E-30	3,32367E-01	0,27	0,183	1,02343E-25
Macro_ISG15	NCOR2	1,21925E-41	3,31620E-01	0,376	0,263	2,24550E-37
Macro_ISG15	STX10	8,49826E-32	3,31550E-01	0,406	0,304	1,56512E-27
Macro_ISG15	SAMSN1	7,72278E-07	3,31226E-01	0,541	0,459	1,42230E-02
Macro_ISG15	HMGA1	1,43579E-25	3,26530E-01	0,496	0,398	2,64429E-21

Macro_ISG15	PLXNB2	8,91600E-18	3,25330E-01	0,331	0,261	1,64206E-13
Macro_ISG15	PDIA4	2,59505E-19	3,24099E-01	0,401	0,316	4,77930E-15
Macro_ISG15	CSRNP1	2,44140E-15	3,24079E-01	0,36	0,287	4,49632E-11
Macro_ISG15	SRP72	4,39903E-07	3,23100E-01	0,314	0,349	8,10170E-03
Macro_ISG15	CHMP1B	8,15849E-22	3,20724E-01	0,474	0,402	1,50255E-17
Macro_ISG15	RPL8	4,69959E-30	3,20575E-01	0,553	0,574	8,65523E-26
Macro_ISG15	STAT6	4,16131E-16	3,19820E-01	0,373	0,301	7,66389E-12
Macro_ISG15	CYBA	5,42056E-33	3,19010E-01	0,945	0,895	9,98304E-29
Macro_ISG15	GNAS	1,64316E-16	3,18877E-01	0,681	0,605	3,02620E-12
Macro_ISG15	DHRSX	2,28125E-23	3,17515E-01	0,284	0,207	4,20138E-19
Macro_ISG15	SGPL1	6,27721E-40	3,16711E-01	0,26	0,163	1,15607E-35
Macro_ISG15	ABRACL	1,36612E-28	3,16367E-01	0,594	0,492	2,51599E-24
Macro_ISG15	BRMS1	7,52929E-08	3,13927E-01	0,274	0,23	1,38667E-03
Macro_ISG15	AAK1	5,58976E-16	3,13426E-01	0,267	0,204	1,02947E-11
Macro_ISG15	RIN3	6,62733E-15	3,13015E-01	0,479	0,4	1,22055E-10
Macro_ISG15	MTCH1	5,61154E-14	3,12364E-01	0,521	0,452	1,03348E-09
Macro_ISG15	MANBA	1,15854E-17	3,11209E-01	0,412	0,332	2,13368E-13
Macro_ISG15	BCAS2	8,97425E-07	3,09508E-01	0,3	0,261	1,65279E-02
Macro_ISG15	ADORA3	2,77401E-08	3,07492E-01	0,311	0,26	5,10889E-04
Macro_ISG15	FRMD4B	7,22270E-08	3,05867E-01	0,248	0,287	1,33020E-03
Macro_ISG15	AP2A2	6,34896E-08	3,05600E-01	0,273	0,224	1,16929E-03
Macro_ISG15	TM6SF1	1,96377E-07	3,04281E-01	0,24	0,274	3,61668E-03
Macro_ISG15	TRADD	2,89529E-07	3,04176E-01	0,286	0,243	5,33225E-03
Macro_ISG15	GGA2	1,97170E-23	3,04027E-01	0,351	0,265	3,63128E-19
Macro_ISG15	HCFC1R1	1,71473E-30	3,02385E-01	0,409	0,31	3,15802E-26
Macro_ISG15	CLEC2B	1,50480E-09	3,00011E-01	0,528	0,466	2,77139E-05
Macro_ISG15	HADHA	5,02984E-07	2,99496E-01	0,498	0,522	9,26346E-03
Macro_ISG15	UXT	2,69981E-16	2,98443E-01	0,499	0,544	4,97225E-12
Macro_ISG15	RILPL2	5,90159E-11	2,98184E-01	0,512	0,452	1,08690E-06
Macro_ISG15	NDUFAF3	8,64134E-08	2,98166E-01	0,519	0,468	1,59147E-03
Macro_ISG15	COX17	4,25853E-12	2,96627E-01	0,562	0,485	7,84293E-08
Macro_ISG15	SPNS1	1,68231E-11	2,96150E-01	0,305	0,245	3,09830E-07
Macro_ISG15	IGF2R	1,05088E-27	2,95769E-01	0,297	0,211	1,93540E-23
Macro_ISG15	DNAJC15	2,82285E-35	2,95395E-01	0,597	0,493	5,19883E-31
Macro_ISG15	PKN1	5,23508E-13	2,94624E-01	0,265	0,208	9,64145E-09
Macro_ISG15	SLC25A39	9,35974E-07	2,94314E-01	0,455	0,405	1,72378E-02
Macro_ISG15	TRPM2	2,90705E-67	2,92394E-01	0,307	0,178	5,35391E-63
Macro_ISG15	ETV6	1,70453E-23	2,92327E-01	0,375	0,285	3,13924E-19
Macro_ISG15	CNPY3	6,28116E-09	2,92120E-01	0,682	0,624	1,15680E-04
Macro_ISG15	CELF1	2,47465E-06	2,91365E-01	0,324	0,283	4,55756E-02
Macro_ISG15	BRK1	3,28863E-21	2,91034E-01	0,701	0,727	6,05666E-17
Macro_ISG15	SKIL	1,36854E-08	2,90812E-01	0,389	0,328	2,52044E-04
Macro_ISG15	PSMB2	8,15637E-11	2,90562E-01	0,499	0,438	1,50216E-06

Macro_ISG15	VASP	8,47389E-09	2,90327E-01	0,542	0,465	1,56064E-04
Macro_ISG15	CCT8	1,19270E-14	2,89802E-01	0,355	0,416	2,19659E-10
Macro_ISG15	GDI1	5,57784E-10	2,89149E-01	0,364	0,306	1,02727E-05
Macro_ISG15	FGD4	2,53460E-11	2,88879E-01	0,283	0,226	4,66798E-07
Macro_ISG15	SNAP23	3,33634E-24	2,88583E-01	0,32	0,404	6,14454E-20
Macro_ISG15	PPP2R2A	6,40064E-25	2,87919E-01	0,298	0,215	1,17881E-20
Macro_ISG15	MRPL41	2,38360E-25	2,87854E-01	0,568	0,469	4,38988E-21
Macro_ISG15	GPRIN3	6,77296E-12	2,87634E-01	0,29	0,234	1,24738E-07
Macro_ISG15	RAB9A	8,49423E-09	2,87371E-01	0,291	0,238	1,56438E-04
Macro_ISG15	HNRNPA2B1	1,62475E-19	2,85336E-01	0,828	0,832	2,99231E-15
Macro_ISG15	EIF1	1,90071E-135	2,84326E-01	0,939	0,944	3,50053E-131
Macro_ISG15	CD55	8,70957E-17	2,83934E-01	0,367	0,433	1,60404E-12
Macro_ISG15	GLG1	3,64930E-07	2,83434E-01	0,337	0,288	6,72092E-03
Macro_ISG15	GSTP1	2,55231E-11	2,82943E-01	0,845	0,822	4,70059E-07
Macro_ISG15	RUNX1	9,47052E-28	2,82400E-01	0,37	0,273	1,74419E-23
Macro_ISG15	ADA2	6,57870E-38	2,82115E-01	0,321	0,223	1,21160E-33
Macro_ISG15	BAG1	2,45709E-13	2,81286E-01	0,437	0,371	4,52523E-09
Macro_ISG15	SYF2	1,75958E-06	2,79477E-01	0,396	0,426	3,24062E-02
Macro_ISG15	CCDC107	6,93848E-54	2,78886E-01	0,425	0,299	1,27786E-49
Macro_ISG15	AMPD2	1,77530E-49	2,78845E-01	0,289	0,177	3,26957E-45
Macro_ISG15	DPM2	1,74837E-06	2,78289E-01	0,254	0,214	3,21997E-02
Macro_ISG15	ARHGDIA	3,15134E-18	2,77906E-01	0,678	0,597	5,80383E-14
Macro_ISG15	TMEM160	1,19157E-19	2,77609E-01	0,458	0,378	2,19451E-15
Macro_ISG15	PHPT1	4,40074E-16	2,76616E-01	0,515	0,442	8,10484E-12
Macro_ISG15	CCDC124	2,23080E-06	2,75878E-01	0,393	0,35	4,10847E-02
Macro_ISG15	B4GALT1	2,13059E-08	2,74692E-01	0,376	0,322	3,92391E-04
Macro_ISG15	MT-ND4	1,02181E-06	2,74508E-01	0,557	0,575	1,88187E-02
Macro_ISG15	CTDNEP1	3,05323E-21	2,73821E-01	0,335	0,257	5,62313E-17
Macro_ISG15	TLN1	5,63893E-13	2,73309E-01	0,622	0,551	1,03852E-08
Macro_ISG15	TSEN34	2,27623E-19	2,72173E-01	0,362	0,287	4,19214E-15
Macro_ISG15	MT-ND3	5,32851E-08	2,72009E-01	0,552	0,57	9,81351E-04
Macro_ISG15	AGPAT2	1,02818E-40	2,71319E-01	0,401	0,285	1,89360E-36
Macro_ISG15	PTDSS1	1,42280E-12	2,70539E-01	0,218	0,273	2,62037E-08
Macro_ISG15	RAB1B	3,92219E-07	2,68686E-01	0,401	0,353	7,22349E-03
Macro_ISG15	GAK	1,05891E-17	2,66143E-01	0,285	0,215	1,95019E-13
Macro_ISG15	HSPA4	6,38711E-10	2,64045E-01	0,353	0,298	1,17631E-05
Macro_ISG15	MCL1	1,35917E-16	2,63212E-01	0,832	0,765	2,50318E-12
Macro_ISG15	IFI27L2	1,77956E-18	2,62914E-01	0,492	0,404	3,27742E-14
Macro_ISG15	NUB1	3,02008E-14	2,62328E-01	0,383	0,309	5,56208E-10
Macro_ISG15	PFDN6	1,46943E-16	2,61430E-01	0,278	0,212	2,70626E-12
Macro_ISG15	ZFYVE16	8,55771E-08	2,61132E-01	0,25	0,206	1,57607E-03
Macro_ISG15	PDK4	8,49800E-24	2,60504E-01	0,246	0,315	1,56508E-19
Macro_ISG15	RELT	1,43739E-10	2,60220E-01	0,274	0,22	2,64724E-06

Macro_ISG15	AURKAIP1	9,34893E-10	2,58970E-01	0,573	0,515	1,72179E-05
Macro_ISG15	PHACTR4	1,40359E-38	2,56858E-01	0,257	0,164	2,58499E-34
Macro_ISG15	TIMM8B	1,70078E-27	2,56437E-01	0,512	0,411	3,13233E-23
Macro_ISG15	DNPEP	5,77758E-11	2,55221E-01	0,273	0,22	1,06406E-06
Macro_ISG15	UQCR11	2,32086E-07	2,55163E-01	0,797	0,752	4,27433E-03
Macro_ISG15	STAC3	2,82279E-56	2,51511E-01	0,296	0,18	5,19874E-52
Macro_ISG15	MAP7D1	1,31235E-12	2,50370E-01	0,339	0,277	2,41696E-08
Macro_ISG15	ATP5F1A	3,87323E-07	-2,56604E-01	0,226	0,256	7,13333E-03
Macro_ISG15	TOMM7	2,19656E-63	-2,57635E-01	0,762	0,793	4,04541E-59
Macro_ISG15	STARD7	4,05175E-07	-2,59275E-01	0,269	0,308	7,46210E-03
Macro_ISG15	RPS2	1,12285E-36	-2,64713E-01	0,536	0,564	2,06795E-32
Macro_ISG15	METTL7A	4,06256E-15	-2,66820E-01	0,19	0,251	7,48202E-11
Macro_ISG15	AKAP9	5,26798E-15	-2,75313E-01	0,327	0,389	9,70204E-11
Macro_ISG15	RPS14	6,15033E-48	-2,82619E-01	0,552	0,577	1,13271E-43
Macro_ISG15	RPLP1	3,34856E-44	-2,82812E-01	0,577	0,599	6,16705E-40
Macro_ISG15	RPS18	1,30824E-36	-2,86307E-01	0,535	0,561	2,40938E-32
Macro_ISG15	IL18	1,92022E-33	-2,97939E-01	0,364	0,447	3,53648E-29
Macro_ISG15	KRT10	2,57192E-33	-3,00897E-01	0,229	0,331	4,73670E-29
Macro_ISG15	RPL21	1,52384E-38	-3,03092E-01	0,517	0,543	2,80645E-34
Macro_ISG15	APEX1	1,21897E-30	-3,07067E-01	0,357	0,447	2,24497E-26
Macro_ISG15	NPM1	3,34518E-118	-3,10381E-01	0,681	0,771	6,16081E-114
Macro_ISG15	RPS3	1,37567E-44	-3,14873E-01	0,549	0,568	2,53357E-40
Macro_ISG15	RPL5	8,15896E-68	-3,16380E-01	0,453	0,521	1,50263E-63
Macro_ISG15	EEF1A1	0,00000E+00	-3,17815E-01	0,976	0,985	0,00000E+00
Macro_ISG15	RPL18	2,42427E-45	-3,19269E-01	0,539	0,564	4,46479E-41
Macro_ISG15	CCNI	2,21160E-103	-3,19570E-01	0,72	0,79	4,07311E-99
Macro_ISG15	GSN	1,44814E-15	-3,26478E-01	0,631	0,634	2,66704E-11
Macro_ISG15	RPS5	1,55743E-41	-3,29240E-01	0,484	0,52	2,86831E-37
Macro_ISG15	AKR1B1	8,32784E-09	-3,29806E-01	0,441	0,47	1,53374E-04
Macro_ISG15	RPL37	4,74059E-29	-3,30609E-01	0,553	0,569	8,73074E-25
Macro_ISG15	DUT	4,06829E-24	-3,31242E-01	0,326	0,406	7,49258E-20
Macro_ISG15	RPS6	2,37070E-40	-3,33129E-01	0,52	0,546	4,36612E-36
Macro_ISG15	ADI1	4,43140E-37	-3,33973E-01	0,206	0,31	8,16131E-33
Macro_ISG15	RPL7	1,12281E-38	-3,34090E-01	0,444	0,5	2,06787E-34
Macro_ISG15	RPS26	9,91197E-08	-3,38415E-01	0,527	0,524	1,82549E-03
Macro_ISG15	CSF2RA	1,52775E-32	-3,40963E-01	0,328	0,423	2,81365E-28
Macro_ISG15	RPL23	2,97030E-31	-3,41036E-01	0,389	0,457	5,47040E-27
Macro_ISG15	RSL24D1	1,57725E-38	-3,42590E-01	0,335	0,44	2,90482E-34
Macro_ISG15	FOXP1	2,31388E-25	-3,43502E-01	0,267	0,35	4,26147E-21
Macro_ISG15	NAP1L1	7,94684E-110	-3,45813E-01	0,48	0,628	1,46357E-105
Macro_ISG15	ZFP36L2	6,15361E-12	-3,51189E-01	0,697	0,694	1,13331E-07
Macro_ISG15	RPS27	3,09712E-26	-3,51498E-01	0,554	0,567	5,70397E-22
Macro_ISG15	RPS13	3,01928E-51	-3,52864E-01	0,544	0,573	5,56060E-47

Macro_ISG15	EIF2A	2,02852E-14	-3,53358E-01	0,252	0,311	3,73593E-10
Macro_ISG15	CD74	1,48813E-61	-3,60720E-01	0,987	0,962	2,74068E-57
Macro_ISG15	LIMD2	1,01718E-12	-3,62715E-01	0,44	0,484	1,87334E-08
Macro_ISG15	RPS3A	1,96434E-61	-3,63389E-01	0,541	0,568	3,61772E-57
Macro_ISG15	SPCS1	4,02586E-24	-3,64368E-01	0,556	0,611	7,41442E-20
Macro_ISG15	RPL35A	8,73800E-44	-3,66954E-01	0,54	0,563	1,60928E-39
Macro_ISG15	RPL6	1,36832E-50	-3,72861E-01	0,526	0,555	2,52003E-46
Macro_ISG15	RASSF2	4,30958E-20	-3,79517E-01	0,194	0,269	7,93696E-16
Macro_ISG15	RPS7	1,05663E-41	-3,80263E-01	0,547	0,561	1,94599E-37
Macro_ISG15	RPL10A	6,19512E-49	-3,81035E-01	0,441	0,5	1,14096E-44
Macro_ISG15	RPS15A	1,42315E-52	-3,89256E-01	0,547	0,57	2,62102E-48
Macro_ISG15	RPSA	4,38725E-32	-3,89758E-01	0,458	0,498	8,07999E-28
Macro_ISG15	RPL32	1,62991E-40	-3,97549E-01	0,556	0,58	3,00180E-36
Macro_ISG15	LDHB	8,60233E-40	-4,07615E-01	0,414	0,517	1,58429E-35
Macro_ISG15	RPS12	1,05081E-44	-4,11836E-01	0,559	0,584	1,93527E-40
Macro_ISG15	PKIB	3,96843E-32	-4,12393E-01	0,156	0,251	7,30866E-28
Macro_ISG15	HLA-DQB1	3,70586E-60	-4,13374E-01	0,844	0,814	6,82507E-56
Macro_ISG15	SLC25A6	1,52330E-145	-4,15607E-01	0,757	0,82	2,80546E-141
Macro_ISG15	RPL13A	3,68978E-31	-4,16081E-01	0,486	0,523	6,79547E-27
Macro_ISG15	NOP53	2,25392E-16	-4,21860E-01	0,234	0,287	4,15104E-12
Macro_ISG15	CD83	1,09232E-09	-4,32109E-01	0,459	0,49	2,01173E-05
Macro_ISG15	RPS8	2,02515E-53	-4,51581E-01	0,554	0,58	3,72971E-49
Macro_ISG15	AHNAK	8,55521E-79	-4,52561E-01	0,485	0,611	1,57561E-74
Macro_ISG15	HLA-DPA1	1,43532E-91	-4,54233E-01	0,932	0,895	2,64343E-87
Macro_ISG15	RPL22	3,70690E-38	-4,63758E-01	0,495	0,532	6,82699E-34
Macro_ISG15	EEF2	2,27115E-146	-4,64106E-01	0,76	0,833	4,18277E-142
Macro_ISG15	EIF4B	4,40188E-91	-4,66514E-01	0,364	0,527	8,10694E-87
Macro_ISG15	RPS27A	5,09036E-66	-4,90372E-01	0,547	0,577	9,37492E-62
Macro_ISG15	DCK	3,17245E-09	-4,98775E-01	0,239	0,282	5,84271E-05
Macro_ISG15	RPL23A	4,21309E-27	-5,05896E-01	0,508	0,527	7,75925E-23
Macro_ISG15	PLAC8	2,75770E-08	-5,20066E-01	0,251	0,202	5,07885E-04
Macro_ISG15	MTRNR2L12	2,53953E-06	-5,22023E-01	0,833	0,762	4,67705E-02
Macro_ISG15	ATP5MC2	5,88458E-13	-5,29564E-01	0,43	0,434	1,08376E-08
Macro_ISG15	EEF1B2	5,18859E-251	-5,32145E-01	0,754	0,847	9,55583E-247
Macro_ISG15	RPL34	9,00473E-47	-5,36635E-01	0,554	0,577	1,65840E-42
Macro_ISG15	RAB32	1,48247E-43	-5,45061E-01	0,274	0,381	2,73027E-39
Macro_ISG15	RPS4X	7,81361E-49	-5,50811E-01	0,542	0,567	1,43903E-44
Macro_ISG15	RPS17	1,17503E-11	-5,67498E-01	0,38	0,409	2,16404E-07
Macro_ISG15	HLA-DPB1	2,22421E-141	-5,68641E-01	0,907	0,882	4,09633E-137
Macro_ISG15	EIF3L	1,18307E-164	-5,86246E-01	0,404	0,612	2,17885E-160
Macro_ISG15	RPS23	1,24030E-57	-5,97064E-01	0,539	0,57	2,28426E-53
Macro_ISG15	RPLP0	3,27944E-39	-6,00318E-01	0,508	0,545	6,03975E-35
Macro_ISG15	GAPT	8,62115E-09	-6,16666E-01	0,213	0,257	1,58776E-04

Macro_ISG15	PTRHD1	1,66517E-44	-6,27653E-01	0,235	0,356	3,06675E-40
Macro_ISG15	RACK1	1,64112E-23	-6,72663E-01	0,468	0,467	3,02244E-19
Macro_ISG15	AREG	4,25614E-08	-6,73581E-01	0,331	0,272	7,83853E-04
Macro_ISG15	HERPUD1	4,24187E-27	-6,81234E-01	0,641	0,684	7,81226E-23
Macro_ISG15	HLA-DQA1	7,04072E-121	-7,33859E-01	0,723	0,787	1,29669E-116
Macro_ISG15	A2M	1,50171E-30	-7,73103E-01	0,32	0,394	2,76569E-26
Macro_ISG15	PEBP1	1,10319E-63	-7,93232E-01	0,46	0,571	2,03174E-59
Macro_ISG15	CCDC50	3,63679E-23	-8,29568E-01	0,205	0,278	6,69788E-19
Macro_ISG15	STMN1	9,75236E-50	-8,76926E-01	0,178	0,293	1,79609E-45
Macro_ISG15	HLA-DOA	5,60988E-67	-9,33268E-01	0,254	0,401	1,03317E-62
Macro_ISG15	LTC4S	9,33831E-40	-1,00149E+00	0,188	0,29	1,71984E-35
Macro_ISG15	HLA-DQB2	6,07160E-82	-1,05539E+00	0,167	0,326	1,11821E-77
Macro_ISG15	GPR183	1,16142E-09	-1,49311E+00	0,42	0,459	2,13898E-05
Macro_ISG15	HLA-DQA2	3,76934E-134	-1,59819E+00	0,342	0,523	6,94198E-130
Macro_ISG15	PLD4	6,68813E-35	-1,82200E+00	0,232	0,338	1,23175E-30
Macro_ISG15	SERPINF1	2,70468E-71	-2,26308E+00	0,256	0,414	4,98121E-67

Macro_LYVE1

cluster	gene	p_val	avg_log2FC	pct.1	pct.2	p_val_adj
Macro_LYVE1	SELENOP	0,00000E+00	3,11386E+00	0,417	0,075	0,00000E+00
Macro_LYVE1	FOLR2	0,00000E+00	2,95945E+00	0,773	0,149	0,00000E+00
Macro_LYVE1	SLC40A1	0,00000E+00	2,90270E+00	0,778	0,202	0,00000E+00
Macro_LYVE1	PLTP	0,00000E+00	2,43401E+00	0,845	0,302	0,00000E+00
Macro_LYVE1	RNASE1	0,00000E+00	2,35079E+00	0,761	0,247	0,00000E+00
Macro_LYVE1	F13A1	0,00000E+00	2,34528E+00	0,686	0,209	0,00000E+00
Macro_LYVE1	DAB2	0,00000E+00	2,12040E+00	0,89	0,41	0,00000E+00
Macro_LYVE1	MS4A4A	0,00000E+00	1,98739E+00	0,858	0,397	0,00000E+00
Macro_LYVE1	LGMN	0,00000E+00	1,85045E+00	0,801	0,389	0,00000E+00
Macro_LYVE1	STAB1	0,00000E+00	1,84690E+00	0,788	0,322	0,00000E+00
Macro_LYVE1	LILRB5	0,00000E+00	1,81760E+00	0,381	0,059	0,00000E+00
Macro_LYVE1	PDK4	0,00000E+00	1,69446E+00	0,655	0,293	0,00000E+00
Macro_LYVE1	MAF	0,00000E+00	1,67860E+00	0,654	0,141	0,00000E+00
Macro_LYVE1	C1QA	0,00000E+00	1,56799E+00	0,983	0,545	0,00000E+00
Macro_LYVE1	C2	1,10321E-253	1,55357E+00	0,404	0,15	2,03179E-249
Macro_LYVE1	SLCO2B1	0,00000E+00	1,53273E+00	0,821	0,346	0,00000E+00
Macro_LYVE1	C1QB	0,00000E+00	1,52430E+00	0,976	0,523	0,00000E+00
Macro_LYVE1	CD163L1	0,00000E+00	1,47657E+00	0,278	0,02	0,00000E+00
Macro_LYVE1	C1QC	0,00000E+00	1,46438E+00	0,985	0,523	0,00000E+00
Macro_LYVE1	GPR34	0,00000E+00	1,43543E+00	0,66	0,287	0,00000E+00
Macro_LYVE1	GLA	1,69488E-06	1,38106E+00	0,345	0,309	3,12147E-02
Macro_LYVE1	IGF1	0,00000E+00	1,33774E+00	0,422	0,063	0,00000E+00
Macro_LYVE1	FUCA1	0,00000E+00	1,33513E+00	0,582	0,233	0,00000E+00
Macro_LYVE1	MS4A7	0,00000E+00	1,32069E+00	0,948	0,648	0,00000E+00
Macro_LYVE1	PLD3	0,00000E+00	1,31019E+00	0,815	0,495	0,00000E+00
Macro_LYVE1	MRC1	0,00000E+00	1,28948E+00	0,365	0,111	0,00000E+00
Macro_LYVE1	CXCL12	0,00000E+00	1,26714E+00	0,385	0,071	0,00000E+00
Macro_LYVE1	CD209	0,00000E+00	1,24506E+00	0,301	0,044	0,00000E+00
Macro_LYVE1	A2M	0,00000E+00	1,23203E+00	0,736	0,371	0,00000E+00
Macro_LYVE1	BLVRB	0,00000E+00	1,21596E+00	0,75	0,477	0,00000E+00
Macro_LYVE1	WWP1	1,11047E-277	1,20259E+00	0,287	0,08	2,04514E-273
Macro_LYVE1	CD163	0,00000E+00	1,20235E+00	0,849	0,426	0,00000E+00
Macro_LYVE1	TMEM37	0,00000E+00	1,14370E+00	0,388	0,088	0,00000E+00
Macro_LYVE1	VSIG4	2,45895E-226	1,13889E+00	0,636	0,333	4,52864E-222
Macro_LYVE1	ADORA3	7,48047E-60	1,10794E+00	0,4	0,256	1,37768E-55
Macro_LYVE1	SIGLEC1	9,32832E-261	1,09573E+00	0,437	0,172	1,71800E-256
Macro_LYVE1	NPL	2,59926E-271	1,09417E+00	0,536	0,244	4,78705E-267
Macro_LYVE1	LIPA	6,52235E-210	1,08704E+00	0,756	0,488	1,20122E-205
Macro_LYVE1	FCGRT	0,00000E+00	1,05867E+00	0,948	0,727	0,00000E+00
Macro_LYVE1	TSPAN4	0,00000E+00	1,03407E+00	0,6	0,277	0,00000E+00
Macro_LYVE1	CREG1	1,25940E-220	1,02547E+00	0,759	0,5	2,31944E-216
Macro_LYVE1	GPNMB	1,03437E-198	1,00092E+00	0,608	0,32	1,90500E-194
Macro_LYVE1	CPM	3,38302E-286	9,99301E-01	0,663	0,354	6,23050E-282
Macro_LYVE1	OTOA	9,75107E-233	9,92013E-01	0,251	0,072	1,79586E-228
Macro_LYVE1	FRMD4B	1,03650E-215	9,58843E-01	0,532	0,272	1,90892E-211
Macro_LYVE1	MSR1	0,00000E+00	9,46660E-01	0,709	0,363	0,00000E+00
Macro_LYVE1	SNX6	2,62324E-284	9,33602E-01	0,758	0,503	4,83122E-280
Macro_LYVE1	TMEM176B	4,18532E-210	9,11373E-01	0,812	0,52	7,70810E-206
Macro_LYVE1	AP1B1	1,11061E-172	8,93001E-01	0,617	0,382	2,04541E-168
Macro_LYVE1	SDC3	0,00000E+00	8,87574E-01	0,414	0,138	0,00000E+00
Macro_LYVE1	ADAP2	1,29996E-178	8,86237E-01	0,679	0,433	2,39413E-174
Macro_LYVE1	CD14	0,00000E+00	8,84703E-01	0,925	0,608	0,00000E+00
Macro_LYVE1	GATM	5,83106E-133	8,79163E-01	0,328	0,151	1,07391E-128

Macro_LYVE1	PIK3IP1	2,97422E-78	8,76235E-01	0,332	0,185	5,47762E-74
Macro_LYVE1	OLFML2B	0,00000E+00	8,69962E-01	0,412	0,138	0,00000E+00
Macro_LYVE1	MS4A6A	0,00000E+00	8,59485E-01	0,968	0,702	0,00000E+00
Macro_LYVE1	TMEM176A	2,13016E-186	8,58042E-01	0,711	0,435	3,92312E-182
Macro_LYVE1	LRP1	5,06757E-205	8,56915E-01	0,675	0,409	9,33294E-201
Macro_LYVE1	CTSD	8,00732E-185	8,55926E-01	0,936	0,76	1,47471E-180
Macro_LYVE1	SLC7A8	0,00000E+00	8,55205E-01	0,265	0,059	0,00000E+00
Macro_LYVE1	MERTK	1,49556E-304	8,54129E-01	0,467	0,178	2,75438E-300
Macro_LYVE1	ABCC5	1,09190E-206	8,37129E-01	0,321	0,115	2,01096E-202
Macro_LYVE1	ITSN1	4,95096E-216	8,31637E-01	0,302	0,101	9,11818E-212
Macro_LYVE1	CSF1R	4,04617E-294	8,28908E-01	0,841	0,557	7,45183E-290
Macro_LYVE1	AP2A2	1,37028E-291	8,23234E-01	0,499	0,213	2,52365E-287
Macro_LYVE1	MKNK1	3,54447E-76	8,20145E-01	0,345	0,203	6,52785E-72
Macro_LYVE1	GYPC	4,98891E-194	8,16580E-01	0,535	0,289	9,18807E-190
Macro_LYVE1	RGL1	7,40323E-232	8,09089E-01	0,359	0,133	1,36345E-227
Macro_LYVE1	NCF4	9,57043E-123	8,04149E-01	0,605	0,413	1,76259E-118
Macro_LYVE1	ALDH1A1	2,41933E-70	8,02590E-01	0,338	0,192	4,45568E-66
Macro_LYVE1	CTSZ	9,08601E-177	7,97298E-01	0,822	0,643	1,67337E-172
Macro_LYVE1	ME1	0,00000E+00	7,87081E-01	0,323	0,086	0,00000E+00
Macro_LYVE1	CD59	2,46886E-227	7,84736E-01	0,598	0,327	4,54691E-223
Macro_LYVE1	MAFB	1,11476E-249	7,71201E-01	0,893	0,578	2,05306E-245
Macro_LYVE1	CMKLR1	3,25503E-207	7,64159E-01	0,357	0,137	5,99478E-203
Macro_LYVE1	LPAR6	8,18225E-96	7,57750E-01	0,492	0,313	1,50693E-91
Macro_LYVE1	CALU	6,25491E-31	7,53152E-01	0,312	0,221	1,15197E-26
Macro_LYVE1	MGST3	3,58219E-33	7,51780E-01	0,618	0,538	6,59731E-29
Macro_LYVE1	DENND4C	1,89437E-32	7,44817E-01	0,261	0,171	3,48887E-28
Macro_LYVE1	TCN2	6,33665E-70	7,14596E-01	0,357	0,212	1,16702E-65
Macro_LYVE1	ARHGAP18	2,70986E-138	7,02634E-01	0,676	0,466	4,99075E-134
Macro_LYVE1	PMP22	3,37377E-158	7,02296E-01	0,508	0,265	6,21348E-154
Macro_LYVE1	EPHX1	9,23129E-252	6,99359E-01	0,358	0,125	1,70013E-247
Macro_LYVE1	MARCKS	1,44371E-211	6,98937E-01	0,799	0,519	2,65889E-207
Macro_LYVE1	LY96	2,98662E-168	6,94085E-01	0,661	0,446	5,50046E-164
Macro_LYVE1	FCHO2	3,10429E-238	6,93746E-01	0,465	0,202	5,71718E-234
Macro_LYVE1	C3AR1	1,56418E-123	6,91096E-01	0,626	0,405	2,88076E-119
Macro_LYVE1	MTRNR2L12	8,42822E-60	6,75608E-01	0,822	0,763	1,55222E-55
Macro_LYVE1	TTYH3	2,50989E-183	6,67366E-01	0,439	0,212	4,62247E-179
Macro_LYVE1	HMOX1	2,50503E-77	6,66320E-01	0,714	0,536	4,61351E-73
Macro_LYVE1	MGLL	6,21842E-77	6,59605E-01	0,288	0,151	1,14525E-72
Macro_LYVE1	FCGR3A	5,36733E-110	6,59485E-01	0,837	0,561	9,88502E-106
Macro_LYVE1	HNMT	4,52864E-163	6,58077E-01	0,601	0,369	8,34039E-159
Macro_LYVE1	FTL	2,41352E-249	6,56964E-01	0,999	0,991	4,44498E-245
Macro_LYVE1	AKR1B1	7,09447E-161	6,53407E-01	0,702	0,456	1,30659E-156
Macro_LYVE1	RASSF4	2,47032E-103	6,46871E-01	0,691	0,497	4,54958E-99
Macro_LYVE1	ARL4C	1,65013E-154	6,41572E-01	0,545	0,321	3,03905E-150
Macro_LYVE1	CD33	5,25332E-11	6,39981E-01	0,359	0,305	9,67505E-07
Macro_LYVE1	MFSD1	1,25436E-231	6,39799E-01	0,813	0,58	2,31016E-227
Macro_LYVE1	GIMAP5	1,16468E-48	6,37648E-01	0,259	0,152	2,14499E-44
Macro_LYVE1	DRAM2	4,24308E-94	6,26807E-01	0,548	0,382	7,81449E-90
Macro_LYVE1	CTSB	3,23154E-302	6,22303E-01	0,987	0,866	5,95153E-298
Macro_LYVE1	KCTD12	4,89938E-142	6,21336E-01	0,693	0,48	9,02319E-138
Macro_LYVE1	SLC1A3	1,07900E-78	6,20976E-01	0,528	0,354	1,98719E-74
Macro_LYVE1	NAIP	4,93993E-190	6,15589E-01	0,618	0,358	9,09788E-186
Macro_LYVE1	ACP5	7,73361E-92	6,08582E-01	0,552	0,392	1,42430E-87
Macro_LYVE1	MCOLN1	6,97964E-142	6,07633E-01	0,469	0,264	1,28544E-137

Macro_LYVE1	GPR155	6,53486E-171	6,05822E-01	0,329	0,134	1,20353E-166
Macro_LYVE1	EPB41L2	1,77777E-50	6,04688E-01	0,308	0,193	3,27411E-46
Macro_LYVE1	TIMP2	2,88678E-160	6,01544E-01	0,573	0,35	5,31658E-156
Macro_LYVE1	SGMS1	1,91638E-128	6,01322E-01	0,273	0,114	3,52940E-124
Macro_LYVE1	NRP1	0,00000E+00	5,97490E-01	0,473	0,148	0,00000E+00
Macro_LYVE1	ABCA1	1,72064E-126	5,95816E-01	0,523	0,304	3,16891E-122
Macro_LYVE1	LAMP1	8,81933E-137	5,95325E-01	0,594	0,387	1,62426E-132
Macro_LYVE1	SMS	1,16526E-39	5,89524E-01	0,529	0,421	2,14606E-35
Macro_LYVE1	IDH1	1,01861E-183	5,87194E-01	0,465	0,235	1,87597E-179
Macro_LYVE1	SMPDL3A	1,13361E-83	5,83469E-01	0,276	0,138	2,08777E-79
Macro_LYVE1	DNPH1	4,90238E-73	5,78900E-01	0,489	0,34	9,02872E-69
Macro_LYVE1	ITM2B	1,31350E-252	5,77633E-01	0,989	0,902	2,41907E-248
Macro_LYVE1	GAL3ST4	1,23229E-115	5,76117E-01	0,329	0,16	2,26950E-111
Macro_LYVE1	CD81	4,46374E-161	5,72048E-01	0,691	0,45	8,22087E-157
Macro_LYVE1	ADAM9	7,12125E-123	5,71103E-01	0,405	0,219	1,31152E-118
Macro_LYVE1	CYFIP1	3,80428E-95	5,68450E-01	0,489	0,315	7,00635E-91
Macro_LYVE1	FRMD4A	5,25183E-141	5,57372E-01	0,369	0,176	9,67229E-137
Macro_LYVE1	KCNMA1	1,78386E-85	5,53007E-01	0,426	0,252	3,28534E-81
Macro_LYVE1	DSC2	2,58069E-123	5,51651E-01	0,326	0,152	4,75286E-119
Macro_LYVE1	CTSC	0,00000E+00	5,51635E-01	0,915	0,665	0,00000E+00
Macro_LYVE1	PLXND1	1,84339E-186	5,51124E-01	0,457	0,221	3,39497E-182
Macro_LYVE1	GAS6	5,82201E-154	5,47764E-01	0,402	0,197	1,07224E-149
Macro_LYVE1	GOLGB1	9,24843E-25	5,44583E-01	0,358	0,278	1,70328E-20
Macro_LYVE1	VAT1	1,48559E-52	5,41510E-01	0,361	0,23	2,73601E-48
Macro_LYVE1	GOLIM4	3,06232E-88	5,36499E-01	0,37	0,214	5,63987E-84
Macro_LYVE1	PLA2G7	2,83641E-108	5,30431E-01	0,366	0,187	5,22382E-104
Macro_LYVE1	ADAM28	8,72621E-25	5,28790E-01	0,31	0,235	1,60711E-20
Macro_LYVE1	CD63	1,68110E-134	5,26726E-01	0,942	0,811	3,09608E-130
Macro_LYVE1	ZFHX3	7,17049E-116	5,25662E-01	0,409	0,223	1,32059E-111
Macro_LYVE1	APMAP	1,23988E-42	5,19533E-01	0,402	0,295	2,28349E-38
Macro_LYVE1	SPRED1	7,17710E-99	5,14010E-01	0,278	0,133	1,32181E-94
Macro_LYVE1	ASPH	6,31719E-25	5,13481E-01	0,301	0,219	1,16344E-20
Macro_LYVE1	CFD	3,03801E-151	5,13301E-01	0,792	0,55	5,59511E-147
Macro_LYVE1	BCAT1	2,22488E-11	5,04344E-01	0,287	0,235	4,09755E-07
Macro_LYVE1	SESN1	2,13810E-105	5,03860E-01	0,374	0,201	3,93773E-101
Macro_LYVE1	SPATS2L	1,04617E-51	5,01836E-01	0,346	0,226	1,92674E-47
Macro_LYVE1	SRGAP1	1,02082E-52	4,98028E-01	0,28	0,165	1,88004E-48
Macro_LYVE1	RB1	9,64936E-93	4,85867E-01	0,559	0,394	1,77712E-88
Macro_LYVE1	ATP2B1	1,41334E-25	4,84619E-01	0,446	0,344	2,60294E-21
Macro_LYVE1	FKBP15	7,06263E-08	4,82739E-01	0,31	0,27	1,30073E-03
Macro_LYVE1	RAB9A	3,69046E-09	4,79846E-01	0,288	0,238	6,79672E-05
Macro_LYVE1	AAK1	1,91305E-62	4,76375E-01	0,331	0,201	3,52327E-58
Macro_LYVE1	MAN1A1	2,86623E-76	4,74984E-01	0,27	0,142	5,27874E-72
Macro_LYVE1	DUSP23	6,08299E-14	4,74597E-01	0,44	0,38	1,12030E-09
Macro_LYVE1	STMN1	4,82968E-132	4,74559E-01	0,494	0,276	8,89483E-128
Macro_LYVE1	PTPN18	1,26790E-14	4,70603E-01	0,409	0,348	2,33508E-10
Macro_LYVE1	CRYL1	4,14876E-103	4,69110E-01	0,39	0,218	7,64078E-99
Macro_LYVE1	MYO5A	4,66230E-132	4,57908E-01	0,377	0,19	8,58655E-128
Macro_LYVE1	CREBL2	9,06399E-85	4,55564E-01	0,441	0,283	1,66932E-80
Macro_LYVE1	LGALS3BP	1,04051E-27	4,51943E-01	0,429	0,314	1,91631E-23
Macro_LYVE1	SLC4A7	2,75267E-66	4,50762E-01	0,358	0,222	5,06959E-62
Macro_LYVE1	IQGAP2	3,05689E-122	4,47577E-01	0,487	0,294	5,62987E-118
Macro_LYVE1	CTSA	6,56600E-41	4,46323E-01	0,612	0,5	1,20926E-36
Macro_LYVE1	GAA	1,29930E-72	4,45920E-01	0,534	0,379	2,39293E-68

Macro_LYVE1	SNX2	7,76298E-50	4,44980E-01	0,552	0,435	1,42971E-45
Macro_LYVE1	CD84	1,96507E-155	4,44972E-01	0,615	0,381	3,61907E-151
Macro_LYVE1	TECR	1,55687E-27	4,41233E-01	0,458	0,375	2,86729E-23
Macro_LYVE1	RAP2B	9,40285E-105	4,36435E-01	0,489	0,306	1,73172E-100
Macro_LYVE1	SCARB2	2,10001E-76	4,31610E-01	0,53	0,363	3,86758E-72
Macro_LYVE1	ATP1B1	4,99809E-36	4,31227E-01	0,314	0,216	9,20499E-32
Macro_LYVE1	MITF	3,29998E-28	4,29777E-01	0,251	0,166	6,07757E-24
Macro_LYVE1	NFIC	1,05651E-79	4,26537E-01	0,443	0,287	1,94577E-75
Macro_LYVE1	SGPL1	3,53666E-79	4,24746E-01	0,299	0,162	6,51346E-75
Macro_LYVE1	FPR3	4,97740E-99	4,18984E-01	0,522	0,329	9,16689E-95
Macro_LYVE1	CD68	1,89181E-139	4,15577E-01	0,901	0,774	3,48415E-135
Macro_LYVE1	RENBP	2,78989E-54	4,14086E-01	0,428	0,291	5,13813E-50
Macro_LYVE1	TBC1D14	7,97248E-75	4,09101E-01	0,311	0,174	1,46829E-70
Macro_LYVE1	FCGR2A	5,04893E-204	4,03558E-01	0,842	0,62	9,29861E-200
Macro_LYVE1	PABPC4	1,08908E-13	4,02862E-01	0,48	0,421	2,00577E-09
Macro_LYVE1	KLF2	3,16981E-30	3,97689E-01	0,37	0,272	5,83784E-26
Macro_LYVE1	BLVRA	9,29655E-30	3,97628E-01	0,472	0,378	1,71215E-25
Macro_LYVE1	BMP2K	2,41763E-88	3,97617E-01	0,448	0,283	4,45256E-84
Macro_LYVE1	CEBPD	1,30332E-65	3,97545E-01	0,734	0,559	2,40033E-61
Macro_LYVE1	SORBS3	3,08494E-54	3,95971E-01	0,265	0,154	5,68154E-50
Macro_LYVE1	MTRNR2L8	8,96600E-11	3,93317E-01	0,498	0,437	1,65127E-06
Macro_LYVE1	NRP2	2,65869E-117	3,90143E-01	0,389	0,2	4,89652E-113
Macro_LYVE1	MTSS1	6,76873E-191	3,87961E-01	0,428	0,187	1,24660E-186
Macro_LYVE1	CLTC	7,08724E-106	3,87270E-01	0,581	0,412	1,30526E-101
Macro_LYVE1	MMP14	1,32761E-56	3,85415E-01	0,348	0,218	2,44506E-52
Macro_LYVE1	ATM	5,75128E-37	3,82940E-01	0,357	0,254	1,05921E-32
Macro_LYVE1	FMN1	1,51151E-68	3,77365E-01	0,271	0,146	2,78374E-64
Macro_LYVE1	LMNA	8,26085E-15	3,71403E-01	0,447	0,365	1,52140E-10
Macro_LYVE1	TLR4	4,52806E-77	3,65660E-01	0,371	0,217	8,33933E-73
Macro_LYVE1	CD93	2,34873E-62	3,65251E-01	0,408	0,272	4,32566E-58
Macro_LYVE1	AKR1A1	1,07671E-62	3,65243E-01	0,708	0,57	1,98299E-58
Macro_LYVE1	KLF4	3,68404E-17	3,64504E-01	0,516	0,443	6,78490E-13
Macro_LYVE1	WASF2	1,49894E-90	3,59449E-01	0,701	0,562	2,76059E-86
Macro_LYVE1	CHD1	9,38682E-08	3,59246E-01	0,245	0,3	1,72877E-03
Macro_LYVE1	IRF2	6,21845E-08	3,57272E-01	0,366	0,326	1,14525E-03
Macro_LYVE1	RAB1A	1,13194E-08	3,50439E-01	0,434	0,394	2,08469E-04
Macro_LYVE1	ASAH1	1,46340E-167	3,44021E-01	0,859	0,68	2,69514E-163
Macro_LYVE1	PEPD	4,58199E-29	3,43491E-01	0,45	0,358	8,43866E-25
Macro_LYVE1	SERPING1	2,69868E-76	3,42999E-01	0,571	0,388	4,97015E-72
Macro_LYVE1	CYTH4	3,68720E-14	3,38904E-01	0,371	0,312	6,79073E-10
Macro_LYVE1	DST	1,12379E-63	3,37147E-01	0,338	0,208	2,06968E-59
Macro_LYVE1	ACAP2	1,86785E-14	3,36515E-01	0,487	0,428	3,44002E-10
Macro_LYVE1	PDE4DIP	1,03170E-08	3,35929E-01	0,377	0,319	1,90007E-04
Macro_LYVE1	DDX3X	4,10736E-07	3,23534E-01	0,513	0,476	7,56452E-03
Macro_LYVE1	AIF1	1,75290E-116	3,23443E-01	0,958	0,815	3,22832E-112
Macro_LYVE1	EPS15	1,89413E-70	3,19942E-01	0,385	0,241	3,48841E-66
Macro_LYVE1	TCEAL4	3,04077E-21	3,19103E-01	0,353	0,276	5,60018E-17
Macro_LYVE1	GBA	4,21471E-14	3,17637E-01	0,254	0,198	7,76224E-10
Macro_LYVE1	FGFR1OP2	2,71834E-16	3,17087E-01	0,365	0,302	5,00637E-12
Macro_LYVE1	PSAP	2,62150E-180	3,12306E-01	0,994	0,912	4,82802E-176
Macro_LYVE1	ITPR2	4,16306E-76	3,12236E-01	0,417	0,27	7,66710E-72
Macro_LYVE1	VPS13C	2,12254E-19	3,11998E-01	0,44	0,367	3,90908E-15
Macro_LYVE1	C5AR1	1,24875E-91	3,11918E-01	0,61	0,397	2,29982E-87
Macro_LYVE1	NEU1	1,04163E-42	3,10368E-01	0,408	0,299	1,91837E-38

Macro_LYVE1	SLC38A6	1,29677E-92	3,10154E-01	0,283	0,139	2,38827E-88
Macro_LYVE1	ARRDC2	1,24117E-44	3,04215E-01	0,306	0,2	2,28587E-40
Macro_LYVE1	ABHD12	1,17417E-37	2,95435E-01	0,479	0,374	2,16247E-33
Macro_LYVE1	FEZ2	1,34717E-50	2,93269E-01	0,465	0,344	2,48109E-46
Macro_LYVE1	PBRM1	1,67975E-06	2,92478E-01	0,265	0,233	3,09360E-02
Macro_LYVE1	EEA1	2,26789E-13	2,91307E-01	0,286	0,231	4,17678E-09
Macro_LYVE1	RAB13	8,87506E-16	2,90787E-01	0,374	0,301	1,63452E-11
Macro_LYVE1	FAM13A	5,51510E-118	2,85994E-01	0,254	0,106	1,01572E-113
Macro_LYVE1	PITHD1	7,66899E-21	2,85476E-01	0,297	0,225	1,41240E-16
Macro_LYVE1	MPP1	4,15927E-42	2,84666E-01	0,52	0,407	7,66012E-38
Macro_LYVE1	HEXA	2,54050E-107	2,82326E-01	0,658	0,459	4,67883E-103
Macro_LYVE1	ADA2	4,46706E-09	2,81457E-01	0,264	0,227	8,22698E-05
Macro_LYVE1	PARP1	4,28288E-10	2,80123E-01	0,382	0,332	7,88778E-06
Macro_LYVE1	LPIN2	1,23065E-09	2,80045E-01	0,264	0,222	2,26648E-05
Macro_LYVE1	LTC4S	4,65594E-57	2,79831E-01	0,428	0,276	8,57484E-53
Macro_LYVE1	HLA-DMB	1,48788E-127	2,77911E-01	0,837	0,628	2,74023E-123
Macro_LYVE1	CITED2	9,55573E-22	2,74803E-01	0,414	0,33	1,75988E-17
Macro_LYVE1	DEGS1	5,74297E-12	2,72604E-01	0,377	0,324	1,05768E-07
Macro_LYVE1	ACP2	2,01230E-65	2,71817E-01	0,397	0,25	3,70606E-61
Macro_LYVE1	RHOB	1,97217E-27	2,70808E-01	0,653	0,536	3,63214E-23
Macro_LYVE1	FMNL2	8,87329E-23	2,68011E-01	0,302	0,223	1,63419E-18
Macro_LYVE1	IGSF6	1,03747E-44	2,67578E-01	0,669	0,551	1,91072E-40
Macro_LYVE1	TPCN1	2,44774E-98	2,64990E-01	0,288	0,139	4,50800E-94
Macro_LYVE1	TEX264	5,51834E-07	2,61958E-01	0,34	0,306	1,01631E-02
Macro_LYVE1	DOCK4	1,78130E-25	2,60137E-01	0,373	0,281	3,28062E-21
Macro_LYVE1	DICER1	3,22205E-32	2,59461E-01	0,362	0,265	5,93405E-28
Macro_LYVE1	LAIR1	8,95266E-89	2,59019E-01	0,695	0,51	1,64881E-84
Macro_LYVE1	BRI3	5,49091E-25	2,58779E-01	0,489	0,398	1,01126E-20
Macro_LYVE1	RNF13	1,14965E-82	2,58524E-01	0,664	0,51	2,11730E-78
Macro_LYVE1	PIK3R1	1,10779E-21	2,57312E-01	0,4	0,317	2,04021E-17
Macro_LYVE1	TNFSF12	3,01662E-27	2,55640E-01	0,289	0,205	5,55572E-23
Macro_LYVE1	SNX9	6,09841E-41	2,53134E-01	0,394	0,283	1,12314E-36
Macro_LYVE1	ATP2A2	1,08436E-14	2,52158E-01	0,29	0,231	1,99706E-10
Macro_LYVE1	ZFP36L1	2,39685E-67	2,50276E-01	0,86	0,718	4,41428E-63
Macro_LYVE1	STOM	1,19905E-44	2,50075E-01	0,46	0,34	2,20828E-40
Macro_LYVE1	HPS1	2,71197E-06	-2,50925E-01	0,386	0,35	4,99463E-02
Macro_LYVE1	LRRC59	3,51536E-11	-2,53439E-01	0,22	0,286	6,47425E-07
Macro_LYVE1	UCP2	1,44775E-11	-2,53995E-01	0,695	0,631	2,66632E-07
Macro_LYVE1	TMED4	1,42593E-08	-2,54162E-01	0,334	0,291	2,62614E-04
Macro_LYVE1	ETNK1	6,06458E-13	-2,56005E-01	0,302	0,245	1,11691E-08
Macro_LYVE1	IL6ST	7,64140E-10	-2,56100E-01	0,255	0,209	1,40732E-05
Macro_LYVE1	TM9SF2	2,65280E-10	-2,57332E-01	0,474	0,425	4,88567E-06
Macro_LYVE1	SERF2	7,62746E-12	-2,58611E-01	0,961	0,953	1,40475E-07
Macro_LYVE1	ELOC	1,05869E-12	-2,59314E-01	0,194	0,258	1,94979E-08
Macro_LYVE1	COX4I1	4,98957E-09	-2,59345E-01	0,91	0,896	9,18929E-05
Macro_LYVE1	SNRPF	2,24737E-06	-2,59555E-01	0,34	0,396	4,13898E-02
Macro_LYVE1	HERC1	3,38584E-19	-2,60434E-01	0,259	0,191	6,23569E-15
Macro_LYVE1	SELENOW	7,23887E-09	-2,60673E-01	0,241	0,294	1,33318E-04
Macro_LYVE1	GLIPR1	1,93228E-36	-2,62592E-01	0,689	0,578	3,55868E-32
Macro_LYVE1	C20orf27	9,93451E-07	-2,63448E-01	0,304	0,347	1,82964E-02
Macro_LYVE1	PLXNB2	3,73920E-11	-2,63949E-01	0,313	0,262	6,88649E-07
Macro_LYVE1	PSMD8	3,69394E-08	-2,64731E-01	0,476	0,528	6,80314E-04
Macro_LYVE1	MYH9	6,80552E-09	-2,65680E-01	0,526	0,581	1,25337E-04
Macro_LYVE1	PRKCD	3,36262E-07	-2,66363E-01	0,209	0,259	6,19294E-03

Macro_LYVE1	HP1BP3	2,71338E-07	-2,66633E-01	0,441	0,405	4,99723E-03
Macro_LYVE1	TMED9	9,06025E-12	-2,66659E-01	0,57	0,521	1,66863E-07
Macro_LYVE1	KRT10	1,27477E-15	-2,67552E-01	0,25	0,329	2,34775E-11
Macro_LYVE1	WDR1	2,77314E-25	-2,68146E-01	0,451	0,546	5,10729E-21
Macro_LYVE1	SNF8	7,77475E-10	-2,71237E-01	0,233	0,297	1,43188E-05
Macro_LYVE1	BAX	3,20482E-08	-2,72929E-01	0,485	0,541	5,90231E-04
Macro_LYVE1	SRSF7	2,86308E-11	-2,74429E-01	0,602	0,549	5,27294E-07
Macro_LYVE1	SKIL	5,61334E-13	-2,76038E-01	0,259	0,335	1,03381E-08
Macro_LYVE1	GPI	1,65535E-07	-2,76087E-01	0,361	0,419	3,04866E-03
Macro_LYVE1	ATP6V0E1	5,84636E-07	-2,77249E-01	0,835	0,793	1,07672E-02
Macro_LYVE1	HSPA6	5,60761E-12	-2,78957E-01	0,353	0,283	1,03275E-07
Macro_LYVE1	ANKRD12	3,66353E-14	-2,79652E-01	0,524	0,467	6,74712E-10
Macro_LYVE1	DDX24	4,38288E-09	-2,80235E-01	0,516	0,475	8,07196E-05
Macro_LYVE1	CLTB	1,49611E-09	-2,81094E-01	0,297	0,364	2,75539E-05
Macro_LYVE1	TRIR	2,17747E-08	-2,82298E-01	0,292	0,338	4,01024E-04
Macro_LYVE1	LRRC25	2,44476E-09	-2,84143E-01	0,387	0,33	4,50251E-05
Macro_LYVE1	AURKAIP1	1,45395E-07	-2,84537E-01	0,463	0,521	2,67773E-03
Macro_LYVE1	RPL22L1	5,00000E-13	-2,86110E-01	0,213	0,277	9,20850E-09
Macro_LYVE1	AKAP9	4,74695E-26	-2,87225E-01	0,467	0,382	8,74245E-22
Macro_LYVE1	ARPC5L	4,51626E-11	-2,88457E-01	0,209	0,274	8,31759E-07
Macro_LYVE1	LYN	6,98915E-10	-2,88691E-01	0,404	0,455	1,28719E-05
Macro_LYVE1	ZYX	2,40400E-12	-2,90998E-01	0,619	0,646	4,42744E-08
Macro_LYVE1	ATP5F1D	2,83767E-09	-2,91087E-01	0,292	0,342	5,22614E-05
Macro_LYVE1	WTAP	1,05827E-12	-2,91573E-01	0,297	0,369	1,94901E-08
Macro_LYVE1	CDC26	7,90770E-11	-2,92105E-01	0,207	0,272	1,45636E-06
Macro_LYVE1	TPT1	1,54609E-42	-2,92484E-01	0,985	0,973	2,84744E-38
Macro_LYVE1	PLCB2	8,19102E-07	-2,92604E-01	0,278	0,24	1,50854E-02
Macro_LYVE1	PIN1	4,09002E-10	-2,93985E-01	0,281	0,35	7,53260E-06
Macro_LYVE1	PNPLA2	9,98713E-20	-2,95186E-01	0,214	0,306	1,83933E-15
Macro_LYVE1	JPT1	5,63400E-08	-2,97019E-01	0,24	0,285	1,03761E-03
Macro_LYVE1	CHMP4B	7,91898E-21	-2,97432E-01	0,319	0,413	1,45844E-16
Macro_LYVE1	HNRNPA0	2,45973E-14	-2,97995E-01	0,439	0,513	4,53009E-10
Macro_LYVE1	ZNFX1	3,64541E-09	-2,98757E-01	0,202	0,26	6,71374E-05
Macro_LYVE1	SMCHD1	2,72678E-12	-3,02034E-01	0,258	0,328	5,02191E-08
Macro_LYVE1	KRAS	5,50491E-08	-3,03993E-01	0,267	0,323	1,01384E-03
Macro_LYVE1	DAD1	1,36516E-10	-3,04525E-01	0,639	0,598	2,51421E-06
Macro_LYVE1	FCHSD2	2,28591E-08	-3,05816E-01	0,268	0,222	4,20995E-04
Macro_LYVE1	APBB1IP	1,41752E-07	-3,08198E-01	0,439	0,481	2,61064E-03
Macro_LYVE1	PSTPIP2	1,14878E-12	-3,08662E-01	0,187	0,254	2,11570E-08
Macro_LYVE1	GPSM3	4,36881E-14	-3,08900E-01	0,644	0,686	8,04603E-10
Macro_LYVE1	LACTB	5,37817E-14	-3,09300E-01	0,254	0,327	9,90497E-10
Macro_LYVE1	VDAC2	4,32030E-10	-3,10479E-01	0,497	0,556	7,95670E-06
Macro_LYVE1	B2M	1,08339E-10	-3,11051E-01	0,999	0,995	1,99528E-06
Macro_LYVE1	ATP5MG	6,15578E-09	-3,11965E-01	0,385	0,421	1,13371E-04
Macro_LYVE1	ATP6V0B	7,48111E-17	-3,13126E-01	0,842	0,788	1,37780E-12
Macro_LYVE1	C4orf3	8,06826E-08	-3,14518E-01	0,633	0,672	1,48593E-03
Macro_LYVE1	NMI	1,86071E-10	-3,15397E-01	0,242	0,303	3,42688E-06
Macro_LYVE1	EFHD2	2,84537E-12	-3,17899E-01	0,363	0,434	5,24031E-08
Macro_LYVE1	SEC13	7,14651E-10	-3,18555E-01	0,333	0,405	1,31617E-05
Macro_LYVE1	PPT1	1,42889E-11	-3,18616E-01	0,732	0,647	2,63159E-07
Macro_LYVE1	SNX3	2,90442E-08	-3,18618E-01	0,666	0,691	5,34907E-04
Macro_LYVE1	BTK	5,17169E-08	-3,19512E-01	0,257	0,313	9,52469E-04
Macro_LYVE1	FGD2	2,48812E-12	-3,22355E-01	0,394	0,342	4,58237E-08
Macro_LYVE1	ISCU	3,47656E-14	-3,25957E-01	0,596	0,534	6,40278E-10

Macro_LYVE1	LGALS1	4,14691E-23	-3,27438E-01	0,785	0,838	7,63737E-19
Macro_LYVE1	FOS	3,91883E-16	-3,28702E-01	0,908	0,84	7,21730E-12
Macro_LYVE1	PKIB	2,22009E-35	-3,30450E-01	0,137	0,251	4,08873E-31
Macro_LYVE1	PSMB2	9,91038E-08	-3,36181E-01	0,386	0,444	1,82519E-03
Macro_LYVE1	POLD4	1,31504E-12	-3,36917E-01	0,528	0,572	2,42191E-08
Macro_LYVE1	VAMP5	2,31763E-12	-3,37299E-01	0,442	0,482	4,26837E-08
Macro_LYVE1	EVI2A	9,79819E-09	-3,38258E-01	0,313	0,272	1,80453E-04
Macro_LYVE1	TCIRG1	1,52679E-11	-3,38466E-01	0,401	0,471	2,81190E-07
Macro_LYVE1	BAG1	9,85299E-20	-3,39655E-01	0,285	0,379	1,81462E-15
Macro_LYVE1	FKBP2	9,69609E-12	-3,40606E-01	0,458	0,407	1,78573E-07
Macro_LYVE1	DOCK10	5,80809E-11	-3,40653E-01	0,256	0,326	1,06968E-06
Macro_LYVE1	ATP5F1B	4,38973E-11	-3,41036E-01	0,267	0,316	8,08457E-07
Macro_LYVE1	QSOX1	1,27587E-09	-3,44853E-01	0,233	0,291	2,34977E-05
Macro_LYVE1	RNF7	7,69863E-09	-3,45370E-01	0,491	0,547	1,41786E-04
Macro_LYVE1	SRSF9	4,94074E-19	-3,46053E-01	0,293	0,384	9,09937E-15
Macro_LYVE1	IST1	6,16750E-07	-3,50283E-01	0,297	0,262	1,13587E-02
Macro_LYVE1	MRPL4	6,23414E-09	-3,51563E-01	0,222	0,28	1,14814E-04
Macro_LYVE1	SLC25A5	6,78689E-07	-3,52330E-01	0,73	0,745	1,24994E-02
Macro_LYVE1	GHITM	6,08853E-16	-3,52543E-01	0,426	0,509	1,12132E-11
Macro_LYVE1	RNPS1	4,01277E-17	-3,57201E-01	0,318	0,409	7,39031E-13
Macro_LYVE1	PILRA	1,48756E-13	-3,57620E-01	0,57	0,489	2,73964E-09
Macro_LYVE1	ATP5MC3	4,76405E-10	-3,58048E-01	0,312	0,355	8,77394E-06
Macro_LYVE1	CYB5R3	5,36255E-13	-3,58770E-01	0,337	0,414	9,87621E-09
Macro_LYVE1	ELOB	1,03766E-10	-3,61118E-01	0,347	0,401	1,91105E-06
Macro_LYVE1	VDAC1	3,29549E-09	-3,61873E-01	0,483	0,536	6,06931E-05
Macro_LYVE1	MLF2	2,06177E-11	-3,63496E-01	0,38	0,456	3,79716E-07
Macro_LYVE1	TIMM17A	6,15370E-07	-3,64488E-01	0,222	0,274	1,13333E-02
Macro_LYVE1	COPE	2,66862E-13	-3,64836E-01	0,55	0,61	4,91480E-09
Macro_LYVE1	SPI1	1,52220E-26	-3,65709E-01	0,747	0,768	2,80343E-22
Macro_LYVE1	MPG	4,34038E-07	-3,70030E-01	0,272	0,325	7,99368E-03
Macro_LYVE1	ERICH1	4,66270E-07	-3,70243E-01	0,272	0,324	8,58729E-03
Macro_LYVE1	RALY	1,26233E-16	-3,70426E-01	0,406	0,497	2,32483E-12
Macro_LYVE1	HPRT1	1,51693E-07	-3,72769E-01	0,214	0,266	2,79373E-03
Macro_LYVE1	CASP4	1,20551E-08	-3,73240E-01	0,386	0,446	2,22019E-04
Macro_LYVE1	OAS1	1,24737E-08	-3,75681E-01	0,281	0,329	2,29728E-04
Macro_LYVE1	PTK2B	1,69578E-08	-3,77376E-01	0,202	0,256	3,12313E-04
Macro_LYVE1	CSNK1D	1,15282E-08	-3,78043E-01	0,281	0,346	2,12315E-04
Macro_LYVE1	MTPN	3,40706E-07	-3,78786E-01	0,478	0,529	6,27478E-03
Macro_LYVE1	FIBP	1,13183E-09	-3,79050E-01	0,304	0,371	2,08448E-05
Macro_LYVE1	RPL36A	1,78548E-21	-3,79959E-01	0,38	0,457	3,28832E-17
Macro_LYVE1	TMSB10	3,33947E-26	-3,84138E-01	0,993	0,984	6,15030E-22
Macro_LYVE1	HSD17B10	1,24634E-11	-3,86088E-01	0,243	0,31	2,29538E-07
Macro_LYVE1	SON	9,75703E-26	-3,87244E-01	0,741	0,671	1,79695E-21
Macro_LYVE1	FDFT1	7,67915E-07	-3,87830E-01	0,215	0,262	1,41427E-02
Macro_LYVE1	NUB1	1,58223E-06	-3,89388E-01	0,269	0,316	2,91400E-02
Macro_LYVE1	THEMIS2	2,71689E-07	-3,89685E-01	0,536	0,493	5,00370E-03
Macro_LYVE1	PSMB1	1,56160E-07	-3,89737E-01	0,586	0,631	2,87601E-03
Macro_LYVE1	EDF1	1,47927E-12	-3,90355E-01	0,736	0,75	2,72437E-08
Macro_LYVE1	MX2	5,09184E-21	-3,93507E-01	0,188	0,274	9,37763E-17
Macro_LYVE1	SCIMP	1,92305E-06	-3,94103E-01	0,234	0,28	3,54168E-02
Macro_LYVE1	POLE4	9,71745E-12	-3,94378E-01	0,268	0,334	1,78966E-07
Macro_LYVE1	TOM1	7,18324E-08	-3,94705E-01	0,235	0,286	1,32294E-03
Macro_LYVE1	SMARCB1	6,54578E-14	-3,95063E-01	0,221	0,299	1,20554E-09
Macro_LYVE1	UQCRFS1	4,93755E-17	-3,95582E-01	0,416	0,497	9,09348E-13

Macro_LYVE1	ENSA	2,74245E-15	-3,98824E-01	0,397	0,476	5,05078E-11
Macro_LYVE1	HNRNPM	1,35232E-11	-3,99140E-01	0,392	0,461	2,49057E-07
Macro_LYVE1	MSN	5,71630E-10	-4,00224E-01	0,586	0,64	1,05277E-05
Macro_LYVE1	ARF6	6,30289E-15	-4,01961E-01	0,34	0,423	1,16080E-10
Macro_LYVE1	MICAL1	1,03454E-12	-4,02633E-01	0,224	0,297	1,90531E-08
Macro_LYVE1	ACTN1	7,86665E-22	-4,03143E-01	0,231	0,323	1,44880E-17
Macro_LYVE1	TMED10	4,29523E-13	-4,03160E-01	0,625	0,564	7,91052E-09
Macro_LYVE1	ATXN10	2,09276E-11	-4,04143E-01	0,213	0,28	3,85424E-07
Macro_LYVE1	HLA-B	1,11427E-14	-4,04820E-01	0,992	0,967	2,05214E-10
Macro_LYVE1	GSTO1	6,53078E-09	-4,04981E-01	0,585	0,621	1,20277E-04
Macro_LYVE1	UBE2A	1,34864E-08	-4,06523E-01	0,346	0,405	2,48378E-04
Macro_LYVE1	FXYD5	1,48140E-08	-4,06541E-01	0,826	0,825	2,72830E-04
Macro_LYVE1	GLRX	1,30474E-06	-4,07885E-01	0,52	0,549	2,40294E-02
Macro_LYVE1	TNFAIP2	3,45870E-29	-4,08972E-01	0,509	0,4	6,36989E-25
Macro_LYVE1	PTMA	3,56963E-15	-4,09035E-01	0,977	0,961	6,57418E-11
Macro_LYVE1	SELENOK	1,57684E-16	-4,09824E-01	0,206	0,282	2,90406E-12
Macro_LYVE1	IFI35	1,23814E-14	-4,10772E-01	0,277	0,35	2,28028E-10
Macro_LYVE1	EIF6	4,47304E-14	-4,11395E-01	0,328	0,409	8,23800E-10
Macro_LYVE1	TMBIM4	1,36360E-07	-4,11554E-01	0,371	0,329	2,51134E-03
Macro_LYVE1	HMGA1	1,63148E-48	-4,12368E-01	0,269	0,411	3,00469E-44
Macro_LYVE1	UBE2D2	4,17457E-17	-4,12433E-01	0,487	0,569	7,68830E-13
Macro_LYVE1	DRAP1	1,61538E-18	-4,12458E-01	0,61	0,66	2,97505E-14
Macro_LYVE1	PSMD6	4,51116E-09	-4,14244E-01	0,221	0,281	8,30820E-05
Macro_LYVE1	SSNA1	8,68957E-07	-4,14357E-01	0,356	0,407	1,60036E-02
Macro_LYVE1	PPM1G	6,29306E-11	-4,15003E-01	0,299	0,372	1,15899E-06
Macro_LYVE1	DOCK2	1,74605E-10	-4,16200E-01	0,398	0,346	3,21571E-06
Macro_LYVE1	DDX21	6,31793E-22	-4,20064E-01	0,34	0,442	1,16357E-17
Macro_LYVE1	LIMS1	1,02321E-20	-4,20200E-01	0,474	0,559	1,88445E-16
Macro_LYVE1	LRRFIP1	1,10948E-19	-4,23649E-01	0,625	0,687	2,04332E-15
Macro_LYVE1	FLOT1	2,43401E-09	-4,23653E-01	0,47	0,527	4,48272E-05
Macro_LYVE1	STRAP	3,10389E-07	-4,24817E-01	0,304	0,358	5,71644E-03
Macro_LYVE1	COX8A	1,00739E-08	-4,26024E-01	0,765	0,778	1,85532E-04
Macro_LYVE1	TOMM22	1,18133E-09	-4,26251E-01	0,362	0,427	2,17566E-05
Macro_LYVE1	RHBDD2	3,35746E-07	-4,27495E-01	0,214	0,26	6,18344E-03
Macro_LYVE1	CUX1	2,90865E-11	-4,27525E-01	0,286	0,354	5,35686E-07
Macro_LYVE1	ARPC3	7,73800E-08	-4,27694E-01	0,908	0,891	1,42511E-03
Macro_LYVE1	PHB2	2,96488E-12	-4,29667E-01	0,429	0,496	5,46041E-08
Macro_LYVE1	LYSMD2	3,61848E-20	-4,29858E-01	0,204	0,295	6,66415E-16
Macro_LYVE1	PCBP2	1,09939E-17	-4,32174E-01	0,799	0,798	2,02475E-13
Macro_LYVE1	CLEC10A	2,73075E-15	-4,32348E-01	0,296	0,22	5,02922E-11
Macro_LYVE1	RGS19	2,58915E-09	-4,37284E-01	0,447	0,498	4,76843E-05
Macro_LYVE1	POU2F2	3,30386E-08	-4,41060E-01	0,221	0,273	6,08472E-04
Macro_LYVE1	PAK1	3,30890E-18	-4,45581E-01	0,358	0,441	6,09401E-14
Macro_LYVE1	GUK1	9,87532E-24	-4,45970E-01	0,66	0,713	1,81874E-19
Macro_LYVE1	GABARAPL1	6,97237E-08	-4,47238E-01	0,245	0,294	1,28410E-03
Macro_LYVE1	LSM10	3,60184E-08	-4,48089E-01	0,316	0,379	6,63351E-04
Macro_LYVE1	EIF3H	8,40905E-08	-4,49311E-01	0,631	0,659	1,54870E-03
Macro_LYVE1	ETV6	2,44293E-10	-4,49419E-01	0,238	0,293	4,49915E-06
Macro_LYVE1	GLRX3	3,71332E-07	-4,49835E-01	0,2	0,25	6,83882E-03
Macro_LYVE1	SF1	2,28434E-06	-4,52263E-01	0,46	0,506	4,20706E-02
Macro_LYVE1	SYAP1	6,80537E-14	-4,52679E-01	0,272	0,348	1,25334E-09
Macro_LYVE1	PPDPF	1,20450E-37	-4,52980E-01	0,69	0,772	2,21833E-33
Macro_LYVE1	RPS17	2,30250E-08	-4,54952E-01	0,357	0,41	4,24052E-04
Macro_LYVE1	STOML2	7,63605E-08	-4,55022E-01	0,223	0,278	1,40633E-03

Macro_LYVE1	MDH2	1,75736E-16	-4,55179E-01	0,451	0,526	3,23654E-12
Macro_LYVE1	CLN8	5,17797E-13	-4,58272E-01	0,289	0,233	9,53626E-09
Macro_LYVE1	CORO1C	9,52741E-10	-4,61400E-01	0,287	0,347	1,75466E-05
Macro_LYVE1	TNFSF13	5,66550E-12	-4,61734E-01	0,505	0,44	1,04342E-07
Macro_LYVE1	RHOG	2,43951E-10	-4,61823E-01	0,715	0,727	4,49284E-06
Macro_LYVE1	BASP1	5,58098E-16	-4,63341E-01	0,28	0,35	1,02785E-11
Macro_LYVE1	DDX5	3,36557E-08	-4,63750E-01	0,883	0,83	6,19837E-04
Macro_LYVE1	RHOA	4,43212E-18	-4,64119E-01	0,892	0,878	8,16264E-14
Macro_LYVE1	PCBP1	9,61200E-10	-4,64133E-01	0,577	0,612	1,77024E-05
Macro_LYVE1	SNX17	1,81572E-08	-4,64675E-01	0,376	0,435	3,34401E-04
Macro_LYVE1	CCNI	2,32651E-27	-4,64790E-01	0,759	0,787	4,28473E-23
Macro_LYVE1	HVCN1	7,44139E-07	-4,65800E-01	0,21	0,256	1,37048E-02
Macro_LYVE1	SHISA5	4,30264E-11	-4,66000E-01	0,309	0,374	7,92417E-07
Macro_LYVE1	RBCK1	7,64366E-11	-4,66282E-01	0,308	0,377	1,40773E-06
Macro_LYVE1	PSMC3	2,13406E-08	-4,66865E-01	0,262	0,322	3,93030E-04
Macro_LYVE1	HEXB	1,97421E-17	-4,68219E-01	0,576	0,511	3,63590E-13
Macro_LYVE1	BAZ1A	2,41549E-39	-4,68241E-01	0,292	0,425	4,44860E-35
Macro_LYVE1	TES	7,71974E-31	-4,68982E-01	0,176	0,285	1,42174E-26
Macro_LYVE1	RAB8A	1,50487E-25	-4,69891E-01	0,325	0,431	2,77153E-21
Macro_LYVE1	ATP1A1	2,52191E-06	-4,70057E-01	0,382	0,432	4,64459E-02
Macro_LYVE1	CSNK1A1	7,31183E-07	-4,71271E-01	0,41	0,465	1,34662E-02
Macro_LYVE1	LUC7L3	8,18837E-08	-4,71921E-01	0,398	0,357	1,50805E-03
Macro_LYVE1	DGUOK	1,90391E-09	-4,72979E-01	0,306	0,374	3,50643E-05
Macro_LYVE1	ATP5MC2	7,62279E-14	-4,74069E-01	0,388	0,436	1,40389E-09
Macro_LYVE1	TMA7	2,60652E-11	-4,74334E-01	0,755	0,795	4,80043E-07
Macro_LYVE1	OAZ1	1,54312E-11	-4,74386E-01	0,963	0,935	2,84197E-07
Macro_LYVE1	IKZF1	1,08514E-13	-4,74764E-01	0,216	0,286	1,99850E-09
Macro_LYVE1	ACTR3	2,99250E-24	-4,75032E-01	0,605	0,667	5,51129E-20
Macro_LYVE1	TPM4	1,38167E-24	-4,75543E-01	0,546	0,622	2,54463E-20
Macro_LYVE1	PSMD9	8,88846E-07	-4,78762E-01	0,207	0,257	1,63699E-02
Macro_LYVE1	USP15	7,87284E-09	-4,81003E-01	0,358	0,428	1,44994E-04
Macro_LYVE1	STAT1	4,07496E-07	-4,83013E-01	0,487	0,519	7,50485E-03
Macro_LYVE1	SH3KBP1	3,66782E-08	-4,83932E-01	0,357	0,417	6,75503E-04
Macro_LYVE1	CCT2	9,28925E-11	-4,83943E-01	0,274	0,344	1,71080E-06
Macro_LYVE1	HNRNPH1	1,67608E-06	-4,87506E-01	0,438	0,403	3,08684E-02
Macro_LYVE1	PSMA7	2,38389E-08	-4,89308E-01	0,728	0,746	4,39041E-04
Macro_LYVE1	SLC25A6	5,87303E-25	-4,90777E-01	0,832	0,815	1,08164E-20
Macro_LYVE1	CSRP1	3,79404E-07	-4,92809E-01	0,214	0,26	6,98749E-03
Macro_LYVE1	PNRC1	8,23348E-15	-4,93931E-01	0,776	0,779	1,51636E-10
Macro_LYVE1	ECHDC1	6,84270E-09	-4,94426E-01	0,214	0,272	1,26022E-04
Macro_LYVE1	ANP32B	2,10846E-07	-4,99911E-01	0,528	0,574	3,88314E-03
Macro_LYVE1	PEA15	3,33600E-28	-5,00016E-01	0,414	0,523	6,14392E-24
Macro_LYVE1	PPP4C	2,71554E-19	-5,02967E-01	0,431	0,517	5,00121E-15
Macro_LYVE1	TPM3	2,01071E-06	-5,03345E-01	0,807	0,803	3,70312E-02
Macro_LYVE1	DDT	2,39752E-11	-5,06069E-01	0,377	0,444	4,41552E-07
Macro_LYVE1	S100A11	1,63396E-68	-5,07175E-01	0,952	0,937	3,00927E-64
Macro_LYVE1	PSMB3	8,47667E-07	-5,07612E-01	0,61	0,645	1,56115E-02
Macro_LYVE1	CD58	3,08774E-15	-5,08013E-01	0,24	0,317	5,68669E-11
Macro_LYVE1	SNX10	1,96858E-37	-5,11217E-01	0,369	0,492	3,62553E-33
Macro_LYVE1	ADRM1	5,50037E-15	-5,12509E-01	0,348	0,433	1,01300E-10
Macro_LYVE1	LSM7	5,03890E-16	-5,13217E-01	0,44	0,518	9,28015E-12
Macro_LYVE1	CSRNP1	2,40612E-16	-5,14474E-01	0,221	0,295	4,43135E-12
Macro_LYVE1	GRSF1	1,56349E-14	-5,14552E-01	0,267	0,344	2,87948E-10
Macro_LYVE1	PTDSS1	3,58856E-12	-5,15689E-01	0,205	0,273	6,60905E-08

Macro_LYVE1	ARPC1B	8,06468E-29	-5,15809E-01	0,783	0,797	1,48527E-24
Macro_LYVE1	MYADM	1,27791E-35	-5,16027E-01	0,222	0,34	2,35353E-31
Macro_LYVE1	UQCRC1	5,69626E-13	-5,16997E-01	0,439	0,509	1,04908E-08
Macro_LYVE1	TUFM	4,56675E-07	-5,17833E-01	0,46	0,512	8,41059E-03
Macro_LYVE1	SAMD9L	1,48781E-08	-5,17917E-01	0,227	0,282	2,74009E-04
Macro_LYVE1	SLC25A3	1,73489E-17	-5,18660E-01	0,673	0,716	3,19516E-13
Macro_LYVE1	ZFP36	2,38613E-08	-5,18927E-01	0,851	0,844	4,39453E-04
Macro_LYVE1	SMDT1	1,42393E-10	-5,24272E-01	0,422	0,486	2,62246E-06
Macro_LYVE1	MYL6	2,25994E-46	-5,24951E-01	0,948	0,941	4,16214E-42
Macro_LYVE1	GNA15	3,26877E-18	-5,25354E-01	0,23	0,31	6,02009E-14
Macro_LYVE1	PPP1CA	2,03713E-09	-5,25461E-01	0,585	0,624	3,75178E-05
Macro_LYVE1	HSP90B1	4,23848E-18	-5,25518E-01	0,767	0,696	7,80602E-14
Macro_LYVE1	ARF5	1,89928E-20	-5,25702E-01	0,553	0,617	3,49791E-16
Macro_LYVE1	PRMT1	1,20720E-17	-5,29041E-01	0,223	0,311	2,22330E-13
Macro_LYVE1	SEC61G	7,12531E-10	-5,29963E-01	0,543	0,603	1,31227E-05
Macro_LYVE1	CBX3	1,93765E-11	-5,32341E-01	0,342	0,411	3,56857E-07
Macro_LYVE1	DOCK8	1,87344E-12	-5,32419E-01	0,382	0,451	3,45032E-08
Macro_LYVE1	APRT	1,43230E-10	-5,32751E-01	0,583	0,625	2,63786E-06
Macro_LYVE1	FMNL1	1,33713E-16	-5,33810E-01	0,322	0,407	2,46260E-12
Macro_LYVE1	MX1	6,39387E-18	-5,34043E-01	0,262	0,337	1,17756E-13
Macro_LYVE1	PTRHD1	9,21650E-07	-5,35018E-01	0,297	0,351	1,69740E-02
Macro_LYVE1	PSMB10	3,68151E-24	-5,36955E-01	0,491	0,571	6,78023E-20
Macro_LYVE1	RACK1	9,50541E-22	-5,40368E-01	0,414	0,47	1,75061E-17
Macro_LYVE1	ACTN4	3,00937E-15	-5,41777E-01	0,276	0,352	5,54236E-11
Macro_LYVE1	FBP1	8,46689E-55	-5,43211E-01	0,211	0,357	1,55935E-50
Macro_LYVE1	EMD	1,28431E-28	-5,43969E-01	0,311	0,422	2,36532E-24
Macro_LYVE1	ZNF331	5,39451E-14	-5,44047E-01	0,409	0,341	9,93507E-10
Macro_LYVE1	CYC1	9,75935E-14	-5,44553E-01	0,359	0,438	1,79738E-09
Macro_LYVE1	ARPC2	1,81287E-70	-5,45536E-01	0,848	0,86	3,33877E-66
Macro_LYVE1	EEF2	2,76909E-39	-5,47335E-01	0,824	0,829	5,09982E-35
Macro_LYVE1	XRCC6	1,91492E-09	-5,47990E-01	0,434	0,498	3,52671E-05
Macro_LYVE1	PSMD2	3,21727E-10	-5,48146E-01	0,256	0,323	5,92525E-06
Macro_LYVE1	MVP	2,54565E-16	-5,51553E-01	0,291	0,378	4,68833E-12
Macro_LYVE1	NME2	4,60736E-28	-5,52058E-01	0,27	0,364	8,48537E-24
Macro_LYVE1	EIF1	1,73026E-10	-5,54396E-01	0,956	0,943	3,18662E-06
Macro_LYVE1	B4GALT1	2,41784E-08	-5,54789E-01	0,37	0,323	4,45293E-04
Macro_LYVE1	SNRPA	8,30261E-18	-5,55411E-01	0,214	0,3	1,52909E-13
Macro_LYVE1	YWHAQ	9,92591E-07	-5,55502E-01	0,38	0,433	1,82805E-02
Macro_LYVE1	TALDO1	3,28739E-08	-5,57338E-01	0,618	0,664	6,05439E-04
Macro_LYVE1	LSM6	4,17806E-08	-5,58136E-01	0,299	0,353	7,69473E-04
Macro_LYVE1	TRABD	7,28049E-34	-5,65709E-01	0,241	0,363	1,34085E-29
Macro_LYVE1	COMMD6	2,25147E-17	-5,65808E-01	0,696	0,738	4,14653E-13
Macro_LYVE1	SLC16A3	9,66103E-48	-5,67261E-01	0,381	0,519	1,77927E-43
Macro_LYVE1	PLIN3	4,56170E-13	-5,70783E-01	0,3	0,375	8,40128E-09
Macro_LYVE1	HMGN2	6,70637E-07	-5,70903E-01	0,725	0,734	1,23511E-02
Macro_LYVE1	FOSL2	4,69930E-07	-5,72826E-01	0,252	0,298	8,65470E-03
Macro_LYVE1	NAPA	1,44437E-18	-5,73160E-01	0,424	0,499	2,66011E-14
Macro_LYVE1	EIF3I	1,95827E-10	-5,73501E-01	0,4	0,469	3,60655E-06
Macro_LYVE1	CFL1	5,12709E-51	-5,74826E-01	0,94	0,921	9,44256E-47
Macro_LYVE1	BTF3	2,17028E-18	-5,74950E-01	0,853	0,846	3,99701E-14
Macro_LYVE1	ZNF385A	1,69466E-15	-5,75400E-01	0,34	0,416	3,12105E-11
Macro_LYVE1	ILF3	2,91042E-07	-5,79927E-01	0,313	0,369	5,36012E-03
Macro_LYVE1	CSK	1,75295E-09	-5,80071E-01	0,289	0,35	3,22841E-05
Macro_LYVE1	FKBP1A	7,37696E-29	-5,80664E-01	0,632	0,698	1,35862E-24

Macro_LYVE1	GBP2	1,36125E-30	-5,82508E-01	0,273	0,378	2,50701E-26
Macro_LYVE1	STK4	1,25304E-08	-5,85859E-01	0,375	0,432	2,30773E-04
Macro_LYVE1	TNFAIP3	3,09272E-42	-5,87231E-01	0,414	0,55	5,69586E-38
Macro_LYVE1	BHLHE40	4,22937E-20	-5,88438E-01	0,26	0,344	7,78922E-16
Macro_LYVE1	NUMB	6,30789E-09	-5,88761E-01	0,281	0,336	1,16172E-04
Macro_LYVE1	EEF1D	6,11822E-35	-5,90806E-01	0,856	0,872	1,12679E-30
Macro_LYVE1	LILRB1	1,85405E-08	-5,92845E-01	0,329	0,379	3,41461E-04
Macro_LYVE1	CSNK2B	3,96044E-08	-5,92870E-01	0,291	0,34	7,29394E-04
Macro_LYVE1	UBE2N	3,26222E-09	-5,92904E-01	0,312	0,377	6,00803E-05
Macro_LYVE1	RPL12	8,46099E-27	-5,96007E-01	0,493	0,58	1,55826E-22
Macro_LYVE1	COX7A2L	5,48094E-07	-5,97538E-01	0,436	0,493	1,00942E-02
Macro_LYVE1	ARFGAP3	3,78207E-14	-6,01265E-01	0,203	0,271	6,96545E-10
Macro_LYVE1	LAT2	7,14462E-11	-6,03217E-01	0,394	0,457	1,31582E-06
Macro_LYVE1	HIGD2A	8,44261E-08	-6,03516E-01	0,698	0,716	1,55488E-03
Macro_LYVE1	UBE2I	4,72803E-12	-6,04751E-01	0,465	0,53	8,70761E-08
Macro_LYVE1	MTHFD2	4,45139E-32	-6,07061E-01	0,295	0,41	8,19812E-28
Macro_LYVE1	SRSF2	1,04165E-19	-6,08224E-01	0,478	0,562	1,91840E-15
Macro_LYVE1	SERP1	5,21549E-50	-6,10956E-01	0,781	0,821	9,60536E-46
Macro_LYVE1	TPI1	2,56896E-83	-6,12529E-01	0,783	0,828	4,73125E-79
Macro_LYVE1	SRSF3	1,79174E-09	-6,15718E-01	0,524	0,573	3,29984E-05
Macro_LYVE1	ID2	1,93959E-09	-6,16658E-01	0,529	0,559	3,57215E-05
Macro_LYVE1	ACSL4	4,80020E-28	-6,18026E-01	0,181	0,281	8,84053E-24
Macro_LYVE1	FPR1	6,90303E-20	-6,18063E-01	0,38	0,452	1,27133E-15
Macro_LYVE1	HNRNPDL	3,44361E-08	-6,20525E-01	0,652	0,676	6,34210E-04
Macro_LYVE1	EMP3	7,97899E-54	-6,21093E-01	0,639	0,733	1,46949E-49
Macro_LYVE1	PGD	8,11718E-08	-6,28827E-01	0,417	0,468	1,49494E-03
Macro_LYVE1	PPIA	4,48976E-09	-6,29218E-01	0,93	0,893	8,26879E-05
Macro_LYVE1	CCDC12	3,30965E-14	-6,29866E-01	0,216	0,291	6,09539E-10
Macro_LYVE1	TIMM17B	1,54450E-12	-6,30110E-01	0,254	0,328	2,84450E-08
Macro_LYVE1	MBOAT7	7,64628E-24	-6,31960E-01	0,165	0,254	1,40822E-19
Macro_LYVE1	PTPN2	8,92697E-12	-6,32581E-01	0,319	0,389	1,64408E-07
Macro_LYVE1	ENO1	3,26999E-64	-6,35263E-01	0,679	0,765	6,02234E-60
Macro_LYVE1	STX11	1,53643E-31	-6,35393E-01	0,224	0,333	2,82965E-27
Macro_LYVE1	IDS	7,39667E-21	-6,38442E-01	0,312	0,402	1,36224E-16
Macro_LYVE1	RALA	3,31748E-26	-6,38446E-01	0,282	0,379	6,10980E-22
Macro_LYVE1	TNFSF10	2,04877E-19	-6,38522E-01	0,29	0,364	3,77322E-15
Macro_LYVE1	RILPL2	1,21192E-25	-6,40084E-01	0,356	0,461	2,23199E-21
Macro_LYVE1	KDM6B	2,40015E-13	-6,40409E-01	0,278	0,344	4,42036E-09
Macro_LYVE1	IFI30	3,39486E-49	-6,42167E-01	0,445	0,575	6,25232E-45
Macro_LYVE1	SHKBP1	6,85951E-21	-6,42487E-01	0,279	0,374	1,26332E-16
Macro_LYVE1	AK2	9,94355E-14	-6,42928E-01	0,269	0,347	1,83130E-09
Macro_LYVE1	FAU	4,26418E-100	-6,43301E-01	0,961	0,957	7,85334E-96
Macro_LYVE1	COX7A2	8,27850E-17	-6,43558E-01	0,668	0,723	1,52465E-12
Macro_LYVE1	GNG5	6,55601E-60	-6,43780E-01	0,74	0,796	1,20742E-55
Macro_LYVE1	RPS28	1,39184E-31	-6,44418E-01	0,497	0,586	2,56336E-27
Macro_LYVE1	HMGN1	3,60942E-19	-6,45005E-01	0,578	0,628	6,64746E-15
Macro_LYVE1	DYNLT1	5,99522E-07	-6,45377E-01	0,463	0,511	1,10414E-02
Macro_LYVE1	SUPT4H1	2,49519E-10	-6,47070E-01	0,401	0,464	4,59540E-06
Macro_LYVE1	SASH3	2,80232E-14	-6,51215E-01	0,213	0,288	5,16103E-10
Macro_LYVE1	IMP4	1,03697E-07	-6,51662E-01	0,215	0,269	1,90979E-03
Macro_LYVE1	SNRPB	1,42361E-22	-6,52143E-01	0,42	0,513	2,62186E-18
Macro_LYVE1	RELB	2,81229E-44	-6,55379E-01	0,13	0,251	5,17939E-40
Macro_LYVE1	UNC119	1,07116E-20	-6,55677E-01	0,185	0,272	1,97276E-16
Macro_LYVE1	RBM3	1,27752E-10	-6,57501E-01	0,646	0,676	2,35281E-06

Macro_LYVE1	RPL35	5,16504E-15	-6,58066E-01	0,472	0,534	9,51246E-11
Macro_LYVE1	RBMX	2,43658E-07	-6,60152E-01	0,374	0,428	4,48744E-03
Macro_LYVE1	OTUB1	3,37058E-08	-6,64098E-01	0,375	0,433	6,20759E-04
Macro_LYVE1	UBE2J1	9,67717E-43	-6,66633E-01	0,181	0,308	1,78225E-38
Macro_LYVE1	PNP	3,39385E-33	-6,68201E-01	0,158	0,264	6,25045E-29
Macro_LYVE1	LDHB	9,59486E-08	-6,68623E-01	0,473	0,513	1,76709E-03
Macro_LYVE1	PSMA4	2,29395E-11	-6,69015E-01	0,402	0,472	4,22477E-07
Macro_LYVE1	PTPN1	3,74846E-34	-6,69963E-01	0,29	0,407	6,90353E-30
Macro_LYVE1	RAP1B	2,01771E-08	-6,71583E-01	0,539	0,581	3,71601E-04
Macro_LYVE1	EIF3F	1,51100E-24	-6,72543E-01	0,617	0,669	2,78281E-20
Macro_LYVE1	ATF5	3,03948E-25	-6,73823E-01	0,272	0,366	5,59781E-21
Macro_LYVE1	BRK1	3,62750E-17	-6,76118E-01	0,686	0,728	6,68077E-13
Macro_LYVE1	PARK7	5,30650E-22	-6,76686E-01	0,611	0,661	9,77297E-18
Macro_LYVE1	NFKBIA	9,11209E-26	-6,78169E-01	0,776	0,81	1,67817E-21
Macro_LYVE1	GBP4	6,25036E-25	-6,78199E-01	0,19	0,282	1,15113E-20
Macro_LYVE1	PSMA5	7,34511E-25	-6,78391E-01	0,334	0,44	1,35275E-20
Macro_LYVE1	GK	1,34975E-28	-6,82668E-01	0,269	0,369	2,48584E-24
Macro_LYVE1	EIF2S3	1,20922E-06	-6,84177E-01	0,402	0,454	2,22702E-02
Macro_LYVE1	PLK3	3,98905E-12	-6,87561E-01	0,195	0,255	7,34664E-08
Macro_LYVE1	PFN1	2,23131E-152	-6,88736E-01	0,957	0,947	4,10940E-148
Macro_LYVE1	MYL12B	2,60977E-31	-6,89205E-01	0,74	0,778	4,80641E-27
Macro_LYVE1	PDIA3	4,26136E-21	-6,89265E-01	0,635	0,678	7,84815E-17
Macro_LYVE1	HMGN4	1,06313E-17	-6,90272E-01	0,171	0,252	1,95797E-13
Macro_LYVE1	PRR13	2,39910E-17	-6,91692E-01	0,523	0,583	4,41843E-13
Macro_LYVE1	ARF1	3,17299E-12	-6,92593E-01	0,658	0,701	5,84370E-08
Macro_LYVE1	CHCHD2	4,81036E-36	-6,94833E-01	0,794	0,817	8,85925E-32
Macro_LYVE1	CLIC1	3,90008E-66	-6,95531E-01	0,846	0,876	7,18277E-62
Macro_LYVE1	FTH1	4,37720E-52	-6,95579E-01	0,997	0,997	8,06148E-48
Macro_LYVE1	PSMB8	1,20504E-43	-6,95960E-01	0,485	0,582	2,21933E-39
Macro_LYVE1	RBM8A	8,16590E-11	-6,97119E-01	0,476	0,537	1,50391E-06
Macro_LYVE1	EIF5A	2,98482E-17	-6,98630E-01	0,503	0,56	5,49714E-13
Macro_LYVE1	ALOX5	9,70907E-19	-6,99054E-01	0,395	0,478	1,78812E-14
Macro_LYVE1	CYCS	2,97146E-08	-6,99650E-01	0,473	0,527	5,47254E-04
Macro_LYVE1	RPL36AL	4,46205E-27	-6,99813E-01	0,425	0,497	8,21775E-23
Macro_LYVE1	TNIP1	2,29091E-32	-6,99905E-01	0,242	0,352	4,21918E-28
Macro_LYVE1	CIB1	2,59310E-31	-7,00574E-01	0,527	0,617	4,77571E-27
Macro_LYVE1	HCK	5,23130E-09	-7,02036E-01	0,482	0,523	9,63449E-05
Macro_LYVE1	VASP	1,33703E-60	-7,03781E-01	0,322	0,477	2,46241E-56
Macro_LYVE1	TYMP	1,56341E-62	-7,09818E-01	0,748	0,795	2,87934E-58
Macro_LYVE1	UBE2L6	5,36810E-12	-7,12121E-01	0,535	0,572	9,88643E-08
Macro_LYVE1	UBA52	9,17837E-62	-7,12698E-01	0,94	0,919	1,69038E-57
Macro_LYVE1	VIM	2,13196E-232	-7,14980E-01	0,856	0,948	3,92643E-228
Macro_LYVE1	TMEM109	1,16979E-28	-7,15823E-01	0,264	0,374	2,15441E-24
Macro_LYVE1	EIF3G	1,91586E-13	-7,17592E-01	0,56	0,617	3,52843E-09
Macro_LYVE1	CPPED1	2,92781E-20	-7,20950E-01	0,189	0,275	5,39214E-16
Macro_LYVE1	PSMA6	1,21256E-21	-7,24081E-01	0,385	0,47	2,23317E-17
Macro_LYVE1	RPL36	8,49164E-18	-7,24735E-01	0,484	0,556	1,56390E-13
Macro_LYVE1	HAX1	1,69195E-06	-7,25315E-01	0,295	0,349	3,11606E-02
Macro_LYVE1	STXBP2	7,55567E-60	-7,25987E-01	0,275	0,427	1,39153E-55
Macro_LYVE1	CCT8	2,39516E-19	-7,32572E-01	0,322	0,417	4,41116E-15
Macro_LYVE1	RPL22	2,00417E-31	-7,32693E-01	0,463	0,534	3,69108E-27
Macro_LYVE1	BZW1	8,92933E-28	-7,33415E-01	0,342	0,448	1,64451E-23
Macro_LYVE1	LILRA2	3,40630E-63	-7,34289E-01	0,103	0,255	6,27338E-59
Macro_LYVE1	CYSTM1	4,17512E-12	-7,34293E-01	0,289	0,356	7,68932E-08

Macro_LYVE1	SP110	3,33372E-09	-7,37119E-01	0,331	0,393	6,13970E-05
Macro_LYVE1	RPL37	4,51758E-36	-7,38986E-01	0,492	0,572	8,32003E-32
Macro_LYVE1	NLRP3	6,97461E-07	-7,43821E-01	0,223	0,265	1,28451E-02
Macro_LYVE1	APEX1	3,71824E-12	-7,48931E-01	0,379	0,445	6,84788E-08
Macro_LYVE1	HERPUD1	6,54792E-33	-7,49461E-01	0,813	0,674	1,20593E-28
Macro_LYVE1	MAP2K3	7,40664E-34	-7,50120E-01	0,165	0,276	1,36408E-29
Macro_LYVE1	RHOC	3,22867E-08	-7,56075E-01	0,262	0,312	5,94624E-04
Macro_LYVE1	CKLF	1,92746E-16	-7,60048E-01	0,45	0,509	3,54981E-12
Macro_LYVE1	MYO1G	2,24903E-52	-7,60062E-01	0,151	0,29	4,14203E-48
Macro_LYVE1	DCXR	3,55571E-13	-7,62392E-01	0,296	0,371	6,54855E-09
Macro_LYVE1	FLNA	5,59627E-88	-7,63955E-01	0,271	0,461	1,03066E-83
Macro_LYVE1	CSTA	1,33499E-36	-7,64412E-01	0,323	0,435	2,45866E-32
Macro_LYVE1	PEF1	6,07453E-07	-7,64790E-01	0,201	0,25	1,11875E-02
Macro_LYVE1	RPS26	1,55777E-21	-7,65783E-01	0,455	0,528	2,86894E-17
Macro_LYVE1	PPP1R2	2,39882E-06	-7,66159E-01	0,344	0,396	4,41790E-02
Macro_LYVE1	EIF3K	2,35714E-17	-7,66446E-01	0,77	0,788	4,34115E-13
Macro_LYVE1	PQBP1	4,91362E-13	-7,72866E-01	0,245	0,318	9,04941E-09
Macro_LYVE1	EEF1B2	5,75555E-30	-7,73548E-01	0,858	0,841	1,06000E-25
Macro_LYVE1	HLA-F	1,09759E-11	-7,73911E-01	0,451	0,519	2,02143E-07
Macro_LYVE1	PIM3	7,52664E-30	-7,75105E-01	0,192	0,291	1,38618E-25
Macro_LYVE1	NR4A3	3,86556E-10	-7,75362E-01	0,241	0,3	7,11921E-06
Macro_LYVE1	COX6A1	3,23730E-19	-7,79120E-01	0,639	0,682	5,96214E-15
Macro_LYVE1	EIF3M	4,33656E-23	-7,81212E-01	0,409	0,504	7,98664E-19
Macro_LYVE1	HNRNPK	1,22439E-06	-7,81332E-01	0,693	0,71	2,25496E-02
Macro_LYVE1	ACTG1	1,24888E-108	-7,82285E-01	0,92	0,922	2,30007E-104
Macro_LYVE1	S100A4	3,37949E-141	-7,84784E-01	0,735	0,831	6,22401E-137
Macro_LYVE1	ILF2	2,18786E-13	-7,86262E-01	0,318	0,395	4,02939E-09
Macro_LYVE1	PTPN6	2,41636E-18	-7,88765E-01	0,437	0,514	4,45021E-14
Macro_LYVE1	RPS24	9,89391E-43	-7,91123E-01	0,496	0,582	1,82216E-38
Macro_LYVE1	RPLP2	5,73418E-36	-7,91336E-01	0,49	0,574	1,05606E-31
Macro_LYVE1	RPS2	1,39751E-34	-7,92153E-01	0,488	0,566	2,57380E-30
Macro_LYVE1	TRIM22	7,50534E-08	-7,93697E-01	0,364	0,42	1,38226E-03
Macro_LYVE1	MAPK1IP1L	2,44920E-08	-7,94071E-01	0,288	0,347	4,51069E-04
Macro_LYVE1	OLR1	1,57036E-76	-7,96057E-01	0,179	0,356	2,89214E-72
Macro_LYVE1	SUB1	7,27822E-30	-7,96852E-01	0,683	0,725	1,34043E-25
Macro_LYVE1	NFKB1	1,25886E-39	-8,03354E-01	0,177	0,294	2,31845E-35
Macro_LYVE1	ATG3	1,16835E-12	-8,03620E-01	0,463	0,526	2,15176E-08
Macro_LYVE1	DBNL	8,36528E-16	-8,04877E-01	0,416	0,496	1,54063E-11
Macro_LYVE1	RAN	1,37166E-17	-8,04969E-01	0,606	0,66	2,52619E-13
Macro_LYVE1	ELOVL5	1,82293E-28	-8,05931E-01	0,187	0,293	3,35728E-24
Macro_LYVE1	ACAA1	3,46672E-28	-8,06454E-01	0,27	0,384	6,38466E-24
Macro_LYVE1	PRELID1	5,85381E-78	-8,09618E-01	0,578	0,69	1,07810E-73
Macro_LYVE1	NCF2	3,66845E-22	-8,10285E-01	0,392	0,468	6,75618E-18
Macro_LYVE1	RASSF5	3,82530E-37	-8,12079E-01	0,162	0,277	7,04506E-33
Macro_LYVE1	GABARAP	6,66862E-42	-8,14239E-01	0,799	0,831	1,22816E-37
Macro_LYVE1	RPL37A	1,83940E-17	-8,15679E-01	0,479	0,541	3,38763E-13
Macro_LYVE1	CREM	2,79631E-28	-8,16416E-01	0,486	0,372	5,14997E-24
Macro_LYVE1	RNASEK	2,52977E-23	-8,17717E-01	0,389	0,47	4,65908E-19
Macro_LYVE1	FYTTD1	3,15120E-14	-8,27730E-01	0,183	0,252	5,80356E-10
Macro_LYVE1	RGS1	1,01839E-20	-8,27959E-01	0,747	0,637	1,87558E-16
Macro_LYVE1	GABARAPL2	2,82028E-16	-8,32003E-01	0,514	0,581	5,19411E-12
Macro_LYVE1	CSF3R	2,37593E-09	-8,32039E-01	0,341	0,396	4,37575E-05
Macro_LYVE1	SERPINB1	4,04497E-78	-8,32136E-01	0,535	0,67	7,44962E-74
Macro_LYVE1	NAP1L1	2,79146E-10	-8,39050E-01	0,575	0,622	5,14103E-06

Macro_LYVE1	SRGN	1,29725E-52	-8,39225E-01	0,957	0,942	2,38914E-48
Macro_LYVE1	SH3BGRL3	1,16563E-187	-8,40108E-01	0,901	0,934	2,14674E-183
Macro_LYVE1	LST1	2,62290E-13	-8,41561E-01	0,727	0,714	4,83060E-09
Macro_LYVE1	IGFLR1	2,83245E-16	-8,43709E-01	0,282	0,363	5,21652E-12
Macro_LYVE1	TKT	4,74203E-14	-8,43879E-01	0,56	0,61	8,73339E-10
Macro_LYVE1	NFKBIZ	3,22867E-25	-8,45061E-01	0,365	0,459	5,94624E-21
Macro_LYVE1	RPLP1	6,48363E-55	-8,47130E-01	0,506	0,603	1,19409E-50
Macro_LYVE1	MYD88	7,85564E-63	-8,47932E-01	0,203	0,365	1,44677E-58
Macro_LYVE1	PLEK	7,18310E-07	-8,48393E-01	0,543	0,563	1,32291E-02
Macro_LYVE1	MIF	1,82353E-42	-8,49077E-01	0,654	0,728	3,35840E-38
Macro_LYVE1	RPL34	1,37653E-36	-8,54306E-01	0,494	0,58	2,53515E-32
Macro_LYVE1	PTPRE	3,83747E-09	-8,56710E-01	0,394	0,442	7,06746E-05
Macro_LYVE1	RIPK2	9,03816E-29	-8,57043E-01	0,175	0,279	1,66456E-24
Macro_LYVE1	RPL21	3,11899E-11	-8,58284E-01	0,48	0,544	5,74424E-07
Macro_LYVE1	DNAJC4	9,09823E-12	-8,63382E-01	0,3	0,365	1,67562E-07
Macro_LYVE1	CAPG	8,77842E-32	-8,68748E-01	0,659	0,745	1,61672E-27
Macro_LYVE1	ITGAX	1,45336E-19	-8,70638E-01	0,313	0,404	2,67666E-15
Macro_LYVE1	GBP5	1,79179E-32	-8,71316E-01	0,151	0,251	3,29994E-28
Macro_LYVE1	LY6E	2,57762E-45	-8,72277E-01	0,436	0,553	4,74719E-41
Macro_LYVE1	RPS21	2,88391E-32	-8,72857E-01	0,47	0,547	5,31131E-28
Macro_LYVE1	RPL15	2,02113E-20	-8,75186E-01	0,487	0,556	3,72231E-16
Macro_LYVE1	CLEC12A	5,95629E-26	-8,81561E-01	0,167	0,265	1,09697E-21
Macro_LYVE1	NHP2	7,21053E-18	-8,86754E-01	0,329	0,417	1,32796E-13
Macro_LYVE1	RAB24	1,69137E-42	-8,87480E-01	0,151	0,277	3,11500E-38
Macro_LYVE1	CD37	4,67260E-45	-8,88181E-01	0,674	0,736	8,60553E-41
Macro_LYVE1	LPXN	7,99581E-14	-8,89997E-01	0,283	0,351	1,47259E-09
Macro_LYVE1	YWHAZ	4,98897E-48	-8,91822E-01	0,637	0,716	9,18819E-44
Macro_LYVE1	EEF1A1	5,92347E-104	-8,92336E-01	0,995	0,984	1,09092E-99
Macro_LYVE1	CDK2AP2	3,18989E-17	-8,92526E-01	0,319	0,401	5,87482E-13
Macro_LYVE1	PKM	2,37454E-109	-8,97413E-01	0,694	0,788	4,37319E-105
Macro_LYVE1	PPIF	1,73246E-47	-8,98816E-01	0,23	0,365	3,19067E-43
Macro_LYVE1	SOCS3	6,42354E-28	-8,99773E-01	0,266	0,362	1,18302E-23
Macro_LYVE1	SNRPD2	2,00424E-12	-9,00155E-01	0,631	0,68	3,69121E-08
Macro_LYVE1	PPP1R15A	4,13712E-33	-9,02083E-01	0,587	0,661	7,61934E-29
Macro_LYVE1	SMCO4	3,01876E-25	-9,02660E-01	0,327	0,423	5,55965E-21
Macro_LYVE1	ACTB	1,45466E-178	-9,04888E-01	0,994	0,991	2,67904E-174
Macro_LYVE1	TAGLN2	8,45586E-31	-9,07554E-01	0,714	0,756	1,55732E-26
Macro_LYVE1	PSME2	4,49175E-79	-9,11576E-01	0,639	0,719	8,27246E-75
Macro_LYVE1	RPS9	1,52337E-30	-9,13392E-01	0,486	0,558	2,80559E-26
Macro_LYVE1	PSMB9	2,12011E-91	-9,19259E-01	0,539	0,664	3,90460E-87
Macro_LYVE1	BTG1	3,04384E-111	-9,19531E-01	0,715	0,815	5,60583E-107
Macro_LYVE1	RPS27A	6,71724E-40	-9,20576E-01	0,49	0,58	1,23711E-35
Macro_LYVE1	RPL13A	9,80749E-07	-9,22941E-01	0,472	0,523	1,80625E-02
Macro_LYVE1	PDE4B	5,94211E-22	-9,23080E-01	0,28	0,372	1,09436E-17
Macro_LYVE1	CDC37	1,45309E-23	-9,28326E-01	0,565	0,642	2,67616E-19
Macro_LYVE1	RPL18	5,46028E-54	-9,29486E-01	0,483	0,566	1,00562E-49
Macro_LYVE1	LCP1	2,45390E-34	-9,30809E-01	0,63	0,708	4,51935E-30
Macro_LYVE1	MRPS6	6,03872E-08	-9,32682E-01	0,275	0,327	1,11215E-03
Macro_LYVE1	KYNU	1,70778E-29	-9,33812E-01	0,218	0,321	3,14522E-25
Macro_LYVE1	RPL30	2,96098E-58	-9,35246E-01	0,491	0,585	5,45324E-54
Macro_LYVE1	PSME1	9,44552E-54	-9,36732E-01	0,703	0,757	1,73958E-49
Macro_LYVE1	FBL	6,27391E-27	-9,42343E-01	0,243	0,353	1,15547E-22
Macro_LYVE1	ALDH2	7,61559E-37	-9,47934E-01	0,462	0,555	1,40256E-32
Macro_LYVE1	PABPC1	1,50230E-84	-9,49920E-01	0,905	0,892	2,76678E-80

Macro_LYVE1	TCF4	7,11006E-26	-9,54562E-01	0,363	0,268	1,30946E-21
Macro_LYVE1	DUSP2	7,67429E-37	-9,55695E-01	0,193	0,307	1,41337E-32
Macro_LYVE1	NACA	3,98791E-53	-9,59958E-01	0,925	0,919	7,34454E-49
Macro_LYVE1	MXD1	8,79686E-22	-9,60462E-01	0,219	0,306	1,62012E-17
Macro_LYVE1	GAPDH	0,00000E+00	-9,70686E-01	0,945	0,961	0,00000E+00
Macro_LYVE1	GLIPR2	2,00757E-74	-9,70802E-01	0,209	0,386	3,69733E-70
Macro_LYVE1	LTA4H	4,69947E-32	-9,74449E-01	0,348	0,453	8,65501E-28
Macro_LYVE1	GAPT	5,12294E-69	-9,81158E-01	0,102	0,263	9,43493E-65
Macro_LYVE1	RPS25	4,34186E-22	-9,85236E-01	0,475	0,551	7,99640E-18
Macro_LYVE1	RPS15	1,47912E-36	-9,85889E-01	0,489	0,578	2,72409E-32
Macro_LYVE1	EIF3L	3,60930E-17	-9,87023E-01	0,551	0,603	6,64724E-13
Macro_LYVE1	RPL32	3,42698E-48	-9,95489E-01	0,491	0,583	6,31147E-44
Macro_LYVE1	C1QBP	1,13385E-22	-9,95941E-01	0,332	0,435	2,08822E-18
Macro_LYVE1	HNRNPA1	7,01654E-30	-9,98469E-01	0,808	0,801	1,29224E-25
Macro_LYVE1	RPL27	4,32151E-13	-1,00082E+00	0,46	0,507	7,95892E-09
Macro_LYVE1	MYL12A	9,78015E-32	-1,00499E+00	0,81	0,826	1,80121E-27
Macro_LYVE1	GBP1	5,48261E-54	-1,00728E+00	0,214	0,353	1,00973E-49
Macro_LYVE1	RPS18	9,08462E-31	-1,00782E+00	0,487	0,563	1,67312E-26
Macro_LYVE1	ALDOA	1,13774E-60	-1,01095E+00	0,687	0,764	2,09538E-56
Macro_LYVE1	RPL41	8,64856E-28	-1,01878E+00	0,491	0,578	1,59281E-23
Macro_LYVE1	RPS13	5,40958E-49	-1,02269E+00	0,489	0,575	9,96282E-45
Macro_LYVE1	RPL14	1,49837E-37	-1,02289E+00	0,471	0,546	2,75954E-33
Macro_LYVE1	UBL7	1,83069E-08	-1,02325E+00	0,202	0,256	3,37158E-04
Macro_LYVE1	RPL10	1,91143E-42	-1,02338E+00	0,504	0,599	3,52029E-38
Macro_LYVE1	MT2A	7,26473E-56	-1,02602E+00	0,52	0,634	1,33794E-51
Macro_LYVE1	RPS27	8,27815E-22	-1,02838E+00	0,49	0,571	1,52459E-17
Macro_LYVE1	STK17B	1,53244E-27	-1,03037E+00	0,348	0,449	2,82229E-23
Macro_LYVE1	LDHA	9,12384E-78	-1,03631E+00	0,625	0,719	1,68034E-73
Macro_LYVE1	S100A10	6,34374E-154	-1,04083E+00	0,746	0,842	1,16833E-149
Macro_LYVE1	RPS14	1,24685E-34	-1,04530E+00	0,497	0,58	2,29632E-30
Macro_LYVE1	PGAM1	4,97746E-73	-1,04906E+00	0,414	0,573	9,16699E-69
Macro_LYVE1	RPL19	1,52278E-51	-1,04972E+00	0,492	0,585	2,80451E-47
Macro_LYVE1	RPL39	1,36796E-43	-1,05366E+00	0,492	0,586	2,51937E-39
Macro_LYVE1	RPS12	1,63339E-42	-1,05573E+00	0,497	0,587	3,00821E-38
Macro_LYVE1	RPS19	8,44386E-42	-1,06303E+00	0,494	0,579	1,55511E-37
Macro_LYVE1	RPL8	3,82028E-70	-1,06783E+00	0,483	0,577	7,03581E-66
Macro_LYVE1	RPS4X	9,52603E-28	-1,07953E+00	0,489	0,569	1,75441E-23
Macro_LYVE1	IRF1	1,45297E-51	-1,08300E+00	0,307	0,445	2,67593E-47
Macro_LYVE1	TUBA1A	7,10219E-14	-1,08345E+00	0,403	0,465	1,30801E-09
Macro_LYVE1	RPL35A	2,53734E-52	-1,08867E+00	0,476	0,567	4,67302E-48
Macro_LYVE1	PGK1	2,04249E-21	-1,09597E+00	0,663	0,708	3,76166E-17
Macro_LYVE1	RPL13	5,04113E-34	-1,09977E+00	0,498	0,586	9,28424E-30
Macro_LYVE1	RPL31	4,51093E-15	-1,09984E+00	0,434	0,5	8,30778E-11
Macro_LYVE1	RPS15A	7,39841E-42	-1,10641E+00	0,485	0,573	1,36257E-37
Macro_LYVE1	RPS23	4,17524E-34	-1,10867E+00	0,495	0,572	7,68955E-30
Macro_LYVE1	RPL28	1,23492E-88	-1,10927E+00	0,5	0,599	2,27435E-84
Macro_LYVE1	RPS6	1,60299E-20	-1,11378E+00	0,484	0,547	2,95224E-16
Macro_LYVE1	RPL24	6,55145E-38	-1,12480E+00	0,464	0,542	1,20658E-33
Macro_LYVE1	SOD2	1,56118E-09	-1,12792E+00	0,645	0,652	2,87522E-05
Macro_LYVE1	RPS29	9,23466E-11	-1,12845E+00	0,444	0,505	1,70075E-06
Macro_LYVE1	S100A6	1,01747E-258	-1,13643E+00	0,711	0,883	1,87387E-254
Macro_LYVE1	SERPINA1	2,21539E-84	-1,14766E+00	0,684	0,723	4,08009E-80
Macro_LYVE1	RPS7	9,30097E-60	-1,14824E+00	0,476	0,565	1,71296E-55
Macro_LYVE1	RPS3	1,80324E-55	-1,14940E+00	0,481	0,572	3,32103E-51

Macro_LYVE1	CDKN1A	5,20752E-48	-1,15137E+00	0,417	0,531	9,59069E-44
Macro_LYVE1	UPP1	8,04181E-63	-1,15174E+00	0,28	0,439	1,48106E-58
Macro_LYVE1	LIMD2	9,85337E-110	-1,15893E+00	0,283	0,492	1,81469E-105
Macro_LYVE1	RPS5	5,11803E-32	-1,16467E+00	0,455	0,521	9,42588E-28
Macro_LYVE1	RPL29	1,42410E-48	-1,17036E+00	0,476	0,562	2,62277E-44
Macro_LYVE1	RPL3	7,28120E-17	-1,18134E+00	0,479	0,536	1,34098E-12
Macro_LYVE1	SLC11A1	1,29666E-73	-1,20239E+00	0,322	0,475	2,38805E-69
Macro_LYVE1	RPL7A	2,92464E-54	-1,20865E+00	0,473	0,563	5,38631E-50
Macro_LYVE1	ISG15	4,00317E-27	-1,20973E+00	0,343	0,432	7,37263E-23
Macro_LYVE1	TAP1	5,54241E-85	-1,21647E+00	0,241	0,432	1,02075E-80
Macro_LYVE1	RPL7	1,59258E-15	-1,21870E+00	0,433	0,5	2,93306E-11
Macro_LYVE1	EZR	1,04723E-13	-1,23078E+00	0,331	0,403	1,92868E-09
Macro_LYVE1	CYTIP	6,84482E-66	-1,23362E+00	0,233	0,399	1,26061E-61
Macro_LYVE1	RPL11	4,96648E-57	-1,23445E+00	0,491	0,585	9,14677E-53
Macro_LYVE1	CD52	7,50787E-80	-1,25371E+00	0,214	0,399	1,38272E-75
Macro_LYVE1	LYZ	7,19149E-14	-1,25612E+00	0,873	0,805	1,32446E-09
Macro_LYVE1	RPS16	1,79072E-36	-1,25955E+00	0,474	0,548	3,29797E-32
Macro_LYVE1	IFITM1	1,20148E-68	-1,25994E+00	0,108	0,262	2,21276E-64
Macro_LYVE1	RPL5	2,78354E-31	-1,26233E+00	0,455	0,521	5,12645E-27
Macro_LYVE1	RPS11	1,03346E-13	-1,27446E+00	0,455	0,506	1,90333E-09
Macro_LYVE1	RPL9	2,20442E-25	-1,27947E+00	0,476	0,547	4,05989E-21
Macro_LYVE1	SEC61B	6,87171E-23	-1,28478E+00	0,702	0,742	1,26556E-18
Macro_LYVE1	RPS8	3,99740E-51	-1,29466E+00	0,494	0,582	7,36202E-47
Macro_LYVE1	CCND3	4,42048E-38	-1,30128E+00	0,195	0,318	8,14120E-34
Macro_LYVE1	COTL1	1,09609E-106	-1,30909E+00	0,599	0,718	2,01866E-102
Macro_LYVE1	RPL18A	1,80782E-45	-1,31864E+00	0,483	0,567	3,32947E-41
Macro_LYVE1	RPSA	5,00186E-36	-1,32848E+00	0,435	0,498	9,21193E-32
Macro_LYVE1	RPLP0	5,16987E-44	-1,34166E+00	0,467	0,547	9,52135E-40
Macro_LYVE1	EEF1G	4,97462E-40	-1,35354E+00	0,182	0,302	9,16175E-36
Macro_LYVE1	PLAUR	1,09961E-59	-1,35891E+00	0,519	0,647	2,02516E-55
Macro_LYVE1	RPL17	1,28166E-27	-1,36411E+00	0,355	0,437	2,36044E-23
Macro_LYVE1	CD48	1,07528E-125	-1,36725E+00	0,259	0,491	1,98035E-121
Macro_LYVE1	SLC2A3	2,89769E-53	-1,37173E+00	0,303	0,44	5,33667E-49
Macro_LYVE1	DDIT4	1,39939E-32	-1,37657E+00	0,394	0,501	2,57726E-28
Macro_LYVE1	RPL26	1,00351E-27	-1,37818E+00	0,468	0,546	1,84816E-23
Macro_LYVE1	TXN	1,87619E-10	-1,37951E+00	0,628	0,669	3,45537E-06
Macro_LYVE1	CNN2	8,65213E-50	-1,38448E+00	0,276	0,409	1,59346E-45
Macro_LYVE1	SLC25A37	3,43945E-71	-1,38582E+00	0,136	0,299	6,33443E-67
Macro_LYVE1	RPL10A	9,88050E-25	-1,39422E+00	0,444	0,5	1,81969E-20
Macro_LYVE1	RPS3A	5,50875E-32	-1,40875E+00	0,484	0,571	1,01455E-27
Macro_LYVE1	PLP2	1,07355E-105	-1,41802E+00	0,203	0,415	1,97715E-101
Macro_LYVE1	RPL6	4,02799E-50	-1,42243E+00	0,472	0,558	7,41834E-46
Macro_LYVE1	RPL23A	1,75778E-23	-1,43116E+00	0,463	0,529	3,23731E-19
Macro_LYVE1	CD44	3,20618E-53	-1,46910E+00	0,592	0,681	5,90483E-49
Macro_LYVE1	LSP1	1,94560E-188	-1,47468E+00	0,447	0,683	3,58322E-184
Macro_LYVE1	MARCKSL1	5,42851E-107	-1,48261E+00	0,076	0,275	9,99769E-103
Macro_LYVE1	CD55	1,17788E-86	-1,48355E+00	0,25	0,439	2,16930E-82
Macro_LYVE1	CORO1A	1,23633E-123	-1,51247E+00	0,485	0,655	2,27694E-119
Macro_LYVE1	FCN1	1,16438E-69	-1,51562E+00	0,107	0,258	2,14445E-65
Macro_LYVE1	IL2RG	1,78018E-53	-1,54072E+00	0,183	0,331	3,27857E-49
Macro_LYVE1	BID	3,37470E-53	-1,56931E+00	0,275	0,422	6,21518E-49
Macro_LYVE1	RPL4	3,50380E-25	-1,57213E+00	0,422	0,48	6,45295E-21
Macro_LYVE1	FGR	5,39653E-122	-1,59364E+00	0,228	0,466	9,93878E-118
Macro_LYVE1	BCL2A1	1,76006E-122	-1,61152E+00	0,257	0,481	3,24150E-118

Macro_LYVE1	GPR183	1,09627E-07	-1,67226E+00	0,537	0,453	2,01899E-03
Macro_LYVE1	ALOX5AP	5,73762E-45	-1,67562E+00	0,391	0,511	1,05670E-40
Macro_LYVE1	SERPINB9	5,78900E-68	-1,67748E+00	0,217	0,383	1,06616E-63
Macro_LYVE1	ISG20	1,08434E-97	-1,67872E+00	0,122	0,321	1,99702E-93
Macro_LYVE1	RAC2	2,07045E-131	-1,77716E+00	0,273	0,508	3,81314E-127
Macro_LYVE1	IL1B	3,23251E-74	-1,78701E+00	0,174	0,344	5,95331E-70
Macro_LYVE1	PPA1	5,16849E-128	-1,81106E+00	0,25	0,472	9,51882E-124
Macro_LYVE1	LGALS2	1,31807E-55	-1,81518E+00	0,126	0,264	2,42749E-51
Macro_LYVE1	TIMP1	3,71616E-65	-1,84294E+00	0,565	0,655	6,84406E-61
Macro_LYVE1	PLD4	1,90296E-53	-1,93421E+00	0,191	0,339	3,50468E-49
Macro_LYVE1	IRF7	8,20674E-70	-1,98062E+00	0,242	0,407	1,51144E-65
Macro_LYVE1	C15orf48	5,62786E-99	-2,10376E+00	0,16	0,354	1,03648E-94
Macro_LYVE1	AREG	9,14967E-79	-2,60262E+00	0,113	0,284	1,68510E-74
Macro_LYVE1	S100A8	5,33167E-123	-3,85718E+00	0,146	0,363	9,81934E-119

cluster	gene	p_val	avg_log2FC	pct.1	pct.2	p_val_adj
Macro_NLRP3	IL1B	0,00000E+00	2,01209E+00	0,7	0,299	0,00000E+00
Macro_NLRP3	EREG	0,00000E+00	1,93880E+00	0,514	0,122	0,00000E+00
Macro_NLRP3	G0S2	0,00000E+00	1,86478E+00	0,468	0,158	0,00000E+00
Macro_NLRP3	NLRP3	0,00000E+00	1,65177E+00	0,553	0,234	0,00000E+00
Macro_NLRP3	THBS1	0,00000E+00	1,62819E+00	0,498	0,148	0,00000E+00
Macro_NLRP3	S100A9	0,00000E+00	1,44085E+00	0,742	0,428	0,00000E+00
Macro_NLRP3	TIMP1	0,00000E+00	1,43194E+00	0,855	0,63	0,00000E+00
Macro_NLRP3	PPIF	0,00000E+00	1,42410E+00	0,58	0,336	0,00000E+00
Macro_NLRP3	S100A12	0,00000E+00	1,39189E+00	0,324	0,094	0,00000E+00
Macro_NLRP3	CXCL2	0,00000E+00	1,38366E+00	0,538	0,196	0,00000E+00
Macro_NLRP3	PLAUR	0,00000E+00	1,32511E+00	0,898	0,616	0,00000E+00
Macro_NLRP3	CXCL3	0,00000E+00	1,31327E+00	0,366	0,142	0,00000E+00
Macro_NLRP3	S100A8	0,00000E+00	1,29406E+00	0,703	0,318	0,00000E+00
Macro_NLRP3	CXCL8	7,51346E-201	1,22544E+00	0,356	0,178	1,38375E-196
Macro_NLRP3	VEGFA	0,00000E+00	1,20466E+00	0,484	0,191	0,00000E+00
Macro_NLRP3	VCAN	0,00000E+00	1,18670E+00	0,608	0,188	0,00000E+00
Macro_NLRP3	KDM6B	0,00000E+00	1,16954E+00	0,63	0,312	0,00000E+00
Macro_NLRP3	C5AR1	0,00000E+00	1,16214E+00	0,642	0,385	0,00000E+00
Macro_NLRP3	SOD2	0,00000E+00	1,15688E+00	0,836	0,633	0,00000E+00
Macro_NLRP3	HBEGF	0,00000E+00	1,14144E+00	0,572	0,246	0,00000E+00
Macro_NLRP3	BCL2A1	0,00000E+00	1,12581E+00	0,75	0,443	0,00000E+00
Macro_NLRP3	AQP9	0,00000E+00	1,11909E+00	0,292	0,098	0,00000E+00
Macro_NLRP3	OSM	6,00290E-229	1,09903E+00	0,303	0,129	1,10555E-224
Macro_NLRP3	OLR1	0,00000E+00	1,08256E+00	0,582	0,325	0,00000E+00
Macro_NLRP3	IER3	0,00000E+00	1,06145E+00	0,786	0,508	0,00000E+00
Macro_NLRP3	EHD1	6,19293E-288	1,05738E+00	0,342	0,144	1,14055E-283
Macro_NLRP3	NAMPT	0,00000E+00	1,02673E+00	0,84	0,547	0,00000E+00
Macro_NLRP3	NFKBIA	0,00000E+00	1,01637E+00	0,91	0,798	0,00000E+00
Macro_NLRP3	TREM1	0,00000E+00	1,01284E+00	0,504	0,211	0,00000E+00
Macro_NLRP3	GABARAPL1	2,81523E-263	9,91185E-01	0,473	0,274	5,18481E-259
Macro_NLRP3	CD300E	0,00000E+00	9,72213E-01	0,406	0,143	0,00000E+00
Macro_NLRP3	ATP13A3	1,90627E-295	9,61778E-01	0,403	0,192	3,51077E-291
Macro_NLRP3	PTGS2	6,37449E-233	9,44543E-01	0,353	0,163	1,17399E-228
Macro_NLRP3	ATP2B1	1,19489E-194	9,38666E-01	0,499	0,334	2,20064E-190
Macro_NLRP3	ANPEP	0,00000E+00	9,32972E-01	0,424	0,188	0,00000E+00
Macro_NLRP3	BTG1	0,00000E+00	9,31568E-01	0,896	0,802	0,00000E+00
Macro_NLRP3	ACSL1	3,41054E-215	9,18897E-01	0,552	0,373	6,28120E-211
Macro_NLRP3	CCL4L2	2,76951E-110	9,15626E-01	0,317	0,182	5,10060E-106
Macro_NLRP3	IL1RN	0,00000E+00	9,13978E-01	0,435	0,198	0,00000E+00
Macro_NLRP3	SLC11A1	0,00000E+00	9,13035E-01	0,75	0,439	0,00000E+00
Macro_NLRP3	SAMSN1	5,85079E-246	9,09725E-01	0,645	0,446	1,07754E-241
Macro_NLRP3	RGCC	1,95048E-279	9,07501E-01	0,429	0,207	3,59219E-275

Macro_NLRP3	FCN1	0,00000E+00	8,98642E-01	0,608	0,216	0,00000E+00
Macro_NLRP3	CLEC4E	2,03378E-167	8,93321E-01	0,447	0,277	3,74562E-163
Macro_NLRP3	CD55	0,00000E+00	8,93203E-01	0,638	0,409	0,00000E+00
Macro_NLRP3	TNFAIP3	0,00000E+00	8,83634E-01	0,742	0,523	0,00000E+00
Macro_NLRP3	SLC25A37	0,00000E+00	8,70332E-01	0,534	0,267	0,00000E+00
Macro_NLRP3	GK	0,00000E+00	8,68475E-01	0,594	0,342	0,00000E+00
Macro_NLRP3	ARL4A	1,59794E-107	8,62873E-01	0,355	0,229	2,94293E-103
Macro_NLRP3	PNRC1	0,00000E+00	8,58399E-01	0,866	0,77	0,00000E+00
Macro_NLRP3	UPP1	0,00000E+00	8,50891E-01	0,632	0,411	0,00000E+00
Macro_NLRP3	SLC2A3	0,00000E+00	8,49654E-01	0,694	0,408	0,00000E+00
Macro_NLRP3	AREG	0,00000E+00	8,35322E-01	0,531	0,25	0,00000E+00
Macro_NLRP3	YBX3	2,33746E-239	8,11172E-01	0,523	0,331	4,30490E-235
Macro_NLRP3	CEBPB	5,84773E-239	8,11118E-01	0,649	0,454	1,07698E-234
Macro_NLRP3	PLK3	1,24287E-76	8,07877E-01	0,347	0,243	2,28899E-72
Macro_NLRP3	APOBEC3A	5,57136E-151	7,93204E-01	0,269	0,125	1,02608E-146
Macro_NLRP3	PHLDA1	3,17331E-160	7,91150E-01	0,277	0,133	5,84428E-156
Macro_NLRP3	AMPD2	1,83965E-153	7,74644E-01	0,317	0,171	3,38809E-149
Macro_NLRP3	PNP	6,32765E-265	7,69643E-01	0,445	0,241	1,16536E-260
Macro_NLRP3	CFP	4,38986E-149	7,65436E-01	0,36	0,192	8,08481E-145
Macro_NLRP3	ITGAX	1,89307E-241	7,49702E-01	0,577	0,382	3,48646E-237
Macro_NLRP3	FLNA	1,68790E-307	7,48127E-01	0,656	0,431	3,10861E-303
Macro_NLRP3	CCL3L1	2,01508E-208	7,42877E-01	0,325	0,146	3,71117E-204
Macro_NLRP3	IRAK3	4,98972E-119	7,38384E-01	0,379	0,242	9,18957E-115
Macro_NLRP3	CCRL2	2,23096E-89	7,38308E-01	0,28	0,176	4,10877E-85
Macro_NLRP3	METRNL	2,19078E-141	7,35976E-01	0,392	0,241	4,03475E-137
Macro_NLRP3	MCL1	0,00000E+00	7,26813E-01	0,89	0,757	0,00000E+00
Macro_NLRP3	ETS2	1,38970E-203	7,26441E-01	0,582	0,411	2,55941E-199
Macro_NLRP3	CD93	6,84980E-131	7,21720E-01	0,413	0,266	1,26153E-126
Macro_NLRP3	IVNS1ABP	8,45157E-246	7,13649E-01	0,541	0,349	1,55653E-241
Macro_NLRP3	DUSP2	4,00231E-242	7,12215E-01	0,508	0,281	7,37105E-238
Macro_NLRP3	TNFRSF1B	3,58720E-307	7,11012E-01	0,71	0,523	6,60655E-303
Macro_NLRP3	CCNH	1,02667E-50	7,10844E-01	0,432	0,365	1,89081E-46
Macro_NLRP3	STX11	4,18982E-165	7,05454E-01	0,471	0,314	7,71639E-161
Macro_NLRP3	RBKS	4,60971E-127	6,95105E-01	0,27	0,141	8,48970E-123
Macro_NLRP3	OXSR1	1,20336E-47	6,91943E-01	0,254	0,178	2,21623E-43
Macro_NLRP3	WTAP	1,36696E-96	6,90846E-01	0,461	0,356	2,51753E-92
Macro_NLRP3	TNIP1	1,36061E-36	6,89413E-01	0,391	0,342	2,50583E-32
Macro_NLRP3	CD44	0,00000E+00	6,78188E-01	0,839	0,661	0,00000E+00
Macro_NLRP3	ITGA5	2,99997E-131	6,75860E-01	0,32	0,185	5,52505E-127
Macro_NLRP3	DENND5A	1,21478E-68	6,72731E-01	0,254	0,163	2,23726E-64
Macro_NLRP3	ELL2	1,78859E-206	6,52147E-01	0,453	0,268	3,29405E-202
Macro_NLRP3	LYZ	0,00000E+00	6,49021E-01	0,938	0,796	0,00000E+00

Macro_NLRP3	FPR1	5,21845E-109	6,48945E-01	0,56	0,437	9,61082E-105
Macro_NLRP3	CHMP1B	6,34650E-213	6,47800E-01	0,568	0,391	1,16883E-208
Macro_NLRP3	RILPL2	8,88372E-225	6,45944E-01	0,602	0,441	1,63611E-220
Macro_NLRP3	SAT1	0,00000E+00	6,42231E-01	0,992	0,944	0,00000E+00
Macro_NLRP3	NFKB1	9,96293E-113	6,34376E-01	0,407	0,276	1,83487E-108
Macro_NLRP3	UBXN11	1,58502E-61	6,27276E-01	0,291	0,198	2,91913E-57
Macro_NLRP3	THBD	2,25921E-289	6,21524E-01	0,399	0,189	4,16078E-285
Macro_NLRP3	SOCS3	2,36835E-236	6,12291E-01	0,556	0,338	4,36180E-232
Macro_NLRP3	GPCPD1	1,73351E-136	6,07044E-01	0,409	0,268	3,19261E-132
Macro_NLRP3	SYAP1	3,55283E-117	6,06610E-01	0,455	0,334	6,54326E-113
Macro_NLRP3	TRIB1	6,33356E-63	6,04740E-01	0,325	0,233	1,16645E-58
Macro_NLRP3	CCL4	2,03748E-113	6,03401E-01	0,448	0,296	3,75242E-109
Macro_NLRP3	MMP19	1,02870E-162	5,92453E-01	0,253	0,115	1,89455E-158
Macro_NLRP3	FOSL2	0,00000E+00	5,90526E-01	0,516	0,274	0,00000E+00
Macro_NLRP3	NFKBIZ	5,48524E-174	5,90195E-01	0,617	0,439	1,01022E-169
Macro_NLRP3	FOSB	4,56724E-292	5,87437E-01	0,768	0,561	8,41149E-288
Macro_NLRP3	CSTA	1,78101E-179	5,84409E-01	0,578	0,415	3,28008E-175
Macro_NLRP3	DDX21	2,70514E-128	5,83795E-01	0,544	0,426	4,98206E-124
Macro_NLRP3	SPAG9	8,58398E-89	5,83479E-01	0,398	0,294	1,58091E-84
Macro_NLRP3	PNPLA8	1,15482E-107	5,70743E-01	0,353	0,232	2,12684E-103
Macro_NLRP3	C15orf48	2,45786E-136	5,67387E-01	0,489	0,33	4,52665E-132
Macro_NLRP3	FGD4	3,61223E-50	5,66682E-01	0,3	0,223	6,65265E-46
Macro_NLRP3	VMP1	2,26406E-23	5,65744E-01	0,575	0,548	4,16971E-19
Macro_NLRP3	SLC43A2	2,13646E-100	5,61007E-01	0,469	0,358	3,93472E-96
Macro_NLRP3	INSIG1	7,10760E-144	5,56602E-01	0,433	0,282	1,30901E-139
Macro_NLRP3	SRGN	0,00000E+00	5,56191E-01	0,975	0,94	0,00000E+00
Macro_NLRP3	VPS37B	4,69728E-173	5,54036E-01	0,263	0,12	8,65098E-169
Macro_NLRP3	NUMB	3,41649E-97	5,53579E-01	0,437	0,323	6,29214E-93
Macro_NLRP3	PELI1	4,82789E-23	5,51342E-01	0,304	0,258	8,89153E-19
Macro_NLRP3	SPHK1	8,46013E-109	5,47869E-01	0,287	0,169	1,55810E-104
Macro_NLRP3	MXD1	0,00000E+00	5,45414E-01	0,524	0,28	0,00000E+00
Macro_NLRP3	C9orf72	6,00600E-106	5,44061E-01	0,338	0,214	1,10613E-101
Macro_NLRP3	FTH1	0,00000E+00	5,34877E-01	0,999	0,997	0,00000E+00
Macro_NLRP3	DSE	6,59137E-102	5,31077E-01	0,527	0,43	1,21393E-97
Macro_NLRP3	PPP1R15B	7,66186E-65	5,27512E-01	0,294	0,198	1,41108E-60
Macro_NLRP3	CLEC4A	3,42670E-47	5,27157E-01	0,368	0,29	6,31095E-43
Macro_NLRP3	MAP2K1	5,58154E-66	5,27083E-01	0,398	0,314	1,02795E-61
Macro_NLRP3	CSRNP1	1,35631E-196	5,26729E-01	0,458	0,275	2,49791E-192
Macro_NLRP3	S100A4	0,00000E+00	5,21159E-01	0,932	0,816	0,00000E+00
Macro_NLRP3	CTNNB1	2,15104E-102	5,17985E-01	0,482	0,369	3,96157E-98
Macro_NLRP3	FGR	5,53574E-92	5,17914E-01	0,562	0,443	1,01952E-87
Macro_NLRP3	FNDC3B	4,17773E-87	5,09534E-01	0,38	0,269	7,69413E-83

Macro_NLRP3	MIDN	1,15937E-93	5,08755E-01	0,385	0,272	2,13522E-89
Macro_NLRP3	CSF3R	2,52047E-50	5,05628E-01	0,461	0,387	4,64194E-46
Macro_NLRP3	BEST1	7,04569E-34	5,03868E-01	0,285	0,224	1,29760E-29
Macro_NLRP3	PIM3	1,04982E-45	4,92660E-01	0,353	0,279	1,93346E-41
Macro_NLRP3	TGIF1	2,17828E-80	4,90289E-01	0,416	0,315	4,01175E-76
Macro_NLRP3	CCL3	0,00000E+00	4,85796E-01	0,625	0,351	0,00000E+00
Macro_NLRP3	PDE4B	4,52683E-258	4,82748E-01	0,544	0,35	8,33706E-254
Macro_NLRP3	SERPINB9	4,75431E-260	4,82668E-01	0,564	0,356	8,75600E-256
Macro_NLRP3	S100A6	0,00000E+00	4,82579E-01	0,949	0,867	0,00000E+00
Macro_NLRP3	SH3BP5	3,92028E-80	4,80755E-01	0,437	0,327	7,21999E-76
Macro_NLRP3	LIMS1	2,77365E-101	4,79176E-01	0,634	0,547	5,10822E-97
Macro_NLRP3	PDE4A	1,07785E-64	4,77674E-01	0,257	0,167	1,98508E-60
Macro_NLRP3	KLF4	6,94621E-167	4,74785E-01	0,602	0,432	1,27928E-162
Macro_NLRP3	B3GNT5	4,77188E-85	4,69368E-01	0,252	0,15	8,78837E-81
Macro_NLRP3	RAB20	4,84060E-46	4,64637E-01	0,416	0,34	8,91493E-42
Macro_NLRP3	DUSP6	4,95654E-58	4,64451E-01	0,375	0,28	9,12846E-54
Macro_NLRP3	RARA	2,13731E-68	4,63131E-01	0,286	0,19	3,93628E-64
Macro_NLRP3	JUND	8,39292E-104	4,63028E-01	0,564	0,444	1,54572E-99
Macro_NLRP3	PHLDA2	6,64185E-98	4,62561E-01	0,27	0,157	1,22323E-93
Macro_NLRP3	PHACTR1	2,72212E-213	4,60363E-01	0,477	0,284	5,01333E-209
Macro_NLRP3	ARL8B	1,73130E-49	4,59980E-01	0,459	0,398	3,18854E-45
Macro_NLRP3	LCP2	1,62239E-91	4,56805E-01	0,653	0,569	2,98796E-87
Macro_NLRP3	ZFAND5	5,01015E-61	4,54049E-01	0,636	0,58	9,22719E-57
Macro_NLRP3	PID1	5,99949E-290	4,53547E-01	0,329	0,131	1,10493E-285
Macro_NLRP3	EFHD2	9,95454E-46	4,51355E-01	0,48	0,426	1,83333E-41
Macro_NLRP3	NDEL1	4,05055E-48	4,51325E-01	0,26	0,183	7,45989E-44
Macro_NLRP3	BASP1	2,61022E-21	4,50152E-01	0,374	0,344	4,80723E-17
Macro_NLRP3	NCOR2	1,83618E-12	4,48118E-01	0,294	0,267	3,38169E-08
Macro_NLRP3	ASGR1	2,25118E-49	4,44253E-01	0,268	0,186	4,14600E-45
Macro_NLRP3	APLP2	2,12719E-141	4,40113E-01	0,703	0,584	3,91765E-137
Macro_NLRP3	CDKN1A	0,00000E+00	4,36811E-01	0,74	0,504	0,00000E+00
Macro_NLRP3	ICAM1	1,82255E-153	4,33483E-01	0,5	0,348	3,35658E-149
Macro_NLRP3	ZC3H12A	8,12020E-102	4,24394E-01	0,262	0,148	1,49550E-97
Macro_NLRP3	NR4A2	0,00000E+00	4,20134E-01	0,676	0,429	0,00000E+00
Macro_NLRP3	IRS2	5,01803E-72	4,00289E-01	0,323	0,219	9,24170E-68
Macro_NLRP3	RNF144B	1,58693E-49	4,00098E-01	0,422	0,351	2,92266E-45
Macro_NLRP3	DDX3X	3,77913E-143	3,99732E-01	0,597	0,466	6,96002E-139
Macro_NLRP3	NINJ1	9,44991E-97	3,97434E-01	0,648	0,551	1,74039E-92
Macro_NLRP3	TSPO	9,69672E-111	3,93725E-01	0,83	0,759	1,78584E-106
Macro_NLRP3	VASP	2,53199E-70	3,93085E-01	0,542	0,463	4,66317E-66
Macro_NLRP3	SERTAD1	6,17622E-128	3,92537E-01	0,451	0,313	1,13748E-123
Macro_NLRP3	EMILIN2	4,06269E-61	3,88858E-01	0,383	0,293	7,48226E-57

Macro_NLRP3	PFKFB3	2,08608E-105	3,85071E-01	0,446	0,323	3,84193E-101
Macro_NLRP3	NFKB2	1,61708E-56	3,82882E-01	0,305	0,219	2,97818E-52
Macro_NLRP3	MAP3K8	8,54873E-161	3,77150E-01	0,604	0,47	1,57442E-156
Macro_NLRP3	REL	1,11160E-80	3,76875E-01	0,659	0,592	2,04723E-76
Macro_NLRP3	TNFAIP2	4,33478E-43	3,74511E-01	0,469	0,4	7,98336E-39
Macro_NLRP3	NOTCH2	6,17372E-26	3,71858E-01	0,36	0,313	1,13701E-21
Macro_NLRP3	CEBPD	9,81718E-54	3,69276E-01	0,643	0,561	1,80803E-49
Macro_NLRP3	C19orf38	2,19071E-39	3,66690E-01	0,299	0,227	4,03462E-35
Macro_NLRP3	ZNF267	4,32141E-43	3,63570E-01	0,297	0,228	7,95874E-39
Macro_NLRP3	LILRB2	1,45677E-45	3,61969E-01	0,526	0,45	2,68294E-41
Macro_NLRP3	SERPINA1	4,53749E-171	3,58841E-01	0,879	0,706	8,35669E-167
Macro_NLRP3	JARID2	9,58423E-49	3,57735E-01	0,283	0,206	1,76513E-44
Macro_NLRP3	CAPN2	8,50140E-52	3,51031E-01	0,313	0,226	1,56570E-47
Macro_NLRP3	JOSD1	1,76577E-96	3,44457E-01	0,288	0,176	3,25202E-92
Macro_NLRP3	STX4	1,59235E-06	3,42844E-01	0,317	0,312	2,93263E-02
Macro_NLRP3	LILRA5	4,54934E-62	3,42364E-01	0,273	0,177	8,37851E-58
Macro_NLRP3	TOM1	7,81283E-99	3,41482E-01	0,392	0,273	1,43889E-94
Macro_NLRP3	BCL3	2,02953E-72	3,41153E-01	0,311	0,209	3,73779E-68
Macro_NLRP3	GMFG	4,73689E-33	3,38732E-01	0,691	0,671	8,72392E-29
Macro_NLRP3	S100A10	2,87790E-278	3,37177E-01	0,925	0,829	5,30023E-274
Macro_NLRP3	STXBP2	1,06229E-138	3,36705E-01	0,545	0,407	1,95642E-134
Macro_NLRP3	MT2A	1,34046E-07	3,36434E-01	0,635	0,627	2,46873E-03
Macro_NLRP3	NCF2	1,36517E-117	3,34092E-01	0,581	0,453	2,51423E-113
Macro_NLRP3	HMGB2	3,53434E-24	3,32798E-01	0,433	0,395	6,50919E-20
Macro_NLRP3	ACTN1	7,39883E-69	3,31397E-01	0,403	0,311	1,36264E-64
Macro_NLRP3	ZEB2	1,37426E-126	3,30876E-01	0,695	0,596	2,53098E-122
Macro_NLRP3	CHMP4B	1,35813E-19	3,30155E-01	0,426	0,407	2,50126E-15
Macro_NLRP3	TET2	7,17314E-115	3,24879E-01	0,359	0,229	1,32108E-110
Macro_NLRP3	SSH2	2,02137E-12	3,23235E-01	0,335	0,309	3,72275E-08
Macro_NLRP3	CHD1	4,49864E-111	3,21287E-01	0,41	0,286	8,28515E-107
Macro_NLRP3	DMXL2	2,67481E-30	3,20762E-01	0,324	0,262	4,92619E-26
Macro_NLRP3	SFPQ	1,29607E-95	3,19068E-01	0,591	0,499	2,38696E-91
Macro_NLRP3	QSOX1	6,28403E-26	3,14004E-01	0,334	0,284	1,15733E-21
Macro_NLRP3	CLEC7A	3,95057E-115	3,13240E-01	0,632	0,514	7,27577E-111
Macro_NLRP3	BACH1	2,02961E-69	3,13185E-01	0,396	0,304	3,73794E-65
Macro_NLRP3	KYNU	1,00031E-52	3,12445E-01	0,383	0,309	1,84226E-48
Macro_NLRP3	EMP3	1,44282E-129	3,09482E-01	0,815	0,72	2,65723E-125
Macro_NLRP3	JMJD1C	4,95195E-94	3,06444E-01	0,515	0,419	9,12001E-90
Macro_NLRP3	TOP1	1,96323E-21	3,04539E-01	0,526	0,52	3,61568E-17
Macro_NLRP3	CD300C	2,29901E-14	3,03475E-01	0,269	0,234	4,23408E-10
Macro_NLRP3	IRF1	1,55852E-15	2,99919E-01	0,461	0,436	2,87032E-11
Macro_NLRP3	NR4A1	3,87270E-219	2,96378E-01	0,625	0,4	7,13234E-215

Macro_NLRP3	SIPA1L1	4,89132E-81	2,95957E-01	0,307	0,203	9,00834E-77
Macro_NLRP3	ADAM17	3,01056E-34	2,95347E-01	0,282	0,221	5,54454E-30
Macro_NLRP3	METTL9	1,51370E-21	2,94655E-01	0,386	0,356	2,78778E-17
Macro_NLRP3	USP3	1,12924E-12	2,92514E-01	0,257	0,225	2,07972E-08
Macro_NLRP3	IL6R	2,26685E-28	2,92306E-01	0,289	0,237	4,17486E-24
Macro_NLRP3	TLR2	5,86049E-59	2,91931E-01	0,465	0,378	1,07933E-54
Macro_NLRP3	NFE2L2	3,22020E-113	2,89897E-01	0,607	0,511	5,93065E-109
Macro_NLRP3	CSGALNACT2	2,00781E-38	2,88404E-01	0,251	0,183	3,69778E-34
Macro_NLRP3	NBPF10	4,74366E-67	2,87704E-01	0,287	0,193	8,73640E-63
Macro_NLRP3	ITGB1	2,98267E-36	2,87267E-01	0,511	0,471	5,49319E-32
Macro_NLRP3	HIF1A	1,27135E-264	2,77523E-01	0,701	0,534	2,34144E-260
Macro_NLRP3	CLEC5A	5,03860E-121	2,72214E-01	0,311	0,179	9,27958E-117
Macro_NLRP3	GADD45B	2,11611E-116	2,71873E-01	0,753	0,632	3,89724E-112
Macro_NLRP3	UBE2R2	6,79264E-17	2,70467E-01	0,294	0,262	1,25100E-12
Macro_NLRP3	JMJD6	1,27309E-20	2,70357E-01	0,252	0,207	2,34465E-16
Macro_NLRP3	PRNP	3,14345E-32	2,70281E-01	0,439	0,397	5,78928E-28
Macro_NLRP3	LYST	1,05247E-10	2,69677E-01	0,262	0,227	1,93833E-06
Macro_NLRP3	ZFP36	2,47733E-168	2,68713E-01	0,915	0,837	4,56249E-164
Macro_NLRP3	ZFYVE16	3,05902E-46	2,68111E-01	0,277	0,202	5,63380E-42
Macro_NLRP3	MYO9B	2,67791E-12	2,66728E-01	0,404	0,386	4,93190E-08
Macro_NLRP3	ROCK1	2,38639E-20	2,61405E-01	0,522	0,51	4,39501E-16
Macro_NLRP3	GNA13	5,88800E-72	2,60110E-01	0,419	0,326	1,08439E-67
Macro_NLRP3	CCNL1	1,07346E-102	2,58600E-01	0,611	0,517	1,97699E-98
Macro_NLRP3	FOXO3	4,60647E-15	2,57804E-01	0,28	0,244	8,48374E-11
Macro_NLRP3	AGTRAP	2,34649E-43	2,57065E-01	0,43	0,354	4,32153E-39
Macro_NLRP3	SRA1	1,01106E-06	2,56313E-01	0,381	0,387	1,86207E-02
Macro_NLRP3	EIF1	0,00000E+00	2,53868E-01	0,965	0,942	0,00000E+00
Macro_NLRP3	ZMIZ1	4,44937E-19	2,52953E-01	0,302	0,261	8,19440E-15
Macro_NLRP3	SERP1	1,85179E-135	2,52723E-01	0,855	0,816	3,41044E-131
Macro_NLRP3	RAB31	3,59326E-75	2,51736E-01	0,707	0,636	6,61770E-71
Macro_NLRP3	RIPK2	6,57506E-86	2,51640E-01	0,372	0,264	1,21093E-81
Macro_NLRP3	MYO1G	6,62437E-47	2,51443E-01	0,36	0,276	1,22001E-42
Macro_NLRP3	TNF	3,49604E-57	2,50420E-01	0,253	0,165	6,43865E-53
Macro_NLRP3	PUF60	7,10051E-16	-2,50665E-01	0,247	0,327	1,30770E-11
Macro_NLRP3	BUB3	3,10656E-30	-2,50778E-01	0,174	0,266	5,72135E-26
Macro_NLRP3	MRPL14	4,08272E-12	-2,51245E-01	0,272	0,351	7,51914E-08
Macro_NLRP3	IMP3	2,07654E-60	-2,51366E-01	0,19	0,327	3,82437E-56
Macro_NLRP3	UNC93B1	4,91969E-65	-2,51383E-01	0,209	0,349	9,06060E-61
Macro_NLRP3	ATP5F1A	4,84770E-42	-2,51496E-01	0,165	0,263	8,92800E-38
Macro_NLRP3	MLF2	1,29000E-21	-2,51689E-01	0,355	0,462	2,37579E-17
Macro_NLRP3	GSTP1	2,75784E-11	-2,51933E-01	0,822	0,824	5,07912E-07
Macro_NLRP3	EMC6	1,00085E-25	-2,52020E-01	0,223	0,322	1,84326E-21

Macro_NLRP3	PHF3	9,07687E-22	-2,52530E-01	0,222	0,307	1,67169E-17
Macro_NLRP3	COX6A1	2,74859E-08	-2,52563E-01	0,623	0,685	5,06208E-04
Macro_NLRP3	ATRX	6,01163E-10	-2,53129E-01	0,318	0,396	1,10716E-05
Macro_NLRP3	SLC25A3	1,41703E-12	-2,54576E-01	0,705	0,714	2,60974E-08
Macro_NLRP3	GTF2I	4,47275E-31	-2,55407E-01	0,219	0,321	8,23746E-27
Macro_NLRP3	CWC15	4,07261E-30	-2,56997E-01	0,233	0,341	7,50053E-26
Macro_NLRP3	KDM5A	4,10176E-09	-2,57252E-01	0,253	0,317	7,55421E-05
Macro_NLRP3	B4GALT1	1,60734E-08	-2,57666E-01	0,341	0,323	2,96024E-04
Macro_NLRP3	RPS27L	6,49974E-55	-2,58114E-01	0,254	0,373	1,19706E-50
Macro_NLRP3	LRP10	3,62996E-40	-2,58262E-01	0,193	0,304	6,68530E-36
Macro_NLRP3	MTMR14	8,05826E-24	-2,59261E-01	0,227	0,318	1,48409E-19
Macro_NLRP3	IRF5	1,53095E-36	-2,59900E-01	0,171	0,271	2,81956E-32
Macro_NLRP3	DERL1	1,05684E-21	-2,59935E-01	0,197	0,281	1,94639E-17
Macro_NLRP3	FUOM	1,86813E-22	-2,59948E-01	0,287	0,385	3,44053E-18
Macro_NLRP3	NCKAP1L	7,19884E-40	-2,60036E-01	0,31	0,441	1,32581E-35
Macro_NLRP3	SNRPB2	6,55274E-11	-2,60077E-01	0,331	0,414	1,20682E-06
Macro_NLRP3	RSBN1L	2,80511E-58	-2,60168E-01	0,171	0,3	5,16618E-54
Macro_NLRP3	CHCHD1	2,13762E-25	-2,60277E-01	0,19	0,278	3,93686E-21
Macro_NLRP3	ATP5MC3	1,29717E-28	-2,60431E-01	0,274	0,361	2,38900E-24
Macro_NLRP3	SDF4	3,66728E-22	-2,60492E-01	0,336	0,441	6,75404E-18
Macro_NLRP3	DNAJA1	7,33828E-44	-2,60933E-01	0,641	0,601	1,35149E-39
Macro_NLRP3	HAVCR2	3,03092E-16	-2,61638E-01	0,352	0,443	5,58204E-12
Macro_NLRP3	KDELR1	6,89198E-27	-2,62064E-01	0,436	0,549	1,26930E-22
Macro_NLRP3	FBXW5	1,24246E-22	-2,62246E-01	0,233	0,322	2,28824E-18
Macro_NLRP3	DRAM2	7,58641E-28	-2,62411E-01	0,292	0,4	1,39719E-23
Macro_NLRP3	GPAA1	1,95812E-33	-2,62914E-01	0,168	0,265	3,60626E-29
Macro_NLRP3	SRSF3	2,21557E-39	-2,64006E-01	0,605	0,567	4,08041E-35
Macro_NLRP3	APBB1IP	4,15952E-31	-2,64664E-01	0,374	0,489	7,66059E-27
Macro_NLRP3	NKTR	1,69632E-25	-2,65382E-01	0,203	0,292	3,12412E-21
Macro_NLRP3	NT5C	1,04365E-52	-2,66010E-01	0,228	0,363	1,92209E-48
Macro_NLRP3	ALCAM	5,80882E-22	-2,66711E-01	0,194	0,275	1,06981E-17
Macro_NLRP3	NDUFB7	6,02648E-13	-2,67411E-01	0,443	0,538	1,10990E-08
Macro_NLRP3	MRPL3	1,72316E-23	-2,67755E-01	0,203	0,291	3,17355E-19
Macro_NLRP3	PFDN2	7,40893E-09	-2,67996E-01	0,363	0,45	1,36450E-04
Macro_NLRP3	BMP2K	4,91037E-28	-2,68166E-01	0,208	0,3	9,04343E-24
Macro_NLRP3	ABHD14B	3,56319E-22	-2,69016E-01	0,188	0,27	6,56233E-18
Macro_NLRP3	MIF	3,57703E-19	-2,70212E-01	0,74	0,722	6,58781E-15
Macro_NLRP3	BLOC1S2	6,89756E-19	-2,70429E-01	0,25	0,339	1,27032E-14
Macro_NLRP3	SF3B4	9,88645E-16	-2,70958E-01	0,229	0,306	1,82079E-11
Macro_NLRP3	LDHA	6,55816E-61	-2,71916E-01	0,75	0,711	1,20782E-56
Macro_NLRP3	ANXA11	2,31164E-34	-2,72425E-01	0,435	0,553	4,25734E-30
Macro_NLRP3	DNAJC1	2,30151E-14	-2,73071E-01	0,223	0,296	4,23869E-10

Macro_NLRP3	UNC119	2,75918E-16	-2,73112E-01	0,203	0,274	5,08158E-12
Macro_NLRP3	RNF5	4,90161E-46	-2,73614E-01	0,178	0,293	9,02730E-42
Macro_NLRP3	TADA3	3,05146E-41	-2,74481E-01	0,198	0,311	5,61988E-37
Macro_NLRP3	ZMAT2	2,20491E-06	-2,74675E-01	0,272	0,33	4,06078E-02
Macro_NLRP3	ANXA2	1,46035E-31	-2,74691E-01	0,811	0,736	2,68952E-27
Macro_NLRP3	WBP2	1,05968E-28	-2,76486E-01	0,281	0,394	1,95162E-24
Macro_NLRP3	SH3GLB1	2,64198E-07	-2,76548E-01	0,359	0,433	4,86573E-03
Macro_NLRP3	PARP14	3,07991E-18	-2,76936E-01	0,3	0,385	5,67227E-14
Macro_NLRP3	TNFSF13B	2,28817E-17	-2,77499E-01	0,508	0,586	4,21413E-13
Macro_NLRP3	CCAR1	7,21967E-13	-2,78393E-01	0,208	0,273	1,32965E-08
Macro_NLRP3	PLEKHO1	5,40676E-49	-2,79106E-01	0,244	0,372	9,95763E-45
Macro_NLRP3	CCDC47	3,70046E-25	-2,79108E-01	0,22	0,312	6,81514E-21
Macro_NLRP3	OTUB1	4,80159E-20	-2,79277E-01	0,337	0,439	8,84309E-16
Macro_NLRP3	ATOX1	6,17121E-42	-2,79322E-01	0,478	0,585	1,13655E-37
Macro_NLRP3	OST4	3,26926E-08	-2,79515E-01	0,768	0,778	6,02099E-04
Macro_NLRP3	EIF3D	6,97423E-20	-2,79819E-01	0,373	0,484	1,28444E-15
Macro_NLRP3	FIS1	3,61821E-10	-2,80688E-01	0,442	0,534	6,66366E-06
Macro_NLRP3	RPN1	1,92192E-23	-2,80797E-01	0,221	0,302	3,53959E-19
Macro_NLRP3	RNF130	1,65070E-07	-2,80809E-01	0,674	0,713	3,04009E-03
Macro_NLRP3	SLC25A11	8,98523E-26	-2,81862E-01	0,218	0,313	1,65481E-21
Macro_NLRP3	SAFB	1,45035E-06	-2,81884E-01	0,204	0,251	2,67112E-02
Macro_NLRP3	RB1	1,19410E-20	-2,81899E-01	0,316	0,411	2,19918E-16
Macro_NLRP3	NEU1	2,37805E-09	-2,82343E-01	0,246	0,31	4,37965E-05
Macro_NLRP3	UQCC2	1,90735E-30	-2,82438E-01	0,216	0,319	3,51276E-26
Macro_NLRP3	DUSP23	1,12651E-14	-2,82702E-01	0,305	0,391	2,07470E-10
Macro_NLRP3	RPL36	2,13010E-06	-2,83540E-01	0,51	0,557	3,92300E-02
Macro_NLRP3	EPS15	5,32053E-17	-2,84093E-01	0,187	0,254	9,79882E-13
Macro_NLRP3	SHARPIN	4,64995E-34	-2,84928E-01	0,165	0,261	8,56380E-30
Macro_NLRP3	RHOG	2,26781E-11	-2,86528E-01	0,672	0,732	4,17662E-07
Macro_NLRP3	TMEM243	4,31798E-14	-2,86550E-01	0,206	0,275	7,95242E-10
Macro_NLRP3	SMCO4	3,26003E-07	-2,86920E-01	0,361	0,424	6,00400E-03
Macro_NLRP3	DPY30	5,14217E-17	-2,86972E-01	0,18	0,251	9,47033E-13
Macro_NLRP3	ADPGK	2,79408E-11	-2,87118E-01	0,326	0,408	5,14585E-07
Macro_NLRP3	C1QBP	2,30059E-45	-2,87305E-01	0,304	0,442	4,23700E-41
Macro_NLRP3	MPG	5,08478E-24	-2,87468E-01	0,236	0,331	9,36463E-20
Macro_NLRP3	RPL24	2,82421E-08	-2,87913E-01	0,493	0,543	5,20135E-04
Macro_NLRP3	THOC2	3,84286E-14	-2,88356E-01	0,194	0,262	7,07740E-10
Macro_NLRP3	GYG1	1,70772E-09	-2,89208E-01	0,218	0,278	3,14511E-05
Macro_NLRP3	SELENOW	2,70374E-39	-2,90153E-01	0,201	0,3	4,97948E-35
Macro_NLRP3	SYNCRIP	9,88269E-07	-2,90181E-01	0,268	0,328	1,82009E-02
Macro_NLRP3	PTDSS1	3,20779E-12	-2,90910E-01	0,21	0,276	5,90778E-08
Macro_NLRP3	BHLHE40	6,35748E-42	-2,90958E-01	0,418	0,332	1,17086E-37

Macro_NLRP3	FXR1	3,38109E-10	-2,90971E-01	0,23	0,293	6,22695E-06
Macro_NLRP3	ACTB	1,88580E-13	-2,91078E-01	0,992	0,991	3,47308E-09
Macro_NLRP3	PSMD2	4,23831E-26	-2,91093E-01	0,231	0,329	7,80570E-22
Macro_NLRP3	XRCC5	1,73578E-11	-2,91422E-01	0,397	0,483	3,19678E-07
Macro_NLRP3	NAAA	5,74149E-14	-2,92223E-01	0,298	0,374	1,05741E-09
Macro_NLRP3	SFT2D2	2,63828E-45	-2,93081E-01	0,188	0,304	4,85892E-41
Macro_NLRP3	SSNA1	2,39898E-24	-2,93303E-01	0,305	0,414	4,41820E-20
Macro_NLRP3	THRAP3	3,36053E-19	-2,93504E-01	0,326	0,429	6,18909E-15
Macro_NLRP3	YWHAZ	2,79298E-94	-2,93688E-01	0,764	0,707	5,14383E-90
Macro_NLRP3	TRIM38	4,99944E-10	-2,94494E-01	0,228	0,29	9,20747E-06
Macro_NLRP3	SNX29	4,23694E-22	-2,94685E-01	0,187	0,267	7,80318E-18
Macro_NLRP3	SLC25A39	3,11852E-34	-2,95272E-01	0,296	0,419	5,74337E-30
Macro_NLRP3	SON	1,37734E-25	-2,95600E-01	0,681	0,674	2,53664E-21
Macro_NLRP3	TMBIM4	1,74024E-08	-2,95807E-01	0,277	0,336	3,20500E-04
Macro_NLRP3	RAB2A	1,38066E-07	-2,95818E-01	0,412	0,494	2,54277E-03
Macro_NLRP3	CYTH4	5,99162E-07	-2,95977E-01	0,264	0,32	1,10348E-02
Macro_NLRP3	APPL1	1,88447E-49	-2,96846E-01	0,239	0,374	3,47063E-45
Macro_NLRP3	COA3	7,08782E-24	-2,97191E-01	0,233	0,328	1,30536E-19
Macro_NLRP3	CAMLG	9,54856E-14	-2,97306E-01	0,227	0,299	1,75856E-09
Macro_NLRP3	PSMB7	7,33456E-09	-2,97743E-01	0,299	0,369	1,35081E-04
Macro_NLRP3	IGSF6	6,32939E-07	-2,97800E-01	0,527	0,56	1,16568E-02
Macro_NLRP3	GUK1	1,41062E-23	-2,98583E-01	0,715	0,71	2,59794E-19
Macro_NLRP3	PTAFR	2,45382E-12	-2,99279E-01	0,311	0,382	4,51920E-08
Macro_NLRP3	EIF5A	4,44822E-09	-2,99480E-01	0,488	0,564	8,19228E-05
Macro_NLRP3	MEF2C	1,56028E-06	-2,99687E-01	0,358	0,423	2,87357E-02
Macro_NLRP3	CCDC90B	3,45548E-28	-2,99713E-01	0,163	0,251	6,36396E-24
Macro_NLRP3	LMBRD1	2,56797E-45	-2,99796E-01	0,167	0,28	4,72943E-41
Macro_NLRP3	LPCAT2	9,69194E-24	-3,00055E-01	0,23	0,317	1,78496E-19
Macro_NLRP3	U2AF1	9,10346E-21	-3,00334E-01	0,315	0,265	1,67658E-16
Macro_NLRP3	SIGLEC10	3,49293E-42	-3,00761E-01	0,218	0,331	6,43293E-38
Macro_NLRP3	IRF2	1,56296E-42	-3,01350E-01	0,216	0,339	2,87849E-38
Macro_NLRP3	HNRNPH1	3,19932E-28	-3,01746E-01	0,449	0,4	5,89219E-24
Macro_NLRP3	ACTG1	3,83828E-19	-3,01876E-01	0,944	0,92	7,06896E-15
Macro_NLRP3	FOXN2	1,70274E-33	-3,01971E-01	0,174	0,271	3,13593E-29
Macro_NLRP3	MFSD1	4,02937E-13	-3,02119E-01	0,524	0,598	7,42089E-09
Macro_NLRP3	ENO1	6,43605E-21	-3,02474E-01	0,775	0,76	1,18533E-16
Macro_NLRP3	FNTA	3,28754E-39	-3,02639E-01	0,155	0,258	6,05466E-35
Macro_NLRP3	CLIC1	4,16093E-09	-3,03922E-01	0,875	0,874	7,66319E-05
Macro_NLRP3	IFNAR2	1,09902E-08	-3,03991E-01	0,218	0,273	2,02407E-04
Macro_NLRP3	RAB34	6,83315E-07	-3,04149E-01	0,225	0,277	1,25846E-02
Macro_NLRP3	MTDH	8,93482E-17	-3,04857E-01	0,62	0,702	1,64553E-12
Macro_NLRP3	STK4	4,37620E-18	-3,04920E-01	0,346	0,437	8,05966E-14

Macro_NLRP3	NDUFA5	4,31192E-22	-3,05218E-01	0,214	0,301	7,94127E-18
Macro_NLRP3	NDUFS7	1,44968E-10	-3,05458E-01	0,454	0,55	2,66988E-06
Macro_NLRP3	RWDD1	5,68764E-12	-3,05519E-01	0,375	0,465	1,04749E-07
Macro_NLRP3	SUMO3	5,28407E-19	-3,05642E-01	0,383	0,491	9,73167E-15
Macro_NLRP3	PHF11	8,62540E-28	-3,06798E-01	0,201	0,296	1,58854E-23
Macro_NLRP3	PFKL	2,29170E-31	-3,07008E-01	0,289	0,403	4,22063E-27
Macro_NLRP3	STX12	9,09973E-13	-3,07010E-01	0,244	0,319	1,67590E-08
Macro_NLRP3	UBXN4	2,42170E-12	-3,07450E-01	0,437	0,529	4,46005E-08
Macro_NLRP3	RTRAF	5,70613E-49	-3,07473E-01	0,166	0,275	1,05090E-44
Macro_NLRP3	ETNK1	1,73452E-08	-3,07498E-01	0,2	0,253	3,19447E-04
Macro_NLRP3	GINM1	2,63569E-17	-3,07693E-01	0,189	0,262	4,85414E-13
Macro_NLRP3	HMGN4	1,50126E-33	-3,09069E-01	0,161	0,257	2,76488E-29
Macro_NLRP3	ATP5MC2	3,24661E-19	-3,09328E-01	0,367	0,44	5,97929E-15
Macro_NLRP3	TRADD	1,11282E-50	-3,09661E-01	0,144	0,256	2,04949E-46
Macro_NLRP3	MFF	2,35390E-13	-3,10185E-01	0,249	0,326	4,33519E-09
Macro_NLRP3	PSMD3	5,71039E-39	-3,10243E-01	0,183	0,289	1,05168E-34
Macro_NLRP3	MRPL28	1,86511E-15	-3,10436E-01	0,202	0,274	3,43497E-11
Macro_NLRP3	CHCHD2	2,16870E-12	-3,11652E-01	0,816	0,815	3,99409E-08
Macro_NLRP3	NUCKS1	1,52579E-58	-3,12006E-01	0,356	0,515	2,81005E-54
Macro_NLRP3	NCF4	1,08486E-51	-3,12321E-01	0,298	0,435	1,99799E-47
Macro_NLRP3	NME3	1,88195E-40	-3,12450E-01	0,247	0,371	3,46598E-36
Macro_NLRP3	RRP7A	8,27697E-14	-3,12519E-01	0,228	0,301	1,52437E-09
Macro_NLRP3	SNX5	2,83839E-77	-3,12537E-01	0,247	0,413	5,22747E-73
Macro_NLRP3	CD2BP2	1,06771E-23	-3,13055E-01	0,175	0,258	1,96641E-19
Macro_NLRP3	TOX4	6,84211E-14	-3,13605E-01	0,218	0,29	1,26011E-09
Macro_NLRP3	MPC1	6,38015E-07	-3,14274E-01	0,261	0,322	1,17503E-02
Macro_NLRP3	CCDC85B	4,88481E-65	-3,15403E-01	0,256	0,411	8,99635E-61
Macro_NLRP3	VAMP3	8,18911E-12	-3,15548E-01	0,282	0,357	1,50819E-07
Macro_NLRP3	NME2	3,16430E-18	-3,15651E-01	0,3	0,365	5,82770E-14
Macro_NLRP3	RPL35	1,08551E-15	-3,16139E-01	0,476	0,536	1,99918E-11
Macro_NLRP3	NDUFB3	2,64279E-14	-3,16679E-01	0,381	0,476	4,86722E-10
Macro_NLRP3	ME2	5,94839E-36	-3,16806E-01	0,209	0,315	1,09552E-31
Macro_NLRP3	TNFRSF14	9,98741E-21	-3,17146E-01	0,356	0,458	1,83938E-16
Macro_NLRP3	MRPL16	2,27153E-54	-3,17474E-01	0,151	0,269	4,18347E-50
Macro_NLRP3	HSD17B4	3,97880E-25	-3,17785E-01	0,182	0,266	7,32776E-21
Macro_NLRP3	DDRGK1	1,66948E-52	-3,18577E-01	0,204	0,333	3,07469E-48
Macro_NLRP3	SERPINB6	1,32470E-19	-3,19684E-01	0,337	0,431	2,43970E-15
Macro_NLRP3	LMAN1	2,44118E-55	-3,20269E-01	0,176	0,3	4,49591E-51
Macro_NLRP3	JPT1	9,76893E-32	-3,21289E-01	0,206	0,29	1,79914E-27
Macro_NLRP3	PMPCB	1,64999E-27	-3,22299E-01	0,163	0,25	3,03879E-23
Macro_NLRP3	SWAP70	2,62588E-34	-3,22348E-01	0,239	0,344	4,83608E-30
Macro_NLRP3	HLA-B	1,38745E-07	-3,22521E-01	0,985	0,966	2,55526E-03

Macro_NLRP3	GOLGA4	8,61879E-24	-3,23188E-01	0,246	0,344	1,58732E-19
Macro_NLRP3	IKZF1	7,82063E-45	-3,23561E-01	0,181	0,293	1,44032E-40
Macro_NLRP3	G3BP1	1,23703E-22	-3,23660E-01	0,227	0,316	2,27825E-18
Macro_NLRP3	ARPC2	5,32933E-16	-3,23990E-01	0,873	0,858	9,81502E-12
Macro_NLRP3	ENSA	1,10480E-09	-3,24133E-01	0,399	0,48	2,03472E-05
Macro_NLRP3	KRCC1	3,33193E-26	-3,24166E-01	0,186	0,274	6,13641E-22
Macro_NLRP3	ITPR2	1,77267E-12	-3,25099E-01	0,22	0,283	3,26473E-08
Macro_NLRP3	SNW1	5,90007E-15	-3,26228E-01	0,221	0,296	1,08662E-10
Macro_NLRP3	PRPF38B	2,86319E-25	-3,26694E-01	0,334	0,446	5,27314E-21
Macro_NLRP3	SMS	6,46384E-09	-3,26909E-01	0,365	0,433	1,19045E-04
Macro_NLRP3	ST14	1,06618E-55	-3,27156E-01	0,159	0,277	1,96359E-51
Macro_NLRP3	LRPAP1	5,16061E-13	-3,27186E-01	0,416	0,503	9,50429E-09
Macro_NLRP3	ADH5	5,14782E-68	-3,27880E-01	0,124	0,253	9,48074E-64
Macro_NLRP3	CCDC107	1,63729E-21	-3,28734E-01	0,224	0,315	3,01540E-17
Macro_NLRP3	HSPB11	4,40824E-29	-3,28773E-01	0,181	0,275	8,11865E-25
Macro_NLRP3	LYSMD2	4,78161E-43	-3,29190E-01	0,19	0,3	8,80629E-39
Macro_NLRP3	CLPTM1	4,43605E-31	-3,29750E-01	0,176	0,267	8,16987E-27
Macro_NLRP3	PIK3R5	2,46099E-06	-3,32739E-01	0,271	0,256	4,53241E-02
Macro_NLRP3	ATP5F1C	9,77551E-45	-3,33050E-01	0,193	0,297	1,80036E-40
Macro_NLRP3	TCEAL4	7,59044E-39	-3,33838E-01	0,183	0,289	1,39793E-34
Macro_NLRP3	SASH3	8,54088E-10	-3,33944E-01	0,228	0,289	1,57297E-05
Macro_NLRP3	GOLGA7	2,03755E-12	-3,34118E-01	0,23	0,299	3,75255E-08
Macro_NLRP3	EMC3	9,12149E-17	-3,34347E-01	0,245	0,327	1,67990E-12
Macro_NLRP3	DDX5	2,17550E-86	-3,35190E-01	0,868	0,829	4,00662E-82
Macro_NLRP3	ABRACL	1,23313E-16	-3,36219E-01	0,411	0,506	2,27106E-12
Macro_NLRP3	UVRAG	2,56669E-41	-3,36923E-01	0,19	0,301	4,72707E-37
Macro_NLRP3	RBMX	2,62339E-08	-3,37015E-01	0,361	0,431	4,83151E-04
Macro_NLRP3	RPL32	5,11142E-08	-3,37304E-01	0,531	0,583	9,41370E-04
Macro_NLRP3	ATXN10	1,69655E-26	-3,38111E-01	0,195	0,284	3,12454E-22
Macro_NLRP3	RACK1	6,60652E-22	-3,38319E-01	0,408	0,473	1,21672E-17
Macro_NLRP3	ENG	1,36131E-08	-3,38407E-01	0,288	0,353	2,50712E-04
Macro_NLRP3	ANAPC5	3,36439E-26	-3,39438E-01	0,203	0,296	6,19620E-22
Macro_NLRP3	RAB32	1,68160E-42	-3,40578E-01	0,268	0,385	3,09701E-38
Macro_NLRP3	GIT2	5,98228E-42	-3,42073E-01	0,192	0,304	1,10176E-37
Macro_NLRP3	MS4A7	8,71327E-07	-3,42426E-01	0,669	0,662	1,60472E-02
Macro_NLRP3	TGFBR2	5,01195E-48	-3,42589E-01	0,223	0,346	9,23050E-44
Macro_NLRP3	PHF5A	2,47276E-15	-3,42936E-01	0,194	0,265	4,55408E-11
Macro_NLRP3	ADAR	3,53154E-32	-3,43064E-01	0,32	0,438	6,50404E-28
Macro_NLRP3	MIIP	6,64888E-47	-3,43799E-01	0,147	0,255	1,22452E-42
Macro_NLRP3	HADHB	6,25357E-18	-3,43891E-01	0,279	0,368	1,15172E-13
Macro_NLRP3	APH1A	5,69390E-30	-3,43894E-01	0,389	0,509	1,04865E-25
Macro_NLRP3	MYL6	6,03874E-36	-3,44493E-01	0,948	0,941	1,11215E-31

Macro_NLRP3	SRSF1	2,01014E-13	-3,44607E-01	0,207	0,274	3,70207E-09
Macro_NLRP3	ATP5IF1	1,48025E-64	-3,44801E-01	0,153	0,276	2,72618E-60
Macro_NLRP3	GNB4	1,18162E-62	-3,44995E-01	0,21	0,349	2,17620E-58
Macro_NLRP3	MTCH2	3,06338E-18	-3,45530E-01	0,242	0,331	5,64183E-14
Macro_NLRP3	DCXR	2,55546E-35	-3,45758E-01	0,264	0,377	4,70639E-31
Macro_NLRP3	LAMTOR3	6,56797E-18	-3,45777E-01	0,191	0,265	1,20962E-13
Macro_NLRP3	RALY	4,30305E-26	-3,46191E-01	0,388	0,503	7,92493E-22
Macro_NLRP3	AKR7A2	4,67604E-30	-3,46464E-01	0,167	0,258	8,61186E-26
Macro_NLRP3	POLR2K	2,90477E-44	-3,46676E-01	0,213	0,337	5,34972E-40
Macro_NLRP3	PTP4A1	2,83285E-12	-3,47260E-01	0,262	0,228	5,21726E-08
Macro_NLRP3	DEGS1	3,29177E-47	-3,47543E-01	0,213	0,337	6,06245E-43
Macro_NLRP3	SRSF6	3,43437E-21	-3,48999E-01	0,252	0,205	6,32508E-17
Macro_NLRP3	TRMT112	2,34216E-21	-3,49356E-01	0,521	0,628	4,31355E-17
Macro_NLRP3	TMEM50A	5,16712E-30	-3,49999E-01	0,495	0,613	9,51628E-26
Macro_NLRP3	ARHGAP15	7,79091E-40	-3,51043E-01	0,159	0,264	1,43485E-35
Macro_NLRP3	DAPK1	1,27769E-15	-3,51500E-01	0,262	0,342	2,35312E-11
Macro_NLRP3	RNPS1	1,53332E-08	-3,51539E-01	0,334	0,412	2,82391E-04
Macro_NLRP3	HAX1	3,60392E-26	-3,51874E-01	0,252	0,355	6,63734E-22
Macro_NLRP3	NDUFV1	3,08037E-46	-3,52846E-01	0,282	0,42	5,67311E-42
Macro_NLRP3	NDUFA11	7,81394E-12	-3,52933E-01	0,38	0,462	1,43909E-07
Macro_NLRP3	RANBP1	2,41765E-13	-3,53087E-01	0,291	0,375	4,45259E-09
Macro_NLRP3	GRHPR	1,71106E-10	-3,53275E-01	0,231	0,297	3,15125E-06
Macro_NLRP3	PRDX4	1,46606E-59	-3,54435E-01	0,171	0,298	2,70005E-55
Macro_NLRP3	YIPF3	2,72367E-31	-3,54469E-01	0,227	0,334	5,01619E-27
Macro_NLRP3	VAMP5	2,19281E-09	-3,54953E-01	0,434	0,485	4,03850E-05
Macro_NLRP3	SMARCB1	1,09438E-21	-3,55103E-01	0,217	0,302	2,01552E-17
Macro_NLRP3	ERH	1,28708E-25	-3,56388E-01	0,42	0,539	2,37041E-21
Macro_NLRP3	C9orf78	2,37161E-23	-3,57560E-01	0,246	0,345	4,36780E-19
Macro_NLRP3	VCP	4,12821E-07	-3,57883E-01	0,398	0,473	7,60292E-03
Macro_NLRP3	TMEM14B	1,16095E-19	-3,58016E-01	0,327	0,432	2,13811E-15
Macro_NLRP3	RPS19BP1	8,25105E-30	-3,58393E-01	0,179	0,267	1,51960E-25
Macro_NLRP3	SH3BGRL	1,74578E-06	-3,58664E-01	0,659	0,726	3,21520E-02
Macro_NLRP3	EVL	8,33065E-78	-3,58715E-01	0,214	0,371	1,53426E-73
Macro_NLRP3	PAK1	2,16227E-09	-3,58844E-01	0,373	0,443	3,98226E-05
Macro_NLRP3	PSMA1	1,62262E-17	-3,58922E-01	0,283	0,369	2,98838E-13
Macro_NLRP3	RNF114	4,11964E-27	-3,60884E-01	0,274	0,38	7,58713E-23
Macro_NLRP3	NOL7	4,26184E-14	-3,61100E-01	0,329	0,423	7,84903E-10
Macro_NLRP3	HNRNPM	5,65048E-17	-3,61863E-01	0,467	0,457	1,04065E-12
Macro_NLRP3	NDUFB10	3,04201E-17	-3,62089E-01	0,461	0,568	5,60247E-13
Macro_NLRP3	SUMO2	2,17221E-07	-3,62884E-01	0,737	0,788	4,00057E-03
Macro_NLRP3	GNPTG	1,74653E-21	-3,63529E-01	0,338	0,438	3,21659E-17
Macro_NLRP3	TMEM147	7,99881E-21	-3,63557E-01	0,339	0,443	1,47314E-16

Macro_NLRP3	SIVA1	6,12425E-11	-3,64267E-01	0,373	0,463	1,12790E-06
Macro_NLRP3	GGNBP2	3,51086E-29	-3,64751E-01	0,243	0,35	6,46596E-25
Macro_NLRP3	FAM50A	9,31659E-12	-3,64764E-01	0,242	0,311	1,71584E-07
Macro_NLRP3	ALDOA	2,22255E-49	-3,65153E-01	0,798	0,756	4,09328E-45
Macro_NLRP3	NDUFAB1	1,79275E-12	-3,65812E-01	0,388	0,484	3,30171E-08
Macro_NLRP3	PNISR	3,87821E-27	-3,65821E-01	0,383	0,504	7,14250E-23
Macro_NLRP3	PRDX5	5,49874E-19	-3,66105E-01	0,51	0,61	1,01270E-14
Macro_NLRP3	MDH2	5,59113E-09	-3,66315E-01	0,445	0,529	1,02972E-04
Macro_NLRP3	MLX	1,89855E-13	-3,66551E-01	0,284	0,366	3,49656E-09
Macro_NLRP3	SPSB3	4,93026E-34	-3,66935E-01	0,195	0,299	9,08006E-30
Macro_NLRP3	COX20	2,29426E-07	-3,67418E-01	0,212	0,264	4,22533E-03
Macro_NLRP3	PRMT1	1,47018E-32	-3,67908E-01	0,213	0,316	2,70764E-28
Macro_NLRP3	FEZ2	1,24766E-66	-3,68003E-01	0,216	0,363	2,29782E-62
Macro_NLRP3	VDAC1	3,17136E-09	-3,68132E-01	0,455	0,541	5,84070E-05
Macro_NLRP3	TNFAIP8L2	1,02588E-66	-3,69052E-01	0,142	0,272	1,88937E-62
Macro_NLRP3	LSP1	3,85690E-08	-3,69638E-01	0,726	0,666	7,10325E-04
Macro_NLRP3	SDCCAG8	2,42002E-18	-3,69830E-01	0,259	0,345	4,45696E-14
Macro_NLRP3	DCAF7	1,70827E-30	-3,69860E-01	0,218	0,321	3,14612E-26
Macro_NLRP3	MEAF6	1,40587E-39	-3,70496E-01	0,21	0,326	2,58919E-35
Macro_NLRP3	RBBP4	5,35535E-14	-3,71617E-01	0,297	0,378	9,86294E-10
Macro_NLRP3	CLTC	7,85519E-15	-3,72471E-01	0,341	0,428	1,44669E-10
Macro_NLRP3	THOC7	8,33208E-12	-3,73571E-01	0,27	0,345	1,53452E-07
Macro_NLRP3	FUCA2	1,30327E-46	-3,73581E-01	0,201	0,319	2,40023E-42
Macro_NLRP3	PA2G4	2,94080E-10	-3,73711E-01	0,357	0,441	5,41606E-06
Macro_NLRP3	GCC2	4,72930E-34	-3,74192E-01	0,186	0,29	8,70995E-30
Macro_NLRP3	NDUFB8	7,18043E-22	-3,74428E-01	0,215	0,294	1,32242E-17
Macro_NLRP3	SDF2L1	1,22166E-07	-3,74688E-01	0,294	0,36	2,24993E-03
Macro_NLRP3	ILK	3,87322E-23	-3,75460E-01	0,321	0,424	7,13331E-19
Macro_NLRP3	ACAA1	1,16182E-12	-3,75666E-01	0,304	0,385	2,13973E-08
Macro_NLRP3	SMIM19	1,18059E-31	-3,75681E-01	0,174	0,268	2,17429E-27
Macro_NLRP3	CSF2RA	9,91556E-26	-3,77196E-01	0,325	0,426	1,82615E-21
Macro_NLRP3	COMMD9	7,37133E-16	-3,77812E-01	0,241	0,321	1,35758E-11
Macro_NLRP3	PSMB2	1,81834E-36	-3,78423E-01	0,328	0,452	3,34884E-32
Macro_NLRP3	NDUFS8	3,32103E-43	-3,78663E-01	0,366	0,508	6,11635E-39
Macro_NLRP3	CD163	4,27777E-14	-3,80448E-01	0,489	0,443	7,87837E-10
Macro_NLRP3	PPIG	3,32365E-09	-3,80962E-01	0,376	0,46	6,12116E-05
Macro_NLRP3	TMED3	3,98609E-51	-3,81552E-01	0,166	0,285	7,34119E-47
Macro_NLRP3	U2SURP	9,76883E-17	-3,81565E-01	0,25	0,334	1,79912E-12
Macro_NLRP3	IFNAR1	3,11427E-09	-3,81936E-01	0,254	0,319	5,73555E-05
Macro_NLRP3	MRPS18B	9,32536E-58	-3,82066E-01	0,16	0,289	1,71745E-53
Macro_NLRP3	ACTR1A	9,67085E-16	-3,82209E-01	0,219	0,295	1,78108E-11
Macro_NLRP3	MLEC	6,23848E-52	-3,82407E-01	0,228	0,359	1,14894E-47

Macro_NLRP3	NDUFA8	2,83273E-48	-3,82596E-01	0,18	0,302	5,21705E-44
Macro_NLRP3	C19orf53	1,08196E-07	-3,82697E-01	0,506	0,592	1,99265E-03
Macro_NLRP3	ATF6B	2,61799E-29	-3,83173E-01	0,199	0,296	4,82155E-25
Macro_NLRP3	LCP1	4,61835E-73	-3,84481E-01	0,758	0,698	8,50561E-69
Macro_NLRP3	VDAC3	4,02786E-47	-3,84494E-01	0,201	0,323	7,41812E-43
Macro_NLRP3	TBCB	6,89829E-28	-3,84865E-01	0,366	0,478	1,27046E-23
Macro_NLRP3	OXA1L	5,25180E-07	-3,85419E-01	0,385	0,462	9,67224E-03
Macro_NLRP3	GTF3C6	1,06981E-10	-3,85596E-01	0,301	0,378	1,97027E-06
Macro_NLRP3	ANXA7	7,97756E-26	-3,85844E-01	0,279	0,382	1,46923E-21
Macro_NLRP3	RAB9A	4,56391E-43	-3,86333E-01	0,147	0,25	8,40535E-39
Macro_NLRP3	KXD1	1,34112E-08	-3,86405E-01	0,286	0,357	2,46993E-04
Macro_NLRP3	GPR137B	4,15789E-67	-3,86752E-01	0,177	0,31	7,65759E-63
Macro_NLRP3	LRRC25	1,00724E-17	-3,87994E-01	0,259	0,341	1,85503E-13
Macro_NLRP3	GLRX3	1,58476E-10	-3,88156E-01	0,193	0,253	2,91865E-06
Macro_NLRP3	RAB6A	2,70934E-17	-3,88360E-01	0,229	0,307	4,98978E-13
Macro_NLRP3	HDLBP	1,38141E-10	-3,88398E-01	0,338	0,417	2,54414E-06
Macro_NLRP3	PLEKHJ1	2,64986E-23	-3,88934E-01	0,176	0,257	4,88025E-19
Macro_NLRP3	LAMTOR1	4,12142E-13	-3,89076E-01	0,57	0,655	7,59042E-09
Macro_NLRP3	PYURF	3,66468E-11	-3,89886E-01	0,333	0,418	6,74923E-07
Macro_NLRP3	TMBIM1	9,47588E-19	-3,90109E-01	0,282	0,369	1,74517E-14
Macro_NLRP3	TNPO1	1,34988E-21	-3,90136E-01	0,179	0,257	2,48608E-17
Macro_NLRP3	TPI1	5,52786E-20	-3,91355E-01	0,84	0,825	1,01807E-15
Macro_NLRP3	PDCD10	1,12093E-17	-3,92268E-01	0,238	0,322	2,06442E-13
Macro_NLRP3	ERGIC3	2,69788E-12	-3,93327E-01	0,414	0,506	4,96869E-08
Macro_NLRP3	CALM3	1,57469E-07	-3,93751E-01	0,45	0,513	2,90011E-03
Macro_NLRP3	DAD1	1,81201E-27	-3,93983E-01	0,5	0,61	3,33717E-23
Macro_NLRP3	TMEM141	4,69169E-29	-3,94813E-01	0,178	0,269	8,64069E-25
Macro_NLRP3	DDX24	4,26964E-20	-3,94964E-01	0,378	0,487	7,86340E-16
Macro_NLRP3	NACA	1,20218E-09	-3,95165E-01	0,915	0,919	2,21405E-05
Macro_NLRP3	ETFB	4,00994E-28	-3,95414E-01	0,218	0,316	7,38510E-24
Macro_NLRP3	ARL6IP4	1,62711E-08	-3,95529E-01	0,556	0,641	2,99666E-04
Macro_NLRP3	ECH1	6,15416E-41	-3,95721E-01	0,189	0,298	1,13341E-36
Macro_NLRP3	DRAP1	3,15475E-50	-3,95843E-01	0,546	0,668	5,81010E-46
Macro_NLRP3	MCTS1	7,69878E-19	-3,96495E-01	0,217	0,303	1,41789E-14
Macro_NLRP3	IDH3G	6,75267E-08	-3,97301E-01	0,296	0,366	1,24364E-03
Macro_NLRP3	TUFM	5,02592E-14	-3,97498E-01	0,419	0,518	9,25623E-10
Macro_NLRP3	LAMTOR2	1,24750E-14	-3,97629E-01	0,455	0,551	2,29751E-10
Macro_NLRP3	CYC1	6,69660E-16	-3,97888E-01	0,347	0,443	1,23331E-11
Macro_NLRP3	CNPPD1	3,65726E-53	-3,98631E-01	0,161	0,282	6,73558E-49
Macro_NLRP3	RASSF4	2,52167E-38	-3,99278E-01	0,401	0,517	4,64415E-34
Macro_NLRP3	NUDT22	2,21067E-34	-4,00015E-01	0,182	0,281	4,07140E-30
Macro_NLRP3	ZFAND6	5,01527E-17	-4,01173E-01	0,236	0,319	9,23663E-13

Macro_NLRP3	RPL7	4,28410E-09	-4,02396E-01	0,444	0,502	7,89003E-05
Macro_NLRP3	MRPL4	1,68492E-44	-4,02836E-01	0,173	0,288	3,10311E-40
Macro_NLRP3	CUTA	5,27658E-10	-4,02924E-01	0,408	0,493	9,71788E-06
Macro_NLRP3	NSL1	1,30989E-44	-4,03281E-01	0,149	0,256	2,41242E-40
Macro_NLRP3	LSM5	1,15466E-09	-4,04263E-01	0,277	0,35	2,12654E-05
Macro_NLRP3	RPS14	1,79382E-08	-4,04543E-01	0,528	0,58	3,30369E-04
Macro_NLRP3	LHFPL2	1,35513E-54	-4,04570E-01	0,175	0,294	2,49575E-50
Macro_NLRP3	UGP2	3,71637E-20	-4,04718E-01	0,222	0,308	6,84444E-16
Macro_NLRP3	TMEM179B	2,63702E-24	-4,04762E-01	0,375	0,492	4,85660E-20
Macro_NLRP3	KDELR2	1,71781E-13	-4,04860E-01	0,42	0,515	3,16370E-09
Macro_NLRP3	TTC1	8,76664E-44	-4,04935E-01	0,153	0,26	1,61455E-39
Macro_NLRP3	COPB2	1,36369E-09	-4,04945E-01	0,202	0,259	2,51150E-05
Macro_NLRP3	PCMT1	1,06191E-19	-4,05036E-01	0,256	0,346	1,95572E-15
Macro_NLRP3	XPO1	2,89107E-25	-4,05237E-01	0,177	0,263	5,32449E-21
Macro_NLRP3	HNRNPR	3,10226E-08	-4,05387E-01	0,299	0,367	5,71343E-04
Macro_NLRP3	CISD2	1,03595E-34	-4,05726E-01	0,266	0,38	1,90791E-30
Macro_NLRP3	CYFIP1	5,62980E-33	-4,06301E-01	0,231	0,333	1,03684E-28
Macro_NLRP3	UBE2L3	2,52784E-27	-4,06593E-01	0,396	0,512	4,65552E-23
Macro_NLRP3	SNRPB	9,11634E-14	-4,06802E-01	0,511	0,508	1,67896E-09
Macro_NLRP3	ITPA	1,03706E-21	-4,06980E-01	0,198	0,282	1,90996E-17
Macro_NLRP3	PCBD1	1,20272E-53	-4,06984E-01	0,235	0,373	2,21505E-49
Macro_NLRP3	TMEM219	5,21566E-13	-4,07989E-01	0,551	0,645	9,60569E-09
Macro_NLRP3	TFPT	2,07017E-96	-4,08558E-01	0,109	0,257	3,81264E-92
Macro_NLRP3	CSRP1	3,30162E-24	-4,08704E-01	0,185	0,265	6,08060E-20
Macro_NLRP3	GAA	7,73025E-33	-4,09418E-01	0,291	0,396	1,42368E-28
Macro_NLRP3	MEA1	1,69540E-24	-4,09484E-01	0,295	0,399	3,12242E-20
Macro_NLRP3	COPS5	3,90719E-15	-4,10978E-01	0,215	0,286	7,19586E-11
Macro_NLRP3	CTBS	5,73168E-14	-4,11858E-01	0,209	0,275	1,05560E-09
Macro_NLRP3	TMEM123	1,26768E-18	-4,14035E-01	0,363	0,464	2,33468E-14
Macro_NLRP3	MRPS7	2,59500E-35	-4,14092E-01	0,213	0,32	4,77921E-31
Macro_NLRP3	TNFSF13	1,36837E-20	-4,14201E-01	0,374	0,45	2,52013E-16
Macro_NLRP3	ATP6AP2	4,07418E-11	-4,14408E-01	0,522	0,598	7,50341E-07
Macro_NLRP3	HMGN2	1,17912E-09	-4,14573E-01	0,664	0,741	2,17159E-05
Macro_NLRP3	RGS10	4,50712E-49	-4,14591E-01	0,673	0,747	8,30077E-45
Macro_NLRP3	CCDC12	4,74286E-37	-4,15196E-01	0,19	0,296	8,73493E-33
Macro_NLRP3	RENBP	5,75993E-92	-4,15396E-01	0,157	0,311	1,06081E-87
Macro_NLRP3	GBP5	1,15596E-11	-4,15447E-01	0,198	0,251	2,12893E-07
Macro_NLRP3	MRPS16	4,09549E-29	-4,15491E-01	0,211	0,31	7,54267E-25
Macro_NLRP3	RPL36AL	6,66278E-23	-4,16242E-01	0,431	0,499	1,22709E-18
Macro_NLRP3	STX7	6,19494E-51	-4,16243E-01	0,285	0,427	1,14092E-46
Macro_NLRP3	EIF6	2,49884E-19	-4,16623E-01	0,317	0,414	4,60211E-15
Macro_NLRP3	IFI16	6,16435E-39	-4,16974E-01	0,38	0,512	1,13529E-34

Macro_NLRP3	STARD7	6,31003E-45	-4,17388E-01	0,198	0,316	1,16212E-40
Macro_NLRP3	RNASEH2B	1,83958E-42	-4,18363E-01	0,197	0,313	3,38795E-38
Macro_NLRP3	AHCY	4,15881E-15	-4,19538E-01	0,197	0,268	7,65929E-11
Macro_NLRP3	LYPLA1	4,73196E-17	-4,19693E-01	0,251	0,335	8,71485E-13
Macro_NLRP3	ITM2B	1,73042E-64	-4,19707E-01	0,883	0,909	3,18692E-60
Macro_NLRP3	TMEM9B	8,09811E-37	-4,19878E-01	0,263	0,383	1,49143E-32
Macro_NLRP3	PARP9	2,56903E-49	-4,21288E-01	0,153	0,264	4,73139E-45
Macro_NLRP3	DECR1	5,41832E-35	-4,21564E-01	0,243	0,357	9,97892E-31
Macro_NLRP3	SCAMP2	2,77853E-70	-4,21682E-01	0,354	0,518	5,11722E-66
Macro_NLRP3	POLR2F	3,41850E-11	-4,21849E-01	0,248	0,319	6,29586E-07
Macro_NLRP3	DYNLRB1	2,59506E-21	-4,22112E-01	0,374	0,471	4,77933E-17
Macro_NLRP3	ITSN2	1,34949E-10	-4,22331E-01	0,318	0,393	2,48536E-06
Macro_NLRP3	FDFT1	3,79589E-30	-4,22999E-01	0,177	0,268	6,99088E-26
Macro_NLRP3	SERINC1	2,04546E-08	-4,23429E-01	0,335	0,411	3,76713E-04
Macro_NLRP3	LARP7	6,12625E-20	-4,25594E-01	0,201	0,284	1,12827E-15
Macro_NLRP3	NMI	1,30438E-39	-4,26383E-01	0,2	0,31	2,40227E-35
Macro_NLRP3	INTS10	4,76575E-26	-4,26436E-01	0,167	0,25	8,77708E-22
Macro_NLRP3	TMEM134	2,26079E-30	-4,26691E-01	0,186	0,282	4,16370E-26
Macro_NLRP3	AP2M1	1,23293E-12	-4,27188E-01	0,49	0,592	2,27069E-08
Macro_NLRP3	MTIF3	3,31509E-30	-4,27282E-01	0,203	0,304	6,10540E-26
Macro_NLRP3	LEPROTL1	1,12789E-34	-4,29417E-01	0,288	0,41	2,07724E-30
Macro_NLRP3	SEC13	7,90613E-20	-4,29633E-01	0,312	0,411	1,45607E-15
Macro_NLRP3	DUT	3,30642E-38	-4,29737E-01	0,287	0,413	6,08943E-34
Macro_NLRP3	CLNS1A	1,63461E-14	-4,30068E-01	0,34	0,429	3,01046E-10
Macro_NLRP3	TMX1	3,05516E-35	-4,30337E-01	0,234	0,346	5,62669E-31
Macro_NLRP3	SNRPC	4,68202E-50	-4,30618E-01	0,288	0,431	8,62287E-46
Macro_NLRP3	MED10	2,39582E-19	-4,30947E-01	0,177	0,253	4,41239E-15
Macro_NLRP3	ARHGAP4	1,46041E-16	-4,31333E-01	0,31	0,392	2,68963E-12
Macro_NLRP3	TMBIM6	1,63617E-18	-4,31435E-01	0,738	0,782	3,01334E-14
Macro_NLRP3	SMIM14	2,62074E-18	-4,32852E-01	0,199	0,276	4,82662E-14
Macro_NLRP3	MICAL1	1,60096E-16	-4,33166E-01	0,222	0,3	2,94850E-12
Macro_NLRP3	ST13	1,72302E-18	-4,33836E-01	0,51	0,615	3,17328E-14
Macro_NLRP3	MRPS34	3,68238E-40	-4,34029E-01	0,277	0,406	6,78184E-36
Macro_NLRP3	ZFP36L2	1,83243E-78	-4,34732E-01	0,558	0,708	3,37479E-74
Macro_NLRP3	MRPS12	9,36713E-31	-4,34884E-01	0,191	0,292	1,72514E-26
Macro_NLRP3	REEP5	8,43959E-13	-4,35210E-01	0,481	0,571	1,55432E-08
Macro_NLRP3	PSMC5	1,67530E-17	-4,35249E-01	0,326	0,42	3,08540E-13
Macro_NLRP3	LPAR6	4,55411E-81	-4,35565E-01	0,183	0,336	8,38730E-77
Macro_NLRP3	RPS15	6,32928E-09	-4,35869E-01	0,524	0,578	1,16566E-04
Macro_NLRP3	SYPL1	1,68531E-17	-4,36228E-01	0,229	0,31	3,10384E-13
Macro_NLRP3	EID1	2,04095E-12	-4,39013E-01	0,493	0,588	3,75881E-08
Macro_NLRP3	NOSIP	4,83451E-34	-4,39166E-01	0,226	0,338	8,90371E-30

Macro_NLRP3	ARL4C	1,58703E-80	-4,39209E-01	0,194	0,346	2,92283E-76
Macro_NLRP3	UBE2K	4,04746E-07	-4,39251E-01	0,234	0,291	7,45420E-03
Macro_NLRP3	NAGA	7,45245E-27	-4,40043E-01	0,267	0,37	1,37252E-22
Macro_NLRP3	EMC7	1,25227E-23	-4,40244E-01	0,237	0,329	2,30631E-19
Macro_NLRP3	NUTF2	5,66473E-14	-4,42241E-01	0,304	0,392	1,04327E-09
Macro_NLRP3	PPP1R7	6,98374E-24	-4,42316E-01	0,199	0,287	1,28620E-19
Macro_NLRP3	MBD4	4,65701E-37	-4,42676E-01	0,16	0,259	8,57682E-33
Macro_NLRP3	MRPL34	3,15977E-78	-4,42831E-01	0,224	0,388	5,81935E-74
Macro_NLRP3	AP1M1	7,41411E-18	-4,42984E-01	0,183	0,254	1,36546E-13
Macro_NLRP3	SHISA5	6,45189E-32	-4,43120E-01	0,27	0,381	1,18825E-27
Macro_NLRP3	PHB2	3,00925E-11	-4,43413E-01	0,41	0,501	5,54214E-07
Macro_NLRP3	MPV17	2,59095E-36	-4,44508E-01	0,209	0,319	4,77175E-32
Macro_NLRP3	GLB1	1,78222E-46	-4,45727E-01	0,186	0,302	3,28231E-42
Macro_NLRP3	PRR13	2,87871E-14	-4,46000E-01	0,5	0,588	5,30173E-10
Macro_NLRP3	PSAP	1,25863E-13	-4,46014E-01	0,954	0,913	2,31803E-09
Macro_NLRP3	ESD	7,60622E-26	-4,46048E-01	0,269	0,373	1,40084E-21
Macro_NLRP3	RGS19	1,51568E-19	-4,46201E-01	0,402	0,505	2,79143E-15
Macro_NLRP3	SDHB	1,71890E-19	-4,48064E-01	0,313	0,408	3,16570E-15
Macro_NLRP3	ARHGAP18	7,79018E-72	-4,48094E-01	0,333	0,49	1,43472E-67
Macro_NLRP3	CLTA	1,00613E-18	-4,48585E-01	0,573	0,656	1,85298E-14
Macro_NLRP3	CCT2	8,32095E-24	-4,50199E-01	0,253	0,349	1,53247E-19
Macro_NLRP3	GSDMD	1,20309E-18	-4,51611E-01	0,226	0,308	2,21572E-14
Macro_NLRP3	TMCO1	5,47777E-17	-4,51705E-01	0,275	0,367	1,00884E-12
Macro_NLRP3	CALHM6	3,81281E-34	-4,51803E-01	0,185	0,269	7,02206E-30
Macro_NLRP3	M6PR	2,77067E-24	-4,54351E-01	0,464	0,573	5,10274E-20
Macro_NLRP3	NDUFS4	1,63713E-24	-4,54526E-01	0,205	0,295	3,01511E-20
Macro_NLRP3	CTSZ	3,14026E-69	-4,56175E-01	0,553	0,662	5,78341E-65
Macro_NLRP3	OCIAD1	2,17296E-24	-4,57107E-01	0,332	0,444	4,00193E-20
Macro_NLRP3	NDUFA12	2,60585E-16	-4,57514E-01	0,401	0,503	4,79919E-12
Macro_NLRP3	PTPMT1	1,15412E-39	-4,58602E-01	0,156	0,258	2,12554E-35
Macro_NLRP3	MAX	7,27741E-56	-4,59766E-01	0,268	0,409	1,34028E-51
Macro_NLRP3	SNRPA	1,47662E-20	-4,59874E-01	0,22	0,304	2,71949E-16
Macro_NLRP3	STOM	1,29275E-55	-4,60198E-01	0,226	0,358	2,38085E-51
Macro_NLRP3	SRSF11	2,23749E-08	-4,60817E-01	0,437	0,523	4,12079E-04
Macro_NLRP3	EEF1B2	2,02228E-08	-4,60950E-01	0,846	0,841	3,72443E-04
Macro_NLRP3	PSMD4	5,28648E-35	-4,61118E-01	0,301	0,423	9,73611E-31
Macro_NLRP3	SDHC	2,56664E-27	-4,61914E-01	0,337	0,451	4,72699E-23
Macro_NLRP3	PARL	1,40348E-22	-4,62623E-01	0,211	0,298	2,58480E-18
Macro_NLRP3	MGST2	1,58169E-44	-4,63356E-01	0,243	0,371	2,91299E-40
Macro_NLRP3	IK	4,75737E-15	-4,63537E-01	0,262	0,346	8,76165E-11
Macro_NLRP3	TAGLN2	6,04467E-41	-4,63683E-01	0,78	0,752	1,11325E-36
Macro_NLRP3	TFG	1,53314E-35	-4,64560E-01	0,17	0,27	2,82358E-31

Macro_NLRP3	HSD17B12	4,19473E-43	-4,64921E-01	0,153	0,256	7,72544E-39
Macro_NLRP3	CAPZB	2,67313E-52	-4,64987E-01	0,693	0,767	4,92311E-48
Macro_NLRP3	SCP2	3,76726E-41	-4,65399E-01	0,335	0,473	6,93817E-37
Macro_NLRP3	CYB5R3	8,52302E-36	-4,65619E-01	0,302	0,421	1,56969E-31
Macro_NLRP3	BBX	2,75673E-30	-4,65779E-01	0,208	0,306	5,07708E-26
Macro_NLRP3	MT-ND6	7,55050E-17	-4,65926E-01	0,239	0,308	1,39057E-12
Macro_NLRP3	CMC2	1,32581E-10	-4,65941E-01	0,218	0,281	2,44174E-06
Macro_NLRP3	CDK5RAP3	6,83205E-13	-4,66302E-01	0,245	0,313	1,25826E-08
Macro_NLRP3	PSMB9	5,23099E-23	-4,66480E-01	0,589	0,665	9,63391E-19
Macro_NLRP3	JUN	4,21202E-09	-4,67126E-01	0,684	0,712	7,75729E-05
Macro_NLRP3	RSU1	7,11994E-47	-4,67185E-01	0,222	0,347	1,31128E-42
Macro_NLRP3	ELMO1	1,82089E-21	-4,67485E-01	0,255	0,347	3,35353E-17
Macro_NLRP3	HLA-E	8,05705E-22	-4,67498E-01	0,91	0,904	1,48387E-17
Macro_NLRP3	RRBP1	1,10176E-30	-4,67873E-01	0,285	0,395	2,02910E-26
Macro_NLRP3	STOML2	1,71982E-28	-4,68227E-01	0,19	0,284	3,16738E-24
Macro_NLRP3	RPL6	6,16800E-13	-4,69024E-01	0,501	0,559	1,13596E-08
Macro_NLRP3	AIP	3,49346E-37	-4,69092E-01	0,232	0,35	6,43390E-33
Macro_NLRP3	ILF2	7,18075E-12	-4,69502E-01	0,316	0,399	1,32248E-07
Macro_NLRP3	PSMD13	1,01452E-15	-4,69720E-01	0,25	0,331	1,86843E-11
Macro_NLRP3	CAPN1	2,97246E-24	-4,69750E-01	0,189	0,275	5,47437E-20
Macro_NLRP3	SNAP23	5,86970E-22	-4,70164E-01	0,309	0,408	1,08102E-17
Macro_NLRP3	AP1B1	2,65108E-54	-4,70208E-01	0,27	0,406	4,88250E-50
Macro_NLRP3	NUDT1	1,76201E-21	-4,70624E-01	0,189	0,27	3,24510E-17
Macro_NLRP3	SSBP1	1,52360E-06	-4,71331E-01	0,414	0,494	2,80601E-02
Macro_NLRP3	PIH1D1	1,60367E-21	-4,72260E-01	0,183	0,261	2,95349E-17
Macro_NLRP3	SND1	1,06267E-08	-4,72721E-01	0,284	0,351	1,95713E-04
Macro_NLRP3	OAZ2	2,64292E-16	-4,72846E-01	0,215	0,29	4,86747E-12
Macro_NLRP3	IL18	1,34922E-70	-4,73321E-01	0,302	0,456	2,48486E-66
Macro_NLRP3	PGK1	4,85977E-33	-4,74197E-01	0,725	0,704	8,95023E-29
Macro_NLRP3	CD84	8,60898E-85	-4,74203E-01	0,243	0,407	1,58552E-80
Macro_NLRP3	SUGT1	1,92857E-16	-4,75873E-01	0,214	0,291	3,55185E-12
Macro_NLRP3	CTSA	1,31696E-22	-4,75994E-01	0,437	0,513	2,42545E-18
Macro_NLRP3	COMMD1	1,65917E-46	-4,76074E-01	0,208	0,334	3,05570E-42
Macro_NLRP3	SARAF	1,38122E-50	-4,77133E-01	0,336	0,437	2,54380E-46
Macro_NLRP3	HEXB	2,07638E-10	-4,78337E-01	0,443	0,521	3,82408E-06
Macro_NLRP3	PIN1	1,00885E-40	-4,78390E-01	0,236	0,357	1,85800E-36
Macro_NLRP3	HDAC2	8,45569E-30	-4,78643E-01	0,182	0,276	1,55728E-25
Macro_NLRP3	ABI1	5,85077E-30	-4,78762E-01	0,244	0,349	1,07754E-25
Macro_NLRP3	BCAP31	5,14988E-70	-4,80347E-01	0,445	0,597	9,48453E-66
Macro_NLRP3	CAPRIN1	1,22211E-11	-4,81505E-01	0,27	0,342	2,25076E-07
Macro_NLRP3	DPP7	6,94805E-66	-4,81996E-01	0,418	0,57	1,27962E-61
Macro_NLRP3	STAB1	5,56431E-35	-4,82808E-01	0,266	0,353	1,02478E-30

Macro_NLRP3	UBXN1	6,89033E-07	-4,83402E-01	0,555	0,636	1,26899E-02
Macro_NLRP3	ARHGAP9	1,78442E-07	-4,83494E-01	0,202	0,25	3,28637E-03
Macro_NLRP3	RPL31	1,54103E-09	-4,83924E-01	0,454	0,501	2,83812E-05
Macro_NLRP3	MRPL51	5,36710E-30	-4,84157E-01	0,331	0,451	9,88459E-26
Macro_NLRP3	RPL12	1,78511E-17	-4,84526E-01	0,522	0,581	3,28764E-13
Macro_NLRP3	TEX264	1,76065E-46	-4,85620E-01	0,196	0,319	3,24260E-42
Macro_NLRP3	N4BP2L2	2,83807E-36	-4,85750E-01	0,343	0,47	5,22688E-32
Macro_NLRP3	BECN1	3,14651E-11	-4,86389E-01	0,205	0,266	5,79493E-07
Macro_NLRP3	SLC39A1	6,54941E-29	-4,86418E-01	0,194	0,285	1,20620E-24
Macro_NLRP3	RPS26	4,15216E-16	-4,86807E-01	0,465	0,53	7,64704E-12
Macro_NLRP3	ERLEC1	2,73421E-57	-4,87689E-01	0,16	0,287	5,03559E-53
Macro_NLRP3	NSFL1C	9,98918E-24	-4,88968E-01	0,196	0,282	1,83971E-19
Macro_NLRP3	SRP9	5,30391E-17	-4,89332E-01	0,376	0,474	9,76821E-13
Macro_NLRP3	HSP90AA1	8,46473E-07	-4,89798E-01	0,888	0,879	1,55895E-02
Macro_NLRP3	SPAG7	4,19612E-28	-4,90100E-01	0,232	0,335	7,72800E-24
Macro_NLRP3	PSMC1	9,96460E-19	-4,90306E-01	0,249	0,338	1,83518E-14
Macro_NLRP3	PRDX3	1,59795E-17	-4,90311E-01	0,389	0,489	2,94295E-13
Macro_NLRP3	MRPL40	1,26956E-51	-4,90962E-01	0,163	0,283	2,33814E-47
Macro_NLRP3	GYPC	3,31386E-46	-4,91906E-01	0,202	0,31	6,10314E-42
Macro_NLRP3	FAM162A	3,09238E-45	-4,92122E-01	0,229	0,357	5,69524E-41
Macro_NLRP3	CORO1B	1,78993E-61	-4,92221E-01	0,328	0,487	3,29651E-57
Macro_NLRP3	NAA20	1,12785E-46	-4,92236E-01	0,197	0,318	2,07716E-42
Macro_NLRP3	FCGRT	2,16790E-16	-4,92404E-01	0,741	0,738	3,99262E-12
Macro_NLRP3	NDUFS3	8,16604E-59	-4,92900E-01	0,214	0,353	1,50394E-54
Macro_NLRP3	SDHD	1,52992E-42	-4,93160E-01	0,233	0,355	2,81766E-38
Macro_NLRP3	PSMD11	3,44365E-13	-4,93455E-01	0,242	0,315	6,34218E-09
Macro_NLRP3	SNAP29	3,18733E-42	-4,93669E-01	0,183	0,295	5,87010E-38
Macro_NLRP3	RPL30	5,91780E-10	-4,94449E-01	0,531	0,585	1,08988E-05
Macro_NLRP3	PSMD7	6,72354E-18	-4,94685E-01	0,319	0,414	1,23827E-13
Macro_NLRP3	DAP3	5,54586E-18	-4,94875E-01	0,24	0,325	1,02138E-13
Macro_NLRP3	YIF1B	5,16304E-22	-4,95037E-01	0,264	0,359	9,50878E-18
Macro_NLRP3	GPS1	3,77083E-23	-4,95233E-01	0,189	0,273	6,94474E-19
Macro_NLRP3	GTF3A	9,08845E-21	-4,95664E-01	0,381	0,49	1,67382E-16
Macro_NLRP3	NDUFB5	4,17394E-17	-4,95879E-01	0,36	0,463	7,68715E-13
Macro_NLRP3	YTHDF2	1,48084E-27	-4,96824E-01	0,196	0,289	2,72726E-23
Macro_NLRP3	ACP1	5,98645E-20	-4,96914E-01	0,25	0,343	1,10253E-15
Macro_NLRP3	UBE2I	9,44121E-26	-4,97454E-01	0,422	0,537	1,73879E-21
Macro_NLRP3	FNBP4	3,10156E-23	-4,97743E-01	0,22	0,308	5,71214E-19
Macro_NLRP3	C18orf32	3,15314E-15	-4,99145E-01	0,214	0,287	5,80714E-11
Macro_NLRP3	NUDC	2,53351E-32	-5,00438E-01	0,276	0,391	4,66596E-28
Macro_NLRP3	PEPD	6,90011E-35	-5,01460E-01	0,262	0,373	1,27079E-30
Macro_NLRP3	CCT4	1,14917E-14	-5,01531E-01	0,347	0,441	2,11642E-10

Macro_NLRP3	HPS1	1,00978E-15	-5,02251E-01	0,276	0,359	1,85971E-11
Macro_NLRP3	GNPTAB	7,64802E-92	-5,02692E-01	0,138	0,293	1,40854E-87
Macro_NLRP3	PPP2R1A	2,49170E-17	-5,04362E-01	0,31	0,405	4,58897E-13
Macro_NLRP3	PRCP	6,64349E-21	-5,04461E-01	0,299	0,399	1,22353E-16
Macro_NLRP3	LPP	1,25593E-12	-5,04617E-01	0,207	0,273	2,31305E-08
Macro_NLRP3	SPOP	6,25695E-25	-5,04622E-01	0,189	0,275	1,15234E-20
Macro_NLRP3	PSMG2	1,10740E-39	-5,05548E-01	0,302	0,436	2,03950E-35
Macro_NLRP3	CCT7	5,12324E-27	-5,07145E-01	0,254	0,359	9,43546E-23
Macro_NLRP3	NDUFA10	1,68615E-18	-5,07156E-01	0,247	0,336	3,10539E-14
Macro_NLRP3	ETFA	6,09284E-12	-5,07485E-01	0,268	0,342	1,12212E-07
Macro_NLRP3	NUDT21	9,08506E-09	-5,07769E-01	0,215	0,272	1,67320E-04
Macro_NLRP3	PSMB10	8,75680E-13	-5,08845E-01	0,496	0,574	1,61274E-08
Macro_NLRP3	SAP18	6,90398E-18	-5,09046E-01	0,559	0,657	1,27151E-13
Macro_NLRP3	NDUFV2	2,38472E-13	-5,09303E-01	0,45	0,542	4,39193E-09
Macro_NLRP3	CD63	4,61364E-62	-5,09393E-01	0,797	0,82	8,49694E-58
Macro_NLRP3	MVP	3,05045E-23	-5,09438E-01	0,284	0,383	5,61802E-19
Macro_NLRP3	MRPL18	8,95934E-07	-5,10525E-01	0,291	0,355	1,65004E-02
Macro_NLRP3	GIMAP4	3,26025E-84	-5,10737E-01	0,291	0,441	6,00441E-80
Macro_NLRP3	DNAJC4	2,63289E-17	-5,11442E-01	0,281	0,37	4,84899E-13
Macro_NLRP3	IDH2	1,03902E-67	-5,12536E-01	0,271	0,425	1,91356E-63
Macro_NLRP3	RPL29	1,01478E-06	-5,13520E-01	0,512	0,563	1,86892E-02
Macro_NLRP3	PRMT2	2,01433E-73	-5,14037E-01	0,279	0,442	3,70979E-69
Macro_NLRP3	CDC42SE2	3,38406E-15	-5,14904E-01	0,21	0,28	6,23243E-11
Macro_NLRP3	IDH3B	1,08033E-11	-5,15060E-01	0,203	0,267	1,98964E-07
Macro_NLRP3	RPS27A	2,97385E-23	-5,15088E-01	0,524	0,581	5,47695E-19
Macro_NLRP3	PHYKPL	7,50354E-25	-5,16802E-01	0,182	0,266	1,38193E-20
Macro_NLRP3	MAP4	4,65031E-27	-5,18106E-01	0,179	0,266	8,56447E-23
Macro_NLRP3	UQCRC2	2,82226E-10	-5,19032E-01	0,34	0,422	5,19776E-06
Macro_NLRP3	CCND3	1,05776E-08	-5,19169E-01	0,262	0,317	1,94807E-04
Macro_NLRP3	PKIB	1,41453E-71	-5,19172E-01	0,129	0,257	2,60514E-67
Macro_NLRP3	PSMB6	5,77223E-15	-5,19180E-01	0,488	0,585	1,06307E-10
Macro_NLRP3	IMP4	6,74789E-32	-5,19307E-01	0,177	0,275	1,24276E-27
Macro_NLRP3	SNX1	2,42587E-33	-5,19423E-01	0,21	0,316	4,46773E-29
Macro_NLRP3	CTNND1	1,10515E-14	-5,19845E-01	0,211	0,278	2,03535E-10
Macro_NLRP3	NEMF	3,65046E-21	-5,19885E-01	0,21	0,296	6,72305E-17
Macro_NLRP3	ZCRB1	2,17302E-34	-5,20258E-01	0,182	0,281	4,00205E-30
Macro_NLRP3	DCTN2	1,12409E-32	-5,20499E-01	0,231	0,339	2,07023E-28
Macro_NLRP3	PSMB8	1,13007E-23	-5,20901E-01	0,493	0,585	2,08125E-19
Macro_NLRP3	PMP22	5,94473E-32	-5,20927E-01	0,193	0,285	1,09484E-27
Macro_NLRP3	RPL27A	5,93932E-20	-5,21456E-01	0,401	0,47	1,09384E-15
Macro_NLRP3	RPS7	6,52422E-13	-5,21544E-01	0,509	0,566	1,20157E-08
Macro_NLRP3	DBNL	1,70302E-18	-5,21954E-01	0,402	0,501	3,13644E-14

Macro_NLRP3	TCP1	3,77095E-09	-5,23154E-01	0,276	0,343	6,94497E-05
Macro_NLRP3	ATP6AP1	2,50723E-28	-5,23578E-01	0,448	0,553	4,61756E-24
Macro_NLRP3	CLPP	2,55900E-39	-5,24502E-01	0,169	0,275	4,71291E-35
Macro_NLRP3	HMGN3	3,02196E-26	-5,25431E-01	0,414	0,534	5,56554E-22
Macro_NLRP3	ATP6V0E1	1,33726E-34	-5,26102E-01	0,743	0,801	2,46284E-30
Macro_NLRP3	VKORC1	2,94422E-21	-5,26415E-01	0,175	0,251	5,42236E-17
Macro_NLRP3	TPM3	1,94668E-22	-5,26529E-01	0,819	0,802	3,58521E-18
Macro_NLRP3	PPM1G	8,55598E-26	-5,26735E-01	0,276	0,377	1,57575E-21
Macro_NLRP3	TXN2	8,55546E-20	-5,28079E-01	0,307	0,405	1,57566E-15
Macro_NLRP3	CD68	7,48964E-20	-5,29056E-01	0,821	0,777	1,37937E-15
Macro_NLRP3	KCNMA1	7,09570E-121	-5,29255E-01	0,107	0,275	1,30682E-116
Macro_NLRP3	TMSB4X	8,31371E-46	-5,30530E-01	0,986	0,984	1,53114E-41
Macro_NLRP3	MGAT4A	2,57078E-18	-5,31270E-01	0,278	0,365	4,73461E-14
Macro_NLRP3	MAN2B1	2,33951E-30	-5,32394E-01	0,388	0,483	4,30867E-26
Macro_NLRP3	DNMT1	2,45243E-36	-5,32878E-01	0,218	0,327	4,51664E-32
Macro_NLRP3	FCGR2B	2,24397E-14	-5,32898E-01	0,362	0,431	4,13272E-10
Macro_NLRP3	STAT2	7,67011E-16	-5,33698E-01	0,229	0,301	1,41260E-11
Macro_NLRP3	CPNE3	4,58407E-24	-5,34646E-01	0,172	0,25	8,44248E-20
Macro_NLRP3	CIAO1	1,93877E-27	-5,35261E-01	0,168	0,254	3,57063E-23
Macro_NLRP3	MRPS18C	4,93727E-36	-5,36851E-01	0,203	0,311	9,09298E-32
Macro_NLRP3	NDUFAF3	6,50283E-39	-5,37397E-01	0,351	0,483	1,19763E-34
Macro_NLRP3	SDHA	1,19810E-50	-5,38392E-01	0,148	0,261	2,20654E-46
Macro_NLRP3	ARL6IP5	4,79444E-59	-5,38484E-01	0,535	0,666	8,82991E-55
Macro_NLRP3	CNOT7	3,47724E-18	-5,40391E-01	0,234	0,315	6,40403E-14
Macro_NLRP3	HLA-DRB1	3,07412E-38	-5,40671E-01	0,945	0,914	5,66161E-34
Macro_NLRP3	DOK1	2,07747E-74	-5,40874E-01	0,119	0,25	3,82607E-70
Macro_NLRP3	PSME1	2,01605E-18	-5,41583E-01	0,703	0,759	3,71295E-14
Macro_NLRP3	TMED4	3,79299E-45	-5,41606E-01	0,186	0,303	6,98554E-41
Macro_NLRP3	APMAP	3,01011E-69	-5,42674E-01	0,172	0,313	5,54373E-65
Macro_NLRP3	DNPH1	1,61535E-78	-5,43399E-01	0,206	0,361	2,97499E-74
Macro_NLRP3	ERP29	5,24490E-16	-5,43990E-01	0,536	0,632	9,65953E-12
Macro_NLRP3	RPS19	3,13933E-20	-5,44240E-01	0,529	0,58	5,78170E-16
Macro_NLRP3	COX6C	4,85961E-15	-5,44641E-01	0,659	0,725	8,94994E-11
Macro_NLRP3	IST1	7,18425E-09	-5,45328E-01	0,212	0,269	1,32312E-04
Macro_NLRP3	FTL	2,08402E-15	-5,45619E-01	0,996	0,991	3,83815E-11
Macro_NLRP3	PSMC3	1,44467E-25	-5,45963E-01	0,231	0,328	2,66065E-21
Macro_NLRP3	UFC1	9,13810E-22	-5,45973E-01	0,403	0,512	1,68296E-17
Macro_NLRP3	LAPTM4A	2,04604E-60	-5,46663E-01	0,494	0,641	3,76818E-56
Macro_NLRP3	TXNL1	1,81484E-31	-5,46778E-01	0,276	0,391	3,34240E-27
Macro_NLRP3	HVCN1	2,15971E-66	-5,47332E-01	0,137	0,265	3,97754E-62
Macro_NLRP3	EMC4	2,42631E-39	-5,47522E-01	0,25	0,372	4,46853E-35
Macro_NLRP3	UBE2V2	3,00659E-12	-5,49466E-01	0,2	0,265	5,53723E-08

Macro_NLRP3	AAMP	1,97766E-22	-5,51863E-01	0,213	0,301	3,64226E-18
Macro_NLRP3	RNASET2	8,60806E-38	-5,53479E-01	0,692	0,745	1,58535E-33
Macro_NLRP3	RPL18	1,91230E-16	-5,54083E-01	0,512	0,567	3,52188E-12
Macro_NLRP3	ALDH2	3,10518E-25	-5,54412E-01	0,47	0,558	5,71881E-21
Macro_NLRP3	PPCS	5,43615E-32	-5,54963E-01	0,284	0,401	1,00118E-27
Macro_NLRP3	CD4	1,25939E-27	-5,55304E-01	0,436	0,549	2,31942E-23
Macro_NLRP3	RPS18	8,43912E-16	-5,57109E-01	0,507	0,565	1,55423E-11
Macro_NLRP3	RPL23	2,38800E-13	-5,57507E-01	0,395	0,459	4,39797E-09
Macro_NLRP3	CIRBP	4,46755E-34	-5,58518E-01	0,605	0,712	8,22788E-30
Macro_NLRP3	RER1	1,58391E-22	-5,59824E-01	0,358	0,471	2,91710E-18
Macro_NLRP3	TMED2	2,74297E-12	-5,61967E-01	0,418	0,507	5,05173E-08
Macro_NLRP3	SSB	1,00674E-32	-5,62199E-01	0,343	0,463	1,85412E-28
Macro_NLRP3	EIF2AK2	1,40962E-59	-5,62509E-01	0,15	0,272	2,59610E-55
Macro_NLRP3	CTSH	3,60461E-07	-5,62923E-01	0,701	0,711	6,63860E-03
Macro_NLRP3	TM9SF2	3,10521E-15	-5,63403E-01	0,348	0,435	5,71886E-11
Macro_NLRP3	HNRNPA1	1,16934E-08	-5,63915E-01	0,795	0,802	2,15358E-04
Macro_NLRP3	SCARB2	6,40988E-67	-5,63987E-01	0,244	0,384	1,18051E-62
Macro_NLRP3	PDIA6	2,34020E-33	-5,65572E-01	0,412	0,531	4,30994E-29
Macro_NLRP3	PSMA5	2,94540E-18	-5,66215E-01	0,349	0,443	5,42455E-14
Macro_NLRP3	ZNF638	1,08938E-16	-5,66505E-01	0,187	0,255	2,00632E-12
Macro_NLRP3	CHURC1	1,13626E-08	-5,67382E-01	0,261	0,323	2,09264E-04
Macro_NLRP3	FCGR1A	1,93402E-16	-5,69906E-01	0,355	0,416	3,56189E-12
Macro_NLRP3	PSMD6	1,10992E-22	-5,70632E-01	0,2	0,286	2,04414E-18
Macro_NLRP3	SPPL2A	4,26095E-34	-5,71143E-01	0,242	0,35	7,84740E-30
Macro_NLRP3	MMADHC	4,55944E-53	-5,71602E-01	0,226	0,36	8,39712E-49
Macro_NLRP3	LSM10	7,83772E-23	-5,71963E-01	0,282	0,385	1,44347E-18
Macro_NLRP3	RPS8	4,86211E-07	-5,72303E-01	0,528	0,583	8,95455E-03
Macro_NLRP3	MPC2	6,05074E-79	-5,75039E-01	0,213	0,374	1,11436E-74
Macro_NLRP3	KRTCAP2	6,51603E-08	-5,76087E-01	0,509	0,584	1,20006E-03
Macro_NLRP3	ZDHHC12	6,38976E-52	-5,78134E-01	0,153	0,272	1,17680E-47
Macro_NLRP3	ATRAID	4,69334E-59	-5,79892E-01	0,324	0,48	8,64373E-55
Macro_NLRP3	PRDX6	1,47679E-18	-5,80111E-01	0,461	0,568	2,71980E-14
Macro_NLRP3	SPCS2	2,25921E-27	-5,82154E-01	0,383	0,503	4,16078E-23
Macro_NLRP3	PTMS	1,90156E-133	-5,82250E-01	0,187	0,38	3,50211E-129
Macro_NLRP3	ISG20	1,24932E-06	-5,82271E-01	0,331	0,309	2,30087E-02
Macro_NLRP3	HMOX1	9,37405E-47	-5,83522E-01	0,441	0,555	1,72642E-42
Macro_NLRP3	IFITM3	2,15295E-13	-5,83888E-01	0,731	0,768	3,96508E-09
Macro_NLRP3	FBXO7	9,78766E-41	-5,84679E-01	0,218	0,332	1,80259E-36
Macro_NLRP3	ATP6V1D	1,75295E-18	-5,84729E-01	0,181	0,254	3,22841E-14
Macro_NLRP3	CIITA	2,98886E-27	-5,85733E-01	0,237	0,333	5,50458E-23
Macro_NLRP3	ARL6IP1	3,56028E-23	-5,86334E-01	0,322	0,424	6,55696E-19
Macro_NLRP3	CST3	6,09775E-30	-5,86954E-01	0,961	0,948	1,12302E-25

Macro_NLRP3	FKBP2	7,71120E-19	-5,87428E-01	0,331	0,417	1,42017E-14
Macro_NLRP3	TRAM1	6,19149E-15	-5,89125E-01	0,369	0,458	1,14029E-10
Macro_NLRP3	C11orf58	1,47477E-13	-5,89627E-01	0,473	0,568	2,71608E-09
Macro_NLRP3	DNASE2	4,61194E-86	-5,90073E-01	0,226	0,381	8,49381E-82
Macro_NLRP3	RPS15A	2,50200E-08	-5,91148E-01	0,521	0,574	4,60792E-04
Macro_NLRP3	RABAC1	2,21004E-20	-5,92155E-01	0,428	0,534	4,07023E-16
Macro_NLRP3	RPL37A	2,37597E-08	-5,92598E-01	0,494	0,542	4,37582E-04
Macro_NLRP3	RPS12	1,37364E-09	-5,94341E-01	0,532	0,587	2,52983E-05
Macro_NLRP3	CREG1	1,08013E-75	-5,95344E-01	0,389	0,525	1,98927E-71
Macro_NLRP3	PLP2	3,20662E-23	-5,95931E-01	0,454	0,399	5,90563E-19
Macro_NLRP3	SSR3	3,56997E-29	-5,95953E-01	0,49	0,607	6,57482E-25
Macro_NLRP3	UBE2E2	1,71788E-26	-5,96247E-01	0,214	0,308	3,16383E-22
Macro_NLRP3	CD47	2,34436E-14	-5,96907E-01	0,342	0,429	4,31761E-10
Macro_NLRP3	TAP1	5,57910E-35	-5,96927E-01	0,324	0,432	1,02750E-30
Macro_NLRP3	MGST3	1,67696E-47	-5,96973E-01	0,425	0,554	3,08847E-43
Macro_NLRP3	CD300A	1,01367E-22	-5,97883E-01	0,372	0,469	1,86687E-18
Macro_NLRP3	TMEM14C	9,22357E-38	-5,98830E-01	0,409	0,541	1,69870E-33
Macro_NLRP3	ATP6V1E1	6,39020E-28	-5,99794E-01	0,239	0,34	1,17688E-23
Macro_NLRP3	RPL14	1,62282E-36	-6,01116E-01	0,482	0,549	2,98875E-32
Macro_NLRP3	NME1-NME2	9,57918E-20	-6,01633E-01	0,287	0,234	1,76420E-15
Macro_NLRP3	NECAP2	2,39311E-49	-6,01801E-01	0,234	0,364	4,40739E-45
Macro_NLRP3	CHMP5	1,82510E-52	-6,01803E-01	0,279	0,42	3,36129E-48
Macro_NLRP3	CYB5A	8,12751E-66	-6,01964E-01	0,127	0,251	1,49684E-61
Macro_NLRP3	SRI	2,08660E-31	-6,02356E-01	0,329	0,444	3,84289E-27
Macro_NLRP3	PARP1	3,21136E-115	-6,02501E-01	0,166	0,351	5,91437E-111
Macro_NLRP3	RAB11A	2,92399E-33	-6,02766E-01	0,295	0,412	5,38511E-29
Macro_NLRP3	RNH1	1,64169E-13	-6,04138E-01	0,544	0,625	3,02350E-09
Macro_NLRP3	CACYBP	1,14703E-42	-6,05481E-01	0,285	0,413	2,11248E-38
Macro_NLRP3	NPC2	4,60428E-70	-6,06663E-01	0,871	0,885	8,47971E-66
Macro_NLRP3	SNX17	2,11326E-40	-6,07171E-01	0,313	0,444	3,89199E-36
Macro_NLRP3	PTGS1	1,04028E-45	-6,08180E-01	0,162	0,266	1,91588E-41
Macro_NLRP3	XAF1	2,36380E-32	-6,09655E-01	0,237	0,337	4,35340E-28
Macro_NLRP3	HLA-A	1,25662E-91	-6,09853E-01	0,962	0,949	2,31431E-87
Macro_NLRP3	NUCB1	3,07424E-46	-6,10109E-01	0,416	0,555	5,66182E-42
Macro_NLRP3	NSMCE1	2,36252E-37	-6,10928E-01	0,18	0,28	4,35105E-33
Macro_NLRP3	HNRNPC	9,18441E-22	-6,12721E-01	0,653	0,647	1,69149E-17
Macro_NLRP3	YIF1A	1,33894E-29	-6,21047E-01	0,197	0,293	2,46592E-25
Macro_NLRP3	AK2	2,71720E-21	-6,21511E-01	0,257	0,351	5,00427E-17
Macro_NLRP3	SP100	5,34020E-20	-6,21790E-01	0,375	0,475	9,83504E-16
Macro_NLRP3	IDH1	8,19256E-102	-6,21805E-01	0,107	0,26	1,50882E-97
Macro_NLRP3	RBM6	1,93934E-09	-6,22376E-01	0,213	0,268	3,57169E-05
Macro_NLRP3	PSMC6	9,60860E-31	-6,22618E-01	0,17	0,263	1,76962E-26

Macro_NLRP3	RPS11	1,15472E-09	-6,25809E-01	0,455	0,509	2,12666E-05
Macro_NLRP3	PSMB5	4,82913E-58	-6,27343E-01	0,192	0,327	8,89381E-54
Macro_NLRP3	RHOC	3,55804E-42	-6,28302E-01	0,207	0,319	6,55283E-38
Macro_NLRP3	SCAMP3	1,31219E-54	-6,29171E-01	0,144	0,262	2,41666E-50
Macro_NLRP3	CD59	1,03837E-106	-6,31210E-01	0,179	0,356	1,91237E-102
Macro_NLRP3	LMAN2	3,99058E-25	-6,33545E-01	0,411	0,521	7,34945E-21
Macro_NLRP3	RPL10	1,11661E-29	-6,34098E-01	0,541	0,599	2,05645E-25
Macro_NLRP3	CCT8	2,14690E-20	-6,37441E-01	0,324	0,421	3,95394E-16
Macro_NLRP3	GBP4	1,51453E-61	-6,39733E-01	0,167	0,288	2,78931E-57
Macro_NLRP3	GNAS	3,44897E-13	-6,39748E-01	0,524	0,618	6,35196E-09
Macro_NLRP3	RTCB	3,35703E-69	-6,40114E-01	0,169	0,307	6,18264E-65
Macro_NLRP3	ACP2	3,78520E-128	-6,41994E-01	0,101	0,273	6,97120E-124
Macro_NLRP3	TMEM230	4,05385E-32	-6,42779E-01	0,344	0,462	7,46597E-28
Macro_NLRP3	CALR	1,18136E-18	-6,44304E-01	0,661	0,73	2,17570E-14
Macro_NLRP3	LAMP2	4,15748E-21	-6,44809E-01	0,314	0,41	7,65683E-17
Macro_NLRP3	MSR1	1,45309E-55	-6,45816E-01	0,259	0,392	2,67615E-51
Macro_NLRP3	LSM4	6,84707E-23	-6,48509E-01	0,333	0,441	1,26102E-18
Macro_NLRP3	APEX1	1,65528E-28	-6,48812E-01	0,336	0,452	3,04852E-24
Macro_NLRP3	ELOVL1	1,68804E-18	-6,48971E-01	0,277	0,369	3,10886E-14
Macro_NLRP3	ABHD12	1,54387E-172	-6,49128E-01	0,175	0,4	2,84335E-168
Macro_NLRP3	NDUFS2	2,39280E-47	-6,49222E-01	0,242	0,372	4,40682E-43
Macro_NLRP3	RPL11	9,14460E-21	-6,51119E-01	0,53	0,586	1,68416E-16
Macro_NLRP3	CCT3	2,41075E-34	-6,51233E-01	0,3	0,421	4,43989E-30
Macro_NLRP3	HMGN1	8,46927E-42	-6,52286E-01	0,502	0,637	1,55979E-37
Macro_NLRP3	RPL13A	3,24952E-14	-6,53031E-01	0,472	0,525	5,98464E-10
Macro_NLRP3	GIMAP7	7,48892E-111	-6,53655E-01	0,11	0,27	1,37923E-106
Macro_NLRP3	RPL22L1	9,95280E-16	-6,54539E-01	0,211	0,28	1,83301E-11
Macro_NLRP3	PDK4	2,72927E-48	-6,54850E-01	0,212	0,32	5,02650E-44
Macro_NLRP3	TMEM176A	1,89415E-98	-6,56206E-01	0,31	0,462	3,48846E-94
Macro_NLRP3	HINT1	9,22868E-25	-6,58584E-01	0,666	0,728	1,69965E-20
Macro_NLRP3	PTGES3	1,71449E-20	-6,59250E-01	0,547	0,653	3,15758E-16
Macro_NLRP3	TMEM59	1,17489E-17	-6,60763E-01	0,53	0,626	2,16379E-13
Macro_NLRP3	SUMF2	3,13719E-55	-6,63597E-01	0,179	0,306	5,77777E-51
Macro_NLRP3	OSTC	4,17446E-14	-6,64880E-01	0,416	0,507	7,68811E-10
Macro_NLRP3	TMEM176B	9,04243E-130	-6,66835E-01	0,407	0,547	1,66534E-125
Macro_NLRP3	CD9	1,82601E-80	-6,67334E-01	0,347	0,499	3,36296E-76
Macro_NLRP3	VOPP1	2,09915E-88	-6,68068E-01	0,202	0,361	3,86601E-84
Macro_NLRP3	P4HB	1,46407E-16	-6,70205E-01	0,549	0,637	2,69638E-12
Macro_NLRP3	HLA-DMA	2,61348E-40	-6,71911E-01	0,756	0,784	4,81324E-36
Macro_NLRP3	RPL15	5,76419E-21	-6,72371E-01	0,502	0,558	1,06159E-16
Macro_NLRP3	TGFBI	1,83664E-12	-6,72966E-01	0,608	0,598	3,38253E-08
Macro_NLRP3	VAMP8	1,98754E-31	-6,73021E-01	0,704	0,788	3,66046E-27

Macro_NLRP3	IMPDH2	5,12722E-08	-6,73622E-01	0,224	0,278	9,44280E-04
Macro_NLRP3	DCK	6,85165E-56	-6,73702E-01	0,166	0,291	1,26187E-51
Macro_NLRP3	CALM2	3,91402E-36	-6,73913E-01	0,704	0,79	7,20844E-32
Macro_NLRP3	RPS29	5,29057E-09	-6,74689E-01	0,463	0,506	9,74365E-05
Macro_NLRP3	POLR2G	1,86440E-21	-6,75333E-01	0,281	0,381	3,43366E-17
Macro_NLRP3	GABARAPL2	6,93564E-08	-6,76443E-01	0,511	0,584	1,27734E-03
Macro_NLRP3	ABI3	1,27299E-104	-6,77016E-01	0,232	0,409	2,34447E-100
Macro_NLRP3	MRPL55	3,52280E-29	-6,77255E-01	0,191	0,286	6,48795E-25
Macro_NLRP3	TECR	1,02218E-75	-6,78441E-01	0,231	0,394	1,88255E-71
Macro_NLRP3	RAN	1,47472E-10	-6,78522E-01	0,585	0,665	2,71599E-06
Macro_NLRP3	GNPDA1	2,63346E-55	-6,79160E-01	0,151	0,269	4,85005E-51
Macro_NLRP3	MS4A6A	1,10369E-38	-6,80855E-01	0,707	0,716	2,03266E-34
Macro_NLRP3	FYTTD1	3,12567E-17	-6,81190E-01	0,188	0,254	5,75654E-13
Macro_NLRP3	RPL23A	1,37253E-12	-6,81221E-01	0,483	0,53	2,52780E-08
Macro_NLRP3	RPS20	3,15647E-17	-6,88001E-01	0,387	0,457	5,81328E-13
Macro_NLRP3	PTPN6	7,56818E-25	-6,89043E-01	0,41	0,52	1,39383E-20
Macro_NLRP3	RAB8A	1,32639E-48	-6,89796E-01	0,301	0,438	2,44281E-44
Macro_NLRP3	FGD2	4,51833E-66	-6,91504E-01	0,214	0,357	8,32141E-62
Macro_NLRP3	CBR1	2,61305E-97	-6,93027E-01	0,166	0,331	4,81245E-93
Macro_NLRP3	TMED9	1,10508E-76	-6,93675E-01	0,373	0,539	2,03523E-72
Macro_NLRP3	BTK	2,20364E-38	-6,93742E-01	0,21	0,321	4,05845E-34
Macro_NLRP3	CAT	4,97153E-30	-6,95042E-01	0,303	0,419	9,15607E-26
Macro_NLRP3	C14orf119	5,33712E-45	-6,95650E-01	0,185	0,303	9,82937E-41
Macro_NLRP3	EPSTI1	4,05439E-81	-6,96474E-01	0,267	0,423	7,46698E-77
Macro_NLRP3	LUC7L3	1,55068E-23	-6,96609E-01	0,268	0,368	2,85589E-19
Macro_NLRP3	CD164	1,02791E-09	-6,97348E-01	0,482	0,565	1,89310E-05
Macro_NLRP3	SNX6	5,63319E-76	-6,97962E-01	0,368	0,53	1,03747E-71
Macro_NLRP3	MAT2B	1,71400E-65	-7,00938E-01	0,203	0,348	3,15667E-61
Macro_NLRP3	MIF4GD	1,82519E-47	-7,03459E-01	0,201	0,323	3,36146E-43
Macro_NLRP3	TXNIP	2,60653E-149	-7,03927E-01	0,533	0,715	4,80045E-145
Macro_NLRP3	CCNDBP1	3,51013E-33	-7,04211E-01	0,183	0,282	6,46460E-29
Macro_NLRP3	RPL7A	7,32909E-28	-7,04230E-01	0,501	0,564	1,34980E-23
Macro_NLRP3	YPEL5	1,68178E-13	-7,05350E-01	0,443	0,433	3,09733E-09
Macro_NLRP3	SNX3	1,01426E-44	-7,05542E-01	0,592	0,699	1,86796E-40
Macro_NLRP3	ADAP2	7,73000E-114	-7,05690E-01	0,277	0,461	1,42363E-109
Macro_NLRP3	RPL9	6,55949E-11	-7,06294E-01	0,5	0,548	1,20806E-06
Macro_NLRP3	TUBA1A	1,85125E-13	-7,10095E-01	0,387	0,47	3,40945E-09
Macro_NLRP3	PSMD8	7,29568E-23	-7,14823E-01	0,422	0,535	1,34365E-18
Macro_NLRP3	DBI	8,98814E-15	-7,15329E-01	0,665	0,73	1,65535E-10
Macro_NLRP3	GRN	2,79500E-78	-7,15773E-01	0,806	0,825	5,14756E-74
Macro_NLRP3	GM2A	7,40723E-129	-7,17502E-01	0,199	0,392	1,36419E-124
Macro_NLRP3	CD81	2,01450E-83	-7,18354E-01	0,323	0,475	3,71011E-79

Macro_NLRP3	RPL19	1,59690E-18	-7,19996E-01	0,529	0,585	2,94102E-14
Macro_NLRP3	LAP3	5,11483E-51	-7,21414E-01	0,478	0,596	9,41997E-47
Macro_NLRP3	CNOT2	6,76192E-10	-7,22214E-01	0,215	0,276	1,24534E-05
Macro_NLRP3	VTI1B	3,47951E-18	-7,25272E-01	0,291	0,386	6,40821E-14
Macro_NLRP3	LGALS9	3,16671E-44	-7,25558E-01	0,435	0,559	5,83214E-40
Macro_NLRP3	RPS3	6,36758E-25	-7,25723E-01	0,512	0,573	1,17272E-20
Macro_NLRP3	ORMDL1	1,09969E-16	-7,27219E-01	0,239	0,323	2,02531E-12
Macro_NLRP3	PSMB4	4,79953E-09	-7,29637E-01	0,308	0,379	8,83929E-05
Macro_NLRP3	CAPG	2,95717E-112	-7,30414E-01	0,637	0,751	5,44622E-108
Macro_NLRP3	DDAH2	5,12014E-56	-7,34321E-01	0,286	0,432	9,42976E-52
Macro_NLRP3	NUB1	1,24901E-85	-7,34584E-01	0,173	0,327	2,30031E-81
Macro_NLRP3	TNFSF10	6,51668E-78	-7,35267E-01	0,23	0,373	1,20018E-73
Macro_NLRP3	PSMA3	9,02330E-55	-7,38086E-01	0,248	0,384	1,66182E-50
Macro_NLRP3	UBL7	4,51606E-25	-7,39141E-01	0,177	0,261	8,31723E-21
Macro_NLRP3	RPS25	5,30567E-21	-7,39496E-01	0,496	0,553	9,77145E-17
Macro_NLRP3	HEXA	1,16839E-71	-7,44028E-01	0,327	0,483	2,15182E-67
Macro_NLRP3	SOD1	2,51357E-70	-7,44241E-01	0,412	0,578	4,62925E-66
Macro_NLRP3	PSMF1	1,06932E-31	-7,44538E-01	0,309	0,432	1,96936E-27
Macro_NLRP3	CCS	5,86106E-27	-7,47021E-01	0,181	0,27	1,07943E-22
Macro_NLRP3	SNAPIN	7,99995E-56	-7,48334E-01	0,173	0,299	1,47335E-51
Macro_NLRP3	SNX2	3,23543E-53	-7,51481E-01	0,308	0,454	5,95869E-49
Macro_NLRP3	SRP14	1,52031E-43	-7,54180E-01	0,797	0,84	2,79996E-39
Macro_NLRP3	RPS23	4,68095E-17	-7,57279E-01	0,522	0,572	8,62090E-13
Macro_NLRP3	RPS4X	7,06016E-32	-7,57418E-01	0,506	0,571	1,30027E-27
Macro_NLRP3	COX5A	1,59306E-15	-7,58903E-01	0,621	0,701	2,93395E-11
Macro_NLRP3	GPR34	2,96557E-137	-7,62826E-01	0,133	0,323	5,46169E-133
Macro_NLRP3	LAIR1	6,29184E-22	-7,63670E-01	0,445	0,527	1,15877E-17
Macro_NLRP3	SAMD9L	1,02813E-95	-7,64496E-01	0,137	0,294	1,89352E-91
Macro_NLRP3	ISCU	3,15340E-92	-7,64669E-01	0,379	0,553	5,80762E-88
Macro_NLRP3	CNDP2	2,09501E-70	-7,65274E-01	0,306	0,469	3,85838E-66
Macro_NLRP3	SSR4	5,79797E-23	-7,68894E-01	0,696	0,759	1,06781E-18
Macro_NLRP3	ADORA3	2,48482E-157	-7,70388E-01	0,091	0,28	4,57629E-153
Macro_NLRP3	TMEM208	3,73543E-20	-7,73305E-01	0,25	0,339	6,87953E-16
Macro_NLRP3	RPL10A	3,70401E-16	-7,78541E-01	0,448	0,502	6,82168E-12
Macro_NLRP3	PDIA4	1,17486E-49	-7,78606E-01	0,21	0,332	2,16375E-45
Macro_NLRP3	PARK7	5,77647E-28	-7,79850E-01	0,572	0,667	1,06385E-23
Macro_NLRP3	PPIA	3,10751E-25	-7,82877E-01	0,876	0,896	5,72309E-21
Macro_NLRP3	B2M	4,30609E-173	-7,85896E-01	0,994	0,996	7,93052E-169
Macro_NLRP3	DHRS7	3,62757E-66	-7,86517E-01	0,34	0,499	6,68090E-62
Macro_NLRP3	ANXA4	1,00658E-67	-7,88267E-01	0,184	0,321	1,85382E-63
Macro_NLRP3	CD40	2,01716E-66	-7,88371E-01	0,172	0,304	3,71500E-62
Macro_NLRP3	RPS27	5,87954E-07	-7,88496E-01	0,526	0,57	1,08284E-02

Macro_NLRP3	AKR1B1	2,04633E-144	-7,91355E-01	0,284	0,486	3,76873E-140
Macro_NLRP3	STAT1	1,59223E-71	-7,92987E-01	0,394	0,53	2,93241E-67
Macro_NLRP3	MYL12A	1,58197E-49	-7,94318E-01	0,773	0,83	2,91351E-45
Macro_NLRP3	RGS2	1,00471E-121	-7,94615E-01	0,727	0,616	1,85037E-117
Macro_NLRP3	SP110	1,68662E-38	-7,95717E-01	0,281	0,401	3,10625E-34
Macro_NLRP3	BST2	1,55360E-165	-7,97690E-01	0,582	0,739	2,86126E-161
Macro_NLRP3	PLD3	1,89428E-138	-7,99679E-01	0,355	0,526	3,48869E-134
Macro_NLRP3	PSMA4	1,01943E-23	-7,99982E-01	0,374	0,478	1,87749E-19
Macro_NLRP3	PHB	1,06973E-53	-8,01024E-01	0,258	0,402	1,97012E-49
Macro_NLRP3	MDH1	6,97290E-89	-8,02484E-01	0,229	0,401	1,28420E-84
Macro_NLRP3	EIF4A2	9,55563E-11	-8,02671E-01	0,477	0,57	1,75986E-06
Macro_NLRP3	CHD9	2,05517E-70	-8,08295E-01	0,208	0,356	3,78501E-66
Macro_NLRP3	IFI35	1,17429E-46	-8,10544E-01	0,237	0,357	2,16268E-42
Macro_NLRP3	GRSF1	6,29913E-74	-8,15064E-01	0,205	0,353	1,16011E-69
Macro_NLRP3	SEC11C	3,07982E-50	-8,17574E-01	0,211	0,337	5,67211E-46
Macro_NLRP3	AKR1A1	1,59383E-123	-8,18752E-01	0,412	0,593	2,93535E-119
Macro_NLRP3	FUCA1	2,25244E-164	-8,21918E-01	0,077	0,267	4,14833E-160
Macro_NLRP3	PSME2	1,12060E-43	-8,23858E-01	0,645	0,722	2,06381E-39
Macro_NLRP3	PDIA3	1,77100E-23	-8,24087E-01	0,608	0,682	3,26164E-19
Macro_NLRP3	RPS6	2,51893E-17	-8,25886E-01	0,496	0,549	4,63911E-13
Macro_NLRP3	HLA-DOA	8,38108E-99	-8,26655E-01	0,225	0,409	1,54354E-94
Macro_NLRP3	HLA-DQB1	1,68273E-68	-8,30483E-01	0,788	0,819	3,09909E-64
Macro_NLRP3	UBC	4,73178E-27	-8,30972E-01	0,889	0,9	8,71452E-23
Macro_NLRP3	SLAMF8	4,85891E-87	-8,33933E-01	0,185	0,339	8,94865E-83
Macro_NLRP3	PSMB1	2,14551E-16	-8,34526E-01	0,546	0,637	3,95139E-12
Macro_NLRP3	BEX4	1,39140E-103	-8,35154E-01	0,17	0,344	2,56255E-99
Macro_NLRP3	TWF2	1,45624E-25	-8,35572E-01	0,25	0,342	2,68195E-21
Macro_NLRP3	NAGK	7,89813E-73	-8,38660E-01	0,336	0,491	1,45460E-68
Macro_NLRP3	LTC4S	7,22056E-99	-8,38925E-01	0,144	0,298	1,32981E-94
Macro_NLRP3	CDK2AP2	3,32871E-40	-8,52533E-01	0,286	0,408	6,13049E-36
Macro_NLRP3	RPLP0	1,39305E-13	-8,55146E-01	0,49	0,548	2,56558E-09
Macro_NLRP3	RNASE6	7,94170E-56	-8,57481E-01	0,428	0,55	1,46262E-51
Macro_NLRP3	RPN2	4,09723E-42	-8,61940E-01	0,19	0,294	7,54588E-38
Macro_NLRP3	RABGAP1L	2,29950E-60	-8,61983E-01	0,142	0,263	4,23500E-56
Macro_NLRP3	LIPA	2,14762E-70	-8,68290E-01	0,382	0,513	3,95527E-66
Macro_NLRP3	PSMC2	6,60994E-89	-8,69658E-01	0,147	0,3	1,21735E-84
Macro_NLRP3	COPZ1	1,25220E-37	-8,70458E-01	0,269	0,393	2,30617E-33
Macro_NLRP3	ACP5	3,28370E-61	-8,70747E-01	0,284	0,411	6,04758E-57
Macro_NLRP3	CORO1C	4,69287E-10	-8,74144E-01	0,282	0,35	8,64285E-06
Macro_NLRP3	AHSA1	1,02107E-22	-8,76641E-01	0,219	0,307	1,88051E-18
Macro_NLRP3	PPIB	2,98478E-56	-8,84671E-01	0,732	0,798	5,49707E-52
Macro_NLRP3	RPS3A	1,63260E-15	-8,85118E-01	0,518	0,571	3,00676E-11

Macro_NLRP3	NPM1	5,50126E-31	-8,85319E-01	0,687	0,774	1,01317E-26
Macro_NLRP3	TUBB	8,78140E-98	-8,92176E-01	0,507	0,676	1,61727E-93
Macro_NLRP3	HLA-DPB1	2,61896E-101	-8,96687E-01	0,859	0,886	4,82335E-97
Macro_NLRP3	C3	2,43305E-64	-8,99094E-01	0,272	0,395	4,48096E-60
Macro_NLRP3	AXL	1,08733E-125	-8,99171E-01	0,115	0,296	2,00253E-121
Macro_NLRP3	IGFLR1	3,58031E-44	-9,08947E-01	0,255	0,37	6,59385E-40
Macro_NLRP3	TMED10	2,07535E-44	-9,11946E-01	0,443	0,58	3,82217E-40
Macro_NLRP3	HLA-DRA	4,93161E-49	-9,15534E-01	0,97	0,92	9,08254E-45
Macro_NLRP3	LY6E	2,89757E-98	-9,15998E-01	0,404	0,562	5,33646E-94
Macro_NLRP3	HLA-DRB5	1,61010E-93	-9,23570E-01	0,858	0,846	2,96531E-89
Macro_NLRP3	LDHB	3,34309E-49	-9,25713E-01	0,388	0,523	6,15697E-45
Macro_NLRP3	HLA-DQA1	4,65659E-99	-9,31020E-01	0,68	0,793	8,57604E-95
Macro_NLRP3	UBE2L6	6,52671E-77	-9,41730E-01	0,435	0,584	1,20202E-72
Macro_NLRP3	NDUFC2	4,84214E-38	-9,52945E-01	0,213	0,314	8,91777E-34
Macro_NLRP3	RPL5	2,28220E-47	-9,67546E-01	0,447	0,524	4,20312E-43
Macro_NLRP3	FCGR3A	6,55350E-46	-9,73877E-01	0,519	0,58	1,20696E-41
Macro_NLRP3	RPL3	2,04405E-26	-9,89083E-01	0,474	0,539	3,76452E-22
Macro_NLRP3	LILRB4	2,48287E-110	-1,01100E+00	0,41	0,563	4,57270E-106
Macro_NLRP3	RPS5	7,32772E-23	-1,01506E+00	0,465	0,523	1,34955E-18
Macro_NLRP3	HSP90B1	2,45579E-15	-1,01804E+00	0,637	0,706	4,52282E-11
Macro_NLRP3	FABP5	8,04657E-49	-1,02385E+00	0,328	0,458	1,48194E-44
Macro_NLRP3	PEBP1	7,49802E-220	-1,03033E+00	0,33	0,587	1,38091E-215
Macro_NLRP3	TUBA1B	2,53595E-89	-1,03636E+00	0,652	0,767	4,67045E-85
Macro_NLRP3	PRDX1	6,02275E-167	-1,03754E+00	0,627	0,759	1,10921E-162
Macro_NLRP3	RPL4	6,91863E-16	-1,03803E+00	0,422	0,482	1,27420E-11
Macro_NLRP3	DSTN	2,58794E-50	-1,04297E+00	0,23	0,358	4,76622E-46
Macro_NLRP3	HLA-DMB	3,00106E-37	-1,04396E+00	0,562	0,646	5,52706E-33
Macro_NLRP3	OAS1	1,00807E-104	-1,04672E+00	0,175	0,342	1,85656E-100
Macro_NLRP3	UBB	4,08266E-66	-1,04797E+00	0,733	0,818	7,51903E-62
Macro_NLRP3	HSPA8	3,72501E-25	-1,05361E+00	0,777	0,824	6,86036E-21
Macro_NLRP3	CD74	2,89446E-169	-1,05495E+00	0,99	0,96	5,33073E-165
Macro_NLRP3	C1orf54	2,61832E-130	-1,05781E+00	0,121	0,298	4,82215E-126
Macro_NLRP3	HLA-DQB2	6,07767E-209	-1,06326E+00	0,109	0,337	1,11932E-204
Macro_NLRP3	VSIG4	6,36387E-09	-1,06896E+00	0,303	0,352	1,17203E-04
Macro_NLRP3	IFI6	3,57530E-83	-1,06997E+00	0,357	0,501	6,58463E-79
Macro_NLRP3	TMEM109	5,73714E-64	-1,07429E+00	0,235	0,382	1,05661E-59
Macro_NLRP3	DDOST	2,17301E-41	-1,08114E+00	0,345	0,475	4,00204E-37
Macro_NLRP3	HLA-DPA1	1,66964E-121	-1,08906E+00	0,876	0,9	3,07498E-117
Macro_NLRP3	HSPA1A	4,06235E-13	-1,09011E+00	0,618	0,668	7,48163E-09
Macro_NLRP3	DAB2	7,29233E-158	-1,09071E+00	0,246	0,452	1,34303E-153
Macro_NLRP3	CCDC50	1,05024E-75	-1,09385E+00	0,15	0,286	1,93423E-71
Macro_NLRP3	SPINT2	9,70155E-112	-1,10608E+00	0,327	0,515	1,78673E-107

Macro_NLRP3	CXCR4	2,99609E-33	-1,10941E+00	0,599	0,578	5,51790E-29
Macro_NLRP3	TRIM22	3,75667E-33	-1,14976E+00	0,311	0,427	6,91867E-29
Macro_NLRP3	SLCO2B1	3,03845E-190	-1,15078E+00	0,161	0,39	5,59592E-186
Macro_NLRP3	SELPLG	1,54567E-54	-1,15208E+00	0,228	0,355	2,84665E-50
Macro_NLRP3	STMN1	1,93003E-131	-1,15263E+00	0,122	0,303	3,55454E-127
Macro_NLRP3	RPSA	5,56254E-38	-1,15291E+00	0,427	0,502	1,02445E-33
Macro_NLRP3	TSPAN3	4,18257E-64	-1,15504E+00	0,193	0,331	7,70304E-60
Macro_NLRP3	LGALS3BP	3,80300E-187	-1,18194E+00	0,123	0,339	7,00398E-183
Macro_NLRP3	TREM2	2,42002E-132	-1,20098E+00	0,244	0,423	4,45696E-128
Macro_NLRP3	HERPUD1	7,42418E-16	-1,21507E+00	0,618	0,687	1,36731E-11
Macro_NLRP3	GSN	1,58064E-171	-1,22049E+00	0,454	0,652	2,91106E-167
Macro_NLRP3	MS4A4A	5,18380E-136	-1,23464E+00	0,25	0,437	9,54700E-132
Macro_NLRP3	SPCS1	3,38052E-49	-1,24699E+00	0,483	0,62	6,22591E-45
Macro_NLRP3	CTSD	2,67667E-152	-1,24826E+00	0,718	0,774	4,92962E-148
Macro_NLRP3	HLA-DQA2	2,57693E-186	-1,26676E+00	0,316	0,532	4,74594E-182
Macro_NLRP3	GPR65	4,44277E-69	-1,28111E+00	0,197	0,34	8,18225E-65
Macro_NLRP3	IFITM1	1,62669E-84	-1,28164E+00	0,135	0,266	2,99588E-80
Macro_NLRP3	SYNGR2	1,00240E-213	-1,31520E+00	0,567	0,744	1,84613E-209
Macro_NLRP3	A2M	8,87628E-212	-1,33014E+00	0,172	0,411	1,63474E-207
Macro_NLRP3	MX1	5,88245E-98	-1,38980E+00	0,187	0,347	1,08337E-93
Macro_NLRP3	TXN	3,71022E-32	-1,39980E+00	0,598	0,674	6,83310E-28
Macro_NLRP3	IFI44L	2,13659E-109	-1,41219E+00	0,111	0,268	3,93495E-105
Macro_NLRP3	UCP2	3,59467E-46	-1,42893E+00	0,534	0,644	6,62031E-42
Macro_NLRP3	IL2RG	4,66475E-63	-1,44364E+00	0,205	0,336	8,59107E-59
Macro_NLRP3	ISG15	5,64428E-56	-1,45203E+00	0,316	0,439	1,03951E-51
Macro_NLRP3	HSPA6	6,72730E-42	-1,45734E+00	0,199	0,295	1,23897E-37
Macro_NLRP3	SERPING1	3,09008E-225	-1,46089E+00	0,172	0,419	5,69101E-221
Macro_NLRP3	GPR183	3,41263E-58	-1,49729E+00	0,55	0,448	6,28503E-54
Macro_NLRP3	CTSC	1,58557E-194	-1,57533E+00	0,523	0,693	2,92015E-190
Macro_NLRP3	HSPB1	1,37181E-157	-1,58596E+00	0,484	0,675	2,52647E-153
Macro_NLRP3	HSPA1B	6,22863E-53	-1,59413E+00	0,399	0,515	1,14713E-48
Macro_NLRP3	GPNMB	1,86295E-185	-1,60963E+00	0,137	0,354	3,43099E-181
Macro_NLRP3	TCF4	9,11588E-77	-1,61774E+00	0,149	0,285	1,67887E-72
Macro_NLRP3	PLTP	4,03324E-202	-1,71376E+00	0,126	0,349	7,42802E-198
Macro_NLRP3	MTRNR2L8	7,57483E-129	-1,71986E+00	0,289	0,455	1,39506E-124
Macro_NLRP3	ALOX5AP	1,18660E-71	-1,85303E+00	0,386	0,517	2,18535E-67
Macro_NLRP3	LGMN	3,56836E-214	-1,86572E+00	0,191	0,431	6,57184E-210
Macro_NLRP3	IRF7	2,41265E-07	-1,88257E+00	0,357	0,403	4,44339E-03
Macro_NLRP3	PLD4	1,20705E-81	-1,96220E+00	0,201	0,345	2,22302E-77
Macro_NLRP3	RGS1	7,49297E-167	-2,15845E+00	0,434	0,663	1,37998E-162
Macro_NLRP3	APOC1	1,69009E-300	-2,24331E+00	0,258	0,532	3,11263E-296
Macro_NLRP3	IRF8	2,18664E-35	-2,25969E+00	0,281	0,388	4,02714E-31

Macro_NLRP3	C1QA	0,00000E+00	-2,32382E+00	0,316	0,591	0,00000E+00
Macro_NLRP3	APOE	0,00000E+00	-2,40854E+00	0,253	0,532	0,00000E+00
Macro_NLRP3	C1QC	0,00000E+00	-2,57854E+00	0,269	0,573	0,00000E+00
Macro_NLRP3	C1QB	0,00000E+00	-2,73704E+00	0,25	0,575	0,00000E+00
Macro_NLRP3	SERPINF1	2,84451E-265	-2,82360E+00	0,153	0,429	5,23874E-261
Macro_NLRP3	RNASE1	4,04401E-165	-3,20145E+00	0,104	0,289	7,44785E-161

cluster	gene	p_val	avg_log2FC	pct.1	pct.2	p_val_adj
Macro_OLFML3	CXCL9	0,00000E+00	2,32046E+00	0,576	0,111	0,00000E+00
Macro_OLFML3	CXCL10	0,00000E+00	2,24891E+00	0,551	0,107	0,00000E+00
Macro_OLFML3	FN1	0,00000E+00	1,61111E+00	0,609	0,176	0,00000E+00
Macro_OLFML3	CXCL11	0,00000E+00	1,60540E+00	0,286	0,034	0,00000E+00
Macro_OLFML3	GBP5	0,00000E+00	1,60137E+00	0,767	0,186	0,00000E+00
Macro_OLFML3	GBP1	0,00000E+00	1,59439E+00	0,857	0,287	0,00000E+00
Macro_OLFML3	GBP4	0,00000E+00	1,55396E+00	0,822	0,215	0,00000E+00
Macro_OLFML3	C3	0,00000E+00	1,55037E+00	0,777	0,339	0,00000E+00
Macro_OLFML3	OLFML3	0,00000E+00	1,42574E+00	0,634	0,179	0,00000E+00
Macro_OLFML3	ANKRD22	0,00000E+00	1,37343E+00	0,697	0,172	0,00000E+00
Macro_OLFML3	C1QB	0,00000E+00	1,26422E+00	0,887	0,507	0,00000E+00
Macro_OLFML3	C1QA	0,00000E+00	1,16693E+00	0,897	0,529	0,00000E+00
Macro_OLFML3	SERPING1	0,00000E+00	1,15526E+00	0,877	0,342	0,00000E+00
Macro_OLFML3	SCIN	0,00000E+00	1,15377E+00	0,407	0,051	0,00000E+00
Macro_OLFML3	HLA-DQA2	2,14134E-285	1,15292E+00	0,664	0,495	3,94370E-281
Macro_OLFML3	CALHM6	1,49705E-216	1,12405E+00	0,409	0,244	2,75712E-212
Macro_OLFML3	VAMP5	0,00000E+00	1,08650E+00	0,908	0,432	0,00000E+00
Macro_OLFML3	APOL3	0,00000E+00	1,08115E+00	0,56	0,139	0,00000E+00
Macro_OLFML3	FCGR1A	0,00000E+00	1,08102E+00	0,829	0,363	0,00000E+00
Macro_OLFML3	C1QC	0,00000E+00	1,07706E+00	0,889	0,507	0,00000E+00
Macro_OLFML3	MDK	0,00000E+00	1,04179E+00	0,362	0,064	0,00000E+00
Macro_OLFML3	IDO1	1,15860E-242	9,40685E-01	0,256	0,093	2,13379E-238
Macro_OLFML3	STAT1	0,00000E+00	9,23243E-01	0,916	0,472	0,00000E+00
Macro_OLFML3	HLA-DRB5	0,00000E+00	9,15927E-01	0,951	0,835	0,00000E+00
Macro_OLFML3	TMEM176B	0,00000E+00	8,98539E-01	0,915	0,491	0,00000E+00
Macro_OLFML3	LGALS3BP	0,00000E+00	8,93492E-01	0,748	0,271	0,00000E+00
Macro_OLFML3	GBP2	0,00000E+00	8,87890E-01	0,815	0,322	0,00000E+00
Macro_OLFML3	PPA1	0,00000E+00	8,82158E-01	0,843	0,418	0,00000E+00
Macro_OLFML3	TNFSF10	0,00000E+00	8,62799E-01	0,743	0,317	0,00000E+00
Macro_OLFML3	FGL2	0,00000E+00	8,25408E-01	0,948	0,576	0,00000E+00
Macro_OLFML3	LAP3	0,00000E+00	7,98953E-01	0,936	0,545	0,00000E+00
Macro_OLFML3	HLA-DQA1	0,00000E+00	7,70360E-01	0,987	0,76	0,00000E+00
Macro_OLFML3	SLAMF8	0,00000E+00	7,55630E-01	0,717	0,28	0,00000E+00
Macro_OLFML3	GIMAP4	0,00000E+00	7,46718E-01	0,841	0,381	0,00000E+00
Macro_OLFML3	CD40	0,00000E+00	6,97598E-01	0,639	0,252	0,00000E+00
Macro_OLFML3	HAPLN3	0,00000E+00	6,91518E-01	0,358	0,086	0,00000E+00
Macro_OLFML3	GIMAP7	0,00000E+00	6,83541E-01	0,637	0,212	0,00000E+00
Macro_OLFML3	IL4I1	0,00000E+00	6,79855E-01	0,499	0,204	0,00000E+00
Macro_OLFML3	GIMAP5	0,00000E+00	6,75650E-01	0,417	0,128	0,00000E+00
Macro_OLFML3	TMEM176A	0,00000E+00	6,67816E-01	0,831	0,405	0,00000E+00
Macro_OLFML3	HLA-DQB1	0,00000E+00	6,64424E-01	0,989	0,796	0,00000E+00
Macro_OLFML3	GSN	0,00000E+00	6,64090E-01	0,933	0,6	0,00000E+00
Macro_OLFML3	SNX10	0,00000E+00	6,61403E-01	0,821	0,448	0,00000E+00
Macro_OLFML3	CD72	0,00000E+00	6,59310E-01	0,511	0,172	0,00000E+00

Macro_OLFML3	HLA-DRB1	0,00000E+00	6,57248E-01	0,998	0,908	0,00000E+00
Macro_OLFML3	PSME2	0,00000E+00	6,49833E-01	0,971	0,686	0,00000E+00
Macro_OLFML3	FCGR3A	0,00000E+00	6,31095E-01	0,939	0,533	0,00000E+00
Macro_OLFML3	HLA-DPB1	0,00000E+00	6,27067E-01	0,996	0,87	0,00000E+00
Macro_OLFML3	PSTPIP2	0,00000E+00	6,22923E-01	0,539	0,218	0,00000E+00
Macro_OLFML3	SLC1A3	0,00000E+00	6,04964E-01	0,704	0,324	0,00000E+00
Macro_OLFML3	HLA-DPA1	0,00000E+00	6,03815E-01	0,998	0,886	0,00000E+00
Macro_OLFML3	TYMP	0,00000E+00	5,98775E-01	0,977	0,772	0,00000E+00
Macro_OLFML3	EBI3	0,00000E+00	5,97165E-01	0,443	0,127	0,00000E+00
Macro_OLFML3	APOC1	0,00000E+00	5,93977E-01	0,808	0,473	0,00000E+00
Macro_OLFML3	HLA-A	0,00000E+00	5,89828E-01	0,998	0,944	0,00000E+00
Macro_OLFML3	YWHAH	5,59555E-272	5,69482E-01	0,786	0,503	1,03053E-267
Macro_OLFML3	FCGR1B	0,00000E+00	5,65102E-01	0,539	0,166	0,00000E+00
Macro_OLFML3	MARCKS	0,00000E+00	5,62477E-01	0,87	0,494	0,00000E+00
Macro_OLFML3	APBB1IP	0,00000E+00	5,57406E-01	0,783	0,444	0,00000E+00
Macro_OLFML3	APOE	0,00000E+00	5,51094E-01	0,792	0,474	0,00000E+00
Macro_OLFML3	BHLHE41	0,00000E+00	5,38524E-01	0,37	0,091	0,00000E+00
Macro_OLFML3	SLC8A1	0,00000E+00	5,30725E-01	0,669	0,331	0,00000E+00
Macro_OLFML3	HLA-DRA	0,00000E+00	5,18375E-01	0,999	0,916	0,00000E+00
Macro_OLFML3	CAMK2D	0,00000E+00	5,14918E-01	0,432	0,139	0,00000E+00
Macro_OLFML3	EPSTI1	0,00000E+00	5,12209E-01	0,776	0,368	0,00000E+00
Macro_OLFML3	GIMAP1	0,00000E+00	5,08342E-01	0,585	0,23	0,00000E+00
Macro_OLFML3	PRDX1	0,00000E+00	5,04149E-01	0,956	0,723	0,00000E+00
Macro_OLFML3	SAMD9L	0,00000E+00	5,02150E-01	0,591	0,244	0,00000E+00
Macro_OLFML3	APOL4	0,00000E+00	5,01920E-01	0,289	0,066	0,00000E+00
Macro_OLFML3	CD38	0,00000E+00	4,93418E-01	0,484	0,152	0,00000E+00
Macro_OLFML3	P2RY13	0,00000E+00	4,92693E-01	0,423	0,17	0,00000E+00
Macro_OLFML3	SFMBT2	0,00000E+00	4,89142E-01	0,46	0,186	0,00000E+00
Macro_OLFML3	PSMB9	0,00000E+00	4,80385E-01	0,946	0,625	0,00000E+00
Macro_OLFML3	IL18	0,00000E+00	4,74198E-01	0,75	0,407	0,00000E+00
Macro_OLFML3	AXL	9,35255E-277	4,73104E-01	0,527	0,252	1,72246E-272
Macro_OLFML3	CX3CR1	0,00000E+00	4,70376E-01	0,33	0,119	0,00000E+00
Macro_OLFML3	CTSH	0,00000E+00	4,69621E-01	0,967	0,681	0,00000E+00
Macro_OLFML3	CCND1	0,00000E+00	4,68769E-01	0,469	0,13	0,00000E+00
Macro_OLFML3	PAK1	0,00000E+00	4,66719E-01	0,746	0,402	0,00000E+00
Macro_OLFML3	P2RY6	0,00000E+00	4,58512E-01	0,45	0,165	0,00000E+00
Macro_OLFML3	REEP4	0,00000E+00	4,54216E-01	0,491	0,212	0,00000E+00
Macro_OLFML3	TLR1	0,00000E+00	4,51903E-01	0,426	0,179	0,00000E+00
Macro_OLFML3	ADORA3	0,00000E+00	4,49298E-01	0,551	0,23	0,00000E+00
Macro_OLFML3	TNFSF13B	0,00000E+00	4,46529E-01	0,903	0,542	0,00000E+00
Macro_OLFML3	DNASE2	0,00000E+00	4,45766E-01	0,685	0,331	0,00000E+00
Macro_OLFML3	FYB1	3,39807E-69	4,45671E-01	0,405	0,308	6,25822E-65
Macro_OLFML3	SLAMF7	0,00000E+00	4,36579E-01	0,492	0,178	0,00000E+00
Macro_OLFML3	TAP1	0,00000E+00	4,35024E-01	0,778	0,382	0,00000E+00
Macro_OLFML3	STOM	0,00000E+00	4,33981E-01	0,643	0,312	0,00000E+00

Macro_OLFML3	ATF5	0,00000E+00	4,33307E-01	0,673	0,325	0,00000E+00
Macro_OLFML3	RASSF4	0,00000E+00	4,31260E-01	0,823	0,471	0,00000E+00
Macro_OLFML3	MYOF	0,00000E+00	4,28877E-01	0,509	0,185	0,00000E+00
Macro_OLFML3	SCIMP	0,00000E+00	4,27423E-01	0,583	0,243	0,00000E+00
Macro_OLFML3	JAK2	0,00000E+00	4,22604E-01	0,491	0,211	0,00000E+00
Macro_OLFML3	GPX1	3,41775E-213	4,20254E-01	0,72	0,53	6,29448E-209
Macro_OLFML3	CD74	0,00000E+00	4,10504E-01	1	0,959	0,00000E+00
Macro_OLFML3	LPCAT2	0,00000E+00	4,06777E-01	0,621	0,274	0,00000E+00
Macro_OLFML3	HMOX1	0,00000E+00	4,05900E-01	0,823	0,513	0,00000E+00
Macro_OLFML3	TUBA1B	0,00000E+00	4,03172E-01	0,957	0,733	0,00000E+00
Macro_OLFML3	CYBA	0,00000E+00	4,02357E-01	0,99	0,888	0,00000E+00
Macro_OLFML3	HLA-C	0,00000E+00	4,01487E-01	0,996	0,945	0,00000E+00
Macro_OLFML3	MNDA	0,00000E+00	3,99651E-01	0,852	0,497	0,00000E+00
Macro_OLFML3	FMNL2	0,00000E+00	3,97529E-01	0,475	0,199	0,00000E+00
Macro_OLFML3	PTAFR	0,00000E+00	3,97276E-01	0,698	0,339	0,00000E+00
Macro_OLFML3	IFIT2	0,00000E+00	3,89731E-01	0,437	0,165	0,00000E+00
Macro_OLFML3	ETV5	0,00000E+00	3,88076E-01	0,432	0,172	0,00000E+00
Macro_OLFML3	VSIR	1,31749E-72	3,79939E-01	0,37	0,262	2,42642E-68
Macro_OLFML3	IL32	5,34286E-271	3,75594E-01	0,299	0,113	9,83994E-267
Macro_OLFML3	APOL6	0,00000E+00	3,71359E-01	0,524	0,23	0,00000E+00
Macro_OLFML3	PLEKHO1	0,00000E+00	3,70365E-01	0,646	0,328	0,00000E+00
Macro_OLFML3	SIGLEC10	0,00000E+00	3,69918E-01	0,624	0,286	0,00000E+00
Macro_OLFML3	NMI	0,00000E+00	3,64600E-01	0,584	0,267	0,00000E+00
Macro_OLFML3	RGS10	2,62909E-296	3,64159E-01	0,944	0,717	4,84199E-292
Macro_OLFML3	LPAR6	0,00000E+00	3,63367E-01	0,647	0,285	0,00000E+00
Macro_OLFML3	IFI27	1,15351E-169	3,62659E-01	0,323	0,154	2,12441E-165
Macro_OLFML3	GALM	0,00000E+00	3,56621E-01	0,47	0,165	0,00000E+00
Macro_OLFML3	GLRX	0,00000E+00	3,49810E-01	0,825	0,516	0,00000E+00
Macro_OLFML3	SERPINA1	0,00000E+00	3,47323E-01	0,965	0,694	0,00000E+00
Macro_OLFML3	IRF1	6,11923E-253	3,45914E-01	0,69	0,41	1,12698E-248
Macro_OLFML3	UBE2L6	0,00000E+00	3,43326E-01	0,899	0,533	0,00000E+00
Macro_OLFML3	ISOC2	9,67140E-306	3,41130E-01	0,436	0,193	1,78118E-301
Macro_OLFML3	AIM2	0,00000E+00	3,40590E-01	0,306	0,061	0,00000E+00
Macro_OLFML3	LPIN2	0,00000E+00	3,39728E-01	0,447	0,199	0,00000E+00
Macro_OLFML3	IL15RA	0,00000E+00	3,37323E-01	0,327	0,095	0,00000E+00
Macro_OLFML3	KCNMB1	0,00000E+00	3,35951E-01	0,368	0,145	0,00000E+00
Macro_OLFML3	SSPN	0,00000E+00	3,35506E-01	0,251	0,055	0,00000E+00
Macro_OLFML3	MEF2A	0,00000E+00	3,33977E-01	0,695	0,39	0,00000E+00
Macro_OLFML3	CTSZ	1,26060E-221	3,31077E-01	0,89	0,625	2,32164E-217
Macro_OLFML3	ADAP2	0,00000E+00	3,30907E-01	0,751	0,41	0,00000E+00
Macro_OLFML3	ASCL2	0,00000E+00	3,28333E-01	0,379	0,128	0,00000E+00
Macro_OLFML3	SMCO4	0,00000E+00	3,25903E-01	0,757	0,379	0,00000E+00
Macro_OLFML3	DRAM1	1,50796E-242	3,25720E-01	0,417	0,196	2,77721E-238
Macro_OLFML3	TIFA	0,00000E+00	3,25188E-01	0,288	0,087	0,00000E+00
Macro_OLFML3	SLC31A2	1,96080E-304	3,24629E-01	0,721	0,41	3,61120E-300

Macro_OLFML3	A2M	5,76875E-259	3,23713E-01	0,652	0,359	1,06243E-254
Macro_OLFML3	IFIT3	0,00000E+00	3,23682E-01	0,451	0,184	0,00000E+00
Macro_OLFML3	HLA-DQB2	0,00000E+00	3,23326E-01	0,575	0,288	0,00000E+00
Macro_OLFML3	TMEM109	0,00000E+00	3,21998E-01	0,678	0,334	0,00000E+00
Macro_OLFML3	LHFPL2	0,00000E+00	3,20937E-01	0,543	0,254	0,00000E+00
Macro_OLFML3	GIMAP8	2,73187E-209	3,19982E-01	0,3	0,127	5,03128E-205
Macro_OLFML3	SAMHD1	0,00000E+00	3,15132E-01	0,907	0,616	0,00000E+00
Macro_OLFML3	PRCP	0,00000E+00	3,14939E-01	0,657	0,359	0,00000E+00
Macro_OLFML3	EPB41L2	7,91004E-281	3,11438E-01	0,398	0,176	1,45679E-276
Macro_OLFML3	RCN1	0,00000E+00	3,11429E-01	0,378	0,153	0,00000E+00
Macro_OLFML3	VASH1	9,48772E-214	3,09362E-01	0,384	0,183	1,74735E-209
Macro_OLFML3	SNX6	0,00000E+00	3,07444E-01	0,79	0,485	0,00000E+00
Macro_OLFML3	PLXDC2	1,74677E-303	3,05974E-01	0,838	0,51	3,21703E-299
Macro_OLFML3	MT2A	2,54724E-277	3,01326E-01	0,885	0,599	4,69124E-273
Macro_OLFML3	RGS1	1,26793E-279	3,00579E-01	0,899	0,613	2,33514E-275
Macro_OLFML3	HLA-DMA	0,00000E+00	2,99406E-01	0,978	0,759	0,00000E+00
Macro_OLFML3	GNB4	1,01418E-294	2,99071E-01	0,589	0,307	1,86781E-290
Macro_OLFML3	SGK1	6,81312E-76	2,94459E-01	0,796	0,583	1,25477E-71
Macro_OLFML3	UBE2E2	0,00000E+00	2,93892E-01	0,556	0,27	0,00000E+00
Macro_OLFML3	CUL1	0,00000E+00	2,92883E-01	0,455	0,191	0,00000E+00
Macro_OLFML3	LIPA	2,58004E-265	2,92510E-01	0,782	0,469	4,75167E-261
Macro_OLFML3	GIMAP6	0,00000E+00	2,91064E-01	0,347	0,114	0,00000E+00
Macro_OLFML3	HLA-G	3,93712E-250	2,85790E-01	0,35	0,151	7,25099E-246
Macro_OLFML3	PARP14	4,87158E-302	2,84504E-01	0,649	0,346	8,97198E-298
Macro_OLFML3	HLA-DOA	0,00000E+00	2,84170E-01	0,728	0,355	0,00000E+00
Macro_OLFML3	LMNB1	3,08320E-107	2,79552E-01	0,271	0,142	5,67833E-103
Macro_OLFML3	GPR155	0,00000E+00	2,79380E-01	0,332	0,122	0,00000E+00
Macro_OLFML3	TRIM69	1,99999E-221	2,79184E-01	0,405	0,195	3,68338E-217
Macro_OLFML3	CBR1	0,00000E+00	2,78812E-01	0,616	0,282	0,00000E+00
Macro_OLFML3	CHCHD10	2,87844E-162	2,75655E-01	0,721	0,493	5,30123E-158
Macro_OLFML3	SCO2	2,66864E-76	2,75576E-01	0,332	0,205	4,91484E-72
Macro_OLFML3	AKR1A1	0,00000E+00	2,73301E-01	0,89	0,541	0,00000E+00
Macro_OLFML3	C2	0,00000E+00	2,71475E-01	0,358	0,14	0,00000E+00
Macro_OLFML3	TFEC	0,00000E+00	2,68594E-01	0,556	0,265	0,00000E+00
Macro_OLFML3	MRPL55	3,07163E-87	2,67639E-01	0,424	0,261	5,65701E-83
Macro_OLFML3	LIMK1	8,53668E-227	2,66501E-01	0,316	0,136	1,57220E-222
Macro_OLFML3	AOAH	0,00000E+00	2,59775E-01	0,693	0,338	0,00000E+00
Macro_OLFML3	NPC2	0,00000E+00	2,59620E-01	0,991	0,872	0,00000E+00
Macro_OLFML3	DOCK4	1,75388E-214	2,58428E-01	0,497	0,261	3,23011E-210
Macro_OLFML3	RASA4	4,96899E-209	2,57726E-01	0,296	0,128	9,15139E-205
Macro_OLFML3	STAC3	3,59714E-262	2,56260E-01	0,379	0,165	6,62485E-258
Macro_OLFML3	ITM2B	1,88552E-265	2,56145E-01	0,99	0,897	3,47256E-261
Macro_OLFML3	ALDH2	0,00000E+00	2,55416E-01	0,836	0,518	0,00000E+00
Macro_OLFML3	FPR3	1,39342E-201	2,55321E-01	0,567	0,313	2,56625E-197
Macro_OLFML3	LILRB1	2,69860E-300	2,51272E-01	0,653	0,345	4,97002E-296

Macro_OLFML3	ABCG1	4,74274E-28	-2,50111E-01	0,257	0,178	8,73470E-24
Macro_OLFML3	PSMD12	3,47117E-53	-2,50289E-01	0,305	0,197	6,39285E-49
Macro_OLFML3	PSMD11	9,73479E-69	-2,50418E-01	0,446	0,293	1,79286E-64
Macro_OLFML3	NUMA1	6,77542E-08	-2,51085E-01	0,28	0,228	1,24783E-03
Macro_OLFML3	TPR	4,74188E-55	-2,51228E-01	0,537	0,372	8,73313E-51
Macro_OLFML3	MRFAP1	1,36439E-61	-2,51476E-01	0,558	0,382	2,51279E-57
Macro_OLFML3	CSF3R	1,54835E-70	-2,51554E-01	0,57	0,373	2,85160E-66
Macro_OLFML3	ACTR1A	1,82266E-81	-2,51672E-01	0,432	0,272	3,35679E-77
Macro_OLFML3	FNBP4	3,80713E-26	-2,51720E-01	0,391	0,29	7,01159E-22
Macro_OLFML3	HNRNPH2	1,13057E-19	-2,52106E-01	0,285	0,211	2,08217E-15
Macro_OLFML3	RAB1B	2,01510E-43	-2,52109E-01	0,483	0,342	3,71120E-39
Macro_OLFML3	PPP1R12A	1,80972E-83	-2,52149E-01	0,546	0,358	3,33296E-79
Macro_OLFML3	YY1AP1	3,89699E-34	-2,52329E-01	0,261	0,177	7,17709E-30
Macro_OLFML3	MGST3	1,18353E-73	-2,53077E-01	0,743	0,519	2,17971E-69
Macro_OLFML3	BAG6	1,23528E-07	-2,53332E-01	0,327	0,267	2,27502E-03
Macro_OLFML3	IVD	2,23547E-126	-2,53407E-01	0,276	0,137	4,11707E-122
Macro_OLFML3	COPE	4,59134E-87	-2,53509E-01	0,799	0,585	8,45587E-83
Macro_OLFML3	GRHPR	1,63016E-54	-2,53650E-01	0,415	0,277	3,00226E-50
Macro_OLFML3	TRMT1	4,14632E-32	-2,54187E-01	0,371	0,263	7,63628E-28
Macro_OLFML3	PIAS1	3,91754E-112	-2,54432E-01	0,491	0,302	7,21493E-108
Macro_OLFML3	NDUFB1	2,68564E-58	-2,54582E-01	0,64	0,448	4,94615E-54
Macro_OLFML3	XRCC5	6,79853E-127	-2,54749E-01	0,693	0,45	1,25208E-122
Macro_OLFML3	TMED4	1,04604E-40	-2,54826E-01	0,405	0,28	1,92649E-36
Macro_OLFML3	FUNDC2	6,46545E-103	-2,55131E-01	0,538	0,339	1,19074E-98
Macro_OLFML3	CHMP2A	1,62543E-104	-2,55180E-01	0,693	0,469	2,99356E-100
Macro_OLFML3	ARID4B	5,38617E-41	-2,55519E-01	0,509	0,358	9,91971E-37
Macro_OLFML3	SLTM	6,13779E-38	-2,55914E-01	0,479	0,341	1,13040E-33
Macro_OLFML3	UBXN4	6,96798E-115	-2,56223E-01	0,739	0,496	1,28329E-110
Macro_OLFML3	SLC38A10	1,86357E-20	-2,56471E-01	0,297	0,22	3,43214E-16
Macro_OLFML3	TAF1D	4,17913E-33	-2,56626E-01	0,514	0,37	7,69670E-29
Macro_OLFML3	PLEKHO2	3,35538E-38	-2,56778E-01	0,296	0,198	6,17960E-34
Macro_OLFML3	SPCS2	2,70779E-55	-2,57188E-01	0,673	0,472	4,98694E-51
Macro_OLFML3	RBBP7	1,10008E-52	-2,57258E-01	0,301	0,194	2,02603E-48
Macro_OLFML3	ARPC1A	3,73286E-103	-2,57557E-01	0,425	0,254	6,87480E-99
Macro_OLFML3	APIP	1,87678E-48	-2,57705E-01	0,334	0,222	3,45646E-44
Macro_OLFML3	HSD17B4	4,80077E-77	-2,57866E-01	0,39	0,243	8,84158E-73
Macro_OLFML3	AP2B1	8,55940E-28	-2,58059E-01	0,255	0,178	1,57638E-23
Macro_OLFML3	ITGAL	5,56119E-71	-2,58082E-01	0,259	0,149	1,02420E-66
Macro_OLFML3	CLEC4A	1,76452E-77	-2,58196E-01	0,438	0,281	3,24972E-73
Macro_OLFML3	COX7C	8,30809E-39	-2,58327E-01	0,947	0,815	1,53010E-34
Macro_OLFML3	HSPA5	1,21775E-54	-2,58488E-01	0,74	0,541	2,24273E-50
Macro_OLFML3	SPCS3	2,70868E-50	-2,58597E-01	0,6	0,427	4,98857E-46
Macro_OLFML3	LSM6	1,05734E-64	-2,58779E-01	0,5	0,333	1,94731E-60
Macro_OLFML3	NDUFAF3	3,55477E-91	-2,58815E-01	0,666	0,449	6,54681E-87
Macro_OLFML3	C11orf58	1,80983E-52	-2,58838E-01	0,742	0,539	3,33315E-48

Macro_OLFML3	CAPG	1,50779E-97	-2,59289E-01	0,912	0,721	2,77689E-93
Macro_OLFML3	CDK4	3,37521E-93	-2,59984E-01	0,365	0,216	6,21612E-89
Macro_OLFML3	FKBP1A	1,86039E-37	-2,60007E-01	0,849	0,677	3,42627E-33
Macro_OLFML3	RDH11	1,25245E-80	-2,60184E-01	0,292	0,17	2,30663E-76
Macro_OLFML3	DDX27	5,48647E-25	-2,60224E-01	0,317	0,228	1,01044E-20
Macro_OLFML3	SECISBP2L	2,00134E-44	-2,60749E-01	0,277	0,182	3,68587E-40
Macro_OLFML3	THOC7	3,82961E-82	-2,60999E-01	0,494	0,321	7,05300E-78
Macro_OLFML3	STX10	5,12298E-44	-2,61047E-01	0,433	0,296	9,43500E-40
Macro_OLFML3	COPB1	3,73115E-35	-2,61454E-01	0,318	0,222	6,87167E-31
Macro_OLFML3	GLG1	3,01939E-29	-2,61701E-01	0,385	0,28	5,56081E-25
Macro_OLFML3	CLPTM1	6,19022E-34	-2,61720E-01	0,356	0,248	1,14005E-29
Macro_OLFML3	PICALM	1,67546E-09	-2,61745E-01	0,529	0,417	3,08570E-05
Macro_OLFML3	RMDN1	2,86901E-109	-2,61891E-01	0,27	0,141	5,28386E-105
Macro_OLFML3	CRTAP	1,73120E-58	-2,61987E-01	0,693	0,501	3,18834E-54
Macro_OLFML3	POLR1D	3,35837E-20	-2,61998E-01	0,6	0,453	6,18511E-16
Macro_OLFML3	MZT2B	1,41844E-40	-2,62480E-01	0,484	0,342	2,61235E-36
Macro_OLFML3	RBM8A	2,33647E-14	-2,62686E-01	0,674	0,518	4,30308E-10
Macro_OLFML3	DDX21	3,93281E-11	-2,62696E-01	0,547	0,424	7,24305E-07
Macro_OLFML3	PPP1CA	7,46904E-75	-2,62809E-01	0,819	0,599	1,37557E-70
Macro_OLFML3	STARD3NL	4,91372E-47	-2,63470E-01	0,379	0,258	9,04960E-43
Macro_OLFML3	CMTM7	6,05353E-39	-2,63508E-01	0,558	0,394	1,11488E-34
Macro_OLFML3	CNIH4	3,27320E-36	-2,63539E-01	0,395	0,274	6,02825E-32
Macro_OLFML3	TBC1D5	1,15299E-51	-2,63588E-01	0,293	0,189	2,12346E-47
Macro_OLFML3	MPDU1	2,75821E-109	-2,63772E-01	0,369	0,211	5,07979E-105
Macro_OLFML3	BCAP29	5,42922E-26	-2,63945E-01	0,28	0,202	9,99899E-22
Macro_OLFML3	AP2S1	2,81386E-29	-2,64051E-01	0,88	0,701	5,18228E-25
Macro_OLFML3	NASP	3,39805E-165	-2,64790E-01	0,464	0,254	6,25819E-161
Macro_OLFML3	TIMM17B	1,71921E-42	-2,64924E-01	0,447	0,31	3,16628E-38
Macro_OLFML3	ZFP36L2	1,62792E-20	-2,65182E-01	0,88	0,673	2,99814E-16
Macro_OLFML3	NSD1	5,77269E-59	-2,65295E-01	0,335	0,215	1,06316E-54
Macro_OLFML3	PGD	2,17923E-44	-2,65488E-01	0,625	0,447	4,01348E-40
Macro_OLFML3	EIF3H	2,30737E-35	-2,65536E-01	0,84	0,637	4,24948E-31
Macro_OLFML3	HIGD2A	2,04184E-31	-2,65765E-01	0,881	0,696	3,76046E-27
Macro_OLFML3	PPIE	1,88795E-98	-2,66153E-01	0,294	0,163	3,47703E-94
Macro_OLFML3	GANAB	7,23492E-38	-2,66374E-01	0,283	0,192	1,33245E-33
Macro_OLFML3	GPBP1L1	3,54247E-11	-2,66432E-01	0,258	0,203	6,52416E-07
Macro_OLFML3	COMMD1	3,78677E-104	-2,66496E-01	0,49	0,303	6,97410E-100
Macro_OLFML3	RNF167	6,23111E-33	-2,66509E-01	0,397	0,282	1,14758E-28
Macro_OLFML3	UBE2J2	3,78918E-20	-2,66743E-01	0,264	0,194	6,97852E-16
Macro_OLFML3	CCT3	5,13032E-162	-2,66832E-01	0,638	0,385	9,44850E-158
Macro_OLFML3	TANK	8,85029E-31	-2,66921E-01	0,391	0,282	1,62996E-26
Macro_OLFML3	UPF2	1,60108E-23	-2,67404E-01	0,295	0,214	2,94870E-19
Macro_OLFML3	HMGB1	1,37559E-11	-2,67574E-01	0,928	0,778	2,53343E-07
Macro_OLFML3	COX20	1,86408E-33	-2,67576E-01	0,351	0,248	3,43307E-29
Macro_OLFML3	PTRHD1	3,56856E-26	-2,67710E-01	0,461	0,336	6,57222E-22

Macro_OLFML3	CMPK1	1,64271E-06	-2,67814E-01	0,333	0,271	3,02538E-02
Macro_OLFML3	LMF2	6,61005E-46	-2,67866E-01	0,296	0,195	1,21737E-41
Macro_OLFML3	MYO1F	5,87454E-26	-2,67913E-01	0,607	0,443	1,08191E-21
Macro_OLFML3	OAZ2	4,21630E-88	-2,68444E-01	0,426	0,267	7,76515E-84
Macro_OLFML3	SUMO2	2,91464E-15	-2,68802E-01	0,927	0,767	5,36788E-11
Macro_OLFML3	MTF2	1,65132E-66	-2,68898E-01	0,278	0,167	3,04124E-62
Macro_OLFML3	SCAMP3	1,86313E-114	-2,69060E-01	0,402	0,234	3,43133E-110
Macro_OLFML3	MSL3	6,34076E-37	-2,69073E-01	0,294	0,199	1,16778E-32
Macro_OLFML3	HMGN3	5,03161E-149	-2,69751E-01	0,763	0,496	9,26672E-145
Macro_OLFML3	CIAO1	5,34879E-56	-2,69806E-01	0,355	0,234	9,85086E-52
Macro_OLFML3	LTBR	3,31968E-59	-2,69910E-01	0,493	0,333	6,11386E-55
Macro_OLFML3	DAPK1	4,12829E-43	-2,69920E-01	0,454	0,322	7,60307E-39
Macro_OLFML3	CHURC1	5,54069E-40	-2,70047E-01	0,435	0,304	1,02043E-35
Macro_OLFML3	VKORC1	3,06001E-43	-2,70107E-01	0,341	0,233	5,63562E-39
Macro_OLFML3	SYS1	2,85204E-54	-2,70623E-01	0,257	0,159	5,25261E-50
Macro_OLFML3	FIS1	6,18566E-84	-2,70672E-01	0,726	0,503	1,13921E-79
Macro_OLFML3	SAMM50	2,52697E-89	-2,70743E-01	0,263	0,145	4,65392E-85
Macro_OLFML3	INTS10	1,12322E-124	-2,70762E-01	0,398	0,225	2,06863E-120
Macro_OLFML3	TRAPPC4	2,23683E-88	-2,70841E-01	0,331	0,194	4,11956E-84
Macro_OLFML3	CFLAR	2,26662E-12	-2,70901E-01	0,64	0,497	4,17444E-08
Macro_OLFML3	NDUFB6	1,45728E-56	-2,71286E-01	0,516	0,351	2,68387E-52
Macro_OLFML3	SEC23B	1,26868E-49	-2,71331E-01	0,26	0,166	2,33653E-45
Macro_OLFML3	CTSS	9,26718E-165	-2,71391E-01	0,975	0,798	1,70674E-160
Macro_OLFML3	FNDC3B	2,32343E-14	-2,71721E-01	0,352	0,271	4,27906E-10
Macro_OLFML3	DIAPH1	5,48417E-11	-2,71980E-01	0,302	0,236	1,01002E-06
Macro_OLFML3	DHX15	3,94115E-09	-2,72254E-01	0,268	0,216	7,25841E-05
Macro_OLFML3	NCF1	3,23979E-120	-2,72573E-01	0,695	0,478	5,96672E-116
Macro_OLFML3	NKIRAS2	1,64514E-24	-2,72775E-01	0,275	0,197	3,02985E-20
Macro_OLFML3	SUPT16H	2,24525E-74	-2,73221E-01	0,313	0,19	4,13508E-70
Macro_OLFML3	ENG	4,56507E-40	-2,73868E-01	0,485	0,332	8,40748E-36
Macro_OLFML3	COX4I1	3,12634E-14	-2,73927E-01	0,975	0,888	5,75778E-10
Macro_OLFML3	ARPC5	4,28778E-18	-2,73950E-01	0,912	0,745	7,89680E-14
Macro_OLFML3	CHMP2B	4,36404E-25	-2,73957E-01	0,391	0,288	8,03724E-21
Macro_OLFML3	TOMM7	1,43972E-09	-2,73988E-01	0,917	0,776	2,65153E-05
Macro_OLFML3	ENOPH1	8,54917E-86	-2,74183E-01	0,271	0,152	1,57450E-81
Macro_OLFML3	RPS4Y1	2,74169E-41	-2,74397E-01	0,251	0,167	5,04937E-37
Macro_OLFML3	TPI1	1,24780E-60	-2,74794E-01	0,95	0,812	2,29807E-56
Macro_OLFML3	PRKCB	1,97607E-38	-2,74878E-01	0,321	0,216	3,63932E-34
Macro_OLFML3	FIP1L1	6,02650E-45	-2,74960E-01	0,289	0,19	1,10990E-40
Macro_OLFML3	MLF2	3,14615E-42	-2,75382E-01	0,609	0,434	5,79426E-38
Macro_OLFML3	ST13	6,69742E-20	-2,75577E-01	0,77	0,586	1,23346E-15
Macro_OLFML3	BLNK	2,15532E-301	-2,75825E-01	0,369	0,148	3,96946E-297
Macro_OLFML3	BRI3	1,45078E-09	-2,75968E-01	0,478	0,394	2,67191E-05
Macro_OLFML3	JMJD6	1,19467E-07	-2,76180E-01	0,255	0,206	2,20023E-03
Macro_OLFML3	MED10	8,48065E-30	-2,76975E-01	0,331	0,237	1,56188E-25

Macro_OLFML3	TECR	6,73756E-85	-2,77062E-01	0,553	0,359	1,24086E-80
Macro_OLFML3	MAD2L2	3,32722E-20	-2,77086E-01	0,291	0,216	6,12774E-16
Macro_OLFML3	TM2D2	8,14999E-127	-2,77218E-01	0,344	0,185	1,50098E-122
Macro_OLFML3	NUDT21	1,63859E-51	-2,77883E-01	0,379	0,254	3,01779E-47
Macro_OLFML3	GORASP2	2,09860E-89	-2,78179E-01	0,34	0,2	3,86499E-85
Macro_OLFML3	UNC119	5,47576E-24	-2,78343E-01	0,354	0,258	1,00847E-19
Macro_OLFML3	YBX1	1,48984E-24	-2,78362E-01	0,942	0,853	2,74384E-20
Macro_OLFML3	SSR3	7,64043E-82	-2,78957E-01	0,803	0,573	1,40714E-77
Macro_OLFML3	NADK	2,48315E-55	-2,79490E-01	0,344	0,223	4,57322E-51
Macro_OLFML3	PHKB	1,62771E-76	-2,79956E-01	0,314	0,188	2,99776E-72
Macro_OLFML3	SMARCB1	6,90767E-48	-2,80456E-01	0,414	0,281	1,27219E-43
Macro_OLFML3	MKNK1	2,87800E-46	-2,80585E-01	0,304	0,199	5,30040E-42
Macro_OLFML3	PAFAH1B1	2,07022E-13	-2,80974E-01	0,425	0,332	3,81273E-09
Macro_OLFML3	RNASEH2C	1,52962E-61	-2,81215E-01	0,494	0,329	2,81711E-57
Macro_OLFML3	TM2D1	1,59556E-33	-2,81223E-01	0,278	0,191	2,93855E-29
Macro_OLFML3	APEH	1,62608E-96	-2,81852E-01	0,325	0,184	2,99475E-92
Macro_OLFML3	NMRAL1	1,63964E-45	-2,81913E-01	0,25	0,159	3,01973E-41
Macro_OLFML3	NCBP2	8,79855E-60	-2,82624E-01	0,308	0,193	1,62043E-55
Macro_OLFML3	CD46	4,30125E-105	-2,83039E-01	0,483	0,298	7,92161E-101
Macro_OLFML3	FNDC3A	1,93021E-26	-2,83082E-01	0,321	0,231	3,55486E-22
Macro_OLFML3	SRSF10	1,06613E-12	-2,83104E-01	0,348	0,271	1,96349E-08
Macro_OLFML3	SHOC2	1,44251E-07	-2,83134E-01	0,318	0,258	2,65666E-03
Macro_OLFML3	STX8	2,52793E-87	-2,83285E-01	0,37	0,223	4,65569E-83
Macro_OLFML3	MATR3	3,95797E-87	-2,83487E-01	0,354	0,213	7,28939E-83
Macro_OLFML3	ZNF511	3,02281E-21	-2,83620E-01	0,29	0,214	5,56710E-17
Macro_OLFML3	NCL	7,97522E-40	-2,84031E-01	0,804	0,605	1,46880E-35
Macro_OLFML3	ATG4B	3,31134E-21	-2,84518E-01	0,253	0,182	6,09849E-17
Macro_OLFML3	GNB1	1,27552E-11	-2,84588E-01	0,48	0,378	2,34913E-07
Macro_OLFML3	ARPC3	4,60897E-69	-2,85009E-01	0,978	0,882	8,48834E-65
Macro_OLFML3	DCTN2	1,65949E-118	-2,85057E-01	0,507	0,309	3,05629E-114
Macro_OLFML3	ITGB1BP1	6,78643E-26	-2,85148E-01	0,332	0,241	1,24986E-21
Macro_OLFML3	APH1B	3,64376E-36	-2,85537E-01	0,261	0,174	6,71072E-32
Macro_OLFML3	MAGOH	1,26303E-70	-2,85873E-01	0,504	0,331	2,32612E-66
Macro_OLFML3	NSRP1	5,21304E-87	-2,86105E-01	0,338	0,199	9,60085E-83
Macro_OLFML3	ARMC8	2,42856E-40	-2,86267E-01	0,252	0,165	4,47268E-36
Macro_OLFML3	VCP	2,22598E-21	-2,86347E-01	0,598	0,451	4,09958E-17
Macro_OLFML3	BIRC6	3,33043E-10	-2,87084E-01	0,302	0,243	6,13365E-06
Macro_OLFML3	IKZF1	3,98282E-19	-2,87449E-01	0,364	0,274	7,33516E-15
Macro_OLFML3	ELF2	2,55956E-11	-2,87699E-01	0,306	0,241	4,71395E-07
Macro_OLFML3	ZNHIT1	1,38574E-38	-2,87762E-01	0,633	0,461	2,55212E-34
Macro_OLFML3	TACC1	3,93088E-36	-2,87876E-01	0,561	0,405	7,23951E-32
Macro_OLFML3	POP4	1,15155E-96	-2,88210E-01	0,337	0,195	2,12080E-92
Macro_OLFML3	TRABD	2,02969E-16	-2,88218E-01	0,457	0,346	3,73809E-12
Macro_OLFML3	NRBP1	1,53689E-29	-2,88354E-01	0,408	0,296	2,83049E-25
Macro_OLFML3	FAM50A	1,04549E-39	-2,88600E-01	0,413	0,292	1,92549E-35

Macro_OLFML3	TMEM256	3,15574E-26	-2,88722E-01	0,539	0,397	5,81192E-22
Macro_OLFML3	TNRC6B	3,09217E-20	-2,88943E-01	0,353	0,263	5,69485E-16
Macro_OLFML3	EIF2AK4	4,07933E-16	-2,88946E-01	0,306	0,234	7,51290E-12
Macro_OLFML3	NIPBL	1,85320E-22	-2,89086E-01	0,457	0,339	3,41304E-18
Macro_OLFML3	MBP	8,34571E-19	-2,89418E-01	0,41	0,308	1,53703E-14
Macro_OLFML3	P2RX4	2,44218E-86	-2,89602E-01	0,366	0,218	4,49776E-82
Macro_OLFML3	MBNL1	9,00287E-54	-2,90157E-01	0,689	0,506	1,65806E-49
Macro_OLFML3	MS4A7	4,58248E-61	-2,90249E-01	0,891	0,637	8,43956E-57
Macro_OLFML3	PEF1	1,25129E-70	-2,90705E-01	0,371	0,233	2,30449E-66
Macro_OLFML3	ACSL3	4,82890E-10	-2,90946E-01	0,312	0,247	8,89338E-06
Macro_OLFML3	SERPINB1	1,55290E-31	-2,91129E-01	0,837	0,643	2,85997E-27
Macro_OLFML3	POLR2J	3,15255E-49	-2,91595E-01	0,427	0,292	5,80605E-45
Macro_OLFML3	ETV6	5,16116E-08	-2,91760E-01	0,349	0,284	9,50531E-04
Macro_OLFML3	SLC15A3	6,19915E-36	-2,91891E-01	0,389	0,271	1,14170E-31
Macro_OLFML3	LAMTOR3	7,05765E-33	-2,91907E-01	0,349	0,248	1,29981E-28
Macro_OLFML3	RAB10	1,08547E-56	-2,92058E-01	0,663	0,47	1,99911E-52
Macro_OLFML3	GLUL	1,60089E-07	-2,92076E-01	0,952	0,787	2,94835E-03
Macro_OLFML3	SLBP	1,44667E-15	-2,92172E-01	0,297	0,226	2,66433E-11
Macro_OLFML3	C19orf53	7,61727E-51	-2,92207E-01	0,768	0,564	1,40287E-46
Macro_OLFML3	PGK1	1,95652E-71	-2,92537E-01	0,886	0,686	3,60333E-67
Macro_OLFML3	LRPAP1	1,50002E-58	-2,92567E-01	0,682	0,474	2,76259E-54
Macro_OLFML3	PLD3	1,18328E-78	-2,92583E-01	0,722	0,487	2,17925E-74
Macro_OLFML3	MITD1	5,11262E-80	-2,92633E-01	0,263	0,149	9,41592E-76
Macro_OLFML3	CPQ	4,39799E-21	-2,92803E-01	0,292	0,214	8,09977E-17
Macro_OLFML3	CYB5R4	4,28617E-32	-2,92869E-01	0,353	0,251	7,89384E-28
Macro_OLFML3	GGA2	2,99355E-138	-2,93530E-01	0,44	0,251	5,51321E-134
Macro_OLFML3	POLE3	2,35490E-45	-2,93530E-01	0,316	0,209	4,33702E-41
Macro_OLFML3	TBL1XR1	4,93860E-19	-2,93928E-01	0,258	0,189	9,09541E-15
Macro_OLFML3	NR1H2	4,55869E-14	-2,94033E-01	0,413	0,321	8,39574E-10
Macro_OLFML3	UBE2D2	1,88327E-06	-2,94183E-01	0,704	0,549	3,46842E-02
Macro_OLFML3	MIEN1	3,84393E-46	-2,94295E-01	0,544	0,38	7,07937E-42
Macro_OLFML3	SLU7	3,13631E-63	-2,94730E-01	0,356	0,228	5,77615E-59
Macro_OLFML3	MAPRE1	5,65435E-47	-2,94911E-01	0,53	0,373	1,04136E-42
Macro_OLFML3	ENO1	3,71398E-78	-2,95402E-01	0,917	0,743	6,84004E-74
Macro_OLFML3	GHITM	2,10429E-135	-2,95439E-01	0,72	0,48	3,87547E-131
Macro_OLFML3	TDG	1,03735E-09	-2,96310E-01	0,267	0,213	1,91048E-05
Macro_OLFML3	HBP1	1,12049E-64	-2,96561E-01	0,357	0,226	2,06360E-60
Macro_OLFML3	AP3S1	3,98960E-09	-2,96980E-01	0,426	0,337	7,34765E-05
Macro_OLFML3	SUMF2	3,11717E-72	-2,97226E-01	0,432	0,279	5,74089E-68
Macro_OLFML3	EIF3I	5,59386E-86	-2,97844E-01	0,652	0,444	1,03022E-81
Macro_OLFML3	CDV3	2,61375E-13	-2,98212E-01	0,342	0,267	4,81374E-09
Macro_OLFML3	LSM4	3,29075E-98	-2,98612E-01	0,624	0,41	6,06057E-94
Macro_OLFML3	PDCD6	1,67737E-68	-2,99021E-01	0,579	0,408	3,08921E-64
Macro_OLFML3	DDX18	2,81158E-52	-2,99260E-01	0,513	0,354	5,17809E-48
Macro_OLFML3	TUBGCP2	1,50943E-10	-2,99276E-01	0,374	0,295	2,77992E-06

Macro_OLFML3	RAB1A	3,50730E-55	-2,99671E-01	0,547	0,379	6,45940E-51
Macro_OLFML3	SNRNP40	2,46400E-67	-2,99975E-01	0,307	0,189	4,53794E-63
Macro_OLFML3	DCK	6,53536E-105	-3,00183E-01	0,436	0,262	1,20362E-100
Macro_OLFML3	VPS41	1,42260E-41	-3,00696E-01	0,25	0,163	2,62001E-37
Macro_OLFML3	SSNA1	6,89384E-62	-3,00702E-01	0,563	0,386	1,26964E-57
Macro_OLFML3	USP15	1,21646E-55	-3,00703E-01	0,584	0,406	2,24035E-51
Macro_OLFML3	WDR1	1,14233E-29	-3,00736E-01	0,706	0,522	2,10383E-25
Macro_OLFML3	MPP1	3,15286E-92	-3,00774E-01	0,609	0,39	5,80662E-88
Macro_OLFML3	SAT1	2,90471E-17	-3,01383E-01	0,997	0,942	5,34961E-13
Macro_OLFML3	LRP1	9,77899E-20	-3,01823E-01	0,557	0,407	1,80100E-15
Macro_OLFML3	LMO4	1,69215E-36	-3,01874E-01	0,333	0,226	3,11644E-32
Macro_OLFML3	DHX9	3,25700E-27	-3,03010E-01	0,281	0,2	5,99842E-23
Macro_OLFML3	TRAPPC2L	2,34962E-92	-3,03368E-01	0,415	0,253	4,32729E-88
Macro_OLFML3	USP16	9,82528E-35	-3,03472E-01	0,373	0,263	1,80952E-30
Macro_OLFML3	ARHGAP17	8,44604E-45	-3,03581E-01	0,273	0,18	1,55551E-40
Macro_OLFML3	PNN	7,17501E-27	-3,03739E-01	0,414	0,304	1,32142E-22
Macro_OLFML3	G3BP2	2,87947E-15	-3,03888E-01	0,349	0,269	5,30312E-11
Macro_OLFML3	TRIP12	3,68048E-20	-3,04013E-01	0,354	0,267	6,77834E-16
Macro_OLFML3	RALA	2,20857E-09	-3,04114E-01	0,46	0,365	4,06752E-05
Macro_OLFML3	SKIL	2,35136E-33	-3,05040E-01	0,445	0,318	4,33050E-29
Macro_OLFML3	CCAR1	1,09754E-68	-3,05074E-01	0,394	0,253	2,02134E-64
Macro_OLFML3	ROMO1	2,38180E-22	-3,05182E-01	0,669	0,514	4,38656E-18
Macro_OLFML3	GRSF1	4,80301E-62	-3,05484E-01	0,487	0,323	8,84571E-58
Macro_OLFML3	PEBP1	4,14302E-84	-3,05735E-01	0,778	0,54	7,63020E-80
Macro_OLFML3	CHMP1A	1,30108E-32	-3,05770E-01	0,262	0,18	2,39620E-28
Macro_OLFML3	PHF3	2,65750E-10	-3,05781E-01	0,364	0,292	4,89432E-06
Macro_OLFML3	DNAJC1	1,42501E-24	-3,06007E-01	0,381	0,279	2,62445E-20
Macro_OLFML3	HNRNPR	4,18666E-86	-3,06048E-01	0,526	0,342	7,71057E-82
Macro_OLFML3	C9orf78	5,98197E-83	-3,06138E-01	0,495	0,318	1,10170E-78
Macro_OLFML3	TUBA1C	7,09096E-08	-3,06159E-01	0,469	0,369	1,30594E-03
Macro_OLFML3	PSMD2	4,99182E-92	-3,06426E-01	0,476	0,302	9,19343E-88
Macro_OLFML3	FBL	1,49713E-50	-3,06464E-01	0,485	0,332	2,75727E-46
Macro_OLFML3	SF3B5	1,48092E-51	-3,06540E-01	0,726	0,532	2,72740E-47
Macro_OLFML3	DNAJC7	2,38796E-63	-3,06683E-01	0,518	0,348	4,39792E-59
Macro_OLFML3	GPS1	3,18334E-63	-3,06906E-01	0,39	0,251	5,86275E-59
Macro_OLFML3	PPIB	8,88207E-138	-3,06941E-01	0,956	0,774	1,63581E-133
Macro_OLFML3	BSG	4,05982E-59	-3,07223E-01	0,759	0,541	7,47697E-55
Macro_OLFML3	PDCD10	1,48454E-56	-3,07564E-01	0,442	0,3	2,73408E-52
Macro_OLFML3	IL16	3,95049E-98	-3,07659E-01	0,356	0,206	7,27562E-94
Macro_OLFML3	ACP1	6,77575E-69	-3,07678E-01	0,478	0,318	1,24789E-64
Macro_OLFML3	EMC4	3,49609E-87	-3,07705E-01	0,528	0,342	6,43876E-83
Macro_OLFML3	SNRNP25	1,13900E-41	-3,07851E-01	0,263	0,173	2,09770E-37
Macro_OLFML3	UBA1	2,19628E-10	-3,07920E-01	0,372	0,295	4,04488E-06
Macro_OLFML3	BCAP31	9,53945E-68	-3,08140E-01	0,78	0,561	1,75688E-63
Macro_OLFML3	UFM1	4,38618E-15	-3,08437E-01	0,409	0,317	8,07804E-11

Macro_OLFML3	DHX36	2,59238E-30	-3,08950E-01	0,385	0,274	4,77439E-26
Macro_OLFML3	SRP14	2,82016E-119	-3,09032E-01	0,96	0,822	5,19389E-115
Macro_OLFML3	NDUFC2	4,74851E-88	-3,09164E-01	0,448	0,289	8,74534E-84
Macro_OLFML3	IDH3B	2,83642E-82	-3,09240E-01	0,397	0,246	5,22384E-78
Macro_OLFML3	DRAM2	5,45662E-147	-3,09694E-01	0,605	0,366	1,00494E-142
Macro_OLFML3	ARCN1	1,44155E-35	-3,10136E-01	0,299	0,206	2,65491E-31
Macro_OLFML3	SNX5	5,68915E-97	-3,10139E-01	0,585	0,377	1,04777E-92
Macro_OLFML3	FAM162A	7,20681E-41	-3,10164E-01	0,474	0,331	1,32728E-36
Macro_OLFML3	DUSP1	1,03804E-105	-3,10232E-01	0,913	0,843	1,91176E-101
Macro_OLFML3	SYPL1	1,78937E-64	-3,10267E-01	0,442	0,287	3,29549E-60
Macro_OLFML3	PDK4	3,45297E-09	-3,10817E-01	0,367	0,304	6,35934E-05
Macro_OLFML3	CSNK1D	5,30722E-07	-3,11691E-01	0,415	0,335	9,77431E-03
Macro_OLFML3	NKTR	2,92478E-19	-3,11827E-01	0,365	0,275	5,38657E-15
Macro_OLFML3	MPC1	2,63506E-66	-3,11945E-01	0,457	0,301	4,85299E-62
Macro_OLFML3	RFXANK	8,62493E-87	-3,12202E-01	0,318	0,185	1,58845E-82
Macro_OLFML3	PSMG2	1,70950E-117	-3,12450E-01	0,628	0,401	3,14838E-113
Macro_OLFML3	CD93	8,43388E-17	-3,12561E-01	0,363	0,269	1,55327E-12
Macro_OLFML3	PLCB2	3,16676E-24	-3,12708E-01	0,322	0,233	5,83222E-20
Macro_OLFML3	LYPLA2	1,47080E-57	-3,13048E-01	0,331	0,212	2,70877E-53
Macro_OLFML3	NEU1	4,18944E-36	-3,13239E-01	0,418	0,291	7,71570E-32
Macro_OLFML3	MOB1A	8,35760E-59	-3,13814E-01	0,761	0,563	1,53922E-54
Macro_OLFML3	STIP1	9,14087E-42	-3,14057E-01	0,392	0,269	1,68347E-37
Macro_OLFML3	RSL24D1	2,87770E-18	-3,14196E-01	0,559	0,419	5,29987E-14
Macro_OLFML3	ANAPC5	1,20139E-69	-3,14212E-01	0,423	0,272	2,21260E-65
Macro_OLFML3	SRSF2	7,06244E-27	-3,14229E-01	0,719	0,539	1,30069E-22
Macro_OLFML3	TCP1	1,91176E-47	-3,14808E-01	0,467	0,322	3,52089E-43
Macro_OLFML3	DDX24	5,20615E-41	-3,15054E-01	0,632	0,459	9,58817E-37
Macro_OLFML3	BIN2	1,53511E-129	-3,15305E-01	0,501	0,292	2,82721E-125
Macro_OLFML3	ANAPC15	1,55171E-13	-3,15437E-01	0,258	0,197	2,85779E-09
Macro_OLFML3	LRRC25	3,38810E-93	-3,16479E-01	0,508	0,313	6,23987E-89
Macro_OLFML3	NDUFA11	4,71154E-22	-3,16896E-01	0,57	0,441	8,67724E-18
Macro_OLFML3	CCT2	1,13972E-106	-3,17010E-01	0,516	0,32	2,09903E-102
Macro_OLFML3	UBE2F	2,97950E-45	-3,17354E-01	0,362	0,248	5,48735E-41
Macro_OLFML3	DCTN4	1,22353E-38	-3,17523E-01	0,317	0,216	2,25337E-34
Macro_OLFML3	AIP	3,07770E-62	-3,17683E-01	0,486	0,323	5,66821E-58
Macro_OLFML3	CYCS	8,66623E-91	-3,18242E-01	0,715	0,502	1,59606E-86
Macro_OLFML3	COX6A1	9,73885E-42	-3,18463E-01	0,848	0,661	1,79360E-37
Macro_OLFML3	TATDN3	3,84778E-81	-3,18621E-01	0,266	0,148	7,08646E-77
Macro_OLFML3	NDUFS5	8,27405E-13	-3,18730E-01	0,804	0,637	1,52383E-08
Macro_OLFML3	MCTP1	2,54694E-60	-3,18925E-01	0,363	0,235	4,69070E-56
Macro_OLFML3	AUP1	2,04352E-39	-3,18971E-01	0,692	0,506	3,76355E-35
Macro_OLFML3	ABCE1	4,80235E-69	-3,18985E-01	0,262	0,154	8,84448E-65
Macro_OLFML3	PSMB7	1,57211E-129	-3,19230E-01	0,551	0,342	2,89536E-125
Macro_OLFML3	IGBP1	1,48430E-38	-3,19388E-01	0,465	0,33	2,73364E-34
Macro_OLFML3	TMX2	5,53405E-81	-3,19625E-01	0,288	0,167	1,01921E-76

Macro_OLFML3	SMDT1	7,98794E-37	-3,19894E-01	0,647	0,464	1,47114E-32
Macro_OLFML3	DYNLL1	3,21044E-21	-3,20104E-01	0,867	0,69	5,91267E-17
Macro_OLFML3	NDUFA10	8,92837E-95	-3,20445E-01	0,49	0,309	1,64434E-90
Macro_OLFML3	NHP2	6,02743E-81	-3,20844E-01	0,591	0,392	1,11007E-76
Macro_OLFML3	CAPZA1	1,94310E-42	-3,21002E-01	0,656	0,476	3,57860E-38
Macro_OLFML3	MFSD10	1,55743E-17	-3,21188E-01	0,347	0,261	2,86832E-13
Macro_OLFML3	RNMT	3,92388E-09	-3,21459E-01	0,302	0,242	7,22661E-05
Macro_OLFML3	SDCCAG8	5,00622E-88	-3,21709E-01	0,497	0,319	9,21996E-84
Macro_OLFML3	CNOT2	2,37349E-62	-3,22524E-01	0,394	0,257	4,37126E-58
Macro_OLFML3	EIF3M	8,38638E-51	-3,22940E-01	0,675	0,479	1,54452E-46
Macro_OLFML3	RABAC1	6,39693E-34	-3,22986E-01	0,688	0,506	1,17812E-29
Macro_OLFML3	SF3A1	5,54844E-07	-3,23980E-01	0,344	0,283	1,02186E-02
Macro_OLFML3	UXT	3,82491E-22	-3,23995E-01	0,694	0,524	7,04434E-18
Macro_OLFML3	TUFM	6,56801E-83	-3,24014E-01	0,708	0,487	1,20963E-78
Macro_OLFML3	ANXA5	9,56471E-15	-3,24152E-01	0,934	0,755	1,76153E-10
Macro_OLFML3	CD4	8,79070E-164	-3,24822E-01	0,785	0,51	1,61898E-159
Macro_OLFML3	EIF4A1	2,76127E-20	-3,25356E-01	0,774	0,663	5,08544E-16
Macro_OLFML3	ZFP36L1	1,27170E-30	-3,25699E-01	0,897	0,706	2,34208E-26
Macro_OLFML3	FBXL5	4,93478E-49	-3,26422E-01	0,39	0,261	9,08839E-45
Macro_OLFML3	ARHGAP4	1,76104E-34	-3,26656E-01	0,516	0,37	3,24330E-30
Macro_OLFML3	TOMM5	1,61735E-10	-3,26933E-01	0,395	0,319	2,97867E-06
Macro_OLFML3	SH3BGRL	1,30663E-34	-3,26935E-01	0,888	0,701	2,40642E-30
Macro_OLFML3	RAN	3,58983E-77	-3,27063E-01	0,86	0,634	6,61138E-73
Macro_OLFML3	GABARAP	1,57450E-19	-3,27113E-01	0,879	0,824	2,89975E-15
Macro_OLFML3	FXR1	2,57392E-16	-3,27199E-01	0,364	0,279	4,74039E-12
Macro_OLFML3	ELOF1	6,35938E-16	-3,27296E-01	0,275	0,21	1,17121E-11
Macro_OLFML3	DAP	7,02238E-23	-3,27356E-01	0,302	0,221	1,29331E-18
Macro_OLFML3	SNRPG	2,98077E-42	-3,27603E-01	0,731	0,537	5,48969E-38
Macro_OLFML3	ACAP2	1,39898E-17	-3,27625E-01	0,541	0,418	2,57649E-13
Macro_OLFML3	PHF20	2,02011E-08	-3,27858E-01	0,333	0,268	3,72044E-04
Macro_OLFML3	CDK2AP2	2,53175E-55	-3,27887E-01	0,554	0,38	4,66273E-51
Macro_OLFML3	EMC7	4,39898E-114	-3,27910E-01	0,493	0,301	8,10160E-110
Macro_OLFML3	NUMB	1,30586E-16	-3,28114E-01	0,422	0,323	2,40501E-12
Macro_OLFML3	ITSN2	1,39980E-43	-3,28875E-01	0,517	0,372	2,57801E-39
Macro_OLFML3	EVI2A	3,18841E-53	-3,29300E-01	0,391	0,26	5,87210E-49
Macro_OLFML3	AMZ2	1,47315E-26	-3,29496E-01	0,304	0,218	2,71310E-22
Macro_OLFML3	KDM5A	2,50263E-22	-3,29534E-01	0,404	0,301	4,60909E-18
Macro_OLFML3	RNF13	1,67153E-32	-3,29788E-01	0,685	0,498	3,07846E-28
Macro_OLFML3	MACF1	3,86139E-13	-3,29830E-01	0,396	0,312	7,11153E-09
Macro_OLFML3	CORO1A	8,15277E-65	-3,30075E-01	0,838	0,624	1,50149E-60
Macro_OLFML3	TMEM230	3,75099E-79	-3,30762E-01	0,636	0,43	6,90820E-75
Macro_OLFML3	RABGGTB	5,96326E-36	-3,30843E-01	0,35	0,246	1,09825E-31
Macro_OLFML3	FCGRT	6,91022E-71	-3,31240E-01	0,936	0,716	1,27265E-66
Macro_OLFML3	HMGB2	2,73983E-13	-3,31973E-01	0,503	0,387	5,04595E-09
Macro_OLFML3	TPD52L2	2,23541E-14	-3,32276E-01	0,34	0,261	4,11696E-10

Macro_OLFML3	SSR4	6,15439E-75	-3,32639E-01	0,919	0,734	1,13345E-70
Macro_OLFML3	FUS	2,93770E-19	-3,33443E-01	0,747	0,569	5,41036E-15
Macro_OLFML3	PCM1	3,14666E-24	-3,33453E-01	0,41	0,305	5,79521E-20
Macro_OLFML3	DMXL2	2,09730E-07	-3,33464E-01	0,325	0,261	3,86259E-03
Macro_OLFML3	DGUOK	2,15040E-55	-3,33532E-01	0,517	0,354	3,96039E-51
Macro_OLFML3	C19orf38	1,23227E-64	-3,33560E-01	0,358	0,22	2,26947E-60
Macro_OLFML3	C4orf48	8,08700E-08	-3,33756E-01	0,656	0,513	1,48938E-03
Macro_OLFML3	NME2	1,71479E-22	-3,33805E-01	0,304	0,366	3,15812E-18
Macro_OLFML3	ANAPC13	2,73180E-30	-3,33854E-01	0,315	0,22	5,03116E-26
Macro_OLFML3	SLC35C2	9,77125E-47	-3,34341E-01	0,259	0,165	1,79957E-42
Macro_OLFML3	TMBIM6	3,26782E-100	-3,34744E-01	0,942	0,759	6,01835E-96
Macro_OLFML3	PARVG	9,99655E-95	-3,35147E-01	0,648	0,427	1,84106E-90
Macro_OLFML3	ATP6V0B	1,62123E-48	-3,35157E-01	0,939	0,774	2,98583E-44
Macro_OLFML3	DCP2	4,48074E-40	-3,35171E-01	0,367	0,254	8,25218E-36
Macro_OLFML3	SUGT1	8,78501E-51	-3,35270E-01	0,402	0,27	1,61794E-46
Macro_OLFML3	FLOT2	3,39333E-12	-3,35629E-01	0,303	0,235	6,24950E-08
Macro_OLFML3	REXO2	6,55073E-37	-3,35726E-01	0,258	0,172	1,20645E-32
Macro_OLFML3	TOMM20	1,11528E-10	-3,35747E-01	0,668	0,519	2,05400E-06
Macro_OLFML3	PSMA1	4,22522E-69	-3,35940E-01	0,51	0,344	7,78159E-65
Macro_OLFML3	PRPF31	9,94613E-82	-3,36064E-01	0,335	0,2	1,83178E-77
Macro_OLFML3	CCRL2	1,39857E-35	-3,36271E-01	0,265	0,176	2,57575E-31
Macro_OLFML3	NDUFC1	8,55028E-69	-3,36313E-01	0,638	0,437	1,57470E-64
Macro_OLFML3	SNX29	4,94647E-15	-3,36394E-01	0,327	0,252	9,10991E-11
Macro_OLFML3	LTA4H	1,79187E-25	-3,36896E-01	0,585	0,432	3,30009E-21
Macro_OLFML3	SH3GLB1	4,74520E-26	-3,36979E-01	0,555	0,412	8,73923E-22
Macro_OLFML3	SEC31A	4,48056E-28	-3,37111E-01	0,392	0,288	8,25185E-24
Macro_OLFML3	ZNF655	2,24743E-12	-3,37272E-01	0,271	0,211	4,13909E-08
Macro_OLFML3	CNOT7	8,18194E-36	-3,37372E-01	0,416	0,296	1,50687E-31
Macro_OLFML3	PDCD6IP	1,50192E-20	-3,38019E-01	0,47	0,357	2,76609E-16
Macro_OLFML3	SYF2	1,17973E-25	-3,38086E-01	0,555	0,41	2,17270E-21
Macro_OLFML3	SAR1B	2,20995E-66	-3,38691E-01	0,359	0,227	4,07007E-62
Macro_OLFML3	PSMD6	1,27117E-100	-3,38737E-01	0,434	0,261	2,34112E-96
Macro_OLFML3	LSM14A	1,16040E-09	-3,38983E-01	0,386	0,309	2,13711E-05
Macro_OLFML3	MS4A4A	2,50524E-76	-3,39009E-01	0,603	0,399	4,61389E-72
Macro_OLFML3	UTRN	8,43532E-15	-3,39770E-01	0,364	0,28	1,55353E-10
Macro_OLFML3	VDAC2	1,52674E-75	-3,39919E-01	0,752	0,53	2,81180E-71
Macro_OLFML3	FDFT1	8,13736E-18	-3,40222E-01	0,335	0,251	1,49866E-13
Macro_OLFML3	SRSF1	7,26253E-26	-3,40517E-01	0,353	0,259	1,33754E-21
Macro_OLFML3	HLA-F	4,17506E-263	-3,40523E-01	0,793	0,484	7,68922E-259
Macro_OLFML3	NEMF	1,42520E-99	-3,40833E-01	0,441	0,271	2,62479E-95
Macro_OLFML3	SNX1	1,26220E-48	-3,41046E-01	0,425	0,293	2,32459E-44
Macro_OLFML3	SURF4	3,83215E-28	-3,41112E-01	0,388	0,28	7,05768E-24
Macro_OLFML3	CALM2	2,47003E-98	-3,41322E-01	0,941	0,764	4,54906E-94
Macro_OLFML3	SLA	1,33496E-92	-3,41340E-01	0,617	0,394	2,45860E-88
Macro_OLFML3	RNF5	1,68456E-36	-3,41503E-01	0,389	0,271	3,10245E-32

Macro_OLFML3	PSMA7	3,04452E-58	-3,41519E-01	0,9	0,728	5,60710E-54
Macro_OLFML3	AFF1	4,69009E-12	-3,41836E-01	0,28	0,221	8,63774E-08
Macro_OLFML3	GPAA1	1,74087E-64	-3,42161E-01	0,379	0,242	3,20616E-60
Macro_OLFML3	GGA1	5,32599E-12	-3,42782E-01	0,297	0,233	9,80887E-08
Macro_OLFML3	GPATCH2L	1,27430E-37	-3,44008E-01	0,253	0,167	2,34687E-33
Macro_OLFML3	DHRS7	2,93979E-84	-3,44067E-01	0,675	0,463	5,41420E-80
Macro_OLFML3	ENY2	6,32085E-37	-3,44495E-01	0,763	0,576	1,16411E-32
Macro_OLFML3	TMEM205	3,11775E-61	-3,44757E-01	0,413	0,27	5,74195E-57
Macro_OLFML3	GGNBP2	5,48400E-61	-3,44852E-01	0,485	0,324	1,00999E-56
Macro_OLFML3	KTN1	2,74920E-16	-3,44866E-01	0,627	0,478	5,06320E-12
Macro_OLFML3	UBE2N	3,63303E-75	-3,45005E-01	0,535	0,356	6,69095E-71
Macro_OLFML3	CCT8	1,68820E-142	-3,45038E-01	0,622	0,389	3,10916E-138
Macro_OLFML3	ATF6B	1,48542E-45	-3,45345E-01	0,4	0,274	2,73570E-41
Macro_OLFML3	ARHGAP15	2,29918E-152	-3,45461E-01	0,427	0,234	4,23440E-148
Macro_OLFML3	PDE4B	2,76106E-11	-3,45841E-01	0,462	0,356	5,08504E-07
Macro_OLFML3	COA5	1,88184E-51	-3,45910E-01	0,289	0,183	3,46579E-47
Macro_OLFML3	HMGN1	7,32662E-58	-3,46189E-01	0,83	0,602	1,34934E-53
Macro_OLFML3	SPAG7	2,13218E-103	-3,46450E-01	0,497	0,307	3,92683E-99
Macro_OLFML3	CNPY3	7,23248E-65	-3,46647E-01	0,826	0,605	1,33201E-60
Macro_OLFML3	DERL2	7,86851E-105	-3,46653E-01	0,375	0,219	1,44914E-100
Macro_OLFML3	ARPC4	1,52209E-72	-3,46942E-01	0,566	0,392	2,80324E-68
Macro_OLFML3	RPS2	7,73659E-09	-3,47171E-01	0,485	0,571	1,42485E-04
Macro_OLFML3	UBXN1	3,23530E-69	-3,47333E-01	0,822	0,606	5,95845E-65
Macro_OLFML3	CAST	5,76384E-29	-3,47563E-01	0,691	0,513	1,06153E-24
Macro_OLFML3	LRRFIP2	2,25940E-09	-3,48204E-01	0,31	0,245	4,16114E-05
Macro_OLFML3	PITHD1	5,14270E-31	-3,48338E-01	0,313	0,219	9,47131E-27
Macro_OLFML3	AKAP9	2,91098E-80	-3,48931E-01	0,552	0,367	5,36115E-76
Macro_OLFML3	PGLS	3,95414E-21	-3,48984E-01	0,664	0,529	7,28234E-17
Macro_OLFML3	NAA10	3,19502E-84	-3,49961E-01	0,299	0,173	5,88426E-80
Macro_OLFML3	DNTTIP2	5,34149E-15	-3,50187E-01	0,351	0,267	9,83742E-11
Macro_OLFML3	MIS18BP1	1,46921E-39	-3,50218E-01	0,49	0,346	2,70585E-35
Macro_OLFML3	SNRNP200	7,71088E-13	-3,50870E-01	0,321	0,252	1,42011E-08
Macro_OLFML3	TRAM1	5,84039E-61	-3,51974E-01	0,622	0,431	1,07562E-56
Macro_OLFML3	HP1BP3	5,79647E-19	-3,52607E-01	0,517	0,394	1,06754E-14
Macro_OLFML3	IST1	1,27138E-47	-3,53427E-01	0,372	0,252	2,34149E-43
Macro_OLFML3	ADAM9	3,42909E-18	-3,53429E-01	0,296	0,221	6,31535E-14
Macro_OLFML3	NDUFB4	4,52167E-33	-3,54178E-01	0,765	0,574	8,32757E-29
Macro_OLFML3	RBM23	1,86190E-50	-3,54238E-01	0,29	0,186	3,42907E-46
Macro_OLFML3	CPNE1	3,99670E-35	-3,55056E-01	0,321	0,224	7,36072E-31
Macro_OLFML3	NDUFB8	3,79685E-66	-3,55263E-01	0,405	0,274	6,99265E-62
Macro_OLFML3	NUBP2	6,41061E-58	-3,55267E-01	0,3	0,188	1,18064E-53
Macro_OLFML3	COX7A2	1,07719E-42	-3,55622E-01	0,87	0,703	1,98386E-38
Macro_OLFML3	CAMLG	8,06221E-18	-3,56507E-01	0,373	0,283	1,48482E-13
Macro_OLFML3	BEX4	8,93190E-79	-3,56541E-01	0,481	0,311	1,64499E-74
Macro_OLFML3	ECH1	6,28281E-77	-3,56918E-01	0,424	0,273	1,15711E-72

Macro_OLFML3	CACYBP	8,29048E-70	-3,56978E-01	0,572	0,382	1,52686E-65
Macro_OLFML3	TNFAIP8	2,13695E-53	-3,57038E-01	0,476	0,32	3,93563E-49
Macro_OLFML3	PPP2CA	4,89343E-13	-3,57041E-01	0,422	0,324	9,01223E-09
Macro_OLFML3	TBCB	1,42190E-87	-3,57205E-01	0,663	0,446	2,61871E-83
Macro_OLFML3	DNAJC19	1,91740E-30	-3,57391E-01	0,317	0,223	3,53127E-26
Macro_OLFML3	AP1S2	2,76791E-19	-3,57659E-01	0,732	0,561	5,09766E-15
Macro_OLFML3	RHOC	1,11698E-14	-3,57729E-01	0,393	0,3	2,05715E-10
Macro_OLFML3	HSPA9	3,14612E-52	-3,58339E-01	0,515	0,354	5,79421E-48
Macro_OLFML3	PRPF8	1,60006E-24	-3,58877E-01	0,394	0,298	2,94683E-20
Macro_OLFML3	UFC1	3,79076E-68	-3,58907E-01	0,688	0,481	6,98144E-64
Macro_OLFML3	CALU	3,90365E-24	-3,59037E-01	0,302	0,217	7,18936E-20
Macro_OLFML3	GUK1	8,05571E-58	-3,59196E-01	0,874	0,691	1,48362E-53
Macro_OLFML3	IFNAR1	2,54879E-99	-3,60397E-01	0,472	0,295	4,69411E-95
Macro_OLFML3	SUMO1	2,81478E-32	-3,60540E-01	0,62	0,451	5,18399E-28
Macro_OLFML3	LGALS8	4,62525E-22	-3,60609E-01	0,262	0,188	8,51833E-18
Macro_OLFML3	BCCIP	5,61147E-51	-3,60922E-01	0,307	0,199	1,03346E-46
Macro_OLFML3	REEP5	2,78117E-108	-3,61143E-01	0,775	0,539	5,12207E-104
Macro_OLFML3	CHD4	2,16800E-16	-3,61605E-01	0,351	0,269	3,99280E-12
Macro_OLFML3	RNPS1	3,31079E-25	-3,61731E-01	0,527	0,391	6,09748E-21
Macro_OLFML3	SHISA5	3,04269E-78	-3,62287E-01	0,537	0,352	5,60371E-74
Macro_OLFML3	PPIA	6,23773E-104	-3,62409E-01	0,984	0,884	1,14880E-99
Macro_OLFML3	CHCHD2	9,71556E-08	-3,62472E-01	0,938	0,802	1,78932E-03
Macro_OLFML3	EWSR1	1,73777E-31	-3,62753E-01	0,607	0,446	3,20046E-27
Macro_OLFML3	PTMA	5,20268E-13	-3,62766E-01	0,995	0,958	9,58178E-09
Macro_OLFML3	PTPN2	1,05361E-74	-3,63054E-01	0,547	0,367	1,94043E-70
Macro_OLFML3	SEC11C	3,86677E-86	-3,63337E-01	0,483	0,308	7,12143E-82
Macro_OLFML3	ABRACL	1,06807E-26	-3,63577E-01	0,653	0,48	1,96706E-22
Macro_OLFML3	HDLBP	2,10248E-18	-3,63749E-01	0,521	0,397	3,87214E-14
Macro_OLFML3	CCDC107	2,47800E-70	-3,64124E-01	0,452	0,29	4,56374E-66
Macro_OLFML3	PNISR	3,36356E-40	-3,64412E-01	0,651	0,476	6,19467E-36
Macro_OLFML3	EIF1AX	5,72761E-09	-3,64814E-01	0,493	0,389	1,05485E-04
Macro_OLFML3	KPNA2	2,89147E-107	-3,65289E-01	0,343	0,194	5,32521E-103
Macro_OLFML3	MED15	2,05087E-13	-3,65737E-01	0,276	0,213	3,77709E-09
Macro_OLFML3	DAD1	2,68158E-49	-3,65785E-01	0,781	0,58	4,93866E-45
Macro_OLFML3	ADIPOR1	4,55798E-11	-3,65847E-01	0,574	0,449	8,39444E-07
Macro_OLFML3	SEC11A	3,66333E-40	-3,66138E-01	0,838	0,647	6,74676E-36
Macro_OLFML3	ARL6IP4	4,66634E-08	-3,67432E-01	0,766	0,619	8,59400E-04
Macro_OLFML3	OTUB1	1,73683E-41	-3,67598E-01	0,575	0,414	3,19873E-37
Macro_OLFML3	PIH1D1	1,09724E-68	-3,67832E-01	0,379	0,24	2,02078E-64
Macro_OLFML3	GPBP1	8,24158E-08	-3,67977E-01	0,42	0,335	1,51785E-03
Macro_OLFML3	CNOT8	1,60532E-19	-3,67989E-01	0,277	0,206	2,95651E-15
Macro_OLFML3	POP5	1,57916E-78	-3,68614E-01	0,304	0,178	2,90833E-74
Macro_OLFML3	PPCS	5,63303E-128	-3,69034E-01	0,595	0,367	1,03744E-123
Macro_OLFML3	PPP6C	4,53334E-18	-3,69179E-01	0,34	0,257	8,34905E-14
Macro_OLFML3	ORMDL1	5,64155E-77	-3,69466E-01	0,464	0,298	1,03900E-72

Macro_OLFML3	PSMA2	7,28665E-36	-3,69558E-01	0,307	0,215	1,34198E-31
Macro_OLFML3	FOXO3	1,01993E-11	-3,69669E-01	0,227	0,25	1,87841E-07
Macro_OLFML3	DDOST	2,17313E-100	-3,69808E-01	0,663	0,44	4,00225E-96
Macro_OLFML3	TM9SF2	1,65727E-71	-3,69945E-01	0,595	0,408	3,05219E-67
Macro_OLFML3	DNAJC8	1,72875E-79	-3,70008E-01	0,59	0,393	3,18384E-75
Macro_OLFML3	SPINT2	4,10299E-33	-3,70013E-01	0,667	0,478	7,55647E-29
Macro_OLFML3	NOTCH2	1,76541E-09	-3,70083E-01	0,384	0,31	3,25136E-05
Macro_OLFML3	VDAC3	1,05554E-66	-3,70265E-01	0,454	0,296	1,94399E-62
Macro_OLFML3	ANAPC11	4,24846E-63	-3,70456E-01	0,796	0,587	7,82439E-59
Macro_OLFML3	MAPKAPK3	4,29186E-20	-3,70643E-01	0,374	0,279	7,90432E-16
Macro_OLFML3	PCBP1	9,03317E-09	-3,70990E-01	0,682	0,602	1,66364E-04
Macro_OLFML3	MORF4L2	5,70808E-19	-3,71103E-01	0,354	0,265	1,05126E-14
Macro_OLFML3	CPPED1	5,52589E-07	-3,71402E-01	0,326	0,264	1,01770E-02
Macro_OLFML3	ASAH1	2,99887E-19	-3,71636E-01	0,865	0,669	5,52302E-15
Macro_OLFML3	NEDD8	1,45716E-67	-3,71747E-01	0,762	0,567	2,68365E-63
Macro_OLFML3	BCLAF1	5,79698E-34	-3,71920E-01	0,509	0,366	1,06763E-29
Macro_OLFML3	FKBP2	7,15983E-73	-3,72422E-01	0,565	0,391	1,31863E-68
Macro_OLFML3	FGFR1OP2	7,40117E-13	-3,72631E-01	0,38	0,297	1,36307E-08
Macro_OLFML3	SRSF11	6,34948E-33	-3,72633E-01	0,679	0,497	1,16938E-28
Macro_OLFML3	MIDN	3,51102E-10	-3,73107E-01	0,268	0,283	6,46624E-06
Macro_OLFML3	MLX	6,36850E-41	-3,73357E-01	0,488	0,344	1,17289E-36
Macro_OLFML3	UQCRQ	5,26544E-20	-3,73976E-01	0,816	0,649	9,69736E-16
Macro_OLFML3	TMEM50A	1,30077E-42	-3,75173E-01	0,786	0,582	2,39564E-38
Macro_OLFML3	STK4	3,42017E-14	-3,75663E-01	0,546	0,415	6,29893E-10
Macro_OLFML3	DNAJB11	5,70819E-72	-3,75967E-01	0,454	0,294	1,05128E-67
Macro_OLFML3	NDUFA13	1,77895E-36	-3,76274E-01	0,574	0,416	3,27629E-32
Macro_OLFML3	PQBP1	4,39248E-35	-3,77434E-01	0,426	0,302	8,08963E-31
Macro_OLFML3	TIMM13	1,08694E-83	-3,78135E-01	0,568	0,375	2,00181E-79
Macro_OLFML3	PLXNB2	9,60193E-29	-3,78175E-01	0,353	0,255	1,76839E-24
Macro_OLFML3	SFT2D1	9,88687E-30	-3,78312E-01	0,605	0,443	1,82087E-25
Macro_OLFML3	MRPL33	3,50465E-11	-3,78536E-01	0,462	0,362	6,45452E-07
Macro_OLFML3	FBXW5	2,89609E-20	-3,78604E-01	0,407	0,304	5,33372E-16
Macro_OLFML3	POLR2I	7,45568E-34	-3,79123E-01	0,398	0,283	1,37311E-29
Macro_OLFML3	FOS	3,56583E-110	-3,79792E-01	0,914	0,835	6,56719E-106
Macro_OLFML3	SLC25A3	1,15699E-44	-3,80089E-01	0,893	0,693	2,13084E-40
Macro_OLFML3	HPS1	5,00456E-11	-3,80798E-01	0,431	0,343	9,21691E-07
Macro_OLFML3	HNRNPM	2,57217E-25	-3,81311E-01	0,593	0,442	4,73716E-21
Macro_OLFML3	MPG	7,02539E-76	-3,81632E-01	0,473	0,305	1,29387E-71
Macro_OLFML3	SOD1	5,02651E-81	-3,82498E-01	0,759	0,541	9,25732E-77
Macro_OLFML3	JTB	1,99616E-17	-3,82648E-01	0,708	0,544	3,67632E-13
Macro_OLFML3	UCP2	2,16434E-241	-3,82828E-01	0,882	0,606	3,98606E-237
Macro_OLFML3	PSMA6	1,04085E-46	-3,82887E-01	0,606	0,45	1,91693E-42
Macro_OLFML3	EIF2A	7,84936E-26	-3,83539E-01	0,406	0,297	1,44562E-21
Macro_OLFML3	GAPT	9,75424E-116	-3,85230E-01	0,413	0,237	1,79644E-111
Macro_OLFML3	TMEM59	1,09802E-79	-3,85235E-01	0,816	0,595	2,02223E-75

Macro_OLFML3	ORMDL2	3,57080E-60	-3,85572E-01	0,427	0,284	6,57634E-56
Macro_OLFML3	SERINC3	1,47017E-10	-3,85626E-01	0,421	0,331	2,70762E-06
Macro_OLFML3	OS9	8,89567E-86	-3,86068E-01	0,734	0,514	1,63832E-81
Macro_OLFML3	ATP6V1H	3,03609E-56	-3,86559E-01	0,314	0,2	5,59157E-52
Macro_OLFML3	PYURF	8,83332E-68	-3,87796E-01	0,577	0,391	1,62683E-63
Macro_OLFML3	NDUFB9	6,72237E-39	-3,87894E-01	0,72	0,531	1,23806E-34
Macro_OLFML3	CHORDC1	1,06297E-09	-3,87908E-01	0,287	0,228	1,95767E-05
Macro_OLFML3	ARAP1	1,11317E-24	-3,88405E-01	0,384	0,281	2,05013E-20
Macro_OLFML3	ERCC1	1,01253E-128	-3,88443E-01	0,584	0,358	1,86478E-124
Macro_OLFML3	UBL7	1,06103E-65	-3,88794E-01	0,377	0,24	1,95410E-61
Macro_OLFML3	PSMD13	1,01999E-131	-3,89435E-01	0,509	0,302	1,87852E-127
Macro_OLFML3	CHID1	2,20212E-49	-3,90993E-01	0,304	0,196	4,05565E-45
Macro_OLFML3	MRPS22	1,61240E-69	-3,90996E-01	0,259	0,151	2,96956E-65
Macro_OLFML3	SNRNP27	9,39759E-39	-3,91762E-01	0,261	0,173	1,73075E-34
Macro_OLFML3	HNRNPK	2,20204E-19	-3,92036E-01	0,872	0,69	4,05549E-15
Macro_OLFML3	NDUFV1	4,19966E-133	-3,92626E-01	0,616	0,384	7,73452E-129
Macro_OLFML3	CYB5R3	2,13375E-20	-3,92704E-01	0,53	0,396	3,92973E-16
Macro_OLFML3	CCT4	8,08989E-65	-3,93022E-01	0,601	0,413	1,48991E-60
Macro_OLFML3	LTC4S	3,82925E-122	-3,93539E-01	0,463	0,263	7,05234E-118
Macro_OLFML3	TMEM50B	8,75731E-40	-3,94516E-01	0,285	0,191	1,61283E-35
Macro_OLFML3	FAM200B	9,27457E-10	-3,95014E-01	0,265	0,207	1,70810E-05
Macro_OLFML3	EMB	9,00010E-30	-3,95417E-01	0,29	0,202	1,65755E-25
Macro_OLFML3	C1orf43	2,36286E-26	-3,95727E-01	0,7	0,529	4,35167E-22
Macro_OLFML3	FAM177A1	6,88531E-24	-3,96075E-01	0,413	0,305	1,26807E-19
Macro_OLFML3	ATP6V0A1	4,90284E-33	-3,96094E-01	0,264	0,179	9,02956E-29
Macro_OLFML3	FLOT1	1,92254E-57	-3,96263E-01	0,706	0,504	3,54074E-53
Macro_OLFML3	KRT10	2,18032E-18	-3,96449E-01	0,304	0,327	4,01550E-14
Macro_OLFML3	CCNDBP1	2,78530E-52	-3,96490E-01	0,389	0,26	5,12969E-48
Macro_OLFML3	GTF2B	3,56326E-28	-3,96536E-01	0,373	0,268	6,56246E-24
Macro_OLFML3	TSPAN3	3,62800E-89	-3,96635E-01	0,475	0,3	6,68169E-85
Macro_OLFML3	ATF3	3,38911E-19	-3,96914E-01	0,41	0,436	6,24172E-15
Macro_OLFML3	SRA1	8,57231E-53	-3,97205E-01	0,538	0,369	1,57876E-48
Macro_OLFML3	ARPP19	5,71893E-17	-3,97542E-01	0,344	0,26	1,05326E-12
Macro_OLFML3	RAB6A	1,02816E-15	-3,97636E-01	0,378	0,291	1,89357E-11
Macro_OLFML3	BRK1	1,47789E-11	-3,97887E-01	0,879	0,708	2,72183E-07
Macro_OLFML3	MORF4L1	2,26751E-36	-3,98237E-01	0,826	0,631	4,17607E-32
Macro_OLFML3	CSNK1A1	4,01041E-07	-3,98690E-01	0,568	0,45	7,38598E-03
Macro_OLFML3	RAB9A	2,88413E-26	-3,99010E-01	0,325	0,231	5,31170E-22
Macro_OLFML3	CPSF6	4,31951E-24	-3,99134E-01	0,252	0,18	7,95524E-20
Macro_OLFML3	LSM5	2,50296E-76	-3,99279E-01	0,502	0,326	4,60970E-72
Macro_OLFML3	RNF10	1,72070E-16	-3,99428E-01	0,349	0,266	3,16901E-12
Macro_OLFML3	N4BP2L1	1,77391E-57	-4,00317E-01	0,299	0,188	3,26701E-53
Macro_OLFML3	NFYC	2,47643E-55	-4,00443E-01	0,372	0,245	4,56084E-51
Macro_OLFML3	DCXR	2,01595E-54	-4,01026E-01	0,514	0,351	3,71278E-50
Macro_OLFML3	TSSC4	3,90362E-28	-4,01207E-01	0,269	0,189	7,18929E-24

Macro_OLFML3	SNRPE	1,98944E-72	-4,01659E-01	0,491	0,325	3,66394E-68
Macro_OLFML3	TMEM18	2,45151E-25	-4,02010E-01	0,262	0,186	4,51494E-21
Macro_OLFML3	FES	6,12692E-25	-4,02768E-01	0,339	0,245	1,12840E-20
Macro_OLFML3	LAMTOR5	2,32895E-45	-4,03289E-01	0,669	0,489	4,28923E-41
Macro_OLFML3	RBPJ	6,81597E-32	-4,04524E-01	0,621	0,449	1,25530E-27
Macro_OLFML3	LUC7L3	1,08279E-69	-4,04651E-01	0,511	0,342	1,99417E-65
Macro_OLFML3	RIC8A	2,41796E-35	-4,04809E-01	0,263	0,178	4,45316E-31
Macro_OLFML3	HEXB	2,62080E-48	-4,05047E-01	0,699	0,493	4,82673E-44
Macro_OLFML3	SERINC1	9,66921E-27	-4,05123E-01	0,531	0,39	1,78078E-22
Macro_OLFML3	ITGB1	9,12881E-15	-4,05564E-01	0,601	0,46	1,68125E-10
Macro_OLFML3	OFD1	8,29764E-73	-4,06215E-01	0,29	0,17	1,52818E-68
Macro_OLFML3	RAP1B	9,22094E-13	-4,06617E-01	0,724	0,563	1,69822E-08
Macro_OLFML3	DPP7	4,82766E-91	-4,06628E-01	0,77	0,532	8,89111E-87
Macro_OLFML3	BNIP2	2,61039E-34	-4,06899E-01	0,532	0,386	4,80755E-30
Macro_OLFML3	RNF149	1,95902E-23	-4,07728E-01	0,792	0,608	3,60793E-19
Macro_OLFML3	EDF1	7,38867E-43	-4,07787E-01	0,909	0,731	1,36077E-38
Macro_OLFML3	GNPTG	1,40200E-50	-4,07824E-01	0,582	0,411	2,58206E-46
Macro_OLFML3	TPST2	5,40193E-09	-4,08149E-01	0,26	0,206	9,94873E-05
Macro_OLFML3	DOCK2	6,82070E-61	-4,08962E-01	0,492	0,332	1,25617E-56
Macro_OLFML3	ATG12	1,03933E-23	-4,09274E-01	0,346	0,254	1,91413E-19
Macro_OLFML3	ACSL1	3,21523E-08	-4,09315E-01	0,483	0,378	5,92150E-04
Macro_OLFML3	PGAM1	7,01141E-75	-4,10054E-01	0,763	0,543	1,29129E-70
Macro_OLFML3	ALG5	4,40588E-49	-4,11099E-01	0,293	0,189	8,11430E-45
Macro_OLFML3	RSL1D1	1,80021E-38	-4,12033E-01	0,626	0,452	3,31544E-34
Macro_OLFML3	NDUFS4	3,96773E-124	-4,12116E-01	0,456	0,268	7,30738E-120
Macro_OLFML3	HAX1	1,11652E-57	-4,12479E-01	0,49	0,33	2,05629E-53
Macro_OLFML3	EFCAB14	2,52240E-09	-4,12529E-01	0,325	0,261	4,64551E-05
Macro_OLFML3	STK25	7,84609E-39	-4,12991E-01	0,252	0,167	1,44501E-34
Macro_OLFML3	COPS6	3,58051E-100	-4,13408E-01	0,514	0,323	6,59422E-96
Macro_OLFML3	STRAP	1,62917E-26	-4,13941E-01	0,466	0,343	3,00045E-22
Macro_OLFML3	PCBP2	2,37902E-23	-4,14293E-01	0,905	0,786	4,38144E-19
Macro_OLFML3	RTN3	3,47573E-21	-4,14561E-01	0,54	0,404	6,40124E-17
Macro_OLFML3	TIMMDC1	2,85088E-100	-4,14765E-01	0,363	0,212	5,25047E-96
Macro_OLFML3	DCTN3	1,79502E-30	-4,14827E-01	0,478	0,347	3,30589E-26
Macro_OLFML3	HERPUD1	5,15301E-153	-4,15221E-01	0,902	0,656	9,49029E-149
Macro_OLFML3	HNRNPH3	7,99942E-45	-4,15638E-01	0,482	0,337	1,47325E-40
Macro_OLFML3	SAFB2	6,49252E-13	-4,16562E-01	0,287	0,22	1,19573E-08
Macro_OLFML3	NPL	1,40647E-55	-4,17097E-01	0,386	0,244	2,59030E-51
Macro_OLFML3	SNRPA1	5,01268E-98	-4,17219E-01	0,335	0,191	9,23185E-94
Macro_OLFML3	MAP3K8	6,69530E-07	-4,17940E-01	0,597	0,469	1,23307E-02
Macro_OLFML3	PNKD	4,07912E-42	-4,18352E-01	0,445	0,308	7,51252E-38
Macro_OLFML3	NOP56	2,66210E-79	-4,19369E-01	0,329	0,196	4,90278E-75
Macro_OLFML3	OSTC	4,37192E-49	-4,20359E-01	0,677	0,479	8,05176E-45
Macro_OLFML3	NCF2	4,68771E-13	-4,20448E-01	0,596	0,45	8,63336E-09
Macro_OLFML3	UBC	1,06186E-35	-4,20532E-01	0,985	0,889	1,95563E-31

Macro_OLFML3	PPP2R3C	6,29146E-47	-4,20919E-01	0,314	0,206	1,15870E-42
Macro_OLFML3	ARL8B	2,59035E-09	-4,21412E-01	0,493	0,393	4,77065E-05
Macro_OLFML3	HMGN4	5,33103E-35	-4,21791E-01	0,34	0,238	9,81817E-31
Macro_OLFML3	TINF2	2,55923E-39	-4,21792E-01	0,318	0,217	4,71333E-35
Macro_OLFML3	RABGAP1L	2,54148E-114	-4,22823E-01	0,407	0,235	4,68064E-110
Macro_OLFML3	UNC50	3,18235E-38	-4,22856E-01	0,253	0,169	5,86093E-34
Macro_OLFML3	RBM3	3,85980E-17	-4,23460E-01	0,835	0,656	7,10859E-13
Macro_OLFML3	TSPO	5,33926E-30	-4,23914E-01	0,922	0,748	9,83332E-26
Macro_OLFML3	SDF2	2,31858E-40	-4,25045E-01	0,332	0,226	4,27013E-36
Macro_OLFML3	NME1-NME2	2,41307E-158	-4,26237E-01	0,405	0,22	4,44415E-154
Macro_OLFML3	EEF1B2	1,92053E-11	-4,26263E-01	0,956	0,829	3,53704E-07
Macro_OLFML3	TRMT112	6,48142E-29	-4,26351E-01	0,792	0,598	1,19368E-24
Macro_OLFML3	BID	8,84823E-21	-4,26765E-01	0,543	0,4	1,62958E-16
Macro_OLFML3	UBAC2	3,20227E-78	-4,26833E-01	0,457	0,292	5,89762E-74
Macro_OLFML3	CUTA	4,66533E-53	-4,27587E-01	0,656	0,466	8,59215E-49
Macro_OLFML3	DUSP22	2,41990E-19	-4,28224E-01	0,29	0,215	4,45672E-15
Macro_OLFML3	CCS	8,09500E-38	-4,28545E-01	0,363	0,25	1,49086E-33
Macro_OLFML3	CAPNS1	6,23496E-14	-4,29233E-01	0,462	0,359	1,14829E-09
Macro_OLFML3	EIF3F	1,56643E-47	-4,29326E-01	0,849	0,645	2,88489E-43
Macro_OLFML3	FAM204A	3,94990E-25	-4,29594E-01	0,378	0,277	7,27454E-21
Macro_OLFML3	EIF3D	1,93122E-105	-4,30127E-01	0,677	0,451	3,55672E-101
Macro_OLFML3	SNX14	1,95564E-46	-4,30254E-01	0,254	0,162	3,60170E-42
Macro_OLFML3	EIF2S3	4,52842E-07	-4,30699E-01	0,554	0,44	8,33999E-03
Macro_OLFML3	GNAS	1,49871E-06	-4,30880E-01	0,751	0,593	2,76018E-02
Macro_OLFML3	TRAF3IP3	6,83356E-53	-4,31419E-01	0,293	0,185	1,25854E-48
Macro_OLFML3	PIN1	5,26890E-64	-4,31685E-01	0,493	0,329	9,70373E-60
Macro_OLFML3	C6orf62	6,30616E-44	-4,33496E-01	0,496	0,349	1,16141E-39
Macro_OLFML3	SET	2,52539E-79	-4,33520E-01	0,727	0,505	4,65102E-75
Macro_OLFML3	LGMN	9,67346E-82	-4,33740E-01	0,581	0,39	1,78156E-77
Macro_OLFML3	DHPS	7,57181E-14	-4,33910E-01	0,275	0,211	1,39450E-09
Macro_OLFML3	TTC3	2,93914E-11	-4,34275E-01	0,406	0,32	5,41302E-07
Macro_OLFML3	DDAH2	2,65277E-09	-4,34666E-01	0,519	0,407	4,88561E-05
Macro_OLFML3	SMS	5,69301E-11	-4,35807E-01	0,536	0,414	1,04848E-06
Macro_OLFML3	ZC3H15	1,83384E-31	-4,36968E-01	0,574	0,422	3,37738E-27
Macro_OLFML3	TMED2	3,30529E-37	-4,37278E-01	0,664	0,481	6,08736E-33
Macro_OLFML3	RAB20	3,70043E-62	-4,38446E-01	0,505	0,329	6,81509E-58
Macro_OLFML3	ANXA11	4,16949E-07	-4,38850E-01	0,677	0,528	7,67895E-03
Macro_OLFML3	PPP2R5C	6,53421E-13	-4,40264E-01	0,263	0,202	1,20341E-08
Macro_OLFML3	TP53I13	3,60561E-67	-4,41772E-01	0,347	0,219	6,64044E-63
Macro_OLFML3	SEC13	6,87953E-80	-4,41892E-01	0,575	0,382	1,26700E-75
Macro_OLFML3	GMFG	1,31888E-16	-4,42046E-01	0,834	0,655	2,42898E-12
Macro_OLFML3	AGTRAP	3,43341E-50	-4,42262E-01	0,516	0,343	6,32332E-46
Macro_OLFML3	MYL12B	1,55577E-12	-4,42501E-01	0,919	0,76	2,86526E-08
Macro_OLFML3	GLIPR1	3,88222E-19	-4,43765E-01	0,749	0,565	7,14988E-15
Macro_OLFML3	GPR65	0,00000E+00	-4,44006E-01	0,615	0,294	0,00000E+00

Macro_OLFML3	SND1	1,95322E-15	-4,44109E-01	0,434	0,335	3,59725E-11
Macro_OLFML3	HUWE1	8,68445E-08	-4,44434E-01	0,263	0,214	1,59941E-03
Macro_OLFML3	ERGIC2	7,33203E-41	-4,44888E-01	0,307	0,206	1,35034E-36
Macro_OLFML3	CCNI	7,48481E-21	-4,45061E-01	0,886	0,774	1,37848E-16
Macro_OLFML3	TMC6	5,24156E-10	-4,45105E-01	0,278	0,221	9,65339E-06
Macro_OLFML3	SNX3	5,30609E-07	-4,46201E-01	0,839	0,673	9,77223E-03
Macro_OLFML3	TCIRG1	2,13021E-13	-4,46615E-01	0,587	0,454	3,92321E-09
Macro_OLFML3	ATP5MC2	1,52987E-16	-4,47798E-01	0,409	0,436	2,81756E-12
Macro_OLFML3	GABARAPL2	2,75519E-07	-4,48419E-01	0,717	0,562	5,07423E-03
Macro_OLFML3	TKT	9,01620E-09	-4,48804E-01	0,767	0,589	1,66051E-04
Macro_OLFML3	HBEGF	5,27625E-23	-4,49021E-01	0,236	0,28	9,71727E-19
Macro_OLFML3	RACK1	5,59586E-28	-4,49071E-01	0,423	0,472	1,03059E-23
Macro_OLFML3	FCHSD2	6,06528E-26	-4,49085E-01	0,305	0,215	1,11704E-21
Macro_OLFML3	LAPTM4A	1,21562E-46	-4,49613E-01	0,818	0,606	2,23881E-42
Macro_OLFML3	LGALS2	3,42357E-11	-4,49945E-01	0,318	0,25	6,30518E-07
Macro_OLFML3	FYTTD1	1,09329E-10	-4,50052E-01	0,307	0,242	2,01352E-06
Macro_OLFML3	RSRC2	3,81752E-26	-4,50374E-01	0,51	0,377	7,03073E-22
Macro_OLFML3	KLF4	5,84544E-68	-4,51167E-01	0,377	0,455	1,07656E-63
Macro_OLFML3	THEMIS2	8,62275E-46	-4,51409E-01	0,667	0,475	1,58805E-41
Macro_OLFML3	FKBP4	8,40205E-18	-4,52055E-01	0,295	0,22	1,54741E-13
Macro_OLFML3	YWHAQ	1,73859E-13	-4,52530E-01	0,547	0,417	3,20196E-09
Macro_OLFML3	MT-ND4	2,57737E-27	-4,53137E-01	0,478	0,585	4,74674E-23
Macro_OLFML3	PSMD4	4,81646E-154	-4,53202E-01	0,63	0,387	8,87048E-150
Macro_OLFML3	TMEM258	2,19947E-07	-4,53414E-01	0,816	0,65	4,05076E-03
Macro_OLFML3	AP1M1	2,24620E-49	-4,54613E-01	0,353	0,235	4,13682E-45
Macro_OLFML3	LDHB	5,87385E-88	-4,54646E-01	0,707	0,489	1,08179E-83
Macro_OLFML3	SIGIRR	8,56386E-11	-4,55168E-01	0,317	0,25	1,57721E-06
Macro_OLFML3	EIF3G	1,41925E-37	-4,55633E-01	0,79	0,594	2,61383E-33
Macro_OLFML3	SH3BP5	8,12155E-20	-4,55659E-01	0,314	0,34	1,49575E-15
Macro_OLFML3	PPP1R10	1,20884E-15	-4,55752E-01	0,393	0,299	2,22633E-11
Macro_OLFML3	IAH1	1,25055E-44	-4,55969E-01	0,428	0,296	2,30313E-40
Macro_OLFML3	HNRNPA1	4,84271E-14	-4,56733E-01	0,942	0,785	8,91882E-10
Macro_OLFML3	RIOK3	7,60928E-08	-4,56928E-01	0,327	0,265	1,40140E-03
Macro_OLFML3	SNX9	2,47151E-18	-4,57090E-01	0,262	0,291	4,55178E-14
Macro_OLFML3	ZSWIM7	1,59274E-19	-4,57528E-01	0,326	0,241	2,93335E-15
Macro_OLFML3	GLA	2,99444E-22	-4,57886E-01	0,404	0,301	5,51486E-18
Macro_OLFML3	GTF3A	5,90039E-67	-4,58101E-01	0,663	0,459	1,08668E-62
Macro_OLFML3	CAMTA1	2,85225E-38	-4,58251E-01	0,491	0,35	5,25299E-34
Macro_OLFML3	IVNS1ABP	8,62816E-07	-4,58877E-01	0,45	0,357	1,58905E-02
Macro_OLFML3	BAZ2B	7,82169E-23	-4,58887E-01	0,341	0,252	1,44052E-18
Macro_OLFML3	FERMT3	2,64535E-105	-4,59561E-01	0,688	0,459	4,87194E-101
Macro_OLFML3	HINT2	5,53656E-29	-4,59786E-01	0,31	0,221	1,01967E-24
Macro_OLFML3	EIF4E	1,43907E-17	-4,60393E-01	0,383	0,288	2,65033E-13
Macro_OLFML3	AZI2	3,21829E-09	-4,60981E-01	0,373	0,294	5,92713E-05
Macro_OLFML3	TSG101	1,13828E-57	-4,64263E-01	0,344	0,223	2,09637E-53

Macro_OLFML3	RAB11A	1,42634E-58	-4,64545E-01	0,564	0,383	2,62689E-54
Macro_OLFML3	ARF1	5,47313E-26	-4,65241E-01	0,862	0,68	1,00799E-21
Macro_OLFML3	CNIH1	1,28319E-08	-4,66038E-01	0,473	0,376	2,36325E-04
Macro_OLFML3	FABP5	3,32172E-55	-4,66321E-01	0,621	0,426	6,11762E-51
Macro_OLFML3	PAPOLA	3,96449E-19	-4,68840E-01	0,583	0,441	7,30140E-15
Macro_OLFML3	EEF2	1,62350E-15	-4,69008E-01	0,946	0,815	2,99000E-11
Macro_OLFML3	PAIP1	2,47141E-15	-4,70051E-01	0,256	0,194	4,55160E-11
Macro_OLFML3	RBBP4	3,32491E-56	-4,70361E-01	0,514	0,354	6,12349E-52
Macro_OLFML3	NABP1	7,96861E-11	-4,70747E-01	0,421	0,33	1,46758E-06
Macro_OLFML3	ADD1	1,13265E-06	-4,70981E-01	0,271	0,222	2,08599E-02
Macro_OLFML3	MAP2K1	6,89757E-08	-4,71109E-01	0,324	0,321	1,27033E-03
Macro_OLFML3	EIF4A2	3,17065E-18	-4,71687E-01	0,711	0,545	5,83938E-14
Macro_OLFML3	TUBB4B	8,62550E-10	-4,71849E-01	0,39	0,379	1,58856E-05
Macro_OLFML3	TMEM123	3,72905E-09	-4,72773E-01	0,566	0,442	6,86780E-05
Macro_OLFML3	KRTCAP2	9,31999E-11	-4,73116E-01	0,71	0,562	1,71646E-06
Macro_OLFML3	COPZ1	4,27582E-170	-4,74167E-01	0,601	0,357	7,87477E-166
Macro_OLFML3	TPP1	4,07075E-55	-4,75704E-01	0,784	0,574	7,49709E-51
Macro_OLFML3	LGALS3	8,06708E-14	-4,77092E-01	0,797	0,716	1,48571E-09
Macro_OLFML3	SMARCE1	1,34143E-17	-4,78187E-01	0,291	0,222	2,47051E-13
Macro_OLFML3	HSP90AB1	3,76956E-35	-4,80230E-01	0,962	0,81	6,94241E-31
Macro_OLFML3	ERP29	8,64565E-47	-4,80614E-01	0,816	0,601	1,59227E-42
Macro_OLFML3	SSR2	9,59193E-56	-4,80692E-01	0,832	0,632	1,76655E-51
Macro_OLFML3	RSRP1	1,22366E-23	-4,80906E-01	0,241	0,285	2,25361E-19
Macro_OLFML3	EIF1B	1,92004E-14	-4,80965E-01	0,584	0,446	3,53614E-10
Macro_OLFML3	PDCD4	4,61415E-20	-4,81998E-01	0,336	0,25	8,49787E-16
Macro_OLFML3	MT1X	3,52458E-16	-4,83675E-01	0,298	0,228	6,49121E-12
Macro_OLFML3	HM13	2,00339E-63	-4,83787E-01	0,737	0,517	3,68965E-59
Macro_OLFML3	FCGR2A	1,03449E-56	-4,83922E-01	0,862	0,605	1,90522E-52
Macro_OLFML3	LPP	9,17285E-30	-4,83949E-01	0,355	0,257	1,68936E-25
Macro_OLFML3	SRSF3	6,15875E-13	-4,84584E-01	0,723	0,553	1,13426E-08
Macro_OLFML3	SH3KBP1	6,39083E-27	-4,85812E-01	0,539	0,4	1,17700E-22
Macro_OLFML3	QSOX1	3,80768E-21	-4,85905E-01	0,379	0,278	7,01260E-17
Macro_OLFML3	SUB1	5,92500E-08	-4,86145E-01	0,874	0,706	1,09121E-03
Macro_OLFML3	POLR2J3	2,41424E-42	-4,87615E-01	0,283	0,186	4,44631E-38
Macro_OLFML3	SAR1A	7,37382E-44	-4,88253E-01	0,344	0,232	1,35804E-39
Macro_OLFML3	NCSTN	1,02851E-54	-4,88394E-01	0,34	0,221	1,89420E-50
Macro_OLFML3	NMT1	3,11326E-81	-4,89162E-01	0,281	0,162	5,73369E-77
Macro_OLFML3	CPNE3	1,05505E-26	-4,90168E-01	0,328	0,233	1,94309E-22
Macro_OLFML3	MXD1	1,46489E-20	-4,91147E-01	0,269	0,305	2,69788E-16
Macro_OLFML3	HSP90AA1	2,31137E-25	-4,91255E-01	0,98	0,868	4,25685E-21
Macro_OLFML3	DNAJB6	6,80275E-07	-4,91257E-01	0,649	0,508	1,25286E-02
Macro_OLFML3	ERGIC3	1,37290E-35	-4,91263E-01	0,656	0,48	2,52848E-31
Macro_OLFML3	BCAS2	2,05409E-16	-4,91353E-01	0,336	0,255	3,78302E-12
Macro_OLFML3	TSTD1	2,17911E-07	-4,91882E-01	0,289	0,233	4,01326E-03
Macro_OLFML3	FTL	3,18322E-23	-4,92010E-01	1	0,991	5,86254E-19

Macro_OLFML3	PHYKPL	5,09268E-20	-4,92747E-01	0,336	0,25	9,37919E-16
Macro_OLFML3	NINJ1	5,72438E-10	-4,93384E-01	0,703	0,543	1,05426E-05
Macro_OLFML3	CD14	5,15608E-165	-4,94132E-01	0,869	0,596	9,49595E-161
Macro_OLFML3	PFDN5	7,47945E-23	-4,94639E-01	0,972	0,894	1,37749E-18
Macro_OLFML3	ASPH	2,25621E-38	-4,95667E-01	0,315	0,212	4,15525E-34
Macro_OLFML3	ZBTB8OS	2,03273E-33	-4,96163E-01	0,307	0,213	3,74367E-29
Macro_OLFML3	LAMTOR4	2,46108E-27	-4,96626E-01	0,877	0,695	4,53257E-23
Macro_OLFML3	UQCRH	1,48037E-17	-4,98524E-01	0,848	0,685	2,72639E-13
Macro_OLFML3	RBM17	1,30265E-18	-4,98892E-01	0,53	0,397	2,39908E-14
Macro_OLFML3	EVI2B	2,99303E-15	-4,98985E-01	0,482	0,364	5,51226E-11
Macro_OLFML3	PARK7	4,89240E-112	-4,99692E-01	0,866	0,635	9,01032E-108
Macro_OLFML3	AHSA1	6,23775E-103	-5,01253E-01	0,463	0,28	1,14881E-98
Macro_OLFML3	BUB3	3,39158E-48	-5,01637E-01	0,364	0,246	6,24627E-44
Macro_OLFML3	MED29	1,66655E-22	-5,02657E-01	0,307	0,226	3,06929E-18
Macro_OLFML3	NUP214	3,59372E-13	-5,03207E-01	0,397	0,305	6,61855E-09
Macro_OLFML3	ATP1B3	1,89736E-44	-5,03879E-01	0,719	0,525	3,49437E-40
Macro_OLFML3	RAP1GDS1	3,76762E-65	-5,05477E-01	0,288	0,176	6,93882E-61
Macro_OLFML3	CD164	7,80590E-12	-5,05604E-01	0,698	0,541	1,43761E-07
Macro_OLFML3	FAU	4,16125E-59	-5,06283E-01	0,99	0,953	7,66377E-55
Macro_OLFML3	NUDT5	2,35425E-52	-5,06434E-01	0,334	0,219	4,33582E-48
Macro_OLFML3	CCDC59	1,13958E-08	-5,07816E-01	0,303	0,243	2,09877E-04
Macro_OLFML3	NDUFV2	5,72802E-16	-5,07888E-01	0,664	0,519	1,05493E-11
Macro_OLFML3	EMC3	6,49961E-63	-5,08159E-01	0,457	0,304	1,19703E-58
Macro_OLFML3	IQGAP2	3,19238E-21	-5,08935E-01	0,275	0,307	5,87940E-17
Macro_OLFML3	CHMP3	2,20124E-36	-5,09662E-01	0,324	0,223	4,05402E-32
Macro_OLFML3	NCOR1	5,82726E-39	-5,10979E-01	0,535	0,383	1,07321E-34
Macro_OLFML3	SH3BGRL3	2,98904E-65	-5,11007E-01	0,98	0,927	5,50492E-61
Macro_OLFML3	GTF2A2	2,35630E-42	-5,11201E-01	0,552	0,39	4,33960E-38
Macro_OLFML3	COPA	1,34480E-15	-5,11590E-01	0,426	0,328	2,47673E-11
Macro_OLFML3	TMCO1	2,07340E-117	-5,11662E-01	0,546	0,337	3,81858E-113
Macro_OLFML3	PPDPF	1,94301E-52	-5,15022E-01	0,836	0,76	3,57844E-48
Macro_OLFML3	HMGN2	9,48378E-40	-5,17033E-01	0,902	0,715	1,74663E-35
Macro_OLFML3	ADPGK	2,08501E-60	-5,18838E-01	0,556	0,383	3,83996E-56
Macro_OLFML3	NONO	4,63710E-30	-5,18909E-01	0,566	0,416	8,54014E-26
Macro_OLFML3	NAP1L1	7,75725E-09	-5,19859E-01	0,77	0,602	1,42865E-04
Macro_OLFML3	SF3B1	1,81199E-13	-5,20422E-01	0,615	0,48	3,33715E-09
Macro_OLFML3	CSNK2B	5,08021E-18	-5,22003E-01	0,429	0,327	9,35622E-14
Macro_OLFML3	U2AF1	2,00053E-55	-5,22471E-01	0,392	0,255	3,68437E-51
Macro_OLFML3	SPSB3	1,01241E-13	-5,22848E-01	0,364	0,282	1,86455E-09
Macro_OLFML3	SRSF7	8,14866E-12	-5,24148E-01	0,697	0,535	1,50074E-07
Macro_OLFML3	PRDX6	1,98498E-06	-5,27309E-01	0,697	0,543	3,65573E-02
Macro_OLFML3	MT-CO3	1,39511E-41	-5,27676E-01	0,483	0,602	2,56938E-37
Macro_OLFML3	SERPINB9	2,52881E-45	-5,33080E-01	0,527	0,358	4,65732E-41
Macro_OLFML3	FUCA1	1,08422E-17	-5,33534E-01	0,33	0,241	1,99680E-13
Macro_OLFML3	COMT	1,23723E-28	-5,35817E-01	0,728	0,538	2,27861E-24

Macro_OLFML3	CHMP1B	3,41305E-15	-5,36252E-01	0,402	0,407	6,28582E-11
Macro_OLFML3	CTSB	6,72002E-109	-5,36890E-01	0,991	0,858	1,23763E-104
Macro_OLFML3	ESD	2,64212E-63	-5,37448E-01	0,515	0,346	4,86600E-59
Macro_OLFML3	SMIM14	2,92573E-21	-5,37576E-01	0,353	0,259	5,38831E-17
Macro_OLFML3	MTRNR2L8	1,50739E-53	-5,37730E-01	0,344	0,452	2,77617E-49
Macro_OLFML3	GADD45B	4,20842E-36	-5,41720E-01	0,666	0,64	7,75065E-32
Macro_OLFML3	RASGEF1B	2,94729E-07	-5,41935E-01	0,258	0,27	5,42802E-03
Macro_OLFML3	FLII	4,20118E-14	-5,41979E-01	0,403	0,315	7,73731E-10
Macro_OLFML3	CSRNP1	1,35280E-59	-5,42192E-01	0,211	0,301	2,49144E-55
Macro_OLFML3	DNAJC4	1,09666E-38	-5,42596E-01	0,491	0,347	2,01972E-34
Macro_OLFML3	PRNP	2,20074E-10	-5,42985E-01	0,5	0,39	4,05309E-06
Macro_OLFML3	HSPB1	2,01107E-56	-5,44703E-01	0,846	0,637	3,70378E-52
Macro_OLFML3	ILF2	1,19220E-64	-5,46149E-01	0,553	0,373	2,19567E-60
Macro_OLFML3	LPXN	2,76065E-30	-5,46276E-01	0,463	0,335	5,08428E-26
Macro_OLFML3	PSMB4	1,89188E-95	-5,46838E-01	0,549	0,353	3,48427E-91
Macro_OLFML3	ARID5A	1,22353E-24	-5,50883E-01	0,379	0,277	2,25337E-20
Macro_OLFML3	OAZ1	1,11043E-07	-5,51160E-01	0,99	0,931	2,04509E-03
Macro_OLFML3	COX7A2L	9,47024E-17	-5,52064E-01	0,626	0,475	1,74413E-12
Macro_OLFML3	DDX6	2,07552E-14	-5,52751E-01	0,294	0,228	3,82249E-10
Macro_OLFML3	TALDO1	2,40771E-09	-5,53981E-01	0,811	0,645	4,43428E-05
Macro_OLFML3	NPM1	2,80895E-22	-5,54745E-01	0,92	0,748	5,17324E-18
Macro_OLFML3	ZNF207	7,86392E-29	-5,54830E-01	0,531	0,391	1,44830E-24
Macro_OLFML3	NSMCE1	3,63075E-34	-5,56343E-01	0,373	0,259	6,68675E-30
Macro_OLFML3	VMP1	5,83423E-24	-5,57910E-01	0,707	0,533	1,07449E-19
Macro_OLFML3	MRPL36	7,34985E-38	-5,58722E-01	0,322	0,22	1,35362E-33
Macro_OLFML3	SERTAD1	1,13364E-13	-5,60694E-01	0,316	0,327	2,08782E-09
Macro_OLFML3	CEBPB	3,84473E-12	-5,61211E-01	0,489	0,47	7,08084E-08
Macro_OLFML3	SDCBP	5,41350E-15	-5,61601E-01	0,883	0,705	9,97005E-11
Macro_OLFML3	RPL28	6,35700E-50	-5,62386E-01	0,488	0,607	1,17077E-45
Macro_OLFML3	RPN2	2,21519E-23	-5,62758E-01	0,357	0,276	4,07971E-19
Macro_OLFML3	HSP90B1	5,06293E-82	-5,63239E-01	0,889	0,678	9,32440E-78
Macro_OLFML3	RBMX	9,03739E-57	-5,63884E-01	0,588	0,407	1,66442E-52
Macro_OLFML3	HNRNPDL	3,41026E-09	-5,63979E-01	0,831	0,657	6,28068E-05
Macro_OLFML3	CORO1C	4,20391E-44	-5,64673E-01	0,469	0,33	7,74234E-40
Macro_OLFML3	PIM3	1,34348E-13	-5,65181E-01	0,26	0,289	2,47428E-09
Macro_OLFML3	JUNB	2,99913E-109	-5,65299E-01	0,872	0,821	5,52350E-105
Macro_OLFML3	CYTH4	6,65518E-26	-5,65395E-01	0,418	0,303	1,22569E-21
Macro_OLFML3	ANXA2	4,19839E-08	-5,67993E-01	0,831	0,733	7,73218E-04
Macro_OLFML3	MYL6	5,40098E-22	-5,71695E-01	0,987	0,936	9,94698E-18
Macro_OLFML3	ZFAND6	3,23207E-26	-5,73680E-01	0,41	0,3	5,95250E-22
Macro_OLFML3	SIVA1	1,70828E-17	-5,73904E-01	0,581	0,441	3,14614E-13
Macro_OLFML3	RPLP2	2,25150E-33	-5,76417E-01	0,483	0,58	4,14658E-29
Macro_OLFML3	MT-ATP6	1,52950E-50	-5,79492E-01	0,478	0,589	2,81687E-46
Macro_OLFML3	SLC40A1	5,37634E-26	-5,80522E-01	0,315	0,221	9,90160E-22
Macro_OLFML3	ARF4	5,20599E-55	-5,80739E-01	0,609	0,427	9,58788E-51

Macro_OLFML3	PER1	8,00454E-13	-5,82356E-01	0,306	0,321	1,47420E-08
Macro_OLFML3	MT-ND6	1,28909E-13	-5,84307E-01	0,273	0,305	2,37411E-09
Macro_OLFML3	UBB	1,53768E-19	-5,86583E-01	0,928	0,797	2,83195E-15
Macro_OLFML3	GYPC	1,84740E-19	-5,86604E-01	0,274	0,304	3,40235E-15
Macro_OLFML3	SPG7	4,19057E-17	-5,88020E-01	0,284	0,214	7,71778E-13
Macro_OLFML3	RPL38	1,11832E-06	-5,88254E-01	0,461	0,496	2,05962E-02
Macro_OLFML3	HNRNPC	3,68978E-25	-5,88949E-01	0,82	0,628	6,79547E-21
Macro_OLFML3	ACP5	1,22817E-09	-5,91259E-01	0,496	0,389	2,26191E-05
Macro_OLFML3	CALM1	2,21776E-07	-5,93844E-01	0,908	0,782	4,08446E-03
Macro_OLFML3	TMA7	1,23585E-25	-5,96341E-01	0,894	0,782	2,27606E-21
Macro_OLFML3	TPT1	1,26329E-68	-5,96341E-01	0,996	0,972	2,32661E-64
Macro_OLFML3	RNASE6	6,10419E-42	-5,97505E-01	0,727	0,518	1,12421E-37
Macro_OLFML3	KLF2	1,12881E-24	-5,97744E-01	0,24	0,281	2,07894E-20
Macro_OLFML3	EIF3L	3,73394E-18	-6,06973E-01	0,757	0,582	6,87680E-14
Macro_OLFML3	MRPS6	2,93913E-21	-6,07393E-01	0,427	0,313	5,41300E-17
Macro_OLFML3	OLR1	1,01073E-89	-6,08002E-01	0,533	0,326	1,86146E-85
Macro_OLFML3	RPS19	2,47498E-18	-6,08647E-01	0,486	0,585	4,55817E-14
Macro_OLFML3	ARHGAP9	3,27236E-21	-6,09108E-01	0,321	0,237	6,02670E-17
Macro_OLFML3	IFITM2	6,70349E-43	-6,11921E-01	0,692	0,641	1,23458E-38
Macro_OLFML3	ETS2	6,70305E-17	-6,18449E-01	0,435	0,425	1,23450E-12
Macro_OLFML3	AKAP13	1,32426E-09	-6,20528E-01	0,723	0,622	2,43889E-05
Macro_OLFML3	PAIP2	1,03513E-34	-6,20638E-01	0,677	0,493	1,90639E-30
Macro_OLFML3	KYNU	5,74690E-21	-6,21197E-01	0,412	0,304	1,05841E-16
Macro_OLFML3	VMO1	7,78333E-43	-6,22654E-01	0,324	0,216	1,43346E-38
Macro_OLFML3	ZFAND5	2,47661E-52	-6,22734E-01	0,586	0,585	4,56118E-48
Macro_OLFML3	MT-CO1	4,51129E-79	-6,26033E-01	0,485	0,612	8,30845E-75
Macro_OLFML3	ILF3	8,39610E-12	-6,35603E-01	0,454	0,356	1,54631E-07
Macro_OLFML3	ZDHHC4	5,32581E-76	-6,39303E-01	0,293	0,173	9,80855E-72
Macro_OLFML3	ELL2	1,10273E-06	-6,40677E-01	0,288	0,284	2,03090E-02
Macro_OLFML3	EIF3E	5,23394E-10	-6,41883E-01	0,758	0,588	9,63935E-06
Macro_OLFML3	HSPE1	4,36345E-47	-6,41990E-01	0,779	0,583	8,03617E-43
Macro_OLFML3	KLF6	4,11444E-106	-6,44888E-01	0,847	0,791	7,57756E-102
Macro_OLFML3	RPL8	5,59308E-85	-6,45822E-01	0,48	0,583	1,03008E-80
Macro_OLFML3	ABCA1	2,83411E-08	-6,46823E-01	0,315	0,315	5,21957E-04
Macro_OLFML3	BHLHE40	1,03827E-72	-6,48407E-01	0,246	0,351	1,91219E-68
Macro_OLFML3	BLVRB	7,02108E-11	-6,48564E-01	0,631	0,474	1,29307E-06
Macro_OLFML3	DDX3X	3,31323E-25	-6,48776E-01	0,477	0,478	6,10197E-21
Macro_OLFML3	RPL37A	1,59863E-29	-6,48896E-01	0,475	0,545	2,94420E-25
Macro_OLFML3	PSEN1	1,30236E-08	-6,49138E-01	0,269	0,216	2,39856E-04
Macro_OLFML3	TAF9	2,95359E-45	-6,51779E-01	0,42	0,288	5,43962E-41
Macro_OLFML3	SFPQ	1,16702E-11	-6,52032E-01	0,545	0,503	2,14930E-07
Macro_OLFML3	SEC61A1	5,01238E-18	-6,53461E-01	0,398	0,301	9,23130E-14
Macro_OLFML3	SLC3A2	2,30323E-08	-6,56227E-01	0,581	0,45	4,24185E-04
Macro_OLFML3	RBM6	1,08249E-25	-6,56629E-01	0,347	0,253	1,99362E-21
Macro_OLFML3	PPIF	2,84011E-13	-6,58670E-01	0,46	0,346	5,23064E-09

Macro_OLFML3	TMEM167A	3,96951E-15	-6,59833E-01	0,509	0,388	7,31064E-11
Macro_OLFML3	CAT	3,97035E-49	-6,60024E-01	0,557	0,392	7,31220E-45
Macro_OLFML3	P4HA1	5,97869E-10	-6,60873E-01	0,27	0,213	1,10109E-05
Macro_OLFML3	RPS9	1,83769E-25	-6,61181E-01	0,481	0,562	3,38447E-21
Macro_OLFML3	HIF1A	1,69012E-10	-6,62588E-01	0,609	0,542	3,11269E-06
Macro_OLFML3	HSPD1	2,71014E-57	-6,63572E-01	0,728	0,513	4,99126E-53
Macro_OLFML3	DDX5	1,12867E-18	-6,65694E-01	0,942	0,82	2,07867E-14
Macro_OLFML3	NACA	3,35583E-16	-6,69910E-01	0,979	0,912	6,18044E-12
Macro_OLFML3	MAPK1IP1L	8,17523E-09	-6,71461E-01	0,419	0,335	1,50563E-04
Macro_OLFML3	RPL35	8,67932E-15	-6,73097E-01	0,476	0,537	1,59847E-10
Macro_OLFML3	RPS15	4,10027E-61	-6,78186E-01	0,483	0,584	7,55148E-57
Macro_OLFML3	LRRFIP1	7,78274E-69	-6,79347E-01	0,731	0,678	1,43335E-64
Macro_OLFML3	NARF	5,07361E-17	-6,80621E-01	0,265	0,199	9,34406E-13
Macro_OLFML3	SOD2	1,27751E-27	-6,82302E-01	0,813	0,633	2,35279E-23
Macro_OLFML3	SEC62	4,40338E-11	-6,84631E-01	0,692	0,539	8,10971E-07
Macro_OLFML3	S100A10	9,64783E-162	-6,86420E-01	0,882	0,833	1,77684E-157
Macro_OLFML3	CD9	6,89409E-15	-6,87283E-01	0,612	0,471	1,26968E-10
Macro_OLFML3	MYL12A	4,96600E-07	-6,87537E-01	0,932	0,813	9,14588E-03
Macro_OLFML3	EEF1D	6,58419E-35	-6,89458E-01	0,954	0,862	1,21261E-30
Macro_OLFML3	ZFP36	1,20744E-117	-6,89639E-01	0,909	0,837	2,22374E-113
Macro_OLFML3	RNASEK	6,91685E-14	-6,89948E-01	0,469	0,465	1,27388E-09
Macro_OLFML3	GABARAPL1	3,22660E-10	-6,90102E-01	0,286	0,292	5,94243E-06
Macro_OLFML3	RPL36AL	3,62170E-30	-6,92134E-01	0,454	0,497	6,67009E-26
Macro_OLFML3	IER2	1,91118E-79	-7,00241E-01	0,588	0,637	3,51982E-75
Macro_OLFML3	PSMD9	9,76327E-08	-7,00888E-01	0,307	0,248	1,79810E-03
Macro_OLFML3	SAP30BP	1,36722E-19	-7,01632E-01	0,351	0,262	2,51802E-15
Macro_OLFML3	MT-ND2	1,83726E-25	-7,03443E-01	0,475	0,572	3,38369E-21
Macro_OLFML3	ACTN4	2,19837E-06	-7,05672E-01	0,36	0,347	4,04873E-02
Macro_OLFML3	COLGALT1	8,89950E-10	-7,06598E-01	0,287	0,224	1,63902E-05
Macro_OLFML3	SRGN	9,34515E-123	-7,06763E-01	0,983	0,938	1,72110E-118
Macro_OLFML3	METRNL	1,61915E-24	-7,08118E-01	0,211	0,259	2,98198E-20
Macro_OLFML3	MIF	2,25187E-16	-7,08283E-01	0,798	0,716	4,14727E-12
Macro_OLFML3	SON	1,52449E-10	-7,11246E-01	0,823	0,657	2,80765E-06
Macro_OLFML3	DNAJA1	1,39828E-17	-7,13435E-01	0,768	0,586	2,57521E-13
Macro_OLFML3	NOP58	6,41163E-45	-7,13617E-01	0,337	0,226	1,18083E-40
Macro_OLFML3	MT-ATP8	6,31402E-37	-7,14416E-01	0,307	0,393	1,16285E-32
Macro_OLFML3	MYO1G	4,64324E-49	-7,19510E-01	0,214	0,291	8,55146E-45
Macro_OLFML3	CCL3	4,73950E-18	-7,20764E-01	0,346	0,379	8,72874E-14
Macro_OLFML3	RPS18	1,28996E-22	-7,22642E-01	0,481	0,569	2,37572E-18
Macro_OLFML3	CCNL1	1,54273E-08	-7,27959E-01	0,573	0,52	2,84125E-04
Macro_OLFML3	RPL36	6,15177E-66	-7,35549E-01	0,478	0,561	1,13297E-61
Macro_OLFML3	CLN8	5,65985E-15	-7,38583E-01	0,3	0,228	1,04237E-10
Macro_OLFML3	STK17B	2,45853E-59	-7,38599E-01	0,39	0,45	4,52788E-55
Macro_OLFML3	RPL37	2,78706E-99	-7,39868E-01	0,479	0,578	5,13293E-95
Macro_OLFML3	MT-ND3	1,25924E-91	-7,40053E-01	0,477	0,58	2,31915E-87

Macro_OLFML3	RPS24	1,05581E-84	-7,42525E-01	0,483	0,588	1,94448E-80
Macro_OLFML3	PLD4	0,00000E+00	-7,42709E-01	0,603	0,301	0,00000E+00
Macro_OLFML3	RPSA	4,53207E-08	-7,42975E-01	0,468	0,498	8,34671E-04
Macro_OLFML3	TGFBI	2,09562E-144	-7,44081E-01	0,836	0,572	3,85951E-140
Macro_OLFML3	PLAU	5,52935E-34	-7,53909E-01	0,277	0,184	1,01834E-29
Macro_OLFML3	RPL27	5,02212E-07	-7,55883E-01	0,47	0,509	9,24924E-03
Macro_OLFML3	MT-ND5	9,29943E-58	-7,60233E-01	0,468	0,545	1,71268E-53
Macro_OLFML3	RPL13A	6,46650E-07	-7,61999E-01	0,473	0,526	1,19094E-02
Macro_OLFML3	RPS21	1,20112E-83	-7,62659E-01	0,472	0,551	2,21210E-79
Macro_OLFML3	RPL29	5,37396E-66	-7,62713E-01	0,476	0,567	9,89722E-62
Macro_OLFML3	ACADVL	4,15684E-09	-7,62914E-01	0,579	0,456	7,65566E-05
Macro_OLFML3	CCDC50	1,74874E-58	-7,65042E-01	0,404	0,259	3,22066E-54
Macro_OLFML3	RPL19	6,68371E-65	-7,65300E-01	0,484	0,591	1,23094E-60
Macro_OLFML3	SLC38A2	2,20693E-29	-7,65967E-01	0,264	0,314	4,06451E-25
Macro_OLFML3	RPL7A	3,63138E-72	-7,67887E-01	0,481	0,567	6,68792E-68
Macro_OLFML3	VAPA	7,77990E-12	-7,69953E-01	0,687	0,594	1,43282E-07
Macro_OLFML3	CD82	1,46128E-39	-7,77402E-01	0,343	0,228	2,69124E-35
Macro_OLFML3	KDM6B	6,13433E-77	-7,77443E-01	0,25	0,351	1,12976E-72
Macro_OLFML3	IER3	2,25915E-06	-7,80230E-01	0,595	0,525	4,16068E-02
Macro_OLFML3	PDE4DIP	1,20428E-29	-7,81888E-01	0,281	0,326	2,21792E-25
Macro_OLFML3	BTG1	1,57990E-97	-7,85798E-01	0,864	0,804	2,90970E-93
Macro_OLFML3	RPS16	1,81556E-35	-7,90840E-01	0,479	0,552	3,34373E-31
Macro_OLFML3	FOSL2	7,47270E-89	-8,04402E-01	0,193	0,307	1,37625E-84
Macro_OLFML3	MCL1	2,52763E-89	-8,06189E-01	0,84	0,761	4,65513E-85
Macro_OLFML3	RPS3	3,18616E-51	-8,08540E-01	0,482	0,577	5,86795E-47
Macro_OLFML3	RPL23A	7,22277E-16	-8,10086E-01	0,475	0,531	1,33022E-11
Macro_OLFML3	NFKBIA	1,92713E-12	-8,15962E-01	0,924	0,795	3,54919E-08
Macro_OLFML3	EEF1G	2,45547E-15	-8,20434E-01	0,273	0,298	4,52224E-11
Macro_OLFML3	CYTIP	2,77750E-21	-8,21745E-01	0,381	0,392	5,11532E-17
Macro_OLFML3	PABPC1	7,05152E-71	-8,23016E-01	0,968	0,884	1,29868E-66
Macro_OLFML3	C12orf57	3,54885E-29	-8,25401E-01	0,291	0,338	6,53592E-25
Macro_OLFML3	PNRC1	1,68693E-143	-8,26872E-01	0,818	0,775	3,10682E-139
Macro_OLFML3	RPL18	1,88168E-67	-8,31896E-01	0,48	0,572	3,46550E-63
Macro_OLFML3	RPL24	1,36276E-69	-8,32713E-01	0,472	0,546	2,50980E-65
Macro_OLFML3	RPL11	9,97740E-82	-8,32725E-01	0,485	0,591	1,83754E-77
Macro_OLFML3	ARL6IP1	1,44166E-17	-8,33328E-01	0,535	0,401	2,65511E-13
Macro_OLFML3	RPS10	2,33019E-22	-8,34482E-01	0,338	0,277	4,29151E-18
Macro_OLFML3	MT-ND1	1,08840E-36	-8,36167E-01	0,477	0,579	2,00450E-32
Macro_OLFML3	SOCS3	3,16288E-32	-8,38758E-01	0,31	0,363	5,82507E-28
Macro_OLFML3	RPL4	3,00971E-12	-8,39094E-01	0,461	0,479	5,54298E-08
Macro_OLFML3	MT-CO2	8,89723E-94	-8,40590E-01	0,484	0,609	1,63860E-89
Macro_OLFML3	PET100	1,29363E-16	-8,43987E-01	0,516	0,393	2,38248E-12
Macro_OLFML3	EIF1	2,73899E-263	-8,46633E-01	0,984	0,94	5,04439E-259
Macro_OLFML3	LSP1	7,34521E-130	-8,47194E-01	0,646	0,674	1,35277E-125
Macro_OLFML3	RPL26	3,19212E-41	-8,49552E-01	0,474	0,55	5,87893E-37

Macro_OLFML3	SAMSN1	1,15508E-16	-8,51462E-01	0,497	0,46	2,12731E-12
Macro_OLFML3	RPL32	9,45889E-97	-8,51549E-01	0,485	0,589	1,74204E-92
Macro_OLFML3	RPL22	9,90006E-90	-8,52145E-01	0,47	0,537	1,82329E-85
Macro_OLFML3	RPL14	7,38746E-67	-8,52154E-01	0,476	0,55	1,36055E-62
Macro_OLFML3	RPL15	7,56066E-37	-8,54149E-01	0,481	0,561	1,39245E-32
Macro_OLFML3	YWHAZ	1,22437E-59	-8,55028E-01	0,776	0,705	2,25493E-55
Macro_OLFML3	NAMPT	3,25984E-86	-8,55967E-01	0,532	0,578	6,00365E-82
Macro_OLFML3	RPL18A	9,47433E-63	-8,57141E-01	0,482	0,572	1,74489E-58
Macro_OLFML3	RPS27A	2,59963E-78	-8,58461E-01	0,483	0,586	4,78774E-74
Macro_OLFML3	CFD	1,70798E-09	-8,58569E-01	0,658	0,552	3,14559E-05
Macro_OLFML3	CDKN1A	3,79739E-75	-8,59781E-01	0,452	0,533	6,99366E-71
Macro_OLFML3	RPL31	1,58330E-11	-8,60198E-01	0,455	0,501	2,91596E-07
Macro_OLFML3	RPLP1	1,07392E-101	-8,61396E-01	0,488	0,61	1,97784E-97
Macro_OLFML3	JUND	1,40419E-112	-8,61412E-01	0,352	0,467	2,58609E-108
Macro_OLFML3	CTSD	4,47751E-27	-8,61734E-01	0,925	0,751	8,24623E-23
Macro_OLFML3	RPS14	5,08000E-56	-8,62856E-01	0,483	0,586	9,35583E-52
Macro_OLFML3	SELL	7,88120E-84	-8,63356E-01	0,331	0,187	1,45148E-79
Macro_OLFML3	AHNAK	3,26999E-133	-8,63958E-01	0,54	0,61	6,02235E-129
Macro_OLFML3	RPL10A	1,21458E-15	-8,65074E-01	0,465	0,5	2,23689E-11
Macro_OLFML3	ANXA1	1,08798E-37	-8,66363E-01	0,788	0,677	2,00374E-33
Macro_OLFML3	LMNA	7,55904E-84	-8,67049E-01	0,279	0,379	1,39215E-79
Macro_OLFML3	FLNA	1,63143E-166	-8,73767E-01	0,3	0,469	3,00461E-162
Macro_OLFML3	RPL12	4,57242E-87	-8,77853E-01	0,483	0,586	8,42103E-83
Macro_OLFML3	RPL5	2,09156E-57	-8,79060E-01	0,466	0,523	3,85202E-53
Macro_OLFML3	RPL9	6,20129E-30	-8,83712E-01	0,477	0,551	1,14209E-25
Macro_OLFML3	BTG2	4,75841E-12	-8,88403E-01	0,536	0,494	8,76356E-08
Macro_OLFML3	DUSP2	3,63554E-29	-8,88987E-01	0,247	0,307	6,69557E-25
Macro_OLFML3	MYADM	5,77317E-67	-8,93757E-01	0,257	0,343	1,06324E-62
Macro_OLFML3	NUCB2	3,01877E-58	-8,97713E-01	0,306	0,19	5,55967E-54
Macro_OLFML3	RPL13	4,02770E-66	-8,99039E-01	0,487	0,592	7,41782E-62
Macro_OLFML3	RPL30	8,18456E-112	-9,06514E-01	0,483	0,591	1,50735E-107
Macro_OLFML3	RPS23	3,23624E-82	-9,11263E-01	0,484	0,578	5,96019E-78
Macro_OLFML3	PHACTR1	4,55201E-73	-9,13356E-01	0,207	0,312	8,38344E-69
Macro_OLFML3	MT-CYB	5,59867E-90	-9,16498E-01	0,48	0,59	1,03111E-85
Macro_OLFML3	FCN1	1,90590E-80	-9,17609E-01	0,153	0,262	3,51009E-76
Macro_OLFML3	RPL41	5,54126E-80	-9,18014E-01	0,479	0,585	1,02053E-75
Macro_OLFML3	RPS5	8,18512E-30	-9,23581E-01	0,47	0,523	1,50745E-25
Macro_OLFML3	GPNMB	4,03173E-08	-9,24412E-01	0,425	0,324	7,42523E-04
Macro_OLFML3	NR3C1	3,26909E-17	-9,24674E-01	0,492	0,376	6,02068E-13
Macro_OLFML3	RPS26	2,77096E-58	-9,24918E-01	0,469	0,531	5,10328E-54
Macro_OLFML3	MAP2K3	1,73785E-18	-9,26209E-01	0,243	0,273	3,20060E-14
Macro_OLFML3	FTH1	4,78342E-185	-9,37225E-01	1	0,997	8,80963E-181
Macro_OLFML3	INSIG1	3,06695E-14	-9,39390E-01	0,276	0,297	5,64839E-10
Macro_OLFML3	TAGLN2	1,15558E-142	-9,48607E-01	0,794	0,75	2,12824E-138
Macro_OLFML3	MT-ND4L	1,02336E-98	-9,52344E-01	0,404	0,527	1,88473E-94

Macro_OLFML3	SPCS1	9,07157E-49	-9,54789E-01	0,799	0,586	1,67071E-44
Macro_OLFML3	RPL21	1,83006E-33	-9,55623E-01	0,476	0,548	3,37043E-29
Macro_OLFML3	LGALS1	2,52080E-185	-9,58159E-01	0,805	0,839	4,64256E-181
Macro_OLFML3	RPS6	3,42861E-36	-9,60202E-01	0,476	0,552	6,31447E-32
Macro_OLFML3	RPS13	1,94081E-85	-9,60798E-01	0,483	0,581	3,57440E-81
Macro_OLFML3	VIM	0,00000E+00	-9,60991E-01	0,954	0,942	0,00000E+00
Macro_OLFML3	STMN1	1,13912E-30	-9,62297E-01	0,244	0,292	2,09791E-26
Macro_OLFML3	RPS12	4,27510E-98	-9,65336E-01	0,484	0,593	7,87345E-94
Macro_OLFML3	RPS15A	9,32215E-98	-9,75113E-01	0,479	0,579	1,71686E-93
Macro_OLFML3	RPL10	1,22753E-117	-9,77951E-01	0,487	0,606	2,26074E-113
Macro_OLFML3	NR4A2	2,82432E-64	-9,78703E-01	0,381	0,459	5,20155E-60
Macro_OLFML3	SDS	2,15845E-22	-9,80292E-01	0,298	0,21	3,97523E-18
Macro_OLFML3	NR4A1	3,85599E-133	-9,80506E-01	0,291	0,435	7,10158E-129
Macro_OLFML3	RPL35A	3,76588E-124	-9,90034E-01	0,476	0,572	6,93563E-120
Macro_OLFML3	NFKBIZ	5,87614E-114	-9,90677E-01	0,345	0,467	1,08221E-109
Macro_OLFML3	RPS25	2,57896E-87	-9,92573E-01	0,477	0,556	4,74967E-83
Macro_OLFML3	PMP22	6,86014E-71	-1,00071E+00	0,192	0,287	1,26343E-66
Macro_OLFML3	TNFRSF1B	1,44486E-55	-1,01040E+00	0,546	0,539	2,66099E-51
Macro_OLFML3	RPL39	1,31608E-141	-1,01222E+00	0,482	0,592	2,42382E-137
Macro_OLFML3	RPL34	1,51907E-129	-1,01399E+00	0,482	0,586	2,79768E-125
Macro_OLFML3	RPS7	7,01325E-91	-1,01643E+00	0,479	0,57	1,29163E-86
Macro_OLFML3	RPL7	3,61016E-27	-1,01689E+00	0,458	0,502	6,64883E-23
Macro_OLFML3	CD44	1,75573E-82	-1,01833E+00	0,701	0,674	3,23353E-78
Macro_OLFML3	CXCR4	2,27682E-06	-1,02715E+00	0,654	0,572	4,19322E-02
Macro_OLFML3	RPS8	3,85973E-110	-1,02941E+00	0,486	0,589	7,10846E-106
Macro_OLFML3	C5AR1	6,49837E-86	-1,02988E+00	0,335	0,416	1,19681E-81
Macro_OLFML3	AREG	5,18051E-143	-1,03353E+00	0,129	0,292	9,54095E-139
Macro_OLFML3	RPS27	1,18017E-76	-1,03478E+00	0,482	0,576	2,17352E-72
Macro_OLFML3	EMP3	5,97746E-134	-1,04231E+00	0,727	0,729	1,10087E-129
Macro_OLFML3	RPL36A	9,63041E-36	-1,04321E+00	0,427	0,457	1,77363E-31
Macro_OLFML3	TCF4	8,90776E-91	-1,04913E+00	0,428	0,256	1,64054E-86
Macro_OLFML3	SLC2A3	1,00582E-86	-1,05177E+00	0,358	0,442	1,85241E-82
Macro_OLFML3	RPS4X	4,83496E-92	-1,05410E+00	0,482	0,575	8,90455E-88
Macro_OLFML3	RPS28	5,95279E-138	-1,06676E+00	0,486	0,593	1,09633E-133
Macro_OLFML3	RPLP0	6,14061E-68	-1,06794E+00	0,479	0,55	1,13092E-63
Macro_OLFML3	NR4A3	5,64056E-66	-1,08467E+00	0,208	0,307	1,03882E-61
Macro_OLFML3	S100A4	2,18022E-211	-1,09961E+00	0,815	0,828	4,01531E-207
Macro_OLFML3	CD83	5,90723E-73	-1,10168E+00	0,421	0,496	1,08793E-68
Macro_OLFML3	RAC2	9,25580E-12	-1,10326E+00	0,55	0,49	1,70464E-07
Macro_OLFML3	RPL3	1,08304E-33	-1,10699E+00	0,475	0,54	1,99464E-29
Macro_OLFML3	RPS3A	9,54871E-104	-1,11106E+00	0,48	0,576	1,75859E-99
Macro_OLFML3	HIGD1A	1,88279E-64	-1,11190E+00	0,328	0,203	3,46753E-60
Macro_OLFML3	HNRNPH1	3,11883E-09	-1,11414E+00	0,418	0,403	5,74396E-05
Macro_OLFML3	CD99	5,33993E-235	-1,12377E+00	0,525	0,631	9,83456E-231
Macro_OLFML3	EIF4A3	6,10027E-14	-1,14566E+00	0,51	0,39	1,12349E-09

Macro_OLFML3	PLP2	6,25140E-59	-1,16096E+00	0,354	0,41	1,15132E-54
Macro_OLFML3	CRIP1	2,92982E-78	-1,17241E+00	0,191	0,304	5,39584E-74
Macro_OLFML3	TXN	2,69394E-06	-1,17967E+00	0,753	0,657	4,96142E-02
Macro_OLFML3	RPL6	4,66053E-101	-1,18928E+00	0,476	0,562	8,58330E-97
Macro_OLFML3	YPEL5	2,74263E-08	-1,19408E+00	0,473	0,43	5,05109E-04
Macro_OLFML3	S100A6	0,00000E+00	-1,19998E+00	0,782	0,885	0,00000E+00
Macro_OLFML3	BCL2A1	2,02245E-21	-1,21336E+00	0,473	0,47	3,72474E-17
Macro_OLFML3	PPP1R15A	1,00661E-214	-1,25898E+00	0,545	0,671	1,85387E-210
Macro_OLFML3	FOSB	2,52534E-260	-1,27636E+00	0,419	0,598	4,65093E-256
Macro_OLFML3	RPL17	1,30423E-59	-1,28961E+00	0,375	0,44	2,40200E-55
Macro_OLFML3	EGR1	6,74395E-32	-1,29535E+00	0,235	0,292	1,24203E-27
Macro_OLFML3	PLAUR	2,92337E-49	-1,31514E+00	0,699	0,634	5,38397E-45
Macro_OLFML3	IRF7	6,10354E-20	-1,34874E+00	0,516	0,386	1,12409E-15
Macro_OLFML3	PLAC8	8,28494E-20	-1,35648E+00	0,269	0,197	1,52584E-15
Macro_OLFML3	CD55	1,63362E-155	-1,39344E+00	0,305	0,443	3,00865E-151
Macro_OLFML3	EZR	2,86097E-74	-1,42141E+00	0,33	0,408	5,26905E-70
Macro_OLFML3	TREM1	1,55451E-104	-1,45792E+00	0,125	0,25	2,86294E-100
Macro_OLFML3	SLC11A1	1,70317E-98	-1,53236E+00	0,41	0,474	3,13672E-94
Macro_OLFML3	ALOX5AP	4,89856E-39	-1,53600E+00	0,492	0,506	9,02167E-35
Macro_OLFML3	CTSL	1,02096E-96	-1,56502E+00	0,779	0,49	1,88030E-92
Macro_OLFML3	IRF8	1,64162E-44	-1,59459E+00	0,53	0,361	3,02337E-40
Macro_OLFML3	S100A9	2,71866E-108	-1,69685E+00	0,349	0,468	5,00696E-104
Macro_OLFML3	CD52	3,70277E-135	-1,71652E+00	0,269	0,404	6,81939E-131
Macro_OLFML3	SERPINF1	9,52565E-119	-1,71764E+00	0,617	0,38	1,75434E-114
Macro_OLFML3	CREM	2,85625E-63	-1,75726E+00	0,318	0,384	5,26036E-59
Macro_OLFML3	PLIN2	5,42860E-27	-1,84773E+00	0,537	0,493	9,99785E-23
Macro_OLFML3	GPR183	6,93541E-46	-1,86193E+00	0,418	0,461	1,27729E-41
Macro_OLFML3	CYB561A3	7,50856E-46	-1,87659E+00	0,316	0,206	1,38285E-41
Macro_OLFML3	IL1B	5,02130E-42	-2,03618E+00	0,28	0,342	9,24773E-38
Macro_OLFML3	TIMP1	7,64286E-92	-2,12471E+00	0,67	0,648	1,40759E-87
Macro_OLFML3	S100A8	5,49730E-87	-2,35565E+00	0,251	0,364	1,01244E-82
Macro_OLFML3	RNASE1	1,08677E-121	-2,45971E+00	0,149	0,286	2,00151E-117

Mast

cluster	gene	p_val	avg_log2FC	pct.1	pct.2	p_val_adj
Mast	TPSAB1	0,00000E+00	7,65551E+00	0,966	0,015	0,00000E+00
Mast	TPSB2	0,00000E+00	6,86636E+00	0,867	0,009	0,00000E+00
Mast	CPA3	0,00000E+00	6,22512E+00	0,919	0,002	0,00000E+00
Mast	TPSD1	0,00000E+00	4,68456E+00	0,597	0,006	0,00000E+00
Mast	HPGD	0,00000E+00	4,67164E+00	0,684	0,015	0,00000E+00
Mast	CLU	0,00000E+00	4,21890E+00	0,771	0,073	0,00000E+00
Mast	GATA2	0,00000E+00	4,21465E+00	0,783	0,001	0,00000E+00
Mast	KIT	0,00000E+00	4,12636E+00	0,72	0,005	0,00000E+00
Mast	MS4A2	0,00000E+00	3,82729E+00	0,528	0	0,00000E+00
Mast	HDC	0,00000E+00	3,74998E+00	0,472	0	0,00000E+00
Mast	VWA5A	0,00000E+00	3,68416E+00	0,733	0,042	0,00000E+00
Mast	HPGDS	0,00000E+00	3,55254E+00	0,552	0,043	0,00000E+00
Mast	LTC4S	0,00000E+00	3,12505E+00	0,77	0,25	0,00000E+00
Mast	SLC18A2	0,00000E+00	3,01300E+00	0,502	0,004	0,00000E+00
Mast	CSF1	0,00000E+00	2,96637E+00	0,385	0,018	0,00000E+00
Mast	IL1RL1	0,00000E+00	2,86154E+00	0,452	0,001	0,00000E+00
Mast	CD69	0,00000E+00	2,69906E+00	0,642	0,187	0,00000E+00
Mast	RHEX	0,00000E+00	2,67103E+00	0,513	0,015	0,00000E+00
Mast	TIMP3	0,00000E+00	2,53410E+00	0,398	0,013	0,00000E+00
Mast	BACE2	0,00000E+00	2,44222E+00	0,498	0,011	0,00000E+00
Mast	SOCS1	0,00000E+00	2,41367E+00	0,497	0,183	0,00000E+00
Mast	LAT	0,00000E+00	2,31875E+00	0,45	0,035	0,00000E+00
Mast	RAB27B	0,00000E+00	2,14899E+00	0,399	0,002	0,00000E+00
Mast	SIGLEC6	0,00000E+00	2,13655E+00	0,381	0,006	0,00000E+00
Mast	RGS13	0,00000E+00	2,12027E+00	0,362	0,011	0,00000E+00
Mast	LMO4	0,00000E+00	2,10055E+00	0,496	0,219	0,00000E+00
Mast	GRAP2	0,00000E+00	2,04557E+00	0,36	0,007	0,00000E+00
Mast	NSMCE1	0,00000E+00	2,02576E+00	0,505	0,255	0,00000E+00
Mast	TNIK	0,00000E+00	1,94843E+00	0,376	0,025	0,00000E+00
Mast	MAOB	0,00000E+00	1,94240E+00	0,358	0,003	0,00000E+00
Mast	CNRIP1	0,00000E+00	1,90758E+00	0,429	0,057	0,00000E+00
Mast	MLPH	0,00000E+00	1,89565E+00	0,352	0,013	0,00000E+00
Mast	HS3ST1	0,00000E+00	1,87766E+00	0,396	0,097	0,00000E+00
Mast	JUN	0,00000E+00	1,82751E+00	0,813	0,702	0,00000E+00
Mast	SLC45A3	0,00000E+00	1,80351E+00	0,366	0,006	0,00000E+00
Mast	TESPA1	0,00000E+00	1,76990E+00	0,363	0,018	0,00000E+00
Mast	ATP6V0A	0,00000E+00	1,72244E+00	0,403	0,103	0,00000E+00
Mast	SYTL3	0,00000E+00	1,67617E+00	0,373	0,1	0,00000E+00
Mast	PTGS1	0,00000E+00	1,67529E+00	0,495	0,24	0,00000E+00
Mast	FOSB	0,00000E+00	1,63505E+00	0,756	0,568	0,00000E+00

Mast	SKAP1	0,00000E+00	1,61571E+00	0,277	0,008	0,00000E+00
Mast	ATP5MC:	0,00000E+00	1,60287E+00	0,736	0,412	0,00000E+00
Mast	LIF	0,00000E+00	1,57963E+00	0,26	0,006	0,00000E+00
Mast	KLRG1	0,00000E+00	1,57658E+00	0,261	0,018	0,00000E+00
Mast	PLPP1	0,00000E+00	1,53944E+00	0,27	0,042	0,00000E+00
Mast	ACSL4	0,00000E+00	1,53832E+00	0,483	0,261	0,00000E+00
Mast	MAST4	0,00000E+00	1,53238E+00	0,296	0,018	0,00000E+00
Mast	ITM2A	0,00000E+00	1,50481E+00	0,28	0,012	0,00000E+00
Mast	CTTNBP2	0,00000E+00	1,47920E+00	0,261	0,047	0,00000E+00
Mast	SMYD3	0,00000E+00	1,47208E+00	0,368	0,042	0,00000E+00
Mast	RHOH	0,00000E+00	1,43539E+00	0,415	0,166	0,00000E+00
Mast	CTSW	0,00000E+00	1,43497E+00	0,308	0,035	0,00000E+00
Mast	DLC1	0,00000E+00	1,42983E+00	0,275	0,009	0,00000E+00
Mast	GALC	0,00000E+00	1,42104E+00	0,378	0,155	0,00000E+00
Mast	EGR1	0,00000E+00	1,41420E+00	0,566	0,267	0,00000E+00
Mast	ADRB2	0,00000E+00	1,40862E+00	0,342	0,082	0,00000E+00
Mast	TENT5A	0,00000E+00	1,40480E+00	0,299	0,099	0,00000E+00
Mast	SELENOK	3,05619E-181	1,38123E+00	0,436	0,267	5,62858E-177
Mast	LEO1	0,00000E+00	1,37031E+00	0,323	0,087	0,00000E+00
Mast	CD82	0,00000E+00	1,36133E+00	0,509	0,221	0,00000E+00
Mast	FOXP1	1,37229E-274	1,36120E+00	0,522	0,333	2,52735E-270
Mast	STXBP6	0,00000E+00	1,32215E+00	0,264	0,004	0,00000E+00
Mast	NDST2	3,26986E-288	1,31575E+00	0,27	0,086	6,02211E-284
Mast	SARAF	0,00000E+00	1,30489E+00	0,623	0,414	0,00000E+00
Mast	ARHGEF6	2,00999E-275	1,29764E+00	0,422	0,21	3,70180E-271
Mast	LMNA	0,00000E+00	1,29172E+00	0,58	0,354	0,00000E+00
Mast	ABCC4	0,00000E+00	1,28926E+00	0,287	0,072	0,00000E+00
Mast	RACK1	0,00000E+00	1,27230E+00	0,765	0,446	0,00000E+00
Mast	ARMH1	0,00000E+00	1,24593E+00	0,277	0,046	0,00000E+00
Mast	RHOBTB3	1,18149E-290	1,24187E+00	0,35	0,139	2,17595E-286
Mast	NDFIP2	0,00000E+00	1,23312E+00	0,259	0,012	0,00000E+00
Mast	AREG	2,73912E-243	1,23287E+00	0,482	0,261	5,04464E-239
Mast	RAC2	0,00000E+00	1,22684E+00	0,745	0,479	0,00000E+00
Mast	NTRK1	0,00000E+00	1,22666E+00	0,251	0	0,00000E+00
Mast	SEPTIN2	4,25283E-226	1,21419E+00	0,269	0,101	7,83244E-222
Mast	STX3	3,70981E-230	1,19855E+00	0,292	0,115	6,83235E-226
Mast	SAMSN1	1,71659E-240	1,19234E+00	0,601	0,455	3,16144E-236
Mast	P2RX1	0,00000E+00	1,17525E+00	0,343	0,061	0,00000E+00
Mast	CD9	0,00000E+00	1,16411E+00	0,687	0,471	0,00000E+00
Mast	ANKRD28	1,53449E-243	1,12242E+00	0,29	0,108	2,82607E-239

Mast	TESC	1,94018E-270	1,11790E+00	0,272	0,088	3,57322E-266
Mast	TMEM15	0,00000E+00	1,07014E+00	0,28	0,082	0,00000E+00
Mast	BTG2	0,00000E+00	1,05376E+00	0,65	0,488	0,00000E+00
Mast	GNPTAB	5,26322E-112	1,05142E+00	0,388	0,271	9,69327E-108
Mast	SQSTM1	6,18546E-161	1,04670E+00	0,68	0,674	1,13918E-156
Mast	RPS26	5,17584E-307	1,04281E+00	0,714	0,511	9,53235E-303
Mast	TSC22D1	9,28248E-271	1,03301E+00	0,36	0,148	1,70956E-266
Mast	TSEN54	0,00000E+00	1,02298E+00	0,295	0,08	0,00000E+00
Mast	CDC42EP	3,86876E-141	1,00226E+00	0,317	0,165	7,12510E-137
Mast	GMPR	0,00000E+00	9,96807E-01	0,275	0,058	0,00000E+00
Mast	ATP5F1C	9,73074E-99	9,96579E-01	0,395	0,281	1,79211E-94
Mast	RHBDD2	1,65955E-150	9,94275E-01	0,392	0,248	3,05639E-146
Mast	RSRP1	9,63194E-53	9,82926E-01	0,351	0,276	1,77391E-48
Mast	AHR	2,58066E-62	9,70919E-01	0,411	0,351	4,75279E-58
Mast	NME2	3,12814E-128	9,70505E-01	0,494	0,35	5,76109E-124
Mast	SLC44A1	4,57398E-130	9,62529E-01	0,317	0,176	8,42389E-126
Mast	MSRA	5,43222E-82	9,62073E-01	0,316	0,213	1,00045E-77
Mast	SELENOV	5,43124E-99	9,59275E-01	0,403	0,284	1,00027E-94
Mast	C12orf57	2,02996E-192	9,43599E-01	0,486	0,323	3,73858E-188
Mast	ADGRE2	1,63574E-54	9,42386E-01	0,27	0,184	3,01255E-50
Mast	IER2	0,00000E+00	9,38870E-01	0,753	0,624	0,00000E+00
Mast	TRIR	4,97059E-102	9,28739E-01	0,443	0,329	9,15433E-98
Mast	TSPYL2	1,55536E-150	9,17384E-01	0,313	0,16	2,86451E-146
Mast	ACOT7	0,00000E+00	9,10509E-01	0,299	0,074	0,00000E+00
Mast	PRKX	2,04685E-91	9,05563E-01	0,265	0,154	3,76968E-87
Mast	ATP5F1D	7,89928E-86	8,93931E-01	0,442	0,332	1,45481E-81
Mast	BATF	2,62032E-132	8,93448E-01	0,26	0,126	4,82584E-128
Mast	VAT1	8,63596E-121	8,79572E-01	0,365	0,227	1,59049E-116
Mast	ATP5MC1	6,89738E-50	8,73081E-01	0,296	0,22	1,27029E-45
Mast	ALOX5	5,54219E-202	8,72677E-01	0,571	0,467	1,02070E-197
Mast	AHNAK	3,17496E-88	8,63433E-01	0,616	0,602	5,84732E-84
Mast	RPL22	0,00000E+00	8,51633E-01	0,771	0,513	0,00000E+00
Mast	ANXA1	0,00000E+00	8,26854E-01	0,789	0,681	0,00000E+00
Mast	SMIM3	8,68942E-83	8,14993E-01	0,254	0,146	1,60033E-78
Mast	IDS	3,08422E-85	8,12132E-01	0,453	0,394	5,68021E-81
Mast	ELL2	1,07882E-41	7,95913E-01	0,343	0,28	1,98687E-37
Mast	LAPTM4A	7,30675E-200	7,88350E-01	0,648	0,627	1,34568E-195
Mast	PPDPF	1,48888E-97	7,76066E-01	0,686	0,773	2,74207E-93
Mast	STMP1	4,31365E-46	7,67487E-01	0,322	0,252	7,94445E-42
Mast	RTRAF	1,75095E-44	7,61832E-01	0,331	0,26	3,22472E-40

Mast	RAB32	8,18538E-15	7,56789E-01	0,366	0,375	1,50750E-10
Mast	NR4A1	4,88665E-228	7,56358E-01	0,585	0,408	8,99974E-224
Mast	RPL30	0,00000E+00	7,47600E-01	0,876	0,56	0,00000E+00
Mast	MAPK1	1,15615E-28	7,39598E-01	0,273	0,227	2,12928E-24
Mast	ARHGAP:	4,77181E-35	7,34941E-01	0,472	0,477	8,78823E-31
Mast	BMP2K	1,57452E-09	7,26478E-01	0,292	0,292	2,89979E-05
Mast	BTK	1,87423E-101	7,24271E-01	0,401	0,304	3,45177E-97
Mast	SELENOH	2,63841E-52	7,07618E-01	0,409	0,325	4,85917E-48
Mast	RPL14	0,00000E+00	6,99283E-01	0,829	0,523	0,00000E+00
Mast	NENF	3,59271E-13	6,97859E-01	0,272	0,256	6,61670E-09
Mast	RENBP	1,18134E-13	6,97813E-01	0,307	0,297	2,17567E-09
Mast	RPL36AL	0,00000E+00	6,90014E-01	0,697	0,479	0,00000E+00
Mast	RPS4Y1	2,16211E-239	6,65021E-01	0,354	0,163	3,98195E-235
Mast	MBOAT7	1,01952E-24	6,64363E-01	0,289	0,246	1,87766E-20
Mast	ATP5IF1	4,42162E-21	6,48519E-01	0,301	0,262	8,14330E-17
Mast	CCDC85E	2,63735E-06	6,40337E-01	0,362	0,4	4,85721E-02
Mast	ATP5PB	3,12497E-25	6,30287E-01	0,324	0,28	5,75525E-21
Mast	PTGS2	9,65984E-111	6,25074E-01	0,316	0,17	1,77905E-106
Mast	NOP53	1,35530E-22	6,22155E-01	0,323	0,282	2,49605E-18
Mast	HSD17B1	2,61510E-47	6,22040E-01	0,309	0,242	4,81623E-43
Mast	NFKBIZ	5,13869E-240	6,16800E-01	0,603	0,444	9,46393E-236
Mast	BHLHE40	5,18247E-184	6,12601E-01	0,491	0,33	9,54455E-180
Mast	TNFSF10	1,02809E-80	6,09324E-01	0,442	0,355	1,89343E-76
Mast	ATP5PF	1,60576E-26	6,05557E-01	0,369	0,32	2,95733E-22
Mast	RPL32	0,00000E+00	6,03478E-01	0,87	0,558	0,00000E+00
Mast	PTPN7	2,05957E-147	6,02429E-01	0,275	0,132	3,79310E-143
Mast	FXYD5	0,00000E+00	6,02154E-01	0,816	0,826	0,00000E+00
Mast	PLGRKT	1,89477E-23	5,99898E-01	0,251	0,209	3,48960E-19
Mast	SOX4	1,30897E-116	5,97292E-01	0,322	0,182	2,41073E-112
Mast	REX1BD	1,15179E-11	5,66219E-01	0,281	0,259	2,12125E-07
Mast	STMN1	3,79239E-73	5,62238E-01	0,379	0,28	6,98444E-69
Mast	OSBPL8	6,16475E-14	5,60095E-01	0,397	0,431	1,13536E-09
Mast	ELF1	2,65369E-24	5,55943E-01	0,457	0,49	4,88731E-20
Mast	MT-ND6	5,83405E-147	5,51265E-01	0,457	0,291	1,07446E-142
Mast	TNFAIP3	1,85086E-151	5,37324E-01	0,598	0,539	3,40873E-147
Mast	RPL34	0,00000E+00	5,30396E-01	0,867	0,555	0,00000E+00
Mast	ATP5MF	5,50421E-16	5,19027E-01	0,391	0,354	1,01371E-11
Mast	RPL11	0,00000E+00	5,17416E-01	0,877	0,56	0,00000E+00
Mast	BEX4	9,42077E-22	5,16680E-01	0,344	0,328	1,73502E-17
Mast	ELOB	3,10826E-38	5,10292E-01	0,476	0,393	5,72447E-34

Mast	TSTD1	5,22058E-32	5,10145E-01	0,288	0,236	9,61474E-28
Mast	PRDX6	5,36980E-37	5,07135E-01	0,502	0,562	9,88956E-33
Mast	RPL24	0,00000E+00	5,06846E-01	0,79	0,521	0,00000E+00
Mast	PTMA	0,00000E+00	5,00293E-01	0,98	0,961	0,00000E+00
Mast	SEM1	9,11020E-13	4,82838E-01	0,343	0,321	1,67783E-08
Mast	RPS27A	0,00000E+00	4,76739E-01	0,874	0,555	0,00000E+00
Mast	RPS7	0,00000E+00	4,67534E-01	0,838	0,541	0,00000E+00
Mast	ATP5MG	4,92980E-48	4,66960E-01	0,522	0,412	9,07922E-44
Mast	VIM	0,00000E+00	4,56983E-01	0,957	0,942	0,00000E+00
Mast	FHL3	6,89691E-34	4,55129E-01	0,26	0,201	1,27020E-29
Mast	MT-CYB	0,00000E+00	4,23721E-01	0,872	0,558	0,00000E+00
Mast	RPS4X	0,00000E+00	4,12805E-01	0,853	0,545	0,00000E+00
Mast	PEBP1	9,16648E-22	4,11872E-01	0,504	0,569	1,68819E-17
Mast	ATP5MC3	2,59444E-14	4,01265E-01	0,391	0,351	4,77819E-10
Mast	CCNI	8,34700E-62	3,97843E-01	0,672	0,793	1,53727E-57
Mast	C4orf48	5,20825E-11	3,93369E-01	0,457	0,533	9,59203E-07
Mast	DNAJC19	1,05788E-27	3,87024E-01	0,274	0,23	1,94829E-23
Mast	NDUFA4	2,65563E-09	3,82774E-01	0,593	0,755	4,89087E-05
Mast	RPL12	0,00000E+00	3,59696E-01	0,862	0,555	0,00000E+00
Mast	RBBP6	2,05268E-07	3,56053E-01	0,219	0,294	3,78042E-03
Mast	SLC2A3	6,73098E-256	3,54084E-01	0,627	0,42	1,23964E-251
Mast	PHF20	3,21537E-07	3,53480E-01	0,276	0,275	5,92174E-03
Mast	RPL7A	0,00000E+00	3,32230E-01	0,856	0,538	0,00000E+00
Mast	RPS27L	3,89122E-24	3,24216E-01	0,397	0,36	7,16647E-20
Mast	LAGE3	2,79994E-12	3,19468E-01	0,224	0,326	5,15664E-08
Mast	RASSF5	2,07538E-40	3,11946E-01	0,327	0,268	3,82222E-36
Mast	CNIH1	6,60227E-27	3,09019E-01	0,387	0,385	1,21594E-22
Mast	SMAP1	3,52064E-10	2,99666E-01	0,234	0,33	6,48396E-06
Mast	RPS8	0,00000E+00	2,93009E-01	0,875	0,557	0,00000E+00
Mast	REL	8,08232E-07	2,90457E-01	0,435	0,61	1,48852E-02
Mast	MLEC	2,76649E-10	2,80920E-01	0,248	0,354	5,09504E-06
Mast	IFITM1	2,41070E-62	2,79775E-01	0,361	0,247	4,43979E-58
Mast	CKLF	1,62916E-09	2,70473E-01	0,448	0,51	3,00042E-05
Mast	RSBN1L	2,22483E-20	2,66509E-01	0,188	0,296	4,09746E-16
Mast	KHDRBS1	3,61471E-09	2,54563E-01	0,325	0,464	6,65722E-05
Mast	RPL35A	0,00000E+00	2,54307E-01	0,817	0,544	0,00000E+00
Mast	PPP1R18	5,70679E-102	-2,50379E-01	0,256	0,518	1,05102E-97
Mast	C9orf78	5,12127E-35	-2,51473E-01	0,203	0,345	9,43184E-31
Mast	MYCBP2	3,15023E-73	-2,52629E-01	0,161	0,349	5,80177E-69
Mast	RAB2A	1,41940E-80	-2,53002E-01	0,258	0,502	2,61411E-76

Mast	PTBP3	1,25195E-46	-2,53376E-01	0,181	0,336	2,30572E-42
Mast	PNISR	2,78600E-34	-2,55146E-01	0,316	0,506	5,13098E-30
Mast	PDIA6	6,38313E-24	-2,55574E-01	0,348	0,532	1,17558E-19
Mast	PPIG	4,03221E-38	-2,56237E-01	0,279	0,465	7,42612E-34
Mast	UHMK1	9,60316E-68	-2,56287E-01	0,105	0,255	1,76861E-63
Mast	PACSIN2	8,90238E-56	-2,57148E-01	0,132	0,279	1,63955E-51
Mast	ARF6	5,98379E-80	-2,57200E-01	0,216	0,433	1,10203E-75
Mast	VPS4B	4,25530E-65	-2,58994E-01	0,127	0,286	7,83699E-61
Mast	FNBP4	3,37395E-14	-2,59272E-01	0,209	0,307	6,21380E-10
Mast	RBCK1	4,91291E-49	-2,59657E-01	0,212	0,385	9,04810E-45
Mast	NDFIP1	5,80354E-136	-2,59918E-01	0,23	0,523	1,06884E-131
Mast	MBD4	4,60095E-47	-2,61603E-01	0,126	0,258	8,47357E-43
Mast	NIPBL	8,01308E-48	-2,61704E-01	0,198	0,361	1,47577E-43
Mast	TOMM7	4,28950E-31	-2,61945E-01	0,56	0,807	7,89997E-27
Mast	TMEM25	1,50429E-32	-2,62188E-01	0,438	0,683	2,77045E-28
Mast	CDKN1B	8,42380E-90	-2,64553E-01	0,084	0,258	1,55141E-85
Mast	AFF4	1,34556E-20	-2,65643E-01	0,252	0,386	2,47812E-16
Mast	SAFB	1,87867E-25	-2,65966E-01	0,15	0,253	3,45994E-21
Mast	GTF2F1	1,00404E-30	-2,66361E-01	0,15	0,262	1,84914E-26
Mast	SNAP29	9,59979E-63	-2,67702E-01	0,136	0,295	1,76799E-58
Mast	ELMO1	9,70325E-18	-2,68567E-01	0,232	0,347	1,78705E-13
Mast	KLF3	1,26837E-75	-2,68973E-01	0,102	0,264	2,33596E-71
Mast	IPO7	9,82616E-70	-2,70029E-01	0,098	0,25	1,80968E-65
Mast	GYPC	1,06576E-41	-2,70994E-01	0,174	0,31	1,96281E-37
Mast	APOL6	2,45163E-86	-2,71436E-01	0,1	0,271	4,51517E-82
Mast	SSU72	2,99056E-43	-2,71809E-01	0,225	0,394	5,50772E-39
Mast	FIS1	1,71681E-18	-2,73212E-01	0,356	0,538	3,16184E-14
Mast	SOD1	5,30770E-09	-2,73434E-01	0,409	0,574	9,77520E-05
Mast	HSPB11	8,88961E-45	-2,73504E-01	0,139	0,276	1,63720E-40
Mast	FCER1A	1,45872E-88	-2,73732E-01	0,259	0,13	2,68653E-84
Mast	NFKBIA	4,59921E-39	-2,73794E-01	0,732	0,814	8,47036E-35
Mast	NDUFB6	2,03280E-52	-2,73886E-01	0,201	0,38	3,74381E-48
Mast	MRPL43	1,76070E-52	-2,74054E-01	0,191	0,361	3,24269E-48
Mast	RPN1	2,32929E-23	-2,74294E-01	0,329	0,292	4,28986E-19
Mast	GDI1	4,05643E-68	-2,74677E-01	0,147	0,321	7,47072E-64
Mast	MORF4L1	4,28556E-12	-2,76133E-01	0,455	0,665	7,89272E-08
Mast	SH3KBP1	4,11293E-20	-2,76519E-01	0,279	0,424	7,57478E-16
Mast	EPS15	7,49867E-36	-2,77572E-01	0,14	0,256	1,38103E-31
Mast	MRPL52	3,59558E-81	-2,77701E-01	0,241	0,475	6,62199E-77
Mast	NUDT21	6,15066E-46	-2,77815E-01	0,14	0,276	1,13277E-41

Mast	CD46	2,45939E-07	-2,77992E-01	0,238	0,322	4,52947E-03
Mast	SF3B2	1,72960E-76	-2,78588E-01	0,282	0,532	3,18540E-72
Mast	URM1	7,99506E-74	-2,79244E-01	0,1	0,257	1,47245E-69
Mast	CAMK1	3,47641E-69	-2,79537E-01	0,111	0,266	6,40251E-65
Mast	STARD3N	1,06151E-46	-2,80171E-01	0,141	0,28	1,95498E-42
Mast	RPS13	0,00000E+00	-2,82302E-01	0,842	0,552	0,00000E+00
Mast	PRKCSH	1,20265E-94	-2,83092E-01	0,132	0,333	2,21492E-90
Mast	HNRNPA	4,05505E-38	-2,84083E-01	0,407	0,645	7,46818E-34
Mast	RPS24	1,08417E-290	-2,84162E-01	0,853	0,559	1,99671E-286
Mast	MRPS18	1,12519E-21	-2,85395E-01	0,194	0,309	2,07227E-17
Mast	DNAJC15	3,66381E-127	-2,85460E-01	0,235	0,518	6,74764E-123
Mast	MRPL54	2,28406E-62	-2,86208E-01	0,217	0,415	4,20656E-58
Mast	MIF	4,15003E-14	-2,88157E-01	0,614	0,732	7,64311E-10
Mast	TAOK3	8,38191E-37	-2,88622E-01	0,281	0,463	1,54370E-32
Mast	TXNL1	1,70048E-43	-2,91518E-01	0,22	0,392	3,13177E-39
Mast	C1D	7,18872E-64	-2,93265E-01	0,126	0,283	1,32395E-59
Mast	TMEM25	1,45334E-62	-2,95072E-01	0,221	0,425	2,67662E-58
Mast	SCAF11	1,33984E-52	-2,95143E-01	0,288	0,503	2,46758E-48
Mast	B3GAT3	1,27850E-63	-2,95875E-01	0,104	0,251	2,35461E-59
Mast	PTMS	7,58639E-68	-2,95875E-01	0,192	0,375	1,39719E-63
Mast	TMUB1	1,58696E-71	-2,96343E-01	0,111	0,272	2,92270E-67
Mast	DNAJB12	3,95635E-52	-2,96640E-01	0,117	0,253	7,28640E-48
Mast	MFSD10	7,61549E-34	-2,97224E-01	0,155	0,278	1,40255E-29
Mast	VPS36	3,39827E-56	-2,97604E-01	0,143	0,297	6,25860E-52
Mast	EIF2AK2	2,24799E-47	-2,98132E-01	0,135	0,27	4,14012E-43
Mast	TMEM18	9,98492E-51	-2,98932E-01	0,117	0,251	1,83892E-46
Mast	PEF1	1,19622E-17	-2,99200E-01	0,162	0,254	2,20308E-13
Mast	PDAP1	2,59819E-90	-3,00023E-01	0,145	0,347	4,78509E-86
Mast	HNRNPD	4,78026E-73	-3,01826E-01	0,164	0,356	8,80380E-69
Mast	BRD4	1,18902E-125	-3,02130E-01	0,117	0,347	2,18981E-121
Mast	PDIA3	8,42527E-07	-3,02405E-01	0,55	0,684	1,55168E-02
Mast	SH3GLB1	6,84750E-36	-3,02732E-01	0,266	0,438	1,26110E-31
Mast	CCDC59	1,05545E-27	-3,04273E-01	0,148	0,256	1,94383E-23
Mast	CD33	1,20742E-11	-3,04489E-01	0,223	0,314	2,22370E-07
Mast	CD2BP2	2,51609E-31	-3,04883E-01	0,145	0,258	4,63387E-27
Mast	ZMAT2	3,45203E-73	-3,05746E-01	0,154	0,337	6,35761E-69
Mast	LRRFIP1	1,18285E-31	-3,05863E-01	0,459	0,699	2,17845E-27
Mast	CHCHD1	7,86497E-122	-3,07537E-01	0,264	0,534	1,44849E-117
Mast	DENR	1,83332E-35	-3,08284E-01	0,139	0,256	3,37642E-31
Mast	IL18	6,15481E-70	-3,08418E-01	0,255	0,455	1,13353E-65

Mast	UGP2	1,71812E-55	-3,09867E-01	0,154	0,311	3,16427E-51
Mast	POLE4	2,34070E-71	-3,10850E-01	0,158	0,343	4,31086E-67
Mast	ZFR	4,86806E-32	-3,11206E-01	0,142	0,256	8,96550E-28
Mast	PNPLA8	1,63730E-43	-3,12641E-01	0,126	0,251	3,01542E-39
Mast	DPY30	5,79989E-51	-3,12792E-01	0,118	0,253	1,06817E-46
Mast	SETX	5,70472E-90	-3,13793E-01	0,109	0,292	1,05064E-85
Mast	JPT1	8,36236E-46	-3,15274E-01	0,167	0,291	1,54010E-41
Mast	FOXN2	8,89686E-88	-3,15501E-01	0,097	0,274	1,63853E-83
Mast	RSL24D1	3,86787E-56	-3,15692E-01	0,247	0,446	7,12345E-52
Mast	MRPS18I	3,47490E-61	-3,15779E-01	0,131	0,287	6,39972E-57
Mast	OCIAD1	4,73506E-28	-3,15856E-01	0,278	0,445	8,72056E-24
Mast	SNRPF	4,84557E-109	-3,16314E-01	0,171	0,409	8,92409E-105
Mast	XPO1	9,74795E-62	-3,18194E-01	0,116	0,265	1,79528E-57
Mast	BIRC6	6,93166E-38	-3,19062E-01	0,136	0,257	1,27660E-33
Mast	SYNCRIP	1,45632E-93	-3,19135E-01	0,134	0,336	2,68211E-89
Mast	NDUFA8	4,91435E-67	-3,20151E-01	0,136	0,301	9,05075E-63
Mast	ARIH2	2,31716E-28	-3,20187E-01	0,156	0,266	4,26751E-24
Mast	CAPRIN1	3,68026E-47	-3,21472E-01	0,187	0,346	6,77793E-43
Mast	NUDT3	1,53161E-150	-3,21612E-01	0,099	0,346	2,82076E-146
Mast	NDUFS3	2,23625E-88	-3,23816E-01	0,149	0,354	4,11851E-84
Mast	HSP90AA	5,47780E-17	-3,23983E-01	0,724	0,89	1,00885E-12
Mast	LRRC59	1,30252E-106	-3,24007E-01	0,099	0,296	2,39885E-102
Mast	CCNDBP1	5,06490E-20	-3,25290E-01	0,178	0,28	9,32802E-16
Mast	ZC3H15	5,28000E-56	-3,25336E-01	0,249	0,451	9,72418E-52
Mast	SHOC2	4,73659E-60	-3,26107E-01	0,124	0,274	8,72339E-56
Mast	ARFGAP3	2,30309E-54	-3,26750E-01	0,133	0,277	4,24161E-50
Mast	STAU1	2,51471E-71	-3,27180E-01	0,131	0,301	4,63134E-67
Mast	TRA2B	7,07263E-34	-3,27899E-01	0,295	0,483	1,30257E-29
Mast	SNX3	2,97801E-40	-3,29118E-01	0,462	0,706	5,48460E-36
Mast	ITSN2	4,13747E-50	-3,29430E-01	0,22	0,398	7,61997E-46
Mast	UBE2R2	5,74529E-105	-3,30164E-01	0,087	0,277	1,05811E-100
Mast	GPAA1	3,95969E-13	-3,30908E-01	0,178	0,261	7,29257E-09
Mast	CANX	6,10218E-10	-3,31632E-01	0,456	0,652	1,12384E-05
Mast	CHMP4B	6,89234E-158	-3,33631E-01	0,151	0,427	1,26936E-153
Mast	EIF2S2	4,99191E-101	-3,33734E-01	0,227	0,479	9,19360E-97
Mast	MAPRE1	1,77095E-23	-3,34112E-01	0,253	0,399	3,26156E-19
Mast	SUMO1	5,83944E-72	-3,34651E-01	0,256	0,483	1,07545E-67
Mast	RPL19	0,00000E+00	-3,35332E-01	0,86	0,561	0,00000E+00
Mast	GOLGA7	1,97774E-50	-3,35345E-01	0,152	0,302	3,64240E-46
Mast	FUCA2	1,07573E-64	-3,35775E-01	0,152	0,319	1,98117E-60

Mast	TPRKB	1,66211E-46	-3,38370E-01	0,121	0,25	3,06110E-42
Mast	MT-ATP6	4,08443E-93	-3,38443E-01	0,821	0,561	7,52229E-89
Mast	APPL1	1,00100E-125	-3,38709E-01	0,137	0,377	1,84353E-121
Mast	OGFR	1,63457E-140	-3,39773E-01	0,11	0,35	3,01038E-136
Mast	METAP2	6,92575E-66	-3,43564E-01	0,138	0,304	1,27552E-61
Mast	SYF2	1,22335E-66	-3,44212E-01	0,23	0,438	2,25305E-62
Mast	IFITM2	5,31217E-187	-3,44486E-01	0,698	0,643	9,78343E-183
Mast	EIF4EBP2	2,39644E-91	-3,44574E-01	0,083	0,252	4,41352E-87
Mast	PRMT1	1,54316E-36	-3,46561E-01	0,178	0,316	2,84204E-32
Mast	MCTP1	8,63675E-25	-3,47700E-01	0,153	0,254	1,59063E-20
Mast	PAK1	2,31064E-37	-3,47816E-01	0,274	0,448	4,25551E-33
Mast	PTPN18	2,74409E-88	-3,47902E-01	0,159	0,364	5,05378E-84
Mast	USF2	7,49605E-93	-3,49539E-01	0,154	0,359	1,38055E-88
Mast	ROMO1	2,93012E-44	-3,49542E-01	0,33	0,544	5,39641E-40
Mast	IL6R	4,41706E-60	-3,50794E-01	0,108	0,251	8,13490E-56
Mast	ARHGEF2	2,71552E-41	-3,52580E-01	0,131	0,258	5,00118E-37
Mast	TBCA	5,38361E-69	-3,52693E-01	0,344	0,606	9,91499E-65
Mast	PFDN2	3,34695E-83	-3,52840E-01	0,225	0,457	6,16408E-79
Mast	CPPED1	4,02612E-24	-3,53331E-01	0,172	0,277	7,41491E-20
Mast	STX10	5,69752E-69	-3,54495E-01	0,149	0,321	1,04931E-64
Mast	ATM	4,85702E-96	-3,54541E-01	0,089	0,271	8,94517E-92
Mast	PTPN1	1,97877E-85	-3,55819E-01	0,2	0,415	3,64430E-81
Mast	CCDC12	6,88942E-13	-3,56305E-01	0,202	0,293	1,26882E-08
Mast	NCOA4	2,05256E-44	-3,58516E-01	0,322	0,545	3,78019E-40
Mast	CHMP2B	3,48388E-53	-3,58653E-01	0,154	0,309	6,41625E-49
Mast	TCF25	1,19949E-20	-3,59096E-01	0,377	0,575	2,20909E-16
Mast	ANKRD44	4,70648E-07	-3,61044E-01	0,221	0,296	8,66793E-03
Mast	CWC15	4,94126E-80	-3,61108E-01	0,152	0,344	9,10031E-76
Mast	MRPL34	6,54117E-53	-3,62573E-01	0,206	0,385	1,20469E-48
Mast	DICER1	8,80448E-99	-3,65418E-01	0,095	0,282	1,62152E-94
Mast	FEZ2	3,00699E-142	-3,65557E-01	0,118	0,366	5,53796E-138
Mast	FAM162A	1,80813E-74	-3,66139E-01	0,165	0,358	3,33003E-70
Mast	AIMP1	1,12655E-77	-3,66657E-01	0,126	0,303	2,07477E-73
Mast	SNF8	1,09940E-101	-3,67102E-01	0,11	0,307	2,02476E-97
Mast	BCLAF1	4,66256E-31	-3,67114E-01	0,237	0,391	8,58704E-27
Mast	CNTRL	1,45763E-61	-3,68581E-01	0,108	0,25	2,68451E-57
Mast	PCNP	2,82331E-39	-3,68730E-01	0,188	0,332	5,19970E-35
Mast	UBL3	2,42960E-133	-3,69147E-01	0,055	0,258	4,47460E-129
Mast	FAM120A	9,90416E-119	-3,69509E-01	0,097	0,308	1,82405E-114
Mast	RAD23A	1,35646E-88	-3,69686E-01	0,266	0,521	2,49818E-84

Mast	RBM25	2,84485E-61	-3,70202E-01	0,283	0,509	5,23937E-57
Mast	PKIB	4,52337E-77	-3,70670E-01	0,094	0,256	8,33069E-73
Mast	TUBGCP2	1,12106E-27	-3,72737E-01	0,188	0,311	2,06465E-23
Mast	EID1	1,74236E-26	-3,73564E-01	0,38	0,594	3,20891E-22
Mast	ANAPC5	2,09809E-25	-3,73588E-01	0,18	0,295	3,86405E-21
Mast	MFF	4,81105E-62	-3,73710E-01	0,16	0,331	8,86052E-58
Mast	SLC25A1	8,08478E-66	-3,74347E-01	0,146	0,315	1,48897E-61
Mast	THOC7	8,17181E-38	-3,75288E-01	0,197	0,348	1,50500E-33
Mast	TM6SF1	2,96656E-96	-3,75471E-01	0,102	0,284	5,46352E-92
Mast	MYO9B	1,09453E-83	-3,76164E-01	0,19	0,402	2,01579E-79
Mast	UBB	1,76437E-184	-3,76419E-01	0,764	0,814	3,24944E-180
Mast	ANXA7	1,38013E-24	-3,76731E-01	0,241	0,382	2,54179E-20
Mast	MED28	6,07328E-90	-3,77226E-01	0,119	0,307	1,11852E-85
Mast	MANBA	9,44287E-51	-3,77393E-01	0,184	0,347	1,73909E-46
Mast	G3BP1	1,53445E-44	-3,78163E-01	0,17	0,318	2,82600E-40
Mast	RNASEH2	2,56215E-65	-3,78452E-01	0,176	0,358	4,71871E-61
Mast	RSRC2	1,12569E-42	-3,78765E-01	0,23	0,402	2,07318E-38
Mast	GINM1	8,67985E-80	-3,80547E-01	0,1	0,266	1,59857E-75
Mast	PSMD3	7,97633E-92	-3,80764E-01	0,108	0,292	1,46900E-87
Mast	SNRPA	3,90414E-51	-3,80826E-01	0,154	0,306	7,19026E-47
Mast	DDIT4	2,89559E-68	-3,81426E-01	0,532	0,493	5,33281E-64
Mast	JTB	7,62502E-71	-3,81686E-01	0,322	0,577	1,40430E-66
Mast	SPOP	4,35096E-73	-3,81732E-01	0,114	0,278	8,01316E-69
Mast	DDT	3,51182E-66	-3,81794E-01	0,242	0,454	6,46771E-62
Mast	OSTC	8,78984E-31	-3,82033E-01	0,321	0,512	1,61882E-26
Mast	NDUFV3	9,61953E-120	-3,82034E-01	0,087	0,294	1,77163E-115
Mast	MED10	6,68960E-40	-3,82259E-01	0,132	0,254	1,23202E-35
Mast	CCDC124	5,98547E-102	-3,83315E-01	0,149	0,367	1,10234E-97
Mast	EIF5	7,50750E-16	-3,83355E-01	0,409	0,605	1,38266E-11
Mast	ITPR2	1,58274E-68	-3,83918E-01	0,126	0,288	2,91494E-64
Mast	PUM1	1,27670E-65	-3,85239E-01	0,103	0,253	2,35129E-61
Mast	CMTM3	6,83570E-68	-3,85422E-01	0,19	0,374	1,25893E-63
Mast	RPS14	1,36797E-200	-3,86071E-01	0,839	0,557	2,51940E-196
Mast	PRRC2C	2,07565E-93	-3,86368E-01	0,324	0,612	3,82272E-89
Mast	CHCHD1	2,74434E-90	-3,87234E-01	0,102	0,282	5,05425E-86
Mast	SNRNP2(	3,40410E-52	-3,87274E-01	0,127	0,268	6,26932E-48
Mast	FOXN3	2,06395E-102	-3,88020E-01	0,154	0,375	3,80118E-98
Mast	DDX39A	4,49496E-16	-3,88273E-01	0,199	0,298	8,27837E-12
Mast	HPRT1	2,58908E-36	-3,88339E-01	0,149	0,271	4,76831E-32
Mast	ERGIC1	9,84210E-75	-3,90524E-01	0,132	0,302	1,81262E-70

Mast	BBX	1,30392E-70	-3,93393E-01	0,137	0,309	2,40143E-66
Mast	SMARCB	3,64697E-70	-3,93935E-01	0,136	0,306	6,71663E-66
Mast	LSM5	2,40701E-61	-3,94238E-01	0,178	0,355	4,43298E-57
Mast	SUGT1	5,38299E-66	-3,94538E-01	0,131	0,295	9,91385E-62
Mast	UQCRFS1	2,66234E-75	-3,94951E-01	0,267	0,508	4,90323E-71
Mast	TFG	8,47963E-21	-3,95181E-01	0,169	0,268	1,56169E-16
Mast	DHRS4	1,32508E-73	-3,97218E-01	0,117	0,282	2,44039E-69
Mast	FAM50A	1,68598E-50	-3,97678E-01	0,163	0,314	3,10508E-46
Mast	OLA1	7,51561E-61	-3,97891E-01	0,154	0,32	1,38415E-56
Mast	TFPT	3,74057E-93	-3,97918E-01	0,081	0,255	6,88900E-89
Mast	COMMD	6,54438E-50	-3,97999E-01	0,167	0,322	1,20528E-45
Mast	SEC61G	1,98124E-12	-3,98557E-01	0,425	0,612	3,64885E-08
Mast	CRTAP	7,87097E-19	-3,99246E-01	0,364	0,532	1,44960E-14
Mast	CCT2	5,49422E-22	-3,99295E-01	0,224	0,348	1,01187E-17
Mast	ETV6	2,72611E-73	-3,99428E-01	0,131	0,302	5,02068E-69
Mast	PDCD6	4,35601E-30	-3,99490E-01	0,272	0,436	8,02246E-26
Mast	ATP6V0C	2,35424E-91	-3,99818E-01	0,486	0,713	4,33581E-87
Mast	BZW1	6,10068E-14	-4,00510E-01	0,311	0,452	1,12356E-09
Mast	MRPS34	3,16795E-54	-4,01285E-01	0,218	0,407	5,83441E-50
Mast	DERL1	7,37013E-53	-4,01293E-01	0,137	0,283	1,35736E-48
Mast	DIAPH1	4,96386E-60	-4,01827E-01	0,11	0,252	9,14193E-56
Mast	SRSF11	2,22028E-37	-4,02125E-01	0,323	0,529	4,08909E-33
Mast	BANF1	2,83163E-33	-4,02701E-01	0,35	0,562	5,21501E-29
Mast	PSMB5	6,13601E-48	-4,03362E-01	0,169	0,326	1,13007E-43
Mast	NFYC	4,79925E-51	-4,03634E-01	0,129	0,267	8,83879E-47
Mast	COA3	6,10409E-77	-4,04706E-01	0,146	0,332	1,12419E-72
Mast	SPSB3	6,52331E-44	-4,05612E-01	0,159	0,299	1,20140E-39
Mast	ZSWIM7	8,06905E-55	-4,06536E-01	0,119	0,259	1,48608E-50
Mast	CTDNEP1	5,96150E-137	-4,06594E-01	0,062	0,275	1,09793E-132
Mast	MIIP	3,58012E-21	-4,07121E-01	0,153	0,251	6,59351E-17
Mast	CYB5R3	4,84796E-33	-4,07582E-01	0,257	0,421	8,92848E-29
Mast	HNRNPU	8,20490E-126	-4,07717E-01	0,173	0,428	1,51110E-121
Mast	SF3A1	7,18962E-57	-4,08585E-01	0,144	0,299	1,32411E-52
Mast	CLPTM1	2,31742E-91	-4,08603E-01	0,093	0,271	4,26800E-87
Mast	DCTN3	4,90891E-35	-4,08774E-01	0,218	0,37	9,04074E-31
Mast	G6PD	6,75466E-86	-4,08803E-01	0,101	0,272	1,24401E-81
Mast	UBE2I	1,68398E-53	-4,09066E-01	0,312	0,542	3,10138E-49
Mast	CIAO1	2,87386E-58	-4,09322E-01	0,113	0,256	5,29279E-54
Mast	TRMT112	8,12043E-11	-4,09469E-01	0,433	0,631	1,49554E-06
Mast	DSTN	6,39923E-17	-4,09956E-01	0,236	0,354	1,17855E-12

Mast	EML4	7,22844E-87	-4,10880E-01	0,167	0,369	1,33126E-82
Mast	RABAC1	9,36651E-41	-4,10896E-01	0,328	0,538	1,72503E-36
Mast	PPM1G	4,58971E-82	-4,12073E-01	0,175	0,381	8,45286E-78
Mast	FIBP	1,61016E-60	-4,12648E-01	0,194	0,38	2,96544E-56
Mast	IFI27L2	4,28559E-97	-4,14499E-01	0,192	0,424	7,89276E-93
Mast	TMBIM4	1,43062E-13	-4,15255E-01	0,235	0,338	2,63478E-09
Mast	TRIP12	1,86370E-70	-4,15582E-01	0,123	0,287	3,43237E-66
Mast	TRAPPC1	5,49985E-124	-4,15648E-01	0,319	0,63	1,01291E-119
Mast	SRRM1	1,36489E-39	-4,15764E-01	0,352	0,579	2,51372E-35
Mast	XBP1	6,34025E-47	-4,17592E-01	0,253	0,437	1,16768E-42
Mast	TNPO1	1,12732E-90	-4,18125E-01	0,087	0,261	2,07618E-86
Mast	COMMD	1,87118E-59	-4,18384E-01	0,164	0,333	3,44614E-55
Mast	C7orf50	2,33634E-26	-4,18588E-01	0,215	0,346	4,30284E-22
Mast	PRKAR1A	2,40739E-19	-4,19162E-01	0,324	0,491	4,43370E-15
Mast	TAF1D	2,17357E-32	-4,20187E-01	0,238	0,395	4,00306E-28
Mast	TOMM20	3,11434E-76	-4,20207E-01	0,297	0,551	5,73568E-72
Mast	HK1	1,14417E-79	-4,20519E-01	0,106	0,273	2,10721E-75
Mast	ITGB1BP1	1,18962E-22	-4,20592E-01	0,157	0,256	2,19093E-18
Mast	RBMX	5,35099E-08	-4,20707E-01	0,309	0,433	9,85491E-04
Mast	CAMTA1	4,50386E-111	-4,20887E-01	0,148	0,379	8,29476E-107
Mast	CSK	5,99032E-55	-4,21719E-01	0,19	0,358	1,10324E-50
Mast	UBE2L3	6,05671E-87	-4,22058E-01	0,262	0,518	1,11546E-82
Mast	CISD3	1,67415E-118	-4,23419E-01	0,106	0,319	3,08329E-114
Mast	TMED4	1,04550E-36	-4,23512E-01	0,168	0,302	1,92550E-32
Mast	UBE2E1	7,05982E-148	-4,24079E-01	0,088	0,325	1,30021E-143
Mast	ITGA4	2,49674E-44	-4,24758E-01	0,22	0,379	4,59824E-40
Mast	DOCK10	1,48350E-26	-4,25202E-01	0,204	0,331	2,73216E-22
Mast	CHURC1	9,41957E-15	-4,25599E-01	0,221	0,325	1,73480E-10
Mast	RAB5A	1,32369E-43	-4,25963E-01	0,172	0,319	2,43785E-39
Mast	PER1	2,98432E-52	-4,26209E-01	0,175	0,33	5,49622E-48
Mast	SZRD1	1,44048E-104	-4,26707E-01	0,108	0,307	2,65294E-100
Mast	PSTPIP2	1,39818E-54	-4,27070E-01	0,124	0,259	2,57503E-50
Mast	NDUFA11	3,30918E-21	-4,27444E-01	0,307	0,465	6,09452E-17
Mast	WIPF1	4,13857E-121	-4,27519E-01	0,217	0,483	7,62201E-117
Mast	HDLBP	1,90070E-28	-4,27549E-01	0,26	0,42	3,50052E-24
Mast	DGCR6L	7,18529E-51	-4,27640E-01	0,128	0,271	1,32331E-46
Mast	GLUL	1,07777E-09	-4,27696E-01	0,719	0,81	1,98492E-05
Mast	VSIR	1,40395E-56	-4,28514E-01	0,151	0,282	2,58565E-52
Mast	COPS5	2,06728E-37	-4,28551E-01	0,159	0,288	3,80730E-33
Mast	FGFR1OP	5,77525E-69	-4,29671E-01	0,144	0,317	1,06363E-64

Mast	NUDC	9,24676E-50	-4,30298E-01	0,213	0,392	1,70298E-45
Mast	UBXN4	5,71315E-34	-4,30491E-01	0,329	0,534	1,05219E-29
Mast	DNAJB6	2,00167E-62	-4,31562E-01	0,304	0,537	3,68648E-58
Mast	YIPF4	3,31144E-76	-4,32719E-01	0,109	0,275	6,09868E-72
Mast	SMIM12	9,90021E-78	-4,33205E-01	0,11	0,279	1,82332E-73
Mast	SEC11A	4,11648E-25	-4,33789E-01	0,455	0,682	7,58131E-21
Mast	SP100	1,39732E-75	-4,34526E-01	0,25	0,481	2,57344E-71
Mast	RPS28	7,86426E-173	-4,35085E-01	0,834	0,564	1,44836E-168
Mast	TIMM17I	9,08530E-81	-4,35219E-01	0,146	0,337	1,67324E-76
Mast	TSC22D3	5,23874E-07	-4,35545E-01	0,544	0,705	9,64818E-03
Mast	GM2A	1,86783E-61	-4,37882E-01	0,207	0,387	3,43999E-57
Mast	CHCHD5	2,16866E-104	-4,38467E-01	0,11	0,312	3,99402E-100
Mast	TMEM9E	7,45873E-45	-4,39091E-01	0,213	0,384	1,37367E-40
Mast	PIM3	2,99350E-59	-4,40057E-01	0,142	0,296	5,51312E-55
Mast	BST2	5,79752E-23	-4,40365E-01	0,517	0,74	1,06773E-18
Mast	PHYKPL	1,01340E-12	-4,41510E-01	0,182	0,264	1,86638E-08
Mast	SMG1	1,24541E-41	-4,41746E-01	0,184	0,328	2,29366E-37
Mast	HTATIP2	2,41594E-80	-4,41907E-01	0,125	0,303	4,44943E-76
Mast	RPS2	1,87395E-64	-4,42696E-01	0,775	0,548	3,45125E-60
Mast	SNRPD1	1,15533E-57	-4,43244E-01	0,215	0,404	2,12776E-53
Mast	FRG1	3,63135E-50	-4,43403E-01	0,118	0,25	6,68787E-46
Mast	COA6	1,09811E-104	-4,43474E-01	0,079	0,265	2,02239E-100
Mast	RAP1A	6,47030E-172	-4,43985E-01	0,256	0,587	1,19164E-167
Mast	ADAR	3,02884E-111	-4,44011E-01	0,195	0,444	5,57822E-107
Mast	GLO1	1,05712E-51	-4,44089E-01	0,118	0,252	1,94690E-47
Mast	POLR2F	1,90665E-62	-4,44355E-01	0,155	0,324	3,51148E-58
Mast	CHCHD3	3,40477E-50	-4,46884E-01	0,124	0,258	6,27056E-46
Mast	CLNS1A	1,05373E-43	-4,48250E-01	0,254	0,433	1,94066E-39
Mast	CSNK1D	1,52078E-62	-4,48563E-01	0,178	0,355	2,80082E-58
Mast	GCC2	1,93353E-65	-4,48947E-01	0,129	0,291	3,56098E-61
Mast	SPCS2	3,21601E-21	-4,49388E-01	0,33	0,504	5,92293E-17
Mast	MAP7D1	2,36783E-147	-4,49665E-01	0,069	0,295	4,36084E-143
Mast	NDUFB1(	1,65778E-47	-4,49717E-01	0,341	0,574	3,05313E-43
Mast	CCAR1	1,83498E-52	-4,50131E-01	0,132	0,277	3,37948E-48
Mast	KIF5B	7,03964E-157	-4,50238E-01	0,207	0,507	1,29649E-152
Mast	TIMM8B	9,39686E-129	-4,51153E-01	0,176	0,434	1,73062E-124
Mast	PTDSS1	5,16392E-14	-4,51195E-01	0,188	0,276	9,51040E-10
Mast	UQCRQ	8,06506E-15	-4,51240E-01	0,462	0,68	1,48534E-10
Mast	ARPC5L	1,55752E-108	-4,51476E-01	0,089	0,284	2,86848E-104
Mast	APH1A	2,33623E-146	-4,52937E-01	0,222	0,518	4,30263E-142

Mast	CCDC88A	8,08648E-111	-4,54290E-01	0,252	0,519	1,48929E-106
Mast	DNAJA2	1,20489E-82	-4,54712E-01	0,14	0,329	2,21905E-78
Mast	TXNDC12	3,30193E-108	-4,54810E-01	0,137	0,355	6,08117E-104
Mast	QKI	2,10981E-153	-4,55453E-01	0,121	0,385	3,88564E-149
Mast	MAP4	1,29760E-55	-4,57538E-01	0,124	0,267	2,38979E-51
Mast	TMEM13	2,14401E-47	-4,57606E-01	0,141	0,282	3,94862E-43
Mast	RTN4	1,04835E-153	-4,57685E-01	0,332	0,672	1,93074E-149
Mast	TOMM22	2,65540E-63	-4,58236E-01	0,232	0,437	4,89046E-59
Mast	EIF1B	1,18567E-39	-4,58674E-01	0,283	0,473	2,18365E-35
Mast	USP8	4,87451E-66	-4,58996E-01	0,122	0,282	8,97738E-62
Mast	PCBP2	9,82221E-37	-4,59410E-01	0,551	0,815	1,80896E-32
Mast	POLR2J	9,35065E-69	-4,59591E-01	0,144	0,317	1,72211E-64
Mast	TRAPPC6	1,29621E-104	-4,59855E-01	0,071	0,253	2,38722E-100
Mast	POLD4	6,34278E-37	-4,60280E-01	0,376	0,583	1,16815E-32
Mast	TGOLN2	5,13870E-115	-4,60539E-01	0,255	0,532	9,46395E-111
Mast	NDUFA5	7,59293E-51	-4,60844E-01	0,153	0,303	1,39839E-46
Mast	ZNFX1	3,03785E-89	-4,61060E-01	0,093	0,268	5,59481E-85
Mast	CDK5RAP	2,02078E-46	-4,61098E-01	0,168	0,317	3,72167E-42
Mast	FUNDC2	8,51107E-31	-4,61343E-01	0,222	0,369	1,56748E-26
Mast	CYB5A	2,36204E-71	-4,62481E-01	0,096	0,25	4,35017E-67
Mast	TMED3	1,38831E-58	-4,64969E-01	0,131	0,284	2,55684E-54
Mast	CSDE1	2,77116E-24	-4,65180E-01	0,404	0,618	5,10364E-20
Mast	SRSF1	9,97724E-28	-4,65433E-01	0,162	0,276	1,83751E-23
Mast	ACP1	3,32060E-77	-4,66098E-01	0,156	0,347	6,11554E-73
Mast	TSPAN4	3,40286E-62	-4,67154E-01	0,144	0,304	6,26704E-58
Mast	PIN1	5,13887E-49	-4,67182E-01	0,19	0,357	9,46426E-45
Mast	DNM2	2,20956E-92	-4,67521E-01	0,119	0,309	4,06936E-88
Mast	CDK2AP2	7,17711E-37	-4,67900E-01	0,246	0,408	1,32181E-32
Mast	NOSIP	2,47503E-70	-4,69786E-01	0,158	0,34	4,55827E-66
Mast	SMC1A	2,43508E-105	-4,70796E-01	0,079	0,265	4,48469E-101
Mast	CISD2	2,51242E-123	-4,70988E-01	0,146	0,386	4,62713E-119
Mast	PPP6C	1,30261E-27	-4,71469E-01	0,162	0,273	2,39902E-23
Mast	SRSF7	6,32538E-32	-4,71944E-01	0,357	0,565	1,16495E-27
Mast	UFM1	1,65673E-47	-4,72812E-01	0,18	0,336	3,05120E-43
Mast	VMA21	1,33842E-121	-4,73134E-01	0,148	0,391	2,46496E-117
Mast	IRF2	6,20024E-105	-4,74810E-01	0,131	0,341	1,14190E-100
Mast	BACH1	3,15629E-52	-4,75396E-01	0,17	0,322	5,81295E-48
Mast	MRPS16	1,02265E-59	-4,76403E-01	0,149	0,312	1,88341E-55
Mast	TIMM17A	1,03139E-58	-4,76586E-01	0,129	0,281	1,89950E-54
Mast	PMVK	1,48974E-86	-4,76594E-01	0,085	0,252	2,74366E-82

Mast	SKP1	4,37943E-10	-4,76599E-01	0,558	0,698	8,06560E-06
Mast	UBE2N	2,44531E-67	-4,76677E-01	0,193	0,387	4,50353E-63
Mast	IRAK3	2,57195E-74	-4,76726E-01	0,107	0,265	4,73677E-70
Mast	MRPL20	8,92562E-85	-4,76900E-01	0,243	0,484	1,64383E-80
Mast	AKAP9	5,07054E-35	-4,77482E-01	0,239	0,396	9,33841E-31
Mast	TMEM23	6,46650E-59	-4,78408E-01	0,255	0,465	1,19093E-54
Mast	MRPL11	4,05412E-86	-4,78897E-01	0,122	0,306	7,46648E-82
Mast	NDUFA1	1,01321E-85	-4,79234E-01	0,418	0,706	1,86602E-81
Mast	CREBL2	4,24261E-181	-4,79896E-01	0,054	0,307	7,81361E-177
Mast	KDM5A	1,81433E-83	-4,81054E-01	0,134	0,324	3,34145E-79
Mast	PNKD	7,32353E-71	-4,81471E-01	0,154	0,334	1,34877E-66
Mast	EMC6	1,16401E-57	-4,82468E-01	0,16	0,324	2,14375E-53
Mast	HSPE1	5,74938E-07	-4,83605E-01	0,454	0,613	1,05886E-02
Mast	GNS	2,81969E-112	-4,84690E-01	0,197	0,447	5,19302E-108
Mast	ID2	9,05491E-62	-4,84985E-01	0,353	0,571	1,66764E-57
Mast	BAG6	1,94673E-62	-4,85042E-01	0,129	0,283	3,58529E-58
Mast	CCS	1,96029E-40	-4,85292E-01	0,143	0,27	3,61027E-36
Mast	MAT2B	1,12340E-48	-4,86199E-01	0,184	0,346	2,06896E-44
Mast	UBE2J1	2,15096E-59	-4,86391E-01	0,157	0,312	3,96142E-55
Mast	PRPF38B	6,70730E-135	-4,86685E-01	0,187	0,453	1,23528E-130
Mast	RBMS1	1,80726E-99	-4,86793E-01	0,123	0,323	3,32844E-95
Mast	MRPL27	9,33818E-79	-4,86902E-01	0,123	0,299	1,71981E-74
Mast	EPN1	1,40722E-195	-4,87173E-01	0,095	0,378	2,59168E-191
Mast	CHD1	6,25197E-35	-4,87654E-01	0,176	0,306	1,15143E-30
Mast	LENG8	1,34861E-45	-4,87967E-01	0,153	0,293	2,48374E-41
Mast	SUPT4H1	4,27612E-46	-4,89914E-01	0,279	0,474	7,87533E-42
Mast	NEMF	1,66386E-44	-4,90230E-01	0,157	0,298	3,06432E-40
Mast	USP9X	1,50437E-101	-4,90514E-01	0,096	0,286	2,77060E-97
Mast	MT-ND2	2,54240E-218	-4,91253E-01	0,822	0,543	4,68233E-214
Mast	CYSTM1	1,29535E-16	-4,91415E-01	0,247	0,36	2,38564E-12
Mast	LUC7L3	2,67853E-17	-4,91729E-01	0,246	0,367	4,93305E-13
Mast	MRPL40	4,06137E-110	-4,92028E-01	0,087	0,285	7,47983E-106
Mast	RBM17	3,54252E-63	-4,92274E-01	0,225	0,424	6,52426E-59
Mast	RPS25	2,79535E-176	-4,92411E-01	0,754	0,533	5,14819E-172
Mast	TSEN34	7,46247E-141	-4,92541E-01	0,079	0,306	1,37436E-136
Mast	SNX9	7,50274E-136	-4,93583E-01	0,083	0,302	1,38178E-131
Mast	YWHAE	2,36187E-45	-4,93754E-01	0,359	0,596	4,34986E-41
Mast	MRPL14	4,54249E-113	-4,94163E-01	0,134	0,359	8,36591E-109
Mast	BTF3L4	1,50099E-116	-4,94196E-01	0,155	0,394	2,76437E-112
Mast	RNPEP	8,50297E-133	-4,94446E-01	0,113	0,347	1,56599E-128

Mast	DDRGK1	1,85302E-97	-4,94760E-01	0,131	0,334	3,41270E-93
Mast	MT-ATP8	7,95259E-81	-4,94996E-01	0,526	0,374	1,46463E-76
Mast	TAGLN2	4,33052E-41	-4,95058E-01	0,671	0,76	7,97552E-37
Mast	WNK1	7,77631E-131	-4,96404E-01	0,131	0,372	1,43216E-126
Mast	STX6	3,40732E-174	-4,99137E-01	0,033	0,266	6,27527E-170
Mast	ROCK1	3,63972E-183	-4,99817E-01	0,212	0,532	6,70328E-179
Mast	XRN2	1,80440E-129	-5,00078E-01	0,16	0,413	3,32317E-125
Mast	BSG	8,45501E-80	-5,00086E-01	0,313	0,58	1,55716E-75
Mast	OSTF1	3,93339E-29	-5,01313E-01	0,357	0,563	7,24412E-25
Mast	PLEC	8,74533E-177	-5,01338E-01	0,106	0,382	1,61063E-172
Mast	FLOT1	4,71292E-11	-5,01364E-01	0,372	0,535	8,67978E-07
Mast	KLF10	2,99016E-33	-5,01517E-01	0,173	0,298	5,50698E-29
Mast	N4BP2L2	6,46871E-22	-5,01524E-01	0,309	0,469	1,19134E-17
Mast	PSMC1	1,28011E-53	-5,02406E-01	0,176	0,34	2,35759E-49
Mast	RNPS1	3,74527E-39	-5,02484E-01	0,245	0,416	6,89767E-35
Mast	COX7C	2,08257E-34	-5,02997E-01	0,605	0,844	3,83548E-30
Mast	DGUOK	1,51608E-58	-5,03548E-01	0,197	0,383	2,79217E-54
Mast	TOX4	1,41361E-39	-5,03563E-01	0,159	0,292	2,60345E-35
Mast	CCT8	8,81478E-20	-5,04381E-01	0,277	0,422	1,62342E-15
Mast	ARHGDIA	2,83168E-193	-5,04433E-01	0,272	0,624	5,21510E-189
Mast	DEGS1	1,69603E-89	-5,05098E-01	0,141	0,339	3,12358E-85
Mast	PPP1R7	5,33736E-82	-5,05257E-01	0,115	0,291	9,82981E-78
Mast	SEC31A	5,17654E-49	-5,05588E-01	0,16	0,308	9,53363E-45
Mast	WBP2	1,16294E-86	-5,06029E-01	0,182	0,398	2,14178E-82
Mast	CNPPD1	1,19587E-106	-5,06136E-01	0,091	0,283	2,20243E-102
Mast	UQCRH	1,20952E-14	-5,06281E-01	0,484	0,717	2,22757E-10
Mast	CLPP	3,89724E-69	-5,07229E-01	0,115	0,276	7,17754E-65
Mast	RASSF2	7,57017E-70	-5,07519E-01	0,115	0,275	1,39420E-65
Mast	UFC1	5,37936E-51	-5,08341E-01	0,298	0,517	9,90718E-47
Mast	ATRAID	2,50378E-73	-5,08492E-01	0,251	0,481	4,61122E-69
Mast	C4orf3	6,48774E-129	-5,08817E-01	0,37	0,691	1,19485E-124
Mast	TMED5	5,82939E-76	-5,08852E-01	0,184	0,386	1,07360E-71
Mast	ETFB	7,72410E-35	-5,09532E-01	0,181	0,316	1,42255E-30
Mast	DHRS7	6,51853E-16	-5,09705E-01	0,338	0,495	1,20052E-11
Mast	METTL9	1,53240E-101	-5,10845E-01	0,157	0,373	2,82221E-97
Mast	TECR	3,04211E-95	-5,12464E-01	0,172	0,394	5,60265E-91
Mast	NDUFA3	1,19375E-51	-5,13689E-01	0,332	0,561	2,19852E-47
Mast	ZEB2	1,80241E-11	-5,14202E-01	0,448	0,616	3,31949E-07
Mast	PDXK	1,40223E-128	-5,14589E-01	0,097	0,316	2,58248E-124
Mast	ZDHHC12	3,00963E-79	-5,14606E-01	0,105	0,273	5,54284E-75

Mast	PABPC4	1,17787E-72	-5,14981E-01	0,226	0,437	2,16929E-68
Mast	DNAJC8	2,51768E-70	-5,16269E-01	0,217	0,427	4,63680E-66
Mast	CNOT7	9,74105E-38	-5,16915E-01	0,18	0,317	1,79401E-33
Mast	ILK	6,61614E-52	-5,17636E-01	0,235	0,428	1,21850E-47
Mast	MTHFD2	5,75649E-23	-5,18093E-01	0,265	0,414	1,06017E-18
Mast	PDCD10	9,31090E-70	-5,18241E-01	0,148	0,326	1,71479E-65
Mast	SUPT5H	6,10471E-82	-5,18920E-01	0,091	0,253	1,12430E-77
Mast	TMEM20	2,89076E-94	-5,19117E-01	0,109	0,297	5,32391E-90
Mast	BAZ1A	8,85096E-112	-5,19252E-01	0,19	0,434	1,63008E-107
Mast	RAB13	3,95692E-105	-5,19369E-01	0,118	0,318	7,28746E-101
Mast	IRF2BP2	2,31243E-77	-5,20031E-01	0,149	0,335	4,25881E-73
Mast	PPCS	1,82516E-73	-5,20170E-01	0,199	0,404	3,36139E-69
Mast	UQCR11	3,16417E-88	-5,20363E-01	0,48	0,774	5,82746E-84
Mast	USP15	2,73786E-54	-5,21006E-01	0,244	0,437	5,04231E-50
Mast	CD81	6,99700E-120	-5,21372E-01	0,22	0,478	1,28864E-115
Mast	CCT4	3,96627E-38	-5,21571E-01	0,264	0,444	7,30469E-34
Mast	SAMD9L	2,69308E-73	-5,21680E-01	0,124	0,291	4,95985E-69
Mast	POLR2I	1,99899E-64	-5,22458E-01	0,139	0,305	3,68153E-60
Mast	LEPROT	1,03559E-117	-5,22872E-01	0,138	0,37	1,90725E-113
Mast	AIP	6,75071E-33	-5,23176E-01	0,206	0,349	1,24328E-28
Mast	SNAPIN	5,75129E-68	-5,23386E-01	0,131	0,299	1,05921E-63
Mast	RNF5	1,58078E-69	-5,23791E-01	0,126	0,294	2,91132E-65
Mast	BCAS2	2,47493E-36	-5,23855E-01	0,149	0,271	4,55808E-32
Mast	SNRPB2	7,88020E-79	-5,26156E-01	0,206	0,42	1,45130E-74
Mast	SLC25A3	7,82492E-80	-5,26372E-01	0,206	0,422	1,44112E-75
Mast	HAGH	8,97310E-81	-5,27580E-01	0,093	0,256	1,65258E-76
Mast	IQGAP1	6,68823E-113	-5,27801E-01	0,294	0,581	1,23177E-108
Mast	RHOG	8,37785E-51	-5,28617E-01	0,483	0,744	1,54295E-46
Mast	SCAND1	6,74799E-154	-5,31427E-01	0,243	0,555	1,24278E-149
Mast	EMC7	1,74472E-65	-5,32065E-01	0,156	0,332	3,21325E-61
Mast	AGPAT2	2,18312E-78	-5,33935E-01	0,127	0,303	4,02066E-74
Mast	FKBP2	2,85646E-77	-5,34839E-01	0,207	0,423	5,26074E-73
Mast	LSM4	2,38764E-56	-5,35084E-01	0,244	0,445	4,39732E-52
Mast	BNIP3L	2,35514E-53	-5,35588E-01	0,351	0,578	4,33746E-49
Mast	FBXL5	2,41681E-25	-5,35678E-01	0,173	0,281	4,45105E-21
Mast	CUEDC2	2,65751E-82	-5,36774E-01	0,125	0,307	4,89434E-78
Mast	STARD3	1,84633E-59	-5,39573E-01	0,108	0,251	3,40039E-55
Mast	PSMC3	1,50563E-67	-5,40855E-01	0,155	0,331	2,77291E-63
Mast	CAPN1	3,68896E-54	-5,41119E-01	0,132	0,276	6,79396E-50
Mast	NSL1	4,21598E-103	-5,42012E-01	0,076	0,258	7,76457E-99

Mast	DYNC1I2	7,63647E-72	-5,42151E-01	0,104	0,263	1,40641E-67
Mast	HSPD1	2,21850E-29	-5,42599E-01	0,355	0,548	4,08580E-25
Mast	PSMG2	5,83985E-81	-5,43602E-01	0,215	0,439	1,07553E-76
Mast	IFI44L	8,69383E-96	-5,44727E-01	0,087	0,265	1,60114E-91
Mast	ARL5A	1,22904E-153	-5,44966E-01	0,173	0,447	2,26353E-149
Mast	ARPP19	6,71440E-64	-5,45056E-01	0,125	0,279	1,23659E-59
Mast	PRDX4	2,10460E-46	-5,46161E-01	0,153	0,296	3,87604E-42
Mast	SH3BGRL	4,07270E-19	-5,46536E-01	0,864	0,938	7,50069E-15
Mast	GPX4	3,03415E-30	-5,47575E-01	0,649	0,858	5,58799E-26
Mast	SSNA1	1,01330E-99	-5,47638E-01	0,184	0,42	1,86619E-95
Mast	JAK2	3,79011E-122	-5,47728E-01	0,058	0,252	6,98024E-118
Mast	KPNB1	1,87478E-137	-5,48476E-01	0,186	0,458	3,45278E-133
Mast	FAM204A	1,23071E-66	-5,51582E-01	0,134	0,298	2,26660E-62
Mast	GNB1	1,11248E-145	-5,51651E-01	0,143	0,406	2,04886E-141
Mast	ST14	1,04100E-162	-5,51783E-01	0,051	0,282	1,91722E-158
Mast	HADHA	7,70001E-73	-5,52225E-01	0,293	0,537	1,41811E-68
Mast	IGBP1	2,51788E-90	-5,52535E-01	0,155	0,357	4,63718E-86
Mast	EIF2A	1,26929E-30	-5,53466E-01	0,188	0,316	2,33765E-26
Mast	HNRNPR	3,27604E-63	-5,54780E-01	0,187	0,373	6,03349E-59
Mast	ZC3HAV1	8,34411E-15	-5,54789E-01	0,19	0,276	1,53674E-10
Mast	CCNH	6,19445E-15	-5,55626E-01	0,262	0,379	1,14083E-10
Mast	CACUL1	1,61828E-118	-5,55787E-01	0,103	0,317	2,98039E-114
Mast	SDHD	1,19799E-49	-5,56942E-01	0,19	0,355	2,20634E-45
Mast	EIF4E2	1,30031E-95	-5,57417E-01	0,167	0,386	2,39479E-91
Mast	SLU7	1,21941E-70	-5,57755E-01	0,098	0,251	2,24579E-66
Mast	PCBD1	4,62794E-124	-5,57964E-01	0,139	0,376	8,52327E-120
Mast	HEBP1	2,32630E-162	-5,58262E-01	0,078	0,326	4,28435E-158
Mast	SET	1,61021E-40	-5,58633E-01	0,327	0,542	2,96552E-36
Mast	RAB8B	1,36943E-67	-5,58859E-01	0,108	0,26	2,52207E-63
Mast	SNRPD3	4,16363E-107	-5,59490E-01	0,151	0,385	7,66816E-103
Mast	SPINT2	2,77401E-09	-5,59795E-01	0,366	0,507	5,10890E-05
Mast	PHF5A	1,53824E-97	-5,59882E-01	0,088	0,27	2,83298E-93
Mast	CDV3	2,59384E-119	-5,60620E-01	0,084	0,288	4,77707E-115
Mast	PCMT1	5,67371E-26	-5,61001E-01	0,215	0,347	1,04493E-21
Mast	ACAP2	4,31397E-56	-5,61009E-01	0,247	0,444	7,94505E-52
Mast	CHD9	2,82411E-42	-5,61877E-01	0,196	0,353	5,20117E-38
Mast	CLTC	1,74331E-139	-5,61993E-01	0,169	0,437	3,21066E-135
Mast	ATF6B	7,06107E-89	-5,62017E-01	0,114	0,299	1,30044E-84
Mast	NDUFB2	8,24397E-68	-5,63242E-01	0,459	0,732	1,51829E-63
Mast	RANBP1	1,33266E-86	-5,64571E-01	0,171	0,381	2,45436E-82

Mast	HSD17B1	2,04092E-89	-5,64720E-01	0,198	0,419	3,75876E-85
Mast	GNAI3	6,98060E-125	-5,65566E-01	0,127	0,361	1,28562E-120
Mast	TUBB	5,22848E-36	-5,65583E-01	0,456	0,675	9,62929E-32
Mast	GYG1	1,51917E-102	-5,65631E-01	0,094	0,285	2,79786E-98
Mast	TRADD	3,23006E-105	-5,66321E-01	0,075	0,258	5,94880E-101
Mast	NDUFS6	9,62401E-171	-5,66937E-01	0,214	0,528	1,77245E-166
Mast	RNF130	1,55866E-49	-5,66968E-01	0,469	0,726	2,87059E-45
Mast	IDH1	1,54899E-111	-5,67014E-01	0,07	0,259	2,85277E-107
Mast	CSNK1A1	8,67063E-44	-5,67167E-01	0,279	0,475	1,59687E-39
Mast	POLR2L	7,65858E-160	-5,67802E-01	0,323	0,648	1,41048E-155
Mast	TUBB4B	8,18737E-46	-5,68068E-01	0,224	0,391	1,50787E-41
Mast	SH2B3	1,14163E-79	-5,68671E-01	0,107	0,276	2,10254E-75
Mast	BAZ2B	2,31191E-37	-5,69545E-01	0,145	0,269	4,25784E-33
Mast	LSM7	3,05560E-94	-5,69798E-01	0,27	0,531	5,62750E-90
Mast	TMEM16	8,15230E-110	-5,70190E-01	0,173	0,408	1,50141E-105
Mast	GPR34	7,14847E-97	-5,70648E-01	0,13	0,318	1,31653E-92
Mast	MT-ND4	1,54784E-104	-5,70648E-01	0,834	0,556	2,85066E-100
Mast	VAPA	1,10264E-28	-5,70960E-01	0,394	0,618	2,03073E-24
Mast	MRPS5	7,22455E-81	-5,71836E-01	0,099	0,266	1,33055E-76
Mast	AMD1	1,37803E-47	-5,72067E-01	0,151	0,295	2,53791E-43
Mast	RHEB	2,13140E-111	-5,73244E-01	0,221	0,479	3,92541E-107
Mast	WAC	7,94641E-70	-5,73854E-01	0,134	0,301	1,46349E-65
Mast	PTAFR	2,00004E-130	-5,74569E-01	0,146	0,392	3,68348E-126
Mast	CBR1	8,99779E-92	-5,74641E-01	0,135	0,329	1,65712E-87
Mast	YBX1	9,48151E-139	-5,75231E-01	0,624	0,878	1,74621E-134
Mast	SPAG7	1,21543E-57	-5,75337E-01	0,169	0,337	2,23845E-53
Mast	RNF7	3,33512E-82	-5,75590E-01	0,298	0,562	6,14230E-78
Mast	RAB9A	6,00812E-68	-5,75760E-01	0,102	0,251	1,10651E-63
Mast	SNRPG	5,33084E-66	-5,77030E-01	0,327	0,573	9,81780E-62
Mast	C18orf32	1,21293E-08	-5,77549E-01	0,209	0,285	2,23385E-04
Mast	DAPK1	4,31830E-93	-5,78686E-01	0,147	0,348	7,95301E-89
Mast	IDH3G	4,38101E-62	-5,78997E-01	0,187	0,372	8,06851E-58
Mast	TUBA1C	6,45409E-122	-5,79129E-01	0,155	0,395	1,18865E-117
Mast	CUX1	4,21721E-111	-5,79235E-01	0,142	0,365	7,76684E-107
Mast	CSNK2B	6,39666E-07	-5,80487E-01	0,324	0,338	1,17807E-02
Mast	PCGF5	7,07826E-91	-5,81582E-01	0,104	0,285	1,30360E-86
Mast	COPS6	8,70859E-31	-5,81734E-01	0,212	0,352	1,60386E-26
Mast	TRAPPC2	4,08123E-103	-5,82296E-01	0,09	0,282	7,51640E-99
Mast	ANKRD1[illegible]	3,47954E-79	-5,83674E-01	0,253	0,485	6,40826E-75
Mast	SAP18	6,38211E-30	-5,83881E-01	0,431	0,663	1,17539E-25

Mast	RRP7A	3,55134E-73	-5,84116E-01	0,134	0,305	6,54050E-69
Mast	SERINC1	7,60908E-59	-5,85174E-01	0,222	0,417	1,40137E-54
Mast	HSPA9	5,51263E-34	-5,86383E-01	0,225	0,381	1,01526E-29
Mast	MRPS12	9,09112E-83	-5,87066E-01	0,117	0,294	1,67431E-78
Mast	NELFE	2,11927E-78	-5,87321E-01	0,125	0,301	3,90305E-74
Mast	NR1H2	1,07415E-68	-5,87985E-01	0,162	0,342	1,97827E-64
Mast	SAT2	2,73011E-109	-5,88488E-01	0,198	0,45	5,02804E-105
Mast	C19orf53	3,62888E-85	-5,88495E-01	0,327	0,603	6,68330E-81
Mast	BLOC1S2	1,53786E-93	-5,89083E-01	0,141	0,344	2,83228E-89
Mast	BUB3	8,02113E-38	-5,89084E-01	0,142	0,266	1,47725E-33
Mast	NDUFB9	6,70606E-66	-5,90270E-01	0,316	0,567	1,23506E-61
Mast	EEF1D	2,51981E-16	-5,90823E-01	0,738	0,881	4,64073E-12
Mast	MYH9	9,95976E-167	-5,90906E-01	0,268	0,6	1,83429E-162
Mast	NUCB1	6,87054E-72	-5,91479E-01	0,312	0,559	1,26535E-67
Mast	NUB1	6,96372E-95	-5,93045E-01	0,128	0,326	1,28251E-90
Mast	SF3B1	1,79259E-57	-5,93238E-01	0,288	0,508	3,30141E-53
Mast	GGA1	7,36533E-101	-5,93325E-01	0,073	0,251	1,35647E-96
Mast	EEF1A1	8,35613E-14	-5,93335E-01	0,974	0,985	1,53895E-09
Mast	ZNHIT1	2,26394E-144	-5,93405E-01	0,209	0,498	4,16950E-140
Mast	ETFA	3,66079E-109	-5,93461E-01	0,133	0,35	6,74207E-105
Mast	KXD1	1,73601E-109	-5,93808E-01	0,143	0,366	3,19721E-105
Mast	ERICH1	5,81249E-95	-5,96282E-01	0,135	0,334	1,07049E-90
Mast	FNBP1	7,29517E-190	-5,96491E-01	0,147	0,453	1,34355E-185
Mast	PTK2B	7,21898E-84	-5,97746E-01	0,098	0,265	1,32952E-79
Mast	NDUFB1	4,22431E-79	-5,97750E-01	0,25	0,483	7,77992E-75
Mast	PDIA4	1,25427E-62	-5,98392E-01	0,162	0,332	2,30998E-58
Mast	PIGT	1,20135E-60	-5,98518E-01	0,141	0,301	2,21253E-56
Mast	RIOK3	6,73174E-61	-5,98695E-01	0,128	0,281	1,23978E-56
Mast	XRCC5	8,68584E-81	-6,00740E-01	0,255	0,491	1,59967E-76
Mast	JOSD2	4,45684E-120	-6,01211E-01	0,122	0,343	8,20816E-116
Mast	MRPS15	1,45651E-104	-6,01797E-01	0,124	0,331	2,68245E-100
Mast	DNTTIP2	9,98108E-66	-6,04332E-01	0,128	0,285	1,83822E-61
Mast	NCOR1	3,42450E-19	-6,04646E-01	0,27	0,408	6,30690E-15
Mast	DUSP3	1,15670E-112	-6,05364E-01	0,081	0,276	2,13029E-108
Mast	RPL26	1,24759E-211	-6,06098E-01	0,764	0,527	2,29769E-207
Mast	TMEM24	1,81984E-128	-6,06554E-01	0,073	0,283	3,35160E-124
Mast	LYN	6,85931E-90	-6,06873E-01	0,24	0,467	1,26328E-85
Mast	GNB2	1,86398E-160	-6,07127E-01	0,27	0,577	3,43289E-156
Mast	TEX264	1,63844E-81	-6,07152E-01	0,134	0,32	3,01752E-77
Mast	CACYBP	3,08282E-78	-6,07239E-01	0,203	0,415	5,67763E-74

Mast	TMEM14	4,76860E-96	-6,10087E-01	0,287	0,546	8,78234E-92
Mast	GRHPR	4,42725E-92	-6,10246E-01	0,115	0,303	8,15367E-88
Mast	RPL13	2,43226E-156	-6,10527E-01	0,838	0,564	4,47950E-152
Mast	EFHD2	3,08427E-193	-6,10788E-01	0,146	0,45	5,68030E-189
Mast	GTF2B	2,78603E-27	-6,11434E-01	0,171	0,286	5,13102E-23
Mast	PIAS1	8,15741E-68	-6,11588E-01	0,155	0,333	1,50235E-63
Mast	NDUFAB1	1,34575E-102	-6,12056E-01	0,235	0,492	2,47847E-98
Mast	IL10RB	5,66964E-72	-6,12501E-01	0,107	0,265	1,04418E-67
Mast	SND1	3,68520E-62	-6,12511E-01	0,178	0,357	6,78703E-58
Mast	MTIF3	4,53605E-86	-6,13245E-01	0,122	0,307	8,35404E-82
Mast	PPP4C	1,87220E-156	-6,13565E-01	0,222	0,533	3,44804E-152
Mast	MPC1	1,57917E-86	-6,13691E-01	0,137	0,329	2,90836E-82
Mast	MRPL21	8,38262E-90	-6,13896E-01	0,098	0,278	1,54383E-85
Mast	RABGGTI	6,17770E-12	-6,14841E-01	0,181	0,262	1,13775E-07
Mast	TMX1	2,66377E-68	-6,14874E-01	0,166	0,348	4,90587E-64
Mast	METTL7A	3,52478E-138	-6,15299E-01	0,054	0,261	6,49159E-134
Mast	RSU1	2,88340E-81	-6,15344E-01	0,156	0,349	5,31035E-77
Mast	SSB	2,66576E-98	-6,15948E-01	0,223	0,468	4,90953E-94
Mast	PPP2CA	1,19711E-26	-6,16605E-01	0,212	0,343	2,20471E-22
Mast	YIF1A	4,00703E-96	-6,16609E-01	0,105	0,297	7,37975E-92
Mast	PRDX1	2,07373E-37	-6,16793E-01	0,544	0,761	3,81919E-33
Mast	ECHS1	4,36692E-106	-6,16920E-01	0,157	0,384	8,04256E-102
Mast	SNW1	1,83931E-75	-6,16993E-01	0,128	0,301	3,38746E-71
Mast	NRBF2	1,45945E-111	-6,17093E-01	0,068	0,256	2,68787E-107
Mast	PTBP1	3,10304E-56	-6,18193E-01	0,157	0,317	5,71487E-52
Mast	COA4	8,65874E-77	-6,18317E-01	0,123	0,297	1,59468E-72
Mast	ATG12	2,49448E-25	-6,18779E-01	0,163	0,27	4,59408E-21
Mast	KIAA093(	3,71938E-192	-6,19190E-01	0,018	0,258	6,84999E-188
Mast	NDUFA12	4,81244E-65	-6,19266E-01	0,279	0,509	8,86307E-61
Mast	RAB24	6,57163E-37	-6,19396E-01	0,158	0,278	1,21030E-32
Mast	SURF4	3,36399E-48	-6,20173E-01	0,154	0,3	6,19546E-44
Mast	STARD7	2,85351E-114	-6,20271E-01	0,108	0,319	5,25532E-110
Mast	TTC3	4,40104E-93	-6,20347E-01	0,139	0,342	8,10540E-89
Mast	GNPTG	8,14517E-140	-6,20828E-01	0,177	0,446	1,50010E-135
Mast	TNIP1	6,02725E-91	-6,21459E-01	0,16	0,359	1,11004E-86
Mast	ACSL3	4,73439E-83	-6,21466E-01	0,097	0,264	8,71932E-79
Mast	TALDO1	7,23119E-70	-6,21574E-01	0,406	0,68	1,33177E-65
Mast	EEF2	5,96654E-39	-6,21657E-01	0,599	0,844	1,09886E-34
Mast	RAB34	4,02178E-25	-6,22165E-01	0,169	0,28	7,40691E-21
Mast	PDCD5	1,79396E-78	-6,22857E-01	0,167	0,364	3,30394E-74

Mast	JAK1	5,37039E-182	-6,22918E-01	0,217	0,543	9,89065E-178
Mast	MMADH	1,07177E-79	-6,23296E-01	0,164	0,361	1,97388E-75
Mast	COPS3	8,37976E-38	-6,24157E-01	0,138	0,259	1,54330E-33
Mast	RNF114	4,34166E-139	-6,24252E-01	0,135	0,387	7,99604E-135
Mast	MAF1	1,63100E-132	-6,24612E-01	0,152	0,405	3,00382E-128
Mast	SPCS3	9,33554E-78	-6,24984E-01	0,233	0,46	1,71933E-73
Mast	RTN3	1,26917E-91	-6,25359E-01	0,204	0,433	2,33743E-87
Mast	PTGES3	7,86013E-52	-6,25433E-01	0,412	0,66	1,44760E-47
Mast	KLHDC3	7,11187E-104	-6,25891E-01	0,072	0,254	1,30979E-99
Mast	EIF3A	2,03896E-187	-6,26160E-01	0,19	0,511	3,75515E-183
Mast	DNAJC4	2,17972E-20	-6,26502E-01	0,242	0,37	4,01440E-16
Mast	SUMF2	2,36228E-67	-6,26960E-01	0,14	0,305	4,35061E-63
Mast	SUCLG1	1,11959E-86	-6,27164E-01	0,131	0,323	2,06195E-82
Mast	MRPL23	1,57135E-201	-6,27371E-01	0,089	0,375	2,89396E-197
Mast	SCP2	3,49904E-44	-6,28816E-01	0,279	0,474	6,44417E-40
Mast	SSBP1	3,52638E-63	-6,29205E-01	0,275	0,502	6,49453E-59
Mast	ATP6V1D	2,60265E-53	-6,29718E-01	0,118	0,256	4,79330E-49
Mast	FBXO7	5,70151E-55	-6,29850E-01	0,168	0,333	1,05005E-50
Mast	FYTTD1	3,49154E-30	-6,29895E-01	0,147	0,256	6,43038E-26
Mast	IK	3,33402E-86	-6,30510E-01	0,152	0,351	6,14027E-82
Mast	NSFL1C	2,22498E-71	-6,30591E-01	0,119	0,285	4,09774E-67
Mast	TMEM50	2,50299E-75	-6,30601E-01	0,351	0,62	4,60976E-71
Mast	NRBP1	4,10407E-55	-6,31957E-01	0,159	0,318	7,55846E-51
Mast	BECN1	4,31910E-52	-6,32218E-01	0,129	0,27	7,95449E-48
Mast	HNRNPH	4,56367E-46	-6,33091E-01	0,199	0,363	8,40492E-42
Mast	EIF4G1	7,27756E-95	-6,33361E-01	0,143	0,346	1,34031E-90
Mast	ATP6V1G	6,61841E-22	-6,33785E-01	0,501	0,734	1,21891E-17
Mast	METRNL	1,92248E-111	-6,35306E-01	0,074	0,267	3,54063E-107
Mast	SDCCAG8	9,11652E-88	-6,36180E-01	0,151	0,35	1,67899E-83
Mast	ST8SIA4	3,08251E-137	-6,38469E-01	0,087	0,312	5,67706E-133
Mast	UVRAG	1,19020E-97	-6,40136E-01	0,11	0,303	2,19200E-93
Mast	RPLP2	7,90520E-123	-6,41240E-01	0,817	0,553	1,45590E-118
Mast	PELI1	8,21153E-89	-6,41411E-01	0,1	0,273	1,51232E-84
Mast	RPL35	1,78691E-43	-6,41622E-01	0,683	0,52	3,29096E-39
Mast	IMPDH2	1,95529E-85	-6,42015E-01	0,107	0,285	3,60105E-81
Mast	TIMM10	2,09541E-67	-6,42271E-01	0,11	0,263	3,85912E-63
Mast	SERP1	5,12670E-85	-6,43144E-01	0,559	0,838	9,44185E-81
Mast	EMC3	3,37566E-87	-6,44534E-01	0,138	0,332	6,21695E-83
Mast	ACO2	5,88105E-98	-6,44903E-01	0,096	0,282	1,08311E-93
Mast	MCTS1	4,01913E-95	-6,45112E-01	0,116	0,308	7,40204E-91

Mast	ARF3	8,55959E-127	-6,45995E-01	0,12	0,352	1,57642E-122
Mast	RAB11A	8,92971E-57	-6,46520E-01	0,221	0,414	1,64458E-52
Mast	GIMAP1	1,62156E-182	-6,48267E-01	0,04	0,282	2,98642E-178
Mast	TRAPPC3	9,45941E-98	-6,48378E-01	0,152	0,367	1,74214E-93
Mast	DPP7	1,59021E-77	-6,48498E-01	0,315	0,573	2,92868E-73
Mast	PPP1R2	2,32643E-37	-6,48534E-01	0,237	0,404	4,28459E-33
Mast	UQCRB	3,23337E-88	-6,48997E-01	0,505	0,792	5,95489E-84
Mast	ABI1	2,02271E-116	-6,50025E-01	0,129	0,355	3,72523E-112
Mast	PSMC4	1,76490E-140	-6,50430E-01	0,112	0,352	3,25042E-136
Mast	ARHGDIE	1,89016E-11	-6,50756E-01	0,705	0,831	3,48110E-07
Mast	ECHDC1	1,84035E-81	-6,51262E-01	0,108	0,281	3,38937E-77
Mast	SIGLEC1(	3,22154E-108	-6,51440E-01	0,126	0,334	5,93311E-104
Mast	CDC42SE	1,51996E-112	-6,51488E-01	0,166	0,4	2,79931E-108
Mast	COX7B	1,38237E-229	-6,52331E-01	0,316	0,691	2,54591E-225
Mast	AURKAIP	2,49857E-121	-6,53121E-01	0,247	0,537	4,60161E-117
Mast	TBCB	3,68279E-44	-6,53563E-01	0,283	0,481	6,78259E-40
Mast	SIVA1	1,08896E-63	-6,54780E-01	0,252	0,469	2,00554E-59
Mast	ME2	1,81121E-147	-6,55065E-01	0,087	0,321	3,33570E-143
Mast	CSRP1	1,25541E-63	-6,56994E-01	0,117	0,268	2,31209E-59
Mast	HADHB	3,81623E-108	-6,58214E-01	0,149	0,375	7,02836E-104
Mast	ITM2B	1,06852E-27	-6,58217E-01	0,819	0,912	1,96789E-23
Mast	ARF4	3,17675E-101	-6,58797E-01	0,214	0,462	5,85062E-97
Mast	SAR1B	2,13368E-65	-6,61118E-01	0,102	0,25	3,92960E-61
Mast	POLR2G	6,41948E-96	-6,61782E-01	0,168	0,386	1,18228E-91
Mast	VASP	1,39615E-169	-6,62979E-01	0,196	0,489	2,57129E-165
Mast	AHCY	3,28478E-125	-6,63129E-01	0,072	0,275	6,04958E-121
Mast	PIH1D1	2,40310E-50	-6,63385E-01	0,124	0,263	4,42578E-46
Mast	NCKAP1L	5,49992E-149	-6,63986E-01	0,171	0,447	1,01292E-144
Mast	SLC3A2	1,91163E-80	-6,64441E-01	0,247	0,478	3,52066E-76
Mast	SCAMP3	3,81498E-63	-6,65026E-01	0,112	0,261	7,02605E-59
Mast	PRKCD	9,75738E-86	-6,66414E-01	0,099	0,267	1,79702E-81
Mast	IL17RA	4,99897E-189	-6,67143E-01	0,058	0,319	9,20661E-185
Mast	TMED2	6,97533E-43	-6,67415E-01	0,305	0,513	1,28465E-38
Mast	PSMD6	1,52864E-37	-6,67883E-01	0,157	0,287	2,81529E-33
Mast	SRA1	1,32251E-93	-6,69656E-01	0,176	0,401	2,43567E-89
Mast	LSM10	6,03426E-107	-6,71463E-01	0,162	0,39	1,11133E-102
Mast	SRGN	1,19921E-21	-6,72255E-01	0,882	0,947	2,20859E-17
Mast	POLR1D	4,17715E-136	-6,72371E-01	0,208	0,486	7,69306E-132
Mast	SHISA5	5,51072E-52	-6,72565E-01	0,209	0,382	1,01491E-47
Mast	SF3B5	1,17017E-102	-6,73437E-01	0,288	0,57	2,15510E-98

Mast	DYNLRB1	1,11483E-130	-6,73609E-01	0,198	0,481	2,05319E-126
Mast	PHPT1	5,50817E-126	-6,73672E-01	0,198	0,464	1,01444E-121
Mast	STX11	6,02900E-63	-6,76226E-01	0,17	0,339	1,11036E-58
Mast	NACA	6,02762E-72	-6,76292E-01	0,859	0,923	1,11011E-67
Mast	PSMD8	2,69583E-96	-6,76981E-01	0,274	0,543	4,96491E-92
Mast	SEC11C	5,60240E-65	-6,78996E-01	0,162	0,337	1,03179E-60
Mast	GPRIN3	5,90223E-198	-6,79003E-01	0,012	0,253	1,08701E-193
Mast	WAS	1,45648E-276	-6,80013E-01	0,176	0,554	2,68240E-272
Mast	DNPH1	1,92477E-142	-6,80055E-01	0,117	0,363	3,54485E-138
Mast	PARL	8,29588E-81	-6,80305E-01	0,124	0,302	1,52785E-76
Mast	ZFAND6	2,14017E-56	-6,80316E-01	0,161	0,322	3,94155E-52
Mast	NFE2L2	3,13718E-73	-6,80615E-01	0,287	0,535	5,77774E-69
Mast	PPP2R1A	1,26295E-115	-6,81652E-01	0,171	0,412	2,32598E-111
Mast	NDUFA2	1,23579E-223	-6,81935E-01	0,218	0,575	2,27595E-219
Mast	CCT6A	3,88773E-101	-6,82016E-01	0,217	0,464	7,16004E-97
Mast	WSB1	4,61235E-74	-6,82106E-01	0,387	0,658	8,49456E-70
Mast	DCP2	1,76554E-114	-6,83477E-01	0,079	0,279	3,25159E-110
Mast	TMEM70	7,60428E-176	-6,84593E-01	0,058	0,306	1,40048E-171
Mast	AP1M1	5,74510E-80	-6,86187E-01	0,094	0,258	1,05807E-75
Mast	PSMA1	3,90194E-18	-6,87284E-01	0,246	0,369	7,18620E-14
Mast	MIS18BP	2,80108E-173	-6,89142E-01	0,107	0,378	5,15874E-169
Mast	INTS10	8,49096E-73	-6,90852E-01	0,097	0,252	1,56378E-68
Mast	BRK1	6,71796E-55	-6,91271E-01	0,467	0,744	1,23725E-50
Mast	CNOT2	4,51426E-35	-6,91293E-01	0,155	0,279	8,31391E-31
Mast	NDUFB7	5,78057E-135	-6,93521E-01	0,252	0,549	1,06461E-130
Mast	ACP2	5,88935E-185	-6,93685E-01	0,033	0,273	1,08464E-180
Mast	DSE	3,12462E-247	-6,94171E-01	0,12	0,461	5,75461E-243
Mast	C14orf11	2,56359E-92	-6,94651E-01	0,115	0,304	4,72136E-88
Mast	COX6A1	1,18710E-18	-6,94658E-01	0,489	0,693	2,18629E-14
Mast	STX4	7,60570E-104	-6,95120E-01	0,121	0,326	1,40074E-99
Mast	SNX27	7,38434E-149	-6,96092E-01	0,045	0,259	1,35997E-144
Mast	HSD17B1	8,11908E-122	-6,96761E-01	0,103	0,32	1,49529E-117
Mast	CEBPB	2,40562E-154	-6,96940E-01	0,219	0,489	4,43043E-150
Mast	FNDC3B	7,53576E-112	-6,97044E-01	0,096	0,292	1,38786E-107
Mast	PFDN1	2,21976E-94	-6,97179E-01	0,11	0,299	4,08813E-90
Mast	YIPF3	5,23469E-87	-6,97339E-01	0,142	0,337	9,64073E-83
Mast	ARHGEF1	5,33831E-45	-6,97745E-01	0,145	0,28	9,83156E-41
Mast	NUTF2	6,63649E-150	-6,98941E-01	0,14	0,402	1,22224E-145
Mast	SFT2D1	3,83417E-71	-6,99403E-01	0,25	0,474	7,06139E-67
Mast	ERGIC3	5,23030E-70	-6,99708E-01	0,276	0,514	9,63264E-66

Mast	CCT7	7,43038E-53	-7,00560E-01	0,19	0,36	1,36845E-48
Mast	MDH2	1,11354E-112	-7,00795E-01	0,262	0,54	2,05080E-108
Mast	RNF135	1,02880E-125	-7,01812E-01	0,055	0,251	1,89475E-121
Mast	GPR137E	9,35698E-168	-7,02030E-01	0,071	0,314	1,72328E-163
Mast	RAPGEF1	1,28278E-153	-7,02289E-01	0,051	0,274	2,36250E-149
Mast	COX14	9,93735E-154	-7,03810E-01	0,188	0,481	1,83016E-149
Mast	TRIM38	8,53595E-139	-7,05474E-01	0,078	0,299	1,57207E-134
Mast	GNPDA1	5,83649E-99	-7,06395E-01	0,086	0,271	1,07491E-94
Mast	RAB1B	1,78623E-115	-7,06654E-01	0,142	0,371	3,28970E-111
Mast	CELF1	2,33771E-92	-7,08487E-01	0,111	0,297	4,30535E-88
Mast	TMEM30	1,03262E-89	-7,10127E-01	0,127	0,317	1,90177E-85
Mast	PLEKHB2	3,90984E-251	-7,10190E-01	0,121	0,462	7,20075E-247
Mast	TMEM14	4,18408E-164	-7,10795E-01	0,165	0,452	7,70581E-160
Mast	UBE2B	3,00456E-92	-7,11090E-01	0,253	0,505	5,53349E-88
Mast	MRPS7	1,42320E-128	-7,12089E-01	0,102	0,325	2,62111E-124
Mast	UBE2A	5,95548E-138	-7,12145E-01	0,159	0,419	1,09682E-133
Mast	TMEM14	3,41520E-87	-7,13030E-01	0,211	0,437	6,28978E-83
Mast	ATP6V1A	8,34563E-131	-7,14251E-01	0,063	0,268	1,53701E-126
Mast	TPM4	8,97136E-40	-7,16666E-01	0,406	0,633	1,65226E-35
Mast	YBX3	1,72118E-186	-7,16961E-01	0,088	0,366	3,16990E-182
Mast	PHB2	9,70717E-89	-7,16962E-01	0,258	0,509	1,78777E-84
Mast	RPS3A	0,00000E+00	-7,16963E-01	0,834	0,548	0,00000E+00
Mast	SON	3,35307E-11	-7,17125E-01	0,481	0,688	6,17534E-07
Mast	PDCD6IP	5,81640E-57	-7,17198E-01	0,201	0,38	1,07121E-52
Mast	ATP6V1C	6,10184E-127	-7,17260E-01	0,078	0,287	1,12378E-122
Mast	FBL	3,48409E-58	-7,17371E-01	0,185	0,359	6,41665E-54
Mast	MAP3K8	8,53670E-77	-7,17860E-01	0,267	0,497	1,57220E-72
Mast	APMAP	1,32040E-127	-7,18015E-01	0,095	0,315	2,43178E-123
Mast	SNAP23	1,07008E-47	-7,18233E-01	0,233	0,411	1,97077E-43
Mast	MRFAP1	4,18861E-76	-7,19538E-01	0,202	0,414	7,71416E-72
Mast	MRPL18	5,56103E-115	-7,19744E-01	0,136	0,364	1,02417E-110
Mast	SSR2	3,62840E-20	-7,20282E-01	0,441	0,667	6,68242E-16
Mast	UXT	6,79598E-92	-7,21729E-01	0,293	0,559	1,25162E-87
Mast	AK2	8,33985E-61	-7,22062E-01	0,178	0,354	1,53595E-56
Mast	PSMB1	2,60727E-49	-7,22191E-01	0,392	0,646	4,80181E-45
Mast	ILF3	2,39090E-20	-7,22961E-01	0,245	0,375	4,40332E-16
Mast	UQCRC2	3,27958E-121	-7,23082E-01	0,176	0,431	6,04000E-117
Mast	TUFM	3,02045E-126	-7,23125E-01	0,243	0,528	5,56275E-122
Mast	C11orf58	4,83695E-48	-7,24353E-01	0,343	0,575	8,90821E-44
Mast	RPL18A	1,12782E-116	-7,24370E-01	0,779	0,547	2,07711E-112

Mast	HSPA1A	2,72578E-48	-7,24625E-01	0,451	0,678	5,02006E-44
Mast	RNF166	4,82019E-89	-7,25738E-01	0,086	0,255	8,87734E-85
Mast	VKORC1	2,03487E-22	-7,26287E-01	0,155	0,25	3,74762E-18
Mast	TOP1	2,06626E-292	-7,27060E-01	0,159	0,546	3,80543E-288
Mast	DBI	3,25560E-21	-7,27165E-01	0,516	0,739	5,99583E-17
Mast	PRPF40A	2,17243E-103	-7,27387E-01	0,221	0,472	4,00097E-99
Mast	PPIB	2,63099E-23	-7,27786E-01	0,576	0,807	4,84550E-19
Mast	ERP44	1,96236E-193	-7,28882E-01	0,144	0,449	3,61409E-189
Mast	TMEM21	6,71547E-80	-7,29058E-01	0,375	0,655	1,23679E-75
Mast	PAPOLA	1,33087E-69	-7,29080E-01	0,248	0,47	2,45107E-65
Mast	COX6B1	4,79428E-124	-7,29521E-01	0,514	0,832	8,82963E-120
Mast	CYB5R4	5,97481E-95	-7,29616E-01	0,093	0,274	1,10038E-90
Mast	TMEM17	2,78274E-109	-7,30221E-01	0,235	0,463	5,12498E-105
Mast	SNRPE	5,14613E-60	-7,30494E-01	0,178	0,354	9,47763E-56
Mast	ATP6V1E	4,42305E-96	-7,30763E-01	0,139	0,345	8,14593E-92
Mast	ARPC1A	1,46264E-107	-7,31822E-01	0,09	0,284	2,69374E-103
Mast	PHC2	1,92022E-190	-7,33755E-01	0,02	0,26	3,53647E-186
Mast	MBNL1	1,61985E-43	-7,34121E-01	0,328	0,539	2,98328E-39
Mast	PSMC6	1,70027E-37	-7,35571E-01	0,141	0,262	3,13139E-33
Mast	FMNL1	4,82504E-153	-7,35585E-01	0,15	0,42	8,88627E-149
Mast	DNAJB11	6,28821E-45	-7,35974E-01	0,17	0,321	1,15810E-40
Mast	RALY	2,18144E-147	-7,37629E-01	0,218	0,512	4,01756E-143
Mast	ATP6V1F	4,03164E-44	-7,38753E-01	0,542	0,779	7,42506E-40
Mast	SPCS1	9,15520E-07	-7,40178E-01	0,503	0,615	1,68611E-02
Mast	GABARAI	4,22646E-64	-7,41963E-01	0,351	0,593	7,78388E-60
Mast	ALKBH7	1,72844E-150	-7,43109E-01	0,215	0,511	3,18326E-146
Mast	NDUFV1	3,21088E-49	-7,44030E-01	0,235	0,42	5,91347E-45
Mast	ADAM10	1,35690E-109	-7,44035E-01	0,1	0,3	2,49901E-105
Mast	FBXW5	2,27919E-101	-7,44769E-01	0,126	0,328	4,19759E-97
Mast	CNBP	7,63128E-09	-7,45764E-01	0,519	0,73	1,40545E-04
Mast	PTP4A2	5,00106E-51	-7,47406E-01	0,234	0,416	9,21045E-47
Mast	ARHGAP:	2,71848E-143	-7,48458E-01	0,107	0,348	5,00662E-139
Mast	UBL5	7,56411E-70	-7,48545E-01	0,494	0,773	1,39308E-65
Mast	IMP4	1,42619E-70	-7,48901E-01	0,116	0,277	2,62662E-66
Mast	FLOT2	1,94182E-85	-7,49163E-01	0,088	0,253	3,57625E-81
Mast	SMIM20	4,43952E-140	-7,51341E-01	0,046	0,251	8,17627E-136
Mast	C1QBP	8,86644E-72	-7,51426E-01	0,232	0,444	1,63293E-67
Mast	GLRX3	1,12827E-85	-7,52321E-01	0,09	0,259	2,07794E-81
Mast	USP4	1,35026E-103	-7,52345E-01	0,109	0,309	2,48678E-99
Mast	MORF4L:	5,79083E-47	-7,52479E-01	0,143	0,284	1,06650E-42

Mast	PNRC1	8,70387E-161	-7,52609E-01	0,5	0,798	1,60299E-156
Mast	MAP1LC3	1,08901E-125	-7,57003E-01	0,28	0,577	2,00562E-121
Mast	RNASEK	3,63904E-54	-7,57165E-01	0,507	0,463	6,70201E-50
Mast	IRF5	9,82594E-179	-7,57913E-01	0,04	0,278	1,80964E-174
Mast	RPSA	4,48544E-109	-7,58370E-01	0,635	0,486	8,26084E-105
Mast	WTAP	8,03149E-92	-7,58578E-01	0,17	0,379	1,47916E-87
Mast	COX6C	1,22406E-63	-7,59317E-01	0,465	0,737	2,25434E-59
Mast	PSMD11	3,25300E-91	-7,61113E-01	0,128	0,321	5,99106E-87
Mast	ABHD12	1,02800E-213	-7,61138E-01	0,101	0,399	1,89327E-209
Mast	PSMD7	1,77741E-85	-7,61462E-01	0,2	0,42	3,27346E-81
Mast	ADAM28	2,47833E-146	-7,61852E-01	0,043	0,252	4,56435E-142
Mast	PSMC5	4,38293E-67	-7,63429E-01	0,218	0,425	8,07204E-63
Mast	SRP72	1,13579E-84	-7,63520E-01	0,16	0,36	2,09179E-80
Mast	DYNLT1	3,56367E-98	-7,63607E-01	0,267	0,526	6,56321E-94
Mast	COX5B	3,86391E-109	-7,65279E-01	0,494	0,792	7,11617E-105
Mast	ATP1A1	2,90375E-151	-7,65392E-01	0,171	0,448	5,34784E-147
Mast	BASP1	5,51968E-215	-7,65694E-01	0,074	0,366	1,01656E-210
Mast	ARL6IP1	1,51497E-43	-7,66798E-01	0,248	0,427	2,79013E-39
Mast	SMS	8,53086E-214	-7,67531E-01	0,132	0,447	1,57113E-209
Mast	GNAS	1,61595E-96	-7,67726E-01	0,338	0,629	2,97610E-92
Mast	OAZ2	4,41636E-65	-7,68069E-01	0,132	0,294	8,13361E-61
Mast	GPR108	1,09201E-96	-7,68286E-01	0,089	0,27	2,01116E-92
Mast	COX7A2	9,34535E-56	-7,68482E-01	0,465	0,738	1,72113E-51
Mast	SLC39A1	8,77824E-99	-7,68735E-01	0,101	0,289	1,61669E-94
Mast	DAP3	2,31311E-103	-7,71439E-01	0,125	0,331	4,26005E-99
Mast	RAC1	3,91043E-138	-7,72253E-01	0,422	0,707	7,20184E-134
Mast	MAP2K1	6,61253E-224	-7,72779E-01	0,052	0,34	1,21783E-219
Mast	ESD	7,98048E-62	-7,73383E-01	0,191	0,376	1,46976E-57
Mast	RCSD1	1,00947E-191	-7,74169E-01	0,109	0,395	1,85913E-187
Mast	SRP9	6,09644E-57	-7,74173E-01	0,266	0,479	1,12278E-52
Mast	STIP1	7,25712E-64	-7,74509E-01	0,131	0,292	1,33654E-59
Mast	LAMTOR	5,63779E-92	-7,74682E-01	0,442	0,733	1,03831E-87
Mast	RBBP4	1,26226E-58	-7,74832E-01	0,201	0,383	2,32471E-54
Mast	POMP	9,63244E-172	-7,75212E-01	0,4	0,743	1,77401E-167
Mast	NDUFA6	1,99231E-154	-7,75423E-01	0,236	0,548	3,66924E-150
Mast	DCTN2	1,12335E-58	-7,77644E-01	0,17	0,34	2,06887E-54
Mast	ILF2	5,76127E-31	-7,77698E-01	0,245	0,401	1,06105E-26
Mast	FRMD4B	9,67872E-155	-7,78304E-01	0,068	0,3	1,78253E-150
Mast	GTF3C6	6,89006E-104	-7,78378E-01	0,161	0,386	1,26894E-99
Mast	GTF2A2	1,32627E-60	-7,79220E-01	0,223	0,42	2,44258E-56

Mast	HLA-E	1,82797E-35	-7,80633E-01	0,721	0,917	3,36657E-31
Mast	TMEM17	3,03986E-121	-7,81022E-01	0,226	0,5	5,59851E-117
Mast	HSPA8	2,99577E-08	-7,83397E-01	0,71	0,828	5,51730E-04
Mast	PHACTR1	8,67464E-105	-7,83498E-01	0,114	0,315	1,59761E-100
Mast	NOTCH2	4,81830E-128	-7,83988E-01	0,109	0,332	8,87387E-124
Mast	SNX17	5,19024E-98	-7,84601E-01	0,207	0,448	9,55886E-94
Mast	ACTR2	2,70060E-238	-7,84861E-01	0,266	0,646	4,97370E-234
Mast	NMI	1,16139E-84	-7,85137E-01	0,128	0,312	2,13893E-80
Mast	COPA	8,54796E-68	-7,85846E-01	0,169	0,349	1,57428E-63
Mast	ITPA	5,13010E-116	-7,87861E-01	0,086	0,288	9,44811E-112
Mast	HMGA1	3,26852E-240	-7,89359E-01	0,103	0,425	6,01964E-236
Mast	GHITM	2,27421E-104	-7,89688E-01	0,254	0,522	4,18842E-100
Mast	PSMC2	1,53111E-58	-7,90041E-01	0,141	0,297	2,81985E-54
Mast	LYSMD2	4,45866E-165	-7,90761E-01	0,065	0,306	8,21151E-161
Mast	RAB18	6,43571E-98	-7,91324E-01	0,102	0,293	1,18527E-93
Mast	HNRNPM	2,98196E-77	-7,91777E-01	0,243	0,473	5,49188E-73
Mast	ACTR1A	8,04757E-88	-7,92188E-01	0,116	0,3	1,48212E-83
Mast	RPL37A	1,16236E-40	-7,92353E-01	0,677	0,528	2,14073E-36
Mast	PSMD9	4,86734E-47	-7,93552E-01	0,129	0,263	8,96418E-43
Mast	NDUFS4	8,99121E-90	-7,93645E-01	0,112	0,299	1,65591E-85
Mast	NDUFS8	6,27772E-106	-7,94191E-01	0,248	0,512	1,15617E-101
Mast	TRA2A	6,18661E-12	-7,94223E-01	0,181	0,261	1,13939E-07
Mast	GPCPD1	4,71527E-111	-7,94689E-01	0,093	0,293	8,68412E-107
Mast	MRPL3	3,89960E-95	-7,94803E-01	0,109	0,295	7,18190E-91
Mast	NHP2	5,50256E-75	-7,94852E-01	0,213	0,426	1,01341E-70
Mast	SSH2	2,47351E-161	-7,95274E-01	0,082	0,327	4,55546E-157
Mast	FES	5,50247E-88	-7,95391E-01	0,094	0,265	1,01339E-83
Mast	NDUFB3	7,43616E-116	-7,95921E-01	0,221	0,485	1,36952E-111
Mast	VDAC1	2,44323E-132	-7,98112E-01	0,256	0,553	4,49970E-128
Mast	CHCHD7	2,81678E-121	-7,98510E-01	0,072	0,27	5,18765E-117
Mast	CCDC107	3,43708E-115	-7,98525E-01	0,109	0,321	6,33008E-111
Mast	MAPKAP	1,36307E-78	-7,99606E-01	0,126	0,3	2,51037E-74
Mast	NUDT22	2,23187E-92	-8,01039E-01	0,104	0,284	4,11043E-88
Mast	GNG10	1,89007E-130	-8,02048E-01	0,126	0,352	3,48094E-126
Mast	GNA15	3,79043E-73	-8,02364E-01	0,145	0,318	6,98083E-69
Mast	GNA13	8,03447E-177	-8,02696E-01	0,088	0,351	1,47971E-172
Mast	UBE2V2	3,41961E-80	-8,02909E-01	0,102	0,27	6,29790E-76
Mast	CALCOCO	9,93738E-31	-8,03390E-01	0,225	0,368	1,83017E-26
Mast	UBE2D1	3,68939E-199	-8,03539E-01	0,092	0,381	6,79475E-195
Mast	GTF3A	1,84040E-115	-8,04998E-01	0,23	0,498	3,38946E-111

Mast	SRSF5	5,73607E-12	-8,05188E-01	0,407	0,591	1,05641E-07
Mast	CEP170	3,02879E-164	-8,06412E-01	0,102	0,361	5,57813E-160
Mast	SNX6	2,96908E-164	-8,07006E-01	0,229	0,536	5,46815E-160
Mast	SNRPC	2,38649E-93	-8,07371E-01	0,204	0,434	4,39520E-89
Mast	CIB1	3,16910E-227	-8,07571E-01	0,263	0,637	5,83654E-223
Mast	PSMA6	6,96577E-32	-8,08803E-01	0,306	0,477	1,28289E-27
Mast	PTEN	1,04444E-148	-8,10433E-01	0,055	0,276	1,92354E-144
Mast	TAX1BP1	3,99554E-157	-8,10968E-01	0,213	0,518	7,35859E-153
Mast	VAMP3	1,99803E-117	-8,11084E-01	0,137	0,366	3,67977E-113
Mast	TM9SF2	2,54798E-101	-8,11383E-01	0,202	0,443	4,69261E-97
Mast	PHB	5,00415E-115	-8,12109E-01	0,165	0,404	9,21614E-111
Mast	MT-ND3	6,34553E-69	-8,12525E-01	0,774	0,555	1,16866E-64
Mast	MDH1	3,90373E-77	-8,12997E-01	0,19	0,399	7,18950E-73
Mast	KDELR2	7,18490E-181	-8,13053E-01	0,206	0,527	1,32324E-176
Mast	ICAM1	5,37065E-44	-8,13471E-01	0,214	0,372	9,89112E-40
Mast	NDUFS2	2,10474E-111	-8,14101E-01	0,147	0,376	3,87631E-107
Mast	RSL1D1	4,95900E-122	-8,14158E-01	0,215	0,487	9,13299E-118
Mast	DUSP23	2,83567E-146	-8,14190E-01	0,141	0,4	5,22245E-142
Mast	EIF4E	2,63400E-120	-8,14241E-01	0,1	0,312	4,85104E-116
Mast	TCP1	3,33910E-27	-8,15386E-01	0,213	0,345	6,14961E-23
Mast	VPS35	2,25682E-196	-8,15411E-01	0,148	0,46	4,15638E-192
Mast	RAP2B	8,67152E-205	-8,16108E-01	0,058	0,333	1,59703E-200
Mast	SLIRP	3,66670E-175	-8,16946E-01	0,155	0,446	6,75295E-171
Mast	YIF1B	1,80821E-140	-8,17335E-01	0,122	0,366	3,33019E-136
Mast	UBE2K	7,70458E-120	-8,17637E-01	0,09	0,299	1,41895E-115
Mast	ODF3B	3,73778E-218	-8,18030E-01	0,071	0,365	6,88387E-214
Mast	VDAC2	4,04412E-92	-8,18514E-01	0,302	0,57	7,44806E-88
Mast	SPG21	1,08349E-198	-8,18742E-01	0,148	0,458	1,99547E-194
Mast	RER1	1,05075E-103	-8,19920E-01	0,221	0,477	1,93517E-99
Mast	DEK	4,19589E-206	-8,20065E-01	0,246	0,597	7,72758E-202
Mast	EIF6	2,81041E-113	-8,20483E-01	0,176	0,421	5,17594E-109
Mast	MPP1	3,14910E-50	-8,20701E-01	0,243	0,424	5,79969E-46
Mast	IFNAR2	2,90855E-87	-8,21539E-01	0,103	0,279	5,35667E-83
Mast	CDC42SE	2,43684E-56	-8,21859E-01	0,134	0,284	4,48792E-52
Mast	EIF4EBP1	9,74297E-228	-8,23110E-01	0,11	0,43	1,79436E-223
Mast	EDF1	6,44126E-52	-8,23239E-01	0,484	0,768	1,18629E-47
Mast	PHF11	8,04679E-96	-8,23393E-01	0,108	0,3	1,48198E-91
Mast	MTCH1	1,11709E-230	-8,27775E-01	0,139	0,478	2,05734E-226
Mast	LAMTOR	2,37058E-95	-8,29304E-01	0,262	0,524	4,36590E-91
Mast	ACTR3	2,33833E-13	-8,29647E-01	0,465	0,678	4,30649E-09

Mast	TMEM51	1,71027E-249	-8,30056E-01	0,001	0,28	3,14981E-245
Mast	REEP4	2,08671E-141	-8,30446E-01	0,048	0,254	3,84309E-137
Mast	STX7	5,18131E-215	-8,32540E-01	0,119	0,434	9,54241E-211
Mast	PARP9	1,85660E-103	-8,33426E-01	0,082	0,266	3,41930E-99
Mast	LIMS1	2,45021E-161	-8,33794E-01	0,266	0,575	4,51255E-157
Mast	IFRD1	3,43836E-21	-8,33800E-01	0,192	0,298	6,33243E-17
Mast	NONO	8,01630E-68	-8,33830E-01	0,232	0,445	1,47636E-63
Mast	EIF4A2	1,87979E-21	-8,34417E-01	0,381	0,575	3,46201E-17
Mast	NASP	1,65608E-67	-8,34559E-01	0,124	0,286	3,05001E-63
Mast	OGFRL1	8,95979E-279	-8,34684E-01	0,015	0,33	1,65012E-274
Mast	TRIB1	8,19593E-175	-8,35259E-01	0,028	0,256	1,50944E-170
Mast	SEC13	4,20286E-87	-8,37231E-01	0,195	0,416	7,74041E-83
Mast	OTUB1	1,96799E-127	-8,37654E-01	0,188	0,447	3,62446E-123
Mast	SRSF2	9,16372E-100	-8,37945E-01	0,298	0,576	1,68768E-95
Mast	EIF4H	1,37610E-131	-8,38129E-01	0,199	0,47	2,53436E-127
Mast	DNMT1	4,71396E-176	-8,38418E-01	0,076	0,334	8,68169E-172
Mast	GBP4	5,89490E-141	-8,40983E-01	0,073	0,292	1,08566E-136
Mast	RPL41	9,94879E-34	-8,42375E-01	0,749	0,562	1,83227E-29
Mast	CCND3	1,18168E-27	-8,42607E-01	0,201	0,32	2,17630E-23
Mast	STK10	2,47640E-81	-8,43709E-01	0,09	0,254	4,56079E-77
Mast	XAF1	2,54605E-104	-8,44046E-01	0,132	0,342	4,68905E-100
Mast	RPS18	8,23462E-78	-8,44633E-01	0,775	0,545	1,51657E-73
Mast	SH3BP2	3,73350E-175	-8,45273E-01	0,063	0,311	6,87598E-171
Mast	HMGB2	6,03402E-146	-8,46286E-01	0,157	0,416	1,11129E-141
Mast	TNFRSF1	7,36146E-123	-8,47291E-01	0,203	0,466	1,35576E-118
Mast	TGIF1	3,32647E-127	-8,48419E-01	0,111	0,338	6,12635E-123
Mast	MYL12B	3,65107E-14	-8,48541E-01	0,553	0,792	6,72418E-10
Mast	SDHA	8,47219E-52	-8,48637E-01	0,123	0,26	1,56032E-47
Mast	NDUFB5	9,68380E-158	-8,48787E-01	0,181	0,473	1,78347E-153
Mast	RALA	8,28593E-151	-8,49346E-01	0,136	0,391	1,52602E-146
Mast	UNC119	7,60015E-128	-8,49418E-01	0,074	0,281	1,39972E-123
Mast	POU2F2	5,22168E-154	-8,50128E-01	0,061	0,285	9,61678E-150
Mast	ANAPC1	1,41905E-133	-8,50884E-01	0,313	0,629	2,61347E-129
Mast	STAT1	5,53775E-78	-8,51347E-01	0,293	0,533	1,01989E-73
Mast	HPS1	1,12577E-133	-8,51861E-01	0,127	0,367	2,07334E-129
Mast	RBX1	6,79618E-246	-8,54863E-01	0,261	0,644	1,25165E-241
Mast	PTPN6	3,63161E-07	-8,55168E-01	0,376	0,52	6,68834E-03
Mast	EMC4	1,81872E-60	-8,55518E-01	0,19	0,373	3,34954E-56
Mast	ADORA3	1,59342E-190	-8,56253E-01	0,034	0,279	2,93459E-186
Mast	PTRHD1	1,54457E-162	-8,56722E-01	0,107	0,366	2,84463E-158

Mast	LAMTOR	1,95440E-73	-8,59149E-01	0,108	0,269	3,59942E-69
Mast	GUSB	5,33387E-106	-8,59312E-01	0,143	0,36	9,82339E-102
Mast	PARP14	3,02846E-208	-8,59670E-01	0,095	0,397	5,57751E-204
Mast	SNX1	9,78452E-111	-8,60109E-01	0,111	0,32	1,80201E-106
Mast	NDUFS7	7,76362E-130	-8,60514E-01	0,269	0,56	1,42983E-125
Mast	TPD52L2	2,13389E-111	-8,61902E-01	0,087	0,282	3,92999E-107
Mast	CMTM6	4,74954E-179	-8,62367E-01	0,302	0,638	8,74723E-175
Mast	IGFLR1	2,04435E-11	-8,62549E-01	0,26	0,366	3,76509E-07
Mast	CCT3	9,88057E-84	-8,63349E-01	0,204	0,425	1,81970E-79
Mast	EIF4G2	1,02831E-88	-8,64431E-01	0,359	0,637	1,89383E-84
Mast	SRSF3	8,78272E-114	-8,64906E-01	0,299	0,59	1,61751E-109
Mast	PICALM	3,43183E-210	-8,65736E-01	0,136	0,449	6,32039E-206
Mast	RPLP0	7,30749E-186	-8,67812E-01	0,75	0,529	1,34582E-181
Mast	BNIP2	1,20173E-62	-8,67820E-01	0,214	0,414	2,21323E-58
Mast	PNP	1,73755E-84	-8,68309E-01	0,101	0,27	3,20004E-80
Mast	MRPL28	2,39476E-113	-8,68632E-01	0,083	0,28	4,41042E-109
Mast	TNFAIP8I	1,40260E-160	-8,68684E-01	0,048	0,275	2,58317E-156
Mast	LMAN2	3,53432E-122	-8,69690E-01	0,249	0,529	6,50916E-118
Mast	TAF9	7,63236E-45	-8,70430E-01	0,167	0,311	1,40565E-40
Mast	STRAP	3,12235E-34	-8,71106E-01	0,218	0,365	5,75044E-30
Mast	ALCAM	6,18928E-208	-8,72252E-01	0,026	0,284	1,13988E-203
Mast	ARRDC1	2,74864E-111	-8,72737E-01	0,102	0,308	5,06216E-107
Mast	TMCO1	1,63604E-91	-8,75447E-01	0,16	0,372	3,01310E-87
Mast	UBA1	8,10326E-124	-8,76116E-01	0,1	0,317	1,49238E-119
Mast	TOM1	9,70775E-138	-8,78403E-01	0,077	0,298	1,78788E-133
Mast	SLC16A3	5,30751E-216	-8,78758E-01	0,2	0,534	9,77484E-212
Mast	PIK3AP1	5,34938E-204	-8,79347E-01	0,053	0,322	9,85196E-200
Mast	VTI1B	1,75935E-75	-8,79452E-01	0,187	0,391	3,24019E-71
Mast	NEDD8	1,18247E-73	-8,79509E-01	0,337	0,604	2,17775E-69
Mast	STX12	1,57849E-188	-8,80340E-01	0,066	0,329	2,90711E-184
Mast	PLP2	1,98627E-07	-8,80340E-01	0,315	0,41	3,65812E-03
Mast	GAPT	7,10942E-34	-8,80446E-01	0,151	0,262	1,30934E-29
Mast	NAA20	6,85076E-117	-8,81068E-01	0,108	0,321	1,26170E-112
Mast	SPPL2A	8,78337E-118	-8,81568E-01	0,13	0,355	1,61763E-113
Mast	APEX1	2,98158E-62	-8,83762E-01	0,251	0,455	5,49117E-58
Mast	PLEKHJ1	1,00713E-123	-8,84894E-01	0,065	0,263	1,85483E-119
Mast	M6PR	9,45321E-72	-8,85325E-01	0,328	0,58	1,74100E-67
Mast	PSMD2	2,39732E-75	-8,85403E-01	0,148	0,332	4,41515E-71
Mast	UQCR10	2,21512E-239	-8,87144E-01	0,375	0,751	4,07958E-235
Mast	GPS1	1,22892E-85	-8,88852E-01	0,102	0,277	2,26330E-81

Mast	CTNNA1	5,84995E-161	-8,89328E-01	0,115	0,38	1,07738E-156
Mast	ATP6V0E	8,30516E-63	-8,90383E-01	0,547	0,813	1,52956E-58
Mast	IFNAR1	2,19711E-156	-8,90594E-01	0,085	0,329	4,04641E-152
Mast	LIMD2	3,40517E-130	-8,90691E-01	0,236	0,499	6,27130E-126
Mast	PTPMT1	1,88291E-128	-8,90798E-01	0,059	0,262	3,46776E-124
Mast	TXNDC17	6,77413E-127	-8,91036E-01	0,188	0,451	1,24759E-122
Mast	TMEM17	2,13708E-73	-8,91296E-01	0,352	0,547	3,93587E-69
Mast	TMEM59	2,54857E-105	-8,91689E-01	0,338	0,637	4,69369E-101
Mast	MSN	1,60832E-196	-8,92351E-01	0,304	0,661	2,96205E-192
Mast	COX7A2L	3,69082E-97	-8,92579E-01	0,25	0,507	6,79739E-93
Mast	UNC93B1	2,61845E-277	-8,94139E-01	0,033	0,357	4,82241E-273
Mast	RB1	6,35259E-242	-8,96131E-01	0,097	0,424	1,16996E-237
Mast	EIF2S3	3,85212E-42	-8,97294E-01	0,277	0,463	7,09445E-38
Mast	C20orf27	3,11864E-227	-8,97710E-01	0,068	0,364	5,74360E-223
Mast	CCNL1	2,21277E-82	-8,98058E-01	0,29	0,542	4,07527E-78
Mast	TXN2	4,91537E-139	-9,01144E-01	0,153	0,414	9,05263E-135
Mast	ACOT9	1,48794E-114	-9,01984E-01	0,091	0,292	2,74033E-110
Mast	DPYD	1,28455E-199	-9,03696E-01	0,094	0,385	2,36576E-195
Mast	TIMM13	5,24342E-151	-9,04927E-01	0,145	0,412	9,65680E-147
Mast	CD58	5,62572E-51	-9,06849E-01	0,169	0,323	1,03609E-46
Mast	OXA1L	2,52526E-115	-9,08347E-01	0,216	0,472	4,65077E-111
Mast	LAMTOR	4,19265E-184	-9,10088E-01	0,324	0,67	7,72160E-180
Mast	UBAC2	5,73361E-77	-9,10217E-01	0,14	0,321	1,05596E-72
Mast	RAB5C	7,40274E-153	-9,10664E-01	0,194	0,479	1,36336E-148
Mast	SPTLC2	7,06005E-116	-9,11976E-01	0,111	0,324	1,30025E-111
Mast	RABGAP1	1,66545E-83	-9,12554E-01	0,096	0,263	3,06726E-79
Mast	MYO1G	1,14880E-131	-9,13180E-01	0,087	0,297	2,11575E-127
Mast	KCNMA1	3,79102E-230	-9,14231E-01	0,01	0,278	6,98192E-226
Mast	BTF3	1,37988E-33	-9,14312E-01	0,654	0,859	2,54132E-29
Mast	NABP1	4,74382E-77	-9,15983E-01	0,161	0,352	8,73669E-73
Mast	BCAT1	2,48467E-161	-9,16070E-01	0,035	0,252	4,57602E-157
Mast	TES	1,35031E-144	-9,16138E-01	0,071	0,294	2,48687E-140
Mast	RPS5	6,70574E-156	-9,16336E-01	0,695	0,505	1,23500E-151
Mast	PUF60	8,23368E-89	-9,16815E-01	0,137	0,333	1,51640E-84
Mast	PTGER4	1,87249E-148	-9,17347E-01	0,058	0,284	3,44857E-144
Mast	AKR7A2	1,72513E-154	-9,17845E-01	0,044	0,264	3,17718E-150
Mast	TMED10	1,56971E-58	-9,17918E-01	0,341	0,583	2,89093E-54
Mast	UBL7	2,51685E-105	-9,18009E-01	0,081	0,266	4,63528E-101
Mast	BIN2	6,85643E-110	-9,18712E-01	0,118	0,327	1,26275E-105
Mast	STXBP2	7,09951E-80	-9,21620E-01	0,228	0,433	1,30752E-75

Mast	EIF3E	3,86027E-76	-9,22477E-01	0,36	0,623	7,10947E-72
Mast	TRABD	4,45016E-155	-9,23075E-01	0,116	0,374	8,19586E-151
Mast	GLG1	1,21654E-243	-9,23220E-01	0,022	0,31	2,24049E-239
Mast	CHMP5	1,48537E-135	-9,23308E-01	0,164	0,424	2,73561E-131
Mast	ASAH1	1,41628E-75	-9,23887E-01	0,47	0,704	2,60837E-71
Mast	MTCH2	2,22901E-125	-9,24648E-01	0,113	0,337	4,10516E-121
Mast	TMEM20	3,15742E-122	-9,25330E-01	0,119	0,346	5,81503E-118
Mast	HIGD2A	9,63480E-124	-9,29060E-01	0,413	0,736	1,77444E-119
Mast	GNB4	8,23807E-268	-9,30575E-01	0,039	0,357	1,51721E-263
Mast	HNRNPK	9,95736E-69	-9,30778E-01	0,439	0,728	1,83385E-64
Mast	FAM32A	4,13113E-104	-9,31569E-01	0,123	0,331	7,60831E-100
Mast	ITGB1	8,62191E-247	-9,32297E-01	0,147	0,498	1,58790E-242
Mast	COX8A	1,95374E-138	-9,32961E-01	0,467	0,799	3,59820E-134
Mast	UBXN1	7,85698E-154	-9,32965E-01	0,316	0,65	1,44702E-149
Mast	EVL	1,07455E-227	-9,33123E-01	0,075	0,377	1,97901E-223
Mast	GIT2	2,16670E-144	-9,33173E-01	0,08	0,309	3,99041E-140
Mast	SCNM1	5,17156E-145	-9,33844E-01	0,106	0,351	9,52446E-141
Mast	ETHE1	8,59145E-182	-9,36869E-01	0,09	0,359	1,58229E-177
Mast	MLX	1,30470E-163	-9,37830E-01	0,112	0,376	2,40287E-159
Mast	ORMDL2	1,90381E-129	-9,38329E-01	0,093	0,313	3,50624E-125
Mast	XRCC6	1,45154E-90	-9,39895E-01	0,259	0,512	2,67329E-86
Mast	ISCU	9,50820E-178	-9,40463E-01	0,237	0,558	1,75112E-173
Mast	FKBP15	8,21747E-191	-9,41072E-01	0,038	0,289	1,51341E-186
Mast	DECR1	1,26132E-96	-9,42437E-01	0,147	0,361	2,32298E-92
Mast	HSBP1	5,11551E-266	-9,44487E-01	0,24	0,626	9,42123E-262
Mast	CTSD	4,72119E-10	-9,45967E-01	0,658	0,777	8,69502E-06
Mast	QSOX1	2,20919E-168	-9,46362E-01	0,065	0,304	4,06866E-164
Mast	GRSF1	3,98645E-129	-9,46387E-01	0,122	0,355	7,34184E-125
Mast	VDAC3	1,75107E-73	-9,46389E-01	0,146	0,324	3,22495E-69
Mast	CHCHD2	2,98450E-58	-9,47679E-01	0,567	0,833	5,49655E-54
Mast	SNX10	1,84738E-266	-9,48315E-01	0,154	0,509	3,40232E-262
Mast	ETF1	6,91364E-98	-9,48502E-01	0,132	0,332	1,27329E-93
Mast	ATF4	2,45331E-83	-9,50146E-01	0,203	0,42	4,51826E-79
Mast	MYL12A	8,51073E-11	-9,50481E-01	0,677	0,835	1,56742E-06
Mast	GNAI2	0,00000E+00	-9,53275E-01	0,235	0,704	0,00000E+00
Mast	LHFPL2	1,09162E-242	-9,53432E-01	0,018	0,302	2,01044E-238
Mast	PREX1	0,00000E+00	-9,53437E-01	0,013	0,353	0,00000E+00
Mast	C1orf43	9,90601E-137	-9,54343E-01	0,263	0,566	1,82439E-132
Mast	CD302	1,16094E-229	-9,55287E-01	0,066	0,369	2,13810E-225
Mast	AUP1	1,11613E-131	-9,55675E-01	0,252	0,544	2,05557E-127

Mast	FUCA1	1,90344E-165	-9,56096E-01	0,04	0,265	3,50557E-161
Mast	SASH3	8,22510E-80	-9,56333E-01	0,122	0,295	1,51482E-75
Mast	VPS29	6,31219E-104	-9,57712E-01	0,278	0,553	1,16252E-99
Mast	RAP1B	7,72462E-161	-9,57823E-01	0,282	0,6	1,42264E-156
Mast	NUDT16	0,00000E+00	-9,58966E-01	0,064	0,432	0,00000E+00
Mast	LAIR1	1,53814E-148	-9,59971E-01	0,252	0,538	2,83280E-144
Mast	COX4I1	1,18982E-15	-9,60775E-01	0,745	0,907	2,19129E-11
Mast	PSMA5	1,68595E-93	-9,64550E-01	0,215	0,45	3,10502E-89
Mast	DDOST	6,78839E-54	-9,67453E-01	0,268	0,477	1,25022E-49
Mast	SDHC	5,46558E-194	-9,69245E-01	0,154	0,461	1,00660E-189
Mast	SOAT1	3,01500E-188	-9,69282E-01	0,047	0,302	5,55272E-184
Mast	RPL23	1,37935E-34	-9,69706E-01	0,531	0,447	2,54035E-30
Mast	CKS2	1,85794E-42	-9,69945E-01	0,129	0,251	3,42177E-38
Mast	CMTM7	4,87161E-208	-9,70161E-01	0,118	0,432	8,97204E-204
Mast	ORMDL1	3,32031E-63	-9,71869E-01	0,154	0,327	6,11502E-59
Mast	HVCN1	2,24215E-177	-9,72063E-01	0,033	0,269	4,12936E-173
Mast	DMXL2	3,39539E-117	-9,72368E-01	0,083	0,28	6,25330E-113
Mast	ACP5	4,09048E-84	-9,72699E-01	0,217	0,412	7,53344E-80
Mast	BCKDK	7,77847E-194	-9,72712E-01	0,068	0,337	1,43256E-189
Mast	CD48	1,66728E-93	-9,73267E-01	0,255	0,495	3,07063E-89
Mast	CLK1	3,85752E-15	-9,73846E-01	0,268	0,39	7,10439E-11
Mast	SCAMP2	5,84059E-154	-9,74896E-01	0,224	0,523	1,07566E-149
Mast	BRI3	8,77020E-249	-9,74965E-01	0,097	0,424	1,61521E-244
Mast	AAMP	6,45002E-99	-9,75789E-01	0,111	0,306	1,18790E-94
Mast	SH3BGRL	3,64935E-140	-9,76286E-01	0,413	0,741	6,72101E-136
Mast	RGS19	4,76487E-178	-9,76756E-01	0,209	0,516	8,77547E-174
Mast	IDH2	7,71165E-196	-9,77286E-01	0,133	0,431	1,42026E-191
Mast	DCK	9,00438E-142	-9,78569E-01	0,074	0,294	1,65834E-137
Mast	MTRNR2	1,63381E-108	-9,79312E-01	0,239	0,455	3,00898E-104
Mast	STAT6	4,85088E-103	-9,82897E-01	0,117	0,318	8,93386E-99
Mast	MCOLN1	4,31066E-188	-9,83677E-01	0,04	0,291	7,93895E-184
Mast	ATOX1	1,62277E-209	-9,84706E-01	0,256	0,598	2,98866E-205
Mast	RPL15	1,35878E-72	-9,84723E-01	0,737	0,54	2,50247E-68
Mast	MAT2A	8,85809E-60	-9,85372E-01	0,256	0,46	1,63140E-55
Mast	MLF2	3,53283E-98	-9,85867E-01	0,223	0,468	6,50641E-94
Mast	ADIPOR1	2,62939E-150	-9,86146E-01	0,191	0,48	4,84254E-146
Mast	DAZAP2	1,54666E-14	-9,87025E-01	0,523	0,757	2,84849E-10
Mast	PQBP1	1,91044E-117	-9,87361E-01	0,111	0,329	3,51846E-113
Mast	RPL36A	5,07282E-50	-9,87981E-01	0,525	0,449	9,34262E-46
Mast	PKM	4,99969E-241	-9,88306E-01	0,437	0,808	9,20794E-237

Mast	PDE4DIP	2,59430E-185	-9,88713E-01	0,076	0,339	4,77792E-181
Mast	CCT5	5,75311E-128	-9,91631E-01	0,152	0,397	1,05955E-123
Mast	EIF1	1,86714E-51	-9,93605E-01	0,844	0,951	3,43871E-47
Mast	LAMTOR	2,74456E-211	-9,94554E-01	0,221	0,565	5,05466E-207
Mast	CORO1B	9,07232E-272	-9,95101E-01	0,138	0,496	1,67085E-267
Mast	HAVCR2	2,49088E-152	-9,95970E-01	0,177	0,453	4,58745E-148
Mast	SORL1	9,01980E-272	-9,96395E-01	0,055	0,385	1,66118E-267
Mast	PSMF1	7,96695E-103	-9,96521E-01	0,197	0,437	1,46727E-98
Mast	EWSR1	5,08052E-78	-9,97218E-01	0,247	0,478	9,35680E-74
Mast	PSMD13	2,05137E-88	-1,00020E+00	0,138	0,336	3,77800E-84
Mast	CYFIP1	2,01198E-229	-1,00046E+00	0,051	0,343	3,70546E-225
Mast	CYTH4	5,92250E-115	-1,00126E+00	0,116	0,329	1,09075E-110
Mast	PRDX3	2,82595E-164	-1,00133E+00	0,195	0,5	5,20456E-160
Mast	SLC25A6	2,84298E-209	-1,00282E+00	0,512	0,837	5,23592E-205
Mast	DNAJA1	1,49639E-53	-1,00288E+00	0,378	0,62	2,75590E-49
Mast	GSTK1	6,33632E-117	-1,00386E+00	0,366	0,664	1,16696E-112
Mast	ENY2	1,13662E-158	-1,00388E+00	0,286	0,617	2,09332E-154
Mast	GLB1	1,27200E-108	-1,00488E+00	0,104	0,305	2,34264E-104
Mast	SIRPA	5,86150E-295	-1,00544E+00	0,035	0,372	1,07951E-290
Mast	NDUFV2	6,34613E-95	-1,00699E+00	0,29	0,551	1,16877E-90
Mast	GCA	1,37917E-242	-1,00714E+00	0,096	0,423	2,54002E-238
Mast	GIMAP7	9,16520E-196	-1,00781E+00	0,025	0,272	1,68796E-191
Mast	SEC61B	9,35709E-16	-1,00798E+00	0,535	0,754	1,72329E-11
Mast	CAP1	1,01277E-217	-1,00844E+00	0,394	0,757	1,86521E-213
Mast	CALHM6	8,13264E-141	-1,00934E+00	0,07	0,274	1,49779E-136
Mast	PSENEN	1,08634E-142	-1,01018E+00	0,081	0,309	2,00071E-138
Mast	SLCO2B1	9,11484E-175	-1,01143E+00	0,115	0,387	1,67868E-170
Mast	EIF5A	1,71070E-106	-1,01218E+00	0,301	0,575	3,15059E-102
Mast	MTMR14	4,12163E-152	-1,01272E+00	0,085	0,326	7,59081E-148
Mast	CAPNS1	9,81972E-184	-1,01302E+00	0,107	0,388	1,80850E-179
Mast	CTNNB1	1,40773E-121	-1,01312E+00	0,156	0,395	2,59261E-117
Mast	AHSA1	1,72338E-87	-1,01731E+00	0,124	0,311	3,17395E-83
Mast	MIF4GD	7,67451E-133	-1,01893E+00	0,102	0,327	1,41341E-128
Mast	NOP10	5,32049E-153	-1,02069E+00	0,327	0,653	9,79875E-149
Mast	VOPP1	2,64097E-205	-1,02381E+00	0,083	0,366	4,86387E-201
Mast	ZNF207	7,45640E-81	-1,02412E+00	0,203	0,419	1,37325E-76
Mast	SEC61A1	5,62582E-54	-1,02442E+00	0,164	0,321	1,03611E-49
Mast	MPV17	1,51004E-80	-1,02455E+00	0,136	0,322	2,78104E-76
Mast	TRAM1	3,26664E-151	-1,02521E+00	0,185	0,469	6,01617E-147
Mast	HNRNPF	1,17489E-134	-1,02557E+00	0,282	0,585	2,16380E-130

Mast	EVI2A	3,15349E-135	-1,02581E+00	0,071	0,288	5,80778E-131
Mast	GPSM3	0,00000E+00	-1,02671E+00	0,264	0,713	0,00000E+00
Mast	AZI2	1,81425E-164	-1,02679E+00	0,073	0,318	3,34131E-160
Mast	CLEC2B	0,00000E+00	-1,02780E+00	0,102	0,496	0,00000E+00
Mast	STOM	4,91292E-226	-1,02835E+00	0,066	0,365	9,04812E-222
Mast	PRDX5	1,33067E-146	-1,02912E+00	0,305	0,621	2,45069E-142
Mast	CHMP2A	4,90779E-167	-1,02953E+00	0,205	0,511	9,03867E-163
Mast	NAPA	9,60422E-158	-1,02957E+00	0,219	0,514	1,76881E-153
Mast	CASP1	6,94942E-167	-1,02965E+00	0,187	0,482	1,27987E-162
Mast	BCAP31	2,33739E-168	-1,02992E+00	0,275	0,605	4,30476E-164
Mast	GGA2	4,70587E-178	-1,03025E+00	0,043	0,286	8,66680E-174
Mast	AKR1A1	3,26295E-119	-1,03087E+00	0,314	0,595	6,00938E-115
Mast	KLF4	2,62432E-217	-1,03102E+00	0,139	0,468	4,83321E-213
Mast	AKIRIN2	1,98888E-216	-1,03128E+00	0,128	0,442	3,66292E-212
Mast	ADRM1	6,07375E-141	-1,03144E+00	0,173	0,446	1,11860E-136
Mast	APBB1IP	0,00000E+00	-1,03166E+00	0,1	0,505	0,00000E+00
Mast	MRPL55	7,59987E-94	-1,03181E+00	0,104	0,29	1,39967E-89
Mast	P4HB	1,07019E-98	-1,03298E+00	0,353	0,648	1,97097E-94
Mast	DOCK8	3,80975E-220	-1,03458E+00	0,146	0,469	7,01642E-216
Mast	SMIM14	3,72544E-101	-1,03467E+00	0,092	0,281	6,86114E-97
Mast	BUD31	3,86675E-103	-1,03695E+00	0,153	0,375	7,12139E-99
Mast	DOCK4	6,13392E-270	-1,03710E+00	0,005	0,305	1,12968E-265
Mast	PTTG1IP	7,59014E-199	-1,03775E+00	0,19	0,524	1,39788E-194
Mast	FYB1	6,20125E-159	-1,03871E+00	0,105	0,332	1,14208E-154
Mast	CD83	8,67945E-19	-1,03914E+00	0,361	0,497	1,59849E-14
Mast	ZNF106	1,44614E-220	-1,04062E+00	0,069	0,363	2,66335E-216
Mast	RTCB	4,25872E-142	-1,04147E+00	0,083	0,31	7,84328E-138
Mast	NDUFA1(	6,50229E-84	-1,04350E+00	0,144	0,341	1,19753E-79
Mast	SKAP2	2,22641E-196	-1,04567E+00	0,126	0,425	4,10037E-192
Mast	ARF1	6,37704E-98	-1,04679E+00	0,412	0,719	1,17446E-93
Mast	MT-ND1	4,83033E-74	-1,04859E+00	0,788	0,554	8,89602E-70
Mast	HIF1A	1,87615E-41	-1,04871E+00	0,358	0,563	3,45531E-37
Mast	TBXAS1	4,10240E-228	-1,04955E+00	0,196	0,541	7,55539E-224
Mast	PFKL	3,94008E-165	-1,05097E+00	0,135	0,411	7,25645E-161
Mast	BLOC1S1	1,43074E-206	-1,05282E+00	0,179	0,493	2,63500E-202
Mast	CARD16	1,19100E-124	-1,05473E+00	0,279	0,571	2,19346E-120
Mast	MICAL1	1,36639E-164	-1,05692E+00	0,068	0,309	2,51648E-160
Mast	EIF3H	1,00407E-162	-1,05843E+00	0,343	0,68	1,84920E-158
Mast	SUMO3	3,97407E-300	-1,06001E+00	0,126	0,506	7,31905E-296
Mast	ARF5	3,12271E-181	-1,06025E+00	0,302	0,635	5,75110E-177

Mast	KCNAB2	4,53465E-177	-1,06128E+00	0,05	0,297	8,35147E-173
Mast	TANK	3,43078E-135	-1,06299E+00	0,087	0,308	6,31847E-131
Mast	NPM1	2,08569E-54	-1,06391E+00	0,541	0,782	3,84122E-50
Mast	LRPAP1	3,51393E-196	-1,06400E+00	0,194	0,517	6,47160E-192
Mast	RNF213	2,54869E-212	-1,06422E+00	0,2	0,526	4,69392E-208
Mast	CDC37	3,15993E-121	-1,06543E+00	0,352	0,658	5,81965E-117
Mast	GMFG	7,00390E-165	-1,06752E+00	0,358	0,695	1,28991E-160
Mast	PGLS	4,37506E-218	-1,06752E+00	0,225	0,565	8,05755E-214
Mast	ERP29	5,82072E-202	-1,06918E+00	0,297	0,646	1,07200E-197
Mast	ATP6AP1	6,49403E-196	-1,06964E+00	0,23	0,566	1,19601E-191
Mast	ARRB2	0,00000E+00	-1,07111E+00	0,222	0,697	0,00000E+00
Mast	IDH3B	1,09241E-89	-1,07161E+00	0,096	0,273	2,01189E-85
Mast	RIPK2	6,90218E-100	-1,07182E+00	0,1	0,286	1,27117E-95
Mast	GSDMD	3,36246E-103	-1,07352E+00	0,113	0,314	6,19265E-99
Mast	CDC42	1,11133E-211	-1,07386E+00	0,346	0,721	2,04674E-207
Mast	MEF2A	0,00000E+00	-1,07389E+00	0,07	0,446	0,00000E+00
Mast	FUOM	5,13260E-215	-1,07546E+00	0,096	0,396	9,45271E-211
Mast	EIF3M	3,12275E-54	-1,07546E+00	0,293	0,514	5,75117E-50
Mast	MAP2K3	4,73796E-80	-1,07551E+00	0,113	0,281	8,72590E-76
Mast	RBM6	1,39523E-19	-1,07584E+00	0,173	0,269	2,56959E-15
Mast	RNF10	2,91952E-53	-1,07698E+00	0,139	0,284	5,37687E-49
Mast	SAP30BP	4,98911E-56	-1,07715E+00	0,133	0,281	9,18844E-52
Mast	PLIN3	2,24366E-167	-1,07838E+00	0,12	0,389	4,13216E-163
Mast	CD55	3,70688E-13	-1,07887E+00	0,325	0,437	6,82697E-09
Mast	CAPZB	1,75710E-288	-1,07933E+00	0,387	0,786	3,23605E-284
Mast	PSMB9	1,10747E-127	-1,08095E+00	0,372	0,678	2,03963E-123
Mast	U2AF1	4,85963E-117	-1,08104E+00	0,079	0,283	8,94999E-113
Mast	EPB41L3	6,11302E-299	-1,08179E+00	0,003	0,325	1,12584E-294
Mast	RPS19	2,57234E-07	-1,08190E+00	0,765	0,562	4,73748E-03
Mast	EIF3I	5,62641E-153	-1,08300E+00	0,194	0,484	1,03622E-148
Mast	B4GALT1	6,63910E-104	-1,08387E+00	0,13	0,339	1,22272E-99
Mast	ZFAND5	1,17954E-179	-1,08476E+00	0,28	0,607	2,17237E-175
Mast	PSMB6	3,78253E-147	-1,08645E+00	0,281	0,597	6,96629E-143
Mast	DNASE2	1,83381E-269	-1,08668E+00	0,063	0,388	3,37734E-265
Mast	DNAJC7	1,19331E-103	-1,08866E+00	0,156	0,38	2,19772E-99
Mast	REEP5	3,28025E-174	-1,08994E+00	0,256	0,584	6,04123E-170
Mast	IFITM3	1,07013E-15	-1,09016E+00	0,592	0,776	1,97086E-11
Mast	CYTH1	7,71403E-168	-1,09134E+00	0,088	0,344	1,42069E-163
Mast	ARPC3	1,35581E-87	-1,09156E+00	0,682	0,907	2,49699E-83
Mast	PARK7	3,32285E-65	-1,09328E+00	0,411	0,676	6,11968E-61

Mast	TPI1	1,58847E-09	-1,09353E+00	0,635	0,839	2,92548E-05
Mast	PLCB2	3,02942E-61	-1,09379E+00	0,107	0,251	5,57929E-57
Mast	UBE2D3	3,58355E-106	-1,09587E+00	0,445	0,757	6,59983E-102
Mast	CD93	6,21627E-269	-1,09682E+00	0,002	0,298	1,14485E-264
Mast	PET100	1,37505E-97	-1,09786E+00	0,189	0,42	2,53244E-93
Mast	TWF2	2,49982E-85	-1,09822E+00	0,157	0,347	4,60392E-81
Mast	GLIPR2	2,34081E-120	-1,09972E+00	0,164	0,392	4,31108E-116
Mast	TOR3A	1,77033E-133	-1,09984E+00	0,051	0,251	3,26042E-129
Mast	LPP	6,07524E-141	-1,10010E+00	0,065	0,282	1,11888E-136
Mast	PSMB7	4,54909E-135	-1,10197E+00	0,133	0,379	8,37806E-131
Mast	WASF2	3,91481E-291	-1,10358E+00	0,2	0,594	7,20990E-287
Mast	SNRPB	8,83124E-171	-1,10666E+00	0,215	0,529	1,62645E-166
Mast	SAR1A	4,57754E-99	-1,10954E+00	0,078	0,255	8,43045E-95
Mast	NUMB	2,72644E-171	-1,11072E+00	0,091	0,35	5,02128E-167
Mast	RNF181	7,94017E-207	-1,11120E+00	0,234	0,585	1,46234E-202
Mast	SEC14L1	9,56805E-212	-1,11247E+00	0,109	0,415	1,76215E-207
Mast	CALR	7,36739E-196	-1,11436E+00	0,409	0,745	1,35685E-191
Mast	EIF3D	6,28073E-96	-1,11513E+00	0,241	0,49	1,15672E-91
Mast	SMAP2	0,00000E+00	-1,11535E+00	0,097	0,497	0,00000E+00
Mast	PSMB8	5,34528E-124	-1,11561E+00	0,303	0,596	9,84441E-120
Mast	WDR1	2,08877E-237	-1,11682E+00	0,204	0,564	3,84688E-233
Mast	CSTB	7,56634E-156	-1,11955E+00	0,524	0,808	1,39349E-151
Mast	RAN	3,55899E-84	-1,12191E+00	0,389	0,676	6,55459E-80
Mast	INPP5D	3,28138E-105	-1,12192E+00	0,118	0,325	6,04332E-101
Mast	ARL8B	3,71931E-137	-1,12261E+00	0,163	0,42	6,84986E-133
Mast	EIF3G	1,43564E-158	-1,12262E+00	0,306	0,635	2,64402E-154
Mast	SLC43A2	0,00000E+00	-1,12455E+00	0,03	0,392	0,00000E+00
Mast	TIMP2	0,00000E+00	-1,12622E+00	0,008	0,385	0,00000E+00
Mast	RAB7A	1,33860E-183	-1,12708E+00	0,31	0,656	2,46530E-179
Mast	GNG5	5,63968E-171	-1,12730E+00	0,512	0,813	1,03866E-166
Mast	MAPK1IF	9,70757E-84	-1,12875E+00	0,158	0,357	1,78784E-79
Mast	SNX2	1,12087E-198	-1,13201E+00	0,149	0,461	2,06431E-194
Mast	FTH1	4,53137E-55	-1,13316E+00	0,989	0,998	8,34543E-51
Mast	RASGEF1	2,04736E-25	-1,13400E+00	0,17	0,276	3,77062E-21
Mast	SH3TC1	4,33728E-161	-1,13545E+00	0,084	0,329	7,98798E-157
Mast	TFEC	1,38267E-289	-1,13556E+00	0,002	0,316	2,54646E-285
Mast	PFDN5	6,09358E-70	-1,13777E+00	0,71	0,916	1,12225E-65
Mast	SGK1	5,69937E-130	-1,13797E+00	0,353	0,622	1,04965E-125
Mast	ARAP1	4,86522E-137	-1,13842E+00	0,085	0,306	8,96027E-133
Mast	SP110	6,88728E-211	-1,14062E+00	0,107	0,41	1,26843E-206

Mast	LY6E	3,88124E-165	-1,14070E+00	0,283	0,566	7,14808E-161
Mast	RHOC	2,23001E-67	-1,14191E+00	0,152	0,32	4,10700E-63
Mast	SHKBP1	9,19406E-141	-1,14304E+00	0,136	0,386	1,69327E-136
Mast	PSMD4	8,34350E-104	-1,14347E+00	0,19	0,428	1,53662E-99
Mast	MGAT4A	9,19435E-236	-1,14490E+00	0,072	0,377	1,69332E-231
Mast	C3AR1	2,22104E-216	-1,14510E+00	0,127	0,436	4,09049E-212
Mast	LPXN	4,70844E-64	-1,14511E+00	0,182	0,36	8,67153E-60
Mast	CD164	2,67567E-140	-1,14519E+00	0,274	0,577	4,92777E-136
Mast	AP1B1	0,00000E+00	-1,14532E+00	0,05	0,418	0,00000E+00
Mast	DOCK2	6,14676E-138	-1,14667E+00	0,12	0,364	1,13205E-133
Mast	SFT2D2	6,05296E-135	-1,14785E+00	0,085	0,308	1,11477E-130
Mast	TAGAP	6,18627E-117	-1,14792E+00	0,084	0,285	1,13933E-112
Mast	PGD	8,29181E-181	-1,14797E+00	0,18	0,485	1,52710E-176
Mast	ATP2B1	4,95121E-235	-1,15094E+00	0,066	0,368	9,11864E-231
Mast	TSPAN14	6,31020E-225	-1,15386E+00	0,061	0,352	1,16215E-220
Mast	NECAP2	6,74830E-206	-1,15402E+00	0,081	0,372	1,24283E-201
Mast	POLR2E	1,05511E-212	-1,15799E+00	0,208	0,552	1,94320E-208
Mast	COPE	1,51356E-185	-1,16162E+00	0,284	0,629	2,78752E-181
Mast	SERF2	4,34705E-290	-1,16206E+00	0,8	0,964	8,00595E-286
Mast	UTRN	5,08016E-155	-1,16276E+00	0,07	0,304	9,35614E-151
Mast	MOB1A	1,34371E-161	-1,16309E+00	0,279	0,604	2,47471E-157
Mast	CD300A	3,43388E-218	-1,16328E+00	0,152	0,482	6,32418E-214
Mast	IFNGR2	4,07903E-219	-1,16394E+00	0,24	0,587	7,51235E-215
Mast	TAPBP	1,68748E-127	-1,16604E+00	0,294	0,599	3,10784E-123
Mast	COMT	2,62872E-233	-1,16831E+00	0,223	0,581	4,84131E-229
Mast	IL13RA1	0,00000E+00	-1,17083E+00	0,005	0,415	0,00000E+00
Mast	ARID5A	1,37059E-150	-1,17083E+00	0,074	0,303	2,52422E-146
Mast	CNN2	9,22714E-92	-1,17212E+00	0,203	0,416	1,69936E-87
Mast	PARVG	5,67077E-175	-1,17262E+00	0,172	0,469	1,04439E-170
Mast	RPS6	2,83730E-41	-1,17473E+00	0,706	0,533	5,22545E-37
Mast	NAP1L1	7,29412E-98	-1,17506E+00	0,363	0,637	1,34336E-93
Mast	ATF5	3,90127E-288	-1,17736E+00	0,049	0,383	7,18496E-284
Mast	GABARAI	2,73826E-168	-1,17755E+00	0,065	0,308	5,04306E-164
Mast	ELOVL1	2,27316E-139	-1,17783E+00	0,13	0,377	4,18648E-135
Mast	SH3BP5	0,00000E+00	-1,17872E+00	0,009	0,36	0,00000E+00
Mast	RPL9	5,54698E-38	-1,17916E+00	0,698	0,533	1,02159E-33
Mast	CYCS	1,19963E-199	-1,18387E+00	0,215	0,545	2,20935E-195
Mast	JMJD1C	3,80369E-201	-1,18509E+00	0,137	0,448	7,00525E-197
Mast	RPL17	1,89181E-234	-1,19034E+00	0,619	0,42	3,48414E-230
Mast	EIF3F	8,37791E-132	-1,19076E+00	0,375	0,686	1,54296E-127

Mast	PLD3	6,51179E-184	-1,19295E+00	0,236	0,53	1,19928E-179
Mast	ARHGAP4	3,78618E-196	-1,19561E+00	0,111	0,404	6,97301E-192
Mast	CLTA	6,83453E-259	-1,19630E+00	0,288	0,674	1,25872E-254
Mast	C19orf38	2,54482E-205	-1,19703E+00	0,007	0,25	4,68680E-201
Mast	CAT	1,75985E-283	-1,19775E+00	0,083	0,432	3,24111E-279
Mast	NANS	4,69822E-226	-1,20478E+00	0,098	0,41	8,65272E-222
Mast	HLA-F	2,89818E-120	-1,20510E+00	0,253	0,534	5,33758E-116
Mast	RILPL2	2,80788E-245	-1,20700E+00	0,138	0,478	5,17127E-241
Mast	HMGN2	4,22188E-88	-1,20708E+00	0,462	0,753	7,77544E-84
Mast	HNRNPC	2,99259E-143	-1,20755E+00	0,334	0,669	5,51145E-139
Mast	CLEC12A	1,66214E-105	-1,20850E+00	0,089	0,272	3,06117E-101
Mast	ATF3	2,90359E-125	-1,20979E+00	0,192	0,45	5,34755E-121
Mast	PTPN2	3,28546E-163	-1,21694E+00	0,133	0,403	6,05084E-159
Mast	NAAA	2,76315E-246	-1,21959E+00	0,071	0,388	5,08889E-242
Mast	CPNE3	1,23820E-207	-1,22238E+00	0,011	0,259	2,28039E-203
Mast	RPL21	3,54802E-14	-1,22302E+00	0,674	0,532	6,53439E-10
Mast	TSPAN3	1,62710E-154	-1,22341E+00	0,091	0,334	2,99664E-150
Mast	COPZ1	7,00185E-119	-1,22476E+00	0,158	0,398	1,28953E-114
Mast	YWHAB	3,47731E-280	-1,22564E+00	0,426	0,813	6,40417E-276
Mast	DDX5	3,86131E-42	-1,22614E+00	0,593	0,849	7,11138E-38
Mast	COMMD	7,54020E-214	-1,22727E+00	0,052	0,332	1,38868E-209
Mast	STAT2	9,00744E-165	-1,22933E+00	0,067	0,31	1,65890E-160
Mast	ARL4C	7,93450E-264	-1,22971E+00	0,036	0,353	1,46130E-259
Mast	NUP214	9,26658E-205	-1,22975E+00	0,058	0,332	1,70663E-200
Mast	SYK	1,55002E-298	-1,22986E+00	0,098	0,467	2,85467E-294
Mast	TXNIP	3,76256E-158	-1,23031E+00	0,46	0,716	6,92952E-154
Mast	PPP1CB	1,29210E-113	-1,23130E+00	0,177	0,413	2,37967E-109
Mast	IFI35	1,47659E-180	-1,23423E+00	0,096	0,364	2,71944E-176
Mast	ACADVL	6,80774E-62	-1,23619E+00	0,265	0,483	1,25378E-57
Mast	AP1S2	4,94546E-207	-1,23661E+00	0,262	0,601	9,10806E-203
Mast	ACTN1	3,76914E-280	-1,23685E+00	0,023	0,339	6,94162E-276
Mast	SLC25A3	8,37418E-64	-1,23843E+00	0,458	0,731	1,54227E-59
Mast	IVNS1AB	2,75805E-174	-1,23987E+00	0,113	0,384	5,07950E-170
Mast	EIF4A3	1,36527E-90	-1,24051E+00	0,197	0,417	2,51443E-86
Mast	RHOB	2,06471E-235	-1,24477E+00	0,229	0,564	3,80257E-231
Mast	PSMA3	1,08878E-79	-1,24516E+00	0,182	0,385	2,00520E-75
Mast	RAB8A	5,71296E-210	-1,24774E+00	0,133	0,446	1,05216E-205
Mast	VMP1	1,83710E-93	-1,24854E+00	0,305	0,568	3,38339E-89
Mast	IST1	2,78481E-61	-1,25132E+00	0,122	0,274	5,12879E-57
Mast	LPAR6	7,87116E-298	-1,25391E+00	0,014	0,344	1,44963E-293

Mast	ZNF331	3,00592E-30	-1,25402E+00	0,219	0,353	5,53600E-26
Mast	RPL10A	8,52299E-39	-1,25453E+00	0,601	0,49	1,56968E-34
Mast	MYL6	1,02820E-64	-1,25612E+00	0,812	0,951	1,89363E-60
Mast	PSMB10	8,78571E-123	-1,25797E+00	0,298	0,585	1,61806E-118
Mast	CALM1	3,86089E-73	-1,25821E+00	0,545	0,812	7,11060E-69
Mast	GUK1	3,73222E-113	-1,26006E+00	0,415	0,731	6,87363E-109
Mast	PSMA7	1,59293E-153	-1,26090E+00	0,432	0,767	2,93370E-149
Mast	SCARB2	1,21181E-292	-1,26178E+00	0,05	0,394	2,23179E-288
Mast	NAIP	0,00000E+00	-1,26271E+00	0,035	0,394	0,00000E+00
Mast	HCST	5,98490E-201	-1,26359E+00	0,332	0,676	1,10224E-196
Mast	CTNND1	2,35568E-146	-1,26430E+00	0,066	0,286	4,33846E-142
Mast	SLC8A1	0,00000E+00	-1,26671E+00	0,003	0,39	0,00000E+00
Mast	MGST3	0,00000E+00	-1,27016E+00	0,152	0,569	0,00000E+00
Mast	PEA15	0,00000E+00	-1,27055E+00	0,102	0,546	0,00000E+00
Mast	ZNF706	6,13077E-180	-1,27124E+00	0,275	0,613	1,12910E-175
Mast	RPN2	4,55342E-31	-1,27368E+00	0,178	0,292	8,38604E-27
Mast	PPP1CA	2,14785E-219	-1,27388E+00	0,277	0,646	3,95570E-215
Mast	MVP	2,45446E-181	-1,27547E+00	0,113	0,392	4,52038E-177
Mast	IFI6	2,98283E-195	-1,27567E+00	0,202	0,508	5,49348E-191
Mast	TMEM12	5,54743E-242	-1,27735E+00	0,136	0,477	1,02167E-237
Mast	MARCKSI	5,39613E-137	-1,27760E+00	0,072	0,278	9,93805E-133
Mast	AKR1B1	7,21389E-290	-1,27807E+00	0,132	0,491	1,32858E-285
Mast	MGST2	3,46243E-281	-1,27848E+00	0,047	0,381	6,37675E-277
Mast	FKBP1A	2,49102E-142	-1,27929E+00	0,389	0,716	4,58771E-138
Mast	HLA-C	0,00000E+00	-1,28470E+00	0,797	0,961	0,00000E+00
Mast	CREG1	0,00000E+00	-1,28556E+00	0,152	0,538	0,00000E+00
Mast	CYC1	3,29240E-188	-1,28579E+00	0,151	0,454	6,06361E-184
Mast	ANKRD1(	2,58693E-70	-1,28605E+00	0,162	0,344	4,76435E-66
Mast	TSPO	3,58220E-202	-1,28953E+00	0,482	0,785	6,59734E-198
Mast	RPS27	2,12362E-15	-1,28967E+00	0,73	0,555	3,91107E-11
Mast	PAIP2	9,54147E-166	-1,29350E+00	0,22	0,532	1,75725E-161
Mast	DPYSL2	3,44497E-235	-1,29376E+00	0,047	0,343	6,34461E-231
Mast	PLEKHO1	0,00000E+00	-1,29603E+00	0,015	0,385	0,00000E+00
Mast	UBE2F	1,22302E-103	-1,29717E+00	0,087	0,271	2,25243E-99
Mast	PFKFB3	6,54883E-236	-1,29764E+00	0,054	0,354	1,20610E-231
Mast	TAP1	3,54185E-116	-1,30093E+00	0,195	0,438	6,52303E-112
Mast	RPL23A	2,24446E-13	-1,30236E+00	0,633	0,518	4,13362E-09
Mast	COX5A	1,16042E-168	-1,30680E+00	0,376	0,716	2,13715E-164
Mast	RBM3	9,43583E-199	-1,31208E+00	0,338	0,698	1,73780E-194
Mast	HINT1	0,00000E+00	-1,31400E+00	0,262	0,755	0,00000E+00

Mast	PIK3R5	9,11739E-225	-1,31481E+00	0,011	0,275	1,67915E-220
Mast	PYCARD	3,13246E-219	-1,31482E+00	0,384	0,729	5,76905E-215
Mast	ENTPD1	3,97483E-288	-1,31733E+00	0,012	0,33	7,32045E-284
Mast	TMED9	6,08601E-250	-1,31809E+00	0,186	0,547	1,12086E-245
Mast	HEXA	1,50939E-250	-1,31842E+00	0,145	0,492	2,77983E-246
Mast	SDCBP	2,56667E-183	-1,31871E+00	0,4	0,746	4,72704E-179
Mast	ATP6V0D	2,05768E-287	-1,32377E+00	0,2	0,597	3,78963E-283
Mast	RHBDF2	1,94820E-292	-1,32674E+00	0,018	0,346	3,58800E-288
Mast	GK	1,53373E-213	-1,32740E+00	0,09	0,384	2,82468E-209
Mast	TMEM10	2,02682E-154	-1,32844E+00	0,127	0,386	3,73279E-150
Mast	PSMA4	1,06173E-146	-1,32933E+00	0,2	0,487	1,95539E-142
Mast	CD47	1,17712E-122	-1,33635E+00	0,186	0,438	2,16789E-118
Mast	SSR1	3,74840E-211	-1,33726E+00	0,16	0,484	6,90342E-207
Mast	HNRNPA	2,30820E-36	-1,33898E+00	0,571	0,818	4,25101E-32
Mast	UBE2E2	3,47062E-265	-1,33911E+00	0,016	0,319	6,39185E-261
Mast	SLA	3,07109E-259	-1,33938E+00	0,101	0,439	5,65602E-255
Mast	ERCC1	1,52059E-141	-1,34059E+00	0,143	0,398	2,80048E-137
Mast	OAS1	1,17200E-259	-1,34077E+00	0,037	0,347	2,15848E-255
Mast	GDI2	3,56526E-228	-1,34467E+00	0,308	0,675	6,56613E-224
Mast	RHOA	0,00000E+00	-1,34762E+00	0,584	0,899	0,00000E+00
Mast	NPL	7,33427E-227	-1,34866E+00	0,01	0,276	1,35075E-222
Mast	OS9	1,78651E-255	-1,34974E+00	0,191	0,561	3,29021E-251
Mast	YWHAH	0,00000E+00	-1,35264E+00	0,151	0,558	0,00000E+00
Mast	SDHB	4,04762E-188	-1,35322E+00	0,125	0,418	7,45451E-184
Mast	LGALS3B	2,84116E-264	-1,35480E+00	0,032	0,34	5,23257E-260
Mast	VAMP8	0,00000E+00	-1,35541E+00	0,455	0,803	0,00000E+00
Mast	RPL3	1,86740E-56	-1,36252E+00	0,678	0,523	3,43919E-52
Mast	PSME1	2,69264E-32	-1,36259E+00	0,527	0,77	4,95904E-28
Mast	EPSTI1	1,28327E-264	-1,36369E+00	0,095	0,431	2,36341E-260
Mast	UBE2L6	2,18723E-156	-1,36465E+00	0,284	0,59	4,02823E-152
Mast	IFI16	0,00000E+00	-1,36537E+00	0,134	0,525	0,00000E+00
Mast	CDKN1A	3,21789E-150	-1,36721E+00	0,252	0,544	5,92639E-146
Mast	ADPGK	3,55979E-195	-1,36737E+00	0,121	0,42	6,55607E-191
Mast	MTRNR2	1,78246E-280	-1,36810E+00	0,495	0,785	3,28275E-276
Mast	SLC15A3	8,73100E-246	-1,36851E+00	0,016	0,301	1,60799E-241
Mast	ETS2	2,64973E-278	-1,36894E+00	0,096	0,449	4,88001E-274
Mast	RAB1A	3,01386E-184	-1,37343E+00	0,124	0,415	5,55063E-180
Mast	GSTO1	1,53217E-282	-1,37491E+00	0,252	0,645	2,82180E-278
Mast	ENG	2,28817E-279	-1,37510E+00	0,04	0,369	4,21413E-275
Mast	MYO1F	1,53644E-304	-1,37534E+00	0,106	0,484	2,82966E-300

Mast	EVI2B	3,28145E-141	-1,37693E+00	0,139	0,393	6,04344E-137
Mast	NCF1	2,24626E-136	-1,38069E+00	0,26	0,517	4,13693E-132
Mast	CAPZA2	7,63520E-199	-1,38119E+00	0,229	0,571	1,40617E-194
Mast	LAPTM5	1,82566E-261	-1,38174E+00	0,666	0,914	3,36233E-257
Mast	CCDC50	2,37524E-210	-1,38508E+00	0,029	0,291	4,37448E-206
Mast	GAA	0,00000E+00	-1,38616E+00	0,038	0,411	0,00000E+00
Mast	FKBP5	0,00000E+00	-1,38696E+00	0,13	0,545	0,00000E+00
Mast	PTPRC	1,56420E-248	-1,38890E+00	0,334	0,715	2,88079E-244
Mast	RAB20	1,17098E-201	-1,38983E+00	0,081	0,365	2,15659E-197
Mast	FBP1	1,95210E-114	-1,39106E+00	0,149	0,363	3,59518E-110
Mast	RBM47	0,00000E+00	-1,39551E+00	0,006	0,383	0,00000E+00
Mast	FLII	2,74688E-126	-1,39795E+00	0,112	0,339	5,05892E-122
Mast	SLC25A5	2,86157E-207	-1,39979E+00	0,417	0,767	5,27015E-203
Mast	ARHGAP!	1,37360E-104	-1,40361E+00	0,076	0,258	2,52975E-100
Mast	MX2	5,66088E-145	-1,40490E+00	0,066	0,284	1,04256E-140
Mast	CNPY3	2,31068E-292	-1,40501E+00	0,253	0,654	4,25558E-288
Mast	ACAA1	2,71233E-172	-1,40571E+00	0,121	0,396	4,99530E-168
Mast	EEF1G	2,73591E-163	-1,40842E+00	0,454	0,285	5,03873E-159
Mast	UBA52	3,37159E-244	-1,40881E+00	0,72	0,934	6,20945E-240
Mast	PLXNB2	5,57540E-230	-1,41086E+00	0,013	0,283	1,02682E-225
Mast	CD40	7,57167E-256	-1,41198E+00	0,016	0,311	1,39447E-251
Mast	SSR3	1,45891E-213	-1,41996E+00	0,262	0,62	2,68687E-209
Mast	SCIMP	5,21422E-265	-1,42350E+00	0,003	0,297	9,60302E-261
Mast	CHMP1B	4,46389E-190	-1,42480E+00	0,137	0,425	8,22114E-186
Mast	RPS11	7,37361E-13	-1,42688E+00	0,585	0,498	1,35800E-08
Mast	RPL7	8,66802E-125	-1,43197E+00	0,657	0,486	1,59639E-120
Mast	LRRC25	7,05791E-298	-1,43867E+00	0,022	0,355	1,29986E-293
Mast	RPL31	8,13434E-42	-1,44375E+00	0,598	0,49	1,49810E-37
Mast	GAPDH	2,63730E-214	-1,44745E+00	0,823	0,97	4,85711E-210
Mast	ARPC5	4,70677E-296	-1,44847E+00	0,386	0,789	8,66845E-292
Mast	SLC25A3	1,45842E-183	-1,45357E+00	0,057	0,307	2,68598E-179
Mast	GPI	2,15655E-115	-1,45520E+00	0,187	0,432	3,97172E-111
Mast	SLC1A3	0,00000E+00	-1,46072E+00	0,003	0,388	0,00000E+00
Mast	MT-ND4L	0,00000E+00	-1,46432E+00	0,846	0,491	0,00000E+00
Mast	GRINA	0,00000E+00	-1,46488E+00	0,174	0,718	0,00000E+00
Mast	FCER1G	4,64141E-137	-1,46785E+00	0,778	0,922	8,54809E-133
Mast	PGAM1	5,93019E-187	-1,47058E+00	0,253	0,587	1,09216E-182
Mast	CREM	3,95903E-60	-1,47097E+00	0,213	0,389	7,29134E-56
Mast	APRT	1,40969E-257	-1,47318E+00	0,262	0,648	2,59623E-253
Mast	GRB2	3,52260E-300	-1,47806E+00	0,299	0,706	6,48756E-296

Mast	HLA-A	0,00000E+00	-1,47981E+00	0,736	0,965	0,00000E+00
Mast	TMEM16	7,06966E-205	-1,48327E+00	0,116	0,42	1,30202E-200
Mast	HNMT	0,00000E+00	-1,48746E+00	0,005	0,407	0,00000E+00
Mast	LTBR	1,37730E-296	-1,48788E+00	0,033	0,371	2,53658E-292
Mast	CAPZA1	4,55756E-197	-1,49039E+00	0,189	0,515	8,39366E-193
Mast	FERMT3	5,94860E-192	-1,49369E+00	0,186	0,503	1,09555E-187
Mast	TXN	1,72491E-63	-1,49421E+00	0,456	0,682	3,17677E-59
Mast	LCP1	9,79751E-143	-1,49541E+00	0,412	0,724	1,80441E-138
Mast	PRCP	0,00000E+00	-1,50079E+00	0,057	0,413	0,00000E+00
Mast	APP	6,27929E-284	-1,50142E+00	0,013	0,329	1,15646E-279
Mast	DOK2	1,45141E-226	-1,50328E+00	0,071	0,364	2,67305E-222
Mast	C1orf54	9,12212E-263	-1,50545E+00	0,007	0,302	1,68002E-258
Mast	GBP5	6,47174E-190	-1,50616E+00	0,023	0,262	1,19190E-185
Mast	ATP6V0B	1,19405E-287	-1,50689E+00	0,432	0,816	2,19908E-283
Mast	ITGAX	9,71731E-183	-1,50784E+00	0,134	0,418	1,78964E-178
Mast	STAB1	3,29940E-175	-1,51308E+00	0,105	0,362	6,07650E-171
Mast	CASP4	2,50420E-166	-1,51325E+00	0,173	0,462	4,61199E-162
Mast	DRAM2	1,90697E-266	-1,52301E+00	0,077	0,412	3,51206E-262
Mast	INSIG1	3,77656E-96	-1,52359E+00	0,121	0,307	6,95529E-92
Mast	NAGA	1,18650E-290	-1,52734E+00	0,044	0,383	2,18517E-286
Mast	HEXB	1,40650E-244	-1,52806E+00	0,184	0,537	2,59036E-240
Mast	TPT1	0,00000E+00	-1,52930E+00	0,871	0,981	0,00000E+00
Mast	TPM3	2,13715E-200	-1,53431E+00	0,483	0,826	3,93598E-196
Mast	TLR2	0,00000E+00	-1,53565E+00	0,005	0,413	0,00000E+00
Mast	MX1	1,41959E-229	-1,53580E+00	0,058	0,352	2,61446E-225
Mast	DBNL	2,00935E-247	-1,53664E+00	0,162	0,515	3,70062E-243
Mast	KCTD12	0,00000E+00	-1,54018E+00	0,006	0,525	0,00000E+00
Mast	TMBIM6	1,61255E-237	-1,54165E+00	0,431	0,802	2,96984E-233
Mast	ATP6AP2	0,00000E+00	-1,54470E+00	0,187	0,619	0,00000E+00
Mast	LGALS1	0,00000E+00	-1,54676E+00	0,514	0,858	0,00000E+00
Mast	IER3	4,34988E-192	-1,54768E+00	0,249	0,552	8,01117E-188
Mast	FPR3	0,00000E+00	-1,54951E+00	0,005	0,362	0,00000E+00
Mast	FLNA	4,07805E-205	-1,55324E+00	0,168	0,471	7,51055E-201
Mast	CLEC4E	6,66850E-285	-1,55497E+00	0,002	0,312	1,22814E-280
Mast	AXL	1,10624E-264	-1,55678E+00	0,004	0,299	2,03736E-260
Mast	SLAMF8	0,00000E+00	-1,56011E+00	0,003	0,348	0,00000E+00
Mast	YPEL5	2,85730E-123	-1,56209E+00	0,195	0,451	5,26229E-119
Mast	ISG15	1,81408E-108	-1,56367E+00	0,224	0,442	3,34099E-104
Mast	SPI1	0,00000E+00	-1,56972E+00	0,296	0,8	0,00000E+00
Mast	SOCS3	6,19115E-242	-1,57002E+00	0,073	0,377	1,14022E-237

Mast	RAB10	0,00000E+00	-1,57720E+00	0,129	0,515	0,00000E+00
Mast	HNRNPH	1,24298E-53	-1,57973E+00	0,228	0,417	2,28920E-49
Mast	LILRA2	1,11485E-228	-1,58003E+00	0,003	0,264	2,05321E-224
Mast	HSPB1	7,63484E-148	-1,58771E+00	0,404	0,676	1,40611E-143
Mast	GABARAI	1,04575E-65	-1,59537E+00	0,673	0,84	1,92596E-61
Mast	ATP1B3	1,44062E-302	-1,59666E+00	0,172	0,57	2,65319E-298
Mast	LRP1	0,00000E+00	-1,60664E+00	0,008	0,451	0,00000E+00
Mast	GLA	1,75100E-80	-1,61188E+00	0,139	0,323	3,22482E-76
Mast	LGALS9	0,00000E+00	-1,61382E+00	0,171	0,574	0,00000E+00
Mast	ZNF385A	0,00000E+00	-1,61428E+00	0,004	0,441	0,00000E+00
Mast	ARPC1B	2,10424E-268	-1,61484E+00	0,439	0,822	3,87538E-264
Mast	ZYX	0,00000E+00	-1,61624E+00	0,175	0,678	0,00000E+00
Mast	RNF13	0,00000E+00	-1,62334E+00	0,129	0,545	0,00000E+00
Mast	RGS2	3,22406E-22	-1,62427E+00	0,467	0,637	5,93775E-18
Mast	GLRX	0,00000E+00	-1,62983E+00	0,119	0,577	0,00000E+00
Mast	RPL13A	3,42874E-17	-1,63120E+00	0,624	0,513	6,31472E-13
Mast	ZFP36L1	4,89608E-108	-1,63367E+00	0,479	0,743	9,01710E-104
Mast	GBP2	3,43461E-250	-1,63456E+00	0,074	0,393	6,32552E-246
Mast	ABI3	0,00000E+00	-1,63574E+00	0,005	0,42	0,00000E+00
Mast	RPS9	1,44669E-18	-1,63947E+00	0,611	0,55	2,66436E-14
Mast	CXCL16	0,00000E+00	-1,64287E+00	0,261	0,678	0,00000E+00
Mast	LAMP2	0,00000E+00	-1,64782E+00	0,04	0,427	0,00000E+00
Mast	CLEC4A	8,01174E-291	-1,65220E+00	0,002	0,318	1,47552E-286
Mast	CYBA	0,00000E+00	-1,65340E+00	0,529	0,924	0,00000E+00
Mast	PRELID1	0,00000E+00	-1,65885E+00	0,291	0,712	0,00000E+00
Mast	ARPC4	9,32290E-166	-1,66326E+00	0,153	0,428	1,71700E-161
Mast	ADAP2	0,00000E+00	-1,66327E+00	0,007	0,476	0,00000E+00
Mast	PSMB4	4,36852E-120	-1,66538E+00	0,15	0,388	8,04551E-116
Mast	CSTA	0,00000E+00	-1,66895E+00	0,005	0,459	0,00000E+00
Mast	RNH1	5,40502E-284	-1,67184E+00	0,251	0,643	9,95443E-280
Mast	MYD88	7,80746E-189	-1,68713E+00	0,098	0,375	1,43790E-184
Mast	CFLAR	0,00000E+00	-1,68794E+00	0,14	0,538	0,00000E+00
Mast	PSMB3	2,52704E-304	-1,68998E+00	0,258	0,67	4,65405E-300
Mast	CORO1C	4,02472E-254	-1,69103E+00	0,051	0,365	7,41232E-250
Mast	LITAF	0,00000E+00	-1,69710E+00	0,346	0,769	0,00000E+00
Mast	PABPC1	5,47913E-25	-1,69997E+00	0,716	0,905	1,00909E-20
Mast	SYNGR2	1,03897E-206	-1,70782E+00	0,421	0,749	1,91346E-202
Mast	HLA-B	0,00000E+00	-1,70851E+00	0,789	0,981	0,00000E+00
Mast	PFN1	0,00000E+00	-1,71289E+00	0,696	0,965	0,00000E+00
Mast	HM13	0,00000E+00	-1,71336E+00	0,16	0,566	0,00000E+00

Mast	UQCRC1	1,57185E-199	-1,71416E+00	0,199	0,527	2,89487E-195
Mast	RBPJ	0,00000E+00	-1,73479E+00	0,083	0,493	0,00000E+00
Mast	EIF3L	7,22939E-160	-1,73508E+00	0,308	0,62	1,33144E-155
Mast	PLBD1	0,00000E+00	-1,73890E+00	0,003	0,42	0,00000E+00
Mast	RNF144B	0,00000E+00	-1,73966E+00	0,006	0,382	0,00000E+00
Mast	CSF2RA	0,00000E+00	-1,74742E+00	0,005	0,446	0,00000E+00
Mast	MEF2C	0,00000E+00	-1,74872E+00	0,008	0,446	0,00000E+00
Mast	ACTB	0,00000E+00	-1,75164E+00	0,943	0,994	0,00000E+00
Mast	PPIA	2,85546E-304	-1,75252E+00	0,66	0,911	5,25891E-300
Mast	C1orf162	0,00000E+00	-1,75307E+00	0,354	0,732	0,00000E+00
Mast	AP2S1	0,00000E+00	-1,75354E+00	0,246	0,753	0,00000E+00
Mast	RPS16	4,33095E-07	-1,75432E+00	0,616	0,54	7,97630E-03
Mast	GSN	0,00000E+00	-1,75582E+00	0,244	0,661	0,00000E+00
Mast	DAB2	0,00000E+00	-1,75910E+00	0,034	0,461	0,00000E+00
Mast	VAMP5	1,92102E-260	-1,76055E+00	0,165	0,502	3,53795E-256
Mast	MXD1	1,76263E-226	-1,76161E+00	0,038	0,32	3,24624E-222
Mast	TKT	0,00000E+00	-1,76224E+00	0,22	0,634	0,00000E+00
Mast	CNDP2	0,00000E+00	-1,76586E+00	0,083	0,481	0,00000E+00
Mast	PLIN2	8,08999E-18	-1,76964E+00	0,372	0,507	1,48993E-13
Mast	PLXDC2	0,00000E+00	-1,78219E+00	0,075	0,577	0,00000E+00
Mast	ACTG1	0,00000E+00	-1,79319E+00	0,649	0,941	0,00000E+00
Mast	EZR	2,67539E-137	-1,80037E+00	0,164	0,416	4,92726E-133
Mast	RNF149	0,00000E+00	-1,80248E+00	0,197	0,657	0,00000E+00
Mast	NLRP3	8,83004E-216	-1,80651E+00	0,019	0,28	1,62623E-211
Mast	CALM2	1,76218E-288	-1,81641E+00	0,436	0,806	3,24541E-284
Mast	HLA-DOA	0,00000E+00	-1,81877E+00	0,013	0,419	0,00000E+00
Mast	HSPA6	2,37489E-221	-1,82239E+00	0,03	0,304	4,37383E-217
Mast	ATG3	0,00000E+00	-1,82406E+00	0,14	0,55	0,00000E+00
Mast	SCPEP1	0,00000E+00	-1,82882E+00	0,036	0,434	0,00000E+00
Mast	ARPC2	0,00000E+00	-1,82962E+00	0,497	0,884	0,00000E+00
Mast	KYNU	0,00000E+00	-1,83171E+00	0,005	0,337	0,00000E+00
Mast	TUBA1B	0,00000E+00	-1,83362E+00	0,416	0,78	0,00000E+00
Mast	S100A11	0,00000E+00	-1,84184E+00	0,677	0,956	0,00000E+00
Mast	A2M	0,00000E+00	-1,84283E+00	0,027	0,415	0,00000E+00
Mast	LY96	0,00000E+00	-1,84508E+00	0,016	0,488	0,00000E+00
Mast	LILRB1	0,00000E+00	-1,84611E+00	0,003	0,403	0,00000E+00
Mast	HLA-DQB	4,51009E-307	-1,85777E+00	0,008	0,339	8,30623E-303
Mast	RPL4	2,20857E-21	-1,85823E+00	0,544	0,472	4,06752E-17
Mast	MFSD1	0,00000E+00	-1,86123E+00	0,158	0,622	0,00000E+00
Mast	LDHA	3,18700E-269	-1,87015E+00	0,351	0,739	5,86950E-265

Mast	GSTP1	8,50258E-308	-1,87331E+00	0,47	0,848	1,56592E-303
Mast	RPL27A	6,69488E-36	-1,87827E+00	0,381	0,47	1,23300E-31
Mast	ACSL1	0,00000E+00	-1,87874E+00	0,024	0,414	0,00000E+00
Mast	BLVRB	0,00000E+00	-1,89407E+00	0,067	0,52	0,00000E+00
Mast	CYTIP	0,00000E+00	-1,89520E+00	0,036	0,416	0,00000E+00
Mast	ALDOA	1,43361E-232	-1,90051E+00	0,459	0,781	2,64028E-228
Mast	UPP1	0,00000E+00	-1,90698E+00	0,052	0,458	0,00000E+00
Mast	PPIF	7,51251E-288	-1,90777E+00	0,047	0,38	1,38358E-283
Mast	TNFRSF1	0,00000E+00	-1,91841E+00	0,006	0,479	0,00000E+00
Mast	SMCO4	0,00000E+00	-1,91853E+00	0,015	0,446	0,00000E+00
Mast	MAN2B1	2,71390E-259	-1,92280E+00	0,152	0,497	4,99820E-255
Mast	HCLS1	0,00000E+00	-1,93079E+00	0,244	0,757	0,00000E+00
Mast	CFL1	0,00000E+00	-1,93929E+00	0,627	0,942	0,00000E+00
Mast	GIMAP4	0,00000E+00	-1,95549E+00	0,007	0,457	0,00000E+00
Mast	OAZ1	0,00000E+00	-1,95914E+00	0,689	0,954	0,00000E+00
Mast	GBP1	3,33843E-229	-1,96142E+00	0,071	0,365	6,14838E-225
Mast	APLP2	0,00000E+00	-1,96446E+00	0,093	0,63	0,00000E+00
Mast	TFRC	2,16031E-160	-1,96691E+00	0,035	0,251	3,97865E-156
Mast	CD53	0,00000E+00	-1,97237E+00	0,251	0,708	0,00000E+00
Mast	NAGK	4,79220E-299	-1,97260E+00	0,126	0,502	8,82580E-295
Mast	CTSZ	0,00000E+00	-1,97661E+00	0,055	0,694	0,00000E+00
Mast	IFNGR1	0,00000E+00	-1,97716E+00	0,163	0,611	0,00000E+00
Mast	PPT1	0,00000E+00	-1,97978E+00	0,187	0,683	0,00000E+00
Mast	MSR1	0,00000E+00	-1,98323E+00	0,004	0,407	0,00000E+00
Mast	TMSB10	0,00000E+00	-1,98908E+00	0,901	0,99	0,00000E+00
Mast	ENO1	8,84399E-294	-1,99159E+00	0,396	0,786	1,62880E-289
Mast	FGD2	0,00000E+00	-1,99383E+00	0,005	0,368	0,00000E+00
Mast	PLSCR1	0,00000E+00	-2,00077E+00	0,127	0,571	0,00000E+00
Mast	MS4A4A	0,00000E+00	-2,02746E+00	0,017	0,448	0,00000E+00
Mast	CCL4	2,52011E-130	-2,03148E+00	0,109	0,324	4,64129E-126
Mast	IL4I1	5,43571E-203	-2,03178E+00	0,008	0,25	1,00109E-198
Mast	TIMP1	5,20213E-25	-2,03600E+00	0,651	0,65	9,58077E-21
Mast	C5AR1	0,00000E+00	-2,04084E+00	0,009	0,435	0,00000E+00
Mast	HBEGF	1,33284E-255	-2,04236E+00	0,006	0,294	2,45469E-251
Mast	SLC7A7	0,00000E+00	-2,04481E+00	0,008	0,498	0,00000E+00
Mast	SLC31A2	0,00000E+00	-2,04685E+00	0,007	0,473	0,00000E+00
Mast	SERPING	0,00000E+00	-2,04976E+00	0,024	0,423	0,00000E+00
Mast	PDE4B	0,00000E+00	-2,05214E+00	0,033	0,391	0,00000E+00
Mast	MARCKS	0,00000E+00	-2,05324E+00	0,014	0,569	0,00000E+00
Mast	ATP6V1B	0,00000E+00	-2,06623E+00	0,059	0,568	0,00000E+00

Mast	PLAUR	4,88271E-195	-2,07743E+00	0,347	0,661	8,99250E-191
Mast	BID	0,00000E+00	-2,08387E+00	0,074	0,438	0,00000E+00
Mast	SELPLG	2,77478E-182	-2,08579E+00	0,095	0,361	5,11031E-178
Mast	UBC	0,00000E+00	-2,09360E+00	0,611	0,919	0,00000E+00
Mast	MGAT1	0,00000E+00	-2,09555E+00	0,121	0,659	0,00000E+00
Mast	CSF3R	0,00000E+00	-2,09800E+00	0,004	0,421	0,00000E+00
Mast	RNASET2	0,00000E+00	-2,10297E+00	0,26	0,774	0,00000E+00
Mast	ISG20	6,13425E-257	-2,10358E+00	0,029	0,331	1,12974E-252
Mast	TRIM22	0,00000E+00	-2,10726E+00	0,066	0,441	0,00000E+00
Mast	TCF4	5,68539E-222	-2,11717E+00	0,022	0,291	1,04708E-217
Mast	LTA4H	0,00000E+00	-2,11977E+00	0,046	0,476	0,00000E+00
Mast	IL10RA	0,00000E+00	-2,13822E+00	0,036	0,494	0,00000E+00
Mast	TCIRG1	2,36541E-293	-2,13907E+00	0,123	0,491	4,35637E-289
Mast	AOAH	0,00000E+00	-2,15009E+00	0,004	0,4	0,00000E+00
Mast	LY86	0,00000E+00	-2,15187E+00	0,006	0,524	0,00000E+00
Mast	PLTP	0,00000E+00	-2,15791E+00	0,011	0,351	0,00000E+00
Mast	PDK4	1,35121E-299	-2,15846E+00	0,007	0,332	2,48852E-295
Mast	TNFAIP2	0,00000E+00	-2,18502E+00	0,026	0,432	0,00000E+00
Mast	CIITA	5,52045E-269	-2,18544E+00	0,031	0,345	1,01670E-264
Mast	LIPA	0,00000E+00	-2,19233E+00	0,094	0,529	0,00000E+00
Mast	PSME2	0,00000E+00	-2,19662E+00	0,349	0,741	0,00000E+00
Mast	CEBPD	0,00000E+00	-2,19853E+00	0,013	0,607	0,00000E+00
Mast	PGK1	2,58206E-301	-2,19972E+00	0,32	0,733	4,75539E-297
Mast	TNFSF13	0,00000E+00	-2,21478E+00	0,045	0,471	0,00000E+00
Mast	TPP1	0,00000E+00	-2,21705E+00	0,172	0,625	0,00000E+00
Mast	CD86	0,00000E+00	-2,22257E+00	0,008	0,529	0,00000E+00
Mast	COTL1	0,00000E+00	-2,23030E+00	0,332	0,738	0,00000E+00
Mast	OLR1	0,00000E+00	-2,23576E+00	0,004	0,372	0,00000E+00
Mast	RPS10	3,74252E-65	-2,24368E+00	0,146	0,293	6,89260E-61
Mast	LILRB2	0,00000E+00	-2,24530E+00	0,012	0,488	0,00000E+00
Mast	SERPINB!	9,95206E-237	-2,24717E+00	0,084	0,395	1,83287E-232
Mast	PTPRE	0,00000E+00	-2,26713E+00	0,032	0,468	0,00000E+00
Mast	RASSF4	0,00000E+00	-2,27568E+00	0,008	0,542	0,00000E+00
Mast	NINJ1	0,00000E+00	-2,27647E+00	0,119	0,59	0,00000E+00
Mast	CTSA	0,00000E+00	-2,30232E+00	0,071	0,536	0,00000E+00
Mast	ALDH2	0,00000E+00	-2,30867E+00	0,054	0,585	0,00000E+00
Mast	PPA1	0,00000E+00	-2,31146E+00	0,075	0,488	0,00000E+00
Mast	NCF2	0,00000E+00	-2,31312E+00	0,004	0,497	0,00000E+00
Mast	FABP5	0,00000E+00	-2,32142E+00	0,057	0,473	0,00000E+00
Mast	RAB31	0,00000E+00	-2,32524E+00	0,018	0,686	0,00000E+00

Mast	CD4	0,00000E+00	-2,32758E+00	0,067	0,571	0,00000E+00
Mast	FCGR2B	0,00000E+00	-2,33211E+00	0,017	0,454	0,00000E+00
Mast	ANXA5	0,00000E+00	-2,33559E+00	0,271	0,808	0,00000E+00
Mast	FCGR1A	0,00000E+00	-2,33668E+00	0,004	0,439	0,00000E+00
Mast	LGALS2	4,67074E-242	-2,34075E+00	0,002	0,275	8,60210E-238
Mast	C6orf62	8,13716E-206	-2,35306E+00	0,091	0,383	1,49862E-201
Mast	NME1-NI	3,32549E-194	-2,35670E+00	0,015	0,255	6,12456E-190
Mast	TNFRSF1	0,00000E+00	-2,38689E+00	0,012	0,577	0,00000E+00
Mast	PLEK	0,00000E+00	-2,38817E+00	0,121	0,592	0,00000E+00
Mast	S100A10	0,00000E+00	-2,39630E+00	0,506	0,861	0,00000E+00
Mast	FGL2	0,00000E+00	-2,40051E+00	0,084	0,651	0,00000E+00
Mast	GLIPR1	0,00000E+00	-2,40307E+00	0,021	0,623	0,00000E+00
Mast	TREM1	2,30818E-219	-2,40643E+00	0,001	0,254	4,25098E-215
Mast	BCL2A1	3,65689E-212	-2,40976E+00	0,187	0,49	6,73490E-208
Mast	FPR1	0,00000E+00	-2,42277E+00	0,006	0,479	0,00000E+00
Mast	NPC2	0,00000E+00	-2,42569E+00	0,523	0,909	0,00000E+00
Mast	CLEC7A	0,00000E+00	-2,43712E+00	0,006	0,56	0,00000E+00
Mast	EIF4A1	0,00000E+00	-2,44935E+00	0,295	0,701	0,00000E+00
Mast	TREM2	0,00000E+00	-2,45010E+00	0,004	0,435	0,00000E+00
Mast	FGR	0,00000E+00	-2,48098E+00	0,039	0,483	0,00000E+00
Mast	HLA-DQA	0,00000E+00	-2,48844E+00	0,079	0,543	0,00000E+00
Mast	MPEG1	0,00000E+00	-2,48852E+00	0,002	0,399	0,00000E+00
Mast	NAMPT	0,00000E+00	-2,49123E+00	0,201	0,599	0,00000E+00
Mast	TYMP	0,00000E+00	-2,49179E+00	0,214	0,834	0,00000E+00
Mast	C3	0,00000E+00	-2,49882E+00	0,009	0,41	0,00000E+00
Mast	LAP3	0,00000E+00	-2,51409E+00	0,118	0,618	0,00000E+00
Mast	MNDA	0,00000E+00	-2,51545E+00	0,016	0,57	0,00000E+00
Mast	TYROBP	0,00000E+00	-2,53712E+00	0,721	0,963	0,00000E+00
Mast	PILRA	0,00000E+00	-2,53713E+00	0,012	0,527	0,00000E+00
Mast	MAFB	0,00000E+00	-2,55621E+00	0,013	0,634	0,00000E+00
Mast	THEMIS2	0,00000E+00	-2,56298E+00	0,032	0,527	0,00000E+00
Mast	CORO1A	0,00000E+00	-2,56705E+00	0,184	0,678	0,00000E+00
Mast	IRF7	7,03550E-212	-2,56804E+00	0,119	0,419	1,29573E-207
Mast	FCGR2A	0,00000E+00	-2,57103E+00	0,095	0,668	0,00000E+00
Mast	HMOX1	0,00000E+00	-2,57764E+00	0,017	0,582	0,00000E+00
Mast	VSIG4	0,00000E+00	-2,60138E+00	0,002	0,372	0,00000E+00
Mast	GPNMB	0,00000E+00	-2,61307E+00	0,008	0,358	0,00000E+00
Mast	SLC11A1	0,00000E+00	-2,63506E+00	0,038	0,497	0,00000E+00
Mast	HCK	0,00000E+00	-2,63949E+00	0,007	0,556	0,00000E+00
Mast	CST3	0,00000E+00	-2,64201E+00	0,773	0,962	0,00000E+00

Mast	SAMHD1	0,00000E+00	-2,64633E+00	0,028	0,689	0,00000E+00
Mast	TMSB4X	0,00000E+00	-2,70197E+00	0,86	0,992	0,00000E+00
Mast	TNFSF13	0,00000E+00	-2,70625E+00	0,013	0,619	0,00000E+00
Mast	CYBB	0,00000E+00	-2,73662E+00	0,005	0,667	0,00000E+00
Mast	CSF1R	0,00000E+00	-2,76250E+00	0,006	0,611	0,00000E+00
Mast	CTSC	0,00000E+00	-2,81989E+00	0,163	0,714	0,00000E+00
Mast	UCP2	0,00000E+00	-2,82598E+00	0,21	0,664	0,00000E+00
Mast	CFD	0,00000E+00	-2,83209E+00	0,022	0,6	0,00000E+00
Mast	LGMN	0,00000E+00	-2,86347E+00	0,007	0,437	0,00000E+00
Mast	LILRB4	0,00000E+00	-2,87656E+00	0,006	0,587	0,00000E+00
Mast	IGSF6	0,00000E+00	-2,88041E+00	0,003	0,596	0,00000E+00
Mast	PSAP	0,00000E+00	-2,91408E+00	0,593	0,939	0,00000E+00
Mast	PLD4	0,00000E+00	-2,95527E+00	0,002	0,355	0,00000E+00
Mast	SAT1	0,00000E+00	-2,98589E+00	0,623	0,97	0,00000E+00
Mast	HERPUD1	0,00000E+00	-3,00484E+00	0,266	0,71	0,00000E+00
Mast	CD68	0,00000E+00	-3,05917E+00	0,209	0,821	0,00000E+00
Mast	RNASE6	0,00000E+00	-3,08544E+00	0,004	0,577	0,00000E+00
Mast	CD163	0,00000E+00	-3,09902E+00	0,005	0,478	0,00000E+00
Mast	LSP1	0,00000E+00	-3,10657E+00	0,059	0,714	0,00000E+00
Mast	MS4A7	0,00000E+00	-3,18198E+00	0,016	0,708	0,00000E+00
Mast	SERPINF1	0,00000E+00	-3,18364E+00	0,06	0,428	0,00000E+00
Mast	FTL	0,00000E+00	-3,21465E+00	0,934	0,996	0,00000E+00
Mast	C15orf48	0,00000E+00	-3,22039E+00	0,02	0,367	0,00000E+00
Mast	FCGRT	0,00000E+00	-3,23527E+00	0,1	0,783	0,00000E+00
Mast	APOC1	0,00000E+00	-3,25711E+00	0,209	0,528	0,00000E+00
Mast	ITGB2	0,00000E+00	-3,27601E+00	0,114	0,809	0,00000E+00
Mast	CPVL	0,00000E+00	-3,28179E+00	0,01	0,603	0,00000E+00
Mast	LST1	0,00000E+00	-3,34217E+00	0,057	0,761	0,00000E+00
Mast	HLA-DMA	0,00000E+00	-3,42020E+00	0,177	0,824	0,00000E+00
Mast	GPX1	0,00000E+00	-3,43841E+00	0,075	0,582	0,00000E+00
Mast	HLA-DMB	0,00000E+00	-3,54161E+00	0,033	0,681	0,00000E+00
Mast	CTSH	0,00000E+00	-3,60000E+00	0,035	0,757	0,00000E+00
Mast	SOD2	0,00000E+00	-3,63187E+00	0,128	0,688	0,00000E+00
Mast	GRN	0,00000E+00	-3,63299E+00	0,234	0,864	0,00000E+00
Mast	CTSL	0,00000E+00	-3,67841E+00	0,04	0,553	0,00000E+00
Mast	GPR183	5,24884E-200	-3,68143E+00	0,184	0,476	9,66680E-196
Mast	HLA-DRB	0,00000E+00	-3,73741E+00	0,245	0,889	0,00000E+00
Mast	ANXA2	0,00000E+00	-3,83337E+00	0,093	0,788	0,00000E+00
Mast	FCGR3A	0,00000E+00	-3,86646E+00	0,01	0,614	0,00000E+00
Mast	CTSB	0,00000E+00	-3,89512E+00	0,419	0,903	0,00000E+00

Mast	HLA-DRB	0,00000E+00	-3,90526E+00	0,378	0,955	0,00000E+00
Mast	SERPINA	0,00000E+00	-3,93821E+00	0,015	0,771	0,00000E+00
Mast	TGFBI	0,00000E+00	-3,98315E+00	0,006	0,64	0,00000E+00
Mast	IRF8	0,00000E+00	-3,99038E+00	0,005	0,404	0,00000E+00
Mast	CTSS	0,00000E+00	-4,00782E+00	0,15	0,863	0,00000E+00
Mast	CD14	0,00000E+00	-4,01318E+00	0,014	0,666	0,00000E+00
Mast	FCN1	2,25673E-235	-4,02082E+00	0,001	0,268	4,15622E-231
Mast	CXCR4	0,00000E+00	-4,08529E+00	0,075	0,615	0,00000E+00
Mast	HLA-DQA	0,00000E+00	-4,09657E+00	0,15	0,827	0,00000E+00
Mast	AIF1	0,00000E+00	-4,09685E+00	0,052	0,876	0,00000E+00
Mast	RNASE1	1,42611E-245	-4,18672E+00	0,011	0,291	2,62646E-241
Mast	APOE	0,00000E+00	-4,23768E+00	0,19	0,529	0,00000E+00
Mast	MS4A6A	0,00000E+00	-4,25003E+00	0,011	0,764	0,00000E+00
Mast	HLA-DPB	0,00000E+00	-4,26421E+00	0,323	0,922	0,00000E+00
Mast	HLA-DQE	0,00000E+00	-4,34479E+00	0,135	0,863	0,00000E+00
Mast	CCL3	0,00000E+00	-4,37615E+00	0,019	0,401	0,00000E+00
Mast	IL1B	1,46476E-302	-4,48063E+00	0,025	0,357	2,69765E-298
Mast	HLA-DPA	0,00000E+00	-4,59244E+00	0,359	0,935	0,00000E+00
Mast	S100A8	0,00000E+00	-4,70019E+00	0,01	0,376	0,00000E+00
Mast	CD74	0,00000E+00	-4,73901E+00	0,584	0,989	0,00000E+00
Mast	C1QA	0,00000E+00	-4,77451E+00	0,019	0,605	0,00000E+00
Mast	C1QC	0,00000E+00	-4,79183E+00	0,016	0,583	0,00000E+00
Mast	IFI30	0,00000E+00	-4,80537E+00	0,151	0,598	0,00000E+00
Mast	S100A9	0,00000E+00	-4,82971E+00	0,008	0,487	0,00000E+00
Mast	C1QB	0,00000E+00	-4,96609E+00	0,017	0,583	0,00000E+00
Mast	HLA-DRA	0,00000E+00	-5,61336E+00	0,266	0,971	0,00000E+00
Mast	LYZ	0,00000E+00	-5,94783E+00	0,037	0,862	0,00000E+00

Mono_CD14

cluster	gene	p_val	avg_log2FC	pct.1	pct.2	p_val_adj
Mono_CD14	S100A8	0,00000E+00	3,96649E+00	0,953	0,308	0,00000E+00
Mono_CD14	S100A12	0,00000E+00	3,39503E+00	0,66	0,075	0,00000E+00
Mono_CD14	S100A9	0,00000E+00	3,19466E+00	0,96	0,419	0,00000E+00
Mono_CD14	FCN1	0,00000E+00	2,75847E+00	0,822	0,209	0,00000E+00
Mono_CD14	VCAN	0,00000E+00	2,35624E+00	0,805	0,183	0,00000E+00
Mono_CD14	CLEC12A	8,77912E-85	1,91505E+00	0,365	0,252	1,61685E-80
Mono_CD14	CSF3R	2,55756E-235	1,81720E+00	0,56	0,381	4,71026E-231
Mono_CD14	LYZ	0,00000E+00	1,79562E+00	0,944	0,799	0,00000E+00
Mono_CD14	CSTA	0,00000E+00	1,78106E+00	0,667	0,412	0,00000E+00
Mono_CD14	DUSP6	1,39114E-123	1,77921E+00	0,433	0,278	2,56206E-119
Mono_CD14	MYO1F	3,87890E-121	1,75202E+00	0,541	0,454	7,14376E-117
Mono_CD14	FGR	0,00000E+00	1,70666E+00	0,647	0,44	0,00000E+00
Mono_CD14	MNDA	0,00000E+00	1,70608E+00	0,701	0,521	0,00000E+00
Mono_CD14	CDA	0,00000E+00	1,67640E+00	0,373	0,068	0,00000E+00
Mono_CD14	LGALS2	0,00000E+00	1,67162E+00	0,501	0,239	0,00000E+00
Mono_CD14	UBXN11	1,59015E-148	1,65784E+00	0,355	0,196	2,92859E-144
Mono_CD14	RBP7	0,00000E+00	1,64815E+00	0,308	0,085	0,00000E+00
Mono_CD14	LYST	5,85547E-158	1,57041E+00	0,387	0,219	1,07840E-153
Mono_CD14	CFP	0,00000E+00	1,56598E+00	0,567	0,181	0,00000E+00
Mono_CD14	NUP214	2,05862E-152	1,55379E+00	0,455	0,304	3,79137E-148
Mono_CD14	AGTRAP	3,33503E-242	1,54163E+00	0,537	0,348	6,14213E-238
Mono_CD14	TSPO	0,00000E+00	1,52684E+00	0,848	0,76	0,00000E+00
Mono_CD14	SULT1A1	2,81775E-166	1,52519E+00	0,308	0,149	5,18945E-162
Mono_CD14	S100A4	0,00000E+00	1,49663E+00	0,949	0,817	0,00000E+00
Mono_CD14	TKT	0,00000E+00	1,49595E+00	0,709	0,6	0,00000E+00
Mono_CD14	MGST1	3,16588E-263	1,48486E+00	0,284	0,1	5,83060E-259
Mono_CD14	NCF2	2,72940E-218	1,48125E+00	0,604	0,454	5,02673E-214
Mono_CD14	USP15	1,26499E-36	1,46008E+00	0,448	0,423	2,32973E-32
Mono_CD14	S100A6	0,00000E+00	1,45770E+00	0,972	0,867	0,00000E+00
Mono_CD14	STXBP2	0,00000E+00	1,43659E+00	0,666	0,402	0,00000E+00
Mono_CD14	C19orf38	8,13373E-227	1,43371E+00	0,418	0,22	1,49799E-222
Mono_CD14	CTSS	0,00000E+00	1,38102E+00	0,88	0,812	0,00000E+00
Mono_CD14	ATG16L2	8,91558E-34	1,37614E+00	0,297	0,235	1,64198E-29
Mono_CD14	EVI2B	1,11390E-86	1,36008E+00	0,46	0,37	2,05146E-82
Mono_CD14	RAB24	3,62176E-69	1,35516E+00	0,364	0,264	6,67020E-65
Mono_CD14	CD300E	0,00000E+00	1,33480E+00	0,441	0,146	0,00000E+00
Mono_CD14	SLC25A3	8,02031E-124	1,33026E+00	0,43	0,281	1,47710E-119
Mono_CD14	CAPNS1	5,39940E-18	1,30861E+00	0,38	0,369	9,94407E-14
Mono_CD14	GMFG	5,72151E-216	1,30302E+00	0,724	0,67	1,05373E-211

Mono_CD14	C9orf72	1,79100E-25	1,28998E+00	0,276	0,222	3,29848E-21
Mono_CD14	BACH1	9,22314E-14	1,27453E+00	0,332	0,311	1,69863E-09
Mono_CD14	THBS1	0,00000E+00	1,26620E+00	0,443	0,16	0,00000E+00
Mono_CD14	TREM1	2,62935E-118	1,24277E+00	0,381	0,227	4,84248E-114
Mono_CD14	PRAM1	2,99336E-155	1,23626E+00	0,327	0,168	5,51286E-151
Mono_CD14	VMP1	2,11822E-76	1,23277E+00	0,587	0,548	3,90113E-72
Mono_CD14	CD52	1,91916E-137	1,22284E+00	0,528	0,38	3,53451E-133
Mono_CD14	COTL1	1,20572E-286	1,20665E+00	0,822	0,704	2,22058E-282
Mono_CD14	LILRA2	8,94322E-68	1,20541E+00	0,343	0,24	1,64707E-63
Mono_CD14	CPPED1	3,86226E-74	1,20169E+00	0,363	0,264	7,11313E-70
Mono_CD14	ICAM3	1,47572E-143	1,20012E+00	0,344	0,186	2,71784E-139
Mono_CD14	MT-ND4L	2,09347E-28	1,19377E+00	0,444	0,519	3,85554E-24
Mono_CD14	ASGR1	7,44646E-209	1,18852E+00	0,369	0,181	1,37141E-204
Mono_CD14	POU2F2	5,03537E-75	1,18772E+00	0,367	0,263	9,27364E-71
Mono_CD14	TNFAIP2	6,94554E-73	1,16792E+00	0,487	0,4	1,27916E-68
Mono_CD14	CLEC4E	2,14721E-180	1,16530E+00	0,458	0,28	3,95452E-176
Mono_CD14	CAST	4,90229E-64	1,15981E+00	0,551	0,53	9,02855E-60
Mono_CD14	CD1D	2,38673E-82	1,15363E+00	0,268	0,157	4,39565E-78
Mono_CD14	ANXA1	7,67287E-88	1,14130E+00	0,706	0,687	1,41311E-83
Mono_CD14	PSTPIP1	1,07295E-101	1,13555E+00	0,288	0,164	1,97604E-97
Mono_CD14	DPYD	8,03106E-21	1,13158E+00	0,388	0,364	1,47908E-16
Mono_CD14	TRAPPC5	3,53118E-49	1,13061E+00	0,31	0,233	6,50338E-45
Mono_CD14	MEGF9	1,17909E-225	1,12920E+00	0,312	0,131	2,17153E-221
Mono_CD14	CD36	0,00000E+00	1,12714E+00	0,457	0,182	0,00000E+00
Mono_CD14	AP1S2	6,14885E-125	1,12044E+00	0,617	0,576	1,13243E-120
Mono_CD14	STK10	1,51100E-17	1,11487E+00	0,28	0,241	2,78280E-13
Mono_CD14	LST1	0,00000E+00	1,10996E+00	0,829	0,706	0,00000E+00
Mono_CD14	CORO1A	6,84083E-204	1,10868E+00	0,72	0,641	1,25988E-199
Mono_CD14	FAM200B	2,09429E-57	1,10840E+00	0,294	0,207	3,85706E-53
Mono_CD14	FBXL5	7,92101E-51	1,10822E+00	0,343	0,269	1,45881E-46
Mono_CD14	LIMD2	3,87124E-143	1,10360E+00	0,585	0,474	7,12967E-139
Mono_CD14	FLNA	2,82567E-197	1,09282E+00	0,621	0,439	5,20403E-193
Mono_CD14	USP3	1,56679E-66	1,09204E+00	0,318	0,221	2,88555E-62
Mono_CD14	ACADVL	4,31024E-25	1,08832E+00	0,331	0,479	7,93818E-21
Mono_CD14	BST1	8,72341E-126	1,08384E+00	0,256	0,125	1,60659E-121
Mono_CD14	NCF1	1,41055E-66	1,08199E+00	0,545	0,497	2,59781E-62
Mono_CD14	STX10	4,38035E-29	1,07755E+00	0,348	0,307	8,06729E-25
Mono_CD14	PLBD1	6,57112E-59	1,07731E+00	0,445	0,389	1,21020E-54
Mono_CD14	SLC11A1	1,09653E-231	1,07400E+00	0,663	0,453	2,01948E-227
Mono_CD14	ACAP2	1,79019E-15	1,06094E+00	0,426	0,431	3,29700E-11

Mono_CD14	CARD16	7,57728E-153	1,05951E+00	0,629	0,546	1,39551E-148
Mono_CD14	ARHGEF1	3,85263E-16	1,05187E+00	0,3	0,269	7,09540E-12
Mono_CD14	METTL9	1,43393E-72	1,04666E+00	0,428	0,354	2,64088E-68
Mono_CD14	CLEC4A	5,18936E-37	1,04263E+00	0,355	0,293	9,55724E-33
Mono_CD14	CLEC7A	1,92594E-48	1,02553E+00	0,55	0,522	3,54700E-44
Mono_CD14	CFD	2,85403E-145	1,01703E+00	0,712	0,552	5,25627E-141
Mono_CD14	SOCS3	1,53949E-192	1,01267E+00	0,526	0,345	2,83527E-188
Mono_CD14	NAIP	1,20478E-44	1,00651E+00	0,426	0,367	2,21885E-40
Mono_CD14	HRH2	2,57163E-103	1,00185E+00	0,34	0,211	4,73617E-99
Mono_CD14	OSCAR	8,75617E-33	9,97723E-01	0,283	0,222	1,61262E-28
Mono_CD14	CEBPD	2,82334E-138	9,95743E-01	0,65	0,562	5,19974E-134
Mono_CD14	HMGB2	4,72745E-53	9,92640E-01	0,452	0,395	8,70654E-49
Mono_CD14	FOS	1,64866E-239	9,90639E-01	0,886	0,84	3,03635E-235
Mono_CD14	GCA	3,39174E-127	9,87321E-01	0,505	0,394	6,24657E-123
Mono_CD14	GIMAP7	2,96116E-12	9,85873E-01	0,283	0,254	5,45356E-08
Mono_CD14	SH3BGRL	0,00000E+00	9,79173E-01	0,943	0,932	0,00000E+00
Mono_CD14	LILRA5	0,00000E+00	9,78131E-01	0,431	0,167	0,00000E+00
Mono_CD14	CASP1	1,68747E-09	9,70386E-01	0,445	0,464	3,10781E-05
Mono_CD14	ITGB2	1,38158E-104	9,68253E-01	0,783	0,762	2,54446E-100
Mono_CD14	CXCL8	3,06196E-63	9,66895E-01	0,3	0,186	5,63921E-59
Mono_CD14	LILRB3	1,01832E-60	9,63122E-01	0,319	0,223	1,87545E-56
Mono_CD14	SULF2	2,24708E-69	9,57895E-01	0,255	0,152	4,13844E-65
Mono_CD14	ERICH1	2,34965E-11	9,56300E-01	0,332	0,32	4,32736E-07
Mono_CD14	IDH3B	1,89621E-08	9,50078E-01	0,199	0,266	3,49224E-04
Mono_CD14	SCPEP1	2,77663E-10	9,44411E-01	0,321	0,414	5,11372E-06
Mono_CD14	TCIRG1	1,92876E-19	9,41978E-01	0,465	0,467	3,55221E-15
Mono_CD14	SERPINA	8,94063E-164	9,39442E-01	0,834	0,713	1,64660E-159
Mono_CD14	FPR1	5,64460E-172	9,39160E-01	0,58	0,438	1,03957E-167
Mono_CD14	MT-ND5	3,89349E-11	9,37196E-01	0,469	0,542	7,17065E-07
Mono_CD14	IMPDH1	2,91599E-21	9,33795E-01	0,259	0,214	5,37038E-17
Mono_CD14	DMXL2	2,03152E-36	9,32857E-01	0,33	0,263	3,74146E-32
Mono_CD14	PFDN5	1,51089E-252	9,32380E-01	0,87	0,905	2,78261E-248
Mono_CD14	PRELID1	1,12227E-160	9,27032E-01	0,718	0,682	2,06688E-156
Mono_CD14	ARHGAP1	3,05525E-07	9,22757E-01	0,327	0,332	5,62686E-03
Mono_CD14	APLP2	1,84787E-143	9,20314E-01	0,658	0,59	3,40321E-139
Mono_CD14	PYCARD	1,74625E-75	9,13865E-01	0,699	0,707	3,21606E-71
Mono_CD14	NOTCH2	1,00315E-12	9,09150E-01	0,334	0,316	1,84749E-08
Mono_CD14	LILRB2	3,62044E-68	8,93351E-01	0,529	0,452	6,66776E-64
Mono_CD14	MSRB1	2,08078E-50	8,91223E-01	0,265	0,186	3,83218E-46
Mono_CD14	GABARAI	1,10091E-238	8,89930E-01	0,844	0,828	2,02754E-234

Mono_CD14	PGLS	1,79157E-44	8,88737E-01	0,552	0,542	3,29954E-40
Mono_CD14	GLIPR2	5,35888E-85	8,88558E-01	0,474	0,37	9,86944E-81
Mono_CD14	CD302	1,19930E-26	8,87246E-01	0,378	0,347	2,20874E-22
Mono_CD14	LTA4H	7,03168E-129	8,86237E-01	0,552	0,44	1,29502E-124
Mono_CD14	CHD4	2,02180E-14	8,85795E-01	0,197	0,283	3,72355E-10
Mono_CD14	LAMTOR	2,19719E-162	8,70559E-01	0,735	0,713	4,04657E-158
Mono_CD14	IRS2	6,10008E-85	8,69933E-01	0,332	0,22	1,12345E-80
Mono_CD14	ITGAM	3,12836E-16	8,69886E-01	0,277	0,239	5,76149E-12
Mono_CD14	ANKRD4	7,35331E-10	8,68983E-01	0,22	0,297	1,35426E-05
Mono_CD14	ZYX	9,73281E-43	8,68776E-01	0,624	0,646	1,79249E-38
Mono_CD14	MYO1G	4,49768E-72	8,68527E-01	0,384	0,276	8,28338E-68
Mono_CD14	NAMPT	0,00000E+00	8,67121E-01	0,754	0,56	0,00000E+00
Mono_CD14	IQGAP1	3,78665E-22	8,65781E-01	0,533	0,564	6,97386E-18
Mono_CD14	CEBPB	1,37162E-105	8,59518E-01	0,574	0,464	2,52612E-101
Mono_CD14	TNFRSF1	1,29035E-104	8,53407E-01	0,608	0,535	2,37643E-100
Mono_CD14	N4BP2L2	4,16807E-40	8,52970E-01	0,3	0,471	7,67634E-36
Mono_CD14	AHNAK	1,55546E-80	8,49716E-01	0,632	0,601	2,86470E-76
Mono_CD14	LRRK2	2,24566E-58	8,47581E-01	0,295	0,205	4,13583E-54
Mono_CD14	IL17RA	1,66288E-70	8,42369E-01	0,387	0,296	3,06252E-66
Mono_CD14	UPP1	2,59308E-89	8,40579E-01	0,523	0,425	4,77567E-85
Mono_CD14	HCK	4,53178E-60	8,39526E-01	0,55	0,518	8,34618E-56
Mono_CD14	PELI1	2,18901E-09	8,38463E-01	0,278	0,261	4,03149E-05
Mono_CD14	NAGK	8,58869E-08	8,36913E-01	0,373	0,485	1,58178E-03
Mono_CD14	FES	5,91508E-14	8,36303E-01	0,279	0,253	1,08938E-09
Mono_CD14	SF3B1	2,46582E-10	8,34921E-01	0,377	0,502	4,54130E-06
Mono_CD14	PTP4A2	7,88995E-11	8,33596E-01	0,397	0,405	1,45309E-06
Mono_CD14	RILPL2	6,60963E-85	8,32751E-01	0,521	0,451	1,21729E-80
Mono_CD14	JUND	3,63702E-85	8,31678E-01	0,534	0,449	6,69831E-81
Mono_CD14	PTPRC	5,08285E-42	8,31625E-01	0,66	0,692	9,36109E-38
Mono_CD14	NDUFS3	1,32269E-45	8,30218E-01	0,2	0,351	2,43600E-41
Mono_CD14	NBPF10	8,51275E-29	8,29992E-01	0,259	0,197	1,56779E-24
Mono_CD14	MT-ATP8	2,68423E-29	8,25369E-01	0,293	0,391	4,94356E-25
Mono_CD14	DUSP1	1,42741E-193	8,24965E-01	0,89	0,847	2,62885E-189
Mono_CD14	SLC7A7	7,41761E-09	8,22880E-01	0,45	0,467	1,36610E-04
Mono_CD14	CAPN2	5,84987E-55	8,22026E-01	0,32	0,228	1,07737E-50
Mono_CD14	OXA1L	4,23730E-15	8,18542E-01	0,335	0,464	7,80383E-11
Mono_CD14	SMAP2	3,42653E-67	8,16722E-01	0,513	0,468	6,31065E-63
Mono_CD14	SLC2A3	1,05818E-173	8,14445E-01	0,6	0,422	1,94885E-169
Mono_CD14	DOK2	1,55409E-26	8,13935E-01	0,382	0,342	2,86217E-22
Mono_CD14	GPSM3	1,09559E-88	8,11595E-01	0,674	0,684	2,01776E-84

Mono_CD14	LRP1	3,63810E-18	8,11361E-01	0,438	0,421	6,70029E-14
Mono_CD14	SRRM2	8,34973E-12	8,08379E-01	0,517	0,573	1,53777E-07
Mono_CD14	AIF1	1,31197E-122	8,07786E-01	0,891	0,817	2,41626E-118
Mono_CD14	CHD1	4,22961E-08	8,05655E-01	0,303	0,297	7,78967E-04
Mono_CD14	TET2	3,63972E-09	7,98930E-01	0,26	0,239	6,70326E-05
Mono_CD14	STK17B	6,95276E-95	7,98089E-01	0,534	0,438	1,28049E-90
Mono_CD14	ZEB2	1,25589E-45	7,93921E-01	0,596	0,606	2,31298E-41
Mono_CD14	HSD17B1	1,31783E-06	7,89628E-01	0,383	0,406	2,42706E-02
Mono_CD14	C5AR1	5,26424E-50	7,89228E-01	0,48	0,402	9,69516E-46
Mono_CD14	POLE4	1,43068E-34	7,85551E-01	0,374	0,328	2,63488E-30
Mono_CD14	STAT6	2,54618E-12	7,77865E-01	0,321	0,304	4,68930E-08
Mono_CD14	MCL1	2,22227E-203	7,76189E-01	0,8	0,767	4,09276E-199
Mono_CD14	BIN2	2,96881E-07	7,75236E-01	0,315	0,314	5,46766E-03
Mono_CD14	SMCHD1	1,42122E-17	7,72869E-01	0,347	0,323	2,61746E-13
Mono_CD14	C4orf48	6,25411E-20	7,72537E-01	0,502	0,53	1,15182E-15
Mono_CD14	CD55	1,56648E-262	7,69535E-01	0,612	0,416	2,88499E-258
Mono_CD14	TRMT1	1,02041E-07	7,66351E-01	0,281	0,274	1,87928E-03
Mono_CD14	CALM2	4,19331E-08	7,64049E-01	0,693	0,788	7,72281E-04
Mono_CD14	IRF1	3,78433E-43	7,59229E-01	0,478	0,436	6,96960E-39
Mono_CD14	TALDO1	9,47307E-156	7,57305E-01	0,68	0,661	1,74465E-151
Mono_CD14	PHC2	1,02593E-12	7,55726E-01	0,267	0,243	1,88945E-08
Mono_CD14	CD14	6,74412E-69	7,55579E-01	0,728	0,616	1,24206E-64
Mono_CD14	KLF4	4,56221E-51	7,51835E-01	0,51	0,442	8,40222E-47
Mono_CD14	ENTPD1	6,50059E-17	7,51030E-01	0,219	0,316	1,19721E-12
Mono_CD14	S100A10	2,87844E-232	7,46802E-01	0,876	0,835	5,30122E-228
Mono_CD14	NUMB	8,78025E-37	7,43871E-01	0,383	0,33	1,61706E-32
Mono_CD14	BLVRB	1,89990E-94	7,42505E-01	0,557	0,485	3,49905E-90
Mono_CD14	IFI30	2,08731E-95	7,40867E-01	0,659	0,562	3,84420E-91
Mono_CD14	CD48	2,78084E-91	7,38752E-01	0,546	0,474	5,12147E-87
Mono_CD14	SERPINB1	2,13180E-110	7,36236E-01	0,676	0,662	3,92614E-106
Mono_CD14	TMEM20	8,67950E-14	7,35468E-01	0,303	0,283	1,59850E-09
Mono_CD14	NKTR	2,38070E-15	7,30463E-01	0,203	0,29	4,38454E-11
Mono_CD14	SERP1	2,19836E-154	7,28848E-01	0,807	0,82	4,04873E-150
Mono_CD14	CCDC69	1,36520E-86	7,26053E-01	0,279	0,164	2,51428E-82
Mono_CD14	JAML	2,72649E-56	7,25991E-01	0,285	0,193	5,02137E-52
Mono_CD14	PER1	6,06769E-27	7,25124E-01	0,361	0,317	1,11749E-22
Mono_CD14	EFHD2	6,91902E-46	7,23432E-01	0,471	0,427	1,27428E-41
Mono_CD14	BIRC6	3,13291E-09	7,21397E-01	0,188	0,253	5,76989E-05
Mono_CD14	IFITM2	1,46817E-127	7,09273E-01	0,731	0,64	2,70392E-123
Mono_CD14	MRPS12	4,02301E-28	7,07185E-01	0,178	0,29	7,40918E-24

Mono_CD14	AMPD2	1,52995E-36	7,02991E-01	0,25	0,179	2,81770E-32
Mono_CD14	MPEG1	2,61376E-136	7,01606E-01	0,51	0,364	4,81376E-132
Mono_CD14	PABPC1	1,20590E-120	7,00079E-01	0,862	0,895	2,22091E-116
Mono_CD14	UBE2D1	1,16340E-16	6,98579E-01	0,372	0,362	2,14263E-12
Mono_CD14	CD93	1,32346E-35	6,98477E-01	0,341	0,275	2,43741E-31
Mono_CD14	GPCPD1	2,11117E-27	6,95599E-01	0,325	0,277	3,88815E-23
Mono_CD14	ARPC1B	1,83403E-106	6,93143E-01	0,782	0,798	3,37773E-102
Mono_CD14	GNAI2	1,83848E-40	6,93056E-01	0,639	0,676	3,38592E-36
Mono_CD14	PARL	1,66818E-16	6,87827E-01	0,204	0,296	3,07229E-12
Mono_CD14	SPSB3	7,16721E-10	6,85981E-01	0,216	0,296	1,31998E-05
Mono_CD14	PTEN	2,28774E-09	6,78300E-01	0,277	0,261	4,21334E-05
Mono_CD14	SHKBP1	3,38240E-10	6,76717E-01	0,36	0,37	6,22937E-06
Mono_CD14	SLC15A3	5,46063E-30	6,76210E-01	0,18	0,29	1,00568E-25
Mono_CD14	TLR4	5,70533E-18	6,74568E-01	0,262	0,221	1,05075E-13
Mono_CD14	KLF2	4,13819E-42	6,74116E-01	0,355	0,271	7,62131E-38
Mono_CD14	INPP5D	1,72553E-28	6,71013E-01	0,201	0,319	3,17791E-24
Mono_CD14	DIAPH1	1,46259E-08	6,68212E-01	0,258	0,242	2,69365E-04
Mono_CD14	TMA7	3,92402E-94	6,65990E-01	0,772	0,795	7,22686E-90
Mono_CD14	CMTM7	3,71586E-08	6,62164E-01	0,316	0,418	6,84350E-04
Mono_CD14	SPNS1	4,83998E-07	6,59287E-01	0,194	0,253	8,91379E-03
Mono_CD14	MIDN	4,01030E-29	6,58749E-01	0,329	0,278	7,38577E-25
Mono_CD14	ARF5	1,44818E-13	6,58687E-01	0,555	0,618	2,66711E-09
Mono_CD14	MYH9	1,34037E-06	6,52133E-01	0,519	0,583	2,46855E-02
Mono_CD14	CCND3	4,18566E-08	6,48692E-01	0,319	0,312	7,70872E-04
Mono_CD14	FMNL1	7,35192E-29	6,48452E-01	0,426	0,401	1,35400E-24
Mono_CD14	CKLF	1,67920E-08	6,47313E-01	0,485	0,507	3,09259E-04
Mono_CD14	EIF2A	1,97873E-15	6,47279E-01	0,22	0,314	3,64423E-11
Mono_CD14	PCGF5	1,38676E-20	6,45374E-01	0,182	0,279	2,55399E-16
Mono_CD14	PFDN1	1,12736E-47	6,43639E-01	0,156	0,296	2,07626E-43
Mono_CD14	WBP2	5,70985E-50	6,43103E-01	0,229	0,395	1,05158E-45
Mono_CD14	CD37	4,03862E-46	6,42729E-01	0,697	0,735	7,43793E-42
Mono_CD14	EEF1D	5,67076E-114	6,42528E-01	0,849	0,873	1,04438E-109
Mono_CD14	GLIPR1	3,85755E-19	6,41632E-01	0,556	0,585	7,10445E-15
Mono_CD14	ATRX	7,48393E-28	6,41593E-01	0,261	0,398	1,37832E-23
Mono_CD14	PTPRE	3,93155E-105	6,41175E-01	0,536	0,433	7,24073E-101
Mono_CD14	IER2	3,09992E-136	6,41115E-01	0,707	0,627	5,70913E-132
Mono_CD14	PPIF	1,43372E-09	6,40626E-01	0,356	0,358	2,64049E-05
Mono_CD14	FKBP1A	6,02652E-37	6,39820E-01	0,653	0,697	1,10990E-32
Mono_CD14	FGD2	6,88391E-20	6,37898E-01	0,242	0,352	1,26781E-15
Mono_CD14	TYROBP	1,18403E-76	6,35951E-01	0,969	0,945	2,18063E-72

Mono_CD14	OAZ1	2,20564E-307	6,35014E-01	0,936	0,937	4,06212E-303
Mono_CD14	CCNL1	4,35316E-70	6,34325E-01	0,548	0,524	8,01721E-66
Mono_CD14	VSIR	8,47505E-23	6,33231E-01	0,312	0,271	1,56085E-18
Mono_CD14	CRIP1	9,18172E-61	6,32124E-01	0,399	0,285	1,69100E-56
Mono_CD14	LRRFIP2	1,03156E-10	6,31724E-01	0,186	0,257	1,89983E-06
Mono_CD14	RAB11FII	8,03924E-40	6,31411E-01	0,291	0,216	1,48059E-35
Mono_CD14	EMILIN2	5,89176E-11	6,31379E-01	0,317	0,3	1,08509E-06
Mono_CD14	FOSB	4,39372E-139	6,30961E-01	0,687	0,572	8,09192E-135
Mono_CD14	COMMD	5,98644E-46	6,30915E-01	0,185	0,332	1,10252E-41
Mono_CD14	MT-ND6	1,74736E-46	6,28730E-01	0,179	0,311	3,21811E-42
Mono_CD14	SSH2	8,92640E-23	6,27940E-01	0,348	0,308	1,64398E-18
Mono_CD14	IRAK3	1,72496E-63	6,27226E-01	0,346	0,248	3,17685E-59
Mono_CD14	PPIG	4,09838E-29	6,26915E-01	0,304	0,463	7,54799E-25
Mono_CD14	ADPGK	2,79389E-25	6,26056E-01	0,272	0,41	5,14550E-21
Mono_CD14	PIAS1	2,55288E-29	6,25237E-01	0,207	0,33	4,70164E-25
Mono_CD14	VASP	1,16274E-18	6,21390E-01	0,467	0,47	2,14142E-14
Mono_CD14	PRKCB	5,42999E-28	6,19140E-01	0,279	0,223	1,00004E-23
Mono_CD14	CCAR1	3,48088E-35	6,18740E-01	0,157	0,275	6,41074E-31
Mono_CD14	MTPN	5,22359E-19	6,14443E-01	0,494	0,529	9,62029E-15
Mono_CD14	RBM39	1,39006E-07	6,14231E-01	0,555	0,628	2,56008E-03
Mono_CD14	TPP1	3,71441E-27	6,13154E-01	0,439	0,607	6,84082E-23
Mono_CD14	CTDNEP1	1,42615E-10	6,12933E-01	0,192	0,266	2,62655E-06
Mono_CD14	SCAND1	1,41460E-06	6,12879E-01	0,478	0,539	2,60526E-02
Mono_CD14	RAC2	6,86714E-44	6,12461E-01	0,543	0,493	1,26472E-39
Mono_CD14	RNF10	5,98706E-25	6,11748E-01	0,176	0,282	1,10264E-20
Mono_CD14	WAS	2,35692E-28	6,07666E-01	0,51	0,53	4,34074E-24
Mono_CD14	WNK1	1,08545E-10	6,07483E-01	0,266	0,363	1,99908E-06
Mono_CD14	RASSF2	4,59887E-16	6,07031E-01	0,295	0,262	8,46974E-12
Mono_CD14	SPI1	2,83465E-61	6,03401E-01	0,761	0,768	5,22057E-57
Mono_CD14	ANPEP	1,62066E-58	6,03326E-01	0,304	0,202	2,98477E-54
Mono_CD14	NDUFB7	4,94368E-08	6,03077E-01	0,41	0,538	9,10478E-04
Mono_CD14	PNPLA2	8,58897E-22	6,01926E-01	0,332	0,299	1,58183E-17
Mono_CD14	EREG	1,67552E-152	6,01784E-01	0,315	0,146	3,08581E-148
Mono_CD14	RPS9	7,17543E-23	6,01247E-01	0,541	0,555	1,32150E-18
Mono_CD14	GNB2	5,28599E-21	5,99156E-01	0,525	0,559	9,73522E-17
Mono_CD14	ARRB2	6,56803E-43	5,97787E-01	0,637	0,668	1,20963E-38
Mono_CD14	PABPN1	1,09677E-06	5,97754E-01	0,223	0,292	2,01991E-02
Mono_CD14	ARL4A	1,33889E-36	5,97187E-01	0,304	0,235	2,46583E-32
Mono_CD14	HIGD2A	1,75679E-13	5,96780E-01	0,635	0,72	3,23548E-09
Mono_CD14	MBOAT7	1,25650E-19	5,95731E-01	0,286	0,247	2,31410E-15

Mono_CD14	RPL39	6,15660E-29	5,93551E-01	0,553	0,583	1,13386E-24
Mono_CD14	STX11	3,27962E-08	5,92729E-01	0,334	0,328	6,04008E-04
Mono_CD14	CD44	1,58402E-116	5,88793E-01	0,732	0,673	2,91729E-112
Mono_CD14	LRRFIP1	1,43397E-37	5,86899E-01	0,645	0,686	2,64095E-33
Mono_CD14	TRAPPC2	1,66650E-14	5,86531E-01	0,19	0,275	3,06919E-10
Mono_CD14	BAG6	1,65905E-14	5,85307E-01	0,195	0,279	3,05547E-10
Mono_CD14	AAMP	7,27261E-41	5,85243E-01	0,169	0,302	1,33940E-36
Mono_CD14	MYO9B	1,06771E-09	5,81288E-01	0,298	0,395	1,96640E-05
Mono_CD14	ARPC3	1,19725E-50	5,80331E-01	0,852	0,895	2,20497E-46
Mono_CD14	SH3BGRL	1,46366E-25	5,79524E-01	0,556	0,732	2,69561E-21
Mono_CD14	SRSF11	3,79340E-22	5,79261E-01	0,367	0,526	6,98631E-18
Mono_CD14	ILF3	3,48934E-16	5,78887E-01	0,265	0,374	6,42632E-12
Mono_CD14	COPS5	1,79112E-31	5,76676E-01	0,17	0,287	3,29870E-27
Mono_CD14	RNPEP	1,82720E-10	5,76665E-01	0,248	0,337	3,36516E-06
Mono_CD14	MRPL33	2,43165E-06	5,76468E-01	0,354	0,373	4,47838E-02
Mono_CD14	CTNNA1	6,28851E-29	5,76162E-01	0,238	0,372	1,15815E-24
Mono_CD14	EIF1B	4,48868E-09	5,74242E-01	0,347	0,468	8,26680E-05
Mono_CD14	HBEGF	5,58536E-33	5,73215E-01	0,339	0,271	1,02866E-28
Mono_CD14	YBX3	2,99885E-30	5,72398E-01	0,387	0,345	5,52298E-26
Mono_CD14	ASAH1	1,00371E-22	5,71782E-01	0,58	0,697	1,84854E-18
Mono_CD14	FXYD5	1,37976E-23	5,70427E-01	0,769	0,829	2,54111E-19
Mono_CD14	MRPS18(	7,50872E-43	5,70175E-01	0,176	0,311	1,38288E-38
Mono_CD14	TRIB1	8,84071E-20	5,68268E-01	0,279	0,238	1,62819E-15
Mono_CD14	LCP1	5,55204E-121	5,67919E-01	0,731	0,702	1,02252E-116
Mono_CD14	DAPK1	9,06583E-64	5,66080E-01	0,182	0,346	1,66965E-59
Mono_CD14	PSMD13	8,84586E-20	5,61853E-01	0,221	0,331	1,62914E-15
Mono_CD14	LUC7L3	6,27726E-22	5,61048E-01	0,248	0,367	1,15608E-17
Mono_CD14	TMEM20	2,49972E-49	5,60973E-01	0,188	0,341	4,60374E-45
Mono_CD14	BRI3	4,39163E-07	5,54918E-01	0,395	0,403	8,08806E-03
Mono_CD14	CYBB	5,41129E-38	5,54710E-01	0,632	0,623	9,96598E-34
Mono_CD14	DAZAP2	1,50841E-10	5,47400E-01	0,659	0,748	2,77804E-06
Mono_CD14	PRPF40A	8,05667E-38	5,43775E-01	0,297	0,467	1,48380E-33
Mono_CD14	RPL26	3,57309E-09	5,43133E-01	0,511	0,545	6,58055E-05
Mono_CD14	RAD23A	6,11791E-46	5,41595E-01	0,325	0,518	1,12674E-41
Mono_CD14	YWHAB	1,30407E-15	5,41167E-01	0,71	0,794	2,40171E-11
Mono_CD14	TIMP1	2,85010E-169	5,40072E-01	0,773	0,641	5,24903E-165
Mono_CD14	IVNS1AB	8,63060E-28	5,39477E-01	0,398	0,364	1,58950E-23
Mono_CD14	S100A11	1,58351E-28	5,39451E-01	0,957	0,936	2,91635E-24
Mono_CD14	FYB1	1,85871E-14	5,38443E-01	0,346	0,316	3,42319E-10
Mono_CD14	ELOB	1,33740E-08	5,37362E-01	0,385	0,4	2,46308E-04

Mono_CD14	CCT6A	8,88621E-47	5,37159E-01	0,283	0,46	1,63657E-42
Mono_CD14	ASH1L	3,15873E-15	5,36592E-01	0,174	0,253	5,81743E-11
Mono_CD14	CCT5	3,09263E-22	5,35034E-01	0,265	0,39	5,69570E-18
Mono_CD14	SLC25A6	1,49853E-59	5,31594E-01	0,764	0,82	2,75984E-55
Mono_CD14	FDFT1	5,19271E-16	5,31212E-01	0,182	0,266	9,56341E-12
Mono_CD14	SPG21	2,35932E-09	5,29890E-01	0,333	0,445	4,34517E-05
Mono_CD14	ZNF207	4,91019E-11	5,29021E-01	0,303	0,412	9,04310E-07
Mono_CD14	BCL3	1,21466E-33	5,29003E-01	0,279	0,214	2,23703E-29
Mono_CD14	RPS24	2,13157E-21	5,25634E-01	0,547	0,58	3,92571E-17
Mono_CD14	TNFAIP8L	5,85971E-21	5,25095E-01	0,172	0,267	1,07918E-16
Mono_CD14	CTNND1	2,29716E-78	5,24406E-01	0,119	0,283	4,23068E-74
Mono_CD14	RNH1	1,18469E-10	5,23093E-01	0,482	0,628	2,18185E-06
Mono_CD14	HSD17B1	6,61044E-08	5,22800E-01	0,232	0,312	1,21744E-03
Mono_CD14	EMP3	4,02386E-91	5,19493E-01	0,754	0,727	7,41074E-87
Mono_CD14	RPL34	5,36617E-20	5,18816E-01	0,551	0,577	9,88287E-16
Mono_CD14	CNOT2	1,47736E-22	5,18165E-01	0,177	0,278	2,72086E-18
Mono_CD14	PSMA4	1,46703E-21	5,16609E-01	0,334	0,478	2,70183E-17
Mono_CD14	RNASEK	9,02971E-19	5,16185E-01	0,472	0,465	1,66300E-14
Mono_CD14	EWSR1	6,14425E-27	5,14650E-01	0,321	0,473	1,13159E-22
Mono_CD14	GLB1	1,05588E-52	5,14546E-01	0,157	0,302	1,94462E-48
Mono_CD14	PLIN3	2,48208E-11	5,13745E-01	0,276	0,379	4,57124E-07
Mono_CD14	GIT2	3,64944E-26	5,13013E-01	0,192	0,302	6,72118E-22
Mono_CD14	CD46	9,63722E-15	5,11716E-01	0,229	0,323	1,77489E-10
Mono_CD14	CIAO1	1,27442E-17	5,11034E-01	0,166	0,252	2,34710E-13
Mono_CD14	APOBEC3	0,00000E+00	5,10793E-01	0,355	0,122	0,00000E+00
Mono_CD14	TMEM30	7,41809E-32	5,07956E-01	0,19	0,313	1,36619E-27
Mono_CD14	OAZ2	2,01234E-27	5,02818E-01	0,18	0,291	3,70612E-23
Mono_CD14	MIER1	6,63413E-12	5,01829E-01	0,184	0,258	1,22181E-07
Mono_CD14	SEC14L1	1,55663E-23	5,00890E-01	0,275	0,404	2,86685E-19
Mono_CD14	UBE2R2	1,63027E-07	4,99571E-01	0,271	0,264	3,00247E-03
Mono_CD14	NDUFB1(	1,20818E-07	4,99439E-01	0,428	0,568	2,22511E-03
Mono_CD14	DGCR6L	1,55230E-15	4,97785E-01	0,182	0,267	2,85888E-11
Mono_CD14	UBA52	3,81564E-169	4,97039E-01	0,897	0,922	7,02727E-165
Mono_CD14	PPP1R12	1,73855E-18	4,96120E-01	0,267	0,385	3,20189E-14
Mono_CD14	SAT1	4,48593E-28	4,90631E-01	0,969	0,946	8,26174E-24
Mono_CD14	NUDT16	2,14902E-08	4,89137E-01	0,391	0,409	3,95785E-04
Mono_CD14	NLRP3	5,02141E-98	4,88764E-01	0,39	0,254	9,24793E-94
Mono_CD14	PTPN2	2,72067E-20	4,88601E-01	0,273	0,394	5,01065E-16
Mono_CD14	JUNB	2,30273E-124	4,88374E-01	0,84	0,825	4,24094E-120
Mono_CD14	EIF1	7,36028E-257	4,86329E-01	0,942	0,944	1,35554E-252

Mono_CD14	RBM6	5,64809E-13	4,85642E-01	0,19	0,268	1,04021E-08
Mono_CD14	CHP1	1,07086E-08	4,85405E-01	0,256	0,236	1,97219E-04
Mono_CD14	BTF3	1,89139E-46	4,85358E-01	0,784	0,851	3,48337E-42
Mono_CD14	RSF1	1,41414E-37	4,82573E-01	0,212	0,353	2,60443E-33
Mono_CD14	CEP170	6,76191E-60	4,80508E-01	0,189	0,355	1,24534E-55
Mono_CD14	ZFP36L1	5,95696E-25	4,80136E-01	0,59	0,735	1,09709E-20
Mono_CD14	UBE2V2	6,45521E-46	4,78644E-01	0,139	0,268	1,18886E-41
Mono_CD14	ZMAT2	1,27046E-19	4,78462E-01	0,223	0,333	2,33981E-15
Mono_CD14	SLTM	4,08201E-14	4,75315E-01	0,258	0,362	7,51784E-10
Mono_CD14	C7orf50	4,12191E-61	4,73113E-01	0,183	0,348	7,59132E-57
Mono_CD14	ORMDL1	3,66348E-43	4,72558E-01	0,184	0,325	6,74702E-39
Mono_CD14	EHBP1L1	8,02268E-17	4,71915E-01	0,267	0,229	1,47754E-12
Mono_CD14	ITPA	8,18340E-39	4,71484E-01	0,157	0,283	1,50714E-34
Mono_CD14	CHD2	1,89193E-20	4,71213E-01	0,205	0,305	3,48437E-16
Mono_CD14	RPLP2	6,70572E-07	4,70771E-01	0,538	0,573	1,23499E-02
Mono_CD14	TNFSF13	4,55909E-19	4,68071E-01	0,348	0,45	8,39648E-15
Mono_CD14	CSNK1D	1,22233E-22	4,67435E-01	0,232	0,351	2,25117E-18
Mono_CD14	NDUFC1	8,34913E-25	4,65960E-01	0,317	0,468	1,53766E-20
Mono_CD14	COX4I1	3,19549E-130	4,64938E-01	0,855	0,9	5,88514E-126
Mono_CD14	SH3TC1	6,92473E-23	4,63520E-01	0,212	0,321	1,27533E-18
Mono_CD14	PPP1R18	9,97631E-14	4,62614E-01	0,374	0,51	1,83734E-09
Mono_CD14	LSP1	1,10830E-92	4,62188E-01	0,75	0,665	2,04115E-88
Mono_CD14	NOSIP	3,42642E-26	4,61884E-01	0,213	0,336	6,31043E-22
Mono_CD14	RPL28	4,44722E-25	4,61439E-01	0,574	0,596	8,19045E-21
Mono_CD14	LRPAP1	3,12260E-18	4,61304E-01	0,364	0,505	5,75090E-14
Mono_CD14	CDK5RAF	2,69009E-11	4,60985E-01	0,228	0,313	4,95433E-07
Mono_CD14	COX8A	1,71123E-13	4,57601E-01	0,702	0,783	3,15157E-09
Mono_CD14	DDX21	5,28012E-18	4,54933E-01	0,434	0,437	9,72439E-14
Mono_CD14	CDC42	4,97039E-08	4,53504E-01	0,613	0,703	9,15398E-04
Mono_CD14	COPA	5,75580E-11	4,51990E-01	0,254	0,344	1,06005E-06
Mono_CD14	PGD	6,85080E-56	4,51500E-01	0,492	0,464	1,26171E-51
Mono_CD14	ITPR2	2,14801E-54	4,51308E-01	0,144	0,288	3,95599E-50
Mono_CD14	GSTP1	2,69538E-108	4,49741E-01	0,798	0,825	4,96409E-104
Mono_CD14	SENP6	1,43161E-06	4,48955E-01	0,193	0,25	2,63660E-02
Mono_CD14	MYD88	3,98753E-10	4,48064E-01	0,358	0,357	7,34384E-06
Mono_CD14	ITSN2	3,44381E-33	4,47988E-01	0,252	0,396	6,34247E-29
Mono_CD14	ZC3H15	2,30975E-18	4,47201E-01	0,315	0,447	4,25387E-14
Mono_CD14	EIF3K	1,91667E-16	4,46422E-01	0,709	0,792	3,52993E-12
Mono_CD14	NFKBIZ	2,05534E-95	4,46306E-01	0,557	0,447	3,78533E-91
Mono_CD14	CUTA	3,81053E-10	4,46303E-01	0,368	0,494	7,01786E-06

Mono_CD14	NELFE	8,39047E-12	4,45659E-01	0,211	0,296	1,54527E-07
Mono_CD14	COMMD	1,80847E-21	4,44051E-01	0,212	0,321	3,33065E-17
Mono_CD14	ALDOA	2,82796E-73	4,43505E-01	0,745	0,761	5,20825E-69
Mono_CD14	GK	3,27633E-08	4,43102E-01	0,369	0,364	6,03402E-04
Mono_CD14	SERF2	3,20022E-144	4,41933E-01	0,943	0,955	5,89385E-140
Mono_CD14	TWF2	1,12504E-11	4,39539E-01	0,256	0,34	2,07199E-07
Mono_CD14	PAK1	1,45704E-10	4,38978E-01	0,337	0,444	2,68343E-06
Mono_CD14	ARF3	1,45308E-14	4,38851E-01	0,244	0,344	2,67614E-10
Mono_CD14	PQBP1	1,57258E-10	4,38439E-01	0,232	0,32	2,89621E-06
Mono_CD14	MIIP	3,46495E-10	4,37479E-01	0,181	0,25	6,38140E-06
Mono_CD14	DNM2	9,88249E-12	4,37125E-01	0,218	0,302	1,82006E-07
Mono_CD14	FCGRT	1,00168E-16	4,37099E-01	0,659	0,744	1,84480E-12
Mono_CD14	HNRNPU	1,03658E-22	4,34406E-01	0,576	0,627	1,90906E-18
Mono_CD14	BCL2A1	1,94996E-134	4,34194E-01	0,618	0,46	3,59125E-130
Mono_CD14	POLD4	1,13885E-21	4,33317E-01	0,436	0,579	2,09742E-17
Mono_CD14	BCKDK	1,59599E-19	4,33298E-01	0,219	0,326	2,93934E-15
Mono_CD14	ERCC1	2,15487E-38	4,33134E-01	0,241	0,392	3,96862E-34
Mono_CD14	ETV6	1,52024E-06	4,32945E-01	0,232	0,295	2,79983E-02
Mono_CD14	HSPB11	1,31150E-63	4,32120E-01	0,125	0,277	2,41538E-59
Mono_CD14	REEP5	2,77055E-19	4,31398E-01	0,412	0,574	5,10253E-15
Mono_CD14	CNPY3	1,71967E-36	4,30406E-01	0,606	0,629	3,16712E-32
Mono_CD14	RNF149	2,00075E-06	4,30123E-01	0,56	0,632	3,68478E-02
Mono_CD14	TRABD	2,10332E-07	4,28904E-01	0,279	0,363	3,87368E-03
Mono_CD14	RPS13	1,65271E-21	4,25526E-01	0,54	0,573	3,04379E-17
Mono_CD14	STK4	8,21484E-28	4,25499E-01	0,301	0,438	1,51293E-23
Mono_CD14	MS4A6A	1,24346E-24	4,24905E-01	0,697	0,716	2,29008E-20
Mono_CD14	GAPDH	2,68139E-182	4,24735E-01	0,951	0,961	4,93832E-178
Mono_CD14	GIMAP1	2,59265E-07	4,24109E-01	0,278	0,265	4,77489E-03
Mono_CD14	PPP1R7	3,16303E-26	4,21267E-01	0,178	0,287	5,82536E-22
Mono_CD14	MLX	2,22046E-21	4,20909E-01	0,246	0,367	4,08943E-17
Mono_CD14	TNRC6B	8,45426E-08	4,20529E-01	0,212	0,276	1,55702E-03
Mono_CD14	ERP29	2,51717E-12	4,18380E-01	0,571	0,627	4,63587E-08
Mono_CD14	HCST	3,23545E-39	4,17408E-01	0,648	0,654	5,95873E-35
Mono_CD14	TYMP	1,55481E-29	4,17063E-01	0,793	0,793	2,86350E-25
Mono_CD14	MXD1	6,69443E-67	4,17043E-01	0,395	0,295	1,23291E-62
Mono_CD14	BECN1	1,42869E-14	4,15953E-01	0,183	0,266	2,63122E-10
Mono_CD14	WASF2	2,26099E-12	4,15019E-01	0,438	0,578	4,16406E-08
Mono_CD14	DDX3X	2,82449E-07	4,14677E-01	0,449	0,48	5,20186E-03
Mono_CD14	MRPL52	6,65572E-07	4,12411E-01	0,355	0,467	1,22578E-02
Mono_CD14	GNAI3	5,79865E-16	4,11508E-01	0,246	0,353	1,06794E-11

Mono_CD14	IFI27L2	2,39846E-08	4,10684E-01	0,316	0,416	4,41724E-04
Mono_CD14	PSMC5	1,46277E-39	4,10263E-01	0,262	0,422	2,69399E-35
Mono_CD14	PSMD6	6,88832E-43	4,08137E-01	0,155	0,287	1,26862E-38
Mono_CD14	ALOX5	1,07136E-20	4,07536E-01	0,357	0,483	1,97312E-16
Mono_CD14	POLR2L	1,27106E-14	4,07515E-01	0,575	0,631	2,34091E-10
Mono_CD14	AP1M1	1,24713E-10	4,07477E-01	0,18	0,252	2,29683E-06
Mono_CD14	GRHPR	4,35796E-17	4,07395E-01	0,203	0,298	8,02606E-13
Mono_CD14	RNASEH2	2,31711E-06	4,06875E-01	0,27	0,352	4,26742E-02
Mono_CD14	TAF1D	8,83929E-26	4,06546E-01	0,258	0,394	1,62793E-21
Mono_CD14	ETS2	7,01619E-19	4,06340E-01	0,44	0,425	1,29217E-14
Mono_CD14	NUDT21	5,24514E-29	4,03962E-01	0,164	0,274	9,65998E-25
Mono_CD14	BTK	1,05743E-33	4,03946E-01	0,195	0,319	1,94747E-29
Mono_CD14	OS9	8,77359E-25	4,03808E-01	0,381	0,548	1,61583E-20
Mono_CD14	IMP4	2,41318E-52	4,02979E-01	0,137	0,276	4,44435E-48
Mono_CD14	ZFP36	7,47578E-74	4,02676E-01	0,841	0,844	1,37681E-69
Mono_CD14	ACTB	2,64962E-55	4,02250E-01	0,993	0,991	4,87980E-51
Mono_CD14	NAA20	4,78678E-85	4,01948E-01	0,138	0,319	8,81582E-81
Mono_CD14	PNRC1	6,67410E-36	4,01023E-01	0,749	0,781	1,22917E-31
Mono_CD14	SNW1	6,04386E-24	4,00388E-01	0,189	0,297	1,11310E-19
Mono_CD14	ATP6V1E	5,85417E-54	3,99984E-01	0,182	0,342	1,07816E-49
Mono_CD14	CD99	1,47708E-11	3,99143E-01	0,574	0,624	2,72034E-07
Mono_CD14	CLTA	1,26382E-20	3,97991E-01	0,49	0,661	2,32758E-16
Mono_CD14	SNRNP2(	4,70577E-32	3,97526E-01	0,156	0,266	8,66661E-28
Mono_CD14	VPS35	1,17337E-29	3,97082E-01	0,294	0,45	2,16100E-25
Mono_CD14	TXNDC12	8,17143E-55	3,96437E-01	0,19	0,352	1,50493E-50
Mono_CD14	ATP5F1E	3,16153E-12	3,95575E-01	0,471	0,478	5,82260E-08
Mono_CD14	KXD1	3,40384E-23	3,95490E-01	0,238	0,359	6,26884E-19
Mono_CD14	CLIC1	1,17962E-19	3,95419E-01	0,811	0,879	2,17251E-15
Mono_CD14	RBBP6	6,13829E-15	3,91080E-01	0,208	0,295	1,13049E-10
Mono_CD14	COX5B	5,59950E-07	3,90632E-01	0,672	0,779	1,03126E-02
Mono_CD14	SHISA5	7,28904E-11	3,90588E-01	0,279	0,378	1,34242E-06
Mono_CD14	USP4	2,04289E-20	3,88655E-01	0,201	0,302	3,76240E-16
Mono_CD14	ARHGEF2	2,00993E-14	3,87540E-01	0,176	0,255	3,70169E-10
Mono_CD14	FIBP	3,59469E-25	3,85759E-01	0,248	0,376	6,62034E-21
Mono_CD14	PLP2	1,47653E-102	3,83683E-01	0,508	0,397	2,71933E-98
Mono_CD14	DNAJC7	1,20039E-18	3,83479E-01	0,257	0,373	2,21076E-14
Mono_CD14	SCNM1	2,59557E-13	3,83126E-01	0,242	0,341	4,78027E-09
Mono_CD14	FGFR1OP	2,47415E-30	3,82584E-01	0,191	0,314	4,55665E-26
Mono_CD14	PTBP1	5,35281E-29	3,82215E-01	0,196	0,315	9,85827E-25
Mono_CD14	HSF1	9,76042E-19	3,82036E-01	0,186	0,279	1,79758E-14

Mono_CD14	SELL	5,90865E-274	3,80699E-01	0,41	0,187	1,08820E-269
Mono_CD14	PPCS	1,61706E-25	3,78615E-01	0,263	0,4	2,97814E-21
Mono_CD14	FAU	4,45053E-248	3,78504E-01	0,944	0,958	8,19654E-244
Mono_CD14	SCP2	2,59076E-66	3,77278E-01	0,273	0,475	4,77140E-62
Mono_CD14	UBXN1	2,06337E-11	3,77221E-01	0,482	0,639	3,80011E-07
Mono_CD14	HP1BP3	4,58237E-08	3,76949E-01	0,312	0,414	8,43935E-04
Mono_CD14	PYURF	1,00925E-33	3,76591E-01	0,267	0,421	1,85873E-29
Mono_CD14	PIK3R5	1,25101E-13	3,76525E-01	0,184	0,263	2,30398E-09
Mono_CD14	RPS14	1,57596E-08	3,76160E-01	0,54	0,578	2,90244E-04
Mono_CD14	CBX3	1,10518E-06	3,75381E-01	0,316	0,414	2,03542E-02
Mono_CD14	POLR2F	1,25266E-19	3,74573E-01	0,214	0,32	2,30703E-15
Mono_CD14	BCLAF1	3,65532E-18	3,74065E-01	0,271	0,389	6,73201E-14
Mono_CD14	GTF3A	4,18966E-33	3,73630E-01	0,328	0,491	7,71609E-29
Mono_CD14	IDH3G	2,61009E-17	3,73393E-01	0,253	0,367	4,80701E-13
Mono_CD14	CCDC59	3,61850E-12	3,72724E-01	0,179	0,254	6,66419E-08
Mono_CD14	PTAFR	2,38684E-10	3,72665E-01	0,292	0,382	4,39585E-06
Mono_CD14	NDUFV3	1,94419E-27	3,71766E-01	0,178	0,288	3,58061E-23
Mono_CD14	UQCR11	1,93278E-28	3,69783E-01	0,702	0,759	3,55959E-24
Mono_CD14	LSM10	5,69937E-10	3,69459E-01	0,281	0,382	1,04965E-05
Mono_CD14	DYNC1H1	1,19839E-31	3,67884E-01	0,252	0,389	2,20708E-27
Mono_CD14	TMEM16	2,49221E-10	3,67552E-01	0,384	0,401	4,58990E-06
Mono_CD14	MRFAP1	3,09272E-10	3,67491E-01	0,297	0,408	5,69586E-06
Mono_CD14	RAB6A	1,24543E-47	3,67259E-01	0,169	0,31	2,29370E-43
Mono_CD14	DNMT1	2,93648E-76	3,66956E-01	0,153	0,329	5,40812E-72
Mono_CD14	LAT2	4,52365E-09	3,66951E-01	0,363	0,461	8,33121E-05
Mono_CD14	OTUB1	9,31709E-15	3,66183E-01	0,314	0,439	1,71593E-10
Mono_CD14	UQCRFS1	3,58070E-13	3,65304E-01	0,367	0,502	6,59457E-09
Mono_CD14	COX6B1	7,13451E-21	3,63634E-01	0,741	0,816	1,31396E-16
Mono_CD14	NDUFB1	1,97418E-07	3,62727E-01	0,436	0,47	3,63585E-03
Mono_CD14	ESD	8,83443E-33	3,61116E-01	0,232	0,373	1,62704E-28
Mono_CD14	PRDX4	5,96911E-98	3,58787E-01	0,115	0,299	1,09933E-93
Mono_CD14	NSFL1C	1,99711E-17	3,58457E-01	0,187	0,281	3,67807E-13
Mono_CD14	SPAG7	9,54711E-35	3,58366E-01	0,2	0,335	1,75829E-30
Mono_CD14	MDH2	4,09542E-18	3,57751E-01	0,382	0,532	7,54254E-14
Mono_CD14	NCF4	2,86185E-10	3,54917E-01	0,322	0,43	5,27067E-06
Mono_CD14	TXNDC17	2,15487E-07	3,54885E-01	0,336	0,441	3,96862E-03
Mono_CD14	RAB10	6,24587E-21	3,54401E-01	0,355	0,5	1,15030E-16
Mono_CD14	KDM6B	2,07062E-35	3,54193E-01	0,402	0,336	3,81346E-31
Mono_CD14	TPM3	9,46440E-28	3,54157E-01	0,743	0,808	1,74306E-23
Mono_CD14	RPL32	4,01925E-09	3,53677E-01	0,543	0,581	7,40225E-05

Mono_CD14	IFNGR2	2,02496E-18	3,53256E-01	0,425	0,574	3,72936E-14
Mono_CD14	SNX2	1,18663E-24	3,51560E-01	0,309	0,45	2,18542E-20
Mono_CD14	MAP2K3	1,25495E-12	3,51313E-01	0,297	0,268	2,31125E-08
Mono_CD14	CYBA	5,31393E-30	3,51284E-01	0,88	0,9	9,78666E-26
Mono_CD14	ACIN1	2,09382E-12	3,51277E-01	0,202	0,282	3,85618E-08
Mono_CD14	HPS1	5,62484E-18	3,51268E-01	0,248	0,359	1,03593E-13
Mono_CD14	CFL1	8,63748E-10	3,50142E-01	0,878	0,925	1,59077E-05
Mono_CD14	RPLP1	8,89676E-15	3,49969E-01	0,568	0,6	1,63852E-10
Mono_CD14	SET	6,11919E-11	3,49887E-01	0,398	0,537	1,12697E-06
Mono_CD14	CHCHD7	5,98231E-09	3,49854E-01	0,194	0,262	1,10176E-04
Mono_CD14	C1orf162	2,21895E-08	3,48718E-01	0,685	0,709	4,08665E-04
Mono_CD14	RAB2A	6,71894E-24	3,48401E-01	0,337	0,497	1,23743E-19
Mono_CD14	VTI1B	1,50362E-28	3,48051E-01	0,248	0,387	2,76921E-24
Mono_CD14	POLR2G	3,99917E-26	3,47761E-01	0,25	0,381	7,36527E-22
Mono_CD14	MANBA	4,14622E-08	3,44304E-01	0,261	0,342	7,63609E-04
Mono_CD14	CYTH4	8,25198E-07	3,43963E-01	0,247	0,32	1,51977E-02
Mono_CD14	RPL38	9,76377E-08	3,43641E-01	0,411	0,498	1,79819E-03
Mono_CD14	ETFA	4,32098E-35	3,42970E-01	0,209	0,345	7,95794E-31
Mono_CD14	NONO	1,13031E-13	3,42431E-01	0,319	0,439	2,08169E-09
Mono_CD14	NCOR1	1,86779E-15	3,42111E-01	0,285	0,407	3,43991E-11
Mono_CD14	MPC1	1,90987E-37	3,41299E-01	0,191	0,326	3,51741E-33
Mono_CD14	STAB1	5,16483E-16	3,40846E-01	0,275	0,35	9,51207E-12
Mono_CD14	MBP	5,08077E-10	3,39782E-01	0,241	0,324	9,35726E-06
Mono_CD14	USP9X	1,48573E-40	3,39618E-01	0,157	0,282	2,73626E-36
Mono_CD14	NEMF	9,41017E-43	3,38091E-01	0,164	0,297	1,73307E-38
Mono_CD14	SSR2	4,91025E-16	3,36314E-01	0,499	0,663	9,04320E-12
Mono_CD14	ACOT9	2,03875E-34	3,35624E-01	0,169	0,287	3,75477E-30
Mono_CD14	ELF2	4,33715E-07	3,35170E-01	0,191	0,252	7,98772E-03
Mono_CD14	HM13	4,86531E-27	3,34412E-01	0,387	0,55	8,96044E-23
Mono_CD14	ACP1	1,22841E-37	3,34393E-01	0,203	0,344	2,26236E-33
Mono_CD14	KPNB1	5,69225E-45	3,33501E-01	0,278	0,452	1,04834E-40
Mono_CD14	HNRNPL	2,34686E-14	3,32777E-01	0,208	0,295	4,32221E-10
Mono_CD14	VPS13C	2,99234E-23	3,32034E-01	0,257	0,379	5,51099E-19
Mono_CD14	PSMD4	2,74424E-31	3,31821E-01	0,271	0,422	5,05406E-27
Mono_CD14	NDUFA1(	6,17832E-28	3,31645E-01	0,213	0,336	1,13786E-23
Mono_CD14	UBXN4	2,60788E-31	3,31573E-01	0,358	0,533	4,80293E-27
Mono_CD14	C1D	2,74528E-35	3,30863E-01	0,16	0,281	5,05598E-31
Mono_CD14	UBE2E1	3,86882E-38	3,29734E-01	0,184	0,318	7,12521E-34
Mono_CD14	PSENEN	4,12194E-30	3,29281E-01	0,184	0,302	7,59137E-26
Mono_CD14	CSDE1	2,77564E-08	3,28630E-01	0,473	0,614	5,11189E-04

Mono_CD14	FIS1	4,04502E-11	3,28507E-01	0,399	0,535	7,44971E-07
Mono_CD14	PDCD5	1,54958E-37	3,27356E-01	0,217	0,361	2,85386E-33
Mono_CD14	ATP6V1A	5,87559E-08	3,27152E-01	0,193	0,259	1,08211E-03
Mono_CD14	C18orf32	5,21054E-28	3,26822E-01	0,18	0,288	9,59624E-24
Mono_CD14	CCNDBP1	3,60903E-12	3,25809E-01	0,198	0,279	6,64675E-08
Mono_CD14	HNRNPM	5,47690E-12	3,25670E-01	0,342	0,466	1,00868E-07
Mono_CD14	VAMP3	9,20005E-46	3,25082E-01	0,208	0,361	1,69437E-41
Mono_CD14	VKORC1	1,47442E-35	3,23237E-01	0,142	0,251	2,71544E-31
Mono_CD14	PDCD6	8,70121E-23	3,22736E-01	0,299	0,434	1,60250E-18
Mono_CD14	PSMD2	1,19820E-37	3,22528E-01	0,193	0,329	2,20672E-33
Mono_CD14	HADHB	1,12432E-28	3,21787E-01	0,237	0,369	2,07066E-24
Mono_CD14	EPS15	3,39460E-20	3,21265E-01	0,165	0,254	6,25184E-16
Mono_CD14	BASP1	9,35391E-32	3,20230E-01	0,22	0,356	1,72271E-27
Mono_CD14	PNISR	6,55702E-37	3,16278E-01	0,331	0,505	1,20761E-32
Mono_CD14	PLXNC1	1,66308E-15	3,14115E-01	0,173	0,252	3,06290E-11
Mono_CD14	TNFRSF1	3,26374E-28	3,13509E-01	0,311	0,459	6,01083E-24
Mono_CD14	COA4	4,61693E-29	3,13346E-01	0,181	0,294	8,50301E-25
Mono_CD14	MCOLN1	2,78159E-27	3,12620E-01	0,176	0,282	5,12285E-23
Mono_CD14	UQCC2	1,18971E-87	3,12155E-01	0,139	0,323	2,19108E-83
Mono_CD14	RNF181	2,35760E-08	3,10774E-01	0,436	0,571	4,34198E-04
Mono_CD14	TPT1	1,49169E-72	3,10546E-01	0,969	0,974	2,74725E-68
Mono_CD14	DNAJA2	7,20489E-24	3,10289E-01	0,208	0,324	1,32692E-19
Mono_CD14	RPS28	3,44078E-13	3,09231E-01	0,551	0,584	6,33689E-09
Mono_CD14	PRRC2C	8,23820E-18	3,07692E-01	0,444	0,604	1,51723E-13
Mono_CD14	RAB34	7,29630E-30	3,07137E-01	0,169	0,28	1,34376E-25
Mono_CD14	VIM	5,39377E-31	3,06288E-01	0,928	0,944	9,93370E-27
Mono_CD14	NCKAP1L	1,29627E-34	3,05941E-01	0,284	0,44	2,38735E-30
Mono_CD14	TPR	6,35773E-23	3,05702E-01	0,267	0,398	1,17090E-18
Mono_CD14	DECR1	1,01517E-29	3,05082E-01	0,225	0,356	1,86964E-25
Mono_CD14	NUFIP2	1,86930E-08	3,04804E-01	0,191	0,254	3,44270E-04
Mono_CD14	DUSP23	2,35149E-29	3,04220E-01	0,256	0,393	4,33073E-25
Mono_CD14	PSMB4	3,01174E-08	3,03842E-01	0,285	0,379	5,54671E-04
Mono_CD14	ZNF638	4,69077E-26	3,03492E-01	0,156	0,255	8,63900E-22
Mono_CD14	DCTN2	1,51673E-39	3,03322E-01	0,199	0,339	2,79337E-35
Mono_CD14	RBM25	1,35362E-26	3,02855E-01	0,343	0,505	2,49296E-22
Mono_CD14	KHDRBS1	1,14732E-16	3,02801E-01	0,33	0,464	2,11302E-12
Mono_CD14	CWC15	1,79032E-56	3,02414E-01	0,18	0,343	3,29724E-52
Mono_CD14	AGPAT2	8,29976E-16	3,01944E-01	0,205	0,298	1,52857E-11
Mono_CD14	PARP14	3,31213E-28	3,00900E-01	0,255	0,386	6,09995E-24
Mono_CD14	SRGN	7,71970E-134	3,00564E-01	0,96	0,941	1,42174E-129

Mono_CD14	NDUFA3	2,95231E-07	3,00247E-01	0,427	0,555	5,43727E-03
Mono_CD14	GTF2I	6,05834E-22	2,99724E-01	0,212	0,319	1,11576E-17
Mono_CD14	SYNCRIP	6,68906E-25	2,99657E-01	0,213	0,33	1,23192E-20
Mono_CD14	U2SURP	1,09340E-28	2,99642E-01	0,213	0,335	2,01371E-24
Mono_CD14	HEXB	2,41574E-64	2,99131E-01	0,328	0,528	4,44907E-60
Mono_CD14	QKI	1,22739E-09	2,98764E-01	0,282	0,374	2,26048E-05
Mono_CD14	TMSB10	3,61190E-49	2,97880E-01	0,966	0,986	6,65204E-45
Mono_CD14	PFN1	6,17860E-31	2,97660E-01	0,94	0,948	1,13791E-26
Mono_CD14	FAM133I	3,57393E-13	2,97517E-01	0,215	0,301	6,58210E-09
Mono_CD14	ANKRD1(	1,21510E-46	2,96887E-01	0,192	0,342	2,23785E-42
Mono_CD14	SEC13	3,02694E-43	2,96235E-01	0,253	0,413	5,57472E-39
Mono_CD14	PJA2	5,38176E-18	2,96168E-01	0,188	0,282	9,91159E-14
Mono_CD14	CELF2	7,05473E-11	2,94756E-01	0,41	0,549	1,29927E-06
Mono_CD14	STX12	1,98516E-53	2,94749E-01	0,17	0,323	3,65607E-49
Mono_CD14	NIPBL	1,59283E-10	2,94683E-01	0,265	0,357	2,93352E-06
Mono_CD14	CACUL1	2,81500E-62	2,94449E-01	0,155	0,314	5,18439E-58
Mono_CD14	GLG1	7,06903E-08	2,94116E-01	0,223	0,296	1,30190E-03
Mono_CD14	SAFB	8,22573E-15	2,93960E-01	0,171	0,252	1,51493E-10
Mono_CD14	RBM17	2,08112E-22	2,92215E-01	0,284	0,42	3,83279E-18
Mono_CD14	NDUFA1:	2,54945E-35	2,91075E-01	0,334	0,505	4,69532E-31
Mono_CD14	CD53	2,08311E-26	2,90630E-01	0,518	0,69	3,83646E-22
Mono_CD14	TOMM7	5,57505E-14	2,89206E-01	0,705	0,797	1,02676E-09
Mono_CD14	KLHDC3	6,72003E-39	2,87898E-01	0,134	0,25	1,23763E-34
Mono_CD14	SF3A1	1,24451E-18	2,87319E-01	0,198	0,296	2,29201E-14
Mono_CD14	PTBP3	6,90823E-09	2,87170E-01	0,249	0,331	1,27229E-04
Mono_CD14	FAM50A	7,20251E-32	2,87011E-01	0,19	0,313	1,32649E-27
Mono_CD14	SH2B3	2,94041E-25	2,86932E-01	0,171	0,271	5,41536E-21
Mono_CD14	PLEKHO1	1,09016E-27	2,86739E-01	0,247	0,369	2,00774E-23
Mono_CD14	NCOR2	3,87663E-21	2,86046E-01	0,18	0,276	7,13959E-17
Mono_CD14	TMEM21	6,16095E-11	2,85915E-01	0,498	0,647	1,13466E-06
Mono_CD14	GYG1	1,43124E-11	2,85894E-01	0,197	0,278	2,63591E-07
Mono_CD14	SLC8A1	1,24410E-35	2,85643E-01	0,236	0,375	2,29126E-31
Mono_CD14	CTSA	4,74859E-29	2,84834E-01	0,372	0,516	8,74547E-25
Mono_CD14	CLTB	1,20306E-13	2,83399E-01	0,265	0,368	2,21568E-09
Mono_CD14	RHBDD2	1,38346E-11	2,83186E-01	0,191	0,262	2,54792E-07
Mono_CD14	RAC1	7,55996E-17	2,83085E-01	0,554	0,698	1,39232E-12
Mono_CD14	OSTF1	1,80443E-18	2,82637E-01	0,402	0,56	3,32322E-14
Mono_CD14	ARF1	5,92927E-22	2,82311E-01	0,527	0,711	1,09199E-17
Mono_CD14	ARPC5	1,81881E-08	2,82242E-01	0,669	0,769	3,34971E-04
Mono_CD14	MRPS5	4,41062E-11	2,82159E-01	0,187	0,26	8,12304E-07

Mono_CD14	ARL8B	3,75124E-22	2,81897E-01	0,284	0,412	6,90866E-18
Mono_CD14	PSMB7	4,74926E-30	2,81830E-01	0,237	0,372	8,74672E-26
Mono_CD14	CMTM6	4,13212E-16	2,81347E-01	0,475	0,627	7,61013E-12
Mono_CD14	STARD3	1,48883E-43	2,81065E-01	0,129	0,25	2,74198E-39
Mono_CD14	NOP10	3,21837E-17	2,80194E-01	0,589	0,635	5,92728E-13
Mono_CD14	PSMB9	2,18415E-06	2,80015E-01	0,558	0,665	4,02255E-02
Mono_CD14	MAP7D1	4,59853E-15	2,79041E-01	0,198	0,287	8,46911E-11
Mono_CD14	LGALS1	4,67172E-10	2,78064E-01	0,819	0,837	8,60391E-06
Mono_CD14	MRPL54	1,28691E-07	2,77232E-01	0,306	0,41	2,37009E-03
Mono_CD14	MYL6	4,99103E-31	2,76567E-01	0,929	0,942	9,19197E-27
Mono_CD14	PDCD10	7,41519E-50	2,76210E-01	0,175	0,324	1,36565E-45
Mono_CD14	EIF3M	1,25272E-06	2,74315E-01	0,45	0,503	2,30713E-02
Mono_CD14	FNBP4	2,93627E-30	2,73783E-01	0,19	0,308	5,40773E-26
Mono_CD14	SLIRP	4,83417E-23	2,73474E-01	0,299	0,436	8,90308E-19
Mono_CD14	SRSF1	1,07827E-31	2,72600E-01	0,164	0,276	1,98584E-27
Mono_CD14	RPL35A	1,75864E-14	2,71522E-01	0,529	0,565	3,23890E-10
Mono_CD14	RAB1B	2,30088E-07	2,71187E-01	0,276	0,362	4,23754E-03
Mono_CD14	RPL30	9,53492E-10	2,71006E-01	0,551	0,582	1,75605E-05
Mono_CD14	IK	3,28243E-22	2,70982E-01	0,229	0,346	6,04525E-18
Mono_CD14	CLK1	2,72848E-09	2,70224E-01	0,297	0,389	5,02505E-05
Mono_CD14	PHYKPL	1,44628E-18	2,69434E-01	0,177	0,265	2,66361E-14
Mono_CD14	CSRNP1	1,46730E-08	2,69217E-01	0,306	0,29	2,70233E-04
Mono_CD14	FKBP15	2,37662E-07	2,68609E-01	0,211	0,277	4,37702E-03
Mono_CD14	IFNGR1	3,52529E-36	2,67882E-01	0,423	0,593	6,49252E-32
Mono_CD14	CUEDC2	1,06604E-16	2,67808E-01	0,204	0,301	1,96333E-12
Mono_CD14	LY96	4,50120E-12	2,67115E-01	0,345	0,465	8,28987E-08
Mono_CD14	MKRN1	1,96528E-08	2,66589E-01	0,22	0,295	3,61946E-04
Mono_CD14	TNPO1	1,42706E-66	2,64855E-01	0,112	0,26	2,62822E-62
Mono_CD14	RBPJ	5,90755E-27	2,64435E-01	0,326	0,477	1,08799E-22
Mono_CD14	WIPF1	4,63237E-29	2,63996E-01	0,318	0,476	8,53143E-25
Mono_CD14	PRPF4B	2,44351E-23	2,63675E-01	0,188	0,293	4,50021E-19
Mono_CD14	MED28	3,41317E-13	2,63584E-01	0,211	0,301	6,28603E-09
Mono_CD14	GOLGA4	9,58124E-54	2,63561E-01	0,189	0,346	1,76458E-49
Mono_CD14	ETHE1	1,05252E-15	2,63529E-01	0,243	0,348	1,93843E-11
Mono_CD14	UBE2L3	1,94900E-51	2,63254E-01	0,322	0,515	3,58947E-47
Mono_CD14	PEF1	1,18661E-42	2,62430E-01	0,133	0,256	2,18538E-38
Mono_CD14	NDUFB4	1,46815E-40	2,62220E-01	0,411	0,607	2,70389E-36
Mono_CD14	UBL5	7,99513E-11	2,61691E-01	0,678	0,761	1,47246E-06
Mono_CD14	PSAP	2,53527E-15	2,61298E-01	0,889	0,918	4,66920E-11
Mono_CD14	IRF2	1,94045E-29	2,60400E-01	0,211	0,336	3,57373E-25

Mono_CD14	PHIP	3,14293E-07	2,60058E-01	0,255	0,334	5,78834E-03
Mono_CD14	COX6A1	2,31243E-19	2,60056E-01	0,531	0,691	4,25880E-15
Mono_CD14	SNRPB	5,28522E-17	2,59524E-01	0,373	0,518	9,73380E-13
Mono_CD14	MPHOSP	5,57278E-23	2,59113E-01	0,254	0,378	1,02634E-18
Mono_CD14	PLEKHJ1	1,54445E-15	2,58739E-01	0,173	0,255	2,84441E-11
Mono_CD14	C19orf53	1,78813E-20	2,57420E-01	0,427	0,596	3,29321E-16
Mono_CD14	MYADM	2,13436E-40	2,57276E-01	0,395	0,33	3,93084E-36
Mono_CD14	HADHA	5,05847E-12	2,57233E-01	0,39	0,53	9,31618E-08
Mono_CD14	NACA	3,70765E-76	2,56825E-01	0,869	0,923	6,82839E-72
Mono_CD14	SIGLEC10	1,08626E-105	2,56673E-01	0,135	0,334	2,00056E-101
Mono_CD14	CAMK1	2,95222E-10	2,56030E-01	0,193	0,26	5,43710E-06
Mono_CD14	ELOVL5	1,19657E-27	2,55056E-01	0,185	0,295	2,20373E-23
Mono_CD14	AKAP9	2,74038E-21	2,54260E-01	0,273	0,394	5,04695E-17
Mono_CD14	SH3GLB1	6,40828E-23	2,54162E-01	0,294	0,437	1,18021E-18
Mono_CD14	SNRPF	6,07039E-16	2,53552E-01	0,285	0,401	1,11798E-11
Mono_CD14	TAX1BP1	3,92771E-15	2,53187E-01	0,366	0,508	7,23367E-11
Mono_CD14	AUP1	5,83819E-54	2,52866E-01	0,336	0,539	1,07522E-49
Mono_CD14	PNN	6,40692E-26	2,52633E-01	0,207	0,323	1,17996E-21
Mono_CD14	ADAR	1,59173E-22	2,52593E-01	0,302	0,437	2,93148E-18
Mono_CD14	PRPF6	2,82082E-11	2,52229E-01	0,202	0,279	5,19511E-07
Mono_CD14	MAP4	5,01785E-75	2,51156E-01	0,11	0,269	9,24138E-71
Mono_CD14	ACSL1	2,13173E-11	2,50811E-01	0,388	0,389	3,92601E-07
Mono_CD14	TSC22D3	1,60472E-13	2,50408E-01	0,647	0,698	2,95542E-09
Mono_CD14	PLAUR	2,91324E-60	2,50383E-01	0,677	0,638	5,36531E-56
Mono_CD14	PRCP	2,51199E-61	2,50268E-01	0,225	0,402	4,62633E-57
Mono_CD14	GPX1	4,13484E-31	-2,50004E-01	0,512	0,552	7,61513E-27
Mono_CD14	CAPG	1,04747E-175	-2,50698E-01	0,532	0,756	1,92912E-171
Mono_CD14	FYTTD1	1,01657E-57	-2,51414E-01	0,122	0,258	1,87222E-53
Mono_CD14	RALY	6,83740E-48	-2,52544E-01	0,317	0,506	1,25924E-43
Mono_CD14	NENF	1,47572E-71	-2,53609E-01	0,119	0,267	2,71784E-67
Mono_CD14	YWHAQ	7,08871E-65	-2,54292E-01	0,251	0,443	1,30553E-60
Mono_CD14	GTF2A2	3,42705E-11	-2,54774E-01	0,305	0,414	6,31160E-07
Mono_CD14	UBC	1,98493E-43	-2,54861E-01	0,816	0,905	3,65564E-39
Mono_CD14	IFI35	1,03446E-35	-2,55337E-01	0,224	0,355	1,90517E-31
Mono_CD14	HSPA9	1,86331E-55	-2,55870E-01	0,212	0,382	3,43166E-51
Mono_CD14	G3BP2	1,35182E-41	-2,58050E-01	0,16	0,286	2,48965E-37
Mono_CD14	ABCA1	4,31237E-118	-2,59466E-01	0,121	0,329	7,94209E-114
Mono_CD14	UGP2	4,58452E-67	-2,60455E-01	0,148	0,312	8,44332E-63
Mono_CD14	IMP3	1,23667E-56	-2,60547E-01	0,17	0,326	2,27757E-52
Mono_CD14	MT2A	2,76108E-25	-2,61856E-01	0,529	0,635	5,08509E-21

Mono_CD14	SELENOV	3,58618E-39	-2,62818E-01	0,185	0,299	6,60466E-35
Mono_CD14	CD47	1,53116E-75	-2,64021E-01	0,233	0,435	2,81993E-71
Mono_CD14	OAS1	1,38121E-43	-2,65729E-01	0,2	0,336	2,54378E-39
Mono_CD14	NDUFA4	1,55031E-92	-2,66703E-01	0,523	0,761	2,85521E-88
Mono_CD14	PTDSS1	1,55983E-38	-2,68479E-01	0,156	0,278	2,87274E-34
Mono_CD14	ENG	7,81356E-133	-2,68680E-01	0,137	0,363	1,43902E-128
Mono_CD14	IDH2	2,26299E-92	-2,68926E-01	0,216	0,426	4,16774E-88
Mono_CD14	CHCHD1(	3,06586E-77	-2,69106E-01	0,323	0,53	5,64640E-73
Mono_CD14	RANBP2	2,93483E-21	-2,69259E-01	0,216	0,319	5,40507E-17
Mono_CD14	DNAJC3	1,12083E-50	-2,69463E-01	0,213	0,375	2,06424E-46
Mono_CD14	DEGS1	7,72302E-124	-2,70191E-01	0,123	0,341	1,42235E-119
Mono_CD14	GYPC	4,42728E-69	-2,71493E-01	0,154	0,311	8,15372E-65
Mono_CD14	PPP1R15	1,51349E-39	-2,73559E-01	0,678	0,656	2,78740E-35
Mono_CD14	PARP1	2,72334E-111	-2,75921E-01	0,138	0,349	5,01557E-107
Mono_CD14	ARMCX3	8,84450E-65	-2,76926E-01	0,12	0,268	1,62889E-60
Mono_CD14	GLUL	2,85668E-62	-2,77084E-01	0,696	0,812	5,26114E-58
Mono_CD14	VOPP1	3,07065E-166	-2,77352E-01	0,111	0,364	5,65521E-162
Mono_CD14	CSF2RA	1,78755E-125	-2,78269E-01	0,198	0,433	3,29213E-121
Mono_CD14	COX20	1,69136E-61	-2,79031E-01	0,125	0,269	3,11497E-57
Mono_CD14	CLPTM1	1,32601E-73	-2,79463E-01	0,114	0,27	2,44211E-69
Mono_CD14	ATP1B3	1,79244E-08	-2,80671E-01	0,426	0,553	3,30113E-04
Mono_CD14	FEZ2	2,87648E-98	-2,82237E-01	0,161	0,364	5,29761E-94
Mono_CD14	STOML2	1,78903E-59	-2,83268E-01	0,136	0,286	3,29485E-55
Mono_CD14	SPCS1	1,95331E-54	-2,83666E-01	0,42	0,621	3,59742E-50
Mono_CD14	CNDP2	2,05465E-164	-2,84560E-01	0,197	0,473	3,78405E-160
Mono_CD14	RPL22L1	6,08395E-18	-2,86923E-01	0,196	0,28	1,12048E-13
Mono_CD14	VDAC3	5,16812E-49	-2,89668E-01	0,176	0,322	9,51812E-45
Mono_CD14	APPL1	1,51496E-80	-2,89703E-01	0,183	0,375	2,79010E-76
Mono_CD14	CYCS	3,87288E-62	-2,89725E-01	0,334	0,538	7,13268E-58
Mono_CD14	SPCS2	1,81355E-102	-2,91440E-01	0,266	0,509	3,34002E-98
Mono_CD14	LAGE3	2,04955E-96	-2,94004E-01	0,138	0,333	3,77466E-92
Mono_CD14	ARL5A	7,80270E-113	-2,94184E-01	0,218	0,444	1,43702E-108
Mono_CD14	B4GALT1	4,93100E-27	-2,94358E-01	0,218	0,333	9,08143E-23
Mono_CD14	FRMD4B	4,05607E-88	-2,95259E-01	0,119	0,297	7,47007E-84
Mono_CD14	PTPN1	1,02552E-74	-2,95397E-01	0,223	0,414	1,88870E-70
Mono_CD14	C12orf57	2,87911E-28	-2,96200E-01	0,219	0,342	5,30245E-24
Mono_CD14	AREG	8,89344E-30	-2,98400E-01	0,346	0,27	1,63791E-25
Mono_CD14	APMAP	1,05513E-113	-2,98956E-01	0,109	0,314	1,94323E-109
Mono_CD14	GNB4	1,21570E-124	-2,99744E-01	0,133	0,351	2,23895E-120
Mono_CD14	TGFBI	8,93688E-44	-3,00599E-01	0,433	0,611	1,64590E-39

Mono_CD14	SLC39A1	2,72785E-71	-3,01269E-01	0,129	0,288	5,02388E-67
Mono_CD14	BHLHE40	3,82612E-39	-3,01557E-01	0,218	0,349	7,04657E-35
Mono_CD14	ARIH2	4,17783E-33	-3,03272E-01	0,155	0,266	7,69431E-29
Mono_CD14	TMEM17	2,51934E-81	-3,05155E-01	0,366	0,547	4,63987E-77
Mono_CD14	OSTC	8,89719E-68	-3,06126E-01	0,306	0,513	1,63860E-63
Mono_CD14	SNX3	1,18710E-45	-3,06288E-01	0,509	0,703	2,18627E-41
Mono_CD14	CALHM6	1,02018E-19	-3,07875E-01	0,192	0,266	1,87887E-15
Mono_CD14	YIPF3	2,17410E-36	-3,15790E-01	0,198	0,333	4,00405E-32
Mono_CD14	SQSTM1	7,49678E-44	-3,19173E-01	0,498	0,687	1,38068E-39
Mono_CD14	SBDS	2,99001E-40	-3,19421E-01	0,164	0,292	5,50670E-36
Mono_CD14	LITAF	1,39810E-95	-3,20156E-01	0,517	0,758	2,57488E-91
Mono_CD14	EIF4A3	1,44745E-08	-3,20302E-01	0,31	0,409	2,66577E-04
Mono_CD14	TRAM1	1,58697E-82	-3,25297E-01	0,25	0,465	2,92272E-78
Mono_CD14	TMED9	2,08769E-143	-3,26008E-01	0,265	0,543	3,84491E-139
Mono_CD14	CANX	1,25085E-98	-3,26136E-01	0,406	0,656	2,30370E-94
Mono_CD14	SSR4	1,75711E-32	-3,26329E-01	0,588	0,765	3,23606E-28
Mono_CD14	IGFLR1	3,10646E-44	-3,26809E-01	0,225	0,369	5,72117E-40
Mono_CD14	CYB5A	3,45426E-79	-3,27570E-01	0,094	0,251	6,36170E-75
Mono_CD14	PDIA6	3,31328E-148	-3,27675E-01	0,266	0,539	6,10207E-144
Mono_CD14	LAPTM4/	2,41656E-153	-3,31174E-01	0,361	0,648	4,45058E-149
Mono_CD14	PFDN2	1,41704E-63	-3,33608E-01	0,259	0,455	2,60976E-59
Mono_CD14	DDAH2	7,41784E-168	-3,36935E-01	0,163	0,437	1,36614E-163
Mono_CD14	TFPT	2,62240E-123	-3,37394E-01	0,065	0,257	4,82968E-119
Mono_CD14	BCAS2	3,83002E-48	-3,37850E-01	0,141	0,272	7,05374E-44
Mono_CD14	CDK2AP2	1,11320E-52	-3,38060E-01	0,245	0,408	2,05018E-48
Mono_CD14	RAPGEF1	9,06166E-69	-3,38666E-01	0,118	0,269	1,66889E-64
Mono_CD14	DAP3	9,46065E-61	-3,40228E-01	0,167	0,329	1,74237E-56
Mono_CD14	IMPDH2	2,11149E-68	-3,43134E-01	0,129	0,284	3,88873E-64
Mono_CD14	SDF2L1	3,99935E-98	-3,43802E-01	0,166	0,368	7,36560E-94
Mono_CD14	MGST3	9,15566E-102	-3,45466E-01	0,322	0,558	1,68620E-97
Mono_CD14	CREG1	3,30605E-98	-3,46801E-01	0,315	0,527	6,08874E-94
Mono_CD14	DNPH1	1,69764E-132	-3,47331E-01	0,135	0,362	3,12655E-128
Mono_CD14	TMEM13	7,62459E-74	-3,47370E-01	0,122	0,284	1,40422E-69
Mono_CD14	NPL	9,32125E-57	-3,47668E-01	0,132	0,268	1,71670E-52
Mono_CD14	TCP1	5,49029E-45	-3,48397E-01	0,2	0,347	1,01115E-40
Mono_CD14	ATP5MC	3,83728E-37	-3,48488E-01	0,247	0,361	7,06713E-33
Mono_CD14	RNASE6	3,75188E-45	-3,48504E-01	0,391	0,55	6,90984E-41
Mono_CD14	RPSA	4,64378E-32	-3,48848E-01	0,401	0,502	8,55245E-28
Mono_CD14	PCBD1	5,23517E-132	-3,49458E-01	0,144	0,376	9,64161E-128
Mono_CD14	ELL2	2,46065E-62	-3,51630E-01	0,144	0,294	4,53178E-58

Mono_CD14	REL	1,00335E-42	-3,54523E-01	0,437	0,61	1,84786E-38
Mono_CD14	HLA-DMI	3,90934E-190	-3,55665E-01	0,389	0,657	7,19984E-186
Mono_CD14	GTF2B	5,26987E-43	-3,55726E-01	0,158	0,288	9,70552E-39
Mono_CD14	RENBP	1,74796E-68	-3,59766E-01	0,151	0,308	3,21921E-64
Mono_CD14	PDIA4	6,92699E-107	-3,63180E-01	0,133	0,335	1,27574E-102
Mono_CD14	TM6SF1	7,71241E-137	-3,63412E-01	0,075	0,286	1,42039E-132
Mono_CD14	ARFGAP3	1,96859E-49	-3,63701E-01	0,143	0,277	3,62556E-45
Mono_CD14	RNF5	3,28751E-67	-3,67791E-01	0,135	0,294	6,05461E-63
Mono_CD14	MDH1	2,68424E-118	-3,68126E-01	0,173	0,401	4,94357E-114
Mono_CD14	TMED10	1,07447E-135	-3,71093E-01	0,313	0,586	1,97886E-131
Mono_CD14	ISCU	7,43280E-124	-3,73423E-01	0,297	0,555	1,36890E-119
Mono_CD14	C1QBP	2,20825E-85	-3,74088E-01	0,232	0,444	4,06694E-81
Mono_CD14	APH1A	4,50162E-104	-3,77550E-01	0,274	0,515	8,29064E-100
Mono_CD14	MARCKSI	1,69971E-13	-3,78407E-01	0,202	0,27	3,13035E-09
Mono_CD14	NUB1	1,20421E-105	-3,78868E-01	0,13	0,327	2,21780E-101
Mono_CD14	RIPK2	1,13229E-06	-3,79449E-01	0,217	0,278	2,08533E-02
Mono_CD14	G3BP1	1,07667E-102	-3,80997E-01	0,126	0,321	1,98290E-98
Mono_CD14	MIF	4,52776E-57	-3,81269E-01	0,55	0,737	8,33878E-53
Mono_CD14	DYNLRB1	3,19981E-92	-3,82078E-01	0,248	0,478	5,89310E-88
Mono_CD14	HSPA1A	9,47971E-137	-3,84421E-01	0,425	0,681	1,74588E-132
Mono_CD14	HEXA	5,89643E-148	-3,84651E-01	0,23	0,487	1,08595E-143
Mono_CD14	IFITM1	2,80394E-60	-3,86195E-01	0,127	0,264	5,16402E-56
Mono_CD14	SKIL	1,19804E-91	-3,90375E-01	0,151	0,344	2,20643E-87
Mono_CD14	RNASET2	4,56998E-67	-3,91576E-01	0,595	0,751	8,41654E-63
Mono_CD14	RPS27L	2,54888E-81	-3,93685E-01	0,204	0,374	4,69428E-77
Mono_CD14	SLC25A3	6,41148E-162	-3,94257E-01	0,158	0,427	1,18080E-157
Mono_CD14	AHCY	1,22051E-80	-3,96007E-01	0,11	0,273	2,24781E-76
Mono_CD14	TMEM12	3,82606E-37	-3,96008E-01	0,301	0,466	7,04646E-33
Mono_CD14	TRIM22	2,80871E-42	-4,01428E-01	0,267	0,428	5,17280E-38
Mono_CD14	MORF4L1	6,41280E-36	-4,04170E-01	0,163	0,282	1,18104E-31
Mono_CD14	DSTN	2,50289E-90	-4,04327E-01	0,164	0,36	4,60956E-86
Mono_CD14	MYDGF	1,58954E-71	-4,05242E-01	0,166	0,321	2,92745E-67
Mono_CD14	CD63	2,23605E-305	-4,05397E-01	0,582	0,835	4,11813E-301
Mono_CD14	SARAF	1,14816E-48	-4,09892E-01	0,306	0,437	2,11456E-44
Mono_CD14	LSM2	1,22501E-81	-4,09907E-01	0,174	0,364	2,25610E-77
Mono_CD14	TMED3	4,09451E-66	-4,10058E-01	0,13	0,285	7,54085E-62
Mono_CD14	GNA15	2,34336E-31	-4,12353E-01	0,196	0,314	4,31577E-27
Mono_CD14	CCT7	1,00477E-77	-4,12430E-01	0,176	0,362	1,85048E-73
Mono_CD14	P4HB	1,02243E-73	-4,13504E-01	0,428	0,644	1,88300E-69
Mono_CD14	PKIB	5,39780E-206	-4,13998E-01	0,019	0,262	9,94112E-202

Mono_CD14	CMC2	5,59090E-55	-4,15074E-01	0,142	0,285	1,02968E-50
Mono_CD14	PPIB	1,58222E-122	-4,15588E-01	0,593	0,807	2,91398E-118
Mono_CD14	MTHFD2	2,11440E-69	-4,15935E-01	0,231	0,417	3,89409E-65
Mono_CD14	MS4A7	1,14762E-223	-4,16992E-01	0,41	0,681	2,11357E-219
Mono_CD14	HAVCR2	7,03329E-166	-4,18537E-01	0,177	0,454	1,29532E-161
Mono_CD14	TMEM10	8,14009E-157	-4,18807E-01	0,137	0,386	1,49916E-152
Mono_CD14	FKBP2	3,09081E-86	-4,19698E-01	0,213	0,423	5,69234E-82
Mono_CD14	EIF5	1,29428E-46	-4,20860E-01	0,397	0,606	2,38367E-42
Mono_CD14	SWAP70	1,41210E-128	-4,21859E-01	0,126	0,35	2,60066E-124
Mono_CD14	C3AR1	1,31691E-156	-4,25850E-01	0,17	0,434	2,42536E-152
Mono_CD14	MLEC	2,08394E-112	-4,26473E-01	0,149	0,361	3,83799E-108
Mono_CD14	TMEM17	1,25201E-74	-4,26583E-01	0,283	0,461	2,30582E-70
Mono_CD14	ZFAND6	1,69555E-50	-4,30008E-01	0,171	0,322	3,12270E-46
Mono_CD14	CORO1C	2,23048E-52	-4,30412E-01	0,197	0,355	4,10788E-48
Mono_CD14	RAB9A	6,83805E-84	-4,31337E-01	0,092	0,252	1,25936E-79
Mono_CD14	LDHA	4,53265E-65	-4,31938E-01	0,534	0,727	8,34779E-61
Mono_CD14	SEC61B	2,24765E-12	-4,32130E-01	0,611	0,749	4,13949E-08
Mono_CD14	ADAP2	2,57016E-177	-4,32899E-01	0,19	0,464	4,73347E-173
Mono_CD14	CHMP5	9,22561E-81	-4,34016E-01	0,215	0,421	1,69908E-76
Mono_CD14	CREBL2	2,42770E-119	-4,36049E-01	0,101	0,305	4,47109E-115
Mono_CD14	TAF7	1,18931E-35	-4,36334E-01	0,184	0,31	2,19035E-31
Mono_CD14	DBI	3,40350E-78	-4,37516E-01	0,538	0,738	6,26823E-74
Mono_CD14	ELMO1	1,07068E-94	-4,38788E-01	0,154	0,353	1,97188E-90
Mono_CD14	HMOX1	4,49900E-173	-4,39735E-01	0,296	0,563	8,28581E-169
Mono_CD14	RRBP1	1,54109E-79	-4,40395E-01	0,204	0,398	2,83822E-75
Mono_CD14	LAIR1	1,32920E-195	-4,49493E-01	0,244	0,539	2,44800E-191
Mono_CD14	ZC3HAV1	3,40176E-38	-4,49683E-01	0,161	0,279	6,26502E-34
Mono_CD14	SNX9	1,73433E-140	-4,49731E-01	0,083	0,303	3,19411E-136
Mono_CD14	M6PR	5,91519E-111	-4,51688E-01	0,329	0,581	1,08940E-106
Mono_CD14	ST14	2,00380E-102	-4,53542E-01	0,098	0,279	3,69039E-98
Mono_CD14	SDSL	1,39497E-133	-4,56213E-01	0,056	0,252	2,56912E-129
Mono_CD14	APP	7,00672E-19	-4,56716E-01	0,219	0,315	1,29043E-14
Mono_CD14	TUBB4B	4,48597E-34	-4,59736E-01	0,252	0,389	8,26181E-30
Mono_CD14	ADAM28	1,01095E-154	-4,59871E-01	0,043	0,253	1,86186E-150
Mono_CD14	TSTD1	1,87651E-91	-4,60145E-01	0,083	0,251	3,45596E-87
Mono_CD14	NUCKS1	6,07533E-157	-4,60507E-01	0,235	0,52	1,11889E-152
Mono_CD14	MMADH	6,14812E-74	-4,69623E-01	0,179	0,36	1,13230E-69
Mono_CD14	TSPAN3	6,29178E-178	-4,70802E-01	0,082	0,336	1,15876E-173
Mono_CD14	LY6E	3,15235E-55	-4,73407E-01	0,386	0,559	5,80568E-51
Mono_CD14	RAN	1,66333E-81	-4,76041E-01	0,44	0,673	3,06335E-77

Mono_CD14	SYNGR2	2,72699E-239	-4,78820E-01	0,465	0,747	5,02230E-235
Mono_CD14	STOM	1,23737E-158	-4,81084E-01	0,114	0,363	2,27886E-154
Mono_CD14	TMEM14	1,97933E-148	-4,83513E-01	0,272	0,548	3,64533E-144
Mono_CD14	HMGN4	1,70528E-65	-4,86200E-01	0,112	0,258	3,14061E-61
Mono_CD14	MPC2	8,89244E-123	-4,91126E-01	0,149	0,375	1,63772E-118
Mono_CD14	HSP90AA	4,23196E-105	-4,91929E-01	0,717	0,891	7,79400E-101
Mono_CD14	SFT2D2	1,07883E-56	-4,92956E-01	0,154	0,303	1,98689E-52
Mono_CD14	PSMB5	1,26988E-75	-4,94018E-01	0,152	0,327	2,33873E-71
Mono_CD14	TCEAL4	4,78490E-112	-4,94131E-01	0,099	0,293	8,81235E-108
Mono_CD14	RAB13	3,47171E-94	-4,97763E-01	0,132	0,317	6,39385E-90
Mono_CD14	CISD2	3,33794E-158	-4,97800E-01	0,129	0,388	6,14749E-154
Mono_CD14	SERPINB!	1,82395E-24	-4,97842E-01	0,27	0,383	3,35917E-20
Mono_CD14	MARCKS	4,75881E-164	-4,98826E-01	0,275	0,551	8,76430E-160
Mono_CD14	GBP5	4,50124E-65	-5,02989E-01	0,116	0,256	8,28994E-61
Mono_CD14	TMEM51	1,00941E-135	-5,07137E-01	0,071	0,276	1,85903E-131
Mono_CD14	PTGS1	6,35339E-143	-5,07457E-01	0,064	0,271	1,17010E-138
Mono_CD14	ATF5	1,38152E-139	-5,11258E-01	0,145	0,377	2,54434E-135
Mono_CD14	MRPS6	9,47061E-53	-5,11690E-01	0,186	0,335	1,74420E-48
Mono_CD14	GM2A	1,57902E-123	-5,15197E-01	0,166	0,39	2,90808E-119
Mono_CD14	APEX1	6,25297E-89	-5,18866E-01	0,24	0,456	1,15161E-84
Mono_CD14	MFSD1	6,83891E-101	-5,20091E-01	0,374	0,607	1,25952E-96
Mono_CD14	LDHB	2,16877E-79	-5,21970E-01	0,314	0,525	3,99422E-75
Mono_CD14	NPC2	3,68349E-306	-5,23516E-01	0,751	0,894	6,78389E-302
Mono_CD14	ATF3	6,78756E-17	-5,25554E-01	0,339	0,44	1,25006E-12
Mono_CD14	NASP	6,33470E-72	-5,25824E-01	0,126	0,286	1,16666E-67
Mono_CD14	DNAJA1	2,98857E-25	-5,27372E-01	0,459	0,615	5,50405E-21
Mono_CD14	ABHD12	1,19895E-252	-5,29129E-01	0,088	0,401	2,20812E-248
Mono_CD14	COPZ1	7,09471E-64	-5,31060E-01	0,21	0,395	1,30663E-59
Mono_CD14	FPR3	5,97009E-224	-5,33901E-01	0,071	0,358	1,09951E-219
Mono_CD14	CHCHD3	1,87804E-48	-5,36351E-01	0,13	0,258	3,45878E-44
Mono_CD14	LMAN1	1,00876E-142	-5,41008E-01	0,086	0,304	1,85784E-138
Mono_CD14	AKR1B1	5,71748E-297	-5,43366E-01	0,143	0,492	1,05299E-292
Mono_CD14	DCK	1,52282E-103	-5,44870E-01	0,106	0,292	2,80458E-99
Mono_CD14	CLPP	1,89085E-52	-5,45685E-01	0,136	0,275	3,48238E-48
Mono_CD14	TCF4	4,27230E-141	-5,46092E-01	0,077	0,287	7,86830E-137
Mono_CD14	ARID5A	6,97607E-29	-5,52963E-01	0,182	0,295	1,28478E-24
Mono_CD14	SGK1	6,59097E-130	-5,61170E-01	0,392	0,62	1,21386E-125
Mono_CD14	ALCAM	7,95569E-158	-5,61351E-01	0,06	0,283	1,46520E-153
Mono_CD14	PDIA3	4,00422E-209	-5,62863E-01	0,391	0,696	7,37458E-205
Mono_CD14	CTSZ	1,08971E-100	-5,67864E-01	0,452	0,667	2,00692E-96

Mono_CD14	ARL6IP1	7,29816E-129	-5,74951E-01	0,186	0,432	1,34410E-124
Mono_CD14	RALA	2,62205E-123	-5,82592E-01	0,165	0,39	4,82903E-119
Mono_CD14	FBL	6,73516E-23	-5,84145E-01	0,235	0,356	1,24041E-18
Mono_CD14	PMP22	3,17364E-190	-5,86449E-01	0,048	0,294	5,84489E-186
Mono_CD14	PTGER4	6,04705E-91	-5,87149E-01	0,109	0,281	1,11369E-86
Mono_CD14	CXCL2	2,54213E-08	-5,91402E-01	0,265	0,224	4,68183E-04
Mono_CD14	AKR1A1	2,77707E-185	-5,92048E-01	0,304	0,597	5,11453E-181
Mono_CD14	GLO1	1,19040E-75	-5,93532E-01	0,102	0,254	2,19237E-71
Mono_CD14	ACP2	1,78033E-134	-5,93894E-01	0,07	0,271	3,27883E-130
Mono_CD14	MGAT4A	4,13596E-179	-5,94646E-01	0,111	0,375	7,61720E-175
Mono_CD14	EPSTI1	1,40388E-144	-5,96013E-01	0,18	0,426	2,58552E-140
Mono_CD14	AXL	8,63873E-267	-5,97697E-01	0,009	0,3	1,59100E-262
Mono_CD14	GBP1	1,40685E-63	-6,03112E-01	0,199	0,356	2,59100E-59
Mono_CD14	TAP1	3,03804E-69	-6,03908E-01	0,251	0,435	5,59516E-65
Mono_CD14	BEX4	1,22607E-203	-6,06577E-01	0,075	0,347	2,25806E-199
Mono_CD14	MANF	9,24056E-108	-6,10898E-01	0,113	0,308	1,70183E-103
Mono_CD14	CACYBP	5,64903E-116	-6,20432E-01	0,185	0,417	1,04038E-111
Mono_CD14	LGALS3B	2,75402E-247	-6,21213E-01	0,048	0,34	5,07208E-243
Mono_CD14	FAM162/	1,96183E-158	-6,22568E-01	0,114	0,362	3,61310E-154
Mono_CD14	RGS10	1,38601E-282	-6,42876E-01	0,449	0,761	2,55261E-278
Mono_CD14	FBP1	6,20658E-46	-6,46597E-01	0,221	0,359	1,14307E-41
Mono_CD14	LHFPL2	1,31739E-160	-6,56968E-01	0,069	0,299	2,42624E-156
Mono_CD14	BST2	1,58904E-186	-6,58555E-01	0,476	0,743	2,92654E-182
Mono_CD14	LMNA	1,45460E-160	-6,60127E-01	0,133	0,386	2,67894E-156
Mono_CD14	STAT1	1,65218E-107	-6,61256E-01	0,313	0,533	3,04282E-103
Mono_CD14	CXCL16	0,00000E+00	-6,61634E-01	0,306	0,676	0,00000E+00
Mono_CD14	IL18	2,68804E-266	-6,72519E-01	0,135	0,464	4,95056E-262
Mono_CD14	VSIG4	1,33784E-229	-6,72817E-01	0,08	0,367	2,46391E-225
Mono_CD14	KCNMA1	1,47363E-210	-6,74656E-01	0,026	0,277	2,71398E-206
Mono_CD14	RASGEF1	1,61284E-41	-6,76071E-01	0,156	0,277	2,97037E-37
Mono_CD14	NR3C1	5,89692E-51	-6,78657E-01	0,237	0,399	1,08604E-46
Mono_CD14	PEBP1	0,00000E+00	-6,82594E-01	0,2	0,591	0,00000E+00
Mono_CD14	GPR137E	2,94658E-182	-6,88452E-01	0,068	0,315	5,42672E-178
Mono_CD14	DNASE2	2,58735E-178	-6,90791E-01	0,125	0,385	4,76512E-174
Mono_CD14	DOCK4	1,13590E-188	-6,92005E-01	0,053	0,302	2,09199E-184
Mono_CD14	CTSB	1,86841E-283	-6,92032E-01	0,729	0,882	3,44105E-279
Mono_CD14	NPM1	7,69272E-187	-6,97216E-01	0,517	0,784	1,41677E-182
Mono_CD14	CSTB	1,16804E-94	-7,08749E-01	0,626	0,801	2,15119E-90
Mono_CD14	GBP4	2,85332E-126	-7,10662E-01	0,09	0,291	5,25496E-122
Mono_CD14	IL2RG	8,06151E-98	-7,11452E-01	0,146	0,337	1,48469E-93

Mono_CD14	LAP3	1,75908E-143	-7,12542E-01	0,343	0,603	3,23970E-139
Mono_CD14	PPA1	3,39655E-81	-7,13291E-01	0,287	0,474	6,25543E-77
Mono_CD14	TUBA1C	3,69402E-147	-7,17924E-01	0,15	0,396	6,80328E-143
Mono_CD14	CTSD	2,34012E-92	-7,29369E-01	0,68	0,776	4,30980E-88
Mono_CD14	TUBA1B	0,00000E+00	-7,31816E-01	0,404	0,782	0,00000E+00
Mono_CD14	SLC1A3	8,88173E-137	-7,32886E-01	0,144	0,379	1,63575E-132
Mono_CD14	PLAC8	3,24490E-28	-7,34423E-01	0,276	0,199	5,97614E-24
Mono_CD14	PLIN2	3,62053E-44	-7,35102E-01	0,345	0,509	6,66792E-40
Mono_CD14	DNAJB1	5,54528E-84	-7,39824E-01	0,371	0,572	1,02127E-79
Mono_CD14	HSPH1	3,16142E-134	-7,40337E-01	0,182	0,432	5,82238E-130
Mono_CD14	ISG20	3,51251E-72	-7,47426E-01	0,158	0,322	6,46898E-68
Mono_CD14	HMGN1	1,17625E-282	-7,59089E-01	0,286	0,65	2,16630E-278
Mono_CD14	HSPA5	3,86369E-129	-7,59762E-01	0,321	0,579	7,11575E-125
Mono_CD14	GLA	5,26611E-62	-7,65640E-01	0,166	0,322	9,69860E-58
Mono_CD14	UBB	4,57520E-151	-7,65750E-01	0,582	0,827	8,42614E-147
Mono_CD14	HSPE1	1,54536E-169	-7,66720E-01	0,329	0,623	2,84609E-165
Mono_CD14	FCGR3A	0,00000E+00	-7,70206E-01	0,186	0,603	0,00000E+00
Mono_CD14	HLA-DRA	0,00000E+00	-7,71686E-01	0,841	0,931	0,00000E+00
Mono_CD14	CPM	8,38788E-145	-7,79195E-01	0,147	0,386	1,54480E-140
Mono_CD14	CALR	0,00000E+00	-7,82840E-01	0,4	0,747	0,00000E+00
Mono_CD14	C15orf48	1,88107E-124	-7,84159E-01	0,141	0,359	3,46437E-120
Mono_CD14	PHB	2,07484E-113	-7,85485E-01	0,18	0,404	3,82123E-109
Mono_CD14	LILRB4	8,48673E-245	-8,02605E-01	0,262	0,57	1,56300E-240
Mono_CD14	HLA-DRB	0,00000E+00	-8,04518E-01	0,812	0,925	0,00000E+00
Mono_CD14	INSIG1	5,28527E-55	-8,12324E-01	0,163	0,305	9,73388E-51
Mono_CD14	ARHGAP	0,00000E+00	-8,16307E-01	0,125	0,502	0,00000E+00
Mono_CD14	HSPA8	1,91192E-285	-8,33476E-01	0,556	0,839	3,52119E-281
Mono_CD14	EZR	1,54468E-11	-8,37355E-01	0,309	0,406	2,84483E-07
Mono_CD14	CTSC	0,00000E+00	-8,40992E-01	0,346	0,702	0,00000E+00
Mono_CD14	PRDX1	0,00000E+00	-8,50535E-01	0,453	0,768	0,00000E+00
Mono_CD14	ARL4C	9,73037E-214	-8,61882E-01	0,077	0,351	1,79204E-209
Mono_CD14	HSP90B1	1,37023E-201	-8,67518E-01	0,422	0,72	2,52356E-197
Mono_CD14	PTMS	2,68012E-271	-8,70370E-01	0,066	0,384	4,93597E-267
Mono_CD14	LIPA	5,92044E-229	-8,71795E-01	0,228	0,521	1,09037E-224
Mono_CD14	CCDC50	2,92825E-91	-8,78666E-01	0,113	0,286	5,39295E-87
Mono_CD14	TUBB	0,00000E+00	-8,79637E-01	0,298	0,687	0,00000E+00
Mono_CD14	MX1	7,88098E-94	-8,80940E-01	0,159	0,345	1,45144E-89
Mono_CD14	HLA-DRB	0,00000E+00	-8,84692E-01	0,653	0,861	0,00000E+00
Mono_CD14	IFI6	4,58540E-92	-8,86936E-01	0,293	0,503	8,44494E-88
Mono_CD14	HLA-DQB	0,00000E+00	-8,91833E-01	0,559	0,835	0,00000E+00

Mono_CD14	PDK4	1,52957E-97	-9,01699E-01	0,15	0,322	2,81701E-93
Mono_CD14	CD40	2,35993E-149	-9,08364E-01	0,085	0,307	4,34629E-145
Mono_CD14	MSR1	0,00000E+00	-9,10738E-01	0,058	0,404	0,00000E+00
Mono_CD14	ADORA3	2,14685E-184	-9,33258E-01	0,043	0,279	3,95384E-180
Mono_CD14	SLAMF8	6,36487E-255	-9,35084E-01	0,046	0,345	1,17222E-250
Mono_CD14	YWHAH	5,62134E-261	-9,40143E-01	0,222	0,554	1,03528E-256
Mono_CD14	HSPD1	3,65295E-128	-9,50624E-01	0,292	0,553	6,72764E-124
Mono_CD14	GPR65	7,47708E-79	-9,54788E-01	0,161	0,339	1,37705E-74
Mono_CD14	CD74	0,00000E+00	-9,57076E-01	0,937	0,965	0,00000E+00
Mono_CD14	MTRNR2	2,14340E-40	-9,67043E-01	0,337	0,448	3,94750E-36
Mono_CD14	NR4A3	7,24846E-46	-9,72876E-01	0,179	0,305	1,33495E-41
Mono_CD14	DAB2	0,00000E+00	-1,00110E+00	0,085	0,459	0,00000E+00
Mono_CD14	HSP90AE	2,85892E-265	-1,01325E+00	0,559	0,845	5,26527E-261
Mono_CD14	GPR34	9,28251E-257	-1,02316E+00	0,032	0,326	1,70956E-252
Mono_CD14	GSN	0,00000E+00	-1,03100E+00	0,259	0,661	0,00000E+00
Mono_CD14	CD59	5,41871E-249	-1,05511E+00	0,06	0,361	9,97963E-245
Mono_CD14	FCGR2B	8,99521E-270	-1,07688E+00	0,117	0,448	1,65665E-265
Mono_CD14	HERPUD1	4,28496E-220	-1,09244E+00	0,44	0,699	7,89161E-216
Mono_CD14	DDIT4	3,51482E-37	-1,11926E+00	0,35	0,507	6,47324E-33
Mono_CD14	HLA-DPA	0,00000E+00	-1,12365E+00	0,684	0,913	0,00000E+00
Mono_CD14	CD81	0,00000E+00	-1,13895E+00	0,119	0,486	0,00000E+00
Mono_CD14	MS4A4A	3,55903E-239	-1,14506E+00	0,136	0,441	6,55467E-235
Mono_CD14	IFI44L	1,93078E-112	-1,16752E+00	0,082	0,266	3,55592E-108
Mono_CD14	FABP5	5,92566E-277	-1,18762E+00	0,136	0,469	1,09133E-272
Mono_CD14	CKS2	3,97839E-69	-1,18935E+00	0,107	0,253	7,32700E-65
Mono_CD14	OLR1	2,65345E-155	-1,20695E+00	0,119	0,364	4,88687E-151
Mono_CD14	SERPING	0,00000E+00	-1,20848E+00	0,068	0,421	0,00000E+00
Mono_CD14	C1orf54	6,79093E-216	-1,23509E+00	0,038	0,3	1,25069E-211
Mono_CD14	TXN	3,57361E-28	-1,24889E+00	0,544	0,676	6,58152E-24
Mono_CD14	ACP5	3,01936E-209	-1,26530E+00	0,133	0,419	5,56076E-205
Mono_CD14	HLA-DOA	0,00000E+00	-1,28948E+00	0,049	0,418	0,00000E+00
Mono_CD14	CD83	1,03752E-41	-1,30979E+00	0,364	0,498	1,91079E-37
Mono_CD14	HLA-DPB	0,00000E+00	-1,31061E+00	0,643	0,901	0,00000E+00
Mono_CD14	STMN1	6,73873E-238	-1,32886E+00	0,028	0,305	1,24107E-233
Mono_CD14	ZNF331	4,25480E-50	-1,34019E+00	0,205	0,355	7,83606E-46
Mono_CD14	HLA-DQE	5,96858E-229	-1,37038E+00	0,057	0,336	1,09923E-224
Mono_CD14	CXCR4	2,65957E-35	-1,39063E+00	0,432	0,591	4,89813E-31
Mono_CD14	LTC4S	8,87809E-259	-1,41750E+00	0,018	0,303	1,63508E-254
Mono_CD14	IL4I1	1,82445E-180	-1,46646E+00	0,026	0,25	3,36009E-176
Mono_CD14	CD9	0,00000E+00	-1,49140E+00	0,09	0,514	0,00000E+00

Mono_CD14	IRF7	1,27348E-27	-1,51582E+00	0,29	0,407	2,34538E-23
Mono_CD14	CCL4	7,34661E-50	-1,52943E+00	0,19	0,319	1,35302E-45
Mono_CD14	SLCO2B1	0,00000E+00	-1,54967E+00	0,02	0,395	0,00000E+00
Mono_CD14	HLA-DQA	5,11341E-250	-1,55290E+00	0,213	0,534	9,41737E-246
Mono_CD14	ALOX5AP	2,54252E-230	-1,58425E+00	0,211	0,526	4,68256E-226
Mono_CD14	ISG15	1,08150E-98	-1,59292E+00	0,237	0,442	1,99180E-94
Mono_CD14	CREM	3,93634E-83	-1,60302E+00	0,2	0,391	7,24956E-79
Mono_CD14	CCL3	8,54086E-30	-1,64003E+00	0,285	0,382	1,57297E-25
Mono_CD14	IRF8	8,87340E-55	-1,64508E+00	0,231	0,389	1,63421E-50
Mono_CD14	HSPB1	0,00000E+00	-1,65121E+00	0,262	0,687	0,00000E+00
Mono_CD14	PLTP	0,00000E+00	-1,67442E+00	0,018	0,351	0,00000E+00
Mono_CD14	HLA-DQA	0,00000E+00	-1,79484E+00	0,394	0,812	0,00000E+00
Mono_CD14	CTSL	4,67500E-286	-1,98226E+00	0,188	0,543	8,60994E-282
Mono_CD14	A2M	0,00000E+00	-2,02753E+00	0,016	0,417	0,00000E+00
Mono_CD14	LGMN	0,00000E+00	-2,04120E+00	0,031	0,437	0,00000E+00
Mono_CD14	PLD4	2,86766E-270	-2,06844E+00	0,048	0,353	5,28138E-266
Mono_CD14	TREM2	0,00000E+00	-2,21583E+00	0,021	0,435	0,00000E+00
Mono_CD14	GPR183	4,04012E-177	-2,22813E+00	0,21	0,475	7,44068E-173
Mono_CD14	C3	0,00000E+00	-2,32249E+00	0,038	0,409	0,00000E+00
Mono_CD14	GPNMB	0,00000E+00	-2,39430E+00	0,018	0,358	0,00000E+00
Mono_CD14	C1QA	0,00000E+00	-2,93380E+00	0,086	0,601	0,00000E+00
Mono_CD14	SERPINF1	0,00000E+00	-2,96333E+00	0,047	0,43	0,00000E+00
Mono_CD14	RGS1	0,00000E+00	-3,04647E+00	0,075	0,684	0,00000E+00
Mono_CD14	RNASE1	9,49378E-185	-3,33975E+00	0,058	0,288	1,74847E-180
Mono_CD14	C1QB	0,00000E+00	-3,44870E+00	0,055	0,582	0,00000E+00
Mono_CD14	C1QC	0,00000E+00	-3,64591E+00	0,046	0,582	0,00000E+00
Mono_CD14	APOC1	0,00000E+00	-4,17146E+00	0,046	0,541	0,00000E+00
Mono_CD14	APOE	0,00000E+00	-5,08328E+00	0,065	0,539	0,00000E+00

Mono_CD16

cluster	gene	p_val	avg_log2FC	pct.1	pct.2	p_val_adj
Mono_CD16	LST1	0,00000E+00	2,22620E+00	0,918	0,707	0,00000E+00
Mono_CD16	CD79B	0,00000E+00	2,10581E+00	0,266	0,02	0,00000E+00
Mono_CD16	IFITM2	0,00000E+00	2,04939E+00	0,917	0,636	0,00000E+00
Mono_CD16	MTSS1	0,00000E+00	1,96963E+00	0,608	0,183	0,00000E+00
Mono_CD16	TCF7L2	0,00000E+00	1,82905E+00	0,476	0,069	0,00000E+00
Mono_CD16	GPBAR1	0,00000E+00	1,75885E+00	0,363	0,084	0,00000E+00
Mono_CD16	FCGR3A	2,42720E-292	1,75262E+00	0,818	0,565	4,47017E-288
Mono_CD16	C19orf38	0,00000E+00	1,72522E+00	0,587	0,22	0,00000E+00
Mono_CD16	LILRB2	0,00000E+00	1,70392E+00	0,765	0,445	0,00000E+00
Mono_CD16	LILRA1	0,00000E+00	1,69241E+00	0,425	0,101	0,00000E+00
Mono_CD16	FAM110A	4,13980E-215	1,66570E+00	0,408	0,158	7,62427E-211
Mono_CD16	FCN1	0,00000E+00	1,66079E+00	0,813	0,229	0,00000E+00
Mono_CD16	RHOC	1,45941E-126	1,65566E+00	0,486	0,302	2,68780E-122
Mono_CD16	CD52	0,00000E+00	1,61898E+00	0,755	0,376	0,00000E+00
Mono_CD16	STXBP2	0,00000E+00	1,60301E+00	0,769	0,406	0,00000E+00
Mono_CD16	LILRA5	0,00000E+00	1,59931E+00	0,681	0,166	0,00000E+00
Mono_CD16	PILRA	1,25837E-201	1,58746E+00	0,703	0,485	2,31755E-197
Mono_CD16	CLEC12A	1,38322E-149	1,55099E+00	0,473	0,251	2,54748E-145
Mono_CD16	SERPINA1	0,00000E+00	1,54654E+00	0,899	0,714	0,00000E+00
Mono_CD16	CDKN1C	0,00000E+00	1,53035E+00	0,366	0,05	0,00000E+00
Mono_CD16	CDA	0,00000E+00	1,51850E+00	0,366	0,078	0,00000E+00
Mono_CD16	LRRC25	1,03575E-261	1,48455E+00	0,6	0,323	1,90755E-257
Mono_CD16	CYTIP	1,05363E-302	1,46915E+00	0,679	0,38	1,94046E-298
Mono_CD16	LYST	0,00000E+00	1,45515E+00	0,572	0,217	0,00000E+00
Mono_CD16	LTA4H	2,29535E-173	1,44957E+00	0,66	0,439	4,22734E-169
Mono_CD16	CUX1	2,63628E-199	1,44229E+00	0,568	0,342	4,85524E-195
Mono_CD16	CTSS	0,00000E+00	1,44039E+00	0,897	0,813	0,00000E+00
Mono_CD16	ABI3	9,67903E-139	1,43315E+00	0,582	0,385	1,78259E-134
Mono_CD16	ICAM2	2,85247E-282	1,43232E+00	0,438	0,155	5,25339E-278
Mono_CD16	SPN	1,88203E-239	1,41792E+00	0,418	0,157	3,46614E-235
Mono_CD16	SYTL1	0,00000E+00	1,40710E+00	0,28	0,032	0,00000E+00
Mono_CD16	MS4A7	1,59598E-174	1,40402E+00	0,812	0,657	2,93932E-170
Mono_CD16	HK3	1,48064E-209	1,38754E+00	0,351	0,119	2,72689E-205
Mono_CD16	NAAA	9,50995E-233	1,37828E+00	0,611	0,358	1,75145E-228
Mono_CD16	AIF1	0,00000E+00	1,37624E+00	0,947	0,817	0,00000E+00
Mono_CD16	TESC	0,00000E+00	1,37265E+00	0,395	0,089	0,00000E+00
Mono_CD16	FGR	5,79520E-240	1,36200E+00	0,692	0,444	1,06730E-235
Mono_CD16	LIMD2	0,00000E+00	1,35855E+00	0,746	0,471	0,00000E+00
Mono_CD16	ITGAL	3,13144E-270	1,34885E+00	0,425	0,15	5,76717E-266
Mono_CD16	COTL1	0,00000E+00	1,33254E+00	0,9	0,704	0,00000E+00
Mono_CD16	TSPAN14	1,78333E-71	1,32000E+00	0,459	0,328	3,28435E-67

Mono_CD16	CFD	1,44628E-222	1,31553E+00	0,81	0,553	2,66361E-218
Mono_CD16	NAP1L1	4,39941E-240	1,31530E+00	0,769	0,613	8,10240E-236
Mono_CD16	S100A4	0,00000E+00	1,31508E+00	0,948	0,822	0,00000E+00
Mono_CD16	GNG2	5,10371E-187	1,30886E+00	0,363	0,137	9,39951E-183
Mono_CD16	SIDT2	1,33046E-230	1,30859E+00	0,422	0,166	2,45031E-226
Mono_CD16	CPPED1	5,37450E-186	1,30020E+00	0,5	0,261	9,89822E-182
Mono_CD16	MAPKAPK3	3,97930E-152	1,29199E+00	0,491	0,281	7,32868E-148
Mono_CD16	POU2F2	4,72265E-308	1,28903E+00	0,583	0,258	8,69771E-304
Mono_CD16	FPR2	3,45070E-269	1,25566E+00	0,309	0,079	6,35515E-265
Mono_CD16	CFP	0,00000E+00	1,24951E+00	0,606	0,191	0,00000E+00
Mono_CD16	PTPN6	2,80408E-162	1,24941E+00	0,669	0,504	5,16428E-158
Mono_CD16	EVL	1,00130E-64	1,23613E+00	0,474	0,353	1,84409E-60
Mono_CD16	RNF144B	7,05553E-153	1,22685E+00	0,559	0,35	1,29942E-148
Mono_CD16	HCK	4,66081E-158	1,21697E+00	0,679	0,514	8,58382E-154
Mono_CD16	LILRA3	0,00000E+00	1,20216E+00	0,289	0,027	0,00000E+00
Mono_CD16	ABRACL	7,09151E-165	1,20029E+00	0,669	0,491	1,30604E-160
Mono_CD16	RAB24	3,42287E-152	1,19691E+00	0,481	0,262	6,30390E-148
Mono_CD16	LILRA2	3,50616E-140	1,19512E+00	0,457	0,239	6,45729E-136
Mono_CD16	CX3CR1	1,10650E-90	1,18420E+00	0,292	0,135	2,03783E-86
Mono_CD16	BIN2	1,10014E-181	1,18383E+00	0,535	0,305	2,02612E-177
Mono_CD16	DPEP2	2,41275E-147	1,18143E+00	0,362	0,156	4,44356E-143
Mono_CD16	CSK	5,34747E-58	1,17689E+00	0,452	0,343	9,84843E-54
Mono_CD16	IFITM3	0,00000E+00	1,17412E+00	0,963	0,757	0,00000E+00
Mono_CD16	PRAM1	6,28702E-195	1,17247E+00	0,412	0,169	1,15788E-190
Mono_CD16	CASP1	7,99530E-139	1,17147E+00	0,639	0,456	1,47249E-134
Mono_CD16	TKT	1,93724E-221	1,16152E+00	0,762	0,601	3,56781E-217
Mono_CD16	CD300LF	2,33847E-85	1,15344E+00	0,323	0,17	4,30677E-81
Mono_CD16	PRELID1	8,73234E-226	1,14901E+00	0,832	0,679	1,60824E-221
Mono_CD16	UTRN	8,82264E-99	1,14665E+00	0,455	0,282	1,62487E-94
Mono_CD16	APOBEC3A	0,00000E+00	1,13379E+00	0,466	0,125	0,00000E+00
Mono_CD16	TNFRSF1B	6,43301E-193	1,13149E+00	0,727	0,533	1,18477E-188
Mono_CD16	UNC119	1,26154E-141	1,12601E+00	0,463	0,26	2,32338E-137
Mono_CD16	SEC14L1	9,35120E-82	1,12427E+00	0,529	0,39	1,72221E-77
Mono_CD16	AMPD2	3,92503E-30	1,11413E+00	0,27	0,18	7,22873E-26
Mono_CD16	SCIMP	1,85513E-145	1,09693E+00	0,488	0,27	3,41659E-141
Mono_CD16	PTPRC	3,55460E-231	1,08619E+00	0,812	0,685	6,54651E-227
Mono_CD16	NCF2	2,62188E-156	1,08117E+00	0,666	0,457	4,82871E-152
Mono_CD16	CD48	3,62925E-242	1,08087E+00	0,711	0,47	6,68400E-238
Mono_CD16	CARD16	3,40900E-285	1,07565E+00	0,775	0,543	6,27836E-281
Mono_CD16	MYO1G	4,46644E-191	1,07550E+00	0,539	0,273	8,22583E-187
Mono_CD16	ICAM3	1,96070E-266	1,07135E+00	0,479	0,185	3,61102E-262
Mono_CD16	TSC22D3	4,84254E-104	1,06861E+00	0,789	0,69	8,91850E-100

Mono_CD16	SIGLEC10	2,69235E-83	1,06369E+00	0,471	0,315	4,95850E-79
Mono_CD16	SLC2A6	5,68994E-167	1,05462E+00	0,329	0,123	1,04792E-162
Mono_CD16	TCIRG1	3,17510E-63	1,05073E+00	0,565	0,463	5,84758E-59
Mono_CD16	TRAF3IP3	1,66830E-82	1,04115E+00	0,346	0,19	3,07251E-78
Mono_CD16	SLC7A7	5,80524E-82	1,03242E+00	0,589	0,461	1,06915E-77
Mono_CD16	CDH23	0,00000E+00	1,02229E+00	0,283	0,038	0,00000E+00
Mono_CD16	TBC1D10C	7,41469E-159	1,02223E+00	0,271	0,092	1,36556E-154
Mono_CD16	CNIH4	2,05214E-160	1,01403E+00	0,498	0,278	3,77943E-156
Mono_CD16	SMAP2	3,77016E-132	9,98693E-01	0,64	0,464	6,94351E-128
Mono_CD16	PAG1	1,97609E-128	9,84798E-01	0,466	0,263	3,63937E-124
Mono_CD16	TBC1D8	6,27063E-172	9,79767E-01	0,398	0,172	1,15486E-167
Mono_CD16	AGTRAP	1,96735E-72	9,78621E-01	0,501	0,356	3,62328E-68
Mono_CD16	PPP1CA	1,76206E-77	9,77987E-01	0,683	0,62	3,24518E-73
Mono_CD16	MTPN	7,41059E-173	9,77950E-01	0,68	0,52	1,36481E-168
Mono_CD16	DOK2	5,46841E-83	9,76014E-01	0,499	0,339	1,00712E-78
Mono_CD16	CORO1A	0,00000E+00	9,63132E-01	0,847	0,638	0,00000E+00
Mono_CD16	RALB	2,30055E-42	9,62502E-01	0,329	0,225	4,23693E-38
Mono_CD16	ARL4A	6,52695E-181	9,62042E-01	0,474	0,231	1,20207E-176
Mono_CD16	GIMAP7	1,76537E-72	9,58241E-01	0,408	0,25	3,25129E-68
Mono_CD16	OAS1	2,43890E-147	9,55288E-01	0,54	0,319	4,49172E-143
Mono_CD16	SLC44A2	9,14571E-237	9,54847E-01	0,32	0,093	1,68436E-232
Mono_CD16	TES	5,29754E-56	9,52174E-01	0,398	0,275	9,75648E-52
Mono_CD16	MYO1F	5,77287E-85	9,51663E-01	0,586	0,455	1,06319E-80
Mono_CD16	STX11	3,44933E-49	9,42791E-01	0,439	0,324	6,35262E-45
Mono_CD16	IFITM1	3,25360E-184	9,23393E-01	0,535	0,243	5,99215E-180
Mono_CD16	FLNA	2,36679E-124	9,20975E-01	0,664	0,443	4,35891E-120
Mono_CD16	SLA	1,18781E-20	9,13660E-01	0,47	0,415	2,18760E-16
Mono_CD16	RAC2	3,78743E-187	9,11983E-01	0,712	0,488	6,97532E-183
Mono_CD16	UBXN11	1,89570E-51	9,09832E-01	0,326	0,202	3,49130E-47
Mono_CD16	LILRB1	4,84681E-95	9,06083E-01	0,531	0,371	8,92638E-91
Mono_CD16	CASP4	2,97007E-18	9,03503E-01	0,478	0,442	5,46998E-14
Mono_CD16	RXRA	9,72722E-69	8,90630E-01	0,293	0,157	1,79146E-64
Mono_CD16	GCH1	1,06993E-93	8,81795E-01	0,314	0,153	1,97049E-89
Mono_CD16	ZBTB16	6,96951E-88	8,80464E-01	0,288	0,136	1,28357E-83
Mono_CD16	ARRB1	9,97709E-52	8,70150E-01	0,276	0,161	1,83748E-47
Mono_CD16	S1PR4	5,43152E-159	8,67545E-01	0,27	0,091	1,00032E-154
Mono_CD16	HES4	9,30216E-149	8,63156E-01	0,275	0,093	1,71318E-144
Mono_CD16	C5AR1	1,82252E-84	8,61330E-01	0,581	0,401	3,35654E-80
Mono_CD16	ZFAND5	1,27911E-111	8,46897E-01	0,703	0,581	2,35574E-107
Mono_CD16	MRPS35	4,20664E-27	8,45524E-01	0,285	0,211	7,74736E-23
Mono_CD16	ARHGEF1	1,38046E-45	8,43762E-01	0,376	0,267	2,54239E-41
Mono_CD16	FGD2	4,65443E-34	8,38885E-01	0,426	0,341	8,57206E-30

Mono_CD16	MSN	6,21448E-81	8,36381E-01	0,703	0,635	1,14452E-76
Mono_CD16	RHOB	1,39054E-67	8,35723E-01	0,655	0,537	2,56096E-63
Mono_CD16	TXNIP	8,51853E-67	8,34729E-01	0,806	0,695	1,56886E-62
Mono_CD16	GIMAP2	1,04848E-85	8,31356E-01	0,344	0,189	1,93098E-81
Mono_CD16	FKBP1A	7,68619E-154	8,29868E-01	0,796	0,69	1,41556E-149
Mono_CD16	CD55	5,41896E-174	8,29263E-01	0,656	0,42	9,98010E-170
Mono_CD16	FAM204A	3,87163E-15	8,28467E-01	0,331	0,286	7,13038E-11
Mono_CD16	RAP1B	2,16618E-105	8,24815E-01	0,678	0,575	3,98945E-101
Mono_CD16	DUSP6	1,24615E-72	8,23738E-01	0,451	0,282	2,29503E-68
Mono_CD16	CD37	1,72627E-209	8,20940E-01	0,835	0,729	3,17927E-205
Mono_CD16	ZNF106	1,92038E-29	8,20126E-01	0,412	0,341	3,53677E-25
Mono_CD16	TMPO	4,48328E-101	8,19540E-01	0,381	0,209	8,25686E-97
Mono_CD16	ERICH1	9,81352E-51	8,12976E-01	0,433	0,317	1,80736E-46
Mono_CD16	RASGRP2	4,22848E-219	8,12565E-01	0,388	0,141	7,78758E-215
Mono_CD16	ASAH1	1,94169E-90	8,07012E-01	0,786	0,685	3,57602E-86
Mono_CD16	EVI2B	3,94783E-96	8,06980E-01	0,524	0,37	7,27073E-92
Mono_CD16	WSB1	3,45701E-86	7,95824E-01	0,708	0,638	6,36678E-82
Mono_CD16	C9orf72	3,56920E-61	7,92725E-01	0,357	0,22	6,57340E-57
Mono_CD16	VASP	3,44619E-134	7,91580E-01	0,632	0,463	6,34685E-130
Mono_CD16	TMEM134	3,77778E-09	7,84960E-01	0,3	0,272	6,95754E-05
Mono_CD16	STK17B	4,24075E-152	7,80834E-01	0,642	0,437	7,81018E-148
Mono_CD16	VCL	2,29549E-101	7,74620E-01	0,25	0,102	4,22761E-97
Mono_CD16	SPI1	7,13308E-126	7,74305E-01	0,822	0,765	1,31370E-121
Mono_CD16	CSF1R	8,95382E-30	7,73614E-01	0,634	0,569	1,64903E-25
Mono_CD16	HIGD2A	2,06231E-103	7,73181E-01	0,786	0,712	3,79815E-99
Mono_CD16	PDLIM2	1,24724E-63	7,70732E-01	0,268	0,142	2,29704E-59
Mono_CD16	ARHGDIB	2,53598E-154	7,66502E-01	0,894	0,82	4,67051E-150
Mono_CD16	BCL2A1	2,88421E-232	7,58303E-01	0,777	0,458	5,31184E-228
Mono_CD16	HSBP1	2,17661E-98	7,56518E-01	0,697	0,597	4,00867E-94
Mono_CD16	GIMAP4	4,30398E-41	7,52770E-01	0,536	0,423	7,92664E-37
Mono_CD16	CEBPB	1,57507E-46	7,49032E-01	0,565	0,468	2,90081E-42
Mono_CD16	SHKBP1	1,34906E-12	7,46875E-01	0,392	0,369	2,48457E-08
Mono_CD16	NUDT16	5,50152E-100	7,45278E-01	0,56	0,402	1,01321E-95
Mono_CD16	WAS	2,38911E-99	7,42510E-01	0,641	0,525	4,40002E-95
Mono_CD16	SLC25A6	1,51413E-182	7,40497E-01	0,88	0,814	2,78858E-178
Mono_CD16	MYD88	1,42600E-99	7,32800E-01	0,516	0,351	2,62627E-95
Mono_CD16	DUSP1	1,89589E-39	7,32534E-01	0,874	0,849	3,49167E-35
Mono_CD16	LCP1	3,30643E-73	7,30876E-01	0,768	0,701	6,08946E-69
Mono_CD16	PYCARD	1,51136E-122	7,30718E-01	0,816	0,702	2,78346E-118
Mono_CD16	RHOG	8,85438E-98	7,27334E-01	0,798	0,724	1,63071E-93
Mono_CD16	LRRFIP1	1,11259E-88	7,20167E-01	0,747	0,681	2,04905E-84
Mono_CD16	KLF2	5,08409E-54	7,18480E-01	0,411	0,272	9,36337E-50

Mono_CD16	CSTA	3,36621E-79	7,15784E-01	0,574	0,424	6,19955E-75
Mono_CD16	TBCB	6,27734E-18	7,12332E-01	0,487	0,467	1,15610E-13
Mono_CD16	RNF141	1,08991E-36	7,11939E-01	0,257	0,161	2,00728E-32
Mono_CD16	GMFG	2,94324E-279	7,11615E-01	0,834	0,667	5,42056E-275
Mono_CD16	CCND3	2,06391E-85	7,10037E-01	0,463	0,306	3,80110E-81
Mono_CD16	LYN	3,10779E-67	7,07639E-01	0,558	0,448	5,72361E-63
Mono_CD16	NFKBIZ	1,02705E-74	7,06870E-01	0,612	0,448	1,89152E-70
Mono_CD16	SLC31A2	6,41365E-89	7,03778E-01	0,571	0,437	1,18120E-84
Mono_CD16	NADK	1,52246E-20	7,01830E-01	0,298	0,233	2,80391E-16
Mono_CD16	ITGA4	6,84275E-108	7,01306E-01	0,54	0,362	1,26023E-103
Mono_CD16	VMO1	8,04038E-17	6,99900E-01	0,274	0,225	1,48080E-12
Mono_CD16	CDC42	6,21620E-93	6,97810E-01	0,766	0,694	1,14484E-88
Mono_CD16	YPEL2	5,59532E-62	6,93533E-01	0,307	0,177	1,03049E-57
Mono_CD16	RGS19	2,86122E-118	6,92747E-01	0,639	0,49	5,26950E-114
Mono_CD16	TMC6	1,06031E-07	6,90149E-01	0,255	0,225	1,95278E-03
Mono_CD16	UQCRB	4,82872E-156	6,89397E-01	0,821	0,771	8,89306E-152
Mono_CD16	SNX18	1,09432E-78	6,85572E-01	0,285	0,146	2,01540E-74
Mono_CD16	PPDPF	3,74360E-114	6,83577E-01	0,836	0,765	6,89458E-110
Mono_CD16	PRKCB	3,77585E-59	6,82646E-01	0,356	0,222	6,95399E-55
Mono_CD16	CMTM7	9,72485E-09	6,82551E-01	0,416	0,411	1,79103E-04
Mono_CD16	ACAA1	2,31333E-22	6,77756E-01	0,423	0,376	4,26046E-18
Mono_CD16	ARPC5	3,29031E-89	6,76828E-01	0,816	0,76	6,05976E-85
Mono_CD16	DOCK8	2,11903E-36	6,76335E-01	0,511	0,445	3,90262E-32
Mono_CD16	SELPLG	4,76499E-182	6,71907E-01	0,576	0,335	8,77568E-178
Mono_CD16	PKN1	2,23879E-43	6,71681E-01	0,313	0,207	4,12318E-39
Mono_CD16	RILPL2	5,25432E-54	6,71364E-01	0,555	0,452	9,67688E-50
Mono_CD16	PSAP	1,73765E-46	6,70385E-01	0,923	0,916	3,20022E-42
Mono_CD16	OAZ1	0,00000E+00	6,69689E-01	0,966	0,936	0,00000E+00
Mono_CD16	RAB10	6,43839E-36	6,68951E-01	0,543	0,488	1,18576E-31
Mono_CD16	VSIR	2,38490E-14	6,67628E-01	0,311	0,272	4,39227E-10
Mono_CD16	FCER1G	2,96429E-256	6,63386E-01	0,948	0,912	5,45934E-252
Mono_CD16	KLF3	1,80838E-18	6,60766E-01	0,31	0,251	3,33049E-14
Mono_CD16	HK1	8,54228E-10	6,60546E-01	0,294	0,261	1,57323E-05
Mono_CD16	CCT5	1,92056E-14	6,60058E-01	0,407	0,38	3,53710E-10
Mono_CD16	S100A6	1,02272E-246	6,59145E-01	0,96	0,871	1,88354E-242
Mono_CD16	MT2A	5,31389E-137	6,53765E-01	0,831	0,62	9,78658E-133
Mono_CD16	RGS18	2,56157E-116	6,52775E-01	0,375	0,191	4,71764E-112
Mono_CD16	ARRB2	4,54953E-98	6,51514E-01	0,748	0,663	8,37888E-94
Mono_CD16	VAMP5	1,11924E-126	6,50128E-01	0,687	0,472	2,06130E-122
Mono_CD16	CMTM6	7,97222E-84	6,48304E-01	0,702	0,613	1,46824E-79
Mono_CD16	CLEC7A	1,26143E-65	6,46909E-01	0,63	0,52	2,32318E-61
Mono_CD16	AP1S2	5,47498E-65	6,46580E-01	0,662	0,575	1,00833E-60

Mono_CD16	NFAM1	2,25397E-31	6,45483E-01	0,252	0,164	4,15113E-27
Mono_CD16	EIF4A2	4,52837E-11	6,43784E-01	0,554	0,562	8,33990E-07
Mono_CD16	CHST15	5,12406E-120	6,42694E-01	0,279	0,11	9,43699E-116
Mono_CD16	BID	6,35926E-104	6,41823E-01	0,572	0,408	1,17119E-99
Mono_CD16	ABHD5	5,22068E-50	6,41157E-01	0,288	0,173	9,61492E-46
Mono_CD16	GIMAP1	2,28464E-70	6,39552E-01	0,41	0,26	4,20762E-66
Mono_CD16	PLXNB2	4,53624E-13	6,39043E-01	0,308	0,263	8,35439E-09
Mono_CD16	GIMAP8	1,05613E-57	6,38397E-01	0,26	0,14	1,94508E-53
Mono_CD16	RNH1	5,02806E-26	6,37224E-01	0,627	0,617	9,26017E-22
Mono_CD16	ARPC3	5,21332E-225	6,36208E-01	0,933	0,89	9,60137E-221
Mono_CD16	CD300E	9,62875E-212	6,30624E-01	0,435	0,156	1,77333E-207
Mono_CD16	IRF1	2,64114E-24	6,29778E-01	0,517	0,436	4,86419E-20
Mono_CD16	CAMK1	1,58716E-48	6,28868E-01	0,368	0,251	2,92307E-44
Mono_CD16	TBXAS1	2,93390E-47	6,25331E-01	0,596	0,515	5,40336E-43
Mono_CD16	ZNF706	5,96435E-84	6,23972E-01	0,688	0,587	1,09846E-79
Mono_CD16	SSH2	1,30553E-96	6,23028E-01	0,477	0,305	2,40439E-92
Mono_CD16	LY6E	9,93815E-155	6,21891E-01	0,755	0,539	1,83031E-150
Mono_CD16	DRAP1	9,67096E-106	6,20951E-01	0,739	0,654	1,78110E-101
Mono_CD16	PFN1	1,55783E-160	6,20578E-01	0,979	0,946	2,86905E-156
Mono_CD16	PCGF5	6,60194E-71	6,19036E-01	0,402	0,268	1,21588E-66
Mono_CD16	SH3BP2	7,98067E-58	6,18882E-01	0,417	0,29	1,46980E-53
Mono_CD16	ARAP1	8,92880E-22	6,18025E-01	0,358	0,289	1,64442E-17
Mono_CD16	IFI30	1,03760E-20	6,15188E-01	0,643	0,566	1,91095E-16
Mono_CD16	GPSM3	1,67566E-115	6,13412E-01	0,762	0,68	3,08607E-111
Mono_CD16	C20orf27	1,96229E-40	6,13213E-01	0,435	0,341	3,61394E-36
Mono_CD16	C9orf78	1,81019E-16	6,06184E-01	0,373	0,334	3,33382E-12
Mono_CD16	NOTCH2	5,58835E-56	6,06151E-01	0,44	0,312	1,02921E-51
Mono_CD16	FKBP5	1,76476E-15	6,05049E-01	0,537	0,517	3,25016E-11
Mono_CD16	TNFSF10	1,33905E-75	5,97906E-01	0,52	0,354	2,46612E-71
Mono_CD16	TMBIM4	2,46732E-08	5,97564E-01	0,352	0,33	4,54407E-04
Mono_CD16	MLX	1,43055E-18	5,93989E-01	0,396	0,357	2,63464E-14
Mono_CD16	IRAK3	1,41125E-108	5,91779E-01	0,439	0,247	2,59911E-104
Mono_CD16	SH3BGRL	3,96832E-51	5,90544E-01	0,738	0,719	7,30846E-47
Mono_CD16	STK38	2,13696E-74	5,87160E-01	0,313	0,17	3,93564E-70
Mono_CD16	PELI1	1,86817E-16	5,84278E-01	0,32	0,26	3,44060E-12
Mono_CD16	RHOA	4,32468E-75	5,82519E-01	0,897	0,878	7,96476E-71
Mono_CD16	BLOC1S1	4,46486E-23	5,81137E-01	0,512	0,471	8,22294E-19
Mono_CD16	AP2A1	6,15672E-57	5,80818E-01	0,335	0,213	1,13388E-52
Mono_CD16	ARPC2	1,37720E-153	5,80451E-01	0,909	0,857	2,53639E-149
Mono_CD16	NAA38	1,85963E-20	5,75262E-01	0,454	0,415	3,42488E-16
Mono_CD16	SH3BGRL3	7,91472E-230	5,75061E-01	0,963	0,932	1,45765E-225
Mono_CD16	NACA	4,20825E-275	5,74820E-01	0,929	0,919	7,75033E-271

Mono_CD16	TPM3	2,46713E-83	5,73865E-01	0,828	0,802	4,54371E-79
Mono_CD16	PTGES3	4,12467E-17	5,72952E-01	0,634	0,644	7,59641E-13
Mono_CD16	CDC42EP3	5,50499E-104	5,72563E-01	0,353	0,168	1,01385E-99
Mono_CD16	ERGIC1	1,40420E-10	5,71517E-01	0,323	0,29	2,58612E-06
Mono_CD16	RARA	6,19036E-103	5,70139E-01	0,369	0,192	1,14008E-98
Mono_CD16	LGALS9	1,10765E-15	5,69830E-01	0,566	0,547	2,03995E-11
Mono_CD16	LRRK2	2,97381E-43	5,69248E-01	0,318	0,207	5,47686E-39
Mono_CD16	PLCB2	8,81159E-26	5,62004E-01	0,318	0,239	1,62283E-21
Mono_CD16	YWHAZ	1,91003E-49	5,61635E-01	0,745	0,711	3,51771E-45
Mono_CD16	SAT1	6,91724E-158	5,58706E-01	0,972	0,947	1,27395E-153
Mono_CD16	STK10	3,02204E-70	5,57169E-01	0,382	0,238	5,56569E-66
Mono_CD16	RSL24D1	6,16388E-26	5,51926E-01	0,478	0,432	1,13520E-21
Mono_CD16	RIN3	1,28104E-22	5,51906E-01	0,457	0,402	2,35929E-18
Mono_CD16	RNF149	1,34715E-100	5,51636E-01	0,721	0,623	2,48105E-96
Mono_CD16	EMP3	1,37171E-119	5,50861E-01	0,836	0,724	2,52628E-115
Mono_CD16	PSMB9	1,37224E-118	5,50452E-01	0,792	0,653	2,52725E-114
Mono_CD16	DOCK5	4,93488E-45	5,48680E-01	0,264	0,155	9,08858E-41
Mono_CD16	HIPK3	5,57649E-20	5,48668E-01	0,28	0,214	1,02702E-15
Mono_CD16	ITSN2	5,24170E-07	5,48651E-01	0,39	0,386	9,65363E-03
Mono_CD16	SCLT1	2,28398E-61	5,46381E-01	0,285	0,158	4,20641E-57
Mono_CD16	CD300A	3,29829E-25	5,45208E-01	0,51	0,458	6,07445E-21
Mono_CD16	CDKN1B	4,29124E-33	5,42846E-01	0,332	0,243	7,90319E-29
Mono_CD16	CALCOCO2	4,88758E-21	5,42571E-01	0,416	0,356	9,00145E-17
Mono_CD16	VPS29	8,20842E-20	5,35600E-01	0,551	0,535	1,51175E-15
Mono_CD16	CAP1	3,45694E-38	5,35182E-01	0,752	0,733	6,36664E-34
Mono_CD16	HSD17B11	2,03697E-17	5,34879E-01	0,443	0,403	3,75148E-13
Mono_CD16	VMP1	1,81821E-80	5,33858E-01	0,662	0,546	3,34860E-76
Mono_CD16	MBD2	2,20466E-49	5,32287E-01	0,311	0,199	4,06032E-45
Mono_CD16	SOD1	1,21310E-34	5,32168E-01	0,595	0,562	2,23417E-30
Mono_CD16	HMGN2	6,59602E-75	5,30304E-01	0,784	0,732	1,21479E-70
Mono_CD16	SERP1	1,06131E-169	5,27721E-01	0,88	0,817	1,95461E-165
Mono_CD16	GSTK1	1,48855E-53	5,27319E-01	0,701	0,642	2,74147E-49
Mono_CD16	PIK3AP1	1,46581E-27	5,26580E-01	0,381	0,301	2,69958E-23
Mono_CD16	MIEN1	7,16824E-31	5,25703E-01	0,459	0,394	1,32018E-26
Mono_CD16	HCLS1	1,27156E-33	5,25373E-01	0,742	0,723	2,34184E-29
Mono_CD16	FGL2	6,11569E-32	5,24279E-01	0,716	0,61	1,12633E-27
Mono_CD16	VPS35	2,47541E-27	5,24220E-01	0,486	0,438	4,55897E-23
Mono_CD16	SNX2	3,05794E-07	5,21452E-01	0,44	0,441	5,63182E-03
Mono_CD16	MOB1A	2,20350E-09	5,19921E-01	0,572	0,584	4,05818E-05
Mono_CD16	ADD3	5,33709E-45	5,19471E-01	0,257	0,15	9,82932E-41
Mono_CD16	COX17	5,23669E-44	5,19000E-01	0,564	0,487	9,64441E-40
Mono_CD16	CCPG1	1,60445E-08	5,16993E-01	0,262	0,23	2,95491E-04

Mono_CD16	ATP6V0C	1,75801E-38	5,16665E-01	0,7	0,698	3,23773E-34
Mono_CD16	PRKAR1A	2,12950E-16	5,08605E-01	0,503	0,479	3,92190E-12
Mono_CD16	ATG3	7,26035E-54	5,08232E-01	0,598	0,52	1,33714E-49
Mono_CD16	PGAM1	1,96140E-26	5,06961E-01	0,587	0,564	3,61232E-22
Mono_CD16	GDI2	2,89174E-60	5,05933E-01	0,702	0,649	5,32572E-56
Mono_CD16	EIF1	3,14589E-240	5,02656E-01	0,96	0,943	5,79379E-236
Mono_CD16	CAPZA1	3,97044E-27	5,02085E-01	0,527	0,493	7,31235E-23
Mono_CD16	CELF2	2,02134E-43	5,00606E-01	0,599	0,537	3,72270E-39
Mono_CD16	ELF1	1,00048E-15	4,98017E-01	0,505	0,487	1,84259E-11
Mono_CD16	SP110	1,56091E-53	4,97594E-01	0,504	0,386	2,87474E-49
Mono_CD16	RASSF5	2,28612E-31	4,97403E-01	0,36	0,268	4,21035E-27
Mono_CD16	C15orf39	9,15095E-79	4,95354E-01	0,27	0,129	1,68533E-74
Mono_CD16	EFHD2	6,11126E-07	4,95295E-01	0,442	0,43	1,12551E-02
Mono_CD16	ADAM10	2,16237E-19	4,95215E-01	0,346	0,285	3,98244E-15
Mono_CD16	ADK	1,30822E-48	4,95093E-01	0,263	0,155	2,40935E-44
Mono_CD16	TPD52L2	7,01733E-10	4,94306E-01	0,187	0,272	1,29238E-05
Mono_CD16	IMPDH1	5,46346E-26	4,93569E-01	0,289	0,214	1,00621E-21
Mono_CD16	GCA	2,64883E-58	4,92329E-01	0,514	0,397	4,87834E-54
Mono_CD16	SMCO4	4,19933E-12	4,92063E-01	0,443	0,417	7,73391E-08
Mono_CD16	WASF2	2,55863E-39	4,89477E-01	0,604	0,567	4,71223E-35
Mono_CD16	LSM6	7,56292E-56	4,89225E-01	0,455	0,346	1,39286E-51
Mono_CD16	SHISA5	2,14242E-13	4,89144E-01	0,399	0,37	3,94569E-09
Mono_CD16	EHBP1L1	3,17938E-10	4,87905E-01	0,272	0,23	5,85547E-06
Mono_CD16	PGLS	4,71636E-34	4,87012E-01	0,579	0,541	8,68612E-30
Mono_CD16	SMCHD1	1,74885E-39	4,86451E-01	0,416	0,321	3,22086E-35
Mono_CD16	SECTM1	1,18276E-77	4,86120E-01	0,305	0,16	2,17829E-73
Mono_CD16	RCSD1	2,45628E-25	4,85653E-01	0,44	0,374	4,52373E-21
Mono_CD16	SYF2	1,89136E-22	4,85607E-01	0,462	0,423	3,48331E-18
Mono_CD16	PSMA4	5,55319E-22	4,81551E-01	0,509	0,467	1,02273E-17
Mono_CD16	ARHGAP30	1,76028E-23	4,81309E-01	0,392	0,329	3,24191E-19
Mono_CD16	FLI1	2,35858E-30	4,79612E-01	0,344	0,259	4,34379E-26
Mono_CD16	VPS28	2,39781E-09	4,78627E-01	0,595	0,613	4,41604E-05
Mono_CD16	DOK3	6,28416E-07	4,78614E-01	0,256	0,227	1,15735E-02
Mono_CD16	FPR1	1,12411E-41	4,77972E-01	0,547	0,444	2,07027E-37
Mono_CD16	KCNAB2	4,91294E-51	4,77874E-01	0,395	0,276	9,04816E-47
Mono_CD16	PTPN18	1,34134E-33	4,76756E-01	0,429	0,348	2,47034E-29
Mono_CD16	EIF4E2	5,04301E-11	4,75554E-01	0,389	0,371	9,28770E-07
Mono_CD16	NUP214	2,06266E-51	4,74220E-01	0,433	0,31	3,79881E-47
Mono_CD16	SLC11A1	8,81939E-88	4,73183E-01	0,672	0,459	1,62427E-83
Mono_CD16	S100A11	6,60588E-122	4,72781E-01	0,981	0,936	1,21660E-117
Mono_CD16	EIF3E	7,05367E-35	4,70094E-01	0,653	0,604	1,29908E-30
Mono_CD16	ACAP2	8,02266E-22	4,69805E-01	0,47	0,429	1,47753E-17

Mono_CD16	MAP3K1	1,65851E-41	4,69482E-01	0,287	0,183	3,05448E-37
Mono_CD16	C1orf162	4,52256E-50	4,67736E-01	0,805	0,704	8,32920E-46
Mono_CD16	SLC9A3R1	6,65591E-42	4,66208E-01	0,263	0,161	1,22582E-37
Mono_CD16	GYG1	2,32631E-08	4,66162E-01	0,294	0,272	4,28436E-04
Mono_CD16	PFDN5	4,85847E-238	4,65183E-01	0,908	0,902	8,94784E-234
Mono_CD16	CACUL1	7,72569E-10	4,64848E-01	0,33	0,302	1,42284E-05
Mono_CD16	SH3BP1	9,70315E-32	4,63143E-01	0,329	0,247	1,78703E-27
Mono_CD16	ST3GAL1	1,62533E-09	4,61724E-01	0,265	0,228	2,99337E-05
Mono_CD16	HLA-E	5,88668E-59	4,59529E-01	0,918	0,904	1,08415E-54
Mono_CD16	SNAP29	5,95396E-10	4,58071E-01	0,312	0,284	1,09654E-05
Mono_CD16	UBE2D3	1,33969E-08	4,57339E-01	0,717	0,737	2,46730E-04
Mono_CD16	HRH2	1,67110E-35	4,56544E-01	0,32	0,216	3,07767E-31
Mono_CD16	NAMPT	9,64962E-36	4,54128E-01	0,663	0,57	1,77717E-31
Mono_CD16	DMXL2	1,80412E-12	4,53551E-01	0,321	0,265	3,32264E-08
Mono_CD16	ARHGAP4	4,33267E-15	4,53008E-01	0,42	0,383	7,97949E-11
Mono_CD16	DOCK2	5,84442E-17	4,52924E-01	0,39	0,347	1,07637E-12
Mono_CD16	RABGAP1L	1,13446E-79	4,48636E-01	0,398	0,247	2,08933E-75
Mono_CD16	LILRB3	3,77134E-26	4,47628E-01	0,31	0,227	6,94568E-22
Mono_CD16	MAPK1	7,59465E-10	4,45843E-01	0,266	0,229	1,39871E-05
Mono_CD16	DBNL	1,51363E-12	4,44252E-01	0,501	0,492	2,78765E-08
Mono_CD16	STAT6	8,97198E-18	4,44188E-01	0,356	0,303	1,65237E-13
Mono_CD16	SSNA1	1,51127E-07	4,39691E-01	0,405	0,404	2,78331E-03
Mono_CD16	OTUB1	2,05014E-08	4,39299E-01	0,43	0,43	3,77575E-04
Mono_CD16	FTH1	1,33506E-142	4,38888E-01	0,998	0,997	2,45879E-138
Mono_CD16	ZEB2	2,37937E-84	4,38677E-01	0,691	0,602	4,38208E-80
Mono_CD16	ACTR3	6,32360E-28	4,35251E-01	0,686	0,663	1,16462E-23
Mono_CD16	CALM2	2,74279E-101	4,35199E-01	0,836	0,78	5,05140E-97
Mono_CD16	NR4A1	2,21286E-11	4,33309E-01	0,477	0,418	4,07542E-07
Mono_CD16	ATP1A1	1,89592E-16	4,32773E-01	0,455	0,429	3,49172E-12
Mono_CD16	CLIC1	9,91073E-18	4,31501E-01	0,887	0,874	1,82526E-13
Mono_CD16	CCNI	1,16590E-45	4,30514E-01	0,8	0,785	2,14725E-41
Mono_CD16	PDCD6IP	1,88191E-08	4,29392E-01	0,38	0,368	3,46591E-04
Mono_CD16	PTP4A2	2,48556E-78	4,27264E-01	0,532	0,399	4,57766E-74
Mono_CD16	LSP1	1,90472E-96	4,26044E-01	0,809	0,666	3,50793E-92
Mono_CD16	TUBA1A	5,31805E-57	4,25624E-01	0,574	0,458	9,79426E-53
Mono_CD16	SUPT4H1	1,91152E-12	4,24545E-01	0,471	0,461	3,52045E-08
Mono_CD16	ARPC1B	1,47872E-53	4,15769E-01	0,828	0,795	2,72336E-49
Mono_CD16	SULT1A1	5,49646E-49	4,15601E-01	0,268	0,156	1,01228E-44
Mono_CD16	FMNL1	5,65332E-07	4,13237E-01	0,409	0,402	1,04117E-02
Mono_CD16	YBX1	3,51823E-51	4,11780E-01	0,857	0,862	6,47952E-47
Mono_CD16	ATG16L2	4,83824E-25	4,11651E-01	0,315	0,236	8,91059E-21
Mono_CD16	NBPF10	2,39161E-44	4,10776E-01	0,311	0,197	4,40463E-40

Mono_CD16	TMA7	3,79703E-75	4,09676E-01	0,829	0,792	6,99299E-71
Mono_CD16	RBX1	1,62197E-32	4,09078E-01	0,659	0,617	2,98718E-28
Mono_CD16	NUMB	1,09797E-15	4,08946E-01	0,38	0,332	2,02213E-11
Mono_CD16	TSPO	1,39631E-39	4,08875E-01	0,817	0,764	2,57159E-35
Mono_CD16	TMOD3	9,19215E-09	4,07841E-01	0,363	0,344	1,69292E-04
Mono_CD16	CAPN2	1,26388E-13	4,07419E-01	0,29	0,232	2,32768E-09
Mono_CD16	PTDSS1	7,77994E-09	4,06286E-01	0,193	0,273	1,43283E-04
Mono_CD16	CALHM6	3,23428E-09	4,05642E-01	0,3	0,26	5,95657E-05
Mono_CD16	SRSF5	4,07131E-33	4,05126E-01	0,602	0,578	7,49814E-29
Mono_CD16	UBE2R2	1,85023E-08	4,04954E-01	0,292	0,264	3,40757E-04
Mono_CD16	CLEC2B	2,16967E-18	4,04948E-01	0,505	0,469	3,99589E-14
Mono_CD16	LAMTOR4	3,53549E-111	4,03409E-01	0,787	0,711	6,51132E-107
Mono_CD16	NKTR	3,88076E-24	4,01518E-01	0,355	0,281	7,14720E-20
Mono_CD16	CSGALNACT2	8,97581E-23	4,01361E-01	0,26	0,187	1,65308E-18
Mono_CD16	YWHAB	1,94275E-36	3,99276E-01	0,803	0,787	3,57796E-32
Mono_CD16	AKIRIN2	3,78257E-22	3,98150E-01	0,46	0,42	6,96637E-18
Mono_CD16	CCNDBP1	5,63529E-15	3,98020E-01	0,315	0,271	1,03785E-10
Mono_CD16	P2RY13	1,30439E-24	3,97393E-01	0,274	0,193	2,40230E-20
Mono_CD16	TRIM38	4,04787E-10	3,92561E-01	0,315	0,284	7,45496E-06
Mono_CD16	CDC40	1,46301E-09	3,90309E-01	0,268	0,232	2,69442E-05
Mono_CD16	ZYX	7,69710E-14	3,89884E-01	0,638	0,645	1,41757E-09
Mono_CD16	MAP2K3	1,53061E-13	3,88591E-01	0,324	0,268	2,81893E-09
Mono_CD16	CTNND1	7,54218E-26	3,83450E-01	0,15	0,277	1,38904E-21
Mono_CD16	PAIP2	2,28898E-17	3,82927E-01	0,524	0,511	4,21561E-13
Mono_CD16	AP1M1	2,73857E-09	3,79151E-01	0,277	0,246	5,04363E-05
Mono_CD16	PPP1CC	3,49231E-07	3,77823E-01	0,361	0,351	6,43178E-03
Mono_CD16	ANP32B	1,52814E-32	3,76391E-01	0,604	0,571	2,81437E-28
Mono_CD16	SASH3	1,17879E-12	3,74378E-01	0,323	0,282	2,17097E-08
Mono_CD16	ELF2	9,80242E-09	3,72717E-01	0,277	0,247	1,80531E-04
Mono_CD16	UBXN1	7,46474E-38	3,69528E-01	0,659	0,627	1,37478E-33
Mono_CD16	RELT	1,25796E-20	3,69134E-01	0,289	0,221	2,31679E-16
Mono_CD16	ACTB	9,81606E-107	3,69014E-01	0,998	0,991	1,80782E-102
Mono_CD16	METTL7A	2,52954E-07	3,67475E-01	0,276	0,246	4,65865E-03
Mono_CD16	APH1B	5,44501E-27	3,66704E-01	0,257	0,18	1,00281E-22
Mono_CD16	MPC1	1,47766E-19	3,64414E-01	0,365	0,315	2,72141E-15
Mono_CD16	POLR1D	1,62187E-19	3,64321E-01	0,491	0,467	2,98699E-15
Mono_CD16	TYROBP	1,89605E-85	3,61854E-01	0,977	0,946	3,49195E-81
Mono_CD16	EIF3G	2,75104E-10	3,61765E-01	0,6	0,614	5,06659E-06
Mono_CD16	COX6B1	1,86081E-97	3,61199E-01	0,845	0,81	3,42705E-93
Mono_CD16	ALDOA	6,99178E-83	3,57617E-01	0,785	0,759	1,28768E-78
Mono_CD16	CCDC115	1,75734E-14	3,56826E-01	0,311	0,264	3,23649E-10
Mono_CD16	EEF1A1	9,41939E-107	3,56631E-01	0,969	0,985	1,73477E-102

Mono_CD16	TLE4	1,34360E-60	3,55679E-01	0,259	0,135	2,47450E-56
Mono_CD16	ENY2	2,20322E-33	3,55378E-01	0,625	0,594	4,05766E-29
Mono_CD16	NPL	4,08081E-07	3,53595E-01	0,197	0,261	7,51564E-03
Mono_CD16	FGD3	2,59303E-38	3,52286E-01	0,253	0,153	4,77559E-34
Mono_CD16	TAX1BP1	9,82938E-15	3,51405E-01	0,502	0,498	1,81028E-10
Mono_CD16	PHF3	1,95091E-09	3,49606E-01	0,327	0,298	3,59300E-05
Mono_CD16	STX10	2,34863E-29	3,49377E-01	0,385	0,307	4,32548E-25
Mono_CD16	UXT	5,62324E-08	3,47172E-01	0,521	0,542	1,03563E-03
Mono_CD16	USP3	3,12274E-19	3,45821E-01	0,29	0,225	5,75115E-15
Mono_CD16	DICER1	1,35155E-24	3,44722E-01	0,336	0,267	2,48916E-20
Mono_CD16	GABARAP	1,40177E-41	3,42868E-01	0,845	0,829	2,58164E-37
Mono_CD16	FBXL5	7,97968E-09	3,41990E-01	0,304	0,273	1,46962E-04
Mono_CD16	IGBP1	1,29663E-09	3,41554E-01	0,365	0,343	2,38801E-05
Mono_CD16	AKNA	5,89643E-16	3,41432E-01	0,31	0,253	1,08594E-11
Mono_CD16	POLD4	1,33472E-29	3,34419E-01	0,609	0,568	2,45815E-25
Mono_CD16	TOM1	2,93766E-11	3,31579E-01	0,197	0,287	5,41030E-07
Mono_CD16	TNFRSF14	3,22446E-15	3,31156E-01	0,473	0,448	5,93848E-11
Mono_CD16	MKRN1	5,40229E-25	3,30713E-01	0,356	0,288	9,94940E-21
Mono_CD16	BCKDK	8,20334E-09	3,30006E-01	0,232	0,323	1,51081E-04
Mono_CD16	ITGB2	2,68223E-18	3,29383E-01	0,791	0,762	4,93986E-14
Mono_CD16	LNPEP	8,23796E-24	3,27634E-01	0,289	0,215	1,51718E-19
Mono_CD16	ARL6IP4	4,21576E-33	3,27433E-01	0,655	0,633	7,76417E-29
Mono_CD16	TMEM167A	7,39025E-08	3,25084E-01	0,406	0,4	1,36106E-03
Mono_CD16	COX7B	5,91403E-21	3,24369E-01	0,685	0,666	1,08919E-16
Mono_CD16	PET100	7,67773E-14	3,21000E-01	0,43	0,404	1,41401E-09
Mono_CD16	ANKRD44	1,16605E-10	3,20866E-01	0,325	0,29	2,14752E-06
Mono_CD16	UPP1	5,81011E-17	3,20016E-01	0,489	0,429	1,07005E-12
Mono_CD16	TOMM7	1,19350E-41	3,17900E-01	0,812	0,79	2,19808E-37
Mono_CD16	ACTR2	1,01171E-14	3,15040E-01	0,621	0,622	1,86327E-10
Mono_CD16	SLC25A5	2,33158E-28	3,14732E-01	0,78	0,743	4,29407E-24
Mono_CD16	BLVRA	8,98959E-27	3,13995E-01	0,453	0,38	1,65561E-22
Mono_CD16	UBE2J1	1,31080E-30	3,13585E-01	0,385	0,298	2,41411E-26
Mono_CD16	ARHGAP27	1,04479E-27	3,13164E-01	0,276	0,193	1,92418E-23
Mono_CD16	ITM2B	2,92953E-14	3,13036E-01	0,922	0,906	5,39532E-10
Mono_CD16	EIF3F	8,28526E-46	3,12507E-01	0,718	0,664	1,52590E-41
Mono_CD16	POLE4	2,81577E-25	3,12014E-01	0,395	0,329	5,18581E-21
Mono_CD16	EIF2S3	8,00405E-21	3,08787E-01	0,489	0,45	1,47411E-16
Mono_CD16	SIGIRR	2,09781E-20	3,07310E-01	0,319	0,254	3,86355E-16
Mono_CD16	SDHD	1,04722E-06	3,05184E-01	0,259	0,347	1,92867E-02
Mono_CD16	DDX5	3,19747E-21	3,02992E-01	0,831	0,833	5,88877E-17
Mono_CD16	MRPL54	2,02913E-06	2,98597E-01	0,402	0,403	3,73704E-02
Mono_CD16	CAPZA2	1,36129E-12	2,98399E-01	0,539	0,549	2,50709E-08

Mono_CD16	MYL12B	7,21953E-45	2,95368E-01	0,782	0,776	1,32962E-40
Mono_CD16	WDR1	2,72829E-08	2,87300E-01	0,522	0,542	5,02470E-04
Mono_CD16	EIF4EBP2	3,69373E-08	2,85780E-01	0,27	0,24	6,80273E-04
Mono_CD16	KMT2C	1,56318E-06	2,84782E-01	0,345	0,33	2,87890E-02
Mono_CD16	FAM32A	1,85803E-12	2,84413E-01	0,346	0,316	3,42194E-08
Mono_CD16	CAPZB	3,04786E-21	2,83861E-01	0,767	0,76	5,61325E-17
Mono_CD16	EIF4B	4,81172E-10	2,83064E-01	0,518	0,517	8,86174E-06
Mono_CD16	CCDC12	9,10617E-15	2,82719E-01	0,33	0,285	1,67708E-10
Mono_CD16	NDUFS6	6,86279E-09	2,82294E-01	0,507	0,507	1,26392E-04
Mono_CD16	RBM42	1,21574E-06	2,81360E-01	0,219	0,299	2,23903E-02
Mono_CD16	MIS18BP1	6,53786E-43	2,80895E-01	0,454	0,357	1,20408E-38
Mono_CD16	RIOK3	7,34072E-12	2,78410E-01	0,308	0,269	1,35194E-07
Mono_CD16	SIPA1L1	1,44099E-08	2,76120E-01	0,25	0,21	2,65388E-04
Mono_CD16	ARF5	4,93940E-10	2,75021E-01	0,605	0,614	9,09689E-06
Mono_CD16	MNDA	8,42217E-10	2,71865E-01	0,556	0,533	1,55111E-05
Mono_CD16	CYBB	1,49807E-20	2,70824E-01	0,676	0,622	2,75900E-16
Mono_CD16	PSME1	3,56059E-67	2,68681E-01	0,813	0,752	6,55753E-63
Mono_CD16	DCP2	8,16957E-13	2,68654E-01	0,308	0,264	1,50459E-08
Mono_CD16	GNB2	1,89578E-11	2,67036E-01	0,556	0,557	3,49145E-07
Mono_CD16	EIF3K	7,10861E-68	2,63748E-01	0,827	0,785	1,30919E-63
Mono_CD16	GNAI2	5,97151E-12	2,63377E-01	0,658	0,674	1,09977E-07
Mono_CD16	KLF4	1,83078E-07	2,56690E-01	0,486	0,445	3,37174E-03
Mono_CD16	UBA52	4,45974E-148	2,55556E-01	0,942	0,92	8,21351E-144
Mono_CD16	TMSB4X	8,53802E-66	2,55215E-01	0,995	0,983	1,57245E-61
Mono_CD16	PDLIM5	1,05234E-28	2,54240E-01	0,314	0,232	1,93809E-24
Mono_CD16	TRAPPC1	4,65414E-16	2,53956E-01	0,611	0,61	8,57154E-12
Mono_CD16	CFL1	6,00371E-94	2,53792E-01	0,944	0,921	1,10570E-89
Mono_CD16	IRF2	4,68169E-09	2,53439E-01	0,35	0,327	8,62227E-05
Mono_CD16	KYNU	1,44560E-09	2,52773E-01	0,35	0,314	2,66236E-05
Mono_CD16	PAK2	2,88110E-13	2,51765E-01	0,527	0,529	5,30612E-09
Mono_CD16	PGK1	7,92461E-50	2,51469E-01	0,728	0,705	1,45947E-45
Mono_CD16	DERL1	1,78061E-11	2,50064E-01	0,186	0,277	3,27936E-07
Mono_CD16	SORL1	1,76015E-32	-2,50248E-01	0,217	0,369	3,24166E-28
Mono_CD16	DNAJB6	2,23628E-20	-2,50416E-01	0,375	0,528	4,11856E-16
Mono_CD16	TNPO1	1,02639E-24	-2,50781E-01	0,133	0,255	1,89031E-20
Mono_CD16	TFG	1,27809E-21	-2,51780E-01	0,15	0,266	2,35386E-17
Mono_CD16	HNMT	3,10323E-21	-2,53280E-01	0,253	0,386	5,71521E-17
Mono_CD16	KDM6B	5,17198E-07	-2,54319E-01	0,274	0,343	9,52524E-03
Mono_CD16	BASP1	5,91691E-59	-2,57644E-01	0,152	0,354	1,08972E-54
Mono_CD16	LAMTOR1	3,92782E-07	-2,57709E-01	0,554	0,651	7,23386E-03
Mono_CD16	SELENOW	1,96099E-19	-2,57967E-01	0,19	0,295	3,61156E-15
Mono_CD16	TMEM259	9,87199E-18	-2,57985E-01	0,15	0,251	1,81812E-13

Mono_CD16	ATOX1	2,94870E-28	-2,58331E-01	0,431	0,581	5,43063E-24
Mono_CD16	TIMP2	6,24662E-32	-2,59009E-01	0,214	0,367	1,15044E-27
Mono_CD16	MT-ATP6	2,20712E-22	-2,62910E-01	0,454	0,583	4,06485E-18
Mono_CD16	ATP6V1A	1,29092E-15	-2,63028E-01	0,159	0,258	2,37749E-11
Mono_CD16	SPCS2	2,49744E-23	-2,64734E-01	0,338	0,499	4,59954E-19
Mono_CD16	TMED3	4,54106E-11	-2,64854E-01	0,187	0,278	8,36327E-07
Mono_CD16	TECR	2,08122E-16	-2,65369E-01	0,257	0,384	3,83299E-12
Mono_CD16	KDELR2	3,24055E-38	-2,66553E-01	0,318	0,514	5,96812E-34
Mono_CD16	MT-ND4L	7,34815E-51	-2,67488E-01	0,339	0,521	1,35331E-46
Mono_CD16	TMEM205	7,67384E-12	-2,68555E-01	0,194	0,288	1,41329E-07
Mono_CD16	HNRNPUL1	6,38979E-18	-2,70565E-01	0,283	0,416	1,17681E-13
Mono_CD16	EMC3	3,58140E-12	-2,72567E-01	0,218	0,324	6,59587E-08
Mono_CD16	GPX4	2,62178E-43	-2,72717E-01	0,781	0,847	4,82853E-39
Mono_CD16	ANKRD11	9,55293E-09	-2,73049E-01	0,23	0,316	1,75936E-04
Mono_CD16	CLTC	2,95080E-14	-2,74207E-01	0,298	0,425	5,43450E-10
Mono_CD16	MEF2A	3,56984E-40	-2,74771E-01	0,251	0,428	6,57457E-36
Mono_CD16	YPEL5	3,33434E-09	-2,74921E-01	0,452	0,433	6,14085E-05
Mono_CD16	STARD7	5,79850E-21	-2,74985E-01	0,187	0,31	1,06791E-16
Mono_CD16	ACADVL	1,75884E-23	-2,75282E-01	0,319	0,475	3,23925E-19
Mono_CD16	RPL22	1,82910E-12	-2,76506E-01	0,423	0,534	3,36864E-08
Mono_CD16	STAU1	2,84208E-12	-2,76676E-01	0,195	0,294	5,23426E-08
Mono_CD16	LAGE3	3,53265E-25	-2,77191E-01	0,189	0,325	6,50609E-21
Mono_CD16	CNDP2	2,54912E-34	-2,77301E-01	0,287	0,461	4,69471E-30
Mono_CD16	GPAA1	7,69830E-20	-2,78537E-01	0,152	0,26	1,41780E-15
Mono_CD16	FAM177A1	3,20768E-12	-2,79634E-01	0,217	0,32	5,90759E-08
Mono_CD16	KDELR1	1,37402E-26	-2,79789E-01	0,374	0,546	2,53054E-22
Mono_CD16	GGA2	8,19489E-53	-2,79996E-01	0,102	0,277	1,50925E-48
Mono_CD16	CHD2	1,55286E-09	-2,80763E-01	0,216	0,302	2,85990E-05
Mono_CD16	NLRP3	4,28868E-23	-2,82328E-01	0,158	0,267	7,89845E-19
Mono_CD16	CDC37	8,44459E-13	-2,83189E-01	0,507	0,643	1,55524E-08
Mono_CD16	RPS8	2,03522E-09	-2,83930E-01	0,461	0,583	3,74827E-05
Mono_CD16	DUSP3	6,03053E-35	-2,89421E-01	0,125	0,268	1,11064E-30
Mono_CD16	FERMT3	5,60136E-12	-2,89583E-01	0,364	0,487	1,03160E-07
Mono_CD16	MBD4	4,27596E-20	-2,89645E-01	0,144	0,254	7,87503E-16
Mono_CD16	PABPN1	5,66797E-16	-2,90180E-01	0,184	0,291	1,04387E-11
Mono_CD16	PCBD1	2,39272E-44	-2,92673E-01	0,183	0,367	4,40668E-40
Mono_CD16	MZT2B	6,43873E-15	-2,92955E-01	0,247	0,361	1,18582E-10
Mono_CD16	DNM2	1,89235E-07	-2,93598E-01	0,224	0,299	3,48514E-03
Mono_CD16	NAIP	4,19158E-57	-2,94055E-01	0,175	0,378	7,71963E-53
Mono_CD16	DNAJB1	1,76414E-08	-2,95218E-01	0,581	0,557	3,24901E-04
Mono_CD16	STAT1	2,15141E-33	-2,95374E-01	0,374	0,523	3,96224E-29
Mono_CD16	UQCC2	2,61468E-12	-2,95686E-01	0,214	0,314	4,81545E-08

Mono_CD16	RNF145	4,30601E-20	-2,95695E-01	0,168	0,28	7,93038E-16
Mono_CD16	NME3	6,60215E-08	-2,95791E-01	0,269	0,363	1,21592E-03
Mono_CD16	PRDX4	2,73400E-16	-2,97415E-01	0,18	0,291	5,03521E-12
Mono_CD16	PDIA6	4,87878E-34	-2,98918E-01	0,358	0,526	8,98525E-30
Mono_CD16	CHST11	1,63679E-34	-3,00384E-01	0,138	0,283	3,01448E-30
Mono_CD16	PRDX6	4,17396E-15	-3,01390E-01	0,415	0,564	7,68718E-11
Mono_CD16	RPS26	8,76685E-13	-3,02037E-01	0,418	0,528	1,61459E-08
Mono_CD16	C6orf62	7,06126E-14	-3,02849E-01	0,254	0,369	1,30047E-09
Mono_CD16	TUBB	5,37810E-18	-3,04719E-01	0,553	0,665	9,90484E-14
Mono_CD16	NANS	1,08123E-12	-3,04810E-01	0,275	0,394	1,99129E-08
Mono_CD16	RAB8B	6,83297E-07	-3,05213E-01	0,187	0,253	1,25843E-02
Mono_CD16	IFRD1	8,25303E-10	-3,07430E-01	0,214	0,294	1,51996E-05
Mono_CD16	SAT2	5,71845E-16	-3,07648E-01	0,3	0,439	1,05317E-11
Mono_CD16	STX6	4,58783E-38	-3,08192E-01	0,111	0,257	8,44941E-34
Mono_CD16	RAP2B	9,56070E-18	-3,08257E-01	0,208	0,319	1,76079E-13
Mono_CD16	HDLBP	1,65617E-11	-3,08342E-01	0,299	0,414	3,05016E-07
Mono_CD16	SEC62	9,94193E-08	-3,09779E-01	0,436	0,559	1,83100E-03
Mono_CD16	CSNK1D	6,51918E-14	-3,10811E-01	0,236	0,347	1,20064E-09
Mono_CD16	IQGAP2	1,40605E-13	-3,11578E-01	0,21	0,307	2,58952E-09
Mono_CD16	TMX1	1,89372E-18	-3,12722E-01	0,214	0,341	3,48766E-14
Mono_CD16	CLEC4E	4,31214E-22	-3,14442E-01	0,18	0,296	7,94167E-18
Mono_CD16	MORF4L2	5,53072E-27	-3,14778E-01	0,147	0,279	1,01859E-22
Mono_CD16	GNAS	2,95755E-32	-3,14834E-01	0,443	0,616	5,44692E-28
Mono_CD16	CHD4	1,28635E-21	-3,15686E-01	0,163	0,281	2,36907E-17
Mono_CD16	RPS12	7,88439E-08	-3,15741E-01	0,459	0,587	1,45207E-03
Mono_CD16	RPL37	7,36142E-08	-3,16079E-01	0,45	0,573	1,35575E-03
Mono_CD16	PTPN2	2,17980E-10	-3,16787E-01	0,285	0,39	4,01455E-06
Mono_CD16	METAP2	6,88334E-11	-3,17101E-01	0,203	0,296	1,26770E-06
Mono_CD16	STARD3NL	3,27529E-12	-3,17886E-01	0,181	0,274	6,03211E-08
Mono_CD16	NENF	4,27551E-34	-3,17946E-01	0,127	0,262	7,87420E-30
Mono_CD16	SBDS	1,75162E-12	-3,19567E-01	0,19	0,287	3,22596E-08
Mono_CD16	CUEDC2	1,53521E-10	-3,22004E-01	0,204	0,298	2,82740E-06
Mono_CD16	RPLP1	3,56667E-18	-3,22254E-01	0,479	0,602	6,56874E-14
Mono_CD16	HCFC1R1	2,26099E-26	-3,22539E-01	0,185	0,321	4,16407E-22
Mono_CD16	RPN1	4,58136E-29	-3,22671E-01	0,17	0,3	8,43749E-25
Mono_CD16	RPS5	1,06908E-11	-3,23135E-01	0,412	0,522	1,96893E-07
Mono_CD16	TMEM243	2,59017E-09	-3,23426E-01	0,188	0,272	4,77031E-05
Mono_CD16	RPS27L	4,61860E-18	-3,24907E-01	0,252	0,367	8,50608E-14
Mono_CD16	TMED4	6,58821E-14	-3,25611E-01	0,193	0,297	1,21335E-09
Mono_CD16	DUSP23	2,32974E-35	-3,26282E-01	0,217	0,39	4,29069E-31
Mono_CD16	PARP1	8,54981E-28	-3,27804E-01	0,194	0,34	1,57462E-23
Mono_CD16	PLK3	4,42386E-17	-3,28009E-01	0,158	0,256	8,14741E-13

Mono_CD16	RPS27A	1,27470E-07	-3,29699E-01	0,461	0,58	2,34761E-03
Mono_CD16	LMAN2	2,86164E-08	-3,30521E-01	0,398	0,516	5,27029E-04
Mono_CD16	RPL5	6,42739E-16	-3,30945E-01	0,408	0,522	1,18373E-11
Mono_CD16	PLIN3	3,02100E-24	-3,31197E-01	0,231	0,377	5,56377E-20
Mono_CD16	PIM3	2,13582E-33	-3,32441E-01	0,151	0,291	3,93353E-29
Mono_CD16	EIF4G1	4,41889E-14	-3,32931E-01	0,227	0,337	8,13827E-10
Mono_CD16	ATP6AP1	4,10541E-66	-3,34971E-01	0,326	0,552	7,56093E-62
Mono_CD16	RPL35	2,01714E-06	-3,36057E-01	0,431	0,535	3,71498E-02
Mono_CD16	NPC2	7,81315E-45	-3,36871E-01	0,889	0,884	1,43895E-40
Mono_CD16	SSR3	4,46368E-07	-3,37580E-01	0,494	0,6	8,22076E-03
Mono_CD16	SLC25A39	3,73543E-50	-3,37916E-01	0,213	0,416	6,87955E-46
Mono_CD16	AGPAT2	8,93542E-21	-3,37947E-01	0,178	0,296	1,64564E-16
Mono_CD16	TMUB1	1,08277E-29	-3,40835E-01	0,133	0,267	1,99414E-25
Mono_CD16	FBXO7	5,06786E-19	-3,41205E-01	0,202	0,327	9,33347E-15
Mono_CD16	GNB4	5,99348E-33	-3,41236E-01	0,189	0,342	1,10382E-28
Mono_CD16	GAPDH	2,21201E-26	-3,42285E-01	0,955	0,96	4,07386E-22
Mono_CD16	TIMM13	2,39199E-14	-3,44850E-01	0,275	0,399	4,40532E-10
Mono_CD16	HSP90AB1	7,03709E-19	-3,45182E-01	0,748	0,829	1,29602E-14
Mono_CD16	RPL7A	1,59869E-12	-3,45459E-01	0,449	0,563	2,94432E-08
Mono_CD16	NASP	9,61223E-19	-3,46082E-01	0,169	0,28	1,77028E-14
Mono_CD16	LAPTM4A	3,28204E-17	-3,48753E-01	0,507	0,633	6,04453E-13
Mono_CD16	NUCKS1	1,51064E-44	-3,49318E-01	0,305	0,509	2,78214E-40
Mono_CD16	DPP7	6,63061E-67	-3,49388E-01	0,333	0,565	1,22116E-62
Mono_CD16	ZNF428	3,21647E-62	-3,50801E-01	0,106	0,302	5,92377E-58
Mono_CD16	LIMS1	2,05011E-36	-3,56222E-01	0,385	0,562	3,77568E-32
Mono_CD16	PRDX1	1,67518E-55	-3,56368E-01	0,63	0,752	3,08518E-51
Mono_CD16	PLXDC2	1,81433E-76	-3,56641E-01	0,335	0,552	3,34145E-72
Mono_CD16	RRBP1	6,63666E-18	-3,57000E-01	0,26	0,39	1,22227E-13
Mono_CD16	PARVB	1,13720E-41	-3,58067E-01	0,142	0,301	2,09438E-37
Mono_CD16	SNRPA	1,29948E-18	-3,59152E-01	0,183	0,3	2,39325E-14
Mono_CD16	ANXA1	3,50316E-10	-3,61111E-01	0,585	0,692	6,45178E-06
Mono_CD16	AKR1A1	3,67437E-69	-3,62491E-01	0,364	0,585	6,76710E-65
Mono_CD16	CYFIP1	2,03141E-80	-3,62493E-01	0,106	0,332	3,74125E-76
Mono_CD16	SNX29	1,57759E-32	-3,62694E-01	0,13	0,265	2,90545E-28
Mono_CD16	SDCCAG8	6,51084E-38	-3,62973E-01	0,178	0,343	1,19910E-33
Mono_CD16	PIK3R1	6,14515E-24	-3,62999E-01	0,198	0,326	1,13175E-19
Mono_CD16	TAP1	3,85853E-28	-3,63067E-01	0,282	0,428	7,10626E-24
Mono_CD16	SRSF7	4,49728E-10	-3,64305E-01	0,446	0,556	8,28265E-06
Mono_CD16	LUC7L3	1,01679E-06	-3,64712E-01	0,274	0,362	1,87262E-02
Mono_CD16	NUB1	3,36907E-11	-3,66189E-01	0,223	0,317	6,20482E-07
Mono_CD16	MAT2A	1,58662E-36	-3,67242E-01	0,273	0,453	2,92207E-32
Mono_CD16	PSMC2	1,92816E-12	-3,69452E-01	0,191	0,29	3,55110E-08

Mono_CD16	RPS19BP1	1,68928E-24	-3,71383E-01	0,149	0,264	3,11116E-20
Mono_CD16	DNMT1	3,06913E-25	-3,72263E-01	0,187	0,322	5,65241E-21
Mono_CD16	DAD1	6,93561E-18	-3,72516E-01	0,459	0,606	1,27733E-13
Mono_CD16	FYTTD1	2,93804E-16	-3,74225E-01	0,154	0,252	5,41099E-12
Mono_CD16	VAMP8	1,72397E-27	-3,75598E-01	0,707	0,783	3,17504E-23
Mono_CD16	PHACTR1	2,61472E-16	-3,76614E-01	0,204	0,305	4,81552E-12
Mono_CD16	ISCU	7,47821E-09	-3,77074E-01	0,434	0,541	1,37726E-04
Mono_CD16	RAB5A	1,20901E-19	-3,77718E-01	0,192	0,314	2,22663E-15
Mono_CD16	YWHAE	2,02203E-20	-3,78736E-01	0,428	0,587	3,72397E-16
Mono_CD16	DDIT4	4,10310E-14	-3,79185E-01	0,386	0,5	7,55668E-10
Mono_CD16	RPS28	1,14104E-07	-3,80081E-01	0,468	0,586	2,10145E-03
Mono_CD16	ADPGK	1,09874E-10	-3,81199E-01	0,292	0,405	2,02355E-06
Mono_CD16	RTCB	5,98718E-27	-3,81779E-01	0,162	0,3	1,10266E-22
Mono_CD16	GCC2	2,49498E-10	-3,83204E-01	0,193	0,284	4,59501E-06
Mono_CD16	LRP1	4,26884E-12	-3,83402E-01	0,329	0,426	7,86193E-08
Mono_CD16	PLAC8	5,29602E-232	-3,85598E-01	0,498	0,193	9,75369E-228
Mono_CD16	RPL36AL	1,01504E-19	-3,85785E-01	0,375	0,498	1,86939E-15
Mono_CD16	SOCS3	7,74373E-29	-3,86235E-01	0,218	0,363	1,42616E-24
Mono_CD16	AHSA1	1,14601E-11	-3,87643E-01	0,204	0,303	2,11061E-07
Mono_CD16	YTHDF2	4,39966E-10	-3,88629E-01	0,194	0,284	8,10286E-06
Mono_CD16	SDHA	2,73910E-17	-3,91249E-01	0,153	0,255	5,04460E-13
Mono_CD16	SDF2L1	1,11603E-55	-3,91501E-01	0,167	0,362	2,05540E-51
Mono_CD16	DDOST	2,56271E-31	-3,91743E-01	0,304	0,469	4,71975E-27
Mono_CD16	PSMA1	4,65547E-07	-3,94011E-01	0,274	0,364	8,57398E-03
Mono_CD16	RBPJ	7,17459E-10	-3,94218E-01	0,357	0,471	1,32134E-05
Mono_CD16	LTB	4,97048E-112	-3,94289E-01	0,322	0,139	9,15414E-108
Mono_CD16	CANX	4,85794E-69	-3,94584E-01	0,417	0,648	8,94688E-65
Mono_CD16	FCGR2A	2,01058E-35	-3,96251E-01	0,504	0,636	3,70289E-31
Mono_CD16	MLF2	8,50798E-21	-3,97111E-01	0,309	0,458	1,56692E-16
Mono_CD16	S100A9	6,25588E-137	-3,97555E-01	0,752	0,444	1,15215E-132
Mono_CD16	CTSH	5,85488E-106	-3,97586E-01	0,538	0,717	1,07829E-101
Mono_CD16	ARL8B	1,21128E-22	-3,98292E-01	0,266	0,409	2,23081E-18
Mono_CD16	CEP170	4,24557E-45	-3,99170E-01	0,17	0,351	7,81907E-41
Mono_CD16	MYDGF	2,34521E-41	-4,00003E-01	0,165	0,317	4,31917E-37
Mono_CD16	PTTG1IP	5,05525E-36	-4,02567E-01	0,323	0,509	9,31025E-32
Mono_CD16	KRTCAP2	1,21980E-12	-4,03104E-01	0,451	0,582	2,24651E-08
Mono_CD16	PRPF8	9,38062E-09	-4,03182E-01	0,223	0,311	1,72763E-04
Mono_CD16	RPSA	1,99777E-15	-4,03803E-01	0,4	0,499	3,67930E-11
Mono_CD16	DSE	2,60130E-61	-4,04014E-01	0,225	0,447	4,79082E-57
Mono_CD16	PSMD7	1,94925E-17	-4,05466E-01	0,273	0,411	3,58994E-13
Mono_CD16	MTDH	9,32852E-28	-4,05678E-01	0,565	0,7	1,71803E-23
Mono_CD16	SQSTM1	5,97481E-55	-4,08566E-01	0,48	0,682	1,10038E-50

Mono_CD16	LY86	8,20619E-59	-4,09290E-01	0,285	0,498	1,51133E-54
Mono_CD16	LAMP1	2,39427E-37	-4,10697E-01	0,245	0,403	4,40952E-33
Mono_CD16	TFPT	3,50238E-71	-4,11698E-01	0,059	0,251	6,45033E-67
Mono_CD16	KCNMA1	4,75924E-44	-4,12006E-01	0,116	0,266	8,76509E-40
Mono_CD16	ERCC1	4,08008E-09	-4,12736E-01	0,284	0,385	7,51428E-05
Mono_CD16	BTG2	4,36888E-10	-4,14531E-01	0,399	0,502	8,04616E-06
Mono_CD16	ARMCX3	4,25614E-23	-4,15436E-01	0,145	0,263	7,83853E-19
Mono_CD16	RPL18	1,17591E-06	-4,15956E-01	0,45	0,567	2,16568E-02
Mono_CD16	RHBDF2	5,64985E-23	-4,16343E-01	0,201	0,329	1,04053E-18
Mono_CD16	RBM17	1,08287E-07	-4,16845E-01	0,314	0,414	1,99433E-03
Mono_CD16	COMT	8,78885E-71	-4,17297E-01	0,319	0,567	1,61864E-66
Mono_CD16	AP1B1	1,11766E-55	-4,18124E-01	0,199	0,401	2,05839E-51
Mono_CD16	ARF3	2,23378E-20	-4,18762E-01	0,213	0,342	4,11396E-16
Mono_CD16	CKLF	3,22765E-12	-4,19411E-01	0,402	0,51	5,94437E-08
Mono_CD16	STX4	1,05608E-25	-4,22791E-01	0,183	0,318	1,94499E-21
Mono_CD16	ARF4	1,25953E-16	-4,22828E-01	0,305	0,451	2,31968E-12
Mono_CD16	RPS18	1,50197E-09	-4,24046E-01	0,445	0,564	2,76618E-05
Mono_CD16	TMEM51	8,33256E-92	-4,25614E-01	0,049	0,27	1,53461E-87
Mono_CD16	LYZ	2,50685E-20	-4,25919E-01	0,856	0,807	4,61687E-16
Mono_CD16	GNPTAB	7,74546E-53	-4,26178E-01	0,113	0,285	1,42648E-48
Mono_CD16	APEX1	9,28271E-48	-4,27530E-01	0,25	0,449	1,70960E-43
Mono_CD16	GAA	2,78385E-45	-4,32869E-01	0,212	0,393	5,12702E-41
Mono_CD16	SRSF10	2,85497E-19	-4,33786E-01	0,17	0,283	5,25800E-15
Mono_CD16	RPL24	7,31371E-08	-4,35203E-01	0,425	0,543	1,34697E-03
Mono_CD16	RPN2	2,57016E-48	-4,35873E-01	0,129	0,291	4,73346E-44
Mono_CD16	PSMA6	1,71636E-07	-4,36089E-01	0,374	0,47	3,16102E-03
Mono_CD16	ZDHHC12	7,54998E-14	-4,36329E-01	0,167	0,266	1,39048E-09
Mono_CD16	TRMT112	6,52844E-09	-4,37649E-01	0,496	0,623	1,20234E-04
Mono_CD16	ORMDL2	1,49025E-08	-4,37843E-01	0,214	0,302	2,74459E-04
Mono_CD16	G3BP1	4,98589E-16	-4,41811E-01	0,199	0,312	9,18252E-12
Mono_CD16	MFSD1	2,43888E-35	-4,43846E-01	0,439	0,597	4,49169E-31
Mono_CD16	CD164	3,33286E-17	-4,45782E-01	0,416	0,563	6,13813E-13
Mono_CD16	ETV6	4,64654E-35	-4,46937E-01	0,149	0,296	8,55753E-31
Mono_CD16	FOSB	1,82666E-15	-4,47123E-01	0,486	0,583	3,36416E-11
Mono_CD16	NR4A2	2,80322E-18	-4,47147E-01	0,346	0,455	5,16269E-14
Mono_CD16	GLB1	1,13884E-24	-4,47298E-01	0,167	0,297	2,09740E-20
Mono_CD16	HAVCR2	6,23125E-87	-4,49702E-01	0,187	0,445	1,14761E-82
Mono_CD16	ARL5A	8,82425E-34	-4,52113E-01	0,267	0,435	1,62516E-29
Mono_CD16	HDAC2	5,08174E-13	-4,53207E-01	0,178	0,271	9,35905E-09
Mono_CD16	ETHE1	6,34700E-62	-4,56164E-01	0,14	0,349	1,16893E-57
Mono_CD16	MPC2	1,36980E-28	-4,57119E-01	0,212	0,366	2,52277E-24
Mono_CD16	GLUL	1,16281E-10	-4,61641E-01	0,768	0,805	2,14154E-06

Mono_CD16	LITAF	9,17384E-92	-4,61712E-01	0,505	0,751	1,68955E-87
Mono_CD16	KLF10	4,04426E-30	-4,64445E-01	0,159	0,295	7,44831E-26
Mono_CD16	GYPC	5,04703E-19	-4,64806E-01	0,199	0,305	9,29512E-15
Mono_CD16	SCAMP3	4,25688E-14	-4,65460E-01	0,158	0,255	7,83989E-10
Mono_CD16	TRAM1	7,44152E-13	-4,65612E-01	0,326	0,455	1,37051E-08
Mono_CD16	GOLGA4	1,29331E-21	-4,66710E-01	0,209	0,34	2,38189E-17
Mono_CD16	ARFGAP3	7,65548E-16	-4,67091E-01	0,171	0,271	1,40991E-11
Mono_CD16	BNIP3L	1,79206E-42	-4,68234E-01	0,38	0,571	3,30044E-38
Mono_CD16	CD83	8,18457E-18	-4,72486E-01	0,393	0,492	1,50735E-13
Mono_CD16	LSM2	9,58677E-35	-4,73602E-01	0,192	0,357	1,76560E-30
Mono_CD16	NSMCE1	6,18874E-14	-4,74043E-01	0,179	0,275	1,13978E-09
Mono_CD16	MAP3K8	2,69536E-12	-4,74816E-01	0,378	0,486	4,96404E-08
Mono_CD16	MAX	4,38366E-31	-4,76435E-01	0,24	0,402	8,07339E-27
Mono_CD16	TAGLN2	5,06313E-13	-4,76822E-01	0,63	0,759	9,32476E-09
Mono_CD16	RPS3A	6,19492E-08	-4,77378E-01	0,453	0,571	1,14092E-03
Mono_CD16	RNASEH2B	1,67625E-14	-4,77409E-01	0,2	0,306	3,08715E-10
Mono_CD16	OSTC	6,42077E-26	-4,77413E-01	0,336	0,506	1,18251E-21
Mono_CD16	GADD45GIP1	1,17767E-21	-4,81208E-01	0,292	0,443	2,16891E-17
Mono_CD16	OCIAD1	3,75006E-08	-4,81385E-01	0,327	0,438	6,90649E-04
Mono_CD16	TAPBP	2,74421E-18	-4,81473E-01	0,436	0,585	5,05401E-14
Mono_CD16	PTGS1	2,36026E-78	-4,81520E-01	0,064	0,265	4,34689E-74
Mono_CD16	ANKRD10	1,00040E-39	-4,81633E-01	0,173	0,338	1,84243E-35
Mono_CD16	NFIC	8,46826E-48	-4,83103E-01	0,132	0,302	1,55960E-43
Mono_CD16	RPL17	1,54646E-07	-4,84477E-01	0,34	0,437	2,84812E-03
Mono_CD16	CDKN1A	9,71949E-29	-4,85599E-01	0,369	0,531	1,79004E-24
Mono_CD16	DNPH1	8,95472E-26	-4,86401E-01	0,21	0,352	1,64919E-21
Mono_CD16	NCF1	6,06260E-66	-4,90036E-01	0,278	0,509	1,11655E-61
Mono_CD16	MPG	1,13423E-09	-4,91532E-01	0,23	0,326	2,08892E-05
Mono_CD16	HINT1	5,74235E-07	-4,92365E-01	0,674	0,725	1,05757E-02
Mono_CD16	RPL37A	2,65620E-09	-4,92951E-01	0,429	0,542	4,89193E-05
Mono_CD16	NCF4	1,34059E-51	-4,93159E-01	0,227	0,43	2,46897E-47
Mono_CD16	SLC38A2	4,49179E-27	-4,94177E-01	0,179	0,314	8,27253E-23
Mono_CD16	HSPA9	1,36350E-21	-4,96115E-01	0,239	0,376	2,51116E-17
Mono_CD16	LMAN1	2,26476E-64	-4,98009E-01	0,101	0,297	4,17101E-60
Mono_CD16	ATP6V1F	2,60593E-64	-4,99524E-01	0,616	0,77	4,79933E-60
Mono_CD16	BRD2	4,65102E-18	-5,00550E-01	0,346	0,493	8,56579E-14
Mono_CD16	GNPDA1	4,98845E-38	-5,01377E-01	0,118	0,264	9,18723E-34
Mono_CD16	ACTN1	2,04032E-75	-5,01713E-01	0,108	0,327	3,75766E-71
Mono_CD16	TTC3	7,55828E-24	-5,03045E-01	0,197	0,334	1,39201E-19
Mono_CD16	GTF2I	7,26913E-50	-5,03430E-01	0,139	0,318	1,33876E-45
Mono_CD16	PEF1	7,36393E-08	-5,05005E-01	0,174	0,25	1,35621E-03
Mono_CD16	CXCR4	8,86148E-11	-5,06450E-01	0,603	0,579	1,63202E-06

Mono_CD16	IRF2BP2	2,31954E-51	-5,06746E-01	0,145	0,329	4,27190E-47
Mono_CD16	TSPAN3	1,35294E-65	-5,07082E-01	0,123	0,326	2,49171E-61
Mono_CD16	AFF4	9,45594E-10	-5,07583E-01	0,277	0,381	1,74150E-05
Mono_CD16	PTPMT1	3,07494E-24	-5,07858E-01	0,133	0,253	5,66311E-20
Mono_CD16	EIF2AK2	8,54367E-24	-5,08934E-01	0,147	0,266	1,57349E-19
Mono_CD16	ADAP2	4,21610E-51	-5,12771E-01	0,26	0,452	7,76479E-47
Mono_CD16	SLC15A3	1,10727E-36	-5,13381E-01	0,141	0,288	2,03926E-32
Mono_CD16	IL13RA1	4,62519E-111	-5,16109E-01	0,119	0,399	8,51822E-107
Mono_CD16	RPS21	5,85089E-11	-5,16251E-01	0,427	0,547	1,07756E-06
Mono_CD16	UVRAG	3,40034E-12	-5,16735E-01	0,202	0,294	6,26241E-08
Mono_CD16	TUBB4B	9,83598E-15	-5,16880E-01	0,268	0,384	1,81149E-10
Mono_CD16	PDE4B	4,55811E-27	-5,16926E-01	0,227	0,373	8,39467E-23
Mono_CD16	FNDC3B	1,31048E-59	-5,17534E-01	0,104	0,286	2,41352E-55
Mono_CD16	TUBA1C	3,35090E-85	-5,20180E-01	0,143	0,389	6,17135E-81
Mono_CD16	RPL3	1,44022E-10	-5,24065E-01	0,424	0,537	2,65245E-06
Mono_CD16	ATF5	5,13868E-78	-5,33007E-01	0,141	0,37	9,46390E-74
Mono_CD16	MLEC	1,48610E-67	-5,33070E-01	0,144	0,355	2,73696E-63
Mono_CD16	SLC2A3	2,71654E-71	-5,33978E-01	0,222	0,442	5,00306E-67
Mono_CD16	SEC61A1	4,65729E-21	-5,37819E-01	0,19	0,315	8,57734E-17
Mono_CD16	UBE2E2	9,19801E-15	-5,37891E-01	0,197	0,304	1,69400E-10
Mono_CD16	KTN1	9,04163E-43	-5,41642E-01	0,298	0,501	1,66520E-38
Mono_CD16	HMGA1	3,57156E-77	-5,43665E-01	0,173	0,413	6,57775E-73
Mono_CD16	XBP1	8,39800E-34	-5,48054E-01	0,267	0,431	1,54666E-29
Mono_CD16	HSD17B12	7,79615E-52	-5,49720E-01	0,087	0,253	1,43582E-47
Mono_CD16	MRPL55	2,61833E-07	-5,50364E-01	0,202	0,281	4,82218E-03
Mono_CD16	MIF	6,54586E-28	-5,53361E-01	0,604	0,729	1,20555E-23
Mono_CD16	MAN2B1	3,96735E-42	-5,56146E-01	0,302	0,481	7,30666E-38
Mono_CD16	ISG15	1,35272E-13	-5,58091E-01	0,49	0,425	2,49130E-09
Mono_CD16	CYB5R3	7,43833E-13	-5,58238E-01	0,293	0,415	1,36992E-08
Mono_CD16	TCEAL4	1,73598E-91	-5,60545E-01	0,061	0,289	3,19715E-87
Mono_CD16	ENTPD1	3,35620E-63	-5,61645E-01	0,117	0,317	6,18111E-59
Mono_CD16	SSR4	3,19114E-28	-5,62472E-01	0,635	0,758	5,87712E-24
Mono_CD16	LAP3	2,16699E-51	-5,66514E-01	0,401	0,592	3,99094E-47
Mono_CD16	RAB32	3,39390E-58	-5,67490E-01	0,183	0,382	6,25054E-54
Mono_CD16	NPM1	2,04759E-28	-5,71791E-01	0,667	0,77	3,77105E-24
Mono_CD16	TRABD	3,92840E-24	-5,72857E-01	0,222	0,363	7,23494E-20
Mono_CD16	PNP	1,13638E-07	-5,73922E-01	0,195	0,261	2,09288E-03
Mono_CD16	YIF1B	2,38693E-29	-5,75783E-01	0,205	0,356	4,39600E-25
Mono_CD16	PRCP	4,50750E-33	-5,76635E-01	0,234	0,396	8,30146E-29
Mono_CD16	SIRPA	1,40517E-102	-5,77162E-01	0,099	0,36	2,58790E-98
Mono_CD16	DCK	6,00996E-22	-5,77985E-01	0,165	0,284	1,10685E-17
Mono_CD16	ID2	4,48009E-13	-5,78045E-01	0,463	0,561	8,25099E-09

Mono_CD16	ANXA7	6,27570E-24	-5,79028E-01	0,235	0,378	1,15580E-19
Mono_CD16	PDIA3	2,16120E-53	-5,79840E-01	0,51	0,682	3,98029E-49
Mono_CD16	ITPR2	2,67582E-37	-5,82835E-01	0,135	0,283	4,92806E-33
Mono_CD16	CCDC88A	4,50891E-46	-5,84511E-01	0,304	0,51	8,30407E-42
Mono_CD16	CRTAP	2,70525E-98	-5,85975E-01	0,25	0,532	4,98226E-94
Mono_CD16	LAT2	7,19012E-57	-5,86590E-01	0,25	0,462	1,32420E-52
Mono_CD16	AHR	2,13482E-49	-5,91898E-01	0,179	0,362	3,93171E-45
Mono_CD16	MIF4GD	1,93012E-58	-5,93683E-01	0,126	0,319	3,55470E-54
Mono_CD16	HLA-A	2,57719E-51	-5,94137E-01	0,944	0,95	4,74642E-47
Mono_CD16	SMIM19	1,63246E-32	-5,96235E-01	0,127	0,265	3,00651E-28
Mono_CD16	TMEM14C	9,49491E-45	-5,97298E-01	0,336	0,537	1,74868E-40
Mono_CD16	BST2	1,23486E-28	-5,97600E-01	0,625	0,729	2,27424E-24
Mono_CD16	PDIA4	3,89935E-43	-5,98089E-01	0,157	0,327	7,18143E-39
Mono_CD16	S100A8	4,23601E-52	-6,04832E-01	0,542	0,345	7,80147E-48
Mono_CD16	UBE2K	4,38849E-13	-6,05968E-01	0,189	0,289	8,08228E-09
Mono_CD16	ST14	2,88353E-98	-6,07380E-01	0,045	0,275	5,31059E-94
Mono_CD16	SLC3A2	5,73404E-57	-6,08024E-01	0,254	0,471	1,05604E-52
Mono_CD16	EPSTI1	1,88691E-41	-6,13485E-01	0,237	0,416	3,47512E-37
Mono_CD16	ELMO1	1,05318E-57	-6,16733E-01	0,149	0,347	1,93964E-53
Mono_CD16	RPL22L1	2,07594E-21	-6,19667E-01	0,168	0,278	3,82325E-17
Mono_CD16	RPLP0	6,38572E-25	-6,20519E-01	0,43	0,547	1,17606E-20
Mono_CD16	BMP2K	8,27593E-80	-6,23797E-01	0,086	0,3	1,52418E-75
Mono_CD16	DHRS7	4,55741E-34	-6,24025E-01	0,315	0,492	8,39339E-30
Mono_CD16	ATF3	3,86384E-32	-6,24161E-01	0,294	0,439	7,11603E-28
Mono_CD16	PRNP	2,57772E-56	-6,32024E-01	0,202	0,409	4,74739E-52
Mono_CD16	VOPP1	1,66328E-77	-6,32300E-01	0,13	0,356	3,06327E-73
Mono_CD16	PEA15	1,26128E-89	-6,32810E-01	0,26	0,527	2,32289E-85
Mono_CD16	NFKB1	8,98419E-27	-6,35065E-01	0,174	0,293	1,65462E-22
Mono_CD16	MANF	6,83875E-52	-6,38341E-01	0,125	0,301	1,25949E-47
Mono_CD16	ARID5A	1,58119E-40	-6,41214E-01	0,135	0,294	2,91207E-36
Mono_CD16	DBI	3,02806E-32	-6,42710E-01	0,622	0,728	5,57678E-28
Mono_CD16	EGR1	1,77015E-33	-6,45019E-01	0,158	0,291	3,26009E-29
Mono_CD16	CSF2RA	1,51610E-97	-6,47778E-01	0,164	0,427	2,79220E-93
Mono_CD16	PLSCR1	2,18014E-33	-6,47996E-01	0,362	0,549	4,01516E-29
Mono_CD16	HSPA6	6,51926E-07	-6,51223E-01	0,227	0,289	1,20065E-02
Mono_CD16	SSR1	3,18122E-24	-6,51295E-01	0,309	0,469	5,85885E-20
Mono_CD16	HIF1A	9,59997E-41	-6,51635E-01	0,371	0,556	1,76803E-36
Mono_CD16	DEGS1	1,19287E-57	-6,51985E-01	0,138	0,334	2,19691E-53
Mono_CD16	PSMB5	3,32724E-30	-6,54247E-01	0,169	0,321	6,12777E-26
Mono_CD16	FUCA1	5,27340E-63	-6,54476E-01	0,081	0,257	9,71201E-59
Mono_CD16	RANBP2	3,20406E-26	-6,56938E-01	0,185	0,317	5,90092E-22
Mono_CD16	HMGN1	1,66265E-101	-6,57542E-01	0,359	0,636	3,06210E-97

Mono_CD16	ELL2	3,55299E-67	-6,58155E-01	0,097	0,291	6,54354E-63
Mono_CD16	TMED10	2,59086E-71	-6,58951E-01	0,33	0,577	4,77159E-67
Mono_CD16	CAMTA1	5,27552E-30	-6,62178E-01	0,213	0,37	9,71593E-26
Mono_CD16	LAIR1	4,23337E-66	-6,65252E-01	0,318	0,527	7,79660E-62
Mono_CD16	GRSF1	5,37432E-15	-6,66080E-01	0,233	0,344	9,89788E-11
Mono_CD16	HSPD1	2,63202E-14	-6,69201E-01	0,414	0,54	4,84740E-10
Mono_CD16	MTRNR2L8	1,10303E-15	-6,72324E-01	0,343	0,444	2,03144E-11
Mono_CD16	NR3C1	4,76641E-09	-6,72672E-01	0,296	0,391	8,77829E-05
Mono_CD16	DOCK10	3,32060E-47	-6,77119E-01	0,151	0,329	6,11555E-43
Mono_CD16	SERPINB9	2,60231E-18	-6,78145E-01	0,263	0,379	4,79267E-14
Mono_CD16	PSMA3	4,60031E-12	-6,79285E-01	0,267	0,376	8,47239E-08
Mono_CD16	P4HB	7,22347E-136	-6,85703E-01	0,336	0,64	1,33035E-131
Mono_CD16	MGST2	1,51473E-99	-6,91610E-01	0,11	0,369	2,78967E-95
Mono_CD16	PPIB	1,01668E-86	-6,92845E-01	0,663	0,797	1,87241E-82
Mono_CD16	GNA15	3,93270E-57	-6,98597E-01	0,125	0,313	7,24285E-53
Mono_CD16	IFI6	4,24125E-08	-7,04099E-01	0,416	0,491	7,81111E-04
Mono_CD16	TMEM208	4,52016E-10	-7,12821E-01	0,234	0,335	8,32478E-06
Mono_CD16	ACSL3	1,92780E-36	-7,16720E-01	0,116	0,259	3,55043E-32
Mono_CD16	VIM	3,45966E-69	-7,30616E-01	0,92	0,944	6,37166E-65
Mono_CD16	RAB20	1,04360E-65	-7,33029E-01	0,148	0,355	1,92200E-61
Mono_CD16	SRSF2	1,66004E-14	-7,39402E-01	0,436	0,562	3,05729E-10
Mono_CD16	CCS	7,06477E-15	-7,40126E-01	0,168	0,265	1,30112E-10
Mono_CD16	GK	9,85437E-35	-7,51040E-01	0,217	0,37	1,81488E-30
Mono_CD16	FCGR1A	1,17062E-119	-7,55030E-01	0,136	0,422	2,15594E-115
Mono_CD16	MARCKS	2,01600E-100	-7,57205E-01	0,293	0,542	3,71286E-96
Mono_CD16	RALA	2,28984E-53	-7,57322E-01	0,189	0,382	4,21719E-49
Mono_CD16	CCDC107	3,19781E-43	-7,57413E-01	0,143	0,313	5,88941E-39
Mono_CD16	TFEC	5,79611E-85	-7,63288E-01	0,078	0,304	1,06747E-80
Mono_CD16	TMED9	1,89426E-37	-7,64261E-01	0,351	0,531	3,48865E-33
Mono_CD16	C1QBP	3,33885E-25	-7,68514E-01	0,281	0,436	6,14916E-21
Mono_CD16	TSPAN4	1,18221E-63	-7,93591E-01	0,112	0,3	2,17727E-59
Mono_CD16	ADORA3	5,98837E-102	-7,96413E-01	0,04	0,272	1,10288E-97
Mono_CD16	PTAFR	1,01002E-74	-7,98822E-01	0,153	0,385	1,86016E-70
Mono_CD16	ALCAM	6,81353E-110	-7,98839E-01	0,033	0,277	1,25485E-105
Mono_CD16	NFKBIA	9,77501E-34	-8,00900E-01	0,695	0,813	1,80026E-29
Mono_CD16	CD40	1,52081E-44	-8,02524E-01	0,139	0,298	2,80088E-40
Mono_CD16	TNFAIP3	2,19582E-58	-8,05519E-01	0,347	0,551	4,04405E-54
Mono_CD16	PTMS	1,54299E-152	-8,07549E-01	0,058	0,375	2,84173E-148
Mono_CD16	TM6SF1	2,42348E-103	-8,11233E-01	0,044	0,281	4,46333E-99
Mono_CD16	IDH1	1,53540E-63	-8,14275E-01	0,072	0,253	2,82774E-59
Mono_CD16	NDUFS8	1,64546E-28	-8,16768E-01	0,325	0,502	3,03045E-24
Mono_CD16	NDUFC2	2,42252E-14	-8,18921E-01	0,213	0,309	4,46155E-10

Mono_CD16	RGS10	1,97457E-92	-8,19881E-01	0,566	0,747	3,63657E-88
Mono_CD16	HEXB	4,85833E-117	-8,21485E-01	0,225	0,525	8,94758E-113
Mono_CD16	APP	5,55307E-39	-8,25251E-01	0,157	0,315	1,02271E-34
Mono_CD16	HLA-DPA1	5,83099E-130	-8,25455E-01	0,927	0,896	1,07389E-125
Mono_CD16	ABCA1	5,15098E-74	-8,30543E-01	0,112	0,323	9,48655E-70
Mono_CD16	FRMD4B	2,18797E-98	-8,38004E-01	0,058	0,294	4,02959E-94
Mono_CD16	CALR	5,30766E-96	-8,41861E-01	0,53	0,731	9,77511E-92
Mono_CD16	GRN	2,03090E-231	-8,43799E-01	0,634	0,83	3,74031E-227
Mono_CD16	LHFPL2	6,17492E-114	-8,49022E-01	0,041	0,293	1,13724E-109
Mono_CD16	ABHD12	1,87820E-114	-8,50082E-01	0,114	0,39	3,45908E-110
Mono_CD16	CTSZ	4,10789E-97	-8,51971E-01	0,436	0,661	7,56551E-93
Mono_CD16	RAB13	4,27264E-124	-8,58122E-01	0,044	0,315	7,86892E-120
Mono_CD16	BHLHE40	3,59801E-102	-8,61943E-01	0,098	0,35	6,62646E-98
Mono_CD16	ENG	1,66180E-73	-8,70234E-01	0,133	0,356	3,06054E-69
Mono_CD16	PPA1	6,44958E-53	-8,81321E-01	0,278	0,468	1,18782E-48
Mono_CD16	OLR1	2,91161E-136	-8,81558E-01	0,067	0,359	5,36232E-132
Mono_CD16	DOCK4	3,39808E-109	-8,88850E-01	0,048	0,295	6,25825E-105
Mono_CD16	HSPA5	2,74762E-70	-8,94522E-01	0,349	0,57	5,06029E-66
Mono_CD16	YWHAH	8,93374E-111	-8,95435E-01	0,264	0,542	1,64533E-106
Mono_CD16	TMEM176A	2,00113E-74	-9,05289E-01	0,238	0,457	3,68548E-70
Mono_CD16	ARL4C	1,40449E-102	-9,06965E-01	0,091	0,342	2,58664E-98
Mono_CD16	B4GALT1	4,25603E-31	-9,08035E-01	0,185	0,33	7,83834E-27
Mono_CD16	TMEM176B	2,19345E-50	-9,11846E-01	0,373	0,541	4,03968E-46
Mono_CD16	PKIB	1,45426E-105	-9,11878E-01	0,024	0,254	2,67831E-101
Mono_CD16	GADD45B	1,48386E-66	-9,13556E-01	0,457	0,65	2,73282E-62
Mono_CD16	BEX4	5,08919E-97	-9,20794E-01	0,089	0,338	9,37276E-93
Mono_CD16	SLC1A3	4,58184E-131	-9,20937E-01	0,083	0,374	8,43837E-127
Mono_CD16	GLA	1,85659E-21	-9,21666E-01	0,193	0,316	3,41929E-17
Mono_CD16	CIITA	2,58810E-66	-9,25208E-01	0,124	0,333	4,76651E-62
Mono_CD16	PDE4DIP	1,69598E-81	-9,26847E-01	0,105	0,33	3,12348E-77
Mono_CD16	VCAN	2,45670E-06	-9,30626E-01	0,286	0,223	4,52451E-02
Mono_CD16	SEC61B	8,77100E-22	-9,33447E-01	0,639	0,744	1,61536E-17
Mono_CD16	LIPA	6,08666E-83	-9,34214E-01	0,295	0,509	1,12098E-78
Mono_CD16	GPR34	5,15939E-88	-9,60786E-01	0,089	0,315	9,50205E-84
Mono_CD16	CREG1	2,79303E-138	-9,60948E-01	0,222	0,524	5,14392E-134
Mono_CD16	MSR1	1,44738E-97	-9,62034E-01	0,138	0,39	2,66564E-93
Mono_CD16	HSPB1	3,20561E-57	-9,63151E-01	0,48	0,665	5,90378E-53
Mono_CD16	HLA-DRB5	9,31186E-143	-9,66571E-01	0,823	0,848	1,71497E-138
Mono_CD16	HEXA	1,55495E-174	-9,71440E-01	0,129	0,483	2,86375E-170
Mono_CD16	HSD17B4	2,46347E-40	-9,77813E-01	0,113	0,264	4,53697E-36
Mono_CD16	SLAMF8	2,48475E-136	-9,86855E-01	0,047	0,336	4,57616E-132
Mono_CD16	HLA-DRA	1,38282E-244	-9,96059E-01	0,937	0,924	2,54673E-240

Mono_CD16	LPXN	5,15882E-69	-1,00747E+00	0,142	0,356	9,50100E-65
Mono_CD16	JUN	4,39603E-83	-1,00860E+00	0,501	0,718	8,09617E-79
Mono_CD16	ICAM1	2,25535E-49	-1,00979E+00	0,188	0,369	4,15367E-45
Mono_CD16	CD81	6,04706E-188	-1,02789E+00	0,108	0,475	1,11369E-183
Mono_CD16	HLA-DOA	1,95769E-183	-1,02854E+00	0,052	0,406	3,60548E-179
Mono_CD16	MX1	1,53994E-16	-1,02882E+00	0,231	0,337	2,83610E-12
Mono_CD16	HLA-DMB	3,22484E-182	-1,04152E+00	0,322	0,651	5,93918E-178
Mono_CD16	IGFLR1	7,31889E-58	-1,04199E+00	0,168	0,367	1,34792E-53
Mono_CD16	HLA-DQA2	1,41100E-38	-1,04993E+00	0,377	0,518	2,59865E-34
Mono_CD16	PEBP1	9,96823E-159	-1,05050E+00	0,239	0,577	1,83585E-154
Mono_CD16	CD59	8,41111E-157	-1,05731E+00	0,038	0,352	1,54907E-152
Mono_CD16	CCDC50	8,24256E-63	-1,06661E+00	0,094	0,281	1,51803E-58
Mono_CD16	IER3	8,66711E-118	-1,09136E+00	0,241	0,544	1,59622E-113
Mono_CD16	CD63	7,08735E-247	-1,09235E+00	0,531	0,829	1,30528E-242
Mono_CD16	FLOT1	3,01599E-109	-1,10461E+00	0,231	0,536	5,55454E-105
Mono_CD16	TGFBI	1,94412E-87	-1,10566E+00	0,363	0,608	3,58048E-83
Mono_CD16	HLA-DRB1	6,63423E-222	-1,11212E+00	0,921	0,917	1,22183E-217
Mono_CD16	TMEM109	2,87110E-98	-1,13843E+00	0,122	0,379	5,28771E-94
Mono_CD16	IL18	3,94991E-144	-1,14087E+00	0,132	0,454	7,27455E-140
Mono_CD16	HSP90B1	1,44053E-102	-1,14277E+00	0,474	0,709	2,65303E-98
Mono_CD16	ARHGAP18	1,02516E-198	-1,14971E+00	0,104	0,491	1,88803E-194
Mono_CD16	GM2A	7,45188E-166	-1,15736E+00	0,057	0,387	1,37241E-161
Mono_CD16	TGIF1	6,07072E-79	-1,16342E+00	0,108	0,332	1,11804E-74
Mono_CD16	LGALS3BP	2,43836E-134	-1,18003E+00	0,051	0,33	4,49074E-130
Mono_CD16	FPR3	6,50227E-146	-1,18036E+00	0,047	0,35	1,19752E-141
Mono_CD16	HLA-DMA	1,30730E-250	-1,18072E+00	0,547	0,791	2,40766E-246
Mono_CD16	CPM	8,85003E-123	-1,19067E+00	0,096	0,38	1,62991E-118
Mono_CD16	AREG	1,67574E-23	-1,19664E+00	0,173	0,28	3,08620E-19
Mono_CD16	HERPUD1	8,96198E-74	-1,19738E+00	0,547	0,686	1,65053E-69
Mono_CD16	MGAT4A	1,53779E-144	-1,20358E+00	0,062	0,369	2,83214E-140
Mono_CD16	HLA-DPB1	5,29102E-203	-1,22953E+00	0,893	0,883	9,74447E-199
Mono_CD16	CTSD	6,69774E-151	-1,23618E+00	0,627	0,775	1,23352E-146
Mono_CD16	IFI44L	4,24333E-31	-1,24593E+00	0,129	0,259	7,81494E-27
Mono_CD16	EIF4A3	1,59331E-19	-1,24992E+00	0,272	0,408	2,93440E-15
Mono_CD16	AKR1B1	1,50281E-195	-1,25430E+00	0,118	0,482	2,76773E-191
Mono_CD16	CD84	5,03170E-145	-1,25949E+00	0,091	0,404	9,26688E-141
Mono_CD16	TPM4	1,16775E-137	-1,26010E+00	0,299	0,631	2,15064E-133
Mono_CD16	ALDH2	8,41116E-170	-1,27667E+00	0,217	0,564	1,54908E-165
Mono_CD16	CD74	1,69372E-285	-1,29933E+00	0,972	0,963	3,11933E-281
Mono_CD16	LILRB4	2,97197E-142	-1,33525E+00	0,26	0,561	5,47348E-138
Mono_CD16	FABP5	4,02187E-159	-1,34697E+00	0,121	0,459	7,40708E-155
Mono_CD16	CTSL	1,40205E-49	-1,35821E+00	0,379	0,525	2,58215E-45

Mono_CD16	PMP22	1,19241E-113	-1,36987E+00	0,039	0,286	2,19606E-109
Mono_CD16	STMN1	1,09869E-118	-1,37098E+00	0,04	0,296	2,02347E-114
Mono_CD16	NR4A3	6,87249E-49	-1,38231E+00	0,142	0,303	1,26571E-44
Mono_CD16	CTSB	1,05331E-258	-1,38881E+00	0,732	0,877	1,93987E-254
Mono_CD16	HLA-DQB1	7,57211E-271	-1,39801E+00	0,649	0,822	1,39456E-266
Mono_CD16	C1orf54	9,82592E-123	-1,40198E+00	0,032	0,292	1,80964E-118
Mono_CD16	SERPING1	3,98284E-156	-1,40242E+00	0,089	0,409	7,33520E-152
Mono_CD16	IRF8	6,94945E-21	-1,41255E+00	0,26	0,383	1,27988E-16
Mono_CD16	SPINT2	1,50844E-119	-1,41622E+00	0,208	0,509	2,77810E-115
Mono_CD16	ACP5	1,88918E-91	-1,43101E+00	0,168	0,409	3,47931E-87
Mono_CD16	CD99	8,50087E-245	-1,45929E+00	0,203	0,637	1,56561E-240
Mono_CD16	AXL	9,65575E-143	-1,46717E+00	0,011	0,291	1,77830E-138
Mono_CD16	HLA-DQA1	1,14617E-243	-1,47260E+00	0,633	0,789	2,11090E-239
Mono_CD16	LTC4S	1,45301E-147	-1,48863E+00	0,009	0,295	2,67601E-143
Mono_CD16	HLA-DQB2	6,72418E-127	-1,48940E+00	0,056	0,327	1,23839E-122
Mono_CD16	SGK1	1,08207E-173	-1,49781E+00	0,278	0,618	1,99284E-169
Mono_CD16	CREM	1,53633E-61	-1,51316E+00	0,179	0,385	2,82946E-57
Mono_CD16	STAB1	7,25153E-133	-1,51327E+00	0,071	0,356	1,33551E-128
Mono_CD16	FCGR2B	7,97789E-73	-1,51452E+00	0,22	0,433	1,46929E-68
Mono_CD16	C15orf48	3,40426E-96	-1,55369E+00	0,106	0,354	6,26963E-92
Mono_CD16	LDHB	7,99022E-82	-1,60247E+00	0,272	0,52	1,47156E-77
Mono_CD16	CCL3	6,52386E-97	-1,61897E+00	0,143	0,385	1,20150E-92
Mono_CD16	SLCO2B1	3,09143E-208	-1,69376E+00	0,014	0,383	5,69349E-204
Mono_CD16	PLD3	5,06079E-167	-1,69565E+00	0,199	0,523	9,32045E-163
Mono_CD16	DAB2	9,45616E-208	-1,71901E+00	0,067	0,448	1,74154E-203
Mono_CD16	C1QA	1,79088E-144	-1,73293E+00	0,374	0,574	3,29827E-140
Mono_CD16	CD14	8,22014E-201	-1,73846E+00	0,272	0,638	1,51390E-196
Mono_CD16	MS4A6A	1,10138E-263	-1,75386E+00	0,376	0,728	2,02842E-259
Mono_CD16	GPX1	1,72561E-69	-1,78511E+00	0,568	0,548	3,17806E-65
Mono_CD16	TXN	1,45524E-78	-1,82845E+00	0,462	0,675	2,68012E-74
Mono_CD16	VSIG4	1,78151E-163	-1,85151E+00	0,04	0,36	3,28101E-159
Mono_CD16	CAPG	0,00000E+00	-1,88817E+00	0,278	0,759	0,00000E+00
Mono_CD16	GSN	0,00000E+00	-1,93751E+00	0,16	0,652	0,00000E+00
Mono_CD16	A2M	4,42460E-222	-1,96154E+00	0,017	0,404	8,14879E-218
Mono_CD16	RNASE6	5,54103E-239	-1,97744E+00	0,124	0,556	1,02049E-234
Mono_CD16	TCF4	2,95382E-91	-2,01123E+00	0,059	0,282	5,44005E-87
Mono_CD16	CD163	6,26586E-158	-2,06422E+00	0,127	0,459	1,15398E-153
Mono_CD16	LMNA	7,44923E-154	-2,06575E+00	0,062	0,381	1,37192E-149
Mono_CD16	CCL4	4,85043E-47	-2,07952E+00	0,159	0,316	8,93304E-43
Mono_CD16	ALOX5AP	1,48339E-259	-2,08850E+00	0,071	0,522	2,73196E-255
Mono_CD16	PLTP	3,02058E-157	-2,11164E+00	0,034	0,34	5,56301E-153
Mono_CD16	PLD4	5,61917E-77	-2,15271E+00	0,136	0,34	1,03488E-72

Mono_CD16	ZNF331	9,10160E-79	-2,21835E+00	0,131	0,353	1,67624E-74
Mono_CD16	C3	2,03397E-181	-2,29067E+00	0,059	0,397	3,74596E-177
Mono_CD16	TREM2	2,17317E-236	-2,31495E+00	0,019	0,422	4,00233E-232
Mono_CD16	GPNMB	2,81082E-167	-2,38301E+00	0,029	0,347	5,17668E-163
Mono_CD16	LGMN	9,38548E-214	-2,44212E+00	0,042	0,424	1,72852E-209
Mono_CD16	C1QB	2,36270E-220	-2,47490E+00	0,21	0,559	4,35138E-216
Mono_CD16	CD9	4,48872E-282	-2,65587E+00	0,047	0,502	8,26688E-278
Mono_CD16	SERPINF1	1,04673E-221	-3,24192E+00	0,029	0,419	1,92777E-217
Mono_CD16	APOC1	1,05584E-262	-3,26145E+00	0,093	0,524	1,94454E-258
Mono_CD16	RGS1	0,00000E+00	-3,53021E+00	0,098	0,664	0,00000E+00
Mono_CD16	C1QC	1,48396E-298	-3,72412E+00	0,123	0,562	2,73301E-294
Mono_CD16	RNASE1	4,40981E-118	-3,73158E+00	0,034	0,282	8,12154E-114
Mono_CD16	GPR183	2,30509E-194	-3,86255E+00	0,098	0,471	4,24528E-190
Mono_CD16	APOE	6,91554E-250	-4,64116E+00	0,129	0,522	1,27363E-245

cluster	gene	p_val	avg_log2FC	pct.1	pct.2	p_val_adj
Mono_INHBA	CXCL8	0,00000E+00	3,64763E+00	0,686	0,164	0,00000E+00
Mono_INHBA	CCL20	0,00000E+00	3,45965E+00	0,345	0,048	0,00000E+00
Mono_INHBA	CXCL3	0,00000E+00	3,00619E+00	0,569	0,137	0,00000E+00
Mono_INHBA	INHBA	0,00000E+00	2,89517E+00	0,469	0,04	0,00000E+00
Mono_INHBA	IL1B	0,00000E+00	2,89355E+00	0,697	0,313	0,00000E+00
Mono_INHBA	CCL3L1	0,00000E+00	2,89189E+00	0,483	0,142	0,00000E+00
Mono_INHBA	CXCL1	0,00000E+00	2,89117E+00	0,255	0,037	0,00000E+00
Mono_INHBA	PTGS2	0,00000E+00	2,87956E+00	0,53	0,158	0,00000E+00
Mono_INHBA	IL6	0,00000E+00	2,76744E+00	0,329	0,029	0,00000E+00
Mono_INHBA	CXCL2	0,00000E+00	2,52169E+00	0,633	0,202	0,00000E+00
Mono_INHBA	PLIN2	6,74768E-168	2,50357E+00	0,648	0,489	1,24272E-163
Mono_INHBA	CCL3	0,00000E+00	2,47252E+00	0,768	0,352	0,00000E+00
Mono_INHBA	IL1RN	0,00000E+00	2,44833E+00	0,676	0,191	0,00000E+00
Mono_INHBA	CCL4	0,00000E+00	2,43221E+00	0,634	0,29	0,00000E+00
Mono_INHBA	C15orf48	0,00000E+00	2,42543E+00	0,807	0,316	0,00000E+00
Mono_INHBA	G0S2	0,00000E+00	2,35449E+00	0,564	0,162	0,00000E+00
Mono_INHBA	CCL4L2	0,00000E+00	2,30601E+00	0,446	0,179	0,00000E+00
Mono_INHBA	TIMP1	0,00000E+00	2,27967E+00	0,878	0,636	0,00000E+00
Mono_INHBA	TNIP3	0,00000E+00	2,26109E+00	0,4	0,021	0,00000E+00
Mono_INHBA	SPP1	0,00000E+00	2,17219E+00	0,487	0,21	0,00000E+00
Mono_INHBA	CSTB	0,00000E+00	2,12323E+00	0,943	0,78	0,00000E+00
Mono_INHBA	SOD2	0,00000E+00	2,11037E+00	0,884	0,637	0,00000E+00
Mono_INHBA	EREG	0,00000E+00	1,98130E+00	0,485	0,137	0,00000E+00
Mono_INHBA	TNF	2,30516E-123	1,94099E+00	0,323	0,163	4,24541E-119
Mono_INHBA	IL1A	0,00000E+00	1,90789E+00	0,278	0,032	0,00000E+00
Mono_INHBA	TREM1	0,00000E+00	1,86433E+00	0,593	0,216	0,00000E+00
Mono_INHBA	TNFAIP6	0,00000E+00	1,85652E+00	0,34	0,048	0,00000E+00
Mono_INHBA	PLAUR	0,00000E+00	1,77407E+00	0,924	0,623	0,00000E+00
Mono_INHBA	ADM	0,00000E+00	1,73165E+00	0,406	0,128	0,00000E+00
Mono_INHBA	AQP9	0,00000E+00	1,67023E+00	0,428	0,097	0,00000E+00
Mono_INHBA	BCL2A1	0,00000E+00	1,66597E+00	0,796	0,45	0,00000E+00
Mono_INHBA	MMP19	0,00000E+00	1,65728E+00	0,459	0,107	0,00000E+00
Mono_INHBA	SLC25A37	6,13291E-267	1,63134E+00	0,538	0,276	1,12950E-262
Mono_INHBA	MIF	2,80022E-259	1,61956E+00	0,847	0,716	5,15717E-255
Mono_INHBA	VCAN	1,54293E-238	1,60770E+00	0,489	0,209	2,84162E-234
Mono_INHBA	IER3	0,00000E+00	1,60093E+00	0,785	0,517	0,00000E+00
Mono_INHBA	MMP9	2,75106E-218	1,58893E+00	0,281	0,097	5,06662E-214
Mono_INHBA	MARCO	4,27614E-153	1,58203E+00	0,288	0,12	7,87536E-149
Mono_INHBA	BNIP3	0,00000E+00	1,58194E+00	0,338	0,098	0,00000E+00
Mono_INHBA	CD300E	2,22340E-251	1,53066E+00	0,394	0,152	4,09484E-247

Mono_INHBA	HK2	0,00000E+00	1,52947E+00	0,37	0,125	0,00000E+00
Mono_INHBA	ANPEP	0,00000E+00	1,52467E+00	0,546	0,188	0,00000E+00
Mono_INHBA	FTH1	0,00000E+00	1,50949E+00	1	0,997	0,00000E+00
Mono_INHBA	NLRP3	1,01992E-72	1,48914E+00	0,39	0,255	1,87840E-68
Mono_INHBA	IVNS1ABP	3,99619E-112	1,46454E+00	0,519	0,357	7,35978E-108
Mono_INHBA	NFKBIA	9,14811E-231	1,45466E+00	0,895	0,803	1,68481E-226
Mono_INHBA	SLC2A3	0,00000E+00	1,43802E+00	0,752	0,414	0,00000E+00
Mono_INHBA	LDHA	1,03553E-232	1,43304E+00	0,822	0,707	1,90714E-228
Mono_INHBA	CD44	0,00000E+00	1,42550E+00	0,889	0,664	0,00000E+00
Mono_INHBA	P4HA1	3,63129E-70	1,40577E+00	0,333	0,212	6,68775E-66
Mono_INHBA	SLC11A1	2,84194E-273	1,39456E+00	0,739	0,45	5,23401E-269
Mono_INHBA	CLEC5A	9,62128E-136	1,37032E+00	0,357	0,18	1,77195E-131
Mono_INHBA	NINJ1	9,89639E-303	1,36119E+00	0,769	0,547	1,82262E-298
Mono_INHBA	GAPDH	0,00000E+00	1,33547E+00	0,989	0,958	0,00000E+00
Mono_INHBA	SLC39A8	0,00000E+00	1,32339E+00	0,365	0,109	0,00000E+00
Mono_INHBA	UPP1	1,00521E-304	1,31958E+00	0,693	0,415	1,85130E-300
Mono_INHBA	SDC4	1,59503E-301	1,31590E+00	0,287	0,083	2,93757E-297
Mono_INHBA	SDC2	0,00000E+00	1,31575E+00	0,359	0,086	0,00000E+00
Mono_INHBA	EHD1	0,00000E+00	1,29739E+00	0,443	0,145	0,00000E+00
Mono_INHBA	SOCS3	4,29603E-269	1,26413E+00	0,606	0,342	7,91200E-265
Mono_INHBA	NDRG1	0,00000E+00	1,25444E+00	0,443	0,183	0,00000E+00
Mono_INHBA	NAMPT	0,00000E+00	1,25122E+00	0,8	0,56	0,00000E+00
Mono_INHBA	RGCC	2,04705E-205	1,24470E+00	0,446	0,213	3,77006E-201
Mono_INHBA	GK	2,40153E-279	1,23951E+00	0,614	0,349	4,42290E-275
Mono_INHBA	IL7R	1,48521E-284	1,19860E+00	0,35	0,118	2,73531E-280
Mono_INHBA	EMP1	0,00000E+00	1,19671E+00	0,361	0,065	0,00000E+00
Mono_INHBA	FNDC3B	7,41033E-272	1,18684E+00	0,529	0,264	1,36476E-267
Mono_INHBA	ATP13A3	0,00000E+00	1,17967E+00	0,504	0,193	0,00000E+00
Mono_INHBA	ERO1A	0,00000E+00	1,17782E+00	0,399	0,116	0,00000E+00
Mono_INHBA	ICAM1	4,15081E-174	1,16873E+00	0,566	0,35	7,64455E-170
Mono_INHBA	PDE4DIP	1,77557E-301	1,16848E+00	0,579	0,306	3,27008E-297
Mono_INHBA	LGALS3	1,96044E-178	1,16261E+00	0,854	0,716	3,61055E-174
Mono_INHBA	FBP1	4,17191E-07	1,16174E+00	0,357	0,349	7,68340E-03
Mono_INHBA	HPCAL1	8,73705E-26	1,15734E+00	0,31	0,243	1,60910E-21
Mono_INHBA	TNFRSF1B	5,70211E-222	1,15211E+00	0,708	0,53	1,05016E-217
Mono_INHBA	P2RX4	1,00447E-72	1,14305E+00	0,353	0,226	1,84994E-68
Mono_INHBA	CTSL	0,00000E+00	1,14010E+00	0,834	0,5	0,00000E+00
Mono_INHBA	PNP	1,21942E-110	1,13840E+00	0,415	0,249	2,24580E-106
Mono_INHBA	VEGFA	0,00000E+00	1,12407E+00	0,537	0,198	0,00000E+00
Mono_INHBA	MT2A	1,13755E-109	1,11986E+00	0,724	0,622	2,09502E-105
Mono_INHBA	ALDOA	7,16692E-145	1,11067E+00	0,842	0,755	1,31993E-140

Mono_INHBA	TNFAIP3	1,31884E-155	1,10457E+00	0,71	0,533	2,42890E-151
Mono_INHBA	S100A10	0,00000E+00	1,09823E+00	0,963	0,83	0,00000E+00
Mono_INHBA	ETS2	2,79250E-193	1,09801E+00	0,63	0,413	5,14295E-189
Mono_INHBA	TPI1	7,49110E-226	1,08683E+00	0,896	0,822	1,37964E-221
Mono_INHBA	HBEGF	3,23016E-114	1,08319E+00	0,45	0,265	5,94899E-110
Mono_INHBA	TRAF1	0,00000E+00	1,08041E+00	0,368	0,118	0,00000E+00
Mono_INHBA	GPI	3,76141E-17	1,05966E+00	0,442	0,414	6,92739E-13
Mono_INHBA	ENO1	2,90097E-136	1,05925E+00	0,843	0,756	5,34271E-132
Mono_INHBA	GPNMB	2,14489E-25	1,05349E+00	0,411	0,33	3,95024E-21
Mono_INHBA	PPIF	1,71457E-174	1,04655E+00	0,547	0,346	3,15772E-170
Mono_INHBA	DNAJB1	1,29045E-11	1,04372E+00	0,486	0,562	2,37662E-07
Mono_INHBA	ABL2	2,35503E-156	1,03393E+00	0,362	0,177	4,33727E-152
Mono_INHBA	HSPB1	1,36923E-36	1,03171E+00	0,541	0,666	2,52171E-32
Mono_INHBA	PHLDA1	5,90132E-206	1,02894E+00	0,337	0,134	1,08685E-201
Mono_INHBA	TNIP1	9,78514E-219	1,02793E+00	0,551	0,334	1,80213E-214
Mono_INHBA	ITGA5	3,48444E-190	1,02183E+00	0,396	0,185	6,41729E-186
Mono_INHBA	DENND5A	9,58647E-96	1,02110E+00	0,305	0,163	1,76554E-91
Mono_INHBA	PIM3	3,36976E-119	1,02005E+00	0,455	0,276	6,20609E-115
Mono_INHBA	SLC16A10	0,00000E+00	1,01852E+00	0,312	0,07	0,00000E+00
Mono_INHBA	CEBPB	2,89398E-239	1,01804E+00	0,692	0,458	5,32985E-235
Mono_INHBA	OLR1	2,98791E-59	1,01482E+00	0,461	0,341	5,50284E-55
Mono_INHBA	PID1	1,03678E-90	1,00916E+00	0,273	0,141	1,90943E-86
Mono_INHBA	KYNU	1,83113E-83	1,00594E+00	0,443	0,308	3,37239E-79
Mono_INHBA	RALA	1,07303E-145	1,00557E+00	0,543	0,364	1,97619E-141
Mono_INHBA	ANXA5	0,00000E+00	9,97329E-01	0,909	0,765	0,00000E+00
Mono_INHBA	BNIP3L	3,39650E-194	9,90993E-01	0,71	0,554	6,25533E-190
Mono_INHBA	RAB13	9,23220E-169	9,56025E-01	0,494	0,293	1,70029E-164
Mono_INHBA	SPHK1	9,39714E-221	9,49517E-01	0,385	0,167	1,73067E-216
Mono_INHBA	THBS1	1,24318E-35	9,33970E-01	0,263	0,174	2,28956E-31
Mono_INHBA	ANXA1	4,10533E-49	9,25559E-01	0,743	0,685	7,56079E-45
Mono_INHBA	HIF1A	1,50695E-128	9,17008E-01	0,672	0,542	2,77535E-124
Mono_INHBA	RNF19B	8,62603E-196	9,16537E-01	0,339	0,145	1,58866E-191
Mono_INHBA	DUSP4	9,01814E-230	9,09972E-01	0,303	0,105	1,66087E-225
Mono_INHBA	BCAT1	2,92937E-185	9,05081E-01	0,438	0,226	5,39502E-181
Mono_INHBA	ADAM8	1,23669E-191	9,02980E-01	0,435	0,214	2,27762E-187
Mono_INHBA	PKM	2,30001E-248	9,01422E-01	0,888	0,777	4,23593E-244
Mono_INHBA	PNRC1	3,95229E-154	8,99576E-01	0,855	0,774	7,27893E-150
Mono_INHBA	CD82	3,60103E-50	8,98382E-01	0,346	0,233	6,63202E-46
Mono_INHBA	CCRL2	9,65219E-65	8,85201E-01	0,293	0,178	1,77764E-60
Mono_INHBA	ABCA1	2,29648E-161	8,84535E-01	0,512	0,303	4,22943E-157
Mono_INHBA	WTAP	4,22875E-165	8,84424E-01	0,546	0,354	7,78809E-161

Mono_INHBA	EGR1	7,56583E-13	8,82280E-01	0,342	0,283	1,39340E-08
Mono_INHBA	LGALS1	0,00000E+00	8,79203E-01	0,944	0,829	0,00000E+00
Mono_INHBA	ATP2B1	2,26364E-36	8,79203E-01	0,423	0,344	4,16894E-32
Mono_INHBA	GSTO1	2,66508E-155	8,79002E-01	0,753	0,611	4,90827E-151
Mono_INHBA	PIM1	1,97453E-97	8,78190E-01	0,267	0,134	3,63649E-93
Mono_INHBA	VIM	0,00000E+00	8,76081E-01	0,985	0,941	0,00000E+00
Mono_INHBA	ACSL1	3,29297E-59	8,73449E-01	0,489	0,383	6,06467E-55
Mono_INHBA	MGAT1	4,51671E-11	8,68978E-01	0,637	0,623	8,31842E-07
Mono_INHBA	SDCBP	1,89982E-52	8,66043E-01	0,792	0,719	3,49890E-48
Mono_INHBA	PGK1	1,38109E-118	8,55702E-01	0,779	0,702	2,54355E-114
Mono_INHBA	GPCPD1	1,85009E-53	8,50552E-01	0,389	0,274	3,40731E-49
Mono_INHBA	APLP2	2,93763E-57	8,45328E-01	0,686	0,589	5,41023E-53
Mono_INHBA	DMXL2	1,23128E-106	8,38743E-01	0,427	0,257	2,26765E-102
Mono_INHBA	BASP1	7,79610E-108	8,38519E-01	0,49	0,338	1,43581E-103
Mono_INHBA	MT1X	2,31474E-39	8,37931E-01	0,314	0,23	4,26306E-35
Mono_INHBA	DUSP2	7,81550E-65	8,33544E-01	0,429	0,293	1,43938E-60
Mono_INHBA	NFKB1	3,20396E-127	8,29458E-01	0,453	0,278	5,90073E-123
Mono_INHBA	TOM1	4,78061E-83	8,20997E-01	0,421	0,275	8,80445E-79
Mono_INHBA	FCGR2A	2,74423E-67	8,15176E-01	0,75	0,624	5,05405E-63
Mono_INHBA	ZC3H12A	2,81140E-100	8,05190E-01	0,294	0,15	5,17776E-96
Mono_INHBA	RIPK2	7,93865E-42	8,02060E-01	0,366	0,268	1,46206E-37
Mono_INHBA	INSIG1	1,84302E-108	7,83776E-01	0,447	0,286	3,39429E-104
Mono_INHBA	PFKFB3	4,93700E-143	7,79956E-01	0,507	0,324	9,09248E-139
Mono_INHBA	METRNL	1,43298E-169	7,74633E-01	0,454	0,242	2,63912E-165
Mono_INHBA	PLK3	1,52261E-20	7,71141E-01	0,314	0,249	2,80420E-16
Mono_INHBA	CYSTM1	2,64975E-213	7,60202E-01	0,567	0,34	4,88005E-209
Mono_INHBA	PDLIM7	1,35374E-120	7,57575E-01	0,353	0,187	2,49318E-116
Mono_INHBA	MMP14	8,65902E-96	7,55269E-01	0,36	0,216	1,59473E-91
Mono_INHBA	PTPN12	7,38077E-94	7,54979E-01	0,366	0,219	1,35932E-89
Mono_INHBA	DRAM1	1,48605E-161	7,53704E-01	0,398	0,207	2,73687E-157
Mono_INHBA	C1orf122	2,52689E-137	7,52829E-01	0,38	0,21	4,65377E-133
Mono_INHBA	MTF1	3,78630E-144	7,52508E-01	0,26	0,107	6,97323E-140
Mono_INHBA	ZFYVE16	2,56105E-144	7,50431E-01	0,379	0,198	4,71669E-140
Mono_INHBA	DUSP6	3,85402E-21	7,45480E-01	0,36	0,284	7,09795E-17
Mono_INHBA	SRGN	9,13722E-140	7,41485E-01	0,964	0,941	1,68280E-135
Mono_INHBA	SERPINB9	2,86609E-83	7,40149E-01	0,491	0,368	5,27848E-79
Mono_INHBA	ADAM9	2,47677E-45	7,30765E-01	0,323	0,223	4,56146E-41
Mono_INHBA	FLNA	0,00000E+00	7,30706E-01	0,751	0,433	0,00000E+00
Mono_INHBA	OTUD1	2,91151E-104	7,28777E-01	0,28	0,139	5,36213E-100
Mono_INHBA	SLC16A3	2,47114E-77	7,27551E-01	0,623	0,505	4,55110E-73
Mono_INHBA	SLC43A3	9,36354E-127	7,23245E-01	0,409	0,232	1,72448E-122

Mono_INHBA	BIRC3	1,89546E-24	7,20548E-01	0,296	0,218	3,49086E-20
Mono_INHBA	NFKBIZ	3,17461E-63	7,20330E-01	0,579	0,447	5,84667E-59
Mono_INHBA	FTL	1,00532E-114	7,20252E-01	0,999	0,991	1,85151E-110
Mono_INHBA	BTG1	8,13564E-131	7,18950E-01	0,893	0,805	1,49834E-126
Mono_INHBA	LIMS1	1,03003E-83	7,17214E-01	0,664	0,548	1,89700E-79
Mono_INHBA	RNASE1	9,98227E-18	7,15788E-01	0,342	0,268	1,83844E-13
Mono_INHBA	FABP5	1,06816E-24	7,14260E-01	0,491	0,444	1,96722E-20
Mono_INHBA	ARID5B	1,44119E-58	7,08036E-01	0,25	0,146	2,65424E-54
Mono_INHBA	IFI30	1,52261E-145	7,06061E-01	0,736	0,558	2,80420E-141
Mono_INHBA	QSOX1	2,17813E-47	7,05356E-01	0,383	0,283	4,01146E-43
Mono_INHBA	VKORC1	1,32895E-35	7,01686E-01	0,32	0,239	2,44753E-31
Mono_INHBA	RNF145	7,21281E-93	7,01456E-01	0,414	0,267	1,32838E-88
Mono_INHBA	TNFAIP8	3,09096E-75	7,00348E-01	0,453	0,329	5,69261E-71
Mono_INHBA	MYADM	1,36302E-55	6,97572E-01	0,459	0,326	2,51028E-51
Mono_INHBA	LILRB1	1,31368E-26	6,95577E-01	0,425	0,374	2,41941E-22
Mono_INHBA	ACTN1	6,24812E-98	6,95499E-01	0,468	0,31	1,15072E-93
Mono_INHBA	EVI2A	3,92378E-11	6,88391E-01	0,212	0,277	7,22643E-07
Mono_INHBA	SAMSN1	1,28475E-50	6,79039E-01	0,565	0,458	2,36613E-46
Mono_INHBA	FNIP2	2,05725E-99	6,76124E-01	0,36	0,213	3,78884E-95
Mono_INHBA	MYO1G	5,57319E-161	6,73947E-01	0,484	0,271	1,02641E-156
Mono_INHBA	BTG3	1,82570E-70	6,72084E-01	0,276	0,159	3,36240E-66
Mono_INHBA	IL4I1	5,91130E-85	6,68799E-01	0,369	0,226	1,08868E-80
Mono_INHBA	NECTIN2	8,31010E-132	6,67810E-01	0,334	0,168	1,53047E-127
Mono_INHBA	PHLDA2	3,59771E-70	6,64579E-01	0,281	0,16	6,62590E-66
Mono_INHBA	JAK1	3,01607E-64	6,55855E-01	0,605	0,517	5,55470E-60
Mono_INHBA	NUMB	6,39162E-13	6,53901E-01	0,373	0,331	1,17714E-08
Mono_INHBA	CD93	4,85310E-72	6,53080E-01	0,402	0,272	8,93796E-68
Mono_INHBA	DDIT4	2,75641E-11	6,50661E-01	0,541	0,493	5,07649E-07
Mono_INHBA	NOP10	4,91816E-114	6,44365E-01	0,74	0,625	9,05778E-110
Mono_INHBA	THBD	2,78567E-13	6,44072E-01	0,255	0,205	5,13036E-09
Mono_INHBA	LILRB2	2,20884E-26	6,41158E-01	0,51	0,454	4,06801E-22
Mono_INHBA	DDX60L	3,97312E-105	6,37603E-01	0,309	0,162	7,31729E-101
Mono_INHBA	ADAM10	1,42196E-07	6,35751E-01	0,318	0,285	2,61881E-03
Mono_INHBA	PIK3AP1	1,14826E-114	6,34232E-01	0,458	0,295	2,11475E-110
Mono_INHBA	BACH1	2,58245E-79	6,31819E-01	0,44	0,305	4,75610E-75
Mono_INHBA	CD58	8,25477E-08	6,31246E-01	0,258	0,317	1,52028E-03
Mono_INHBA	SQOR	1,21408E-94	6,30701E-01	0,324	0,184	2,23598E-90
Mono_INHBA	ACSL4	1,83126E-79	6,29295E-01	0,405	0,268	3,37264E-75
Mono_INHBA	CARD19	2,38306E-155	6,25042E-01	0,328	0,155	4,38888E-151
Mono_INHBA	GNA15	3,40484E-80	6,24753E-01	0,439	0,298	6,27070E-76
Mono_INHBA	CD9	3,80658E-22	6,24577E-01	0,393	0,491	7,01059E-18

Mono_INHBA	PLIN3	6,00621E-13	6,24564E-01	0,406	0,37	1,10616E-08
Mono_INHBA	PLA2G7	1,55592E-22	6,24463E-01	0,265	0,192	2,86554E-18
Mono_INHBA	LPCAT1	4,42563E-143	6,22768E-01	0,305	0,138	8,15068E-139
Mono_INHBA	EPB41L3	3,77928E-35	6,17500E-01	0,38	0,299	6,96030E-31
Mono_INHBA	CALM1	2,55453E-245	6,16516E-01	0,875	0,79	4,70469E-241
Mono_INHBA	MRPS6	1,27233E-09	6,13612E-01	0,349	0,323	2,34325E-05
Mono_INHBA	KIFC3	3,82091E-189	6,12468E-01	0,264	0,095	7,03697E-185
Mono_INHBA	C4orf3	2,34856E-113	6,10263E-01	0,749	0,666	4,32535E-109
Mono_INHBA	TFRC	8,72635E-110	6,08800E-01	0,39	0,228	1,60713E-105
Mono_INHBA	FLOT1	2,29194E-06	6,00736E-01	0,536	0,524	4,22106E-02
Mono_INHBA	RALGDS	1,20677E-84	5,99593E-01	0,315	0,182	2,22251E-80
Mono_INHBA	SAT1	2,84840E-166	5,95273E-01	0,991	0,945	5,24589E-162
Mono_INHBA	RIN2	5,66810E-81	5,92332E-01	0,255	0,134	1,04389E-76
Mono_INHBA	TANK	5,41314E-07	5,92099E-01	0,318	0,292	9,96938E-03
Mono_INHBA	DUSP5	8,52380E-166	5,91055E-01	0,299	0,124	1,56983E-161
Mono_INHBA	AREG	2,20535E-20	5,83154E-01	0,338	0,272	4,06159E-16
Mono_INHBA	CPD	1,35708E-107	5,81383E-01	0,259	0,122	2,49933E-103
Mono_INHBA	TUBB4B	2,17140E-17	5,70220E-01	0,437	0,376	3,99908E-13
Mono_INHBA	PPP1R15B	1,43990E-38	5,69360E-01	0,293	0,201	2,65187E-34
Mono_INHBA	ELOC	1,14518E-94	5,63809E-01	0,391	0,247	2,10908E-90
Mono_INHBA	SEC62	1,53460E-17	5,57971E-01	0,578	0,553	2,82628E-13
Mono_INHBA	SIRPA	1,01450E-33	5,56239E-01	0,43	0,345	1,86840E-29
Mono_INHBA	ERGIC1	2,73712E-30	5,53476E-01	0,362	0,287	5,04095E-26
Mono_INHBA	SIPA1L1	5,96312E-29	5,52849E-01	0,288	0,207	1,09823E-24
Mono_INHBA	IRAK1	6,22797E-64	5,48935E-01	0,274	0,162	1,14701E-59
Mono_INHBA	PMP22	2,94581E-100	5,48267E-01	0,434	0,268	5,42529E-96
Mono_INHBA	CD59	1,65242E-30	5,47972E-01	0,406	0,336	3,04325E-26
Mono_INHBA	SLC43A2	4,44284E-42	5,46762E-01	0,447	0,363	8,18237E-38
Mono_INHBA	CIB1	3,90348E-18	5,46454E-01	0,634	0,611	7,18904E-14
Mono_INHBA	EMP3	4,32804E-45	5,43070E-01	0,774	0,726	7,97096E-41
Mono_INHBA	SNX9	8,55625E-41	5,41550E-01	0,373	0,283	1,57581E-36
Mono_INHBA	CTNNB1	1,39763E-18	5,40398E-01	0,429	0,376	2,57402E-14
Mono_INHBA	RAB20	9,19937E-09	5,37333E-01	0,383	0,345	1,69425E-04
Mono_INHBA	SMS	2,78503E-26	5,34705E-01	0,484	0,423	5,12919E-22
Mono_INHBA	CD55	7,26619E-16	5,34671E-01	0,482	0,426	1,33821E-11
Mono_INHBA	RAB1A	2,87134E-09	5,31763E-01	0,417	0,395	5,28814E-05
Mono_INHBA	PDXK	1,25275E-126	5,30583E-01	0,468	0,291	2,30718E-122
Mono_INHBA	BZW1	2,02765E-63	5,22696E-01	0,547	0,436	3,73432E-59
Mono_INHBA	GRAMD1A	7,42387E-95	5,20215E-01	0,309	0,175	1,36725E-90
Mono_INHBA	ARL8B	1,59586E-93	5,18997E-01	0,54	0,395	2,93909E-89
Mono_INHBA	HSPD1	4,23013E-75	5,13941E-01	0,351	0,547	7,79063E-71

Mono_INHBA	LILRB3	2,08898E-27	5,13239E-01	0,301	0,225	3,84728E-23
Mono_INHBA	PPP1R15A	1,27144E-85	5,12281E-01	0,781	0,65	2,34161E-81
Mono_INHBA	SH3BGRL3	3,36112E-82	5,06695E-01	0,956	0,931	6,19018E-78
Mono_INHBA	CFLAR	8,92141E-73	5,05125E-01	0,602	0,506	1,64306E-68
Mono_INHBA	ITGB1	1,84531E-60	5,02827E-01	0,564	0,469	3,39851E-56
Mono_INHBA	GLUL	8,85061E-07	5,02045E-01	0,816	0,803	1,63002E-02
Mono_INHBA	SQSTM1	4,56398E-32	5,01393E-01	0,723	0,671	8,40548E-28
Mono_INHBA	OASL	3,09560E-163	4,99084E-01	0,26	0,102	5,70116E-159
Mono_INHBA	ITGAX	2,17546E-150	4,98320E-01	0,59	0,388	4,00654E-146
Mono_INHBA	BEST1	1,86549E-24	4,96857E-01	0,295	0,225	3,43567E-20
Mono_INHBA	RYBP	2,99057E-96	4,92857E-01	0,26	0,128	5,50773E-92
Mono_INHBA	VTI1B	2,85552E-14	4,91432E-01	0,294	0,383	5,25902E-10
Mono_INHBA	TLR2	1,62034E-07	4,90405E-01	0,405	0,385	2,98419E-03
Mono_INHBA	CALM3	6,04567E-12	4,89741E-01	0,537	0,506	1,11343E-07
Mono_INHBA	TNFAIP2	1,38760E-41	4,86577E-01	0,486	0,401	2,55554E-37
Mono_INHBA	BCL3	1,82072E-37	4,84993E-01	0,305	0,213	3,35321E-33
Mono_INHBA	PTTG1IP	4,19994E-19	4,80110E-01	0,538	0,5	7,73503E-15
Mono_INHBA	HSPH1	1,82536E-32	4,77117E-01	0,291	0,423	3,36177E-28
Mono_INHBA	PGAM1	3,38126E-26	4,68520E-01	0,593	0,563	6,22726E-22
Mono_INHBA	CTSB	8,01659E-150	4,67118E-01	0,941	0,868	1,47642E-145
Mono_INHBA	HEXA	4,72186E-60	4,65315E-01	0,313	0,479	8,69624E-56
Mono_INHBA	CD83	4,19726E-78	4,63238E-01	0,63	0,48	7,73009E-74
Mono_INHBA	ELL2	9,13587E-107	4,62977E-01	0,449	0,274	1,68255E-102
Mono_INHBA	ANXA2	9,08103E-158	4,61320E-01	0,872	0,735	1,67245E-153
Mono_INHBA	JARID2	3,18084E-39	4,60721E-01	0,297	0,207	5,85816E-35
Mono_INHBA	SLC7A5	6,37560E-79	4,60557E-01	0,35	0,209	1,17419E-74
Mono_INHBA	BRI3	1,66510E-66	4,59764E-01	0,525	0,395	3,06661E-62
Mono_INHBA	TXN	1,03031E-125	4,59672E-01	0,786	0,66	1,89753E-121
Mono_INHBA	STARD3NL	8,33262E-16	4,51429E-01	0,316	0,268	1,53462E-11
Mono_INHBA	CD63	7,06720E-23	4,51056E-01	0,867	0,815	1,30157E-18
Mono_INHBA	FAM107B	2,25592E-48	4,49553E-01	0,274	0,175	4,15472E-44
Mono_INHBA	NAA50	6,02331E-09	4,49349E-01	0,253	0,218	1,10931E-04
Mono_INHBA	SLC44A1	9,22380E-44	4,49114E-01	0,274	0,18	1,69875E-39
Mono_INHBA	UBE2B	3,64072E-17	4,48689E-01	0,52	0,487	6,70511E-13
Mono_INHBA	SPAG9	3,88247E-56	4,48069E-01	0,41	0,297	7,15034E-52
Mono_INHBA	S100A11	4,07280E-56	4,43588E-01	0,965	0,936	7,50088E-52
Mono_INHBA	PLEKHB2	4,03608E-56	4,42535E-01	0,527	0,435	7,43326E-52
Mono_INHBA	GRINA	4,74386E-62	4,40541E-01	0,751	0,679	8,73677E-58
Mono_INHBA	SERTAD1	2,54329E-13	4,38159E-01	0,37	0,323	4,68398E-09
Mono_INHBA	SERPINA1	2,91555E-12	4,36335E-01	0,81	0,716	5,36957E-08
Mono_INHBA	S100A6	6,49183E-92	4,34424E-01	0,937	0,871	1,19560E-87

Mono_INHBA	EMD	4,10382E-32	4,33775E-01	0,484	0,412	7,55801E-28
Mono_INHBA	POR	9,20732E-40	4,33254E-01	0,266	0,177	1,69571E-35
Mono_INHBA	STK10	4,23836E-13	4,32336E-01	0,289	0,241	7,80579E-09
Mono_INHBA	NFKB2	1,19068E-81	4,29854E-01	0,358	0,218	2,19287E-77
Mono_INHBA	NABP1	4,43757E-11	4,29686E-01	0,374	0,337	8,17268E-07
Mono_INHBA	ATF3	3,66830E-62	4,24280E-01	0,581	0,424	6,75591E-58
Mono_INHBA	RIOK3	3,53945E-31	4,24144E-01	0,346	0,266	6,51860E-27
Mono_INHBA	MAP2K3	4,64537E-118	4,24103E-01	0,433	0,26	8,55537E-114
Mono_INHBA	GRB2	2,40300E-60	4,22750E-01	0,735	0,676	4,42560E-56
Mono_INHBA	FOSL2	4,70124E-193	4,20874E-01	0,522	0,282	8,65827E-189
Mono_INHBA	SH3BP5	6,18838E-47	4,19582E-01	0,445	0,331	1,13971E-42
Mono_INHBA	TRAPPC5	6,73099E-18	4,17439E-01	0,297	0,234	1,23965E-13
Mono_INHBA	CREG1	5,11522E-30	4,16424E-01	0,573	0,509	9,42069E-26
Mono_INHBA	CDC42EP3	1,76028E-44	4,14421E-01	0,266	0,17	3,24192E-40
Mono_INHBA	TCIRG1	3,67357E-50	4,13113E-01	0,553	0,462	6,76561E-46
Mono_INHBA	FCER1G	2,78514E-84	4,12519E-01	0,959	0,91	5,12940E-80
Mono_INHBA	DNM2	2,09461E-20	4,11163E-01	0,356	0,293	3,85765E-16
Mono_INHBA	FAM50A	1,54520E-11	4,10376E-01	0,343	0,302	2,84579E-07
Mono_INHBA	MMP24OS	1,83814E-53	4,09339E-01	0,294	0,19	3,38530E-49
Mono_INHBA	SLA	3,98691E-39	4,09110E-01	0,287	0,425	7,34270E-35
Mono_INHBA	SELENOK	1,85952E-58	4,08814E-01	0,395	0,271	3,42468E-54
Mono_INHBA	IRAK3	3,68465E-19	4,08768E-01	0,317	0,25	6,78602E-15
Mono_INHBA	PDE4A	8,90049E-65	4,08574E-01	0,286	0,168	1,63920E-60
Mono_INHBA	PTPN1	9,99339E-58	4,04703E-01	0,502	0,395	1,84048E-53
Mono_INHBA	TPM4	4,20073E-123	4,04313E-01	0,743	0,61	7,73648E-119
Mono_INHBA	CRIP1	2,74581E-40	4,03937E-01	0,395	0,286	5,05697E-36
Mono_INHBA	NFYC	3,15521E-22	4,03724E-01	0,172	0,264	5,81095E-18
Mono_INHBA	RAC1	6,86058E-42	4,02880E-01	0,779	0,683	1,26351E-37
Mono_INHBA	EMILIN2	9,63772E-67	3,99170E-01	0,424	0,294	1,77498E-62
Mono_INHBA	TPD52L2	1,78865E-06	3,97862E-01	0,295	0,267	3,29415E-02
Mono_INHBA	RAPGEF1	5,72492E-49	3,95569E-01	0,357	0,253	1,05436E-44
Mono_INHBA	DDX39A	1,63110E-13	3,92851E-01	0,22	0,296	3,00400E-09
Mono_INHBA	CD300A	3,57115E-42	3,92341E-01	0,322	0,469	6,57698E-38
Mono_INHBA	ST3GAL1	9,25278E-39	3,91289E-01	0,315	0,224	1,70408E-34
Mono_INHBA	IL10RB	1,06173E-13	3,91268E-01	0,301	0,252	1,95539E-09
Mono_INHBA	PLEK	1,97310E-68	3,84226E-01	0,657	0,556	3,63385E-64
Mono_INHBA	MXD1	1,18084E-116	3,82280E-01	0,471	0,291	2,17476E-112
Mono_INHBA	ATP6V1F	2,66306E-39	3,80786E-01	0,803	0,762	4,90456E-35
Mono_INHBA	EML4	1,12372E-65	3,80032E-01	0,47	0,349	2,06955E-61
Mono_INHBA	SLC3A2	1,95229E-21	3,79398E-01	0,509	0,46	3,59554E-17
Mono_INHBA	DUSP1	1,62159E-11	3,75330E-01	0,863	0,849	2,98648E-07

Mono_INHBA	CD99	6,72124E-76	3,70796E-01	0,725	0,614	1,23785E-71
Mono_INHBA	WSB1	3,34569E-08	3,69338E-01	0,57	0,645	6,16176E-04
Mono_INHBA	CSGALNACT2	1,77853E-99	3,69039E-01	0,331	0,181	3,27552E-95
Mono_INHBA	CAPN2	4,26595E-28	3,67627E-01	0,313	0,229	7,85660E-24
Mono_INHBA	PRDM1	8,49827E-19	3,67485E-01	0,252	0,194	1,56513E-14
Mono_INHBA	LAT2	8,16839E-43	3,67337E-01	0,321	0,462	1,50437E-38
Mono_INHBA	RAB8B	6,90777E-51	3,67175E-01	0,357	0,244	1,27220E-46
Mono_INHBA	CYTH1	1,10737E-14	3,64510E-01	0,373	0,324	2,03945E-10
Mono_INHBA	MARCKS	9,89297E-18	3,63976E-01	0,595	0,529	1,82199E-13
Mono_INHBA	TNS3	1,29074E-21	3,63653E-01	0,254	0,19	2,37716E-17
Mono_INHBA	BCKDK	1,09801E-28	3,63318E-01	0,386	0,315	2,02221E-24
Mono_INHBA	SLC6A6	6,60848E-30	3,61858E-01	0,258	0,18	1,21708E-25
Mono_INHBA	PNPLA8	2,21171E-20	3,61676E-01	0,301	0,239	4,07330E-16
Mono_INHBA	OPTN	1,62573E-135	3,53001E-01	0,259	0,11	2,99412E-131
Mono_INHBA	UBALD2	7,94775E-49	3,51362E-01	0,269	0,17	1,46374E-44
Mono_INHBA	NCF2	5,32806E-11	3,51150E-01	0,505	0,462	9,81268E-07
Mono_INHBA	C3AR1	1,60839E-15	3,49544E-01	0,337	0,421	2,96218E-11
Mono_INHBA	MAP1LC3B	5,82159E-49	3,48075E-01	0,623	0,554	1,07216E-44
Mono_INHBA	ASPH	5,76132E-25	3,44493E-01	0,286	0,219	1,06106E-20
Mono_INHBA	GPR137B	9,58233E-09	3,43512E-01	0,33	0,296	1,76478E-04
Mono_INHBA	PPP4C	4,43623E-16	3,41797E-01	0,537	0,511	8,17021E-12
Mono_INHBA	ZNF267	7,66889E-53	3,40809E-01	0,337	0,228	1,41238E-48
Mono_INHBA	YBX3	7,09684E-38	3,37936E-01	0,431	0,343	1,30702E-33
Mono_INHBA	ZEB2	7,10958E-16	3,37130E-01	0,643	0,603	1,30937E-11
Mono_INHBA	MYO9B	6,18430E-27	3,36817E-01	0,455	0,384	1,13896E-22
Mono_INHBA	ZMIZ1	2,83545E-18	3,33976E-01	0,322	0,262	5,22204E-14
Mono_INHBA	UBA1	3,71702E-14	3,30375E-01	0,346	0,3	6,84564E-10
Mono_INHBA	RELB	4,51602E-38	3,29175E-01	0,338	0,239	8,31716E-34
Mono_INHBA	KDM6B	7,17362E-56	3,28054E-01	0,463	0,333	1,32116E-51
Mono_INHBA	GTF2A2	1,48650E-32	3,27868E-01	0,289	0,414	2,73770E-28
Mono_INHBA	GLIPR2	3,95118E-69	3,24305E-01	0,5	0,369	7,27690E-65
Mono_INHBA	AHSA1	2,38346E-44	3,22258E-01	0,173	0,307	4,38962E-40
Mono_INHBA	ATP6V1B2	5,95301E-19	3,22214E-01	0,583	0,532	1,09637E-14
Mono_INHBA	ELOB	2,04410E-47	3,20207E-01	0,529	0,391	3,76462E-43
Mono_INHBA	CMC2	7,25810E-12	3,20182E-01	0,209	0,28	1,33672E-07
Mono_INHBA	RHBDD2	1,07540E-06	3,15498E-01	0,282	0,256	1,98057E-02
Mono_INHBA	VAT1	4,09701E-08	3,10477E-01	0,273	0,234	7,54547E-04
Mono_INHBA	TUBA1A	2,63299E-56	3,08336E-01	0,316	0,471	4,84917E-52
Mono_INHBA	SEC61G	1,06566E-141	3,07685E-01	0,724	0,592	1,96262E-137
Mono_INHBA	PLEC	4,57334E-30	3,04700E-01	0,443	0,359	8,42272E-26
Mono_INHBA	CDKN1A	5,87038E-59	3,02357E-01	0,659	0,517	1,08115E-54

Mono_INHBA	TUBB6	4,82096E-35	3,01677E-01	0,253	0,169	8,87876E-31
Mono_INHBA	CLIC4	5,01098E-23	2,99973E-01	0,292	0,226	9,22872E-19
Mono_INHBA	METTL9	8,41371E-12	2,99387E-01	0,395	0,357	1,54955E-07
Mono_INHBA	MAPKAPK2	3,63590E-51	2,97661E-01	0,29	0,184	6,69624E-47
Mono_INHBA	RAB31	1,01194E-36	2,97566E-01	0,7	0,639	1,86369E-32
Mono_INHBA	FPR1	9,47261E-20	2,96677E-01	0,353	0,454	1,74457E-15
Mono_INHBA	PPDPF	1,13044E-19	2,95885E-01	0,771	0,767	2,08194E-15
Mono_INHBA	SLC38A2	1,09823E-26	2,94346E-01	0,383	0,304	2,02260E-22
Mono_INHBA	CITED2	8,00238E-08	2,89409E-01	0,363	0,333	1,47380E-03
Mono_INHBA	DSE	3,17903E-19	2,87832E-01	0,485	0,436	5,85481E-15
Mono_INHBA	PHC2	2,42236E-25	2,87824E-01	0,313	0,24	4,46126E-21
Mono_INHBA	RBMS1	1,33928E-25	2,86733E-01	0,377	0,306	2,46654E-21
Mono_INHBA	UBE2J1	1,22360E-40	2,86697E-01	0,391	0,296	2,25350E-36
Mono_INHBA	LRRFIP1	1,10468E-25	2,83799E-01	0,712	0,682	2,03449E-21
Mono_INHBA	PSMA6	7,01649E-71	2,80216E-01	0,573	0,459	1,29223E-66
Mono_INHBA	VASP	5,08232E-30	2,79826E-01	0,541	0,465	9,36010E-26
Mono_INHBA	VSIG4	1,02400E-49	2,78889E-01	0,217	0,356	1,88591E-45
Mono_INHBA	CXCL16	1,04020E-09	2,78096E-01	0,603	0,654	1,91573E-05
Mono_INHBA	PIK3R5	1,22347E-10	2,77300E-01	0,296	0,255	2,25327E-06
Mono_INHBA	MAP2K1	7,82146E-22	2,76406E-01	0,383	0,318	1,44048E-17
Mono_INHBA	NPTN	1,94792E-49	2,76175E-01	0,322	0,222	3,58748E-45
Mono_INHBA	CSNK1D	2,17558E-10	2,75996E-01	0,277	0,347	4,00676E-06
Mono_INHBA	PAPSS1	8,72843E-09	2,73239E-01	0,27	0,234	1,60751E-04
Mono_INHBA	EFHD2	1,83925E-26	2,72417E-01	0,498	0,426	3,38734E-22
Mono_INHBA	ADGRE2	2,61439E-30	2,70314E-01	0,264	0,185	4,81493E-26
Mono_INHBA	NDUFAF3	2,33238E-21	2,69965E-01	0,37	0,477	4,29554E-17
Mono_INHBA	HAVCR2	4,41901E-39	2,69707E-01	0,306	0,443	8,13849E-35
Mono_INHBA	RSRC2	3,14286E-12	2,69419E-01	0,313	0,395	5,78821E-08
Mono_INHBA	SERPINB1	9,44679E-16	2,67860E-01	0,677	0,662	1,73981E-11
Mono_INHBA	DNAJC3	1,88623E-15	2,67854E-01	0,4	0,362	3,47387E-11
Mono_INHBA	LYN	6,98381E-40	2,65734E-01	0,527	0,447	1,28621E-35
Mono_INHBA	CSNK2B	4,22355E-13	2,65073E-01	0,392	0,334	7,77852E-09
Mono_INHBA	GADD45B	2,76753E-22	2,61759E-01	0,712	0,638	5,09696E-18
Mono_INHBA	HSPA1B	5,50122E-18	2,61307E-01	0,415	0,51	1,01316E-13
Mono_INHBA	UBE2D1	1,22367E-09	2,55991E-01	0,391	0,361	2,25363E-05
Mono_INHBA	HCFC1R1	4,68893E-07	2,53956E-01	0,331	0,315	8,63560E-03
Mono_INHBA	NCOR2	2,28669E-22	2,51953E-01	0,332	0,266	4,21139E-18
Mono_INHBA	ELF2	1,15289E-24	-2,50687E-01	0,162	0,253	2,12328E-20
Mono_INHBA	SHISA5	4,77753E-33	-2,51467E-01	0,256	0,378	8,79877E-29
Mono_INHBA	ANAPC16	1,56539E-12	-2,52211E-01	0,393	0,486	2,88298E-08
Mono_INHBA	AAMP	4,49469E-39	-2,52586E-01	0,177	0,3	8,27787E-35

Mono_INHBA	TMEM141	9,30341E-28	-2,52889E-01	0,168	0,267	1,71341E-23
Mono_INHBA	SMARCB1	1,59082E-38	-2,53060E-01	0,18	0,302	2,92982E-34
Mono_INHBA	UQCRQ	2,42269E-16	-2,53375E-01	0,566	0,672	4,46186E-12
Mono_INHBA	RBM6	1,14204E-22	-2,53983E-01	0,178	0,268	2,10330E-18
Mono_INHBA	TSPO	2,23672E-31	-2,55000E-01	0,694	0,77	4,11936E-27
Mono_INHBA	AKR1A1	1,47019E-150	-2,55189E-01	0,339	0,592	2,70765E-146
Mono_INHBA	PDIA6	1,32206E-15	-2,56564E-01	0,434	0,525	2,43484E-11
Mono_INHBA	MRPS5	1,75653E-30	-2,56972E-01	0,157	0,261	3,23500E-26
Mono_INHBA	UQCRC2	2,88779E-50	-2,59051E-01	0,269	0,423	5,31845E-46
Mono_INHBA	GPS1	1,08595E-39	-2,59082E-01	0,152	0,272	1,99999E-35
Mono_INHBA	MRPL43	2,74187E-43	-2,59090E-01	0,218	0,358	5,04970E-39
Mono_INHBA	SNX27	4,76774E-21	-2,59105E-01	0,165	0,25	8,78074E-17
Mono_INHBA	MRPL14	2,02018E-06	-2,59250E-01	0,288	0,347	3,72057E-02
Mono_INHBA	ILF3	4,80692E-23	-2,59482E-01	0,264	0,372	8,85291E-19
Mono_INHBA	LASP1	2,25748E-12	-2,59553E-01	0,238	0,314	4,15760E-08
Mono_INHBA	MED28	5,08018E-20	-2,60431E-01	0,21	0,3	9,35616E-16
Mono_INHBA	ARF4	7,26058E-11	-2,60544E-01	0,368	0,45	1,33718E-06
Mono_INHBA	MRPL34	2,41556E-68	-2,61282E-01	0,21	0,383	4,44873E-64
Mono_INHBA	ISG15	1,22866E-13	-2,61346E-01	0,451	0,426	2,26282E-09
Mono_INHBA	MRPL54	1,66149E-29	-2,61440E-01	0,286	0,41	3,05996E-25
Mono_INHBA	SNRNP70	1,67038E-35	-2,62513E-01	0,259	0,391	3,07635E-31
Mono_INHBA	NFIC	9,24110E-28	-2,62530E-01	0,195	0,301	1,70193E-23
Mono_INHBA	SENP6	5,85028E-41	-2,62878E-01	0,135	0,253	1,07745E-36
Mono_INHBA	C17orf49	1,83469E-19	-2,62987E-01	0,292	0,226	3,37895E-15
Mono_INHBA	TKT	1,92850E-119	-2,63010E-01	0,391	0,62	3,55172E-115
Mono_INHBA	PCMT1	3,80036E-17	-2,63338E-01	0,255	0,343	6,99913E-13
Mono_INHBA	NUDT21	1,82742E-14	-2,64023E-01	0,197	0,271	3,36556E-10
Mono_INHBA	PIN4	3,50899E-20	-2,64033E-01	0,181	0,268	6,46251E-16
Mono_INHBA	LAMTOR5	7,34669E-08	-2,64231E-01	0,432	0,512	1,35304E-03
Mono_INHBA	UBA52	3,69536E-46	-2,64403E-01	0,913	0,921	6,80574E-42
Mono_INHBA	SFPQ	3,84078E-10	-2,64878E-01	0,438	0,512	7,07356E-06
Mono_INHBA	GAA	1,16032E-50	-2,64944E-01	0,245	0,395	2,13697E-46
Mono_INHBA	MAFB	7,74351E-36	-2,66731E-01	0,507	0,599	1,42612E-31
Mono_INHBA	EMC4	1,31986E-26	-2,67093E-01	0,252	0,367	2,43079E-22
Mono_INHBA	SOD1	8,42043E-62	-2,67274E-01	0,399	0,574	1,55079E-57
Mono_INHBA	MRPL23	7,32450E-56	-2,67600E-01	0,204	0,366	1,34895E-51
Mono_INHBA	CAMLG	2,71462E-36	-2,67844E-01	0,182	0,299	4,99951E-32
Mono_INHBA	RRP7A	5,98764E-25	-2,67882E-01	0,2	0,3	1,10274E-20
Mono_INHBA	TERF2IP	1,38827E-29	-2,67905E-01	0,23	0,344	2,55677E-25
Mono_INHBA	REEP5	1,03121E-30	-2,68286E-01	0,446	0,57	1,89918E-26
Mono_INHBA	PPM1G	1,18678E-32	-2,68622E-01	0,254	0,375	2,18570E-28

Mono_INHBA	ARF5	1,63224E-31	-2,69006E-01	0,515	0,62	3,00610E-27
Mono_INHBA	TRAPPC2L	4,97457E-09	-2,69284E-01	0,212	0,273	9,16167E-05
Mono_INHBA	FOXN3	1,15079E-34	-2,69375E-01	0,242	0,368	2,11942E-30
Mono_INHBA	ARID1A	3,45863E-23	-2,69472E-01	0,181	0,274	6,36976E-19
Mono_INHBA	MPHOSPH8	2,67901E-31	-2,70354E-01	0,255	0,377	4,93394E-27
Mono_INHBA	SRP14	1,80529E-103	-2,70424E-01	0,759	0,841	3,32480E-99
Mono_INHBA	GADD45GIP1	3,15761E-24	-2,70933E-01	0,326	0,445	5,81536E-20
Mono_INHBA	IL13RA1	1,02945E-41	-2,71255E-01	0,26	0,396	1,89595E-37
Mono_INHBA	GRHPR	2,02176E-53	-2,71416E-01	0,155	0,299	3,72347E-49
Mono_INHBA	RPS16	7,32890E-32	-2,72023E-01	0,726	0,534	1,34976E-27
Mono_INHBA	SNRPD3	7,60860E-33	-2,72480E-01	0,254	0,377	1,40128E-28
Mono_INHBA	COA6	2,26605E-10	-2,72807E-01	0,193	0,256	4,17338E-06
Mono_INHBA	LPCAT2	6,18967E-62	-2,73226E-01	0,163	0,319	1,13995E-57
Mono_INHBA	EMC10	4,80410E-44	-2,73932E-01	0,218	0,356	8,84771E-40
Mono_INHBA	CNDP2	6,39221E-23	-2,74718E-01	0,35	0,461	1,17725E-18
Mono_INHBA	RNH1	4,69871E-43	-2,75916E-01	0,491	0,626	8,65361E-39
Mono_INHBA	SPSB3	1,91324E-41	-2,76896E-01	0,172	0,297	3,52362E-37
Mono_INHBA	NDFIP1	1,00749E-30	-2,76940E-01	0,382	0,511	1,85549E-26
Mono_INHBA	YY1	4,81509E-23	-2,77361E-01	0,249	0,351	8,86795E-19
Mono_INHBA	SPI1	8,59528E-36	-2,77531E-01	0,713	0,77	1,58299E-31
Mono_INHBA	TMUB1	6,06240E-19	-2,77931E-01	0,182	0,267	1,11651E-14
Mono_INHBA	DCAF7	3,06599E-31	-2,78547E-01	0,205	0,319	5,64663E-27
Mono_INHBA	CREBL2	1,02960E-38	-2,79421E-01	0,177	0,298	1,89622E-34
Mono_INHBA	NDUFC1	3,79237E-30	-2,82164E-01	0,331	0,465	6,98441E-26
Mono_INHBA	KCTD12	3,42283E-41	-2,82586E-01	0,351	0,499	6,30383E-37
Mono_INHBA	CMTM3	7,33892E-39	-2,83839E-01	0,237	0,369	1,35161E-34
Mono_INHBA	TTC1	1,07640E-18	-2,83981E-01	0,174	0,255	1,98241E-14
Mono_INHBA	BTG2	1,45235E-53	-2,85163E-01	0,359	0,507	2,67479E-49
Mono_INHBA	PSMD7	1,01115E-14	-2,85241E-01	0,324	0,411	1,86224E-10
Mono_INHBA	TCEA1	1,13181E-13	-2,85869E-01	0,388	0,479	2,08445E-09
Mono_INHBA	SRSF2	1,38263E-40	-2,85924E-01	0,428	0,566	2,54640E-36
Mono_INHBA	SNX5	4,81603E-84	-2,86246E-01	0,215	0,409	8,86969E-80
Mono_INHBA	HSD17B12	3,99253E-24	-2,86297E-01	0,163	0,251	7,35305E-20
Mono_INHBA	ATP5F1C	4,63438E-08	-2,87414E-01	0,25	0,29	8,53513E-04
Mono_INHBA	CHCHD2	6,04825E-07	-2,87447E-01	0,787	0,817	1,11391E-02
Mono_INHBA	RNASE6	6,95467E-152	-2,87985E-01	0,292	0,555	1,28084E-147
Mono_INHBA	RTRAF	4,12647E-18	-2,88290E-01	0,199	0,269	7,59972E-14
Mono_INHBA	CNPPD1	1,86177E-52	-2,88601E-01	0,14	0,279	3,42882E-48
Mono_INHBA	SSR2	1,09336E-46	-2,89837E-01	0,525	0,66	2,01364E-42
Mono_INHBA	NDUFA11	2,70582E-07	-2,90473E-01	0,403	0,458	4,98330E-03
Mono_INHBA	NT5C	1,07461E-52	-2,90784E-01	0,209	0,36	1,97912E-48

Mono_INHBA	COMMD9	6,79700E-46	-2,90896E-01	0,184	0,322	1,25180E-41
Mono_INHBA	KLF3	4,43808E-31	-2,91668E-01	0,155	0,259	8,17361E-27
Mono_INHBA	EIF5B	2,76599E-41	-2,91954E-01	0,231	0,367	5,09411E-37
Mono_INHBA	ATP5PB	8,71275E-15	-2,91966E-01	0,224	0,286	1,60463E-10
Mono_INHBA	CAPRIN1	7,38346E-33	-2,91984E-01	0,226	0,342	1,35981E-28
Mono_INHBA	TMEM243	1,95821E-67	-2,92098E-01	0,123	0,278	3,60644E-63
Mono_INHBA	LSM14A	8,57301E-26	-2,92291E-01	0,217	0,323	1,57889E-21
Mono_INHBA	COMMD7	8,91802E-50	-2,92481E-01	0,177	0,32	1,64243E-45
Mono_INHBA	HNRNPUL1	8,97895E-38	-2,92598E-01	0,284	0,419	1,65365E-33
Mono_INHBA	PARP9	1,73657E-31	-2,93647E-01	0,153	0,26	3,19825E-27
Mono_INHBA	TALDO1	2,77847E-09	-2,94718E-01	0,591	0,666	5,11711E-05
Mono_INHBA	MPC2	1,15535E-42	-2,96080E-01	0,228	0,368	2,12782E-38
Mono_INHBA	FKBP1A	7,29496E-36	-2,96251E-01	0,598	0,7	1,34351E-31
Mono_INHBA	DDX46	1,70457E-60	-2,96295E-01	0,23	0,403	3,13930E-56
Mono_INHBA	TRABD	6,66750E-37	-2,96919E-01	0,239	0,364	1,22795E-32
Mono_INHBA	MX2	4,36257E-07	-2,97083E-01	0,22	0,273	8,03455E-03
Mono_INHBA	RHOG	3,98612E-37	-2,97293E-01	0,648	0,732	7,34123E-33
Mono_INHBA	SPG21	1,00978E-16	-2,97787E-01	0,348	0,443	1,85972E-12
Mono_INHBA	SNF8	1,30715E-10	-2,99375E-01	0,232	0,298	2,40738E-06
Mono_INHBA	SUMO3	5,01078E-23	-2,99426E-01	0,37	0,488	9,22835E-19
Mono_INHBA	HSPB11	1,20397E-16	-2,99470E-01	0,192	0,271	2,21734E-12
Mono_INHBA	FOXN2	3,76021E-54	-2,99658E-01	0,133	0,27	6,92517E-50
Mono_INHBA	FBXO7	3,71370E-35	-3,00749E-01	0,207	0,329	6,83952E-31
Mono_INHBA	COA3	1,86936E-37	-3,00812E-01	0,2	0,327	3,44281E-33
Mono_INHBA	AP1M1	1,33421E-19	-3,00993E-01	0,17	0,252	2,45721E-15
Mono_INHBA	BLOC1S2	3,99208E-14	-3,01107E-01	0,252	0,336	7,35221E-10
Mono_INHBA	RNF167	4,52908E-48	-3,01271E-01	0,166	0,301	8,34121E-44
Mono_INHBA	PPP1CA	3,21787E-41	-3,01910E-01	0,494	0,63	5,92635E-37
Mono_INHBA	SPTLC2	4,52083E-61	-3,03204E-01	0,165	0,319	8,32601E-57
Mono_INHBA	HP1BP3	6,93102E-27	-3,03327E-01	0,298	0,413	1,27649E-22
Mono_INHBA	ZC3H15	9,66776E-27	-3,03844E-01	0,325	0,445	1,78051E-22
Mono_INHBA	GINM1	6,40995E-17	-3,04313E-01	0,183	0,26	1,18052E-12
Mono_INHBA	C19orf53	6,87761E-23	-3,04531E-01	0,473	0,592	1,26665E-18
Mono_INHBA	SF3B5	4,00755E-30	-3,04702E-01	0,43	0,559	7,38070E-26
Mono_INHBA	TOMM5	5,01663E-18	-3,04723E-01	0,241	0,332	9,23913E-14
Mono_INHBA	VMA21	2,32639E-22	-3,05073E-01	0,275	0,381	4,28452E-18
Mono_INHBA	ITPR2	9,28454E-30	-3,05140E-01	0,178	0,284	1,70993E-25
Mono_INHBA	SNRNP200	1,01173E-15	-3,05787E-01	0,188	0,263	1,86331E-11
Mono_INHBA	FEZ2	2,53660E-52	-3,06110E-01	0,21	0,359	4,67165E-48
Mono_INHBA	COX4I1	6,15503E-110	-3,06276E-01	0,855	0,899	1,13357E-105
Mono_INHBA	RAC2	7,73506E-10	-3,06598E-01	0,545	0,493	1,42457E-05

Mono_INHBA	HLA-B	1,42052E-141	-3,06675E-01	0,986	0,967	2,61617E-137
Mono_INHBA	ELF1	2,49458E-44	-3,08023E-01	0,347	0,496	4,59427E-40
Mono_INHBA	RAP1A	2,81271E-28	-3,09066E-01	0,456	0,572	5,18017E-24
Mono_INHBA	CDK2AP2	1,53763E-42	-3,09163E-01	0,268	0,405	2,83185E-38
Mono_INHBA	EIF4E2	1,01885E-08	-3,09465E-01	0,305	0,376	1,87641E-04
Mono_INHBA	SRI	1,25541E-54	-3,09489E-01	0,286	0,443	2,31208E-50
Mono_INHBA	TUBB	6,44151E-13	-3,10376E-01	0,589	0,665	1,18633E-08
Mono_INHBA	TMEM179B	5,51123E-40	-3,10748E-01	0,344	0,49	1,01500E-35
Mono_INHBA	SYNCRIP	4,58507E-28	-3,11374E-01	0,22	0,329	8,44432E-24
Mono_INHBA	C11orf58	7,86105E-39	-3,11557E-01	0,433	0,568	1,44777E-34
Mono_INHBA	SMC1A	1,27441E-35	-3,11656E-01	0,145	0,26	2,34707E-31
Mono_INHBA	YIF1A	8,06960E-16	-3,12587E-01	0,208	0,289	1,48618E-11
Mono_INHBA	UBE2V2	1,33518E-07	-3,12627E-01	0,208	0,262	2,45901E-03
Mono_INHBA	RNASEH2C	3,75319E-09	-3,12698E-01	0,28	0,35	6,91225E-05
Mono_INHBA	PRDX1	5,82590E-35	-3,13279E-01	0,682	0,751	1,07296E-30
Mono_INHBA	PIK3R1	2,91129E-48	-3,13334E-01	0,188	0,329	5,36173E-44
Mono_INHBA	PRDX3	5,46849E-53	-3,13630E-01	0,33	0,489	1,00713E-48
Mono_INHBA	RHOB	2,98546E-23	-3,13972E-01	0,461	0,547	5,49831E-19
Mono_INHBA	NCOR1	9,70989E-48	-3,14183E-01	0,257	0,407	1,78827E-43
Mono_INHBA	PTMA	1,17619E-191	-3,14766E-01	0,912	0,965	2,16620E-187
Mono_INHBA	CLEC4A	1,13702E-27	-3,15209E-01	0,199	0,303	2,09405E-23
Mono_INHBA	ZNF207	4,04359E-09	-3,16069E-01	0,338	0,409	7,44708E-05
Mono_INHBA	PPP1R7	9,85303E-37	-3,16277E-01	0,17	0,286	1,81463E-32
Mono_INHBA	NOP53	3,92964E-20	-3,16501E-01	0,212	0,289	7,23722E-16
Mono_INHBA	ZSWIM7	5,39312E-44	-3,16953E-01	0,132	0,257	9,93251E-40
Mono_INHBA	EIF3G	5,05835E-136	-3,17381E-01	0,391	0,627	9,31597E-132
Mono_INHBA	ZNHIT1	1,11241E-12	-3,17846E-01	0,394	0,484	2,04873E-08
Mono_INHBA	ADAP2	2,59770E-82	-3,18816E-01	0,261	0,456	4,78419E-78
Mono_INHBA	TMEM160	2,95576E-58	-3,18826E-01	0,226	0,392	5,44362E-54
Mono_INHBA	ATF6B	3,57650E-50	-3,19109E-01	0,157	0,295	6,58685E-46
Mono_INHBA	CD84	3,86668E-68	-3,19175E-01	0,225	0,403	7,12127E-64
Mono_INHBA	FCGR1A	9,76947E-108	-3,19261E-01	0,213	0,423	1,79924E-103
Mono_INHBA	CANX	1,22942E-06	-3,19536E-01	0,582	0,642	2,26422E-02
Mono_INHBA	PSMB3	2,35618E-22	-3,19641E-01	0,555	0,649	4,33937E-18
Mono_INHBA	C1orf162	2,65123E-195	-3,19737E-01	0,425	0,725	4,88277E-191
Mono_INHBA	STARD7	1,17310E-53	-3,20524E-01	0,168	0,314	2,16049E-49
Mono_INHBA	HMGB2	1,89268E-34	-3,20535E-01	0,281	0,406	3,48574E-30
Mono_INHBA	OSTC	6,18957E-73	-3,21836E-01	0,327	0,51	1,13993E-68
Mono_INHBA	NDUFA10	1,01597E-58	-3,22435E-01	0,183	0,337	1,87110E-54
Mono_INHBA	CUTA	1,28606E-36	-3,22505E-01	0,355	0,493	2,36853E-32
Mono_INHBA	CAP1	9,36183E-12	-3,22551E-01	0,686	0,736	1,72417E-07

Mono_INHBA	TMEM14C	9,06664E-106	-3,22824E-01	0,314	0,543	1,66980E-101
Mono_INHBA	ARGLU1	1,80616E-56	-3,23062E-01	0,276	0,443	3,32640E-52
Mono_INHBA	ILF2	1,52690E-23	-3,23403E-01	0,289	0,398	2,81209E-19
Mono_INHBA	U2SURP	2,53884E-35	-3,23455E-01	0,209	0,334	4,67577E-31
Mono_INHBA	NOSIP	4,30136E-17	-3,23789E-01	0,246	0,333	7,92182E-13
Mono_INHBA	ADAR	2,98021E-28	-3,24439E-01	0,316	0,435	5,48866E-24
Mono_INHBA	HNRNPR	1,35481E-48	-3,24453E-01	0,22	0,369	2,49515E-44
Mono_INHBA	RENBP	4,71647E-25	-3,24581E-01	0,207	0,303	8,68633E-21
Mono_INHBA	CAPNS1	1,65085E-07	-3,24745E-01	0,31	0,374	3,04037E-03
Mono_INHBA	TAF15	4,61587E-36	-3,24791E-01	0,194	0,317	8,50105E-32
Mono_INHBA	GCC2	8,38105E-21	-3,24841E-01	0,196	0,286	1,54354E-16
Mono_INHBA	GMFG	1,02526E-62	-3,24980E-01	0,54	0,681	1,88822E-58
Mono_INHBA	NCL	1,38202E-104	-3,25207E-01	0,422	0,638	2,54526E-100
Mono_INHBA	GPSM3	6,11447E-34	-3,25425E-01	0,579	0,69	1,12610E-29
Mono_INHBA	SP100	7,82135E-16	-3,25487E-01	0,381	0,472	1,44046E-11
Mono_INHBA	HNRNPU	8,21985E-13	-3,25659E-01	0,553	0,628	1,51385E-08
Mono_INHBA	NDUFA8	3,51002E-42	-3,25776E-01	0,171	0,298	6,46439E-38
Mono_INHBA	SDHD	5,60504E-38	-3,26081E-01	0,222	0,351	1,03228E-33
Mono_INHBA	PSMD2	3,02375E-14	-3,26504E-01	0,245	0,325	5,56883E-10
Mono_INHBA	NUCKS1	8,50038E-47	-3,26686E-01	0,352	0,51	1,56551E-42
Mono_INHBA	NUDT3	6,38737E-54	-3,27385E-01	0,186	0,339	1,17636E-49
Mono_INHBA	USP8	5,00381E-14	-3,27550E-01	0,201	0,276	9,21551E-10
Mono_INHBA	CD47	1,86004E-09	-3,27616E-01	0,35	0,426	3,42563E-05
Mono_INHBA	HDAC2	2,74268E-27	-3,27697E-01	0,173	0,273	5,05119E-23
Mono_INHBA	NDUFA12	5,26621E-52	-3,28059E-01	0,346	0,503	9,69878E-48
Mono_INHBA	MTDH	5,92918E-80	-3,28545E-01	0,549	0,703	1,09198E-75
Mono_INHBA	DCTN3	3,51877E-26	-3,28649E-01	0,255	0,367	6,48052E-22
Mono_INHBA	PPP1CC	5,57371E-56	-3,28821E-01	0,205	0,361	1,02651E-51
Mono_INHBA	ASH1L	1,57832E-10	-3,29600E-01	0,192	0,251	2,90678E-06
Mono_INHBA	OSTF1	4,62982E-36	-3,29885E-01	0,432	0,557	8,52675E-32
Mono_INHBA	PTMS	2,17981E-43	-3,30177E-01	0,242	0,37	4,01455E-39
Mono_INHBA	MS4A4A	1,39018E-52	-3,30977E-01	0,289	0,428	2,56029E-48
Mono_INHBA	HNRNPA0	4,00755E-48	-3,31123E-01	0,372	0,518	7,38070E-44
Mono_INHBA	LSM2	8,74923E-63	-3,31271E-01	0,195	0,36	1,61135E-58
Mono_INHBA	TMEM123	1,71205E-38	-3,31539E-01	0,326	0,462	3,15309E-34
Mono_INHBA	SLC25A39	1,22446E-44	-3,32566E-01	0,271	0,417	2,25509E-40
Mono_INHBA	PSMA2	2,18174E-06	-3,33213E-01	0,256	0,223	4,01810E-02
Mono_INHBA	CCAR1	5,57227E-38	-3,33299E-01	0,157	0,274	1,02624E-33
Mono_INHBA	ITGB2	6,75971E-67	-3,33388E-01	0,675	0,769	1,24494E-62
Mono_INHBA	CAPG	2,55246E-13	-3,33839E-01	0,684	0,744	4,70087E-09
Mono_INHBA	LSM6	1,09734E-38	-3,34556E-01	0,227	0,358	2,02097E-34

Mono_INHBA	PSMB2	7,34131E-28	-3,34585E-01	0,333	0,448	1,35205E-23
Mono_INHBA	TRMT112	9,02090E-21	-3,34672E-01	0,511	0,625	1,66138E-16
Mono_INHBA	NCF4	2,02434E-100	-3,34940E-01	0,224	0,435	3,72822E-96
Mono_INHBA	FBXL5	1,73613E-23	-3,35385E-01	0,187	0,279	3,19743E-19
Mono_INHBA	LIPA	3,92142E-60	-3,35915E-01	0,342	0,511	7,22207E-56
Mono_INHBA	RNF10	1,82580E-09	-3,35938E-01	0,22	0,278	3,36258E-05
Mono_INHBA	GLIPR1	3,28686E-134	-3,36590E-01	0,345	0,598	6,05341E-130
Mono_INHBA	FXYD5	2,63299E-82	-3,37319E-01	0,739	0,83	4,84918E-78
Mono_INHBA	TRA2B	5,98602E-08	-3,37376E-01	0,406	0,475	1,10244E-03
Mono_INHBA	FBXW5	2,99932E-08	-3,37569E-01	0,253	0,318	5,52384E-04
Mono_INHBA	EWSR1	1,42186E-21	-3,37579E-01	0,362	0,469	2,61864E-17
Mono_INHBA	HNRNPL	1,50508E-19	-3,38342E-01	0,204	0,294	2,77191E-15
Mono_INHBA	CIR1	3,23842E-23	-3,39197E-01	0,184	0,283	5,96419E-19
Mono_INHBA	TXN2	2,01423E-76	-3,39406E-01	0,221	0,407	3,70961E-72
Mono_INHBA	NONO	1,09605E-24	-3,39623E-01	0,326	0,437	2,01860E-20
Mono_INHBA	UBE2L3	3,35532E-15	-3,39674E-01	0,416	0,507	6,17949E-11
Mono_INHBA	TNFAIP8L2	1,15996E-77	-3,39853E-01	0,106	0,27	2,13629E-73
Mono_INHBA	MEF2A	5,75301E-82	-3,39890E-01	0,238	0,433	1,05953E-77
Mono_INHBA	TRAM1	8,95101E-19	-3,40207E-01	0,359	0,456	1,64851E-14
Mono_INHBA	RSL1D1	3,18751E-88	-3,40450E-01	0,268	0,482	5,87043E-84
Mono_INHBA	CFL1	1,63776E-37	-3,40462E-01	0,915	0,922	3,01626E-33
Mono_INHBA	HNRNPA3	6,40603E-36	-3,40881E-01	0,507	0,637	1,17980E-31
Mono_INHBA	HNRNPD	1,20916E-16	-3,41223E-01	0,26	0,348	2,22691E-12
Mono_INHBA	GLB1	1,43385E-54	-3,41887E-01	0,156	0,3	2,64073E-50
Mono_INHBA	BAG1	5,15735E-26	-3,42340E-01	0,272	0,381	9,49830E-22
Mono_INHBA	HLA-E	5,43796E-70	-3,43141E-01	0,885	0,905	1,00151E-65
Mono_INHBA	EZR	2,74806E-10	-3,43204E-01	0,338	0,404	5,06110E-06
Mono_INHBA	PRKDC	7,39946E-47	-3,43352E-01	0,133	0,261	1,36276E-42
Mono_INHBA	CMTM7	6,76166E-73	-3,43590E-01	0,236	0,422	1,24530E-68
Mono_INHBA	PNISR	9,43198E-104	-3,44072E-01	0,284	0,506	1,73709E-99
Mono_INHBA	DHX36	1,97197E-36	-3,44165E-01	0,174	0,292	3,63177E-32
Mono_INHBA	USP15	4,26702E-17	-3,44439E-01	0,339	0,43	7,85857E-13
Mono_INHBA	TMEM30A	7,05391E-07	-3,45306E-01	0,253	0,308	1,29912E-02
Mono_INHBA	LUC7L3	7,89425E-49	-3,45795E-01	0,218	0,368	1,45388E-44
Mono_INHBA	EIF3I	1,17209E-16	-3,46911E-01	0,374	0,471	2,15864E-12
Mono_INHBA	HNRNPK	1,72366E-24	-3,47314E-01	0,625	0,714	3,17446E-20
Mono_INHBA	BIRC6	1,89417E-10	-3,48540E-01	0,192	0,252	3,48848E-06
Mono_INHBA	UBXN1	9,68664E-91	-3,49134E-01	0,454	0,639	1,78399E-86
Mono_INHBA	SNAP29	5,68014E-24	-3,49548E-01	0,196	0,291	1,04611E-19
Mono_INHBA	SUMO2	1,83668E-90	-3,50001E-01	0,672	0,79	3,38262E-86
Mono_INHBA	GYG1	2,93819E-41	-3,50599E-01	0,158	0,28	5,41127E-37

Mono_INHBA	HNRNPF	1,88295E-44	-3,51239E-01	0,435	0,573	3,46784E-40
Mono_INHBA	URM1	3,65361E-52	-3,51327E-01	0,12	0,255	6,72886E-48
Mono_INHBA	TMED9	3,75293E-50	-3,51749E-01	0,378	0,533	6,91178E-46
Mono_INHBA	POLE4	3,02738E-23	-3,52034E-01	0,236	0,337	5,57552E-19
Mono_INHBA	SERBP1	7,35374E-84	-3,53059E-01	0,411	0,606	1,35434E-79
Mono_INHBA	UBE2K	2,05289E-07	-3,53249E-01	0,232	0,289	3,78081E-03
Mono_INHBA	CRTAP	8,32999E-101	-3,53795E-01	0,313	0,534	1,53413E-96
Mono_INHBA	NAA38	1,35691E-46	-3,53952E-01	0,279	0,424	2,49902E-42
Mono_INHBA	SNX29	1,38344E-40	-3,53968E-01	0,148	0,267	2,54788E-36
Mono_INHBA	HVCN1	1,77859E-83	-3,54388E-01	0,094	0,264	3,27562E-79
Mono_INHBA	NDUFV3	1,53133E-27	-3,54450E-01	0,181	0,287	2,82025E-23
Mono_INHBA	ANKRD44	2,45113E-72	-3,54451E-01	0,137	0,301	4,51425E-68
Mono_INHBA	NCOA4	2,97056E-51	-3,54801E-01	0,388	0,539	5,47088E-47
Mono_INHBA	ARRDC1	3,12417E-73	-3,56109E-01	0,133	0,304	5,75379E-69
Mono_INHBA	COX5B	1,30867E-27	-3,56184E-01	0,698	0,777	2,41019E-23
Mono_INHBA	NDUFB2	1,72388E-41	-3,57332E-01	0,59	0,722	3,17486E-37
Mono_INHBA	C1orf43	3,87107E-25	-3,57367E-01	0,431	0,553	7,12935E-21
Mono_INHBA	PTPN6	1,46719E-24	-3,57402E-01	0,408	0,517	2,70212E-20
Mono_INHBA	DDX18	3,31524E-20	-3,58320E-01	0,277	0,376	6,10568E-16
Mono_INHBA	TGFBR2	2,28106E-72	-3,58576E-01	0,173	0,345	4,20103E-68
Mono_INHBA	LYSMD2	4,63774E-18	-3,58994E-01	0,21	0,295	8,54133E-14
Mono_INHBA	TMEM258	1,62372E-10	-3,59571E-01	0,589	0,672	2,99041E-06
Mono_INHBA	PDIA3	1,66997E-20	-3,59911E-01	0,616	0,679	3,07558E-16
Mono_INHBA	SMCO4	3,47146E-38	-3,60491E-01	0,297	0,425	6,39338E-34
Mono_INHBA	TNFSF13B	3,60764E-97	-3,60494E-01	0,382	0,591	6,64418E-93
Mono_INHBA	COX5A	8,75949E-97	-3,61072E-01	0,531	0,704	1,61323E-92
Mono_INHBA	CHCHD3	6,59524E-31	-3,63073E-01	0,152	0,256	1,21464E-26
Mono_INHBA	FUCA2	1,04234E-45	-3,63262E-01	0,182	0,316	1,91968E-41
Mono_INHBA	FNTA	3,60757E-45	-3,63645E-01	0,132	0,256	6,64405E-41
Mono_INHBA	RNF213	2,27726E-08	-3,63839E-01	0,448	0,508	4,19403E-04
Mono_INHBA	HLA-A	1,63974E-150	-3,63923E-01	0,965	0,949	3,01991E-146
Mono_INHBA	CYBA	1,73958E-72	-3,64664E-01	0,875	0,9	3,20379E-68
Mono_INHBA	DGCR6L	5,27915E-27	-3,65837E-01	0,168	0,267	9,72261E-23
Mono_INHBA	PTGES3	7,43157E-59	-3,66259E-01	0,493	0,653	1,36867E-54
Mono_INHBA	EPS15	8,77901E-31	-3,66315E-01	0,152	0,254	1,61683E-26
Mono_INHBA	NDUFS7	1,20208E-103	-3,66330E-01	0,326	0,554	2,21387E-99
Mono_INHBA	SSR3	5,61236E-50	-3,66635E-01	0,45	0,605	1,03363E-45
Mono_INHBA	DDX17	4,85852E-21	-3,66811E-01	0,431	0,538	8,94794E-17
Mono_INHBA	MTCH2	9,18227E-24	-3,66937E-01	0,227	0,329	1,69110E-19
Mono_INHBA	LPAR6	6,73330E-92	-3,67491E-01	0,142	0,333	1,24007E-87
Mono_INHBA	NDUFB6	5,84297E-25	-3,67769E-01	0,266	0,374	1,07610E-20

Mono_INHBA	ACTG1	1,49449E-34	-3,68681E-01	0,907	0,923	2,75241E-30
Mono_INHBA	MTRNR2L12	8,99982E-25	-3,68972E-01	0,774	0,766	1,65750E-20
Mono_INHBA	VPS36	1,71396E-53	-3,69882E-01	0,153	0,295	3,15659E-49
Mono_INHBA	PRMT2	2,89864E-54	-3,70532E-01	0,277	0,437	5,33842E-50
Mono_INHBA	KPNB1	2,92281E-14	-3,70845E-01	0,356	0,445	5,38295E-10
Mono_INHBA	N4BP2L2	3,10870E-75	-3,71138E-01	0,278	0,47	5,72529E-71
Mono_INHBA	CCT2	4,15072E-42	-3,71301E-01	0,214	0,348	7,64439E-38
Mono_INHBA	SNRPC	2,07290E-58	-3,71911E-01	0,259	0,428	3,81766E-54
Mono_INHBA	TMEM176A	1,50533E-116	-3,71944E-01	0,246	0,461	2,77236E-112
Mono_INHBA	C14orf119	2,40784E-15	-3,72093E-01	0,217	0,297	4,43451E-11
Mono_INHBA	MGMT	1,27484E-56	-3,72548E-01	0,125	0,264	2,34788E-52
Mono_INHBA	TMED10	4,06620E-46	-3,73110E-01	0,433	0,576	7,48871E-42
Mono_INHBA	MPC1	2,25312E-32	-3,73178E-01	0,206	0,324	4,14957E-28
Mono_INHBA	ACO2	3,96205E-36	-3,73756E-01	0,161	0,277	7,29690E-32
Mono_INHBA	TADA3	2,87601E-37	-3,73842E-01	0,185	0,308	5,29674E-33
Mono_INHBA	C9orf78	1,72955E-52	-3,74232E-01	0,192	0,345	3,18531E-48
Mono_INHBA	NDUFS5	2,75123E-27	-3,75279E-01	0,544	0,661	5,06695E-23
Mono_INHBA	FYTTD1	4,15198E-07	-3,75431E-01	0,203	0,251	7,64671E-03
Mono_INHBA	PCNP	1,54961E-15	-3,75512E-01	0,247	0,328	2,85392E-11
Mono_INHBA	BPTF	1,76767E-26	-3,75959E-01	0,193	0,295	3,25552E-22
Mono_INHBA	WDR33	2,73291E-28	-3,75979E-01	0,164	0,262	5,03321E-24
Mono_INHBA	UNC119	1,53551E-19	-3,76549E-01	0,187	0,272	2,82794E-15
Mono_INHBA	SH3BGRL	3,26799E-122	-3,76600E-01	0,539	0,731	6,01865E-118
Mono_INHBA	SRP72	2,08960E-46	-3,77351E-01	0,212	0,355	3,84841E-42
Mono_INHBA	MRPS36	6,70711E-18	-3,78327E-01	0,241	0,334	1,23525E-13
Mono_INHBA	TSPAN4	1,22628E-42	-3,79853E-01	0,177	0,3	2,25844E-38
Mono_INHBA	LY6E	5,29112E-36	-3,81016E-01	0,423	0,555	9,74466E-32
Mono_INHBA	SON	2,73074E-20	-3,81325E-01	0,581	0,68	5,02920E-16
Mono_INHBA	SURF1	3,64993E-52	-3,81642E-01	0,187	0,335	6,72208E-48
Mono_INHBA	PTPRC	5,36356E-53	-3,81988E-01	0,567	0,697	9,87808E-49
Mono_INHBA	PHF20	1,02411E-26	-3,84276E-01	0,182	0,281	1,88610E-22
Mono_INHBA	PSMG2	5,03910E-95	-3,84350E-01	0,227	0,436	9,28050E-91
Mono_INHBA	APMAP	1,50405E-50	-3,84489E-01	0,169	0,309	2,77000E-46
Mono_INHBA	THRAP3	2,57927E-52	-3,85015E-01	0,27	0,429	4,75024E-48
Mono_INHBA	ARPC3	5,95539E-91	-3,85049E-01	0,85	0,895	1,09680E-86
Mono_INHBA	NDUFB11	2,91453E-32	-3,85406E-01	0,548	0,669	5,36770E-28
Mono_INHBA	CSTA	2,19781E-12	-3,86461E-01	0,366	0,433	4,04772E-08
Mono_INHBA	ATP6V0E1	1,29507E-31	-3,86831E-01	0,741	0,799	2,38514E-27
Mono_INHBA	NKTR	6,33381E-39	-3,87167E-01	0,171	0,291	1,16650E-34
Mono_INHBA	RSBN1L	3,26723E-59	-3,87328E-01	0,146	0,297	6,01726E-55
Mono_INHBA	SNRPF	2,32651E-49	-3,88077E-01	0,247	0,402	4,28474E-45

Mono_INHBA	PCBD1	3,78421E-42	-3,88128E-01	0,229	0,368	6,96937E-38
Mono_INHBA	RB1	1,48180E-47	-3,88164E-01	0,259	0,411	2,72902E-43
Mono_INHBA	DNAJC4	1,34443E-88	-3,88915E-01	0,178	0,373	2,47604E-84
Mono_INHBA	ECHDC1	2,95127E-42	-3,89047E-01	0,152	0,277	5,43536E-38
Mono_INHBA	CIAO1	6,29915E-20	-3,89550E-01	0,166	0,251	1,16011E-15
Mono_INHBA	MRPL4	5,02281E-73	-3,89708E-01	0,121	0,287	9,25050E-69
Mono_INHBA	ATM	3,56845E-67	-3,90080E-01	0,115	0,268	6,57201E-63
Mono_INHBA	CCDC12	4,18782E-59	-3,90487E-01	0,146	0,295	7,71271E-55
Mono_INHBA	SERINC3	4,01014E-25	-3,92349E-01	0,244	0,346	7,38548E-21
Mono_INHBA	ZNF428	4,88721E-76	-3,92631E-01	0,133	0,304	9,00078E-72
Mono_INHBA	CBR1	1,11012E-76	-3,92634E-01	0,154	0,327	2,04452E-72
Mono_INHBA	BTF3	9,06983E-163	-3,92642E-01	0,723	0,854	1,67039E-158
Mono_INHBA	NUTF2	7,33870E-30	-3,92797E-01	0,271	0,391	1,35157E-25
Mono_INHBA	TRAPPC1	1,18181E-54	-3,93004E-01	0,464	0,619	2,17654E-50
Mono_INHBA	B2M	2,64501E-262	-3,93072E-01	0,998	0,995	4,87132E-258
Mono_INHBA	PSMC4	6,04247E-33	-3,95594E-01	0,227	0,343	1,11284E-28
Mono_INHBA	PSMB6	1,90444E-49	-3,95823E-01	0,439	0,584	3,50740E-45
Mono_INHBA	DDRGK1	3,78352E-61	-3,95927E-01	0,174	0,33	6,96811E-57
Mono_INHBA	MRPL11	2,57284E-48	-3,96309E-01	0,162	0,302	4,73839E-44
Mono_INHBA	WDR83OS	7,29305E-55	-3,96323E-01	0,49	0,628	1,34316E-50
Mono_INHBA	PSMC2	1,00827E-41	-3,96511E-01	0,168	0,294	1,85693E-37
Mono_INHBA	SLC8A1	2,17903E-51	-3,96675E-01	0,224	0,374	4,01312E-47
Mono_INHBA	GLRX3	1,55165E-10	-3,96766E-01	0,189	0,251	2,85768E-06
Mono_INHBA	CNTRL	7,98966E-64	-3,97144E-01	0,103	0,25	1,47146E-59
Mono_INHBA	CPPED1	2,14748E-27	-3,97156E-01	0,178	0,276	3,95501E-23
Mono_INHBA	PHPT1	2,37366E-29	-3,98129E-01	0,325	0,454	4,37157E-25
Mono_INHBA	TUFM	2,18204E-112	-3,99259E-01	0,293	0,523	4,01866E-108
Mono_INHBA	FUNDC2	4,82969E-38	-3,99748E-01	0,234	0,367	8,89485E-34
Mono_INHBA	TEX264	6,77532E-52	-3,99897E-01	0,17	0,316	1,24781E-47
Mono_INHBA	IKZF1	7,39946E-69	-4,00926E-01	0,133	0,292	1,36276E-64
Mono_INHBA	APPL1	1,49049E-53	-4,01233E-01	0,217	0,37	2,74503E-49
Mono_INHBA	CLTB	3,15312E-33	-4,01351E-01	0,249	0,367	5,80710E-29
Mono_INHBA	UQCRB	3,14971E-58	-4,02492E-01	0,666	0,78	5,80083E-54
Mono_INHBA	CORO1B	9,63824E-79	-4,02573E-01	0,292	0,484	1,77507E-74
Mono_INHBA	SDHC	1,53792E-70	-4,03698E-01	0,272	0,452	2,83238E-66
Mono_INHBA	SFT2D2	1,39368E-46	-4,03913E-01	0,168	0,301	2,56674E-42
Mono_INHBA	DRAP1	6,04404E-67	-4,04196E-01	0,516	0,666	1,11313E-62
Mono_INHBA	RAB5A	3,17036E-10	-4,05018E-01	0,246	0,314	5,83886E-06
Mono_INHBA	UQCRH	4,17519E-55	-4,05864E-01	0,578	0,709	7,68945E-51
Mono_INHBA	DNAJB12	3,36114E-33	-4,06071E-01	0,143	0,25	6,19021E-29
Mono_INHBA	WASF2	1,44197E-57	-4,07782E-01	0,41	0,578	2,65568E-53

Mono_INHBA	MCTS1	1,09259E-36	-4,08735E-01	0,183	0,302	2,01221E-32
Mono_INHBA	SCPEP1	2,38731E-92	-4,08765E-01	0,229	0,419	4,39671E-88
Mono_INHBA	TMEM256	1,59654E-23	-4,09082E-01	0,305	0,418	2,94035E-19
Mono_INHBA	RWDD1	9,28181E-56	-4,09482E-01	0,3	0,467	1,70943E-51
Mono_INHBA	DBNL	5,01149E-46	-4,09630E-01	0,353	0,501	9,22966E-42
Mono_INHBA	GRSF1	1,00926E-65	-4,10189E-01	0,187	0,349	1,85875E-61
Mono_INHBA	UBXN4	1,77908E-35	-4,12309E-01	0,399	0,528	3,27653E-31
Mono_INHBA	ACTB	1,22941E-35	-4,14362E-01	0,99	0,991	2,26421E-31
Mono_INHBA	PHYKPL	4,33819E-52	-4,14824E-01	0,132	0,266	7,98964E-48
Mono_INHBA	COX6B1	7,62337E-34	-4,15492E-01	0,751	0,815	1,40400E-29
Mono_INHBA	UTRN	4,08011E-35	-4,16152E-01	0,181	0,295	7,51435E-31
Mono_INHBA	FAU	2,61187E-114	-4,17407E-01	0,951	0,957	4,81029E-110
Mono_INHBA	CAPZB	2,89498E-81	-4,18649E-01	0,655	0,767	5,33169E-77
Mono_INHBA	EIF2S3	4,10842E-46	-4,18773E-01	0,312	0,46	7,56649E-42
Mono_INHBA	CCNI	4,11574E-63	-4,20101E-01	0,695	0,791	7,57997E-59
Mono_INHBA	STK4	7,39392E-39	-4,20630E-01	0,304	0,436	1,36174E-34
Mono_INHBA	OXA1L	7,90306E-114	-4,21463E-01	0,243	0,468	1,45551E-109
Mono_INHBA	SNX6	5,32319E-67	-4,22167E-01	0,355	0,526	9,80371E-63
Mono_INHBA	POLR2K	1,00285E-46	-4,22724E-01	0,193	0,334	1,84694E-42
Mono_INHBA	HADHA	5,04241E-59	-4,23455E-01	0,368	0,53	9,28660E-55
Mono_INHBA	SNRPE	1,67272E-32	-4,24685E-01	0,228	0,349	3,08065E-28
Mono_INHBA	DDX5	6,81229E-69	-4,25767E-01	0,738	0,839	1,25462E-64
Mono_INHBA	IL18	2,82014E-157	-4,26190E-01	0,196	0,457	5,19385E-153
Mono_INHBA	CCNDBP1	4,82907E-44	-4,26587E-01	0,154	0,28	8,89370E-40
Mono_INHBA	PPIG	4,00918E-49	-4,26808E-01	0,301	0,462	7,38370E-45
Mono_INHBA	MRPS34	3,16684E-67	-4,26994E-01	0,226	0,405	5,83237E-63
Mono_INHBA	ZDHHC12	1,18931E-38	-4,27457E-01	0,15	0,269	2,19035E-34
Mono_INHBA	SSNA1	1,62550E-33	-4,27985E-01	0,281	0,412	2,99368E-29
Mono_INHBA	RSL24D1	1,68007E-52	-4,28225E-01	0,279	0,443	3,09419E-48
Mono_INHBA	KRTCAP2	3,43656E-27	-4,28497E-01	0,463	0,584	6,32911E-23
Mono_INHBA	RNF166	8,64138E-77	-4,28893E-01	0,092	0,253	1,59148E-72
Mono_INHBA	PQBP1	9,32367E-46	-4,29951E-01	0,187	0,322	1,71714E-41
Mono_INHBA	PRPF38B	2,15818E-108	-4,31527E-01	0,226	0,449	3,97473E-104
Mono_INHBA	NDUFA1	2,75760E-22	-4,31601E-01	0,605	0,692	5,07868E-18
Mono_INHBA	HMGB1	4,28319E-118	-4,31714E-01	0,635	0,803	7,88834E-114
Mono_INHBA	POLR2I	1,67275E-12	-4,31742E-01	0,224	0,299	3,08071E-08
Mono_INHBA	FAM32A	1,55252E-29	-4,32530E-01	0,212	0,323	2,85927E-25
Mono_INHBA	SNRPA	2,80693E-63	-4,33318E-01	0,148	0,305	5,16951E-59
Mono_INHBA	HNRNPA2B1	6,10698E-56	-4,33589E-01	0,744	0,837	1,12472E-51
Mono_INHBA	STX7	1,46897E-33	-4,34180E-01	0,291	0,421	2,70540E-29
Mono_INHBA	RBM25	9,60985E-48	-4,34205E-01	0,348	0,503	1,76985E-43

Mono_INHBA	CLPP	1,66580E-28	-4,34336E-01	0,168	0,271	3,06791E-24
Mono_INHBA	SEC14L1	3,73265E-34	-4,34504E-01	0,278	0,402	6,87443E-30
Mono_INHBA	TNRC6B	2,55927E-45	-4,35461E-01	0,15	0,279	4,71340E-41
Mono_INHBA	SKAP2	1,40882E-41	-4,35959E-01	0,274	0,413	2,59463E-37
Mono_INHBA	PPP6C	3,99455E-13	-4,36366E-01	0,2	0,27	7,35676E-09
Mono_INHBA	ARHGAP9	5,94211E-46	-4,36631E-01	0,126	0,253	1,09436E-41
Mono_INHBA	NEMF	1,42982E-39	-4,36879E-01	0,173	0,295	2,63329E-35
Mono_INHBA	DRAM2	2,04867E-63	-4,37079E-01	0,233	0,4	3,77304E-59
Mono_INHBA	POLR2J	8,44836E-07	-4,37136E-01	0,255	0,309	1,55593E-02
Mono_INHBA	CCT3	9,74713E-87	-4,37158E-01	0,225	0,422	1,79513E-82
Mono_INHBA	NSMCE1	1,36713E-57	-4,37202E-01	0,138	0,279	2,51784E-53
Mono_INHBA	ELOVL5	3,07078E-31	-4,37525E-01	0,186	0,294	5,65545E-27
Mono_INHBA	SMCHD1	4,64560E-19	-4,37692E-01	0,244	0,329	8,55581E-15
Mono_INHBA	ALKBH7	9,74846E-80	-4,39373E-01	0,304	0,503	1,79537E-75
Mono_INHBA	PARVG	1,98778E-90	-4,39655E-01	0,252	0,462	3,66089E-86
Mono_INHBA	PLD3	1,50532E-35	-4,40861E-01	0,395	0,518	2,77235E-31
Mono_INHBA	GSDMD	1,22640E-58	-4,42192E-01	0,157	0,31	2,25866E-54
Mono_INHBA	SMIM19	1,41540E-41	-4,42525E-01	0,145	0,267	2,60675E-37
Mono_INHBA	CUEDC2	6,40549E-39	-4,42630E-01	0,178	0,302	1,17970E-34
Mono_INHBA	NDUFS6	3,37843E-67	-4,44190E-01	0,329	0,518	6,22206E-63
Mono_INHBA	DPYSL2	7,90732E-22	-4,45011E-01	0,229	0,329	1,45629E-17
Mono_INHBA	BRD2	1,33805E-12	-4,45535E-01	0,417	0,492	2,46428E-08
Mono_INHBA	ARHGAP15	2,70077E-97	-4,45674E-01	0,081	0,265	4,97402E-93
Mono_INHBA	FLII	7,98076E-09	-4,47704E-01	0,263	0,328	1,46982E-04
Mono_INHBA	NPC2	1,41534E-92	-4,48460E-01	0,845	0,886	2,60664E-88
Mono_INHBA	ARHGEF1	6,34142E-35	-4,51389E-01	0,163	0,278	1,16790E-30
Mono_INHBA	GBP4	3,60471E-61	-4,51679E-01	0,138	0,286	6,63879E-57
Mono_INHBA	POLR3GL	3,39663E-59	-4,52143E-01	0,156	0,306	6,25557E-55
Mono_INHBA	MRPS12	2,09857E-49	-4,52367E-01	0,154	0,291	3,86494E-45
Mono_INHBA	LSM7	5,13562E-48	-4,52578E-01	0,362	0,524	9,45827E-44
Mono_INHBA	YIF1B	5,82192E-41	-4,53520E-01	0,225	0,358	1,07222E-36
Mono_INHBA	WAS	3,45561E-74	-4,53650E-01	0,353	0,54	6,36419E-70
Mono_INHBA	CTSS	2,40544E-123	-4,53763E-01	0,719	0,822	4,43009E-119
Mono_INHBA	OLA1	1,42109E-46	-4,54048E-01	0,182	0,317	2,61721E-42
Mono_INHBA	EMC7	1,36035E-29	-4,55110E-01	0,217	0,327	2,50535E-25
Mono_INHBA	PLXNB2	1,62219E-06	-4,55219E-01	0,219	0,268	2,98760E-02
Mono_INHBA	YWHAB	1,08437E-30	-4,55562E-01	0,719	0,792	1,99708E-26
Mono_INHBA	DDX24	5,01152E-33	-4,56265E-01	0,356	0,485	9,22971E-29
Mono_INHBA	IFRD1	1,43967E-14	-4,57098E-01	0,222	0,296	2,65145E-10
Mono_INHBA	NDUFV1	7,54188E-105	-4,57565E-01	0,204	0,42	1,38899E-100
Mono_INHBA	ARL6IP4	1,60980E-82	-4,58013E-01	0,454	0,645	2,96476E-78

Mono_INHBA	CCDC85B	2,20055E-94	-4,58116E-01	0,202	0,409	4,05275E-90
Mono_INHBA	C1QBP	1,02774E-122	-4,58165E-01	0,204	0,444	1,89279E-118
Mono_INHBA	AUP1	3,76141E-49	-4,58315E-01	0,38	0,534	6,92738E-45
Mono_INHBA	PLTP	1,24711E-18	-4,59356E-01	0,245	0,334	2,29679E-14
Mono_INHBA	RAB8A	2,18513E-50	-4,59489E-01	0,282	0,435	4,02435E-46
Mono_INHBA	SARAF	2,06317E-09	-4,60477E-01	0,433	0,427	3,79974E-05
Mono_INHBA	MRPL16	7,22142E-54	-4,62010E-01	0,129	0,267	1,32997E-49
Mono_INHBA	CSK	3,52843E-57	-4,62826E-01	0,2	0,356	6,49830E-53
Mono_INHBA	MRPS15	1,69049E-54	-4,63036E-01	0,176	0,327	3,11338E-50
Mono_INHBA	BIN2	2,31629E-74	-4,63258E-01	0,152	0,324	4,26592E-70
Mono_INHBA	ERGIC3	5,24692E-44	-4,63279E-01	0,357	0,507	9,66326E-40
Mono_INHBA	AKAP9	6,10308E-91	-4,65490E-01	0,196	0,397	1,12400E-86
Mono_INHBA	SORL1	3,12366E-65	-4,65534E-01	0,201	0,373	5,75284E-61
Mono_INHBA	SIGLEC10	2,92561E-123	-4,66621E-01	0,116	0,333	5,38809E-119
Mono_INHBA	MAT2B	2,30652E-69	-4,66976E-01	0,179	0,345	4,24792E-65
Mono_INHBA	CEBPD	3,86860E-45	-4,68370E-01	0,452	0,575	7,12480E-41
Mono_INHBA	DNASE2	8,48065E-74	-4,68599E-01	0,206	0,377	1,56188E-69
Mono_INHBA	TACC1	2,17974E-22	-4,71229E-01	0,32	0,427	4,01443E-18
Mono_INHBA	C20orf27	6,32118E-101	-4,71295E-01	0,151	0,356	1,16417E-96
Mono_INHBA	PSMC3	1,21485E-28	-4,71360E-01	0,214	0,326	2,23739E-24
Mono_INHBA	UNC93B1	3,82537E-55	-4,71995E-01	0,196	0,345	7,04519E-51
Mono_INHBA	ABHD14B	7,25681E-68	-4,72421E-01	0,117	0,272	1,33649E-63
Mono_INHBA	SEC11A	4,33559E-100	-4,73163E-01	0,487	0,678	7,98485E-96
Mono_INHBA	VPS35	4,15038E-24	-4,73393E-01	0,338	0,446	7,64375E-20
Mono_INHBA	IDH2	5,02974E-75	-4,73427E-01	0,241	0,422	9,26327E-71
Mono_INHBA	PSMA3	2,42202E-50	-4,73706E-01	0,233	0,38	4,46064E-46
Mono_INHBA	SLC1A3	1,49446E-64	-4,74137E-01	0,213	0,372	2,75234E-60
Mono_INHBA	THEMIS2	4,57370E-45	-4,74433E-01	0,356	0,503	8,42338E-41
Mono_INHBA	ANXA11	2,24429E-46	-4,74679E-01	0,404	0,551	4,13331E-42
Mono_INHBA	RPL27A	1,57622E-08	-4,74836E-01	0,482	0,463	2,90293E-04
Mono_INHBA	AKAP13	4,84823E-56	-4,75150E-01	0,485	0,642	8,92899E-52
Mono_INHBA	RNF5	6,56583E-49	-4,76340E-01	0,154	0,291	1,20923E-44
Mono_INHBA	EIF4B	1,52587E-74	-4,77038E-01	0,338	0,528	2,81020E-70
Mono_INHBA	SSB	1,18910E-38	-4,77158E-01	0,322	0,46	2,18996E-34
Mono_INHBA	MIEN1	7,75500E-10	-4,77688E-01	0,322	0,401	1,42824E-05
Mono_INHBA	SPOP	7,80159E-64	-4,79243E-01	0,126	0,276	1,43682E-59
Mono_INHBA	EPSTI1	5,77271E-46	-4,79555E-01	0,276	0,418	1,06316E-41
Mono_INHBA	SMDT1	7,46949E-65	-4,80608E-01	0,319	0,493	1,37566E-60
Mono_INHBA	UQCR10	9,99392E-65	-4,80653E-01	0,599	0,735	1,84058E-60
Mono_INHBA	RTCB	1,61415E-29	-4,80841E-01	0,193	0,301	2,97277E-25
Mono_INHBA	COX7A2L	1,77129E-39	-4,82167E-01	0,354	0,498	3,26219E-35

Mono_INHBA	CALHM6	1,37991E-15	-4,82286E-01	0,205	0,264	2,54139E-11
Mono_INHBA	NME2	1,27472E-11	-4,82421E-01	0,328	0,361	2,34765E-07
Mono_INHBA	MICAL1	6,88742E-20	-4,82546E-01	0,209	0,298	1,26846E-15
Mono_INHBA	MDH2	7,97852E-88	-4,82877E-01	0,329	0,534	1,46940E-83
Mono_INHBA	GUSB	1,20982E-48	-4,83296E-01	0,213	0,354	2,22813E-44
Mono_INHBA	COX6C	1,71154E-57	-4,84370E-01	0,595	0,727	3,15214E-53
Mono_INHBA	LYZ	6,91454E-45	-4,84716E-01	0,784	0,81	1,27345E-40
Mono_INHBA	RBCK1	6,15600E-44	-4,85911E-01	0,243	0,381	1,13375E-39
Mono_INHBA	ATP5F1A	6,32137E-54	-4,87293E-01	0,137	0,262	1,16421E-49
Mono_INHBA	DECR1	6,59131E-54	-4,87363E-01	0,205	0,356	1,21392E-49
Mono_INHBA	MRPS21	1,69362E-89	-4,87848E-01	0,329	0,539	3,11913E-85
Mono_INHBA	SRSF4	3,90810E-41	-4,89783E-01	0,226	0,361	7,19755E-37
Mono_INHBA	CYBB	7,61837E-137	-4,89985E-01	0,405	0,637	1,40307E-132
Mono_INHBA	SCNM1	4,37850E-53	-4,90124E-01	0,194	0,343	8,06389E-49
Mono_INHBA	MTMR14	5,62899E-28	-4,90260E-01	0,21	0,316	1,03669E-23
Mono_INHBA	OS9	1,81679E-40	-4,90579E-01	0,405	0,545	3,34598E-36
Mono_INHBA	IGBP1	2,35527E-57	-4,91225E-01	0,2	0,353	4,33770E-53
Mono_INHBA	TTC3	1,76503E-33	-4,91488E-01	0,216	0,335	3,25065E-29
Mono_INHBA	TOMM22	2,36625E-73	-4,92861E-01	0,247	0,435	4,35793E-69
Mono_INHBA	AHCY	8,39477E-54	-4,93574E-01	0,129	0,27	1,54606E-49
Mono_INHBA	BBX	2,04646E-66	-4,93585E-01	0,147	0,307	3,76897E-62
Mono_INHBA	ENSA	1,03637E-70	-4,94258E-01	0,304	0,483	1,90867E-66
Mono_INHBA	SET	2,07704E-73	-4,94406E-01	0,353	0,539	3,82529E-69
Mono_INHBA	EEF1D	4,57818E-99	-4,95383E-01	0,806	0,875	8,43164E-95
Mono_INHBA	NMI	8,81158E-56	-4,95408E-01	0,161	0,308	1,62283E-51
Mono_INHBA	SDHA	8,68841E-18	-4,95748E-01	0,177	0,256	1,60014E-13
Mono_INHBA	ANXA7	3,60962E-29	-4,96479E-01	0,261	0,379	6,64783E-25
Mono_INHBA	PSMA1	7,07681E-07	-4,96530E-01	0,305	0,364	1,30334E-02
Mono_INHBA	VOPP1	5,44817E-51	-4,97281E-01	0,214	0,355	1,00339E-46
Mono_INHBA	AKNA	1,55208E-58	-4,98695E-01	0,123	0,264	2,85846E-54
Mono_INHBA	POLR1D	8,05427E-27	-5,00598E-01	0,354	0,475	1,48336E-22
Mono_INHBA	CDK5RAP3	1,25101E-51	-5,01376E-01	0,171	0,315	2,30399E-47
Mono_INHBA	PTPMT1	1,77669E-34	-5,01851E-01	0,145	0,255	3,27214E-30
Mono_INHBA	VPS13C	1,34542E-68	-5,02429E-01	0,205	0,381	2,47787E-64
Mono_INHBA	METTL7A	4,20700E-95	-5,02990E-01	0,077	0,258	7,74804E-91
Mono_INHBA	FBL	9,77883E-67	-5,03171E-01	0,188	0,357	1,80097E-62
Mono_INHBA	NDUFAB1	1,36349E-33	-5,03405E-01	0,352	0,483	2,51114E-29
Mono_INHBA	TOMM7	1,94858E-83	-5,06960E-01	0,675	0,798	3,58870E-79
Mono_INHBA	TBCA	3,19865E-14	-5,07594E-01	0,497	0,595	5,89095E-10
Mono_INHBA	PRPF8	5,60817E-13	-5,09314E-01	0,24	0,312	1,03286E-08
Mono_INHBA	MYCBP2	8,21617E-54	-5,09987E-01	0,193	0,345	1,51317E-49

Mono_INHBA	RAN	1,72938E-40	-5,10918E-01	0,533	0,665	3,18500E-36
Mono_INHBA	TSC22D3	1,50039E-104	-5,11383E-01	0,535	0,704	2,76327E-100
Mono_INHBA	NUDT1	1,12574E-39	-5,13683E-01	0,15	0,269	2,07327E-35
Mono_INHBA	RCSD1	3,66419E-115	-5,15072E-01	0,161	0,389	6,74833E-111
Mono_INHBA	CHCHD10	1,06990E-78	-5,15919E-01	0,348	0,526	1,97044E-74
Mono_INHBA	NSL1	2,57909E-41	-5,16773E-01	0,135	0,253	4,74991E-37
Mono_INHBA	APH1A	4,49444E-78	-5,17746E-01	0,325	0,509	8,27741E-74
Mono_INHBA	BUB3	2,04943E-26	-5,18171E-01	0,166	0,263	3,77443E-22
Mono_INHBA	ZCRB1	8,32095E-26	-5,18691E-01	0,18	0,278	1,53247E-21
Mono_INHBA	HLA-F	3,52215E-38	-5,18873E-01	0,386	0,523	6,48674E-34
Mono_INHBA	BEX4	8,37427E-61	-5,20894E-01	0,181	0,338	1,54229E-56
Mono_INHBA	UQCC2	5,79479E-57	-5,22499E-01	0,164	0,319	1,06723E-52
Mono_INHBA	UQCRFS1	3,52583E-36	-5,22595E-01	0,366	0,5	6,49351E-32
Mono_INHBA	RNASEH2B	1,59616E-108	-5,24068E-01	0,109	0,314	2,93964E-104
Mono_INHBA	SRSF11	2,15198E-44	-5,24073E-01	0,374	0,524	3,96331E-40
Mono_INHBA	SRP9	1,37727E-36	-5,25619E-01	0,342	0,473	2,53653E-32
Mono_INHBA	KCNMA1	1,37442E-70	-5,26214E-01	0,113	0,269	2,53127E-66
Mono_INHBA	BCLAF1	1,08887E-22	-5,27340E-01	0,281	0,387	2,00538E-18
Mono_INHBA	NPM1	6,49427E-130	-5,27504E-01	0,584	0,777	1,19605E-125
Mono_INHBA	FUCA1	1,18493E-81	-5,28589E-01	0,096	0,26	2,18229E-77
Mono_INHBA	DHRS4	6,80737E-53	-5,29092E-01	0,139	0,279	1,25371E-48
Mono_INHBA	SLC25A5	9,90108E-189	-5,29150E-01	0,534	0,757	1,82348E-184
Mono_INHBA	JUN	6,82115E-31	-5,29576E-01	0,647	0,713	1,25625E-26
Mono_INHBA	AKR7A2	5,53383E-44	-5,29950E-01	0,137	0,256	1,01916E-39
Mono_INHBA	RPL14	2,22335E-20	-5,30084E-01	0,597	0,539	4,09474E-16
Mono_INHBA	ISCU	8,55518E-82	-5,31220E-01	0,363	0,548	1,57561E-77
Mono_INHBA	ABRACL	1,44291E-46	-5,31361E-01	0,358	0,506	2,65741E-42
Mono_INHBA	VSIR	7,53372E-25	-5,33057E-01	0,204	0,278	1,38749E-20
Mono_INHBA	SNRPD1	1,45580E-40	-5,33875E-01	0,261	0,399	2,68114E-36
Mono_INHBA	FKBP2	4,76145E-27	-5,33962E-01	0,303	0,416	8,76917E-23
Mono_INHBA	FIBP	9,35728E-48	-5,34155E-01	0,23	0,376	1,72333E-43
Mono_INHBA	PARL	5,02115E-67	-5,34175E-01	0,139	0,299	9,24745E-63
Mono_INHBA	DNMT1	9,56879E-43	-5,34369E-01	0,192	0,325	1,76228E-38
Mono_INHBA	FCGRT	1,05340E-128	-5,35548E-01	0,575	0,748	1,94004E-124
Mono_INHBA	RASSF5	2,01975E-18	-5,35804E-01	0,195	0,276	3,71978E-14
Mono_INHBA	ADI1	1,33379E-80	-5,36236E-01	0,136	0,314	2,45645E-76
Mono_INHBA	MIIP	1,98047E-47	-5,36500E-01	0,126	0,252	3,64742E-43
Mono_INHBA	PLCB2	1,14217E-74	-5,37344E-01	0,092	0,251	2,10353E-70
Mono_INHBA	LY96	5,80344E-36	-5,40248E-01	0,329	0,465	1,06882E-31
Mono_INHBA	SLAMF8	7,38665E-55	-5,41735E-01	0,189	0,333	1,36040E-50
Mono_INHBA	TMEM219	5,93917E-92	-5,42491E-01	0,437	0,649	1,09382E-87

Mono_INHBA	TPR	1,37219E-31	-5,43019E-01	0,274	0,396	2,52715E-27
Mono_INHBA	PPIB	6,94293E-33	-5,44683E-01	0,736	0,796	1,27868E-28
Mono_INHBA	NUB1	1,40041E-55	-5,44789E-01	0,175	0,322	2,57914E-51
Mono_INHBA	RBBP4	2,12493E-63	-5,45737E-01	0,214	0,38	3,91348E-59
Mono_INHBA	NCKAP1L	6,17886E-69	-5,46235E-01	0,261	0,439	1,13796E-64
Mono_INHBA	U2AF1	1,24998E-30	-5,47612E-01	0,167	0,276	2,30208E-26
Mono_INHBA	RPS3	2,72830E-09	-5,47685E-01	0,692	0,56	5,02472E-05
Mono_INHBA	PPIA	1,86087E-139	-5,47766E-01	0,832	0,898	3,42717E-135
Mono_INHBA	LSM4	8,18949E-76	-5,49111E-01	0,252	0,443	1,50826E-71
Mono_INHBA	PDK4	5,32081E-70	-5,49662E-01	0,16	0,32	9,79933E-66
Mono_INHBA	GIMAP1	1,26258E-108	-5,50390E-01	0,08	0,277	2,32529E-104
Mono_INHBA	GIT2	3,21861E-36	-5,56487E-01	0,181	0,301	5,92771E-32
Mono_INHBA	TMSB4X	1,16904E-216	-5,57368E-01	0,98	0,984	2,15301E-212
Mono_INHBA	PSMD9	1,36177E-12	-5,59209E-01	0,19	0,258	2,50796E-08
Mono_INHBA	FUOM	7,37766E-41	-5,59306E-01	0,247	0,384	1,35874E-36
Mono_INHBA	GSTP1	1,56577E-142	-5,59351E-01	0,662	0,833	2,88367E-138
Mono_INHBA	COX14	3,94718E-60	-5,59439E-01	0,296	0,472	7,26951E-56
Mono_INHBA	DDOST	2,79497E-86	-5,60199E-01	0,281	0,474	5,14749E-82
Mono_INHBA	TAPBP	2,48372E-19	-5,60568E-01	0,489	0,585	4,57427E-15
Mono_INHBA	MFSD1	3,57107E-123	-5,60601E-01	0,373	0,605	6,57685E-119
Mono_INHBA	HIGD2A	2,07528E-143	-5,60606E-01	0,514	0,727	3,82205E-139
Mono_INHBA	PSMA5	2,18516E-50	-5,61030E-01	0,291	0,444	4,02441E-46
Mono_INHBA	RPS27A	2,70880E-06	-5,62037E-01	0,728	0,566	4,98880E-02
Mono_INHBA	PDIA4	6,64179E-25	-5,62491E-01	0,224	0,327	1,22322E-20
Mono_INHBA	NDUFA3	3,16482E-52	-5,63723E-01	0,39	0,556	5,82865E-48
Mono_INHBA	NAIP	7,26681E-76	-5,64662E-01	0,202	0,381	1,33833E-71
Mono_INHBA	ATP5MC2	7,65855E-14	-5,65212E-01	0,434	0,433	1,41047E-09
Mono_INHBA	CELF2	6,39323E-161	-5,65224E-01	0,276	0,555	1,17744E-156
Mono_INHBA	PHB2	3,28881E-88	-5,65792E-01	0,305	0,505	6,05700E-84
Mono_INHBA	COMT	3,88971E-76	-5,66212E-01	0,374	0,569	7,16368E-72
Mono_INHBA	ENTPD1	2,27825E-67	-5,69140E-01	0,15	0,319	4,19585E-63
Mono_INHBA	HNRNPA1	1,84933E-200	-5,69346E-01	0,625	0,812	3,40591E-196
Mono_INHBA	DHRS7	8,44932E-47	-5,69828E-01	0,347	0,493	1,55611E-42
Mono_INHBA	PCGF5	2,65754E-31	-5,69862E-01	0,171	0,279	4,89439E-27
Mono_INHBA	COPS6	1,13076E-43	-5,71038E-01	0,216	0,35	2,08252E-39
Mono_INHBA	RGS19	4,42040E-100	-5,71207E-01	0,297	0,508	8,14105E-96
Mono_INHBA	ERH	1,05958E-56	-5,72974E-01	0,376	0,538	1,95143E-52
Mono_INHBA	HMGN2	1,84640E-158	-5,73186E-01	0,523	0,747	3,40051E-154
Mono_INHBA	EVL	4,85713E-127	-5,73210E-01	0,139	0,371	8,94539E-123
Mono_INHBA	EIF4A3	1,22218E-15	-5,76203E-01	0,316	0,408	2,25089E-11
Mono_INHBA	RPL17	1,74314E-12	-5,76477E-01	0,54	0,427	3,21035E-08

Mono_INHBA	SMAP2	6,68129E-51	-5,76780E-01	0,327	0,48	1,23049E-46
Mono_INHBA	PTPN18	9,01809E-73	-5,77174E-01	0,185	0,361	1,66086E-68
Mono_INHBA	TMEM14B	3,80758E-41	-5,78749E-01	0,286	0,431	7,01241E-37
Mono_INHBA	AOAH	1,04544E-114	-5,79204E-01	0,163	0,387	1,92538E-110
Mono_INHBA	NSA2	4,53370E-74	-5,81088E-01	0,301	0,498	8,34971E-70
Mono_INHBA	GPX1	6,34709E-112	-5,81877E-01	0,335	0,562	1,16894E-107
Mono_INHBA	NAGA	4,89871E-67	-5,82260E-01	0,198	0,37	9,02195E-63
Mono_INHBA	EIF3K	5,30524E-159	-5,84253E-01	0,616	0,797	9,77066E-155
Mono_INHBA	PGLS	3,36577E-122	-5,85257E-01	0,327	0,556	6,19874E-118
Mono_INHBA	TMED3	7,12410E-56	-5,86132E-01	0,137	0,283	1,31205E-51
Mono_INHBA	TIMM10	1,74838E-36	-5,89105E-01	0,146	0,26	3,21999E-32
Mono_INHBA	IRF1	8,64313E-17	-5,89455E-01	0,353	0,444	1,59181E-12
Mono_INHBA	PUF60	2,27024E-35	-5,89719E-01	0,206	0,327	4,18110E-31
Mono_INHBA	UVRAG	6,97146E-76	-5,89900E-01	0,132	0,301	1,28393E-71
Mono_INHBA	RANBP1	9,12921E-51	-5,91293E-01	0,224	0,376	1,68133E-46
Mono_INHBA	NDUFB10	2,06366E-81	-5,92252E-01	0,374	0,57	3,80064E-77
Mono_INHBA	TAF9	1,60154E-11	-5,93028E-01	0,232	0,306	2,94956E-07
Mono_INHBA	EEF2	1,75474E-154	-5,93469E-01	0,702	0,836	3,23171E-150
Mono_INHBA	CYC1	7,55932E-60	-5,93926E-01	0,28	0,444	1,39220E-55
Mono_INHBA	RBX1	1,13692E-75	-5,94144E-01	0,446	0,63	2,09386E-71
Mono_INHBA	COX7C	5,46780E-154	-5,94625E-01	0,699	0,837	1,00701E-149
Mono_INHBA	RPL18A	4,48897E-07	-5,96097E-01	0,676	0,556	8,26734E-03
Mono_INHBA	CCNH	5,68026E-54	-5,98856E-01	0,229	0,38	1,04613E-49
Mono_INHBA	NDUFA4	5,71421E-117	-5,99001E-01	0,594	0,754	1,05239E-112
Mono_INHBA	ATRX	2,76463E-83	-5,99608E-01	0,21	0,4	5,09161E-79
Mono_INHBA	YWHAH	6,14757E-62	-5,99673E-01	0,394	0,54	1,13220E-57
Mono_INHBA	PYCARD	9,23771E-204	-6,02826E-01	0,464	0,721	1,70131E-199
Mono_INHBA	CNOT7	2,10273E-40	-6,03415E-01	0,188	0,316	3,87260E-36
Mono_INHBA	EIF3H	1,18536E-94	-6,03879E-01	0,475	0,669	2,18308E-90
Mono_INHBA	ARPC1B	5,14823E-74	-6,04119E-01	0,716	0,802	9,48149E-70
Mono_INHBA	XRCC5	1,44864E-46	-6,05215E-01	0,332	0,484	2,66796E-42
Mono_INHBA	SEC11C	4,43101E-52	-6,05952E-01	0,187	0,334	8,16059E-48
Mono_INHBA	RPS18	5,97328E-07	-6,07770E-01	0,668	0,553	1,10010E-02
Mono_INHBA	RPL35	7,72913E-08	-6,09009E-01	0,601	0,527	1,42347E-03
Mono_INHBA	RPS2	1,36264E-09	-6,13754E-01	0,679	0,555	2,50958E-05
Mono_INHBA	TXNIP	5,05454E-193	-6,14476E-01	0,464	0,713	9,30895E-189
Mono_INHBA	MRPL3	9,94877E-77	-6,14564E-01	0,123	0,293	1,83227E-72
Mono_INHBA	RPL11	2,19290E-09	-6,14712E-01	0,727	0,572	4,03866E-05
Mono_INHBA	CLEC12A	5,91598E-47	-6,15650E-01	0,137	0,267	1,08955E-42
Mono_INHBA	SEC13	5,68677E-38	-6,16432E-01	0,278	0,409	1,04733E-33
Mono_INHBA	SNX3	3,29393E-63	-6,17478E-01	0,564	0,697	6,06643E-59

Mono_INHBA	PARK7	9,23778E-101	-6,18039E-01	0,48	0,67	1,70132E-96
Mono_INHBA	RPS6	3,19646E-12	-6,19253E-01	0,628	0,539	5,88693E-08
Mono_INHBA	TRMT1	6,71063E-56	-6,20716E-01	0,137	0,282	1,23590E-51
Mono_INHBA	RASSF2	2,93401E-69	-6,21763E-01	0,12	0,273	5,40356E-65
Mono_INHBA	LST1	1,66805E-153	-6,22503E-01	0,537	0,726	3,07204E-149
Mono_INHBA	PMPCB	9,10017E-54	-6,25160E-01	0,115	0,25	1,67598E-49
Mono_INHBA	PA2G4	1,06325E-68	-6,28642E-01	0,263	0,444	1,95819E-64
Mono_INHBA	GGNBP2	8,09335E-31	-6,28671E-01	0,23	0,347	1,49055E-26
Mono_INHBA	VAMP8	9,46255E-233	-6,32531E-01	0,501	0,798	1,74272E-228
Mono_INHBA	EIF3E	2,60265E-128	-6,34236E-01	0,371	0,62	4,79331E-124
Mono_INHBA	RPL3	3,57331E-07	-6,34447E-01	0,622	0,527	6,58097E-03
Mono_INHBA	PHB	1,21746E-88	-6,35524E-01	0,196	0,401	2,24219E-84
Mono_INHBA	APRT	2,15092E-89	-6,35540E-01	0,437	0,635	3,96136E-85
Mono_INHBA	CYCS	2,34936E-21	-6,35555E-01	0,423	0,53	4,32682E-17
Mono_INHBA	HNRNPDL	7,63476E-78	-6,35962E-01	0,514	0,685	1,40609E-73
Mono_INHBA	FES	4,59893E-48	-6,38003E-01	0,132	0,262	8,46985E-44
Mono_INHBA	RPS5	1,06363E-09	-6,39663E-01	0,571	0,514	1,95889E-05
Mono_INHBA	RPL6	5,91476E-09	-6,44078E-01	0,665	0,547	1,08932E-04
Mono_INHBA	LAMTOR1	9,92391E-79	-6,44466E-01	0,482	0,658	1,82769E-74
Mono_INHBA	PSMB5	4,99355E-44	-6,45417E-01	0,187	0,323	9,19663E-40
Mono_INHBA	LMAN2	4,02109E-27	-6,46376E-01	0,4	0,518	7,40565E-23
Mono_INHBA	ERICH1	8,90887E-57	-6,46388E-01	0,18	0,33	1,64075E-52
Mono_INHBA	GPR65	5,09286E-92	-6,48824E-01	0,148	0,338	9,37952E-88
Mono_INHBA	DCK	2,42593E-75	-6,48857E-01	0,125	0,289	4,46783E-71
Mono_INHBA	RPL7A	4,43933E-08	-6,49047E-01	0,676	0,551	8,17592E-04
Mono_INHBA	OAS1	4,64972E-52	-6,49171E-01	0,19	0,335	8,56339E-48
Mono_INHBA	CD37	1,01068E-239	-6,50118E-01	0,463	0,749	1,86137E-235
Mono_INHBA	ARHGAP4	2,93146E-67	-6,54717E-01	0,22	0,395	5,39888E-63
Mono_INHBA	EIF2A	2,66953E-41	-6,56068E-01	0,186	0,315	4,91648E-37
Mono_INHBA	INTS10	1,26947E-64	-6,56614E-01	0,104	0,251	2,33799E-60
Mono_INHBA	NUDT16	3,14897E-123	-6,58699E-01	0,184	0,421	5,79946E-119
Mono_INHBA	SRSF7	9,42168E-115	-6,59038E-01	0,333	0,565	1,73519E-110
Mono_INHBA	LSM10	2,18775E-51	-6,59152E-01	0,232	0,384	4,02918E-47
Mono_INHBA	PRMT1	2,19496E-53	-6,60021E-01	0,17	0,315	4,04245E-49
Mono_INHBA	GRN	1,61487E-150	-6,60564E-01	0,718	0,829	2,97411E-146
Mono_INHBA	CD302	3,40741E-120	-6,62078E-01	0,133	0,362	6,27542E-116
Mono_INHBA	SLC25A3	2,35378E-114	-6,63759E-01	0,554	0,723	4,33496E-110
Mono_INHBA	MDH1	4,68646E-62	-6,65431E-01	0,23	0,395	8,63106E-58
Mono_INHBA	CIRBP	2,54380E-87	-6,65874E-01	0,542	0,712	4,68492E-83
Mono_INHBA	COPS3	3,98519E-62	-6,66292E-01	0,113	0,26	7,33952E-58
Mono_INHBA	SLCO2B1	6,54036E-77	-6,66973E-01	0,202	0,38	1,20454E-72

Mono_INHBA	EID1	9,00331E-94	-6,67878E-01	0,379	0,592	1,65814E-89
Mono_INHBA	SUMF2	3,53553E-83	-6,68257E-01	0,127	0,305	6,51139E-79
Mono_INHBA	TMEM147	2,17433E-59	-6,70327E-01	0,272	0,443	4,00446E-55
Mono_INHBA	AP1S2	2,14688E-50	-6,70742E-01	0,424	0,588	3,95392E-46
Mono_INHBA	PSMA4	1,46033E-48	-6,71316E-01	0,33	0,477	2,68950E-44
Mono_INHBA	RBMX	6,21252E-99	-6,71740E-01	0,224	0,437	1,14416E-94
Mono_INHBA	PTRHD1	2,20549E-71	-6,72112E-01	0,185	0,359	4,06185E-67
Mono_INHBA	RANBP2	3,33634E-14	-6,73077E-01	0,242	0,316	6,14454E-10
Mono_INHBA	PPT1	6,46722E-131	-6,73645E-01	0,447	0,664	1,19107E-126
Mono_INHBA	VDAC3	7,69321E-52	-6,74752E-01	0,176	0,321	1,41686E-47
Mono_INHBA	GDI2	8,91350E-131	-6,76234E-01	0,449	0,663	1,64160E-126
Mono_INHBA	ERCC1	9,80511E-29	-6,80039E-01	0,27	0,388	1,80581E-24
Mono_INHBA	DCTN2	6,90103E-43	-6,80289E-01	0,202	0,337	1,27096E-38
Mono_INHBA	HMGN1	1,87521E-65	-6,81726E-01	0,481	0,634	3,45357E-61
Mono_INHBA	PCBP2	9,75079E-100	-6,82279E-01	0,703	0,804	1,79580E-95
Mono_INHBA	INPP5D	1,54838E-122	-6,82979E-01	0,101	0,324	2,85166E-118
Mono_INHBA	COMMD6	1,76265E-67	-6,87884E-01	0,601	0,744	3,24628E-63
Mono_INHBA	ITPA	8,69267E-51	-6,88769E-01	0,144	0,283	1,60093E-46
Mono_INHBA	IMP4	2,51161E-56	-6,90076E-01	0,133	0,274	4,62563E-52
Mono_INHBA	NME3	2,41871E-80	-6,91097E-01	0,183	0,37	4,45454E-76
Mono_INHBA	MMADHC	2,65560E-45	-6,91189E-01	0,217	0,356	4,89082E-41
Mono_INHBA	NDUFB5	1,47519E-90	-6,94645E-01	0,26	0,465	2,71686E-86
Mono_INHBA	HINT1	5,26636E-80	-6,95261E-01	0,595	0,731	9,69906E-76
Mono_INHBA	ARHGDIB	0,00000E+00	-6,97545E-01	0,562	0,839	0,00000E+00
Mono_INHBA	LY86	4,06068E-139	-6,98096E-01	0,251	0,505	7,47856E-135
Mono_INHBA	LEPROTL1	2,58556E-81	-6,98397E-01	0,22	0,41	4,76182E-77
Mono_INHBA	ARL6IP5	1,07533E-104	-7,03799E-01	0,473	0,666	1,98043E-100
Mono_INHBA	TBCB	1,35309E-71	-7,04534E-01	0,296	0,478	2,49199E-67
Mono_INHBA	TM9SF2	8,50139E-51	-7,04975E-01	0,281	0,436	1,56570E-46
Mono_INHBA	RPS3A	4,56992E-09	-7,05395E-01	0,69	0,559	8,41642E-05
Mono_INHBA	NDUFS4	9,28324E-44	-7,09097E-01	0,165	0,294	1,70970E-39
Mono_INHBA	PARP1	3,57778E-116	-7,09381E-01	0,132	0,347	6,58919E-112
Mono_INHBA	FYB1	1,58663E-37	-7,13454E-01	0,232	0,323	2,92209E-33
Mono_INHBA	PSMB4	1,04194E-09	-7,15449E-01	0,301	0,377	1,91894E-05
Mono_INHBA	PLBD1	2,75083E-104	-7,16183E-01	0,19	0,405	5,06621E-100
Mono_INHBA	CNN2	6,24431E-13	-7,17717E-01	0,338	0,406	1,15001E-08
Mono_INHBA	RPL7	2,32487E-08	-7,19500E-01	0,523	0,496	4,28171E-04
Mono_INHBA	PSMB10	3,01793E-136	-7,19731E-01	0,346	0,58	5,55812E-132
Mono_INHBA	NAP1L1	4,64351E-73	-7,19734E-01	0,468	0,629	8,55196E-69
Mono_INHBA	NACA	6,14388E-267	-7,20399E-01	0,828	0,925	1,13152E-262
Mono_INHBA	ITM2B	0,00000E+00	-7,20872E-01	0,789	0,913	0,00000E+00

Mono_INHBA	PDCD4	2,14847E-57	-7,22822E-01	0,126	0,267	3,95685E-53
Mono_INHBA	IFITM2	8,93588E-59	-7,24483E-01	0,502	0,655	1,64572E-54
Mono_INHBA	ELMO1	9,08383E-138	-7,24682E-01	0,112	0,353	1,67297E-133
Mono_INHBA	SLTM	3,25509E-38	-7,25561E-01	0,235	0,363	5,99489E-34
Mono_INHBA	ABI3	2,43529E-165	-7,25858E-01	0,137	0,409	4,48507E-161
Mono_INHBA	UBE2E2	1,44082E-38	-7,27353E-01	0,184	0,307	2,65356E-34
Mono_INHBA	PRCP	4,84318E-125	-7,28272E-01	0,17	0,403	8,91968E-121
Mono_INHBA	CLNS1A	6,23644E-130	-7,29209E-01	0,191	0,435	1,14857E-125
Mono_INHBA	XRCC6	4,29793E-53	-7,31090E-01	0,344	0,504	7,91549E-49
Mono_INHBA	PABPC1	4,82344E-167	-7,35270E-01	0,827	0,897	8,88334E-163
Mono_INHBA	PIGT	8,97601E-61	-7,36041E-01	0,148	0,3	1,65311E-56
Mono_INHBA	UFC1	6,81735E-119	-7,36834E-01	0,276	0,516	1,25555E-114
Mono_INHBA	AP1B1	9,01253E-76	-7,37210E-01	0,222	0,404	1,65984E-71
Mono_INHBA	NDUFS3	2,55645E-58	-7,37356E-01	0,192	0,35	4,70821E-54
Mono_INHBA	CHD9	2,89046E-86	-7,39832E-01	0,164	0,354	5,32336E-82
Mono_INHBA	HLA-C	0,00000E+00	-7,41402E-01	0,929	0,952	0,00000E+00
Mono_INHBA	ZNF331	2,60317E-17	-7,45523E-01	0,265	0,349	4,79426E-13
Mono_INHBA	BTK	8,87328E-94	-7,47530E-01	0,132	0,322	1,63419E-89
Mono_INHBA	ADORA3	3,78065E-106	-7,51517E-01	0,081	0,274	6,96283E-102
Mono_INHBA	SSR4	1,60149E-61	-7,52472E-01	0,636	0,76	2,94947E-57
Mono_INHBA	ATXN10	7,77151E-75	-7,52584E-01	0,121	0,286	1,43128E-70
Mono_INHBA	SLC25A6	4,36637E-232	-7,53759E-01	0,633	0,827	8,04154E-228
Mono_INHBA	BST2	3,32667E-177	-7,54286E-01	0,511	0,738	6,12673E-173
Mono_INHBA	RPL4	1,03695E-31	-7,54633E-01	0,454	0,478	1,90976E-27
Mono_INHBA	IFI16	1,69169E-110	-7,56244E-01	0,285	0,513	3,11559E-106
Mono_INHBA	ARL5A	8,70424E-133	-7,60187E-01	0,198	0,443	1,60306E-128
Mono_INHBA	NHP2	6,06957E-66	-7,63187E-01	0,241	0,423	1,11783E-61
Mono_INHBA	SAMD9L	1,17629E-68	-7,63299E-01	0,13	0,289	2,16637E-64
Mono_INHBA	ITGA4	6,10587E-136	-7,64922E-01	0,141	0,382	1,12452E-131
Mono_INHBA	RGS10	2,66882E-168	-7,68575E-01	0,582	0,75	4,91517E-164
Mono_INHBA	CALM2	1,85253E-247	-7,69897E-01	0,558	0,796	3,41180E-243
Mono_INHBA	MEF2C	1,27768E-128	-7,72417E-01	0,185	0,432	2,35310E-124
Mono_INHBA	VAMP5	2,57958E-163	-7,73723E-01	0,24	0,495	4,75081E-159
Mono_INHBA	TRIM22	2,48385E-105	-7,76330E-01	0,206	0,43	4,57450E-101
Mono_INHBA	RPL10A	2,54581E-22	-7,78954E-01	0,504	0,496	4,68862E-18
Mono_INHBA	MRPS7	2,08178E-69	-7,80071E-01	0,153	0,32	3,83401E-65
Mono_INHBA	RBM3	5,58208E-61	-7,82128E-01	0,531	0,683	1,02805E-56
Mono_INHBA	HLA-DQA2	6,06655E-37	-7,88081E-01	0,43	0,517	1,11728E-32
Mono_INHBA	SMIM14	1,91165E-43	-7,92675E-01	0,152	0,276	3,52069E-39
Mono_INHBA	SNX17	9,39989E-91	-7,93382E-01	0,242	0,444	1,73118E-86
Mono_INHBA	GTF3A	2,42547E-73	-7,96778E-01	0,3	0,491	4,46698E-69

Mono_INHBA	EEF1A1	0,00000E+00	-7,99930E-01	0,974	0,985	0,00000E+00
Mono_INHBA	EIF3F	1,86082E-183	-8,04064E-01	0,421	0,681	3,42707E-179
Mono_INHBA	RPS4X	2,56631E-09	-8,04138E-01	0,68	0,558	4,72637E-05
Mono_INHBA	SP110	1,23771E-102	-8,09471E-01	0,184	0,403	2,27950E-98
Mono_INHBA	GSTK1	2,30233E-234	-8,11912E-01	0,343	0,663	4,24020E-230
Mono_INHBA	NUDT22	1,99168E-37	-8,13424E-01	0,162	0,279	3,66807E-33
Mono_INHBA	IFI35	9,37556E-50	-8,13714E-01	0,21	0,355	1,72670E-45
Mono_INHBA	CORO1A	8,45670E-140	-8,16526E-01	0,438	0,659	1,55747E-135
Mono_INHBA	EEF1B2	1,86397E-243	-8,19128E-01	0,655	0,853	3,43287E-239
Mono_INHBA	CCT7	5,70059E-51	-8,21532E-01	0,208	0,358	1,04988E-46
Mono_INHBA	FKBP5	5,65091E-154	-8,22060E-01	0,254	0,534	1,04073E-149
Mono_INHBA	TMEM176B	3,48755E-211	-8,26909E-01	0,277	0,55	6,42302E-207
Mono_INHBA	GSN	6,37643E-104	-8,27685E-01	0,467	0,644	1,17435E-99
Mono_INHBA	HLA-DRA	3,45960E-209	-8,29182E-01	0,954	0,923	6,37155E-205
Mono_INHBA	MIS18BP1	2,48637E-106	-8,32721E-01	0,157	0,373	4,57915E-102
Mono_INHBA	RPL23A	1,07003E-06	-8,36076E-01	0,613	0,52	1,97067E-02
Mono_INHBA	PDCD6	7,13909E-48	-8,36299E-01	0,286	0,434	1,31481E-43
Mono_INHBA	LGALS3BP	2,95470E-88	-8,38093E-01	0,153	0,33	5,44168E-84
Mono_INHBA	SELPLG	9,05683E-74	-8,47816E-01	0,184	0,354	1,66800E-69
Mono_INHBA	TNFSF13	2,26596E-108	-8,51325E-01	0,242	0,456	4,17322E-104
Mono_INHBA	GPR183	9,91384E-13	-8,53667E-01	0,391	0,461	1,82583E-08
Mono_INHBA	TAP1	1,95997E-35	-8,55003E-01	0,312	0,429	3,60968E-31
Mono_INHBA	SCAMP2	1,58122E-118	-8,55169E-01	0,284	0,517	2,91213E-114
Mono_INHBA	LGALS9	4,27905E-125	-8,57307E-01	0,323	0,561	7,88073E-121
Mono_INHBA	SAMHD1	2,27681E-143	-8,61802E-01	0,421	0,66	4,19319E-139
Mono_INHBA	MAN2B1	1,23864E-62	-8,63068E-01	0,325	0,483	2,28121E-58
Mono_INHBA	UBL7	2,22536E-64	-8,64331E-01	0,113	0,262	4,09845E-60
Mono_INHBA	DNPH1	4,25918E-54	-8,66288E-01	0,203	0,356	7,84414E-50
Mono_INHBA	ALDH2	4,09304E-163	-8,68037E-01	0,305	0,566	7,53816E-159
Mono_INHBA	TECR	1,13535E-76	-8,73215E-01	0,208	0,39	2,09098E-72
Mono_INHBA	CCND3	2,36496E-57	-8,76329E-01	0,175	0,321	4,35554E-53
Mono_INHBA	IGFLR1	1,40115E-54	-8,81253E-01	0,216	0,368	2,58049E-50
Mono_INHBA	APEX1	1,21162E-164	-8,88587E-01	0,182	0,457	2,23143E-160
Mono_INHBA	GIMAP7	3,09103E-135	-8,91055E-01	0,055	0,268	5,69276E-131
Mono_INHBA	GPR34	2,17742E-137	-8,92710E-01	0,092	0,319	4,01015E-133
Mono_INHBA	EIF3L	6,40989E-191	-8,93962E-01	0,334	0,616	1,18051E-186
Mono_INHBA	YPEL5	1,59158E-23	-8,95408E-01	0,341	0,44	2,93120E-19
Mono_INHBA	RGS2	2,00541E-19	-8,97302E-01	0,526	0,632	3,69337E-15
Mono_INHBA	HLA-DRB1	8,36004E-242	-8,98719E-01	0,905	0,918	1,53967E-237
Mono_INHBA	DUT	5,74948E-124	-9,05412E-01	0,179	0,415	1,05888E-119
Mono_INHBA	MGST2	1,02180E-141	-9,09874E-01	0,126	0,373	1,88184E-137

Mono_INHBA	TUBA1B	2,35668E-185	-9,18525E-01	0,569	0,768	4,34029E-181
Mono_INHBA	HLA-DMA	1,10349E-296	-9,22746E-01	0,572	0,794	2,03229E-292
Mono_INHBA	CXCR4	4,26546E-48	-9,27205E-01	0,451	0,588	7,85570E-44
Mono_INHBA	RPS23	6,57925E-15	-9,27564E-01	0,686	0,561	1,21170E-10
Mono_INHBA	NDUFB7	2,28943E-67	-9,31812E-01	0,352	0,54	4,21645E-63
Mono_INHBA	FGD2	1,16629E-129	-9,39193E-01	0,122	0,358	2,14796E-125
Mono_INHBA	RASSF4	2,97796E-88	-9,44122E-01	0,337	0,517	5,48450E-84
Mono_INHBA	LGALS2	6,02451E-65	-9,49553E-01	0,123	0,265	1,10953E-60
Mono_INHBA	ERP29	2,04426E-207	-9,58396E-01	0,342	0,64	3,76491E-203
Mono_INHBA	PAK1	8,95934E-130	-9,61441E-01	0,21	0,451	1,65004E-125
Mono_INHBA	STAT1	6,82544E-83	-9,61703E-01	0,357	0,528	1,25704E-78
Mono_INHBA	SERPING1	8,58975E-99	-9,65420E-01	0,218	0,408	1,58197E-94
Mono_INHBA	EIF3M	4,02392E-101	-9,67834E-01	0,293	0,512	7,41085E-97
Mono_INHBA	MTRNR2L8	1,94850E-63	-9,69758E-01	0,302	0,449	3,58855E-59
Mono_INHBA	CCT6A	3,41083E-89	-9,70278E-01	0,257	0,46	6,28173E-85
Mono_INHBA	LIMD2	1,54765E-210	-9,74162E-01	0,194	0,499	2,85031E-206
Mono_INHBA	IFI44L	1,51393E-62	-9,74446E-01	0,118	0,262	2,78821E-58
Mono_INHBA	PSME1	3,73159E-170	-9,74724E-01	0,572	0,765	6,87247E-166
Mono_INHBA	IFITM1	1,48750E-10	-9,76718E-01	0,203	0,257	2,73952E-06
Mono_INHBA	UCP2	6,23802E-92	-9,80609E-01	0,471	0,644	1,14886E-87
Mono_INHBA	ORMDL1	2,34701E-52	-9,81640E-01	0,178	0,324	4,32249E-48
Mono_INHBA	CST3	0,00000E+00	-9,82844E-01	0,886	0,953	0,00000E+00
Mono_INHBA	MIF4GD	1,60404E-105	-9,89237E-01	0,121	0,324	2,95417E-101
Mono_INHBA	LAP3	1,32311E-104	-9,91032E-01	0,407	0,596	2,43678E-100
Mono_INHBA	CNPY3	1,16783E-188	-9,93367E-01	0,356	0,644	2,15080E-184
Mono_INHBA	HLA-DRB5	7,03172E-196	-9,98186E-01	0,791	0,851	1,29503E-191
Mono_INHBA	CAT	2,46693E-124	-1,00337E+00	0,187	0,423	4,54334E-120
Mono_INHBA	SYNGR2	9,93920E-190	-1,00679E+00	0,531	0,74	1,83050E-185
Mono_INHBA	RPSA	1,34513E-36	-1,00681E+00	0,484	0,496	2,47732E-32
Mono_INHBA	PEBP1	3,91038E-190	-1,01817E+00	0,277	0,582	7,20175E-186
Mono_INHBA	C1orf54	9,95133E-77	-1,01895E+00	0,128	0,292	1,83274E-72
Mono_INHBA	TREM2	1,99433E-87	-1,02744E+00	0,238	0,417	3,67296E-83
Mono_INHBA	HLA-DQB2	1,59295E-113	-1,02787E+00	0,124	0,329	2,93374E-109
Mono_INHBA	RABGAP1L	1,21932E-77	-1,02880E+00	0,1	0,262	2,24563E-73
Mono_INHBA	SCP2	7,40069E-94	-1,03280E+00	0,261	0,473	1,36299E-89
Mono_INHBA	UBE2L6	1,39025E-130	-1,03399E+00	0,35	0,584	2,56043E-126
Mono_INHBA	CCDC50	4,71741E-68	-1,04022E+00	0,129	0,283	8,68806E-64
Mono_INHBA	RPL5	2,20870E-39	-1,04595E+00	0,509	0,518	4,06776E-35
Mono_INHBA	TMEM109	2,21736E-108	-1,05776E+00	0,167	0,381	4,08372E-104
Mono_INHBA	MNDA	6,31376E-195	-1,06088E+00	0,25	0,551	1,16280E-190
Mono_INHBA	ZFP36L2	3,16024E-261	-1,06572E+00	0,397	0,713	5,82022E-257

Mono_INHBA	CD4	7,05937E-121	-1,07096E+00	0,314	0,552	1,30012E-116
Mono_INHBA	MPEG1	4,56501E-99	-1,07118E+00	0,184	0,385	8,40739E-95
Mono_INHBA	RGS1	1,70958E-145	-1,07517E+00	0,438	0,655	3,14854E-141
Mono_INHBA	AKR1B1	2,92926E-141	-1,08181E+00	0,242	0,482	5,39481E-137
Mono_INHBA	MS4A6A	0,00000E+00	-1,08797E+00	0,373	0,736	0,00000E+00
Mono_INHBA	HLA-DMB	1,72132E-286	-1,09630E+00	0,328	0,658	3,17016E-282
Mono_INHBA	SPCS1	1,30884E-124	-1,09783E+00	0,395	0,621	2,41048E-120
Mono_INHBA	MX1	6,07116E-26	-1,10209E+00	0,234	0,339	1,11813E-21
Mono_INHBA	PSME2	6,98682E-169	-1,10253E+00	0,526	0,727	1,28676E-164
Mono_INHBA	NAAA	4,29495E-123	-1,10266E+00	0,156	0,38	7,91001E-119
Mono_INHBA	PSMB9	2,46454E-132	-1,10302E+00	0,463	0,67	4,53894E-128
Mono_INHBA	GIMAP4	4,92915E-184	-1,13227E+00	0,162	0,444	9,07801E-180
Mono_INHBA	CIITA	4,24905E-106	-1,16028E+00	0,129	0,337	7,82547E-102
Mono_INHBA	STMN1	4,00233E-104	-1,16344E+00	0,105	0,298	7,37108E-100
Mono_INHBA	SPINT2	1,58114E-131	-1,17058E+00	0,274	0,512	2,91199E-127
Mono_INHBA	HMGN3	1,72320E-218	-1,17591E+00	0,214	0,542	3,17361E-214
Mono_INHBA	CD74	0,00000E+00	-1,19754E+00	0,975	0,962	0,00000E+00
Mono_INHBA	PPA1	1,12743E-92	-1,20059E+00	0,281	0,472	2,07639E-88
Mono_INHBA	TNFSF10	5,84253E-92	-1,20865E+00	0,177	0,371	1,07602E-87
Mono_INHBA	AXL	2,22692E-139	-1,24794E+00	0,069	0,293	4,10132E-135
Mono_INHBA	CPVL	1,17720E-193	-1,25213E+00	0,296	0,58	2,16804E-189
Mono_INHBA	HLA-DOA	4,60702E-198	-1,26297E+00	0,116	0,41	8,48475E-194
Mono_INHBA	HLA-DQB1	6,25265E-300	-1,26832E+00	0,684	0,824	1,15155E-295
Mono_INHBA	APOC1	5,55071E-86	-1,27879E+00	0,342	0,517	1,02227E-81
Mono_INHBA	LTC4S	8,71931E-149	-1,29758E+00	0,066	0,297	1,60584E-144
Mono_INHBA	FGL2	0,00000E+00	-1,30482E+00	0,276	0,635	0,00000E+00
Mono_INHBA	CTSC	3,08713E-204	-1,30518E+00	0,436	0,692	5,68556E-200
Mono_INHBA	LDHB	2,05309E-193	-1,30966E+00	0,23	0,528	3,78118E-189
Mono_INHBA	GAPT	2,09684E-99	-1,32542E+00	0,08	0,265	3,86175E-95
Mono_INHBA	HLA-DPA1	0,00000E+00	-1,33157E+00	0,825	0,902	0,00000E+00
Mono_INHBA	A2M	9,19637E-134	-1,36043E+00	0,175	0,402	1,69370E-129
Mono_INHBA	HLA-DPB1	0,00000E+00	-1,36224E+00	0,784	0,889	0,00000E+00
Mono_INHBA	PSMB8	2,82006E-133	-1,39865E+00	0,354	0,59	5,19370E-129
Mono_INHBA	HLA-DQA1	6,86084E-292	-1,58503E+00	0,618	0,793	1,26356E-287
Mono_INHBA	C3	1,69357E-124	-1,58963E+00	0,185	0,396	3,11904E-120
Mono_INHBA	TCF4	5,92845E-57	-1,61396E+00	0,14	0,281	1,09184E-52
Mono_INHBA	HERPUD1	2,47333E-232	-1,69122E+00	0,428	0,697	4,55512E-228
Mono_INHBA	IRF8	1,71794E-15	-1,78753E+00	0,295	0,383	3,16394E-11
Mono_INHBA	C1QA	5,51402E-240	-1,79259E+00	0,313	0,582	1,01552E-235
Mono_INHBA	APOE	1,33225E-119	-1,82023E+00	0,332	0,517	2,45361E-115
Mono_INHBA	C1QB	2,82366E-261	-1,83067E+00	0,279	0,562	5,20034E-257

Mono_INHBA	C1QC	1,37485E-217	-2,03491E+00	0,322	0,56	2,53206E-213
Mono_INHBA	PLD4	7,89895E-194	-2,21991E+00	0,069	0,348	1,45475E-189
Mono_INHBA	SERPINF1	7,50223E-238	-2,51576E+00	0,1	0,423	1,38169E-233

cluster	gene	p_val	avg_log2FC	pct.1	pct.2	p_val_adj
pDC_LILRA4	GZMB	0,00000E+00	8,63013E+00	0,783	0,014	0,00000E+00
pDC_LILRA4	PTGDS	0,00000E+00	7,47997E+00	0,385	0,014	0,00000E+00
pDC_LILRA4	LILRA4	0,00000E+00	6,77358E+00	0,68	0,032	0,00000E+00
pDC_LILRA4	TCL1A	0,00000E+00	6,60341E+00	0,442	0,001	0,00000E+00
pDC_LILRA4	CLIC3	0,00000E+00	5,73688E+00	0,688	0,021	0,00000E+00
pDC_LILRA4	SERPINF1	0,00000E+00	5,53967E+00	0,742	0,386	0,00000E+00
pDC_LILRA4	CYB561A3	0,00000E+00	5,51880E+00	0,709	0,19	0,00000E+00
pDC_LILRA4	BCL11A	0,00000E+00	5,34374E+00	0,646	0,054	0,00000E+00
pDC_LILRA4	ITM2C	0,00000E+00	5,33298E+00	0,779	0,105	0,00000E+00
pDC_LILRA4	PLAC8	0,00000E+00	5,33269E+00	0,742	0,176	0,00000E+00
pDC_LILRA4	IRF7	0,00000E+00	5,22599E+00	0,804	0,378	0,00000E+00
pDC_LILRA4	IRF8	0,00000E+00	5,17455E+00	0,766	0,357	0,00000E+00
pDC_LILRA4	MZB1	0,00000E+00	5,15391E+00	0,695	0,007	0,00000E+00
pDC_LILRA4	TSPAN13	0,00000E+00	5,12853E+00	0,761	0,039	0,00000E+00
pDC_LILRA4	IRF4	0,00000E+00	5,07232E+00	0,648	0,063	0,00000E+00
pDC_LILRA4	SPIB	0,00000E+00	5,06068E+00	0,59	0,044	0,00000E+00
pDC_LILRA4	IGKC	0,00000E+00	5,01357E+00	0,785	0,124	0,00000E+00
pDC_LILRA4	C12orf75	0,00000E+00	4,99592E+00	0,725	0,054	0,00000E+00
pDC_LILRA4	GPR183	0,00000E+00	4,92749E+00	0,777	0,44	0,00000E+00
pDC_LILRA4	PLD4	0,00000E+00	4,86136E+00	0,756	0,309	0,00000E+00
pDC_LILRA4	SMPD3	0,00000E+00	4,72145E+00	0,638	0,006	0,00000E+00
pDC_LILRA4	TCF4	0,00000E+00	4,68314E+00	0,8	0,245	0,00000E+00
pDC_LILRA4	CXCR3	0,00000E+00	4,65319E+00	0,571	0,025	0,00000E+00
pDC_LILRA4	IGHM	0,00000E+00	4,46025E+00	0,447	0,023	0,00000E+00
pDC_LILRA4	DERL3	0,00000E+00	4,43940E+00	0,638	0,014	0,00000E+00
pDC_LILRA4	ZFAT	0,00000E+00	4,40569E+00	0,569	0,021	0,00000E+00
pDC_LILRA4	IL3RA	0,00000E+00	4,37963E+00	0,728	0,1	0,00000E+00
pDC_LILRA4	POLB	0,00000E+00	4,35232E+00	0,551	0,123	0,00000E+00
pDC_LILRA4	LTB	0,00000E+00	4,34107E+00	0,628	0,12	0,00000E+00
pDC_LILRA4	EGLN3	0,00000E+00	4,32612E+00	0,388	0,035	0,00000E+00
pDC_LILRA4	SELL	0,00000E+00	4,28256E+00	0,698	0,175	0,00000E+00
pDC_LILRA4	HIGD1A	1,17747E-87	4,27488E+00	0,368	0,208	2,16854E-83
pDC_LILRA4	CXCR4	1,90872E-262	4,24415E+00	0,784	0,569	3,51528E-258
pDC_LILRA4	LIME1	0,00000E+00	4,18084E+00	0,517	0,01	0,00000E+00
pDC_LILRA4	JCHAIN	0,00000E+00	4,17941E+00	0,726	0,019	0,00000E+00
pDC_LILRA4	IDH3A	0,00000E+00	4,16546E+00	0,59	0,139	0,00000E+00
pDC_LILRA4	CLEC4C	0,00000E+00	4,15288E+00	0,569	0,004	0,00000E+00
pDC_LILRA4	PIM2	0,00000E+00	4,06440E+00	0,404	0,084	0,00000E+00
pDC_LILRA4	NR3C1	2,38990E-290	4,05014E+00	0,645	0,374	4,40148E-286

pDC_LILRA4	TPM2	0,00000E+00	4,04889E+00	0,539	0,026	0,00000E+00
pDC_LILRA4	SEL1L3	0,00000E+00	4,04577E+00	0,604	0,085	0,00000E+00
pDC_LILRA4	PTPRCAP	0,00000E+00	4,04409E+00	0,344	0,024	0,00000E+00
pDC_LILRA4	PTPRS	0,00000E+00	4,01827E+00	0,605	0,002	0,00000E+00
pDC_LILRA4	UGCG	0,00000E+00	3,97433E+00	0,757	0,158	0,00000E+00
pDC_LILRA4	SPCS1	0,00000E+00	3,93537E+00	0,788	0,598	0,00000E+00
pDC_LILRA4	NUCB2	0,00000E+00	3,86757E+00	0,557	0,183	0,00000E+00
pDC_LILRA4	CCDC50	0,00000E+00	3,86134E+00	0,756	0,248	0,00000E+00
pDC_LILRA4	SEC61B	0,00000E+00	3,85403E+00	0,834	0,735	0,00000E+00
pDC_LILRA4	RASD1	0,00000E+00	3,83174E+00	0,324	0,033	0,00000E+00
pDC_LILRA4	KRT5	0,00000E+00	3,82795E+00	0,37	0,004	0,00000E+00
pDC_LILRA4	CREM	2,08061E-122	3,82627E+00	0,568	0,367	3,83186E-118
pDC_LILRA4	SLC20A1	0,00000E+00	3,81465E+00	0,626	0,208	0,00000E+00
pDC_LILRA4	HERPUD1	0,00000E+00	3,79427E+00	0,838	0,673	0,00000E+00
pDC_LILRA4	PTCRA	0,00000E+00	3,77116E+00	0,526	0,012	0,00000E+00
pDC_LILRA4	SIDT1	0,00000E+00	3,76397E+00	0,516	0,027	0,00000E+00
pDC_LILRA4	LY9	0,00000E+00	3,73763E+00	0,295	0,024	0,00000E+00
pDC_LILRA4	ALOX5AP	0,00000E+00	3,73577E+00	0,805	0,489	0,00000E+00
pDC_LILRA4	SELPLG	1,57566E-259	3,72758E+00	0,606	0,33	2,90189E-255
pDC_LILRA4	GNL3	0,00000E+00	3,72358E+00	0,46	0,158	0,00000E+00
pDC_LILRA4	YPEL5	6,36808E-177	3,67677E+00	0,648	0,423	1,17281E-172
pDC_LILRA4	PLP2	0,00000E+00	3,65170E+00	0,767	0,385	0,00000E+00
pDC_LILRA4	SLC44A2	0,00000E+00	3,64203E+00	0,494	0,081	0,00000E+00
pDC_LILRA4	BLNK	0,00000E+00	3,61935E+00	0,599	0,148	0,00000E+00
pDC_LILRA4	SLC15A4	0,00000E+00	3,60944E+00	0,638	0,101	0,00000E+00
pDC_LILRA4	LDLRAD4	0,00000E+00	3,59282E+00	0,574	0,193	0,00000E+00
pDC_LILRA4	LAMP5	0,00000E+00	3,54216E+00	0,531	0,009	0,00000E+00
pDC_LILRA4	PPP1R14B	0,00000E+00	3,53923E+00	0,679	0,116	0,00000E+00
pDC_LILRA4	RGS2	3,43876E-23	3,53882E+00	0,642	0,625	6,33316E-19
pDC_LILRA4	ERN1	0,00000E+00	3,52924E+00	0,607	0,149	0,00000E+00
pDC_LILRA4	HSP90B1	0,00000E+00	3,51718E+00	0,833	0,693	0,00000E+00
pDC_LILRA4	SOX4	0,00000E+00	3,50736E+00	0,562	0,171	0,00000E+00
pDC_LILRA4	DNASE1L3	0,00000E+00	3,49469E+00	0,587	0,039	0,00000E+00
pDC_LILRA4	GPR65	8,54674E-65	3,49268E+00	0,451	0,32	1,57405E-60
pDC_LILRA4	EZR	2,14840E-199	3,46113E+00	0,615	0,388	3,95671E-195
pDC_LILRA4	MAP2K6	0,00000E+00	3,45054E+00	0,304	0,035	0,00000E+00
pDC_LILRA4	ST6GALNAC4	0,00000E+00	3,43438E+00	0,587	0,098	0,00000E+00
pDC_LILRA4	NPIPB5	6,18521E-122	3,42482E+00	0,257	0,106	1,13913E-117
pDC_LILRA4	EIF4A3	6,23056E-99	3,40746E+00	0,574	0,393	1,14748E-94
pDC_LILRA4	RPLP0	0,00000E+00	3,39383E+00	0,912	0,523	0,00000E+00

pDC_LILRA4	APP	0,00000E+00	3,38067E+00	0,758	0,285	0,00000E+00
pDC_LILRA4	CLN8	8,46126E-250	3,37861E+00	0,488	0,222	1,55831E-245
pDC_LILRA4	GAPT	2,49381E-281	3,36515E+00	0,544	0,239	4,59285E-277
pDC_LILRA4	ZDHHC17	0,00000E+00	3,36365E+00	0,419	0,073	0,00000E+00
pDC_LILRA4	MYBL2	0,00000E+00	3,35256E+00	0,481	0,003	0,00000E+00
pDC_LILRA4	RPS3A	0,00000E+00	3,34320E+00	0,926	0,547	0,00000E+00
pDC_LILRA4	RPL3	0,00000E+00	3,29875E+00	0,92	0,512	0,00000E+00
pDC_LILRA4	MPEG1	0,00000E+00	3,29185E+00	0,7	0,356	0,00000E+00
pDC_LILRA4	TSPYL2	0,00000E+00	3,26979E+00	0,456	0,155	0,00000E+00
pDC_LILRA4	EEF1G	1,44234E-130	3,26774E+00	0,467	0,286	2,65636E-126
pDC_LILRA4	ADAM19	0,00000E+00	3,25756E+00	0,472	0,09	0,00000E+00
pDC_LILRA4	SMIM5	0,00000E+00	3,25446E+00	0,416	0,002	0,00000E+00
pDC_LILRA4	RPL23A	0,00000E+00	3,23720E+00	0,892	0,506	0,00000E+00
pDC_LILRA4	RPS10	1,44530E-174	3,21130E+00	0,544	0,269	2,66182E-170
pDC_LILRA4	RPL17	0,00000E+00	3,20467E+00	0,757	0,416	0,00000E+00
pDC_LILRA4	N4BP2L1	0,00000E+00	3,20451E+00	0,493	0,183	0,00000E+00
pDC_LILRA4	RGS1	1,02040E-07	3,20034E+00	0,689	0,64	1,87927E-03
pDC_LILRA4	IFI44L	4,58860E-197	3,19656E+00	0,465	0,242	8,45083E-193
pDC_LILRA4	SEPHS1	0,00000E+00	3,17066E+00	0,288	0,049	0,00000E+00
pDC_LILRA4	BCL2L11	1,80539E-173	3,16578E+00	0,316	0,127	3,32499E-169
pDC_LILRA4	SRSF6	9,21889E-172	3,15877E+00	0,419	0,198	1,69784E-167
pDC_LILRA4	LDHB	3,32334E-129	3,15025E+00	0,692	0,501	6,12060E-125
pDC_LILRA4	CNP	0,00000E+00	3,14898E+00	0,479	0,147	0,00000E+00
pDC_LILRA4	NOP58	4,59261E-174	3,14316E+00	0,453	0,226	8,45821E-170
pDC_LILRA4	ZC3HAV1	2,13386E-111	3,13693E+00	0,451	0,261	3,92993E-107
pDC_LILRA4	CD4	2,04255E-40	3,13665E+00	0,609	0,535	3,76177E-36
pDC_LILRA4	RPL10A	0,00000E+00	3,12871E+00	0,89	0,476	0,00000E+00
pDC_LILRA4	OGT	0,00000E+00	3,12691E+00	0,614	0,182	0,00000E+00
pDC_LILRA4	IGFLR1	1,37188E-242	3,11310E+00	0,615	0,346	2,52660E-238
pDC_LILRA4	SUSD1	0,00000E+00	3,11218E+00	0,386	0,072	0,00000E+00
pDC_LILRA4	TSPAN3	3,83146E-214	3,11150E+00	0,57	0,305	7,05640E-210
pDC_LILRA4	RPS5	0,00000E+00	3,10932E+00	0,9	0,497	0,00000E+00
pDC_LILRA4	CYFIP2	0,00000E+00	3,10847E+00	0,526	0,048	0,00000E+00
pDC_LILRA4	PARK7	0,00000E+00	3,09962E+00	0,798	0,651	0,00000E+00
pDC_LILRA4	NPC1	0,00000E+00	3,09304E+00	0,378	0,114	0,00000E+00
pDC_LILRA4	RPS27	0,00000E+00	3,09217E+00	0,942	0,546	0,00000E+00
pDC_LILRA4	RPSA	0,00000E+00	3,09138E+00	0,879	0,475	0,00000E+00
pDC_LILRA4	B4GALT1	7,39273E-264	3,08948E+00	0,605	0,31	1,36152E-259
pDC_LILRA4	TP53I11	0,00000E+00	3,08540E+00	0,347	0,075	0,00000E+00
pDC_LILRA4	ZDHHC4	4,91326E-143	3,07086E+00	0,367	0,175	9,04875E-139

pDC_LILRA4	OFD1	0,00000E+00	3,06598E+00	0,578	0,161	0,00000E+00
pDC_LILRA4	SGSM3	3,46810E-248	3,06509E+00	0,323	0,105	6,38721E-244
pDC_LILRA4	SLAMF7	0,00000E+00	3,05640E+00	0,525	0,193	0,00000E+00
pDC_LILRA4	DCK	0,00000E+00	3,03891E+00	0,669	0,259	0,00000E+00
pDC_LILRA4	RPS6	0,00000E+00	3,03204E+00	0,92	0,524	0,00000E+00
pDC_LILRA4	TP53I13	3,17054E-144	3,02810E+00	0,43	0,221	5,83918E-140
pDC_LILRA4	CORO1C	5,28124E-168	3,02732E+00	0,562	0,332	9,72646E-164
pDC_LILRA4	NR4A3	4,57347E-82	3,01665E+00	0,453	0,288	8,42296E-78
pDC_LILRA4	RPL9	0,00000E+00	3,01595E+00	0,902	0,524	0,00000E+00
pDC_LILRA4	RPL4	0,00000E+00	3,00573E+00	0,854	0,457	0,00000E+00
pDC_LILRA4	RAB11FIP1	0,00000E+00	2,98512E+00	0,533	0,204	0,00000E+00
pDC_LILRA4	RPL13A	0,00000E+00	2,98512E+00	0,93	0,499	0,00000E+00
pDC_LILRA4	SLA2	0,00000E+00	2,98466E+00	0,393	0,004	0,00000E+00
pDC_LILRA4	TRAF4	0,00000E+00	2,98399E+00	0,681	0,037	0,00000E+00
pDC_LILRA4	EPHB1	0,00000E+00	2,98230E+00	0,359	0,002	0,00000E+00
pDC_LILRA4	TRIM22	6,02926E-247	2,97406E+00	0,67	0,403	1,11041E-242
pDC_LILRA4	HNRNPH1	6,46097E-149	2,97345E+00	0,622	0,393	1,18992E-144
pDC_LILRA4	SLC7A5	0,00000E+00	2,96941E+00	0,66	0,194	0,00000E+00
pDC_LILRA4	SLC38A1	0,00000E+00	2,96814E+00	0,451	0,06	0,00000E+00
pDC_LILRA4	FCHSD2	0,00000E+00	2,96055E+00	0,695	0,199	0,00000E+00
pDC_LILRA4	RPL31	0,00000E+00	2,96050E+00	0,888	0,476	0,00000E+00
pDC_LILRA4	RPL7	0,00000E+00	2,96043E+00	0,888	0,476	0,00000E+00
pDC_LILRA4	NRP1	4,30848E-61	2,95942E+00	0,28	0,157	7,93493E-57
pDC_LILRA4	UBL7	6,66329E-118	2,95064E+00	0,435	0,244	1,22718E-113
pDC_LILRA4	PDCD4	2,42160E-145	2,94929E+00	0,46	0,248	4,45986E-141
pDC_LILRA4	DNAJB9	0,00000E+00	2,94708E+00	0,507	0,19	0,00000E+00
pDC_LILRA4	P2RY14	0,00000E+00	2,93233E+00	0,386	0,061	0,00000E+00
pDC_LILRA4	USP11	4,57655E-228	2,93018E+00	0,396	0,156	8,42863E-224
pDC_LILRA4	RUNX2	0,00000E+00	2,92492E+00	0,488	0,068	0,00000E+00
pDC_LILRA4	STMN1	0,00000E+00	2,92182E+00	0,662	0,267	0,00000E+00
pDC_LILRA4	RPS20	0,00000E+00	2,92045E+00	0,835	0,43	0,00000E+00
pDC_LILRA4	RNASE6	6,29584E-19	2,92010E+00	0,619	0,535	1,15950E-14
pDC_LILRA4	RPN2	0,00000E+00	2,91959E+00	0,621	0,267	0,00000E+00
pDC_LILRA4	CARD11	0,00000E+00	2,90696E+00	0,467	0,058	0,00000E+00
pDC_LILRA4	NFKBID	5,74490E-104	2,90671E+00	0,383	0,205	1,05804E-99
pDC_LILRA4	DSTN	4,38396E-192	2,90640E+00	0,572	0,334	8,07394E-188
pDC_LILRA4	RPL5	0,00000E+00	2,90377E+00	0,896	0,497	0,00000E+00
pDC_LILRA4	ADA	0,00000E+00	2,89856E+00	0,373	0,096	0,00000E+00
pDC_LILRA4	RPS11	0,00000E+00	2,89685E+00	0,876	0,484	0,00000E+00
pDC_LILRA4	PFKFB2	0,00000E+00	2,89154E+00	0,303	0,058	0,00000E+00

pDC_LILRA4	SSR4	1,32723E-300	2,88743E+00	0,823	0,749	2,44436E-296
pDC_LILRA4	MYL12A	9,72569E-271	2,88538E+00	0,843	0,824	1,79118E-266
pDC_LILRA4	ALG2	0,00000E+00	2,86621E+00	0,514	0,172	0,00000E+00
pDC_LILRA4	UCP2	3,23777E-62	2,85689E+00	0,696	0,631	5,96300E-58
pDC_LILRA4	IL2RG	2,40067E-224	2,85346E+00	0,583	0,31	4,42131E-220
pDC_LILRA4	RPL6	0,00000E+00	2,85279E+00	0,907	0,535	0,00000E+00
pDC_LILRA4	KPNA2	7,69757E-121	2,85193E+00	0,381	0,2	1,41766E-116
pDC_LILRA4	RPS23	0,00000E+00	2,84886E+00	0,932	0,548	0,00000E+00
pDC_LILRA4	RPS12	0,00000E+00	2,84199E+00	0,947	0,563	0,00000E+00
pDC_LILRA4	RPL27	0,00000E+00	2,84150E+00	0,875	0,485	0,00000E+00
pDC_LILRA4	RPL23	0,00000E+00	2,84006E+00	0,833	0,433	0,00000E+00
pDC_LILRA4	RABGAP1L	1,98910E-183	2,83848E+00	0,485	0,24	3,66333E-179
pDC_LILRA4	IDI1	2,02930E-71	2,83138E+00	0,331	0,19	3,73737E-67
pDC_LILRA4	RPS29	0,00000E+00	2,81760E+00	0,895	0,481	0,00000E+00
pDC_LILRA4	P2RY6	0,00000E+00	2,81658E+00	0,588	0,173	0,00000E+00
pDC_LILRA4	NPM1	1,15241E-55	2,81332E+00	0,821	0,763	2,12240E-51
pDC_LILRA4	LILRB4	3,37768E-164	2,81251E+00	0,736	0,539	6,22067E-160
pDC_LILRA4	PHC3	4,46107E-229	2,81040E+00	0,381	0,144	8,21596E-225
pDC_LILRA4	TLR9	0,00000E+00	2,80735E+00	0,295	0,001	0,00000E+00
pDC_LILRA4	AHI1	0,00000E+00	2,80728E+00	0,458	0,085	0,00000E+00
pDC_LILRA4	DNAJC4	2,93598E-60	2,80630E+00	0,5	0,355	5,40720E-56
pDC_LILRA4	RPS4X	0,00000E+00	2,80366E+00	0,932	0,546	0,00000E+00
pDC_LILRA4	FYTTD1	1,05148E-233	2,80228E+00	0,488	0,236	1,93651E-229
pDC_LILRA4	RPL21	0,00000E+00	2,79846E+00	0,923	0,521	0,00000E+00
pDC_LILRA4	CSF2RB	0,00000E+00	2,79690E+00	0,544	0,183	0,00000E+00
pDC_LILRA4	THEMIS2	1,55159E-95	2,79349E+00	0,644	0,487	2,85757E-91
pDC_LILRA4	RPS8	0,00000E+00	2,79025E+00	0,926	0,559	0,00000E+00
pDC_LILRA4	BTG2	6,10041E-278	2,78873E+00	0,742	0,485	1,12351E-273
pDC_LILRA4	RPS3	0,00000E+00	2,78180E+00	0,928	0,548	0,00000E+00
pDC_LILRA4	PPIB	1,34745E-215	2,77460E+00	0,819	0,791	2,48159E-211
pDC_LILRA4	NREP	0,00000E+00	2,76923E+00	0,319	0,051	0,00000E+00
pDC_LILRA4	GPM6B	0,00000E+00	2,76290E+00	0,289	0,007	0,00000E+00
pDC_LILRA4	CTSC	7,06850E-150	2,75621E+00	0,789	0,672	1,30181E-145
pDC_LILRA4	PPM1K	0,00000E+00	2,75585E+00	0,593	0,132	0,00000E+00
pDC_LILRA4	PPP2R5C	9,14955E-172	2,75266E+00	0,417	0,197	1,68507E-167
pDC_LILRA4	SEMA7A	0,00000E+00	2,75037E+00	0,379	0,038	0,00000E+00
pDC_LILRA4	RPS28	0,00000E+00	2,75000E+00	0,941	0,563	0,00000E+00
pDC_LILRA4	RANBP2	4,93881E-112	2,74688E+00	0,5	0,302	9,09581E-108
pDC_LILRA4	RBM6	4,80658E-106	2,74631E+00	0,441	0,253	8,85228E-102
pDC_LILRA4	RPS13	0,00000E+00	2,73770E+00	0,922	0,552	0,00000E+00

pDC_LILRA4	RPL13	0,00000E+00	2,73712E+00	0,96	0,561	0,00000E+00
pDC_LILRA4	SFT2D2	0,00000E+00	2,72615E+00	0,594	0,277	0,00000E+00
pDC_LILRA4	APEX1	1,38351E-166	2,72436E+00	0,644	0,431	2,54801E-162
pDC_LILRA4	MORF4L2	3,24507E-154	2,71939E+00	0,479	0,263	5,97645E-150
pDC_LILRA4	SEC11C	6,11196E-280	2,71864E+00	0,599	0,311	1,12564E-275
pDC_LILRA4	USP36	4,72037E-33	2,71260E+00	0,264	0,172	8,69350E-29
pDC_LILRA4	PRMT7	0,00000E+00	2,71185E+00	0,282	0,056	0,00000E+00
pDC_LILRA4	TAGLN2	4,82865E-110	2,71110E+00	0,805	0,752	8,89292E-106
pDC_LILRA4	RPS15A	0,00000E+00	2,70713E+00	0,937	0,549	0,00000E+00
pDC_LILRA4	RAC2	3,10754E-144	2,70456E+00	0,666	0,487	5,72315E-140
pDC_LILRA4	RPL37A	0,00000E+00	2,70383E+00	0,912	0,518	0,00000E+00
pDC_LILRA4	SIVA1	2,72070E-181	2,69809E+00	0,656	0,444	5,01072E-177
pDC_LILRA4	TNFRSF21	0,00000E+00	2,69690E+00	0,567	0,147	0,00000E+00
pDC_LILRA4	RPL15	0,00000E+00	2,69079E+00	0,913	0,533	0,00000E+00
pDC_LILRA4	EIF3L	9,91776E-49	2,68621E+00	0,712	0,594	1,82655E-44
pDC_LILRA4	RPS25	0,00000E+00	2,68505E+00	0,902	0,528	0,00000E+00
pDC_LILRA4	IVD	1,74918E-186	2,67757E+00	0,351	0,141	3,22146E-182
pDC_LILRA4	VDAC3	2,63169E-112	2,67653E+00	0,484	0,303	4,84678E-108
pDC_LILRA4	DDX5	1,75915E-139	2,66918E+00	0,887	0,83	3,23983E-135
pDC_LILRA4	TM9SF2	1,73421E-115	2,66560E+00	0,608	0,418	3,19389E-111
pDC_LILRA4	PSMA2	1,70602E-98	2,66148E+00	0,374	0,217	3,14197E-94
pDC_LILRA4	RPS18	0,00000E+00	2,66089E+00	0,944	0,539	0,00000E+00
pDC_LILRA4	RPL19	0,00000E+00	2,65634E+00	0,933	0,561	0,00000E+00
pDC_LILRA4	SEC61A1	4,28827E-196	2,65575E+00	0,55	0,298	7,89771E-192
pDC_LILRA4	MX1	3,55747E-302	2,65551E+00	0,621	0,317	6,55180E-298
pDC_LILRA4	RPS16	0,00000E+00	2,65490E+00	0,911	0,525	0,00000E+00
pDC_LILRA4	SLC12A3	0,00000E+00	2,65482E+00	0,26	0,007	0,00000E+00
pDC_LILRA4	ISG20	7,07390E-222	2,65230E+00	0,563	0,297	1,30280E-217
pDC_LILRA4	SLC7A6	0,00000E+00	2,65081E+00	0,342	0,075	0,00000E+00
pDC_LILRA4	MCOLN2	0,00000E+00	2,64855E+00	0,419	0,101	0,00000E+00
pDC_LILRA4	PDIA4	5,19207E-245	2,64811E+00	0,58	0,307	9,56224E-241
pDC_LILRA4	ST3GAL4	0,00000E+00	2,64417E+00	0,37	0,07	0,00000E+00
pDC_LILRA4	DHRS7	1,08717E-50	2,64348E+00	0,607	0,478	2,00225E-46
pDC_LILRA4	ERCC1	6,46867E-36	2,64257E+00	0,475	0,376	1,19133E-31
pDC_LILRA4	LUC7L3	1,81637E-114	2,64080E+00	0,549	0,349	3,34520E-110
pDC_LILRA4	RPL7A	0,00000E+00	2,63830E+00	0,902	0,54	0,00000E+00
pDC_LILRA4	CLK1	1,03416E-28	2,63811E+00	0,481	0,377	1,90462E-24
pDC_LILRA4	DCPS	8,12244E-291	2,63778E+00	0,413	0,151	1,49591E-286
pDC_LILRA4	TRAM1	4,40985E-260	2,63509E+00	0,684	0,438	8,12162E-256
pDC_LILRA4	RPL36A	0,00000E+00	2,63439E+00	0,788	0,436	0,00000E+00

pDC_LILRA4	CD164	3,04102E-110	2,63404E+00	0,697	0,55	5,60065E-106
pDC_LILRA4	LPXN	2,93746E-223	2,62999E+00	0,593	0,335	5,40992E-219
pDC_LILRA4	MAP1A	0,00000E+00	2,62967E+00	0,677	0,008	0,00000E+00
pDC_LILRA4	TMEM109	8,37496E-92	2,62809E+00	0,534	0,36	1,54242E-87
pDC_LILRA4	SRP14	8,16639E-273	2,62735E+00	0,849	0,836	1,50400E-268
pDC_LILRA4	SMG1	4,53577E-76	2,62414E+00	0,474	0,31	8,35353E-72
pDC_LILRA4	GAS6	0,00000E+00	2,62086E+00	0,6	0,186	0,00000E+00
pDC_LILRA4	ERP29	1,26155E-194	2,61978E+00	0,76	0,616	2,32339E-190
pDC_LILRA4	SUB1	0,00000E+00	2,61969E+00	0,816	0,718	0,00000E+00
pDC_LILRA4	SPON2	0,00000E+00	2,61748E+00	0,314	0,03	0,00000E+00
pDC_LILRA4	RPS27A	0,00000E+00	2,61493E+00	0,94	0,556	0,00000E+00
pDC_LILRA4	HNRNPA1	1,40831E-163	2,61080E+00	0,856	0,799	2,59369E-159
pDC_LILRA4	P4HB	1,32256E-139	2,60825E+00	0,764	0,622	2,43577E-135
pDC_LILRA4	RPL27A	0,00000E+00	2,60359E+00	0,869	0,442	0,00000E+00
pDC_LILRA4	AFF3	0,00000E+00	2,60178E+00	0,344	0,032	0,00000E+00
pDC_LILRA4	SAP30BP	1,35013E-163	2,60162E+00	0,486	0,259	2,48654E-159
pDC_LILRA4	RBBP4	9,96015E-139	2,59996E+00	0,57	0,36	1,83436E-134
pDC_LILRA4	TUBG1	3,75228E-218	2,59469E+00	0,258	0,079	6,91058E-214
pDC_LILRA4	SCAMP5	0,00000E+00	2,59346E+00	0,463	0,009	0,00000E+00
pDC_LILRA4	HINT1	0,00000E+00	2,59045E+00	0,847	0,716	0,00000E+00
pDC_LILRA4	CDK2AP2	2,07594E-283	2,58587E+00	0,665	0,383	3,82326E-279
pDC_LILRA4	DDIT4	1,51686E-91	2,58238E+00	0,637	0,488	2,79359E-87
pDC_LILRA4	HYOU1	0,00000E+00	2,57994E+00	0,484	0,155	0,00000E+00
pDC_LILRA4	MAGED1	0,00000E+00	2,57715E+00	0,307	0,049	0,00000E+00
pDC_LILRA4	RPL10	0,00000E+00	2,57463E+00	0,961	0,574	0,00000E+00
pDC_LILRA4	ETV3	1,81762E-58	2,57180E+00	0,256	0,14	3,34751E-54
pDC_LILRA4	MRPS6	4,51513E-94	2,57085E+00	0,487	0,316	8,31552E-90
pDC_LILRA4	TXN	0,00000E+00	2,56855E+00	0,821	0,659	0,00000E+00
pDC_LILRA4	WDFY4	0,00000E+00	2,56184E+00	0,48	0,146	0,00000E+00
pDC_LILRA4	NKG7	0,00000E+00	2,55894E+00	0,391	0,113	0,00000E+00
pDC_LILRA4	C6orf62	1,28015E-38	2,55798E+00	0,472	0,358	2,35766E-34
pDC_LILRA4	RPS14	0,00000E+00	2,55309E+00	0,941	0,556	0,00000E+00
pDC_LILRA4	SLC35C2	3,43053E-179	2,55250E+00	0,376	0,164	6,31801E-175
pDC_LILRA4	ILF2	5,03834E-135	2,54970E+00	0,578	0,381	9,27911E-131
pDC_LILRA4	PACSIN1	0,00000E+00	2,54873E+00	0,532	0	0,00000E+00
pDC_LILRA4	ERGIC2	3,91013E-136	2,54848E+00	0,407	0,207	7,20129E-132
pDC_LILRA4	SHD	0,00000E+00	2,54838E+00	0,387	0	0,00000E+00
pDC_LILRA4	MAPK1IP1L	1,02747E-109	2,54793E+00	0,529	0,334	1,89229E-105
pDC_LILRA4	PAIP1	0,00000E+00	2,54500E+00	0,518	0,183	0,00000E+00
pDC_LILRA4	RPL41	0,00000E+00	2,54472E+00	0,946	0,554	0,00000E+00

pDC_LILRA4	OSTC	2,03555E-199	2,54450E+00	0,69	0,489	3,74887E-195
pDC_LILRA4	EMB	3,87894E-146	2,54354E+00	0,409	0,201	7,14384E-142
pDC_LILRA4	HNRNPC	7,11246E-62	2,54164E+00	0,731	0,643	1,30990E-57
pDC_LILRA4	RBMX	5,86294E-112	2,54058E+00	0,598	0,416	1,07978E-107
pDC_LILRA4	SYNGR2	1,01785E-28	2,53936E+00	0,761	0,726	1,87457E-24
pDC_LILRA4	TMED2	2,25022E-178	2,53881E+00	0,693	0,489	4,14423E-174
pDC_LILRA4	ORMDL1	1,60159E-84	2,53762E+00	0,475	0,307	2,94964E-80
pDC_LILRA4	MAP4K1	2,42845E-291	2,53535E+00	0,318	0,091	4,47247E-287
pDC_LILRA4	CCS	1,81422E-103	2,53309E+00	0,431	0,253	3,34125E-99
pDC_LILRA4	ITGAE	0,00000E+00	2,53279E+00	0,447	0,15	0,00000E+00
pDC_LILRA4	RPL18A	0,00000E+00	2,53212E+00	0,941	0,542	0,00000E+00
pDC_LILRA4	TMX3	1,90876E-92	2,52892E+00	0,265	0,127	3,51537E-88
pDC_LILRA4	CTNS	0,00000E+00	2,52867E+00	0,351	0,091	0,00000E+00
pDC_LILRA4	SMIM14	1,44694E-210	2,52643E+00	0,509	0,256	2,66482E-206
pDC_LILRA4	CHD9	1,13230E-184	2,52501E+00	0,579	0,33	2,08536E-180
pDC_LILRA4	NACA	5,92364E-88	2,52054E+00	0,907	0,92	1,09096E-83
pDC_LILRA4	RPS19	0,00000E+00	2,51473E+00	0,957	0,555	0,00000E+00
pDC_LILRA4	FLNB	0,00000E+00	2,51427E+00	0,454	0,064	0,00000E+00
pDC_LILRA4	STT3A	1,75611E-146	2,51358E+00	0,335	0,149	3,23423E-142
pDC_LILRA4	SRSF7	1,99598E-148	2,51304E+00	0,723	0,542	3,67600E-144
pDC_LILRA4	UBC	6,64059E-21	2,51107E+00	0,886	0,899	1,22300E-16
pDC_LILRA4	FERMT3	5,47901E-91	2,50791E+00	0,626	0,475	1,00907E-86
pDC_LILRA4	TMED10	1,85070E-72	2,50603E+00	0,691	0,561	3,40843E-68
pDC_LILRA4	UBB	1,97202E-23	2,50307E+00	0,812	0,81	3,63187E-19
pDC_LILRA4	GNA15	1,65320E-160	2,50299E+00	0,523	0,295	3,04470E-156
pDC_LILRA4	SPINT2	9,47796E-165	2,49954E+00	0,692	0,487	1,74556E-160
pDC_LILRA4	NGLY1	0,00000E+00	2,49543E+00	0,488	0,164	0,00000E+00
pDC_LILRA4	EEF1B2	3,79882E-172	2,49507E+00	0,866	0,84	6,99629E-168
pDC_LILRA4	PABPC1	8,87554E-154	2,49442E+00	0,892	0,893	1,63461E-149
pDC_LILRA4	ARHGAP9	3,39661E-79	2,49417E+00	0,391	0,238	6,25553E-75
pDC_LILRA4	RPL26	4,15102E-303	2,49335E+00	0,903	0,523	7,64494E-299
pDC_LILRA4	PSMB4	2,14675E-42	2,49194E+00	0,487	0,367	3,95366E-38
pDC_LILRA4	PHB	7,59276E-117	2,48958E+00	0,566	0,379	1,39836E-112
pDC_LILRA4	CNPY3	9,78831E-96	2,48809E+00	0,735	0,622	1,80271E-91
pDC_LILRA4	MDM4	4,57077E-129	2,48654E+00	0,382	0,192	8,41798E-125
pDC_LILRA4	CDC42SE2	3,61317E-181	2,48644E+00	0,502	0,262	6,65437E-177
pDC_LILRA4	PSMA3	1,41571E-117	2,48108E+00	0,555	0,362	2,60731E-113
pDC_LILRA4	DDX24	1,84853E-83	2,47934E+00	0,626	0,469	3,40444E-79
pDC_LILRA4	YWHAZ	7,88048E-89	2,47515E+00	0,772	0,709	1,45135E-84
pDC_LILRA4	SPN	0,00000E+00	2,47370E+00	0,45	0,152	0,00000E+00

pDC_LILRA4	RNF5	2,13589E-233	2,47286E+00	0,535	0,269	3,93367E-229
pDC_LILRA4	EIF4A2	8,11431E-27	2,47201E+00	0,651	0,557	1,49441E-22
pDC_LILRA4	PPIA	3,44751E-91	2,47189E+00	0,879	0,895	6,34929E-87
pDC_LILRA4	COMMD3	1,25959E-67	2,46896E+00	0,272	0,148	2,31978E-63
pDC_LILRA4	GPX7	0,00000E+00	2,46690E+00	0,316	0,051	0,00000E+00
pDC_LILRA4	NHP2	8,14803E-107	2,46427E+00	0,586	0,403	1,50062E-102
pDC_LILRA4	EIF3F	9,37302E-31	2,46403E+00	0,731	0,663	1,72623E-26
pDC_LILRA4	U2AF1	6,95024E-32	2,46189E+00	0,162	0,275	1,28003E-27
pDC_LILRA4	SEC61G	6,09156E-278	2,46131E+00	0,762	0,591	1,12188E-273
pDC_LILRA4	NIPA2	3,70186E-205	2,46095E+00	0,437	0,199	6,81771E-201
pDC_LILRA4	CHAF1A	0,00000E+00	2,45973E+00	0,47	0,063	0,00000E+00
pDC_LILRA4	RPL29	0,00000E+00	2,45396E+00	0,916	0,539	0,00000E+00
pDC_LILRA4	MAD2L2	1,03336E-183	2,44736E+00	0,438	0,212	1,90314E-179
pDC_LILRA4	AKAP9	2,93361E-120	2,44577E+00	0,582	0,375	5,40283E-116
pDC_LILRA4	ZNF706	3,87518E-201	2,44350E+00	0,739	0,583	7,13691E-197
pDC_LILRA4	CCND3	5,52999E-108	2,43906E+00	0,483	0,303	1,01846E-103
pDC_LILRA4	NEK8	0,00000E+00	2,43752E+00	0,367	0,013	0,00000E+00
pDC_LILRA4	RPL18	0,00000E+00	2,43596E+00	0,916	0,543	0,00000E+00
pDC_LILRA4	COPZ1	9,11241E-21	2,43580E+00	0,463	0,378	1,67823E-16
pDC_LILRA4	RAP1GDS1	1,95984E-159	2,43247E+00	0,383	0,177	3,60943E-155
pDC_LILRA4	MRPL36	7,59442E-284	2,42586E+00	0,499	0,216	1,39866E-279
pDC_LILRA4	MAN2B1	1,42341E-81	2,42582E+00	0,634	0,466	2,62150E-77
pDC_LILRA4	RPS7	0,00000E+00	2,42470E+00	0,909	0,542	0,00000E+00
pDC_LILRA4	MLF2	1,94813E-137	2,42459E+00	0,639	0,442	3,58787E-133
pDC_LILRA4	RPN1	0,00000E+00	2,42431E+00	0,629	0,277	0,00000E+00
pDC_LILRA4	GUK1	2,79302E-81	2,42416E+00	0,781	0,706	5,14390E-77
pDC_LILRA4	SLC2A1	0,00000E+00	2,42182E+00	0,349	0,052	0,00000E+00
pDC_LILRA4	TOR3A	6,44953E-154	2,42069E+00	0,443	0,227	1,18781E-149
pDC_LILRA4	OCIAD2	0,00000E+00	2,41740E+00	0,422	0,062	0,00000E+00
pDC_LILRA4	RPA2	1,26176E-272	2,41530E+00	0,351	0,117	2,32379E-268
pDC_LILRA4	PSMB8	6,44030E-20	2,41347E+00	0,635	0,574	1,18611E-15
pDC_LILRA4	CD38	1,35882E-158	2,41281E+00	0,378	0,175	2,50254E-154
pDC_LILRA4	TATDN3	2,75455E-171	2,41106E+00	0,353	0,15	5,07305E-167
pDC_LILRA4	CD47	1,41319E-171	2,40969E+00	0,63	0,41	2,60267E-167
pDC_LILRA4	DDOST	3,20547E-38	2,40929E+00	0,577	0,457	5,90351E-34
pDC_LILRA4	TBL1XR1	6,24300E-106	2,40678E+00	0,353	0,188	1,14977E-101
pDC_LILRA4	SRP19	1,36202E-156	2,40424E+00	0,37	0,172	2,50843E-152
pDC_LILRA4	HS3ST3B1	0,00000E+00	2,40373E+00	0,333	0,061	0,00000E+00
pDC_LILRA4	FKBP2	1,35972E-72	2,40257E+00	0,536	0,402	2,50420E-68
pDC_LILRA4	SDR39U1	7,86652E-138	2,40230E+00	0,344	0,157	1,44878E-133

pDC_LILRA4	RPL35	0,00000E+00	2,40159E+00	0,902	0,511	0,00000E+00
pDC_LILRA4	SCT	0,00000E+00	2,40121E+00	0,582	0,001	0,00000E+00
pDC_LILRA4	RPS9	0,00000E+00	2,40033E+00	0,92	0,534	0,00000E+00
pDC_LILRA4	TRA2A	9,10282E-62	2,39748E+00	0,388	0,248	1,67647E-57
pDC_LILRA4	TUBB6	2,05164E-161	2,39732E+00	0,368	0,163	3,77850E-157
pDC_LILRA4	SSR1	2,12375E-104	2,39632E+00	0,629	0,454	3,91131E-100
pDC_LILRA4	TMCO1	4,49447E-37	2,38792E+00	0,465	0,353	8,27747E-33
pDC_LILRA4	HVCN1	3,43037E-147	2,38735E+00	0,453	0,243	6,31770E-143
pDC_LILRA4	ABCE1	2,12641E-205	2,38602E+00	0,377	0,154	3,91621E-201
pDC_LILRA4	RPLP1	0,00000E+00	2,38510E+00	0,956	0,579	0,00000E+00
pDC_LILRA4	CPNE1	5,84143E-43	2,38463E+00	0,341	0,228	1,07582E-38
pDC_LILRA4	UCK2	0,00000E+00	2,38382E+00	0,319	0,085	0,00000E+00
pDC_LILRA4	RPL11	0,00000E+00	2,38209E+00	0,947	0,561	0,00000E+00
pDC_LILRA4	XBP1	1,78395E-258	2,38099E+00	0,668	0,412	3,28550E-254
pDC_LILRA4	TMEM19	1,21358E-248	2,37868E+00	0,36	0,127	2,23506E-244
pDC_LILRA4	UBAC2	1,23133E-31	2,37764E+00	0,408	0,304	2,26774E-27
pDC_LILRA4	PHYKPL	2,57841E-185	2,37678E+00	0,486	0,247	4,74865E-181
pDC_LILRA4	RAN	5,37755E-105	2,37569E+00	0,756	0,652	9,90384E-101
pDC_LILRA4	PI4KA	1,59238E-205	2,37276E+00	0,386	0,156	2,93269E-201
pDC_LILRA4	SND1	2,40383E-123	2,37262E+00	0,539	0,334	4,42713E-119
pDC_LILRA4	PFKP	3,56873E-185	2,37063E+00	0,293	0,106	6,57253E-181
pDC_LILRA4	HIVEP1	0,00000E+00	2,36928E+00	0,361	0,079	0,00000E+00
pDC_LILRA4	ARL6IP1	3,71703E-59	2,36598E+00	0,552	0,408	6,84566E-55
pDC_LILRA4	TARBP1	0,00000E+00	2,36315E+00	0,254	0,054	0,00000E+00
pDC_LILRA4	PLEK	2,25032E-178	2,36306E+00	0,731	0,553	4,14441E-174
pDC_LILRA4	LPIN1	7,60740E-160	2,36301E+00	0,273	0,103	1,40106E-155
pDC_LILRA4	SSR3	6,42743E-76	2,36086E+00	0,704	0,59	1,18374E-71
pDC_LILRA4	RPLP2	0,00000E+00	2,35943E+00	0,934	0,551	0,00000E+00
pDC_LILRA4	EIF3M	2,11330E-27	2,35943E+00	0,599	0,494	3,89207E-23
pDC_LILRA4	NOP56	7,31813E-70	2,35907E+00	0,342	0,202	1,34778E-65
pDC_LILRA4	SRSF5	4,93688E-52	2,35862E+00	0,687	0,573	9,09225E-48
pDC_LILRA4	SPCS2	2,11078E-97	2,35676E+00	0,648	0,484	3,88742E-93
pDC_LILRA4	C11orf58	5,16301E-110	2,35590E+00	0,701	0,552	9,50871E-106
pDC_LILRA4	NMT1	2,34003E-118	2,35490E+00	0,337	0,166	4,30962E-114
pDC_LILRA4	DNAJA1	1,06357E-88	2,35150E+00	0,724	0,598	1,95877E-84
pDC_LILRA4	SNRNP25	1,89944E-255	2,35131E+00	0,426	0,17	3,49819E-251
pDC_LILRA4	NDUFC2	6,83223E-25	2,35118E+00	0,38	0,301	1,25829E-20
pDC_LILRA4	SH2D3C	0,00000E+00	2,35111E+00	0,334	0,059	0,00000E+00
pDC_LILRA4	RBM39	1,24490E-84	2,34888E+00	0,744	0,616	2,29274E-80
pDC_LILRA4	ANKRD10	4,28916E-13	2,34847E+00	0,39	0,329	7,89934E-09

pDC_LILRA4	SUMF2	1,82250E-22	2,34846E+00	0,371	0,291	3,35649E-18
pDC_LILRA4	ENPP2	1,73823E-196	2,34717E+00	0,256	0,081	3,20130E-192
pDC_LILRA4	NONO	7,60161E-46	2,34554E+00	0,548	0,425	1,39999E-41
pDC_LILRA4	RPL38	4,41185E-226	2,34445E+00	0,842	0,473	8,12530E-222
pDC_LILRA4	HSPE1	1,23712E-50	2,34163E+00	0,706	0,597	2,27840E-46
pDC_LILRA4	RPL24	0,00000E+00	2,33981E+00	0,899	0,519	0,00000E+00
pDC_LILRA4	PPP1R2	8,78379E-120	2,33827E+00	0,581	0,383	1,61771E-115
pDC_LILRA4	LRRC26	0,00000E+00	2,33696E+00	0,502	0,002	0,00000E+00
pDC_LILRA4	GRSF1	2,21675E-215	2,33663E+00	0,587	0,327	4,08259E-211
pDC_LILRA4	RPL22L1	0,00000E+00	2,33398E+00	0,63	0,255	0,00000E+00
pDC_LILRA4	SKP1	2,13297E-22	2,32973E+00	0,718	0,687	3,92828E-18
pDC_LILRA4	RPL35A	0,00000E+00	2,32593E+00	0,926	0,543	0,00000E+00
pDC_LILRA4	GTF3A	2,10768E-64	2,32415E+00	0,612	0,473	3,88171E-60
pDC_LILRA4	CHMP4A	2,11600E-28	2,32326E+00	0,258	0,175	3,89704E-24
pDC_LILRA4	ARF4	2,17391E-64	2,31851E+00	0,573	0,439	4,00369E-60
pDC_LILRA4	CD320	0,00000E+00	2,31656E+00	0,364	0,033	0,00000E+00
pDC_LILRA4	RPS21	0,00000E+00	2,31647E+00	0,894	0,524	0,00000E+00
pDC_LILRA4	INPP4A	0,00000E+00	2,31647E+00	0,518	0,116	0,00000E+00
pDC_LILRA4	CYTH4	4,77465E-90	2,31506E+00	0,476	0,306	8,79348E-86
pDC_LILRA4	TIMM13	1,98294E-146	2,31334E+00	0,603	0,383	3,65198E-142
pDC_LILRA4	SH3KBP1	8,02160E-18	2,31312E+00	0,488	0,41	1,47734E-13
pDC_LILRA4	NUDT5	1,15163E-80	2,31286E+00	0,38	0,223	2,12095E-76
pDC_LILRA4	PIP5K1A	9,80876E-224	2,31276E+00	0,317	0,107	1,80648E-219
pDC_LILRA4	VEGFB	0,00000E+00	2,31229E+00	0,399	0,123	0,00000E+00
pDC_LILRA4	EEF1D	2,85711E-63	2,30721E+00	0,871	0,871	5,26194E-59
pDC_LILRA4	FAM214A	0,00000E+00	2,30658E+00	0,293	0,05	0,00000E+00
pDC_LILRA4	PDE4B	3,49334E-54	2,30595E+00	0,501	0,36	6,43368E-50
pDC_LILRA4	C12orf57	1,01676E-121	2,30491E+00	0,535	0,322	1,87257E-117
pDC_LILRA4	FAM53C	5,52036E-153	2,30339E+00	0,357	0,164	1,01668E-148
pDC_LILRA4	PPP1R15A	8,28200E-12	2,30336E+00	0,732	0,654	1,52530E-07
pDC_LILRA4	IFNAR2	1,10017E-104	2,30240E+00	0,438	0,259	2,02619E-100
pDC_LILRA4	RBM5	1,88929E-42	2,29925E+00	0,306	0,197	3,47950E-38
pDC_LILRA4	IRF9	2,75120E-132	2,29832E+00	0,356	0,172	5,06689E-128
pDC_LILRA4	ANKRD12	1,29443E-236	2,29738E+00	0,698	0,458	2,38394E-232
pDC_LILRA4	CIITA	1,33222E-83	2,29724E+00	0,484	0,316	2,45355E-79
pDC_LILRA4	SMC6	0,00000E+00	2,29520E+00	0,485	0,078	0,00000E+00
pDC_LILRA4	RPL30	0,00000E+00	2,29414E+00	0,936	0,561	0,00000E+00
pDC_LILRA4	CHST12	0,00000E+00	2,29364E+00	0,453	0,113	0,00000E+00
pDC_LILRA4	RBM4	1,52498E-131	2,29313E+00	0,324	0,147	2,80855E-127
pDC_LILRA4	DUSP5	0,00000E+00	2,29268E+00	0,563	0,111	0,00000E+00

pDC_LILRA4	SON	6,63221E-48	2,29165E+00	0,753	0,67	1,22145E-43
pDC_LILRA4	TMED9	1,52037E-21	2,29107E+00	0,602	0,52	2,80006E-17
pDC_LILRA4	HNRNPDL	1,59269E-44	2,29079E+00	0,75	0,671	2,93325E-40
pDC_LILRA4	MIF4GD	1,19662E-28	2,29076E+00	0,4	0,307	2,20381E-24
pDC_LILRA4	CYB5B	5,78566E-88	2,28934E+00	0,337	0,183	1,06554E-83
pDC_LILRA4	TMIGD2	0,00000E+00	2,28680E+00	0,274	0,003	0,00000E+00
pDC_LILRA4	RPL34	0,00000E+00	2,28646E+00	0,933	0,556	0,00000E+00
pDC_LILRA4	SSR2	6,16670E-35	2,28566E+00	0,715	0,649	1,13572E-30
pDC_LILRA4	COX7A2L	1,66525E-53	2,28232E+00	0,612	0,483	3,06690E-49
pDC_LILRA4	PMAIP1	2,66069E-54	2,28223E+00	0,297	0,18	4,90019E-50
pDC_LILRA4	DNAJB11	1,18905E-72	2,28196E+00	0,458	0,303	2,18988E-68
pDC_LILRA4	PAIP2	2,96967E-66	2,28055E+00	0,63	0,505	5,46924E-62
pDC_LILRA4	NDUFA9	5,13674E-130	2,27992E+00	0,31	0,142	9,46033E-126
pDC_LILRA4	NUDT22	1,74030E-187	2,27904E+00	0,496	0,261	3,20510E-183
pDC_LILRA4	CAT	1,38450E-43	2,27243E+00	0,529	0,403	2,54984E-39
pDC_LILRA4	PMEPA1	0,00000E+00	2,27135E+00	0,415	0,062	0,00000E+00
pDC_LILRA4	IRF2BP2	0,00000E+00	2,26943E+00	0,636	0,306	0,00000E+00
pDC_LILRA4	AREG	8,39318E-69	2,26773E+00	0,447	0,266	1,54577E-64
pDC_LILRA4	SRSF3	2,70218E-145	2,26689E+00	0,719	0,563	4,97661E-141
pDC_LILRA4	SDHA	3,22018E-37	2,26607E+00	0,351	0,246	5,93061E-33
pDC_LILRA4	STRAP	1,52148E-94	2,26538E+00	0,519	0,347	2,80210E-90
pDC_LILRA4	EIF4G2	2,21258E-167	2,26505E+00	0,76	0,611	4,07491E-163
pDC_LILRA4	AMZ2	3,50468E-101	2,26425E+00	0,386	0,219	6,45458E-97
pDC_LILRA4	UCHL3	1,75384E-19	2,26356E+00	0,286	0,215	3,23005E-15
pDC_LILRA4	CNOT2	1,55395E-47	2,26333E+00	0,388	0,265	2,86191E-43
pDC_LILRA4	COBLL1	0,00000E+00	2,26175E+00	0,329	0,014	0,00000E+00
pDC_LILRA4	CHCHD2	6,38973E-167	2,25972E+00	0,831	0,815	1,17680E-162
pDC_LILRA4	ARHGAP17	5,28645E-300	2,25859E+00	0,456	0,175	9,73606E-296
pDC_LILRA4	C17orf49	5,47896E-235	2,25713E+00	0,492	0,216	1,00906E-230
pDC_LILRA4	B2M	7,03819E-30	2,25623E+00	0,995	0,995	1,29622E-25
pDC_LILRA4	BST2	2,80157E-22	2,25330E+00	0,727	0,725	5,15966E-18
pDC_LILRA4	C1QBP	6,50289E-134	2,25173E+00	0,629	0,419	1,19764E-129
pDC_LILRA4	EIF5	5,64684E-12	2,25017E+00	0,645	0,589	1,03998E-07
pDC_LILRA4	ERGIC3	2,05184E-31	2,24580E+00	0,591	0,493	3,77888E-27
pDC_LILRA4	PSMD9	1,47715E-84	2,24544E+00	0,409	0,246	2,72046E-80
pDC_LILRA4	CCT8	5,14573E-42	2,24324E+00	0,53	0,406	9,47689E-38
pDC_LILRA4	IL16	1,70446E-133	2,23961E+00	0,406	0,211	3,13910E-129
pDC_LILRA4	ARF1	1,63610E-49	2,23525E+00	0,745	0,696	3,01321E-45
pDC_LILRA4	BANP	1,29511E-97	2,23505E+00	0,274	0,129	2,38520E-93
pDC_LILRA4	CD36	1,22597E-99	2,23451E+00	0,376	0,191	2,25788E-95

pDC_LILRA4	EIF3E	6,14617E-07	2,23297E+00	0,658	0,603	1,13194E-02
pDC_LILRA4	DBNL	5,05696E-34	2,23294E+00	0,576	0,488	9,31340E-30
pDC_LILRA4	PSMA6	1,69307E-46	2,23036E+00	0,591	0,459	3,11813E-42
pDC_LILRA4	FBL	3,33772E-43	2,22867E+00	0,467	0,341	6,14708E-39
pDC_LILRA4	TAX1BP3	2,64030E-82	2,22819E+00	0,308	0,167	4,86264E-78
pDC_LILRA4	TMEM59	3,24890E-36	2,22758E+00	0,703	0,613	5,98350E-32
pDC_LILRA4	DDX6	3,22507E-64	2,22727E+00	0,371	0,227	5,93960E-60
pDC_LILRA4	SNRPD2	6,16732E-60	2,22637E+00	0,745	0,674	1,13584E-55
pDC_LILRA4	PPP6R1	0,00000E+00	2,22422E+00	0,418	0,133	0,00000E+00
pDC_LILRA4	COMMD6	5,22582E-130	2,22323E+00	0,795	0,732	9,62439E-126
pDC_LILRA4	PTP4A3	0,00000E+00	2,22160E+00	0,275	0,019	0,00000E+00
pDC_LILRA4	CCT7	1,58176E-27	2,21775E+00	0,441	0,344	2,91313E-23
pDC_LILRA4	NARF	2,89964E-97	2,21716E+00	0,362	0,198	5,34027E-93
pDC_LILRA4	SMARCE1	3,83371E-127	2,21710E+00	0,416	0,219	7,06054E-123
pDC_LILRA4	TOMM6	5,47549E-110	2,21595E+00	0,283	0,127	1,00842E-105
pDC_LILRA4	RPL39	0,00000E+00	2,21523E+00	0,934	0,562	0,00000E+00
pDC_LILRA4	ATPAF2	1,31015E-61	2,21227E+00	0,259	0,142	2,41291E-57
pDC_LILRA4	ZNF207	1,25902E-81	2,21146E+00	0,562	0,397	2,31873E-77
pDC_LILRA4	TAPBP	3,22885E-76	2,20285E+00	0,703	0,572	5,94658E-72
pDC_LILRA4	RNF138	4,66668E-155	2,20234E+00	0,34	0,148	8,59463E-151
pDC_LILRA4	GMPPB	3,48302E-244	2,20218E+00	0,307	0,099	6,41468E-240
pDC_LILRA4	PLVAP	0,00000E+00	2,19814E+00	0,279	0,017	0,00000E+00
pDC_LILRA4	SEC31A	6,54277E-113	2,19553E+00	0,484	0,289	1,20498E-108
pDC_LILRA4	SP110	2,29877E-40	2,19426E+00	0,49	0,385	4,23365E-36
pDC_LILRA4	IFI35	2,53120E-36	2,19026E+00	0,444	0,341	4,66172E-32
pDC_LILRA4	CCT3	1,28222E-60	2,19023E+00	0,542	0,403	2,36146E-56
pDC_LILRA4	EDEM1	3,85446E-161	2,18689E+00	0,391	0,182	7,09876E-157
pDC_LILRA4	THBD	3,23438E-63	2,18648E+00	0,34	0,201	5,95675E-59
pDC_LILRA4	ATP13A2	0,00000E+00	2,18635E+00	0,344	0,07	0,00000E+00
pDC_LILRA4	ARHGAP24	4,38452E-13	2,18541E+00	0,254	0,195	8,07496E-09
pDC_LILRA4	SNRPN	0,00000E+00	2,18213E+00	0,432	0,106	0,00000E+00
pDC_LILRA4	MYL12B	4,96200E-71	2,18072E+00	0,809	0,775	9,13851E-67
pDC_LILRA4	AHCY	1,00806E-117	2,18029E+00	0,444	0,252	1,85654E-113
pDC_LILRA4	DBI	1,27647E-26	2,17985E+00	0,762	0,722	2,35087E-22
pDC_LILRA4	ACAP1	4,39361E-233	2,17797E+00	0,35	0,125	8,09172E-229
pDC_LILRA4	ATF7IP	1,30163E-88	2,17764E+00	0,338	0,18	2,39721E-84
pDC_LILRA4	SFT2D1	1,62679E-46	2,17757E+00	0,573	0,454	2,99607E-42
pDC_LILRA4	RBBP7	5,12242E-85	2,17530E+00	0,353	0,197	9,43395E-81
pDC_LILRA4	OST4	2,92332E-45	2,17515E+00	0,799	0,776	5,38388E-41
pDC_LILRA4	SLC25A3	2,70096E-25	2,17465E+00	0,763	0,711	4,97436E-21

pDC_LILRA4	STAT2	4,02150E-96	2,17200E+00	0,466	0,285	7,40639E-92
pDC_LILRA4	SLC41A2	1,16849E-295	2,17115E+00	0,263	0,066	2,15201E-291
pDC_LILRA4	NDUFB8	1,44021E-29	2,16612E+00	0,368	0,283	2,65243E-25
pDC_LILRA4	AP3S1	3,45795E-113	2,16428E+00	0,532	0,336	6,36850E-109
pDC_LILRA4	SPCS3	8,53627E-131	2,16297E+00	0,633	0,435	1,57212E-126
pDC_LILRA4	ARHGAP27	9,34667E-131	2,16253E+00	0,377	0,186	1,72138E-126
pDC_LILRA4	PARP10	4,08647E-297	2,15890E+00	0,486	0,2	7,52606E-293
pDC_LILRA4	ANAPC5	1,19025E-91	2,15767E+00	0,455	0,279	2,19209E-87
pDC_LILRA4	NAPA	4,65688E-124	2,15517E+00	0,665	0,486	8,57658E-120
pDC_LILRA4	XPNPEP1	7,93508E-116	2,15131E+00	0,305	0,143	1,46140E-111
pDC_LILRA4	NR4A2	4,09372E-28	2,15009E+00	0,327	0,458	7,53940E-24
pDC_LILRA4	CYCS	3,15330E-34	2,14976E+00	0,622	0,519	5,80744E-30
pDC_LILRA4	RASSF2	3,86304E-243	2,14926E+00	0,53	0,25	7,11457E-239
pDC_LILRA4	DHPS	4,09020E-20	2,14895E+00	0,291	0,214	7,53291E-16
pDC_LILRA4	ACTR10	1,50253E-13	2,14690E+00	0,254	0,197	2,76720E-09
pDC_LILRA4	PTPRE	2,10371E-154	2,14107E+00	0,657	0,428	3,87440E-150
pDC_LILRA4	HNRNPK	1,82791E-48	2,14101E+00	0,768	0,706	3,36646E-44
pDC_LILRA4	CCNC	3,45124E-60	2,13743E+00	0,277	0,157	6,35615E-56
pDC_LILRA4	RABAC1	8,46469E-19	2,13691E+00	0,591	0,521	1,55894E-14
pDC_LILRA4	PWP1	2,55065E-28	2,13672E+00	0,298	0,207	4,69754E-24
pDC_LILRA4	TMX1	3,92213E-109	2,13589E+00	0,514	0,327	7,22339E-105
pDC_LILRA4	TAP1	9,69708E-119	2,13550E+00	0,607	0,412	1,78591E-114
pDC_LILRA4	ANAPC16	1,04150E-82	2,13390E+00	0,631	0,473	1,91813E-78
pDC_LILRA4	RPL36AL	0,00000E+00	2,13358E+00	0,828	0,475	0,00000E+00
pDC_LILRA4	RPL14	0,00000E+00	2,13307E+00	0,895	0,524	0,00000E+00
pDC_LILRA4	OAS1	6,08191E-42	2,13139E+00	0,44	0,321	1,12010E-37
pDC_LILRA4	SUPT5H	1,46351E-196	2,13130E+00	0,472	0,23	2,69534E-192
pDC_LILRA4	ISCU	5,15267E-28	2,13051E+00	0,632	0,532	9,48968E-24
pDC_LILRA4	LMAN2	1,57078E-11	2,13021E+00	0,574	0,508	2,89290E-07
pDC_LILRA4	HSPA8	8,76323E-09	2,12962E+00	0,815	0,82	1,61392E-04
pDC_LILRA4	HMGN1	1,02634E-150	2,12929E+00	0,773	0,617	1,89022E-146
pDC_LILRA4	TASP1	0,00000E+00	2,12921E+00	0,258	0,039	0,00000E+00
pDC_LILRA4	TBCC	8,77466E-169	2,12672E+00	0,281	0,105	1,61603E-164
pDC_LILRA4	NAPSA	0,00000E+00	2,12606E+00	0,533	0,161	0,00000E+00
pDC_LILRA4	RPS26	0,00000E+00	2,12542E+00	0,863	0,506	0,00000E+00
pDC_LILRA4	RNF126	0,00000E+00	2,12320E+00	0,49	0,173	0,00000E+00
pDC_LILRA4	HLA-F	1,66597E-13	2,12190E+00	0,569	0,513	3,06821E-09
pDC_LILRA4	TRABD	0,00000E+00	2,12183E+00	0,65	0,342	0,00000E+00
pDC_LILRA4	NEMF	2,86069E-24	2,12167E+00	0,372	0,284	5,26854E-20
pDC_LILRA4	PEBP1	3,58083E-64	2,12120E+00	0,705	0,557	6,59481E-60

pDC_LILRA4	SULF2	0,00000E+00	2,12103E+00	0,532	0,139	0,00000E+00
pDC_LILRA4	RPL32	0,00000E+00	2,12069E+00	0,941	0,559	0,00000E+00
pDC_LILRA4	COX5A	4,67506E-67	2,11794E+00	0,744	0,692	8,61005E-63
pDC_LILRA4	SRSF2	6,56163E-140	2,11719E+00	0,715	0,549	1,20846E-135
pDC_LILRA4	ARID5A	1,58934E-33	2,11643E+00	0,39	0,282	2,92709E-29
pDC_LILRA4	RHOF	0,00000E+00	2,11422E+00	0,438	0,143	0,00000E+00
pDC_LILRA4	HMGN3	1,91166E-96	2,11397E+00	0,69	0,514	3,52071E-92
pDC_LILRA4	SLC3A2	4,85914E-61	2,11259E+00	0,585	0,457	8,94908E-57
pDC_LILRA4	FUT7	0,00000E+00	2,11233E+00	0,259	0,034	0,00000E+00
pDC_LILRA4	MAP2K3	3,13089E-75	2,11140E+00	0,421	0,262	5,76617E-71
pDC_LILRA4	EDF1	3,66807E-28	2,10860E+00	0,784	0,748	6,75548E-24
pDC_LILRA4	NDUFV2	3,57216E-126	2,10768E+00	0,688	0,526	6,57884E-122
pDC_LILRA4	HINT2	1,79987E-77	2,10524E+00	0,374	0,222	3,31482E-73
pDC_LILRA4	RPS17	2,28195E-294	2,10276E+00	0,766	0,388	4,20266E-290
pDC_LILRA4	CBX6	0,00000E+00	2,10123E+00	0,399	0,116	0,00000E+00
pDC_LILRA4	EEF1A1	2,07636E-89	2,10106E+00	0,98	0,984	3,82403E-85
pDC_LILRA4	GAK	7,26990E-61	2,09977E+00	0,345	0,213	1,33890E-56
pDC_LILRA4	TTC3	3,93321E-79	2,09923E+00	0,482	0,32	7,24379E-75
pDC_LILRA4	RNASET2	6,16206E-24	2,09835E+00	0,794	0,737	1,13487E-19
pDC_LILRA4	DNAJC7	6,11019E-104	2,09636E+00	0,54	0,356	1,12531E-99
pDC_LILRA4	ABHD15	0,00000E+00	2,09542E+00	0,379	0,074	0,00000E+00
pDC_LILRA4	RPS15	0,00000E+00	2,09378E+00	0,938	0,554	0,00000E+00
pDC_LILRA4	NASP	2,85946E-45	2,09347E+00	0,391	0,269	5,26626E-41
pDC_LILRA4	AHSA1	2,39660E-52	2,09010E+00	0,423	0,292	4,41382E-48
pDC_LILRA4	HSPA9	1,58589E-44	2,08625E+00	0,49	0,364	2,92074E-40
pDC_LILRA4	RBM3	1,34594E-19	2,08611E+00	0,725	0,672	2,47881E-15
pDC_LILRA4	SNRPB	8,67038E-60	2,08570E+00	0,632	0,502	1,59682E-55
pDC_LILRA4	BTAF1	2,47026E-271	2,08523E+00	0,409	0,148	4,54947E-267
pDC_LILRA4	DNAJC19	1,42723E-39	2,07824E+00	0,338	0,227	2,62852E-35
pDC_LILRA4	SCFD1	1,42273E-54	2,07516E+00	0,263	0,151	2,62024E-50
pDC_LILRA4	SET	2,81813E-51	2,07451E+00	0,638	0,522	5,19014E-47
pDC_LILRA4	N4BP2L2	9,49195E-100	2,07328E+00	0,638	0,449	1,74813E-95
pDC_LILRA4	TMED4	4,86992E-45	2,07235E+00	0,409	0,287	8,96894E-41
pDC_LILRA4	PNISR	1,59446E-146	2,07227E+00	0,689	0,483	2,93651E-142
pDC_LILRA4	ACTN4	9,65890E-145	2,07216E+00	0,555	0,337	1,77888E-140
pDC_LILRA4	RNASEK	1,60313E-25	2,07168E+00	0,535	0,462	2,95249E-21
pDC_LILRA4	PHEX	0,00000E+00	2,07160E+00	0,259	0,001	0,00000E+00
[truncated: 168,151 more chars]
