# Supplementary material for: A single-cell pan-cancer analysis to show the variability of tumor-infiltrating myeloid cells in immune checkpoint blockade
Source: Nat Commun. 2024 Jul 21;15:6142. doi: 10.1038/s41467-024-50478-8 (PMC11271490; doi:10.1038/s41467-024-50478-8)
Supplement: Supplementary file 6 — Supplementary Data 3 [file 41467_2024_50478_MOESM6_ESM.pdf]

Macro_Mono

Cell_Type_Class	Response	State	pathway	pval	padj	log2err	ES	NES	size	leadingEdge
Macro/Mono	Responder	3	HALLMARK_TNFA_SIGNALING_VIA_NFKB	4,50854E-46	2,25427E-44	1,76830E+00	8,33304E-01	5,39428E+00	72	c("CXCL3", "G0S2", "PNRC1", "SOD2", "SOCS3", "PTGS2", "BCL2A1", "CCL4", "CXCL2", "CEBPB", "PLEK", "IL1B", "NFKB1", "PLAUR", "BCL3", "DUSP2", "PDE4B", "EHD1", "KLF2", "ETS2", "PPP1R15A", "NAMPT", "VEGFA", "AREG", "KDM6B", "ICAM1", "NFKBIA", "IER2", "BTG1", "MXD1", "IER3", "MCL1", "TRIB1", "TNFAIP3", "ABCA1", "RNF19B", "NFKB2", "PFKFB3", "SQSTM1", "TNIP1", "CD44", "SLC2A3", "FOSL2", "MAP2K3", "NINJ1", "SPHK1", "JUN", "KLF6", "EGR1", "JUNB", "FOSB", "CFLAR", "IFIT2", "CD83", "CDKN1A", "RELB", "BHLHE40", "NR4A1", "ZFP36", "HBEGF", "CCL5", "ATP2B1", "LITAF", "NR4A3")
Macro/Mono	Responder	1	HALLMARK_TNFA_SIGNALING_VIA_NFKB	2,09307E-25	1,04654E-23	1,30309E+00	-7,89416E-01	-3,02921E+00	61	c("IL1B", "NAMPT", "FOS", "CXCL2", "SOD2", "TNFAIP3", "BCL2A1", "NFKBIA", "ZFP36", "CCL4", "PLAUR", "NR4A1", "TNFAIP2", "CD83", "KLF6", "PPP1R15A", "CD44", "FOSB", "PLEK", "CDKN1A", "IER3", "ATF3", "SAT1", "SGK1", "PTPRE", "ICAM1", "HBEGF", "KDM6B", "NR4A2", "NR4A3", "PDE4B", "ATP2B1", "GADD45B", "MAP3K8", "CCNL1", "REL", "ETS2", "MCL1", "MXD1", "PER1", "KLF4", "EIF1", "LITAF", "CFLAR", "NFE2L2", "SQSTM1", "JUNB", "PFKFB3", "PNRC1")
Macro/Mono	Non-responder	1	HALLMARK_TNFA_SIGNALING_VIA_NFKB	9,22241E-25	4,61121E-23	1,28710E+00	-7,55158E-01	-2,88177E+00	70	c("IL1B", "CXCL2", "BCL2A1", "PLAUR", "DUSP2", "AREG", "SLC2A3", "NAMPT", "SOD2", "SAT1", "TNFAIP3", "CCL4", "NR4A1", "CD44", "NFKBIA", "CD83", "EGR1", "PPP1R15A", "ICAM1", "IER3", "PLEK", "HBEGF", "MXD1", "ZFP36", "TNFAIP2", "SOCS3", "PTPRE", "DUSP1", "IER2", "PDE4B", "NR4A2", "CFLAR", "ATF3", "IRF1", "RIPK2", "FOSL2", "BTG2", "CDKN1A", "KYNU", "CCNL1", "JUNB", "OLR1", "SGK1", "FOSB", "IFNGR2", "BHLHE40", "KDM6B", "MAP2K3", "ID2")
Macro/Mono	Non-responder	3	HALLMARK_TNFA_SIGNALING_VIA_NFKB	2,61270E-24	1,30635E-22	1,27903E+00	7,33894E-01	2,87578E+00	72	c("IL1B", "CXCL2", "DUSP2", "PLAUR", "BCL2A1", "SLC2A3", "AREG", "NAMPT", "SOD2", "SAT1", "CCL4", "TNFAIP3", "CD44", "NR4A1", "NFKBIA", "CD83", "EGR1", "IER3", "PPP1R15A", "ICAM1", "PLEK", "HBEGF", "MXD1", "TNFAIP2", "ZFP36", "PTPRE", "PDE4B", "SOCS3", "IER2", "DUSP1", "CFLAR", "NR4A2", "ATF3", "IRF1", "BTG2", "FOSL2", "CDKN1A", "KYNU", "OLR1", "SGK1", "CCNL1", "JUNB", "IFNGR2", "BHLHE40", "FOSB", "FOS", "ID2", "KDM6B", "MAP2K3", "PFKFB3")
Macro/Mono	Responder	3	HALLMARK_INFLAMMATORY_RESPONSE	4,45539E-11	1,11385E-09	8,51339E-01	5,35479E-01	3,29272E+00	60	c("CXCL8", "IFITM1", "EREG", "IL1B", "NFKB1", "PLAUR", "PDE4B", "NAMPT", "ICAM1", "NFKBIA", "MXD1", "ABCA1", "BEST1", "AQP9", "NLRP3", "SPHK1", "CD55", "LYN", "KLF6", "TNFRSF1B", "ITGA5", "IL4R", "TIMP1", "GNA15", "AHR", "CDKN1A", "IRF7", "HIF1A", "C5AR1", "HBEGF", "CCL5", "ATP2B1")
Macro/Mono	Non-responder	3	HALLMARK_INTERFERON_GAMMA_RESPONSE	5,11812E-11	1,27953E-09	8,51339E-01	5,98268E-01	2,32200E+00	66	c("IFI30", "ISG15", "NAMPT", "SOD2", "TNFAIP3", "IFITM2", "NFKBIA", "PSME1", "IFITM3", "ICAM1", "PTPN6", "B2M", "HIF1A", "TNFAIP2", "PSMA2", "PDE4B", "SOCS3", "MT2A", "PSMB8", "LAP3", "PSMA3", "IRF1", "CDKN1A", "PSMB10", "MX2", "CASP1", "IRF7", "PSMB9", "UBE2L6", "IL18BP", "CD86", "FPR1", "FCGR1A", "MTHFD2", "SRI")

Macro_Mono

Macro/Mono	Non-responder	1	HALLMARK_INTERFERON_GAMMA_RESPONSE	2,81574E-10	7,03935E-09	8,14036E-01	-5,94748E-01	-2,26963E+00	70	c("IFI30", "ISG15", "IFITM2", "NAMPT", "SOD2", "TNFAIP3", "NFKBIA", "MYD88", "IFITM3", "PSME1", "MT2A", "ICAM1", "B2M", "HIF1A", "TNFAIP2", "SOCS3", "TNFSF10", "PSMB8", "VAMP5", "PDE4B", "LAP3", "IRF1", "RIPK2", "PSMA3", "CDKN1A", "UPP1", "PSMB10", "MX2", "CASP1", "IRF7", "UBE2L6", "PNP", "FPR1", "IL18BP", "MTHFD2", "CD86", "SRI", "FCGR1A")
Macro/Mono	Responder	1	HALLMARK_INFLAMMATORY_RESPONSE	2,02635E-09	3,37725E-08	7,74939E-01	-6,68123E-01	-2,41306E+00	40	c("IL1B", "TIMP1", "NAMPT", "NFKBIA", "PLAUR", "CD55", "KLF6", "CDKN1A", "C5AR1", "PTPRE", "LCP2", "ICAM1", "HBEGF", "HIF1A", "NLRP3", "TNFRSF1B", "PDE4B", "ATP2B1", "CSF3R", "MXD1", "EMP3")
Macro/Mono	Responder	1	HALLMARK_OXIDATIVE_PHOSPHORYLATION	1,56615E-09	3,37725E-08	7,88187E-01	5,16154E-01	2,82674E+00	47	c("ATP5MC3", "ATP5F1E", "ATP5F1B", "ATP5PO", "ATP5MG", "ATP5MF", "ATP5F1A", "ATP5PF", "ATP5MC2", "ATP5PD", "ATP5F1D", "ATP5PB", "ATP5F1C", "ATP5MC1", "ATP5ME", "NDUFB8", "NDUFC2")
Macro/Mono	Non-responder	3	HALLMARK_HYPOXIA	2,99253E-09	4,98755E-08	7,74939E-01	6,13223E-01	2,31991E+00	53	c("PLAUR", "SLC2A3", "TNFAIP3", "PGK1", "ALDOA", "IER3", "PPP1R15A", "PLIN2", "TGFBI", "NAGK", "LDHA", "CXCR4", "ZFP36", "MT2A", "TPI1", "MIF", "DUSP1", "DDIT4", "ATF3", "GPI", "FOSL2", "CDKN1A", "FBP1", "BHLHE40", "FOS", "WSB1", "PFKFB3", "RBPJ", "HSPA5")
Macro/Mono	Non-responder	1	HALLMARK_INFLAMMATORY_RESPONSE	4,54419E-09	7,57365E-08	7,61461E-01	-6,16533E-01	-2,29816E+00	53	c("TIMP1", "IL1B", "PLAUR", "NAMPT", "CXCL8", "NFKBIA", "CD55", "CD48", "ICAM1", "HIF1A", "HBEGF", "MXD1", "NLRP3", "TNFSF10", "PTPRE", "PDE4B", "IRF1", "RIPK2", "RGS1", "BTG2", "CDKN1A", "C5AR1", "CSF3R", "IRF7", "OLR1", "IFNGR2", "FPR1", "TNFRSF1B", "SRI", "ADRM1", "P2RX4", "SLC31A2", "EMP3", "LYN", "NFKB1", "RHOG", "PTAFR")
Macro/Mono	Non-responder	1	HALLMARK_HYPOXIA	1,06878E-08	1,33598E-07	7,47740E-01	-6,19845E-01	-2,26617E+00	46	c("PLAUR", "SLC2A3", "TNFAIP3", "PGK1", "PPP1R15A", "MT2A", "IER3", "PLIN2", "CXCR4", "NAGK", "TGFBI", "ZFP36", "DUSP1", "TPI1", "DDIT4", "MIF", "ATF3", "GPI", "FOSL2", "CDKN1A", "FBP1", "BHLHE40", "WSB1", "PFKFB3", "RBPJ", "HSPA5", "PRDX5", "S100A4")
Macro/Mono	Non-responder	3	HALLMARK_INFLAMMATORY_RESPONSE	1,29754E-07	1,62193E-06	6,90132E-01	5,83990E-01	2,19986E+00	51	c("IL1B", "TIMP1", "PLAUR", "NAMPT", "CXCL8", "NFKBIA", "CD55", "ICAM1", "HIF1A", "HBEGF", "CD48", "MXD1", "NLRP3", "PTPRE", "PDE4B", "RGS1", "IRF1", "BTG2", "CDKN1A", "OLR1", "CSF3R", "C5AR1", "IFNGR2", "IRF7")
Macro/Mono	Non-responder	3	HALLMARK_MTORC1_SIGNALING	1,37895E-06	1,14912E-05	6,43552E-01	4,95664E-01	1,95198E+00	76	c("IFI30", "SLC2A3", "NAMPT", "CFP", "TFRC", "PGK1", "ALDOA", "RPN1", "PPP1R15A", "LDHA", "CXCR4", "INSIG1", "CORO1A", "TPI1", "DDIT4", "PPA1", "PSMA3", "GPI", "ACTR3", "BTG2", "PPIA", "CDKN1A", "PSMD13", "BHLHE40", "M6PR", "MAP2K3", "STIP1", "HSPA9", "LTA4H", "MTHFD2", "DDX39A", "HSPA5")
Macro/Mono	Responder	2	HALLMARK_OXIDATIVE_PHOSPHORYLATION	3,69201E-07	1,84601E-05	6,74963E-01	-3,32049E-01	-2,43674E+00	94	c("ATP5F1E", "ATP5MC2", "ATP5MG", "ATP5MC3", "ATP5MF", "ATP5F1D", "ATP5F1B", "ATP5PO", "ATP5PD", "ATP5PF", "ATP5F1A", "ATP5F1C", "ATP5PB", "ATP5ME")
Macro/Mono	Non-responder	1	HALLMARK_INTERFERON_ALPHA_RESPONSE	4,10140E-06	2,92957E-05	6,10527E-01	-6,09988E-01	-2,14184E+00	33	c("IFI30", "ISG15", "IFITM2", "IFITM3", "PSME1", "B2M", "PSMB8", "LAP3", "IRF1", "RIPK2", "PSMA3", "CASP1", "IRF7", "UBE2L6")

Macro_Mono

Macro/Mono	Non-responder	1	HALLMARK_APOPTOSIS	5,45382E-06	3,40864E-05	6,10527E-01	-5,61618E-01	-2,04164E+00	44	c("TIMP1", "IL1B", "SOD2", "SAT1", "CD44", "IFITM3", "IER3", "ANXA1", "BID", "TNFSF10", "CFLAR", "ATF3", "IRF1", "BTG2", "CDKN1A", "CASP1")
Macro/Mono	Responder	1	HALLMARK_HYPOXIA	3,27437E-06	4,09296E-05	6,27257E-01	-6,39928E-01	-2,18842E+00	31	c("ALDOA", "FOS", "TNFAIP3", "ZFP36", "PLAUR", "KLF6", "PPP1R15A", "CDKN1A", "IER3", "ATF3", "S100A4", "WSB1", "MIF", "PLIN2", "MYH9", "PFKFB3", "PNRC1", "FOSL2", "BNIP3L", "HDLBP", "NR3C1")
Macro/Mono	Non-responder	3	HALLMARK_APOPTOSIS	1,24850E-05	7,80310E-05	5,93325E-01	5,44009E-01	2,00707E+00	44	c("IL1B", "TIMP1", "SOD2", "SAT1", "CD44", "IER3", "IFITM3", "ANXA1", "LMNA", "CFLAR", "ATF3", "IRF1", "BTG2", "PLCB2", "CDKN1A", "CASP1")
Macro/Mono	Non-responder	3	HALLMARK_INTERFERON_ALPHA_RESPONSE	1,09725E-05	7,80310E-05	5,93325E-01	5,91277E-01	2,12183E+00	34	c("IFI30", "ISG15", "IFITM2", "PSME1", "IFITM3", "B2M", "PSMB8", "LAP3", "PSMA3", "IRF1", "CASP1", "IRF7", "PSMB9", "UBE2L6")
Macro/Mono	Non-responder	1	HALLMARK_MTORC1_SIGNALING	1,82910E-05	8,31410E-05	5,75610E-01	-4,77625E-01	-1,84435E+00	77	c("IFI30", "CFP", "SLC2A3", "NAMPT", "TFRC", "PGK1", "RPN1", "PPP1R15A", "CXCR4", "CORO1A", "INSIG1", "TPI1", "DDIT4", "PPA1", "PSMA3", "GPI", "BTG2", "PPIA", "CDKN1A", "PSMD13", "BHLHE40", "PNP", "MAP2K3", "M6PR", "LTA4H", "STIP1", "MTHFD2", "HSPA9", "DDX39A", "HSPA5", "CTSC", "IFRD1", "ITGB2", "SSR1", "HSPE1", "PRDX1", "SDF2L1", "PSMC2", "PSMC6", "TES", "HSPD1", "PFKL", "CCT6A", "CALR")
Macro/Mono	Responder	1	HALLMARK_APOPTOSIS	1,09036E-05	1,09036E-04	5,93325E-01	-6,03982E-01	-2,10268E+00	33	c("IL1B", "TIMP1", "SOD2", "CD44", "CDKN1A", "IER3", "ATF3", "SAT1", "DNAJA1", "GADD45B", "MCL1", "CFLAR", "SQSTM1", "DPYD", "ANXA1", "IFITM3", "CTNNB1")
Macro/Mono	Non-responder	1	HALLMARK_KRAS_SIGNALING_UP	1,24940E-04	4,80539E-04	5,18848E-01	-5,60375E-01	-1,98214E+00	34	c("IL1B", "PLAUR", "TNFAIP3", "CTSS", "PPP1R15A", "DUSP6", "LCP1", "CXCR4", "HBEGF", "FCER1G", "PSMB8", "DOCK2", "TNFRSF1B", "ID2")
Macro/Mono	Responder	1	HALLMARK_KRAS_SIGNALING_UP	2,12689E-04	1,32931E-03	5,18848E-01	-5,86831E-01	-1,97045E+00	29	c("IL1B", "TNFAIP3", "DUSP6", "PLAUR", "PPP1R15A", "LCP1", "HBEGF", "TNFRSF1B", "DOCK2", "CLEC4A", "F13A1", "KLF4")
Macro/Mono	Non-responder	3	HALLMARK_TGF_BETA_SIGNALING	2,75014E-03	9,82195E-03	4,31708E-01	6,32281E-01	1,77985E+00	11	c("THBS1", "PPP1R15A", "JUNB", "IFNGR2", "ID2", "PPP1CA", "TGIF1")
Macro/Mono	Non-responder	3	HALLMARK_KRAS_SIGNALING_UP	4,68507E-03	1,56169E-02	4,07018E-01	4,83685E-01	1,72460E+00	33	c("IL1B", "PLAUR", "TNFAIP3", "PPP1R15A", "DUSP6", "CXCR4", "HBEGF", "LCP1", "PSMB8", "ID2", "DOCK2", "CLEC4A", "ITGB2", "SPP1", "LAT2")
Macro/Mono	Non-responder	3	HALLMARK_GLYCOLYSIS	5,49303E-03	1,61560E-02	4,07018E-01	4,38964E-01	1,63348E+00	47	c("VCAN", "CD44", "PGK1", "ALDOA", "IER3", "TGFBI", "LDHA", "CXCR4", "TPI1", "MIF", "DDIT4", "PPIA")
Macro/Mono	Responder	3	HALLMARK_HYPOXIA	2,11626E-03	1,76355E-02	4,31708E-01	3,58360E-01	1,97346E+00	41	c("PNRC1", "PLAUR", "PPP1R15A", "VEGFA", "BTG1", "IER3", "TNFAIP3", "PFKFB3", "SLC2A3", "FOSL2", "JUN", "KLF6", "CDKN1A", "BHLHE40", "ZFP36")
Macro/Mono	Non-responder	1	HALLMARK_TGF_BETA_SIGNALING	7,68765E-03	2,26107E-02	4,07018E-01	-6,08837E-01	-1,73327E+00	12	c("THBS1", "PPP1R15A", "FKBP1A", "JUNB", "IFNGR2", "ID2", "PPP1CA", "TGIF1")
Macro/Mono	Responder	3	HALLMARK_INTERFERON_ALPHA_RESPONSE	5,05835E-03	2,29925E-02	4,07018E-01	-4,27868E-01	-1,84532E+00	42	c("CD74", "TXNIP", "CXCL10", "PSME1", "PSME2", "HLA-C", "B2M", "LGALS3BP", "BST2", "LAP3", "PSMB8", "UBE2L6", "GBP4", "PSMB9", "LPAR6", "SP110", "CASP1", "OAS1", "PSMA3")

Macro/Mono	Responder	3	HALLMARK_INTERFERON_GAMMA_RESPONSE	4,25816E-03	2,29925E-02	4,07018E-01	-3,41130E-01	-1,71673E+00	83	c("CD74", "HLA-DMA", "TXNIP", "HLA-DQA1", "HLA-DRB1", "CXCL10", "PSME1", "PSME2", "FCGR1A", "B2M", "HLA-A", "LGALS3BP", "BST2", "LAP3", "VAMP8", "PSMB8", "SERPING1", "FGL2", "UBE2L6", "GBP4", "CIITA", "CXCL9", "VAMP5", "HLA-B", "PSMB9", "IRF8")
Macro/Mono	Responder	1	HALLMARK_INTERFERON_GAMMA_RESPONSE	5,37857E-03	2,68929E-02	4,07018E-01	-4,15498E-01	-1,59438E+00	61	c("NAMPT", "IFITM2", "SOD2", "TNFAIP3", "NFKBIA", "RNF213", "TNFAIP2", "CDKN1A", "LCP2", "PSMB10", "ICAM1", "HIF1A", "PDE4B", "MYD88", "SAMHD1", "PTPN6", "IFITM3", "CD74")
Macro/Mono	Responder	3	HALLMARK_OXIDATIVE_PHOSPHORYLATION	9,59464E-03	3,69024E-02	3,80730E-01	-2,97567E-01	-1,56643E+00	119	c("PDK4", "SLC25A5", "COX7C", "LDHB", "IDH3B", "NDUFA4", "SLC25A3", "COX6C", "NDUFB2", "NDUFS7", "ACADVL", "SDHB", "COX5A", "UQCRC1", "NDUFA3", "NDUFS3", "OXA1L", "MDH1", "PRDX3", "COX4I1", "SDHC", "HSD17B10", "GPX4", "ISCU", "UQCRB", "MGST3", "NDUFS8", "SDHA", "ATP6V0E1", "HADHA", "NDUFB5", "NDUFB4", "VDAC1", "NDUFB7", "SLC25A6", "NDUFV1", "UQCR10", "ETFA", "IDH3G", "HADHB", "NDUFB1", "ECHS1", "MDH2", "DECR1", "UQCRH", "PDHA1", "ATP6V1H", "NDUFC1", "NDUFS4", "UQCRC2", "NDUFB3", "COX6B1", "ATP6V1F", "MPC1", "NDUFA6", "VDAC2", "NDUFS2", "COX7A2L", "COX7B", "CYC1", "NDUFA5", "ATP6AP1", "COX5B", "CYCS", "NDUFA1", "SUCLG1", "TIMM13", "COX7A2", "ACAT1", "PDHB", "IDH2", "TOMM22", "COX8A", "NDUFA2", "NDUFS6", "GPI", "MRPS12", "HSPA9")
Macro/Mono	Responder	2	HALLMARK_KRAS_SIGNALING_UP	2,43772E-03	4,06287E-02	4,31708E-01	4,45727E-01	1,89441E+00	32	c("DOCK2", "DUSP6", "F13A1", "CXCL10", "IRF8", "CLEC4A", "CXCR4", "LAT2", "CD37", "CTSS", "ID2", "ITGB2", "LCP1", "PSMB8", "IL10RA", "RABGAP1L", "FCER1G")
Macro/Mono	Responder	2	HALLMARK_TNFA_SIGNALING_VIA_NFKB	1,65451E-03	4,06287E-02	4,55060E-01	-3,07393E-01	-1,94002E+00	53	c("CEBPB", "PNRC1", "SOCS3", "CEBPD", "G0S2", "BCL3", "BTG1", "SOD2", "NFKB1", "PLAU", "BCL2A1", "CCL4", "KLF2", "DUSP2", "CXCL2", "PDE4B", "NFKBIE", "CD69", "TNIP1", "ETS2", "IER2", "IER5", "PLAUR")
Macro/Mono	Responder	3	HALLMARK_MYC_TARGETS_V1	1,19720E-02	4,27572E-02	3,80730E-01	-2,93249E-01	-1,54120E+00	114	c("HNRNPA1", "PPIA", "NPM1", "CLNS1A", "EEF1B2", "EIF3D", "U2AF1", "EIF4A1", "APEX1", "PRDX4", "RSL1D1", "NAP1L1", "SLC25A3", "COX5A", "SSBP1", "PSMA4", "PRDX3", "SSB", "PTGES3", "TUFM", "CNBP", "XRCC6", "RAN", "SET", "COPS5", "SNRPD3", "DUT", "NHP2", "PSMD8", "VDAC1", "SNRPD2", "CCT2", "C1QBP", "SNRPA1", "PSMA7", "PGK1", "PHB", "SERBP1", "GLO1", "PSMC4", "SRSF7", "MCM5", "LSM7", "SNRPD1", "CANX", "BUB3", "ERH", "CCT3", "PSMB3", "SNRPG", "MRPS18B", "CYC1", "HSPE1", "NOP56", "CCT7", "PSMD1", "PSMB2", "HSP90AB1", "SNRPB2", "PA2G4", "CCT5")

DC

Cell_Type_Class	Response	State	pathway	pval	padj	log2err	ES	NES	size	leadingEdge
DC	Non-responder	1	HALLMARK_INTERFERON_GAMMA_RESPONSE	2,44600E-09	1,22300E-07	7,74939E-01	-5,26889E-01	-2,07418E+00	81	c("TXNIP", "PTPN6", "IDO1", "UBE2L6", "ISG15", "FPR1", "VAMP5", "SLAMF7", "IFI44L", "HLA-DMA", "PSME1", "VAMP8", "IFITM2", "FGL2", "B2M", "IRF8", "SRI", "PSMB9", "TNFAIP3", "CASP4", "STAT2", "LCP2", "SAMHD1", "P2RY14", "LAP3", "HLA-DQA1", "CD74", "EPSTI1", "MYD88", "IFI35", "IRF9", "HLA-DRB1", "HIF1A", "PSMA3", "IFNAR2", "IL18BP", "TNFAIP2", "NAMPT", "RIPK2", "PDE4B", "OAS2", "PFKP", "IFI30", "ISG20", "UPP1", "HLA-A", "TAPBP", "FCGR1A", "CIITA", "IRF1")
DC	Non-responder	1	HALLMARK_KRAS_SIGNALING_UP	7,22766E-06	1,20461E-04	6,10527E-01	-5,81498E-01	-2,07061E+00	35	c("FCER1G", "IL1B", "CTSS", "LAPTM5", "ITGB2", "IRF8", "CSF2RA", "TNFAIP3", "LAT2", "F13A1", "CLEC4A", "SDCCAG8", "MAFB", "TMEM176B", "LCP1", "TMEM176A", "RABGAP1L", "RBM4", "IL2RG", "KLF4", "C3AR1")
DC	Non-responder	4	HALLMARK_INTERFERON_GAMMA_RESPONSE	8,96163E-06	1,49361E-04	5,93325E-01	4,33293E-01	1,81431E+00	87	c("PSMB8", "TXNIP", "UBE2L6", "PTPN6", "IRF8", "HLA-DMA", "PSME1", "VAMP8", "SP110", "IFITM2", "SLAMF7", "PSMB10", "IRF9", "BST2", "PSMA3", "ISG15", "CASP4", "PSMB9", "SRI", "SAMHD1", "CD74", "CIITA", "FPR1", "PSMA2", "ISG20", "MX1", "IRF7", "XAF1", "HIF1A", "IRF4", "HLA-DQA1", "IDO1", "TAPBP", "FGL2", "PDE4B", "SERPING1", "HLA-A", "CD38", "TAP1", "IFI30", "HLA-DRB1", "RNF213", "PFKP", "CASP1", "ICAM1", "CSF2RB", "RBCK1")
DC	Non-responder	4	HALLMARK_KRAS_SIGNALING_UP	7,59502E-06	1,49361E-04	6,10527E-01	5,15900E-01	2,00018E+00	45	c("FCER1G", "PSMB8", "CXCR4", "CTSS", "IRF8", "LAPTM5", "CD37", "ITGB2", "MAP4K1", "PPP1R15A", "RABGAP1L", "LCP1", "SDCCAG8", "HDAC9", "IL1B", "F13A1", "DOCK2", "LAT2", "IL2RG", "CLEC4A", "GLRX", "RBM4")
DC	Non-responder	3	HALLMARK_OXIDATIVE_PHOSPHORYLATION	1,30243E-05	6,38192E-04	5,93325E-01	5,60201E-01	2,82998E+00	21	c("ATP5F1E", "ATP5MC2", "ATP5MG", "ATP5MC3", "ATP5MF", "ATP5F1D", "ATP5PF", "ATP5PD", "ATP5ME", "ATP5F1A", "ATP5F1B", "ATP5PO", "ATP5PB", "ATP5F1C", "ATP5MC1")
DC	Non-responder	1	HALLMARK_INFLAMMATORY_RESPONSE	1,14613E-04	9,55106E-04	5,38434E-01	-4,69928E-01	-1,78956E+00	56	c("TIMP1", "IL1B", "RGS1", "CD48", "CD14", "FPR1", "AXL", "SELL", "SRI", "SLC31A2", "NLRP3", "MXD1", "LCP2", "IL18", "CSF3R", "PTPRE", "KLF6", "HIF1A", "RHOG", "NAMPT", "RNF144B", "IFNGR2", "RIPK2", "PDE4B", "GPR183", "PTAFR", "TAPBP", "C3AR1", "IRF1")
DC	Non-responder	1	HALLMARK_INTERFERON_ALPHA_RESPONSE	9,57038E-05	9,55106E-04	5,38434E-01	-5,06940E-01	-1,85778E+00	42	c("TXNIP", "UBE2L6", "ISG15", "IFI44L", "PSME1", "IFITM2", "SELL", "B2M", "PSMB9", "STAT2", "OAS1", "LAP3", "CD74", "EPSTI1", "IFI35", "HLA-C", "IRF9", "PSMA3", "RIPK2", "IFI30", "ISG20", "CNP", "IRF1", "GBP2", "LPAR6", "CASP1", "MX1", "TAP1", "CD47")
DC	Responder	3	HALLMARK_MTORC1_SIGNALING	3,94211E-05	1,85279E-03	5,57332E-01	-6,94809E-01	-2,03628E+00	17	c("IFI30", "PPP1R15A", "RPN1", "LGMN", "MAP2K3", "SLC7A5", "SQSTM1", "ALDOA", "EDEM1", "PPIA", "XBP1")
DC	Responder	2	HALLMARK_TNFA_SIGNALING_VIA_NFKB	7,62207E-05	2,14422E-03	5,38434E-01	-4,13422E-01	-1,90949E+00	66	c("GPR183", "AREG", "PLAUR", "NR4A3", "SLC2A3", "CD83", "NINJ1", "PDE4B", "NR4A2", "CDKN1A", "NFE2L2", "NAMPT", "MAP2K3", "ZFP36", "LITAF", "PLEK", "CD44", "PTPRE", "ICAM1", "PPP1R15A", "KDM6B", "EIF1", "BHLHE40", "CFLAR", "FOSL2", "B4GALT1")
DC	Responder	1	HALLMARK_MTORC1_SIGNALING	1,14498E-04	2,62626E-03	5,38434E-01	4,01882E-01	1,76253E+00	82	c("IFI30", "GLA", "EGLN3", "INSIG1", "IFRD1", "ALDOA", "CDKN1A", "NAMPT", "PGK1", "MAP2K3", "LGMN", "PPP1R15A", "LDHA", "HSP90B1", "IDI1", "CORO1A", "CFP", "ETF1", "SLC7A5", "SSR1", "HSPA9", "RPN1", "EDEM1", "BHLHE40", "CALR", "MTHFD2", "PRDX1", "HSPA5", "SQSTM1", "DDX39A", "BTG2", "UBE2D3", "STIP1", "TFRC")
DC	Responder	1	HALLMARK_TNFA_SIGNALING_VIA_NFKB	1,57576E-04	2,62626E-03	5,18848E-01	3,98019E-01	1,74118E+00	81	c("GPR183", "NR4A3", "DUSP2", "NINJ1", "PDE4B", "CDKN1A", "NR4A2", "NAMPT", "CD83", "MAP2K3", "NFE2L2", "PPP1R15A", "ICAM1", "CD44", "FOSL2", "LITAF", "PLEK", "ZFP36", "KDM6B", "JUNB", "B4GALT1", "BHLHE40", "CFLAR", "NR4A1", "SQSTM1", "BTG2", "NFKBIA", "PMEPA1", "SOD2", "AREG", "BCL2A1", "IL1B", "MAP3K8", "IL6ST", "KLF10", "PFKFB3", "TAP1", "RELB", "BIRC3", "BCL3", "CCNL1", "DENND5A", "ZC3H12A", "TNFAIP3")
DC	Non-responder	1	HALLMARK_HYPOXIA	5,27692E-04	3,76923E-03	4,77271E-01	-4,71634E-01	-1,76013E+00	47	c("DUSP1", "SLC2A3", "FOS", "PLAC8", "TGFBI", "PLIN2", "FBP1", "ENO1", "ATF3", "NAGK", "HEXA", "RBPJ", "TNFAIP3", "S100A4", "ANXA2", "KLF6", "GALK1", "GRHPR", "PFKP", "GPI", "TPI1", "XPNPEP1", "ISG20")
DC	Responder	2	HALLMARK_MTORC1_SIGNALING	2,48603E-04	4,14338E-03	4,98493E-01	-3,99813E-01	-1,84104E+00	65	c("INSIG1", "GLA", "ALDOA", "SLC2A3", "PPIA", "CDKN1A", "HSP90B1", "CFP", "PGK1", "NAMPT", "CORO1A", "MAP2K3", "LGMN", "ETF1", "SSR1", "PPP1R15A", "FKBP2", "BHLHE40", "HSPA9", "EDEM1", "SLC7A5", "CALR", "PRDX1", "HSPA5", "MTHFD2", "ELOVL5", "DDX39A", "PSMA3", "UBE2D3", "RPN1", "CANX", "STIP1", "M6PR", "ACTR3", "PSMA4")

DC	Responder	1	HALLMARK_MYC_TARGETS_V1	4,12402E-04	4,33662E-03	4,98493E-01	3,86455E-01	1,68991E+00	80	c("GNL3", "PABPC1", "IFRD1", "RPLP0", "KPNA2", "EIF4A1", "PGK1", "SRSF2", "HNRNPC", "PSMA6", "SLC25A3", "LDHA", "ETF1", "ILF2", "EIF4G2", "CNBP", "PSMA2", "CCT7", "VDAC3", "XRCC6", "TCP1", "RAN", "EIF4H", "C1QBP", "BUB3", "SRSF3", "SET", "YWHAE", "CANX", "TARDBP", "SRSF1", "AP3S1", "MCM5", "EIF2S1", "TRIM28", "TUFM", "HSP90AB1", "PSMA1", "ABCE1", "HDAC2", "PSMC6", "YWHAQ", "HNRNPA2B1", "HSPD1", "HNRNPR", "NOLC1", "SF3A1", "PPM1G", "RNPS1", "PCBP1", "TRA2B", "HNRNPU", "G3BP1", "EIF4E")
DC	Non-responder	4	HALLMARK_INTERFERON_ALPHA_RESPONSE	4,63918E-04	5,79897E-03	4,98493E-01	4,69968E-01	1,81526E+00	44	c("PSMB8", "TXNIP", "UBE2L6", "SELL", "PSME1", "SP110", "IFITM2", "IRF9", "BST2", "PSMA3", "ISG15", "PSMB9", "CNP", "CD74", "ISG20", "CD47", "HLA-C", "MX1", "IRF7")
DC	Non-responder	4	HALLMARK_HYPOXIA	6,10286E-04	6,10286E-03	4,77271E-01	4,38867E-01	1,72623E+00	51	c("TGFBI", "CXCR4", "FOS", "DUSP1", "PLIN2", "DDIT4", "PGK1", "ALDOA", "PPP1R15A", "HEXA", "FBP1", "ENO1", "ATF3", "PRDX5", "SLC2A3", "ISG20", "GPI", "GLRX", "KLF6", "ANXA2", "GALK1", "TPI1", "NR3C1", "ZFP36", "XPNPEP1", "RBPJ", "PFKP")
DC	Responder	2	HALLMARK_HYPOXIA	8,48369E-04	7,06974E-03	4,77271E-01	-4,74121E-01	-1,90588E+00	32	c("ALDOA", "PLAUR", "NR3C1", "SLC2A3", "CDKN1A", "TGFBI", "PGK1", "ZFP36", "PPP1R15A", "ANXA2", "BHLHE40", "FOSL2", "NAGK", "HSPA5", "MIF", "FOS", "HDLBP")
DC	Responder	2	HALLMARK_KRAS_SIGNALING_UP	1,05214E-03	7,51531E-03	4,55060E-01	-4,79230E-01	-1,87685E+00	29	c("IRF8", "PLAUR", "IL2RG", "FCER1G", "LCP1", "PPP1R15A", "TSPAN13", "CLEC4A", "IL10RA", "MAP4K1", "TNFRSF1B", "DOCK2", "RBM4", "BIRC3", "RABGAP1L", "ADAM8", "IL1B", "CD37")
DC	Non-responder	5	HALLMARK_HYPOXIA	7,59399E-04	9,30264E-03	4,77271E-01	-4,74926E-01	-1,67392E+00	47	c("PLAC8", "DDIT4", "TGFBI", "FOS", "PPP1R15A", "ALDOA", "PLAUR", "PGK1", "NAGK", "SLC2A3", "HEXA", "PLIN2", "ZFP36", "DUSP1", "TPI1", "HSPA5", "PRDX5", "GPI", "GLRX", "ATF3", "FBP1", "S100A4", "ANXA2", "ISG20", "TNFAIP3", "WSB1", "RBPJ")
DC	Non-responder	5	HALLMARK_INTERFERON_ALPHA_RESPONSE	5,66101E-04	9,30264E-03	4,77271E-01	-4,91987E-01	-1,69037E+00	39	c("SELL", "UBE2L6", "IFITM2", "PSMB8", "TXNIP", "CD47", "SP110", "MX1", "B2M", "OAS1", "CNP", "PSME2", "IFI30", "BST2", "STAT2", "PSMB9", "CD74", "PLSCR1")
DC	Non-responder	5	HALLMARK_KRAS_SIGNALING_DN	6,78915E-04	9,30264E-03	4,77271E-01	-8,12397E-01	-1,87370E+00	6	c("BTG2", "MX1", "NR4A2", "SGK1")
DC	Non-responder	4	HALLMARK_MYC_TARGETS_V1	1,44504E-03	1,03217E-02	4,55060E-01	3,68079E-01	1,54593E+00	90	c("PSMA6", "IFRD1", "U2AF1", "PGK1", "HNRNPA1", "EIF4A1", "SLC25A3", "PSMA4", "NPM1", "NHP2", "XRCC6", "APEX1", "EEF1B2", "HNRNPC", "CLNS1A", "PSMD8", "VDAC3", "PABPC1", "PSMA2", "NAP1L1", "TCP1", "PHB", "RPLP0", "CNBP", "PHB2", "PSMA7", "ILF2", "EIF3D", "IMPDH2", "RAN", "COX5A", "PRDX3", "BUB3", "RSL1D1", "FBL", "EIF2S1", "LSM7", "SRSF3", "CBX3", "TUFM", "HNRNPR", "SRSF2", "SRSF1", "EIF4H", "GLO1", "ABCE1", "HPRT1", "PSMD14", "EIF4G2", "AP3S1", "SNRPD3", "DUT", "HSP90AB1", "CCT2", "NDUFAB1", "VDAC1", "SERBP1", "GNL3", "PSMC4", "PTGES3", "LSM2", "HDAC2", "HSPE1", "SNRPG", "SNRPD1")
DC	Non-responder	4	HALLMARK_OXIDATIVE_PHOSPHORYLATION	1,44504E-03	1,03217E-02	4,55060E-01	3,61620E-01	1,53233E+00	99	c("IDH3A", "LDHB", "SLC25A3", "SLC25A5", "TCIRG1", "NDUFC2", "ACADVL", "ATP6AP1", "UQCRC2", "NDUFV1", "VDAC3", "NDUFS7", "NDUFB5", "ATP6V0B", "DECR1", "NDUFA9", "NDUFB8", "ATP6V1F", "IDH3B", "GPI", "PHB2", "OXA1L", "COX8A", "HADHB", "NDUFS2", "NDUFA3", "COX7A2L", "IDH2", "NDUFS8", "ETFA", "MDH2", "TOMM22", "UQCRH", "COX5A", "IDH3G", "COX7A2", "PRDX3", "COX4I1", "MRPS15", "UQCRC1", "HADHA", "PDK4", "SDHC", "NDUFB3", "MGST3", "ATP6V1D", "ACADM", "NDUFB6", "COX5B", "NDUFC1", "CYB5R3", "COX6A1", "VDAC2", "NDUFB4", "SLC25A6", "NDUFV2", "HCCS", "NDUFAB1", "NDUFA5", "VDAC1", "ATP6V1G1")
DC	Responder	2	HALLMARK_INFLAMMATORY_RESPONSE	1,78111E-03	1,11319E-02	4,55060E-01	-4,28259E-01	-1,77691E+00	37	c("GPR183", "IRF7", "TIMP1", "PLAUR", "SELL", "PDE4B", "CDKN1A", "NAMPT", "GNA15", "PTPRE", "CD55", "ICAM1", "TAPBP", "IL10RA", "HIF1A", "TNFRSF1B", "MXD1", "NFKBIA", "PIK3R5", "EMP3", "IL1B", "ADRM1")
DC	Non-responder	5	HALLMARK_MYC_TARGETS_V1	2,46531E-03	2,01334E-02	4,31708E-01	-4,27070E-01	-1,54301E+00	57	c("EIF4A1", "APEX1", "PGK1", "U2AF1", "NPM1", "HNRNPA1", "NHP2", "PSMB3", "SRSF7", "EEF1B2", "RPLP0", "C1QBP", "CCT7", "SNRPD2", "COX5A", "PHB", "PABPC1", "EIF3D", "FBL", "SSBP1", "XRCC6", "PSMD8", "SET", "CLNS1A", "SSB", "PA2G4", "CANX", "TUFM", "PHB2", "PRDX3", "IMPDH2", "RSL1D1", "LSM7", "HNRNPA2B1", "EIF4G2", "CDK4", "SNRPD3", "PPM1G", "RANBP1", "SNRPA", "SERBP1", "PRDX4", "GLO1")

DC

DC	Non-responder	1	HALLMARK_APOPTOSIS	4,95002E-03	2,25001E-02	4,07018E-01	-4,13349E-01	-1,56909E+00	55	c("TXNIP", "TIMP1", "IL1B", "PPT1", "GSN", "CD14", "GPX1", "PLCB2", "TSPO", "ATF3", "CD44", "ANXA1", "CASP4", "SAT1", "IL18", "PAK1", "GPX4", "HMGB2", "GADD45B", "BCL2L11", "ISG20", "SLC20A1", "IRF1", "IFNGR1", "RHOB", "CASP1", "BTG2", "TAP1", "DNAJA1", "CD38", "VDAC2", "ADD1", "IFITM3", "DPYD", "JUN", "LGALS3", "CFLAR", "IER3", "PEA15", "BNIP3L", "XIAP")
DC	Non-responder	1	HALLMARK_TNFA_SIGNALING_VIA_NFKB	8,44443E-03	3,51851E-02	3,80730E-01	-3,86594E-01	-1,50443E+00	72	c("SGK1", "IL1B", "DUSP1", "SLC2A3", "FOS", "NR4A2", "ATF3", "CD44", "TNFAIP3", "MXD1", "SAT1", "IL18", "JUNB", "MAP3K8", "PTPRE", "KLF6", "PER1", "TNFAIP2", "NAMPT", "LITAF", "IFNGR2", "RIPK2", "PDE4B", "CEBPD", "GPR183", "CCNL1", "FOSB", "GADD45B", "ETS2", "KLF4", "NR4A1", "FOSL2", "IRF1")
DC	Non-responder	3	HALLMARK_GLYCOLYSIS	1,44589E-03	3,54243E-02	4,55060E-01	-8,54460E-01	-1,73927E+00	5	c("CXCR4", "ALDOA", "ISG20", "PPIA", "STMN1")
DC	Responder	2	HALLMARK_INTERFERON_GAMMA_RESPONSE	9,81896E-03	3,77652E-02	3,80730E-01	-3,51758E-01	-1,57906E+00	55	c("IRF7", "IRF8", "IRF4", "IFITM2", "PDE4B", "CDKN1A", "NAMPT", "CD74", "PSMB10", "CIITA", "SLAMF7", "ICAM1", "PLSCR1", "MYD88", "HLA-DMA", "MTHFD2", "TAPBP", "IL10RA", "MX2", "HIF1A")
DC	Non-responder	4	HALLMARK_KRAS_SIGNALING_DN	6,07379E-03	3,79612E-02	4,07018E-01	6,32679E-01	1,73920E+00	9	c("SGK1", "NR4A2", "MX1", "BTG2", "SIDT1")

Mast

Cell_Type_Class	Response	State	pathway	pval	padj	log2err	ES	NES	size	leadingEdge
Mast	Responder	7	HALLMARK_TNFA_SIGNALING_VIA_NFKB	2,04211E-10	9,59793E-09	8,26657E-01	9,35484E-01	3,64257E+00	10	c("NR4A1", "TNFAIP3", "CD69", "PPP1R15A", "FOSB", "BTG2", "KLF6", "CD44", "ZFP36", "JUN")
Mast	Responder	1	HALLMARK_INTERFERON_ALPHA_RESPONSE	2,48889E-09	1,14489E-07	7,74939E-01	8,05634E-01	2,70202E+00	17	c("IFITM1", "CD74", "IFITM3", "PSMB9", "B2M", "HLA-C", "ISG15", "LY6E", "IFITM2", "BST2", "PSME2", "UBE2L6", "PSMB8", "TAP1", "PSME1")
Mast	Responder	1	HALLMARK_INTERFERON_GAMMA_RESPONSE	7,63312E-07	1,75562E-05	6,59444E-01	6,20200E-01	2,46146E+00	33	c("CD74", "HLA-DRB1", "IFITM3", "PSMB9", "MT2A", "B2M", "HLA-B", "ISG15", "LY6E", "IFITM2", "TNFSF10", "BST2", "PSME2", "NFKB1", "HLA-A", "UBE2L6", "PSMB8", "TAP1", "PSME1")
Mast	Non-responder	2	HALLMARK_TNFA_SIGNALING_VIA_NFKB	8,94784E-06	4,11601E-04	5,93325E-01	-5,32736E-01	-2,10310E+00	39	c("PLAUR", "CD69", "NFKBIA", "NR4A2", "GPR183", "BIRC3", "MCL1", "PPP1R15A", "SLC2A3", "BHLHE40", "TNFAIP3", "CD44", "PER1", "NR4A3", "ZFP36", "NR4A1", "REL", "SAT1", "RHOB", "KLF6", "BTG2", "JUNB", "LIF", "CCNL1")
Mast	Responder	1	HALLMARK_TNFA_SIGNALING_VIA_NFKB	7,81823E-05	8,99097E-04	5,38434E-01	-4,81752E-01	-2,34838E+00	25	c("JUN", "FOSB", "SQSTM1", "KLF2", "EGR1", "PPP1R15A", "NR4A1", "FOS", "DUSP1", "ZFP36", "IER2")
Mast	Non-responder	4	HALLMARK_INTERFERON_GAMMA_RESPONSE	6,29426E-05	1,51062E-03	5,38434E-01	4,54788E-01	2,41818E+00	35	c("HLA-DRB1", "CD74", "STAT1", "NFKB1", "CD69", "HLA-DQA1", "CD38", "CASP1", "P2RY14", "SOCS1", "PSME2", "HLA-B", "ISG15", "HLA-A", "TAPBP", "TAP1", "HLA-DMA", "PSMA2", "IRF7")
Mast	Non-responder	1	HALLMARK_OXIDATIVE_PHOSPHORYLATION	6,74788E-05	3,23898E-03	5,38434E-01	-4,70813E-01	-2,22428E+00	51	c("ATP5F1E", "ATP5PO", "NDUFC2", "ATP5MC2", "ATP5MG", "ATP5MC3", "ATP5F1C", "COX6C", "UQCR11", "ATP5MC1", "ATP5PB", "ATP5MF", "ATP5ME", "ATP6V0C", "NDUFB8", "FDX1", "ATP5F1D", "NDUFAB1", "COX5A", "NDUFA7", "NDUFA6", "ECH1", "ATP5PD", "COX17", "NDUFA4", "ATP5PF", "LDHB", "NDUFB2", "UQCR10")
Mast	Responder	1	HALLMARK_INFLAMMATORY_RESPONSE	5,26325E-04	4,84219E-03	4,77271E-01	6,54436E-01	2,12603E+00	15	c("IFITM1", "LY6E", "TIMP1", "TNFSF10", "BST2", "NFKB1", "RGS16", "PLAUR", "IFNGR2")
Mast	Non-responder	5	HALLMARK_TNFA_SIGNALING_VIA_NFKB	1,54969E-04	5,88883E-03	5,18848E-01	-7,84802E-01	-2,11813E+00	8	c("SQSTM1", "SAT1", "PPP1R15A", "FOSB", "JUN", "JUNB", "EIF1")
Mast	Non-responder	1	HALLMARK_TNFA_SIGNALING_VIA_NFKB	3,48138E-04	8,35532E-03	4,98493E-01	5,67837E-01	2,22876E+00	21	c("PLAUR", "SAT1", "PPP1R15A", "SQSTM1", "JUN", "FOSB", "NFKBIA", "SLC2A3", "DUSP1", "PNRC1", "IFNGR2", "GPR183")
Mast	Responder	1	HALLMARK_KRAS_SIGNALING_UP	1,58130E-03	1,21233E-02	4,55060E-01	6,66434E-01	1,92365E+00	11	c("TMEM176B", "BIRC3", "IL2RG", "FCER1G", "TMEM176A", "RGS16", "PLAUR", "PSMB8")
Mast	Non-responder	1	HALLMARK_APOPTOSIS	1,01543E-03	1,62469E-02	4,55060E-01	6,62444E-01	2,12327E+00	12	c("SAT1", "TIMP3", "SQSTM1", "JUN", "GSN", "TSPO")
Mast	Responder	7	HALLMARK_INTERFERON_ALPHA_RESPONSE	1,10044E-03	2,58604E-02	4,55060E-01	-6,86575E-01	-2,08018E+00	8	c("B2M", "IFITM3", "PSME1", "BST2", "IFITM2", "PSMB9")
