# Supplementary material for: A single-cell pan-cancer analysis to show the variability of tumor-infiltrating myeloid cells in immune checkpoint blockade
Source: Nat Commun. 2024 Jul 21;15:6142. doi: 10.1038/s41467-024-50478-8 (PMC11271490; doi:10.1038/s41467-024-50478-8)
Supplement: Supplementary file 7 — Supplementary Data 4 [file 41467_2024_50478_MOESM7_ESM.pdf]

Cell_Type_Class	Cancer	Response	Regulon	Cell_Type	AUC
Mast	ccRCC	Post(NR)	MAX	Mast	8,58679E-01
Mast	BCC	Post(NR)	BHLHE40	Mast	8,52060E-01
DC	BCC	Post(NR)	BHLHE40	pDC_LILRA4	8,51705E-01
Macro/Mono	BCC	Post(NR)	BHLHE40	Macro_IER3	8,47205E-01
Macro/Mono	BCC	Post(NR)	BHLHE40	Macro_LYVE1	8,46481E-01
DC	BCC	Post(NR)	BHLHE40	cDC(CD1C)	8,44173E-01
Macro/Mono	BCC	Post(NR)	BHLHE40	Mono_CD16	8,42116E-01
DC	BCC	Post(NR)	BHLHE40	cDC_LAMP3	8,40943E-01
Macro/Mono	BCC	Post(NR)	BHLHE40	Macro_IFI27	8,36904E-01
DC	ccRCC	Post(NR)	MAX	pDC_LILRA4	8,36158E-01
Macro/Mono	BCC	Post(NR)	BHLHE40	Mono_CD14	8,35485E-01
DC	BCC	Post(NR)	BHLHE40	cDC_CLEC9A	8,35284E-01
Macro/Mono	BCC	Post(NR)	BHLHE40	Macro_FOLR2-APOE+	8,32634E-01
Macro/Mono	BCC	Post(NR)	BHLHE40	Macro_FOLR2+APOE-	8,28944E-01
Macro/Mono	BCC	Post(NR)	BHLHE40	Macro_NLRP3	8,21762E-01
Macro/Mono	BCC	Post(NR)	BHLHE40	Macro_OLFML3	8,21685E-01
Mast	Melanoma	Post(NR)	BHLHE40	Mast	8,21332E-01
Macro/Mono	ccRCC	Post(NR)	MAX	Mono_CD16	8,20478E-01
Macro/Mono	ccRCC	Post(NR)	MAX	Macro_NLRP3	8,19880E-01
DC	ccRCC	Post(NR)	MAX	cDC_CLEC9A	8,18906E-01
Macro/Mono	ccRCC	Post(NR)	MAX	Mono_CD14	8,16862E-01
Macro/Mono	ccRCC	Post(NR)	MAX	Macro_IER3	8,13731E-01
Macro/Mono	ccRCC	Post(NR)	MAX	Mono_INHBA	8,12605E-01
DC	ccRCC	Post(NR)	MAX	cDC_LAMP3	8,11099E-01
DC	Melanoma	Post(NR)	BHLHE40	cDC_LAMP3	8,09379E-01
DC	ccRCC	Post(NR)	MAX	cDC(CD1C)	8,08768E-01
DC	Melanoma	Post(NR)	BHLHE40	cDC_CLEC9A	8,07819E-01
Macro/Mono	BCC	Post(NR)	BHLHE40	Macro_FOLR2+APOE+	8,06602E-01
DC	Melanoma	Post(NR)	BHLHE40	pDC_LILRA4	8,05294E-01
Macro/Mono	BCC	Post(NR)	BHLHE40	Macro_ISG15	8,04932E-01
DC	Melanoma	Post(NR)	BHLHE40	cDC(CD1C)	8,02637E-01
Macro/Mono	ccRCC	Post(NR)	MAX	Macro_FOLR2+APOE-	8,02178E-01
Macro/Mono	ccRCC	Post(NR)	ELF1	Mono_CD16	8,01987E-01
DC	Melanoma	Post(NR)	POLR2A	pDC_LILRA4	8,01483E-01
Macro/Mono	BCC	Post(NR)	BHLHE40	Mono_INHBA	8,01396E-01
Macro/Mono	Melanoma	Post(NR)	BHLHE40	Mono_INHBA	8,00085E-01
Macro/Mono	ccRCC	Post(NR)	MAX	Macro_IFI27	7,98994E-01
DC	Melanoma	Post(NR)	POLR2A	cDC_CLEC9A	7,98894E-01
Macro/Mono	Melanoma	Post(NR)	BHLHE40	Mono_CD16	7,98740E-01
Macro/Mono	ccRCC	Post(NR)	MAX	Macro_LYVE1	7,97177E-01
Macro/Mono	ccRCC	Post(NR)	MAX	Macro_FOLR2-APOE+	7,96474E-01
Mast	Melanoma	Post(NR)	POLR2A	Mast	7,95475E-01

Mast	ccRCC	Post(NR)	ELF1	Mast	7,95258E-01
Macro/Mono	Melanoma	Post(NR)	BHLHE40	Mono_CD14	7,93196E-01
DC	Melanoma	Post(NR)	POLR2A	cDC_LAMP3	7,92508E-01
Macro/Mono	ccRCC	Post(NR)	MAX	Macro_OLFML3	7,92379E-01
Macro/Mono	Melanoma	Post(NR)	BHLHE40	Macro_NLRP3	7,91965E-01
Macro/Mono	Melanoma	Post(NR)	BHLHE40	Macro_IER3	7,91684E-01
Macro/Mono	ccRCC	Post(NR)	ELF1	Mono_CD14	7,89772E-01
Macro/Mono	Melanoma	Post(NR)	BHLHE40	Macro_FOLR2+APOE+	7,89744E-01
Macro/Mono	Melanoma	Post(NR)	BHLHE40	Macro_OLFML3	7,89738E-01
DC	Melanoma	Post(NR)	POLR2A	cDC(CD1C)	7,86562E-01
Macro/Mono	Melanoma	Post(NR)	BHLHE40	Macro_FOLR2-APOE+	7,86558E-01
Macro/Mono	BCC	Post(R)	ELF1	Mono_INHBA	7,85677E-01
Macro/Mono	BCC	Post(R)	ELF1	Mono_CD14	7,84791E-01
Macro/Mono	Melanoma	Post(NR)	BHLHE40	Macro_LYVE1	7,84002E-01
Macro/Mono	ccRCC	Post(NR)	ELF1	Macro_NLRP3	7,83224E-01
Mast	CRC	Post(R)	ELF1	Mast	7,82664E-01
Macro/Mono	Melanoma	Post(NR)	BHLHE40	Macro_ISG15	7,82081E-01
Macro/Mono	ccRCC	Post(NR)	MAX	Macro_ISG15	7,81981E-01
Macro/Mono	BCC	Post(R)	ELF1	Macro_NLRP3	7,80440E-01
Macro/Mono	BCC	Post(R)	ELF1	Mono_CD16	7,79741E-01
Macro/Mono	ccRCC	Post(NR)	MAX	Macro_FOLR2+APOE+	7,79463E-01
Macro/Mono	Melanoma	Post(NR)	POLR2A	Mono_INHBA	7,78118E-01
Macro/Mono	BCC	Post(R)	ELF1	Macro_IER3	7,77482E-01
DC	ccRCC	Post(NR)	ELF1	cDC_CLEC9A	7,77163E-01
Macro/Mono	ccRCC	Post(NR)	ELF1	Mono_INHBA	7,76840E-01
Macro/Mono	ccRCC	Post(R)	MAX	Macro_FOLR2+APOE-	7,76465E-01
DC	BCC	Post(R)	ELF1	cDC_CLEC9A	7,76445E-01
Macro/Mono	Melanoma	Post(NR)	POLR2A	Mono_CD16	7,76272E-01
Mast	ccRCC	Post(NR)	POLR2A	Mast	7,75970E-01
DC	BCC	Post(R)	ELF1	cDC(CD1C)	7,75753E-01
DC	ccRCC	Post(NR)	ELF1	cDC(CD1C)	7,75559E-01
Macro/Mono	Melanoma	Post(NR)	POLR2A	Macro_NLRP3	7,73923E-01
Macro/Mono	ccRCC	Post(R)	MAX	Mono_INHBA	7,73822E-01
Macro/Mono	Melanoma	Post(NR)	POLR2A	Mono_CD14	7,72719E-01
DC	ccRCC	Post(NR)	ELF1	cDC_LAMP3	7,72553E-01
Macro/Mono	BCC	Post(R)	ELF1	Macro_FOLR2-APOE+	7,72468E-01
Macro/Mono	Melanoma	Post(NR)	POLR2A	Macro_FOLR2-APOE+	7,71517E-01
Macro/Mono	BCC	Post(R)	ELF1	Macro_ISG15	7,71314E-01
Macro/Mono	ccRCC	Post(NR)	ELF1	Macro_IER3	7,69746E-01
Mast	ccRCC	Post(R)	MAX	Mast	7,69640E-01
Macro/Mono	Melanoma	Post(NR)	POLR2A	Macro_OLFML3	7,69366E-01
Macro/Mono	ccRCC	Post(R)	ELF1	Mono_CD16	7,68483E-01

Macro/Mono	Melanoma	Post(NR)	BHLHE40	Macro_IFI27	7,68308E-01
Macro/Mono	Melanoma	Post(NR)	POLR2A	Macro_IER3	7,68303E-01
Macro/Mono	ccRCC	Post(R)	MAX	Mono_CD16	7,68253E-01
DC	ccRCC	Post(NR)	ELF1	pDC_LILRA4	7,66320E-01
Macro/Mono	ccRCC	Post(R)	MAX	Macro_LYVE1	7,65816E-01
DC	BCC	Post(R)	ELF1	pDC_LILRA4	7,65264E-01
Macro/Mono	Melanoma	Post(NR)	POLR2A	Macro_LYVE1	7,63646E-01
Mast	BCC	Post(R)	ELF1	Mast	7,63407E-01
Macro/Mono	ccRCC	Post(NR)	ELF1	Macro_FOLR2-APOE+	7,62650E-01
Macro/Mono	BCC	Post(R)	ELF1	Macro_OLFML3	7,62633E-01
Macro/Mono	ccRCC	Post(NR)	ELF1	Macro_FOLR2+APOE-	7,62275E-01
Macro/Mono	Melanoma	Post(NR)	POLR2A	Macro_FOLR2+APOE+	7,61458E-01
Macro/Mono	ccRCC	Post(NR)	ELF1	Macro_LYVE1	7,60096E-01
Macro/Mono	ccRCC	Post(NR)	ELF1	Macro_IFI27	7,60061E-01
Macro/Mono	Melanoma	Post(NR)	POLR2A	Macro_ISG15	7,59274E-01
DC	BCC	Post(R)	ELF1	cDC_LAMP3	7,58813E-01
Macro/Mono	BCC	Post(R)	ELF1	Macro_LYVE1	7,58709E-01
Macro/Mono	ccRCC	Post(NR)	ELF1	Macro_ISG15	7,58554E-01
Macro/Mono	ccRCC	Post(NR)	ELF1	Macro_OLFML3	7,58245E-01
Macro/Mono	BCC	Post(R)	ELF1	Macro_IFI27	7,56882E-01
Macro/Mono	BCC	Post(R)	ELF1	Macro_FOLR2+APOE+	7,55939E-01
Macro/Mono	ccRCC	Post(R)	MAX	Mono_CD14	7,51516E-01
Mast	HNSCC	Post(NR)	BHLHE40	Mast	7,50675E-01
Macro/Mono	BCC	Post(R)	ELF1	Macro_FOLR2+APOE-	7,48576E-01
Macro/Mono	ccRCC	Post(R)	MAX	Macro_IFI27	7,48572E-01
Macro/Mono	CRC	Post(R)	ELF1	Mono_CD14	7,48293E-01
Macro/Mono	ccRCC	Post(R)	MAX	Macro_NLRP3	7,47964E-01
Macro/Mono	ccRCC	Post(NR)	ELF1	Macro_FOLR2+APOE+	7,45238E-01
DC	BCC	Post(NR)	POLR2A	pDC_LILRA4	7,44638E-01
Macro/Mono	ccRCC	Post(R)	ELF1	Mono_INHBA	7,44352E-01
DC	CRC	Post(R)	ELF1	pDC_LILRA4	7,43587E-01
Macro/Mono	CRC	Post(R)	ELF1	Macro_NLRP3	7,41942E-01
Macro/Mono	ccRCC	Post(R)	ELF1	Mono_CD14	7,36296E-01
Macro/Mono	Melanoma	Post(NR)	POLR2A	Macro_IFI27	7,35669E-01
DC	BCC	Post(NR)	RAD21	cDC_LAMP3	7,32795E-01
Macro/Mono	BCC	Post(NR)	RAD21	Macro_IER3	7,30963E-01
Macro/Mono	ccRCC	Post(R)	ELF1	Macro_FOLR2+APOE-	7,30962E-01
Macro/Mono	BCC	Post(NR)	RAD21	Macro_LYVE1	7,29394E-01
Mast	HNSCC	Post(R)	ELF1	Mast	7,28878E-01
Macro/Mono	ccRCC	Post(R)	ELF1	Macro_NLRP3	7,28765E-01
Macro/Mono	CRC	Post(R)	ELF1	Mono_CD16	7,28168E-01
Macro/Mono	BCC	Post(NR)	RAD21	Mono_CD16	7,27166E-01

Macro/Mono	CRC	Post(R)	ELF1	Macro_ISG15	7,26683E-01
Mast	BCC	Post(NR)	RAD21	Mast	7,26576E-01
Macro/Mono	ccRCC	Post(R)	ELF1	Macro_LYVE1	7,25985E-01
DC	BCC	Post(NR)	RAD21	cDC_CLEC9A	7,24688E-01
Macro/Mono	BCC	Post(NR)	RAD21	Macro_IFI27	7,22222E-01
DC	ccRCC	Post(R)	MAX	cDC_CLEC9A	7,21683E-01
DC	BCC	Post(NR)	RAD21	cDC(CD1C)	7,21458E-01
DC	CRC	Post(R)	ELF1	cDC_LAMP3	7,21369E-01
Macro/Mono	BCC	Post(NR)	RAD21	Mono_CD14	7,18860E-01
DC	BCC	Post(NR)	RAD21	pDC_LILRA4	7,18241E-01
Mast	BCC	Post(NR)	POLR2A	Mast	7,17387E-01
Macro/Mono	CRC	Post(R)	ELF1	Mono_INHBA	7,16312E-01
Macro/Mono	BCC	Post(NR)	RAD21	Macro_NLRP3	7,12513E-01
Macro/Mono	ccRCC	Post(R)	MAX	Macro_FOLR2-APOE+	7,10725E-01
Macro/Mono	BCC	Post(NR)	RAD21	Macro_FOLR2-APOE+	7,09958E-01
Macro/Mono	BCC	Post(NR)	RAD21	Macro_FOLR2+APOE-	7,09840E-01
DC	ccRCC	Post(R)	MAX	cDC_LAMP3	7,08581E-01
Macro/Mono	BCC	Post(NR)	RAD21	Macro_OLFML3	7,06550E-01
Mast	ccRCC	Post(R)	ELF1	Mast	7,06231E-01
Mast	HNSCC	Post(NR)	ETV6	Mast	7,05894E-01
DC	BCC	Post(NR)	ELF1	cDC_CLEC9A	7,05713E-01
DC	BCC	Post(NR)	ELF1	cDC(CD1C)	7,04856E-01
Macro/Mono	BCC	Post(NR)	ELF1	Macro_LYVE1	7,03884E-01
Mast	CRC	Post(R)	ETV6	Mast	6,99702E-01
DC	Melanoma	Post(R)	POLR2A	cDC_CLEC9A	6,99430E-01
DC	CRC	Post(R)	ETV6	cDC(CD1C)	6,97280E-01
Macro/Mono	ccRCC	Post(R)	ELF1	Macro_IFI27	6,95428E-01
Macro/Mono	HNSCC	Post(R)	ELF1	Mono_CD14	6,95064E-01
DC	HNSCC	Post(NR)	BHLHE40	pDC_LILRA4	6,94674E-01
DC	BCC	Post(NR)	ELF1	pDC_LILRA4	6,93956E-01
Macro/Mono	HNSCC	Post(R)	ELF1	Macro_NLRP3	6,93889E-01
Macro/Mono	BCC	Post(NR)	ELF1	Mono_CD16	6,93183E-01
Macro/Mono	BCC	Post(NR)	RAD21	Mono_INHBA	6,92990E-01
Macro/Mono	BCC	Post(NR)	ELF1	Mono_CD14	6,92405E-01
Macro/Mono	BCC	Post(NR)	RAD21	Macro_ISG15	6,91617E-01
Macro/Mono	BCC	Post(NR)	RAD21	Macro_FOLR2+APOE+	6,91531E-01
DC	HNSCC	Post(NR)	ETV6	cDC_LAMP3	6,91523E-01
DC	Melanoma	Post(R)	POLR2A	pDC_LILRA4	6,88992E-01
Macro/Mono	CRC	Post(R)	ELF1	Macro_IER3	6,88044E-01
Macro/Mono	BCC	Post(NR)	ELF1	Macro_FOLR2-APOE+	6,88018E-01
Macro/Mono	BCC	Post(NR)	ELF1	Macro_NLRP3	6,87591E-01
Mast	BCC	Post(NR)	ELF1	Mast	6,86940E-01

Macro/Mono	ccRCC	Post(R)	MAX	Macro_OLFML3	6,85672E-01
DC	CRC	Post(R)	ETV6	cDC_CLEC9A	6,85625E-01
DC	HNSCC	Post(NR)	ETV6	cDC_CLEC9A	6,84856E-01
Macro/Mono	BCC	Post(NR)	ELF1	Macro_IFI27	6,84225E-01
DC	HNSCC	Post(NR)	BHLHE40	cDC_LAMP3	6,83801E-01
Macro/Mono	BCC	Post(NR)	ELF1	Macro_IER3	6,83435E-01
Macro/Mono	CRC	Post(R)	ETV6	Macro_IER3	6,83033E-01
Macro/Mono	HNSCC	Post(NR)	ETV6	Mono_CD14	6,81430E-01
Macro/Mono	Melanoma	Post(R)	POLR2A	Macro_NLRP3	6,81418E-01
Macro/Mono	HNSCC	Post(NR)	ETV6	Mono_CD16	6,81343E-01
DC	ccRCC	Post(R)	ELF1	cDC_CLEC9A	6,81245E-01
Mast	CRC	Post(NR)	ELF1	Mast	6,80683E-01
Macro/Mono	BCC	Post(NR)	ZMIZ1	Macro_NLRP3	6,80378E-01
Macro/Mono	BCC	Post(NR)	ZMIZ1	Mono_CD14	6,79474E-01
DC	HNSCC	Post(NR)	ETV6	cDC(CD1C)	6,79167E-01
DC	Melanoma	Post(R)	POLR2A	cDC(CD1C)	6,78629E-01
DC	HNSCC	Post(NR)	ETV6	pDC_LILRA4	6,78539E-01
DC	CRC	Post(R)	ETV6	cDC_LAMP3	6,78529E-01
Macro/Mono	Melanoma	Post(R)	POLR2A	Mono_INHBA	6,78207E-01
Macro/Mono	BCC	Post(NR)	ZMIZ1	Macro_IER3	6,77781E-01
Macro/Mono	BCC	Post(NR)	ZMIZ1	Mono_INHBA	6,77780E-01
Mast	HNSCC	Post(R)	ETV6	Mast	6,77685E-01
Macro/Mono	CRC	Post(R)	ETV6	Macro_FOLR2+APOE-	6,75306E-01
Macro/Mono	BCC	Post(NR)	POLR2A	Macro_FOLR2+APOE-	6,73581E-01
DC	CRC	Post(R)	ETV6	pDC_LILRA4	6,72838E-01
Macro/Mono	BCC	Post(NR)	ELF1	Macro_FOLR2+APOE-	6,72831E-01
DC	BCC	Post(NR)	ELF1	cDC_LAMP3	6,72669E-01
Macro/Mono	BCC	Post(NR)	ELF1	Macro_OLFML3	6,72310E-01
DC	BCC	Post(R)	ETV3	pDC_LILRA4	6,71502E-01
Macro/Mono	HNSCC	Post(NR)	BHLHE40	Mono_CD14	6,70921E-01
Macro/Mono	HNSCC	Post(NR)	BHLHE40	Macro_NLRP3	6,70920E-01
Macro/Mono	BCC	Post(NR)	ZMIZ1	Mono_CD16	6,70677E-01
DC	ccRCC	Post(NR)	POLR2A	pDC_LILRA4	6,69350E-01
Macro/Mono	CRC	Post(R)	ETV6	Macro_OLFML3	6,68106E-01
Macro/Mono	CRC	Post(R)	ETV6	Macro_NLRP3	6,67963E-01
DC	CRC	Post(R)	ELF1	cDC(CD1C)	6,67344E-01
DC	BCC	Post(NR)	POLR2A	cDC_CLEC9A	6,66886E-01
Macro/Mono	BCC	Post(NR)	POLR2A	Macro_LYVE1	6,66885E-01
Macro/Mono	BCC	Post(NR)	ELF1	Macro_ISG15	6,66874E-01
DC	BCC	Post(R)	ETV3	cDC_CLEC9A	6,66545E-01
Macro/Mono	BCC	Post(NR)	ELF1	Macro_FOLR2+APOE+	6,66340E-01
DC	BCC	Post(R)	ETV3	cDC_LAMP3	6,66329E-01

Macro/Mono	ccRCC	Post(R)	MAX	Macro_IER3	6,66288E-01
DC	HNSCC	Post(R)	ETV6	pDC_LILRA4	6,65088E-01
DC	ccRCC	Post(NR)	POLR2A	cDC_CLEC9A	6,65028E-01
DC	BCC	Post(R)	ETV3	cDC(CD1C)	6,64310E-01
Macro/Mono	HNSCC	Post(R)	ELF1	Mono_CD16	6,63635E-01
Macro/Mono	ccRCC	Post(NR)	POLR2A	Mono_CD14	6,63219E-01
Macro/Mono	BCC	Post(NR)	ELF1	Mono_INHBA	6,61912E-01
Macro/Mono	Melanoma	Post(R)	POLR2A	Macro_OLFML3	6,61773E-01
Macro/Mono	ccRCC	Post(R)	ELF1	Macro_FOLR2-APOE+	6,61450E-01
Macro/Mono	HNSCC	Post(NR)	ETV6	Macro_IER3	6,61326E-01
Macro/Mono	HNSCC	Post(NR)	BHLHE40	Macro_IFI27	6,61223E-01
DC	ccRCC	Post(R)	ELF1	cDC_LAMP3	6,60998E-01
Macro/Mono	BCC	Post(NR)	ZMIZ1	Macro_FOLR2-APOE+	6,60435E-01
Macro/Mono	BCC	Post(NR)	POLR2A	Macro_IER3	6,60097E-01
Macro/Mono	BCC	Post(NR)	ZMIZ1	Macro_IFI27	6,60047E-01
Macro/Mono	HNSCC	Post(NR)	ETV6	Macro_NLRP3	6,59903E-01
Macro/Mono	BCC	Post(NR)	ZMIZ1	Macro_LYVE1	6,59244E-01
Macro/Mono	HNSCC	Post(R)	ELF1	Mono_INHBA	6,59039E-01
Macro/Mono	Melanoma	Post(R)	POLR2A	Mono_CD14	6,58866E-01
DC	BCC	Post(NR)	ETV3	cDC_CLEC9A	6,58456E-01
Macro/Mono	CRC	Post(R)	ETV6	Macro_LYVE1	6,57348E-01
Macro/Mono	BCC	Post(NR)	POLR2A	Mono_CD16	6,57198E-01
DC	HNSCC	Post(NR)	BHLHE40	cDC_CLEC9A	6,56574E-01
Macro/Mono	CRC	Post(R)	ETV6	Mono_CD14	6,56230E-01
Macro/Mono	ccRCC	Post(NR)	POLR2A	Macro_NLRP3	6,54251E-01
Macro/Mono	HNSCC	Post(NR)	BHLHE40	Mono_CD16	6,54018E-01
Macro/Mono	BCC	Post(NR)	ZMIZ1	Macro_ISG15	6,53061E-01
Macro/Mono	BCC	Post(R)	ETV3	Macro_IER3	6,52181E-01
Macro/Mono	BCC	Post(R)	ETV3	Mono_CD16	6,51793E-01
Macro/Mono	Melanoma	Post(R)	POLR2A	Macro_FOLR2-APOE+	6,51745E-01
Macro/Mono	BCC	Post(R)	ETV3	Mono_CD14	6,51386E-01
DC	BCC	Post(NR)	ETV3	pDC_LILRA4	6,51103E-01
Macro/Mono	CRC	Post(R)	ETV6	Macro_FOLR2+APOE+	6,50900E-01
Macro/Mono	CRC	Post(R)	ELF1	Macro_FOLR2+APOE-	6,50109E-01
DC	BCC	Post(NR)	ETV3	cDC(CD1C)	6,49825E-01
Macro/Mono	CRC	Post(R)	ETV6	Macro_FOLR2-APOE+	6,48299E-01
DC	ccRCC	Post(NR)	POLR2A	cDC_LAMP3	6,48196E-01
Macro/Mono	CRC	Post(R)	ETV6	Mono_CD16	6,47854E-01
Macro/Mono	HNSCC	Post(NR)	ETV6	Macro_OLFML3	6,47659E-01
Macro/Mono	BCC	Post(R)	ETV3	Macro_FOLR2-APOE+	6,47631E-01
Macro/Mono	ccRCC	Post(NR)	POLR2A	Mono_CD16	6,47421E-01
DC	ccRCC	Post(R)	MAX	cDC(CD1C)	6,47380E-01

Macro/Mono	HNSCC	Post(NR)	BHLHE40	Macro_FOLR2+APOE-	6,46993E-01
Macro/Mono	BCC	Post(R)	ETV3	Macro_NLRP3	6,46981E-01
Macro/Mono	ccRCC	Post(NR)	POLR2A	Mono_INHBA	6,46950E-01
DC	BCC	Post(NR)	ETV6	cDC_CLEC9A	6,46859E-01
Macro/Mono	HNSCC	Post(NR)	BHLHE40	Mono_INHBA	6,46558E-01
Macro/Mono	ccRCC	Post(NR)	POLR2A	Macro_FOLR2+APOE-	6,46456E-01
Macro/Mono	Melanoma	Post(R)	ZMIZ1	Mono_INHBA	6,46399E-01
Macro/Mono	HNSCC	Post(NR)	BHLHE40	Macro_IER3	6,46291E-01
Mast	HNSCC	Post(R)	BHLHE40	Mast	6,46085E-01
Macro/Mono	BCC	Post(NR)	POLR2A	Macro_IFI27	6,45664E-01
Macro/Mono	BCC	Post(R)	ETV3	Macro_OLFML3	6,44945E-01
Macro/Mono	BCC	Post(NR)	POLR2A	Mono_CD14	6,44384E-01
Macro/Mono	BCC	Post(NR)	ETV3	Macro_IER3	6,44199E-01
DC	HNSCC	Post(R)	ETV6	cDC_LAMP3	6,44179E-01
DC	BCC	Post(NR)	ZMIZ1	cDC(CD1C)	6,43898E-01
Mast	BCC	Post(NR)	ETV6	Mast	6,42978E-01
Macro/Mono	CRC	Post(R)	ETV6	Macro_IFI27	6,42973E-01
Macro/Mono	BCC	Post(NR)	ETV3	Macro_LYVE1	6,42489E-01
DC	CRC	Post(R)	ELF1	cDC_CLEC9A	6,41884E-01
Macro/Mono	CRC	Post(R)	ETV6	Mono_INHBA	6,41342E-01
Macro/Mono	BCC	Post(R)	ETV3	Macro_ISG15	6,40536E-01
DC	HNSCC	Post(NR)	BHLHE40	cDC(CD1C)	6,40107E-01
DC	BCC	Post(NR)	POLR2A	cDC(CD1C)	6,39655E-01
Mast	CRC	Post(R)	EGR1	Mast	6,39217E-01
Macro/Mono	CRC	Post(R)	ELF1	Macro_LYVE1	6,39109E-01
Mast	ccRCC	Post(NR)	ETV5	Mast	6,37666E-01
Macro/Mono	ccRCC	Post(R)	ELF1	Macro_OLFML3	6,37610E-01
Macro/Mono	BCC	Post(R)	ETV3	Macro_FOLR2+APOE-	6,37159E-01
Macro/Mono	BCC	Post(NR)	ZMIZ1	Macro_FOLR2+APOE+	6,36764E-01
Macro/Mono	BCC	Post(R)	ETV3	Macro_LYVE1	6,36562E-01
Macro/Mono	BCC	Post(R)	ETV3	Macro_IFI27	6,36328E-01
Macro/Mono	CRC	Post(R)	ELF1	Macro_FOLR2-APOE+	6,35835E-01
Mast	BCC	Post(R)	ETV3	Mast	6,35682E-01
Macro/Mono	BCC	Post(NR)	ETV3	Macro_IFI27	6,35477E-01
Macro/Mono	HNSCC	Post(NR)	ETV6	Macro_LYVE1	6,35424E-01
Macro/Mono	BCC	Post(NR)	ETV3	Mono_CD16	6,35420E-01
DC	BCC	Post(NR)	ETV6	cDC(CD1C)	6,35402E-01
Macro/Mono	CRC	Post(R)	ZMIZ1	Mono_CD16	6,35153E-01
Macro/Mono	BCC	Post(NR)	ETV3	Mono_CD14	6,35065E-01
Macro/Mono	BCC	Post(NR)	ETV6	Macro_IER3	6,34223E-01
Mast	BCC	Post(NR)	ETV3	Mast	6,33955E-01
Macro/Mono	BCC	Post(NR)	ZMIZ1	Macro_FOLR2+APOE-	6,33035E-01

DC	BCC	Post(NR)	ETV6	pDC_LILRA4	6,33005E-01
Macro/Mono	Melanoma	Post(R)	POLR2A	Macro_ISG15	6,32523E-01
Macro/Mono	ccRCC	Post(NR)	POLR2A	Macro_LYVE1	6,32129E-01
Macro/Mono	CRC	Post(R)	ETV6	Macro_ISG15	6,31780E-01
Mast	BCC	Post(R)	ETV6	Mast	6,31661E-01
Macro/Mono	BCC	Post(R)	ETV3	Mono_INHBA	6,31602E-01
Macro/Mono	BCC	Post(NR)	ETV3	Macro_FOLR2-APOE+	6,31598E-01
DC	BCC	Post(NR)	ETV3	cDC_LAMP3	6,31471E-01
Macro/Mono	BCC	Post(R)	ETV3	Macro_FOLR2+APOE+	6,31263E-01
Macro/Mono	BCC	Post(NR)	ETV6	Mono_CD14	6,29956E-01
Mast	Melanoma	Post(NR)	ELF1	Mast	6,29875E-01
Macro/Mono	BCC	Post(NR)	ETV6	Mono_CD16	6,29452E-01
DC	BCC	Post(NR)	ZMIZ1	cDC_CLEC9A	6,29406E-01
Macro/Mono	CRC	Post(R)	ZMIZ1	Mono_INHBA	6,29374E-01
Macro/Mono	BCC	Post(NR)	ETV6	Macro_LYVE1	6,29188E-01
DC	BCC	Post(NR)	POLR2A	cDC_LAMP3	6,28911E-01
Macro/Mono	Melanoma	Post(NR)	ETV6	Mono_CD16	6,28819E-01
Macro/Mono	BCC	Post(R)	ETV6	Mono_CD14	6,28761E-01
Macro/Mono	ccRCC	Post(NR)	POLR2A	Macro_IER3	6,28636E-01
DC	ccRCC	Post(NR)	POLR2A	cDC(CD1C)	6,28542E-01
DC	BCC	Post(R)	ETV6	pDC_LILRA4	6,28311E-01
DC	BCC	Post(R)	ETV6	cDC(CD1C)	6,28225E-01
DC	HNSCC	Post(R)	ETV6	cDC_CLEC9A	6,28002E-01
Macro/Mono	CRC	Post(R)	ELF1	Macro_OLFML3	6,27704E-01
Mast	Melanoma	Post(NR)	ETV6	Mast	6,27173E-01
DC	Melanoma	Post(NR)	ETV6	cDC_CLEC9A	6,27071E-01
Macro/Mono	HNSCC	Post(NR)	ETV6	Macro_FOLR2-APOE+	6,25996E-01
Macro/Mono	HNSCC	Post(NR)	ETV6	Macro_IFI27	6,25775E-01
Macro/Mono	ccRCC	Post(R)	ELF1	Macro_IER3	6,25771E-01
DC	Melanoma	Post(NR)	ETV6	cDC_LAMP3	6,25706E-01
DC	CRC	Post(NR)	ETV6	cDC_CLEC9A	6,25576E-01
DC	Melanoma	Post(R)	ZMIZ1	cDC(CD1C)	6,25569E-01
DC	Melanoma	Post(NR)	ETV6	cDC(CD1C)	6,25491E-01
Macro/Mono	HNSCC	Post(NR)	ETV6	Macro_FOLR2+APOE-	6,25136E-01
Macro/Mono	BCC	Post(R)	ETV6	Macro_IER3	6,24901E-01
Macro/Mono	HNSCC	Post(R)	ELF1	Macro_IER3	6,24897E-01
Mast	HNSCC	Post(NR)	ELF1	Mast	6,24732E-01
Macro/Mono	BCC	Post(NR)	ETV6	Macro_IFI27	6,24524E-01
Macro/Mono	BCC	Post(NR)	ETV3	Macro_NLRP3	6,24416E-01
Macro/Mono	HNSCC	Post(NR)	BHLHE40	Macro_LYVE1	6,24412E-01
Macro/Mono	BCC	Post(R)	ETV6	Mono_INHBA	6,24213E-01
Macro/Mono	ccRCC	Post(NR)	POLR2A	Macro_IFI27	6,24038E-01

Macro/Mono	BCC	Post(NR)	ETV3	Macro_OLFML3	6,24022E-01
DC	BCC	Post(R)	ETV6	cDC_LAMP3	6,23925E-01
DC	Melanoma	Post(NR)	ETV6	pDC_LILRA4	6,23918E-01
Macro/Mono	BCC	Post(R)	ETV6	Mono_CD16	6,23749E-01
Mast	BCC	Post(NR)	ZMIZ1	Mast	6,23595E-01
Macro/Mono	CRC	Post(R)	ZMIZ1	Mono_CD14	6,23453E-01
Macro/Mono	Melanoma	Post(NR)	ELF1	Mono_CD16	6,23436E-01
Macro/Mono	BCC	Post(R)	ETV6	Macro_NLRP3	6,23338E-01
Macro/Mono	BCC	Post(NR)	ETV3	Macro_FOLR2+APOE-	6,23316E-01
Macro/Mono	BCC	Post(NR)	POLR2A	Macro_FOLR2-APOE+	6,23281E-01
Macro/Mono	HNSCC	Post(NR)	BHLHE40	Macro_FOLR2-APOE+	6,23186E-01
Macro/Mono	Melanoma	Post(NR)	ETV6	Mono_CD14	6,23175E-01
Macro/Mono	Melanoma	Post(R)	ZMIZ1	Macro_NLRP3	6,22747E-01
Macro/Mono	HNSCC	Post(NR)	ETV6	Mono_INHBA	6,22376E-01
Mast	Melanoma	Post(NR)	ZMIZ1	Mast	6,22069E-01
Macro/Mono	BCC	Post(R)	ETV6	Macro_OLFML3	6,21874E-01
Macro/Mono	BCC	Post(NR)	ETV6	Macro_NLRP3	6,21770E-01
Macro/Mono	Melanoma	Post(NR)	ELF1	Macro_NLRP3	6,20934E-01
Macro/Mono	HNSCC	Post(R)	ETV6	Mono_CD14	6,20645E-01
Macro/Mono	BCC	Post(R)	ETV6	Macro_ISG15	6,20547E-01
Macro/Mono	Melanoma	Post(NR)	ELF1	Macro_ISG15	6,20146E-01
Macro/Mono	BCC	Post(NR)	POLR2A	Macro_NLRP3	6,20126E-01
Macro/Mono	Melanoma	Post(NR)	ETV6	Macro_IER3	6,20042E-01
Macro/Mono	Melanoma	Post(NR)	ETV6	Macro_OLFML3	6,19980E-01
Macro/Mono	BCC	Post(R)	ETV6	Macro_FOLR2-APOE+	6,19885E-01
Macro/Mono	BCC	Post(NR)	POLR2A	Mono_INHBA	6,18070E-01
DC	CRC	Post(R)	EGR1	pDC_LILRA4	6,18035E-01
DC	ccRCC	Post(R)	ELF1	cDC(CD1C)	6,17759E-01
Macro/Mono	HNSCC	Post(NR)	BHLHE40	Macro_OLFML3	6,17700E-01
DC	Melanoma	Post(NR)	ZMIZ1	cDC_CLEC9A	6,17673E-01
Macro/Mono	Melanoma	Post(NR)	ELF1	Macro_OLFML3	6,17615E-01
DC	Melanoma	Post(NR)	ELF1	cDC(CD1C)	6,17574E-01
Macro/Mono	HNSCC	Post(NR)	ETV6	Macro_FOLR2+APOE+	6,17279E-01
Macro/Mono	HNSCC	Post(NR)	ETV6	Macro_ISG15	6,16995E-01
Macro/Mono	BCC	Post(NR)	ETV6	Macro_FOLR2-APOE+	6,16978E-01
Macro/Mono	BCC	Post(NR)	POLR2A	Macro_OLFML3	6,16976E-01
DC	HNSCC	Post(R)	ELF1	pDC_LILRA4	6,16562E-01
Mast	ccRCC	Post(NR)	RAD21	Mast	6,16495E-01
Macro/Mono	Melanoma	Post(R)	ZMIZ1	Macro_FOLR2-APOE+	6,16147E-01
Macro/Mono	Melanoma	Post(NR)	ETV6	Macro_ISG15	6,15854E-01
Macro/Mono	Melanoma	Post(NR)	ELF1	Macro_FOLR2+APOE+	6,15803E-01
Macro/Mono	HNSCC	Post(R)	ELF1	Macro_ISG15	6,15787E-01

Macro/Mono	Melanoma	Post(NR)	ETV6	Macro_NLRP3	6,15615E-01
Macro/Mono	BCC	Post(NR)	ETV6	Macro_FOLR2+APOE-	6,15576E-01
DC	BCC	Post(R)	ETV6	cDC_CLEC9A	6,15507E-01
DC	Melanoma	Post(R)	ZMIZ1	cDC_CLEC9A	6,15505E-01
Macro/Mono	ccRCC	Post(NR)	POLR2A	Macro_FOLR2-APOE+	6,15461E-01
DC	BCC	Post(NR)	ETV6	cDC_LAMP3	6,15456E-01
Macro/Mono	Melanoma	Post(NR)	ETV6	Mono_INHBA	6,15286E-01
Macro/Mono	HNSCC	Post(R)	ETV6	Mono_CD16	6,14903E-01
Macro/Mono	Melanoma	Post(NR)	ELF1	Macro_IER3	6,14754E-01
Macro/Mono	Melanoma	Post(NR)	ETV6	Macro_FOLR2+APOE+	6,14620E-01
Macro/Mono	CRC	Post(NR)	ETV6	Mono_CD14	6,14517E-01
Macro/Mono	Melanoma	Post(NR)	ELF1	Mono_INHBA	6,14351E-01
Macro/Mono	CRC	Post(R)	ZMIZ1	Macro_ISG15	6,14254E-01
Macro/Mono	Melanoma	Post(NR)	ETV6	Macro_LYVE1	6,14152E-01
Macro/Mono	CRC	Post(NR)	ELF1	Mono_CD16	6,13966E-01
Macro/Mono	HNSCC	Post(R)	ETV6	Macro_NLRP3	6,13399E-01
DC	ccRCC	Post(R)	MAX	pDC_LILRA4	6,12860E-01
DC	CRC	Post(NR)	ETV6	cDC(CD1C)	6,12817E-01
Macro/Mono	BCC	Post(NR)	ETV6	Macro_OLFML3	6,12545E-01
Macro/Mono	Melanoma	Post(NR)	ZMIZ1	Mono_CD16	6,12209E-01
Macro/Mono	Melanoma	Post(NR)	ELF1	Mono_CD14	6,12062E-01
Macro/Mono	Melanoma	Post(NR)	ZMIZ1	Macro_FOLR2-APOE+	6,11935E-01
Mast	ccRCC	Post(NR)	EGR1	Mast	6,11439E-01
Macro/Mono	Melanoma	Post(NR)	ZMIZ1	Mono_INHBA	6,11418E-01
Macro/Mono	Melanoma	Post(NR)	ELF1	Macro_LYVE1	6,11348E-01
Macro/Mono	Melanoma	Post(NR)	ETV6	Macro_FOLR2-APOE+	6,11151E-01
Macro/Mono	Melanoma	Post(NR)	ZMIZ1	Macro_NLRP3	6,10548E-01
DC	CRC	Post(R)	EGR1	cDC_CLEC9A	6,10337E-01
DC	HNSCC	Post(R)	ETV6	cDC(CD1C)	6,10038E-01
Macro/Mono	BCC	Post(NR)	ZMIZ1	Macro_OLFML3	6,09463E-01
DC	Melanoma	Post(NR)	ELF1	cDC_LAMP3	6,09430E-01
Mast	CRC	Post(NR)	ETV6	Mast	6,09364E-01
Macro/Mono	Melanoma	Post(R)	POLR2A	Macro_IFI27	6,08930E-01
Macro/Mono	BCC	Post(R)	ETV6	Macro_IFI27	6,08879E-01
Macro/Mono	BCC	Post(R)	ETV6	Macro_LYVE1	6,08683E-01
Macro/Mono	Melanoma	Post(NR)	ELF1	Macro_FOLR2-APOE+	6,08555E-01
Macro/Mono	BCC	Post(NR)	POLR2A	Macro_ISG15	6,08491E-01
Macro/Mono	BCC	Post(NR)	POLR2A	Macro_FOLR2+APOE+	6,08452E-01
Macro/Mono	BCC	Post(NR)	ETV3	Macro_FOLR2+APOE+	6,08033E-01
DC	Melanoma	Post(NR)	ZMIZ1	cDC_LAMP3	6,07821E-01
Macro/Mono	BCC	Post(R)	ETV6	Macro_FOLR2+APOE-	6,07694E-01
DC	HNSCC	Post(R)	ELF1	cDC_LAMP3	6,07304E-01

Macro/Mono	BCC	Post(NR)	ETV3	Macro_ISG15	6,07059E-01
DC	Melanoma	Post(NR)	ZMIZ1	cDC(CD1C)	6,06972E-01
Macro/Mono	ccRCC	Post(NR)	POLR2A	Macro_OLFML3	6,06930E-01
DC	CRC	Post(NR)	ETV6	cDC_LAMP3	6,06873E-01
DC	Melanoma	Post(NR)	ELF1	cDC_CLEC9A	6,06069E-01
Macro/Mono	Melanoma	Post(NR)	ZMIZ1	Mono_CD14	6,05989E-01
Macro/Mono	CRC	Post(NR)	ELF1	Macro_LYVE1	6,05908E-01
Macro/Mono	BCC	Post(NR)	ETV6	Macro_ISG15	6,05901E-01
DC	CRC	Post(NR)	ETV6	pDC_LILRA4	6,05227E-01
Macro/Mono	CRC	Post(NR)	ETV6	Macro_NLRP3	6,04727E-01
Macro/Mono	BCC	Post(R)	ETV6	Macro_FOLR2+APOE+	6,03469E-01
Macro/Mono	Melanoma	Post(NR)	ZMIZ1	Macro_IER3	6,02816E-01
Macro/Mono	Melanoma	Post(R)	ZMIZ1	Mono_CD14	6,02218E-01
DC	CRC	Post(NR)	ELF1	cDC_CLEC9A	6,00922E-01
Macro/Mono	BCC	Post(NR)	ETV3	Mono_INHBA	6,00720E-01
Macro/Mono	Melanoma	Post(NR)	ZMIZ1	Macro_ISG15	6,00683E-01
Macro/Mono	Melanoma	Post(NR)	ZMIZ1	Macro_LYVE1	6,00351E-01
Macro/Mono	BCC	Post(NR)	ETV6	Mono_INHBA	5,99377E-01
DC	BCC	Post(NR)	ZMIZ1	cDC_LAMP3	5,99276E-01
Macro/Mono	ccRCC	Post(NR)	POLR2A	Macro_FOLR2+APOE+	5,98519E-01
DC	HNSCC	Post(R)	ELF1	cDC(CD1C)	5,98428E-01
Mast	ccRCC	Post(NR)	FLI1	Mast	5,97529E-01
Macro/Mono	CRC	Post(R)	ELF1	Macro_IFI27	5,97493E-01
Macro/Mono	ccRCC	Post(NR)	POLR2A	Macro_ISG15	5,97460E-01
Macro/Mono	Melanoma	Post(NR)	ZMIZ1	Macro_OLFML3	5,96816E-01
Macro/Mono	CRC	Post(NR)	SPI1	Macro_ISG15	5,96069E-01
Macro/Mono	CRC	Post(NR)	ETV6	Mono_CD16	5,96020E-01
Macro/Mono	Melanoma	Post(NR)	ZMIZ1	Macro_FOLR2+APOE+	5,95690E-01
DC	Melanoma	Post(NR)	ZMIZ1	pDC_LILRA4	5,95399E-01
Macro/Mono	BCC	Post(NR)	ETV6	Macro_FOLR2+APOE+	5,95102E-01
Macro/Mono	HNSCC	Post(NR)	BHLHE40	Macro_FOLR2+APOE+	5,94614E-01
DC	Melanoma	Post(NR)	ELF1	pDC_LILRA4	5,93857E-01
Macro/Mono	ccRCC	Post(NR)	MAFB	Macro_FOLR2+APOE+	5,93028E-01
Macro/Mono	Melanoma	Post(R)	ZMIZ1	Macro_ISG15	5,92219E-01
Macro/Mono	ccRCC	Post(R)	SAP30	Macro_FOLR2+APOE+	5,91430E-01
Macro/Mono	HNSCC	Post(R)	ELF1	Macro_FOLR2-APOE+	5,90808E-01
Macro/Mono	CRC	Post(NR)	SPI1	Mono_INHBA	5,90543E-01
DC	CRC	Post(NR)	ELF1	pDC_LILRA4	5,90359E-01
DC	CRC	Post(R)	EGR1	cDC(CD1C)	5,90126E-01
Macro/Mono	HNSCC	Post(R)	ELF1	Macro_FOLR2+APOE-	5,89907E-01
Macro/Mono	CRC	Post(NR)	ETV6	Macro_ISG15	5,89420E-01
Macro/Mono	ccRCC	Post(NR)	JUN	Macro_IER3	5,89003E-01

Mast	Melanoma	Post(NR)	BCLAF1	Mast	5,88901E-01
DC	BCC	Post(NR)	ZMIZ1	pDC_LILRA4	5,88776E-01
Macro/Mono	HNSCC	Post(R)	ETV6	Macro_IER3	5,87922E-01
Mast	ccRCC	Post(NR)	BCLAF1	Mast	5,87777E-01
DC	Melanoma	Post(NR)	BCLAF1	cDC_CLEC9A	5,87757E-01
Mast	CRC	Post(NR)	ETS2	Mast	5,87143E-01
Macro/Mono	Melanoma	Post(R)	ZMIZ1	Macro_OLFML3	5,87115E-01
Macro/Mono	CRC	Post(R)	EGR1	Macro_FOLR2+APOE-	5,86386E-01
DC	Melanoma	Post(NR)	BCLAF1	pDC_LILRA4	5,86221E-01
Macro/Mono	CRC	Post(NR)	ETV6	Macro_OLFML3	5,85890E-01
Macro/Mono	CRC	Post(NR)	ETS2	Mono_CD14	5,83702E-01
DC	HNSCC	Post(R)	ELF1	cDC_CLEC9A	5,82896E-01
Macro/Mono	HNSCC	Post(R)	ELF1	Macro_IFI27	5,82421E-01
Macro/Mono	CRC	Post(R)	ZMIZ1	Macro_FOLR2-APOE+	5,82371E-01
Macro/Mono	Melanoma	Post(NR)	ELF1	Macro_IFI27	5,82201E-01
Mast	CRC	Post(R)	ETS2	Mast	5,81584E-01
Macro/Mono	ccRCC	Post(NR)	MAFB	Macro_LYVE1	5,81563E-01
Macro/Mono	HNSCC	Post(NR)	BHLHE40	Macro_ISG15	5,81105E-01
DC	Melanoma	Post(NR)	BCLAF1	cDC(CD1C)	5,80862E-01
DC	Melanoma	Post(NR)	BCLAF1	cDC_LAMP3	5,80410E-01
DC	CRC	Post(R)	EGR1	cDC_LAMP3	5,80374E-01
Macro/Mono	CRC	Post(NR)	ETS2	Macro_NLRP3	5,80217E-01
DC	Melanoma	Post(R)	ZMIZ1	pDC_LILRA4	5,80083E-01
Macro/Mono	ccRCC	Post(NR)	ETV5	Macro_IER3	5,79734E-01
Macro/Mono	Melanoma	Post(NR)	ETV6	Macro_IFI27	5,78930E-01
DC	ccRCC	Post(NR)	ETV5	pDC_LILRA4	5,78668E-01
Macro/Mono	Melanoma	Post(NR)	BCLAF1	Mono_CD16	5,77948E-01
Macro/Mono	CRC	Post(R)	EGR1	Mono_CD16	5,77756E-01
Macro/Mono	ccRCC	Post(NR)	ETV5	Macro_FOLR2+APOE-	5,77687E-01
Macro/Mono	CRC	Post(NR)	ETS2	Mono_INHBA	5,77597E-01
Macro/Mono	CRC	Post(R)	EGR1	Mono_CD14	5,75033E-01
Macro/Mono	ccRCC	Post(NR)	ETV5	Mono_CD16	5,74627E-01
Macro/Mono	CRC	Post(R)	EGR1	Macro_IER3	5,74433E-01
Macro/Mono	CRC	Post(NR)	SPI1	Macro_NLRP3	5,73925E-01
Macro/Mono	Melanoma	Post(NR)	BCLAF1	Mono_CD14	5,73805E-01
DC	CRC	Post(NR)	ETS2	pDC_LILRA4	5,72934E-01
DC	BCC	Post(R)	REST	pDC_LILRA4	5,72651E-01
Macro/Mono	CRC	Post(NR)	SPI1	Macro_OLFML3	5,72368E-01
Macro/Mono	CRC	Post(NR)	ETV6	Macro_IER3	5,71867E-01
Macro/Mono	ccRCC	Post(NR)	ETV5	Macro_NLRP3	5,71794E-01
Macro/Mono	Melanoma	Post(NR)	BCLAF1	Macro_NLRP3	5,71671E-01
Macro/Mono	HNSCC	Post(NR)	ATF3	Mono_INHBA	5,71638E-01

DC	CRC	Post(NR)	ELF1	cDC_LAMP3	5,71129E-01
Macro/Mono	Melanoma	Post(NR)	BCLAF1	Macro_IER3	5,70936E-01
Macro/Mono	CRC	Post(R)	EGR1	Macro_LYVE1	5,70617E-01
Macro/Mono	CRC	Post(R)	ZMIZ1	Macro_NLRP3	5,70232E-01
Macro/Mono	CRC	Post(NR)	ETV6	Macro_IFI27	5,69958E-01
Macro/Mono	ccRCC	Post(R)	MAX	Macro_ISG15	5,69370E-01
Macro/Mono	ccRCC	Post(NR)	ETV5	Mono_CD14	5,69284E-01
Macro/Mono	ccRCC	Post(NR)	ETV5	Macro_LYVE1	5,68783E-01
Macro/Mono	ccRCC	Post(NR)	ETV5	Mono_INHBA	5,68749E-01
Macro/Mono	Melanoma	Post(NR)	BCLAF1	Macro_OLFML3	5,68643E-01
Macro/Mono	HNSCC	Post(R)	ELF1	Macro_OLFML3	5,68215E-01
Macro/Mono	Melanoma	Post(NR)	BCLAF1	Mono_INHBA	5,67590E-01
DC	CRC	Post(R)	ETS2	pDC_LILRA4	5,67526E-01
DC	ccRCC	Post(NR)	ETV5	cDC(CD1C)	5,67199E-01
Macro/Mono	CRC	Post(R)	ZMIZ1	Macro_LYVE1	5,67034E-01
Mast	ccRCC	Post(NR)	CEBPB	Mast	5,66862E-01
Macro/Mono	ccRCC	Post(NR)	BCLAF1	Mono_CD16	5,66851E-01
DC	CRC	Post(R)	ZMIZ1	cDC_LAMP3	5,66824E-01
DC	ccRCC	Post(NR)	ETV5	cDC_LAMP3	5,66684E-01
Mast	ccRCC	Post(NR)	ELF2	Mast	5,64956E-01
Macro/Mono	ccRCC	Post(NR)	ETV5	Macro_IFI27	5,64163E-01
Macro/Mono	CRC	Post(NR)	ETS2	Macro_ISG15	5,64096E-01
Macro/Mono	CRC	Post(NR)	ELF1	Macro_IER3	5,63541E-01
Macro/Mono	Melanoma	Post(NR)	ZMIZ1	Macro_IFI27	5,63095E-01
Macro/Mono	ccRCC	Post(NR)	MAFB	Macro_FOLR2-APOE+	5,63070E-01
Macro/Mono	CRC	Post(R)	ETS2	Mono_CD14	5,62806E-01
Macro/Mono	CRC	Post(R)	EGR1	Macro_NLRP3	5,62579E-01
Macro/Mono	HNSCC	Post(R)	BHLHE40	Mono_CD14	5,62114E-01
Macro/Mono	HNSCC	Post(R)	ETV6	Mono_INHBA	5,62054E-01
Macro/Mono	Melanoma	Post(NR)	BCLAF1	Macro_FOLR2-APOE+	5,61883E-01
DC	ccRCC	Post(R)	ELF1	pDC_LILRA4	5,61111E-01
Macro/Mono	Melanoma	Post(NR)	BCLAF1	Macro_LYVE1	5,61097E-01
Macro/Mono	HNSCC	Post(R)	BHLHE40	Mono_INHBA	5,61094E-01
Macro/Mono	Melanoma	Post(NR)	BCLAF1	Macro_FOLR2+APOE+	5,60629E-01
DC	ccRCC	Post(NR)	BCLAF1	cDC_CLEC9A	5,60494E-01
Macro/Mono	CRC	Post(NR)	ETV6	Mono_INHBA	5,59915E-01
Mast	ccRCC	Post(NR)	ETS2	Mast	5,59388E-01
Macro/Mono	CRC	Post(R)	EGR1	Macro_IFI27	5,59050E-01
DC	CRC	Post(NR)	ETS2	cDC(CD1C)	5,58853E-01
Macro/Mono	CRC	Post(R)	ELF1	Macro_FOLR2+APOE+	5,58678E-01
DC	ccRCC	Post(NR)	ETV5	cDC_CLEC9A	5,58432E-01
Macro/Mono	Melanoma	Post(NR)	BCLAF1	Macro_ISG15	5,57091E-01

Macro/Mono	ccRCC	Post(NR)	MAFB	Macro_IFI27	5,56672E-01
Macro/Mono	ccRCC	Post(NR)	ETV5	Macro_FOLR2-APOE+	5,55910E-01
Mast	CRC	Post(R)	POLR2A	Mast	5,55658E-01
Mast	BCC	Post(NR)	REST	Mast	5,55134E-01
Macro/Mono	BCC	Post(R)	ETS2	Mono_INHBA	5,55036E-01
DC	CRC	Post(NR)	ETS2	cDC_CLEC9A	5,54899E-01
DC	CRC	Post(R)	ETS2	cDC_CLEC9A	5,54604E-01
Macro/Mono	ccRCC	Post(NR)	RAD21	Mono_CD16	5,53933E-01
Macro/Mono	CRC	Post(NR)	ETS2	Mono_CD16	5,53690E-01
Macro/Mono	CRC	Post(R)	EGR1	Macro_OLFML3	5,53623E-01
DC	BCC	Post(NR)	REL	cDC_LAMP3	5,53240E-01
Macro/Mono	HNSCC	Post(R)	ELF1	Macro_LYVE1	5,52943E-01
Macro/Mono	CRC	Post(NR)	ETS2	Macro_OLFML3	5,52938E-01
Macro/Mono	BCC	Post(R)	ETS2	Mono_CD14	5,51759E-01
Macro/Mono	ccRCC	Post(NR)	ETV5	Macro_OLFML3	5,51744E-01
Macro/Mono	CRC	Post(NR)	SPI1	Mono_CD14	5,51545E-01
DC	BCC	Post(R)	ETS2	cDC_CLEC9A	5,51322E-01
DC	ccRCC	Post(NR)	BCLAF1	pDC_LILRA4	5,51302E-01
Mast	ccRCC	Post(R)	ETV5	Mast	5,51175E-01
Macro/Mono	CRC	Post(R)	ETS2	Macro_NLRP3	5,50395E-01
DC	CRC	Post(R)	ETS2	cDC(CD1C)	5,50282E-01
Macro/Mono	HNSCC	Post(R)	ETV6	Macro_IFI27	5,50173E-01
Macro/Mono	CRC	Post(NR)	ETS2	Macro_IER3	5,50148E-01
DC	BCC	Post(NR)	REST	pDC_LILRA4	5,50129E-01
Macro/Mono	ccRCC	Post(NR)	ETS2	Mono_CD16	5,49878E-01
Macro/Mono	HNSCC	Post(R)	ETV6	Macro_FOLR2-APOE+	5,49850E-01
Macro/Mono	HNSCC	Post(R)	ETV6	Macro_OLFML3	5,49664E-01
Macro/Mono	CRC	Post(NR)	ETV6	Macro_FOLR2+APOE-	5,49393E-01
DC	ccRCC	Post(NR)	CEBPB	cDC_CLEC9A	5,49217E-01
Macro/Mono	ccRCC	Post(NR)	CEBPB	Mono_CD14	5,48898E-01
Macro/Mono	ccRCC	Post(R)	ETV5	Macro_FOLR2+APOE-	5,48633E-01
Macro/Mono	ccRCC	Post(NR)	BCLAF1	Macro_NLRP3	5,48256E-01
Macro/Mono	HNSCC	Post(R)	ETV6	Macro_ISG15	5,48180E-01
Macro/Mono	CRC	Post(NR)	ETV6	Macro_FOLR2+APOE+	5,48033E-01
Macro/Mono	CRC	Post(NR)	ELF1	Macro_FOLR2+APOE-	5,47515E-01
DC	ccRCC	Post(NR)	RAD21	cDC_CLEC9A	5,47367E-01
Macro/Mono	ccRCC	Post(NR)	FOS	Macro_IER3	5,47281E-01
Macro/Mono	ccRCC	Post(NR)	CEBPB	Mono_CD16	5,47228E-01
DC	ccRCC	Post(NR)	BCLAF1	cDC(CD1C)	5,47126E-01
Macro/Mono	HNSCC	Post(R)	ETV6	Macro_FOLR2+APOE-	5,46794E-01
Macro/Mono	BCC	Post(R)	ETS2	Mono_CD16	5,46527E-01
Macro/Mono	BCC	Post(R)	ETS2	Macro_NLRP3	5,45970E-01

Macro/Mono	ccRCC	Post(NR)	ETV5	Macro_ISG15	5,45692E-01
Macro/Mono	ccRCC	Post(NR)	RAD21	Mono_CD14	5,45288E-01
Macro/Mono	ccRCC	Post(NR)	BCLAF1	Mono_CD14	5,45144E-01
DC	BCC	Post(R)	REST	cDC_CLEC9A	5,44746E-01
Macro/Mono	CRC	Post(R)	EGR1	Macro_ISG15	5,44332E-01
DC	BCC	Post(NR)	REST	cDC(CD1C)	5,44277E-01
Macro/Mono	BCC	Post(NR)	ETS2	Mono_CD14	5,43620E-01
DC	BCC	Post(R)	ETS2	cDC(CD1C)	5,43613E-01
DC	CRC	Post(NR)	ETS2	cDC_LAMP3	5,43596E-01
DC	ccRCC	Post(NR)	BCLAF1	cDC_LAMP3	5,43563E-01
DC	BCC	Post(NR)	ETS2	cDC(CD1C)	5,43177E-01
Macro/Mono	CRC	Post(R)	ETS2	Mono_CD16	5,43149E-01
Macro/Mono	CRC	Post(R)	EGR1	Macro_FOLR2-APOE+	5,42830E-01
DC	CRC	Post(R)	ETS2	cDC_LAMP3	5,42744E-01
DC	BCC	Post(NR)	REST	cDC_CLEC9A	5,42638E-01
Macro/Mono	BCC	Post(NR)	ETS2	Macro_IFI27	5,42240E-01
Macro/Mono	BCC	Post(R)	ETS2	Macro_IER3	5,42111E-01
Macro/Mono	CRC	Post(NR)	ETV6	Macro_LYVE1	5,42088E-01
Macro/Mono	BCC	Post(R)	ETS2	Macro_ISG15	5,41887E-01
DC	BCC	Post(NR)	ETS2	cDC_CLEC9A	5,41427E-01
Macro/Mono	ccRCC	Post(NR)	FLI1	Mono_CD16	5,40876E-01
Macro/Mono	CRC	Post(R)	ETS2	Macro_ISG15	5,40656E-01
Macro/Mono	CRC	Post(R)	ETS2	Macro_FOLR2+APOE-	5,40561E-01
DC	BCC	Post(NR)	BCLAF1	pDC_LILRA4	5,40353E-01
Macro/Mono	Melanoma	Post(R)	ZMIZ1	Macro_IFI27	5,40315E-01
Macro/Mono	HNSCC	Post(R)	BHLHE40	Macro_NLRP3	5,39803E-01
Macro/Mono	BCC	Post(NR)	REL	Macro_OLFML3	5,39452E-01
Macro/Mono	ccRCC	Post(NR)	RAD21	Macro_NLRP3	5,39060E-01
Macro/Mono	ccRCC	Post(NR)	BCLAF1	Mono_INHBA	5,38340E-01
Macro/Mono	BCC	Post(R)	ETS2	Macro_FOLR2-APOE+	5,38278E-01
DC	ccRCC	Post(NR)	ETS2	cDC(CD1C)	5,38137E-01
Macro/Mono	ccRCC	Post(NR)	CEBPB	Macro_NLRP3	5,38051E-01
Macro/Mono	HNSCC	Post(R)	MAX	Macro_FOLR2+APOE-	5,38009E-01
Macro/Mono	BCC	Post(NR)	ETS2	Macro_IER3	5,37995E-01
Macro/Mono	HNSCC	Post(R)	BHLHE40	Macro_FOLR2+APOE-	5,37985E-01
Macro/Mono	BCC	Post(NR)	ETS2	Macro_NLRP3	5,37839E-01
Macro/Mono	ccRCC	Post(R)	ELF1	Macro_ISG15	5,37795E-01
Macro/Mono	CRC	Post(R)	EGR1	Mono_INHBA	5,37704E-01
Macro/Mono	BCC	Post(NR)	ETS2	Mono_CD16	5,36925E-01
Mast	BCC	Post(R)	ETS2	Mast	5,36887E-01
Macro/Mono	ccRCC	Post(R)	MAX	Macro_FOLR2+APOE+	5,36062E-01
Macro/Mono	ccRCC	Post(NR)	ETS2	Mono_CD14	5,35676E-01

Macro/Mono	BCC	Post(R)	ETS2	Macro_OLFML3	5,35546E-01
Macro/Mono	Melanoma	Post(NR)	BCLAF1	Macro_IFI27	5,35416E-01
Macro/Mono	ccRCC	Post(NR)	BCLAF1	Macro_IER3	5,35381E-01
Macro/Mono	BCC	Post(NR)	REST	Macro_IFI27	5,35146E-01
Macro/Mono	HNSCC	Post(NR)	ATF3	Macro_NLRP3	5,34809E-01
Macro/Mono	ccRCC	Post(NR)	BCLAF1	Macro_FOLR2+APOE-	5,34806E-01
Macro/Mono	BCC	Post(NR)	ETS2	Macro_FOLR2-APOE+	5,34395E-01
DC	ccRCC	Post(NR)	RAD21	pDC_LILRA4	5,34217E-01
DC	ccRCC	Post(NR)	ETS2	cDC_CLEC9A	5,34138E-01
Macro/Mono	HNSCC	Post(R)	ELF1	Macro_FOLR2+APOE+	5,33863E-01
Macro/Mono	ccRCC	Post(NR)	CEBPB	Mono_INHBA	5,33749E-01
Macro/Mono	ccRCC	Post(NR)	ETV5	Macro_FOLR2+APOE+	5,33571E-01
Macro/Mono	ccRCC	Post(NR)	ETS2	Macro_NLRP3	5,33488E-01
Macro/Mono	ccRCC	Post(NR)	FLI1	Mono_CD14	5,33238E-01
DC	CRC	Post(NR)	ELF1	cDC(CD1C)	5,33184E-01
Macro/Mono	BCC	Post(NR)	REST	Mono_CD16	5,32999E-01
Macro/Mono	BCC	Post(NR)	ETS2	Macro_LYVE1	5,32808E-01
Macro/Mono	CRC	Post(R)	ETS2	Mono_INHBA	5,32792E-01
DC	BCC	Post(NR)	REL	cDC(CD1C)	5,32784E-01
Macro/Mono	ccRCC	Post(R)	ETV5	Macro_IFI27	5,32378E-01
Macro/Mono	CRC	Post(NR)	ETV6	Macro_FOLR2-APOE+	5,32253E-01
Macro/Mono	CRC	Post(NR)	ELF1	Macro_IFI27	5,31946E-01
DC	ccRCC	Post(NR)	ETS2	pDC_LILRA4	5,31270E-01
DC	BCC	Post(R)	ETS2	pDC_LILRA4	5,31229E-01
DC	BCC	Post(NR)	REST	cDC_LAMP3	5,30431E-01
Macro/Mono	BCC	Post(R)	ETS2	Macro_IFI27	5,30366E-01
Macro/Mono	BCC	Post(R)	ETS2	Macro_LYVE1	5,30149E-01
Macro/Mono	BCC	Post(R)	ETS2	Macro_FOLR2+APOE-	5,29350E-01
DC	ccRCC	Post(NR)	RAD21	cDC_LAMP3	5,28508E-01
Macro/Mono	BCC	Post(R)	ELF2	Mono_INHBA	5,28494E-01
Macro/Mono	BCC	Post(NR)	REST	Macro_LYVE1	5,28467E-01
Macro/Mono	BCC	Post(NR)	REST	Mono_CD14	5,28319E-01
Macro/Mono	ccRCC	Post(NR)	BCLAF1	Macro_IFI27	5,28258E-01
Macro/Mono	CRC	Post(NR)	ETS2	Macro_FOLR2+APOE+	5,28055E-01
Macro/Mono	ccRCC	Post(NR)	MAFB	Macro_FOLR2+APOE-	5,27711E-01
Macro/Mono	HNSCC	Post(R)	BHLHE40	Macro_IER3	5,27629E-01
Macro/Mono	CRC	Post(R)	ETS2	Macro_IER3	5,27364E-01
Mast	CRC	Post(NR)	EGR1	Mast	5,27169E-01
Macro/Mono	ccRCC	Post(NR)	BCLAF1	Macro_LYVE1	5,27083E-01
DC	ccRCC	Post(R)	SAP30	pDC_LILRA4	5,26625E-01
Macro/Mono	ccRCC	Post(NR)	RAD21	Mono_INHBA	5,25596E-01
Macro/Mono	ccRCC	Post(NR)	MAFB	Macro_IER3	5,25301E-01

Macro/Mono	ccRCC	Post(R)	HMGB1	Mono_INHBA	5,25243E-01
DC	ccRCC	Post(NR)	FLI1	pDC_LILRA4	5,24935E-01
Macro/Mono	BCC	Post(NR)	REL	Macro_LYVE1	5,24540E-01
Macro/Mono	ccRCC	Post(NR)	BCLAF1	Macro_FOLR2-APOE+	5,24359E-01
Macro/Mono	CRC	Post(NR)	ETS2	Macro_IFI27	5,23718E-01
Macro/Mono	ccRCC	Post(R)	HMGB1	Macro_OLFML3	5,23445E-01
DC	BCC	Post(R)	ETS2	cDC_LAMP3	5,23249E-01
DC	ccRCC	Post(NR)	RAD21	cDC(CD1C)	5,22863E-01
Macro/Mono	HNSCC	Post(R)	ETV6	Macro_LYVE1	5,22652E-01
DC	CRC	Post(R)	POLR2A	pDC_LILRA4	5,22367E-01
DC	ccRCC	Post(NR)	ETS2	cDC_LAMP3	5,22209E-01
Macro/Mono	CRC	Post(R)	ATF3	Mono_INHBA	5,21749E-01
DC	BCC	Post(NR)	REL	cDC_CLEC9A	5,21548E-01
Macro/Mono	CRC	Post(R)	ZMIZ1	Macro_IER3	5,21417E-01
Macro/Mono	CRC	Post(NR)	POLR2A	Macro_LYVE1	5,21245E-01
DC	ccRCC	Post(NR)	CEBPB	cDC_LAMP3	5,21237E-01
Macro/Mono	BCC	Post(NR)	REST	Macro_IER3	5,21228E-01
Mast	BCC	Post(NR)	BCLAF1	Mast	5,20747E-01
DC	Melanoma	Post(NR)	ETV3	pDC_LILRA4	5,20460E-01
DC	ccRCC	Post(NR)	CEBPB	pDC_LILRA4	5,19201E-01
Macro/Mono	BCC	Post(NR)	ETS2	Macro_FOLR2+APOE-	5,18745E-01
Mast	BCC	Post(NR)	REL	Mast	5,18733E-01
Mast	BCC	Post(NR)	ETS2	Mast	5,18656E-01
DC	HNSCC	Post(R)	BHLHE40	cDC_LAMP3	5,18280E-01
Macro/Mono	HNSCC	Post(R)	BHLHE40	Mono_CD16	5,18101E-01
Macro/Mono	ccRCC	Post(NR)	MAFB	Macro_OLFML3	5,18062E-01
Macro/Mono	BCC	Post(NR)	REL	Macro_IER3	5,18059E-01
Macro/Mono	ccRCC	Post(NR)	RAD21	Macro_FOLR2+APOE-	5,18002E-01
DC	ccRCC	Post(R)	HMGB1	cDC(CD1C)	5,17743E-01
Macro/Mono	BCC	Post(NR)	ETS2	Macro_OLFML3	5,17527E-01
Macro/Mono	ccRCC	Post(NR)	BCLAF1	Macro_OLFML3	5,17075E-01
DC	BCC	Post(NR)	ETS2	cDC_LAMP3	5,17063E-01
Macro/Mono	ccRCC	Post(R)	ETV5	Mono_CD16	5,16929E-01
Macro/Mono	BCC	Post(NR)	ETS2	Macro_ISG15	5,16724E-01
Macro/Mono	ccRCC	Post(NR)	FLI1	Macro_FOLR2+APOE-	5,16639E-01
Macro/Mono	ccRCC	Post(NR)	ETS2	Macro_IER3	5,16346E-01
Macro/Mono	BCC	Post(NR)	REST	Macro_FOLR2-APOE+	5,16320E-01
Macro/Mono	CRC	Post(R)	SPI1	Macro_FOLR2+APOE+	5,15832E-01
Macro/Mono	ccRCC	Post(R)	HMGB1	Macro_LYVE1	5,15591E-01
Macro/Mono	BCC	Post(R)	REST	Mono_CD14	5,15175E-01
Macro/Mono	BCC	Post(NR)	REL	Mono_CD16	5,15083E-01
Macro/Mono	ccRCC	Post(NR)	ETS2	Mono_INHBA	5,15063E-01

Macro/Mono	HNSCC	Post(NR)	ATF3	Macro_IER3	5,14954E-01
Macro/Mono	HNSCC	Post(NR)	ATF3	Macro_FOLR2-APOE+	5,14432E-01
DC	BCC	Post(R)	REST	cDC(CD1C)	5,14418E-01
DC	ccRCC	Post(NR)	CEBPB	cDC(CD1C)	5,14344E-01
Macro/Mono	ccRCC	Post(R)	HMGB1	Macro_NLRP3	5,14273E-01
Macro/Mono	BCC	Post(R)	REST	Mono_CD16	5,13954E-01
Macro/Mono	BCC	Post(NR)	REST	Macro_OLFML3	5,13822E-01
Macro/Mono	ccRCC	Post(NR)	RAD21	Macro_IER3	5,13721E-01
Macro/Mono	ccRCC	Post(R)	HMGB1	Mono_CD16	5,13513E-01
Macro/Mono	BCC	Post(NR)	REL	Macro_FOLR2-APOE+	5,13410E-01
Macro/Mono	BCC	Post(NR)	REL	Mono_CD14	5,13339E-01
Macro/Mono	ccRCC	Post(R)	ETV5	Mono_INHBA	5,13045E-01
Macro/Mono	BCC	Post(NR)	REST	Macro_FOLR2+APOE-	5,12889E-01
Macro/Mono	BCC	Post(NR)	REL	Macro_NLRP3	5,12760E-01
Mast	CRC	Post(R)	ZMIZ1	Mast	5,12632E-01
Macro/Mono	HNSCC	Post(NR)	ATF3	Macro_ISG15	5,12395E-01
DC	Melanoma	Post(NR)	ETV3	cDC_LAMP3	5,12374E-01
Macro/Mono	ccRCC	Post(NR)	BCLAF1	Macro_ISG15	5,11950E-01
Macro/Mono	HNSCC	Post(R)	MAX	Macro_IFI27	5,11949E-01
Macro/Mono	ccRCC	Post(R)	ETV5	Mono_CD14	5,11847E-01
Mast	Melanoma	Post(NR)	ETS2	Mast	5,11619E-01
Macro/Mono	CRC	Post(R)	ETS2	Macro_OLFML3	5,11593E-01
Macro/Mono	ccRCC	Post(NR)	FOS	Macro_NLRP3	5,11573E-01
Macro/Mono	BCC	Post(R)	ETS2	Macro_FOLR2+APOE+	5,11552E-01
Macro/Mono	HNSCC	Post(R)	ETV6	Macro_FOLR2+APOE+	5,11272E-01
Macro/Mono	ccRCC	Post(NR)	ETS2	Macro_FOLR2+APOE-	5,11134E-01
Macro/Mono	BCC	Post(NR)	REST	Macro_NLRP3	5,10821E-01
Macro/Mono	CRC	Post(NR)	ETS2	Macro_LYVE1	5,10202E-01
DC	BCC	Post(NR)	ETS2	pDC_LILRA4	5,09955E-01
Macro/Mono	HNSCC	Post(R)	BHLHE40	Macro_ISG15	5,09721E-01
DC	HNSCC	Post(R)	BHLHE40	pDC_LILRA4	5,09365E-01
Macro/Mono	CRC	Post(R)	ATF3	Macro_NLRP3	5,09353E-01
Macro/Mono	BCC	Post(NR)	ETS2	Macro_FOLR2+APOE+	5,09258E-01
Macro/Mono	HNSCC	Post(NR)	ATF3	Macro_FOLR2+APOE+	5,08806E-01
DC	Melanoma	Post(NR)	ETS2	cDC(CD1C)	5,08806E-01
Macro/Mono	BCC	Post(R)	REST	Macro_IER3	5,08696E-01
Macro/Mono	CRC	Post(NR)	ETS2	Macro_FOLR2-APOE+	5,08558E-01
Macro/Mono	ccRCC	Post(NR)	RAD21	Macro_IFI27	5,08381E-01
Macro/Mono	BCC	Post(NR)	REL	Macro_IFI27	5,07932E-01
DC	ccRCC	Post(R)	HMGB1	cDC_CLEC9A	5,07868E-01
Macro/Mono	ccRCC	Post(NR)	ETS2	Macro_IFI27	5,07722E-01
Macro/Mono	BCC	Post(NR)	ETS2	Mono_INHBA	5,07677E-01

Macro/Mono	Melanoma	Post(NR)	ETS2	Macro_IER3	5,07544E-01
Macro/Mono	HNSCC	Post(R)	BHLHE40	Macro_IFI27	5,07246E-01
DC	Melanoma	Post(NR)	ETV3	cDC_CLEC9A	5,07240E-01
Macro/Mono	Melanoma	Post(NR)	ETS2	Mono_CD14	5,07062E-01
DC	Melanoma	Post(NR)	ETV3	cDC(CD1C)	5,07033E-01
Macro/Mono	Melanoma	Post(NR)	ETS2	Mono_CD16	5,06996E-01
Macro/Mono	CRC	Post(R)	EGR1	Macro_FOLR2+APOE+	5,06561E-01
DC	Melanoma	Post(R)	ETV3	pDC_LILRA4	5,06545E-01
Macro/Mono	ccRCC	Post(NR)	FLI1	Macro_NLRP3	5,05677E-01
Macro/Mono	ccRCC	Post(R)	HMGB1	Macro_FOLR2-APOE+	5,05532E-01
Macro/Mono	HNSCC	Post(NR)	ATF3	Macro_LYVE1	5,05343E-01
Macro/Mono	CRC	Post(NR)	POLR2A	Mono_CD16	5,05302E-01
Macro/Mono	Melanoma	Post(NR)	ETS2	Macro_NLRP3	5,05046E-01
Macro/Mono	BCC	Post(R)	REST	Macro_IFI27	5,04986E-01
Macro/Mono	ccRCC	Post(R)	ETV5	Macro_LYVE1	5,04856E-01
Macro/Mono	CRC	Post(R)	ZMIZ1	Macro_IFI27	5,04767E-01
Macro/Mono	ccRCC	Post(R)	SAP30	Macro_ISG15	5,04604E-01
DC	Melanoma	Post(NR)	ETS2	cDC_CLEC9A	5,04568E-01
Macro/Mono	BCC	Post(R)	REST	Macro_ISG15	5,04396E-01
Macro/Mono	ccRCC	Post(R)	BCLAF1	Mono_CD16	5,04280E-01
Macro/Mono	BCC	Post(NR)	REL	Mono_INHBA	5,03863E-01
Macro/Mono	BCC	Post(NR)	REL	Macro_ISG15	5,03656E-01
Macro/Mono	ccRCC	Post(NR)	RAD21	Macro_LYVE1	5,03453E-01
DC	Melanoma	Post(NR)	ETS2	cDC_LAMP3	5,03048E-01
Macro/Mono	BCC	Post(R)	REST	Macro_NLRP3	5,02983E-01
Macro/Mono	ccRCC	Post(NR)	CEBPB	Macro_FOLR2+APOE-	5,02982E-01
Macro/Mono	ccRCC	Post(NR)	ETS2	Macro_LYVE1	5,02875E-01
Macro/Mono	BCC	Post(R)	REST	Macro_FOLR2+APOE+	5,02791E-01
Macro/Mono	ccRCC	Post(NR)	CEBPB	Macro_FOLR2-APOE+	5,02743E-01
Macro/Mono	CRC	Post(NR)	ETS2	Macro_FOLR2+APOE-	5,02651E-01
Macro/Mono	ccRCC	Post(NR)	SPI1	Mono_CD16	5,02628E-01
Macro/Mono	ccRCC	Post(NR)	FLI1	Macro_LYVE1	5,02435E-01
DC	BCC	Post(NR)	BCLAF1	cDC_CLEC9A	5,02302E-01
DC	CRC	Post(R)	POLR2A	cDC_LAMP3	5,02127E-01
DC	BCC	Post(NR)	REL	pDC_LILRA4	5,01983E-01
Mast	Melanoma	Post(NR)	ETV3	Mast	5,01522E-01
Macro/Mono	BCC	Post(NR)	REL	Macro_FOLR2+APOE-	5,01423E-01
Macro/Mono	ccRCC	Post(NR)	ETS2	Macro_FOLR2-APOE+	5,01392E-01
Macro/Mono	Melanoma	Post(NR)	ETS2	Mono_INHBA	5,01223E-01
Macro/Mono	ccRCC	Post(NR)	FLI1	Macro_IER3	5,00948E-01
Macro/Mono	ccRCC	Post(NR)	BCLAF1	Macro_FOLR2+APOE+	5,00654E-01
Macro/Mono	ccRCC	Post(NR)	MAFB	Macro_ISG15	5,00634E-01

Macro/Mono	ccRCC	Post(R)	HMGB1	Macro_IFI27	5,00522E-01
Macro/Mono	ccRCC	Post(NR)	CEBPB	Macro_IER3	5,00514E-01
Macro/Mono	ccRCC	Post(R)	ETV5	Macro_FOLR2-APOE+	5,00387E-01
Macro/Mono	ccRCC	Post(NR)	RAD21	Macro_FOLR2-APOE+	5,00155E-01
Macro/Mono	HNSCC	Post(NR)	ELF1	Macro_NLRP3	4,99997E-01
DC	Melanoma	Post(NR)	ETS2	pDC_LILRA4	4,99516E-01
Macro/Mono	HNSCC	Post(NR)	ELF1	Mono_INHBA	4,99017E-01
Macro/Mono	CRC	Post(R)	ATF3	Macro_IER3	4,98919E-01
Macro/Mono	BCC	Post(NR)	BCLAF1	Mono_CD16	4,98762E-01
Macro/Mono	BCC	Post(NR)	REST	Macro_ISG15	4,98703E-01
Macro/Mono	BCC	Post(R)	REST	Macro_FOLR2-APOE+	4,98539E-01
DC	ccRCC	Post(NR)	FLI1	cDC(CD1C)	4,98521E-01
Mast	BCC	Post(R)	REST	Mast	4,98479E-01
Macro/Mono	ccRCC	Post(NR)	CEBPB	Macro_IFI27	4,98366E-01
Macro/Mono	Melanoma	Post(NR)	ETV3	Macro_NLRP3	4,98324E-01
DC	ccRCC	Post(NR)	FLI1	cDC_LAMP3	4,98280E-01
Macro/Mono	CRC	Post(NR)	ELF1	Macro_FOLR2-APOE+	4,97944E-01
Macro/Mono	BCC	Post(NR)	BCLAF1	Mono_CD14	4,97889E-01
Macro/Mono	ccRCC	Post(NR)	ETS2	Macro_OLFML3	4,97725E-01
Macro/Mono	ccRCC	Post(NR)	SPI1	Macro_FOLR2+APOE+	4,97551E-01
Macro/Mono	Melanoma	Post(NR)	ETV3	Macro_IER3	4,96847E-01
Macro/Mono	Melanoma	Post(NR)	ETV3	Macro_OLFML3	4,96803E-01
Macro/Mono	ccRCC	Post(R)	ETV5	Macro_NLRP3	4,96685E-01
Macro/Mono	BCC	Post(NR)	BCLAF1	Macro_LYVE1	4,96334E-01
Macro/Mono	Melanoma	Post(NR)	ETV3	Mono_CD16	4,96294E-01
Macro/Mono	Melanoma	Post(NR)	ETS2	Macro_LYVE1	4,96219E-01
Macro/Mono	Melanoma	Post(NR)	ETS2	Macro_FOLR2+APOE+	4,96149E-01
Macro/Mono	ccRCC	Post(R)	ELF1	Macro_FOLR2+APOE+	4,96005E-01
Macro/Mono	Melanoma	Post(R)	ETV3	Macro_NLRP3	4,95333E-01
Macro/Mono	CRC	Post(R)	ETS2	Macro_IFI27	4,95249E-01
Macro/Mono	CRC	Post(R)	SPI1	Macro_OLFML3	4,95190E-01
Macro/Mono	Melanoma	Post(NR)	ETS2	Macro_FOLR2-APOE+	4,95105E-01
Macro/Mono	ccRCC	Post(NR)	RAD21	Macro_OLFML3	4,94926E-01
Mast	BCC	Post(NR)	ELF2	Mast	4,94900E-01
DC	BCC	Post(NR)	BCLAF1	cDC(CD1C)	4,94836E-01
Macro/Mono	Melanoma	Post(NR)	ETS2	Macro_ISG15	4,94717E-01
DC	BCC	Post(R)	REST	cDC_LAMP3	4,94501E-01
Macro/Mono	Melanoma	Post(NR)	ETS2	Macro_OLFML3	4,94369E-01
DC	ccRCC	Post(NR)	FLI1	cDC_CLEC9A	4,94251E-01
Macro/Mono	BCC	Post(NR)	BCLAF1	Macro_FOLR2+APOE-	4,94219E-01
Macro/Mono	BCC	Post(NR)	BCLAF1	Macro_IFI27	4,94128E-01
DC	Melanoma	Post(R)	ETV3	cDC(CD1C)	4,93964E-01

Macro/Mono	ccRCC	Post(NR)	ETS2	Macro_ISG15	4,93805E-01
Macro/Mono	BCC	Post(R)	REST	Macro_OLFML3	4,93617E-01
Macro/Mono	CRC	Post(R)	ZMIZ1	Macro_FOLR2+APOE+	4,93381E-01
Macro/Mono	ccRCC	Post(NR)	FLI1	Macro_IFI27	4,93161E-01
Macro/Mono	Melanoma	Post(NR)	ETV3	Macro_FOLR2+APOE+	4,92863E-01
DC	ccRCC	Post(R)	ETV5	cDC_LAMP3	4,92789E-01
Macro/Mono	CRC	Post(R)	ETS2	Macro_LYVE1	4,91830E-01
Macro/Mono	Melanoma	Post(NR)	ETV3	Mono_INHBA	4,91618E-01
DC	BCC	Post(NR)	GABPB1	cDC_LAMP3	4,91526E-01
Macro/Mono	Melanoma	Post(NR)	ETV3	Mono_CD14	4,91416E-01
Macro/Mono	ccRCC	Post(NR)	FLI1	Mono_INHBA	4,91340E-01
DC	Melanoma	Post(R)	ETV3	cDC_CLEC9A	4,91036E-01
Macro/Mono	BCC	Post(NR)	REL	Macro_FOLR2+APOE+	4,91001E-01
DC	CRC	Post(NR)	EGR1	pDC_LILRA4	4,90661E-01
Macro/Mono	HNSCC	Post(NR)	ATF3	Macro_IFI27	4,90220E-01
Macro/Mono	Melanoma	Post(NR)	ETV3	Macro_FOLR2-APOE+	4,90190E-01
DC	ccRCC	Post(R)	ETV5	cDC_CLEC9A	4,89737E-01
Macro/Mono	CRC	Post(R)	ETS2	Macro_FOLR2-APOE+	4,89719E-01
Macro/Mono	ccRCC	Post(NR)	CEBPB	Macro_LYVE1	4,89291E-01
Macro/Mono	Melanoma	Post(NR)	ETV3	Macro_ISG15	4,88246E-01
Macro/Mono	ccRCC	Post(NR)	RAD21	Macro_ISG15	4,88201E-01
Macro/Mono	ccRCC	Post(R)	CEBPD	Macro_FOLR2+APOE+	4,87824E-01
Mast	BCC	Post(NR)	GABPB1	Mast	4,87787E-01
Macro/Mono	ccRCC	Post(NR)	CEBPB	Macro_OLFML3	4,87289E-01
Macro/Mono	Melanoma	Post(NR)	ETV3	Macro_LYVE1	4,86608E-01
Macro/Mono	BCC	Post(NR)	REST	Macro_FOLR2+APOE+	4,86252E-01
Macro/Mono	BCC	Post(NR)	REST	Mono_INHBA	4,86230E-01
Macro/Mono	HNSCC	Post(NR)	ELF1	Mono_CD14	4,86207E-01
Macro/Mono	CRC	Post(NR)	ELF1	Macro_FOLR2+APOE+	4,85779E-01
Macro/Mono	CRC	Post(NR)	ELF1	Mono_CD14	4,85046E-01
Mast	ccRCC	Post(R)	BCLAF1	Mast	4,85008E-01
Macro/Mono	HNSCC	Post(R)	MAX	Macro_LYVE1	4,84759E-01
Macro/Mono	CRC	Post(R)	POLR2A	Mono_CD14	4,84603E-01
Macro/Mono	HNSCC	Post(R)	MAX	Macro_FOLR2+APOE+	4,84129E-01
Macro/Mono	ccRCC	Post(R)	ETV5	Macro_OLFML3	4,83535E-01
Macro/Mono	HNSCC	Post(R)	BHLHE40	Macro_FOLR2-APOE+	4,83523E-01
DC	CRC	Post(NR)	EGR1	cDC_CLEC9A	4,83396E-01
Macro/Mono	CRC	Post(NR)	SPI1	Macro_FOLR2-APOE+	4,83318E-01
Macro/Mono	HNSCC	Post(NR)	ATF3	Mono_CD14	4,83118E-01
Macro/Mono	ccRCC	Post(NR)	SPI1	Macro_FOLR2-APOE+	4,82495E-01
Macro/Mono	ccRCC	Post(NR)	CEBPB	Macro_ISG15	4,81831E-01
Macro/Mono	BCC	Post(R)	ELF2	Mono_CD14	4,80523E-01

Macro/Mono	BCC	Post(R)	REST	Macro_LYVE1	4,80515E-01
Macro/Mono	ccRCC	Post(NR)	SPI1	Mono_INHBA	4,80434E-01
Macro/Mono	BCC	Post(R)	POLR2A	Macro_FOLR2+APOE-	4,79445E-01
Macro/Mono	BCC	Post(NR)	BCLAF1	Macro_IER3	4,79255E-01
Macro/Mono	BCC	Post(R)	ELF2	Macro_NLRP3	4,78074E-01
Macro/Mono	ccRCC	Post(R)	HMGB1	Mono_CD14	4,77593E-01
DC	BCC	Post(NR)	GABPB1	cDC_CLEC9A	4,77244E-01
DC	BCC	Post(NR)	BCLAF1	cDC_LAMP3	4,76870E-01
Macro/Mono	ccRCC	Post(NR)	SPI1	Macro_OLFML3	4,76820E-01
Macro/Mono	ccRCC	Post(NR)	JUN	Macro_NLRP3	4,76669E-01
DC	ccRCC	Post(R)	HMGB1	cDC_LAMP3	4,76433E-01
Macro/Mono	ccRCC	Post(NR)	FLI1	Macro_FOLR2-APOE+	4,76378E-01
Macro/Mono	HNSCC	Post(NR)	ATF3	Macro_OLFML3	4,76322E-01
Macro/Mono	CRC	Post(R)	POLR2A	Mono_CD16	4,76163E-01
Macro/Mono	BCC	Post(NR)	ELF2	Mono_CD16	4,76008E-01
Macro/Mono	BCC	Post(NR)	ELF2	Mono_CD14	4,75654E-01
Macro/Mono	CRC	Post(R)	POLR2A	Mono_INHBA	4,75325E-01
Macro/Mono	ccRCC	Post(NR)	FLI1	Macro_ISG15	4,75294E-01
Macro/Mono	Melanoma	Post(R)	ETV3	Macro_OLFML3	4,75172E-01
Macro/Mono	ccRCC	Post(NR)	ETS2	Macro_FOLR2+APOE+	4,75078E-01
DC	ccRCC	Post(NR)	SPI1	cDC_CLEC9A	4,75029E-01
Macro/Mono	ccRCC	Post(R)	BCLAF1	Mono_CD14	4,74818E-01
Macro/Mono	Melanoma	Post(NR)	ETS2	Macro_IFI27	4,74406E-01
Mast	ccRCC	Post(R)	POLR2A	Mast	4,74316E-01
Mast	HNSCC	Post(R)	MAX	Mast	4,73639E-01
DC	Melanoma	Post(R)	ETV6	cDC_CLEC9A	4,73567E-01
Macro/Mono	HNSCC	Post(NR)	ELF1	Macro_IER3	4,73079E-01
Macro/Mono	CRC	Post(NR)	EGR1	Macro_FOLR2+APOE+	4,73066E-01
Macro/Mono	BCC	Post(R)	REST	Macro_FOLR2+APOE-	4,72992E-01
Mast	HNSCC	Post(NR)	EGR1	Mast	4,72504E-01
Macro/Mono	ccRCC	Post(NR)	RAD21	Macro_FOLR2+APOE+	4,72502E-01
Macro/Mono	CRC	Post(NR)	SPI1	Macro_FOLR2+APOE+	4,72370E-01
DC	BCC	Post(NR)	RELB	cDC_LAMP3	4,72018E-01
Macro/Mono	BCC	Post(R)	POLR2A	Mono_INHBA	4,71793E-01
Macro/Mono	BCC	Post(NR)	BCLAF1	Macro_FOLR2-APOE+	4,71677E-01
Macro/Mono	BCC	Post(NR)	BCLAF1	Macro_NLRP3	4,71512E-01
DC	BCC	Post(NR)	GABPB1	cDC(CD1C)	4,71348E-01
DC	CRC	Post(NR)	SPI1	cDC(CD1C)	4,70927E-01
DC	CRC	Post(NR)	EGR1	cDC(CD1C)	4,70892E-01
Macro/Mono	BCC	Post(R)	ELF2	Macro_FOLR2+APOE+	4,70741E-01
Macro/Mono	BCC	Post(NR)	BCLAF1	Macro_OLFML3	4,70256E-01
Macro/Mono	ccRCC	Post(NR)	CEBPB	Macro_FOLR2+APOE+	4,69371E-01

Macro/Mono	BCC	Post(NR)	ELF2	Macro_IER3	4,69209E-01
Mast	Melanoma	Post(NR)	EGR1	Mast	4,68909E-01
Macro/Mono	BCC	Post(R)	REST	Mono_INHBA	4,68836E-01
Macro/Mono	BCC	Post(NR)	GABPB1	Mono_CD16	4,68830E-01
Macro/Mono	CRC	Post(R)	ETS2	Macro_FOLR2+APOE+	4,67997E-01
Macro/Mono	BCC	Post(NR)	GABPB1	Macro_OLFML3	4,67992E-01
Macro/Mono	BCC	Post(NR)	ELF2	Macro_LYVE1	4,67689E-01
Mast	BCC	Post(R)	POLR2A	Mast	4,67418E-01
Macro/Mono	ccRCC	Post(NR)	FLI1	Macro_OLFML3	4,67371E-01
Macro/Mono	ccRCC	Post(NR)	SPI1	Macro_NLRP3	4,67224E-01
Macro/Mono	ccRCC	Post(R)	BCLAF1	Mono_INHBA	4,67095E-01
Mast	CRC	Post(NR)	POLR2A	Mast	4,67084E-01
Macro/Mono	ccRCC	Post(R)	ATF3	Mono_INHBA	4,66993E-01
Macro/Mono	Melanoma	Post(R)	ETV3	Mono_INHBA	4,66796E-01
Macro/Mono	Melanoma	Post(R)	ETV6	Mono_INHBA	4,66758E-01
DC	CRC	Post(R)	ZMIZ1	pDC_LILRA4	4,66628E-01
DC	Melanoma	Post(R)	ETV6	cDC(CD1C)	4,66570E-01
Macro/Mono	ccRCC	Post(R)	ATF3	Macro_LYVE1	4,66175E-01
Macro/Mono	Melanoma	Post(NR)	ETV3	Macro_IFI27	4,66143E-01
Macro/Mono	CRC	Post(NR)	EGR1	Mono_CD16	4,66064E-01
Macro/Mono	BCC	Post(NR)	ELF2	Macro_IFI27	4,66044E-01
Macro/Mono	BCC	Post(R)	POLR2A	Macro_NLRP3	4,65908E-01
Macro/Mono	BCC	Post(NR)	BCLAF1	Macro_ISG15	4,65279E-01
Macro/Mono	ccRCC	Post(NR)	SPI1	Macro_ISG15	4,65130E-01
Macro/Mono	BCC	Post(NR)	ELF2	Macro_NLRP3	4,65004E-01
Macro/Mono	ccRCC	Post(R)	BCLAF1	Macro_FOLR2+APOE-	4,64120E-01
Mast	HNSCC	Post(NR)	ATF3	Mast	4,64085E-01
Macro/Mono	BCC	Post(NR)	GABPB1	Mono_CD14	4,64008E-01
Macro/Mono	ccRCC	Post(NR)	JUN	Mono_CD14	4,63989E-01
DC	HNSCC	Post(R)	MAX	cDC_CLEC9A	4,63340E-01
Macro/Mono	ccRCC	Post(R)	BCLAF1	Macro_NLRP3	4,63286E-01
Macro/Mono	BCC	Post(R)	ELF2	Macro_ISG15	4,63069E-01
Macro/Mono	BCC	Post(R)	POLR2A	Mono_CD14	4,62309E-01
Macro/Mono	BCC	Post(R)	POLR2A	Macro_IFI27	4,61566E-01
DC	Melanoma	Post(NR)	EGR1	pDC_LILRA4	4,61488E-01
Macro/Mono	HNSCC	Post(R)	MAX	Macro_FOLR2-APOE+	4,61151E-01
Macro/Mono	ccRCC	Post(NR)	KLF4	Macro_IER3	4,60994E-01
DC	HNSCC	Post(NR)	ELF1	pDC_LILRA4	4,60576E-01
DC	Melanoma	Post(NR)	EGR1	cDC_CLEC9A	4,60546E-01
Macro/Mono	BCC	Post(R)	POLR2A	Macro_ISG15	4,60353E-01
Macro/Mono	Melanoma	Post(R)	ETV6	Macro_ISG15	4,59935E-01
Macro/Mono	CRC	Post(NR)	EGR1	Macro_IER3	4,59851E-01

DC	BCC	Post(NR)	ELF2	cDC_CLEC9A	4,59594E-01
Macro/Mono	BCC	Post(NR)	ELF2	Macro_FOLR2+APOE-	4,59406E-01
Macro/Mono	Melanoma	Post(NR)	EGR1	Macro_FOLR2-APOE+	4,59185E-01
DC	BCC	Post(NR)	GABPB1	pDC_LILRA4	4,59096E-01
Macro/Mono	CRC	Post(R)	SPI1	Mono_CD16	4,58905E-01
DC	Melanoma	Post(NR)	EGR1	cDC(CD1C)	4,58881E-01
DC	BCC	Post(NR)	ELF2	pDC_LILRA4	4,58711E-01
DC	ccRCC	Post(NR)	MAFB	pDC_LILRA4	4,58596E-01
Macro/Mono	HNSCC	Post(R)	BHLHE40	Macro_OLFML3	4,58465E-01
Macro/Mono	CRC	Post(NR)	ELF1	Macro_OLFML3	4,58454E-01
Macro/Mono	BCC	Post(R)	ELF2	Mono_CD16	4,58220E-01
Macro/Mono	HNSCC	Post(R)	MAX	Macro_OLFML3	4,58115E-01
DC	BCC	Post(NR)	ELF2	cDC(CD1C)	4,57839E-01
Macro/Mono	Melanoma	Post(NR)	EGR1	Macro_IER3	4,57676E-01
Macro/Mono	Melanoma	Post(R)	BCLAF1	Macro_NLRP3	4,57539E-01
Macro/Mono	BCC	Post(NR)	BCLAF1	Macro_FOLR2+APOE+	4,57238E-01
Macro/Mono	BCC	Post(NR)	GABPB1	Macro_IER3	4,57230E-01
Macro/Mono	HNSCC	Post(R)	BHLHE40	Macro_LYVE1	4,56938E-01
Macro/Mono	BCC	Post(NR)	GABPB1	Macro_IFI27	4,56839E-01
Macro/Mono	ccRCC	Post(R)	ATF3	Macro_NLRP3	4,56681E-01
Macro/Mono	Melanoma	Post(NR)	EGR1	Mono_CD14	4,56280E-01
Mast	CRC	Post(R)	RAD21	Mast	4,55588E-01
Macro/Mono	BCC	Post(NR)	GABPB1	Macro_LYVE1	4,55487E-01
Macro/Mono	Melanoma	Post(NR)	EGR1	Mono_CD16	4,55242E-01
Macro/Mono	BCC	Post(NR)	ELF2	Mono_INHBA	4,55137E-01
Macro/Mono	Melanoma	Post(R)	ETV6	Macro_OLFML3	4,55081E-01
Macro/Mono	Melanoma	Post(R)	BHLHE41	Macro_IFI27	4,55028E-01
DC	CRC	Post(R)	ATF3	cDC_LAMP3	4,55002E-01
DC	BCC	Post(NR)	BCL11A	pDC_LILRA4	4,54846E-01
Macro/Mono	BCC	Post(NR)	BCLAF1	Mono_INHBA	4,54844E-01
Macro/Mono	CRC	Post(R)	POLR2A	Macro_ISG15	4,54600E-01
Macro/Mono	BCC	Post(R)	POLR2A	Macro_FOLR2+APOE+	4,54580E-01
Macro/Mono	Melanoma	Post(R)	ETV6	Macro_NLRP3	4,54327E-01
Macro/Mono	BCC	Post(R)	ELF2	Macro_FOLR2-APOE+	4,54324E-01
Macro/Mono	HNSCC	Post(R)	BHLHE40	Macro_FOLR2+APOE+	4,54246E-01
DC	HNSCC	Post(R)	MAX	pDC_LILRA4	4,54125E-01
Macro/Mono	CRC	Post(R)	ATF3	Mono_CD14	4,54018E-01
DC	ccRCC	Post(NR)	EGR1	pDC_LILRA4	4,53600E-01
Macro/Mono	Melanoma	Post(R)	ELF1	Mono_INHBA	4,53087E-01
DC	Melanoma	Post(NR)	EGR1	cDC_LAMP3	4,52967E-01
DC	ccRCC	Post(NR)	ELF2	pDC_LILRA4	4,52965E-01
DC	HNSCC	Post(R)	BCLAF1	pDC_LILRA4	4,52939E-01

Macro/Mono	HNSCC	Post(R)	ETS2	Mono_CD14	4,52844E-01
DC	HNSCC	Post(R)	BHLHE40	cDC(CD1C)	4,52828E-01
Macro/Mono	HNSCC	Post(NR)	ATF3	Macro_FOLR2+APOE-	4,52567E-01
Macro/Mono	Melanoma	Post(R)	ETV3	Mono_CD14	4,52318E-01
Macro/Mono	ccRCC	Post(R)	ETV5	Macro_IER3	4,52151E-01
Macro/Mono	BCC	Post(NR)	ELF2	Macro_ISG15	4,52104E-01
DC	ccRCC	Post(R)	BCLAF1	cDC_CLEC9A	4,52012E-01
DC	Melanoma	Post(R)	BCLAF1	cDC_CLEC9A	4,51644E-01
Macro/Mono	ccRCC	Post(NR)	JUN	Mono_CD16	4,51547E-01
DC	CRC	Post(NR)	POLR2A	cDC_LAMP3	4,51477E-01
Macro/Mono	HNSCC	Post(R)	MAX	Macro_IER3	4,51418E-01
DC	ccRCC	Post(NR)	JUN	cDC(CD1C)	4,51276E-01
Macro/Mono	Melanoma	Post(R)	ETV6	Macro_FOLR2-APOE+	4,51254E-01
Macro/Mono	Melanoma	Post(R)	ELF1	Mono_CD14	4,51152E-01
DC	Melanoma	Post(R)	ETV6	pDC_LILRA4	4,51093E-01
Macro/Mono	BCC	Post(NR)	GABPB1	Macro_FOLR2-APOE+	4,51055E-01
Macro/Mono	Melanoma	Post(NR)	EGR1	Macro_OLFML3	4,50547E-01
Macro/Mono	BCC	Post(R)	POLR2A	Macro_LYVE1	4,50484E-01
Macro/Mono	CRC	Post(NR)	ELF1	Macro_ISG15	4,50299E-01
Macro/Mono	BCC	Post(NR)	ELF2	Macro_FOLR2-APOE+	4,50094E-01
Macro/Mono	Melanoma	Post(NR)	EGR1	Macro_LYVE1	4,50072E-01
Macro/Mono	BCC	Post(R)	POLR2A	Mono_CD16	4,49917E-01
DC	HNSCC	Post(NR)	ATF3	cDC_LAMP3	4,49873E-01
Macro/Mono	Melanoma	Post(NR)	EGR1	Mono_INHBA	4,49239E-01
DC	HNSCC	Post(NR)	REL	cDC_LAMP3	4,49063E-01
Macro/Mono	CRC	Post(R)	SPI1	Mono_CD14	4,49061E-01
Macro/Mono	Melanoma	Post(R)	ETV6	Mono_CD14	4,48865E-01
Macro/Mono	Melanoma	Post(NR)	EGR1	Macro_NLRP3	4,48552E-01
Macro/Mono	HNSCC	Post(NR)	ELF1	Mono_CD16	4,48428E-01
Macro/Mono	BCC	Post(NR)	GABPB1	Macro_FOLR2+APOE-	4,47954E-01
Mast	ccRCC	Post(NR)	NELFE	Mast	4,47619E-01
DC	HNSCC	Post(R)	MAX	cDC_LAMP3	4,47568E-01
Macro/Mono	BCC	Post(R)	ELF2	Macro_IER3	4,47317E-01
Macro/Mono	HNSCC	Post(R)	MAX	Macro_ISG15	4,47224E-01
Macro/Mono	ccRCC	Post(NR)	FLI1	Macro_FOLR2+APOE+	4,47165E-01
Macro/Mono	Melanoma	Post(R)	ETV3	Macro_ISG15	4,47104E-01
Macro/Mono	HNSCC	Post(NR)	ATF3	Mono_CD16	4,46755E-01
Macro/Mono	CRC	Post(NR)	ELF1	Macro_NLRP3	4,46353E-01
Macro/Mono	CRC	Post(R)	SPI1	Macro_FOLR2-APOE+	4,46311E-01
DC	HNSCC	Post(R)	MAX	cDC(CD1C)	4,46197E-01
Macro/Mono	CRC	Post(NR)	EGR1	Mono_CD14	4,45987E-01
Mast	HNSCC	Post(R)	BCLAF1	Mast	4,45712E-01

Macro/Mono	CRC	Post(R)	ZMIZ1	Macro_FOLR2+APOE-	4,45358E-01
Macro/Mono	CRC	Post(NR)	SPI1	Macro_IFI27	4,44973E-01
DC	HNSCC	Post(NR)	ATF3	cDC(CD1C)	4,44492E-01
Macro/Mono	Melanoma	Post(R)	ELF1	Macro_IFI27	4,44467E-01
Macro/Mono	BCC	Post(NR)	GABPB1	Macro_NLRP3	4,44434E-01
Macro/Mono	BCC	Post(R)	POLR2A	Macro_IER3	4,44250E-01
Macro/Mono	ccRCC	Post(R)	ATF3	Mono_CD16	4,44215E-01
Macro/Mono	ccRCC	Post(NR)	ELF2	Macro_FOLR2+APOE-	4,44171E-01
Macro/Mono	Melanoma	Post(NR)	EGR1	Macro_FOLR2+APOE+	4,43898E-01
Macro/Mono	Melanoma	Post(R)	ELF1	Macro_OLFML3	4,43672E-01
Macro/Mono	CRC	Post(R)	POLR2A	Macro_NLRP3	4,43505E-01
Macro/Mono	HNSCC	Post(NR)	ELF1	Macro_FOLR2+APOE-	4,42892E-01
Macro/Mono	HNSCC	Post(NR)	ELF1	Macro_LYVE1	4,42766E-01
Macro/Mono	BCC	Post(NR)	RELB	Macro_OLFML3	4,42636E-01
Macro/Mono	BCC	Post(R)	ELF2	Macro_OLFML3	4,42632E-01
Macro/Mono	ccRCC	Post(NR)	FOS	Mono_CD14	4,42590E-01
Macro/Mono	CRC	Post(R)	SPI1	Macro_ISG15	4,42428E-01
Macro/Mono	BCC	Post(R)	POLR2A	Macro_FOLR2-APOE+	4,42362E-01
Macro/Mono	Melanoma	Post(NR)	EGR1	Macro_ISG15	4,42139E-01
DC	ccRCC	Post(NR)	SPI1	cDC(CD1C)	4,42079E-01
DC	Melanoma	Post(R)	BCLAF1	pDC_LILRA4	4,41669E-01
Macro/Mono	ccRCC	Post(R)	BCLAF1	Macro_LYVE1	4,41289E-01
DC	HNSCC	Post(NR)	ATF3	cDC_CLEC9A	4,40178E-01
Macro/Mono	Melanoma	Post(R)	ELF1	Macro_ISG15	4,40089E-01
DC	HNSCC	Post(NR)	ATF3	pDC_LILRA4	4,39868E-01
DC	Melanoma	Post(R)	BCLAF1	cDC(CD1C)	4,39517E-01
DC	Melanoma	Post(R)	ELF1	cDC(CD1C)	4,39383E-01
DC	CRC	Post(R)	REL	cDC(CD1C)	4,39047E-01
Macro/Mono	BCC	Post(NR)	GABPB1	Macro_ISG15	4,38760E-01
Macro/Mono	BCC	Post(R)	ELF2	Macro_LYVE1	4,38323E-01
Macro/Mono	ccRCC	Post(NR)	ELF2	Mono_CD16	4,37927E-01
Macro/Mono	ccRCC	Post(NR)	ELF2	Mono_CD14	4,37635E-01
DC	HNSCC	Post(R)	BHLHE40	cDC_CLEC9A	4,37595E-01
Macro/Mono	Melanoma	Post(R)	ETV3	Macro_FOLR2-APOE+	4,37223E-01
Macro/Mono	HNSCC	Post(NR)	ELF1	Macro_IFI27	4,37195E-01
Macro/Mono	CRC	Post(R)	SPI1	Macro_IFI27	4,37098E-01
Macro/Mono	ccRCC	Post(NR)	JUN	Mono_INHBA	4,37095E-01
DC	Melanoma	Post(R)	ELF1	cDC_CLEC9A	4,36694E-01
Macro/Mono	Melanoma	Post(R)	ELF1	Macro_NLRP3	4,36570E-01
DC	BCC	Post(R)	POLR2A	cDC_CLEC9A	4,35965E-01
Macro/Mono	CRC	Post(NR)	EGR1	Macro_NLRP3	4,35938E-01
Macro/Mono	CRC	Post(NR)	POLR2A	Macro_IER3	4,35756E-01

Macro/Mono	CRC	Post(R)	ATF3	Macro_FOLR2-APOE+	4,35590E-01
Mast	BCC	Post(NR)	RELB	Mast	4,34894E-01
Macro/Mono	CRC	Post(NR)	EGR1	Macro_ISG15	4,34677E-01
DC	CRC	Post(NR)	POLR2A	cDC_CLEC9A	4,34243E-01
DC	CRC	Post(NR)	EGR1	cDC_LAMP3	4,34191E-01
Macro/Mono	ccRCC	Post(NR)	FOS	Mono_INHBA	4,33344E-01
Macro/Mono	HNSCC	Post(R)	ETS2	Macro_NLRP3	4,33206E-01
Macro/Mono	CRC	Post(NR)	EGR1	Macro_LYVE1	4,32030E-01
DC	BCC	Post(R)	POLR2A	pDC_LILRA4	4,31355E-01
DC	ccRCC	Post(NR)	JUN	pDC_LILRA4	4,31342E-01
Macro/Mono	Melanoma	Post(R)	BCLAF1	Macro_OLFML3	4,30387E-01
DC	CRC	Post(NR)	ATF3	cDC_CLEC9A	4,30263E-01
Macro/Mono	BCC	Post(NR)	GABPB1	Macro_FOLR2+APOE+	4,30184E-01
Macro/Mono	Melanoma	Post(R)	ETV3	Macro_IFI27	4,30040E-01
Macro/Mono	ccRCC	Post(NR)	JUN	Macro_ISG15	4,30030E-01
DC	CRC	Post(R)	REL	cDC_CLEC9A	4,29915E-01
DC	BCC	Post(NR)	ELF2	cDC_LAMP3	4,29848E-01
Macro/Mono	BCC	Post(NR)	ELF2	Macro_OLFML3	4,29818E-01
Macro/Mono	BCC	Post(R)	ATF3	Mono_CD16	4,29558E-01
Macro/Mono	BCC	Post(NR)	GABPB1	Mono_INHBA	4,29457E-01
Macro/Mono	BCC	Post(R)	ELF2	Macro_IFI27	4,29435E-01
Macro/Mono	ccRCC	Post(R)	ATF3	Mono_CD14	4,29427E-01
DC	ccRCC	Post(R)	ETV5	cDC(CD1C)	4,29051E-01
Macro/Mono	BCC	Post(NR)	ELF2	Macro_FOLR2+APOE+	4,28720E-01
DC	CRC	Post(R)	REL	pDC_LILRA4	4,28563E-01
DC	HNSCC	Post(NR)	REL	pDC_LILRA4	4,28487E-01
Macro/Mono	ccRCC	Post(NR)	SPI1	Mono_CD14	4,28375E-01
Macro/Mono	CRC	Post(R)	REL	Macro_IER3	4,27716E-01
DC	CRC	Post(R)	RAD21	pDC_LILRA4	4,27647E-01
Macro/Mono	ccRCC	Post(NR)	JUN	Macro_OLFML3	4,27572E-01
DC	BCC	Post(NR)	RELB	cDC(CD1C)	4,26399E-01
Mast	HNSCC	Post(R)	REL	Mast	4,26114E-01
Mast	HNSCC	Post(R)	ETS2	Mast	4,25954E-01
DC	BCC	Post(R)	RELB	cDC_LAMP3	4,25611E-01
Macro/Mono	BCC	Post(R)	ATF3	Macro_FOLR2+APOE+	4,25440E-01
Macro/Mono	ccRCC	Post(NR)	JUNB	Macro_IER3	4,24954E-01
Macro/Mono	CRC	Post(NR)	ATF3	Macro_IER3	4,24871E-01
DC	HNSCC	Post(NR)	ELF1	cDC_LAMP3	4,24792E-01
DC	BCC	Post(NR)	RELB	cDC_CLEC9A	4,24656E-01
Macro/Mono	BCC	Post(R)	ATF3	Mono_CD14	4,24297E-01
Macro/Mono	CRC	Post(NR)	EGR1	Macro_FOLR2+APOE-	4,23928E-01
Macro/Mono	BCC	Post(R)	ATF3	Macro_LYVE1	4,23917E-01

Mast	HNSCC	Post(NR)	BCLAF1	Mast	4,23815E-01
Mast	HNSCC	Post(NR)	REL	Mast	4,23650E-01
Macro/Mono	Melanoma	Post(R)	GTF2F1	Mono_INHBA	4,23646E-01
Macro/Mono	HNSCC	Post(NR)	EGR1	Mono_CD14	4,23473E-01
Macro/Mono	CRC	Post(R)	ATF3	Macro_ISG15	4,23262E-01
Macro/Mono	CRC	Post(NR)	EGR1	Macro_OLFML3	4,23250E-01
Macro/Mono	ccRCC	Post(NR)	JUN	Macro_LYVE1	4,23249E-01
Macro/Mono	BCC	Post(R)	ATF3	Macro_NLRP3	4,23218E-01
Macro/Mono	HNSCC	Post(NR)	SPI1	Macro_ISG15	4,22998E-01
Macro/Mono	CRC	Post(R)	REL	Macro_NLRP3	4,22750E-01
Macro/Mono	Melanoma	Post(NR)	EGR1	Macro_IFI27	4,22715E-01
Macro/Mono	ccRCC	Post(R)	BCLAF1	Macro_IFI27	4,21972E-01
Mast	ccRCC	Post(R)	ATF3	Mast	4,21956E-01
DC	ccRCC	Post(R)	BCLAF1	cDC_LAMP3	4,21946E-01
Macro/Mono	CRC	Post(R)	RAD21	Mono_CD16	4,21913E-01
Macro/Mono	ccRCC	Post(NR)	MAFB	Mono_INHBA	4,21865E-01
Macro/Mono	BCC	Post(R)	ATF3	Macro_IER3	4,21861E-01
Macro/Mono	CRC	Post(R)	RAD21	Mono_CD14	4,21746E-01
Macro/Mono	ccRCC	Post(R)	HMGB1	Macro_IER3	4,21572E-01
Macro/Mono	Melanoma	Post(R)	NFIL3	Macro_IFI27	4,21219E-01
Macro/Mono	HNSCC	Post(R)	MAX	Mono_CD16	4,21171E-01
DC	BCC	Post(R)	POLR2A	cDC(CD1C)	4,20861E-01
Macro/Mono	ccRCC	Post(NR)	JUN	Macro_FOLR2-APOE+	4,20803E-01
DC	CRC	Post(NR)	ATF3	cDC_LAMP3	4,20795E-01
Macro/Mono	ccRCC	Post(R)	ATF3	Macro_IER3	4,20284E-01
DC	HNSCC	Post(NR)	BCLAF1	pDC_LILRA4	4,20206E-01
Macro/Mono	CRC	Post(R)	ATF3	Mono_CD16	4,20107E-01
Macro/Mono	Melanoma	Post(R)	ETV6	Macro_IFI27	4,19965E-01
Macro/Mono	CRC	Post(R)	SPI1	Macro_LYVE1	4,19928E-01
Macro/Mono	Melanoma	Post(R)	BCLAF1	Mono_CD14	4,19704E-01
DC	ccRCC	Post(NR)	SPI1	cDC_LAMP3	4,19406E-01
DC	ccRCC	Post(R)	ETV5	pDC_LILRA4	4,18947E-01
Macro/Mono	ccRCC	Post(NR)	SPI1	Macro_IFI27	4,18656E-01
DC	BCC	Post(R)	ELF2	cDC(CD1C)	4,18558E-01
Mast	CRC	Post(R)	REL	Mast	4,18448E-01
Macro/Mono	HNSCC	Post(R)	MAX	Mono_CD14	4,18447E-01
Macro/Mono	HNSCC	Post(R)	ETS2	Mono_CD16	4,18417E-01
Macro/Mono	HNSCC	Post(NR)	ETS2	Mono_INHBA	4,17774E-01
Mast	ccRCC	Post(NR)	GTF2F1	Mast	4,17722E-01
Mast	CRC	Post(R)	ATF3	Mast	4,17518E-01
DC	ccRCC	Post(NR)	FOS	cDC(CD1C)	4,16700E-01
Macro/Mono	Melanoma	Post(R)	EGR1	Mono_INHBA	4,16170E-01

Macro/Mono	BCC	Post(R)	POLR2A	Macro_OLFML3	4,16090E-01
DC	BCC	Post(NR)	RELB	pDC_LILRA4	4,16089E-01
Macro/Mono	BCC	Post(R)	ATF3	Macro_ISG15	4,15922E-01
DC	BCC	Post(R)	POLR2A	cDC_LAMP3	4,15773E-01
Macro/Mono	CRC	Post(NR)	EGR1	Macro_IFI27	4,15405E-01
Macro/Mono	ccRCC	Post(NR)	ELF2	Macro_LYVE1	4,15404E-01
Macro/Mono	BCC	Post(NR)	RELB	Macro_IER3	4,15223E-01
DC	CRC	Post(NR)	REL	pDC_LILRA4	4,15085E-01
Macro/Mono	Melanoma	Post(R)	BCLAF1	Macro_ISG15	4,14834E-01
Macro/Mono	HNSCC	Post(NR)	ELF1	Macro_ISG15	4,14821E-01
Macro/Mono	CRC	Post(R)	SPI1	Macro_IER3	4,14291E-01
Macro/Mono	BCC	Post(R)	ELF2	Macro_FOLR2+APOE-	4,13973E-01
DC	ccRCC	Post(NR)	FOS	pDC_LILRA4	4,13881E-01
DC	Melanoma	Post(NR)	HDAC2	cDC_CLEC9A	4,13663E-01
DC	ccRCC	Post(NR)	JUN	cDC_LAMP3	4,13313E-01
DC	HNSCC	Post(R)	BCLAF1	cDC_CLEC9A	4,13238E-01
Macro/Mono	Melanoma	Post(R)	BCLAF1	Macro_FOLR2-APOE+	4,13176E-01
Macro/Mono	Melanoma	Post(R)	ELF1	Macro_FOLR2-APOE+	4,12853E-01
DC	CRC	Post(R)	REL	cDC_LAMP3	4,12743E-01
Macro/Mono	BCC	Post(NR)	RELB	Macro_LYVE1	4,12738E-01
Macro/Mono	ccRCC	Post(R)	ATF3	Macro_FOLR2+APOE-	4,12647E-01
DC	ccRCC	Post(NR)	NELFE	cDC_CLEC9A	4,12518E-01
Macro/Mono	BCC	Post(R)	ATF3	Mono_INHBA	4,12103E-01
DC	CRC	Post(R)	SPI1	cDC_CLEC9A	4,11718E-01
DC	HNSCC	Post(R)	REL	pDC_LILRA4	4,11497E-01
DC	Melanoma	Post(NR)	HDAC2	pDC_LILRA4	4,11327E-01
DC	BCC	Post(R)	ELF2	cDC_CLEC9A	4,11294E-01
Macro/Mono	HNSCC	Post(NR)	EGR1	Macro_IFI27	4,10681E-01
Macro/Mono	ccRCC	Post(R)	CEBPD	Macro_ISG15	4,10590E-01
DC	Melanoma	Post(NR)	NELFE	cDC_CLEC9A	4,10507E-01
Macro/Mono	Melanoma	Post(R)	SIX6	Macro_IFI27	4,10468E-01
Macro/Mono	BCC	Post(NR)	RELB	Mono_CD16	4,10420E-01
DC	BCC	Post(R)	ATF3	cDC(CD1C)	4,10272E-01
Macro/Mono	CRC	Post(NR)	SPI1	Macro_IER3	4,09870E-01
Mast	CRC	Post(NR)	REL	Mast	4,09676E-01
Macro/Mono	BCC	Post(R)	ATF3	Macro_IFI27	4,09607E-01
Macro/Mono	HNSCC	Post(R)	ETS2	Mono_INHBA	4,09556E-01
Macro/Mono	ccRCC	Post(R)	HMGB1	Macro_FOLR2+APOE-	4,09439E-01
Macro/Mono	CRC	Post(R)	ZMIZ1	Macro_OLFML3	4,09361E-01
Macro/Mono	CRC	Post(NR)	REL	Macro_NLRP3	4,09053E-01
Macro/Mono	BCC	Post(NR)	RELB	Mono_CD14	4,08748E-01
DC	CRC	Post(NR)	REL	cDC(CD1C)	4,08606E-01

Macro/Mono	HNSCC	Post(NR)	EGR1	Macro_NLRP3	4,08243E-01
Macro/Mono	HNSCC	Post(R)	MAX	Mono_INHBA	4,07876E-01
Macro/Mono	BCC	Post(R)	ATF3	Macro_FOLR2-APOE+	4,07807E-01
Mast	Melanoma	Post(NR)	NELFE	Mast	4,07691E-01
Macro/Mono	CRC	Post(NR)	MAFB	Macro_FOLR2+APOE+	4,07533E-01
Macro/Mono	BCC	Post(R)	EP300	Macro_FOLR2+APOE+	4,07410E-01
Macro/Mono	HNSCC	Post(NR)	SPI1	Macro_FOLR2+APOE+	4,07283E-01
Macro/Mono	HNSCC	Post(NR)	ELF1	Macro_OLFML3	4,07109E-01
DC	HNSCC	Post(NR)	ELF1	cDC_CLEC9A	4,06889E-01
Mast	BCC	Post(R)	ATF3	Mast	4,06831E-01
DC	ccRCC	Post(R)	ATF3	cDC_CLEC9A	4,06806E-01
Macro/Mono	BCC	Post(NR)	RELB	Macro_IFI27	4,06501E-01
DC	Melanoma	Post(R)	ELF1	pDC_LILRA4	4,05579E-01
Mast	BCC	Post(R)	ELF2	Mast	4,05338E-01
DC	BCC	Post(R)	ATF3	cDC_CLEC9A	4,04862E-01
Macro/Mono	Melanoma	Post(R)	BCLAF1	Mono_INHBA	4,04673E-01
DC	HNSCC	Post(R)	BCLAF1	cDC_LAMP3	4,04627E-01
Mast	BCC	Post(R)	MAX	Mast	4,04485E-01
Macro/Mono	ccRCC	Post(NR)	ELF2	Macro_IFI27	4,04383E-01
DC	BCC	Post(R)	RELB	cDC(CD1C)	4,04194E-01
Macro/Mono	CRC	Post(R)	POLR2A	Macro_IER3	4,04006E-01
Macro/Mono	BCC	Post(NR)	RELB	Macro_NLRP3	4,03976E-01
Macro/Mono	ccRCC	Post(NR)	EGR1	Macro_FOLR2+APOE-	4,03915E-01
Macro/Mono	CRC	Post(R)	ATF3	Macro_LYVE1	4,03860E-01
Macro/Mono	Melanoma	Post(R)	TFDP2	Macro_IFI27	4,03720E-01
DC	Melanoma	Post(NR)	NELFE	cDC_LAMP3	4,03696E-01
DC	ccRCC	Post(R)	ATF3	cDC_LAMP3	4,03588E-01
Macro/Mono	BCC	Post(NR)	RELB	Macro_FOLR2-APOE+	4,03561E-01
DC	HNSCC	Post(NR)	ELF1	cDC(CD1C)	4,03532E-01
Macro/Mono	HNSCC	Post(NR)	SPI1	Macro_OLFML3	4,03079E-01
DC	CRC	Post(R)	SPI1	cDC(CD1C)	4,03071E-01
DC	HNSCC	Post(NR)	REL	cDC_CLEC9A	4,02791E-01
Macro/Mono	CRC	Post(NR)	EGR1	Macro_FOLR2-APOE+	4,02744E-01
DC	ccRCC	Post(NR)	MAFB	cDC(CD1C)	4,02642E-01
DC	Melanoma	Post(NR)	HDAC2	cDC_LAMP3	4,02122E-01
Macro/Mono	CRC	Post(R)	POLR2A	Macro_LYVE1	4,01662E-01
Macro/Mono	HNSCC	Post(NR)	EGR1	Macro_IER3	4,01494E-01
Macro/Mono	CRC	Post(R)	SPI1	Macro_NLRP3	4,01482E-01
Macro/Mono	BCC	Post(R)	RELB	Macro_IER3	4,01037E-01
DC	ccRCC	Post(R)	CEBPD	pDC_LILRA4	4,00924E-01
Macro/Mono	HNSCC	Post(NR)	ELF1	Macro_FOLR2-APOE+	4,00546E-01
DC	BCC	Post(R)	RELB	cDC_CLEC9A	4,00358E-01

Macro/Mono	HNSCC	Post(NR)	EGR1	Mono_INHBA	3,99865E-01
Mast	ccRCC	Post(R)	FLI1	Mast	3,99596E-01
Macro/Mono	BCC	Post(NR)	RELB	Mono_INHBA	3,99579E-01
Macro/Mono	BCC	Post(NR)	RELB	Macro_ISG15	3,98745E-01
DC	CRC	Post(R)	RAD21	cDC_LAMP3	3,98678E-01
Macro/Mono	Melanoma	Post(R)	HCFC1	Macro_NLRP3	3,98663E-01
Macro/Mono	BCC	Post(R)	RELB	Macro_NLRP3	3,98653E-01
Macro/Mono	BCC	Post(R)	RELB	Macro_OLFML3	3,98555E-01
Macro/Mono	Melanoma	Post(NR)	HDAC2	Macro_FOLR2-APOE+	3,98525E-01
Macro/Mono	Melanoma	Post(R)	HCFC1	Mono_INHBA	3,98509E-01
Macro/Mono	HNSCC	Post(NR)	EGR1	Mono_CD16	3,98433E-01
DC	Melanoma	Post(NR)	NELFE	pDC_LILRA4	3,98208E-01
Macro/Mono	CRC	Post(NR)	ELF1	Mono_INHBA	3,97860E-01
DC	CRC	Post(R)	ATF3	cDC(CD1C)	3,97713E-01
Macro/Mono	BCC	Post(R)	RELB	Mono_CD16	3,97371E-01
DC	CRC	Post(NR)	REL	cDC_CLEC9A	3,97197E-01
DC	Melanoma	Post(NR)	PML	cDC_CLEC9A	3,97052E-01
Macro/Mono	BCC	Post(R)	RELB	Mono_CD14	3,97005E-01
Macro/Mono	HNSCC	Post(NR)	EGR1	Macro_FOLR2+APOE-	3,96039E-01
Mast	HNSCC	Post(R)	RELB	Mast	3,96009E-01
DC	Melanoma	Post(NR)	HCFC1	pDC_LILRA4	3,95989E-01
Macro/Mono	BCC	Post(R)	RELB	Macro_FOLR2-APOE+	3,95815E-01
DC	BCC	Post(R)	RELB	pDC_LILRA4	3,95796E-01
Macro/Mono	ccRCC	Post(NR)	FOS	Mono_CD16	3,95777E-01
DC	Melanoma	Post(NR)	NELFE	cDC(CD1C)	3,95747E-01
Macro/Mono	BCC	Post(R)	EP300	Mono_CD14	3,95743E-01
DC	CRC	Post(NR)	SPI1	cDC_LAMP3	3,95722E-01
Macro/Mono	Melanoma	Post(NR)	HDAC2	Mono_CD14	3,95532E-01
Macro/Mono	CRC	Post(NR)	EGR1	Mono_INHBA	3,95275E-01
DC	HNSCC	Post(NR)	BCLAF1	cDC_CLEC9A	3,95243E-01
DC	BCC	Post(R)	MAX	pDC_LILRA4	3,95022E-01
Macro/Mono	CRC	Post(NR)	REL	Mono_CD14	3,94105E-01
DC	Melanoma	Post(NR)	PML	pDC_LILRA4	3,93970E-01
DC	Melanoma	Post(NR)	HCFC1	cDC_CLEC9A	3,93854E-01
Macro/Mono	HNSCC	Post(NR)	EGR1	Macro_LYVE1	3,93837E-01
DC	Melanoma	Post(NR)	HDAC2	cDC(CD1C)	3,93788E-01
Macro/Mono	BCC	Post(R)	MAX	Mono_CD16	3,93538E-01
Macro/Mono	CRC	Post(NR)	POLR2A	Macro_FOLR2+APOE-	3,92944E-01
Macro/Mono	Melanoma	Post(R)	ATF3	Macro_NLRP3	3,92905E-01
Macro/Mono	ccRCC	Post(NR)	JUN	Macro_FOLR2+APOE+	3,92275E-01
DC	BCC	Post(R)	NR3C1	pDC_LILRA4	3,92178E-01
Macro/Mono	ccRCC	Post(NR)	SPI1	Macro_IER3	3,92091E-01

DC	HNSCC	Post(NR)	REL	cDC(CD1C)	3,92055E-01
DC	HNSCC	Post(R)	REL	cDC_LAMP3	3,91983E-01
Macro/Mono	Melanoma	Post(NR)	HDAC2	Mono_CD16	3,91796E-01
Macro/Mono	BCC	Post(R)	ATF3	Macro_OLFML3	3,91748E-01
Macro/Mono	HNSCC	Post(NR)	SPI1	Macro_FOLR2-APOE+	3,91722E-01
Macro/Mono	CRC	Post(R)	SPI1	Mono_INHBA	3,91630E-01
Mast	Melanoma	Post(NR)	HDAC2	Mast	3,91371E-01
Macro/Mono	HNSCC	Post(NR)	SPI1	Mono_CD16	3,91252E-01
DC	Melanoma	Post(NR)	HCFC1	cDC_LAMP3	3,91179E-01
Macro/Mono	ccRCC	Post(NR)	NELFE	Mono_CD14	3,91153E-01
Macro/Mono	BCC	Post(NR)	RELB	Macro_FOLR2+APOE-	3,90889E-01
Macro/Mono	CRC	Post(R)	REL	Mono_INHBA	3,90543E-01
Macro/Mono	CRC	Post(NR)	RAD21	Mono_CD16	3,90540E-01
Macro/Mono	ccRCC	Post(NR)	NELFE	Mono_CD16	3,90369E-01
Macro/Mono	BCC	Post(R)	MAX	Mono_CD14	3,90318E-01
Macro/Mono	ccRCC	Post(NR)	EGR1	Mono_CD14	3,90225E-01
Mast	BCC	Post(R)	EP300	Mast	3,90108E-01
Macro/Mono	BCC	Post(R)	ATF3	Macro_FOLR2+APOE-	3,90071E-01
Mast	Melanoma	Post(NR)	HCFC1	Mast	3,90061E-01
DC	BCC	Post(R)	REL	cDC_LAMP3	3,89491E-01
Macro/Mono	BCC	Post(R)	EP300	Mono_CD16	3,89459E-01
Macro/Mono	BCC	Post(R)	EP300	Macro_IER3	3,89195E-01
Macro/Mono	BCC	Post(R)	EP300	Macro_NLRP3	3,89158E-01
Macro/Mono	Melanoma	Post(R)	ATF3	Mono_INHBA	3,89072E-01
Macro/Mono	CRC	Post(R)	SPI1	Macro_FOLR2+APOE-	3,89046E-01
Macro/Mono	HNSCC	Post(R)	REL	Macro_NLRP3	3,88851E-01
Macro/Mono	CRC	Post(R)	RAD21	Mono_INHBA	3,88818E-01
Macro/Mono	HNSCC	Post(NR)	ELF1	Macro_FOLR2+APOE+	3,88793E-01
Macro/Mono	BCC	Post(R)	RELB	Mono_INHBA	3,88637E-01
Macro/Mono	BCC	Post(R)	EP300	Macro_ISG15	3,88623E-01
DC	Melanoma	Post(NR)	PML	cDC_LAMP3	3,88510E-01
Macro/Mono	HNSCC	Post(NR)	REL	Macro_NLRP3	3,88460E-01
Macro/Mono	BCC	Post(R)	RELB	Macro_ISG15	3,88352E-01
DC	ccRCC	Post(NR)	NELFE	pDC_LILRA4	3,88254E-01
Macro/Mono	ccRCC	Post(NR)	ELF2	Macro_IER3	3,88096E-01
Macro/Mono	HNSCC	Post(R)	SPI1	Macro_FOLR2+APOE+	3,87946E-01
Macro/Mono	ccRCC	Post(NR)	ELF2	Macro_NLRP3	3,87936E-01
DC	Melanoma	Post(NR)	EWSR1	cDC(CD1C)	3,87726E-01
DC	HNSCC	Post(NR)	EGR1	pDC_LILRA4	3,87643E-01
DC	Melanoma	Post(NR)	HCFC1	cDC(CD1C)	3,87452E-01
Macro/Mono	Melanoma	Post(NR)	NELFE	Mono_CD16	3,87363E-01
Macro/Mono	CRC	Post(NR)	REL	Mono_INHBA	3,87319E-01

Macro/Mono	BCC	Post(NR)	RELB	Macro_FOLR2+APOE+	3,86993E-01
Macro/Mono	ccRCC	Post(NR)	KLF4	Macro_NLRP3	3,86865E-01
DC	BCC	Post(R)	EP300	cDC_CLEC9A	3,86802E-01
Macro/Mono	CRC	Post(R)	POLR2A	Macro_FOLR2-APOE+	3,86745E-01
Macro/Mono	ccRCC	Post(NR)	JUNB	Macro_NLRP3	3,86734E-01
Macro/Mono	Melanoma	Post(NR)	NELFE	Mono_CD14	3,86228E-01
Macro/Mono	Melanoma	Post(R)	BCLAF1	Macro_IFI27	3,86190E-01
DC	BCC	Post(R)	ENO1	cDC_LAMP3	3,86024E-01
DC	Melanoma	Post(NR)	PML	cDC(CD1C)	3,86010E-01
DC	ccRCC	Post(NR)	ELF2	cDC_LAMP3	3,85969E-01
Macro/Mono	ccRCC	Post(R)	ATF3	Macro_FOLR2-APOE+	3,85903E-01
Macro/Mono	Melanoma	Post(R)	ELK1	Mono_INHBA	3,85871E-01
Macro/Mono	Melanoma	Post(NR)	EWSR1	Macro_OLFML3	3,85725E-01
Macro/Mono	CRC	Post(NR)	SPI1	Macro_FOLR2+APOE-	3,85702E-01
Mast	ccRCC	Post(NR)	REST	Mast	3,85662E-01
Macro/Mono	HNSCC	Post(NR)	REL	Macro_IER3	3,85185E-01
DC	ccRCC	Post(NR)	NELFE	cDC_LAMP3	3,85138E-01
DC	ccRCC	Post(NR)	REL	cDC_LAMP3	3,84708E-01
Macro/Mono	ccRCC	Post(R)	ATF3	Macro_IFI27	3,84698E-01
Macro/Mono	Melanoma	Post(R)	HCFC1	Macro_FOLR2-APOE+	3,84535E-01
Macro/Mono	Melanoma	Post(NR)	HDAC2	Macro_OLFML3	3,84400E-01
Macro/Mono	BCC	Post(R)	RELB	Macro_IFI27	3,83989E-01
Macro/Mono	HNSCC	Post(NR)	REL	Mono_CD14	3,83920E-01
Macro/Mono	HNSCC	Post(R)	SPI1	Macro_OLFML3	3,83737E-01
Macro/Mono	BCC	Post(R)	RELB	Macro_LYVE1	3,83679E-01
Macro/Mono	CRC	Post(NR)	REL	Macro_ISG15	3,83379E-01
Macro/Mono	Melanoma	Post(NR)	HDAC2	Macro_LYVE1	3,82914E-01
DC	HNSCC	Post(NR)	RELB	cDC_LAMP3	3,82715E-01
DC	HNSCC	Post(NR)	EGR1	cDC_LAMP3	3,82673E-01
Mast	Melanoma	Post(NR)	PML	Mast	3,82658E-01
Mast	CRC	Post(NR)	RAD21	Mast	3,82601E-01
Macro/Mono	ccRCC	Post(NR)	SPI1	Macro_LYVE1	3,82592E-01
DC	ccRCC	Post(NR)	REL	pDC_LILRA4	3,82536E-01
Macro/Mono	HNSCC	Post(R)	ETS2	Macro_IFI27	3,82510E-01
Macro/Mono	ccRCC	Post(R)	BCLAF1	Macro_FOLR2-APOE+	3,82415E-01
Macro/Mono	ccRCC	Post(NR)	NELFE	Macro_NLRP3	3,82332E-01
Macro/Mono	HNSCC	Post(R)	ETS2	Macro_FOLR2+APOE-	3,82270E-01
Mast	BCC	Post(R)	RELB	Mast	3,82257E-01
Macro/Mono	Melanoma	Post(NR)	HCFC1	Mono_CD16	3,81783E-01
DC	CRC	Post(R)	ATF3	pDC_LILRA4	3,81141E-01
Macro/Mono	Melanoma	Post(NR)	NELFE	Macro_FOLR2-APOE+	3,81122E-01
Macro/Mono	ccRCC	Post(R)	POLR2A	Macro_FOLR2+APOE-	3,81053E-01

Macro/Mono	ccRCC	Post(NR)	REL	Macro_IER3	3,81043E-01
DC	BCC	Post(R)	ATF3	cDC_LAMP3	3,81037E-01
Macro/Mono	CRC	Post(R)	RAD21	Macro_ISG15	3,80945E-01
Macro/Mono	Melanoma	Post(NR)	HCFC1	Mono_CD14	3,80903E-01
Macro/Mono	CRC	Post(NR)	REL	Macro_IER3	3,80898E-01
DC	ccRCC	Post(R)	BCLAF1	cDC(CD1C)	3,80893E-01
Macro/Mono	HNSCC	Post(R)	MAX	Macro_NLRP3	3,80886E-01
Macro/Mono	HNSCC	Post(R)	ETS2	Macro_IER3	3,80852E-01
Macro/Mono	Melanoma	Post(NR)	HDAC2	Macro_IER3	3,80756E-01
Macro/Mono	Melanoma	Post(NR)	NELFE	Macro_OLFML3	3,80688E-01
DC	ccRCC	Post(NR)	FOS	cDC_LAMP3	3,80550E-01
DC	ccRCC	Post(NR)	REL	cDC(CD1C)	3,80538E-01
DC	HNSCC	Post(R)	REL	cDC_CLEC9A	3,80421E-01
Macro/Mono	ccRCC	Post(R)	ETV5	Macro_ISG15	3,80417E-01
Macro/Mono	Melanoma	Post(NR)	PML	Mono_CD16	3,80415E-01
Macro/Mono	BCC	Post(R)	RELB	Macro_FOLR2+APOE+	3,80279E-01
DC	HNSCC	Post(R)	BCLAF1	cDC(CD1C)	3,80086E-01
DC	Melanoma	Post(R)	ATF3	pDC_LILRA4	3,79762E-01
Macro/Mono	Melanoma	Post(NR)	HDAC2	Macro_NLRP3	3,79551E-01
Macro/Mono	Melanoma	Post(NR)	NELFE	Macro_NLRP3	3,79489E-01
Macro/Mono	CRC	Post(R)	REL	Macro_OLFML3	3,79357E-01
Macro/Mono	CRC	Post(R)	REL	Macro_ISG15	3,78901E-01
Macro/Mono	HNSCC	Post(R)	REL	Mono_CD14	3,78891E-01
Macro/Mono	BCC	Post(R)	EP300	Mono_INHBA	3,78839E-01
DC	HNSCC	Post(R)	RELB	pDC_LILRA4	3,78828E-01
Macro/Mono	HNSCC	Post(R)	SPI1	Macro_ISG15	3,78778E-01
DC	BCC	Post(R)	ENO1	cDC_CLEC9A	3,78725E-01
Macro/Mono	BCC	Post(R)	MAX	Macro_ISG15	3,78498E-01
Macro/Mono	ccRCC	Post(NR)	ELF2	Mono_INHBA	3,78426E-01
DC	BCC	Post(R)	ATF3	pDC_LILRA4	3,78410E-01
Macro/Mono	BCC	Post(R)	MAX	Macro_FOLR2+APOE+	3,78013E-01
Macro/Mono	CRC	Post(R)	ATF3	Macro_FOLR2+APOE+	3,77854E-01
Macro/Mono	ccRCC	Post(NR)	NELFE	Mono_INHBA	3,77842E-01
Macro/Mono	BCC	Post(R)	EP300	Macro_FOLR2-APOE+	3,77575E-01
Mast	ccRCC	Post(NR)	JUN	Mast	3,77352E-01
DC	BCC	Post(R)	ELF2	cDC_LAMP3	3,76798E-01
Macro/Mono	Melanoma	Post(NR)	NELFE	Mono_INHBA	3,76455E-01
Macro/Mono	ccRCC	Post(R)	BCLAF1	Macro_IER3	3,76432E-01
Macro/Mono	Melanoma	Post(NR)	PML	Mono_CD14	3,76419E-01
Macro/Mono	CRC	Post(R)	ATF3	Macro_OLFML3	3,76399E-01
Macro/Mono	Melanoma	Post(R)	HCFC1	Macro_OLFML3	3,75499E-01
Macro/Mono	HNSCC	Post(NR)	REL	Mono_CD16	3,75277E-01

Macro/Mono	Melanoma	Post(NR)	EWSR1	Mono_CD16	3,75215E-01
Macro/Mono	Melanoma	Post(NR)	NELFE	Macro_IER3	3,75177E-01
Macro/Mono	BCC	Post(R)	MAX	Macro_IER3	3,75066E-01
Macro/Mono	HNSCC	Post(R)	BCLAF1	Macro_FOLR2+APOE-	3,74997E-01
Macro/Mono	ccRCC	Post(NR)	NELFE	Macro_FOLR2+APOE-	3,74912E-01
Macro/Mono	Melanoma	Post(NR)	EWSR1	Mono_CD14	3,74904E-01
Macro/Mono	HNSCC	Post(R)	BCLAF1	Mono_CD14	3,74894E-01
DC	Melanoma	Post(R)	HCFC1	cDC_CLEC9A	3,74809E-01
Macro/Mono	ccRCC	Post(NR)	ELF2	Macro_ISG15	3,74570E-01
DC	ccRCC	Post(NR)	NELFE	cDC(CD1C)	3,74422E-01
DC	Melanoma	Post(R)	ATF3	cDC(CD1C)	3,74413E-01
Macro/Mono	Melanoma	Post(NR)	HCFC1	Macro_NLRP3	3,74351E-01
Macro/Mono	Melanoma	Post(NR)	PML	Macro_OLFML3	3,74122E-01
Macro/Mono	Melanoma	Post(NR)	HDAC2	Macro_ISG15	3,73944E-01
Macro/Mono	CRC	Post(R)	REL	Mono_CD14	3,73796E-01
DC	BCC	Post(R)	MAX	cDC_CLEC9A	3,73619E-01
Macro/Mono	HNSCC	Post(R)	SPI1	Macro_FOLR2-APOE+	3,73529E-01
DC	CRC	Post(NR)	SPI1	cDC_CLEC9A	3,73226E-01
Macro/Mono	HNSCC	Post(R)	ETS2	Macro_ISG15	3,73193E-01
Macro/Mono	Melanoma	Post(NR)	HDAC2	Macro_FOLR2+APOE+	3,73140E-01
Mast	HNSCC	Post(R)	EGR1	Mast	3,73139E-01
Mast	ccRCC	Post(NR)	ATF3	Mast	3,72912E-01
DC	ccRCC	Post(R)	ATF3	cDC(CD1C)	3,72873E-01
Macro/Mono	Melanoma	Post(NR)	EWSR1	Macro_FOLR2+APOE+	3,72803E-01
DC	BCC	Post(R)	EP300	cDC(CD1C)	3,72685E-01
Macro/Mono	BCC	Post(R)	EP300	Macro_OLFML3	3,72560E-01
Macro/Mono	Melanoma	Post(R)	EGR1	Macro_NLRP3	3,72514E-01
Macro/Mono	ccRCC	Post(NR)	EGR1	Macro_LYVE1	3,72490E-01
Macro/Mono	ccRCC	Post(NR)	REL	Macro_NLRP3	3,72392E-01
Macro/Mono	Melanoma	Post(NR)	NELFE	Macro_LYVE1	3,72178E-01
Macro/Mono	Melanoma	Post(NR)	HCFC1	Macro_IER3	3,71939E-01
DC	ccRCC	Post(NR)	ELF2	cDC(CD1C)	3,71932E-01
Macro/Mono	HNSCC	Post(R)	ETS2	Macro_FOLR2-APOE+	3,71620E-01
Macro/Mono	Melanoma	Post(NR)	HDAC2	Mono_INHBA	3,71479E-01
Macro/Mono	BCC	Post(R)	MAX	Macro_NLRP3	3,71369E-01
DC	BCC	Post(R)	ELF2	pDC_LILRA4	3,71095E-01
Macro/Mono	Melanoma	Post(NR)	NELFE	Macro_FOLR2+APOE+	3,70641E-01
Macro/Mono	BCC	Post(R)	MAX	Macro_LYVE1	3,70543E-01
DC	HNSCC	Post(NR)	SPI1	cDC(CD1C)	3,70298E-01
Macro/Mono	Melanoma	Post(NR)	HCFC1	Macro_OLFML3	3,70243E-01
Macro/Mono	HNSCC	Post(NR)	EGR1	Macro_FOLR2-APOE+	3,70208E-01
Macro/Mono	Melanoma	Post(R)	GTF2F1	Macro_FOLR2-APOE+	3,70126E-01

Macro/Mono	CRC	Post(NR)	ATF3	Mono_CD16	3,70067E-01
Macro/Mono	ccRCC	Post(NR)	FOS	Macro_OLFML3	3,69994E-01
Macro/Mono	CRC	Post(R)	REL	Macro_FOLR2+APOE-	3,69880E-01
DC	HNSCC	Post(R)	RELB	cDC_LAMP3	3,69868E-01
Macro/Mono	Melanoma	Post(NR)	HCFC1	Mono_INHBA	3,69770E-01
DC	Melanoma	Post(R)	EGR1	cDC_CLEC9A	3,69487E-01
Macro/Mono	BCC	Post(R)	EP300	Macro_IFI27	3,69183E-01
Macro/Mono	HNSCC	Post(NR)	EGR1	Macro_OLFML3	3,69091E-01
Macro/Mono	HNSCC	Post(R)	ATF3	Macro_IER3	3,69074E-01
DC	HNSCC	Post(R)	REL	cDC(CD1C)	3,68592E-01
Macro/Mono	Melanoma	Post(NR)	NELFE	Macro_ISG15	3,68545E-01
DC	Melanoma	Post(NR)	EWSR1	cDC_CLEC9A	3,68486E-01
Macro/Mono	Melanoma	Post(NR)	PML	Macro_IER3	3,68475E-01
Macro/Mono	CRC	Post(NR)	ATF3	Macro_LYVE1	3,68400E-01
DC	Melanoma	Post(NR)	EWSR1	pDC_LILRA4	3,68351E-01
Macro/Mono	Melanoma	Post(NR)	HDAC2	Macro_IFI27	3,68098E-01
Macro/Mono	Melanoma	Post(NR)	EWSR1	Macro_NLRP3	3,67988E-01
Macro/Mono	ccRCC	Post(R)	HMGB1	Macro_ISG15	3,67935E-01
Mast	CRC	Post(NR)	ATF3	Mast	3,67475E-01
DC	HNSCC	Post(NR)	BCLAF1	cDC_LAMP3	3,67429E-01
Macro/Mono	Melanoma	Post(NR)	PML	Macro_NLRP3	3,67254E-01
Macro/Mono	HNSCC	Post(R)	BCLAF1	Mono_CD16	3,66756E-01
Macro/Mono	BCC	Post(R)	MAX	Mono_INHBA	3,66697E-01
Macro/Mono	Melanoma	Post(NR)	PML	Macro_ISG15	3,66546E-01
Macro/Mono	Melanoma	Post(NR)	NELFE	Macro_IFI27	3,66506E-01
Macro/Mono	HNSCC	Post(R)	SPI1	Macro_IFI27	3,66327E-01
Macro/Mono	HNSCC	Post(R)	ATF3	Mono_INHBA	3,65992E-01
DC	BCC	Post(R)	REL	cDC(CD1C)	3,65985E-01
Macro/Mono	HNSCC	Post(R)	REL	Mono_CD16	3,65886E-01
Macro/Mono	HNSCC	Post(R)	SPI1	Mono_CD16	3,65884E-01
DC	ccRCC	Post(R)	BCLAF1	pDC_LILRA4	3,65831E-01
Macro/Mono	Melanoma	Post(NR)	PML	Macro_LYVE1	3,65663E-01
Macro/Mono	HNSCC	Post(NR)	SPI1	Macro_IFI27	3,65549E-01
Macro/Mono	Melanoma	Post(NR)	PML	Mono_INHBA	3,65435E-01
DC	HNSCC	Post(R)	ETS2	cDC(CD1C)	3,65342E-01
Macro/Mono	Melanoma	Post(NR)	HCFC1	Macro_ISG15	3,65284E-01
Macro/Mono	BCC	Post(R)	MAX	Macro_FOLR2-APOE+	3,65208E-01
DC	BCC	Post(R)	MAX	cDC(CD1C)	3,65190E-01
DC	Melanoma	Post(R)	EGR1	cDC(CD1C)	3,65111E-01
Macro/Mono	Melanoma	Post(NR)	HCFC1	Macro_FOLR2-APOE+	3,65048E-01
DC	ccRCC	Post(R)	REL	pDC_LILRA4	3,64887E-01
Macro/Mono	ccRCC	Post(NR)	NELFE	Macro_IFI27	3,64689E-01

Macro/Mono	CRC	Post(NR)	REL	Macro_FOLR2+APOE+	3,64673E-01
Macro/Mono	Melanoma	Post(NR)	HCFC1	Macro_LYVE1	3,64654E-01
Macro/Mono	BCC	Post(R)	MAX	Macro_IFI27	3,64542E-01
Macro/Mono	HNSCC	Post(R)	SPI1	Macro_LYVE1	3,64402E-01
Mast	ccRCC	Post(NR)	REL	Mast	3,64324E-01
Macro/Mono	Melanoma	Post(NR)	EWSR1	Macro_ISG15	3,64178E-01
DC	ccRCC	Post(NR)	MAFB	cDC_LAMP3	3,64158E-01
Macro/Mono	HNSCC	Post(R)	RELB	Macro_NLRP3	3,64037E-01
Macro/Mono	ccRCC	Post(NR)	ELF2	Macro_FOLR2-APOE+	3,63793E-01
Macro/Mono	BCC	Post(R)	EP300	Macro_LYVE1	3,63535E-01
Macro/Mono	CRC	Post(NR)	REL	Macro_OLFML3	3,63420E-01
Macro/Mono	HNSCC	Post(NR)	EGR1	Macro_ISG15	3,63378E-01
DC	Melanoma	Post(R)	HCFC1	cDC(CD1C)	3,63278E-01
Macro/Mono	ccRCC	Post(NR)	FOS	Macro_ISG15	3,63226E-01
Macro/Mono	Melanoma	Post(NR)	PML	Macro_IFI27	3,63198E-01
Macro/Mono	CRC	Post(R)	POLR2A	Macro_FOLR2+APOE-	3,63180E-01
DC	CRC	Post(NR)	REL	cDC_LAMP3	3,63121E-01
Macro/Mono	ccRCC	Post(NR)	ATF3	Macro_NLRP3	3,63063E-01
Macro/Mono	ccRCC	Post(NR)	JUN	Macro_FOLR2+APOE-	3,62895E-01
Macro/Mono	CRC	Post(NR)	POLR2A	Macro_IFI27	3,62693E-01
Macro/Mono	BCC	Post(R)	ENO1	Macro_OLFML3	3,62474E-01
DC	Melanoma	Post(NR)	REL	cDC_LAMP3	3,62457E-01
Macro/Mono	ccRCC	Post(NR)	ATF3	Macro_IER3	3,62430E-01
Macro/Mono	BCC	Post(R)	RELB	Macro_FOLR2+APOE-	3,62367E-01
DC	Melanoma	Post(R)	HCFC1	pDC_LILRA4	3,62325E-01
Macro/Mono	CRC	Post(NR)	RAD21	Macro_LYVE1	3,62036E-01
DC	ccRCC	Post(NR)	ELF2	cDC_CLEC9A	3,62025E-01
DC	HNSCC	Post(NR)	EGR1	cDC(CD1C)	3,61672E-01
Macro/Mono	Melanoma	Post(R)	EGR1	Mono_CD14	3,61598E-01
Macro/Mono	CRC	Post(R)	RAD21	Macro_NLRP3	3,61296E-01
Macro/Mono	Melanoma	Post(R)	ATF3	Macro_FOLR2-APOE+	3,61207E-01
Macro/Mono	HNSCC	Post(NR)	REL	Mono_INHBA	3,61056E-01
Macro/Mono	HNSCC	Post(NR)	SPI1	Mono_CD14	3,60981E-01
Macro/Mono	Melanoma	Post(R)	ELK4	Macro_NLRP3	3,60628E-01
Macro/Mono	HNSCC	Post(NR)	ETS2	Macro_NLRP3	3,60570E-01
Macro/Mono	HNSCC	Post(NR)	EGR1	Macro_FOLR2+APOE+	3,60562E-01
Macro/Mono	Melanoma	Post(NR)	EWSR1	Macro_IER3	3,60511E-01
Mast	ccRCC	Post(R)	ELF2	Mast	3,60469E-01
Macro/Mono	ccRCC	Post(NR)	REL	Mono_CD14	3,60404E-01
DC	ccRCC	Post(NR)	REL	cDC_CLEC9A	3,60029E-01
Macro/Mono	ccRCC	Post(NR)	NELFE	Macro_IER3	3,59664E-01
DC	BCC	Post(R)	EP300	pDC_LILRA4	3,59652E-01

Macro/Mono	HNSCC	Post(R)	REL	Macro_IER3	3,59496E-01
Macro/Mono	HNSCC	Post(R)	RELB	Mono_CD14	3,59418E-01
DC	HNSCC	Post(NR)	EGR1	cDC_CLEC9A	3,59416E-01
Macro/Mono	ccRCC	Post(R)	ATF3	Macro_OLFML3	3,59402E-01
DC	Melanoma	Post(R)	ATF3	cDC_CLEC9A	3,59210E-01
Macro/Mono	ccRCC	Post(R)	BCLAF1	Macro_OLFML3	3,59148E-01
DC	ccRCC	Post(NR)	SPI1	pDC_LILRA4	3,59144E-01
Macro/Mono	Melanoma	Post(NR)	HCFC1	Macro_FOLR2+APOE+	3,58838E-01
Macro/Mono	Melanoma	Post(NR)	PML	Macro_FOLR2-APOE+	3,58795E-01
Macro/Mono	ccRCC	Post(NR)	REL	Mono_CD16	3,58583E-01
Macro/Mono	BCC	Post(R)	MAX	Macro_FOLR2+APOE-	3,58559E-01
Macro/Mono	HNSCC	Post(NR)	ETS2	Macro_ISG15	3,58521E-01
Macro/Mono	Melanoma	Post(NR)	PML	Macro_FOLR2+APOE+	3,58419E-01
Macro/Mono	Melanoma	Post(NR)	EWSR1	Macro_FOLR2-APOE+	3,58183E-01
Macro/Mono	HNSCC	Post(NR)	SPI1	Macro_IER3	3,58063E-01
Macro/Mono	ccRCC	Post(NR)	EGR1	Macro_IER3	3,58053E-01
Macro/Mono	HNSCC	Post(NR)	SPI1	Macro_LYVE1	3,57679E-01
Macro/Mono	HNSCC	Post(R)	ETS2	Macro_OLFML3	3,57559E-01
Macro/Mono	CRC	Post(NR)	POLR2A	Macro_FOLR2+APOE+	3,57369E-01
Macro/Mono	CRC	Post(R)	REL	Macro_LYVE1	3,56837E-01
Macro/Mono	CRC	Post(NR)	REL	Mono_CD16	3,56828E-01
Macro/Mono	ccRCC	Post(NR)	NELFE	Macro_LYVE1	3,56717E-01
DC	CRC	Post(NR)	ATF3	cDC(CD1C)	3,56420E-01
DC	ccRCC	Post(R)	ATF3	pDC_LILRA4	3,56387E-01
Macro/Mono	ccRCC	Post(NR)	SPI1	Macro_FOLR2+APOE-	3,56291E-01
Macro/Mono	HNSCC	Post(NR)	BCLAF1	Mono_CD14	3,55494E-01
DC	BCC	Post(R)	EP300	cDC_LAMP3	3,55395E-01
Macro/Mono	ccRCC	Post(NR)	REL	Mono_INHBA	3,54970E-01
DC	CRC	Post(R)	ZMIZ1	cDC(CD1C)	3,54901E-01
Macro/Mono	HNSCC	Post(R)	REL	Mono_INHBA	3,54682E-01
Macro/Mono	CRC	Post(R)	REL	Macro_FOLR2-APOE+	3,54569E-01
Macro/Mono	BCC	Post(R)	ENO1	Macro_NLRP3	3,54549E-01
Macro/Mono	ccRCC	Post(NR)	NELFE	Macro_FOLR2-APOE+	3,54539E-01
Mast	HNSCC	Post(R)	ATF3	Mast	3,54510E-01
Macro/Mono	HNSCC	Post(R)	BCLAF1	Macro_IFI27	3,54337E-01
DC	Melanoma	Post(NR)	REL	cDC_CLEC9A	3,54156E-01
Mast	ccRCC	Post(R)	ETS2	Mast	3,54123E-01
Macro/Mono	BCC	Post(R)	REL	Macro_OLFML3	3,54117E-01
Macro/Mono	ccRCC	Post(NR)	REL	Macro_OLFML3	3,53947E-01
Macro/Mono	HNSCC	Post(R)	BCLAF1	Macro_IER3	3,53441E-01
DC	ccRCC	Post(NR)	JUNB	cDC(CD1C)	3,53379E-01
Macro/Mono	HNSCC	Post(R)	ETS2	Macro_FOLR2+APOE+	3,53357E-01

Mast	BCC	Post(R)	REL	Mast	3,53060E-01
Macro/Mono	HNSCC	Post(NR)	REL	Macro_OLFML3	3,52978E-01
Macro/Mono	ccRCC	Post(NR)	REL	Macro_FOLR2+APOE-	3,52892E-01
DC	BCC	Post(R)	NR3C1	cDC_CLEC9A	3,51998E-01
Macro/Mono	HNSCC	Post(R)	ETS2	Macro_LYVE1	3,51964E-01
Macro/Mono	ccRCC	Post(NR)	ELF2	Macro_OLFML3	3,51823E-01
Macro/Mono	CRC	Post(R)	REL	Mono_CD16	3,51708E-01
Macro/Mono	Melanoma	Post(NR)	HCFC1	Macro_IFI27	3,51564E-01
Macro/Mono	ccRCC	Post(NR)	JUNB	Mono_CD16	3,51393E-01
Macro/Mono	HNSCC	Post(NR)	SPI1	Mono_INHBA	3,51185E-01
DC	Melanoma	Post(NR)	REL	cDC(CD1C)	3,51074E-01
Macro/Mono	HNSCC	Post(NR)	ETS2	Macro_IFI27	3,50695E-01
Macro/Mono	ccRCC	Post(NR)	JUN	Macro_IFI27	3,50692E-01
Macro/Mono	ccRCC	Post(NR)	ATF3	Mono_CD14	3,50663E-01
DC	HNSCC	Post(NR)	BCLAF1	cDC(CD1C)	3,50659E-01
Macro/Mono	HNSCC	Post(R)	SPI1	Mono_CD14	3,50413E-01
Macro/Mono	CRC	Post(R)	POLR2A	Macro_IFI27	3,50292E-01
Mast	ccRCC	Post(NR)	FOS	Mast	3,50138E-01
Macro/Mono	Melanoma	Post(R)	ATF3	Macro_OLFML3	3,50110E-01
Macro/Mono	ccRCC	Post(NR)	REL	Macro_LYVE1	3,49970E-01
Macro/Mono	ccRCC	Post(NR)	NELFE	Macro_OLFML3	3,49811E-01
Macro/Mono	CRC	Post(NR)	ATF3	Macro_NLRP3	3,49741E-01
Macro/Mono	Melanoma	Post(R)	PPARG	Macro_IFI27	3,49654E-01
Macro/Mono	Melanoma	Post(R)	ATF3	Mono_CD14	3,49087E-01
DC	ccRCC	Post(NR)	ATF3	pDC_LILRA4	3,48451E-01
Macro/Mono	HNSCC	Post(NR)	SPI1	Macro_FOLR2+APOE-	3,48291E-01
Macro/Mono	HNSCC	Post(NR)	SPI1	Macro_NLRP3	3,48238E-01
Macro/Mono	BCC	Post(R)	MAX	Macro_OLFML3	3,48222E-01
Macro/Mono	Melanoma	Post(R)	EGR1	Macro_ISG15	3,48121E-01
Macro/Mono	ccRCC	Post(NR)	FOS	Macro_FOLR2-APOE+	3,48118E-01
DC	Melanoma	Post(R)	EGR1	pDC_LILRA4	3,47909E-01
DC	HNSCC	Post(R)	ETS2	pDC_LILRA4	3,47807E-01
Macro/Mono	Melanoma	Post(R)	EGR1	Macro_OLFML3	3,47496E-01
Macro/Mono	BCC	Post(R)	ENO1	Mono_INHBA	3,47471E-01
Macro/Mono	Melanoma	Post(NR)	REL	Macro_OLFML3	3,47451E-01
Macro/Mono	ccRCC	Post(R)	HMGB1	Macro_FOLR2+APOE+	3,47365E-01
Macro/Mono	ccRCC	Post(R)	FLI1	Macro_FOLR2+APOE-	3,47302E-01
DC	Melanoma	Post(NR)	EWSR1	cDC_LAMP3	3,47128E-01
Macro/Mono	HNSCC	Post(R)	RELB	Mono_CD16	3,46842E-01
Macro/Mono	CRC	Post(R)	REL	Macro_IFI27	3,46814E-01
Macro/Mono	ccRCC	Post(NR)	ATF3	Mono_INHBA	3,46492E-01
DC	ccRCC	Post(R)	SAP30	cDC(CD1C)	3,46432E-01

Mast	Melanoma	Post(NR)	ELF2	Mast	3,46355E-01
Macro/Mono	ccRCC	Post(NR)	REL	Macro_IFI27	3,46351E-01
Macro/Mono	ccRCC	Post(NR)	EGR1	Macro_NLRP3	3,46311E-01
DC	BCC	Post(R)	ENO1	cDC(CD1C)	3,46306E-01
Macro/Mono	HNSCC	Post(R)	KLF4	Macro_NLRP3	3,45971E-01
Macro/Mono	ccRCC	Post(R)	SPI1	Macro_OLFML3	3,45497E-01
DC	BCC	Post(NR)	IKZF1	cDC_CLEC9A	3,45243E-01
Macro/Mono	ccRCC	Post(NR)	REL	Macro_ISG15	3,45117E-01
DC	Melanoma	Post(NR)	REL	pDC_LILRA4	3,45033E-01
Macro/Mono	ccRCC	Post(NR)	MAFB	Macro_NLRP3	3,44898E-01
Macro/Mono	ccRCC	Post(NR)	EGR1	Mono_INHBA	3,44867E-01
Macro/Mono	ccRCC	Post(R)	POLR2A	Mono_CD16	3,44589E-01
Macro/Mono	ccRCC	Post(R)	ETV5	Macro_FOLR2+APOE+	3,44569E-01
Macro/Mono	CRC	Post(NR)	POLR2A	Macro_FOLR2-APOE+	3,44550E-01
Macro/Mono	HNSCC	Post(NR)	REL	Macro_LYVE1	3,44404E-01
Macro/Mono	CRC	Post(NR)	SPI1	Macro_LYVE1	3,44394E-01
Macro/Mono	ccRCC	Post(R)	POLR2A	Mono_CD14	3,44309E-01
Macro/Mono	BCC	Post(R)	EP300	Macro_FOLR2+APOE-	3,44027E-01
Mast	CRC	Post(R)	FOS	Mast	3,43944E-01
Macro/Mono	HNSCC	Post(NR)	BCLAF1	Mono_CD16	3,43804E-01
DC	HNSCC	Post(R)	RELB	cDC(CD1C)	3,43499E-01
DC	HNSCC	Post(NR)	SPI1	cDC_CLEC9A	3,43339E-01
DC	CRC	Post(NR)	POLR2A	pDC_LILRA4	3,43323E-01
DC	BCC	Post(R)	REL	cDC_CLEC9A	3,43312E-01
Macro/Mono	CRC	Post(NR)	ATF3	Mono_INHBA	3,43275E-01
Mast	Melanoma	Post(NR)	REL	Mast	3,43150E-01
DC	BCC	Post(NR)	IKZF1	cDC(CD1C)	3,43146E-01
Macro/Mono	HNSCC	Post(R)	BCLAF1	Macro_NLRP3	3,43141E-01
DC	HNSCC	Post(R)	RELB	cDC_CLEC9A	3,43111E-01
Macro/Mono	ccRCC	Post(NR)	EGR1	Mono_CD16	3,43091E-01
Macro/Mono	Melanoma	Post(NR)	REL	Mono_INHBA	3,42975E-01
Macro/Mono	Melanoma	Post(NR)	ELF2	Mono_CD16	3,42812E-01
Macro/Mono	BCC	Post(R)	ENO1	Mono_CD16	3,42580E-01
DC	BCC	Post(R)	REL	pDC_LILRA4	3,42514E-01
Macro/Mono	CRC	Post(NR)	REL	Macro_IFI27	3,42269E-01
Mast	Melanoma	Post(NR)	JUNB	Mast	3,41697E-01
DC	ccRCC	Post(R)	REL	cDC(CD1C)	3,41607E-01
DC	ccRCC	Post(NR)	EGR1	cDC_LAMP3	3,41515E-01
DC	HNSCC	Post(R)	ETS2	cDC_LAMP3	3,41495E-01
DC	Melanoma	Post(R)	GTF2F1	cDC(CD1C)	3,41100E-01
Macro/Mono	ccRCC	Post(NR)	NELFE	Macro_ISG15	3,40842E-01
Macro/Mono	ccRCC	Post(NR)	REL	Macro_FOLR2-APOE+	3,40736E-01

Macro/Mono	Melanoma	Post(NR)	ELF2	Macro_NLRP3	3,40727E-01
Macro/Mono	CRC	Post(NR)	SPI1	Mono_CD16	3,40683E-01
DC	CRC	Post(NR)	RAD21	cDC_LAMP3	3,40630E-01
Macro/Mono	HNSCC	Post(R)	ATF3	Macro_NLRP3	3,40455E-01
DC	ccRCC	Post(NR)	ATF3	cDC_LAMP3	3,39797E-01
Macro/Mono	HNSCC	Post(R)	ATF3	Macro_ISG15	3,39790E-01
Macro/Mono	HNSCC	Post(R)	SPI1	Macro_IER3	3,39692E-01
Macro/Mono	Melanoma	Post(NR)	ELF2	Macro_LYVE1	3,39677E-01
Macro/Mono	ccRCC	Post(NR)	EGR1	Macro_IFI27	3,39479E-01
DC	Melanoma	Post(NR)	GTF2F1	pDC_LILRA4	3,39477E-01
Macro/Mono	Melanoma	Post(R)	EGR1	Macro_FOLR2-APOE+	3,39398E-01
Macro/Mono	HNSCC	Post(R)	SPI1	Mono_INHBA	3,39395E-01
Macro/Mono	CRC	Post(NR)	REL	Macro_FOLR2+APOE-	3,39207E-01
DC	Melanoma	Post(NR)	GTF2F1	cDC_CLEC9A	3,39172E-01
Macro/Mono	Melanoma	Post(NR)	REL	Macro_NLRP3	3,39126E-01
Macro/Mono	BCC	Post(R)	REL	Macro_IER3	3,39124E-01
Macro/Mono	Melanoma	Post(NR)	REL	Macro_ISG15	3,39080E-01
Macro/Mono	BCC	Post(R)	REL	Macro_FOLR2-APOE+	3,38920E-01
Macro/Mono	ccRCC	Post(R)	REL	Macro_ISG15	3,38874E-01
Macro/Mono	HNSCC	Post(R)	SPI1	Macro_NLRP3	3,38573E-01
DC	CRC	Post(NR)	RAD21	cDC_CLEC9A	3,38447E-01
Macro/Mono	ccRCC	Post(NR)	JUNB	Mono_CD14	3,38226E-01
Macro/Mono	Melanoma	Post(R)	EGR1	Macro_IFI27	3,38032E-01
Macro/Mono	Melanoma	Post(NR)	REL	Mono_CD16	3,37987E-01
Macro/Mono	Melanoma	Post(NR)	ELF2	Macro_IER3	3,37931E-01
Macro/Mono	Melanoma	Post(NR)	REL	Macro_FOLR2+APOE+	3,37862E-01
Macro/Mono	Melanoma	Post(NR)	EWSR1	Mono_INHBA	3,37798E-01
Macro/Mono	HNSCC	Post(NR)	BCLAF1	Macro_IER3	3,37640E-01
Macro/Mono	BCC	Post(NR)	ATF3	Macro_IER3	3,37590E-01
Macro/Mono	Melanoma	Post(NR)	REL	Macro_FOLR2-APOE+	3,37487E-01
Macro/Mono	Melanoma	Post(R)	FOSB	Macro_NLRP3	3,37159E-01
DC	HNSCC	Post(R)	ETS2	cDC_CLEC9A	3,37037E-01
Macro/Mono	CRC	Post(R)	REL	Macro_FOLR2+APOE+	3,36515E-01
DC	Melanoma	Post(NR)	ELF2	cDC_LAMP3	3,36326E-01
Macro/Mono	CRC	Post(NR)	ATF3	Mono_CD14	3,36162E-01
Macro/Mono	Melanoma	Post(NR)	REL	Macro_IER3	3,35972E-01
Macro/Mono	Melanoma	Post(NR)	ELF2	Mono_INHBA	3,35960E-01
Macro/Mono	HNSCC	Post(R)	RELB	Mono_INHBA	3,35595E-01
DC	HNSCC	Post(R)	SPI1	cDC(CD1C)	3,35556E-01
DC	Melanoma	Post(NR)	GTF2F1	cDC_LAMP3	3,35410E-01
Macro/Mono	CRC	Post(R)	RAD21	Macro_FOLR2+APOE-	3,35404E-01
Macro/Mono	HNSCC	Post(NR)	ETS2	Mono_CD14	3,35162E-01

Macro/Mono	ccRCC	Post(NR)	ELF2	Macro_FOLR2+APOE+	3,35059E-01
Macro/Mono	BCC	Post(R)	REL	Mono_CD16	3,35029E-01
Macro/Mono	CRC	Post(NR)	MAX	Macro_LYVE1	3,34763E-01
Macro/Mono	HNSCC	Post(NR)	REL	Macro_FOLR2+APOE-	3,34632E-01
Mast	Melanoma	Post(NR)	GTF2F1	Mast	3,34580E-01
Macro/Mono	ccRCC	Post(R)	REL	Macro_IER3	3,34550E-01
Macro/Mono	HNSCC	Post(R)	SPI1	Macro_FOLR2+APOE-	3,34540E-01
Macro/Mono	HNSCC	Post(NR)	REL	Macro_FOLR2-APOE+	3,34454E-01
DC	BCC	Post(R)	MAX	cDC_LAMP3	3,34425E-01
Macro/Mono	BCC	Post(NR)	ATF3	Mono_INHBA	3,34402E-01
Macro/Mono	BCC	Post(NR)	IKZF1	Macro_IFI27	3,34104E-01
Macro/Mono	Melanoma	Post(R)	HCFC1	Mono_CD14	3,33621E-01
Macro/Mono	HNSCC	Post(NR)	BCLAF1	Macro_NLRP3	3,33451E-01
Mast	BCC	Post(R)	NR3C1	Mast	3,33307E-01
Macro/Mono	ccRCC	Post(NR)	FOS	Macro_LYVE1	3,33239E-01
Mast	HNSCC	Post(NR)	ETS2	Mast	3,33024E-01
Macro/Mono	Melanoma	Post(NR)	ELF2	Mono_CD14	3,32951E-01
Macro/Mono	Melanoma	Post(R)	GTF2F1	Macro_NLRP3	3,32695E-01
Macro/Mono	BCC	Post(R)	NR3C1	Mono_CD16	3,32598E-01
Macro/Mono	Melanoma	Post(NR)	REL	Mono_CD14	3,32437E-01
DC	ccRCC	Post(R)	HMGB1	pDC_LILRA4	3,32327E-01
Macro/Mono	BCC	Post(R)	CREB1	Macro_FOLR2+APOE+	3,32231E-01
Macro/Mono	ccRCC	Post(NR)	FOS	Macro_FOLR2+APOE+	3,32171E-01
Macro/Mono	Melanoma	Post(R)	GTF2F1	Mono_CD14	3,32003E-01
Macro/Mono	ccRCC	Post(NR)	ATF3	Mono_CD16	3,31961E-01
DC	Melanoma	Post(NR)	ELF2	cDC_CLEC9A	3,31953E-01
Macro/Mono	ccRCC	Post(R)	REL	Macro_FOLR2+APOE+	3,31792E-01
DC	Melanoma	Post(NR)	GTF2F1	cDC(CD1C)	3,31723E-01
Mast	BCC	Post(NR)	IKZF1	Mast	3,31707E-01
Macro/Mono	HNSCC	Post(R)	ATF3	Mono_CD14	3,31653E-01
DC	BCC	Post(NR)	IKZF1	pDC_LILRA4	3,31647E-01
Macro/Mono	Melanoma	Post(NR)	ELF2	Macro_ISG15	3,31639E-01
Macro/Mono	ccRCC	Post(NR)	REL	Macro_FOLR2+APOE+	3,31635E-01
Macro/Mono	BCC	Post(NR)	IKZF1	Mono_CD14	3,31383E-01
Macro/Mono	ccRCC	Post(R)	POLR2A	Macro_NLRP3	3,31332E-01
DC	BCC	Post(R)	CREB1	cDC_LAMP3	3,30750E-01
Macro/Mono	HNSCC	Post(R)	KLF4	Mono_CD14	3,30733E-01
Macro/Mono	Melanoma	Post(R)	HCFC1	Macro_IFI27	3,30570E-01
DC	ccRCC	Post(R)	REL	cDC_LAMP3	3,30457E-01
Macro/Mono	HNSCC	Post(NR)	REL	Macro_IFI27	3,30215E-01
Macro/Mono	HNSCC	Post(R)	RELB	Macro_IER3	3,30189E-01
Macro/Mono	CRC	Post(R)	ATF3	Macro_IFI27	3,29970E-01

Macro/Mono	HNSCC	Post(NR)	JUNB	Macro_IER3	3,29951E-01
DC	ccRCC	Post(NR)	JUNB	cDC_LAMP3	3,29878E-01
DC	Melanoma	Post(R)	GTF2F1	cDC_CLEC9A	3,29714E-01
Macro/Mono	ccRCC	Post(R)	ETS2	Mono_CD16	3,29647E-01
Macro/Mono	Melanoma	Post(R)	GTF2F1	Macro_OLFML3	3,29619E-01
Macro/Mono	HNSCC	Post(R)	KLF4	Mono_CD16	3,29566E-01
Macro/Mono	ccRCC	Post(NR)	NELFE	Macro_FOLR2+APOE+	3,29557E-01
Macro/Mono	Melanoma	Post(NR)	ELF2	Macro_FOLR2+APOE+	3,29519E-01
Macro/Mono	HNSCC	Post(R)	ATF3	Macro_FOLR2-APOE+	3,29427E-01
Macro/Mono	Melanoma	Post(NR)	REL	Macro_LYVE1	3,29282E-01
Macro/Mono	ccRCC	Post(R)	POLR2A	Mono_INHBA	3,29230E-01
Macro/Mono	ccRCC	Post(NR)	KLF4	Mono_CD14	3,29064E-01
DC	Melanoma	Post(NR)	ELF2	cDC(CD1C)	3,29060E-01
Macro/Mono	ccRCC	Post(R)	FOS	Macro_IER3	3,28986E-01
Macro/Mono	CRC	Post(R)	RAD21	Macro_IFI27	3,28949E-01
DC	ccRCC	Post(NR)	ATF3	cDC(CD1C)	3,28877E-01
DC	ccRCC	Post(NR)	GTF2F1	pDC_LILRA4	3,28787E-01
DC	BCC	Post(NR)	IKZF1	cDC_LAMP3	3,28645E-01
Mast	ccRCC	Post(NR)	CREB1	Mast	3,28644E-01
Mast	CRC	Post(NR)	SPI1	Mast	3,28624E-01
Macro/Mono	CRC	Post(NR)	REL	Macro_LYVE1	3,28215E-01
Macro/Mono	HNSCC	Post(NR)	BCLAF1	Macro_FOLR2+APOE-	3,27977E-01
Macro/Mono	Melanoma	Post(NR)	ELF2	Macro_OLFML3	3,27865E-01
Macro/Mono	Melanoma	Post(R)	ATF3	Macro_ISG15	3,27811E-01
Macro/Mono	Melanoma	Post(NR)	ELF2	Macro_FOLR2-APOE+	3,27506E-01
Macro/Mono	Melanoma	Post(NR)	ELF2	Macro_IFI27	3,27388E-01
Macro/Mono	BCC	Post(NR)	IKZF1	Macro_FOLR2-APOE+	3,27124E-01
Macro/Mono	BCC	Post(R)	ENO1	Macro_FOLR2-APOE+	3,27021E-01
Macro/Mono	BCC	Post(R)	CREB1	Mono_INHBA	3,26951E-01
Macro/Mono	BCC	Post(NR)	IKZF1	Macro_NLRP3	3,26905E-01
Macro/Mono	BCC	Post(NR)	IKZF1	Mono_CD16	3,26872E-01
DC	CRC	Post(R)	SPI1	cDC_LAMP3	3,26719E-01
Macro/Mono	HNSCC	Post(NR)	ETS2	Macro_FOLR2+APOE+	3,26636E-01
Macro/Mono	HNSCC	Post(R)	KLF4	Macro_IER3	3,26546E-01
DC	CRC	Post(NR)	SPI1	pDC_LILRA4	3,26479E-01
Macro/Mono	BCC	Post(R)	CREB1	Mono_CD16	3,26379E-01
Macro/Mono	CRC	Post(R)	RAD21	Macro_LYVE1	3,26280E-01
Mast	CRC	Post(R)	BCLAF1	Mast	3,26247E-01
Macro/Mono	HNSCC	Post(R)	BCLAF1	Macro_FOLR2-APOE+	3,25835E-01
Macro/Mono	BCC	Post(NR)	NFE2L2	Macro_IER3	3,25767E-01
Macro/Mono	BCC	Post(R)	REL	Macro_LYVE1	3,25524E-01
Macro/Mono	CRC	Post(R)	RAD21	Macro_FOLR2-APOE+	3,25293E-01

DC	ccRCC	Post(R)	REL	cDC_CLEC9A	3,25034E-01
Macro/Mono	BCC	Post(R)	NR3C1	Macro_IER3	3,24951E-01
Macro/Mono	HNSCC	Post(R)	BCLAF1	Macro_LYVE1	3,24535E-01
Macro/Mono	HNSCC	Post(R)	ATF3	Mono_CD16	3,24359E-01
Macro/Mono	BCC	Post(NR)	ATF3	Macro_NLRP3	3,24259E-01
Macro/Mono	Melanoma	Post(NR)	TBL1XR1	Macro_FOLR2-APOE+	3,24149E-01
Macro/Mono	BCC	Post(R)	REL	Macro_ISG15	3,24138E-01
Macro/Mono	BCC	Post(R)	NR3C1	Mono_CD14	3,23785E-01
Macro/Mono	BCC	Post(R)	REL	Macro_NLRP3	3,23590E-01
Macro/Mono	CRC	Post(NR)	BCLAF1	Mono_CD16	3,23472E-01
Macro/Mono	HNSCC	Post(R)	REL	Macro_FOLR2-APOE+	3,23320E-01
Macro/Mono	HNSCC	Post(R)	REL	Macro_OLFML3	3,23297E-01
Macro/Mono	BCC	Post(NR)	IKZF1	Macro_OLFML3	3,23097E-01
Macro/Mono	BCC	Post(R)	REL	Mono_CD14	3,23093E-01
Macro/Mono	Melanoma	Post(NR)	GTF2F1	Mono_CD16	3,22970E-01
Macro/Mono	Melanoma	Post(NR)	CREB1	Mono_CD16	3,22912E-01
DC	BCC	Post(R)	NR3C1	cDC(CD1C)	3,22866E-01
DC	Melanoma	Post(NR)	ELF2	pDC_LILRA4	3,22617E-01
DC	Melanoma	Post(NR)	TBL1XR1	pDC_LILRA4	3,22397E-01
Macro/Mono	Melanoma	Post(NR)	GTF2F1	Macro_OLFML3	3,22215E-01
Macro/Mono	ccRCC	Post(R)	REL	Macro_NLRP3	3,22077E-01
Macro/Mono	HNSCC	Post(NR)	ETS2	Macro_IER3	3,22035E-01
DC	ccRCC	Post(NR)	JUN	cDC_CLEC9A	3,21446E-01
Macro/Mono	Melanoma	Post(NR)	TBL1XR1	Mono_INHBA	3,21445E-01
Macro/Mono	ccRCC	Post(R)	FLI1	Mono_CD16	3,21216E-01
Macro/Mono	ccRCC	Post(R)	ETS2	Mono_CD14	3,20988E-01
DC	HNSCC	Post(R)	ATF3	cDC_LAMP3	3,20627E-01
Macro/Mono	BCC	Post(NR)	IKZF1	Macro_LYVE1	3,20548E-01
Macro/Mono	HNSCC	Post(R)	BCLAF1	Macro_OLFML3	3,20488E-01
Macro/Mono	HNSCC	Post(R)	EGR1	Mono_CD14	3,20367E-01
Macro/Mono	Melanoma	Post(NR)	TBL1XR1	Macro_NLRP3	3,20113E-01
DC	CRC	Post(NR)	ETV3	cDC_LAMP3	3,20066E-01
DC	BCC	Post(R)	CREB1	cDC_CLEC9A	3,20048E-01
DC	CRC	Post(R)	POLR2A	cDC(CD1C)	3,20009E-01
DC	ccRCC	Post(NR)	GTF2F1	cDC_CLEC9A	3,19906E-01
Macro/Mono	HNSCC	Post(NR)	ETS2	Macro_FOLR2-APOE+	3,19890E-01
DC	ccRCC	Post(NR)	ATF3	cDC_CLEC9A	3,19803E-01
Macro/Mono	ccRCC	Post(R)	ATF3	Macro_ISG15	3,19745E-01
Macro/Mono	HNSCC	Post(NR)	ETS2	Macro_LYVE1	3,19530E-01
Macro/Mono	ccRCC	Post(NR)	JUNB	Mono_INHBA	3,19524E-01
Macro/Mono	ccRCC	Post(NR)	GTF2F1	Mono_CD14	3,19475E-01
DC	Melanoma	Post(NR)	CREB1	cDC_CLEC9A	3,19426E-01

Mast	HNSCC	Post(NR)	RELB	Mast	3,19350E-01
DC	BCC	Post(NR)	SPI1	cDC(CD1C)	3,19305E-01
Macro/Mono	CRC	Post(NR)	RELB	Macro_IER3	3,19191E-01
Macro/Mono	BCC	Post(R)	CREB1	Macro_IFI27	3,18969E-01
Macro/Mono	BCC	Post(R)	CREB1	Macro_ISG15	3,18849E-01
Macro/Mono	Melanoma	Post(R)	MAZ	Macro_NLRP3	3,18781E-01
Macro/Mono	HNSCC	Post(R)	ATF3	Macro_OLFML3	3,18768E-01
Macro/Mono	CRC	Post(NR)	ATF3	Macro_ISG15	3,18701E-01
DC	CRC	Post(NR)	RAD21	pDC_LILRA4	3,18446E-01
Mast	Melanoma	Post(NR)	EWSR1	Mast	3,18443E-01
Mast	ccRCC	Post(R)	MYC	Mast	3,18408E-01
Macro/Mono	HNSCC	Post(R)	EGR1	Macro_FOLR2+APOE-	3,18365E-01
Macro/Mono	CRC	Post(NR)	BCLAF1	Macro_LYVE1	3,18248E-01
Macro/Mono	BCC	Post(NR)	IKZF1	Macro_IER3	3,18146E-01
Macro/Mono	HNSCC	Post(NR)	REL	Macro_ISG15	3,18101E-01
DC	Melanoma	Post(NR)	ELK4	pDC_LILRA4	3,18016E-01
Macro/Mono	BCC	Post(R)	NR3C1	Macro_NLRP3	3,17958E-01
Macro/Mono	BCC	Post(NR)	IKZF1	Macro_ISG15	3,17912E-01
Macro/Mono	Melanoma	Post(R)	GTF2F1	Macro_ISG15	3,17769E-01
Mast	ccRCC	Post(R)	REL	Mast	3,17756E-01
Macro/Mono	BCC	Post(R)	REL	Macro_IFI27	3,17752E-01
Macro/Mono	HNSCC	Post(R)	REL	Macro_ISG15	3,17258E-01
Macro/Mono	BCC	Post(R)	CREB1	Mono_CD14	3,17199E-01
Macro/Mono	BCC	Post(R)	NR3C1	Macro_FOLR2-APOE+	3,17176E-01
DC	Melanoma	Post(NR)	CREB1	pDC_LILRA4	3,17150E-01
Macro/Mono	ccRCC	Post(R)	REL	Mono_CD16	3,17143E-01
Macro/Mono	ccRCC	Post(R)	POLR2A	Macro_LYVE1	3,17114E-01
Macro/Mono	ccRCC	Post(R)	SPI1	Macro_FOLR2-APOE+	3,17064E-01
DC	Melanoma	Post(R)	GTF2F1	pDC_LILRA4	3,16939E-01
Macro/Mono	CRC	Post(NR)	RELB	Mono_INHBA	3,16553E-01
DC	Melanoma	Post(NR)	CREB1	cDC(CD1C)	3,16384E-01
Macro/Mono	BCC	Post(R)	NR3C1	Macro_ISG15	3,16332E-01
Macro/Mono	Melanoma	Post(NR)	TBL1XR1	Mono_CD14	3,16236E-01
Macro/Mono	BCC	Post(R)	ENO1	Macro_IER3	3,16179E-01
Macro/Mono	Melanoma	Post(R)	HCFC1	Macro_ISG15	3,16160E-01
Macro/Mono	HNSCC	Post(R)	REL	Macro_FOLR2+APOE-	3,16130E-01
Macro/Mono	BCC	Post(R)	REL	Macro_FOLR2+APOE+	3,15838E-01
Macro/Mono	HNSCC	Post(R)	EGR1	Mono_INHBA	3,15562E-01
Macro/Mono	HNSCC	Post(R)	REL	Macro_IFI27	3,15306E-01
Macro/Mono	Melanoma	Post(NR)	GTF2F1	Macro_IER3	3,15255E-01
Macro/Mono	CRC	Post(NR)	ATF3	Macro_FOLR2-APOE+	3,15126E-01
Mast	CRC	Post(NR)	JUNB	Mast	3,15036E-01

Macro/Mono	ccRCC	Post(R)	SAP30	Macro_IER3	3,15017E-01
Macro/Mono	Melanoma	Post(NR)	TBL1XR1	Mono_CD16	3,14949E-01
Macro/Mono	ccRCC	Post(R)	REL	Mono_CD14	3,14727E-01
Macro/Mono	ccRCC	Post(NR)	ATF3	Macro_LYVE1	3,14606E-01
Macro/Mono	BCC	Post(NR)	IKZF1	Macro_FOLR2+APOE+	3,14535E-01
DC	CRC	Post(R)	BCLAF1	cDC_LAMP3	3,14532E-01
Macro/Mono	HNSCC	Post(NR)	BCLAF1	Macro_IFI27	3,14401E-01
Macro/Mono	BCC	Post(NR)	ATF3	Macro_LYVE1	3,14391E-01
DC	Melanoma	Post(NR)	TBL1XR1	cDC_LAMP3	3,14357E-01
Mast	Melanoma	Post(NR)	CREB1	Mast	3,14321E-01
Macro/Mono	Melanoma	Post(NR)	CREB1	Mono_CD14	3,14279E-01
Macro/Mono	Melanoma	Post(NR)	GTF2F1	Mono_CD14	3,14155E-01
Macro/Mono	Melanoma	Post(NR)	TBL1XR1	Macro_OLFML3	3,14090E-01
Macro/Mono	BCC	Post(R)	NR3C1	Macro_FOLR2+APOE+	3,14065E-01
Macro/Mono	Melanoma	Post(R)	ATF3	Macro_IFI27	3,13982E-01
DC	Melanoma	Post(NR)	CREB1	cDC_LAMP3	3,13891E-01
Macro/Mono	CRC	Post(NR)	ATF3	Macro_OLFML3	3,13875E-01
Macro/Mono	Melanoma	Post(NR)	TBL1XR1	Macro_ISG15	3,13782E-01
Macro/Mono	Melanoma	Post(NR)	GTF2F1	Macro_ISG15	3,13580E-01
Macro/Mono	Melanoma	Post(NR)	TBL1XR1	Macro_IER3	3,13541E-01
DC	Melanoma	Post(NR)	TBL1XR1	cDC_CLEC9A	3,13453E-01
Macro/Mono	HNSCC	Post(NR)	REL	Macro_FOLR2+APOE+	3,13226E-01
Macro/Mono	HNSCC	Post(R)	EGR1	Macro_IER3	3,13187E-01
DC	Melanoma	Post(NR)	ELK4	cDC_CLEC9A	3,13109E-01
Macro/Mono	ccRCC	Post(R)	ETS2	Macro_FOLR2+APOE-	3,13089E-01
Macro/Mono	Melanoma	Post(NR)	REL	Macro_IFI27	3,13082E-01
DC	Melanoma	Post(NR)	TBL1XR1	cDC(CD1C)	3,12955E-01
Macro/Mono	ccRCC	Post(NR)	KLF4	Mono_CD16	3,12953E-01
Macro/Mono	Melanoma	Post(NR)	EWSR1	Macro_LYVE1	3,12847E-01
DC	HNSCC	Post(NR)	RELB	pDC_LILRA4	3,12790E-01
Macro/Mono	BCC	Post(R)	REL	Mono_INHBA	3,12736E-01
Macro/Mono	BCC	Post(NR)	ATF3	Macro_FOLR2-APOE+	3,12535E-01
Macro/Mono	HNSCC	Post(R)	ATF3	Macro_FOLR2+APOE+	3,12504E-01
DC	BCC	Post(R)	NR3C1	cDC_LAMP3	3,12367E-01
Macro/Mono	BCC	Post(R)	NR3C1	Macro_LYVE1	3,12336E-01
Macro/Mono	ccRCC	Post(NR)	GTF2F1	Mono_CD16	3,12329E-01
Macro/Mono	ccRCC	Post(NR)	KLF4	Mono_INHBA	3,12260E-01
Macro/Mono	CRC	Post(NR)	REL	Macro_FOLR2-APOE+	3,12219E-01
Macro/Mono	BCC	Post(R)	CREB1	Macro_FOLR2+APOE-	3,11969E-01
Macro/Mono	ccRCC	Post(NR)	EGR1	Macro_ISG15	3,11834E-01
DC	HNSCC	Post(R)	ATF3	pDC_LILRA4	3,11719E-01
Macro/Mono	BCC	Post(R)	CREB1	Macro_OLFML3	3,11714E-01

Macro/Mono	Melanoma	Post(NR)	TBL1XR1	Macro_FOLR2+APOE+	3,11695E-01
Macro/Mono	BCC	Post(NR)	NFE2L2	Mono_INHBA	3,11601E-01
Macro/Mono	BCC	Post(R)	NR3C1	Macro_OLFML3	3,11458E-01
Macro/Mono	CRC	Post(NR)	MAX	Mono_CD16	3,11044E-01
Macro/Mono	CRC	Post(NR)	JUNB	Macro_IER3	3,10907E-01
Macro/Mono	ccRCC	Post(NR)	GTF2F1	Macro_FOLR2+APOE-	3,10605E-01
Macro/Mono	HNSCC	Post(NR)	ETS2	Macro_FOLR2+APOE-	3,10598E-01
Macro/Mono	Melanoma	Post(NR)	CREB1	Macro_IER3	3,10557E-01
Macro/Mono	ccRCC	Post(NR)	ATF3	Macro_FOLR2-APOE+	3,10402E-01
Macro/Mono	Melanoma	Post(NR)	GTF2F1	Macro_NLRP3	3,10379E-01
DC	ccRCC	Post(NR)	FOS	cDC_CLEC9A	3,10147E-01
Macro/Mono	Melanoma	Post(NR)	GTF2F1	Mono_INHBA	3,10101E-01
DC	Melanoma	Post(R)	FOS	pDC_LILRA4	3,10084E-01
Macro/Mono	Melanoma	Post(R)	HES1	Mono_INHBA	3,09939E-01
Macro/Mono	CRC	Post(R)	CEBPB	Macro_ISG15	3,09867E-01
Macro/Mono	BCC	Post(R)	CREB1	Macro_LYVE1	3,09752E-01
DC	Melanoma	Post(R)	ELK4	cDC(CD1C)	3,09623E-01
Macro/Mono	ccRCC	Post(NR)	FOXN3	Macro_LYVE1	3,09583E-01
Macro/Mono	Melanoma	Post(NR)	ELK4	Mono_CD16	3,09404E-01
Mast	Melanoma	Post(NR)	MAZ	Mast	3,09316E-01
DC	CRC	Post(R)	ATF3	cDC_CLEC9A	3,09312E-01
Macro/Mono	CRC	Post(R)	CEBPB	Macro_NLRP3	3,09298E-01
DC	CRC	Post(NR)	POLR2A	cDC(CD1C)	3,09145E-01
DC	BCC	Post(R)	CREB1	cDC(CD1C)	3,09037E-01
Macro/Mono	ccRCC	Post(NR)	ATF3	Macro_OLFML3	3,09019E-01
Macro/Mono	BCC	Post(R)	NR3C1	Macro_IFI27	3,09001E-01
DC	CRC	Post(NR)	RELB	cDC_CLEC9A	3,08988E-01
Macro/Mono	ccRCC	Post(R)	SPI1	Macro_IFI27	3,08979E-01
Macro/Mono	Melanoma	Post(NR)	CREB1	Macro_NLRP3	3,08877E-01
Macro/Mono	BCC	Post(R)	CREB1	Macro_NLRP3	3,08783E-01
Macro/Mono	BCC	Post(R)	CREB1	Macro_IER3	3,08763E-01
Macro/Mono	Melanoma	Post(R)	SUPT20H	Mono_INHBA	3,08395E-01
Macro/Mono	ccRCC	Post(NR)	ATF3	Macro_ISG15	3,08358E-01
Macro/Mono	CRC	Post(NR)	ATF3	Macro_FOLR2+APOE+	3,08331E-01
Macro/Mono	Melanoma	Post(NR)	CREB1	Mono_INHBA	3,07964E-01
DC	Melanoma	Post(R)	ELK4	cDC_CLEC9A	3,07929E-01
DC	CRC	Post(NR)	BCLAF1	cDC_CLEC9A	3,07790E-01
Macro/Mono	Melanoma	Post(NR)	CREB1	Macro_OLFML3	3,07601E-01
Macro/Mono	ccRCC	Post(NR)	ATF3	Macro_FOLR2+APOE-	3,07554E-01
Mast	ccRCC	Post(R)	EGR1	Mast	3,07076E-01
Macro/Mono	CRC	Post(NR)	RAD21	Macro_IER3	3,06832E-01
DC	CRC	Post(NR)	RELB	cDC_LAMP3	3,06818E-01

DC	Melanoma	Post(NR)	ELK4	cDC(CD1C)	3,06699E-01
Macro/Mono	Melanoma	Post(NR)	GTF2F1	Macro_FOLR2+APOE+	3,06639E-01
DC	Melanoma	Post(NR)	ELK4	cDC_LAMP3	3,06585E-01
Macro/Mono	ccRCC	Post(NR)	EGR1	Macro_FOLR2-APOE+	3,06575E-01
Macro/Mono	ccRCC	Post(NR)	KLF6	Macro_IER3	3,06529E-01
DC	BCC	Post(R)	GTF2F1	cDC_CLEC9A	3,06516E-01
Macro/Mono	HNSCC	Post(R)	ATF3	Macro_FOLR2+APOE-	3,06302E-01
Mast	CRC	Post(NR)	BCLAF1	Mast	3,06211E-01
Macro/Mono	Melanoma	Post(NR)	GTF2F1	Macro_LYVE1	3,05632E-01
Macro/Mono	ccRCC	Post(R)	ATF3	Macro_FOLR2+APOE+	3,05481E-01
Macro/Mono	HNSCC	Post(R)	RELB	Macro_ISG15	3,05294E-01
Macro/Mono	BCC	Post(NR)	ATF3	Mono_CD14	3,05118E-01
Macro/Mono	ccRCC	Post(R)	SPI1	Macro_FOLR2+APOE+	3,04844E-01
DC	CRC	Post(NR)	ATF3	pDC_LILRA4	3,04747E-01
Macro/Mono	ccRCC	Post(R)	FLI1	Mono_CD14	3,04570E-01
Macro/Mono	ccRCC	Post(R)	ETS2	Macro_NLRP3	3,04478E-01
Macro/Mono	BCC	Post(NR)	ATF3	Mono_CD16	3,04246E-01
Macro/Mono	HNSCC	Post(NR)	ZMIZ1	Mono_INHBA	3,04192E-01
Macro/Mono	Melanoma	Post(NR)	TBL1XR1	Macro_IFI27	3,04182E-01
Macro/Mono	CRC	Post(R)	FOS	Macro_IER3	3,04182E-01
DC	ccRCC	Post(NR)	JUNB	pDC_LILRA4	3,04119E-01
Macro/Mono	CRC	Post(NR)	ETV3	Mono_CD16	3,04104E-01
DC	HNSCC	Post(R)	ATF3	cDC(CD1C)	3,04073E-01
DC	CRC	Post(R)	BCLAF1	pDC_LILRA4	3,04029E-01
Macro/Mono	HNSCC	Post(R)	ATF3	Macro_LYVE1	3,03991E-01
Macro/Mono	HNSCC	Post(R)	BCLAF1	Mono_INHBA	3,03978E-01
Macro/Mono	Melanoma	Post(NR)	GTF2F1	Macro_FOLR2-APOE+	3,03955E-01
Macro/Mono	Melanoma	Post(NR)	TBL1XR1	Macro_LYVE1	3,03898E-01
DC	ccRCC	Post(NR)	REST	pDC_LILRA4	3,03687E-01
Mast	Melanoma	Post(NR)	ELK4	Mast	3,03506E-01
Macro/Mono	HNSCC	Post(R)	RELB	Macro_FOLR2-APOE+	3,03370E-01
DC	ccRCC	Post(R)	POLR2A	cDC_CLEC9A	3,03299E-01
Macro/Mono	Melanoma	Post(R)	GTF2F1	Macro_IFI27	3,03279E-01
Macro/Mono	Melanoma	Post(R)	FOS	Macro_NLRP3	3,03278E-01
Macro/Mono	HNSCC	Post(R)	ATF3	Macro_IFI27	3,03166E-01
Macro/Mono	HNSCC	Post(R)	RELB	Macro_OLFML3	3,03164E-01
Macro/Mono	ccRCC	Post(NR)	JUNB	Macro_OLFML3	3,03154E-01
Macro/Mono	ccRCC	Post(NR)	JUNB	Macro_ISG15	3,03147E-01
Mast	HNSCC	Post(R)	JUN	Mast	3,03110E-01
Macro/Mono	HNSCC	Post(NR)	BCLAF1	Macro_LYVE1	3,02984E-01
Macro/Mono	BCC	Post(R)	ENO1	Macro_IFI27	3,02980E-01
Macro/Mono	HNSCC	Post(NR)	ETS2	Macro_OLFML3	3,02824E-01

Macro/Mono	BCC	Post(NR)	ELK1	Mono_INHBA	3,02735E-01
Macro/Mono	BCC	Post(NR)	SPI1	Macro_ISG15	3,02597E-01
Macro/Mono	BCC	Post(NR)	SPI1	Macro_FOLR2-APOE+	3,02361E-01
Macro/Mono	CRC	Post(R)	BCLAF1	Mono_CD14	3,02060E-01
Macro/Mono	HNSCC	Post(R)	REL	Macro_LYVE1	3,02008E-01
Macro/Mono	BCC	Post(R)	CREB1	Macro_FOLR2-APOE+	3,01950E-01
Macro/Mono	ccRCC	Post(R)	REL	Mono_INHBA	3,01937E-01
DC	Melanoma	Post(R)	FOSB	pDC_LILRA4	3,01877E-01
Macro/Mono	Melanoma	Post(NR)	JUNB	Macro_IER3	3,01629E-01
Macro/Mono	CRC	Post(NR)	RELB	Macro_NLRP3	3,01308E-01
Mast	ccRCC	Post(NR)	PML	Mast	3,01243E-01
Macro/Mono	Melanoma	Post(R)	ELK4	Mono_CD14	3,01147E-01
Macro/Mono	Melanoma	Post(NR)	ELK4	Macro_NLRP3	3,01087E-01
Macro/Mono	Melanoma	Post(NR)	FLI1	Mono_CD14	3,01062E-01
DC	ccRCC	Post(NR)	EGR1	cDC(CD1C)	3,00927E-01
Macro/Mono	Melanoma	Post(NR)	FLI1	Macro_NLRP3	3,00899E-01
DC	CRC	Post(R)	POLR2A	cDC_CLEC9A	3,00898E-01
DC	BCC	Post(R)	CREB1	pDC_LILRA4	3,00888E-01
Macro/Mono	HNSCC	Post(R)	KLF4	Mono_INHBA	3,00819E-01
Macro/Mono	BCC	Post(NR)	SPI1	Macro_NLRP3	3,00752E-01
Macro/Mono	Melanoma	Post(NR)	CREB1	Macro_ISG15	3,00683E-01
DC	CRC	Post(R)	ZMIZ1	cDC_CLEC9A	3,00543E-01
Mast	ccRCC	Post(NR)	MYC	Mast	3,00369E-01
Macro/Mono	ccRCC	Post(R)	REL	Macro_OLFML3	3,00317E-01
Macro/Mono	CRC	Post(R)	RAD21	Macro_IER3	3,00314E-01
Macro/Mono	CRC	Post(R)	BCLAF1	Mono_CD16	3,00215E-01
Macro/Mono	BCC	Post(NR)	SPI1	Macro_IER3	3,00085E-01
DC	ccRCC	Post(NR)	GTF2F1	cDC_LAMP3	2,99926E-01
Macro/Mono	CRC	Post(NR)	JUNB	Macro_NLRP3	2,99508E-01
Macro/Mono	Melanoma	Post(NR)	ELK4	Macro_FOLR2-APOE+	2,99412E-01
Macro/Mono	HNSCC	Post(R)	CEBPB	Mono_INHBA	2,99410E-01
Macro/Mono	ccRCC	Post(NR)	MAFB	Mono_CD16	2,99213E-01
Macro/Mono	BCC	Post(NR)	NFE2L2	Macro_FOLR2-APOE+	2,99088E-01
Macro/Mono	ccRCC	Post(NR)	FOXN3	Macro_FOLR2+APOE-	2,98963E-01
Macro/Mono	BCC	Post(R)	NR3C1	Mono_INHBA	2,98705E-01
Macro/Mono	HNSCC	Post(NR)	RELB	Macro_NLRP3	2,98243E-01
Macro/Mono	ccRCC	Post(NR)	ATF3	Macro_IFI27	2,98088E-01
Macro/Mono	HNSCC	Post(R)	EGR1	Macro_ISG15	2,98073E-01
Macro/Mono	Melanoma	Post(NR)	ELK4	Mono_CD14	2,97969E-01
DC	BCC	Post(NR)	NFKB2	cDC_LAMP3	2,97949E-01
Mast	Melanoma	Post(NR)	TBL1XR1	Mast	2,97896E-01
Macro/Mono	Melanoma	Post(NR)	FLI1	Macro_IER3	2,97893E-01

Macro/Mono	ccRCC	Post(NR)	ATF3	Macro_FOLR2+APOE+	2,97809E-01
Macro/Mono	HNSCC	Post(R)	BCLAF1	Macro_FOLR2+APOE+	2,97724E-01
Macro/Mono	CRC	Post(NR)	RAD21	Macro_IFI27	2,97693E-01
Macro/Mono	Melanoma	Post(NR)	ELK4	Macro_OLFML3	2,97165E-01
DC	CRC	Post(NR)	RELB	cDC(CD1C)	2,96658E-01
DC	CRC	Post(NR)	ETV3	cDC_CLEC9A	2,96635E-01
Macro/Mono	Melanoma	Post(NR)	ELK4	Mono_INHBA	2,96607E-01
DC	Melanoma	Post(R)	ELK4	pDC_LILRA4	2,96469E-01
Macro/Mono	HNSCC	Post(NR)	ETS2	Mono_CD16	2,96426E-01
Macro/Mono	HNSCC	Post(R)	RELB	Macro_IFI27	2,96232E-01
Macro/Mono	Melanoma	Post(NR)	ELK4	Macro_ISG15	2,96192E-01
Macro/Mono	BCC	Post(NR)	IKZF1	Macro_FOLR2+APOE-	2,96147E-01
Macro/Mono	BCC	Post(NR)	ATF3	Macro_ISG15	2,95858E-01
Macro/Mono	Melanoma	Post(NR)	ELK4	Macro_IER3	2,95542E-01
Macro/Mono	HNSCC	Post(NR)	RELB	Mono_CD14	2,95488E-01
Macro/Mono	HNSCC	Post(R)	RELB	Macro_FOLR2+APOE-	2,95475E-01
Macro/Mono	BCC	Post(NR)	IKZF1	Mono_INHBA	2,95455E-01
DC	BCC	Post(NR)	IRF4	pDC_LILRA4	2,95436E-01
Macro/Mono	Melanoma	Post(R)	FOS	Macro_OLFML3	2,95398E-01
Macro/Mono	Melanoma	Post(R)	ELK4	Macro_OLFML3	2,95375E-01
Macro/Mono	ccRCC	Post(R)	ETS2	Mono_INHBA	2,95173E-01
Macro/Mono	Melanoma	Post(NR)	JUNB	Macro_NLRP3	2,95100E-01
Macro/Mono	ccRCC	Post(NR)	GTF2F1	Macro_NLRP3	2,94990E-01
Macro/Mono	Melanoma	Post(NR)	FLI1	Mono_INHBA	2,94614E-01
Macro/Mono	Melanoma	Post(NR)	CREB1	Macro_LYVE1	2,94465E-01
DC	Melanoma	Post(R)	FOSB	cDC(CD1C)	2,94416E-01
DC	ccRCC	Post(NR)	KLF4	cDC(CD1C)	2,94392E-01
Macro/Mono	Melanoma	Post(NR)	FLI1	Mono_CD16	2,94181E-01
Macro/Mono	Melanoma	Post(NR)	ELK4	Macro_LYVE1	2,94108E-01
Macro/Mono	CRC	Post(R)	CEBPB	Mono_CD14	2,94018E-01
DC	BCC	Post(R)	TCF4	pDC_LILRA4	2,93954E-01
Macro/Mono	BCC	Post(R)	ENO1	Macro_LYVE1	2,93832E-01
Macro/Mono	CRC	Post(NR)	RAD21	Macro_FOLR2+APOE-	2,93809E-01
Macro/Mono	Melanoma	Post(NR)	GTF2F1	Macro_IFI27	2,93708E-01
Macro/Mono	BCC	Post(NR)	SPI1	Mono_INHBA	2,93604E-01
Macro/Mono	Melanoma	Post(R)	FOS	Macro_FOLR2-APOE+	2,93373E-01
DC	HNSCC	Post(NR)	JUNB	pDC_LILRA4	2,93330E-01
Macro/Mono	Melanoma	Post(NR)	EWSR1	Macro_IFI27	2,93045E-01
Macro/Mono	CRC	Post(NR)	RELB	Mono_CD16	2,92959E-01
Macro/Mono	Melanoma	Post(NR)	ELK4	Macro_IFI27	2,92928E-01
Macro/Mono	Melanoma	Post(R)	FOS	Macro_ISG15	2,92925E-01
Macro/Mono	HNSCC	Post(NR)	BCLAF1	Mono_INHBA	2,92785E-01

Macro/Mono	Melanoma	Post(NR)	CREB1	Macro_FOLR2-APOE+	2,92709E-01
Macro/Mono	CRC	Post(NR)	BCL3	Macro_NLRP3	2,92551E-01
Macro/Mono	HNSCC	Post(R)	EGR1	Macro_NLRP3	2,92492E-01
Macro/Mono	BCC	Post(NR)	ATF3	Macro_IFI27	2,92296E-01
Macro/Mono	Melanoma	Post(R)	FOSB	Mono_INHBA	2,92204E-01
DC	HNSCC	Post(NR)	RELB	cDC_CLEC9A	2,92172E-01
DC	Melanoma	Post(R)	FOS	cDC(CD1C)	2,92137E-01
Macro/Mono	ccRCC	Post(NR)	GTF2F1	Mono_INHBA	2,91563E-01
Macro/Mono	ccRCC	Post(R)	REL	Macro_FOLR2-APOE+	2,91498E-01
DC	BCC	Post(R)	NFE2L2	pDC_LILRA4	2,91388E-01
DC	ccRCC	Post(R)	POLR2A	cDC_LAMP3	2,91246E-01
Macro/Mono	CRC	Post(R)	AKR1A1	Macro_FOLR2+APOE+	2,91186E-01
Macro/Mono	ccRCC	Post(R)	BCLAF1	Macro_ISG15	2,91068E-01
DC	Melanoma	Post(R)	CREB5	pDC_LILRA4	2,91063E-01
DC	HNSCC	Post(R)	ATF3	cDC_CLEC9A	2,90957E-01
Macro/Mono	HNSCC	Post(R)	REL	Macro_FOLR2+APOE+	2,90956E-01
DC	HNSCC	Post(R)	SPI1	cDC_CLEC9A	2,90625E-01
Macro/Mono	BCC	Post(R)	NFKB2	Mono_INHBA	2,90260E-01
Macro/Mono	ccRCC	Post(NR)	GTF2F1	Macro_LYVE1	2,90256E-01
Macro/Mono	HNSCC	Post(NR)	RELB	Macro_IER3	2,90167E-01
Macro/Mono	HNSCC	Post(NR)	CEBPB	Mono_INHBA	2,89786E-01
Macro/Mono	CRC	Post(NR)	BCL3	Mono_INHBA	2,89731E-01
Macro/Mono	BCC	Post(NR)	SPI1	Macro_FOLR2+APOE+	2,89385E-01
Macro/Mono	Melanoma	Post(NR)	FLI1	Macro_LYVE1	2,89175E-01
Macro/Mono	BCC	Post(NR)	ELK1	Macro_NLRP3	2,89143E-01
Macro/Mono	BCC	Post(NR)	NFE2L2	Macro_NLRP3	2,89012E-01
Macro/Mono	Melanoma	Post(NR)	CREB1	Macro_FOLR2+APOE+	2,88963E-01
DC	ccRCC	Post(NR)	GTF2F1	cDC(CD1C)	2,88793E-01
DC	Melanoma	Post(NR)	FLI1	cDC(CD1C)	2,88784E-01
Macro/Mono	BCC	Post(NR)	ATF3	Macro_FOLR2+APOE+	2,88426E-01
Mast	BCC	Post(R)	CREB1	Mast	2,88411E-01
Macro/Mono	Melanoma	Post(NR)	FLI1	Macro_ISG15	2,88381E-01
Macro/Mono	Melanoma	Post(NR)	ELK4	Macro_FOLR2+APOE+	2,88284E-01
Macro/Mono	ccRCC	Post(R)	EGR1	Mono_CD16	2,88280E-01
Macro/Mono	ccRCC	Post(NR)	FOXN3	Macro_FOLR2+APOE+	2,88229E-01
Macro/Mono	ccRCC	Post(NR)	GTF2F1	Macro_IFI27	2,88211E-01
DC	ccRCC	Post(NR)	KLF4	pDC_LILRA4	2,87826E-01
Macro/Mono	CRC	Post(R)	ATF3	Macro_FOLR2+APOE-	2,87803E-01
DC	ccRCC	Post(R)	POLR2A	pDC_LILRA4	2,87756E-01
Macro/Mono	CRC	Post(NR)	ATF3	Macro_IFI27	2,87714E-01
Macro/Mono	HNSCC	Post(R)	EGR1	Mono_CD16	2,87643E-01
Macro/Mono	CRC	Post(NR)	MAF	Macro_FOLR2+APOE+	2,87640E-01

Macro/Mono	BCC	Post(NR)	NFE2L2	Macro_FOLR2+APOE+	2,87616E-01
Macro/Mono	CRC	Post(NR)	RELB	Mono_CD14	2,87597E-01
Mast	CRC	Post(NR)	RELB	Mast	2,87506E-01
Macro/Mono	Melanoma	Post(R)	ELK4	Macro_ISG15	2,87443E-01
Macro/Mono	HNSCC	Post(R)	BCLAF1	Macro_ISG15	2,87412E-01
Macro/Mono	ccRCC	Post(R)	REL	Macro_IFI27	2,87341E-01
Macro/Mono	Melanoma	Post(NR)	JUNB	Mono_INHBA	2,87302E-01
Macro/Mono	ccRCC	Post(R)	FOS	Macro_LYVE1	2,87054E-01
Macro/Mono	BCC	Post(NR)	SPI1	Macro_IFI27	2,87054E-01
Macro/Mono	CRC	Post(NR)	ETV3	Macro_IER3	2,86816E-01
Macro/Mono	BCC	Post(NR)	SPI1	Macro_OLFML3	2,86696E-01
DC	BCC	Post(R)	GTF2F1	pDC_LILRA4	2,86482E-01
Macro/Mono	HNSCC	Post(NR)	RELB	Mono_CD16	2,86288E-01
Macro/Mono	Melanoma	Post(NR)	FLI1	Macro_FOLR2+APOE+	2,86015E-01
Macro/Mono	CRC	Post(NR)	RAD21	Macro_FOLR2+APOE+	2,85996E-01
Macro/Mono	ccRCC	Post(R)	FLI1	Macro_NLRP3	2,85924E-01
Macro/Mono	HNSCC	Post(R)	CEBPB	Macro_NLRP3	2,85806E-01
DC	HNSCC	Post(NR)	RELB	cDC(CD1C)	2,85513E-01
Macro/Mono	Melanoma	Post(NR)	FLI1	Macro_OLFML3	2,85475E-01
Macro/Mono	ccRCC	Post(NR)	GTF2F1	Macro_IER3	2,85336E-01
Macro/Mono	BCC	Post(R)	GTF2F1	Mono_CD16	2,85136E-01
DC	ccRCC	Post(NR)	JUNB	cDC_CLEC9A	2,85035E-01
Macro/Mono	HNSCC	Post(R)	EGR1	Macro_IFI27	2,84908E-01
DC	HNSCC	Post(R)	KLF4	cDC(CD1C)	2,84878E-01
Macro/Mono	ccRCC	Post(NR)	MAFB	Mono_CD14	2,84863E-01
DC	BCC	Post(R)	GTF2F1	cDC(CD1C)	2,84839E-01
DC	BCC	Post(R)	YY1	pDC_LILRA4	2,84774E-01
Macro/Mono	ccRCC	Post(NR)	FOXN3	Macro_IFI27	2,84467E-01
Macro/Mono	HNSCC	Post(NR)	BCLAF1	Macro_OLFML3	2,84360E-01
Macro/Mono	Melanoma	Post(R)	CREB5	Macro_IFI27	2,84348E-01
Macro/Mono	ccRCC	Post(R)	REL	Macro_LYVE1	2,84297E-01
Mast	Melanoma	Post(NR)	FLI1	Mast	2,84258E-01
Macro/Mono	ccRCC	Post(R)	SPI1	Macro_ISG15	2,84091E-01
Macro/Mono	HNSCC	Post(R)	KLF4	Macro_ISG15	2,83515E-01
Macro/Mono	ccRCC	Post(NR)	JUNB	Macro_FOLR2-APOE+	2,83445E-01
DC	BCC	Post(NR)	NFKB1	cDC_LAMP3	2,83410E-01
DC	CRC	Post(NR)	BCLAF1	cDC_LAMP3	2,83381E-01
Macro/Mono	Melanoma	Post(R)	ETS2	Mono_INHBA	2,83229E-01
Macro/Mono	CRC	Post(R)	YBX1	Macro_FOLR2+APOE+	2,83025E-01
DC	BCC	Post(NR)	SPI1	cDC_CLEC9A	2,82739E-01
Macro/Mono	BCC	Post(R)	GTF2F1	Mono_CD14	2,82493E-01
Macro/Mono	Melanoma	Post(NR)	MAFB	Macro_FOLR2-APOE+	2,82475E-01

DC	CRC	Post(R)	RAD21	cDC_CLEC9A	2,82194E-01
Macro/Mono	ccRCC	Post(R)	EGR1	Mono_CD14	2,81423E-01
DC	Melanoma	Post(NR)	FLI1	cDC_CLEC9A	2,81379E-01
DC	Melanoma	Post(NR)	JUNB	cDC_LAMP3	2,81357E-01
DC	ccRCC	Post(NR)	EGR1	cDC_CLEC9A	2,81264E-01
Macro/Mono	ccRCC	Post(R)	CEBPD	Macro_IER3	2,81141E-01
Macro/Mono	Melanoma	Post(R)	SREBF1	Macro_NLRP3	2,80987E-01
Macro/Mono	HNSCC	Post(NR)	RELB	Mono_INHBA	2,80982E-01
Macro/Mono	BCC	Post(R)	GTF2F1	Macro_FOLR2+APOE-	2,80912E-01
DC	HNSCC	Post(R)	KLF4	cDC_LAMP3	2,80797E-01
Macro/Mono	BCC	Post(R)	GTF2F1	Macro_IER3	2,80608E-01
Macro/Mono	BCC	Post(NR)	NFE2L2	Macro_LYVE1	2,80581E-01
DC	Melanoma	Post(NR)	FLI1	cDC_LAMP3	2,80440E-01
Macro/Mono	ccRCC	Post(NR)	EGR1	Macro_OLFML3	2,80396E-01
DC	CRC	Post(R)	RAD21	cDC(CD1C)	2,80265E-01
Macro/Mono	HNSCC	Post(R)	RELB	Macro_LYVE1	2,80221E-01
Macro/Mono	CRC	Post(NR)	MAX	Macro_FOLR2+APOE-	2,79805E-01
Macro/Mono	Melanoma	Post(NR)	JUNB	Macro_FOLR2+APOE+	2,79738E-01
Macro/Mono	ccRCC	Post(R)	REL	Macro_FOLR2+APOE-	2,79654E-01
Macro/Mono	CRC	Post(NR)	MAFB	Macro_FOLR2-APOE+	2,79621E-01
Macro/Mono	Melanoma	Post(NR)	MAFB	Macro_LYVE1	2,79332E-01
Macro/Mono	ccRCC	Post(NR)	FOXN3	Macro_IER3	2,79301E-01
Macro/Mono	ccRCC	Post(NR)	FOXN3	Macro_OLFML3	2,79283E-01
Macro/Mono	BCC	Post(NR)	ELK1	Mono_CD14	2,79196E-01
DC	BCC	Post(NR)	ELK1	cDC_LAMP3	2,79111E-01
Macro/Mono	ccRCC	Post(R)	ELF2	Macro_FOLR2+APOE-	2,78992E-01
Macro/Mono	BCC	Post(R)	GTF2F1	Macro_ISG15	2,78967E-01
Macro/Mono	BCC	Post(NR)	SPI1	Mono_CD14	2,78957E-01
Macro/Mono	BCC	Post(R)	GTF2F1	Macro_NLRP3	2,78857E-01
Macro/Mono	Melanoma	Post(NR)	JUNB	Mono_CD16	2,78781E-01
DC	HNSCC	Post(R)	NR3C1	pDC_LILRA4	2,78740E-01
Macro/Mono	HNSCC	Post(R)	RELB	Macro_FOLR2+APOE+	2,78626E-01
Macro/Mono	BCC	Post(NR)	SPI1	Mono_CD16	2,78343E-01
DC	Melanoma	Post(R)	CREB5	cDC(CD1C)	2,78213E-01
Macro/Mono	Melanoma	Post(NR)	FLI1	Macro_FOLR2-APOE+	2,78167E-01
Macro/Mono	ccRCC	Post(NR)	JUNB	Macro_LYVE1	2,78151E-01
Macro/Mono	CRC	Post(R)	CEBPB	Mono_INHBA	2,77999E-01
Mast	Melanoma	Post(NR)	SMARCA4	Mast	2,77982E-01
Macro/Mono	BCC	Post(R)	GTF2F1	Macro_FOLR2+APOE+	2,77824E-01
Macro/Mono	BCC	Post(R)	REL	Macro_FOLR2+APOE-	2,77597E-01
Macro/Mono	HNSCC	Post(R)	KLF4	Macro_OLFML3	2,77502E-01
Macro/Mono	CRC	Post(NR)	RELB	Macro_ISG15	2,77224E-01

Macro/Mono	Melanoma	Post(NR)	CREB1	Macro_IFI27	2,77142E-01
Macro/Mono	BCC	Post(NR)	SPI1	Macro_LYVE1	2,76855E-01
Macro/Mono	Melanoma	Post(NR)	MAFB	Macro_FOLR2+APOE+	2,76849E-01
DC	Melanoma	Post(R)	CREB5	cDC_CLEC9A	2,76809E-01
DC	ccRCC	Post(R)	FLI1	cDC_CLEC9A	2,76744E-01
Macro/Mono	BCC	Post(R)	GTF2F1	Macro_FOLR2-APOE+	2,76705E-01
Macro/Mono	ccRCC	Post(NR)	REST	Mono_CD14	2,76657E-01
Macro/Mono	ccRCC	Post(R)	FLI1	Macro_IFI27	2,76503E-01
Macro/Mono	BCC	Post(NR)	ATF3	Macro_FOLR2+APOE-	2,76478E-01
DC	HNSCC	Post(NR)	SPI1	cDC_LAMP3	2,76370E-01
Macro/Mono	HNSCC	Post(NR)	JUNB	Macro_LYVE1	2,76297E-01
Macro/Mono	HNSCC	Post(NR)	JUNB	Macro_NLRP3	2,76137E-01
Macro/Mono	HNSCC	Post(R)	KLF4	Macro_FOLR2-APOE+	2,76075E-01
Macro/Mono	ccRCC	Post(NR)	REST	Mono_CD16	2,76055E-01
Macro/Mono	ccRCC	Post(R)	FLI1	Mono_INHBA	2,75837E-01
DC	Melanoma	Post(R)	BACH1	cDC(CD1C)	2,75731E-01
Macro/Mono	BCC	Post(NR)	ELK1	Mono_CD16	2,75718E-01
Macro/Mono	BCC	Post(NR)	JUNB	Macro_IER3	2,75320E-01
Mast	BCC	Post(R)	GTF2F1	Mast	2,75293E-01
DC	ccRCC	Post(R)	FLI1	cDC_LAMP3	2,75049E-01
Macro/Mono	ccRCC	Post(NR)	FOS	Macro_FOLR2+APOE-	2,75022E-01
Macro/Mono	BCC	Post(R)	GTF2F1	Macro_IFI27	2,74886E-01
DC	ccRCC	Post(R)	ETS2	cDC_CLEC9A	2,74828E-01
Macro/Mono	Melanoma	Post(R)	FOS	Mono_CD14	2,74543E-01
DC	BCC	Post(R)	GTF2F1	cDC_LAMP3	2,74475E-01
Macro/Mono	CRC	Post(R)	BCLAF1	Macro_ISG15	2,74441E-01
Macro/Mono	BCC	Post(R)	NR3C1	Macro_FOLR2+APOE-	2,74383E-01
DC	ccRCC	Post(NR)	KLF4	cDC_LAMP3	2,74239E-01
Macro/Mono	ccRCC	Post(NR)	REST	Macro_FOLR2+APOE-	2,74196E-01
Macro/Mono	CRC	Post(NR)	RELB	Macro_LYVE1	2,74147E-01
Macro/Mono	CRC	Post(NR)	BCLAF1	Macro_IER3	2,73852E-01
Macro/Mono	BCC	Post(R)	SPI1	Mono_INHBA	2,73643E-01
Mast	ccRCC	Post(R)	HMGB1	Mast	2,73638E-01
Macro/Mono	BCC	Post(NR)	ELK1	Macro_IER3	2,73591E-01
Mast	BCC	Post(R)	ENO1	Mast	2,73181E-01
Macro/Mono	ccRCC	Post(R)	ELF2	Mono_CD16	2,73043E-01
DC	BCC	Post(R)	IRF4	pDC_LILRA4	2,73016E-01
Macro/Mono	BCC	Post(NR)	FOSL2	Macro_NLRP3	2,72911E-01
Macro/Mono	CRC	Post(R)	BCLAF1	Mono_INHBA	2,72908E-01
Mast	ccRCC	Post(NR)	JUNB	Mast	2,72554E-01
Macro/Mono	Melanoma	Post(R)	ELK4	Macro_FOLR2-APOE+	2,72383E-01
Macro/Mono	Melanoma	Post(R)	ELK4	Mono_INHBA	2,72082E-01

Macro/Mono	CRC	Post(R)	POLR2A	Macro_OLFML3	2,72070E-01
Macro/Mono	CRC	Post(NR)	ATF3	Macro_FOLR2+APOE-	2,71883E-01
DC	BCC	Post(NR)	ATF3	cDC(CD1C)	2,71381E-01
Macro/Mono	BCC	Post(NR)	EP300	Mono_CD14	2,71326E-01
Mast	ccRCC	Post(R)	NELFE	Mast	2,71268E-01
Macro/Mono	ccRCC	Post(R)	EGR1	Macro_NLRP3	2,71266E-01
DC	CRC	Post(NR)	JUNB	cDC(CD1C)	2,71224E-01
Macro/Mono	CRC	Post(NR)	RELB	Macro_FOLR2+APOE+	2,71196E-01
Macro/Mono	CRC	Post(NR)	BCL3	Mono_CD14	2,71129E-01
Macro/Mono	BCC	Post(R)	GTF2F1	Macro_OLFML3	2,70970E-01
Macro/Mono	BCC	Post(NR)	NELFE	Macro_FOLR2+APOE-	2,70763E-01
Macro/Mono	ccRCC	Post(R)	ETS2	Macro_LYVE1	2,70752E-01
Mast	BCC	Post(NR)	NELFE	Mast	2,70675E-01
Macro/Mono	CRC	Post(NR)	MAFB	Mono_INHBA	2,70616E-01
Macro/Mono	ccRCC	Post(NR)	FOXN3	Macro_FOLR2-APOE+	2,70444E-01
Macro/Mono	ccRCC	Post(NR)	GTF2F1	Macro_FOLR2-APOE+	2,70401E-01
DC	Melanoma	Post(NR)	FLI1	pDC_LILRA4	2,70342E-01
Macro/Mono	ccRCC	Post(NR)	FOS	Macro_IFI27	2,70068E-01
Macro/Mono	Melanoma	Post(R)	FOSB	Macro_IFI27	2,69964E-01
Macro/Mono	CRC	Post(NR)	BCL3	Macro_ISG15	2,69745E-01
Macro/Mono	BCC	Post(NR)	ELK1	Macro_LYVE1	2,69561E-01
Macro/Mono	BCC	Post(R)	GTF2F1	Macro_LYVE1	2,69450E-01
DC	ccRCC	Post(R)	CEBPD	cDC(CD1C)	2,69216E-01
Macro/Mono	HNSCC	Post(R)	EGR1	Macro_LYVE1	2,69047E-01
Mast	BCC	Post(NR)	EP300	Mast	2,68960E-01
Macro/Mono	ccRCC	Post(NR)	JUNB	Macro_FOLR2+APOE+	2,68894E-01
Macro/Mono	BCC	Post(NR)	EP300	Mono_CD16	2,68779E-01
Macro/Mono	BCC	Post(NR)	EP300	Macro_IER3	2,68672E-01
Macro/Mono	BCC	Post(R)	ENO1	Mono_CD14	2,68649E-01
Macro/Mono	Melanoma	Post(R)	ELK4	Macro_IFI27	2,67868E-01
Macro/Mono	ccRCC	Post(NR)	CEBPD	Mono_CD14	2,67801E-01
Macro/Mono	ccRCC	Post(R)	FLI1	Macro_LYVE1	2,67771E-01
Macro/Mono	HNSCC	Post(NR)	JUNB	Mono_CD14	2,67569E-01
Macro/Mono	Melanoma	Post(R)	SMAD5	Macro_IFI27	2,67487E-01
Macro/Mono	BCC	Post(NR)	EP300	Macro_NLRP3	2,67456E-01
Macro/Mono	ccRCC	Post(R)	POLR2A	Macro_IER3	2,67263E-01
Macro/Mono	Melanoma	Post(R)	FOSB	Mono_CD14	2,67076E-01
DC	CRC	Post(NR)	RAD21	cDC(CD1C)	2,66950E-01
Macro/Mono	CRC	Post(R)	BCLAF1	Macro_LYVE1	2,66802E-01
Macro/Mono	ccRCC	Post(R)	ELF2	Mono_CD14	2,66647E-01
Macro/Mono	ccRCC	Post(R)	FOS	Macro_NLRP3	2,66440E-01
DC	Melanoma	Post(R)	FOSB	cDC_CLEC9A	2,66423E-01

Macro/Mono	BCC	Post(NR)	EP300	Macro_LYVE1	2,66386E-01
Macro/Mono	ccRCC	Post(NR)	EGR1	Macro_FOLR2+APOE+	2,65713E-01
Macro/Mono	Melanoma	Post(NR)	JUNB	Macro_OLFML3	2,65439E-01
Macro/Mono	HNSCC	Post(NR)	RELB	Macro_OLFML3	2,65395E-01
Macro/Mono	CRC	Post(R)	CEBPB	Mono_CD16	2,65300E-01
Macro/Mono	CRC	Post(NR)	RAD21	Macro_FOLR2-APOE+	2,65042E-01
Macro/Mono	Melanoma	Post(R)	FLI1	Mono_INHBA	2,64905E-01
Macro/Mono	BCC	Post(R)	GTF2F1	Mono_INHBA	2,64738E-01
Macro/Mono	CRC	Post(NR)	RELB	Macro_OLFML3	2,64729E-01
Macro/Mono	BCC	Post(NR)	JUNB	Mono_INHBA	2,64600E-01
Macro/Mono	BCC	Post(NR)	ATF3	Macro_OLFML3	2,64578E-01
Macro/Mono	ccRCC	Post(NR)	GTF2F1	Macro_OLFML3	2,64303E-01
Macro/Mono	CRC	Post(R)	YBX1	Macro_IFI27	2,64263E-01
DC	CRC	Post(NR)	RELB	pDC_LILRA4	2,63945E-01
Macro/Mono	Melanoma	Post(NR)	JUNB	Macro_LYVE1	2,63893E-01
DC	ccRCC	Post(R)	ETS2	cDC_LAMP3	2,63848E-01
Macro/Mono	ccRCC	Post(NR)	FOXN3	Macro_ISG15	2,63597E-01
Macro/Mono	CRC	Post(NR)	FOSB	Macro_IER3	2,63289E-01
Macro/Mono	ccRCC	Post(R)	EGR1	Mono_INHBA	2,63265E-01
Macro/Mono	HNSCC	Post(NR)	BCLAF1	Macro_FOLR2-APOE+	2,63248E-01
Macro/Mono	Melanoma	Post(NR)	FLI1	Macro_IFI27	2,63059E-01
DC	HNSCC	Post(R)	KLF4	cDC_CLEC9A	2,62885E-01
DC	Melanoma	Post(R)	ETS2	cDC(CD1C)	2,62852E-01
Mast	CRC	Post(NR)	FOSB	Mast	2,62699E-01
Macro/Mono	HNSCC	Post(R)	EGR1	Macro_FOLR2+APOE+	2,62443E-01
Macro/Mono	ccRCC	Post(R)	MYC	Macro_FOLR2+APOE-	2,62032E-01
Mast	HNSCC	Post(R)	KLF4	Mast	2,61978E-01
Macro/Mono	ccRCC	Post(R)	STAT1	Macro_OLFML3	2,61837E-01
Macro/Mono	BCC	Post(NR)	JUNB	Macro_NLRP3	2,61770E-01
Macro/Mono	CRC	Post(NR)	MAX	Macro_IER3	2,61550E-01
Macro/Mono	ccRCC	Post(NR)	GTF2F1	Macro_ISG15	2,61517E-01
DC	ccRCC	Post(NR)	REST	cDC_CLEC9A	2,61261E-01
Mast	BCC	Post(NR)	ELK1	Mast	2,61119E-01
Macro/Mono	CRC	Post(NR)	MAF	Macro_FOLR2-APOE+	2,61002E-01
Macro/Mono	CRC	Post(NR)	MAFB	Macro_ISG15	2,60965E-01
Macro/Mono	CRC	Post(NR)	CREM	Mono_INHBA	2,60712E-01
Macro/Mono	Melanoma	Post(R)	CREB5	Macro_FOLR2-APOE+	2,60644E-01
Macro/Mono	CRC	Post(NR)	ETV3	Macro_LYVE1	2,60503E-01
Macro/Mono	ccRCC	Post(R)	EGR1	Macro_FOLR2+APOE-	2,60485E-01
Macro/Mono	BCC	Post(NR)	NELFE	Mono_CD14	2,60341E-01
DC	Melanoma	Post(R)	SMAD5	pDC_LILRA4	2,59792E-01
Macro/Mono	ccRCC	Post(R)	FOS	Mono_INHBA	2,59791E-01

Macro/Mono	ccRCC	Post(R)	NELFE	Mono_CD16	2,59607E-01
Macro/Mono	CRC	Post(R)	IRF8	Macro_FOLR2+APOE+	2,59487E-01
Macro/Mono	HNSCC	Post(R)	EGR1	Macro_FOLR2-APOE+	2,59411E-01
Macro/Mono	HNSCC	Post(NR)	RELB	Macro_LYVE1	2,59404E-01
Macro/Mono	Melanoma	Post(R)	ATF4	Mono_INHBA	2,59313E-01
Macro/Mono	BCC	Post(NR)	NFE2L2	Macro_OLFML3	2,59118E-01
DC	BCC	Post(NR)	NFE2L2	cDC(CD1C)	2,59103E-01
Macro/Mono	ccRCC	Post(NR)	NFIC	Macro_LYVE1	2,58865E-01
Macro/Mono	Melanoma	Post(R)	MAZ	Macro_FOLR2-APOE+	2,58727E-01
DC	HNSCC	Post(NR)	ETS2	cDC(CD1C)	2,58577E-01
DC	BCC	Post(NR)	EP300	pDC_LILRA4	2,58398E-01
DC	BCC	Post(R)	NFKB2	cDC_LAMP3	2,58290E-01
DC	Melanoma	Post(R)	FOS	cDC_CLEC9A	2,57763E-01
Macro/Mono	Melanoma	Post(R)	NFYC	Mono_INHBA	2,57719E-01
DC	BCC	Post(R)	YY1	cDC_CLEC9A	2,57644E-01
Macro/Mono	BCC	Post(NR)	ELK1	Macro_ISG15	2,57531E-01
DC	ccRCC	Post(NR)	FOXN3	cDC(CD1C)	2,57475E-01
Macro/Mono	BCC	Post(NR)	NFKB1	Macro_OLFML3	2,57455E-01
Macro/Mono	CRC	Post(NR)	POLR2A	Mono_CD14	2,57439E-01
Macro/Mono	BCC	Post(R)	ENO1	Macro_ISG15	2,57011E-01
Macro/Mono	ccRCC	Post(R)	POLR2A	Macro_IFI27	2,56973E-01
DC	CRC	Post(NR)	MAX	cDC_CLEC9A	2,56885E-01
Macro/Mono	BCC	Post(NR)	ELK1	Macro_FOLR2-APOE+	2,56850E-01
Macro/Mono	BCC	Post(NR)	ELK1	Macro_IFI27	2,56820E-01
Macro/Mono	Melanoma	Post(R)	SMARCA4	Mono_CD14	2,56792E-01
Macro/Mono	ccRCC	Post(NR)	KLF4	Macro_ISG15	2,56696E-01
DC	Melanoma	Post(R)	BACH1	cDC_CLEC9A	2,56561E-01
Macro/Mono	CRC	Post(NR)	MAX	Macro_FOLR2+APOE+	2,56511E-01
Macro/Mono	Melanoma	Post(NR)	SMARCA4	Macro_NLRP3	2,56380E-01
Macro/Mono	BCC	Post(NR)	ELK1	Macro_FOLR2+APOE-	2,56214E-01
Macro/Mono	CRC	Post(R)	POLR2A	Macro_FOLR2+APOE+	2,56160E-01
DC	ccRCC	Post(R)	SPI1	cDC_LAMP3	2,55912E-01
Macro/Mono	Melanoma	Post(R)	RUNX1	Mono_INHBA	2,55860E-01
Macro/Mono	BCC	Post(NR)	FOSL2	Mono_INHBA	2,55743E-01
Macro/Mono	Melanoma	Post(R)	RUNX1	Mono_CD14	2,55525E-01
DC	Melanoma	Post(R)	ETS2	cDC_CLEC9A	2,55382E-01
Macro/Mono	BCC	Post(NR)	EP300	Mono_INHBA	2,55200E-01
Macro/Mono	CRC	Post(R)	IRF1	Macro_ISG15	2,55157E-01
Macro/Mono	Melanoma	Post(R)	FOSB	Macro_FOLR2-APOE+	2,54769E-01
Macro/Mono	Melanoma	Post(R)	EZH2	Macro_ISG15	2,54718E-01
Macro/Mono	BCC	Post(NR)	CEBPB	Macro_NLRP3	2,54490E-01
Macro/Mono	BCC	Post(NR)	NFE2L2	Macro_ISG15	2,54268E-01

Macro/Mono	Melanoma	Post(R)	BACH1	Macro_NLRP3	2,54249E-01
Macro/Mono	Melanoma	Post(R)	ETS2	Macro_FOLR2-APOE+	2,54102E-01
DC	BCC	Post(NR)	EP300	cDC(CD1C)	2,53892E-01
DC	Melanoma	Post(R)	BACH1	pDC_LILRA4	2,53870E-01
DC	ccRCC	Post(R)	NELFE	cDC_CLEC9A	2,53783E-01
DC	ccRCC	Post(R)	SPI1	cDC(CD1C)	2,53775E-01
Mast	ccRCC	Post(R)	GTF2F1	Mast	2,53721E-01
Macro/Mono	BCC	Post(NR)	EP300	Macro_FOLR2-APOE+	2,53684E-01
DC	Melanoma	Post(NR)	JUNB	cDC(CD1C)	2,53554E-01
Macro/Mono	ccRCC	Post(NR)	KLF4	Macro_OLFML3	2,53516E-01
Macro/Mono	CRC	Post(R)	BACH1	Mono_INHBA	2,53501E-01
Macro/Mono	ccRCC	Post(R)	NELFE	Mono_CD14	2,53303E-01
Macro/Mono	BCC	Post(NR)	EP300	Macro_IFI27	2,53266E-01
DC	ccRCC	Post(NR)	KLF6	cDC(CD1C)	2,53044E-01
Macro/Mono	CRC	Post(R)	BCLAF1	Macro_NLRP3	2,52835E-01
DC	ccRCC	Post(NR)	REST	cDC_LAMP3	2,52758E-01
Macro/Mono	Melanoma	Post(R)	BACH1	Macro_FOLR2-APOE+	2,52754E-01
Macro/Mono	ccRCC	Post(R)	SPI1	Macro_IER3	2,52011E-01
Macro/Mono	CRC	Post(NR)	JUNB	Macro_FOLR2+APOE+	2,51990E-01
Macro/Mono	CRC	Post(NR)	MAFB	Macro_NLRP3	2,51964E-01
Macro/Mono	BCC	Post(NR)	BACH1	Macro_NLRP3	2,51803E-01
Macro/Mono	ccRCC	Post(R)	ETS2	Macro_IFI27	2,51759E-01
Macro/Mono	BCC	Post(NR)	NELFE	Mono_CD16	2,51651E-01
Macro/Mono	ccRCC	Post(NR)	KLF6	Macro_NLRP3	2,51500E-01
Macro/Mono	CRC	Post(R)	BCLAF1	Macro_FOLR2-APOE+	2,51425E-01
Macro/Mono	Melanoma	Post(R)	RUNX1	Macro_NLRP3	2,50994E-01
Macro/Mono	CRC	Post(NR)	RELB	Macro_FOLR2+APOE-	2,50931E-01
DC	HNSCC	Post(R)	SPI1	cDC_LAMP3	2,50818E-01
Macro/Mono	HNSCC	Post(NR)	RELB	Macro_FOLR2+APOE-	2,50625E-01
Macro/Mono	BCC	Post(NR)	JUNB	Macro_FOLR2-APOE+	2,50455E-01
Macro/Mono	BCC	Post(NR)	CEBPD	Macro_FOLR2-APOE+	2,50262E-01
Mast	ccRCC	Post(R)	REST	Mast	2,49878E-01
Macro/Mono	ccRCC	Post(NR)	KLF4	Macro_FOLR2-APOE+	2,49628E-01
DC	BCC	Post(NR)	NELFE	pDC_LILRA4	2,49623E-01
Macro/Mono	ccRCC	Post(NR)	NFIC	Macro_FOLR2+APOE+	2,49578E-01
Mast	BCC	Post(NR)	GTF2F1	Mast	2,49554E-01
DC	ccRCC	Post(R)	FLI1	pDC_LILRA4	2,49413E-01
DC	Melanoma	Post(R)	SMAD5	cDC(CD1C)	2,49346E-01
DC	ccRCC	Post(NR)	FOXN3	pDC_LILRA4	2,49023E-01
Macro/Mono	CRC	Post(NR)	RELB	Macro_IFI27	2,48801E-01
Macro/Mono	CRC	Post(NR)	MAX	Macro_IFI27	2,48798E-01
Macro/Mono	CRC	Post(R)	IRF8	Macro_LYVE1	2,48661E-01

Macro/Mono	ccRCC	Post(R)	EGR1	Macro_LYVE1	2,48634E-01
DC	BCC	Post(NR)	EP300	cDC_CLEC9A	2,48570E-01
Macro/Mono	HNSCC	Post(R)	EGR1	Macro_OLFML3	2,48257E-01
Mast	HNSCC	Post(NR)	JUNB	Mast	2,48221E-01
Macro/Mono	Melanoma	Post(R)	RUNX1	Macro_FOLR2-APOE+	2,48134E-01
Macro/Mono	BCC	Post(NR)	FOSL2	Mono_CD14	2,48077E-01
Macro/Mono	Melanoma	Post(R)	ETS2	Macro_NLRP3	2,48023E-01
Macro/Mono	HNSCC	Post(NR)	ZMIZ1	Macro_IFI27	2,47968E-01
Macro/Mono	CRC	Post(NR)	JUNB	Mono_CD14	2,47722E-01
Macro/Mono	CRC	Post(NR)	ELF2	Mono_CD16	2,47608E-01
DC	CRC	Post(NR)	BCLAF1	pDC_LILRA4	2,47546E-01
DC	Melanoma	Post(NR)	JUND	cDC(CD1C)	2,47538E-01
Macro/Mono	HNSCC	Post(NR)	RELB	Macro_IFI27	2,47427E-01
Macro/Mono	BCC	Post(NR)	ELK1	Macro_OLFML3	2,47198E-01
Macro/Mono	CRC	Post(R)	YBX1	Macro_OLFML3	2,47159E-01
Macro/Mono	ccRCC	Post(R)	NELFE	Macro_FOLR2+APOE-	2,47056E-01
DC	HNSCC	Post(R)	EGR1	cDC_LAMP3	2,46930E-01
Macro/Mono	ccRCC	Post(NR)	REST	Macro_LYVE1	2,46909E-01
DC	BCC	Post(R)	ENO1	pDC_LILRA4	2,46822E-01
DC	Melanoma	Post(R)	RUNX1	cDC_CLEC9A	2,46685E-01
Macro/Mono	BCC	Post(R)	SPI1	Macro_OLFML3	2,46557E-01
DC	BCC	Post(NR)	GTF2F1	pDC_LILRA4	2,46506E-01
Mast	Melanoma	Post(NR)	POU2F1	Mast	2,46489E-01
Mast	ccRCC	Post(NR)	KLF4	Mast	2,46343E-01
Macro/Mono	BCC	Post(NR)	BACH1	Mono_INHBA	2,46237E-01
Macro/Mono	HNSCC	Post(R)	KLF4	Macro_FOLR2+APOE+	2,45880E-01
Macro/Mono	Melanoma	Post(R)	BACH1	Mono_INHBA	2,45646E-01
Macro/Mono	ccRCC	Post(NR)	GTF2F1	Macro_FOLR2+APOE+	2,45467E-01
Macro/Mono	BCC	Post(NR)	FOSL2	Macro_ISG15	2,45063E-01
DC	ccRCC	Post(R)	ETS2	pDC_LILRA4	2,45021E-01
Macro/Mono	BCC	Post(NR)	JUNB	Macro_OLFML3	2,45004E-01
DC	BCC	Post(R)	YY1	cDC_LAMP3	2,44994E-01
Macro/Mono	BCC	Post(NR)	EP300	Macro_ISG15	2,44928E-01
Macro/Mono	ccRCC	Post(NR)	REST	Macro_IFI27	2,44821E-01
Macro/Mono	CRC	Post(NR)	ELF2	Macro_LYVE1	2,44752E-01
Macro/Mono	ccRCC	Post(NR)	JUNB	Macro_IFI27	2,44746E-01
Macro/Mono	BCC	Post(NR)	JUNB	Macro_ISG15	2,44745E-01
Macro/Mono	BCC	Post(NR)	FOSL2	Macro_IER3	2,44689E-01
Macro/Mono	HNSCC	Post(NR)	RELB	Macro_FOLR2-APOE+	2,44520E-01
Mast	BCC	Post(NR)	KDM5B	Mast	2,44453E-01
Macro/Mono	ccRCC	Post(NR)	KLF4	Macro_LYVE1	2,44220E-01
Macro/Mono	Melanoma	Post(R)	ZNF407	Mono_INHBA	2,44050E-01

Mast	BCC	Post(R)	GABPB1	Mast	2,43997E-01
Macro/Mono	Melanoma	Post(NR)	SMARCA4	Mono_CD14	2,43934E-01
Macro/Mono	HNSCC	Post(R)	CEBPB	Mono_CD14	2,43932E-01
Mast	Melanoma	Post(NR)	FOSB	Mast	2,43862E-01
Macro/Mono	Melanoma	Post(R)	NFE2L2	Macro_NLRP3	2,43823E-01
Macro/Mono	ccRCC	Post(NR)	REST	Macro_NLRP3	2,43664E-01
Macro/Mono	ccRCC	Post(R)	NELFE	Mono_INHBA	2,43623E-01
Macro/Mono	BCC	Post(NR)	NELFE	Macro_IFI27	2,43555E-01
Macro/Mono	ccRCC	Post(R)	SPI1	Macro_FOLR2+APOE-	2,43385E-01
DC	BCC	Post(NR)	CREM	cDC(CD1C)	2,43328E-01
Macro/Mono	BCC	Post(NR)	EP300	Macro_FOLR2+APOE-	2,43280E-01
Macro/Mono	Melanoma	Post(R)	MAZ	Macro_OLFML3	2,43126E-01
Macro/Mono	Melanoma	Post(R)	RUNX1	Macro_OLFML3	2,43063E-01
DC	ccRCC	Post(NR)	KLF6	pDC_LILRA4	2,43020E-01
Mast	BCC	Post(R)	SUPT20H	Mast	2,42921E-01
Macro/Mono	Melanoma	Post(R)	EZH2	Macro_NLRP3	2,42913E-01
Macro/Mono	ccRCC	Post(R)	SPI1	Macro_LYVE1	2,42846E-01
Macro/Mono	Melanoma	Post(R)	CREB5	Macro_OLFML3	2,42716E-01
DC	ccRCC	Post(NR)	FOXN3	cDC_LAMP3	2,42499E-01
Macro/Mono	ccRCC	Post(R)	NELFE	Macro_NLRP3	2,42461E-01
Macro/Mono	CRC	Post(NR)	CREM	Macro_NLRP3	2,42453E-01
Macro/Mono	CRC	Post(R)	RAD21	Macro_OLFML3	2,42284E-01
Macro/Mono	HNSCC	Post(NR)	JUNB	Mono_CD16	2,42210E-01
Macro/Mono	Melanoma	Post(R)	RUNX1	Macro_ISG15	2,42142E-01
Macro/Mono	CRC	Post(NR)	BCLAF1	Macro_FOLR2+APOE+	2,42136E-01
DC	Melanoma	Post(R)	ETS2	pDC_LILRA4	2,42116E-01
Macro/Mono	HNSCC	Post(NR)	CEBPB	Macro_NLRP3	2,42032E-01
Macro/Mono	Melanoma	Post(R)	ETS2	Macro_OLFML3	2,41579E-01
DC	ccRCC	Post(R)	SPI1	cDC_CLEC9A	2,41579E-01
Macro/Mono	BCC	Post(NR)	NFE2L2	Macro_IFI27	2,41525E-01
Macro/Mono	ccRCC	Post(R)	ELF2	Macro_NLRP3	2,41410E-01
DC	Melanoma	Post(NR)	SMARCA4	cDC_CLEC9A	2,41355E-01
Macro/Mono	BCC	Post(R)	YY1	Macro_FOLR2+APOE+	2,41231E-01
DC	BCC	Post(R)	YY1	cDC(CD1C)	2,41072E-01
Macro/Mono	Melanoma	Post(NR)	SMARCA4	Macro_FOLR2-APOE+	2,40972E-01
Macro/Mono	CRC	Post(NR)	MAX	Macro_FOLR2-APOE+	2,40867E-01
Macro/Mono	BCC	Post(R)	YY1	Macro_IER3	2,40716E-01
Macro/Mono	Melanoma	Post(NR)	MAZ	Macro_IFI27	2,40677E-01
DC	Melanoma	Post(NR)	ESRRA	cDC_CLEC9A	2,40653E-01
DC	Melanoma	Post(R)	RUNX1	cDC(CD1C)	2,40642E-01
Macro/Mono	HNSCC	Post(R)	KLF4	Macro_IFI27	2,40634E-01
Macro/Mono	Melanoma	Post(R)	SMAD5	Macro_FOLR2-APOE+	2,40532E-01

Macro/Mono	BCC	Post(R)	YY1	Mono_CD16	2,40478E-01
Macro/Mono	Melanoma	Post(NR)	SMARCA4	Mono_INHBA	2,40464E-01
DC	Melanoma	Post(R)	NELFE	pDC_LILRA4	2,40342E-01
DC	CRC	Post(NR)	JUNB	cDC_CLEC9A	2,40279E-01
Macro/Mono	BCC	Post(NR)	NELFE	Macro_IER3	2,40206E-01
DC	Melanoma	Post(NR)	JUNB	cDC_CLEC9A	2,40108E-01
DC	Melanoma	Post(NR)	SMARCA4	cDC(CD1C)	2,40087E-01
DC	HNSCC	Post(NR)	SPI1	pDC_LILRA4	2,39979E-01
Macro/Mono	Melanoma	Post(R)	ETS2	Macro_ISG15	2,39831E-01
Macro/Mono	ccRCC	Post(NR)	JUNB	Macro_FOLR2+APOE-	2,39830E-01
Macro/Mono	BCC	Post(NR)	SPI1	Macro_FOLR2+APOE-	2,39813E-01
Macro/Mono	BCC	Post(NR)	FOS	Macro_NLRP3	2,39604E-01
Macro/Mono	BCC	Post(NR)	FOSL2	Macro_FOLR2-APOE+	2,39310E-01
DC	Melanoma	Post(R)	NELFE	cDC_CLEC9A	2,39256E-01
Macro/Mono	ccRCC	Post(R)	BCLAF1	Macro_FOLR2+APOE+	2,39062E-01
Macro/Mono	Melanoma	Post(NR)	SMARCA4	Macro_OLFML3	2,39035E-01
Macro/Mono	CRC	Post(R)	FOS	Macro_NLRP3	2,38842E-01
Macro/Mono	Melanoma	Post(NR)	SMARCA4	Mono_CD16	2,38778E-01
Macro/Mono	CRC	Post(R)	MAF	Macro_FOLR2+APOE+	2,38754E-01
Macro/Mono	BCC	Post(NR)	BACH1	Macro_IER3	2,38636E-01
DC	Melanoma	Post(R)	EZH2	pDC_LILRA4	2,38559E-01
Macro/Mono	ccRCC	Post(NR)	REST	Mono_INHBA	2,38465E-01
Macro/Mono	Melanoma	Post(NR)	MAFB	Macro_IFI27	2,38350E-01
Macro/Mono	Melanoma	Post(R)	MAZ	Mono_CD14	2,38176E-01
DC	CRC	Post(NR)	FOSB	cDC_CLEC9A	2,38095E-01
Macro/Mono	BCC	Post(NR)	JUNB	Macro_FOLR2+APOE+	2,37697E-01
DC	ccRCC	Post(NR)	KLF6	cDC_LAMP3	2,37579E-01
Macro/Mono	Melanoma	Post(NR)	JUND	Macro_OLFML3	2,37518E-01
Macro/Mono	ccRCC	Post(NR)	NFIC	Macro_FOLR2-APOE+	2,37482E-01
Mast	HNSCC	Post(R)	NR3C1	Mast	2,37199E-01
Macro/Mono	HNSCC	Post(R)	KLF4	Macro_LYVE1	2,37129E-01
Macro/Mono	Melanoma	Post(NR)	JUNB	Macro_ISG15	2,37107E-01
Macro/Mono	CRC	Post(NR)	JUNB	Macro_ISG15	2,36803E-01
DC	CRC	Post(R)	YBX1	cDC_CLEC9A	2,36795E-01
DC	BCC	Post(NR)	ATF3	cDC_LAMP3	2,36769E-01
Macro/Mono	Melanoma	Post(R)	ELK1	Macro_NLRP3	2,36760E-01
Macro/Mono	HNSCC	Post(NR)	RELB	Macro_ISG15	2,36529E-01
DC	ccRCC	Post(NR)	REST	cDC(CD1C)	2,36520E-01
Macro/Mono	BCC	Post(NR)	NELFE	Macro_NLRP3	2,36473E-01
DC	CRC	Post(NR)	MAX	cDC_LAMP3	2,36462E-01
DC	CRC	Post(R)	YBX1	cDC(CD1C)	2,36068E-01
Macro/Mono	CRC	Post(NR)	RELB	Macro_FOLR2-APOE+	2,36049E-01

Macro/Mono	ccRCC	Post(NR)	NFIC	Macro_IFI27	2,35958E-01
Macro/Mono	Melanoma	Post(R)	CREB5	Mono_CD14	2,35898E-01
DC	Melanoma	Post(NR)	ESRRA	cDC_LAMP3	2,35892E-01
Macro/Mono	BCC	Post(R)	SPI1	Macro_FOLR2-APOE+	2,35809E-01
DC	Melanoma	Post(R)	MAZ	cDC(CD1C)	2,35739E-01
DC	BCC	Post(NR)	NELFE	cDC_CLEC9A	2,35696E-01
Macro/Mono	Melanoma	Post(R)	ETS2	Mono_CD14	2,35538E-01
Mast	HNSCC	Post(R)	JUNB	Mast	2,35398E-01
DC	BCC	Post(NR)	MYB	pDC_LILRA4	2,35275E-01
DC	BCC	Post(NR)	ATF3	cDC_CLEC9A	2,35144E-01
Macro/Mono	CRC	Post(R)	BCLAF1	Macro_IER3	2,35067E-01
Macro/Mono	BCC	Post(R)	SPI1	Macro_NLRP3	2,35029E-01
Macro/Mono	Melanoma	Post(R)	BCL11A	Mono_INHBA	2,34932E-01
Macro/Mono	BCC	Post(R)	YY1	Mono_CD14	2,34873E-01
Macro/Mono	BCC	Post(NR)	BACH1	Macro_FOLR2-APOE+	2,34862E-01
DC	Melanoma	Post(NR)	ESRRA	cDC(CD1C)	2,34606E-01
DC	HNSCC	Post(NR)	ETS2	cDC_LAMP3	2,34577E-01
DC	BCC	Post(NR)	SUZ12	cDC_LAMP3	2,34573E-01
Macro/Mono	BCC	Post(NR)	NELFE	Macro_LYVE1	2,34503E-01
DC	Melanoma	Post(R)	MAZ	cDC_CLEC9A	2,34161E-01
Macro/Mono	HNSCC	Post(NR)	RELB	Macro_FOLR2+APOE+	2,33892E-01
DC	BCC	Post(NR)	JUNB	cDC(CD1C)	2,33890E-01
Macro/Mono	BCC	Post(NR)	EP300	Macro_OLFML3	2,33747E-01
Macro/Mono	Melanoma	Post(R)	TRPS1	Mono_INHBA	2,33481E-01
DC	Melanoma	Post(NR)	JUND	cDC_LAMP3	2,33291E-01
Macro/Mono	ccRCC	Post(NR)	KLF6	Mono_CD16	2,32883E-01
Macro/Mono	Melanoma	Post(R)	BACH1	Mono_CD14	2,32815E-01
Macro/Mono	BCC	Post(NR)	JUNB	Macro_IFI27	2,32809E-01
Macro/Mono	Melanoma	Post(R)	FOSB	Macro_OLFML3	2,32740E-01
Macro/Mono	ccRCC	Post(R)	FLI1	Macro_IER3	2,32642E-01
Macro/Mono	ccRCC	Post(R)	ETS2	Macro_IER3	2,32560E-01
DC	Melanoma	Post(NR)	SMARCA4	cDC_LAMP3	2,32480E-01
DC	BCC	Post(NR)	NFE2L2	cDC_CLEC9A	2,32469E-01
Macro/Mono	Melanoma	Post(R)	SMAD5	Macro_NLRP3	2,32461E-01
Macro/Mono	BCC	Post(NR)	CEBPB	Mono_INHBA	2,32459E-01
DC	Melanoma	Post(NR)	HMGB1	cDC_LAMP3	2,32402E-01
Macro/Mono	Melanoma	Post(NR)	SMARCA4	Macro_IER3	2,32353E-01
Macro/Mono	BCC	Post(R)	YY1	Macro_FOLR2-APOE+	2,32260E-01
Macro/Mono	BCC	Post(NR)	EP300	Macro_FOLR2+APOE+	2,32181E-01
Macro/Mono	Melanoma	Post(NR)	SMARCA4	Macro_LYVE1	2,31976E-01
DC	Melanoma	Post(R)	NELFE	cDC(CD1C)	2,31866E-01
Macro/Mono	HNSCC	Post(NR)	BCLAF1	Macro_FOLR2+APOE+	2,31720E-01

Macro/Mono	BCC	Post(NR)	NFE2L2	Mono_CD14	2,31718E-01
Macro/Mono	BCC	Post(NR)	NFE2L2	Mono_CD16	2,31673E-01
DC	BCC	Post(NR)	ELK1	cDC(CD1C)	2,31635E-01
DC	Melanoma	Post(R)	SMAD5	cDC_CLEC9A	2,31522E-01
DC	ccRCC	Post(R)	NELFE	cDC_LAMP3	2,31462E-01
DC	Melanoma	Post(NR)	SMARCA4	pDC_LILRA4	2,31455E-01
Macro/Mono	CRC	Post(NR)	MAFB	Macro_IER3	2,31453E-01
Macro/Mono	CRC	Post(NR)	ETV3	Mono_INHBA	2,31415E-01
Macro/Mono	Melanoma	Post(NR)	JUND	Macro_FOLR2+APOE+	2,31413E-01
Macro/Mono	Melanoma	Post(NR)	JUND	Macro_NLRP3	2,31382E-01
Macro/Mono	CRC	Post(NR)	NFKB1	Mono_INHBA	2,31342E-01
Macro/Mono	CRC	Post(NR)	POLR2A	Macro_OLFML3	2,31321E-01
Macro/Mono	ccRCC	Post(NR)	CEBPD	Macro_NLRP3	2,31303E-01
DC	BCC	Post(NR)	UBTF	pDC_LILRA4	2,31234E-01
Macro/Mono	Melanoma	Post(R)	ETS2	Macro_IFI27	2,31173E-01
Macro/Mono	Melanoma	Post(NR)	SMARCA4	Macro_IFI27	2,31011E-01
Macro/Mono	BCC	Post(NR)	NELFE	Macro_ISG15	2,30841E-01
Macro/Mono	Melanoma	Post(NR)	JUNB	Macro_FOLR2-APOE+	2,30670E-01
Macro/Mono	Melanoma	Post(NR)	SMARCA4	Macro_ISG15	2,30661E-01
Mast	HNSCC	Post(NR)	SPI1	Mast	2,30595E-01
Mast	BCC	Post(NR)	PML	Mast	2,30405E-01
Macro/Mono	BCC	Post(R)	YY1	Macro_ISG15	2,30400E-01
Macro/Mono	ccRCC	Post(NR)	NFIC	Macro_FOLR2+APOE-	2,30316E-01
DC	BCC	Post(NR)	EP300	cDC_LAMP3	2,30280E-01
DC	ccRCC	Post(NR)	CEBPD	cDC(CD1C)	2,30234E-01
Macro/Mono	CRC	Post(NR)	JUNB	Mono_INHBA	2,30042E-01
Macro/Mono	HNSCC	Post(NR)	CEBPB	Macro_ISG15	2,29780E-01
DC	ccRCC	Post(R)	EGR1	cDC_CLEC9A	2,29743E-01
Mast	CRC	Post(NR)	MAX	Mast	2,29739E-01
Macro/Mono	BCC	Post(NR)	NFKB2	Macro_OLFML3	2,29600E-01
Macro/Mono	BCC	Post(NR)	NELFE	Macro_FOLR2-APOE+	2,29550E-01
DC	BCC	Post(NR)	GTF2F1	cDC_CLEC9A	2,29476E-01
Mast	BCC	Post(R)	YY1	Mast	2,29355E-01
Macro/Mono	Melanoma	Post(NR)	ESRRA	Macro_OLFML3	2,29305E-01
DC	CRC	Post(NR)	ELF2	cDC_LAMP3	2,29184E-01
Mast	BCC	Post(NR)	SUZ12	Mast	2,29157E-01
Macro/Mono	BCC	Post(NR)	NELFE	Mono_INHBA	2,28960E-01
DC	BCC	Post(NR)	SOX4	pDC_LILRA4	2,28947E-01
DC	BCC	Post(R)	SOX4	pDC_LILRA4	2,28945E-01
Macro/Mono	BCC	Post(NR)	JUNB	Mono_CD16	2,28828E-01
Mast	Melanoma	Post(NR)	JUND	Mast	2,28755E-01
Macro/Mono	BCC	Post(NR)	NFKB1	Mono_INHBA	2,28732E-01

Macro/Mono	ccRCC	Post(NR)	REST	Macro_IER3	2,28585E-01
Macro/Mono	Melanoma	Post(R)	SMARCA4	Macro_OLFML3	2,28453E-01
Macro/Mono	ccRCC	Post(R)	SPI1	Mono_INHBA	2,28452E-01
Macro/Mono	ccRCC	Post(NR)	KLF6	Mono_INHBA	2,28428E-01
DC	BCC	Post(NR)	IRF8	pDC_LILRA4	2,28377E-01
DC	BCC	Post(NR)	ELK1	cDC_CLEC9A	2,28163E-01
Mast	BCC	Post(NR)	UBTF	Mast	2,28141E-01
Macro/Mono	BCC	Post(R)	YY1	Macro_NLRP3	2,28018E-01
Macro/Mono	Melanoma	Post(R)	CREB5	Macro_ISG15	2,27877E-01
Macro/Mono	BCC	Post(NR)	JUNB	Mono_CD14	2,27851E-01
Macro/Mono	CRC	Post(R)	MAF	Macro_LYVE1	2,27810E-01
Macro/Mono	BCC	Post(R)	YY1	Macro_IFI27	2,27724E-01
DC	HNSCC	Post(R)	KLF4	pDC_LILRA4	2,27723E-01
Macro/Mono	Melanoma	Post(NR)	JUNB	Mono_CD14	2,27630E-01
Macro/Mono	BCC	Post(NR)	CEBPB	Macro_IER3	2,27605E-01
Macro/Mono	Melanoma	Post(R)	FOSB	Macro_ISG15	2,27543E-01
Macro/Mono	BCC	Post(NR)	ELK1	Macro_FOLR2+APOE+	2,27462E-01
Macro/Mono	Melanoma	Post(NR)	JUND	Macro_IER3	2,27321E-01
DC	ccRCC	Post(NR)	MAFB	cDC_CLEC9A	2,27234E-01
DC	Melanoma	Post(NR)	TCF4	pDC_LILRA4	2,27077E-01
Macro/Mono	BCC	Post(R)	YY1	Macro_OLFML3	2,27075E-01
DC	Melanoma	Post(R)	RUNX1	pDC_LILRA4	2,27028E-01
Macro/Mono	Melanoma	Post(NR)	SMARCA4	Macro_FOLR2+APOE+	2,26896E-01
DC	Melanoma	Post(NR)	ESRRA	pDC_LILRA4	2,26879E-01
Macro/Mono	ccRCC	Post(R)	NELFE	Macro_IFI27	2,26701E-01
Macro/Mono	Melanoma	Post(R)	ZNF274	Macro_NLRP3	2,26607E-01
Macro/Mono	BCC	Post(R)	GABPB1	Mono_CD16	2,26567E-01
DC	ccRCC	Post(R)	ELF2	cDC_CLEC9A	2,26331E-01
Macro/Mono	ccRCC	Post(NR)	KLF6	Macro_OLFML3	2,26279E-01
Macro/Mono	ccRCC	Post(NR)	CEBPD	Mono_CD16	2,26216E-01
Mast	ccRCC	Post(R)	CREB1	Mast	2,26207E-01
Macro/Mono	Melanoma	Post(R)	RELB	Mono_INHBA	2,26081E-01
DC	Melanoma	Post(R)	MAZ	pDC_LILRA4	2,25967E-01
DC	HNSCC	Post(NR)	ETS2	pDC_LILRA4	2,25946E-01
Macro/Mono	BCC	Post(NR)	CEBPB	Macro_ISG15	2,25914E-01
Macro/Mono	Melanoma	Post(R)	MAZ	Mono_INHBA	2,25894E-01
Macro/Mono	CRC	Post(R)	BCLAF1	Macro_IFI27	2,25834E-01
Macro/Mono	Melanoma	Post(NR)	JUND	Macro_ISG15	2,25750E-01
Mast	ccRCC	Post(R)	PML	Mast	2,25641E-01
Mast	Melanoma	Post(NR)	ESRRA	Mast	2,25596E-01
Macro/Mono	BCC	Post(R)	YY1	Macro_LYVE1	2,25447E-01
Macro/Mono	BCC	Post(NR)	GTF2F1	Mono_CD14	2,25397E-01

Macro/Mono	Melanoma	Post(R)	JUN	Mono_INHBA	2,25314E-01
Macro/Mono	BCC	Post(R)	GABPB1	Macro_LYVE1	2,25193E-01
Macro/Mono	BCC	Post(NR)	GTF2F1	Mono_CD16	2,25169E-01
Macro/Mono	Melanoma	Post(R)	BACH1	Macro_IFI27	2,25164E-01
Macro/Mono	Melanoma	Post(R)	NELFE	Macro_OLFML3	2,25032E-01
DC	Melanoma	Post(R)	EZH2	cDC(CD1C)	2,25023E-01
DC	Melanoma	Post(NR)	JUND	pDC_LILRA4	2,24962E-01
Macro/Mono	ccRCC	Post(R)	STAT1	Macro_IFI27	2,24913E-01
Macro/Mono	Melanoma	Post(R)	MXI1	Mono_CD14	2,24882E-01
Macro/Mono	BCC	Post(NR)	CEBPB	Mono_CD14	2,24828E-01
Macro/Mono	CRC	Post(R)	YBX1	Macro_FOLR2+APOE-	2,24803E-01
Macro/Mono	HNSCC	Post(NR)	JUNB	Mono_INHBA	2,24642E-01
Mast	CRC	Post(R)	SPI1	Mast	2,24559E-01
DC	BCC	Post(R)	SPI1	cDC(CD1C)	2,24508E-01
Macro/Mono	HNSCC	Post(NR)	JUNB	Macro_ISG15	2,24299E-01
DC	HNSCC	Post(R)	EGR1	pDC_LILRA4	2,24103E-01
Macro/Mono	ccRCC	Post(NR)	KLF6	Mono_CD14	2,24064E-01
Macro/Mono	BCC	Post(R)	GABPB1	Mono_CD14	2,23845E-01
Macro/Mono	BCC	Post(NR)	GTF2F1	Macro_LYVE1	2,23838E-01
DC	Melanoma	Post(NR)	JUND	cDC_CLEC9A	2,23759E-01
Macro/Mono	CRC	Post(NR)	MAFB	Macro_OLFML3	2,23755E-01
Macro/Mono	HNSCC	Post(NR)	ZMIZ1	Macro_NLRP3	2,23662E-01
Macro/Mono	HNSCC	Post(NR)	NFE2L2	Mono_INHBA	2,23473E-01
Macro/Mono	BCC	Post(NR)	FOS	Mono_CD14	2,23462E-01
Macro/Mono	Melanoma	Post(R)	CEBPZ	Macro_OLFML3	2,23433E-01
Macro/Mono	Melanoma	Post(R)	MAZ	Macro_ISG15	2,23269E-01
Macro/Mono	Melanoma	Post(NR)	MAFB	Macro_IER3	2,23055E-01
Macro/Mono	ccRCC	Post(R)	ELF2	Mono_INHBA	2,22941E-01
Macro/Mono	ccRCC	Post(NR)	FOXN3	Mono_INHBA	2,22801E-01
DC	ccRCC	Post(R)	ELF2	cDC_LAMP3	2,22789E-01
Macro/Mono	CRC	Post(NR)	CREM	Macro_ISG15	2,22605E-01
Macro/Mono	BCC	Post(R)	ENO1	Macro_FOLR2+APOE-	2,22592E-01
DC	CRC	Post(NR)	ETV3	cDC(CD1C)	2,22553E-01
Macro/Mono	BCC	Post(NR)	CEBPD	Macro_NLRP3	2,22509E-01
Macro/Mono	BCC	Post(NR)	GTF2F1	Macro_NLRP3	2,22448E-01
DC	ccRCC	Post(R)	ETS2	cDC(CD1C)	2,22318E-01
Macro/Mono	Melanoma	Post(NR)	MAFB	Macro_ISG15	2,22308E-01
DC	BCC	Post(NR)	SPI1	cDC_LAMP3	2,22231E-01
DC	BCC	Post(NR)	NELFE	cDC(CD1C)	2,22176E-01
Macro/Mono	BCC	Post(NR)	FOS	Macro_IER3	2,22158E-01
Macro/Mono	CRC	Post(R)	BACH1	Macro_NLRP3	2,22048E-01
DC	BCC	Post(NR)	CEBPD	cDC(CD1C)	2,22001E-01

Mast	CRC	Post(R)	REST	Mast	2,21920E-01
Macro/Mono	ccRCC	Post(NR)	CEBPD	Macro_IER3	2,21809E-01
Macro/Mono	ccRCC	Post(NR)	KLF10	Macro_IER3	2,21671E-01
Macro/Mono	Melanoma	Post(NR)	ESRRA	Macro_FOLR2+APOE+	2,21667E-01
Macro/Mono	Melanoma	Post(R)	RUNX1	Macro_IFI27	2,21499E-01
DC	ccRCC	Post(NR)	CREB1	pDC_LILRA4	2,21456E-01
Macro/Mono	Melanoma	Post(R)	NELFE	Macro_ISG15	2,21306E-01
Macro/Mono	CRC	Post(NR)	ETV3	Mono_CD14	2,21142E-01
Macro/Mono	ccRCC	Post(R)	SPI1	Mono_CD16	2,21123E-01
Macro/Mono	CRC	Post(NR)	ELK3	Mono_CD16	2,21053E-01
DC	Melanoma	Post(NR)	JUNB	pDC_LILRA4	2,21040E-01
Macro/Mono	ccRCC	Post(R)	STAT1	Macro_FOLR2-APOE+	2,20872E-01
Macro/Mono	Melanoma	Post(R)	RELA	Macro_IFI27	2,20811E-01
Macro/Mono	Melanoma	Post(R)	NELFE	Mono_CD14	2,20768E-01
Macro/Mono	HNSCC	Post(NR)	JUNB	Macro_OLFML3	2,20690E-01
Macro/Mono	ccRCC	Post(NR)	KLF6	Macro_LYVE1	2,20641E-01
Macro/Mono	Melanoma	Post(R)	EZH2	Macro_OLFML3	2,20598E-01
Macro/Mono	BCC	Post(NR)	CREM	Macro_LYVE1	2,20501E-01
DC	Melanoma	Post(NR)	SIN3A	pDC_LILRA4	2,20440E-01
Mast	Melanoma	Post(NR)	ZNF250	Mast	2,20438E-01
Macro/Mono	ccRCC	Post(NR)	MAF	Macro_LYVE1	2,20437E-01
Macro/Mono	CRC	Post(R)	RAD21	Macro_FOLR2+APOE+	2,20418E-01
Macro/Mono	ccRCC	Post(R)	FLI1	Macro_FOLR2-APOE+	2,20410E-01
DC	BCC	Post(NR)	JUNB	cDC_LAMP3	2,20379E-01
Macro/Mono	HNSCC	Post(R)	CEBPB	Macro_ISG15	2,20364E-01
Macro/Mono	CRC	Post(R)	IRF8	Macro_FOLR2-APOE+	2,20329E-01
DC	BCC	Post(NR)	GTF2F1	cDC(CD1C)	2,20150E-01
Macro/Mono	CRC	Post(NR)	BCLAF1	Macro_FOLR2+APOE-	2,20148E-01
Macro/Mono	Melanoma	Post(R)	BACH1	Macro_OLFML3	2,20102E-01
Macro/Mono	BCC	Post(NR)	CEBPB	Macro_FOLR2+APOE+	2,19967E-01
Macro/Mono	Melanoma	Post(R)	BACH1	Macro_ISG15	2,19772E-01
Macro/Mono	Melanoma	Post(NR)	ESRRA	Mono_CD16	2,19691E-01
DC	HNSCC	Post(R)	NR3C1	cDC_LAMP3	2,19455E-01
Macro/Mono	BCC	Post(NR)	BACH1	Mono_CD14	2,19427E-01
Macro/Mono	CRC	Post(NR)	BCLAF1	Macro_IFI27	2,19390E-01
Macro/Mono	BCC	Post(NR)	FOS	Macro_ISG15	2,19277E-01
DC	ccRCC	Post(R)	SAP30	cDC_LAMP3	2,19258E-01
DC	HNSCC	Post(NR)	JUNB	cDC_LAMP3	2,19134E-01
Macro/Mono	BCC	Post(R)	GABPB1	Macro_ISG15	2,18943E-01
Macro/Mono	Melanoma	Post(NR)	ESRRA	Mono_CD14	2,18738E-01
Macro/Mono	Melanoma	Post(R)	EZH2	Macro_IFI27	2,18559E-01
Macro/Mono	BCC	Post(NR)	JUNB	Macro_LYVE1	2,18496E-01

DC	BCC	Post(NR)	NFE2L2	cDC_LAMP3	2,18449E-01
Macro/Mono	ccRCC	Post(R)	NELFE	Macro_LYVE1	2,18362E-01
Macro/Mono	ccRCC	Post(R)	SPI1	Macro_NLRP3	2,18290E-01
Macro/Mono	BCC	Post(NR)	BACH1	Macro_ISG15	2,17987E-01
Macro/Mono	CRC	Post(NR)	MAFB	Macro_LYVE1	2,17881E-01
Macro/Mono	Melanoma	Post(NR)	MAFB	Mono_INHBA	2,17833E-01
DC	BCC	Post(NR)	NELFE	cDC_LAMP3	2,17823E-01
Macro/Mono	ccRCC	Post(R)	KLF6	Macro_LYVE1	2,17786E-01
Macro/Mono	BCC	Post(R)	GABPB1	Mono_INHBA	2,17713E-01
Macro/Mono	BCC	Post(R)	SPI1	Mono_CD14	2,17609E-01
Macro/Mono	BCC	Post(NR)	CREM	Macro_FOLR2-APOE+	2,17573E-01
Macro/Mono	ccRCC	Post(NR)	KLF6	Macro_ISG15	2,17462E-01
Macro/Mono	BCC	Post(NR)	CEBPB	Macro_FOLR2-APOE+	2,17446E-01
DC	ccRCC	Post(R)	EGR1	cDC_LAMP3	2,17211E-01
Macro/Mono	CRC	Post(R)	IRF1	Mono_CD14	2,17129E-01
Mast	ccRCC	Post(NR)	SPI1	Mast	2,17022E-01
Macro/Mono	Melanoma	Post(NR)	JUND	Mono_CD16	2,16976E-01
Macro/Mono	BCC	Post(R)	SPI1	Macro_FOLR2+APOE+	2,16964E-01
Macro/Mono	CRC	Post(R)	CEBPB	Macro_IER3	2,16763E-01
DC	ccRCC	Post(R)	NELFE	pDC_LILRA4	2,16606E-01
Macro/Mono	BCC	Post(R)	SPI1	Macro_IER3	2,16490E-01
Macro/Mono	CRC	Post(NR)	BCL3	Macro_OLFML3	2,16400E-01
Macro/Mono	BCC	Post(R)	GABPB1	Macro_IFI27	2,16362E-01
Macro/Mono	BCC	Post(NR)	NELFE	Macro_FOLR2+APOE+	2,16163E-01
Mast	ccRCC	Post(R)	ZMIZ1	Mast	2,16157E-01
Macro/Mono	BCC	Post(NR)	FOSL2	Macro_FOLR2+APOE+	2,15994E-01
Macro/Mono	HNSCC	Post(NR)	NFE2L2	Macro_NLRP3	2,15882E-01
Macro/Mono	BCC	Post(NR)	GTF2F1	Macro_IFI27	2,15870E-01
Macro/Mono	Melanoma	Post(NR)	ESRRA	Macro_ISG15	2,15813E-01
Mast	CRC	Post(R)	JUN	Mast	2,15719E-01
Macro/Mono	ccRCC	Post(NR)	CREB1	Macro_FOLR2+APOE-	2,15478E-01
Macro/Mono	HNSCC	Post(NR)	NFE2L2	Macro_IER3	2,15429E-01
Macro/Mono	CRC	Post(NR)	RAD21	Macro_OLFML3	2,15423E-01
Macro/Mono	CRC	Post(NR)	BCLAF1	Macro_FOLR2-APOE+	2,15419E-01
Macro/Mono	CRC	Post(R)	BCLAF1	Macro_FOLR2+APOE-	2,15380E-01
Macro/Mono	ccRCC	Post(NR)	KLF6	Macro_FOLR2-APOE+	2,15246E-01
DC	CRC	Post(R)	SPI1	pDC_LILRA4	2,15195E-01
Macro/Mono	CRC	Post(NR)	GTF2F1	Macro_LYVE1	2,15149E-01
Macro/Mono	Melanoma	Post(NR)	ESRRA	Macro_IER3	2,14839E-01
Macro/Mono	CRC	Post(R)	IRF8	Macro_IER3	2,14832E-01
Macro/Mono	ccRCC	Post(R)	SPI1	Mono_CD14	2,14774E-01
DC	CRC	Post(NR)	ELF2	cDC_CLEC9A	2,14764E-01

Macro/Mono	Melanoma	Post(NR)	JUND	Mono_INHBA	2,14697E-01
Macro/Mono	BCC	Post(R)	ENO1	Macro_FOLR2+APOE+	2,14692E-01
DC	Melanoma	Post(R)	EZH2	cDC_CLEC9A	2,14616E-01
Macro/Mono	HNSCC	Post(NR)	BCLAF1	Macro_ISG15	2,14526E-01
Macro/Mono	Melanoma	Post(NR)	ESRRA	Macro_FOLR2-APOE+	2,14481E-01
Macro/Mono	ccRCC	Post(NR)	REST	Macro_FOLR2-APOE+	2,14452E-01
Macro/Mono	ccRCC	Post(NR)	KLF4	Macro_FOLR2+APOE+	2,14359E-01
Macro/Mono	CRC	Post(NR)	RAD21	Mono_CD14	2,14351E-01
Macro/Mono	BCC	Post(NR)	FOSL2	Mono_CD16	2,14133E-01
Macro/Mono	ccRCC	Post(R)	ETS2	Macro_FOLR2-APOE+	2,14044E-01
Macro/Mono	CRC	Post(NR)	CEBPB	Macro_NLRP3	2,13818E-01
DC	Melanoma	Post(R)	CEBPZ	cDC_CLEC9A	2,13817E-01
Mast	BCC	Post(NR)	ATF3	Mast	2,13373E-01
Macro/Mono	BCC	Post(NR)	PML	Mono_CD14	2,13370E-01
Macro/Mono	CRC	Post(NR)	CREB1	Macro_LYVE1	2,13335E-01
Macro/Mono	CRC	Post(NR)	CREM	Mono_CD14	2,13236E-01
DC	BCC	Post(NR)	ELK1	pDC_LILRA4	2,13163E-01
Macro/Mono	BCC	Post(NR)	GTF2F1	Macro_IER3	2,12988E-01
Macro/Mono	Melanoma	Post(R)	CREB5	Macro_NLRP3	2,12874E-01
Macro/Mono	Melanoma	Post(NR)	ESRRA	Macro_NLRP3	2,12824E-01
Macro/Mono	BCC	Post(R)	GABPB1	Macro_NLRP3	2,12817E-01
Macro/Mono	BCC	Post(NR)	GTF2F1	Macro_FOLR2-APOE+	2,12786E-01
Macro/Mono	CRC	Post(NR)	ETV3	Macro_NLRP3	2,12722E-01
DC	ccRCC	Post(R)	ELF2	pDC_LILRA4	2,12704E-01
Macro/Mono	Melanoma	Post(NR)	ESRRA	Mono_INHBA	2,12703E-01
Macro/Mono	BCC	Post(NR)	PML	Mono_CD16	2,12613E-01
DC	Melanoma	Post(R)	ELK1	cDC_CLEC9A	2,12365E-01
Macro/Mono	Melanoma	Post(R)	NELFE	Macro_FOLR2-APOE+	2,12213E-01
DC	CRC	Post(R)	REST	pDC_LILRA4	2,12072E-01
DC	Melanoma	Post(R)	EP300	cDC_CLEC9A	2,11965E-01
DC	Melanoma	Post(NR)	MAZ	cDC_CLEC9A	2,11875E-01
Macro/Mono	ccRCC	Post(R)	EGR1	Macro_IER3	2,11589E-01
Macro/Mono	BCC	Post(NR)	RARA	Macro_FOLR2+APOE-	2,11497E-01
Macro/Mono	CRC	Post(NR)	CUX1	Mono_CD16	2,11322E-01
DC	BCC	Post(R)	NFKB2	cDC(CD1C)	2,11298E-01
Macro/Mono	Melanoma	Post(R)	KLF13	Macro_IFI27	2,11295E-01
Macro/Mono	BCC	Post(NR)	FOS	Macro_FOLR2-APOE+	2,10824E-01
Macro/Mono	Melanoma	Post(R)	EZH2	Mono_CD14	2,10580E-01
Macro/Mono	HNSCC	Post(R)	JUNB	Macro_IER3	2,10281E-01
Macro/Mono	Melanoma	Post(R)	NELFE	Macro_NLRP3	2,10160E-01
Macro/Mono	Melanoma	Post(NR)	MAZ	Mono_CD14	2,10135E-01
Macro/Mono	BCC	Post(NR)	BACH1	Macro_FOLR2+APOE+	2,10005E-01

Macro/Mono	ccRCC	Post(NR)	REST	Macro_ISG15	2,09930E-01
Macro/Mono	BCC	Post(NR)	NELFE	Macro_OLFML3	2,09923E-01
Macro/Mono	HNSCC	Post(R)	KLF4	Macro_FOLR2+APOE-	2,09660E-01
DC	ccRCC	Post(R)	SPI1	pDC_LILRA4	2,09580E-01
Macro/Mono	CRC	Post(NR)	CREM	Macro_OLFML3	2,09533E-01
Macro/Mono	Melanoma	Post(NR)	MAZ	Macro_NLRP3	2,09443E-01
DC	BCC	Post(NR)	SUZ12	pDC_LILRA4	2,09379E-01
DC	ccRCC	Post(NR)	MYC	pDC_LILRA4	2,09321E-01
Macro/Mono	ccRCC	Post(R)	SAP30	Macro_OLFML3	2,09183E-01
Macro/Mono	BCC	Post(NR)	CREM	Macro_IER3	2,08990E-01
Macro/Mono	BCC	Post(R)	NFKB2	Macro_OLFML3	2,08909E-01
Macro/Mono	Melanoma	Post(R)	ELK1	Macro_OLFML3	2,08905E-01
Macro/Mono	BCC	Post(NR)	GTF2F1	Macro_ISG15	2,08882E-01
DC	Melanoma	Post(NR)	MAZ	cDC(CD1C)	2,08860E-01
Macro/Mono	CRC	Post(R)	FOS	Mono_INHBA	2,08786E-01
DC	Melanoma	Post(NR)	MAZ	pDC_LILRA4	2,08751E-01
DC	HNSCC	Post(R)	EGR1	cDC(CD1C)	2,08741E-01
Macro/Mono	Melanoma	Post(NR)	ESRRA	Macro_LYVE1	2,08684E-01
DC	CRC	Post(NR)	CUX1	cDC_LAMP3	2,08536E-01
Macro/Mono	BCC	Post(R)	SPI1	Macro_ISG15	2,08450E-01
Macro/Mono	BCC	Post(NR)	FOS	Mono_INHBA	2,08293E-01
Macro/Mono	ccRCC	Post(NR)	CEBPD	Macro_OLFML3	2,08280E-01
Macro/Mono	HNSCC	Post(R)	JUNB	Macro_NLRP3	2,08126E-01
Macro/Mono	CRC	Post(R)	IRF1	Mono_CD16	2,08047E-01
Macro/Mono	BCC	Post(NR)	KDM5B	Macro_FOLR2+APOE-	2,08002E-01
Macro/Mono	CRC	Post(R)	IRF8	Macro_OLFML3	2,07905E-01
Macro/Mono	Melanoma	Post(NR)	MAFB	Macro_NLRP3	2,07825E-01
DC	Melanoma	Post(NR)	SIN3A	cDC_CLEC9A	2,07804E-01
DC	BCC	Post(NR)	PML	pDC_LILRA4	2,07801E-01
DC	Melanoma	Post(NR)	MAZ	cDC_LAMP3	2,07795E-01
Macro/Mono	BCC	Post(NR)	JUNB	Macro_FOLR2+APOE-	2,07697E-01
Macro/Mono	BCC	Post(R)	NFKB2	Macro_FOLR2-APOE+	2,07665E-01
DC	ccRCC	Post(NR)	PML	pDC_LILRA4	2,07521E-01
Mast	HNSCC	Post(R)	SPI1	Mast	2,07517E-01
Macro/Mono	Melanoma	Post(NR)	FOSB	Macro_NLRP3	2,07507E-01
Macro/Mono	Melanoma	Post(NR)	CEBPB	Macro_NLRP3	2,07326E-01
DC	ccRCC	Post(R)	NELFE	cDC(CD1C)	2,07304E-01
DC	Melanoma	Post(R)	SREBF1	cDC_CLEC9A	2,07262E-01
DC	BCC	Post(NR)	GTF2F1	cDC_LAMP3	2,07247E-01
DC	Melanoma	Post(R)	CEBPZ	pDC_LILRA4	2,07197E-01
Macro/Mono	Melanoma	Post(R)	E2F4	Macro_ISG15	2,07158E-01
Macro/Mono	BCC	Post(R)	YY1	Mono_INHBA	2,07119E-01

Macro/Mono	ccRCC	Post(NR)	FOSB	Macro_IER3	2,07071E-01
Macro/Mono	Melanoma	Post(R)	CEBPZ	Macro_ISG15	2,07033E-01
Macro/Mono	Melanoma	Post(NR)	IRF4	Macro_NLRP3	2,07028E-01
Macro/Mono	HNSCC	Post(NR)	CEBPD	Macro_IER3	2,06778E-01
Macro/Mono	HNSCC	Post(NR)	CEBPB	Mono_CD14	2,06635E-01
Macro/Mono	Melanoma	Post(NR)	MAZ	Mono_INHBA	2,06631E-01
Mast	Melanoma	Post(NR)	EP300	Mast	2,06585E-01
Macro/Mono	BCC	Post(NR)	CREM	Macro_OLFML3	2,06329E-01
Macro/Mono	CRC	Post(NR)	CEBPB	Macro_ISG15	2,06328E-01
DC	BCC	Post(NR)	JUNB	cDC_CLEC9A	2,06054E-01
Macro/Mono	Melanoma	Post(NR)	MAZ	Mono_CD16	2,05954E-01
DC	ccRCC	Post(R)	EGR1	pDC_LILRA4	2,05946E-01
Macro/Mono	HNSCC	Post(NR)	CEBPD	Macro_LYVE1	2,05755E-01
Macro/Mono	CRC	Post(NR)	CEBPB	Mono_INHBA	2,05732E-01
Macro/Mono	Melanoma	Post(R)	FOS	Mono_INHBA	2,05502E-01
DC	BCC	Post(NR)	ETS1	pDC_LILRA4	2,05417E-01
Macro/Mono	BCC	Post(NR)	BACH1	Macro_LYVE1	2,05372E-01
DC	BCC	Post(NR)	BATF3	cDC_LAMP3	2,05352E-01
Macro/Mono	BCC	Post(R)	GABPB1	Macro_IER3	2,05333E-01
DC	Melanoma	Post(R)	SMARCA4	cDC(CD1C)	2,05330E-01
Macro/Mono	Melanoma	Post(R)	EP300	Mono_INHBA	2,05266E-01
Macro/Mono	Melanoma	Post(NR)	JUND	Macro_LYVE1	2,05161E-01
Macro/Mono	HNSCC	Post(NR)	CEBPD	Macro_ISG15	2,05156E-01
Macro/Mono	Melanoma	Post(NR)	MAZ	Macro_LYVE1	2,05152E-01
DC	HNSCC	Post(R)	NR3C1	cDC_CLEC9A	2,05142E-01
DC	HNSCC	Post(NR)	JUNB	cDC(CD1C)	2,05089E-01
Macro/Mono	Melanoma	Post(R)	NELFE	Macro_IFI27	2,04984E-01
Macro/Mono	Melanoma	Post(R)	EP300	Mono_CD14	2,04953E-01
Macro/Mono	Melanoma	Post(R)	SMARCA4	Macro_FOLR2-APOE+	2,04791E-01
Macro/Mono	Melanoma	Post(R)	RELA	Macro_NLRP3	2,04790E-01
Macro/Mono	ccRCC	Post(R)	NELFE	Macro_FOLR2-APOE+	2,04641E-01
DC	Melanoma	Post(R)	BCL11A	pDC_LILRA4	2,04304E-01
DC	BCC	Post(NR)	CREM	cDC_CLEC9A	2,04280E-01
Mast	ccRCC	Post(R)	FOS	Mast	2,04255E-01
Macro/Mono	Melanoma	Post(R)	EZH2	Macro_FOLR2-APOE+	2,04179E-01
Macro/Mono	BCC	Post(NR)	GTF2F1	Mono_INHBA	2,04084E-01
Mast	ccRCC	Post(NR)	TAF1	Mast	2,03925E-01
Macro/Mono	ccRCC	Post(R)	FOS	Mono_CD16	2,03876E-01
Macro/Mono	Melanoma	Post(NR)	JUND	Mono_CD14	2,03839E-01
Macro/Mono	ccRCC	Post(NR)	REST	Macro_OLFML3	2,03817E-01
Macro/Mono	BCC	Post(NR)	CREM	Macro_NLRP3	2,03801E-01
DC	Melanoma	Post(NR)	EP300	cDC_CLEC9A	2,03782E-01

Macro/Mono	ccRCC	Post(R)	POLR2A	Macro_FOLR2-APOE+	2,03777E-01
Macro/Mono	BCC	Post(NR)	BACH1	Macro_IFI27	2,03701E-01
Macro/Mono	Melanoma	Post(NR)	MAZ	Macro_FOLR2-APOE+	2,03660E-01
Macro/Mono	BCC	Post(NR)	CEBPD	Mono_CD14	2,03524E-01
Macro/Mono	Melanoma	Post(NR)	MAZ	Macro_IER3	2,03513E-01
DC	CRC	Post(R)	HDAC2	pDC_LILRA4	2,03465E-01
DC	CRC	Post(NR)	CREM	cDC(CD1C)	2,03463E-01
Macro/Mono	BCC	Post(R)	SPI1	Macro_LYVE1	2,03457E-01
Macro/Mono	BCC	Post(NR)	KDM5B	Mono_CD14	2,03442E-01
DC	HNSCC	Post(NR)	ETS2	cDC_CLEC9A	2,03401E-01
Macro/Mono	HNSCC	Post(NR)	CEBPB	Macro_FOLR2+APOE+	2,03306E-01
Macro/Mono	ccRCC	Post(NR)	FOXN3	Macro_NLRP3	2,03126E-01
Mast	BCC	Post(NR)	MAZ	Mast	2,03093E-01
Macro/Mono	Melanoma	Post(NR)	MAZ	Macro_OLFML3	2,03064E-01
Macro/Mono	BCC	Post(NR)	CREM	Mono_INHBA	2,02954E-01
Macro/Mono	BCC	Post(NR)	PML	Macro_NLRP3	2,02921E-01
Macro/Mono	BCC	Post(NR)	GTF2F1	Macro_FOLR2+APOE+	2,02912E-01
Macro/Mono	BCC	Post(NR)	GTF2F1	Macro_FOLR2+APOE-	2,02857E-01
DC	Melanoma	Post(R)	EP300	cDC(CD1C)	2,02838E-01
DC	Melanoma	Post(R)	SREBF1	cDC(CD1C)	2,02795E-01
Macro/Mono	Melanoma	Post(NR)	MAZ	Macro_FOLR2+APOE+	2,02785E-01
Macro/Mono	ccRCC	Post(NR)	NFIC	Macro_IER3	2,02752E-01
Macro/Mono	Melanoma	Post(R)	SMAD5	Mono_CD14	2,02690E-01
Macro/Mono	HNSCC	Post(NR)	JUNB	Macro_FOLR2+APOE+	2,02604E-01
DC	Melanoma	Post(R)	ELK1	cDC(CD1C)	2,02599E-01
Macro/Mono	ccRCC	Post(R)	MYC	Macro_IFI27	2,02573E-01
Macro/Mono	BCC	Post(NR)	KDM5B	Mono_INHBA	2,02504E-01
Macro/Mono	Melanoma	Post(R)	EP300	Macro_NLRP3	2,02501E-01
Mast	Melanoma	Post(NR)	KLF6	Mast	2,02469E-01
DC	Melanoma	Post(NR)	SIN3A	cDC_LAMP3	2,02299E-01
Macro/Mono	BCC	Post(NR)	FOSL2	Macro_IFI27	2,02164E-01
DC	Melanoma	Post(NR)	EP300	cDC_LAMP3	2,01965E-01
Macro/Mono	CRC	Post(NR)	CEBPB	Mono_CD14	2,01959E-01
Mast	BCC	Post(NR)	SPI1	Mast	2,01943E-01
Macro/Mono	ccRCC	Post(R)	GTF2F1	Macro_FOLR2+APOE-	2,01793E-01
Mast	BCC	Post(R)	CREB3L2	Mast	2,01740E-01
Mast	CRC	Post(NR)	ELF2	Mast	2,01611E-01
Macro/Mono	BCC	Post(NR)	SUZ12	Mono_CD16	2,01590E-01
DC	ccRCC	Post(R)	FLI1	cDC(CD1C)	2,01572E-01
DC	BCC	Post(NR)	SPIB	pDC_LILRA4	2,01532E-01
DC	Melanoma	Post(R)	SREBF1	pDC_LILRA4	2,01497E-01
Macro/Mono	ccRCC	Post(NR)	MAF	Macro_FOLR2+APOE-	2,01440E-01

Macro/Mono	CRC	Post(NR)	ETV3	Macro_IFI27	2,01249E-01
Macro/Mono	ccRCC	Post(R)	FOS	Mono_CD14	2,01233E-01
Macro/Mono	Melanoma	Post(R)	ELK1	Macro_ISG15	2,01227E-01
Macro/Mono	Melanoma	Post(NR)	MAZ	Macro_ISG15	2,01185E-01
Macro/Mono	Melanoma	Post(R)	CEBPZ	Mono_CD14	2,01054E-01
Macro/Mono	BCC	Post(NR)	FOSL2	Macro_LYVE1	2,01012E-01
Mast	ccRCC	Post(NR)	KLF6	Mast	2,00980E-01
Macro/Mono	ccRCC	Post(NR)	KLF6	Macro_FOLR2+APOE+	2,00778E-01
DC	BCC	Post(R)	NFE2L2	cDC(CD1C)	2,00776E-01
DC	BCC	Post(NR)	KDM5B	cDC_LAMP3	2,00763E-01
DC	ccRCC	Post(R)	POLR2A	cDC(CD1C)	2,00736E-01
Macro/Mono	BCC	Post(R)	SPI1	Mono_CD16	2,00586E-01
Macro/Mono	BCC	Post(NR)	SUZ12	Mono_CD14	2,00147E-01
Macro/Mono	Melanoma	Post(NR)	IRF4	Macro_ISG15	2,00140E-01
Macro/Mono	BCC	Post(NR)	PML	Macro_ISG15	2,00130E-01
Macro/Mono	Melanoma	Post(R)	KLF6	Mono_INHBA	2,00079E-01
