# Supplementary material for: A single-cell pan-cancer analysis to show the variability of tumor-infiltrating myeloid cells in immune checkpoint blockade
Source: Nat Commun. 2024 Jul 21;15:6142. doi: 10.1038/s41467-024-50478-8 (PMC11271490; doi:10.1038/s41467-024-50478-8)
Supplement: Supplementary file 8 — Supplementary Data 5 [file 41467_2024_50478_MOESM8_ESM.pdf]

Post_NR_Myeloid_Myeloid_CellCell

Response	source	target	ligand.complex	receptor.complex	aggregate_rank	mean_rank	natmi.edge_specificity	natmi.rank	connectome.weight_sc	connectome.rank	logfc.logfc_comb	logfc.rank	sca.LRscore	sca.rank	cellphonedb.pvalue	cellphonedb.rank
Non-responder	cDC_LAMP3	cDC_LAMP3	CCL19	CCR7	1,57382E-11	4198,7	5,92054E-01	4	3,66789E+00	1	5,07407E+00	5	9,27748E-01	3453	0	17530,5
Non-responder	cDC_LAMP3	pDC_LILRA4	CCL19	CXCR3	6,44599E-11	5104,1	5,16069E-01	8	2,88254E+00	3	5,13471E+00	4	9,05833E-01	7975	0	17530,5
Non-responder	pDC_LILRA4	pDC_LILRA4	SCT	VIPR2	1,00694E-09	10488,9	9,91012E-01	2	2,40030E+00	11	4,61381E+00	20	7,95701E-01	34881	0	17530,5
Non-responder	Macro_OLFML3	pDC_LILRA4	CXCL10	CXCR3	3,06921E-09	4630,9	1,88113E-01	29	2,09595E+00	22	5,04613E+00	6	9,16876E-01	5567	0	17530,5
Non-responder	Mono_CD14	Mast	S100A8	CD69	4,27597E-09	3594,9	7,53538E-02	266	2,04575E+00	27	3,61481E+00	69	9,54012E-01	82	0	17530,5
Non-responder	pDC_LILRA4	Macro_FOLR2+APOE+	SCT	GPR84	4,94568E-09	8101,5	2,09128E-01	23	2,07406E+00	25	4,13737E+00	34	8,45243E-01	22895	0	17530,5
Non-responder	Macro_FOLR2+APOE+	pDC_LILRA4	CXCL9	CXCR3	5,87055E-09	4897,1	1,76948E-01	36	2,07916E+00	24	4,79362E+00	12	9,10713E-01	6883	0	17530,5
Non-responder	pDC_LILRA4	pDC_LILRA4	GZMB	IGF2R	6,90405E-09	4338,5	1,74170E-01	38	2,44791E+00	9	6,42113E+00	1	9,24299E-01	4114	0	17530,5
Non-responder	Macro_OLFML3	pDC_LILRA4	CXCL9	CXCR3	8,67118E-09	4996,7	1,67415E-01	41	2,01986E+00	30	4,77974E+00	13	9,08436E-01	7369	0	17530,5
Non-responder	Macro_ISG15	pDC_LILRA4	CXCL10	CXCR3	1,07166E-08	4894,7	1,61367E-01	44	1,93867E+00	37	4,85964E+00	9	9,10842E-01	6853	0	17530,5
Non-responder	Macro_OLFML3	pDC_LILRA4	CXCL11	CXCR3	1,14638E-08	7318,7	2,68136E-01	17	1,88109E+00	45	4,29693E+00	25	8,61730E-01	18976	0	17530,5
Non-responder	cDC_LAMP3	pDC_LILRA4	CCL22	DPP4	1,22449E-08	8428,9	3,39773E-01	14	2,31805E+00	13	3,93202E+00	46	8,38390E-01	24541	0	17530,5
Non-responder	pDC_LILRA4	Macro_ISG15	SCT	GPR84	1,76864E-08	9239,5	1,50017E-01	52	1,91243E+00	41	3,95088E+00	44	8,22250E-01	28530	0	17530,5
Non-responder	Mono_INHBA	pDC_LILRA4	TNF	PTPRS	2,71651E-08	5814,3	1,41104E-01	60	1,89192E+00	43	3,78659E+00	55	8,91464E-01	11383	0	17530,5
Non-responder	Mono_INHBA	pDC_LILRA4	CCL20	CXCR3	3,29657E-08	6624,7	2,00572E-01	26	1,76386E+00	64	4,19630E+00	30	8,75930E-01	15473	0	17530,5
Non-responder	cDC_LAMP3	cDC_CLEC9A	CCL22	DPP4	3,45346E-08	8170,5	3,68556E-01	11	2,36521E+00	12	3,66660E+00	65	8,43823E-01	23234	0	17530,5
Non-responder	pDC_LILRA4	Macro_OLFML3	SCT	GPR84	3,95379E-08	9606,1	1,35797E-01	68	1,87355E+00	47	3,78038E+00	56	8,14856E-01	30329	0	17530,5
Non-responder	pDC_LILRA4	pDC_LILRA4	HSP90B1	TLR9	4,50024E-08	4778,1	1,34011E-01	71	1,79422E+00	59	4,02024E+00	39	9,13885E-01	6191	0	17530,5
Non-responder	Macro_NLRP3	pDC_LILRA4	TNF	PTPRS	5,73957E-08	6052,1	1,28878E-01	77	1,83777E+00	50	3,73777E+00	59	8,87002E-01	12544	0	17530,5
Non-responder	cDC_LAMP3	pDC_LILRA4	CCL19	CCR7	6,19828E-08	7595,5	1,27041E-01	79	2,09135E+00	23	3,80999E+00	50	8,56074E-01	20295	0	17530,5
Non-responder	pDC_LILRA4	Mast	SCT	ADRB2	6,43652E-08	8300,1	2,35077E-01	20	2,02453E+00	29	3,56055E+00	80	8,41265E-01	23841	0	17530,5
Non-responder	Macro_NLRP3	pDC_LILRA4	CCL20	CXCR3	7,45046E-08	7145,7	1,67457E-01	40	1,64160E+00	84	3,93555E+00	45	8,65788E-01	18029	0	17530,5
Non-responder	Macro_FOLR2+APOE+	cDC(CD1C)	B2M	CD1C	7,70411E-08	3867,1	3,77787E-02	1063	1,20375E+00	430	3,13894E+00	192	9,52275E-01	120	0	17530,5
Non-responder	Macro_NLRP3	Mast	S100A8	CD69	7,73488E-08	3786,9	5,95630E-02	467	1,76255E+00	65	2,65506E+00	549	9,48569E-01	323	0	17530,5
Non-responder	pDC_LILRA4	Macro_IER3	SCT	GPR84	9,16262E-08	10088,9	1,19324E-01	90	1,82850E+00	55	3,59736E+00	72	8,04902E-01	32697	0	17530,5
Non-responder	cDC_LAMP3	Macro_ISG15	CCL19	CCRL2	1,07750E-07	7247,7	1,23089E-01	86	2,12367E+00	20	3,50008E+00	95	8,63666E-01	18507	0	17530,5
Non-responder	pDC_LILRA4	Macro_FOLR2+APOE+	GZMB	IGF2R	1,07750E-07	4979,7	1,16561E-01	95	2,19311E+00	17	5,45790E+00	2	9,08995E-01	7254	0	17530,5
Non-responder	Macro_LYVE1	pDC_LILRA4	CXCL12	CXCR3	1,11187E-07	7569,1	1,89618E-01	28	1,76240E+00	66	3,48806E+00	96	8,56788E-01	20125	0	17530,5
Non-responder	Macro_FOLR2+APOE+	pDC_LILRA4	MMP9	TLR9	1,14695E-07	8532,3	2,44471E-01	18	1,56763E+00	97	3,73447E+00	61	8,36686E-01	24955	0	17530,5
Non-responder	pDC_LILRA4	Macro_NLRP3	SCT	GPR84	1,25662E-07	10321,1	1,12404E-01	100	1,80958E+00	58	3,57590E+00	75	8,00168E-01	33842	0	17530,5
Non-responder	cDC_LAMP3	cDC_CLEC9A	CCL19	CXCR3	1,29467E-07	7797,1	1,84403E-01	32	2,10519E+00	21	3,45800E+00	101	8,51856E-01	21301	0	17530,5
Non-responder	Macro_ISG15	pDC_LILRA4	CXCL11	CXCR3	1,37306E-07	8931,9	1,61405E-01	43	1,55040E+00	103	4,00959E+00	40	8,28630E-01	26943	0	17530,5
Non-responder	cDC_LAMP3	Macro_FOLR2+APOE+	CCL19	CCRL2	1,62706E-07	7012,9	1,34122E-01	70	2,17001E+00	18	3,42282E+00	109	8,68641E-01	17337	0	17530,5
Non-responder	Macro_IER3	pDC_LILRA4	TNF	PTPRS	1,76503E-07	6582,9	1,06829E-01	112	1,74110E+00	67	3,56585E+00	77	8,77252E-01	15128	0	17530,5
Non-responder	Mono_CD14	Macro_FOLR2+APOE+	S100A8	CD68	1,76503E-07	4014,9	2,32265E-02	2329	1,52165E+00	112	3,44633E+00	102	9,63248E-01	1	0	17530,5
Non-responder	Macro_FOLR2+APOE+	Mono_CD16	HLA-A	LILRB2	1,92703E-07	5626,3	1,03262E-02	9371	1,14630E+00	593	2,66081E+00	539	9,53145E-01	98	0	17530,5
Non-responder	pDC_LILRA4	cDC_CLEC9A	SCT	ADRB3	1,96081E-07	16294,3	5,60931E-01	7	2,00456E+00	31	3,38979E+00	116	6,40106E-01	63787	0	17530,5
Non-responder	Macro_OLFML3	pDC_LILRA4	CXCL10	DPP4	2,39473E-07	8824,9	1,25296E-01	83	1,48844E+00	124	3,55462E+00	82	8,31140E-01	26305	0	17530,5
Non-responder	Macro_FOLR2+APOE+	pDC_LILRA4	BST2	LILRA4	2,50323E-07	4065,5	6,64572E-02	367	1,59464E+00	91	4,32498E+00	24	9,33388E-01	2315	0	17530,5
Non-responder	pDC_LILRA4	Macro_NLRP3	SPON2	ITGA5	2,63381E-07	8117,1	1,04056E-01	119	1,47491E+00	128	3,59923E+00	71	8,45912E-01	22737	0	17530,5
Non-responder	cDC_CLEC9A	pDC_LILRA4	TNF	PTPRS	2,69597E-07	6702,5	1,02137E-01	129	1,71932E+00	72	3,88877E+00	49	8,74813E-01	15732	0	17530,5
Non-responder	pDC_LILRA4	Mono_INHBA	SCT	GPR84	2,95438E-07	10717,5	1,01502E-01	133	1,77977E+00	62	3,46272E+00	100	7,91886E-01	35762	0	17530,5
Non-responder	pDC_LILRA4	pDC_LILRA4	APP	RPSA	2,98590E-07	3763,7	4,53345E-02	770	1,92049E+00	38	4,73684E+00	15	9,46384E-01	465	0	17530,5
Non-responder	cDC_LAMP3	Macro_NLRP3	CCL19	CCRL2	3,08954E-07	7833,5	1,00983E-01	135	2,03081E+00	28	3,40543E+00	111	8,51586E-01	21363	0	17530,5
Non-responder	Macro_FOLR2+APOE+	pDC_LILRA4	CXCL9	DPP4	3,15865E-07	9397,9	1,17859E-01	91	1,47164E+00	129	3,30211E+00	136	8,19870E-01	29103	0	17530,5
Non-responder	Macro_NLRP3	cDC_CLEC9A	CXCL2	XCR1	3,22877E-07	5268,3	1,82192E-01	33	3,03203E+00	2	3,29954E+00	137	9,02868E-01	8639	0	17530,5
Non-responder	Macro_FOLR2+APOE+	Mono_CD16	HLA-B	LILRB2	3,37504E-07	5914,9	9,62832E-03	10443	1,09303E+00	794	2,52233E+00	736	9,54388E-01	71	0	17530,5
Non-responder	pDC_LILRA4	cDC_CLEC9A	APP	CD74	3,44534E-07	4111,3	2,09592E-02	2819	1,44280E+00	140	3,67110E+00	64	9,61954E-01	3	0	17530,5
Non-responder	Macro_OLFML3	cDC_CLEC9A	CXCL10	DPP4	3,51963E-07	8556,3	1,35910E-01	67	1,53559E+00	109	3,28920E+00	141	8,36770E-01	24934	0	17530,5
Non-responder	Macro_FOLR2+APOE+	pDC_LILRA4	CXCL12	CXCR3	3,51963E-07	9356,5	1,10633E-01	104	1,44259E+00	141	3,73514E+00	60	8,20461E-01	28947	0	17530,5
Non-responder	Mast	pDC_LILRA4	COL18A1	PTPRS	4,15267E-07	8571,1	5,65585E-01	5	2,13472E+00	19	3,26330E+00	149	8,35869E-01	25152	0	17530,5
Non-responder	Macro_FOLR2+APOE+	pDC_LILRA4	CCL5	CXCR3	4,15267E-07	9894,7	1,12110E-01	101	1,41755E+00	149	3,62185E+00	68	8,09464E-01	31625	0	17530,5
Non-responder	Macro_OLFML3	pDC_LILRA4	CXCL9	DPP4	4,32197E-07	9608,9	1,11510E-01	102	1,41235E+00	151	3,28823E+00	142	8,15744E-01	30119	0	17530,5
Non-responder	Macro_FOLR2+APOE+	Mono_CD16	B2M	LILRB2	4,34741E-07	5964,3	9,44925E-03	10766	1,08164E+00	846	2,57334E+00	657	9,58793E-01	22	0	17530,5
Non-responder	Macro_FOLR2+APOE+	pDC_LILRA4	CXCL10	CXCR3	4,85732E-07	6077,9	9,51195E-02	157	1,54907E+00	104	4,15061E+00	32	8,86923E-01	12566	0	17530,5
Non-responder	Macro_FOLR2+APOE+	pDC_LILRA4	TNF	PTPRS	5,43510E-07	6982,5	9,34236E-02	163	1,68073E+00	77	3,38432E+00	117	8,69848E-01	17025	0	17530,5
Non-responder	Macro_FOLR2+APOE+	Mast	LGALS1	CD69	5,57006E-07	4551,7	2,20634E-02	2568	1,15049E+00	580	2,22468E+00	1309	9,42674E-01	771	0	17530,5
Non-responder	Macro_FOLR2+APOE+	pDC_LILRA4	C1QB	C1QBP	5,85516E-07	4349,7	2,01483E-02	3020	1,13232E+00	643	3,52972E+00	88	9,46356E-01	467	0	17530,5
Non-responder	pDC_LILRA4	Macro_FOLR2+APOE+	APP	CD74	6,49672E-07	4152,5	2,03627E-02	2977	1,38461E+00	173	3,56948E+00	76	9,61422E-01	6	0	17530,5
Non-responder	Macro_FOLR2+APOE+	Mono_CD16	HLA-A	LILRA1	6,90196E-07	4954,7	2,11115E-02	2787	1,11308E+00	722	2,60955E+00	613	9,29368E-01	3121	0	17530,5
Non-responder	pDC_LILRA4	Macro_NLRP3	SCT	ADRB2	7,07573E-07	11959,5	8,94287E-02	178	1,68618E+00	76	3,42665E+00	107	7,65744E-01	41906	0	17530,5
Non-responder	Macro_FOLR2+APOE+	Mono_CD16	HLA-B	LILRA1	7,24012E-07	5027,3	1,96847E-02	3151	1,05981E+00	935	2,47107E+00	808	9,31196E-01	2712	0	17530,5
Non-responder	pDC_LILRA4	Macro_ISG15	GZMB	IGF2R	7,56293E-07	5541,3	8,87457E-02	182	2,07009E+00	26	5,32389E+00	3	8,97072E-01	9965	0	17530,5
Non-responder	Macro_FOLR2+APOE+	cDC_CLEC9A	C1QB	C1QBP	7,73568E-07	4397,9	1,99296E-02	3079	1,12309E+00	681	3,10959E+00	205	9,46079E-01	494	0	17530,5
Non-responder	Macro_FOLR2+APOE+	Mono_CD16	HLA-C	LILRA1	7,74780E-07	5152,5	2,02897E-02	2989	9,90296E-01	1249	2,47479E+00	800	9,28992E-01	3194	0	17530,5
Non-responder	Macro_ISG15	pDC_LILRA4	TNF	PTPRS	7,94263E-07	7163,9	8,75700E-02	185	1,65480E+00	80	3,52416E+00	90	8,66141E-01	17934	0	17530,5
Non-responder	pDC_LILRA4	pDC_LILRA4	APP	TNFRSF21	8,07196E-07	4548,1	8,74881E-02	186	1,97512E+00	33	4,73260E+00	16	9,19827E-01	4975	0	17530,5
Non-responder	Macro_FOLR2+APOE+	Macro_NLRP3	MMP12	PLAUR	8,17244E-07	5718,5	3,93288E-02	991	1,19535E+00	449	3,32564E+00	132	8,98985E-01	9490	0	17530,5
Non-responder	Mono_CD14	Macro_FOLR2+APOE+	S100A9	CD68	8,60331E-07	4285,5	1,83118E-02	3552	1,41051E+00	153	3,14190E+00	190	9,62635E-01	2	0	17530,5
Non-responder	Mono_CD14	pDC_LILRA4	VCAN	SELL	9,15745E-07	3833,9	8,63533E-02	194	1,99414E+00	32	4,26698E+00	28	9,37860E-01	1385	0	17530,5

Non-responder	Macro_FOLR2+APOE+	Macro_NLRP3	C1QA	CD93	1,06682E-06	5112,3	1,87704E-02	3405	9,97528E-01	1215	2,64454E+00	560	9,30522E-01	2851	0	17530,5
Non-responder	Macro_ISG15	cDC_CLEC9A	CXCL10	DPP4	1,11215E-06	9117,9	1,16587E-01	94	1,37830E+00	180	3,10271E+00	207	8,26024E-01	27578	0	17530,5
Non-responder	Macro_FOLR2+APOE+	Macro_FOLR2+APOE+	APOE	TREM2	1,11790E-06	4999,5	2,03620E-02	2978	7,15821E-01	3437	2,42056E+00	897	9,51408E-01	155	0	17530,5
Non-responder	pDC_LILRA4	cDC(CD1C)	B2M	CD1C	1,11802E-06	3857,5	3,75958E-02	1072	1,19037E+00	467	3,50613E+00	94	9,52165E-01	124	0	17530,5
Non-responder	Mono_CD14	Macro_FOLR2+APOE+	S100A8	ITGB2	1,14466E-06	4205,7	2,00159E-02	3059	1,34693E+00	201	3,09954E+00	209	9,57295E-01	29	0	17530,5
Non-responder	Macro_FOLR2+APOE+	Mono_CD16	HLA-C	LILRB2	1,23910E-06	5887,1	9,92427E-03	9971	1,02352E+00	1100	2,52605E+00	729	9,52889E-01	105	0	17530,5
Non-responder	cDC_LAMP3	pDC_LILRA4	CXCL9	CXCR3	1,24594E-06	6796,7	8,18286E-02	215	1,48757E+00	125	3,19376E+00	168	8,73996E-01	15945	0	17530,5
Non-responder	pDC_LILRA4	cDC(CD1C)	APP	CD74	1,37145E-06	4177,5	2,04162E-02	2964	1,38983E+00	166	3,07798E+00	222	9,61471E-01	5	0	17530,5
Non-responder	Macro_ISG15	pDC_LILRA4	CCL13	CXCR3	1,39004E-06	13877,5	1,46184E-01	57	1,35375E+00	196	3,07739E+00	223	7,21685E-01	51381	0	17530,5
Non-responder	Macro_FOLR2+APOE+	Macro_FOLR2+APOE+	APOE	ABCA1	1,40153E-06	5558,5	1,81733E-02	3596	7,73631E-01	2780	2,14049E+00	1543	9,33256E-01	2343	0	17530,5
Non-responder	Macro_ISG15	pDC_LILRA4	CXCL10	DPP4	1,40879E-06	9405,5	1,07482E-01	110	1,33115E+00	224	3,36812E+00	121	8,20104E-01	29042	0	17530,5
Non-responder	Macro_FOLR2+APOE+	Macro_FOLR2+APOE+	LILRB4	LAIR1	1,48327E-06	4738,9	1,80727E-02	3637	1,13992E+00	611	3,02410E+00	252	9,36487E-01	1664	0	17530,5
Non-responder	Macro_ISG15	cDC(CD1C)	B2M	CD1C	1,50003E-06	4026,9	3,58778E-02	1154	1,06474E+00	914	2,85820E+00	368	9,51088E-01	168	0	17530,5
Non-responder	Macro_FOLR2+APOE+	cDC_CLEC9A	CXCL2	XCR1	1,58522E-06	6585,3	1,08303E-01	107	2,66305E+00	6	3,05212E+00	233	8,77552E-01	15050	0	17530,5
Non-responder	Macro_FOLR2+APOE+	cDC_CLEC9A	CXCL9	DPP4	1,73219E-06	9121,1	1,27844E-01	78	1,51879E+00	115	3,03669E+00	240	8,25796E-01	27642	0	17530,5
Non-responder	Macro_FOLR2+APOE+	Macro_ISG15	CXCL9	FCGR2A	1,73539E-06	4648,1	2,91641E-02	1634	1,35901E+00	192	3,32719E+00	131	9,26148E-01	3753	0	17530,5
Non-responder	Macro_FOLR2+APOE+	Macro_FOLR2+APOE+	RARRES1	NRP2	1,81197E-06	11152,7	8,34719E-02	212	1,22635E+00	380	2,26096E+00	1210	7,88875E-01	36431	0	17530,5
Non-responder	Macro_FOLR2+APOE+	Macro_FOLR2-APOE+	APOE	TREM2	1,84681E-06	5108,5	2,42779E-02	2184	8,62683E-01	1976	1,69754E+00	3800	9,55317E-01	52	0	17530,5
Non-responder	Macro_FOLR2+APOE+	Macro_FOLR2+APOE+	CXCL9	FCGR2A	1,86515E-06	4750,5	2,77600E-02	1748	1,31300E+00	246	3,29434E+00	139	9,24443E-01	4089	0	17530,5
Non-responder	pDC_LILRA4	pDC_LILRA4	TNFSF9	ADGRG5	1,93410E-06	15596,7	2,05684E-01	24	1,31087E+00	249	3,05613E+00	230	6,70503E-01	59950	0	17530,5
Non-responder	Mono_CD14	Mono_CD14	S100A12	CD36	2,02865E-06	5980,5	7,66121E-02	253	1,52468E+00	110	3,14396E+00	187	8,89694E-01	11822	0	17530,5
Non-responder	Macro_OLFML3	cDC_CLEC9A	CXCL9	DPP4	2,07705E-06	9332,5	1,20956E-01	87	1,45950E+00	135	3,02281E+00	255	8,21777E-01	28655	0	17530,5
Non-responder	Mono_INHBA	pDC_LILRA4	CCL5	CXCR3	2,07705E-06	11268,5	7,96496E-02	235	1,30273E+00	255	3,15099E+00	185	7,81702E-01	38137	0	17530,5
Non-responder	Macro_FOLR2+APOE+	pDC_LILRA4	CXCL11	CXCR3	2,33065E-06	11673,1	8,01288E-02	231	1,29858E+00	265	3,42442E+00	108	7,73083E-01	40231	0	17530,5
Non-responder	Mono_CD14	Macro_ISG15	S100A8	CD68	2,35708E-06	4256,9	1,94682E-02	3210	1,29855E+00	266	3,01217E+00	266	9,59992E-01	12	0	17530,5
Non-responder	Mono_CD14	pDC_LILRA4	S100A12	CD36	2,46482E-06	6013,7	7,50180E-02	270	1,51393E+00	119	3,69535E+00	62	8,88658E-01	12087	0	17530,5
Non-responder	Macro_OLFML3	cDC(CD1C)	B2M	CD1C	2,48665E-06	4199,5	3,46897E-02	1220	9,77863E-01	1310	2,53077E+00	723	9,50299E-01	214	0	17530,5
Non-responder	Macro_NLRP3	pDC_LILRA4	VCAN	SELL	2,49226E-06	4034,5	7,47793E-02	271	1,83036E+00	53	4,03991E+00	38	9,33532E-01	2280	0	17530,5
Non-responder	Macro_NLRP3	Macro_FOLR2+APOE+	S100A8	CD68	2,61904E-06	4445,1	1,83592E-02	3539	1,23845E+00	354	2,48658E+00	783	9,58851E-01	19	0	17530,5
Non-responder	Macro_NLRP3	Macro_FOLR2+APOE+	VCAN	CD44	2,68394E-06	5670,3	1,69416E-02	4061	1,01650E+00	1132	2,05374E+00	1873	9,26147E-01	3755	0	17530,5
Non-responder	pDC_LILRA4	cDC_CLEC9A	APP	RPSA	2,77858E-06	4058,5	3,34725E-02	1285	1,63071E+00	87	3,96029E+00	43	9,38146E-01	1347	0	17530,5
Non-responder	Macro_IER3	pDC_LILRA4	CCL20	CXCR3	3,11636E-06	9994,7	7,27148E-02	292	1,29180E+00	275	3,00700E+00	268	8,09556E-01	31608	0	17530,5
Non-responder	pDC_LILRA4	Macro_OLFML3	APP	CD74	3,24592E-06	4266,3	1,92280E-02	3268	1,27392E+00	296	3,06989E+00	226	9,60344E-01	11	0	17530,5
Non-responder	Macro_NLRP3	Macro_NLRP3	VCAN	CD44	3,32412E-06	4275,9	2,59898E-02	1957	1,37562E+00	182	2,64737E+00	557	9,39513E-01	1153	0	17530,5
Non-responder	Macro_NLRP3	Macro_NLRP3	HBEGF	CD44	3,38681E-06	4905,7	1,87757E-02	3402	1,18180E+00	493	2,46263E+00	822	9,33526E-01	2281	0	17530,5
Non-responder	pDC_LILRA4	Mono_INHBA	SCT	ADRB2	3,65602E-06	12949,5	7,15008E-02	306	1,64453E+00	83	2,95035E+00	308	7,45085E-01	46520	0	17530,5
Non-responder	Macro_ISG15	pDC_LILRA4	MMP9	TLR9	3,72755E-06	9912,9	1,69624E-01	39	1,34007E+00	207	2,94959E+00	310	8,10155E-01	31478	0	17530,5
Non-responder	pDC_LILRA4	pDC_LILRA4	VEGFB	RET	3,76366E-06	12997,7	2,10759E-01	21	1,26519E+00	311	3,15403E+00	183	7,43043E-01	46943	0	17530,5
Non-responder	Macro_FOLR2+APOE+	pDC_LILRA4	CD14	TLR9	3,83659E-06	5313,5	1,03454E-01	123	1,40107E+00	157	2,94578E+00	313	9,03849E-01	8444	0	17530,5
Non-responder	Macro_NLRP3	Macro_FOLR2+APOE+	TIMP1	CD63	3,87340E-06	4869,1	1,32289E-02	6190	1,28197E+00	285	2,94394E+00	314	9,58324E-01	26	0	17530,5
Non-responder	pDC_LILRA4	Macro_FOLR2+APOE+	SCT	ADRB2	4,02299E-06	12693,5	7,58156E-02	259	1,65455E+00	81	2,93981E+00	318	7,50609E-01	45279	0	17530,5
Non-responder	cDC_LAMP3	cDC(CD1C)	B2M	CD1C	4,14238E-06	4387,3	3,42002E-02	1248	9,42070E-01	1489	2,17832E+00	1443	9,49962E-01	226	0	17530,5
Non-responder	Macro_FOLR2+APOE+	Macro_LYVE1	CXCL9	FCGR2A	4,16882E-06	4986,3	2,62340E-02	1923	1,26298E+00	315	2,55173E+00	691	9,22445E-01	4472	0	17530,5
Non-responder	Macro_FOLR2+APOE-	pDC_LILRA4	CXCL12	CXCR3	4,29393E-06	8380,7	1,48269E-01	53	1,59497E+00	90	2,93263E+00	325	8,41025E-01	23905	0	17530,5
Non-responder	pDC_LILRA4	cDC_LAMP3	LTB	CD40	4,53561E-06	4976,9	6,91462E-02	331	1,72632E+00	69	4,12103E+00	36	9,10573E-01	6918	0	17530,5
Non-responder	Macro_FOLR2+APOE+	Macro_ISG15	C3	IFITM1	4,56530E-06	4837,9	3,10426E-02	1456	1,23003E+00	370	2,98506E+00	279	9,21948E-01	4554	0	17530,5
Non-responder	Macro_FOLR2+APOE+	Mono_INHBA	C1QA	CD93	4,60554E-06	5882,7	1,58537E-02	4562	8,91673E-01	1784	2,14854E+00	1522	9,24860E-01	4015	0	17530,5
Non-responder	Macro_NLRP3	Macro_FOLR2+APOE+	S100A8	ITGB2	4,66643E-06	4937,9	1,58215E-02	4574	1,06373E+00	917	2,13979E+00	1546	9,52221E-01	122	0	17530,5
Non-responder	Mono_CD14	Macro_NLRP3	VCAN	CD44	4,74932E-06	4046,9	3,00124E-02	1541	1,53940E+00	108	2,87444E+00	355	9,43475E-01	700	0	17530,5
Non-responder	Macro_FOLR2+APOE+	Mono_CD16	LYZ	ITGAL	4,76934E-06	5822,9	1,57964E-02	4594	8,44217E-01	2139	2,49098E+00	778	9,24533E-01	4073	0	17530,5
Non-responder	Macro_NLRP3	Macro_NLRP3	VEGFA	CD44	4,79536E-06	5010,7	2,55478E-02	2012	1,29807E+00	269	2,58642E+00	643	9,21722E-01	4599	0	17530,5
Non-responder	Macro_IER3	cDC_CLEC9A	CXCL2	XCR1	4,87169E-06	5762,3	1,48189E-01	54	2,86223E+00	4	2,91237E+00	339	8,93426E-01	10884	0	17530,5
Non-responder	Macro_NLRP3	Macro_FOLR2+APOE+	HBEGF	CD9	5,15499E-06	5831,1	1,85707E-02	3479	8,64165E-01	1962	2,15043E+00	1518	9,21403E-01	4666	0	17530,5
Non-responder	cDC_LAMP3	Macro_IER3	CCL19	CCRL2	5,31457E-06	8956,9	7,32012E-02	289	1,91411E+00	40	2,88257E+00	349	8,30085E-01	26576	0	17530,5
Non-responder	cDC_LAMP3	Macro_OLFML3	CCL19	CCRL2	5,36028E-06	9256,7	6,74922E-02	350	1,89013E+00	44	2,93685E+00	320	8,24281E-01	28039	0	17530,5
Non-responder	Macro_FOLR2+APOE+	Macro_FOLR2+APOE+	MMP9	EPHB2	5,39338E-06	13044,1	5,80408E-02	494	9,23700E-01	1591	2,66514E+00	537	7,51632E-01	45068	0	17530,5
Non-responder	Mono_INHBA	Macro_NLRP3	IL6	F3	5,49896E-06	12420,9	1,78918E-01	34	1,49580E+00	121	2,87666E+00	353	7,55808E-01	44066	0	17530,5
Non-responder	pDC_LILRA4	Mono_CD14	GZMB	IGF2R	5,49896E-06	6277,3	6,72399E-02	353	1,97497E+00	34	4,93814E+00	8	8,83537E-01	13461	0	17530,5
Non-responder	Macro_NLRP3	Macro_FOLR2+APOE+	S100A9	CD68	5,52419E-06	4654,3	1,55130E-02	4731	1,21098E+00	408	2,62770E+00	587	9,59535E-01	15	0	17530,5
Non-responder	Macro_OLFML3	cDC_CLEC9A	CXCL10	CXCR3	5,59273E-06	7131,1	6,72170E-02	355	1,31860E+00	239	3,36941E+00	120	8,68309E-01	17411	0	17530,5
Non-responder	Macro_NLRP3	Mono_CD16	VCAN	CD44	5,67766E-06	6265,9	1,54469E-02	4757	9,57177E-01	1410	1,77682E+00	3249	9,22926E-01	4383	0	17530,5
Non-responder	Macro_FOLR2+APOE+	cDC_LAMP3	TNFSF13	TNFRSF11B	5,93941E-06	9127,3	8,58807E-02	198	1,25499E+00	328	2,18535E+00	1424	8,31697E-01	26156	0	17530,5
Non-responder	Macro_FOLR2+APOE+	Macro_FOLR2+APOE+	C1QB	LRP1	5,94514E-06	5264,5	1,53709E-02	4801	8,70919E-01	1912	2,56469E+00	672	9,37771E-01	1407	0	17530,5
Non-responder	Mast	pDC_LILRA4	HSPG2	PTPRS	5,97843E-06	12404,9	3,45126E-01	13	1,72232E+00	71	2,86822E+00	363	7,55895E-01	44047	0	17530,5
Non-responder	Macro_FOLR2+APOE+	Macro_FOLR2+APOE+	APOE	LRP1	6,21619E-06	6001,9	1,52924E-02	4844	6,50628E-01	4386	2,12042E+00	1620	9,36629E-01	1629	0	17530,5
Non-responder	Macro_NLRP3	pDC_LILRA4	VEGFA	RET	6,33010E-06	10605,9	1,75547E-01	37	1,46124E+00	133	2,85637E+00	370	7,95364E-01	34959	0	17530,5
Non-responder	pDC_LILRA4	Macro_IER3	SCT	ADRB2	6,33010E-06	13249,3	6,63530E-02	370	1,63257E+00	85	3,22149E+00	163	7,37924E-01	48098	0	17530,5
Non-responder	Macro_NLRP3	Mono_CD16	LYZ	ITGAL	6,44403E-06	6359,1	1,52192E-02	4879	7,99168E-01	2565	1,91236E+00	2498	9,23224E-01	4323	0	17530,5
Non-responder	Macro_FOLR2+APOE+	Macro_NLRP3	MMP9	CD44	6,49467E-06	5669,5	2,88329E-02	1667	1,15272E+00	574	3,23432E+00	157	9,03995E-01	8419	0	17530,5
Non-responder	cDC_LAMP3	Mono_INHBA	CCL19	CCRL2	6,58953E-06	9001,5	7,24817E-02	296	1,91109E+00	42	2,85186E+00	375	8,29387E-01	26764	0	17530,5
Non-responder	Macro_FOLR2+APOE+	Macro_NLRP3	PLAU	ITGA5	6,68282E-06	9201,7	3,45481E-02	1232	9,08349E-01	1679	2,37684E+00	977	8,38192E-01	24590	0	17530,5

Post_NR_Myeloid_Myeloid_CellCell

Non-responder	pDC_LILRA4	Mono_INHBA	SPON2	ITGA5	6,96448E-06	9851,9	6,56929E-02	382	1,25020E+00	337	2,86889E+00	361	8,13500E-01	30649	0	17530,5
Non-responder	Macro_FOLR2+APOE+	cDC_LAMP3	SPP1	S1PR1	7,07415E-06	8501,7	1,14704E-01	98	1,34751E+00	200	2,84535E+00	384	8,39404E-01	24296	0	17530,5
Non-responder	Macro_FOLR2+APOE+	cDC_LAMP3	ADM	RAMP1	7,10443E-06	10984,7	6,71298E-02	357	1,42888E+00	145	2,18601E+00	1419	7,93175E-01	35472	0	17530,5
Non-responder	Mono_CD14	Mono_CD16	S100A8	ITGB2	7,12942E-06	4395,9	1,80219E-02	3654	1,23839E+00	355	2,84534E+00	385	9,55097E-01	55	0	17530,5
Non-responder	Macro_FOLR2+APOE+	cDC_LAMP3	CD72	CD5	7,28867E-06	11644,1	1,40075E-01	61	1,19394E+00	453	2,13516E+00	1563	7,79680E-01	38613	0	17530,5
Non-responder	pDC_LILRA4	Mono_CD16	SPON2	ITGA4	7,35335E-06	7019,9	6,51218E-02	389	1,38707E+00	171	3,54762E+00	86	8,70209E-01	16923	0	17530,5
Non-responder	Macro_FOLR2+APOE+	pDC_LILRA4	LGALS9	CD47	7,39364E-06	5526,3	1,72012E-02	3969	1,05897E+00	940	3,16971E+00	177	9,19614E-01	5015	0	17530,5
Non-responder	Macro_FOLR2+APOE+	Macro_ISG15	APOE	ABCA1	7,43798E-06	6407,5	1,49855E-02	5021	6,58875E-01	4234	2,11224E+00	1651	9,26992E-01	3601	0	17530,5
Non-responder	pDC_LILRA4	Mono_CD16	APP	FPR2	7,58190E-06	6407,9	6,42068E-02	393	1,63085E+00	86	3,79365E+00	54	8,81592E-01	13976	0	17530,5
Non-responder	pDC_LILRA4	pDC_LILRA4	HMGB1	TLR9	7,63977E-06	5859,5	7,63879E-02	255	1,21930E+00	394	2,97956E+00	287	8,93637E-01	10831	0	17530,5
Non-responder	Macro_ISG15	pDC_LILRA4	BST2	LILRA4	7,81514E-06	4116,5	6,39683E-02	397	1,55691E+00	100	4,21013E+00	29	9,32191E-01	2526	0	17530,5
Non-responder	pDC_LILRA4	pDC_LILRA4	APP	CD74	7,87418E-06	4297,3	1,86326E-02	3455	1,21585E+00	398	3,52547E+00	89	9,59741E-01	14	0	17530,5
Non-responder	Macro_FOLR2+APOE+	Macro_LYVE1	C1QB	LRP1	7,97895E-06	5607,5	1,48621E-02	5092	8,49795E-01	2091	2,09397E+00	1729	9,36781E-01	1595	0	17530,5
Non-responder	Macro_FOLR2+APOE+	cDC_LAMP3	ADM	CALCRL	8,06153E-06	10258,5	3,68968E-02	1109	1,04205E+00	1016	2,08393E+00	1760	8,16731E-01	29877	0	17530,5
Non-responder	Macro_FOLR2+APOE+	cDC_LAMP3	RARRES1	NRP2	8,26396E-06	9367,7	1,29895E-01	74	1,58735E+00	94	2,42652E+00	888	8,23358E-01	28252	0	17530,5
Non-responder	Macro_FOLR2+APOE+	Macro_LYVE1	APOE	LRP1	8,29730E-06	6687,1	1,47862E-02	5132	6,29504E-01	4733	1,64970E+00	4197	9,35622E-01	1843	0	17530,5
Non-responder	Macro_ISG15	pDC_LILRA4	CD14	TLR9	8,35724E-06	5493,3	9,60995E-02	152	1,32820E+00	227	2,81014E+00	406	9,00596E-01	9151	0	17530,5
Non-responder	Macro_FOLR2+APOE+	cDC_CLEC9A	CXCL9	CXCR3	8,54335E-06	7597,5	6,32276E-02	409	1,30180E+00	257	3,11691E+00	200	8,59098E-01	19591	0	17530,5
Non-responder	Macro_NLRP3	Mono_CD16	HLA-B	LILRA1	8,60075E-06	7284,9	1,59511E-02	4505	6,06711E-01	5169	1,54653E+00	5075	9,24145E-01	4145	0	17530,5
Non-responder	Macro_NLRP3	Mono_INHBA	VCAN	CD44	8,71786E-06	4643,7	2,35921E-02	2277	1,28046E+00	287	2,15231E+00	1511	9,36703E-01	1613	0	17530,5
Non-responder	Macro_ISG15	pDC_LILRA4	ADM	CALCRL	8,73219E-06	7869,7	6,25495E-02	412	1,27516E+00	294	3,58433E+00	73	8,52993E-01	21039	0	17530,5
Non-responder	Mono_CD14	Mono_INHBA	VCAN	CD44	8,75751E-06	4284,7	2,72436E-02	1797	1,44424E+00	139	2,37938E+00	969	9,40838E-01	988	0	17530,5
Non-responder	Macro_NLRP3	Mono_INHBA	HBEGF	CD44	8,84474E-06	5491,9	1,70436E-02	4023	1,08664E+00	824	1,96757E+00	2220	9,30459E-01	2862	0	17530,5
Non-responder	pDC_LILRA4	Macro_NLRP3	GZMB	IGF2R	8,92379E-06	6490,9	6,25073E-02	415	1,95404E+00	35	5,02882E+00	7	8,79729E-01	14467	0	17530,5
Non-responder	Macro_OLFML3	pDC_LILRA4	CXCL11	DPP4	8,98827E-06	13378,3	1,78597E-01	35	1,27357E+00	297	2,80542E+00	416	7,35522E-01	48613	0	17530,5
Non-responder	cDC_CLEC9A	cDC(CD1C)	B2M	CD1C	9,05209E-06	4294,9	3,34225E-02	1290	8,85202E-01	1812	2,63281E+00	577	9,49413E-01	265	0	17530,5
Non-responder	pDC_LILRA4	Macro_ISG15	SPON2	ITGA5	9,18356E-06	10027,1	6,22150E-02	419	1,22983E+00	371	3,23843E+00	156	8,09339E-01	31659	0	17530,5
Non-responder	Macro_NLRP3	pDC_LILRA4	THBS1	CD47	9,26956E-06	4632,9	3,76179E-02	1071	1,41318E+00	150	3,55431E+00	83	9,23187E-01	4330	0	17530,5
Non-responder	Macro_FOLR2+APOE+	cDC(CD1C)	B2M	CD1A	9,47687E-06	5311,9	5,32921E-02	582	9,02725E-01	1718	2,47114E+00	807	9,15167E-01	5922	0	17530,5
Non-responder	Mono_CD14	Macro_FOLR2+APOE+	S100A9	ITGB2	9,51530E-06	4591,9	1,57806E-02	4608	1,23579E+00	362	2,79511E+00	424	9,56587E-01	35	0	17530,5
Non-responder	Macro_NLRP3	pDC_LILRA4	S100A9	CD36	9,60103E-06	5251,7	2,45585E-02	2145	1,06920E+00	898	2,81535E+00	403	9,18378E-01	5282	0	17530,5
Non-responder	Macro_ISG15	pDC_LILRA4	CCL5	CXCR3	9,65019E-06	12385,9	6,15359E-02	426	1,23866E+00	353	3,02308E+00	254	7,58890E-01	43366	0	17530,5
Non-responder	Macro_FOLR2+APOE+	Macro_NLRP3	PLAU	PLAUR	9,91637E-06	5239,7	2,67569E-02	1854	1,18983E+00	468	2,79897E+00	420	9,15141E-01	5926	0	17530,5
Non-responder	Macro_NLRP3	Mono_INHBA	VEGFA	CD44	9,96061E-06	5472,7	2,31909E-02	2336	1,20290E+00	433	2,09136E+00	1741	9,18158E-01	5323	0	17530,5
Non-responder	Macro_ISG15	cDC_CLEC9A	CXCL2	XCR1	9,99296E-06	6739,9	1,03995E-01	121	2,64154E+00	7	2,78452E+00	431	8,75354E-01	15610	0	17530,5
Non-responder	Macro_FOLR2+APOE+	Macro_FOLR2+APOE+	C1QB	CD33	1,03116E-05	6359,9	1,44503E-02	5331	7,79311E-01	2733	2,44946E+00	845	9,17987E-01	5360	0	17530,5
Non-responder	pDC_LILRA4	Macro_OLFML3	GZMB	IGF2R	1,05581E-05	6587,5	6,05728E-02	439	1,94548E+00	36	4,85205E+00	10	8,78056E-01	14922	0	17530,5
Non-responder	Macro_FOLR2+APOE+	Mono_CD16	HLA-C	LILRA3	1,06133E-05	5440,9	2,40251E-02	2215	9,30996E-01	1550	2,68666E+00	518	9,17852E-01	5391	0	17530,5
Non-responder	Macro_NLRP3	Macro_OLFML3	VCAN	CD44	1,06725E-05	6690,7	1,43710E-02	5397	9,14474E-01	1642	1,67134E+00	4012	9,20318E-01	4872	0	17530,5
Non-responder	pDC_LILRA4	Macro_FOLR2+APOE+	SPON2	ITGA5	1,09218E-05	10172,3	6,03137E-02	444	1,21869E+00	395	2,97551E+00	293	8,06933E-01	32199	0	17530,5
Non-responder	Macro_FOLR2+APOE+	Macro_FOLR2-APOE+	C1QB	LRP1	1,10943E-05	6343,9	1,43027E-02	5439	8,26571E-01	2310	1,59929E+00	4600	9,35636E-01	1840	0	17530,5
Non-responder	cDC_CLEC9A	Macro_FOLR2+APOE+	HLA-DOB	CD4	1,13692E-05	5593,1	6,00571E-02	450	1,65046E+00	82	2,79022E+00	428	8,99059E-01	9475	0	17530,5
Non-responder	Macro_OLFML3	pDC_LILRA4	BST2	LILRA4	1,14450E-05	4221,3	5,99331E-02	451	1,49573E+00	122	3,92102E+00	48	9,30103E-01	2955	0	17530,5
Non-responder	Macro_NLRP3	Mono_CD16	S100A8	ITGB2	1,14768E-05	5458,9	1,42453E-02	5476	9,55185E-01	1422	1,88559E+00	2627	9,49776E-01	239	0	17530,5
Non-responder	Macro_ISG15	cDC_LAMP3	ADM	RAMP1	1,15210E-05	8607,3	1,20309E-01	88	1,71307E+00	73	2,75450E+00	452	8,36975E-01	24893	0	17530,5
Non-responder	Macro_FOLR2+APOE+	Macro_FOLR2+APOE+	HLA-A	LILRB2	1,17074E-05	7314,3	7,65937E-03	15132	8,68173E-01	1933	2,16613E+00	1476	9,46004E-01	500	0	17530,5
Non-responder	pDC_LILRA4	Macro_LYVE1	APP	CD74	1,17512E-05	4260,5	1,98698E-02	3098	1,33653E+00	211	2,75287E+00	455	9,60965E-01	8	0	17530,5
Non-responder	Macro_FOLR2+APOE+	Macro_IER3	CXCL9	FCGR2A	1,18161E-05	5382,3	2,27384E-02	2421	1,14842E+00	586	2,44010E+00	866	9,17172E-01	5508	0	17530,5
Non-responder	Macro_NLRP3	Mast	LGALS1	CD69	1,18268E-05	5260,1	2,04714E-02	2951	1,07301E+00	887	1,68137E+00	3917	9,40616E-01	1015	0	17530,5
Non-responder	Macro_OLFML3	cDC_CLEC9A	CXCL9	CXCR3	1,18286E-05	7783,1	5,98212E-02	456	1,24251E+00	348	3,10302E+00	206	8,55713E-01	20375	0	17530,5
Non-responder	Macro_NLRP3	pDC_LILRA4	S100A8	CD36	1,21304E-05	5204,7	2,90643E-02	1646	1,09668E+00	779	2,67422E+00	531	9,17059E-01	5537	0	17530,5
Non-responder	Mono_INHBA	pDC_LILRA4	IL6	IL6R_IL6ST	1,24601E-05	8880,9	5,96828E-02	464	1,33427E+00	216	2,99557E+00	275	8,32647E-01	25919	0	17530,5
Non-responder	Macro_FOLR2+APOE+	Macro_FOLR2+APOE+	MMP9	LRP1	1,34871E-05	8088,3	2,71148E-02	1807	8,59974E-01	2003	2,81159E+00	404	8,62843E-01	18697	0	17530,5
Non-responder	Macro_NLRP3	Mono_CD14	S100A8	ITGB2	1,38406E-05	5688,1	1,40872E-02	5580	9,44298E-01	1476	1,72480E+00	3593	9,49509E-01	261	0	17530,5
Non-responder	Macro_NLRP3	Mono_CD14	S100A8	CD36	1,38528E-05	5369,9	2,96819E-02	1576	1,10743E+00	742	2,12283E+00	1612	9,17856E-01	5389	0	17530,5
Non-responder	Macro_NLRP3	Mono_CD14	S100A9	CD36	1,39138E-05	5354,9	2,50804E-02	2074	1,07995E+00	856	2,26396E+00	1199	9,19163E-01	5115	0	17530,5
Non-responder	Macro_NLRP3	Mono_CD14	VCAN	CD44	1,40488E-05	5683,1	1,77507E-02	3758	1,04862E+00	980	1,87544E+00	2689	9,27727E-01	3458	0	17530,5
Non-responder	pDC_LILRA4	Mono_INHBA	GZMB	IGF2R	1,40622E-05	6938,3	5,39767E-02	567	1,91631E+00	39	4,64579E+00	18	8,71747E-01	16537	0	17530,5
Non-responder	pDC_LILRA4	Macro_FOLR2+APOE+	SPON2	ITGB1	1,46665E-05	7362,7	5,80867E-02	490	1,29484E+00	273	3,39485E+00	115	8,64137E-01	18405	0	17530,5
Non-responder	Macro_FOLR2+APOE+	Macro_FOLR2+APOE-	CXCL9	FCGR2A	1,49585E-05	5800,9	2,19817E-02	2580	1,12362E+00	676	1,92306E+00	2444	9,15878E-01	5774	0	17530,5
Non-responder	Macro_FOLR2+APOE+	Macro_FOLR2+APOE+	MMP9	IFNAR1	1,51087E-05	9741,7	2,64192E-02	1899	8,53909E-01	2061	2,86464E+00	366	8,28992E-01	26852	0	17530,5
Non-responder	cDC_LAMP3	cDC(CD1C)	CCL19	CXCR3	1,53019E-05	11808,3	6,36308E-02	405	1,82212E+00	57	2,70910E+00	497	7,71573E-01	40552	0	17530,5
Non-responder	Macro_FOLR2+APOE+	Mono_CD16	GRN	TNFRSF1B	1,53728E-05	6494,1	9,43197E-03	10808	8,52188E-01	2070	2,24434E+00	1242	9,42208E-01	820	0	17530,5
Non-responder	Macro_NLRP3	pDC_LILRA4	ICAM1	IL2RG	1,54174E-05	5540,1	1,89357E-02	3351	1,09816E+00	774	3,04708E+00	236	9,15700E-01	5809	0	17530,5
Non-responder	Macro_NLRP3	Macro_OLFML3	S100A8	ITGB2	1,54174E-05	5856,9	1,37683E-02	5809	9,22333E-01	1601	1,66785E+00	4051	9,48958E-01	293	0	17530,5
Non-responder	Mono_CD14	Macro_ISG15	S100A9	CD68	1,54867E-05	4667,3	1,53487E-02	4814	1,18741E+00	476	2,70774E+00	499	9,59327E-01	17	0	17530,5
Non-responder	Macro_NLRP3	Macro_ISG15	S100A8	CD68	1,58196E-05	5078,9	1,53885E-02	4797	1,01535E+00	1137	2,05241E+00	1877	9,55224E-01	53	0	17530,5
Non-responder	Macro_ISG15	cDC_LAMP3	TNFSF10	TNFRSF11B	1,58608E-05	8587,7	1,46512E-01	55	1,67140E+00	78	2,70245E+00	503	8,37505E-01	24772	0	17530,5
Non-responder	Macro_FOLR2+APOE+	Macro_NLRP3	APOC2	LDLR	1,58810E-05	13462,1	5,50124E-02	545	8,50196E-01	2087	2,61386E+00	608	7,44984E-01	46540	0	17530,5
Non-responder	Macro_FOLR2+APOE+	Macro_FOLR2+APOE+	C1QA	CD33	1,58875E-05	6688,9	1,37182E-02	5844	7,32520E-01	3231	2,29499E+00	1130	9,16134E-01	5709	0	17530,5
Non-responder	Macro_NLRP3	Macro_ISG15	VCAN	CD44	1,60924E-05	5209,1	1,89288E-02	3355	1,09537E+00	786	2,20682E+00	1353	9,29852E-01	3021	0	17530,5

Post_NR_Myeloid_Myeloid_CellCell

Non-responder	Mono_CD14	cDC_CLEC9A	S100A8	ITGB2	1,61453E-05	4537,7	1,68957E-02	4081	1,17708E+00	506	2,72999E+00	482	9,53693E-01	89	0	17530,5
Non-responder	pDC_LILRA4	pDC_LILRA4	BST2	LILRA4	1,63368E-05	4285,3	5,76179E-02	508	1,46064E+00	134	4,64205E+00	19	9,28811E-01	3235	0	17530,5
Non-responder	Macro_NLRP3	Macro_ISG15	HBEGF	CD44	1,63968E-05	6299,5	1,36747E-02	5872	9,01553E-01	1725	2,02208E+00	1998	9,22988E-01	4372	0	17530,5
Non-responder	Mono_INHBA	cDC_CLEC9A	CXCL2	XCR1	1,65299E-05	5937,1	1,39994E-01	62	2,82131E+00	5	2,69155E+00	510	8,90687E-01	11578	0	17530,5
Non-responder	Mono_CD14	Mono_CD14	S100A8	ITGB2	1,76187E-05	4443,3	1,78219E-02	3728	1,22750E+00	376	2,68456E+00	521	9,54857E-01	61	0	17530,5
Non-responder	Macro_NLRP3	cDC(CD1C)	B2M	CD1C	1,80037E-05	5637,1	2,91021E-02	1643	5,69267E-01	5992	1,90828E+00	2517	9,45983E-01	503	0	17530,5
Non-responder	Mast	Macro_FOLR2+APOE+	CSF1	SIRPA	1,84398E-05	6455,9	1,06666E-01	113	1,54756E+00	105	2,67582E+00	529	8,81462E-01	14002	0	17530,5
Non-responder	Macro_FOLR2+APOE+	Macro_FOLR2+APOE+	CCL18	CCR1	1,84432E-05	11251,9	3,97511E-02	973	8,41564E-01	2167	2,78029E+00	435	7,94537E-01	35154	0	17530,5
Non-responder	Macro_FOLR2+APOE+	Mono_CD14	C1QA	CD93	1,86282E-05	6453,5	1,34495E-02	6033	8,04421E-01	2506	2,34433E+00	1037	9,18943E-01	5161	0	17530,5
Non-responder	Macro_FOLR2+APOE+	Macro_FOLR2+APOE+	PLAU	ITGB5	1,90599E-05	12165,9	4,97458E-02	660	8,39716E-01	2185	2,13524E+00	1562	7,78483E-01	38892	0	17530,5
Non-responder	Macro_FOLR2+APOE+	Macro_OLFML3	CXCL9	FCGR2A	1,92698E-05	5537,1	2,12151E-02	2757	1,09849E+00	770	2,65120E+00	554	9,14500E-01	6074	0	17530,5
Non-responder	Macro_FOLR2+APOE+	Macro_LYVE1	MMP9	LRP1	1,94090E-05	8374,9	2,62173E-02	1928	8,38850E-01	2195	2,34086E+00	1044	8,60839E-01	19177	0	17530,5
Non-responder	Macro_NLRP3	Macro_FOLR2+APOE+	S100A9	ITGB2	1,94929E-05	5183,7	1,33687E-02	6088	1,03626E+00	1037	2,28091E+00	1161	9,53009E-01	102	0	17530,5
Non-responder	pDC_LILRA4	Macro_FOLR2-APOE+	GZMB	IGF2R	1,95594E-05	7496,1	4,49885E-02	779	1,87655E+00	46	4,09475E+00	37	8,61216E-01	19088	0	17530,5
Non-responder	Macro_NLRP3	cDC_CLEC9A	S100A8	ITGB2	1,96858E-05	5805,9	1,33551E-02	6100	8,93877E-01	1768	1,77024E+00	3289	9,48215E-01	342	0	17530,5
Non-responder	pDC_LILRA4	Macro_NLRP3	PROC	THBD	1,98277E-05	9849,1	8,98917E-02	174	1,16419E+00	542	3,08570E+00	215	8,12915E-01	30784	0	17530,5
Non-responder	Macro_NLRP3	Macro_OLFML3	S100A8	CD68	1,98639E-05	6113,5	1,33397E-02	6111	8,61484E-01	1989	1,57441E+00	4809	9,52067E-01	128	0	17530,5
Non-responder	Macro_FOLR2+APOE+	Mono_CD16	HLA-A	LILRB1	1,99058E-05	6944,5	8,79898E-03	12047	8,37233E-01	2209	2,20170E+00	1370	9,36908E-01	1566	0	17530,5
Non-responder	pDC_LILRA4	Macro_ISG15	APP	FPR2	2,02683E-05	6876,9	5,49677E-02	546	1,54185E+00	107	3,77200E+00	57	8,73239E-01	16144	0	17530,5
Non-responder	Macro_NLRP3	Macro_IER3	VCAN	CD44	2,04393E-05	7110,1	1,32890E-02	6146	8,71531E-01	1907	1,61119E+00	4499	9,17401E-01	5468	0	17530,5
Non-responder	Macro_FOLR2+APOE+	Mono_INHBA	CXCL9	FCGR2A	2,04726E-05	5643,5	2,09963E-02	2812	1,09133E+00	805	2,40507E+00	922	9,14094E-01	6148	0	17530,5
Non-responder	Macro_ISG15	cDC_CLEC9A	CXCL10	CXCR3	2,04910E-05	7659,9	5,76603E-02	506	1,16131E+00	548	3,18292E+00	172	8,59290E-01	19543	0	17530,5
Non-responder	Mono_CD16	Macro_FOLR2+APOE+	CD52	SIGLEC10	2,05393E-05	5476,5	2,11554E-02	2776	1,09441E+00	788	2,52874E+00	725	9,16889E-01	5563	0	17530,5
Non-responder	pDC_LILRA4	Mono_CD14	SPON2	ITGAM	2,07153E-05	9358,5	5,71790E-02	516	1,16066E+00	550	2,96870E+00	302	8,24836E-01	27894	0	17530,5
Non-responder	Mono_CD16	cDC(CD1C)	B2M	CD1C	2,07796E-05	4463,9	3,27323E-02	1336	8,34726E-01	2233	2,40367E+00	923	9,48909E-01	297	0	17530,5
Non-responder	Macro_ISG15	cDC_LAMP3	ADM	CALCRL	2,08281E-05	7779,7	6,61260E-02	376	1,32624E+00	230	2,65241E+00	551	8,56445E-01	20211	0	17530,5
Non-responder	pDC_LILRA4	cDC_CLEC9A	GZMB	IGF2R	2,12960E-05	7637,9	4,29554E-02	853	1,86756E+00	48	4,83337E+00	11	8,58429E-01	19747	0	17530,5
Non-responder	Macro_FOLR2+APOE+	Mono_CD16	CD38	PECAM1	2,13400E-05	10622,3	2,38268E-02	2248	9,23262E-01	1594	2,13170E+00	1581	8,15551E-01	30158	0	17530,5
Non-responder	Macro_FOLR2+APOE+	Macro_NLRP3	GRN	TNFRSF1B	2,13777E-05	6660,3	9,22623E-03	11178	8,32897E-01	2249	2,17838E+00	1442	9,41605E-01	902	0	17530,5
Non-responder	pDC_LILRA4	pDC_LILRA4	PROC	THBD	2,13980E-05	9869,9	8,89191E-02	180	1,15751E+00	556	3,51940E+00	91	8,12087E-01	30992	0	17530,5
Non-responder	Mono_CD14	Macro_NLRP3	S100A12	TLR4	2,18614E-05	7261,7	5,43821E-02	560	1,41842E+00	148	3,04987E+00	234	8,66544E-01	17836	0	17530,5
Non-responder	Macro_FOLR2+APOE+	Macro_LYVE1	CD59	STAB1	2,20660E-05	5641,1	2,40298E-02	2213	1,13638E+00	624	2,21832E+00	1321	9,12464E-01	6517	0	17530,5
Non-responder	pDC_LILRA4	Mono_CD16	GZMB	IGF2R	2,21920E-05	7676,3	4,24212E-02	871	1,86520E+00	49	4,77357E+00	14	8,57667E-01	19917	0	17530,5
Non-responder	Macro_FOLR2+APOE+	Macro_LYVE1	C1QA	CD93	2,23172E-05	6862,1	1,31668E-02	6255	7,94160E-01	2603	1,89190E+00	2595	9,18148E-01	5327	0	17530,5
Non-responder	Macro_ISG15	Mast	LGALS1	CD69	2,23380E-05	4451,9	2,36218E-02	2274	1,22634E+00	381	2,17032E+00	1461	9,44490E-01	613	0	17530,5
Non-responder	Macro_FOLR2+APOE+	Macro_FOLR2+APOE+	APOE	SCARB1	2,29669E-05	6204,7	2,48343E-02	2111	7,13935E-01	3460	2,11798E+00	1631	9,13407E-01	6291	0	17530,5
Non-responder	Mono_INHBA	pDC_LILRA4	ADM	CALCRL	2,30488E-05	8446,9	5,38056E-02	570	1,18528E+00	484	2,97461E+00	294	8,43299E-01	23356	0	17530,5
Non-responder	Macro_ISG15	Mast	S100A8	CD69	2,36545E-05	4526,7	3,44087E-02	1238	1,31142E+00	248	2,22463E+00	1310	9,33414E-01	2307	0	17530,5
Non-responder	Mono_CD16	Mono_CD16	HLA-B	LILRA1	2,38001E-05	6120,7	1,72208E-02	3958	7,60801E-01	2913	1,89675E+00	2565	9,26786E-01	3637	0	17530,5
Non-responder	Mono_CD16	Mono_CD16	HLA-A	LILRA1	2,38754E-05	7046,1	1,62716E-02	4354	6,17260E-01	4977	1,73696E+00	3502	9,20329E-01	4867	0	17530,5
Non-responder	Macro_FOLR2+APOE+	Macro_NLRP3	CXCL9	FCGR2A	2,39130E-05	5638,3	2,05249E-02	2934	1,07588E+00	874	2,69150E+00	511	9,13198E-01	6342	0	17530,5
Non-responder	pDC_LILRA4	Macro_IER3	GZMB	IGF2R	2,40393E-05	8265,9	3,55944E-02	1169	1,83500E+00	51	4,65255E+00	17	8,46618E-01	22562	0	17530,5
Non-responder	Mono_CD16	Mono_CD16	HLA-C	LILRA1	2,41972E-05	6205,3	1,76613E-02	3785	7,23315E-01	3331	1,95839E+00	2259	9,24277E-01	4121	0	17530,5
Non-responder	Macro_ISG15	pDC_LILRA4	TNFSF13B	TNFRSF17	2,45294E-05	8689,1	1,06421E-01	115	1,14915E+00	582	2,72747E+00	486	8,37632E-01	24732	0	17530,5
Non-responder	Mono_CD14	Macro_FOLR2+APOE+	S100A12	TLR4	2,46555E-05	7359,3	5,32476E-02	583	1,40893E+00	154	2,84519E+00	386	8,65320E-01	18143	0	17530,5
Non-responder	pDC_LILRA4	Macro_LYVE1	GZMB	IGF2R	2,49905E-05	8368,7	3,45911E-02	1227	1,83057E+00	52	4,36567E+00	23	8,44752E-01	23011	0	17530,5
Non-responder	Macro_NLRP3	Macro_ISG15	S100A9	CD68	2,50463E-05	5326,9	1,30029E-02	6401	9,87879E-01	1260	2,19354E+00	1400	9,55965E-01	43	0	17530,5
Non-responder	Mono_CD14	Macro_OLFML3	S100A8	ITGB2	2,52928E-05	4500,9	1,74184E-02	3888	1,20553E+00	426	2,62760E+00	588	9,54361E-01	72	0	17530,5
Non-responder	cDC_CLEC9A	pDC_LILRA4	HLA-DOB	CD4	2,54215E-05	5868,9	5,30781E-02	589	1,55977E+00	98	3,08899E+00	213	8,93314E-01	10914	0	17530,5
Non-responder	Macro_FOLR2+APOE+	Mono_INHBA	APOE	SDC2	2,54988E-05	6385,5	2,80760E-02	1720	8,25215E-01	2317	2,11591E+00	1636	9,02529E-01	8724	0	17530,5
Non-responder	Macro_FOLR2+APOE+	Macro_NLRP3	SPP1	CD44	2,55989E-05	6051,3	1,95873E-02	3179	7,78630E-01	2740	2,85102E+00	378	9,12829E-01	6429	0	17530,5
Non-responder	Macro_FOLR2+APOE+	Macro_FOLR2+APOE+	ADAM10	GPNMB	2,57584E-05	7379,7	2,48917E-02	2101	8,21156E-01	2357	2,13587E+00	1560	8,83966E-01	13350	0	17530,5
Non-responder	Macro_FOLR2+APOE+	Macro_LYVE1	APOE	ABCA1	2,58587E-05	7805,9	1,29461E-02	6442	5,85462E-01	5614	1,56676E+00	4876	9,21884E-01	4567	0	17530,5
Non-responder	Macro_FOLR2+APOE+	pDC_LILRA4	HLA-C	NOTCH4	2,62626E-05	5680,3	2,99718E-02	1545	8,73908E-01	1884	2,37543E+00	980	9,12676E-01	6462	0	17530,5
Non-responder	pDC_LILRA4	Macro_IFI27	GZMB	IGF2R	2,69484E-05	8415,9	3,41458E-02	1252	1,82860E+00	54	4,28267E+00	27	8,43901E-01	23216	0	17530,5
Non-responder	pDC_LILRA4	Macro_FOLR2+APOE+	LTB	CD40	2,72698E-05	5572,9	5,24422E-02	603	1,51649E+00	116	4,14365E+00	33	8,98658E-01	9582	0	17530,5
Non-responder	Mono_CD16	Mono_CD16	HLA-C	LILRA3	2,73785E-05	6500,1	2,09128E-02	2832	6,64015E-01	4160	2,17026E+00	1462	9,12467E-01	6516	0	17530,5
Non-responder	Mast	pDC_LILRA4	ADCYAP1	VIPR2	2,74052E-05	13731,9	9,92827E-01	1	1,65892E+00	79	2,61549E+00	604	7,26567E-01	50445	0	17530,5
Non-responder	pDC_LILRA4	Macro_OLFML3	LTB	CD40	2,74052E-05	5576,7	5,24286E-02	604	1,51631E+00	117	3,93108E+00	47	8,98646E-01	9585	0	17530,5
Non-responder	Macro_NLRP3	pDC_LILRA4	CXCL2	DPP4	2,75409E-05	8831,3	7,83137E-02	240	1,20890E+00	414	2,61436E+00	605	8,34956E-01	25367	0	17530,5
Non-responder	Macro_FOLR2+APOE+	Macro_FOLR2+APOE+	HLA-B	LILRB2	2,79528E-05	7853,7	7,14171E-03	16958	8,14906E-01	2406	2,02765E+00	1980	9,47426E-01	394	0	17530,5
Non-responder	Macro_FOLR2+APOE+	Macro_NLRP3	APOE	ABCA1	2,80360E-05	7261,9	1,28258E-02	6547	5,81134E-01	5708	2,05035E+00	1888	9,21548E-01	4636	0	17530,5
Non-responder	Macro_ISG15	Macro_ISG15	CCL8	CCR1	2,80885E-05	10603,7	8,38382E-02	206	1,14014E+00	609	3,08038E+00	220	7,97598E-01	34453	0	17530,5
Non-responder	Macro_NLRP3	Mono_CD14	HBEGF	CD44	2,81003E-05	6953,5	1,28236E-02	6550	8,54795E-01	2056	1,69070E+00	3847	9,20673E-01	4784	0	17530,5
Non-responder	Macro_LYVE1	pDC_LILRA4	IGF1	IGF2R	2,82265E-05	8741,7	5,20269E-02	610	1,17736E+00	505	2,66905E+00	532	8,38423E-01	24531	0	17530,5
Non-responder	Macro_FOLR2+APOE+	Mono_INHBA	MMP9	CD44	2,83239E-05	6045,1	2,61730E-02	1936	1,05756E+00	943	2,73926E+00	473	8,99712E-01	9343	0	17530,5
Non-responder	Macro_FOLR2+APOE+	Macro_FOLR2+APOE+	HLA-DMB	CD4	2,84668E-05	6009,1	1,28091E-02	6567	8,58004E-01	2022	2,44103E+00	863	9,29645E-01	3063	0	17530,5
Non-responder	Macro_FOLR2+APOE+	Macro_FOLR2+APOE+	SEMA4A	PLXND1	2,87457E-05	9323,1	2,43325E-02	2178	8,43344E-01	2145	1,92813E+00	2423	8,47530E-01	22339	0	17530,5
Non-responder	Macro_FOLR2+APOE+	Mono_INHBA	MMP12	PLAUR	2,89821E-05	6445,5	3,27254E-02	1337	1,00167E+00	1201	2,70850E+00	498	8,90328E-01	11661	0	17530,5
Non-responder	Macro_IER3	pDC_LILRA4	IL10	IFNLR1	2,92053E-05	11028,7	1,24737E-01	84	1,13822E+00	617	2,76304E+00	446	7,88750E-01	36466	0	17530,5
Non-responder	Macro_LYVE1	pDC_LILRA4	BST2	LILRA4	2,93469E-05	4479,7	5,17817E-02	618	1,37216E+00	186	3,36470E+00	122	9,25198E-01	3942	0	17530,5

Post_NR_Myeloid_Myeloid_CellCell

Non-responder	Macro_FOLR2+APOE+	cDC(CD1C)	C1QB	CD33	2,95895E-05	7172,9	1,27407E-02	6618	7,29602E-01	3250	1,99818E+00	2106	9,13121E-01	6360	0	17530,5
Non-responder	Mast	Macro_NLRP3	TIMP3	CD44	2,96315E-05	5126,5	9,79226E-02	145	1,54254E+00	106	2,60111E+00	620	9,09116E-01	7231	0	17530,5
Non-responder	pDC_LILRA4	cDC_LAMP3	APP	RPSA	2,97483E-05	4417,5	2,79001E-02	1737	1,49459E+00	123	3,02333E+00	253	9,32648E-01	2444	0	17530,5
Non-responder	pDC_LILRA4	pDC_LILRA4	SELPLG	SELL	2,97745E-05	4355,7	5,16842E-02	621	1,61538E+00	89	4,54696E+00	21	9,27493E-01	3517	0	17530,5
Non-responder	Macro_NLRP3	Macro_ISG15	S100A8	ITGB2	3,00846E-05	5991,3	1,27136E-02	6640	8,49697E-01	2092	1,77114E+00	3278	9,46992E-01	416	0	17530,5
Non-responder	cDC_LAMP3	Mono_CD16	CCL19	CCRL2	3,03511E-05	10318,3	5,13508E-02	625	1,82233E+00	56	2,85236E+00	373	8,03602E-01	33007	0	17530,5
Non-responder	Macro_FOLR2+APOE+	Mono_INHBA	APOE	ABCA1	3,05863E-05	7831,5	1,26951E-02	6662	5,76429E-01	5813	1,61781E+00	4443	9,21177E-01	4709	0	17530,5
Non-responder	Macro_FOLR2+APOE+	Macro_FOLR2+APOE+	HLA-DQA1	CD4	3,06777E-05	6277,9	1,00533E-02	9774	8,09297E-01	2463	2,39149E+00	944	9,43723E-01	678	0	17530,5
Non-responder	Macro_FOLR2+APOE+	cDC(CD1C)	CXCL9	FCGR2A	3,12812E-05	5894,5	1,97431E-02	3135	1,05025E+00	970	2,28755E+00	1145	9,11647E-01	6692	0	17530,5
Non-responder	pDC_LILRA4	Macro_NLRP3	SPON2	ITGAM	3,13778E-05	9645,3	5,32342E-02	584	1,13479E+00	632	2,95224E+00	307	8,19612E-01	29173	0	17530,5
Non-responder	Macro_NLRP3	Mono_CD16	S100A8	CD68	3,13982E-05	6278,1	1,26478E-02	6697	8,09520E-01	2461	1,60887E+00	4520	9,50837E-01	182	0	17530,5
Non-responder	pDC_LILRA4	Macro_ISG15	SPON2	ITGB1	3,15264E-05	7789,7	5,10277E-02	633	1,22168E+00	388	3,20375E+00	166	8,56350E-01	20231	0	17530,5
Non-responder	Mono_CD14	Mono_CD14	S100A12	TLR4	3,16754E-05	7490,9	5,10123E-02	634	1,39022E+00	165	2,80115E+00	419	8,62802E-01	18706	0	17530,5
Non-responder	Macro_IFI27	pDC_LILRA4	CXCL12	CXCR3	3,18248E-05	12246,1	5,59704E-02	533	1,22127E+00	390	2,59362E+00	635	7,64727E-01	42142	0	17530,5
Non-responder	pDC_LILRA4	Macro_ISG15	APP	CD74	3,21252E-05	4442,5	1,77963E-02	3740	1,13428E+00	637	2,98064E+00	285	9,58845E-01	20	0	17530,5
Non-responder	Macro_FOLR2+APOE+	pDC_LILRA4	TNFSF13	TNFRSF17	3,21891E-05	9538,3	9,81652E-02	144	9,64449E-01	1374	2,61593E+00	603	8,24280E-01	28040	0	17530,5
Non-responder	Macro_NLRP3	Macro_NLRP3	S100A8	ITGB2	3,22273E-05	6320,1	1,26109E-02	6732	8,42626E-01	2153	1,58159E+00	4753	9,46789E-01	432	0	17530,5
Non-responder	Macro_NLRP3	pDC_LILRA4	ADM	CALCRL	3,22761E-05	8633,9	5,07840E-02	638	1,15422E+00	566	3,26440E+00	148	8,39442E-01	24287	0	17530,5
Non-responder	Macro_FOLR2+APOE+	pDC_LILRA4	TNF	TNFRSF21	3,22918E-05	7937,1	2,86331E-02	1684	1,08000E+00	855	2,82431E+00	398	8,60683E-01	19218	0	17530,5
Non-responder	Macro_NLRP3	Mono_CD16	VCAN	ITGA4	3,23712E-05	5548,1	2,47762E-02	2118	1,17378E+00	514	2,45381E+00	840	9,11438E-01	6738	0	17530,5
Non-responder	Mono_CD14	Macro_ISG15	S100A8	ITGB2	3,25792E-05	4639,9	1,60841E-02	4439	1,13290E+00	640	2,73089E+00	479	9,52594E-01	111	0	17530,5
Non-responder	Macro_FOLR2+APOE+	Macro_IFI27	CXCL9	FCGR2A	3,29031E-05	6225,5	1,95953E-02	3176	1,04541E+00	994	1,87941E+00	2667	9,11344E-01	6760	0	17530,5
Non-responder	Macro_FOLR2+APOE+	Macro_FOLR2+APOE+	C3	CD81	3,29518E-05	6789,5	1,67341E-02	4135	6,71975E-01	4033	2,16165E+00	1487	9,11327E-01	6762	0	17530,5
Non-responder	Macro_FOLR2+APOE+	cDC(CD1C)	PSAP	CD1B	3,31222E-05	7254,5	3,34245E-02	1289	8,03763E-01	2511	2,00881E+00	2053	8,85660E-01	12889	0	17530,5
Non-responder	Macro_FOLR2+APOE+	Macro_FOLR2+APOE+	B2M	LILRB2	3,32797E-05	7887,3	7,00890E-03	17497	8,03517E-01	2514	2,07867E+00	1781	9,52469E-01	114	0	17530,5
Non-responder	pDC_LILRA4	Macro_IER3	APP	CD74	3,35000E-05	4451,3	1,82709E-02	3567	1,18057E+00	497	2,58443E+00	646	9,59361E-01	16	0	17530,5
Non-responder	Macro_FOLR2+APOE+	Mono_INHBA	PLAU	PLAUR	3,36492E-05	6047,3	2,22644E-02	2521	9,96151E-01	1222	2,18183E+00	1434	9,07727E-01	7529	0	17530,5
Non-responder	pDC_LILRA4	Macro_FOLR2+APOE+	SEMA7A	PLXNC1	3,36551E-05	9370,5	5,68551E-02	523	1,13178E+00	647	2,72273E+00	488	8,25712E-01	27664	0	17530,5
Non-responder	pDC_LILRA4	cDC_CLEC9A	SPON2	ITGA4	3,38107E-05	7849,1	5,04194E-02	648	1,23745E+00	357	3,19444E+00	167	8,55062E-01	20543	0	17530,5
Non-responder	pDC_LILRA4	Macro_FOLR2+APOE+	SPON2	ITGB2	3,41233E-05	5016,1	5,03357E-02	650	1,34863E+00	199	3,50693E+00	93	9,12023E-01	6608	0	17530,5
Non-responder	pDC_LILRA4	cDC(CD1C)	GZMB	IGF2R	3,43813E-05	9833,9	2,39174E-02	2229	1,78336E+00	61	4,28967E+00	26	8,18991E-01	29323	0	17530,5
Non-responder	Macro_FOLR2+APOE+	pDC_LILRA4	SIRPA	CD47	3,48307E-05	5933,5	2,29642E-02	2378,5	1,11693E+00	706,5	3,19201E+00	169,5	9,01739E-01	8882,5	0	17530,5
Non-responder	Macro_FOLR2+APOE+	Macro_FOLR2-APOE+	CXCL9	FCGR2A	3,51012E-05	6726,1	1,93643E-02	3234	1,03784E+00	1031	1,55495E+00	4987	9,10863E-01	6848	0	17530,5
Non-responder	Mast	Mono_CD14	CSF1	CSF3R	3,53930E-05	6208,7	1,04055E-01	120	1,47498E+00	127	2,57291E+00	658	8,86771E-01	12608	0	17530,5
Non-responder	Macro_FOLR2+APOE+	pDC_LILRA4	SEMA4A	NRP1_PLXNA4	3,55432E-05	10034,9	3,48517E-02	1210	9,84713E-01	1280	2,30478E+00	1110	8,20100E-01	29044	0	17530,5
Non-responder	Macro_FOLR2+APOE+	pDC_LILRA4	TNFSF13B	TNFRSF17	3,59314E-05	9144,7	9,57878E-02	155	1,06280E+00	919	2,60020E+00	622	8,30346E-01	26497	0	17530,5
Non-responder	Macro_FOLR2+APOE+	pDC_LILRA4	LAMB2	RPSA	3,60987E-05	11120,7	2,39837E-02	2219	9,14503E-01	1640	2,57172E+00	661	8,01364E-01	33553	0	17530,5
Non-responder	Macro_FOLR2+APOE+	pDC_LILRA4	CCL24	CCR2	3,61546E-05	12881,3	8,65416E-02	191	9,86585E-01	1269	3,47155E+00	99	7,50442E-01	45317	0	17530,5
Non-responder	pDC_LILRA4	Macro_IER3	RPS19	C5AR1	3,65294E-05	5005,9	1,40237E-02	5626	1,12578E+00	665	2,63116E+00	579	9,44321E-01	629	0	17530,5
Non-responder	pDC_LILRA4	cDC_LAMP3	GZMB	IGF2R	3,66708E-05	10267,5	2,17277E-02	2641	1,77367E+00	63	4,17900E+00	31	8,11765E-01	31072	0	17530,5
Non-responder	Macro_FOLR2+APOE+	pDC_LILRA4	IGF1	IGF2R	3,67738E-05	11677,1	2,75932E-02	1764	8,03549E-01	2513	2,63200E+00	578	7,90749E-01	36000	0	17530,5
Non-responder	Macro_NLRP3	Macro_LYVE1	S100A9	CD68	3,68257E-05	6485,5	1,24267E-02	6914	9,36671E-01	1517	1,42012E+00	6408	9,55001E-01	58	0	17530,5
Non-responder	cDC_CLEC9A	pDC_LILRA4	CXCL9	CXCR3	3,70237E-05	8399,3	4,92144E-02	668	1,28473E+00	281	3,26695E+00	146	8,43241E-01	23371	0	17530,5
Non-responder	Macro_FOLR2-APOE+	pDC_LILRA4	BST2	LILRA4	3,71895E-05	4657,3	4,91787E-02	669	1,33269E+00	219	2,63337E+00	576	9,23394E-01	4292	0	17530,5
Non-responder	pDC_LILRA4	Macro_LYVE1	SERPINF1	PLXDC1	3,78575E-05	9718,7	7,41501E-02	278	1,12382E+00	673	2,86792E+00	364	8,17233E-01	29748	0	17530,5
Non-responder	Macro_FOLR2+APOE+	pDC_LILRA4	ADM	CALCRL	3,80940E-05	10223,7	3,49012E-02	1206	9,90971E-01	1244	3,01584E+00	263	8,12533E-01	30875	0	17530,5
Non-responder	Macro_ISG15	pDC_LILRA4	CXCL9	CXCR3	3,81944E-05	8405,1	4,90192E-02	675	1,28351E+00	283	3,43269E+00	105	8,42978E-01	23432	0	17530,5
Non-responder	Mono_CD14	Macro_ISG15	VCAN	CD44	3,82687E-05	4684,7	2,18585E-02	2604	1,25916E+00	320	2,43389E+00	878	9,34402E-01	2091	0	17530,5
Non-responder	Macro_FOLR2+APOE+	pDC_LILRA4	CXCL12	CXCR4	3,83271E-05	8009,3	2,34459E-02	2299	8,93703E-01	1769	3,40010E+00	114	8,64501E-01	18334	0	17530,5
Non-responder	Macro_FOLR2+APOE+	pDC_LILRA4	VEGFB	RET	3,84441E-05	17213,5	7,77612E-02	246	8,10817E-01	2447	2,08319E+00	1765	6,37217E-01	64079	0	17530,5
Non-responder	Macro_FOLR2+APOE+	Mono_CD16	HLA-F	LILRB2	3,84515E-05	6448,7	1,23696E-02	6974	9,79532E-01	1302	2,32181E+00	1086	9,18027E-01	5351	0	17530,5
Non-responder	Macro_FOLR2+APOE+	pDC_LILRA4	COPA	P2RY6	3,85613E-05	6271,7	3,27085E-02	1340	1,35287E+00	197	3,47156E+00	98	8,88304E-01	12193	0	17530,5
Non-responder	Macro_NLRP3	Macro_ISG15	HBEGF	CD9	3,86726E-05	7454,5	1,39214E-02	5704	6,83019E-01	3855	1,78542E+00	3201	9,10315E-01	6982	0	17530,5
Non-responder	Macro_FOLR2+APOE+	cDC(CD1C)	CXCL9	CXCR3	3,89738E-05	12077,7	2,18175E-02	2616	1,01874E+00	1121	2,36801E+00	994	7,81735E-01	38127	0	17530,5
Non-responder	Macro_FOLR2+APOE+	pDC_LILRA4	CXCL10	DPP4	3,90923E-05	11808,3	6,33560E-02	407	9,41551E-01	1491	2,65910E+00	542	7,77780E-01	39071	0	17530,5
Non-responder	Macro_FOLR2+APOE+	pDC_LILRA4	CXCL2	DPP4	3,92110E-05	11253,7	4,65530E-02	737	8,39923E-01	2183	2,36694E+00	995	7,95939E-01	34823	0	17530,5
Non-responder	Macro_FOLR2+APOE+	Macro_IER3	MMP12	PLAUR	3,92110E-05	7631,5	2,56085E-02	2002	7,92928E-01	2620	2,35932E+00	1007	8,77770E-01	14998	0	17530,5
Non-responder	Macro_FOLR2+APOE+	pDC_LILRA4	CXCL12	DPP4	3,93300E-05	16233,1	7,36891E-02	284	8,35074E-01	2226	2,24363E+00	1247	6,70966E-01	59878	0	17530,5
Non-responder	cDC_CLEC9A	Mast	LGALS1	CD69	3,95089E-05	4681,9	2,17760E-02	2625	1,13650E+00	623	2,07054E+00	1820	9,42318E-01	811	0	17530,5
Non-responder	Macro_NLRP3	cDC_LAMP3	THBS1	TNFRSF11B	3,99090E-05	8237,9	1,87102E-01	30	1,70490E+00	74	2,55433E+00	685	8,45341E-01	22870	0	17530,5
Non-responder	pDC_LILRA4	Macro_LYVE1	SPON2	ITGAM	3,99090E-05	9864,3	5,12845E-02	629	1,12200E+00	685	2,59825E+00	627	8,16837E-01	29850	0	17530,5
Non-responder	Macro_NLRP3	cDC_LAMP3	VEGFA	NRP2	4,00832E-05	6228,1	5,97825E-02	459	1,58078E+00	95	2,55383E+00	686	8,87678E-01	12370	0	17530,5
Non-responder	Mono_CD14	Macro_FOLR2+APOE+	S100A12	CD36	4,00832E-05	7391,3	4,86564E-02	686	1,33612E+00	212	2,82191E+00	399	8,65371E-01	18129	0	17530,5
Non-responder	Macro_FOLR2+APOE+	pDC_LILRA4	PLAU	IGF2R	4,07177E-05	8574,9	3,73456E-02	1088	1,04359E+00	1001	3,14225E+00	189	8,44501E-01	23066	0	17530,5
Non-responder	Macro_FOLR2+APOE+	Macro_FOLR2+APOE+	SEMA4A	NRP1_PLXNA1	4,07177E-05	10927,9	2,90745E-02	1645	8,41685E-01	2166	1,88355E+00	2645	8,13490E-01	30653	0	17530,5
Non-responder	Macro_FOLR2+APOE+	Macro_FOLR2+APOE+	PLAU	IGF2R	4,10857E-05	10739,1	2,49929E-02	2088	7,88784E-01	2651	2,17903E+00	1439	8,16272E-01	29987	0	17530,5
Non-responder	Macro_OLFML3	pDC_LILRA4	TNF	PTPRS	4,14951E-05	9165,1	4,84034E-02	694	1,48131E+00	126	2,89470E+00	345	8,27901E-01	27130	0	17530,5
Non-responder	Mono_INHBA	Mono_INHBA	IL6	F3	4,20330E-05	12004,9	2,01789E-01	25	1,55623E+00	101	2,55008E+00	697	7,66738E-01	41671	0	17530,5
Non-responder	Macro_FOLR2+APOE+	pDC_LILRA4	HSP90B1	TLR9	4,20790E-05	6322,5	7,55085E-02	262	1,16137E+00	547	2,29456E+00	1131	8,88467E-01	12142	0	17530,5
Non-responder	Macro_FOLR2+APOE+	pDC_LILRA4	ICAM1	SPN	4,22044E-05	7388,9	2,15946E-02	2669	9,23220E-01	1595	2,59192E+00	639	8,79580E-01	14511	0	17530,5
Non-responder	pDC_LILRA4	Macro_FOLR2+APOE+	SPON2	ITGAM	4,22133E-05	9858,7	5,07252E-02	641	1,11833E+00	698	2,85456E+00	372	8,16016E-01	30052	0	17530,5

Post_NR_Myeloid_Myeloid_CellCell

Non-responder	Macro_FOLR2+APOE+	pDC_LILRA4	GNAI2	CXCR3	4,22673E-05	5610,1	4,23843E-02	874	1,20379E+00	429	3,23202E+00	158	9,01019E-01	9059	0	17530,5
Non-responder	Macro_FOLR2+APOE+	pDC_LILRA4	HMGB1	TLR9	4,24561E-05	6873,9	6,11715E-02	432	1,00618E+00	1182	2,15209E+00	1512	8,82609E-01	13713	0	17530,5
Non-responder	Macro_NLRP3	Macro_FOLR2+APOE+	HBEGF	CD44	4,24691E-05	6974,3	1,22390E-02	7114	8,22681E-01	2346	1,86900E+00	2722	9,18952E-01	5159	0	17530,5
Non-responder	Mono_CD16	pDC_LILRA4	BST2	LILRA4	4,25288E-05	5159,7	3,68992E-02	1108	1,14653E+00	592	3,55281E+00	84	9,12596E-01	6484	0	17530,5
Non-responder	Mono_CD16	pDC_LILRA4	SELPLG	SELL	4,25587E-05	5006,9	3,61486E-02	1139	1,33465E+00	215	3,55721E+00	81	9,14514E-01	6069	0	17530,5
Non-responder	Macro_NLRP3	cDC(CD1C)	S100A8	ITGB2	4,26485E-05	6523,5	1,35525E-02	5959	9,07476E-01	1686	1,36137E+00	7120	9,48574E-01	322	0	17530,5
Non-responder	pDC_LILRA4	cDC_CLEC9A	LTB	CD40	4,27573E-05	5797,7	4,80932E-02	701	1,46185E+00	132	3,98474E+00	41	8,94647E-01	10584	0	17530,5
Non-responder	Mono_CD14	Macro_NLRP3	S100A8	ITGB2	4,33060E-05	4703,5	1,59542E-02	4504	1,12583E+00	664	2,54135E+00	704	9,52410E-01	115	0	17530,5
Non-responder	Macro_NLRP3	Macro_ISG15	VEGFA	CD44	4,33722E-05	6158,7	1,86069E-02	3465	1,01782E+00	1126	2,14587E+00	1528	9,09494E-01	7144	0	17530,5
Non-responder	Mono_CD16	Mono_CD16	CD52	SIGLEC10	4,34633E-05	6146,3	1,75460E-02	3827	9,80039E-01	1300	2,40099E+00	927	9,09478E-01	7147	0	17530,5
Non-responder	Macro_FOLR2+APOE+	pDC_LILRA4	MMP9	IFNAR1	4,34740E-05	9584,7	2,69718E-02	1821	8,64035E-01	1966	3,31677E+00	133	8,30455E-01	26473	0	17530,5
Non-responder	pDC_LILRA4	pDC_LILRA4	FARP2	PLXNA4	4,34899E-05	13444,7	1,56663E-01	49	1,11708E+00	705	2,74013E+00	470	7,36196E-01	48469	0	17530,5
Non-responder	Macro_FOLR2+APOE+	Macro_FOLR2+APOE+	LGALS3	ENG	4,36460E-05	6894,9	1,21905E-02	7153	7,16043E-01	3434	2,12045E+00	1619	9,20933E-01	4738	0	17530,5
Non-responder	Mono_CD14	Mono_CD16	S100A9	ITGB2	4,36743E-05	4893,7	1,42085E-02	5500	1,12724E+00	659	2,54092E+00	706	9,54355E-01	73	0	17530,5
Non-responder	Macro_FOLR2+APOE+	Mono_CD16	HLA-B	LILRB1	4,37313E-05	7381,3	8,20430E-03	13547	7,83965E-01	2693	2,06322E+00	1847	9,38554E-01	1289	0	17530,5
Non-responder	Mono_CD16	cDC(CD1C)	B2M	CD1A	4,38600E-05	7176,3	4,61734E-02	745	5,33704E-01	6938	1,73588E+00	3508	9,09433E-01	7160	0	17530,5
Non-responder	Macro_FOLR2+APOE+	pDC_LILRA4	CCL5	DPP4	4,41194E-05	16773,5	7,46727E-02	273	8,10036E-01	2455	2,13034E+00	1586	6,54668E-01	62023	0	17530,5
Non-responder	Macro_FOLR2+APOE+	Macro_FOLR2+APOE+	SERPING1	LRP1	4,41364E-05	6072,1	1,82925E-02	3561	9,88474E-01	1256	2,45255E+00	844	9,09397E-01	7169	0	17530,5
Non-responder	Macro_FOLR2+APOE+	pDC_LILRA4	IL10	IFNLR1	4,41843E-05	13071,7	8,13523E-02	220	9,47569E-01	1460	2,39854E+00	931	7,50952E-01	45217	0	17530,5
Non-responder	pDC_LILRA4	Macro_FOLR2+APOE+	SPON2	ITGA4	4,44172E-05	8045,9	4,76591E-02	710	1,20936E+00	413	3,11021E+00	202	8,51538E-01	21374	0	17530,5
Non-responder	Macro_FOLR2+APOE+	Macro_FOLR2+APOE-	C1QB	LRP1	4,47244E-05	7080,5	1,21517E-02	7188	7,37268E-01	3176	1,59268E+00	4665	9,30550E-01	2843	0	17530,5
Non-responder	Macro_OLFML3	cDC_CLEC9A	CXCL11	DPP4	4,47918E-05	13079,1	1,93726E-01	27	1,32073E+00	235	2,54000E+00	712	7,43355E-01	46891	0	17530,5
Non-responder	pDC_LILRA4	cDC_CLEC9A	SPON2	ITGB1	4,49798E-05	8041,1	4,75708E-02	713	1,18586E+00	482	3,09737E+00	210	8,51981E-01	21270	0	17530,5
Non-responder	Macro_FOLR2+APOE+	Macro_ISG15	HLA-DRA	LAG3	4,50051E-05	5792,3	2,14124E-02	2709	1,11515E+00	712	2,46962E+00	813	9,09255E-01	7197	0	17530,5
Non-responder	cDC(CD1C)	pDC_LILRA4	BST2	LILRA4	4,57374E-05	4629,7	4,74475E-02	717	1,30645E+00	252	3,35373E+00	123	9,22117E-01	4526	0	17530,5
Non-responder	Macro_FOLR2+APOE+	Macro_OLFML3	C1QB	CD33	4,57605E-05	7360,7	1,21111E-02	7221	7,11297E-01	3498	2,08937E+00	1746	9,11090E-01	6808	0	17530,5
Non-responder	Mono_CD14	Macro_OLFML3	S100A8	CD68	4,59281E-05	4592,9	1,68762E-02	4087	1,14468E+00	599	2,53417E+00	718	9,57157E-01	30	0	17530,5
Non-responder	Macro_FOLR2+APOE+	cDC(CD1C)	C1QA	CD33	4,63017E-05	7655,9	1,20952E-02	7238	6,82811E-01	3857	1,84370E+00	2859	9,11169E-01	6795	0	17530,5
Non-responder	Mono_CD14	cDC_LAMP3	THBS1	TNFRSF11B	4,65033E-05	8814,1	1,57107E-01	48	1,55801E+00	99	2,53265E+00	721	8,33571E-01	25672	0	17530,5
Non-responder	Macro_FOLR2+APOE+	Macro_FOLR2+APOE+	B2M	TFRC	4,71806E-05	6747,1	9,03093E-03	11562	7,77676E-01	2745	2,37308E+00	983	9,41484E-01	915	0	17530,5
Non-responder	Macro_FOLR2+APOE+	Mono_INHBA	SPP1	CD44	4,74320E-05	6681,3	1,77802E-02	3747	6,83467E-01	3845	2,35596E+00	1011	9,08900E-01	7273	0	17530,5
Non-responder	Macro_NLRP3	pDC_LILRA4	TGFB1	CXCR4	4,79232E-05	6509,7	1,20492E-02	7288	8,10121E-01	2453	2,98039E+00	286	9,19748E-01	4991	0	17530,5
Non-responder	pDC_LILRA4	pDC_LILRA4	COPA	P2RY6	4,82577E-05	5321,1	4,67795E-02	730	1,55546E+00	102	4,39639E+00	22	9,04861E-01	8221	0	17530,5
Non-responder	Mono_CD16	Mast	LGALS1	CD69	4,82859E-05	5264,9	1,99231E-02	3083	1,04632E+00	990	1,72450E+00	3600	9,39853E-01	1121	0	17530,5
Non-responder	Macro_NLRP3	Mono_CD16	S100A9	ITGB2	4,83521E-05	5715,1	1,20369E-02	7301	9,27713E-01	1566	2,02672E+00	1985	9,50603E-01	193	0	17530,5
Non-responder	cDC_CLEC9A	pDC_LILRA4	HMGB1	TLR9	4,88521E-05	6158,9	7,14440E-02	307	1,15006E+00	581	2,52290E+00	733	8,90415E-01	11643	0	17530,5
Non-responder	Mono_CD16	Mast	S100A8	CD69	4,89511E-05	5385,5	2,77143E-02	1752	1,19136E+00	460	1,74184E+00	3471	9,26367E-01	3714	0	17530,5
Non-responder	Macro_FOLR2+APOE+	Macro_OLFML3	HLA-A	LILRB1	4,91902E-05	7684,1	7,98430E-03	14183	7,74737E-01	2774	2,09185E+00	1737	9,33975E-01	2196	0	17530,5
Non-responder	pDC_LILRA4	Macro_ISG15	RPS19	C5AR1	4,92510E-05	5003,5	1,37320E-02	5836	1,11005E+00	735	3,03268E+00	242	9,43765E-01	674	0	17530,5
Non-responder	Macro_FOLR2+APOE+	Macro_NLRP3	LGALS9	CD44	4,94723E-05	6351,5	1,11520E-02	8287	8,96231E-01	1753	2,18851E+00	1409	9,30879E-01	2778	0	17530,5
Non-responder	pDC_LILRA4	Mono_CD16	HLA-B	LILRB2	4,96521E-05	5794,3	9,69446E-03	10352	1,10944E+00	737	2,98155E+00	283	9,54537E-01	69	0	17530,5
Non-responder	Macro_FOLR2+APOE-	pDC_LILRA4	BST2	LILRA4	4,98535E-05	4711,7	4,65410E-02	738	1,29271E+00	274	2,87547E+00	354	9,21421E-01	4662	0	17530,5
Non-responder	Macro_FOLR2+APOE+	Mono_CD16	B2M	LILRB1	5,03975E-05	7311,7	8,05172E-03	13973	7,72576E-01	2791	2,11423E+00	1643	9,44398E-01	621	0	17530,5
Non-responder	pDC_LILRA4	Mono_CD16	RPS19	C5AR1	5,04608E-05	5007,7	1,37098E-02	5849	1,10885E+00	741	3,04140E+00	239	9,43722E-01	679	0	17530,5
Non-responder	Macro_OLFML3	Mono_INHBA	CXCL10	SDC4	5,06643E-05	8292,1	4,63259E-02	742	1,28881E+00	277	3,04292E+00	238	8,46162E-01	22673	0	17530,5
Non-responder	pDC_LILRA4	Macro_NLRP3	RPS19	C5AR1	5,10730E-05	5036,7	1,36606E-02	5878	1,10620E+00	744	2,90836E+00	342	9,43627E-01	689	0	17530,5
Non-responder	pDC_LILRA4	pDC_LILRA4	SEMA3C	NRP1_PLXNA4	5,12782E-05	12087,3	8,63874E-02	192	1,10616E+00	745	2,70992E+00	496	7,67621E-01	41473	0	17530,5
Non-responder	Macro_NLRP3	Mast	VCAN	CD44	5,13025E-05	6543,9	1,81782E-02	3594	1,06558E+00	909	1,34235E+00	7388	9,28521E-01	3298	0	17530,5
Non-responder	Macro_IFI27	pDC_LILRA4	BST2	LILRA4	5,16901E-05	4722,1	4,61161E-02	747	1,28626E+00	279	2,91981E+00	334	9,21089E-01	4720	0	17530,5
Non-responder	Macro_OLFML3	cDC_CLEC9A	CXCL11	CXCR3	5,23122E-05	11117,5	9,58112E-02	154	1,10374E+00	750	2,62021E+00	600	7,88378E-01	36553	0	17530,5
Non-responder	Mono_CD16	pDC_LILRA4	LGALS9	CD47	5,23879E-05	6788,9	1,28556E-02	6522	8,57176E-01	2031	2,77177E+00	442	9,08172E-01	7419	0	17530,5
Non-responder	Macro_NLRP3	pDC_LILRA4	GNAI2	CXCR3	5,25206E-05	5416,9	4,60455E-02	751	1,26756E+00	307	3,06597E+00	228	9,04653E-01	8268	0	17530,5
Non-responder	Mast	Macro_ISG15	CSF1	SIRPA	5,31493E-05	6893,9	9,32210E-02	165	1,45136E+00	137	2,51160E+00	754	8,74241E-01	15883	0	17530,5
Non-responder	Macro_NLRP3	Mono_CD14	S100A9	ITGB2	5,32050E-05	5911,1	1,19033E-02	7442	9,16826E-01	1633	1,86593E+00	2740	9,50340E-01	210	0	17530,5
Non-responder	Macro_ISG15	Macro_ISG15	CCL2	CCR1	5,33599E-05	7845,1	4,90434E-02	674	1,10325E+00	755	3,34443E+00	126	8,56721E-01	20140	0	17530,5
Non-responder	Macro_FOLR2+APOE+	Macro_LYVE1	SERPING1	LRP1	5,39599E-05	6458,9	1,76869E-02	3775	9,67350E-01	1360	1,98182E+00	2166	9,08001E-01	7463	0	17530,5
Non-responder	Macro_ISG15	cDC_CLEC9A	NECTIN2	CD226	5,39952E-05	10167,3	8,37489E-02	209	1,52073E+00	113	2,50906E+00	758	8,06822E-01	32226	0	17530,5
Non-responder	Macro_FOLR2+APOE+	pDC_LILRA4	APOE	SORL1	5,48697E-05	7345,5	1,29024E-02	6479	5,15378E-01	7488	2,50570E+00	761	9,22462E-01	4469	0	17530,5
Non-responder	pDC_LILRA4	Macro_OLFML3	SPON2	ITGA4	5,61490E-05	8228,7	4,55584E-02	768	1,18798E+00	474	2,97349E+00	298	8,48666E-01	22073	0	17530,5
Non-responder	Macro_FOLR2+APOE+	Macro_FOLR2-APOE+	MMP9	LRP1	5,64563E-05	8908,1	2,52305E-02	2047	8,15626E-01	2402	1,84618E+00	2838	8,58525E-01	19723	0	17530,5
Non-responder	pDC_LILRA4	Mono_CD16	SPON2	ITGB2	5,68061E-05	5267,5	4,53212E-02	771	1,24009E+00	351	3,25273E+00	154	9,07721E-01	7531	0	17530,5
Non-responder	pDC_LILRA4	Mono_CD16	APP	PTGER2	5,74682E-05	7638,3	4,51804E-02	774	1,43688E+00	142	3,43537E+00	103	8,58904E-01	19642	0	17530,5
Non-responder	Macro_FOLR2+APOE+	pDC_LILRA4	HSP90B1	TLR7	5,75561E-05	7227,1	2,13144E-02	2735	7,63240E-01	2886	2,25787E+00	1219	8,89906E-01	11765	0	17530,5
Non-responder	Macro_NLRP3	Macro_FOLR2+APOE+	CXCL8	SDC3	5,79125E-05	6657,3	4,50745E-02	776	1,23770E+00	356	2,56088E+00	677	8,81702E-01	13947	0	17530,5
Non-responder	Macro_FOLR2+APOE+	pDC_LILRA4	HLA-B	CANX	5,81120E-05	8015,7	6,73063E-03	18672	7,62579E-01	2893	2,46850E+00	816	9,51091E-01	167	0	17530,5
Non-responder	cDC(CD1C)	cDC(CD1C)	B2M	CD1C	5,83514E-05	4956,5	3,17446E-02	1398	7,62504E-01	2896	1,88862E+00	2612	9,48161E-01	346	0	17530,5
Non-responder	cDC_CLEC9A	Mono_CD16	ICAM3	ITGAL	5,83590E-05	6840,5	4,50095E-02	778	1,31336E+00	245	2,61429E+00	606	8,77593E-01	15043	0	17530,5
Non-responder	Mono_CD14	pDC_LILRA4	GNAI2	CXCR3	5,88078E-05	5482,9	4,49741E-02	780	1,24889E+00	338	2,98875E+00	277	9,03632E-01	8489	0	17530,5
Non-responder	Macro_FOLR2+APOE+	Macro_NLRP3	APOE	LDLR	5,89130E-05	7137,9	2,91335E-02	1640	7,61617E-01	2903	2,21583E+00	1328	8,87997E-01	12288	0	17530,5
Non-responder	pDC_LILRA4	Mono_CD14	SPON2	ITGB2	5,97123E-05	5309,5	4,48182E-02	784	1,22920E+00	373	3,09195E+00	212	9,07252E-01	7648	0	17530,5
Non-responder	Macro_NLRP3	cDC_LAMP3	ICAM1	IL2RG	6,03921E-05	6971,7	1,53712E-02	4800	9,31218E-01	1548	1,76143E+00	3347	9,07293E-01	7633	0	17530,5

Post_NR_Myeloid_Myeloid_CellCell

Non-responder	Macro_FOLR2+APOE+	Macro_NLRP3	C1QB	LRP1	6,09480E-05	6596,5	1,17026E-02	7647	7,18624E-01	3394	2,23547E+00	1281	9,29324E-01	3130	0	17530,5
Non-responder	Macro_FOLR2+APOE+	Macro_FOLR2+APOE+	C1QB	C1QBP	6,10343E-05	6414,9	1,15100E-02	7860	7,68034E-01	2836	2,40627E+00	919	9,30235E-01	2929	0	17530,5
Non-responder	Macro_FOLR2+APOE+	Mono_CD16	SEMA4A	LILRB2	6,10862E-05	6411,3	1,99264E-02	3081	1,10511E+00	746	2,48130E+00	790	8,97299E-01	9909	0	17530,5
Non-responder	Macro_ISG15	Macro_ISG15	CCL18	CCR1	6,17810E-05	9346,1	5,97575E-02	460	1,09348E+00	793	2,94210E+00	315	8,25825E-01	27632	0	17530,5
Non-responder	pDC_LILRA4	Macro_FOLR2+APOE-	APP	CD74	6,19487E-05	4334,7	2,05125E-02	2940	1,39923E+00	158	2,34240E+00	1041	9,61558E-01	4	0	17530,5
Non-responder	pDC_LILRA4	Macro_IER3	SPON2	ITGAM	6,22470E-05	10235,1	4,68520E-02	729	1,09293E+00	795	2,61390E+00	607	8,09978E-01	31514	0	17530,5
Non-responder	Macro_NLRP3	Macro_FOLR2+APOE+	VCAN	ITGB1	6,23962E-05	5947,5	2,20996E-02	2559	1,08154E+00	847	2,30104E+00	1118	9,07089E-01	7683	0	17530,5
Non-responder	Macro_NLRP3	Macro_ISG15	CCL3L1	CCR1	6,24810E-05	5618,1	4,45030E-02	796	1,33309E+00	217	2,91204E+00	340	9,00392E-01	9207	0	17530,5
Non-responder	Mono_CD14	Mono_CD14	VCAN	CD44	6,26201E-05	5011,7	2,04981E-02	2948	1,21240E+00	406	2,10251E+00	1684	9,32405E-01	2490	0	17530,5
Non-responder	Mono_CD14	Mono_CD16	S100A8	CD68	6,27154E-05	4703,5	1,60008E-02	4479	1,09272E+00	797	2,56862E+00	669	9,56051E-01	42	0	17530,5
Non-responder	Macro_FOLR2+APOE+	Macro_FOLR2+APOE+	A2M	LRP1	6,29258E-05	7139,5	1,63426E-02	4323	6,83903E-01	3835	1,95057E+00	2313	9,07037E-01	7696	0	17530,5
Non-responder	Macro_NLRP3	Macro_ISG15	CCL3	CCR1	6,31305E-05	5783,3	2,65586E-02	1875	1,05004E+00	973	2,45433E+00	837	9,07029E-01	7701	0	17530,5
Non-responder	Mono_CD16	pDC_LILRA4	HLA-C	NOTCH4	6,34591E-05	7024,3	2,60891E-02	1944	6,06927E-01	5165	1,85903E+00	2773	9,06987E-01	7709	0	17530,5
Non-responder	Macro_NLRP3	Macro_OLFML3	S100A9	ITGB2	6,35003E-05	6060,7	1,16338E-02	7710	8,94861E-01	1764	1,80897E+00	3061	9,49797E-01	238	0	17530,5
Non-responder	Macro_FOLR2+APOE+	Macro_ISG15	LILRB4	LAIR1	6,36239E-05	6882,1	1,16328E-02	7713	7,40653E-01	3136	2,16110E+00	1488	9,22056E-01	4543	0	17530,5
Non-responder	Macro_FOLR2+APOE+	Mono_CD16	CD14	ITGA4	6,36652E-05	6833,7	1,16317E-02	7714	7,84316E-01	2690	2,25208E+00	1231	9,19664E-01	5003	0	17530,5
Non-responder	Mono_CD16	pDC_LILRA4	TNF	PTPRS	6,50925E-05	9497,5	4,41935E-02	807	1,46266E+00	131	3,01492E+00	264	8,21322E-01	28755	0	17530,5
Non-responder	Macro_FOLR2+APOE+	Mast	GRN	NTRK1	6,52731E-05	6504,7	8,70109E-02	188	1,32175E+00	233	1,83230E+00	2934	8,90429E-01	11638	0	17530,5
Non-responder	Mono_CD14	Macro_ISG15	S100A12	CD36	6,53334E-05	7702,9	4,41622E-02	808	1,30580E+00	253	2,74612E+00	462	8,59625E-01	19461	0	17530,5
Non-responder	Macro_FOLR2+APOE+	Macro_OLFML3	HLA-A	LILRB2	6,53600E-05	8649,9	6,56697E-03	19424	7,54247E-01	2980	1,91857E+00	2467	9,41936E-01	848	0	17530,5
Non-responder	Macro_NLRP3	Mono_CD14	VEGFA	CD44	6,55439E-05	6707,1	1,74489E-02	3872	9,71061E-01	1343	1,81449E+00	3031	9,06814E-01	7759	0	17530,5
Non-responder	Mono_INHBA	Macro_FOLR2+APOE+	TIMP1	CD63	6,55749E-05	5477,9	1,11312E-02	8316	1,09072E+00	809	2,56605E+00	671	9,54736E-01	63	0	17530,5
Non-responder	Mono_INHBA	Macro_FOLR2+APOE+	INHBA	ENG	6,58171E-05	7890,1	5,03105E-02	651	1,10200E+00	759	2,46973E+00	810	8,58602E-01	19700	0	17530,5
Non-responder	Macro_FOLR2+APOE+	Macro_LYVE1	MMP9	EPHB2	6,72949E-05	15635,5	3,46458E-02	1225	7,52100E-01	3002	2,27764E+00	1168	7,00431E-01	55252	0	17530,5
Non-responder	cDC_CLEC9A	pDC_LILRA4	BST2	LILRA4	6,80228E-05	4764,9	4,39589E-02	819	1,25356E+00	331	3,67441E+00	63	9,19330E-01	5081	0	17530,5
Non-responder	Macro_FOLR2+APOE+	Mast	MMP9	CD44	6,84582E-05	7516,3	2,01668E-02	3015	8,42683E-01	2150	1,92930E+00	2419	8,87323E-01	12467	0	17530,5
Non-responder	Macro_FOLR2-APOE+	Macro_ISG15	C3	IFITM1	6,85483E-05	4695,5	3,91254E-02	1004	1,38635E+00	172	2,08485E+00	1755	9,29879E-01	3016	0	17530,5
Non-responder	pDC_LILRA4	Macro_OLFML3	SPON2	ITGB2	6,87688E-05	5378,7	4,38035E-02	822	1,20724E+00	418	3,03499E+00	241	9,06284E-01	7882	0	17530,5
Non-responder	Macro_OLFML3	Macro_FOLR2+APOE+	LILRB4	LAIR1	6,93903E-05	6186,3	1,28188E-02	6555	8,68147E-01	1934	2,35119E+00	1024	9,25473E-01	3888	0	17530,5
Non-responder	Mono_CD16	pDC_LILRA4	GNAI2	CXCR3	6,95202E-05	5533,9	4,37930E-02	825	1,22832E+00	375	3,13752E+00	195	9,02467E-01	8744	0	17530,5
Non-responder	Macro_FOLR2+APOE+	Macro_FOLR2-APOE+	SERPING1	LRP1	7,00114E-05	7315,3	1,70212E-02	4033	9,44126E-01	1479	1,48714E+00	5672	9,06386E-01	7862	0	17530,5
Non-responder	Macro_FOLR2+APOE+	Macro_LYVE1	C1QB	CD33	7,05026E-05	7820,1	1,14980E-02	7873	6,93470E-01	3699	1,86577E+00	2741	9,08963E-01	7257	0	17530,5
Non-responder	Macro_FOLR2+APOE+	Macro_OLFML3	C1QA	CD33	7,05474E-05	7835,3	1,14975E-02	7874	6,64506E-01	4150	1,93490E+00	2385	9,09097E-01	7237	0	17530,5
Non-responder	pDC_LILRA4	Mono_CD14	SPON2	ITGA5	7,07846E-05	11557,7	4,36092E-02	830	1,12085E+00	690	2,96980E+00	301	7,80410E-01	38437	0	17530,5
Non-responder	pDC_LILRA4	Macro_NLRP3	APP	FPR2	7,10393E-05	7609,7	4,36032E-02	831	1,43237E+00	143	3,33857E+00	127	8,59857E-01	19417	0	17530,5
Non-responder	Macro_FOLR2+APOE+	cDC_CLEC9A	HLA-DQA1	LAG3	7,12926E-05	6985,1	2,18332E-02	2611	1,09379E+00	792	2,51298E+00	752	8,84311E-01	13240	0	17530,5
Non-responder	Macro_FOLR2+APOE+	cDC_CLEC9A	HLA-DQB1	LAG3	7,16646E-05	7185,5	2,02430E-02	2998	1,01162E+00	1154	2,32139E+00	1087	8,84569E-01	13158	0	17530,5
Non-responder	Mono_CD14	Macro_FOLR2-APOE+	S100A8	CD68	7,19445E-05	4638,3	2,00444E-02	3053	1,33276E+00	218	1,93590E+00	2380	9,60549E-01	10	0	17530,5
Non-responder	Macro_FOLR2+APOE+	cDC_CLEC9A	ITGB2	CD226	7,22253E-05	6680,7	3,49003E-02	1207	1,10341E+00	752	2,08863E+00	1749	8,88404E-01	12165	0	17530,5
Non-responder	Macro_FOLR2+APOE+	Macro_ISG15	PLAU	PLAUR	7,26323E-05	6059,5	2,14147E-02	2708	9,59519E-01	1398	2,52043E+00	741	9,06084E-01	7920	0	17530,5
Non-responder	Macro_NLRP3	cDC(CD1C)	S100A9	ITGB2	7,28159E-05	6617,3	1,14515E-02	7924	8,80004E-01	1854	1,50250E+00	5514	9,49419E-01	264	0	17530,5
Non-responder	Macro_FOLR2+APOE+	cDC(CD1C)	B2M	CD1B	7,28835E-05	6993,7	2,69712E-02	1822	7,46799E-01	3063	1,84392E+00	2857	8,98185E-01	9696	0	17530,5
Non-responder	Macro_FOLR2+APOE+	Macro_IFI27	APOE	TREM2	7,34151E-05	6395,3	2,04365E-02	2961	7,18616E-01	3395	1,30288E+00	7937	9,51493E-01	153	0	17530,5
Non-responder	Macro_OLFML3	Macro_FOLR2+APOE+	CXCL9	FCGR2A	7,35077E-05	4876,7	2,62644E-02	1919	1,25370E+00	330	3,28045E+00	143	9,22486E-01	4461	0	17530,5
Non-responder	Macro_FOLR2+APOE+	Macro_FOLR2+APOE+	CD14	ITGB2	7,40224E-05	6810,1	8,99067E-03	11650	7,45875E-01	3075	2,21138E+00	1339	9,46524E-01	456	0	17530,5
Non-responder	Macro_OLFML3	Macro_FOLR2+APOE+	CXCL10	SDC4	7,41432E-05	8527,7	4,32665E-02	843	1,26093E+00	318	3,13514E+00	197	8,41662E-01	23750	0	17530,5
Non-responder	Macro_FOLR2+APOE+	Macro_FOLR2+APOE+	HLA-C	LILRB2	7,44050E-05	7846,7	7,36123E-03	16136	7,45391E-01	3079	2,03138E+00	1966	9,45711E-01	522	0	17530,5
Non-responder	Mono_INHBA	pDC_LILRA4	TNF	TNFRSF21	7,44058E-05	6451,5	4,32464E-02	844	1,29119E+00	276	3,22658E+00	162	8,83619E-01	13445	0	17530,5
Non-responder	Macro_FOLR2+APOE+	Macro_NLRP3	CXCL12	ITGA5	7,46929E-05	13013,7	3,03339E-02	1513	7,45155E-01	3082	2,16078E+00	1490	7,67687E-01	41453	0	17530,5
Non-responder	pDC_LILRA4	cDC(CD1C)	SPON2	ITGB2	7,49330E-05	5468,5	4,31172E-02	846	1,19238E+00	457	2,72851E+00	484	9,05611E-01	8025	0	17530,5
Non-responder	Macro_FOLR2+APOE+	cDC_CLEC9A	NECTIN2	CD226	7,53678E-05	12083,5	5,41776E-02	563	1,21003E+00	410	2,29262E+00	1135	7,70602E-01	40779	0	17530,5
Non-responder	Mono_INHBA	pDC_LILRA4	VCAN	SELL	7,54625E-05	4995,1	4,31161E-02	848	1,38230E+00	176	2,94985E+00	309	9,14271E-01	6112	0	17530,5
Non-responder	Mono_CD16	Macro_OLFML3	CD52	SIGLEC10	7,56621E-05	6668,3	1,60689E-02	4447	9,33235E-01	1541	2,06656E+00	1838	9,05792E-01	7985	0	17530,5
Non-responder	pDC_LILRA4	Macro_FOLR2+APOE+	RPS19	C5AR1	7,59946E-05	5130,7	1,31997E-02	6224	1,08135E+00	850	2,98157E+00	282	9,42707E-01	767	0	17530,5
Non-responder	pDC_LILRA4	Macro_IFI27	APP	CD74	7,61448E-05	4363,3	1,98717E-02	3097	1,33672E+00	210	2,37782E+00	972	9,60967E-01	7	0	17530,5
Non-responder	Macro_FOLR2+APOE+	cDC_CLEC9A	TNFSF13B	HLA-DPB1	7,61448E-05	7331,7	7,88403E-03	14469	7,51416E-01	3011	2,20559E+00	1359	9,49013E-01	289	0	17530,5
Non-responder	Macro_FOLR2+APOE+	Macro_NLRP3	TIMP2	CD44	7,63754E-05	7079,1	1,35804E-02	5937	8,19219E-01	2378	2,13883E+00	1550	9,05711E-01	8000	0	17530,5
Non-responder	pDC_LILRA4	Mono_INHBA	SPON2	ITGAM	7,65292E-05	10401,3	4,50495E-02	777	1,08111E+00	852	2,58056E+00	652	8,06941E-01	32195	0	17530,5
Non-responder	Macro_FOLR2+APOE+	Macro_ISG15	HLA-DRB1	LAG3	7,66623E-05	5929,5	2,16342E-02	2661	1,13436E+00	636	2,46926E+00	814	9,05698E-01	8006	0	17530,5
Non-responder	Macro_FOLR2+APOE+	cDC_CLEC9A	CXCL10	CXCR3	7,72228E-05	10088,9	3,39884E-02	1255	7,71714E-01	2800	2,47389E+00	801	8,24209E-01	28058	0	17530,5
Non-responder	Macro_FOLR2+APOE+	Macro_LYVE1	C1QA	CR1	7,72228E-05	9651,3	1,98503E-02	3108	7,82618E-01	2700	2,05323E+00	1874	8,44626E-01	23044	0	17530,5
Non-responder	Macro_FOLR2+APOE+	Macro_LYVE1	A2M	LRP1	7,76246E-05	8016,5	1,58017E-02	4590	6,62780E-01	4183	1,47985E+00	5753	9,05608E-01	8026	0	17530,5
Non-responder	pDC_LILRA4	Macro_FOLR2+APOE+	GAS6	MERTK	7,78764E-05	7042,9	4,28577E-02	857	1,22300E+00	387	3,11556E+00	201	8,72875E-01	16239	0	17530,5
Non-responder	pDC_LILRA4	Mono_CD16	HLA-B	LILRA1	7,80139E-05	4902,9	1,98199E-02	3116	1,07622E+00	872	2,93029E+00	328	9,31415E-01	2668	0	17530,5
Non-responder	cDC_LAMP3	pDC_LILRA4	CD70	TNFRSF17	7,81397E-05	15452,5	7,42476E-01	3	1,59400E+00	92	2,36239E+00	1001	6,79400E-01	58636	0	17530,5
Non-responder	Macro_IER3	pDC_LILRA4	BST2	LILRA4	7,81477E-05	4837,7	4,28508E-02	858	1,23676E+00	360	3,23086E+00	159	9,18378E-01	5281	0	17530,5
Non-responder	Macro_FOLR2+APOE+	Macro_NLRP3	APOE	LRP1	7,84502E-05	7965,7	1,16428E-02	7703	4,98333E-01	8043	1,79120E+00	3162	9,28038E-01	3390	0	17530,5
Non-responder	pDC_LILRA4	Mono_CD14	RPS19	C5AR1	7,86923E-05	5164,9	1,31551E-02	6264	1,07894E+00	860	2,82839E+00	396	9,42616E-01	774	0	17530,5
Non-responder	Macro_FOLR2+APOE+	Mono_INHBA	CXCL10	SDC4	7,88111E-05	11842,9	2,34248E-02	2302	7,41926E-01	3124	2,14739E+00	1526	7,96386E-01	34732	0	17530,5
Non-responder	Macro_FOLR2+APOE+	cDC_CLEC9A	LGALS3	LAG3	7,89111E-05	7878,1	2,38589E-02	2237	1,05944E+00	938	2,39979E+00	929	8,66841E-01	17756	0	17530,5
Non-responder	Macro_FOLR2+APOE+	Macro_ISG15	HLA-DPA1	LAG3	7,89881E-05	6019,5	2,17924E-02	2621	1,07767E+00	867	2,35001E+00	1025	9,05493E-01	8054	0	17530,5

Non-responder	Macro_FOLR2+APOE+	cDC_CLEC9A	FLT3LG	FLT3	7,90113E-05	17264,5	2,62431E-02	1922	1,08522E+00	830	1,79759E+00	3126	6,47417E-01	62914	0	17530,5
Non-responder	Macro_FOLR2+APOE+	pDC_LILRA4	HRAS	TLR9	7,93124E-05	17509,1	7,97348E-02	234	1,01931E+00	1119	1,79673E+00	3129	6,23386E-01	65533	0	17530,5
Non-responder	Macro_FOLR2+APOE+	pDC_LILRA4	HLA-DMB	CD4	7,93812E-05	6556,3	1,13206E-02	8062	7,67318E-01	2842	2,73981E+00	471	9,25497E-01	3876	0	17530,5
Non-responder	Macro_FOLR2+APOE+	cDC_CLEC9A	CALR	SCARF1	7,96143E-05	8621,9	2,22900E-02	2514	8,83459E-01	1827	2,02549E+00	1989	8,60566E-01	19249	0	17530,5
Non-responder	cDC_LAMP3	Mono_CD14	CCL19	CCRL2	8,00646E-05	11093,7	4,25124E-02	865	1,78520E+00	60	2,77830E+00	436	7,88269E-01	36577	0	17530,5
Non-responder	Macro_ISG15	pDC_LILRA4	GNAI2	CXCR3	8,03409E-05	5609,5	4,24955E-02	866	1,20572E+00	425	3,12622E+00	198	9,01136E-01	9028	0	17530,5
Non-responder	pDC_LILRA4	cDC_CLEC9A	SPON2	ITGB2	8,06179E-05	5455,9	4,24889E-02	867	1,17878E+00	502	3,13738E+00	196	9,04982E-01	8184	0	17530,5
Non-responder	Mast	Mono_CD14	HDC	HRH2	8,08955E-05	7563,1	1,29514E-01	76	1,56858E+00	96	2,43905E+00	868	8,60580E-01	19245	0	17530,5
Non-responder	Macro_FOLR2+APOE+	cDC_CLEC9A	CXCL10	DPP4	8,14438E-05	11497,9	6,87230E-02	338	9,88706E-01	1255	2,39368E+00	939	7,84728E-01	37427	0	17530,5
Non-responder	Macro_FOLR2+APOE+	cDC_LAMP3	GNAI2	S1PR1	8,15464E-05	8344,5	4,46764E-02	790	1,18924E+00	470	1,79394E+00	3151	8,58280E-01	19781	0	17530,5
Non-responder	Macro_FOLR2+APOE+	cDC_CLEC9A	CXCL2	DPP4	8,16490E-05	10990,7	5,04966E-02	644	8,87079E-01	1808	2,10152E+00	1691	8,02463E-01	33280	0	17530,5
Non-responder	Macro_FOLR2+APOE+	Macro_FOLR2+APOE+	APOC2	LRP1	8,16490E-05	9214,9	2,88765E-02	1664	7,39207E-01	3152	2,51846E+00	745	8,44862E-01	22983	0	17530,5
Non-responder	Macro_NLRP3	cDC_CLEC9A	S100A9	ITGB2	8,17222E-05	6075,7	1,12847E-02	8109	8,66405E-01	1949	1,91137E+00	2503	9,49066E-01	287	0	17530,5
Non-responder	Macro_FOLR2+APOE+	cDC_CLEC9A	CXCL12	DPP4	8,18546E-05	16070,1	7,99314E-02	232	8,82229E-01	1841	1,97821E+00	2176	6,79879E-01	58571	0	17530,5
Non-responder	Mono_INHBA	pDC_LILRA4	GNAI2	CXCR3	8,22931E-05	5684,9	4,24051E-02	873	1,20415E+00	428	2,65936E+00	541	9,01041E-01	9052	0	17530,5
Non-responder	Macro_NLRP3	Macro_OLFML3	S100A9	CD68	8,23794E-05	6332,7	1,12717E-02	8122	8,34012E-01	2239	1,71554E+00	3666	9,52858E-01	106	0	17530,5
Non-responder	Macro_FOLR2+APOE+	Macro_NLRP3	LRPAP1	LDLR	8,26808E-05	11001,5	2,31497E-02	2344	7,38324E-01	3162	1,82256E+00	2983	8,20277E-01	28988	0	17530,5
Non-responder	Macro_FOLR2+APOE+	Mono_CD14	C1QB	LRP1	8,27350E-05	6857,5	1,12627E-02	8129	7,00361E-01	3625	2,12177E+00	1616	9,28055E-01	3387	0	17530,5
Non-responder	Macro_NLRP3	Macro_ISG15	TIMP1	CD63	8,28565E-05	5454,5	1,12252E-02	8166	1,07567E+00	875	2,58937E+00	642	9,54918E-01	59	0	17530,5
Non-responder	Macro_FOLR2+APOE+	Macro_FOLR2+APOE+	MDK	SDC3	8,29922E-05	15555,9	5,81448E-02	488	8,49001E-01	2098	1,79061E+00	3165	7,04864E-01	54498	0	17530,5
Non-responder	Macro_NLRP3	cDC_LAMP3	ADM	RAMP1	8,31392E-05	9434,7	9,76792E-02	146	1,59214E+00	93	2,43457E+00	876	8,22255E-01	28528	0	17530,5
Non-responder	pDC_LILRA4	Macro_ISG15	LTB	CD40	8,39911E-05	6157,3	4,22895E-02	879	1,38895E+00	167	3,80618E+00	52	8,88431E-01	12158	0	17530,5
Non-responder	Macro_FOLR2+APOE+	cDC_LAMP3	TNFSF13B	CD40	8,40666E-05	6624,3	1,57845E-02	4605	9,91598E-01	1240	2,12938E+00	1591	9,05099E-01	8155	0	17530,5
Non-responder	Macro_FOLR2+APOE+	pDC_LILRA4	ICAM1	IL2RG	8,42730E-05	6605,1	1,45920E-02	5244	9,22051E-01	1603	2,72156E+00	489	9,05082E-01	8159	0	17530,5
Non-responder	Macro_LYVE1	cDC(CD1C)	B2M	CD1C	8,44571E-05	4975,9	3,23493E-02	1368	8,06722E-01	2482	1,78889E+00	3179	9,48623E-01	320	0	17530,5
Non-responder	Mono_CD14	Macro_FOLR2+APOE+	VCAN	CD44	8,51967E-05	5028,9	1,95637E-02	3186	1,18028E+00	498	2,28081E+00	1162	9,30920E-01	2768	0	17530,5
Non-responder	pDC_LILRA4	Macro_FOLR2-APOE+	SERPINF1	PLXDC2	8,57279E-05	5364,7	1,95469E-02	3191	9,62016E-01	1387	2,07465E+00	1793	9,30265E-01	2922	0	17530,5
Non-responder	pDC_LILRA4	cDC_LAMP3	TNFSF9	TNFRSF9	8,60014E-05	12051,5	1,41998E-01	59	1,34681E+00	202	2,42777E+00	886	7,67073E-01	41580	0	17530,5
Non-responder	Macro_FOLR2+APOE+	Macro_ISG15	CCL18	CCR1	8,60014E-05	9470,1	5,77621E-02	501	1,07304E+00	886	3,14323E+00	188	8,23369E-01	28245	0	17530,5
Non-responder	Macro_OLFML3	Macro_NLRP3	CXCL9	FCGR2A	8,60436E-05	5847,5	1,94191E-02	3219	1,01658E+00	1130	2,67762E+00	527	9,10978E-01	6831	0	17530,5
Non-responder	Macro_FOLR2+APOE+	Macro_FOLR2+APOE+	ICAM1	IL2RA	8,60479E-05	11627,1	1,97376E-02	3137	7,35253E-01	3194	1,79519E+00	3140	8,11495E-01	31134	0	17530,5
Non-responder	Macro_NLRP3	Mast	HBEGF	CD82	8,65700E-05	5859,7	3,83740E-02	1033	1,26525E+00	310	1,96745E+00	2222	9,04919E-01	8203	0	17530,5
Non-responder	Macro_FOLR2+APOE+	cDC_CLEC9A	ADAM10	CADM1	8,69054E-05	8600,1	4,44179E-02	800	1,23960E+00	352	2,15250E+00	1510	8,45616E-01	22808	0	17530,5
Non-responder	Macro_FOLR2+APOE+	pDC_LILRA4	MDK	NCL	8,74445E-05	11239,3	2,58940E-02	1971	7,34231E-01	3207	2,21192E+00	1336	8,07131E-01	32152	0	17530,5
Non-responder	Macro_FOLR2+APOE+	Macro_OLFML3	CCL24	CCR2	8,75527E-05	16749,1	3,63074E-02	1132	7,34189E-01	3208	2,57052E+00	666	6,60758E-01	61209	0	17530,5
Non-responder	Macro_OLFML3	Macro_NLRP3	C1QA	CD93	8,77371E-05	8740,3	1,15053E-02	7867	5,40256E-01	6760	1,53959E+00	5139	9,12934E-01	6405	0	17530,5
Non-responder	pDC_LILRA4	Macro_FOLR2-APOE+	APP	CD74	8,79862E-05	4581,9	1,94534E-02	3212	1,29591E+00	271	2,05035E+00	1887	9,60566E-01	9	0	17530,5
Non-responder	Macro_NLRP3	Macro_FOLR2+APOE+	VEGFA	CD44	8,81111E-05	6713,9	1,66535E-02	4175	9,38946E-01	1502	1,99279E+00	2130	9,04824E-01	8232	0	17530,5
Non-responder	pDC_LILRA4	Macro_FOLR2+APOE+	LILRB4	LAIR1	8,83124E-05	4499,9	1,94414E-02	3215	1,21072E+00	409	3,61178E+00	70	9,38624E-01	1275	0	17530,5
Non-responder	Macro_NLRP3	Macro_FOLR2+APOE+	THBS1	CD47	8,88629E-05	6032,3	2,34963E-02	2291	1,04501E+00	998	2,31388E+00	1096	9,04749E-01	8246	0	17530,5
Non-responder	Macro_NLRP3	Mast	VEGFA	CD44	8,92948E-05	7652,9	1,78690E-02	3713	9,88026E-01	1259	1,28140E+00	8254	9,07815E-01	7508	0	17530,5
Non-responder	Macro_FOLR2+APOE+	Mono_CD14	C1QB	CD33	8,95656E-05	7726,9	1,11700E-02	8259	6,83932E-01	3834	2,16175E+00	1486	9,07759E-01	7525	0	17530,5
Non-responder	Macro_FOLR2+APOE+	cDC_LAMP3	FN1	TNFRSF11B	8,96261E-05	14123,7	1,03446E-01	124	1,09019E+00	810	1,78071E+00	3227	7,33947E-01	48927	0	17530,5
Non-responder	Macro_NLRP3	Mono_CD16	HBEGF	CD44	9,03819E-05	7843,7	1,11593E-02	8274	7,63357E-01	2885	1,59208E+00	4672	9,15445E-01	5857	0	17530,5
Non-responder	pDC_LILRA4	Mono_CD16	B2M	LILRB2	9,04159E-05	5925,3	9,40351E-03	10856	1,06827E+00	901	2,94054E+00	316	9,58697E-01	23	0	17530,5
Non-responder	Macro_FOLR2+APOE+	cDC_CLEC9A	CCL5	DPP4	9,06210E-05	16672,9	8,09983E-02	223	8,57191E-01	2030	1,86492E+00	2746	6,63801E-01	60835	0	17530,5
Non-responder	Macro_NLRP3	Macro_LYVE1	S100A8	CD68	9,08745E-05	6488,3	1,47066E-02	5175	9,64143E-01	1377	1,27900E+00	8283	9,54245E-01	76	0	17530,5
Non-responder	Macro_FOLR2+APOE+	Mono_CD16	C1QB	CD33	9,09843E-05	7664,3	1,11526E-02	8285	6,83428E-01	3846	2,30113E+00	1117	9,07693E-01	7543	0	17530,5
Non-responder	Mast	Mono_CD16	HDC	HRH2	9,10156E-05	7884,7	1,16519E-01	96	1,51172E+00	120	2,41761E+00	903	8,54115E-01	20774	0	17530,5
Non-responder	Macro_OLFML3	Mono_CD16	HLA-B	LILRA1	9,11491E-05	5785,9	1,79978E-02	3662	8,55102E-01	2052	1,94481E+00	2333	9,28270E-01	3352	0	17530,5
Non-responder	Macro_NLRP3	Macro_NLRP3	S100A8	CD69	9,14795E-05	7972,5	1,62792E-02	4348	8,95588E-01	1757	1,27819E+00	8294	9,06034E-01	7933	0	17530,5
Non-responder	Macro_OLFML3	Mono_CD16	HLA-A	LILRA1	9,16451E-05	5768,5	1,87605E-02	3410	8,72239E-01	1898	1,99953E+00	2096	9,25393E-01	3908	0	17530,5
Non-responder	Macro_FOLR2+APOE+	cDC_LAMP3	TNFSF10	TNFRSF11B	9,18481E-05	11906,9	7,13260E-02	308	1,18732E+00	477	1,77688E+00	3247	7,82425E-01	37972	0	17530,5
Non-responder	Macro_LYVE1	pDC_LILRA4	GNAI2	CXCR3	9,19200E-05	5764,7	4,16462E-02	906	1,19093E+00	463	2,55184E+00	689	9,00233E-01	9235	0	17530,5
Non-responder	Macro_FOLR2+APOE+	Macro_FOLR2+APOE+	PLAU	LRP1	9,20725E-05	8712,9	2,13289E-02	2732	7,29607E-01	3249	2,09606E+00	1716	8,64484E-01	18337	0	17530,5
Non-responder	pDC_LILRA4	Macro_LYVE1	APP	TSPAN15	9,22228E-05	8192,7	4,15248E-02	907	1,29495E+00	272	2,85129E+00	377	8,49485E-01	21877	0	17530,5
Non-responder	Mono_CD14	cDC_CLEC9A	S100A9	ITGB2	9,22228E-05	5111,7	1,33206E-02	6126	1,06593E+00	907	2,42557E+00	892	9,52928E-01	103	0	17530,5
Non-responder	pDC_LILRA4	cDC_LAMP3	SPON2	ITGA4	9,25263E-05	8609,1	4,15128E-02	908	1,14681E+00	590	2,72105E+00	490	8,42597E-01	23527	0	17530,5
Non-responder	Macro_OLFML3	pDC_LILRA4	GNAI2	CXCR3	9,28304E-05	5688,7	4,15093E-02	909	1,18854E+00	471	3,00036E+00	273	9,00085E-01	9260	0	17530,5
Non-responder	Macro_OLFML3	Mono_CD16	HLA-C	LILRA1	9,29224E-05	5991,9	1,84297E-02	3520	8,01366E-01	2542	1,90133E+00	2544	9,25754E-01	3823	0	17530,5
Non-responder	Macro_OLFML3	Mono_CD16	HLA-C	LILRA3	9,30341E-05	6210,3	2,18226E-02	2612	7,42066E-01	3121	2,11320E+00	1649	9,14153E-01	6139	0	17530,5
Non-responder	Mono_INHBA	Mono_CD16	IL6	FCGR3B	9,31352E-05	11232,1	9,72985E-02	147	1,37424E+00	184	2,41259E+00	910	7,84909E-01	37389	0	17530,5
Non-responder	Macro_NLRP3	Mono_CD16	HLA-C	LILRA1	9,34261E-05	8419,5	1,53630E-02	4806	4,89866E-01	8329	1,42602E+00	6331	9,19252E-01	5101	0	17530,5
Non-responder	Macro_FOLR2+APOE+	Macro_FOLR2+APOE+	PLAU	ITGB1	9,38827E-05	9108,3	1,92856E-02	3253	7,28274E-01	3265	2,17247E+00	1455	8,57176E-01	20038	0	17530,5
Non-responder	Macro_NLRP3	Macro_FOLR2+APOE+	THBS1	LRP1	9,40446E-05	6236,3	2,29354E-02	2385	9,57694E-01	1407	2,15037E+00	1519	9,04331E-01	8340	0	17530,5
Non-responder	pDC_LILRA4	cDC_CLEC9A	PROC	THBD	9,40536E-05	10650,5	7,54195E-02	263	1,06486E+00	913	2,73176E+00	477	7,99199E-01	34069	0	17530,5
Non-responder	Macro_FOLR2+APOE+	Macro_IER3	CCL24	CCR2	9,47976E-05	16960,7	3,50232E-02	1198	7,27736E-01	3273	2,34017E+00	1047	6,56710E-01	61755	0	17530,5
Non-responder	Macro_FOLR2+APOE+	Macro_NLRP3	HLA-A	LILRB2	9,53728E-05	9084,9	6,31051E-03	20683	7,27501E-01	3278	1,83101E+00	2944	9,40837E-01	989	0	17530,5
Non-responder	Macro_FOLR2+APOE+	Macro_FOLR2+APOE+	HLA-DQB1	CD4	9,61825E-05	6770,9	9,32111E-03	10995	7,27129E-01	3285	2,19990E+00	1379	9,43857E-01	665	0	17530,5
Non-responder	pDC_LILRA4	Macro_ISG15	SPON2	ITGAM	9,62197E-05	10633,3	4,22012E-02	884	1,06243E+00	920	2,83741E+00	391	8,01802E-01	33441	0	17530,5
Non-responder	Mono_CD14	Macro_ISG15	S100A12	TLR4	9,77869E-05	8200,3	4,10634E-02	925	1,30697E+00	251	2,80677E+00	413	8,49449E-01	21882	0	17530,5

Post_NR_Myeloid_Myeloid_CellCell

Non-responder	Macro_FOLR2+APOE+	Macro_FOLR2+APOE+	TNFSF13	TNFRSF14	9,78834E-05	7688,1	1,21059E-02	7226	7,10271E-01	3514	2,08332E+00	1763	9,04044E-01	8407	0	17530,5
Non-responder	Macro_FOLR2+APOE+	cDC(CD1C)	C1QB	C1QBP	9,85241E-05	6928,3	1,09136E-02	8600	7,42881E-01	3112	1,99968E+00	2094	9,28489E-01	3305	0	17530,5
Non-responder	Macro_OLFML3	Mono_CD16	LYZ	ITGAL	9,89945E-05	6327,1	1,49482E-02	5045	7,78010E-01	2742	2,05282E+00	1876	9,22585E-01	4442	0	17530,5
Non-responder	pDC_LILRA4	Macro_OLFML3	SPON2	ITGAM	9,90529E-05	10690,3	4,19525E-02	894	1,06080E+00	929	2,66384E+00	538	8,01332E-01	33560	0	17530,5
Non-responder	pDC_LILRA4	pDC_LILRA4	NUCB2	ERAP1	1,00650E-04	9235,9	4,29020E-02	855	1,05990E+00	934	3,08689E+00	214	8,29820E-01	26646	0	17530,5
Non-responder	Macro_NLRP3	Macro_FOLR2+APOE+	VEGFA	NRP1	1,00972E-04	7452,1	4,08217E-02	935	1,17851E+00	503	2,54921E+00	698	8,67519E-01	17594	0	17530,5
Non-responder	Macro_ISG15	Mono_CD16	HLA-B	LILRA1	1,01510E-04	5240,5	1,90195E-02	3330	9,79084E-01	1304	2,32571E+00	1075	9,30086E-01	2963	0	17530,5
Non-responder	Macro_FOLR2+APOE+	Mono_CD14	CCL24	CCR2	1,01873E-04	15748,7	4,66484E-02	736	7,86146E-01	2675	2,66055E+00	540	6,88256E-01	57262	0	17530,5
Non-responder	Macro_NLRP3	pDC_LILRA4	VEGFA	NRP1	1,01941E-04	7441,1	4,07482E-02	938	1,17746E+00	504	2,61275E+00	610	8,67416E-01	17623	0	17530,5
Non-responder	Macro_FOLR2+APOE+	Mono_CD14	APOC2	LDLR	1,02359E-04	14766,3	4,30418E-02	851	7,52817E-01	2992	2,38682E+00	951	7,20983E-01	51507	0	17530,5
Non-responder	Macro_FOLR2+APOE+	Macro_NLRP3	CD38	PECAM1	1,02968E-04	11545,5	2,13572E-02	2725	8,60999E-01	1994	1,76251E+00	3342	8,07179E-01	32136	0	17530,5
Non-responder	Macro_NLRP3	Mono_CD16	THBS1	ITGA4	1,03175E-04	6059,5	2,32499E-02	2323	1,04860E+00	981	2,37955E+00	967	9,03612E-01	8496	0	17530,5
Non-responder	Macro_FOLR2+APOE+	Mono_INHBA	GRN	TNFRSF1B	1,03334E-04	7837,7	8,04858E-03	13984	7,22474E-01	3345	1,83447E+00	2919	9,37736E-01	1410	0	17530,5
Non-responder	Macro_FOLR2+APOE+	Macro_OLFML3	HLA-B	LILRB1	1,04440E-04	8172,3	7,44468E-03	15862	7,21469E-01	3354	1,95337E+00	2288	9,35692E-01	1827	0	17530,5
Non-responder	pDC_LILRA4	Macro_ISG15	SPON2	ITGB2	1,04884E-04	5590,7	4,04480E-02	947	1,13460E+00	634	3,13828E+00	194	9,02844E-01	8648	0	17530,5
Non-responder	Macro_FOLR2+APOE+	Macro_FOLR2+APOE+	HLA-A	LILRB1	1,06679E-04	8397,5	7,27575E-03	16444	7,20382E-01	3372	2,04886E+00	1899	9,31051E-01	2742	0	17530,5
Non-responder	Mono_CD14	Mono_INHBA	S100A12	TLR4	1,07212E-04	8338,7	4,03284E-02	954	1,30082E+00	260	2,48853E+00	781	8,48290E-01	22168	0	17530,5
Non-responder	Macro_FOLR2+APOE+	Mono_CD14	CD38	PECAM1	1,07685E-04	11141,1	2,30296E-02	2368	9,03163E-01	1713	1,76479E+00	3327	8,12978E-01	30767	0	17530,5
Non-responder	Mono_CD14	Mono_CD16	S100A12	TLR4	1,08219E-04	8290,1	4,02339E-02	957	1,30003E+00	261	2,70588E+00	501	8,48139E-01	22201	0	17530,5
Non-responder	Macro_OLFML3	Macro_OLFML3	CXCL9	FCGR2A	1,08504E-04	5744,7	2,00721E-02	3045	1,03920E+00	1027	2,63732E+00	570	9,12310E-01	6551	0	17530,5
Non-responder	Macro_FOLR2+APOE+	cDC_CLEC9A	HLA-DQB2	LAG3	1,08953E-04	11934,1	2,11790E-02	2770	7,50946E-01	3013	1,75328E+00	3390	8,03758E-01	32967	0	17530,5
Non-responder	Mono_INHBA	Macro_ISG15	CCL2	CCR1	1,09234E-04	8208,3	4,53101E-02	772	1,05372E+00	960	2,74701E+00	461	8,51792E-01	21318	0	17530,5
Non-responder	Macro_LYVE1	pDC_LILRA4	CXCL12	CXCR4	1,09234E-04	6008,9	4,01848E-02	960	1,21351E+00	404	3,15302E+00	184	8,93079E-01	10966	0	17530,5
Non-responder	Macro_FOLR2+APOE+	Macro_LYVE1	C1QA	CD33	1,09392E-04	8399,1	1,09155E-02	8596	6,46679E-01	4443	1,71130E+00	3702	9,06927E-01	7724	0	17530,5
Non-responder	Macro_FOLR2+APOE+	Macro_FOLR2+APOE+	MMP9	CD44	1,09719E-04	7501,7	1,87949E-02	3396	7,93604E-01	2614	2,64069E+00	565	8,83753E-01	13403	0	17530,5
Non-responder	Macro_FOLR2+APOE+	pDC_LILRA4	HLA-DQA1	CD4	1,09719E-04	6871,3	8,88506E-03	11864	7,18610E-01	3396	2,69026E+00	513	9,40351E-01	1053	0	17530,5
Non-responder	pDC_LILRA4	Macro_ISG15	SNCA	LAG3	1,09913E-04	13052,5	4,42416E-02	804	1,05309E+00	962	2,75343E+00	454	7,49636E-01	45512	0	17530,5
Non-responder	pDC_LILRA4	Macro_NLRP3	SPON2	ITGB2	1,09913E-04	5640,3	4,01213E-02	962	1,12753E+00	658	2,94873E+00	311	9,02488E-01	8740	0	17530,5
Non-responder	Macro_FOLR2+APOE+	Mono_CD14	MMP9	CD44	1,09975E-04	7318,5	1,96926E-02	3150	8,25718E-01	2315	2,46240E+00	824	8,86128E-01	12773	0	17530,5
Non-responder	Macro_NLRP3	Macro_ISG15	S100A8	CD69	1,10094E-04	8132,1	1,58975E-02	4540	8,87941E-01	1806	1,25630E+00	8607	9,05019E-01	8177	0	17530,5
Non-responder	Macro_OLFML3	pDC_LILRA4	TNFSF13B	TNFRSF17	1,10254E-04	9271,1	9,45608E-02	158	1,05284E+00	963	2,38541E+00	953	8,29436E-01	26751	0	17530,5
Non-responder	Macro_FOLR2+APOE+	Macro_FOLR2+APOE+	C3	C3AR1	1,10414E-04	7770,9	1,56318E-02	4675	5,76905E-01	5801	1,96455E+00	2236	9,03014E-01	8612	0	17530,5
Non-responder	Macro_FOLR2+APOE+	Macro_LYVE1	APOC2	LRP1	1,10617E-04	9616,9	2,79206E-02	1734	7,18083E-01	3403	2,04774E+00	1902	8,42643E-01	23515	0	17530,5
Non-responder	pDC_LILRA4	Macro_FOLR2+APOE+	LTB	TNFRSF1A	1,10938E-04	5444,3	3,99837E-02	965	1,34413E+00	203	3,80963E+00	51	9,03732E-01	8472	0	17530,5
Non-responder	Mono_CD14	Mono_CD14	S100A9	ITGB2	1,10938E-04	4977,3	1,40508E-02	5605	1,11635E+00	709	2,38013E+00	965	9,54111E-01	77	0	17530,5
Non-responder	Macro_FOLR2+APOE+	cDC(CD1C)	CCL24	CCR2	1,11133E-04	17175,9	3,30414E-02	1315	7,17779E-01	3407	2,35123E+00	1023	6,50114E-01	62604	0	17530,5
Non-responder	cDC_CLEC9A	pDC_LILRA4	SELPLG	SELL	1,11281E-04	4804,1	3,99833E-02	966	1,40394E+00	155	3,55217E+00	85	9,18374E-01	5284	0	17530,5
Non-responder	Macro_FOLR2+APOE+	cDC_CLEC9A	CCL4	CNR2	1,11392E-04	14402,3	7,41486E-02	279	8,73625E-01	1886	1,75108E+00	3409	7,34052E-01	48907	0	17530,5
Non-responder	Macro_FOLR2+APOE+	Macro_FOLR2-APOE+	MMP9	EPHB2	1,11781E-04	16417,9	2,99469E-02	1549	7,17634E-01	3412	2,07295E+00	1805	6,84920E-01	57793	0	17530,5
Non-responder	Macro_FOLR2+APOE+	Macro_IER3	C1QB	CD33	1,12156E-04	8144,7	1,08834E-02	8639	6,75598E-01	3969	1,85459E+00	2792	9,06664E-01	7793	0	17530,5
Non-responder	Macro_FOLR2+APOE+	Mono_CD14	MMP12	PLAUR	1,12171E-04	7296,9	2,69230E-02	1832	8,31483E-01	2268	2,62438E+00	594	8,80430E-01	14260	0	17530,5
Non-responder	Macro_NLRP3	Macro_LYVE1	THBS1	LRP1	1,12286E-04	6832,5	2,21762E-02	2538	9,36570E-01	1520	1,67965E+00	3933	9,02865E-01	8641	0	17530,5
Non-responder	Mono_CD16	Macro_FOLR2+APOE+	SERPINA1	LRP1	1,12286E-04	7145,5	1,08820E-02	8641	7,35513E-01	3192	1,93778E+00	2369	9,24948E-01	3995	0	17530,5
Non-responder	Macro_FOLR2+APOE+	Macro_FOLR2+APOE+	MMP12	PLAUR	1,12301E-04	8057,7	2,30326E-02	2365	7,17374E-01	3416	2,71179E+00	494	8,71968E-01	16483	0	17530,5
Non-responder	Mono_CD14	Macro_NLRP3	S100A12	CD36	1,12660E-04	8095,3	3,98232E-02	970	1,27654E+00	293	2,55176E+00	690	8,53268E-01	20993	0	17530,5
Non-responder	Mono_INHBA	pDC_LILRA4	CXCL10	CXCR3	1,13006E-04	8884,9	3,98072E-02	971	1,22378E+00	385	3,01380E+00	265	8,35366E-01	25273	0	17530,5
Non-responder	Macro_ISG15	Mono_INHBA	CXCL10	SDC4	1,14050E-04	8933,7	3,97394E-02	974	1,13152E+00	648	2,85642E+00	369	8,35914E-01	25147	0	17530,5
Non-responder	Macro_FOLR2+APOE+	Macro_ISG15	CCL3	CCR1	1,15439E-04	6091,7	2,39918E-02	2217	9,70367E-01	1346	2,56132E+00	676	9,02654E-01	8689	0	17530,5
Non-responder	pDC_LILRA4	Mono_CD16	SPON2	ITGB1	1,15451E-04	8734,9	3,96083E-02	978	1,10334E+00	753	3,01613E+00	262	8,40055E-01	24151	0	17530,5
Non-responder	Macro_FOLR2+APOE+	Mono_CD14	APOE	LRP1	1,16305E-04	8403,7	1,12052E-02	8197	4,80070E-01	8702	1,67750E+00	3948	9,26748E-01	3641	0	17530,5
Non-responder	Macro_FOLR2+APOE+	Mono_CD14	MMP9	ITGAM	1,17197E-04	10869,3	2,41341E-02	2201	7,24465E-01	3314	2,46184E+00	825	8,14191E-01	30476	0	17530,5
Non-responder	Macro_FOLR2+APOE+	Mono_CD16	HLA-C	LILRB1	1,17197E-04	7468,1	8,45648E-03	12880	7,14450E-01	3453	2,06694E+00	1834	9,36569E-01	1643	0	17530,5
Non-responder	Macro_OLFML3	Macro_IFI27	CXCL9	FCGR2A	1,17446E-04	6454,9	1,85396E-02	3489	9,86118E-01	1274	1,86552E+00	2742	9,09081E-01	7239	0	17530,5
Non-responder	Macro_NLRP3	pDC_LILRA4	TNF	TNFRSF21	1,17575E-04	6751,3	3,94993E-02	984	1,23704E+00	359	3,17776E+00	174	8,78877E-01	14709	0	17530,5
Non-responder	Macro_FOLR2+APOE+	Macro_FOLR2+APOE+	CXCL10	SDC4	1,18006E-04	12166,9	2,18777E-02	2596	7,14045E-01	3459	2,23962E+00	1260	7,90790E-01	35989	0	17530,5
Non-responder	cDC_CLEC9A	Macro_FOLR2+APOE+	BTLA	TNFRSF14	1,18288E-04	7647,5	7,42162E-02	276	1,45950E+00	136	2,37162E+00	986	8,60285E-01	19309	0	17530,5
Non-responder	pDC_LILRA4	Macro_IER3	SPON2	ITGB2	1,18646E-04	5754,3	3,94103E-02	987	1,11214E+00	725	2,59079E+00	641	9,01698E-01	8888	0	17530,5
Non-responder	Mono_CD14	Macro_LYVE1	S100A8	CD68	1,18954E-04	4524,1	1,86055E-02	3466	1,24734E+00	342	2,23875E+00	1264	9,59113E-01	18	0	17530,5
Non-responder	Macro_NLRP3	pDC_LILRA4	S100A8	CD68	1,19890E-04	6814,5	1,07892E-02	8755	6,69945E-01	4067	1,76851E+00	3303	9,46987E-01	417	0	17530,5
Non-responder	Mono_INHBA	cDC_LAMP3	SPP1	S1PR1	1,19907E-04	7706,7	1,60225E-01	46	1,51942E+00	114	2,12029E+00	1622	8,60676E-01	19221	0	17530,5
Non-responder	Macro_FOLR2+APOE+	Macro_ISG15	C1QB	LRP1	1,20027E-04	7006,9	1,07876E-02	8757	6,80635E-01	3901	2,27029E+00	1180	9,26603E-01	3666	0	17530,5
Non-responder	Macro_FOLR2+APOE+	Mono_CD14	C1QA	CR1	1,20046E-04	8089,7	2,76457E-02	1758	8,96669E-01	1749	2,25208E+00	1230	8,65143E-01	18181	0	17530,5
Non-responder	Macro_NLRP3	Macro_FOLR2+APOE+	VCAN	TLR2	1,20233E-04	6252,3	2,29791E-02	2375	1,03623E+00	1040	2,13645E+00	1556	9,02398E-01	8760	0	17530,5
Non-responder	Macro_FOLR2+APOE+	Macro_FOLR2+APOE+	HEBP1	FPR3	1,21558E-04	9566,3	1,85458E-02	3485	7,20492E-01	3370	1,92495E+00	2433	8,53152E-01	21013	0	17530,5
Non-responder	Macro_FOLR2+APOE+	pDC_LILRA4	PSEN1	NOTCH4	1,21835E-04	12259,1	4,07471E-02	939	7,11845E-01	3487	1,88361E+00	2644	7,87795E-01	36695	0	17530,5
Non-responder	pDC_LILRA4	Macro_IER3	SPON2	ITGA4	1,22629E-04	8841,7	3,92131E-02	998	1,12341E+00	678	2,65605E+00	548	8,38781E-01	24454	0	17530,5
Non-responder	Macro_FOLR2+APOE+	pDC_LILRA4	FAM3C	ADGRG5	1,22945E-04	19199,5	5,27240E-02	596	7,77214E-01	2750	1,73788E+00	3495	5,32119E-01	71626	0	17530,5
Non-responder	pDC_LILRA4	cDC(CD1C)	SPON2	ITGA4	1,24100E-04	8849,7	3,91642E-02	1002	1,12291E+00	682	2,64207E+00	563	8,38697E-01	24471	0	17530,5
Non-responder	Macro_FOLR2+APOE+	Macro_ISG15	HLA-B	LILRB2	1,24345E-04	6980,5	7,97546E-03	14205	9,08160E-01	1681	2,23907E+00	1263	9,50109E-01	223	0	17530,5
Non-responder	Macro_FOLR2+APOE+	Macro_ISG15	HLA-B	LILRB1	1,24485E-04	6604,9	9,13821E-03	11339	8,60801E-01	1995	2,24057E+00	1255	9,41590E-01	905	0	17530,5
Non-responder	Macro_FOLR2+APOE+	Macro_ISG15	HLA-DQA2	LAG3	1,24908E-04	9627,7	1,95092E-02	3201	8,41740E-01	2164	2,05226E+00	1878	8,43263E-01	23365	0	17530,5

Post_NR_Myeloid_Myeloid_CellCell

Non-responder	Macro_NLRP3	Macro_ISG15	S100A9	ITGB2	1,25114E-04	6315,3	1,07426E-02	8830	8,22225E-01	2350	1,91227E+00	2501	9,47862E-01	365	0	17530,5
Non-responder	Macro_FOLR2+APOE+	Macro_ISG15	HLA-DQA1	LAG3	1,25190E-04	6191,9	2,65551E-02	1876	1,25274E+00	333	2,74215E+00	467	8,93955E-01	10753	0	17530,5
Non-responder	Macro_ISG15	pDC_LILRA4	SEMA6B	PLXNA4	1,25210E-04	14757,9	1,66617E-01	42	1,09848E+00	771	2,36057E+00	1005	7,05140E-01	54441	0	17530,5
Non-responder	Macro_NLRP3	pDC_LILRA4	CXCL8	CD79A	1,25581E-04	11465,7	1,04696E-01	118	1,10278E+00	756	2,36005E+00	1006	7,82626E-01	37918	0	17530,5
Non-responder	Macro_FOLR2+APOE+	Macro_ISG15	HLA-DQB1	LAG3	1,25755E-04	6316,9	2,46211E-02	2136	1,17057E+00	531	2,55056E+00	695	8,94195E-01	10692	0	17530,5
Non-responder	Macro_FOLR2+APOE+	Macro_OLFML3	B2M	LILRB1	1,26323E-04	8066,5	7,30623E-03	16342	7,10080E-01	3519	2,00438E+00	2070	9,41792E-01	871	0	17530,5
Non-responder	Macro_FOLR2+APOE+	Macro_ISG15	HLA-A	LILRB2	1,26465E-04	6566,3	8,55355E-03	12626	9,61427E-01	1392	2,37755E+00	973	9,48756E-01	310	0	17530,5
Non-responder	Macro_FOLR2+APOE+	Macro_ISG15	HLA-A	LILRB1	1,26608E-04	6285,5	9,80058E-03	10180	9,14069E-01	1647	2,37905E+00	970	9,40020E-01	1100	0	17530,5
Non-responder	Macro_FOLR2+APOE+	Macro_ISG15	HLA-DPB1	LAG3	1,26893E-04	6224,5	2,18684E-02	2599	1,07476E+00	879	2,30575E+00	1108	9,01199E-01	9006	0	17530,5
Non-responder	Macro_OLFML3	Macro_IER3	CXCL9	FCGR2A	1,27039E-04	5574,7	2,15134E-02	2688	1,08913E+00	815	2,42622E+00	891	9,15044E-01	5949	0	17530,5
Non-responder	Macro_FOLR2+APOE+	Mono_CD16	FABP5	RXRA	1,27321E-04	9322,1	1,98931E-02	3089	7,09444E-01	3526	1,89989E+00	2551	8,57673E-01	19914	0	17530,5
Non-responder	Mono_INHBA	Macro_FOLR2+APOE+	IL6	FCGR3B	1,28580E-04	11283,5	9,65102E-02	151	1,37103E+00	187	2,35433E+00	1014	7,84222E-01	37535	0	17530,5
Non-responder	Macro_FOLR2+APOE+	Macro_FOLR2+APOE+	PLTP	ABCA1	1,29046E-04	6893,1	1,83644E-02	3538	7,95496E-01	2596	2,07734E+00	1783	9,01169E-01	9018	0	17530,5
Non-responder	Macro_NLRP3	Mono_CD16	S100A9	CD68	1,29569E-04	6538,9	1,06870E-02	8892	7,82048E-01	2708	1,74999E+00	3419	9,51647E-01	145	0	17530,5
Non-responder	Macro_FOLR2+APOE+	Macro_ISG15	HLA-C	LILRB2	1,29769E-04	6962,1	8,22061E-03	13505	8,38645E-01	2198	2,24279E+00	1250	9,48477E-01	327	0	17530,5
Non-responder	Mono_CD14	Macro_IFI27	S100A8	CD68	1,29915E-04	4799,3	1,83394E-02	3544	1,23155E+00	368	1,90357E+00	2533	9,58830E-01	21	0	17530,5
Non-responder	Macro_FOLR2+APOE+	Macro_ISG15	HLA-C	LILRB1	1,29915E-04	6675,5	9,41910E-03	10830	7,91266E-01	2634	2,24429E+00	1243	9,39697E-01	1140	0	17530,5
Non-responder	pDC_LILRA4	Macro_ISG15	SPN	SIGLEC1	1,30097E-04	8387,1	4,24540E-02	868	1,04122E+00	1018	2,79413E+00	425	8,48582E-01	22094	0	17530,5
Non-responder	Mono_INHBA	Macro_FOLR2+APOE+	CXCL8	SDC3	1,30097E-04	7176,7	3,97292E-02	975	1,12792E+00	655	2,35351E+00	1018	8,74959E-01	15705	0	17530,5
Non-responder	cDC(CD1C)	pDC_LILRA4	GNAI2	CXCR3	1,30097E-04	5963,5	3,89091E-02	1018	1,14325E+00	602	2,53937E+00	714	8,97138E-01	9953	0	17530,5
Non-responder	Macro_FOLR2+APOE+	Macro_NLRP3	MMP9	LRP1	1,30205E-04	9503,3	2,06438E-02	2910	7,07679E-01	3546	2,48237E+00	788	8,45896E-01	22742	0	17530,5
Non-responder	Macro_FOLR2+APOE+	cDC(CD1C)	TNFSF13B	HLA-DPB1	1,30642E-04	7891,1	7,76855E-03	14805	7,29381E-01	3252	1,73055E+00	3549	9,48655E-01	319	0	17530,5
Non-responder	pDC_LILRA4	cDC(CD1C)	SPON2	ITGA5	1,30860E-04	12183,3	3,87097E-02	1020	1,09215E+00	801	2,58089E+00	651	7,70028E-01	40914	0	17530,5
Non-responder	Macro_FOLR2+APOE+	Mono_CD14	PLAU	PLAUR	1,30934E-04	6908,3	1,83168E-02	3551	8,25962E-01	2314	2,09771E+00	1706	8,99221E-01	9440	0	17530,5
Non-responder	Macro_FOLR2+APOE+	Macro_ISG15	LGALS9	HAVCR2	1,31253E-04	8088,7	1,11972E-02	8210	6,86150E-01	3807	2,02738E+00	1981	9,01582E-01	8915	0	17530,5
Non-responder	Macro_IER3	cDC(CD1C)	B2M	CD1C	1,31519E-04	5159,9	3,09823E-02	1458	7,06760E-01	3555	1,84245E+00	2867	9,47561E-01	389	0	17530,5
Non-responder	Mono_CD14	Macro_LYVE1	S100A12	TLR4	1,32010E-04	8519,3	3,85799E-02	1023	1,28619E+00	280	2,41534E+00	907	8,45416E-01	22856	0	17530,5
Non-responder	Mono_INHBA	Macro_ISG15	CCL3L1	CCR1	1,32394E-04	6065,3	3,85721E-02	1024	1,20675E+00	419	2,63840E+00	568	8,93792E-01	10785	0	17530,5
Non-responder	Macro_FOLR2+APOE+	Macro_FOLR2-APOE+	MMP9	ITGAM	1,32400E-04	11491,3	2,38032E-02	2252	7,19323E-01	3386	1,72883E+00	3561	8,13144E-01	30727	0	17530,5
Non-responder	Macro_NLRP3	Macro_NLRP3	S100A9	TLR4	1,32658E-04	6598,7	1,78030E-02	3738	9,73696E-01	1325	2,16987E+00	1466	9,01513E-01	8934	0	17530,5
Non-responder	Macro_NLRP3	Macro_NLRP3	S100A9	ITGB2	1,32732E-04	6573,1	1,06559E-02	8935	8,15154E-01	2405	1,72272E+00	3614	9,47662E-01	381	0	17530,5
Non-responder	Macro_FOLR2+APOE+	Macro_ISG15	B2M	LILRB2	1,32842E-04	7022,7	7,82714E-03	14633	8,96771E-01	1748	2,29008E+00	1142	9,54907E-01	60	0	17530,5
Non-responder	Macro_FOLR2+APOE+	Macro_ISG15	B2M	LILRB1	1,32990E-04	6574,7	8,96826E-03	11702	8,49412E-01	2096	2,29158E+00	1139	9,47162E-01	406	0	17530,5
Non-responder	Macro_FOLR2+APOE+	Mono_INHBA	TIMP2	CD44	1,33030E-04	8209,9	1,23275E-02	7021	7,24055E-01	3321	1,64377E+00	4238	9,01497E-01	8939	0	17530,5
Non-responder	Macro_FOLR2+APOE+	Macro_IER3	APOE	ABCA1	1,33030E-04	8910,1	1,06539E-02	8939	5,02951E-01	7881	1,65567E+00	4146	9,14573E-01	6054	0	17530,5
Non-responder	Macro_ISG15	Macro_NLRP3	MMP12	PLAUR	1,33166E-04	5783,9	3,85598E-02	1026	1,18416E+00	489	3,25742E+00	150	8,98085E-01	9724	0	17530,5
Non-responder	Macro_NLRP3	Macro_ISG15	VCAN	ITGB1	1,33253E-04	6505,1	1,94140E-02	3223	1,00838E+00	1170	2,10993E+00	1660	9,01483E-01	8942	0	17530,5
Non-responder	pDC_LILRA4	cDC_LAMP3	APP	CD74	1,33553E-04	4625,1	1,75608E-02	3817	1,11130E+00	727	2,34983E+00	1027	9,58581E-01	24	0	17530,5
Non-responder	Macro_FOLR2+APOE+	Macro_NLRP3	CCL24	CCR2	1,34175E-04	17444,5	3,05557E-02	1494	7,05290E-01	3573	2,38310E+00	958	6,41168E-01	63667	0	17530,5
Non-responder	Macro_NLRP3	cDC_CLEC9A	CXCL2	DPP4	1,34329E-04	8624,9	8,49478E-02	202	1,25605E+00	327	2,34894E+00	1029	8,40483E-01	24036	0	17530,5
Non-responder	pDC_LILRA4	Macro_NLRP3	SPON2	ITGB1	1,34329E-04	8864,1	3,84809E-02	1029	1,09166E+00	804	2,91407E+00	337	8,38105E-01	24620	0	17530,5
Non-responder	Macro_FOLR2+APOE+	Macro_FOLR2+APOE+	CCL5	CCRL2	1,34473E-04	14360,9	2,91363E-02	1638	7,05028E-01	3575	1,90996E+00	2511	7,44928E-01	46550	0	17530,5
Non-responder	pDC_LILRA4	Mast	APP	RPSA	1,34622E-04	4884,9	2,35652E-02	2285	1,38869E+00	168	2,44075E+00	865	9,27146E-01	3576	0	17530,5
Non-responder	Macro_FOLR2+APOE+	Macro_FOLR2+APOE+	IL18	IL18BP	1,34622E-04	8639,7	1,98460E-02	3110	7,05022E-01	3576	2,13716E+00	1555	8,68217E-01	17427	0	17530,5
Non-responder	Macro_NLRP3	Mono_CD16	HLA-A	LILRA1	1,34675E-04	8768,3	1,48589E-02	5093	4,72541E-01	8961	1,39496E+00	6705	9,16935E-01	5552	0	17530,5
Non-responder	Macro_OLFML3	cDC_LAMP3	TNFSF13B	CD40	1,34825E-04	6859,9	1,55823E-02	4704	9,81635E-01	1294	1,91459E+00	2484	9,04544E-01	8287	0	17530,5
Non-responder	Macro_FOLR2+APOE+	Macro_FOLR2+APOE+	HLA-DRA	CD4	1,35059E-04	7426,3	8,10636E-03	13816	6,71715E-01	4037	2,11896E+00	1627	9,52226E-01	121	0	17530,5
Non-responder	Macro_FOLR2+APOE+	Macro_ISG15	HLA-DRB5	LAG3	1,35070E-04	6374,7	2,16317E-02	2663	1,05598E+00	947	2,30264E+00	1113	8,98511E-01	9620	0	17530,5
Non-responder	Macro_NLRP3	Macro_FOLR2+APOE+	VEGFA	NRP2	1,35109E-04	7681,9	3,84169E-02	1031	1,21978E+00	393	2,38827E+00	949	8,63672E-01	18506	0	17530,5
Non-responder	Macro_IER3	pDC_LILRA4	GNAI2	CXCR3	1,36283E-04	6002,3	3,82990E-02	1034	1,13263E+00	641	2,56886E+00	667	8,96407E-01	10139	0	17530,5
Non-responder	Mono_INHBA	cDC_LAMP3	ADM	RAMP1	1,37297E-04	9351,3	1,03491E-01	122	1,62319E+00	88	2,14478E+00	1530	8,26439E-01	27486	0	17530,5
Non-responder	pDC_LILRA4	Macro_ISG15	LTB	TNFRSF1A	1,37464E-04	5548,1	3,82355E-02	1037	1,31624E+00	242	3,74260E+00	58	9,01770E-01	8873	0	17530,5
Non-responder	pDC_LILRA4	cDC_LAMP3	TNFSF13	TNFRSF11B	1,37860E-04	9683,7	7,33145E-02	288	1,14685E+00	588	2,34424E+00	1038	8,20332E-01	28974	0	17530,5
Non-responder	Macro_NLRP3	Mono_CD16	ICAM1	ITGAL_ITGB2	1,38014E-04	7331,9	1,06079E-02	9005	8,31947E-01	2263	2,12549E+00	1604	9,13516E-01	6257	0	17530,5
Non-responder	Macro_FOLR2+APOE+	Mono_CD14	C1QA	CD33	1,38397E-04	8239,3	1,06041E-02	9010	6,37141E-01	4594	2,00728E+00	2058	9,05698E-01	8004	0	17530,5
Non-responder	Macro_FOLR2+APOE+	Macro_FOLR2+APOE+	LGALS9	MRC2	1,38539E-04	11052,5	2,12658E-02	2746	7,15257E-01	3444	1,72445E+00	3602	8,24693E-01	27940	0	17530,5
Non-responder	Macro_FOLR2+APOE+	Macro_ISG15	APOE	SDC2	1,38551E-04	6321,7	2,72361E-02	1798	8,09122E-01	2465	2,47283E+00	803	9,01185E-01	9012	0	17530,5
Non-responder	pDC_LILRA4	Macro_LYVE1	SPON2	ITGB2	1,39050E-04	5911,7	3,81358E-02	1041	1,08455E+00	834	2,40814E+00	916	9,00232E-01	9237	0	17530,5
Non-responder	cDC_CLEC9A	Mono_CD16	HLA-B	LILRA1	1,39455E-04	5535,5	1,81440E-02	3608	8,72840E-01	1891	2,20680E+00	1355	9,28539E-01	3293	0	17530,5
Non-responder	Macro_FOLR2+APOE+	Macro_OLFML3	CCL18	CCR1	1,39608E-04	13034,7	2,88741E-02	1665	7,01777E-01	3609	2,46629E+00	818	7,67214E-01	41551	0	17530,5
Non-responder	Macro_FOLR2+APOE+	Mono_CD16	C1QA	CD33	1,40018E-04	8141,9	1,05876E-02	9031	6,36637E-01	4601	2,14666E+00	1527	9,05632E-01	8020	0	17530,5
Non-responder	pDC_LILRA4	Macro_FOLR2+APOE+	SERPINF1	PLXDC2	1,40529E-04	5300,9	1,81330E-02	3615	9,07601E-01	1684	3,05546E+00	231	9,27790E-01	3444	0	17530,5
Non-responder	Macro_FOLR2+APOE+	Macro_OLFML3	HLA-B	LILRB2	1,40992E-04	9351,7	6,12314E-03	21676	7,00979E-01	3618	1,78009E+00	3233	9,43459E-01	701	0	17530,5
Non-responder	Macro_FOLR2+APOE+	Macro_FOLR2+APOE+	HLA-DMA	CD4	1,41146E-04	7007,9	9,51574E-03	10657	7,00839E-01	3619	2,09668E+00	1712	9,37211E-01	1521	0	17530,5
Non-responder	Macro_FOLR2+APOE+	Macro_ISG15	HLA-DQB2	LAG3	1,41455E-04	10544,1	2,57595E-02	1982	9,09894E-01	1668	1,98245E+00	2163	8,18742E-01	29377	0	17530,5
Non-responder	Macro_FOLR2+APOE+	pDC_LILRA4	FARP2	PLXNA4	1,41610E-04	18130,1	5,21226E-02	609	7,81222E-01	2717	1,72206E+00	3622	6,16812E-01	66172	0	17530,5
Non-responder	cDC_CLEC9A	pDC_LILRA4	GNAI2	CXCR3	1,42661E-04	5946,9	3,79685E-02	1050	1,12687E+00	661	3,02484E+00	251	8,96004E-01	10242	0	17530,5
Non-responder	Macro_OLFML3	cDC(CD1C)	B2M	CD1A	1,43146E-04	6314,7	4,89346E-02	679	6,76840E-01	3949	1,86297E+00	2754	9,11796E-01	6661	0	17530,5
Non-responder	Macro_FOLR2+APOE+	Macro_ISG15	CCL4	CCR1	1,43476E-04	6254,7	2,49914E-02	2089	9,76956E-01	1315	2,63025E+00	581	8,97951E-01	9758	0	17530,5
Non-responder	Macro_NLRP3	Macro_IER3	S100A8	ITGB2	1,43778E-04	7265,9	1,23874E-02	6948	8,27235E-01	2304	1,22365E+00	9079	9,46336E-01	468	0	17530,5
Non-responder	Macro_FOLR2+APOE+	Macro_NLRP3	C1QA	CR1	1,43789E-04	10090,7	1,80783E-02	3636	7,56693E-01	2955	2,07497E+00	1792	8,38391E-01	24540	0	17530,5

Post_NR_Myeloid_Myeloid_CellCell

Non-responder	Macro_FOLR2+APOE+	Macro_ISG15	CCL5	CCR1	1,44259E-04	12496,5	3,58335E-02	1157	8,54248E-01	2060	2,24739E+00	1238	7,71850E-01	40497	0	17530,5
Non-responder	Macro_FOLR2+APOE+	cDC_CLEC9A	HLA-DRA	LAG3	1,44652E-04	6618,7	1,76049E-02	3803	9,56207E-01	1414	2,24045E+00	1256	9,00848E-01	9090	0	17530,5
Non-responder	pDC_LILRA4	cDC_LAMP3	APP	TSPAN15	1,44694E-04	8505,7	3,79285E-02	1055	1,26350E+00	313	2,89064E+00	348	8,43602E-01	23282	0	17530,5
Non-responder	pDC_LILRA4	Macro_OLFML3	SPON2	ITGB1	1,45103E-04	8937,1	3,79110E-02	1056	1,08575E+00	827	2,80917E+00	409	8,37090E-01	24863	0	17530,5
Non-responder	Macro_FOLR2+APOE+	Macro_NLRP3	MMP9	ITGAM	1,45203E-04	11259,7	2,24691E-02	2475	6,98593E-01	3645	2,44539E+00	851	8,08723E-01	31797	0	17530,5
Non-responder	Macro_NLRP3	Mono_CD16	VEGFA	CD44	1,45769E-04	7410,5	1,51842E-02	4897	8,79623E-01	1858	1,71587E+00	3663	9,00772E-01	9104	0	17530,5
Non-responder	Mono_CD16	Macro_LYVE1	SERPINA1	LRP1	1,46250E-04	8039,1	1,05218E-02	9110	7,14389E-01	3455	1,46706E+00	5873	9,23771E-01	4227	0	17530,5
Non-responder	Macro_OLFML3	Mast	LGALS1	CD69	1,46310E-04	5363,7	1,92600E-02	3258	1,01405E+00	1143	1,71700E+00	3652	9,38889E-01	1235	0	17530,5
Non-responder	Macro_FOLR2+APOE+	Macro_ISG15	CCL3L1	CCR1	1,46469E-04	8047,1	2,39839E-02	2218	8,95995E-01	1755	2,16284E+00	1484	8,69041E-01	17248	0	17530,5
Non-responder	Macro_NLRP3	cDC_LAMP3	ADM	CALCRL	1,46746E-04	8596,3	5,36878E-02	573	1,20531E+00	427	2,33248E+00	1060	8,43154E-01	23391	0	17530,5
Non-responder	pDC_LILRA4	pDC_LILRA4	HSP90B1	TLR7	1,46746E-04	4949,5	3,78284E-02	1060	1,39609E+00	159	3,98355E+00	42	9,15027E-01	5956	0	17530,5
Non-responder	Macro_FOLR2+APOE+	Macro_ISG15	LGALS3	LAG3	1,46787E-04	7033,9	2,90190E-02	1649	1,21839E+00	396	2,62895E+00	585	8,77741E-01	15009	0	17530,5
Non-responder	Macro_OLFML3	cDC(CD1C)	CXCL9	FCGR2A	1,47216E-04	6111,9	1,86794E-02	3439	9,90960E-01	1245	2,27367E+00	1174	9,09391E-01	7171	0	17530,5
Non-responder	Macro_FOLR2+APOE+	Macro_ISG15	MDK	SDC2	1,47264E-04	17109,3	4,87688E-02	683	7,42021E-01	3122	1,81327E+00	3037	6,61052E-01	61174	0	17530,5
Non-responder	Macro_NLRP3	Macro_ISG15	CXCL8	SDC2	1,47572E-04	7906,9	3,78061E-02	1062	1,13072E+00	650	2,58354E+00	647	8,58885E-01	19645	0	17530,5
Non-responder	Macro_NLRP3	cDC_CLEC9A	VCAN	SELL	1,47700E-04	5878,9	3,11676E-02	1447	1,16322E+00	545	2,51892E+00	744	9,00669E-01	9128	0	17530,5
Non-responder	Macro_NLRP3	Macro_ISG15	THBS1	CD47	1,48024E-04	6377,5	2,13974E-02	2714	9,90287E-01	1250	2,23959E+00	1261	9,00640E-01	9132	0	17530,5
Non-responder	Macro_FOLR2+APOE+	Mono_CD14	HLA-A	APLP2	1,48543E-04	8737,5	6,68774E-03	18850	6,96740E-01	3666	1,81818E+00	3013	9,44328E-01	628	0	17530,5
Non-responder	Macro_FOLR2+APOE+	cDC_CLEC9A	HLA-DPB1	LAG3	1,48704E-04	7166,7	1,79798E-02	3667	9,15808E-01	1637	2,07659E+00	1785	8,92133E-01	11214	0	17530,5
Non-responder	Mono_CD14	Macro_IER3	S100A12	TLR4	1,48817E-04	8617,1	3,77684E-02	1065	1,27940E+00	291	2,35299E+00	1019	8,44022E-01	23180	0	17530,5
Non-responder	Macro_NLRP3	Macro_FOLR2+APOE+	S100A9	CD36	1,49000E-04	7063,5	1,59286E-02	4514	8,91393E-01	1785	1,94191E+00	2344	9,00612E-01	9144	0	17530,5
Non-responder	Macro_FOLR2+APOE+	Macro_ISG15	SNCA	LAG3	1,49025E-04	15240,9	3,05392E-02	1497	9,14134E-01	1646	1,89600E+00	2567	7,13275E-01	52964	0	17530,5
Non-responder	pDC_LILRA4	cDC(CD1C)	SPON2	ITGB1	1,49233E-04	8994,7	3,77508E-02	1066	1,08409E+00	835	2,60301E+00	617	8,36801E-01	24925	0	17530,5
Non-responder	Macro_NLRP3	Macro_FOLR2+APOE+	S100A9	TLR4	1,49816E-04	6834,1	1,74316E-02	3881	9,64203E-01	1376	1,96518E+00	2229	9,00573E-01	9154	0	17530,5
Non-responder	Macro_FOLR2+APOE+	cDC(CD1C)	APOE	SORL1	1,49980E-04	9037,5	1,15454E-02	7831	4,67659E-01	9156	1,51540E+00	5394	9,18393E-01	5276	0	17530,5
Non-responder	pDC_LILRA4	Mono_INHBA	SPON2	ITGB1	1,50069E-04	8994,7	3,76701E-02	1068	1,08325E+00	839	2,63794E+00	569	8,36655E-01	24967	0	17530,5
Non-responder	Macro_NLRP3	cDC_CLEC9A	VCAN	ITGA4	1,50226E-04	6553,3	1,91826E-02	3286	1,02415E+00	1096	2,10063E+00	1695	9,00553E-01	9159	0	17530,5
Non-responder	Mono_INHBA	Mast	LGALS1	CD69	1,50315E-04	4914,3	2,33073E-02	2313	1,21103E+00	407	1,71418E+00	3677	9,44138E-01	644	0	17530,5
Non-responder	Macro_NLRP3	Mast	IL1B	ADRB2	1,50718E-04	5758,5	4,77968E-02	706	1,31798E+00	240	2,28575E+00	1151	9,00529E-01	9165	0	17530,5
Non-responder	Mono_CD14	Mono_CD14	S100A8	CD36	1,52171E-04	4542,5	3,75509E-02	1073	1,39063E+00	163	3,08258E+00	218	9,26296E-01	3728	0	17530,5
Non-responder	Mono_CD16	Mono_CD16	LYZ	ITGAL	1,52287E-04	9654,9	1,09606E-02	8533	4,66746E-01	9184	1,43947E+00	6156	9,10752E-01	6871	0	17530,5
Non-responder	Macro_FOLR2+APOE+	cDC_CLEC9A	HLA-DPA1	LAG3	1,53085E-04	6899,7	1,79174E-02	3694	9,18725E-01	1623	2,12084E+00	1617	8,96776E-01	10034	0	17530,5
Non-responder	Macro_FOLR2+APOE+	Macro_FOLR2+APOE+	LGALS9	CD47	1,53785E-04	8343,3	1,07439E-02	8828	6,90794E-01	3736	1,92928E+00	2420	9,00411E-01	9202	0	17530,5
Non-responder	Macro_NLRP3	Macro_IER3	S100A9	ITGB2	1,53785E-04	7353,7	1,04670E-02	9202	7,99763E-01	2557	1,36478E+00	7076	9,47216E-01	403	0	17530,5
Non-responder	Macro_NLRP3	Macro_ISG15	CCL3L1	CCR5	1,54292E-04	9070,7	3,74562E-02	1078	1,03013E+00	1067	2,37885E+00	971	8,37731E-01	24707	0	17530,5
Non-responder	Mono_CD14	Macro_OLFML3	S100A9	ITGB2	1,54718E-04	5064,9	1,37327E-02	5835	1,09439E+00	789	2,32317E+00	1079	9,53607E-01	91	0	17530,5
Non-responder	Macro_FOLR2+APOE+	Macro_ISG15	CD38	PECAM1	1,54732E-04	11134,3	2,23044E-02	2510	8,84880E-01	1817	1,92417E+00	2436	8,10533E-01	31378	0	17530,5
Non-responder	Macro_NLRP3	Macro_FOLR2+APOE+	ICAM1	ITGAX_ITGB2	1,54875E-04	7335,5	1,04566E-02	9215	7,76908E-01	2754	2,01488E+00	2026	9,18994E-01	5152	0	17530,5
Non-responder	Macro_FOLR2+APOE+	Macro_FOLR2+APOE-	CD59	STAB1	1,55464E-04	7900,5	1,80370E-02	3650	8,48450E-01	2102	1,37173E+00	6998	9,00309E-01	9222	0	17530,5
Non-responder	Macro_FOLR2+APOE+	Mono_CD16	TNFSF12	TNFRSF8	1,56392E-04	15844,9	5,10759E-02	632	7,50002E-01	3024	1,70966E+00	3714	7,05827E-01	54324	0	17530,5
Non-responder	pDC_LILRA4	cDC_LAMP3	TNFSF9	PVR	1,56433E-04	13556,1	8,66180E-02	190	1,02687E+00	1083	2,32602E+00	1074	7,38781E-01	47903	0	17530,5
Non-responder	Mono_CD14	Mono_CD16	VCAN	CD44	1,57227E-04	5486,9	1,78377E-02	3719	1,12096E+00	689	2,00389E+00	2073	9,27891E-01	3423	0	17530,5
Non-responder	Macro_FOLR2+APOE+	Macro_OLFML3	LILRB4	LAIR1	1,57242E-04	7796,5	1,04318E-02	9243	6,66188E-01	4120	1,86550E+00	2743	9,18048E-01	5346	0	17530,5
Non-responder	pDC_LILRA4	Mono_CD14	APP	FPR2	1,57294E-04	8122,7	3,73768E-02	1085	1,37239E+00	185	3,26919E+00	144	8,50313E-01	21669	0	17530,5
Non-responder	Macro_FOLR2+APOE+	Macro_ISG15	MMP9	CD44	1,58065E-04	6916,7	2,09995E-02	2811	8,72476E-01	1896	2,79378E+00	426	8,89330E-01	11920	0	17530,5
Non-responder	Macro_FOLR2+APOE+	Macro_ISG15	MMP9	EPHB2	1,58233E-04	14523,3	4,30780E-02	850	8,13949E-01	2413	2,57150E+00	663	7,22775E-01	51160	0	17530,5
Non-responder	Mono_CD14	cDC(CD1C)	S100A8	ITGB2	1,58593E-04	4630,5	1,71455E-02	3988	1,19068E+00	465	2,32113E+00	1088	9,54016E-01	81	0	17530,5
Non-responder	Macro_FOLR2+APOE+	pDC_LILRA4	CXCL11	DPP4	1,59244E-04	18333,7	5,33712E-02	580	6,91066E-01	3731	1,93291E+00	2395	6,03218E-01	67432	0	17530,5
Non-responder	Macro_NLRP3	Macro_NLRP3	IL1B	IL1R1_IL1RAP	1,59463E-04	7107,9	3,73251E-02	1090	1,21405E+00	401	2,51280E+00	753	8,74705E-01	15765	0	17530,5
Non-responder	cDC_LAMP3	Mast	LGALS1	CD69	1,59582E-04	5093,3	2,15137E-02	2687	1,12374E+00	674	1,70770E+00	3733	9,41988E-01	842	0	17530,5
Non-responder	Macro_FOLR2+APOE+	Macro_FOLR2+APOE+	HLA-DRB1	CD4	1,59752E-04	7338,3	8,19034E-03	13581	6,90917E-01	3734	2,11860E+00	1628	9,50261E-01	218	0	17530,5
Non-responder	Macro_FOLR2+APOE+	Macro_FOLR2+APOE+	LGALS9	LRP1	1,60847E-04	8907,9	1,04875E-02	9163	6,03480E-01	5240	1,76577E+00	3321	8,99976E-01	9285	0	17530,5
Non-responder	Macro_FOLR2+APOE+	Macro_ISG15	MMP12	PLAUR	1,61281E-04	6542,5	3,14765E-02	1416	9,65040E-01	1368	3,04710E+00	235	8,88414E-01	12163	0	17530,5
Non-responder	Macro_FOLR2+APOE+	cDC_CLEC9A	HLA-DRB1	LAG3	1,61281E-04	6767,1	1,77873E-02	3743	9,75409E-01	1320	2,24009E+00	1258	8,96998E-01	9984	0	17530,5
Non-responder	Macro_NLRP3	Macro_ISG15	VCAN	TLR2	1,61367E-04	6404,3	2,17508E-02	2631	1,00760E+00	1175	2,19484E+00	1394	8,99952E-01	9291	0	17530,5
Non-responder	Macro_FOLR2+APOE+	cDC_CLEC9A	HLA-DRB5	LAG3	1,61451E-04	7354,7	1,77852E-02	3744	8,97030E-01	1746	2,07348E+00	1800	8,89230E-01	11953	0	17530,5
Non-responder	Macro_NLRP3	Macro_NLRP3	S100A8	TLR4	1,61454E-04	6559,5	2,10693E-02	2797	1,00117E+00	1202	2,02874E+00	1976	8,99951E-01	9292	0	17530,5
Non-responder	Macro_OLFML3	Macro_LYVE1	CXCL9	FCGR2A	1,61541E-04	5124,1	2,48206E-02	2113	1,20369E+00	431	2,53785E+00	715	9,20440E-01	4831	0	17530,5
Non-responder	Macro_NLRP3	Macro_OLFML3	VCAN	TLR2	1,61715E-04	6623,7	2,17408E-02	2633	1,00737E+00	1177	1,91491E+00	2483	8,99931E-01	9295	0	17530,5
Non-responder	Macro_NLRP3	pDC_LILRA4	BST2	LILRA4	1,62238E-04	6004,3	2,73497E-02	1787	1,00176E+00	1200	3,11017E+00	203	8,99890E-01	9301	0	17530,5
Non-responder	Macro_NLRP3	Macro_OLFML3	HBEGF	CD44	1,62238E-04	8471,7	1,03820E-02	9301	7,20654E-01	3366	1,48660E+00	5681	9,12609E-01	6480	0	17530,5
Non-responder	Mono_CD16	cDC(CD1C)	CD52	SIGLEC10	1,62325E-04	7522,9	1,40428E-02	5611	8,69033E-01	1925	1,77709E+00	3246	8,99882E-01	9302	0	17530,5
Non-responder	Macro_NLRP3	Macro_ISG15	CCL4	CCR1	1,62412E-04	6210,7	2,60765E-02	1947	1,00665E+00	1181	2,31752E+00	1092	8,99882E-01	9303	0	17530,5
Non-responder	Macro_FOLR2+APOE+	Macro_OLFML3	B2M	LILRB2	1,62649E-04	9370,7	6,00927E-03	22327	6,89590E-01	3751	1,83110E+00	2943	9,48862E-01	302	0	17530,5
Non-responder	Macro_FOLR2+APOE+	Macro_FOLR2+APOE+	CD14	ITGB1	1,62849E-04	7613,5	1,03751E-02	9308	6,92079E-01	3717	2,09930E+00	1701	9,15681E-01	5811	0	17530,5
Non-responder	Macro_FOLR2+APOE+	Mono_CD14	MMP9	LRP1	1,62993E-04	9741,1	1,98678E-02	3099	6,89416E-01	3753	2,36866E+00	993	8,43383E-01	23330	0	17530,5
Non-responder	pDC_LILRA4	Macro_FOLR2+APOE+	APP	NCSTN	1,63415E-04	6176,9	3,71702E-02	1099	1,38454E+00	174	3,38014E+00	118	8,89173E-01	11963	0	17530,5
Non-responder	Mono_INHBA	Macro_FOLR2+APOE+	IL6	IL6R_IL6ST	1,63858E-04	9732,1	5,00091E-02	656	1,27335E+00	298	2,31185E+00	1100	8,19961E-01	29076	0	17530,5
Non-responder	Macro_ISG15	Macro_FOLR2+APOE+	CXCL10	SDC4	1,64747E-04	9197,5	3,71149E-02	1102	1,10364E+00	751	2,94865E+00	312	8,31174E-01	26292	0	17530,5
Non-responder	pDC_LILRA4	Macro_FOLR2+APOE+	GAS6	AXL	1,65192E-04	6997,7	3,70467E-02	1103	1,11475E+00	715	3,00583E+00	270	8,76314E-01	15370	0	17530,5
Non-responder	Mast	cDC_LAMP3	CSF1	CSF2RA	1,65580E-04	5882,3	1,01291E-01	134	1,52166E+00	111	2,30355E+00	1112	8,94912E-01	10524	0	17530,5

Non-responder	Macro_FOLR2+APOE+	Macro_FOLR2+APOE+	CXCL12	CD4	1,66457E-04	10388,5	1,95713E-02	3183	6,88290E-01	3773	2,18638E+00	1417	8,32207E-01	26039	0	17530,5
Non-responder	Macro_FOLR2+APOE+	Macro_OLFML3	MMP9	IFNAR1	1,66457E-04	12014,5	1,76890E-02	3773	6,93932E-01	3691	2,42019E+00	898	7,98658E-01	34180	0	17530,5
Non-responder	pDC_LILRA4	Macro_LYVE1	SPON2	ITGB1	1,66982E-04	9110,5	3,69362E-02	1107	1,07565E+00	876	2,51094E+00	755	8,35306E-01	25284	0	17530,5
Non-responder	Macro_FOLR2+APOE+	Macro_NLRP3	C3	ITGAX	1,67360E-04	7912,1	1,47840E-02	5134	5,86708E-01	5585	2,03523E+00	1952	8,99616E-01	9359	0	17530,5
Non-responder	Mono_CD14	Macro_ISG15	S100A9	ITGB2	1,67431E-04	5268,9	1,26807E-02	6678	1,02175E+00	1108	2,42647E+00	889	9,51812E-01	139	0	17530,5
Non-responder	Macro_FOLR2+APOE+	Macro_IER3	C1QA	CD33	1,67807E-04	8742,3	1,03320E-02	9364	6,28807E-01	4750	1,70012E+00	3786	9,04582E-01	8281	0	17530,5
Non-responder	Macro_ISG15	Mono_CD16	HLA-A	LILRA1	1,68562E-04	5521,1	1,90872E-02	3308	9,05706E-01	1698	2,23407E+00	1284	9,25987E-01	3785	0	17530,5
Non-responder	pDC_LILRA4	cDC_CLEC9A	ADA	DPP4	1,68785E-04	13302,9	1,00390E-01	137	1,02116E+00	1111	2,33152E+00	1063	7,44370E-01	46673	0	17530,5
Non-responder	Mono_CD14	pDC_LILRA4	S100A8	CD36	1,69691E-04	4549,9	3,67695E-02	1113	1,37988E+00	178	3,63397E+00	67	9,25575E-01	3861	0	17530,5
Non-responder	Macro_FOLR2+APOE+	Macro_NLRP3	PKM	CD44	1,70154E-04	8098,3	7,76132E-03	14821	6,86800E-01	3794	1,85102E+00	2809	9,37112E-01	1537	0	17530,5
Non-responder	Macro_IFI27	Macro_ISG15	C3	IFITM1	1,70509E-04	5009,9	3,47496E-02	1215	1,30172E+00	258	1,96131E+00	2250	9,25912E-01	3796	0	17530,5
Non-responder	pDC_LILRA4	Macro_NLRP3	APP	PTGER2	1,70600E-04	8347,3	3,67501E-02	1115	1,35699E+00	193	3,21665E+00	164	8,45920E-01	22734	0	17530,5
Non-responder	pDC_LILRA4	Macro_FOLR2-APOE+	APP	TSPAN15	1,71513E-04	8693,3	3,66869E-02	1117	1,25264E+00	334	2,56357E+00	675	8,41394E-01	23810	0	17530,5
Non-responder	Macro_FOLR2+APOE+	Macro_ISG15	SPP1	CD44	1,71787E-04	8271,9	1,42657E-02	5468	4,98381E-01	8039	2,41047E+00	914	8,99363E-01	9408	0	17530,5
Non-responder	Macro_FOLR2+APOE+	Macro_ISG15	APOE	LRP1	1,72153E-04	8535,3	1,07325E-02	8841	4,60344E-01	9412	1,82602E+00	2964	9,25272E-01	3929	0	17530,5
Non-responder	Macro_FOLR2+APOE+	Macro_ISG15	MMP9	IFNAR1	1,72831E-04	10713,5	2,20910E-02	2563	7,74597E-01	2775	2,59435E+00	633	8,15934E-01	30066	0	17530,5
Non-responder	Mono_INHBA	pDC_LILRA4	VEGFA	RET	1,72888E-04	11495,5	1,46200E-01	56	1,29926E+00	264	2,29986E+00	1120	7,80075E-01	38507	0	17530,5
Non-responder	cDC_LAMP3	Macro_FOLR2+APOE+	IL15	IL2RA	1,73348E-04	10823,1	6,64826E-02	364	1,31445E+00	243	2,29972E+00	1121	7,95789E-01	34857	0	17530,5
Non-responder	Macro_FOLR2+APOE+	Macro_LYVE1	MMP9	ITGAM	1,73370E-04	11641,1	2,16462E-02	2655	6,85806E-01	3812	2,09139E+00	1739	8,05820E-01	32469	0	17530,5
Non-responder	Macro_FOLR2+APOE+	pDC_LILRA4	SELPLG	SELL	1,74091E-04	7375,5	1,75630E-02	3816	9,98799E-01	1210	2,84318E+00	388	8,81751E-01	13933	0	17530,5
Non-responder	pDC_LILRA4	Macro_LYVE1	SPON2	ITGA4	1,74270E-04	9152,3	3,65199E-02	1123	1,09600E+00	781	2,52162E+00	737	8,33912E-01	25590	0	17530,5
Non-responder	Macro_FOLR2+APOE+	Macro_FOLR2+APOE-	SERPING1	LRP1	1,74359E-04	8017,5	1,44613E-02	5323	8,54823E-01	2055	1,48054E+00	5743	8,99239E-01	9436	0	17530,5
Non-responder	Macro_FOLR2+APOE+	Mono_INHBA	LGALS9	CD44	1,75177E-04	7406,9	1,01232E-02	9669	8,01068E-01	2547	1,69345E+00	3822	9,27700E-01	3466	0	17530,5
Non-responder	pDC_LILRA4	Macro_ISG15	APP	PTGER2	1,75660E-04	8372,5	3,64948E-02	1126	1,35457E+00	195	3,22816E+00	160	8,45465E-01	22851	0	17530,5
Non-responder	Macro_FOLR2+APOE+	Macro_ISG15	RARRES1	NRP2	1,75722E-04	14609,3	4,29125E-02	854	9,10945E-01	1664	1,84282E+00	2862	7,28194E-01	50136	0	17530,5
Non-responder	Mono_CD14	Macro_OLFML3	S100A12	TLR4	1,76124E-04	8682,3	3,64711E-02	1127	1,26854E+00	304	2,54036E+00	710	8,41707E-01	23740	0	17530,5
Non-responder	Macro_OLFML3	Mono_INHBA	CXCL9	FCGR2A	1,76307E-04	5855,7	1,98652E-02	3101	1,03203E+00	1061	2,39119E+00	946	9,11895E-01	6640	0	17530,5
Non-responder	Macro_FOLR2+APOE+	Macro_IFI27	APOE	SORL1	1,76307E-04	8836,9	1,34209E-02	6055	5,33614E-01	6940	1,20028E+00	9457	9,23859E-01	4202	0	17530,5
Non-responder	Macro_FOLR2+APOE+	Macro_FOLR2+APOE+	PSAP	LRP1	1,77913E-04	7420,3	8,85121E-03	11925	6,83827E-01	3837	1,99099E+00	2135	9,36423E-01	1674	0	17530,5
Non-responder	Macro_FOLR2+APOE+	Macro_FOLR2+APOE+	CCL4	CCR5	1,78096E-04	10311,9	2,16329E-02	2662	6,83765E-01	3838	1,93275E+00	2398	8,35970E-01	25131	0	17530,5
Non-responder	Macro_NLRP3	Macro_FOLR2+APOE+	S100A8	CD36	1,78556E-04	7023,3	1,88510E-02	3381	9,18865E-01	1621	1,80078E+00	3103	8,99037E-01	9481	0	17530,5
Non-responder	Macro_NLRP3	Macro_FOLR2+APOE+	S100A8	TLR4	1,79216E-04	6829,1	2,06298E-02	2913	9,91675E-01	1239	1,82406E+00	2975	8,98998E-01	9488	0	17530,5
Non-responder	Macro_OLFML3	Macro_FOLR2+APOE+	C3	CD81	1,79594E-04	9103,1	1,25471E-02	6790	5,21763E-01	7270	1,61889E+00	4433	8,98983E-01	9492	0	17530,5
Non-responder	Macro_FOLR2+APOE+	Macro_ISG15	HLA-F	LILRB2	1,79689E-04	7691,7	1,02461E-02	9493	7,94662E-01	2600	2,03855E+00	1934	9,10655E-01	6901	0	17530,5
Non-responder	Macro_OLFML3	Macro_ISG15	CXCL11	CCR5	1,81291E-04	13543,7	7,21332E-02	298	1,01483E+00	1138	2,38928E+00	948	7,39205E-01	47804	0	17530,5
Non-responder	Macro_NLRP3	Macro_FOLR2+APOE+	NAMPT	ITGA5_ITGB1	1,81494E-04	8021,3	1,18185E-02	7517	8,14097E-01	2412	1,79626E+00	3135	8,98913E-01	9512	0	17530,5
Non-responder	pDC_LILRA4	cDC(CD1C)	SERPINF1	PLXDC2	1,81610E-04	5504,7	1,74821E-02	3857	8,82547E-01	1838	2,60038E+00	621	9,26556E-01	3677	0	17530,5
Non-responder	Macro_OLFML3	pDC_LILRA4	LGALS9	CD47	1,81685E-04	6243,9	1,44624E-02	5322	9,31789E-01	1546	2,79679E+00	422	9,12966E-01	6399	0	17530,5
Non-responder	Macro_NLRP3	Mono_CD14	VCAN	SELL	1,82834E-04	6044,1	2,99331E-02	1552	1,14434E+00	600	2,35580E+00	1012	8,98846E-01	9526	0	17530,5
Non-responder	Macro_FOLR2+APOE+	pDC_LILRA4	SEMA6B	PLXNA4	1,82918E-04	19289,1	4,26087E-02	864	7,37328E-01	3175	1,68794E+00	3864	5,47377E-01	71012	0	17530,5
Non-responder	pDC_LILRA4	Macro_LYVE1	GAS6	MERTK	1,83194E-04	7728,9	3,60921E-02	1142	1,11444E+00	718	2,62495E+00	592	8,63033E-01	18662	0	17530,5
Non-responder	Macro_FOLR2+APOE+	pDC_LILRA4	PDCD1LG2	PDCD2	1,83480E-04	15160,7	2,54690E-02	2023	6,82449E-01	3867	2,17861E+00	1440	7,23943E-01	50943	0	17530,5
Non-responder	Mast	pDC_LILRA4	BST2	LILRA4	1,83672E-04	5284,1	3,60898E-02	1143	1,13426E+00	638	2,78601E+00	430	9,11707E-01	6679	0	17530,5
Non-responder	Macro_LYVE1	pDC_LILRA4	C1QB	C1QBP	1,84044E-04	4942,1	1,74535E-02	3870	9,69647E-01	1350	2,26953E+00	1181	9,42593E-01	779	0	17530,5
Non-responder	Macro_FOLR2+APOE+	Mono_INHBA	TNFSF13	SDC2	1,84232E-04	10282,3	1,93278E-02	3240	6,82376E-01	3871	1,77688E+00	3248	8,42622E-01	23522	0	17530,5
Non-responder	pDC_LILRA4	pDC_LILRA4	APP	NCSTN	1,84630E-04	6259,7	3,60304E-02	1145	1,37040E+00	188	3,79481E+00	53	8,87629E-01	12382	0	17530,5
Non-responder	Macro_OLFML3	pDC_LILRA4	HLA-C	NOTCH4	1,84761E-04	6708,3	2,72242E-02	1801	6,84978E-01	3821	1,80197E+00	3095	9,08768E-01	7294	0	17530,5
Non-responder	Macro_FOLR2+APOE+	Macro_FOLR2+APOE+	MMP9	ITGAM	1,85553E-04	11564,9	2,14101E-02	2711	6,82138E-01	3878	2,34770E+00	1030	8,04961E-01	32675	0	17530,5
Non-responder	Mono_CD14	cDC_CLEC9A	VCAN	SELL	1,85592E-04	5418,7	3,59916E-02	1147	1,32701E+00	228	2,74599E+00	463	9,06923E-01	7725	0	17530,5
Non-responder	Macro_FOLR2+APOE+	Macro_FOLR2+APOE+	CD99	CD81	1,86412E-04	8700,9	1,01965E-02	9563	5,55939E-01	6335	1,72232E+00	3619	9,12691E-01	6457	0	17530,5
Non-responder	Macro_NLRP3	Macro_IER3	S100A9	CD68	1,86802E-04	8002,1	1,01994E-02	9559	7,38712E-01	3159	1,19369E+00	9567	9,50562E-01	195	0	17530,5
Non-responder	Macro_FOLR2+APOE+	Macro_IER3	PLAU	PLAUR	1,86882E-04	7402,9	1,74225E-02	3885	7,87407E-01	2663	1,83265E+00	2933	8,96931E-01	10003	0	17530,5
Non-responder	Macro_ISG15	Mono_CD16	HLA-B	LILRB2	1,87040E-04	6154,3	9,30294E-03	11026	1,01230E+00	1150	2,37697E+00	975	9,53634E-01	90	0	17530,5
Non-responder	Macro_NLRP3	Mono_CD14	S100A9	TLR4	1,88960E-04	7038,1	1,66998E-02	4148	9,45498E-01	1470	1,92115E+00	2453	8,98636E-01	9589	0	17530,5
Non-responder	Macro_FOLR2+APOE+	Macro_FOLR2+APOE+	IL15RA	AXL	1,89368E-04	12245,3	2,51946E-02	2052	6,81030E-01	3898	1,70996E+00	3711	7,99323E-01	34035	0	17530,5
Non-responder	pDC_LILRA4	Macro_IER3	SPON2	ITGB1	1,89960E-04	9222,9	3,58416E-02	1156	1,06431E+00	916	2,51334E+00	751	8,33227E-01	25761	0	17530,5
Non-responder	Mast	cDC_CLEC9A	ADCYAP1	DPP4	1,91164E-04	13818,3	4,48257E-01	9	1,42970E+00	144	1,87831E+00	2673	7,34958E-01	48735	0	17530,5
Non-responder	Macro_FOLR2+APOE+	Mono_INHBA	MMP9	IFNAR1	1,91684E-04	12230,7	1,73481E-02	3910	6,87685E-01	3780	2,20356E+00	1367	7,97089E-01	34566	0	17530,5
Non-responder	Macro_ISG15	cDC_LAMP3	SPP1	S1PR1	1,91923E-04	10238,5	7,50769E-02	268	1,19787E+00	445	2,28100E+00	1160	8,08746E-01	31789	0	17530,5
Non-responder	Macro_FOLR2+APOE+	Macro_FOLR2+APOE+	LGALS9	HAVCR2	1,92334E-04	8706,9	1,04569E-02	9214	6,41464E-01	4521	1,88345E+00	2646	8,98505E-01	9623	0	17530,5
Non-responder	Macro_FOLR2+APOE+	Macro_FOLR2+APOE-	MMP9	LRP1	1,92461E-04	9713,1	2,14360E-02	2702	7,26323E-01	3291	1,83958E+00	2885	8,48335E-01	22157	0	17530,5
Non-responder	Macro_OLFML3	pDC_LILRA4	C1QB	C1QBP	1,94140E-04	6403,9	1,23887E-02	6946	6,63903E-01	4163	2,40262E+00	925	9,32585E-01	2455	0	17530,5
Non-responder	Macro_FOLR2+APOE+	cDC_LAMP3	ENTPD1	ADORA2A	1,94218E-04	11671,9	4,81560E-02	699	9,64468E-01	1373	1,68089E+00	3923	7,95883E-01	34834	0	17530,5
Non-responder	Macro_FOLR2+APOE+	Macro_ISG15	GRN	TNFRSF1B	1,94218E-04	8076,3	7,58458E-03	15371	6,78967E-01	3923	2,07545E+00	1790	9,35979E-01	1767	0	17530,5
Non-responder	Macro_FOLR2+APOE+	Macro_LYVE1	LGALS9	LRP1	1,94543E-04	10108,1	1,01403E-02	9645	5,82356E-01	5681	1,29504E+00	8045	8,98451E-01	9639	0	17530,5
Non-responder	pDC_LILRA4	pDC_LILRA4	TNF	PTPRS	1,95888E-04	10318,9	3,56316E-02	1168	1,42474E+00	146	3,56545E+00	78	8,04971E-01	32672	0	17530,5
Non-responder	Macro_NLRP3	cDC_CLEC9A	VCAN	ITGB1	1,96060E-04	6844,9	1,80988E-02	3630	9,72560E-01	1329	2,00355E+00	2075	8,98324E-01	9660	0	17530,5
Non-responder	Mono_INHBA	Macro_NLRP3	SPP1	CD44	1,96381E-04	5258,5	2,73607E-02	1785	9,50540E-01	1441	2,12596E+00	1602	9,25241E-01	3934	0	17530,5
Non-responder	Macro_FOLR2+APOE+	Macro_FOLR2+APOE+	ADM	GPR84	1,97766E-04	13668,9	2,31558E-02	2343	6,77570E-01	3941	1,88673E+00	2621	7,65735E-01	41909	0	17530,5
Non-responder	Macro_NLRP3	Macro_FOLR2+APOE+	ICAM1	ITGAL_ITGB2	1,98303E-04	7990,9	1,01134E-02	9682	7,27581E-01	3275	1,86139E+00	2766	9,11612E-01	6701	0	17530,5
Non-responder	Macro_FOLR2+APOE+	Macro_FOLR2+APOE+	CCL3	CCR5	1,98561E-04	10104,7	2,07676E-02	2877	6,77175E-01	3945	1,86383E+00	2748	8,43030E-01	23423	0	17530,5

Post_NR_Myeloid_Myeloid_CellCell

Non-responder	Macro_LYVE1	cDC_CLEC9A	C1QB	C1QBP	1,98561E-04	5300,1	1,72641E-02	3945	9,60424E-01	1396	1,84941E+00	2816	9,42297E-01	813	0	17530,5
Non-responder	Macro_FOLR2+APOE+	Macro_ISG15	CCL2	CCR1	1,99359E-04	13515,7	1,72512E-02	3949	6,81422E-01	3888	1,71269E+00	3694	7,80041E-01	38517	0	17530,5
Non-responder	Macro_NLRP3	Macro_FOLR2-APOE+	THBS1	LRP1	2,00876E-04	8127,5	2,13415E-02	2730	9,13347E-01	1654	1,18497E+00	9707	9,01170E-01	9016	0	17530,5
Non-responder	Macro_NLRP3	Macro_NLRP3	NAMPT	ITGA5_ITGB1	2,01187E-04	7974,7	1,16175E-02	7726	8,40617E-01	2178	1,86772E+00	2729	8,98130E-01	9710	0	17530,5
Non-responder	Mono_CD14	Mono_CD14	S100A9	CD36	2,01564E-04	4759,9	2,96051E-02	1582	1,27948E+00	290	2,77816E+00	437	9,25114E-01	3960	0	17530,5
Non-responder	Macro_FOLR2+APOE+	Mono_CD14	CXCL9	FCGR2A	2,02850E-04	7149,7	1,43993E-02	5374	8,75114E-01	1878	2,24548E+00	1240	8,98082E-01	9726	0	17530,5
Non-responder	Macro_FOLR2+APOE+	Macro_FOLR2+APOE+	CCL4	CCR1	2,03991E-04	8059,3	1,71987E-02	3972	7,45484E-01	3078	2,26731E+00	1187	8,79512E-01	14529	0	17530,5
Non-responder	Macro_FOLR2+APOE+	Macro_IER3	C1QB	LRP1	2,04000E-04	7750,3	1,00868E-02	9737	6,51543E-01	4373	1,82151E+00	2992	9,24286E-01	4119	0	17530,5
Non-responder	Macro_NLRP3	Macro_FOLR2+APOE+	VCAN	ITGA4	2,04629E-04	6827,1	1,81323E-02	3616	9,96060E-01	1225	2,01640E+00	2021	8,98003E-01	9743	0	17530,5
Non-responder	Macro_NLRP3	Mast	HBEGF	CD9	2,05260E-04	7544,5	1,82203E-02	3585	8,50513E-01	2084	1,18242E+00	9749	9,20711E-01	4774	0	17530,5
Non-responder	Macro_IER3	Macro_ISG15	CCL3L1	CCR1	2,05524E-04	6383,3	3,52785E-02	1187	1,13659E+00	622	2,55079E+00	693	8,89481E-01	11884	0	17530,5
Non-responder	Macro_NLRP3	Macro_LYVE1	S100A9	ITGB2	2,05892E-04	8041,3	1,01285E-02	9663	7,72175E-01	2795	1,18212E+00	9755	9,46389E-01	463	0	17530,5
Non-responder	Mast	Macro_FOLR2+APOE+	CSF1	CSF2RA	2,06039E-04	7110,3	6,63513E-02	371	1,25401E+00	329	2,26711E+00	1188	8,73294E-01	16133	0	17530,5
Non-responder	Macro_FOLR2+APOE+	Macro_FOLR2+APOE+	HLA-B	CANX	2,06849E-04	9337,3	6,08644E-03	21890	6,74547E-01	3986	1,82595E+00	2965	9,48697E-01	315	0	17530,5
Non-responder	Macro_FOLR2+APOE+	Macro_IER3	APOE	SORL1	2,07586E-04	9541,9	1,10940E-02	8365	4,51785E-01	9771	1,41439E+00	6478	9,16886E-01	5565	0	17530,5
Non-responder	Macro_FOLR2+APOE+	Mono_CD16	MMP9	CD44	2,07877E-04	8084,1	1,71367E-02	3991	7,34280E-01	3205	2,36377E+00	999	8,78924E-01	14695	0	17530,5
Non-responder	Macro_FOLR2+APOE+	Macro_NLRP3	HLA-B	LILRB2	2,07877E-04	9844,9	5,88402E-03	23067	6,74233E-01	3991	1,69253E+00	3834	9,42387E-01	802	0	17530,5
Non-responder	cDC_CLEC9A	pDC_LILRA4	SEMA4A	NRP1_PLXNA4	2,08110E-04	9882,7	3,59429E-02	1151	1,00334E+00	1192	2,34640E+00	1032	8,22363E-01	28508	0	17530,5
Non-responder	Macro_OLFML3	Macro_FOLR2-APOE+	CXCL9	FCGR2A	2,08757E-04	6968,9	1,83211E-02	3548	9,78546E-01	1306	1,54106E+00	5124	9,08589E-01	7336	0	17530,5
Non-responder	Macro_NLRP3	Macro_FOLR2+APOE+	ICAM1	IL2RA	2,08909E-04	9847,9	2,56130E-02	2001	9,11358E-01	1662	2,12071E+00	1618	8,30622E-01	26428	0	17530,5
Non-responder	Macro_FOLR2+APOE+	Macro_ISG15	CCL4	CCR5	2,08909E-04	10321,3	2,10342E-02	2803	6,73997E-01	3996	2,09706E+00	1709	8,34037E-01	25568	0	17530,5
Non-responder	Macro_FOLR2+APOE+	Macro_LYVE1	PLAU	ITGB5	2,09116E-04	14277,5	3,65147E-02	1124	7,13220E-01	3468	1,67292E+00	3997	7,50680E-01	45268	0	17530,5
Non-responder	pDC_LILRA4	Macro_ISG15	SPON2	ITGA4	2,09151E-04	9225,9	3,50769E-02	1194	1,08131E+00	851	3,04475E+00	237	8,31101E-01	26317	0	17530,5
Non-responder	Macro_FOLR2+APOE+	Mono_CD16	LGALS9	PTPRC	2,09323E-04	7441,9	9,26103E-03	11101	7,70561E-01	2814	2,08242E+00	1766	9,24943E-01	3998	0	17530,5
Non-responder	Macro_NLRP3	Macro_FOLR2+APOE+	THBS1	CD36	2,09945E-04	8439,1	2,14120E-02	2710	8,65293E-01	1956	1,99985E+00	2092	8,66245E-01	17907	0	17530,5
Non-responder	Macro_FOLR2+APOE+	Mono_CD14	HLA-A	LILRB2	2,09945E-04	10094,1	5,79603E-03	23637	6,73845E-01	3999	1,67242E+00	4001	9,38426E-01	1303	0	17530,5
Non-responder	Macro_NLRP3	Macro_FOLR2+APOE+	THBS1	ITGA6	2,10152E-04	12390,9	4,35363E-02	832	9,01492E-01	1726	1,69888E+00	3795	7,82011E-01	38071	0	17530,5
Non-responder	Macro_NLRP3	Macro_FOLR2+APOE+	THBS1	PTPRJ	2,10776E-04	9419,5	2,72443E-02	1796	9,64238E-01	1375	2,00857E+00	2054	8,39212E-01	24342	0	17530,5
Non-responder	Macro_NLRP3	Macro_FOLR2+APOE+	THBS1	SCARB1	2,10984E-04	7600,9	3,72461E-02	1093	1,02100E+00	1112	2,14793E+00	1524	8,70906E-01	16745	0	17530,5
Non-responder	Macro_NLRP3	cDC(CD1C)	HBEGF	CD9	2,12086E-04	9175,3	1,29499E-02	6439	6,45168E-01	4468	1,17900E+00	9813	9,07318E-01	7626	0	17530,5
Non-responder	Macro_OLFML3	cDC_LAMP3	TNFSF10	TNFRSF11B	2,12948E-04	10044,7	1,06198E-01	116	1,41184E+00	152	2,02419E+00	1992	8,14403E-01	30433	0	17530,5
Non-responder	Mono_CD16	cDC_CLEC9A	CD52	SIGLEC10	2,13495E-04	7495,9	1,33713E-02	6087	8,47755E-01	2106	2,03939E+00	1930	8,97653E-01	9826	0	17530,5
Non-responder	Macro_FOLR2+APOE+	Macro_NLRP3	HGF	CD44	2,13915E-04	10437,9	2,27194E-02	2423	7,58374E-01	2939	1,67083E+00	4020	8,35337E-01	25277	0	17530,5
Non-responder	Macro_OLFML3	Mono_CD16	HLA-F	LILRB2	2,14039E-04	7766,7	1,00093E-02	9831	8,56635E-01	2036	1,94584E+00	2330	9,09700E-01	7106	0	17530,5
Non-responder	pDC_LILRA4	Macro_LYVE1	SPN	SIGLEC1	2,14406E-04	8736,7	3,97689E-02	972	1,00099E+00	1204	2,43663E+00	872	8,44337E-01	23105	0	17530,5
Non-responder	Macro_FOLR2+APOE+	Macro_NLRP3	SERPING1	LRP1	2,16225E-04	7381,7	1,39269E-02	5698	8,36179E-01	2218	2,12333E+00	1611	8,97520E-01	9851	0	17530,5
Non-responder	cDC_CLEC9A	Mono_CD16	HLA-C	LILRA1	2,16239E-04	6030,1	1,79181E-02	3693	7,49401E-01	3030	2,05571E+00	1866	9,24781E-01	4031	0	17530,5
Non-responder	Macro_FOLR2+APOE+	Mast	SPP1	CD44	2,16334E-04	9489,1	1,37000E-02	5856	4,68588E-01	9123	1,54600E+00	5084	8,97517E-01	9852	0	17530,5
Non-responder	Macro_NLRP3	Macro_OLFML3	VEGFA	CD44	2,16664E-04	7930,7	1,41266E-02	5549	8,36920E-01	2214	1,61039E+00	4505	8,97498E-01	9855	0	17530,5
Non-responder	Macro_NLRP3	Macro_FOLR2+APOE+	THBS1	ITGA4	2,17088E-04	7558,3	1,70153E-02	4035	8,70882E-01	1913	1,94214E+00	2343	8,89134E-01	11970	0	17530,5
Non-responder	Mono_CD16	cDC_CLEC9A	AGTRAP	RACK1	2,17545E-04	7893,7	1,24985E-02	6843	7,57747E-01	2942	1,95332E+00	2290	8,97477E-01	9863	0	17530,5
Non-responder	Macro_OLFML3	Macro_FOLR2+APOE+	TNFSF13	TNFRSF14	2,17766E-04	9371,7	1,04500E-02	9224	6,09172E-01	5120	1,54165E+00	5119	8,97471E-01	9865	0	17530,5
Non-responder	Mono_CD16	Mono_CD16	ICAM4	ITGAL	2,18672E-04	12060,9	6,92466E-02	329	1,04055E+00	1023	2,26021E+00	1212	7,73160E-01	40210	0	17530,5
Non-responder	Macro_FOLR2+APOE+	pDC_LILRA4	APP	TNFRSF21	2,19009E-04	9986,1	1,69865E-02	4044	8,59035E-01	2015	2,39125E+00	945	8,34859E-01	25396	0	17530,5
Non-responder	Macro_FOLR2+APOE+	Macro_OLFML3	C1QB	C1QBP	2,20512E-04	7438,5	9,74688E-03	10274	6,93680E-01	3696	2,11466E+00	1641	9,24643E-01	4051	0	17530,5
Non-responder	Macro_FOLR2+APOE+	cDC_CLEC9A	CXCL11	DPP4	2,21374E-04	18356,9	5,78923E-02	496	7,38221E-01	3165	1,66749E+00	4055	6,12907E-01	66538	0	17530,5
Non-responder	Macro_FOLR2+APOE+	Macro_NLRP3	HLA-A	APLP2	2,21590E-04	9146,7	6,44954E-03	20024	6,70502E-01	4056	1,75044E+00	3414	9,43367E-01	709	0	17530,5
Non-responder	Macro_FOLR2+APOE+	cDC_CLEC9A	SNCA	LAG3	2,21806E-04	16631,3	2,51088E-02	2068	7,55186E-01	2967	1,66684E+00	4057	6,92844E-01	56534	0	17530,5
Non-responder	cDC_CLEC9A	pDC_LILRA4	FARP2	PLXNA4	2,21908E-04	12787,9	1,86729E-01	31	1,21367E+00	403	2,25789E+00	1218	7,52887E-01	44757	0	17530,5
Non-responder	pDC_LILRA4	Mono_CD14	SPON2	ITGA4	2,22451E-04	9309,5	3,46915E-02	1219	1,07739E+00	869	2,79657E+00	423	8,30324E-01	26506	0	17530,5
Non-responder	Macro_NLRP3	cDC_CLEC9A	S100A8	CD69	2,23453E-04	8980,7	1,37994E-02	5786	8,45916E-01	2120	1,17331E+00	9916	8,98759E-01	9551	0	17530,5
Non-responder	Macro_OLFML3	Mast	S100A8	CD69	2,23679E-04	6776,5	1,93411E-02	3237	1,04119E+00	1019	1,48120E+00	5735	9,13118E-01	6361	0	17530,5
Non-responder	Macro_NLRP3	Mono_INHBA	HBEGF	CD9	2,23904E-04	10155,1	1,11244E-02	8328	5,74042E-01	5885	1,17314E+00	9920	9,00728E-01	9112	0	17530,5
Non-responder	Macro_FOLR2+APOE+	Macro_IER3	LILRB4	LAIR1	2,25035E-04	8482,7	9,94868E-03	9930	6,36236E-01	4611	1,59434E+00	4651	9,16247E-01	5691	0	17530,5
Non-responder	Macro_FOLR2+APOE+	Macro_ISG15	MMP9	LRP1	2,25066E-04	9940,3	1,90296E-02	3327	6,69690E-01	4072	2,51719E+00	747	8,40515E-01	24025	0	17530,5
Non-responder	Macro_OLFML3	cDC_CLEC9A	HLA-DRA	LAG3	2,25148E-04	7308,3	1,66623E-02	4170	8,69193E-01	1924	1,77408E+00	3263	8,98363E-01	9654	0	17530,5
Non-responder	Macro_NLRP3	Mono_CD16	IL1B	SIGIRR	2,25489E-04	6569,5	1,85450E-02	3486	1,04919E+00	977	2,40604E+00	920	8,97205E-01	9934	0	17530,5
Non-responder	pDC_LILRA4	Macro_LYVE1	LTB	TNFRSF1A	2,26272E-04	5833,9	3,46287E-02	1226	1,25869E+00	321	3,15653E+00	181	8,97293E-01	9911	0	17530,5
Non-responder	Macro_FOLR2+APOE+	Macro_FOLR2-APOE+	C1QB	CD33	2,26626E-04	9712,7	9,94047E-03	9944	6,48183E-01	4419	1,29772E+00	8008	9,02759E-01	8662	0	17530,5
Non-responder	Macro_NLRP3	Macro_FOLR2+APOE+	CXCL8	SDC2	2,27700E-04	9569,5	2,59801E-02	1961	9,67476E-01	1359	2,14082E+00	1541	8,34587E-01	25456	0	17530,5
Non-responder	Macro_NLRP3	Macro_FOLR2+APOE+	CCL3	CCR1	2,27920E-04	7391,5	1,82772E-02	3564	8,18569E-01	2385	2,09139E+00	1740	8,90029E-01	11738	0	17530,5
Non-responder	Macro_NLRP3	Macro_FOLR2+APOE+	CCL3	CCR5	2,28141E-04	9613,1	2,29894E-02	2371	7,56850E-01	2951	1,75683E+00	3372	8,49639E-01	21841	0	17530,5
Non-responder	Macro_NLRP3	Macro_FOLR2+APOE+	CCL4	CCR1	2,28362E-04	8039,5	1,79455E-02	3684	7,75179E-01	2768	1,95458E+00	2281	8,81746E-01	13934	0	17530,5
Non-responder	Macro_NLRP3	Mono_INHBA	S100A9	CD68	2,28455E-04	7960,1	9,93216E-03	9960	7,14957E-01	3449	1,25470E+00	8634	9,49934E-01	227	0	17530,5
Non-responder	Mono_CD14	Mono_CD14	VCAN	SELL	2,29029E-04	5557,1	3,45660E-02	1231	1,30812E+00	250	2,58287E+00	648	9,05203E-01	8126	0	17530,5
Non-responder	Macro_NLRP3	Mono_CD14	S100A8	TLR4	2,29950E-04	7038,7	1,97638E-02	3129	9,72971E-01	1327	1,78002E+00	3234	8,97034E-01	9973	0	17530,5
Non-responder	Macro_FOLR2+APOE+	Macro_LYVE1	LILRB4	LAIR1	2,30180E-04	8757,5	9,92328E-03	9975	6,34662E-01	4635	1,46056E+00	5941	9,16149E-01	5706	0	17530,5
Non-responder	Macro_NLRP3	Macro_FOLR2+APOE+	VCAN	TLR1	2,30356E-04	7622,3	2,95965E-02	1584	1,09451E+00	787	2,15468E+00	1507	8,71044E-01	16703	0	17530,5
Non-responder	Macro_FOLR2+APOE+	Mono_INHBA	C1QB	LRP1	2,30873E-04	7809,9	9,91881E-03	9981	6,44567E-01	4482	1,84991E+00	2814	9,23696E-01	4242	0	17530,5
Non-responder	Mono_CD14	pDC_LILRA4	S100A9	CD36	2,31247E-04	4743,1	2,89891E-02	1653	1,26873E+00	303	3,32955E+00	129	9,24383E-01	4100	0	17530,5
Non-responder	Macro_FOLR2+APOE+	Macro_ISG15	CCL3	CCR5	2,31470E-04	10097,1	2,01928E-02	3006	6,67407E-01	4101	2,02813E+00	1978	8,41164E-01	23870	0	17530,5

Post_NR_Myeloid_Myeloid_CellCell

Non-responder	Macro_NLRP3	Macro_FOLR2+APOE+	VEGFA	ITGB1	2,31693E-04	7350,5	2,17238E-02	2643	1,00398E+00	1190	2,24009E+00	1257	8,80970E-01	14132	0	17530,5
Non-responder	Macro_NLRP3	Macro_FOLR2+APOE+	VEGFA	ITGAV	2,31917E-04	10219,1	2,44062E-02	2165	9,26123E-01	1576	2,05767E+00	1859	8,24593E-01	27965	0	17530,5
Non-responder	Macro_NLRP3	Macro_FOLR2+APOE+	VEGFA	EPHB2	2,32140E-04	11403,5	5,14278E-02	624	1,06904E+00	899	2,01723E+00	2015	7,90988E-01	35949	0	17530,5
Non-responder	Macro_NLRP3	Macro_FOLR2+APOE+	VEGFA	SIRPA	2,32364E-04	6709,7	3,00621E-02	1535	1,18594E+00	481	2,49242E+00	775	8,84345E-01	13227	0	17530,5
Non-responder	pDC_LILRA4	Mono_INHBA	SPON2	ITGB2	2,32365E-04	6214,1	3,44578E-02	1237	1,00494E+00	1187	2,49204E+00	776	8,95584E-01	10340	0	17530,5
Non-responder	Macro_NLRP3	Macro_FOLR2+APOE+	VEGFA	ITGA9	2,32588E-04	13675,9	5,54619E-02	540	1,05231E+00	967	1,89407E+00	2582	7,44004E-01	46760	0	17530,5
Non-responder	Macro_NLRP3	Macro_FOLR2+APOE+	CCL3L1	CCR1	2,32812E-04	6852,9	3,06263E-02	1482	1,10162E+00	762	2,54910E+00	699	8,82336E-01	13791	0	17530,5
Non-responder	Macro_FOLR2+APOE+	Macro_FOLR2+APOE+	HLA-B	LILRB1	2,32812E-04	8998,9	6,78402E-03	18433	6,67115E-01	4107	1,91039E+00	2508	9,32839E-01	2416	0	17530,5
Non-responder	Macro_NLRP3	Macro_FOLR2+APOE+	CCL3L1	CCR5	2,33036E-04	9030,7	3,85223E-02	1027	1,03990E+00	1025	2,21454E+00	1330	8,39629E-01	24241	0	17530,5
Non-responder	Macro_FOLR2+APOE+	Macro_FOLR2+APOE+	LGALS3BP	ITGB1	2,33260E-04	8078,7	1,68020E-02	4109	7,47593E-01	3052	2,09247E+00	1733	8,81624E-01	13969	0	17530,5
Non-responder	Macro_NLRP3	Macro_FOLR2+APOE+	ADM	GPR84	2,33934E-04	11392,1	3,36935E-02	1272	8,40823E-01	2175	2,13528E+00	1561	7,97689E-01	34422	0	17530,5
Non-responder	pDC_LILRA4	Macro_IFI27	SPON2	ITGAM	2,34607E-04	11443,9	3,63679E-02	1131	1,02417E+00	1095	2,24536E+00	1241	7,89716E-01	36222	0	17530,5
Non-responder	pDC_LILRA4	Macro_FOLR2+APOE+	NUCB2	ERAP1	2,34607E-04	10149,3	3,52284E-02	1190	9,91527E-01	1241	2,61943E+00	601	8,15449E-01	30184	0	17530,5
Non-responder	Macro_FOLR2+APOE-	Macro_ISG15	C3	IFITM1	2,35287E-04	5157,7	3,31586E-02	1307	1,27095E+00	301	1,90360E+00	2532	9,24288E-01	4118	0	17530,5
Non-responder	Macro_FOLR2+APOE+	Macro_ISG15	TNFSF13	SDC2	2,35287E-04	10128,9	1,87496E-02	3414	6,66283E-01	4118	2,13379E+00	1572	8,40597E-01	24010	0	17530,5
Non-responder	Macro_OLFML3	Macro_ISG15	CXCL10	SDC4	2,36298E-04	9459,5	3,42999E-02	1244	1,17922E+00	500	2,94012E+00	317	8,25567E-01	27706	0	17530,5
Non-responder	cDC_CLEC9A	Macro_ISG15	HLA-DOB	CD4	2,36863E-04	7267,5	3,58152E-02	1159	1,33546E+00	213	2,24405E+00	1245	8,73067E-01	16190	0	17530,5
Non-responder	Macro_FOLR2+APOE+	pDC_LILRA4	SEMA3C	NRP1_PLXNA4	2,37099E-04	17789,7	2,87277E-02	1677	6,90910E-01	3735	1,65877E+00	4126	6,55756E-01	61880	0	17530,5
Non-responder	Mono_CD16	Mono_CD14	S100A9	CD36	2,37187E-04	9227,9	1,46412E-02	5210	6,19615E-01	4927	1,26748E+00	8437	8,96776E-01	10035	0	17530,5
Non-responder	Macro_OLFML3	Macro_ISG15	CXCL9	FCGR2A	2,37326E-04	4763,7	2,75928E-02	1765	1,29972E+00	262	3,31330E+00	134	9,24232E-01	4127	0	17530,5
Non-responder	Macro_FOLR2+APOE+	Macro_ISG15	PLAU	IGF2R	2,37553E-04	12390,3	1,90289E-02	3328	6,65761E-01	4128	2,04502E+00	1910	7,94942E-01	35055	0	17530,5
Non-responder	Macro_OLFML3	cDC_CLEC9A	C1QB	C1QBP	2,37897E-04	6725,5	1,22542E-02	7101	6,54680E-01	4317	1,98249E+00	2162	9,32241E-01	2517	0	17530,5
Non-responder	Mono_CD16	Macro_FOLR2+APOE+	TIMP1	CD63	2,38846E-04	6238,9	9,87731E-03	10049	9,76391E-01	1317	1,97954E+00	2172	9,52083E-01	126	0	17530,5
Non-responder	Macro_FOLR2+APOE+	cDC_LAMP3	PDGFB	S1PR1	2,38921E-04	13691,5	1,29524E-01	75	1,39027E+00	164	1,65745E+00	4134	7,44910E-01	46554	0	17530,5
Non-responder	Macro_NLRP3	Macro_FOLR2+APOE+	THBS1	SDC4	2,39378E-04	9385,3	3,11639E-02	1449	9,98848E-01	1208	2,09453E+00	1726	8,36481E-01	25013	0	17530,5
Non-responder	Mono_CD14	Macro_FOLR2+APOE+	VCAN	TLR1	2,39704E-04	6952,5	3,41773E-02	1250	1,25829E+00	322	2,38175E+00	963	8,78913E-01	14697	0	17530,5
Non-responder	Macro_ISG15	cDC_CLEC9A	CXCL11	DPP4	2,40275E-04	15352,5	1,16614E-01	93	9,90043E-01	1251	2,25266E+00	1229	6,92044E-01	56659	0	17530,5
Non-responder	Macro_FOLR2+APOE+	cDC_CLEC9A	CXCL12	CXCR3	2,40524E-04	14769,9	3,95317E-02	983	6,65236E-01	4141	2,05842E+00	1857	7,32023E-01	49338	0	17530,5
Non-responder	Mast	Macro_FOLR2+APOE+	CSF1	CSF3R	2,41419E-04	6905,1	8,53108E-02	200	1,35595E+00	194	2,24203E+00	1253	8,76410E-01	15348	0	17530,5
Non-responder	cDC_LAMP3	cDC_LAMP3	CD274	CD80	2,41823E-04	10350,7	9,36836E-02	162	1,51411E+00	118	1,91745E+00	2471	8,10182E-01	31472	0	17530,5
Non-responder	Mono_CD16	Macro_ISG15	CD52	SIGLEC10	2,42072E-04	7569,3	1,30762E-02	6328	8,38404E-01	2201	2,09677E+00	1711	8,96624E-01	10076	0	17530,5
Non-responder	Macro_NLRP3	Macro_FOLR2+APOE+	ICAM1	ITGAM_ITGB2	2,43155E-04	8345,5	9,85289E-03	10085	6,65991E-01	4126	1,75067E+00	3412	9,12190E-01	6574	0	17530,5
Non-responder	Macro_FOLR2+APOE+	Macro_ISG15	HLA-A	APLP2	2,43291E-04	9094,9	6,39357E-03	20273	6,64336E-01	4153	1,85566E+00	2786	9,43133E-01	732	0	17530,5
Non-responder	Macro_FOLR2+APOE+	Mono_CD14	APOE	LDLR	2,43986E-04	8377,5	2,27941E-02	2408	6,64237E-01	4156	1,98878E+00	2145	8,75201E-01	15648	0	17530,5
Non-responder	cDC_LAMP3	pDC_LILRA4	BST2	LILRA4	2,46613E-04	5417,9	3,38437E-02	1262	1,10021E+00	764	2,97438E+00	295	9,09086E-01	7238	0	17530,5
Non-responder	Macro_FOLR2+APOE+	Macro_ISG15	PLAU	ITGA5	2,47720E-04	12091,9	2,06562E-02	2905	6,63265E-01	4172	2,01605E+00	2023	8,00219E-01	33829	0	17530,5
Non-responder	Macro_FOLR2+APOE+	Mono_CD14	SPP1	CD44	2,49366E-04	9061,7	1,33779E-02	6083	4,51623E-01	9780	2,07909E+00	1779	8,96418E-01	10136	0	17530,5
Non-responder	pDC_LILRA4	Mono_CD14	APP	PTGER2	2,49530E-04	8655,7	3,37562E-02	1267	1,32861E+00	226	3,18176E+00	173	8,40300E-01	24082	0	17530,5
Non-responder	Macro_FOLR2+APOE+	Macro_NLRP3	B2M	LILRB2	2,49603E-04	9859,7	5,77459E-03	23767	6,62844E-01	4180	1,74354E+00	3461	9,47887E-01	360	0	17530,5
Non-responder	Macro_NLRP3	Macro_FOLR2+APOE+	NRG1	MS4A4A	2,50075E-04	11640,9	2,77896E-02	1747	7,56278E-01	2957	1,83080E+00	2945	8,03497E-01	33025	0	17530,5
Non-responder	Macro_FOLR2+APOE+	Macro_NLRP3	CXCL10	TLR4	2,50075E-04	10752,1	1,68006E-02	4111	6,62792E-01	4182	2,37290E+00	984	8,28593E-01	26953	0	17530,5
Non-responder	Macro_FOLR2+APOE+	Macro_LYVE1	HLA-DMB	CD4	2,50598E-04	8154,7	9,81470E-03	10146	6,75569E-01	3971	1,63741E+00	4288	9,20423E-01	4838	0	17530,5
Non-responder	Macro_FOLR2+APOE+	pDC_LILRA4	B2M	TFRC	2,51259E-04	8398,1	7,25942E-03	16504	6,62410E-01	4187	2,06327E+00	1846	9,35171E-01	1923	0	17530,5
Non-responder	Mono_INHBA	cDC_CLEC9A	CCL20	CXCR3	2,51291E-04	10343,5	7,16691E-02	303	9,86504E-01	1270	2,51958E+00	743	8,08437E-01	31871	0	17530,5
Non-responder	pDC_LILRA4	Macro_OLFML3	LTB	TNFRSF1A	2,51291E-04	5895,3	3,37305E-02	1270	1,24436E+00	345	3,42226E+00	110	8,96076E-01	10221	0	17530,5
Non-responder	Mono_CD16	Macro_NLRP3	VCAN	CD44	2,51836E-04	9034,7	9,81017E-03	10156	7,16861E-01	3424	1,46248E+00	5921	9,05148E-01	8142	0	17530,5
Non-responder	Macro_NLRP3	Mono_CD14	THBS1	CD36	2,51880E-04	6496,1	3,37143E-02	1271	1,05385E+00	958	2,32190E+00	1085	8,90431E-01	11636	0	17530,5
Non-responder	Mono_INHBA	pDC_LILRA4	INHBA	ACTR2	2,53060E-04	7497,9	3,36743E-02	1273	1,02953E+00	1069	2,83472E+00	393	8,69153E-01	17224	0	17530,5
Non-responder	Mono_CD14	Macro_NLRP3	S100A9	ITGB2	2,53060E-04	5370,5	1,25783E-02	6763	1,01468E+00	1140	2,23692E+00	1273	9,51626E-01	146	0	17530,5
Non-responder	pDC_LILRA4	Macro_IER3	SERPINF1	PLXDC2	2,53639E-04	5711,1	1,66057E-02	4197	8,48818E-01	2100	2,54853E+00	700	9,24787E-01	4028	0	17530,5
Non-responder	cDC(CD1C)	Macro_ISG15	HLA-DQB2	LAG3	2,53652E-04	8590,1	3,84303E-02	1030	1,16579E+00	539	2,23687E+00	1274	8,46560E-01	22577	0	17530,5
Non-responder	Macro_NLRP3	Mast	HBEGF	CD44	2,53826E-04	8106,7	1,31324E-02	6290	8,71760E-01	1903	1,15761E+00	10172	9,21537E-01	4638	0	17530,5
Non-responder	Macro_OLFML3	pDC_LILRA4	TGFB1	CXCR4	2,54200E-04	7782,5	9,80330E-03	10175	6,79160E-01	3918	2,59736E+00	629	9,11798E-01	6660	0	17530,5
Non-responder	Mono_CD14	Macro_OLFML3	VCAN	CD44	2,54357E-04	5808,5	1,65953E-02	4200	1,07826E+00	861	1,89841E+00	2557	9,25438E-01	3894	0	17530,5
Non-responder	Macro_FOLR2+APOE+	Macro_IER3	C1QA	CD93	2,54575E-04	8478,1	9,80103E-03	10178	6,72010E-01	4031	1,85644E+00	2781	9,06348E-01	7870	0	17530,5
Non-responder	Macro_FOLR2+APOE+	Macro_FOLR2+APOE+	APOE	SORL1	2,55954E-04	9155,9	1,07825E-02	8769	4,40830E-01	10189	1,73606E+00	3507	9,15794E-01	5784	0	17530,5
Non-responder	cDC_LAMP3	pDC_LILRA4	TNFSF13B	TNFRSF17	2,56027E-04	9701,1	8,62127E-02	195	9,85054E-01	1278	2,31102E+00	1102	8,22798E-01	28400	0	17530,5
Non-responder	pDC_LILRA4	Macro_FOLR2-APOE+	SPON2	ITGAM	2,56027E-04	9607,3	5,63951E-02	528	1,15552E+00	562	2,23568E+00	1278	8,23837E-01	28138	0	17530,5
Non-responder	Macro_ISG15	Mono_CD16	HLA-C	LILRA1	2,56277E-04	6339,9	1,74418E-02	3877	7,01023E-01	3616	1,91841E+00	2468	9,23838E-01	4208	0	17530,5
Non-responder	Macro_NLRP3	Macro_ISG15	S100A9	CD36	2,56708E-04	7557,3	1,44573E-02	5329	8,61080E-01	1993	1,86611E+00	2739	8,96189E-01	10195	0	17530,5
Non-responder	Macro_FOLR2+APOE+	Macro_FOLR2+APOE+	CD59	STAB1	2,57212E-04	7269,9	1,64714E-02	4260	7,73228E-01	2783	2,13255E+00	1577	8,96159E-01	10199	0	17530,5
Non-responder	Mono_CD16	pDC_LILRA4	VCAN	SELL	2,57843E-04	6067,9	2,82264E-02	1711	1,17160E+00	523	2,85502E+00	371	8,96146E-01	10204	0	17530,5
Non-responder	Macro_NLRP3	Macro_NLRP3	THBS1	CD36	2,58450E-04	9630,7	1,75248E-02	3835	8,05712E-01	2490	1,72970E+00	3555	8,54208E-01	20743	0	17530,5
Non-responder	Macro_FOLR2+APOE+	Mono_CD16	TGM2	ITGA4	2,58450E-04	11670,3	1,68586E-02	4094	6,59751E-01	4217	1,69674E+00	3804	8,21528E-01	28706	0	17530,5
Non-responder	Macro_FOLR2+APOE+	Macro_ISG15	HLA-F	LILRB1	2,58476E-04	8065,5	1,17399E-02	7602	7,47303E-01	3057	2,04005E+00	1929	8,96137E-01	10209	0	17530,5
Non-responder	Macro_NLRP3	Macro_NLRP3	THBS1	LRP1	2,58692E-04	7634,7	1,74618E-02	3866	8,05399E-01	2495	1,82115E+00	2997	8,91868E-01	11285	0	17530,5
Non-responder	Macro_FOLR2+APOE+	Macro_ISG15	LGALS9	CD47	2,58729E-04	9071,5	9,78420E-03	10211	6,36073E-01	4614	1,85498E+00	2791	8,96136E-01	10211	0	17530,5
Non-responder	Macro_FOLR2+APOE+	Macro_FOLR2-APOE+	APOE	LRP1	2,59236E-04	8097,7	1,42297E-02	5484	6,06280E-01	5179	1,15502E+00	10215	9,34457E-01	2080	0	17530,5
Non-responder	Macro_FOLR2+APOE+	Macro_FOLR2+APOE+	CALR	LRP1	2,59363E-04	8073,3	9,78065E-03	10216	6,34040E-01	4649	1,90667E+00	2523	9,17508E-01	5448	0	17530,5
Non-responder	Macro_FOLR2+APOE+	Mono_INHBA	PLAU	ITGA5	2,60149E-04	12200,7	2,18109E-02	2617	6,83637E-01	3840	1,64651E+00	4224	8,04532E-01	32792	0	17530,5
Non-responder	Macro_OLFML3	Macro_FOLR2+APOE+	SERPING1	LRP1	2,62168E-04	8144,7	1,34839E-02	6010	7,03184E-01	3597	1,76103E+00	3348	8,96024E-01	10238	0	17530,5

Non-responder	Macro_FOLR2+APOE+	Macro_FOLR2+APOE+	CCL3	CCR1	2,62347E-04	7874,5	1,65108E-02	4233	7,38894E-01	3158	2,19838E+00	1385	8,84955E-01	13066	0	17530,5
Non-responder	cDC_CLEC9A	pDC_LILRA4	BTLA	CD79A	2,63123E-04	15795,7	3,18319E-01	15	1,38758E+00	169	2,19862E+00	1384	6,70916E-01	59880	0	17530,5
Non-responder	Macro_FOLR2+APOE+	Mono_CD16	MMP12	PLAUR	2,63819E-04	8700,3	2,10327E-02	2804	6,58715E-01	4239	2,27862E+00	1165	8,66812E-01	17763	0	17530,5
Non-responder	Macro_FOLR2+APOE+	Macro_ISG15	SEMA4A	LILRB2	2,63819E-04	7393,5	1,65057E-02	4239	9,20241E-01	1611	2,19804E+00	1388	8,88290E-01	12199	0	17530,5
Non-responder	pDC_LILRA4	Macro_FOLR2+APOE+	VEGFB	NRP1	2,63849E-04	9311,7	4,90099E-02	676	9,82450E-01	1291	2,84687E+00	382	8,29696E-01	26679	0	17530,5
Non-responder	Macro_NLRP3	Macro_FOLR2+APOE+	HBEGF	CD82	2,64065E-04	9382,1	1,65055E-02	4240	7,93341E-01	2616	1,72387E+00	3606	8,61913E-01	18918	0	17530,5
Non-responder	Macro_FOLR2+APOE+	Macro_FOLR2+APOE+	CCL3L1	CCR1	2,64311E-04	10336,3	1,65054E-02	4241	6,64523E-01	4148	1,79990E+00	3113	8,46272E-01	22649	0	17530,5
Non-responder	pDC_LILRA4	Macro_IER3	SPON2	ITGA5	2,64457E-04	12831,1	3,34034E-02	1292	1,06107E+00	927	2,63424E+00	572	7,56715E-01	43834	0	17530,5
Non-responder	Macro_FOLR2+APOE+	Macro_ISG15	CCL5	CCRL2	2,64558E-04	14828,7	2,67395E-02	1856	6,58682E-01	4242	1,98721E+00	2147	7,36687E-01	48368	0	17530,5
Non-responder	Macro_NLRP3	Macro_OLFML3	VCAN	ITGA4	2,64739E-04	7138,5	1,73331E-02	3916	9,74681E-01	1322	1,87968E+00	2666	8,95920E-01	10258	0	17530,5
Non-responder	Macro_NLRP3	Macro_NLRP3	LAMB3	CD44	2,65051E-04	11334,3	2,86376E-02	1683	8,28712E-01	2291	1,72447E+00	3601	8,09770E-01	31566	0	17530,5
Non-responder	Mono_CD14	Macro_OLFML3	S100A9	CD68	2,65066E-04	5209,7	1,33052E-02	6134	1,03354E+00	1054	2,22974E+00	1293	9,56447E-01	37	0	17530,5
Non-responder	Mono_CD14	Mono_CD16	S100A9	CD68	2,66287E-04	5360,1	1,26151E-02	6726	9,81576E-01	1295	2,26420E+00	1198	9,55324E-01	51	0	17530,5
Non-responder	Macro_NLRP3	Macro_NLRP3	IL1B	IL1R2_IL1RAP	2,66535E-04	6727,5	2,82952E-02	1707	1,11727E+00	704	2,52142E+00	738	8,85383E-01	12958	0	17530,5
Non-responder	Macro_NLRP3	Macro_NLRP3	IL1B	ADRB2	2,66783E-04	9229,3	1,81830E-02	3593	9,79619E-01	1301	2,15185E+00	1514	8,48113E-01	22208	0	17530,5
Non-responder	pDC_LILRA4	pDC_LILRA4	VEGFB	NRP1	2,66899E-04	9312,7	4,89216E-02	680	9,81399E-01	1296	2,91041E+00	341	8,29568E-01	26716	0	17530,5
Non-responder	Macro_OLFML3	Macro_ISG15	HLA-DRA	LAG3	2,66940E-04	6279,3	2,02659E-02	2993	1,02814E+00	1079	2,00325E+00	2077	9,06959E-01	7717	0	17530,5
Non-responder	Macro_FOLR2+APOE+	Macro_FOLR2+APOE+	LGALS3	LAG3	2,67031E-04	9649,7	1,64926E-02	4252	8,32530E-01	2258	2,34578E+00	1034	8,44052E-01	23174	0	17530,5
Non-responder	Macro_OLFML3	pDC_LILRA4	MMP9	TLR9	2,67512E-04	13563,1	7,50414E-02	269	1,05252E+00	965	2,22892E+00	1297	7,39476E-01	47754	0	17530,5
Non-responder	Macro_OLFML3	Macro_ISG15	HLA-DRB1	LAG3	2,67720E-04	6516,9	2,01493E-02	3019	1,01843E+00	1122	1,96940E+00	2213	9,02617E-01	8700	0	17530,5
Non-responder	Mono_CD16	pDC_LILRA4	S100A9	CD36	2,68111E-04	8275,3	1,43365E-02	5423	6,08863E-01	5129	1,81887E+00	3010	8,95798E-01	10284	0	17530,5
Non-responder	pDC_LILRA4	Macro_FOLR2+APOE-	SPON2	ITGAM	2,68126E-04	11093,5	3,95785E-02	979	1,04523E+00	996	2,22877E+00	1298	7,96655E-01	34664	0	17530,5
Non-responder	Macro_NLRP3	Macro_NLRP3	IL6	F3	2,68523E-04	17437,7	5,94627E-02	469	7,19789E-01	3379	1,99731E+00	2113	6,40847E-01	63697	0	17530,5
Non-responder	Macro_FOLR2+APOE+	Macro_ISG15	APOC2	LDLR	2,68523E-04	16433,1	3,13753E-02	1426	6,57911E-01	4258	2,10568E+00	1673	6,88103E-01	57278	0	17530,5
Non-responder	Mono_INHBA	Macro_ISG15	CXCL8	SDC2	2,68740E-04	8481,1	3,33227E-02	1299	1,02094E+00	1113	2,37617E+00	978	8,51061E-01	21485	0	17530,5
Non-responder	Macro_OLFML3	Macro_ISG15	HLA-DPB1	LAG3	2,69024E-04	6715,9	2,07769E-02	2874	1,00550E+00	1186	1,91688E+00	2474	8,98896E-01	9515	0	17530,5
Non-responder	Macro_FOLR2+APOE+	Macro_NLRP3	HBEGF	CD44	2,69417E-04	8159,3	9,72913E-03	10294	7,37646E-01	3172	1,86332E+00	2752	9,09984E-01	7048	0	17530,5
Non-responder	Macro_FOLR2+APOE+	Mono_CD14	SERPING1	LRP1	2,70728E-04	7668,1	1,34034E-02	6067	8,17916E-01	2389	2,00962E+00	2050	8,95744E-01	10304	0	17530,5
Non-responder	pDC_LILRA4	Macro_FOLR2+APOE+	APP	RPSA	2,70772E-04	4993,1	2,12218E-02	2755	1,33144E+00	222	3,14090E+00	191	9,23529E-01	4267	0	17530,5
Non-responder	Macro_FOLR2+APOE+	Mono_CD16	CXCL12	ITGA4	2,70772E-04	12067,3	1,89839E-02	3341	6,57318E-01	4267	2,10918E+00	1663	8,01424E-01	33535	0	17530,5
Non-responder	Macro_FOLR2+APOE+	cDC_LAMP3	ICAM1	IL2RG	2,70991E-04	8902,9	1,18452E-02	7493	7,55113E-01	2970	1,43590E+00	6215	8,95738E-01	10306	0	17530,5
Non-responder	Macro_FOLR2+APOE+	Macro_FOLR2+APOE+	LGALS3BP	CD33	2,71023E-04	9766,3	1,74693E-02	3862	6,57317E-01	4268	1,90084E+00	2546	8,54649E-01	20625	0	17530,5
Non-responder	Macro_OLFML3	Macro_NLRP3	CXCL10	TLR4	2,71208E-04	7184,3	3,32256E-02	1303	1,20968E+00	412	3,26843E+00	145	8,71763E-01	16531	0	17530,5
Non-responder	Macro_IER3	pDC_LILRA4	CXCL2	DPP4	2,71827E-04	9853,7	6,36976E-02	404	1,03910E+00	1028	2,22719E+00	1304	8,20226E-01	29002	0	17530,5
Non-responder	Macro_NLRP3	Mast	PTGS2	ALOX5	2,71912E-04	8169,7	2,02582E-02	2996	8,51077E-01	2078	1,30363E+00	7931	8,95704E-01	10313	0	17530,5
Non-responder	Macro_NLRP3	Macro_NLRP3	VCAN	SELL	2,72280E-04	8248,5	1,82645E-02	3571	9,65843E-01	1365	1,84549E+00	2847	8,74074E-01	15929	0	17530,5
Non-responder	pDC_LILRA4	Mono_CD16	HLA-A	LILRA1	2,72280E-04	5839,7	1,77683E-02	3751	7,70587E-01	2813	2,45894E+00	831	9,23496E-01	4273	0	17530,5
Non-responder	Macro_FOLR2+APOE+	Macro_FOLR2+APOE-	APOE	LRP1	2,72308E-04	9131,3	1,20896E-02	7240	5,16977E-01	7433	1,14841E+00	10316	9,29286E-01	3137	0	17530,5
Non-responder	Macro_FOLR2+APOE+	Macro_IER3	MMP9	ITGAM	2,72784E-04	12156,5	1,97753E-02	3127	6,56735E-01	4275	2,10704E+00	1669	7,98650E-01	34181	0	17530,5
Non-responder	Macro_NLRP3	Mono_INHBA	CXCL8	SDC2	2,73069E-04	7929,7	3,89719E-02	1015	1,14681E+00	589	2,22662E+00	1306	8,60716E-01	19208	0	17530,5
Non-responder	Macro_OLFML3	Macro_ISG15	HLA-DPA1	LAG3	2,73366E-04	6565,5	2,05135E-02	2939	9,93433E-01	1232	1,91400E+00	2488	9,02873E-01	8638	0	17530,5
Non-responder	pDC_LILRA4	Mast	VEGFB	ADRB2	2,73691E-04	10902,9	6,46828E-02	391	9,78524E-01	1307	2,24347E+00	1249	7,99316E-01	34037	0	17530,5
Non-responder	Macro_NLRP3	Macro_NLRP3	VEGFA	ITGAV	2,74300E-04	11271,7	2,01380E-02	3027	8,61877E-01	1986	1,93923E+00	2363	8,10255E-01	31452	0	17530,5
Non-responder	Macro_OLFML3	Macro_FOLR2+APOE+	CXCL11	CCR5	2,74314E-04	13480,1	7,41865E-02	277	1,02460E+00	1090	2,22497E+00	1308	7,41901E-01	47195	0	17530,5
Non-responder	Macro_FOLR2+APOE+	Macro_LYVE1	APOE	TREM2	2,75357E-04	8997,7	1,29232E-02	6462	4,36835E-01	10339	1,19640E+00	9521	9,39753E-01	1136	0	17530,5
Non-responder	Macro_OLFML3	Macro_ISG15	HLA-DRB5	LAG3	2,76157E-04	6926,9	2,04066E-02	2967	9,75779E-01	1318	1,90197E+00	2540	8,95822E-01	10279	0	17530,5
Non-responder	Macro_OLFML3	Mono_CD14	CXCL9	FCGR2A	2,77360E-04	7496,7	1,36235E-02	5912	8,15823E-01	2399	2,23160E+00	1288	8,95520E-01	10354	0	17530,5
Non-responder	pDC_LILRA4	Macro_FOLR2+APOE+	SPN	SIGLEC1	2,78072E-04	8815,1	3,81683E-02	1039	9,77012E-01	1314	2,78305E+00	433	8,41618E-01	23759	0	17530,5
Non-responder	Macro_NLRP3	cDC(CD1C)	IL1B	IL1R2_IL1RAP	2,78072E-04	6333,1	3,30457E-02	1314	1,13220E+00	644	2,26540E+00	1195	8,93025E-01	10982	0	17530,5
Non-responder	Macro_NLRP3	Macro_NLRP3	TIMP1	CD63	2,78629E-04	8220,3	7,47343E-03	15767	6,89392E-01	3755	1,73785E+00	3496	9,45305E-01	553	0	17530,5
Non-responder	Macro_NLRP3	Macro_NLRP3	THBS1	SDC4	2,79398E-04	11662,3	1,98852E-02	3093	8,56152E-01	2041	1,89316E+00	2589	8,03392E-01	33058	0	17530,5
Non-responder	Macro_FOLR2+APOE+	Macro_FOLR2+APOE+	B2M	LILRB1	2,79398E-04	8850,5	6,65785E-03	18987	6,55725E-01	4301	1,96140E+00	2249	9,39192E-01	1185	0	17530,5
Non-responder	Macro_NLRP3	pDC_LILRA4	THBS1	CD36	2,79963E-04	6422,1	3,30128E-02	1317	1,04310E+00	1005	2,87329E+00	357	8,89401E-01	11901	0	17530,5
Non-responder	Macro_FOLR2+APOE+	Macro_FOLR2+APOE-	APOE	SORL1	2,80185E-04	9418,9	1,25954E-02	6749	5,04582E-01	7814	1,14514E+00	10375	9,21596E-01	4626	0	17530,5
Non-responder	Macro_FOLR2+APOE+	Mast	CALR	ITGA2B	2,80426E-04	8945,3	8,16488E-02	217	1,07942E+00	859	1,63498E+00	4305	8,49718E-01	21815	0	17530,5
Non-responder	Macro_FOLR2+APOE+	Macro_ISG15	PLAU	ITGB1	2,81199E-04	10004,9	1,69419E-02	4060	6,55120E-01	4308	1,98136E+00	2167	8,49060E-01	21959	0	17530,5
Non-responder	Mast	pDC_LILRA4	ADCYAP1	DPP4	2,82092E-04	13925,1	4,13250E-01	10	1,38255E+00	175	2,14373E+00	1532	7,26963E-01	50378	0	17530,5
Non-responder	Macro_NLRP3	Macro_OLFML3	TIMP1	CD63	2,82216E-04	6285,1	9,67084E-03	10390	9,15635E-01	1638	2,09511E+00	1720	9,51598E-01	147	0	17530,5
Non-responder	pDC_LILRA4	pDC_LILRA4	ADA	DPP4	2,84410E-04	13610,7	9,25497E-02	167	9,74003E-01	1324	2,59694E+00	630	7,36557E-01	48402	0	17530,5
Non-responder	Macro_FOLR2+APOE+	Macro_FOLR2+APOE+	TNFSF13B	TFRC	2,84532E-04	7679,7	1,29889E-02	6413	7,56957E-01	2949	2,31264E+00	1099	8,95316E-01	10407	0	17530,5
Non-responder	Macro_NLRP3	Macro_FOLR2+APOE+	TNF	TRPM2	2,84566E-04	11585,7	1,93007E-02	3248	6,54316E-01	4321	1,89633E+00	2566	8,15163E-01	30263	0	17530,5
Non-responder	Macro_NLRP3	Macro_ISG15	NAMPT	ITGA5_ITGB1	2,85216E-04	8431,5	1,09278E-02	8580	7,83088E-01	2698	1,83217E+00	2937	8,95296E-01	10412	0	17530,5
Non-responder	Mast	Macro_ISG15	CSF1	CSF3R	2,86330E-04	7709,9	6,59786E-02	378	1,23319E+00	365	2,21604E+00	1327	8,61807E-01	18949	0	17530,5
Non-responder	Mono_CD16	cDC_CLEC9A	FLT3LG	FLT3	2,87615E-04	12377,5	8,01743E-02	230	1,33515E+00	214	2,21563E+00	1329	7,62440E-01	42584	0	17530,5
Non-responder	pDC_LILRA4	Macro_FOLR2+APOE+	APP	LRP10	2,88561E-04	6388,3	3,13812E-02	1425	1,38124E+00	177	3,43366E+00	104	8,86378E-01	12705	0	17530,5
Non-responder	Macro_FOLR2+APOE+	Mono_CD16	CXCL9	FCGR2A	2,88657E-04	7446,1	1,35302E-02	5979	8,46631E-01	2111	2,27376E+00	1173	8,95198E-01	10437	0	17530,5
Non-responder	Macro_FOLR2+APOE+	Mono_CD14	GRN	TNFRSF1B	2,88752E-04	8704,3	7,31372E-03	16312	6,53569E-01	4337	1,76030E+00	3353	9,34881E-01	1989	0	17530,5
Non-responder	Macro_NLRP3	Mono_CD16	TNF	FFAR2	2,89278E-04	15024,7	3,58105E-02	1160	7,27136E-01	3283	1,80051E+00	3108	7,28559E-01	50042	0	17530,5
Non-responder	Mono_CD14	Macro_FOLR2+APOE-	S100A8	CD68	2,89541E-04	5328,3	1,67622E-02	4120	1,13792E+00	618	1,62988E+00	4340	9,57018E-01	33	0	17530,5
Non-responder	Macro_FOLR2+APOE+	Macro_FOLR2+APOE+	CXCL10	TLR4	2,89541E-04	10987,5	1,64501E-02	4275	6,53299E-01	4340	2,16822E+00	1467	8,27091E-01	27325	0	17530,5
Non-responder	Macro_IER3	pDC_LILRA4	TNF	TNFRSF21	2,91492E-04	7422,9	3,27418E-02	1335	1,13938E+00	614	3,00584E+00	269	8,68530E-01	17366	0	17530,5

Non-responder	pDC_LILRA4	Macro_OLFML3	SPON2	ITGA5	2,93443E-04	12920,3	3,27226E-02	1338	1,05708E+00	945	2,73898E+00	474	7,54814E-01	44314	0	17530,5
Non-responder	Macro_NLRP3	Mono_CD16	ICAM1	SPN	2,94581E-04	7973,3	1,95996E-02	3175	8,65066E-01	1957	2,20682E+00	1354	8,74352E-01	15850	0	17530,5
Non-responder	Macro_FOLR2+APOE+	Macro_FOLR2+APOE+	GRN	TNFRSF1A	2,94581E-04	8123,1	8,33775E-03	13184	6,52319E-01	4359	2,00487E+00	2066	9,27662E-01	3476	0	17530,5
Non-responder	pDC_LILRA4	cDC_CLEC9A	NUCB2	ERAP1	2,94749E-04	10481,9	3,29490E-02	1319	9,71218E-01	1340	2,47652E+00	798	8,10363E-01	31422	0	17530,5
Non-responder	Mono_CD14	Mono_CD16	LYZ	ITGAL	2,95383E-04	5930,5	1,62569E-02	4362	8,80165E-01	1851	2,01238E+00	2039	9,25529E-01	3870	0	17530,5
Non-responder	Macro_FOLR2+APOE+	Macro_FOLR2+APOE+	PLAU	ITGA5	2,95650E-04	12550,5	2,00250E-02	3056	6,52129E-01	4363	1,75313E+00	3391	7,97727E-01	34412	0	17530,5
Non-responder	Macro_OLFML3	Macro_ISG15	C3	IFITM1	2,95779E-04	5681,5	2,32755E-02	2319	1,07982E+00	857	2,44230E+00	860	9,10938E-01	6841	0	17530,5
Non-responder	pDC_LILRA4	Macro_FOLR2+APOE-	SERPINF1	PLXDC2	2,95918E-04	6069,1	1,62499E-02	4364	8,35123E-01	2225	2,00475E+00	2067	9,24030E-01	4159	0	17530,5
Non-responder	Macro_NLRP3	Mono_CD16	THBS1	PTPRJ	2,96186E-04	11721,3	1,75988E-02	3806	8,23934E-01	2328	1,84132E+00	2872	8,07504E-01	32070	0	17530,5
Non-responder	Macro_NLRP3	Macro_IER3	HBEGF	CD44	2,96626E-04	9089,9	9,60033E-03	10494	6,77711E-01	3940	1,42645E+00	6328	9,09437E-01	7157	0	17530,5
Non-responder	Macro_FOLR2+APOE+	Macro_OLFML3	HLA-C	LILRB1	2,96723E-04	8302,9	7,67351E-03	15092	6,51954E-01	4367	1,95709E+00	2267	9,33621E-01	2258	0	17530,5
Non-responder	pDC_LILRA4	Mono_CD14	APP	APLP2	2,98336E-04	4815,1	2,69416E-02	1831	1,37003E+00	189	3,25358E+00	152	9,22985E-01	4373	0	17530,5
Non-responder	pDC_LILRA4	Macro_IFI27	SERPINF1	PLXDC2	2,99415E-04	6037,5	1,62152E-02	4377	8,33787E-01	2244	2,06058E+00	1855	9,23955E-01	4181	0	17530,5
Non-responder	Macro_FOLR2+APOE+	Macro_LYVE1	PLAU	LRP1	2,99686E-04	9427,7	2,06228E-02	2915	7,08483E-01	3540	1,62533E+00	4378	8,62500E-01	18775	0	17530,5
Non-responder	pDC_LILRA4	Macro_FOLR2+APOE+	SEMA3C	NRP2_PLXNA1	3,00672E-04	12937,9	6,90415E-02	334	9,83768E-01	1284	2,20821E+00	1349	7,55300E-01	44192	0	17530,5
Non-responder	Mono_CD16	Mono_CD16	HLA-F	LILRB2	3,00747E-04	7941,3	9,58421E-03	10523	8,34500E-01	2236	2,03932E+00	1931	9,07901E-01	7486	0	17530,5
Non-responder	Macro_FOLR2+APOE+	Macro_NLRP3	ADAM10	CD44	3,01176E-04	8131,9	1,25738E-02	6765	7,77494E-01	2747	1,80352E+00	3091	8,94910E-01	10526	0	17530,5
Non-responder	Mast	Mono_INHBA	TIMP3	CD44	3,01717E-04	5538,9	8,88888E-02	181	1,44737E+00	138	2,10605E+00	1672	9,05037E-01	8173	0	17530,5
Non-responder	pDC_LILRA4	Mono_CD16	APP	NOTCH2	3,01717E-04	6003,5	3,04717E-02	1505	1,37638E+00	181	3,37895E+00	119	8,94226E-01	10682	0	17530,5
Non-responder	Macro_FOLR2+APOE+	Macro_NLRP3	A2M	LRP1	3,01892E-04	9271,3	1,24424E-02	6893	5,31609E-01	6995	1,62135E+00	4407	8,94886E-01	10531	0	17530,5
Non-responder	Macro_NLRP3	Mono_CD16	ICAM3	ITGAL	3,02399E-04	11622,3	1,74195E-02	3887	6,87677E-01	3782	1,80764E+00	3069	8,16855E-01	29843	0	17530,5
Non-responder	Macro_ISG15	Macro_NLRP3	TNFSF10	TNFRSF10D	3,02663E-04	10448,3	3,27976E-02	1331	9,69258E-01	1352	2,26548E+00	1193	8,12741E-01	30835	0	17530,5
Non-responder	Macro_OLFML3	Macro_FOLR2+APOE+	CXCL10	TLR4	3,03996E-04	7276,1	3,25325E-02	1354	1,20019E+00	439	3,06374E+00	229	8,70580E-01	16828	0	17530,5
Non-responder	Macro_FOLR2+APOE+	Macro_FOLR2+APOE+	LRPAP1	LRP1	3,04048E-04	8723,9	1,21515E-02	7189	6,27335E-01	4779	1,72716E+00	3575	8,94830E-01	10546	0	17530,5
Non-responder	pDC_LILRA4	cDC_CLEC9A	ADAM10	CADM1	3,06002E-04	9680,3	3,24698E-02	1357	1,10903E+00	738	2,55262E+00	687	8,24039E-01	28089	0	17530,5
Non-responder	Macro_FOLR2+APOE+	Macro_IER3	APOE	LRP1	3,06362E-04	9840,3	1,00353E-02	9800	4,31252E-01	10562	1,37724E+00	6923	9,22916E-01	4386	0	17530,5
Non-responder	Macro_OLFML3	Macro_FOLR2+APOE-	CXCL9	FCGR2A	3,07233E-04	6008,3	2,07974E-02	2865	1,06433E+00	915	1,90917E+00	2514	9,13720E-01	6217	0	17530,5
Non-responder	Macro_OLFML3	Macro_FOLR2+APOE+	TNFSF13B	TFRC	3,08252E-04	7884,1	1,28225E-02	6551	7,46994E-01	3059	2,09785E+00	1705	8,94710E-01	10575	0	17530,5
Non-responder	Macro_FOLR2+APOE+	Mono_CD14	CCL18	CCR1	3,09815E-04	13941,7	2,47241E-02	2125	6,48442E-01	4415	2,39800E+00	932	7,53072E-01	44706	0	17530,5
Non-responder	Macro_NLRP3	Mono_CD16	HLA-C	LILRA3	3,10151E-04	8753,5	1,81913E-02	3591	4,30565E-01	10588	1,63789E+00	4284	9,06737E-01	7774	0	17530,5
Non-responder	Macro_FOLR2+APOE+	Macro_ISG15	ANOS1	SDC2	3,11203E-04	18840,9	7,79113E-02	243	9,27343E-01	1570	1,62035E+00	4420	5,59087E-01	70441	0	17530,5
Non-responder	Macro_NLRP3	Macro_NLRP3	ICAM1	ITGAX_ITGB2	3,11325E-04	7986,5	9,54816E-03	10596	7,22357E-01	3346	1,88665E+00	2622	9,15546E-01	5838	0	17530,5
Non-responder	Macro_FOLR2+APOE+	Macro_ISG15	B2M	TFRC	3,11481E-04	8669,1	7,03588E-03	17378	6,47864E-01	4421	2,05287E+00	1875	9,34216E-01	2141	0	17530,5
Non-responder	Macro_NLRP3	Macro_ISG15	S100A8	CD36	3,11913E-04	7504,5	1,71098E-02	4000	8,88552E-01	1801	1,72498E+00	3591	8,94553E-01	10600	0	17530,5
Non-responder	Macro_NLRP3	Macro_FOLR2+APOE+	CCL4	CCR5	3,12038E-04	10460,7	2,25721E-02	2457	7,13460E-01	3464	1,62003E+00	4423	8,38864E-01	24429	0	17530,5
Non-responder	Macro_FOLR2+APOE+	Macro_FOLR2+APOE+	ALCAM	NRP1	3,12317E-04	10834,1	2,11866E-02	2766	6,47631E-01	4424	1,85558E+00	2787	8,29764E-01	26663	0	17530,5
Non-responder	pDC_LILRA4	Macro_FOLR2+APOE+	SEMA3C	NRP1_NRP2_PLXND1	3,12752E-04	11141,1	6,11021E-02	434	9,65336E-01	1367	2,40726E+00	918	7,93266E-01	35456	0	17530,5
Non-responder	Macro_FOLR2+APOE+	cDC_CLEC9A	LGALS9	HAVCR2	3,13978E-04	9450,5	9,59014E-03	10512	5,89138E-01	5538	1,80947E+00	3058	8,94491E-01	10614	0	17530,5
Non-responder	Macro_LYVE1	Macro_NLRP3	C1QA	CD93	3,13993E-04	6189,1	1,73602E-02	3904	9,08767E-01	1674	1,61926E+00	4430	9,27955E-01	3407	0	17530,5
Non-responder	pDC_LILRA4	Macro_LYVE1	SERPINF1	PLXDC2	3,14834E-04	5894,3	1,60924E-02	4433	8,29062E-01	2288	2,37709E+00	974	9,23687E-01	4246	0	17530,5
Non-responder	Macro_OLFML3	Macro_LYVE1	SERPING1	LRP1	3,16203E-04	9303,5	1,30375E-02	6368	6,82060E-01	3879	1,29031E+00	8111	8,94445E-01	10629	0	17530,5
Non-responder	Macro_FOLR2+APOE+	pDC_LILRA4	ALCAM	NRP1	3,17930E-04	10781,1	2,11484E-02	2777	6,46579E-01	4444	1,91911E+00	2463	8,29637E-01	26691	0	17530,5
Non-responder	Macro_FOLR2+APOE+	Macro_ISG15	C3	C3AR1	3,18141E-04	9569,5	1,29309E-02	6456	4,45627E-01	9996	1,78134E+00	3223	8,94384E-01	10642	0	17530,5
Non-responder	Macro_FOLR2+APOE+	Macro_FOLR2+APOE+	ADAM9	ITGB5	3,20196E-04	14296,1	3,77767E-02	1064	6,86411E-01	3802	1,61640E+00	4452	7,53369E-01	44632	0	17530,5
Non-responder	Macro_FOLR2+APOE+	Macro_FOLR2+APOE+	APOE	SDC2	3,20480E-04	8189,5	1,87165E-02	3428	6,45881E-01	4453	2,03011E+00	1970	8,83180E-01	13566	0	17530,5
Non-responder	Macro_IFI27	Macro_FOLR2+APOE+	LILRB4	LAIR1	3,21594E-04	7910,1	1,07175E-02	8853	7,59452E-01	2930	1,54408E+00	5101	9,19059E-01	5136	0	17530,5
Non-responder	Mono_INHBA	pDC_LILRA4	MMP9	TLR9	3,22367E-04	12321,3	9,95760E-02	139	1,12711E+00	660	2,19961E+00	1381	7,65789E-01	41896	0	17530,5
Non-responder	Macro_FOLR2+APOE+	cDC_CLEC9A	HLA-DQA2	LAG3	3,23046E-04	11092,5	1,60402E-02	4462	6,82792E-01	3858	1,82309E+00	2981	8,29885E-01	26631	0	17530,5
Non-responder	pDC_LILRA4	Macro_FOLR2+APOE+	SEMA3C	NRP1_PLXNA1	3,23061E-04	12714,7	7,20676E-02	300	9,63132E-01	1382	2,28869E+00	1143	7,59648E-01	43218	0	17530,5
Non-responder	Macro_ISG15	Mono_INHBA	MMP12	PLAUR	3,24452E-04	6528,9	3,20855E-02	1384	9,90478E-01	1248	2,64028E+00	567	8,89360E-01	11915	0	17530,5
Non-responder	Macro_OLFML3	cDC(CD1C)	B2M	CD1B	3,24773E-04	9306,5	2,47658E-02	2120	5,20914E-01	7298	1,23575E+00	8898	8,94218E-01	10686	0	17530,5
Non-responder	Macro_FOLR2+APOE+	Mono_INHBA	MMP9	ITGAM	3,26490E-04	12412,7	1,90145E-02	3331	6,44914E-01	4474	2,07370E+00	1798	7,95477E-01	34930	0	17530,5
Non-responder	Macro_NLRP3	Mono_INHBA	S100A8	ITGB2	3,28590E-04	8216,9	1,08308E-02	8705	7,20033E-01	3377	1,12489E+00	10711	9,42823E-01	761	0	17530,5
Non-responder	Macro_ISG15	Macro_FOLR2+APOE+	ADM	GPR84	3,28649E-04	10239,3	4,14995E-02	911	9,61756E-01	1390	2,45522E+00	834	8,13983E-01	30531	0	17530,5
Non-responder	Macro_NLRP3	Macro_FOLR2-APOE+	VCAN	TLR2	3,29819E-04	8664,5	1,91537E-02	3290	9,47080E-01	1464	1,14834E+00	10319	8,94080E-01	10719	0	17530,5
Non-responder	Macro_FOLR2+APOE+	Macro_FOLR2+APOE+	HLA-A	APLP2	3,31125E-04	9402,9	6,21042E-03	21186	6,44161E-01	4490	1,81989E+00	3003	9,42349E-01	805	0	17530,5
Non-responder	Macro_NLRP3	Macro_OLFML3	THBS1	ITGA4	3,32000E-04	7927,7	1,62653E-02	4356	8,49504E-01	2094	1,80542E+00	3083	8,86893E-01	12575	0	17530,5
Non-responder	Macro_NLRP3	Macro_OLFML3	THBS1	CD47	3,32876E-04	7327,3	1,79229E-02	3688	8,99703E-01	1733	1,90064E+00	2547	8,92425E-01	11138	0	17530,5
Non-responder	cDC_CLEC9A	Mono_CD16	HLA-A	LILRA1	3,35221E-04	6340,1	1,71269E-02	3995	7,04885E-01	3578	1,99975E+00	2093	9,22187E-01	4504	0	17530,5
Non-responder	Macro_FOLR2+APOE+	Macro_LYVE1	CALR	LRP1	3,35239E-04	9046,3	9,45689E-03	10754	6,12916E-01	5039	1,43595E+00	6213	9,16226E-01	5695	0	17530,5
Non-responder	pDC_LILRA4	Macro_FOLR2+APOE+	APP	LRP1	3,35886E-04	5176,1	3,14430E-02	1419	1,36481E+00	191	3,34897E+00	125	9,11988E-01	6615	0	17530,5
Non-responder	Macro_IFI27	Macro_FOLR2+APOE+	C3	CD81	3,36019E-04	8066,3	1,87324E-02	3420	7,43666E-01	3102	1,13790E+00	10494	9,15781E-01	5785	0	17530,5
Non-responder	Macro_OLFML3	cDC_CLEC9A	HLA-DPA1	LAG3	3,36019E-04	7701,7	1,68659E-02	4091	8,34485E-01	2237	1,68483E+00	3891	8,93943E-01	10759	0	17530,5
Non-responder	Macro_OLFML3	Macro_FOLR2+APOE+	HLA-DMB	CD4	3,36644E-04	8453,1	9,45160E-03	10763	5,98787E-01	5332	1,73764E+00	3498	9,19032E-01	5142	0	17530,5
Non-responder	Macro_FOLR2+APOE+	Macro_OLFML3	MMP9	CD44	3,37282E-04	8533,3	1,59431E-02	4511	6,91577E-01	3724	2,25830E+00	1216	8,75029E-01	15685	0	17530,5
Non-responder	Macro_NLRP3	Macro_IER3	VCAN	TLR2	3,37897E-04	7494,7	1,90713E-02	3312	9,45159E-01	1472	1,62356E+00	4388	8,93876E-01	10771	0	17530,5
Non-responder	Macro_ISG15	pDC_LILRA4	VCAN	SELL	3,38582E-04	5678,5	3,16944E-02	1404	1,22067E+00	391	3,43179E+00	106	9,01416E-01	8961	0	17530,5
Non-responder	Macro_NLRP3	Macro_IER3	VEGFA	CD44	3,39153E-04	8459,9	1,30630E-02	6340	7,93976E-01	2609	1,55024E+00	5041	8,93842E-01	10779	0	17530,5
Non-responder	Macro_FOLR2+APOE+	Macro_FOLR2+APOE+	LGALS9	PTPRC	3,40122E-04	9485,3	7,51942E-03	15604	5,88003E-01	5560	1,77444E+00	3261	9,17383E-01	5471	0	17530,5
Non-responder	Macro_FOLR2+APOE+	Macro_FOLR2-APOE+	C1QA	CD33	3,42311E-04	10634,3	9,43683E-03	10799	6,01392E-01	5279	1,14324E+00	10413	9,00599E-01	9150	0	17530,5
Non-responder	Macro_FOLR2+APOE+	Macro_NLRP3	FN1	PLAUR	3,42926E-04	7635,3	1,59078E-02	4530	8,03214E-01	2515	2,07966E+00	1777	8,89690E-01	11824	0	17530,5

Post_NR_Myeloid_Myeloid_CellCell

Non-responder	Macro_ISG15	pDC_LILRA4	CCL8	CCR2	3,42938E-04	13774,1	9,93692E-02	141	9,23988E-01	1589	3,13829E+00	193	7,31572E-01	49417	0	17530,5
Non-responder	Mono_CD14	pDC_LILRA4	THBS1	CD47	3,43622E-04	4985,3	3,15872E-02	1411	1,26629E+00	308	3,53263E+00	87	9,16758E-01	5590	0	17530,5
Non-responder	Macro_NLRP3	Mono_CD16	NAMPT	INSR	3,43824E-04	10052,1	1,59040E-02	4533	7,92545E-01	2624	1,75259E+00	3397	8,48266E-01	22176	0	17530,5
Non-responder	Macro_FOLR2+APOE+	Macro_ISG15	SERPING1	LRP1	3,43899E-04	7790,1	1,28379E-02	6534	7,98190E-01	2575	2,15814E+00	1502	8,93714E-01	10809	0	17530,5
Non-responder	Macro_FOLR2+APOE+	Macro_IER3	MMP9	LRP1	3,45324E-04	10554,9	1,77935E-02	3741	6,40598E-01	4538	2,06841E+00	1829	8,35962E-01	25136	0	17530,5
Non-responder	Macro_NLRP3	Macro_FOLR2+APOE-	THBS1	LRP1	3,45333E-04	8822,7	1,81319E-02	3617	8,24043E-01	2323	1,17836E+00	9825	8,93671E-01	10818	0	17530,5
Non-responder	Macro_OLFML3	cDC_CLEC9A	HLA-DRB1	LAG3	3,45972E-04	7610,1	1,65664E-02	4210	8,59484E-01	2008	1,74023E+00	3480	8,93666E-01	10822	0	17530,5
Non-responder	Macro_NLRP3	Macro_OLFML3	CXCL8	SDC3	3,46828E-04	11022,7	1,75411E-02	3829	8,21403E-01	2355	1,80992E+00	3053	8,22993E-01	28346	0	17530,5
Non-responder	Macro_NLRP3	Macro_OLFML3	CCL3	CCR5	3,47431E-04	10754,3	1,89418E-02	3348	6,94709E-01	3683	1,63672E+00	4294	8,36845E-01	24916	0	17530,5
Non-responder	Macro_OLFML3	cDC_LAMP3	CXCL10	SDC4	3,47981E-04	9855,9	3,14684E-02	1417	1,15342E+00	572	2,68828E+00	516	8,19276E-01	29244	0	17530,5
Non-responder	Macro_FOLR2+APOE+	Macro_NLRP3	LAMB2	CD44	3,48035E-04	13751,9	1,76886E-02	3774	6,51577E-01	4371	1,60658E+00	4547	7,79972E-01	38537	0	17530,5
Non-responder	Macro_OLFML3	Macro_ISG15	LGALS9	HAVCR2	3,48377E-04	9923,3	9,41434E-03	10835	5,58974E-01	6255	1,65445E+00	4159	8,93615E-01	10837	0	17530,5
Non-responder	Mono_INHBA	pDC_LILRA4	INHBA	SMAD3	3,49442E-04	15786,9	5,91439E-02	472	9,55555E-01	1419	2,25553E+00	1225	6,81716E-01	58288	0	17530,5
Non-responder	Macro_FOLR2+APOE+	cDC_CLEC9A	CCL5	CXCR3	3,49549E-04	15503,9	4,00594E-02	963	6,40199E-01	4552	1,94513E+00	2332	7,17476E-01	52142	0	17530,5
Non-responder	Macro_NLRP3	Macro_FOLR2-APOE+	S100A9	CD68	3,49665E-04	7118,3	1,33878E-02	6074	1,02209E+00	1106	1,11727E+00	10845	9,56575E-01	36	0	17530,5
Non-responder	Macro_NLRP3	Macro_OLFML3	VCAN	TLR1	3,50763E-04	8413,1	2,51291E-02	2063	1,02894E+00	1072	1,93356E+00	2390	8,61572E-01	19010	0	17530,5
Non-responder	Macro_FOLR2+APOE-	pDC_LILRA4	CXCL12	CXCR4	3,50907E-04	6950,9	3,14217E-02	1421	1,04609E+00	991	2,59759E+00	628	8,80754E-01	14184	0	17530,5
Non-responder	cDC_CLEC9A	Macro_OLFML3	HLA-DOB	CD4	3,51641E-04	6863,9	4,09948E-02	928	1,40276E+00	156	2,18543E+00	1422	8,80365E-01	14283	0	17530,5
Non-responder	Macro_NLRP3	Macro_OLFML3	VEGFA	SIRPA	3,53201E-04	9188,5	1,80794E-02	3635	8,81739E-01	1846	1,89981E+00	2552	8,55696E-01	20379	0	17530,5
Non-responder	Macro_FOLR2+APOE+	Mono_INHBA	APOE	LRP1	3,53388E-04	9905,7	9,86816E-03	10061	4,24276E-01	10868	1,40564E+00	6580	9,22317E-01	4489	0	17530,5
Non-responder	Macro_NLRP3	Macro_OLFML3	CCL3L1	CCR1	3,53506E-04	8201,9	2,22461E-02	2527	9,61831E-01	1389	2,23510E+00	1282	8,64701E-01	18281	0	17530,5
Non-responder	Macro_FOLR2+APOE+	Macro_IFI27	C1QB	LRP1	3,53713E-04	8792,7	9,39600E-03	10870	6,22862E-01	4868	1,44478E+00	6103	9,21766E-01	4592	0	17530,5
Non-responder	Macro_NLRP3	Macro_OLFML3	CCL3L1	CCR5	3,53812E-04	9902,5	3,17400E-02	1399	9,77759E-01	1311	2,09443E+00	1727	8,26158E-01	27545	0	17530,5
Non-responder	Macro_NLRP3	Macro_OLFML3	ADM	GPR84	3,54731E-04	14183,1	2,18789E-02	2595	6,40307E-01	4547	1,77830E+00	3240	7,60610E-01	43003	0	17530,5
Non-responder	Macro_FOLR2+APOE+	Macro_FOLR2+APOE+	HGF	NRP1	3,55958E-04	15113,5	3,63022E-02	1133	6,38815E-01	4573	1,63363E+00	4315	7,38301E-01	48016	0	17530,5
Non-responder	Mono_CD14	Macro_IER3	S100A8	ITGB2	3,56808E-04	4897,1	1,56714E-02	4662	1,11044E+00	733	2,18341E+00	1429	9,52004E-01	131	0	17530,5
Non-responder	pDC_LILRA4	Macro_FOLR2+APOE+	APP	TSPAN15	3,58294E-04	9200,7	3,13369E-02	1431	1,20584E+00	424	3,15427E+00	182	8,30591E-01	26436	0	17530,5
Non-responder	Mono_CD14	pDC_LILRA4	S100A8	CD68	3,58294E-04	5088,7	1,36496E-02	5885	9,53146E-01	1431	2,72827E+00	485	9,52589E-01	112	0	17530,5
Non-responder	Mono_INHBA	Mono_INHBA	SPP1	CD44	3,58423E-04	6121,3	2,48365E-02	2110	8,55376E-01	2050	1,63090E+00	4335	9,21824E-01	4581	0	17530,5
Non-responder	Macro_OLFML3	Macro_NLRP3	LGALS9	CD44	3,58786E-04	7649,7	9,37637E-03	10901	7,69055E-01	2827	1,81558E+00	3026	9,25087E-01	3964	0	17530,5
Non-responder	Macro_IFI27	Mono_CD16	HLA-C	LILRA3	3,59115E-04	8406,3	2,05611E-02	2930	6,33849E-01	4652	1,15280E+00	10255	9,11788E-01	6664	0	17530,5
Non-responder	Macro_FOLR2+APOE+	pDC_LILRA4	HGF	NRP1	3,59660E-04	15021,5	3,62368E-02	1136	6,37763E-01	4585	1,69716E+00	3803	7,38127E-01	48053	0	17530,5
Non-responder	Mono_CD14	Macro_FOLR2-APOE+	S100A9	CD68	3,60279E-04	5370,5	1,58031E-02	4587	1,22162E+00	389	1,63147E+00	4333	9,59893E-01	13	0	17530,5
Non-responder	Mono_CD16	cDC_LAMP3	TNFSF10	TNFRSF11B	3,60888E-04	10367,9	9,65749E-02	150	1,34989E+00	198	2,07254E+00	1808	8,07117E-01	32153	0	17530,5
Non-responder	Macro_NLRP3	Macro_OLFML3	THBS1	SDC4	3,60900E-04	12152,1	1,84056E-02	3528	8,37433E-01	2207	1,82951E+00	2949	7,97214E-01	34546	0	17530,5
Non-responder	Macro_FOLR2+APOE+	Mono_CD16	CD14	ITGB2	3,61521E-04	7782,1	8,09500E-03	13853	6,37329E-01	4591	1,95718E+00	2266	9,43805E-01	670	0	17530,5
Non-responder	Mast	pDC_LILRA4	HDC	HRH2	3,62025E-04	10731,5	5,46636E-02	552	1,24105E+00	350	2,17991E+00	1436	8,00404E-01	33789	0	17530,5
Non-responder	cDC_CLEC9A	pDC_LILRA4	TNF	TNFRSF21	3,62025E-04	7556,5	3,13037E-02	1436	1,11860E+00	697	3,32876E+00	130	8,65944E-01	17989	0	17530,5
Non-responder	Mono_CD16	Macro_FOLR2+APOE-	CD52	SIGLEC10	3,62255E-04	9233,3	1,21787E-02	7160	8,09962E-01	2457	1,29135E+00	8097	8,93281E-01	10922	0	17530,5
Non-responder	Macro_FOLR2+APOE+	Macro_LYVE1	LRPAP1	LRP1	3,63251E-04	9967,3	1,17492E-02	7594	6,06211E-01	5180	1,25644E+00	8604	8,93235E-01	10928	0	17530,5
Non-responder	Mono_INHBA	Macro_NLRP3	IL6	IL6R_IL6ST	3,63525E-04	10975,5	3,76454E-02	1069	1,17515E+00	511	2,17921E+00	1438	7,98040E-01	34329	0	17530,5
Non-responder	Macro_NLRP3	Mono_INHBA	S100A8	CD68	3,63584E-04	7890,7	1,17544E-02	7590	7,42430E-01	3118	1,11358E+00	10930	9,49097E-01	285	0	17530,5
Non-responder	Macro_FOLR2+APOE+	Macro_FOLR2+APOE+	TNFSF13	TNFRSF1A	3,64083E-04	9957,9	9,35391E-03	10933	5,11051E-01	7638	1,83372E+00	2925	8,93926E-01	10763	0	17530,5
Non-responder	Macro_FOLR2+APOE+	pDC_LILRA4	HLA-DQB1	CD4	3,65892E-04	7477,3	8,23795E-03	13450	6,36443E-01	4605	2,49867E+00	769	9,40493E-01	1032	0	17530,5
Non-responder	pDC_LILRA4	Macro_NLRP3	APP	APLP2	3,67149E-04	4894,9	2,59820E-02	1960	1,34380E+00	204	3,18584E+00	171	9,21686E-01	4609	0	17530,5
Non-responder	Macro_NLRP3	Mono_CD16	HLA-B	LILRB2	3,68093E-04	8332,9	7,80210E-03	14709	6,39931E-01	4555	1,59779E+00	4612	9,49585E-01	258	0	17530,5
Non-responder	Macro_FOLR2+APOE+	Mono_CD14	A2M	LRP1	3,70454E-04	9779,3	1,19747E-02	7366	5,13345E-01	7555	1,50765E+00	5474	8,93071E-01	10971	0	17530,5
Non-responder	Macro_FOLR2+APOE+	Mono_INHBA	CXCL12	SDC4	3,70619E-04	16728,1	2,72452E-02	1795	6,35448E-01	4620	1,73193E+00	3541	6,95008E-01	56154	0	17530,5
Non-responder	Macro_OLFML3	Mono_CD14	CXCL10	TLR4	3,71085E-04	7436,7	3,11668E-02	1448	1,18148E+00	495	3,01971E+00	259	8,68145E-01	17451	0	17530,5
Non-responder	Macro_FOLR2+APOE+	cDC_LAMP3	IL10	CD70	3,71888E-04	15858,7	1,04803E-01	117	1,13416E+00	639	1,59676E+00	4624	6,93719E-01	56383	0	17530,5
Non-responder	Macro_FOLR2+APOE+	Macro_FOLR2+APOE+	TNFSF13B	HLA-DPB1	3,73159E-04	8260,9	7,27262E-03	16453	6,34763E-01	4628	1,95504E+00	2280	9,47025E-01	413	0	17530,5
Non-responder	Mono_CD14	cDC_CLEC9A	CXCL2	XCR1	3,73373E-04	8100,1	7,02616E-02	324	2,47309E+00	8	2,17378E+00	1451	8,52342E-01	21187	0	17530,5
Non-responder	Macro_FOLR2+APOE+	Mono_CD14	CXCL10	TLR4	3,75711E-04	11298,1	1,57595E-02	4618	6,34595E-01	4636	2,12419E+00	1609	8,24002E-01	28097	0	17530,5
Non-responder	pDC_LILRA4	Mono_INHBA	APP	CD74	3,76352E-04	5100,1	1,57255E-02	4638	9,32278E-01	1544	2,08697E+00	1750	9,56333E-01	38	0	17530,5
Non-responder	Macro_NLRP3	Macro_NLRP3	S100A8	CD68	3,76744E-04	8629,3	1,02972E-02	9425	6,32994E-01	4670	1,10895E+00	11008	9,45803E-01	513	0	17530,5
Non-responder	Macro_FOLR2+APOE+	Mono_CD16	C1QB	LRP1	3,76915E-04	7995,5	9,31294E-03	11009	6,19414E-01	4933	2,06358E+00	1845	9,21445E-01	4660	0	17530,5
Non-responder	Macro_FOLR2+APOE+	Mono_CD14	LRPAP1	LDLR	3,76992E-04	12770,5	1,81124E-02	3626	6,40944E-01	4532	1,59552E+00	4640	8,01474E-01	33524	0	17530,5
Non-responder	Macro_FOLR2+APOE+	Macro_FOLR2+APOE+	C3	LRP1	3,77771E-04	9983,9	1,25932E-02	6751	4,30708E-01	10584	1,66898E+00	4040	8,92911E-01	11014	0	17530,5
Non-responder	Macro_FOLR2+APOE+	Macro_FOLR2+APOE+	HLA-DPA1	CD4	3,79242E-04	7583,1	8,25024E-03	13417	6,34233E-01	4647	1,99935E+00	2099	9,50147E-01	222	0	17530,5
Non-responder	Mono_INHBA	pDC_LILRA4	BST2	LILRA4	3,81842E-04	5664,1	3,08999E-02	1462	1,05558E+00	950	3,01867E+00	260	9,05255E-01	8118	0	17530,5
Non-responder	Macro_FOLR2+APOE+	Macro_FOLR2+APOE+	SERPINA1	LRP1	3,81905E-04	8774,9	9,29595E-03	11038	5,62597E-01	6158	1,66687E+00	4056	9,19294E-01	5092	0	17530,5
Non-responder	Macro_FOLR2+APOE+	Mono_INHBA	MMP9	LRP1	3,82473E-04	10631,3	1,74971E-02	3849	6,33622E-01	4657	2,09681E+00	1710	8,34807E-01	25410	0	17530,5
Non-responder	Macro_ISG15	Macro_ISG15	MMP12	PLAUR	3,82618E-04	6629,1	3,08610E-02	1463	9,53845E-01	1427	2,97888E+00	288	8,87431E-01	12437	0	17530,5
Non-responder	Mono_INHBA	Macro_ISG15	IL6	FCGR3B	3,84174E-04	11688,1	8,95511E-02	177	1,34278E+00	206	2,17018E+00	1465	7,77822E-01	39062	0	17530,5
Non-responder	pDC_LILRA4	Mono_CD14	SPON2	ITGB1	3,84174E-04	9823,9	3,08454E-02	1465	1,01253E+00	1149	2,69069E+00	512	8,22534E-01	28463	0	17530,5
Non-responder	Macro_FOLR2+APOE+	Macro_FOLR2+APOE+	PLAU	PLAUR	3,84422E-04	7674,9	1,56700E-02	4663	7,11854E-01	3486	2,18511E+00	1427	8,91926E-01	11268	0	17530,5
Non-responder	Macro_FOLR2+APOE+	Macro_FOLR2+APOE+	HBEGF	CD9	3,84507E-04	10958,7	9,62291E-03	10453	4,20009E-01	11053	1,55112E+00	5028	8,94055E-01	10729	0	17530,5
Non-responder	pDC_LILRA4	Macro_OLFML3	SERPINF1	PLXDC2	3,85073E-04	5908,3	1,56661E-02	4665	8,12652E-01	2425	2,69406E+00	507	9,22736E-01	4414	0	17530,5
Non-responder	Macro_NLRP3	pDC_LILRA4	ICAM1	SPN	3,85726E-04	6283,5	2,80228E-02	1726	1,09933E+00	766	2,91744E+00	335	8,92712E-01	11060	0	17530,5
Non-responder	Macro_ISG15	Macro_FOLR2+APOE+	TIMP1	CD63	3,85733E-04	6087,3	9,54925E-03	10593	9,46480E-01	1467	2,55189E+00	688	9,51306E-01	158	0	17530,5
Non-responder	Macro_OLFML3	pDC_LILRA4	CD14	TLR9	3,86514E-04	6601,5	7,06569E-02	317	1,07611E+00	873	2,16797E+00	1468	8,85957E-01	12819	0	17530,5

Non-responder	pDC_LILRA4	Macro_ISG15	APP	APLP2	3,86704E-04	4906,5	2,57565E-02	1984	1,33763E+00	208	3,29106E+00	140	9,21371E-01	4670	0	17530,5
Non-responder	Macro_NLRP3	pDC_LILRA4	CD14	TLR9	3,87297E-04	6508,1	7,26129E-02	293	1,09549E+00	784	2,16732E+00	1469	8,87329E-01	12464	0	17530,5
Non-responder	Macro_FOLR2+APOE+	Macro_FOLR2+APOE+	SPP1	CD44	3,87824E-04	9420,9	1,27681E-02	6601	4,19509E-01	11072	2,25739E+00	1221	8,94231E-01	10680	0	17530,5
Non-responder	Macro_FOLR2+APOE+	Macro_ISG15	CXCL10	SDC4	3,89654E-04	13747,9	1,73438E-02	3912	6,32335E-01	4679	2,04460E+00	1915	7,70931E-01	40703	0	17530,5
Non-responder	Macro_FOLR2+APOE+	cDC_CLEC9A	LYZ	ITGAL	3,90106E-04	9123,7	9,26907E-03	11085	5,74031E-01	5886	1,88396E+00	2641	9,03701E-01	8476	0	17530,5
Non-responder	Macro_NLRP3	Macro_LYVE1	TIMP1	CD63	3,91339E-04	7065,5	9,26531E-03	11092	8,73881E-01	1885	1,59658E+00	4626	9,50602E-01	194	0	17530,5
Non-responder	Macro_FOLR2+APOE+	Macro_FOLR2-APOE+	APOE	SCARB1	3,92575E-04	9718,1	1,54077E-02	4785	5,22967E-01	7242	1,30320E+00	7934	8,92572E-01	11099	0	17530,5
Non-responder	Mono_CD14	cDC(CD1C)	B2M	CD1C	3,92620E-04	5382,1	2,99588E-02	1547	6,31914E-01	4688	1,87213E+00	2708	9,46720E-01	437	0	17530,5
Non-responder	Macro_OLFML3	Mono_CD16	CXCL9	FCGR2A	3,92752E-04	7816,3	1,28013E-02	6574	7,87340E-01	2664	2,25987E+00	1213	8,92571E-01	11100	0	17530,5
Non-responder	pDC_LILRA4	Macro_FOLR2+APOE-	GAS6	MERTK	3,92803E-04	8498,5	3,06820E-02	1476	1,02763E+00	1081	2,19672E+00	1391	8,53149E-01	21014	0	17530,5
Non-responder	Macro_FOLR2+APOE-	pDC_LILRA4	C1QB	C1QBP	3,93281E-04	5768,5	1,67427E-02	4131	9,26737E-01	1573	1,58948E+00	4690	9,41458E-01	918	0	17530,5
Non-responder	Macro_FOLR2+APOE+	pDC_LILRA4	APOE	SCARB1	3,93460E-04	8628,9	1,54056E-02	4788	5,22925E-01	7243	1,91609E+00	2479	8,92566E-01	11104	0	17530,5
Non-responder	Mono_CD16	Macro_FOLR2+APOE+	SELPLG	ITGB2	3,94170E-04	7966,5	9,25752E-03	11108	6,39619E-01	4561	1,93042E+00	2409	9,23782E-01	4224	0	17530,5
Non-responder	Macro_OLFML3	Macro_NLRP3	FN1	PLAUR	3,94347E-04	7424,1	1,68714E-02	4088	8,20238E-01	2365	2,01418E+00	2028	8,92543E-01	11109	0	17530,5
Non-responder	Macro_FOLR2+APOE+	Macro_FOLR2+APOE+	TNFSF13B	CD40	3,94525E-04	8047,5	1,19714E-02	7373	7,81763E-01	2711	2,15200E+00	1513	8,92540E-01	11110	0	17530,5
Non-responder	Macro_FOLR2+APOE+	pDC_LILRA4	PLAU	ST14	3,94938E-04	10790,5	1,99492E-02	3073	6,31495E-01	4695	2,63355E+00	575	8,24095E-01	28079	0	17530,5
Non-responder	Macro_FOLR2+APOE+	Macro_OLFML3	HLA-C	LILRB2	3,95270E-04	9401,1	6,31135E-03	20678	6,31464E-01	4696	1,78381E+00	3206	9,41623E-01	895	0	17530,5
Non-responder	Macro_FOLR2+APOE+	Macro_OLFML3	TNFSF13B	CD40	3,95591E-04	8219,9	1,19683E-02	7378	7,81592E-01	2714	1,93944E+00	2361	8,92528E-01	11116	0	17530,5
Non-responder	Macro_FOLR2+APOE+	Macro_FOLR2+APOE+	HLA-DPB1	CD4	3,95935E-04	7643,7	8,27900E-03	13337	6,31316E-01	4698	1,95509E+00	2279	9,47765E-01	374	0	17530,5
Non-responder	pDC_LILRA4	pDC_LILRA4	SPON2	ITGA4	3,95972E-04	9794,9	3,06409E-02	1480	1,03617E+00	1041	3,16629E+00	179	8,21398E-01	28744	0	17530,5
Non-responder	Macro_NLRP3	Mono_CD16	S100A8	TLR4	3,96268E-04	8174,9	1,55879E-02	4699	8,82778E-01	1832	1,68475E+00	3892	8,85545E-01	12921	0	17530,5
Non-responder	Macro_LYVE1	pDC_LILRA4	LAMB2	RPSA	3,96767E-04	10047,9	3,06350E-02	1481	9,72000E-01	1332	2,44269E+00	858	8,20129E-01	29038	0	17530,5
Non-responder	Macro_ISG15	pDC_LILRA4	CXCL11	DPP4	3,99958E-04	15582,3	1,07507E-01	109	9,42887E-01	1485	2,51808E+00	746	6,83312E-01	58041	0	17530,5
Non-responder	Mono_INHBA	cDC(CD1C)	B2M	CD1C	4,00611E-04	5675,9	2,99422E-02	1550	6,30703E-01	4712	1,65533E+00	4149	9,46706E-01	438	0	17530,5
Non-responder	Macro_ISG15	Mono_CD16	B2M	LILRB2	4,00758E-04	6373,3	8,97379E-03	11686	9,42635E-01	1486	2,29261E+00	1136	9,57761E-01	28	0	17530,5
Non-responder	Mono_CD16	Mono_CD16	ICAM3	ITGAL	4,02362E-04	8325,3	3,05910E-02	1488	9,86377E-01	1271	2,44297E+00	856	8,55294E-01	20481	0	17530,5
Non-responder	Macro_FOLR2+APOE+	Macro_OLFML3	C1QB	LRP1	4,03302E-04	8121,7	9,23511E-03	11159	6,16182E-01	4999	1,97034E+00	2206	9,21141E-01	4714	0	17530,5
Non-responder	Macro_ISG15	Macro_FOLR2+APOE+	LILRB4	LAIR1	4,03639E-04	5278,9	1,55257E-02	4721	1,00817E+00	1172	2,85187E+00	374	9,31817E-01	2597	0	17530,5
Non-responder	Macro_NLRP3	Macro_FOLR2+APOE+	CD52	SIGLEC10	4,04931E-04	9309,3	1,19321E-02	7421	6,30489E-01	4718	1,48406E+00	5709	8,92302E-01	11168	0	17530,5
Non-responder	Mast	Macro_ISG15	TIMP3	CD44	4,05582E-04	6021,1	7,13187E-02	309	1,26229E+00	316	2,16056E+00	1492	8,95142E-01	10458	0	17530,5
Non-responder	pDC_LILRA4	pDC_LILRA4	TNFSF9	TRAF2	4,05582E-04	13385,9	6,99616E-02	328	9,41432E-01	1492	2,93213E+00	326	7,41600E-01	47253	0	17530,5
Non-responder	pDC_LILRA4	cDC(CD1C)	GAS6	AXL	4,06389E-04	7774,1	3,05583E-02	1493	1,00928E+00	1164	2,62081E+00	598	8,65496E-01	18085	0	17530,5
Non-responder	Mono_CD16	Macro_LYVE1	CD52	SIGLEC10	4,06565E-04	8759,9	1,19251E-02	7425	8,01927E-01	2532	1,53992E+00	5135	8,92274E-01	11177	0	17530,5
Non-responder	pDC_LILRA4	Macro_ISG15	APP	RPSA	4,07023E-04	5189,7	1,97813E-02	3126	1,29625E+00	270	2,97639E+00	291	9,21009E-01	4731	0	17530,5
Non-responder	Mono_CD16	Macro_NLRP3	AGTRAP	RACK1	4,07840E-04	9586,3	1,11837E-02	8234	6,79294E-01	3913	1,36524E+00	7070	8,92249E-01	11184	0	17530,5
Non-responder	Macro_FOLR2+APOE+	Macro_ISG15	PLTP	ABCA1	4,08387E-04	7887,3	1,51430E-02	4923	6,80741E-01	3900	2,04909E+00	1896	8,92241E-01	11187	0	17530,5
Non-responder	Macro_NLRP3	Macro_NLRP3	CCL3L1	CCR1	4,09746E-04	9996,3	1,54855E-02	4739	8,49059E-01	2097	2,03192E+00	1962	8,42077E-01	23653	0	17530,5
Non-responder	Macro_FOLR2+APOE+	Macro_ISG15	LGALS3	ENG	4,10765E-04	8916,3	9,21792E-03	11200	5,52524E-01	6429	1,93109E+00	2406	9,10139E-01	7016	0	17530,5
Non-responder	pDC_LILRA4	Macro_FOLR2+APOE+	APP	PTGER2	4,11257E-04	9051,3	3,05350E-02	1499	1,29809E+00	268	3,01842E+00	261	8,33456E-01	25698	0	17530,5
Non-responder	Macro_NLRP3	cDC_CLEC9A	IL1B	SIGIRR	4,12418E-04	7149,9	1,66565E-02	4172	1,00979E+00	1160	2,10490E+00	1678	8,92146E-01	11209	0	17530,5
Non-responder	Macro_NLRP3	Macro_IER3	CXCL8	SDC3	4,13855E-04	11695,3	1,59724E-02	4498	7,97686E-01	2581	1,69253E+00	3833	8,16066E-01	30034	0	17530,5
Non-responder	Macro_ISG15	Mono_CD16	HLA-A	LILRB2	4,14524E-04	6267,7	9,33609E-03	10971	9,38925E-01	1503	2,28533E+00	1153	9,50842E-01	181	0	17530,5
Non-responder	Macro_FOLR2+APOE+	Macro_ISG15	C3	ITGAX	4,15370E-04	9390,7	1,25857E-02	6758	4,84873E-01	8517	1,83400E+00	2923	8,92110E-01	11225	0	17530,5
Non-responder	Macro_NLRP3	Macro_IER3	VCAN	TLR1	4,17302E-04	10739,5	1,62209E-02	4374	8,98189E-01	1738	1,63242E+00	4324	8,33348E-01	25731	0	17530,5
Non-responder	Mono_INHBA	Macro_ISG15	INHBA	ENG	4,17808E-04	9042,7	3,80425E-02	1045	9,38486E-01	1507	2,28037E+00	1163	8,40770E-01	23968	0	17530,5
Non-responder	pDC_LILRA4	Macro_LYVE1	APP	LRP1	4,18632E-04	5301,1	3,04022E-02	1508	1,34369E+00	205	2,87824E+00	351	9,10628E-01	6911	0	17530,5
Non-responder	Mono_INHBA	pDC_LILRA4	CXCL8	CD79A	4,19456E-04	12192,9	9,22806E-02	168	9,93004E-01	1233	2,15269E+00	1509	7,71698E-01	40524	0	17530,5
Non-responder	cDC_LAMP3	pDC_LILRA4	PDCD1LG2	PDCD2	4,19456E-04	12674,9	3,93995E-02	988	9,38266E-01	1509	2,20919E+00	1347	7,65353E-01	42000	0	17530,5
Non-responder	Macro_NLRP3	Macro_IER3	CCL3L1	CCR1	4,20075E-04	9985,1	1,61129E-02	4422	8,59525E-01	2007	1,83123E+00	2942	8,44700E-01	23024	0	17530,5
Non-responder	Macro_NLRP3	Mono_INHBA	TIMP1	CD63	4,22449E-04	6887,7	9,18246E-03	11263	8,65351E-01	1955	1,73936E+00	3483	9,50391E-01	207	0	17530,5
Non-responder	Macro_NLRP3	Macro_FOLR2+APOE+	IL1B	ADRB2	4,23212E-04	10537,7	1,54151E-02	4778	9,47994E-01	1459	1,66501E+00	4074	8,37168E-01	24847	0	17530,5
Non-responder	Macro_OLFML3	Macro_FOLR2+APOE+	TNFSF13B	CD40	4,23575E-04	8297,9	1,18180E-02	7519	7,71800E-01	2798	1,93721E+00	2373	8,91920E-01	11269	0	17530,5
Non-responder	Macro_OLFML3	Macro_OLFML3	TNFSF13B	CD40	4,24704E-04	8546,1	1,18149E-02	7524	7,71629E-01	2803	1,72465E+00	3598	8,91908E-01	11275	0	17530,5
Non-responder	Macro_OLFML3	Mono_CD16	HLA-A	LILRB2	4,24893E-04	6519,5	9,17630E-03	11276	9,05458E-01	1700	2,05079E+00	1886	9,50437E-01	205	0	17530,5
Non-responder	Macro_FOLR2+APOE+	Macro_FOLR2+APOE+	HLA-F	LILRB2	4,25458E-04	8661,3	9,17501E-03	11279	7,01408E-01	3612	1,82713E+00	2958	9,06061E-01	7927	0	17530,5
Non-responder	Macro_NLRP3	Macro_NLRP3	IL1A	IL1R1_IL1RAP	4,26014E-04	18334,7	3,89921E-02	1014	6,27033E-01	4786	1,62878E+00	4351	6,37931E-01	63992	0	17530,5
Non-responder	Macro_NLRP3	cDC_CLEC9A	THBS1	ITGA4	4,26024E-04	7239,1	1,80008E-02	3660	8,98974E-01	1735	2,02637E+00	1988	8,91879E-01	11282	0	17530,5
Non-responder	pDC_LILRA4	Mast	TNFSF9	TNFRSF9	4,26092E-04	13627,3	1,03020E-01	126	1,11735E+00	703	2,15101E+00	1517	7,37190E-01	48260	0	17530,5
Non-responder	Macro_FOLR2+APOE+	Macro_LYVE1	HLA-DQA1	CD4	4,27421E-04	8747,5	7,70312E-03	15000	6,26862E-01	4790	1,58786E+00	4705	9,36219E-01	1712	0	17530,5
Non-responder	Mono_INHBA	Mast	S100A8	CD69	4,27762E-04	5049,9	3,90150E-02	1013	1,39403E+00	161	1,55129E+00	5026	9,37213E-01	1519	0	17530,5
Non-responder	cDC_LAMP3	pDC_LILRA4	TNFSF4	TNFRSF4	4,28599E-04	17282,5	1,52194E-01	50	1,00595E+00	1183	2,15032E+00	1520	6,17347E-01	66129	0	17530,5
Non-responder	Macro_FOLR2+APOE+	Mono_CD16	C3	IFITM1	4,28674E-04	8354,1	1,51198E-02	4941	5,95691E-01	5406	1,89166E+00	2597	8,91818E-01	11296	0	17530,5
Non-responder	cDC_CLEC9A	Mono_CD16	LYZ	ITGAL	4,28830E-04	6030,9	1,53987E-02	4794	8,13180E-01	2419	2,28102E+00	1159	9,23639E-01	4252	0	17530,5
Non-responder	Macro_FOLR2+APOE+	Mono_CD14	CD14	ITGB2	4,30952E-04	8057,9	8,00517E-03	14127	6,26442E-01	4800	1,79640E+00	3134	9,43508E-01	698	0	17530,5
Non-responder	Mono_CD16	Mono_CD16	PTPN6	CLEC12A	4,31719E-04	7471,5	1,69698E-02	4052	7,62694E-01	2892	2,13410E+00	1571	8,91745E-01	11312	0	17530,5
Non-responder	Macro_NLRP3	Mono_INHBA	S100A9	ITGB2	4,32100E-04	8338,7	9,15169E-03	11314	6,92561E-01	3713	1,26602E+00	8461	9,43757E-01	675	0	17530,5
Non-responder	Macro_IFI27	cDC(CD1C)	B2M	CD1C	4,32674E-04	5522,9	3,26392E-02	1346	8,27923E-01	2296	1,44045E+00	6137	9,48840E-01	305	0	17530,5
Non-responder	Macro_NLRP3	Macro_LYVE1	VCAN	TLR2	4,33056E-04	7751,7	1,82275E-02	3581	9,25495E-01	1578	1,58285E+00	4750	8,91710E-01	11319	0	17530,5
Non-responder	Macro_FOLR2+APOE+	Macro_ISG15	MMP9	ITGAM	4,33080E-04	12654,9	1,78123E-02	3735	6,26233E-01	4806	2,33056E+00	1065	7,90113E-01	36138	0	17530,5
Non-responder	Macro_FOLR2+APOE+	pDC_LILRA4	HMGB1	CXCR4	4,34861E-04	7490,9	8,49421E-03	12778	6,25813E-01	4811	2,74931E+00	456	9,35378E-01	1879	0	17530,5
Non-responder	Macro_NLRP3	Macro_FOLR2+APOE+	SEMA4D	CD72	4,35574E-04	14272,9	1,90942E-02	3306	6,88312E-01	3772	1,57386E+00	4813	7,65595E-01	41943	0	17530,5

Non-responder	Macro_FOLR2+APOE+	Macro_LYVE1	C3	C3AR1	4,35933E-04	11279,9	1,22837E-02	7069	4,14170E-01	11334	1,21634E+00	9201	8,91934E-01	11265	0	17530,5
Non-responder	Mono_CD14	pDC_LILRA4	CD14	TLR9	4,36176E-04	6268,1	7,84694E-02	239	1,15352E+00	570	2,14540E+00	1529	8,91149E-01	11472	0	17530,5
Non-responder	pDC_LILRA4	Macro_LYVE1	APP	TNFRSF21	4,36176E-04	7321,5	3,01018E-02	1529	1,27964E+00	288	2,75940E+00	449	8,70631E-01	16811	0	17530,5
Non-responder	Mono_CD14	Macro_IER3	VCAN	CD44	4,37720E-04	6141,5	1,53458E-02	4819	1,03531E+00	1045	1,83826E+00	2891	9,22692E-01	4422	0	17530,5
Non-responder	cDC_LAMP3	pDC_LILRA4	CXCL10	CXCR3	4,37872E-04	9878,3	3,30784E-02	1309	1,18421E+00	487	2,14429E+00	1531	8,22235E-01	28534	0	17530,5
Non-responder	Macro_NLRP3	Mono_CD14	ICAM1	ITGAM_ITGB2	4,38439E-04	9096,3	9,13394E-03	11347	6,27438E-01	4778	1,60025E+00	4591	9,09108E-01	7235	0	17530,5
Non-responder	Macro_FOLR2+APOE+	Macro_ISG15	HEBP1	FPR3	4,39514E-04	10465,5	1,63054E-02	4335	6,25323E-01	4824	1,88106E+00	2662	8,44902E-01	22976	0	17530,5
Non-responder	Macro_NLRP3	cDC_LAMP3	VCAN	ITGA4	4,42511E-04	7880,3	1,57939E-02	4596	9,33509E-01	1539	1,62724E+00	4368	8,91504E-01	11368	0	17530,5
Non-responder	Macro_FOLR2+APOE+	cDC_CLEC9A	LAMB2	RPSA	4,43481E-04	13740,7	1,77082E-02	3767	6,24726E-01	4835	1,79517E+00	3141	7,76115E-01	39430	0	17530,5
Non-responder	Macro_NLRP3	Mono_CD16	TNF	TNFRSF1B	4,43485E-04	7681,1	1,42688E-02	5464	8,29868E-01	2284	2,08593E+00	1754	8,91490E-01	11373	0	17530,5
Non-responder	Macro_FOLR2+APOE+	Macro_OLFML3	MMP9	ITGAM	4,44930E-04	12777,9	1,77073E-02	3770	6,24603E-01	4839	2,15698E+00	1504	7,89623E-01	36246	0	17530,5
Non-responder	Macro_ISG15	pDC_LILRA4	LGALS9	CD47	4,44930E-04	5450,9	1,75836E-02	3811	1,07672E+00	870	3,10987E+00	204	9,20423E-01	4839	0	17530,5
Non-responder	Mast	Macro_FOLR2+APOE+	ADCYAP1	GPR84	4,45274E-04	11172,7	2,09511E-01	22	1,33268E+00	220	2,13905E+00	1549	7,88416E-01	36542	0	17530,5
Non-responder	Macro_NLRP3	pDC_LILRA4	S100A9	CD68	4,45438E-04	7260,5	9,11662E-03	11383	6,42473E-01	4511	1,90964E+00	2512	9,47857E-01	366	0	17530,5
Non-responder	Macro_FOLR2+APOE+	Macro_ISG15	CD14	ITGB1	4,45830E-04	8662,3	9,11426E-03	11385	6,18925E-01	4949	1,90820E+00	2518	9,10543E-01	6929	0	17530,5
Non-responder	Macro_FOLR2+APOE+	Macro_ISG15	GRN	TNFRSF1A	4,46382E-04	8541,1	7,97319E-03	14210	6,24425E-01	4843	1,93784E+00	2368	9,26148E-01	3754	0	17530,5
Non-responder	Macro_FOLR2+APOE+	Mono_CD14	HLA-A	LILRA1	4,46417E-04	9358,1	9,11272E-03	11388	6,62277E-01	4190	1,73381E+00	3521	8,96316E-01	10161	0	17530,5
Non-responder	Macro_FOLR2+APOE+	Macro_ISG15	HGF	SDC2	4,48567E-04	17422,1	3,80525E-02	1044	6,24117E-01	4849	1,60050E+00	4589	6,76517E-01	59098	0	17530,5
Non-responder	Macro_NLRP3	Mono_CD16	ICAM1	ITGAX_ITGB2	4,50746E-04	8545,1	9,09767E-03	11410	6,64266E-01	4154	1,75146E+00	3407	9,13658E-01	6224	0	17530,5
Non-responder	Mono_CD14	Macro_IER3	S100A8	CD68	4,51127E-04	5092,1	1,52708E-02	4856	1,04938E+00	976	2,01232E+00	2041	9,55060E-01	57	0	17530,5
Non-responder	pDC_LILRA4	cDC(CD1C)	LTB	TNFRSF1A	4,52461E-04	6277,5	2,99471E-02	1548	1,18399E+00	490	3,17495E+00	175	8,90405E-01	11644	0	17530,5
Non-responder	Mono_CD14	Mast	VCAN	CD44	4,52962E-04	5588,3	2,09917E-02	2814	1,22936E+00	372	1,56941E+00	4861	9,33151E-01	2364	0	17530,5
Non-responder	Mast	cDC(CD1C)	CSF1	CSF2RA	4,53382E-04	6647,1	8,10352E-02	222	1,36649E+00	190	2,03714E+00	1940	8,83949E-01	13353	0	17530,5
Non-responder	Macro_NLRP3	cDC_LAMP3	CCL20	CCR6	4,53697E-04	17775,9	6,24137E-02	416	7,22735E-01	3340	1,63832E+00	4281	6,44202E-01	63312	0	17530,5
Non-responder	Macro_NLRP3	Macro_NLRP3	S100A9	CD36	4,53717E-04	8319,9	1,30369E-02	6370	8,31813E-01	2267	1,67176E+00	4007	8,91279E-01	11425	0	17530,5
Non-responder	Macro_FOLR2+APOE+	Macro_ISG15	TIMP2	CD44	4,53915E-04	9914,7	9,89083E-03	10026	5,38969E-01	6793	1,69828E+00	3798	8,91277E-01	11426	0	17530,5
Non-responder	Macro_NLRP3	Macro_IFI27	S100A9	CD68	4,54512E-04	7547,1	1,22490E-02	7103	9,20873E-01	1607	1,08495E+00	11429	9,54691E-01	66	0	17530,5
Non-responder	Macro_FOLR2+APOE+	Macro_LYVE1	APOE	SORL1	4,54512E-04	10653,5	9,95902E-03	9912	4,11871E-01	11429	1,30303E+00	7935	9,12680E-01	6461	0	17530,5
Non-responder	Macro_FOLR2+APOE+	Macro_FOLR2+APOE+	C3	ITGAX	4,54909E-04	9669,9	1,23662E-02	6978	4,74705E-01	8886	1,73351E+00	3524	8,91260E-01	11431	0	17530,5
Non-responder	Macro_NLRP3	cDC_LAMP3	THBS1	SDC4	4,55540E-04	11320,9	2,26660E-02	2434	8,91334E-01	1786	1,64766E+00	4215	8,13524E-01	30639	0	17530,5
Non-responder	Macro_FOLR2+APOE+	Macro_FOLR2+APOE+	CCL5	CCR1	4,56279E-04	15080,3	2,46601E-02	2132	6,22776E-01	4870	1,88445E+00	2637	7,37292E-01	48232	0	17530,5
Non-responder	pDC_LILRA4	cDC(CD1C)	SPON2	ITGAM	4,56812E-04	12368,3	2,99312E-02	1553	9,81957E-01	1292	2,24903E+00	1236	7,73087E-01	40230	0	17530,5
Non-responder	Macro_NLRP3	cDC_LAMP3	NAMPT	ADORA2A	4,57018E-04	7504,3	4,79898E-02	703	1,27804E+00	292	2,05430E+00	1872	8,69520E-01	17124	0	17530,5
Non-responder	pDC_LILRA4	Macro_FOLR2+APOE+	APP	APLP2	4,57758E-04	4975,7	2,50187E-02	2082	1,31745E+00	241	3,25529E+00	151	9,20312E-01	4874	0	17530,5
Non-responder	pDC_LILRA4	Macro_ISG15	PROC	THBD	4,58560E-04	12021,7	5,58041E-02	535	9,30243E-01	1555	2,73008E+00	481	7,73939E-01	40007	0	17530,5
Non-responder	Macro_FOLR2+APOE+	Macro_NLRP3	HMGB1	THBD	4,59103E-04	10351,9	9,07627E-03	11452	4,78668E-01	8756	1,73650E+00	3505	8,94931E-01	10516	0	17530,5
Non-responder	Macro_NLRP3	cDC_LAMP3	GNAI2	S1PR1	4,59242E-04	8304,1	4,85356E-02	688	1,25302E+00	332	1,62788E+00	4362	8,63244E-01	18608	0	17530,5
Non-responder	Mono_CD14	Macro_FOLR2-APOE+	S100A8	ITGB2	4,59613E-04	5399,7	1,70505E-02	4020	1,18551E+00	483	1,56638E+00	4879	9,53894E-01	86	0	17530,5
Non-responder	Macro_FOLR2+APOE+	Macro_LYVE1	LGALS3	ENG	4,59705E-04	9921,1	9,07512E-03	11455	5,44669E-01	6637	1,38388E+00	6840	9,09498E-01	7143	0	17530,5
Non-responder	Macro_FOLR2+APOE+	Macro_IFI27	C1QB	CD33	4,60307E-04	9802,1	9,07323E-03	11458	6,22967E-01	4867	1,49531E+00	5577	8,98678E-01	9578	0	17530,5
Non-responder	Macro_FOLR2+APOE+	Mono_CD14	FABP5	RXRA	4,60729E-04	10534,1	1,66973E-02	4151	6,22145E-01	4882	1,72968E+00	3556	8,46646E-01	22551	0	17530,5
Non-responder	Macro_FOLR2+APOE+	Macro_OLFML3	CCL4	CCR5	4,63714E-04	11542,9	1,78241E-02	3727	6,21624E-01	4890	1,81264E+00	3040	8,22257E-01	28527	0	17530,5
Non-responder	pDC_LILRA4	pDC_LILRA4	APP	NOTCH2	4,65680E-04	6293,7	2,76566E-02	1757	1,33003E+00	225	3,50759E+00	92	8,89554E-01	11864	0	17530,5
Non-responder	Mono_CD16	cDC_LAMP3	TNFSF13B	CD40	4,65959E-04	8594,7	1,16153E-02	7730	7,86156E-01	2674	1,73003E+00	3553	8,91083E-01	11486	0	17530,5
Non-responder	Macro_OLFML3	pDC_LILRA4	ICAM1	IL2RG	4,67177E-04	8349,1	1,07336E-02	8838	7,65621E-01	2863	2,35176E+00	1022	8,91046E-01	11492	0	17530,5
Non-responder	Mono_CD16	Macro_FOLR2+APOE+	S100A9	CD68	4,67991E-04	7351,5	9,05604E-03	11496	7,50642E-01	3018	1,63122E+00	4334	9,47692E-01	379	0	17530,5
Non-responder	Macro_FOLR2+APOE+	Mono_CD16	HLA-F	LILRB1	4,68398E-04	8985,9	1,05401E-02	9086	6,70467E-01	4057	1,86270E+00	2758	8,91011E-01	11498	0	17530,5
Non-responder	Macro_NLRP3	Macro_ISG15	ICAM1	ITGAX_ITGB2	4,68602E-04	8387,7	9,05486E-03	11499	6,74975E-01	3978	1,88080E+00	2664	9,13472E-01	6267	0	17530,5
Non-responder	Macro_FOLR2+APOE+	Macro_ISG15	A2M	LRP1	4,69214E-04	9858,7	1,14695E-02	7906	4,93619E-01	8212	1,65617E+00	4143	8,90995E-01	11502	0	17530,5
Non-responder	Mono_CD14	Macro_LYVE1	S100A8	ITGB2	4,70482E-04	5105,7	1,51646E-02	4908	1,08285E+00	841	2,00075E+00	2088	9,51247E-01	161	0	17530,5
Non-responder	Macro_FOLR2+APOE+	cDC_CLEC9A	ENTPD1	ADORA2B	4,73133E-04	13589,3	4,12085E-02	922	8,11941E-01	2434	1,56275E+00	4915	7,64714E-01	42145	0	17530,5
Non-responder	Mono_CD14	Macro_IFI27	S100A8	ITGB2	4,73893E-04	5453,5	1,51536E-02	4917	1,08225E+00	845	1,69501E+00	3813	9,51230E-01	162	0	17530,5
Non-responder	Macro_FOLR2+APOE+	Macro_LYVE1	C3	LRP1	4,76603E-04	11430,1	1,21763E-02	7164	4,09584E-01	11538	1,19825E+00	9495	8,91291E-01	11423	0	17530,5
Non-responder	Macro_NLRP3	Macro_OLFML3	THBS1	PTPRJ	4,76941E-04	12777,7	1,51416E-02	4925	7,88191E-01	2659	1,68807E+00	3861	7,95545E-01	34913	0	17530,5
Non-responder	Macro_FOLR2+APOE+	Mast	APOE	ABCA1	4,77843E-04	10953,9	9,03618E-03	11544	4,44716E-01	10031	1,28603E+00	8181	9,07916E-01	7483	0	17530,5
Non-responder	Macro_NLRP3	Macro_NLRP3	PKM	CD44	4,78088E-04	8883,3	7,46721E-03	15787	6,49770E-01	4400	1,56118E+00	4928	9,35964E-01	1771	0	17530,5
Non-responder	Macro_IFI27	pDC_LILRA4	C1QB	C1QBP	4,78257E-04	6145,7	1,53388E-02	4820	8,41987E-01	2159	1,55400E+00	4996	9,38997E-01	1223	0	17530,5
Non-responder	Macro_NLRP3	cDC(CD1C)	IL1B	IL1R1_IL1RAP	4,79236E-04	10921,9	1,62954E-02	4340	9,70108E-01	1348	1,86493E+00	2745	8,21834E-01	28646	0	17530,5
Non-responder	Macro_NLRP3	cDC(CD1C)	IL1B	SIGIRR	4,80003E-04	7687,7	1,54238E-02	4769	9,84063E-01	1282	1,87627E+00	2687	8,88390E-01	12170	0	17530,5
Non-responder	Macro_OLFML3	Macro_FOLR2+APOE+	LGALS9	CD47	4,80124E-04	10270,1	9,03328E-03	11555	5,63617E-01	6138	1,55636E+00	4971	8,92361E-01	11156	0	17530,5
Non-responder	Macro_FOLR2+APOE+	cDC_CLEC9A	PLAU	ITGB1	4,81540E-04	10563,3	1,57942E-02	4595	6,19296E-01	4937	1,87498E+00	2692	8,44510E-01	23062	0	17530,5
Non-responder	pDC_LILRA4	Macro_OLFML3	SEMA7A	PLXNC1	4,81689E-04	11771,9	3,41822E-02	1249	9,24736E-01	1581	2,19864E+00	1383	7,86026E-01	37116	0	17530,5
Non-responder	Macro_LYVE1	pDC_LILRA4	CD14	TLR9	4,82332E-04	5774,7	9,59268E-02	153	1,32649E+00	229	2,07526E+00	1791	9,00516E-01	9170	0	17530,5
Non-responder	Macro_FOLR2+APOE+	Mono_CD16	C1QA	CR1	4,82695E-04	11109,1	1,51217E-02	4940	7,13436E-01	3466	2,02106E+00	2002	8,25924E-01	27607	0	17530,5
Non-responder	Mono_CD14	pDC_LILRA4	ADM	CALCRL	4,83500E-04	10989,1	2,96020E-02	1583	9,36503E-01	1521	2,89168E+00	347	7,99667E-01	33964	0	17530,5
Non-responder	pDC_LILRA4	Macro_ISG15	HLA-B	LILRB2	4,83500E-04	6776,3	8,03025E-03	14046	9,24572E-01	1583	2,69829E+00	506	9,50271E-01	216	0	17530,5
Non-responder	Macro_FOLR2+APOE+	Mono_CD16	ICAM1	SPN	4,85010E-04	9686,7	1,51036E-02	4946	6,88961E-01	3762	1,88129E+00	2661	8,59327E-01	19534	0	17530,5
Non-responder	Macro_FOLR2+APOE+	Macro_FOLR2+APOE+	HLA-DQA1	LAG3	4,86559E-04	8736,9	1,50923E-02	4950	8,66880E-01	1947	2,45898E+00	830	8,64042E-01	18427	0	17530,5
Non-responder	Macro_FOLR2+APOE+	Macro_OLFML3	RARRES1	NRP2	4,88889E-04	16348,3	3,21160E-02	1382	8,26988E-01	2306	1,55765E+00	4956	6,98587E-01	55567	0	17530,5
Non-responder	Macro_OLFML3	Mono_CD16	HLA-C	LILRB2	4,89124E-04	6769,1	9,01448E-03	11598	8,34586E-01	2235	1,95259E+00	2293	9,50683E-01	189	0	17530,5
Non-responder	Macro_NLRP3	cDC(CD1C)	CCL20	CCR6	4,92008E-04	17303,5	6,99902E-02	327	7,54555E-01	2976	1,67206E+00	4003	6,57221E-01	61681	0	17530,5

Post_NR_Myeloid_Myeloid_CellCell

Non-responder	Macro_NLRP3	Mono_CD16	VCAN	ITGB1	4,92008E-04	7714,3	1,50694E-02	4964	8,90042E-01	1792	1,92232E+00	2450	8,89648E-01	11835	0	17530,5
Non-responder	Macro_FOLR2+APOE+	cDC_CLEC9A	CD14	ITGA4	4,92932E-04	8689,5	9,00562E-03	11616	6,34692E-01	4634	1,89889E+00	2556	9,09689E-01	7111	0	17530,5
Non-responder	Macro_NLRP3	Macro_NLRP3	VIM	CD44	4,93573E-04	8337,9	7,49536E-03	15694	7,17358E-01	3417	1,55669E+00	4968	9,54017E-01	80	0	17530,5
Non-responder	Macro_FOLR2+APOE+	Macro_IER3	SERPING1	LRP1	4,94844E-04	8607,3	1,20040E-02	7335	7,69098E-01	2826	1,70936E+00	3720	8,90482E-01	11625	0	17530,5
Non-responder	Macro_IER3	cDC_LAMP3	IL10	CD70	4,95012E-04	13664,9	1,60694E-01	45	1,32482E+00	232	1,96126E+00	2251	7,37163E-01	48266	0	17530,5
Non-responder	Macro_OLFML3	Macro_FOLR2+APOE+	SERPINA1	LRP1	4,95909E-04	9654,9	9,00059E-03	11630	5,30397E-01	7036	1,39240E+00	6741	9,18088E-01	5337	0	17530,5
Non-responder	pDC_LILRA4	Mono_CD16	APP	CD74	4,95927E-04	5168,5	1,50563E-02	4974	8,67004E-01	1944	2,21008E+00	1344	9,55416E-01	50	0	17530,5
Non-responder	Macro_NLRP3	Macro_FOLR2+APOE-	VCAN	TLR2	4,97404E-04	8511,5	1,77529E-02	3756	9,14433E-01	1643	1,29899E+00	7991	8,90430E-01	11637	0	17530,5
Non-responder	pDC_LILRA4	Macro_NLRP3	APP	RPSA	4,97896E-04	5306,1	1,91505E-02	3291	1,28084E+00	286	2,76707E+00	444	9,19823E-01	4979	0	17530,5
Non-responder	Macro_NLRP3	Macro_NLRP3	TNF	TNFRSF1B	4,98045E-04	7861,3	1,39576E-02	5680	8,10576E-01	2450	2,01997E+00	2006	8,90418E-01	11640	0	17530,5
Non-responder	Macro_FOLR2+APOE+	Macro_NLRP3	HSPA1A	TLR4	4,99544E-04	10648,3	8,99206E-03	11647	4,70802E-01	9038	1,68801E+00	3863	8,92318E-01	11163	0	17530,5
Non-responder	Macro_FOLR2+APOE+	Macro_LYVE1	SERPINA1	LRP1	5,01047E-04	10156,5	8,98824E-03	11654	5,41474E-01	6723	1,19614E+00	9525	9,18036E-01	5350	0	17530,5
Non-responder	Macro_ISG15	Macro_ISG15	CXCL10	SDC4	5,02779E-04	10231,5	2,94233E-02	1604	1,02193E+00	1107	2,75363E+00	453	8,14248E-01	30463	0	17530,5
Non-responder	Macro_FOLR2+APOE+	Macro_ISG15	LGALS9	PTPRC	5,04234E-04	8827,3	8,00477E-03	14130	6,38878E-01	4571	1,83565E+00	2910	9,19723E-01	4995	0	17530,5
Non-responder	Mono_CD16	Macro_IER3	CD52	SIGLEC10	5,05578E-04	8927,3	1,14473E-02	7928	7,86789E-01	2670	1,57200E+00	4833	8,90293E-01	11675	0	17530,5
Non-responder	Macro_FOLR2+APOE+	pDC_LILRA4	HMGB1	THBD	5,05578E-04	10057,3	8,97807E-03	11675	4,71993E-01	8983	2,17020E+00	1463	8,94418E-01	10635	0	17530,5
Non-responder	Mono_CD14	Macro_LYVE1	S100A12	CD36	5,06508E-04	9323,3	2,93717E-02	1608	1,20604E+00	422	2,23567E+00	1279	8,33170E-01	25777	0	17530,5
Non-responder	Macro_FOLR2+APOE+	Mono_INHBA	ADAM10	CD44	5,07096E-04	9784,5	1,14138E-02	7966	6,82331E-01	3873	1,30846E+00	7871	8,90271E-01	11682	0	17530,5
Non-responder	Macro_FOLR2+APOE+	Macro_ISG15	LGALS9	CD44	5,07825E-04	8939,3	8,12219E-03	13779	6,15982E-01	5004	1,74796E+00	3434	9,19957E-01	4949	0	17530,5
Non-responder	Mono_CD14	Mono_CD16	S100A12	CD36	5,08379E-04	9205,5	2,93506E-02	1610	1,20590E+00	423	2,56748E+00	670	8,33120E-01	25794	0	17530,5
Non-responder	Macro_FOLR2+APOE+	Macro_FOLR2-APOE+	APOC2	LRP1	5,09026E-04	10441,3	2,68697E-02	1842	6,94859E-01	3681	1,55306E+00	5007	8,40083E-01	24146	0	17530,5
Non-responder	pDC_LILRA4	Macro_FOLR2+APOE+	PROC	PROCR	5,12136E-04	16222,3	9,92042E-02	142	9,19651E-01	1614	2,26340E+00	1203	6,65438E-01	60622	0	17530,5
Non-responder	Macro_NLRP3	Macro_LYVE1	ICAM1	IL2RA	5,12240E-04	10263,5	2,51915E-02	2053	9,02685E-01	1719	1,77338E+00	3268	8,29452E-01	26747	0	17530,5
Non-responder	Macro_FOLR2+APOE+	Macro_OLFML3	CCL3	CCR5	5,13046E-04	11331,5	1,71112E-02	3999	6,15034E-01	5017	1,74372E+00	3458	8,29787E-01	26653	0	17530,5
Non-responder	Mono_INHBA	Macro_ISG15	CCL4	CCR1	5,14020E-04	5736,3	2,93267E-02	1616	1,09560E+00	783	2,62939E+00	584	9,05051E-01	8168	0	17530,5
Non-responder	Macro_FOLR2+APOE+	Macro_FOLR2+APOE+	PLAU	ST14	5,14256E-04	11298,9	1,92111E-02	3277	6,14645E-01	5020	2,04358E+00	1918	8,21346E-01	28749	0	17530,5
Non-responder	Macro_NLRP3	cDC_CLEC9A	ICAM1	ITGAL_ITGB2	5,14518E-04	8899,3	8,96312E-03	11716	6,66200E-01	4119	1,76431E+00	3330	9,06625E-01	7801	0	17530,5
Non-responder	Macro_NLRP3	Macro_LYVE1	THBS1	PTPRJ	5,14660E-04	12547,3	1,63288E-02	4328	8,05460E-01	2493	1,56561E+00	4887	8,01615E-01	33498	0	17530,5
Non-responder	Mono_INHBA	Macro_ISG15	INHBA	ACTR2	5,14965E-04	8255,9	2,93225E-02	1617	9,34597E-01	1533	2,16343E+00	1483	8,61081E-01	19116	0	17530,5
Non-responder	Macro_NLRP3	Macro_LYVE1	THBS1	SCARB1	5,15065E-04	10732,5	2,04454E-02	2957	7,94064E-01	2605	1,57294E+00	4822	8,33287E-01	25748	0	17530,5
Non-responder	Mono_INHBA	Macro_NLRP3	VCAN	CD44	5,15469E-04	6731,5	1,49851E-02	5023	9,27562E-01	1567	1,55730E+00	4960	9,21839E-01	4577	0	17530,5
Non-responder	Mono_CD16	Mast	AGTRAP	RACK1	5,17599E-04	8515,3	1,59443E-02	4510	9,63356E-01	1379	1,07000E+00	11730	9,08149E-01	7427	0	17530,5
Non-responder	Macro_NLRP3	cDC(CD1C)	VCAN	TLR1	5,17901E-04	11226,7	1,49749E-02	5029	8,79902E-01	1856	1,60797E+00	4530	8,27724E-01	27188	0	17530,5
Non-responder	Macro_OLFML3	Macro_FOLR2+APOE+	HLA-DQA2	CD4	5,18483E-04	8983,5	8,94978E-03	11734	5,36613E-01	6856	1,64885E+00	4204	9,21759E-01	4593	0	17530,5
Non-responder	Macro_IER3	Macro_ISG15	CCL4	CCR1	5,19703E-04	5869,1	2,92822E-02	1622	1,09438E+00	790	2,25762E+00	1220	9,04985E-01	8183	0	17530,5
Non-responder	Macro_IFI27	cDC_CLEC9A	C1QB	C1QBP	5,20474E-04	7300,5	1,51723E-02	4903	8,32764E-01	2252	1,13387E+00	10557	9,38684E-01	1260	0	17530,5
Non-responder	Macro_NLRP3	Mono_CD14	THBS1	LRP1	5,21139E-04	7958,1	1,68054E-02	4107	7,87136E-01	2668	1,70745E+00	3739	8,90007E-01	11746	0	17530,5
Non-responder	Macro_FOLR2+APOE+	Macro_ISG15	TNFSF13	TNFRSF1A	5,21361E-04	10495,7	8,94492E-03	11747	4,83157E-01	8581	1,76669E+00	3316	8,91788E-01	11304	0	17530,5
Non-responder	pDC_LILRA4	Macro_FOLR2-APOE+	APP	LRP1	5,21606E-04	5518,1	2,92579E-02	1624	1,32047E+00	236	2,38356E+00	957	9,09054E-01	7243	0	17530,5
Non-responder	Macro_FOLR2+APOE+	Macro_OLFML3	C1QA	CD93	5,24252E-04	8956,9	8,93588E-03	11760	6,40612E-01	4537	1,97603E+00	2186	9,02352E-01	8771	0	17530,5
Non-responder	Macro_FOLR2+APOE+	Macro_FOLR2+APOE+	HLA-DRB5	CD4	5,25246E-04	7787,3	8,18940E-03	13585	6,12537E-01	5047	1,95198E+00	2300	9,46269E-01	474	0	17530,5
Non-responder	Macro_NLRP3	cDC_LAMP3	ICAM1	IL2RA	5,25657E-04	12492,3	1,77548E-02	3755	7,49670E-01	3028	1,54947E+00	5048	8,03264E-01	33100	0	17530,5
Non-responder	Mono_CD16	Mono_CD14	AGTRAP	RACK1	5,26038E-04	10074,1	1,06537E-02	8940	6,47666E-01	4423	1,31962E+00	7709	8,89893E-01	11768	0	17530,5
Non-responder	Macro_NLRP3	cDC_CLEC9A	LYZ	ITGAL	5,26933E-04	10620,9	8,93042E-03	11772	5,28981E-01	7068	1,30534E+00	7912	9,02070E-01	8822	0	17530,5
Non-responder	Macro_LYVE1	pDC_LILRA4	CXCL12	CD4	5,27343E-04	8312,7	2,96460E-02	1581	9,17408E-01	1630	2,23808E+00	1265	8,59239E-01	19557	0	17530,5
Non-responder	Macro_FOLR2+APOE+	Macro_IFI27	MMP9	LRP1	5,28948E-04	11394,5	1,65749E-02	4208	6,11917E-01	5056	1,69167E+00	3841	8,31039E-01	26337	0	17530,5
Non-responder	Macro_NLRP3	Macro_IER3	VCAN	ITGA4	5,28948E-04	8248,9	1,49190E-02	5056	9,10107E-01	1665	1,56224E+00	4920	8,88717E-01	12073	0	17530,5
Non-responder	Macro_NLRP3	Macro_LYVE1	CXCL8	SDC3	5,30600E-04	7827,5	3,42785E-02	1246	1,07447E+00	880	2,10527E+00	1675	8,66660E-01	17806	0	17530,5
Non-responder	Macro_NLRP3	pDC_LILRA4	S100A8	CD69	5,30973E-04	8443,1	1,14185E-02	7955	7,98228E-01	2573	1,93853E+00	2367	8,89811E-01	11790	0	17530,5
Non-responder	Macro_NLRP3	cDC(CD1C)	S100A9	CD68	5,30973E-04	9113,3	9,11885E-03	11376	6,42671E-01	4506	1,06664E+00	11790	9,47863E-01	364	0	17530,5
Non-responder	pDC_LILRA4	cDC_CLEC9A	TNFSF9	HLA-DPA1	5,31841E-04	5286,1	2,54616E-02	2024	9,49051E-01	1448	2,86631E+00	365	9,19414E-01	5063	0	17530,5
Non-responder	Macro_FOLR2+APOE+	pDC_LILRA4	FN1	DPP4	5,32669E-04	16237,3	4,98969E-02	657	6,11648E-01	5065	1,74038E+00	3478	7,05075E-01	54456	0	17530,5
Non-responder	Macro_FOLR2+APOE+	Macro_NLRP3	HSPA8	LDLR	5,34329E-04	10259,3	1,49617E-02	5038	6,11441E-01	5069	1,61062E+00	4504	8,60926E-01	19155	0	17530,5
Non-responder	Macro_NLRP3	Macro_LYVE1	VEGFA	NRP1	5,35161E-04	8606,7	3,16803E-02	1405	1,04779E+00	985	2,04616E+00	1908	8,52260E-01	21205	0	17530,5
Non-responder	Macro_ISG15	Mono_CD16	LYZ	ITGAL	5,35161E-04	6267,5	1,49006E-02	5071	7,74299E-01	2777	2,16024E+00	1493	9,22471E-01	4466	0	17530,5
Non-responder	Macro_NLRP3	Macro_LYVE1	VEGFA	NRP2	5,35577E-04	10795,5	2,11720E-02	2772	9,28406E-01	1561	1,65347E+00	4167	8,24657E-01	27947	0	17530,5
Non-responder	Macro_NLRP3	cDC(CD1C)	VCAN	ITGA4	5,35577E-04	8284,5	1,49004E-02	5072	9,09608E-01	1670	1,54826E+00	5062	8,88655E-01	12088	0	17530,5
Non-responder	Macro_NLRP3	Macro_LYVE1	VEGFA	ITGAV	5,36410E-04	10978,3	2,30772E-02	2356	9,06118E-01	1695	1,62554E+00	4376	8,20507E-01	28934	0	17530,5
Non-responder	Macro_NLRP3	Macro_LYVE1	VEGFA	EPHB2	5,36826E-04	14319,3	3,06984E-02	1474	8,97443E-01	1745	1,62973E+00	4341	7,45149E-01	46506	0	17530,5
Non-responder	Macro_NLRP3	Macro_LYVE1	VEGFA	ITGA9	5,37661E-04	14558,5	4,84985E-02	691	1,00712E+00	1178	1,68825E+00	3857	7,31020E-01	49536	0	17530,5
Non-responder	Macro_FOLR2+APOE+	Mono_INHBA	SERPING1	LRP1	5,39586E-04	8656,9	1,18041E-02	7532	7,62122E-01	2897	1,73777E+00	3497	8,89660E-01	11828	0	17530,5
Non-responder	Macro_OLFML3	Macro_FOLR2+APOE+	C3	C3AR1	5,40270E-04	10828,5	1,17206E-02	7632	4,26693E-01	10760	1,42179E+00	6389	8,89652E-01	11831	0	17530,5
Non-responder	Macro_FOLR2+APOE+	Mono_INHBA	CCL5	SDC4	5,41008E-04	17491,5	2,76089E-02	1761	6,10410E-01	5085	1,61864E+00	4438	6,79331E-01	58643	0	17530,5
Non-responder	Macro_OLFML3	cDC_CLEC9A	HLA-DPB1	LAG3	5,41413E-04	7872,3	1,70824E-02	4012	8,46550E-01	2113	1,68771E+00	3870	8,89645E-01	11836	0	17530,5
Non-responder	Mono_CD14	Mono_INHBA	S100A8	CD68	5,41847E-04	5115,7	1,48706E-02	5087	1,02563E+00	1088	2,07333E+00	1803	9,54486E-01	70	0	17530,5
Non-responder	Macro_FOLR2+APOE+	Macro_FOLR2+APOE+	GAS6	MERTK	5,42687E-04	12499,3	1,62456E-02	4365	6,10286E-01	5089	1,71049E+00	3707	8,08700E-01	31805	0	17530,5
Non-responder	Macro_NLRP3	Macro_FOLR2+APOE+	SERPINA1	LRP1	5,42787E-04	9924,5	8,89389E-03	11842	5,18763E-01	7369	1,33742E+00	7459	9,17638E-01	5422	0	17530,5
Non-responder	pDC_LILRA4	Macro_FOLR2+APOE+	HSP90B1	LRP1	5,43528E-04	5381,7	1,48635E-02	5091	1,08657E+00	825	3,09735E+00	211	9,28731E-01	3251	0	17530,5
Non-responder	Macro_FOLR2+APOE+	pDC_LILRA4	HLA-DMA	CD4	5,43528E-04	7768,5	8,40995E-03	12995	6,10152E-01	5091	2,39545E+00	936	9,33477E-01	2290	0	17530,5
Non-responder	Macro_FOLR2+APOE+	cDC_CLEC9A	LGALS9	MRC2	5,43949E-04	12107,9	1,87056E-02	3431	6,58684E-01	4241	1,54486E+00	5092	8,15227E-01	30245	0	17530,5
Non-responder	Macro_FOLR2+APOE+	Mono_INHBA	B2M	TFRC	5,43949E-04	9851,1	6,45620E-03	19998	6,10146E-01	5092	1,67374E+00	3987	9,31525E-01	2648	0	17530,5

Post_NR_Myeloid_Myeloid_CellCell

Non-responder	Macro_NLRP3	Macro_NLRP3	S100A8	CD36	5,46003E-04	8282,3	1,54287E-02	4767	8,59285E-01	2010	1,53063E+00	5248	8,89575E-01	11856	0	17530,5
Non-responder	Mono_CD14	Macro_OLFML3	VCAN	TLR1	5,46767E-04	7624,7	2,90184E-02	1650	1,19272E+00	455	2,16063E+00	1491	8,69932E-01	16997	0	17530,5
Non-responder	Macro_IFI27	Macro_ISG15	HLA-DRA	LAG3	5,46924E-04	8031,7	2,01027E-02	3036	1,01575E+00	1134	1,12904E+00	10654	9,06617E-01	7804	0	17530,5
Non-responder	Macro_IFI27	Macro_ISG15	HLA-DRB1	LAG3	5,48309E-04	8297,5	2,00649E-02	3048	1,01184E+00	1153	1,10900E+00	11003	9,02432E-01	8753	0	17530,5
Non-responder	pDC_LILRA4	Mast	PROC	PROCR	5,48735E-04	15081,5	1,38011E-01	64	1,03509E+00	1046	2,11212E+00	1652	7,01133E-01	55115	0	17530,5
Non-responder	Mono_INHBA	Macro_NLRP3	IL1B	IL1R1_IL1RAP	5,48735E-04	8311,9	2,89912E-02	1652	9,49612E-01	1444	2,12419E+00	1608	8,60191E-01	19325	0	17530,5
Non-responder	Macro_OLFML3	Macro_FOLR2+APOE+	C1QB	LRP1	5,49465E-04	10180,7	9,45122E-03	10765	4,02504E-01	11871	1,43759E+00	6185	9,21977E-01	4552	0	17530,5
Non-responder	Macro_FOLR2+APOE+	cDC_LAMP3	THBS1	TNFRSF11B	5,49720E-04	14513,1	5,18211E-02	616	1,04242E+00	1011	1,43322E+00	6239	7,42038E-01	47169	0	17530,5
Non-responder	Macro_OLFML3	Macro_ISG15	HLA-DQB1	LAG3	5,50392E-04	6972,1	2,23440E-02	2500	1,05406E+00	956	2,02184E+00	1999	8,89516E-01	11875	0	17530,5
Non-responder	Macro_IFI27	Macro_ISG15	HLA-DPB1	LAG3	5,50392E-04	8285,1	2,10746E-02	2796	1,02439E+00	1092	1,13019E+00	10630	8,99540E-01	9377	0	17530,5
Non-responder	Macro_ISG15	cDC_CLEC9A	ADAM10	CADM1	5,51695E-04	9092,1	3,92662E-02	993	1,18330E+00	492	2,11130E+00	1655	8,37397E-01	24790	0	17530,5
Non-responder	pDC_LILRA4	Macro_IFI27	SPON2	ITGB1	5,53675E-04	10281,5	2,89477E-02	1657	9,92862E-01	1234	2,18967E+00	1406	8,17852E-01	29580	0	17530,5
Non-responder	Macro_IFI27	Macro_ISG15	HLA-DPA1	LAG3	5,54343E-04	7787,1	2,12672E-02	2745	1,04307E+00	1006	1,20667E+00	9338	9,04444E-01	8316	0	17530,5
Non-responder	Macro_NLRP3	cDC_LAMP3	THBS1	ITGA4	5,54551E-04	8791,9	1,48209E-02	5117	8,08332E-01	2471	1,55298E+00	5008	8,82144E-01	13833	0	17530,5
Non-responder	Macro_NLRP3	cDC_CLEC9A	ICAM1	IL2RG	5,55042E-04	9131,3	1,03863E-02	9297	6,97758E-01	3655	1,77104E+00	3279	8,89439E-01	11895	0	17530,5
Non-responder	Macro_NLRP3	cDC(CD1C)	PTGS2	ALOX5	5,55276E-04	8841,3	1,77743E-02	3748	7,65216E-01	2870	1,28727E+00	8162	8,89434E-01	11896	0	17530,5
Non-responder	Macro_FOLR2+APOE+	Mono_CD14	B2M	LILRB2	5,55405E-04	10981,1	5,30380E-03	27007	6,09188E-01	5119	1,58495E+00	4733	9,45746E-01	516	0	17530,5
Non-responder	Macro_NLRP3	Mono_CD16	VEGFA	ITGB1	5,56689E-04	9473,1	1,48131E-02	5122	8,12488E-01	2427	1,86137E+00	2767	8,59386E-01	19519	0	17530,5
Non-responder	Macro_IFI27	Macro_ISG15	HLA-DRB5	LAG3	5,57613E-04	8695,7	2,05125E-02	2941	9,82706E-01	1289	1,08162E+00	11493	8,96063E-01	10225	0	17530,5
Non-responder	Macro_FOLR2+APOE+	Macro_FOLR2+APOE-	MMP9	ITGAM	5,58404E-04	13569,7	1,67053E-02	4146	6,09033E-01	5126	1,72191E+00	3624	7,84743E-01	37422	0	17530,5
Non-responder	Macro_NLRP3	Macro_NLRP3	CD55	ADGRE2	5,60124E-04	9394,3	1,47895E-02	5130	7,71583E-01	2804	1,72668E+00	3578	8,66158E-01	17929	0	17530,5
Non-responder	cDC_CLEC9A	Mono_CD16	HLA-DOB	CD4	5,60640E-04	7722,5	3,20837E-02	1385	1,28697E+00	278	2,10912E+00	1664	8,66844E-01	17755	0	17530,5
Non-responder	pDC_LILRA4	Mast	COPA	NTRK1	5,60862E-04	9727,5	1,34775E-01	69	1,31264E+00	247	2,10239E+00	1687	8,19868E-01	29104	0	17530,5
Non-responder	Mono_INHBA	cDC_CLEC9A	IL6	IL6R_IL6ST	5,61640E-04	11462,7	3,39340E-02	1260	1,14667E+00	591	2,10878E+00	1665	7,89546E-01	36267	0	17530,5
Non-responder	Mono_CD14	pDC_LILRA4	BST2	LILRA4	5,62641E-04	5856,7	2,88399E-02	1666	1,02435E+00	1093	3,09971E+00	208	9,02255E-01	8786	0	17530,5
Non-responder	Macro_FOLR2+APOE+	Mono_CD16	MMP9	LRP1	5,64006E-04	10910,3	1,64284E-02	4293	6,08469E-01	5139	2,31048E+00	1103	8,30415E-01	26486	0	17530,5
Non-responder	Macro_NLRP3	Macro_OLFML3	VEGFA	NRP2	5,64006E-04	12695,3	1,47810E-02	5139	8,20421E-01	2362	1,68495E+00	3888	7,97146E-01	34557	0	17530,5
Non-responder	Macro_FOLR2+APOE+	Macro_FOLR2+APOE+	SEMA4A	LILRB2	5,64439E-04	8164,1	1,47802E-02	5140	8,26987E-01	2307	1,98663E+00	2153	8,82693E-01	13690	0	17530,5
Non-responder	pDC_LILRA4	Macro_NLRP3	LTB	TNFRSF1A	5,66656E-04	6399,5	2,87862E-02	1670	1,16547E+00	540	3,40153E+00	113	8,88461E-01	12144	0	17530,5
Non-responder	Macro_NLRP3	Macro_OLFML3	VEGFA	ITGAV	5,67040E-04	13449,7	1,47668E-02	5146	7,81029E-01	2719	1,60432E+00	4561	7,85254E-01	37292	0	17530,5
Non-responder	Macro_NLRP3	Mono_CD16	TNF	VSIR	5,67474E-04	10004,3	1,47663E-02	5147	7,69790E-01	2822	2,13638E+00	1557	8,44936E-01	22965	0	17530,5
Non-responder	Macro_NLRP3	Macro_FOLR2+APOE+	ICAM1	IL2RG	5,69421E-04	9016,5	1,03381E-02	9356	6,95502E-01	3676	1,89701E+00	2564	8,89210E-01	11956	0	17530,5
Non-responder	Macro_FOLR2+APOE+	Macro_ISG15	LGALS3BP	ITGB1	5,70521E-04	8997,1	1,47602E-02	5154	6,74439E-01	3989	1,90137E+00	2543	8,74694E-01	15769	0	17530,5
Non-responder	Macro_FOLR2+APOE+	Macro_FOLR2+APOE+	CXCL12	SDC4	5,70958E-04	17006,9	2,54459E-02	2026	6,07568E-01	5155	1,82415E+00	2974	6,87719E-01	57349	0	17530,5
Non-responder	Macro_ISG15	Macro_FOLR2+APOE+	CCL8	CCR1	5,71702E-04	12420,9	5,76963E-02	503	9,08672E-01	1675	2,71744E+00	492	7,65756E-01	41904	0	17530,5
Non-responder	pDC_LILRA4	pDC_LILRA4	SPON2	ITGB1	5,71702E-04	10063,7	2,87394E-02	1675	9,90704E-01	1247	3,29475E+00	138	8,17313E-01	29728	0	17530,5
Non-responder	Macro_FOLR2+APOE+	Mono_INHBA	HBEGF	CD44	5,71806E-04	9798,7	8,83158E-03	11966	6,42483E-01	4510	1,36826E+00	7031	9,05940E-01	7956	0	17530,5
Non-responder	Macro_NLRP3	Mono_CD14	ICAM1	ITGAL_ITGB2	5,73002E-04	9256,5	8,83022E-03	11971	6,29022E-01	4744	1,66215E+00	4095	9,05991E-01	7942	0	17530,5
Non-responder	Macro_NLRP3	Mono_INHBA	IL1B	IL1R1_IL1RAP	5,73143E-04	8576,7	2,63466E-02	1910	1,09122E+00	807	2,04202E+00	1922	8,54340E-01	20714	0	17530,5
Non-responder	Macro_NLRP3	Mono_INHBA	IL1B	IL1R2_IL1RAP	5,73581E-04	8380,7	1,99683E-02	3070	1,00888E+00	1167	1,95333E+00	2289	8,66476E-01	17847	0	17530,5
Non-responder	Macro_FOLR2+APOE+	Macro_IER3	MMP9	CD44	5,73581E-04	9056,7	1,47428E-02	5161	6,48634E-01	4411	2,19814E+00	1387	8,70686E-01	16794	0	17530,5
Non-responder	Mono_INHBA	Mono_CD16	INHBA	ACTR2	5,74743E-04	8367,1	2,87166E-02	1678	9,21381E-01	1605	2,12697E+00	1598	8,59828E-01	19424	0	17530,5
Non-responder	Macro_OLFML3	Macro_OLFML3	CXCL11	CCR5	5,75760E-04	14430,9	6,11250E-02	433	9,62455E-01	1385	2,10486E+00	1679	7,22930E-01	51127	0	17530,5
Non-responder	Mono_CD14	pDC_LILRA4	TNF	PTPRS	5,76777E-04	11386,3	2,86754E-02	1680	1,39393E+00	162	2,51535E+00	749	7,87356E-01	36810	0	17530,5
Non-responder	Macro_NLRP3	Mono_INHBA	IL6	F3	5,77092E-04	17332,7	6,70638E-02	358	7,80220E-01	2724	1,67073E+00	4022	6,54571E-01	62029	0	17530,5
Non-responder	Macro_NLRP3	Mono_CD14	IL1B	SIGIRR	5,78769E-04	7436,9	1,56312E-02	4677	9,88392E-01	1258	2,09467E+00	1724	8,89051E-01	11995	0	17530,5
Non-responder	Mono_INHBA	Macro_ISG15	IL6	IL6R_IL6ST	5,78815E-04	11946,5	3,06000E-02	1486	1,11759E+00	702	2,10314E+00	1682	7,80825E-01	38332	0	17530,5
Non-responder	Macro_FOLR2+APOE+	cDC_LAMP3	CXCL10	SDC4	5,78854E-04	14560,1	1,59120E-02	4524	6,06531E-01	5173	1,79276E+00	3156	7,63235E-01	42417	0	17530,5
Non-responder	Macro_OLFML3	Macro_FOLR2+APOE+	LGALS9	LRP1	5,80701E-04	11277,7	8,81765E-03	12003	4,76304E-01	8841	1,39285E+00	6737	8,91895E-01	11277	0	17530,5
Non-responder	Macro_NLRP3	Macro_IER3	THBS1	PTPRJ	5,81062E-04	12998,5	1,46940E-02	5178	7,81679E-01	2713	1,66294E+00	4088	7,93094E-01	35483	0	17530,5
Non-responder	Macro_NLRP3	Mono_INHBA	VCAN	TLR2	5,81504E-04	8790,3	1,48130E-02	5123	8,45922E-01	2119	1,54089E+00	5126	8,81281E-01	14053	0	17530,5
Non-responder	pDC_LILRA4	Macro_IFI27	SPON2	ITGB2	5,82906E-04	6067,3	3,81080E-02	1043	1,08395E+00	836	2,10240E+00	1686	9,00199E-01	9241	0	17530,5
Non-responder	Mono_CD14	Mono_CD16	VCAN	ITGA4	5,82906E-04	5096,3	2,86110E-02	1686	1,33756E+00	209	2,68088E+00	525	9,17077E-01	5531	0	17530,5
Non-responder	Macro_FOLR2+APOE+	Mono_CD16	APOE	LRP1	5,85312E-04	10000,3	9,26538E-03	11090	3,99123E-01	12022	1,61931E+00	4428	9,20029E-01	4931	0	17530,5
Non-responder	Mono_CD14	Macro_LYVE1	S100A9	CD68	5,85941E-04	5151,7	1,46686E-02	5189	1,13620E+00	627	1,93433E+00	2387	9,58434E-01	25	0	17530,5
Non-responder	Macro_NLRP3	Mono_INHBA	VEGFA	SIRPA	5,86831E-04	9925,5	1,73321E-02	3917	8,62769E-01	1975	1,53671E+00	5175	8,53071E-01	21030	0	17530,5
Non-responder	cDC(CD1C)	Macro_ISG15	HLA-DQA2	LAG3	5,87016E-04	7552,3	3,08005E-02	1468	1,21979E+00	392	2,10184E+00	1690	8,71135E-01	16681	0	17530,5
Non-responder	Macro_FOLR2+APOE+	Macro_FOLR2+APOE+	HSPA1A	TLR4	5,87506E-04	11215,1	8,80448E-03	12031	4,61309E-01	9380	1,48333E+00	5716	8,91301E-01	11418	0	17530,5
Non-responder	Macro_OLFML3	Mono_CD16	HLA-B	LILRB2	5,88728E-04	6720,5	8,80324E-03	12036	8,88321E-01	1803	1,99607E+00	2117	9,52398E-01	116	0	17530,5
Non-responder	Mono_CD14	Macro_FOLR2+APOE-	S100A8	ITGB2	5,89061E-04	5721,1	1,46607E-02	5196	1,05542E+00	951	1,58610E+00	4727	9,50457E-01	201	0	17530,5
Non-responder	Macro_FOLR2+APOE+	Macro_FOLR2+APOE-	APOC2	LRP1	5,89508E-04	11419,1	2,28287E-02	2402	6,05556E-01	5197	1,54645E+00	5076	8,28830E-01	26890	0	17530,5
Non-responder	Macro_IER3	Macro_FOLR2+APOE+	TNFSF13	TNFRSF14	5,90197E-04	11721,9	9,08477E-03	11435	5,25822E-01	7155	1,11242E+00	10949	8,90848E-01	11540	0	17530,5
Non-responder	Macro_ISG15	Macro_NLRP3	CXCL10	TLR4	5,91145E-04	7817,7	2,85017E-02	1694	1,05239E+00	966	3,08193E+00	219	8,62944E-01	18679	0	17530,5
Non-responder	Macro_FOLR2+APOE+	Macro_OLFML3	MMP9	LRP1	5,93541E-04	11009,3	1,62911E-02	4343	6,05237E-01	5206	2,21724E+00	1324	8,29823E-01	26643	0	17530,5
Non-responder	Macro_NLRP3	Mono_INHBA	THBS1	SDC4	5,93990E-04	9173,9	3,33676E-02	1294	1,02673E+00	1085	2,00230E+00	2082	8,41100E-01	23878	0	17530,5
Non-responder	pDC_LILRA4	cDC(CD1C)	TNFSF9	HLA-DPA1	5,93990E-04	5485,1	2,49826E-02	2090	9,20589E-01	1609	2,37068E+00	989	9,18708E-01	5207	0	17530,5
Non-responder	cDC_CLEC9A	Mono_CD16	HLA-B	LILRB2	5,94254E-04	6487,9	8,87473E-03	11882	9,06059E-01	1697	2,25806E+00	1217	9,52581E-01	113	0	17530,5
Non-responder	Macro_FOLR2+APOE+	pDC_LILRA4	SEMA4A	PLXNB2	5,94440E-04	11575,3	1,46416E-02	5208	6,06357E-01	5178	1,84274E+00	2863	8,28017E-01	27097	0	17530,5
Non-responder	Macro_OLFML3	Macro_FOLR2+APOE+	LGALS9	HAVCR2	5,95361E-04	10844,1	8,79197E-03	12063	5,14287E-01	7521	1,51052E+00	5444	8,90320E-01	11662	0	17530,5
Non-responder	Macro_NLRP3	Macro_NLRP3	VCAN	ITGB1	5,95790E-04	7966,5	1,46404E-02	5211	8,78359E-01	1864	1,82026E+00	3002	8,88222E-01	12225	0	17530,5
Non-responder	Macro_FOLR2+APOE+	Macro_NLRP3	HLA-C	LILRB2	5,95790E-04	9917,9	6,06488E-03	22008	6,04718E-01	5211	1,69625E+00	3810	9,40518E-01	1030	0	17530,5

Post_NR_Myeloid_Myeloid_CellCell

Non-responder	Macro_FOLR2+APOE+	Macro_NLRP3	CD14	TLR4	5,96102E-04	8502,7	1,19728E-02	7372	6,83313E-01	3848	2,10033E+00	1697	8,88750E-01	12066	0	17530,5
Non-responder	Macro_NLRP3	Macro_IFI27	HBEGF	CD9	5,96240E-04	8540,9	1,72435E-02	3954	8,12455E-01	2428	9,79055E-01	13580	9,18676E-01	5212	0	17530,5
Non-responder	Macro_FOLR2+APOE+	Mono_CD14	HLA-B	LILRB2	5,97142E-04	11004,7	5,40430E-03	26271	6,20577E-01	4911	1,53394E+00	5214	9,40034E-01	1097	0	17530,5
Non-responder	Macro_NLRP3	Mono_INHBA	NAMPT	ADORA2A	5,97594E-04	9460,3	3,06744E-02	1477	1,01432E+00	1142	1,74190E+00	3470	8,41968E-01	23682	0	17530,5
Non-responder	Macro_FOLR2+APOE+	Macro_OLFML3	CD14	ITGB2	5,98045E-04	8325,1	7,82393E-03	14643	6,04478E-01	5216	1,73944E+00	3482	9,42895E-01	754	0	17530,5
Non-responder	Mono_CD14	Mono_INHBA	S100A12	CD36	5,99460E-04	9495,1	2,83357E-02	1702	1,19905E+00	443	2,19918E+00	1382	8,30659E-01	26418	0	17530,5
Non-responder	Macro_NLRP3	Mast	TIMP1	CD63	6,01058E-04	7963,1	8,78437E-03	12086	8,24364E-01	2321	1,32577E+00	7605	9,49336E-01	273	0	17530,5
Non-responder	Macro_IER3	Macro_ISG15	CCL3	CCR1	6,01551E-04	5674,9	2,83178E-02	1704	1,10465E+00	748	2,23387E+00	1285	9,09698E-01	7107	0	17530,5
Non-responder	Macro_FOLR2+APOE+	Macro_IER3	C1QA	CR1	6,02121E-04	11501,5	1,46252E-02	5225	7,06173E-01	3564	1,82462E+00	2971	8,23511E-01	28217	0	17530,5
Non-responder	pDC_LILRA4	cDC_CLEC9A	APP	NOTCH2	6,02331E-04	6530,1	2,59913E-02	1956	1,30262E+00	256	3,07616E+00	225	8,86466E-01	12683	0	17530,5
Non-responder	Mono_CD14	Mono_CD14	THBS1	CD36	6,02598E-04	7203,9	2,83095E-02	1705	9,06967E-01	1693	2,30022E+00	1119	8,81612E-01	13972	0	17530,5
Non-responder	pDC_LILRA4	Macro_OLFML3	APP	RPSA	6,03484E-04	5365,9	1,85363E-02	3490	1,26584E+00	309	3,00370E+00	272	9,18612E-01	5228	0	17530,5
Non-responder	Macro_NLRP3	Macro_IER3	S100A8	CD68	6,04798E-04	7999,1	1,20707E-02	7264	7,66184E-01	2857	1,05257E+00	12101	9,49734E-01	243	0	17530,5
Non-responder	Macro_NLRP3	Macro_NLRP3	VEGFA	SIRPA	6,04850E-04	10462,1	1,46225E-02	5231	7,93978E-01	2608	1,77006E+00	3292	8,42094E-01	23649	0	17530,5
Non-responder	Macro_FOLR2+APOE+	Macro_FOLR2+APOE+	C3	NRP1	6,05761E-04	8656,3	2,13971E-02	2715	6,03900E-01	5233	2,05451E+00	1871	8,74063E-01	15932	0	17530,5
Non-responder	Macro_NLRP3	Macro_NLRP3	THBS1	PTPRJ	6,06674E-04	12966,3	1,46149E-02	5235	7,80528E-01	2723	1,70654E+00	3744	7,92650E-01	35599	0	17530,5
Non-responder	pDC_LILRA4	pDC_LILRA4	MIF	CD74_CXCR4	6,07030E-04	7950,7	6,81764E-03	18301	7,09390E-01	3528	3,01976E+00	257	9,51837E-01	137	0	17530,5
Non-responder	Macro_NLRP3	pDC_LILRA4	APP	TNFRSF21	6,07587E-04	10827,5	1,49232E-02	5054	8,26370E-01	2311	2,13058E+00	1585	8,25737E-01	27657	0	17530,5
Non-responder	Mono_INHBA	Macro_ISG15	CCL3L1	CCR5	6,07852E-04	9888,9	3,24644E-02	1358	9,03792E-01	1710	2,10520E+00	1676	8,27773E-01	27170	0	17530,5
Non-responder	Macro_OLFML3	cDC_CLEC9A	LYZ	ITGAL	6,08304E-04	10492,7	8,77138E-03	12115	5,07823E-01	7737	1,44580E+00	6085	9,01273E-01	8996	0	17530,5
Non-responder	Macro_FOLR2+APOE+	Macro_ISG15	RPS19	C5AR1	6,10793E-04	9337,7	7,47828E-03	15755	6,03398E-01	5244	1,64449E+00	4233	9,25289E-01	3926	0	17530,5
Non-responder	Macro_NLRP3	Macro_FOLR2+APOE+	IL1B	SIGIRR	6,11252E-04	7712,5	1,45899E-02	5245	9,66663E-01	1362	2,14819E+00	1523	8,85605E-01	12902	0	17530,5
Non-responder	Macro_NLRP3	pDC_LILRA4	THBS1	PTPRJ	6,12630E-04	11809,1	1,66912E-02	4157	8,10731E-01	2449	2,06643E+00	1839	8,03355E-01	33070	0	17530,5
Non-responder	Macro_NLRP3	pDC_LILRA4	THBS1	SCARB1	6,13090E-04	9650,5	2,31052E-02	2351	8,29991E-01	2281	1,94603E+00	2329	8,41609E-01	23761	0	17530,5
Non-responder	Macro_FOLR2+APOE+	pDC_LILRA4	C3	NRP1	6,13551E-04	8617,9	2,13586E-02	2723	6,02848E-01	5250	2,11804E+00	1630	8,73963E-01	15956	0	17530,5
Non-responder	Macro_IER3	Macro_FOLR2+APOE+	LILRB4	LAIR1	6,13848E-04	8216,9	9,57800E-03	10540	7,00507E-01	3624	1,75464E+00	3384	9,14779E-01	6006	0	17530,5
Non-responder	Macro_NLRP3	Mono_CD14	ICAM1	ITGAX_ITGB2	6,14100E-04	9047,1	8,76327E-03	12138	6,34337E-01	4644	1,62946E+00	4345	9,12170E-01	6578	0	17530,5
Non-responder	Mono_INHBA	Macro_ISG15	CCL3	CCR1	6,14197E-04	5588,1	2,81442E-02	1716	1,09926E+00	767	2,49282E+00	773	9,09445E-01	7154	0	17530,5
Non-responder	Macro_FOLR2-APOE+	Macro_FOLR2+APOE+	APOE	TREM2	6,16780E-04	5596,1	2,22099E-02	2532	7,98947E-01	2568	1,52964E+00	5257	9,53377E-01	93	0	17530,5
Non-responder	Macro_FOLR2+APOE+	Mono_CD14	HLA-C	LILRA1	6,17142E-04	10270,5	8,75801E-03	12150	5,39494E-01	6776	1,59905E+00	4604	8,95783E-01	10292	0	17530,5
Non-responder	Macro_NLRP3	Macro_FOLR2+APOE+	IL1B	IL1R2_IL1RAP	6,18167E-04	9689,7	1,45643E-02	5260	9,35223E-01	1526	2,09892E+00	1702	8,47143E-01	22430	0	17530,5
Non-responder	Macro_FOLR2+APOE+	Mono_CD16	RPS19	C5AR1	6,20485E-04	9338,7	7,46619E-03	15790	6,02201E-01	5265	1,65321E+00	4172	9,25233E-01	3936	0	17530,5
Non-responder	cDC_LAMP3	Macro_LYVE1	CCL19	CCRL2	6,20585E-04	13096,1	2,80610E-02	1722	1,72450E+00	70	2,33268E+00	1059	7,51534E-01	45099	0	17530,5
Non-responder	Macro_FOLR2+APOE+	pDC_LILRA4	JAG1	NOTCH4	6,20950E-04	19474,5	3,34211E-02	1291	6,02182E-01	5266	1,62250E+00	4398	5,84425E-01	68887	0	17530,5
Non-responder	Macro_NLRP3	pDC_LILRA4	SEMA6B	PLXNA4	6,23275E-04	16702,5	1,08185E-01	108	9,28307E-01	1563	1,86095E+00	2770	6,58354E-01	61541	0	17530,5
Non-responder	Macro_NLRP3	pDC_LILRA4	TNFSF13B	TNFRSF17	6,24208E-04	12740,3	5,07285E-02	640	6,96920E-01	3663	1,73310E+00	3528	7,80787E-01	38340	0	17530,5
Non-responder	Macro_IER3	Macro_FOLR2+APOE+	CCL3	CCR1	6,24286E-04	7257,5	1,94879E-02	3207	8,73176E-01	1889	1,87093E+00	2715	8,93129E-01	10946	0	17530,5
Non-responder	Macro_NLRP3	Mono_INHBA	IL1B	ADRB2	6,25141E-04	10814,9	1,45378E-02	5275	9,37971E-01	1511	1,67555E+00	3972	8,33135E-01	25786	0	17530,5
Non-responder	Mast	Macro_OLFML3	HDC	HRH2	6,26004E-04	9543,3	7,55325E-02	261	1,33237E+00	221	2,06295E+00	1848	8,24986E-01	27856	0	17530,5
Non-responder	pDC_LILRA4	Macro_NLRP3	APP	CD74	6,26075E-04	5390,5	1,45369E-02	5277	8,16344E-01	2398	2,10379E+00	1680	9,54662E-01	67	0	17530,5
Non-responder	Macro_NLRP3	Macro_ISG15	S100A9	TLR4	6,26339E-04	8031,9	1,34429E-02	6037	8,62246E-01	1979	1,92676E+00	2427	8,88319E-01	12186	0	17530,5
Non-responder	Macro_FOLR2+APOE+	cDC_CLEC9A	TNFSF13B	CD40	6,26853E-04	8728,3	1,09786E-02	8512	7,27131E-01	3284	1,99309E+00	2127	8,88317E-01	12188	0	17530,5
Non-responder	Macro_FOLR2+APOE+	Mono_CD16	MMP9	IFNAR1	6,27010E-04	13301,7	1,45340E-02	5279	6,36120E-01	4613	2,30930E+00	1104	7,82399E-01	37982	0	17530,5
Non-responder	Macro_FOLR2+APOE+	Macro_OLFML3	HLA-DMB	CD4	6,27625E-04	8687,5	8,74346E-03	12191	6,10304E-01	5088	1,83625E+00	2903	9,16087E-01	5725	0	17530,5
Non-responder	Macro_FOLR2+APOE+	pDC_LILRA4	TGFB1	CXCR4	6,28398E-04	8566,9	8,74123E-03	12194	6,17230E-01	4979	2,76519E+00	445	9,07076E-01	7686	0	17530,5
Non-responder	Macro_FOLR2+APOE+	cDC_CLEC9A	COPA	P2RY6	6,28415E-04	10493,5	1,66131E-02	4191	6,91482E-01	3726	1,52772E+00	5282	8,50027E-01	21738	0	17530,5
Non-responder	Macro_FOLR2+APOE+	Macro_OLFML3	APOE	LRP1	6,28913E-04	10253,9	9,18795E-03	11253	3,95891E-01	12196	1,52607E+00	5294	9,19719E-01	4996	0	17530,5
Non-responder	cDC_CLEC9A	Macro_NLRP3	VIM	CD44	6,30248E-04	6867,1	8,27109E-03	13359	9,00314E-01	1731	2,10535E+00	1674	9,56130E-01	41	0	17530,5
Non-responder	pDC_LILRA4	Macro_IER3	PROC	THBD	6,33492E-04	12556,7	5,13178E-02	626	8,99453E-01	1734	2,27602E+00	1171	7,66523E-01	41722	0	17530,5
Non-responder	Macro_FOLR2+APOE+	Macro_LYVE1	RARRES1	NRP2	6,33585E-04	14765,1	4,60024E-02	755	9,34973E-01	1529	1,52617E+00	5293	7,35021E-01	48718	0	17530,5
Non-responder	Macro_ISG15	Macro_FOLR2+APOE+	CXCL10	TLR4	6,34575E-04	7922,5	2,79071E-02	1735	1,04290E+00	1009	2,87725E+00	352	8,61693E-01	18986	0	17530,5
Non-responder	Macro_FOLR2-APOE+	cDC_LAMP3	SPP1	S1PR1	6,35601E-04	10493,3	1,02016E-01	130	1,29960E+00	263	1,27842E+00	8292	8,31345E-01	26251	0	17530,5
Non-responder	Mono_CD14	Macro_OLFML3	S100A12	CD36	6,35660E-04	9455,7	2,79062E-02	1736	1,19616E+00	448	2,44507E+00	853	8,29582E-01	26711	0	17530,5
Non-responder	Macro_FOLR2+APOE+	pDC_LILRA4	HLA-DRB1	CD4	6,36892E-04	8145,9	7,23857E-03	16595	6,00230E-01	5300	2,41737E+00	904	9,47259E-01	400	0	17530,5
Non-responder	Macro_NLRP3	pDC_LILRA4	CCL3L1	CCR5	6,37839E-04	12213,9	1,87874E-02	3399	8,59085E-01	2014	2,46119E+00	827	7,85236E-01	37299	0	17530,5
Non-responder	Macro_NLRP3	Macro_IFI27	S100A8	CD68	6,39262E-04	7756,1	1,44963E-02	5305	9,48345E-01	1454	9,43821E-01	14406	9,53929E-01	85	0	17530,5
Non-responder	Macro_NLRP3	pDC_LILRA4	COPA	P2RY6	6,40212E-04	8032,5	2,01505E-02	3018	1,17206E+00	520	3,16802E+00	178	8,61920E-01	18916	0	17530,5
Non-responder	Macro_IER3	Macro_FOLR2+APOE+	HBEGF	CD9	6,40864E-04	8560,5	1,30417E-02	6361	5,89711E-01	5527	1,47143E+00	5825	9,07615E-01	7559	0	17530,5
Non-responder	pDC_LILRA4	Mono_CD16	SEMA7A	PLXNC1	6,41102E-04	12143,7	3,12263E-02	1443	8,97744E-01	1741	2,32540E+00	1077	7,78322E-01	38927	0	17530,5
Non-responder	Macro_NLRP3	pDC_LILRA4	S100A12	CD36	6,41163E-04	10500,7	2,63548E-02	1909	6,57437E-01	4263	2,32262E+00	1081	8,25501E-01	27720	0	17530,5
Non-responder	pDC_LILRA4	cDC_CLEC9A	SPON2	ITGA5	6,42194E-04	13734,5	2,78360E-02	1742	1,02845E+00	1078	2,62894E+00	586	7,39542E-01	47736	0	17530,5
Non-responder	Macro_IFI27	pDC_LILRA4	APOE	SORL1	6,42436E-04	9912,1	1,25431E-02	6795	4,89871E-01	8328	1,04420E+00	12248	9,21445E-01	4659	0	17530,5
Non-responder	Macro_NLRP3	Mono_CD16	THBS1	CD47	6,42591E-04	8464,5	1,44822E-02	5312	8,10000E-01	2456	1,80225E+00	3093	8,81757E-01	13931	0	17530,5
Non-responder	Macro_ISG15	pDC_LILRA4	VEGFA	RET	6,43287E-04	14729,5	7,34302E-02	287	8,97581E-01	1743	2,15167E+00	1516	7,15406E-01	52571	0	17530,5
Non-responder	Mast	Macro_ISG15	HDC	HRH2	6,43287E-04	9738,5	7,13083E-02	310	1,31389E+00	244	2,09091E+00	1743	8,20793E-01	28865	0	17530,5
Non-responder	Macro_FOLR2+APOE+	Mono_CD14	TIMP2	CD44	6,43485E-04	11231,5	9,27525E-03	11075	4,92212E-01	8251	1,36690E+00	7049	8,88124E-01	12252	0	17530,5
Non-responder	Macro_OLFML3	Mono_INHBA	C1QA	CD93	6,46116E-04	11698,3	9,71752E-03	10312	4,34401E-01	10444	1,04359E+00	12262	9,05984E-01	7943	0	17530,5
Non-responder	Macro_NLRP3	pDC_LILRA4	PSEN1	NOTCH4	6,46411E-04	14218,9	2,97155E-02	1570	6,09706E-01	5106	1,69916E+00	3791	7,60209E-01	43097	0	17530,5
Non-responder	Macro_FOLR2+APOE+	Macro_IER3	CCL18	CCR1	6,46890E-04	15159,1	2,09136E-02	2831	5,99471E-01	5321	2,06242E+00	1850	7,37182E-01	48263	0	17530,5
Non-responder	Macro_NLRP3	cDC_LAMP3	VEGFA	SIRPA	6,46890E-04	10368,1	1,60075E-02	4474	8,29140E-01	2287	1,52290E+00	5321	8,48018E-01	22228	0	17530,5
Non-responder	Macro_NLRP3	pDC_LILRA4	HBEGF	CD82	6,47369E-04	8227,1	1,87907E-02	3397	8,42655E-01	2152	2,41139E+00	912	8,69450E-01	17144	0	17530,5

Post_NR_Myeloid_Myeloid_CellCell

Non-responder	Macro_OLFML3	Macro_ISG15	HLA-A	LILRB1	6,49019E-04	7750,1	8,70920E-03	12273	6,73228E-01	4016	1,76903E+00	3297	9,36604E-01	1634	0	17530,5
Non-responder	Mono_CD14	Macro_IFI27	S100A9	CD68	6,50248E-04	5635,7	1,44588E-02	5328	1,12040E+00	691	1,59915E+00	4602	9,58146E-01	27	0	17530,5
Non-responder	Macro_FOLR2+APOE+	cDC_CLEC9A	LGALS9	SLC1A5	6,51402E-04	8477,3	1,31364E-02	6283	6,82515E-01	3865	1,92678E+00	2426	8,88027E-01	12282	0	17530,5
Non-responder	Macro_IER3	Macro_FOLR2+APOE+	C3	CD81	6,52464E-04	10787,7	1,12164E-02	8178	4,74024E-01	8905	1,26120E+00	8535	8,93777E-01	10790	0	17530,5
Non-responder	Macro_NLRP3	Macro_NLRP3	S100A9	CD68	6,52464E-04	8831,9	8,70087E-03	12286	6,05522E-01	5198	1,25007E+00	8702	9,46691E-01	443	0	17530,5
Non-responder	Macro_FOLR2+APOE+	Macro_FOLR2+APOE+	SEMA4A	PLXNB2	6,52654E-04	12038,1	1,44498E-02	5333	6,00769E-01	5288	1,58756E+00	4709	8,27076E-01	27330	0	17530,5
Non-responder	Macro_NLRP3	pDC_LILRA4	HMGB1	TLR9	6,54102E-04	8386,1	4,78640E-02	705	8,19796E-01	2373	1,65700E+00	4136	8,69292E-01	17186	0	17530,5
Non-responder	Mast	cDC(CD1C)	HDC	HRH2	6,55010E-04	9944,9	7,52620E-02	267	1,33119E+00	223	1,70081E+00	3779	8,24727E-01	27925	0	17530,5
Non-responder	Mono_CD14	pDC_LILRA4	THBS1	CD36	6,55393E-04	7130,3	2,77204E-02	1751	8,96215E-01	1754	2,85161E+00	376	8,80510E-01	14240	0	17530,5
Non-responder	Macro_NLRP3	pDC_LILRA4	TNF	TRAF2	6,57003E-04	12412,5	2,90058E-02	1651	7,28169E-01	3268	2,22850E+00	1301	7,80898E-01	38312	0	17530,5
Non-responder	Macro_FOLR2+APOE+	Macro_LYVE1	PSAP	LRP1	6,57487E-04	8309,7	8,55822E-03	12616	6,62703E-01	4185	1,52026E+00	5343	9,35413E-01	1874	0	17530,5
Non-responder	Mono_CD14	Mono_INHBA	S100A8	ITGB2	6,57611E-04	5327,5	1,37021E-02	5854	1,00323E+00	1193	2,08465E+00	1756	9,48841E-01	304	0	17530,5
Non-responder	Macro_FOLR2+APOE+	Macro_NLRP3	RPS19	C5AR1	6,57972E-04	9605,7	7,43944E-03	15873	5,99551E-01	5320	1,52017E+00	5344	9,25109E-01	3961	0	17530,5
Non-responder	Macro_FOLR2+APOE+	cDC_LAMP3	NAMPT	ADORA2A	6,58943E-04	10140,1	2,93408E-02	1613	8,85189E-01	1813	1,51978E+00	5346	8,38988E-01	24398	0	17530,5
Non-responder	Macro_NLRP3	Macro_ISG15	THBS1	LRP1	6,59397E-04	7979,9	1,60964E-02	4432	7,67410E-01	2841	1,85597E+00	2784	8,87879E-01	12312	0	17530,5
Non-responder	Macro_NLRP3	Macro_LYVE1	S100A8	ITGB2	6,59397E-04	8057,9	1,19868E-02	7348	7,99647E-01	2560	1,04099E+00	12312	9,45495E-01	539	0	17530,5
Non-responder	Macro_NLRP3	Mono_CD16	IL6	FCGR3B	6,61373E-04	17672,7	3,23368E-02	1369	5,98230E-01	5351	1,53323E+00	5220	6,77809E-01	58893	0	17530,5
Non-responder	Macro_NLRP3	pDC_LILRA4	IL10	IFNLR1	6,61860E-04	15726,5	4,73235E-02	720	7,98031E-01	2577	2,03510E+00	1953	6,96948E-01	55852	0	17530,5
Non-responder	Macro_FOLR2+APOE+	pDC_LILRA4	HLA-A	LILRB1	6,62834E-04	10687,7	5,68216E-03	24384	5,98134E-01	5354	2,09052E+00	1745	9,22681E-01	4425	0	17530,5
Non-responder	Macro_NLRP3	Macro_OLFML3	VCAN	ITGB1	6,63810E-04	8174,7	1,44236E-02	5356	8,72452E-01	1897	1,71536E+00	3667	8,87479E-01	12423	0	17530,5
Non-responder	Macro_OLFML3	Macro_NLRP3	CXCL10	SDC4	6,64292E-04	10391,3	2,76076E-02	1762	1,11823E+00	699	2,93378E+00	323	8,09383E-01	31642	0	17530,5
Non-responder	Macro_FOLR2+APOE+	Macro_OLFML3	LGALS9	PTPRC	6,66254E-04	9512,5	7,63875E-03	15189	6,00510E-01	5297	1,65172E+00	4185	9,17978E-01	5361	0	17530,5
Non-responder	Mast	Macro_OLFML3	CSF1	SIRPA	6,66529E-04	8250,5	6,41490E-02	394	1,24335E+00	346	2,08320E+00	1764	8,52218E-01	21218	0	17530,5
Non-responder	Macro_OLFML3	Mono_CD16	B2M	LILRB2	6,68011E-04	6836,1	8,67662E-03	12344	8,55756E-01	2044	1,96517E+00	2230	9,57074E-01	32	0	17530,5
Non-responder	Macro_FOLR2+APOE+	pDC_LILRA4	CXCL12	CD4	6,68704E-04	11170,3	1,72970E-02	3934	5,97603E-01	5366	2,48516E+00	785	8,23404E-01	28236	0	17530,5
Non-responder	pDC_LILRA4	cDC_CLEC9A	SNCA	LAG3	6,68771E-04	14148,7	3,63747E-02	1130	8,94143E-01	1766	2,52426E+00	730	7,30818E-01	49587	0	17530,5
Non-responder	Macro_FOLR2+APOE+	Macro_FOLR2+APOE+	HLA-C	LILRB1	6,69195E-04	9151,3	6,99254E-03	17560	5,97599E-01	5367	1,91411E+00	2486	9,30683E-01	2813	0	17530,5
Non-responder	Macro_NLRP3	Macro_FOLR2+APOE+	INHBA	ENG	6,71653E-04	12423,1	2,15344E-02	2681	5,97149E-01	5372	1,93154E+00	2404	7,98901E-01	34128	0	17530,5
Non-responder	cDC_LAMP3	Macro_FOLR2-APOE+	CCL19	CCRL2	6,72143E-04	12668,1	3,16240E-02	1409	1,73946E+00	68	2,08161E+00	1769	7,62526E-01	42564	0	17530,5
Non-responder	Macro_FOLR2+APOE+	Macro_FOLR2+APOE+	CD14	TLR4	6,73167E-04	8818,1	1,17230E-02	7627	6,73820E-01	4001	1,89565E+00	2569	8,87703E-01	12363	0	17530,5
Non-responder	Macro_FOLR2+APOE+	Macro_IER3	A2M	LRP1	6,73985E-04	11465,9	1,07245E-02	8849	4,64527E-01	9259	1,20739E+00	9325	8,87690E-01	12366	0	17530,5
Non-responder	Macro_OLFML3	cDC_CLEC9A	TNFSF13B	CD40	6,75348E-04	9051,3	1,08380E-02	8697	7,17168E-01	3419	1,77830E+00	3239	8,87676E-01	12371	0	17530,5
Non-responder	Mono_CD14	Macro_IER3	S100A12	CD36	6,75526E-04	9661,3	2,74964E-02	1772	1,19339E+00	454	2,13205E+00	1579	8,28534E-01	26971	0	17530,5
Non-responder	Macro_NLRP3	Macro_NLRP3	VEGFA	ITGB1	6,76095E-04	9749,9	1,43915E-02	5381	8,00805E-01	2549	1,75931E+00	3362	8,57633E-01	19927	0	17530,5
Non-responder	Macro_IER3	Macro_FOLR2+APOE+	CCL4	CCR1	6,77261E-04	7494,9	2,01516E-02	3017	8,62908E-01	1973	1,89468E+00	2576	8,87658E-01	12378	0	17530,5
Non-responder	pDC_LILRA4	Mono_CD16	APP	RPSA	6,78077E-04	5428,5	1,81727E-02	3598	1,25696E+00	324	2,95753E+00	305	9,17869E-01	5385	0	17530,5
Non-responder	Macro_FOLR2+APOE+	Macro_FOLR2+APOE+	LGALS9	CD44	6,80226E-04	10254,3	7,26949E-03	16465	5,37110E-01	6841	1,59487E+00	4648	9,15778E-01	5787	0	17530,5
Non-responder	pDC_LILRA4	Macro_LYVE1	HSP90B1	LRP1	6,83048E-04	5583,3	1,43715E-02	5395	1,06544E+00	910	2,62663E+00	589	9,27608E-01	3492	0	17530,5
Non-responder	Macro_IER3	Macro_NLRP3	VCAN	CD44	6,83852E-04	9706,7	8,97116E-03	11693	6,82700E-01	3861	1,41720E+00	6450	9,01240E-01	8999	0	17530,5
Non-responder	pDC_LILRA4	Macro_FOLR2+APOE-	SPON2	ITGA4	6,84603E-04	10591,3	2,73970E-02	1780	1,00315E+00	1194	2,09805E+00	1704	8,13042E-01	30748	0	17530,5
Non-responder	pDC_LILRA4	Macro_FOLR2+APOE-	TNFSF9	HLA-DPA1	6,85544E-04	6451,7	2,44589E-02	2156	8,89467E-01	1795	1,51492E+00	5400	9,17913E-01	5377	0	17530,5
Non-responder	Macro_IER3	Macro_NLRP3	HBEGF	CD44	6,87721E-04	6655,7	1,31856E-02	6242	9,07348E-01	1688	1,78362E+00	3208	9,21683E-01	4610	0	17530,5
Non-responder	Macro_NLRP3	Macro_FOLR2-APOE+	S100A8	CD68	6,88047E-04	7352,5	1,58440E-02	4565	1,04956E+00	975	9,76142E-01	13647	9,55844E-01	45	0	17530,5
Non-responder	Macro_NLRP3	Mono_INHBA	VEGFA	ITGAV	6,88549E-04	12470,7	1,80356E-02	3651	8,30232E-01	2276	1,51409E+00	5406	8,01634E-01	33490	0	17530,5
Non-responder	Mono_CD16	Mono_CD16	HLA-C	LILRB2	6,89384E-04	7040,9	8,63863E-03	12422	7,56535E-01	2956	2,00965E+00	2049	9,49675E-01	247	0	17530,5
Non-responder	Macro_FOLR2+APOE+	pDC_LILRA4	VEGFA	RET	6,89552E-04	17799,7	4,21969E-02	885	7,25179E-01	3303	1,51405E+00	5408	6,55836E-01	61872	0	17530,5
Non-responder	Macro_FOLR2+APOE+	Mono_CD16	PLAU	PLAUR	6,90217E-04	8627,3	1,43094E-02	5436	6,53195E-01	4342	1,75194E+00	3403	8,87470E-01	12425	0	17530,5
Non-responder	Macro_FOLR2+APOE+	Macro_FOLR2+APOE+	SIRPA	CD47	6,93577E-04	8602,5	1,43435E-02	5416,5	7,48764E-01	3037,5	1,95158E+00	2305,5	8,78828E-01	14722,5	0	17530,5
Non-responder	pDC_LILRA4	Macro_IFI27	APP	TSPAN15	6,93760E-04	9904,1	2,73449E-02	1788	1,17093E+00	528	2,47117E+00	806	8,20787E-01	28868	0	17530,5
Non-responder	Macro_NLRP3	Macro_IER3	ADM	GPR84	6,94082E-04	15315,1	1,92248E-02	3270	5,95263E-01	5417	1,59528E+00	4645	7,48639E-01	45713	0	17530,5
Non-responder	Macro_FOLR2+APOE+	Macro_FOLR2+APOE+	CD47	SIRPA	6,94082E-04	8602,5	1,43435E-02	5416,5	7,48764E-01	3037,5	1,95158E+00	2305,5	8,78828E-01	14722,5	0	17530,5
Non-responder	Mast	Macro_FOLR2+APOE+	HDC	HRH2	6,94910E-04	9944,5	6,77927E-02	347	1,29850E+00	267	2,07546E+00	1789	8,17044E-01	29789	0	17530,5
Non-responder	Macro_NLRP3	Mono_INHBA	S100A9	TLR4	6,95231E-04	8552,3	1,32023E-02	6220	8,56096E-01	2042	1,60853E+00	4526	8,87420E-01	12443	0	17530,5
Non-responder	Macro_NLRP3	Macro_FOLR2-APOE+	TIMP1	CD63	6,97189E-04	8538,9	9,44794E-03	10768	8,92684E-01	1776	1,03501E+00	12450	9,51058E-01	170	0	17530,5
Non-responder	Macro_NLRP3	Mono_INHBA	VCAN	ITGB1	6,97620E-04	8495,1	1,43319E-02	5424	8,69956E-01	1917	1,54413E+00	5098	8,87161E-01	12506	0	17530,5
Non-responder	Macro_IER3	Macro_NLRP3	VEGFA	CD44	7,01680E-04	9228,1	1,15150E-02	7856	7,65827E-01	2860	1,50061E+00	5532	8,87707E-01	12362	0	17530,5
Non-responder	Macro_IER3	Macro_NLRP3	HMGB1	THBD	7,01962E-04	12377,5	8,61716E-03	12467	4,35330E-01	10404	1,14606E+00	10360	8,92465E-01	11126	0	17530,5
Non-responder	Mono_CD16	Macro_IFI27	CD52	SIGLEC10	7,02807E-04	10222,1	1,07769E-02	8780	7,65545E-01	2864	1,19977E+00	9466	8,87311E-01	12470	0	17530,5
Non-responder	Macro_NLRP3	Mono_CD16	S100A9	TLR4	7,03935E-04	8254,5	1,31713E-02	6251	8,55306E-01	2051	1,82588E+00	2966	8,87303E-01	12474	0	17530,5
Non-responder	Macro_FOLR2+APOE+	Macro_FOLR2+APOE+	TNFSF12	CD163	7,04226E-04	9655,1	1,43093E-02	5437	6,14010E-01	5023	2,07342E+00	1801	8,63753E-01	18484	0	17530,5
Non-responder	pDC_LILRA4	cDC(CD1C)	B2M	CD1A	7,05319E-04	5255,9	5,30341E-02	592	8,89351E-01	1798	2,83834E+00	390	9,14978E-01	5969	0	17530,5
Non-responder	Macro_FOLR2+APOE+	Mono_CD14	PLAU	ITGAM	7,05757E-04	11946,7	1,89842E-02	3340	5,94098E-01	5440	1,74631E+00	3440	8,16290E-01	29983	0	17530,5
Non-responder	Macro_IER3	Mono_CD16	HLA-B	LILRA1	7,06195E-04	7744,3	1,60999E-02	4429	6,24774E-01	4834	1,30996E+00	7847	9,24470E-01	4081	0	17530,5
Non-responder	Macro_NLRP3	cDC(CD1C)	VCAN	ITGB1	7,06195E-04	8558,3	1,43626E-02	5406	8,70792E-01	1914	1,50919E+00	5459	8,87268E-01	12482	0	17530,5
Non-responder	Macro_NLRP3	Macro_FOLR2-APOE+	HBEGF	CD9	7,06268E-04	7867,5	2,03464E-02	2981	9,33349E-01	1540	9,94634E-01	13238	9,24647E-01	4048	0	17530,5
Non-responder	Macro_FOLR2+APOE+	Macro_IFI27	C1QA	CD33	7,06478E-04	10674,1	8,61353E-03	12483	5,76176E-01	5819	1,34084E+00	7409	8,96438E-01	10129	0	17530,5
Non-responder	pDC_LILRA4	Mono_INHBA	APP	APLP2	7,06779E-04	5201,9	2,32240E-02	2330	1,26838E+00	305	2,81610E+00	402	9,17539E-01	5442	0	17530,5
Non-responder	Macro_FOLR2+APOE+	pDC_LILRA4	IRAK4	TLR7	7,07291E-04	14968,1	1,88933E-02	3366	5,94025E-01	5443	1,89588E+00	2568	7,47664E-01	45933	0	17530,5
Non-responder	Macro_IER3	Mono_CD16	HLA-A	LILRA1	7,08177E-04	7441,5	1,67504E-02	4127	6,66310E-01	4117	1,39025E+00	6764	9,21385E-01	4669	0	17530,5
Non-responder	Macro_FOLR2+APOE+	pDC_LILRA4	GRN	TNFRSF1B	7,08827E-04	9055,5	6,67714E-03	18894	5,93880E-01	5446	2,44430E+00	854	9,32054E-01	2553	0	17530,5
Non-responder	Macro_FOLR2+APOE+	Macro_FOLR2+APOE+	CD99	PILRA	7,09880E-04	10062,9	8,60875E-03	12495	4,82603E-01	8604	1,57830E+00	4777	9,10631E-01	6908	0	17530,5

Post_NR_Myeloid_Myeloid_CellCell

Non-responder	cDC(CD1C)	pDC_LILRA4	TNF	PTPRS	7,11146E-04	11737,3	2,71556E-02	1803	1,38720E+00	170	2,22851E+00	1300	7,82761E-01	37883	0	17530,5
Non-responder	Mono_INHBA	cDC_CLEC9A	INHBA	ACTR2	7,13485E-04	8597,1	2,71841E-02	1802	8,87950E-01	1805	2,11353E+00	1648	8,56490E-01	20200	0	17530,5
Non-responder	pDC_LILRA4	Macro_FOLR2+APOE+	TNFSF9	HLA-DPA1	7,13965E-04	5513,3	2,41571E-02	2196	8,71533E-01	1906	2,73139E+00	478	9,17444E-01	5456	0	17530,5
Non-responder	Macro_IER3	Mono_CD16	CD14	ITGA4	7,14722E-04	9539,9	9,39005E-03	10881	5,86776E-01	5583	1,37482E+00	6958	9,11392E-01	6747	0	17530,5
Non-responder	Macro_NLRP3	Mono_INHBA	NAMPT	ITGA5_ITGB1	7,14722E-04	10319,5	9,23518E-03	11157	7,24060E-01	3320	1,36449E+00	7078	8,87142E-01	12512	0	17530,5
Non-responder	Macro_IER3	Mono_CD16	HLA-C	LILRA1	7,15580E-04	7900,9	1,64844E-02	4255	6,03770E-01	5235	1,30605E+00	7904	9,21828E-01	4580	0	17530,5
Non-responder	Macro_ISG15	pDC_LILRA4	CCL2	CCR2	7,15830E-04	10506,9	5,81287E-02	489	8,87094E-01	1807	3,40233E+00	112	8,05273E-01	32596	0	17530,5
Non-responder	Macro_IER3	Mono_CD16	HLA-C	LILRA3	7,16152E-04	7970,7	1,95192E-02	3198	5,44470E-01	6644	1,51792E+00	5366	9,09674E-01	7115	0	17530,5
Non-responder	Macro_FOLR2+APOE+	Macro_ISG15	CCL3L1	CCR5	7,19648E-04	13249,9	2,01861E-02	3008	5,93036E-01	5467	1,62964E+00	4344	7,91229E-01	35900	0	17530,5
Non-responder	Macro_FOLR2+APOE+	Mast	HSPA8	ADRB2	7,21028E-04	9189,5	1,76111E-02	3798	6,69717E-01	4071	1,29735E+00	8014	8,87046E-01	12534	0	17530,5
Non-responder	Macro_NLRP3	Macro_IER3	VEGFA	SIRPA	7,21204E-04	10310,5	1,62593E-02	4360	8,35534E-01	2223	1,50800E+00	5470	8,49021E-01	21969	0	17530,5
Non-responder	Macro_IER3	Macro_FOLR2+APOE+	HBEGF	CD44	7,21891E-04	10895,5	8,59512E-03	12537	5,48226E-01	6540	1,18999E+00	9628	9,04778E-01	8242	0	17530,5
Non-responder	pDC_LILRA4	Mono_CD16	SPON2	ITGA5	7,25259E-04	13884,9	2,70264E-02	1815	1,02371E+00	1098	2,60444E+00	615	7,36689E-01	48366	0	17530,5
Non-responder	Macro_FOLR2+APOE+	Macro_IFI27	SERPING1	LRP1	7,26220E-04	9796,9	1,11819E-02	8241	7,40417E-01	3140	1,33263E+00	7521	8,86974E-01	12552	0	17530,5
Non-responder	cDC_CLEC9A	Mono_CD16	BTLA	TNFRSF14	7,27629E-04	8984,9	5,24818E-02	602	1,23521E+00	363	2,07101E+00	1817	8,38133E-01	24612	0	17530,5
Non-responder	Macro_ISG15	cDC_LAMP3	CXCL10	SDC4	7,30004E-04	10674,7	2,69943E-02	1819	9,96129E-01	1223	2,50179E+00	767	8,07644E-01	32034	0	17530,5
Non-responder	Mono_CD14	Macro_NLRP3	S100A8	TLR4	7,30375E-04	5398,7	2,66550E-02	1867	1,28437E+00	282	2,98849E+00	278	9,10051E-01	7036	0	17530,5
Non-responder	Macro_LYVE1	Macro_ISG15	C3	IFITM1	7,31194E-04	5376,1	2,69818E-02	1820	1,15150E+00	578	2,20517E+00	1361	9,16753E-01	5591	0	17530,5
Non-responder	Mono_INHBA	Macro_ISG15	TIMP1	CD63	7,31194E-04	6327,3	9,44521E-03	10777	8,84412E-01	1820	2,21148E+00	1338	9,51052E-01	171	0	17530,5
Non-responder	Macro_FOLR2+APOE+	Mono_INHBA	A2M	LRP1	7,36109E-04	11520,9	1,05459E-02	9074	4,57552E-01	9517	1,23579E+00	8897	8,86850E-01	12586	0	17530,5
Non-responder	Mono_CD14	Mono_CD16	HLA-B	LILRA1	7,37428E-04	7330,1	1,60103E-02	4471	6,13903E-01	5025	1,50485E+00	5501	9,24275E-01	4123	0	17530,5
Non-responder	Mono_CD14	Mast	LGALS1	CD69	7,37956E-04	5737,5	1,92309E-02	3267	1,01263E+00	1148	1,50471E+00	5502	9,38846E-01	1240	0	17530,5
Non-responder	Macro_NLRP3	Mast	ADM	ADRB2	7,39012E-04	12345,7	3,78744E-02	1059	7,91293E-01	2633	1,55846E+00	4948	7,92789E-01	35558	0	17530,5
Non-responder	Mono_CD14	Macro_NLRP3	S100A8	CD68	7,39555E-04	5504,3	1,30271E-02	6379	9,16195E-01	1635	2,06870E+00	1827	9,51523E-01	150	0	17530,5
Non-responder	Macro_IER3	Mono_CD16	LYZ	ITGAL	7,39919E-04	8513,9	1,26746E-02	6679	6,00538E-01	5296	1,33977E+00	7424	9,16484E-01	5640	0	17530,5
Non-responder	Mast	Mast	ADCYAP1	ADRB2	7,40731E-04	12098,5	2,35508E-01	19	1,28315E+00	284	1,56223E+00	4921	7,83352E-01	37738	0	17530,5
Non-responder	Macro_IER3	pDC_LILRA4	CD14	TLR9	7,40755E-04	6141,1	8,35168E-02	211	1,20353E+00	432	2,06852E+00	1828	8,94136E-01	10704	0	17530,5
Non-responder	pDC_LILRA4	Macro_ISG15	B2M	LILRB2	7,40755E-04	6942,1	7,78924E-03	14744	8,83396E-01	1828	2,65727E+00	546	9,54802E-01	62	0	17530,5
Non-responder	pDC_LILRA4	Mono_CD16	CD38	PECAM1	7,41956E-04	9746,7	2,69464E-02	1829	1,00900E+00	1165	3,02524E+00	250	8,24626E-01	27959	0	17530,5
Non-responder	Macro_IFI27	Macro_ISG15	HLA-DQB1	LAG3	7,42271E-04	9041,9	2,11455E-02	2779	9,92740E-01	1235	1,10626E+00	11058	8,86778E-01	12607	0	17530,5
Non-responder	Macro_OLFML3	Macro_NLRP3	HMGB1	THBD	7,43449E-04	11658,3	8,56130E-03	12611	4,30057E-01	10609	1,42596E+00	6334	8,92153E-01	11207	0	17530,5
Non-responder	Macro_FOLR2+APOE+	Mono_CD16	TNFSF13	TNFRSF14	7,43744E-04	10826,3	8,56068E-03	12612	4,85981E-01	8474	1,78270E+00	3213	8,87926E-01	12302	0	17530,5
Non-responder	Macro_NLRP3	Macro_OLFML3	VEGFA	ITGB1	7,45375E-04	9989,5	1,41783E-02	5516	7,94898E-01	2598	1,65441E+00	4160	8,56719E-01	20143	0	17530,5
Non-responder	pDC_LILRA4	Macro_IER3	APP	RPSA	7,46439E-04	5553,1	1,78257E-02	3725	1,24848E+00	339	2,57968E+00	653	9,17139E-01	5518	0	17530,5
Non-responder	Macro_OLFML3	Macro_ISG15	HLA-C	LILRB1	7,46697E-04	8199,7	8,55562E-03	12622	6,02356E-01	5261	1,67083E+00	4021	9,36915E-01	1564	0	17530,5
Non-responder	Macro_FOLR2+APOE-	pDC_LILRA4	IGF1	IGF2R	7,51610E-04	10486,9	3,70428E-02	1104	9,48118E-01	1458	2,06663E+00	1837	8,14073E-01	30505	0	17530,5
Non-responder	Macro_FOLR2+APOE+	Macro_ISG15	C1QB	C1QBP	7,53921E-04	8832,5	7,91808E-03	14367	6,16558E-01	4991	2,09095E+00	1742	9,17077E-01	5532	0	17530,5
Non-responder	Macro_NLRP3	cDC_CLEC9A	ITGB2	CD226	7,54994E-04	8329,9	2,78181E-02	1744	8,82307E-01	1840	1,53043E+00	5252	8,76656E-01	15283	0	17530,5
Non-responder	Macro_OLFML3	Macro_NLRP3	HBEGF	CD44	7,56510E-04	9218,9	8,54418E-03	12655	6,79469E-01	3910	1,71127E+00	3703	9,04521E-01	8296	0	17530,5
Non-responder	Macro_NLRP3	Macro_OLFML3	CCL4	CCR5	7,56607E-04	11735,3	1,85980E-02	3469	6,51319E-01	4377	1,49991E+00	5537	8,25342E-01	27763	0	17530,5
Non-responder	Macro_NLRP3	cDC_CLEC9A	ICAM1	SPN	7,56607E-04	9130,7	1,60826E-02	4440	7,67251E-01	2843	1,97467E+00	2193	8,63080E-01	18647	0	17530,5
Non-responder	Macro_FOLR2+APOE+	Macro_OLFML3	LGALS3BP	CD33	7,56607E-04	11350,3	1,46414E-02	5209	5,89304E-01	5537	1,54075E+00	5127	8,43334E-01	23348	0	17530,5
Non-responder	Macro_FOLR2+APOE+	pDC_LILRA4	SIRPB2	CD47	7,56607E-04	11553,9	1,41394E-02	5537	7,49714E-01	3027	2,43381E+00	879	8,12866E-01	30796	0	17530,5
Non-responder	Macro_NLRP3	Macro_ISG15	S100A8	TLR4	7,56809E-04	7940,9	1,59092E-02	4526	8,89719E-01	1794	1,78564E+00	3198	8,86575E-01	12656	0	17530,5
Non-responder	Mono_CD16	Macro_FOLR2+APOE+	S100A8	CD68	7,57407E-04	8020,5	8,54244E-03	12658	6,67262E-01	4104	1,57336E+00	4817	9,40810E-01	993	0	17530,5
Non-responder	Macro_NLRP3	Macro_FOLR2+APOE+	TGFB1	ITGB5	7,57683E-04	12930,9	2,24467E-02	2480	5,92940E-01	5471	1,49947E+00	5539	8,01078E-01	33634	0	17530,5
Non-responder	pDC_LILRA4	Mono_CD14	APP	RPSA	7,58221E-04	5534,9	1,77816E-02	3746	1,24740E+00	341	2,68782E+00	517	9,17045E-01	5540	0	17530,5
Non-responder	Macro_NLRP3	cDC_CLEC9A	THBS1	CD47	7,58221E-04	8522,5	1,43943E-02	5377	8,07707E-01	2476	1,78131E+00	3224	8,81439E-01	14005	0	17530,5
Non-responder	Macro_FOLR2+APOE+	Macro_NLRP3	CCL18	CCR1	7,58760E-04	15275,9	2,00992E-02	3038	5,89005E-01	5541	2,26311E+00	1204	7,33316E-01	49066	0	17530,5
Non-responder	Macro_NLRP3	cDC_CLEC9A	THBS1	PTPRJ	7,58760E-04	12584,5	1,58766E-02	4552	7,98881E-01	2569	1,64326E+00	4247	7,99373E-01	34024	0	17530,5
Non-responder	Macro_FOLR2+APOE+	Macro_LYVE1	APOE	SCARB1	7,59204E-04	9927,5	1,36322E-02	5902	4,86998E-01	8431	1,54300E+00	5110	8,86560E-01	12664	0	17530,5
Non-responder	cDC(CD1C)	Mono_CD16	HLA-C	LILRA1	7,60378E-04	6829,7	1,77524E-02	3757	7,32566E-01	3230	1,49895E+00	5544	9,24457E-01	4087	0	17530,5
Non-responder	Macro_NLRP3	Macro_OLFML3	S100A8	TLR4	7,60918E-04	8950,5	1,41300E-02	5545	8,51291E-01	2077	1,51922E+00	5352	8,80474E-01	14248	0	17530,5
Non-responder	Macro_FOLR2+APOE+	Mono_CD16	SERPING1	LRP1	7,61605E-04	8815,1	1,10830E-02	8384	7,36969E-01	3181	1,95144E+00	2308	8,86529E-01	12672	0	17530,5
Non-responder	Macro_ISG15	pDC_LILRA4	TNF	TNFRSF21	7,62568E-04	8153,5	2,68390E-02	1846	1,05407E+00	955	2,96415E+00	304	8,56759E-01	20132	0	17530,5
Non-responder	Mono_CD14	Macro_ISG15	VCAN	TLR1	7,65018E-04	7820,1	2,68322E-02	1848	1,16493E+00	541	2,32205E+00	1084	8,65436E-01	18097	0	17530,5
Non-responder	Macro_ISG15	Macro_FOLR2+APOE+	S100A9	CD68	7,65018E-04	5864,1	1,08915E-02	8626	8,81495E-01	1848	2,26711E+00	1189	9,52081E-01	127	0	17530,5
Non-responder	Macro_FOLR2+APOE+	Mono_CD16	CD99	PILRA	7,65218E-04	10147,1	8,52978E-03	12684	4,75093E-01	8871	1,59421E+00	4653	9,10256E-01	6997	0	17530,5
Non-responder	Macro_NLRP3	cDC_CLEC9A	NRG1	HLA-DPB1	7,65791E-04	9036,1	1,64712E-02	4261	6,70990E-01	4046	1,72115E+00	3631	8,74908E-01	15712	0	17530,5
Non-responder	Macro_NLRP3	cDC_CLEC9A	NECTIN2	CD226	7,67963E-04	14744,5	3,47196E-02	1217	1,00559E+00	1185	1,69473E+00	3814	7,28936E-01	49976	0	17530,5
Non-responder	pDC_LILRA4	Macro_FOLR2-APOE+	SPON2	ITGA5	7,68702E-04	13985,1	2,78419E-02	1740	1,02849E+00	1076	2,06221E+00	1851	7,39562E-01	47728	0	17530,5
Non-responder	Macro_NLRP3	Macro_IFI27	VCAN	TLR2	7,69147E-04	9007,3	1,63658E-02	4313	8,82108E-01	1842	1,25350E+00	8654	8,86398E-01	12697	0	17530,5
Non-responder	Mono_CD16	pDC_LILRA4	TGFB1	CXCR4	7,69147E-04	8801,5	8,52626E-03	12697	6,04695E-01	5212	2,59597E+00	632	9,06022E-01	7936	0	17530,5
Non-responder	Macro_FOLR2+APOE+	Macro_IFI27	MMP9	ITGAM	7,69596E-04	14105,7	1,53502E-02	4812	5,87976E-01	5561	1,73850E+00	3490	7,77512E-01	39135	0	17530,5
Non-responder	Mono_CD14	cDC_LAMP3	ADM	RAMP1	7,71164E-04	11753,5	5,69373E-02	520	1,37442E+00	183	2,06185E+00	1853	7,79342E-01	38681	0	17530,5
Non-responder	Macro_NLRP3	Macro_IER3	RPS19	C5AR1	7,72485E-04	9927,7	8,55036E-03	12640	6,91577E-01	3725	1,02168E+00	12708	9,29790E-01	3035	0	17530,5
Non-responder	Macro_FOLR2+APOE+	pDC_LILRA4	LYZ	ITGAL	7,73397E-04	9507,3	8,51873E-03	12711	5,42971E-01	6689	2,21989E+00	1319	8,99965E-01	9287	0	17530,5
Non-responder	Macro_NLRP3	cDC_LAMP3	TNF	FAS	7,73416E-04	14096,3	2,55341E-02	2013	6,93430E-01	3700	1,49596E+00	5568	7,66742E-01	41670	0	17530,5
Non-responder	Macro_NLRP3	cDC_CLEC9A	CXCL8	SDC2	7,75605E-04	12348,1	1,56300E-02	4678	8,24608E-01	2320	1,91035E+00	2509	7,96478E-01	34703	0	17530,5
Non-responder	Macro_OLFML3	Macro_FOLR2+APOE+	HLA-DQA1	CD4	7,75834E-04	8055,5	8,51510E-03	12719	6,15290E-01	5013	1,69433E+00	3819	9,39147E-01	1196	0	17530,5
Non-responder	Macro_ISG15	Mono_CD14	CXCL10	TLR4	7,76104E-04	8091,7	2,67356E-02	1857	1,02419E+00	1094	2,83321E+00	394	8,59117E-01	19583	0	17530,5

Non-responder	Macro_OLFML3	cDC_CLEC9A	HLA-DRB5	LAG3	7,76749E-04	8152,5	1,67780E-02	4115	8,16831E-01	2396	1,67280E+00	3999	8,86326E-01	12722	0	17530,5
Non-responder	Macro_FOLR2+APOE+	Macro_FOLR2+APOE+	CD14	ITGA4	7,77054E-04	9214,5	8,51258E-03	12723	6,06600E-01	5170	1,81467E+00	3029	9,07350E-01	7620	0	17530,5
Non-responder	Macro_FOLR2+APOE+	Mono_CD16	SEMA4A	PLXNB2	7,77249E-04	11965,5	1,40946E-02	5575	5,90419E-01	5516	1,74843E+00	3431	8,25289E-01	27775	0	17530,5
Non-responder	Macro_NLRP3	cDC_CLEC9A	VCAN	TLR1	7,78897E-04	11363,9	1,41557E-02	5529	8,67879E-01	1938	1,71950E+00	3638	8,23676E-01	28184	0	17530,5
Non-responder	Macro_OLFML3	Mono_INHBA	LGALS9	CD44	7,79194E-04	9314,5	8,51136E-03	12730	6,73892E-01	3998	1,32052E+00	7701	9,21664E-01	4613	0	17530,5
Non-responder	Macro_NLRP3	Macro_OLFML3	ICAM1	ITGAM_ITGB2	7,80113E-04	10131,1	8,50844E-03	12733	5,66525E-01	6063	1,41934E+00	6420	9,06134E-01	7909	0	17530,5
Non-responder	Macro_FOLR2+APOE+	Macro_NLRP3	APOC2	LRP1	7,80546E-04	10929,7	2,19851E-02	2579	5,86912E-01	5581	2,18924E+00	1408	8,26142E-01	27550	0	17530,5
Non-responder	cDC_LAMP3	Mono_CD16	HLA-B	LILRA1	7,80546E-04	6802,7	1,70323E-02	4027	7,37932E-01	3169	1,49507E+00	5581	9,26412E-01	3706	0	17530,5
Non-responder	Macro_NLRP3	cDC_CLEC9A	VEGFA	ITGB1	7,81097E-04	8465,9	1,77910E-02	3742	8,95006E-01	1762	1,94260E+00	2339	8,70094E-01	16956	0	17530,5
Non-responder	pDC_LILRA4	Macro_FOLR2+APOE+	SELPLG	ITGB2	7,82309E-04	5503,7	1,32361E-02	6183	9,20351E-01	1610	2,92016E+00	333	9,35453E-01	1862	0	17530,5
Non-responder	Macro_NLRP3	Macro_OLFML3	ICAM1	ITGAL_ITGB2	7,83181E-04	9892,5	8,50589E-03	12743	6,00579E-01	5294	1,49593E+00	5569	9,04385E-01	8326	0	17530,5
Non-responder	Macro_NLRP3	cDC_CLEC9A	ADM	RAMP1	7,83302E-04	15136,5	3,16362E-02	1407	7,15899E-01	3436	1,90609E+00	2524	7,24722E-01	50785	0	17530,5
Non-responder	Mono_INHBA	pDC_LILRA4	VEGFA	NRP1	7,83554E-04	8395,1	3,39362E-02	1259	1,01547E+00	1136	2,05624E+00	1863	8,56539E-01	20187	0	17530,5
Non-responder	Macro_NLRP3	cDC_CLEC9A	ADM	CALCRL	7,84406E-04	14173,7	2,03949E-02	2971	6,19606E-01	4928	1,66189E+00	4097	7,68158E-01	41342	0	17530,5
Non-responder	cDC_CLEC9A	Macro_ISG15	HLA-DQA1	LAG3	7,84800E-04	6184,9	2,66648E-02	1864	1,25798E+00	323	2,70049E+00	504	8,94151E-01	10703	0	17530,5
Non-responder	Macro_FOLR2-APOE+	pDC_LILRA4	C1QB	C1QBP	7,85512E-04	6157,7	1,57782E-02	4609	8,68513E-01	1929	1,49467E+00	5590	9,39801E-01	1130	0	17530,5
Non-responder	Macro_FOLR2+APOE+	cDC_LAMP3	MMP9	IFNAR1	7,85512E-04	13773,3	1,40760E-02	5590	6,27727E-01	4768	1,94005E+00	2357	7,79661E-01	38621	0	17530,5
Non-responder	Macro_FOLR2+APOE+	Macro_OLFML3	HLA-F	LILRB1	7,85951E-04	9877,7	9,56423E-03	10564	6,07971E-01	5147	1,75285E+00	3395	8,86203E-01	12752	0	17530,5
Non-responder	Macro_NLRP3	cDC_CLEC9A	CCL20	CXCR3	7,86065E-04	11287,3	5,98363E-02	455	8,64241E-01	1961	2,25883E+00	1214	7,94074E-01	35276	0	17530,5
Non-responder	Macro_NLRP3	cDC_CLEC9A	CCL20	CCR6	7,86618E-04	17357,1	6,79319E-02	346	7,45911E-01	3074	1,70962E+00	3715	6,53851E-01	62120	0	17530,5
Non-responder	Macro_FOLR2-APOE+	pDC_LILRA4	APOE	SORL1	7,87726E-04	7360,9	1,40733E-02	5594	5,98504E-01	5340	1,61479E+00	4466	9,25512E-01	3874	0	17530,5
Non-responder	Macro_NLRP3	Macro_LYVE1	VCAN	ITGB1	7,88110E-04	8864,3	1,40527E-02	5603	8,62350E-01	1978	1,41712E+00	6451	8,86172E-01	12759	0	17530,5
Non-responder	Macro_FOLR2+APOE+	cDC_CLEC9A	AGTRAP	RACK1	7,89656E-04	9681,3	9,88349E-03	10034	6,28110E-01	4765	1,76716E+00	3313	8,86163E-01	12764	0	17530,5
Non-responder	pDC_LILRA4	Macro_FOLR2-APOE+	GAS6	MERTK	7,89798E-04	9010,7	2,76430E-02	1759	9,78866E-01	1305	2,05535E+00	1868	8,46494E-01	22591	0	17530,5
Non-responder	Macro_FOLR2+APOE+	Mono_CD14	HLA-B	LILRA1	7,89965E-04	9915,7	8,49684E-03	12765	6,09009E-01	5127	1,59533E+00	4643	8,98906E-01	9513	0	17530,5
Non-responder	Macro_FOLR2+APOE+	cDC_CLEC9A	CD14	ITGB1	7,90274E-04	9322,1	8,49682E-03	12766	5,83101E-01	5663	1,80182E+00	3096	9,07644E-01	7555	0	17530,5
Non-responder	Macro_FOLR2+APOE+	Macro_FOLR2+APOE+	MIF	TNFRSF14	7,90584E-04	9526,9	8,49672E-03	12767	6,10527E-01	5082	1,61447E+00	4467	9,06696E-01	7788	0	17530,5
Non-responder	pDC_LILRA4	Macro_ISG15	HLA-B	LILRB1	7,91050E-04	6401,3	9,20099E-03	11229	8,77213E-01	1869	2,69979E+00	505	9,41778E-01	873	0	17530,5
Non-responder	Macro_IER3	Macro_NLRP3	HSPA1A	TLR4	7,91204E-04	12663,1	8,49648E-03	12769	4,17524E-01	11172	1,16939E+00	9982	8,89564E-01	11862	0	17530,5
Non-responder	Macro_FOLR2+APOE+	Macro_FOLR2+APOE-	APOE	ABCA1	7,91513E-04	11384,1	9,61823E-03	10464	4,65668E-01	9215	1,01865E+00	12770	9,10492E-01	6941	0	17530,5
Non-responder	Macro_FOLR2+APOE+	Macro_OLFML3	SERPING1	LRP1	7,94617E-04	8958,3	1,09904E-02	8494	7,33737E-01	3211	1,85819E+00	2776	8,86106E-01	12780	0	17530,5
Non-responder	Mono_INHBA	Macro_FOLR2+APOE+	INHBA	ACTR2	7,94816E-04	8649,9	2,66597E-02	1866	8,76510E-01	1872	2,15858E+00	1497	8,55289E-01	20484	0	17530,5
Non-responder	Macro_NLRP3	Mono_CD16	GNAI2	S1PR4	7,95512E-04	11017,3	1,71000E-02	4006	6,25637E-01	4817	1,49311E+00	5608	8,44247E-01	23125	0	17530,5
Non-responder	pDC_LILRA4	Macro_OLFML3	NUCB2	ERAP1	7,96074E-04	11484,5	2,65655E-02	1873	9,14340E-01	1644	2,40796E+00	917	7,93261E-01	35458	0	17530,5
Non-responder	Macro_NLRP3	cDC_CLEC9A	ADAM10	CADM1	7,96629E-04	11727,1	2,38271E-02	2247	1,01457E+00	1141	1,67817E+00	3941	8,00467E-01	33776	0	17530,5
Non-responder	Macro_FOLR2+APOE+	pDC_LILRA4	APP	RPSA	7,98043E-04	9160,5	8,80206E-03	12039	8,04405E-01	2507	2,39549E+00	935	8,86076E-01	12791	0	17530,5
Non-responder	Macro_FOLR2+APOE+	Macro_ISG15	C1QA	CD93	7,99916E-04	9270,9	8,48873E-03	12797	6,24384E-01	4845	2,04298E+00	1920	9,00067E-01	9262	0	17530,5
Non-responder	Macro_NLRP3	cDC(CD1C)	ICAM1	IL2RG	8,01480E-04	10226,5	9,69882E-03	10341	6,65561E-01	4135	1,42687E+00	6324	8,86026E-01	12802	0	17530,5
Non-responder	Mono_INHBA	Macro_FOLR2+APOE+	CCL3L1	CCR1	8,02383E-04	7533,1	2,65447E-02	1878	9,75279E-01	1321	2,27546E+00	1172	8,74706E-01	15764	0	17530,5
Non-responder	Macro_NLRP3	cDC_CLEC9A	TNF	TRADD	8,02793E-04	11708,7	1,46222E-02	5232	6,35964E-01	4615	1,72704E+00	3577	8,25976E-01	27589	0	17530,5
Non-responder	Mono_CD14	Macro_FOLR2+APOE+	VCAN	TLR2	8,04916E-04	5639,1	2,65357E-02	1880	1,20001E+00	440	2,36352E+00	1000	9,08555E-01	7345	0	17530,5
Non-responder	Mono_CD14	Macro_FOLR2+APOE+	THBS1	SCARB1	8,07454E-04	8328,9	3,12751E-02	1440	8,74114E-01	1882	2,12625E+00	1599	8,60762E-01	19193	0	17530,5
Non-responder	Macro_FOLR2+APOE+	Mono_CD16	MDK	NOTCH2	8,09558E-04	16407,5	2,65366E-02	1879	5,95094E-01	5421	1,49085E+00	5633	7,20571E-01	51574	0	17530,5
Non-responder	pDC_LILRA4	Macro_IFI27	TNFSF9	HLA-DPA1	8,09558E-04	6472,3	2,35748E-02	2283	8,36935E-01	2213	1,58806E+00	4702	9,16516E-01	5633	0	17530,5
Non-responder	pDC_LILRA4	Macro_FOLR2-APOE+	SPON2	ITGB1	8,09997E-04	9162,3	3,85627E-02	1025	1,09251E+00	798	2,05101E+00	1884	8,38249E-01	24574	0	17530,5
Non-responder	Macro_NLRP3	Mono_CD14	TNF	VSIR	8,11822E-04	8935,3	1,80354E-02	3652	9,03375E-01	1711	2,06271E+00	1849	8,57590E-01	19934	0	17530,5
Non-responder	Macro_FOLR2-APOE+	Macro_FOLR2-APOE+	APOE	TREM2	8,13822E-04	7738,7	2,64812E-02	1887	9,45808E-01	1469	8,06625E-01	17776	9,57134E-01	31	0	17530,5
Non-responder	Macro_OLFML3	pDC_LILRA4	HMGB1	THBD	8,14714E-04	11077,3	8,46867E-03	12844	4,23382E-01	10901	1,85966E+00	2771	8,91628E-01	11340	0	17530,5
Non-responder	Macro_OLFML3	Macro_ISG15	HLA-F	LILRB1	8,15031E-04	9996,3	9,49981E-03	10680	6,24405E-01	4844	1,66408E+00	4082	8,85862E-01	12845	0	17530,5
Non-responder	Macro_OLFML3	Macro_NLRP3	C3	ITGAX	8,15984E-04	10946,5	1,10849E-02	8380	4,36496E-01	10358	1,49247E+00	5616	8,85845E-01	12848	0	17530,5
Non-responder	Macro_FOLR2+APOE+	Macro_FOLR2+APOE+	HLA-DQB1	LAG3	8,17503E-04	9082,5	1,39931E-02	5647	7,84712E-01	2687	2,26739E+00	1186	8,64339E-01	18362	0	17530,5
Non-responder	Macro_OLFML3	Macro_ISG15	HLA-DQA1	LAG3	8,18209E-04	7141,9	2,24920E-02	2472	1,05873E+00	941	2,04499E+00	1911	8,85823E-01	12855	0	17530,5
Non-responder	Macro_FOLR2+APOE+	Macro_FOLR2+APOE+	TIMP2	CD44	8,21396E-04	11365,5	8,85245E-03	11922	4,60097E-01	9420	1,54520E+00	5090	8,85785E-01	12865	0	17530,5
Non-responder	Macro_FOLR2+APOE+	Macro_NLRP3	PSEN1	CD44	8,23313E-04	9287,9	1,08444E-02	8689	6,99425E-01	3636	1,70972E+00	3713	8,85751E-01	12871	0	17530,5
Non-responder	Macro_OLFML3	Macro_FOLR2+APOE+	HLA-DQB1	CD4	8,23953E-04	8087,7	8,45904E-03	12873	6,10621E-01	5079	1,67118E+00	4016	9,41230E-01	940	0	17530,5
Non-responder	cDC_CLEC9A	pDC_LILRA4	HSP90B1	TLR9	8,24080E-04	6932,1	6,61747E-02	375	1,06041E+00	932	2,04932E+00	1895	8,81760E-01	13928	0	17530,5
Non-responder	Macro_NLRP3	Macro_OLFML3	ICAM1	IL2RG	8,24594E-04	9771,3	9,64231E-03	10429	6,62914E-01	4177	1,69102E+00	3845	8,85731E-01	12875	0	17530,5
Non-responder	Macro_IFI27	pDC_LILRA4	GNAI2	CXCR3	8,25875E-04	7043,1	3,07076E-02	1473	1,00039E+00	1205	1,99292E+00	2128	8,85691E-01	12879	0	17530,5
Non-responder	Macro_NLRP3	Macro_IER3	THBS1	ITGA4	8,26654E-04	9228,7	1,39999E-02	5641	7,84929E-01	2682	1,48797E+00	5663	8,79149E-01	14627	0	17530,5
Non-responder	Macro_NLRP3	Macro_OLFML3	ICAM1	ITGAX_ITGB2	8,27159E-04	9580,1	8,45597E-03	12883	6,08504E-01	5135	1,50792E+00	5471	9,10729E-01	6881	0	17530,5
Non-responder	Macro_NLRP3	pDC_LILRA4	LGALS9	CD47	8,28122E-04	9575,3	8,45428E-03	12886	6,52798E-01	4347	2,28815E+00	1144	8,89138E-01	11969	0	17530,5
Non-responder	Macro_NLRP3	Mono_INHBA	S100A8	TLR4	8,28765E-04	8558,9	1,56245E-02	4682	8,83568E-01	1825	1,46740E+00	5869	8,85663E-01	12888	0	17530,5
Non-responder	pDC_LILRA4	Macro_FOLR2+APOE+	HSP90B1	TLR2	8,28954E-04	5852,9	1,39744E-02	5667	1,03992E+00	1024	3,00917E+00	267	9,20701E-01	4776	0	17530,5
Non-responder	Macro_ISG15	Macro_FOLR2+APOE+	CCL2	CCR1	8,33125E-04	9403,7	3,37510E-02	1268	8,71778E-01	1902	2,98149E+00	284	8,32223E-01	26034	0	17530,5
Non-responder	Macro_FOLR2+APOE+	Macro_FOLR2+APOE+	CCL5	SDC4	8,35302E-04	17731,9	2,57855E-02	1977	5,82530E-01	5678	1,71086E+00	3705	6,71844E-01	59769	0	17530,5
Non-responder	pDC_LILRA4	Macro_ISG15	APP	LRP10	8,37192E-04	7176,5	2,50485E-02	2077	1,27081E+00	302	3,21553E+00	165	8,74525E-01	15808	0	17530,5
Non-responder	Macro_NLRP3	Mono_CD14	IL1B	IL1R2_IL1RAP	8,37620E-04	8357,1	1,92744E-02	3256	9,86154E-01	1272	2,20040E+00	1375	8,64417E-01	18352	0	17530,5
Non-responder	Macro_FOLR2+APOE+	Macro_IER3	MMP9	IFNAR1	8,37620E-04	13756,3	1,39560E-02	5682	6,25528E-01	4818	2,03165E+00	1964	7,78925E-01	38787	0	17530,5
Non-responder	Macro_NLRP3	pDC_LILRA4	MAML2	NOTCH4	8,38780E-04	16362,5	3,13009E-02	1437	5,82302E-01	5684	1,70196E+00	3772	7,10857E-01	53389	0	17530,5
Non-responder	Macro_FOLR2+APOE+	Mono_CD14	CD14	TLR4	8,39105E-04	9145,1	1,12309E-02	8159	6,55115E-01	4309	1,85162E+00	2807	8,85548E-01	12920	0	17530,5
Non-responder	Macro_FOLR2+APOE+	cDC_CLEC9A	TOR2A	ATP5F1B	8,39361E-04	12084,7	1,39493E-02	5685	7,36051E-01	3189	1,92954E+00	2413	8,09577E-01	31606	0	17530,5

Post_NR_Myeloid_Myeloid_CellCell

Non-responder	Macro_IFI27	Macro_NLRP3	C3	ITGAX	8,41055E-04	9440,7	1,65494E-02	4217	6,58399E-01	4250	1,01148E+00	12926	9,04596E-01	8280	0	17530,5
Non-responder	Macro_FOLR2+APOE+	Macro_LYVE1	TNFSF12	CD163	8,41687E-04	9922,3	1,53142E-02	4832	6,73806E-01	4003	1,48567E+00	5689	8,67697E-01	17557	0	17530,5
Non-responder	Macro_IFI27	Macro_ISG15	APOE	SDC2	8,42357E-04	8868,3	2,64777E-02	1888	7,83615E-01	2696	1,01132E+00	12930	8,99921E-01	9297	0	17530,5
Non-responder	Macro_FOLR2+APOE+	Mono_CD14	CIRBP	TREM1	8,45293E-04	12138,7	9,80464E-03	10170	4,03060E-01	11853	1,28462E+00	8201	8,85470E-01	12939	0	17530,5
Non-responder	Macro_FOLR2+APOE+	Mono_CD14	HSPA1A	TLR4	8,45620E-04	11742,3	8,43487E-03	12940	4,42605E-01	10122	1,43929E+00	6161	8,89206E-01	11958	0	17530,5
Non-responder	Macro_NLRP3	Mono_CD14	VCAN	TLR2	8,46937E-04	8695,5	1,42166E-02	5495	8,32021E-01	2262	1,73028E+00	3552	8,79114E-01	14638	0	17530,5
Non-responder	cDC_CLEC9A	Macro_ISG15	HLA-DQB1	LAG3	8,47469E-04	6037,9	2,63086E-02	1913	1,25691E+00	325	2,69277E+00	508	8,97291E-01	9913	0	17530,5
Non-responder	pDC_LILRA4	Macro_LYVE1	TNFSF9	HLA-DPA1	8,50451E-04	6052,1	2,33551E-02	2311	8,23879E-01	2329	1,93467E+00	2386	9,16157E-01	5704	0	17530,5
Non-responder	Macro_NLRP3	cDC(CD1C)	CCL20	CXCR3	8,52212E-04	17595,9	2,06473E-02	2909	5,81176E-01	5707	1,50994E+00	5452	6,93736E-01	56381	0	17530,5
Non-responder	Macro_NLRP3	Mono_CD14	CCL3L1	CCR1	8,53975E-04	8891,7	1,90487E-02	3319	9,08497E-01	1677	2,16682E+00	1471	8,55365E-01	20461	0	17530,5
Non-responder	Macro_NLRP3	Macro_ISG15	VEGFA	SIRPA	8,54043E-04	7290,3	2,62729E-02	1918	1,08975E+00	811	2,32821E+00	1068	8,77275E-01	15124	0	17530,5
Non-responder	Macro_FOLR2+APOE+	pDC_LILRA4	HLA-DRA	CD4	8,54564E-04	8256,7	7,16436E-03	16868	5,81028E-01	5711	2,41774E+00	902	9,49336E-01	272	0	17530,5
Non-responder	Mono_CD16	Mono_CD16	HLA-B	LILRB2	8,54808E-04	7116,3	8,42316E-03	12968	7,94020E-01	2607	1,94801E+00	2320	9,51387E-01	156	0	17530,5
Non-responder	Macro_NLRP3	Mono_CD14	S100A12	CD36	8,55742E-04	10814,1	2,69148E-02	1834	6,68189E-01	4087	1,77123E+00	3276	8,27010E-01	27343	0	17530,5
Non-responder	Macro_NLRP3	Mono_INHBA	THBS1	PTPRJ	8,55742E-04	13544,1	1,39054E-02	5713	7,70209E-01	2819	1,53941E+00	5141	7,88531E-01	36517	0	17530,5
Non-responder	Mono_INHBA	cDC_LAMP3	ADM	CALCRL	8,58003E-04	8561,7	5,68821E-02	522	1,23636E+00	361	2,04269E+00	1921	8,46938E-01	22474	0	17530,5
Non-responder	Macro_NLRP3	Mono_CD16	THBS1	LRP1	8,58692E-04	9059,3	1,38961E-02	5718	7,06189E-01	3561	1,64926E+00	4200	8,80352E-01	14287	0	17530,5
Non-responder	Macro_NLRP3	Macro_ISG15	ANXA1	FPR2_FPR3	8,58771E-04	10271,1	1,16032E-02	7748	5,35327E-01	6885	1,43597E+00	6212	8,85271E-01	12980	0	17530,5
Non-responder	Macro_NLRP3	Mono_INHBA	VEGFA	ITGB1	8,59283E-04	10338,5	1,40882E-02	5579	7,92402E-01	2625	1,48318E+00	5719	8,56327E-01	20239	0	17530,5
Non-responder	Macro_FOLR2+APOE+	Macro_FOLR2+APOE+	CD14	TLR1	8,59283E-04	8570,7	1,38946E-02	5719	7,05046E-01	3574	1,95295E+00	2292	8,82538E-01	13738	0	17530,5
Non-responder	pDC_LILRA4	cDC_CLEC9A	APP	LRP10	8,59422E-04	7196,1	2,48953E-02	2100	1,26814E+00	306	3,26662E+00	147	8,74188E-01	15897	0	17530,5
Non-responder	Macro_NLRP3	Macro_LYVE1	S100A9	TLR4	8,63744E-04	8920,9	1,26299E-02	6713	8,41465E-01	2171	1,53534E+00	5195	8,85187E-01	12995	0	17530,5
Non-responder	Macro_IER3	cDC_LAMP3	ICAM1	IL2RG	8,64409E-04	10004,3	1,11673E-02	8264	7,21256E-01	3357	1,17594E+00	9865	8,92953E-01	11005	0	17530,5
Non-responder	Macro_ISG15	Macro_ISG15	LGALS3	LAG3	8,64631E-04	7534,1	2,62265E-02	1926	1,11060E+00	731	2,32658E+00	1073	8,72208E-01	16410	0	17530,5
Non-responder	Macro_NLRP3	Macro_LYVE1	VCAN	ITGA4	8,68741E-04	8882,3	1,38943E-02	5720	8,82697E-01	1836	1,42781E+00	6315	8,85149E-01	13010	0	17530,5
Non-responder	Macro_FOLR2+APOE+	Macro_FOLR2-APOE+	LGALS3	ENG	8,71750E-04	10819,1	9,47517E-03	10712	5,66676E-01	6060	1,00630E+00	13019	9,11258E-01	6774	0	17530,5
Non-responder	Macro_FOLR2+APOE+	Macro_OLFML3	C3	IFITM1	8,72420E-04	9404,9	1,32081E-02	6210	5,19533E-01	7352	1,83515E+00	2911	8,85122E-01	13021	0	17530,5
Non-responder	pDC_LILRA4	Macro_ISG15	NUCB2	ERAP1	8,76644E-04	11524,9	2,61924E-02	1935	9,11016E-01	1663	2,48092E+00	791	7,92099E-01	35705	0	17530,5
Non-responder	Macro_FOLR2+APOE+	Macro_OLFML3	APOE	ABCA1	8,78466E-04	11043,9	8,39426E-03	13039	4,21609E-01	10979	1,51144E+00	5432	9,04789E-01	8239	0	17530,5
Non-responder	Macro_FOLR2+APOE+	Macro_IER3	APOC2	LDLR	8,79552E-04	18133,9	2,16963E-02	2649	5,79173E-01	5753	2,07351E+00	1799	6,47216E-01	62938	0	17530,5
Non-responder	Macro_NLRP3	pDC_LILRA4	CCL2	CCR2	8,81358E-04	14309,9	3,06057E-02	1485	5,78988E-01	5756	2,20540E+00	1360	7,50043E-01	45418	0	17530,5
Non-responder	Macro_FOLR2+APOE+	Macro_LYVE1	PLTP	ABCA1	8,81502E-04	9513,5	1,30822E-02	6323	6,07327E-01	5157	1,50360E+00	5509	8,85004E-01	13048	0	17530,5
Non-responder	Mast	pDC_LILRA4	CSF1	CSF2RA	8,82019E-04	8605,7	4,43441E-02	802	1,08542E+00	829	2,03718E+00	1939	8,49274E-01	21928	0	17530,5
Non-responder	Macro_NLRP3	Macro_ISG15	TNF	VSIR	8,83769E-04	8883,7	1,78125E-02	3734	8,94269E-01	1765	2,23646E+00	1275	8,56829E-01	20114	0	17530,5
Non-responder	Mono_CD14	Macro_FOLR2+APOE+	S100A8	TLR4	8,86064E-04	5482,5	2,60990E-02	1942	1,27488E+00	295	2,78381E+00	432	9,09185E-01	7213	0	17530,5
Non-responder	pDC_LILRA4	Macro_FOLR2-APOE+	APP	APLP2	8,86185E-04	5577,1	2,22523E-02	2525	1,24181E+00	349	2,09589E+00	1717	9,15907E-01	5764	0	17530,5
Non-responder	pDC_LILRA4	Mono_CD14	LTB	TNFRSF1A	8,87415E-04	6751,9	2,60963E-02	1943	1,12255E+00	684	3,33544E+00	128	8,83506E-01	13474	0	17530,5
Non-responder	Mono_CD16	Macro_ISG15	TIMP1	CD63	8,87939E-04	7629,1	8,38121E-03	13067	7,70086E-01	2821	1,62497E+00	4383	9,48194E-01	344	0	17530,5
Non-responder	pDC_LILRA4	Macro_FOLR2-APOE+	HSP90B1	LRP1	8,88001E-04	5923,1	1,38306E-02	5767	1,04222E+00	1012	2,13195E+00	1580	9,26310E-01	3726	0	17530,5
Non-responder	pDC_LILRA4	cDC_LAMP3	SPON2	ITGB1	8,90121E-04	10721,3	2,60884E-02	1945	9,63231E-01	1381	2,25045E+00	1234	8,09977E-01	31516	0	17530,5
Non-responder	Mono_CD16	cDC(CD1C)	B2M	CD1B	8,93730E-04	11065,3	2,33684E-02	2307	3,77778E-01	13084	1,10865E+00	11013	8,91440E-01	11392	0	17530,5
Non-responder	Macro_IER3	Macro_FOLR2+APOE+	CD14	ITGB1	8,94413E-04	11118,3	8,37564E-03	13086	4,94538E-01	8171	1,22204E+00	9109	9,07040E-01	7695	0	17530,5
Non-responder	Macro_NLRP3	Macro_ISG15	THBS1	CD36	8,94681E-04	8935,1	1,94343E-02	3217	8,34979E-01	2228	1,92405E+00	2440	8,60531E-01	19260	0	17530,5
Non-responder	Macro_IFI27	Mono_CD16	HLA-A	LILRB2	8,94755E-04	9347,5	8,46782E-03	12846	7,57072E-01	2948	1,00229E+00	13087	9,48510E-01	326	0	17530,5
Non-responder	pDC_LILRA4	Mono_CD14	CD38	PECAM1	8,95549E-04	9967,5	2,60448E-02	1949	9,88897E-01	1253	2,65833E+00	545	8,22152E-01	28560	0	17530,5
Non-responder	Macro_FOLR2+APOE+	Macro_FOLR2+APOE+	HSP90B1	LRP1	8,96124E-04	10988,1	8,37482E-03	13091	4,53718E-01	9670	1,37167E+00	7000	9,07250E-01	7649	0	17530,5
Non-responder	Macro_NLRP3	Macro_ISG15	THBS1	PTPRJ	8,96509E-04	12448,3	1,57809E-02	4607	7,97490E-01	2585	1,75389E+00	3387	7,98888E-01	34132	0	17530,5
Non-responder	Macro_NLRP3	Mono_CD14	THBS1	PTPRJ	8,97119E-04	13379,7	1,38048E-02	5782	7,68745E-01	2833	1,66293E+00	4089	7,87925E-01	36664	0	17530,5
Non-responder	Macro_NLRP3	Macro_ISG15	CD55	ADGRE2	8,98340E-04	9596,1	1,43224E-02	5427	7,52164E-01	3001	1,71708E+00	3651	8,64287E-01	18371	0	17530,5
Non-responder	Macro_NLRP3	Macro_ISG15	RPS19	C5AR1	8,98865E-04	8827,5	8,37251E-03	13099	6,75846E-01	3963	1,42320E+00	6371	9,29101E-01	3174	0	17530,5
Non-responder	Macro_NLRP3	Macro_FOLR2+APOE+	IL6	FCGR3B	8,99563E-04	17834,7	3,20747E-02	1386	5,95029E-01	5424	1,47498E+00	5786	6,76920E-01	59047	0	17530,5
Non-responder	Macro_FOLR2+APOE+	cDC_CLEC9A	FN1	DPP4	9,00786E-04	16209,3	5,41238E-02	565	6,58803E-01	4237	1,47496E+00	5788	7,13458E-01	52926	0	17530,5
Non-responder	Mono_CD16	Macro_ISG15	LGALS9	HAVCR2	9,01613E-04	11165,3	8,36839E-03	13107	4,84361E-01	8531	1,62944E+00	4347	8,87886E-01	12311	0	17530,5
Non-responder	Macro_FOLR2+APOE+	cDC_CLEC9A	SPP1	PTGER4	9,02624E-04	9093,7	2,11849E-02	2768	5,77573E-01	5791	2,86871E+00	362	8,61539E-01	19017	0	17530,5
Non-responder	Macro_FOLR2+APOE+	Macro_NLRP3	PLAU	LRP1	9,04464E-04	10661,3	1,62387E-02	4366	5,77312E-01	5794	1,76684E+00	3314	8,47704E-01	22302	0	17530,5
Non-responder	Mast	cDC_CLEC9A	ADCYAP1	ADRB3	9,04749E-04	19113,5	5,61958E-01	6	1,26318E+00	314	1,39147E+00	6753	5,48215E-01	70964	0	17530,5
Non-responder	Macro_FOLR2+APOE+	Macro_IFI27	APOE	LRP1	9,06093E-04	11664,3	9,34801E-03	10946	4,02571E-01	11869	1,00051E+00	13120	9,20355E-01	4856	0	17530,5
Non-responder	Macro_NLRP3	cDC(CD1C)	THBS1	ITGA4	9,06922E-04	9264,9	1,39824E-02	5660	7,84431E-01	2689	1,47400E+00	5798	8,79082E-01	14647	0	17530,5
Non-responder	Mono_CD16	cDC_CLEC9A	ADAM10	CADM1	9,07843E-04	9918,5	3,26042E-02	1348	1,11049E+00	732	2,03357E+00	1958	8,24339E-01	28024	0	17530,5
Non-responder	Macro_NLRP3	Macro_OLFML3	THBS1	LRP1	9,08153E-04	9262,9	1,37800E-02	5800	7,02958E-01	3599	1,55602E+00	4976	8,79909E-01	14409	0	17530,5
Non-responder	Macro_FOLR2+APOE+	Macro_LYVE1	MMP9	IFNAR1	9,10002E-04	13909,3	1,37792E-02	5803	6,22288E-01	4878	1,95596E+00	2275	7,77825E-01	39060	0	17530,5
Non-responder	Macro_FOLR2+APOE+	Macro_ISG15	APOE	TREM2	9,10245E-04	8638,5	1,13274E-02	8053	3,76986E-01	13132	1,87438E+00	2693	9,35912E-01	1784	0	17530,5
Non-responder	pDC_LILRA4	cDC_CLEC9A	CDH1	ITGAE_ITGB7	9,10590E-04	14908,1	1,01616E-01	132	9,07419E-01	1687	2,03341E+00	1960	7,11840E-01	53231	0	17530,5
Non-responder	Macro_NLRP3	Mono_CD16	RPS19	C5AR1	9,10938E-04	8816,7	8,35897E-03	13134	6,74648E-01	3984	1,43192E+00	6250	9,29048E-01	3185	0	17530,5
Non-responder	Macro_NLRP3	Macro_IER3	VCAN	ITGB1	9,12326E-04	9013,1	1,36363E-02	5899	8,51007E-01	2080	1,41953E+00	6418	8,84646E-01	13138	0	17530,5
Non-responder	Macro_NLRP3	cDC(CD1C)	VCAN	TLR2	9,12471E-04	8865,3	1,49936E-02	5018	8,50129E-01	2089	1,47336E+00	5807	8,81914E-01	13882	0	17530,5
Non-responder	Macro_FOLR2+APOE+	pDC_LILRA4	TNFSF13B	TFRC	9,14063E-04	9300,9	1,04410E-02	9233	6,41691E-01	4519	2,00283E+00	2079	8,84633E-01	13143	0	17530,5
Non-responder	pDC_LILRA4	pDC_LILRA4	FAM3C	ADGRG5	9,14721E-04	18062,5	7,20864E-02	299	8,64133E-01	1963	2,50628E+00	759	5,70784E-01	69761	0	17530,5
Non-responder	Macro_OLFML3	Macro_ISG15	HLA-B	LILRB1	9,15107E-04	7970,9	8,35513E-03	13146	6,56092E-01	4292	1,71431E+00	3675	9,39077E-01	1211	0	17530,5
Non-responder	Macro_OLFML3	pDC_LILRA4	HLA-DMB	CD4	9,16500E-04	9292,1	8,35327E-03	13150	5,08101E-01	7730	2,03641E+00	1946	9,14315E-01	6104	0	17530,5
Non-responder	Macro_NLRP3	Macro_NLRP3	ADM	GPR84	9,17424E-04	15749,7	1,81099E-02	3629	5,76341E-01	5815	1,57382E+00	4814	7,42976E-01	46960	0	17530,5

Post_NR_Myeloid_Myeloid_CellCell

Non-responder	Macro_FOLR2+APOE+	cDC_CLEC9A	LGALS3BP	ITGB1	9,18045E-04	9566,5	1,37602E-02	5816	6,38614E-01	4576	1,79499E+00	3143	8,70799E-01	16767	0	17530,5
Non-responder	Macro_FOLR2+APOE+	Macro_ISG15	HLA-A	LILRA1	9,19291E-04	9962,9	8,34900E-03	13158	6,33584E-01	4658	1,77382E+00	3266	8,92178E-01	11202	0	17530,5
Non-responder	Macro_FOLR2+APOE+	Macro_FOLR2+APOE+	ANXA2	TLR2	9,20690E-04	10170,1	8,34638E-03	13162	4,72974E-01	8949	1,66981E+00	4030	9,09346E-01	7179	0	17530,5
Non-responder	Macro_ISG15	pDC_LILRA4	IL10	IFNLR1	9,21632E-04	14271,5	6,22432E-02	418	8,63595E-01	1968	2,52774E+00	727	7,25085E-01	50714	0	17530,5
Non-responder	Mast	cDC_LAMP3	TNFSF10	TNFRSF11B	9,22045E-04	12307,1	8,27693E-02	213	1,26100E+00	317	1,27052E+00	8396	7,94824E-01	35079	0	17530,5
Non-responder	Macro_FOLR2+APOE+	Macro_NLRP3	PLTP	ABCA1	9,23491E-04	8905,3	1,29607E-02	6430	6,02999E-01	5247	1,98719E+00	2149	8,84529E-01	13170	0	17530,5
Non-responder	Macro_NLRP3	Macro_ISG15	TGFB1	SDC2	9,25516E-04	11701,9	1,71409E-02	3989	6,41326E-01	4524	1,81269E+00	3038	8,18531E-01	29428	0	17530,5
Non-responder	Macro_OLFML3	pDC_LILRA4	TNFSF13	TNFRSF17	9,25796E-04	10437,7	8,47374E-02	203	8,63350E-01	1971	2,07426E+00	1795	8,13372E-01	30689	0	17530,5
Non-responder	Mono_CD14	Macro_NLRP3	S100A8	CD69	9,26141E-04	5609,7	2,05950E-02	2921	1,17879E+00	501	2,23795E+00	1267	9,15578E-01	5829	0	17530,5
Non-responder	Macro_FOLR2+APOE+	cDC_CLEC9A	CD14	ITGB2	9,26766E-04	8483,7	7,58912E-03	15357	5,76022E-01	5830	1,84183E+00	2868	9,42069E-01	833	0	17530,5
Non-responder	Macro_NLRP3	Macro_IER3	THBS1	LRP1	9,28058E-04	9083,7	1,50509E-02	4980	7,38318E-01	3163	1,40719E+00	6562	8,84493E-01	13183	0	17530,5
Non-responder	Macro_NLRP3	Macro_ISG15	IL1B	IL1R1_IL1RAP	9,28642E-04	11324,1	1,44420E-02	5339	9,49147E-01	1447	2,15892E+00	1495	8,12823E-01	30809	0	17530,5
Non-responder	Macro_NLRP3	Macro_ISG15	IL1B	IL1R2_IL1RAP	9,29268E-04	8451,7	1,87425E-02	3416	9,78164E-01	1308	2,23319E+00	1287	8,62768E-01	18717	0	17530,5
Non-responder	Mono_CD16	Mono_CD16	SELPLG	ITGB2	9,31583E-04	9328,9	8,33527E-03	13193	5,31073E-01	7017	1,67622E+00	3963	9,20004E-01	4941	0	17530,5
Non-responder	Macro_IER3	pDC_LILRA4	VCAN	SELL	9,32762E-04	6361,3	2,58123E-02	1976	1,13744E+00	620	2,80975E+00	407	8,91911E-01	11273	0	17530,5
Non-responder	Macro_LYVE1	cDC_LAMP3	PDGFB	S1PR1	9,33665E-04	15676,7	8,93245E-02	179	1,26093E+00	319	1,41633E+00	6458	7,08033E-01	53897	0	17530,5
Non-responder	Mono_CD16	Macro_NLRP3	LGALS9	CD44	9,33703E-04	8465,1	8,33463E-03	13199	6,94442E-01	3686	1,79057E+00	3167	9,20903E-01	4743	0	17530,5
Non-responder	cDC(CD1C)	Mono_CD16	LYZ	ITGAL	9,34289E-04	7400,9	1,37204E-02	5842	6,82171E-01	3877	1,58758E+00	4707	9,19468E-01	5048	0	17530,5
Non-responder	Macro_NLRP3	Macro_OLFML3	HBEGF	CD9	9,36182E-04	13020,1	8,33116E-03	13206	4,65215E-01	9234	1,02798E+00	12592	8,87032E-01	12538	0	17530,5
Non-responder	Macro_NLRP3	Macro_ISG15	CXCL8	SDC3	9,37437E-04	10406,7	1,92588E-02	3259	8,47374E-01	2108	1,92076E+00	2456	8,29696E-01	26680	0	17530,5
Non-responder	Macro_NLRP3	Macro_NLRP3	RPS19	C5AR1	9,38311E-04	9196,1	8,32902E-03	13212	6,71999E-01	4032	1,29889E+00	7995	9,28929E-01	3211	0	17530,5
Non-responder	Macro_FOLR2+APOE+	Macro_IER3	RARRES1	NRP2	9,38699E-04	16990,7	2,89302E-02	1659	8,02215E-01	2528	1,46901E+00	5849	6,87475E-01	57387	0	17530,5
Non-responder	Macro_NLRP3	Macro_ISG15	CCL3	CCR5	9,39330E-04	9562,5	2,23531E-02	2496	7,47082E-01	3058	1,92114E+00	2454	8,47837E-01	22274	0	17530,5
Non-responder	Macro_NLRP3	Macro_ISG15	CCL4	CCR5	9,40593E-04	10360,1	2,19474E-02	2585	7,03692E-01	3586	1,78433E+00	3203	8,36958E-01	24896	0	17530,5
Non-responder	Macro_NLRP3	Macro_ISG15	CCL2	CCR1	9,41226E-04	10894,7	2,58222E-02	1975	7,95144E-01	2597	2,14750E+00	1525	8,12689E-01	30846	0	17530,5
Non-responder	Macro_ISG15	Macro_FOLR2+APOE+	CCL18	CCR1	9,42573E-04	11123,1	4,11243E-02	923	8,62005E-01	1983	2,57916E+00	654	7,97295E-01	34525	0	17530,5
Non-responder	Macro_FOLR2+APOE+	Macro_FOLR2+APOE+	APOC2	LDLR	9,43757E-04	18317,5	2,11874E-02	2765	5,75033E-01	5857	1,98555E+00	2157	6,44502E-01	63278	0	17530,5
Non-responder	Macro_NLRP3	Macro_NLRP3	CXCL8	SDC2	9,43757E-04	13247,1	1,36992E-02	5857	7,97957E-01	2578	1,80960E+00	3056	7,85582E-01	37214	0	17530,5
Non-responder	Macro_ISG15	cDC_LAMP3	THBS1	TNFRSF11B	9,43980E-04	11031,3	9,37128E-02	161	1,24756E+00	340	2,02679E+00	1984	7,94588E-01	35141	0	17530,5
Non-responder	Macro_NLRP3	Macro_ISG15	VCAN	SELL	9,44391E-04	8444,1	1,71072E-02	4001	9,48139E-01	1457	1,93866E+00	2366	8,70427E-01	16866	0	17530,5
Non-responder	Macro_NLRP3	Macro_ISG15	VCAN	TLR1	9,45659E-04	8578,5	2,32358E-02	2325	1,00115E+00	1203	2,09498E+00	1721	8,56834E-01	20113	0	17530,5
Non-responder	Macro_FOLR2+APOE+	Macro_FOLR2+APOE+	ADAM9	ITGB1	9,46294E-04	11423,1	1,46453E-02	5201	5,74969E-01	5861	1,65362E+00	4166	8,39140E-01	24357	0	17530,5
Non-responder	Mono_CD16	Macro_IER3	AGTRAP	RACK1	9,46864E-04	12054,1	9,53048E-03	10627	5,80646E-01	5722	9,98844E-01	13155	8,84315E-01	13236	0	17530,5
Non-responder	Macro_NLRP3	Macro_ISG15	VEGFA	NRP1	9,48200E-04	9465,3	2,51832E-02	2055	9,54882E-01	1423	2,16505E+00	1480	8,37219E-01	24838	0	17530,5
Non-responder	Macro_ISG15	pDC_LILRA4	HMGB1	TLR9	9,48210E-04	7301,5	5,60849E-02	531	9,34938E-01	1530	2,02661E+00	1987	8,78036E-01	14929	0	17530,5
Non-responder	Macro_NLRP3	Macro_ISG15	VEGFA	NRP2	9,48836E-04	10751,1	1,97499E-02	3132	9,04377E-01	1706	1,97012E+00	2207	8,19573E-01	29180	0	17530,5
Non-responder	Macro_NLRP3	Macro_ISG15	VEGFA	ITGB1	9,49472E-04	8045,5	1,90838E-02	3310	9,30830E-01	1551	2,04899E+00	1897	8,74008E-01	15939	0	17530,5
Non-responder	Macro_NLRP3	Macro_ISG15	VEGFA	ITGAV	9,50109E-04	12596,9	1,63758E-02	4307	8,05248E-01	2499	1,76640E+00	3319	7,93846E-01	35329	0	17530,5
Non-responder	Macro_FOLR2+APOE+	Macro_FOLR2+APOE+	RPS19	C5AR1	9,50109E-04	9802,1	7,18840E-03	16768	5,74692E-01	5867	1,59338E+00	4656	9,23911E-01	4189	0	17530,5
Non-responder	Macro_NLRP3	Macro_ISG15	VEGFA	EPHB2	9,50746E-04	12885,9	3,81698E-02	1038	9,59292E-01	1401	1,92359E+00	2443	7,65276E-01	42017	0	17530,5
Non-responder	Macro_FOLR2+APOE-	pDC_LILRA4	GNAI2	CXCR3	9,52452E-04	6507,1	3,55642E-02	1172	1,08499E+00	831	2,02488E+00	1990	8,92916E-01	11012	0	17530,5
Non-responder	Macro_FOLR2+APOE+	Macro_ISG15	CIRBP	TREM1	9,52960E-04	12018,7	9,57769E-03	10541	3,89914E-01	12475	1,42922E+00	6294	8,84277E-01	13253	0	17530,5
Non-responder	Macro_FOLR2+APOE+	cDC_LAMP3	TNFSF13	FAS	9,54573E-04	12755,3	1,89356E-02	3352	5,74483E-01	5874	1,48322E+00	5718	8,10844E-01	31302	0	17530,5
Non-responder	Macro_NLRP3	Macro_ISG15	ADM	GPR84	9,55213E-04	13394,1	2,41699E-02	2194	6,79190E-01	3916	1,94880E+00	2318	7,69558E-01	41012	0	17530,5
Non-responder	Mono_CD14	cDC(CD1C)	S100A8	CD68	9,59695E-04	5516,1	1,36529E-02	5882	9,53344E-01	1429	1,88527E+00	2629	9,52594E-01	110	0	17530,5
Non-responder	Mono_CD14	Macro_FOLR2+APOE+	NRG1	MS4A4A	9,60973E-04	10234,7	3,55914E-02	1170	8,60743E-01	1996	2,03373E+00	1956	8,22301E-01	28521	0	17530,5
Non-responder	Macro_IFI27	Mono_CD16	HLA-C	LILRB2	9,62707E-04	9431,5	8,49337E-03	12781	7,26369E-01	3290	9,92191E-01	13280	9,49269E-01	276	0	17530,5
Non-responder	Macro_ISG15	pDC_LILRA4	COPA	P2RY6	9,63824E-04	7104,1	2,56540E-02	1998	1,25130E+00	336	3,34917E+00	124	8,75672E-01	15532	0	17530,5
Non-responder	Macro_NLRP3	cDC(CD1C)	VCAN	CD44	9,65974E-04	10134,3	1,03737E-02	9309	7,55824E-01	2962	9,91898E-01	13289	9,07519E-01	7581	0	17530,5
Non-responder	Macro_FOLR2+APOE+	Mono_INHBA	C3	ITGAX	9,66337E-04	11943,5	1,07198E-02	8852	3,98435E-01	12058	1,29976E+00	7987	8,84140E-01	13290	0	17530,5
Non-responder	Macro_FOLR2+APOE+	Macro_FOLR2+APOE+	CCL3L1	CCR5	9,68707E-04	13390,5	2,07607E-02	2879	6,02804E-01	5252	1,46534E+00	5896	7,93538E-01	35395	0	17530,5
Non-responder	Macro_NLRP3	Macro_ISG15	THBS1	SDC4	9,69353E-04	10582,9	2,47055E-02	2126	9,17138E-01	1632	1,89951E+00	2554	8,19972E-01	29072	0	17530,5
Non-responder	Macro_OLFML3	Macro_FOLR2+APOE+	B2M	TFRC	9,70708E-04	8362,1	8,29250E-03	13302	5,51790E-01	6447	1,76491E+00	3326	9,39089E-01	1205	0	17530,5
Non-responder	Macro_NLRP3	Macro_IER3	S100A9	TLR4	9,71803E-04	9172,5	1,23642E-02	6982	8,34675E-01	2234	1,47299E+00	5811	8,84102E-01	13305	0	17530,5
Non-responder	Macro_OLFML3	Macro_ISG15	HLA-F	LILRB2	9,71803E-04	9572,5	8,29105E-03	13305	6,71764E-01	4036	1,66258E+00	4091	9,01660E-01	8900	0	17530,5
Non-responder	Macro_NLRP3	Macro_ISG15	HBEGF	CD82	9,73884E-04	9111,1	1,73705E-02	3902	8,12007E-01	2433	1,74465E+00	3455	8,64925E-01	18235	0	17530,5
Non-responder	pDC_LILRA4	Mono_CD16	HLA-F	LILRB2	9,75182E-04	5894,5	1,36299E-02	5906	1,04516E+00	997	2,80593E+00	415	9,21604E-01	4624	0	17530,5
Non-responder	Macro_FOLR2+APOE+	Mono_CD14	APOE	SORL1	9,75460E-04	10996,9	8,86605E-03	11894	3,73435E-01	13315	1,58052E+00	4764	9,07935E-01	7481	0	17530,5
Non-responder	Macro_FOLR2+APOE+	cDC(CD1C)	PLAU	ST14	9,77131E-04	12607,1	1,73942E-02	3895	5,73168E-01	5909	1,53787E+00	5162	8,13940E-01	30539	0	17530,5
Non-responder	Macro_IER3	cDC_LAMP3	TNFSF13B	CD40	9,77293E-04	10933,9	1,00881E-02	9736	7,10899E-01	3503	1,13279E+00	10580	8,84052E-01	13320	0	17530,5
Non-responder	Mono_INHBA	Mono_INHBA	CXCL8	SDC2	9,78165E-04	8572,3	3,43503E-02	1242	1,03703E+00	1036	2,01926E+00	2008	8,52976E-01	21045	0	17530,5
Non-responder	Mono_CD16	Macro_NLRP3	SERPINA1	LRP1	9,78394E-04	9402,5	8,28497E-03	13323	5,83218E-01	5659	1,60856E+00	4523	9,14918E-01	5977	0	17530,5
Non-responder	cDC_CLEC9A	Macro_LYVE1	CD59	STAB1	9,79607E-04	5527,9	2,55607E-02	2009	1,18082E+00	496	2,12011E+00	1623	9,14899E-01	5981	0	17530,5
Non-responder	Macro_IER3	Macro_FOLR2+APOE+	C3	C3AR1	9,80232E-04	12985,7	1,04776E-02	9185	3,78955E-01	13036	1,06410E+00	11849	8,84028E-01	13328	0	17530,5
Non-responder	Mono_CD16	Mono_CD14	S100A8	CD36	9,81335E-04	10561,1	1,38108E-02	5777	5,36236E-01	6866	1,20961E+00	9301	8,84015E-01	13331	0	17530,5
Non-responder	Macro_FOLR2+APOE+	Mono_INHBA	PLTP	ABCA1	9,81703E-04	9549,1	1,28286E-02	6544	5,98295E-01	5348	1,55466E+00	4991	8,84005E-01	13332	0	17530,5
Non-responder	Macro_OLFML3	Macro_OLFML3	CXCL10	SDC4	9,82495E-04	10747,9	2,55535E-02	2011	1,09952E+00	765	2,87012E+00	360	8,03347E-01	33073	0	17530,5
Non-responder	Macro_OLFML3	pDC_LILRA4	TNFSF13B	TFRC	9,86869E-04	9630,7	1,03072E-02	9403	6,31728E-01	4690	1,78804E+00	3184	8,83973E-01	13346	0	17530,5
Non-responder	Macro_NLRP3	Macro_ISG15	NRG1	MS4A4A	9,88229E-04	13469,9	2,29102E-02	2389	6,04103E-01	5224	1,50219E+00	5516	7,87807E-01	36690	0	17530,5
Non-responder	Mono_CD14	Macro_FOLR2+APOE+	VCAN	ITGB1	9,88287E-04	5402,3	2,55201E-02	2015	1,24532E+00	344	2,52811E+00	726	9,12978E-01	6396	0	17530,5
Non-responder	Macro_FOLR2+APOE+	Macro_FOLR2+APOE+	ADAM10	AXL	9,88884E-04	11374,5	1,72958E-02	3935	5,72475E-01	5927	1,55651E+00	4970	8,38506E-01	24510	0	17530,5

Post_NR_Myeloid_Myeloid_CellCell

Non-responder	pDC_LILRA4	Macro_OLFML3	TNFSF9	HLA-DPA1	9,89541E-04	5934,5	2,27394E-02	2420	7,87293E-01	2666	2,29539E+00	1128	9,15125E-01	5928	0	17530,5
Non-responder	pDC_LILRA4	pDC_LILRA4	HLA-B	CANX	9,92849E-04	7842,3	6,77687E-03	18456	7,78991E-01	2736	2,92772E+00	329	9,51250E-01	160	0	17530,5
Non-responder	Macro_FOLR2+APOE+	Macro_FOLR2+APOE+	MDK	LRP1	9,93483E-04	14823,1	2,73825E-02	1782	5,83527E-01	5654	1,46086E+00	5934	7,59656E-01	43215	0	17530,5
Non-responder	Macro_FOLR2+APOE+	Macro_NLRP3	MMP9	IFNAR1	9,93483E-04	13756,9	1,35888E-02	5934	6,18799E-01	4952	2,34173E+00	1043	7,76620E-01	39325	0	17530,5
Non-responder	Mono_CD14	cDC(CD1C)	S100A9	ITGB2	9,94101E-04	5298,5	1,35175E-02	5989	1,07953E+00	858	2,01670E+00	2019	9,53256E-01	96	0	17530,5
Non-responder	Macro_NLRP3	Macro_FOLR2+APOE+	TNF	VSIR	9,96778E-04	10510,9	1,35769E-02	5939	7,21185E-01	3360	2,18843E+00	1410	8,39355E-01	24315	0	17530,5
Non-responder	Macro_IER3	Mono_INHBA	HBEGF	CD44	9,99877E-04	8160,9	1,19692E-02	7376	8,12184E-01	2431	1,28856E+00	8137	9,18118E-01	5330	0	17530,5
Non-responder	Macro_OLFML3	Macro_FOLR2+APOE+	HLA-DMA	CD4	1,00062E-03	8957,3	8,26149E-03	13383	5,45419E-01	6616	1,56947E+00	4858	9,32921E-01	2399	0	17530,5
Non-responder	Macro_FOLR2+APOE+	Macro_NLRP3	CXCL10	SDC4	1,00273E-03	15253,5	1,39598E-02	5679	5,71349E-01	5948	2,03826E+00	1936	7,51205E-01	45174	0	17530,5
Non-responder	Macro_NLRP3	cDC_CLEC9A	AGTRAP	RACK1	1,00399E-03	10387,5	9,43747E-03	10797	6,05999E-01	5187	1,55089E+00	5031	8,83813E-01	13392	0	17530,5
Non-responder	Macro_FOLR2+APOE+	pDC_LILRA4	TNF	TRAF2	1,00538E-03	14783,1	2,10263E-02	2806	5,71127E-01	5952	1,87505E+00	2690	7,52137E-01	44937	0	17530,5
Non-responder	Mono_CD16	pDC_LILRA4	TNFSF13B	TNFRSF17	1,00727E-03	10658,3	7,04873E-02	322	8,57363E-01	2028	2,20084E+00	1373	8,07637E-01	32038	0	17530,5
Non-responder	Macro_NLRP3	Macro_FOLR2+APOE-	CXCL8	SDC3	1,01004E-03	11309,9	1,79312E-02	3686	8,27302E-01	2302	1,54796E+00	5065	8,24590E-01	27966	0	17530,5
Non-responder	Macro_OLFML3	Macro_FOLR2+APOE+	LGALS3	ENG	1,01038E-03	12403,9	8,44382E-03	12916	3,71768E-01	13409	1,14773E+00	10330	9,06487E-01	7834	0	17530,5
Non-responder	Macro_FOLR2+APOE+	Mono_CD14	PLAU	IGF2R	1,01204E-03	14712,3	1,44176E-02	5361	5,70643E-01	5962	1,65927E+00	4121	7,71398E-01	40587	0	17530,5
Non-responder	Macro_OLFML3	Macro_ISG15	LILRB4	LAIR1	1,01264E-03	10601,3	8,25105E-03	13415	4,68877E-01	9109	1,48819E+00	5659	9,08783E-01	7293	0	17530,5
Non-responder	Macro_IER3	pDC_LILRA4	LGALS9	CD47	1,01453E-03	9038,1	9,25267E-03	11124	6,89871E-01	3750	2,04181E+00	1926	8,93508E-01	10860	0	17530,5
Non-responder	Mono_INHBA	Mono_CD14	IL6	IL6R_IL6ST	1,01611E-03	11861,7	3,16295E-02	1408	1,12758E+00	656	2,01293E+00	2034	7,83644E-01	37680	0	17530,5
Non-responder	Macro_NLRP3	Mono_INHBA	VEGFA	NRP1	1,01873E-03	13364,9	1,35372E-02	5972	7,88348E-01	2657	1,59954E+00	4597	7,90396E-01	36068	0	17530,5
Non-responder	Macro_FOLR2+APOE+	cDC_CLEC9A	PDCD1LG2	PDCD2	1,01940E-03	17261,1	2,11272E-02	2786	5,89821E-01	5524	1,45755E+00	5973	7,04882E-01	54492	0	17530,5
Non-responder	Mono_CD16	Macro_ISG15	HLA-F	LILRB1	1,01984E-03	10201,7	9,09635E-03	11413	6,02270E-01	5263	1,75756E+00	3368	8,83650E-01	13434	0	17530,5
Non-responder	Mono_CD16	Mono_CD14	SELPLG	ITGB2	1,02022E-03	9739,1	8,24277E-03	13435	5,20186E-01	7319	1,51543E+00	5393	9,19593E-01	5018	0	17530,5
Non-responder	Macro_FOLR2+APOE+	Mono_CD16	A2M	LRP1	1,02060E-03	11506,1	9,90170E-03	10008	4,32398E-01	10516	1,44946E+00	6040	8,83649E-01	13436	0	17530,5
Non-responder	Macro_NLRP3	pDC_LILRA4	NAMPT	INSR	1,02074E-03	11144,1	1,35348E-02	5975	7,45022E-01	3085	1,62410E+00	4386	8,37591E-01	24744	0	17530,5
Non-responder	Macro_FOLR2+APOE+	Macro_FOLR2-APOE+	A2M	LRP1	1,02098E-03	9768,7	1,52069E-02	4887	6,39556E-01	4563	9,85166E-01	13437	9,03956E-01	8426	0	17530,5
Non-responder	Macro_NLRP3	Mono_INHBA	THBS1	LRP1	1,02250E-03	9111,3	1,48002E-02	5127	7,31343E-01	3240	1,43559E+00	6218	8,83632E-01	13441	0	17530,5
Non-responder	Macro_IER3	pDC_LILRA4	ICAM1	IL2RG	1,02288E-03	6941,5	1,37569E-02	5819	8,88194E-01	1804	2,46160E+00	826	9,02520E-01	8728	0	17530,5
Non-responder	Macro_FOLR2+APOE+	Mono_INHBA	MDK	SDC2	1,02477E-03	17571,1	5,02726E-02	654	7,58114E-01	2940	1,45635E+00	5981	6,64446E-01	60750	0	17530,5
Non-responder	pDC_LILRA4	pDC_LILRA4	TNFSF13	TNFRSF17	1,02500E-03	10219,3	8,38014E-02	208	8,56303E-01	2040	2,77483E+00	440	8,12527E-01	30878	0	17530,5
Non-responder	Macro_NLRP3	Macro_NLRP3	ICAM1	ITGAM_ITGB2	1,02554E-03	10140,5	8,23824E-03	13449	5,63666E-01	6137	1,52042E+00	5341	9,04752E-01	8245	0	17530,5
Non-responder	Macro_IER3	pDC_LILRA4	HLA-C	NOTCH4	1,02707E-03	9173,3	2,43506E-02	2175	4,87382E-01	8416	1,20669E+00	9336	9,04037E-01	8409	0	17530,5
Non-responder	Macro_FOLR2+APOE+	Macro_FOLR2-APOE+	PSAP	LRP1	1,02707E-03	10063,5	8,23610E-03	13453	6,39479E-01	4564	1,02558E+00	12634	9,34245E-01	2136	0	17530,5
Non-responder	Macro_NLRP3	Mono_INHBA	TNF	TNFRSF1B	1,02783E-03	9148,9	1,21760E-02	7165	7,00153E-01	3627	1,67606E+00	3967	8,83577E-01	13455	0	17530,5
Non-responder	Macro_OLFML3	Macro_ISG15	B2M	LILRB1	1,02783E-03	8064,5	8,23496E-03	13455	6,23526E-01	4859	1,68341E+00	3904	9,44986E-01	574	0	17530,5
Non-responder	Macro_NLRP3	Mono_INHBA	TGFB1	SDC2	1,02882E-03	12086,5	1,76694E-02	3780	6,57418E-01	4264	1,45578E+00	5987	8,20776E-01	28871	0	17530,5
Non-responder	pDC_LILRA4	Macro_OLFML3	APP	FPR2	1,02922E-03	9757,1	2,48228E-02	2112	1,25146E+00	335	2,97076E+00	299	8,22360E-01	28509	0	17530,5
Non-responder	cDC_CLEC9A	pDC_LILRA4	LGALS9	CD47	1,02950E-03	6021,9	1,51773E-02	4900	9,64982E-01	1370	2,93669E+00	321	9,14864E-01	5988	0	17530,5
Non-responder	Mono_INHBA	Macro_NLRP3	VEGFA	CD44	1,03017E-03	5771,9	2,12769E-02	2741	1,13608E+00	628	2,02991E+00	1971	9,14863E-01	5989	0	17530,5
Non-responder	Macro_FOLR2+APOE+	Macro_FOLR2+APOE+	SPP1	ITGAV_ITGB5	1,03095E-03	12430,5	2,52635E-02	2044	5,01337E-01	7946	2,39487E+00	937	8,00827E-01	33695	0	17530,5
Non-responder	Macro_FOLR2+APOE+	Macro_ISG15	APOE	LDLR	1,03152E-03	10309,3	1,66158E-02	4189	5,69332E-01	5991	1,70765E+00	3735	8,56888E-01	20101	0	17530,5
Non-responder	Mono_CD16	Mono_CD14	CIRBP	TREM1	1,03319E-03	12529,1	9,43819E-03	10793	3,76080E-01	13178	1,32215E+00	7675	8,83524E-01	13469	0	17530,5
Non-responder	pDC_LILRA4	Macro_ISG15	CD38	PECAM1	1,03693E-03	10091,1	2,52246E-02	2048	9,70614E-01	1345	2,81771E+00	400	8,19800E-01	29132	0	17530,5
Non-responder	Macro_FOLR2+APOE+	Macro_FOLR2+APOE+	CD38	PECAM1	1,03831E-03	14523,5	1,34966E-02	6001	6,62822E-01	4181	1,70766E+00	3734	7,68935E-01	41171	0	17530,5
Non-responder	Macro_OLFML3	Macro_FOLR2+APOE+	MIF	TNFRSF14	1,03857E-03	9977,5	8,22759E-03	13483	5,87899E-01	5562	1,53415E+00	5212	9,05326E-01	8100	0	17530,5
Non-responder	Macro_FOLR2+APOE+	Mono_CD14	APOC2	LRP1	1,03967E-03	11267,5	2,11586E-02	2774	5,68649E-01	6003	2,07554E+00	1788	8,23373E-01	28242	0	17530,5
Non-responder	Macro_NLRP3	Macro_IER3	IL1B	ADRB2	1,03967E-03	10914,7	1,34911E-02	6003	9,26012E-01	1577	1,94669E+00	2326	8,27876E-01	27137	0	17530,5
Non-responder	Macro_FOLR2+APOE+	Macro_OLFML3	MMP12	PLAUR	1,04035E-03	9784,3	1,79613E-02	3677	5,68630E-01	6004	2,09221E+00	1736	8,57433E-01	19974	0	17530,5
Non-responder	Macro_OLFML3	Macro_ISG15	LGALS9	CD47	1,04050E-03	11358,9	8,22636E-03	13488	5,08897E-01	7705	1,48206E+00	5727	8,87783E-01	12344	0	17530,5
Non-responder	Macro_FOLR2+APOE+	Macro_NLRP3	FN1	CD44	1,04376E-03	9283,9	1,34842E-02	6009	6,35739E-01	4617	1,79948E+00	3116	8,77164E-01	15147	0	17530,5
Non-responder	Macro_NLRP3	Macro_IFI27	TIMP1	CD63	1,04397E-03	8839,5	8,22433E-03	13497	7,66704E-01	2850	1,17182E+00	9944	9,47728E-01	376	0	17530,5
Non-responder	pDC_LILRA4	Macro_LYVE1	APP	APLP2	1,04512E-03	5438,1	2,16045E-02	2668	1,22410E+00	384	2,62163E+00	597	9,14763E-01	6011	0	17530,5
Non-responder	Macro_NLRP3	Mono_CD16	HLA-F	LILRB2	1,04514E-03	9177,5	8,22328E-03	13500	7,63637E-01	2884	1,82231E+00	2985	9,01295E-01	8988	0	17530,5
Non-responder	Macro_OLFML3	Mono_INHBA	FN1	PLAUR	1,04668E-03	9625,9	1,40386E-02	5615	6,26556E-01	4798	1,39704E+00	6682	8,83405E-01	13504	0	17530,5
Non-responder	Macro_NLRP3	Macro_LYVE1	S100A8	TLR4	1,04746E-03	8945,3	1,49470E-02	5046	8,68937E-01	1926	1,39421E+00	6718	8,83400E-01	13506	0	17530,5
Non-responder	Macro_OLFML3	Macro_FOLR2+APOE+	CALR	LRP1	1,04746E-03	10737,9	8,22049E-03	13506	4,67692E-01	9155	1,40347E+00	6609	9,10689E-01	6889	0	17530,5
Non-responder	cDC(CD1C)	cDC_CLEC9A	HLA-DQB2	LAG3	1,04895E-03	9577,3	3,15968E-02	1410	1,00684E+00	1180	2,00770E+00	2056	8,33408E-01	25710	0	17530,5
Non-responder	Macro_NLRP3	Macro_FOLR2-APOE+	S100A8	ITGB2	1,04923E-03	9794,3	1,34775E-02	6017	9,02306E-01	1721	6,06624E-01	23373	9,48438E-01	330	0	17530,5
Non-responder	Mast	Macro_FOLR2+APOE+	TIMP3	CD44	1,05046E-03	6453,5	6,38314E-02	400	1,18341E+00	491	2,00748E+00	2057	8,89821E-01	11789	0	17530,5
Non-responder	Macro_FOLR2+APOE+	Macro_NLRP3	PLAU	ITGAM	1,05197E-03	12435,1	1,76745E-02	3777	5,68226E-01	6021	1,72986E+00	3554	8,10869E-01	31293	0	17530,5
Non-responder	cDC_CLEC9A	cDC_CLEC9A	ICAM3	ITGAL	1,05349E-03	9043,3	2,64109E-02	1901	1,04317E+00	1003	2,00727E+00	2059	8,45963E-01	22723	0	17530,5
Non-responder	Macro_IER3	pDC_LILRA4	HMGB1	THBD	1,05407E-03	11387,9	8,52392E-03	12701	4,28655E-01	10677	1,57976E+00	4767	8,91942E-01	11264	0	17530,5
Non-responder	Mono_INHBA	pDC_LILRA4	CXCL2	DPP4	1,05652E-03	10261,5	6,01751E-02	448	9,98176E-01	1213	2,00637E+00	2061	8,15994E-01	30055	0	17530,5
Non-responder	Macro_NLRP3	Macro_LYVE1	VCAN	TLR1	1,05748E-03	12083,9	1,34546E-02	6029	8,57589E-01	2027	1,47975E+00	5755	8,19957E-01	29078	0	17530,5
Non-responder	Macro_IER3	pDC_LILRA4	C1QB	C1QBP	1,05797E-03	8407,5	1,02120E-02	9542	5,32502E-01	6973	1,64204E+00	4255	9,26251E-01	3737	0	17530,5
Non-responder	Macro_NLRP3	pDC_LILRA4	LYZ	ITGAL	1,05836E-03	10611,7	8,20750E-03	13534	4,97922E-01	8059	1,64127E+00	4263	8,98277E-01	9672	0	17530,5
Non-responder	Macro_NLRP3	Macro_OLFML3	S100A8	CD69	1,05993E-03	9539,5	1,43417E-02	5420	8,56778E-01	2033	9,80568E-01	13538	9,00499E-01	9176	0	17530,5
Non-responder	Mono_CD14	Macro_ISG15	S100A8	CD69	1,06162E-03	5689,7	2,01121E-02	3031	1,17114E+00	526	2,21605E+00	1326	9,14656E-01	6035	0	17530,5
Non-responder	Mono_INHBA	Macro_OLFML3	INHBA	ACTR2	1,06259E-03	8281,5	2,99649E-02	1546	9,48611E-01	1451	2,00517E+00	2065	8,62372E-01	18815	0	17530,5
Non-responder	Mono_CD16	Macro_FOLR2+APOE+	ICAM2	ITGAM_ITGB2	1,06369E-03	9068,3	1,60815E-02	4441	6,16630E-01	4989	1,74104E+00	3474	8,78117E-01	14907	0	17530,5
Non-responder	Macro_FOLR2+APOE+	cDC(CD1C)	LGALS3BP	CD33	1,06369E-03	11211,9	1,54025E-02	4790	6,07608E-01	5152	1,44955E+00	6038	8,46652E-01	22549	0	17530,5
Non-responder	pDC_LILRA4	Macro_FOLR2+APOE+	PROC	THBD	1,06411E-03	13195,3	4,45644E-02	795	8,53105E-01	2066	2,36198E+00	1003	7,53660E-01	44582	0	17530,5

Post_NR_Myeloid_Myeloid_CellCell

Non-responder	cDC(CD1C)	Macro_ISG15	HLA-DQA1	LAG3	1,06411E-03	6663,3	2,51197E-02	2066	1,18420E+00	488	2,07242E+00	1811	8,91293E-01	11421	0	17530,5
Non-responder	Mono_CD16	Macro_FOLR2+APOE+	ICAM2	ITGAL_ITGB2	1,06439E-03	8721,9	1,65068E-02	4237	6,78219E-01	3935	1,85176E+00	2805	8,77346E-01	15102	0	17530,5
Non-responder	Macro_FOLR2+APOE+	Macro_FOLR2+APOE+	TGM2	ITGB1	1,06439E-03	12851,5	1,50374E-02	4989	5,67513E-01	6039	1,54396E+00	5104	8,13665E-01	30595	0	17530,5
Non-responder	Mono_CD14	Macro_ISG15	VCAN	TLR2	1,06564E-03	5780,9	2,51173E-02	2067	1,17139E+00	524	2,42191E+00	894	9,06247E-01	7889	0	17530,5
Non-responder	Mono_CD16	Macro_FOLR2+APOE+	SPN	SIGLEC1	1,06577E-03	10858,1	2,66956E-02	1861	7,42752E-01	3116	2,07242E+00	1810	8,16313E-01	29973	0	17530,5
Non-responder	Macro_NLRP3	cDC_CLEC9A	THBS1	SCARB1	1,06577E-03	12698,1	1,34402E-02	6041	6,99440E-01	3635	1,83571E+00	2909	8,02080E-01	33375	0	17530,5
Non-responder	Macro_FOLR2+APOE+	Macro_OLFML3	A2M	LRP1	1,06778E-03	11817,1	9,81895E-03	10140	4,29167E-01	10654	1,35622E+00	7203	8,83217E-01	13558	0	17530,5
Non-responder	Mono_CD16	Macro_ISG15	HLA-C	LILRB1	1,06896E-03	8741,7	8,19890E-03	13561	5,24305E-01	7208	1,72789E+00	3571	9,35644E-01	1838	0	17530,5
Non-responder	Mono_CD16	Macro_FOLR2+APOE+	ICAM3	ITGAL	1,06994E-03	12405,9	1,57471E-02	4623	6,69098E-01	4075	1,66058E+00	4109	8,09185E-01	31692	0	17530,5
Non-responder	Mono_CD14	Macro_OLFML3	VCAN	TLR2	1,07022E-03	5911,3	2,51058E-02	2070	1,17115E+00	525	2,14198E+00	1539	9,06227E-01	7892	0	17530,5
Non-responder	Mast	Macro_IER3	HDC	HRH2	1,07248E-03	10476,3	6,84675E-02	342	1,30146E+00	259	1,59403E+00	4654	8,17783E-01	29596	0	17530,5
Non-responder	Macro_OLFML3	Macro_ISG15	CXCL10	TLR4	1,07328E-03	8224,5	2,50883E-02	2072	1,09823E+00	772	3,02532E+00	249	8,55225E-01	20499	0	17530,5
Non-responder	Mono_CD14	Macro_FOLR2+APOE+	THBS1	SDC4	1,07481E-03	10281,7	2,61679E-02	1937	8,51961E-01	2073	2,07285E+00	1806	8,24177E-01	28062	0	17530,5
Non-responder	pDC_LILRA4	Mono_CD14	APP	CD74	1,07481E-03	6115,1	1,34217E-02	6054	7,07559E-01	3547	1,76261E+00	3340	9,52903E-01	104	0	17530,5
Non-responder	Macro_FOLR2+APOE+	Macro_OLFML3	LGALS9	CD47	1,07489E-03	11098,3	8,19546E-03	13576	5,45489E-01	6613	1,51604E+00	5384	8,87596E-01	12388	0	17530,5
Non-responder	Macro_IER3	Mono_CD16	HLA-A	LILRB2	1,07568E-03	8248,7	8,19308E-03	13578	6,99530E-01	3633	1,44151E+00	6124	9,47699E-01	378	0	17530,5
Non-responder	Macro_NLRP3	cDC(CD1C)	VEGFA	ITGB1	1,07621E-03	10392,3	1,41184E-02	5554	7,93238E-01	2618	1,44825E+00	6056	8,56459E-01	20203	0	17530,5
Non-responder	pDC_LILRA4	Macro_LYVE1	VEGFB	NRP1	1,07788E-03	10588,1	3,80348E-02	1047	8,51730E-01	2075	2,34382E+00	1039	8,11029E-01	31249	0	17530,5
Non-responder	Mono_CD16	cDC_CLEC9A	ITGB2	CD226	1,07806E-03	7336,9	3,14234E-02	1420	9,94865E-01	1230	1,83443E+00	2920	8,83095E-01	13584	0	17530,5
Non-responder	Macro_NLRP3	Mono_CD16	ICAM1	ITGAM_ITGB2	1,07806E-03	10587,7	8,18947E-03	13584	5,20750E-01	7301	1,43562E+00	6217	9,04496E-01	8306	0	17530,5
Non-responder	Macro_FOLR2+APOE+	Macro_LYVE1	ICAM1	IL2RA	1,07831E-03	12312,9	1,94128E-02	3224	7,26580E-01	3288	1,44786E+00	6059	8,10223E-01	31463	0	17530,5
Non-responder	Macro_FOLR2+APOE+	Macro_FOLR2+APOE+	HLA-DRA	LAG3	1,07845E-03	8591,9	1,21695E-02	7172	7,29298E-01	3256	2,18645E+00	1416	8,83093E-01	13585	0	17530,5
Non-responder	pDC_LILRA4	Mono_CD14	APP	NOTCH2	1,07873E-03	7031,7	2,25762E-02	2456	1,24639E+00	343	3,08277E+00	217	8,79182E-01	14612	0	17530,5
Non-responder	Macro_FOLR2+APOE+	Macro_FOLR2+APOE-	A2M	LRP1	1,07965E-03	10830,9	1,29199E-02	6465	5,50252E-01	6499	9,78563E-01	13588	8,96644E-01	10072	0	17530,5
Non-responder	Macro_NLRP3	Macro_ISG15	VCAN	ITGA4	1,08004E-03	8294,5	1,33454E-02	6107	8,68013E-01	1936	1,95094E+00	2310	8,83084E-01	13589	0	17530,5
Non-responder	Mono_CD16	Mono_CD16	B2M	LILRB2	1,08084E-03	7506,7	8,18703E-03	13591	7,12620E-01	3476	1,83808E+00	2892	9,55865E-01	44	0	17530,5
Non-responder	Macro_NLRP3	Macro_IER3	TNF	VSIR	1,08111E-03	11421,9	1,34111E-02	6063	7,14409E-01	3454	1,49756E+00	5557	8,38525E-01	24505	0	17530,5
Non-responder	Mono_CD16	Macro_FOLR2+APOE+	ICAM4	ITGB2	1,08321E-03	8806,3	2,89113E-02	1661	8,30391E-01	2274	1,90875E+00	2515	8,57124E-01	20051	0	17530,5
Non-responder	Mono_CD16	Macro_FOLR2+APOE+	ICAM4	ITGB1	1,08391E-03	12351,1	3,33632E-02	1295	7,76594E-01	2756	1,79667E+00	3130	7,86358E-01	37044	0	17530,5
Non-responder	Mono_CD16	Macro_FOLR2+APOE+	ICAM4	ITGAV	1,08461E-03	16395,7	3,74827E-02	1077	6,98733E-01	3643	1,61425E+00	4470	7,00407E-01	55258	0	17530,5
Non-responder	Macro_FOLR2+APOE+	Mono_CD16	LGALS1	PTPRC	1,08461E-03	9346,3	6,60493E-03	19239	5,66288E-01	6068	1,83757E+00	2894	9,40726E-01	1000	0	17530,5
Non-responder	Macro_FOLR2+APOE+	Mono_CD16	TNF	FFAR2	1,08532E-03	17522,7	2,59590E-02	1965	5,70094E-01	5973	1,44706E+00	6069	6,95606E-01	56076	0	17530,5
Non-responder	Mono_CD16	Macro_FOLR2+APOE+	ICAM4	ITGA4	1,08602E-03	13948,7	2,73739E-02	1784	6,91115E-01	3730	1,51203E+00	5424	7,68479E-01	41275	0	17530,5
Non-responder	Mono_CD16	Macro_FOLR2+APOE+	ICAM4	ITGAL	1,08672E-03	16276,3	3,56456E-02	1167	7,23270E-01	3332	1,47782E+00	5765	7,09760E-01	53587	0	17530,5
Non-responder	cDC(CD1C)	Macro_ISG15	HLA-DQB1	LAG3	1,08712E-03	6436,7	2,50263E-02	2081	1,19131E+00	461	2,12383E+00	1610	8,94965E-01	10501	0	17530,5
Non-responder	Macro_FOLR2+APOE+	Macro_ISG15	TNFSF13B	TFRC	1,08762E-03	9545,9	1,01195E-02	9675	6,27146E-01	4783	1,99243E+00	2133	8,83027E-01	13608	0	17530,5
Non-responder	Macro_IFI27	cDC_CLEC9A	HLA-DPA1	LAG3	1,08881E-03	9428,7	1,74855E-02	3856	8,84127E-01	1822	9,77504E-01	13611	8,95641E-01	10324	0	17530,5
Non-responder	Macro_FOLR2+APOE+	Macro_NLRP3	CCL5	CCRL2	1,09165E-03	16204,7	2,19372E-02	2586	5,65824E-01	6078	1,89257E+00	2593	7,17043E-01	52236	0	17530,5
Non-responder	Macro_LYVE1	pDC_LILRA4	TNF	PTPRS	1,09176E-03	11604,3	2,89693E-02	1654	1,39523E+00	160	2,00200E+00	2084	7,88208E-01	36593	0	17530,5
Non-responder	Mono_CD14	Mono_CD14	S100A8	TLR4	1,09331E-03	5604,7	2,50033E-02	2085	1,25617E+00	326	2,73978E+00	472	9,07398E-01	7610	0	17530,5
Non-responder	Macro_NLRP3	Mono_CD16	CD52	SIGLEC10	1,09362E-03	11166,3	9,89635E-03	10017	5,16118E-01	7461	1,35631E+00	7200	8,82979E-01	13623	0	17530,5
Non-responder	Macro_OLFML3	pDC_LILRA4	APP	RPSA	1,09483E-03	9770,5	8,28112E-03	13329	7,88490E-01	2654	2,09647E+00	1713	8,82960E-01	13626	0	17530,5
Non-responder	Macro_FOLR2+APOE+	Macro_OLFML3	TNFSF13B	HLA-DPB1	1,09660E-03	9383,1	6,90962E-03	17899	5,65504E-01	6085	1,56616E+00	4882	9,45726E-01	519	0	17530,5
Non-responder	Macro_FOLR2+APOE+	Mono_CD16	ICAM1	ITGAL_ITGB2	1,09684E-03	9452,5	8,17450E-03	13631	6,55842E-01	4299	1,79997E+00	3112	9,02653E-01	8690	0	17530,5
Non-responder	Mono_CD16	pDC_LILRA4	S100A8	CD36	1,09805E-03	9533,5	1,35234E-02	5985	5,25484E-01	7169	1,76100E+00	3349	8,82933E-01	13634	0	17530,5
Non-responder	pDC_LILRA4	Macro_FOLR2-APOE+	LTB	TNFRSF1A	1,10264E-03	7082,7	2,49767E-02	2091	1,10468E+00	747	2,37372E+00	982	8,81231E-01	14063	0	17530,5
Non-responder	Mono_INHBA	cDC_LAMP3	FN1	TNFRSF11B	1,10392E-03	12056,9	1,59764E-01	47	1,24321E+00	347	1,92907E+00	2421	7,74180E-01	39939	0	17530,5
Non-responder	Macro_FOLR2+APOE+	Macro_FOLR2+APOE+	ADAM10	TREM2	1,10572E-03	9247,7	1,57445E-02	4627	5,49937E-01	6506	1,68092E+00	3922	8,82861E-01	13653	0	17530,5
Non-responder	Macro_NLRP3	Mono_CD16	CD14	ITGA4	1,10653E-03	10753,7	8,16409E-03	13655	4,78740E-01	8749	1,47362E+00	5802	9,05578E-01	8032	0	17530,5
Non-responder	Macro_FOLR2+APOE+	Macro_FOLR2+APOE+	CXCL12	ITGB1	1,10655E-03	13098,1	1,69331E-02	4064	5,65081E-01	6099	1,95640E+00	2271	7,92900E-01	35526	0	17530,5
Non-responder	Macro_NLRP3	Macro_FOLR2+APOE+	ADAM10	GPNMB	1,10797E-03	11070,5	1,33527E-02	6101	5,96128E-01	5390	1,66154E+00	4101	8,48017E-01	22230	0	17530,5
Non-responder	Macro_IER3	pDC_LILRA4	TGFB1	CXCR4	1,10856E-03	9233,3	8,16339E-03	13660	5,83535E-01	5653	2,39244E+00	942	9,04154E-01	8381	0	17530,5
Non-responder	pDC_LILRA4	Macro_ISG15	GAS6	MERTK	1,11045E-03	9165,5	2,49524E-02	2096	9,35694E-01	1524	2,76041E+00	448	8,39722E-01	24229	0	17530,5
Non-responder	Macro_IER3	Mono_INHBA	VEGFA	CD44	1,11180E-03	11500,9	1,04527E-02	9221	6,70664E-01	4053	1,00555E+00	13032	8,82791E-01	13668	0	17530,5
Non-responder	Macro_FOLR2+APOE+	Macro_LYVE1	SEMA4A	PLXND1	1,11513E-03	12101,1	1,69912E-02	4042	6,47052E-01	4438	1,44359E+00	6111	8,22853E-01	28384	0	17530,5
Non-responder	pDC_LILRA4	Mono_CD14	PROC	THBD	1,11516E-03	13293,9	4,39594E-02	818	8,48953E-01	2099	2,28626E+00	1149	7,52389E-01	44873	0	17530,5
Non-responder	Macro_OLFML3	pDC_LILRA4	HSP90B1	TLR7	1,11669E-03	8320,7	1,84820E-02	3507	6,54699E-01	4316	1,89531E+00	2570	8,82724E-01	13680	0	17530,5
Non-responder	pDC_LILRA4	Mono_CD14	PROC	ITGAM	1,11830E-03	12629,7	5,98089E-02	457	8,48533E-01	2101	2,22866E+00	1299	7,66385E-01	41761	0	17530,5
Non-responder	pDC_LILRA4	pDC_LILRA4	LTB	TNFRSF1A	1,11987E-03	6906,9	2,48916E-02	2102	1,10333E+00	754	4,13449E+00	35	8,81052E-01	14113	0	17530,5
Non-responder	Mono_CD16	Macro_FOLR2+APOE+	SEMA4D	CD72	1,12520E-03	14036,1	1,83427E-02	3542	6,78418E-01	3929	1,91394E+00	2489	7,61973E-01	42690	0	17530,5
Non-responder	Macro_FOLR2+APOE+	Macro_ISG15	ANXA1	FPR2_FPR3	1,12735E-03	10527,7	1,10193E-02	8451	4,99109E-01	8017	1,56061E+00	4934	8,82623E-01	13706	0	17530,5
Non-responder	pDC_LILRA4	cDC_LAMP3	TNFSF9	TRAF2	1,12777E-03	14771,1	5,52565E-02	542	8,47716E-01	2107	2,09991E+00	1699	7,18355E-01	51977	0	17530,5
Non-responder	pDC_LILRA4	Macro_FOLR2+APOE-	APP	LRP1	1,12777E-03	5960,1	2,48577E-02	2107	1,23116E+00	369	2,37696E+00	976	9,02088E-01	8818	0	17530,5
Non-responder	Macro_FOLR2+APOE+	Macro_LYVE1	HMGB1	CD163	1,12858E-03	10947,7	8,14647E-03	13709	5,02617E-01	7894	1,07674E+00	11593	9,24876E-01	4012	0	17530,5
Non-responder	Macro_OLFML3	Macro_NLRP3	SERPING1	LRP1	1,12899E-03	10686,9	1,02659E-02	9464	5,50889E-01	6477	1,43181E+00	6253	8,82619E-01	13710	0	17530,5
Non-responder	Macro_NLRP3	cDC_LAMP3	VCAN	CD44	1,12899E-03	10616,3	9,70653E-03	10326	7,29343E-01	3253	9,73241E-01	13710	9,04691E-01	8262	0	17530,5
Non-responder	Macro_NLRP3	Macro_IER3	VEGFA	NRP2	1,12953E-03	13478,3	1,33148E-02	6131	7,95647E-01	2595	1,59631E+00	4629	7,88569E-01	36506	0	17530,5
Non-responder	pDC_LILRA4	Macro_ISG15	SEMA7A	PLXNC1	1,13094E-03	13193,7	2,56815E-02	1993	8,47111E-01	2109	2,18552E+00	1421	7,61000E-01	42915	0	17530,5
Non-responder	Macro_NLRP3	Macro_FOLR2+APOE+	CCL2	CCR1	1,13243E-03	13710,1	1,77704E-02	3749	5,63672E-01	6135	1,78456E+00	3202	7,82574E-01	37934	0	17530,5
Non-responder	Macro_ISG15	Macro_FOLR2+APOE+	CCL8	CCR5	1,13252E-03	15402,1	7,25714E-02	295	8,46953E-01	2110	2,38289E+00	959	6,95346E-01	56116	0	17530,5
Non-responder	Macro_NLRP3	Mono_CD14	GNAI2	FPR1	1,13436E-03	11135,3	8,14051E-03	13723	4,72373E-01	8967	1,31293E+00	7796	9,07193E-01	7660	0	17530,5

Post_NR_Myeloid_Myeloid_CellCell

Non-responder	Mono_INHBA	cDC_LAMP3	VEGFA	NRP2	1,13570E-03	7065,9	4,97885E-02	659	1,41879E+00	147	1,99731E+00	2112	8,78230E-01	14881	0	17530,5
Non-responder	Macro_IER3	Mast	LGALS1	CD69	1,13601E-03	7440,1	1,59403E-02	4512	8,52479E-01	2069	1,12342E+00	10740	9,33231E-01	2349	0	17530,5
Non-responder	Macro_NLRP3	Macro_IER3	THBS1	SCARB1	1,13606E-03	13356,9	1,32940E-02	6140	6,97466E-01	3658	1,46806E+00	5859	8,01211E-01	33597	0	17530,5
Non-responder	Macro_FOLR2+APOE+	Mono_CD14	RPS19	C5AR1	1,13606E-03	10138,1	7,16415E-03	16870	5,72290E-01	5930	1,44021E+00	6140	9,23792E-01	4220	0	17530,5
Non-responder	Mono_CD16	Macro_FOLR2-APOE+	SERPINA1	LRP1	1,13808E-03	9827,1	1,01257E-02	9666	6,91165E-01	3729	9,72376E-01	13732	9,22409E-01	4478	0	17530,5
Non-responder	Macro_FOLR2+APOE+	Macro_OLFML3	CD14	ITGA4	1,13849E-03	9780,3	8,13736E-03	13733	5,85221E-01	5619	1,67795E+00	3942	9,05437E-01	8077	0	17530,5
Non-responder	Macro_LYVE1	pDC_LILRA4	CXCL12	DPP4	1,13888E-03	14113,3	1,26299E-01	81	1,15488E+00	565	1,99655E+00	2114	7,27496E-01	50276	0	17530,5
Non-responder	Macro_IER3	Mast	HBEGF	CD82	1,13891E-03	8169,3	2,69489E-02	1827	9,90798E-01	1246	1,28844E+00	8140	8,88588E-01	12103	0	17530,5
Non-responder	Macro_NLRP3	cDC_CLEC9A	ICAM1	ITGAX_ITGB2	1,13932E-03	9535,5	8,13730E-03	13735	5,82510E-01	5679	1,73452E+00	3516	9,09155E-01	7217	0	17530,5
Non-responder	Macro_NLRP3	Macro_LYVE1	CCL3L1	CCR1	1,13970E-03	11159,9	1,32892E-02	6145	8,12424E-01	2429	1,73362E+00	3523	8,31639E-01	26172	0	17530,5
Non-responder	Macro_NLRP3	Macro_FOLR2+APOE+	PTGS2	ALOX5	1,14262E-03	10013,7	1,35972E-02	5927	6,20829E-01	4906	1,43984E+00	6149	8,75559E-01	15556	0	17530,5
Non-responder	Mono_CD16	Macro_FOLR2+APOE+	LTB	CD40	1,14335E-03	12276,1	1,45697E-02	5254	6,10099E-01	5093	1,52038E+00	5342	8,23757E-01	28161	0	17530,5
Non-responder	Macro_NLRP3	Mono_CD14	VCAN	ITGA4	1,14348E-03	8646,9	1,31987E-02	6225	8,64090E-01	1965	1,70276E+00	3769	8,82512E-01	13745	0	17530,5
Non-responder	Macro_NLRP3	Macro_OLFML3	CCL3	CCR1	1,14481E-03	9392,1	1,32761E-02	6152	6,78782E-01	3924	1,77739E+00	3244	8,73381E-01	16110	0	17530,5
Non-responder	cDC_CLEC9A	pDC_LILRA4	HRAS	TLR9	1,14686E-03	17327,1	7,90766E-02	237	1,01787E+00	1125	1,99568E+00	2119	6,22412E-01	65624	0	17530,5
Non-responder	Macro_LYVE1	Mono_CD16	F13A1	ITGA4	1,14686E-03	5814,3	2,47677E-02	2119	1,13576E+00	630	2,41959E+00	901	9,06240E-01	7891	0	17530,5
Non-responder	Macro_FOLR2+APOE+	Mono_CD16	HLA-G	LILRB2	1,14700E-03	9013,3	1,32713E-02	6155	8,23826E-01	2331	2,03628E+00	1948	8,69579E-01	17102	0	17530,5
Non-responder	Macro_FOLR2+APOE+	Macro_FOLR2+APOE+	LYZ	ITGAL	1,14723E-03	10383,5	8,13139E-03	13754	5,26938E-01	7123	1,70859E+00	3726	8,97851E-01	9784	0	17530,5
Non-responder	Macro_FOLR2+APOE+	Macro_IER3	HLA-A	LILRB2	1,14847E-03	11812,5	5,13996E-03	28181	6,05424E-01	5200	1,43939E+00	6157	9,34862E-01	1994	0	17530,5
Non-responder	Macro_IER3	cDC_CLEC9A	HLA-DRA	LAG3	1,14848E-03	8902,5	1,55019E-02	4735	7,62080E-01	2898	1,23853E+00	8865	8,95019E-01	10484	0	17530,5
Non-responder	Macro_IER3	cDC_CLEC9A	HLA-DRB1	LAG3	1,14973E-03	9585,1	1,51355E-02	4929	7,23623E-01	3328	1,15556E+00	10205	8,89297E-01	11933	0	17530,5
Non-responder	Mono_CD16	Macro_FOLR2+APOE+	VCL	ITGB5	1,14993E-03	14698,3	5,16920E-02	620	7,59495E-01	2929	1,78384E+00	3205	7,32602E-01	49207	0	17530,5
Non-responder	Macro_IER3	cDC_CLEC9A	HLA-DPB1	LAG3	1,15057E-03	9522,1	1,56755E-02	4660	7,37976E-01	3168	1,21022E+00	9291	8,85354E-01	12961	0	17530,5
Non-responder	Macro_NLRP3	pDC_LILRA4	HSP90B1	TLR9	1,15067E-03	9617,9	4,07602E-02	937	7,85490E-01	2679	1,43935E+00	6160	8,54073E-01	20783	0	17530,5
Non-responder	Mono_CD16	Macro_FOLR2+APOE+	ADAM10	GPNMB	1,15140E-03	8905,5	1,82714E-02	3566	6,92049E-01	3718	2,01694E+00	2018	8,67144E-01	17695	0	17530,5
Non-responder	Macro_FOLR2+APOE+	Macro_ISG15	CD99	PILRA	1,15266E-03	10883,5	8,12670E-03	13767	4,36762E-01	10344	1,51723E+00	5374	9,08259E-01	7402	0	17530,5
Non-responder	pDC_LILRA4	Macro_FOLR2+APOE-	SPON2	ITGB2	1,15488E-03	6255,7	3,68685E-02	1111	1,05712E+00	944	1,99349E+00	2124	8,98704E-01	9569	0	17530,5
Non-responder	cDC(CD1C)	Mono_CD16	CD1D	LILRB2	1,15488E-03	6851,9	2,47274E-02	2124	1,02727E+00	1082	2,16658E+00	1473	8,88784E-01	12050	0	17530,5
Non-responder	Macro_IFI27	Macro_ISG15	HLA-DQA1	LAG3	1,15727E-03	9403,7	2,10285E-02	2805	9,88847E-01	1254	1,07397E+00	11651	8,82376E-01	13778	0	17530,5
Non-responder	Macro_OLFML3	Macro_ISG15	CCL3	CCR1	1,15811E-03	8528,7	1,57018E-02	4648	7,13036E-01	3471	1,78257E+00	3214	8,82373E-01	13780	0	17530,5
Non-responder	Macro_IER3	cDC_CLEC9A	HLA-DPA1	LAG3	1,15853E-03	9599,9	1,53179E-02	4829	7,10468E-01	3508	1,15588E+00	10198	8,89292E-01	11934	0	17530,5
Non-responder	Macro_NLRP3	cDC_CLEC9A	CCL4	CNR2	1,15876E-03	14739,9	7,73679E-02	247	9,03320E-01	1712	1,43835E+00	6171	7,38180E-01	48039	0	17530,5
Non-responder	Macro_NLRP3	Macro_FOLR2+APOE-	S100A8	CD68	1,15876E-03	9479,3	1,32496E-02	6171	8,54720E-01	2057	6,70127E-01	21504	9,51913E-01	134	0	17530,5
Non-responder	Macro_OLFML3	Macro_ISG15	TNFSF13B	TFRC	1,15937E-03	9879,5	9,98984E-03	9862	6,17183E-01	4980	1,77764E+00	3242	8,82359E-01	13783	0	17530,5
Non-responder	Macro_FOLR2+APOE+	Mono_CD14	LGALS3BP	CD33	1,16024E-03	11767,3	1,35036E-02	5997	5,61939E-01	6173	1,61313E+00	4482	8,37915E-01	24654	0	17530,5
Non-responder	Macro_NLRP3	Mono_CD14	S100A9	CD68	1,16063E-03	9890,5	8,11865E-03	13786	5,53774E-01	6393	1,10000E+00	11162	9,44916E-01	581	0	17530,5
Non-responder	Macro_NLRP3	Macro_NLRP3	S100A12	TLR4	1,16097E-03	13262,3	1,91051E-02	3303	5,61930E-01	6174	1,67714E+00	3954	7,93754E-01	35350	0	17530,5
Non-responder	Macro_NLRP3	cDC_CLEC9A	NAMPT	ITGA5_ITGB1	1,16190E-03	10854,7	8,40492E-03	13007	6,64489E-01	4151	1,47423E+00	5796	8,82339E-01	13789	0	17530,5
Non-responder	Macro_IER3	cDC_CLEC9A	HLA-DRB5	LAG3	1,16274E-03	9215,3	1,60606E-02	4450	7,59709E-01	2928	1,30875E+00	7866	8,84106E-01	13302	0	17530,5
Non-responder	Macro_FOLR2+APOE+	cDC_LAMP3	CD274	CD80	1,16393E-03	17174,3	2,43048E-02	2182	8,39031E-01	2191	1,43798E+00	6178	6,84939E-01	57790	0	17530,5
Non-responder	Macro_FOLR2+APOE+	Mono_CD16	GNAI2	S1PR4	1,16393E-03	11384,7	1,57403E-02	4628	5,61864E-01	6178	1,65916E+00	4122	8,38721E-01	24465	0	17530,5
Non-responder	Macro_NLRP3	Macro_IER3	S100A8	TLR4	1,16612E-03	9211,7	1,46327E-02	5219	8,62147E-01	1980	1,33186E+00	7530	8,82301E-01	13799	0	17530,5
Non-responder	Mono_CD16	Macro_FOLR2+APOE+	PTPN6	CD300LF	1,16615E-03	10085,9	1,50750E-02	4963	6,34762E-01	4629	1,98734E+00	2146	8,52489E-01	21161	0	17530,5
Non-responder	Mono_INHBA	Macro_FOLR2+APOE+	VEGFA	NRP1	1,16616E-03	8441,7	3,39974E-02	1254	1,01652E+00	1131	1,99270E+00	2131	8,56649E-01	20162	0	17530,5
Non-responder	Macro_NLRP3	Macro_OLFML3	S100A9	TLR4	1,16654E-03	9037,7	1,19395E-02	7414	8,23819E-01	2332	1,66035E+00	4112	8,82300E-01	13800	0	17530,5
Non-responder	Mono_CD14	Mono_CD16	HLA-A	LILRA1	1,16689E-03	7887,5	1,57300E-02	4635	5,61774E-01	6182	1,45878E+00	5960	9,19079E-01	5130	0	17530,5
Non-responder	Macro_FOLR2+APOE+	Mono_INHBA	FN1	PLAUR	1,16689E-03	9806,1	1,32369E-02	6182	6,09532E-01	5110	1,46252E+00	5919	8,80342E-01	14289	0	17530,5
Non-responder	Macro_FOLR2+APOE+	Macro_OLFML3	HLA-DQA1	CD4	1,16986E-03	9489,7	6,86235E-03	18098	5,61596E-01	6186	1,78670E+00	3191	9,32681E-01	2443	0	17530,5
Non-responder	Macro_OLFML3	Mono_INHBA	CXCL10	TLR4	1,17102E-03	8348,3	2,46393E-02	2134	1,09208E+00	802	2,70709E+00	500	8,54103E-01	20775	0	17530,5
Non-responder	Macro_IER3	Macro_FOLR2+APOE+	HLA-DQA2	CD4	1,17120E-03	10321,5	8,10844E-03	13811	4,62205E-01	9345	1,49458E+00	5592	9,18124E-01	5329	0	17530,5
Non-responder	Macro_NLRP3	Macro_ISG15	PTGS2	ALOX5	1,17209E-03	9914,7	1,38233E-02	5770	6,28644E-01	4755	1,43736E+00	6189	8,76454E-01	15329	0	17530,5
Non-responder	Macro_FOLR2+APOE+	Mono_CD14	MMP9	IFNAR1	1,17209E-03	13967,1	1,32301E-02	6189	6,12225E-01	5051	2,28396E+00	1157	7,74291E-01	39908	0	17530,5
Non-responder	Macro_FOLR2+APOE+	Mono_CD16	LGALS3BP	CD33	1,17358E-03	11563,7	1,34827E-02	6011	5,61434E-01	6191	1,75251E+00	3398	8,37809E-01	24688	0	17530,5
Non-responder	pDC_LILRA4	Macro_ISG15	HSP90B1	TLR2	1,17358E-03	6063,3	1,32274E-02	6191	1,01130E+00	1155	3,06756E+00	227	9,18672E-01	5213	0	17530,5
Non-responder	Macro_FOLR2+APOE+	Mono_CD16	ADAM28	ITGA4	1,17433E-03	11232,5	1,32257E-02	6192	5,73861E-01	5889	1,82147E+00	2993	8,42476E-01	23558	0	17530,5
Non-responder	Macro_FOLR2+APOE-	cDC_LAMP3	PDGFB	S1PR1	1,17466E-03	16658,1	8,19058E-02	214	1,23706E+00	358	1,18646E+00	9681	6,98991E-01	55507	0	17530,5
Non-responder	Macro_FOLR2+APOE+	Macro_OLFML3	TNFSF13	TNFRSF14	1,17502E-03	11790,5	8,10316E-03	13820	4,57036E-01	9541	1,54859E+00	5057	8,85164E-01	13004	0	17530,5
Non-responder	Macro_FOLR2+APOE+	Macro_LYVE1	LGALS9	COLEC12	1,17731E-03	14291,9	2,91747E-02	1631	6,13866E-01	5026	1,43700E+00	6196	7,69259E-01	41076	0	17530,5
Non-responder	pDC_LILRA4	Macro_OLFML3	HSP90B1	TLR2	1,17731E-03	6106,1	1,32214E-02	6196	1,01107E+00	1157	2,78763E+00	429	9,18655E-01	5218	0	17530,5
Non-responder	Macro_FOLR2+APOE+	Macro_LYVE1	TNFSF13	TNFRSF1A	1,17885E-03	12902,7	8,10115E-03	13829	4,25609E-01	10809	1,18061E+00	9777	8,86914E-01	12568	0	17530,5
Non-responder	Macro_OLFML3	Mono_CD16	CXCL10	TLR4	1,17915E-03	8323,3	2,45816E-02	2139	1,09129E+00	806	2,92444E+00	332	8,53957E-01	20809	0	17530,5
Non-responder	Macro_FOLR2+APOE+	Macro_NLRP3	FABP5	RXRA	1,18030E-03	11679,9	1,44653E-02	5320	5,61175E-01	6200	1,61245E+00	4488	8,37097E-01	24861	0	17530,5
Non-responder	Macro_NLRP3	Macro_LYVE1	VEGFA	SIRPA	1,18104E-03	10757,3	1,53931E-02	4795	8,13541E-01	2415	1,43669E+00	6201	8,45478E-01	22845	0	17530,5
Non-responder	Macro_OLFML3	Mono_CD14	HLA-A	LILRA1	1,18183E-03	12930,5	8,09794E-03	13836	4,21437E-01	10983	1,12379E+00	10730	8,90700E-01	11573	0	17530,5
Non-responder	Macro_OLFML3	pDC_LILRA4	CXCL11	CCR5	1,18241E-03	16391,9	3,61808E-02	1138	8,43781E-01	2141	2,47162E+00	804	6,67489E-01	60346	0	17530,5
Non-responder	Macro_IER3	cDC_CLEC9A	C1QB	C1QBP	1,18397E-03	9475,9	1,01011E-02	9703	5,23278E-01	7238	1,22191E+00	9111	9,25878E-01	3797	0	17530,5
Non-responder	Macro_FOLR2+APOE+	Macro_FOLR2+APOE+	CCL5	CCR5	1,18404E-03	18257,9	3,10179E-02	1457	5,61057E-01	6205	1,54989E+00	5043	6,62101E-01	61054	0	17530,5
Non-responder	Mono_CD16	Mono_INHBA	VCAN	CD44	1,18525E-03	11433,5	8,90514E-03	11825	6,21698E-01	4889	9,67417E-01	13844	9,00911E-01	9079	0	17530,5
Non-responder	Mast	cDC_CLEC9A	CSF1	CSF2RA	1,18568E-03	8349,3	4,81952E-02	698	1,11492E+00	714	1,98898E+00	2143	8,54527E-01	20661	0	17530,5
Non-responder	Mono_INHBA	Macro_NLRP3	FN1	PLAUR	1,18568E-03	5916,5	2,45684E-02	2143	9,56230E-01	1413	2,22802E+00	1303	9,09282E-01	7193	0	17530,5
Non-responder	Macro_IER3	Mono_CD16	TNF	TNFRSF1B	1,18697E-03	8921,9	1,18277E-02	7510	7,32205E-01	3234	1,91401E+00	2487	8,82076E-01	13848	0	17530,5

Post_NR_Myeloid_Myeloid_CellCell

Non-responder	Macro_OLFML3	Macro_LYVE1	C1QB	LRP1	1,19255E-03	12080,7	9,13837E-03	11338	3,81381E-01	12909	9,66867E-01	13861	9,20757E-01	4765	0	17530,5
Non-responder	Macro_NLRP3	cDC_LAMP3	TNF	TRADD	1,19457E-03	11961,5	1,52246E-02	4877	6,55940E-01	4298	1,43536E+00	6219	8,28858E-01	26883	0	17530,5
Non-responder	Mono_CD16	Macro_NLRP3	CD55	ADGRE2	1,19532E-03	8805,1	1,58675E-02	4557	8,21520E-01	2354	1,88207E+00	2655	8,70184E-01	16929	0	17530,5
Non-responder	Mono_CD16	Macro_FOLR2+APOE-	SERPINA1	LRP1	1,19988E-03	10970,3	8,60289E-03	12508	6,01862E-01	5272	9,65773E-01	13878	9,16372E-01	5663	0	17530,5
Non-responder	Mono_INHBA	Macro_OLFML3	IL6	IL6R_IL6ST	1,20045E-03	11850,3	3,18780E-02	1393	1,12487E+00	668	1,98666E+00	2152	7,84307E-01	37508	0	17530,5
Non-responder	Macro_NLRP3	Macro_IER3	GNAI2	C5AR1	1,20248E-03	11410,9	8,08496E-03	13884	5,35445E-01	6884	1,08459E+00	11436	9,08662E-01	7320	0	17530,5
Non-responder	cDC_CLEC9A	Macro_LYVE1	HLA-DOB	CD4	1,20375E-03	6658,7	4,60174E-02	753	1,46802E+00	130	1,98660E+00	2154	8,86319E-01	12726	0	17530,5
Non-responder	Macro_IER3	pDC_LILRA4	THBS1	CD47	1,20508E-03	8018,1	1,45135E-02	5295	8,50432E-01	2085	2,23041E+00	1290	8,81870E-01	13890	0	17530,5
Non-responder	Mono_CD16	Macro_NLRP3	TNFSF10	TNFRSF10D	1,20516E-03	13538,9	2,16189E-02	2665	6,47741E-01	4422	1,63556E+00	4300	7,78945E-01	38777	0	17530,5
Non-responder	Mono_CD16	Macro_ISG15	CIRBP	TREM1	1,20595E-03	12457,3	9,21973E-03	11193	3,62934E-01	13892	1,46675E+00	5877	8,82313E-01	13794	0	17530,5
Non-responder	Macro_IER3	Macro_NLRP3	C1QA	CD93	1,20725E-03	11053,9	1,07956E-02	8745	4,95587E-01	8134	9,65111E-01	13895	9,10371E-01	6965	0	17530,5
Non-responder	Mono_CD14	pDC_LILRA4	S100A9	CD68	1,20872E-03	5904,5	1,07614E-02	8802	8,42001E-01	2157	2,42384E+00	893	9,51807E-01	140	0	17530,5
Non-responder	Macro_FOLR2+APOE+	cDC(CD1C)	CD14	ITGB2	1,21125E-03	9019,3	7,70133E-03	15003	5,89621E-01	5529	1,43297E+00	6241	9,42468E-01	793	0	17530,5
Non-responder	pDC_LILRA4	Macro_ISG15	APP	NCSTN	1,21203E-03	7599,5	2,44443E-02	2159	1,22670E+00	378	3,25030E+00	155	8,66778E-01	17775	0	17530,5
Non-responder	Macro_IER3	Macro_FOLR2+APOE+	HSPA1A	TLR4	1,21247E-03	13681,1	8,31924E-03	13231	4,08031E-01	11615	9,64701E-01	13907	8,88524E-01	12122	0	17530,5
Non-responder	Macro_NLRP3	Macro_OLFML3	PTGS2	ALOX5	1,21552E-03	9509,1	1,52827E-02	4851	6,79092E-01	3920	1,34717E+00	7330	8,81786E-01	13914	0	17530,5
Non-responder	Macro_OLFML3	Macro_FOLR2+APOE+	TNFSF13	TNFRSF1A	1,21596E-03	12733,7	8,07441E-03	13915	4,09952E-01	11519	1,29205E+00	8090	8,86748E-01	12614	0	17530,5
Non-responder	Mono_CD16	Macro_NLRP3	ICAM4	ITGAV	1,21659E-03	17555,1	3,09276E-02	1461	6,34487E-01	4641	1,49581E+00	5570	6,79859E-01	58573	0	17530,5
Non-responder	Macro_FOLR2+APOE+	Macro_ISG15	TNFSF13B	CD40	1,21859E-03	9844,5	9,65373E-03	10417	6,54225E-01	4324	1,81454E+00	3030	8,81777E-01	13921	0	17530,5
Non-responder	Macro_FOLR2+APOE+	Mono_CD14	PLAU	LRP1	1,21965E-03	11107,7	1,56283E-02	4679	5,59049E-01	6252	1,65313E+00	4176	8,45215E-01	22901	0	17530,5
Non-responder	Macro_FOLR2+APOE+	Macro_ISG15	C1QB	CD33	1,22209E-03	9962,5	8,06654E-03	13929	5,93696E-01	5453	2,03165E+00	1965	8,93196E-01	10935	0	17530,5
Non-responder	Macro_IER3	pDC_LILRA4	HMGB1	CXCR4	1,22385E-03	8175,5	8,06454E-03	13933	5,82475E-01	5680	2,15887E+00	1496	9,33791E-01	2238	0	17530,5
Non-responder	Macro_OLFML3	cDC_CLEC9A	LGALS9	HAVCR2	1,22516E-03	11966,7	8,06319E-03	13936	4,61961E-01	9357	1,43655E+00	6207	8,86023E-01	12803	0	17530,5
Non-responder	Macro_IER3	Mono_CD16	HLA-C	LILRB2	1,22560E-03	8719,9	8,06297E-03	13937	6,36989E-01	4597	1,35731E+00	7185	9,48002E-01	350	0	17530,5
Non-responder	Mono_INHBA	Macro_ISG15	SPP1	CD44	1,22579E-03	6963,5	1,99272E-02	3080	6,70290E-01	4060	1,68541E+00	3887	9,13511E-01	6260	0	17530,5
Non-responder	Mast	Mast	TIMP3	CD44	1,22748E-03	7439,3	6,84905E-02	341	1,23249E+00	366	1,29609E+00	8028	8,93228E-01	10931	0	17530,5
Non-responder	Mono_CD14	Mono_INHBA	THBS1	SDC4	1,22869E-03	10023,5	2,80183E-02	1727	8,79841E-01	1857	1,98062E+00	2169	8,29073E-01	26834	0	17530,5
Non-responder	Macro_OLFML3	pDC_LILRA4	LYZ	ITGAL	1,22913E-03	10678,1	8,06132E-03	13945	4,76764E-01	8824	1,78173E+00	3222	8,97453E-01	9869	0	17530,5
Non-responder	Macro_IER3	Mono_CD14	HBEGF	CD44	1,23221E-03	11111,9	9,00563E-03	11615	5,80341E-01	5727	1,01170E+00	12919	9,06768E-01	7768	0	17530,5
Non-responder	Macro_FOLR2+APOE+	Macro_FOLR2+APOE+	ICAM1	ITGAX_ITGB2	1,23354E-03	9585,5	8,05794E-03	13955	6,00802E-01	5285	1,68936E+00	3851	9,08750E-01	7306	0	17530,5
Non-responder	Macro_OLFML3	Macro_FOLR2+APOE+	HBEGF	CD9	1,23575E-03	12679,9	8,45089E-03	12895	3,61832E-01	13960	1,39907E+00	6662	8,87745E-01	12352	0	17530,5
Non-responder	Macro_IER3	Macro_FOLR2+APOE+	HLA-DMB	CD4	1,23575E-03	10934,5	8,05716E-03	13960	4,91129E-01	8281	1,26388E+00	8485	9,12891E-01	6416	0	17530,5
Non-responder	Macro_NLRP3	pDC_LILRA4	IL6	IL6R_IL6ST	1,23582E-03	15166,3	1,98353E-02	3111	5,58268E-01	6273	2,11622E+00	1635	7,41488E-01	47282	0	17530,5
Non-responder	Mono_CD16	Macro_OLFML3	SELPLG	ITGB2	1,23708E-03	10140,1	8,05615E-03	13963	4,98222E-01	8047	1,45848E+00	5962	9,18742E-01	5198	0	17530,5
Non-responder	Macro_FOLR2+APOE+	Mono_INHBA	HLA-A	LILRB1	1,24124E-03	12001,9	5,47121E-03	25824	5,81951E-01	5692	1,42986E+00	6280	9,21320E-01	4683	0	17530,5
Non-responder	Mono_CD16	Macro_NLRP3	RPS19	C5AR1	1,24202E-03	9602,7	7,20186E-03	16722	5,80203E-01	5733	1,68929E+00	3852	9,23977E-01	4176	0	17530,5
Non-responder	pDC_LILRA4	Mono_CD14	SEMA7A	PLXNC1	1,24213E-03	13461,9	2,49720E-02	2092	8,40632E-01	2177	2,01204E+00	2043	7,58443E-01	43467	0	17530,5
Non-responder	Macro_ISG15	pDC_LILRA4	HSP90B1	TLR9	1,24382E-03	7344,1	5,99035E-02	454	9,92570E-01	1236	1,97795E+00	2178	8,76471E-01	15322	0	17530,5
Non-responder	Macro_NLRP3	Macro_FOLR2+APOE+	RPS19	C5AR1	1,24775E-03	9278,9	8,04796E-03	13987	6,47139E-01	4435	1,37209E+00	6995	9,27788E-01	3447	0	17530,5
Non-responder	Macro_FOLR2+APOE+	Macro_NLRP3	ARPC5	LDLR	1,24980E-03	11911,7	1,49062E-02	5066	5,57458E-01	6291	1,51451E+00	5404	8,35392E-01	25267	0	17530,5
Non-responder	Mono_CD16	Macro_FOLR2+APOE+	NAMPT	ITGA5_ITGB1	1,25087E-03	11975,7	8,27073E-03	13360	5,10626E-01	7652	1,34644E+00	7342	8,81502E-01	13994	0	17530,5
Non-responder	Macro_IER3	Macro_FOLR2+APOE+	CCL3L1	CCR1	1,25228E-03	7978,1	2,42782E-02	2183	9,05120E-01	1704	2,18785E+00	1412	8,69733E-01	17061	0	17530,5
Non-responder	cDC_CLEC9A	pDC_LILRA4	ICAM3	ITGAL	1,25737E-03	9193,7	2,42729E-02	2186	1,01211E+00	1151	2,34320E+00	1040	8,40382E-01	24061	0	17530,5
Non-responder	Macro_FOLR2+APOE+	Macro_FOLR2+APOE+	HLA-F	LILRB1	1,25804E-03	10782,3	8,71548E-03	12261	5,53616E-01	6398	1,70987E+00	3712	8,81433E-01	14010	0	17530,5
Non-responder	Macro_NLRP3	Mono_CD14	ANXA1	FPR1	1,25849E-03	11743,1	8,04206E-03	14011	4,39648E-01	10240	1,10510E+00	11074	9,15436E-01	5860	0	17530,5
Non-responder	Mono_INHBA	Macro_NLRP3	INHBA	ACTR2	1,25907E-03	8699,3	2,72294E-02	1800	8,88939E-01	1800	1,97547E+00	2187	8,56592E-01	20179	0	17530,5
Non-responder	Mono_CD16	Macro_FOLR2+APOE+	S100A4	CCR5	1,26154E-03	9380,9	1,64492E-02	4276	6,88789E-01	3765	1,42836E+00	6306	8,77659E-01	15027	0	17530,5
Non-responder	Macro_FOLR2+APOE+	Macro_OLFML3	APOE	SORL1	1,26299E-03	11667,9	8,50560E-03	12744	3,60760E-01	14021	1,43985E+00	6147	9,06186E-01	7897	0	17530,5
Non-responder	Macro_OLFML3	Macro_ISG15	FN1	PLAUR	1,26479E-03	9317,3	1,35029E-02	6000	5,89923E-01	5521	1,73564E+00	3510	8,81386E-01	14025	0	17530,5
Non-responder	Macro_IER3	Macro_ISG15	HLA-DRA	LAG3	1,26569E-03	7363,7	1,88545E-02	3378	9,21027E-01	1606	1,46770E+00	5866	9,03868E-01	8438	0	17530,5
Non-responder	Macro_IER3	Macro_ISG15	HLA-DRB1	LAG3	1,26840E-03	7863,5	1,84089E-02	3525	8,82570E-01	1837	1,38473E+00	6826	8,98574E-01	9599	0	17530,5
Non-responder	pDC_LILRA4	pDC_LILRA4	HMGB1	CXCR4	1,26930E-03	5935,7	1,06072E-02	9006	8,38934E-01	2193	3,57678E+00	74	9,41776E-01	875	0	17530,5
Non-responder	Macro_IER3	Macro_ISG15	HLA-DQB1	LAG3	1,26930E-03	7850,3	2,09343E-02	2826	9,81935E-01	1293	1,56833E+00	4867	8,86273E-01	12735	0	17530,5
Non-responder	Macro_IFI27	Macro_FOLR2+APOE+	APOE	TREM2	1,27021E-03	7722,7	1,97950E-02	3120	6,90313E-01	3741	9,59062E-01	14037	9,50751E-01	185	0	17530,5
Non-responder	Macro_NLRP3	cDC(CD1C)	ICAM1	ITGAM_ITGB2	1,27066E-03	11873,1	8,03358E-03	14038	5,19675E-01	7342	1,05870E+00	11974	9,03663E-01	8481	0	17530,5
Non-responder	Macro_OLFML3	cDC_CLEC9A	ITGB2	CD226	1,27202E-03	7784,1	3,03712E-02	1510	9,62014E-01	1388	1,61669E+00	4451	8,81325E-01	14041	0	17530,5
Non-responder	Macro_NLRP3	Mono_CD14	S100A8	CD68	1,27202E-03	9682,7	9,60818E-03	10482	5,81246E-01	5705	9,58870E-01	14041	9,44000E-01	655	0	17530,5
Non-responder	pDC_LILRA4	Macro_FOLR2-APOE+	SPON2	ITGB2	1,27272E-03	5829,5	4,28783E-02	856	1,18721E+00	478	1,97377E+00	2195	9,05374E-01	8088	0	17530,5
Non-responder	Macro_IER3	Macro_ISG15	HLA-DPB1	LAG3	1,27293E-03	7853,5	1,90657E-02	3313	8,96923E-01	1747	1,43938E+00	6158	8,94922E-01	10519	0	17530,5
Non-responder	Macro_FOLR2+APOE+	Mono_CD16	SPP1	CD44	1,27338E-03	10648,9	1,16416E-02	7704	3,60185E-01	14044	1,98047E+00	2170	8,89783E-01	11796	0	17530,5
Non-responder	Mono_CD16	Mono_CD16	LGALS9	PTPRC	1,27495E-03	10280,7	6,92139E-03	17849	5,68772E-01	6001	1,68449E+00	3895	9,14188E-01	6128	0	17530,5
Non-responder	Mono_CD16	Macro_FOLR2+APOE+	LGALS9	CD47	1,27519E-03	11566,5	8,02966E-03	14048	4,89004E-01	8355	1,53135E+00	5241	8,86572E-01	12658	0	17530,5
Non-responder	Macro_FOLR2+APOE+	Macro_NLRP3	LRPAP1	LRP1	1,27565E-03	11650,5	9,25150E-03	11127	4,75040E-01	8875	1,39794E+00	6671	8,81292E-01	14049	0	17530,5
Non-responder	pDC_LILRA4	Macro_NLRP3	CD38	PECAM1	1,27615E-03	10330,7	2,41534E-02	2197	9,46733E-01	1465	2,65605E+00	547	8,16573E-01	29914	0	17530,5
Non-responder	Macro_FOLR2+APOE+	Mono_CD14	TNF	VSIR	1,28129E-03	11116,7	1,30739E-02	6331	7,46333E-01	3071	1,70926E+00	3723	8,36793E-01	24928	0	17530,5
Non-responder	Macro_FOLR2+APOE+	Macro_ISG15	HLA-C	LILRA1	1,28293E-03	10971,3	8,02402E-03	14065	5,10801E-01	7644	1,63906E+00	4275	8,91627E-01	11342	0	17530,5
Non-responder	Macro_OLFML3	pDC_LILRA4	HSP90B1	TLR9	1,28430E-03	7069,7	6,54745E-02	384	1,05283E+00	964	1,93200E+00	2402	8,81205E-01	14068	0	17530,5
Non-responder	pDC_LILRA4	Mono_INHBA	PROC	THBD	1,28475E-03	13585,9	4,32863E-02	842	8,44333E-01	2137	1,97167E+00	2202	7,50949E-01	45218	0	17530,5
Non-responder	Macro_FOLR2+APOE+	Macro_FOLR2-APOE+	ADAM10	TREM2	1,28704E-03	10000,9	1,87723E-02	3404	6,96798E-01	3665	9,57901E-01	14074	8,91655E-01	11331	0	17530,5
Non-responder	Macro_NLRP3	Mono_CD14	RPS19	C5AR1	1,28795E-03	9743,7	8,02081E-03	14076	6,44737E-01	4479	1,21892E+00	9160	9,27674E-01	3473	0	17530,5
Non-responder	Macro_IER3	Macro_ISG15	HLA-DPA1	LAG3	1,28841E-03	7866,9	1,86308E-02	3456	8,69416E-01	1922	1,38505E+00	6824	8,98569E-01	9602	0	17530,5

Post_NR_Myeloid_Myeloid_CellCell

Non-responder	Macro_FOLR2+APOE+	Mono_CD16	PTPN6	CLEC12A	1,28926E-03	9472,7	1,30627E-02	6341	5,94198E-01	5438	1,78122E+00	3225	8,78452E-01	14829	0	17530,5
Non-responder	Mono_CD16	Mono_CD16	HLA-A	LILRB2	1,28926E-03	7954,3	7,95888E-03	14250	6,50480E-01	4390	1,78822E+00	3182	9,46975E-01	419	0	17530,5
Non-responder	Mono_INHBA	cDC_CLEC9A	CCL20	CCR6	1,28993E-03	16271,3	8,13657E-02	219	8,68174E-01	1932	1,97037E+00	2205	6,73978E-01	59470	0	17530,5
Non-responder	Macro_ISG15	cDC_CLEC9A	TNFSF13B	HLA-DPB1	1,28993E-03	6618,3	8,75921E-03	12146	8,37757E-01	2205	2,33286E+00	1058	9,51501E-01	152	0	17530,5
Non-responder	Macro_FOLR2+APOE+	Macro_ISG15	SIRPA	CD47	1,29165E-03	9252,1	1,30622E-02	6344,5	6,94043E-01	3688,5	1,87729E+00	2680,5	8,73757E-01	16016,5	0	17530,5
Non-responder	Mono_INHBA	pDC_LILRA4	CCL2	CCR2	1,29166E-03	10966,3	5,37038E-02	572	8,37559E-01	2206	2,80492E+00	417	7,98990E-01	34106	0	17530,5
Non-responder	Macro_NLRP3	Macro_FOLR2-APOE+	VEGFA	EPHB2	1,29405E-03	15451,1	2,65348E-02	1881	8,62977E-01	1972	1,42505E+00	6347	7,31064E-01	49525	0	17530,5
Non-responder	Macro_NLRP3	Macro_FOLR2-APOE+	VCAN	ITGB1	1,29621E-03	10171,1	1,46716E-02	5186	8,79206E-01	1860	9,57202E-01	14094	8,88328E-01	12185	0	17530,5
Non-responder	Macro_FOLR2+APOE+	Macro_IFI27	APOE	SCARB1	1,29621E-03	11183,1	1,22610E-02	7096	4,59220E-01	9455	1,31741E+00	7740	8,81119E-01	14094	0	17530,5
Non-responder	Macro_NLRP3	Mono_CD16	VCAN	TLR1	1,29646E-03	11723,1	1,30579E-02	6350	8,51766E-01	2074	1,81029E+00	3049	8,17737E-01	29612	0	17530,5
Non-responder	Mono_CD16	Macro_FOLR2+APOE+	RPS19	C5AR1	1,29646E-03	9867,7	6,95884E-03	17706	5,55343E-01	6350	1,76249E+00	3343	9,22763E-01	4409	0	17530,5
Non-responder	Macro_IER3	Macro_ISG15	HLA-DRB5	LAG3	1,29667E-03	7661,3	1,95341E-02	3196	9,18657E-01	1624	1,53791E+00	5161	8,93765E-01	10795	0	17530,5
Non-responder	Macro_OLFML3	Macro_ISG15	TNFSF13B	CD40	1,29805E-03	10267,5	9,53007E-03	10628	6,44262E-01	4485	1,59975E+00	4596	8,81103E-01	14098	0	17530,5
Non-responder	Macro_ISG15	Macro_ISG15	CCL8	CCR5	1,29859E-03	15473,9	7,05629E-02	320	8,37185E-01	2210	2,54719E+00	702	6,92365E-01	56607	0	17530,5
Non-responder	cDC_CLEC9A	Macro_FOLR2+APOE+	SEMA4A	PLXND1	1,29859E-03	9133,3	2,50944E-02	2071	8,61966E-01	1984	1,96975E+00	2210	8,49512E-01	21871	0	17530,5
Non-responder	Macro_ISG15	cDC(CD1C)	B2M	CD1A	1,29967E-03	5762,5	5,06106E-02	642	7,63719E-01	2882	2,19041E+00	1404	9,13141E-01	6354	0	17530,5
Non-responder	Macro_OLFML3	pDC_LILRA4	HMGB1	CXCR4	1,29989E-03	8112,3	8,01226E-03	14102	5,77202E-01	5797	2,43877E+00	869	9,33590E-01	2263	0	17530,5
Non-responder	Macro_FOLR2+APOE+	Macro_NLRP3	HLA-A	LILRB1	1,30047E-03	12313,3	5,12285E-03	28326	5,55228E-01	6355	1,65103E+00	4189	9,18903E-01	5166	0	17530,5
Non-responder	Mono_CD16	Mono_CD16	CD48	CD244	1,30127E-03	11185,1	3,05797E-02	1490	8,48138E-01	2104	1,92211E+00	2451	8,06313E-01	32350	0	17530,5
Non-responder	Mono_INHBA	cDC_LAMP3	THBS1	TNFRSF11B	1,30198E-03	11791,5	8,95542E-02	176	1,22720E+00	377	1,56406E+00	4900	7,90859E-01	35974	0	17530,5
Non-responder	Macro_LYVE1	pDC_LILRA4	IL10	IFNLR1	1,30206E-03	14496,3	6,33377E-02	408	8,68405E-01	1930	1,96946E+00	2212	7,26819E-01	50401	0	17530,5
Non-responder	Macro_NLRP3	Macro_ISG15	ICAM1	ITGAL_ITGB2	1,30266E-03	10057,3	8,01095E-03	14108	5,73978E-01	5887	1,68553E+00	3885	9,01762E-01	8876	0	17530,5
Non-responder	Mono_CD16	Mono_CD16	ICAM2	ITGAL_ITGB2	1,30610E-03	8043,1	1,73137E-02	3928	7,82586E-01	2702	2,11586E+00	1637	8,79891E-01	14418	0	17530,5
Non-responder	Macro_FOLR2+APOE+	Mono_CD16	PSAP	SORT1	1,30636E-03	10933,5	8,30550E-03	13269	5,57601E-01	6288	1,74324E+00	3464	8,81035E-01	14116	0	17530,5
Non-responder	Macro_FOLR2+APOE+	Mono_CD16	TIMP2	CD44	1,30728E-03	13188,9	8,07144E-03	13918	4,00774E-01	11952	1,26828E+00	8426	8,81029E-01	14118	0	17530,5
Non-responder	Macro_ISG15	cDC_CLEC9A	ADM	RAMP1	1,30903E-03	13719,9	3,89655E-02	1016	8,36832E-01	2216	2,22602E+00	1307	7,45015E-01	46530	0	17530,5
Non-responder	pDC_LILRA4	Macro_ISG15	PROC	PROCR	1,31077E-03	17261,9	7,12894E-02	311	8,36617E-01	2217	2,26544E+00	1194	6,27710E-01	65057	0	17530,5
Non-responder	Macro_NLRP3	Macro_OLFML3	CCL4	CCR1	1,31337E-03	10243,7	1,30351E-02	6371	6,35391E-01	4621	1,64059E+00	4268	8,64035E-01	18428	0	17530,5
Non-responder	pDC_LILRA4	Macro_LYVE1	SEMA7A	PLXNC1	1,31427E-03	11724,5	3,57513E-02	1163	9,39065E-01	1500	1,96763E+00	2219	7,89776E-01	36210	0	17530,5
Non-responder	pDC_LILRA4	Mast	CDH1	KLRG1	1,31576E-03	15080,7	3,66816E-01	12	1,22662E+00	379	1,95001E+00	2315	7,00868E-01	55167	0	17530,5
Non-responder	pDC_LILRA4	Macro_ISG15	B2M	LILRB1	1,31602E-03	6498,7	8,92485E-03	11788	8,36037E-01	2220	2,65878E+00	543	9,47040E-01	412	0	17530,5
Non-responder	Macro_IFI27	Macro_NLRP3	TIMP2	CD44	1,31657E-03	12772,7	8,06101E-03	13947	5,70058E-01	5974	1,04290E+00	12274	8,80961E-01	14138	0	17530,5
Non-responder	Mono_CD16	Mono_CD16	ICAM4	ITGB2	1,31661E-03	9718,5	2,60311E-02	1951	7,21845E-01	3350	1,65455E+00	4158	8,50578E-01	21603	0	17530,5
Non-responder	Mono_CD16	Mono_CD16	ICAM4	ITGA4	1,31823E-03	11575,7	3,74039E-02	1081	8,68832E-01	1927	1,94945E+00	2317	7,95082E-01	35023	0	17530,5
Non-responder	Macro_IER3	Macro_NLRP3	TNF	TNFRSF1B	1,31890E-03	9155,1	1,15697E-02	7802	7,12914E-01	3473	1,84805E+00	2827	8,80924E-01	14143	0	17530,5
Non-responder	cDC_CLEC9A	Mono_CD16	HLA-C	LILRA3	1,31986E-03	6318,9	2,12169E-02	2756	6,90101E-01	3744	2,26758E+00	1185	9,13042E-01	6379	0	17530,5
Non-responder	Macro_FOLR2+APOE+	Macro_FOLR2+APOE+	ADAM10	CADM1	1,32229E-03	14407,1	1,80155E-02	3656	5,54382E-01	6382	1,52883E+00	5271	7,77199E-01	39196	0	17530,5
Non-responder	Macro_NLRP3	Mono_CD16	VCAN	TLR2	1,32229E-03	9300,9	1,30216E-02	6382	8,04173E-01	2509	1,64388E+00	4237	8,74371E-01	15846	0	17530,5
Non-responder	Macro_LYVE1	Macro_FOLR2+APOE+	PLTP	ABCA1	1,32311E-03	5879,3	2,43471E-02	2176	1,06073E+00	930	1,93618E+00	2377	9,13036E-01	6383	0	17530,5
Non-responder	Macro_FOLR2+APOE+	cDC_CLEC9A	FN1	ITGA4_ITGB7	1,32311E-03	13476,5	1,49122E-02	5061	5,54338E-01	6383	1,59622E+00	4631	8,00467E-01	33777	0	17530,5
Non-responder	Macro_NLRP3	Macro_FOLR2+APOE+	NRG1	HLA-DPB1	1,32392E-03	10301,5	1,51938E-02	4891	5,54337E-01	6384	1,47059E+00	5834	8,70424E-01	16868	0	17530,5
Non-responder	Macro_FOLR2+APOE+	Mono_CD14	PLAU	ITGA5	1,32474E-03	14705,3	1,44788E-02	5314	5,54282E-01	6385	1,74741E+00	3438	7,70299E-01	40859	0	17530,5
Non-responder	pDC_LILRA4	Macro_IER3	LTB	TNFRSF1A	1,32479E-03	7107,3	2,39417E-02	2225	1,08817E+00	817	2,97620E+00	292	8,78998E-01	14672	0	17530,5
Non-responder	Mono_CD16	Macro_ISG15	HLA-B	LILRB1	1,32497E-03	8666,9	7,99440E-03	14156	5,61790E-01	6181	1,66625E+00	4065	9,37802E-01	1402	0	17530,5
Non-responder	pDC_LILRA4	Macro_NLRP3	APP	LRP1	1,32655E-03	5924,1	2,39391E-02	2226	1,21252E+00	405	3,01975E+00	258	9,00413E-01	9201	0	17530,5
Non-responder	pDC_LILRA4	Mono_CD16	APP	APLP2	1,32800E-03	5501,7	2,06674E-02	2902	1,19847E+00	444	3,03179E+00	243	9,13018E-01	6389	0	17530,5
Non-responder	pDC_LILRA4	Mast	SPON2	ITGA4	1,32831E-03	11209,5	2,39190E-02	2227	9,67758E-01	1358	2,10937E+00	1662	8,02504E-01	33270	0	17530,5
Non-responder	Macro_NLRP3	Macro_IFI27	THBS1	LRP1	1,32872E-03	10677,1	1,40200E-02	5627	7,09637E-01	3525	1,03046E+00	12539	8,80819E-01	14164	0	17530,5
Non-responder	Mast	Macro_IER3	CSF1	CSF3R	1,32961E-03	8092,9	6,58471E-02	381	1,23235E+00	367	1,78572E+00	3197	8,61688E-01	18989	0	17530,5
Non-responder	Macro_IER3	Macro_ISG15	HBEGF	CD44	1,32966E-03	9465,9	9,60331E-03	10491	6,27098E-01	4784	1,34307E+00	7372	9,09449E-01	7152	0	17530,5
Non-responder	Mast	Macro_OLFML3	CSF1	CSF2RA	1,33008E-03	7621,5	6,03500E-02	441	1,20804E+00	416	1,96528E+00	2228	8,67956E-01	17492	0	17530,5
Non-responder	pDC_LILRA4	cDC(CD1C)	APP	TSPAN15	1,33361E-03	10451,7	2,39143E-02	2230	1,14092E+00	608	2,65084E+00	555	8,10715E-01	31335	0	17530,5
Non-responder	Macro_IFI27	pDC_LILRA4	CD14	TLR9	1,33389E-03	8674,5	6,39258E-02	399	1,00942E+00	1163	1,16168E+00	10105	8,80801E-01	14175	0	17530,5
Non-responder	Macro_IER3	Macro_ISG15	HBEGF	CD9	1,33624E-03	12188,3	9,77656E-03	10222	4,08564E-01	11585	1,10641E+00	11053	8,94803E-01	10551	0	17530,5
Non-responder	Macro_OLFML3	pDC_LILRA4	COPA	P2RY6	1,33657E-03	7370,9	2,38579E-02	2239	1,22544E+00	382	3,25312E+00	153	8,71667E-01	16550	0	17530,5
Non-responder	Macro_FOLR2+APOE+	Macro_NLRP3	LGALS9	LRP1	1,33718E-03	12051,5	7,98462E-03	14182	4,51186E-01	9798	1,43655E+00	6206	8,87017E-01	12541	0	17530,5
Non-responder	Macro_FOLR2+APOE+	Mono_INHBA	PSEN1	CD44	1,33766E-03	11253,7	9,84392E-03	10099	6,04262E-01	5223	1,21466E+00	9233	8,80761E-01	14183	0	17530,5
Non-responder	Mono_CD16	Mono_CD16	RPS19	C5AR1	1,34356E-03	9396,3	7,22776E-03	16641	5,82852E-01	5674	1,82232E+00	2984	9,24103E-01	4152	0	17530,5
Non-responder	Macro_FOLR2+APOE+	Macro_LYVE1	C3	CD81	1,34522E-03	12822,1	1,09711E-02	8524	3,57586E-01	14199	1,01777E+00	12800	8,92723E-01	11057	0	17530,5
Non-responder	Macro_NLRP3	cDC(CD1C)	ICAM1	ITGAX_ITGB2	1,34664E-03	10945,5	7,97570E-03	14202	5,65412E-01	6089	1,19664E+00	9515	9,08324E-01	7391	0	17530,5
Non-responder	Macro_IER3	Macro_ISG15	C3	IFITM1	1,34854E-03	6218,1	2,08071E-02	2862	1,03208E+00	1060	2,08462E+00	1757	9,06284E-01	7881	0	17530,5
Non-responder	Mono_CD16	Mono_CD14	SERPINA1	LRP1	1,34949E-03	9942,9	7,97353E-03	14208	5,64955E-01	6104	1,49486E+00	5585	9,13415E-01	6287	0	17530,5
Non-responder	Mast	Macro_NLRP3	CSF1	CSF3R	1,35313E-03	8321,9	5,78830E-02	498	1,18178E+00	494	1,96389E+00	2241	8,53826E-01	20846	0	17530,5
Non-responder	Macro_OLFML3	Mono_CD14	SERPING1	LRP1	1,35472E-03	11298,3	9,88002E-03	10043	5,32626E-01	6969	1,31810E+00	7730	8,80620E-01	14219	0	17530,5
Non-responder	Mono_CD14	Macro_FOLR2+APOE-	S100A12	TLR4	1,35491E-03	9868,9	2,91741E-02	1632	1,20748E+00	417	1,96385E+00	2242	8,26262E-01	27523	0	17530,5
Non-responder	Mono_CD14	Macro_FOLR2+APOE+	S100A8	CD36	1,35491E-03	5571,3	2,38486E-02	2242	1,20207E+00	434	2,76054E+00	447	9,09221E-01	7203	0	17530,5
Non-responder	Mono_CD16	Macro_NLRP3	NAMPT	ITGA5_ITGB1	1,35662E-03	11759,3	8,13006E-03	13758	5,37147E-01	6840	1,41791E+00	6445	8,80603E-01	14223	0	17530,5
Non-responder	Macro_FOLR2+APOE+	cDC_CLEC9A	ADM	RAMP1	1,35678E-03	17676,1	2,17419E-02	2632	5,52646E-01	6424	1,65753E+00	4130	6,85783E-01	57664	0	17530,5
Non-responder	cDC_LAMP3	Macro_LYVE1	IL15	IL2RA	1,35753E-03	11128,9	6,53885E-02	385	1,30577E+00	254	1,95239E+00	2296	7,94437E-01	35179	0	17530,5
Non-responder	Macro_OLFML3	Macro_NLRP3	PKM	CD44	1,35949E-03	8189,7	7,96488E-03	14229	7,12430E-01	3478	1,63169E+00	4329	9,37870E-01	1382	0	17530,5
Non-responder	Macro_FOLR2+APOE+	Macro_OLFML3	GRN	TNFRSF1A	1,36010E-03	10078,1	7,03378E-03	17391	5,52546E-01	6428	1,61750E+00	4446	9,21744E-01	4595	0	17530,5

Non-responder	Macro_IER3	cDC_CLEC9A	CXCL2	DPP4	1,36206E-03	9696,9	6,90936E-02	333	1,08625E+00	826	1,96177E+00	2246	8,26143E-01	27549	0	17530,5
Non-responder	Macro_IER3	Macro_ISG15	CCL3L1	CCR5	1,36206E-03	10424,1	2,96923E-02	1574	8,33633E-01	2246	2,01759E+00	2011	8,21317E-01	28759	0	17530,5
Non-responder	Mono_CD16	Mast	ICAM4	ITGA2B	1,36455E-03	16921,5	3,08024E-01	16	1,22331E+00	386	1,44857E+00	6051	6,65421E-01	60624	0	17530,5
Non-responder	Macro_NLRP3	Macro_FOLR2+APOE+	S100A12	TLR4	1,36509E-03	13786,5	1,87066E-02	3430	5,52438E-01	6434	1,47246E+00	5814	7,92023E-01	35724	0	17530,5
Non-responder	Macro_IFI27	Mono_CD16	HLA-A	LILRA1	1,36523E-03	8694,3	1,73121E-02	3930	7,23853E-01	3324	9,51034E-01	14241	9,22572E-01	4446	0	17530,5
Non-responder	Mono_CD16	Mono_CD16	PTPN6	CD300LF	1,36592E-03	9718,7	1,61567E-02	4396	6,67599E-01	4095	1,92234E+00	2449	8,56793E-01	20123	0	17530,5
Non-responder	Macro_OLFML3	cDC_LAMP3	ICAM1	IL2RG	1,36619E-03	12236,7	8,71307E-03	12268	5,98683E-01	5335	1,06610E+00	11807	8,80502E-01	14243	0	17530,5
Non-responder	Macro_NLRP3	Macro_ISG15	ICAM1	ITGAM_ITGB2	1,36619E-03	10461,1	7,96071E-03	14243	5,31022E-01	7019	1,55778E+00	4953	9,03266E-01	8560	0	17530,5
Non-responder	Macro_FOLR2+APOE+	Macro_LYVE1	CCL18	CCR1	1,36759E-03	16433,9	1,72486E-02	3952	5,52370E-01	6437	1,96481E+00	2233	7,18096E-01	52017	0	17530,5
Non-responder	Mono_CD16	Mono_CD16	GNAI2	S1PR4	1,36759E-03	11260,9	1,62635E-02	4358	5,86400E-01	5593	1,56466E+00	4896	8,40920E-01	23927	0	17530,5
Non-responder	Macro_NLRP3	Macro_ISG15	TNF	TNFRSF1B	1,36811E-03	9285,3	1,14741E-02	7900	6,56646E-01	4277	1,91704E+00	2472	8,80488E-01	14247	0	17530,5
Non-responder	pDC_LILRA4	Macro_IFI27	SPON2	ITGA4	1,36924E-03	11294,1	2,38253E-02	2250	9,66805E-01	1361	2,02803E+00	1979	8,02193E-01	33350	0	17530,5
Non-responder	Mono_CD16	Mono_CD16	ICAM4	ITGB1	1,37343E-03	15375,3	2,27498E-02	2417	5,85098E-01	5623	1,41795E+00	6444	7,52438E-01	44862	0	17530,5
Non-responder	Macro_FOLR2+APOE+	Macro_NLRP3	AGTRAP	RACK1	1,37388E-03	12008,9	8,84379E-03	11937	5,49657E-01	6510	1,17908E+00	9808	8,80435E-01	14259	0	17530,5
Non-responder	Macro_NLRP3	cDC(CD1C)	ICAM1	ITGAL_ITGB2	1,37436E-03	11626,9	7,95498E-03	14260	5,42623E-01	6696	1,12573E+00	10698	9,01451E-01	8950	0	17530,5
Non-responder	Mono_CD14	cDC_LAMP3	ADM	CALCRL	1,37644E-03	11074,1	3,12946E-02	1439	9,87587E-01	1262	1,95976E+00	2254	8,04084E-01	32885	0	17530,5
Non-responder	Macro_FOLR2+APOE+	Macro_FOLR2+APOE+	PLAU	ITGAM	1,37762E-03	12910,7	1,68415E-02	4099	5,51771E-01	6449	1,63217E+00	4326	8,07140E-01	32149	0	17530,5
Non-responder	Mono_CD16	Macro_ISG15	AGTRAP	RACK1	1,37774E-03	11723,7	8,84036E-03	11946	5,39467E-01	6777	1,29125E+00	8098	8,80414E-01	14267	0	17530,5
Non-responder	Macro_ISG15	pDC_LILRA4	TNFSF13	TNFRSF17	1,37824E-03	10555,3	8,06664E-02	225	8,32699E-01	2255	2,27858E+00	1166	8,09606E-01	31600	0	17530,5
Non-responder	Mono_CD14	pDC_LILRA4	TNFSF13B	TNFRSF17	1,38186E-03	11043,7	6,74408E-02	351	8,32625E-01	2257	1,96781E+00	2218	8,04182E-01	32862	0	17530,5
Non-responder	pDC_LILRA4	cDC_CLEC9A	COPA	P2RY6	1,38186E-03	7800,9	2,37600E-02	2257	8,94070E-01	1767	2,45255E+00	843	8,71436E-01	16607	0	17530,5
Non-responder	Macro_ISG15	Mono_CD16	HLA-F	LILRB2	1,38266E-03	6226,1	1,29338E-02	6455	1,00891E+00	1166	2,37597E+00	979	9,19690E-01	5000	0	17530,5
Non-responder	Macro_FOLR2+APOE+	Mono_CD14	LGALS9	CD44	1,38350E-03	10135,7	7,61669E-03	15262	5,69225E-01	5993	1,41658E+00	6456	9,17559E-01	5437	0	17530,5
Non-responder	Macro_NLRP3	Macro_NLRP3	VCAN	TLR2	1,38602E-03	9348,1	1,29251E-02	6459	8,01923E-01	2533	1,64172E+00	4259	8,73962E-01	15959	0	17530,5
Non-responder	Macro_OLFML3	Mono_CD16	CD14	ITGA4	1,38839E-03	11077,1	7,94417E-03	14289	4,59360E-01	9450	1,47426E+00	5795	9,04404E-01	8321	0	17530,5
Non-responder	Macro_FOLR2+APOE+	Macro_OLFML3	CXCL10	SDC4	1,39024E-03	15879,1	1,29211E-02	6464	5,52630E-01	6425	1,97460E+00	2194	7,43910E-01	46782	0	17530,5
Non-responder	cDC(CD1C)	Mono_CD16	HLA-C	LILRA3	1,39108E-03	6904,3	2,10206E-02	2807	6,73266E-01	4013	1,71082E+00	3706	9,12672E-01	6465	0	17530,5
Non-responder	cDC_LAMP3	Macro_FOLR2+APOE+	HLA-DQA2	CD4	1,39131E-03	10921,9	8,40447E-03	13010	4,88386E-01	8376	1,12962E+00	10641	9,19462E-01	5052	0	17530,5
Non-responder	cDC_CLEC9A	Macro_ISG15	HLA-DPB1	LAG3	1,39273E-03	5836,3	2,37069E-02	2263	1,19141E+00	459	2,55631E+00	680	9,04735E-01	8249	0	17530,5
Non-responder	Macro_FOLR2+APOE+	Macro_ISG15	CCL5	CCR5	1,39362E-03	18133,1	3,01594E-02	1523	5,51288E-01	6468	1,71420E+00	3676	6,58955E-01	61468	0	17530,5
Non-responder	Macro_NLRP3	Mono_CD16	THBS1	CD36	1,39362E-03	11235,7	1,29162E-02	6468	7,35074E-01	3197	1,74541E+00	3445	8,34163E-01	25538	0	17530,5
Non-responder	Macro_OLFML3	Mono_CD16	HLA-F	LILRB1	1,39472E-03	11350,5	8,52895E-03	12687	5,47570E-01	6553	1,48673E+00	5680	8,80298E-01	14302	0	17530,5
Non-responder	Macro_OLFML3	Macro_NLRP3	FN1	CD44	1,39521E-03	9028,1	1,43009E-02	5440	6,52763E-01	4348	1,73401E+00	3519	8,80297E-01	14303	0	17530,5
Non-responder	Mono_CD16	Macro_ISG15	HLA-F	LILRB2	1,39521E-03	9790,5	7,93892E-03	14303	6,49629E-01	4403	1,75606E+00	3375	8,99719E-01	9341	0	17530,5
Non-responder	Macro_FOLR2+APOE+	Macro_ISG15	TNF	VSIR	1,39616E-03	10993,3	1,29123E-02	6471	7,37226E-01	3177	1,88301E+00	2649	8,35942E-01	25139	0	17530,5
Non-responder	Macro_FOLR2+APOE+	Mono_CD14	HLA-C	LILRB2	1,39700E-03	11133,5	5,57042E-03	25146	5,51062E-01	6472	1,53766E+00	5164	9,38094E-01	1355	0	17530,5
Non-responder	Macro_ISG15	Macro_NLRP3	CXCL10	SDC4	1,39819E-03	11236,3	2,36824E-02	2266	9,60947E-01	1393	2,74728E+00	459	7,97271E-01	34533	0	17530,5
Non-responder	pDC_LILRA4	Macro_FOLR2+APOE-	LTB	TNFRSF1A	1,40366E-03	7228,1	2,36562E-02	2269	1,08362E+00	837	2,58168E+00	650	8,78359E-01	14854	0	17530,5
Non-responder	pDC_LILRA4	Macro_FOLR2+APOE+	HLA-B	LILRB2	1,40366E-03	7545,7	7,19078E-03	16761	8,31318E-01	2269	2,48687E+00	782	9,47596E-01	386	0	17530,5
Non-responder	Mono_CD16	Macro_OLFML3	ICAM2	ITGAL_ITGB2	1,40464E-03	10592,5	1,38830E-02	5729	5,51218E-01	6469	1,48630E+00	5685	8,67724E-01	17549	0	17530,5
Non-responder	Macro_IER3	Macro_ISG15	ANXA1	FPR2_FPR3	1,40499E-03	12553,5	1,05275E-02	9104	4,68607E-01	9122	1,02271E+00	12688	8,80237E-01	14323	0	17530,5
Non-responder	Macro_NLRP3	Macro_LYVE1	THBS1	CD36	1,40719E-03	11837,9	1,29255E-02	6458	7,35217E-01	3195	1,41361E+00	6484	8,34213E-01	25522	0	17530,5
Non-responder	Macro_OLFML3	Macro_ISG15	HLA-DQA2	LAG3	1,40732E-03	8722,9	2,36402E-02	2271	9,80053E-01	1299	1,99951E+00	2097	8,55541E-01	20417	0	17530,5
Non-responder	Macro_OLFML3	Macro_FOLR2+APOE+	HLA-DRA	LAG3	1,40745E-03	9571,9	1,15179E-02	7851	6,42284E-01	4514	1,72008E+00	3636	8,80222E-01	14328	0	17530,5
Non-responder	cDC_LAMP3	Macro_FOLR2+APOE+	B2M	TFRC	1,40892E-03	9276,9	8,17550E-03	13629	5,15998E-01	7465	1,41247E+00	6499	9,38682E-01	1261	0	17530,5
Non-responder	Mono_CD16	cDC(CD1C)	SELPLG	ITGB2	1,40941E-03	11206,7	7,92992E-03	14332	4,83364E-01	8574	1,15200E+00	10272	9,18151E-01	5325	0	17530,5
Non-responder	Macro_NLRP3	Macro_IER3	ICAM1	ITGAM_ITGB2	1,41138E-03	11459,3	7,92879E-03	14336	5,35042E-01	6898	1,17228E+00	9934	9,03090E-01	8598	0	17530,5
Non-responder	Mast	Mono_CD16	CSF1	CSF3R	1,41282E-03	8941,9	4,85885E-02	687	1,12276E+00	683	1,95612E+00	2274	8,42561E-01	23535	0	17530,5
Non-responder	Macro_IFI27	Macro_FOLR2-APOE+	APOE	TREM2	1,41465E-03	11571,9	2,36018E-02	2275	8,37175E-01	2211	2,36042E-01	35779	9,54710E-01	64	0	17530,5
Non-responder	Macro_IER3	Macro_NLRP3	FN1	PLAUR	1,41483E-03	8945,1	1,31927E-02	6233	7,55241E-01	2966	1,71678E+00	3653	8,80166E-01	14343	0	17530,5
Non-responder	Macro_IER3	Macro_FOLR2+APOE+	HLA-DQB1	CD4	1,41779E-03	9807,3	7,92537E-03	14349	5,38495E-01	6808	1,21767E+00	9180	9,39401E-01	1169	0	17530,5
Non-responder	Macro_IFI27	Macro_FOLR2+APOE+	C3	NRP1	1,41878E-03	10121,3	2,39523E-02	2223	6,75591E-01	3970	1,03076E+00	12532	8,80142E-01	14351	0	17530,5
Non-responder	Mono_CD16	Macro_OLFML3	ICAM4	ITGB2	1,41916E-03	10338,5	2,51594E-02	2059	6,88993E-01	3761	1,43681E+00	6200	8,48400E-01	22142	0	17530,5
Non-responder	Macro_FOLR2+APOE+	Macro_FOLR2-APOE+	APOE	SORL1	1,41977E-03	10285,7	1,24706E-02	6862	5,00193E-01	7986	9,46313E-01	14353	9,21235E-01	4697	0	17530,5
Non-responder	cDC_LAMP3	Macro_FOLR2+APOE+	TNFSF13B	TFRC	1,42175E-03	8554,7	1,16905E-02	7655	6,79206E-01	3915	2,02346E+00	1994	8,90277E-01	11679	0	17530,5
Non-responder	cDC_LAMP3	Macro_FOLR2+APOE+	TNFSF13B	CD40	1,42274E-03	9020,5	1,07747E-02	8783	7,04012E-01	3582	1,86283E+00	2757	8,87384E-01	12450	0	17530,5
Non-responder	pDC_LILRA4	Macro_LYVE1	GAS6	AXL	1,42385E-03	8913,5	2,35791E-02	2280	8,95830E-01	1756	2,27364E+00	1175	8,49677E-01	21826	0	17530,5
Non-responder	Macro_IER3	Macro_NLRP3	PLAU	PLAUR	1,42472E-03	8910,1	1,23872E-02	6950	8,21884E-01	2352	1,76023E+00	3355	8,80063E-01	14363	0	17530,5
Non-responder	Macro_IER3	Macro_NLRP3	C3	ITGAX	1,42571E-03	12993,7	9,90934E-03	9997	3,88757E-01	12535	1,13478E+00	10541	8,80053E-01	14365	0	17530,5
Non-responder	Mono_CD16	Macro_FOLR2+APOE+	TNFSF13B	TFRC	1,42720E-03	10286,5	9,55811E-03	10580	5,51515E-01	6460	1,91328E+00	2494	8,80047E-01	14368	0	17530,5
Non-responder	pDC_LILRA4	Macro_FOLR2+APOE+	FARP2	PLXNA1	1,42754E-03	15328,5	5,91215E-02	473	8,29969E-01	2282	1,96120E+00	2252	7,06999E-01	54105	0	17530,5
Non-responder	pDC_LILRA4	Mono_CD16	LTB	TNFRSF1A	1,42754E-03	7135,9	2,35778E-02	2282	1,08236E+00	843	3,30538E+00	135	8,78181E-01	14889	0	17530,5
Non-responder	Macro_NLRP3	Macro_ISG15	GNAI2	C5AR1	1,42770E-03	10488,9	7,91679E-03	14369	5,19715E-01	7341	1,48611E+00	5686	9,07786E-01	7518	0	17530,5
Non-responder	Macro_IFI27	pDC_LILRA4	C3	NRP1	1,42820E-03	9876,3	2,39091E-02	2231	6,74540E-01	3987	1,09429E+00	11263	8,80046E-01	14370	0	17530,5
Non-responder	Macro_FOLR2+APOE+	Macro_NLRP3	PLAU	IGF2R	1,42860E-03	15125,9	1,34028E-02	6068	5,49710E-01	6509	1,74994E+00	3420	7,64899E-01	42102	0	17530,5
Non-responder	Macro_OLFML3	Macro_LYVE1	CXCL10	TLR4	1,43124E-03	8538,1	2,35710E-02	2284	1,07745E+00	868	2,63390E+00	574	8,51320E-01	21434	0	17530,5
Non-responder	cDC_LAMP3	Macro_FOLR2+APOE+	CXCL9	FCGR2A	1,43218E-03	8460,1	1,28374E-02	6535	7,21407E-01	3355	1,69447E+00	3816	8,92706E-01	11064	0	17530,5
Non-responder	Macro_FOLR2+APOE+	Macro_FOLR2+APOE+	TGM2	SDC4	1,43378E-03	16838,9	2,25972E-02	2451	6,10001E-01	5096	1,41171E+00	6515	7,15243E-01	52602	0	17530,5
Non-responder	Macro_OLFML3	pDC_LILRA4	HLA-DQA2	CD4	1,43566E-03	9945,3	7,90976E-03	14385	4,45926E-01	9983	1,94763E+00	2323	9,17187E-01	5505	0	17530,5
Non-responder	Mast	Macro_NLRP3	CSF1	SIRPA	1,43680E-03	9108,3	5,18832E-02	614	1,15559E+00	561	1,95346E+00	2287	8,38349E-01	24549	0	17530,5
Non-responder	cDC_LAMP3	Macro_FOLR2+APOE+	RPS19	C5AR1	1,43816E-03	7571,5	9,79332E-03	10189	7,94245E-01	2602	1,51813E+00	5364	9,34093E-01	2172	0	17530,5

Post_NR_Myeloid_Myeloid_CellCell

Non-responder	Macro_FOLR2+APOE+	Macro_ISG15	APOC2	LRP1	1,44330E-03	11473,3	2,02660E-02	2992	5,48923E-01	6526	2,22406E+00	1311	8,20217E-01	29007	0	17530,5
Non-responder	Macro_NLRP3	Mono_CD16	GNAI2	C5AR1	1,44567E-03	10486,5	7,90399E-03	14405	5,18517E-01	7377	1,49483E+00	5587	9,07718E-01	7533	0	17530,5
Non-responder	Macro_FOLR2+APOE+	pDC_LILRA4	MDK	NOTCH2	1,44677E-03	16822,9	2,40851E-02	2205	5,48746E-01	6530	1,61949E+00	4426	7,10709E-01	53423	0	17530,5
Non-responder	Macro_FOLR2+APOE+	Macro_FOLR2+APOE+	IGF1	IGF2R	1,44764E-03	15147,1	1,84663E-02	3513	5,48745E-01	6531	1,66877E+00	4044	7,55586E-01	44117	0	17530,5
Non-responder	Mast	Macro_ISG15	ADCYAP1	GPR84	1,44982E-03	12734,5	1,50292E-01	51	1,17105E+00	527	1,95256E+00	2294	7,59384E-01	43270	0	17530,5
Non-responder	pDC_LILRA4	Mast	SPON2	ITGAM	1,44982E-03	13619,3	2,34809E-02	2294	9,39653E-01	1498	2,13230E+00	1578	7,51096E-01	45196	0	17530,5
Non-responder	Macro_IER3	Mast	IL1B	ADRB2	1,45070E-03	8764,7	3,12998E-02	1438	9,09204E-01	1672	1,24512E+00	8768	8,79895E-01	14415	0	17530,5
Non-responder	Macro_FOLR2+APOE+	Macro_ISG15	ANXA2	TLR2	1,45070E-03	10650,3	7,90026E-03	14415	4,44350E-01	10047	1,72820E+00	3569	9,07056E-01	7690	0	17530,5
Non-responder	Macro_IER3	pDC_LILRA4	HMGB1	TLR9	1,45170E-03	7749,9	5,80772E-02	491	9,62842E-01	1384	1,56165E+00	4927	8,79893E-01	14417	0	17530,5
Non-responder	Macro_LYVE1	Mono_CD16	HLA-C	LILRA3	1,45374E-03	7315,7	2,08578E-02	2847	6,59300E-01	4226	1,51085E+00	5437	9,12362E-01	6538	0	17530,5
Non-responder	Macro_NLRP3	Macro_IFI27	CXCL8	SDC3	1,45549E-03	13031,3	1,39246E-02	5701	7,66724E-01	2849	1,40928E+00	6540	8,05545E-01	32536	0	17530,5
Non-responder	Macro_FOLR2+APOE+	Macro_OLFML3	ANXA2	TLR2	1,45624E-03	11153,3	7,89664E-03	14426	4,44118E-01	10058	1,44827E+00	6055	9,07037E-01	7697	0	17530,5
Non-responder	pDC_LILRA4	Macro_FOLR2+APOE-	GAS6	AXL	1,45729E-03	9095,1	2,34475E-02	2298	8,93692E-01	1770	2,03346E+00	1959	8,49319E-01	21918	0	17530,5
Non-responder	Mast	Mono_CD14	TIMP3	CD44	1,45750E-03	6494,3	6,68800E-02	361	1,21553E+00	399	1,82919E+00	2950	8,92088E-01	11231	0	17530,5
Non-responder	Macro_FOLR2+APOE+	Mast	C1QB	CD33	1,45826E-03	10920,5	7,89492E-03	14430	5,88706E-01	5547	1,46591E+00	5891	8,92166E-01	11204	0	17530,5
Non-responder	Macro_FOLR2+APOE+	Macro_FOLR2+APOE-	C1QA	CD93	1,46028E-03	10918,3	7,89411E-03	14434	6,02804E-01	5254	1,34708E+00	7331	8,96753E-01	10042	0	17530,5
Non-responder	Macro_ISG15	Macro_ISG15	HLA-B	LILRB2	1,46104E-03	7363,9	7,70594E-03	14985	8,27432E-01	2300	2,09370E+00	1730	9,49288E-01	274	0	17530,5
Non-responder	Macro_FOLR2+APOE+	Macro_OLFML3	TNFSF13	TNFRSF1A	1,46687E-03	12482,9	7,89103E-03	14447	4,11279E-01	11450	1,44635E+00	6080	8,85589E-01	12907	0	17530,5
Non-responder	Macro_FOLR2+APOE+	Macro_FOLR2-APOE+	CALR	LRP1	1,47450E-03	10988,3	9,10095E-03	11405	5,89692E-01	5528	9,41268E-01	14462	9,14741E-01	6016	0	17530,5
Non-responder	cDC(CD1C)	Macro_ISG15	HLA-DPB1	LAG3	1,47609E-03	6106,9	2,33596E-02	2308	1,16937E+00	534	2,08127E+00	1770	9,04097E-01	8392	0	17530,5
Non-responder	Mono_CD16	Macro_FOLR2+APOE+	TNFSF10	TNFRSF10B	1,47655E-03	13001,5	1,43607E-02	5407	6,11672E-01	5063	1,40698E+00	6564	8,14334E-01	30443	0	17530,5
Non-responder	Macro_IFI27	Mono_CD16	HLA-C	LILRA1	1,47910E-03	8771,3	1,73643E-02	3903	6,93150E-01	3705	9,40930E-01	14471	9,23682E-01	4247	0	17530,5
Non-responder	Macro_IFI27	Macro_FOLR2+APOE+	C3	C3AR1	1,47961E-03	9552,3	1,74985E-02	3848	6,48597E-01	4413	9,40803E-01	14472	9,07843E-01	7498	0	17530,5
Non-responder	Macro_FOLR2+APOE+	Mono_CD16	C3	ITGAX	1,48063E-03	12442,9	9,84620E-03	10098	3,57968E-01	14177	1,46086E+00	5935	8,79715E-01	14474	0	17530,5
Non-responder	cDC_LAMP3	Macro_NLRP3	RPS19	C5AR1	1,48370E-03	7518,5	1,01353E-02	9654	8,19104E-01	2379	1,44493E+00	6101	9,35142E-01	1928	0	17530,5
Non-responder	Macro_FOLR2+APOE+	Macro_NLRP3	CIRBP	TREM1	1,48832E-03	13431,5	8,93518E-03	11762	3,52697E-01	14489	1,21810E+00	9171	8,80676E-01	14205	0	17530,5
Non-responder	Macro_NLRP3	Macro_NLRP3	GNAI2	C5AR1	1,48883E-03	10834,1	7,87566E-03	14490	5,15868E-01	7470	1,36179E+00	7108	9,07567E-01	7572	0	17530,5
Non-responder	Macro_IER3	Mono_CD16	HLA-B	LILRB2	1,49037E-03	8727,9	7,87491E-03	14493	6,57993E-01	4257	1,36123E+00	7123	9,49807E-01	236	0	17530,5
Non-responder	Macro_NLRP3	Mono_INHBA	ADM	GPR84	1,49161E-03	16694,5	1,63534E-02	4315	5,46529E-01	6581	1,46064E+00	5939	7,33114E-01	49107	0	17530,5
Non-responder	Macro_FOLR2+APOE+	Macro_FOLR2+APOE+	HSP90B1	TLR2	1,49398E-03	12377,1	7,87385E-03	14500	4,07076E-01	11659	1,28349E+00	8230	8,97068E-01	9966	0	17530,5
Non-responder	Macro_FOLR2+APOE+	cDC_CLEC9A	LGALS9	PTPRC	1,49874E-03	10161,9	7,12001E-03	17035	5,46136E-01	6589	1,70448E+00	3757	9,15291E-01	5898	0	17530,5
Non-responder	Macro_FOLR2+APOE+	Macro_OLFML3	HLA-F	LILRB2	1,50069E-03	10361,3	7,86644E-03	14513	5,87481E-01	5569	1,57957E+00	4768	8,99304E-01	9426	0	17530,5
Non-responder	cDC_LAMP3	Mono_CD16	HLA-B	LILRB2	1,50120E-03	7758,7	8,33098E-03	13207	7,71151E-01	2809	1,54633E+00	5081	9,51132E-01	166	0	17530,5
Non-responder	Macro_OLFML3	pDC_LILRA4	HMGB1	TLR9	1,50224E-03	7365,1	5,77007E-02	502	9,57569E-01	1408	1,84155E+00	2869	8,79549E-01	14516	0	17530,5
Non-responder	Macro_OLFML3	Macro_FOLR2+APOE+	HLA-DPB1	CD4	1,50224E-03	8710,7	7,86577E-03	14516	5,62057E-01	6170	1,56622E+00	4880	9,46483E-01	457	0	17530,5
Non-responder	Macro_FOLR2+APOE+	Macro_FOLR2+APOE+	MDK	SDC4	1,50231E-03	18958,3	3,72065E-02	1095	6,24681E-01	4836	1,40502E+00	6593	6,31059E-01	64737	0	17530,5
Non-responder	Macro_NLRP3	pDC_LILRA4	TGFB1	TGFBR1_TGFBR2	1,50231E-03	10134,5	1,27811E-02	6593	5,86589E-01	5588	2,21762E+00	1323	8,58928E-01	19638	0	17530,5
Non-responder	cDC_LAMP3	Mono_CD16	HLA-A	LILRA1	1,50327E-03	8454,1	1,57250E-02	4639	5,61271E-01	6194	1,24477E+00	8774	9,19067E-01	5133	0	17530,5
Non-responder	Macro_NLRP3	Macro_FOLR2+APOE+	CD99	CD81	1,50483E-03	12366,3	7,86283E-03	14521	3,88371E-01	12555	1,27363E+00	8350	9,01766E-01	8875	0	17530,5
Non-responder	Macro_FOLR2+APOE+	Macro_FOLR2+APOE+	CXCL8	SDC3	1,50768E-03	13630,3	1,27723E-02	6599	5,74286E-01	5878	1,67526E+00	3975	7,98690E-01	34169	0	17530,5
Non-responder	Macro_OLFML3	cDC_CLEC9A	HLA-DQB1	LAG3	1,50794E-03	8101,9	1,83708E-02	3535	8,95114E-01	1760	1,79267E+00	3157	8,79522E-01	14527	0	17530,5
Non-responder	cDC_LAMP3	Mono_CD16	HLA-C	LILRA1	1,51053E-03	9727,3	1,51514E-02	4919	4,68376E-01	9131	1,06386E+00	11855	9,18736E-01	5201	0	17530,5
Non-responder	Mono_CD16	Macro_NLRP3	ADAM10	CD44	1,51418E-03	10310,1	9,22956E-03	11171	6,48388E-01	4416	1,68458E+00	3894	8,79458E-01	14539	0	17530,5
Non-responder	cDC_LAMP3	Mono_CD16	HLA-F	LILRB2	1,51470E-03	8208,9	9,90178E-03	10007	8,51036E-01	2079	1,64511E+00	4230	9,09255E-01	7198	0	17530,5
Non-responder	cDC_LAMP3	Mono_CD16	HLA-F	LILRB1	1,51522E-03	12267,9	8,43732E-03	12936	5,41971E-01	6713	1,18600E+00	9691	8,79727E-01	14469	0	17530,5
Non-responder	cDC_LAMP3	Mono_CD16	B2M	LILRB2	1,51730E-03	7408,5	8,55420E-03	12625	8,19964E-01	2368	1,61273E+00	4485	9,56781E-01	34	0	17530,5
Non-responder	Macro_ISG15	Macro_FOLR2+APOE+	CXCL8	SDC3	1,51801E-03	9253,3	2,49249E-02	2098	8,23870E-01	2330	2,05173E+00	1880	8,47150E-01	22428	0	17530,5
Non-responder	cDC_LAMP3	pDC_LILRA4	GNAI2	CXCR3	1,51993E-03	8124,1	2,32227E-02	2331	8,70018E-01	1916	2,00606E+00	2062	8,70769E-01	16781	0	17530,5
Non-responder	Macro_NLRP3	Macro_NLRP3	IL1B	SIGIRR	1,52117E-03	8511,7	1,27453E-02	6614	9,28173E-01	1564	2,00472E+00	2068	8,78578E-01	14782	0	17530,5
Non-responder	Mono_CD16	Macro_NLRP3	CD55	ADGRE5	1,52297E-03	9490,5	1,27421E-02	6616	7,32656E-01	3229	1,76895E+00	3298	8,70774E-01	16779	0	17530,5
Non-responder	Macro_NLRP3	Macro_LYVE1	ICAM1	ITGAM_ITGB2	1,52305E-03	11864,9	7,85113E-03	14556	5,35783E-01	6876	1,07312E+00	11674	9,02658E-01	8688	0	17530,5
Non-responder	Macro_NLRP3	Macro_FOLR2+APOE-	TIMP1	CD63	1,52462E-03	9838,3	7,84988E-03	14559	7,28150E-01	3270	9,87854E-01	13380	9,46561E-01	452	0	17530,5
Non-responder	Macro_NLRP3	Macro_ISG15	LGALS3	LAG3	1,52478E-03	10702,7	1,72091E-02	3966	7,62518E-01	2895	1,40287E+00	6618	8,46830E-01	22504	0	17530,5
Non-responder	Macro_FOLR2+APOE+	Macro_OLFML3	PLAU	PLAUR	1,52724E-03	10051,1	1,22198E-02	7128	5,63110E-01	6145	1,56553E+00	4888	8,79343E-01	14564	0	17530,5
Non-responder	Macro_ISG15	Mono_CD16	HLA-C	LILRA3	1,52749E-03	6632,7	2,06529E-02	2907	6,41722E-01	4518	2,13028E+00	1587	9,11966E-01	6621	0	17530,5
Non-responder	Macro_OLFML3	cDC(CD1C)	CXCL10	CXCR3	1,52765E-03	11319,9	2,31941E-02	2335	1,03553E+00	1044	2,62052E+00	599	7,94795E-01	35091	0	17530,5
Non-responder	Macro_FOLR2+APOE+	Macro_ISG15	FN1	PLAUR	1,53021E-03	9609,3	1,27317E-02	6624	5,72900E-01	5919	1,80112E+00	3100	8,78278E-01	14873	0	17530,5
Non-responder	cDC_LAMP3	Mono_CD16	RPS19	C5AR1	1,53091E-03	7235,5	1,01718E-02	9602	8,21754E-01	2353	1,57796E+00	4780	9,35251E-01	1912	0	17530,5
Non-responder	Macro_FOLR2+APOE+	Macro_ISG15	SEMA4A	PLXNB2	1,53838E-03	13243,3	1,27186E-02	6633	5,50323E-01	6496	1,45924E+00	5953	8,17759E-01	29604	0	17530,5
Non-responder	Macro_FOLR2+APOE+	pDC_LILRA4	HLA-B	LILRB1	1,53838E-03	11510,7	5,29814E-03	27045	5,44867E-01	6633	1,95204E+00	2299	9,24668E-01	4046	0	17530,5
Non-responder	Macro_FOLR2+APOE+	Mono_CD14	LRPAP1	LRP1	1,53881E-03	12342,5	8,90373E-03	11828	4,56777E-01	9552	1,28424E+00	8216	8,79273E-01	14586	0	17530,5
Non-responder	Macro_IER3	Macro_FOLR2+APOE+	NAMPT	ITGA5_ITGB1	1,54356E-03	13756,5	7,92229E-03	14359	4,80821E-01	8669	9,76900E-01	13629	8,79235E-01	14595	0	17530,5
Non-responder	Mono_CD16	Macro_FOLR2+APOE+	LGALS9	LRP1	1,54621E-03	12773,7	7,83799E-03	14600	4,01691E-01	11911	1,36783E+00	7038	8,86085E-01	12789	0	17530,5
Non-responder	pDC_LILRA4	Macro_NLRP3	PROC	ITGAM	1,55093E-03	12981,1	5,56826E-02	537	8,22661E-01	2347	2,21220E+00	1335	7,59925E-01	43156	0	17530,5
Non-responder	Macro_NLRP3	Macro_FOLR2+APOE+	LYZ	ITGAL	1,55257E-03	12324,1	7,83431E-03	14612	4,81889E-01	8628	1,12997E+00	10637	8,96131E-01	10213	0	17530,5
Non-responder	Mono_INHBA	Macro_FOLR2+APOE+	CCL2	CCR1	1,55483E-03	9942,9	3,11818E-02	1445	8,22243E-01	2349	2,38407E+00	955	8,26623E-01	27435	0	17530,5
Non-responder	Macro_NLRP3	Mono_CD14	CD55	CR1	1,55574E-03	13456,1	2,08465E-02	2853	6,59817E-01	4215	1,40006E+00	6652	7,90614E-01	36030	0	17530,5
Non-responder	Macro_OLFML3	Mono_CD14	HLA-C	LILRA1	1,56109E-03	14099,9	7,95514E-03	14259	3,50564E-01	14628	1,02559E+00	12633	8,91209E-01	11449	0	17530,5
Non-responder	Mono_INHBA	Macro_FOLR2+APOE+	CCL3L1	CCR5	1,56462E-03	9898,3	3,33884E-02	1293	9,13560E-01	1652	1,94090E+00	2354	8,29764E-01	26662	0	17530,5
Non-responder	Macro_OLFML3	cDC_CLEC9A	LGALS9	SLC1A5	1,56697E-03	10388,3	1,10448E-02	8423	5,55339E-01	6351	1,55385E+00	4998	8,79110E-01	14639	0	17530,5
Non-responder	cDC_LAMP3	Macro_OLFML3	TNFSF13B	CD40	1,56697E-03	9310,3	1,07719E-02	8785	7,03841E-01	3585	1,65026E+00	4195	8,87371E-01	12456	0	17530,5

Post_NR_Myeloid_Myeloid_CellCell

Non-responder	Mono_CD16	Macro_FOLR2+APOE+	B2M	TFRC	1,56697E-03	9903,5	7,82459E-03	14639	4,08654E-01	11577	1,63781E+00	4285	9,37407E-01	1486	0	17530,5
Non-responder	Macro_NLRP3	Macro_FOLR2+APOE+	ANXA1	FPR2_FPR3	1,56858E-03	13289,9	1,03049E-02	9409	4,16180E-01	11225	9,76261E-01	13643	8,79106E-01	14642	0	17530,5
Non-responder	Macro_OLFML3	Macro_IER3	CXCL10	TLR4	1,57051E-03	8637,9	2,30752E-02	2357	1,07066E+00	894	2,57155E+00	662	8,49969E-01	21746	0	17530,5
Non-responder	Macro_ISG15	Macro_NLRP3	HBEGF	CD44	1,57231E-03	6481,9	1,26860E-02	6670	8,82816E-01	1831	2,15845E+00	1500	9,20278E-01	4878	0	17530,5
Non-responder	cDC_LAMP3	Macro_OLFML3	CXCL9	FCGR2A	1,57287E-03	12439,7	9,81078E-03	10153	5,06905E-01	7761	1,05134E+00	12120	8,79133E-01	14634	0	17530,5
Non-responder	Macro_FOLR2+APOE+	Macro_ISG15	CXCL10	TLR4	1,57324E-03	12856,1	1,26859E-02	6671	5,51343E-01	6466	2,12980E+00	1590	8,07715E-01	32023	0	17530,5
Non-responder	Macro_OLFML3	Macro_LYVE1	CALR	LRP1	1,57448E-03	12720,7	7,94838E-03	14276	4,46568E-01	9954	9,32744E-01	14653	9,09311E-01	7190	0	17530,5
Non-responder	Macro_FOLR2+APOE+	pDC_LILRA4	HLA-DPA1	CD4	1,57509E-03	8424,7	7,29152E-03	16389	5,43546E-01	6673	2,29812E+00	1123	9,47139E-01	408	0	17530,5
Non-responder	Macro_OLFML3	Mono_CD16	HLA-A	LILRB1	1,57555E-03	8915,9	7,81914E-03	14655	5,96393E-01	5389	1,59168E+00	4675	9,33327E-01	2330	0	17530,5
Non-responder	pDC_LILRA4	Macro_FOLR2+APOE+	APP	FPR2	1,57604E-03	10685,7	2,03509E-02	2980	1,20838E+00	415	2,80869E+00	410	8,07384E-01	32093	0	17530,5
Non-responder	Macro_FOLR2+APOE+	cDC(CD1C)	LGALS3	ENG	1,57609E-03	11352,7	7,81896E-03	14656	4,75571E-01	8857	1,35931E+00	7144	9,03177E-01	8576	0	17530,5
Non-responder	Macro_FOLR2+APOE+	Macro_ISG15	ADAM10	CD44	1,57717E-03	11734,3	9,15768E-03	11305	4,97245E-01	8086	1,36297E+00	7092	8,79043E-01	14658	0	17530,5
Non-responder	Macro_FOLR2-APOE+	cDC(CD1C)	B2M	CD1C	1,57839E-03	6508,9	3,25397E-02	1352	8,20646E-01	2361	1,10963E+00	10992	9,48766E-01	309	0	17530,5
Non-responder	pDC_LILRA4	Mono_CD14	APP	LRP1	1,57839E-03	6052,3	2,30392E-02	2361	1,19426E+00	452	2,90604E+00	343	8,98682E-01	9575	0	17530,5
Non-responder	Macro_NLRP3	Macro_FOLR2+APOE+	TGFB1	ENG	1,57986E-03	10318,1	1,11607E-02	8273	5,45803E-01	6603	1,60883E+00	4521	8,79023E-01	14663	0	17530,5
Non-responder	Macro_LYVE1	Macro_FOLR2+APOE+	CXCL12	CD4	1,58036E-03	8035,7	3,35440E-02	1279	1,00809E+00	1173	1,93930E+00	2362	8,66545E-01	17834	0	17530,5
Non-responder	Macro_FOLR2+APOE+	Macro_OLFML3	SEMA4A	LILRB2	1,58250E-03	9397,5	1,26722E-02	6681	7,13060E-01	3470	1,73906E+00	3485	8,74489E-01	15821	0	17530,5
Non-responder	Mono_CD16	Macro_ISG15	ANXA1	FPR2_FPR3	1,58255E-03	11328,7	1,02870E-02	9434	4,53688E-01	9675	1,52105E+00	5336	8,79013E-01	14668	0	17530,5
Non-responder	Mono_CD16	Macro_FOLR2+APOE+	LGALS9	HAVCR2	1,58255E-03	12265,5	7,81515E-03	14668	4,39674E-01	10238	1,48551E+00	5690	8,84436E-01	13201	0	17530,5
Non-responder	Macro_ISG15	Mono_CD14	MMP12	PLAUR	1,58431E-03	7407,1	2,63966E-02	1904	8,20288E-01	2364	2,55616E+00	681	8,79387E-01	14556	0	17530,5
Non-responder	Mono_CD16	cDC_CLEC9A	SELPLG	ITGB2	1,58525E-03	10328,5	7,81437E-03	14673	4,69765E-01	9076	1,56087E+00	4932	9,17597E-01	5431	0	17530,5
Non-responder	Macro_NLRP3	Mono_CD16	CCL3L1	CCR1	1,58715E-03	11226,1	1,26641E-02	6686	8,01996E-01	2531	1,94236E+00	2341	8,28239E-01	27042	0	17530,5
Non-responder	Mast	Macro_OLFML3	CSF1	CSF3R	1,59119E-03	8156,1	6,22789E-02	417	1,20970E+00	411	1,88222E+00	2654	8,58335E-01	19768	0	17530,5
Non-responder	Macro_FOLR2+APOE+	Macro_FOLR2-APOE+	C1QB	C1QBP	1,59175E-03	10767,9	7,81049E-03	14685	6,12021E-01	5054	1,11265E+00	10945	9,16555E-01	5625	0	17530,5
Non-responder	Macro_NLRP3	cDC(CD1C)	VEGFA	CD44	1,59229E-03	11940,5	1,01973E-02	9560	6,78270E-01	3933	9,30949E-01	14686	8,81506E-01	13993	0	17530,5
Non-responder	Macro_FOLR2+APOE+	Macro_NLRP3	B2M	TFRC	1,59275E-03	11512,5	5,42234E-03	26169	5,42876E-01	6692	1,76210E+00	3345	9,25745E-01	3826	0	17530,5
Non-responder	Macro_FOLR2+APOE+	Macro_ISG15	PLAU	ITGB5	1,59368E-03	18210,5	1,86908E-02	3434	5,42815E-01	6693	1,52519E+00	5306	6,82958E-01	58089	0	17530,5
Non-responder	Macro_ISG15	Mono_CD14	TNFSF10	TNFRSF10D	1,59422E-03	12211,7	2,30226E-02	2369	8,54930E-01	2053	2,12610E+00	1601	7,84313E-01	37505	0	17530,5
Non-responder	Macro_NLRP3	cDC_LAMP3	TNF	TRAF2	1,59461E-03	14877,9	2,29091E-02	2391	6,34453E-01	4642	1,39628E+00	6694	7,60045E-01	43132	0	17530,5
Non-responder	Macro_NLRP3	Macro_ISG15	ICAM1	IL2RG	1,59500E-03	10891,9	8,46096E-03	12866	6,07587E-01	5153	1,64742E+00	4219	8,78948E-01	14691	0	17530,5
Non-responder	Macro_FOLR2+APOE+	Macro_LYVE1	HLA-DQB1	CD4	1,59555E-03	9900,1	7,14209E-03	16956	5,44694E-01	6636	1,39628E+00	6695	9,36370E-01	1683	0	17530,5
Non-responder	Macro_LYVE1	Mono_CD16	HLA-A	LILRA1	1,59835E-03	6950,7	1,79547E-02	3680	7,89686E-01	2644	1,39581E+00	6698	9,23864E-01	4201	0	17530,5
Non-responder	Mono_CD16	Macro_FOLR2+APOE+	S100A9	ITGB2	1,60316E-03	9489,5	7,80425E-03	14706	5,75919E-01	5832	1,28443E+00	8208	9,39377E-01	1171	0	17530,5
Non-responder	Mono_CD16	Macro_IER3	RPS19	C5AR1	1,60398E-03	9879,3	7,39324E-03	16036	5,99781E-01	5312	1,41208E+00	6508	9,24893E-01	4010	0	17530,5
Non-responder	pDC_LILRA4	Mono_CD16	SPON2	ITGAM	1,60417E-03	13570,7	2,29844E-02	2374	9,36397E-01	1522	2,47864E+00	795	7,49093E-01	45632	0	17530,5
Non-responder	cDC_CLEC9A	pDC_LILRA4	BTLA	TNFRSF14	1,60616E-03	11584,5	2,92571E-02	1626	9,95538E-01	1228	1,93703E+00	2375	7,94494E-01	35163	0	17530,5
Non-responder	cDC_CLEC9A	Macro_ISG15	HLA-DPA1	LAG3	1,60816E-03	5757,5	2,29693E-02	2376	1,15519E+00	563	2,48493E+00	786	9,07719E-01	7532	0	17530,5
Non-responder	Macro_FOLR2+APOE+	cDC(CD1C)	MMP9	ITGAM	1,60961E-03	15482,3	1,26334E-02	6710	5,45761E-01	6606	1,74218E+00	3469	7,60210E-01	43096	0	17530,5
Non-responder	Macro_FOLR2+APOE+	Mono_INHBA	ANOS1	SDC2	1,61016E-03	19592,1	8,03139E-02	227	9,43436E-01	1481	1,26343E+00	8494	5,62827E-01	70228	0	17530,5
Non-responder	pDC_LILRA4	Macro_NLRP3	SPON2	ITGA4	1,61016E-03	11214,7	2,29672E-02	2377	9,58072E-01	1405	2,54043E+00	709	7,99266E-01	34052	0	17530,5
Non-responder	Macro_FOLR2+APOE+	Mono_CD16	CCL18	CCR1	1,61338E-03	16592,7	1,64372E-02	4286	5,41942E-01	6714	2,17355E+00	1452	7,13194E-01	52981	0	17530,5
Non-responder	Mono_INHBA	Macro_FOLR2+APOE+	VEGFA	SIRPA	1,61416E-03	7772,9	2,50365E-02	2079	1,02396E+00	1097	1,93591E+00	2379	8,74657E-01	15779	0	17530,5
Non-responder	pDC_LILRA4	Macro_FOLR2+APOE+	CD47	SIRPA	1,61416E-03	5933,5	2,29642E-02	2378,5	1,11693E+00	706,5	3,19201E+00	169,5	9,01739E-01	8882,5	0	17530,5
Non-responder	Macro_FOLR2+APOE+	Macro_FOLR2+APOE+	ICAM1	ITGAL_ITGB2	1,61793E-03	10615,1	7,79349E-03	14733	5,51476E-01	6462	1,53587E+00	5186	9,00536E-01	9164	0	17530,5
Non-responder	Mast	Macro_LYVE1	HDC	HRH2	1,62168E-03	11070,9	6,19605E-02	421	1,27298E+00	299	1,48974E+00	5642	8,10225E-01	31462	0	17530,5
Non-responder	Macro_FOLR2+APOE+	Mono_CD16	C1QA	CD93	1,62343E-03	9956,7	7,78938E-03	14743	5,99003E-01	5330	2,03098E+00	1968	8,96133E-01	10212	0	17530,5
Non-responder	cDC_LAMP3	Macro_IER3	RPS19	C5AR1	1,62618E-03	8157,7	1,04047E-02	9273	8,38682E-01	2197	1,16772E+00	10007	9,35933E-01	1781	0	17530,5
Non-responder	Macro_NLRP3	Macro_FOLR2+APOE+	TNF	TNFRSF1A	1,62661E-03	9958,1	1,26135E-02	6728	6,29998E-01	4726	1,84646E+00	2834	8,65993E-01	17972	0	17530,5
Non-responder	Macro_ISG15	pDC_LILRA4	CXCL2	DPP4	1,62821E-03	11601,5	4,47013E-02	787	8,18412E-01	2386	2,09934E+00	1700	7,92623E-01	35604	0	17530,5
Non-responder	Macro_FOLR2+APOE+	Macro_OLFML3	PLAU	IGF2R	1,62851E-03	15632,5	1,29880E-02	6414	5,41155E-01	6730	1,57317E+00	4821	7,62060E-01	42667	0	17530,5
Non-responder	Macro_OLFML3	Mono_CD16	LGALS9	PTPRC	1,63005E-03	9142,3	7,78649E-03	14755	6,43385E-01	4498	1,70950E+00	3717	9,18696E-01	5211	0	17530,5
Non-responder	Macro_FOLR2+APOE+	Macro_ISG15	HLA-B	LILRA1	1,63392E-03	10572,3	7,78474E-03	14762	5,80316E-01	5728	1,63534E+00	4301	8,94859E-01	10540	0	17530,5
Non-responder	cDC_CLEC9A	Macro_ISG15	BTLA	TNFRSF14	1,63426E-03	9623,5	4,58533E-02	759	1,16680E+00	536	1,93425E+00	2389	8,28764E-01	26903	0	17530,5
Non-responder	Macro_FOLR2+APOE+	Mono_CD14	HLA-A	LILRB1	1,63611E-03	12985,7	4,93613E-03	29835	5,40904E-01	6738	1,51690E+00	5376	9,17508E-01	5449	0	17530,5
Non-responder	Mono_CD16	cDC_LAMP3	TNFSF13	TNFRSF11B	1,63801E-03	12370,9	5,02746E-02	653	9,48563E-01	1452	1,43301E+00	6240	7,90836E-01	35979	0	17530,5
Non-responder	Macro_OLFML3	cDC_CLEC9A	TNFSF13B	HLA-DPB1	1,63835E-03	7575,7	7,78304E-03	14770	7,41452E-01	3128	1,99080E+00	2136	9,48701E-01	314	0	17530,5
Non-responder	Macro_FOLR2+APOE+	Macro_FOLR2+APOE+	HLA-DRB1	LAG3	1,63890E-03	8764,5	1,22955E-02	7060	7,48500E-01	3043	2,18609E+00	1418	8,78647E-01	14771	0	17530,5
Non-responder	Mono_INHBA	Macro_FOLR2+APOE+	CXCL8	SDC2	1,64032E-03	10401,9	2,28992E-02	2392	8,57696E-01	2026	1,93345E+00	2392	8,25689E-01	27669	0	17530,5
Non-responder	Mono_CD16	cDC_LAMP3	THBS1	TNFRSF11B	1,64183E-03	12855,7	6,77382E-02	349	1,12037E+00	692	1,66638E+00	4064	7,66833E-01	41643	0	17530,5
Non-responder	cDC_CLEC9A	pDC_LILRA4	MIF	CD74_CXCR4	1,64437E-03	7151,1	7,61044E-03	15280	8,17140E-01	2394	2,73391E+00	476	9,54297E-01	75	0	17530,5
Non-responder	Mono_CD16	cDC_LAMP3	FLT3LG	FLT3	1,64565E-03	17112,3	3,66794E-02	1118	6,90015E-01	3747	1,52395E+00	5316	6,84625E-01	57850	0	17530,5
Non-responder	Macro_FOLR2+APOE+	Macro_ISG15	HLA-G	LILRB1	1,64565E-03	11605,5	1,25957E-02	6748	5,91596E-01	5491	1,75452E+00	3385	8,37048E-01	24873	0	17530,5
Non-responder	Macro_FOLR2+APOE+	pDC_LILRA4	HLA-DPB1	CD4	1,64565E-03	8481,5	7,31693E-03	16297	5,40629E-01	6748	2,25387E+00	1228	9,44621E-01	604	0	17530,5
Non-responder	Mono_CD14	Macro_FOLR2+APOE+	THBS1	PTPRJ	1,64640E-03	10360,5	2,28766E-02	2395	8,17351E-01	2393	1,98690E+00	2151	8,27071E-01	27333	0	17530,5
Non-responder	cDC_LAMP3	cDC_LAMP3	TNFSF13B	CD40	1,64668E-03	7349,1	1,42067E-02	5501	9,13847E-01	1650	1,84021E+00	2881	9,00478E-01	9183	0	17530,5
Non-responder	Macro_IER3	Macro_ISG15	HLA-A	LILRB1	1,64836E-03	10797,3	7,77603E-03	14788	4,67300E-01	9170	1,15975E+00	10136	9,33155E-01	2362	0	17530,5
Non-responder	Mono_INHBA	pDC_LILRA4	IL10	IFNLR1	1,64842E-03	15186,1	5,47226E-02	550	8,30546E-01	2273	1,93290E+00	2396	7,12066E-01	53181	0	17530,5
Non-responder	Macro_NLRP3	Mono_CD14	TNF	TNFRSF1B	1,64947E-03	9999,9	1,10643E-02	8401	6,31248E-01	4702	1,60189E+00	4576	8,78561E-01	14790	0	17530,5
Non-responder	Macro_OLFML3	Mono_CD16	PTPN6	CLEC12A	1,65003E-03	9780,1	1,30888E-02	6319	5,95321E-01	5416	1,57085E+00	4844	8,78559E-01	14791	0	17530,5
Non-responder	Mono_INHBA	cDC(CD1C)	CCL20	CCR6	1,65046E-03	16199,1	8,38309E-02	207	8,76818E-01	1871	1,93280E+00	2397	6,77249E-01	58990	0	17530,5
Non-responder	Macro_NLRP3	cDC(CD1C)	S100A8	CD68	1,65338E-03	9111,3	1,07919E-02	8752	6,70144E-01	4062	9,25519E-01	14797	9,46993E-01	415	0	17530,5

Post_NR_Myeloid_Myeloid_CellCell

Non-responder	Macro_OLFML3	Mono_CD14	C1QA	CD93	1,65450E-03	12834,1	8,24389E-03	13431	3,47149E-01	14799	1,23938E+00	8853	8,98743E-01	9557	0	17530,5
Non-responder	Macro_NLRP3	Mono_CD16	GNAI2	FPR1	1,65505E-03	11570,3	7,77114E-03	14800	4,39214E-01	10261	1,35985E+00	7138	9,05219E-01	8122	0	17530,5
Non-responder	Mono_CD16	Macro_ISG15	B2M	LILRB1	1,65561E-03	9337,9	7,77029E-03	14801	4,80390E-01	8683	1,55632E+00	4973	9,43457E-01	702	0	17530,5
Non-responder	Macro_OLFML3	Mono_CD14	HLA-B	LILRA1	1,65729E-03	13285,5	7,76872E-03	14804	4,04300E-01	11781	1,06907E+00	11749	8,94762E-01	10563	0	17530,5
Non-responder	Mono_CD16	cDC_LAMP3	ADM	RAMP1	1,66004E-03	13739,9	3,91949E-02	1001	1,27960E+00	289	1,73892E+00	3486	7,45572E-01	46393	0	17530,5
Non-responder	Mono_CD16	pDC_LILRA4	THBS1	CD47	1,66065E-03	8217,1	1,36191E-02	5918	8,28647E-01	2293	2,66636E+00	534	8,78516E-01	14810	0	17530,5
Non-responder	Macro_OLFML3	Macro_FOLR2+APOE+	HLA-DPA1	CD4	1,66065E-03	8813,5	7,76607E-03	14810	5,49993E-01	6505	1,56334E+00	4906	9,48695E-01	316	0	17530,5
Non-responder	Mono_CD16	cDC_LAMP3	ADM	CALCRL	1,66100E-03	13305,1	2,15428E-02	2677	8,92773E-01	1775	1,63684E+00	4293	7,72998E-01	40250	0	17530,5
Non-responder	Macro_FOLR2+APOE+	Mono_INHBA	TNFSF13B	TFRC	1,66234E-03	10681,3	9,28572E-03	11049	5,89428E-01	5534	1,61330E+00	4480	8,78513E-01	14813	0	17530,5
Non-responder	pDC_LILRA4	Macro_IER3	TNFSF9	HLA-DPA1	1,66293E-03	6938,7	2,06523E-02	2908	6,63276E-01	4171	1,76643E+00	3318	9,11311E-01	6766	0	17530,5
Non-responder	Mono_CD16	cDC_LAMP3	LTB	CD40	1,66679E-03	10436,1	1,92104E-02	3278	8,19934E-01	2369	1,49776E+00	5555	8,42940E-01	23448	0	17530,5
Non-responder	Mono_CD16	cDC_LAMP3	NAMPT	ADORA2A	1,66775E-03	9390,3	3,35838E-02	1276	9,74569E-01	1323	1,60449E+00	4560	8,47903E-01	22262	0	17530,5
Non-responder	Mast	Mono_INHBA	CSF1	SIRPA	1,66796E-03	8767,9	6,14976E-02	427	1,22438E+00	383	1,72010E+00	3635	8,49540E-01	21864	0	17530,5
Non-responder	cDC_LAMP3	cDC(CD1C)	B2M	CD1A	1,67077E-03	6996,5	4,82442E-02	697	6,41048E-01	4529	1,51053E+00	5443	9,11223E-01	6783	0	17530,5
Non-responder	Mono_CD16	cDC_LAMP3	GNAI2	S1PR1	1,67258E-03	8356,3	4,61612E-02	746	1,21378E+00	402	1,69944E+00	3789	8,60256E-01	19314	0	17530,5
Non-responder	cDC_CLEC9A	Macro_FOLR2+APOE+	HLA-DQA1	CD4	1,67290E-03	6269,3	1,00948E-02	9715	8,14535E-01	2408	2,34983E+00	1026	9,43832E-01	667	0	17530,5
Non-responder	Macro_FOLR2+APOE+	Macro_FOLR2+APOE+	MDK	ITGA4_ITGB1	1,67452E-03	16265,7	2,25467E-02	2461	5,39454E-01	6778	1,39495E+00	6706	7,38998E-01	47853	0	17530,5
Non-responder	Macro_NLRP3	Macro_ISG15	IL1B	SIGIRR	1,67549E-03	8509,7	1,25544E-02	6779	9,24188E-01	1586	2,11100E+00	1656	8,77770E-01	14997	0	17530,5
Non-responder	Macro_FOLR2+APOE+	Macro_ISG15	PLAU	LRP1	1,67646E-03	11204,7	1,49690E-02	5033	5,39323E-01	6780	1,80166E+00	3097	8,42374E-01	23583	0	17530,5
Non-responder	Macro_NLRP3	Macro_FOLR2+APOE+	TNF	NOTCH1	1,67840E-03	15658,5	1,72500E-02	3951	5,74660E-01	5871	1,38828E+00	6782	7,55430E-01	44158	0	17530,5
Non-responder	Macro_OLFML3	Mono_INHBA	HBEGF	CD44	1,67867E-03	11289,7	7,75594E-03	14842	5,84305E-01	5642	1,21621E+00	9204	9,00259E-01	9230	0	17530,5
Non-responder	Macro_ISG15	cDC_LAMP3	FN1	TNFRSF11B	1,67905E-03	14511,3	9,08080E-02	170	1,05585E+00	948	1,93002E+00	2411	7,21032E-01	51497	0	17530,5
Non-responder	Macro_FOLR2+APOE+	Macro_FOLR2+APOE+	HLA-DPA1	LAG3	1,67980E-03	8977,1	1,23855E-02	6955	6,91816E-01	3721	2,06684E+00	1835	8,78391E-01	14844	0	17530,5
Non-responder	pDC_LILRA4	Macro_IFI27	GAS6	MERTK	1,68111E-03	9895,7	2,27686E-02	2412	9,00652E-01	1730	1,99768E+00	2110	8,33462E-01	25696	0	17530,5
Non-responder	Macro_NLRP3	Mono_CD14	VCAN	TLR1	1,68228E-03	12171,7	1,25499E-02	6786	8,44310E-01	2138	1,66772E+00	4052	8,14761E-01	30352	0	17530,5
Non-responder	Macro_NLRP3	cDC_CLEC9A	ADM	ADRB3	1,68325E-03	19514,9	9,03741E-02	172	7,71323E-01	2808	1,38771E+00	6787	5,62170E-01	70277	0	17530,5
Non-responder	Macro_NLRP3	Macro_OLFML3	CD52	SIGLEC10	1,68604E-03	13133,5	9,06325E-03	11479	4,69313E-01	9100	1,02188E+00	12703	8,78358E-01	14855	0	17530,5
Non-responder	Macro_OLFML3	Macro_ISG15	SERPING1	LRP1	1,68888E-03	11316,1	9,46320E-03	10736	5,12900E-01	7576	1,46663E+00	5878	8,78335E-01	14860	0	17530,5
Non-responder	Macro_IER3	Mono_CD16	B2M	LILRB2	1,68888E-03	9278,7	7,74933E-03	14860	5,84653E-01	5630	1,27685E+00	8308	9,54691E-01	65	0	17530,5
Non-responder	cDC_CLEC9A	Macro_FOLR2+APOE+	HLA-DQB1	CD4	1,68934E-03	6285,1	9,96000E-03	9908	8,13474E-01	2416	2,34211E+00	1042	9,45588E-01	529	0	17530,5
Non-responder	Macro_IER3	Macro_NLRP3	NAMPT	ITGA5_ITGB1	1,69172E-03	13411,3	7,78754E-03	14750	5,07341E-01	7747	1,04837E+00	12164	8,78321E-01	14865	0	17530,5
Non-responder	Macro_FOLR2+APOE-	cDC(CD1C)	B2M	CD1C	1,69347E-03	5955,7	3,24410E-02	1360	8,13431E-01	2418	1,28779E+00	8153	9,48692E-01	317	0	17530,5
Non-responder	Macro_FOLR2+APOE+	cDC_CLEC9A	MMP9	IFNAR1	1,69495E-03	14372,3	1,25393E-02	6799	5,99567E-01	5319	2,26131E+00	1208	7,69571E-01	41005	0	17530,5
Non-responder	Macro_FOLR2+APOE+	Macro_ISG15	CD47	SIRPA	1,69789E-03	9693,5	1,25356E-02	6802,5	6,52567E-01	4350,5	1,78736E+00	3187,5	8,71470E-01	16596,5	0	17530,5
Non-responder	Macro_NLRP3	Macro_FOLR2-APOE+	CXCL8	SDC3	1,69886E-03	11621,9	1,80427E-02	3643	8,28988E-01	2289	1,38666E+00	6803	8,25038E-01	27844	0	17530,5
Non-responder	Macro_FOLR2+APOE+	pDC_LILRA4	LGALS9	SLC1A5	1,70199E-03	9641,1	1,08621E-02	8667	5,80309E-01	5729	2,19471E+00	1396	8,78221E-01	14883	0	17530,5
Non-responder	Mono_CD16	Mono_CD14	GNAI2	FPR1	1,70199E-03	11580,5	7,74227E-03	14883	4,33136E-01	10492	1,38448E+00	6831	9,05060E-01	8166	0	17530,5
Non-responder	Macro_IER3	Mono_INHBA	VCAN	CD44	1,70256E-03	12344,7	8,14353E-03	13718	5,87537E-01	5568	9,22140E-01	14884	8,96848E-01	10023	0	17530,5
Non-responder	Macro_OLFML3	Macro_LYVE1	LGALS9	LRP1	1,70313E-03	13282,7	8,52577E-03	12699	4,55180E-01	9615	9,22122E-01	14885	8,90262E-01	11684	0	17530,5
Non-responder	Macro_ISG15	cDC_CLEC9A	TNFSF10	CCR6	1,70590E-03	13778,7	4,30410E-02	852	9,04691E-01	1705	1,92771E+00	2424	7,45608E-01	46382	0	17530,5
Non-responder	Mast	cDC(CD1C)	B2M	CD1C	1,70671E-03	5835,1	3,15269E-02	1412	7,46581E-01	3069	1,38606E+00	6811	9,47992E-01	353	0	17530,5
Non-responder	Macro_OLFML3	Macro_LYVE1	SERPINA1	LRP1	1,70714E-03	11594,9	8,70266E-03	12282	5,09273E-01	7689	9,21680E-01	14892	9,16813E-01	5581	0	17530,5
Non-responder	Macro_FOLR2+APOE+	Macro_FOLR2-APOE+	MMP9	IFNAR1	1,70867E-03	15204,5	1,25304E-02	6813	5,99405E-01	5322	1,52120E+00	5334	7,69508E-01	41023	0	17530,5
Non-responder	Macro_NLRP3	Macro_ISG15	THBS1	ITGA4	1,71458E-03	9265,3	1,25232E-02	6819	7,42836E-01	3113	1,87668E+00	2684	8,73101E-01	16180	0	17530,5
Non-responder	Mono_INHBA	Mono_INHBA	VEGFA	CD44	1,71557E-03	6763,5	1,93140E-02	3243	1,04092E+00	1021	1,53485E+00	5203	9,11017E-01	6820	0	17530,5
Non-responder	Macro_NLRP3	pDC_LILRA4	APP	RPSA	1,71633E-03	10272,9	7,73288E-03	14908	7,71741E-01	2799	2,13482E+00	1567	8,79374E-01	14560	0	17530,5
Non-responder	Macro_FOLR2+APOE+	Macro_FOLR2+APOE-	CALR	LRP1	1,71806E-03	12489,3	7,73222E-03	14911	5,00389E-01	7977	9,34665E-01	14606	9,08167E-01	7422	0	17530,5
Non-responder	cDC_LAMP3	Macro_LYVE1	CXCL9	FCGR2A	1,72036E-03	10949,9	1,21318E-02	7199	6,71395E-01	4041	9,51865E-01	14226	8,89969E-01	11753	0	17530,5
Non-responder	pDC_LILRA4	Macro_FOLR2+APOE-	APP	APLP2	1,72149E-03	5885,3	1,96549E-02	3162	1,17079E+00	530	2,20014E+00	1378	9,11003E-01	6826	0	17530,5
Non-responder	Macro_OLFML3	Mast	GRN	NTRK1	1,72209E-03	9181,1	6,83172E-02	343	1,03930E+00	1026	1,05337E+00	12088	8,78061E-01	14918	0	17530,5
Non-responder	Macro_FOLR2+APOE+	cDC(CD1C)	LGALS9	HAVCR2	1,72209E-03	12653,9	7,72846E-03	14918	4,76752E-01	8826	1,25581E+00	8616	8,83865E-01	13379	0	17530,5
Non-responder	cDC_CLEC9A	Macro_FOLR2+APOE+	SEMA4A	NRP1_PLXNA1	1,72255E-03	10719,7	2,99849E-02	1544	8,60307E-01	2000	1,92517E+00	2432	8,15818E-01	30092	0	17530,5
Non-responder	Macro_FOLR2+APOE+	Macro_NLRP3	APOE	SORL1	1,72267E-03	12192,3	8,05777E-03	13957	3,45011E-01	14919	1,44306E+00	6114	9,03861E-01	8441	0	17530,5
Non-responder	Macro_IER3	Mono_CD14	HSPA1A	TLR4	1,72383E-03	14375,9	7,97001E-03	14217	3,89326E-01	12507	9,20667E-01	14921	8,86383E-01	12704	0	17530,5
Non-responder	pDC_LILRA4	Macro_ISG15	LILRB4	LAIR1	1,72446E-03	6262,5	1,25138E-02	6829	8,11450E-01	2444	2,74878E+00	457	9,24639E-01	4052	0	17530,5
Non-responder	Macro_OLFML3	Macro_FOLR2+APOE+	HLA-DRB5	CD4	1,72903E-03	9011,1	7,72561E-03	14930	5,32339E-01	6979	1,55131E+00	5025	9,44767E-01	591	0	17530,5
Non-responder	Macro_LYVE1	cDC_CLEC9A	CXCL12	DPP4	1,73067E-03	14039,1	1,36998E-01	65	1,20203E+00	435	1,73113E+00	3545	7,35481E-01	48620	0	17530,5
Non-responder	Mono_CD16	Mono_CD16	HLA-F	LILRB1	1,73251E-03	11610,5	8,16672E-03	13650	5,25435E-01	7171	1,58021E+00	4765	8,77992E-01	14936	0	17530,5
Non-responder	Macro_NLRP3	Macro_NLRP3	AGTRAP	RACK1	1,73483E-03	13287,7	8,44469E-03	12913	5,27546E-01	7105	9,62808E-01	13950	8,77983E-01	14940	0	17530,5
Non-responder	cDC_CLEC9A	cDC_CLEC9A	CRTAM	CADM1	1,73859E-03	14629,1	7,12705E-02	312,5	1,20201E+00	436,5	1,83748E+00	2895,5	7,18370E-01	51970,5	0	17530,5
Non-responder	Macro_OLFML3	Macro_ISG15	TNFSF13	TNFRSF1A	1,73890E-03	13518,5	7,72137E-03	14947	3,82058E-01	12879	1,22502E+00	9050	8,84483E-01	13186	0	17530,5
Non-responder	Macro_FOLR2+APOE+	cDC_CLEC9A	QDPR	DYSF	1,74234E-03	19415,7	1,61374E-02	4407	5,49312E-01	6518	1,38348E+00	6847	6,56587E-01	61776	0	17530,5
Non-responder	Macro_FOLR2+APOE+	Macro_FOLR2+APOE+	IL10	IL10RA_IL10RB	1,74234E-03	13473,3	1,39122E-02	5708	5,36926E-01	6847	1,59283E+00	4663	8,05186E-01	32618	0	17530,5
Non-responder	Macro_FOLR2+APOE+	Macro_OLFML3	CCL4	CCR1	1,74633E-03	10178,5	1,24927E-02	6851	6,05697E-01	5194	1,95331E+00	2291	8,61520E-01	19026	0	17530,5
Non-responder	cDC_CLEC9A	cDC_CLEC9A	CADM1	CRTAM	1,74652E-03	14629,1	7,12705E-02	312,5	1,20201E+00	436,5	1,83748E+00	2895,5	7,18370E-01	51970,5	0	17530,5
Non-responder	Mono_CD14	Macro_IER3	S100A8	CD69	1,74733E-03	6263,5	1,82758E-02	3565	1,14207E+00	606	1,86166E+00	2764	9,10844E-01	6852	0	17530,5
Non-responder	Macro_FOLR2+APOE+	Mono_CD16	PLAU	ITGB1	1,74933E-03	11975,3	1,31505E-02	6272	5,36778E-01	6854	1,79374E+00	3152	8,32100E-01	26068	0	17530,5
Non-responder	Macro_FOLR2+APOE+	Mono_CD16	MRC1	PTPRC	1,74933E-03	9295,1	1,24913E-02	6854	6,88578E-01	3768	2,11158E+00	1653	8,71174E-01	16670	0	17530,5
Non-responder	Macro_NLRP3	Mono_INHBA	ICAM1	ITGAX_ITGB2	1,74998E-03	10876,9	7,71337E-03	14966	5,66924E-01	6054	1,29056E+00	8108	9,06922E-01	7726	0	17530,5
Non-responder	Macro_FOLR2+APOE+	Mono_CD14	AGTRAP	RACK1	1,75290E-03	12685,1	8,42464E-03	12964	5,18029E-01	7393	1,13345E+00	10567	8,77855E-01	14971	0	17530,5
Non-responder	pDC_LILRA4	Macro_NLRP3	APP	NOTCH2	1,75448E-03	7560,9	1,98638E-02	3103	1,20173E+00	438	2,93348E+00	324	8,72217E-01	16409	0	17530,5

Post_NR_Myeloid_Myeloid_CellCell

Non-responder	Macro_FOLR2+APOE+	Macro_IER3	APOE	TREM2	1,75642E-03	10710,1	1,04452E-02	9229	3,43902E-01	14977	1,19673E+00	9513	9,33437E-01	2301	0	17530,5
Non-responder	Macro_OLFML3	Mono_INHBA	TNFSF13B	TFRC	1,75876E-03	11243,1	9,16678E-03	11288	5,79464E-01	5749	1,39851E+00	6667	8,77823E-01	14981	0	17530,5
Non-responder	Macro_IER3	Mono_CD16	ICAM1	ITGAL_ITGB2	1,75876E-03	10359,7	7,70667E-03	14981	6,21985E-01	4884	1,54001E+00	5134	9,00033E-01	9269	0	17530,5
Non-responder	Mono_CD16	cDC(CD1C)	PSAP	CD1B	1,76036E-03	9592,5	2,80739E-02	1721	5,40916E-01	6737	1,39906E+00	6663	8,76526E-01	15311	0	17530,5
Non-responder	cDC_LAMP3	pDC_LILRA4	LGALS9	CD47	1,76229E-03	9553,3	8,64190E-03	12414	6,61510E-01	4199	2,04111E+00	1927	8,90215E-01	11696	0	17530,5
Non-responder	cDC_LAMP3	pDC_LILRA4	APP	RPSA	1,76759E-03	9497,1	8,79727E-03	12050	8,04258E-01	2508	1,89133E+00	2599	8,86048E-01	12798	0	17530,5
Non-responder	Macro_ISG15	Macro_FOLR2+APOE+	CCL2	CCR5	1,76891E-03	12177,3	4,24526E-02	869	8,10059E-01	2454	2,64693E+00	558	7,75947E-01	39475	0	17530,5
Non-responder	Macro_IFI27	pDC_LILRA4	HMGB1	THBD	1,76936E-03	14191,1	7,70314E-03	14999	3,50328E-01	14641	1,09633E+00	11231	8,86965E-01	12554	0	17530,5
Non-responder	Macro_FOLR2+APOE+	Macro_ISG15	HLA-DMB	CD4	1,76943E-03	9770,3	7,63876E-03	15188	5,43000E-01	6686	1,89487E+00	2573	9,10747E-01	6874	0	17530,5
Non-responder	Macro_NLRP3	cDC(CD1C)	IL1A	IL1R2_IL1RAP	1,77043E-03	18220,7	3,45215E-02	1234	5,45184E-01	6627	1,38137E+00	6875	6,78132E-01	58837	0	17530,5
Non-responder	cDC_LAMP3	pDC_LILRA4	ICAM1	IL2RG	1,77054E-03	8114,5	1,16190E-02	7725	8,01520E-01	2539	1,96475E+00	2234	8,94834E-01	10544	0	17530,5
Non-responder	Macro_LYVE1	Macro_FOLR2+APOE+	F13A1	ITGA9	1,77104E-03	11846,3	5,64018E-02	527	1,09185E+00	803	1,92080E+00	2455	7,82632E-01	37916	0	17530,5
Non-responder	pDC_LILRA4	Macro_FOLR2+APOE+	CD99	CD81	1,77144E-03	6717,1	1,24595E-02	6876	7,18428E-01	3400	2,40531E+00	921	9,20354E-01	4858	0	17530,5
Non-responder	Macro_FOLR2+APOE+	Mono_INHBA	CXCL10	TLR4	1,77346E-03	13286,3	1,24589E-02	6878	5,45192E-01	6626	1,81157E+00	3044	8,06308E-01	32353	0	17530,5
Non-responder	Macro_IER3	Macro_NLRP3	CD14	TLR4	1,77763E-03	12102,1	9,66542E-03	10398	4,85773E-01	8478	1,22308E+00	9091	8,77718E-01	15013	0	17530,5
Non-responder	pDC_LILRA4	Macro_IER3	APP	PTGER2	1,77845E-03	10513,9	2,20036E-02	2575	1,21724E+00	397	2,77267E+00	441	8,09457E-01	31626	0	17530,5
Non-responder	cDC_LAMP3	pDC_LILRA4	SELPLG	SELL	1,77941E-03	7158,7	1,88208E-02	3389	1,02153E+00	1109	2,47776E+00	797	8,85310E-01	12968	0	17530,5
Non-responder	cDC_LAMP3	pDC_LILRA4	TNFSF13B	TFRC	1,78237E-03	10567,7	9,39728E-03	10867	5,63940E-01	6131	1,71365E+00	3682	8,79149E-01	14628	0	17530,5
Non-responder	Macro_FOLR2+APOE+	Mono_INHBA	HLA-A	APLP2	1,78258E-03	10945,7	5,76493E-03	23830	5,95088E-01	5422	1,38070E+00	6887	9,40293E-01	1059	0	17530,5
Non-responder	pDC_LILRA4	cDC(CD1C)	SEMA7A	PLXNC1	1,78383E-03	12751,7	2,92970E-02	1620	8,80126E-01	1852	1,91948E+00	2461	7,72772E-01	40295	0	17530,5
Non-responder	Macro_FOLR2+APOE+	pDC_LILRA4	ADA	DPP4	1,78562E-03	19269,3	3,29084E-02	1323	6,02804E-01	5253	1,38048E+00	6890	6,25074E-01	65350	0	17530,5
Non-responder	Macro_OLFML3	Macro_FOLR2+APOE+	LYZ	ITGAL	1,78593E-03	12153,5	7,69478E-03	15027	4,60731E-01	9399	1,27043E+00	8397	8,95292E-01	10414	0	17530,5
Non-responder	Macro_NLRP3	cDC(CD1C)	VEGFA	SIRPA	1,78664E-03	11854,7	1,30386E-02	6365	7,53768E-01	2984	1,38042E+00	6891	8,34321E-01	25503	0	17530,5
Non-responder	cDC(CD1C)	Macro_ISG15	HLA-DPA1	LAG3	1,78811E-03	6104,7	2,25372E-02	2463	1,12673E+00	662	1,98930E+00	2141	9,06921E-01	7727	0	17530,5
Non-responder	cDC(CD1C)	Macro_ISG15	HLA-DRB5	LAG3	1,79025E-03	6470,9	2,25329E-02	2464	1,11497E+00	713	1,92431E+00	2435	9,00357E-01	9212	0	17530,5
Non-responder	cDC_LAMP3	Mono_CD16	HLA-A	LILRB2	1,79248E-03	9305,1	7,69156E-03	15038	5,94490E-01	5435	1,29603E+00	8032	9,46111E-01	490	0	17530,5
Non-responder	Macro_NLRP3	Macro_IER3	IL1B	SIGIRR	1,79274E-03	8982,9	1,24409E-02	6897	9,21821E-01	1604	1,70350E+00	3763	8,77283E-01	15120	0	17530,5
Non-responder	Mono_CD14	Mono_CD14	S100A8	CD68	1,79454E-03	5873,7	1,21554E-02	7183	8,64447E-01	1960	1,91862E+00	2466	9,49901E-01	229	0	17530,5
Non-responder	Mono_CD16	Macro_IER3	GNAI2	C5AR1	1,79486E-03	11742,1	7,68943E-03	15042	4,96209E-01	8121	1,15614E+00	10196	9,06559E-01	7821	0	17530,5
Non-responder	pDC_LILRA4	Mast	LGALS1	CD69	1,79784E-03	6996,9	1,24386E-02	6902	6,82049E-01	3880	1,87243E+00	2705	9,25076E-01	3967	0	17530,5
Non-responder	Macro_OLFML3	Mono_CD14	S100A9	CD36	1,79904E-03	12740,9	9,96727E-03	9896	4,13510E-01	11360	1,17576E+00	9869	8,77572E-01	15049	0	17530,5
Non-responder	Macro_NLRP3	Macro_FOLR2-APOE+	THBS1	ITGA6	1,80193E-03	14715,5	3,06875E-02	1475	8,15423E-01	2404	1,37867E+00	6906	7,50739E-01	45262	0	17530,5
Non-responder	Macro_FOLR2+APOE+	Mono_CD14	LGALS9	LRP1	1,80323E-03	12754,3	7,68447E-03	15056	4,32922E-01	10498	1,32284E+00	7659	8,85083E-01	13028	0	17530,5
Non-responder	Macro_FOLR2+APOE+	Mono_CD16	CXCL10	TLR4	1,80500E-03	13091,5	1,24297E-02	6909	5,44402E-01	6647	2,02892E+00	1975	8,06125E-01	32396	0	17530,5
Non-responder	Mono_CD16	Macro_NLRP3	CD55	CR1	1,80602E-03	15765,3	1,46258E-02	5224	5,69778E-01	5977	1,37834E+00	6910	7,59772E-01	43185	0	17530,5
Non-responder	Macro_FOLR2+APOE+	Macro_FOLR2+APOE+	HLA-DPB1	LAG3	1,80704E-03	9279,5	1,24286E-02	6911	6,88899E-01	3764	2,02258E+00	1997	8,73038E-01	16195	0	17530,5
Non-responder	Macro_OLFML3	Mono_CD16	HLA-C	LILRB1	1,80983E-03	9524,5	7,68125E-03	15067	5,25520E-01	7166	1,49348E+00	5605	9,33653E-01	2254	0	17530,5
Non-responder	Macro_FOLR2+APOE+	Macro_FOLR2+APOE+	IL15	IL2RA	1,81012E-03	17060,1	2,48393E-02	2109	6,64068E-01	4159	1,37802E+00	6914	7,04313E-01	54588	0	17530,5
Non-responder	Mono_CD16	Macro_LYVE1	SPN	SIGLEC1	1,81320E-03	10985,3	2,78151E-02	1745	7,66732E-01	2848	1,72601E+00	3582	8,19372E-01	29221	0	17530,5
Non-responder	Macro_OLFML3	Macro_NLRP3	TIMP2	CD44	1,81584E-03	12081,9	7,67866E-03	15077	5,52798E-01	6418	1,40931E+00	6539	8,78390E-01	14845	0	17530,5
Non-responder	Macro_NLRP3	Mono_CD16	ANXA1	FPR1	1,81765E-03	12160,9	7,67716E-03	15080	4,06488E-01	11691	1,15202E+00	10271	9,13621E-01	6232	0	17530,5
Non-responder	pDC_LILRA4	Macro_FOLR2+APOE+	IL16	CCR5	1,81824E-03	11776,1	3,30540E-02	1312	8,07426E-01	2477	2,32425E+00	1078	7,88660E-01	36483	0	17530,5
Non-responder	Macro_LYVE1	cDC_CLEC9A	CXCL2	XCR1	1,81876E-03	8821,3	6,02552E-02	446	2,42312E+00	10	1,90020E+00	2550	8,42411E-01	23570	0	17530,5
Non-responder	pDC_LILRA4	Macro_OLFML3	APP	LRP10	1,81876E-03	7917,9	2,08359E-02	2858	1,19735E+00	446	2,91420E+00	336	8,64069E-01	18419	0	17530,5
Non-responder	Macro_NLRP3	Mono_INHBA	THBS1	CD36	1,82143E-03	12148,5	1,24696E-02	6865	7,28228E-01	3267	1,37712E+00	6925	8,31715E-01	26155	0	17530,5
Non-responder	Mono_CD14	Macro_OLFML3	S100A8	CD69	1,82349E-03	6206,3	1,81438E-02	3609	1,13998E+00	610	1,94032E+00	2355	9,10549E-01	6927	0	17530,5
Non-responder	Macro_IER3	Mono_CD16	HBEGF	CD44	1,82369E-03	12939,9	7,83681E-03	14605	4,88903E-01	8360	9,13071E-01	15090	9,00724E-01	9114	0	17530,5
Non-responder	Macro_IFI27	pDC_LILRA4	C3	IFITM1	1,82429E-03	11173,1	1,13941E-02	7986	4,70534E-01	9047	1,43603E+00	6211	8,77395E-01	15091	0	17530,5
Non-responder	Mono_CD16	Macro_ISG15	SPN	SIGLEC1	1,82474E-03	10283,1	2,96931E-02	1573	8,06961E-01	2480	2,08350E+00	1762	8,24157E-01	28070	0	17530,5
Non-responder	Macro_OLFML3	Macro_FOLR2+APOE+	HLA-DRA	CD4	1,82731E-03	8522,1	7,67232E-03	15096	5,84701E-01	5629	1,65259E+00	4178	9,50958E-01	177	0	17530,5
Non-responder	Macro_NLRP3	Macro_NLRP3	ICAM1	ITGAL_ITGB2	1,82792E-03	10986,5	7,67201E-03	15097	5,37431E-01	6837	1,43955E+00	6152	8,99830E-01	9316	0	17530,5
Non-responder	cDC_LAMP3	Macro_NLRP3	CXCL9	FCGR2A	1,82852E-03	12631,9	9,49164E-03	10689	4,84287E-01	8537	1,09163E+00	11305	8,77365E-01	15098	0	17530,5
Non-responder	Macro_IER3	Macro_FOLR2+APOE+	HLA-DMA	CD4	1,82974E-03	10904,7	7,67054E-03	15100	4,72191E-01	8974	1,16395E+00	10078	9,30561E-01	2841	0	17530,5
Non-responder	Macro_OLFML3	cDC(CD1C)	TNFSF13B	HLA-DPB1	1,83277E-03	8348,9	7,66904E-03	15105	7,19418E-01	3384	1,51576E+00	5389	9,48340E-01	336	0	17530,5
Non-responder	Macro_NLRP3	Mono_CD14	S100A12	TLR4	1,83279E-03	14198,3	1,79213E-02	3690	5,33733E-01	6936	1,42842E+00	6303	7,88469E-01	36532	0	17530,5
Non-responder	Macro_OLFML3	cDC_CLEC9A	NECTIN2	CD226	1,83561E-03	13353,3	4,37933E-02	824	1,10092E+00	763	1,91457E+00	2485	7,51255E-01	45164	0	17530,5
Non-responder	Macro_FOLR2+APOE+	Macro_LYVE1	PLAU	ITGAM	1,83590E-03	13360,7	1,70271E-02	4028	5,55439E-01	6347	1,37586E+00	6939	8,07992E-01	31959	0	17530,5
Non-responder	Macro_FOLR2+APOE+	cDC_CLEC9A	ICAM1	SPN	1,83590E-03	11328,9	1,23934E-02	6939	5,91146E-01	5501	1,64914E+00	4201	8,46943E-01	22473	0	17530,5
Non-responder	Macro_NLRP3	cDC_LAMP3	VEGFA	CD44	1,83702E-03	12511,5	9,54147E-03	10608	6,51789E-01	4369	9,12293E-01	15112	8,77990E-01	14938	0	17530,5
Non-responder	pDC_LILRA4	Macro_FOLR2+APOE+	PROC	ITGAM	1,83779E-03	13278,1	5,30583E-02	590	8,06206E-01	2486	2,11452E+00	1642	7,55493E-01	44142	0	17530,5
Non-responder	Mono_CD14	Macro_ISG15	VCAN	ITGB1	1,83779E-03	5826,1	2,24188E-02	2486	1,17217E+00	519	2,33700E+00	1051	9,07691E-01	7544	0	17530,5
Non-responder	Macro_FOLR2+APOE+	cDC_LAMP3	TNFSF9	TNFRSF9	1,83997E-03	22087,5	3,15090E-02	1413	8,06177E-01	2487	6,53792E-01	22006	6,08042E-01	67001	0	17530,5
Non-responder	pDC_LILRA4	Macro_ISG15	SERPINF1	PLXDC2	1,84213E-03	7016,3	1,23893E-02	6945	6,86538E-01	3799	2,59922E+00	625	9,13945E-01	6182	0	17530,5
Non-responder	Macro_FOLR2+APOE+	pDC_LILRA4	B2M	LILRB1	1,84213E-03	11377,1	5,19960E-03	27717	5,33478E-01	6945	2,00306E+00	2078	9,31737E-01	2615	0	17530,5
Non-responder	Macro_IER3	Macro_ISG15	HLA-DQA1	LAG3	1,84371E-03	8793,1	1,90943E-02	3305	8,96495E-01	1750	1,43150E+00	6257	8,77276E-01	15123	0	17530,5
Non-responder	cDC_CLEC9A	Macro_ISG15	HLA-DQA2	LAG3	1,84433E-03	8774,1	2,23955E-02	2489	9,38378E-01	1508	2,30231E+00	1114	8,52167E-01	21229	0	17530,5
Non-responder	Macro_LYVE1	Macro_NLRP3	CXCL12	ITGA5	1,84652E-03	10499,3	5,19905E-02	612	1,06496E+00	912	1,91371E+00	2490	8,12250E-01	30952	0	17530,5
Non-responder	Macro_IER3	Macro_FOLR2+APOE+	CD99	CD81	1,84737E-03	13781,1	7,66050E-03	15129	3,73843E-01	13294	9,69435E-01	13804	9,00605E-01	9148	0	17530,5
Non-responder	Mono_CD16	Macro_OLFML3	ICAM4	ITGA4	1,84837E-03	14535,7	2,61673E-02	1938	6,69737E-01	4070	1,37531E+00	6951	7,64444E-01	42189	0	17530,5
Non-responder	Mono_CD14	Mono_CD16	HLA-C	LILRA1	1,84942E-03	8060,7	1,57892E-02	4603	5,33158E-01	6952	1,42588E+00	6338	9,20262E-01	4880	0	17530,5
Non-responder	Macro_NLRP3	Mono_CD14	THBS1	ITGA4	1,85150E-03	9662,3	1,23856E-02	6954	7,38913E-01	3156	1,62850E+00	4354	8,72488E-01	16317	0	17530,5

Post_NR_Myeloid_Myeloid_CellCell

Non-responder	Macro_FOLR2+APOE+	Macro_ISG15	C1QA	CD33	1,85165E-03	10690,9	7,65785E-03	15136	5,46906E-01	6567	1,87718E+00	2682	8,90850E-01	11539	0	17530,5
Non-responder	pDC_LILRA4	Macro_ISG15	APP	TNFRSF21	1,85528E-03	8367,3	2,23794E-02	2494	1,18605E+00	480	2,97395E+00	296	8,53000E-01	21036	0	17530,5
Non-responder	Macro_FOLR2+APOE+	Macro_IER3	C1QB	C1QBP	1,85568E-03	10803,9	6,68279E-03	18875	5,64464E-01	6114	1,60703E+00	4542	9,10395E-01	6958	0	17530,5
Non-responder	Macro_IER3	Macro_ISG15	HLA-C	LILRB1	1,85962E-03	11668,5	7,65254E-03	15149	4,04760E-01	11767	1,07555E+00	11619	9,33536E-01	2277	0	17530,5
Non-responder	Mono_CD16	Macro_NLRP3	PKM	CD44	1,85986E-03	10530,7	6,53852E-03	19562	5,32841E-01	6962	1,45325E+00	6008	9,31867E-01	2591	0	17530,5
Non-responder	Macro_NLRP3	cDC(CD1C)	NAMPT	ITGA5_ITGB1	1,86146E-03	12331,5	7,65205E-03	15152	6,45452E-01	4466	1,20302E+00	9415	8,77380E-01	15094	0	17530,5
Non-responder	Macro_NLRP3	Macro_IER3	TIMP1	CD63	1,86195E-03	8938,5	7,44861E-03	15847	6,86836E-01	3793	1,37380E+00	6964	9,45219E-01	558	0	17530,5
Non-responder	Macro_OLFML3	Mono_CD16	C3	IFITM1	1,86699E-03	11610,3	1,13367E-02	8043	4,45479E-01	10007	1,34889E+00	7310	8,77123E-01	15161	0	17530,5
Non-responder	Mast	Macro_NLRP3	HDC	HRH2	1,86772E-03	10625,5	5,99191E-02	452	1,26405E+00	312	1,85432E+00	2793	8,07636E-01	32040	0	17530,5
Non-responder	Macro_NLRP3	Macro_OLFML3	VEGFA	NRP1	1,86825E-03	13810,7	1,23735E-02	6970	7,71707E-01	2801	1,68487E+00	3890	7,82853E-01	37862	0	17530,5
Non-responder	Macro_FOLR2+APOE+	Mono_CD16	MDK	ITGA4_ITGB1	1,86930E-03	16294,3	2,22963E-02	2511	5,32564E-01	6971	1,42430E+00	6359	7,37919E-01	48100	0	17530,5
Non-responder	cDC_LAMP3	cDC_CLEC9A	HLA-DPB1	LAG3	1,87131E-03	10562,3	1,49115E-02	5062	6,79018E-01	3922	1,02588E+00	12630	8,82794E-01	13667	0	17530,5
Non-responder	Mono_CD16	Macro_ISG15	NAMPT	ITGA5_ITGB1	1,87254E-03	12675,9	7,64738E-03	15170	4,79617E-01	8718	1,38235E+00	6860	8,77347E-01	15101	0	17530,5
Non-responder	Macro_IFI27	Macro_FOLR2+APOE+	HLA-DPA1	LAG3	1,87439E-03	11812,9	1,20869E-02	7242	6,57218E-01	4271	9,23503E-01	14848	8,77082E-01	15173	0	17530,5
Non-responder	Macro_FOLR2+APOE+	Macro_OLFML3	LGALS9	HAVCR2	1,88306E-03	12334,1	7,63910E-03	15187	4,71357E-01	9018	1,42154E+00	6393	8,83267E-01	13542	0	17530,5
Non-responder	Macro_FOLR2-APOE+	Mast	LGALS1	CD69	1,88393E-03	7299,3	2,23178E-02	2507	1,16287E+00	546	9,10280E-01	15170	9,42982E-01	743	0	17530,5
Non-responder	cDC_LAMP3	cDC_CLEC9A	TNFSF13B	CD40	1,88678E-03	9872,3	9,88115E-03	10040	6,49380E-01	4407	1,70392E+00	3762	8,82985E-01	13622	0	17530,5
Non-responder	Macro_FOLR2+APOE+	Mono_CD14	ANXA1	FPR1	1,88927E-03	11964,7	7,63734E-03	15197	4,03430E-01	11832	1,22974E+00	8978	9,13415E-01	6286	0	17530,5
Non-responder	cDC_CLEC9A	cDC(CD1C)	B2M	CD1A	1,88934E-03	6623,5	4,71471E-02	723	5,84179E-01	5643	1,96501E+00	2231	9,10288E-01	6990	0	17530,5
Non-responder	Macro_FOLR2+APOE+	Macro_ISG15	CD14	ITGB2	1,88934E-03	9008,1	7,22457E-03	16654	5,31842E-01	6990	1,84273E+00	2864	9,40711E-01	1002	0	17530,5
Non-responder	Mono_CD16	Macro_ISG15	SERPINA1	LRP1	1,88989E-03	10056,3	7,63715E-03	15198	5,45228E-01	6624	1,64338E+00	4244	9,11695E-01	6685	0	17530,5
Non-responder	Macro_FOLR2+APOE+	Macro_IER3	RPS19	C5AR1	1,89051E-03	10049,9	7,63714E-03	15199	6,19129E-01	4942	1,24297E+00	8795	9,26013E-01	3783	0	17530,5
Non-responder	pDC_LILRA4	Mono_CD16	HLA-A	LILRB2	1,89058E-03	6678,1	8,69095E-03	12311	8,03806E-01	2510	2,51020E+00	757	9,49142E-01	282	0	17530,5
Non-responder	Macro_FOLR2+APOE+	Macro_ISG15	LRPAP1	LRP1	1,89175E-03	12398,1	8,52811E-03	12690	4,37051E-01	10325	1,43276E+00	6244	8,76966E-01	15201	0	17530,5
Non-responder	Mono_CD14	Macro_IER3	S100A9	ITGB2	1,89252E-03	5712,5	1,23554E-02	6993	9,99291E-01	1207	1,87898E+00	2669	9,51212E-01	163	0	17530,5
Non-responder	Mono_CD16	pDC_LILRA4	ADA	DPP4	1,89503E-03	15529,3	6,51915E-02	387	8,03730E-01	2512	1,99796E+00	2109	7,01184E-01	55108	0	17530,5
Non-responder	pDC_LILRA4	Mono_CD14	HSP90B1	ASGR1	1,90071E-03	5819,7	2,18647E-02	2600	1,19271E+00	456	3,15932E+00	180	9,04375E-01	8332	0	17530,5
Non-responder	Macro_FOLR2+APOE+	Macro_NLRP3	PSAP	LRP1	1,90101E-03	10141,5	6,73885E-03	18636	5,31532E-01	7001	1,66177E+00	4099	9,27807E-01	3441	0	17530,5
Non-responder	Macro_OLFML3	Macro_OLFML3	CXCL10	TLR4	1,90170E-03	8745,9	2,22826E-02	2515	1,05980E+00	936	2,75891E+00	450	8,47727E-01	22298	0	17530,5
Non-responder	Mono_CD16	Macro_FOLR2+APOE+	TNFSF13B	CD40	1,90173E-03	10795,5	8,80936E-03	12018	5,76321E-01	5816	1,75265E+00	3396	8,76922E-01	15217	0	17530,5
Non-responder	Macro_FOLR2+APOE+	pDC_LILRA4	IL10	IL10RA_IL10RB	1,90208E-03	12567,9	1,48327E-02	5108	5,31509E-01	7002	2,09484E+00	1722	8,10163E-01	31477	0	17530,5
Non-responder	Mono_INHBA	Mono_OLFML3	IL6	FCGR3B	1,90393E-03	15347,5	4,01886E-02	959	1,14236E+00	604	1,90844E+00	2516	7,01072E-01	55128	0	17530,5
Non-responder	Macro_FOLR2+APOE+	pDC_LILRA4	MAML2	NOTCH4	1,90421E-03	18049,3	2,48074E-02	2114	5,31470E-01	7004	1,45070E+00	6029	6,86390E-01	57569	0	17530,5
Non-responder	Mono_CD16	Macro_OLFML3	TNFSF13B	CD40	1,90423E-03	11146,3	8,80707E-03	12026	5,76150E-01	5821	1,54008E+00	5133	8,76908E-01	15221	0	17530,5
Non-responder	Macro_IER3	Macro_OLFML3	CCL3	CCR1	1,90611E-03	9292,7	1,41555E-02	5530	7,33389E-01	3215	1,55693E+00	4964	8,76885E-01	15224	0	17530,5
Non-responder	pDC_LILRA4	pDC_LILRA4	IRAK4	TLR7	1,90840E-03	10985,5	3,55298E-02	1174	8,02705E-01	2518	2,78221E+00	434	8,02497E-01	33271	0	17530,5
Non-responder	Macro_FOLR2+APOE+	Macro_FOLR2+APOE+	MDK	SDC2	1,90954E-03	19534,5	3,35136E-02	1283	5,78780E-01	5765	1,37055E+00	7009	6,17846E-01	66085	0	17530,5
Non-responder	Macro_OLFML3	Macro_FOLR2+APOE+	HLA-DRB1	CD4	1,90987E-03	8675,7	7,62816E-03	15230	5,74991E-01	5858	1,61874E+00	4435	9,48553E-01	325	0	17530,5
Non-responder	Mono_CD14	Mono_CD16	ICAM3	ITGAL	1,91511E-03	9996,7	2,24892E-02	2473	8,02646E-01	2521	1,98717E+00	2150	8,35196E-01	25309	0	17530,5
Non-responder	pDC_LILRA4	Macro_IER3	SEMA7A	PLXNC1	1,91735E-03	13151,9	2,70821E-02	1810	8,59900E-01	2004	1,90717E+00	2522	7,65795E-01	41893	0	17530,5
Non-responder	Macro_OLFML3	pDC_LILRA4	SIRPA	CD47	1,91740E-03	8319,5	1,38107E-02	5778,5	8,12730E-01	2422,5	2,59940E+00	623,5	8,76798E-01	15242,5	0	17530,5
Non-responder	Mono_INHBA	Macro_FOLR2+APOE+	CCL4	CCR5	1,91959E-03	9408,7	2,53856E-02	2035	8,02408E-01	2523	1,93189E+00	2403	8,46646E-01	22552	0	17530,5
Non-responder	pDC_LILRA4	Mono_CD16	SELPLG	ITGB2	1,91959E-03	6091,7	1,19175E-02	7432	8,11805E-01	2438	2,66596E+00	535	9,32211E-01	2523	0	17530,5
Non-responder	Macro_NLRP3	Mono_CD14	VCAN	ITGB1	1,92559E-03	9514,9	1,17354E-02	7605	7,99230E-01	2563	1,59688E+00	4621	8,76762E-01	15255	0	17530,5
Non-responder	Macro_ISG15	cDC_CLEC9A	TNFSF10	TNFRSF10B	1,92632E-03	9282,9	2,22477E-02	2526	9,41399E-01	1493	2,03368E+00	1957	8,45180E-01	22908	0	17530,5
Non-responder	Macro_FOLR2+APOE+	Mono_INHBA	ENTPD1	ADORA2A	1,92667E-03	14679,3	3,07807E-02	1469	7,00748E-01	3621	1,36850E+00	7025	7,57124E-01	43751	0	17530,5
Non-responder	pDC_LILRA4	Macro_IFI27	LTB	TNFRSF1A	1,93082E-03	7455,9	2,22441E-02	2528	1,06108E+00	926	2,61090E+00	611	8,75032E-01	15684	0	17530,5
Non-responder	Macro_FOLR2+APOE+	cDC_CLEC9A	HMGB1	THBD	1,93381E-03	13072,9	7,61502E-03	15268	3,79345E-01	13007	1,38255E+00	6858	8,86387E-01	12701	0	17530,5
Non-responder	Macro_FOLR2+APOE+	Mast	HBEGF	CD82	1,93420E-03	9260,1	1,98845E-02	3094	8,21096E-01	2359	1,36813E+00	7032	8,72628E-01	16285	0	17530,5
Non-responder	Macro_FOLR2+APOE+	Mono_CD14	HSP90B1	ASGR1	1,93420E-03	10467,5	1,23196E-02	7032	5,59858E-01	6230	1,43364E+00	6235	8,76530E-01	15310	0	17530,5
Non-responder	pDC_LILRA4	Macro_FOLR2+APOE-	APP	TSPAN15	1,93533E-03	10860,5	2,22297E-02	2530	1,12618E+00	663	2,39992E+00	928	8,05046E-01	32651	0	17530,5
Non-responder	Macro_FOLR2+APOE+	Macro_FOLR2+APOE+	HMGB1	CD163	1,93698E-03	10289,5	7,61192E-03	15273	4,42821E-01	10103	1,66449E+00	4079	9,22484E-01	4462	0	17530,5
Non-responder	Macro_NLRP3	Macro_NLRP3	IL1A	IL1R2_IL1RAP	1,94067E-03	18326,3	2,95589E-02	1589	5,30249E-01	7038	1,63740E+00	4289	6,60966E-01	61185	0	17530,5
Non-responder	cDC_LAMP3	Mono_CD14	RPS19	C5AR1	1,94142E-03	7935,1	9,76028E-03	10248	7,91843E-01	2629	1,36496E+00	7074	9,33989E-01	2194	0	17530,5
Non-responder	Macro_NLRP3	Macro_FOLR2+APOE+	GNAI2	C5AR1	1,94333E-03	11046,3	7,60991E-03	15283	4,91008E-01	8285	1,43500E+00	6222	9,06117E-01	7911	0	17530,5
Non-responder	pDC_LILRA4	Mono_CD16	HLA-C	LILRA1	1,94608E-03	7193,5	1,57599E-02	4617	5,30179E-01	7043	2,05154E+00	1881	9,20193E-01	4896	0	17530,5
Non-responder	Macro_FOLR2-APOE+	Macro_IFI27	APOE	TREM2	1,94888E-03	10467,5	2,22912E-02	2513	8,01742E-01	2536	4,11958E-01	29667	9,53459E-01	92	0	17530,5
Non-responder	Macro_IER3	Macro_FOLR2+APOE+	CD14	TLR4	1,95353E-03	13036,5	9,46379E-03	10732	4,76280E-01	8842	1,01839E+00	12779	8,76582E-01	15299	0	17530,5
Non-responder	cDC_CLEC9A	Macro_FOLR2+APOE+	MIF	TNFRSF14	1,95584E-03	6450,7	1,23017E-02	7052	9,30457E-01	1554	2,18754E+00	1414	9,21215E-01	4703	0	17530,5
Non-responder	Macro_LYVE1	Macro_ISG15	CCL18	CCR1	1,95796E-03	12436,7	3,12551E-02	1441	8,01500E-01	2540	2,52066E+00	739	7,74215E-01	39933	0	17530,5
Non-responder	Macro_OLFML3	Macro_ISG15	HLA-A	LILRB2	1,95928E-03	8005,1	7,60104E-03	15308	7,20587E-01	3367	1,76753E+00	3309	9,45808E-01	511	0	17530,5
Non-responder	cDC_LAMP3	Macro_ISG15	HLA-B	LILRB1	1,96312E-03	9734,5	7,90692E-03	14395	5,38921E-01	6796	1,26457E+00	8480	9,37480E-01	1471	0	17530,5
Non-responder	cDC_LAMP3	Macro_ISG15	HLA-DQA1	LAG3	1,96569E-03	8568,5	1,95905E-02	3178	9,20188E-01	1612	1,47980E+00	5754	8,78650E-01	14768	0	17530,5
Non-responder	Macro_NLRP3	cDC(CD1C)	TGFB1	CXCR4	1,96569E-03	12995,3	7,59693E-03	15318	4,35978E-01	10372	1,02234E+00	12693	9,00992E-01	9063	0	17530,5
Non-responder	Macro_IER3	Macro_FOLR2+APOE+	ICAM1	ITGAX_ITGB2	1,96697E-03	10615,3	7,59678E-03	15320	5,66945E-01	6052	1,42940E+00	6290	9,06277E-01	7884	0	17530,5
Non-responder	cDC_LAMP3	Macro_ISG15	HLA-DPB1	LAG3	1,96761E-03	8616,9	1,81365E-02	3614	8,37966E-01	2204	1,25505E+00	8629	8,92550E-01	11107	0	17530,5
Non-responder	Macro_FOLR2+APOE+	Macro_FOLR2+APOE+	HLA-DRB5	LAG3	1,96782E-03	9547,1	1,22941E-02	7063	6,70120E-01	4063	2,01948E+00	2007	8,69696E-01	17072	0	17530,5
Non-responder	Mono_CD14	cDC_CLEC9A	VCAN	ITGA4	1,96934E-03	5876,1	2,21516E-02	2545	1,18793E+00	475	2,32770E+00	1070	9,06814E-01	7760	0	17530,5
Non-responder	Macro_FOLR2+APOE+	Mast	C3	IFITM1	1,97340E-03	13012,1	1,11965E-02	8212	4,39392E-01	10251	9,72166E-01	13737	8,76451E-01	15330	0	17530,5
Non-responder	Macro_OLFML3	pDC_LILRA4	S100A9	CD36	1,97920E-03	11711,9	9,75988E-03	10250	4,02758E-01	11864	1,72715E+00	3576	8,76438E-01	15339	0	17530,5
Non-responder	Macro_FOLR2+APOE+	Macro_FOLR2+APOE+	ICAM1	ITGAM_ITGB2	1,97920E-03	11311,3	7,59271E-03	15339	4,89886E-01	8327	1,42515E+00	6346	9,01178E-01	9014	0	17530,5

Post_NR_Myeloid_Myeloid_CellCell

Non-responder	Macro_NLRP3	Macro_OLFML3	THBS1	CD36	1,98094E-03	11744,9	1,22805E-02	7075	7,25331E-01	3302	1,62301E+00	4392	8,30643E-01	26425	0	17530,5
Non-responder	cDC_LAMP3	Macro_ISG15	HLA-F	LILRB2	1,98178E-03	10265,1	8,20198E-03	13553	6,66165E-01	4122	1,36185E+00	7107	9,01180E-01	9013	0	17530,5
Non-responder	cDC_LAMP3	Macro_ISG15	HLA-F	LILRB1	1,98242E-03	10679,5	9,39776E-03	10864	6,18806E-01	4951	1,36335E+00	7085	8,85315E-01	12967	0	17530,5
Non-responder	pDC_LILRA4	Mono_CD16	HLA-B	LILRB1	1,98305E-03	7090,9	8,26067E-03	13389	8,00378E-01	2551	2,52244E+00	735	9,38751E-01	1249	0	17530,5
Non-responder	Macro_IER3	pDC_LILRA4	ICAM1	SPN	1,98307E-03	7742,5	2,03587E-02	2979	8,89363E-01	1797	2,33196E+00	1061	8,76424E-01	15345	0	17530,5
Non-responder	Macro_FOLR2+APOE+	cDC(CD1C)	HLA-DMB	CD4	1,98314E-03	10726,7	7,47045E-03	15775	5,32746E-01	6966	1,42964E+00	6285	9,09837E-01	7077	0	17530,5
Non-responder	cDC_LAMP3	Macro_ISG15	B2M	LILRB1	1,98436E-03	9007,1	8,11877E-03	13785	5,87734E-01	5565	1,33097E+00	7549	9,44616E-01	606	0	17530,5
Non-responder	Macro_ISG15	Macro_ISG15	CCL2	CCR5	1,98534E-03	12304,7	4,12777E-02	919	8,00291E-01	2552	2,81123E+00	405	7,73497E-01	40117	0	17530,5
Non-responder	Macro_FOLR2+APOE+	pDC_LILRA4	HLA-DRB5	CD4	1,98534E-03	8673,7	7,23774E-03	16601	5,21851E-01	7266	2,25076E+00	1232	9,43041E-01	739	0	17530,5
Non-responder	Mono_CD16	Macro_ISG15	S100A9	CD68	1,98566E-03	10044,3	7,59067E-03	15349	5,27539E-01	7106	1,19706E+00	9507	9,43140E-01	729	0	17530,5
Non-responder	Macro_FOLR2+APOE+	Macro_FOLR2-APOE+	PLAU	ITGB5	1,98643E-03	17429,3	2,29568E-02	2381	5,83600E-01	5650	1,36435E+00	7080	7,04786E-01	54505	0	17530,5
Non-responder	Mono_CD16	Macro_FOLR2+APOE+	ADM	GPR84	1,98973E-03	17912,1	1,35199E-02	5987	5,28288E-01	7083	1,43964E+00	6151	7,14092E-01	52809	0	17530,5
Non-responder	Macro_IER3	Macro_FOLR2+APOE+	HLA-DRA	LAG3	1,99149E-03	11670,9	1,07158E-02	8856	5,35170E-01	6894	1,18453E+00	9716	8,76365E-01	15358	0	17530,5
Non-responder	Macro_FOLR2+APOE+	Macro_FOLR2+APOE+	SNCA	LAG3	1,99193E-03	19066,7	1,73566E-02	3905	5,28277E-01	7085	1,61283E+00	4484	6,52223E-01	62329	0	17530,5
Non-responder	Mono_INHBA	cDC_LAMP3	CCL20	CCR6	1,99222E-03	16643,1	7,47562E-02	272	8,44998E-01	2132	1,89907E+00	2555	6,64603E-01	60726	0	17530,5
Non-responder	Macro_ISG15	Macro_ISG15	ADM	GPR84	1,99222E-03	11920,7	2,97696E-02	1565	8,00123E-01	2555	2,26873E+00	1182	7,87514E-01	36771	0	17530,5
Non-responder	Mono_CD16	pDC_LILRA4	TNFSF13	TNFRSF17	1,99303E-03	12619,9	5,74659E-02	510	6,58020E-01	4256	1,86359E+00	2749	7,82090E-01	38054	0	17530,5
Non-responder	cDC_LAMP3	Macro_ISG15	TNFSF13B	TFRC	1,99343E-03	10854,1	9,10790E-03	11394	5,49395E-01	6514	1,70325E+00	3765	8,77477E-01	15067	0	17530,5
Non-responder	Macro_NLRP3	Macro_IER3	S100A8	CD69	1,99408E-03	9868,5	1,44460E-02	5337	8,58868E-01	2017	9,01902E-01	15362	9,00823E-01	9096	0	17530,5
Non-responder	Mono_CD16	pDC_LILRA4	TNF	TNFRSF21	1,99413E-03	11587,1	1,35447E-02	5966	8,61938E-01	1985	2,45491E+00	835	8,09489E-01	31619	0	17530,5
Non-responder	Macro_FOLR2+APOE+	Macro_FOLR2+APOE+	ANXA1	FPR2_FPR3	1,99603E-03	13445,7	9,78628E-03	10204	3,79961E-01	12977	1,10090E+00	11152	8,76335E-01	15365	0	17530,5
Non-responder	Macro_FOLR2+APOE+	Mono_CD16	ADAM10	TSPAN14	1,99965E-03	12215,9	1,30582E-02	6349	5,28099E-01	7092	1,53696E+00	5172	8,36768E-01	24936	0	17530,5
Non-responder	Macro_FOLR2+APOE+	Macro_FOLR2+APOE+	VEGFB	NRP1	2,00075E-03	15498,1	1,80826E-02	3634	5,28081E-01	7093	1,77604E+00	3252	7,47427E-01	45981	0	17530,5
Non-responder	Macro_NLRP3	Mono_CD14	GNAI2	C5AR1	2,00123E-03	11494,5	7,58423E-03	15373	4,88606E-01	8372	1,28182E+00	8250	9,05974E-01	7947	0	17530,5
Non-responder	Mono_INHBA	Macro_LYVE1	CXCL8	SDC3	2,00143E-03	8508,5	3,02135E-02	1519	9,64685E-01	1371	1,89791E+00	2559	8,59196E-01	19563	0	17530,5
Non-responder	Macro_NLRP3	cDC_CLEC9A	VCAN	CD44	2,00188E-03	12303,5	7,58419E-03	15374	6,45108E-01	4470	9,92065E-01	13285	8,93510E-01	10858	0	17530,5
Non-responder	cDC_LAMP3	Macro_ISG15	CXCL9	FCGR2A	2,00449E-03	8085,7	1,34867E-02	6005	7,67424E-01	2840	1,72732E+00	3574	8,95047E-01	10479	0	17530,5
Non-responder	Macro_LYVE1	Macro_FOLR2+APOE+	F13A1	ITGB1	2,00835E-03	6236,7	2,20920E-02	2562	1,04352E+00	1002	2,26682E+00	1190	9,01663E-01	8899	0	17530,5
Non-responder	cDC_LAMP3	Macro_ISG15	RPS19	C5AR1	2,00905E-03	7242,1	1,01883E-02	9579	8,22951E-01	2339	1,56924E+00	4863	9,35300E-01	1899	0	17530,5
Non-responder	Mono_INHBA	Mast	IL1B	ADRB2	2,01066E-03	6849,5	3,71248E-02	1100	1,05354E+00	961	1,89713E+00	2563	8,88625E-01	12093	0	17530,5
Non-responder	Macro_IFI27	cDC_CLEC9A	HLA-DPB1	LAG3	2,01101E-03	10090,3	1,73272E-02	3921	8,65439E-01	1954	9,01023E-01	15388	8,90341E-01	11658	0	17530,5
Non-responder	Macro_FOLR2+APOE+	Macro_LYVE1	HSP90B1	LRP1	2,01232E-03	13049,3	8,09760E-03	13838	4,32595E-01	10511	9,00945E-01	15390	9,05824E-01	7977	0	17530,5
Non-responder	cDC_LAMP3	Macro_ISG15	CIRBP	TREM1	2,01624E-03	13285,3	9,58895E-03	10516	3,90763E-01	12427	1,02101E+00	12723	8,84337E-01	13230	0	17530,5
Non-responder	pDC_LILRA4	Macro_ISG15	APP	LRP1	2,01760E-03	6178,1	2,20672E-02	2566	1,17453E+00	512	3,05457E+00	232	8,96702E-01	10050	0	17530,5
Non-responder	Mono_CD16	pDC_LILRA4	ICAM3	ITGAL	2,01960E-03	11577,3	1,64973E-02	4248	6,85131E-01	3818	2,17188E+00	1457	8,12751E-01	30833	0	17530,5
Non-responder	Mono_CD16	Macro_FOLR2-APOE+	CD52	SIGLEC10	2,02018E-03	11999,1	9,81881E-03	10141	7,35185E-01	3196	9,00577E-01	15402	8,82572E-01	13726	0	17530,5
Non-responder	cDC_LAMP3	Macro_ISG15	TNFSF13B	CD40	2,02214E-03	11273,3	8,68873E-03	12316	5,76474E-01	5812	1,52536E+00	5303	8,76176E-01	15405	0	17530,5
Non-responder	cDC_LAMP3	Mast	HSPA8	ADRB2	2,02411E-03	10793,5	1,73287E-02	3919	6,52063E-01	4364	9,00341E-01	15408	8,86234E-01	12746	0	17530,5
Non-responder	Macro_FOLR2+APOE+	pDC_LILRA4	VEGFB	NRP1	2,02963E-03	15439,3	1,80500E-02	3640	5,27030E-01	7119	1,83957E+00	2886	7,47257E-01	46021	0	17530,5
Non-responder	Mono_CD16	Macro_ISG15	S100A4	CCR5	2,03069E-03	9204,1	1,59939E-02	4485	6,79020E-01	3921	1,59266E+00	4666	8,76145E-01	15418	0	17530,5
Non-responder	Mono_CD16	pDC_LILRA4	ICAM4	ITGAL	2,03075E-03	15325,3	3,73436E-02	1089	7,39303E-01	3151	1,98912E+00	2142	7,14530E-01	52714	0	17530,5
Non-responder	Macro_FOLR2+APOE+	Macro_FOLR2+APOE+	FAM3C	LAMP1	2,03075E-03	15410,9	1,22308E-02	7120	6,09902E-01	5100	1,85683E+00	2780	7,53901E-01	44524	0	17530,5
Non-responder	Mono_CD16	pDC_LILRA4	SEMA4A	NRP1_PLXNA4	2,03298E-03	16688,5	1,26355E-02	6706	6,05603E-01	5196	1,56837E+00	4866	7,32967E-01	49144	0	17530,5
Non-responder	Macro_IFI27	cDC_CLEC9A	HLA-DRA	LAG3	2,03333E-03	9791,1	1,65281E-02	4225	8,56806E-01	2032	8,99877E-01	15422	8,97993E-01	9746	0	17530,5
Non-responder	Macro_NLRP3	pDC_LILRA4	SEMA4A	NRP1_PLXNA4	2,03857E-03	17155,7	1,22199E-02	7127	5,98511E-01	5339	1,46035E+00	5944	7,29681E-01	49838	0	17530,5
Non-responder	Mono_CD16	Mono_INHBA	LGALS9	CD44	2,04390E-03	10364,1	7,56573E-03	15438	5,99279E-01	5324	1,29551E+00	8037	9,17305E-01	5491	0	17530,5
Non-responder	Macro_FOLR2-APOE+	pDC_LILRA4	GNAI2	CXCR3	2,04553E-03	6129,5	4,18398E-02	897	1,19430E+00	451	1,89448E+00	2578	9,00441E-01	9191	0	17530,5
Non-responder	Macro_IER3	cDC_CLEC9A	HLA-DQB1	LAG3	2,04588E-03	9341,1	1,72118E-02	3963	8,22987E-01	2338	1,33916E+00	7433	8,76026E-01	15441	0	17530,5
Non-responder	pDC_LILRA4	Macro_IER3	APP	APLP2	2,04755E-03	5843,9	1,89778E-02	3343	1,15227E+00	575	2,59353E+00	636	9,09571E-01	7135	0	17530,5
Non-responder	Macro_IFI27	Macro_LYVE1	CD59	STAB1	2,04853E-03	11942,7	1,10397E-02	8430	7,59300E-01	2931	9,01296E-01	15377	8,76012E-01	15445	0	17530,5
Non-responder	Mono_INHBA	Macro_NLRP3	IL1B	IL1R2_IL1RAP	2,05255E-03	8050,3	2,19775E-02	2581	8,52828E-01	2068	2,13281E+00	1576	8,71925E-01	16496	0	17530,5
Non-responder	Macro_ISG15	cDC_LAMP3	TNFSF13B	CD40	2,05317E-03	6117,7	1,75367E-02	3831	1,07794E+00	865	2,25666E+00	1222	9,09524E-01	7140	0	17530,5
Non-responder	Macro_FOLR2+APOE+	Macro_IER3	PLAU	ITGAM	2,05430E-03	13962,9	1,55555E-02	4714	5,26368E-01	7141	1,39151E+00	6752	8,00882E-01	33677	0	17530,5
Non-responder	Macro_IER3	Mono_CD16	IL1B	SIGIRR	2,05451E-03	10356,9	1,21442E-02	7192	6,40421E-01	4544	1,36542E+00	7064	8,75977E-01	15454	0	17530,5
Non-responder	cDC(CD1C)	Macro_FOLR2+APOE+	TNFSF13	TNFRSF14	2,05451E-03	13741,5	7,71213E-03	14968	4,42019E-01	10150	1,03902E+00	12356	8,82626E-01	13703	0	17530,5
Non-responder	Macro_FOLR2+APOE+	Macro_NLRP3	HLA-F	LILRB2	2,05518E-03	10934,7	7,55924E-03	15455	5,60735E-01	6211	1,49201E+00	5620	8,97486E-01	9857	0	17530,5
Non-responder	pDC_LILRA4	Macro_ISG15	IL16	CCR5	2,05724E-03	11865,5	3,21392E-02	1381	7,97658E-01	2583	2,48856E+00	780	7,86312E-01	37053	0	17530,5
Non-responder	Mono_CD16	pDC_LILRA4	ADM	CALCRL	2,05768E-03	12938,5	2,03776E-02	2973	8,41689E-01	2165	2,56875E+00	668	7,68082E-01	41356	0	17530,5
Non-responder	cDC(CD1C)	Macro_FOLR2+APOE+	HLA-DRA	CD4	2,05784E-03	8130,7	8,04045E-03	14016	6,58501E-01	4248	1,58590E+00	4730	9,52040E-01	129	0	17530,5
Non-responder	cDC(CD1C)	Macro_FOLR2+APOE+	HLA-DRA	LAG3	2,05850E-03	9218,5	1,20705E-02	7266	7,16084E-01	3433	1,65339E+00	4169	8,82671E-01	13694	0	17530,5
Non-responder	Mono_CD16	pDC_LILRA4	COPA	P2RY6	2,05881E-03	8598,7	1,76615E-02	3784	1,13623E+00	626	3,07724E+00	224	8,53886E-01	20829	0	17530,5
Non-responder	cDC(CD1C)	Macro_FOLR2+APOE+	HLA-DQA2	CD4	2,05917E-03	6835,5	1,16605E-02	7684	7,76352E-01	2757	1,75118E+00	3408	9,30783E-01	2798	0	17530,5
Non-responder	Macro_FOLR2+APOE+	cDC_CLEC9A	ICAM1	IL2RG	2,05917E-03	12098,3	8,00377E-03	14133	5,21653E-01	7274	1,44552E+00	6093	8,75962E-01	15461	0	17530,5
Non-responder	cDC(CD1C)	Macro_FOLR2+APOE+	HLA-DQA1	CD4	2,06050E-03	7154,3	9,50990E-03	10664	7,40759E-01	3134	1,72176E+00	3626	9,42229E-01	817	0	17530,5
Non-responder	cDC(CD1C)	Macro_FOLR2+APOE+	HLA-DRB1	CD4	2,06183E-03	8314,5	8,00585E-03	14124	6,52873E-01	4345	1,52188E+00	5328	9,49720E-01	245	0	17530,5
Non-responder	Mono_CD16	Macro_NLRP3	ARPC5	LDLR	2,06220E-03	12350,5	1,43705E-02	5398	5,26059E-01	7148	1,47331E+00	5808	8,32861E-01	25868	0	17530,5
Non-responder	cDC(CD1C)	Macro_FOLR2+APOE+	HLA-DRB1	LAG3	2,06250E-03	9626,5	1,20186E-02	7319	7,10456E-01	3509	1,58937E+00	4692	8,77427E-01	15082	0	17530,5
Non-responder	cDC(CD1C)	Macro_FOLR2+APOE+	HLA-DQB1	CD4	2,06317E-03	7039,7	9,47454E-03	10715	7,47865E-01	3050	1,77318E+00	3269	9,44288E-01	634	0	17530,5
Non-responder	Mono_CD16	pDC_LILRA4	SIRPB2	CD47	2,06333E-03	10403,7	1,71262E-02	3996	8,00237E-01	2553	2,62389E+00	595	8,27007E-01	27344	0	17530,5
Non-responder	Macro_FOLR2+APOE+	cDC(CD1C)	CCL18	CCR1	2,06333E-03	17312,7	1,51985E-02	4889	5,26023E-01	7149	1,90126E+00	2545	7,05114E-01	54450	0	17530,5
Non-responder	Macro_IER3	Mono_CD16	MRC1	PTPRC	2,06383E-03	9168,1	1,36199E-02	5915	7,29306E-01	3255	1,71494E+00	3672	8,75951E-01	15468	0	17530,5

Post_NR_Myeloid_Myeloid_CellCell

Non-responder	Mono_CD16	Macro_ISG15	HLA-A	LILRB1	2,06650E-03	10427,1	7,55376E-03	15472	4,18250E-01	11137	1,50646E+00	5481	9,32245E-01	2515	0	17530,5
Non-responder	cDC_CLEC9A	cDC_CLEC9A	HLA-DQA1	LAG3	2,06664E-03	6973,5	2,19234E-02	2587	1,09903E+00	768	2,47133E+00	805	8,84522E-01	13177	0	17530,5
Non-responder	Macro_NLRP3	pDC_LILRA4	VCAN	ITGA4	2,06784E-03	8968,9	1,16576E-02	7690	8,22868E-01	2341	2,07248E+00	1809	8,75921E-01	15474	0	17530,5
Non-responder	cDC(CD1C)	Macro_FOLR2+APOE+	HLA-DPB1	CD4	2,06784E-03	7315,9	8,84355E-03	11938	7,25935E-01	3296	1,73061E+00	3547	9,49374E-01	268	0	17530,5
Non-responder	cDC(CD1C)	Macro_FOLR2+APOE+	HLA-DPB1	LAG3	2,06851E-03	8957,1	1,32762E-02	6151	7,83518E-01	2697	1,79810E+00	3120	8,76650E-01	15287	0	17530,5
Non-responder	Macro_FOLR2+APOE+	Macro_ISG15	CXCL12	SDC4	2,06898E-03	18615,5	2,01725E-02	3013	5,25858E-01	7154	1,62913E+00	4350	6,62255E-01	61030	0	17530,5
Non-responder	Mono_CD16	Macro_LYVE1	LGALS9	LRP1	2,06918E-03	14914,5	7,57854E-03	15390	3,80567E-01	12957	8,97111E-01	15476	8,84375E-01	13219	0	17530,5
Non-responder	Mono_CD16	pDC_LILRA4	PSEN1	NOTCH4	2,07012E-03	14085,9	2,96628E-02	1579	6,09219E-01	5118	1,80730E+00	3071	7,60047E-01	43131	0	17530,5
Non-responder	Macro_NLRP3	Mono_INHBA	VCAN	TLR1	2,07125E-03	12739,7	1,21875E-02	7156	8,38990E-01	2192	1,46023E+00	5947	8,12540E-01	30873	0	17530,5
Non-responder	Macro_ISG15	Macro_OLFML3	CXCL10	SDC4	2,07135E-03	11617,7	2,19203E-02	2589	9,42228E-01	1488	2,68363E+00	523	7,90951E-01	35958	0	17530,5
Non-responder	Mono_CD16	Macro_NLRP3	HBEGF	CD44	2,07319E-03	10389,3	7,55030E-03	15482	6,30673E-01	4713	1,58415E+00	4741	8,99046E-01	9480	0	17530,5
Non-responder	Macro_FOLR2+APOE+	cDC_CLEC9A	CD86	CTLA4	2,07579E-03	17158,9	1,63684E-02	4312	5,25674E-01	7160	1,43062E+00	6267	7,26059E-01	50525	0	17530,5
Non-responder	Mono_INHBA	Macro_ISG15	CCL3	CCR5	2,07606E-03	9203,1	2,36877E-02	2265	7,96301E-01	2591	1,95962E+00	2256	8,51540E-01	21373	0	17530,5
Non-responder	Macro_FOLR2+APOE+	pDC_LILRA4	MIF	CD74_CXCR4	2,07606E-03	10995,5	5,25647E-03	27333	4,97210E-01	8088	2,16085E+00	1489	9,45514E-01	537	0	17530,5
Non-responder	Macro_NLRP3	Macro_IER3	VEGFA	ITGB1	2,07692E-03	10907,5	1,34044E-02	6066	7,73452E-01	2782	1,35858E+00	7161	8,53239E-01	20998	0	17530,5
Non-responder	pDC_LILRA4	Mono_CD14	SELPLG	ITGB2	2,07843E-03	6196,7	1,17853E-02	7551	8,00918E-01	2548	2,50518E+00	762	9,31858E-01	2592	0	17530,5
Non-responder	Mast	Macro_FOLR2-APOE+	CSF1	SIRPA	2,07862E-03	10045,5	5,89782E-02	477	1,20635E+00	420	1,20954E+00	9302	8,46847E-01	22498	0	17530,5
Non-responder	Macro_FOLR2+APOE+	Macro_NLRP3	SEMA4A	LILRB2	2,07919E-03	9814,1	1,21774E-02	7163	6,86315E-01	3804	1,65150E+00	4188	8,72287E-01	16385	0	17530,5
Non-responder	Macro_FOLR2+APOE+	cDC(CD1C)	PLAU	ITGA5	2,08147E-03	16322,1	1,28521E-02	6524	5,25584E-01	7165	1,35850E+00	7164	7,59586E-01	43227	0	17530,5
Non-responder	cDC(CD1C)	Macro_FOLR2+APOE+	HLA-DPA1	CD4	2,08594E-03	7703,1	8,53219E-03	12678	6,83289E-01	3849	1,63864E+00	4279	9,50937E-01	179	0	17530,5
Non-responder	cDC(CD1C)	Macro_FOLR2+APOE+	HLA-DPA1	LAG3	2,08662E-03	9063,9	1,28087E-02	6568	7,40872E-01	3133	1,70613E+00	3747	8,80175E-01	14341	0	17530,5
Non-responder	cDC(CD1C)	Macro_FOLR2+APOE+	HLA-DMA	CD4	2,08796E-03	8822,3	8,64377E-03	12407	5,92789E-01	5473	1,40539E+00	6586	9,34323E-01	2115	0	17530,5
Non-responder	cDC(CD1C)	Macro_FOLR2+APOE+	HLA-DMB	CD4	2,08864E-03	10224,1	8,40138E-03	13020	5,17705E-01	7405	1,36205E+00	7104	9,14540E-01	6061	0	17530,5
Non-responder	Mono_CD16	pDC_LILRA4	HMGB1	TLR9	2,09058E-03	7928,3	5,04247E-02	647	8,55662E-01	2046	1,81866E+00	3012	8,72225E-01	16406	0	17530,5
Non-responder	cDC(CD1C)	Macro_FOLR2+APOE+	B2M	TFRC	2,09201E-03	12142,3	7,58849E-03	15358	3,36431E-01	15412	1,12276E+00	10751	9,36502E-01	1660	0	17530,5
Non-responder	Macro_NLRP3	Macro_IER3	PTGS2	ALOX5	2,09403E-03	11412,5	1,36405E-02	5893	6,22325E-01	4876	9,93946E-01	13250	8,75732E-01	15513	0	17530,5
Non-responder	Mast	cDC(CD1C)	CSF1	CSF3R	2,09597E-03	8606,5	5,88754E-02	479	1,18808E+00	473	1,67619E+00	3965	8,54883E-01	20585	0	17530,5
Non-responder	Macro_FOLR2+APOE+	Mono_CD16	HLA-A	APLP2	2,09629E-03	11831,7	5,13029E-03	28260	5,25178E-01	7178	1,59639E+00	4628	9,36934E-01	1562	0	17530,5
Non-responder	Macro_FOLR2+APOE+	Macro_FOLR2+APOE+	ICAM1	IL2RG	2,09741E-03	11893,1	7,96664E-03	14224	5,19397E-01	7355	1,57148E+00	4838	8,75709E-01	15518	0	17530,5
Non-responder	Mono_CD16	pDC_LILRA4	GSTP1	TRAF2	2,09858E-03	10381,5	1,54879E-02	4738	5,57207E-01	6300	1,84927E+00	2819	8,55166E-01	20520	0	17530,5
Non-responder	Macro_FOLR2+APOE+	Macro_NLRP3	PLAU	ITGB1	2,09973E-03	12345,1	1,27762E-02	6596	5,25095E-01	7181	1,69168E+00	3840	8,30073E-01	26578	0	17530,5
Non-responder	Macro_OLFML3	Macro_ISG15	C3	ITGAX	2,10011E-03	13404,9	9,43666E-03	10800	3,34661E-01	15522	1,29123E+00	8099	8,77449E-01	15073	0	17530,5
Non-responder	Mono_CD14	cDC_LAMP3	NAMPT	ADORA2A	2,10213E-03	8110,7	4,20172E-02	892	1,15222E+00	576	1,89096E+00	2602	8,61793E-01	18953	0	17530,5
Non-responder	Macro_FOLR2+APOE+	Macro_NLRP3	LPL	CD44	2,10316E-03	12274,3	1,24773E-02	6859	5,44313E-01	6651	1,35745E+00	7184	8,44166E-01	23147	0	17530,5
Non-responder	Macro_IER3	cDC_CLEC9A	ITGB2	CD226	2,10350E-03	9344,9	2,73251E-02	1789	8,66915E-01	1946	1,17250E+00	9932	8,75686E-01	15527	0	17530,5
Non-responder	cDC(CD1C)	Macro_FOLR2+APOE+	HLA-DRB5	CD4	2,10417E-03	7893,5	8,53055E-03	12683	6,71530E-01	4039	1,57365E+00	4816	9,47297E-01	399	0	17530,5
Non-responder	Mono_CD16	pDC_LILRA4	IRAK4	TLR7	2,10431E-03	15680,7	1,68827E-02	4086	5,68804E-01	6000	1,91891E+00	2465	7,36903E-01	48322	0	17530,5
Non-responder	cDC(CD1C)	Macro_FOLR2+APOE+	LILRB4	LAIR1	2,10553E-03	10001,5	7,72422E-03	14936	6,04615E-01	5215	1,62359E+00	4387	9,06011E-01	7939	0	17530,5
Non-responder	Macro_NLRP3	Mono_CD16	TNFSF8	TNFRSF8	2,10660E-03	18863,1	5,34366E-02	577	6,66819E-01	4110	1,35720E+00	7187	6,29327E-01	64911	0	17530,5
Non-responder	Macro_NLRP3	Macro_LYVE1	S100A9	CD36	2,11028E-03	10731,1	9,61539E-03	10468	7,61317E-01	2906	1,35567E+00	7214	8,75628E-01	15537	0	17530,5
Non-responder	Macro_ISG15	cDC(CD1C)	TNFSF10	CCR6	2,11167E-03	13668,1	4,43451E-02	801	9,13335E-01	1655	1,89014E+00	2606	7,48428E-01	45748	0	17530,5
Non-responder	Macro_NLRP3	cDC_CLEC9A	THBS1	SDC4	2,11235E-03	14843,3	1,34826E-02	6012	7,75148E-01	2770	1,35703E+00	7192	7,70890E-01	40712	0	17530,5
Non-responder	Macro_FOLR2+APOE+	Macro_NLRP3	CD14	ITGB2	2,11465E-03	9359,3	7,16622E-03	16862	5,24771E-01	7194	1,65319E+00	4175	9,40485E-01	1035	0	17530,5
Non-responder	Macro_FOLR2+APOE+	Macro_IER3	HLA-DMB	CD4	2,11580E-03	10860,5	7,36721E-03	16118	5,26456E-01	7136	1,42695E+00	6323	9,09265E-01	7195	0	17530,5
Non-responder	Macro_NLRP3	pDC_LILRA4	INHBA	ACTR2	2,11695E-03	12398,7	1,44136E-02	5362	5,24673E-01	7196	2,29654E+00	1125	8,12937E-01	30780	0	17530,5
Non-responder	Macro_NLRP3	Mono_CD16	S100A9	CD36	2,11708E-03	10068,1	9,60846E-03	10481	7,61175E-01	2910	1,68747E+00	3872	8,75589E-01	15547	0	17530,5
Non-responder	Mono_CD16	Macro_LYVE1	CD55	CR1	2,11810E-03	15181,7	1,60594E-02	4451	5,95703E-01	5405	1,35660E+00	7197	7,68201E-01	41325	0	17530,5
Non-responder	Macro_FOLR2+APOE+	Mono_CD14	ADAM10	CD44	2,11912E-03	13593,1	8,58773E-03	12550	4,50488E-01	9820	1,03159E+00	12515	8,75585E-01	15550	0	17530,5
Non-responder	Macro_NLRP3	Macro_LYVE1	VEGFA	ITGB1	2,12618E-03	10744,9	1,38138E-02	5773	7,84796E-01	2685	1,35618E+00	7204	8,55113E-01	20532	0	17530,5
Non-responder	Macro_NLRP3	Macro_ISG15	HLA-F	LILRB1	2,12731E-03	11986,9	7,80470E-03	14704	5,31408E-01	7009	1,54055E+00	5129	8,75543E-01	15562	0	17530,5
Non-responder	Macro_OLFML3	cDC_CLEC9A	HLA-DQA1	LAG3	2,12799E-03	8270,9	1,84926E-02	3504	8,99783E-01	1732	1,81582E+00	3025	8,75542E-01	15563	0	17530,5
Non-responder	Macro_NLRP3	Macro_IER3	HBEGF	CD9	2,12799E-03	12503,5	9,44556E-03	10775	5,08633E-01	7711	8,93632E-01	15563	8,93171E-01	10938	0	17530,5
Non-responder	Mono_CD14	Macro_FOLR2+APOE+	CXCL8	SDC3	2,12842E-03	9208,3	2,59586E-02	1966	8,45100E-01	2131	1,88854E+00	2613	8,49762E-01	21801	0	17530,5
Non-responder	Macro_FOLR2+APOE+	Macro_FOLR2+APOE+	SEMA3C	NRP1_NRP2_PLXND1	2,12849E-03	18284,7	2,03192E-02	2983	5,50086E-01	6502	1,35611E+00	7206	6,88740E-01	57202	0	17530,5
Non-responder	Macro_NLRP3	Mono_CD16	PTGS2	ALOX5	2,12965E-03	10817,5	1,24670E-02	6869	5,81761E-01	5695	1,35607E+00	7207	8,70754E-01	16786	0	17530,5
Non-responder	pDC_LILRA4	cDC(CD1C)	APP	LRP10	2,13089E-03	8070,1	2,02006E-02	3004	1,18627E+00	479	2,72914E+00	483	8,62240E-01	18854	0	17530,5
Non-responder	Macro_FOLR2+APOE+	Mono_INHBA	PKM	CD44	2,13196E-03	9945,5	7,04530E-03	17349	5,91637E-01	5490	1,35596E+00	7209	9,34199E-01	2149	0	17530,5
Non-responder	pDC_LILRA4	Macro_FOLR2+APOE-	SPON2	ITGB1	2,13322E-03	11734,5	2,18177E-02	2615	9,18973E-01	1620	2,00560E+00	2064	7,95837E-01	34843	0	17530,5
Non-responder	Mono_CD16	Macro_ISG15	GNAI2	C5AR1	2,13347E-03	10950,3	7,52949E-03	15571	4,80478E-01	8681	1,55766E+00	4955	9,05665E-01	8014	0	17530,5
Non-responder	Macro_FOLR2+APOE+	Macro_FOLR2+APOE+	HLA-DQB2	LAG3	2,13776E-03	14799,3	1,46401E-02	5213	5,24037E-01	7214	1,69928E+00	3790	7,73000E-01	40249	0	17530,5
Non-responder	Macro_OLFML3	cDC_CLEC9A	AGTRAP	RACK1	2,13964E-03	12046,1	8,06181E-03	13944	5,37802E-01	6828	1,42491E+00	6348	8,75476E-01	15580	0	17530,5
Non-responder	Macro_OLFML3	pDC_LILRA4	HLA-DQA1	CD4	2,14102E-03	8858,9	7,52559E-03	15582	5,24604E-01	7198	1,99310E+00	2126	9,35520E-01	1858	0	17530,5
Non-responder	Macro_FOLR2+APOE+	Mono_INHBA	CCL18	CCR1	2,14472E-03	17362,5	1,50329E-02	4995	5,23894E-01	7220	1,92949E+00	2416	7,03974E-01	54651	0	17530,5
Non-responder	cDC(CD1C)	Macro_FOLR2+APOE+	C3	C3AR1	2,14514E-03	14139,3	9,61153E-03	10472	3,45693E-01	14890	9,92163E-01	13281	8,79531E-01	14523	0	17530,5
Non-responder	Macro_FOLR2+APOE+	cDC_CLEC9A	MDK	NCL	2,15055E-03	14154,1	2,01803E-02	3010	5,23783E-01	7225	1,44445E+00	6107	7,86981E-01	36898	0	17530,5
Non-responder	Mono_CD16	Mono_CD16	CD55	ADGRE5	2,15055E-03	9774,7	1,21062E-02	7225	7,05440E-01	3571	1,81436E+00	3032	8,67866E-01	17515	0	17530,5
Non-responder	pDC_LILRA4	Macro_ISG15	TNFSF9	HLA-DPA1	2,15171E-03	7019,7	1,95733E-02	3181	5,99160E-01	5328	2,06712E+00	1833	9,09118E-01	7226	0	17530,5
Non-responder	Mono_INHBA	Macro_ISG15	CCL4	CCR5	2,15250E-03	9398,5	2,46830E-02	2130	7,92640E-01	2623	2,09619E+00	1715	8,44815E-01	22994	0	17530,5
Non-responder	Macro_ISG15	Macro_FOLR2+APOE+	TNFSF10	TNFRSF10B	2,15250E-03	9375,7	2,17863E-02	2623	9,33188E-01	1542	2,03690E+00	1941	8,43804E-01	23242	0	17530,5
Non-responder	Macro_IER3	pDC_LILRA4	APP	RPSA	2,15410E-03	10805,3	7,52083E-03	15601	7,65262E-01	2869	1,80883E+00	3062	8,77891E-01	14964	0	17530,5
Non-responder	Macro_NLRP3	Mono_CD14	AGTRAP	RACK1	2,15963E-03	14051,9	8,04445E-03	14000	4,95918E-01	8125	9,17182E-01	14995	8,75358E-01	15609	0	17530,5

Post_NR_Myeloid_Myeloid_CellCell

Non-responder	cDC(CD1C)	Macro_FOLR2+APOE+	CD99	CD81	2,16240E-03	14202,3	7,51797E-03	15613	3,63609E-01	13862	9,31606E-01	14674	8,99761E-01	9332	0	17530,5
Non-responder	Mono_CD16	Mono_CD16	GNAI2	C5AR1	2,16378E-03	10956,5	7,51732E-03	15615	4,79280E-01	8730	1,56638E+00	4878	9,05595E-01	8029	0	17530,5
Non-responder	Mono_CD16	Macro_NLRP3	ICAM4	ITGAM	2,16456E-03	16418,5	3,05760E-02	1491	6,16545E-01	4992	1,35406E+00	7237	7,24470E-01	50842	0	17530,5
Non-responder	Macro_NLRP3	Macro_LYVE1	THBS1	ITGA4	2,16725E-03	9942,7	1,30384E-02	6366	7,57520E-01	2944	1,35355E+00	7253	8,75318E-01	15620	0	17530,5
Non-responder	Mono_INHBA	Mono_CD14	SPP1	CD44	2,16808E-03	7980,7	1,86870E-02	3435	6,23533E-01	4858	1,35403E+00	7240	9,10939E-01	6840	0	17530,5
Non-responder	Macro_NLRP3	Mono_CD16	HLA-C	LILRB2	2,16808E-03	9327,9	7,51446E-03	15623	5,23085E-01	7240	1,47728E+00	5769	9,46238E-01	477	0	17530,5
Non-responder	Macro_NLRP3	Macro_IER3	VEGFA	ITGAV	2,17747E-03	13541,1	1,59226E-02	4518	7,98427E-01	2570	1,35373E+00	7248	7,91540E-01	35839	0	17530,5
Non-responder	Mono_INHBA	Macro_FOLR2+APOE+	SPP1	CD44	2,17864E-03	7845,1	1,78352E-02	3721	5,91418E-01	5495	1,53233E+00	5230	9,09028E-01	7249	0	17530,5
Non-responder	Macro_ISG15	Macro_NLRP3	LGALS9	CD44	2,17919E-03	6277,7	1,13999E-02	7981	9,13985E-01	1649	2,12867E+00	1594	9,31583E-01	2634	0	17530,5
Non-responder	cDC(CD1C)	Macro_FOLR2+APOE+	C3	CD81	2,17977E-03	11727,9	1,02893E-02	9432	4,40762E-01	10195	1,18926E+00	9642	8,89611E-01	11840	0	17530,5
Non-responder	Macro_FOLR2+APOE+	Macro_OLFML3	TIMP2	CD44	2,18325E-03	14515,5	7,50924E-03	15643	3,58071E-01	14171	1,16280E+00	10097	8,77193E-01	15136	0	17530,5
Non-responder	cDC_CLEC9A	cDC_CLEC9A	BTLA	TNFRSF14	2,18407E-03	10063,1	4,17816E-02	901	1,12479E+00	669	1,88452E+00	2636	8,22065E-01	28579	0	17530,5
Non-responder	Mono_CD14	cDC_CLEC9A	S100A8	CD69	2,18571E-03	6176,5	1,74577E-02	3869	1,12912E+00	653	2,13306E+00	1575	9,08967E-01	7255	0	17530,5
Non-responder	Macro_NLRP3	cDC(CD1C)	THBS1	SCARB1	2,18807E-03	13859,3	1,28321E-02	6538	6,91227E-01	3728	1,35320E+00	7257	7,98380E-01	34243	0	17530,5
Non-responder	Macro_NLRP3	pDC_LILRA4	TGFB1	ACVR1_TGFBR1_TGFBR2	2,19043E-03	11644,7	1,28344E-02	6536	5,22133E-01	7259	1,95881E+00	2258	8,37971E-01	24640	0	17530,5
Non-responder	pDC_LILRA4	Macro_FOLR2+APOE+	B2M	LILRB2	2,19384E-03	7754,9	6,97496E-03	17637	7,90142E-01	2640	2,44586E+00	849	9,52359E-01	118	0	17530,5
Non-responder	cDC(CD1C)	Macro_FOLR2+APOE+	HMGB1	CD163	2,19444E-03	10162,3	8,22792E-03	13482	5,12155E-01	7594	1,28005E+00	8267	9,25221E-01	3938	0	17530,5
Non-responder	Macro_OLFML3	Macro_FOLR2+APOE+	HLA-DPA1	LAG3	2,19654E-03	10073,9	1,16586E-02	7687	6,07576E-01	5154	1,63083E+00	4336	8,75124E-01	15662	0	17530,5
Non-responder	Macro_OLFML3	Mono_INHBA	FN1	CD44	2,19935E-03	10952,3	1,29816E-02	6418	5,57599E-01	6289	1,23895E+00	8858	8,75103E-01	15666	0	17530,5
Non-responder	Macro_NLRP3	Macro_IER3	THBS1	SDC4	2,19990E-03	15207,1	1,20698E-02	7267	7,57274E-01	2947	1,51777E+00	5367	7,60969E-01	42924	0	17530,5
Non-responder	Macro_NLRP3	Macro_OLFML3	IL1B	SIGIRR	2,20146E-03	8987,5	1,19500E-02	7404	9,11578E-01	1660	1,87805E+00	2674	8,75099E-01	15669	0	17530,5
Non-responder	pDC_LILRA4	Mono_INHBA	APP	FPR2	2,20158E-03	11130,5	1,86807E-02	3437	1,19229E+00	458	2,72001E+00	491	8,00637E-01	33736	0	17530,5
Non-responder	cDC(CD1C)	Macro_ISG15	HLA-DRA	LAG3	2,20227E-03	6137,5	2,12383E-02	2752	1,10194E+00	760	1,93656E+00	2376	9,08918E-01	7269	0	17530,5
Non-responder	Macro_FOLR2+APOE+	Mono_CD16	CD48	CD244	2,20227E-03	15479,3	1,88114E-02	3393	5,21774E-01	7269	1,35390E+00	7245	7,65539E-01	41959	0	17530,5
Non-responder	pDC_LILRA4	cDC_CLEC9A	APP	TSPAN15	2,20364E-03	10862,1	2,17161E-02	2644	1,12169E+00	687	2,91404E+00	338	8,03206E-01	33111	0	17530,5
Non-responder	Macro_OLFML3	Macro_OLFML3	HLA-F	LILRB1	2,20497E-03	12705,9	7,73927E-03	14891	4,85073E-01	8508	1,37688E+00	6926	8,75083E-01	15674	0	17530,5
Non-responder	Macro_NLRP3	Macro_ISG15	TNF	TNFRSF1A	2,20583E-03	10377,3	1,20620E-02	7272	6,02104E-01	5268	1,77943E+00	3235	8,63378E-01	18581	0	17530,5
Non-responder	Macro_OLFML3	Mono_CD16	HLA-B	LILRB1	2,20638E-03	9182,7	7,50125E-03	15676	5,79256E-01	5751	1,53696E+00	5173	9,35919E-01	1783	0	17530,5
Non-responder	cDC(CD1C)	Mono_CD16	HLA-A	LILRA1	2,20701E-03	7641,9	1,65509E-02	4215	6,45879E-01	4454	1,35237E+00	7273	9,20951E-01	4737	0	17530,5
Non-responder	Macro_IER3	Mast	S100A8	CD69	2,20779E-03	8991,1	1,80420E-02	3644	1,01789E+00	1124	8,88218E-01	15678	9,10320E-01	6979	0	17530,5
Non-responder	Macro_IER3	Mono_CD16	HLA-F	LILRB2	2,20920E-03	10502,5	7,50007E-03	15680	7,25980E-01	3295	1,44872E+00	6050	8,97124E-01	9957	0	17530,5
Non-responder	Macro_FOLR2+APOE+	Macro_IER3	B2M	LILRB2	2,21177E-03	12857,1	4,70345E-03	31952	5,40767E-01	6745	1,35192E+00	7277	9,42580E-01	781	0	17530,5
Non-responder	Macro_FOLR2+APOE+	Macro_LYVE1	GRN	TNFRSF1A	2,21534E-03	10390,5	7,22108E-03	16664	5,66877E-01	6055	1,35176E+00	7280	9,22687E-01	4423	0	17530,5
Non-responder	Macro_FOLR2+APOE+	Mast	C1QA	CD33	2,22120E-03	11911,9	7,49492E-03	15697	5,41915E-01	6715	1,31144E+00	7824	8,89800E-01	11793	0	17530,5
Non-responder	Macro_FOLR2+APOE+	Macro_OLFML3	IL18	IL18BP	2,22608E-03	11277,3	1,44261E-02	5354	5,21260E-01	7289	1,64960E+00	4198	8,48874E-01	22015	0	17530,5
Non-responder	Macro_FOLR2+APOE+	Mono_CD14	GNAI2	FPR1	2,22757E-03	11812,5	7,49324E-03	15706	4,08600E-01	11580	1,47899E+00	5760	9,03646E-01	8486	0	17530,5
Non-responder	Macro_FOLR2+APOE+	cDC_CLEC9A	CXCL11	CXCR3	2,22967E-03	17973,1	2,86318E-02	1685	5,21229E-01	7292	1,74770E+00	3435	6,70676E-01	59923	0	17530,5
Non-responder	Mono_CD16	Macro_NLRP3	ICAM4	ITGB2	2,23086E-03	11161,3	2,30444E-02	2359	6,09286E-01	5117	1,35055E+00	7293	8,42667E-01	23507	0	17530,5
Non-responder	Macro_FOLR2+APOE+	Mono_CD16	TNF	TNFRSF1B	2,23112E-03	10029,3	1,03435E-02	9349	6,72825E-01	4023	1,73248E+00	3533	8,74921E-01	15711	0	17530,5
Non-responder	Mono_CD16	Macro_NLRP3	S100A9	TLR4	2,23254E-03	12000,3	1,03928E-02	9289	5,13356E-01	7554	1,17339E+00	9915	8,74903E-01	15713	0	17530,5
Non-responder	Mono_CD14	Macro_IER3	S100A9	CD68	2,23446E-03	6028,5	1,20395E-02	7296	9,38240E-01	1510	1,70790E+00	3732	9,54316E-01	74	0	17530,5
Non-responder	Mono_CD16	Macro_NLRP3	GNAI2	C5AR1	2,23538E-03	11277,1	7,49038E-03	15717	4,76631E-01	8828	1,43334E+00	6237	9,05442E-01	8073	0	17530,5
Non-responder	Mono_CD14	Macro_ISG15	S100A8	CD36	2,23570E-03	5874,5	2,16458E-02	2657	1,17175E+00	521	2,68474E+00	520	9,05141E-01	8144	0	17530,5
Non-responder	pDC_LILRA4	Macro_LYVE1	RPS19	C5AR1	2,23570E-03	7207,1	9,01526E-03	11594	8,55683E-01	2045	1,96987E+00	2209	9,31499E-01	2657	0	17530,5
Non-responder	Macro_FOLR2+APOE+	Macro_NLRP3	ADAM17	ITGA5	2,24166E-03	15269,3	1,42193E-02	5493	5,20726E-01	7302	1,48501E+00	5698	7,72669E-01	40323	0	17530,5
Non-responder	Macro_OLFML3	Macro_ISG15	ANXA1	FPR2_FPR3	2,24250E-03	13232,3	9,51990E-03	10645	4,06111E-01	11703	1,13391E+00	10556	8,74832E-01	15727	0	17530,5
Non-responder	cDC_CLEC9A	Macro_NLRP3	PKM	CD44	2,24562E-03	7217,1	8,56254E-03	12609	7,87679E-01	2661	1,97833E+00	2174	9,39945E-01	1111	0	17530,5
Non-responder	Macro_OLFML3	Macro_FOLR2+APOE+	HLA-DRB1	LAG3	2,24750E-03	9948,5	1,14516E-02	7923	6,32574E-01	4676	1,68623E+00	3879	8,74805E-01	15734	0	17530,5
Non-responder	Macro_FOLR2+APOE+	Macro_OLFML3	APOE	TREM2	2,24821E-03	10282,1	1,01033E-02	9696	3,31081E-01	15735	1,45894E+00	5957	9,32396E-01	2492	0	17530,5
Non-responder	Macro_NLRP3	Macro_LYVE1	NRG1	MS4A4A	2,25008E-03	11943,7	3,06074E-02	1484	8,44160E-01	2140	1,34895E+00	7309	8,11010E-01	31255	0	17530,5
Non-responder	cDC_LAMP3	Macro_ISG15	CD274	CD80	2,25060E-03	12682,9	5,43438E-02	561	1,17170E+00	522	1,88097E+00	2663	7,64749E-01	42138	0	17530,5
Non-responder	Macro_FOLR2+APOE+	Mono_INHBA	CXCL12	ITGA5	2,25249E-03	17061,1	1,91504E-02	3292	5,20444E-01	7311	1,43044E+00	6271	7,24187E-01	50901	0	17530,5
Non-responder	cDC_CLEC9A	cDC_CLEC9A	HLA-DQB1	LAG3	2,25308E-03	6819,7	2,16305E-02	2664	1,09797E+00	776	2,46360E+00	820	8,87911E-01	12308	0	17530,5
Non-responder	Macro_FOLR2+APOE+	Mono_INHBA	HLA-A	LILRB2	2,25490E-03	12362,1	5,00254E-03	29278	5,91092E-01	5503	1,34868E+00	7313	9,34032E-01	2186	0	17530,5
Non-responder	Macro_ISG15	Macro_FOLR2+APOE+	S100A8	CD68	2,25558E-03	6304,3	1,06059E-02	9008	7,87322E-01	2665	2,05615E+00	1865	9,46555E-01	453	0	17530,5
Non-responder	Mono_INHBA	Macro_FOLR2+APOE+	S100A8	CD68	2,25611E-03	6773,3	1,20257E-02	7314	8,69934E-01	1918	1,38281E+00	6854	9,49645E-01	250	0	17530,5
Non-responder	cDC_CLEC9A	Macro_ISG15	HLA-DRB1	LAG3	2,25807E-03	5943,3	2,16116E-02	2666	1,13259E+00	642	2,44230E+00	861	9,05653E-01	8017	0	17530,5
Non-responder	Macro_FOLR2+APOE+	Macro_ISG15	TNFSF13	TNFRSF14	2,25823E-03	12558,1	7,47945E-03	15749	4,17577E-01	11166	1,64594E+00	4228	8,81030E-01	14117	0	17530,5
Non-responder	Macro_OLFML3	pDC_LILRA4	HLA-DQB1	CD4	2,26541E-03	8848,3	7,47606E-03	15759	5,19935E-01	7332	1,96995E+00	2208	9,37718E-01	1412	0	17530,5
Non-responder	Macro_FOLR2+APOE+	Macro_IER3	PLTP	ABCA1	2,26757E-03	10789,5	1,07660E-02	8796	5,24817E-01	7192	1,59251E+00	4667	8,74711E-01	15762	0	17530,5
Non-responder	Macro_FOLR2+APOE+	Mono_INHBA	APOE	SORL1	2,26901E-03	13389,5	7,64972E-03	15162	3,30661E-01	15764	1,19312E+00	9574	9,01580E-01	8917	0	17530,5
Non-responder	Macro_IER3	Macro_ISG15	HLA-B	LILRB1	2,26901E-03	11488,1	7,47405E-03	15764	4,25763E-01	10799	1,07947E+00	11542	9,35810E-01	1805	0	17530,5
Non-responder	cDC(CD1C)	Macro_NLRP3	C3	ITGAX	2,26973E-03	14150,1	9,09025E-03	11426	3,55495E-01	14322	1,06284E+00	11879	8,75424E-01	15593	0	17530,5
Non-responder	Macro_NLRP3	Macro_IFI27	THBS1	SCARB1	2,27063E-03	11777,5	1,83890E-02	3530	7,66286E-01	2856	1,34735E+00	7326	8,25793E-01	27645	0	17530,5
Non-responder	Macro_OLFML3	Macro_IER3	SERPING1	LRP1	2,27117E-03	13316,5	8,84850E-03	11930	4,83808E-01	8558	1,01785E+00	12797	8,74701E-01	15767	0	17530,5
Non-responder	Macro_FOLR2+APOE+	Mast	AGTRAP	RACK1	2,27189E-03	10410,9	1,26083E-02	6738	8,33719E-01	2245	8,83841E-01	15768	8,97879E-01	9773	0	17530,5
Non-responder	cDC(CD1C)	Macro_NLRP3	HMGB1	THBD	2,27261E-03	10223,3	9,81077E-03	10154	5,48002E-01	6546	1,35206E+00	7275	8,98534E-01	9611	0	17530,5
Non-responder	Mono_CD14	Macro_NLRP3	S100A9	TLR4	2,27306E-03	5740,9	2,10148E-02	2809	1,17322E+00	515	2,68407E+00	522	9,08635E-01	7328	0	17530,5
Non-responder	Macro_ISG15	Mast	ADM	ADRB2	2,27306E-03	10847,5	4,66490E-02	735	9,12226E-01	1659	1,87840E+00	2672	8,09383E-01	31641	0	17530,5
Non-responder	Mono_CD16	Mast	HSPA8	ADRB2	2,27477E-03	12779,9	1,41928E-02	5509	4,56013E-01	9583	8,83715E-01	15772	8,75775E-01	15505	0	17530,5
Non-responder	Macro_ISG15	cDC_CLEC9A	LGALS3	LAG3	2,27557E-03	8502,3	2,15630E-02	2673	9,51650E-01	1436	2,09741E+00	1707	8,60893E-01	19165	0	17530,5

Non-responder	cDC(CD1C)	Macro_NLRP3	CIRBP	TREM1	2,27838E-03	13503,9	9,75873E-03	10253	4,19232E-01	11088	8,88588E-01	15664	8,85231E-01	12984	0	17530,5
Non-responder	Macro_FOLR2+APOE+	Mast	LGALS9	CD44	2,27910E-03	11775,7	7,80010E-03	14710	5,86190E-01	5599	8,83483E-01	15778	9,18455E-01	5261	0	17530,5
Non-responder	cDC_LAMP3	cDC(CD1C)	B2M	CD1B	2,28055E-03	10965,5	2,44164E-02	2164	4,85122E-01	8506	8,83307E-01	15780	8,93544E-01	10847	0	17530,5
Non-responder	Macro_FOLR2+APOE+	Macro_IER3	APOC2	LRP1	2,28157E-03	12344,3	1,89496E-02	3347	5,19831E-01	7335	1,77528E+00	3259	8,15212E-01	30250	0	17530,5
Non-responder	Macro_NLRP3	Macro_ISG15	CD55	ADGRE5	2,28278E-03	9984,7	1,20001E-02	7336	6,88397E-01	3771	1,71758E+00	3646	8,67361E-01	17640	0	17530,5
Non-responder	Macro_OLFML3	Macro_ISG15	HLA-C	LILRB2	2,28633E-03	8450,1	7,46700E-03	15788	6,49715E-01	4401	1,66933E+00	4036	9,46076E-01	495	0	17530,5
Non-responder	pDC_LILRA4	cDC_CLEC9A	SELPLG	SELL	2,28812E-03	6633,1	2,15417E-02	2678	9,48240E-01	1455	3,02596E+00	248	8,91989E-01	11254	0	17530,5
Non-responder	cDC(CD1C)	Macro_NLRP3	PKM	CD44	2,28995E-03	8968,7	7,77898E-03	14778	6,89024E-01	3759	1,35368E+00	7250	9,37179E-01	1526	0	17530,5
Non-responder	Mono_CD16	cDC_CLEC9A	ICAM2	ITGAL_ITGB2	2,29010E-03	9582,1	1,46293E-02	5221	6,16839E-01	4985	1,75468E+00	3383	8,70700E-01	16791	0	17530,5
Non-responder	Macro_FOLR2+APOE+	Macro_OLFML3	CCL3	CCR1	2,29010E-03	10081,9	1,19930E-02	7342	5,99107E-01	5329	1,88439E+00	2638	8,67654E-01	17570	0	17530,5
Non-responder	Mono_CD16	cDC_CLEC9A	ICAM3	ITGAL	2,29132E-03	11367,9	1,79504E-02	3682	7,16190E-01	3430	1,83595E+00	2907	8,19090E-01	29290	0	17530,5
Non-responder	Mono_CD16	cDC_CLEC9A	TNFSF10	TNFRSF10B	2,29376E-03	12864,9	1,46648E-02	5192	6,19883E-01	4925	1,40377E+00	6606	8,15913E-01	30071	0	17530,5
Non-responder	Macro_FOLR2+APOE+	Macro_OLFML3	CCL3L1	CCR1	2,29376E-03	13158,3	1,19890E-02	7345	5,24736E-01	7195	1,48590E+00	5688	8,24307E-01	28033	0	17530,5
Non-responder	Macro_FOLR2+APOE+	cDC_CLEC9A	ANXA1	DYSF	2,29498E-03	11417,5	1,63161E-02	4334	6,11349E-01	5071	1,34613E+00	7346	8,45620E-01	22806	0	17530,5
Non-responder	Macro_NLRP3	Macro_OLFML3	TNF	VSIR	2,29498E-03	11680,5	1,19883E-02	7346	6,56270E-01	4286	1,84513E+00	2853	8,30787E-01	26387	0	17530,5
Non-responder	Mono_CD16	cDC_CLEC9A	SELPLG	SELL	2,29988E-03	8924,3	1,50665E-02	4967	6,67508E-01	4097	2,03622E+00	1949	8,73521E-01	16078	0	17530,5
Non-responder	Macro_FOLR2+APOE+	cDC(CD1C)	CXCL12	CXCR4	2,29988E-03	12291,7	1,47824E-02	5138	5,19560E-01	7350	1,44205E+00	6120	8,35148E-01	25320	0	17530,5
Non-responder	Mono_INHBA	cDC(CD1C)	IL1B	IL1R2_IL1RAP	2,30072E-03	7687,3	2,56673E-02	1995	8,67763E-01	1940	1,87678E+00	2683	8,80343E-01	14288	0	17530,5
Non-responder	Mono_CD16	cDC_CLEC9A	ICAM4	ITGB2	2,30110E-03	10331,3	2,44043E-02	2166	6,60537E-01	4211	1,53920E+00	5145	8,46430E-01	22604	0	17530,5
Non-responder	cDC(CD1C)	Mono_CD16	HLA-B	LILRA1	2,30110E-03	7498,5	1,63495E-02	4317	6,55070E-01	4311	1,34551E+00	7351	9,25005E-01	3983	0	17530,5
Non-responder	Mono_CD16	cDC_CLEC9A	ICAM4	ITGB1	2,30233E-03	14016,9	2,73232E-02	1790	6,67616E-01	4094	1,49919E+00	5541	7,69102E-01	41129	0	17530,5
Non-responder	Macro_ISG15	Macro_OLFML3	CCL8	CCR5	2,30325E-03	16256,1	5,97942E-02	458	7,84812E-01	2684	2,26277E+00	1207	6,74454E-01	59401	0	17530,5
Non-responder	Macro_ISG15	Macro_ISG15	CXCL10	TLR4	2,30325E-03	8989,5	2,15213E-02	2684	9,40941E-01	1496	2,83883E+00	389	8,45470E-01	22848	0	17530,5
Non-responder	Mono_CD16	cDC_CLEC9A	ICAM4	ITGA4	2,30355E-03	13468,1	2,89593E-02	1655	7,19207E-01	3389	1,59626E+00	4630	7,73450E-01	40136	0	17530,5
Non-responder	Mono_CD16	cDC_CLEC9A	ICAM4	ITGAL	2,30478E-03	15312,9	4,06329E-02	943	7,70362E-01	2816	1,65319E+00	4174	7,23061E-01	51101	0	17530,5
Non-responder	Macro_FOLR2+APOE+	Macro_OLFML3	CCL3L1	CCR5	2,30478E-03	14965,3	1,71055E-02	4004	5,40663E-01	6747	1,34523E+00	7354	7,77223E-01	39191	0	17530,5
Non-responder	Mono_CD16	cDC_CLEC9A	TNFSF13B	HLA-DPB1	2,30601E-03	10354,1	5,80162E-03	23604	5,45973E-01	6598	1,80623E+00	3079	9,41061E-01	959	0	17530,5
Non-responder	cDC(CD1C)	Mono_CD16	HLA-B	LILRB2	2,30668E-03	8472,7	7,99701E-03	14153	6,88289E-01	3774	1,39677E+00	6686	9,50173E-01	220	0	17530,5
Non-responder	cDC_LAMP3	Macro_OLFML3	HLA-F	LILRB1	2,30887E-03	13763,3	7,65612E-03	15142	4,79474E-01	8723	1,07615E+00	11602	8,74492E-01	15819	0	17530,5
Non-responder	Macro_FOLR2+APOE+	Macro_OLFML3	PLAU	ITGB1	2,31215E-03	12642,9	1,25870E-02	6756	5,19188E-01	7360	1,58678E+00	4721	8,29018E-01	26847	0	17530,5
Non-responder	cDC(CD1C)	Mono_CD16	HLA-A	LILRB2	2,31325E-03	8460,5	8,09552E-03	13851	6,79098E-01	3919	1,40363E+00	6607	9,47401E-01	395	0	17530,5
Non-responder	Macro_FOLR2+APOE+	Macro_ISG15	HSP90B1	TLR2	2,31544E-03	12884,9	7,45298E-03	15828	3,78451E-01	13059	1,34187E+00	7395	8,94504E-01	10612	0	17530,5
Non-responder	Macro_NLRP3	Macro_IFI27	S100A8	ITGB2	2,31584E-03	9531,3	1,19781E-02	7363	7,99046E-01	2567	7,35254E-01	19656	9,45477E-01	540	0	17530,5
Non-responder	Macro_IFI27	pDC_LILRA4	HMGB1	TLR9	2,31837E-03	9469,7	5,24848E-02	601	8,84515E-01	1819	1,07822E+00	11566	8,74439E-01	15832	0	17530,5
Non-responder	Mast	Macro_IER3	CSF1	SIRPA	2,31888E-03	9036,3	5,76911E-02	504	1,19714E+00	447	1,69139E+00	3843	8,45411E-01	22857	0	17530,5
Non-responder	Mono_CD16	cDC_CLEC9A	LGALS3	LAG3	2,32323E-03	11833,7	1,49295E-02	5050	6,40214E-01	4550	1,35386E+00	7246	8,37386E-01	24792	0	17530,5
Non-responder	Mono_CD16	cDC_CLEC9A	CXCL2	XCR1	2,32446E-03	11960,1	2,88303E-02	1668	2,26620E+00	15	1,70948E+00	3718	7,87127E-01	36869	0	17530,5
Non-responder	cDC(CD1C)	Mono_CD16	HLA-C	LILRB2	2,32497E-03	7600,3	8,68318E-03	12331	7,65785E-01	2861	1,55021E+00	5042	9,49798E-01	237	0	17530,5
Non-responder	Macro_FOLR2+APOE+	Macro_OLFML3	HSP90B1	TLR2	2,32643E-03	13791,1	7,44957E-03	15843	3,78219E-01	13068	1,06195E+00	11898	8,94482E-01	10616	0	17530,5
Non-responder	Macro_FOLR2+APOE+	Macro_NLRP3	CALR	LRP1	2,33600E-03	10924,3	7,44648E-03	15856	4,81745E-01	8634	1,57745E+00	4786	9,06585E-01	7815	0	17530,5
Non-responder	cDC(CD1C)	Mono_CD16	B2M	LILRB2	2,33673E-03	8816,9	7,94000E-03	14300	6,40397E-01	4546	1,32302E+00	7654	9,55214E-01	54	0	17530,5
Non-responder	Macro_IFI27	cDC_CLEC9A	HLA-DRB1	LAG3	2,34042E-03	10116,1	1,64970E-02	4249	8,52895E-01	2067	8,79835E-01	15862	8,93467E-01	10872	0	17530,5
Non-responder	Mono_CD16	Mono_CD16	AGTRAP	RACK1	2,34042E-03	12722,3	7,89091E-03	14448	4,82814E-01	8598	1,35811E+00	7173	8,74303E-01	15862	0	17530,5
Non-responder	cDC_CLEC9A	Mono_CD16	HLA-C	LILRB2	2,34134E-03	6852,1	8,76425E-03	12136	7,82620E-01	2699	2,10697E+00	1670	9,50019E-01	225	0	17530,5
Non-responder	Macro_FOLR2+APOE+	Macro_IER3	GNAI2	C5AR1	2,34337E-03	11844,9	7,44210E-03	15866	4,71672E-01	8998	1,25065E+00	8693	9,05165E-01	8137	0	17530,5
Non-responder	Mono_CD16	cDC_CLEC9A	LTB	CD40	2,34427E-03	13358,1	1,33614E-02	6092	5,55467E-01	6346	1,36147E+00	7115	8,17385E-01	29707	0	17530,5
Non-responder	cDC(CD1C)	cDC_CLEC9A	HLA-DQA2	LAG3	2,34645E-03	8527,9	2,53237E-02	2040	1,06084E+00	928	1,87268E+00	2701	8,59741E-01	19440	0	17530,5
Non-responder	Mono_CD16	Macro_ISG15	SELPLG	ITGB2	2,35151E-03	10989,5	7,43901E-03	15877	4,25586E-01	10813	1,56177E+00	4925	9,15717E-01	5802	0	17530,5
Non-responder	Macro_FOLR2+APOE+	Macro_LYVE1	SPP1	ITGAV_ITGB5	2,35157E-03	14013,9	2,14327E-02	2703	4,28086E-01	10712	1,94764E+00	2322	7,87388E-01	36802	0	17530,5
Non-responder	Mono_INHBA	pDC_LILRA4	TNF	TRAF2	2,35413E-03	11862,1	3,17573E-02	1397	7,82322E-01	2704	2,27732E+00	1169	7,88552E-01	36510	0	17530,5
Non-responder	Mono_CD14	Macro_LYVE1	S100A9	ITGB2	2,35422E-03	6053,7	1,19558E-02	7394	9,71703E-01	1334	1,69632E+00	3808	9,50444E-01	202	0	17530,5
Non-responder	Macro_IER3	Mono_CD14	CD14	TLR4	2,35521E-03	13617,1	9,06651E-03	11470	4,57575E-01	9515	9,74357E-01	13688	8,74244E-01	15882	0	17530,5
Non-responder	cDC_CLEC9A	pDC_LILRA4	GSTP1	TRAF2	2,35670E-03	7638,9	2,14252E-02	2705	9,52435E-01	1435	2,61078E+00	612	8,74128E-01	15912	0	17530,5
Non-responder	Mono_CD16	Mono_INHBA	ADAM10	CD44	2,35744E-03	12506,5	8,37809E-03	13075	5,53225E-01	6410	1,18952E+00	9632	8,74233E-01	15885	0	17530,5
Non-responder	Macro_FOLR2+APOE+	Mast	GPI	NTRK1	2,35796E-03	11144,5	9,33786E-02	164	1,13133E+00	649	1,34183E+00	7397	8,16295E-01	29982	0	17530,5
Non-responder	cDC_CLEC9A	Macro_ISG15	HLA-DRA	LAG3	2,35796E-03	5931,5	2,09194E-02	2828	1,07774E+00	866	2,34472E+00	1036	9,08289E-01	7397	0	17530,5
Non-responder	Macro_NLRP3	Mono_INHBA	VEGFA	NRP2	2,35796E-03	14728,3	1,19545E-02	7397	7,72664E-01	2790	1,35264E+00	7265	7,79445E-01	38659	0	17530,5
Non-responder	Mono_CD16	pDC_LILRA4	ICAM4	ITGA4	2,36170E-03	16798,5	1,75992E-02	3805	5,17923E-01	7400	1,56811E+00	4869	7,26884E-01	50388	0	17530,5
Non-responder	Mono_CD16	Macro_FOLR2+APOE+	PSAP	LRP1	2,36189E-03	10811,7	7,43431E-03	15891	4,20980E-01	11008	1,38124E+00	6881	9,31028E-01	2748	0	17530,5
Non-responder	cDC_CLEC9A	pDC_LILRA4	COPA	P2RY6	2,36480E-03	7993,9	2,03979E-02	2970	1,17563E+00	509	3,08564E+00	216	8,62645E-01	18744	0	17530,5
Non-responder	Macro_NLRP3	Macro_NLRP3	TNF	NOTCH1	2,36920E-03	16735,5	1,42305E-02	5483	5,17665E-01	7406	1,55312E+00	5006	7,37221E-01	48252	0	17530,5
Non-responder	Mono_CD16	cDC_CLEC9A	ADA	DPP4	2,37421E-03	15413,3	7,07140E-02	315	8,50885E-01	2081	1,73254E+00	3532	7,09632E-01	53608	0	17530,5
Non-responder	Mono_CD14	Macro_IFI27	S100A9	ITGB2	2,37421E-03	6649,9	1,19471E-02	7410	9,71102E-01	1342	1,39058E+00	6761	9,50427E-01	206	0	17530,5
Non-responder	Macro_OLFML3	cDC(CD1C)	CXCL11	CXCR3	2,37469E-03	16297,1	3,30609E-02	1311	8,20672E-01	2360	1,87132E+00	2712	6,86361E-01	57572	0	17530,5
Non-responder	Macro_ISG15	Macro_IER3	MMP12	PLAUR	2,37469E-03	7743,9	2,51078E-02	2069	7,81733E-01	2712	2,29110E+00	1141	8,76707E-01	15267	0	17530,5
Non-responder	Macro_FOLR2+APOE+	Mono_INHBA	FN1	SDC2	2,37547E-03	15965,7	2,32810E-02	2317	5,17575E-01	7411	1,37224E+00	6986	7,49304E-01	45584	0	17530,5
Non-responder	Macro_FOLR2+APOE+	cDC(CD1C)	PLAU	ITGB1	2,37672E-03	13110,7	1,25338E-02	6805	5,17528E-01	7412	1,38062E+00	6889	8,28718E-01	26917	0	17530,5
Non-responder	Mono_CD16	Macro_NLRP3	HMGB1	THBD	2,37679E-03	13745,7	7,48173E-03	15743	3,28150E-01	15911	1,40307E+00	6614	8,85495E-01	12930	0	17530,5
Non-responder	cDC_LAMP3	pDC_LILRA4	HLA-DQA2	CD4	2,37754E-03	11571,5	7,42783E-03	15912	3,97699E-01	12100	1,42840E+00	6305	9,14768E-01	6010	0	17530,5
Non-responder	Macro_FOLR2+APOE+	Macro_FOLR2+APOE+	ADAM10	TSPAN15	2,38047E-03	20154,7	1,17846E-02	7552	3,25772E-01	16060	1,18608E+00	9688	7,29140E-01	49943	0	17530,5
Non-responder	Macro_OLFML3	Macro_FOLR2+APOE+	HLA-F	LILRB2	2,38277E-03	11062,3	7,42431E-03	15919	5,78510E-01	5772	1,45117E+00	6024	8,96655E-01	10066	0	17530,5

Post_NR_Myeloid_Myeloid_CellCell

Non-responder	Macro_NLRP3	cDC(CD1C)	NRG1	HLA-DPB1	2,38427E-03	10196,7	1,62299E-02	4372	6,48956E-01	4409	1,24610E+00	8751	8,74099E-01	15921	0	17530,5
Non-responder	Macro_FOLR2+APOE+	Mono_INHBA	B2M	LILRB1	2,38552E-03	12886,5	5,00656E-03	29246	5,17295E-01	7419	1,34239E+00	7387	9,30524E-01	2850	0	17530,5
Non-responder	Macro_FOLR2+APOE+	Macro_ISG15	CD14	TLR4	2,38652E-03	10740,5	9,04053E-03	11534	5,71863E-01	5935	1,85723E+00	2779	8,74086E-01	15924	0	17530,5
Non-responder	Macro_NLRP3	cDC_LAMP3	TNFSF13B	CD40	2,39177E-03	11983,9	8,35936E-03	13131	6,25713E-01	4813	1,26228E+00	8514	8,74064E-01	15931	0	17530,5
Non-responder	Mono_CD16	Macro_OLFML3	ICAM2	ITGAM_ITGB2	2,39433E-03	10837,9	1,38871E-02	5727	5,17164E-01	7426	1,40971E+00	6536	8,70046E-01	16970	0	17530,5
Non-responder	Mono_CD14	cDC_CLEC9A	ADAM10	CADM1	2,39537E-03	10335,1	3,06458E-02	1479	1,08909E+00	816	1,86904E+00	2720	8,19809E-01	29130	0	17530,5
Non-responder	Macro_IFI27	cDC_CLEC9A	HLA-DQB1	LAG3	2,39703E-03	10982,3	1,73855E-02	3898	8,33792E-01	2243	8,77089E-01	15938	8,76570E-01	15302	0	17530,5
Non-responder	pDC_LILRA4	Macro_IER3	PROC	ITGAM	2,39796E-03	13890,3	4,90069E-02	677	7,80804E-01	2721	1,87386E+00	2696	7,48083E-01	45827	0	17530,5
Non-responder	Mono_CD16	Mono_CD14	ICAM2	ITGAM_ITGB2	2,39928E-03	9800,3	1,49081E-02	5064	5,78077E-01	5781	1,59062E+00	4685	8,74004E-01	15941	0	17530,5
Non-responder	Macro_FOLR2+APOE+	Macro_LYVE1	CXCL10	TLR4	2,39938E-03	13737,7	1,19187E-02	7430	5,30561E-01	7031	1,73838E+00	3491	8,02823E-01	33206	0	17530,5
Non-responder	Macro_OLFML3	Macro_ISG15	HLA-A	LILRA1	2,40004E-03	13717,7	7,41927E-03	15942	3,92744E-01	12327	1,16380E+00	10081	8,86366E-01	12708	0	17530,5
Non-responder	Mono_CD16	cDC_CLEC9A	QDPR	DYSF	2,40065E-03	19768,9	1,54549E-02	4752	5,41029E-01	6734	1,33938E+00	7431	6,51699E-01	62397	0	17530,5
Non-responder	Macro_FOLR2+APOE+	cDC(CD1C)	MMP9	IFNAR1	2,40065E-03	14969,9	1,19186E-02	7431	5,88194E-01	5553	1,95911E+00	2257	7,65039E-01	42078	0	17530,5
Non-responder	cDC(CD1C)	Macro_OLFML3	HLA-DQA2	CD4	2,40079E-03	10933,5	7,95944E-03	14249	5,28651E-01	7074	1,14639E+00	10354	9,17424E-01	5460	0	17530,5
Non-responder	Macro_OLFML3	Mast	HSPA8	ADRB2	2,40230E-03	11683,5	1,57893E-02	4602	5,55825E-01	6337	8,76632E-01	15945	8,81459E-01	14003	0	17530,5
Non-responder	cDC_LAMP3	Macro_FOLR2+APOE+	HLA-DQA1	CD4	2,40305E-03	10980,7	7,41664E-03	15946	4,76748E-01	8827	1,12914E+00	10652	9,35079E-01	1948	0	17530,5
Non-responder	cDC_LAMP3	Mono_CD14	CIRBP	TREM1	2,40531E-03	13670,1	9,81617E-03	10145	4,03909E-01	11802	8,76409E-01	15949	8,85529E-01	12924	0	17530,5
Non-responder	pDC_LILRA4	cDC_CLEC9A	CALR	SCARF1	2,40575E-03	8710,5	2,13585E-02	2724	8,39879E-01	2184	2,23801E+00	1266	8,57985E-01	19848	0	17530,5
Non-responder	Macro_FOLR2+APOE+	cDC_LAMP3	CD14	ITGA4	2,40606E-03	11090,5	7,41476E-03	15950	5,44050E-01	6663	1,42550E+00	6342	9,01381E-01	8967	0	17530,5
Non-responder	Macro_FOLR2+APOE+	Macro_LYVE1	ALCAM	NRP1	2,40697E-03	13549,9	1,64421E-02	4284	5,16911E-01	7436	1,35252E+00	7268	8,11103E-01	31231	0	17530,5
Non-responder	Macro_NLRP3	Macro_IFI27	S100A9	ITGB2	2,40757E-03	9285,1	1,01211E-02	9672	7,71574E-01	2805	8,76380E-01	15952	9,46370E-01	466	0	17530,5
Non-responder	Mono_CD16	Mono_CD14	CD55	CR1	2,40824E-03	12652,5	2,23661E-02	2495	7,09754E-01	3521	1,55545E+00	4982	7,96378E-01	34734	0	17530,5
Non-responder	Macro_ISG15	Macro_ISG15	HLA-B	LILRB1	2,40835E-03	6991,9	8,82940E-03	11973	7,80073E-01	2725	2,09521E+00	1719	9,40637E-01	1012	0	17530,5
Non-responder	Mast	Macro_LYVE1	CSF1	CSF2RA	2,41115E-03	8226,1	5,72788E-02	514	1,18451E+00	485	1,62713E+00	4369	8,64934E-01	18232	0	17530,5
Non-responder	Mono_CD16	Mono_CD14	CD48	CD244	2,41204E-03	16585,1	1,33764E-02	6084	6,31286E-01	4699	1,49391E+00	5599	7,33569E-01	49013	0	17530,5
Non-responder	cDC(CD1C)	Macro_FOLR2+APOE+	HLA-DQB2	LAG3	2,41356E-03	11507,3	2,18414E-02	2608	7,79933E-01	2727	1,95370E+00	2286	8,06176E-01	32385	0	17530,5
Non-responder	cDC_LAMP3	Mono_CD16	HLA-C	LILRB2	2,41513E-03	10567,5	7,41098E-03	15962	5,01595E-01	7935	1,11512E+00	10903	9,45884E-01	507	0	17530,5
Non-responder	Mono_CD16	Mono_CD14	ICAM2	ITGAL_ITGB2	2,41839E-03	9967,9	1,44124E-02	5363	5,79660E-01	5744	1,65252E+00	4180	8,69857E-01	17022	0	17530,5
Non-responder	pDC_LILRA4	Macro_OLFML3	PROC	THBD	2,42138E-03	14668,1	3,38791E-02	1261	7,79771E-01	2730	2,15840E+00	1501	7,27339E-01	50318	0	17530,5
Non-responder	Mono_CD16	Mono_CD14	ICAM3	ITGAL	2,42602E-03	14163,9	1,21127E-02	7219	5,91413E-01	5496	1,67708E+00	3955	7,88101E-01	36619	0	17530,5
Non-responder	Macro_IFI27	pDC_LILRA4	HLA-DQA2	CD4	2,42650E-03	13473,1	7,40680E-03	15977	3,95595E-01	12210	8,90640E-01	15614	9,14657E-01	6034	0	17530,5
Non-responder	Mono_CD16	Mono_CD14	TNFSF10	TNFRSF10D	2,42857E-03	16206,5	1,51756E-02	4901	5,33414E-01	6948	1,49618E+00	5565	7,46984E-01	46088	0	17530,5
Non-responder	Macro_FOLR2+APOE+	Mono_INHBA	PLAU	ITGB1	2,42857E-03	13049,5	1,25070E-02	6833	5,16692E-01	7453	1,41556E+00	6470	8,28566E-01	26961	0	17530,5
Non-responder	pDC_LILRA4	Macro_OLFML3	GAS6	AXL	2,42922E-03	9326,7	2,13269E-02	2733	8,59221E-01	2012	2,37985E+00	966	8,43153E-01	23392	0	17530,5
Non-responder	Macro_FOLR2+APOE+	Mono_INHBA	RARRES1	NRP2	2,43183E-03	18096,3	2,59747E-02	1963	7,79232E-01	2734	1,22534E+00	9048	6,75783E-01	59206	0	17530,5
Non-responder	Macro_OLFML3	Mono_CD16	GRN	TNFRSF1B	2,43258E-03	9459,9	7,40558E-03	15985	5,69738E-01	5978	1,46541E+00	5895	9,35260E-01	1911	0	17530,5
Non-responder	Mono_CD16	Mono_CD14	CD55	ADGRE5	2,43495E-03	10174,7	1,18860E-02	7458	6,96016E-01	3673	1,61685E+00	4448	8,66810E-01	17764	0	17530,5
Non-responder	Macro_IFI27	Mono_CD16	B2M	LILRB2	2,43562E-03	10157,7	8,16376E-03	13657	7,05816E-01	3566	8,74853E-01	15989	9,55805E-01	46	0	17530,5
Non-responder	cDC_LAMP3	cDC_LAMP3	CCL19	CCRL2	2,43707E-03	14478,9	2,13133E-02	2736	1,69615E+00	75	2,21904E+00	1320	7,24977E-01	50733	0	17530,5
Non-responder	cDC_LAMP3	Macro_LYVE1	CD59	STAB1	2,43715E-03	11691,9	1,06089E-02	9003	7,46794E-01	3064	1,01406E+00	12871	8,73834E-01	15991	0	17530,5
Non-responder	Macro_NLRP3	Macro_ISG15	HLA-B	LILRB1	2,43715E-03	10951,5	7,40496E-03	15991	4,07701E-01	11630	1,31603E+00	7753	9,35531E-01	1853	0	17530,5
Non-responder	Mono_CD16	Mono_CD14	SELPLG	SELL	2,43878E-03	9320,7	1,44698E-02	5319	6,48623E-01	4412	1,87310E+00	2698	8,71272E-01	16644	0	17530,5
Non-responder	Mono_CD14	Macro_ISG15	S100A8	TLR4	2,43918E-03	6263,3	2,01269E-02	3030	1,17292E+00	517	2,74539E+00	464	8,97872E-01	9775	0	17530,5
Non-responder	pDC_LILRA4	Macro_OLFML3	SELPLG	ITGB2	2,43969E-03	6337,3	1,15185E-02	7848	7,78954E-01	2737	2,44822E+00	847	9,31127E-01	2724	0	17530,5
Non-responder	Mono_CD16	Mono_CD14	ICAM4	ITGB2	2,44006E-03	10078,5	2,57422E-02	1987	7,10958E-01	3502	1,49377E+00	5603	8,49867E-01	21770	0	17530,5
Non-responder	Mono_CD16	Mono_CD14	ICAM4	ITGAM	2,44262E-03	15960,3	3,28418E-02	1327	6,42417E-01	4512	1,37052E+00	7010	7,31548E-01	49422	0	17530,5
Non-responder	Mono_CD16	Mono_CD14	ICAM4	ITGAL	2,44518E-03	17518,5	2,74185E-02	1779	6,45585E-01	4460	1,49432E+00	5594	6,82008E-01	58229	0	17530,5
Non-responder	Mono_CD16	Macro_FOLR2+APOE+	S100A9	CD36	2,44631E-03	13901,3	9,29862E-03	11032	4,31054E-01	10569	9,45431E-01	14372	8,73793E-01	16003	0	17530,5
Non-responder	Macro_FOLR2+APOE+	Macro_ISG15	ADM	GPR84	2,44646E-03	16352,7	1,66107E-02	4193	5,15937E-01	7467	1,70025E+00	3784	7,34639E-01	48789	0	17530,5
Non-responder	Mono_INHBA	Mono_CD16	IL6	IL6R_IL6ST	2,44757E-03	13861,1	2,12775E-02	2740	1,04302E+00	1007	1,96602E+00	2225	7,48157E-01	45803	0	17530,5
Non-responder	Macro_NLRP3	Macro_NLRP3	CD55	ADGRE5	2,44774E-03	10223,5	1,18764E-02	7468	6,82718E-01	3860	1,61356E+00	4476	8,66764E-01	17783	0	17530,5
Non-responder	Macro_FOLR2+APOE+	Macro_OLFML3	CD14	TLR1	2,44784E-03	9835,7	1,17973E-02	7535	6,39477E-01	4565	1,73183E+00	3543	8,73788E-01	16005	0	17530,5
Non-responder	Mono_CD16	Macro_FOLR2+APOE+	HLA-B	LILRB2	2,44903E-03	10528,1	6,24780E-03	20977	5,15895E-01	7469	1,45334E+00	6007	9,43994E-01	657	0	17530,5
Non-responder	Macro_OLFML3	Mono_INHBA	SERPING1	LRP1	2,45013E-03	13370,5	8,70111E-03	12285	4,76832E-01	8823	1,04625E+00	12206	8,73778E-01	16008	0	17530,5
Non-responder	Macro_OLFML3	Macro_OLFML3	LILRB4	LAIR1	2,45319E-03	12752,9	7,39915E-03	16012	3,94413E-01	12259	1,19259E+00	9584	9,04164E-01	8379	0	17530,5
Non-responder	cDC(CD1C)	Mono_CD16	HLA-C	LILRB1	2,45396E-03	11376,7	7,39895E-03	16013	4,56720E-01	9554	1,09110E+00	11312	9,32483E-01	2474	0	17530,5
Non-responder	Macro_FOLR2+APOE+	Macro_FOLR2+APOE+	SEMA4D	CD72	2,45416E-03	17531,3	1,18715E-02	7473	5,93220E-01	5463	1,49819E+00	5553	7,20306E-01	51637	0	17530,5
Non-responder	Mono_CD16	Macro_FOLR2+APOE+	S100A9	TLR4	2,46009E-03	12963,3	1,01760E-02	9598	5,03863E-01	7850	9,68703E-01	13817	8,73744E-01	16021	0	17530,5
Non-responder	Macro_FOLR2+APOE+	Macro_FOLR2+APOE+	GRN	TNFRSF1B	2,46188E-03	11129,5	5,84173E-03	23341	5,15546E-01	7479	1,69291E+00	3830	9,27697E-01	3467	0	17530,5
Non-responder	Macro_NLRP3	Macro_LYVE1	S100A8	CD36	2,46394E-03	10688,1	1,13795E-02	7999	7,88790E-01	2650	1,21454E+00	9235	8,73713E-01	16026	0	17530,5
Non-responder	Macro_IER3	Macro_FOLR2+APOE+	HLA-DRB5	CD4	2,46624E-03	10557,3	7,39527E-03	16029	4,75217E-01	8869	1,18725E+00	9668	9,43616E-01	690	0	17530,5
Non-responder	Macro_FOLR2+APOE+	Macro_NLRP3	TNF	TNFRSF1B	2,46701E-03	10327,7	1,01179E-02	9677	6,53533E-01	4339	1,66651E+00	4062	8,73709E-01	16030	0	17530,5
Non-responder	Macro_NLRP3	Mono_CD16	S100A8	CD36	2,46932E-03	9859,9	1,13713E-02	8003	7,88647E-01	2653	1,54634E+00	5080	8,73674E-01	16033	0	17530,5
Non-responder	Macro_OLFML3	Mono_CD16	B2M	LILRB1	2,47086E-03	9290,9	7,39336E-03	16035	5,46691E-01	6575	1,50606E+00	5484	9,42116E-01	830	0	17530,5
Non-responder	Macro_NLRP3	Mono_INHBA	S100A9	CD36	2,47549E-03	11067,9	9,27621E-03	11074	7,54329E-01	2978	1,31918E+00	7716	8,73659E-01	16041	0	17530,5
Non-responder	Macro_FOLR2+APOE+	cDC_CLEC9A	COPA	CD74	2,47607E-03	9895,3	6,77440E-03	18473	5,15333E-01	7490	1,61993E+00	4424	9,36953E-01	1559	0	17530,5
Non-responder	Macro_FOLR2+APOE+	Macro_FOLR2+APOE-	HLA-DMB	CD4	2,47626E-03	11799,3	7,39211E-03	16042	5,27973E-01	7095	1,09994E+00	11164	9,09404E-01	7165	0	17530,5
Non-responder	Macro_FOLR2+APOE+	Macro_LYVE1	MDK	SDC3	2,47736E-03	17775,9	4,42182E-02	806	6,85769E-01	3814	1,33500E+00	7491	6,75610E-01	59238	0	17530,5
Non-responder	Mono_CD16	Mono_CD14	RPS19	C5AR1	2,47995E-03	10136,9	6,93536E-03	17787	5,52941E-01	6416	1,60932E+00	4516	9,22642E-01	4435	0	17530,5
Non-responder	Macro_NLRP3	pDC_LILRA4	TNF	TNFRSF1B	2,48399E-03	10075,5	1,01013E-02	9702	5,71559E-01	5943	2,28589E+00	1150	8,73619E-01	16052	0	17530,5
Non-responder	Mono_CD16	Mono_CD16	GNAI2	FPR1	2,48399E-03	12086,5	7,39096E-03	16052	3,99977E-01	11988	1,43140E+00	6258	9,03046E-01	8604	0	17530,5

Post_NR_Myeloid_Myeloid_CellCell

Non-responder	Macro_OLFML3	Mono_CD16	TNFSF13	TNFRSF14	2,48708E-03	13879,9	7,38969E-03	16056	3,84882E-01	12716	1,24103E+00	8822	8,80395E-01	14275	0	17530,5
Non-responder	Mono_CD16	Macro_NLRP3	CIRBP	TREM1	2,49174E-03	13897,9	8,60123E-03	12514	3,25717E-01	16062	1,25563E+00	8619	8,78660E-01	14764	0	17530,5
Non-responder	Macro_FOLR2+APOE+	Mono_CD16	APP	FPR2	2,49292E-03	15935,9	1,24663E-02	6871	5,14760E-01	7503	1,45229E+00	6015	7,66392E-01	41760	0	17530,5
Non-responder	Macro_IER3	Mono_INHBA	TNF	TNFRSF1B	2,49329E-03	10815,5	1,00929E-02	9719	6,02490E-01	5260	1,50415E+00	5504	8,73573E-01	16064	0	17530,5
Non-responder	Macro_FOLR2+APOE+	Macro_FOLR2+APOE+	HLA-DQA2	CD4	2,49406E-03	11099,5	7,38586E-03	16065	3,98299E-01	12067	1,70160E+00	3775	9,14546E-01	6060	0	17530,5
Non-responder	Macro_LYVE1	Mono_CD16	CXCL12	ITGA4	2,50051E-03	9381,1	3,25373E-02	1353	9,77124E-01	1313	1,86210E+00	2760	8,40857E-01	23949	0	17530,5
Non-responder	Macro_FOLR2+APOE+	Mono_INHBA	PLAU	ITGAM	2,50203E-03	14331,9	1,49570E-02	5039	5,14547E-01	7510	1,35817E+00	7171	7,97735E-01	34409	0	17530,5
Non-responder	pDC_LILRA4	pDC_LILRA4	GNAI2	CXCR3	2,50318E-03	8174,1	2,12043E-02	2761	8,34860E-01	2231	2,99270E+00	276	8,65566E-01	18072	0	17530,5
Non-responder	Macro_NLRP3	Macro_FOLR2-APOE+	THBS1	SCARB1	2,50464E-03	10685,5	2,31084E-02	2350	8,30034E-01	2278	1,33314E+00	7512	8,41618E-01	23757	0	17530,5
Non-responder	Mast	cDC_LAMP3	CSF1	SIRPA	2,50520E-03	9070,5	5,67975E-02	524	1,19075E+00	464	1,70629E+00	3745	8,44388E-01	23089	0	17530,5
Non-responder	Macro_FOLR2+APOE+	Macro_IFI27	LGALS3	ENG	2,50651E-03	13273,3	7,38019E-03	16081	4,51435E-01	9788	9,68207E-01	13828	9,00622E-01	9139	0	17530,5
Non-responder	Mono_CD14	Macro_FOLR2+APOE+	S100A9	CD36	2,50855E-03	6015,9	1,88023E-02	3394	1,09092E+00	808	2,45611E+00	832	9,07792E-01	7515	0	17530,5
Non-responder	Macro_NLRP3	Macro_LYVE1	IL1B	SIGIRR	2,51119E-03	9507,9	1,16033E-02	7747	9,04342E-01	1707	1,61441E+00	4468	8,73481E-01	16087	0	17530,5
Non-responder	Mono_CD16	Macro_NLRP3	SELPLG	ITGB2	2,51197E-03	11519,9	7,37893E-03	16088	4,18514E-01	11127	1,37222E+00	6988	9,15404E-01	5866	0	17530,5
Non-responder	Macro_IER3	pDC_LILRA4	S100A9	CD36	2,51275E-03	13755,9	9,24537E-03	11138	3,79588E-01	13002	1,10794E+00	11020	8,73476E-01	16089	0	17530,5
Non-responder	Mono_CD16	Macro_NLRP3	ICAM2	ITGAM_ITGB2	2,51378E-03	10790,5	1,34461E-02	6035	5,14305E-01	7519	1,51079E+00	5439	8,68211E-01	17429	0	17530,5
Non-responder	Macro_ISG15	Mono_CD16	TGM2	ITGA4	2,51386E-03	9880,7	2,13091E-02	2737	7,75362E-01	2765	2,08893E+00	1747	8,38060E-01	24624	0	17530,5
Non-responder	Macro_NLRP3	pDC_LILRA4	ADA	DPP4	2,52294E-03	20722,1	2,24568E-02	2478	5,37755E-01	6831	1,33197E+00	7526	5,79342E-01	69245	0	17530,5
Non-responder	Mono_CD14	Macro_FOLR2+APOE+	S100A9	TLR4	2,52425E-03	5863,7	2,05764E-02	2924	1,16373E+00	544	2,47938E+00	793	9,07756E-01	7527	0	17530,5
Non-responder	Macro_FOLR2+APOE+	Mono_INHBA	CIRBP	TREM1	2,52448E-03	15269,7	8,62232E-03	12457	3,34575E-01	15524	8,70493E-01	16104	8,78791E-01	14733	0	17530,5
Non-responder	Macro_NLRP3	Macro_IER3	IL1B	IL1R2_IL1RAP	2,52950E-03	11154,5	1,18071E-02	7531	9,09992E-01	1667	1,77902E+00	3236	8,33054E-01	25808	0	17530,5
Non-responder	pDC_LILRA4	Macro_LYVE1	PROC	ITGAM	2,54070E-03	13444,9	5,36432E-02	574	8,09874E-01	2458	1,85821E+00	2775	7,56505E-01	43887	0	17530,5
Non-responder	Macro_FOLR2+APOE+	Macro_IER3	LRPAP1	LRP1	2,54572E-03	14589,5	7,97414E-03	14207	4,07959E-01	11620	9,83980E-01	13459	8,73297E-01	16131	0	17530,5
Non-responder	Mono_CD14	Macro_FOLR2+APOE+	VSTM1	LAMP1	2,54609E-03	15086,9	4,20807E-02	890	8,21240E-01	2356	1,85783E+00	2777	7,18896E-01	51881	0	17530,5
Non-responder	Mono_CD16	cDC(CD1C)	TNFSF13B	HLA-DPB1	2,54662E-03	11489,7	5,71663E-03	24145	5,23939E-01	7218	1,33119E+00	7544	9,40651E-01	1011	0	17530,5
Non-responder	cDC(CD1C)	Macro_ISG15	HLA-DRB1	LAG3	2,54879E-03	6408,9	2,11469E-02	2778	1,09631E+00	780	1,87254E+00	2702	9,04720E-01	8254	0	17530,5
Non-responder	Macro_ISG15	cDC(CD1C)	TNFSF13B	HLA-DPB1	2,54879E-03	7062,1	8,63090E-03	12437	8,15723E-01	2400	1,85782E+00	2778	9,51159E-01	165	0	17530,5
Non-responder	Macro_NLRP3	Macro_IFI27	VCAN	ITGB1	2,54888E-03	11217,9	1,10134E-02	8458	7,79564E-01	2731	1,09586E+00	11235	8,73291E-01	16135	0	17530,5
Non-responder	Mono_CD16	Macro_FOLR2+APOE+	S100A8	ITGB2	2,54888E-03	10719,5	7,36164E-03	16135	4,92540E-01	8245	1,22657E+00	9028	9,31481E-01	2659	0	17530,5
Non-responder	Macro_OLFML3	Macro_FOLR2+APOE+	C3	ITGAX	2,55125E-03	13934,9	9,27207E-03	11078	3,24492E-01	16138	1,19075E+00	9612	8,76500E-01	15316	0	17530,5
Non-responder	Mono_CD16	Mono_CD16	HLA-C	LILRB1	2,55125E-03	10226,1	7,36099E-03	16138	4,47469E-01	9923	1,55054E+00	5037	9,32321E-01	2502	0	17530,5
Non-responder	Macro_FOLR2+APOE+	Macro_ISG15	LGALS9	LRP1	2,55283E-03	12905,1	7,36029E-03	16140	4,13196E-01	11377	1,47137E+00	5828	8,82873E-01	13650	0	17530,5
Non-responder	Macro_OLFML3	cDC_LAMP3	PDGFB	S1PR1	2,55289E-03	17167,3	6,13155E-02	429	1,17081E+00	529	1,36741E+00	7040	6,67684E-01	60308	0	17530,5
Non-responder	Mono_CD16	Macro_ISG15	HLA-B	LILRB2	2,55587E-03	8955,9	6,97719E-03	17623	6,09149E-01	5122	1,66475E+00	4076	9,46842E-01	428	0	17530,5
Non-responder	Macro_ISG15	Mono_INHBA	CXCL10	TLR4	2,55690E-03	9139,3	2,11361E-02	2781	9,34790E-01	1531	2,52060E+00	740	8,44286E-01	23114	0	17530,5
Non-responder	Macro_OLFML3	cDC(CD1C)	PSAP	CD1B	2,55916E-03	11202,7	2,64252E-02	1896	4,59925E-01	9427	1,10869E+00	11012	8,73213E-01	16148	0	17530,5
Non-responder	Macro_ISG15	cDC_LAMP3	TNFSF10	CCR6	2,55960E-03	14264,7	3,95447E-02	981	8,81515E-01	1847	1,85641E+00	2782	7,37490E-01	48183	0	17530,5
Non-responder	pDC_LILRA4	Macro_FOLR2+APOE+	IL16	CD4	2,55960E-03	6432,5	2,11344E-02	2782	9,09621E-01	1669	2,84745E+00	380	8,97763E-01	9801	0	17530,5
Non-responder	Macro_FOLR2+APOE+	Macro_NLRP3	ICAM1	ITGAX_ITGB2	2,56075E-03	10680,7	7,35789E-03	16150	5,46252E-01	6588	1,56112E+00	4929	9,04911E-01	8206	0	17530,5
Non-responder	Macro_IER3	Macro_ISG15	CD14	ITGB1	2,56233E-03	13230,9	7,35779E-03	16152	4,21384E-01	10988	1,03094E+00	12528	9,01432E-01	8956	0	17530,5
Non-responder	Macro_FOLR2+APOE+	Mono_CD14	PSAP	LRP1	2,56382E-03	10736,1	6,48553E-03	19841	5,13269E-01	7557	1,54806E+00	5064	9,26513E-01	3688	0	17530,5
Non-responder	Macro_FOLR2+APOE+	Macro_NLRP3	PIGF	FLT1	2,56515E-03	19228,1	2,74898E-02	1774	5,13130E-01	7558	1,58446E+00	4740	6,32910E-01	64538	0	17530,5
Non-responder	Macro_ISG15	cDC_CLEC9A	CXCL11	CXCR3	2,56773E-03	13768,5	5,76738E-02	505	7,73050E-01	2785	2,33287E+00	1057	7,42956E-01	46965	0	17530,5
Non-responder	Macro_IER3	Macro_ISG15	B2M	LILRB1	2,57027E-03	12453,1	7,35487E-03	16162	3,52424E-01	14501	9,95091E-01	13229	9,41973E-01	843	0	17530,5
Non-responder	Macro_OLFML3	Macro_ISG15	CCL4	CCR1	2,57266E-03	8937,5	1,52942E-02	4842	7,11575E-01	3491	1,88144E+00	2659	8,73153E-01	16165	0	17530,5
Non-responder	cDC_CLEC9A	pDC_LILRA4	HLA-C	NOTCH4	2,57312E-03	6785,1	2,64685E-02	1891	6,33013E-01	4668	1,95635E+00	2272	9,07594E-01	7564	0	17530,5
Non-responder	Macro_ISG15	pDC_LILRA4	SEMA4A	NRP1_PLXNA4	2,57316E-03	12323,1	2,24404E-02	2482	7,72920E-01	2787	2,14337E+00	1534	7,85314E-01	37282	0	17530,5
Non-responder	cDC(CD1C)	Macro_FOLR2+APOE+	CD1D	LILRB2	2,57505E-03	8856,3	1,83414E-02	3543	7,49142E-01	3035	1,67191E+00	4005	8,73139E-01	16168	0	17530,5
Non-responder	Macro_NLRP3	Macro_IER3	ICAM1	ITGAX_ITGB2	2,57584E-03	12026,9	7,35313E-03	16169	5,18069E-01	7391	1,11807E+00	10828	9,04883E-01	8216	0	17530,5
Non-responder	Mono_CD16	Macro_ISG15	CD55	ADGRE5	2,57978E-03	9305,1	1,28748E-02	6506	7,38334E-01	3161	1,87298E+00	2699	8,71356E-01	16629	0	17530,5
Non-responder	Macro_IFI27	Mono_CD16	C3	IFITM1	2,57983E-03	10365,3	1,69254E-02	4068	6,67383E-01	4102	8,67907E-01	16174	8,97140E-01	9952	0	17530,5
Non-responder	Mono_CD16	Macro_ISG15	CD55	ADGRE2	2,58111E-03	8993,7	1,53664E-02	4803	8,02101E-01	2530	1,87247E+00	2703	8,68361E-01	17402	0	17530,5
Non-responder	pDC_LILRA4	Macro_FOLR2+APOE+	APP	NOTCH2	2,58172E-03	8007,3	1,79633E-02	3675	1,17044E+00	532	2,74843E+00	458	8,66507E-01	17841	0	17530,5
Non-responder	Mono_CD16	Macro_ISG15	CD48	CD244	2,58245E-03	13045,3	2,21495E-02	2547	7,41873E-01	3125	1,74288E+00	3465	7,79880E-01	38559	0	17530,5
Non-responder	Mono_CD14	cDC_CLEC9A	NRG1	HLA-DPB1	2,58404E-03	7570,3	2,10954E-02	2791	7,75455E-01	2764	1,92408E+00	2439	8,87833E-01	12327	0	17530,5
Non-responder	Mono_CD16	Macro_ISG15	HLA-C	LILRB2	2,58512E-03	8905,7	7,15567E-03	16905	5,71664E-01	5938	1,72639E+00	3580	9,44979E-01	575	0	17530,5
Non-responder	Macro_FOLR2+APOE+	Mono_INHBA	CD14	TLR4	2,58622E-03	11362,9	8,87871E-03	11873	5,65713E-01	6080	1,53900E+00	5149	8,73088E-01	16182	0	17530,5
Non-responder	Mono_CD14	Macro_NLRP3	VCAN	SELL	2,58676E-03	7339,5	2,10915E-02	2792	1,12963E+00	652	2,07256E+00	1807	8,81783E-01	13916	0	17530,5
Non-responder	Mono_CD16	Macro_ISG15	ICAM2	ITGAL_ITGB2	2,58912E-03	10681,9	1,30752E-02	6329	5,24616E-01	7197	1,67590E+00	3969	8,64245E-01	18384	0	17530,5
Non-responder	Macro_FOLR2+APOE+	Mono_INHBA	APOC2	LRP1	2,59046E-03	12437,1	1,86339E-02	3452	5,12856E-01	7577	1,80368E+00	3088	8,13943E-01	30538	0	17530,5
Non-responder	Macro_ISG15	Mono_CD16	CXCL10	TLR4	2,59222E-03	9098,3	2,10866E-02	2794	9,34000E-01	1537	2,73794E+00	475	8,44132E-01	23155	0	17530,5
Non-responder	Macro_NLRP3	Macro_FOLR2+APOE+	TGFB1	ITGB1	2,59342E-03	11965,3	8,70219E-03	12284	4,81498E-01	8644	1,53670E+00	5177	8,73053E-01	16191	0	17530,5
Non-responder	Macro_IER3	Macro_FOLR2+APOE+	ICAM1	ITGAL_ITGB2	2,59342E-03	11846,3	7,34747E-03	16191	5,17619E-01	7410	1,27591E+00	8322	8,97865E-01	9778	0	17530,5
Non-responder	Macro_NLRP3	Macro_FOLR2+APOE+	S100A8	CD69	2,59582E-03	13058,7	8,53573E-03	12670	7,40487E-01	3139	8,67246E-01	16194	8,74717E-01	15760	0	17530,5
Non-responder	Macro_FOLR2+APOE+	Macro_FOLR2+APOE+	ADAM10	CD44	2,59983E-03	13545,9	8,19627E-03	13572	4,18373E-01	11131	1,20989E+00	9297	8,73022E-01	16199	0	17530,5
Non-responder	cDC_LAMP3	Macro_FOLR2+APOE+	MIF	TNFRSF14	2,60143E-03	13297,7	7,34536E-03	16201	5,13719E-01	7543	8,74284E-01	16001	9,00352E-01	9213	0	17530,5
Non-responder	Macro_IER3	Macro_FOLR2+APOE+	CCL3L1	CCR5	2,60315E-03	10442,9	3,05375E-02	1498	8,43401E-01	2142	1,85329E+00	2798	8,23368E-01	28246	0	17530,5
Non-responder	cDC_LAMP3	Macro_FOLR2+APOE+	HLA-F	LILRB2	2,60384E-03	12029,5	7,34456E-03	16204	5,72911E-01	5917	1,15044E+00	10294	8,96153E-01	10202	0	17530,5
Non-responder	Mono_CD16	Macro_ISG15	B2M	LILRB2	2,60385E-03	9639,5	6,78160E-03	18436	5,27749E-01	7100	1,55481E+00	4989	9,51717E-01	142	0	17530,5
Non-responder	Macro_NLRP3	Macro_LYVE1	SERPINA1	LRP1	2,60706E-03	11998,3	8,59948E-03	12519	4,97640E-01	8066	8,66701E-01	16208	9,16357E-01	5668	0	17530,5
Non-responder	Macro_OLFML3	Mono_CD14	FN1	PLAUR	2,61028E-03	11785,5	1,15495E-02	7822	4,56367E-01	9566	1,31292E+00	7797	8,72972E-01	16212	0	17530,5

Post_NR_Myeloid_Myeloid_CellCell

Non-responder	cDC(CD1C)	pDC_LILRA4	COPA	P2RY6	2,61070E-03	8144,7	1,98120E-02	3117	1,16719E+00	535	2,84723E+00	381	8,60909E-01	19160	0	17530,5
Non-responder	pDC_LILRA4	Macro_FOLR2+APOE-	HSP90B1	LRP1	2,61191E-03	6592,5	1,17505E-02	7593	9,52915E-01	1433	2,12534E+00	1605	9,20550E-01	4801	0	17530,5
Non-responder	pDC_LILRA4	Mast	GNAS	ADRB2	2,61411E-03	8783,9	2,42083E-02	2191	7,71635E-01	2802	1,90282E+00	2536	8,62196E-01	18860	0	17530,5
Non-responder	Macro_FOLR2+APOE+	Mono_CD16	CD14	TLR4	2,61431E-03	11027,1	8,85792E-03	11909	5,64923E-01	6105	1,75635E+00	3374	8,72958E-01	16217	0	17530,5
Non-responder	Mono_CD16	Macro_FOLR2+APOE+	LGALS9	MRC2	2,61999E-03	14093,5	1,58933E-02	4543	5,13468E-01	7550	1,32652E+00	7599	8,02640E-01	33245	0	17530,5
Non-responder	Mast	Macro_IER3	CSF1	CSF2RA	2,62040E-03	8286,5	5,57501E-02	536	1,17280E+00	518	1,64183E+00	4258	8,63346E-01	18590	0	17530,5
Non-responder	Mono_CD16	Macro_ISG15	ICAM4	ITGB2	2,62134E-03	10673,1	2,32320E-02	2327	6,16357E-01	4994	1,54010E+00	5132	8,43204E-01	23382	0	17530,5
Non-responder	Macro_FOLR2+APOE+	Macro_FOLR2-APOE+	ADAM10	GPNMB	2,62157E-03	10646,3	2,02343E-02	3000	6,50111E-01	4395	1,05379E+00	12080	8,72913E-01	16226	0	17530,5
Non-responder	Mono_CD16	Macro_ISG15	ICAM4	ITGB1	2,62269E-03	13388,1	2,93087E-02	1618	7,03440E-01	3592	1,60557E+00	4553	7,75272E-01	39647	0	17530,5
Non-responder	cDC_CLEC9A	Macro_NLRP3	ADAM15	ITGA5	2,62510E-03	11013,3	2,76072E-02	1763	7,71524E-01	2806	1,93959E+00	2359	8,13621E-01	30608	0	17530,5
Non-responder	Macro_NLRP3	pDC_LILRA4	VCAN	ITGB1	2,62561E-03	9290,5	1,09342E-02	8571	7,77405E-01	2748	2,20094E+00	1372	8,72891E-01	16231	0	17530,5
Non-responder	Macro_OLFML3	Mono_CD14	GNAI2	FPR1	2,62561E-03	12694,1	7,33854E-03	16231	3,93359E-01	12304	1,24732E+00	8735	9,02734E-01	8670	0	17530,5
Non-responder	Mono_CD16	Macro_ISG15	ICAM4	ITGA4	2,62674E-03	16079,9	2,01471E-02	3021	5,63068E-01	6146	1,44657E+00	6077	7,40098E-01	47625	0	17530,5
Non-responder	Mono_CD16	Macro_ISG15	ICAM4	ITGAL	2,62809E-03	17821,1	2,57783E-02	1978	6,30097E-01	4723	1,49474E+00	5588	6,75282E-01	59286	0	17530,5
Non-responder	Macro_FOLR2+APOE+	pDC_LILRA4	LGALS9	PTPRC	2,63485E-03	10338,1	6,79080E-03	18405	5,11627E-01	7610	2,05480E+00	1869	9,13438E-01	6276	0	17530,5
Non-responder	cDC(CD1C)	cDC_LAMP3	TNFSF13B	CD40	2,63614E-03	11366,9	9,26196E-03	11099	6,70190E-01	4061	1,18929E+00	9640	8,79600E-01	14504	0	17530,5
Non-responder	Mono_CD14	Macro_FOLR2+APOE-	S100A9	CD68	2,63620E-03	6493,3	1,32154E-02	6202	1,02678E+00	1084	1,32545E+00	7611	9,56305E-01	39	0	17530,5
Non-responder	Mono_CD14	Macro_IER3	VCAN	TLR2	2,63888E-03	6564,9	2,20231E-02	2572	1,10894E+00	740	1,85063E+00	2811	9,00511E-01	9171	0	17530,5
Non-responder	Macro_NLRP3	Macro_OLFML3	S100A9	CD36	2,64752E-03	10606,7	9,13561E-03	11343	7,51432E-01	3010	1,56506E+00	4892	8,72814E-01	16258	0	17530,5
Non-responder	Macro_FOLR2+APOE+	cDC(CD1C)	IL15RA	AXL	2,64841E-03	14317,9	2,07820E-02	2871	5,75562E-01	5842	1,32493E+00	7620	7,83435E-01	37726	0	17530,5
Non-responder	cDC_LAMP3	Mono_INHBA	TNFSF13B	TFRC	2,64915E-03	12434,5	8,35750E-03	13139	5,11677E-01	7609	1,32412E+00	7634	8,72780E-01	16260	0	17530,5
Non-responder	Mono_CD16	Mono_CD16	CD55	CR1	2,65521E-03	17268,1	1,22338E-02	7118	5,26521E-01	7134	1,32443E+00	7625	7,43099E-01	46933	0	17530,5
Non-responder	Mono_CD14	Mono_INHBA	S100A9	CD68	2,65521E-03	6036,9	1,17240E-02	7625	9,14486E-01	1641	1,76891E+00	3300	9,53734E-01	88	0	17530,5
Non-responder	Macro_IER3	cDC_CLEC9A	ICAM1	IL2RG	2,65731E-03	13478,7	7,54572E-03	15500	4,87796E-01	8394	1,18556E+00	9699	8,72725E-01	16270	0	17530,5
Non-responder	Macro_FOLR2+APOE+	pDC_LILRA4	TNFSF9	ADGRG5	2,65825E-03	20427,5	4,56408E-02	763	7,70237E-01	2818	1,28215E+00	8248	4,89424E-01	72778	0	17530,5
Non-responder	Mono_INHBA	Mono_CD16	TNF	FFAR2	2,65825E-03	14452,7	3,92076E-02	999	7,81290E-01	2715	1,84933E+00	2818	7,37427E-01	48201	0	17530,5
Non-responder	Mono_CD16	cDC_CLEC9A	LGALS9	SLC1A5	2,66057E-03	11578,5	9,81769E-03	10143	4,80726E-01	8675	1,52884E+00	5270	8,72711E-01	16274	0	17530,5
Non-responder	Macro_IER3	Macro_ISG15	NAMPT	ITGA5_ITGB1	2,66057E-03	14446,9	7,32520E-03	16274	4,49812E-01	9843	1,01281E+00	12895	8,75013E-01	15692	0	17530,5
Non-responder	Mono_CD16	Macro_OLFML3	HLA-F	LILRB1	2,66221E-03	12986,5	7,41057E-03	15963	4,62938E-01	9325	1,47036E+00	5838	8,72692E-01	16276	0	17530,5
Non-responder	Macro_FOLR2+APOE+	Macro_LYVE1	C1QB	C1QBP	2,66339E-03	11691,3	6,20120E-03	21243	5,44155E-01	6661	1,51557E+00	5391	9,07297E-01	7631	0	17530,5
Non-responder	Macro_ISG15	cDC_LAMP3	VEGFA	NRP2	2,66658E-03	9720,1	2,50067E-02	2084	1,01712E+00	1128	1,84912E+00	2821	8,36369E-01	25037	0	17530,5
Non-responder	Macro_FOLR2+APOE+	Macro_FOLR2+APOE+	CALR	SCARF1	2,66885E-03	12786,7	1,17163E-02	7635	6,03489E-01	5239	1,69516E+00	3811	8,17338E-01	29718	0	17530,5
Non-responder	Macro_NLRP3	Macro_OLFML3	NAMPT	ITGA5_ITGB1	2,67040E-03	12217,9	7,32100E-03	16286	6,28747E-01	4751	1,38515E+00	6821	8,74981E-01	15701	0	17530,5
Non-responder	cDC_CLEC9A	pDC_LILRA4	HMGB1	CXCR4	2,67215E-03	6328,5	9,92065E-03	9977	7,69690E-01	2823	3,12012E+00	199	9,39914E-01	1113	0	17530,5
Non-responder	Macro_NLRP3	cDC(CD1C)	CCL3L1	CCR1	2,67296E-03	12064,3	1,17097E-02	7638	7,86077E-01	2676	1,67007E+00	4026	8,22594E-01	28451	0	17530,5
Non-responder	Mono_CD14	Macro_FOLR2+APOE+	VCAN	ITGA4	2,67493E-03	6094,7	2,09388E-02	2824	1,15984E+00	551	2,24347E+00	1248	9,04407E-01	8320	0	17530,5
Non-responder	pDC_LILRA4	Mono_INHBA	RPS19	C5AR1	2,67493E-03	7273,5	8,77370E-03	12109	8,42656E-01	2151	2,08620E+00	1753	9,30627E-01	2824	0	17530,5
Non-responder	Mono_CD16	Macro_ISG15	LGALS3	LAG3	2,67706E-03	10025,3	1,81584E-02	3604	7,99162E-01	2566	1,58303E+00	4749	8,50280E-01	21677	0	17530,5
Non-responder	Macro_FOLR2+APOE+	Mono_CD14	HSPA8	LDLR	2,67706E-03	12474,1	1,17060E-02	7641	5,14061E-01	7530	1,38358E+00	6845	8,45575E-01	22824	0	17530,5
Non-responder	Macro_ISG15	pDC_LILRA4	ICAM1	SPN	2,67772E-03	7530,7	2,09356E-02	2825	9,05166E-01	1703	2,58501E+00	645	8,77929E-01	14950	0	17530,5
Non-responder	Macro_FOLR2+APOE+	pDC_LILRA4	LRPAP1	SORL1	2,67778E-03	10642,3	1,02523E-02	9481	4,92085E-01	8255	2,11244E+00	1650	8,72589E-01	16295	0	17530,5
Non-responder	Mono_CD16	Macro_FOLR2+APOE+	ANXA1	FPR2_FPR3	2,68354E-03	14523,3	9,13590E-03	11341	3,34540E-01	15527	1,06134E+00	11916	8,72560E-01	16302	0	17530,5
Non-responder	cDC(CD1C)	cDC(CD1C)	B2M	CD1A	2,68436E-03	8849,5	4,47802E-02	786	4,61481E-01	9375	1,22082E+00	9132	9,08163E-01	7424	0	17530,5
Non-responder	Mono_CD14	Macro_FOLR2+APOE+	TIMP1	CD63	2,68609E-03	7031,9	8,60676E-03	12499	8,60550E-01	1999	1,84800E+00	2828	9,48842E-01	303	0	17530,5
Non-responder	pDC_LILRA4	Macro_OLFML3	APP	PTGER2	2,68878E-03	12021,5	1,63776E-02	4305	1,16392E+00	543	2,67480E+00	530	7,85640E-01	37199	0	17530,5
Non-responder	Macro_ISG15	cDC_LAMP3	TNFSF13	TNFRSF11B	2,68888E-03	10206,3	7,05718E-02	319	1,12324E+00	679	1,84800E+00	2829	8,17505E-01	29674	0	17530,5
Non-responder	Macro_ISG15	Macro_OLFML3	CCL8	CCR1	2,69168E-03	14144,1	4,19090E-02	895	7,68884E-01	2830	2,40345E+00	924	7,35878E-01	48541	0	17530,5
Non-responder	Mono_CD16	Macro_ISG15	LGALS9	CD47	2,69426E-03	12837,5	7,31239E-03	16315	4,34284E-01	10446	1,45705E+00	5977	8,81781E-01	13919	0	17530,5
Non-responder	cDC(CD1C)	Macro_FOLR2+APOE+	HMGB1	HAVCR2	2,69508E-03	13577,1	7,31236E-03	16316	4,05841E-01	11718	9,62441E-01	13966	9,04251E-01	8355	0	17530,5
Non-responder	Mono_CD16	Macro_ISG15	ICAM4	ITGAV	2,69629E-03	18949,1	2,51497E-02	2060	5,77858E-01	5784	1,32298E+00	7655	6,56947E-01	61716	0	17530,5
Non-responder	pDC_LILRA4	Mast	CALR	ITGA2B	2,69728E-03	8822,9	7,82367E-02	241	1,03584E+00	1043	1,84749E+00	2832	8,46972E-01	22468	0	17530,5
Non-responder	Mono_CD16	Macro_ISG15	RPS19	C5AR1	2,69904E-03	9389,3	7,23946E-03	16594	5,84050E-01	5646	1,81360E+00	3036	9,24160E-01	4140	0	17530,5
Non-responder	cDC(CD1C)	pDC_LILRA4	HLA-C	NOTCH4	2,70042E-03	7754,3	2,62236E-02	1927	6,16178E-01	5000	1,39959E+00	6656	9,07204E-01	7658	0	17530,5
Non-responder	Mono_CD16	pDC_LILRA4	HMGB1	THBD	2,70252E-03	13167,1	7,40078E-03	16007	3,21475E-01	16325	1,83677E+00	2902	8,84942E-01	13071	0	17530,5
Non-responder	Macro_FOLR2+APOE+	Macro_LYVE1	PLAU	ST14	2,70318E-03	14093,1	1,51538E-02	4916	5,22024E-01	7261	1,32267E+00	7660	8,03272E-01	33098	0	17530,5
Non-responder	Mono_CD14	cDC_CLEC9A	VCAN	ITGB1	2,70569E-03	6106,9	2,09000E-02	2835	1,13634E+00	625	2,23062E+00	1289	9,04710E-01	8255	0	17530,5
Non-responder	Macro_IER3	Macro_FOLR2+APOE+	ICAM1	IL2RG	2,70667E-03	13161,7	7,51071E-03	15637	4,85540E-01	8490	1,31152E+00	7821	8,72466E-01	16330	0	17530,5
Non-responder	pDC_LILRA4	Macro_FOLR2-APOE+	GAS6	AXL	2,70850E-03	9365,7	2,29267E-02	2387	8,85226E-01	1811	1,84642E+00	2836	8,47876E-01	22264	0	17530,5
Non-responder	Macro_FOLR2+APOE+	Macro_IER3	PLAU	LRP1	2,71146E-03	12555,1	1,39966E-02	5644	5,10231E-01	7666	1,35288E+00	7262	8,37864E-01	24673	0	17530,5
Non-responder	Macro_FOLR2+APOE+	cDC_CLEC9A	TGM2	ITGA4	2,71561E-03	14463,7	1,30525E-02	6355	5,10127E-01	7669	1,34355E+00	7364	8,01992E-01	33400	0	17530,5
Non-responder	Macro_FOLR2+APOE+	pDC_LILRA4	MDK	SORL1	2,71693E-03	16936,3	2,31029E-02	2352	4,48277E-01	9896	1,84614E+00	2839	7,17844E-01	52064	0	17530,5
Non-responder	Macro_IER3	Macro_FOLR2+APOE+	TNFSF13B	TFRC	2,71995E-03	12750,7	8,30136E-03	13281	4,76258E-01	8844	1,31604E+00	7752	8,72405E-01	16346	0	17530,5
Non-responder	Macro_FOLR2+APOE+	Macro_FOLR2+APOE+	ADAM10	IL6R	2,72046E-03	15659,7	1,16109E-02	7740	4,79473E-01	8724	1,20090E+00	9443	7,95768E-01	34861	0	17530,5
Non-responder	Mono_INHBA	cDC_CLEC9A	INHBA	SMAD3	2,72538E-03	16178,3	5,80771E-02	492	9,51348E-01	1437	1,84598E+00	2842	6,79738E-01	58590	0	17530,5
Non-responder	Macro_OLFML3	pDC_LILRA4	HLA-DMA	CD4	2,72661E-03	9889,7	7,30146E-03	16354	4,54732E-01	9633	1,86825E+00	2725	9,28951E-01	3206	0	17530,5
Non-responder	cDC_LAMP3	Macro_ISG15	HLA-A	LILRB1	2,72995E-03	12682,5	7,30004E-03	16358	3,62260E-01	13935	1,01427E+00	12868	9,31158E-01	2721	0	17530,5
Non-responder	Macro_FOLR2+APOE+	Mono_INHBA	LRPAP1	LRP1	2,73079E-03	14663,5	7,84132E-03	14584	4,00983E-01	11937	1,01238E+00	12907	8,72365E-01	16359	0	17530,5
Non-responder	Macro_FOLR2+APOE+	Mono_CD16	BSG	SLC16A7	2,73087E-03	15934,5	2,31997E-02	2333	5,87793E-01	5563	1,32170E+00	7680	7,44829E-01	46566	0	17530,5
Non-responder	Macro_FOLR2+APOE+	Macro_IER3	CXCL10	TLR4	2,73087E-03	14004,1	1,16680E-02	7680	5,23771E-01	7226	1,67603E+00	3968	8,01135E-01	33616	0	17530,5
Non-responder	Mono_CD16	Macro_LYVE1	VCL	ITGB5	2,73226E-03	17206,1	3,79432E-02	1052	6,32999E-01	4669	1,32152E+00	7681	7,01251E-01	55098	0	17530,5
Non-responder	Mono_INHBA	Macro_FOLR2+APOE+	ADM	GPR84	2,73667E-03	11353,1	3,56982E-02	1165	8,71880E-01	1901	1,84549E+00	2846	8,02313E-01	33323	0	17530,5

Post_NR_Myeloid_Myeloid_CellCell

Non-responder	Macro_FOLR2+APOE+	Macro_IFI27	C1QB	C1QBP	2,74061E-03	12165,3	6,16710E-03	21411	5,42717E-01	6694	1,33365E+00	7504	9,07065E-01	7687	0	17530,5
Non-responder	Mono_CD16	Macro_ISG15	PTPN6	CD300LF	2,74619E-03	10438,1	1,44383E-02	5343	6,15436E-01	5010	1,91128E+00	2504	8,49755E-01	21803	0	17530,5
Non-responder	cDC(CD1C)	Macro_LYVE1	HLA-DQA2	CD4	2,74752E-03	10735,3	8,93463E-03	11764	5,93917E-01	5444	9,47560E-01	14332	9,21698E-01	4606	0	17530,5
Non-responder	Macro_OLFML3	Macro_FOLR2+APOE+	ANXA2	TLR2	2,74752E-03	13670,9	7,29470E-03	16379	3,49055E-01	14694	1,09419E+00	11264	9,03640E-01	8487	0	17530,5
Non-responder	Macro_IER3	pDC_LILRA4	CXCL10	CXCR3	2,74800E-03	11917,9	2,08495E-02	2850	1,11229E+00	724	2,20655E+00	1356	7,85968E-01	37129	0	17530,5
Non-responder	Macro_IER3	Macro_ISG15	HLA-DQA2	LAG3	2,75367E-03	9340,3	2,14179E-02	2706	9,05645E-01	1699	1,84524E+00	2852	8,49333E-01	21914	0	17530,5
Non-responder	Macro_OLFML3	Macro_ISG15	HLA-B	LILRB2	2,75508E-03	8311,7	7,29202E-03	16388	7,03450E-01	3591	1,71281E+00	3693	9,47942E-01	356	0	17530,5
Non-responder	pDC_LILRA4	Macro_FOLR2-APOE+	HSP90B1	TLR2	2,75598E-03	6974,5	1,16481E-02	7698	9,50775E-01	1439	2,02106E+00	2001	9,13794E-01	6204	0	17530,5
Non-responder	Macro_FOLR2+APOE+	Macro_LYVE1	HLA-DMA	CD4	2,75676E-03	10517,5	7,29122E-03	16390	5,18404E-01	7379	1,29305E+00	8075	9,28905E-01	3213	0	17530,5
Non-responder	Macro_FOLR2+APOE+	cDC_LAMP3	C1QB	C1QBP	2,75737E-03	11778,7	6,16252E-03	21434	5,42524E-01	6700	1,50067E+00	5530	9,07034E-01	7699	0	17530,5
Non-responder	Macro_FOLR2+APOE+	Mono_CD16	ANXA1	FPR1	2,75928E-03	12489,1	7,29080E-03	16393	3,70270E-01	13498	1,27665E+00	8312	9,11561E-01	6712	0	17530,5
Non-responder	cDC_LAMP3	Mono_CD16	B2M	LILRB1	2,76433E-03	10538,3	7,28904E-03	16399	5,10898E-01	7639	1,15362E+00	10244	9,41727E-01	879	0	17530,5
Non-responder	Macro_NLRP3	Macro_FOLR2+APOE-	VEGFA	NRP1	2,76438E-03	14116,3	1,32531E-02	6167	7,84285E-01	2691	1,31999E+00	7704	7,88633E-01	36489	0	17530,5
Non-responder	Macro_OLFML3	Macro_IER3	GNAI2	C5AR1	2,76518E-03	12922,1	7,28846E-03	16400	4,56431E-01	9565	1,01898E+00	12763	9,04265E-01	8352	0	17530,5
Non-responder	Macro_OLFML3	Macro_ISG15	HLA-C	LILRA1	2,76602E-03	14923,9	7,28844E-03	16401	3,21871E-01	16296	1,06560E+00	11818	8,86893E-01	12574	0	17530,5
Non-responder	Macro_IFI27	pDC_LILRA4	HMGB1	CXCR4	2,76855E-03	9715,7	7,28799E-03	16404	5,04149E-01	7834	1,67544E+00	3974	9,30592E-01	2836	0	17530,5
Non-responder	Macro_FOLR2+APOE+	Macro_FOLR2+APOE+	SPP1	ITGA9_ITGB1	2,77140E-03	11131,1	1,81287E-02	3618	5,08710E-01	7709	2,33168E+00	1062	8,33339E-01	25736	0	17530,5
Non-responder	Mono_INHBA	Macro_NLRP3	FN1	CD44	2,77359E-03	6984,9	2,08252E-02	2859	7,88755E-01	2652	1,94785E+00	2321	8,98728E-01	9562	0	17530,5
Non-responder	Macro_FOLR2+APOE+	Macro_ISG15	GNAI2	C5AR1	2,77446E-03	11212,7	7,28730E-03	16411	4,55942E-01	9585	1,65217E+00	4183	9,04259E-01	8354	0	17530,5
Non-responder	Macro_FOLR2+APOE+	Mono_INHBA	TGM2	SDC4	2,77562E-03	16657,1	2,41951E-02	2193	6,37881E-01	4581	1,31948E+00	7712	7,22150E-01	51269	0	17530,5
Non-responder	Macro_FOLR2+APOE+	cDC(CD1C)	ICAM1	IL2RG	2,77615E-03	13838,7	7,47399E-03	15765	4,89456E-01	8341	1,10135E+00	11144	8,72193E-01	16413	0	17530,5
Non-responder	cDC(CD1C)	Macro_LYVE1	HLA-DQA1	CD4	2,77700E-03	11450,1	7,28675E-03	16414	5,58324E-01	6270	9,18133E-01	14974	9,34540E-01	2062	0	17530,5
Non-responder	cDC(CD1C)	Mono_CD16	GRN	TNFRSF1B	2,77785E-03	10514,3	7,28649E-03	16415	5,53140E-01	6411	1,15540E+00	10209	9,34768E-01	2006	0	17530,5
Non-responder	Mono_CD16	cDC_CLEC9A	TNFSF13B	CD40	2,78377E-03	11956,5	8,07880E-03	13902	5,21689E-01	7273	1,59373E+00	4655	8,72174E-01	16422	0	17530,5
Non-responder	Macro_LYVE1	Mono_CD14	C1QA	CD93	2,78548E-03	8267,3	1,24390E-02	6901	7,15660E-01	3440	1,31905E+00	7719	9,15986E-01	5746	0	17530,5
Non-responder	pDC_LILRA4	cDC_CLEC9A	APP	PTGER2	2,78796E-03	12191,5	1,58194E-02	4577	1,15863E+00	553	2,81763E+00	401	7,82705E-01	37896	0	17530,5
Non-responder	Macro_NLRP3	Macro_FOLR2+APOE+	CD14	ITGB1	2,78971E-03	12671,3	7,28212E-03	16429	3,86502E-01	12640	1,32084E+00	7693	9,00972E-01	9064	0	17530,5
Non-responder	Macro_NLRP3	Mono_CD16	B2M	LILRB2	2,79736E-03	10274,9	7,27904E-03	16438	4,47160E-01	9932	1,34268E+00	7380	9,53318E-01	94	0	17530,5
Non-responder	Mast	Macro_LYVE1	CSF1	SIRPA	2,79798E-03	9347,7	5,46174E-02	554	1,17515E+00	510	1,62008E+00	4422	8,41799E-01	23722	0	17530,5
Non-responder	Macro_FOLR2+APOE+	Mono_CD16	GNAI2	C5AR1	2,80332E-03	11216,1	7,27552E-03	16445	4,54744E-01	9631	1,66089E+00	4105	9,04189E-01	8369	0	17530,5
Non-responder	Macro_FOLR2-APOE+	cDC_LAMP3	PDGFB	S1PR1	2,80801E-03	18281,3	5,73829E-02	512	1,15816E+00	555	1,08004E+00	11529	6,60290E-01	61280	0	17530,5
Non-responder	pDC_LILRA4	Macro_IFI27	GAS6	AXL	2,81083E-03	9732,3	2,07803E-02	2872	8,50337E-01	2086	1,93763E+00	2371	8,41428E-01	23802	0	17530,5
Non-responder	Macro_FOLR2+APOE+	cDC_CLEC9A	CXCL12	ITGA4	2,81237E-03	14457,5	1,46980E-02	5177	5,07694E-01	7738	1,75599E+00	3376	7,80277E-01	38466	0	17530,5
Non-responder	Macro_ISG15	Macro_FOLR2+APOE+	ADAM10	GPNMB	2,81371E-03	7952,7	2,20047E-02	2574	7,64855E-01	2873	2,09467E+00	1723	8,77493E-01	15063	0	17530,5
Non-responder	Mono_INHBA	pDC_LILRA4	FN1	DPP4	2,81659E-03	13811,7	7,70617E-02	248	7,64663E-01	2874	1,88874E+00	2610	7,48175E-01	45796	0	17530,5
Non-responder	Macro_IER3	cDC_CLEC9A	CD14	ITGA4	2,81699E-03	13238,9	7,27008E-03	16461	4,37152E-01	10319	1,02163E+00	12710	9,00501E-01	9174	0	17530,5
Non-responder	Macro_FOLR2+APOE+	Macro_LYVE1	LGALS3BP	CD33	2,81805E-03	12224,5	1,39002E-02	5716	5,71477E-01	5944	1,31714E+00	7742	8,39871E-01	24190	0	17530,5
Non-responder	Macro_IFI27	Macro_FOLR2+APOE+	HLA-DPA1	CD4	2,82212E-03	10709,1	8,05139E-03	13974	5,99635E-01	5315	8,56012E-01	16467	9,49566E-01	259	0	17530,5
Non-responder	cDC(CD1C)	Macro_FOLR2+APOE+	HLA-DRB5	LAG3	2,82470E-03	9618,9	1,28063E-02	6571	7,29113E-01	3257	1,64114E+00	4266	8,71991E-01	16470	0	17530,5
Non-responder	pDC_LILRA4	Mono_INHBA	PROC	ITGAM	2,82524E-03	14127,1	4,71215E-02	725	7,68982E-01	2828	1,84052E+00	2877	7,44368E-01	46675	0	17530,5
Non-responder	pDC_LILRA4	Macro_FOLR2+APOE+	B2M	TFRC	2,82524E-03	6691,9	8,98720E-03	11657	7,64301E-01	2877	2,74027E+00	469	9,41350E-01	926	0	17530,5
Non-responder	Macro_NLRP3	Macro_IER3	S100A9	CD36	2,82555E-03	11468,3	9,00147E-03	11625	7,48668E-01	3040	1,25204E+00	8675	8,71991E-01	16471	0	17530,5
Non-responder	Macro_NLRP3	Mono_CD16	HLA-A	LILRB2	2,82555E-03	9695,1	7,26791E-03	16471	5,05761E-01	7793	1,44622E+00	6082	9,44648E-01	599	0	17530,5
Non-responder	Macro_OLFML3	Macro_FOLR2+APOE+	LGALS9	MRC2	2,82660E-03	13027,1	1,78798E-02	3706	5,88081E-01	5556	1,35153E+00	7284	8,11802E-01	31059	0	17530,5
Non-responder	Macro_IER3	Mono_CD16	F13A1	ITGA4	2,82813E-03	10631,5	1,22997E-02	7053	5,93741E-01	5452	1,40042E+00	6648	8,71980E-01	16474	0	17530,5
Non-responder	pDC_LILRA4	Mono_INHBA	APP	TNFRSF21	2,82813E-03	8886,7	1,99939E-02	3066	1,15714E+00	557	2,68881E+00	515	8,45791E-01	22765	0	17530,5
Non-responder	Macro_FOLR2-APOE+	Macro_FOLR2+APOE+	LILRB4	LAIR1	2,83088E-03	7945,7	1,17698E-02	7569	8,13887E-01	2414	1,31623E+00	7751	9,22475E-01	4464	0	17530,5
Non-responder	Macro_FOLR2+APOE+	Macro_FOLR2+APOE+	ANOS1	SDC2	2,83391E-03	20617,1	5,35401E-02	576	7,64102E-01	2880	1,17763E+00	9837	5,12469E-01	72262	0	17530,5
Non-responder	Mono_CD16	Macro_NLRP3	ICAM4	ITGB1	2,83659E-03	15847,1	2,21022E-02	2558	5,73415E-01	5904	1,31589E+00	7755	7,49739E-01	45488	0	17530,5
Non-responder	pDC_LILRA4	Macro_IER3	HSP90B1	TLR2	2,83659E-03	6747,5	1,15979E-02	7755	9,48854E-01	1449	2,49628E+00	772	9,13624E-01	6231	0	17530,5
Non-responder	cDC_CLEC9A	Macro_ISG15	HLA-DRB5	LAG3	2,83680E-03	6599,9	2,07548E-02	2881	9,98570E-01	1212	2,22974E+00	1294	8,96609E-01	10082	0	17530,5
Non-responder	pDC_LILRA4	cDC(CD1C)	SELPLG	ITGB2	2,83680E-03	6564,1	1,13380E-02	8041	7,64097E-01	2881	2,14175E+00	1540	9,30619E-01	2828	0	17530,5
Non-responder	Mono_CD14	Macro_ISG15	THBS1	SDC4	2,83970E-03	11631,7	2,07449E-02	2882	7,70250E-01	2817	1,87783E+00	2677	8,06713E-01	32252	0	17530,5
Non-responder	Macro_OLFML3	Macro_FOLR2+APOE+	HSP90B1	LRP1	2,84534E-03	14187,1	7,26193E-03	16494	3,45177E-01	14906	1,00911E+00	12963	9,01074E-01	9042	0	17530,5
Non-responder	pDC_LILRA4	Mono_INHBA	APP	PTGER2	2,84832E-03	10963,7	2,01414E-02	3023	1,19959E+00	442	2,64493E+00	559	8,02544E-01	33264	0	17530,5
Non-responder	Macro_OLFML3	Macro_FOLR2+APOE+	HLA-B	LILRB2	2,84948E-03	9663,9	6,52972E-03	19612	6,10196E-01	5090	1,50139E+00	5524	9,45149E-01	563	0	17530,5
Non-responder	cDC(CD1C)	Macro_LYVE1	HLA-DQB1	CD4	2,85138E-03	11099,5	7,25965E-03	16501	5,65430E-01	6088	9,69551E-01	13800	9,36855E-01	1578	0	17530,5
Non-responder	cDC_CLEC9A	Mono_CD16	B2M	LILRB2	2,85421E-03	7083,9	8,35968E-03	13130	7,63095E-01	2887	2,06721E+00	1832	9,56303E-01	40	0	17530,5
Non-responder	Macro_OLFML3	Macro_FOLR2+APOE+	HLA-DQA2	LAG3	2,85665E-03	12494,1	1,34356E-02	6044	5,94196E-01	5439	1,71634E+00	3657	8,17010E-01	29800	0	17530,5
Non-responder	Macro_FOLR2+APOE+	Macro_LYVE1	HLA-DRB1	CD4	2,85809E-03	10913,1	6,27566E-03	20854	5,08482E-01	7719	1,31497E+00	7770	9,43577E-01	692	0	17530,5
Non-responder	Macro_IER3	Macro_FOLR2+APOE+	CD14	ITGB2	2,85830E-03	9812,9	7,25801E-03	16509	5,48335E-01	6538	1,33412E+00	7500	9,40840E-01	987	0	17530,5
Non-responder	Macro_FOLR2+APOE+	Macro_OLFML3	ICAM1	IL2RG	2,85916E-03	13088,5	7,43044E-03	15901	4,86809E-01	8438	1,36550E+00	7063	8,71867E-01	16510	0	17530,5
Non-responder	cDC_LAMP3	Macro_NLRP3	PKM	CD44	2,85916E-03	10235,5	7,25767E-03	16510	6,23387E-01	4861	1,14726E+00	10337	9,35106E-01	1939	0	17530,5
Non-responder	Macro_OLFML3	Macro_FOLR2+APOE+	HLA-DQA1	LAG3	2,85953E-03	10456,7	1,27831E-02	6590	6,72873E-01	4021	1,76182E+00	3346	8,53990E-01	20796	0	17530,5
Non-responder	Macro_NLRP3	Macro_FOLR2+APOE-	S100A8	ITGB2	2,86241E-03	10302,5	1,15885E-02	7773	7,72215E-01	2794	6,26343E-01	22810	9,44618E-01	605	0	17530,5
Non-responder	Macro_FOLR2+APOE+	Mono_INHBA	FN1	CD44	2,86263E-03	11165,7	1,22402E-02	7111	5,40576E-01	6751	1,30442E+00	7922	8,71854E-01	16514	0	17530,5
Non-responder	pDC_LILRA4	Macro_FOLR2+APOE+	IL16	CD9	2,86293E-03	6597,9	2,12695E-02	2744	7,62860E-01	2890	2,65161E+00	553	9,00024E-01	9272	0	17530,5
Non-responder	Macro_OLFML3	Macro_FOLR2+APOE+	HLA-DQB1	LAG3	2,86528E-03	10294,7	1,26989E-02	6655	6,68204E-01	4086	1,73867E+00	3487	8,58548E-01	19715	0	17530,5
Non-responder	Mono_CD14	Mono_CD16	S100A8	TLR4	2,86858E-03	6354,5	1,97204E-02	3141	1,16598E+00	538	2,64450E+00	561	8,96933E-01	10002	0	17530,5
Non-responder	Macro_FOLR2+APOE+	Macro_ISG15	LRPAP1	LDLR	2,87105E-03	15562,1	1,32030E-02	6218	5,46039E-01	6596	1,31438E+00	7779	7,75121E-01	39687	0	17530,5
Non-responder	Macro_OLFML3	Macro_FOLR2+APOE+	HLA-A	LILRB2	2,87105E-03	9277,1	6,80644E-03	18348	6,27333E-01	4780	1,55612E+00	4974	9,42908E-01	753	0	17530,5

Post_NR_Myeloid_Myeloid_CellCell

Non-responder	Macro_OLFML3	Macro_FOLR2+APOE+	HLA-DPB1	LAG3	2,87538E-03	10246,1	1,18083E-02	7528	6,19640E-01	4926	1,63371E+00	4314	8,70173E-01	16932	0	17530,5
Non-responder	Macro_FOLR2+APOE+	Mono_CD14	S100A9	CD36	2,87565E-03	15050,7	8,96605E-03	11708	3,69359E-01	13545	8,76900E-01	15941	8,71771E-01	16529	0	17530,5
Non-responder	Macro_NLRP3	Mono_INHBA	CCL3L1	CCR1	2,87682E-03	12091,3	1,15821E-02	7783	7,83948E-01	2694	1,69830E+00	3797	8,21793E-01	28652	0	17530,5
Non-responder	pDC_LILRA4	Mast	HSPA8	ADRB2	2,87752E-03	6836,5	2,07005E-02	2895	8,62853E-01	1974	2,24189E+00	1254	8,94894E-01	10529	0	17530,5
Non-responder	pDC_LILRA4	Mono_CD14	SELPLG	SELL	2,88336E-03	6818,5	2,06885E-02	2897	9,29356E-01	1557	2,86284E+00	367	8,90026E-01	11741	0	17530,5
Non-responder	Macro_FOLR2+APOE+	Macro_NLRP3	GNAI2	C5AR1	2,88436E-03	11502,3	7,24945E-03	16539	4,52094E-01	9751	1,52785E+00	5280	9,04033E-01	8411	0	17530,5
Non-responder	Macro_NLRP3	Mono_INHBA	S100A8	CD36	2,88611E-03	11024,9	1,09781E-02	8513	7,81801E-01	2710	1,17805E+00	9830	8,71719E-01	16541	0	17530,5
Non-responder	Macro_FOLR2+APOE+	Macro_LYVE1	CXCL12	CD4	2,88695E-03	13602,3	1,49960E-02	5017	5,05854E-01	7790	1,38276E+00	6855	8,12785E-01	30819	0	17530,5
Non-responder	Macro_OLFML3	Macro_FOLR2+APOE+	ICAM1	IL2RA	2,88984E-03	14369,1	1,45186E-02	5290	5,78822E-01	5763	1,42539E+00	6343	7,86878E-01	36919	0	17530,5
Non-responder	cDC(CD1C)	Macro_NLRP3	VIM	CD44	2,89135E-03	9470,5	7,24844E-03	16547	6,59119E-01	4230	1,23156E+00	8950	9,53277E-01	95	0	17530,5
Non-responder	Macro_FOLR2+APOE+	Mono_INHBA	PLAU	IGF2R	2,89274E-03	17017,7	1,15737E-02	7794	5,11980E-01	7596	1,36692E+00	7048	7,51450E-01	45120	0	17530,5
Non-responder	Mono_CD16	Macro_IER3	SELPLG	ITGB2	2,89397E-03	12963,3	7,24816E-03	16550	4,03123E-01	11850	1,01428E+00	12866	9,14709E-01	6020	0	17530,5
Non-responder	cDC_CLEC9A	Macro_OLFML3	BTLA	TNFRSF14	2,89508E-03	9400,9	4,96769E-02	662	1,20626E+00	421	1,83690E+00	2901	8,34373E-01	25490	0	17530,5
Non-responder	Macro_FOLR2+APOE+	Mono_INHBA	MDK	SDC4	2,89855E-03	18906,9	3,98374E-02	969	6,52561E-01	4352	1,31279E+00	7798	6,38977E-01	63885	0	17530,5
Non-responder	pDC_LILRA4	Mono_CD16	APP	NCSTN	2,89910E-03	8706,1	1,86745E-02	3443	1,15514E+00	564	2,87251E+00	359	8,50451E-01	21634	0	17530,5
Non-responder	Macro_IFI27	cDC_CLEC9A	HLA-DRB5	LAG3	2,90360E-03	10633,5	1,68650E-02	4092	8,23758E-01	2333	8,52451E-01	16561	8,86586E-01	12651	0	17530,5
Non-responder	cDC(CD1C)	cDC_CLEC9A	HLA-DQA1	LAG3	2,90977E-03	7678,3	2,06530E-02	2906	1,02525E+00	1089	1,84325E+00	2860	8,81438E-01	14006	0	17530,5
Non-responder	Macro_OLFML3	Macro_FOLR2+APOE+	HLA-C	LILRB2	2,91018E-03	9880,1	6,68641E-03	18860	5,56461E-01	6320	1,45791E+00	5969	9,43189E-01	721	0	17530,5
Non-responder	Macro_OLFML3	Macro_NLRP3	GRN	TNFRSF1B	2,91062E-03	9859,5	7,24404E-03	16569	5,50447E-01	6492	1,39945E+00	6657	9,34589E-01	2049	0	17530,5
Non-responder	Macro_FOLR2+APOE+	Macro_ISG15	HMGB1	HAVCR2	2,91238E-03	12220,1	7,24379E-03	16571	3,81194E-01	12917	1,49081E+00	5634	9,03842E-01	8448	0	17530,5
Non-responder	Macro_NLRP3	Macro_FOLR2+APOE+	LAMB3	CD151	2,91310E-03	18040,3	2,73949E-02	1781	5,04971E-01	7808	1,32111E+00	7688	6,99628E-01	55394	0	17530,5
Non-responder	cDC(CD1C)	pDC_LILRA4	LGALS9	CD47	2,91413E-03	8549,9	9,81841E-03	10142	7,16141E-01	3432	2,16553E+00	1479	8,96298E-01	10166	0	17530,5
Non-responder	Macro_ISG15	Macro_OLFML3	ADM	GPR84	2,91566E-03	12541,7	2,69477E-02	1828	7,61240E-01	2908	2,09823E+00	1703	7,79063E-01	38739	0	17530,5
Non-responder	cDC(CD1C)	pDC_LILRA4	HLA-DQA2	CD4	2,92117E-03	7258,9	1,03055E-02	9406	6,85665E-01	3815	2,04996E+00	1889	9,26696E-01	3654	0	17530,5
Non-responder	cDC(CD1C)	pDC_LILRA4	HLA-DQA1	CD4	2,92205E-03	7636,5	8,40480E-03	13008	6,50073E-01	4396	2,02053E+00	2004	9,38773E-01	1244	0	17530,5
Non-responder	Macro_OLFML3	Macro_LYVE1	HLA-DMB	CD4	2,92205E-03	13458,1	7,24208E-03	16582	4,16352E-01	11213	9,34012E-01	14621	9,08556E-01	7344	0	17530,5
Non-responder	cDC(CD1C)	pDC_LILRA4	HLA-DQB1	CD4	2,92382E-03	7536,7	8,37355E-03	13094	6,57179E-01	4272	2,07195E+00	1814	9,40948E-01	973	0	17530,5
Non-responder	Macro_OLFML3	cDC(CD1C)	CXCL9	CXCR3	2,92451E-03	12420,1	2,06421E-02	2911	9,59445E-01	1400	2,35413E+00	1016	7,76973E-01	39243	0	17530,5
Non-responder	Macro_FOLR2+APOE+	Macro_OLFML3	SPP1	CD44	2,92558E-03	11653,7	1,08307E-02	8706	3,17482E-01	16586	1,87499E+00	2691	8,86193E-01	12755	0	17530,5
Non-responder	cDC(CD1C)	pDC_LILRA4	HLA-DPB1	CD4	2,92558E-03	7851,5	7,81588E-03	14664	6,35248E-01	4622	2,02938E+00	1972	9,46322E-01	469	0	17530,5
Non-responder	pDC_LILRA4	Macro_IER3	APP	LRP1	2,92746E-03	6499,5	2,06338E-02	2912	1,14544E+00	596	2,60579E+00	614	8,93550E-01	10845	0	17530,5
Non-responder	cDC(CD1C)	pDC_LILRA4	APP	RPSA	2,92823E-03	9964,5	8,23958E-03	13446	7,87221E-01	2667	1,91371E+00	2491	8,82700E-01	13688	0	17530,5
Non-responder	Mono_CD16	cDC_CLEC9A	VCAN	SELL	2,93209E-03	12541,7	1,17646E-02	7577	5,04462E-01	7821	1,33403E+00	7501	8,47811E-01	22279	0	17530,5
Non-responder	Mono_INHBA	Macro_FOLR2+APOE+	CCL2	CCR5	2,93633E-03	12899,1	3,92210E-02	997	7,60524E-01	2915	2,04951E+00	1894	7,68989E-01	41159	0	17530,5
Non-responder	Macro_OLFML3	Macro_FOLR2+APOE+	TNFSF10	TNFRSF10B	2,93649E-03	12407,3	1,57917E-02	4600	6,73631E-01	4007	1,35863E+00	7160	8,21407E-01	28739	0	17530,5
Non-responder	Macro_NLRP3	cDC_CLEC9A	TGFB1	LPP	2,93649E-03	11625,7	1,15484E-02	7824	5,25475E-01	7170	1,61914E+00	4431	8,52405E-01	21173	0	17530,5
Non-responder	Macro_OLFML3	Macro_FOLR2+APOE+	B2M	LILRB2	2,93942E-03	9887,1	6,43581E-03	20081	5,77631E-01	5789	1,47049E+00	5837	9,50501E-01	198	0	17530,5
Non-responder	Mono_CD16	Macro_FOLR2+APOE+	GNAI2	C5AR1	2,93972E-03	11561,5	7,23762E-03	16602	4,51771E-01	9772	1,50655E+00	5480	9,03962E-01	8423	0	17530,5
Non-responder	Macro_FOLR2-APOE+	cDC_LAMP3	FN1	TNFRSF11B	2,94005E-03	14476,3	1,26897E-01	80	1,15390E+00	568	1,19312E+00	9575	7,53413E-01	44628	0	17530,5
Non-responder	Macro_FOLR2+APOE+	Macro_FOLR2+APOE+	TIMP2	ITGB1	2,94089E-03	11111,3	1,15477E-02	7827	5,25134E-01	7180	1,79250E+00	3158	8,57909E-01	19861	0	17530,5
Non-responder	cDC(CD1C)	pDC_LILRA4	HLA-DPA1	CD4	2,94238E-03	8250,7	7,54070E-03	15521	5,92602E-01	5475	1,93741E+00	2372	9,47974E-01	355	0	17530,5
Non-responder	cDC(CD1C)	pDC_LILRA4	HLA-DMA	CD4	2,94415E-03	9451,5	7,63932E-03	15186	5,02103E-01	7909	1,70417E+00	3759	9,30430E-01	2873	0	17530,5
Non-responder	cDC(CD1C)	pDC_LILRA4	HLA-DMB	CD4	2,94503E-03	11087,7	7,42509E-03	15917	4,27018E-01	10753	1,66082E+00	4106	9,09587E-01	7132	0	17530,5
Non-responder	Macro_OLFML3	cDC_LAMP3	TNFSF13	TNFRSF11B	2,95033E-03	10282,9	7,41333E-02	280	1,15389E+00	569	1,64368E+00	4241	8,21149E-01	28794	0	17530,5
Non-responder	cDC(CD1C)	pDC_LILRA4	SELPLG	SELL	2,95213E-03	8351,9	1,46715E-02	5187	9,46549E-01	1466	2,29628E+00	1126	8,72046E-01	16450	0	17530,5
Non-responder	cDC(CD1C)	pDC_LILRA4	HLA-DRB5	CD4	2,95302E-03	8425,9	7,53925E-03	15530	5,80843E-01	5719	1,87243E+00	2704	9,44127E-01	646	0	17530,5
Non-responder	Macro_NLRP3	Mono_CD14	VEGFA	ITGB1	2,95412E-03	11437,7	1,15359E-02	7836	7,21676E-01	3353	1,53593E+00	5185	8,43589E-01	23284	0	17530,5
Non-responder	Mono_CD16	Mono_CD14	S100A9	TLR4	2,96103E-03	13551,1	9,74885E-03	10271	4,85159E-01	8504	9,24669E-01	14824	8,71360E-01	16626	0	17530,5
Non-responder	Macro_OLFML3	Mono_INHBA	PKM	CD44	2,96192E-03	10323,7	7,23008E-03	16627	6,17266E-01	4976	1,13663E+00	10519	9,34990E-01	1966	0	17530,5
Non-responder	Macro_ISG15	cDC_CLEC9A	CXCL2	DPP4	2,96306E-03	11432,7	4,84881E-02	692	8,65567E-01	1952	1,83393E+00	2924	7,99226E-01	34065	0	17530,5
Non-responder	cDC(CD1C)	pDC_LILRA4	TGFB1	CXCR4	2,96370E-03	9319,7	8,18725E-03	13589	5,84927E-01	5624	2,15546E+00	1506	9,04280E-01	8349	0	17530,5
Non-responder	cDC(CD1C)	cDC_CLEC9A	HLA-DQB1	LAG3	2,96603E-03	7408,5	2,05762E-02	2925	1,03236E+00	1059	1,89467E+00	2577	8,85400E-01	12951	0	17530,5
Non-responder	Macro_NLRP3	Macro_IER3	THBS1	CD36	2,96888E-03	12530,9	1,21002E-02	7232	7,22568E-01	3342	1,30999E+00	7846	8,29600E-01	26704	0	17530,5
Non-responder	Macro_OLFML3	Macro_FOLR2+APOE+	HLA-DRB5	LAG3	2,97036E-03	10624,5	1,15979E-02	7756	5,89922E-01	5522	1,61880E+00	4434	8,66357E-01	17880	0	17530,5
Non-responder	Macro_IER3	Macro_FOLR2+APOE+	HLA-DQA1	CD4	2,97084E-03	11501,3	7,22879E-03	16637	4,53055E-01	9707	1,08084E+00	11513	9,34295E-01	2119	0	17530,5
Non-responder	pDC_LILRA4	Macro_OLFML3	APP	NCSTN	2,97095E-03	8748,3	1,85397E-02	3488	1,15347E+00	571	2,80754E+00	412	8,49990E-01	21740	0	17530,5
Non-responder	Macro_ISG15	pDC_LILRA4	HLA-C	NOTCH4	2,97184E-03	7199,7	2,57649E-02	1980	5,84635E-01	5631	1,81905E+00	3009	9,06458E-01	7848	0	17530,5
Non-responder	Macro_FOLR2+APOE+	Mast	ADM	ADRB2	2,97332E-03	15034,9	2,60291E-02	1952	6,28040E-01	4766	1,30991E+00	7849	7,60293E-01	43077	0	17530,5
Non-responder	Macro_FOLR2+APOE+	Macro_NLRP3	HSP90B1	TLR4	2,97352E-03	12498,9	8,73862E-03	12199	4,43620E-01	10080	1,44911E+00	6045	8,71297E-01	16640	0	17530,5
Non-responder	Macro_IFI27	pDC_LILRA4	C3	CD46	2,97441E-03	13033,5	1,25375E-02	6801	4,45848E-01	9986	9,52357E-01	14209	8,71295E-01	16641	0	17530,5
Non-responder	Macro_OLFML3	Macro_FOLR2+APOE+	SEMA4A	NRP1_PLXNA1	2,97480E-03	15325,7	1,66114E-02	4192	5,86747E-01	5584	1,31397E+00	7781	7,67270E-01	41541	0	17530,5
Non-responder	Macro_IER3	Macro_FOLR2+APOE+	HMGB1	CD163	2,97709E-03	12523,7	7,22688E-03	16644	3,99483E-01	12005	1,07405E+00	11648	9,20608E-01	4791	0	17530,5
Non-responder	Macro_ISG15	Mono_INHBA	HBEGF	CD44	2,97776E-03	7547,1	1,15156E-02	7852	7,87653E-01	2662	1,66339E+00	4085	9,16654E-01	5606	0	17530,5
Non-responder	Macro_OLFML3	Macro_FOLR2+APOE+	SEMA4A	PLXND1	2,97924E-03	13594,5	1,39021E-02	5715	5,88406E-01	5549	1,35856E+00	7163	8,07752E-01	32015	0	17530,5
Non-responder	Macro_FOLR2+APOE+	Macro_FOLR2+APOE+	NAMPT	ITGA5_ITGB1	2,98067E-03	13914,9	7,22581E-03	16648	4,21246E-01	10996	1,26174E+00	8523	8,74264E-01	15877	0	17530,5
Non-responder	Macro_FOLR2+APOE+	Macro_FOLR2+APOE+	PSAP	SORT1	2,98157E-03	12472,3	7,22567E-03	16649	5,19216E-01	7358	1,58269E+00	4751	8,73540E-01	16073	0	17530,5
Non-responder	Macro_NLRP3	Mono_CD16	NAMPT	ITGA5_ITGB1	2,98246E-03	12271,3	7,22557E-03	16650	6,20859E-01	4904	1,42136E+00	6394	8,74262E-01	15878	0	17530,5
Non-responder	Macro_OLFML3	Macro_FOLR2+APOE+	TNFSF13B	HLA-DPB1	2,98517E-03	8617,7	7,17946E-03	16807	6,24799E-01	4833	1,74025E+00	3479	9,46700E-01	439	0	17530,5
Non-responder	Macro_LYVE1	Macro_ISG15	HLA-DRA	LAG3	2,98666E-03	7055,1	2,00062E-02	3061	1,00844E+00	1169	1,48829E+00	5657	9,06414E-01	7858	0	17530,5
Non-responder	pDC_LILRA4	Macro_LYVE1	SEMA3C	NRP1_NRP2_PLXND1	2,98694E-03	13503,7	4,14692E-02	912	7,59207E-01	2932	1,83313E+00	2931	7,59680E-01	43213	0	17530,5
Non-responder	cDC_CLEC9A	Mono_CD16	SEMA4A	LILRB2	2,98694E-03	6286,9	2,05503E-02	2932	1,12373E+00	675	2,52292E+00	732	8,98711E-01	9565	0	17530,5

Post_NR_Myeloid_Myeloid_CellCell

Non-responder	cDC(CD1C)	pDC_LILRA4	HMGB1	THBD	2,98784E-03	9502,5	9,70462E-03	10327	5,41327E-01	6725	1,78576E+00	3195	8,98037E-01	9735	0	17530,5
Non-responder	Macro_NLRP3	Macro_IER3	ICAM1	ITGAL_ITGB2	2,98874E-03	12968,9	7,22414E-03	16657	4,91738E-01	8267	1,03602E+00	12427	8,97087E-01	9963	0	17530,5
Non-responder	pDC_LILRA4	Mono_CD16	B2M	LILRB1	2,98994E-03	7197,7	8,01274E-03	14101	7,59201E-01	2933	2,48142E+00	789	9,44271E-01	635	0	17530,5
Non-responder	Macro_FOLR2+APOE+	cDC(CD1C)	MMP9	CD44	2,99409E-03	11482,9	1,15086E-02	7863	5,32927E-01	6959	1,57885E+00	4772	8,56093E-01	20290	0	17530,5
Non-responder	Macro_FOLR2+APOE+	pDC_LILRA4	C3	IFITM1	2,99412E-03	11330,3	1,01786E-02	9593	3,98843E-01	12037	2,45978E+00	828	8,71197E-01	16663	0	17530,5
Non-responder	Mono_CD16	Macro_OLFML3	TIMP1	CD63	2,99772E-03	10104,7	7,22068E-03	16667	6,10054E-01	5094	1,13070E+00	10614	9,44408E-01	618	0	17530,5
Non-responder	pDC_LILRA4	Macro_ISG15	VEGFB	NRP1	2,99893E-03	11670,9	3,02346E-02	1517	7,58824E-01	2936	2,46271E+00	821	7,92811E-01	35550	0	17530,5
Non-responder	cDC(CD1C)	pDC_LILRA4	C1QB	C1QBP	3,00132E-03	9475,7	9,28267E-03	11053	4,76403E-01	8833	1,49529E+00	5578	9,22925E-01	4384	0	17530,5
Non-responder	Macro_FOLR2+APOE+	Mono_INHBA	PLAU	LRP1	3,00153E-03	12606,9	1,37635E-02	5813	5,03256E-01	7868	1,38128E+00	6880	8,36719E-01	24943	0	17530,5
Non-responder	Macro_FOLR2+APOE+	Mono_CD14	C3	CR1	3,00302E-03	14114,5	2,38586E-02	2238	5,03249E-01	7869	1,51084E+00	5438	7,84349E-01	37497	0	17530,5
Non-responder	cDC(CD1C)	pDC_LILRA4	LYZ	ITGAL	3,00672E-03	13020,9	7,39920E-03	16011	3,80925E-01	12935	1,31649E+00	7748	8,93442E-01	10880	0	17530,5
Non-responder	Macro_IER3	Macro_FOLR2+APOE+	HLA-DPB1	CD4	3,00943E-03	11178,9	7,21795E-03	16680	4,53483E-01	9685	1,08872E+00	11363	9,44264E-01	636	0	17530,5
Non-responder	cDC(CD1C)	pDC_LILRA4	HSP90B1	TLR9	3,01033E-03	8666,3	5,45939E-02	555	9,35134E-01	1528	1,36300E+00	7090	8,71358E-01	16628	0	17530,5
Non-responder	Mono_CD16	Macro_OLFML3	S100A4	CCR5	3,01048E-03	10782,1	1,35531E-02	5958	6,26648E-01	4795	1,30824E+00	7874	8,66877E-01	17753	0	17530,5
Non-responder	cDC_CLEC9A	Macro_FOLR2+APOE+	ICAM3	ITGAL	3,01095E-03	9773,7	2,31693E-02	2340	9,96078E-01	1224	1,83190E+00	2940	8,37236E-01	24834	0	17530,5
Non-responder	cDC(CD1C)	pDC_LILRA4	HSP90B1	TLR7	3,01123E-03	10592,5	1,54107E-02	4783	5,36999E-01	6844	1,32631E+00	7600	8,72985E-01	16205	0	17530,5
Non-responder	Macro_OLFML3	Macro_FOLR2+APOE+	CD1D	LILRB2	3,01347E-03	12481,1	1,15626E-02	7809	5,56626E-01	6313	1,30816E+00	7876	8,45314E-01	22877	0	17530,5
Non-responder	cDC(CD1C)	pDC_LILRA4	HMGB1	TLR9	3,01394E-03	6949,5	6,61218E-02	377	1,07551E+00	877	1,76765E+00	3308	8,86581E-01	12655	0	17530,5
Non-responder	Mono_INHBA	Macro_FOLR2+APOE+	VEGFA	NRP2	3,01396E-03	8791,9	3,19946E-02	1391	1,05779E+00	942	1,83176E+00	2941	8,52540E-01	21155	0	17530,5
Non-responder	Macro_ISG15	Macro_ISG15	B2M	LILRB2	3,01396E-03	7700,7	7,43329E-03	15894	7,57764E-01	2941	2,00935E+00	2051	9,53782E-01	87	0	17530,5
Non-responder	cDC(CD1C)	pDC_LILRA4	HMGB1	CXCR4	3,01484E-03	6979,9	9,18161E-03	11269	6,95147E-01	3680	2,36487E+00	998	9,37690E-01	1422	0	17530,5
Non-responder	Macro_FOLR2+APOE+	Macro_FOLR2+APOE+	TNFSF13	SDC2	3,01646E-03	13261,5	1,28846E-02	6492	5,03042E-01	7878	1,69107E+00	3844	8,13833E-01	30563	0	17530,5
Non-responder	Mono_CD16	Macro_OLFML3	LTB	CD40	3,02095E-03	12787,3	1,45659E-02	5258	6,09928E-01	5099	1,30782E+00	7881	8,23739E-01	28168	0	17530,5
Non-responder	Macro_FOLR2+APOE+	Macro_FOLR2-APOE+	RARRES1	NRP2	3,02300E-03	18641,3	3,09668E-02	1460	8,18052E-01	2387	8,90370E-01	15619	6,94737E-01	56210	0	17530,5
Non-responder	Mono_CD16	Mono_CD14	GNAI2	C5AR1	3,02389E-03	11958,1	7,21320E-03	16696	4,49369E-01	9854	1,35338E+00	7255	9,03815E-01	8455	0	17530,5
Non-responder	Macro_IER3	cDC(CD1C)	IL1B	IL1R2_IL1RAP	3,02570E-03	9854,1	2,16400E-02	2658	7,23428E-01	3330	1,22477E+00	9054	8,71058E-01	16698	0	17530,5
Non-responder	cDC_LAMP3	Macro_NLRP3	VIM	CD44	3,02751E-03	9619,3	7,21144E-03	16700	6,50392E-01	4391	1,20464E+00	9378	9,53163E-01	97	0	17530,5
Non-responder	pDC_LILRA4	Macro_OLFML3	PROC	PROCR	3,02904E-03	18668,3	4,46177E-02	792	7,57280E-01	2946	1,94093E+00	2353	5,71530E-01	69720	0	17530,5
Non-responder	Macro_ISG15	pDC_LILRA4	HRAS	TLR9	3,03509E-03	17847,5	6,88408E-02	335	9,95587E-01	1227	1,83017E+00	2948	6,05990E-01	67197	0	17530,5
Non-responder	Macro_OLFML3	Macro_FOLR2+APOE+	S100A8	CD68	3,03595E-03	11670,7	5,96156E-03	22605	5,17093E-01	7428	1,31272E+00	7799	9,29964E-01	2991	0	17530,5
Non-responder	Mono_CD14	Macro_NLRP3	HBEGF	CD44	3,03746E-03	7089,9	1,14803E-02	7892	8,23624E-01	2335	2,00565E+00	2063	9,16537E-01	5629	0	17530,5
Non-responder	Macro_NLRP3	Macro_ISG15	TNF	FAS	3,03896E-03	18470,9	1,37389E-02	5831	5,02648E-01	7893	1,37348E+00	6969	7,06846E-01	54131	0	17530,5
Non-responder	cDC(CD1C)	Macro_ISG15	CD1D	LILRB2	3,04114E-03	7983,3	2,04826E-02	2950	8,42396E-01	2154	1,88332E+00	2647	8,79130E-01	14635	0	17530,5
Non-responder	Macro_OLFML3	Macro_FOLR2+APOE+	S100A9	CD68	3,04347E-03	10448,1	6,16508E-03	21423	5,44536E-01	6641	1,53951E+00	5140	9,37298E-01	1506	0	17530,5
Non-responder	Macro_IER3	Macro_FOLR2+APOE+	CD14	TLR1	3,04660E-03	12356,7	1,12169E-02	8177	5,07506E-01	7742	1,07569E+00	11613	8,70979E-01	16721	0	17530,5
Non-responder	Mono_INHBA	Mono_CD14	INHBA	ACTR2	3,04720E-03	9513,3	2,35789E-02	2281	8,09308E-01	2462	1,82881E+00	2952	8,47522E-01	22341	0	17530,5
Non-responder	Macro_IFI27	Macro_FOLR2+APOE+	HLA-DPB1	LAG3	3,04842E-03	12579,5	1,19775E-02	7365	6,38529E-01	4577	8,47022E-01	16702	8,70975E-01	16723	0	17530,5
Non-responder	Mono_CD16	cDC_CLEC9A	ANXA1	DYSF	3,04950E-03	12052,1	1,52317E-02	4870	5,65927E-01	6074	1,30658E+00	7900	8,41078E-01	23886	0	17530,5
Non-responder	Macro_FOLR2+APOE+	Macro_ISG15	PLAU	ST14	3,04950E-03	13829,5	1,42917E-02	5448	5,02342E-01	7900	1,66448E+00	4080	7,98603E-01	34189	0	17530,5
Non-responder	Mono_CD14	cDC(CD1C)	S100A12	CD36	3,05327E-03	11083,5	2,04558E-02	2954	1,14590E+00	595	2,01328E+00	2031	8,06492E-01	32307	0	17530,5
Non-responder	pDC_LILRA4	Macro_IER3	APP	NOTCH2	3,05411E-03	8292,7	1,68018E-02	4110	1,15132E+00	579	2,72544E+00	487	8,62593E-01	18757	0	17530,5
Non-responder	Macro_IFI27	Macro_FOLR2+APOE+	HLA-DRA	LAG3	3,05572E-03	12276,3	1,14251E-02	7950	6,29897E-01	4727	8,45876E-01	16731	8,79795E-01	14443	0	17530,5
Non-responder	Macro_NLRP3	cDC(CD1C)	NAMPT	INSR	3,05705E-03	12692,5	1,14710E-02	7905	7,03625E-01	3588	1,38251E+00	6859	8,26021E-01	27580	0	17530,5
Non-responder	Macro_IER3	Mono_CD16	C3	IFITM1	3,05846E-03	13865,1	1,01344E-02	9656	3,97741E-01	12096	9,91209E-01	13309	8,70953E-01	16734	0	17530,5
Non-responder	Macro_FOLR2+APOE+	Macro_IER3	LGALS3BP	CD33	3,05856E-03	12638,7	1,31571E-02	6260	5,53605E-01	6399	1,30596E+00	7906	8,36142E-01	25098	0	17530,5
Non-responder	Macro_IER3	pDC_LILRA4	SIRPA	CD47	3,05937E-03	9077,9	1,24204E-02	6920,5	7,66524E-01	2852,5	2,20758E+00	1350,5	8,70952E-01	16735,5	0	17530,5
Non-responder	Macro_OLFML3	Macro_LYVE1	CD59	STAB1	3,06303E-03	11267,7	1,00688E-02	9751	7,31116E-01	3241	1,22379E+00	9077	8,70926E-01	16739	0	17530,5
Non-responder	pDC_LILRA4	Macro_IER3	APP	FPR2	3,06459E-03	12152,7	1,54506E-02	4755	1,16118E+00	549	2,63090E+00	580	7,85053E-01	37349	0	17530,5
Non-responder	Macro_FOLR2+APOE+	Macro_FOLR2+APOE+	GAS6	AXL	3,06612E-03	13256,7	1,40429E-02	5610	5,02033E-01	7911	1,60077E+00	4587	8,13505E-01	30645	0	17530,5
Non-responder	Macro_IER3	Macro_ISG15	VEGFA	CD44	3,06669E-03	13551,3	8,38656E-03	13051	4,85578E-01	8487	1,06006E+00	11945	8,70909E-01	16743	0	17530,5
Non-responder	cDC_CLEC9A	Macro_ISG15	RPS19	C5AR1	3,06848E-03	7247,5	9,36347E-03	10916	7,56130E-01	2959	1,93299E+00	2394	9,32698E-01	2438	0	17530,5
Non-responder	cDC(CD1C)	Macro_FOLR2+APOE+	IL18	IL18BP	3,06944E-03	8701,1	2,08034E-02	2863	7,35053E-01	3198	1,79020E+00	3168	8,70888E-01	16746	0	17530,5
Non-responder	Macro_FOLR2+APOE+	Macro_NLRP3	HLA-B	LILRB1	3,07066E-03	13374,7	4,77662E-03	31275	5,01961E-01	7914	1,51255E+00	5420	9,20978E-01	4734	0	17530,5
Non-responder	Macro_NLRP3	Macro_FOLR2+APOE+	CCL2	CCR5	3,07218E-03	17311,5	2,23520E-02	2497	5,01953E-01	7915	1,45000E+00	6032	7,15340E-01	52583	0	17530,5
Non-responder	Macro_NLRP3	Macro_OLFML3	S100A8	CD36	3,07219E-03	10420,9	1,08117E-02	8722	7,78904E-01	2738	1,42394E+00	6365	8,70863E-01	16749	0	17530,5
Non-responder	Mast	Macro_FOLR2-APOE+	CSF1	CSF2RA	3,07508E-03	9886,3	5,33248E-02	581	1,15422E+00	567	1,07944E+00	11543	8,60701E-01	19210	0	17530,5
Non-responder	Macro_FOLR2+APOE+	Mono_CD16	LGALS3BP	ITGB1	3,07521E-03	10958,9	1,14570E-02	7917	5,56096E-01	6328	1,71375E+00	3680	8,60139E-01	19339	0	17530,5
Non-responder	Macro_FOLR2+APOE+	Macro_OLFML3	CD14	TLR6	3,07976E-03	13522,3	1,14552E-02	7920	5,67775E-01	6032	1,52919E+00	5266	8,12602E-01	30863	0	17530,5
Non-responder	Macro_LYVE1	Macro_FOLR2+APOE+	C1QB	LRP1	3,08128E-03	7484,3	1,33151E-02	6129	7,08248E-01	3544	1,30451E+00	7921	9,33447E-01	2297	0	17530,5
Non-responder	Macro_FOLR2+APOE+	Macro_ISG15	ADAM9	ITGB1	3,08280E-03	12885,7	1,28656E-02	6517	5,01815E-01	7922	1,46252E+00	5920	8,30202E-01	26539	0	17530,5
Non-responder	Macro_IFI27	cDC_CLEC9A	HLA-DQA1	LAG3	3,08873E-03	11407,9	1,72893E-02	3939	8,29899E-01	2283	8,44807E-01	16767	8,71829E-01	16520	0	17530,5
Non-responder	Mono_CD16	Macro_LYVE1	PSAP	LRP1	3,09058E-03	12890,3	7,18822E-03	16769	3,99856E-01	11992	9,10517E-01	15162	9,29939E-01	2998	0	17530,5
Non-responder	Mono_INHBA	Macro_NLRP3	HBEGF	CD44	3,09346E-03	7709,9	1,14466E-02	7929	8,21967E-01	2351	1,54534E+00	5089	9,16424E-01	5650	0	17530,5
Non-responder	Macro_OLFML3	Macro_ISG15	B2M	LILRB2	3,09611E-03	8473,7	7,18714E-03	16775	6,70885E-01	4049	1,68191E+00	3913	9,53034E-01	101	0	17530,5
Non-responder	Macro_IER3	pDC_LILRA4	COPA	P2RY6	3,09612E-03	8418,9	1,85416E-02	3487	1,14890E+00	583	2,82790E+00	397	8,56893E-01	20097	0	17530,5
Non-responder	pDC_LILRA4	cDC(CD1C)	APP	NCSTN	3,10666E-03	8873,5	1,81606E-02	3601	1,14877E+00	584	2,62995E+00	582	8,48668E-01	22070	0	17530,5
Non-responder	cDC_CLEC9A	Mono_CD16	RPS19	C5AR1	3,10825E-03	7247,7	9,34833E-03	10945	7,54933E-01	2972	1,94171E+00	2346	9,32647E-01	2445	0	17530,5
Non-responder	Macro_FOLR2+APOE+	Macro_ISG15	FN1	SDC2	3,10872E-03	15537,1	2,25846E-02	2454	5,01482E-01	7939	1,72915E+00	3557	7,46441E-01	46205	0	17530,5
Non-responder	Macro_IER3	Macro_OLFML3	CCL4	CCR1	3,10905E-03	9526,3	1,46376E-02	5216	7,23120E-01	3336	1,58069E+00	4760	8,70703E-01	16789	0	17530,5
Non-responder	Macro_OLFML3	cDC_CLEC9A	HMGB1	THBD	3,11090E-03	15055,7	7,18296E-03	16791	3,30734E-01	15760	1,07202E+00	11697	8,83413E-01	13500	0	17530,5
Non-responder	Macro_OLFML3	Macro_FOLR2+APOE+	IL15RA	AXL	3,11331E-03	13851,1	2,08158E-02	2860	5,97137E-01	5374	1,47406E+00	5797	7,83573E-01	37694	0	17530,5

Post_NR_Myeloid_Myeloid_CellCell

Non-responder	pDC_LILRA4	pDC_LILRA4	TNFSF13B	TNFRSF17	3,11748E-03	11519,3	5,78296E-02	499	7,54581E-01	2975	2,46884E+00	815	7,91792E-01	35777	0	17530,5
Non-responder	Macro_ISG15	Macro_FOLR2+APOE+	HBEGF	CD9	3,11791E-03	8239,5	1,25475E-02	6788	5,65179E-01	6097	1,84626E+00	2837	9,05982E-01	7945	0	17530,5
Non-responder	Macro_OLFML3	Macro_FOLR2+APOE+	HMGB1	CD163	3,12297E-03	11740,1	7,18004E-03	16804	3,94210E-01	12269	1,35395E+00	7244	9,20370E-01	4853	0	17530,5
Non-responder	pDC_LILRA4	cDC(CD1C)	PROC	PROCR	3,12364E-03	18639,3	4,81284E-02	700	7,67722E-01	2839	1,82377E+00	2977	5,80778E-01	69150	0	17530,5
Non-responder	Macro_NLRP3	Macro_FOLR2+APOE+	MIF	TNFRSF14	3,12389E-03	11818,9	7,17977E-03	16805	4,99796E-01	8000	1,34653E+00	7339	8,99324E-01	9420	0	17530,5
Non-responder	Macro_IER3	Macro_FOLR2+APOE+	SERPINA1	LRP1	3,12761E-03	13975,9	7,56055E-03	15453	3,73398E-01	13317	8,43316E-01	16809	9,11289E-01	6770	0	17530,5
Non-responder	Macro_NLRP3	Mast	VEGFA	ITGAV	3,13172E-03	13353,1	1,68660E-02	4090	8,12627E-01	2426	1,30184E+00	7954	7,96249E-01	34765	0	17530,5
Non-responder	Mono_CD14	Mono_CD14	S100A9	TLR4	3,13326E-03	6020,1	1,97127E-02	3143	1,14503E+00	598	2,43535E+00	874	9,05944E-01	7955	0	17530,5
Non-responder	Macro_FOLR2+APOE+	Macro_LYVE1	CD14	TLR4	3,13413E-03	11897,9	8,49376E-03	12780	5,51082E-01	6471	1,46581E+00	5892	8,70612E-01	16816	0	17530,5
Non-responder	Macro_OLFML3	Macro_FOLR2+APOE+	SEMA4D	CD72	3,13480E-03	13350,1	2,19932E-02	2576	7,26479E-01	3289	1,62772E+00	4364	7,78039E-01	38991	0	17530,5
Non-responder	Mono_CD16	Mono_CD16	HLA-B	LILRB1	3,13600E-03	10112,1	7,17738E-03	16818	4,84955E-01	8512	1,48890E+00	5647	9,34583E-01	2053	0	17530,5
Non-responder	pDC_LILRA4	Mono_CD14	APP	NCSTN	3,13841E-03	8870,7	1,80389E-02	3646	1,14726E+00	587	2,80785E+00	411	8,48236E-01	22179	0	17530,5
Non-responder	Macro_ISG15	Macro_ISG15	HLA-A	LILRB2	3,13907E-03	7595,5	7,73340E-03	14906	7,54054E-01	2982	2,00207E+00	2083	9,46249E-01	476	0	17530,5
Non-responder	Macro_FOLR2+APOE+	pDC_LILRA4	S100A9	CD36	3,13973E-03	13378,5	8,77949E-03	12099	3,58607E-01	14132	1,42829E+00	6309	8,70591E-01	16822	0	17530,5
Non-responder	Macro_OLFML3	Macro_FOLR2+APOE+	RPS19	C5AR1	3,14096E-03	11204,5	6,43881E-03	20071	5,11514E-01	7615	1,46838E+00	5854	9,19949E-01	4952	0	17530,5
Non-responder	Macro_FOLR2+APOE+	Mast	COPA	NTRK1	3,14217E-03	12803,5	9,42357E-02	159	1,11005E+00	736	1,17756E+00	9840	7,91921E-01	35752	0	17530,5
Non-responder	Macro_NLRP3	Macro_IFI27	IL1B	SIGIRR	3,14346E-03	10165,1	1,10160E-02	8455	8,92087E-01	1782	1,43370E+00	6232	8,70583E-01	16826	0	17530,5
Non-responder	Macro_FOLR2+APOE+	Macro_ISG15	CCL5	SDC4	3,15176E-03	19393,1	2,04418E-02	2959	5,00820E-01	7967	1,51584E+00	5387	6,45752E-01	63122	0	17530,5
Non-responder	Macro_FOLR2+APOE+	Macro_IER3	HLA-B	LILRB2	3,15176E-03	12938,7	4,79258E-03	31108	5,52156E-01	6442	1,30091E+00	7967	9,36558E-01	1646	0	17530,5
Non-responder	cDC(CD1C)	Mast	LGALS1	CD69	3,15188E-03	6508,1	1,78308E-02	3723	9,44488E-01	1475	1,28568E+00	8185	9,36640E-01	1627	0	17530,5
Non-responder	Macro_IFI27	pDC_LILRA4	HLA-C	NOTCH4	3,15375E-03	10011,1	2,56504E-02	1999	5,76762E-01	5803	8,41572E-01	16837	9,06269E-01	7886	0	17530,5
Non-responder	Macro_FOLR2+APOE+	Mast	AIMP1	NTRK1	3,15456E-03	14484,7	8,49824E-02	201	1,03728E+00	1033	1,18306E+00	9737	7,56400E-01	43922	0	17530,5
Non-responder	Macro_ISG15	Macro_ISG15	CCL4	CCR1	3,15456E-03	7212,3	2,02925E-02	2987	8,48363E-01	2103	2,28495E+00	1156	8,88005E-01	12285	0	17530,5
Non-responder	Macro_NLRP3	Macro_OLFML3	TNF	TRPM2	3,15485E-03	15059,3	1,30081E-02	6392	5,00700E-01	7969	1,48351E+00	5713	7,83576E-01	37692	0	17530,5
Non-responder	Macro_NLRP3	cDC(CD1C)	THBS1	CD47	3,15640E-03	10742,1	1,15386E-02	7835	7,33255E-01	3217	1,30060E+00	7970	8,69389E-01	17158	0	17530,5
Non-responder	Macro_OLFML3	Macro_FOLR2+APOE+	TIMP1	CD63	3,15640E-03	10498,5	5,87764E-03	23108	6,11726E-01	5060	1,49865E+00	5546	9,38753E-01	1248	0	17530,5
Non-responder	pDC_LILRA4	Mono_INHBA	APP	LRP1	3,15766E-03	6551,3	2,02901E-02	2988	1,13846E+00	616	2,63419E+00	573	8,92749E-01	11049	0	17530,5
Non-responder	Mono_CD16	Macro_FOLR2+APOE+	HBEGF	CD9	3,16407E-03	14509,3	7,46787E-03	15786	3,13036E-01	16848	1,27195E+00	8374	8,81434E-01	14008	0	17530,5
Non-responder	Macro_FOLR2+APOE+	Mono_CD16	LRPAP1	SORT1	3,16723E-03	14118,9	1,14023E-02	7977	5,01109E-01	7955	1,47942E+00	5757	8,10537E-01	31375	0	17530,5
Non-responder	Mono_CD16	cDC_CLEC9A	LGALS9	HAVCR2	3,17347E-03	13582,7	7,16735E-03	16858	3,87348E-01	12607	1,41153E+00	6519	8,79940E-01	14399	0	17530,5
Non-responder	Macro_FOLR2+APOE+	Mono_CD14	CALR	LRP1	3,17441E-03	11558,9	7,16656E-03	16859	4,63482E-01	9301	1,46375E+00	5912	9,04950E-01	8192	0	17530,5
Non-responder	Macro_NLRP3	Macro_FOLR2+APOE+	S100A4	CCR5	3,17818E-03	12073,9	1,44246E-02	5355	5,23682E-01	7229	9,87490E-01	13392	8,70432E-01	16863	0	17530,5
Non-responder	Macro_IER3	pDC_LILRA4	HLA-DQA2	CD4	3,17818E-03	11456,3	7,16619E-03	16863	3,71518E-01	13429	1,79336E+00	3153	9,13359E-01	6306	0	17530,5
Non-responder	cDC(CD1C)	cDC_CLEC9A	HLA-DRA	LAG3	3,18006E-03	7160,7	1,74618E-02	3867	9,42994E-01	1484	1,70739E+00	3740	9,00482E-01	9182	0	17530,5
Non-responder	cDC(CD1C)	cDC_CLEC9A	HLA-DRB1	LAG3	3,18572E-03	7488,7	1,73866E-02	3897	9,37366E-01	1515	1,64337E+00	4245	8,95941E-01	10256	0	17530,5
Non-responder	Mono_CD16	Macro_OLFML3	AGTRAP	RACK1	3,18667E-03	14115,9	7,35753E-03	16153	4,50987E-01	9807	1,15494E+00	10217	8,70407E-01	16872	0	17530,5
Non-responder	cDC_LAMP3	Macro_IER3	CXCL9	FCGR2A	3,18856E-03	12701,3	1,05152E-02	9122	5,56831E-01	6308	8,40239E-01	16874	8,82769E-01	13672	0	17530,5
Non-responder	Macro_FOLR2+APOE+	Macro_ISG15	CXCL12	ITGA5	3,18900E-03	16832,1	1,81366E-02	3613	5,00072E-01	7991	1,79999E+00	3111	7,18722E-01	51915	0	17530,5
Non-responder	Macro_NLRP3	Macro_FOLR2-APOE+	S100A9	ITGB2	3,18900E-03	9397,3	1,13881E-02	7991	8,74833E-01	1879	7,47750E-01	19311	9,49286E-01	275	0	17530,5
Non-responder	cDC(CD1C)	cDC_CLEC9A	HLA-DPB1	LAG3	3,18950E-03	7038,1	1,92059E-02	3280	1,01043E+00	1158	1,85210E+00	2802	8,95266E-01	10420	0	17530,5
Non-responder	Macro_FOLR2+APOE+	cDC_LAMP3	CXCL12	SDC4	3,19055E-03	19647,1	1,85072E-02	3502	5,00054E-01	7992	1,37729E+00	6922	6,52553E-01	62289	0	17530,5
Non-responder	Macro_LYVE1	Mono_CD16	HLA-C	LILRA1	3,19055E-03	7370,3	1,76149E-02	3797	7,18600E-01	3397	1,29898E+00	7992	9,24185E-01	4135	0	17530,5
Non-responder	Mono_CD16	Macro_ISG15	ICAM3	ITGAL	3,19055E-03	14630,1	1,13881E-02	7992	5,75925E-01	5831	1,67750E+00	3947	7,82905E-01	37850	0	17530,5
Non-responder	cDC(CD1C)	cDC_CLEC9A	ITGB2	CD226	3,19234E-03	8527,1	2,98953E-02	1557	9,47156E-01	1463	1,31022E+00	7841	8,80497E-01	14244	0	17530,5
Non-responder	Mono_INHBA	Macro_OLFML3	CCL3L1	CCR5	3,19505E-03	10903,5	2,75100E-02	1770	8,51419E-01	2076	1,82079E+00	3000	8,15647E-01	30141	0	17530,5
Non-responder	cDC(CD1C)	cDC_CLEC9A	HLA-DPA1	LAG3	3,19707E-03	7079,1	1,85297E-02	3491	9,67781E-01	1357	1,76013E+00	3356	8,98321E-01	9661	0	17530,5
Non-responder	Macro_ISG15	Macro_LYVE1	CXCL10	TLR4	3,19818E-03	9362,9	2,02197E-02	3001	9,20159E-01	1613	2,44741E+00	848	8,41350E-01	23822	0	17530,5
Non-responder	Mono_CD16	Macro_ISG15	S100A8	CD68	3,19896E-03	11352,5	7,16018E-03	16885	4,44160E-01	10054	1,13920E+00	10469	9,35700E-01	1824	0	17530,5
Non-responder	Macro_IFI27	Macro_NLRP3	C1QA	CD93	3,20086E-03	9327,3	1,50556E-02	4975	7,63712E-01	2883	8,39662E-01	16887	9,23046E-01	4361	0	17530,5
Non-responder	Macro_IER3	Macro_FOLR2+APOE+	ICAM1	ITGAM_ITGB2	3,20370E-03	12733,3	7,15819E-03	16890	4,56029E-01	9580	1,16519E+00	10050	8,98523E-01	9616	0	17530,5
Non-responder	cDC(CD1C)	cDC_CLEC9A	HLA-DRB5	LAG3	3,20560E-03	7539,3	1,85261E-02	3495	9,56022E-01	1415	1,69515E+00	3812	8,91224E-01	11444	0	17530,5
Non-responder	Macro_FOLR2+APOE+	cDC_CLEC9A	C1QA	CD93	3,21035E-03	10908,5	7,15655E-03	16897	5,76036E-01	5828	1,80802E+00	3066	8,92123E-01	11221	0	17530,5
Non-responder	pDC_LILRA4	cDC_CLEC9A	APP	NCSTN	3,21309E-03	8874,3	1,79580E-02	3678	1,14625E+00	594	2,93810E+00	319	8,47946E-01	22250	0	17530,5
Non-responder	Mono_CD16	cDC_CLEC9A	TNFSF10	CCR6	3,21400E-03	17504,3	2,83710E-02	1700	5,83175E-01	5662	1,29779E+00	8007	7,04106E-01	54622	0	17530,5
Non-responder	Macro_NLRP3	Mono_CD14	CCL3	CCR1	3,22027E-03	10481,7	1,13679E-02	8011	6,25447E-01	4821	1,70911E+00	3724	8,64550E-01	18322	0	17530,5
Non-responder	Mono_CD16	Macro_FOLR2-APOE+	ADAM10	TREM2	3,22081E-03	12355,3	1,37795E-02	5802	5,67692E-01	6035	8,38966E-01	16908	8,75791E-01	15501	0	17530,5
Non-responder	Macro_OLFML3	Mono_CD16	SERPING1	LRP1	3,22081E-03	13280,9	8,16962E-03	13641	4,51679E-01	9776	1,25992E+00	8549	8,70261E-01	16908	0	17530,5
Non-responder	Macro_IER3	Macro_ISG15	TNF	TNFRSF1B	3,22271E-03	10960,7	9,51109E-03	10660	5,58983E-01	6254	1,74512E+00	3449	8,70258E-01	16910	0	17530,5
Non-responder	Mono_INHBA	Macro_FOLR2+APOE+	CCL4	CCR1	3,22329E-03	7209,9	2,01822E-02	3009	8,64127E-01	1964	2,26645E+00	1191	8,87734E-01	12355	0	17530,5
Non-responder	Macro_FOLR2+APOE+	Mono_CD16	GNAI2	FPR1	3,22653E-03	12373,7	7,15323E-03	16914	3,75441E-01	13216	1,52590E+00	5298	9,01605E-01	8910	0	17530,5
Non-responder	Macro_FOLR2+APOE+	Macro_IFI27	LILRB4	LAIR1	3,22939E-03	13349,3	7,52089E-03	15600	4,85716E-01	8480	8,38590E-01	16917	9,04869E-01	8219	0	17530,5
Non-responder	Macro_FOLR2+APOE+	Mono_CD16	PTPN6	CD300LF	3,23127E-03	12294,9	1,24368E-02	6904	4,99103E-01	8018	1,56945E+00	4859	8,39978E-01	24163	0	17530,5
Non-responder	cDC(CD1C)	cDC_CLEC9A	HMGB1	THBD	3,23130E-03	13146,3	8,23127E-03	13465	4,48679E-01	9881	9,98115E-01	13164	8,90247E-01	11691	0	17530,5
Non-responder	Macro_OLFML3	Macro_FOLR2+APOE+	ADAM10	GPNMB	3,23285E-03	11869,7	1,22711E-02	7090	5,75035E-01	5855	1,52296E+00	5320	8,42493E-01	23553	0	17530,5
Non-responder	Macro_FOLR2+APOE+	Mono_CD14	LGALS9	PTPRC	3,23285E-03	11359,3	6,67121E-03	18928	4,99092E-01	8019	1,46697E+00	5875	9,12733E-01	6444	0	17530,5
Non-responder	Macro_NLRP3	Macro_FOLR2+APOE-	S100A9	TLR4	3,23608E-03	11876,9	9,55069E-03	10591	7,62758E-01	2891	1,08385E+00	11448	8,70205E-01	16924	0	17530,5
Non-responder	cDC(CD1C)	cDC_CLEC9A	C1QB	C1QBP	3,24086E-03	10809,5	9,18191E-03	11268	4,67180E-01	9173	1,07516E+00	11626	9,22536E-01	4450	0	17530,5
Non-responder	Macro_FOLR2+APOE+	Macro_OLFML3	CCL5	CCR5	3,24388E-03	19526,5	2,55568E-02	2010	4,98916E-01	8026	1,42978E+00	6281	6,40110E-01	63785	0	17530,5
Non-responder	Macro_OLFML3	Macro_FOLR2+APOE+	IL18	IL18BP	3,24388E-03	11982,7	1,34128E-02	6060	5,03217E-01	7871	1,52578E+00	5300	8,44143E-01	23152	0	17530,5
Non-responder	cDC(CD1C)	cDC_CLEC9A	LYZ	ITGAL	3,24469E-03	13270,7	8,05093E-03	13977	4,11984E-01	11424	9,80561E-01	13539	8,97394E-01	9883	0	17530,5
Non-responder	Mono_INHBA	Macro_ISG15	CCL2	CCR5	3,24536E-03	12930,7	3,81355E-02	1042	7,50756E-01	3016	2,21382E+00	1332	7,66487E-01	41733	0	17530,5

Post_NR_Myeloid_Myeloid_CellCell

Non-responder	Macro_OLFML3	Macro_IER3	FN1	PLAUR	3,24661E-03	13259,5	1,09856E-02	8502	4,17811E-01	11153	1,04786E+00	12177	8,70170E-01	16935	0	17530,5
Non-responder	Macro_FOLR2+APOE+	pDC_LILRA4	THBS1	CD47	3,24852E-03	9848,9	1,04189E-02	9259	7,50701E-01	3017	2,43320E+00	880	8,63483E-01	18558	0	17530,5
Non-responder	Macro_OLFML3	pDC_LILRA4	SELPLG	SELL	3,24852E-03	8450,1	1,41863E-02	5514	9,37781E-01	1513	2,51076E+00	756	8,70158E-01	16937	0	17530,5
Non-responder	Macro_FOLR2+APOE+	cDC_CLEC9A	CALM1	HMMR	3,25020E-03	13998,1	4,69009E-02	727	7,32316E-01	3233	1,29608E+00	8030	7,71957E-01	40470	0	17530,5
Non-responder	pDC_LILRA4	cDC_CLEC9A	SELPLG	ITGB2	3,25485E-03	6488,1	1,11728E-02	8254	7,50498E-01	3019	2,55061E+00	694	9,30144E-01	2943	0	17530,5
Non-responder	Mono_CD16	Macro_ISG15	VCAN	CD44	3,25524E-03	13823,3	7,14492E-03	16944	4,36612E-01	10351	1,02193E+00	12700	8,90638E-01	11591	0	17530,5
Non-responder	Macro_NLRP3	Macro_FOLR2-APOE+	IL1B	SIGIRR	3,25813E-03	10909,9	1,09282E-02	8579	8,90255E-01	1791	1,18538E+00	9702	8,70131E-01	16947	0	17530,5
Non-responder	Macro_IER3	Mono_INHBA	FN1	PLAUR	3,26005E-03	12070,5	1,09776E-02	8515	5,61559E-01	6188	1,09964E+00	11170	8,70129E-01	16949	0	17530,5
Non-responder	Macro_FOLR2+APOE+	Macro_FOLR2+APOE+	HSP90B1	TLR4	3,26294E-03	13266,7	8,55632E-03	12619	4,34127E-01	10452	1,24442E+00	8780	8,70110E-01	16952	0	17530,5
Non-responder	pDC_LILRA4	Macro_ISG15	PROC	ITGAM	3,26435E-03	14225,5	4,41422E-02	809	7,50302E-01	3022	2,09737E+00	1708	7,38105E-01	48058	0	17530,5
Non-responder	Macro_OLFML3	Macro_FOLR2+APOE+	FAM3C	LAMP1	3,26920E-03	16511,9	1,13376E-02	8042	5,92617E-01	5474	1,51602E+00	5385	7,46798E-01	46128	0	17530,5
Non-responder	Macro_FOLR2+APOE+	Macro_IER3	CD14	ITGB2	3,26920E-03	10345,5	7,03922E-03	17366	5,09380E-01	7684	1,29525E+00	8042	9,39982E-01	1105	0	17530,5
Non-responder	cDC(CD1C)	Mono_CD16	PTPN6	CLEC12A	3,27064E-03	11827,3	1,12156E-02	8179	5,14542E-01	7511	1,23108E+00	8956	8,70076E-01	16960	0	17530,5
Non-responder	Macro_FOLR2+APOE+	Macro_ISG15	PSAP	SORT1	3,27064E-03	12501,7	7,14151E-03	16960	5,16224E-01	7458	1,63151E+00	4331	8,72891E-01	16229	0	17530,5
Non-responder	cDC_LAMP3	pDC_LILRA4	SIRPA	CD47	3,27161E-03	9169,7	1,22280E-02	7121,5	7,60130E-01	2920,5	2,22249E+00	1314,5	8,70072E-01	16961,5	0	17530,5
Non-responder	Mono_CD16	Macro_IER3	SERPINA1	LRP1	3,27161E-03	11752,5	7,14106E-03	16961	5,16137E-01	7460	1,19460E+00	9552	9,08954E-01	7259	0	17530,5
Non-responder	Mast	Macro_FOLR2+APOE-	CSF1	CSF2RA	3,27779E-03	9262,3	5,25960E-02	600	1,14864E+00	585	1,28580E+00	8183	8,59874E-01	19413	0	17530,5
Non-responder	Macro_IER3	Macro_FOLR2+APOE+	HLA-DRA	CD4	3,27933E-03	10885,1	7,13800E-03	16969	4,77587E-01	8797	1,11704E+00	10852	9,49247E-01	277	0	17530,5
Non-responder	Macro_NLRP3	Macro_IER3	S100A8	CD36	3,28223E-03	11435,7	1,06530E-02	8942	7,76141E-01	2758	1,11092E+00	10976	8,70029E-01	16972	0	17530,5
Non-responder	Macro_OLFML3	Macro_FOLR2+APOE+	C1QB	CD33	3,28320E-03	12769,5	8,88516E-03	11863	3,10896E-01	16973	1,32236E+00	7669	8,97720E-01	9812	0	17530,5
Non-responder	Macro_IER3	cDC_CLEC9A	IL1B	SIGIRR	3,28416E-03	12047,7	1,09075E-02	8606	6,01013E-01	5283	1,06428E+00	11845	8,70024E-01	16974	0	17530,5
Non-responder	Macro_OLFML3	Macro_ISG15	GNAI2	C5AR1	3,28416E-03	11929,5	7,13686E-03	16974	4,40700E-01	10199	1,42050E+00	6399	9,03352E-01	8545	0	17530,5
Non-responder	Macro_NLRP3	pDC_LILRA4	TNF	TRADD	3,28510E-03	13292,7	1,13285E-02	8052	5,26742E-01	7128	2,14315E+00	1535	8,06864E-01	32218	0	17530,5
Non-responder	Mono_INHBA	cDC_CLEC9A	SPP1	PTGER4	3,28661E-03	7596,3	2,95924E-02	1585	7,49483E-01	3029	2,14365E+00	1533	8,80297E-01	14304	0	17530,5
Non-responder	Macro_LYVE1	Macro_FOLR2+APOE+	C3	CD81	3,28669E-03	8636,3	1,45450E-02	5269	5,93440E-01	5459	1,38176E+00	6870	9,05497E-01	8053	0	17530,5
Non-responder	Macro_IER3	Mono_INHBA	PLAU	PLAUR	3,28803E-03	11817,9	1,03074E-02	9402	6,28202E-01	4763	1,14309E+00	10416	8,70019E-01	16978	0	17530,5
Non-responder	Macro_NLRP3	Macro_NLRP3	CD14	TLR4	3,28997E-03	13658,3	8,40351E-03	13015	3,77736E-01	13088	1,32187E+00	7678	8,70009E-01	16980	0	17530,5
Non-responder	Macro_IFI27	Mono_CD16	HLA-B	LILRB2	3,30647E-03	10679,3	7,89185E-03	14443	6,62198E-01	4193	8,35320E-01	16997	9,49858E-01	233	0	17530,5
Non-responder	Mono_CD16	Mono_CD14	ANXA1	FPR1	3,30939E-03	13045,7	7,12977E-03	17000	3,58008E-01	14175	1,19018E+00	9623	9,10657E-01	6900	0	17530,5
Non-responder	Macro_FOLR2+APOE+	Macro_NLRP3	VIM	CD44	3,31038E-03	10330,3	6,44905E-03	20026	4,70582E-01	9044	1,56943E+00	4860	9,50605E-01	191	0	17530,5
Non-responder	Macro_OLFML3	Macro_FOLR2+APOE+	NRG1	MS4A4A	3,31066E-03	15200,1	1,55179E-02	4728	5,91965E-01	5486	1,72157E+00	3629	7,53425E-01	44627	0	17530,5
Non-responder	pDC_LILRA4	Macro_NLRP3	HSP90B1	LRP1	3,31386E-03	6538,9	1,13163E-02	8070	9,34271E-01	1535	2,76813E+00	443	9,19162E-01	5116	0	17530,5
Non-responder	Mono_CD16	Mono_CD14	PTPN6	CLEC12A	3,31426E-03	10874,3	1,11832E-02	8235	5,33490E-01	6944	1,59337E+00	4657	8,69912E-01	17005	0	17530,5
Non-responder	cDC(CD1C)	Macro_NLRP3	GRN	TNFRSF1B	3,31524E-03	10997,9	7,12755E-03	17006	5,33848E-01	6931	1,08944E+00	11347	9,34092E-01	2175	0	17530,5
Non-responder	Mono_CD14	pDC_LILRA4	SEMA6B	PLXNA4	3,31857E-03	17527,9	8,44813E-02	205	8,59275E-01	2011	1,81268E+00	3039	6,30023E-01	64854	0	17530,5
Non-responder	pDC_LILRA4	Macro_OLFML3	PROC	ITGAM	3,31857E-03	14401,5	4,38821E-02	821	7,48671E-01	3039	1,92380E+00	2442	7,37533E-01	48175	0	17530,5
Non-responder	Macro_OLFML3	Mono_CD16	GNAI2	C5AR1	3,32207E-03	11927,5	7,12532E-03	17013	4,39503E-01	10245	1,42922E+00	6293	9,03281E-01	8556	0	17530,5
Non-responder	Macro_NLRP3	Macro_FOLR2+APOE+	IL6	IL6R_IL6ST	3,32670E-03	17388,7	1,66203E-02	4187	4,97349E-01	8078	1,43250E+00	6246	7,24181E-01	50902	0	17530,5
Non-responder	Macro_IFI27	pDC_LILRA4	LGALS9	CD47	3,32793E-03	11632,5	7,12389E-03	17019	5,91021E-01	5505	1,69176E+00	3839	8,80414E-01	14269	0	17530,5
Non-responder	Macro_LYVE1	Macro_ISG15	PLTP	ABCA1	3,32820E-03	6544,9	2,00762E-02	3042	9,45977E-01	1468	1,90793E+00	2520	9,05067E-01	8164	0	17530,5
Non-responder	Macro_NLRP3	cDC_CLEC9A	RETN	CAP1	3,32831E-03	13479,9	1,45730E-02	5251	4,97306E-01	8079	1,63346E+00	4318	8,06851E-01	32221	0	17530,5
Non-responder	pDC_LILRA4	Macro_NLRP3	SEMA7A	PLXNC1	3,33141E-03	14737,1	2,00761E-02	3043	7,95924E-01	2593	1,92952E+00	2415	7,37894E-01	48104	0	17530,5
Non-responder	Macro_IER3	cDC_LAMP3	THBS1	TNFRSF11B	3,33219E-03	13551,1	7,21866E-02	297	1,14215E+00	605	1,23043E+00	8966	7,72471E-01	40357	0	17530,5
Non-responder	Macro_FOLR2+APOE+	Macro_FOLR2+APOE+	TNF	TRPM2	3,33475E-03	14521,3	1,39911E-02	5649	4,97274E-01	8083	1,54287E+00	5113	7,89690E-01	36231	0	17530,5
Non-responder	Macro_FOLR2+APOE+	Mono_CD16	HLA-G	LILRB1	3,33636E-03	12925,3	1,13085E-02	8084	5,14760E-01	7502	1,57717E+00	4790	8,29561E-01	26720	0	17530,5
Non-responder	Mono_INHBA	Macro_OLFML3	INHBA	ENG	3,33784E-03	10628,1	2,91574E-02	1636	8,20059E-01	2367	1,81148E+00	3045	8,22148E-01	28562	0	17530,5
Non-responder	Macro_IER3	pDC_LILRA4	HLA-DMB	CD4	3,33968E-03	11788,1	7,12087E-03	17031	4,00443E-01	11966	1,56265E+00	4917	9,07852E-01	7496	0	17530,5
Non-responder	Macro_LYVE1	cDC_CLEC9A	CXCL12	CXCR3	3,34106E-03	12079,9	6,77550E-02	348	9,85042E-01	1279	1,81135E+00	3046	7,81480E-01	38196	0	17530,5
Non-responder	pDC_LILRA4	Macro_ISG15	CD47	SIRPA	3,34106E-03	6454,5	2,00697E-02	3046,5	1,02074E+00	1114,5	3,02779E+00	244,5	8,95607E-01	10336,5	0	17530,5
Non-responder	Mono_CD16	Mono_CD14	HLA-B	LILRA1	3,34262E-03	14850,1	7,43331E-03	15893	3,09999E-01	17034	1,02102E+00	12722	8,92666E-01	11071	0	17530,5
Non-responder	Mono_CD16	Mono_INHBA	NAMPT	ADORA2A	3,34281E-03	12338,9	2,14663E-02	2698	7,10850E-01	3505	1,29209E+00	8088	8,16748E-01	29873	0	17530,5
Non-responder	Macro_ISG15	pDC_LILRA4	SIRPA	CD47	3,34428E-03	6454,5	2,00697E-02	3046,5	1,02074E+00	1114,5	3,02779E+00	244,5	8,95607E-01	10336,5	0	17530,5
Non-responder	cDC(CD1C)	Mono_CD14	CIRBP	TREM1	3,34458E-03	12281,3	1,07083E-02	8866	4,69595E-01	9088	9,55108E-01	14147	8,89865E-01	11775	0	17530,5
Non-responder	cDC_CLEC9A	Macro_FOLR2+APOE+	HLA-DPB1	CD4	3,34751E-03	6772,9	8,97502E-03	11684	7,47969E-01	3048	2,20565E+00	1358	9,49728E-01	244	0	17530,5
Non-responder	Macro_IER3	pDC_LILRA4	SELPLG	SELL	3,34753E-03	8573,7	1,40990E-02	5570	9,36203E-01	1523	2,26287E+00	1206	8,69809E-01	17039	0	17530,5
Non-responder	Macro_FOLR2+APOE+	Macro_FOLR2+APOE+	ADAM9	ITGAV	3,34765E-03	15411,7	1,64537E-02	4268	4,97108E-01	8091	1,47120E+00	5829	7,68164E-01	41340	0	17530,5
Non-responder	Macro_FOLR2+APOE+	Mast	TIMP2	CD44	3,35048E-03	12974,7	9,49860E-03	10682	5,09177E-01	7690	8,33805E-01	17042	8,89301E-01	11929	0	17530,5
Non-responder	Macro_ISG15	Macro_OLFML3	CCL2	CCR5	3,35073E-03	13184,7	3,49783E-02	1203	7,47918E-01	3049	2,52682E+00	728	7,58664E-01	43413	0	17530,5
Non-responder	Macro_IER3	Macro_ISG15	HLA-F	LILRB1	3,35244E-03	13931,5	7,11830E-03	17044	4,93750E-01	8204	1,16696E+00	10017	8,70440E-01	16862	0	17530,5
Non-responder	Macro_FOLR2+APOE+	Mono_INHBA	HLA-B	LILRB1	3,35412E-03	13098,1	5,10144E-03	28490	5,28684E-01	7073	1,29138E+00	8095	9,23339E-01	4302	0	17530,5
Non-responder	Macro_OLFML3	Macro_OLFML3	SERPING1	LRP1	3,35638E-03	13663,9	8,10134E-03	13827	4,48447E-01	9890	1,16668E+00	10024	8,69786E-01	17048	0	17530,5
Non-responder	Macro_OLFML3	Macro_ISG15	HLA-B	LILRA1	3,35638E-03	14077,5	7,11764E-03	17048	3,75607E-01	13203	1,10908E+00	10998	8,90570E-01	11608	0	17530,5
Non-responder	Macro_FOLR2+APOE+	pDC_LILRA4	LGALS1	PTPRC	3,35719E-03	14223,5	4,84317E-03	30648	3,07354E-01	17225	1,80995E+00	3051	9,31461E-01	2663	0	17530,5
Non-responder	Macro_IER3	Macro_FOLR2+APOE+	HLA-DRB1	LAG3	3,36032E-03	12606,9	1,04625E-02	9208	4,96714E-01	8102	1,10156E+00	11142	8,69774E-01	17052	0	17530,5
Non-responder	Mono_CD14	Macro_LYVE1	VCAN	TLR2	3,36042E-03	6765,7	2,10487E-02	2800	1,08928E+00	814	1,80992E+00	3052	8,98465E-01	9632	0	17530,5
Non-responder	Macro_IER3	Macro_FOLR2+APOE+	HLA-DPA1	LAG3	3,36229E-03	12663,5	1,05886E-02	9028	4,83559E-01	8568	1,10188E+00	11137	8,69768E-01	17054	0	17530,5
Non-responder	Macro_IFI27	pDC_LILRA4	HLA-DPA1	CD4	3,36328E-03	10592,9	7,11577E-03	17055	5,08948E-01	7702	1,15479E+00	10222	9,46525E-01	455	0	17530,5
Non-responder	Macro_FOLR2+APOE+	Macro_IER3	PLAU	ITGB1	3,36545E-03	13798,9	1,18999E-02	7446	4,97743E-01	8063	1,29096E+00	8102	8,25003E-01	27853	0	17530,5
Non-responder	pDC_LILRA4	pDC_LILRA4	ASIP	MGRN1	3,36689E-03	15346,1	8,05779E-02	226	7,47563E-01	3054	2,42634E+00	890	7,01666E-01	55030	0	17530,5
Non-responder	Macro_NLRP3	Macro_ISG15	IL6	FCGR3B	3,36869E-03	18686,3	2,97619E-02	1566	5,66774E-01	6057	1,29082E+00	8104	6,68682E-01	60174	0	17530,5
Non-responder	Macro_NLRP3	Macro_NLRP3	ADAM17	ITGA5	3,37032E-03	16163,7	1,31563E-02	6263	4,96661E-01	8105	1,36939E+00	7020	7,65772E-01	41900	0	17530,5

Post_NR_Myeloid_Myeloid_CellCell

Non-responder	Macro_FOLR2+APOE+	Macro_ISG15	CD274	CD80	3,37194E-03	20672,1	1,40987E-02	5571	4,96623E-01	8106	1,40150E+00	6634	6,23462E-01	65519	0	17530,5
Non-responder	pDC_LILRA4	Macro_ISG15	HLA-F	LILRB2	3,37194E-03	6887,5	1,12901E-02	8106	8,60288E-01	2001	2,52267E+00	734	9,14525E-01	6066	0	17530,5
Non-responder	Macro_NLRP3	Macro_FOLR2+APOE-	VCAN	ITGA4	3,37512E-03	11909,9	1,04235E-02	9252	7,89855E-01	2643	1,00423E+00	13057	8,69712E-01	17067	0	17530,5
Non-responder	Macro_NLRP3	Mono_CD16	CD55	ADGRE5	3,38169E-03	10510,5	1,12837E-02	8112	6,55503E-01	4304	1,65897E+00	4124	8,63780E-01	18482	0	17530,5
Non-responder	Macro_FOLR2+APOE+	Mono_CD14	FN1	PLAUR	3,38601E-03	12080,7	1,08899E-02	8630	4,39343E-01	10256	1,37840E+00	6909	8,69675E-01	17078	0	17530,5
Non-responder	Mono_CD14	Macro_OLFML3	VCAN	ITGA4	3,38635E-03	6325,3	2,00159E-02	3060	1,13846E+00	615	2,10675E+00	1671	9,02441E-01	8750	0	17530,5
Non-responder	Macro_FOLR2+APOE+	Mono_CD16	ADAM10	NOTCH2	3,38821E-03	13699,9	1,14592E-02	7916	4,96310E-01	8116	1,41076E+00	6527	8,22750E-01	28410	0	17530,5
Non-responder	cDC(CD1C)	Macro_ISG15	HLA-B	LILRB1	3,38899E-03	11013,5	7,58994E-03	15354	4,56059E-01	9579	1,11501E+00	10906	9,36271E-01	1698	0	17530,5
Non-responder	Macro_FOLR2+APOE+	Macro_OLFML3	B2M	TFRC	3,38984E-03	13369,1	4,70621E-03	31929	4,96280E-01	8117	1,61106E+00	4500	9,20727E-01	4769	0	17530,5
Non-responder	Mono_CD16	Macro_FOLR2+APOE+	HLA-F	LILRB2	3,38998E-03	11323,5	7,10900E-03	17082	5,56375E-01	6322	1,54465E+00	5095	8,94626E-01	10588	0	17530,5
Non-responder	Macro_OLFML3	Macro_OLFML3	C3	IFITM1	3,39097E-03	13250,7	9,90336E-03	10003	3,69321E-01	13551	1,29239E+00	8086	8,69651E-01	17083	0	17530,5
Non-responder	pDC_LILRA4	Macro_FOLR2-APOE+	SELPLG	ITGB2	3,39147E-03	7650,5	1,12752E-02	8118	7,58926E-01	2935	1,38700E+00	6800	9,30440E-01	2869	0	17530,5
Non-responder	pDC_LILRA4	Mono_CD16	PROC	THBD	3,39285E-03	15532,1	2,90777E-02	1644	7,46820E-01	3062	1,96933E+00	2215	7,11924E-01	53209	0	17530,5
Non-responder	Macro_ISG15	Macro_NLRP3	MMP9	CD44	3,39285E-03	7117,1	2,00054E-02	3062	9,25169E-01	1580	2,44944E+00	846	8,86921E-01	12567	0	17530,5
Non-responder	cDC_CLEC9A	Macro_NLRP3	RPS19	C5AR1	3,39610E-03	7412,9	9,31483E-03	11007	7,52283E-01	2997	1,80867E+00	3063	9,32534E-01	2467	0	17530,5
Non-responder	Mast	Macro_ISG15	CSF1	CSF2RA	3,39936E-03	9496,1	3,71908E-02	1096	1,03062E+00	1066	1,80841E+00	3064	8,37665E-01	24724	0	17530,5
Non-responder	cDC(CD1C)	Macro_ISG15	ANXA1	FPR2_FPR3	3,39992E-03	11674,9	1,13125E-02	8078	5,17296E-01	7418	1,05701E+00	12006	8,83976E-01	13342	0	17530,5
Non-responder	Macro_FOLR2+APOE+	Macro_OLFML3	C3	C3AR1	3,39992E-03	12989,3	1,01224E-02	9671	3,09122E-01	17092	1,38391E+00	6839	8,82248E-01	13814	0	17530,5
Non-responder	cDC(CD1C)	pDC_LILRA4	HLA-DRA	CD4	3,39992E-03	8714,3	7,10611E-03	17092	5,67815E-01	6031	1,88468E+00	2635	9,49139E-01	283	0	17530,5
Non-responder	Macro_FOLR2+APOE+	Macro_OLFML3	CXCL10	TLR4	3,40289E-03	14047,1	1,12672E-02	8125	5,12915E-01	7575	1,86339E+00	2751	7,98336E-01	34254	0	17530,5
Non-responder	cDC(CD1C)	Macro_ISG15	HLA-A	LILRB1	3,40489E-03	11146,9	7,68344E-03	15058	4,46868E-01	9946	1,12187E+00	10772	9,32781E-01	2428	0	17530,5
Non-responder	Macro_FOLR2+APOE+	Macro_ISG15	PLAU	ITGAM	3,40780E-03	14278,7	1,40114E-02	5633	4,95867E-01	8128	1,61503E+00	4465	7,92415E-01	35637	0	17530,5
Non-responder	Macro_FOLR2+APOE+	Mono_CD14	C3	ITGAX	3,40888E-03	13937,3	8,78906E-03	12074	3,08997E-01	17101	1,37765E+00	6918	8,73575E-01	16063	0	17530,5
Non-responder	pDC_LILRA4	Macro_ISG15	APP	NOTCH2	3,40909E-03	8456,5	1,60877E-02	4435	1,13956E+00	612	2,92518E+00	331	8,59999E-01	19374	0	17530,5
Non-responder	Macro_OLFML3	Macro_NLRP3	TNFSF10	TNFRSF10B	3,41271E-03	13846,9	1,20566E-02	7279	5,81933E-01	5693	1,55157E+00	5023	8,00748E-01	33709	0	17530,5
Non-responder	Macro_OLFML3	Macro_NLRP3	TNFSF10	TNFRSF10D	3,41435E-03	12982,7	2,37732E-02	2254	7,09700E-01	3524	1,58721E+00	4713	7,87015E-01	36892	0	17530,5
Non-responder	Macro_FOLR2+APOE+	cDC_CLEC9A	APOE	SDC2	3,41435E-03	11475,7	1,12601E-02	8132	5,03013E-01	7880	1,79964E+00	3115	8,54312E-01	20721	0	17530,5
Non-responder	Macro_FOLR2+APOE+	Macro_NLRP3	NAMPT	ITGA5_ITGB1	3,41586E-03	13637,9	7,10291E-03	17108	4,47767E-01	9918	1,33321E+00	7509	8,73318E-01	16124	0	17530,5
Non-responder	Macro_FOLR2+APOE+	cDC(CD1C)	LGALS9	SLC1A5	3,41985E-03	12186,3	9,28171E-03	11059	5,09286E-01	7688	1,33129E+00	7542	8,69560E-01	17112	0	17530,5
Non-responder	pDC_LILRA4	pDC_LILRA4	APP	LRP10	3,42015E-03	8599,9	1,75165E-02	3841	1,13946E+00	613	3,48072E+00	97	8,53552E-01	20918	0	17530,5
Non-responder	cDC(CD1C)	Macro_ISG15	HLA-C	LILRB2	3,42185E-03	9803,9	7,19257E-03	16758	5,80914E-01	5714	1,26695E+00	8450	9,45113E-01	567	0	17530,5
Non-responder	cDC(CD1C)	Macro_ISG15	HLA-C	LILRB1	3,42285E-03	9628,7	8,24118E-03	13442	5,33556E-01	6942	1,26845E+00	8423	9,35799E-01	1806	0	17530,5
Non-responder	Macro_FOLR2+APOE+	Macro_LYVE1	PLAU	ITGB1	3,42419E-03	13554,7	1,22633E-02	7094	5,09086E-01	7696	1,28855E+00	8138	8,27164E-01	27315	0	17530,5
Non-responder	Macro_IER3	cDC_CLEC9A	AGTRAP	RACK1	3,42485E-03	14099,5	7,24728E-03	16555	4,97422E-01	8075	1,09672E+00	11220	8,69553E-01	17117	0	17530,5
Non-responder	Macro_OLFML3	Macro_NLRP3	GNAI2	C5AR1	3,42485E-03	12319,7	7,09978E-03	17117	4,36853E-01	10338	1,29619E+00	8027	9,03124E-01	8586	0	17530,5
Non-responder	cDC_LAMP3	Mono_CD16	HLA-B	LILRB1	3,42786E-03	11505,7	7,09884E-03	17120	4,62086E-01	9352	1,08722E+00	11392	9,34246E-01	2134	0	17530,5
Non-responder	Macro_FOLR2+APOE+	Macro_IER3	B2M	TFRC	3,42912E-03	13946,7	4,69328E-03	32065	4,95439E-01	8141	1,35601E+00	7208	9,20627E-01	4789	0	17530,5
Non-responder	Macro_ISG15	cDC_LAMP3	NAMPT	ADORA2A	3,43203E-03	8822,9	3,58490E-02	1155	1,02229E+00	1105	1,80683E+00	3074	8,52064E-01	21250	0	17530,5
Non-responder	Macro_FOLR2+APOE+	cDC_CLEC9A	MDK	ITGA4_ITGB1	3,43241E-03	17192,7	2,08813E-02	2841	4,99011E-01	8022	1,28832E+00	8143	7,31530E-01	49427	0	17530,5
Non-responder	cDC_LAMP3	cDC_CLEC9A	TNFSF13B	HLA-DPB1	3,43487E-03	8319,9	7,09593E-03	17127	6,73665E-01	4005	1,91641E+00	2477	9,46404E-01	460	0	17530,5
Non-responder	Macro_OLFML3	Macro_OLFML3	HLA-A	LILRB1	3,44089E-03	10050,1	7,09518E-03	17133	5,33897E-01	6929	1,48183E+00	5730	9,30240E-01	2928	0	17530,5
Non-responder	cDC(CD1C)	Macro_ISG15	B2M	LILRB1	3,44189E-03	11553,5	7,53584E-03	15546	4,08167E-01	11610	1,04126E+00	12309	9,42634E-01	772	0	17530,5
Non-responder	Macro_FOLR2+APOE+	Macro_IFI27	A2M	LRP1	3,44893E-03	13644,1	9,99001E-03	9861	4,35846E-01	10385	8,30659E-01	17141	8,84105E-01	13303	0	17530,5
Non-responder	pDC_LILRA4	Macro_OLFML3	IL16	CCR5	3,45502E-03	12842,9	2,72344E-02	1799	7,45285E-01	3081	2,20414E+00	1364	7,72070E-01	40440	0	17530,5
Non-responder	Macro_FOLR2+APOE+	Macro_IER3	CD14	TLR4	3,46001E-03	12238,3	8,31511E-03	13246	5,44291E-01	6653	1,40346E+00	6610	8,69410E-01	17152	0	17530,5
Non-responder	Macro_FOLR2+APOE+	Macro_ISG15	CD14	TLR1	3,46102E-03	10186,9	1,09085E-02	8603	6,11690E-01	5061	1,89325E+00	2587	8,69405E-01	17153	0	17530,5
Non-responder	Mono_CD16	Macro_FOLR2+APOE+	TNFSF13	TNFRSF14	3,46506E-03	13786,7	7,08682E-03	17157	4,03842E-01	11808	1,33098E+00	7548	8,78174E-01	14890	0	17530,5
Non-responder	Macro_OLFML3	Macro_FOLR2+APOE+	CD14	ITGB1	3,47112E-03	13078,9	7,08596E-03	17163	3,67122E-01	13680	1,32149E+00	7682	8,99747E-01	9339	0	17530,5
Non-responder	Macro_FOLR2+APOE+	Macro_ISG15	HBEGF	CD44	3,47213E-03	12161,5	7,08589E-03	17164	4,57397E-01	9523	1,42277E+00	6376	8,96128E-01	10214	0	17530,5
Non-responder	cDC_LAMP3	Macro_ISG15	B2M	LILRB2	3,47416E-03	9400,3	7,08573E-03	17166	6,35093E-01	4624	1,32947E+00	7573	9,52715E-01	108	0	17530,5
Non-responder	pDC_LILRA4	Macro_OLFML3	LILRB4	LAIR1	3,47538E-03	6897,7	1,12218E-02	8169	7,36986E-01	3180	2,45318E+00	842	9,20753E-01	4767	0	17530,5
Non-responder	Macro_ISG15	cDC(CD1C)	CXCL10	CXCR3	3,47811E-03	12286,5	1,98964E-02	3088	8,78247E-01	1865	2,43403E+00	877	7,82006E-01	38072	0	17530,5
Non-responder	Macro_FOLR2+APOE+	Mono_CD16	HLA-DMB	CD4	3,48036E-03	11031,1	6,84289E-03	18194	4,94512E-01	8172	1,75993E+00	3359	9,06173E-01	7900	0	17530,5
Non-responder	Mono_CD14	cDC_LAMP3	TNFSF10	TNFRSF11B	3,48686E-03	13072,5	6,35820E-02	406	1,13746E+00	619	1,41686E+00	6454	7,72484E-01	40353	0	17530,5
Non-responder	Macro_ISG15	Mono_CD14	S100A9	CD36	3,49035E-03	7012,7	1,76086E-02	3801	7,50469E-01	3020	1,90336E+00	2534	9,05010E-01	8178	0	17530,5
Non-responder	Macro_FOLR2+APOE+	Macro_ISG15	TGM2	ITGB1	3,49368E-03	14436,1	1,32100E-02	6207	4,94359E-01	8180	1,35286E+00	7263	8,03643E-01	33000	0	17530,5
Non-responder	Macro_OLFML3	Macro_NLRP3	AGTRAP	RACK1	3,49546E-03	15564,3	7,21374E-03	16694	4,59348E-01	9451	8,36837E-01	16959	8,69289E-01	17187	0	17530,5
Non-responder	Macro_FOLR2-APOE+	Macro_ISG15	APOE	SDC2	3,49701E-03	6762,9	2,97078E-02	1571	8,92248E-01	1779	1,58191E+00	4752	9,04986E-01	8182	0	17530,5
Non-responder	Macro_FOLR2+APOE+	Macro_FOLR2+APOE+	TLN1	ITGB5	3,49868E-03	13924,5	1,85976E-02	3470	4,94307E-01	8183	1,38129E+00	6878	8,01330E-01	33561	0	17530,5
Non-responder	Macro_NLRP3	pDC_LILRA4	CCL3	CCR5	3,49868E-03	13600,1	1,12119E-02	8183	5,76035E-01	5829	2,00348E+00	2076	7,97823E-01	34382	0	17530,5
Non-responder	cDC(CD1C)	Macro_ISG15	HMGB1	HAVCR2	3,49953E-03	12137,9	7,83000E-03	14623	4,50528E-01	9818	1,10637E+00	11054	9,07171E-01	7664	0	17530,5
Non-responder	Macro_FOLR2+APOE+	Macro_ISG15	HLA-DQA1	CD4	3,50035E-03	10862,1	5,99532E-03	22402	4,94293E-01	8184	1,84532E+00	2850	9,28314E-01	3344	0	17530,5
Non-responder	Macro_FOLR2+APOE+	Macro_NLRP3	SERPINA1	LRP1	3,50156E-03	12202,5	7,07745E-03	17193	4,10303E-01	11498	1,33765E+00	7454	9,08583E-01	7337	0	17530,5
Non-responder	Mono_CD16	Mono_INHBA	CIRBP	TREM1	3,50666E-03	15699,3	8,30006E-03	13285	3,07595E-01	17198	9,08020E-01	15226	8,76747E-01	15257	0	17530,5
Non-responder	Macro_FOLR2+APOE+	Macro_OLFML3	PLAU	ITGAM	3,50870E-03	14657,9	1,39288E-02	5695	4,94236E-01	8189	1,44145E+00	6126	7,91928E-01	35749	0	17530,5
Non-responder	cDC_CLEC9A	pDC_LILRA4	TNFSF9	ADGRG5	3,51127E-03	18906,1	6,72038E-02	356	8,43078E-01	2148	1,80161E+00	3098	5,37717E-01	71398	0	17530,5
Non-responder	cDC(CD1C)	pDC_LILRA4	HLA-DRB1	CD4	3,51176E-03	8868,9	7,07552E-03	17203	5,62187E-01	6166	1,82065E+00	3001	9,46687E-01	444	0	17530,5
Non-responder	Macro_FOLR2+APOE+	Macro_FOLR2+APOE+	CALR	ITGAV	3,51380E-03	10832,1	9,93564E-03	9954	5,54846E-01	6366	1,80066E+00	3105	8,69221E-01	17205	0	17530,5
Non-responder	Macro_IFI27	pDC_LILRA4	HLA-DQB1	CD4	3,51584E-03	11605,3	7,07505E-03	17207	4,58613E-01	9481	1,05437E+00	12068	9,36089E-01	1740	0	17530,5
Non-responder	Macro_FOLR2+APOE+	Mono_CD16	CD14	ITGB1	3,51687E-03	11138,5	7,07460E-03	17208	5,00583E-01	7972	1,72058E+00	3632	8,99674E-01	9350	0	17530,5
Non-responder	Macro_OLFML3	Macro_FOLR2+APOE+	TGM2	SDC4	3,51874E-03	19042,3	1,71015E-02	4005	5,03493E-01	7857	1,28482E+00	8195	6,86038E-01	57624	0	17530,5

Post_NR_Myeloid_Myeloid_CellCell

Non-responder	Macro_FOLR2+APOE+	Macro_IER3	CD59	STAB1	3,51993E-03	12568,7	9,76378E-03	10244	4,50953E-01	9811	1,29482E+00	8047	8,69187E-01	17211	0	17530,5
Non-responder	Mast	Macro_FOLR2-APOE+	CSF1	CSF3R	3,52045E-03	10961,3	5,15529E-02	622	1,14159E+00	607	9,84668E-01	13446	8,46449E-01	22601	0	17530,5
Non-responder	cDC(CD1C)	Macro_ISG15	CIRBP	TREM1	3,52505E-03	11909,3	1,04605E-02	9209	4,56449E-01	9564	1,09971E+00	11169	8,88712E-01	12074	0	17530,5
Non-responder	pDC_LILRA4	Macro_NLRP3	HMGB1	THBD	3,52545E-03	7633,9	1,13340E-02	8044	6,91788E-01	3722	2,56397E+00	674	9,04926E-01	8199	0	17530,5
Non-responder	Macro_OLFML3	Macro_NLRP3	RPS19	C5AR1	3,52881E-03	10952,1	6,66367E-03	18960	5,36373E-01	6863	1,39517E+00	6703	9,21204E-01	4704	0	17530,5
Non-responder	Macro_NLRP3	cDC_CLEC9A	TIMP1	CD63	3,52881E-03	11536,9	5,57372E-03	25126	4,93801E-01	8201	1,52524E+00	5305	9,37208E-01	1522	0	17530,5
Non-responder	Macro_FOLR2+APOE+	Macro_FOLR2+APOE+	C3	CD46	3,52914E-03	11721,3	1,20729E-02	7262	4,07037E-01	11661	1,56069E+00	4933	8,69163E-01	17220	0	17530,5
Non-responder	cDC(CD1C)	Macro_NLRP3	HMGB1	TLR4	3,53017E-03	15794,5	7,65229E-03	15150	3,57759E-01	14188	9,22204E-01	14883	8,69163E-01	17221	0	17530,5
Non-responder	Macro_ISG15	pDC_LILRA4	PLAU	IGF2R	3,53127E-03	11446,1	2,09890E-02	2815	7,43512E-01	3104	2,63444E+00	571	8,02817E-01	33210	0	17530,5
Non-responder	cDC(CD1C)	Macro_ISG15	C3	IFITM1	3,53427E-03	6559,5	1,90872E-02	3309	9,98818E-01	1209	2,01267E+00	2036	9,02556E-01	8713	0	17530,5
Non-responder	pDC_LILRA4	cDC_LAMP3	SEMA7A	PLXNC1	3,53795E-03	13339,9	2,67100E-02	1859	8,56502E-01	2037	1,80065E+00	3106	7,64553E-01	42167	0	17530,5
Non-responder	cDC_CLEC9A	cDC_CLEC9A	CALR	SCARF1	3,54129E-03	9470,5	1,99464E-02	3074	7,73813E-01	2779	1,80057E+00	3107	8,53766E-01	20862	0	17530,5
Non-responder	Macro_IER3	Macro_FOLR2+APOE+	TNFSF13B	CD40	3,54351E-03	13617,5	7,65106E-03	15157	5,01064E-01	7958	1,15541E+00	10208	8,69110E-01	17234	0	17530,5
Non-responder	Macro_IER3	Macro_OLFML3	TNFSF13B	CD40	3,54866E-03	14465,1	7,64907E-03	15167	5,00893E-01	7964	9,42840E-01	14425	8,69096E-01	17239	0	17530,5
Non-responder	Macro_NLRP3	Macro_FOLR2+APOE-	S100A9	CD68	3,54900E-03	9162,9	1,11956E-02	8213	8,27248E-01	2303	8,11252E-01	17659	9,52706E-01	109	0	17530,5
Non-responder	Macro_FOLR2+APOE+	Macro_ISG15	PSAP	LRP1	3,55068E-03	10940,3	6,21193E-03	21174	4,93543E-01	8214	1,69659E+00	3806	9,25032E-01	3977	0	17530,5
Non-responder	Macro_OLFML3	Macro_FOLR2+APOE+	HLA-DQB2	LAG3	3,55237E-03	15777,1	1,37772E-02	5805	4,93375E-01	8215	1,46789E+00	5863	7,67626E-01	41472	0	17530,5
Non-responder	Macro_FOLR2-APOE+	Macro_NLRP3	SPP1	CD44	3,55575E-03	8065,5	1,74206E-02	3886	7,30714E-01	3244	1,28409E+00	8217	9,08051E-01	7450	0	17530,5
Non-responder	Macro_NLRP3	Macro_IER3	NAMPT	ITGA5_ITGB1	3,56309E-03	13121,5	7,06717E-03	17253	6,20018E-01	4921	1,18487E+00	9709	8,73038E-01	16194	0	17530,5
Non-responder	Macro_FOLR2+APOE+	Macro_FOLR2+APOE-	PLTP	ABCA1	3,56412E-03	13526,3	9,71937E-03	10310	4,87534E-01	8406	9,55496E-01	14131	8,68999E-01	17254	0	17530,5
Non-responder	Macro_NLRP3	Macro_ISG15	LAMB3	CD44	3,56420E-03	14536,9	2,08572E-02	2848	5,48462E-01	6535	1,28392E+00	8222	7,84149E-01	37549	0	17530,5
Non-responder	Macro_FOLR2-APOE+	Macro_FOLR2+APOE+	APOE	ABCA1	3,56812E-03	6633,7	1,98226E-02	3115	8,56756E-01	2034	1,24958E+00	8704	9,35911E-01	1785	0	17530,5
Non-responder	Macro_FOLR2+APOE+	Macro_OLFML3	LGALS3	ENG	3,57550E-03	12151,5	7,06500E-03	17265	4,34097E-01	10453	1,46220E+00	5925	8,98652E-01	9584	0	17530,5
Non-responder	Macro_OLFML3	Macro_FOLR2+APOE+	HEBP1	FPR3	3,57775E-03	13955,3	1,11851E-02	8230	5,21632E-01	7276	1,34769E+00	7321	8,18573E-01	29419	0	17530,5
Non-responder	Macro_FOLR2+APOE+	Mono_CD14	SEMA4A	LILRB2	3,57944E-03	10730,3	1,11845E-02	8231	6,32659E-01	4674	1,49291E+00	5611	8,67474E-01	17605	0	17530,5
Non-responder	Macro_IFI27	Macro_FOLR2+APOE+	HLA-DRB1	LAG3	3,58379E-03	12677,5	1,14036E-02	7973	6,25986E-01	4809	8,25833E-01	17273	8,74575E-01	15802	0	17530,5
Non-responder	Macro_FOLR2+APOE+	Macro_NLRP3	TNFSF13B	TFRC	3,58587E-03	12109,9	7,79876E-03	14712	5,22158E-01	7258	1,70165E+00	3774	8,68888E-01	17275	0	17530,5
Non-responder	Macro_ISG15	Macro_IER3	CXCL10	TLR4	3,58832E-03	9486,7	1,97944E-02	3121	9,13369E-01	1653	2,38505E+00	954	8,39926E-01	24175	0	17530,5
Non-responder	cDC(CD1C)	Mono_INHBA	PKM	CD44	3,59106E-03	11759,3	7,06134E-03	17280	5,93861E-01	5447	8,58619E-01	16413	9,34268E-01	2126	0	17530,5
Non-responder	Macro_IFI27	Macro_NLRP3	SPP1	CD44	3,59730E-03	14747,3	7,84075E-03	14587	5,18855E-01	7366	8,36639E-01	16967	8,68859E-01	17286	0	17530,5
Non-responder	pDC_LILRA4	Macro_FOLR2+APOE+	APP	TNFRSF21	3,59945E-03	9251,3	1,82471E-02	3577	1,13597E+00	629	2,97371E+00	297	8,39735E-01	24223	0	17530,5
Non-responder	Macro_FOLR2+APOE+	Macro_FOLR2+APOE-	MMP9	IFNAR1	3,59985E-03	15760,1	1,11802E-02	8243	5,74662E-01	5870	1,68818E+00	3858	7,59242E-01	43299	0	17530,5
Non-responder	Macro_NLRP3	Macro_ISG15	S100A4	CCR5	3,60354E-03	11651,1	1,40254E-02	5625	5,13914E-01	7534	1,15179E+00	10274	8,68841E-01	17292	0	17530,5
Non-responder	Macro_FOLR2+APOE+	Mono_CD16	LRPAP1	LRP1	3,60980E-03	14637,9	7,36234E-03	16132	3,75830E-01	13193	1,22605E+00	9036	8,68814E-01	17298	0	17530,5
Non-responder	Macro_NLRP3	Macro_FOLR2+APOE+	CD14	TLR4	3,61189E-03	14553,9	8,22821E-03	13479	3,68243E-01	13613	1,11719E+00	10847	8,68813E-01	17300	0	17530,5
Non-responder	Mono_CD14	Mono_INHBA	S100A8	TLR4	3,61199E-03	6410,7	1,97667E-02	3128	1,16677E+00	537	2,42716E+00	887	8,97041E-01	9971	0	17530,5
Non-responder	Mono_CD14	Macro_FOLR2+APOE-	S100A9	ITGB2	3,61350E-03	7064,5	1,15585E-02	7812	9,44271E-01	1477	1,28167E+00	8251	9,49642E-01	252	0	17530,5
Non-responder	Macro_OLFML3	Macro_IER3	LILRB4	LAIR1	3,61502E-03	14473,1	7,05649E-03	17303	3,64461E-01	13816	9,21427E-01	14899	9,02090E-01	8817	0	17530,5
Non-responder	Macro_LYVE1	Macro_FOLR2+APOE+	TNFSF13	TNFRSF14	3,61607E-03	11923,9	9,01500E-03	11595	5,21562E-01	7277	1,07683E+00	11590	8,90473E-01	11627	0	17530,5
Non-responder	Macro_NLRP3	Macro_ISG15	CCL2	CCR5	3,61691E-03	17199,1	2,17333E-02	2640	4,92184E-01	8253	1,61431E+00	4469	7,12474E-01	53103	0	17530,5
Non-responder	Macro_FOLR2+APOE+	cDC(CD1C)	GRN	TNFRSF1A	3,61862E-03	11855,5	6,24483E-03	20999	4,92179E-01	8254	1,37019E+00	7013	9,17345E-01	5481	0	17530,5
Non-responder	Mono_CD14	Macro_ISG15	VCAN	SELL	3,61877E-03	7545,5	1,97550E-02	3130	1,11192E+00	726	2,16573E+00	1478	8,78328E-01	14863	0	17530,5
Non-responder	Macro_NLRP3	Macro_NLRP3	PSEN1	CD44	3,62025E-03	11980,7	7,90841E-03	14390	5,97286E-01	5371	1,52527E+00	5304	8,68778E-01	17308	0	17530,5
Non-responder	Macro_LYVE1	Macro_FOLR2+APOE+	HLA-DRA	CD4	3,62129E-03	9945,9	7,57402E-03	15406	5,64996E-01	6102	1,13763E+00	10501	9,50657E-01	190	0	17530,5
Non-responder	Macro_NLRP3	Mono_INHBA	ICAM1	ITGAM_ITGB2	3,62129E-03	12907,7	7,05520E-03	17309	4,75530E-01	8860	1,10623E+00	11059	8,97860E-01	9780	0	17530,5
Non-responder	Macro_LYVE1	Macro_FOLR2+APOE+	HLA-DRA	LAG3	3,62234E-03	10857,9	1,13703E-02	8008	6,22579E-01	4872	1,20512E+00	9360	8,79541E-01	14519	0	17530,5
Non-responder	Macro_LYVE1	Macro_FOLR2+APOE+	HLA-DQA2	CD4	3,62339E-03	10113,5	9,05475E-03	11500	5,45897E-01	6601	1,14228E+00	10429	9,22178E-01	4507	0	17530,5
Non-responder	Macro_NLRP3	pDC_LILRA4	SIRPA	CD47	3,62376E-03	9629,5	1,11700E-02	8257,5	7,24968E-01	3307,5	2,46965E+00	811,5	8,64870E-01	18240,5	0	17530,5
Non-responder	Macro_LYVE1	Macro_FOLR2+APOE+	HLA-DQA1	CD4	3,62548E-03	9600,5	8,22810E-03	13481	5,79093E-01	5754	1,17441E+00	9893	9,38160E-01	1344	0	17530,5
Non-responder	pDC_LILRA4	pDC_LILRA4	SEMA4A	NRP1_PLXNA4	3,62556E-03	12776,1	2,05664E-02	2926	7,40942E-01	3132	2,24408E+00	1244	7,77871E-01	39048	0	17530,5
Non-responder	Macro_LYVE1	Macro_FOLR2+APOE+	HLA-DRB1	CD4	3,62757E-03	9689,5	7,66662E-03	15111	5,82921E-01	5670	1,17873E+00	9818	9,48676E-01	318	0	17530,5
Non-responder	Macro_LYVE1	Macro_FOLR2+APOE+	HLA-DRB1	LAG3	3,62862E-03	10871,5	1,15093E-02	7861	6,40504E-01	4541	1,24622E+00	8749	8,75080E-01	15676	0	17530,5
Non-responder	Macro_LYVE1	Macro_FOLR2+APOE+	HLA-DQB1	CD4	3,62967E-03	9992,1	8,04422E-03	14002	5,54558E-01	6379	1,11403E+00	10924	9,39823E-01	1125	0	17530,5
Non-responder	Macro_IER3	Macro_FOLR2+APOE+	HLA-DPA1	CD4	3,62967E-03	11714,7	7,05328E-03	17317	4,25976E-01	10794	1,03439E+00	12460	9,46301E-01	472	0	17530,5
Non-responder	Macro_FOLR2+APOE+	Macro_ISG15	CXCL12	ITGB1	3,63061E-03	14451,3	1,48753E-02	5085	4,91927E-01	8261	1,76530E+00	3323	7,82060E-01	38057	0	17530,5
Non-responder	Mono_INHBA	Mono_CD14	TNF	VSIR	3,63235E-03	8477,7	1,97463E-02	3134	9,57529E-01	1409	2,11153E+00	1654	8,63035E-01	18661	0	17530,5
Non-responder	Macro_FOLR2+APOE+	Macro_ISG15	PSEN1	CD44	3,63281E-03	13755,7	7,89813E-03	14422	4,19176E-01	11093	1,26917E+00	8413	8,68704E-01	17320	0	17530,5
Non-responder	Macro_OLFML3	Macro_FOLR2+APOE+	HLA-F	LILRB1	3,63281E-03	13987,9	7,05246E-03	17320	4,30719E-01	10583	1,33390E+00	7502	8,69915E-01	17004	0	17530,5
Non-responder	Mono_CD16	Macro_NLRP3	PSEN1	CD44	3,63596E-03	11797,3	7,89440E-03	14433	5,96799E-01	5381	1,63342E+00	4319	8,68677E-01	17323	0	17530,5
Non-responder	Macro_LYVE1	Macro_FOLR2+APOE+	HLA-DPB1	CD4	3,63701E-03	10198,7	7,78360E-03	14768	5,48286E-01	6539	1,07288E+00	11676	9,46217E-01	480	0	17530,5
Non-responder	Macro_LYVE1	Macro_FOLR2+APOE+	HLA-DPB1	LAG3	3,63806E-03	11587,1	1,16849E-02	7664	6,05869E-01	5190	1,14037E+00	10448	8,69579E-01	17103	0	17530,5
Non-responder	Macro_ISG15	Macro_ISG15	SNCA	LAG3	3,63916E-03	16273,3	2,50087E-02	2083	8,58050E-01	2021	1,79621E+00	3136	6,92419E-01	56596	0	17530,5
Non-responder	Macro_IFI27	pDC_LILRA4	HLA-DPB1	CD4	3,64226E-03	11084,5	7,05132E-03	17329	4,90260E-01	8314	1,07831E+00	11563	9,43646E-01	686	0	17530,5
Non-responder	Macro_ISG15	cDC_CLEC9A	ADM	CALCRL	3,64597E-03	12393,5	2,51200E-02	2065	7,40538E-01	3138	1,98182E+00	2165	7,86193E-01	37069	0	17530,5
Non-responder	Mono_CD14	Macro_FOLR2+APOE+	THBS1	CD47	3,64597E-03	6708,9	1,97295E-02	3138	8,98120E-01	1739	2,29220E+00	1137	8,96949E-01	10000	0	17530,5
Non-responder	Macro_NLRP3	Mono_CD14	CCL4	CCR1	3,64779E-03	11388,9	1,11616E-02	8271	5,82057E-01	5688	1,57230E+00	4832	8,54661E-01	20623	0	17530,5
Non-responder	Mono_INHBA	Macro_FOLR2+APOE+	CCL3	CCR5	3,64938E-03	9261,3	2,43619E-02	2173	8,06069E-01	2488	1,79532E+00	3139	8,53305E-01	20976	0	17530,5
Non-responder	Mono_INHBA	pDC_LILRA4	S100A9	CD36	3,65123E-03	7297,7	1,74506E-02	3871	7,49103E-01	3036	1,70136E+00	3778	9,04622E-01	8273	0	17530,5
Non-responder	Macro_LYVE1	Macro_FOLR2+APOE+	CD14	TLR4	3,65278E-03	11505,1	1,08700E-02	8652	5,99239E-01	5327	1,02512E+00	12641	8,83883E-01	13375	0	17530,5
Non-responder	Macro_OLFML3	pDC_LILRA4	LGALS9	SLC1A5	3,65278E-03	11781,1	9,13264E-03	11349	4,53133E-01	9700	1,82178E+00	2987	8,68639E-01	17339	0	17530,5
Non-responder	Macro_LYVE1	Macro_FOLR2+APOE+	CD14	ITGB2	3,65384E-03	8556,1	8,33650E-03	13190	6,71294E-01	4044	1,34085E+00	7408	9,44580E-01	608	0	17530,5

Post_NR_Myeloid_Myeloid_CellCell

Non-responder	Macro_LYVE1	Macro_FOLR2+APOE+	CD14	ITGB1	3,65489E-03	9680,7	9,62020E-03	10459	6,17497E-01	4973	1,22878E+00	8994	9,12719E-01	6447	0	17530,5
Non-responder	Macro_LYVE1	Macro_FOLR2+APOE+	CD14	ITGA4	3,65595E-03	12348,7	7,89320E-03	14436	5,32019E-01	6989	9,44140E-01	14402	9,04125E-01	8386	0	17530,5
Non-responder	Macro_LYVE1	Macro_FOLR2+APOE+	CD14	TLR1	3,65700E-03	11000,1	1,28836E-02	6493	6,30465E-01	4719	1,08242E+00	11471	8,78565E-01	14787	0	17530,5
Non-responder	Macro_FOLR2+APOE+	Macro_FOLR2-APOE+	CCL18	CCR1	3,65812E-03	19309,7	1,24851E-02	6856	4,91151E-01	8277	1,45519E+00	5993	6,84265E-01	57892	0	17530,5
Non-responder	Mono_INHBA	Macro_OLFML3	CCL4	CCR5	3,65963E-03	10439,9	2,09161E-02	2830	7,40267E-01	3142	1,81178E+00	3043	8,33647E-01	25654	0	17530,5
Non-responder	Macro_FOLR2+APOE+	Macro_IFI27	APOC2	LRP1	3,65984E-03	13563,7	1,76518E-02	3789	4,91150E-01	8278	1,39855E+00	6665	8,09808E-01	31556	0	17530,5
Non-responder	Macro_IER3	cDC(CD1C)	ICAM1	IL2RG	3,66016E-03	15719,9	7,04625E-03	17346	4,55599E-01	9600	8,41387E-01	16842	8,68872E-01	17281	0	17530,5
Non-responder	Macro_LYVE1	Macro_FOLR2+APOE+	CD99	CD81	3,66122E-03	10176,7	1,01480E-02	9633	5,52459E-01	6433	1,12124E+00	10781	9,12501E-01	6506	0	17530,5
Non-responder	Macro_LYVE1	Macro_FOLR2+APOE+	CD99	PILRA	3,66439E-03	11885,3	8,56784E-03	12590	4,79123E-01	8735	9,77222E-01	13621	9,10437E-01	6950	0	17530,5
Non-responder	Macro_OLFML3	Mono_CD14	HSP90B1	ASGR1	3,66544E-03	13056,5	1,06826E-02	8897	4,51316E-01	9795	1,07108E+00	11709	8,68605E-01	17351	0	17530,5
Non-responder	Macro_LYVE1	Macro_FOLR2+APOE+	HLA-DPA1	CD4	3,66861E-03	9402,1	7,97635E-03	14199	5,86579E-01	5589	1,20263E+00	9421	9,49342E-01	271	0	17530,5
Non-responder	Macro_LYVE1	Macro_FOLR2+APOE+	HLA-DPA1	LAG3	3,66967E-03	10618,1	1,19743E-02	7368	6,44162E-01	4489	1,27012E+00	8403	8,76577E-01	15300	0	17530,5
Non-responder	cDC_CLEC9A	Mono_CD14	HLA-DOB	CD4	3,66989E-03	8674,5	2,65514E-02	1877	1,21508E+00	400	1,79445E+00	3145	8,55537E-01	20420	0	17530,5
Non-responder	Macro_ISG15	Macro_ISG15	TIMP1	CD63	3,66989E-03	7256,9	8,10284E-03	13822	7,40175E-01	3145	2,19732E+00	1389	9,47358E-01	398	0	17530,5
Non-responder	Macro_LYVE1	Macro_FOLR2+APOE+	MRC1	PTPRC	3,67073E-03	10382,1	1,27948E-02	6579	6,23915E-01	4852	1,40148E+00	6636	8,72515E-01	16313	0	17530,5
Non-responder	Macro_LYVE1	Macro_FOLR2+APOE+	HLA-DMA	CD4	3,67178E-03	10019,1	8,27637E-03	13342	5,47263E-01	6560	1,15193E+00	10273	9,32978E-01	2390	0	17530,5
Non-responder	Macro_LYVE1	Macro_FOLR2+APOE+	HLA-DMB	CD4	3,67284E-03	10435,9	8,52749E-03	12692	5,27442E-01	7107	1,23399E+00	8920	9,15120E-01	5930	0	17530,5
Non-responder	Macro_LYVE1	Macro_FOLR2+APOE+	B2M	TFRC	3,67708E-03	11921,7	7,73304E-03	14907	3,80649E-01	12950	1,02303E+00	12680	9,37061E-01	1541	0	17530,5
Non-responder	Macro_NLRP3	Macro_FOLR2+APOE+	NRG1	SIGLEC7	3,67712E-03	19019,3	2,68354E-02	1847	4,90951E-01	8288	1,28337E+00	8233	6,75842E-01	59198	0	17530,5
Non-responder	Macro_FOLR2+APOE+	Macro_NLRP3	C1QB	C1QBP	3,68232E-03	11912,5	5,81116E-03	23536	5,27707E-01	7101	1,80076E+00	3104	9,04529E-01	8291	0	17530,5
Non-responder	Macro_FOLR2+APOE+	Mono_CD14	ICAM1	ITGAM_ITGB2	3,68343E-03	12566,5	7,03869E-03	17368	4,51333E-01	9793	1,27473E+00	8337	8,97753E-01	9804	0	17530,5
Non-responder	Macro_OLFML3	Mono_CD16	SEMA4A	LILRB2	3,68980E-03	9497,5	1,13847E-02	7993	8,50174E-01	2088	1,91173E+00	2502	8,68490E-01	17374	0	17530,5
Non-responder	Macro_IFI27	pDC_LILRA4	HLA-DQA1	CD4	3,69299E-03	11906,7	7,03590E-03	17377	4,54720E-01	9634	1,02209E+00	12698	9,33460E-01	2294	0	17530,5
Non-responder	Macro_NLRP3	Mono_CD16	TGM2	ITGA4	3,69446E-03	14617,7	1,11436E-02	8298	5,11288E-01	7621	1,77393E+00	3265	7,89137E-01	36374	0	17530,5
Non-responder	Macro_OLFML3	Macro_NLRP3	CD14	TLR4	3,69724E-03	14069,9	8,17714E-03	13623	3,58356E-01	14148	1,32252E+00	7667	8,68458E-01	17381	0	17530,5
Non-responder	Macro_LYVE1	Macro_FOLR2+APOE+	HLA-DRB5	CD4	3,69724E-03	9680,3	7,97860E-03	14194	5,76085E-01	5824	1,14781E+00	10326	9,45602E-01	527	0	17530,5
Non-responder	Macro_LYVE1	Macro_FOLR2+APOE+	LILRB4	LAIR1	3,69937E-03	8043,7	9,94068E-03	9943	7,19268E-01	3388	1,71598E+00	3661	9,16216E-01	5696	0	17530,5
Non-responder	Macro_FOLR2+APOE+	Macro_IER3	APOE	LDLR	3,69967E-03	12716,9	1,14899E-02	7882	4,90594E-01	8301	1,67547E+00	3973	8,32749E-01	25898	0	17530,5
Non-responder	pDC_LILRA4	Mono_INHBA	SEMA7A	PLXNC1	3,70078E-03	14418,1	2,17982E-02	2620	8,11650E-01	2440	1,79325E+00	3154	7,45774E-01	46346	0	17530,5
Non-responder	Macro_FOLR2+APOE+	Macro_NLRP3	B2M	LILRB1	3,70489E-03	13239,5	4,68779E-03	32128	4,90571E-01	8304	1,56356E+00	4904	9,28367E-01	3331	0	17530,5
Non-responder	Macro_FOLR2+APOE+	cDC(CD1C)	C1QA	CD93	3,70576E-03	11508,9	7,03462E-03	17389	5,71611E-01	5941	1,52938E+00	5264	8,91293E-01	11420	0	17530,5
Non-responder	Macro_NLRP3	Macro_FOLR2-APOE+	S100A9	TLR4	3,71215E-03	13090,3	9,25209E-03	11126	7,55125E-01	2969	8,57983E-01	16431	8,68400E-01	17395	0	17530,5
Non-responder	pDC_LILRA4	pDC_LILRA4	HMGB1	THBD	3,71883E-03	7624,3	1,12114E-02	8186	6,85113E-01	3819	2,99767E+00	274	9,04457E-01	8312	0	17530,5
Non-responder	cDC_CLEC9A	Macro_ISG15	HLA-DQB2	LAG3	3,72147E-03	11666,1	2,15379E-02	2680	8,24635E-01	2319	1,79194E+00	3160	8,05080E-01	32641	0	17530,5
Non-responder	Mono_CD16	Macro_ISG15	S100A9	CD36	3,72177E-03	15188,9	8,43974E-03	12929	4,00740E-01	11954	8,69631E-01	16127	8,68351E-01	17404	0	17530,5
Non-responder	Macro_FOLR2+APOE+	Macro_OLFML3	LRPAP1	LRP1	3,72284E-03	15045,5	7,30081E-03	16357	3,72598E-01	13356	1,13281E+00	10579	8,68335E-01	17405	0	17530,5
Non-responder	Macro_LYVE1	Macro_FOLR2+APOE+	APOE	TREM2	3,72712E-03	9587,1	1,52632E-02	4860	4,86456E-01	8456	8,56373E-01	16457	9,44296E-01	632	0	17530,5
Non-responder	Macro_NLRP3	cDC_CLEC9A	GNAI2	CXCR3	3,72756E-03	11720,5	1,64531E-02	4270	4,90204E-01	8317	1,38925E+00	6770	8,50110E-01	21715	0	17530,5
Non-responder	Macro_FOLR2+APOE+	Macro_NLRP3	LGALS3BP	ITGB1	3,72931E-03	11348,9	1,11309E-02	8318	5,44413E-01	6646	1,61169E+00	4495	8,58393E-01	19755	0	17530,5
Non-responder	pDC_LILRA4	cDC_LAMP3	SPON2	ITGA5	3,73531E-03	15521,1	1,96424E-02	3164	9,80461E-01	1298	2,32547E+00	1076	7,04593E-01	54537	0	17530,5
Non-responder	Macro_IER3	Mono_CD14	CCL3	CCR1	3,73783E-03	10343,5	1,21209E-02	7212	6,80054E-01	3906	1,48865E+00	5650	8,68262E-01	17419	0	17530,5
Non-responder	Macro_FOLR2+APOE+	Macro_FOLR2+APOE+	ADAM10	NOTCH1	3,74033E-03	19440,9	1,43684E-02	5401	4,32668E-01	10507	9,74929E-01	13675	7,28375E-01	50091	0	17530,5
Non-responder	Mono_CD16	Mono_CD16	S100A9	ITGB2	3,74213E-03	11670,3	7,02678E-03	17423	4,67373E-01	9165	1,03024E+00	12544	9,36319E-01	1689	0	17530,5
Non-responder	Mono_CD14	Macro_ISG15	CXCL8	SDC2	3,74224E-03	10882,9	2,17727E-02	2627	7,38121E-01	3166	1,91120E+00	2505	8,22029E-01	28586	0	17530,5
Non-responder	Macro_OLFML3	Mono_CD16	CD1D	LILRB2	3,74508E-03	9203,9	1,55884E-02	4697	8,34751E-01	2232	1,80284E+00	3092	8,63856E-01	18468	0	17530,5
Non-responder	Macro_NLRP3	Macro_FOLR2+APOE-	S100A8	TLR4	3,74535E-03	12023,3	1,13030E-02	8091	7,90230E-01	2639	9,42723E-01	14430	8,68219E-01	17426	0	17530,5
Non-responder	pDC_LILRA4	Mono_INHBA	LTB	CD40	3,74571E-03	8965,3	1,96352E-02	3167	1,10436E+00	749	2,97837E+00	289	8,44383E-01	23091	0	17530,5
Non-responder	cDC_CLEC9A	Mono_CD16	HLA-A	LILRB2	3,74571E-03	7200,3	8,37725E-03	13079	7,38104E-01	3167	2,05101E+00	1885	9,48247E-01	340	0	17530,5
Non-responder	Macro_LYVE1	Macro_FOLR2+APOE+	HLA-DRB5	LAG3	3,74750E-03	11239,7	1,19777E-02	7364	6,33668E-01	4654	1,21530E+00	9222	8,68211E-01	17428	0	17530,5
Non-responder	Macro_FOLR2+APOE+	Macro_FOLR2-APOE+	HLA-DMB	CD4	3,74965E-03	13037,7	7,40257E-03	16001	5,28610E-01	7077	8,19605E-01	17430	9,09462E-01	7150	0	17530,5
Non-responder	Macro_NLRP3	cDC_CLEC9A	CD52	SIGLEC10	3,75180E-03	15298,3	7,54174E-03	15517	3,83833E-01	12776	9,94707E-01	13236	8,68194E-01	17432	0	17530,5
Non-responder	Macro_OLFML3	Mono_CD16	MRC1	PTPRC	3,75210E-03	9954,7	1,16029E-02	7749	6,56517E-01	4280	1,91560E+00	2481	8,66977E-01	17733	0	17530,5
Non-responder	Macro_IER3	Mono_CD14	TNF	TNFRSF1B	3,75611E-03	11893,7	9,17143E-03	11284	5,33586E-01	6941	1,42997E+00	6277	8,68191E-01	17436	0	17530,5
Non-responder	pDC_LILRA4	cDC_CLEC9A	CALM2	MYLK	3,75613E-03	12871,1	4,14134E-02	913	7,37903E-01	3170	1,95084E+00	2311	7,72143E-01	40431	0	17530,5
Non-responder	pDC_LILRA4	Macro_OLFML3	HLA-B	LILRB1	3,75960E-03	7818,5	7,49583E-03	15693	7,37881E-01	3171	2,41258E+00	911	9,35897E-01	1787	0	17530,5
Non-responder	Macro_FOLR2+APOE+	Macro_OLFML3	HLA-B	CANX	3,76089E-03	13087,9	4,73366E-03	31682	4,89685E-01	8336	1,36497E+00	7073	9,42224E-01	818	0	17530,5
Non-responder	Mono_CD16	Mono_INHBA	SERPINA1	LRP1	3,76905E-03	11833,3	7,02211E-03	17448	5,09161E-01	7691	1,22300E+00	9094	9,08257E-01	7403	0	17530,5
Non-responder	Mono_CD16	Macro_FOLR2+APOE+	AGTRAP	RACK1	3,77013E-03	14120,5	7,07210E-03	17219	4,33956E-01	10464	1,30277E+00	7940	8,68159E-01	17449	0	17530,5
Non-responder	Macro_OLFML3	Macro_NLRP3	TNFSF13B	TFRC	3,77121E-03	12652,3	7,69887E-03	15012	5,12195E-01	7593	1,48686E+00	5676	8,68152E-01	17450	0	17530,5
Non-responder	Macro_NLRP3	Mono_INHBA	TGM2	SDC4	3,77146E-03	19155,5	1,59929E-02	4486	4,89418E-01	8342	1,39668E+00	6688	6,78775E-01	58731	0	17530,5
Non-responder	pDC_LILRA4	Macro_IER3	APP	LRP10	3,77162E-03	8780,7	1,72480E-02	3953	1,13478E+00	633	2,58571E+00	644	8,52584E-01	21143	0	17530,5
Non-responder	Macro_OLFML3	Mono_CD16	ICAM1	SPN	3,77851E-03	12501,9	1,11099E-02	8346	5,32530E-01	6972	1,51149E+00	5431	8,39722E-01	24230	0	17530,5
Non-responder	Macro_FOLR2+APOE+	Macro_LYVE1	HLA-DRA	CD4	3,77851E-03	11066,1	6,21132E-03	21179	4,89280E-01	8346	1,31534E+00	7761	9,45791E-01	514	0	17530,5
Non-responder	Macro_IER3	Macro_FOLR2+APOE+	TNFSF13	TNFRSF1A	3,77986E-03	16362,3	7,01954E-03	17458	3,26602E-01	15997	8,62823E-01	16301	8,79525E-01	14525	0	17530,5
Non-responder	Macro_LYVE1	Macro_FOLR2+APOE+	C3	C3AR1	3,78203E-03	10254,3	1,35869E-02	5935	4,98371E-01	8040	1,18466E+00	9713	8,96699E-01	10053	0	17530,5
Non-responder	Mast	Macro_OLFML3	ADCYAP1	GPR84	3,78324E-03	13369,1	1,36046E-01	66	1,13217E+00	645	1,78206E+00	3217	7,50169E-01	45387	0	17530,5
Non-responder	Macro_FOLR2+APOE+	Macro_FOLR2+APOE+	SPP1	PTGER4	3,78735E-03	10343,5	1,79894E-02	3664	4,89169E-01	8351	2,48328E+00	787	8,51495E-01	21385	0	17530,5
Non-responder	Mono_CD16	Mono_CD16	NAMPT	INSR	3,79266E-03	14056,5	1,11298E-02	8322	4,89075E-01	8354	1,30277E+00	7939	8,23841E-01	28137	0	17530,5
Non-responder	Mono_CD16	pDC_LILRA4	TNFSF13B	TFRC	3,79722E-03	12998,7	7,68319E-03	15059	4,36249E-01	10365	1,60347E+00	4565	8,68035E-01	17474	0	17530,5
Non-responder	Macro_FOLR2+APOE+	Macro_FOLR2+APOE+	MDK	ITGA6_ITGB1	3,79797E-03	18091,9	2,67172E-02	1858	5,54759E-01	6370	1,27332E+00	8357	6,93937E-01	56344	0	17530,5
Non-responder	Macro_FOLR2+APOE+	Macro_FOLR2+APOE+	CXCL12	ITGA5	3,79797E-03	17480,9	1,75823E-02	3812	4,88935E-01	8357	1,53706E+00	5171	7,15574E-01	52534	0	17530,5

Post_NR_Myeloid_Myeloid_CellCell

Non-responder	Macro_NLRP3	Macro_NLRP3	LGALS9	CD44	3,79975E-03	13570,1	5,48113E-03	25749	4,90064E-01	8322	1,30695E+00	7891	9,04229E-01	8358	0	17530,5
Non-responder	Macro_LYVE1	Macro_FOLR2+APOE+	C3	ITGAX	3,80265E-03	13192,5	1,07485E-02	8817	3,96170E-01	12179	9,53620E-01	14184	8,84277E-01	13252	0	17530,5
Non-responder	Macro_LYVE1	Macro_FOLR2+APOE+	F13A1	ITGA4	3,80483E-03	7189,1	1,81261E-02	3619	9,58041E-01	1406	1,98218E+00	2164	8,92109E-01	11226	0	17530,5
Non-responder	Mono_CD16	Macro_LYVE1	SELPLG	ITGB2	3,80483E-03	14323,5	7,01376E-03	17481	3,75535E-01	13209	8,31625E-01	17114	9,13418E-01	6283	0	17530,5
Non-responder	pDC_LILRA4	Mono_INHBA	LTB	TNFRSF1A	3,80499E-03	7943,3	1,95696E-02	3184	1,01841E+00	1123	2,87274E+00	358	8,67858E-01	17521	0	17530,5
Non-responder	Macro_LYVE1	Macro_FOLR2+APOE+	A2M	LRP1	3,81027E-03	9012,3	1,52317E-02	4871	6,24105E-01	4850	1,20413E+00	9397	9,04027E-01	8413	0	17530,5
Non-responder	Macro_NLRP3	Mono_CD16	LGALS1	PTPRC	3,81217E-03	11373,9	6,12833E-03	21639	4,88803E-01	8365	1,29427E+00	8055	9,38603E-01	1280	0	17530,5
Non-responder	Macro_LYVE1	Macro_FOLR2+APOE+	C3	LRP1	3,81463E-03	13812,5	1,09458E-02	8558	3,52173E-01	14520	8,89085E-01	15649	8,86021E-01	12805	0	17530,5
Non-responder	Macro_FOLR2+APOE+	Mono_CD16	ICAM1	ITGAX_ITGB2	3,81572E-03	11679,5	7,01074E-03	17491	4,88161E-01	8385	1,42594E+00	6335	9,02811E-01	8656	0	17530,5
Non-responder	Macro_OLFML3	cDC_CLEC9A	FLT3LG	FLT3	3,82254E-03	14945,1	4,84701E-02	693	1,18823E+00	472	1,78735E+00	3189	7,13915E-01	52841	0	17530,5
Non-responder	Macro_FOLR2+APOE+	cDC(CD1C)	CXCL10	CXCR3	3,82285E-03	17223,9	1,17281E-02	7620	4,88649E-01	8371	1,72500E+00	3590	7,33630E-01	49008	0	17530,5
Non-responder	Macro_FOLR2+APOE+	Macro_IER3	PLAU	ITGA5	3,82285E-03	17383,5	1,10904E-02	8371	4,94502E-01	8175	1,41185E+00	6513	7,45868E-01	46328	0	17530,5
Non-responder	Macro_LYVE1	Macro_FOLR2+APOE+	C1QA	CD33	3,82446E-03	8693,1	1,26875E-02	6667	6,43758E-01	4496	1,26971E+00	8408	9,13085E-01	6364	0	17530,5
Non-responder	Macro_LYVE1	Macro_FOLR2+APOE+	C1QB	CD33	3,82555E-03	9101,9	1,25176E-02	6825	6,16640E-01	4988	1,18928E+00	9641	9,12418E-01	6525	0	17530,5
Non-responder	Macro_IER3	Macro_ISG15	FN1	PLAUR	3,82555E-03	11489,5	1,05586E-02	9058	5,24927E-01	7186	1,43824E+00	6173	8,67914E-01	17500	0	17530,5
Non-responder	Macro_LYVE1	Macro_FOLR2+APOE+	SERPING1	LRP1	3,82993E-03	11253,9	1,07462E-02	8823	5,40758E-01	6746	1,16163E+00	10107	8,84967E-01	13063	0	17530,5
Non-responder	Macro_NLRP3	Mono_CD16	HLA-F	LILRB1	3,83211E-03	13775,7	7,00707E-03	17506	4,54572E-01	9638	1,36320E+00	7086	8,69549E-01	17118	0	17530,5
Non-responder	Macro_LYVE1	Macro_FOLR2+APOE+	C1QB	C1QBP	3,83430E-03	9375,9	9,97061E-03	9893	6,05363E-01	5201	1,14609E+00	10359	9,25430E-01	3896	0	17530,5
Non-responder	Macro_FOLR2+APOE+	cDC(CD1C)	TNFSF13	TNFRSF1A	3,83430E-03	14734,9	7,00591E-03	17508	3,50912E-01	14603	1,19904E+00	9486	8,79422E-01	14547	0	17530,5
Non-responder	Mono_CD16	Mono_CD16	ICAM2	ITGAM_ITGB2	3,83759E-03	11295,7	1,33665E-02	6090	4,71388E-01	9015	1,42599E+00	6332	8,67871E-01	17511	0	17530,5
Non-responder	Macro_OLFML3	Mono_CD16	GNAI2	FPR1	3,83759E-03	13252,7	7,00555E-03	17511	3,60199E-01	14041	1,29424E+00	8056	9,00676E-01	9125	0	17530,5
Non-responder	Macro_IER3	Macro_OLFML3	ICAM1	IL2RG	3,83869E-03	14637,3	7,00520E-03	17512	4,52952E-01	9712	1,10553E+00	11071	8,68539E-01	17361	0	17530,5
Non-responder	cDC_CLEC9A	Macro_NLRP3	MIF	TNFRSF10D	3,84015E-03	10201,1	1,95359E-02	3194	7,73034E-01	2786	1,85259E+00	2799	8,37785E-01	24696	0	17530,5
Non-responder	Macro_FOLR2+APOE+	Macro_FOLR2+APOE+	GNAI2	C5AR1	3,84197E-03	11818,7	7,00482E-03	17515	4,27235E-01	10744	1,60105E+00	4581	9,02534E-01	8723	0	17530,5
Non-responder	Macro_OLFML3	Macro_FOLR2+APOE+	LGALS3	LAG3	3,84247E-03	14062,7	1,14236E-02	7953	4,88255E-01	8382	1,37306E+00	6975	8,18331E-01	29473	0	17530,5
Non-responder	pDC_LILRA4	Macro_LYVE1	HSP90B1	TLR2	3,84247E-03	6991,7	1,10848E-02	8382	9,29189E-01	1559	2,45557E+00	833	9,11821E-01	6654	0	17530,5
Non-responder	Macro_IER3	pDC_LILRA4	HLA-DQB1	CD4	3,84417E-03	10429,9	7,00439E-03	17517	4,47808E-01	9915	1,51645E+00	5380	9,35788E-01	1807	0	17530,5
Non-responder	Macro_FOLR2+APOE+	Macro_IER3	CD14	ITGA4	3,84636E-03	11802,7	7,00402E-03	17519	5,20647E-01	7304	1,36050E+00	7129	8,98818E-01	9531	0	17530,5
Non-responder	Mono_CD14	Mono_CD16	THBS1	ITGA4	3,85073E-03	6753,9	1,95226E-02	3197	9,01711E-01	1724	2,35787E+00	1008	8,95730E-01	10310	0	17530,5
Non-responder	Mono_CD16	Macro_NLRP3	ICAM2	ITGAL_ITGB2	3,85321E-03	11601,3	1,25220E-02	6822	4,88070E-01	8388	1,42992E+00	6278	8,61689E-01	18988	0	17530,5
Non-responder	cDC_LAMP3	cDC_CLEC9A	CD274	PDCD1	3,85332E-03	18818,9	1,30326E-01	73	1,13020E+00	651	1,30038E+00	7974	5,97922E-01	67866	0	17530,5
Non-responder	Macro_ISG15	pDC_LILRA4	S100A9	CD36	3,85500E-03	6771,3	1,72422E-02	3955	7,39717E-01	3146	2,45476E+00	836	9,04102E-01	8389	0	17530,5
Non-responder	Macro_NLRP3	Mono_CD14	CD55	ADGRE5	3,85500E-03	11006,3	1,10785E-02	8389	6,46079E-01	4451	1,46146E+00	5931	8,62696E-01	18730	0	17530,5
Non-responder	Macro_LYVE1	Macro_FOLR2+APOE+	HMGB1	CD163	3,85515E-03	12471,3	7,40684E-03	15976	4,19737E-01	11063	9,99211E-01	13142	9,21502E-01	4645	0	17530,5
Non-responder	Mono_CD16	pDC_LILRA4	HMGB1	CXCR4	3,85515E-03	9593,3	7,00193E-03	17527	4,75295E-01	8867	2,41588E+00	906	9,29287E-01	3136	0	17530,5
Non-responder	Macro_OLFML3	Mono_CD16	RPS19	C5AR1	3,85679E-03	10625,3	6,68764E-03	18852	5,39023E-01	6792	1,52821E+00	5275	9,21334E-01	4677	0	17530,5
Non-responder	Macro_FOLR2+APOE-	Macro_FOLR2-APOE+	APOE	TREM2	3,85780E-03	15256,1	2,08920E-02	2837	7,34937E-01	3199	-2,53935E-01	52581	9,51999E-01	133	0	17530,5
Non-responder	Macro_FOLR2+APOE+	pDC_LILRA4	HGF	ST14	3,85780E-03	18079,1	1,99835E-02	3067	3,67511E-01	13662	1,78559E+00	3199	7,13393E-01	52937	0	17530,5
Non-responder	Mono_CD14	Macro_NLRP3	S100A8	CD36	3,85780E-03	6251,7	1,95191E-02	3199	1,14249E+00	603	2,49039E+00	779	9,00607E-01	9147	0	17530,5
Non-responder	cDC_LAMP3	cDC_CLEC9A	HLA-DQA1	LAG3	3,85845E-03	10217,7	1,61070E-02	4427	7,61241E-01	2907	1,25063E+00	8694	8,67819E-01	17530	0	17530,5
Non-responder	Macro_OLFML3	Mono_CD16	CD38	PECAM1	3,85859E-03	14217,5	1,45209E-02	5286	6,67506E-01	4098	1,60515E+00	4556	7,75369E-01	39617	0	17530,5
Non-responder	Macro_NLRP3	cDC_CLEC9A	TNF	VSIR	3,85859E-03	12422,3	1,10744E-02	8391	6,18925E-01	4948	1,74924E+00	3426	8,25140E-01	27816	0	17530,5
Non-responder	Macro_FOLR2+APOE+	Mono_CD16	ADAM10	CD44	3,85955E-03	15917,5	7,47315E-03	15769	3,59050E-01	14110	9,32965E-01	14647	8,67813E-01	17531	0	17530,5
Non-responder	Macro_IFI27	Mast	LGALS1	CD69	3,86396E-03	8433,3	1,75475E-02	3825	9,30704E-01	1552	8,15660E-01	17535	9,36163E-01	1724	0	17530,5
Non-responder	Macro_IER3	Macro_ISG15	MRC1	PTPRC	3,86396E-03	10770,5	1,17724E-02	7564	5,97622E-01	5365	1,46816E+00	5858	8,67811E-01	17535	0	17530,5
Non-responder	Macro_ISG15	Macro_NLRP3	PKM	CD44	3,86488E-03	7731,9	8,14333E-03	13719	7,34898E-01	3201	1,83479E+00	2916	9,38513E-01	1293	0	17530,5
Non-responder	Macro_IER3	Macro_ISG15	PLAU	PLAUR	3,86727E-03	11256,3	9,91399E-03	9989	5,91569E-01	5492	1,48169E+00	5732	8,67803E-01	17538	0	17530,5
Non-responder	Macro_FOLR2+APOE+	Mono_CD16	APOC2	LRP1	3,86756E-03	12700,7	1,74957E-02	3850	4,87702E-01	8396	2,01735E+00	2014	8,09124E-01	31713	0	17530,5
Non-responder	Macro_OLFML3	Macro_FOLR2+APOE+	PSAP	LRP1	3,86837E-03	12950,3	6,99772E-03	17539	3,39988E-01	15187	1,09087E+00	11315	9,29059E-01	3180	0	17530,5
Non-responder	Mono_INHBA	Macro_ISG15	TNF	VSIR	3,86842E-03	8435,1	1,95023E-02	3202	9,48422E-01	1453	2,28528E+00	1154	8,62299E-01	18836	0	17530,5
Non-responder	Mono_CD16	Macro_FOLR2+APOE+	IL15	IL2RA	3,86936E-03	20312,3	1,35453E-02	5964	4,87679E-01	8397	1,49086E+00	5631	6,37545E-01	64039	0	17530,5
Non-responder	Macro_FOLR2+APOE+	Macro_FOLR2+APOE-	PSAP	LRP1	3,86947E-03	11503,1	6,99744E-03	17540	5,50176E-01	6500	1,01898E+00	12764	9,29058E-01	3181	0	17530,5
Non-responder	Macro_FOLR2+APOE+	Mono_CD14	HLA-B	LILRB1	3,87295E-03	14159,7	4,60252E-03	32947	4,87637E-01	8399	1,37842E+00	6908	9,19617E-01	5014	0	17530,5
Non-responder	Macro_LYVE1	Macro_FOLR2+APOE+	CD59	STAB1	3,87830E-03	11300,1	1,12420E-02	8149	5,51767E-01	6450	1,21782E+00	9177	8,76995E-01	15194	0	17530,5
Non-responder	pDC_LILRA4	Macro_FOLR2-APOE+	IL16	CD9	3,88015E-03	7215,9	2,33032E-02	2314	8,32043E-01	2261	1,49581E+00	5571	9,04058E-01	8403	0	17530,5
Non-responder	cDC_CLEC9A	cDC_CLEC9A	HLA-DPB1	LAG3	3,88261E-03	6623,7	1,94914E-02	3206	1,03246E+00	1057	2,32714E+00	1072	8,95956E-01	10253	0	17530,5
Non-responder	Macro_ISG15	Mono_CD16	HLA-C	LILRB2	3,88261E-03	7179,1	8,53128E-03	12681	7,34242E-01	3206	1,96967E+00	2211	9,49376E-01	267	0	17530,5
Non-responder	Macro_FOLR2+APOE+	cDC(CD1C)	CD14	ITGA4	3,88273E-03	11858,7	6,99527E-03	17552	5,20148E-01	7321	1,34652E+00	7340	8,98762E-01	9550	0	17530,5
Non-responder	Macro_OLFML3	Macro_OLFML3	TNFSF13	TNFRSF14	3,88605E-03	15492,7	6,99475E-03	17555	3,55937E-01	14302	1,00692E+00	13008	8,77473E-01	15068	0	17530,5
Non-responder	Macro_LYVE1	Macro_ISG15	HLA-DPA1	LAG3	3,88916E-03	6961,3	2,10690E-02	2798	1,03002E+00	1068	1,55329E+00	5002	9,04038E-01	8408	0	17530,5
Non-responder	Macro_ISG15	Macro_FOLR2+APOE+	HLA-B	LILRB2	3,89327E-03	8355,9	6,90037E-03	17936	7,34178E-01	3209	1,88229E+00	2653	9,46563E-01	451	0	17530,5
Non-responder	cDC_LAMP3	cDC(CD1C)	TNFSF13B	HLA-DPB1	3,89380E-03	9217,7	6,99199E-03	17562	6,51631E-01	4370	1,44137E+00	6128	9,46029E-01	498	0	17530,5
Non-responder	Macro_FOLR2+APOE+	Mono_CD14	HSP90B1	TLR4	3,89824E-03	13877,1	8,19714E-03	13568	4,15422E-01	11269	1,20039E+00	9452	8,67668E-01	17566	0	17530,5
Non-responder	Macro_IER3	Macro_FOLR2+APOE+	MIF	TNFRSF14	3,89935E-03	13342,5	6,99156E-03	17567	4,83972E-01	8554	9,89832E-01	13343	8,98115E-01	9718	0	17530,5
Non-responder	Macro_ISG15	pDC_LILRA4	CCL24	CCR2	3,90039E-03	15191,3	5,59426E-02	534	7,33552E-01	3212	2,57824E+00	655	7,07407E-01	54025	0	17530,5
Non-responder	Macro_FOLR2+APOE+	Mono_INHBA	C1QB	C1QBP	3,90542E-03	12404,9	5,74292E-03	23976	5,24829E-01	7190	1,56289E+00	4911	9,04018E-01	8417	0	17530,5
Non-responder	Macro_LYVE1	Macro_NLRP3	CD14	TLR4	3,90823E-03	10611,7	1,11016E-02	8356	6,08732E-01	5130	1,22981E+00	8977	8,84960E-01	13065	0	17530,5
Non-responder	Macro_OLFML3	Macro_FOLR2+APOE+	TGFB1	ENG	3,90934E-03	13378,7	9,08041E-03	11444	4,14842E-01	11300	1,22579E+00	9043	8,67620E-01	17576	0	17530,5
Non-responder	pDC_LILRA4	cDC(CD1C)	B2M	CD1B	3,91109E-03	6736,9	2,68406E-02	1844	7,33425E-01	3214	2,21111E+00	1341	8,97963E-01	9755	0	17530,5
Non-responder	pDC_LILRA4	Macro_LYVE1	APP	NCSTN	3,91221E-03	9177,7	1,70498E-02	4021	1,13499E+00	631	2,57635E+00	656	8,44570E-01	23050	0	17530,5
Non-responder	Macro_FOLR2+APOE+	Macro_FOLR2+APOE+	CXCL12	ITGAV	3,91447E-03	17267,9	1,90239E-02	3329	4,87220E-01	8422	1,77398E+00	3264	7,08606E-01	53794	0	17530,5

Post_NR_Myeloid_Myeloid_CellCell

Non-responder	pDC_LILRA4	Macro_IFI27	APP	APLP2	3,91447E-03	6530,3	1,66194E-02	4188	1,08778E+00	818	2,10105E+00	1693	9,03964E-01	8422	0	17530,5
Non-responder	cDC(CD1C)	Macro_FOLR2+APOE+	TNFSF13B	TFRC	3,91714E-03	13549,1	7,62155E-03	15252	4,35549E-01	10401	1,37254E+00	6979	8,67573E-01	17583	0	17530,5
Non-responder	Macro_NLRP3	cDC(CD1C)	TIMP1	CD63	3,91810E-03	10134,7	6,69773E-03	18808	6,09527E-01	5111	1,26838E+00	8424	9,42402E-01	800	0	17530,5
Non-responder	Macro_OLFML3	Mono_CD16	FN1	C5AR1	3,92173E-03	11590,7	1,36381E-02	5896	4,87147E-01	8426	1,59528E+00	4644	8,51188E-01	21457	0	17530,5
Non-responder	Macro_NLRP3	pDC_LILRA4	HBEGF	CD9	3,92382E-03	12811,3	6,98332E-03	17589	4,12700E-01	11399	1,89496E+00	2572	8,77884E-01	14966	0	17530,5
Non-responder	Macro_FOLR2+APOE+	Macro_OLFML3	PLAU	ST14	3,93263E-03	14298,1	1,36192E-02	5917	4,86990E-01	8432	1,61131E+00	4498	7,94699E-01	35113	0	17530,5
Non-responder	Macro_NLRP3	Macro_OLFML3	NRG1	HLA-DPB1	3,93387E-03	12093,5	1,44354E-02	5345	4,85079E-01	8507	1,08172E+00	11487	8,67509E-01	17598	0	17530,5
Non-responder	Macro_FOLR2+APOE+	Macro_ISG15	AGTRAP	RACK1	3,93611E-03	15059,9	6,99073E-03	17569	4,09830E-01	11525	1,10508E+00	11075	8,67495E-01	17600	0	17530,5
Non-responder	Macro_IER3	Mono_CD16	HLA-A	LILRB1	3,93723E-03	12823,1	6,98133E-03	17601	3,90464E-01	12446	9,82399E-01	13490	9,29713E-01	3048	0	17530,5
Non-responder	Macro_FOLR2+APOE+	Mono_CD16	HSP90B1	ASGR1	3,93946E-03	11724,9	1,04760E-02	9186	4,77974E-01	8786	1,50196E+00	5519	8,67486E-01	17603	0	17530,5
Non-responder	Macro_FOLR2+APOE+	Mono_CD14	GNAI2	C5AR1	3,93946E-03	12161,1	6,98119E-03	17603	4,24833E-01	10849	1,44788E+00	6058	9,02385E-01	8765	0	17530,5
Non-responder	Macro_OLFML3	Mono_CD16	TGM2	ITGA4	3,93990E-03	13848,3	1,27586E-02	6608	5,53244E-01	6408	1,56985E+00	4855	8,00178E-01	33840	0	17530,5
Non-responder	Macro_NLRP3	Macro_LYVE1	TNF	TRPM2	3,94173E-03	15860,1	1,24392E-02	6900	4,86814E-01	8437	1,31008E+00	7844	7,79761E-01	38589	0	17530,5
Non-responder	Macro_OLFML3	Mono_CD16	PTPN6	CD300LF	3,94537E-03	12733,9	1,24616E-02	6874	5,00226E-01	7984	1,35909E+00	7146	8,40112E-01	24135	0	17530,5
Non-responder	Macro_OLFML3	Mono_CD16	GNAI2	S1PR4	3,94902E-03	12003,9	1,54154E-02	4777	5,46622E-01	6578	1,42750E+00	6317	8,37305E-01	24817	0	17530,5
Non-responder	Macro_NLRP3	pDC_LILRA4	TNF	TRPM2	3,94902E-03	14810,9	1,24362E-02	6906	4,86740E-01	8441	1,89398E+00	2583	7,79740E-01	38594	0	17530,5
Non-responder	Macro_LYVE1	Macro_ISG15	F13A1	ITGB1	3,95047E-03	6837,9	1,94073E-02	3225	9,70366E-01	1347	2,07572E+00	1787	8,95767E-01	10300	0	17530,5
Non-responder	Macro_FOLR2+APOE+	Macro_IER3	AGTRAP	RACK1	3,95179E-03	15405,3	7,53646E-03	15543	4,51009E-01	9804	8,12680E-01	17614	8,71756E-01	16535	0	17530,5
Non-responder	Macro_IFI27	pDC_LILRA4	HLA-DMA	CD4	3,95291E-03	12531,1	6,97810E-03	17615	4,09395E-01	11544	1,03542E+00	12441	9,27442E-01	3525	0	17530,5
Non-responder	Mono_INHBA	Macro_FOLR2+APOE+	S100A9	CD68	3,95449E-03	6658,5	1,10231E-02	8444	8,90881E-01	1788	1,51371E+00	5411	9,52355E-01	119	0	17530,5
Non-responder	Macro_OLFML3	Macro_FOLR2+APOE+	CD274	CD80	3,95814E-03	19116,3	1,99816E-02	3068	5,83472E-01	5655	1,26718E+00	8446	6,63434E-01	60882	0	17530,5
Non-responder	Macro_FOLR2+APOE+	Macro_ISG15	ICAM1	ITGAX_ITGB2	3,95852E-03	11373,3	6,97775E-03	17620	4,98870E-01	8029	1,55528E+00	4984	9,02604E-01	8703	0	17530,5
Non-responder	Macro_NLRP3	cDC_LAMP3	VCAN	ITGB1	3,96077E-03	11667,1	9,92558E-03	9969	7,49932E-01	3026	1,15664E+00	10188	8,67424E-01	17622	0	17530,5
Non-responder	Macro_LYVE1	Macro_NLRP3	GRN	TNFRSF1B	3,96190E-03	11609,7	7,13957E-03	16964	5,35562E-01	6880	9,38848E-01	14516	9,34144E-01	2158	0	17530,5
Non-responder	Macro_FOLR2+APOE+	Macro_OLFML3	CD14	TLR4	3,96414E-03	12165,9	8,02949E-03	14049	5,33436E-01	6946	1,59082E+00	4679	8,67413E-01	17625	0	17530,5
Non-responder	pDC_LILRA4	Macro_FOLR2+APOE+	MIF	TNFRSF14	3,96546E-03	6929,3	1,10202E-02	8450	8,22707E-01	2344	2,47339E+00	802	9,17130E-01	5520	0	17530,5
Non-responder	cDC_LAMP3	Macro_FOLR2+APOE+	HLA-F	LILRB1	3,96752E-03	15132,5	6,97670E-03	17628	4,25120E-01	10837	1,03317E+00	12484	8,69303E-01	17183	0	17530,5
Non-responder	Mono_CD16	Mono_CD16	B2M	LILRB1	3,97090E-03	10984,3	6,97618E-03	17631	4,03554E-01	11826	1,37896E+00	6903	9,40512E-01	1031	0	17530,5
Non-responder	Macro_ISG15	Macro_FOLR2+APOE+	LGALS3	ENG	3,97095E-03	7929,9	1,10175E-02	8453	6,08253E-01	5142	1,81808E+00	3015	9,17170E-01	5509	0	17530,5
Non-responder	Macro_LYVE1	Macro_NLRP3	PLAU	PLAUR	3,97428E-03	8980,5	1,24594E-02	6877	8,23732E-01	2334	1,68586E+00	3881	8,80369E-01	14280	0	17530,5
Non-responder	Mono_INHBA	Macro_FOLR2+APOE+	CCL3	CCR1	3,97566E-03	7064,5	1,93684E-02	3232	8,67788E-01	1939	2,12988E+00	1589	8,92835E-01	11032	0	17530,5
Non-responder	Mono_CD14	Mono_INHBA	S100A9	ITGB2	3,97566E-03	6324,9	1,08027E-02	8730	8,92089E-01	1781	1,78022E+00	3232	9,48000E-01	351	0	17530,5
Non-responder	Macro_ISG15	Macro_NLRP3	VCAN	CD44	3,97645E-03	7564,7	1,10155E-02	8456	7,65936E-01	2859	2,03925E+00	1932	9,10007E-01	7046	0	17530,5
Non-responder	Macro_FOLR2+APOE+	Macro_FOLR2+APOE+	APOE	LDLR	3,97828E-03	13037,5	1,12204E-02	8170	4,86454E-01	8457	1,58751E+00	4710	8,31090E-01	26320	0	17530,5
Non-responder	Macro_ISG15	Macro_ISG15	HLA-DRB1	LAG3	3,97927E-03	6616,9	1,93644E-02	3233	9,57161E-01	1411	2,06891E+00	1824	9,00857E-01	9086	0	17530,5
Non-responder	Macro_LYVE1	Macro_NLRP3	C3	ITGAX	3,98217E-03	10273,3	1,28500E-02	6526	5,08173E-01	7726	1,25534E+00	8626	8,93106E-01	10958	0	17530,5
Non-responder	Macro_NLRP3	Macro_ISG15	THBS1	SCARB1	3,98378E-03	14234,1	1,10111E-02	8460	6,66629E-01	4113	1,68388E+00	3896	7,85780E-01	37171	0	17530,5
Non-responder	Macro_FOLR2+APOE+	Mono_CD16	GRN	SORT1	3,98556E-03	11641,3	8,80526E-03	12030	5,22831E-01	7246	1,70480E+00	3756	8,67355E-01	17644	0	17530,5
Non-responder	Macro_LYVE1	Macro_NLRP3	F13A1	ITGB1	3,98669E-03	8403,3	1,46354E-02	5217	8,40340E-01	2180	1,78604E+00	3194	8,81838E-01	13895	0	17530,5
Non-responder	Macro_LYVE1	Macro_NLRP3	GNAI2	C5AR1	3,98782E-03	14012,3	7,12320E-03	17022	4,39238E-01	10260	8,47670E-01	16690	9,03268E-01	8559	0	17530,5
Non-responder	Macro_LYVE1	Macro_FOLR2+APOE+	HSPA1A	TLR4	3,98895E-03	13517,9	8,86026E-03	11906	4,67434E-01	9162	8,11565E-01	17647	8,91607E-01	11344	0	17530,5
Non-responder	Macro_LYVE1	Macro_NLRP3	HMGB1	THBD	3,99008E-03	12326,3	8,83173E-03	11965	4,55584E-01	9601	1,07122E+00	11706	8,93640E-01	10829	0	17530,5
Non-responder	Macro_ISG15	Macro_OLFML3	CCL2	CCR1	3,99011E-03	11050,5	2,45158E-02	2152	7,31991E-01	3236	2,66749E+00	533	8,08706E-01	31801	0	17530,5
Non-responder	Macro_LYVE1	Macro_NLRP3	A2M	LRP1	3,99347E-03	12325,7	1,15966E-02	7758	4,71811E-01	8991	8,74910E-01	15988	8,91529E-01	11361	0	17530,5
Non-responder	cDC_CLEC9A	cDC(CD1C)	HLA-DOB	CD4	3,99372E-03	7743,3	3,50261E-02	1197	1,32520E+00	231	1,77883E+00	3237	8,71827E-01	16521	0	17530,5
Non-responder	Macro_IFI27	Macro_OLFML3	C3	IFITM1	3,99573E-03	11476,9	1,47854E-02	5133	5,91225E-01	5499	8,11403E-01	17653	8,90734E-01	11569	0	17530,5
Non-responder	Macro_NLRP3	pDC_LILRA4	CCL4	CCR5	3,99665E-03	14643,9	1,10084E-02	8467	5,32645E-01	6968	1,86667E+00	2737	7,84278E-01	37517	0	17530,5
Non-responder	Macro_OLFML3	Macro_OLFML3	HLA-B	LILRB1	3,99665E-03	10407,9	6,80672E-03	18345	5,16760E-01	7447	1,42711E+00	6320	9,32943E-01	2397	0	17530,5
Non-responder	Macro_LYVE1	Macro_NLRP3	C1QB	LRP1	3,99800E-03	10253,3	1,01374E-02	9650	5,55954E-01	6333	9,75290E-01	13668	9,24461E-01	4085	0	17530,5
Non-responder	Macro_OLFML3	Mono_INHBA	TIMP2	CD44	3,99913E-03	15187,3	6,97027E-03	17656	4,57635E-01	9510	9,14247E-01	15068	8,73125E-01	16172	0	17530,5
Non-responder	Macro_OLFML3	Macro_OLFML3	HLA-C	LILRB1	4,00026E-03	10837,9	6,97006E-03	17657	4,63024E-01	9321	1,38363E+00	6843	9,30579E-01	2838	0	17530,5
Non-responder	Macro_LYVE1	Macro_NLRP3	PLTP	ABCA1	4,00253E-03	7195,5	1,71829E-02	3975	8,68236E-01	1931	1,84603E+00	2841	8,98168E-01	9700	0	17530,5
Non-responder	Macro_IER3	Macro_FOLR2+APOE+	HLA-DRB1	CD4	4,00366E-03	11678,3	6,96931E-03	17660	4,39130E-01	10265	1,03407E+00	12465	9,46304E-01	471	0	17530,5
Non-responder	Macro_ISG15	pDC_LILRA4	ICAM1	IL2RG	4,00402E-03	6747,9	1,41467E-02	5536	9,03998E-01	1709	2,71465E+00	493	9,03743E-01	8471	0	17530,5
Non-responder	Macro_ISG15	Macro_ISG15	HLA-DRA	LAG3	4,00770E-03	6673,9	1,87955E-02	3395	9,16544E-01	1634	1,94312E+00	2337	9,03732E-01	8473	0	17530,5
Non-responder	Macro_LYVE1	Macro_NLRP3	SERPING1	LRP1	4,00933E-03	15531,9	8,18158E-03	13607	3,88464E-01	12550	8,32407E-01	17087	8,70343E-01	16885	0	17530,5
Non-responder	Macro_LYVE1	Macro_NLRP3	TIMP2	CD44	4,01842E-03	9829,3	1,05221E-02	9109	6,81159E-01	3895	1,30293E+00	7936	8,94239E-01	10676	0	17530,5
Non-responder	Macro_OLFML3	Macro_FOLR2+APOE+	CD14	TLR4	4,01956E-03	14973,1	8,00656E-03	14119	3,48863E-01	14709	1,11784E+00	10833	8,67249E-01	17674	0	17530,5
Non-responder	Macro_LYVE1	Macro_FOLR2+APOE+	TNFSF13	TNFRSF1A	4,02297E-03	16667,3	6,96563E-03	17677	3,22343E-01	16263	8,27237E-01	17229	8,79116E-01	14637	0	17530,5
Non-responder	Mono_CD14	Macro_ISG15	S100A9	CD36	4,02432E-03	6384,7	1,70656E-02	4016	1,06061E+00	931	2,38031E+00	964	9,03655E-01	8482	0	17530,5
Non-responder	Macro_OLFML3	Macro_OLFML3	HLA-A	LILRB2	4,02432E-03	11522,7	5,83568E-03	23386	5,13406E-01	7552	1,30855E+00	7869	9,38622E-01	1276	0	17530,5
Non-responder	Macro_IFI27	Macro_ISG15	C3	ITGAX	4,02866E-03	11396,7	1,40886E-02	5578	5,56565E-01	6316	8,10244E-01	17682	8,97420E-01	9877	0	17530,5
Non-responder	Mono_CD14	pDC_LILRA4	COPA	P2RY6	4,03129E-03	8800,7	1,69212E-02	4070	1,12557E+00	666	2,98176E+00	281	8,51195E-01	21456	0	17530,5
Non-responder	cDC_LAMP3	Macro_NLRP3	CIRBP	TREM1	4,04007E-03	15119,1	8,94569E-03	11745	3,53546E-01	14439	8,09888E-01	17692	8,80738E-01	14189	0	17530,5
Non-responder	Mono_INHBA	Macro_ISG15	CCL5	CCR1	4,04093E-03	14701,1	2,54583E-02	2025	7,39425E-01	3149	1,77653E+00	3250	7,40365E-01	47551	0	17530,5
Non-responder	Macro_FOLR2+APOE+	Macro_ISG15	HLA-G	LILRB2	4,04100E-03	10741,5	1,09930E-02	8491	6,38955E-01	4569	1,75302E+00	3392	8,58523E-01	19725	0	17530,5
Non-responder	Macro_LYVE1	Macro_NLRP3	HSPA1A	TLR4	4,04235E-03	12356,1	9,04903E-03	11513	4,76927E-01	8820	1,01625E+00	12830	8,92621E-01	11087	0	17530,5
Non-responder	cDC_CLEC9A	Macro_IER3	HLA-DOB	CD4	4,04457E-03	7790,7	3,45420E-02	1233	1,31891E+00	238	1,77614E+00	3251	8,71048E-01	16701	0	17530,5
Non-responder	Macro_NLRP3	Mono_CD14	NAMPT	ITGA5_ITGB1	4,05035E-03	12525,1	6,96032E-03	17701	6,24020E-01	4851	1,44132E+00	6129	8,72192E-01	16414	0	17530,5
Non-responder	Macro_FOLR2+APOE+	Macro_FOLR2+APOE-	LILRB4	LAIR1	4,05150E-03	14222,1	6,96021E-03	17702	4,50954E-01	9810	8,31191E-01	17125	9,01482E-01	8943	0	17530,5
Non-responder	Mono_INHBA	Macro_OLFML3	CCL3L1	CCR1	4,05552E-03	9107,9	1,92814E-02	3254	8,35491E-01	2224	1,96146E+00	2248	8,56114E-01	20283	0	17530,5

Post_NR_Myeloid_Myeloid_CellCell

Non-responder	Macro_FOLR2+APOE+	Macro_IER3	HLA-A	LILRB1	4,05586E-03	14791,9	4,49663E-03	33992	5,07189E-01	7751	1,26306E+00	8499	9,13910E-01	6187	0	17530,5
Non-responder	Macro_LYVE1	Mono_CD16	HLA-B	LILRA1	4,05722E-03	7761,3	1,65763E-02	4207	6,82586E-01	3863	1,20781E+00	9323	9,25482E-01	3883	0	17530,5
Non-responder	Macro_FOLR2+APOE+	Macro_ISG15	C3	CD81	4,05837E-03	12571,5	9,91196E-03	9992	2,99804E-01	17708	1,52800E+00	5278	8,87764E-01	12349	0	17530,5
Non-responder	Macro_LYVE1	Mono_CD16	HLA-B	LILRB2	4,05837E-03	8708,3	8,10791E-03	13812	7,15805E-01	3438	1,25907E+00	8562	9,50498E-01	199	0	17530,5
Non-responder	Macro_FOLR2+APOE+	Macro_NLRP3	TNFSF10	TNFRSF10D	4,06330E-03	16597,7	1,59668E-02	4501	4,85176E-01	8503	1,33991E+00	7420	7,51756E-01	45034	0	17530,5
Non-responder	Macro_FOLR2+APOE+	Macro_FOLR2+APOE+	CD14	TLR6	4,06517E-03	13706,5	1,09814E-02	8504	5,58308E-01	6271	1,60162E+00	4577	8,09364E-01	31650	0	17530,5
Non-responder	Macro_IFI27	pDC_LILRA4	HSP90B1	TLR7	4,06869E-03	12882,1	1,38730E-02	5735	4,78072E-01	8783	9,33038E-01	14645	8,67042E-01	17717	0	17530,5
Non-responder	Macro_LYVE1	Mono_CD16	HLA-A	LILRB2	4,06984E-03	7659,9	8,78215E-03	12095	8,22905E-01	2340	1,44707E+00	6068	9,49393E-01	266	0	17530,5
Non-responder	Macro_LYVE1	Mono_CD16	HLA-A	LILRB1	4,07099E-03	11351,1	7,48328E-03	15738	5,13840E-01	7538	9,87958E-01	13377	9,31948E-01	2572	0	17530,5
Non-responder	Macro_OLFML3	Macro_FOLR2+APOE+	C1QB	C1QBP	4,07099E-03	13435,3	7,07725E-03	17196	2,99619E-01	17719	1,27917E+00	8281	9,12707E-01	6450	0	17530,5
Non-responder	Macro_FOLR2+APOE+	Macro_FOLR2+APOE+	TIMP1	CD63	4,07449E-03	12043,1	5,38400E-03	26400	5,66720E-01	6059	1,26253E+00	8509	9,36182E-01	1717	0	17530,5
Non-responder	cDC(CD1C)	Macro_ISG15	HLA-DQA2	CD4	4,07673E-03	12115,5	6,95380E-03	17724	4,61348E-01	9379	1,20502E+00	9363	9,12161E-01	6581	0	17530,5
Non-responder	cDC_CLEC9A	Mono_CD16	CD48	CD244	4,07748E-03	11510,3	2,97372E-02	1567	8,24772E-01	2318	1,77469E+00	3260	8,04122E-01	32876	0	17530,5
Non-responder	Mono_CD14	Macro_FOLR2+APOE+	THBS1	LRP1	4,07748E-03	6987,9	1,92585E-02	3260	8,10807E-01	2448	2,12869E+00	1593	8,96501E-01	10108	0	17530,5
Non-responder	Mast	Mono_CD16	TIMP3	CD44	4,07941E-03	7044,1	5,81998E-02	486	1,12409E+00	670	1,73056E+00	3548	8,85212E-01	12986	0	17530,5
Non-responder	Macro_FOLR2+APOE+	Macro_FOLR2+APOE+	ADAM10	NOTCH2	4,08024E-03	22245,5	6,75532E-03	18558	2,90370E-01	18358	7,80240E-01	18468	7,80890E-01	38313	0	17530,5
Non-responder	Macro_LYVE1	Mono_CD16	CD14	TLR4	4,08364E-03	14480,7	8,21342E-03	13519	4,90341E-01	8313	8,85820E-01	15722	8,68710E-01	17319	0	17530,5
Non-responder	cDC_LAMP3	pDC_LILRA4	ICAM1	SPN	4,08479E-03	8933,1	1,71949E-02	3973	8,02688E-01	2519	1,83511E+00	2912	8,66983E-01	17731	0	17530,5
Non-responder	Macro_LYVE1	Mono_CD16	CD14	ITGB2	4,08479E-03	10323,9	7,50601E-03	15654	5,62748E-01	6155	1,08666E+00	11403	9,41768E-01	877	0	17530,5
Non-responder	Macro_OLFML3	pDC_LILRA4	HLA-DPB1	CD4	4,08594E-03	9547,7	6,95172E-03	17732	4,71371E-01	9016	1,86499E+00	2744	9,43267E-01	716	0	17530,5
Non-responder	Macro_LYVE1	Mono_CD16	CD14	ITGA4	4,08710E-03	8452,1	1,07853E-02	8761	7,09735E-01	3523	1,38155E+00	6872	9,16829E-01	5574	0	17530,5
Non-responder	Macro_LYVE1	Mono_CD16	HLA-C	LILRB2	4,09055E-03	8112,5	8,61592E-03	12471	7,51819E-01	3007	1,35024E+00	7298	9,49612E-01	256	0	17530,5
Non-responder	Macro_LYVE1	Mono_CD16	HLA-C	LILRB1	4,09286E-03	12397,7	7,34164E-03	16222	4,42754E-01	10107	8,91125E-01	15610	9,32238E-01	2519	0	17530,5
Non-responder	Mono_CD14	Macro_FOLR2-APOE+	S100A9	ITGB2	4,09319E-03	6613,5	1,34426E-02	6038	1,07436E+00	881	1,26195E+00	8519	9,53133E-01	99	0	17530,5
Non-responder	Macro_LYVE1	Mono_CD16	CD99	PILRA	4,09517E-03	11925,5	8,48923E-03	12795	4,71613E-01	9003	9,93132E-01	13267	9,10061E-01	7032	0	17530,5
Non-responder	Mono_CD16	Mono_CD14	S100A9	ITGB2	4,09632E-03	12545,7	6,94880E-03	17741	4,56486E-01	9563	8,69450E-01	16132	9,35986E-01	1762	0	17530,5
Non-responder	Macro_LYVE1	Mono_CD16	MRC1	PTPRC	4,09979E-03	8356,9	1,57583E-02	4619	8,06473E-01	2483	1,70947E+00	3719	8,83659E-01	13433	0	17530,5
Non-responder	Macro_NLRP3	Macro_ISG15	CD52	SIGLEC10	4,10094E-03	15349,3	7,37530E-03	16100	3,74483E-01	13262	1,05208E+00	12109	8,66912E-01	17745	0	17530,5
Non-responder	Macro_ISG15	Mast	GRN	NTRK1	4,10358E-03	7705,5	7,39133E-02	282	1,12385E+00	672	1,43503E+00	6221	8,82213E-01	13822	0	17530,5
Non-responder	Macro_IER3	Macro_OLFML3	HBEGF	CD44	4,10441E-03	14302,5	7,29095E-03	16391	4,46200E-01	9973	8,07595E-01	17748	8,97449E-01	9870	0	17530,5
Non-responder	Macro_OLFML3	Macro_NLRP3	FN1	C5AR1	4,10444E-03	11886,3	1,35892E-02	5933	4,84498E-01	8525	1,46224E+00	5924	8,50961E-01	21519	0	17530,5
Non-responder	Macro_LYVE1	Mono_CD16	B2M	LILRB2	4,10557E-03	8872,9	8,09125E-03	13872	6,84615E-01	3827	1,22329E+00	9086	9,55616E-01	49	0	17530,5
Non-responder	Macro_FOLR2+APOE+	Macro_OLFML3	APOC2	LRP1	4,10819E-03	12852,3	1,73495E-02	3909	4,84471E-01	8527	1,92411E+00	2437	8,08475E-01	31858	0	17530,5
Non-responder	Macro_FOLR2-APOE+	Macro_FOLR2+APOE+	C3	CD81	4,11195E-03	7206,3	2,10913E-02	2793	8,28294E-01	2295	1,26143E+00	8529	9,20243E-01	4884	0	17530,5
Non-responder	Macro_FOLR2+APOE+	Macro_OLFML3	LGALS3BP	ITGB1	4,11195E-03	11660,7	1,09661E-02	8529	5,38507E-01	6806	1,50679E+00	5479	8,57484E-01	19959	0	17530,5
Non-responder	Macro_FOLR2+APOE+	Macro_IER3	TIMP2	CD44	4,11483E-03	15874,5	6,94388E-03	17757	3,15127E-01	16734	1,10265E+00	11126	8,72914E-01	16225	0	17530,5
Non-responder	Mono_INHBA	cDC_CLEC9A	NECTIN2	CD226	4,11569E-03	13151,1	4,90797E-02	673	1,15647E+00	559	1,64222E+00	4253	7,61750E-01	42740	0	17530,5
Non-responder	Mono_CD16	Macro_NLRP3	TNFSF10	TNFRSF10B	4,11571E-03	14695,1	1,09641E-02	8531	5,19974E-01	7330	1,59992E+00	4593	7,93062E-01	35491	0	17530,5
Non-responder	Macro_NLRP3	cDC(CD1C)	HBEGF	CD44	4,11599E-03	13344,9	7,49426E-03	15698	5,62004E-01	6171	8,07159E-01	17758	8,98707E-01	9567	0	17530,5
Non-responder	Macro_FOLR2+APOE+	Mono_CD14	HLA-F	LILRB2	4,11715E-03	12282,3	6,94295E-03	17759	5,07079E-01	7754	1,33342E+00	7507	8,93507E-01	10861	0	17530,5
Non-responder	Macro_NLRP3	Macro_OLFML3	TNF	TRAF2	4,11759E-03	18960,1	1,32779E-02	6150	4,86408E-01	8460	1,26127E+00	8532	7,06866E-01	54128	0	17530,5
Non-responder	Macro_FOLR2+APOE+	Macro_IER3	FN1	PLAUR	4,12063E-03	13502,3	1,03582E-02	9332	4,00787E-01	11951	1,11334E+00	10936	8,66812E-01	17762	0	17530,5
Non-responder	Macro_FOLR2+APOE+	Mono_INHBA	B2M	LILRB2	4,12135E-03	13448,5	4,57770E-03	33180	5,26435E-01	7139	1,26121E+00	8534	9,41843E-01	859	0	17530,5
Non-responder	pDC_LILRA4	Macro_IFI27	APP	LRP1	4,12164E-03	6904,3	1,92206E-02	3272	1,11676E+00	708	2,22906E+00	1295	8,90129E-01	11716	0	17530,5
Non-responder	Macro_FOLR2+APOE+	Macro_IFI27	LRPAP1	SORL1	4,12179E-03	13525,5	1,06644E-02	8926	5,10321E-01	7663	8,07018E-01	17763	8,74764E-01	15745	0	17530,5
Non-responder	Macro_OLFML3	Macro_OLFML3	TNFSF13B	HLA-DPB1	4,12512E-03	9998,1	6,82111E-03	18286	5,55541E-01	6343	1,35137E+00	7286	9,45394E-01	545	0	17530,5
Non-responder	Mono_INHBA	Macro_ISG15	VEGFA	SIRPA	4,12533E-03	8534,7	2,18808E-02	2594	9,27761E-01	1565	1,77169E+00	3273	8,67083E-01	17711	0	17530,5
Non-responder	Macro_LYVE1	Mono_CD16	GRN	TNFRSF1B	4,13224E-03	11061,9	7,29879E-03	16363	5,54853E-01	6365	1,00481E+00	13050	9,34819E-01	2001	0	17530,5
Non-responder	Macro_OLFML3	Macro_FOLR2+APOE+	LGALS3BP	ITGB1	4,13266E-03	11924,1	1,13029E-02	8092	4,84241E-01	8540	1,46230E+00	5923	8,59323E-01	19535	0	17530,5
Non-responder	Macro_OLFML3	Macro_OLFML3	B2M	LILRB1	4,13454E-03	10538,5	6,70882E-03	18759	4,84194E-01	8541	1,39621E+00	6696	9,39409E-01	1166	0	17530,5
Non-responder	Macro_IER3	Macro_FOLR2+APOE+	IL10	IL10RA_IL10RB	4,13642E-03	10160,1	2,13315E-02	2731	7,27579E-01	3276	1,95733E+00	2265	8,36545E-01	24998	0	17530,5
Non-responder	Macro_IER3	Macro_NLRP3	ICAM1	ITGAX_ITGB2	4,14155E-03	11926,9	6,93680E-03	17780	5,12395E-01	7588	1,30116E+00	7963	9,02345E-01	8773	0	17530,5
Non-responder	Mono_CD16	cDC(CD1C)	ICAM2	ITGAM_ITGB2	4,14271E-03	12565,7	1,31121E-02	6305	4,70314E-01	9055	1,04908E+00	12157	8,66765E-01	17781	0	17530,5
Non-responder	Mono_CD16	cDC(CD1C)	TNFSF10	CCR6	4,14398E-03	17451,3	2,92306E-02	1629	5,91819E-01	5487	1,26023E+00	8546	7,07206E-01	54064	0	17530,5
Non-responder	Macro_FOLR2+APOE+	cDC(CD1C)	HLA-DQA1	CD4	4,14966E-03	11935,7	5,86322E-03	23201	4,84038E-01	8549	1,38010E+00	6894	9,27569E-01	3504	0	17530,5
Non-responder	Macro_IER3	Macro_NLRP3	FN1	CD44	4,15087E-03	11064,7	1,11827E-02	8237	5,87766E-01	5564	1,43660E+00	6204	8,66721E-01	17788	0	17530,5
Non-responder	cDC_CLEC9A	Macro_FOLR2+APOE+	RPS19	C5AR1	4,15124E-03	7552,7	9,00051E-03	11631	7,27423E-01	3280	1,88188E+00	2656	9,31446E-01	2666	0	17530,5
Non-responder	Macro_FOLR2+APOE+	Macro_FOLR2+APOE+	MIF	CD44_CD74	4,15212E-03	13795,7	4,84929E-03	30591	3,84756E-01	12720	1,33736E+00	7461	9,43748E-01	676	0	17530,5
Non-responder	Mono_CD16	Mono_CD16	SERPINA1	LRP1	4,15533E-03	11922,9	6,59318E-03	19302	4,84007E-01	8552	1,43667E+00	6202	9,05597E-01	8028	0	17530,5
Non-responder	Macro_LYVE1	Mono_CD16	F13A1	ITGB1	4,16021E-03	8142,3	1,50642E-02	4969	8,52023E-01	2071	1,88810E+00	2614	8,83334E-01	13527	0	17530,5
Non-responder	Macro_LYVE1	Mono_CD16	GNAI2	C5AR1	4,16138E-03	13336,5	7,14882E-03	16931	4,41887E-01	10155	9,80708E-01	13533	9,03425E-01	8533	0	17530,5
Non-responder	Macro_FOLR2+APOE+	pDC_LILRA4	CCL2	CCR2	4,16238E-03	17298,3	2,04470E-02	2956	4,65266E-01	9233	1,77059E+00	3283	7,10367E-01	53489	0	17530,5
Non-responder	Macro_LYVE1	Mono_CD16	GNAI2	FPR1	4,16255E-03	14938,7	7,02866E-03	17415	3,62584E-01	13915	8,45724E-01	16736	9,00823E-01	9097	0	17530,5
Non-responder	Mono_CD16	Mono_CD14	CD55	ADGRE2	4,16291E-03	11791,9	1,09463E-02	8556	6,30816E-01	4711	1,46749E+00	5868	8,47736E-01	22294	0	17530,5
Non-responder	Macro_IFI27	pDC_LILRA4	SELPLG	SELL	4,16606E-03	9059,3	1,33495E-02	6104	9,22660E-01	1599	1,95764E+00	2262	8,66685E-01	17801	0	17530,5
Non-responder	Macro_IER3	pDC_LILRA4	VEGFA	RET	4,16610E-03	14746,7	7,91230E-02	236	9,29004E-01	1560	1,77056E+00	3284	7,22945E-01	51123	0	17530,5
Non-responder	Macro_LYVE1	Mono_CD16	C1QA	CD93	4,16957E-03	13214,7	7,20416E-03	16715	5,10242E-01	7665	1,00570E+00	13030	8,92441E-01	11133	0	17530,5
Non-responder	Macro_NLRP3	Macro_OLFML3	TNF	FAS	4,17050E-03	18730,1	1,38439E-02	5758	5,04346E-01	7825	1,25932E+00	8560	7,07634E-01	53977	0	17530,5
Non-responder	Macro_OLFML3	cDC_CLEC9A	HLA-DQA2	LAG3	4,17354E-03	9963,1	1,94366E-02	3216	8,21105E-01	2358	1,77034E+00	3286	8,43017E-01	23425	0	17530,5
Non-responder	Macro_LYVE1	Mono_CD16	C1QA	CD33	4,17543E-03	10767,7	9,79214E-03	10191	5,47875E-01	6550	1,12138E+00	10778	9,02241E-01	8789	0	17530,5
Non-responder	Macro_LYVE1	Mono_CD16	C1QB	CD33	4,17660E-03	11297,5	9,66103E-03	10406	5,20757E-01	7300	1,04095E+00	12313	9,01500E-01	8938	0	17530,5

Post_NR_Myeloid_Myeloid_CellCell

Non-responder	cDC_CLEC9A	cDC_LAMP3	LTB	CD40	4,17726E-03	9887,9	1,91797E-02	3287	8,19377E-01	2377	1,85893E+00	2774	8,42834E-01	23471	0	17530,5
Non-responder	Macro_FOLR2+APOE+	Macro_OLFML3	SIRPA	CD47	4,17810E-03	11000,9	1,09412E-02	8564,5	6,03459E-01	5242,5	1,53834E+00	5156,5	8,63657E-01	18510,5	0	17530,5
Non-responder	Macro_OLFML3	Macro_FOLR2+APOE+	CD47	SIRPA	4,18001E-03	11000,9	1,09412E-02	8564,5	6,03459E-01	5242,5	1,53834E+00	5156,5	8,63657E-01	18510,5	0	17530,5
Non-responder	cDC_LAMP3	Mono_INHBA	CXCL9	FCGR2A	4,18012E-03	13689,1	9,70963E-03	10320	4,99737E-01	8002	8,05208E-01	17813	8,78582E-01	14780	0	17530,5
Non-responder	cDC(CD1C)	Macro_FOLR2+APOE+	LYZ	ITGAL	4,18364E-03	15573,9	7,06277E-03	17275	3,64892E-01	13797	8,05191E-01	17816	8,91206E-01	11451	0	17530,5
Non-responder	Macro_NLRP3	pDC_LILRA4	THBS1	ITGA4	4,18381E-03	9992,9	1,09394E-02	8567	6,97690E-01	3656	1,99821E+00	2105	8,65420E-01	18106	0	17530,5
Non-responder	Macro_LYVE1	cDC_CLEC9A	F13A1	ITGA4	4,18472E-03	6897,7	1,91759E-02	3289	9,86133E-01	1273	2,06641E+00	1840	8,94789E-01	10556	0	17530,5
Non-responder	Macro_LYVE1	Mono_CD16	C3	IFITM1	4,18717E-03	11057,7	1,31419E-02	6279	5,17157E-01	7427	1,11177E+00	10963	8,84866E-01	13089	0	17530,5
Non-responder	Macro_FOLR2+APOE+	Macro_IFI27	C3	CD81	4,18834E-03	15099,5	9,89825E-03	10015	2,99056E-01	17767	8,05114E-01	17820	8,87695E-01	12365	0	17530,5
Non-responder	Macro_LYVE1	Mono_CD16	LYZ	ITGAL	4,19187E-03	11019,7	1,12305E-02	8160	4,87812E-01	8392	9,46974E-01	14343	9,11736E-01	6673	0	17530,5
Non-responder	Macro_OLFML3	Macro_NLRP3	ARPC5	LDLR	4,19334E-03	13322,5	1,38282E-02	5768	4,94270E-01	8188	1,25826E+00	8572	8,30166E-01	26554	0	17530,5
Non-responder	Macro_FOLR2+APOE+	Mono_INHBA	CXCL8	SDC2	4,19524E-03	16714,1	1,10431E-02	8426	4,83399E-01	8573	1,34100E+00	7406	7,66872E-01	41635	0	17530,5
Non-responder	Macro_FOLR2+APOE+	Macro_FOLR2+APOE-	APOE	SCARB1	4,19540E-03	13596,9	9,41436E-03	10834	4,01550E-01	11916	1,17513E+00	9878	8,66571E-01	17826	0	17530,5
Non-responder	Macro_ISG15	Macro_FOLR2+APOE+	SEMA4D	CD72	4,19591E-03	13061,7	2,19746E-02	2582	7,26234E-01	3292	1,83874E+00	2889	7,77966E-01	39015	0	17530,5
Non-responder	Macro_FOLR2+APOE+	Macro_IER3	ANXA2	TLR2	4,19658E-03	13478,9	6,92701E-03	17827	3,81905E-01	12886	1,15692E+00	10182	9,01365E-01	8969	0	17530,5
Non-responder	pDC_LILRA4	cDC_LAMP3	TNFSF9	HLA-DPA1	4,19906E-03	14668,6	1,76239E-02	3795	4,83319E-01	8575	1,28450E+00	8205	9,04690E-01	8263	0,176	44505
Non-responder	Mast	Macro_IFI27	CSF1	CSF2RA	4,20094E-03	9585,9	4,92665E-02	665	1,12313E+00	680	1,24757E+00	8730	8,55888E-01	20324	0	17530,5
Non-responder	Macro_ISG15	Macro_ISG15	CCL3	CCR1	4,20339E-03	7171,9	1,91401E-02	3294	8,19766E-01	2375	2,16396E+00	1482	8,92266E-01	11178	0	17530,5
Non-responder	Mono_INHBA	Macro_ISG15	FN1	PLAUR	4,21462E-03	7130,9	1,96631E-02	3160	7,25915E-01	3297	1,94948E+00	2316	8,99668E-01	9351	0	17530,5
Non-responder	pDC_LILRA4	cDC(CD1C)	LTB	CD40	4,22211E-03	9080,9	1,91218E-02	3299	1,09792E+00	777	2,98212E+00	280	8,42634E-01	23518	0	17530,5
Non-responder	Macro_FOLR2+APOE+	Macro_OLFML3	CCL5	CCR1	4,22584E-03	17819,7	1,79124E-02	3696	4,82988E-01	8589	1,57045E+00	4847	7,05181E-01	54436	0	17530,5
Non-responder	Macro_FOLR2+APOE+	cDC(CD1C)	LGALS3BP	ITGB1	4,22775E-03	12193,3	1,09197E-02	8590	5,36847E-01	6850	1,30063E+00	7969	8,57225E-01	20027	0	17530,5
Non-responder	Macro_ISG15	Macro_FOLR2+APOE+	TGM2	SDC4	4,22962E-03	14714,7	2,85625E-02	1690	7,25611E-01	3301	1,80390E+00	3086	7,38488E-01	47966	0	17530,5
Non-responder	Macro_IER3	Macro_NLRP3	SPP1	CD44	4,22964E-03	13333,7	7,51894E-03	15605	5,11738E-01	7604	1,29305E+00	8074	8,66453E-01	17855	0	17530,5
Non-responder	Macro_NLRP3	Mono_INHBA	HBEGF	CD82	4,23159E-03	11043,3	1,45144E-02	5293	7,50374E-01	3021	1,25714E+00	8592	8,54083E-01	20780	0	17530,5
Non-responder	Macro_ISG15	Macro_OLFML3	CXCL10	TLR4	4,23337E-03	9592,9	1,91145E-02	3302	9,02513E-01	1720	2,57242E+00	660	8,37563E-01	24752	0	17530,5
Non-responder	Macro_FOLR2+APOE+	Macro_NLRP3	ADAM15	ITGA5	4,23543E-03	16993,7	1,26572E-02	6690	4,82847E-01	8594	1,44212E+00	6119	7,47210E-01	46035	0	17530,5
Non-responder	Macro_NLRP3	Macro_NLRP3	ADAM15	ITGA5	4,23927E-03	17264,9	1,26570E-02	6691	4,82844E-01	8596	1,33643E+00	7471	7,47209E-01	46036	0	17530,5
Non-responder	Macro_IER3	Mono_CD14	IL1B	SIGIRR	4,24387E-03	12545,3	1,02361E-02	9511	5,79620E-01	5745	1,05404E+00	12073	8,66390E-01	17867	0	17530,5
Non-responder	Macro_LYVE1	Mono_CD16	C1QB	LRP1	4,24743E-03	12920,9	8,06738E-03	13928	4,56743E-01	9553	8,03401E-01	17870	9,16089E-01	5723	0	17530,5
Non-responder	Macro_FOLR2+APOE+	Macro_IER3	HLA-C	LILRB2	4,25082E-03	13184,7	4,93989E-03	29806	4,82641E-01	8602	1,30463E+00	7918	9,34513E-01	2067	0	17530,5
Non-responder	cDC_CLEC9A	Macro_FOLR2+APOE-	CD59	STAB1	4,25274E-03	7908,7	1,91861E-02	3285	8,92889E-01	1774	1,27352E+00	8351	9,03046E-01	8603	0	17530,5
Non-responder	Macro_LYVE1	Macro_OLFML3	CD14	ITGB2	4,25338E-03	11647,5	7,25466E-03	16522	5,29897E-01	7050	8,68913E-01	16145	9,40827E-01	990	0	17530,5
Non-responder	Macro_LYVE1	Macro_OLFML3	CD14	ITGA4	4,25576E-03	13447,9	7,54529E-03	15502	5,10640E-01	7650	8,07420E-01	17753	9,02153E-01	8804	0	17530,5
Non-responder	Macro_LYVE1	Macro_OLFML3	CD14	TLR1	4,25695E-03	13132,3	1,09389E-02	8570	5,64896E-01	6106	8,61301E-01	16346	8,69563E-01	17109	0	17530,5
Non-responder	Mono_CD16	Macro_FOLR2+APOE+	ICAM4	ITGAM	4,25852E-03	16966,9	2,91350E-02	1639	6,00091E-01	5307	1,25638E+00	8606	7,19626E-01	51752	0	17530,5
Non-responder	pDC_LILRA4	Macro_FOLR2+APOE+	ASIP	MGRN1	4,25973E-03	16051,1	7,56785E-02	260	7,24829E-01	3309	1,81924E+00	3008	6,95059E-01	56148	0	17530,5
Non-responder	Macro_NLRP3	Mono_CD14	CD14	TLR4	4,26290E-03	15246,1	7,88279E-03	14473	3,49538E-01	14671	1,07316E+00	11673	8,66349E-01	17883	0	17530,5
Non-responder	Mono_INHBA	cDC_LAMP3	IL6	IL6R_IL6ST	4,26350E-03	11570,7	3,58078E-02	1161	1,15835E+00	554	1,76734E+00	3310	7,93977E-01	35298	0	17530,5
Non-responder	Macro_IER3	cDC_CLEC9A	HLA-DQA1	LAG3	4,26887E-03	10532,9	1,56990E-02	4649	7,37547E-01	3174	1,20233E+00	9423	8,66341E-01	17888	0	17530,5
Non-responder	Macro_LYVE1	Macro_OLFML3	MRC1	PTPRC	4,27245E-03	10587,7	1,29978E-02	6405	6,36422E-01	4607	1,27877E+00	8288	8,73388E-01	16108	0	17530,5
Non-responder	cDC_CLEC9A	Mono_CD16	HLA-F	LILRB2	4,27590E-03	7165,1	1,09028E-02	8615	9,03159E-01	1714	2,12249E+00	1615	9,13151E-01	6351	0	17530,5
Non-responder	Macro_FOLR2+APOE+	cDC(CD1C)	LRPAP1	SORL1	4,28321E-03	13505,3	9,17407E-03	11281	4,44366E-01	10046	1,12214E+00	10769	8,66283E-01	17900	0	17530,5
Non-responder	Macro_FOLR2+APOE+	Mono_INHBA	LGALS3BP	ITGB1	4,28558E-03	12112,7	1,08964E-02	8620	5,36010E-01	6869	1,33556E+00	7486	8,57094E-01	20058	0	17530,5
Non-responder	Macro_FOLR2+APOE+	Macro_FOLR2+APOE+	TGM2	ITGA4	4,28752E-03	15242,1	1,23379E-02	7011	4,82035E-01	8621	1,25933E+00	8559	7,97483E-01	34489	0	17530,5
Non-responder	pDC_LILRA4	Mono_CD16	APP	LRP1	4,28997E-03	6754,1	1,90507E-02	3317	1,11331E+00	721	2,84786E+00	379	8,89694E-01	11823	0	17530,5
Non-responder	Mono_INHBA	Mono_CD16	SPP1	CD44	4,29140E-03	9128,7	1,62617E-02	4359	5,32095E-01	6986	1,25541E+00	8623	9,05136E-01	8145	0	17530,5
Non-responder	Mono_CD16	Macro_ISG15	ICAM2	ITGAM_ITGB2	4,29398E-03	11109,5	1,29932E-02	6409	4,81661E-01	8636	1,54815E+00	5063	8,66238E-01	17909	0	17530,5
Non-responder	Macro_OLFML3	Macro_NLRP3	ADAM15	ITGA5	4,29527E-03	17459,5	1,27125E-02	6643	4,83915E-01	8555	1,25534E+00	8625	7,47622E-01	45944	0	17530,5
Non-responder	Mono_CD16	Macro_ISG15	TNFSF13B	TFRC	4,29758E-03	13385,9	7,44660E-03	15854	4,21704E-01	10974	1,59307E+00	4659	8,66234E-01	17912	0	17530,5
Non-responder	Macro_FOLR2+APOE+	cDC_CLEC9A	ICAM1	ITGAL_ITGB2	4,29758E-03	12083,9	6,90705E-03	17912	4,90095E-01	8320	1,43878E+00	6166	8,94996E-01	10491	0	17530,5
Non-responder	pDC_LILRA4	Mono_CD14	HSP90B1	LRP1	4,29916E-03	6750,9	1,08909E-02	8627	9,16008E-01	1636	2,65443E+00	550	9,17727E-01	5411	0	17530,5
Non-responder	Macro_ISG15	Macro_ISG15	HLA-DQB1	LAG3	4,30135E-03	7866,9	1,90424E-02	3320	8,85134E-01	1814	1,88253E+00	2652	8,81411E-01	14018	0	17530,5
Non-responder	Mono_CD16	Macro_FOLR2+APOE+	LGALS3	ENG	4,30358E-03	14226,3	7,62814E-03	15231	2,96817E-01	17917	1,07453E+00	11637	9,02091E-01	8816	0	17530,5
Non-responder	Mono_INHBA	Macro_ISG15	ANGPTL4	SDC2	4,30895E-03	16484,5	8,58987E-02	197	8,56447E-01	2038	1,76532E+00	3322	6,74970E-01	59335	0	17530,5
Non-responder	Macro_OLFML3	Macro_ISG15	ANXA2	TLR2	4,31079E-03	14223,5	6,90478E-03	17923	3,20431E-01	16401	1,15258E+00	10261	9,01222E-01	9002	0	17530,5
Non-responder	Macro_FOLR2+APOE+	Mono_CD16	CALM3	INSR	4,31471E-03	15084,1	1,30622E-02	6346	4,81697E-01	8635	1,35072E+00	7291	7,92542E-01	35618	0	17530,5
Non-responder	Mono_INHBA	Macro_IER3	IL6	IL6R_IL6ST	4,31655E-03	12770,5	2,76609E-02	1756	1,09235E+00	799	1,76503E+00	3324	7,72063E-01	40443	0	17530,5
Non-responder	pDC_LILRA4	Mono_INHBA	SERPINF1	PLXDC2	4,31861E-03	7911,9	1,08853E-02	8637	6,28651E-01	4754	2,21673E+00	1325	9,08717E-01	7313	0	17530,5
Non-responder	cDC_CLEC9A	pDC_LILRA4	HLA-DQA1	CD4	4,32036E-03	6848,1	8,92176E-03	11793	7,23849E-01	3325	2,64861E+00	556	9,40466E-01	1036	0	17530,5
Non-responder	Macro_OLFML3	Macro_OLFML3	ANXA2	TLR2	4,32163E-03	15386,3	6,90162E-03	17932	3,20199E-01	16421	8,72650E-01	16043	9,01201E-01	9005	0	17530,5
Non-responder	Macro_LYVE1	Macro_FOLR2+APOE+	C3	NRP1	4,32283E-03	10889,3	1,85981E-02	3468	5,25365E-01	7174	1,27462E+00	8341	8,66142E-01	17933	0	17530,5
Non-responder	cDC_LAMP3	Macro_ISG15	HLA-B	LILRB2	4,32404E-03	10002,1	6,90084E-03	17934	5,86280E-01	5598	1,26307E+00	8498	9,46565E-01	450	0	17530,5
Non-responder	Macro_NLRP3	Macro_IFI27	VCAN	TLR1	4,33226E-03	13813,1	1,08781E-02	8644	8,19773E-01	2374	1,33144E+00	7539	8,03730E-01	32978	0	17530,5
Non-responder	Macro_ISG15	Mono_CD14	CCL8	CCR2	4,33560E-03	16586,9	5,35628E-02	575	7,23549E-01	3329	2,32729E+00	1071	6,66772E-01	60429	0	17530,5
Non-responder	cDC(CD1C)	Mono_CD16	HLA-A	LILRB1	4,33611E-03	13301,9	6,89821E-03	17944	3,70033E-01	13512	9,44518E-01	14391	9,29321E-01	3132	0	17530,5
Non-responder	Macro_OLFML3	Macro_OLFML3	HLA-B	LILRB2	4,33812E-03	12044,5	5,59843E-03	24959	4,96270E-01	8119	1,25383E+00	8647	9,41021E-01	967	0	17530,5
Non-responder	Macro_FOLR2+APOE+	Macro_FOLR2-APOE+	LGALS9	LRP1	4,33852E-03	12406,5	9,75866E-03	10254	5,59133E-01	6247	8,00364E-01	17946	8,96687E-01	10055	0	17530,5
Non-responder	Macro_LYVE1	Macro_ISG15	HLA-DRB1	LAG3	4,34203E-03	7105,1	2,02508E-02	2997	1,02636E+00	1086	1,52939E+00	5263	9,02838E-01	8649	0	17530,5
Non-responder	Mono_CD14	Macro_NLRP3	S100A9	CD68	4,34324E-03	6599,9	1,02706E-02	9451	8,05050E-01	2501	1,76428E+00	3331	9,50725E-01	186	0	17530,5
Non-responder	Macro_NLRP3	Macro_FOLR2+APOE+	TNF	TNFRSF1B	4,35184E-03	12174,1	8,83746E-03	11953	4,93225E-01	8221	1,53450E+00	5209	8,66054E-01	17957	0	17530,5

Post_NR_Myeloid_Myeloid_CellCell

Non-responder	Macro_LYVE1	Mono_CD16	HLA-B	LILRB1	4,35184E-03	13476,5	6,90876E-03	17906	4,06740E-01	11678	7,99957E-01	17957	9,33407E-01	2311	0	17530,5
Non-responder	Macro_LYVE1	Macro_OLFML3	F13A1	ITGA4	4,35305E-03	7516,5	1,73271E-02	3922	9,36662E-01	1518	1,84546E+00	2848	8,89921E-01	11764	0	17530,5
Non-responder	Macro_LYVE1	Macro_OLFML3	F13A1	ITGB1	4,35426E-03	8632,1	1,44186E-02	5360	8,34434E-01	2238	1,68114E+00	3921	8,81058E-01	14111	0	17530,5
Non-responder	pDC_LILRA4	Macro_OLFML3	APP	NOTCH2	4,36155E-03	8888,3	1,48549E-02	5099	1,11926E+00	693	2,62547E+00	590	8,55131E-01	20529	0	17530,5
Non-responder	Macro_FOLR2+APOE+	Mono_CD16	C1QB	C1QBP	4,36162E-03	12209,3	5,58253E-03	25064	5,18065E-01	7392	1,93222E+00	2401	9,02782E-01	8659	0	17530,5
Non-responder	cDC(CD1C)	Mono_CD16	C3	IFITM1	4,36275E-03	15060,7	9,29674E-03	11037	3,64479E-01	13815	9,19267E-01	14955	8,66026E-01	17966	0	17530,5
Non-responder	Macro_LYVE1	Macro_OLFML3	C1QA	CD93	4,36275E-03	12224,3	8,26452E-03	13377	5,51851E-01	6446	9,50754E-01	14244	8,98856E-01	9524	0	17530,5
Non-responder	Mono_CD16	Macro_IER3	ICAM2	ITGAM_ITGB2	4,36518E-03	12105,5	1,29411E-02	6448	4,85681E-01	8483	1,16265E+00	10098	8,66005E-01	17968	0	17530,5
Non-responder	Macro_FOLR2+APOE+	Macro_LYVE1	CALR	ITGAV	4,36640E-03	12060,5	9,39460E-03	10873	5,34841E-01	6906	1,36853E+00	7024	8,66005E-01	17969	0	17530,5
Non-responder	Macro_FOLR2+APOE+	Macro_OLFML3	PLAU	ITGA5	4,36751E-03	17331,3	1,08644E-02	8662	4,90515E-01	8305	1,51659E+00	5378	7,43912E-01	46781	0	17530,5
Non-responder	Macro_LYVE1	Macro_OLFML3	PLTP	ABCA1	4,36761E-03	10454,7	1,12459E-02	8146	7,08711E-01	3534	1,30712E+00	7889	8,77081E-01	15174	0	17530,5
Non-responder	Macro_LYVE1	Macro_OLFML3	C1QA	CD33	4,36883E-03	11101,1	1,06337E-02	8973	5,75745E-01	5836	9,09619E-01	15187	9,05817E-01	7979	0	17530,5
Non-responder	Macro_LYVE1	Macro_OLFML3	C1QB	CD33	4,37004E-03	11712,7	1,04913E-02	9161	5,48627E-01	6533	8,29190E-01	17185	9,05101E-01	8154	0	17530,5
Non-responder	Macro_FOLR2+APOE+	Mono_INHBA	TNF	TNFRSF1B	4,37126E-03	12366,3	8,82640E-03	11982	5,43110E-01	6683	1,32261E+00	7663	8,65981E-01	17973	0	17530,5
Non-responder	Macro_IER3	Macro_FOLR2+APOE+	F13A1	ITGB1	4,37369E-03	12139,3	1,09710E-02	8525	5,01503E-01	7937	1,24764E+00	8729	8,65978E-01	17975	0	17530,5
Non-responder	cDC_CLEC9A	pDC_LILRA4	HLA-DQB1	CD4	4,37387E-03	6856,3	8,80259E-03	12038	7,22788E-01	3339	2,64088E+00	564	9,42321E-01	810	0	17530,5
Non-responder	Macro_LYVE1	Macro_OLFML3	C1QB	C1QBP	4,37613E-03	11804,3	8,44329E-03	12918	5,31010E-01	7020	8,54482E-01	16509	9,19486E-01	5044	0	17530,5
Non-responder	Macro_OLFML3	Macro_OLFML3	LGALS9	CD47	4,37613E-03	14364,9	6,89058E-03	17977	4,18313E-01	11133	1,14312E+00	10415	8,78649E-01	14769	0	17530,5
Non-responder	Macro_LYVE1	Macro_OLFML3	C3	IFITM1	4,37856E-03	12527,5	1,14803E-02	7893	4,40998E-01	10184	1,05526E+00	12043	8,77800E-01	14987	0	17530,5
Non-responder	Macro_LYVE1	pDC_LILRA4	COPA	P2RY6	4,38652E-03	8977,7	1,64621E-02	4264	1,11896E+00	695	2,68954E+00	514	8,49444E-01	21885	0	17530,5
Non-responder	Mono_CD16	Macro_OLFML3	SERPINA1	LRP1	4,38916E-03	12250,9	6,53808E-03	19565	4,80776E-01	8673	1,34343E+00	7366	9,05237E-01	8120	0	17530,5
Non-responder	pDC_LILRA4	cDC_LAMP3	APP	NOTCH2	4,39904E-03	8216,9	1,72927E-02	3936	1,15940E+00	552	2,55029E+00	696	8,64291E-01	18370	0	17530,5
Non-responder	Mono_CD16	Macro_FOLR2+APOE+	ADAM10	TREM2	4,40052E-03	11856,3	1,15570E-02	7814	4,20830E-01	11017	1,56199E+00	4923	8,65903E-01	17997	0	17530,5
Non-responder	Mono_CD16	Macro_FOLR2+APOE+	HEBP1	FPR3	4,40298E-03	13657,9	1,08499E-02	8680	5,12575E-01	7585	1,60936E+00	4515	8,16302E-01	29979	0	17530,5
Non-responder	Macro_IFI27	Macro_FOLR2+APOE+	HLA-DRB5	LAG3	4,40664E-03	13281,5	1,16580E-02	7688	5,96849E-01	5378	7,98450E-01	18002	8,66656E-01	17809	0	17530,5
Non-responder	Macro_ISG15	Macro_OLFML3	CCL18	CCR1	4,40850E-03	12900,7	2,98715E-02	1559	7,22217E-01	3348	2,26517E+00	1196	7,70233E-01	40870	0	17530,5
Non-responder	Macro_NLRP3	Mono_CD14	VEGFA	SIRPA	4,40891E-03	13020,7	1,08487E-02	8683	6,98174E-01	3650	1,41611E+00	6462	8,21220E-01	28778	0	17530,5
Non-responder	Macro_IER3	pDC_LILRA4	IL10	IL10RA_IL10RB	4,41236E-03	9614,1	2,27430E-02	2419	7,22163E-01	3349	2,45934E+00	829	8,40878E-01	23943	0	17530,5
Non-responder	Macro_OLFML3	Macro_ISG15	C3	C3AR1	4,41399E-03	13826,3	9,69548E-03	10347	2,95415E-01	18008	1,23857E+00	8863	8,79991E-01	14383	0	17530,5
Non-responder	Macro_LYVE1	Macro_FOLR2+APOE+	GNAI2	C5AR1	4,41521E-03	14130,7	6,88283E-03	18009	4,14378E-01	11323	9,20876E-01	14914	9,01758E-01	8877	0	17530,5
Non-responder	Macro_FOLR2+APOE+	Macro_IER3	LGALS9	LRP1	4,41767E-03	15109,9	6,88218E-03	18011	3,84104E-01	12756	1,02259E+00	12689	8,79355E-01	14563	0	17530,5
Non-responder	Macro_FOLR2+APOE+	Macro_FOLR2+APOE+	ADAM10	TSPAN17	4,42014E-03	21711,5	1,33933E-02	6071	3,95929E-01	12194	1,00144E+00	13101	6,72613E-01	59661	0	17530,5
Non-responder	Mono_CD16	cDC_CLEC9A	TOR2A	ATP5F1B	4,42277E-03	13890,1	1,08442E-02	8690	6,53788E-01	4332	1,89165E+00	2598	7,89409E-01	36300	0	17530,5
Non-responder	Macro_IER3	Macro_ISG15	HMGB1	HAVCR2	4,43609E-03	15042,3	6,87737E-03	18026	3,37855E-01	15325	9,00372E-01	15406	9,01563E-01	8924	0	17530,5
Non-responder	Macro_FOLR2+APOE+	Macro_FOLR2+APOE-	APOE	TREM2	4,43855E-03	10496,1	1,33182E-02	6127	4,51650E-01	9777	7,97449E-01	18028	9,40600E-01	1018	0	17530,5
Non-responder	Macro_FOLR2+APOE+	Macro_IER3	FN1	C5AR1	4,43865E-03	12586,5	1,31536E-02	6265	4,87052E-01	8429	1,25052E+00	8698	8,48883E-01	22010	0	17530,5
Non-responder	Macro_ISG15	pDC_LILRA4	PSEN1	NOTCH4	4,43943E-03	12135,3	4,17694E-02	902	7,21310E-01	3356	1,87393E+00	2695	7,89858E-01	36193	0	17530,5
Non-responder	cDC_CLEC9A	Macro_ISG15	HLA-B	LILRB2	4,45106E-03	7920,9	7,35124E-03	16176	7,21188E-01	3359	1,97480E+00	2192	9,48141E-01	347	0	17530,5
Non-responder	Mono_CD14	Macro_LYVE1	S100A8	TLR4	4,45494E-03	6596,1	1,89097E-02	3360	1,15214E+00	577	2,35397E+00	1017	8,94976E-01	10496	0	17530,5
Non-responder	Macro_FOLR2+APOE+	Macro_NLRP3	CD14	ITGB1	4,45829E-03	11605,5	6,87324E-03	18044	4,88900E-01	8361	1,61852E+00	4439	8,98364E-01	9653	0	17530,5
Non-responder	Macro_NLRP3	pDC_LILRA4	VCAN	TLR1	4,45856E-03	12777,5	1,08263E-02	8708	8,19013E-01	2380	1,97342E+00	2197	8,03353E-01	33072	0	17530,5
Non-responder	Macro_ISG15	Macro_FOLR2+APOE+	TNFSF13B	HLA-DPB1	4,45883E-03	7362,9	8,07993E-03	13899	7,21104E-01	3361	2,08231E+00	1767	9,49604E-01	257	0	17530,5
Non-responder	Mast	cDC_LAMP3	RARRES1	NRP2	4,46186E-03	13749,9	5,81907E-02	487	1,11777E+00	701	1,42828E+00	6310	7,57269E-01	43721	0	17530,5
Non-responder	Macro_NLRP3	Macro_ISG15	TGFB1	ITGB1	4,46447E-03	13940,9	7,64465E-03	15178	4,08344E-01	11597	1,34559E+00	7350	8,65698E-01	18049	0	17530,5
Non-responder	Mono_CD16	Mono_CD16	PSAP	SORT1	4,46447E-03	16068,5	6,97596E-03	17632	2,94755E-01	18049	1,13350E+00	10565	8,71584E-01	16566	0	17530,5
Non-responder	Macro_IER3	Macro_FOLR2+APOE+	CD14	ITGA4	4,46570E-03	14291,5	6,87206E-03	18050	4,09060E-01	11563	9,37407E-01	14555	8,97950E-01	9759	0	17530,5
Non-responder	Macro_LYVE1	Macro_IFI27	F13A1	ITGB1	4,47065E-03	11790,5	1,10096E-02	8462	7,41545E-01	3127	1,06164E+00	11908	8,66182E-01	17925	0	17530,5
Non-responder	Macro_NLRP3	Macro_FOLR2+APOE+	S100A12	CD36	4,47454E-03	14400,5	1,70936E-02	4008	4,79628E-01	8716	1,44918E+00	6041	7,92095E-01	35707	0	17530,5
Non-responder	Macro_IER3	pDC_LILRA4	HSP90B1	TLR7	4,47561E-03	12063,5	1,35441E-02	5969	4,65471E-01	9223	1,19557E+00	9537	8,65654E-01	18058	0	17530,5
Non-responder	Macro_IER3	Mono_CD16	HLA-C	LILRB1	4,47809E-03	13985,1	6,87046E-03	18060	3,27924E-01	15925	8,98195E-01	15458	9,30113E-01	2952	0	17530,5
Non-responder	Macro_ISG15	Macro_ISG15	LGALS9	HAVCR2	4,47854E-03	7996,7	1,14460E-02	7930	7,03904E-01	3584	1,96754E+00	2221	9,02553E-01	8718	0	17530,5
Non-responder	Macro_IER3	cDC(CD1C)	IL1B	SIGIRR	4,48181E-03	13626,9	1,01003E-02	9705	5,75291E-01	5847	8,35647E-01	16989	8,65615E-01	18063	0	17530,5
Non-responder	pDC_LILRA4	Macro_OLFML3	APP	LRP1	4,48218E-03	6804,5	1,88915E-02	3367	1,11008E+00	734	2,75462E+00	451	8,89281E-01	11940	0	17530,5
Non-responder	Macro_FOLR2+APOE+	Macro_FOLR2+APOE+	CXCL12	ITGA4	4,48254E-03	15116,3	1,38933E-02	5721	4,79602E-01	8720	1,67177E+00	4006	7,75412E-01	39604	0	17530,5
Non-responder	Macro_NLRP3	Macro_NLRP3	PTGS2	ALOX5	4,48429E-03	11948,1	1,13938E-02	7987	5,44663E-01	6638	1,19640E+00	9520	8,65603E-01	18065	0	17530,5
Non-responder	Macro_OLFML3	Macro_FOLR2+APOE+	HLA-A	LILRB1	4,48455E-03	11185,1	6,46553E-03	19935	4,79542E-01	8721	1,43885E+00	6165	9,27164E-01	3574	0	17530,5
Non-responder	pDC_LILRA4	cDC_CLEC9A	COPA	CD74	4,48711E-03	6486,7	9,68869E-03	10361	7,17922E-01	3404	2,54476E+00	703	9,46731E-01	435	0	17530,5
Non-responder	Macro_OLFML3	Macro_FOLR2+APOE+	CCL3	CCR1	4,49256E-03	12065,3	1,08057E-02	8725	4,81564E-01	8641	1,41963E+00	6416	8,61553E-01	19014	0	17530,5
Non-responder	Macro_FOLR2+APOE+	Macro_OLFML3	HLA-DQB1	CD4	4,49256E-03	10747,3	6,36256E-03	20420	4,79429E-01	8725	1,59511E+00	4646	9,32839E-01	2415	0	17530,5
Non-responder	cDC_CLEC9A	cDC_CLEC9A	HLA-DPA1	LAG3	4,49389E-03	6558,5	1,88850E-02	3370	9,96243E-01	1221	2,25576E+00	1223	8,99185E-01	9448	0	17530,5
Non-responder	Macro_ISG15	Mono_CD16	CD14	ITGA4	4,49658E-03	7389,1	1,08048E-02	8727	7,11447E-01	3494	2,11644E+00	1634	9,16897E-01	5560	0	17530,5
Non-responder	Macro_FOLR2+APOE+	Mast	ARPC5	ADRB2	4,49671E-03	10775,5	1,75459E-02	3828	6,15734E-01	5009	1,20123E+00	9435	8,65559E-01	18075	0	17530,5
Non-responder	Macro_ISG15	cDC_CLEC9A	FLT3LG	FLT3	4,49780E-03	16681,9	3,13728E-02	1428	1,10899E+00	739	1,75697E+00	3371	6,67516E-01	60341	0	17530,5
Non-responder	Macro_OLFML3	Macro_FOLR2-APOE+	SERPING1	LRP1	4,50418E-03	11545,1	1,25468E-02	6791	6,58836E-01	4235	7,95624E-01	18081	8,92620E-01	11088	0	17530,5
Non-responder	cDC_LAMP3	Macro_FOLR2+APOE+	HLA-DPB1	CD4	4,50667E-03	12785,3	6,86617E-03	18083	3,94525E-01	12255	9,04390E-01	15309	9,42935E-01	749	0	17530,5
Non-responder	Macro_OLFML3	Macro_NLRP3	PLAU	PLAUR	4,51416E-03	10551,7	9,52446E-03	10635	7,48581E-01	3041	1,74353E+00	3463	8,65486E-01	18089	0	17530,5
Non-responder	Mono_CD14	Macro_IFI27	S100A12	TLR4	4,51737E-03	12067,3	1,88583E-02	3376	1,12116E+00	688	1,79421E+00	3148	7,92687E-01	35594	0	17530,5
Non-responder	Macro_FOLR2+APOE+	Macro_ISG15	CALR	LRP1	4,51915E-03	11757,9	6,86422E-03	18093	4,43755E-01	10074	1,61227E+00	4491	9,03080E-01	8601	0	17530,5
Non-responder	Macro_OLFML3	pDC_LILRA4	HLA-DPA1	CD4	4,52165E-03	9672,5	6,86361E-03	18095	4,59306E-01	9452	1,86212E+00	2759	9,45604E-01	526	0	17530,5
Non-responder	Macro_FOLR2+APOE+	Macro_ISG15	MRC1	PTPRC	4,52272E-03	10807,7	1,07969E-02	8740	5,56895E-01	6306	1,86480E+00	2747	8,62770E-01	18715	0	17530,5
Non-responder	Macro_IFI27	pDC_LILRA4	HLA-DRB5	CD4	4,52290E-03	11794,1	6,86325E-03	18096	4,48580E-01	9884	1,02973E+00	12556	9,41597E-01	904	0	17530,5

Post_NR_Myeloid_Myeloid_CellCell

Non-responder	Mono_CD16	Macro_LYVE1	ICAM2	ITGAM_ITGB2	4,52665E-03	12502,5	1,28143E-02	6561	4,86422E-01	8458	1,06349E+00	11864	8,65433E-01	18099	0	17530,5
Non-responder	Mono_CD14	cDC_CLEC9A	AGTRAP	RACK1	4,52675E-03	7231,5	1,39688E-02	5671	8,30637E-01	2272	2,03685E+00	1942	9,02482E-01	8742	0	17530,5
Non-responder	pDC_LILRA4	Macro_FOLR2+APOE-	HSP90B1	TLR2	4,52877E-03	7245,5	1,07962E-02	8743	9,18128E-01	1627	2,17171E+00	1458	9,10755E-01	6869	0	17530,5
Non-responder	Macro_OLFML3	Macro_FOLR2+APOE+	GNAI2	C5AR1	4,54167E-03	12598,3	6,86021E-03	18111	4,11994E-01	11423	1,36939E+00	7021	9,01612E-01	8906	0	17530,5
Non-responder	Macro_ISG15	pDC_LILRA4	THBS1	CD47	4,54485E-03	6625,9	1,88415E-02	3383	9,55846E-01	1416	3,02677E+00	247	8,94801E-01	10553	0	17530,5
Non-responder	Macro_FOLR2+APOE+	Mast	PLTP	ABCA1	4,54795E-03	13056,7	9,13119E-03	11351	4,66581E-01	9188	1,22287E+00	9098	8,65404E-01	18116	0	17530,5
Non-responder	Macro_IER3	cDC_CLEC9A	CD14	ITGB1	4,55046E-03	14568,1	6,85934E-03	18118	3,85560E-01	12689	9,24558E-01	14829	8,98271E-01	9674	0	17530,5
Non-responder	Macro_NLRP3	Mono_CD16	CD48	CD244	4,55303E-03	15554,9	1,93315E-02	3239	5,36198E-01	6867	1,24574E+00	8755	7,67978E-01	41383	0	17530,5
Non-responder	Macro_FOLR2+APOE+	Macro_FOLR2+APOE-	LGALS9	LRP1	4,55423E-03	14011,1	8,29101E-03	13306	4,69829E-01	9072	7,93760E-01	18121	8,88890E-01	12026	0	17530,5
Non-responder	Macro_OLFML3	Macro_IFI27	CXCL10	TLR4	4,55505E-03	12584,7	1,15218E-02	7844	9,12418E-01	1658	2,01277E+00	2035	8,00129E-01	33856	0	17530,5
Non-responder	pDC_LILRA4	Macro_FOLR2+APOE+	SEMA4D	CD72	4,56061E-03	13000,5	2,14460E-02	2701	7,19275E-01	3387	2,04988E+00	1890	7,75856E-01	39494	0	17530,5
Non-responder	Macro_OLFML3	Macro_LYVE1	HLA-DQA2	CD4	4,56429E-03	14702,7	6,85757E-03	18129	3,54177E-01	14400	8,45228E-01	16752	9,11601E-01	6702	0	17530,5
Non-responder	Macro_IFI27	pDC_LILRA4	HSP90B1	TLR9	4,57059E-03	10401,3	4,91464E-02	671	8,76206E-01	1874	9,69726E-01	13797	8,65351E-01	18134	0	17530,5
Non-responder	Macro_ISG15	pDC_LILRA4	C1QB	C1QBP	4,57535E-03	7248,7	1,07831E-02	8766	5,66980E-01	6049	2,68648E+00	519	9,28088E-01	3379	0	17530,5
Non-responder	cDC_LAMP3	pDC_LILRA4	COPA	P2RY6	4,57604E-03	9041,9	1,62652E-02	4357	1,11613E+00	710	2,65851E+00	544	8,48673E-01	22068	0	17530,5
Non-responder	Mono_CD16	Macro_FOLR2+APOE+	HLA-C	LILRB2	4,57942E-03	10548,3	6,40762E-03	20209	4,78410E-01	8768	1,51497E+00	5398	9,42037E-01	836	0	17530,5
Non-responder	Macro_NLRP3	cDC_LAMP3	TGFB1	CXCR4	4,58068E-03	15393,7	6,85481E-03	18142	3,73615E-01	13307	8,04966E-01	17826	8,96312E-01	10163	0	17530,5
Non-responder	Mono_CD16	Mono_INHBA	HBEGF	CD44	4,58573E-03	12901,7	6,85375E-03	18146	5,35510E-01	6883	1,08909E+00	11353	8,94568E-01	10596	0	17530,5
Non-responder	cDC(CD1C)	Macro_FOLR2+APOE+	HLA-DQB1	LAG3	4,58699E-03	9306,9	1,42234E-02	5487	8,05448E-01	2494	1,84067E+00	2876	8,65294E-01	18147	0	17530,5
Non-responder	Macro_OLFML3	Macro_NLRP3	SERPINA1	LRP1	4,59458E-03	13655,9	6,85258E-03	18153	3,78102E-01	13073	1,06319E+00	11869	9,07233E-01	7654	0	17530,5
Non-responder	Macro_LYVE1	Macro_IER3	F13A1	ITGA4	4,60091E-03	8710,3	1,49139E-02	5059	8,72088E-01	1900	1,52802E+00	5277	8,82357E-01	13785	0	17530,5
Non-responder	Macro_FOLR2+APOE+	Mono_CD16	PLAU	LRP1	4,60183E-03	12682,7	1,29228E-02	6463	4,78102E-01	8779	1,59495E+00	4647	8,32368E-01	25994	0	17530,5
Non-responder	Macro_LYVE1	Macro_IER3	F13A1	ITGB1	4,60218E-03	9517,7	1,36316E-02	5904	8,12988E-01	2420	1,38531E+00	6820	8,78086E-01	14914	0	17530,5
Non-responder	Macro_IER3	cDC_CLEC9A	HMGB1	THBD	4,60218E-03	16234,1	7,22983E-03	16630	3,36007E-01	15444	7,92115E-01	18159	8,83747E-01	13407	0	17530,5
Non-responder	Macro_IER3	Macro_FOLR2+APOE+	HLA-DPB1	LAG3	4,60345E-03	12444,1	1,08358E-02	8699	5,11066E-01	7637	1,15622E+00	10194	8,65241E-01	18160	0	17530,5
Non-responder	Macro_OLFML3	Macro_FOLR2+APOE+	CD99	CD81	4,60345E-03	14674,5	6,85077E-03	18160	3,15702E-01	16705	1,13063E+00	10616	8,95492E-01	10361	0	17530,5
Non-responder	Macro_NLRP3	Macro_FOLR2+APOE+	TGFB1	SDC2	4,60796E-03	15458,5	1,17791E-02	7557	4,78085E-01	8782	1,36997E+00	7014	7,88992E-01	36409	0	17530,5
Non-responder	Macro_OLFML3	Mast	HBEGF	CD82	4,60852E-03	10330,9	1,74627E-02	3865	7,62919E-01	2889	1,21608E+00	9206	8,65234E-01	18164	0	17530,5
Non-responder	Mono_INHBA	pDC_LILRA4	ICAM1	SPN	4,61603E-03	8210,1	1,87859E-02	3401	8,46275E-01	2116	2,14240E+00	1537	8,72004E-01	16466	0	17530,5
Non-responder	Macro_LYVE1	Macro_IER3	C1QA	CD93	4,61613E-03	12081,5	9,06468E-03	11476	5,83249E-01	5658	8,31159E-01	17126	9,02980E-01	8617	0	17530,5
Non-responder	Macro_LYVE1	Macro_IER3	PLTP	ABCA1	4,61740E-03	8713,3	1,42732E-02	5460	7,90053E-01	2642	1,45135E+00	6023	8,89364E-01	11911	0	17530,5
Non-responder	Macro_FOLR2+APOE+	Mono_INHBA	HGF	SDC2	4,62228E-03	18088,5	3,92259E-02	996	6,40210E-01	4551	1,24359E+00	8789	6,79831E-01	58576	0	17530,5
Non-responder	Macro_FOLR2+APOE+	Mono_CD16	LGALS9	CD44	4,62228E-03	11954,1	6,62814E-03	19131	4,77786E-01	8789	1,31795E+00	7732	9,12147E-01	6588	0	17530,5
Non-responder	Macro_FOLR2+APOE+	cDC_LAMP3	MMP9	CD44	4,62433E-03	12061,9	1,07684E-02	8790	5,06446E-01	7773	1,56020E+00	4937	8,51949E-01	21279	0	17530,5
Non-responder	Macro_FOLR2+APOE+	Macro_IER3	HLA-DQA1	CD4	4,62433E-03	12107,7	5,78219E-03	23715	4,77748E-01	8790	1,37741E+00	6921	9,27101E-01	3582	0	17530,5
Non-responder	pDC_LILRA4	cDC_CLEC9A	QDPR	DYSF	4,63593E-03	15129,3	3,00294E-02	1538	7,17896E-01	3406	2,01348E+00	2029	7,22850E-01	51143	0	17530,5
Non-responder	Macro_OLFML3	Mono_CD14	AGTRAP	RACK1	4,64160E-03	16468,5	6,87185E-03	18053	4,27721E-01	10727	7,91211E-01	18190	8,66506E-01	17842	0	17530,5
Non-responder	Macro_IER3	Macro_OLFML3	MRC1	PTPRC	4,64415E-03	11667,5	1,12341E-02	8157	5,59255E-01	6243	1,28424E+00	8215	8,65103E-01	18192	0	17530,5
Non-responder	Mono_CD14	cDC(CD1C)	S100A9	CD68	4,64485E-03	6676,5	1,07640E-02	8800	8,42200E-01	2155	1,58085E+00	4759	9,51812E-01	138	0	17530,5
Non-responder	Macro_OLFML3	Macro_ISG15	HLA-DQB2	LAG3	4,65189E-03	11093,1	2,42413E-02	2188	8,79232E-01	1859	1,75106E+00	3410	8,14190E-01	30478	0	17530,5
Non-responder	Macro_FOLR2-APOE+	pDC_LILRA4	CD14	TLR9	4,65294E-03	8455,3	7,45521E-02	274	1,11470E+00	716	1,07340E+00	11666	8,88640E-01	12090	0	17530,5
Non-responder	Macro_OLFML3	Mono_CD14	CIRBP	TREM1	4,65309E-03	15937,1	8,30500E-03	13270	2,92648E-01	18199	8,98917E-01	15439	8,76779E-01	15247	0	17530,5
Non-responder	Macro_NLRP3	Mono_CD16	TNF	FAS	4,65719E-03	19282,1	1,21715E-02	7168	4,77296E-01	8806	1,40481E+00	6597	6,94140E-01	56309	0	17530,5
Non-responder	Macro_OLFML3	Macro_IER3	TNFSF10	TNFRSF10B	4,65925E-03	14719,1	1,14557E-02	7919	5,67179E-01	6046	1,33932E+00	7432	7,96638E-01	34668	0	17530,5
Non-responder	Macro_OLFML3	Macro_IER3	RPS19	C5AR1	4,66076E-03	11482,9	6,84076E-03	18205	5,55951E-01	6334	1,11797E+00	10830	9,22151E-01	4515	0	17530,5
Non-responder	Macro_FOLR2+APOE-	pDC_LILRA4	CD14	TLR9	4,66582E-03	7910,9	7,45464E-02	275	1,11465E+00	717	1,23224E+00	8941	8,88636E-01	12091	0	17530,5
Non-responder	pDC_LILRA4	Macro_OLFML3	HLA-B	LILRB2	4,67189E-03	8863,3	6,16521E-03	21422	7,17391E-01	3415	2,23931E+00	1262	9,43641E-01	687	0	17530,5
Non-responder	cDC(CD1C)	Macro_LYVE1	CD59	STAB1	4,67230E-03	12803,9	9,07919E-03	11445	7,02388E-01	3602	9,95346E-01	13228	8,64998E-01	18214	0	17530,5
Non-responder	Macro_FOLR2+APOE+	Mono_CD14	PSEN1	CD44	4,67486E-03	15927,1	7,40657E-03	15978	3,72419E-01	13367	9,37797E-01	14544	8,64996E-01	18216	0	17530,5
Non-responder	Mono_INHBA	cDC_CLEC9A	CCL4	CNR2	4,67590E-03	13587,7	8,70113E-02	187	9,92268E-01	1237	1,75021E+00	3416	7,49371E-01	45568	0	17530,5
Non-responder	Macro_FOLR2+APOE+	Macro_OLFML3	ADM	GPR84	4,67782E-03	17470,5	1,50362E-02	4991	4,77054E-01	8816	1,52975E+00	5255	7,24819E-01	50760	0	17530,5
Non-responder	Macro_NLRP3	Macro_NLRP3	HMGB1	THBD	4,68128E-03	15068,1	7,10178E-03	17110	2,92285E-01	18221	1,24141E+00	8817	8,82826E-01	13662	0	17530,5
Non-responder	Macro_FOLR2+APOE+	Macro_LYVE1	TNFSF13B	HLA-DPB1	4,68128E-03	10884,7	6,83744E-03	18221	5,51733E-01	6452	1,07283E+00	11677	9,45455E-01	543	0	17530,5
Non-responder	Macro_NLRP3	pDC_LILRA4	VEGFA	ITGB1	4,68195E-03	11189,9	1,07482E-02	8818	6,99851E-01	3629	2,13999E+00	1545	8,38866E-01	24427	0	17530,5
Non-responder	Macro_OLFML3	Mono_CD14	GNAI2	C5AR1	4,68257E-03	13088,3	6,83706E-03	18222	4,09592E-01	11537	1,21622E+00	9203	9,01462E-01	8949	0	17530,5
Non-responder	Macro_ISG15	Macro_FOLR2+APOE+	ICAM1	IL2RA	4,68392E-03	11828,3	1,91353E-02	3297	7,17199E-01	3418	1,78827E+00	3181	8,09114E-01	31715	0	17530,5
Non-responder	Macro_LYVE1	cDC_LAMP3	TNFSF13B	CD40	4,68642E-03	12951,9	8,76296E-03	12139	6,45601E-01	4459	9,02566E-01	15340	8,76637E-01	15291	0	17530,5
Non-responder	Macro_IER3	pDC_LILRA4	LYZ	ITGAL	4,69028E-03	15420,7	6,83522E-03	18228	2,99292E-01	17742	1,06868E+00	11758	8,89608E-01	11845	0	17530,5
Non-responder	Mono_CD14	cDC_LAMP3	GNAI2	S1PR1	4,69163E-03	8516,3	4,74062E-02	719	1,23435E+00	364	1,55067E+00	5034	8,61848E-01	18934	0	17530,5
Non-responder	Mono_CD14	Macro_IER3	VCAN	TLR1	4,69597E-03	9610,9	1,87315E-02	3421	1,06197E+00	923	1,85949E+00	2772	8,43103E-01	23408	0	17530,5
Non-responder	Macro_OLFML3	Macro_ISG15	HMGB1	HAVCR2	4,70187E-03	14038,1	6,83279E-03	18237	3,32582E-01	15645	1,18027E+00	9783	9,01274E-01	8995	0	17530,5
Non-responder	Macro_ISG15	Macro_ISG15	CXCL8	SDC2	4,70401E-03	10977,7	2,09057E-02	2834	7,16891E-01	3423	2,07439E+00	1794	8,19037E-01	29307	0	17530,5
Non-responder	Macro_FOLR2+APOE+	Macro_OLFML3	HLA-A	LILRA1	4,70703E-03	12179,1	6,83113E-03	18241	5,76556E-01	5810	1,50691E+00	5478	8,82140E-01	13836	0	17530,5
Non-responder	Macro_FOLR2+APOE+	Macro_FOLR2+APOE+	S100A9	CD68	4,71096E-03	12366,1	5,54580E-03	25325	5,00386E-01	7978	1,24065E+00	8832	9,34114E-01	2165	0	17530,5
Non-responder	Macro_LYVE1	cDC_LAMP3	F13A1	ITGA4	4,71348E-03	8306,1	1,57885E-02	4604	8,95491E-01	1759	1,59302E+00	4662	8,85283E-01	12975	0	17530,5
Non-responder	Macro_OLFML3	Macro_FOLR2+APOE-	SERPING1	LRP1	4,71478E-03	12770,7	1,06599E-02	8931	5,69533E-01	5982	7,89021E-01	18247	8,84556E-01	13163	0	17530,5
Non-responder	Macro_FOLR2+APOE+	pDC_LILRA4	C3	CD46	4,71607E-03	11888,9	1,12000E-02	8207	3,74156E-01	13274	1,97611E+00	2185	8,64837E-01	18248	0	17530,5
Non-responder	Macro_OLFML3	cDC_CLEC9A	CXCL10	SDC4	4,71609E-03	12406,3	1,87186E-02	3426	1,03723E+00	1035	2,39765E+00	933	7,77597E-01	39107	0	17530,5
Non-responder	Mono_CD16	Macro_ISG15	TNFSF13B	CD40	4,71736E-03	13845,9	7,10388E-03	17104	4,48783E-01	9873	1,41518E+00	6473	8,64832E-01	18249	0	17530,5
Non-responder	Mast	Macro_IFI27	CSF1	CSF3R	4,71751E-03	10429,9	4,72605E-02	721	1,11433E+00	719	1,21641E+00	9197	8,40714E-01	23982	0	17530,5
Non-responder	Macro_OLFML3	Macro_ISG15	LGALS9	CD44	4,72124E-03	11483,1	6,82897E-03	18252	4,88806E-01	8364	1,37503E+00	6955	9,13335E-01	6314	0	17530,5

Post_NR_Myeloid_Myeloid_CellCell

Non-responder	Macro_FOLR2+APOE+	Macro_FOLR2+APOE+	PDGFB	LRP1	4,72343E-03	14212,7	2,08000E-02	2864	5,28636E-01	7075	1,24038E+00	8838	7,96280E-01	34756	0	17530,5
Non-responder	Macro_OLFML3	pDC_LILRA4	HLA-DRB5	CD4	4,72771E-03	9935,7	6,82785E-03	18257	4,41652E-01	10160	1,85008E+00	2812	9,41454E-01	919	0	17530,5
Non-responder	Macro_ISG15	Macro_IER3	ADM	GPR84	4,72819E-03	13429,3	2,36788E-02	2267	7,16196E-01	3429	1,91521E+00	2482	7,67733E-01	41438	0	17530,5
Non-responder	Macro_NLRP3	cDC_LAMP3	HBEGF	CD44	4,73030E-03	14096,5	7,01226E-03	17486	5,35523E-01	6882	7,88502E-01	18259	8,95641E-01	10325	0	17530,5
Non-responder	Macro_OLFML3	Macro_FOLR2+APOE+	HSP90B1	TLR2	4,73289E-03	16035,7	6,82753E-03	18261	2,98535E-01	17801	9,20929E-01	14913	8,90296E-01	11673	0	17530,5
Non-responder	Mono_CD16	pDC_LILRA4	FLT3LG	FLT3	4,73385E-03	19744,9	2,22688E-02	2519	4,76269E-01	8843	1,57062E+00	4845	6,28455E-01	64987	0	17530,5
Non-responder	Macro_FOLR2+APOE+	Mono_CD14	B2M	LILRB1	4,73802E-03	14007,9	4,51693E-03	33798	4,76248E-01	8845	1,42944E+00	6288	9,27123E-01	3578	0	17530,5
Non-responder	pDC_LILRA4	Macro_OLFML3	APP	APLP2	4,74010E-03	6478,1	1,58664E-02	4558	1,06719E+00	904	2,65199E+00	552	9,01932E-01	8846	0	17530,5
Non-responder	Macro_OLFML3	Mono_CD14	CD14	TLR4	4,74197E-03	15669,5	7,67045E-03	15101	3,30158E-01	15792	1,07380E+00	11656	8,64761E-01	18268	0	17530,5
Non-responder	pDC_LILRA4	cDC(CD1C)	APP	NOTCH2	4,75646E-03	8918,9	1,48397E-02	5104	1,11901E+00	694	2,52917E+00	724	8,55067E-01	20542	0	17530,5
Non-responder	Macro_OLFML3	Macro_LYVE1	LILRB4	LAIR1	4,75887E-03	15183,5	7,03847E-03	17370	3,62886E-01	13895	7,87649E-01	18281	9,01977E-01	8841	0	17530,5
Non-responder	Mono_CD16	Macro_ISG15	ICAM4	ITGAM	4,75891E-03	18084,5	2,42391E-02	2189	5,44186E-01	6658	1,23923E+00	8855	7,00697E-01	55190	0	17530,5
Non-responder	Macro_OLFML3	cDC_CLEC9A	CXCL2	XCR1	4,76003E-03	10788,5	3,79592E-02	1051	2,31178E+00	14	1,71444E+00	3674	8,09264E-01	31673	0	17530,5
Non-responder	Macro_LYVE1	cDC(CD1C)	MRC1	PTPRC	4,76668E-03	13695,9	1,13237E-02	8057	5,33294E-01	6950	8,03369E-01	17871	8,65567E-01	18071	0	17530,5
Non-responder	pDC_LILRA4	Macro_LYVE1	HMGB1	CD163	4,76867E-03	7123,7	1,01729E-02	9601	7,15737E-01	3439	1,90422E+00	2530	9,32238E-01	2518	0	17530,5
Non-responder	Macro_ISG15	Macro_ISG15	HLA-F	LILRB2	4,76938E-03	7392,9	1,07135E-02	8860	8,24042E-01	2324	2,09271E+00	1732	9,12454E-01	6518	0	17530,5
Non-responder	Macro_LYVE1	cDC(CD1C)	B2M	CD1A	4,77059E-03	8826,7	4,56332E-02	764	5,05699E-01	7795	1,12109E+00	10783	9,08947E-01	7261	0	17530,5
Non-responder	Macro_OLFML3	Macro_NLRP3	ACTR2	LDLR	4,77147E-03	14512,7	1,47236E-02	5167	5,01389E-01	7943	1,23871E+00	8861	8,03375E-01	33062	0	17530,5
Non-responder	Macro_ISG15	Mono_CD16	CD48	CD244	4,77680E-03	12348,7	2,59886E-02	1958	7,20815E-01	3363	1,74623E+00	3441	7,93293E-01	35451	0	17530,5
Non-responder	pDC_LILRA4	pDC_LILRA4	IL16	CD4	4,77680E-03	6959,1	1,86784E-02	3441	8,18935E-01	2381	3,14622E+00	186	8,91953E-01	11257	0	17530,5
Non-responder	Macro_OLFML3	Macro_LYVE1	C1QA	CD93	4,77712E-03	14986,3	8,07059E-03	13919	3,36888E-01	15387	7,86950E-01	18295	8,97772E-01	9800	0	17530,5
Non-responder	Macro_FOLR2+APOE+	pDC_LILRA4	HLA-C	LILRB1	4,77987E-03	11812,1	5,46099E-03	25895	4,75352E-01	8865	1,95577E+00	2276	9,22272E-01	4494	0	17530,5
Non-responder	Macro_ISG15	Mono_CD14	CCL8	CCR1	4,78086E-03	14953,3	3,58855E-02	1153	7,15550E-01	3442	2,33516E+00	1053	7,20525E-01	51588	0	17530,5
Non-responder	Macro_FOLR2+APOE+	Mono_CD16	TNF	VSIR	4,78827E-03	12617,1	1,07041E-02	8869	6,12748E-01	5043	1,78293E+00	3212	8,22673E-01	28431	0	17530,5
Non-responder	pDC_LILRA4	Macro_IER3	LILRB4	LAIR1	4,79458E-03	7307,5	1,07021E-02	8872	7,07034E-01	3553	2,18202E+00	1433	9,19006E-01	5149	0	17530,5
Non-responder	Macro_OLFML3	Macro_IER3	CXCL10	SDC4	4,79668E-03	12971,5	1,67572E-02	4122	1,01936E+00	1118	2,55839E+00	678	7,67879E-01	41409	0	17530,5
Non-responder	cDC(CD1C)	Mono_CD14	GNAI2	FPR1	4,79673E-03	15584,5	6,87884E-03	18018	3,48066E-01	14753	7,86338E-01	18310	8,99856E-01	9311	0	17530,5
Non-responder	Macro_FOLR2+APOE+	Macro_ISG15	TOR2A	ATP5F1B	4,79879E-03	14976,7	1,07006E-02	8874	5,45589E-01	6609	1,52574E+00	5301	7,88298E-01	36569	0	17530,5
Non-responder	Macro_FOLR2+APOE+	cDC_CLEC9A	TNFSF13	TNFRSF14	4,79935E-03	13822,7	6,81530E-03	18312	3,75559E-01	13206	1,59621E+00	4632	8,76069E-01	15433	0	17530,5
Non-responder	Macro_FOLR2+APOE+	cDC_LAMP3	CCL5	SDC4	4,80300E-03	20508,9	1,87542E-02	3412	4,75016E-01	8876	1,26400E+00	8483	6,35837E-01	64243	0	17530,5
Non-responder	Macro_LYVE1	cDC(CD1C)	CXCL12	CXCR4	4,80329E-03	9712,9	2,53362E-02	2039	8,39365E-01	2187	1,19497E+00	9548	8,68979E-01	17260	0	17530,5
Non-responder	cDC(CD1C)	Mono_CD16	HLA-B	LILRB1	4,80591E-03	13160,9	6,81426E-03	18317	3,79224E-01	13019	9,37655E-01	14549	9,32978E-01	2389	0	17530,5
Non-responder	Macro_FOLR2+APOE+	Mono_CD14	CCL4	CCR1	4,80721E-03	11347,9	1,06972E-02	8878	5,52362E-01	6438	1,88503E+00	2630	8,52001E-01	21263	0	17530,5
Non-responder	pDC_LILRA4	Macro_IER3	APP	NCSTN	4,80864E-03	9776,1	1,51348E-02	4930	1,11124E+00	728	2,54077E+00	707	8,36589E-01	24985	0	17530,5
Non-responder	Macro_FOLR2+APOE+	Macro_FOLR2+APOE+	SEMA3C	NRP1_PLXNA1	4,80932E-03	19661,5	2,39657E-02	2221	5,47882E-01	6549	1,23754E+00	8879	6,45717E-01	63128	0	17530,5
Non-responder	Macro_FOLR2+APOE+	cDC(CD1C)	APOE	TREM2	4,81116E-03	12368,9	9,03105E-03	11561	2,90866E-01	18321	1,09833E+00	11191	9,28773E-01	3241	0	17530,5
Non-responder	Macro_FOLR2+APOE+	Macro_OLFML3	PLAU	LRP1	4,81143E-03	12926,5	1,28148E-02	6560	4,74871E-01	8880	1,50171E+00	5521	8,31782E-01	26141	0	17530,5
Non-responder	Macro_OLFML3	Macro_OLFML3	TNFSF13	TNFRSF1A	4,81247E-03	16624,5	6,81163E-03	18322	3,10180E-01	17021	9,04680E-01	15297	8,77923E-01	14952	0	17530,5
Non-responder	Macro_IER3	Macro_FOLR2+APOE+	VEGFA	CD44	4,81378E-03	15687,1	7,50611E-03	15652	4,06706E-01	11682	9,06980E-01	15248	8,64545E-01	18323	0	17530,5
Non-responder	Macro_NLRP3	Macro_ISG15	HLA-C	LILRB1	4,81378E-03	13013,7	7,13196E-03	16989	2,90855E-01	18323	1,19552E+00	9540	9,31317E-01	2686	0	17530,5
Non-responder	Macro_NLRP3	Macro_ISG15	HLA-F	LILRB2	4,81378E-03	11571,9	6,81162E-03	18323	5,78766E-01	5766	1,53905E+00	5146	8,92595E-01	11094	0	17530,5
Non-responder	Macro_FOLR2+APOE+	Macro_LYVE1	CD14	ITGB2	4,81510E-03	11329,9	6,81158E-03	18324	4,81792E-01	8632	1,11259E+00	10946	9,39048E-01	1217	0	17530,5
Non-responder	Macro_FOLR2+APOE+	Mono_CD14	SERPINA1	LRP1	4,81773E-03	13001,1	6,81141E-03	18326	3,92039E-01	12365	1,22394E+00	9073	9,06979E-01	7711	0	17530,5
Non-responder	pDC_LILRA4	Macro_FOLR2-APOE+	SPON2	ITGA4	4,82163E-03	11046,7	2,67903E-02	1850	9,96979E-01	1219	1,74492E+00	3452	8,11334E-01	31182	0	17530,5
Non-responder	Macro_LYVE1	cDC(CD1C)	F13A1	ITGA4	4,82430E-03	8743,5	1,48952E-02	5073	8,71589E-01	1904	1,51404E+00	5409	8,82292E-01	13801	0	17530,5
Non-responder	Macro_LYVE1	cDC(CD1C)	F13A1	ITGB1	4,82562E-03	9027,7	1,43577E-02	5410	8,32774E-01	2251	1,47497E+00	5787	8,80836E-01	14160	0	17530,5
Non-responder	Mono_INHBA	Macro_ISG15	VEGFA	CD44	4,83467E-03	7578,7	1,54963E-02	4736	8,55831E-01	2043	1,58936E+00	4693	9,01678E-01	8891	0	17530,5
Non-responder	pDC_LILRA4	Mono_CD16	HSP90B1	ASGR1	4,83483E-03	6369,7	1,85926E-02	3472	1,11082E+00	730	3,22764E+00	161	8,97132E-01	9955	0	17530,5
Non-responder	Macro_LYVE1	cDC(CD1C)	C1QA	CD33	4,83879E-03	11227,3	1,11865E-02	8227	5,94049E-01	5442	8,18424E-01	17464	9,07957E-01	7473	0	17530,5
Non-responder	Macro_OLFML3	Macro_NLRP3	CD38	PECAM1	4,84315E-03	15987,5	1,30159E-02	6387	6,05244E-01	5205	1,23597E+00	8895	7,65697E-01	41920	0	17530,5
Non-responder	Macro_FOLR2+APOE+	Macro_IFI27	CD14	ITGB2	4,84407E-03	12704,7	6,80662E-03	18346	4,81191E-01	8659	8,06848E-01	17769	9,39027E-01	1219	0	17530,5
Non-responder	Macro_FOLR2+APOE+	pDC_LILRA4	HLA-F	LILRB1	4,84539E-03	13469,3	6,80655E-03	18347	4,31369E-01	10555	1,75152E+00	3406	8,67894E-01	17508	0	17530,5
Non-responder	Mono_CD16	Mono_CD16	ANXA1	FPR1	4,84804E-03	13639,1	6,80626E-03	18349	3,24849E-01	16124	1,23709E+00	8885	9,08750E-01	7307	0	17530,5
Non-responder	Macro_OLFML3	Macro_FOLR2+APOE+	IL15	IL2RA	4,85375E-03	19377,9	1,77193E-02	3764	5,52868E-01	6417	1,23571E+00	8900	6,67974E-01	60278	0	17530,5
Non-responder	Mono_CD14	Macro_FOLR2+APOE+	HBEGF	CD9	4,85588E-03	9212,3	1,13550E-02	8024	5,05987E-01	7785	1,69346E+00	3821	9,01642E-01	8901	0	17530,5
Non-responder	Macro_FOLR2+APOE+	Mono_CD14	ICAM1	ITGAL_ITGB2	4,85729E-03	12746,9	6,80464E-03	18356	4,52916E-01	9715	1,33663E+00	7469	8,94292E-01	10664	0	17530,5
Non-responder	Mono_CD16	Macro_NLRP3	ACTR2	LDLR	4,85800E-03	14601,9	1,41102E-02	5563	4,74159E-01	8902	1,36051E+00	7128	7,99993E-01	33886	0	17530,5
Non-responder	cDC(CD1C)	Macro_OLFML3	CD1D	LILRB2	4,85994E-03	10301,1	1,57255E-02	4637	6,35215E-01	4623	1,42434E+00	6357	8,64370E-01	18358	0	17530,5
Non-responder	Macro_LYVE1	Macro_FOLR2+APOE+	TNFSF13B	TFRC	4,86126E-03	15095,1	7,21093E-03	16702	4,10960E-01	11470	1,08582E+00	11414	8,64360E-01	18359	0	17530,5
Non-responder	Macro_NLRP3	Mono_INHBA	VIM	CD44	4,86258E-03	10563,3	6,80388E-03	18360	6,22195E-01	4881	1,06163E+00	11909	9,51847E-01	136	0	17530,5
Non-responder	pDC_LILRA4	Macro_LYVE1	LILRB4	LAIR1	4,86863E-03	7414,7	1,06748E-02	8907	7,05459E-01	3570	2,04824E+00	1901	9,18910E-01	5165	0	17530,5
Non-responder	Mono_INHBA	Mono_INHBA	IL6	IL6R_IL6ST	4,87909E-03	12701,9	2,82235E-02	1712	1,09856E+00	769	1,74266E+00	3466	7,73829E-01	40032	0	17530,5
Non-responder	Mono_CD14	Mono_CD16	HLA-C	LILRA3	4,87928E-03	8330,3	1,86960E-02	3433	4,73858E-01	8912	1,63776E+00	4286	9,07888E-01	7490	0	17530,5
Non-responder	Macro_IFI27	Mono_CD16	HLA-B	LILRA1	4,87982E-03	9825,9	1,61345E-02	4411	6,28978E-01	4746	7,84059E-01	18373	9,24545E-01	4069	0	17530,5
Non-responder	Macro_OLFML3	Macro_FOLR2+APOE+	GAS6	MERTK	4,88141E-03	16698,1	1,06699E-02	8913	4,81913E-01	8626	1,26697E+00	8449	7,74061E-01	39972	0	17530,5
Non-responder	Mono_INHBA	Macro_FOLR2+APOE+	ANGPTL4	SDC3	4,88322E-03	14916,3	1,02413E-01	128	9,63427E-01	1378	1,74266E+00	3467	7,17751E-01	52078	0	17530,5
Non-responder	Macro_OLFML3	Mono_CD14	S100A8	CD36	4,88647E-03	14659,7	9,63822E-03	10432	3,86067E-01	12667	9,48971E-01	14291	8,64263E-01	18378	0	17530,5
Non-responder	Macro_FOLR2+APOE+	Macro_FOLR2+APOE+	HSP90B1	TLR1	4,88913E-03	12548,1	1,01413E-02	9644	4,65353E-01	9227	1,30172E+00	7959	8,64259E-01	18380	0	17530,5
Non-responder	Macro_FOLR2+APOE+	Macro_NLRP3	GRN	TNFRSF1A	4,88994E-03	11842,3	6,00275E-03	22362	4,73657E-01	8917	1,59677E+00	4623	9,15833E-01	5779	0	17530,5
Non-responder	Macro_IER3	Macro_NLRP3	TIMP2	CD44	4,89445E-03	14430,9	6,79768E-03	18384	5,13027E-01	7568	1,05073E+00	12133	8,71729E-01	16539	0	17530,5
Non-responder	Macro_ISG15	cDC_CLEC9A	C1QB	C1QBP	4,89635E-03	7477,5	1,06661E-02	8920	5,57756E-01	6285	2,26636E+00	1192	9,27723E-01	3460	0	17530,5

Post_NR_Myeloid_Myeloid_CellCell

Non-responder	Macro_FOLR2+APOE+	pDC_LILRA4	FN1	CD79A	4,90490E-03	19330,7	6,25433E-02	413	4,73549E-01	8924	1,50567E+00	5485	6,35223E-01	64301	0	17530,5
Non-responder	Macro_IER3	Macro_FOLR2+APOE+	MRC1	PTPRC	4,91178E-03	11496,3	1,10586E-02	8415	5,46747E-01	6574	1,40695E+00	6565	8,64182E-01	18397	0	17530,5
Non-responder	cDC(CD1C)	Macro_FOLR2+APOE+	TNFSF13B	CD40	4,91311E-03	14406,9	7,02451E-03	17431	4,60355E-01	9410	1,21191E+00	9265	8,64174E-01	18398	0	17530,5
Non-responder	Mono_INHBA	cDC_CLEC9A	CXCL2	DPP4	4,91627E-03	10195,1	6,52726E-02	386	1,04533E+00	995	1,74095E+00	3475	8,22020E-01	28589	0	17530,5
Non-responder	cDC(CD1C)	Macro_OLFML3	TNFSF13B	CD40	4,91712E-03	15185,9	7,02268E-03	17443	4,60184E-01	9417	9,99339E-01	13138	8,64158E-01	18401	0	17530,5
Non-responder	Macro_NLRP3	cDC_CLEC9A	VEGFA	CD44	4,91979E-03	14495,1	7,45522E-03	15821	5,67554E-01	6037	9,31117E-01	14684	8,64146E-01	18403	0	17530,5
Non-responder	Mono_CD16	Macro_OLFML3	S100A9	ITGB2	4,91979E-03	13178,1	6,79147E-03	18403	4,34521E-01	10438	8,12492E-01	17618	9,35296E-01	1901	0	17530,5
Non-responder	Macro_NLRP3	cDC_LAMP3	IL10	CD70	4,91989E-03	18531,9	6,09653E-02	435	9,84624E-01	1281	1,23333E+00	8931	6,33364E-01	64482	0	17530,5
Non-responder	Macro_IER3	cDC_CLEC9A	TNFSF13B	CD40	4,92514E-03	15313,3	7,01656E-03	17468	4,46432E-01	9962	9,96498E-01	13199	8,64107E-01	18407	0	17530,5
Non-responder	Macro_LYVE1	Macro_OLFML3	HLA-A	LILRB1	4,92514E-03	12974,7	6,79041E-03	18407	4,51344E-01	9791	8,78106E-01	15908	9,28802E-01	3237	0	17530,5
Non-responder	Macro_NLRP3	Macro_ISG15	TNF	TRADD	4,92633E-03	14462,5	1,06577E-02	8934	5,04498E-01	7817	1,59425E+00	4652	8,02063E-01	33379	0	17530,5
Non-responder	Macro_OLFML3	Macro_OLFML3	LGALS9	PTPRC	4,92633E-03	12335,7	6,42250E-03	20141	4,73334E-01	8934	1,27880E+00	8287	9,11208E-01	6786	0	17530,5
Non-responder	Mono_INHBA	Macro_FOLR2+APOE+	HBEGF	CD9	4,92848E-03	10256,5	1,13216E-02	8059	5,04330E-01	7826	1,23314E+00	8932	9,01512E-01	8935	0	17530,5
Non-responder	pDC_LILRA4	pDC_LILRA4	ICAM1	SPN	4,92870E-03	8055,1	1,85726E-02	3478	8,40432E-01	2179	2,74382E+00	465	8,71365E-01	16623	0	17530,5
Non-responder	Macro_FOLR2+APOE+	Macro_ISG15	HSPA1A	TLR4	4,92916E-03	14210,7	6,78982E-03	18410	3,59353E-01	14092	1,44491E+00	6102	8,78060E-01	14919	0	17530,5
Non-responder	Macro_FOLR2+APOE+	Macro_LYVE1	C3	NRP1	4,93922E-03	11038,7	1,66055E-02	4198	4,73180E-01	8940	1,55145E+00	5024	8,59435E-01	19501	0	17530,5
Non-responder	Macro_OLFML3	Macro_FOLR2+APOE+	FN1	PLAUR	4,94256E-03	13541,1	9,88057E-03	10041	3,42258E-01	15064	1,40033E+00	6650	8,64063E-01	18420	0	17530,5
Non-responder	Macro_IER3	Macro_ISG15	HLA-A	LILRB2	4,94256E-03	10874,3	6,78660E-03	18420	5,14659E-01	7504	1,15825E+00	10158	9,42829E-01	759	0	17530,5
Non-responder	Macro_NLRP3	Macro_LYVE1	PTGS2	ALOX5	4,94793E-03	13735,5	1,10950E-02	8362	5,34333E-01	6922	8,19218E-01	17439	8,64050E-01	18424	0	17530,5
Non-responder	Macro_FOLR2+APOE+	Macro_FOLR2+APOE-	LGALS3	ENG	4,95196E-03	14493,5	6,78539E-03	18427	4,18716E-01	11116	9,01799E-01	15363	8,96798E-01	10031	0	17530,5
Non-responder	Macro_NLRP3	Macro_ISG15	TNF	TRPM2	4,95213E-03	15651,9	1,18751E-02	7469	4,73042E-01	8946	1,57730E+00	4788	7,75749E-01	39526	0	17530,5
Non-responder	Macro_FOLR2+APOE+	Macro_NLRP3	SPP1	ITGA5_ITGB1	4,95644E-03	10846,7	1,65565E-02	4213	4,72994E-01	8948	2,36649E+00	996	8,46663E-01	22546	0	17530,5
Non-responder	Mono_CD16	Mono_CD16	HLA-A	LILRB1	4,96272E-03	12379,1	6,78178E-03	18435	3,41414E-01	15110	1,32911E+00	7576	9,28760E-01	3244	0	17530,5
Non-responder	Macro_OLFML3	pDC_LILRA4	HLA-DRA	CD4	4,96810E-03	9365,5	6,78075E-03	18439	4,94014E-01	8197	1,95136E+00	2309	9,47996E-01	352	0	17530,5
Non-responder	cDC_CLEC9A	Macro_FOLR2+APOE+	HLA-DPA1	CD4	4,97029E-03	7009,5	8,69578E-03	12303	7,11751E-01	3488	2,13427E+00	1569	9,51378E-01	157	0	17530,5
Non-responder	Macro_FOLR2+APOE+	Macro_IER3	LRPAP1	SORL1	4,97484E-03	14277,3	8,81538E-03	12007	4,28492E-01	10686	1,02113E+00	12719	8,63956E-01	18444	0	17530,5
Non-responder	Macro_IER3	pDC_LILRA4	HLA-DMA	CD4	4,97619E-03	11698,5	6,77918E-03	18445	3,81504E-01	12903	1,46273E+00	5916	9,26463E-01	3698	0	17530,5
Non-responder	Macro_IER3	pDC_LILRA4	HSP90B1	TLR9	4,97754E-03	9517,5	4,79815E-02	704	8,63605E-01	1967	1,23226E+00	8940	8,63947E-01	18446	0	17530,5
Non-responder	Macro_NLRP3	Mono_INHBA	PKM	CD44	4,98024E-03	11309,1	6,77833E-03	18448	5,54607E-01	6377	1,06612E+00	11803	9,33001E-01	2387	0	17530,5
Non-responder	Macro_OLFML3	cDC_LAMP3	CD274	CD80	4,98236E-03	14529,5	4,10746E-02	924	1,00221E+00	1197	1,54845E+00	5059	7,38642E-01	47937	0	17530,5
Non-responder	Macro_LYVE1	Macro_LYVE1	F13A1	ITGA4	4,98699E-03	9377,7	1,38895E-02	5723	8,44679E-01	2136	1,39359E+00	6724	8,78613E-01	14775	0	17530,5
Non-responder	Macro_LYVE1	Macro_LYVE1	F13A1	ITGB1	4,98834E-03	9358,7	1,40479E-02	5606	8,24332E-01	2322	1,38291E+00	6851	8,79687E-01	14484	0	17530,5
Non-responder	Mono_INHBA	Mono_INHBA	FN1	PLAUR	4,98886E-03	7369,7	2,04433E-02	2958	7,62548E-01	2894	1,61089E+00	4503	9,01411E-01	8963	0	17530,5
Non-responder	Mono_CD16	pDC_LILRA4	ICAM4	ITGB1	4,98886E-03	17195,1	1,65070E-02	4236	4,72461E-01	8963	1,69657E+00	3807	7,21371E-01	51439	0	17530,5
Non-responder	Macro_NLRP3	Macro_OLFML3	TNF	TNFRSF1A	4,98886E-03	11966,9	1,06408E-02	8963	5,30225E-01	7039	1,45909E+00	5955	8,55815E-01	20347	0	17530,5
Non-responder	Macro_LYVE1	pDC_LILRA4	MMP9	TLR9	4,99116E-03	13649,1	8,11139E-02	221	1,07098E+00	893	1,73808E+00	3493	7,46901E-01	46108	0	17530,5
Non-responder	Mono_CD14	Macro_FOLR2-APOE+	S100A12	TLR4	4,99535E-03	10256,9	2,82620E-02	1708	1,19985E+00	441	1,73799E+00	3494	8,23970E-01	28111	0	17530,5
Non-responder	Macro_OLFML3	cDC_LAMP3	FN1	TNFRSF11B	4,99536E-03	13951,9	1,09712E-01	105	1,10721E+00	743	1,71523E+00	3668	7,39649E-01	47713	0	17530,5
Non-responder	Macro_ISG15	Macro_FOLR2+APOE+	LGALS9	CD47	4,99536E-03	8251,1	1,09827E-02	8503	7,08547E-01	3537	1,86944E+00	2719	9,01392E-01	8966	0	17530,5
Non-responder	Macro_LYVE1	Macro_LYVE1	C1QB	LRP1	4,99917E-03	9472,9	1,28744E-02	6507	6,87125E-01	3790	8,33786E-01	17044	9,32394E-01	2493	0	17530,5
Non-responder	cDC(CD1C)	Macro_LYVE1	HLA-DPB1	CD4	5,00052E-03	11641,3	6,77617E-03	18463	5,43500E-01	6675	9,26983E-01	14756	9,42579E-01	782	0	17530,5
Non-responder	Macro_FOLR2+APOE+	Macro_ISG15	HGF	CD44	5,00187E-03	14083,1	1,65469E-02	4218	4,78125E-01	8778	1,23028E+00	8969	8,12361E-01	30920	0	17530,5
Non-responder	pDC_LILRA4	Macro_ISG15	SELPLG	ITGB2	5,00187E-03	6809,1	1,06361E-02	8969	7,06318E-01	3559	2,55151E+00	692	9,28527E-01	3295	0	17530,5
Non-responder	Macro_LYVE1	Macro_LYVE1	C1QA	CD93	5,00187E-03	10079,7	1,21775E-02	7162	7,05399E-01	3572	8,66620E-01	16210	9,15165E-01	5924	0	17530,5
Non-responder	Macro_LYVE1	Macro_LYVE1	PLTP	ABCA1	5,00594E-03	8005,5	1,73440E-02	3911	8,72564E-01	1893	1,36245E+00	7098	8,98594E-01	9595	0	17530,5
Non-responder	Mast	cDC(CD1C)	CSF1	SIRPA	5,00678E-03	10073,5	4,62633E-02	743	1,11538E+00	711	1,56382E+00	4901	8,30429E-01	26482	0	17530,5
Non-responder	Mono_CD16	cDC(CD1C)	ICAM2	ITGAL_ITGB2	5,01408E-03	12303,9	1,29838E-02	6416	4,93262E-01	8219	1,11610E+00	10881	8,63833E-01	18473	0	17530,5
Non-responder	Mono_CD14	Macro_IER3	S100A8	TLR4	5,01629E-03	6702,1	1,85119E-02	3499	1,14535E+00	597	2,29162E+00	1138	8,93973E-01	10746	0	17530,5
Non-responder	Macro_OLFML3	cDC_LAMP3	VEGFA	NRP2	5,01925E-03	13110,5	1,51778E-02	4899	8,57807E-01	2025	1,36673E+00	7051	7,99280E-01	34047	0	17530,5
Non-responder	Macro_IER3	Macro_FOLR2+APOE+	HLA-DRB5	LAG3	5,01951E-03	11991,7	1,11020E-02	8355	5,32800E-01	6963	1,25475E+00	8633	8,63807E-01	18477	0	17530,5
Non-responder	Macro_FOLR2-APOE+	Macro_FOLR2+APOE+	APOE	LRP1	5,02796E-03	7015,7	1,66802E-02	4162	7,33754E-01	3210	1,22950E+00	8981	9,39158E-01	1195	0	17530,5
Non-responder	Macro_OLFML3	cDC_LAMP3	ADM	RAMP1	5,03014E-03	16681,1	2,34106E-02	2304	1,19525E+00	450	1,39292E+00	6736	6,93695E-01	56385	0	17530,5
Non-responder	Macro_IFI27	Macro_FOLR2+APOE+	HLA-DPB1	CD4	5,03038E-03	11269,9	7,97847E-03	14195	5,80946E-01	5712	7,79531E-01	18485	9,46842E-01	427	0	17530,5
Non-responder	Macro_OLFML3	cDC_LAMP3	ADM	CALCRL	5,03232E-03	17083,9	1,28672E-02	6515	8,08423E-01	2470	1,29084E+00	8103	7,24648E-01	50801	0	17530,5
Non-responder	Macro_NLRP3	Mono_INHBA	LAMB3	CD44	5,03450E-03	13005,9	2,59956E-02	1955	7,33548E-01	3213	1,22941E+00	8984	8,02203E-01	33347	0	17530,5
Non-responder	Macro_FOLR2+APOE+	Macro_IFI27	TNFSF13B	HLA-DPB1	5,03583E-03	11929,5	7,00862E-03	17499	5,84393E-01	5636	7,79474E-01	18489	9,46090E-01	493	0	17530,5
Non-responder	Macro_FOLR2+APOE+	Macro_NLRP3	HMGB1	TLR4	5,03719E-03	15879,3	7,07939E-03	17188	2,88425E-01	18490	1,30664E+00	7898	8,64674E-01	18290	0	17530,5
Non-responder	Macro_FOLR2+APOE+	Macro_OLFML3	CD14	ITGB1	5,03719E-03	11968,3	6,77143E-03	18490	4,82993E-01	8588	1,51362E+00	5412	8,97681E-01	9821	0	17530,5
Non-responder	Macro_NLRP3	Macro_NLRP3	SERPINA1	LRP1	5,03855E-03	14096,9	6,77134E-03	18491	3,66469E-01	13706	1,00821E+00	12981	9,06730E-01	7776	0	17530,5
Non-responder	Macro_IER3	Mono_CD14	GNAI2	FPR1	5,04128E-03	15672,1	6,77099E-03	18493	3,37440E-01	15349	8,15822E-01	17527	8,99142E-01	9461	0	17530,5
Non-responder	Macro_LYVE1	Macro_LYVE1	CD59	STAB1	5,04264E-03	8330,7	1,64007E-02	4300	9,14920E-01	1639	1,30358E+00	7932	8,95959E-01	10252	0	17530,5
Non-responder	Macro_OLFML3	cDC_LAMP3	CD72	CD5	5,04323E-03	16551,9	6,32072E-02	410	7,74785E-01	2773	1,34263E+00	7382	7,03896E-01	54664	0	17530,5
Non-responder	Macro_FOLR2+APOE+	cDC_CLEC9A	APOE	SCARB1	5,05356E-03	12635,3	8,96137E-03	11720	3,92374E-01	12344	1,80576E+00	3080	8,63694E-01	18502	0	17530,5
Non-responder	Macro_FOLR2+APOE+	Mono_INHBA	LGALS9	LRP1	5,05766E-03	15222,3	6,76754E-03	18505	3,77129E-01	13124	1,05099E+00	12125	8,78461E-01	14827	0	17530,5
Non-responder	cDC(CD1C)	Macro_FOLR2+APOE+	MIF	TNFRSF14	5,06860E-03	14005,1	6,76602E-03	18513	4,65008E-01	9244	9,32707E-01	14654	8,96605E-01	10084	0	17530,5
Non-responder	Macro_ISG15	Macro_ISG15	B2M	LILRB1	5,07102E-03	7262,3	8,51700E-03	12714	7,10405E-01	3512	2,01085E+00	2046	9,45855E-01	509	0	17530,5
Non-responder	Macro_OLFML3	cDC_LAMP3	NAMPT	ADORA2A	5,07168E-03	11012,7	2,68780E-02	1841	8,33308E-01	2247	1,32540E+00	7613	8,32978E-01	25832	0	17530,5
Non-responder	cDC(CD1C)	Mono_CD16	B2M	LILRB1	5,07408E-03	13834,9	6,76569E-03	18517	3,31332E-01	15718	8,63909E-01	16266	9,39649E-01	1143	0	17530,5
Non-responder	Macro_OLFML3	pDC_LILRA4	SEMA4A	NRP1_PLXNA4	5,07525E-03	13415,9	1,99121E-02	3085	7,29775E-01	3247	1,73520E+00	3513	7,75065E-01	39704	0	17530,5
Non-responder	Macro_IFI27	Macro_FOLR2+APOE+	HLA-DRA	CD4	5,07545E-03	11487,9	7,61053E-03	15278	5,72314E-01	5929	7,78385E-01	18518	9,50769E-01	184	0	17530,5
Non-responder	Macro_FOLR2+APOE+	Macro_FOLR2+APOE+	HMGB1	HAVCR2	5,07682E-03	13577,5	6,76491E-03	18519	3,36507E-01	15408	1,34688E+00	7335	9,00829E-01	9095	0	17530,5

Post_NR_Myeloid_Myeloid_CellCell

Non-responder	Macro_FOLR2+APOE+	Macro_OLFML3	ADAM10	CD44	5,08093E-03	17534,7	6,95262E-03	17729	3,16347E-01	16668	8,27489E-01	17224	8,63617E-01	18522	0	17530,5
Non-responder	Macro_FOLR2+APOE+	Macro_FOLR2+APOE+	LGALS1	ITGB1	5,08642E-03	11701,1	6,76307E-03	18526	3,97874E-01	12084	1,59733E+00	4617	9,15981E-01	5748	0	17530,5
Non-responder	cDC_LAMP3	cDC_LAMP3	IL15	IL2RA	5,08714E-03	12923,5	4,60852E-02	749	1,15276E+00	573	1,72848E+00	3565	7,64400E-01	42200	0	17530,5
Non-responder	Macro_OLFML3	cDC_LAMP3	GNAI2	S1PR1	5,09584E-03	8772,1	4,37541E-02	827	1,17400E+00	513	1,56228E+00	4919	8,57006E-01	20071	0	17530,5
Non-responder	Macro_IER3	Macro_NLRP3	ADAM10	CD44	5,09741E-03	14246,3	6,94785E-03	17742	5,60300E-01	6220	1,09766E+00	11205	8,63577E-01	18534	0	17530,5
Non-responder	Macro_FOLR2+APOE+	Macro_FOLR2+APOE+	SDC2	PTPRJ	5,09804E-03	19701,3	1,62679E-02	4355	4,82620E-01	8603	1,22721E+00	9013	6,77140E-01	59005	0	17530,5
Non-responder	Macro_OLFML3	Mono_CD16	HLA-G	LILRB2	5,09804E-03	10726,5	1,06019E-02	9013	7,46110E-01	3072	1,70142E+00	3776	8,56308E-01	20241	0	17530,5
Non-responder	Macro_FOLR2+APOE+	Macro_ISG15	SEMA4A	PLXND1	5,10024E-03	15849,1	1,06016E-02	9014	4,76209E-01	8846	1,39644E+00	6692	7,85827E-01	37163	0	17530,5
Non-responder	Macro_OLFML3	cDC_LAMP3	ENTPD1	ADORA2A	5,10244E-03	14496,9	3,35779E-02	1277	7,89227E-01	2649	1,23163E+00	8948	7,65032E-01	42080	0	17530,5
Non-responder	Macro_FOLR2-APOE+	Macro_FOLR2+APOE+	APOE	SCARB1	5,10465E-03	7305,9	2,70880E-02	1809	7,97061E-01	2587	1,22706E+00	9016	9,16782E-01	5587	0	17530,5
Non-responder	cDC_CLEC9A	Macro_NLRP3	HMGB1	THBD	5,10685E-03	8384,5	1,06004E-02	9017	6,22545E-01	4873	2,10731E+00	1667	9,02009E-01	8835	0	17530,5
Non-responder	Macro_FOLR2+APOE+	cDC_CLEC9A	TNF	TRADD	5,10905E-03	15150,1	1,05996E-02	9018	4,78922E-01	8745	1,37359E+00	6966	8,01630E-01	33491	0	17530,5
Non-responder	cDC_CLEC9A	cDC_CLEC9A	HLA-DQA2	LAG3	5,11763E-03	9976,9	1,84132E-02	3523	7,79430E-01	2732	2,07314E+00	1804	8,39404E-01	24295	0	17530,5
Non-responder	Macro_FOLR2+APOE+	Macro_ISG15	FN1	C5AR1	5,11788E-03	11915,9	1,28800E-02	6503	4,71321E-01	9022	1,65204E+00	4184	8,47530E-01	22340	0	17530,5
Non-responder	cDC_CLEC9A	cDC_CLEC9A	HLA-DOB	CD4	5,12613E-03	9691,7	2,10562E-02	2799	1,14367E+00	601	1,73338E+00	3525	8,40609E-01	24003	0	17530,5
Non-responder	Macro_IER3	Macro_OLFML3	CCL3L1	CCR5	5,13039E-03	11519,9	2,51610E-02	2058	7,81260E-01	2716	1,73318E+00	3526	8,08842E-01	31769	0	17530,5
Non-responder	Macro_LYVE1	Mono_INHBA	PLAU	PLAUR	5,13326E-03	12041,7	1,03674E-02	9316	6,30050E-01	4724	1,06872E+00	11756	8,70347E-01	16882	0	17530,5
Non-responder	Mono_CD16	cDC_LAMP3	TNFSF10	CCR6	5,13335E-03	18172,9	2,60664E-02	1948	5,59999E-01	6223	1,22649E+00	9029	6,95205E-01	56134	0	17530,5
Non-responder	Mono_INHBA	Macro_LYVE1	INHBA	ENG	5,13465E-03	9583,3	3,74531E-02	1079	9,30631E-01	1553	1,73316E+00	3527	8,39722E-01	24227	0	17530,5
Non-responder	Macro_LYVE1	Mono_INHBA	F13A1	ITGB1	5,14295E-03	8973,5	1,43270E-02	5425	8,31937E-01	2264	1,50991E+00	5454	8,80724E-01	14194	0	17530,5
Non-responder	Macro_FOLR2+APOE+	Mono_CD14	ICAM1	ITGAX_ITGB2	5,14295E-03	12507,7	6,75304E-03	18567	4,58232E-01	9491	1,30394E+00	7928	9,01156E-01	9022	0	17530,5
Non-responder	Mono_CD16	Mono_CD16	VCAN	ITGA4	5,14434E-03	12590,1	9,35210E-03	10936	5,15015E-01	7498	1,26892E+00	8418	8,63443E-01	18568	0	17530,5
Non-responder	Mono_CD16	Macro_FOLR2+APOE+	HLA-F	LILRB1	5,14572E-03	14323,5	6,75294E-03	18569	4,08584E-01	11583	1,42738E+00	6318	8,67440E-01	17617	0	17530,5
Non-responder	cDC_CLEC9A	Macro_ISG15	SPN	SIGLEC1	5,14744E-03	11537,7	2,43648E-02	2171	7,09147E-01	3530	1,85136E+00	2808	8,09364E-01	31649	0	17530,5
Non-responder	pDC_LILRA4	Mono_CD16	HLA-C	LILRA3	5,14885E-03	7745,7	1,86613E-02	3448	4,70879E-01	9036	2,26341E+00	1202	9,07810E-01	7512	0	17530,5
Non-responder	Mast	Macro_ISG15	ANXA1	FPR2_FPR3	5,14885E-03	8007,9	1,61731E-02	4388	8,18767E-01	2384	1,39548E+00	6701	9,01086E-01	9036	0	17530,5
Non-responder	Mono_CD16	Mast	ARPC5	ADRB2	5,15127E-03	11189,7	1,69153E-02	4075	5,84335E-01	5641	1,16004E+00	10129	8,63416E-01	18573	0	17530,5
Non-responder	Macro_LYVE1	Mono_INHBA	C1QA	CD93	5,15543E-03	8103,1	1,46626E-02	5193	8,02912E-01	2517	1,12326E+00	10742	9,22101E-01	4533	0	17530,5
Non-responder	Macro_LYVE1	Mono_INHBA	PLTP	ABCA1	5,15820E-03	7968,9	1,70079E-02	4037	8,63531E-01	1970	1,41350E+00	6490	8,97699E-01	9817	0	17530,5
Non-responder	Macro_OLFML3	Macro_LYVE1	TNFSF13B	HLA-DPB1	5,15959E-03	11964,1	6,74985E-03	18579	5,41769E-01	6717	8,58036E-01	16429	9,45122E-01	565	0	17530,5
Non-responder	Macro_FOLR2+APOE+	Macro_IER3	CD38	PECAM1	5,15994E-03	15216,3	1,42141E-02	5496	6,80911E-01	3899	1,22581E+00	9041	7,73504E-01	40115	0	17530,5
Non-responder	Macro_NLRP3	Macro_ISG15	TGFB1	ENG	5,16793E-03	13665,9	8,43920E-03	12931	3,82285E-01	12864	1,41947E+00	6419	8,63357E-01	18585	0	17530,5
Non-responder	Macro_NLRP3	Macro_NLRP3	TNF	VSIR	5,16883E-03	13107,7	1,05735E-02	9045	5,98455E-01	5342	1,55692E+00	4965	8,21776E-01	28656	0	17530,5
Non-responder	Macro_LYVE1	Mono_INHBA	TIMP2	CD44	5,17489E-03	12665,3	9,55140E-03	10590	5,85996E-01	5606	8,07870E-01	17743	8,89573E-01	11857	0	17530,5
Non-responder	Mono_CD16	Mono_INHBA	TIMP1	CD63	5,17767E-03	12246,7	6,85603E-03	18139	5,59770E-01	6232	7,74952E-01	18592	9,43032E-01	740	0	17530,5
Non-responder	Macro_FOLR2-APOE+	Mono_INHBA	APOE	SDC2	5,18218E-03	7525,5	3,06239E-02	1483	9,08340E-01	1680	1,22500E+00	9051	9,06284E-01	7883	0	17530,5
Non-responder	Macro_IER3	Macro_ISG15	C3	ITGAX	5,18603E-03	16069,1	8,43588E-03	12939	2,86922E-01	18598	9,33546E-01	14636	8,71293E-01	16642	0	17530,5
Non-responder	pDC_LILRA4	cDC_CLEC9A	GNAS	ADRB3	5,19021E-03	16708,5	5,77646E-02	500	7,51665E-01	3009	1,73207E+00	3540	6,77391E-01	58963	0	17530,5
Non-responder	Macro_LYVE1	cDC(CD1C)	PSAP	CD1B	5,19161E-03	11996,5	2,80334E-02	1724	5,38924E-01	6795	7,74691E-01	18602	8,76447E-01	15331	0	17530,5
Non-responder	Macro_FOLR2+APOE+	Macro_OLFML3	LGALS9	SLC1A5	5,19161E-03	13289,1	8,32719E-03	13217	4,66390E-01	9197	1,30663E+00	7899	8,63281E-01	18602	0	17530,5
Non-responder	Mono_INHBA	Macro_FOLR2+APOE+	TNF	TRPM2	5,19450E-03	10962,3	2,11317E-02	2783	7,08470E-01	3541	1,94515E+00	2331	8,21894E-01	28626	0	17530,5
Non-responder	Macro_FOLR2+APOE+	Macro_ISG15	FABP5	RXRA	5,19556E-03	14046,7	1,11377E-02	8306	4,70278E-01	9057	1,46509E+00	5899	8,18479E-01	29441	0	17530,5
Non-responder	Macro_LYVE1	pDC_LILRA4	LGALS9	CD47	5,19999E-03	9265,9	9,04794E-03	11516	6,80365E-01	3905	1,96318E+00	2244	8,92438E-01	11134	0	17530,5
Non-responder	Macro_FOLR2+APOE+	cDC(CD1C)	CD14	ITGB1	5,20558E-03	12507,5	6,74282E-03	18612	4,81333E-01	8651	1,30746E+00	7886	8,97486E-01	9858	0	17530,5
Non-responder	Mast	Macro_NLRP3	COL18A1	ITGA5	5,20884E-03	12524,1	1,00912E-01	136	1,10243E+00	758	1,60045E+00	4590	7,75410E-01	39606	0	17530,5
Non-responder	Macro_LYVE1	pDC_LILRA4	HLA-DQA2	CD4	5,21117E-03	10568,9	8,00254E-03	14141	4,55210E-01	9614	1,44106E+00	6132	9,17628E-01	5427	0	17530,5
Non-responder	Macro_FOLR2+APOE+	Mono_CD16	FN1	C5AR1	5,21120E-03	11916,1	1,28592E-02	6519	4,70123E-01	9064	1,66076E+00	4107	8,47425E-01	22360	0	17530,5
Non-responder	cDC_CLEC9A	pDC_LILRA4	ICAM1	SPN	5,21168E-03	8196,7	1,83320E-02	3545	8,33839E-01	2241	2,44279E+00	857	8,70633E-01	16810	0	17530,5
Non-responder	Macro_LYVE1	pDC_LILRA4	HLA-DQA1	CD4	5,21257E-03	10048,9	7,27195E-03	16456	4,88406E-01	8375	1,47318E+00	5810	9,34478E-01	2073	0	17530,5
Non-responder	Macro_LYVE1	pDC_LILRA4	HLA-DRB1	CD4	5,21397E-03	10108,9	6,77571E-03	18466	4,92235E-01	8250	1,47751E+00	5768	9,45584E-01	530	0	17530,5
Non-responder	Macro_LYVE1	pDC_LILRA4	HLA-DQB1	CD4	5,21537E-03	10420,1	7,10944E-03	17079	4,63872E-01	9289	1,41280E+00	6495	9,36234E-01	1707	0	17530,5
Non-responder	Macro_OLFML3	pDC_LILRA4	HLA-DRB1	CD4	5,21678E-03	9539,7	6,74173E-03	18620	4,84305E-01	8534	1,91751E+00	2470	9,45454E-01	544	0	17530,5
Non-responder	Macro_FOLR2+APOE+	pDC_LILRA4	CCL4	CCR5	5,21792E-03	14858,5	1,05504E-02	9067	5,02950E-01	7882	2,17940E+00	1437	7,80661E-01	38376	0	17530,5
Non-responder	Macro_LYVE1	pDC_LILRA4	HLA-DPB1	CD4	5,21818E-03	10560,9	6,87910E-03	18017	4,57599E-01	9514	1,37166E+00	7001	9,42985E-01	742	0	17530,5
Non-responder	Macro_ISG15	Macro_FOLR2+APOE+	LGALS9	LRP1	5,22016E-03	8819,3	1,07206E-02	8851	6,21234E-01	4898	1,70593E+00	3749	9,00961E-01	9068	0	17530,5
Non-responder	pDC_LILRA4	Macro_NLRP3	SELPLG	ITGB2	5,22016E-03	6918,5	1,05502E-02	9068	6,99247E-01	3637	2,36197E+00	1004	9,28258E-01	3353	0	17530,5
Non-responder	Macro_IER3	Macro_OLFML3	C3	IFITM1	5,22098E-03	15798,7	8,85309E-03	11921	3,21582E-01	16315	9,34705E-01	14604	8,63164E-01	18623	0	17530,5
Non-responder	Macro_LYVE1	pDC_LILRA4	APP	RPSA	5,22098E-03	11308,1	7,12983E-03	16999	7,53317E-01	2988	1,76464E+00	3328	8,75001E-01	15695	0	17530,5
Non-responder	Macro_NLRP3	Mono_INHBA	PSEN1	CD44	5,22519E-03	14684,3	7,17883E-03	16812	5,02123E-01	7907	1,03021E+00	12546	8,63162E-01	18626	0	17530,5
Non-responder	Macro_OLFML3	Macro_FOLR2+APOE+	HLA-B	CANX	5,22688E-03	12047,3	5,56487E-03	25189	4,69837E-01	9071	1,29969E+00	7988	9,46473E-01	458	0	17530,5
Non-responder	Mono_INHBA	cDC_CLEC9A	FN1	ITGA4_ITGB7	5,22890E-03	10549,7	2,30307E-02	2366	7,07353E-01	3549	1,74459E+00	3456	8,32930E-01	25847	0	17530,5
Non-responder	Macro_FOLR2+APOE+	cDC_LAMP3	ICAM1	IL2RA	5,22912E-03	15294,7	1,36820E-02	5869	5,73565E-01	5900	1,22395E+00	9072	7,81859E-01	38102	0	17530,5
Non-responder	Macro_LYVE1	pDC_LILRA4	HLA-C	NOTCH4	5,23923E-03	8391,9	2,60205E-02	1953	6,02212E-01	5264	1,19962E+00	9471	9,06876E-01	7741	0	17530,5
Non-responder	Macro_LYVE1	pDC_LILRA4	HLA-DPA1	CD4	5,24485E-03	9797,5	7,04946E-03	17334	4,95893E-01	8127	1,50140E+00	5523	9,46287E-01	473	0	17530,5
Non-responder	Macro_LYVE1	pDC_LILRA4	MRC1	PTPRC	5,24626E-03	10720,5	1,15550E-02	7817	5,47539E-01	6554	1,68185E+00	3915	8,66739E-01	17786	0	17530,5
Non-responder	Macro_LYVE1	pDC_LILRA4	HLA-DMA	CD4	5,24767E-03	10523,5	7,31461E-03	16307	4,56577E-01	9560	1,45070E+00	6028	9,29011E-01	3192	0	17530,5
Non-responder	Macro_LYVE1	pDC_LILRA4	HLA-DMB	CD4	5,24907E-03	11129,5	7,53655E-03	15542	4,36755E-01	10345	1,53277E+00	5224	9,10198E-01	7006	0	17530,5
Non-responder	Macro_ISG15	Macro_FOLR2+APOE+	S100A9	ITGB2	5,25048E-03	7009,5	9,38598E-03	10888	7,06773E-01	3554	1,92032E+00	2459	9,44424E-01	616	0	17530,5
Non-responder	Macro_FOLR2+APOE+	cDC_CLEC9A	BSG	SLC16A1	5,25158E-03	16594,1	1,50092E-02	5012	4,69686E-01	9082	1,64016E+00	4272	7,42467E-01	47074	0	17530,5
Non-responder	Macro_OLFML3	Macro_NLRP3	HSP90B1	TLR4	5,25189E-03	15695,5	7,57738E-03	15392	3,35079E-01	15504	1,08655E+00	11406	8,63089E-01	18645	0	17530,5
Non-responder	Macro_LYVE1	pDC_LILRA4	SIRPA	CD47	5,25330E-03	9458,5	1,17587E-02	7585,5	7,44531E-01	3091,5	2,13628E+00	1558,5	8,67843E-01	17526,5	0	17530,5

Non-responder	Macro_LYVE1	pDC_LILRA4	SELPLG	SELL	5,25894E-03	7518,9	1,74774E-02	3859	9,97251E-01	1217	2,36892E+00	992	8,81496E-01	13996	0	17530,5
Non-responder	Macro_LYVE1	pDC_LILRA4	HLA-DRB5	CD4	5,26035E-03	10047,7	7,05144E-03	17328	4,85399E-01	8497	1,44659E+00	6076	9,42336E-01	807	0	17530,5
Non-responder	Macro_ISG15	Macro_ISG15	HLA-A	LILRB1	5,26345E-03	7320,1	8,86086E-03	11903	7,06696E-01	3557	2,00357E+00	2074	9,37115E-01	1536	0	17530,5
Non-responder	Mono_CD16	Mono_INHBA	PSEN1	CD44	5,26599E-03	14294,1	7,16611E-03	16864	5,01636E-01	7934	1,13836E+00	10487	8,63057E-01	18655	0	17530,5
Non-responder	Macro_FOLR2+APOE+	Macro_IER3	LGALS3	ENG	5,26740E-03	13357,5	6,73508E-03	18656	4,15948E-01	11246	1,21502E+00	9228	8,96453E-01	10127	0	17530,5
Non-responder	Macro_FOLR2+APOE+	Macro_IER3	APOE	SCARB1	5,26881E-03	13342,1	8,86393E-03	11897	3,90400E-01	12451	1,43811E+00	6175	8,63049E-01	18657	0	17530,5
Non-responder	cDC(CD1C)	Mono_CD16	ANXA1	FPR1	5,26881E-03	14190,9	7,48481E-03	15734	3,88457E-01	12554	7,73051E-01	18657	9,12614E-01	6479	0	17530,5
Non-responder	Macro_FOLR2+APOE+	Mono_CD16	CD1D	LILRB2	5,27185E-03	11769,5	1,05388E-02	9091	7,28381E-01	3264	1,59776E+00	4613	8,39156E-01	24349	0	17530,5
Non-responder	Macro_FOLR2+APOE+	Macro_NLRP3	TNFSF13	TNFRSF1A	5,27305E-03	14660,3	6,73433E-03	18660	3,32389E-01	15657	1,42562E+00	6341	8,77310E-01	15113	0	17530,5
Non-responder	Macro_LYVE1	pDC_LILRA4	TGFB1	CXCR4	5,27588E-03	10704,3	7,13572E-03	16977	5,23611E-01	7232	2,00276E+00	2080	8,98163E-01	9702	0	17530,5
Non-responder	Macro_OLFML3	pDC_LILRA4	S100A8	CD36	5,27729E-03	13148,9	9,43768E-03	10796	3,75315E-01	13221	1,50036E+00	5534	8,63025E-01	18663	0	17530,5
Non-responder	Macro_FOLR2+APOE+	Macro_ISG15	CD14	RIPK1	5,27862E-03	13173,7	1,05352E-02	9094	5,61240E-01	6195	1,56982E+00	4856	8,23639E-01	28193	0	17530,5
Non-responder	Macro_NLRP3	Macro_NLRP3	CD55	CR1	5,28088E-03	16903,3	1,36322E-02	5903	5,19841E-01	7334	1,22295E+00	9095	7,53293E-01	44654	0	17530,5
Non-responder	Macro_LYVE1	cDC_LAMP3	CXCL12	CXCR4	5,28153E-03	10990,5	2,28612E-02	2396	7,77002E-01	2751	9,77597E-01	13609	8,63015E-01	18666	0	17530,5
Non-responder	Macro_IFI27	cDC(CD1C)	B2M	CD1A	5,28295E-03	10251,9	4,60422E-02	752	5,26900E-01	7124	7,72655E-01	18667	9,09315E-01	7186	0	17530,5
Non-responder	Macro_ISG15	Macro_FOLR2+APOE+	MMP12	PLAUR	5,28512E-03	8171,9	2,25822E-02	2455	7,06180E-01	3562	2,64356E+00	562	8,70862E-01	16750	0	17530,5
Non-responder	cDC_CLEC9A	Macro_FOLR2+APOE+	SELPLG	ITGB2	5,28512E-03	7312,5	1,02396E-02	9507	7,08913E-01	3532	1,92538E+00	2431	9,27256E-01	3562	0	17530,5
Non-responder	Mono_CD16	Mono_CD14	THBS1	CD36	5,28540E-03	13306,1	1,22059E-02	7139	4,69323E-01	9097	1,43395E+00	6231	8,30214E-01	26533	0	17530,5
Non-responder	Mono_CD14	pDC_LILRA4	S100A8	CD69	5,28766E-03	6631,7	1,44457E-02	5338	1,08143E+00	848	2,89828E+00	344	9,00822E-01	9098	0	17530,5
Non-responder	pDC_LILRA4	Mono_INHBA	TFPI	F3	5,28946E-03	19111,7	8,63725E-02	193	7,06173E-01	3563	1,75238E+00	3400	5,50544E-01	70872	0	17530,5
Non-responder	cDC_CLEC9A	Mono_CD14	RPS19	C5AR1	5,28946E-03	7755,9	8,97014E-03	11696	7,25022E-01	3306	1,72871E+00	3563	9,31338E-01	2684	0	17530,5
Non-responder	Macro_ISG15	Macro_NLRP3	C1QA	CD93	5,28992E-03	9036,5	1,05316E-02	9099	4,78970E-01	8742	1,88473E+00	2634	9,09355E-01	7177	0	17530,5
Non-responder	pDC_LILRA4	Macro_LYVE1	APP	NOTCH2	5,29076E-03	8867,5	1,50179E-02	5006	1,12195E+00	686	2,50506E+00	764	8,55805E-01	20351	0	17530,5
Non-responder	Macro_OLFML3	Macro_ISG15	LGALS9	PTPRC	5,29144E-03	11218,5	6,73025E-03	18673	5,11701E-01	7607	1,46273E+00	5917	9,13083E-01	6365	0	17530,5
Non-responder	Macro_FOLR2+APOE+	Mono_INHBA	CD14	ITGB1	5,30137E-03	12432,5	6,72841E-03	18680	4,80497E-01	8680	1,34239E+00	7386	8,97387E-01	9886	0	17530,5
Non-responder	Macro_NLRP3	Macro_FOLR2+APOE+	CD14	TLR1	5,30421E-03	13674,7	9,75242E-03	10264	3,99469E-01	12006	1,17448E+00	9891	8,62912E-01	18682	0	17530,5
Non-responder	cDC_CLEC9A	pDC_LILRA4	HLA-DPB1	CD4	5,30448E-03	7466,1	7,93207E-03	14324	6,57282E-01	4269	2,50442E+00	765	9,46695E-01	442	0	17530,5
Non-responder	Macro_OLFML3	Macro_FOLR2+APOE+	CCL4	CCR1	5,30577E-03	12441,3	1,05252E-02	9106	4,80103E-01	8700	1,51850E+00	5357	8,50977E-01	21513	0	17530,5
Non-responder	Macro_IFI27	pDC_LILRA4	HLA-DRA	CD4	5,30989E-03	11361,9	6,72614E-03	18686	4,81627E-01	8638	1,07716E+00	11585	9,47797E-01	370	0	17530,5
Non-responder	Macro_IER3	pDC_LILRA4	TNF	TNFRSF1B	5,31273E-03	11973,7	8,37315E-03	13096	4,73896E-01	8910	2,11397E+00	1644	8,62893E-01	18688	0	17530,5
Non-responder	Macro_LYVE1	pDC_LILRA4	APOE	SORL1	5,32269E-03	13551,9	9,67152E-03	10388	2,86014E-01	18665	9,41513E-01	14456	9,11506E-01	6720	0	17530,5
Non-responder	pDC_LILRA4	Macro_FOLR2+APOE+	CD99	PILRA	5,32393E-03	7516,9	1,05193E-02	9114	6,45092E-01	4471	2,26130E+00	1209	9,18459E-01	5260	0	17530,5
Non-responder	Macro_NLRP3	pDC_LILRA4	HMGB1	THBD	5,32554E-03	14288,7	7,02494E-03	17429	2,85610E-01	18697	1,67511E+00	3977	8,82262E-01	13810	0	17530,5
Non-responder	Macro_LYVE1	pDC_LILRA4	F13A1	ITGA4	5,32839E-03	9421,3	1,16536E-02	7693	7,84849E-01	2683	2,03826E+00	1935	8,68938E-01	17265	0	17530,5
Non-responder	Macro_LYVE1	pDC_LILRA4	F13A1	ITGB1	5,32981E-03	9752,9	1,09304E-02	8576	7,39386E-01	3150	2,16672E+00	1472	8,65763E-01	18036	0	17530,5
Non-responder	Macro_NLRP3	cDC_CLEC9A	ANXA1	DYSF	5,33075E-03	11407,3	1,71807E-02	3976	6,47567E-01	4425	1,22150E+00	9117	8,48961E-01	21988	0	17530,5
Non-responder	Macro_NLRP3	cDC(CD1C)	GNAI2	FPR1	5,33124E-03	15784,7	6,72190E-03	18701	3,45022E-01	14918	7,89393E-01	18239	8,98812E-01	9535	0	17530,5
Non-responder	Macro_LYVE1	pDC_LILRA4	HMGB1	THBD	5,33266E-03	11211,7	8,73617E-03	12205	4,48909E-01	9871	1,50492E+00	5500	8,93122E-01	10952	0	17530,5
Non-responder	Mono_CD14	Macro_FOLR2-APOE+	VCAN	TLR2	5,33757E-03	7376,9	2,21183E-02	2555	1,11086E+00	729	1,37541E+00	6950	9,00704E-01	9120	0	17530,5
Non-responder	Macro_LYVE1	pDC_LILRA4	C3	NRP1	5,34265E-03	10724,9	1,85646E-02	3480	5,24314E-01	7207	1,33815E+00	7445	8,66037E-01	17962	0	17530,5
Non-responder	Macro_FOLR2-APOE+	Macro_ISG15	APOE	ABCA1	5,34669E-03	7423,3	1,63454E-02	4322	7,42001E-01	3123	1,22132E+00	9124	9,29877E-01	3017	0	17530,5
Non-responder	cDC_LAMP3	Macro_NLRP3	TNFSF13B	TFRC	5,34836E-03	14049,1	7,01918E-03	17460	4,44407E-01	10044	1,41248E+00	6498	8,62772E-01	18713	0	17530,5
Non-responder	Macro_LYVE1	pDC_LILRA4	C3	IFITM1	5,34979E-03	13688,1	8,84713E-03	11933	3,20308E-01	16412	1,67989E+00	3932	8,63124E-01	18633	0	17530,5
Non-responder	Macro_LYVE1	pDC_LILRA4	HSP90B1	TLR9	5,35408E-03	8540,9	5,72021E-02	515	9,63347E-01	1380	1,34792E+00	7319	8,73951E-01	15960	0	17530,5
Non-responder	Macro_LYVE1	pDC_LILRA4	HSP90B1	TLR7	5,35551E-03	10282,5	1,61469E-02	4403	5,65213E-01	6094	1,31123E+00	7827	8,75550E-01	15558	0	17530,5
Non-responder	Macro_OLFML3	Macro_NLRP3	HLA-A	LILRB2	5,35581E-03	12294,5	5,60779E-03	24895	4,86661E-01	8444	1,22099E+00	9128	9,37465E-01	1475	0	17530,5
Non-responder	cDC_CLEC9A	Macro_FOLR2+APOE+	MIF	CD44_CD74	5,35923E-03	8235,5	7,02091E-03	17454	7,04686E-01	3579	1,91042E+00	2507	9,52801E-01	107	0	17530,5
Non-responder	Macro_LYVE1	pDC_LILRA4	HMGB1	TLR9	5,35980E-03	7807,5	5,95233E-02	468	9,83096E-01	1287	1,48681E+00	5679	8,81187E-01	14073	0	17530,5
Non-responder	Macro_LYVE1	pDC_LILRA4	HMGB1	CXCR4	5,36124E-03	7995,9	8,26535E-03	13375	6,02730E-01	5256	2,08403E+00	1758	9,34547E-01	2060	0	17530,5
Non-responder	Macro_NLRP3	Macro_OLFML3	GNAI2	FPR1	5,36267E-03	14773,3	6,71784E-03	18723	3,44657E-01	14943	1,00019E+00	13126	8,98784E-01	9544	0	17530,5
Non-responder	Mono_CD14	cDC_LAMP3	VCAN	ITGA4	5,36361E-03	6871,5	1,82385E-02	3580	1,09729E+00	778	1,85431E+00	2794	8,98270E-01	9675	0	17530,5
Non-responder	Macro_ISG15	cDC_LAMP3	IL10	CD70	5,37677E-03	16609,5	8,01859E-02	229	1,05019E+00	972	1,72597E+00	3583	6,64563E-01	60733	0	17530,5
Non-responder	cDC(CD1C)	Macro_OLFML3	HLA-C	LILRB1	5,37844E-03	13034,9	6,71389E-03	18734	3,94224E-01	12266	9,81247E-01	13521	9,29360E-01	3123	0	17530,5
Non-responder	Macro_IFI27	pDC_LILRA4	HLA-DRB1	CD4	5,38418E-03	11523,3	6,71349E-03	18738	4,77716E-01	8793	1,05712E+00	12004	9,45346E-01	551	0	17530,5
Non-responder	Macro_OLFML3	Macro_NLRP3	LGALS9	LRP1	5,38562E-03	15846,7	6,71330E-03	18739	3,24009E-01	16174	1,06363E+00	11860	8,78031E-01	14930	0	17530,5
Non-responder	Macro_FOLR2+APOE+	Macro_LYVE1	GAS6	MERTK	5,39012E-03	15101,3	1,36810E-02	5870	5,01727E-01	7930	1,21988E+00	9143	7,95057E-01	35033	0	17530,5
Non-responder	Macro_IER3	Macro_ISG15	TNFSF13	TNFRSF1A	5,39137E-03	17457,9	6,71262E-03	18743	2,98708E-01	17788	7,95796E-01	18074	8,77136E-01	15154	0	17530,5
Non-responder	Macro_FOLR2+APOE+	Macro_FOLR2+APOE-	TNFSF12	CD163	5,41009E-03	12198,1	1,40326E-02	5621	5,97542E-01	5368	9,73043E-01	13715	8,62600E-01	18756	0	17530,5
Non-responder	Macro_IER3	Mono_CD16	HLA-B	LILRB1	5,41009E-03	13762,5	6,71022E-03	18756	3,48928E-01	14699	9,02113E-01	15354	9,32495E-01	2473	0	17530,5
Non-responder	pDC_LILRA4	Macro_FOLR2+APOE+	HSP90B1	TLR1	5,41481E-03	6563,5	1,79987E-02	3661	1,09820E+00	773	3,02740E+00	246	8,94539E-01	10607	0	17530,5
Non-responder	pDC_LILRA4	Macro_ISG15	HLA-F	LILRB1	5,41999E-03	7258,3	1,29361E-02	6453	8,12929E-01	2421	2,52417E+00	731	9,00566E-01	9156	0	17530,5
Non-responder	Mono_INHBA	cDC(CD1C)	CCL20	CXCR3	5,42076E-03	15924,1	2,47304E-02	2123	7,03439E-01	3593	1,77069E+00	3282	7,12564E-01	53092	0	17530,5
Non-responder	Macro_OLFML3	Macro_ISG15	FN1	CD44	5,42309E-03	13397,7	1,04156E-02	9264	3,72513E-01	13361	1,29346E+00	8068	8,62563E-01	18765	0	17530,5
Non-responder	Mono_CD14	cDC(CD1C)	VCAN	CD44	5,42460E-03	8392,5	1,19793E-02	7358	9,19607E-01	1616	1,21897E+00	9158	9,13383E-01	6300	0	17530,5
Non-responder	cDC(CD1C)	cDC_CLEC9A	HMGB1	HAVCR2	5,42742E-03	15119,7	6,70623E-03	18768	3,53515E-01	14441	8,88464E-01	15667	9,00439E-01	9192	0	17530,5
Non-responder	pDC_LILRA4	Mast	APP	PTGER2	5,42869E-03	11673,9	1,76480E-02	3790	1,17596E+00	508	2,49247E+00	774	7,91863E-01	35767	0	17530,5
Non-responder	Macro_FOLR2+APOE+	Macro_NLRP3	FN1	C5AR1	5,42921E-03	12191,1	1,28131E-02	6563	4,67474E-01	9160	1,52772E+00	5281	8,47193E-01	22421	0	17530,5
Non-responder	cDC(CD1C)	Macro_ISG15	HLA-A	LILRB2	5,43032E-03	11215,9	6,70580E-03	18770	4,94227E-01	8190	1,12037E+00	10799	9,42506E-01	790	0	17530,5
Non-responder	Mono_CD16	Macro_OLFML3	ICAM4	ITGAL	5,43382E-03	18911,1	2,37203E-02	2260	6,10664E-01	5077	1,21883E+00	9162	6,66094E-01	60526	0	17530,5
Non-responder	Macro_ISG15	Mono_CD16	HLA-B	LILRB1	5,43401E-03	7878,5	7,92705E-03	14341	7,03238E-01	3596	1,91786E+00	2469	9,37555E-01	1456	0	17530,5
Non-responder	Macro_OLFML3	Macro_FOLR2+APOE+	PTPN6	CD300LF	5,43613E-03	13207,3	1,16272E-02	7718	4,67389E-01	9163	1,42410E+00	6362	8,35403E-01	25263	0	17530,5

Non-responder	cDC_CLEC9A	Macro_NLRP3	HLA-DOB	CD4	5,43843E-03	9315,7	2,30650E-02	2358	1,16978E+00	533	1,72467E+00	3597	8,46619E-01	22560	0	17530,5
Non-responder	Macro_NLRP3	Macro_FOLR2+APOE+	TGM2	ITGA9	5,43843E-03	21217,9	2,53765E-02	2037	4,67379E-01	9164	1,27514E+00	8332	5,82304E-01	69026	0	17530,5
Non-responder	Macro_FOLR2-APOE+	cDC_CLEC9A	CXCL2	XCR1	5,43973E-03	19139,7	2,11938E-02	2763	2,22806E+00	16	3,35811E-01	32294	7,60211E-01	43095	0	17530,5
Non-responder	Macro_FOLR2+APOE+	Macro_FOLR2+APOE+	TNFSF12	TNFRSF12A	5,43973E-03	22919,7	1,23680E-02	6976	2,99630E-01	17717	1,32578E+00	7604	6,30737E-01	64771	0	17530,5
Non-responder	Macro_FOLR2+APOE+	Macro_ISG15	TNF	TNFRSF1B	5,44045E-03	12490,7	8,31756E-03	13239	4,99603E-01	8004	1,56359E+00	4903	8,62498E-01	18777	0	17530,5
Non-responder	Macro_FOLR2+APOE+	Macro_ISG15	CXCL8	SDC2	5,44536E-03	16321,9	1,07128E-02	8862	4,67306E-01	9167	1,69792E+00	3799	7,64146E-01	42251	0	17530,5
Non-responder	cDC_CLEC9A	pDC_LILRA4	HMGB1	THBD	5,44536E-03	8267,9	1,04858E-02	9167	6,15870E-01	5006	2,54101E+00	705	9,01527E-01	8931	0	17530,5
Non-responder	Macro_FOLR2+APOE+	Macro_LYVE1	CXCL12	ITGAV	5,45692E-03	18522,9	1,79880E-02	3665	4,67215E-01	9172	1,34185E+00	7396	7,02792E-01	54851	0	17530,5
Non-responder	Macro_FOLR2+APOE+	Macro_FOLR2+APOE+	HLA-G	CD4	5,45924E-03	12076,5	1,04833E-02	9173	4,90923E-01	8289	1,59542E+00	4642	8,54197E-01	20748	0	17530,5
Non-responder	Macro_OLFML3	Macro_NLRP3	PLAU	ITGA5	5,46387E-03	17115,1	1,22978E-02	7055	4,67096E-01	9175	1,32141E+00	7686	7,55538E-01	44129	0	17530,5
Non-responder	Macro_IER3	Macro_ISG15	CCL4	CCR5	5,46500E-03	9783,5	2,46455E-02	2133	7,91421E-01	2632	1,72443E+00	3603	8,44715E-01	23019	0	17530,5
Non-responder	Macro_ISG15	Mono_INHBA	MMP9	CD44	5,46500E-03	7912,9	1,81599E-02	3603	8,30006E-01	2279	1,95438E+00	2283	8,81975E-01	13869	0	17530,5
Non-responder	Macro_OLFML3	Macro_FOLR2+APOE+	LRPAP1	LRP1	5,46512E-03	17040,1	7,04937E-03	17336	2,84559E-01	18794	9,76159E-01	13644	8,66319E-01	17896	0	17530,5
Non-responder	Macro_NLRP3	Macro_LYVE1	TGFB1	ENG	5,46949E-03	15780,9	8,30846E-03	13262	3,74430E-01	13264	8,72260E-01	16051	8,62434E-01	18797	0	17530,5
Non-responder	Macro_NLRP3	Macro_FOLR2+APOE-	S100A9	ITGB2	5,47094E-03	10028,9	9,79195E-03	10193	7,44743E-01	3089	7,67469E-01	18798	9,45525E-01	534	0	17530,5
Non-responder	Mono_CD16	Macro_OLFML3	ICAM3	ITGAL	5,47314E-03	15836,5	1,04789E-02	9179	5,56493E-01	6318	1,40159E+00	6630	7,75751E-01	39525	0	17530,5
Non-responder	Macro_OLFML3	Macro_ISG15	RPS19	C5AR1	5,47822E-03	10622,7	6,69847E-03	18803	5,40220E-01	6762	1,51949E+00	5351	9,21393E-01	4667	0	17530,5
Non-responder	Macro_FOLR2+APOE+	Macro_FOLR2+APOE+	SPP1	ITGA4_ITGB1	5,48697E-03	10480,9	1,51671E-02	4907	4,41807E-01	10156	2,36237E+00	1002	8,62396E-01	18809	0	17530,5
Non-responder	Macro_FOLR2+APOE+	Macro_ISG15	TGM2	SDC4	5,49404E-03	18866,7	1,79141E-02	3695	5,28290E-01	7082	1,21669E+00	9188	6,91015E-01	56838	0	17530,5
Non-responder	Mono_CD14	Macro_FOLR2+APOE+	CD52	SIGLEC10	5,49404E-03	7682,3	1,42202E-02	5491	7,45579E-01	3077	1,79760E+00	3125	9,00446E-01	9188	0	17530,5
Non-responder	Macro_OLFML3	Macro_OLFML3	CD274	CD80	5,50102E-03	19391,1	1,95514E-02	3190	5,74932E-01	5864	1,21665E+00	9191	6,61000E-01	61180	0	17530,5
Non-responder	Macro_LYVE1	pDC_LILRA4	HLA-DRA	CD4	5,50449E-03	10367,7	6,69388E-03	18821	4,74310E-01	8899	1,43641E+00	6208	9,47678E-01	380	0	17530,5
Non-responder	Macro_ISG15	Macro_ISG15	HLA-DRB5	LAG3	5,50501E-03	7672,5	1,81388E-02	3612	8,27319E-01	2301	1,78235E+00	3216	8,90196E-01	11703	0	17530,5
Non-responder	Macro_IER3	Macro_FOLR2+APOE+	IL1B	SIGIRR	5,50595E-03	12849,3	9,55421E-03	10586	5,57891E-01	6282	1,10757E+00	11026	8,62349E-01	18822	0	17530,5
Non-responder	Macro_OLFML3	pDC_LILRA4	ICAM1	SPN	5,50888E-03	9012,9	1,58845E-02	4547	7,66790E-01	2847	2,22212E+00	1316	8,62346E-01	18824	0	17530,5
Non-responder	Macro_LYVE1	Macro_FOLR2-APOE+	F13A1	ITGB1	5,51620E-03	10727,1	1,46665E-02	5190	8,41188E-01	2173	9,22983E-01	14866	8,81949E-01	13876	0	17530,5
Non-responder	Macro_OLFML3	Macro_LYVE1	HMGB1	CD163	5,52206E-03	13096,1	7,68425E-03	15057	4,54005E-01	9658	7,66206E-01	18833	9,22821E-01	4402	0	17530,5
Non-responder	cDC_LAMP3	Macro_FOLR2+APOE+	HLA-DPB1	LAG3	5,52353E-03	13851,5	1,03077E-02	9401	4,52109E-01	9750	9,71881E-01	13742	8,62301E-01	18834	0	17530,5
Non-responder	Macro_FOLR2+APOE+	Macro_FOLR2+APOE+	PTPN6	CD300LF	5,52666E-03	12815,7	1,16041E-02	7746	4,66265E-01	9202	1,63446E+00	4310	8,35266E-01	25290	0	17530,5
Non-responder	cDC(CD1C)	Macro_ISG15	GNAI2	C5AR1	5,53380E-03	14358,3	6,68979E-03	18841	3,95408E-01	12219	9,59517E-01	14022	9,00491E-01	9179	0	17530,5
Non-responder	Macro_FOLR2+APOE+	Macro_FOLR2+APOE+	LRPAP1	SORL1	5,53674E-03	13502,3	8,56785E-03	12589	4,17537E-01	11171	1,34279E+00	7378	8,62274E-01	18843	0	17530,5
Non-responder	Macro_FOLR2+APOE+	cDC_CLEC9A	ADAM9	ITGB1	5,54069E-03	13816,1	1,19940E-02	7340	4,65991E-01	9208	1,35614E+00	7205	8,25200E-01	27797	0	17530,5
Non-responder	Macro_OLFML3	Macro_FOLR2+APOE+	POMC	GPR84	5,54537E-03	20307,1	1,53724E-02	4799	4,65823E-01	9210	1,22881E+00	8993	6,62426E-01	61003	0	17530,5
Non-responder	Macro_FOLR2+APOE+	Macro_FOLR2+APOE+	PSEN1	CD44	5,54850E-03	15935,7	7,06895E-03	17244	3,40304E-01	15171	1,11609E+00	10882	8,62248E-01	18851	0	17530,5
Non-responder	Macro_OLFML3	Macro_NLRP3	PSEN1	CD44	5,55145E-03	13297,1	7,06825E-03	17247	5,68058E-01	6028	1,38472E+00	6827	8,62242E-01	18853	0	17530,5
Non-responder	Macro_IFI27	Macro_FOLR2+APOE+	TNFSF13	TNFRSF14	5,55292E-03	13096,1	9,29567E-03	11039	5,38697E-01	6801	7,65360E-01	18854	8,91959E-01	11256	0	17530,5
Non-responder	Macro_FOLR2+APOE+	cDC(CD1C)	C1QB	LRP1	5,56178E-03	13574,5	5,60856E-03	24887	4,65621E-01	9217	1,35779E+00	7178	9,01018E-01	9060	0	17530,5
Non-responder	Mono_CD16	Macro_ISG15	TNFSF10	RIPK1	5,56412E-03	18177,7	1,08694E-02	8654	4,70511E-01	9049	1,21551E+00	9218	7,45413E-01	46437	0	17530,5
Non-responder	Mono_CD16	Macro_ISG15	HLA-A	LILRB2	5,56412E-03	10477,7	6,59261E-03	19306	4,65609E-01	9218	1,50496E+00	5499	9,42043E-01	835	0	17530,5
Non-responder	pDC_LILRA4	Macro_LYVE1	APP	LRP10	5,56834E-03	9058,7	1,63251E-02	4329	1,11868E+00	696	2,48600E+00	784	8,49094E-01	21954	0	17530,5
Non-responder	cDC_LAMP3	Macro_FOLR2+APOE+	CD274	CD80	5,58241E-03	13820,9	4,55743E-02	767	1,09538E+00	785	1,63618E+00	4297	7,48551E-01	45725	0	17530,5
Non-responder	Macro_ISG15	cDC_LAMP3	GNAI2	S1PR1	5,58241E-03	8475,9	4,47936E-02	785	1,19118E+00	462	1,68814E+00	3859	8,58439E-01	19743	0	17530,5
Non-responder	Macro_NLRP3	Macro_IER3	TNF	TNFRSF1B	5,58983E-03	14294,3	8,26283E-03	13381	4,57609E-01	9513	1,04830E+00	12168	8,62106E-01	18879	0	17530,5
Non-responder	pDC_LILRA4	Macro_OLFML3	TNFSF9	TRAF2	5,59009E-03	17219,3	3,20262E-02	1389	6,99670E-01	3631	1,96489E+00	2232	6,60070E-01	61314	0	17530,5
Non-responder	Macro_FOLR2+APOE+	Macro_ISG15	NAMPT	ITGA5_ITGB1	5,59279E-03	14777,7	6,68122E-03	18881	3,90237E-01	12459	1,29765E+00	8009	8,69893E-01	17009	0	17530,5
Non-responder	Macro_LYVE1	cDC_CLEC9A	F13A1	ITGB1	5,59459E-03	7208,3	1,80925E-02	3632	9,34541E-01	1534	1,96933E+00	2214	8,92447E-01	11131	0	17530,5
Non-responder	Macro_LYVE1	Mono_CD16	B2M	LILRB1	5,59724E-03	13731,7	6,89456E-03	17957	3,75550E-01	13207	7,64181E-01	18884	9,40182E-01	1080	0	17530,5
Non-responder	Macro_IER3	Macro_NLRP3	IL1B	IL1R2_IL1RAP	5,59872E-03	9836,9	1,85291E-02	3492	7,08493E-01	3539	1,48080E+00	5738	8,62089E-01	18885	0	17530,5
Non-responder	Mono_CD16	Macro_OLFML3	HLA-C	LILRB1	5,59872E-03	11683,1	6,67945E-03	18885	3,84973E-01	12713	1,44069E+00	6135	9,29191E-01	3152	0	17530,5
Non-responder	cDC(CD1C)	Mono_CD16	GNAI2	C5AR1	5,60168E-03	14342,1	6,67897E-03	18887	3,94210E-01	12268	9,68238E-01	13827	9,00418E-01	9198	0	17530,5
Non-responder	Macro_IER3	Macro_ISG15	HLA-C	LILRB2	5,60317E-03	11708,3	6,67883E-03	18888	4,52118E-01	9749	1,07404E+00	11649	9,43159E-01	725	0	17530,5
Non-responder	Macro_NLRP3	Mono_CD14	TIMP1	CD63	5,60649E-03	12856,3	5,29536E-03	27068	4,65141E-01	9236	1,25566E+00	8618	9,35684E-01	1829	0	17530,5
Non-responder	Macro_ISG15	cDC_CLEC9A	ITGB2	CD226	5,61714E-03	7971,5	2,80446E-02	1723	8,89378E-01	1796	1,71998E+00	3637	8,77094E-01	15171	0	17530,5
Non-responder	cDC(CD1C)	Macro_NLRP3	CD1D	LILRB2	5,61950E-03	10795,9	1,51113E-02	4945	6,08470E-01	5138	1,33678E+00	7467	8,62018E-01	18899	0	17530,5
Non-responder	Macro_FOLR2+APOE+	Macro_IFI27	CCL18	CCR1	5,62067E-03	19983,3	1,04556E-02	9216	4,65068E-01	9242	1,78178E+00	3221	6,64796E-01	60707	0	17530,5
Non-responder	Macro_LYVE1	Mast	LGALS1	CD69	5,62099E-03	9417,3	1,34123E-02	6061	7,29442E-01	3251	8,45025E-01	16760	9,27646E-01	3484	0	17530,5
Non-responder	Macro_NLRP3	Macro_IER3	TGFB1	CXCR4	5,62099E-03	15536,9	6,67619E-03	18900	3,58605E-01	14133	8,48997E-01	16650	8,95078E-01	10471	0	17530,5
Non-responder	Mono_INHBA	cDC_LAMP3	GNAI2	S1PR1	5,62469E-03	9537,7	4,46983E-02	788	1,18961E+00	469	1,22128E+00	9126	8,58310E-01	19775	0	17530,5
Non-responder	pDC_LILRA4	Macro_NLRP3	HSP90B1	TLR4	5,62540E-03	6511,1	1,55092E-02	4734	1,07647E+00	871	3,17479E+00	176	9,00188E-01	9244	0	17530,5
Non-responder	pDC_LILRA4	Macro_ISG15	HSP90B1	LRP1	5,62540E-03	6933,9	1,04315E-02	9244	8,96282E-01	1751	2,80295E+00	418	9,16085E-01	5726	0	17530,5
Non-responder	Macro_FOLR2+APOE+	Mono_INHBA	HLA-C	LILRB2	5,62776E-03	13823,5	4,80782E-03	30958	4,68309E-01	9133	1,21392E+00	9245	9,33679E-01	2251	0	17530,5
Non-responder	Macro_NLRP3	Macro_ISG15	AGTRAP	RACK1	5,62843E-03	16586,5	6,67525E-03	18905	3,87719E-01	12585	8,88811E-01	15658	8,64818E-01	18254	0	17530,5
Non-responder	Macro_FOLR2+APOE+	cDC_CLEC9A	CADM1	CRTAM	5,63013E-03	20369,7	2,89067E-02	1662,5	5,16792E-01	7442,5	1,21381E+00	9246,5	6,18972E-01	65966,5	0	17530,5
Non-responder	cDC_CLEC9A	cDC_CLEC9A	ITGB2	CD226	5,63069E-03	7756,3	2,94597E-02	1601	9,33557E-01	1538	1,71908E+00	3640	8,79722E-01	14472	0	17530,5
Non-responder	Macro_FOLR2+APOE+	Macro_NLRP3	CXCL12	SDC4	5,63250E-03	19915,5	1,62366E-02	4368	4,64872E-01	9247	1,62279E+00	4394	6,37570E-01	64038	0	17530,5
Non-responder	Macro_LYVE1	Mast	PLTP	ABCA1	5,63439E-03	10721,7	1,21059E-02	7227	7,31818E-01	3237	1,08171E+00	11488	8,80998E-01	14126	0	17530,5
Non-responder	Mast	Mono_INHBA	CSF1	CSF3R	5,63882E-03	9837,1	4,46926E-02	789	1,09820E+00	775	1,53546E+00	5192	8,36938E-01	24899	0	17530,5
Non-responder	Mono_CD14	pDC_LILRA4	VEGFA	RET	5,63974E-03	16702,7	5,12860E-02	628	7,75349E-01	2766	1,71873E+00	3642	6,77504E-01	58947	0	17530,5
Non-responder	cDC_CLEC9A	Macro_NLRP3	LGALS9	CD44	5,63974E-03	7217,1	9,83982E-03	10110	8,02248E-01	2526	1,95548E+00	2277	9,26742E-01	3642	0	17530,5
Non-responder	Macro_IER3	Macro_ISG15	RPS19	C5AR1	5,64184E-03	11819,9	6,67338E-03	18914	5,38187E-01	6819	1,10147E+00	11143	9,21257E-01	4693	0	17530,5
Non-responder	Macro_ISG15	Macro_FOLR2+APOE+	TNFSF13B	TFRC	5,64435E-03	7028,7	1,44307E-02	5348	8,43299E-01	2146	2,43991E+00	867	9,00147E-01	9252	0	17530,5

Post_NR_Myeloid_Myeloid_CellCell

Non-responder	Macro_NLRP3	pDC_LILRA4	SELPLG	SELL	5,64672E-03	10245,3	1,04234E-02	9253	8,69784E-01	1919	2,26348E+00	1201	8,51732E-01	21323	0	17530,5
Non-responder	pDC_LILRA4	Mono_CD16	CD99	PILRA	5,64909E-03	7574,7	1,04228E-02	9254	6,37582E-01	4587	2,27721E+00	1170	9,18113E-01	5332	0	17530,5
Non-responder	Mono_CD14	Mono_INHBA	HBEGF	CD44	5,65384E-03	8385,5	1,04212E-02	9256	7,28461E-01	3262	1,51059E+00	5440	9,12759E-01	6439	0	17530,5
Non-responder	Mono_INHBA	Macro_OLFML3	CCL2	CCR5	5,66240E-03	14022,1	3,23156E-02	1371	6,98383E-01	3647	1,92940E+00	2418	7,51342E-01	45144	0	17530,5
Non-responder	Macro_ISG15	Mono_INHBA	CXCL8	SDC2	5,66240E-03	11169,1	2,15503E-02	2675	7,32983E-01	3225	1,71748E+00	3647	8,21277E-01	28768	0	17530,5
Non-responder	Macro_FOLR2+APOE+	Mono_INHBA	TGFB1	SDC2	5,66335E-03	15399,9	1,28185E-02	6556	4,64527E-01	9260	1,24057E+00	8834	7,95945E-01	34819	0	17530,5
Non-responder	Macro_FOLR2+APOE+	cDC_CLEC9A	C3	CD46	5,66425E-03	13611,1	1,06493E-02	8952	3,53410E-01	14450	1,28483E+00	8194	8,61863E-01	18929	0	17530,5
Non-responder	Mono_CD16	Macro_IER3	TNFSF10	TNFRSF10B	5,66573E-03	15563,5	1,04176E-02	9261	5,05220E-01	7801	1,38767E+00	6788	7,88835E-01	36437	0	17530,5
Non-responder	pDC_LILRA4	Macro_NLRP3	APP	NCSTN	5,66714E-03	10259,7	1,37624E-02	5814	1,09422E+00	791	2,64038E+00	566	8,29987E-01	26597	0	17530,5
Non-responder	Macro_LYVE1	cDC_CLEC9A	HLA-DRA	LAG3	5,66724E-03	8452,5	1,64488E-02	4277	8,49488E-01	2095	1,25912E+00	8561	8,97772E-01	9799	0	17530,5
Non-responder	Macro_ISG15	pDC_LILRA4	SELPLG	SELL	5,67148E-03	7263,7	1,80382E-02	3649	1,00739E+00	1176	2,84324E+00	387	8,83136E-01	13576	0	17530,5
Non-responder	Macro_LYVE1	cDC_CLEC9A	HLA-DQA1	LAG3	5,67173E-03	9456,7	1,78693E-02	3712	8,63585E-01	1969	1,29590E+00	8033	8,73661E-01	16039	0	17530,5
Non-responder	Macro_LYVE1	cDC_CLEC9A	HLA-DRB1	LAG3	5,67473E-03	8478,7	1,66499E-02	4177	8,67413E-01	1942	1,30022E+00	7976	8,93905E-01	10768	0	17530,5
Non-responder	Macro_FOLR2+APOE+	Macro_IER3	PSAP	LRP1	5,67525E-03	12700,9	5,80842E-03	23555	4,64451E-01	9265	1,24781E+00	8726	9,22670E-01	4428	0	17530,5
Non-responder	Mono_INHBA	Macro_OLFML3	TIMP1	CD63	5,67602E-03	7724,3	8,13735E-03	13734	7,24380E-01	3315	1,71721E+00	3650	9,47464E-01	392	0	17530,5
Non-responder	Macro_LYVE1	cDC_CLEC9A	HLA-DQB1	LAG3	5,67623E-03	9544,7	1,74700E-02	3861	8,39051E-01	2190	1,23552E+00	8905	8,76832E-01	15237	0	17530,5
Non-responder	Macro_LYVE1	cDC_CLEC9A	HLA-DPB1	LAG3	5,67772E-03	9077,1	1,69039E-02	4080	8,32778E-01	2250	1,19437E+00	9554	8,89128E-01	11971	0	17530,5
Non-responder	Macro_OLFML3	Macro_FOLR2+APOE+	HSP90B1	TLR4	5,67772E-03	16859,7	7,41931E-03	15941	3,25586E-01	16073	8,81867E-01	15816	8,61838E-01	18938	0	17530,5
Non-responder	Macro_LYVE1	cDC_CLEC9A	ITGB2	CD226	5,68222E-03	10186,5	2,64414E-02	1894	8,39327E-01	2188	9,89838E-01	13342	8,73885E-01	15978	0	17530,5
Non-responder	Macro_OLFML3	cDC(CD1C)	TNFSF10	CCR6	5,68239E-03	16954,5	3,21433E-02	1380	6,53778E-01	4333	1,21188E+00	9268	7,16943E-01	52261	0	17530,5
Non-responder	pDC_LILRA4	Macro_OLFML3	SPN	SIGLEC1	5,68512E-03	12356,9	1,95342E-02	3195	6,97834E-01	3652	2,12273E+00	1614	7,91732E-01	35793	0	17530,5
Non-responder	Macro_IER3	Macro_ISG15	CD14	TLR4	5,68672E-03	15934,7	7,29827E-03	16368	3,74323E-01	13271	9,79974E-01	13560	8,61826E-01	18944	0	17530,5
Non-responder	Macro_NLRP3	Mast	VCAN	ITGA4	5,68823E-03	12740,5	9,10019E-03	11407	7,54459E-01	2977	1,01556E+00	12843	8,61825E-01	18945	0	17530,5
Non-responder	Macro_FOLR2+APOE+	Mono_INHBA	HSPA1A	TLR4	5,68823E-03	15358,5	6,66829E-03	18945	3,53202E-01	14463	1,12668E+00	10682	8,77089E-01	15172	0	17530,5
Non-responder	Macro_LYVE1	cDC_CLEC9A	CD14	ITGB2	5,68973E-03	11541,7	7,03693E-03	17373	5,01440E-01	7940	9,71308E-01	13758	9,39973E-01	1107	0	17530,5
Non-responder	Macro_NLRP3	Macro_NLRP3	ADAM10	CD44	5,68973E-03	13816,7	6,74495E-03	18600	5,52467E-01	6432	1,32919E+00	7575	8,61821E-01	18946	0	17530,5
Non-responder	Macro_LYVE1	cDC_CLEC9A	CD14	ITGB1	5,69123E-03	12545,1	7,87859E-03	14480	5,08519E-01	7715	9,31291E-01	14683	9,04429E-01	8317	0	17530,5
Non-responder	Macro_LYVE1	cDC_CLEC9A	CD14	ITGA4	5,69273E-03	11463,1	8,35037E-03	13156	5,60111E-01	6221	1,02837E+00	12583	9,06538E-01	7825	0	17530,5
Non-responder	Macro_FOLR2+APOE+	Macro_FOLR2+APOE+	HGF	ITGB1	5,69432E-03	16103,7	1,93187E-02	3242	4,64290E-01	9273	1,32450E+00	7624	7,61261E-01	42849	0	17530,5
Non-responder	Macro_FOLR2+APOE+	cDC_LAMP3	VEGFA	NRP2	5,69432E-03	13881,9	1,43702E-02	5399	8,44716E-01	2135	1,21150E+00	9273	7,94858E-01	35072	0	17530,5
Non-responder	Macro_FOLR2+APOE+	Macro_FOLR2-APOE+	LRPAP1	LRP1	5,69724E-03	12327,5	1,13070E-02	8086	5,82987E-01	5668	7,61756E-01	18951	8,91392E-01	11402	0	17530,5
Non-responder	Macro_LYVE1	cDC_CLEC9A	HLA-DPA1	LAG3	5,69874E-03	8286,5	1,73226E-02	3924	8,71072E-01	1909	1,32412E+00	7633	8,95203E-01	10436	0	17530,5
Non-responder	Macro_LYVE1	cDC_CLEC9A	MRC1	PTPRC	5,70025E-03	11023,3	1,21152E-02	7217	5,82048E-01	5689	1,33153E+00	7535	8,69449E-01	17145	0	17530,5
Non-responder	Macro_OLFML3	pDC_LILRA4	B2M	TFRC	5,70025E-03	11060,3	6,66585E-03	18953	4,36524E-01	10355	1,45510E+00	5997	9,32537E-01	2466	0	17530,5
Non-responder	Mono_CD16	Macro_OLFML3	ICAM4	ITGB1	5,70388E-03	16247,5	2,17749E-02	2626	5,67508E-01	6040	1,21099E+00	9277	7,48337E-01	45764	0	17530,5
Non-responder	Mono_CD16	pDC_LILRA4	LGALS9	SLC1A5	5,70927E-03	13291,7	8,11798E-03	13789	3,78520E-01	13052	1,79677E+00	3128	8,61772E-01	18959	0	17530,5
Non-responder	Mono_CD14	Macro_OLFML3	S100A8	TLR4	5,70974E-03	6766,9	1,78761E-02	3708	1,13449E+00	635	2,47898E+00	794	8,92305E-01	11167	0	17530,5
Non-responder	Macro_LYVE1	cDC_CLEC9A	HLA-DRB5	LAG3	5,71078E-03	8831,7	1,73274E-02	3920	8,60578E-01	1998	1,26931E+00	8412	8,87939E-01	12298	0	17530,5
Non-responder	Mono_INHBA	pDC_LILRA4	THBS1	CD47	5,71246E-03	6892,7	1,80054E-02	3658	9,35482E-01	1525	2,56404E+00	673	8,92645E-01	11077	0	17530,5
Non-responder	Macro_ISG15	Macro_NLRP3	ADM	GPR84	5,71703E-03	13777,7	2,23056E-02	2509	6,97273E-01	3659	1,89375E+00	2586	7,62364E-01	42604	0	17530,5
Non-responder	Macro_IER3	Mono_CD16	RPS19	C5AR1	5,72586E-03	11807,3	6,66259E-03	18970	5,36990E-01	6845	1,11019E+00	10985	9,21198E-01	4706	0	17530,5
Non-responder	Macro_LYVE1	Macro_OLFML3	HLA-C	LILRB1	5,73190E-03	14216,3	6,66189E-03	18974	3,80258E-01	12963	7,81273E-01	18443	9,29104E-01	3171	0	17530,5
Non-responder	Macro_FOLR2+APOE+	Mono_CD16	SPP1	ITGA4_ITGB1	5,73341E-03	10577,3	1,49986E-02	5016	4,34917E-01	10422	2,39172E+00	943	8,61732E-01	18975	0	17530,5
Non-responder	Macro_FOLR2+APOE+	Macro_NLRP3	TGM2	SDC4	5,73501E-03	20340,7	1,44189E-02	5359	4,67304E-01	9168	1,21034E+00	9290	6,67377E-01	60356	0	17530,5
Non-responder	Macro_FOLR2+APOE+	Macro_ISG15	CALR	SCARF1	5,73741E-03	13782,5	1,03911E-02	9291	5,68400E-01	6017	1,65459E+00	4157	8,08207E-01	31917	0	17530,5
Non-responder	Macro_FOLR2-APOE+	cDC_LAMP3	TNFSF13	TNFRSF11B	5,73822E-03	14403,7	6,70494E-02	360	1,09293E+00	796	6,28902E-01	22733	8,13655E-01	30599	0	17530,5
Non-responder	Macro_IER3	Mono_CD14	CCL4	CCR1	5,74096E-03	10561,3	1,25337E-02	6806	6,69786E-01	4069	1,51240E+00	5421	8,61715E-01	18980	0	17530,5
Non-responder	Macro_OLFML3	Macro_OLFML3	B2M	LILRB2	5,74222E-03	12367,5	5,51791E-03	25483	4,63704E-01	9293	1,22293E+00	9097	9,46753E-01	434	0	17530,5
Non-responder	Macro_FOLR2+APOE+	Mono_INHBA	HLA-B	LILRB2	5,74222E-03	13559,7	4,66444E-03	32333	5,37825E-01	6826	1,21020E+00	9293	9,35748E-01	1816	0	17530,5
Non-responder	Macro_FOLR2+APOE+	Macro_ISG15	MDK	SDC4	5,74702E-03	20502,1	2,94958E-02	1598	5,42971E-01	6690	1,21000E+00	9295	6,03638E-01	67397	0	17530,5
Non-responder	Mono_CD16	Macro_OLFML3	SPN	SIGLEC1	5,74942E-03	16436,9	1,36626E-02	5877	4,63575E-01	9296	1,41210E+00	6507	7,60722E-01	42974	0	17530,5
Non-responder	Mono_CD16	pDC_LILRA4	HLA-B	CANX	5,75183E-03	10562,1	5,88817E-03	23041	4,63568E-01	9297	1,89418E+00	2581	9,47885E-01	361	0	17530,5
Non-responder	pDC_LILRA4	Macro_OLFML3	B2M	LILRB1	5,75364E-03	7906,3	7,27085E-03	16458	6,96705E-01	3667	2,37157E+00	987	9,41659E-01	889	0	17530,5
Non-responder	Mono_CD14	Macro_FOLR2+APOE+	THBS1	CD36	5,75822E-03	9426,1	1,79794E-02	3668	7,18405E-01	3401	1,97818E+00	2177	8,55795E-01	20354	0	17530,5
Non-responder	Mono_CD16	Mono_CD16	CD1D	LILRB2	5,76386E-03	11552,7	1,03819E-02	9302	7,25074E-01	3305	1,81800E+00	3016	8,38141E-01	24610	0	17530,5
Non-responder	Mono_CD16	Macro_ISG15	ADAM10	CD44	5,77128E-03	15526,5	6,72204E-03	18699	3,68139E-01	13618	1,24403E+00	8785	8,61619E-01	19000	0	17530,5
Non-responder	Macro_LYVE1	Macro_LYVE1	F13A1	ITGA9	5,77200E-03	12707,3	4,93205E-02	664	1,04666E+00	988	1,71498E+00	3671	7,71004E-01	40683	0	17530,5
Non-responder	Macro_ISG15	Macro_FOLR2+APOE+	MMP9	EPHB2	5,77200E-03	15455,3	4,02710E-02	955	6,96144E-01	3671	1,88025E+00	2665	7,15974E-01	52455	0	17530,5
Non-responder	Mono_CD14	Macro_ISG15	THBS1	CD47	5,78119E-03	7155,3	1,79671E-02	3673	8,43400E-01	2143	2,21791E+00	1322	8,92543E-01	11108	0	17530,5
Non-responder	Macro_NLRP3	Macro_IFI27	VCAN	ITGA4	5,78496E-03	13123,5	9,06456E-03	11477	7,53506E-01	2985	9,34222E-01	14616	8,61591E-01	19009	0	17530,5
Non-responder	cDC(CD1C)	Macro_NLRP3	GNAI2	C5AR1	5,78648E-03	15032,7	6,65503E-03	19010	3,91560E-01	12391	8,35199E-01	17001	9,00257E-01	9231	0	17530,5
Non-responder	Macro_NLRP3	Macro_FOLR2+APOE+	TNFSF13B	TFRC	5,78800E-03	14612,9	6,87881E-03	18020	3,91072E-01	12412	1,44553E+00	6091	8,61572E-01	19011	0	17530,5
Non-responder	Macro_NLRP3	cDC_CLEC9A	TGFB1	ITGB1	5,78952E-03	15153,7	7,12677E-03	17009	3,72520E-01	13360	1,23921E+00	8857	8,61567E-01	19012	0	17530,5
Non-responder	Macro_FOLR2+APOE+	cDC(CD1C)	LGALS9	PTPRC	5,78952E-03	12191,1	6,65490E-03	19012	4,97382E-01	8077	1,17633E+00	9862	9,12635E-01	6474	0	17530,5
Non-responder	Macro_FOLR2+APOE+	Macro_LYVE1	LGALS3BP	ITGB1	5,79524E-03	12629,1	1,06841E-02	8895	5,28405E-01	7079	1,20856E+00	9315	8,55885E-01	20326	0	17530,5
Non-responder	Macro_FOLR2+APOE+	Macro_IER3	LGALS3BP	ITGB1	5,79524E-03	12868,5	1,03675E-02	9315	5,17061E-01	7429	1,21097E+00	9279	8,54020E-01	20789	0	17530,5
Non-responder	Macro_IER3	Macro_ISG15	CIRBP	TREM1	5,80019E-03	16698,5	8,13743E-03	13731	2,81361E-01	19019	8,10231E-01	17683	8,75674E-01	15529	0	17530,5
Non-responder	Macro_FOLR2+APOE+	Mono_CD16	HSPA1A	TLR4	5,80171E-03	14723,7	6,65267E-03	19020	3,52412E-01	14502	1,34402E+00	7362	8,76963E-01	15204	0	17530,5
Non-responder	Macro_FOLR2+APOE+	Macro_FOLR2+APOE+	ARF1	PLD2	5,80492E-03	13590,5	1,20453E-02	7293	4,63111E-01	9319	1,26352E+00	8491	8,35151E-01	25319	0	17530,5
Non-responder	Mono_CD16	Macro_ISG15	HLA-B	LILRA1	5,80782E-03	15794,9	6,81034E-03	18330	2,81305E-01	19024	1,06103E+00	11923	8,88400E-01	12167	0	17530,5
Non-responder	Mono_CD16	Macro_IFI27	SERPINA1	LRP1	5,80782E-03	14078,7	6,65198E-03	19024	4,87456E-01	8411	8,17869E-01	17482	9,05976E-01	7946	0	17530,5

Post_NR_Myeloid_Myeloid_CellCell

Non-responder	cDC_CLEC9A	cDC_CLEC9A	HLA-DRA	LAG3	5,80976E-03	6816,7	1,71996E-02	3970	9,18788E-01	1622	2,11555E+00	1640	8,99802E-01	9321	0	17530,5
Non-responder	Macro_IFI27	Macro_FOLR2+APOE+	HLA-DRB1	CD4	5,81698E-03	11645,9	7,59621E-03	15325	5,68403E-01	6016	7,58342E-01	19030	9,48451E-01	328	0	17530,5
Non-responder	Macro_IER3	Macro_OLFML3	CD14	TLR1	5,82157E-03	14772,1	9,52378E-03	10637	4,41937E-01	10152	8,54568E-01	16508	8,61503E-01	19033	0	17530,5
Non-responder	Macro_NLRP3	Mono_CD16	HLA-B	LILRB1	5,82157E-03	13064,3	6,64818E-03	19033	3,30865E-01	15751	1,13868E+00	10482	9,32202E-01	2525	0	17530,5
Non-responder	Macro_FOLR2+APOE+	Mono_INHBA	NAMPT	ADORA2A	5,82189E-03	13495,7	1,87542E-02	3411	6,21470E-01	4892	1,20738E+00	9326	8,06424E-01	32319	0	17530,5
Non-responder	pDC_LILRA4	Macro_IER3	SELPLG	ITGB2	5,82189E-03	7248,5	1,03632E-02	9326	6,83856E-01	3836	2,00403E+00	2071	9,27660E-01	3479	0	17530,5
Non-responder	Macro_LYVE1	Macro_IER3	CXCL12	CXCR4	5,83535E-03	10940,5	2,22655E-02	2520	7,61992E-01	2899	1,02163E+00	12711	8,61447E-01	19042	0	17530,5
Non-responder	Macro_NLRP3	pDC_LILRA4	HMGB1	CXCR4	5,83994E-03	10311,3	6,64635E-03	19045	4,39430E-01	10249	2,25422E+00	1227	9,27555E-01	3505	0	17530,5
Non-responder	Macro_FOLR2+APOE+	Macro_IER3	GRN	TNFRSF1B	5,84376E-03	13073,9	5,46188E-03	25888	4,79930E-01	8709	1,20671E+00	9335	9,25410E-01	3907	0	17530,5
Non-responder	Macro_NLRP3	Macro_OLFML3	THBS1	SCARB1	5,84862E-03	15210,9	1,03523E-02	9337	6,57731E-01	4260	1,41152E+00	6521	7,80542E-01	38406	0	17530,5
Non-responder	Macro_LYVE1	Mono_CD14	CD14	TLR4	5,85222E-03	12000,3	1,04137E-02	9266	5,80534E-01	5724	9,81090E-01	13525	8,81664E-01	13956	0	17530,5
Non-responder	Macro_OLFML3	Macro_FOLR2+APOE+	HLA-B	LILRB1	5,85350E-03	11585,9	6,20268E-03	21236	4,62405E-01	9339	1,38413E+00	6836	9,29977E-01	2988	0	17530,5
Non-responder	Macro_LYVE1	Mono_CD14	CD14	ITGB2	5,85376E-03	11121,9	7,42271E-03	15928	5,51861E-01	6445	9,25871E-01	14789	9,41461E-01	917	0	17530,5
Non-responder	Macro_FOLR2+APOE+	Mono_CD14	HBEGF	CD44	5,85376E-03	14067,9	6,64489E-03	19054	4,10639E-01	11485	1,09139E+00	11309	8,93100E-01	10961	0	17530,5
Non-responder	Macro_IFI27	Macro_ISG15	C3	C3AR1	5,85529E-03	11736,5	1,44750E-02	5316	5,17319E-01	7417	7,57588E-01	19055	8,99595E-01	9364	0	17530,5
Non-responder	Macro_LYVE1	Macro_NLRP3	CD14	ITGB2	5,85529E-03	13228,9	6,64481E-03	19055	4,50190E-01	9832	7,82660E-01	18409	9,38335E-01	1318	0	17530,5
Non-responder	Macro_ISG15	Mono_INHBA	LGALS9	CD44	5,85593E-03	7377,5	1,03482E-02	9340	8,18822E-01	2383	1,63361E+00	4316	9,28434E-01	3318	0	17530,5
Non-responder	Macro_ISG15	Macro_FOLR2+APOE+	CXCL11	CCR5	5,86901E-03	16262,5	4,46567E-02	791	6,93908E-01	3692	1,93763E+00	2370	6,90421E-01	56929	0	17530,5
Non-responder	Macro_LYVE1	Mono_CD14	MRC1	PTPRC	5,87067E-03	12354,7	1,13515E-02	8029	5,35004E-01	6901	1,09401E+00	11268	8,65709E-01	18045	0	17530,5
Non-responder	Macro_FOLR2+APOE+	Macro_FOLR2+APOE+	POMC	GPR84	5,87545E-03	19983,7	1,50586E-02	4972	4,62140E-01	9348	1,39106E+00	6759	6,60117E-01	61309	0	17530,5
Non-responder	Mono_CD16	Mono_CD16	LGALS1	PTPRC	5,87789E-03	11665,5	5,96421E-03	22590	4,62120E-01	9349	1,33739E+00	7460	9,37816E-01	1398	0	17530,5
Non-responder	Macro_FOLR2+APOE+	Macro_ISG15	CD59	STAB1	5,87837E-03	13635,3	8,52837E-03	12689	3,91596E-01	12390	1,41269E+00	6497	8,61302E-01	19070	0	17530,5
Non-responder	Macro_OLFML3	Macro_FOLR2+APOE+	CD14	TLR1	5,88146E-03	14027,9	9,48972E-03	10691	3,80089E-01	12969	1,17513E+00	9877	8,61289E-01	19072	0	17530,5
Non-responder	Macro_LYVE1	Macro_FOLR2+APOE+	PSAP	LRP1	5,88300E-03	13276,9	7,42357E-03	15921	4,18988E-01	11103	7,56871E-01	19073	9,30981E-01	2757	0	17530,5
Non-responder	Macro_FOLR2+APOE+	Macro_FOLR2-APOE+	ENTPD1	ADORA3	5,88454E-03	12918,9	1,75608E-02	3818	5,58184E-01	6275	8,02262E-01	17897	8,61277E-01	19074	0	17530,5
Non-responder	cDC_CLEC9A	pDC_LILRA4	VEGFB	RET	5,88761E-03	18103,9	6,58558E-02	380	7,70143E-01	2820	1,71241E+00	3696	6,17800E-01	66093	0	17530,5
Non-responder	cDC(CD1C)	Macro_FOLR2+APOE+	HMGB1	TLR2	5,89226E-03	16262,3	6,89503E-03	17953	3,21215E-01	16340	7,56581E-01	19079	8,95310E-01	10409	0	17530,5
Non-responder	Macro_IER3	Macro_FOLR2+APOE+	B2M	TFRC	5,89844E-03	13199,7	7,40626E-03	15981	2,80688E-01	19083	1,07659E+00	11595	9,35775E-01	1809	0	17530,5
Non-responder	Macro_NLRP3	Macro_FOLR2+APOE+	LAMB3	ITGA9_ITGB1	5,90237E-03	19004,5	2,65051E-02	1885	5,58792E-01	6261	1,20513E+00	9359	6,70251E-01	59987	0	17530,5
Non-responder	Macro_FOLR2+APOE+	cDC_CLEC9A	PLAU	ST14	5,90237E-03	14985,1	1,25213E-02	6823	4,61927E-01	9359	1,60949E+00	4511	7,87758E-01	36702	0	17530,5
Non-responder	Macro_IER3	Macro_NLRP3	RPS19	C5AR1	5,90308E-03	12376,9	6,63871E-03	19086	5,34340E-01	6921	9,77148E-01	13624	9,21068E-01	4723	0	17530,5
Non-responder	Macro_NLRP3	Macro_FOLR2+APOE+	CD99	PILRA	5,90462E-03	14677,9	6,63846E-03	19087	3,15036E-01	16740	1,12962E+00	10642	8,99476E-01	9390	0	17530,5
Non-responder	Macro_ISG15	Mono_INHBA	TGM2	SDC4	5,90625E-03	14446,7	3,05822E-02	1489	7,53492E-01	2986	1,71168E+00	3700	7,45031E-01	46528	0	17530,5
Non-responder	Macro_OLFML3	Macro_LYVE1	NRG1	MS4A4A	5,90972E-03	15383,1	1,70914E-02	4009	6,79846E-01	3907	1,23972E+00	8846	7,62286E-01	42623	0	17530,5
Non-responder	Macro_NLRP3	Macro_FOLR2+APOE-	THBS1	SCARB1	5,90972E-03	13681,7	1,41195E-02	5553	7,08617E-01	3535	1,20508E+00	9362	8,05965E-01	32428	0	17530,5
Non-responder	Macro_NLRP3	Macro_IER3	THBS1	CD47	5,91236E-03	11875,1	1,00243E-02	9817	6,93775E-01	3694	1,21395E+00	9242	8,61192E-01	19092	0	17530,5
Non-responder	Macro_OLFML3	Macro_FOLR2+APOE+	TGFB1	ITGB1	5,91701E-03	15736,9	7,08015E-03	17187	3,50538E-01	14629	1,15366E+00	10243	8,61175E-01	19095	0	17530,5
Non-responder	Macro_NLRP3	Macro_OLFML3	ANXA1	FPR1	5,91856E-03	15846,5	6,63660E-03	19096	3,11932E-01	16914	7,92366E-01	18152	9,07698E-01	7540	0	17530,5
Non-responder	Mono_CD16	Mono_INHBA	TNFSF13B	TFRC	5,92011E-03	15373,7	6,83308E-03	18234	3,83985E-01	12764	1,21394E+00	9243	8,61173E-01	19097	0	17530,5
Non-responder	Macro_NLRP3	Mono_CD14	TNF	NOTCH1	5,92200E-03	19060,9	1,12617E-02	8131	4,61627E-01	9367	1,33823E+00	7444	7,13937E-01	52832	0	17530,5
Non-responder	Macro_OLFML3	Macro_FOLR2+APOE+	C3	LRP1	5,92321E-03	14590,5	9,44229E-03	10779	2,80496E-01	19099	1,12621E+00	10688	8,78345E-01	14856	0	17530,5
Non-responder	Macro_NLRP3	pDC_LILRA4	TGFB1	ACVR1_TGFBR2	5,92446E-03	12635,1	1,10540E-02	8419	4,61556E-01	9368	1,84942E+00	2815	8,36349E-01	25043	0	17530,5
Non-responder	Macro_IER3	Mono_CD14	ICAM1	ITGAM_ITGB2	5,92476E-03	14229,7	6,63587E-03	19100	4,17476E-01	11174	1,01477E+00	12859	8,95016E-01	10485	0	17530,5
Non-responder	pDC_LILRA4	Macro_FOLR2-APOE+	TNFSF9	HLA-DPA1	5,92938E-03	7611,3	2,26142E-02	2448	7,79853E-01	2729	1,20478E+00	9370	9,14910E-01	5979	0	17530,5
Non-responder	Macro_IFI27	Macro_FOLR2+APOE+	HLA-DQB1	CD4	5,93252E-03	11684,3	8,00532E-03	14126	5,49300E-01	6519	7,55597E-01	19105	9,39686E-01	1141	0	17530,5
Non-responder	Macro_IFI27	Macro_IFI27	APOE	TREM2	5,93427E-03	14804,9	1,98674E-02	3100	6,93109E-01	3706	-1,58625E-01	49505	9,50837E-01	183	0	17530,5
Non-responder	Macro_NLRP3	Macro_FOLR2+APOE+	TGM2	SDC4	5,93676E-03	19486,5	1,49367E-02	5047	4,61538E-01	9373	1,48890E+00	5648	6,71282E-01	59834	0	17530,5
Non-responder	Macro_OLFML3	Mast	ARPC5	ADRB2	5,94028E-03	12360,1	1,62769E-02	4351	5,52546E-01	6427	9,44989E-01	14382	8,61131E-01	19110	0	17530,5
Non-responder	Macro_LYVE1	Mono_CD14	F13A1	ITGA4	5,94495E-03	9121,7	1,31941E-02	6230	8,26071E-01	2312	1,66854E+00	4047	8,75848E-01	15489	0	17530,5
Non-responder	Macro_FOLR2+APOE+	Macro_FOLR2-APOE+	CD14	ITGB1	5,94495E-03	14519,3	6,88784E-03	17985	4,89747E-01	8335	7,55466E-01	19113	8,98461E-01	9633	0	17530,5
Non-responder	Macro_LYVE1	Mono_CD14	F13A1	ITGB1	5,94650E-03	10000,5	1,17314E-02	7614	7,61211E-01	2909	1,56266E+00	4916	8,69820E-01	17033	0	17530,5
Non-responder	Macro_LYVE1	Mono_CD14	GNAI2	C5AR1	5,94806E-03	14954,1	6,85961E-03	18115	4,11976E-01	11425	7,67702E-01	18793	9,01608E-01	8907	0	17530,5
Non-responder	Macro_OLFML3	Mono_INHBA	TNFSF13	SDC2	5,94907E-03	12446,1	1,66840E-02	4160	5,81277E-01	5704	1,23521E+00	8907	8,32621E-01	25929	0	17530,5
Non-responder	Macro_LYVE1	Mono_CD14	GNAI2	FPR1	5,94962E-03	14497,9	7,36274E-03	16130	3,95743E-01	12201	7,98808E-01	17991	9,02878E-01	8637	0	17530,5
Non-responder	Macro_LYVE1	Mono_CD14	A2M	LRP1	5,95584E-03	13254,9	1,11607E-02	8272	4,53547E-01	9680	7,61205E-01	18965	8,89662E-01	11827	0	17530,5
Non-responder	Macro_NLRP3	Macro_LYVE1	TNF	TNFRSF21	5,95894E-03	14226,7	1,35904E-02	5931	5,41560E-01	6720	1,20456E+00	9382	8,09749E-01	31570	0	17530,5
Non-responder	Macro_LYVE1	Mono_CD14	C1QB	LRP1	5,96208E-03	11061,3	9,75637E-03	10259	5,37690E-01	6833	8,61585E-01	16339	9,23112E-01	4345	0	17530,5
Non-responder	Macro_ISG15	Macro_FOLR2+APOE+	LGALS9	HAVCR2	5,96635E-03	8602,5	1,06893E-02	8889	6,59217E-01	4228	1,82361E+00	2980	8,99503E-01	9385	0	17530,5
Non-responder	Macro_FOLR2+APOE+	Macro_OLFML3	PLTP	ABCA1	5,96675E-03	13122,1	8,48253E-03	12815	4,43474E-01	10084	1,44828E+00	6054	8,61055E-01	19127	0	17530,5
Non-responder	Macro_FOLR2+APOE+	Macro_FOLR2+APOE-	LRPAP1	LRP1	5,96831E-03	13779,7	9,60651E-03	10486	4,93684E-01	8206	7,55153E-01	19128	8,83247E-01	13548	0	17530,5
Non-responder	pDC_LILRA4	Mono_INHBA	APP	NOTCH2	5,96861E-03	9589,1	1,30509E-02	6356	1,08956E+00	812	2,49778E+00	770	8,46925E-01	22477	0	17530,5
Non-responder	Macro_LYVE1	Mono_CD14	C1QA	CD33	5,97143E-03	11302,1	9,80737E-03	10163	5,48380E-01	6537	9,82003E-01	13500	9,02310E-01	8780	0	17530,5
Non-responder	Mono_CD16	Mono_CD16	S100A8	ITGB2	5,97143E-03	13308,5	6,62826E-03	19130	3,83994E-01	12763	9,72374E-01	13733	9,28055E-01	3386	0	17530,5
Non-responder	Macro_IER3	Mono_INHBA	FN1	CD44	5,97299E-03	13797,5	1,01510E-02	9628	4,92603E-01	8244	9,41542E-01	14454	8,61030E-01	19131	0	17530,5
Non-responder	Macro_LYVE1	Mono_CD14	C1QB	CD33	5,97299E-03	11897,3	9,67606E-03	10380	5,21262E-01	7288	9,01574E-01	15368	9,01569E-01	8920	0	17530,5
Non-responder	Mono_INHBA	pDC_LILRA4	S100A8	CD36	5,97624E-03	7671,7	1,90377E-02	3324	7,28155E-01	3269	1,57046E+00	4846	8,99484E-01	9389	0	17530,5
Non-responder	Macro_OLFML3	Macro_FOLR2+APOE+	TGM2	ITGB1	5,97871E-03	15437,9	1,13803E-02	7998	4,61006E-01	9390	1,41707E+00	6452	7,91613E-01	35819	0	17530,5
Non-responder	Mono_INHBA	pDC_LILRA4	COPA	P2RY6	5,98316E-03	9639,9	1,44057E-02	5371	1,08935E+00	813	2,70286E+00	502	8,40711E-01	23983	0	17530,5
Non-responder	Macro_ISG15	Macro_LYVE1	LGALS9	LRP1	5,98366E-03	10090,5	1,03657E-02	9320	6,00110E-01	5304	1,23521E+00	8906	8,99449E-01	9392	0	17530,5
Non-responder	Macro_OLFML3	Macro_FOLR2+APOE+	LGALS9	PTPRC	5,99357E-03	12217,3	6,32218E-03	20609	4,60826E-01	9396	1,40152E+00	6632	9,10569E-01	6919	0	17530,5
Non-responder	Macro_LYVE1	Macro_FOLR2+APOE+	C3	CD46	5,99488E-03	16036,1	1,04936E-02	9159	3,28502E-01	15893	7,80803E-01	18453	8,60983E-01	19145	0	17530,5

Post_NR_Myeloid_Myeloid_CellCell

Non-responder	Macro_FOLR2+APOE+	Macro_FOLR2+APOE+	MIF	CD74_CXCR4	5,99772E-03	15436,9	4,65075E-03	32473	2,96407E-01	17945	1,26848E+00	8422	9,42273E-01	814	0	17530,5
Non-responder	Macro_FOLR2+APOE+	Macro_NLRP3	C1QB	CD33	5,99958E-03	12041,5	6,62490E-03	19148	5,51779E-01	6448	1,72544E+00	3588	8,83435E-01	13493	0	17530,5
Non-responder	Macro_IER3	cDC_CLEC9A	MRC1	PTPRC	6,00585E-03	12230,1	1,04712E-02	9194	5,04880E-01	7809	1,33700E+00	7465	8,60947E-01	19152	0	17530,5
Non-responder	cDC(CD1C)	Macro_ISG15	HLA-B	LILRB2	6,00585E-03	11202,3	6,62419E-03	19152	5,03418E-01	7862	1,11350E+00	10932	9,45520E-01	535	0	17530,5
Non-responder	Macro_FOLR2+APOE+	cDC(CD1C)	APOE	SCARB1	6,00742E-03	13941,7	8,55595E-03	12620	3,84160E-01	12754	1,32326E+00	7651	8,60946E-01	19153	0	17530,5
Non-responder	Macro_LYVE1	Macro_FOLR2+APOE+	CXCL12	ITGB1	6,00943E-03	10072,9	2,90223E-02	1648	8,84886E-01	1816	1,70933E+00	3722	8,33674E-01	25648	0	17530,5
Non-responder	Macro_FOLR2-APOE+	cDC_LAMP3	GNAI2	S1PR1	6,01230E-03	13394,1	4,41024E-02	815	1,17976E+00	499	4,56395E-01	28173	8,57492E-01	19953	0	17530,5
Non-responder	Macro_IFI27	Macro_NLRP3	APOE	LDLR	6,01526E-03	10846,1	2,83222E-02	1703	7,36110E-01	3187	7,54323E-01	19158	8,86585E-01	12652	0	17530,5
Non-responder	Macro_LYVE1	Mono_CD14	HSPA1A	TLR4	6,01526E-03	14175,5	8,48831E-03	12799	4,48729E-01	9878	7,67530E-01	18796	8,89517E-01	11874	0	17530,5
Non-responder	Macro_FOLR2+APOE+	Macro_NLRP3	MDK	SDC4	6,01591E-03	21437,1	2,37408E-02	2258	4,81985E-01	8623	1,20365E+00	9405	5,77403E-01	69369	0	17530,5
Non-responder	Macro_FOLR2+APOE+	Mono_CD16	LGALS9	CD47	6,01840E-03	13608,3	6,62218E-03	19160	4,55786E-01	9590	1,41765E+00	6447	8,76515E-01	15314	0	17530,5
Non-responder	Mono_INHBA	cDC(CD1C)	INHBA	ENG	6,02359E-03	10273,1	3,22689E-02	1374	8,61533E-01	1988	1,70860E+00	3725	8,29441E-01	26748	0	17530,5
Non-responder	Macro_FOLR2+APOE+	Macro_FOLR2+APOE+	TGFB1	ENG	6,02469E-03	14348,1	8,09665E-03	13845	3,52911E-01	14478	1,39362E+00	6723	8,60895E-01	19164	0	17530,5
Non-responder	Macro_LYVE1	Macro_FOLR2+APOE+	TNFSF13B	CD40	6,02783E-03	16189,3	6,64605E-03	19049	4,35766E-01	10390	9,25185E-01	14811	8,60890E-01	19166	0	17530,5
Non-responder	Macro_OLFML3	Macro_FOLR2+APOE-	CXCL10	TLR4	6,02832E-03	9958,7	1,78244E-02	3726	9,98740E-01	1211	2,18241E+00	1431	8,32753E-01	25895	0	17530,5
Non-responder	Macro_FOLR2+APOE+	Macro_LYVE1	ANXA2	TLR2	6,02941E-03	14185,5	6,62053E-03	19167	3,62241E-01	13938	1,11622E+00	10875	8,99335E-01	9417	0	17530,5
Non-responder	Macro_LYVE1	Macro_ISG15	TNFSF13	TNFRSF1A	6,03098E-03	17765,1	6,66107E-03	18976	2,94449E-01	18066	7,60209E-01	18989	8,76720E-01	15264	0	17530,5
Non-responder	Macro_OLFML3	Macro_ISG15	CIRBP	TREM1	6,03413E-03	15667,3	8,11276E-03	13804	2,79502E-01	19170	1,04352E+00	12263	8,75509E-01	15569	0	17530,5
Non-responder	Macro_FOLR2+APOE+	Mono_INHBA	LILRB4	LAIR1	6,03570E-03	13746,5	6,61980E-03	19171	4,29848E-01	10621	1,05879E+00	11971	8,99233E-01	9439	0	17530,5
Non-responder	Macro_FOLR2+APOE+	Macro_OLFML3	MRC1	PTPRC	6,03582E-03	11525,1	1,03032E-02	9413	5,18528E-01	7376	1,68088E+00	3924	8,59976E-01	19382	0	17530,5
Non-responder	Macro_OLFML3	Macro_FOLR2+APOE+	NAMPT	ITGA5_ITGB1	6,03885E-03	15838,1	6,61928E-03	19173	3,69365E-01	13544	1,06736E+00	11778	8,69365E-01	17165	0	17530,5
Non-responder	Macro_LYVE1	cDC_CLEC9A	C1QA	CD93	6,04042E-03	15144,5	6,61888E-03	19174	4,87275E-01	8420	7,82740E-01	18406	8,88307E-01	12192	0	17530,5
Non-responder	cDC_CLEC9A	Mono_CD14	ICAM3	ITGAL	6,04251E-03	11026,9	1,78217E-02	3729	9,18393E-01	1625	1,84840E+00	2825	8,18557E-01	29425	0	17530,5
Non-responder	Macro_LYVE1	Macro_ISG15	HLA-B	LILRB2	6,04357E-03	11489,5	6,71606E-03	18729	5,30934E-01	7023	9,75806E-01	13657	9,45874E-01	508	0	17530,5
Non-responder	Macro_LYVE1	Macro_ISG15	HLA-B	LILRB1	6,04515E-03	11271,7	7,69520E-03	15026	4,83575E-01	8567	9,77309E-01	13615	9,36680E-01	1620	0	17530,5
Non-responder	Macro_NLRP3	Macro_LYVE1	HBEGF	CD9	6,04830E-03	12749,1	1,00350E-02	9802	5,31599E-01	6997	7,53322E-01	19179	8,96025E-01	10237	0	17530,5
Non-responder	Macro_FOLR2-APOE+	Macro_NLRP3	FN1	PLAUR	6,05079E-03	7542,7	1,95140E-02	3200	8,66928E-01	1945	1,49207E+00	5619	8,99324E-01	9419	0	17530,5
Non-responder	Macro_LYVE1	Macro_ISG15	HLA-DQA1	LAG3	6,05461E-03	7978,1	2,17339E-02	2638	1,02253E+00	1103	1,52507E+00	5308	8,84078E-01	13311	0	17530,5
Non-responder	Macro_FOLR2+APOE+	Mono_CD14	ARPC5	LDLR	6,05578E-03	14475,9	1,16626E-02	7682	4,60078E-01	9421	1,28747E+00	8159	8,17819E-01	29587	0	17530,5
Non-responder	pDC_LILRA4	Macro_FOLR2+APOE+	HLA-B	CANX	6,05672E-03	8871,9	6,12825E-03	21641	6,90959E-01	3732	2,28516E+00	1155	9,48864E-01	301	0	17530,5
Non-responder	Macro_LYVE1	Macro_ISG15	HLA-DQB1	LAG3	6,06093E-03	7987,1	2,12483E-02	2749	9,97998E-01	1214	1,46469E+00	5902	8,87021E-01	12540	0	17530,5
Non-responder	Macro_FOLR2+APOE+	Macro_FOLR2-APOE+	C3	CD81	6,06251E-03	12454,5	1,27633E-02	6607	4,55356E-01	9612	7,52892E-01	19188	8,99756E-01	9335	0	17530,5
Non-responder	Macro_OLFML3	Mono_INHBA	FN1	SDC2	6,06578E-03	15756,7	2,46911E-02	2128	5,34598E-01	6913	1,30676E+00	7895	7,54787E-01	44317	0	17530,5
Non-responder	Macro_FOLR2+APOE+	Macro_OLFML3	C3	ITGAX	6,06725E-03	15439,7	8,14789E-03	13704	2,79295E-01	19191	1,19153E+00	9599	8,69333E-01	17174	0	17530,5
Non-responder	Macro_LYVE1	Macro_ISG15	HLA-A	LILRB2	6,06725E-03	9846,9	7,27455E-03	16447	6,38035E-01	4580	1,16381E+00	10080	9,44672E-01	597	0	17530,5
Non-responder	Macro_LYVE1	Macro_ISG15	HLA-A	LILRB1	6,06883E-03	9638,1	8,33511E-03	13197	5,90676E-01	5513	1,16531E+00	10047	9,35288E-01	1903	0	17530,5
Non-responder	Macro_IFI27	cDC_LAMP3	SPP1	S1PR1	6,07080E-03	15531,9	4,59157E-02	757	1,08774E+00	819	8,30966E-01	17133	7,67817E-01	41420	0	17530,5
Non-responder	Macro_LYVE1	Macro_ISG15	HLA-DPB1	LAG3	6,07199E-03	7542,1	2,05598E-02	2931	9,91726E-01	1238	1,42354E+00	6369	8,98417E-01	9642	0	17530,5
Non-responder	cDC_LAMP3	Macro_OLFML3	B2M	LILRB1	6,07199E-03	12019,5	6,61416E-03	19194	4,48402E-01	9892	1,04376E+00	12259	9,39003E-01	1222	0	17530,5
Non-responder	Macro_IER3	Mono_INHBA	SPP1	CD44	6,07357E-03	16841,7	6,82528E-03	18269	4,16575E-01	11204	7,97995E-01	18010	8,60753E-01	19195	0	17530,5
Non-responder	Macro_ISG15	cDC_CLEC9A	ADM	ADRB3	6,07570E-03	18357,7	1,11312E-01	103	8,92256E-01	1778	1,70764E+00	3736	5,87626E-01	68641	0	17530,5
Non-responder	cDC(CD1C)	Macro_FOLR2+APOE+	HLA-DQA1	LAG3	6,07991E-03	9587,1	1,42765E-02	5458	7,98343E-01	2571	1,78925E+00	3177	8,60745E-01	19199	0	17530,5
Non-responder	Macro_LYVE1	Macro_ISG15	CD14	TLR4	6,08466E-03	13818,5	8,38274E-03	13056	4,97282E-01	8082	9,86707E-01	13407	8,69869E-01	17017	0	17530,5
Non-responder	pDC_LILRA4	Macro_NLRP3	HLA-B	LILRB2	6,08521E-03	9278,1	5,92444E-03	22824	6,90646E-01	3738	2,15175E+00	1515	9,42572E-01	783	0	17530,5
Non-responder	Mast	Macro_IER3	ADCYAP1	GPR84	6,08547E-03	14231,3	1,19543E-01	89	1,08712E+00	820	1,59904E+00	4605	7,37856E-01	48112	0	17530,5
Non-responder	Macro_LYVE1	cDC_LAMP3	GNAI2	S1PR1	6,08547E-03	9963,3	4,38984E-02	820	1,17639E+00	507	1,11376E+00	10929	8,57208E-01	20030	0	17530,5
Non-responder	Macro_LYVE1	Macro_ISG15	CD14	ITGB2	6,08624E-03	12175,7	6,69891E-03	18799	4,57261E-01	9528	9,72208E-01	13735	9,38569E-01	1286	0	17530,5
Non-responder	Macro_FOLR2+APOE+	Macro_FOLR2+APOE+	C1QA	CD93	6,08624E-03	11460,5	6,61130E-03	19203	5,56248E-01	6325	2,01301E+00	2033	8,88250E-01	12211	0	17530,5
Non-responder	Macro_LYVE1	Macro_ISG15	CD14	ITGB1	6,08783E-03	11411,3	8,45111E-03	12893	5,44343E-01	6648	1,03767E+00	12382	9,07418E-01	7603	0	17530,5
Non-responder	pDC_LILRA4	Macro_FOLR2+APOE+	ADAM10	GPNMB	6,08996E-03	8664,7	1,81960E-02	3589	6,90580E-01	3739	2,53599E+00	717	8,66906E-01	17748	0	17530,5
Non-responder	Macro_LYVE1	Macro_ISG15	CD14	TLR1	6,09100E-03	12988,5	1,01148E-02	9681	5,37109E-01	6842	1,02272E+00	12687	8,65056E-01	18202	0	17530,5
Non-responder	Macro_FOLR2+APOE+	Macro_FOLR2+APOE-	LRPAP1	SORL1	6,09258E-03	14374,9	1,00084E-02	9832	4,81289E-01	8654	7,51881E-01	19207	8,71245E-01	16651	0	17530,5
Non-responder	Macro_NLRP3	Macro_LYVE1	CD55	CR1	6,09333E-03	16262,9	1,49684E-02	5034	5,45766E-01	6605	1,20121E+00	9436	7,61878E-01	42709	0	17530,5
Non-responder	Macro_FOLR2+APOE+	Macro_LYVE1	B2M	TFRC	6,09333E-03	15372,3	4,14418E-03	37800	4,59711E-01	9436	1,42526E+00	6344	9,15960E-01	5751	0	17530,5
Non-responder	Macro_LYVE1	Macro_ISG15	HLA-C	LILRB2	6,09417E-03	10584,5	7,13686E-03	16973	5,66948E-01	6051	1,06697E+00	11786	9,44911E-01	582	0	17530,5
Non-responder	Macro_LYVE1	Macro_ISG15	HLA-C	LILRB1	6,09576E-03	10421,9	8,17735E-03	13621	5,19590E-01	7348	1,06848E+00	11760	9,35565E-01	1850	0	17530,5
Non-responder	Macro_IER3	Mono_CD16	ICAM1	ITGAX_ITGB2	6,09576E-03	13133,1	6,60952E-03	19209	4,54304E-01	9645	1,16598E+00	10039	9,00195E-01	9242	0	17530,5
Non-responder	Macro_OLFML3	Macro_FOLR2+APOE+	FN1	C5AR1	6,09584E-03	12099,1	1,31307E-02	6292	4,59638E-01	9437	1,53545E+00	5193	8,48771E-01	22043	0	17530,5
Non-responder	pDC_LILRA4	Macro_FOLR2+APOE+	HSP90B1	TLR4	6,09835E-03	6613,7	1,51856E-02	4895	1,06697E+00	905	2,97011E+00	300	8,99237E-01	9438	0	17530,5
Non-responder	Macro_LYVE1	Macro_ISG15	CD99	PILRA	6,10052E-03	12872,7	8,08807E-03	13878	4,33282E-01	10485	9,16150E-01	15021	9,08060E-01	7449	0	17530,5
Non-responder	Mono_CD16	Macro_NLRP3	ADAM15	ITGA5	6,10337E-03	18175,5	1,14520E-02	7922	4,59575E-01	9440	1,31097E+00	7833	7,37644E-01	48152	0	17530,5
Non-responder	Macro_LYVE1	Macro_ISG15	MRC1	PTPRC	6,10846E-03	9762,1	1,36206E-02	5914	6,74790E-01	3981	1,46269E+00	5918	8,75954E-01	15467	0	17530,5
Non-responder	Macro_FOLR2+APOE+	Macro_FOLR2+APOE+	HMGB1	TLR4	6,10846E-03	16851,3	6,93171E-03	17805	2,78932E-01	19217	1,10196E+00	11135	8,63436E-01	18569	0	17530,5
Non-responder	Macro_FOLR2+APOE+	Macro_IER3	SPP1	PTGER4	6,11343E-03	10977,3	1,69171E-02	4073	4,59503E-01	9444	2,15440E+00	1508	8,47567E-01	22331	0	17530,5
Non-responder	pDC_LILRA4	cDC(CD1C)	APP	RPSA	6,11485E-03	7404,9	1,12120E-02	8182	1,08692E+00	822	2,55815E+00	679	8,97731E-01	9811	0	17530,5
Non-responder	Macro_LYVE1	Macro_ISG15	B2M	LILRB2	6,11800E-03	11791,3	6,70225E-03	18780	4,99744E-01	8001	9,40029E-01	14491	9,51446E-01	154	0	17530,5
Non-responder	Macro_FOLR2+APOE+	Macro_FOLR2+APOE+	TNFSF13	FAS	6,11937E-03	25411,3	5,39002E-03	26359	2,79041E-01	19210	1,30488E+00	7916	6,95775E-01	56041	0	17530,5
Non-responder	Macro_LYVE1	Macro_ISG15	B2M	LILRB1	6,11959E-03	11505,3	7,67938E-03	15074	4,52385E-01	9739	9,41533E-01	14455	9,43142E-01	728	0	17530,5
Non-responder	Mono_INHBA	Mono_CD16	HLA-B	LILRA1	6,12350E-03	8264,9	1,58028E-02	4588	5,88723E-01	5546	1,20072E+00	9448	9,23817E-01	4212	0	17530,5
Non-responder	cDC_LAMP3	Macro_OLFML3	CD274	CD80	6,12957E-03	14013,5	4,45931E-02	793	1,08684E+00	823	1,58566E+00	4731	7,46498E-01	46190	0	17530,5
Non-responder	pDC_LILRA4	cDC_LAMP3	APP	LRP10	6,12957E-03	8984,7	1,66076E-02	4195	1,12361E+00	677	2,46240E+00	823	8,50190E-01	21698	0	17530,5

Post_NR_Myeloid_Myeloid_CellCell

Non-responder	Macro_FOLR2+APOE+	Macro_OLFML3	TNFSF13B	TFRC	6,13074E-03	13831,5	6,76878E-03	18503	4,75562E-01	8858	1,55061E+00	5035	8,60607E-01	19231	0	17530,5
Non-responder	pDC_LILRA4	Mast	IL16	CD9	6,13105E-03	7351,5	2,08682E-02	2842	7,49208E-01	3034	1,68360E+00	3900	8,99164E-01	9451	0	17530,5
Non-responder	Macro_IER3	Mono_CD16	CD14	TLR4	6,13234E-03	16646,9	7,15085E-03	16924	3,67382E-01	13668	8,79087E-01	15880	8,60606E-01	19232	0	17530,5
Non-responder	Macro_LYVE1	Macro_ISG15	HLA-DRB5	LAG3	6,14191E-03	7379,7	2,10749E-02	2795	1,01953E+00	1117	1,49847E+00	5549	8,97316E-01	9907	0	17530,5
Non-responder	Macro_LYVE1	Macro_FOLR2+APOE+	IGF1	IGF2R	6,14244E-03	11149,7	3,48181E-02	1212	9,22553E-01	1600	1,70582E+00	3750	8,09340E-01	31656	0	17530,5
Non-responder	cDC_CLEC9A	cDC_CLEC9A	HLA-DRB1	LAG3	6,14244E-03	6787,9	1,77687E-02	3750	9,73643E-01	1326	2,21313E+00	1334	8,96949E-01	9999	0	17530,5
Non-responder	Mono_CD14	cDC_LAMP3	VCAN	CD44	6,14367E-03	8766,3	1,12089E-02	8190	8,93126E-01	1773	1,20031E+00	9456	9,10717E-01	6882	0	17530,5
Non-responder	Macro_FOLR2+APOE+	Mono_CD14	CCL3	CCR1	6,14367E-03	11269,7	1,02693E-02	9456	5,45772E-01	6604	1,81610E+00	3022	8,58488E-01	19736	0	17530,5
Non-responder	Macro_FOLR2+APOE+	Mono_INHBA	HLA-C	LILRB1	6,14367E-03	13418,9	5,25824E-03	27323	4,59169E-01	9456	1,29510E+00	8043	9,20905E-01	4742	0	17530,5
Non-responder	Mono_INHBA	Macro_LYVE1	IL6	IL6R_IL6ST	6,15201E-03	13005,1	2,68296E-02	1849	1,08317E+00	840	1,70559E+00	3752	7,69366E-01	41054	0	17530,5
Non-responder	Macro_FOLR2+APOE+	Macro_FOLR2+APOE+	FN1	PLAUR	6,15789E-03	13954,9	9,31629E-03	11002	3,25234E-01	16101	1,46580E+00	5893	8,60573E-01	19248	0	17530,5
Non-responder	Mono_CD14	Macro_ISG15	NRG1	MS4A4A	6,16160E-03	11689,3	2,93422E-02	1612	7,08567E-01	3536	1,70513E+00	3754	8,07753E-01	32014	0	17530,5
Non-responder	Macro_LYVE1	Macro_ISG15	APOE	SDC2	6,16589E-03	10767,3	2,04160E-02	2965	5,79757E-01	5742	9,08636E-01	15209	8,87590E-01	12390	0	17530,5
Non-responder	Macro_FOLR2+APOE+	Mono_CD14	CCL3L1	CCR1	6,16641E-03	14654,9	1,02659E-02	9465	4,71401E-01	9013	1,41761E+00	6449	8,12787E-01	30817	0	17530,5
Non-responder	Macro_FOLR2+APOE+	cDC_CLEC9A	IL18	IL18BP	6,17148E-03	12380,9	1,25862E-02	6757	4,58878E-01	9467	1,67557E+00	3971	8,39913E-01	24179	0	17530,5
Non-responder	Macro_LYVE1	Macro_ISG15	CCL3	CCR1	6,17711E-03	11947,9	1,22956E-02	7059	6,07307E-01	5158	1,01970E+00	12749	8,69078E-01	17243	0	17530,5
Non-responder	cDC_CLEC9A	Macro_FOLR2+APOE+	HLA-DRB1	CD4	6,18079E-03	7370,3	8,18177E-03	13606	6,89150E-01	3758	2,09164E+00	1738	9,50236E-01	219	0	17530,5
Non-responder	Macro_LYVE1	Mono_CD14	C1QA	CR1	6,18192E-03	10058,7	2,55686E-02	2006	8,07907E-01	2474	1,22680E+00	9020	8,60522E-01	19263	0	17530,5
Non-responder	Macro_FOLR2+APOE+	Macro_NLRP3	C3	LRP1	6,19477E-03	13873,1	9,58781E-03	10517	2,78413E-01	19271	1,33976E+00	7425	8,79160E-01	14622	0	17530,5
Non-responder	Mono_CD14	Macro_NLRP3	S100A9	CD36	6,19684E-03	6857,7	1,53888E-02	4796	1,03134E+00	1065	2,18596E+00	1420	8,99058E-01	9477	0	17530,5
Non-responder	Mono_CD14	pDC_LILRA4	SELPLG	SELL	6,20002E-03	7362,1	1,77355E-02	3762	1,00192E+00	1198	2,69159E+00	509	8,82260E-01	13811	0	17530,5
Non-responder	Macro_FOLR2+APOE+	cDC(CD1C)	COPA	CD74	6,20281E-03	12095,1	6,59890E-03	19276	4,62369E-01	9340	1,02681E+00	12609	9,36173E-01	1720	0	17530,5
Non-responder	Macro_ISG15	pDC_LILRA4	CXCL8	CD79A	6,20484E-03	14952,7	5,78940E-02	495	6,88958E-01	3763	1,85091E+00	2810	7,28062E-01	50165	0	17530,5
Non-responder	Macro_OLFML3	Mono_CD14	ANXA1	FPR1	6,20925E-03	15857,7	6,59812E-03	19280	3,10431E-01	17004	8,03035E-01	17880	9,07454E-01	7594	0	17530,5
Non-responder	Mono_CD16	pDC_LILRA4	THBS1	CD36	6,20955E-03	12696,7	1,19519E-02	7401	4,58571E-01	9482	1,98534E+00	2158	8,28727E-01	26912	0	17530,5
Non-responder	Macro_FOLR2+APOE+	Macro_IER3	TNFSF13B	TFRC	6,21086E-03	14461,9	6,75018E-03	18577	4,74720E-01	8885	1,29556E+00	8036	8,60442E-01	19281	0	17530,5
Non-responder	Macro_FOLR2+APOE+	cDC_CLEC9A	TGM2	ITGB1	6,21210E-03	15424,3	1,23151E-02	7036	4,58535E-01	9483	1,24648E+00	8746	7,98049E-01	34326	0	17530,5
Non-responder	Macro_FOLR2+APOE+	Macro_LYVE1	CD14	ITGB1	6,21408E-03	13021,1	6,59732E-03	19283	4,72891E-01	8955	1,21539E+00	9221	8,96478E-01	10116	0	17530,5
Non-responder	Macro_FOLR2+APOE+	Macro_IER3	SEMA4A	LILRB2	6,21891E-03	12293,5	9,91855E-03	9982	5,64238E-01	6119	1,25988E+00	8550	8,60415E-01	19286	0	17530,5
Non-responder	cDC_CLEC9A	Macro_FOLR2+APOE+	CD59	STAB1	6,22229E-03	7040,3	1,75207E-02	3839	8,17667E-01	2390	2,03434E+00	1955	8,98998E-01	9487	0	17530,5
Non-responder	Macro_FOLR2+APOE+	Macro_OLFML3	SEMA4A	PLXND1	6,22229E-03	16474,7	1,02501E-02	9487	4,66810E-01	9181	1,27371E+00	8348	7,82976E-01	37827	0	17530,5
Non-responder	Macro_LYVE1	Macro_ISG15	C3	C3AR1	6,22859E-03	12971,5	1,12393E-02	8151	3,67093E-01	13682	1,00145E+00	13099	8,87577E-01	12395	0	17530,5
Non-responder	Macro_ISG15	Macro_ISG15	CCL13	CCR1	6,22894E-03	16835,1	4,67247E-02	731	7,90440E-01	2637	1,70293E+00	3768	6,73727E-01	59509	0	17530,5
Non-responder	Macro_LYVE1	Macro_ISG15	PLAU	PLAUR	6,23021E-03	11379,1	9,97176E-03	9892	5,93418E-01	5460	1,40732E+00	6560	8,68136E-01	17453	0	17530,5
Non-responder	Macro_OLFML3	Mono_CD14	SERPINA1	LRP1	6,23021E-03	14638,5	6,59499E-03	19293	3,59839E-01	14067	9,49479E-01	14275	9,05608E-01	8027	0	17530,5
Non-responder	Macro_LYVE1	Macro_ISG15	HMGB1	HAVCR2	6,23182E-03	15000,5	7,04862E-03	17337	3,58110E-01	14162	8,25528E-01	17281	9,02649E-01	8692	0	17530,5
Non-responder	cDC_LAMP3	Macro_FOLR2+APOE+	LGALS1	ITGB1	6,23344E-03	13551,3	6,59457E-03	19295	3,71120E-01	13451	1,08035E+00	11522	9,15005E-01	5958	0	17530,5
Non-responder	Macro_FOLR2+APOE+	Macro_OLFML3	CXCL12	ITGA4	6,23503E-03	15788,9	1,32809E-02	6148	4,58223E-01	9492	1,53505E+00	5198	7,71463E-01	40576	0	17530,5
Non-responder	Mono_CD16	Mono_CD14	ICAM4	ITGA4	6,23759E-03	16838,7	1,99257E-02	3082	5,59145E-01	6246	1,19839E+00	9493	7,39034E-01	47842	0	17530,5
Non-responder	Macro_NLRP3	Mono_CD14	PTGS2	ALOX5	6,24314E-03	13008,3	1,04221E-02	9255	5,11075E-01	7635	1,09067E+00	11320	8,60333E-01	19301	0	17530,5
Non-responder	cDC_LAMP3	pDC_LILRA4	CXCL9	DPP4	6,24344E-03	13555,5	5,45034E-02	558	8,80050E-01	1853	1,70224E+00	3771	7,55811E-01	44065	0	17530,5
Non-responder	Macro_FOLR2+APOE+	Mono_CD14	TNF	TNFRSF1B	6,24475E-03	13705,5	8,02052E-03	14078	4,74206E-01	8901	1,24843E+00	8716	8,60327E-01	19302	0	17530,5
Non-responder	Macro_LYVE1	Macro_ISG15	C3	ITGAX	6,24961E-03	12574,1	1,09393E-02	8568	4,06339E-01	11701	1,05410E+00	12072	8,85175E-01	12999	0	17530,5
Non-responder	Macro_LYVE1	Macro_ISG15	F13A1	ITGA4	6,25285E-03	8745,5	1,33408E-02	6109	8,29994E-01	2280	1,91672E+00	2476	8,76447E-01	15332	0	17530,5
Non-responder	Macro_FOLR2+APOE+	Macro_FOLR2+APOE+	TGM2	ITGA9	6,25547E-03	19549,7	3,83911E-02	1032	6,15842E-01	5007	1,19794E+00	9500	6,31635E-01	64679	0	17530,5
Non-responder	Macro_LYVE1	Macro_ISG15	GNAI2	C5AR1	6,25609E-03	13353,3	7,16039E-03	16883	4,43085E-01	10095	9,71987E-01	13740	9,03495E-01	8518	0	17530,5
Non-responder	Macro_LYVE1	Macro_ISG15	A2M	LRP1	6,26743E-03	12895,3	1,06899E-02	8887	4,33821E-01	10468	9,09729E-01	15182	8,87529E-01	12409	0	17530,5
Non-responder	Mono_INHBA	pDC_LILRA4	ICAM1	IL2RG	6,26827E-03	7401,3	1,26941E-02	6664	8,45106E-01	2129	2,27204E+00	1178	8,98926E-01	9505	0	17530,5
Non-responder	Macro_LYVE1	Macro_ISG15	C1QB	LRP1	6,27393E-03	10692,3	9,34478E-03	10954	5,17964E-01	7398	1,01011E+00	12946	9,21568E-01	4633	0	17530,5
Non-responder	Macro_IER3	Macro_IER3	HBEGF	CD44	6,27393E-03	15620,3	6,74202E-03	18618	4,03256E-01	11847	7,47443E-01	19320	8,93790E-01	10786	0	17530,5
Non-responder	Macro_FOLR2+APOE+	Macro_FOLR2-APOE+	PLAU	LRP1	6,27555E-03	10878,5	1,98466E-02	3109	6,85260E-01	3817	1,13065E+00	10615	8,60209E-01	19321	0	17530,5
Non-responder	Macro_LYVE1	Macro_ISG15	C1QA	CD93	6,27717E-03	12375,3	7,85097E-03	14557	5,35622E-01	6878	1,01770E+00	12802	8,96499E-01	10109	0	17530,5
Non-responder	pDC_LILRA4	Mono_CD16	APP	LRP10	6,27772E-03	9626,9	1,43682E-02	5402	1,08456E+00	833	2,83484E+00	392	8,40730E-01	23977	0	17530,5
Non-responder	Mono_CD16	cDC_CLEC9A	S100A9	ITGB2	6,28042E-03	13143,3	6,58765E-03	19324	4,06065E-01	11707	9,14887E-01	15053	9,34368E-01	2102	0	17530,5
Non-responder	Macro_NLRP3	cDC_LAMP3	CXCL8	SDC2	6,28109E-03	15946,1	1,02382E-02	9510	7,50182E-01	3023	1,41034E+00	6532	7,60039E-01	43135	0	17530,5
Non-responder	Macro_LYVE1	Macro_ISG15	C1QA	CD33	6,28205E-03	14663,7	7,08251E-03	17176	4,58144E-01	9493	8,51897E-01	16571	8,86994E-01	12548	0	17530,5
Non-responder	Macro_LYVE1	Macro_ISG15	C1QB	CD33	6,28367E-03	15427,9	6,98768E-03	17577	4,31026E-01	10571	7,71468E-01	18694	8,86152E-01	12767	0	17530,5
Non-responder	Macro_LYVE1	Macro_ISG15	CIRBP	TREM1	6,28693E-03	14268,1	9,34630E-03	10952	3,72474E-01	13362	8,78941E-01	15886	8,83020E-01	13610	0	17530,5
Non-responder	Macro_IER3	Macro_ISG15	CCL3	CCR5	6,28705E-03	9475,3	2,38338E-02	2246	8,01689E-01	2537	1,70068E+00	3780	8,51928E-01	21283	0	17530,5
Non-responder	Macro_LYVE1	Macro_ISG15	SERPING1	LRP1	6,28855E-03	16385,1	7,54185E-03	15515	3,50474E-01	14633	8,67226E-01	16195	8,65680E-01	18052	0	17530,5
Non-responder	Macro_NLRP3	cDC_CLEC9A	COPA	P2RY6	6,28879E-03	14738,3	1,02347E-02	9513	5,10676E-01	7647	1,22418E+00	9067	8,16470E-01	29934	0	17530,5
Non-responder	Macro_IER3	Macro_ISG15	GNAI2	C5AR1	6,29018E-03	14452,1	6,58490E-03	19330	3,84782E-01	12718	9,89001E-01	13356	8,99780E-01	9326	0	17530,5
Non-responder	Macro_LYVE1	Macro_ISG15	C1QB	C1QBP	6,29181E-03	13836,7	6,85908E-03	18120	4,53887E-01	9661	8,30772E-01	17137	9,11451E-01	6735	0	17530,5
Non-responder	Macro_IFI27	Macro_FOLR2+APOE+	APOE	ABCA1	6,30163E-03	9620,5	1,76672E-02	3783	7,48123E-01	3046	6,78993E-01	21248	9,32371E-01	2495	0	17530,5
Non-responder	Macro_LYVE1	Macro_ISG15	TIMP2	CD44	6,30321E-03	15156,9	7,66343E-03	15119	4,00910E-01	11945	8,62381E-01	16318	8,78284E-01	14872	0	17530,5
Non-responder	Macro_OLFML3	pDC_LILRA4	TNF	TNFRSF21	6,30421E-03	11097,9	1,48350E-02	5106	8,80585E-01	1850	2,33469E+00	1055	8,16407E-01	29948	0	17530,5
Non-responder	Macro_FOLR2+APOE+	Mono_INHBA	PSAP	LRP1	6,31193E-03	12818,9	5,71167E-03	24183	4,57475E-01	9522	1,27621E+00	8318	9,22068E-01	4541	0	17530,5
Non-responder	Macro_FOLR2+APOE+	cDC_CLEC9A	LGALS9	CD47	6,31462E-03	13730,1	6,58195E-03	19345	4,53493E-01	9684	1,39671E+00	6687	8,76185E-01	15404	0	17530,5
Non-responder	Macro_NLRP3	Mono_INHBA	IL1B	SIGIRR	6,31789E-03	10905,9	9,20272E-03	11226	8,54251E-01	2059	1,62740E+00	4367	8,60109E-01	19347	0	17530,5
Non-responder	Macro_FOLR2+APOE+	Macro_FOLR2+APOE+	COPA	CD74	6,31789E-03	10702,5	6,58159E-03	19347	4,57145E-01	9537	1,51831E+00	5360	9,36095E-01	1738	0	17530,5
Non-responder	pDC_LILRA4	pDC_LILRA4	TGFB1	CXCR4	6,32740E-03	7399,3	1,02217E-02	9528	7,03557E-01	3590	3,56100E+00	79	9,13464E-01	6269	0	17530,5

Post_NR_Myeloid_Myeloid_CellCell

Non-responder	Macro_LYVE1	Macro_ISG15	HSPA1A	TLR4	6,32933E-03	16604,5	6,83284E-03	18236	3,65477E-01	13762	7,73148E-01	18651	8,78397E-01	14843	0	17530,5
Non-responder	Macro_FOLR2+APOE+	Macro_IFI27	HLA-DMB	CD4	6,33096E-03	13372,1	6,58040E-03	19355	4,78519E-01	8762	1,01218E+00	12909	9,04497E-01	8304	0	17530,5
Non-responder	Macro_IFI27	Macro_FOLR2+APOE+	CD99	CD81	6,33260E-03	12861,7	9,14400E-03	11331	4,80365E-01	8685	7,46012E-01	19356	9,08252E-01	7406	0	17530,5
Non-responder	Macro_OLFML3	pDC_LILRA4	APP	TNFRSF21	6,33514E-03	10467,5	1,59812E-02	4496	8,43119E-01	2147	2,09223E+00	1735	8,30610E-01	26429	0	17530,5
Non-responder	Macro_FOLR2+APOE+	cDC_LAMP3	CXCL12	CXCR4	6,34030E-03	13869,7	1,33384E-02	6114	4,57197E-01	9533	1,22467E+00	9057	8,27950E-01	27114	0	17530,5
Non-responder	Macro_LYVE1	cDC_LAMP3	F13A1	ITGB1	6,34242E-03	12222,5	9,92215E-03	9976	7,11913E-01	3485	1,12242E+00	10759	8,60039E-01	19362	0	17530,5
Non-responder	Macro_NLRP3	pDC_LILRA4	VCAN	CD44	6,34569E-03	12416,9	6,57864E-03	19364	6,05198E-01	5207	1,34850E+00	7315	8,86551E-01	12668	0	17530,5
Non-responder	Macro_IER3	Macro_ISG15	ICAM1	ITGAX_ITGB2	6,34733E-03	12691,7	6,57841E-03	19365	4,65013E-01	9243	1,29532E+00	8038	8,99983E-01	9282	0	17530,5
Non-responder	Mono_INHBA	cDC(CD1C)	IL6	IL6R_IL6ST	6,35039E-03	13129,5	2,62074E-02	1931	1,08052E+00	854	1,69901E+00	3793	7,67278E-01	41539	0	17530,5
Non-responder	Macro_IER3	Macro_OLFML3	CCL3L1	CCR1	6,35039E-03	9700,7	1,76350E-02	3793	7,65332E-01	2868	1,87385E+00	2697	8,50529E-01	21615	0	17530,5
Non-responder	Macro_IFI27	cDC_LAMP3	TNFSF13	TNFRSF11B	6,35244E-03	13169,7	6,59444E-02	379	1,08342E+00	838	8,67391E-01	16189	8,12392E-01	30912	0	17530,5
Non-responder	Macro_NLRP3	Macro_OLFML3	TNF	TRADD	6,35323E-03	15243,7	1,02135E-02	9538	4,89769E-01	8333	1,40097E+00	6640	7,98662E-01	34177	0	17530,5
Non-responder	Macro_NLRP3	Mono_CD16	CD99	PILRA	6,35553E-03	14791,1	6,57756E-03	19370	3,07525E-01	17209	1,14553E+00	10370	8,99059E-01	9476	0	17530,5
Non-responder	cDC(CD1C)	Macro_ISG15	B2M	LILRB2	6,35881E-03	11802,9	6,57697E-03	19372	4,55526E-01	9604	1,03976E+00	12335	9,51009E-01	173	0	17530,5
Non-responder	Mono_INHBA	Mono_CD14	VEGFA	CD44	6,36100E-03	8679,1	1,45319E-02	5281	8,09074E-01	2466	1,25798E+00	8577	8,98792E-01	9541	0	17530,5
Non-responder	Macro_ISG15	Mono_CD16	FABP5	RXRA	6,36507E-03	9447,1	1,92514E-02	3263	6,86665E-01	3796	1,95963E+00	2255	8,55660E-01	20391	0	17530,5
Non-responder	Macro_IER3	Macro_FOLR2+APOE+	C3	ITGAX	6,36702E-03	16837,7	8,28875E-03	13311	2,76754E-01	19377	8,33062E-01	17069	8,70303E-01	16901	0	17530,5
Non-responder	Macro_ISG15	Mono_CD14	CCL2	CCR2	6,36996E-03	13661,7	3,13330E-02	1432	6,86656E-01	3797	2,59133E+00	640	7,52239E-01	44909	0	17530,5
Non-responder	Macro_NLRP3	Mast	IL1B	SIGIRR	6,37031E-03	11245,7	9,18406E-03	11261	8,53862E-01	2062	1,45513E+00	5996	8,59987E-01	19379	0	17530,5
Non-responder	Mono_CD16	Macro_NLRP3	ADAM17	ITGA5	6,37655E-03	17463,5	1,13995E-02	7982	4,56891E-01	9547	1,33777E+00	7453	7,52675E-01	44805	0	17530,5
Non-responder	Macro_NLRP3	cDC_LAMP3	THBS1	PTPRJ	6,37914E-03	16154,7	1,02076E-02	9548	7,16419E-01	3427	1,33493E+00	7493	7,61609E-01	42775	0	17530,5
Non-responder	Macro_IER3	Mono_CD16	GNAI2	C5AR1	6,38512E-03	14446,5	6,57426E-03	19388	3,83584E-01	12798	9,97722E-01	13171	8,99708E-01	9345	0	17530,5
Non-responder	Macro_FOLR2+APOE+	Mono_CD14	MDK	NOTCH2	6,38693E-03	19316,1	1,96607E-02	3161	4,65100E-01	9240	1,19467E+00	9551	6,89406E-01	57098	0	17530,5
Non-responder	cDC_LAMP3	Macro_NLRP3	HSPA8	LDLR	6,39006E-03	11358,1	1,47218E-02	5169	5,93787E-01	5451	1,21361E+00	9249	8,59955E-01	19391	0	17530,5
Non-responder	Macro_NLRP3	Mono_CD14	CD55	ADGRE2	6,39473E-03	12801,3	1,02027E-02	9554	5,80879E-01	5716	1,31210E+00	7808	8,43139E-01	23398	0	17530,5
Non-responder	Macro_FOLR2+APOE+	Macro_FOLR2+APOE-	C1QB	CD33	6,39665E-03	12829,1	6,57310E-03	19395	5,50273E-01	6497	1,36145E+00	7117	8,83030E-01	13606	0	17530,5
Non-responder	Macro_LYVE1	Macro_FOLR2+APOE-	F13A1	ITGA4	6,40325E-03	12475,7	1,04199E-02	9258	7,51837E-01	3006	9,70016E-01	13788	8,62434E-01	18796	0	17530,5
Non-responder	cDC_LAMP3	pDC_LILRA4	B2M	TFRC	6,41151E-03	12512,3	6,57179E-03	19404	4,00732E-01	11955	1,10266E+00	11125	9,32088E-01	2547	0	17530,5
Non-responder	Macro_NLRP3	Macro_IFI27	ICAM1	ITGAM_ITGB2	6,41812E-03	14135,5	7,39823E-03	16016	4,86568E-01	8448	7,43807E-01	19408	9,00017E-01	9275	0	17530,5
Non-responder	Macro_LYVE1	Macro_FOLR2+APOE-	PLTP	ABCA1	6,41977E-03	11569,9	1,28857E-02	6490	7,52771E-01	2994	8,14338E-01	17571	8,84232E-01	13264	0	17530,5
Non-responder	Macro_IER3	Macro_OLFML3	CD14	ITGA4	6,42639E-03	15546,7	6,56916E-03	19413	3,87681E-01	12587	8,00687E-01	17936	8,95866E-01	10267	0	17530,5
Non-responder	Macro_FOLR2+APOE+	cDC_CLEC9A	ADM	CALCRL	6,42859E-03	17665,9	1,40164E-02	5629	4,56353E-01	9567	1,41334E+00	6491	7,33100E-01	49112	0	17530,5
Non-responder	Macro_FOLR2-APOE+	Mono_CD16	HLA-C	LILRA1	6,42891E-03	9046,7	1,84452E-02	3516	8,02945E-01	2516	8,03694E-01	17862	9,25783E-01	3809	0	17530,5
Non-responder	pDC_LILRA4	Macro_NLRP3	HSPA8	LDLR	6,42891E-03	8284,1	1,75863E-02	3809	8,04577E-01	2505	2,55516E+00	683	8,70323E-01	16893	0	17530,5
Non-responder	Mono_CD16	Macro_NLRP3	S100A8	TLR4	6,43136E-03	13725,5	9,80342E-03	10173	4,29977E-01	10613	1,11552E+00	10895	8,59861E-01	19416	0	17530,5
Non-responder	Macro_OLFML3	pDC_LILRA4	SEMA6B	PLXNA4	6,43381E-03	19066,5	5,22839E-02	607	7,65506E-01	2865	1,58904E+00	4697	5,72582E-01	69633	0	17530,5
Non-responder	cDC(CD1C)	Mast	GRN	NTRK1	6,43798E-03	10708,9	6,72187E-02	354	1,02270E+00	1102	7,43364E-01	19420	8,77190E-01	15138	0	17530,5
Non-responder	Macro_NLRP3	Macro_LYVE1	TNF	TNFRSF1A	6,43903E-03	12460,1	1,09242E-02	8583	5,44556E-01	6640	1,19335E+00	9571	8,57429E-01	19976	0	17530,5
Non-responder	Mono_CD16	Macro_LYVE1	HMGB1	CD163	6,44130E-03	15118,1	6,71528E-03	18730	3,52099E-01	14526	7,43312E-01	19422	9,17883E-01	5382	0	17530,5
Non-responder	Macro_OLFML3	Macro_OLFML3	TNFSF13B	TFRC	6,44130E-03	14505,7	6,68208E-03	18877	4,65599E-01	9220	1,33582E+00	7479	8,59832E-01	19422	0	17530,5
Non-responder	cDC(CD1C)	Mono_CD16	GNAI2	FPR1	6,44628E-03	16115,5	6,56671E-03	19425	3,14907E-01	16748	8,33253E-01	17065	8,97744E-01	9809	0	17530,5
Non-responder	Macro_OLFML3	pDC_LILRA4	FN1	CD79A	6,44687E-03	19185,3	6,63316E-02	372	4,90573E-01	8303	1,44019E+00	6141	6,42009E-01	63580	0	17530,5
Non-responder	Macro_OLFML3	pDC_LILRA4	EBI3_IL27	IL27RA_IL6ST	6,44949E-03	16678,9	1,68883E-02	4083	4,95218E-01	8148	1,67165E+00	4008	7,30669E-01	49625	0	17530,5
Non-responder	Macro_FOLR2+APOE+	cDC_CLEC9A	CXCL12	ITGB1	6,45472E-03	15286,3	1,38676E-02	5742	4,56103E-01	9577	1,65892E+00	4125	7,76023E-01	39457	0	17530,5
Non-responder	Mono_INHBA	Macro_FOLR2+APOE+	TNF	TNFRSF1A	6,45624E-03	9276,7	1,38100E-02	5780	6,84151E-01	3830	1,89528E+00	2571	8,71165E-01	16672	0	17530,5
Non-responder	Mono_INHBA	Macro_FOLR2+APOE+	TNF	TNFRSF1B	6,45790E-03	11174,7	9,67581E-03	10382	5,47379E-01	6557	1,58332E+00	4747	8,71224E-01	16657	0	17530,5
Non-responder	Macro_FOLR2+APOE+	Macro_OLFML3	HLA-C	LILRA1	6,45790E-03	13520,9	6,56523E-03	19432	4,53773E-01	9666	1,37216E+00	6991	8,81544E-01	13985	0	17530,5
Non-responder	Mono_CD16	pDC_LILRA4	TNFSF10	RIPK1	6,46520E-03	17646,1	1,02386E-02	9509	4,56021E-01	9581	1,68303E+00	3907	7,39699E-01	47703	0	17530,5
Non-responder	Macro_NLRP3	Macro_OLFML3	IL1B	IL1R2_IL1RAP	6,46782E-03	11996,5	1,01861E-02	9582	8,85118E-01	1815	1,89262E+00	2591	8,22531E-01	28464	0	17530,5
Non-responder	Macro_IER3	pDC_LILRA4	TNFSF13B	TFRC	6,46788E-03	16582,3	6,67297E-03	18918	3,60992E-01	14004	1,00623E+00	13021	8,59750E-01	19438	0	17530,5
Non-responder	Macro_OLFML3	Mono_INHBA	TGM2	SDC4	6,47044E-03	18823,3	1,83108E-02	3554	5,31373E-01	7010	1,19260E+00	9583	6,93349E-01	56439	0	17530,5
Non-responder	pDC_LILRA4	pDC_LILRA4	LTB	CD40	6,48326E-03	9437,3	1,75539E-02	3820	1,07822E+00	862	3,65334E+00	66	8,36878E-01	24908	0	17530,5
Non-responder	Macro_FOLR2+APOE+	Macro_FOLR2+APOE+	HLA-DQA2	LAG3	6,48356E-03	14426,9	1,10878E-02	8378	4,55883E-01	9588	1,76909E+00	3296	8,02214E-01	33342	0	17530,5
Non-responder	Mono_INHBA	Macro_FOLR2+APOE+	ICAM1	ITGAM_ITGB2	6,48453E-03	14474,1	6,60518E-03	19236	4,12941E-01	11387	9,75627E-01	13663	8,94798E-01	10554	0	17530,5
Non-responder	Mono_CD16	Macro_NLRP3	ICAM4	ITGAL	6,48619E-03	20094,9	1,87863E-02	3400	5,64075E-01	6127	1,19233E+00	9589	6,39679E-01	63828	0	17530,5
Non-responder	Mono_INHBA	Macro_FOLR2+APOE+	ICAM1	ITGAL_ITGB2	6,48620E-03	13397,1	6,77984E-03	18444	4,74530E-01	8894	1,08635E+00	11409	8,94119E-01	10708	0	17530,5
Non-responder	Mono_INHBA	Macro_FOLR2+APOE+	ICAM1	ITGAX_ITGB2	6,48786E-03	11949,9	7,00989E-03	17495	5,23857E-01	7222	1,23984E+00	8845	9,02806E-01	8657	0	17530,5
Non-responder	Macro_OLFML3	Macro_IER3	TNFSF13B	TFRC	6,49120E-03	15341,3	6,66372E-03	18959	4,64757E-01	9251	1,08077E+00	11514	8,59666E-01	19452	0	17530,5
Non-responder	Macro_OLFML3	pDC_LILRA4	VCAN	SELL	6,49144E-03	9406,3	1,29648E-02	6428	9,55635E-01	1418	2,44524E+00	852	8,53973E-01	20803	0	17530,5
Non-responder	Mono_INHBA	Macro_FOLR2+APOE+	ICAM1	IL2RG	6,49287E-03	14747,3	6,93047E-03	17809	4,42451E-01	10130	1,12196E+00	10770	8,67926E-01	17497	0	17530,5
Non-responder	Macro_ISG15	Macro_ISG15	HLA-DQA1	LAG3	6,49318E-03	8523,5	1,75497E-02	3822	8,22740E-01	2343	1,88387E+00	2643	8,72662E-01	16279	0	17530,5
Non-responder	Mono_INHBA	Macro_FOLR2+APOE+	ANXA2	TLR2	6,49454E-03	14616,5	7,34803E-03	16189	3,55340E-01	14330	8,50710E-01	16608	9,03957E-01	8425	0	17530,5
Non-responder	Macro_OLFML3	pDC_LILRA4	MIF	CD74_CXCR4	6,49670E-03	11475,3	5,08998E-03	28585	4,74581E-01	8893	2,08053E+00	1772	9,44679E-01	596	0	17530,5
Non-responder	Macro_LYVE1	Mono_CD16	CD14	ITGB1	6,49788E-03	14918,5	6,55985E-03	19456	4,26001E-01	10792	8,50053E-01	16625	8,96213E-01	10189	0	17530,5
Non-responder	Mono_INHBA	Macro_FOLR2+APOE+	THBS1	LRP1	6,50122E-03	12502,3	1,09777E-02	8514	4,79997E-01	8706	1,16010E+00	10125	8,67369E-01	17636	0	17530,5
Non-responder	Macro_FOLR2+APOE+	cDC(CD1C)	NECTIN2	CD226	6,50196E-03	22541,9	1,01775E-02	9595	4,83444E-01	8571	1,24565E+00	8757	5,92828E-01	68256	0	17530,5
Non-responder	Macro_IFI27	Mono_CD16	LYZ	ITGAL	6,50289E-03	11104,5	1,23922E-02	6941	5,78490E-01	5773	7,42486E-01	19459	9,15617E-01	5819	0	17530,5
Non-responder	Mono_INHBA	Macro_FOLR2+APOE+	THBS1	CD47	6,50289E-03	11372,9	1,12462E-02	8145	5,67310E-01	6044	1,32361E+00	7647	8,67925E-01	17498	0	17530,5
Non-responder	Macro_IER3	Mono_CD16	B2M	LILRB1	6,50456E-03	14990,7	6,60322E-03	19250	2,75588E-01	19460	8,17739E-01	17487	9,38956E-01	1226	0	17530,5
Non-responder	Macro_OLFML3	pDC_LILRA4	VEGFA	RET	6,50460E-03	17318,5	4,45684E-02	794	7,38269E-01	3164	1,66927E+00	4038	6,61980E-01	61066	0	17530,5
Non-responder	Macro_NLRP3	Mono_CD16	APP	FPR2	6,50460E-03	17729,5	1,09520E-02	8543	4,82096E-01	8619	1,19163E+00	9596	7,54600E-01	44359	0	17530,5

Post_NR_Myeloid_Myeloid_CellCell

Non-responder	Macro_OLFML3	pDC_LILRA4	TGFB1	TGFBR1_TGFBR2	6,50986E-03	12410,7	1,03987E-02	9282	4,55628E-01	9598	1,83458E+00	2918	8,45962E-01	22725	0	17530,5
Non-responder	Macro_OLFML3	pDC_LILRA4	ADM	CALCRL	6,51777E-03	16161,7	1,21713E-02	7170	7,57339E-01	2945	2,22275E+00	1313	7,19066E-01	51850	0	17530,5
Non-responder	Mast	Macro_OLFML3	TIMP3	CD44	6,51834E-03	7464,1	5,41460E-02	564	1,08139E+00	849	1,62509E+00	4380	8,81492E-01	13997	0	17530,5
Non-responder	Mast	Macro_FOLR2+APOE-	HDC	HRH2	6,51834E-03	13514,9	4,30883E-02	849	1,19040E+00	466	1,14568E+00	10367	7,80717E-01	38362	0	17530,5
Non-responder	Macro_FOLR2+APOE+	Macro_OLFML3	ICAM1	ITGAM_ITGB2	6,51962E-03	14269,3	6,55667E-03	19469	3,90420E-01	12450	1,09382E+00	11269	8,94450E-01	10628	0	17530,5
Non-responder	Macro_OLFML3	pDC_LILRA4	VEGFB	RET	6,52304E-03	20102,9	3,51150E-02	1192	6,65121E-01	4143	1,42311E+00	6372	5,41355E-01	71277	0	17530,5
Non-responder	Macro_OLFML3	Macro_FOLR2+APOE+	VEGFA	NRP1	6,52304E-03	17015,5	1,03640E-02	9324	4,55534E-01	9603	1,36212E+00	7103	7,67413E-01	41517	0	17530,5
Non-responder	Mono_INHBA	Macro_FOLR2+APOE+	CD99	CD81	6,52464E-03	11377,3	8,89631E-03	11837	4,62580E-01	9332	1,13732E+00	10508	9,07101E-01	7679	0	17530,5
Non-responder	Mono_CD16	Mono_CD16	CD55	ADGRE2	6,52568E-03	12261,5	1,01710E-02	9604	6,00769E-01	5287	1,51110E+00	5435	8,42933E-01	23451	0	17530,5
Non-responder	Mono_INHBA	Macro_FOLR2+APOE+	CD99	PILRA	6,52799E-03	13428,1	7,51102E-03	15636	3,89244E-01	12511	9,93299E-01	13261	9,04923E-01	8202	0	17530,5
Non-responder	cDC_LAMP3	pDC_LILRA4	HLA-DQA1	CD4	6,53638E-03	11739,1	6,55479E-03	19479	3,86062E-01	12668	1,42791E+00	6314	9,31227E-01	2704	0	17530,5
Non-responder	Macro_ISG15	Macro_ISG15	CXCL11	CCR5	6,53791E-03	16256,1	4,34208E-02	836	6,84140E-01	3831	2,10194E+00	1689	6,87413E-01	57394	0	17530,5
Non-responder	Macro_FOLR2+APOE+	Macro_OLFML3	ICAM1	ITGAL_ITGB2	6,53973E-03	13790,7	6,55471E-03	19481	4,24474E-01	10859	1,17041E+00	9964	8,92510E-01	11119	0	17530,5
Non-responder	Mono_CD16	Mono_CD14	S100A8	ITGB2	6,54141E-03	14291,5	6,55471E-03	19482	3,73107E-01	13330	8,11587E-01	17645	9,27682E-01	3470	0	17530,5
Non-responder	Macro_OLFML3	pDC_LILRA4	SIRPB2	CD47	6,54153E-03	12422,3	1,25293E-02	6814	7,22477E-01	3344	2,19461E+00	1397	8,03497E-01	33026	0	17530,5
Non-responder	Macro_NLRP3	Macro_NLRP3	HBEGF	CD82	6,54153E-03	13188,3	1,01643E-02	9610	6,56502E-01	4281	1,29473E+00	8048	8,30456E-01	26472	0	17530,5
Non-responder	Macro_OLFML3	pDC_LILRA4	FAM3C	ADGRG5	6,54417E-03	19950,9	4,88735E-02	681	7,59929E-01	2924	1,39707E+00	6681	5,22668E-01	71938	0	17530,5
Non-responder	Macro_FOLR2+APOE+	Macro_IFI27	MMP9	IFNAR1	6,54417E-03	16383,7	1,01642E-02	9611	5,56045E-01	6332	1,79801E+00	3123	7,50427E-01	45322	0	17530,5
Non-responder	Macro_OLFML3	Macro_FOLR2+APOE+	C1QA	CD33	6,54645E-03	14009,9	8,40858E-03	12999	2,75248E-01	19485	1,19004E+00	9627	8,95314E-01	10408	0	17530,5
Non-responder	cDC_LAMP3	Mono_CD16	HLA-A	LILRB1	6,54813E-03	15236,5	6,55399E-03	19486	2,85424E-01	18722	8,36922E-01	16956	9,27621E-01	3488	0	17530,5
Non-responder	cDC(CD1C)	Macro_NLRP3	HSPA1A	TLR4	6,55149E-03	17073,3	7,17288E-03	16836	2,75226E-01	19488	8,21114E-01	17378	8,80967E-01	14134	0	17530,5
Non-responder	Macro_FOLR2+APOE+	Mono_INHBA	HLA-F	LILRB1	6,55149E-03	15532,5	6,55385E-03	19488	4,15186E-01	11284	1,09086E+00	11316	8,65710E-01	18044	0	17530,5
Non-responder	Macro_NLRP3	Macro_OLFML3	VCAN	SELL	6,55475E-03	11977,1	1,01600E-02	9615	8,41866E-01	2161	1,45872E+00	5961	8,38108E-01	24618	0	17530,5
Non-responder	Macro_ISG15	Mono_CD16	LGALS9	PTPRC	6,55785E-03	7349,5	9,46688E-03	10728	7,88315E-01	2658	2,02259E+00	1996	9,25702E-01	3835	0	17530,5
Non-responder	Mono_INHBA	Macro_FOLR2+APOE+	LILRB4	LAIR1	6,57000E-03	10830,9	7,04284E-03	17358	5,69368E-01	5988	1,61811E+00	4441	9,02005E-01	8837	0	17530,5
Non-responder	Macro_NLRP3	Macro_ISG15	HLA-B	LILRB2	6,57065E-03	11092,3	6,46275E-03	19950	4,55060E-01	9621	1,31453E+00	7775	9,44881E-01	585	0	17530,5
Non-responder	Macro_IER3	Mono_CD16	PTPN6	CLEC12A	6,57169E-03	14044,1	9,35015E-03	10941	4,34092E-01	10454	1,06648E+00	11795	8,59443E-01	19500	0	17530,5
Non-responder	pDC_LILRA4	Mast	AIMP1	NTRK1	6,57284E-03	12931,7	9,26013E-02	166	1,07229E+00	890	1,69197E+00	3838	7,64223E-01	42234	0	17530,5
Non-responder	Macro_FOLR2+APOE+	Mono_INHBA	LGALS3	ENG	6,57674E-03	13532,9	6,55122E-03	19503	4,05835E-01	11719	1,26613E+00	8459	8,95161E-01	10453	0	17530,5
Non-responder	Macro_IER3	cDC_LAMP3	SPP1	S1PR1	6,57918E-03	13927,5	4,40312E-02	817	1,08062E+00	853	1,28738E+00	8160	7,64061E-01	42277	0	17530,5
Non-responder	Macro_IER3	Macro_NLRP3	GNAI2	C5AR1	6,58012E-03	15119,1	6,55070E-03	19505	3,80935E-01	12934	8,64683E-01	16251	8,99545E-01	9375	0	17530,5
Non-responder	Macro_OLFML3	pDC_LILRA4	PSEN1	NOTCH4	6,58126E-03	15107,5	2,65586E-02	1874	5,80477E-01	5725	1,55861E+00	4945	7,49823E-01	45463	0	17530,5
Non-responder	Macro_OLFML3	Macro_NLRP3	HSPA8	LDLR	6,59189E-03	12404,1	1,34139E-02	6059	4,97549E-01	8069	1,18991E+00	9629	8,54259E-01	20733	0	17530,5
Non-responder	Macro_OLFML3	pDC_LILRA4	HLA-B	CANX	6,59189E-03	9586,1	6,15387E-03	21483	5,57869E-01	6283	1,94224E+00	2342	9,48965E-01	292	0	17530,5
Non-responder	Mono_INHBA	Macro_FOLR2+APOE+	FN1	CD44	6,59362E-03	11280,1	1,35750E-02	5942	4,29633E-01	10638	1,35422E+00	7235	8,77525E-01	15055	0	17530,5
Non-responder	Mono_INHBA	Macro_FOLR2+APOE+	FN1	C5AR1	6,59531E-03	9259,1	1,91211E-02	3301	5,95630E-01	5409	1,74929E+00	3424	8,71346E-01	16631	0	17530,5
Non-responder	Macro_OLFML3	cDC(CD1C)	LGALS9	SLC1A5	6,59531E-03	15736,3	7,80387E-03	14707	3,82110E-01	12873	9,58362E-01	14057	8,59405E-01	19514	0	17530,5
Non-responder	pDC_LILRA4	Macro_FOLR2+APOE+	HLA-B	LILRB1	6,59786E-03	8598,5	6,83063E-03	18248	6,83527E-01	3843	2,36960E+00	991	9,33053E-01	2380	0	17530,5
Non-responder	Mono_CD16	Macro_FOLR2+APOE+	TGFB1	ENG	6,59869E-03	15138,5	7,89754E-03	14424	3,40376E-01	15162	1,22441E+00	9060	8,59397E-01	19516	0	17530,5
Non-responder	Mono_INHBA	Macro_FOLR2+APOE+	FN1	PLAUR	6,60038E-03	9858,3	1,43882E-02	5384	4,78250E-01	8773	1,61417E+00	4472	8,84666E-01	13132	0	17530,5
Non-responder	Macro_NLRP3	Macro_NLRP3	VCAN	ITGA4	6,60038E-03	11682,3	8,73808E-03	12201	7,44773E-01	3088	1,44662E+00	6075	8,59389E-01	19517	0	17530,5
Non-responder	Macro_OLFML3	pDC_LILRA4	PLAU	IGF2R	6,60253E-03	14586,7	1,32936E-02	6141	6,02335E-01	5262	2,08681E+00	1751	7,64163E-01	42249	0	17530,5
Non-responder	cDC(CD1C)	Macro_FOLR2+APOE+	HLA-DQA2	LAG3	6,60789E-03	10351,9	1,75051E-02	3845	8,33935E-01	2240	1,81867E+00	3011	8,35966E-01	25133	0	17530,5
Non-responder	cDC(CD1C)	cDC(CD1C)	HLA-DQA2	CD4	6,60884E-03	14400,7	6,80058E-03	18377	4,51093E-01	9801	7,39793E-01	19522	9,11265E-01	6773	0	17530,5
Non-responder	pDC_LILRA4	Macro_FOLR2+APOE+	COPA	CD74	6,60971E-03	6789,3	9,41294E-03	10839	6,59733E-01	4220	2,44315E+00	855	9,45998E-01	502	0	17530,5
Non-responder	Macro_OLFML3	Macro_FOLR2+APOE+	GRN	TNFRSF1A	6,61053E-03	12946,1	6,54644E-03	19523	3,69868E-01	13516	1,22594E+00	9040	9,19115E-01	5121	0	17530,5
Non-responder	Macro_OLFML3	pDC_LILRA4	IL10	IFNLR1	6,61291E-03	17613,7	3,20656E-02	1387	7,30980E-01	3242	1,69071E+00	3846	6,54345E-01	62063	0	17530,5
Non-responder	Macro_NLRP3	Macro_FOLR2+APOE+	TNF	TRADD	6,61318E-03	15173,5	1,01443E-02	9637	4,87474E-01	8410	1,45633E+00	5982	7,98115E-01	34308	0	17530,5
Non-responder	Macro_OLFML3	pDC_LILRA4	VEGFA	NRP1	6,62384E-03	16880,5	1,03453E-02	9346	4,54482E-01	9641	1,42565E+00	6340	7,67252E-01	41545	0	17530,5
Non-responder	cDC_LAMP3	Macro_FOLR2+APOE+	TNFSF13B	HLA-DPB1	6,62409E-03	9613,1	6,54564E-03	19531	5,57012E-01	6304	1,66586E+00	4070	9,44320E-01	630	0	17530,5
Non-responder	Macro_LYVE1	cDC(CD1C)	C1QB	C1QBP	6,62579E-03	11563,5	9,45394E-03	10759	5,80211E-01	5732	7,39499E-01	19532	9,23573E-01	4264	0	17530,5
Non-responder	Macro_FOLR2+APOE+	Macro_FOLR2+APOE+	MRC1	PTPRC	6,62651E-03	11531,5	1,01422E-02	9642	5,06020E-01	7783	1,80359E+00	3090	8,59026E-01	19612	0	17530,5
Non-responder	Mono_INHBA	Macro_FOLR2+APOE+	IL1B	SIGIRR	6,62918E-03	9793,7	1,13323E-02	8047	7,02226E-01	3604	1,75958E+00	3361	8,72169E-01	16426	0	17530,5
Non-responder	Mono_INHBA	Macro_FOLR2+APOE+	S100A8	ITGB2	6,63257E-03	8771,7	1,03634E-02	9325	6,95211E-01	3679	1,03602E+00	12428	9,41622E-01	896	0	17530,5
Non-responder	pDC_LILRA4	pDC_LILRA4	ICAM1	IL2RG	6,63719E-03	7301,3	1,25500E-02	6785	8,39263E-01	2189	2,87346E+00	356	8,98406E-01	9646	0	17530,5
Non-responder	Mono_INHBA	Macro_FOLR2+APOE+	S100A9	TLR4	6,63937E-03	11767,7	1,23864E-02	6952	6,44103E-01	4492	8,51195E-01	16588	8,84194E-01	13276	0	17530,5
Non-responder	Macro_OLFML3	pDC_LILRA4	TNFSF10	RIPK1	6,63986E-03	16612,9	1,12589E-02	8133	5,17980E-01	7395	1,63468E+00	4308	7,48739E-01	45698	0	17530,5
Non-responder	Mono_INHBA	Macro_FOLR2+APOE+	S100A9	ITGB2	6,64106E-03	8450,9	9,49943E-03	10681	7,16159E-01	3431	1,16693E+00	10018	9,44738E-01	594	0	17530,5
Non-responder	Mono_INHBA	Macro_FOLR2+APOE+	S100A9	CD36	6,64276E-03	12404,3	1,13184E-02	8065	5,71293E-01	5950	8,27922E-01	17214	8,84239E-01	13262	0	17530,5
Non-responder	Macro_ISG15	Macro_FOLR2+APOE+	TGM2	ITGB1	6,64306E-03	10696,7	1,90070E-02	3332	6,83124E-01	3852	1,93615E+00	2378	8,30777E-01	26391	0	17530,5
Non-responder	pDC_LILRA4	Macro_NLRP3	SERPINF1	PLXDC2	6,64521E-03	8320,3	1,01376E-02	9649	5,99871E-01	5311	2,30203E+00	1115	9,05722E-01	7996	0	17530,5
Non-responder	Macro_FOLR2+APOE+	Macro_IER3	SPP1	CD44	6,64616E-03	12750,1	1,00153E-02	9823	2,74539E-01	19544	1,81484E+00	3028	8,82186E-01	13825	0	17530,5
Non-responder	Mono_INHBA	Macro_FOLR2+APOE+	LGALS1	ITGB1	6,64616E-03	12140,7	7,14436E-03	16947	4,58415E-01	9486	1,08683E+00	11398	9,18067E-01	5342	0	17530,5
Non-responder	Mono_CD14	pDC_LILRA4	THBS1	SCARB1	6,64809E-03	10748,9	1,94011E-02	3227	6,83104E-01	3853	1,92436E+00	2434	8,29613E-01	26700	0	17530,5
Non-responder	Macro_OLFML3	pDC_LILRA4	FARP2	PLXNA4	6,65056E-03	18177,3	5,52473E-02	543	7,91260E-01	2635	1,59127E+00	4677	6,23668E-01	65501	0	17530,5
Non-responder	Macro_IFI27	pDC_LILRA4	IGF1	IGF2R	6,65313E-03	14146,7	1,97165E-02	3142	6,83043E-01	3854	1,74850E+00	3430	7,61585E-01	42777	0	17530,5
Non-responder	Mono_INHBA	Macro_FOLR2+APOE+	SPP1	ITGAV_ITGB1	6,65637E-03	9261,9	2,40243E-02	2216	6,17525E-01	4971	1,68842E+00	3856	8,66961E-01	17736	0	17530,5
Non-responder	Macro_OLFML3	cDC(CD1C)	C1QB	C1QBP	6,65637E-03	15759,3	6,71051E-03	18752	2,74467E-01	19550	8,72580E-01	16044	9,10564E-01	6920	0	17530,5
Non-responder	Mono_INHBA	Macro_FOLR2+APOE+	SPP1	ITGA5_ITGB1	6,65807E-03	9417,5	2,35273E-02	2287	6,18383E-01	4958	1,56996E+00	4853	8,68111E-01	17459	0	17530,5
Non-responder	Mono_INHBA	Macro_FOLR2+APOE+	SPP1	ITGA4_ITGB1	6,65978E-03	8746,1	2,11863E-02	2767	6,13716E-01	5031	1,63731E+00	4290	8,81054E-01	14112	0	17530,5
Non-responder	Macro_OLFML3	Mono_CD14	HSP90B1	TLR4	6,65978E-03	17673,7	7,10786E-03	17086	3,06881E-01	17264	8,37833E-01	16936	8,59265E-01	19552	0	17530,5

Post_NR_Myeloid_Myeloid_CellCell

Non-responder	Macro_IER3	Mono_CD16	ANXA1	FPR1	6,66148E-03	15414,5	6,96541E-03	17678	3,39768E-01	15206	7,38756E-01	19553	9,09704E-01	7105	0	17530,5
Non-responder	Macro_FOLR2+APOE+	cDC_CLEC9A	MDK	NOTCH2	6,66395E-03	18301,3	2,26348E-02	2445	5,21328E-01	7286	1,18805E+00	9656	7,04283E-01	54589	0	17530,5
Non-responder	Mono_INHBA	Macro_FOLR2+APOE+	SPP1	PTGER4	6,66659E-03	8755,5	2,51287E-02	2064	6,61078E-01	4204	1,75822E+00	3365	8,71411E-01	16614	0	17530,5
Non-responder	Macro_OLFML3	pDC_LILRA4	FN1	DPP4	6,67467E-03	16040,5	5,29191E-02	594	6,28672E-01	4752	1,67490E+00	3979	7,11152E-01	53347	0	17530,5
Non-responder	pDC_LILRA4	pDC_LILRA4	LGALS9	CD47	6,68004E-03	8031,3	1,02583E-02	9473	7,36566E-01	3185	2,95623E+00	306	8,98317E-01	9662	0	17530,5
Non-responder	Mono_INHBA	Macro_FOLR2+APOE+	VCAN	TLR2	6,68024E-03	11415,9	1,32492E-02	6172	5,88168E-01	5555	1,04638E+00	12203	8,75320E-01	15619	0	17530,5
Non-responder	Mono_INHBA	Macro_FOLR2+APOE+	VCAN	ITGB1	6,68195E-03	10434,5	1,27422E-02	6615	6,33478E-01	4661	1,21098E+00	9278	8,81140E-01	14088	0	17530,5
Non-responder	Mono_INHBA	Macro_FOLR2+APOE+	VCAN	ITGA4	6,68365E-03	13017,1	1,04547E-02	9219	5,47999E-01	6547	9,26339E-01	14776	8,69881E-01	17013	0	17530,5
Non-responder	cDC(CD1C)	Macro_LYVE1	HLA-DPA1	CD4	6,68536E-03	12539,3	6,53760E-03	19567	5,00854E-01	7965	8,35014E-01	17007	9,44339E-01	627	0	17530,5
Non-responder	Macro_OLFML3	pDC_LILRA4	PDCD1LG2	PDCD2	6,68541E-03	18987,3	1,48324E-02	5109	4,87122E-01	8427	1,74531E+00	3446	6,66808E-01	60424	0	17530,5
Non-responder	Macro_FOLR2+APOE+	pDC_LILRA4	CCL3	CCR5	6,68541E-03	14648,3	1,01284E-02	9664	4,96361E-01	8114	2,11047E+00	1659	7,89501E-01	36274	0	17530,5
Non-responder	Mono_INHBA	Macro_FOLR2+APOE+	VCAN	CD44	6,68707E-03	11179,9	9,76814E-03	10236	5,68441E-01	6013	9,63674E-01	13932	9,04964E-01	8188	0	17530,5
Non-responder	Macro_FOLR2+APOE+	Macro_FOLR2-APOE+	HBEGF	CD9	6,69078E-03	14971,3	1,05430E-02	9082	4,89193E-01	8350	3,95320E-01	30228	8,98303E-01	9666	0	17530,5
Non-responder	Mono_INHBA	Macro_FOLR2+APOE+	MIF	TNFRSF14	6,69391E-03	10139,5	8,56631E-03	12598	6,16379E-01	4993	1,30781E+00	7882	9,07040E-01	7694	0	17530,5
Non-responder	Mono_INHBA	Macro_FOLR2+APOE+	VEGFA	ITGB1	6,69904E-03	8785,7	1,80922E-02	3633	8,41996E-01	2158	1,68358E+00	3901	8,71040E-01	16706	0	17530,5
Non-responder	cDC(CD1C)	Macro_LYVE1	HLA-DRB5	CD4	6,69904E-03	13052,1	6,53634E-03	19575	4,89095E-01	8353	7,70030E-01	18735	9,40240E-01	1067	0	17530,5
Non-responder	Macro_IER3	pDC_LILRA4	HLA-DRB5	CD4	6,70075E-03	11318,3	6,53590E-03	19576	3,84530E-01	12729	1,48603E+00	5687	9,40238E-01	1069	0	17530,5
Non-responder	Mast	Macro_LYVE1	CSF1	CSF3R	6,70170E-03	10377,3	4,27529E-02	861	1,08571E+00	828	1,36521E+00	7071	8,33888E-01	25596	0	17530,5
Non-responder	Macro_LYVE1	cDC(CD1C)	C1QB	CD33	6,70246E-03	11848,3	1,10367E-02	8434	5,66931E-01	6053	7,37995E-01	19577	9,07255E-01	7647	0	17530,5
Non-responder	Macro_IER3	Mono_CD16	CD14	ITGB2	6,70418E-03	12052,7	6,53496E-03	19578	4,39789E-01	10232	1,07993E+00	11534	9,37851E-01	1389	0	17530,5
Non-responder	Macro_FOLR2+APOE+	Macro_FOLR2+APOE+	GRN	SORT1	6,70589E-03	13172,7	7,66045E-03	15130	4,84445E-01	8528	1,54424E+00	5096	8,59136E-01	19579	0	17530,5
Non-responder	Macro_FOLR2+APOE+	Macro_IER3	HSP90B1	TLR2	6,70760E-03	16953,3	6,53484E-03	19580	3,16006E-01	16688	7,70596E-01	18720	8,88138E-01	12248	0	17530,5
Non-responder	Macro_NLRP3	Macro_OLFML3	ANXA1	FPR2_FPR3	6,70931E-03	18213,1	7,24899E-03	16541	2,89045E-01	18442	7,61084E-01	18971	8,59133E-01	19581	0	17530,5
Non-responder	Mono_INHBA	Macro_OLFML3	CCL2	CCR1	6,71376E-03	11785,3	2,26496E-02	2439	6,82456E-01	3866	2,07007E+00	1822	8,02506E-01	33269	0	17530,5
Non-responder	Macro_OLFML3	pDC_LILRA4	IRAK4	TLR7	6,71770E-03	16130,9	1,65346E-02	4223	5,64438E-01	6117	1,66950E+00	4034	7,34878E-01	48750	0	17530,5
Non-responder	Macro_OLFML3	Macro_FOLR2+APOE+	C3	NRP1	6,71770E-03	11419,1	1,60434E-02	4459	4,53688E-01	9676	1,51174E+00	5429	8,57342E-01	20001	0	17530,5
Non-responder	Macro_NLRP3	Macro_OLFML3	S100A4	CCR5	6,71788E-03	14027,7	1,18849E-02	7460	4,61541E-01	9372	8,67379E-01	16190	8,59114E-01	19586	0	17530,5
Non-responder	Macro_IER3	Macro_ISG15	VCAN	CD44	6,72131E-03	15075,7	6,53384E-03	19588	4,02451E-01	11874	9,76652E-01	13635	8,86207E-01	12751	0	17530,5
Non-responder	Macro_NLRP3	Macro_NLRP3	TIMP2	CD44	6,72818E-03	13971,5	6,53304E-03	19592	5,01081E-01	7957	1,32355E+00	7648	8,69493E-01	17130	0	17530,5
Non-responder	Mono_CD14	pDC_LILRA4	CXCL8	CD79A	6,72898E-03	14938,3	6,02950E-02	445	7,10188E-01	3517	1,68771E+00	3869	7,32066E-01	49330	0	17530,5
Non-responder	Macro_OLFML3	pDC_LILRA4	MAML2	NOTCH4	6,73118E-03	19410,7	2,04042E-02	2968	4,97003E-01	8093	1,31369E+00	7785	6,64986E-01	60677	0	17530,5
Non-responder	Mono_CD16	Mono_INHBA	ICAM2	ITGAM_ITGB2	6,73162E-03	13397,5	1,15152E-02	7855	4,26169E-01	10784	1,09660E+00	11224	8,59086E-01	19594	0	17530,5
Non-responder	Mono_INHBA	Macro_FOLR2+APOE+	HBEGF	CD44	6,73162E-03	13302,5	7,46152E-03	15806	4,62845E-01	9327	9,51709E-01	14228	8,98508E-01	9621	0	17530,5
Non-responder	Macro_OLFML3	Mono_CD16	HSP90B1	ASGR1	6,73505E-03	14513,9	9,08387E-03	11437	3,69433E-01	13539	1,13940E+00	10467	8,59074E-01	19596	0	17530,5
Non-responder	Macro_FOLR2+APOE+	Macro_LYVE1	HLA-A	APLP2	6,74469E-03	12316,9	5,36292E-03	26552	5,50804E-01	6482	1,18623E+00	9686	9,38232E-01	1334	0	17530,5
Non-responder	cDC(CD1C)	Macro_IER3	HLA-DQA2	CD4	6,74881E-03	14562,7	6,70659E-03	18767	4,44803E-01	10027	7,37102E-01	19604	9,10700E-01	6885	0	17530,5
Non-responder	pDC_LILRA4	Macro_FOLR2+APOE+	HLA-F	LILRB2	6,75010E-03	7637,9	1,01099E-02	9688	7,67034E-01	2844	2,31126E+00	1101	9,10109E-01	7026	0	17530,5
Non-responder	Macro_ISG15	Macro_ISG15	HLA-F	LILRB1	6,75551E-03	7757,1	1,22755E-02	7082	7,76683E-01	2755	2,09421E+00	1728	8,98194E-01	9690	0	17530,5
Non-responder	Macro_FOLR2+APOE+	Macro_ISG15	FN1	CD44	6,75570E-03	13749,3	9,82075E-03	10134	3,55490E-01	14323	1,35894E+00	7151	8,59040E-01	19608	0	17530,5
Non-responder	Mono_INHBA	Macro_FOLR2+APOE+	SERPINA1	LRP1	6,75914E-03	12929,9	8,04712E-03	13994	4,26445E-01	10768	8,68656E-01	16151	9,13778E-01	6206	0	17530,5
Non-responder	Mono_INHBA	Macro_FOLR2+APOE+	TGFB1	ENG	6,76259E-03	16465,9	8,10024E-03	13831	3,53138E-01	14466	8,22553E-01	17345	8,60921E-01	19157	0	17530,5
Non-responder	Macro_OLFML3	Macro_FOLR2+APOE+	CALR	ITGAV	6,76432E-03	14171,9	8,35076E-03	13155	3,88498E-01	12548	1,29746E+00	8013	8,59023E-01	19613	0	17530,5
Non-responder	Macro_NLRP3	Macro_ISG15	HLA-A	LILRB1	6,76949E-03	13658,1	6,89796E-03	17945	2,73531E-01	19616	1,16446E+00	10065	9,29320E-01	3134	0	17530,5
Non-responder	Macro_IFI27	Macro_FOLR2+APOE+	HLA-DMA	CD4	6,77122E-03	12444,5	7,89562E-03	14428	5,00082E-01	7989	7,36640E-01	19617	9,31490E-01	2658	0	17530,5
Non-responder	Mono_INHBA	Macro_FOLR2+APOE+	GNAI2	C5AR1	6,77294E-03	13411,5	7,00825E-03	17500	4,27597E-01	10732	1,02839E+00	12581	9,02555E-01	8714	0	17530,5
Non-responder	cDC(CD1C)	Mono_INHBA	VIM	CD44	6,77640E-03	12561,9	6,57973E-03	19360	5,63956E-01	6130	7,36496E-01	19620	9,51073E-01	169	0	17530,5
Non-responder	Macro_FOLR2+APOE+	pDC_LILRA4	HLA-DQA2	CD4	6,77812E-03	12713,5	6,52758E-03	19621	3,07613E-01	17197	2,00037E+00	2090	9,09594E-01	7129	0	17530,5
Non-responder	Macro_FOLR2+APOE+	Macro_OLFML3	HLA-DMA	CD4	6,77990E-03	11323,1	6,49541E-03	19779	4,53138E-01	9699	1,49189E+00	5622	9,24993E-01	3985	0	17530,5
Non-responder	cDC(CD1C)	Macro_ISG15	HMGB1	TLR2	6,78676E-03	16795,3	6,52648E-03	19626	2,92590E-01	18202	8,14970E-01	17555	8,92707E-01	11063	0	17530,5
Non-responder	Macro_FOLR2+APOE+	Macro_NLRP3	MIF	TNFRSF10D	6,79348E-03	14551,5	1,34933E-02	6002	4,53104E-01	9704	1,27953E+00	8275	8,11044E-01	31246	0	17530,5
Non-responder	Macro_OLFML3	Macro_IER3	FN1	C5AR1	6,79891E-03	12378,7	1,39503E-02	5684	5,04075E-01	7838	1,18504E+00	9706	8,52616E-01	21135	0	17530,5
Non-responder	Macro_ISG15	pDC_LILRA4	HLA-B	CANX	6,80027E-03	8490,5	6,50318E-03	19744	6,81851E-01	3883	2,32314E+00	1080	9,50285E-01	215	0	17530,5
Non-responder	Macro_LYVE1	Mono_CD14	PLAU	PLAUR	6,80407E-03	14545,7	8,52921E-03	12686	4,59861E-01	9428	9,84596E-01	13448	8,58931E-01	19636	0	17530,5
Non-responder	Macro_OLFML3	Macro_LYVE1	HLA-DQA1	CD4	6,80407E-03	13203,3	6,52450E-03	19636	4,32855E-01	10502	8,90705E-01	15613	9,31078E-01	2735	0	17530,5
Non-responder	Macro_ISG15	Macro_ISG15	HLA-DPA1	LAG3	6,80538E-03	7582,9	1,76574E-02	3786	8,05300E-01	2496	1,68574E+00	3884	8,96098E-01	10218	0	17530,5
Non-responder	Mono_INHBA	Macro_FOLR2+APOE+	CD52	SIGLEC10	6,80580E-03	15280,5	7,92253E-03	14358	4,28810E-01	10670	8,31146E-01	17127	8,70987E-01	16717	0	17530,5
Non-responder	Macro_FOLR2+APOE+	Macro_ISG15	SERPINA1	LRP1	6,81274E-03	13133,5	6,52405E-03	19641	3,72313E-01	13372	1,37247E+00	6981	9,05145E-01	8143	0	17530,5
Non-responder	Macro_IER3	Macro_ISG15	HLA-B	LILRB2	6,81968E-03	11649,9	6,52305E-03	19645	4,73122E-01	8942	1,07796E+00	11568	9,45123E-01	564	0	17530,5
Non-responder	Macro_NLRP3	Macro_NLRP3	ADM	ADRB2	6,82069E-03	18778,7	1,44083E-02	5368	4,52936E-01	9714	1,42457E+00	6356	7,02364E-01	54925	0	17530,5
Non-responder	Macro_FOLR2+APOE+	Macro_LYVE1	CD14	ITGA4	6,82315E-03	12956,7	6,52296E-03	19647	4,93238E-01	8220	1,22608E+00	9035	8,95537E-01	10351	0	17530,5
Non-responder	Mono_INHBA	Macro_FOLR2+APOE+	CD14	ITGB1	6,82489E-03	16480,9	6,52253E-03	19648	3,11456E-01	16941	7,97413E-01	18030	8,95948E-01	10255	0	17530,5
Non-responder	Macro_IER3	pDC_LILRA4	S100A8	CD36	6,82662E-03	15797,5	8,80374E-03	12034	3,52015E-01	14535	9,07381E-01	15239	8,58863E-01	19649	0	17530,5
Non-responder	Macro_NLRP3	Macro_NLRP3	VCAN	TLR1	6,84526E-03	13647,1	1,00912E-02	9723	8,08223E-01	2472	1,66102E+00	4102	7,97739E-01	34408	0	17530,5
Non-responder	Mono_INHBA	Macro_FOLR2+APOE+	PTGS2	ALOX5	6,84575E-03	9736,5	1,40837E-02	5582	6,48861E-01	4410	1,44555E+00	6090	8,77461E-01	15070	0	17530,5
Non-responder	Macro_FOLR2+APOE+	Macro_ISG15	ICAM1	IL2RG	6,84750E-03	14754,7	6,52008E-03	19661	4,31482E-01	10550	1,32190E+00	7677	8,64388E-01	18355	0	17530,5
Non-responder	Macro_FOLR2+APOE+	Mono_INHBA	SEMA4A	LILRB2	6,85098E-03	12820,7	9,65336E-03	10418	5,49906E-01	6507	1,16917E+00	9985	8,58780E-01	19663	0	17530,5
Non-responder	Mono_CD14	Mono_CD16	VCAN	ITGB1	6,85149E-03	6801,7	1,74017E-02	3893	1,05382E+00	959	2,14938E+00	1521	8,96516E-01	10105	0	17530,5
Non-responder	Macro_NLRP3	Macro_FOLR2+APOE-	THBS1	ITGA4	6,85446E-03	13252,3	9,78132E-03	10214	6,64678E-01	4145	9,29974E-01	14707	8,58769E-01	19665	0	17530,5
Non-responder	Macro_OLFML3	pDC_LILRA4	C3	NRP1	6,85619E-03	11311,1	1,60145E-02	4469	4,52636E-01	9727	1,57528E+00	4805	8,57232E-01	20024	0	17530,5
Non-responder	pDC_LILRA4	Mono_CD14	CIRBP	TREM1	6,86167E-03	8094,1	1,27305E-02	6625	6,18482E-01	4957	2,11830E+00	1629	8,98060E-01	9729	0	17530,5
Non-responder	Macro_OLFML3	Mono_CD14	FN1	CD44	6,87540E-03	15495,7	9,76735E-03	10237	3,25756E-01	16061	9,62083E-01	13973	8,58709E-01	19677	0	17530,5

Non-responder	Macro_LYVE1	Macro_NLRP3	MRC1	PTPRC	6,87715E-03	14031,3	1,00893E-02	9732	4,57247E-01	9530	9,74559E-01	13686	8,58708E-01	19678	0	17530,5
Non-responder	Macro_LYVE1	Mono_CD14	CIRBP	TREM1	6,87890E-03	14743,3	9,56776E-03	10555	3,85620E-01	12687	7,34339E-01	19679	8,84224E-01	13265	0	17530,5
Non-responder	Mono_INHBA	Macro_FOLR2+APOE+	NAMPT	ITGA5_ITGB1	6,87890E-03	11237,3	9,15537E-03	11309	5,86296E-01	5597	1,22139E+00	9123	8,86707E-01	12627	0	17530,5
Non-responder	Mono_CD16	Macro_ISG15	S100A9	TLR4	6,87890E-03	15674,5	7,84753E-03	14565	4,01907E-01	11899	9,30286E-01	14699	8,58703E-01	19679	0	17530,5
Non-responder	Macro_NLRP3	Mono_CD14	SERPINA1	LRP1	6,87890E-03	15129,9	6,51681E-03	19679	3,48205E-01	14742	8,94500E-01	15541	9,05097E-01	8157	0	17530,5
Non-responder	Mono_INHBA	Macro_FOLR2+APOE+	VEGFA	CD44	6,88239E-03	8460,3	1,38695E-02	5740	7,76959E-01	2752	1,43628E+00	6209	8,96650E-01	10070	0	17530,5
Non-responder	Macro_IER3	Mono_CD14	FN1	PLAUR	6,88239E-03	14802,5	9,03118E-03	11557	3,91370E-01	12399	1,01552E+00	12845	8,58697E-01	19681	0	17530,5
Non-responder	Macro_FOLR2+APOE+	Macro_OLFML3	ICAM1	ITGAX_ITGB2	6,88239E-03	13369,9	6,51624E-03	19681	4,32399E-01	10515	1,18240E+00	9750	8,99555E-01	9373	0	17530,5
Non-responder	Mono_INHBA	Macro_FOLR2+APOE+	LGALS3	ENG	6,88414E-03	11861,1	9,03402E-03	11552	4,25999E-01	10793	1,04471E+00	12241	9,09311E-01	7189	0	17530,5
Non-responder	Mono_CD16	Macro_IFI27	ICAM2	ITGAM_ITGB2	6,88939E-03	14740,9	1,20751E-02	7258	4,37206E-01	10317	7,34177E-01	19685	8,61935E-01	18914	0	17530,5
Non-responder	Macro_OLFML3	Mono_CD16	FN1	PLAUR	6,89464E-03	16300,9	9,02265E-03	11574	2,83599E-01	18861	9,67157E-01	13851	8,58640E-01	19688	0	17530,5
Non-responder	Macro_LYVE1	Macro_FOLR2+APOE+	HLA-A	LILRB2	6,89639E-03	11788,5	6,51408E-03	19689	5,44781E-01	6635	9,52395E-01	14208	9,41715E-01	880	0	17530,5
Non-responder	Mono_CD16	Macro_ISG15	LTB	CD40	6,89732E-03	15104,7	1,17490E-02	7595	4,82561E-01	8605	1,18292E+00	9742	8,07590E-01	32051	0	17530,5
Non-responder	Macro_FOLR2+APOE+	Macro_OLFML3	HLA-G	LILRB1	6,89732E-03	14213,1	1,02614E-02	9468	4,52264E-01	9742	1,46732E+00	5872	8,22582E-01	28453	0	17530,5
Non-responder	Mono_CD16	Macro_FOLR2+APOE+	S100A8	CD36	6,89815E-03	15960,1	8,77126E-03	12116	3,47674E-01	14774	8,87567E-01	15690	8,58639E-01	19690	0	17530,5
Non-responder	Mono_CD16	Mono_CD14	HLA-C	LILRA1	6,89815E-03	15181,3	7,62346E-03	15246	2,72513E-01	19690	1,08265E+00	11468	8,89127E-01	11972	0	17530,5
Non-responder	Mono_CD16	Macro_OLFML3	HLA-B	LILRB1	6,90340E-03	11540,5	6,51284E-03	19693	4,22458E-01	10936	1,37905E+00	6902	9,31549E-01	2641	0	17530,5
Non-responder	Mono_INHBA	Macro_NLRP3	TNF	TNFRSF1B	6,90516E-03	7345,9	1,52817E-02	4853	8,64729E-01	1958	2,06879E+00	1826	8,94762E-01	10562	0	17530,5
Non-responder	Macro_IER3	cDC_CLEC9A	ICAM1	ITGAL_ITGB2	6,91042E-03	13562,7	6,51176E-03	19697	4,56238E-01	9571	1,17882E+00	9817	8,92194E-01	11198	0	17530,5
Non-responder	Macro_LYVE1	Macro_LYVE1	A2M	LRP1	6,91919E-03	11271,5	1,47275E-02	5164	6,02982E-01	5249	7,33405E-01	19702	9,02556E-01	8712	0	17530,5
Non-responder	Mono_CD16	Macro_FOLR2+APOE+	S100A8	TLR4	6,92446E-03	14782,5	9,59892E-03	10495	4,20484E-01	11032	9,10840E-01	15150	8,58586E-01	19705	0	17530,5
Non-responder	Macro_IER3	Mono_CD14	PLAU	PLAUR	6,93149E-03	14305,7	8,47980E-03	12821	4,58012E-01	9500	1,05897E+00	11968	8,58579E-01	19709	0	17530,5
Non-responder	Mono_CD16	Macro_LYVE1	ICAM4	ITGAV	6,93587E-03	17727,1	3,54416E-02	1178	6,78728E-01	3925	1,18212E+00	9756	6,94500E-01	56246	0	17530,5
Non-responder	Macro_FOLR2+APOE-	pDC_LILRA4	CXCL12	CD4	6,93914E-03	10012,7	2,31811E-02	2337	7,49986E-01	3025	1,68264E+00	3910	8,43696E-01	23261	0	17530,5
Non-responder	cDC_LAMP3	Mono_CD16	ICAM1	ITGAL_ITGB2	6,94205E-03	13520,7	6,50903E-03	19715	5,35310E-01	6886	1,04316E+00	12269	8,92174E-01	11203	0	17530,5
Non-responder	Macro_IER3	cDC_CLEC9A	LYZ	ITGAL	6,94381E-03	15945,5	7,43728E-03	15884	3,30352E-01	15784	7,32752E-01	19716	8,93686E-01	10813	0	17530,5
Non-responder	Macro_FOLR2+APOE+	Macro_ISG15	TIMP2	ITGB1	6,94416E-03	12657,5	1,01444E-02	9636	4,51980E-01	9759	1,60139E+00	4578	8,49827E-01	21784	0	17530,5
Non-responder	pDC_LILRA4	pDC_LILRA4	PSEN1	NOTCH4	6,94432E-03	12487,9	3,72429E-02	1094	6,79400E-01	3911	2,18721E+00	1415	7,80181E-01	38489	0	17530,5
Non-responder	Mono_INHBA	Macro_NLRP3	CD14	TLR4	6,94557E-03	17430,3	7,52694E-03	15580	3,02690E-01	17523	7,98445E-01	18003	8,63652E-01	18515	0	17530,5
Non-responder	Macro_FOLR2+APOE+	Macro_FOLR2-APOE+	LGALS3BP	ITGB1	6,96144E-03	14288,9	1,11546E-02	8281	5,45261E-01	6622	7,48637E-01	19285	8,58522E-01	19726	0	17530,5
Non-responder	Macro_OLFML3	Macro_NLRP3	NAMPT	ITGA5_ITGB1	6,96144E-03	15466,1	6,50670E-03	19726	3,95885E-01	12197	1,13883E+00	10479	8,68388E-01	17398	0	17530,5
Non-responder	Macro_FOLR2-APOE+	Mast	GRN	NTRK1	6,96572E-03	14453,5	7,06731E-02	316	1,07490E+00	878	1,39111E-01	39117	8,79864E-01	14426	0	17530,5
Non-responder	Macro_NLRP3	cDC(CD1C)	CD52	SIGLEC10	6,97026E-03	16020,9	7,92048E-03	14360	4,05111E-01	11756	7,32407E-01	19731	8,70973E-01	16727	0	17530,5
Non-responder	Macro_FOLR2+APOE+	Macro_LYVE1	HLA-DPA1	CD4	6,97458E-03	11630,3	6,32156E-03	20615	4,51798E-01	9770	1,19573E+00	9533	9,43449E-01	703	0	17530,5
Non-responder	Macro_NLRP3	Macro_FOLR2-APOE+	VCAN	CD44	6,97735E-03	15232,9	1,00543E-02	9771	7,43147E-01	3107	1,75008E-01	37862	9,06198E-01	7894	0	17530,5
Non-responder	Mono_INHBA	Macro_NLRP3	FN1	C5AR1	6,97910E-03	9134,7	1,97888E-02	3125	6,20489E-01	4913	1,67608E+00	3966	8,73258E-01	16139	0	17530,5
Non-responder	Macro_FOLR2+APOE+	Macro_FOLR2+APOE-	ENTPD1	ADORA3	6,98618E-03	13509,9	1,67593E-02	4121	5,30484E-01	7033	7,55217E-01	19125	8,58463E-01	19740	0	17530,5
Non-responder	Mono_CD16	Mono_CD14	SELPLG	ITGAM	6,99677E-03	13163,7	1,05161E-02	9120	4,51646E-01	9778	1,39219E+00	6745	8,46285E-01	22645	0	17530,5
Non-responder	Mono_INHBA	Macro_NLRP3	IL1B	SIGIRR	6,99857E-03	10902,7	9,89957E-03	10012	6,63736E-01	4167	1,61610E+00	4454	8,64443E-01	18350	0	17530,5
Non-responder	Mono_INHBA	Macro_NLRP3	S100A8	TLR4	7,00034E-03	11481,3	1,38008E-02	5785	6,32649E-01	4675	9,24976E-01	14818	8,79227E-01	14598	0	17530,5
Non-responder	Macro_FOLR2+APOE+	Macro_ISG15	GRN	SORT1	7,00034E-03	13199,7	7,57123E-03	15414	4,81454E-01	8646	1,59307E+00	4660	8,58426E-01	19748	0	17530,5
Non-responder	Macro_FOLR2+APOE+	cDC(CD1C)	SERPING1	LRP1	7,00389E-03	14121,9	6,67458E-03	18910	5,83176E-01	5661	1,24565E+00	8758	8,58417E-01	19750	0	17530,5
Non-responder	Mono_INHBA	Macro_NLRP3	S100A9	TLR4	7,01098E-03	10714,1	1,26503E-02	6695	6,53596E-01	4336	1,05588E+00	12028	8,85269E-01	12981	0	17530,5
Non-responder	cDC_CLEC9A	pDC_LILRA4	HLA-DPA1	CD4	7,01282E-03	7738,9	7,68528E-03	15053	6,21064E-01	4901	2,43304E+00	881	9,48440E-01	329	0	17530,5
Non-responder	Macro_IER3	Macro_IER3	CCL3	CCR1	7,01631E-03	12345,3	1,02528E-02	9479	6,31083E-01	4708	1,15306E+00	10252	8,58391E-01	19757	0	17530,5
Non-responder	Macro_LYVE1	Macro_IER3	MRC1	PTPRC	7,01809E-03	14764,1	1,05665E-02	9052	4,86645E-01	8446	7,31294E-01	19758	8,61489E-01	19034	0	17530,5
Non-responder	Mono_CD14	cDC(CD1C)	VCAN	TLR2	7,02230E-03	7668,5	1,73142E-02	3926	1,01391E+00	1144	1,70043E+00	3783	8,89203E-01	11959	0	17530,5
Non-responder	Mono_INHBA	Macro_NLRP3	SPP1	ITGA5_ITGB1	7,02697E-03	9262,5	2,31271E-02	2347	6,44903E-01	4475	1,64143E+00	4262	8,67126E-01	17698	0	17530,5
Non-responder	Macro_ISG15	Mono_CD14	CCL2	CCR1	7,02751E-03	11923,7	2,09922E-02	2813	6,78656E-01	3927	2,59920E+00	626	7,96415E-01	34722	0	17530,5
Non-responder	pDC_LILRA4	Macro_NLRP3	APP	LRP10	7,02856E-03	9876,9	1,37671E-02	5810	1,07408E+00	882	2,73014E+00	480	8,37848E-01	24682	0	17530,5
Non-responder	Macro_FOLR2+APOE+	cDC_CLEC9A	APP	RPSA	7,03053E-03	13251,7	6,49895E-03	19765	5,14628E-01	7505	1,61894E+00	4432	8,69846E-01	17026	0	17530,5
Non-responder	Macro_OLFML3	Macro_ISG15	MRC1	PTPRC	7,03408E-03	11667,7	1,00290E-02	9810	5,24834E-01	7189	1,66883E+00	4042	8,58344E-01	19767	0	17530,5
Non-responder	Macro_IFI27	Macro_FOLR2+APOE+	HLA-DRB5	CD4	7,03764E-03	11895,5	7,76567E-03	14811	5,39266E-01	6784	7,30959E-01	19769	9,44902E-01	583	0	17530,5
Non-responder	Macro_ISG15	Macro_ISG15	HLA-DPB1	LAG3	7,03796E-03	11753,8	1,73128E-02	3929	7,85699E-01	2677	1,68378E+00	3898	8,90301E-01	11671	0,002	36594
Non-responder	Macro_OLFML3	cDC(CD1C)	LGALS9	HAVCR2	7,04120E-03	16707,9	6,49793E-03	19771	3,49575E-01	14669	8,82889E-01	15792	8,74663E-01	15777	0	17530,5
Non-responder	cDC_CLEC9A	pDC_LILRA4	ICAM1	IL2RG	7,04131E-03	7437,7	1,23874E-02	6949	8,32670E-01	2256	2,57243E+00	659	8,97809E-01	9794	0	17530,5
Non-responder	Macro_NLRP3	Macro_ISG15	GNAI2	FPR1	7,04299E-03	14223,9	6,49741E-03	19772	3,24870E-01	16122	1,31459E+00	7774	8,97256E-01	9921	0	17530,5
Non-responder	Macro_FOLR2+APOE+	cDC_CLEC9A	PDCD1LG2	PDCD1	7,04841E-03	21421,9	7,08363E-02	314	6,78344E-01	3931	1,02849E+00	12579	4,90611E-01	72755	0	17530,5
Non-responder	cDC_LAMP3	cDC_CLEC9A	APP	RPSA	7,05368E-03	14553,9	6,49541E-03	19778	5,14481E-01	7514	1,11478E+00	10910	8,69815E-01	17037	0	17530,5
Non-responder	pDC_LILRA4	cDC(CD1C)	APP	TNFRSF21	7,06009E-03	10658,9	1,42051E-02	5502	1,08698E+00	821	2,42874E+00	884	8,22160E-01	28557	0	17530,5
Non-responder	Macro_IER3	Macro_ISG15	F13A1	ITGB1	7,06081E-03	14091,1	9,63773E-03	10434	4,28349E-01	10695	1,05654E+00	12014	8,58280E-01	19782	0	17530,5
Non-responder	cDC_LAMP3	pDC_LILRA4	GSTP1	TRAF2	7,07868E-03	10282,3	1,62782E-02	4350	6,09811E-01	5101	1,59578E+00	4638	8,58221E-01	19792	0	17530,5
Non-responder	Mono_CD14	cDC(CD1C)	VCAN	TLR1	7,07984E-03	10016,9	1,72926E-02	3937	1,04368E+00	1000	1,83504E+00	2913	8,37744E-01	24704	0	17530,5
Non-responder	Mono_CD16	cDC_CLEC9A	GNAI2	CXCR3	7,08325E-03	12086,9	1,56482E-02	4670	4,50967E-01	9809	1,46080E+00	5936	8,46887E-01	22489	0	17530,5
Non-responder	Mono_INHBA	Macro_NLRP3	PLAU	PLAUR	7,08762E-03	11870,5	8,42942E-03	12955	7,20541E-01	3369	1,48457E+00	5705	8,58217E-01	19793	0	17530,5
Non-responder	pDC_LILRA4	Macro_LYVE1	SELPLG	ITGB2	7,08886E-03	7660,1	1,00281E-02	9811	6,56268E-01	4287	1,82137E+00	2994	9,26549E-01	3678	0	17530,5
Non-responder	Mono_CD16	Macro_ISG15	TNFSF10	TNFRSF10D	7,09447E-03	19063,3	1,05182E-02	9116	4,50775E-01	9813	1,50926E+00	5458	7,10806E-01	53399	0	17530,5
Non-responder	cDC(CD1C)	Macro_OLFML3	HLA-DQA1	CD4	7,09479E-03	11837,5	6,49142E-03	19801	4,93059E-01	8229	1,11697E+00	10858	9,30915E-01	2769	0	17530,5
Non-responder	Macro_NLRP3	pDC_LILRA4	INHBA	SMAD3	7,10008E-03	20396,7	2,53153E-02	2043	4,50699E-01	9815	1,71734E+00	3648	5,83556E-01	68947	0	17530,5
Non-responder	Mono_INHBA	Macro_NLRP3	GNAI2	C5AR1	7,10554E-03	13268,1	7,25300E-03	16528	4,52456E-01	9736	9,55186E-01	14142	9,04054E-01	8404	0	17530,5
Non-responder	Macro_LYVE1	pDC_LILRA4	PLAU	IGF2R	7,10609E-03	12823,9	1,73900E-02	3896	6,77486E-01	3942	2,02914E+00	1974	7,87504E-01	36777	0	17530,5

Post_NR_Myeloid_Myeloid_CellCell

Non-responder	Mono_INHBA	Macro_NLRP3	HMGB1	THBD	7,10913E-03	15649,9	7,19863E-03	16737	3,01426E-01	17598	1,01172E+00	12917	8,83525E-01	13467	0	17530,5
Non-responder	Macro_NLRP3	Macro_LYVE1	ICAM1	ITGAX_ITGB2	7,11093E-03	14641,9	6,48974E-03	19810	4,40723E-01	10197	8,63999E-01	16265	8,99370E-01	9407	0	17530,5
Non-responder	pDC_LILRA4	Macro_IFI27	SELPLG	ITGB2	7,11131E-03	8145,1	1,00208E-02	9819	6,55667E-01	4302	1,51563E+00	5390	9,26525E-01	3684	0	17530,5
Non-responder	cDC_CLEC9A	pDC_LILRA4	CXCL9	DPP4	7,11661E-03	16073,3	3,27801E-02	1333	6,77208E-01	3944	1,77544E+00	3256	7,05916E-01	54303	0	17530,5
Non-responder	Macro_OLFML3	Macro_FOLR2-APOE+	CXCL10	TLR4	7,11661E-03	10287,5	1,72671E-02	3944	9,91108E-01	1242	1,95654E+00	2270	8,30529E-01	26451	0	17530,5
Non-responder	Macro_NLRP3	Macro_ISG15	S100A12	TLR4	7,11975E-03	15978,5	1,44261E-02	5353	4,50481E-01	9822	1,43404E+00	6230	7,69811E-01	40957	0	17530,5
Non-responder	Macro_IER3	pDC_LILRA4	MRC1	PTPRC	7,12170E-03	12027,9	9,98700E-03	9867	4,70372E-01	9053	1,68732E+00	3873	8,58089E-01	19816	0	17530,5
Non-responder	Macro_IFI27	pDC_LILRA4	COPA	P2RY6	7,12334E-03	10163,7	1,32412E-02	6178	1,07259E+00	888	2,44226E+00	862	8,34986E-01	25360	0	17530,5
Non-responder	Macro_IER3	Mono_CD16	VCAN	ITGA4	7,12350E-03	13545,9	8,55226E-03	12634	4,80853E-01	8668	1,22365E+00	9080	8,58086E-01	19817	0	17530,5
Non-responder	Mono_INHBA	Macro_NLRP3	VIM	CD44	7,12350E-03	8675,3	7,67840E-03	15078	7,60528E-01	2914	1,31356E+00	7786	9,54544E-01	68	0	17530,5
Non-responder	Mono_INHBA	Macro_NLRP3	TIMP2	CD44	7,12889E-03	13035,5	7,30495E-03	16345	5,35927E-01	6871	1,23484E+00	8912	8,75700E-01	15519	0	17530,5
Non-responder	Macro_IER3	Mono_CD14	VEGFA	CD44	7,13609E-03	15964,7	7,86461E-03	14517	4,38821E-01	10279	7,28685E-01	19824	8,67253E-01	17673	0	17530,5
Non-responder	Macro_OLFML3	cDC_LAMP3	TNFSF10	CCR6	7,13664E-03	17672,3	2,86638E-02	1681	6,21958E-01	4885	1,17814E+00	9828	7,05175E-01	54437	0	17530,5
Non-responder	Macro_FOLR2+APOE+	Macro_FOLR2+APOE+	TNF	TNFRSF21	7,13868E-03	24458,3	5,97190E-03	22542	2,40846E-01	22383	1,06542E+00	11822	7,38315E-01	48014	0	17530,5
Non-responder	Mono_CD16	Macro_IER3	ICAM2	ITGAL_ITGB2	7,13969E-03	13531,3	1,17910E-02	7545	4,42377E-01	10134	1,02639E+00	12621	8,58066E-01	19826	0	17530,5
Non-responder	Mono_INHBA	Macro_NLRP3	PKM	CD44	7,13969E-03	8453,1	8,20527E-03	13541	7,42696E-01	3117	1,38510E+00	6823	9,38731E-01	1254	0	17530,5
Non-responder	Mono_INHBA	Macro_NLRP3	PTGS2	ALOX5	7,14329E-03	11597,5	1,18015E-02	7533	5,72696E-01	5925	1,20212E+00	9427	8,67635E-01	17572	0	17530,5
Non-responder	cDC(CD1C)	pDC_LILRA4	SIRPA	CD47	7,16313E-03	10574,9	9,96010E-03	9906,5	6,84759E-01	3823,5	2,08001E+00	1774,5	8,58030E-01	19839,5	0	17530,5
Non-responder	Mono_INHBA	Macro_NLRP3	NAMPT	ITGA5_ITGB1	7,16313E-03	11027,1	8,99965E-03	11633	6,12817E-01	5042	1,29285E+00	8080	8,85842E-01	12850	0	17530,5
Non-responder	Mono_CD14	Macro_FOLR2+APOE+	THBS1	ITGA6	7,16407E-03	13448,5	3,65568E-02	1121	7,54604E-01	2974	1,67720E+00	3953	7,66752E-01	41664	0	17530,5
Non-responder	Macro_FOLR2+APOE+	pDC_LILRA4	ADAM10	NOTCH2	7,16486E-03	14389,3	1,04006E-02	9280	4,49963E-01	9838	1,53940E+00	5142	8,15572E-01	30156	0	17530,5
Non-responder	Mono_CD16	Mast	GPI	NTRK1	7,16768E-03	12646,7	7,34907E-02	286	1,01320E+00	1147	1,17759E+00	9839	7,97653E-01	34431	0	17530,5
Non-responder	Macro_FOLR2+APOE+	Macro_FOLR2+APOE+	CCL2	CCR1	7,16768E-03	17674,7	1,18721E-02	7472	4,49950E-01	9839	1,34975E+00	7301	7,46315E-01	46231	0	17530,5
Non-responder	Macro_ISG15	Mono_INHBA	VCAN	CD44	7,17616E-03	8894,1	9,99926E-03	9842	6,70773E-01	4051	1,54419E+00	5097	9,05965E-01	7950	0	17530,5
Non-responder	cDC(CD1C)	Macro_OLFML3	C3	IFITM1	7,17758E-03	17191,9	8,12130E-03	13780	2,88320E-01	18499	8,62763E-01	16303	8,57989E-01	19847	0	17530,5
Non-responder	Macro_FOLR2+APOE+	Macro_IFI27	CD59	STAB1	7,18301E-03	14294,7	1,04251E-02	9249	4,82729E-01	8601	7,27817E-01	19850	8,72868E-01	16243	0	17530,5
Non-responder	Macro_FOLR2+APOE+	Mono_CD16	C3	C5AR2	7,18466E-03	15305,9	2,45785E-02	2141	4,49723E-01	9845	1,53402E+00	5213	7,66227E-01	41800	0	17530,5
Non-responder	Mono_CD14	Macro_IER3	VCAN	ITGA4	7,18523E-03	7174,5	1,72281E-02	3957	1,07389E+00	883	1,78931E+00	3176	8,95636E-01	10326	0	17530,5
Non-responder	pDC_LILRA4	Macro_OLFML3	B2M	LILRB2	7,18523E-03	9137,7	5,98017E-03	22503	6,76215E-01	3957	2,19830E+00	1386	9,48744E-01	312	0	17530,5
Non-responder	Mono_INHBA	Mono_CD16	TNF	TNFRSF1B	7,18663E-03	7178,7	1,56224E-02	4685	8,84021E-01	1823	2,13475E+00	1568	8,95796E-01	10287	0	17530,5
Non-responder	Mono_CD14	Mono_CD16	S100A8	CD36	7,18687E-03	7470,7	1,43860E-02	5386	1,07185E+00	892	2,50610E+00	760	8,86091E-01	12785	0	17530,5
Non-responder	Macro_ISG15	pDC_LILRA4	CXCL9	DPP4	7,19053E-03	15912,1	3,26501E-02	1345	6,75994E-01	3958	1,94117E+00	2350	7,05503E-01	54377	0	17530,5
Non-responder	Mono_INHBA	Mono_CD16	HLA-B	LILRB2	7,19568E-03	9255,7	7,72960E-03	14916	6,21942E-01	4886	1,25198E+00	8677	9,49361E-01	269	0	17530,5
Non-responder	Macro_FOLR2+APOE+	Mast	ST6GAL1	CD22	7,19583E-03	17657,3	6,16787E-02	424	6,75907E-01	3959	1,11333E+00	10937	6,99398E-01	55436	0	17530,5
Non-responder	Mono_INHBA	Mono_CD16	HLA-B	LILRB1	7,19749E-03	14888,7	6,58640E-03	19327	3,12877E-01	16857	7,92868E-01	18143	9,31907E-01	2586	0	17530,5
Non-responder	Mono_INHBA	Mono_CD16	ANXA1	FPR1	7,19931E-03	14358,1	7,34320E-03	16214	3,75182E-01	13229	7,91817E-01	18167	9,11850E-01	6650	0	17530,5
Non-responder	Mono_INHBA	Mono_CD16	HLA-A	LILRA1	7,20112E-03	9165,5	1,52327E-02	4869	5,10837E-01	7641	1,14383E+00	10403	9,17876E-01	5384	0	17530,5
Non-responder	Mast	Macro_IFI27	CSF1	SIRPA	7,20280E-03	11033,7	4,19870E-02	893	1,08478E+00	832	1,32077E+00	7694	8,23491E-01	28219	0	17530,5
Non-responder	Mono_INHBA	Mono_CD16	HLA-A	LILRB2	7,20293E-03	10025,3	7,45076E-03	15836	5,44056E-01	6662	1,19509E+00	9544	9,45294E-01	554	0	17530,5
Non-responder	Macro_OLFML3	Macro_LYVE1	HLA-DQB1	CD4	7,20293E-03	13316,7	6,48155E-03	19861	4,28186E-01	10706	8,67553E-01	16182	9,33417E-01	2304	0	17530,5
Non-responder	Macro_ISG15	Macro_ISG15	LILRB4	LAIR1	7,20450E-03	8061,9	9,99336E-03	9852	6,08898E-01	5128	1,98886E+00	2144	9,16419E-01	5655	0	17530,5
Non-responder	Macro_FOLR2+APOE+	Macro_FOLR2+APOE-	RARRES1	NRP2	7,20644E-03	19476,1	2,18220E-02	2613	7,46939E-01	3061	1,03791E+00	12379	6,56415E-01	61797	0	17530,5
Non-responder	Macro_FOLR2+APOE+	Macro_FOLR2+APOE-	C1QB	C1QBP	7,20733E-03	14127,3	5,36871E-03	26509	5,09048E-01	7697	1,17686E+00	9853	9,01054E-01	9047	0	17530,5
Non-responder	Macro_FOLR2+APOE+	Macro_NLRP3	CALR	ITGAV	7,20837E-03	12633,9	8,19807E-03	13563	4,90600E-01	8300	1,68222E+00	3912	8,57902E-01	19864	0	17530,5
Non-responder	Mono_INHBA	Mono_CD16	ICAM1	ITGAL_ITGB2	7,21563E-03	11561,9	7,11129E-03	17076	5,78897E-01	5759	1,35044E+00	7295	8,96358E-01	10149	0	17530,5
Non-responder	Macro_NLRP3	Macro_ISG15	S100A12	CD36	7,21869E-03	15333,9	1,55148E-02	4730	4,49314E-01	9857	1,37338E+00	6970	7,84002E-01	37582	0	17530,5
Non-responder	Mono_CD16	Mast	HBEGF	CD82	7,22472E-03	11396,7	1,54314E-02	4764	7,14123E-01	3457	1,08897E+00	11359	8,57860E-01	19873	0	17530,5
Non-responder	Mono_INHBA	Mono_CD16	THBS1	ITGA4	7,22472E-03	11288,7	1,11283E-02	8324	5,70902E-01	5959	1,38928E+00	6768	8,66414E-01	17862	0	17530,5
Non-responder	Macro_FOLR2+APOE+	Mono_CD16	MMP2	PECAM1	7,22722E-03	19968,9	1,51222E-02	4938	4,49166E-01	9860	1,39831E+00	6670	6,63646E-01	60846	0	17530,5
Non-responder	Macro_ISG15	Macro_FOLR2+APOE+	TNFSF13B	CD40	7,22722E-03	7324,9	1,33003E-02	6135	8,68105E-01	1935	2,27928E+00	1164	8,97485E-01	9860	0	17530,5
Non-responder	Mono_INHBA	Macro_NLRP3	VCAN	ITGB1	7,23017E-03	16143,7	8,44136E-03	12924	4,30299E-01	10599	7,30193E-01	19789	8,57831E-01	19876	0	17530,5
Non-responder	Macro_FOLR2+APOE+	Macro_IER3	CCL5	CCRL2	7,23291E-03	19398,1	1,59021E-02	4535	4,49125E-01	9862	1,36971E+00	7018	6,83299E-01	58045	0	17530,5
Non-responder	Mono_CD14	Macro_NLRP3	AGTRAP	RACK1	7,23291E-03	8656,3	1,24993E-02	6841	7,52183E-01	2999	1,44877E+00	6049	8,97480E-01	9862	0	17530,5
Non-responder	Macro_IER3	cDC_LAMP3	TNFSF13	TNFRSF11B	7,23470E-03	11881,1	6,44483E-02	392	1,07054E+00	895	1,21445E+00	9237	8,10637E-01	31351	0	17530,5
Non-responder	Macro_FOLR2+APOE+	Macro_ISG15	HLA-B	CANX	7,23576E-03	13848,3	4,43673E-03	34629	4,49108E-01	9863	1,43793E+00	6179	9,40435E-01	1040	0	17530,5
Non-responder	Mono_CD14	cDC(CD1C)	VCAN	ITGA4	7,23831E-03	7196,3	1,72066E-02	3967	1,07339E+00	885	1,77533E+00	3258	8,95578E-01	10341	0	17530,5
Non-responder	Macro_ISG15	Macro_OLFML3	TNFSF13B	CD40	7,23860E-03	7460,9	1,32968E-02	6137	8,67933E-01	1937	2,06671E+00	1836	8,97473E-01	9864	0	17530,5
Non-responder	Macro_FOLR2+APOE+	cDC_CLEC9A	SPP1	ITGA4_ITGB1	7,24291E-03	11233,7	1,40468E-02	5607	4,01364E-01	11924	2,25574E+00	1224	8,57779E-01	19883	0	17530,5
Non-responder	Macro_IER3	pDC_LILRA4	PLAU	IGF2R	7,24363E-03	12801,7	1,72893E-02	3938	6,75638E-01	3968	2,10351E+00	1681	7,87017E-01	36891	0	17530,5
Non-responder	Mono_INHBA	Mono_CD16	CD14	ITGA4	7,24474E-03	13804,7	7,31250E-03	16313	4,03694E-01	11817	9,50187E-01	14255	9,00762E-01	9108	0	17530,5
Non-responder	Mono_INHBA	Mono_CD16	HLA-C	LILRA1	7,24838E-03	9301,3	1,53365E-02	4823	4,87179E-01	8424	1,13053E+00	10620	9,19188E-01	5109	0	17530,5
Non-responder	Macro_FOLR2+APOE+	Mono_INHBA	HGF	CD44	7,25000E-03	12288,7	2,06234E-02	2914	6,63211E-01	4173	1,17577E+00	9868	8,28572E-01	26958	0	17530,5
Non-responder	Mono_INHBA	Mono_CD16	HLA-C	LILRB2	7,25020E-03	10151,9	7,50152E-03	15675	5,20398E-01	7312	1,18179E+00	9759	9,46194E-01	483	0	17530,5
Non-responder	Mono_INHBA	Mono_CD16	HLA-C	LILRA3	7,25203E-03	9406,7	1,81600E-02	3602	4,27878E-01	10722	1,34240E+00	7385	9,06664E-01	7794	0	17530,5
Non-responder	Macro_NLRP3	Macro_FOLR2-APOE+	VEGFA	ITGB1	7,25203E-03	12162,1	1,44221E-02	5357	8,01652E-01	2538	8,96254E-01	15497	8,57762E-01	19888	0	17530,5
Non-responder	Macro_FOLR2+APOE+	Macro_FOLR2-APOE+	APOE	ABCA1	7,25203E-03	12918,1	9,47916E-03	10706	4,60662E-01	9402	7,26332E-01	19888	9,09897E-01	7064	0	17530,5
Non-responder	Macro_ISG15	cDC_CLEC9A	CXCL9	DPP4	7,25428E-03	15772,7	3,54159E-02	1180	7,23149E-01	3335	1,67576E+00	3970	7,13879E-01	52848	0	17530,5
Non-responder	Mono_INHBA	Mono_CD16	CD99	PILRA	7,25932E-03	13507,9	7,44212E-03	15865	3,81734E-01	12891	1,00921E+00	12960	9,04526E-01	8293	0	17530,5
Non-responder	Macro_FOLR2+APOE+	Macro_FOLR2+APOE+	SPP1	ITGAV_ITGB1	7,25961E-03	11000,7	1,71988E-02	3971	4,45616E-01	9998	2,41348E+00	909	8,46477E-01	22595	0	17530,5
Non-responder	pDC_LILRA4	Macro_IER3	APP	TSPAN15	7,26668E-03	12583,5	1,58374E-02	4568	1,07027E+00	897	2,54814E+00	701	7,77060E-01	39221	0	17530,5
Non-responder	cDC(CD1C)	Mono_CD14	ANXA1	FPR1	7,26845E-03	13816,3	7,84058E-03	14590	4,21617E-01	10978	7,26135E-01	19897	9,14448E-01	6086	0	17530,5

Post_NR_Myeloid_Myeloid_CellCell

Non-responder	Mono_INHBA	Mono_CD16	B2M	LILRB2	7,27210E-03	10477,1	7,48918E-03	15721	5,08597E-01	7712	1,08973E+00	11338	9,53947E-01	84	0	17530,5
Non-responder	Mono_INHBA	Macro_OLFML3	CCL3	CCR5	7,28630E-03	10324,3	2,00727E-02	3044	7,43928E-01	3101	1,67521E+00	3976	8,40765E-01	23970	0	17530,5
Non-responder	Mono_INHBA	Mono_CD16	FN1	CD44	7,28856E-03	13178,7	1,23774E-02	6963	3,70310E-01	13497	1,07730E+00	11579	8,72475E-01	16324	0	17530,5
Non-responder	Mono_INHBA	Mono_CD16	FN1	C5AR1	7,29039E-03	8930,9	1,98600E-02	3105	6,23139E-01	4864	1,80912E+00	3060	8,73457E-01	16095	0	17530,5
Non-responder	Mono_INHBA	Mono_CD16	FN1	PLAUR	7,29223E-03	11809,3	1,31389E-02	6282	4,19591E-01	11068	1,18100E+00	9771	8,79950E-01	14395	0	17530,5
Non-responder	Mono_INHBA	Mono_CD16	IL1B	SIGIRR	7,29955E-03	8133,9	1,44043E-02	5372	7,84756E-01	2686	2,01743E+00	2013	8,84955E-01	13068	0	17530,5
Non-responder	Mono_INHBA	Mono_CD16	S100A8	ITGB2	7,30322E-03	10758,9	9,33096E-03	10982	5,86665E-01	5587	7,81825E-01	18431	9,38670E-01	1264	0	17530,5
Non-responder	Macro_IER3	Macro_FOLR2+APOE+	CD99	PILRA	7,30872E-03	16411,7	6,46764E-03	19919	3,00508E-01	17653	8,25417E-01	17285	8,98292E-01	9671	0	17530,5
Non-responder	Mono_INHBA	Mono_CD16	S100A9	ITGB2	7,31239E-03	10252,7	8,55308E-03	12630	6,07613E-01	5151	9,12728E-01	15102	9,41934E-01	850	0	17530,5
Non-responder	Macro_IER3	Macro_ISG15	TNFSF13B	TFRC	7,31239E-03	17076,1	6,46748E-03	19921	3,46447E-01	14841	9,95833E-01	13213	8,57854E-01	19875	0	17530,5
Non-responder	Macro_IER3	cDC_LAMP3	TNFSF10	TNFRSF11B	7,31476E-03	14864,3	5,29035E-02	595	1,06871E+00	900	1,09455E+00	11258	7,55924E-01	44038	0	17530,5
Non-responder	Macro_FOLR2+APOE+	Macro_ISG15	TGFB1	SDC2	7,31577E-03	14871,5	1,24350E-02	6908	4,48434E-01	9891	1,59749E+00	4615	7,93467E-01	35413	0	17530,5
Non-responder	cDC(CD1C)	Macro_OLFML3	HLA-DQB1	CD4	7,31606E-03	11552,7	6,46729E-03	19923	5,00165E-01	7987	1,16839E+00	9997	9,33349E-01	2326	0	17530,5
Non-responder	Mono_INHBA	Mono_CD16	LGALS1	PTPRC	7,31974E-03	9670,3	6,97730E-03	17622	6,26829E-01	4792	1,32708E+00	7591	9,42236E-01	816	0	17530,5
Non-responder	Mono_INHBA	Mono_CD16	SPP1	ITGA4_ITGB1	7,32341E-03	8765,9	2,09510E-02	2822	6,06826E-01	5168	1,66666E+00	4060	8,80467E-01	14249	0	17530,5
Non-responder	cDC_LAMP3	cDC_CLEC9A	TNFSF9	HLA-DPA1	7,32709E-03	14369,5	7,09619E-03	17125	4,47887E-01	9907	1,34513E+00	7356	8,57614E-01	19929	0	17530,5
Non-responder	Macro_LYVE1	Macro_FOLR2+APOE+	IL18	IL18BP	7,33076E-03	10605,9	1,65848E-02	4205	6,02721E-01	5257	1,44449E+00	6106	8,57603E-01	19931	0	17530,5
Non-responder	Mono_INHBA	Mast	PTGS2	ALOX5	7,33588E-03	7992,9	2,09830E-02	2816	8,79110E-01	1862	1,30934E+00	7858	8,97334E-01	9898	0	17530,5
Non-responder	Mono_CD16	Macro_FOLR2+APOE+	TLN1	ITGB5	7,33876E-03	14941,1	1,72129E-02	3962	4,48178E-01	9899	1,27803E+00	8297	7,95099E-01	35017	0	17530,5
Non-responder	Mono_INHBA	Mono_CD16	VCAN	ITGB1	7,33996E-03	15312,3	8,68866E-03	12317	4,41982E-01	10151	8,32252E-01	17092	8,59582E-01	19471	0	17530,5
Non-responder	Mono_INHBA	Mono_CD16	VCAN	ITGA4	7,34180E-03	9206,3	1,42854E-02	5453	7,25716E-01	3300	1,36375E+00	7081	8,86553E-01	12667	0	17530,5
Non-responder	Mast	Macro_NLRP3	ADCYAP1	GPR84	7,34691E-03	14529,9	1,12610E-01	99	1,06820E+00	902	1,57758E+00	4784	7,32037E-01	49334	0	17530,5
Non-responder	Macro_FOLR2+APOE+	cDC_CLEC9A	CALM3	MYLK	7,35027E-03	17744,3	4,61120E-02	748	6,59015E-01	4232	1,17401E+00	9903	6,94141E-01	56308	0	17530,5
Non-responder	Macro_NLRP3	Macro_ISG15	TNF	TNFRSF21	7,35315E-03	16141,9	1,01039E-02	9695	4,47970E-01	9904	1,41911E+00	6423	7,85862E-01	37157	0	17530,5
Non-responder	Macro_IER3	Mono_CD16	GNAI2	FPR1	7,35470E-03	16236,1	6,46375E-03	19944	3,04281E-01	17422	8,62737E-01	16305	8,97017E-01	9979	0	17530,5
Non-responder	Mono_CD14	cDC_CLEC9A	ITGB2	CD226	7,35597E-03	7599,9	3,10747E-02	1454	9,83978E-01	1283	1,67365E+00	3989	8,82518E-01	13743	0	17530,5
Non-responder	Mono_CD16	Mono_INHBA	NAMPT	ITGA5_ITGB1	7,36208E-03	16209,7	6,46288E-03	19948	4,20589E-01	11028	9,14678E-01	15058	8,68001E-01	17484	0	17530,5
Non-responder	pDC_LILRA4	Macro_IER3	LTB	CD40	7,36300E-03	9714,9	1,67429E-02	4130	1,06803E+00	903	2,88126E+00	350	8,33624E-01	25661	0	17530,5
Non-responder	Mono_CD16	Macro_ISG15	VCL	ITGB5	7,36467E-03	21032,5	1,94220E-02	3218	4,62593E-01	9330	1,17379E+00	9908	6,26778E-01	65176	0	17530,5
Non-responder	Macro_NLRP3	cDC_CLEC9A	LGALS3	LAG3	7,36756E-03	12771,9	1,41490E-02	5535	6,03570E-01	5238	1,17370E+00	9909	8,33697E-01	25647	0	17530,5
Non-responder	Macro_OLFML3	Macro_ISG15	HSP90B1	TLR2	7,36762E-03	16670,7	6,46259E-03	19951	2,69910E-01	19903	9,79317E-01	13575	8,87585E-01	12394	0	17530,5
Non-responder	Macro_IER3	Mono_CD14	CD14	ITGB2	7,37501E-03	12913,7	6,46244E-03	19955	4,28902E-01	10664	9,19138E-01	14958	9,37525E-01	1461	0	17530,5
Non-responder	Macro_FOLR2+APOE+	Macro_LYVE1	LRPAP1	SORL1	7,37685E-03	15917,7	7,91351E-03	14375	3,88578E-01	12546	9,09765E-01	15181	8,57488E-01	19956	0	17530,5
Non-responder	pDC_LILRA4	Mono_CD14	HSP90B1	TLR4	7,38488E-03	6805,1	1,45481E-02	5267	1,04827E+00	983	2,92607E+00	330	8,97277E-01	9915	0	17530,5
Non-responder	Mono_INHBA	Mono_CD16	GNAI2	C5AR1	7,38980E-03	12664,5	7,27908E-03	16437	4,55106E-01	9618	1,08822E+00	11373	9,04210E-01	8364	0	17530,5
Non-responder	Mono_CD16	Mono_CD16	S100A9	TLR4	7,39165E-03	16390,5	7,68902E-03	15045	3,94966E-01	12234	8,29399E-01	17179	8,57461E-01	19964	0	17530,5
Non-responder	Mono_INHBA	Mono_CD16	GNAI2	FPR1	7,39165E-03	14143,9	7,15674E-03	16896	3,75803E-01	13196	9,53240E-01	14192	9,01627E-01	8905	0	17530,5
Non-responder	Mast	cDC_CLEC9A	HDC	HRH2	7,39525E-03	12537,9	4,16749E-02	905	1,18422E+00	486	1,58704E+00	4715	7,77848E-01	39053	0	17530,5
Non-responder	Macro_IFI27	Macro_FOLR2+APOE+	HLA-DQA1	CD4	7,39536E-03	11975,7	7,96101E-03	14242	5,45407E-01	6617	7,23314E-01	19966	9,37196E-01	1523	0	17530,5
Non-responder	cDC_LAMP3	Macro_ISG15	HLA-C	LILRB1	7,40277E-03	14946,1	7,03374E-03	17392	2,69366E-01	19970	8,33360E-01	17058	9,30872E-01	2780	0	17530,5
Non-responder	Macro_OLFML3	Mono_CD14	LGALS9	LRP1	7,40277E-03	16908,9	6,46095E-03	19970	3,05746E-01	17325	9,49921E-01	14260	8,75964E-01	15459	0	17530,5
Non-responder	Mono_CD14	Macro_FOLR2+APOE-	VCAN	TLR2	7,40512E-03	7311,3	2,05006E-02	2946	1,07822E+00	863	1,52606E+00	5295	8,97255E-01	9922	0	17530,5
Non-responder	Macro_OLFML3	Macro_ISG15	B2M	TFRC	7,40833E-03	11441,9	6,46058E-03	19973	4,21979E-01	10959	1,44470E+00	6104	9,31546E-01	2643	0	17530,5
Non-responder	Macro_LYVE1	cDC_LAMP3	TNFSF13	TNFRSF11B	7,41140E-03	12030,3	6,39534E-02	398	1,06628E+00	906	1,17887E+00	9814	8,10044E-01	31503	0	17530,5
Non-responder	Mono_INHBA	Mono_CD16	LYZ	ITGAL	7,41204E-03	9673,9	1,20589E-02	7275	5,52480E-01	6431	1,10498E+00	11076	9,14558E-01	6057	0	17530,5
Non-responder	cDC_CLEC9A	Macro_ISG15	HLA-B	LILRB1	7,41527E-03	7572,5	8,42298E-03	12971	6,73829E-01	4000	1,97630E+00	2183	9,39308E-01	1178	0	17530,5
Non-responder	pDC_LILRA4	Macro_IFI27	HSP90B1	TLR2	7,41671E-03	7695,3	9,95262E-03	9926	8,85803E-01	1809	2,12622E+00	1600	9,07393E-01	7611	0	17530,5
Non-responder	Macro_FOLR2+APOE+	Mast	VEGFB	ADRB2	7,42541E-03	18179,3	2,38653E-02	2235	5,24155E-01	7211	1,17263E+00	9929	7,07545E-01	53991	0	17530,5
Non-responder	Mono_INHBA	Mono_CD16	PTGS2	ALOX5	7,42875E-03	10496,7	1,29131E-02	6469	6,09794E-01	5103	1,36178E+00	7109	8,72719E-01	16272	0	17530,5
Non-responder	Mono_CD14	Mono_INHBA	VCAN	TLR2	7,43149E-03	7619,5	1,71057E-02	4003	1,00970E+00	1161	1,76795E+00	3305	8,88605E-01	12098	0	17530,5
Non-responder	cDC_LAMP3	pDC_LILRA4	HMGB1	TLR9	7,43805E-03	10747,3	3,91120E-02	1005	6,97217E-01	3660	1,07909E+00	11552	8,57386E-01	19989	0	17530,5
Non-responder	Mono_INHBA	Mono_CD16	VEGFA	CD44	7,44736E-03	9791,1	1,26458E-02	6698	7,17635E-01	3411	1,15936E+00	10144	8,92292E-01	11172	0	17530,5
Non-responder	Macro_OLFML3	Macro_ISG15	TNFSF13	TNFRSF14	7,45295E-03	16290,9	6,45635E-03	19997	3,16478E-01	16658	1,10427E+00	11090	8,73102E-01	16179	0	17530,5
Non-responder	Macro_OLFML3	Macro_ISG15	SEMA4A	LILRB2	7,45481E-03	11367,5	9,43033E-03	10815	6,65303E-01	4139	1,62846E+00	4355	8,57357E-01	19998	0	17530,5
Non-responder	Macro_OLFML3	cDC_CLEC9A	LGALS9	MRC2	7,45736E-03	14509,9	1,57273E-02	4636	5,31507E-01	7003	1,17194E+00	9940	8,01806E-01	33440	0	17530,5
Non-responder	Macro_FOLR2+APOE+	Mono_CD16	B2M	TFRC	7,45736E-03	15868,1	3,94815E-03	39971	4,46956E-01	9940	1,48041E+00	5747	9,14076E-01	6152	0	17530,5
Non-responder	Macro_NLRP3	Mono_CD16	VCAN	SELL	7,46027E-03	11952,3	9,94133E-03	9941	8,38521E-01	2199	1,54259E+00	5115	8,36627E-01	24976	0	17530,5
Non-responder	Mono_INHBA	Macro_OLFML3	TNF	TNFRSF1A	7,46227E-03	11080,5	1,16502E-02	7696	5,84378E-01	5637	1,50791E+00	5472	8,61317E-01	19067	0	17530,5
Non-responder	Macro_FOLR2+APOE+	Macro_ISG15	PTPN6	CD300LF	7,46319E-03	13357,5	1,11140E-02	8342	4,46939E-01	9942	1,55839E+00	4950	8,32275E-01	26023	0	17530,5
Non-responder	Mono_INHBA	Macro_NLRP3	MMP9	CD44	7,46787E-03	10480,1	1,17440E-02	7598	7,12205E-01	3479	1,69946E+00	3788	8,57336E-01	20005	0	17530,5
Non-responder	Macro_NLRP3	Mono_CD16	TIMP1	CD63	7,46901E-03	12814,9	5,11841E-03	28358	4,46922E-01	9944	1,43618E+00	6210	9,34654E-01	2032	0	17530,5
Non-responder	Macro_ISG15	Macro_ISG15	LGALS9	CD47	7,47484E-03	8957,5	1,00017E-02	9839	6,53827E-01	4330	1,79515E+00	3142	8,97155E-01	9946	0	17530,5
Non-responder	Mono_CD14	Macro_FOLR2+APOE+	SEMA4D	CD72	7,47487E-03	13294,9	2,19496E-02	2584	7,25905E-01	3298	1,67149E+00	4011	7,77868E-01	39051	0	17530,5
Non-responder	Mono_INHBA	Macro_OLFML3	TNF	TNFRSF1B	7,47720E-03	14615,5	7,62882E-03	15227	4,31497E-01	10549	1,18155E+00	9761	8,57292E-01	20010	0	17530,5
Non-responder	Macro_FOLR2+APOE+	cDC(CD1C)	ADAM10	AXL	7,47775E-03	13992,5	1,42666E-02	5467	4,67007E-01	9176	1,17149E+00	9947	8,25041E-01	27842	0	17530,5
Non-responder	Macro_FOLR2+APOE+	Macro_FOLR2+APOE+	TNF	TNFRSF1A	7,47841E-03	13217,9	9,14351E-03	11332	4,72955E-01	8951	1,49301E+00	5609	8,46203E-01	22667	0	17530,5
Non-responder	Macro_FOLR2+APOE+	Macro_NLRP3	PDGFC	FLT1	7,48359E-03	21265,5	2,57063E-02	1989	4,46722E-01	9949	1,50152E+00	5522	5,39538E-01	71337	0	17530,5
Non-responder	Macro_LYVE1	Macro_FOLR2+APOE+	GRN	TNFRSF1A	7,48655E-03	15196,9	6,45204E-03	20015	3,54983E-01	14351	7,65340E-01	18855	9,18574E-01	5233	0	17530,5
Non-responder	Mono_INHBA	Macro_OLFML3	ICAM1	IL2RG	7,49403E-03	16496,3	6,46401E-03	19942	4,09864E-01	11522	9,15972E-01	15026	8,63881E-01	18461	0	17530,5
Non-responder	Mono_CD14	Macro_NLRP3	CD55	ADGRE2	7,49661E-03	8348,1	1,70703E-02	4015	8,77234E-01	1868	1,92274E+00	2447	8,74255E-01	15880	0	17530,5
Non-responder	Macro_OLFML3	Macro_OLFML3	HLA-DMB	CD4	7,49965E-03	14241,5	6,45163E-03	20022	3,51087E-01	14589	1,13285E+00	10578	9,03640E-01	8488	0	17530,5

Post_NR_Myeloid_Myeloid_CellCell

Non-responder	Mono_CD16	Mono_CD14	VCAN	SELL	7,50403E-03	13394,9	1,12986E-02	8095	4,85578E-01	8488	1,17091E+00	9956	8,45186E-01	22905	0	17530,5
Non-responder	Macro_NLRP3	cDC_CLEC9A	INHBA	SMAD3	7,50695E-03	21314,5	2,48587E-02	2106	4,46493E-01	9957	1,30779E+00	7883	5,81343E-01	69096	0	17530,5
Non-responder	cDC_LAMP3	pDC_LILRA4	HRAS	TLR9	7,50750E-03	17357,5	8,99676E-02	173	1,04159E+00	1017	1,67118E+00	4017	6,37450E-01	64050	0	17530,5
Non-responder	Macro_FOLR2+APOE+	Macro_FOLR2+APOE+	SPP1	ITGA5_ITGB1	7,50988E-03	11000,9	1,68430E-02	4098	4,46473E-01	9958	2,29502E+00	1129	8,47774E-01	22289	0	17530,5
Non-responder	Macro_OLFML3	cDC_CLEC9A	TNFSF10	TNFRSF10B	7,50988E-03	12281,5	1,61261E-02	4414	6,81842E-01	3884	1,35542E+00	7220	8,22939E-01	28359	0	17530,5
Non-responder	Macro_IFI27	Macro_FOLR2+APOE+	HLA-DMB	CD4	7,51089E-03	15372,5	6,72735E-03	18682	3,88462E-01	12552	7,21200E-01	20028	9,05446E-01	8070	0	17530,5
Non-responder	Macro_FOLR2+APOE+	Macro_FOLR2+APOE-	ANXA2	TLR2	7,51089E-03	15787,9	6,44814E-03	20028	3,51179E-01	14582	8,32356E-01	17090	8,98134E-01	9709	0	17530,5
Non-responder	cDC_CLEC9A	cDC_CLEC9A	HLA-DRB5	LAG3	7,51840E-03	7665,3	1,70643E-02	4019	8,39623E-01	2186	2,00057E+00	2089	8,87175E-01	12502	0	17530,5
Non-responder	Macro_ISG15	Macro_FOLR2+APOE+	CD14	ITGB2	7,51840E-03	7418,5	8,35152E-03	13154	6,73006E-01	4019	2,07574E+00	1786	9,44627E-01	603	0	17530,5
Non-responder	pDC_LILRA4	Macro_ISG15	CD99	PILRA	7,52451E-03	7975,7	9,93029E-03	9963	5,99251E-01	5325	2,20022E+00	1377	9,16275E-01	5683	0	17530,5
Non-responder	Macro_ISG15	Macro_NLRP3	APOC2	LDLR	7,54570E-03	15674,9	3,90602E-02	1011	6,72572E-01	4024	1,92037E+00	2458	7,11115E-01	53351	0	17530,5
Non-responder	Macro_IFI27	Macro_ISG15	HLA-A	LILRB1	7,54659E-03	12189,5	8,03679E-03	14028	5,24842E-01	7188	7,20535E-01	20047	9,34177E-01	2154	0	17530,5
Non-responder	pDC_LILRA4	Mono_CD16	FABP5	RXRA	7,55117E-03	9221,5	1,88536E-02	3380	6,72542E-01	4025	2,74132E+00	468	8,54366E-01	20704	0	17530,5
Non-responder	cDC(CD1C)	Macro_NLRP3	PSEN1	CD44	7,55224E-03	14790,7	6,49326E-03	19794	5,48054E-01	6545	1,16619E+00	10034	8,57125E-01	20050	0	17530,5
Non-responder	Macro_LYVE1	Macro_OLFML3	CD14	TLR4	7,55601E-03	16316,7	7,44526E-03	15861	4,58854E-01	9471	7,20293E-01	20052	8,63009E-01	18669	0	17530,5
Non-responder	cDC(CD1C)	cDC_CLEC9A	TNFSF13B	CD40	7,55601E-03	16204,1	6,44197E-03	20052	4,05723E-01	11726	1,05300E+00	12094	8,59012E-01	19618	0	17530,5
Non-responder	Macro_FOLR2+APOE+	Macro_OLFML3	CXCL12	SDC4	7,55678E-03	20484,7	1,50285E-02	4998	4,46152E-01	9974	1,55913E+00	4944	6,28590E-01	64977	0	17530,5
Non-responder	Macro_FOLR2+APOE+	pDC_LILRA4	CD14	RIPK1	7,55678E-03	13052,7	9,92381E-03	9974	5,46751E-01	6572	2,03734E+00	1938	8,19255E-01	29249	0	17530,5
Non-responder	Mono_INHBA	Macro_FOLR2+APOE+	S100A8	TLR4	7,55789E-03	12669,9	1,35129E-02	5994	6,23156E-01	4863	7,20292E-01	20053	8,78104E-01	14909	0	17530,5
Non-responder	Macro_FOLR2+APOE+	Mono_INHBA	CCL5	CCRL2	7,55972E-03	19551,5	1,57458E-02	4625	4,46102E-01	9975	1,33900E+00	7435	6,82229E-01	58192	0	17530,5
Non-responder	Mono_CD16	Macro_FOLR2+APOE+	VCAN	ITGB1	7,55977E-03	14511,7	8,34179E-03	13178	4,22777E-01	10918	1,11615E+00	10878	8,57106E-01	20054	0	17530,5
Non-responder	Mono_INHBA	Macro_OLFML3	FN1	CD44	7,56166E-03	14492,7	1,15152E-02	7854	3,27607E-01	15943	9,71821E-01	13744	8,68404E-01	17392	0	17530,5
Non-responder	cDC_LAMP3	Macro_OLFML3	HLA-B	LILRB1	7,56166E-03	13182,1	6,44157E-03	20055	3,99589E-01	12001	9,77366E-01	13613	9,31198E-01	2711	0	17530,5
Non-responder	cDC(CD1C)	Macro_FOLR2+APOE+	GRN	TNFRSF1A	7,56354E-03	14463,7	6,44117E-03	20056	3,53270E-01	14459	9,15933E-01	15028	9,18511E-01	5245	0	17530,5
Non-responder	Mono_INHBA	Macro_OLFML3	FN1	PLAUR	7,56543E-03	14279,9	1,12203E-02	8172	3,29506E-01	15830	9,94589E-01	13240	8,71359E-01	16627	0	17530,5
Non-responder	Macro_OLFML3	cDC_CLEC9A	FN1	ITGA4_ITGB7	7,56560E-03	13186,1	1,58154E-02	4578	5,71362E-01	5947	1,53075E+00	5245	8,05122E-01	32630	0	17530,5
Non-responder	cDC(CD1C)	Macro_FOLR2+APOE+	HLA-C	LILRB2	7,56732E-03	11769,1	6,44067E-03	20058	4,87660E-01	8398	1,05554E+00	12036	9,42178E-01	823	0	17530,5
Non-responder	Macro_FOLR2+APOE+	Macro_FOLR2+APOE+	LRPAP1	SORT1	7,56854E-03	15706,7	9,91983E-03	9978	4,62724E-01	9328	1,31887E+00	7720	7,99611E-01	33977	0	17530,5
Non-responder	cDC_LAMP3	Mono_CD16	LGALS1	PTPRC	7,57109E-03	10633,3	6,44037E-03	20060	5,39534E-01	6775	1,32060E+00	7700	9,40018E-01	1101	0	17530,5
Non-responder	Macro_OLFML3	Macro_NLRP3	PIGF	FLT1	7,57443E-03	21068,9	2,10003E-02	2810	4,45973E-01	9980	1,33986E+00	7422	6,01108E-01	67602	0	17530,5
Non-responder	Mono_INHBA	Macro_OLFML3	IL1B	SIGIRR	7,57675E-03	11579,1	9,28183E-03	11058	6,47141E-01	4434	1,48944E+00	5645	8,60624E-01	19228	0	17530,5
Non-responder	Macro_FOLR2+APOE+	Mono_CD14	PLAU	ITGB1	7,57737E-03	14683,1	1,02411E-02	9502	4,45966E-01	9981	1,46830E+00	5855	8,13900E-01	30547	0	17530,5
Non-responder	Macro_ISG15	Macro_FOLR2+APOE+	SERPING1	LRP1	7,58032E-03	7710,5	1,37707E-02	5808	7,20200E-01	3374	2,05829E+00	1858	8,97000E-01	9982	0	17530,5
Non-responder	Macro_FOLR2+APOE-	cDC_CLEC9A	C1QB	C1QBP	7,58326E-03	6860,1	1,65610E-02	4211	9,17514E-01	1629	1,16936E+00	9983	9,41156E-01	947	0	17530,5
Non-responder	Mono_INHBA	Macro_FOLR2+APOE+	SPP1	ITGAV_ITGB5	7,58402E-03	10861,7	3,52896E-02	1185	6,73246E-01	4015	1,66981E+00	4031	8,26150E-01	27547	0	17530,5
Non-responder	cDC_CLEC9A	Macro_ISG15	SEMA4A	LILRB2	7,58951E-03	7223,7	1,70225E-02	4032	9,38863E-01	1505	2,23966E+00	1259	8,89811E-01	11792	0	17530,5
Non-responder	Macro_OLFML3	cDC_CLEC9A	HLA-DQB2	LAG3	7,59210E-03	12686,3	1,99308E-02	3077	7,20284E-01	3373	1,52189E+00	5327	7,98923E-01	34124	0	17530,5
Non-responder	Macro_IER3	cDC_CLEC9A	F13A1	ITGA4	7,59376E-03	14097,9	9,52284E-03	10638	4,44117E-01	10059	1,04723E+00	12190	8,57006E-01	20072	0	17530,5
Non-responder	cDC_CLEC9A	pDC_LILRA4	ADA	DPP4	7,59499E-03	17365,3	4,64683E-02	739	6,87199E-01	3788	1,66968E+00	4033	6,64555E-01	60736	0	17530,5
Non-responder	Macro_NLRP3	Mono_CD16	CD55	CR1	7,59505E-03	18553,9	1,14027E-02	7975	4,76584E-01	8829	1,16904E+00	9987	7,36326E-01	48448	0	17530,5
Non-responder	Macro_IER3	Mono_CD14	MRC1	PTPRC	7,59565E-03	13687,1	9,81113E-03	10152	4,57836E-01	9505	1,09948E+00	11175	8,57004E-01	20073	0	17530,5
Non-responder	Macro_FOLR2+APOE+	Mono_CD16	HLA-DQA1	CD4	7,59800E-03	12356,7	5,37068E-03	26490	4,45804E-01	9988	1,71038E+00	3708	9,24566E-01	4067	0	17530,5
Non-responder	pDC_LILRA4	Macro_ISG15	CIRBP	TREM1	7,60095E-03	8166,9	1,24359E-02	6907	6,05337E-01	5203	2,26290E+00	1205	8,96983E-01	9989	0	17530,5
Non-responder	Mono_INHBA	Macro_OLFML3	CCL3	CCR1	7,60133E-03	8945,7	1,40687E-02	5598	7,28000E-01	3272	1,81588E+00	3024	8,76553E-01	15304	0	17530,5
Non-responder	pDC_LILRA4	Macro_LYVE1	LTB	CD40	7,60653E-03	9861,7	1,63476E-02	4319	1,06307E+00	918	2,77545E+00	438	8,31960E-01	26103	0	17530,5
Non-responder	Mono_INHBA	Macro_OLFML3	SPP1	ITGA4_ITGB1	7,60701E-03	11100,7	1,67091E-02	4144	4,98484E-01	8037	1,27611E+00	8319	8,68041E-01	17473	0	17530,5
Non-responder	Macro_NLRP3	cDC(CD1C)	TNF	SEMA4C	7,60981E-03	20354,3	1,44395E-02	5342	4,47103E-01	9933	1,16875E+00	9992	6,77302E-01	58974	0	17530,5
Non-responder	Mono_INHBA	Macro_OLFML3	SPP1	CD44	7,61080E-03	9988,3	1,51290E-02	4932	4,89392E-01	8343	1,14993E+00	10298	9,01991E-01	8838	0	17530,5
Non-responder	Mono_INHBA	Macro_OLFML3	SPP1	PTGER4	7,61270E-03	9848,1	2,20803E-02	2564	6,00705E-01	5291	1,51235E+00	5422	8,63990E-01	18433	0	17530,5
Non-responder	cDC(CD1C)	cDC_CLEC9A	LGALS9	SLC1A5	7,61270E-03	16677,9	7,49820E-03	15687	3,39692E-01	15215	9,22603E-01	14875	8,56974E-01	20082	0	17530,5
Non-responder	Mono_INHBA	Macro_OLFML3	CCL4	CCR1	7,61459E-03	9021,7	1,46598E-02	5197	7,24340E-01	3317	1,95245E+00	2295	8,70789E-01	16769	0	17530,5
Non-responder	Macro_FOLR2+APOE+	Mast	MMP9	ITGAM	7,61867E-03	17595,1	9,91080E-03	9995	5,03457E-01	7860	1,62545E+00	4377	7,37395E-01	48213	0	17530,5
Non-responder	Macro_ISG15	Macro_OLFML3	CCL8	CCR2	7,62247E-03	17536,7	4,16891E-02	904	6,71593E-01	4038	2,23726E+00	1269	6,38374E-01	63942	0	17530,5
Non-responder	Macro_OLFML3	cDC_CLEC9A	LGALS3	LAG3	7,62459E-03	10933,7	1,65259E-02	4226	7,15164E-01	3446	1,42706E+00	6321	8,44185E-01	23145	0	17530,5
Non-responder	pDC_LILRA4	Macro_FOLR2+APOE+	TNFSF13	TNFRSF14	7,62459E-03	8680,9	1,03346E-02	9358	6,02124E-01	5267	2,24221E+00	1252	8,96959E-01	9997	0	17530,5
Non-responder	Mono_INHBA	Macro_OLFML3	VCAN	TLR2	7,62977E-03	12853,9	1,25353E-02	6804	5,59312E-01	6241	8,24847E-01	17301	8,72266E-01	16393	0	17530,5
Non-responder	cDC(CD1C)	Macro_FOLR2+APOE+	TGFB1	ENG	7,63167E-03	17552,7	7,58353E-03	15376	3,20609E-01	16389	7,83894E-01	18376	8,56928E-01	20092	0	17530,5
Non-responder	Macro_OLFML3	cDC_CLEC9A	CALR	SCARF1	7,63346E-03	10296,9	1,87344E-02	3419	7,17111E-01	3421	1,52229E+00	5325	8,49810E-01	21789	0	17530,5
Non-responder	Mono_INHBA	Macro_OLFML3	VCAN	ITGA4	7,63357E-03	14080,1	9,99389E-03	9849	5,26621E-01	7130	7,89619E-01	18233	8,67309E-01	17658	0	17530,5
Non-responder	Mono_CD14	pDC_LILRA4	SIRPB2	CD47	7,63898E-03	10467,9	1,70026E-02	4041	7,98146E-01	2576	2,53404E+00	719	8,26488E-01	27473	0	17530,5
Non-responder	Mono_CD16	Macro_OLFML3	S100A9	CD68	7,63927E-03	14294,5	6,58005E-03	19358	3,73672E-01	13301	7,19061E-01	20096	9,39185E-01	1187	0	17530,5
Non-responder	Macro_FOLR2+APOE+	Macro_OLFML3	HLA-DRA	CD4	7,63929E-03	11996,1	5,53337E-03	25398	4,24014E-01	10881	1,51417E+00	5405	9,42751E-01	766	0	17530,5
Non-responder	Macro_OLFML3	cDC_CLEC9A	SNCA	LAG3	7,64235E-03	19859,7	1,54303E-02	4766	6,35809E-01	4616	1,26459E+00	8479	6,38764E-01	63907	0	17530,5
Non-responder	Macro_IFI27	Macro_ISG15	HLA-A	LILRB2	7,64307E-03	12343,3	7,01418E-03	17477	5,72201E-01	5931	7,19031E-01	20098	9,43712E-01	680	0	17530,5
Non-responder	Macro_OLFML3	cDC_CLEC9A	COPA	P2RY6	7,64828E-03	13125,7	1,21178E-02	7215	5,64054E-01	6128	1,30928E+00	7859	8,28787E-01	26896	0	17530,5
Non-responder	cDC(CD1C)	Mono_CD14	CD1D	LILRB2	7,65068E-03	11912,1	1,38793E-02	5733	5,54814E-01	6368	1,17819E+00	9827	8,56882E-01	20102	0	17530,5
Non-responder	Macro_LYVE1	Mono_CD14	SERPING1	LRP1	7,65449E-03	16616,5	7,87403E-03	14499	3,70200E-01	13503	7,18701E-01	20104	8,68166E-01	17446	0	17530,5
Non-responder	cDC(CD1C)	Macro_FOLR2+APOE+	GNAI2	C5AR1	7,65449E-03	15222,3	6,43047E-03	20104	3,66701E-01	13693	9,08406E-01	15216	8,98705E-01	9568	0	17530,5
Non-responder	Macro_IER3	cDC(CD1C)	MRC1	PTPRC	7,66211E-03	15026,9	9,78714E-03	10201	4,56126E-01	9574	8,08840E-01	17721	8,56854E-01	20108	0	17530,5
Non-responder	Macro_FOLR2+APOE+	Macro_IER3	ADAM10	CD44	7,66211E-03	19139,3	6,42917E-03	20108	2,73403E-01	19623	7,67337E-01	18802	8,58942E-01	19633	0	17530,5
Non-responder	cDC_LAMP3	pDC_LILRA4	APP	TNFRSF21	7,67208E-03	10323,3	1,69773E-02	4047	8,58888E-01	2016	1,88709E+00	2618	8,34821E-01	25405	0	17530,5

Post_NR_Myeloid_Myeloid_CellCell

Non-responder	Macro_NLRP3	Macro_ISG15	TGFB1	ACVRL1	7,67796E-03	18931,7	1,74021E-02	3892	4,45160E-01	10015	1,17184E+00	9942	7,11549E-01	53279	0	17530,5
Non-responder	Mono_INHBA	cDC(CD1C)	INHBA	ACTR2	7,68314E-03	9117,7	2,69110E-02	1835	8,81993E-01	1843	1,66816E+00	4049	8,55868E-01	20331	0	17530,5
Non-responder	Mono_INHBA	Macro_ISG15	CXCL8	SDC3	7,68314E-03	11470,3	1,69749E-02	4049	7,37594E-01	3173	1,71339E+00	3684	8,20591E-01	28915	0	17530,5
Non-responder	Macro_NLRP3	Macro_FOLR2+APOE-	VCAN	ITGB1	7,68500E-03	13925,3	8,30077E-03	13283	7,05674E-01	3567	9,11784E-01	15126	8,56803E-01	20120	0	17530,5
Non-responder	Macro_FOLR2+APOE+	Macro_FOLR2+APOE+	PTDSS1	SCARB1	7,68688E-03	15455,1	1,52799E-02	4855	4,45068E-01	10018	1,28220E+00	8246	7,88058E-01	36626	0	17530,5
Non-responder	Macro_FOLR2+APOE+	cDC(CD1C)	MMP9	LRP1	7,68986E-03	15509,7	9,89370E-03	10019	4,54676E-01	9635	1,60469E+00	4557	7,91669E-01	35807	0	17530,5
Non-responder	Macro_IER3	Mono_CD16	TNFSF13	TNFRSF14	7,70411E-03	17828,9	6,42427E-03	20130	3,01532E-01	17590	8,11808E-01	17638	8,72826E-01	16256	0	17530,5
Non-responder	Mono_CD14	Macro_ISG15	S100A9	TLR4	7,70502E-03	6809,5	1,58681E-02	4556	1,06177E+00	924	2,44097E+00	864	8,96286E-01	10173	0	17530,5
Non-responder	Macro_IER3	Macro_ISG15	CD14	TLR1	7,70603E-03	14772,1	8,80625E-03	12028	4,14149E-01	11336	1,01599E+00	12835	8,56764E-01	20131	0	17530,5
Non-responder	Mono_CD14	Macro_FOLR2+APOE+	ADAM10	GPNMB	7,71082E-03	9381,5	1,71739E-02	3978	6,70647E-01	4054	1,85241E+00	2801	8,63535E-01	18544	0	17530,5
Non-responder	Macro_IER3	Macro_FOLR2+APOE+	TGFB1	ENG	7,71369E-03	16466,1	7,56142E-03	15452	3,19217E-01	16489	1,02088E+00	12724	8,56749E-01	20135	0	17530,5
Non-responder	Macro_OLFML3	Macro_OLFML3	LGALS9	HAVCR2	7,71943E-03	16146,1	6,42280E-03	20138	3,44181E-01	14967	1,04862E+00	12162	8,74024E-01	15933	0	17530,5
Non-responder	Macro_IER3	Macro_FOLR2+APOE+	HMGB1	HAVCR2	7,72135E-03	16909,3	6,42271E-03	20139	2,93169E-01	18165	7,56441E-01	19086	8,98486E-01	9626	0	17530,5
Non-responder	Macro_OLFML3	Macro_NLRP3	HLA-B	LILRB2	7,72265E-03	12838,9	5,37980E-03	26429	4,69524E-01	9090	1,16627E+00	10030	9,39906E-01	1115	0	17530,5
Non-responder	cDC(CD1C)	Macro_FOLR2+APOE+	HMGB1	TLR4	7,72327E-03	17121,3	7,49266E-03	15709	3,48266E-01	14737	7,17519E-01	20140	8,67959E-01	17490	0	17530,5
Non-responder	Mono_INHBA	Macro_OLFML3	PTGS2	ALOX5	7,73286E-03	9272,3	1,58296E-02	4571	7,07125E-01	3551	1,35288E+00	7261	8,83606E-01	13448	0	17530,5
Non-responder	Macro_LYVE1	cDC_CLEC9A	HMGB1	THBD	7,73670E-03	16195,1	7,40985E-03	15967	3,56261E-01	14284	7,17272E-01	20147	8,85005E-01	13047	0	17530,5
Non-responder	pDC_LILRA4	pDC_LILRA4	JAG1	NOTCH4	7,73856E-03	17783,1	4,76720E-02	708	6,70294E-01	4059	2,17608E+00	1446	6,26807E-01	65172	0	17530,5
Non-responder	Macro_OLFML3	cDC_CLEC9A	TOR2A	ATP5F1B	7,74058E-03	13888,3	1,12031E-02	8201	6,63296E-01	4169	1,69213E+00	3837	7,92102E-01	35704	0	17530,5
Non-responder	Macro_NLRP3	Macro_NLRP3	RETN	TLR4	7,74357E-03	18844,9	1,91369E-02	3296	4,44564E-01	10037	1,52959E+00	5259	6,82887E-01	58102	0	17530,5
Non-responder	Macro_FOLR2+APOE+	Mono_CD16	PSEN1	CD44	7,75207E-03	18736,3	6,44529E-03	20039	2,80981E-01	19056	8,39172E-01	16901	8,56671E-01	20155	0	17530,5
Non-responder	Macro_IER3	Macro_ISG15	B2M	LILRB2	7,75399E-03	12628,5	6,41903E-03	20156	3,99782E-01	11996	9,93588E-01	13256	9,50439E-01	204	0	17530,5
Non-responder	cDC(CD1C)	cDC_LAMP3	GNAI2	S1PR1	7,75450E-03	10259,1	4,10132E-02	927	1,12871E+00	654	1,10129E+00	11146	8,52997E-01	21038	0	17530,5
Non-responder	Macro_NLRP3	Macro_ISG15	ANXA1	FPR1	7,75592E-03	14968,7	6,41884E-03	20157	2,92144E-01	18233	1,10677E+00	11043	9,06291E-01	7880	0	17530,5
Non-responder	Macro_FOLR2+APOE+	Macro_IER3	CALR	LRP1	7,75977E-03	13667,9	6,41834E-03	20159	4,14663E-01	11305	1,16349E+00	10088	9,00101E-01	9257	0	17530,5
Non-responder	Mono_INHBA	Macro_OLFML3	VEGFA	CD44	7,76362E-03	10643,7	1,17650E-02	7576	6,74932E-01	3979	1,05388E+00	12077	8,88773E-01	12056	0	17530,5
Non-responder	Macro_OLFML3	cDC_CLEC9A	ADAM10	CADM1	7,76453E-03	12373,1	2,18970E-02	2592	9,93480E-01	1231	1,53959E+00	5138	7,93635E-01	35374	0	17530,5
Non-responder	Macro_NLRP3	Macro_FOLR2-APOE+	S100A8	TLR4	7,76940E-03	13361,7	1,09496E-02	8548	7,82598E-01	2701	7,16857E-01	20164	8,66391E-01	17865	0	17530,5
Non-responder	Macro_OLFML3	Mono_CD14	RPS19	C5AR1	7,76940E-03	11626,1	6,41709E-03	20164	5,09112E-01	7694	1,31520E+00	7765	9,19825E-01	4977	0	17530,5
Non-responder	Macro_OLFML3	Macro_ISG15	LGALS3	LAG3	7,77325E-03	9351,7	2,01001E-02	3037	8,74112E-01	1883	1,65623E+00	4142	8,56632E-01	20166	0	17530,5
Non-responder	Macro_NLRP3	Macro_FOLR2+APOE-	VEGFA	NRP2	7,77953E-03	16555,1	1,00433E-02	9791	7,40371E-01	3141	1,16521E+00	10049	7,64108E-01	42264	0	17530,5
Non-responder	cDC_LAMP3	Macro_FOLR2+APOE+	ICAM1	ITGAX_ITGB2	7,78096E-03	14116,1	6,41622E-03	20170	4,80271E-01	8693	9,32554E-01	14662	8,98854E-01	9525	0	17530,5
Non-responder	Macro_OLFML3	cDC_CLEC9A	FN1	DPP4	7,78853E-03	16064,7	5,74020E-02	511	6,75827E-01	3964	1,40949E+00	6537	7,19431E-01	51781	0	17530,5
Non-responder	Macro_LYVE1	cDC_CLEC9A	LAMB2	RPSA	7,78866E-03	12516,7	2,26192E-02	2447	6,82223E-01	3875	1,66614E+00	4068	7,96661E-01	34663	0	17530,5
Non-responder	Macro_IER3	Macro_ISG15	HSPA1A	TLR4	7,78868E-03	17097,5	6,41561E-03	20174	3,06074E-01	17308	9,26284E-01	14778	8,74992E-01	15697	0	17530,5
Non-responder	Macro_IER3	Mono_CD14	ICAM1	ITGAL_ITGB2	7,79061E-03	14355,3	6,41521E-03	20175	4,19059E-01	11098	1,07667E+00	11594	8,91474E-01	11379	0	17530,5
Non-responder	cDC_CLEC9A	cDC_LAMP3	GSTP1	TRAF2	7,79424E-03	9221,3	1,69219E-02	4069	8,58719E-01	2018	1,77856E+00	3238	8,60564E-01	19251	0	17530,5
Non-responder	Macro_NLRP3	Mono_INHBA	PTGS2	ALOX5	7,79447E-03	14840,9	9,80075E-03	10179	4,89596E-01	8338	7,99257E-01	17980	8,56599E-01	20177	0	17530,5
Non-responder	Macro_IER3	Macro_FOLR2+APOE+	RPS19	C5AR1	7,79640E-03	12502,3	6,41469E-03	20178	5,09481E-01	7680	1,05035E+00	12141	9,19811E-01	4982	0	17530,5
Non-responder	Macro_FOLR2+APOE+	Macro_OLFML3	C3	CD81	7,80220E-03	13928,5	9,31245E-03	11011	2,67098E-01	20181	1,31476E+00	7773	8,84618E-01	13147	0	17530,5
Non-responder	pDC_LILRA4	pDC_LILRA4	COPA	CD74	7,80413E-03	7982,9	8,61320E-03	12484	4,90980E-01	8286	2,39913E+00	930	9,43685E-01	684	0	17530,5
Non-responder	Mono_CD16	Macro_FOLR2+APOE+	PTPN6	CLEC12A	7,80414E-03	12990,7	8,92029E-03	11799	4,43856E-01	10071	1,51745E+00	5371	8,56576E-01	20182	0	17530,5
Non-responder	Macro_FOLR2-APOE+	pDC_LILRA4	COPA	P2RY6	7,80541E-03	8982,1	1,69186E-02	4071	1,12553E+00	667	2,26771E+00	1184	8,51185E-01	21458	0	17530,5
Non-responder	Macro_FOLR2+APOE+	Macro_NLRP3	APOE	SDC2	7,80657E-03	12590,3	9,86914E-03	10058	4,76362E-01	8834	1,69889E+00	3794	8,45913E-01	22735	0	17530,5
Non-responder	Macro_FOLR2+APOE+	Macro_FOLR2+APOE+	TNF	TNFRSF1B	7,81813E-03	17120,1	6,40627E-03	20215	3,36183E-01	15435	1,18105E+00	9769	8,46271E-01	22651	0	17530,5
Non-responder	Macro_OLFML3	cDC_CLEC9A	TNFSF10	CCR6	7,83067E-03	16999,1	3,11980E-02	1444	6,45133E-01	4469	1,24945E+00	8707	7,13904E-01	52845	0	17530,5
Non-responder	Mono_CD16	Macro_NLRP3	CD52	SIGLEC10	7,83512E-03	13614,5	6,41069E-03	20198	6,27191E-01	4782	1,46783E+00	5865	8,58616E-01	19697	0	17530,5
Non-responder	Macro_ISG15	Macro_FOLR2+APOE+	LGALS9	MRC2	7,84458E-03	10997,1	2,17385E-02	2634	7,33011E-01	3224	1,66462E+00	4078	8,26277E-01	27519	0	17530,5
Non-responder	Mono_CD14	Macro_NLRP3	VCAN	ITGB1	7,84458E-03	6995,3	1,69064E-02	4078	1,04214E+00	1014	2,04733E+00	1904	8,95169E-01	10450	0	17530,5
Non-responder	cDC(CD1C)	Mono_CD14	GNAI2	C5AR1	7,84676E-03	16057,7	6,40877E-03	20204	3,64299E-01	13824	7,55232E-01	19124	8,98551E-01	9606	0	17530,5
Non-responder	Macro_IER3	Mono_CD16	CD99	PILRA	7,85065E-03	16505,9	6,40831E-03	20206	2,92997E-01	18173	8,41327E-01	16844	8,97870E-01	9776	0	17530,5
Non-responder	pDC_LILRA4	Macro_IFI27	IL16	CD9	7,85181E-03	7996,9	1,97494E-02	3133	7,11150E-01	3499	1,48023E+00	5749	8,96638E-01	10073	0	17530,5
Non-responder	cDC_LAMP3	cDC(CD1C)	TNFSF9	HLA-DPA1	7,85259E-03	16630,7	6,96269E-03	17691	4,19425E-01	11081	8,49498E-01	16644	8,56450E-01	20207	0	17530,5
Non-responder	Macro_ISG15	Mono_CD14	CCL18	CCR1	7,85579E-03	13793,1	2,55782E-02	2005	6,68883E-01	4080	2,19688E+00	1390	7,56216E-01	43960	0	17530,5
Non-responder	Macro_NLRP3	Macro_OLFML3	INHBA	ACTR2	7,85785E-03	14590,9	1,28258E-02	6546	4,43755E-01	10075	1,46698E+00	5874	8,03900E-01	32929	0	17530,5
Non-responder	Macro_OLFML3	pDC_LILRA4	CCL2	CCR2	7,86088E-03	18033,7	1,85233E-02	3496	4,43732E-01	10076	1,70542E+00	3753	7,00098E-01	55313	0	17530,5
Non-responder	Mono_CD16	Macro_OLFML3	S100A8	ITGB2	7,86231E-03	15018,3	6,40631E-03	20212	3,51142E-01	14585	7,54629E-01	19147	9,26910E-01	3617	0	17530,5
Non-responder	Macro_LYVE1	pDC_LILRA4	C3	CD46	7,86815E-03	15110,3	9,73488E-03	10285	2,95622E-01	17997	1,19622E+00	9524	8,56431E-01	20215	0	17530,5
Non-responder	Macro_NLRP3	Macro_NLRP3	THBS1	CD47	7,87594E-03	11844,1	9,26253E-03	11096	6,73915E-01	3997	1,42261E+00	6378	8,56400E-01	20219	0	17530,5
Non-responder	Macro_NLRP3	Mono_CD16	S100A12	TLR4	7,87601E-03	16408,5	1,41347E-02	5540	4,43540E-01	10081	1,33315E+00	7511	7,67998E-01	41380	0	17530,5
Non-responder	Mono_INHBA	Macro_IFI27	TIMP1	CD63	7,87788E-03	12011,3	6,92021E-03	17852	5,75449E-01	5844	7,93923E-01	18117	9,43282E-01	713	0	17530,5
Non-responder	Macro_IER3	Macro_FOLR2+APOE+	PLTP	ABCA1	7,87983E-03	16245,3	8,90018E-03	11831	3,75907E-01	13189	7,14720E-01	20221	8,63905E-01	18455	0	17530,5
Non-responder	Macro_OLFML3	Mono_CD14	LGALS9	CD44	7,87983E-03	13405,5	6,40396E-03	20221	4,42048E-01	10149	1,04366E+00	12260	9,10758E-01	6867	0	17530,5
Non-responder	Macro_OLFML3	Mono_INHBA	PSEN1	CD44	7,88178E-03	16501,7	6,41617E-03	20171	4,72894E-01	8954	8,89664E-01	15631	8,56392E-01	20222	0	17530,5
Non-responder	Macro_ISG15	Mast	HBEGF	CD82	7,89512E-03	7517,1	2,59277E-02	1967	9,66266E-01	1364	1,66327E+00	4087	8,86661E-01	12637	0	17530,5
Non-responder	Mono_CD16	Macro_FOLR2+APOE+	ADAM10	AXL	7,89723E-03	14080,3	1,26957E-02	6661	4,43369E-01	10088	1,43758E+00	6186	8,16461E-01	29936	0	17530,5
Non-responder	Macro_OLFML3	Mono_CD14	HLA-A	APLP2	7,90027E-03	12020,1	5,94300E-03	22714	4,55900E-01	9586	1,20816E+00	9321	9,41142E-01	949	0	17530,5
Non-responder	Macro_FOLR2+APOE+	Macro_OLFML3	HLA-DRB1	CD4	7,90330E-03	11816,5	5,59069E-03	25009	4,43216E-01	10090	1,51381E+00	5410	9,40421E-01	1043	0	17530,5
Non-responder	Macro_FOLR2+APOE+	Macro_IER3	CD14	ITGB1	7,90519E-03	13357,1	6,40181E-03	20234	4,61547E-01	9370	1,21780E+00	9178	8,95074E-01	10473	0	17530,5
Non-responder	Mono_INHBA	Macro_IER3	TNF	TNFRSF1B	7,90715E-03	13098,7	9,04667E-03	11519	5,11762E-01	7603	1,09712E+00	11213	8,67405E-01	17628	0	17530,5
Non-responder	Mono_INHBA	Macro_NLRP3	ICAM1	ITGAX_ITGB2	7,91301E-03	13478,1	6,40090E-03	20238	4,69306E-01	9101	1,11160E+00	10965	8,98745E-01	9556	0	17530,5

Post_NR_Myeloid_Myeloid_CellCell

Non-responder	Macro_FOLR2+APOE+	Macro_FOLR2+APOE+	HLA-G	LILRB2	7,93371E-03	12138,5	9,84383E-03	10100	5,45701E-01	6607	1,54161E+00	5120	8,51683E-01	21335	0	17530,5
Non-responder	Macro_LYVE1	Macro_ISG15	LILRB4	LAIR1	7,93650E-03	16118,1	6,39850E-03	20250	3,19998E-01	16442	8,52977E-01	16548	8,97682E-01	9820	0	17530,5
Non-responder	pDC_LILRA4	Macro_ISG15	HLA-DQA1	LAG3	7,94023E-03	8513,9	1,68540E-02	4095	7,89520E-01	2647	2,18543E+00	1423	8,70398E-01	16874	0	17530,5
Non-responder	Macro_FOLR2+APOE+	Mono_CD14	C1QB	C1QBP	7,94285E-03	14209,1	4,85907E-03	30510	4,87556E-01	8405	1,61143E+00	4497	8,96518E-01	10103	0	17530,5
Non-responder	Macro_LYVE1	Macro_ISG15	HLA-DQA2	LAG3	7,94630E-03	9375,1	2,39175E-02	2228	9,89337E-01	1252	1,49294E+00	5610	8,56261E-01	20255	0	17530,5
Non-responder	Macro_FOLR2+APOE+	Macro_FOLR2+APOE+	TNF	VSIR	7,94895E-03	13321,7	9,84188E-03	10105	5,64142E-01	6124	1,83498E+00	2914	8,16464E-01	29935	0	17530,5
Non-responder	Macro_FOLR2+APOE+	Macro_IFI27	APOE	ABCA1	7,95219E-03	16186,7	6,39722E-03	20258	3,49720E-01	14664	8,22584E-01	17344	8,92426E-01	11137	0	17530,5
Non-responder	Macro_NLRP3	Macro_ISG15	CD14	ITGB1	7,95611E-03	15147,7	6,39716E-03	20260	3,13348E-01	16829	1,12974E+00	10638	8,95040E-01	10481	0	17530,5
Non-responder	Macro_ISG15	Mono_INHBA	ADM	GPR84	7,96284E-03	14521,9	2,01421E-02	3022	6,67462E-01	4099	1,78057E+00	3230	7,52999E-01	44728	0	17530,5
Non-responder	pDC_LILRA4	Macro_FOLR2-APOE+	APP	LRP10	7,96850E-03	9102,3	1,68329E-02	4100	1,12754E+00	657	2,09336E+00	1731	8,51046E-01	21493	0	17530,5
Non-responder	Mono_INHBA	Macro_IER3	FN1	CD44	7,97380E-03	15772,7	1,06483E-02	8954	2,84663E-01	18784	9,11670E-01	15131	8,63867E-01	18464	0	17530,5
Non-responder	Mono_CD16	Macro_FOLR2+APOE+	VCAN	CD44	7,97380E-03	16231,7	6,39481E-03	20269	3,57739E-01	14189	8,68846E-01	16147	8,85118E-01	13023	0	17530,5
Non-responder	pDC_LILRA4	Mono_INHBA	SPON2	ITGA4	7,97416E-03	13042,7	1,68246E-02	4101	8,95560E-01	1758	2,12472E+00	1607	7,73136E-01	40217	0	17530,5
Non-responder	Mono_INHBA	Macro_IER3	FN1	C5AR1	7,97577E-03	9499,7	2,03147E-02	2984	6,40067E-01	4554	1,39888E+00	6664	8,74702E-01	15766	0	17530,5
Non-responder	Mono_CD16	Macro_OLFML3	ICAM4	ITGAV	7,97643E-03	19933,9	2,26786E-02	2432	5,53639E-01	6397	1,16090E+00	10114	6,45200E-01	63196	0	17530,5
Non-responder	Mono_INHBA	Macro_IER3	FN1	PLAUR	7,97774E-03	9736,7	1,59974E-02	4483	5,53803E-01	6392	1,26170E+00	8524	8,89965E-01	11754	0	17530,5
Non-responder	pDC_LILRA4	Macro_IER3	APP	TNFRSF21	7,98743E-03	10500,1	1,46403E-02	5212	1,09226E+00	800	2,39308E+00	941	8,24356E-01	28017	0	17530,5
Non-responder	Mono_CD16	cDC(CD1C)	GNAI2	FPR1	7,98758E-03	16318,1	6,39306E-03	20276	3,05785E-01	17324	8,60944E-01	16353	8,96508E-01	10107	0	17530,5
Non-responder	Macro_IER3	Mono_CD14	RPS19	C5AR1	7,98955E-03	13207,5	6,39305E-03	20277	5,07079E-01	7755	8,97180E-01	15474	9,19686E-01	5001	0	17530,5
Non-responder	Mono_INHBA	Macro_IER3	IL1B	SIGIRR	7,99152E-03	11727,3	9,66312E-03	10404	6,57384E-01	4266	1,31488E+00	7772	8,63021E-01	18664	0	17530,5
Non-responder	Macro_FOLR2+APOE+	Macro_FOLR2+APOE+	FN1	C5AR1	7,99172E-03	12430,7	1,23808E-02	6959	4,42614E-01	10119	1,60093E+00	4584	8,44958E-01	22961	0	17530,5
Non-responder	pDC_LILRA4	Macro_FOLR2+APOE+	SNCA	LAG3	7,99684E-03	16200,7	2,51442E-02	2061	6,67234E-01	4105	2,47026E+00	809	6,92994E-01	56498	0	17530,5
Non-responder	Macro_LYVE1	Macro_OLFML3	TNFSF13B	CD40	7,99743E-03	17288,1	6,64432E-03	19059	4,35595E-01	10399	7,12617E-01	20281	8,60875E-01	19171	0	17530,5
Non-responder	cDC(CD1C)	Macro_ISG15	LGALS9	HAVCR2	8,00335E-03	16304,3	6,39130E-03	20284	3,43327E-01	15011	1,02320E+00	12677	8,73754E-01	16019	0	17530,5
Non-responder	Macro_IER3	Mono_CD16	HLA-F	LILRB1	8,00730E-03	16149,3	6,39082E-03	20286	4,16915E-01	11194	9,89606E-01	13348	8,64239E-01	18388	0	17530,5
Non-responder	Macro_LYVE1	Macro_FOLR2+APOE+	HLA-C	LILRB2	8,00927E-03	12811,7	6,39078E-03	20287	4,73694E-01	8915	8,55561E-01	16482	9,41966E-01	844	0	17530,5
Non-responder	Macro_OLFML3	Mono_CD14	TNFSF10	TNFRSF10D	8,01317E-03	15443,7	1,66878E-02	4159	5,95373E-01	5413	1,44783E+00	6060	7,55854E-01	44056	0	17530,5
Non-responder	Macro_OLFML3	Macro_OLFML3	HLA-C	LILRB2	8,01317E-03	12443,9	5,73277E-03	24043	4,42534E-01	10126	1,21035E+00	9289	9,38923E-01	1231	0	17530,5
Non-responder	Macro_FOLR2+APOE+	Macro_IER3	B2M	LILRB1	8,01624E-03	15966,9	4,11476E-03	38114	4,42533E-01	10127	1,17560E+00	9871	9,23909E-01	4192	0	17530,5
Non-responder	Mono_INHBA	Macro_IER3	CCL3	CCR1	8,01717E-03	11654,7	1,01900E-02	9574	6,25695E-01	4816	1,41201E+00	6510	8,58017E-01	19843	0	17530,5
Non-responder	Mono_CD16	Macro_OLFML3	GNAI2	FPR1	8,01914E-03	15397,1	6,38920E-03	20292	3,05421E-01	17348	1,07175E+00	11700	8,96480E-01	10115	0	17530,5
Non-responder	Mono_INHBA	Macro_IER3	SPP1	ITGA4_ITGB1	8,02112E-03	12974,7	1,50278E-02	4999	4,55474E-01	9608	9,69476E-01	13801	8,61847E-01	18935	0	17530,5
Non-responder	Macro_IER3	pDC_LILRA4	HLA-DQA1	CD4	8,02112E-03	12304,9	6,38876E-03	20293	3,62368E-01	13926	1,37961E+00	6899	9,30401E-01	2876	0	17530,5
Non-responder	Mono_INHBA	Macro_IER3	SPP1	CD44	8,02507E-03	10822,1	1,39900E-02	5653	4,46448E-01	9961	1,08978E+00	11337	8,98476E-01	9629	0	17530,5
Non-responder	Mono_INHBA	Macro_IER3	SPP1	PTGER4	8,02705E-03	9657,7	2,36308E-02	2273	6,31412E-01	4697	1,42934E+00	6292	8,67928E-01	17496	0	17530,5
Non-responder	Mono_INHBA	Mono_CD16	S100A9	TLR4	8,03892E-03	14578,7	9,35918E-03	10924	5,35206E-01	6891	7,11890E-01	20302	8,69056E-01	17246	0	17530,5
Non-responder	cDC(CD1C)	pDC_LILRA4	TNFSF13B	TNFRSF17	8,04801E-03	12319,3	5,62059E-02	530	7,41397E-01	3129	1,66010E+00	4114	7,89435E-01	36293	0	17530,5
Non-responder	Macro_FOLR2+APOE+	Macro_IER3	CXCL12	CXCR4	8,05620E-03	14014,3	1,29908E-02	6410	4,42187E-01	10140	1,26871E+00	8420	8,26061E-01	27571	0	17530,5
Non-responder	Macro_ISG15	Macro_IER3	CCL8	CCR1	8,05942E-03	16001,7	3,03548E-02	1511	6,66579E-01	4116	1,99957E+00	2095	7,03366E-01	54756	0	17530,5
Non-responder	Mono_CD16	Mono_CD14	S100A8	TLR4	8,06271E-03	15437,7	9,19596E-03	11236	4,01779E-01	11905	8,66806E-01	16203	8,55963E-01	20314	0	17530,5
Non-responder	Macro_IER3	Macro_NLRP3	CCL3	CCR1	8,06469E-03	12017,3	9,85359E-03	10084	6,20616E-01	4910	1,35376E+00	7247	8,55959E-01	20315	0	17530,5
Non-responder	Macro_FOLR2-APOE+	Macro_NLRP3	APOE	ABCA1	8,06544E-03	8307,3	1,39898E-02	5654	6,64260E-01	4155	1,15943E+00	10143	9,24631E-01	4054	0	17530,5
Non-responder	Macro_OLFML3	Mono_CD14	FN1	C5AR1	8,07161E-03	12469,7	1,30863E-02	6321	4,57236E-01	9531	1,38228E+00	6862	8,48554E-01	22104	0	17530,5
Non-responder	Macro_FOLR2+APOE+	Macro_FOLR2+APOE+	CXCL11	CCR5	8,07469E-03	20836,7	2,21696E-02	2542	4,42087E-01	10146	1,35246E+00	7270	6,11101E-01	66695	0	17530,5
Non-responder	Macro_FOLR2+APOE-	Macro_NLRP3	C1QA	CD93	8,07654E-03	8192,5	1,67678E-02	4119	8,71480E-01	1908	9,70842E-01	13767	9,26785E-01	3638	0	17530,5
Non-responder	Mono_INHBA	pDC_LILRA4	CCL5	DPP4	8,08225E-03	18449,9	5,30520E-02	591	6,95213E-01	3678	1,65947E+00	4120	6,15076E-01	66330	0	17530,5
Non-responder	Macro_FOLR2+APOE+	Macro_FOLR2+APOE+	VEGFA	NRP1	8,08395E-03	17942,3	9,81249E-03	10149	4,42444E-01	10132	1,20689E+00	9331	7,62497E-01	42569	0	17530,5
Non-responder	cDC_CLEC9A	pDC_LILRA4	HLA-DRB1	CD4	8,08828E-03	8168,3	7,23100E-03	16622	5,98464E-01	5341	2,39042E+00	947	9,47233E-01	401	0	17530,5
Non-responder	Macro_NLRP3	pDC_LILRA4	HSP90B1	TLR7	8,09053E-03	12990,1	1,15057E-02	7865	3,87355E-01	12606	1,40267E+00	6621	8,55883E-01	20328	0	17530,5
Non-responder	Macro_LYVE1	Macro_IER3	PLAU	PLAUR	8,09451E-03	16547,5	8,11278E-03	13803	4,21305E-01	10993	7,19535E-01	20081	8,55871E-01	20330	0	17530,5
Non-responder	Macro_ISG15	pDC_LILRA4	CCL8	CCR5	8,09940E-03	17960,7	3,53931E-02	1181	6,66138E-01	4123	2,62953E+00	583	6,14485E-01	66386	0	17530,5
Non-responder	Mono_INHBA	Macro_ISG15	ADM	GPR84	8,09940E-03	13398,5	2,56080E-02	2003	7,10247E-01	3515	1,65901E+00	4123	7,74643E-01	39821	0	17530,5
Non-responder	Macro_OLFML3	Macro_FOLR2+APOE+	HMGB1	HAVCR2	8,10049E-03	15702,9	6,38108E-03	20333	2,87896E-01	18538	1,03634E+00	12419	8,98189E-01	9694	0	17530,5
Non-responder	cDC_CLEC9A	Macro_ISG15	ICAM3	ITGAL	8,10512E-03	11347,1	1,67556E-02	4124	9,02906E-01	1716	1,84882E+00	2824	8,13931E-01	30541	0	17530,5
Non-responder	Macro_IFI27	Macro_ISG15	HLA-C	LILRB1	8,10646E-03	12387,9	8,06104E-03	13946	4,94139E-01	8194	7,10431E-01	20336	9,35132E-01	1933	0	17530,5
Non-responder	Macro_OLFML3	Macro_FOLR2+APOE+	FN1	CD44	8,10846E-03	15486,5	9,32212E-03	10993	2,93641E-01	18125	1,14038E+00	10447	8,55855E-01	20337	0	17530,5
Non-responder	Macro_FOLR2+APOE+	Macro_FOLR2+APOE+	ADAM10	TSPAN14	8,10868E-03	14464,1	1,11899E-02	8221	4,41727E-01	10157	1,24155E+00	8815	8,25947E-01	27597	0	17530,5
Non-responder	Macro_ISG15	pDC_LILRA4	MDK	NCL	8,11085E-03	12353,1	2,17380E-02	2635	6,66018E-01	4125	2,02130E+00	2000	7,93147E-01	35475	0	17530,5
Non-responder	Macro_NLRP3	Macro_NLRP3	ACTR2	LDLR	8,11177E-03	15591,3	1,33795E-02	6080	4,41717E-01	10158	1,20901E+00	9308	7,95704E-01	34880	0	17530,5
Non-responder	Macro_IER3	pDC_LILRA4	HLA-DPB1	CD4	8,11444E-03	11907,1	6,37918E-03	20340	3,62797E-01	13900	1,38750E+00	6790	9,40923E-01	975	0	17530,5
Non-responder	Macro_OLFML3	Macro_FOLR2+APOE+	TNFSF12	CD163	8,11487E-03	13319,1	9,80838E-03	10159	4,69611E-01	9085	1,48841E+00	5655	8,39967E-01	24166	0	17530,5
Non-responder	Macro_FOLR2+APOE+	Macro_LYVE1	HSPA1A	TLR4	8,11643E-03	16202,5	6,37917E-03	20341	3,38571E-01	15284	1,05349E+00	12084	8,74680E-01	15773	0	17530,5
Non-responder	Macro_FOLR2+APOE+	Macro_IER3	HLA-A	APLP2	8,11796E-03	14084,5	4,71087E-03	31887	4,78977E-01	8741	1,15813E+00	10160	9,34367E-01	2104	0	17530,5
Non-responder	Macro_LYVE1	Macro_OLFML3	C1QB	LRP1	8,12043E-03	13498,3	7,99996E-03	14144	4,53512E-01	9682	7,10159E-01	20343	9,15766E-01	5792	0	17530,5
Non-responder	Macro_FOLR2+APOE+	pDC_LILRA4	GSTP1	TRAF2	8,12726E-03	11854,5	1,37515E-02	5823	4,41616E-01	10163	1,74522E+00	3448	8,47646E-01	22308	0	17530,5
Non-responder	Mono_CD14	Macro_LYVE1	THBS1	LRP1	8,12804E-03	7655,7	1,86210E-02	3458	7,89683E-01	2645	1,65797E+00	4128	8,94929E-01	10517	0	17530,5
Non-responder	Macro_NLRP3	Mono_INHBA	IL1A	IL1R1_IL1RAP	8,13036E-03	21047,5	2,75232E-02	1768	5,04202E-01	7832	1,15800E+00	10164	5,96820E-01	67943	0	17530,5
Non-responder	Macro_IFI27	Macro_FOLR2+APOE+	C3	ITGAX	8,13241E-03	12061,5	1,38429E-02	5760	5,46396E-01	6585	7,09760E-01	20349	8,96607E-01	10083	0	17530,5
Non-responder	Mono_INHBA	Macro_IER3	PTGS2	ALOX5	8,14040E-03	11127,1	1,41286E-02	5547	6,50357E-01	4392	9,99660E-01	13132	8,77632E-01	15034	0	17530,5
Non-responder	cDC_LAMP3	Mono_INHBA	VIM	CD44	8,14040E-03	12788,3	6,54615E-03	19526	5,55229E-01	6354	7,09581E-01	20353	9,50954E-01	178	0	17530,5
Non-responder	Macro_FOLR2+APOE+	Macro_FOLR2+APOE+	HGF	SDC2	8,14276E-03	20687,7	2,61494E-02	1939	4,60876E-01	9393	1,15778E+00	10168	6,34191E-01	64408	0	17530,5

Non-responder	Macro_FOLR2+APOE+	Macro_ISG15	LRPAP1	SORT1	8,15208E-03	15669,9	9,80430E-03	10171	4,59732E-01	9435	1,36769E+00	7039	7,98671E-01	34174	0	17530,5
Non-responder	Macro_FOLR2+APOE+	Macro_NLRP3	HSP90B1	LRP1	8,15241E-03	15646,5	6,37615E-03	20359	3,01424E-01	17600	1,04245E+00	12282	8,95124E-01	10461	0	17530,5
Non-responder	Macro_FOLR2+APOE+	Macro_LYVE1	ENTPD1	ADORA3	8,15441E-03	12167,3	1,60400E-02	4463	5,05625E-01	7796	1,12622E+00	10687	8,55777E-01	20360	0	17530,5
Non-responder	Macro_LYVE1	Macro_FOLR2+APOE+	TIMP2	CD44	8,15641E-03	17740,1	6,85890E-03	18121	3,22038E-01	16287	7,09299E-01	20361	8,72230E-01	16401	0	17530,5
Non-responder	Macro_FOLR2+APOE+	Macro_FOLR2+APOE+	TNF	FAS	8,15783E-03	29775,1	5,26879E-03	27262	2,40946E-01	22373	9,64172E-01	13924	5,98903E-01	67786	0	17530,5
Non-responder	Mono_INHBA	Macro_IER3	VEGFA	CD44	8,16243E-03	11440,9	1,08792E-02	8643	6,31989E-01	4686	9,93728E-01	13254	8,84845E-01	13091	0	17530,5
Non-responder	Macro_OLFML3	Macro_NLRP3	VEGFA	CD44	8,16643E-03	14049,1	6,48617E-03	19835	5,75093E-01	5854	1,39932E+00	6660	8,55764E-01	20366	0	17530,5
Non-responder	Macro_LYVE1	Mono_CD16	TNFSF13	TNFRSF14	8,16643E-03	18139,5	6,37494E-03	20366	2,97273E-01	17889	7,76221E-01	18564	8,72397E-01	16348	0	17530,5
Non-responder	Macro_IFI27	Macro_ISG15	HLA-C	LILRB2	8,16844E-03	12521,9	7,03535E-03	17380	5,41498E-01	6722	7,08928E-01	20367	9,44537E-01	610	0	17530,5
Non-responder	Mono_INHBA	cDC_LAMP3	TNF	TNFRSF1B	8,17646E-03	15939,7	7,49051E-03	15716	4,23667E-01	10892	9,04873E-01	15291	8,56169E-01	20269	0	17530,5
Non-responder	Macro_IER3	Mono_CD16	ICAM1	SPN	8,17847E-03	10420,1	1,42393E-02	5480	6,55104E-01	4310	1,62133E+00	4408	8,55727E-01	20372	0	17530,5
Non-responder	Macro_IER3	pDC_LILRA4	HRAS	TLR9	8,17975E-03	17761,9	7,81537E-02	242	1,01586E+00	1133	1,65689E+00	4137	6,21032E-01	65767	0	17530,5
Non-responder	Macro_FOLR2+APOE+	Macro_FOLR2+APOE+	SEMA3C	NRP2_PLXNA1	8,18007E-03	19972,5	2,29594E-02	2380	5,68518E-01	6010	1,15706E+00	10180	6,40283E-01	63762	0	17530,5
Non-responder	pDC_LILRA4	Macro_ISG15	HLA-DRA	LAG3	8,18007E-03	13627	1,59192E-02	4522	6,98250E-01	3649	1,84593E+00	2843	8,96261E-01	10180	0,372	46941
Non-responder	Macro_LYVE1	Macro_NLRP3	CD14	ITGB1	8,18249E-03	15814,7	6,37314E-03	20374	4,14318E-01	11326	7,47994E-01	19304	8,94863E-01	10539	0	17530,5
Non-responder	Macro_OLFML3	Mono_CD14	CD38	PECAM1	8,18942E-03	15364,3	1,40351E-02	5618	6,47407E-01	4428	1,23825E+00	8870	7,72392E-01	40375	0	17530,5
Non-responder	Macro_LYVE1	cDC_LAMP3	IL10	CD70	8,18975E-03	17835,7	8,15958E-02	218	1,05500E+00	953	1,16769E+00	10008	6,66503E-01	60469	0	17530,5
Non-responder	Macro_IER3	cDC_LAMP3	GNAI2	S1PR1	8,18975E-03	10211,5	4,03702E-02	953	1,11808E+00	700	1,13078E+00	10612	8,52003E-01	21262	0	17530,5
Non-responder	Mono_INHBA	cDC_LAMP3	ICAM1	IL2RG	8,19052E-03	11257,9	1,03045E-02	9410	6,78168E-01	3936	9,86380E-01	13417	8,89050E-01	11996	0	17530,5
Non-responder	Mono_CD16	Macro_FOLR2+APOE+	IL16	CD9	8,19052E-03	14370,3	9,22858E-03	11173	3,36382E-01	15415	1,34519E+00	7355	8,55698E-01	20378	0	17530,5
Non-responder	cDC_CLEC9A	Mono_CD16	F11R	ITGAL_ITGB2	8,19128E-03	9344,7	1,67261E-02	4139	7,71089E-01	2810	1,83330E+00	2929	8,60230E-01	19315	0	17530,5
Non-responder	Macro_FOLR2+APOE+	cDC_CLEC9A	MRC1	PTPRC	8,19655E-03	12239,9	9,60350E-03	10490	4,64153E-01	9276	1,73364E+00	3522	8,55688E-01	20381	0	17530,5
Non-responder	pDC_LILRA4	Macro_NLRP3	MIF	TNFRSF10D	8,19704E-03	10723,9	1,75008E-02	3847	6,65284E-01	4140	2,13844E+00	1551	8,30170E-01	26551	0	17530,5
Non-responder	pDC_LILRA4	Macro_FOLR2+APOE+	FAM3C	LAMP1	8,19704E-03	12809,5	1,67225E-02	4140	6,96821E-01	3664	2,62522E+00	591	7,81755E-01	38122	0	17530,5
Non-responder	Macro_FOLR2+APOE+	pDC_LILRA4	VEGFA	NRP1	8,20189E-03	17779,3	9,79481E-03	10187	4,41392E-01	10170	1,27043E+00	8398	7,62334E-01	42611	0	17530,5
Non-responder	Macro_FOLR2+APOE+	cDC_CLEC9A	HLA-B	CANX	8,20189E-03	13916,7	4,37692E-03	35243	4,40936E-01	10187	1,50094E+00	5528	9,40054E-01	1095	0	17530,5
Non-responder	Macro_OLFML3	Macro_FOLR2+APOE+	HSP90B1	TLR1	8,20460E-03	15747,3	8,79369E-03	12060	3,56812E-01	14252	9,39162E-01	14509	8,55676E-01	20385	0	17530,5
Non-responder	cDC_LAMP3	Macro_ISG15	HLA-A	LILRB2	8,20460E-03	12660,3	6,37118E-03	20385	4,09619E-01	11534	1,01277E+00	12897	9,41103E-01	955	0	17530,5
Non-responder	Macro_ISG15	Macro_IER3	CCL8	CCR2	8,20858E-03	17819,5	4,02145E-02	958	6,65140E-01	4142	2,00690E+00	2060	6,34206E-01	64407	0	17530,5
Non-responder	Mono_INHBA	Macro_IFI27	IL1B	SIGIRR	8,21064E-03	13507,9	8,55633E-03	12618	6,27650E-01	4770	1,04509E+00	12233	8,55670E-01	20388	0	17530,5
Non-responder	Macro_NLRP3	Macro_IER3	TNF	TRPM2	8,21438E-03	17249,9	1,05553E-02	9063	4,40824E-01	10191	1,33727E+00	7462	7,65336E-01	42003	0	17530,5
Non-responder	pDC_LILRA4	cDC(CD1C)	COPA	CD74	8,22012E-03	7053,9	9,43770E-03	10795	6,64958E-01	4144	1,95164E+00	2304	9,46065E-01	496	0	17530,5
Non-responder	Macro_FOLR2+APOE+	Macro_OLFML3	HLA-B	LILRA1	8,22474E-03	13044,7	6,36945E-03	20395	5,23288E-01	7236	1,36844E+00	7027	8,85039E-01	13035	0	17530,5
Non-responder	Macro_ISG15	pDC_LILRA4	VEGFB	RET	8,22590E-03	19378,5	4,05406E-02	945	6,83657E-01	3839	1,65574E+00	4145	5,59131E-01	70433	0	17530,5
Non-responder	Macro_IER3	Macro_IER3	FN1	PLAUR	8,22676E-03	16839,1	8,59024E-03	12547	3,52815E-01	14483	7,50456E-01	19239	8,55633E-01	20396	0	17530,5
Non-responder	cDC_LAMP3	Macro_ISG15	B2M	TFRC	8,22676E-03	12922,9	6,36942E-03	20396	3,86187E-01	12661	1,09226E+00	11296	9,31092E-01	2731	0	17530,5
Non-responder	Macro_FOLR2+APOE+	Macro_IFI27	C3	C3AR1	8,23685E-03	16827,5	9,27519E-03	11076	2,67941E-01	20095	7,07742E-01	20401	8,77631E-01	15035	0	17530,5
Non-responder	Mono_CD14	Macro_OLFML3	S100A8	CD36	8,24072E-03	7749,9	1,36780E-02	5871	1,06210E+00	922	2,38369E+00	956	8,83519E-01	13470	0	17530,5
Non-responder	Macro_IER3	Mono_CD14	ICAM1	ITGAX_ITGB2	8,24493E-03	14135,5	6,36657E-03	20405	4,24375E-01	10864	1,04398E+00	12254	8,98500E-01	9624	0	17530,5
Non-responder	Macro_ISG15	Macro_FOLR2+APOE+	B2M	LILRB2	8,24904E-03	8792,7	6,65622E-03	19001	6,64510E-01	4149	1,79793E+00	3124	9,51287E-01	159	0	17530,5
Non-responder	Macro_OLFML3	Macro_LYVE1	CALR	ITGAV	8,25099E-03	16440,3	7,89603E-03	14427	3,68494E-01	13598	8,65322E-01	16238	8,55598E-01	20408	0	17530,5
Non-responder	cDC(CD1C)	Macro_NLRP3	LGALS9	CD44	8,25099E-03	12200,1	6,36552E-03	20408	5,53408E-01	6405	1,18433E+00	9721	9,10513E-01	6936	0	17530,5
Non-responder	Macro_FOLR2+APOE+	Macro_OLFML3	CXCL12	CD4	8,25192E-03	14305,5	1,33593E-02	6094	4,40589E-01	10203	1,58159E+00	4754	8,03833E-01	32946	0	17530,5
Non-responder	Macro_OLFML3	Macro_OLFML3	HLA-F	LILRB2	8,25301E-03	13706,1	6,36543E-03	20409	4,64583E-01	9257	1,20360E+00	9407	8,89304E-01	11927	0	17530,5
Non-responder	Mono_CD14	Macro_NLRP3	THBS1	SDC4	8,25483E-03	12853,3	1,66973E-02	4150	7,09264E-01	3529	1,87148E+00	2710	7,89226E-01	36347	0	17530,5
Non-responder	pDC_LILRA4	Mono_INHBA	APP	LRP10	8,25774E-03	10453,1	1,26079E-02	6739	1,05386E+00	957	2,42000E+00	900	8,31784E-01	26139	0	17530,5
Non-responder	Mono_INHBA	Macro_NLRP3	CCL3	CCR1	8,25908E-03	11526,5	9,79319E-03	10190	6,15228E-01	5014	1,61270E+00	4486	8,55580E-01	20412	0	17530,5
Non-responder	Macro_NLRP3	pDC_LILRA4	VEGFA	CD44	8,26110E-03	14626,1	6,46677E-03	19927	5,27644E-01	7103	1,28755E+00	8157	8,55579E-01	20413	0	17530,5
Non-responder	Macro_NLRP3	Macro_FOLR2+APOE+	CD14	ITGB2	8,27702E-03	11251,9	6,31041E-03	20685	4,40298E-01	10211	1,43292E+00	6243	9,36824E-01	1590	0	17530,5
Non-responder	cDC(CD1C)	Macro_FOLR2+APOE+	CALR	LRP1	8,27730E-03	17383,5	6,36250E-03	20421	2,69588E-01	19942	7,34488E-01	19678	8,99707E-01	9346	0	17530,5
Non-responder	Macro_OLFML3	Macro_ISG15	CD47	SIRPA	8,27933E-03	12646,9	9,56212E-03	10570,5	5,07263E-01	7748,5	1,37412E+00	6962,5	8,55528E-01	20422,5	0	17530,5
Non-responder	Macro_IER3	Macro_IER3	PLAU	PLAUR	8,28338E-03	16216,3	8,06578E-03	13932	4,19457E-01	11076	7,93910E-01	18119	8,55513E-01	20424	0	17530,5
Non-responder	Macro_FOLR2+APOE+	Mono_CD14	FN1	C5AR1	8,28959E-03	12765,9	1,23390E-02	7009	4,40212E-01	10215	1,44775E+00	6061	8,44736E-01	23014	0	17530,5
Non-responder	Macro_OLFML3	Macro_OLFML3	MRC1	PTPRC	8,30368E-03	12533,1	9,57037E-03	10547	4,86467E-01	8455	1,48490E+00	5699	8,55475E-01	20434	0	17530,5
Non-responder	Macro_LYVE1	Macro_FOLR2+APOE+	HLA-DQB1	LAG3	8,30571E-03	12007,3	1,20762E-02	7256	6,12141E-01	5053	1,18152E+00	9762	8,55468E-01	20435	0	17530,5
Non-responder	Macro_NLRP3	Macro_OLFML3	HLA-F	LILRB1	8,31588E-03	15487,7	6,35830E-03	20440	3,92076E-01	12362	1,25335E+00	8658	8,63939E-01	18448	0	17530,5
Non-responder	Mono_INHBA	cDC_LAMP3	SPP1	ITGA4_ITGB1	8,31995E-03	14251,9	1,35244E-02	5983	4,16638E-01	11202	8,70533E-01	16102	8,55452E-01	20442	0	17530,5
Non-responder	Macro_FOLR2+APOE+	Macro_NLRP3	CCL5	SDC4	8,32739E-03	20659,1	1,64533E-02	4269	4,39834E-01	10227	1,50950E+00	5455	6,20552E-01	65814	0	17530,5
Non-responder	Mono_CD16	Mono_INHBA	CD55	ADGRE2	8,32739E-03	12961,3	1,05207E-02	9113	6,14323E-01	5022	1,15440E+00	10227	8,45159E-01	22914	0	17530,5
Non-responder	Macro_NLRP3	Macro_OLFML3	CCL2	CCR5	8,33370E-03	18996,3	1,84166E-02	3522	4,39812E-01	10229	1,32989E+00	7567	6,95219E-01	56133	0	17530,5
Non-responder	Macro_FOLR2+APOE+	Mono_CD16	LGALS9	LRP1	8,34032E-03	15335,9	6,35416E-03	20452	3,51975E-01	14538	1,26466E+00	8478	8,75056E-01	15681	0	17530,5
Non-responder	Macro_FOLR2+APOE+	Macro_ISG15	HLA-DQB1	CD4	8,34200E-03	12329,3	5,55867E-03	25227	4,12125E-01	11417	1,65373E+00	4165	9,28483E-01	3307	0	17530,5
Non-responder	Macro_FOLR2+APOE+	Macro_LYVE1	ADAM9	ITGB5	8,34318E-03	17350,3	2,77291E-02	1750	5,59915E-01	6225	1,15408E+00	10232	7,23533E-01	51014	0	17530,5
Non-responder	Mono_INHBA	Mono_INHBA	IL1B	IL1R1_IL1RAP	8,35951E-03	10323,3	2,04640E-02	2953	8,26781E-01	2308	1,65341E+00	4168	8,37904E-01	24657	0	17530,5
Non-responder	cDC_CLEC9A	cDC_CLEC9A	SELPLG	SELL	8,35951E-03	8305,3	1,66648E-02	4168	7,36802E-01	3184	2,03118E+00	1967	8,78987E-01	14677	0	17530,5
Non-responder	Macro_OLFML3	Macro_FOLR2+APOE+	HLA-C	LILRB1	8,36482E-03	12098,9	6,35152E-03	20464	4,08669E-01	11576	1,34065E+00	7412	9,27517E-01	3512	0	17530,5
Non-responder	pDC_LILRA4	Macro_ISG15	SEMA3C	NRP1_NRP2_PLXND1	8,37119E-03	14838,7	3,17057E-02	1403	6,63281E-01	4170	1,96259E+00	2245	7,34329E-01	48845	0	17530,5
Non-responder	Macro_FOLR2+APOE+	Mono_CD14	SEMA4A	PLXNB2	8,37165E-03	15640,1	9,76632E-03	10241	4,64292E-01	9272	1,40303E+00	6616	7,97246E-01	34541	0	17530,5
Non-responder	cDC_CLEC9A	cDC_LAMP3	GNAI2	S1PR1	8,37738E-03	9067,3	4,00217E-02	964	1,11233E+00	723	1,58675E+00	4722	8,51456E-01	21397	0	17530,5
Non-responder	Mono_CD14	Mast	AGTRAP	RACK1	8,38432E-03	7800,9	1,78200E-02	3732	1,03625E+00	1038	1,15353E+00	10245	9,12684E-01	6459	0	17530,5
Non-responder	Macro_IFI27	Macro_FOLR2+APOE+	IL18	IL18BP	8,38732E-03	11879,7	1,59762E-02	4497	5,83629E-01	5649	1,09536E+00	11247	8,55305E-01	20475	0	17530,5

Post_NR_Myeloid_Myeloid_CellCell

Non-responder	Mono_CD14	Macro_OLFML3	VCAN	ITGB1	8,38874E-03	7141,3	1,66560E-02	4173	1,03623E+00	1039	1,94243E+00	2340	8,94467E-01	10624	0	17530,5
Non-responder	Macro_FOLR2+APOE+	Macro_NLRP3	ICAM1	ITGAM_ITGB2	8,38937E-03	14231,9	6,34846E-03	20476	3,87561E-01	12593	1,19490E+00	9549	8,92917E-01	11011	0	17530,5
Non-responder	Macro_OLFML3	Mono_CD16	APP	FPR2	8,39384E-03	17273,5	1,17285E-02	7619	4,98845E-01	8030	1,15328E+00	10248	7,60886E-01	42940	0	17530,5
Non-responder	cDC(CD1C)	Macro_FOLR2+APOE+	CD99	PILRA	8,40167E-03	16906,7	6,34731E-03	20482	2,90274E-01	18363	7,87588E-01	18282	8,97430E-01	9876	0	17530,5
Non-responder	Macro_FOLR2+APOE+	pDC_LILRA4	CXCL8	CD79A	8,40972E-03	19368,9	2,96668E-02	1578	4,39374E-01	10253	1,47443E+00	5791	6,57127E-01	61692	0	17530,5
Non-responder	Mono_INHBA	Macro_FOLR2+APOE+	SPP1	ITGA9_ITGB1	8,41193E-03	9701,3	2,53233E-02	2041	6,80620E-01	3902	1,60662E+00	4546	8,55275E-01	20487	0	17530,5
Non-responder	Macro_FOLR2+APOE-	Mono_CD16	HLA-C	LILRA1	8,41217E-03	9478,3	1,75056E-02	3844	7,07497E-01	3548	7,87030E-01	18292	9,23967E-01	4177	0	17530,5
Non-responder	Macro_FOLR2+APOE+	cDC(CD1C)	PLAU	PLAUR	8,42014E-03	16424,9	8,03434E-03	14034	3,82667E-01	12848	8,27585E-01	17221	8,55271E-01	20491	0	17530,5
Non-responder	Mono_INHBA	cDC(CD1C)	IL1B	SIGIRR	8,42425E-03	9914,7	1,19800E-02	7356	7,19626E-01	3383	1,48766E+00	5667	8,75236E-01	15637	0	17530,5
Non-responder	Mono_CD16	Macro_IER3	GNAI2	FPR1	8,42631E-03	17017,3	6,34509E-03	20494	3,01257E-01	17611	7,49963E-01	19251	8,96158E-01	10200	0	17530,5
Non-responder	cDC_LAMP3	Macro_FOLR2+APOE+	B2M	LILRB2	8,42837E-03	11158,3	6,34500E-03	20495	5,41839E-01	6716	1,11805E+00	10829	9,50165E-01	221	0	17530,5
Non-responder	Macro_IFI27	Macro_ISG15	LGALS3	LAG3	8,43454E-03	11272,7	1,96494E-02	3163	8,56717E-01	2035	9,99349E-01	13137	8,55234E-01	20498	0	17530,5
Non-responder	pDC_LILRA4	Macro_IER3	HSP90B1	LRP1	8,43517E-03	7403,5	9,75385E-03	10261	8,67190E-01	1943	2,35417E+00	1015	9,13468E-01	6268	0	17530,5
Non-responder	cDC_LAMP3	pDC_LILRA4	CLCF1	SORL1	8,43564E-03	14765,1	3,50078E-02	1200	6,79713E-01	3908	1,65236E+00	4181	7,42789E-01	47006	0	17530,5
Non-responder	Macro_ISG15	Macro_NLRP3	TNFSF10	TNFRSF10B	8,43564E-03	10594,9	1,66334E-02	4181	8,41491E-01	2170	2,22983E+00	1292	8,25185E-01	27801	0	17530,5
Non-responder	Macro_OLFML3	cDC_CLEC9A	QDPR	DYSF	8,44154E-03	18834,9	2,04257E-02	2963	6,01351E-01	5280	1,15253E+00	10263	6,82644E-01	58138	0	17530,5
Non-responder	cDC_CLEC9A	Macro_ISG15	LGALS9	HAVCR2	8,44154E-03	9293,9	9,87967E-03	10045	5,92167E-01	5484	1,79435E+00	3147	8,95887E-01	10263	0	17530,5
Non-responder	Macro_OLFML3	Macro_FOLR2+APOE+	VEGFA	NRP2	8,44154E-03	17626,9	9,75341E-03	10263	4,96806E-01	8099	1,20117E+00	9438	7,61458E-01	42804	0	17530,5
Non-responder	Macro_OLFML3	Macro_ISG15	TNFSF13	SDC2	8,45111E-03	11829,3	1,61849E-02	4386	5,65184E-01	6096	1,59212E+00	4671	8,30494E-01	26463	0	17530,5
Non-responder	Macro_FOLR2+APOE+	Macro_ISG15	LGALS3BP	CD33	8,45111E-03	14581,3	9,75181E-03	10266	4,71703E-01	8997	1,48302E+00	5720	8,14579E-01	30393	0	17530,5
Non-responder	Macro_FOLR2+APOE+	Macro_LYVE1	HLA-DPB1	CD4	8,45307E-03	11836,7	6,34360E-03	20507	4,48881E-01	9872	1,15147E+00	10277	9,40767E-01	997	0	17530,5
Non-responder	Mono_INHBA	cDC(CD1C)	SPP1	ITGA4_ITGB1	8,45513E-03	12630,9	1,54020E-02	4791	4,65117E-01	9238	1,00732E+00	12999	8,63305E-01	18596	0	17530,5
Non-responder	cDC_LAMP3	Macro_FOLR2+APOE+	ICAM1	IL2RG	8,45513E-03	17269,9	6,34353E-03	20508	3,98865E-01	12033	8,14675E-01	17564	8,62771E-01	18714	0	17530,5
Non-responder	Macro_FOLR2+APOE+	Macro_FOLR2+APOE+	HBEGF	CD44	8,46544E-03	14208,5	6,34199E-03	20513	3,78524E-01	13051	1,26969E+00	8410	8,90852E-01	11538	0	17530,5
Non-responder	Macro_IFI27	Macro_FOLR2+APOE+	HLA-DQB1	LAG3	8,47783E-03	13573,5	1,20178E-02	7320	6,06883E-01	5167	8,23088E-01	17331	8,55168E-01	20519	0	17530,5
Non-responder	Mono_CD16	Macro_NLRP3	LPL	CD44	8,47984E-03	13718,9	9,74481E-03	10275	4,96032E-01	8123	1,51824E+00	5362	8,27208E-01	27304	0	17530,5
Non-responder	Macro_FOLR2+APOE+	Macro_IER3	TNFSF13B	HLA-DPB1	8,48816E-03	11932,5	6,34055E-03	20524	4,56930E-01	9545	1,08867E+00	11364	9,43477E-01	699	0	17530,5
Non-responder	Macro_FOLR2+APOE+	Macro_ISG15	MDK	ITGA4_ITGB1	8,48944E-03	18341,7	1,83660E-02	3537	4,38854E-01	10278	1,26667E+00	8453	7,18740E-01	51910	0	17530,5
Non-responder	cDC_CLEC9A	pDC_LILRA4	HSP90B1	TLR7	8,49451E-03	8146,7	1,86797E-02	3438	6,62274E-01	4191	2,01263E+00	2037	8,83274E-01	13537	0	17530,5
Non-responder	Macro_FOLR2+APOE+	Macro_FOLR2+APOE+	TNF	NOTCH1	8,49750E-03	19763,3	1,25045E-02	6837	4,17618E-01	11165	1,03482E+00	12454	7,24506E-01	50830	0	17530,5
Non-responder	Macro_NLRP3	Macro_FOLR2+APOE+	TNFSF13B	CD40	8,50058E-03	15466,9	6,33995E-03	20530	4,15878E-01	11249	1,28490E+00	8193	8,58043E-01	19832	0	17530,5
Non-responder	Macro_FOLR2+APOE+	Macro_FOLR2-APOE+	C3	LRP1	8,50265E-03	14049,1	1,17180E-02	7634	3,86360E-01	12651	7,03570E-01	20531	8,89419E-01	11899	0	17530,5
Non-responder	Macro_ISG15	cDC(CD1C)	B2M	CD1B	8,50545E-03	7974,1	2,56140E-02	2000	6,07793E-01	5149	1,56319E+00	4908	8,95800E-01	10283	0	17530,5
Non-responder	Macro_FOLR2+APOE+	Mono_CD14	FN1	CD44	8,50679E-03	15797,9	9,20954E-03	11215	3,08732E-01	17116	1,02756E+00	12595	8,55104E-01	20533	0	17530,5
Non-responder	Macro_NLRP3	Macro_OLFML3	TNFSF13B	CD40	8,51094E-03	16170,3	6,33830E-03	20535	4,15707E-01	11256	1,07233E+00	11689	8,58027E-01	19841	0	17530,5
Non-responder	Macro_IER3	Macro_FOLR2+APOE+	THBS1	CD47	8,51508E-03	14298,5	9,06518E-03	11472	4,82260E-01	8615	9,89985E-01	13338	8,55071E-01	20537	0	17530,5
Non-responder	Mono_INHBA	Mono_CD16	CD52	SIGLEC10	8,51508E-03	18719,7	6,57084E-03	19409	3,14438E-01	16774	7,03400E-01	20537	8,60107E-01	19348	0	17530,5
Non-responder	Mono_CD16	Mono_INHBA	SELPLG	ITGB2	8,51715E-03	15653,9	6,33733E-03	20538	2,95921E-01	17977	9,15525E-01	15040	9,09322E-01	7184	0	17530,5
Non-responder	Macro_FOLR2+APOE+	Macro_FOLR2+APOE+	SPARC	ENG	8,53111E-03	18683,9	2,24612E-02	2477	4,38202E-01	10291	1,53885E+00	5154	6,83792E-01	57967	0	17530,5
Non-responder	Macro_FOLR2+APOE+	Macro_ISG15	MDK	SDC3	8,53754E-03	21254,3	2,48433E-02	2108	4,58678E-01	9478	1,15049E+00	10293	6,09543E-01	66862	0	17530,5
Non-responder	Macro_LYVE1	Mono_CD16	A2M	LRP1	8,53999E-03	15399,1	9,22861E-03	11172	3,72600E-01	13355	7,03021E-01	20549	8,79981E-01	14389	0	17530,5
Non-responder	Macro_FOLR2+APOE+	Mono_CD16	FN1	PLAUR	8,54414E-03	16705,7	8,50736E-03	12736	2,66575E-01	20218	1,03263E+00	12493	8,55033E-01	20551	0	17530,5
Non-responder	Macro_IER3	Macro_OLFML3	HLA-A	LILRB1	8,54622E-03	14748,9	6,33494E-03	20552	3,27968E-01	15923	8,72547E-01	16045	9,26472E-01	3694	0	17530,5
Non-responder	Macro_FOLR2+APOE+	Mono_CD16	TGS1	RXRA	8,54718E-03	20126,1	1,27337E-02	6622	4,37993E-01	10296	1,25466E+00	8635	6,86499E-01	57547	0	17530,5
Non-responder	Mono_INHBA	Mono_CD14	S100A9	CD36	8,55040E-03	8505,3	1,78214E-02	3731	7,59855E-01	2925	1,14997E+00	10297	9,05525E-01	8043	0	17530,5
Non-responder	Mono_INHBA	cDC(CD1C)	PTGS2	ALOX5	8,55454E-03	8643,7	1,84102E-02	3524	7,93248E-01	2617	1,29299E+00	8077	8,91150E-01	11470	0	17530,5
Non-responder	Mono_CD16	Macro_FOLR2+APOE+	LTB	TNFRSF1A	8,56005E-03	14397,5	1,11084E-02	8348	4,37743E-01	10300	1,18637E+00	9683	8,31881E-01	26126	0	17530,5
Non-responder	Mono_CD14	cDC(CD1C)	VCAN	ITGB1	8,57144E-03	7391,3	1,65856E-02	4204	1,03457E+00	1049	1,73626E+00	3506	8,94267E-01	10667	0	17530,5
Non-responder	Macro_NLRP3	Mast	AGTRAP	RACK1	8,57616E-03	11828,5	1,20393E-02	7297	8,11608E-01	2441	6,67570E-01	21569	8,95742E-01	10305	0	17530,5
Non-responder	Macro_FOLR2+APOE+	cDC_LAMP3	CALM1	FAS	8,57938E-03	13801,9	1,34309E-02	6045	4,49475E-01	9852	1,14907E+00	10306	8,35341E-01	25276	0	17530,5
Non-responder	Mono_CD16	Macro_NLRP3	VIM	CD44	8,57938E-03	11465,9	6,30888E-03	20690	4,37522E-01	10306	1,25792E+00	8579	9,50086E-01	224	0	17530,5
Non-responder	Macro_NLRP3	Macro_LYVE1	TNF	TNFRSF1B	8,58163E-03	16568,3	7,34409E-03	16211	4,00665E-01	11959	8,51879E-01	16572	8,54949E-01	20569	0	17530,5
Non-responder	Mono_CD16	Macro_OLFML3	B2M	LILRB1	8,58163E-03	12609,3	6,33027E-03	20569	3,41058E-01	15121	1,26911E+00	8415	9,37735E-01	1411	0	17530,5
Non-responder	Macro_OLFML3	Macro_LYVE1	HLA-DMA	CD4	8,58580E-03	14997,7	6,33018E-03	20571	3,62984E-01	13889	7,65850E-01	18844	9,24094E-01	4154	0	17530,5
Non-responder	Mono_INHBA	Macro_LYVE1	TNF	TNFRSF1A	8,59206E-03	11549,3	1,19605E-02	7387	5,98709E-01	5334	1,24217E+00	8806	8,62879E-01	18689	0	17530,5
Non-responder	Macro_IER3	Macro_FOLR2+APOE+	GNAI2	C5AR1	8,59206E-03	15337,9	6,32965E-03	20574	3,56075E-01	14295	9,37890E-01	14540	8,97984E-01	9750	0	17530,5
Non-responder	Mono_INHBA	Macro_LYVE1	TNF	TNFRSF1B	8,59415E-03	15169,1	8,04077E-03	14014	4,54818E-01	9628	9,00699E-01	15397	8,60479E-01	19276	0	17530,5
Non-responder	Macro_ISG15	Macro_ISG15	CCL5	CCR1	8,59519E-03	16308,9	1,96686E-02	3157	6,75350E-01	3975	1,64862E+00	4208	7,14810E-01	52674	0	17530,5
Non-responder	Macro_ISG15	Macro_FOLR2+APOE+	HLA-A	LILRB2	8,59519E-03	8688,5	6,92496E-03	17832	6,60800E-01	4208	1,79065E+00	3164	9,43371E-01	708	0	17530,5
Non-responder	Macro_FOLR2+APOE+	Macro_LYVE1	COPA	CD74	8,60878E-03	14341,1	6,42229E-03	20144	4,09073E-01	11562	7,01696E-01	20582	9,35358E-01	1887	0	17530,5
Non-responder	Macro_ISG15	cDC_CLEC9A	CALR	SCARF1	8,61900E-03	10117,3	1,86179E-02	3460	7,11659E-01	3489	1,64826E+00	4212	8,49411E-01	21895	0	17530,5
Non-responder	pDC_LILRA4	cDC_CLEC9A	APP	TNFRSF21	8,61917E-03	12170,5	1,10638E-02	8403	1,04891E+00	978	2,46803E+00	817	8,03149E-01	33124	0	17530,5
Non-responder	Macro_OLFML3	Macro_ISG15	CD1D	LILRB2	8,62138E-03	10984,9	1,29124E-02	6470	6,49880E-01	4399	1,51958E+00	5350	8,52396E-01	21175	0	17530,5
Non-responder	Macro_NLRP3	Macro_FOLR2+APOE-	VCAN	TLR1	8,62138E-03	14863,5	9,71222E-03	10319	8,02661E-01	2520	1,24120E+00	8820	7,94634E-01	35128	0	17530,5
Non-responder	Macro_OLFML3	Macro_FOLR2+APOE+	CALR	SCARF1	8,62462E-03	16088,7	9,84736E-03	10096	4,37141E-01	10320	1,19195E+00	9591	8,04007E-01	32906	0	17530,5
Non-responder	Mono_INHBA	Macro_NLRP3	INHBA	ENG	8,62495E-03	12673,7	2,04667E-02	2952	7,04224E-01	3581	1,64817E+00	4213	7,94786E-01	35092	0	17530,5
Non-responder	Macro_FOLR2+APOE+	Macro_FOLR2-APOE+	SERPINA1	LRP1	8,62552E-03	12705,9	8,64993E-03	12399	5,18250E-01	7388	7,01462E-01	20590	9,16581E-01	5622	0	17530,5
Non-responder	Macro_OLFML3	Macro_ISG15	HLA-G	LILRB1	8,63109E-03	14007,7	1,00622E-02	9759	5,13880E-01	7537	1,41966E+00	6415	8,21147E-01	28797	0	17530,5
Non-responder	Mono_CD16	Macro_ISG15	LGALS9	PTPRC	8,63109E-03	12783,9	5,98250E-03	22488	4,37088E-01	10322	1,43771E+00	6183	9,08295E-01	7396	0	17530,5
Non-responder	pDC_LILRA4	Mono_CD14	APP	LRP10	8,63657E-03	10547,1	1,23197E-02	7031	1,04883E+00	979	2,59318E+00	637	8,30160E-01	26558	0	17530,5
Non-responder	Mono_CD14	Mono_INHBA	VCAN	ITGB1	8,64284E-03	7354,7	1,65502E-02	4216	1,03374E+00	1053	1,77120E+00	3277	8,94166E-01	10697	0	17530,5

Post_NR_Myeloid_Myeloid_CellCell

Non-responder	Macro_NLRP3	Mono_INHBA	TGFB1	ITGB8	8,64730E-03	16510,7	2,49685E-02	2093	5,77270E-01	5796	1,14781E+00	10327	7,43791E-01	46807	0	17530,5
Non-responder	pDC_LILRA4	Mono_CD16	HLA-F	LILRB1	8,64730E-03	7961,9	1,16141E-02	7733	7,36093E-01	3188	2,34682E+00	1031	8,95634E-01	10327	0	17530,5
Non-responder	Macro_IER3	Macro_FOLR2+APOE+	TNF	TNFRSF1B	8,64859E-03	14742,7	7,32554E-03	16272	3,95563E-01	12213	1,36258E+00	7097	8,54792E-01	20601	0	17530,5
Non-responder	Macro_FOLR2+APOE+	pDC_LILRA4	TNF	TNFRSF1B	8,65069E-03	13625,5	7,32242E-03	16281	4,14516E-01	11314	1,93244E+00	2400	8,54766E-01	20602	0	17530,5
Non-responder	Mono_CD16	Macro_FOLR2-APOE+	ADAM10	GPNMB	8,65699E-03	13026,3	1,48526E-02	5100	5,21005E-01	7295	9,34854E-01	14601	8,54751E-01	20605	0	17530,5
Non-responder	Macro_OLFML3	Macro_ISG15	TNFSF10	TNFRSF10D	8,66028E-03	18089,7	1,15663E-02	7807	5,12734E-01	7580	1,46091E+00	5933	7,20470E-01	51598	0	17530,5
Non-responder	Macro_IER3	Macro_FOLR2+APOE+	TNF	TNFRSF1A	8,66539E-03	11663,5	1,04556E-02	9217	5,32335E-01	6980	1,67454E+00	3981	8,54728E-01	20609	0	17530,5
Non-responder	Macro_FOLR2+APOE+	Mono_CD16	MMP9	ITGAM	8,67002E-03	17341,9	9,70126E-03	10334	5,00201E-01	7985	1,97178E+00	2200	7,35321E-01	48660	0	17530,5
Non-responder	Macro_IER3	pDC_LILRA4	SEMA6B	PLXNA4	8,67270E-03	17245,5	1,00333E-01	138	9,05440E-01	1701	1,64726E+00	4221	6,49831E-01	62637	0	17530,5
Non-responder	Macro_FOLR2+APOE+	Macro_ISG15	HSP90B1	TLR4	8,67802E-03	16488,3	6,59845E-03	19279	3,32170E-01	15670	1,20601E+00	9347	8,54708E-01	20615	0	17530,5
Non-responder	Mono_INHBA	Macro_LYVE1	IL1B	SIGIRR	8,68223E-03	12477,1	9,01251E-03	11603	6,39905E-01	4556	1,22580E+00	9042	8,58848E-01	19654	0	17530,5
Non-responder	Macro_NLRP3	cDC_CLEC9A	CD14	ITGA4	8,68433E-03	15105,7	6,32091E-03	20618	3,29115E-01	15856	1,12043E+00	10798	8,94056E-01	10726	0	17530,5
Non-responder	Mono_CD14	Macro_ISG15	CD55	ADGRE2	8,69066E-03	8518,7	1,65312E-02	4224	8,57815E-01	2024	1,91313E+00	2495	8,72481E-01	16320	0	17530,5
Non-responder	Mono_CD16	Macro_FOLR2+APOE+	SEMA4A	NRP1_PLXNA1	8,69605E-03	19435,7	1,05410E-02	9085	4,62575E-01	9333	1,14714E+00	10342	7,24232E-01	50888	0	17530,5
Non-responder	Macro_OLFML3	Mono_INHBA	GRN	TNFRSF1B	8,69698E-03	12665,3	6,31940E-03	20624	4,40024E-01	10224	1,05554E+00	12035	9,30289E-01	2913	0	17530,5
Non-responder	Macro_OLFML3	Mono_CD14	C1QA	CR1	8,70256E-03	13553,3	1,69455E-02	4058	4,39397E-01	10250	1,14713E+00	10344	8,33958E-01	25584	0	17530,5
Non-responder	Mono_INHBA	Macro_LYVE1	SPP1	ITGA4_ITGB1	8,71386E-03	13452,3	1,47110E-02	5173	4,47441E-01	9924	9,01060E-01	15387	8,60574E-01	19247	0	17530,5
Non-responder	Mono_CD16	cDC_CLEC9A	LGALS9	MRC2	8,71561E-03	15751,3	1,39799E-02	5664	4,56894E-01	9546	1,14693E+00	10348	7,92282E-01	35668	0	17530,5
Non-responder	Macro_IER3	Macro_OLFML3	CD14	TLR4	8,71808E-03	18744,5	6,48207E-03	19857	3,35895E-01	15449	7,13560E-01	20252	8,54612E-01	20634	0	17530,5
Non-responder	Macro_OLFML3	Mono_CD16	CD48	CD244	8,72213E-03	16525,3	1,78847E-02	3704	4,96073E-01	8122	1,14675E+00	10350	7,60975E-01	42920	0	17530,5
Non-responder	pDC_LILRA4	Macro_FOLR2+APOE-	SELPLG	ITGB2	8,72213E-03	8620,9	9,69483E-03	10350	6,28836E-01	4747	1,40672E+00	6568	9,25391E-01	3909	0	17530,5
Non-responder	Mast	pDC_LILRA4	COPA	P2RY6	8,72381E-03	11003,1	1,15417E-02	7834	1,04812E+00	984	2,42076E+00	895	8,25303E-01	27772	0	17530,5
Non-responder	Macro_OLFML3	Macro_ISG15	CD274	CD80	8,72540E-03	17507,9	2,38265E-02	2249	6,59800E-01	4216	1,51197E+00	5425	6,82791E-01	58119	0	17530,5
Non-responder	Macro_FOLR2+APOE+	Macro_OLFML3	TGM2	SDC4	8,72540E-03	21073,1	1,33460E-02	6106	4,48585E-01	9883	1,14669E+00	10351	6,58740E-01	61495	0	17530,5
Non-responder	Macro_IER3	Macro_IER3	RPS19	C5AR1	8,72653E-03	13483,9	6,81513E-03	18314	5,53918E-01	6388	6,99945E-01	20638	9,22016E-01	4549	0	17530,5
Non-responder	Macro_OLFML3	cDC(CD1C)	C1QB	CD33	8,72865E-03	16033,9	7,83396E-03	14614	2,61187E-01	20639	8,71076E-01	16084	8,91793E-01	11302	0	17530,5
Non-responder	Macro_ISG15	cDC_CLEC9A	LGALS9	HAVCR2	8,73193E-03	9329,3	9,80330E-03	10176	6,06891E-01	5166	1,74963E+00	3421	8,95524E-01	10353	0	17530,5
Non-responder	Macro_IER3	pDC_LILRA4	TNFSF13	TNFRSF17	8,73263E-03	11607,9	7,36670E-02	285	7,80000E-01	2726	1,64504E+00	4231	8,02512E-01	33267	0	17530,5
Non-responder	Macro_OLFML3	Macro_ISG15	SERPINA1	LRP1	8,73711E-03	14603,7	6,31676E-03	20643	3,40112E-01	15180	1,09800E+00	11197	9,03750E-01	8468	0	17530,5
Non-responder	Mono_CD16	Mast	CD55	ADGRE2	8,74770E-03	12329,5	1,21946E-02	7151	6,79188E-01	3917	1,03705E+00	12401	8,54575E-01	20648	0	17530,5
Non-responder	Macro_FOLR2+APOE+	Mast	C1QB	C1QBP	8,74827E-03	15413,9	4,75380E-03	31509	4,83117E-01	8584	1,22321E+00	9088	8,95498E-01	10358	0	17530,5
Non-responder	Macro_IER3	Macro_OLFML3	CD14	ITGB2	8,74982E-03	13549,7	6,31613E-03	20649	4,06938E-01	11664	8,62180E-01	16324	9,36851E-01	1581	0	17530,5
Non-responder	Mono_INHBA	Mono_CD14	CCL3L1	CCR1	8,75066E-03	9940,3	1,65101E-02	4234	7,82157E-01	2706	1,89317E+00	2588	8,46290E-01	22643	0	17530,5
Non-responder	Macro_OLFML3	Macro_OLFML3	HSP90B1	TLR2	8,75405E-03	18097,9	6,45963E-03	19976	2,69678E-01	19934	6,99391E-01	20651	8,87562E-01	12398	0	17530,5
Non-responder	Mono_CD16	Macro_FOLR2+APOE+	THBS1	SCARB1	8,75481E-03	15151,7	1,34845E-02	6008	4,36470E-01	10360	1,25998E+00	8548	8,02342E-01	33312	0	17530,5
Non-responder	Macro_OLFML3	Macro_ISG15	FN1	C5AR1	8,75809E-03	11590,5	1,36602E-02	5879	4,88345E-01	8379	1,58656E+00	4724	8,51291E-01	21440	0	17530,5
Non-responder	Macro_NLRP3	cDC(CD1C)	S100A9	CD36	8,75829E-03	14237,1	6,69657E-03	18811	7,01179E-01	3615	1,13328E+00	10576	8,54555E-01	20653	0	17530,5
Non-responder	Macro_OLFML3	Macro_ISG15	FN1	SDC2	8,76136E-03	15239,1	2,39525E-02	2222	5,18506E-01	7378	1,66368E+00	4084	7,51965E-01	44981	0	17530,5
Non-responder	Mono_INHBA	pDC_LILRA4	CD14	TLR9	8,76270E-03	7443,7	6,50387E-02	390	1,02045E+00	1116	1,64389E+00	4236	8,81704E-01	13946	0	17530,5
Non-responder	Macro_IER3	Macro_FOLR2+APOE+	HLA-DQB1	LAG3	8,76678E-03	11843,5	1,18978E-02	7449	5,96078E-01	5391	1,28516E+00	8190	8,54545E-01	20657	0	17530,5
Non-responder	Mono_CD14	Macro_FOLR2+APOE+	NRG1	HLA-DPB1	8,77474E-03	8474,9	1,94594E-02	3211	6,58802E-01	4238	1,67352E+00	3991	8,83750E-01	13404	0	17530,5
Non-responder	Mono_CD14	Mono_INHBA	S100A9	TLR4	8,78103E-03	7032,7	1,55841E-02	4703	1,05562E+00	949	2,12273E+00	1613	8,95443E-01	10368	0	17530,5
Non-responder	Macro_LYVE1	Macro_ISG15	CCL4	CCR1	8,78377E-03	13103,7	1,11341E-02	8313	5,97729E-01	5362	9,76125E-01	13648	8,54508E-01	20665	0	17530,5
Non-responder	Macro_OLFML3	Mono_CD14	PTPN6	CLEC12A	8,78589E-03	15386,3	8,62558E-03	12453	3,66117E-01	13733	1,03013E+00	12549	8,54500E-01	20666	0	17530,5
Non-responder	Mono_INHBA	Macro_LYVE1	TIMP1	CD63	8,78589E-03	9148,5	7,79612E-03	14721	6,82627E-01	3862	1,21868E+00	9165	9,46387E-01	464	0	17530,5
Non-responder	Macro_OLFML3	Macro_FOLR2+APOE+	MRC1	PTPRC	8,78802E-03	12493,3	9,42088E-03	10829	4,73959E-01	8907	1,60762E+00	4533	8,54499E-01	20667	0	17530,5
Non-responder	pDC_LILRA4	cDC_LAMP3	APP	NCSTN	8,79392E-03	11703,9	1,10089E-02	8465	1,06006E+00	933	2,37096E+00	988	8,13652E-01	30603	0	17530,5
Non-responder	Macro_OLFML3	Macro_ISG15	TGFB1	SDC2	8,80072E-03	14077,9	1,39459E-02	5687	5,10365E-01	7662	1,42965E+00	6284	8,02705E-01	33226	0	17530,5
Non-responder	Macro_ISG15	Macro_LYVE1	SERPING1	LRP1	8,80401E-03	8476,7	1,33149E-02	6130	6,99077E-01	3640	1,58756E+00	4708	8,95434E-01	10375	0	17530,5
Non-responder	pDC_LILRA4	Mast	GRN	NTRK1	8,80490E-03	7721,7	6,64055E-02	368	1,01042E+00	1159	1,64338E+00	4243	8,76533E-01	15308	0	17530,5
Non-responder	Macro_FOLR2+APOE+	Mono_INHBA	CALR	LRP1	8,80717E-03	13768,9	6,31143E-03	20676	4,07688E-01	11631	1,19189E+00	9593	8,99343E-01	9414	0	17530,5
Non-responder	Macro_IER3	Macro_FOLR2+APOE+	THBS1	LRP1	8,80930E-03	15923,7	8,84879E-03	11928	3,94947E-01	12235	8,26474E-01	17248	8,54470E-01	20677	0	17530,5
Non-responder	Macro_OLFML3	Macro_NLRP3	ADAM17	ITGA5	8,81387E-03	18448,5	1,09046E-02	8613	4,45686E-01	9991	1,14501E+00	10378	7,48520E-01	45730	0	17530,5
Non-responder	Mono_CD16	Macro_ISG15	TGFB1	SDC2	8,81716E-03	15456,7	1,21292E-02	7201	4,35899E-01	10379	1,42827E+00	6311	7,91420E-01	35862	0	17530,5
Non-responder	Macro_FOLR2+APOE+	Mono_CD16	ICAM1	ITGAM_ITGB2	8,81782E-03	15045,1	6,31087E-03	20681	3,44644E-01	14945	1,11009E+00	10988	8,92633E-01	11081	0	17530,5
Non-responder	Macro_NLRP3	Macro_IER3	CCL3	CCR1	8,81996E-03	12291,5	9,61590E-03	10467	5,76476E-01	5811	1,37352E+00	6967	8,54448E-01	20682	0	17530,5
Non-responder	cDC(CD1C)	Mono_CD16	ICAM3	ITGAL	8,82303E-03	11069,5	1,99317E-02	3076	7,44648E-01	3090	1,64332E+00	4246	8,26719E-01	27405	0	17530,5
Non-responder	Mono_INHBA	Macro_LYVE1	PTGS2	ALOX5	8,82849E-03	13363,7	1,14919E-02	7879	5,62366E-01	6164	8,24933E-01	17299	8,66101E-01	17946	0	17530,5
Non-responder	Macro_OLFML3	Mono_INHBA	PLAU	PLAUR	8,83703E-03	13923,3	7,92528E-03	14350	5,54899E-01	6361	1,12639E+00	10685	8,54423E-01	20690	0	17530,5
Non-responder	Macro_FOLR2+APOE+	Macro_FOLR2+APOE+	TNF	SEMA4C	8,83716E-03	28897,1	6,73991E-03	18634	2,24076E-01	23871	8,77604E-01	15919	5,89147E-01	68531	0	17530,5
Non-responder	Macro_IER3	pDC_LILRA4	HLA-DRA	CD4	8,84557E-03	11559,9	6,30853E-03	20694	3,86901E-01	12625	1,41581E+00	6466	9,46188E-01	484	0	17530,5
Non-responder	Macro_IER3	Mono_CD14	GNAI2	C5AR1	8,84771E-03	16160,5	6,30829E-03	20695	3,53673E-01	14432	7,84716E-01	18357	8,97829E-01	9788	0	17530,5
Non-responder	Macro_ISG15	Macro_ISG15	S100A9	CD68	8,85329E-03	7285,9	9,12911E-03	11357	6,58393E-01	4251	1,83295E+00	2932	9,47891E-01	359	0	17530,5
Non-responder	Macro_FOLR2+APOE+	Macro_FOLR2+APOE-	CD14	ITGB2	8,85413E-03	13712,1	6,58523E-03	19329	4,54360E-01	9644	6,97937E-01	20698	9,38074E-01	1359	0	17530,5
Non-responder	Mono_INHBA	Macro_LYVE1	CD59	STAB1	8,85626E-03	14469,3	7,66624E-03	15113	6,61372E-01	4202	9,21148E-01	14907	8,54813E-01	20594	0	17530,5
Non-responder	Macro_FOLR2+APOE+	Mono_CD14	PTPN6	CLEC12A	8,85840E-03	14670,7	8,60842E-03	12497	3,64994E-01	13791	1,24049E+00	8835	8,54376E-01	20700	0	17530,5
Non-responder	Macro_FOLR2+APOE+	Macro_OLFML3	HSP90B1	TLR1	8,86268E-03	14847,7	8,61055E-03	12492	3,99784E-01	11995	1,08060E+00	11519	8,54372E-01	20702	0	17530,5
Non-responder	cDC_LAMP3	Macro_FOLR2+APOE+	TNFSF9	HLA-DPA1	8,86483E-03	15937,3	6,73261E-03	18666	3,70369E-01	13495	1,21021E+00	9292	8,54372E-01	20703	0	17530,5
Non-responder	Macro_FOLR2+APOE-	Mono_CD16	HLA-B	LILRA1	8,86542E-03	10077,1	1,64913E-02	4253	6,72278E-01	4029	6,99450E-01	20649	9,25304E-01	3924	0	17530,5
Non-responder	Macro_FOLR2+APOE+	Macro_IER3	TNF	VSIR	8,86991E-03	14937,9	9,72169E-03	10307	5,57366E-01	6295	1,14410E+00	10395	8,15542E-01	30162	0	17530,5
Non-responder	Mono_INHBA	Mono_INHBA	TNF	TNFRSF1B	8,87125E-03	8491,9	1,33311E-02	6118	7,54306E-01	2979	1,72488E+00	3592	8,88158E-01	12240	0	17530,5

Non-responder	Macro_ISG15	Macro_ISG15	TNFSF10	RIPK1	8,87148E-03	13021,7	1,64897E-02	4254	7,92027E-01	2626	1,84542E+00	2849	7,82907E-01	37849	0	17530,5
Non-responder	Macro_FOLR2+APOE+	cDC(CD1C)	ANXA1	FPR1	8,87339E-03	17230,1	6,30642E-03	20707	2,76079E-01	19422	7,06200E-01	20450	9,05538E-01	8041	0	17530,5
Non-responder	pDC_LILRA4	Mono_INHBA	APP	NCSTN	8,88194E-03	12426,9	9,84932E-03	10092	1,04568E+00	993	2,43490E+00	875	8,05066E-01	32644	0	17530,5
Non-responder	Macro_OLFML3	Macro_ISG15	CCL2	CCR1	8,88314E-03	14243,7	1,56282E-02	4680	6,59888E-01	4214	1,64751E+00	4216	7,71447E-01	40578	0	17530,5
Non-responder	Mono_INHBA	cDC_LAMP3	NAMPT	ADORA2A	8,88626E-03	9213,5	3,71759E-02	1097	1,05024E+00	971	1,47943E+00	5756	8,54340E-01	20713	0	17530,5
Non-responder	Mono_CD14	Mono_CD16	S100A9	TLR4	8,88976E-03	6929,9	1,55476E-02	4716	1,05483E+00	954	2,34008E+00	1048	8,95333E-01	10401	0	17530,5
Non-responder	Mono_INHBA	Macro_FOLR2+APOE+	S100A8	CD36	8,89913E-03	13328,9	1,23478E-02	7003	5,50346E-01	6494	6,97019E-01	20719	8,78150E-01	14898	0	17530,5
Non-responder	Macro_FOLR2+APOE+	Macro_FOLR2+APOE-	C3	LRP1	8,90343E-03	16031,3	9,95572E-03	9917	2,97057E-01	17904	6,96967E-01	20721	8,81146E-01	14084	0	17530,5
Non-responder	Mono_CD16	Mono_CD14	ADAM10	CD44	8,91203E-03	17916,9	6,30368E-03	20725	3,21381E-01	16331	9,12656E-01	15104	8,57743E-01	19894	0	17530,5
Non-responder	Mono_CD16	Macro_LYVE1	IL15	IL2RA	8,91295E-03	21413,5	1,33224E-02	6125	4,79006E-01	8740	1,14354E+00	10408	6,35626E-01	64264	0	17530,5
Non-responder	Mono_CD14	Mono_CD16	CD1D	LILRB2	8,92011E-03	8708,9	1,64697E-02	4262	8,53315E-01	2065	2,02924E+00	1973	8,67057E-01	17714	0	17530,5
Non-responder	Macro_FOLR2+APOE+	Mono_CD14	HMGB1	TLR4	8,92493E-03	17698,7	6,64072E-03	19076	2,60227E-01	20731	1,05792E+00	11989	8,60888E-01	19167	0	17530,5
Non-responder	Macro_OLFML3	Macro_ISG15	CCL3L1	CCR1	8,92622E-03	12155,9	1,29736E-02	6421	6,61457E-01	4200	1,45205E+00	6016	8,29950E-01	26612	0	17530,5
Non-responder	Mono_INHBA	Mono_INHBA	FN1	CD44	8,92709E-03	8255,5	1,89040E-02	3361	6,93591E-01	3697	1,45279E+00	6012	8,94237E-01	10677	0	17530,5
Non-responder	Macro_FOLR2+APOE+	Macro_OLFML3	ANXA1	FPR1	8,92924E-03	16151,7	6,30260E-03	20733	2,75714E-01	19446	9,17001E-01	14999	9,05512E-01	8050	0	17530,5
Non-responder	Macro_NLRP3	Macro_FOLR2+APOE+	LGALS3	ENG	8,93139E-03	15957,9	7,22935E-03	16633	2,60174E-01	20734	8,94366E-01	15545	8,99694E-01	9347	0	17530,5
Non-responder	Macro_FOLR2+APOE+	Mono_CD14	C3	LRP1	8,93786E-03	14729,3	9,22740E-03	11176	2,60150E-01	20737	1,22605E+00	9037	8,77110E-01	15166	0	17530,5
Non-responder	Macro_FOLR2+APOE+	Macro_OLFML3	LGALS9	LRP1	8,94001E-03	15744,7	6,30106E-03	20738	3,48744E-01	14714	1,17142E+00	9949	8,74597E-01	15792	0	17530,5
Non-responder	Macro_FOLR2+APOE+	Macro_OLFML3	LGALS9	CD44	8,94283E-03	13164,9	6,16647E-03	21414	4,35083E-01	10417	1,21248E+00	9259	9,09210E-01	7204	0	17530,5
Non-responder	Macro_ISG15	Mono_CD16	CD38	PECAM1	8,94449E-03	12967,7	1,64596E-02	4266	7,20787E-01	3364	1,89435E+00	2579	7,86094E-01	37099	0	17530,5
Non-responder	Macro_NLRP3	Macro_FOLR2+APOE+	RETN	TLR4	8,94615E-03	19494,5	1,87377E-02	3417	4,35071E-01	10418	1,32490E+00	7622	6,80600E-01	58485	0	17530,5
Non-responder	Mono_CD14	Macro_FOLR2-APOE+	VCAN	ITGB1	8,94948E-03	8547,7	1,69424E-02	4059	1,04299E+00	1008	1,18427E+00	9722	8,95269E-01	10419	0	17530,5
Non-responder	Mono_INHBA	Macro_FOLR2+APOE+	TGFB1	ITGB1	8,95295E-03	19325,3	6,31590E-03	20653	2,88833E-01	18458	7,50419E-01	19241	8,54206E-01	20744	0	17530,5
Non-responder	Mono_INHBA	Macro_NLRP3	ADAM10	CD44	8,95295E-03	15337,1	6,29929E-03	20744	5,35262E-01	6888	1,07562E+00	11617	8,57701E-01	19906	0	17530,5
Non-responder	Macro_OLFML3	Macro_ISG15	SNCA	LAG3	8,95946E-03	18063,5	1,87674E-02	3407	7,94757E-01	2599	1,49376E+00	5604	6,61033E-01	61177	0	17530,5
Non-responder	Macro_OLFML3	Mono_CD16	ANXA1	FPR1	8,96159E-03	16460,7	6,29873E-03	20748	2,77272E-01	19338	8,49951E-01	16629	9,05485E-01	8058	0	17530,5
Non-responder	Macro_FOLR2+APOE+	cDC_LAMP3	TNF	FAS	8,96612E-03	17334,3	1,85096E-02	3500	5,36388E-01	6862	1,14251E+00	10424	7,36750E-01	48355	0	17530,5
Non-responder	Macro_LYVE1	Mast	F13A1	ITGA4	8,96807E-03	13327,5	9,09705E-03	11411	7,16441E-01	3426	9,81337E-01	13519	8,54180E-01	20751	0	17530,5
Non-responder	Macro_FOLR2+APOE+	Macro_FOLR2+APOE+	TGFB1	ITGB1	8,97239E-03	17021,3	6,31310E-03	20667	2,88607E-01	18473	1,32149E+00	7683	8,54178E-01	20753	0	17530,5
Non-responder	cDC_LAMP3	Macro_OLFML3	HLA-F	LILRB2	8,97239E-03	15027,9	6,29705E-03	20753	4,58984E-01	9461	9,02872E-01	15337	8,88772E-01	12058	0	17530,5
Non-responder	pDC_LILRA4	Macro_FOLR2+APOE-	APP	TNFRSF21	8,97502E-03	9935,9	1,64522E-02	4271	1,11421E+00	720	2,24857E+00	1237	8,32644E-01	25921	0	17530,5
Non-responder	Mono_INHBA	Macro_FOLR2+APOE+	C3	CD81	8,97672E-03	18735,3	6,29670E-03	20755	2,97524E-01	17873	7,64564E-01	18875	8,63095E-01	18643	0	17530,5
Non-responder	Mono_CD14	Mono_CD14	AGTRAP	RACK1	8,98278E-03	9075,5	1,19069E-02	7438	7,20556E-01	3368	1,40315E+00	6612	8,95225E-01	10429	0	17530,5
Non-responder	Macro_FOLR2+APOE+	Macro_FOLR2-APOE+	MMP9	CD44	8,98753E-03	14566,7	1,11542E-02	8282	5,20250E-01	7316	7,61964E-01	18945	8,54156E-01	20760	0	17530,5
Non-responder	pDC_LILRA4	Macro_OLFML3	APP	TSPAN15	8,98812E-03	13806,7	1,29471E-02	6441	1,04499E+00	999	2,52030E+00	742	7,59120E-01	43321	0	17530,5
Non-responder	Macro_ISG15	Macro_FOLR2+APOE+	CD14	ITGB1	9,00615E-03	8316,7	9,63752E-03	10436	6,19209E-01	4940	1,96366E+00	2243	9,12790E-01	6434	0	17530,5
Non-responder	Macro_FOLR2+APOE+	Macro_ISG15	LGALS1	PTPRC	9,00615E-03	11701,5	5,70897E-03	24209	4,34605E-01	10436	1,59080E+00	4681	9,36528E-01	1651	0	17530,5
Non-responder	Macro_IER3	Macro_NLRP3	IL1B	SIGIRR	9,01137E-03	14548,7	8,34630E-03	13163	5,19402E-01	7354	9,64092E-01	13925	8,54129E-01	20771	0	17530,5
Non-responder	Macro_OLFML3	Macro_FOLR2+APOE+	ICAM1	ITGAX_ITGB2	9,01283E-03	13706,3	5,92726E-03	22807	4,44372E-01	10045	1,31956E+00	7711	8,95193E-01	10438	0	17530,5
Non-responder	Mono_INHBA	Mono_INHBA	VCAN	CD44	9,02005E-03	8580,9	1,36027E-02	5923	8,32399E-01	2259	1,06224E+00	11894	9,18280E-01	5298	0	17530,5
Non-responder	Mono_INHBA	Macro_OLFML3	S100A9	ITGB2	9,02005E-03	11702,9	8,26667E-03	13373	5,74761E-01	5866	6,94984E-01	20775	9,40996E-01	970	0	17530,5
Non-responder	Macro_ISG15	Macro_FOLR2+APOE+	TNFSF13	TNFRSF14	9,02287E-03	9423,3	9,94795E-03	9931	5,78521E-01	5771	1,74597E+00	3443	8,95184E-01	10441	0	17530,5
Non-responder	Mono_CD16	Macro_FOLR2+APOE+	B2M	LILRB2	9,02287E-03	11517,1	6,07266E-03	21963	4,34495E-01	10441	1,34340E+00	7367	9,49116E-01	284	0	17530,5
Non-responder	Macro_FOLR2+APOE+	Macro_FOLR2+APOE-	SERPINA1	LRP1	9,02656E-03	14434,1	7,34903E-03	16184	4,28946E-01	10661	6,94858E-01	20778	9,10135E-01	7017	0	17530,5
Non-responder	Mono_CD16	pDC_LILRA4	B2M	TFRC	9,03526E-03	13371,3	6,28972E-03	20782	2,93388E-01	18146	1,32801E+00	7586	9,30687E-01	2812	0	17530,5
Non-responder	Mono_INHBA	cDC(CD1C)	TNF	TNFRSF1A	9,04178E-03	12684,7	1,03435E-02	9350	5,24012E-01	7216	1,26060E+00	8542	8,54057E-01	20785	0	17530,5
Non-responder	Macro_OLFML3	Macro_ISG15	CD38	PECAM1	9,04296E-03	15176,9	1,35931E-02	5929	6,29124E-01	4742	1,39762E+00	6675	7,69567E-01	41008	0	17530,5
Non-responder	Macro_FOLR2+APOE+	Macro_FOLR2+APOE-	CD14	TLR4	9,04613E-03	16056,7	6,42298E-03	20135	4,72375E-01	8966	1,01432E+00	12865	8,54042E-01	20787	0	17530,5
Non-responder	Macro_FOLR2+APOE+	Macro_NLRP3	C1QA	CD33	9,04613E-03	13022,5	6,28925E-03	20787	5,04988E-01	7807	1,57097E+00	4842	8,80903E-01	14146	0	17530,5
Non-responder	Macro_FOLR2+APOE+	pDC_LILRA4	CALM3	INSR	9,04966E-03	16838,9	1,11163E-02	8337	4,34173E-01	10449	1,22223E+00	9106	7,78968E-01	38772	0	17530,5
Non-responder	Macro_ISG15	Macro_NLRP3	TIMP2	CD44	9,04966E-03	8771,5	1,07325E-02	8840	6,90657E-01	3737	1,76887E+00	3301	8,95171E-01	10449	0	17530,5
Non-responder	Mono_INHBA	Mono_INHBA	HBEGF	CD44	9,05048E-03	9744,7	1,03906E-02	9293	7,26804E-01	3287	1,05028E+00	12142	9,12642E-01	6471	0	17530,5
Non-responder	cDC(CD1C)	Mono_CD16	CD99	PILRA	9,05048E-03	17016,9	6,28908E-03	20789	2,82763E-01	18917	8,03498E-01	17867	8,97006E-01	9981	0	17530,5
Non-responder	Macro_IFI27	Macro_NLRP3	FN1	PLAUR	9,05266E-03	13792,7	8,36910E-03	13104	6,70018E-01	4066	9,82987E-01	13473	8,54015E-01	20790	0	17530,5
Non-responder	Macro_IFI27	Macro_FOLR2+APOE+	CD59	STAB1	9,05702E-03	16694,7	7,56727E-03	15433	3,96147E-01	12181	8,15533E-01	17537	8,54005E-01	20792	0	17530,5
Non-responder	Mono_INHBA	Macro_NLRP3	TIMP1	CD63	9,05702E-03	10903,1	6,28838E-03	20792	4,98138E-01	8050	1,35995E+00	7136	9,40666E-01	1007	0	17530,5
Non-responder	Mast	Macro_IFI27	HDC	HRH2	9,05925E-03	13871,3	3,91290E-02	1003	1,17308E+00	516	1,17286E+00	9924	7,72354E-01	40383	0	17530,5
Non-responder	Macro_ISG15	Mono_CD14	S100A8	CD36	9,06087E-03	8043,5	1,71468E-02	3987	6,56296E-01	4285	1,69240E+00	3835	8,94655E-01	10580	0	17530,5
Non-responder	Macro_FOLR2-APOE+	Mono_CD16	HLA-B	LILRA1	9,06087E-03	11293,7	1,64379E-02	4285	6,65794E-01	4127	5,05094E-01	26582	9,25192E-01	3944	0	17530,5
Non-responder	Mono_INHBA	Mono_INHBA	TIMP1	CD63	9,06573E-03	8810,7	7,72641E-03	14927	6,74096E-01	3994	1,36146E+00	7116	9,46159E-01	486	0	17530,5
Non-responder	Macro_FOLR2+APOE+	cDC_CLEC9A	MDK	SDC2	9,06980E-03	22395,5	2,01623E-02	3016	4,35912E-01	10377	1,14008E+00	10455	5,56346E-01	70599	0	17530,5
Non-responder	cDC_CLEC9A	Macro_FOLR2+APOE+	ARF6	PLD1	9,07318E-03	11986,5	3,07530E-02	1471	8,72686E-01	1892	1,63763E+00	4287	7,96298E-01	34752	0	17530,5
Non-responder	Macro_FOLR2+APOE+	Macro_OLFML3	MDK	SDC4	9,07652E-03	21962,1	2,19744E-02	2583	4,63265E-01	9312	1,14000E+00	10457	5,67942E-01	69928	0	17530,5
Non-responder	Mono_CD16	Mast	CD55	ADGRE5	9,07882E-03	14380,1	9,59831E-03	10497	5,98108E-01	5355	8,09039E-01	17716	8,53979E-01	20802	0	17530,5
Non-responder	Mono_CD16	Macro_OLFML3	PTPN6	CD300LF	9,07988E-03	14396,1	9,62030E-03	10458	4,69188E-01	9105	1,42991E+00	6279	8,21959E-01	28608	0	17530,5
Non-responder	Macro_IER3	Mono_CD16	HSPA1A	TLR4	9,08100E-03	17871,5	6,28603E-03	20803	2,99133E-01	17756	8,25396E-01	17287	8,73872E-01	15981	0	17530,5
Non-responder	Macro_NLRP3	Macro_FOLR2-APOE+	CCL3L1	CCR1	9,08660E-03	14415,5	9,61920E-03	10460	7,51205E-01	3012	1,22400E+00	9070	8,07787E-01	32005	0	17530,5
Non-responder	Macro_IER3	cDC_CLEC9A	F13A1	ITGB1	9,08755E-03	15318,3	8,98482E-03	11663	3,92525E-01	12335	9,50157E-01	14257	8,53959E-01	20806	0	17530,5
Non-responder	Mono_INHBA	Mono_INHBA	VIM	CD44	9,09192E-03	11380,5	6,97003E-03	17658	6,65364E-01	4137	8,18496E-01	17460	9,52397E-01	117	0	17530,5
Non-responder	Mono_INHBA	Mono_INHBA	TIMP2	CD44	9,09629E-03	16651,5	6,63103E-03	19120	4,40764E-01	10194	7,39776E-01	19524	8,70335E-01	16889	0	17530,5
Non-responder	Macro_ISG15	Macro_NLRP3	CCL8	CCR1	9,09782E-03	16060,7	2,91728E-02	1633	6,56112E-01	4291	2,20027E+00	1376	6,99206E-01	55473	0	17530,5

Post_NR_Myeloid_Myeloid_CellCell

Non-responder	Mono_INHBA	Mono_INHBA	PKM	CD44	9,10285E-03	11043,9	7,44829E-03	15850	6,47532E-01	4426	8,90035E-01	15624	9,35888E-01	1789	0	17530,5
Non-responder	pDC_LILRA4	cDC_LAMP3	LTB	TNFRSF1A	9,10399E-03	8730,5	1,64304E-02	4292	9,68323E-01	1355	2,68016E+00	526	8,57506E-01	19949	0	17530,5
Non-responder	Macro_NLRP3	Macro_NLRP3	PLAU	PLAUR	9,10399E-03	13538,9	6,65430E-03	19013	6,75088E-01	3976	1,69858E+00	3796	8,43212E-01	23379	0	17530,5
Non-responder	Macro_LYVE1	Macro_IFI27	F13A1	ITGA4	9,10503E-03	13738,5	9,06143E-03	11486	7,15487E-01	3443	9,00003E-01	15419	8,53936E-01	20814	0	17530,5
Non-responder	Mono_INHBA	Mono_INHBA	PTGS2	ALOX5	9,10722E-03	14411,3	1,01514E-02	9625	5,17629E-01	7408	8,04972E-01	17825	8,58744E-01	19668	0	17530,5
Non-responder	cDC(CD1C)	Macro_NLRP3	C1QA	CD93	9,10722E-03	14629,5	8,75252E-03	12163	3,66989E-01	13684	6,93618E-01	20815	9,01435E-01	8955	0	17530,5
Non-responder	pDC_LILRA4	cDC(CD1C)	NUCB2	ERAP1	9,11634E-03	14219,3	1,64229E-02	4294	8,23968E-01	2326	2,09065E+00	1744	7,51052E-01	45202	0	17530,5
Non-responder	Mono_CD14	Mono_CD14	VCAN	TLR2	9,12251E-03	7596,3	1,64170E-02	4295	9,95803E-01	1226	1,95735E+00	2264	8,86555E-01	12666	0	17530,5
Non-responder	Mono_INHBA	Mono_INHBA	NAMPT	ITGA5_ITGB1	9,12474E-03	15363,7	7,15415E-03	16911	4,96260E-01	8120	7,89620E-01	18232	8,73715E-01	16025	0	17530,5
Non-responder	Macro_FOLR2+APOE+	cDC_CLEC9A	ADM	ADRB3	9,12703E-03	21150,7	6,21094E-02	420	6,08070E-01	5145	1,13916E+00	10472	5,15606E-01	72186	0	17530,5
Non-responder	Macro_NLRP3	Macro_ISG15	INHBA	ENG	9,13378E-03	15080,3	1,62833E-02	4347	4,33631E-01	10474	1,74219E+00	3468	7,75509E-01	39582	0	17530,5
Non-responder	pDC_LILRA4	Macro_FOLR2+APOE+	HMGB1	CD163	9,13487E-03	7248,5	9,50539E-03	10672	6,55941E-01	4297	2,49196E+00	777	9,30063E-01	2966	0	17530,5
Non-responder	Mono_INHBA	pDC_LILRA4	LGALS9	CD47	9,13570E-03	11595,7	6,97575E-03	17635	5,84142E-01	5644	1,89257E+00	2592	8,79303E-01	14577	0	17530,5
Non-responder	Macro_FOLR2+APOE+	Macro_OLFML3	CD38	PECAM1	9,14053E-03	18024,7	9,61033E-03	10476	5,64842E-01	6107	1,31270E+00	7800	7,37401E-01	48210	0	17530,5
Non-responder	Mono_INHBA	pDC_LILRA4	TNF	TNFRSF1B	9,14447E-03	9322,7	1,10595E-02	8412	6,25712E-01	4814	2,33471E+00	1054	8,78538E-01	14803	0	17530,5
Non-responder	Macro_IER3	Macro_ISG15	CXCL8	SDC2	9,14725E-03	11089,3	2,23469E-02	2499	7,52180E-01	3000	1,63575E+00	4299	8,23925E-01	28118	0	17530,5
Non-responder	Mono_INHBA	pDC_LILRA4	APP	RPSA	9,15325E-03	9802,1	8,39517E-03	13035	7,91974E-01	2627	1,93431E+00	2388	8,83665E-01	13430	0	17530,5
Non-responder	cDC(CD1C)	Macro_LYVE1	HMGB1	CD163	9,16864E-03	11968,9	8,80573E-03	12029	5,71950E-01	5934	6,92305E-01	20843	9,27535E-01	3508	0	17530,5
Non-responder	Macro_OLFML3	Macro_ISG15	TNFSF10	RIPK1	9,17774E-03	17270,7	1,19525E-02	7400	5,32469E-01	6975	1,16716E+00	10015	7,54320E-01	44433	0	17530,5
Non-responder	pDC_LILRA4	Macro_FOLR2+APOE-	APP	NOTCH2	9,18438E-03	8874,7	1,51442E-02	4922	1,12403E+00	671	2,35705E+00	1010	8,56321E-01	20240	0	17530,5
Non-responder	Macro_FOLR2+APOE+	Macro_LYVE1	IL15	IL2RA	9,18443E-03	18282,9	2,44306E-02	2163	6,55395E-01	4305	1,03069E+00	12536	7,02582E-01	54880	0	17530,5
Non-responder	Macro_NLRP3	Macro_LYVE1	TNF	NOTCH1	9,18791E-03	19425,3	1,16117E-02	7738	4,68235E-01	9136	1,13823E+00	10490	7,17053E-01	52232	0	17530,5
Non-responder	Mono_INHBA	pDC_LILRA4	SIRPA	CD47	9,19286E-03	8682,3	1,32399E-02	6179,5	7,93759E-01	2610,5	2,23629E+00	1276,5	8,74500E-01	15814,5	0	17530,5
Non-responder	Macro_IER3	Mono_CD14	ANXA1	FPR1	9,19286E-03	14960,3	7,29649E-03	16372	3,72927E-01	13340	6,91840E-01	20854	9,11593E-01	6705	0	17530,5
Non-responder	Macro_IER3	Mono_CD16	GRN	TNFRSF1B	9,19506E-03	13613,1	6,27542E-03	20855	4,12211E-01	11414	9,04644E-01	15299	9,30063E-01	2967	0	17530,5
Non-responder	Macro_ISG15	cDC(CD1C)	CCL8	CCR2	9,19684E-03	18000,1	3,79389E-02	1053	6,55183E-01	4307	2,01797E+00	2010	6,27424E-01	65100	0	17530,5
Non-responder	Macro_NLRP3	Macro_FOLR2+APOE+	LGALS1	ITGB1	9,20168E-03	14648,3	6,27506E-03	20858	3,20388E-01	16402	1,05402E+00	12074	9,13053E-01	6377	0	17530,5
Non-responder	Macro_FOLR2+APOE+	Macro_LYVE1	HLA-DRB5	CD4	9,20609E-03	12104,3	6,27494E-03	20860	4,30102E-01	10608	1,14836E+00	10317	9,39083E-01	1206	0	17530,5
Non-responder	Mono_CD16	Macro_FOLR2+APOE+	HMGB1	CD163	9,21492E-03	14011,7	6,27465E-03	20864	2,92303E-01	18218	1,33106E+00	7547	9,15288E-01	5899	0	17530,5
Non-responder	Mono_CD16	Macro_OLFML3	CD55	ADGRE5	9,21934E-03	11672,1	9,56225E-03	10569	5,96564E-01	5386	1,67163E+00	4009	8,53744E-01	20866	0	17530,5
Non-responder	Macro_FOLR2+APOE+	Macro_NLRP3	ADAM9	ITGAV	9,22186E-03	17302,9	1,35762E-02	5940	4,32862E-01	10500	1,35276E+00	7264	7,50608E-01	45280	0	17530,5
Non-responder	Mono_INHBA	pDC_LILRA4	TGFB1	CXCR4	9,22597E-03	8752,7	8,74511E-03	12181	6,17456E-01	4974	2,19412E+00	1398	9,07095E-01	7680	0	17530,5
Non-responder	pDC_LILRA4	Macro_FOLR2+APOE+	ALCAM	NRP1	9,22793E-03	10322,1	2,14933E-02	2694	6,54983E-01	4312	2,55434E+00	684	8,30777E-01	26390	0	17530,5
Non-responder	pDC_LILRA4	Macro_FOLR2+APOE+	LIN7C	ABCA1	9,23415E-03	12776,3	2,27630E-02	2415	6,54959E-01	4313	1,73917E+00	3484	7,90111E-01	36139	0	17530,5
Non-responder	Macro_IER3	cDC_LAMP3	FN1	TNFRSF11B	9,23826E-03	15556,5	8,57899E-02	199	1,04222E+00	1013	1,41782E+00	6446	7,15279E-01	52594	0	17530,5
Non-responder	Mono_INHBA	pDC_LILRA4	S100A9	CD68	9,23924E-03	14601,3	6,47801E-03	19883	3,22373E-01	16261	7,95651E-01	18080	9,38737E-01	1252	0	17530,5
Non-responder	pDC_LILRA4	Macro_ISG15	ASIP	MGRN1	9,24038E-03	17275,1	6,05930E-02	438	6,54829E-01	4314	1,64733E+00	4220	6,71002E-01	59873	0	17530,5
Non-responder	Macro_NLRP3	Macro_IFI27	VEGFA	SIRPA	9,24228E-03	13222,3	1,18334E-02	7505	7,23171E-01	3334	1,13738E+00	10506	8,27508E-01	27236	0	17530,5
Non-responder	Macro_ISG15	Macro_FOLR2+APOE+	HLA-F	LILRB2	9,24228E-03	8283,3	9,59354E-03	10506	7,30788E-01	3243	1,88130E+00	2660	9,07942E-01	7477	0	17530,5
Non-responder	Macro_IER3	Macro_FOLR2+APOE+	CCL3	CCR5	9,24588E-03	9637,1	2,45122E-02	2153	8,11457E-01	2443	1,53637E+00	5181	8,53690E-01	20878	0	17530,5
Non-responder	Mono_CD16	Macro_ISG15	S100A9	ITGB2	9,25031E-03	13962,9	6,27121E-03	20880	3,61886E-01	13957	9,15787E-01	15034	9,32843E-01	2413	0	17530,5
Non-responder	Macro_LYVE1	Macro_LYVE1	SERPING1	LRP1	9,25917E-03	13720,7	1,03905E-02	9295	5,19634E-01	7344	6,90902E-01	20884	8,83242E-01	13550	0	17530,5
Non-responder	Macro_FOLR2+APOE+	cDC_CLEC9A	ICAM1	ITGAX_ITGB2	9,25917E-03	13289,5	6,27067E-03	20884	4,06405E-01	11696	1,40900E+00	6542	8,97806E-01	9795	0	17530,5
Non-responder	Macro_OLFML3	Macro_ISG15	PTPN6	CD300LF	9,25932E-03	13808,3	1,11362E-02	8309	4,48063E-01	9902	1,34803E+00	7318	8,32414E-01	25982	0	17530,5
Non-responder	pDC_LILRA4	Mono_INHBA	HSP90B1	LRP1	9,25932E-03	7487,1	9,59138E-03	10511	8,60215E-01	2002	2,38257E+00	960	9,12801E-01	6432	0	17530,5
Non-responder	Macro_FOLR2+APOE+	Macro_NLRP3	HLA-C	LILRB1	9,26614E-03	13726,1	4,92345E-03	29951	4,32445E-01	10513	1,51627E+00	5382	9,18475E-01	5254	0	17530,5
Non-responder	Mono_INHBA	Macro_ISG15	FN1	SDC2	9,27156E-03	12602,7	3,48800E-02	1209	6,54498E-01	4319	1,87752E+00	2679	7,85337E-01	37276	0	17530,5
Non-responder	Macro_LYVE1	Macro_FOLR2+APOE+	SERPINF1	PLXDC2	9,27248E-03	16163,7	8,30941E-03	13260	2,80104E-01	19124	6,90693E-01	20890	8,96883E-01	10014	0	17530,5
Non-responder	Mono_CD14	cDC_CLEC9A	VCAN	TLR1	9,27780E-03	10167,7	1,63467E-02	4320	1,03166E+00	1063	1,94657E+00	2328	8,33884E-01	25597	0	17530,5
Non-responder	Mono_CD16	Mono_CD14	VCAN	CD44	9,27914E-03	16424,1	6,70024E-03	18793	3,89854E-01	12478	6,90551E-01	20893	8,87469E-01	12426	0	17530,5
Non-responder	Mono_INHBA	Macro_IER3	TIMP1	CD63	9,28136E-03	12157,7	6,26749E-03	20894	4,95581E-01	8135	9,95899E-01	13209	9,40573E-01	1020	0	17530,5
Non-responder	Macro_FOLR2+APOE+	Mono_CD16	PSAP	LRP1	9,28321E-03	13046,3	5,36278E-03	26555	4,32322E-01	10518	1,48988E+00	5640	9,19774E-01	4988	0	17530,5
Non-responder	Macro_FOLR2+APOE+	Macro_ISG15	CXCL11	CCR5	9,28662E-03	20625,9	2,15560E-02	2674	4,32319E-01	10519	1,51677E+00	5377	6,07761E-01	67029	0	17530,5
Non-responder	Macro_IFI27	Macro_FOLR2+APOE+	A2M	LRP1	9,29025E-03	12349,9	1,32657E-02	6157	5,18281E-01	7385	6,90458E-01	20898	8,97862E-01	9779	0	17530,5
Non-responder	Macro_ISG15	Mono_CD16	GRN	TNFRSF1B	9,29030E-03	8046,3	8,01219E-03	14103	6,54291E-01	4322	1,84707E+00	2833	9,37603E-01	1443	0	17530,5
Non-responder	Macro_NLRP3	Macro_ISG15	CD99	PILRA	9,29247E-03	16039,1	6,26674E-03	20899	2,69194E-01	19983	1,06854E+00	11759	8,96841E-01	10024	0	17530,5
Non-responder	Macro_FOLR2+APOE+	Macro_ISG15	CD14	ITGA4	9,29914E-03	12290,7	6,26523E-03	20902	4,78553E-01	8760	1,74920E+00	3428	8,93636E-01	10833	0	17530,5
Non-responder	Macro_OLFML3	Macro_ISG15	NRG1	MS4A4A	9,30030E-03	17958,5	1,27933E-02	6582	4,39789E-01	10231	1,39297E+00	6735	7,35055E-01	48714	0	17530,5
Non-responder	Macro_OLFML3	Macro_ISG15	PKM	CD44	9,30714E-03	12934,9	5,80096E-03	23610	4,32180E-01	10525	1,19114E+00	9607	9,27967E-01	3402	0	17530,5
Non-responder	Macro_FOLR2+APOE+	Mono_INHBA	HSP90B1	TLR4	9,30805E-03	18004,9	6,48034E-03	19863	3,26020E-01	16040	8,87772E-01	15685	8,53583E-01	20906	0	17530,5
Non-responder	cDC_LAMP3	cDC_CLEC9A	NECTIN2	CD226	9,31034E-03	14474,7	3,89382E-02	1017	1,04991E+00	974	1,53177E+00	5234	7,40115E-01	47618	0	17530,5
Non-responder	Mono_CD14	Mono_CD14	S100A9	CD68	9,31742E-03	7160,9	9,58334E-03	10528	7,53303E-01	2989	1,61420E+00	4471	9,49077E-01	286	0	17530,5
Non-responder	Mono_CD16	Macro_FOLR2+APOE+	SIRPB2	CD47	9,32427E-03	15958,9	1,06971E-02	8879	4,32066E-01	10530	1,38346E+00	6848	7,90715E-01	36007	0	17530,5
Non-responder	Macro_LYVE1	Macro_OLFML3	HLA-B	LILRB1	9,32587E-03	15439,9	6,26909E-03	20890	3,44243E-01	14963	6,90105E-01	20914	9,30323E-01	2902	0	17530,5
Non-responder	Mono_INHBA	pDC_LILRA4	HMGB1	THBD	9,33033E-03	14466,7	7,12074E-03	17033	2,94752E-01	18050	1,44542E+00	6095	8,82964E-01	13625	0	17530,5
Non-responder	Macro_OLFML3	Macro_ISG15	GRN	TNFRSF1A	9,33033E-03	13828,1	6,26021E-03	20916	3,41975E-01	15085	1,15891E+00	10151	9,17438E-01	5458	0	17530,5
Non-responder	pDC_LILRA4	pDC_LILRA4	ALCAM	NRP1	9,33412E-03	10316,9	2,14546E-02	2700	6,53931E-01	4329	2,61788E+00	602	8,30650E-01	26423	0	17530,5
Non-responder	Macro_FOLR2+APOE+	Macro_IER3	ADM	GPR84	9,33455E-03	18970,9	1,32122E-02	6205	4,32010E-01	10533	1,34672E+00	7338	7,11737E-01	53248	0	17530,5
Non-responder	Macro_OLFML3	Macro_NLRP3	B2M	LILRB2	9,33455E-03	13186,5	5,30242E-03	27020	4,36959E-01	10331	1,13537E+00	10533	9,45740E-01	518	0	17530,5
Non-responder	cDC(CD1C)	Macro_OLFML3	HLA-A	LILRB1	9,33702E-03	15290,3	6,25951E-03	20919	3,07536E-01	17207	8,34666E-01	17020	9,26063E-01	3775	0	17530,5
Non-responder	cDC_CLEC9A	Macro_FOLR2+APOE+	HLA-DMB	CD4	9,34142E-03	8111,1	9,57945E-03	10535	6,08658E-01	5132	1,94712E+00	2324	9,19530E-01	5034	0	17530,5

Non-responder	Macro_OLFML3	Macro_IER3	TNFSF13B	HLA-DPB1	9,34149E-03	13027,5	6,25933E-03	20921	4,46967E-01	9938	8,73877E-01	16015	9,43132E-01	733	0	17530,5
Non-responder	Macro_ISG15	cDC_CLEC9A	HLA-DRA	LAG3	9,34828E-03	7888,5	1,54533E-02	4754	7,57596E-01	2943	1,71396E+00	3678	8,94872E-01	10537	0	17530,5
Non-responder	Macro_IER3	Macro_FOLR2+APOE+	F13A1	ITGA4	9,35265E-03	15052,7	9,00148E-03	11624	4,16025E-01	11239	9,63006E-01	13944	8,53521E-01	20926	0	17530,5
Non-responder	Mono_CD14	Macro_ISG15	THBS1	CD36	9,35295E-03	9998,7	1,63187E-02	4332	6,88091E-01	3777	1,90238E+00	2538	8,49711E-01	21816	0	17530,5
Non-responder	Macro_OLFML3	Macro_NLRP3	CALR	LRP1	9,35489E-03	15263,9	6,25866E-03	20927	3,15397E-01	16723	1,07425E+00	11645	8,98962E-01	9494	0	17530,5
Non-responder	Macro_FOLR2-APOE+	Macro_NLRP3	C3	ITGAX	9,35859E-03	8352,9	1,86334E-02	3454	7,43027E-01	3110	1,13501E+00	10540	9,09593E-01	7130	0	17530,5
Non-responder	Mono_INHBA	pDC_LILRA4	HSP90B1	TLR9	9,35936E-03	8868,9	5,12249E-02	630	8,98690E-01	1737	1,37957E+00	6900	8,67746E-01	17547	0	17530,5
Non-responder	Mono_INHBA	pDC_LILRA4	HSP90B1	TLR7	9,36160E-03	11071,5	1,44597E-02	5327	5,00556E-01	7973	1,34288E+00	7376	8,69412E-01	17151	0	17530,5
Non-responder	cDC(CD1C)	Macro_ISG15	ANXA1	FPR1	9,36160E-03	17202,9	6,25802E-03	20930	2,74113E-01	19578	7,27800E-01	19852	9,05208E-01	8124	0	17530,5
Non-responder	Mono_INHBA	pDC_LILRA4	HMGB1	TLR9	9,36831E-03	8759,1	4,85167E-02	690	8,28938E-01	2290	1,42731E+00	6319	8,70060E-01	16966	0	17530,5
Non-responder	Mono_INHBA	Macro_LYVE1	THBS1	LRP1	9,36831E-03	15007,5	1,06144E-02	9000	4,58873E-01	9469	6,89377E-01	20933	8,65421E-01	18105	0	17530,5
Non-responder	Mono_INHBA	pDC_LILRA4	HMGB1	CXCR4	9,37055E-03	10289,1	6,73699E-03	18644	4,48572E-01	9886	2,02453E+00	1991	9,28009E-01	3394	0	17530,5
Non-responder	Macro_FOLR2+APOE+	pDC_LILRA4	CD99	PILRB	9,37278E-03	12627,1	1,13755E-02	8000	3,72035E-01	13390	1,77085E+00	3280	8,53468E-01	20935	0	17530,5
Non-responder	Mono_INHBA	pDC_LILRA4	S100A8	CD69	9,37950E-03	15717,5	7,47934E-03	15750	4,29708E-01	10630	8,34765E-01	17015	8,67297E-01	17662	0	17530,5
Non-responder	Macro_IER3	Mono_CD16	THBS1	ITGA4	9,38174E-03	14135,3	8,97012E-03	11697	4,85851E-01	8477	1,05566E+00	12033	8,53437E-01	20939	0	17530,5
Non-responder	Macro_FOLR2+APOE+	Mono_CD16	HSP90B1	TLR4	9,38398E-03	17116,5	6,46517E-03	19937	3,25230E-01	16102	1,10512E+00	11073	8,53437E-01	20940	0	17530,5
Non-responder	Macro_FOLR2+APOE+	Macro_FOLR2+APOE+	PDGFB	ITGAV	9,38611E-03	19201,9	2,11297E-02	2785	4,49442E-01	9853	1,13437E+00	10548	7,00215E-01	55293	0	17530,5
Non-responder	Macro_FOLR2+APOE+	Mono_CD14	LYZ	ITGAL	9,39519E-03	12984,7	6,25464E-03	20945	4,49253E-01	9858	1,72509E+00	3589	8,85174E-01	13001	0	17530,5
Non-responder	pDC_LILRA4	Macro_IFI27	SEMA7A	PLXNC1	9,39696E-03	13673,7	2,62142E-02	1929	8,51975E-01	2072	1,63004E+00	4339	7,62862E-01	42498	0	17530,5
Non-responder	Macro_IER3	Macro_NLRP3	VIM	CD44	9,39968E-03	12738,5	6,25457E-03	20947	4,24714E-01	10853	9,55518E-01	14130	9,49881E-01	232	0	17530,5
Non-responder	pDC_LILRA4	Macro_NLRP3	LTB	CD40	9,40082E-03	10449,7	1,45611E-02	5264	1,04062E+00	1022	3,02211E+00	256	8,23715E-01	28176	0	17530,5
Non-responder	Macro_IFI27	Macro_NLRP3	LPL	CD44	9,40641E-03	12136,5	1,44103E-02	5365	5,78466E-01	5775	1,10591E+00	11062	8,53407E-01	20950	0	17530,5
Non-responder	cDC_LAMP3	cDC_CLEC9A	ICAM1	IL2RG	9,40866E-03	17888,9	6,37309E-03	20375	4,01121E-01	11930	6,88711E-01	20951	8,63046E-01	18658	0	17530,5
Non-responder	Macro_NLRP3	Macro_LYVE1	THBS1	CD47	9,42214E-03	13210,7	8,82120E-03	11990	6,62409E-01	4188	1,08737E+00	11388	8,53372E-01	20957	0	17530,5
Non-responder	Macro_NLRP3	cDC(CD1C)	TGFB1	ENG	9,43338E-03	17884,9	7,15842E-03	16889	3,05331E-01	17354	8,47692E-01	16689	8,53355E-01	20962	0	17530,5
Non-responder	Mono_INHBA	pDC_LILRA4	CCL3L1	CCR5	9,44110E-03	13356,1	1,62836E-02	4346	7,32745E-01	3227	2,18755E+00	1413	7,72930E-01	40264	0	17530,5
Non-responder	Macro_FOLR2+APOE-	Macro_ISG15	HLA-DPA1	LAG3	9,44133E-03	7875,7	2,20647E-02	2567	1,09561E+00	782	1,13354E+00	10564	9,06023E-01	7935	0	17530,5
Non-responder	Mono_INHBA	Mono_INHBA	SPP1	ITGA5_ITGB1	9,44464E-03	11920,1	1,83846E-02	3531	5,28346E-01	7081	1,13820E+00	10491	8,53339E-01	20967	0	17530,5
Non-responder	Macro_FOLR2-APOE+	Mono_CD16	HLA-A	LILRA1	9,44742E-03	9833,7	1,75761E-02	3814	7,50901E-01	3014	7,05844E-01	20463	9,23111E-01	4347	0	17530,5
Non-responder	cDC_LAMP3	Macro_FOLR2+APOE+	HLA-DRA	CD4	9,44914E-03	15164,9	6,25005E-03	20969	2,99579E-01	17724	7,56283E-01	19095	9,45950E-01	506	0	17530,5
Non-responder	cDC_CLEC9A	Macro_IER3	RPS19	C5AR1	9,45518E-03	7691,7	9,56237E-03	10568	7,71861E-01	2797	1,53147E+00	5239	9,33355E-01	2324	0	17530,5
Non-responder	cDC_LAMP3	cDC(CD1C)	CXCL9	FCGR2A	9,46492E-03	14993,3	9,13007E-03	11352	4,58662E-01	9479	6,87686E-01	20976	8,75260E-01	15629	0	17530,5
Non-responder	Macro_IER3	cDC_CLEC9A	LGALS9	SLC1A5	9,47169E-03	18115,7	7,06615E-03	17257	3,13422E-01	16824	7,98880E-01	17988	8,53298E-01	20979	0	17530,5
Non-responder	Macro_NLRP3	Macro_ISG15	CD14	TLR4	9,48751E-03	18162,1	6,34541E-03	20492	2,66286E-01	20244	1,07877E+00	11558	8,53283E-01	20986	0	17530,5
Non-responder	pDC_LILRA4	Macro_FOLR2+APOE+	ICAM1	IL2RA	9,49172E-03	12443,3	1,69755E-02	4048	6,52464E-01	4354	1,94708E+00	2325	7,99693E-01	33959	0	17530,5
Non-responder	Macro_LYVE1	Macro_ISG15	CXCL12	CXCR4	9,49429E-03	10724,9	1,94792E-02	3208	6,91787E-01	3723	1,28645E+00	8174	8,53275E-01	20989	0	17530,5
Non-responder	Macro_FOLR2+APOE+	Macro_LYVE1	HSP90B1	TLR2	9,50334E-03	17821,3	6,24571E-03	20993	2,96342E-01	17947	7,29890E-01	19793	8,85871E-01	12843	0	17530,5
Non-responder	Macro_FOLR2+APOE+	Mono_CD14	GRN	TNFRSF1A	9,50374E-03	13203,9	5,44182E-03	26042	4,30737E-01	10582	1,53067E+00	5247	9,11974E-01	6618	0	17530,5
Non-responder	Mast	Mono_CD14	CSF1	SIRPA	9,50997E-03	10775,5	3,84933E-02	1028	1,05979E+00	937	1,59950E+00	4598	8,17088E-01	29784	0	17530,5
Non-responder	Macro_FOLR2+APOE+	Macro_IER3	HSPA1A	TLR4	9,51240E-03	16721,1	6,24500E-03	20997	3,31781E-01	15685	9,91134E-01	13313	8,73510E-01	16080	0	17530,5
Non-responder	Macro_FOLR2+APOE+	Macro_FOLR2+APOE+	HLA-B	LILRA1	9,51643E-03	24834,5	2,66407E-03	55253	3,73984E-01	13284	1,04626E+00	12205	8,32746E-01	25900	0	17530,5
Non-responder	Mono_INHBA	Macro_FOLR2-APOE+	IL1B	SIGIRR	9,52600E-03	14742,5	8,48813E-03	12800	6,25818E-01	4810	7,96764E-01	18056	8,55175E-01	20516	0	17530,5
Non-responder	Mast	Macro_IER3	TIMP3	CD44	9,52821E-03	7842,3	5,00694E-02	655	1,03844E+00	1029	1,56493E+00	4893	8,77342E-01	15104	0	17530,5
Non-responder	Mono_INHBA	Mono_CD16	VCAN	CD44	9,53053E-03	13425,7	8,90634E-03	11823	5,09117E-01	7693	6,86753E-01	21005	9,00917E-01	9077	0	17530,5
Non-responder	Macro_OLFML3	Macro_NLRP3	MIF	TNFRSF10D	9,53506E-03	15156,1	1,30659E-02	6338	4,30476E-01	10591	1,19921E+00	9484	8,08565E-01	31837	0	17530,5
Non-responder	Macro_IER3	Macro_ISG15	IL1B	SIGIRR	9,53507E-03	14249,3	8,22124E-03	13503	5,15416E-01	7485	1,07038E+00	11721	8,53186E-01	21007	0	17530,5
Non-responder	Macro_LYVE1	pDC_LILRA4	CD99	PILRB	9,54188E-03	14033,9	1,13214E-02	8060	3,68555E-01	13594	1,16977E+00	9975	8,53170E-01	21010	0	17530,5
Non-responder	Mast	Mono_INHBA	ADCYAP1	GPR84	9,54648E-03	15183,5	1,01688E-01	131	1,03839E+00	1030	1,46440E+00	5903	7,21913E-01	51323	0	17530,5
Non-responder	Macro_OLFML3	Mast	CALR	ITGA2B	9,55249E-03	10937,7	6,86246E-02	339	9,13076E-01	1657	1,13177E+00	10596	8,38283E-01	24566	0	17530,5
Non-responder	Macro_NLRP3	Macro_ISG15	SERPINA1	LRP1	9,55324E-03	15056,1	6,24188E-03	21015	3,28479E-01	15895	1,04302E+00	12271	9,03230E-01	8569	0	17530,5
Non-responder	Macro_FOLR2+APOE+	Macro_NLRP3	MDK	LRP1	9,56645E-03	18098,1	2,08476E-02	2852	4,31232E-01	10564	1,13164E+00	10600	7,33892E-01	48944	0	17530,5
Non-responder	Macro_LYVE1	Macro_LYVE1	C1QA	CD33	9,57144E-03	12609,5	1,00954E-02	9713	5,57918E-01	6281	6,86017E-01	21023	9,03578E-01	8500	0	17530,5
Non-responder	Macro_FOLR2+APOE+	Mono_CD14	C3	ITGAM	9,57372E-03	14496,1	1,12088E-02	8191	2,95199E-01	18020	1,31923E+00	7715	8,53109E-01	21024	0	17530,5
Non-responder	Macro_FOLR2+APOE+	Macro_FOLR2+APOE-	C1QA	CD33	9,57599E-03	13997,9	6,24008E-03	21025	5,03482E-01	7858	1,20698E+00	9330	8,80491E-01	14246	0	17530,5
Non-responder	Macro_NLRP3	Macro_LYVE1	VCAN	CD44	9,57827E-03	13639,5	7,78966E-03	14741	6,53263E-01	4341	6,85939E-01	21026	8,94775E-01	10559	0	17530,5
Non-responder	Macro_FOLR2+APOE+	Mono_CD16	AGTRAP	RACK1	9,57827E-03	16427,7	6,23993E-03	21026	3,53177E-01	14465	1,17194E+00	9939	8,60828E-01	19178	0	17530,5
Non-responder	Macro_ISG15	Macro_OLFML3	TNFSF13B	HLA-DPB1	9,58072E-03	8227,7	7,67663E-03	15083	6,51845E-01	4368	1,69344E+00	3823	9,48365E-01	334	0	17530,5
Non-responder	pDC_LILRA4	Macro_NLRP3	APP	TNFRSF21	9,58307E-03	12725,7	1,01364E-02	9652	1,03767E+00	1032	2,59391E+00	634	7,96136E-01	34780	0	17530,5
Non-responder	Macro_FOLR2+APOE+	Macro_IFI27	MMP9	CD44	9,58392E-03	14205,3	9,58307E-03	10530	4,64040E-01	9283	1,13122E+00	10605	8,44443E-01	23078	0	17530,5
Non-responder	Macro_FOLR2-APOE+	pDC_LILRA4	LGALS9	CD47	9,58392E-03	9659,1	9,54243E-03	10605	7,03326E-01	3595	1,44749E+00	6065	8,94966E-01	10500	0	17530,5
Non-responder	Mono_CD16	Mono_CD16	S100A9	CD68	9,58738E-03	15094,9	6,23875E-03	21030	3,21708E-01	16305	7,53515E-01	19174	9,37646E-01	1435	0	17530,5
Non-responder	cDC_LAMP3	pDC_LILRA4	FARP2	PLXNA4	9,59348E-03	17083,1	7,61713E-02	256	8,58482E-01	2020	1,62712E+00	4370	6,60547E-01	61239	0	17530,5
Non-responder	Macro_NLRP3	Macro_FOLR2+APOE+	LAMB3	CD44	9,60142E-03	16100,9	1,86676E-02	3445	4,69590E-01	9089	1,13084E+00	10610	7,74614E-01	39830	0	17530,5
Non-responder	Mono_CD14	cDC_CLEC9A	LYZ	ITGAL	9,60142E-03	9603,9	9,53930E-03	10610	6,09978E-01	5097	1,40536E+00	6588	9,04944E-01	8194	0	17530,5
Non-responder	Mono_CD14	Macro_LYVE1	VCAN	ITGB1	9,61264E-03	7630,9	1,62278E-02	4373	1,02613E+00	1087	1,64419E+00	4234	8,93231E-01	10930	0	17530,5
Non-responder	pDC_LILRA4	pDC_LILRA4	ASIP	ATRN	9,61903E-03	18675,5	8,18077E-02	216	6,51463E-01	4374	2,04183E+00	1925	5,77963E-01	69332	0	17530,5
Non-responder	Macro_IER3	Macro_OLFML3	HLA-C	LILRB1	9,61934E-03	16155,5	6,23434E-03	21044	2,65428E-01	20313	7,88343E-01	18265	9,26888E-01	3625	0	17530,5
Non-responder	pDC_LILRA4	Macro_ISG15	APP	TSPAN15	9,61972E-03	14256,1	1,20607E-02	7274	1,03724E+00	1034	2,60431E+00	616	7,52576E-01	44826	0	17530,5
Non-responder	Mono_CD14	cDC_LAMP3	THBS1	SDC4	9,62542E-03	12445,3	1,90323E-02	3326	7,44447E-01	3093	1,62599E+00	4375	7,99907E-01	33902	0	17530,5
Non-responder	pDC_LILRA4	cDC(CD1C)	APP	APLP2	9,62596E-03	7330,7	1,34791E-02	6016	1,00191E+00	1199	2,22996E+00	1291	8,94480E-01	10617	0	17530,5
Non-responder	Macro_FOLR2+APOE+	Macro_LYVE1	HGF	NRP1	9,63297E-03	18118,5	2,81729E-02	1714	5,08095E-01	7731	1,13057E+00	10619	7,13081E-01	52998	0	17530,5

Post_NR_Myeloid_Myeloid_CellCell

Non-responder	Macro_IER3	pDC_LILRA4	HLA-DPA1	CD4	9,63306E-03	12463,9	6,23365E-03	21050	3,35289E-01	15491	1,33316E+00	7510	9,43075E-01	738	0	17530,5
Non-responder	Macro_FOLR2+APOE+	Macro_OLFML3	CD59	STAB1	9,63535E-03	15897,7	7,44230E-03	15863	3,39415E-01	15235	1,17906E+00	9809	8,52964E-01	21051	0	17530,5
Non-responder	Macro_OLFML3	Mono_CD16	LGALS1	PTPRC	9,64351E-03	12226,9	5,76568E-03	23825	4,29844E-01	10622	1,32989E+00	7566	9,36822E-01	1591	0	17530,5
Non-responder	Macro_NLRP3	Macro_OLFML3	CD14	TLR1	9,64451E-03	16335,1	8,28036E-03	13332	3,33900E-01	15567	9,53366E-01	14191	8,52944E-01	21055	0	17530,5
Non-responder	cDC(CD1C)	Macro_NLRP3	HSPA8	LDLR	9,64680E-03	14082,3	1,31338E-02	6286	4,76938E-01	8819	8,46394E-01	16720	8,52940E-01	21056	0	17530,5
Non-responder	Macro_OLFML3	Macro_FOLR2+APOE+	B2M	LILRB1	9,64702E-03	11752,9	6,11346E-03	21743	4,29840E-01	10623	1,35323E+00	7256	9,36710E-01	1612	0	17530,5
Non-responder	Mono_CD16	cDC(CD1C)	ICAM4	ITGB2	9,65405E-03	11327,5	2,47651E-02	2121	6,74136E-01	3993	1,13033E+00	10625	8,47382E-01	22368	0	17530,5
Non-responder	Macro_NLRP3	Macro_ISG15	INHBA	ACTR2	9,66460E-03	14534,5	1,25509E-02	6783	4,29742E-01	10628	1,62524E+00	4379	8,02186E-01	33352	0	17530,5
Non-responder	Mono_INHBA	pDC_LILRA4	APP	TNFRSF21	9,67026E-03	10524,3	1,62013E-02	4382	8,46604E-01	2112	1,93006E+00	2410	8,31570E-01	26187	0	17530,5
Non-responder	Macro_FOLR2+APOE+	Macro_OLFML3	MIF	CD44_CD74	9,67482E-03	17458,9	4,48522E-03	34105	2,78402E-01	19275	8,96362E-01	15493	9,41640E-01	891	0	17530,5
Non-responder	pDC_LILRA4	Macro_LYVE1	IL16	CD4	9,67667E-03	8038,9	1,61937E-02	4383	7,27186E-01	3282	2,04383E+00	1917	8,84879E-01	13082	0	17530,5
Non-responder	cDC_CLEC9A	Macro_FOLR2+APOE+	IL16	CD4	9,68309E-03	7996,3	1,61886E-02	4384	7,33326E-01	3216	2,08357E+00	1761	8,84863E-01	13090	0	17530,5
Non-responder	cDC(CD1C)	Mono_CD16	HLA-DQA2	CD4	9,68350E-03	13854,9	6,22929E-03	21072	4,12859E-01	11391	1,07008E+00	11727	9,07652E-01	7554	0	17530,5
Non-responder	Macro_ISG15	Macro_NLRP3	SPP1	CD44	9,68573E-03	8121,9	1,28204E-02	6553	6,28981E-01	4745	2,28667E+00	1147	8,94425E-01	10634	0	17530,5
Non-responder	Macro_OLFML3	Macro_NLRP3	HLA-A	APLP2	9,68573E-03	12750,5	5,73133E-03	24055	4,29662E-01	10634	1,14042E+00	10446	9,40129E-01	1087	0	17530,5
Non-responder	Macro_IFI27	cDC_CLEC9A	ITGB2	CD226	9,69270E-03	11737,9	2,64222E-02	1898	8,38726E-01	2196	6,84097E-01	21076	8,73845E-01	15989	0	17530,5
Non-responder	Macro_FOLR2+APOE+	Mono_CD14	CCL5	CCR1	9,69278E-03	19139,7	1,53379E-02	4822	4,29654E-01	10636	1,50217E+00	5517	6,88799E-01	57193	0	17530,5
Non-responder	Macro_FOLR2+APOE+	Macro_OLFML3	MIF	CD74_CXCR4	9,69323E-03	17992,7	4,45107E-03	34486	2,46810E-01	21856	9,11568E-01	15133	9,41068E-01	958	0	17530,5
Non-responder	Mono_INHBA	cDC_CLEC9A	FN1	DPP4	9,72166E-03	13728,5	8,35897E-02	210	8,11819E-01	2437	1,62333E+00	4390	7,55758E-01	44075	0	17530,5
Non-responder	Mono_INHBA	Mono_CD16	TNF	VSIR	9,72166E-03	9445,7	1,61671E-02	4390	8,23943E-01	2327	2,18520E+00	1425	8,50781E-01	21556	0	17530,5
Non-responder	Mono_CD16	Macro_FOLR2+APOE+	TNFSF13B	HLA-DPB1	9,73162E-03	12206,3	5,35170E-03	26631	4,29320E-01	10647	1,55568E+00	4980	9,38782E-01	1243	0	17530,5
Non-responder	Macro_IER3	Mono_CD16	CD1D	LILRB2	9,73185E-03	10450,7	1,29840E-02	6415	7,79887E-01	2728	1,61250E+00	4487	8,52744E-01	21093	0	17530,5
Non-responder	Macro_OLFML3	Macro_ISG15	CD14	ITGB1	9,73185E-03	15628,7	6,22484E-03	21093	2,93968E-01	18099	1,13039E+00	10622	8,93750E-01	10799	0	17530,5
Non-responder	Macro_FOLR2+APOE+	pDC_LILRA4	MRC1	PTPRC	9,73647E-03	12464,5	9,15946E-03	11301	4,29644E-01	10637	2,08396E+00	1759	8,52741E-01	21095	0	17530,5
Non-responder	Macro_FOLR2+APOE+	Macro_NLRP3	ACTR2	LDLR	9,73870E-03	15306,9	1,30995E-02	6312	4,29289E-01	10649	1,39212E+00	6746	7,93980E-01	35297	0	17530,5
Non-responder	Macro_NLRP3	Macro_ISG15	HLA-C	LILRB2	9,73878E-03	12885,7	6,22448E-03	21096	3,38214E-01	15302	1,19402E+00	9561	9,41241E-01	939	0	17530,5
Non-responder	Macro_FOLR2+APOE+	cDC_CLEC9A	C3	ITGAX	9,74339E-03	15397,7	7,63991E-03	15184	2,55763E-01	21098	1,54233E+00	5116	8,65633E-01	18060	0	17530,5
Non-responder	Macro_OLFML3	Macro_OLFML3	LGALS9	SLC1A5	9,74801E-03	17208,1	7,00133E-03	17529	3,39214E-01	15249	9,33708E-01	14632	8,52721E-01	21100	0	17530,5
Non-responder	Macro_NLRP3	Mono_CD16	SEMA4A	LILRB2	9,75388E-03	13485,1	6,98671E-03	17579	7,18910E-01	3393	1,63687E+00	4292	8,38017E-01	24631	0	17530,5
Non-responder	Macro_FOLR2+APOE+	Macro_FOLR2-APOE+	CD59	STAB1	9,75957E-03	13279,1	1,20304E-02	7307	5,59855E-01	6231	6,83276E-01	21105	8,80604E-01	14222	0	17530,5
Non-responder	Macro_FOLR2+APOE+	Macro_OLFML3	PSAP	LRP1	9,76349E-03	13365,5	5,31796E-03	26901	4,29090E-01	10656	1,39664E+00	6690	9,19463E-01	5050	0	17530,5
Non-responder	Macro_ISG15	Macro_ISG15	MDK	SDC2	9,76678E-03	18105,3	4,09415E-02	931	6,73809E-01	4002	1,62265E+00	4397	6,41185E-01	63666	0	17530,5
Non-responder	Macro_OLFML3	Macro_ISG15	HBEGF	CD44	9,76882E-03	14161,5	6,22287E-03	21109	3,99219E-01	12018	1,27072E+00	8391	8,89927E-01	11759	0	17530,5
Non-responder	cDC_CLEC9A	Macro_LYVE1	HMGB1	CD163	9,77058E-03	8331,1	9,51451E-03	10658	6,46493E-01	4446	1,44755E+00	6064	9,30094E-01	2957	0	17530,5
Non-responder	Macro_FOLR2+APOE+	cDC_CLEC9A	ADA	DPP4	9,77324E-03	19675,7	3,56962E-02	1166	6,49959E-01	4398	1,11507E+00	10904	6,34553E-01	64380	0	17530,5
Non-responder	Macro_LYVE1	Mono_CD16	CXCL12	CXCR4	9,77345E-03	10547,3	1,92966E-02	3249	6,87184E-01	3789	1,36624E+00	7057	8,52684E-01	21111	0	17530,5
Non-responder	Mono_INHBA	Mast	SPP1	CD44	9,77808E-03	9877,5	1,91370E-02	3295	6,40498E-01	4542	8,20938E-01	17384	9,11899E-01	6636	0	17530,5
Non-responder	Mono_CD16	Macro_ISG15	S100A8	CD36	9,78734E-03	17425,1	7,96109E-03	14241	3,17361E-01	16596	8,11768E-01	17641	8,52655E-01	21117	0	17530,5
Non-responder	Macro_ISG15	cDC_LAMP3	ICAM1	IL2RG	9,79543E-03	9112,1	1,14837E-02	7890	7,37059E-01	3179	1,42899E+00	6296	8,94282E-01	10665	0	17530,5
Non-responder	pDC_LILRA4	cDC_CLEC9A	HMGB1	THBD	9,79543E-03	8995,9	9,50927E-03	10665	5,92465E-01	5478	2,21003E+00	1345	8,97102E-01	9961	0	17530,5
Non-responder	Macro_NLRP3	Macro_FOLR2-APOE+	VCAN	TLR1	9,79898E-03	13894,7	1,18003E-02	7534	8,33308E-01	2248	1,12830E+00	10666	8,10069E-01	31495	0	17530,5
Non-responder	Mono_CD16	Macro_NLRP3	S100A9	ITGB2	9,80126E-03	15072,1	6,22056E-03	21123	3,54814E-01	14359	7,26239E-01	19894	9,32588E-01	2454	0	17530,5
Non-responder	Macro_OLFML3	Macro_ISG15	TGFB1	ITGB1	9,80590E-03	18588,1	6,21973E-03	21125	2,77384E-01	19327	9,62554E-01	13962	8,53248E-01	20996	0	17530,5
Non-responder	Mono_CD16	Macro_IER3	CD55	CR1	9,80965E-03	18145,9	1,18322E-02	7506	5,19258E-01	7357	1,12799E+00	10669	7,39900E-01	47667	0	17530,5
Non-responder	Macro_ISG15	cDC_CLEC9A	ANXA1	DYSF	9,81204E-03	9603,5	1,97022E-02	3147	7,53187E-01	2990	1,62174E+00	4404	8,57532E-01	19946	0	17530,5
Non-responder	Mono_INHBA	Mast	TIMP1	CD63	9,81286E-03	10627,5	7,39144E-03	16049	6,33110E-01	4665	9,47867E-01	14323	9,45019E-01	570	0	17530,5
Non-responder	Mono_INHBA	Mast	HBEGF	CD82	9,81983E-03	9540,9	2,33947E-02	2305	9,05417E-01	1702	1,05016E+00	12143	8,81392E-01	14024	0	17530,5
Non-responder	cDC_LAMP3	Macro_OLFML3	TNFSF13B	HLA-DPB1	9,81983E-03	11222,1	6,21892E-03	21131	4,87753E-01	8395	1,27699E+00	8306	9,42958E-01	748	0	17530,5
Non-responder	Macro_FOLR2+APOE+	Macro_OLFML3	SPP1	PTGER4	9,82032E-03	11490,5	1,58071E-02	4583	4,28795E-01	10672	2,23741E+00	1268	8,43132E-01	23399	0	17530,5
Non-responder	pDC_LILRA4	Macro_NLRP3	B2M	LILRB2	9,82499E-03	9583,3	5,74663E-03	23950	6,49469E-01	4406	2,11073E+00	1657	9,47767E-01	373	0	17530,5
Non-responder	Macro_OLFML3	Macro_NLRP3	VIM	CD44	9,82680E-03	12051,5	6,21797E-03	21134	4,16080E-01	11234	1,16062E+00	10118	9,49741E-01	241	0	17530,5
Non-responder	cDC(CD1C)	Mono_INHBA	GRN	TNFRSF1B	9,83145E-03	14394,3	6,21778E-03	21136	4,23425E-01	10899	7,45536E-01	19366	9,29762E-01	3040	0	17530,5
Non-responder	Mono_CD16	Mono_CD14	ARPC5	LDLR	9,83457E-03	15076,7	1,12435E-02	8147	4,28680E-01	10676	1,24627E+00	8748	8,15077E-01	30282	0	17530,5
Non-responder	pDC_LILRA4	pDC_LILRA4	B2M	TFRC	9,83796E-03	8286,9	7,22428E-03	16656	6,49035E-01	4408	2,43047E+00	882	9,35024E-01	1958	0	17530,5
Non-responder	Mono_CD16	Macro_FOLR2+APOE+	IL16	CD4	9,84309E-03	12732,9	9,16997E-03	11286	4,83143E-01	8582	1,54104E+00	5125	8,52598E-01	21141	0	17530,5
Non-responder	Macro_FOLR2+APOE+	Mono_CD16	IGF1	INSR	9,84883E-03	19011,1	1,93910E-02	3230	4,28611E-01	10680	1,40887E+00	6546	6,89575E-01	57069	0	17530,5
Non-responder	Mono_INHBA	Mast	VEGFA	CD44	9,85240E-03	10831,3	1,48818E-02	5079	8,26039E-01	2313	7,24885E-01	19927	8,99869E-01	9307	0	17530,5
Non-responder	Macro_IFI27	Macro_FOLR2+APOE+	APOE	LRP1	9,85597E-03	10220,1	1,48666E-02	5089	6,25120E-01	4828	6,58921E-01	21845	9,35785E-01	1808	0	17530,5
Non-responder	Macro_LYVE1	pDC_LILRA4	B2M	TFRC	9,85940E-03	16433,3	6,21613E-03	21148	2,65383E-01	20321	7,13224E-01	20261	9,30306E-01	2906	0	17530,5
Non-responder	Macro_IFI27	Macro_FOLR2+APOE+	APOE	SCARB1	9,85954E-03	10394,5	2,41427E-02	2198	6,88427E-01	3770	6,56480E-01	21919	9,12284E-01	6555	0	17530,5
Non-responder	Macro_IER3	Macro_IER3	MRC1	PTPRC	9,86639E-03	16236,5	9,13265E-03	11348	4,09478E-01	11541	7,36765E-01	19612	8,52556E-01	21151	0	17530,5
Non-responder	Macro_FOLR2+APOE+	Macro_FOLR2-APOE+	ANXA2	TLR2	9,86872E-03	15619,7	6,95696E-03	17714	3,83826E-01	12777	6,81705E-01	21152	9,01556E-01	8925	0	17530,5
Non-responder	Macro_FOLR2-APOE+	Macro_LYVE1	APOE	LRP1	9,87043E-03	9155,5	1,61281E-02	4413	7,12630E-01	3475	7,58780E-01	19022	9,38189E-01	1337	0	17530,5
Non-responder	Mono_INHBA	cDC_CLEC9A	ITGB2	CD226	9,87106E-03	10357,1	2,38913E-02	2233	7,59714E-01	2927	1,07374E+00	11658	8,68190E-01	17437	0	17530,5
Non-responder	Macro_FOLR2+APOE+	Mono_INHBA	APOE	TREM2	9,87339E-03	13679,3	8,07880E-03	13903	2,55153E-01	21154	1,06535E+00	11825	9,24998E-01	3984	0	17530,5
Non-responder	Macro_LYVE1	Macro_FOLR2+APOE+	HMGB1	HAVCR2	9,87572E-03	16842,9	6,58264E-03	19344	3,13423E-01	16823	6,81598E-01	21155	8,99602E-01	9362	0	17530,5
Non-responder	Macro_FOLR2+APOE-	cDC_CLEC9A	CXCL12	DPP4	9,87819E-03	16388,9	1,07123E-01	111	1,03461E+00	1048	1,17570E+00	9870	7,10871E-01	53385	0	17530,5
Non-responder	Mono_INHBA	cDC_CLEC9A	ICAM1	IL2RG	9,88039E-03	15179,1	6,96277E-03	17690	4,44708E-01	10032	9,95996E-01	13208	8,68192E-01	17435	0	17530,5
Non-responder	Macro_OLFML3	Macro_FOLR2+APOE+	CCL2	CCR1	9,88098E-03	18713,5	1,07551E-02	8811	4,28415E-01	10689	1,28457E+00	8203	7,36849E-01	48334	0	17530,5
Non-responder	Macro_FOLR2+APOE+	Macro_OLFML3	COPA	CD74	9,88273E-03	13679,9	6,21483E-03	21158	3,46463E-01	14837	1,01872E+00	12769	9,34358E-01	2105	0	17530,5
Non-responder	Macro_FOLR2+APOE+	Macro_IFI27	ENTPD1	ADORA3	9,88506E-03	14568,3	1,52167E-02	4882	4,77169E-01	8812	7,06028E-01	20458	8,52494E-01	21159	0	17530,5

Post_NR_Myeloid_Myeloid_CellCell

Non-responder	Macro_FOLR2+APOE+	Macro_ISG15	IL10	IL10RA_IL10RB	9,89171E-03	15783,9	1,16131E-02	7736	4,28388E-01	10692	1,37639E+00	6934	7,90628E-01	36027	0	17530,5
Non-responder	Mono_CD16	cDC_CLEC9A	S100A8	ITGB2	9,89441E-03	15036,3	6,21404E-03	21163	3,22686E-01	16244	8,57024E-01	16446	9,25871E-01	3798	0	17530,5
Non-responder	Macro_IER3	Macro_LYVE1	HLA-DQA2	CD4	9,90844E-03	17261,1	6,21291E-03	21169	2,79770E-01	19147	6,90958E-01	20882	9,07542E-01	7577	0	17530,5
Non-responder	Macro_IFI27	Macro_ISG15	LILRB4	LAIR1	9,91079E-03	15944,9	6,89852E-03	17942	3,60182E-01	14045	6,81074E-01	21170	9,01086E-01	9037	0	17530,5
Non-responder	Macro_NLRP3	Macro_FOLR2+APOE-	ICAM1	ITGAM_ITGB2	9,91313E-03	14614,5	7,28964E-03	16396	4,83681E-01	8564	6,81058E-01	21171	8,99349E-01	9411	0	17530,5
Non-responder	Macro_IER3	Macro_ISG15	HLA-F	LILRB2	9,91313E-03	13547,7	6,21256E-03	21171	5,41109E-01	6732	1,16545E+00	10044	8,88102E-01	12261	0	17530,5
Non-responder	Macro_FOLR2+APOE+	Macro_FOLR2+APOE+	ADAM9	ITGA6	9,91678E-03	19181,3	3,07454E-02	1472	5,20100E-01	7324	1,12572E+00	10699	6,77865E-01	58881	0	17530,5
Non-responder	pDC_LILRA4	Macro_FOLR2+APOE-	COPA	CD74	9,91678E-03	8382,3	9,48222E-03	10699	6,74352E-01	3990	1,21607E+00	9207	9,46185E-01	485	0	17530,5
Non-responder	Macro_LYVE1	Mono_CD16	C3	ITGAX	9,92249E-03	17388,1	8,55818E-03	12617	2,79434E-01	19176	6,80970E-01	21175	8,72098E-01	16442	0	17530,5
Non-responder	Macro_NLRP3	Mono_CD16	CD55	ADGRE2	9,93113E-03	13302,1	9,47998E-03	10703	5,50832E-01	6481	1,35571E+00	7213	8,38220E-01	24583	0	17530,5
Non-responder	cDC_CLEC9A	Macro_FOLR2+APOE+	LGALS9	CD47	9,93472E-03	9600,3	9,47977E-03	10704	5,96811E-01	5379	1,69626E+00	3809	8,94656E-01	10579	0	17530,5
Non-responder	Mono_INHBA	cDC_CLEC9A	IL1B	SIGIRR	9,93656E-03	9055,7	1,29374E-02	6449	7,45348E-01	3080	1,71629E+00	3658	8,79374E-01	14561	0	17530,5
Non-responder	Macro_NLRP3	cDC(CD1C)	S100A8	CD36	9,93656E-03	13920,9	7,92519E-03	14351	7,28651E-01	3260	9,92153E-01	13282	8,52370E-01	21181	0	17530,5
Non-responder	Macro_FOLR2+APOE+	Macro_FOLR2+APOE+	LY86	CD180	9,94550E-03	14199,9	1,58554E-02	4561	4,28149E-01	10707	1,35089E+00	7288	8,12385E-01	30913	0	17530,5
Non-responder	Mono_INHBA	cDC_CLEC9A	S100A9	ITGB2	9,94595E-03	11464,5	8,01857E-03	14089	5,46305E-01	6586	7,97379E-01	18032	9,40144E-01	1085	0	17530,5
Non-responder	Mast	Macro_FOLR2+APOE+	COL18A1	ITGB5	9,95265E-03	16194,9	1,45303E-01	58	1,03380E+00	1052	1,35884E+00	7157	7,00804E-01	55177	0	17530,5
Non-responder	pDC_LILRA4	Macro_FOLR2+APOE-	APP	NCSTN	9,95265E-03	11171,1	1,19980E-02	7338	1,07233E+00	889	2,33605E+00	1052	8,20086E-01	29046	0	17530,5
Non-responder	Mono_INHBA	cDC_CLEC9A	SPP1	ITGA4_ITGB1	9,95534E-03	9410,3	1,96214E-02	3169	5,73273E-01	5906	1,53068E+00	5246	8,76974E-01	15200	0	17530,5
Non-responder	Macro_FOLR2+APOE+	Macro_IER3	LGALS9	PTPRC	9,95534E-03	13340,9	6,20987E-03	21189	4,50733E-01	9814	1,10425E+00	11093	9,09836E-01	7078	0	17530,5
Non-responder	Macro_ISG15	Mono_CD16	MMP12	PLAUR	9,96169E-03	8854,5	2,06214E-02	2916	6,47521E-01	4427	2,21039E+00	1343	8,65668E-01	18056	0	17530,5
Non-responder	Macro_OLFML3	Mono_CD16	TNFSF12	TNFRSF8	9,96347E-03	19057,9	3,50101E-02	1199	6,05603E-01	5195	1,12466E+00	10712	6,65157E-01	60653	0	17530,5
Non-responder	Mono_INHBA	cDC_CLEC9A	VCAN	ITGB1	9,96944E-03	13190,5	1,04354E-02	9236	5,24500E-01	7201	9,13490E-01	15080	8,70278E-01	16905	0	17530,5
Non-responder	Mono_INHBA	cDC_CLEC9A	VCAN	ITGA4	9,97179E-03	12180,1	1,10602E-02	8411	5,76092E-01	5822	1,01056E+00	12941	8,73034E-01	16196	0	17530,5
Non-responder	Macro_OLFML3	Macro_NLRP3	S100A9	TLR4	9,97179E-03	16930,9	7,07513E-03	17206	3,07251E-01	17231	1,08167E+00	11491	8,52300E-01	21196	0	17530,5
Non-responder	Mono_INHBA	cDC_CLEC9A	VCAN	SELL	9,97415E-03	9420,5	1,79705E-02	3670	7,15164E-01	3447	1,42886E+00	6298	8,73178E-01	16157	0	17530,5
Non-responder	Macro_FOLR2+APOE+	Macro_IER3	HLA-B	LILRB1	9,97787E-03	16175,3	4,19273E-03	37254	4,53922E-01	9660	1,12458E+00	10716	9,16102E-01	5716	0	17530,5
Non-responder	Macro_IFI27	Macro_FOLR2+APOE+	FAM3C	LAMP1	9,98508E-03	17861,9	1,03200E-02	9377	5,72925E-01	5915	1,27296E+00	8366	7,37804E-01	48121	0	17530,5
Non-responder	Mono_INHBA	cDC_CLEC9A	VEGFA	ITGB1	9,98592E-03	10439,7	1,48168E-02	5120	7,33018E-01	3223	1,38609E+00	6810	8,59402E-01	19515	0	17530,5

Post_R_Myeloid_Myeloid_CellCel

Response	source	target	ligand.complex	receptor.complex	aggregate_rank	mean_rank	natmi.edge_specificity	natmi.rank	connectome.weight_sc	connectome.rank	logfc.logfc_comb	logfc.rank	sca.LRscore	sca.rank	cellphonedb.pvalue	cellphonedb.rank
Responder	Mono_CD14	Mast	S100A8	CD69	2,50519E-10	2281,9	9,20322E-02	113	1,95925E+00	44	3,90217E+00	18	9,53114E-01	26	0	11208,5
Responder	Macro_OLFML3	cDC(CD1C)	B2M	CD1C	1,35515E-09	2599,9	4,51056E-02	583	1,27760E+00	206	1,96140E+00	775	9,43174E-01	227	0	11208,5
Responder	cDC_LAMP3	pDC_LILRA4	CCL19	CXCR3	2,37587E-09	5564,7	4,41761E-01	7	2,94766E+00	4	3,60956E+00	23	8,49361E-01	16581	0	11208,5
Responder	pDC_LILRA4	Mast	SCT	ADRB2	4,76180E-09	5583,9	4,15096E-01	8	2,45195E+00	12	3,46884E+00	29	8,48911E-01	16662	0	11208,5
Responder	cDC_LAMP3	cDC_LAMP3	CCL19	CCR7	8,36988E-09	3828,1	4,53566E-01	5	3,43403E+00	1	3,35406E+00	35	8,94181E-01	7891	0	11208,5
Responder	cDC_LAMP3	pDC_LILRA4	CCL19	CCR7	8,36988E-09	6336,7	1,54250E-01	35	2,34138E+00	19	3,49889E+00	28	8,31304E-01	20393	0	11208,5
Responder	Macro_OLFML3	Mono_CD14	FN1	PLAUR	1,24684E-08	2955,7	3,02329E-02	1208	8,62051E-01	974	2,16453E+00	507	9,34185E-01	881	0	11208,5
Responder	pDC_LILRA4	pDC_LILRA4	GZMB	IGF2R	1,55181E-08	3638,7	1,41566E-01	43	2,58517E+00	9	5,66980E+00	1	8,99165E-01	6932	0	11208,5
Responder	Macro_OLFML3	Macro_NLRP3	FN1	PLAUR	2,14939E-08	2982,3	3,29972E-02	1033	9,46584E-01	712	1,67729E+00	1347	9,36824E-01	611	0	11208,5
Responder	pDC_LILRA4	pDC_LILRA4	SCT	VIPR2	2,43933E-08	10006,7	8,61705E-01	1	2,54806E+00	10	3,16296E+00	50	7,38321E-01	38764	0	11208,5
Responder	Macro_IER3	pDC_LILRA4	TNF	PTPRS	3,80683E-08	4962,7	2,19336E-01	19	2,08173E+00	36	3,04966E+00	58	8,64221E-01	13492	0	11208,5
Responder	Mono_CD14	Macro_LYVE1	S100A8	CD36	4,76254E-08	2401,5	6,51789E-02	249	1,70926E+00	77	3,12333E+00	53	9,39365E-01	420	0	11208,5
Responder	Macro_OLFML3	Macro_ISG15	C3	IFITM1	5,11435E-08	2782,3	4,81623E-02	519	1,15590E+00	320	2,45345E+00	262	9,28363E-01	1602	0	11208,5
Responder	pDC_LILRA4	pDC_LILRA4	PIGA	PIGR	6,13343E-08	8478,3	2,34484E-01	17	1,76102E+00	68	4,36691E+00	5	7,78518E-01	31093	0	11208,5
Responder	Mono_INHBA	cDC_CLEC9A	CXCL2	XCR1	6,13343E-08	3147,9	1,82937E-01	26	3,07478E+00	2	2,99991E+00	68	9,12536E-01	4435	0	11208,5
Responder	Macro_OLFML3	Mono_INHBA	FN1	PLAUR	6,40985E-08	3188,3	3,02503E-02	1206	8,62583E-01	971	1,57007E+00	1676	9,34202E-01	880	0	11208,5
Responder	Macro_OLFML3	Mono_INHBA	FN1	CD44	6,42899E-08	3334,3	2,84212E-02	1375	8,48771E-01	1022	1,56915E+00	1677	9,29855E-01	1389	0	11208,5
Responder	Mono_INHBA	pDC_LILRA4	CCL20	CXCR3	8,22789E-08	6827,7	2,00557E-01	22	1,71909E+00	75	3,35962E+00	34	8,19657E-01	22799	0	11208,5
Responder	Macro_ISG15	pDC_LILRA4	CXCL10	CXCR3	9,61490E-08	4417,9	1,74417E-01	27	1,69778E+00	79	3,30996E+00	37	8,78504E-01	10738	0	11208,5
Responder	cDC_LAMP3	cDC(CD1C)	CCL19	CCR7	1,11495E-07	7508,3	1,06142E-01	79	2,16576E+00	29	2,89487E+00	83	8,03450E-01	26142	0	11208,5
Responder	Macro_FOLR2+APOE-	pDC_LILRA4	CCL13	CXCR3	1,32868E-07	8003,7	3,37025E-01	10	2,26697E+00	23	2,86896E+00	88	7,90553E-01	28689	0	11208,5
Responder	Macro_OLFML3	pDC_LILRA4	BST2	LILRA4	1,40203E-07	2679,3	8,45903E-02	144	2,02330E+00	40	3,19854E+00	44	9,26119E-01	1960	0	11208,5
Responder	Mono_CD14	Macro_LYVE1	S100A8	CD68	1,46914E-07	2457,1	3,55672E-02	911	1,63112E+00	91	2,96688E+00	74	9,63340E-01	1	0	11208,5
Responder	pDC_LILRA4	pDC_LILRA4	HSP90B1	TLR9	2,00818E-07	4603,3	9,60289E-02	101	1,64394E+00	88	3,18119E+00	48	8,74446E-01	11571	0	11208,5
Responder	Mono_INHBA	pDC_LILRA4	TNF	PTPRS	2,12975E-07	5946,3	1,51521E-01	38	1,85072E+00	55	2,80622E+00	103	8,41023E-01	18327	0	11208,5
Responder	pDC_LILRA4	pDC_LILRA4	APP	TNFRSF21	2,12975E-07	4227,7	9,57039E-02	103	1,87452E+00	48	4,02893E+00	12	8,83814E-01	9767	0	11208,5
Responder	Macro_ISG15	cDC(CD1C)	B2M	CD1C	2,37429E-07	2596,9	4,31780E-02	628	1,15185E+00	329	2,15202E+00	532	9,41992E-01	287	0	11208,5
Responder	pDC_LILRA4	Mono_CD16	FAM3D	FPR2	2,52368E-07	10321,7	2,54084E-01	14	1,53243E+00	109	2,81086E+00	102	7,30167E-01	40175	0	11208,5
Responder	Macro_OLFML3	Macro_ISG15	FN1	PLAUR	2,85772E-07	3871,5	2,34786E-02	1953	6,55504E-01	2260	1,49289E+00	1951	9,25972E-01	1985	0	11208,5
Responder	pDC_LILRA4	pDC_LILRA4	MUC2	AGR2	3,36656E-07	12004,5	3,84193E-01	9	1,49941E+00	120	4,97790E+00	2	6,79639E-01	48683	0	11208,5
Responder	Mono_CD14	Macro_LYVE1	S100A12	CD36	3,45134E-07	3467,9	1,17613E-01	60	1,57741E+00	94	2,74764E+00	121	9,04791E-01	5856	0	11208,5
Responder	Mono_CD14	Macro_LYVE1	S100A9	CD36	3,51779E-07	2544,3	4,59626E-02	566	1,55511E+00	103	2,66779E+00	151	9,36038E-01	693	0	11208,5
Responder	cDC(CD1C)	cDC(CD1C)	B2M	CD1C	3,85331E-07	2613,5	4,09088E-02	709	1,00382E+00	571	2,51413E+00	223	9,40499E-01	356	0	11208,5
Responder	cDC_LAMP3	pDC_LILRA4	CCL22	DPP4	3,99008E-07	10562,9	2,87858E-01	13	1,75594E+00	70	2,72087E+00	127	7,23300E-01	41396	0	11208,5
Responder	cDC_CLEC9A	cDC(CD1C)	B2M	CD1C	4,28222E-07	2672,7	4,00776E-02	728	9,49600E-01	703	2,34480E+00	338	9,39922E-01	386	0	11208,5
Responder	Macro_OLFML3	Mono_CD14	FN1	CD44	4,32250E-07	3891,1	2,33879E-02	1968	6,82083E-01	1995	1,52442E+00	1829	9,23226E-01	2455	0	11208,5
Responder	Mast	pDC_LILRA4	COL18A1	PTPRS	4,37868E-07	8702,9	5,74122E-01	2	1,98770E+00	41	2,70855E+00	131	7,73046E-01	32132	0	11208,5
Responder	Macro_ISG15	pDC_LILRA4	CXCL11	CXCR3	4,79170E-07	8445,9	1,97535E-01	24	1,45690E+00	135	2,82033E+00	100	7,80134E-01	30762	0	11208,5
Responder	pDC_LILRA4	cDC(CD1C)	B2M	CD1C	5,49656E-07	2638,7	3,96788E-02	745	9,23587E-01	775	3,05202E+00	57	9,39639E-01	408	0	11208,5
Responder	pDC_LILRA4	Mono_INHBA	SCT	GPR84	5,57547E-07	9726,7	1,39294E-01	44	1,91358E+00	46	2,68511E+00	142	7,46636E-01	37193	0	11208,5
Responder	Macro_OLFML3	Mono_CD16	C3	IFITM1	5,67091E-07	3342,3	4,07216E-02	712	1,02811E+00	516	1,56782E+00	1683	9,22578E-01	2592	0	11208,5
Responder	Macro_OLFML3	Mast	FN1	CD44	5,96109E-07	3687,9	2,64898E-02	1566	7,84809E-01	1328	1,34334E+00	2618	9,27525E-01	1719	0	11208,5
Responder	Macro_OLFML3	Macro_LYVE1	C3	LRP1	6,11057E-07	3500,7	3,45525E-02	959	9,83372E-01	620	1,45753E+00	2085	9,22319E-01	2631	0	11208,5
Responder	Mono_CD14	Mono_CD14	S100A12	CD36	6,70278E-07	4011,5	8,68407E-02	137	1,38635E+00	151	3,00255E+00	65	8,90900E-01	8496	0	11208,5
Responder	Macro_NLRP3	cDC_CLEC9A	CXCL2	XCR1	6,97233E-07	3476,3	1,49611E-01	39	2,91904E+00	5	2,66234E+00	153	9,04171E-01	5976	0	11208,5
Responder	Mono_CD14	cDC_CLEC9A	CXCL2	XCR1	7,10976E-07	4382,5	9,07133E-02	118	2,64379E+00	7	2,65821E+00	154	8,80195E-01	10425	0	11208,5
Responder	Mono_CD14	Macro_IFI27	S100A8	CD68	7,10976E-07	2589,7	2,74951E-02	1449	1,38211E+00	154	2,69699E+00	134	9,58513E-01	3	0	11208,5
Responder	Macro_OLFML3	pDC_LILRA4	CXCL9	CXCR3	7,82408E-07	5627,3	9,11968E-02	115	1,36938E+00	158	2,65188E+00	159	8,49745E-01	16496	0	11208,5
Responder	Macro_FOLR2+APOE-	pDC_LILRA4	CXCL12	CXCR3	8,12267E-07	6918,1	1,42610E-01	40	1,57701E+00	95	2,65040E+00	161	8,18308E-01	23086	0	11208,5
Responder	Macro_IER3	pDC_LILRA4	TNF	TNFRSF21	8,27477E-07	4813,7	8,30402E-02	152	1,57570E+00	96	2,64885E+00	162	8,69334E-01	12450	0	11208,5
Responder	Mono_CD14	Mono_INHBA	S100A8	CD68	8,58463E-07	2598,5	2,68687E-02	1522	1,36279E+00	164	2,83217E+00	94	9,58052E-01	4	0	11208,5
Responder	pDC_LILRA4	Mono_CD16	APP	FPR2	8,90213E-07	5565,9	8,05312E-02	166	1,43762E+00	141	2,76191E+00	115	8,51214E-01	16199	0	11208,5
Responder	pDC_LILRA4	Mono_INHBA	GZMB	IGF2R	9,06377E-07	4736,5	8,03567E-02	167	2,31422E+00	20	4,39919E+00	3	8,70438E-01	12284	0	11208,5
Responder	pDC_LILRA4	Macro_FOLR2+APOE+	GZMB	IGF2R	9,72986E-07	4754,1	7,96826E-02	171	2,31124E+00	21	4,00493E+00	13	8,69962E-01	12357	0	11208,5
Responder	Macro_OLFML3	pDC_LILRA4	CXCL10	CXCR3	1,04278E-06	5926,5	9,55686E-02	105	1,33402E+00	175	2,64134E+00	163	8,42578E-01	17981	0	11208,5
Responder	pDC_LILRA4	Mast	CDH1	KLRG1	1,06073E-06	10878,7	4,48598E-01	6	1,40969E+00	147	2,59945E+00	176	7,15438E-01	42856	0	11208,5
Responder	pDC_LILRA4	Macro_ISG15	SCT	GPR84	1,19220E-06	11087,9	9,57319E-02	102	1,82118E+00	61	2,58605E+00	183	7,09557E-01	43885	0	11208,5
Responder	Macro_OLFML3	Macro_LYVE1	C3	CD81	1,28351E-06	3910,9	2,67482E-02	1535	8,48539E-01	1025	1,26642E+00	3052	9,21786E-01	2734	0	11208,5
Responder	Mast	cDC(CD1C)	CSF1	CSF2RA	1,35523E-06	4131,9	8,99311E-02	120	1,29967E+00	191	2,83468E+00	93	8,87924E-01	9047	0	11208,5
Responder	Macro_FOLR2+APOE+	cDC(CD1C)	B2M	CD1C	1,45373E-06	2664,7	4,41153E-02	612	1,21299E+00	260	1,83662E+00	989	9,42576E-01	254	0	11208,5
Responder	pDC_LILRA4	Macro_NLRP3	SCT	GPR84	1,48679E-06	11220,7	9,24383E-02	112	1,81419E+00	63	2,55953E+00	197	7,05936E-01	44523	0	11208,5
Responder	pDC_LILRA4	Mono_CD16	FAM3D	FPR1	1,60271E-06	7467,3	1,03478E-01	84	1,28352E+00	202	2,56604E+00	193	8,06034E-01	25649	0	11208,5
Responder	Mono_CD14	Macro_FOLR2-APOE+	S100A8	CD68	1,60271E-06	2654,1	2,55066E-02	1673	1,32077E+00	180	2,54993E+00	202	9,56994E-01	7	0	11208,5
Responder	pDC_LILRA4	Macro_LYVE1	LCN2	FCGR2A	1,65071E-06	6226,1	7,90427E-02	178	1,31018E+00	185	2,54623E+00	204	8,36229E-01	19355	0	11208,5

Responder	Macro_FOLR2+APOE+	pDC_LILRA4	BST2	LILRA4	1,65071E-06	2907,3	7,20964E-02	204	1,87179E+00	49	2,89472E+00	84	9,20462E-01	2991	0	11208,5
Responder	Mast	pDC_LILRA4	HSPG2	PTPRS	1,72448E-06	10014,5	5,50762E-01	3	1,93513E+00	45	2,54130E+00	207	7,39174E-01	38609	0	11208,5
Responder	Macro_ISG15	pDC_LILRA4	BST2	LILRA4	1,77487E-06	2911,5	7,14160E-02	209	1,86354E+00	52	3,39413E+00	32	9,20114E-01	3056	0	11208,5
Responder	pDC_LILRA4	Macro_FOLR2-APOE+	GZMB	IGF2R	1,80043E-06	5015,1	7,12992E-02	210	2,27413E+00	22	4,08945E+00	9	8,63544E-01	13626	0	11208,5
Responder	Mono_CD14	Macro_OLFML3	S100A8	ITGB2	1,82623E-06	2592,3	2,82653E-02	1390	1,41185E+00	146	2,53738E+00	211	9,57001E-01	6	0	11208,5
Responder	Mono_CD14	cDC(CD1C)	S100A8	ITGB2	1,87856E-06	2672,1	2,40234E-02	1874	1,26222E+00	213	3,21830E+00	41	9,53529E-01	24	0	11208,5
Responder	Mono_CD14	Macro_FOLR2+APOE-	S100A8	CD68	1,93188E-06	2709,1	2,36026E-02	1938	1,26203E+00	215	2,61583E+00	170	9,55369E-01	14	0	11208,5
Responder	pDC_LILRA4	Macro_ISG15	FAM3D	FPR2	1,98621E-06	12131,7	1,52431E-01	36	1,25977E+00	217	2,67084E+00	149	6,76994E-01	49048	0	11208,5
Responder	Macro_OLFML3	Macro_OLFML3	APOE	TREM2	2,10057E-06	3493,5	2,74287E-02	1460	7,98561E-01	1255	1,21144E+00	3368	9,44652E-01	176	0	11208,5
Responder	Mono_CD14	Mono_INHBA	S100A12	CD36	2,18433E-06	4333,3	7,46127E-02	195	1,31042E+00	184	2,51368E+00	224	8,83303E-01	9855	0	11208,5
Responder	Mono_CD14	pDC_LILRA4	VCAN	SELL	2,18433E-06	2961,9	6,89121E-02	224	1,47647E+00	127	3,58549E+00	24	9,19154E-01	3226	0	11208,5
Responder	Mono_CD14	Macro_LYVE1	S100A9	CD68	2,21366E-06	2658,1	2,50812E-02	1729	1,47697E+00	126	2,51134E+00	225	9,61273E-01	2	0	11208,5
Responder	Mast	Mono_CD14	CSF1	CSF3R	2,24325E-06	4425,5	1,01313E-01	88	1,25067E+00	226	2,78854E+00	106	8,79810E-01	10499	0	11208,5
Responder	pDC_LILRA4	Macro_IER3	GZMB	IGF2R	2,36426E-06	5153,9	6,73736E-02	230	2,25675E+00	24	4,10251E+00	8	8,60173E-01	14299	0	11208,5
Responder	pDC_LILRA4	Macro_FOLR2-APOE+	SCT	GPR84	2,45781E-06	10658,1	1,07808E-01	77	1,84679E+00	57	2,50199E+00	233	7,21644E-01	41715	0	11208,5
Responder	cDC_LAMP3	cDC_CLEC9A	CCL19	CXCR3	2,48953E-06	7314,1	2,48054E-01	15	2,44107E+00	13	2,49398E+00	234	8,08615E-01	25100	0	11208,5
Responder	Macro_NLRP3	Mast	S100A8	CD69	2,68911E-06	2798,1	4,58454E-02	571	1,23660E+00	239	1,75992E+00	1154	9,34844E-01	818	0	11208,5
Responder	Macro_IFI27	cDC(CD1C)	B2M	CD1C	2,71707E-06	2770,1	4,16024E-02	684	1,04907E+00	467	1,75627E+00	1157	9,40968E-01	334	0	11208,5
Responder	Mono_CD14	pDC_LILRA4	S100A12	CD36	2,75318E-06	4569,5	6,57056E-02	242	1,25512E+00	223	3,12627E+00	52	8,76590E-01	11122	0	11208,5
Responder	pDC_LILRA4	cDC_CLEC9A	APP	CD74	2,75318E-06	2836,7	1,93184E-02	2673	1,23471E+00	242	3,40801E+00	31	9,52852E-01	29	0	11208,5
Responder	Macro_OLFML3	cDC_CLEC9A	C1QB	C1QBP	2,77158E-06	3754,3	1,64927E-02	3560	7,81080E-01	1351	1,57670E+00	1652	9,33158E-01	1000	0	11208,5
Responder	pDC_LILRA4	Macro_IER3	SCT	GPR84	2,78738E-06	10596,5	1,09589E-01	75	1,85057E+00	56	2,47771E+00	243	7,23287E-01	41400	0	11208,5
Responder	cDC(CD1C)	Mast	LGALS1	CD69	2,99654E-06	3335,1	1,94194E-02	2647	9,27641E-01	766	2,01790E+00	687	9,30005E-01	1367	0	11208,5
Responder	Macro_OLFML3	Macro_NLRP3	FN1	C5AR1	3,16165E-06	3631,9	3,99361E-02	734	9,00763E-01	843	1,55698E+00	1719	9,16715E-01	3655	0	11208,5
Responder	pDC_LILRA4	Macro_OLFML3	SCT	GPR84	3,22014E-06	11069,3	9,66620E-02	100	1,82315E+00	60	2,45841E+00	255	7,10552E-01	43723	0	11208,5
Responder	Mono_CD14	Mono_INHBA	VCAN	CD44	3,34532E-06	2753,5	3,01204E-02	1219	1,25482E+00	224	2,21148E+00	445	9,36219E-01	671	0	11208,5
Responder	pDC_LILRA4	Mono_CD14	GZMB	IGF2R	3,41290E-06	5289,7	6,40252E-02	260	2,24193E+00	25	4,35285E+00	6	8,57079E-01	14949	0	11208,5
Responder	pDC_LILRA4	Macro_NLRP3	FAM3D	FPR1	3,49210E-06	7799,7	9,31186E-02	110	1,21203E+00	262	2,65380E+00	158	7,97655E-01	27260	0	11208,5
Responder	Macro_OLFML3	Mono_CD14	C3	CR1	3,51237E-06	5754,1	4,64215E-02	552	8,67808E-01	953	1,72527E+00	1234	8,57615E-01	14823	0	11208,5
Responder	Mono_CD14	Mono_CD14	S100A8	ITGB2	3,57251E-06	2734,1	2,25623E-02	2093	1,21068E+00	264	2,99186E+00	71	9,52118E-01	34	0	11208,5
Responder	cDC_LAMP3	cDC_CLEC9A	CCL22	DPP4	3,69543E-06	11826,9	2,03091E-01	21	1,56552E+00	97	2,44914E+00	267	6,87077E-01	47541	0	11208,5
Responder	Macro_OLFML3	Macro_FOLR2+APOE+	C3	CD81	3,80533E-06	4038,1	2,69906E-02	1513	8,55254E-01	1001	1,14547E+00	3793	9,22111E-01	2675	0	11208,5
Responder	Macro_OLFML3	Mast	APOE	ABCA1	3,81537E-06	4226,9	1,85222E-02	2884	7,28056E-01	1656	1,14510E+00	3795	9,28449E-01	1591	0	11208,5
Responder	Macro_OLFML3	Mono_CD14	FN1	C5AR1	3,86511E-06	3773,9	3,47452E-02	951	7,97912E-01	1264	1,96321E+00	772	9,11243E-01	4674	0	11208,5
Responder	pDC_LILRA4	cDC_CLEC9A	CDH1	CDH2	3,99310E-06	14360,3	5,23237E-01	4	1,49646E+00	122	2,43838E+00	274	5,64579E-01	60193	0	11208,5
Responder	pDC_LILRA4	Mono_CD14	FAM3D	FPR2	4,03689E-06	12603,9	1,30685E-01	46	1,20144E+00	275	2,82513E+00	99	6,59941E-01	51391	0	11208,5
Responder	pDC_LILRA4	Macro_LYVE1	GZMB	IGF2R	4,03689E-06	5331,7	6,30432E-02	275	2,23759E+00	26	3,95775E+00	15	8,56130E-01	15134	0	11208,5
Responder	Mono_CD14	Macro_FOLR2+APOE+	S100A8	ITGB2	4,03689E-06	2631,7	2,70446E-02	1505	1,36879E+00	159	2,43434E+00	275	9,56084E-01	11	0	11208,5
Responder	pDC_LILRA4	Macro_OLFML3	APP	CD74	4,26060E-06	2891,3	1,88792E-02	2790	1,19483E+00	280	2,67616E+00	146	9,52333E-01	32	0	11208,5
Responder	Mono_CD14	Macro_NLRP3	S100A8	CD68	4,30631E-06	2757,3	2,28859E-02	2045	1,23992E+00	236	2,42835E+00	281	9,54707E-01	16	0	11208,5
Responder	Mono_CD14	Macro_FOLR2-APOE+	S100A8	ITGB2	4,35235E-06	2780,1	2,24595E-02	2103	1,20705E+00	271	2,42816E+00	282	9,52014E-01	36	0	11208,5
Responder	Macro_IER3	cDC_CLEC9A	CXCL2	XCR1	4,49242E-06	3422,5	1,57066E-01	34	2,95388E+00	3	2,42297E+00	285	9,06257E-01	5582	0	11208,5
Responder	Mono_CD16	cDC(CD1C)	B2M	CD1C	4,49738E-06	2774,7	4,24349E-02	660	1,10338E+00	387	1,68919E+00	1313	9,41516E-01	305	0	11208,5
Responder	Macro_OLFML3	Macro_NLRP3	FN1	CD44	4,49796E-06	4348,1	2,31656E-02	2009	6,74720E-01	2078	1,13038E+00	3922	9,22887E-01	2523	0	11208,5
Responder	Mono_CD14	cDC(CD1C)	B2M	CD1C	4,62143E-06	2951,1	3,75666E-02	824	7,85804E-01	1322	1,88045E+00	900	9,38069E-01	501	0	11208,5
Responder	cDC(CD1C)	pDC_LILRA4	TNF	PTPRS	4,68380E-06	8832,9	6,14797E-02	289	1,54399E+00	106	3,00495E+00	64	7,71156E-01	32497	0	11208,5
Responder	pDC_LILRA4	Mast	VEGFB	ADRB2	4,73247E-06	6863,5	9,54344E-02	106	1,18219E+00	290	2,83051E+00	95	8,20455E-01	22618	0	11208,5
Responder	cDC_CLEC9A	pDC_LILRA4	BTLA	CD79A	4,78149E-06	11292,7	3,05612E-01	12	2,13472E+00	33	2,41844E+00	291	7,03698E-01	44919	0	11208,5
Responder	Mono_INHBA	cDC(CD1C)	B2M	CD1C	4,80511E-06	3034,1	3,76248E-02	821	7,89596E-01	1307	1,68078E+00	1335	9,38114E-01	499	0	11208,5
Responder	Macro_OLFML3	pDC_LILRA4	HLA-C	NOTCH4	4,83385E-06	4762,1	3,52486E-02	923	8,33332E-01	1087	1,77321E+00	1113	8,85359E-01	9479	0	11208,5
Responder	pDC_LILRA4	pDC_LILRA4	APP	RPSA	4,87719E-06	2703,5	3,65869E-02	864	1,55562E+00	101	4,37964E+00	4	9,30192E-01	1340	0	11208,5
Responder	pDC_LILRA4	Macro_FOLR2+APOE+	SCT	GPR84	4,93055E-06	11568,1	8,42170E-02	147	1,79675E+00	66	2,41108E+00	294	6,96177E-01	46125	0	11208,5
Responder	Mono_CD14	Mono_CD14	S100A8	CD36	5,05348E-06	2645,3	4,81254E-02	520	1,51819E+00	113	3,37823E+00	33	9,30129E-01	1352	0	11208,5
Responder	Macro_OLFML3	pDC_LILRA4	FN1	CD79A	5,14339E-06	8738,5	1,13411E-01	66	8,58476E-01	986	1,89694E+00	877	7,81138E-01	30555	0	11208,5
Responder	Mono_CD14	Mono_CD16	S100A8	CD68	5,18579E-06	2658,1	2,61788E-02	1608	1,34150E+00	170	2,40391E+00	299	9,57526E-01	5	0	11208,5
Responder	Macro_OLFML3	pDC_LILRA4	CXCL11	CXCR3	5,18879E-06	10585,9	1,09968E-01	74	1,24620E+00	230	2,18931E+00	473	7,25833E-01	40944	0	11208,5
Responder	Macro_LYVE1	cDC(CD1C)	B2M	CD1C	5,29589E-06	2794,7	4,21198E-02	668	1,08282E+00	418	1,66910E+00	1368	9,41310E-01	311	0	11208,5
Responder	Mono_CD14	Macro_NLRP3	S100A12	CD36	5,34308E-06	4513,5	6,89137E-02	223	1,27504E+00	209	2,40149E+00	302	8,79145E-01	10625	0	11208,5
Responder	pDC_LILRA4	pDC_LILRA4	HMGB1	TLR9	5,34308E-06	5299,3	6,06420E-02	302	1,29393E+00	195	2,55077E+00	201	8,58791E-01	14590	0	11208,5
Responder	Macro_LYVE1	pDC_LILRA4	BST2	LILRA4	5,39620E-06	3172,9	6,05567E-02	303	1,73185E+00	72	2,79130E+00	105	9,13838E-01	4176	0	11208,5
Responder	Macro_OLFML3	pDC_LILRA4	CXCL12	CXCR3	5,48333E-06	9525,3	6,80921E-02	226	1,23494E+00	241	2,04646E+00	646	7,56816E-01	35305	0	11208,5
Responder	Mono_CD14	Macro_OLFML3	S100A8	CD68	5,50350E-06	2696,3	2,47852E-02	1766	1,29851E+00	193	2,40007E+00	305	9,56400E-01	9	0	11208,5
Responder	Macro_NLRP3	cDC(CD1C)	B2M	CD1C	5,54689E-06	2913,5	3,94972E-02	753	9,11743E-01	810	1,66247E+00	1384	9,39509E-01	412	0	11208,5
Responder	Mono_CD14	Mono_CD14	S100A12	TLR4	5,61221E-06	5239,7	5,99771E-02	307	1,17737E+00	294	2,75975E+00	117	8,60277E-01	14272	0	11208,5
Responder	Macro_OLFML3	pDC_LILRA4	C3	NRP1	5,93994E-06	4507,5	4,11397E-02	701	9,47657E-01	709	2,16802E+00	498	8,85709E-01	9421	0	11208,5

Post_R_Myeloid_Myeloid_CellCel

Responder	Mono_CD14	Mast	VCAN	CD44	5,95676E-06	2907,1	2,80735E-02	1409	1,19086E+00	284	1,98567E+00	735	9,34085E-01	899	0	11208,5
Responder	Macro_OLFML3	Mono_INHBA	APOE	ABCA1	6,06052E-06	4248,7	1,88806E-02	2789	7,39049E-01	1588	1,10177E+00	4163	9,29083E-01	1495	0	11208,5
Responder	pDC_LILRA4	Macro_IER3	LCN2	FCGR2A	6,06134E-06	6798,9	6,62190E-02	239	1,16670E+00	305	2,38727E+00	315	8,23744E-01	21927	0	11208,5
Responder	Macro_OLFML3	pDC_LILRA4	FN1	DPP4	6,28309E-06	8014,7	1,25277E-01	50	1,03912E+00	493	1,83904E+00	984	7,97278E-01	27338	0	11208,5
Responder	Mono_CD14	Mono_INHBA	S100A9	CD68	6,29459E-06	2915,5	1,89472E-02	2769	1,20863E+00	268	2,37664E+00	319	9,55702E-01	13	0	11208,5
Responder	Macro_OLFML3	Mono_CD14	C3	IFITM1	6,53196E-06	3938,7	2,75733E-02	1442	8,02288E-01	1241	2,23109E+00	428	9,07455E-01	5374	0	11208,5
Responder	Mono_CD14	Macro_ISG15	S100A8	ITGB2	6,53374E-06	2826,5	2,09444E-02	2361	1,15360E+00	323	2,58492E+00	185	9,50393E-01	55	0	11208,5
Responder	Macro_FOLR2-APOE+	pDC_LILRA4	MMP9	TLR9	6,59446E-06	7105,5	2,44062E-01	16	1,86069E+00	53	2,36663E+00	324	8,14237E-01	23926	0	11208,5
Responder	Macro_OLFML3	Mono_CD14	C3	ITGAX	6,76839E-06	3909,7	2,44833E-02	1808	7,86593E-01	1318	1,85154E+00	958	9,13451E-01	4256	0	11208,5
Responder	pDC_LILRA4	Mono_CD14	FAM3D	FPR1	6,77886E-06	8118,7	8,44657E-02	145	1,15232E+00	327	3,00837E+00	63	7,89670E-01	28850	0	11208,5
Responder	Macro_OLFML3	Macro_FOLR2-APOE+	C3	CD81	6,80026E-06	4643,3	2,14407E-02	2285	7,01530E-01	1838	1,16800E+00	3625	9,13432E-01	4260	0	11208,5
Responder	Mono_CD14	Macro_NLRP3	S100A8	ITGB2	6,84108E-06	2849,7	2,09028E-02	2367	1,15214E+00	328	2,42067E+00	288	9,50347E-01	57	0	11208,5
Responder	pDC_LILRA4	Macro_ISG15	FAM3D	FPR1	7,03001E-06	8156,5	8,40893E-02	148	1,14972E+00	331	2,61073E+00	172	7,89299E-01	28923	0	11208,5
Responder	Macro_OLFML3	Mono_INHBA	C3	ITGAX	7,10902E-06	4133,9	2,43479E-02	1825	7,83402E-01	1333	1,47717E+00	2005	9,13232E-01	4298	0	11208,5
Responder	Mono_CD14	Macro_IFI27	S100A8	ITGB2	7,15788E-06	2849,5	2,08282E-02	2380	1,14951E+00	333	2,44971E+00	266	9,50262E-01	60	0	11208,5
Responder	pDC_LILRA4	Mono_INHBA	LCN2	FCGR2A	7,41822E-06	6860,3	6,44924E-02	255	1,14738E+00	337	2,66534E+00	152	8,21818E-01	22349	0	11208,5
Responder	Mono_INHBA	pDC_LILRA4	TNF	TNFRSF21	7,41822E-06	5816,7	5,73658E-02	337	1,34469E+00	168	2,40541E+00	298	8,46854E-01	17072	0	11208,5
Responder	Macro_FOLR2-APOE+	cDC(CD1C)	B2M	CD1C	7,52133E-06	2908,5	4,00020E-02	733	9,44669E-01	719	1,62541E+00	1494	9,39869E-01	388	0	11208,5
Responder	pDC_LILRA4	Macro_LYVE1	GAS6	MERTK	7,95766E-06	5729,9	5,68458E-02	345	1,38613E+00	152	2,74992E+00	119	8,48077E-01	16825	0	11208,5
Responder	Mono_CD14	Macro_IER3	S100A8	ITGB2	8,23688E-06	2848,9	2,11711E-02	2322	1,16160E+00	315	2,33256E+00	349	9,50647E-01	50	0	11208,5
Responder	Macro_OLFML3	Macro_LYVE1	C1QB	LRP1	8,27916E-06	3931,3	1,88661E-02	2794	8,89454E-01	869	1,06823E+00	4431	9,40540E-01	354	0	11208,5
Responder	Macro_OLFML3	pDC_LILRA4	CCL5	CXCR3	8,33381E-06	9689,5	4,42816E-02	608	1,18168E+00	292	1,61086E+00	1533	7,59546E-01	34806	0	11208,5
Responder	pDC_LILRA4	pDC_LILRA4	TNFSF9	ADGRG5	8,37891E-06	13859,3	1,57539E-01	33	1,13034E+00	351	2,57519E+00	188	6,02160E-01	57516	0	11208,5
Responder	pDC_LILRA4	cDC_LAMP3	APP	TSPAN15	8,37891E-06	9116,9	5,64265E-02	351	1,20426E+00	274	2,50310E+00	231	7,66036E-01	33520	0	11208,5
Responder	Macro_OLFML3	pDC_LILRA4	CXCL9	DPP4	8,46439E-06	9878,7	7,93334E-02	175	8,85657E-01	884	1,60868E+00	1539	7,55171E-01	35587	0	11208,5
Responder	Macro_OLFML3	Mono_CD14	C1QA	CD93	8,47722E-06	4977,7	1,46351E-02	4452	6,45542E-01	2346	1,33909E+00	2639	9,13525E-01	4243	0	11208,5
Responder	Macro_OLFML3	Mono_CD16	FN1	C5AR1	8,55366E-06	4110,1	3,57261E-02	901	8,17346E-01	1167	1,30734E+00	2814	9,12363E-01	4460	0	11208,5
Responder	pDC_LILRA4	Macro_NLRP3	GZMB	IGF2R	9,03506E-06	5990,7	5,01058E-02	464	2,18032E+00	27	3,95248E+00	16	8,41398E-01	18238	0	11208,5
Responder	Macro_IFI27	pDC_LILRA4	BST2	LILRA4	9,18922E-06	3313,1	5,55260E-02	362	1,67084E+00	86	2,93221E+00	77	9,10362E-01	4832	0	11208,5
Responder	Macro_OLFML3	Macro_OLFML3	C3	C3AR1	9,23611E-06	4678,9	2,57402E-02	1653	7,22141E-01	1690	1,08243E+00	4314	9,12019E-01	4529	0	11208,5
Responder	pDC_LILRA4	Macro_NLRP3	FAM3D	FPR2	9,26536E-06	13346,5	1,01021E-01	90	1,12188E+00	363	2,45479E+00	261	6,30485E-01	54810	0	11208,5
Responder	cDC_LAMP3	pDC_LILRA4	LYPD3	AGR2	9,34192E-06	13861,5	1,67427E-01	30	1,12106E+00	364	2,68580E+00	141	6,01753E-01	57564	0	11208,5
Responder	Macro_OLFML3	pDC_LILRA4	CXCL10	DPP4	9,37447E-06	10296,7	8,31365E-02	150	8,50296E-01	1018	1,59815E+00	1579	7,44848E-01	37528	0	11208,5
Responder	pDC_LILRA4	cDC(CD1C)	APP	CD74	9,65238E-06	2939,7	1,80118E-02	3038	1,11607E+00	368	3,20715E+00	42	9,51254E-01	42	0	11208,5
Responder	pDC_LILRA4	Macro_ISG15	GZMB	IGF2R	9,71641E-06	6075,1	4,87551E-02	501	2,17434E+00	28	4,07803E+00	11	8,39566E-01	18627	0	11208,5
Responder	Macro_OLFML3	Mono_CD14	C3	ITGAM	9,83148E-06	5337,3	3,10957E-02	1142	7,37349E-01	1598	1,68344E+00	1324	8,75180E-01	11414	0	11208,5
Responder	Macro_FOLR2+APOE-	cDC(CD1C)	B2M	CD1C	9,95447E-06	2920,1	4,01630E-02	727	9,55175E-01	683	1,59017E+00	1603	9,39983E-01	379	0	11208,5
Responder	Mono_CD14	cDC(CD1C)	S100A9	ITGB2	1,03758E-05	3025,3	1,69407E-02	3379	1,10807E+00	377	2,76277E+00	114	9,50938E-01	48	0	11208,5
Responder	Macro_OLFML3	Macro_NLRP3	C3	ITGAX	1,07087E-05	4377,7	2,32022E-02	1998	7,56411E-01	1494	1,36351E+00	2523	9,11303E-01	4665	0	11208,5
Responder	Macro_OLFML3	cDC(CD1C)	FN1	PLAUR	1,07202E-05	5008,9	1,79720E-02	3060	4,87113E-01	4666	1,39534E+00	2374	9,16274E-01	3736	0	11208,5
Responder	Macro_OLFML3	Macro_LYVE1	FN1	C5AR1	1,09169E-05	4419,9	3,46847E-02	956	7,96713E-01	1268	1,12422E+00	3984	9,11173E-01	4683	0	11208,5
Responder	Macro_NLRP3	pDC_LILRA4	CCL20	CXCR3	1,11344E-05	9971,1	8,09609E-02	163	1,29302E+00	196	2,27813E+00	386	7,42778E-01	37902	0	11208,5
Responder	pDC_LILRA4	cDC_LAMP3	GZMB	IGF2R	1,11533E-05	6202,9	4,68146E-02	540	2,16575E+00	30	4,08413E+00	10	8,36812E-01	19226	0	11208,5
Responder	Macro_FOLR2+APOE+	Macro_OLFML3	APOE	TREM2	1,14393E-05	2849,7	3,57794E-02	898	1,07292E+00	438	1,57498E+00	1660	9,51203E-01	44	0	11208,5
Responder	pDC_LILRA4	pDC_LILRA4	FARP2	PLXNA4	1,14831E-05	12422,9	2,16445E-01	20	1,27697E+00	208	2,27563E+00	390	6,68523E-01	50288	0	11208,5
Responder	Macro_NLRP3	pDC_LILRA4	VCAN	SELL	1,20195E-05	3406,7	5,32600E-02	396	1,23696E+00	238	2,73945E+00	123	9,09049E-01	5068	0	11208,5
Responder	Mono_CD14	cDC_CLEC9A	S100A8	ITGB2	1,20195E-05	2893,5	1,92596E-02	2689	1,09417E+00	396	2,85372E+00	89	9,48379E-01	85	0	11208,5
Responder	Macro_OLFML3	cDC_CLEC9A	FN1	DPP4	1,21116E-05	9447,7	8,83862E-02	127	8,48703E-01	1023	1,56730E+00	1684	7,67628E-01	33196	0	11208,5
Responder	Macro_ISG15	pDC_LILRA4	CXCL10	DPP4	1,22020E-05	7828,7	1,51728E-01	37	1,21406E+00	258	2,26676E+00	398	7,97723E-01	27242	0	11208,5
Responder	cDC(CD1C)	pDC_LILRA4	BST2	LILRA4	1,23863E-05	3373,1	5,31850E-02	400	1,64245E+00	89	3,52957E+00	25	9,08589E-01	5143	0	11208,5
Responder	pDC_LILRA4	Macro_OLFML3	GZMB	IGF2R	1,26892E-05	6509,1	4,25312E-02	653	2,14679E+00	32	3,72198E+00	22	8,30154E-01	20630	0	11208,5
Responder	Macro_OLFML3	Macro_LYVE1	APOE	LRP1	1,27809E-05	3913,9	2,20905E-02	2171	8,75285E-01	920	1,02692E+00	4833	9,39101E-01	437	0	11208,5
Responder	Macro_OLFML3	pDC_LILRA4	C1QB	C1QBP	1,28339E-05	4081,3	1,39951E-02	4837	6,74975E-01	2074	2,07688E+00	609	9,27851E-01	1678	0	11208,5
Responder	Macro_FOLR2-APOE+	pDC_LILRA4	BST2	LILRA4	1,28551E-05	3401,5	5,29820E-02	405	1,63999E+00	90	2,73801E+00	124	9,08430E-01	5180	0	11208,5
Responder	pDC_LILRA4	Macro_NLRP3	RPS19	C5AR1	1,28551E-05	3046,1	1,74840E-02	3203	1,09047E+00	405	2,59445E+00	177	9,43000E-01	237	0	11208,5
Responder	Mono_INHBA	Mono_INHBA	VCAN	CD44	1,28870E-05	5285,7	1,50676E-02	4218	7,27828E-01	1658	1,02618E+00	4841	9,12141E-01	4503	0	11208,5
Responder	pDC_LILRA4	Macro_ISG15	LCN2	FCGR2A	1,29502E-05	7146,5	5,93281E-02	314	1,08960E+00	406	2,67123E+00	148	8,15625E-01	23656	0	11208,5
Responder	Mast	Mono_INHBA	TIMP3	CD44	1,31419E-05	3069,7	8,89712E-02	125	1,44139E+00	140	2,25104E+00	408	9,17717E-01	3467	0	11208,5
Responder	Macro_NLRP3	pDC_LILRA4	TNF	PTPRS	1,37283E-05	7504,5	9,30690E-02	111	1,65160E+00	87	2,24475E+00	414	8,05678E-01	25702	0	11208,5
Responder	Macro_OLFML3	Macro_LYVE1	C3	C3AR1	1,39167E-05	4821,1	2,44299E-02	1814	6,90123E-01	1936	1,09152E+00	4231	9,09900E-01	4916	0	11208,5
Responder	Mono_INHBA	cDC_CLEC9A	CCL20	CXCR3	1,39276E-05	8817,9	1,12615E-01	67	1,21250E+00	261	2,24403E+00	416	7,73023E-01	32137	0	11208,5
Responder	Mono_CD14	Macro_IFI27	S100A9	CD68	1,40279E-05	2908,1	1,93889E-02	2656	1,22796E+00	249	2,24145E+00	417	9,56187E-01	10	0	11208,5
Responder	cDC_LAMP3	cDC(CD1C)	B2M	CD1C	1,41128E-05	3082,1	3,80591E-02	804	8,17927E-01	1163	1,55031E+00	1750	9,38447E-01	485	0	11208,5
Responder	pDC_LILRA4	Mono_CD16	GZMB	IGF2R	1,43240E-05	6896,7	3,77034E-02	818	2,12542E+00	34	3,85142E+00	19	8,21490E-01	22404	0	11208,5
Responder	Mono_CD14	Mono_CD14	VCAN	CD44	1,46001E-05	3045,9	2,47861E-02	1765	1,08813E+00	408	2,16675E+00	501	9,30146E-01	1347	0	11208,5

Responder	Macro_OLFML3	Macro_LYVE1	C3	NRP1	1,46331E-05	4559,5	4,48949E-02	589	9,98599E-01	585	1,54634E+00	1766	8,90057E-01	8649	0	11208,5
Responder	Mono_INHBA	Mast	LGALS1	CD69	1,54058E-05	2982,9	2,45872E-02	1789	1,13534E+00	349	1,83371E+00	995	9,37306E-01	573	0	11208,5
Responder	Macro_OLFML3	cDC_CLEC9A	CXCL9	CXCR3	1,56123E-05	7885,5	5,12079E-02	438	8,62787E-01	968	1,53629E+00	1795	8,09079E-01	25018	0	11208,5
Responder	Mono_CD14	Macro_NLRP3	VCAN	CD44	1,56816E-05	3187,7	2,45505E-02	1797	1,08077E+00	425	1,77270E+00	1114	9,29835E-01	1394	0	11208,5
Responder	Mono_CD14	Macro_NLRP3	S100A12	TLR4	1,58081E-05	5714,7	5,20181E-02	426	1,12565E+00	357	2,22544E+00	434	8,51499E-01	16148	0	11208,5
Responder	Macro_OLFML3	Macro_FOLR2+APOE+	C3	C3AR1	1,61097E-05	4773,9	2,62414E-02	1599	7,34387E-01	1616	1,00300E+00	5062	9,12789E-01	4384	0	11208,5
Responder	Macro_OLFML3	cDC_CLEC9A	CXCL10	CXCR3	1,66398E-05	8257,7	5,36627E-02	391	8,27426E-01	1120	1,52576E+00	1824	8,00429E-01	26745	0	11208,5
Responder	pDC_LILRA4	cDC_CLEC9A	GZMB	IGF2R	1,69617E-05	9197,9	2,04493E-02	2439	2,04904E+00	37	3,93165E+00	17	7,72164E-01	32288	0	11208,5
Responder	pDC_LILRA4	pDC_LILRA4	PROC	THBD	1,72663E-05	10722,7	1,03348E-01	85	1,06147E+00	447	2,80248E+00	104	7,21391E-01	41769	0	11208,5
Responder	Mast	Mono_CD14	TIMP3	CD44	1,74983E-05	3388,9	7,32145E-02	199	1,27470E+00	210	2,20630E+00	449	9,10051E-01	4878	0	11208,5
Responder	Macro_IFI27	pDC_LILRA4	CXCL12	CXCR3	1,77323E-05	8439,1	9,11128E-02	116	1,34061E+00	171	2,20283E+00	451	7,82606E-01	30249	0	11208,5
Responder	Macro_LYVE1	Mast	LGALS1	CD69	1,77549E-05	3057,7	2,41739E-02	1854	1,11873E+00	365	1,71919E+00	1248	9,36806E-01	613	0	11208,5
Responder	pDC_LILRA4	Mono_INHBA	SPON2	ITGA5	1,78501E-05	9671,5	7,60183E-02	190	1,15235E+00	326	2,20131E+00	452	7,51968E-01	36181	0	11208,5
Responder	pDC_LILRA4	cDC(CD1C)	GZMB	IGF2R	1,78904E-05	9223,1	2,03479E-02	2462	2,04859E+00	38	3,96973E+00	14	7,71727E-01	32393	0	11208,5
Responder	Mono_CD14	Macro_ISG15	S100A12	TLR4	1,79684E-05	5783,1	5,05122E-02	453	1,11587E+00	370	2,31343E+00	363	8,49632E-01	16521	0	11208,5
Responder	Macro_IFI27	pDC_LILRA4	MMP9	TLR9	1,80872E-05	8918,1	1,41780E-01	41	1,56302E+00	98	2,19954E+00	454	7,69628E-01	32789	0	11208,5
Responder	Mono_CD14	Mono_CD16	S100A8	ITGB2	1,82066E-05	2969,5	1,92228E-02	2696	1,09287E+00	401	2,19709E+00	455	9,48332E-01	87	0	11208,5
Responder	Mono_CD14	Mono_CD14	S100A9	ITGB2	1,82066E-05	3150,3	1,59104E-02	3809	1,05653E+00	455	2,53633E+00	212	9,49453E-01	67	0	11208,5
Responder	Macro_LYVE1	pDC_LILRA4	CXCL12	CXCR3	1,84468E-05	8297,7	9,50571E-02	107	1,35872E+00	166	2,19552E+00	457	7,86190E-01	29550	0	11208,5
Responder	Mast	cDC_LAMP3	CSF1	CSF2RA	1,88112E-05	4392,7	8,17700E-02	159	1,23843E+00	237	2,19525E+00	460	8,83102E-01	9899	0	11208,5
Responder	Macro_FOLR2+APOE+	Mast	APOE	ABCA1	1,90050E-05	3228,1	2,41613E-02	1857	1,00241E+00	574	1,50865E+00	1886	9,36790E-01	615	0	11208,5
Responder	Macro_ISG15	cDC_CLEC9A	CXCL10	CXCR3	1,90567E-05	5943,5	9,79369E-02	98	1,19119E+00	283	2,19437E+00	462	8,44195E-01	17666	0	11208,5
Responder	pDC_LILRA4	pDC_LILRA4	ADA	DPP4	1,91803E-05	12592,3	1,03608E-01	83	1,08192E+00	422	2,19429E+00	463	6,64995E-01	50785	0	11208,5
Responder	pDC_LILRA4	Mono_CD14	SPON2	ITGAM	1,94290E-05	10079,5	6,56993E-02	243	1,05078E+00	465	2,30706E+00	370	7,41731E-01	38111	0	11208,5
Responder	Macro_OLFML3	Mono_INHBA	FN1	SDC2	2,01517E-05	7232,9	4,82549E-02	517	7,58862E-01	1484	1,50264E+00	1914	8,28045E-01	21041	0	11208,5
Responder	Mono_CD14	Mono_CD14	S100A9	CD36	2,05310E-05	2872,5	3,39369E-02	990	1,36404E+00	163	2,92270E+00	78	9,26334E-01	1923	0	11208,5
Responder	cDC(CD1C)	Mast	HSPA8	ADRB2	2,06749E-05	3967,7	3,22755E-02	1073	8,58842E-01	985	1,71670E+00	1251	9,07713E-01	5321	0	11208,5
Responder	pDC_LILRA4	Macro_IFI27	APP	CD74	2,09663E-05	3075,1	1,72242E-02	3286	1,04456E+00	477	2,34082E+00	343	9,50206E-01	61	0	11208,5
Responder	pDC_LILRA4	Macro_FOLR2-APOE+	LCN2	FCGR2A	2,12302E-05	7433,3	5,52760E-02	367	1,04426E+00	479	2,31709E+00	360	8,10246E-01	24752	0	11208,5
Responder	Macro_ISG15	pDC_LILRA4	TNF	PTPRS	2,12302E-05	9655,7	4,96978E-02	479	1,50386E+00	118	2,44675E+00	268	7,51845E-01	36205	0	11208,5
Responder	Mono_CD14	Mast	IL1B	ADRB2	2,14962E-05	3783,5	6,09777E-02	298	1,13062E+00	350	2,18292E+00	481	9,01065E-01	6580	0	11208,5
Responder	pDC_LILRA4	cDC(CD1C)	FAM3D	FPR1	2,18993E-05	8877,5	6,84856E-02	225	1,04204E+00	484	2,93920E+00	75	7,71725E-01	32395	0	11208,5
Responder	pDC_LILRA4	Macro_FOLR2+APOE-	APP	CD74	2,18993E-05	3079,7	1,74667E-02	3212	1,06658E+00	443	2,17881E+00	484	9,50536E-01	51	0	11208,5
Responder	cDC_CLEC9A	pDC_LILRA4	CCL5	CXCR3	2,23075E-05	8989,1	4,94053E-02	487	1,21551E+00	256	2,65152E+00	160	7,69402E-01	32834	0	11208,5
Responder	Mono_INHBA	Mono_INHBA	HBEGF	CD44	2,24006E-05	5236,1	1,54364E-02	4028	7,79472E-01	1361	1,09923E+00	4176	9,07229E-01	5407	0	11208,5
Responder	Mono_INHBA	Macro_FOLR2+APOE+	TIMP1	CD63	2,24213E-05	3766,1	1,30594E-02	5408	1,10228E+00	390	1,52810E+00	1816	9,56841E-01	8	0	11208,5
Responder	Mono_CD14	Mono_CD14	VCAN	SELL	2,28595E-05	3547,7	4,91949E-02	491	1,22621E+00	251	2,76335E+00	113	9,05713E-01	5675	0	11208,5
Responder	Mono_CD14	Mono_CD16	S100A12	TLR4	2,31389E-05	5553,5	5,50664E-02	370	1,14546E+00	339	2,17089E+00	493	8,55063E-01	15357	0	11208,5
Responder	Mono_CD14	Macro_OLFML3	S100A9	ITGB2	2,31776E-05	2920,3	1,99320E-02	2548	1,25770E+00	221	2,08184E+00	607	9,54595E-01	17	0	11208,5
Responder	Mono_CD14	Macro_OLFML3	S100A9	CD68	2,31989E-05	3114,9	1,74779E-02	3204	1,14436E+00	344	1,94454E+00	797	9,53961E-01	21	0	11208,5
Responder	Macro_IFI27	pDC_LILRA4	CXCL9	CXCR3	2,39906E-05	7643,9	4,87649E-02	499	1,12918E+00	353	2,27363E+00	392	8,05275E-01	25767	0	11208,5
Responder	Mono_INHBA	Mast	S100A8	CD69	2,45501E-05	4504,1	2,72048E-02	1489	9,44952E-01	718	9,61937E-01	5507	9,17029E-01	3598	0	11208,5
Responder	Mono_INHBA	Mono_INHBA	VEGFA	CD44	2,46394E-05	4362,1	2,18513E-02	2211	9,65176E-01	660	1,42897E+00	2220	9,06696E-01	5511	0	11208,5
Responder	Mono_CD14	cDC(CD1C)	S100A8	CD68	2,49765E-05	3590,7	1,30622E-02	5404	9,36873E-01	742	2,45341E+00	263	9,40914E-01	336	0	11208,5
Responder	Mono_CD14	Macro_LYVE1	S100A12	TLR4	2,50103E-05	5176,7	6,33866E-02	268	1,19952E+00	278	2,16540E+00	506	8,63567E-01	13623	0	11208,5
Responder	Mono_CD14	cDC(CD1C)	VCAN	CD44	2,52033E-05	4011,7	1,70500E-02	3341	8,46389E-01	1031	1,90096E+00	870	9,16969E-01	3608	0	11208,5
Responder	Mono_CD14	cDC(CD1C)	S100A8	CD69	2,57313E-05	3757,3	2,22900E-02	2133	1,19474E+00	281	2,76489E+00	112	9,09127E-01	5052	0	11208,5
Responder	Macro_OLFML3	Mono_INHBA	APOE	LRP1	2,59868E-05	5326,9	1,42224E-02	4693	5,83056E-01	3058	9,55642E-01	5570	9,25224E-01	2105	0	11208,5
Responder	cDC_CLEC9A	pDC_LILRA4	HMGB1	TLR9	2,60584E-05	4718,5	7,93782E-02	174	1,55021E+00	105	2,16187E+00	513	8,74341E-01	11592	0	11208,5
Responder	pDC_LILRA4	Macro_ISG15	APP	FPR2	2,62105E-05	7158,9	4,83126E-02	514	1,16496E+00	308	2,62189E+00	168	8,15880E-01	23596	0	11208,5
Responder	Mono_CD14	Macro_FOLR2+APOE-	S100A9	CD68	2,65164E-05	3124,9	1,66440E-02	3495	1,10788E+00	378	2,16030E+00	516	9,52876E-01	27	0	11208,5
Responder	Mono_CD14	Mono_INHBA	S100A8	ITGB2	2,66942E-05	3088,3	1,73541E-02	3243	1,02696E+00	519	2,34488E+00	335	9,45768E-01	136	0	11208,5
Responder	Mono_CD14	Mono_INHBA	S100A8	CD36	2,67181E-05	2851,3	4,13489E-02	690	1,44226E+00	138	2,88936E+00	86	9,25034E-01	2134	0	11208,5
Responder	Macro_FOLR2+APOE+	Mono_INHBA	APOE	ABCA1	2,67300E-05	3233,5	2,46288E-02	1785	1,01341E+00	549	1,46531E+00	2055	9,37355E-01	570	0	11208,5
Responder	Mono_CD14	Mono_INHBA	S100A9	CD36	2,68136E-05	3176,5	2,91583E-02	1309	1,28811E+00	200	2,43383E+00	276	9,20986E-01	2889	0	11208,5
Responder	pDC_LILRA4	Macro_NLRP3	LCN2	FCGR2A	2,77636E-05	7546,9	5,36426E-02	392	1,02599E+00	524	2,32157E+00	356	8,07930E-01	25254	0	11208,5
Responder	cDC_CLEC9A	pDC_LILRA4	CXCL9	CXCR3	2,79222E-05	7739,9	4,75318E-02	525	1,12220E+00	361	2,22747E+00	431	8,03259E-01	26174	0	11208,5
Responder	Macro_FOLR2-APOE+	Mast	LGALS1	CD69	2,82067E-05	3212,7	2,26214E-02	2083	1,05633E+00	456	1,62547E+00	1493	9,34812E-01	823	0	11208,5
Responder	Mast	cDC_CLEC9A	CSF1	CSF2RA	2,82412E-05	5420,9	5,31943E-02	399	1,02401E+00	527	2,23519E+00	422	8,59019E-01	14548	0	11208,5
Responder	cDC(CD1C)	Mono_CD16	HLA-C	LILRA3	2,83048E-05	4899,7	2,91884E-02	1302	6,60488E-01	2198	1,10563E+00	4124	9,05755E-01	5666	0	11208,5
Responder	Mono_CD14	Mono_CD14	S100A8	TLR4	2,86815E-05	3494,5	3,32381E-02	1021	1,30921E+00	186	3,13544E+00	51	9,09397E-01	5006	0	11208,5
Responder	Mono_CD14	Macro_LYVE1	S100A8	ITGB2	2,87242E-05	2938,9	2,00464E-02	2525	1,12193E+00	362	2,15296E+00	530	9,49350E-01	69	0	11208,5
Responder	Mono_CD14	Mono_CD14	S100A8	CD68	2,87573E-05	3216,1	1,56812E-02	3927	1,01767E+00	540	2,44246E+00	270	9,45794E-01	135	0	11208,5
Responder	Macro_OLFML3	cDC(CD1C)	C1QB	CD33	2,94726E-05	5951,9	1,26434E-02	5712	5,15883E-01	4113	1,20673E+00	3393	9,07617E-01	5333	0	11208,5
Responder	Macro_OLFML3	Macro_FOLR2-APOE+	FN1	CD44	2,96536E-05	5283,3	1,98393E-02	2564	5,64566E-01	3347	9,41912E-01	5719	9,17188E-01	3578	0	11208,5

Responder	Mono_INHBA	Mast	IL1B	ADRB2	3,00970E-05	3629,1	6,73512E-02	231	1,20488E+00	273	2,01069E+00	697	9,05409E-01	5736	0	11208,5
Responder	Mast	Mono_CD14	CSF1	SIRPA	3,02060E-05	7029,9	4,99721E-02	467	1,01858E+00	539	2,20707E+00	448	8,21024E-01	22487	0	11208,5
Responder	Mono_INHBA	Mono_INHBA	SPP1	CD44	3,04926E-05	5206,7	1,40404E-02	4809	6,60096E-01	2203	1,46444E+00	2062	9,05303E-01	5751	0	11208,5
Responder	pDC_LILRA4	Macro_OLFML3	CD24	SIGLEC10	3,07110E-05	9592,5	8,77085E-02	131	1,02676E+00	521	2,13779E+00	542	7,55362E-01	35560	0	11208,5
Responder	Mono_CD14	Macro_FOLR2-APOE+	S100A9	ITGB2	3,10534E-05	3269,1	1,58379E-02	3846	1,05290E+00	461	1,97262E+00	760	9,49343E-01	70	0	11208,5
Responder	Mono_CD14	Macro_FOLR2-APOE+	S100A9	CD68	3,11072E-05	3035,7	1,79867E-02	3054	1,16662E+00	306	2,09439E+00	592	9,54587E-01	18	0	11208,5
Responder	Mono_CD14	Macro_FOLR2-APOE+	VCAN	CD44	3,12963E-05	3610,9	2,10254E-02	2349	9,70615E-01	645	1,58424E+00	1628	9,24607E-01	2224	0	11208,5
Responder	cDC(CD1C)	Macro_NLRP3	RPS19	C5AR1	3,14862E-05	4041,7	1,25431E-02	5788	7,87713E-01	1313	1,87249E+00	924	9,33389E-01	975	0	11208,5
Responder	Macro_OLFML3	Macro_IFI27	C3	CD81	3,18414E-05	5321,1	1,74870E-02	3201	5,92020E-01	2939	1,19529E+00	3456	9,05026E-01	5801	0	11208,5
Responder	Macro_OLFML3	Mono_INHBA	C3	LRP1	3,20615E-05	4702,5	2,22458E-02	2143	6,91143E-01	1928	1,38625E+00	2424	9,05005E-01	5809	0	11208,5
Responder	Macro_OLFML3	cDC(CD1C)	FN1	CD44	3,20615E-05	5790,9	1,60882E-02	3729	4,40340E-01	5809	1,25863E+00	3100	9,08872E-01	5108	0	11208,5
Responder	Mast	pDC_LILRA4	TNF	PTPRS	3,22596E-05	9058,5	5,93844E-02	313	1,53685E+00	108	2,12976E+00	551	7,68082E-01	33112	0	11208,5
Responder	pDC_LILRA4	Mono_CD14	SPON2	ITGA5	3,24348E-05	10963,1	5,40986E-02	383	1,01178E+00	552	2,21306E+00	444	7,18909E-01	42228	0	11208,5
Responder	Macro_OLFML3	Mono_INHBA	FN1	C5AR1	3,25055E-05	4567,1	2,98794E-02	1240	7,01502E-01	1839	1,32309E+00	2723	9,04950E-01	5825	0	11208,5
Responder	cDC_LAMP3	Macro_NLRP3	CCL19	CCRL2	3,26106E-05	5973,3	1,20393E-01	55	2,14998E+00	31	2,12907E+00	553	8,42449E-01	18019	0	11208,5
Responder	Mast	Macro_ISG15	CSF1	CSF3R	3,31418E-05	5878,9	5,92046E-02	316	1,01031E+00	554	2,12719E+00	556	8,48389E-01	16760	0	11208,5
Responder	pDC_LILRA4	pDC_LILRA4	BST2	LILRA4	3,31418E-05	3632,9	4,62661E-02	556	1,55854E+00	100	4,23759E+00	7	9,02634E-01	6293	0	11208,5
Responder	Mono_CD14	Macro_ISG15	S100A8	CD68	3,33797E-05	3474,7	1,38883E-02	4900	9,62356E-01	666	2,33496E+00	346	9,42596E-01	253	0	11208,5
Responder	Mono_CD14	Macro_ISG15	S100A9	ITGB2	3,34368E-05	3362,1	1,47695E-02	4373	9,99451E-01	580	2,12939E+00	552	9,47637E-01	97	0	11208,5
Responder	Mono_CD14	Macro_ISG15	VCAN	CD44	3,36657E-05	4227,3	1,66388E-02	3499	8,33538E-01	1086	1,60090E+00	1564	9,16035E-01	3779	0	11208,5
Responder	Mono_CD14	pDC_LILRA4	S100A8	CD69	3,41677E-05	2919,3	3,36822E-02	999	1,31962E+00	182	3,74743E+00	21	9,24801E-01	2186	0	11208,5
Responder	pDC_LILRA4	Mono_CD16	RPS19	C5AR1	3,44037E-05	3287,5	1,56408E-02	3945	1,00705E+00	563	2,34481E+00	337	9,39931E-01	384	0	11208,5
Responder	Mono_CD14	Macro_ISG15	S100A8	CD69	3,44184E-05	3435,3	2,64122E-02	1575	1,23992E+00	235	2,32419E+00	351	9,15897E-01	3807	0	11208,5
Responder	Mast	cDC(CD1C)	CSF1	CSF3R	3,45865E-05	5899,1	5,86204E-02	323	1,00698E+00	564	2,16641E+00	504	8,47750E-01	16896	0	11208,5
Responder	Mono_CD14	Mono_CD14	S100A9	TLR4	3,46527E-05	3907,1	2,34387E-02	1961	1,15506E+00	322	2,67990E+00	144	9,04592E-01	5900	0	11208,5
Responder	cDC_LAMP3	pDC_LILRA4	CCL5	CXCR3	3,49541E-05	8918,3	5,14767E-02	434	1,22919E+00	247	2,11449E+00	566	7,73025E-01	32136	0	11208,5
Responder	Macro_OLFML3	pDC_LILRA4	GNAI2	CXCR3	3,56180E-05	6479,3	3,09606E-02	1151	1,08326E+00	417	1,43173E+00	2209	8,45318E-01	17411	0	11208,5
Responder	Mast	Macro_FOLR2-APOE+	CSF1	SIRPA	3,58844E-05	5271,1	9,02474E-02	119	1,25910E+00	218	2,11171E+00	571	8,60428E-01	14239	0	11208,5
Responder	Mono_CD14	Macro_NLRP3	S100A8	CD36	3,60551E-05	2983,1	3,81906E-02	799	1,40688E+00	148	2,77717E+00	108	9,22232E-01	2652	0	11208,5
Responder	Mono_CD14	Macro_NLRP3	S100A9	ITGB2	3,61462E-05	3409,3	1,47402E-02	4384	9,97987E-01	589	1,96513E+00	767	9,47588E-01	98	0	11208,5
Responder	Mono_CD14	Macro_NLRP3	S100A9	CD36	3,61766E-05	3343,5	2,69311E-02	1518	1,25273E+00	225	2,32164E+00	355	9,18046E-01	3411	0	11208,5
Responder	Mono_CD14	Macro_NLRP3	S100A9	CD68	3,62070E-05	3222,5	1,61386E-02	3700	1,08577E+00	412	1,97281E+00	759	9,52179E-01	33	0	11208,5
Responder	Macro_FOLR2-APOE+	pDC_LILRA4	CCL20	CXCR3	3,64504E-05	11058,1	6,10545E-02	296	1,22211E+00	254	2,11028E+00	574	7,14911E-01	42958	0	11208,5
Responder	pDC_LILRA4	Macro_FOLR2+APOE+	CD24	SIGLEC10	3,72142E-05	9007,7	1,02617E-01	87	1,12622E+00	356	2,10746E+00	578	7,69575E-01	32809	0	11208,5
Responder	Mast	cDC(CD1C)	CSF1	SIRPA	3,72142E-05	7268,3	4,69814E-02	536	1,00072E+00	578	2,13911E+00	541	8,16445E-01	23478	0	11208,5
Responder	cDC_LAMP3	Mono_INHBA	CCL19	CCRL2	3,76000E-05	6259,7	1,09543E-01	76	2,10289E+00	35	2,10631E+00	580	8,36080E-01	19399	0	11208,5
Responder	pDC_LILRA4	Macro_FOLR2-APOE+	APP	CD74	3,77940E-05	3177,1	1,67233E-02	3462	9,99083E-01	581	2,11241E+00	568	9,49504E-01	66	0	11208,5
Responder	Mono_INHBA	Mono_CD14	VCAN	CD44	3,80058E-05	6358,3	1,23992E-02	5904	5,61140E-01	3383	9,81444E-01	5286	9,04011E-01	6010	0	11208,5
Responder	Mono_CD14	cDC_CLEC9A	S100A9	ITGB2	3,81008E-05	3494,1	1,35814E-02	5078	9,40022E-01	735	2,39818E+00	306	9,45517E-01	143	0	11208,5
Responder	Macro_OLFML3	cDC(CD1C)	C1QA	CD33	3,83230E-05	6190,3	1,22574E-02	6020	5,04160E-01	4317	1,14631E+00	3787	9,06013E-01	5619	0	11208,5
Responder	pDC_LILRA4	Macro_LYVE1	APP	CD74	3,87735E-05	3107,3	1,73731E-02	3237	1,05808E+00	452	2,10316E+00	586	9,50410E-01	53	0	11208,5
Responder	Mono_CD14	Mono_INHBA	S100A9	ITGB2	3,89316E-05	3862,5	1,22377E-02	6039	8,72804E-01	934	1,88934E+00	885	9,42771E-01	246	0	11208,5
Responder	Macro_OLFML3	Macro_IER3	C3	CD81	3,96133E-05	5630,5	1,84981E-02	2894	6,20026E-01	2608	9,13364E-01	6060	9,07414E-01	5382	0	11208,5
Responder	Mono_CD14	pDC_LILRA4	S100A8	CD36	3,97442E-05	3048,7	3,64127E-02	873	1,38696E+00	150	3,50195E+00	27	9,20505E-01	2985	0	11208,5
Responder	Mono_CD14	pDC_LILRA4	S100A8	CD68	3,97770E-05	3318,5	1,45705E-02	4497	9,83403E-01	619	2,99762E+00	69	9,43879E-01	199	0	11208,5
Responder	Mono_CD14	pDC_LILRA4	S100A9	CD36	3,98426E-05	3383,1	2,56774E-02	1660	1,23281E+00	244	3,04642E+00	59	9,16235E-01	3744	0	11208,5
Responder	Mast	pDC_LILRA4	CSF1	CSF2RA	4,01730E-05	5580,5	4,94629E-02	486	9,96010E-01	593	2,67595E+00	147	8,54557E-01	15468	0	11208,5
Responder	Mono_INHBA	Mast	VEGFA	CD44	4,02051E-05	4798,9	2,03664E-02	2458	9,01214E-01	837	1,20316E+00	3413	9,03676E-01	6078	0	11208,5
Responder	Macro_OLFML3	Macro_FOLR2+APOE+	APOE	TREM2	4,02450E-05	4544,3	2,37784E-02	1914	6,97294E-01	1876	8,11513E-01	7384	9,40797E-01	339	0	11208,5
Responder	Macro_OLFML3	Macro_ISG15	C3	C3AR1	4,03376E-05	5065,1	2,10705E-02	2340	6,08030E-01	2743	1,28336E+00	2952	9,03649E-01	6082	0	11208,5
Responder	Macro_NLRP3	Mast	LGALS1	CD69	4,03855E-05	3440,7	2,14601E-02	2280	1,00966E+00	555	1,44032E+00	2166	9,33188E-01	994	0	11208,5
Responder	Macro_OLFML3	Mono_CD14	APOE	LRP1	4,04039E-05	5792,7	1,21739E-02	6084	5,06972E-01	4260	1,08708E+00	4272	9,19663E-01	3139	0	11208,5
Responder	Mono_CD16	pDC_LILRA4	BST2	LILRA4	4,05789E-05	3728,5	4,47850E-02	595	1,54058E+00	107	2,60398E+00	174	9,01195E-01	6558	0	11208,5
Responder	pDC_LILRA4	Macro_OLFML3	FAM3D	FPR1	4,07829E-05	8853,5	7,08828E-02	213	1,05858E+00	450	2,09272E+00	596	7,74741E-01	31800	0	11208,5
Responder	Macro_OLFML3	Mono_INHBA	C1QB	LRP1	4,10724E-05	5426,1	1,21465E-02	6104	5,97226E-01	2867	9,96955E-01	5132	9,26966E-01	1819	0	11208,5
Responder	Mono_CD14	Macro_IFI27	S100A9	ITGB2	4,14438E-05	3407,3	1,46875E-02	4412	9,95354E-01	594	1,99418E+00	722	9,47499E-01	100	0	11208,5
Responder	Macro_OLFML3	Macro_NLRP3	C3	LRP1	4,15456E-05	4976,7	2,14776E-02	2275	6,72902E-01	2085	1,24035E+00	3197	9,03483E-01	6118	0	11208,5
Responder	Mono_CD14	Macro_IFI27	VCAN	CD44	4,16476E-05	5480,1	1,26904E-02	5677	7,10157E-01	1775	1,28570E+00	2932	9,05013E-01	5808	0	11208,5
Responder	pDC_LILRA4	cDC(CD1C)	LCN2	FCGR2A	4,18129E-05	7719,5	5,03518E-02	456	9,89168E-01	601	2,83894E+00	92	8,02970E-01	26240	0	11208,5
Responder	Macro_OLFML3	Macro_LYVE1	CXCL9	FCGR2A	4,20231E-05	5697,7	1,79878E-02	3052	6,07693E-01	2748	9,75105E-01	5348	9,03426E-01	6132	0	11208,5
Responder	pDC_LILRA4	cDC_CLEC9A	CDH1	ITGAE_ITGB7	4,22298E-05	12665,3	8,47378E-02	142	9,88597E-01	603	2,36093E+00	327	6,62894E-01	51046	0	11208,5
Responder	Mono_CD14	Macro_NLRP3	S100A8	TLR4	4,23324E-05	3816,7	2,88274E-02	1337	1,25750E+00	222	2,60112E+00	175	9,03359E-01	6141	0	11208,5
Responder	Macro_OLFML3	Macro_ISG15	FN1	CD44	4,24359E-05	6420,3	1,57001E-02	3917	4,27489E-01	6144	9,58568E-01	5540	9,07856E-01	5292	0	11208,5
Responder	Mono_INHBA	pDC_LILRA4	CCL5	CXCR3	4,24392E-05	9429,1	4,44582E-02	604	1,18284E+00	288	2,39496E+00	307	7,59909E-01	34738	0	11208,5
Responder	pDC_LILRA4	Mono_CD14	RPS19	C5AR1	4,26493E-05	3291,3	1,52114E-02	4143	9,87619E-01	605	3,00069E+00	66	9,39140E-01	434	0	11208,5

Responder	Macro_OLFML3	pDC_LILRA4	TNF	PTPRS	4,28300E-05	12716,9	2,51396E-02	1723	1,42020E+00	145	1,40632E+00	2314	6,83027E-01	48194	0	11208,5
Responder	Macro_OLFML3	pDC_LILRA4	APP	TNFRSF21	4,29772E-05	9319,5	2,12226E-02	2316	1,00049E+00	579	1,46531E+00	2056	7,81760E-01	30438	0	11208,5
Responder	Mono_CD14	Macro_LYVE1	S100A8	TLR4	4,36232E-05	3422,5	3,51276E-02	929	1,33137E+00	177	2,54108E+00	208	9,11649E-01	4590	0	11208,5
Responder	Mast	Mono_INHBA	CSF1	SIRPA	4,37103E-05	6207,5	6,52389E-02	248	1,10975E+00	376	2,07474E+00	610	8,39781E-01	18595	0	11208,5
Responder	Mono_CD14	Macro_LYVE1	S100A9	TLR4	4,37646E-05	3868,5	2,47712E-02	1770	1,17721E+00	295	2,08555E+00	603	9,06951E-01	5466	0	11208,5
Responder	Mono_CD14	Macro_LYVE1	S100A9	ITGB2	4,38000E-05	3605,1	1,41363E-02	4752	9,67776E-01	651	1,69742E+00	1294	9,46539E-01	120	0	11208,5
Responder	pDC_LILRA4	Macro_LYVE1	RPS19	C5AR1	4,39246E-05	3387,7	1,51849E-02	4165	9,86420E-01	611	2,16170E+00	514	9,39091E-01	440	0	11208,5
Responder	cDC_LAMP3	Mono_CD14	CCL19	CCRL2	4,41396E-05	8460,5	5,51484E-02	369	1,86684E+00	50	2,07194E+00	612	7,83503E-01	30063	0	11208,5
Responder	pDC_LILRA4	Mono_CD14	LCN2	FCGR2A	4,43552E-05	7742,1	5,00434E-02	466	9,85718E-01	613	2,87798E+00	87	8,02483E-01	26336	0	11208,5
Responder	Macro_OLFML3	Mono_CD16	HLA-A	LILRB2	4,50900E-05	4873,1	1,21334E-02	6116	9,37943E-01	740	9,00481E-01	6219	9,48514E-01	82	0	11208,5
Responder	Macro_FOLR2-APOE+	cDC_CLEC9A	CXCL2	XCR1	4,52249E-05	4285,3	9,99536E-02	92	2,68698E+00	6	2,06690E+00	617	8,85216E-01	9503	0	11208,5
Responder	Mono_CD14	cDC_LAMP3	S100A8	ITGB2	4,61142E-05	3972,7	1,21413E-02	6111	8,43071E-01	1046	1,95900E+00	779	9,35844E-01	719	0	11208,5
Responder	Mono_CD14	cDC_LAMP3	VCAN	CD44	4,64103E-05	4674,9	1,57028E-02	3913	8,04291E-01	1230	1,30370E+00	2828	9,13782E-01	4195	0	11208,5
Responder	Macro_OLFML3	Macro_FOLR2-APOE+	C3	NRP1	4,66233E-05	6127,7	2,65932E-02	1554	7,50325E-01	1521	1,39725E+00	2364	8,61700E-01	13991	0	11208,5
Responder	Macro_OLFML3	cDC_CLEC9A	FLT3LG	FLT3	4,68586E-05	13128,5	4,62932E-02	555	1,30276E+00	188	1,39653E+00	2367	6,60562E-01	51324	0	11208,5
Responder	Mono_INHBA	Mast	INHBA	SMAD3	4,72228E-05	9389,7	8,80128E-02	130	1,09829E+00	392	2,05945E+00	626	7,60773E-01	34592	0	11208,5
Responder	Macro_OLFML3	Mono_INHBA	FN1	ITGA5_ITGB1	4,73318E-05	5669,5	2,72368E-02	1485	6,58443E-01	2222	1,39560E+00	2373	8,76851E-01	11059	0	11208,5
Responder	cDC_CLEC9A	pDC_LILRA4	BST2	LILRA4	4,74484E-05	3766,5	4,33127E-02	627	1,52273E+00	110	3,18593E+00	46	8,99697E-01	6841	0	11208,5
Responder	cDC_LAMP3	pDC_LILRA4	CXCL9	CXCR3	4,79016E-05	6828,7	6,29520E-02	276	1,20949E+00	267	2,05740E+00	629	8,24520E-01	21763	0	11208,5
Responder	pDC_LILRA4	Macro_FOLR2+APOE-	LCN2	FCGR2A	4,79016E-05	10804,3	4,95188E-02	484	9,79848E-01	629	2,34422E+00	340	8,01647E-01	26498	0,012	26070,5
Responder	Macro_NLRP3	pDC_LILRA4	BST2	LILRA4	4,81293E-05	3790,9	4,31546E-02	630	1,52081E+00	111	2,69976E+00	133	8,99532E-01	6872	0	11208,5
Responder	Mono_CD14	Mono_CD16	S100A8	TLR4	4,84101E-05	3689,5	3,05167E-02	1178	1,27730E+00	207	2,54657E+00	203	9,05816E-01	5651	0	11208,5
Responder	Mono_CD14	Mono_CD16	S100A9	ITGB2	4,86023E-05	3675,3	1,35555E-02	5090	9,38723E-01	738	1,74155E+00	1194	9,45468E-01	146	0	11208,5
Responder	Mono_CD14	Mono_CD16	S100A9	CD68	4,86793E-05	3040,3	1,84607E-02	2903	1,18735E+00	286	1,94837E+00	789	9,55148E-01	15	0	11208,5
Responder	Mono_CD14	Mono_CD16	VCAN	CD44	4,89497E-05	4349,5	1,87901E-02	2819	9,00764E-01	842	1,13122E+00	3910	9,20595E-01	2968	0	11208,5
Responder	Macro_OLFML3	Macro_LYVE1	SERPING1	LRP1	4,90660E-05	5243,9	2,11165E-02	2330	8,39353E-01	1066	8,92777E-01	6325	9,07862E-01	5290	0	11208,5
Responder	Macro_FOLR2+APOE+	Macro_LYVE1	APOE	LRP1	4,96696E-05	3081,9	2,88160E-02	1341	1,14964E+00	332	1,39046E+00	2402	9,46272E-01	126	0	11208,5
Responder	Mono_CD14	Mono_CD16	LYZ	ITGAL	4,96897E-05	5091,5	1,48376E-02	4341	6,89982E-01	1938	1,37416E+00	2481	9,06832E-01	5489	0	11208,5
Responder	Mono_INHBA	Mast	SPP1	CD44	5,03990E-05	5808,7	1,30863E-02	5383	5,96134E-01	2886	1,23862E+00	3207	9,02243E-01	6359	0	11208,5
Responder	Mono_CD14	Mast	LGALS1	CD69	5,07965E-05	3690,5	1,76775E-02	3142	8,57632E-01	989	1,70412E+00	1276	9,26883E-01	1837	0	11208,5
Responder	Mono_CD14	Mast	AGTRAP	RACK1	5,11564E-05	6144,9	1,28753E-02	5538	6,51826E-01	2292	9,62249E-01	5504	9,03177E-01	6182	0	11208,5
Responder	Macro_IER3	pDC_LILRA4	BST2	LILRA4	5,13926E-05	3827,7	4,26574E-02	644	1,51478E+00	114	2,53422E+00	213	8,99007E-01	6959	0	11208,5
Responder	Macro_OLFML3	Macro_FOLR2-APOE+	C3	LRP1	5,15184E-05	5223,3	2,08011E-02	2386	6,56838E-01	2242	1,13343E+00	3893	9,02079E-01	6387	0	11208,5
Responder	Mono_CD14	Macro_ISG15	S100A8	TLR4	5,16395E-05	3875,7	2,79929E-02	1415	1,24771E+00	228	2,68912E+00	137	9,02069E-01	6390	0	11208,5
Responder	Macro_OLFML3	Macro_FOLR2-APOE+	FN1	PLAUR	5,17203E-05	5128,5	2,11081E-02	2332	5,83016E-01	3060	8,87006E-01	6392	9,22240E-01	2650	0	11208,5
Responder	Mono_CD14	Mono_INHBA	HBEGF	CD44	5,18013E-05	5369,1	1,36864E-02	5028	7,12101E-01	1759	1,37968E+00	2456	9,02039E-01	6394	0	11208,5
Responder	cDC_LAMP3	Macro_FOLR2-APOE+	CCL19	CCR7	5,23512E-05	8865,3	7,32395E-02	198	2,04565E+00	39	2,04594E+00	648	7,72498E-01	32233	0	11208,5
Responder	Mono_CD14	Macro_IER3	S100A8	CD68	5,24118E-05	3272,1	1,61210E-02	3708	1,03123E+00	508	1,93720E+00	815	9,46498E-01	121	0	11208,5
Responder	Mono_CD14	Macro_IER3	S100A9	ITGB2	5,24936E-05	3412,9	1,49294E-02	4293	1,00745E+00	562	1,87703E+00	910	9,47904E-01	91	0	11208,5
Responder	Mono_CD14	Macro_IER3	VCAN	CD44	5,27808E-05	4107,5	1,84611E-02	2902	8,90482E-01	866	1,37425E+00	2479	9,19946E-01	3082	0	11208,5
Responder	Macro_FOLR2+APOE-	pDC_LILRA4	BST2	LILRA4	5,30779E-05	3811,1	4,25599E-02	651	1,51360E+00	116	2,81355E+00	101	8,98903E-01	6979	0	11208,5
Responder	Macro_OLFML3	cDC_CLEC9A	APOE	SCARB1	5,34663E-05	5744,1	2,10328E-02	2347	6,34732E-01	2447	1,40914E+00	2297	8,80207E-01	10421	0	11208,5
Responder	Mono_CD14	Macro_IER3	S100A8	CD69	5,36083E-05	3240,1	3,02995E-02	1203	1,28254E+00	203	2,00576E+00	707	9,21037E-01	2879	0	11208,5
Responder	Mono_CD14	Macro_LYVE1	SERPINA1	LRP1	5,46996E-05	6070,7	1,22233E-02	6047	5,64967E-01	3339	8,81915E-01	6464	9,18784E-01	3295	0	11208,5
Responder	pDC_LILRA4	cDC_CLEC9A	LTB	CD40	5,57996E-05	5052,9	4,24110E-02	662	1,22871E+00	248	3,30778E+00	38	8,66110E-01	13108	0	11208,5
Responder	pDC_LILRA4	pDC_LILRA4	SEMA3C	NRP1_PLXNA4	5,60515E-05	12904,3	6,94561E-02	218	1,04348E+00	481	2,03211E+00	663	6,55358E-01	51951	0	11208,5
Responder	Mono_CD14	Macro_FOLR2+APOE+	S100A8	CD68	5,61534E-05	3819,1	1,25657E-02	5774	9,21559E-01	780	1,86324E+00	941	9,39828E-01	392	0	11208,5
Responder	Mono_CD14	Macro_FOLR2+APOE+	S100A9	ITGB2	5,62399E-05	2996,9	1,90712E-02	2744	1,21464E+00	257	1,97881E+00	752	9,53628E-01	23	0	11208,5
Responder	Macro_OLFML3	Macro_ISG15	C3	CD81	5,64999E-05	5929,7	1,61104E-02	3718	5,53891E-01	3501	1,04112E+00	4715	9,01443E-01	6506	0	11208,5
Responder	Mono_CD14	cDC_CLEC9A	LYZ	ITGAL	5,68045E-05	5345,3	1,30974E-02	5374	6,29223E-01	2507	1,76977E+00	1124	9,01427E-01	6513	0	11208,5
Responder	Mast	Macro_ISG15	CSF1	SIRPA	5,68118E-05	6232,5	6,48574E-02	253	1,10747E+00	379	2,03175E+00	666	8,39386E-01	18656	0	11208,5
Responder	Mono_CD14	Macro_FOLR2-APOE+	S100A12	TLR4	5,78361E-05	6337,9	4,29628E-02	638	1,06681E+00	442	2,02784E+00	670	8,38996E-01	18731	0	11208,5
Responder	Mono_INHBA	Mast	HBEGF	CD44	5,85704E-05	6011,9	1,43874E-02	4596	7,15510E-01	1735	8,73421E-01	6553	9,04224E-01	5967	0	11208,5
Responder	Macro_OLFML3	cDC(CD1C)	C3	ITGAX	5,86599E-05	4922,7	1,82888E-02	2958	6,40656E-01	2395	1,62469E+00	1497	9,01204E-01	6555	0	11208,5
Responder	Mast	Mast	TIMP3	CD44	5,88725E-05	3232,5	8,29250E-02	155	1,37743E+00	155	2,02523E+00	674	9,15020E-01	3970	0	11208,5
Responder	Macro_FOLR2+APOE-	cDC_CLEC9A	CCL13	CXCR3	5,89287E-05	10228,9	1,89243E-01	25	1,76038E+00	69	1,75338E+00	1164	7,38791E-01	38678	0	11208,5
Responder	Macro_NLRP3	Mono_INHBA	VCAN	CD44	5,97973E-05	3580,3	2,32791E-02	1983	1,01531E+00	543	1,36545E+00	2517	9,28080E-01	1650	0	11208,5
Responder	pDC_LILRA4	Macro_LYVE1	VEGFB	NRP1	6,01853E-05	8306,7	4,17387E-02	679	9,66566E-01	655	2,31465E+00	362	7,90867E-01	28629	0	11208,5
Responder	Mono_CD14	Macro_IER3	S100A12	TLR4	6,07158E-05	6394,7	4,16864E-02	681	1,05852E+00	451	2,21571E+00	440	8,36948E-01	19193	0	11208,5
Responder	Macro_OLFML3	Macro_IER3	FN1	PLAUR	6,09363E-05	5006,1	2,25098E-02	2098	6,25878E-01	2529	8,42847E-01	6954	9,24515E-01	2241	0	11208,5
Responder	Mono_CD14	Mono_CD16	S100A9	TLR4	6,20934E-05	4212,1	2,15197E-02	2266	1,12315E+00	359	2,09104E+00	597	9,00841E-01	6630	0	11208,5
Responder	Macro_OLFML3	Macro_FOLR2-APOE+	APOE	TREM2	6,21402E-05	4794,3	2,00875E-02	2521	5,94899E-01	2898	8,67211E-01	6631	9,35921E-01	713	0	11208,5
Responder	Mono_CD14	pDC_LILRA4	CXCL8	CD79A	6,23259E-05	8334,1	7,19886E-02	206	9,53653E-01	687	2,36730E+00	323	7,87606E-01	29246	0	11208,5
Responder	cDC_CLEC9A	pDC_LILRA4	TNF	PTPRS	6,25970E-05	9635,1	5,10841E-02	443	1,50858E+00	117	2,01774E+00	688	7,54402E-01	35719	0	11208,5
Responder	pDC_LILRA4	cDC_LAMP3	LTB	CD40	6,25970E-05	5132,1	4,14233E-02	688	1,21574E+00	255	2,76884E+00	110	8,64738E-01	13399	0	11208,5

Post_R_Myeloid_Myeloid_CellCel

Responder	pDC_LILRA4	Mono_CD14	APP	FPR2	6,28689E-05	7679,5	4,14202E-02	689	1,10663E+00	380	2,77617E+00	109	8,04037E-01	26011	0	11208,5
Responder	Mono_CD14	Mono_CD16	VCAN	ITGB1	6,29882E-05	4242,7	2,56932E-02	1657	1,02688E+00	520	1,74857E+00	1179	9,00720E-01	6649	0	11208,5
Responder	pDC_LILRA4	Macro_OLFML3	LCN2	FCGR2A	6,31415E-05	7380,3	5,72351E-02	339	1,06618E+00	444	2,01617E+00	690	8,12909E-01	24220	0	11208,5
Responder	pDC_LILRA4	Macro_OLFML3	LTB	CD40	6,39641E-05	5145,1	4,12935E-02	693	1,21404E+00	259	2,68695E+00	139	8,64554E-01	13426	0	11208,5
Responder	Macro_OLFML3	pDC_LILRA4	HLA-DQB1	CD4	6,75960E-05	4949,3	9,29972E-03	9656	6,20811E-01	2596	2,06884E+00	614	9,36195E-01	672	0	11208,5
Responder	Macro_OLFML3	Mono_CD16	C3	ITGAX	6,86778E-05	5834,3	1,78541E-02	3092	6,30414E-01	2489	9,51344E-01	5617	9,00128E-01	6765	0	11208,5
Responder	cDC(CD1C)	Macro_LYVE1	HMGB1	CD163	6,88302E-05	4687,5	1,14444E-02	6768	7,79134E-01	1362	1,30946E+00	2799	9,30482E-01	1300	0	11208,5
Responder	Macro_ISG15	Mast	S100A8	CD69	6,94741E-05	3501,3	3,15084E-02	1122	1,01229E+00	551	1,47429E+00	2011	9,22448E-01	2614	0	11208,5
Responder	Macro_FOLR2+APOE+	pDC_LILRA4	CXCL9	CXCR3	6,96303E-05	5783,1	8,96457E-02	121	1,36060E+00	165	2,00034E+00	713	8,48647E-01	16708	0	11208,5
Responder	pDC_LILRA4	Macro_NLRP3	APP	PTGER2	7,08021E-05	7954,5	4,04591E-02	717	1,10551E+00	384	2,47686E+00	244	7,97846E-01	27219	0	11208,5
Responder	Macro_ISG15	cDC_CLEC9A	CXCL10	DPP4	7,16894E-05	9126,9	1,07048E-01	78	1,02364E+00	529	1,99503E+00	720	7,68119E-01	33099	0	11208,5
Responder	Mono_CD14	Macro_IER3	S100A9	CD68	7,22524E-05	4231,5	1,13681E-02	6834	8,77082E-01	912	1,48166E+00	1991	9,43539E-01	212	0	11208,5
Responder	cDC_CLEC9A	pDC_LILRA4	HLA-DOB	CD4	7,22850E-05	5030,5	4,03122E-02	722	1,24675E+00	229	2,76011E+00	116	8,67323E-01	12877	0	11208,5
Responder	Macro_OLFML3	Macro_NLRP3	C1QB	LRP1	7,23582E-05	5937,7	1,17270E-02	6508	5,78984E-01	3123	8,51052E-01	6836	9,25767E-01	2013	0	11208,5
Responder	pDC_LILRA4	Macro_IFI27	LCN2	FCGR2A	7,28839E-05	8116,1	4,61995E-02	560	9,42709E-01	724	2,16736E+00	500	7,96073E-01	27588	0	11208,5
Responder	Mono_CD14	Mono_INHBA	S100A12	TLR4	7,28839E-05	6522,7	4,02032E-02	724	1,04888E+00	468	2,18149E+00	482	8,34461E-01	19731	0	11208,5
Responder	Macro_OLFML3	pDC_LILRA4	CD14	TLR9	7,29086E-05	5249,5	8,56045E-02	138	1,46469E+00	132	1,33852E+00	2646	8,71433E-01	12123	0	11208,5
Responder	Macro_OLFML3	cDC_CLEC9A	CXCL9	DPP4	7,42286E-05	11571,1	5,59719E-02	353	6,95239E-01	1895	1,33694E+00	2658	7,21513E-01	41741	0	11208,5
Responder	cDC_LAMP3	pDC_LILRA4	TNF	PTPRS	7,47003E-05	10555,3	4,00180E-02	730	1,47088E+00	130	2,03639E+00	660	7,31090E-01	40048	0	11208,5
Responder	Macro_OLFML3	pDC_LILRA4	HLA-DQA1	CD4	7,51184E-05	4951,7	9,38646E-03	9490	6,14569E-01	2666	1,99461E+00	721	9,36183E-01	673	0	11208,5
Responder	pDC_LILRA4	Mono_INHBA	FAM3D	FPR1	7,59278E-05	9874,5	5,37046E-02	389	9,40030E-01	734	2,20994E+00	447	7,49606E-01	36594	0	11208,5
Responder	cDC(CD1C)	Mono_INHBA	VIM	CD44	7,60162E-05	6347,9	7,08961E-03	15149	6,19901E-01	2609	1,33524E+00	2669	9,47279E-01	104	0	11208,5
Responder	Macro_FOLR2-APOE+	Mono_INHBA	SPP1	CD44	7,62499E-05	4133,3	1,95804E-02	2623	8,28381E-01	1112	1,39012E+00	2406	9,18631E-01	3317	0	11208,5
Responder	cDC(CD1C)	Mono_CD16	HLA-B	LILRA1	7,76957E-05	6453,1	1,66841E-02	3476	4,01319E-01	6934	1,06600E+00	4460	9,03153E-01	6187	0	11208,5
Responder	cDC_CLEC9A	cDC_CLEC9A	BTLA	TNFRSF14	7,81079E-05	7109,9	4,49526E-02	586	1,68464E+00	82	1,98337E+00	741	8,18968E-01	22932	0	11208,5
Responder	Macro_FOLR2-APOE+	Mono_INHBA	MMP9	CD44	7,85397E-05	4272,3	2,63568E-02	1583	1,00644E+00	567	1,60154E+00	1562	9,01783E-01	6441	0	11208,5
Responder	pDC_LILRA4	Mast	GNAS	ADRB2	7,87383E-05	4385,9	3,97113E-02	743	1,01411E+00	548	2,58348E+00	186	8,86771E-01	9244	0	11208,5
Responder	Mono_CD14	Macro_OLFML3	VCAN	TLR2	7,96764E-05	4186,1	3,48264E-02	947	1,02910E+00	513	1,69797E+00	1293	8,98964E-01	6969	0	11208,5
Responder	Macro_OLFML3	Macro_FOLR2-APOE+	C3	C3AR1	8,03072E-05	6041,5	1,89376E-02	2773	5,55909E-01	3467	9,36169E-01	5779	8,98902E-01	6980	0	11208,5
Responder	cDC(CD1C)	Mono_CD16	RPS19	C5AR1	8,03072E-05	4580,5	1,12208E-02	6980	7,04296E-01	1818	1,62284E+00	1504	9,29842E-01	1392	0	11208,5
Responder	Macro_OLFML3	cDC_CLEC9A	CXCL10	DPP4	8,03904E-05	12041,7	5,86551E-02	321	6,59878E-01	2207	1,32641E+00	2712	7,10316E-01	43760	0	11208,5
Responder	Mono_CD14	Mast	HBEGF	CD44	8,05376E-05	5971,5	1,27563E-02	5624	6,48139E-01	2321	1,15386E+00	3720	8,98886E-01	6984	0	11208,5
Responder	Macro_LYVE1	pDC_LILRA4	IGF1	IGF2R	8,06497E-05	8567,5	4,48422E-02	593	9,34325E-01	749	2,10741E+00	579	7,85253E-01	29708	0	11208,5
Responder	pDC_LILRA4	cDC_LAMP3	APP	CD74	8,09712E-05	3305,1	1,60477E-02	3743	9,37742E-01	741	1,97983E+00	750	9,48506E-01	83	0	11208,5
Responder	cDC(CD1C)	Mono_CD16	LYZ	ITGAL	8,11739E-05	6411,1	1,23617E-02	5938	4,92716E-01	4544	1,21130E+00	3370	8,98828E-01	6995	0	11208,5
Responder	Macro_OLFML3	Macro_OLFML3	TNFSF13B	HLA-DPB1	8,11773E-05	8619,9	7,61195E-03	13509	5,66535E-01	3322	4,20662E-01	14979	9,48586E-01	81	0	11208,5
Responder	Macro_ISG15	Macro_ISG15	CCL8	CCR1	8,12936E-05	8588,1	6,54034E-02	247	9,33167E-01	751	2,09394E+00	594	7,83134E-01	30140	0	11208,5
Responder	Mono_INHBA	Mono_CD14	HBEGF	CD44	8,24584E-05	6232,7	1,27026E-02	5666	6,12784E-01	2691	1,05450E+00	4581	8,98694E-01	7017	0	11208,5
Responder	Macro_OLFML3	cDC_CLEC9A	FN1	ITGA4_ITGB7	8,32480E-05	7120,1	2,30891E-02	2017	6,08926E-01	2736	1,48553E+00	1975	8,44202E-01	17664	0	11208,5
Responder	Macro_FOLR2-APOE+	Mono_CD14	FN1	PLAUR	8,38782E-05	5058,1	1,66796E-02	3477	5,36398E-01	3752	1,35170E+00	2581	9,13366E-01	4272	0	11208,5
Responder	Macro_FOLR2-APOE+	Mono_CD14	SPP1	CD44	8,45355E-05	4881,9	1,61128E-02	3714	6,61692E-01	2182	1,34538E+00	2607	9,11042E-01	4698	0	11208,5
Responder	Macro_OLFML3	Mono_CD14	C3	LRP1	8,49495E-05	5120,5	1,90417E-02	2750	6,15060E-01	2660	1,51768E+00	1847	8,98106E-01	7137	0	11208,5
Responder	pDC_LILRA4	Macro_FOLR2+APOE+	FAM3D	FPR1	8,52288E-05	9162,1	6,58285E-02	241	1,02370E+00	528	1,97095E+00	763	7,68220E-01	33070	0	11208,5
Responder	Macro_NLRP3	pDC_LILRA4	GNAI2	CXCR3	8,55623E-05	5441,9	3,93781E-02	758	1,24021E+00	234	1,96964E+00	764	8,60397E-01	14245	0	11208,5
Responder	pDC_LILRA4	cDC(CD1C)	SPON2	ITGA4	8,55623E-05	9449,9	3,91320E-02	764	9,29660E-01	761	2,18764E+00	476	7,63502E-01	34040	0	11208,5
Responder	Mast	Macro_NLRP3	CSF1	CSF3R	8,58966E-05	5429,5	7,04181E-02	215	1,07432E+00	437	1,96752E+00	765	8,59210E-01	14522	0	11208,5
Responder	pDC_LILRA4	Mono_INHBA	SPON2	ITGB1	8,62319E-05	8373,9	4,08492E-02	710	9,87248E-01	606	1,96536E+00	766	7,91101E-01	28579	0	11208,5
Responder	cDC(CD1C)	Mono_CD14	RPS19	C5AR1	8,66768E-05	4479,9	1,09128E-02	7322	6,84862E-01	1971	2,27872E+00	385	9,28928E-01	1513	0	11208,5
Responder	Macro_OLFML3	Macro_IFI27	APOE	TREM2	8,70217E-05	5012,7	1,92667E-02	2687	5,72129E-01	3239	8,32706E-01	7093	9,34659E-01	836	0	11208,5
Responder	Mono_CD14	Macro_NLRP3	S100A9	TLR4	8,79457E-05	4341,3	2,03284E-02	2465	1,10334E+00	388	2,14559E+00	537	8,98269E-01	7108	0	11208,5
Responder	pDC_LILRA4	pDC_LILRA4	APP	NCSTN	8,82615E-05	5686,9	3,88922E-02	772	1,15976E+00	316	3,17120E+00	49	8,51694E-01	16089	0	11208,5
Responder	Macro_IER3	pDC_LILRA4	CCL5	CXCR3	8,89450E-05	9961,9	3,87418E-02	774	1,14510E+00	340	2,25266E+00	407	7,47130E-01	37080	0	11208,5
Responder	Mono_INHBA	Mono_CD14	VEGFA	CD44	8,96288E-05	5017,9	1,79815E-02	3057	7,98488E-01	1257	1,38423E+00	2432	8,98118E-01	7135	0	11208,5
Responder	Macro_ISG15	Mono_CD14	CCL8	CCR2	8,96320E-05	11952,1	9,48388E-02	108	9,22864E-01	776	2,11232E+00	569	6,90058E-01	47099	0	11208,5
Responder	pDC_LILRA4	Macro_FOLR2+APOE+	LCN2	FCGR2A	8,99768E-05	7917,7	4,95253E-02	482	9,79921E-01	628	1,96049E+00	777	8,01657E-01	26493	0	11208,5
Responder	Macro_OLFML3	Macro_OLFML3	C3	CD81	9,07651E-05	5887,5	1,83071E-02	2951	6,14737E-01	2664	8,27899E-01	7153	9,06978E-01	5461	0	11208,5
Responder	Macro_OLFML3	cDC_CLEC9A	C3	ITGAX	9,08285E-05	5248,5	1,70368E-02	3348	6,11158E-01	2707	1,52563E+00	1825	8,98002E-01	7154	0	11208,5
Responder	pDC_LILRA4	cDC(CD1C)	SPON2	ITGA5	9,13649E-05	12168,5	4,00043E-02	732	9,21390E-01	781	2,05042E+00	638	6,87433E-01	47483	0	11208,5
Responder	Mast	pDC_LILRA4	BST2	LILRA4	9,13649E-05	4023,1	3,85420E-02	781	1,46487E+00	131	2,69977E+00	132	8,94307E-01	7863	0	11208,5
Responder	Mono_CD14	Mono_CD14	S100A9	CD68	9,15929E-05	4063,3	1,10580E-02	7166	8,63514E-01	965	1,98692E+00	732	9,42797E-01	245	0	11208,5
Responder	Mast	Macro_OLFML3	CSF1	CSF2RA	9,17141E-05	4707,1	7,27444E-02	201	1,17070E+00	302	1,95690E+00	782	8,76929E-01	11042	0	11208,5
Responder	Mono_CD14	Macro_LYVE1	VCAN	CD44	9,35262E-05	5935,5	1,40984E-02	4775	7,54155E-01	1506	8,24525E-01	7196	9,09440E-01	4992	0	11208,5
Responder	cDC(CD1C)	pDC_LILRA4	HMGB1	TLR9	9,38280E-05	5118,5	6,79468E-02	227	1,39385E+00	149	1,95024E+00	788	8,65547E-01	13220	0	11208,5
Responder	Macro_NLRP3	Mono_CD14	VCAN	CD44	9,39809E-05	4101,7	1,91564E-02	2716	8,48620E-01	1024	1,32071E+00	2739	9,21297E-01	2821	0	11208,5
Responder	Mono_INHBA	Macro_NLRP3	VEGFA	CD44	9,43086E-05	5604,1	1,78106E-02	3103	7,91125E-01	1296	9,90192E-01	5205	8,97680E-01	7208	0	11208,5

Responder	Mono_CD14	cDC_LAMP3	S100A8	CD69	9,46362E-05	4481,1	1,71236E-02	3319	1,13810E+00	348	2,38400E+00	317	8,97631E-01	7213	0	11208,5
Responder	Mono_INHBA	pDC_LILRA4	CXCL2	DPP4	9,66964E-05	7536,7	8,46384E-02	143	1,23241E+00	245	1,94518E+00	796	8,07731E-01	25291	0	11208,5
Responder	pDC_LILRA4	pDC_LILRA4	VEGFB	NRP1	9,70590E-05	8585,7	3,82474E-02	795	9,15623E-01	797	2,93633E+00	76	7,83551E-01	30052	0	11208,5
Responder	Mono_INHBA	Macro_FOLR2-APOE+	INHBA	ENG	9,74224E-05	6440,7	5,56259E-02	361	1,09863E+00	391	1,94428E+00	798	8,35845E-01	19445	0	11208,5
Responder	Mono_CD14	Macro_FOLR2-APOE+	VCAN	ITGB1	9,76926E-05	4443,9	2,38796E-02	1891	9,87006E-01	607	1,71580E+00	1254	8,97399E-01	7259	0	11208,5
Responder	Macro_ISG15	Macro_ISG15	CCL8	CCR5	9,99920E-05	12002,9	7,84036E-02	180	9,23971E-01	774	1,94097E+00	805	6,90406E-01	47047	0	11208,5
Responder	Macro_IFI27	pDC_LILRA4	CCL5	CXCR3	1,00363E-04	9637,1	4,31383E-02	633	1,17413E+00	297	1,94053E+00	806	7,57149E-01	35241	0	11208,5
Responder	Mast	cDC(CD1C)	TIMP3	CD44	1,00734E-04	4195,1	5,03632E-02	455	1,03296E+00	505	1,94051E+00	807	8,93519E-01	8000	0	11208,5
Responder	Macro_ISG15	pDC_LILRA4	CXCL9	CXCR3	1,01107E-04	7374,5	5,40451E-02	384	1,15907E+00	317	1,94034E+00	808	8,13210E-01	24155	0	11208,5
Responder	Macro_FOLR2+APOE+	Mono_INHBA	APOE	LRP1	1,01175E-04	3746,3	1,85525E-02	2874	8,57412E-01	990	1,31918E+00	2743	9,33914E-01	916	0	11208,5
Responder	pDC_LILRA4	cDC_CLEC9A	CD24	SIGLEC10	1,01480E-04	10367,7	7,05413E-02	214	9,12231E-01	809	2,62275E+00	167	7,34682E-01	39440	0	11208,5
Responder	Macro_OLFML3	pDC_LILRA4	HLA-DQA2	CD4	1,01728E-04	5472,7	1,09150E-02	7318	5,11950E-01	4183	2,04743E+00	645	9,14777E-01	4009	0	11208,5
Responder	Macro_OLFML3	Mono_CD14	APOE	SORL1	1,02076E-04	7026,5	1,09123E-02	7323	4,03070E-01	6875	1,07515E+00	4375	9,07568E-01	5351	0	11208,5
Responder	pDC_LILRA4	cDC(CD1C)	SPON2	ITGB2	1,02230E-04	5738,1	3,79333E-02	811	9,31532E-01	755	2,38864E+00	313	8,53917E-01	15603	0	11208,5
Responder	Macro_OLFML3	Mono_INHBA	C1QA	CD93	1,02985E-04	6820,9	1,19241E-02	6326	5,57204E-01	3446	8,14124E-01	7336	9,05083E-01	5788	0	11208,5
Responder	cDC(CD1C)	Macro_LYVE1	RPS19	C5AR1	1,03618E-04	4845,9	1,08938E-02	7345	6,83663E-01	1984	1,43973E+00	2170	9,28871E-01	1522	0	11208,5
Responder	Mono_CD14	Mono_INHBA	VCAN	ITGB1	1,03901E-04	4390,3	2,36493E-02	1932	9,81943E-01	621	1,91996E+00	841	8,96952E-01	7349	0	11208,5
Responder	Mono_CD14	Macro_ISG15	S100A9	TLR4	1,04468E-04	4393,7	1,97399E-02	2581	1,09356E+00	397	2,23358E+00	425	8,96919E-01	7357	0	11208,5
Responder	pDC_LILRA4	Macro_LYVE1	APP	LRP1	1,04501E-04	3853,9	3,77221E-02	817	1,33606E+00	174	2,65699E+00	155	8,99275E-01	6915	0	11208,5
Responder	Macro_FOLR2+APOE+	pDC_LILRA4	CD14	TLR9	1,04686E-04	4718,1	1,02694E-01	86	1,62156E+00	92	1,48234E+00	1988	8,81290E-01	10216	0	11208,5
Responder	Mono_CD16	Mast	LGALS1	CD69	1,04851E-04	3548,7	2,19266E-02	2201	1,02841E+00	514	1,29229E+00	2900	9,33855E-01	920	0	11208,5
Responder	pDC_LILRA4	cDC(CD1C)	CDH1	ITGAE_ITGB7	1,07580E-04	13348,1	6,72681E-02	234	9,05374E-01	825	2,31669E+00	361	6,36632E-01	54112	0	11208,5
Responder	Mono_CD14	Macro_LYVE1	VCAN	ITGB1	1,07702E-04	4566,5	2,35629E-02	1941	9,80044E-01	627	1,57651E+00	1654	8,96783E-01	7402	0	11208,5
Responder	Macro_OLFML3	Macro_NLRP3	APOE	LRP1	1,07847E-04	5855,5	1,37313E-02	4994	5,64815E-01	3342	8,09739E-01	7404	9,24000E-01	2329	0	11208,5
Responder	Mono_CD14	Macro_FOLR2+APOE-	S100A8	CD36	1,08139E-04	4303,5	2,04763E-02	2436	1,20841E+00	269	2,56017E+00	196	8,96730E-01	7408	0	11208,5
Responder	pDC_LILRA4	Mono_INHBA	APP	PTGER2	1,08359E-04	8227,9	3,75557E-02	827	1,08054E+00	426	2,49279E+00	236	7,91775E-01	28442	0	11208,5
Responder	Macro_FOLR2-APOE+	Macro_ISG15	C3	IFITM1	1,09018E-04	4654,5	2,43929E-02	1820	7,01121E-01	1843	1,47085E+00	2030	9,02179E-01	6371	0	11208,5
Responder	Macro_OLFML3	Macro_FOLR2+APOE-	C3	NRP1	1,10103E-04	6820,5	2,27054E-02	2074	6,97585E-01	1870	1,28541E+00	2936	8,52011E-01	16014	0	11208,5
Responder	Mono_INHBA	Mono_CD14	SPP1	CD44	1,10124E-04	6420,9	1,15539E-02	6665	4,93408E-01	4537	1,41970E+00	2259	8,96612E-01	7435	0	11208,5
Responder	cDC(CD1C)	Mono_CD16	ANXA1	FPR1	1,11988E-04	6980,9	1,07935E-02	7460	5,05559E-01	4291	8,66247E-01	6647	9,07839E-01	5298	0	11208,5
Responder	Mono_CD14	Mono_INHBA	NAMPT	ITGA5_ITGB1	1,12590E-04	5100,5	1,42044E-02	4707	8,62150E-01	973	1,76174E+00	1146	8,96395E-01	7468	0	11208,5
Responder	pDC_LILRA4	Mono_CD16	LCN2	FCGR2A	1,13918E-04	11226,3	4,24602E-02	657	9,00870E-01	841	2,06174E+00	622	7,89136E-01	28955	0,0060000000000000	25056,5
Responder	Macro_OLFML3	Macro_NLRP3	C1QA	CD93	1,14487E-04	6803,7	1,20396E-02	6208	5,60969E-01	3386	8,03859E-01	7493	9,05496E-01	5723	0	11208,5
Responder	Mast	cDC(CD1C)	B2M	CD1C	1,14627E-04	3681,7	3,50269E-02	934	6,20128E-01	2604	1,28073E+00	2966	9,36005E-01	696	0	11208,5
Responder	Macro_OLFML3	pDC_LILRA4	C3	IFITM1	1,14627E-04	5166,1	1,82601E-02	2966	6,42333E-01	2380	2,32657E+00	350	8,88636E-01	8926	0	11208,5
Responder	Macro_FOLR2-APOE+	Macro_NLRP3	FN1	PLAUR	1,14640E-04	5419,5	1,82046E-02	2979	6,20931E-01	2595	8,64454E-01	6667	9,16766E-01	3648	0	11208,5
Responder	Macro_FOLR2+APOE-	pDC_LILRA4	IGF1	IGF2R	1,15133E-04	8775,1	4,24972E-02	655	9,00402E-01	844	2,15988E+00	518	7,80689E-01	30650	0	11208,5
Responder	Macro_NLRP3	Mono_CD14	VCAN	SELL	1,15133E-04	4274,1	3,80211E-02	807	9,86697E-01	609	1,91731E+00	844	8,94122E-01	7902	0	11208,5
Responder	Mono_INHBA	Macro_NLRP3	SPP1	CD44	1,15407E-04	7003,5	1,14440E-02	6769	4,86046E-01	4690	1,02566E+00	4845	8,96168E-01	7505	0	11208,5
Responder	Macro_ISG15	pDC_LILRA4	CD14	TLR9	1,15540E-04	4736,9	9,16484E-02	114	1,52017E+00	112	1,91720E+00	845	8,75207E-01	11405	0	11208,5
Responder	Macro_FOLR2-APOE+	Macro_NLRP3	SPP1	CD44	1,15792E-04	5532,5	1,59596E-02	3789	6,54330E-01	2273	9,51340E-01	5618	9,10654E-01	4774	0	11208,5
Responder	Mono_CD14	pDC_LILRA4	AGTRAP	RACK1	1,16023E-04	5997,1	1,10097E-02	7218	5,33083E-01	3820	2,51116E+00	226	8,96114E-01	7513	0	11208,5
Responder	cDC(CD1C)	Mono_INHBA	PKM	CD44	1,16630E-04	6088,5	8,03531E-03	12343	5,88688E-01	2979	1,37259E+00	2487	9,29562E-01	1425	0	11208,5
Responder	Mono_CD14	Macro_FOLR2+APOE-	S100A12	CD36	1,16767E-04	6221,3	3,69488E-02	848	1,07656E+00	436	2,18448E+00	480	8,41935E-01	18134	0	11208,5
Responder	Macro_ISG15	cDC(CD1C)	CCL8	CCR2	1,17178E-04	12257,3	8,69169E-02	134	8,98072E-01	849	2,13077E+00	550	6,80653E-01	48545	0	11208,5
Responder	cDC(CD1C)	Mono_INHBA	RPS19	C5AR1	1,17408E-04	5500,5	9,38450E-03	9495	5,88452E-01	2984	1,63860E+00	1456	9,23784E-01	2359	0	11208,5
Responder	Mono_INHBA	Mast	VCAN	CD44	1,17966E-04	6147,7	1,40437E-02	4804	6,63866E-01	2161	8,00369E-01	7538	9,09280E-01	5027	0	11208,5
Responder	Mono_INHBA	pDC_LILRA4	BST2	LILRA4	1,18002E-04	4111,9	3,68632E-02	851	1,44451E+00	137	2,72195E+00	126	8,92184E-01	8237	0	11208,5
Responder	Mono_CD14	Mast	ADM	ADRB2	1,18416E-04	7790,5	5,72303E-02	340	9,13589E-01	802	1,91368E+00	852	8,05359E-01	25750	0	11208,5
Responder	Macro_OLFML3	Mono_CD14	C1QA	CR1	1,18817E-04	5321,7	2,45729E-02	1792	7,62167E-01	1462	1,27556E+00	2993	8,87306E-01	9153	0	11208,5
Responder	Macro_OLFML3	Mono_CD16	C3	C3AR1	1,18830E-04	6229,9	1,77599E-02	3116	5,27131E-01	3910	9,73986E-01	5366	8,95947E-01	7549	0	11208,5
Responder	cDC_LAMP3	Macro_ISG15	CCL19	CCRL2	1,19663E-04	8076,7	6,26570E-02	280	1,89943E+00	47	1,91029E+00	855	7,94133E-01	27993	0	11208,5
Responder	Macro_FOLR2+APOE+	cDC_CLEC9A	C1QB	C1QBP	1,20397E-04	3541,5	1,81316E-02	3003	8,81291E-01	897	1,50354E+00	1908	9,36053E-01	691	0	11208,5
Responder	pDC_LILRA4	cDC_LAMP3	TNFSF9	PVR	1,20918E-04	12979,9	8,43956E-02	146	1,05127E+00	464	1,90805E+00	858	6,52979E-01	52223	0	11208,5
Responder	pDC_LILRA4	Macro_ISG15	SPON2	ITGA5	1,21760E-04	12087,3	4,14353E-02	687	9,30566E-01	760	1,90673E+00	860	6,91196E-01	46921	0	11208,5
Responder	Mast	Macro_ISG15	CSF1	CSF2RA	1,22182E-04	5854,7	4,73743E-02	529	9,80338E-01	625	1,90553E+00	861	8,51855E-01	16050	0	11208,5
Responder	pDC_LILRA4	cDC_LAMP3	TNFSF9	TNFRSF9	1,22605E-04	12096,7	9,95023E-02	95	1,07214E+00	439	1,90409E+00	862	6,84943E-01	47879	0	11208,5
Responder	pDC_LILRA4	cDC_CLEC9A	ADA	DPP4	1,22605E-04	13701,3	7,30985E-02	200	8,91499E-01	862	1,92255E+00	836	6,25095E-01	55400	0	11208,5
Responder	pDC_LILRA4	Mono_INHBA	RPS19	C5AR1	1,23030E-04	3723,9	1,30811E-02	5386	8,91209E-01	863	2,36056E+00	328	9,34683E-01	834	0	11208,5
Responder	Macro_OLFML3	Macro_IER3	APOE	ABCA1	1,23303E-04	5337,9	1,65533E-02	3530	6,67660E-01	2126	7,95758E-01	7605	9,24625E-01	2220	0	11208,5
Responder	Mono_INHBA	pDC_LILRA4	MMP9	TLR9	1,24736E-04	10326,7	9,81056E-02	97	1,43592E+00	142	1,90222E+00	867	7,35380E-01	39319	0	11208,5
Responder	Macro_ISG15	cDC_CLEC9A	CXCL2	XCR1	1,25596E-04	5962,1	5,06430E-02	451	2,45653E+00	11	1,90166E+00	869	8,45904E-01	17271	0	11208,5
Responder	Mono_INHBA	pDC_LILRA4	VEGFA	NRP1	1,25596E-04	6889,7	3,65067E-02	869	9,66140E-01	656	1,96373E+00	769	8,28476E-01	20946	0	11208,5
Responder	Macro_OLFML3	Macro_IER3	CXCL9	FCGR2A	1,26663E-04	7115,1	1,50695E-02	4216	4,64212E-01	5202	8,16148E-01	7303	8,95423E-01	7646	0	11208,5
Responder	Mono_INHBA	pDC_LILRA4	ADM	CALCRL	1,26892E-04	8866,5	4,31438E-02	632	8,88560E-01	872	2,75561E+00	118	7,76316E-01	31502	0	11208,5

Responder	cDC_CLEC9A	cDC(CD1C)	BTLA	TNFRSF14	1,28197E-04	6906,9	4,82059E-02	518	1,71216E+00	76	1,89762E+00	875	8,24090E-01	21857	0	11208,5
Responder	Macro_IER3	cDC(CD1C)	B2M	CD1C	1,30209E-04	6374,3	3,49814E-02	937	6,17162E-01	2642	1,26430E+00	3063	9,35966E-01	704	0,004	24525,5
Responder	Macro_OLFML3	Mono_INHBA	C3	NRP1	1,31222E-04	7764,9	1,81406E-02	3000	6,35660E-01	2438	1,26349E+00	3069	8,37294E-01	19109	0	11208,5
Responder	Macro_OLFML3	pDC_LILRA4	APOE	SCARB1	1,32923E-04	6208,1	1,80002E-02	3047	5,81332E-01	3079	1,58030E+00	1639	8,71753E-01	12067	0	11208,5
Responder	pDC_LILRA4	cDC_LAMP3	TNFSF9	TRAF2	1,33954E-04	12907,7	5,99345E-02	308	9,25602E-01	770	1,88719E+00	888	6,60247E-01	51364	0	11208,5
Responder	Mono_CD14	Macro_OLFML3	S100A8	CD69	1,34295E-04	5083,9	1,61769E-02	3678	1,12772E+00	355	1,38267E+00	2442	8,94989E-01	7736	0	11208,5
Responder	pDC_LILRA4	Macro_OLFML3	GAS6	AXL	1,35760E-04	6551,7	3,58938E-02	892	1,00554E+00	569	2,45932E+00	253	8,33922E-01	19836	0	11208,5
Responder	Mono_CD14	Macro_OLFML3	S100A12	TLR4	1,36669E-04	6468,9	4,18702E-02	673	1,05971E+00	449	1,88380E+00	894	8,37249E-01	19120	0	11208,5
Responder	cDC_LAMP3	pDC_LILRA4	BST2	LILRA4	1,37124E-04	4209,1	3,58247E-02	895	1,43192E+00	143	2,42886E+00	280	8,90801E-01	8519	0	11208,5
Responder	Macro_OLFML3	pDC_LILRA4	HLA-DPA1	CD4	1,38654E-04	5447,3	8,20783E-03	11932	5,79313E-01	3112	1,97733E+00	754	9,43149E-01	230	0	11208,5
Responder	Mono_CD14	Macro_FOLR2-APOE+	S100A8	TLR4	1,39495E-04	4297,7	2,38091E-02	1906	1,19866E+00	279	2,40353E+00	300	8,94682E-01	7795	0	11208,5
Responder	Macro_FOLR2-APOE+	cDC(CD1C)	SPP1	CD44	1,39943E-04	7368,7	1,10837E-02	7133	4,19950E-01	6360	1,07959E+00	4342	8,94670E-01	7800	0	11208,5
Responder	Macro_OLFML3	Macro_FOLR2-APOE+	FN1	SDC2	1,40961E-04	7893,5	4,31603E-02	629	7,12204E-01	1758	1,25403E+00	3125	8,19955E-01	22747	0	11208,5
Responder	pDC_LILRA4	Macro_NLRP3	SPON2	ITGA5	1,41738E-04	11428,1	4,92643E-02	489	9,80774E-01	624	1,87832E+00	905	7,09354E-01	43914	0	11208,5
Responder	pDC_LILRA4	Mono_CD14	SPON2	ITGB2	1,41738E-04	5996,7	3,56262E-02	905	8,79992E-01	899	2,16220E+00	512	8,49959E-01	16459	0	11208,5
Responder	Macro_OLFML3	pDC_LILRA4	HLA-DRB5	CD4	1,42395E-04	5200,3	8,84017E-03	10508	5,78307E-01	3133	2,00925E+00	699	9,38911E-01	453	0	11208,5
Responder	Macro_OLFML3	pDC_LILRA4	MDK	NCL	1,43478E-04	5455,1	2,39142E-02	1888	5,77951E-01	3139	2,17036E+00	494	8,79528E-01	10546	0	11208,5
Responder	pDC_LILRA4	pDC_LILRA4	SELPLG	SELL	1,45029E-04	4166,7	3,55560E-02	912	1,12820E+00	354	3,81110E+00	20	8,91687E-01	8339	0	11208,5
Responder	Macro_FOLR2-APOE+	pDC_LILRA4	APP	TNFRSF21	1,45503E-04	7088,3	3,55292E-02	913	1,16837E+00	304	1,92032E+00	840	8,22532E-01	22176	0	11208,5
Responder	Mono_CD14	pDC_LILRA4	GNAI2	CXCR3	1,46454E-04	5664,1	3,55253E-02	915	1,16838E+00	303	2,35333E+00	332	8,54098E-01	15562	0	11208,5
Responder	Macro_ISG15	Mast	LGALS1	CD69	1,47500E-04	3879,7	1,76170E-02	3161	8,55199E-01	1003	1,43882E+00	2172	9,26767E-01	1854	0	11208,5
Responder	Macro_OLFML3	pDC_LILRA4	HLA-DPB1	CD4	1,47685E-04	5494,3	8,19742E-03	11953	5,76538E-01	3162	1,91584E+00	849	9,41686E-01	299	0	11208,5
Responder	Macro_OLFML3	Mono_INHBA	CXCL9	FCGR2A	1,48393E-04	6683,5	1,46766E-02	4421	4,44893E-01	5680	1,09422E+00	4216	8,94179E-01	7892	0	11208,5
Responder	Macro_FOLR2-APOE+	Macro_OLFML3	APOE	TREM2	1,48581E-04	5117,1	2,03104E-02	2473	5,64694E-01	3345	7,75025E-01	7894	9,36252E-01	665	0	11208,5
Responder	Mono_CD14	Macro_FOLR2+APOE+	S100A12	TLR4	1,49333E-04	6704,1	3,90880E-02	766	1,04163E+00	487	1,87292E+00	921	8,32509E-01	20138	0	11208,5
Responder	cDC(CD1C)	cDC_CLEC9A	ITGB2	CD226	1,49729E-04	6790,3	3,03440E-02	1196	7,34363E-01	1618	1,47875E+00	1999	8,42833E-01	17930	0	11208,5
Responder	Mono_CD14	Macro_FOLR2+APOE-	S100A8	ITGB2	1,50473E-04	4480,5	1,04257E-02	7914	7,82552E-01	1343	1,99262E+00	726	9,31116E-01	1211	0	11208,5
Responder	Macro_OLFML3	Macro_NLRP3	APOE	SORL1	1,50758E-04	6396,9	1,39310E-02	4875	4,98684E-01	4429	7,73292E-01	7917	9,17315E-01	3555	0	11208,5
Responder	pDC_LILRA4	pDC_LILRA4	ASIP	MGRN1	1,50786E-04	12704,7	7,46378E-02	194	8,74546E-01	924	2,27276E+00	393	6,64788E-01	50804	0	11208,5
Responder	Mono_INHBA	Mast	HBEGF	CD9	1,50853E-04	5156,3	2,43645E-02	1823	7,81784E-01	1348	1,19123E+00	3484	8,93973E-01	7918	0	11208,5
Responder	Macro_FOLR2+APOE+	pDC_LILRA4	CXCL13	CXCR3	1,51272E-04	13750,5	5,81694E-02	328	1,16238E+00	311	1,87217E+00	925	6,19287E-01	55980	0	11208,5
Responder	cDC(CD1C)	cDC_CLEC9A	NECTIN2	CD226	1,51606E-04	14145,3	2,68386E-02	1525	7,01645E-01	1837	1,29159E+00	2906	6,44260E-01	53250	0	11208,5
Responder	pDC_LILRA4	Macro_ISG15	LTB	TNFRSF1A	1,51760E-04	5148,1	3,51930E-02	926	1,14410E+00	345	2,85073E+00	90	8,65767E-01	13171	0	11208,5
Responder	Macro_OLFML3	cDC(CD1C)	C3	IFITM1	1,52172E-04	5692,5	1,75276E-02	3186	6,29753E-01	2500	1,41025E+00	2293	8,86594E-01	9275	0	11208,5
Responder	Macro_OLFML3	cDC(CD1C)	C1QB	C1QBP	1,52480E-04	5966,7	1,04131E-02	7935	5,22802E-01	3988	1,25022E+00	3146	9,17308E-01	3556	0	11208,5
Responder	cDC(CD1C)	cDC_CLEC9A	CXCL2	XCR1	1,53690E-04	6886,3	3,79324E-02	812	2,39713E+00	14	1,85391E+00	957	8,26114E-01	21440	0	11208,5
Responder	Macro_OLFML3	Mono_CD14	C1QB	LRP1	1,54702E-04	5982,9	1,03970E-02	7958	5,21142E-01	4023	1,12839E+00	3950	9,21523E-01	2775	0	11208,5
Responder	Macro_OLFML3	cDC(CD1C)	TNFSF13B	HLA-DPB1	1,55411E-04	6202,3	7,65574E-03	13388	5,74334E-01	3203	1,25185E+00	3134	9,48725E-01	78	0	11208,5
Responder	cDC(CD1C)	cDC_CLEC9A	ANXA1	DYSF	1,55603E-04	7077,7	2,00266E-02	2529	5,79949E-01	3098	1,42306E+00	2246	8,50723E-01	16307	0	11208,5
Responder	Mono_CD14	Macro_NLRP3	VCAN	ITGB1	1,55872E-04	4648,5	2,20412E-02	2178	9,46588E-01	711	1,74945E+00	1175	8,93652E-01	7970	0	11208,5
Responder	pDC_LILRA4	cDC_LAMP3	SPON2	ITGA4	1,56195E-04	8583,3	4,99311E-02	472	1,03546E+00	501	1,86724E+00	935	7,84794E-01	29800	0	11208,5
Responder	cDC(CD1C)	cDC_CLEC9A	CALM1	MYLK	1,56374E-04	9000,7	4,12605E-02	697	8,43439E-01	1042	1,64131E+00	1446	7,80906E-01	30610	0	11208,5
Responder	Macro_OLFML3	Macro_NLRP3	C3	C3AR1	1,56558E-04	6312,1	1,69075E-02	3389	5,06302E-01	4270	1,04110E+00	4716	8,93632E-01	7977	0	11208,5
Responder	cDC(CD1C)	cDC_CLEC9A	CALM2	MYLK	1,56567E-04	8680,9	4,30423E-02	636	8,21340E-01	1147	1,68138E+00	1331	7,88522E-01	29082	0	11208,5
Responder	cDC(CD1C)	cDC_CLEC9A	ADAM10	CADM1	1,56760E-04	9566,9	2,22309E-02	2145	7,96835E-01	1267	1,60957E+00	1535	7,75398E-01	31679	0	11208,5
Responder	cDC(CD1C)	cDC_CLEC9A	GNAI2	CXCR3	1,57534E-04	8622,3	1,92465E-02	2693	6,38498E-01	2414	1,40625E+00	2315	8,11632E-01	24481	0	11208,5
Responder	cDC_CLEC9A	pDC_LILRA4	GNAI2	CXCR3	1,58695E-04	5714,5	3,49281E-02	940	1,15724E+00	318	2,42034E+00	289	8,53038E-01	15817	0	11208,5
Responder	Macro_FOLR2-APOE+	Macro_ISG15	SPP1	CD44	1,59027E-04	8246,5	1,08164E-02	7435	4,07099E-01	6754	7,79532E-01	7833	8,93514E-01	8002	0	11208,5
Responder	cDC(CD1C)	pDC_LILRA4	TNF	TNFRSF21	1,59679E-04	8975,5	2,32761E-02	1984	1,03796E+00	496	2,60414E+00	173	7,78876E-01	31016	0	11208,5
Responder	cDC(CD1C)	pDC_LILRA4	HLA-DQA1	CD4	1,60464E-04	5007,7	9,15564E-03	9911	5,85683E-01	3027	2,71030E+00	130	9,35435E-01	762	0	11208,5
Responder	Mono_CD14	Macro_OLFML3	S100A8	TLR4	1,60523E-04	4381,5	2,32037E-02	1997	1,19156E+00	282	2,25949E+00	403	8,93462E-01	8017	0	11208,5
Responder	cDC(CD1C)	pDC_LILRA4	HLA-DPB1	CD4	1,61055E-04	5300,3	8,24457E-03	11837	5,84337E-01	3039	2,74703E+00	122	9,41843E-01	295	0	11208,5
Responder	cDC(CD1C)	pDC_LILRA4	APP	TNFRSF21	1,61252E-04	8627,9	2,32966E-02	1980	1,02482E+00	526	2,12368E+00	560	7,89610E-01	28865	0	11208,5
Responder	cDC_LAMP3	cDC(CD1C)	CCL19	CCRL2	1,61730E-04	9503,5	4,24364E-02	659	1,81168E+00	64	1,86095E+00	946	7,60457E-01	34640	0	11208,5
Responder	Mono_CD14	cDC_LAMP3	VCAN	ITGA4	1,61929E-04	4418,3	2,89072E-02	1328	1,03015E+00	511	1,82184E+00	1013	8,93411E-01	8031	0	11208,5
Responder	pDC_LILRA4	Macro_LYVE1	SPN	SIGLEC1	1,62750E-04	7779,7	5,22788E-02	418	1,06600E+00	445	1,86023E+00	948	8,04664E-01	25879	0	11208,5
Responder	Mono_INHBA	pDC_LILRA4	GNAI2	CXCR3	1,62750E-04	5821,9	3,48220E-02	948	1,15526E+00	321	1,95931E+00	778	8,52847E-01	15854	0	11208,5
Responder	Macro_OLFML3	Macro_ISG15	C3	ITGAX	1,64058E-04	5934,3	1,53942E-02	4050	5,72460E-01	3234	1,25347E+00	3127	8,93264E-01	8052	0	11208,5
Responder	Mono_CD14	Macro_IER3	S100A8	TLR4	1,64160E-04	4348,1	2,31018E-02	2015	1,19036E+00	285	2,59139E+00	179	8,93253E-01	8053	0	11208,5
Responder	cDC(CD1C)	pDC_LILRA4	ALCAM	NRP1	1,64434E-04	10696,5	1,87830E-02	2821	5,85308E-01	3032	1,76229E+00	1144	7,56965E-01	35277	0	11208,5
Responder	Macro_FOLR2-APOE+	Macro_FOLR2-APOE+	SPP1	CD44	1,64772E-04	6783,3	1,36681E-02	5035	5,44176E-01	3632	7,62876E-01	8059	9,04145E-01	5982	0	11208,5
Responder	cDC(CD1C)	Macro_LYVE1	CALR	LRP1	1,65386E-04	6601,7	1,03083E-02	8065	5,30885E-01	3845	1,05827E+00	4535	9,07558E-01	5355	0	11208,5
Responder	cDC_CLEC9A	Mast	GNAS	ADRB2	1,65836E-04	4726,3	3,68429E-02	853	9,53521E-01	688	1,85505E+00	954	8,82953E-01	9928	0	11208,5
Responder	cDC(CD1C)	pDC_LILRA4	HMGB1	THBD	1,66311E-04	5668,7	1,27953E-02	5597	5,75377E-01	3178	2,42258E+00	286	8,93125E-01	8074	0	11208,5
Responder	Macro_FOLR2+APOE+	Macro_FOLR2-APOE+	APOE	TREM2	1,66446E-04	3447,1	2,62032E-02	1605	8,69256E-01	946	1,23075E+00	3259	9,43444E-01	217	0	11208,5

Responder	cDC(CD1C)	pDC_LILRA4	CCL5	CXCR3	1,66648E-04	12461,5	2,13823E-02	2298	1,03046E+00	510	1,98414E+00	739	6,87012E-01	47552	0	11208,5
Responder	Mono_CD14	Mono_CD14	HBEGF	CD44	1,67551E-04	6503,5	1,12625E-02	6939	5,45413E-01	3611	1,33494E+00	2673	8,93084E-01	8086	0	11208,5
Responder	cDC(CD1C)	pDC_LILRA4	CXCL9	CXCR3	1,68272E-04	11493,3	1,90686E-02	2745	9,61081E-01	670	1,81499E+00	1032	7,21138E-01	41811	0	11208,5
Responder	Macro_LYVE1	pDC_LILRA4	CD14	TLR9	1,69178E-04	5060,1	9,09660E-02	117	1,51390E+00	115	1,39665E+00	2366	8,74798E-01	11494	0	11208,5
Responder	cDC(CD1C)	cDC_CLEC9A	LYZ	ITGAL	1,69215E-04	6842,5	1,09119E-02	7325	4,31957E-01	6030	1,60691E+00	1547	8,93013E-01	8102	0	11208,5
Responder	Macro_OLFML3	cDC(CD1C)	FN1	ITGA4_ITGB7	1,69293E-04	7092,9	2,39934E-02	1880	5,69868E-01	3273	1,47949E+00	1997	8,46712E-01	17106	0	11208,5
Responder	cDC(CD1C)	pDC_LILRA4	PSEN1	NOTCH4	1,70113E-04	13291,7	3,63458E-02	875	6,17680E-01	2634	1,85922E+00	949	6,64936E-01	50792	0	11208,5
Responder	Mono_CD14	pDC_LILRA4	S100A9	CD68	1,70262E-04	4194,9	1,02748E-02	8112	8,29251E-01	1107	2,54209E+00	206	9,40784E-01	341	0	11208,5
Responder	Macro_OLFML3	Macro_LYVE1	C3	ITGAX	1,70367E-04	6863,5	1,52831E-02	4109	5,69844E-01	3275	7,95229E-01	7612	8,92918E-01	8113	0	11208,5
Responder	Mast	Macro_LYVE1	CSF1	SIRPA	1,70537E-04	6208,7	6,67254E-02	237	1,11863E+00	366	1,84911E+00	963	8,41291E-01	18269	0	11208,5
Responder	Mono_INHBA	pDC_LILRA4	VCAN	SELL	1,71065E-04	4388,3	3,44730E-02	964	9,49483E-01	704	2,40019E+00	304	8,89395E-01	8761	0	11208,5
Responder	Mast	Macro_IFI27	CSF1	SIRPA	1,71594E-04	7378,1	4,66592E-02	547	9,98792E-01	583	1,84859E+00	965	8,15929E-01	23587	0	11208,5
Responder	pDC_LILRA4	pDC_LILRA4	CCL5	CXCR3	1,71594E-04	10362,9	3,44368E-02	965	1,11667E+00	367	3,34825E+00	36	7,35842E-01	39238	0	11208,5
Responder	Macro_FOLR2-APOE+	Mono_CD14	MMP9	CD44	1,72371E-04	4872,9	2,16890E-02	2240	8,39748E-01	1064	1,55680E+00	1720	8,92807E-01	8132	0	11208,5
Responder	cDC(CD1C)	pDC_LILRA4	HSP90B1	TLR9	1,72384E-04	5903,3	6,19988E-02	286	1,28973E+00	199	1,79640E+00	1066	8,48397E-01	16757	0	11208,5
Responder	Mono_INHBA	cDC(CD1C)	INHBA	ENG	1,72655E-04	8056,1	3,43632E-02	967	9,04286E-01	829	2,19058E+00	469	8,00081E-01	26807	0	11208,5
Responder	cDC(CD1C)	pDC_LILRA4	GNAI2	CXCR3	1,72799E-04	5757,7	3,42763E-02	970	1,14509E+00	341	2,52183E+00	218	8,51854E-01	16051	0	11208,5
Responder	Mono_INHBA	pDC_LILRA4	CXCL8	CD79A	1,73187E-04	8940,7	6,37444E-02	263	8,70752E-01	942	1,84797E+00	968	7,77255E-01	31322	0	11208,5
Responder	Macro_IFI27	pDC_LILRA4	CXCL10	CXCR3	1,73187E-04	9574,9	3,42835E-02	968	1,05128E+00	463	1,87522E+00	913	7,62231E-01	34322	0	11208,5
Responder	Macro_FOLR2-APOE+	Mono_INHBA	FN1	PLAUR	1,73967E-04	6167,9	1,66892E-02	3474	5,36930E-01	3743	7,57236E-01	8147	9,13389E-01	4267	0	11208,5
Responder	Macro_OLFML3	Macro_FOLR2+APOE-	C3	LRP1	1,74287E-04	5950,7	1,69631E-02	3367	5,65702E-01	3329	1,15667E+00	3699	8,92694E-01	8150	0	11208,5
Responder	Mono_CD14	Macro_NLRP3	HBEGF	CD44	1,75037E-04	7174,3	1,11555E-02	7048	5,38050E-01	3728	9,40896E-01	5730	8,92627E-01	8157	0	11208,5
Responder	cDC(CD1C)	pDC_LILRA4	MMP9	TLR9	1,75306E-04	12183,7	6,01569E-02	306	1,32548E+00	178	1,66495E+00	1379	6,85152E-01	47847	0	11208,5
Responder	Macro_FOLR2-APOE+	Mono_INHBA	FN1	CD44	1,75574E-04	6517,5	1,56801E-02	3929	5,23118E-01	3981	7,56318E-01	8162	9,07803E-01	5307	0	11208,5
Responder	pDC_LILRA4	Macro_FOLR2+APOE+	APP	RPSA	1,75937E-04	3373,5	2,63245E-02	1586	1,25853E+00	219	2,13149E+00	549	9,18718E-01	3305	0	11208,5
Responder	cDC(CD1C)	pDC_LILRA4	SEMA4A	NRP1_PLXNA4	1,76570E-04	12951,1	1,75444E-02	3182	6,96268E-01	1885	1,22241E+00	3308	7,02190E-01	45172	0	11208,5
Responder	cDC(CD1C)	pDC_LILRA4	HLA-DQB1	CD4	1,77205E-04	5183,7	8,88556E-03	10421	5,67291E-01	3311	2,65630E+00	157	9,34821E-01	821	0	11208,5
Responder	Mono_CD14	Macro_LYVE1	CXCL8	SDC3	1,77482E-04	4779,1	3,41696E-02	976	9,49725E-01	702	1,92062E+00	839	8,81589E-01	10170	0	11208,5
Responder	cDC(CD1C)	Macro_ISG15	HLA-DQA1	LAG3	1,79118E-04	6521,1	1,71215E-02	3320	6,15042E-01	2661	1,52746E+00	1820	8,63707E-01	13596	0	11208,5
Responder	Macro_OLFML3	pDC_LILRA4	HLA-DRA	CD4	1,79332E-04	5671,3	7,83080E-03	12896	5,67001E-01	3321	1,95369E+00	784	9,45438E-01	147	0	11208,5
Responder	Macro_NLRP3	pDC_LILRA4	TNF	TNFRSF21	1,79656E-04	7549,7	3,52358E-02	924	1,14557E+00	338	1,84394E+00	980	8,12517E-01	24298	0	11208,5
Responder	Macro_FOLR2-APOE+	Macro_NLRP3	MMP9	CD44	1,80248E-04	5286,5	2,14829E-02	2274	8,32385E-01	1089	1,16276E+00	3656	8,92349E-01	8205	0	11208,5
Responder	Macro_FOLR2+APOE+	pDC_LILRA4	MMP9	TLR9	1,80920E-04	9724,3	1,19604E-01	58	1,49849E+00	121	1,62944E+00	1481	7,54204E-01	35753	0	11208,5
Responder	Macro_OLFML3	Macro_IER3	C3	LRP1	1,81128E-04	6288,5	1,68189E-02	3432	5,62278E-01	3375	9,89367E-01	5214	8,92284E-01	8213	0	11208,5
Responder	Mono_INHBA	Mast	HSPA8	ADRB2	1,81239E-04	6455,1	2,28892E-02	2044	5,43306E-01	3645	8,27000E-01	7164	8,92276E-01	8214	0	11208,5
Responder	Macro_OLFML3	Macro_FOLR2-APOE+	C3	ITGAX	1,82234E-04	6684,1	1,50660E-02	4220	5,64728E-01	3344	8,84247E-01	6425	8,92232E-01	8223	0	11208,5
Responder	Macro_OLFML3	Macro_IER3	C3	NRP1	1,83425E-04	6972,5	2,23058E-02	2132	6,92163E-01	1918	1,21683E+00	3340	8,50888E-01	16264	0	11208,5
Responder	cDC(CD1C)	Macro_LYVE1	HLA-DQA1	CD4	1,84296E-04	5178,7	9,60093E-03	9115	6,18027E-01	2631	1,40358E+00	2329	9,36854E-01	610	0	11208,5
Responder	cDC(CD1C)	Macro_LYVE1	HLA-DQB1	CD4	1,84732E-04	5384,9	9,31771E-03	9622	5,99635E-01	2838	1,34958E+00	2592	9,36253E-01	664	0	11208,5
Responder	Mono_INHBA	Macro_OLFML3	TIMP1	CD63	1,84797E-04	4984,1	1,01767E-02	8246	7,90160E-01	1301	1,10553E+00	4125	9,51387E-01	40	0	11208,5
Responder	Macro_FOLR2-APOE+	Mast	SPP1	CD44	1,85133E-04	4612,5	1,82498E-02	2969	7,64419E-01	1440	1,16431E+00	3650	9,15961E-01	3795	0	11208,5
Responder	cDC(CD1C)	Macro_LYVE1	HLA-DPB1	CD4	1,85169E-04	5435,1	8,64555E-03	10919	6,16681E-01	2647	1,44031E+00	2167	9,43130E-01	234	0	11208,5
Responder	Macro_FOLR2-APOE+	Mast	MMP9	CD44	1,86484E-04	4645,1	2,45657E-02	1793	9,42474E-01	727	1,37573E+00	2468	8,98622E-01	7029	0	11208,5
Responder	Macro_OLFML3	Mono_CD14	APOE	LDLR	1,86705E-04	5525,7	2,94111E-02	1284	7,01493E-01	1840	1,21406E+00	3355	8,82890E-01	9941	0	11208,5
Responder	Mast	Macro_NLRP3	CSF1	SIRPA	1,88528E-04	7346,1	4,71517E-02	533	1,00173E+00	576	1,83239E+00	996	8,16716E-01	23417	0	11208,5
Responder	Mono_INHBA	cDC_CLEC9A	LYZ	ITGAL	1,88980E-04	8197,1	1,06760E-02	7601	4,10661E-01	6646	8,20635E-01	7247	8,91965E-01	8283	0	11208,5
Responder	Macro_OLFML3	Mono_CD16	HLA-A	LILRA1	1,89322E-04	5703,3	2,12083E-02	2318	7,64877E-01	1438	7,47976E-01	8286	9,07982E-01	5266	0	11208,5
Responder	Macro_OLFML3	Macro_OLFML3	APOE	SORL1	1,89584E-04	6093,9	1,69593E-02	3368	5,94607E-01	2903	6,02083E-01	10739	9,24475E-01	2251	0	11208,5
Responder	Mono_INHBA	Mono_CD14	S100A9	CD36	1,91157E-04	7023,7	1,46809E-02	4418	5,92043E-01	2938	7,46716E-01	8302	8,92134E-01	8252	0	11208,5
Responder	pDC_LILRA4	Macro_FOLR2+APOE+	APP	CD74	1,91359E-04	3302,5	1,64309E-02	3588	9,72536E-01	642	1,82980E+00	1001	9,49079E-01	73	0	11208,5
Responder	Macro_OLFML3	Mono_CD16	HLA-C	LILRA3	1,93820E-04	5042,3	3,55777E-02	909	9,91236E-01	597	7,45411E-01	8325	9,13871E-01	4172	0	11208,5
Responder	Macro_OLFML3	Macro_LYVE1	A2M	LRP1	1,94870E-04	6186,1	2,17851E-02	2224	7,61480E-01	1469	7,88582E-01	7695	8,91708E-01	8334	0	11208,5
Responder	Macro_OLFML3	pDC_LILRA4	MAML2	NOTCH4	1,95211E-04	15004,9	2,83135E-02	1386	5,60539E-01	3393	1,40470E+00	2318	6,11284E-01	56719	0	11208,5
Responder	pDC_LILRA4	Macro_ISG15	SPN	SIGLEC1	1,95369E-04	9025,5	3,75232E-02	829	8,54103E-01	1008	1,96487E+00	768	7,77281E-01	31314	0	11208,5
Responder	Macro_OLFML3	Macro_FOLR2-APOE+	C1QB	LRP1	1,96277E-04	6396,5	1,13577E-02	6843	5,62920E-01	3370	7,44134E-01	8346	9,24660E-01	2215	0	11208,5
Responder	Macro_OLFML3	Mono_INHBA	C3	CD81	1,97692E-04	7074,3	1,30256E-02	5442	4,68448E-01	5084	9,81805E-01	5279	8,91590E-01	8358	0	11208,5
Responder	pDC_LILRA4	Macro_LYVE1	RARRES2	CMKLR1	1,98850E-04	11753,9	5,39450E-02	386	8,53249E-01	1014	2,23087E+00	429	6,98614E-01	45732	0	11208,5
Responder	Macro_FOLR2+APOE+	pDC_LILRA4	CXCL12	CXCR3	1,99434E-04	11145,1	4,51161E-02	582	1,12946E+00	352	1,82161E+00	1015	7,16972E-01	42568	0	11208,5
Responder	Mono_CD14	Mono_CD16	HLA-C	LILRA3	1,99830E-04	5951,5	2,81144E-02	1406	6,04893E-01	2783	7,42569E-01	8376	9,04143E-01	5984	0	11208,5
Responder	Mono_CD14	Mono_INHBA	S100A8	TLR4	2,00188E-04	4442,9	2,22798E-02	2135	1,18073E+00	293	2,55718E+00	199	8,91513E-01	8379	0	11208,5
Responder	Macro_FOLR2+APOE-	pDC_LILRA4	CXCL9	CXCR3	2,01782E-04	11447,5	3,32540E-02	1019	1,04138E+00	488	1,96178E+00	773	7,73500E-01	32031	0,001	22926,5
Responder	pDC_LILRA4	Macro_OLFML3	SCT	ADRB2	2,02372E-04	12742,5	5,11287E-02	441	1,72421E+00	74	1,82101E+00	1020	6,63516E-01	50969	0	11208,5
Responder	pDC_LILRA4	Macro_FOLR2+APOE+	LTB	TNFRSF1A	2,02372E-04	5347,5	3,32444E-02	1020	1,11594E+00	369	2,40135E+00	303	8,62422E-01	13837	0	11208,5
Responder	Macro_OLFML3	Macro_LYVE1	HLA-DQA2	CD4	2,03313E-04	6736,9	1,14459E-02	6766	5,44294E-01	3630	7,40713E-01	8405	9,16610E-01	3675	0	11208,5
Responder	Mono_CD14	Macro_FOLR2+APOE-	S100A9	CD36	2,03677E-04	5043,3	1,44394E-02	4558	1,05425E+00	460	2,10463E+00	582	8,91333E-01	8408	0	11208,5

Responder	cDC_CLEC9A	Mast	LGALS1	CD69	2,03767E-04	3817,5	1,68220E-02	3430	8,23249E-01	1135	1,74032E+00	1198	9,25185E-01	2116	0	11208,5
Responder	pDC_LILRA4	Macro_FOLR2-APOE+	SPON2	ITGA5	2,06532E-04	11039,9	5,46459E-02	377	1,01529E+00	544	1,81683E+00	1027	7,19924E-01	42043	0	11208,5
Responder	Mono_CD14	Macro_FOLR2-APOE+	NAMPT	ITGA5_ITGB1	2,07336E-04	5721,7	1,27289E-02	5647	7,96152E-01	1270	1,46742E+00	2045	8,91190E-01	8438	0	11208,5
Responder	Macro_FOLR2+APOE+	Macro_IFI27	APOE	TREM2	2,09464E-04	3537,1	2,51325E-02	1724	8,46485E-01	1030	1,19625E+00	3454	9,42321E-01	269	0	11208,5
Responder	Macro_FOLR2-APOE+	Macro_NLRP3	FN1	C5AR1	2,11422E-04	6676,7	2,20329E-02	2179	5,75110E-01	3180	7,44140E-01	8345	8,91016E-01	8471	0	11208,5
Responder	Mast	Macro_NLRP3	TIMP3	CD44	2,12574E-04	3523,1	7,25186E-02	203	1,26734E+00	211	1,81226E+00	1037	9,09660E-01	4956	0	11208,5
Responder	Mast	Macro_FOLR2-APOE+	CSF1	CSF2RA	2,14408E-04	5613,9	5,19197E-02	428	1,01445E+00	546	1,81151E+00	1040	8,57544E-01	14847	0	11208,5
Responder	Mono_CD14	Macro_FOLR2+APOE+	TIMP1	CD63	2,15193E-04	4929,1	9,98930E-03	8501	8,49602E-01	1020	1,13646E+00	3869	9,50956E-01	47	0	11208,5
Responder	Macro_ISG15	Mono_INHBA	CXCL10	SDC4	2,15637E-04	6788,3	6,23447E-02	282	9,25383E-01	771	1,80906E+00	1042	8,30137E-01	20638	0	11208,5
Responder	Macro_FOLR2-APOE+	cDC_CLEC9A	C1QB	C1QBP	2,16971E-04	7217,7	1,09194E-02	7312	4,40295E-01	5811	7,33205E-01	8515	9,19091E-01	3242	0	11208,5
Responder	cDC(CD1C)	Macro_LYVE1	HLA-DRA	CD4	2,17244E-04	6064,5	7,96508E-03	12525	5,54781E-01	3486	1,28029E+00	2970	9,45875E-01	133	0	11208,5
Responder	Mono_INHBA	Macro_ISG15	INHBA	ENG	2,18109E-04	8104,1	3,50026E-02	935	9,10131E-01	813	1,80714E+00	1046	8,01552E-01	26518	0	11208,5
Responder	Macro_OLFML3	Mast	C3	CD81	2,18120E-04	7300,9	1,28050E-02	5588	4,62339E-01	5235	9,22409E-01	5949	8,90762E-01	8524	0	11208,5
Responder	Macro_FOLR2+APOE-	pDC_LILRA4	CD14	TLR9	2,18502E-04	5023,1	8,70350E-02	133	1,47782E+00	125	1,56729E+00	1685	8,72359E-01	11964	0	11208,5
Responder	pDC_LILRA4	Macro_IER3	FAM3D	FPR1	2,19353E-04	15275,5	3,97738E-02	740	8,43893E-01	1039	1,80647E+00	1048	7,20384E-01	41949	0,159	31601,5
Responder	Macro_OLFML3	Macro_IER3	FN1	CD44	2,19917E-04	6440,1	1,74196E-02	3221	4,84433E-01	4722	7,31923E-01	8538	9,12112E-01	4511	0	11208,5
Responder	Macro_ISG15	Mono_CD14	CCL8	CCR1	2,20600E-04	9489,1	5,19282E-02	427	8,42135E-01	1050	2,09394E+00	593	7,62904E-01	34167	0	11208,5
Responder	cDC(CD1C)	Macro_ISG15	HLA-DQB1	LAG3	2,21713E-04	6685,1	1,66164E-02	3504	5,96650E-01	2879	1,47346E+00	2019	8,62511E-01	13815	0	11208,5
Responder	cDC(CD1C)	Mono_CD16	HLA-B	LILRB2	2,23218E-04	5418,5	9,54506E-03	9211	5,74384E-01	3202	1,21851E+00	3333	9,45686E-01	138	0	11208,5
Responder	Mono_INHBA	Macro_FOLR2-APOE+	VEGFA	CD44	2,27092E-04	6690,7	1,52532E-02	4124	6,80971E-01	2008	8,01728E-01	7520	8,90339E-01	8593	0	11208,5
Responder	Macro_FOLR2-APOE+	pDC_LILRA4	APP	RPSA	2,27224E-04	5259,3	1,35825E-02	5077	8,49467E-01	1021	2,27103E+00	396	8,90338E-01	8594	0	11208,5
Responder	pDC_LILRA4	Macro_OLFML3	LTB	TNFRSF1A	2,28185E-04	5414,9	3,24082E-02	1062	1,10386E+00	386	2,43125E+00	278	8,60904E-01	14140	0	11208,5
Responder	cDC(CD1C)	Mono_CD16	CD1D	LILRB2	2,29313E-04	6214,3	2,61849E-02	1607	8,30456E-01	1099	1,44753E+00	2126	8,56649E-01	15031	0	11208,5
Responder	pDC_LILRA4	Macro_FOLR2+APOE+	LTB	CD40	2,29466E-04	5888,1	3,23937E-02	1064	1,09722E+00	394	2,44369E+00	269	8,49703E-01	16505	0	11208,5
Responder	Macro_IFI27	Mast	LGALS1	CD69	2,30084E-04	3941,3	1,65394E-02	3537	8,11889E-01	1199	1,60953E+00	1536	9,24596E-01	2226	0	11208,5
Responder	Macro_ISG15	Macro_OLFML3	APOE	TREM2	2,30281E-04	6105,9	1,49733E-02	4266	3,89347E-01	7316	9,31571E-01	5845	9,26526E-01	1894	0	11208,5
Responder	Macro_OLFML3	Macro_FOLR2+APOE-	APOE	LRP1	2,31218E-04	7304,7	1,08450E-02	7410	4,57615E-01	5355	7,26059E-01	8624	9,15288E-01	3926	0	11208,5
Responder	Macro_OLFML3	Macro_ISG15	CXCL9	FCGR2A	2,31352E-04	7304,7	1,35014E-02	5131	3,87111E-01	7388	1,10011E+00	4171	8,90165E-01	8625	0	11208,5
Responder	pDC_LILRA4	Macro_NLRP3	SPON2	ITGB1	2,31395E-04	8698,7	3,80715E-02	803	9,51894E-01	696	1,79485E+00	1067	7,85222E-01	29719	0	11208,5
Responder	Macro_FOLR2-APOE+	pDC_LILRA4	CXCL12	CXCR3	2,32041E-04	12243,9	3,39345E-02	991	1,07814E+00	430	1,79451E+00	1068	6,87206E-01	47522	0	11208,5
Responder	Mono_CD14	Macro_FOLR2+APOE+	S100A8	TLR4	2,32158E-04	4558,3	2,16618E-02	2243	1,17348E+00	299	2,24861E+00	410	8,90145E-01	8631	0	11208,5
Responder	Mono_CD14	cDC_LAMP3	NAMPT	ADORA2A	2,32688E-04	5754,7	4,80190E-02	522	1,14923E+00	334	1,79443E+00	1069	8,53722E-01	15640	0	11208,5
Responder	Macro_OLFML3	pDC_LILRA4	HLA-DRB1	CD4	2,33185E-04	5822,3	7,75544E-03	13123	5,50904E-01	3549	1,83966E+00	983	9,42723E-01	248	0	11208,5
Responder	pDC_LILRA4	Mono_CD16	SPON2	ITGB1	2,33336E-04	8120,9	4,43795E-02	606	1,03218E+00	506	1,79397E+00	1070	7,97867E-01	27214	0	11208,5
Responder	Mono_INHBA	Macro_LYVE1	TIMP1	CD63	2,33911E-04	5268,1	9,88820E-03	8644	7,58929E-01	1483	1,01416E+00	4956	9,50718E-01	49	0	11208,5
Responder	pDC_LILRA4	Macro_IER3	APP	CD74	2,33985E-04	3402,3	1,58225E-02	3852	9,17290E-01	791	1,79372E+00	1071	9,48160E-01	89	0	11208,5
Responder	cDC(CD1C)	Mono_CD16	HLA-C	LILRA1	2,34996E-04	7348,9	1,67269E-02	3460	3,51909E-01	8652	9,09737E-01	6104	8,97087E-01	7320	0	11208,5
Responder	Macro_NLRP3	Macro_LYVE1	S100A9	CD36	2,35532E-04	4018,5	2,74358E-02	1459	1,00668E+00	565	1,17942E+00	3558	9,18742E-01	3302	0	11208,5
Responder	Macro_OLFML3	Mono_CD16	FN1	PLAUR	2,35793E-04	5930,1	2,00248E-02	2530	5,49888E-01	3559	6,81686E-01	9342	9,20330E-01	3011	0	11208,5
Responder	Macro_ISG15	Macro_ISG15	CCL8	CCR2	2,37249E-04	12683,5	7,91019E-02	177	8,73615E-01	931	1,78935E+00	1076	6,70328E-01	50025	0	11208,5
Responder	pDC_LILRA4	pDC_LILRA4	TNFSF9	TRAF2	2,37249E-04	13667,3	4,45242E-02	603	8,35996E-01	1076	2,63018E+00	166	6,26162E-01	55283	0	11208,5
Responder	Mono_CD14	pDC_LILRA4	CD14	TLR9	2,37906E-04	5341,1	7,19951E-02	205	1,33976E+00	172	1,78913E+00	1077	8,61418E-01	14043	0	11208,5
Responder	cDC(CD1C)	Macro_LYVE1	HLA-DPA1	CD4	2,39481E-04	6002,3	8,21144E-03	11927	5,48039E-01	3573	1,26242E+00	3074	9,43160E-01	229	0	11208,5
Responder	Macro_OLFML3	pDC_LILRA4	HMGB1	THBD	2,40202E-04	6556,3	1,19442E-02	6306	5,13556E-01	4149	1,38536E+00	2428	8,89795E-01	8690	0	11208,5
Responder	Macro_OLFML3	cDC_CLEC9A	C3	CD46	2,40277E-04	6404,5	1,69436E-02	3378	5,47859E-01	3576	1,30373E+00	2827	8,76991E-01	11033	0	11208,5
Responder	Mono_CD14	Macro_NLRP3	GNAI2	C5AR1	2,40755E-04	7045,3	9,85167E-03	8694	5,46297E-01	3598	1,02808E+00	4824	8,99353E-01	6902	0	11208,5
Responder	Macro_OLFML3	Macro_FOLR2-APOE+	FN1	ITGAV_ITGB8	2,41341E-04	7405,9	4,17327E-02	680	6,65716E-01	2138	1,17520E+00	3580	8,35962E-01	19423	0	11208,5
Responder	Macro_FOLR2+APOE+	Macro_FOLR2+APOE+	APOE	TREM2	2,41608E-04	3334,7	3,10178E-02	1147	9,71651E-01	644	1,17506E+00	3581	9,47780E-01	93	0	11208,5
Responder	Macro_FOLR2-APOE+	pDC_LILRA4	CD14	TLR9	2,42054E-04	5114,1	8,49879E-02	140	1,45903E+00	134	1,50427E+00	1901	8,71028E-01	12187	0	11208,5
Responder	Macro_OLFML3	pDC_LILRA4	PSEN1	NOTCH4	2,42142E-04	14764,5	2,62595E-02	1597	5,47495E-01	3583	1,40467E+00	2319	6,27812E-01	55115	0	11208,5
Responder	pDC_LILRA4	Macro_NLRP3	APP	FPR2	2,43867E-04	8656,9	3,20183E-02	1086	1,02706E+00	518	2,40584E+00	297	7,82958E-01	30175	0	11208,5
Responder	pDC_LILRA4	Macro_FOLR2-APOE+	LTB	TNFRSF1A	2,44536E-04	5440,7	3,20142E-02	1087	1,09817E+00	393	2,52686E+00	214	8,60170E-01	14301	0	11208,5
Responder	Macro_FOLR2+APOE+	Macro_NLRP3	APOE	LRP1	2,44555E-04	3994,5	1,79118E-02	3074	8,39171E-01	1068	1,17328E+00	3592	9,32822E-01	1030	0	11208,5
Responder	cDC(CD1C)	cDC_CLEC9A	COPA	P2RY6	2,44824E-04	9592,9	1,64153E-02	3593	6,02345E-01	2811	1,51241E+00	1867	7,91549E-01	28485	0	11208,5
Responder	Macro_ISG15	cDC(CD1C)	SPP1	CD44	2,45922E-04	7589,7	1,02582E-02	8132	3,75647E-01	7781	1,40115E+00	2345	8,90967E-01	8482	0	11208,5
Responder	pDC_LILRA4	cDC(CD1C)	APP	RPSA	2,46715E-04	3361,9	2,51545E-02	1718	1,22466E+00	253	3,45980E+00	30	9,17004E-01	3600	0	11208,5
Responder	Macro_LYVE1	pDC_LILRA4	GNAI2	CXCR3	2,48572E-04	5467,3	3,99256E-02	735	1,25042E+00	227	1,78175E+00	1093	8,61224E-01	14073	0	11208,5
Responder	cDC(CD1C)	Mast	POMC	ADRB2	2,48889E-04	12067,5	4,11274E-02	703	7,69470E-01	1404	1,33681E+00	2660	7,06787E-01	44362	0	11208,5
Responder	cDC(CD1C)	Mast	IL1B	ADRB2	2,49161E-04	5496,7	3,24672E-02	1059	7,98459E-01	1258	1,62802E+00	1485	8,69209E-01	12473	0	11208,5
Responder	Macro_FOLR2+APOE+	Mono_CD14	C1QA	CD93	2,50255E-04	4471,3	1,63620E-02	3613	7,65239E-01	1435	1,33716E+00	2654	9,17830E-01	3446	0	11208,5
Responder	cDC(CD1C)	Mast	IL16	CD9	2,50803E-04	7564,9	1,71381E-02	3315	5,63951E-01	3357	1,21440E+00	3353	8,49288E-01	16591	0	11208,5
Responder	cDC(CD1C)	Mast	GNAS	ADRB2	2,51626E-04	5441,5	2,98746E-02	1241	8,06340E-01	1222	1,63727E+00	1459	8,71677E-01	12077	0	11208,5
Responder	cDC(CD1C)	Mast	ARPC5	ADRB2	2,53279E-04	4737,1	3,08312E-02	1157	8,15774E-01	1179	1,68634E+00	1319	8,89074E-01	8822	0	11208,5
Responder	Mono_CD14	Macro_ISG15	S100A9	CD68	2,53766E-04	4505,5	9,79366E-03	8786	8,08204E-01	1212	1,87943E+00	904	9,39434E-01	417	0	11208,5
Responder	Macro_OLFML3	Mono_INHBA	C3	C3AR1	2,54779E-04	6737,9	1,54345E-02	4030	4,70307E-01	5043	1,05194E+00	4615	8,89221E-01	8793	0	11208,5

Responder	cDC(CD1C)	Macro_ISG15	HLA-DQB2	LAG3	2,55218E-04	10040,9	2,67349E-02	1538	5,44269E-01	3631	1,59966E+00	1572	7,72380E-01	32255	0	11208,5
Responder	Mono_CD14	Macro_FOLR2-APOE+	S100A9	TLR4	2,55649E-04	4944,3	1,67896E-02	3446	1,04450E+00	478	1,94799E+00	790	8,89191E-01	8799	0	11208,5
Responder	pDC_LILRA4	pDC_LILRA4	APP	CD74	2,56051E-04	3494,1	1,42390E-02	4681	7,73521E-01	1382	3,08177E+00	55	9,45506E-01	144	0	11208,5
Responder	cDC(CD1C)	Mast	ACTR2	ADRB2	2,56610E-04	5514,3	3,26163E-02	1049	8,00736E-01	1246	1,64800E+00	1428	8,68394E-01	12640	0	11208,5
Responder	Macro_ISG15	pDC_LILRA4	CXCL11	DPP4	2,56776E-04	12892,3	1,71839E-01	29	9,73180E-01	641	1,77714E+00	1105	6,59312E-01	51478	0	11208,5
Responder	cDC(CD1C)	Macro_LYVE1	SERPINA1	LRP1	2,57543E-04	7895,7	9,78016E-03	8812	3,92177E-01	7230	8,13071E-01	7352	9,10066E-01	4876	0	11208,5
Responder	Macro_OLFML3	Macro_OLFML3	C1QB	CD33	2,58128E-04	6337,3	1,47306E-02	4388	5,80299E-01	3090	7,13385E-01	8816	9,13826E-01	4184	0	11208,5
Responder	Macro_ISG15	Mono_INHBA	SPP1	CD44	2,59302E-04	4129,5	1,81220E-02	3007	7,84078E-01	1330	1,71167E+00	1258	9,15690E-01	3844	0	11208,5
Responder	Macro_FOLR2-APOE+	Mono_INHBA	TIMP2	CD44	2,59302E-04	8070,1	9,85846E-03	8685	5,81115E-01	3082	7,12760E-01	8824	8,90580E-01	8551	0	11208,5
Responder	pDC_LILRA4	cDC(CD1C)	LTB	TNFRSF1A	2,60246E-04	5444,3	3,16436E-02	1110	1,09281E+00	403	3,09373E+00	54	8,59468E-01	14446	0	11208,5
Responder	Macro_FOLR2-APOE+	Mono_CD14	FN1	CD44	2,61217E-04	8194,7	1,29032E-02	5520	3,56430E-01	8503	7,11580E-01	8837	8,99315E-01	6905	0	11208,5
Responder	Mast	cDC_LAMP3	CSF1	SIRPA	2,61643E-04	7815,9	4,15702E-02	685	9,68401E-01	650	1,77404E+00	1112	8,07098E-01	25424	0	11208,5
Responder	pDC_LILRA4	Mono_CD16	APP	PTGER2	2,63044E-04	8908,1	3,16049E-02	1114	1,02937E+00	512	2,30011E+00	376	7,77196E-01	31330	0	11208,5
Responder	Mono_INHBA	Macro_ISG15	CCL3	CCR1	2,63442E-04	4813,7	2,28179E-02	2057	9,17749E-01	789	1,75423E+00	1162	8,88987E-01	8852	0	11208,5
Responder	Mono_INHBA	Macro_FOLR2-APOE+	TIMP1	CD63	2,65083E-04	5182,7	9,75365E-03	8863	7,44361E-01	1553	1,09133E+00	4235	9,50396E-01	54	0	11208,5
Responder	Macro_OLFML3	Macro_LYVE1	HLA-DQB1	CD4	2,65532E-04	6194,1	9,75201E-03	8866	6,53155E-01	2282	7,62122E-01	8069	9,37599E-01	545	0	11208,5
Responder	cDC(CD1C)	Mono_CD16	B2M	LILRB2	2,65651E-04	5528,9	9,50186E-03	9283	5,57021E-01	3450	1,16132E+00	3668	9,52024E-01	35	0	11208,5
Responder	Mono_CD14	Macro_NLRP3	VCAN	TLR2	2,66281E-04	4667,1	2,81705E-02	1399	9,22346E-01	778	1,78828E+00	1079	8,88915E-01	8871	0	11208,5
Responder	Mono_INHBA	Mast	ADM	ADRB2	2,67989E-04	6810,3	7,73904E-02	186	1,04632E+00	473	1,77081E+00	1121	8,27929E-01	21063	0	11208,5
Responder	Macro_FOLR2-APOE+	Mast	APOE	ABCA1	2,68540E-04	6614,1	1,37153E-02	5008	4,94189E-01	4520	7,08693E-01	8886	9,17804E-01	3448	0	11208,5
Responder	Mono_CD16	pDC_LILRA4	GNAI2	CXCR3	2,69413E-04	5677,5	3,72903E-02	836	1,20129E+00	276	1,76989E+00	1123	8,57093E-01	14944	0	11208,5
Responder	Mono_CD14	Macro_FOLR2+APOE+	S100A8	CD69	2,69751E-04	5495,3	1,42377E-02	4682	1,10647E+00	381	1,40706E+00	2311	8,88835E-01	8894	0	11208,5
Responder	Macro_FOLR2+APOE+	Macro_IER3	APOE	ABCA1	2,69970E-04	3770,1	2,15930E-02	2255	9,42017E-01	728	1,15930E+00	3683	9,33380E-01	976	0	11208,5
Responder	Mono_CD14	Mono_CD16	VCAN	ITGA4	2,70662E-04	4746,9	2,62870E-02	1589	9,85814E-01	612	1,64820E+00	1425	8,88801E-01	8900	0	11208,5
Responder	cDC(CD1C)	cDC_CLEC9A	CALR	SCARF1	2,72003E-04	8107,3	2,23279E-02	2128	5,40283E-01	3690	1,37289E+00	2485	8,28132E-01	21025	0	11208,5
Responder	Mono_INHBA	Macro_FOLR2-APOE+	SPP1	CD44	2,72339E-04	8741,1	9,80085E-03	8775	3,75892E-01	7772	8,37194E-01	7039	8,88732E-01	8911	0	11208,5
Responder	Macro_ISG15	pDC_LILRA4	GNAI2	CXCR3	2,74437E-04	6145,7	3,14310E-02	1130	1,09204E+00	404	1,94672E+00	794	8,46302E-01	17192	0	11208,5
Responder	cDC(CD1C)	Mono_CD16	HLA-F	LILRB2	2,74485E-04	7168,9	9,98464E-03	8513	5,93523E-01	2920	1,08646E+00	4278	8,88646E-01	8925	0	11208,5
Responder	Macro_ISG15	Mono_CD14	S100A9	CD36	2,74793E-04	6189,3	1,43232E-02	4637	5,77702E-01	3144	1,19366E+00	3468	8,90941E-01	8489	0	11208,5
Responder	Mast	Macro_IER3	CSF1	SIRPA	2,75883E-04	6957,5	5,31591E-02	401	1,03761E+00	497	1,76682E+00	1132	8,25522E-01	21549	0	11208,5
Responder	Macro_ISG15	Mono_CD14	SPP1	CD44	2,76026E-04	4962,7	1,49126E-02	4299	6,17389E-01	2640	1,66694E+00	1373	9,07855E-01	5293	0	11208,5
Responder	cDC(CD1C)	Macro_NLRP3	ANXA1	FPR1	2,76026E-04	7570,7	9,71298E-03	8935	4,34069E-01	5981	9,54010E-01	5581	9,03330E-01	6148	0	11208,5
Responder	pDC_LILRA4	Macro_FOLR2-APOE+	FAM3D	FPR1	2,76609E-04	15772,3	3,68111E-02	856	8,23446E-01	1133	1,81188E+00	1038	7,12522E-01	43373	0,209	32461,5
Responder	Macro_OLFML3	Macro_NLRP3	C3	CR1	2,77579E-04	8596,7	2,45183E-02	1800	6,51492E-01	2295	1,15566E+00	3709	8,14036E-01	23971	0	11208,5
Responder	Macro_ISG15	Mono_CD14	RPS19	C5AR1	2,78818E-04	4764,5	1,11807E-02	7012	7,03729E-01	1820	1,39413E+00	2380	9,29725E-01	1402	0	11208,5
Responder	Macro_OLFML3	Macro_IER3	C3	ITGAX	2,79909E-04	6950,3	1,39395E-02	4870	5,38188E-01	3724	9,18012E-01	5989	8,88439E-01	8960	0	11208,5
Responder	cDC(CD1C)	Macro_FOLR2+APOE+	HLA-DRA	LAG3	2,79952E-04	5990,1	1,61208E-02	3709	6,04719E-01	2786	1,22877E+00	3268	8,88294E-01	8979	0	11208,5
Responder	Macro_OLFML3	cDC_CLEC9A	C1QA	CD93	2,80222E-04	7224,7	9,69666E-03	8962	4,84626E-01	4718	1,16261E+00	3659	8,95822E-01	7576	0	11208,5
Responder	cDC(CD1C)	Macro_FOLR2+APOE+	HLA-DQA1	LAG3	2,81144E-04	6149,9	1,94317E-02	2644	6,67965E-01	2124	1,35205E+00	2578	8,70986E-01	12195	0	11208,5
Responder	Macro_IER3	pDC_LILRA4	CD14	TLR9	2,81488E-04	5333,3	8,31258E-02	151	1,44193E+00	139	1,31366E+00	2778	8,69778E-01	12390	0	11208,5
Responder	pDC_LILRA4	Macro_LYVE1	FAM3D	FPR1	2,81720E-04	15686,3	3,67191E-02	860	8,22812E-01	1140	1,76748E+00	1130	7,12265E-01	43420	0,175	31881,5
Responder	cDC(CD1C)	Macro_FOLR2+APOE+	HLA-DQB1	LAG3	2,82340E-04	6312,3	1,88585E-02	2797	6,49573E-01	2309	1,29806E+00	2867	8,69845E-01	12380	0	11208,5
Responder	Mono_INHBA	Mono_INHBA	INHBA	ENG	2,82455E-04	8509,3	3,11047E-02	1141	8,74503E-01	925	1,89265E+00	882	7,91994E-01	28390	0	11208,5
Responder	pDC_LILRA4	Mono_CD16	CD24	SIGLEC10	2,83192E-04	11335,7	5,70843E-02	342	8,22453E-01	1142	1,94572E+00	795	7,13547E-01	43191	0	11208,5
Responder	cDC(CD1C)	Macro_FOLR2+APOE+	HLA-DPB1	LAG3	2,83240E-04	5774,3	1,74981E-02	3195	6,66619E-01	2133	1,38878E+00	2414	8,82991E-01	9921	0	11208,5
Responder	Macro_OLFML3	pDC_LILRA4	HLA-DMA	CD4	2,84141E-04	5723,7	8,88088E-03	10429	5,37785E-01	3731	1,71968E+00	1245	9,25834E-01	2005	0	11208,5
Responder	Mono_CD14	Macro_ISG15	AGTRAP	RACK1	2,84944E-04	8357,5	9,68183E-03	8992	4,48568E-01	5585	8,13603E-01	7343	8,89977E-01	8659	0	11208,5
Responder	pDC_LILRA4	Macro_FOLR2-APOE+	SPON2	ITGB1	2,86894E-04	8413,1	4,12471E-02	699	9,92312E-01	596	1,76120E+00	1147	7,91900E-01	28415	0	11208,5
Responder	Macro_OLFML3	Macro_FOLR2-APOE+	APOE	LRP1	2,87009E-04	6311,5	1,32988E-02	5245	5,48750E-01	3569	7,02821E-01	9005	9,22868E-01	2530	0	11208,5
Responder	Mono_CD14	Mast	HBEGF	CD9	2,88127E-04	5247,5	2,16022E-02	2253	7,14412E-01	1740	1,47167E+00	2024	8,88134E-01	9012	0	11208,5
Responder	cDC(CD1C)	Macro_FOLR2+APOE+	HLA-DPA1	LAG3	2,88377E-04	6170,5	1,66195E-02	3503	5,97977E-01	2860	1,21090E+00	3372	8,83049E-01	9909	0	11208,5
Responder	Macro_OLFML3	Mono_CD16	HLA-C	LILRB2	2,88607E-04	5575,7	1,16643E-02	6554	8,55722E-01	998	7,02021E-01	9015	9,47280E-01	103	0	11208,5
Responder	Mono_CD14	pDC_LILRA4	TGFB1	CXCR4	2,89087E-04	6572,3	9,66382E-03	9018	5,49526E-01	3563	2,52051E+00	219	8,88983E-01	8853	0	11208,5
Responder	Macro_LYVE1	pDC_LILRA4	TNF	PTPRS	2,90627E-04	9903,5	4,86870E-02	502	1,50041E+00	119	1,76042E+00	1152	7,49923E-01	36536	0	11208,5
Responder	Macro_OLFML3	pDC_LILRA4	HLA-DMB	CD4	2,92145E-04	5949,3	9,65295E-03	9037	5,20321E-01	4040	1,72935E+00	1227	9,13558E-01	4234	0	11208,5
Responder	cDC(CD1C)	Macro_FOLR2+APOE+	HLA-DQB2	LAG3	2,93274E-04	9524,7	3,03422E-02	1197	5,97192E-01	2869	1,42426E+00	2239	7,83314E-01	30110	0	11208,5
Responder	Macro_ISG15	Macro_FOLR2-APOE+	SPP1	CD44	2,93279E-04	6452,9	1,26500E-02	5707	4,99873E-01	4405	1,08443E+00	4298	9,00738E-01	6646	0	11208,5
Responder	Mono_CD14	Macro_OLFML3	S100A9	TLR4	2,94090E-04	5083,9	1,63627E-02	3612	1,03741E+00	498	1,80395E+00	1052	8,87916E-01	9049	0	11208,5
Responder	pDC_LILRA4	Macro_IER3	CD24	SIGLEC10	2,94392E-04	11418,5	5,64783E-02	350	8,18410E-01	1157	1,83212E+00	997	7,12455E-01	43380	0	11208,5
Responder	pDC_LILRA4	Macro_ISG15	SPON2	ITGB2	2,95149E-04	6397,7	3,30714E-02	1028	8,22916E-01	1139	1,75526E+00	1158	8,45153E-01	17455	0	11208,5
Responder	Macro_OLFML3	Macro_NLRP3	FN1	ITGA5_ITGB1	2,95746E-04	6747,1	2,23641E-02	2123	5,54979E-01	3478	1,14885E+00	3769	8,65807E-01	13157	0	11208,5
Responder	pDC_LILRA4	Macro_ISG15	SPON2	ITGAM	2,96667E-04	12381,3	3,78083E-02	815	8,77047E-01	913	1,75446E+00	1160	6,85401E-01	47810	0	11208,5
Responder	Macro_OLFML3	Macro_LYVE1	APOE	ABCA1	2,97610E-04	6174,1	1,59799E-02	3775	6,50070E-01	2304	5,78452E-01	11153	9,23387E-01	2430	0	11208,5
Responder	Macro_FOLR2+APOE-	Macro_ISG15	CCL13	CCR1	3,00391E-04	9270,7	8,30265E-02	153	1,37701E+00	156	1,42423E+00	2242	7,70652E-01	32594	0	11208,5
Responder	Macro_OLFML3	Mono_INHBA	SERPING1	LRP1	3,00648E-04	7238,1	1,35954E-02	5070	5,47125E-01	3588	8,21500E-01	7235	8,87718E-01	9089	0	11208,5

Responder	Mono_CD14	Macro_IER3	S100A9	TLR4	3,00979E-04	4995,3	1,62908E-02	3634	1,03621E+00	500	2,13586E+00	543	8,87697E-01	9091	0	11208,5
Responder	Macro_OLFML3	pDC_LILRA4	CXCL11	DPP4	3,01679E-04	15001,3	9,56628E-02	104	7,62475E-01	1456	1,14612E+00	3788	5,90823E-01	58450	0	11208,5
Responder	Macro_ISG15	Mono_INHBA	RPS19	C5AR1	3,01973E-04	6695,3	9,61487E-03	9097	6,07319E-01	2756	7,54003E-01	8196	9,24633E-01	2219	0	11208,5
Responder	Mono_CD14	Mono_INHBA	CXCL8	SDC2	3,02020E-04	6816,3	3,06303E-02	1167	8,54039E-01	1009	1,97300E+00	758	8,33422E-01	19939	0	11208,5
Responder	Macro_FOLR2-APOE+	pDC_LILRA4	GNAI2	CXCR3	3,02789E-04	6048,9	3,31724E-02	1023	1,12451E+00	358	1,75115E+00	1168	8,49776E-01	16487	0	11208,5
Responder	Macro_FOLR2+APOE+	pDC_LILRA4	HRAS	TLR9	3,04245E-04	14655,3	1,11609E-01	70	1,37098E+00	157	1,31198E+00	2785	5,82179E-01	59056	0	11208,5
Responder	pDC_LILRA4	Mono_INHBA	APP	CD74	3,05107E-04	3545,1	1,47144E-02	4399	8,16677E-01	1171	1,93160E+00	822	9,46346E-01	125	0	11208,5
Responder	cDC(CD1C)	Macro_LYVE1	HLA-DMB	CD4	3,07996E-04	6551,3	9,58998E-03	9133	5,03939E-01	4322	1,14426E+00	3805	9,13299E-01	4288	0	11208,5
Responder	cDC(CD1C)	Macro_LYVE1	HLA-DQA2	CD4	3,10533E-04	7118,5	9,58262E-03	9148	4,24697E-01	6226	1,11731E+00	4036	9,09563E-01	4974	0	11208,5
Responder	Mono_CD16	pDC_LILRA4	TNF	PTPRS	3,10559E-04	10467,3	4,21644E-02	667	1,47819E+00	124	1,74903E+00	1178	7,36195E-01	39159	0	11208,5
Responder	Macro_ISG15	Macro_ISG15	SPP1	CD44	3,12234E-04	8161,9	1,00107E-02	8479	3,62796E-01	8263	1,10109E+00	4166	8,89775E-01	8693	0	11208,5
Responder	Macro_OLFML3	cDC(CD1C)	APOE	SORL1	3,12746E-04	8076,5	9,57494E-03	9161	3,60709E-01	8335	9,83324E-01	5265	9,01936E-01	6413	0	11208,5
Responder	Macro_FOLR2+APOE+	Mono_CD14	APOE	LRP1	3,14134E-04	4001,7	1,58803E-02	3827	7,81329E-01	1350	1,45062E+00	2112	9,28950E-01	1511	0	11208,5
Responder	cDC(CD1C)	Mono_CD16	HLA-C	LILRB2	3,14628E-04	5819,5	9,56954E-03	9172	5,24974E-01	3946	1,06224E+00	4491	9,42113E-01	280	0	11208,5
Responder	cDC(CD1C)	Macro_LYVE1	ALCAM	NRP1	3,15758E-04	10731,3	2,04975E-02	2433	6,36251E-01	2434	1,14061E+00	3832	7,64909E-01	33749	0	11208,5
Responder	Macro_FOLR2+APOE-	Macro_ISG15	CCL13	CCR5	3,15953E-04	12771,9	9,95297E-02	94	1,36781E+00	160	1,27126E+00	3019	6,74803E-01	49378	0	11208,5
Responder	Mono_CD14	Macro_FOLR2+APOE-	VCAN	CD44	3,16692E-04	7214,1	9,56374E-03	9184	6,12454E-01	2694	1,03757E+00	4733	8,92139E-01	8251	0	11208,5
Responder	Macro_OLFML3	Mono_INHBA	CXCL10	SDC4	3,16736E-04	9897,3	3,41606E-02	977	5,61622E-01	3379	1,14045E+00	3835	7,83435E-01	30087	0	11208,5
Responder	Macro_OLFML3	Mono_CD16	HLA-B	LILRB2	3,18247E-04	5741,7	1,09645E-02	7260	8,61915E-01	975	6,90927E-01	9193	9,49138E-01	72	0	11208,5
Responder	Macro_NLRP3	Mast	VCAN	CD44	3,19025E-04	4001,9	2,16971E-02	2239	9,51346E-01	697	1,13964E+00	3842	9,25696E-01	2023	0	11208,5
Responder	Macro_FOLR2+APOE-	Mono_CD14	CCL13	CCR2	3,19905E-04	12602,3	1,20393E-01	56	1,36671E+00	161	1,44261E+00	2153	6,74446E-01	49433	0	11208,5
Responder	Mono_INHBA	cDC(CD1C)	INHBA	ACTR2	3,20855E-04	4880,3	3,03949E-02	1191	9,59661E-01	673	2,34923E+00	333	8,77211E-01	10996	0	11208,5
Responder	Mast	Macro_FOLR2+APOE+	CSF1	SIRPA	3,21656E-04	6346,9	6,46591E-02	254	1,10629E+00	382	1,74212E+00	1192	8,39179E-01	18698	0	11208,5
Responder	Mono_INHBA	Mast	ARPC5	ADRB2	3,21724E-04	5353,5	2,95293E-02	1275	7,79736E-01	1359	1,15523E+00	3712	8,86929E-01	9213	0	11208,5
Responder	Macro_OLFML3	Mast	HSPA8	ADRB2	3,21898E-04	5605,3	3,17695E-02	1105	8,41833E-01	1051	6,89684E-01	9214	9,07049E-01	5448	0	11208,5
Responder	Mono_CD14	Macro_NLRP3	NAMPT	ITGA5_ITGB1	3,23824E-04	6066,7	1,16632E-02	6557	7,58686E-01	1486	1,51499E+00	1857	8,86878E-01	9225	0	11208,5
Responder	cDC(CD1C)	Mono_CD16	HLA-A	LILRA1	3,24879E-04	7454,5	1,64391E-02	3584	3,37493E-01	9231	9,31591E-01	5844	8,96773E-01	7405	0	11208,5
Responder	Macro_OLFML3	Macro_LYVE1	C1QB	CD33	3,25407E-04	6938,9	1,31218E-02	5358	5,30648E-01	3848	6,88465E-01	9234	9,09162E-01	5046	0	11208,5
Responder	Macro_OLFML3	Macro_LYVE1	HLA-DQA1	CD4	3,26642E-04	6409,5	9,84297E-03	8712	6,46914E-01	2338	6,87892E-01	9241	9,37587E-01	548	0	11208,5
Responder	Macro_FOLR2+APOE+	Macro_NLRP3	APOE	SORL1	3,26968E-04	4257,7	1,81723E-02	2990	7,73041E-01	1384	1,13683E+00	3866	9,26851E-01	1840	0	11208,5
Responder	cDC(CD1C)	Macro_FOLR2+APOE+	HLA-DRB1	LAG3	3,27971E-04	6382,5	1,57969E-02	3869	5,73833E-01	3212	1,16796E+00	3626	8,82537E-01	9997	0	11208,5
Responder	Macro_IER3	Macro_ISG15	CCL3L1	CCR1	3,28113E-04	5008,1	3,59981E-02	885	1,08922E+00	407	1,74023E+00	1200	8,75507E-01	11340	0	11208,5
Responder	Macro_OLFML3	Macro_FOLR2+APOE-	C3	CD81	3,28769E-04	7820,3	1,17966E-02	6432	4,34406E-01	5973	8,99466E-01	6235	8,86707E-01	9253	0	11208,5
Responder	Macro_OLFML3	Mono_INHBA	APOE	SDC2	3,28976E-04	5707,1	2,67294E-02	1540	7,48696E-01	1527	1,13614E+00	3872	8,80326E-01	10388	0	11208,5
Responder	Mono_INHBA	Macro_OLFML3	CCL3	CCR1	3,29312E-04	6427,9	1,58451E-02	3843	7,82731E-01	1341	1,21803E+00	3336	8,69676E-01	12411	0	11208,5
Responder	cDC(CD1C)	pDC_LILRA4	COPA	P2RY6	3,29312E-04	9586,7	1,57834E-02	3873	5,72378E-01	3236	2,17193E+00	491	7,88292E-01	29125	0	11208,5
Responder	Mono_INHBA	Macro_OLFML3	CCL3	CCR5	3,29647E-04	9532,3	1,61128E-02	3713	7,74365E-01	1379	1,29949E+00	2859	7,91471E-01	28502	0	11208,5
Responder	Macro_ISG15	Macro_NLRP3	SPP1	CD44	3,30014E-04	5337,3	1,47708E-02	4372	6,10027E-01	2724	1,27289E+00	3007	9,07455E-01	5375	0	11208,5
Responder	Macro_OLFML3	pDC_LILRA4	APOE	SORL1	3,30014E-04	7407,7	9,51694E-03	9260	3,58872E-01	8414	1,56527E+00	1692	9,01667E-01	6464	0	11208,5
Responder	Macro_OLFML3	Macro_FOLR2-APOE+	CXCL9	FCGR2A	3,30727E-04	8719,3	1,25792E-02	5766	3,41773E-01	9043	7,45966E-01	8315	8,86659E-01	9264	0	11208,5
Responder	Macro_FOLR2+APOE-	pDC_LILRA4	MMP9	TLR9	3,31906E-04	11234,9	8,07576E-02	164	1,38543E+00	153	1,50384E+00	1903	7,16019E-01	42746	0	11208,5
Responder	pDC_LILRA4	Macro_ISG15	CD24	SIGLEC10	3,32192E-04	13775,9	5,52610E-02	368	8,10289E-01	1205	2,08627E+00	602	7,10218E-01	43778	0,001	22926,5
Responder	pDC_LILRA4	Macro_NLRP3	SPON2	ITGAM	3,32192E-04	11651,3	4,59340E-02	567	9,27662E-01	765	1,73900E+00	1205	7,06001E-01	44511	0	11208,5
Responder	cDC(CD1C)	Macro_NLRP3	GNAI2	C5AR1	3,33055E-04	7022,9	9,50531E-03	9277	5,23009E-01	3984	1,19658E+00	3451	8,97721E-01	7194	0	11208,5
Responder	Macro_ISG15	Macro_NLRP3	RPS19	C5AR1	3,33953E-04	4820,7	1,28510E-02	5560	8,06580E-01	1220	9,87893E-01	5226	9,34139E-01	889	0	11208,5
Responder	Macro_OLFML3	Macro_IFI27	C3	C3AR1	3,34313E-04	7467,9	1,46154E-02	4467	4,50291E-01	5547	8,51176E-01	6833	8,86507E-01	9284	0	11208,5
Responder	Macro_FOLR2+APOE+	Macro_FOLR2+APOE-	CP	SLC40A1	3,34655E-04	11580,9	7,03187E-02	216	1,16330E+00	310	1,73763E+00	1208	7,03479E-01	44962	0	11208,5
Responder	Mono_INHBA	Macro_OLFML3	CCL3L1	CCR5	3,40875E-04	12135,7	1,73538E-02	3245	6,12793E-01	2690	1,13147E+00	3907	7,33621E-01	39628	0	11208,5
Responder	Mono_INHBA	Macro_OLFML3	INHBA	ENG	3,42600E-04	7728,7	3,95878E-02	751	9,52040E-01	695	1,65217E+00	1411	8,11161E-01	24578	0	11208,5
Responder	Macro_ISG15	Mono_CD14	S100A8	CD36	3,42678E-04	6597,3	1,64763E-02	3567	5,71228E-01	3249	9,50360E-01	5632	8,86224E-01	9330	0	11208,5
Responder	Mono_INHBA	Macro_IFI27	INHBA	ENG	3,42953E-04	8699,9	3,01240E-02	1218	8,65539E-01	961	1,74085E+00	1197	7,89343E-01	28915	0	11208,5
Responder	Macro_OLFML3	Mast	C3	CD46	3,43292E-04	6927,1	1,58541E-02	3841	5,26843E-01	3914	1,13461E+00	3885	8,73361E-01	11787	0	11208,5
Responder	Macro_OLFML3	Macro_ISG15	C3	ITGAM	3,43639E-04	7946,3	1,78948E-02	3082	5,63615E-01	3360	1,13084E+00	3915	8,41746E-01	18166	0	11208,5
Responder	Mono_CD14	Macro_OLFML3	VCAN	TLR1	3,44628E-04	5310,3	3,80214E-02	806	1,00833E+00	560	1,73387E+00	1220	8,67875E-01	12757	0	11208,5
Responder	cDC(CD1C)	Macro_LYVE1	HLA-DMA	CD4	3,47467E-04	6214,3	8,94693E-03	10313	5,26027E-01	3926	1,16267E+00	3657	9,26088E-01	1967	0	11208,5
Responder	Macro_OLFML3	Macro_FOLR2+APOE+	C3	LRP1	3,47479E-04	7531,7	1,48339E-02	4344	5,15144E-01	4120	7,25441E-01	8630	8,86098E-01	9356	0	11208,5
Responder	Macro_OLFML3	Macro_FOLR2-APOE+	FN1	ITGAV_ITGB1	3,48866E-04	6073,1	2,67482E-02	1536	6,19571E-01	2613	1,12962E+00	3930	8,76782E-01	11078	0	11208,5
Responder	Macro_OLFML3	Macro_ISG15	APOE	TREM2	3,49713E-04	7474,5	1,23109E-02	5983	3,79157E-01	7661	6,80371E-01	9368	9,19577E-01	3152	0	11208,5
Responder	Macro_FOLR2+APOE-	cDC(CD1C)	CCL13	CCR2	3,52398E-04	12865,3	1,10337E-01	73	1,34192E+00	169	1,46106E+00	2073	6,64798E-01	50803	0	11208,5
Responder	Macro_LYVE1	pDC_LILRA4	HSP90B1	TLR9	3,52398E-04	5547,3	7,98201E-02	169	1,47522E+00	129	1,33159E+00	2686	8,63941E-01	13544	0	11208,5
Responder	Mono_INHBA	Macro_OLFML3	IL6	FCGR3B	3,53444E-04	12999,5	7,59928E-02	191	8,42924E-01	1047	1,26235E+00	3076	6,74172E-01	49475	0	11208,5
Responder	Mono_INHBA	Macro_OLFML3	INHBA	ACTR2	3,53798E-04	5489,3	2,84714E-02	1371	9,18604E-01	787	1,40122E+00	2343	8,73647E-01	11737	0	11208,5
Responder	Macro_OLFML3	cDC_CLEC9A	ITGB2	CD226	3,53941E-04	7263,1	3,57019E-02	902	8,83997E-01	890	7,97822E-01	7571	8,53305E-01	15744	0	11208,5
Responder	Mono_CD14	Mono_INHBA	S100A9	TLR4	3,54782E-04	5124,9	1,57112E-02	3910	1,02657E+00	522	2,10164E+00	589	8,85878E-01	9395	0	11208,5
Responder	cDC(CD1C)	Macro_OLFML3	ANXA2	TLR2	3,55727E-04	8281,5	9,43803E-03	9400	4,15359E-01	6514	9,06872E-01	6139	8,92710E-01	8146	0	11208,5

Post_R_Myeloid_Myeloid_CellCel

Responder	pDC_LILRA4	cDC(CD1C)	CD24	SIGLEC10	3,56510E-04	11441,1	5,41768E-02	382	8,03056E-01	1234	2,47170E+00	248	7,08175E-01	44133	0	11208,5
Responder	pDC_LILRA4	Macro_FOLR2+APOE+	GAS6	AXL	3,56510E-04	7238,5	2,99427E-02	1234	9,20789E-01	784	2,18966E+00	472	8,20986E-01	22494	0	11208,5
Responder	cDC_LAMP3	Macro_LYVE1	CCL19	CCRL2	3,56569E-04	7389,1	7,97035E-02	170	1,97340E+00	42	1,67508E+00	1352	8,13109E-01	24173	0	11208,5
Responder	cDC(CD1C)	Mast	PKM	CD44	3,57353E-04	6918,7	7,48926E-03	13882	5,24726E-01	3954	1,14678E+00	3782	9,27223E-01	1767	0	11208,5
Responder	cDC(CD1C)	Macro_FOLR2+APOE+	CD52	SIGLEC10	3,58574E-04	8217,7	1,11714E-02	7022	5,02683E-01	4350	6,77324E-01	9415	8,87692E-01	9093	0	11208,5
Responder	Macro_FOLR2-APOE+	pDC_LILRA4	TNF	PTPRS	3,60818E-04	10937,1	3,72475E-02	837	1,46144E+00	133	1,72262E+00	1239	7,23980E-01	41268	0	11208,5
Responder	Macro_OLFML3	Macro_NLRP3	SERPING1	LRP1	3,63360E-04	7851,3	1,31259E-02	5354	5,28883E-01	3877	6,75597E-01	9440	8,85955E-01	9377	0	11208,5
Responder	Macro_OLFML3	Macro_LYVE1	C3	CR1	3,63818E-04	7745,3	3,06071E-02	1171	7,11625E-01	1764	1,12540E+00	3972	8,30244E-01	20611	0	11208,5
Responder	cDC(CD1C)	Macro_LYVE1	HLA-DRB1	CD4	3,63818E-04	6343,5	7,80502E-03	12970	5,23895E-01	3972	1,21948E+00	3325	9,42894E-01	242	0	11208,5
Responder	Macro_LYVE1	cDC_CLEC9A	CXCL2	XCR1	3,64985E-04	4928,3	7,96029E-02	172	2,59187E+00	8	1,64710E+00	1432	8,73134E-01	11821	0	11208,5
Responder	pDC_LILRA4	Mono_INHBA	SPON2	ITGAM	3,65160E-04	11872,5	4,34693E-02	623	9,12310E-01	808	1,71975E+00	1244	7,00246E-01	45479	0	11208,5
Responder	Mono_INHBA	cDC(CD1C)	IL6	IL6R_IL6ST	3,65629E-04	10874,5	3,12863E-02	1136	7,71932E-01	1389	1,46823E+00	2041	7,39220E-01	38598	0	11208,5
Responder	Mono_CD14	Macro_ISG15	VCAN	TLR2	3,67420E-04	4834,7	2,62830E-02	1592	8,92072E-01	859	1,80393E+00	1053	8,85445E-01	9461	0	11208,5
Responder	cDC(CD1C)	Mono_CD16	HLA-A	LILRB2	3,68003E-04	5894,5	9,40490E-03	9464	5,10559E-01	4207	1,08410E+00	4301	9,41927E-01	292	0	11208,5
Responder	Mono_CD14	pDC_LILRA4	BST2	LILRA4	3,69230E-04	4735,3	2,80799E-02	1408	1,33800E+00	173	2,74919E+00	120	8,78378E-01	10767	0	11208,5
Responder	cDC(CD1C)	Macro_LYVE1	HSP90B1	LRP1	3,71124E-04	7876,5	9,39486E-03	9480	4,72153E-01	4989	8,55576E-01	6785	8,99261E-01	6920	0	11208,5
Responder	cDC(CD1C)	pDC_LILRA4	HLA-DRA	CD4	3,71837E-04	5822,7	7,59566E-03	13552	5,22437E-01	3994	2,58701E+00	182	9,44646E-01	177	0	11208,5
Responder	Macro_FOLR2-APOE+	pDC_LILRA4	HMGB1	THBD	3,72104E-04	7151,1	1,09237E-02	7305	4,39434E-01	5828	1,49800E+00	1929	8,85339E-01	9485	0	11208,5
Responder	Macro_OLFML3	Mono_CD16	FN1	ITGA4_ITGB1	3,72300E-04	5978,7	2,44855E-02	1807	6,00297E-01	2831	1,05606E+00	4561	8,85339E-01	9486	0	11208,5
Responder	Macro_OLFML3	Macro_OLFML3	C3	LRP1	3,72300E-04	7615,7	1,51316E-02	4188	5,22213E-01	4006	6,73136E-01	9486	8,87097E-01	9190	0	11208,5
Responder	Macro_ISG15	Macro_LYVE1	LGALS9	LRP1	3,72693E-04	7669,3	1,21963E-02	6071	6,03768E-01	2799	6,99886E-01	9046	8,86895E-01	9222	0	11208,5
Responder	Macro_FOLR2+APOE-	Macro_ISG15	CCL18	CCR1	3,73062E-04	6969,5	3,58043E-02	897	7,98802E-01	1253	2,16413E+00	509	8,28332E-01	20980	0	11208,5
Responder	Mono_INHBA	cDC(CD1C)	MIF	CD74_CXCR4	3,73309E-04	6035,5	7,09603E-03	15129	6,63254E-01	2170	1,59030E+00	1602	9,49355E-01	68	0	11208,5
Responder	Mono_INHBA	cDC(CD1C)	ADM	CALCRL	3,76266E-04	12223,7	2,19266E-02	2202	5,86775E-01	3009	1,71560E+00	1255	7,12161E-01	43444	0	11208,5
Responder	Macro_OLFML3	Macro_FOLR2-APOE+	FN1	C5AR1	3,76439E-04	7030,1	1,95913E-02	2620	4,97657E-01	4449	8,12640E-01	7366	8,85181E-01	9507	0	11208,5
Responder	cDC_CLEC9A	pDC_LILRA4	BTLA	TNFRSF14	3,77501E-04	8464,1	2,97003E-02	1258	1,55562E+00	102	2,54263E+00	205	7,86196E-01	29547	0	11208,5
Responder	Macro_IER3	pDC_LILRA4	PLAU	IGF2R	3,78393E-04	7379,1	2,96862E-02	1259	8,45661E-01	1033	2,03304E+00	662	8,20022E-01	22733	0	11208,5
Responder	pDC_LILRA4	cDC(CD1C)	GAS6	AXL	3,79286E-04	7201,5	2,96768E-02	1260	9,17003E-01	793	2,82920E+00	96	8,20330E-01	22650	0	11208,5
Responder	Macro_OLFML3	Macro_FOLR2-APOE+	APOE	ABCA1	3,79987E-04	6126,5	1,54612E-02	4016	6,34160E-01	2451	6,27101E-01	10299	9,22212E-01	2658	0	11208,5
Responder	Mast	Mono_INHBA	CSF1	CSF3R	3,80181E-04	7424,3	3,85239E-02	783	8,92269E-01	858	1,71029E+00	1261	8,18640E-01	23011	0	11208,5
Responder	cDC(CD1C)	Mast	VEGFB	ADRB2	3,80734E-04	13084,5	2,36695E-02	1923	6,91924E-01	1921	1,11936E+00	4018	6,94726E-01	46352	0	11208,5
Responder	Macro_ISG15	Macro_LYVE1	APOE	LRP1	3,81615E-04	6808,3	1,20592E-02	6192	4,66070E-01	5147	7,47053E-01	8298	9,19313E-01	3196	0	11208,5
Responder	Macro_ISG15	Macro_LYVE1	S100A8	CD36	3,82216E-04	6115,9	2,23148E-02	2130	7,62296E-01	1460	6,95458E-01	9116	9,00644E-01	6665	0	11208,5
Responder	Macro_ISG15	Macro_LYVE1	S100A9	CD36	3,83018E-04	5374,7	1,93987E-02	2653	7,68770E-01	1410	9,38759E-01	5752	9,04827E-01	5850	0	11208,5
Responder	Macro_ISG15	Macro_LYVE1	S100A9	CD68	3,83219E-04	5788,1	1,05856E-02	7712	6,90636E-01	1931	7,82304E-01	7789	9,41609E-01	300	0	11208,5
Responder	Mono_CD14	Macro_FOLR2-APOE+	HBEGF	CD44	3,83621E-04	8860,9	9,55371E-03	9196	4,27896E-01	6135	7,52432E-01	8222	8,84970E-01	9543	0	11208,5
Responder	pDC_LILRA4	Macro_IER3	SPON2	ITGA4	3,83774E-04	9403,3	4,11393E-02	702	9,49325E-01	705	1,71003E+00	1265	7,67988E-01	33136	0	11208,5
Responder	Macro_FOLR2-APOE+	Macro_ISG15	FN1	PLAUR	3,85232E-04	8377,3	1,29532E-02	5496	3,29851E-01	9551	6,80057E-01	9375	9,02826E-01	6256	0	11208,5
Responder	Macro_ISG15	Mono_INHBA	APOE	ABCA1	3,85433E-04	8325,5	1,03069E-02	8067	3,29835E-01	9552	8,21907E-01	7232	9,06364E-01	5568	0	11208,5
Responder	Macro_OLFML3	Macro_FOLR2+APOE+	C3	NRP1	3,85619E-04	7893,7	1,83997E-02	2920	6,39175E-01	2405	1,11830E+00	4031	8,38258E-01	18904	0	11208,5
Responder	cDC(CD1C)	Macro_ISG15	HLA-DPB1	LAG3	3,88648E-04	6164,1	1,54178E-02	4039	6,13696E-01	2679	1,56419E+00	1701	8,76293E-01	11193	0	11208,5
Responder	Macro_OLFML3	Macro_LYVE1	C3	CD46	3,88672E-04	6381,9	1,96757E-02	2600	6,00559E-01	2829	9,43247E-01	5704	8,84830E-01	9568	0	11208,5
Responder	pDC_LILRA4	Macro_OLFML3	SPON2	ITGB2	3,90114E-04	5417,3	4,46312E-02	599	1,08117E+00	424	1,70772E+00	1272	8,63770E-01	13583	0	11208,5
Responder	Mono_INHBA	Mono_INHBA	TNF	TNFRSF1B	3,90931E-04	6521,9	1,64249E-02	3589	6,91228E-01	1927	1,20068E+00	3429	8,69299E-01	12456	0	11208,5
Responder	Macro_NLRP3	Mast	IL1B	ADRB2	3,91939E-04	3880,7	6,29048E-02	277	1,15308E+00	324	1,70550E+00	1274	9,02444E-01	6320	0	11208,5
Responder	pDC_LILRA4	Macro_FOLR2-APOE+	LTB	CD40	3,91939E-04	6168,9	2,95456E-02	1274	1,05983E+00	448	2,61766E+00	169	8,43732E-01	17745	0	11208,5
Responder	Macro_OLFML3	Mono_INHBA	MDK	SDC2	3,92842E-04	10169,1	4,67438E-02	544	6,78141E-01	2043	1,11603E+00	4050	7,68573E-01	33000	0	11208,5
Responder	Macro_ISG15	cDC_CLEC9A	CXCL11	CXCR3	3,92853E-04	10810,9	1,10918E-01	71	9,50310E-01	700	1,70475E+00	1275	7,26688E-01	40800	0	11208,5
Responder	Macro_OLFML3	Macro_OLFML3	LGALS9	PTPRC	3,93757E-04	21507,5	5,70749E-03	21257	2,03036E-01	16652	-4,73318E-01	49820	8,90302E-01	8600	0	11208,5
Responder	Macro_FOLR2+APOE+	pDC_LILRA4	C1QB	C1QBP	3,93991E-04	3716,3	1,53858E-02	4053	7,75186E-01	1375	2,00372E+00	708	9,30958E-01	1237	0	11208,5
Responder	Macro_FOLR2-APOE+	Macro_FOLR2-APOE+	MMP9	CD44	3,94393E-04	6154,7	1,83983E-02	2921	7,22231E-01	1689	9,74298E-01	5359	8,84674E-01	9596	0	11208,5
Responder	pDC_LILRA4	Macro_OLFML3	SERPINF1	PLXDC2	3,95143E-04	3488,3	2,62282E-02	1600	1,04799E+00	470	2,78744E+00	107	9,14543E-01	4056	0	11208,5
Responder	Mono_CD16	cDC_CLEC9A	FLT3LG	FLT3	3,95211E-04	11155,3	7,88171E-02	179	1,42514E+00	144	1,54774E+00	1760	7,17454E-01	42485	0	11208,5
Responder	Macro_ISG15	Macro_LYVE1	SERPING1	LRP1	3,95833E-04	6066,9	1,67001E-02	3470	6,70110E-01	2114	8,93393E-01	6316	8,97568E-01	7226	0	11208,5
Responder	Macro_OLFML3	Macro_NLRP3	C3	ITGAM	3,95913E-04	7137,7	2,17407E-02	2232	6,14230E-01	2671	1,11537E+00	4058	8,54285E-01	15519	0	11208,5
Responder	Macro_OLFML3	Macro_OLFML3	C3	CD46	3,97692E-04	6741,1	1,95866E-02	2621	5,98840E-01	2849	8,09179E-01	7415	8,84599E-01	9612	0	11208,5
Responder	Macro_ISG15	cDC_LAMP3	CD14	ITGA4	4,00180E-04	8469,7	9,49867E-03	9288	4,41246E-01	5780	7,62471E-01	8062	8,93489E-01	8010	0	11208,5
Responder	Macro_OLFML3	Mast	C3	IFITM1	4,00596E-04	6755,7	1,68244E-02	3428	6,17675E-01	2635	8,47572E-01	6881	8,84519E-01	9626	0	11208,5
Responder	Macro_FOLR2-APOE+	Mono_INHBA	APOE	ABCA1	4,01429E-04	6663,9	1,39807E-02	4847	5,05182E-01	4298	6,65361E-01	9630	9,18524E-01	3336	0	11208,5
Responder	Macro_ISG15	cDC_LAMP3	SPP1	CD44	4,02890E-04	9340,5	9,44762E-03	9390	3,33549E-01	9394	8,03893E-01	7492	8,86904E-01	9218	0	11208,5
Responder	Mono_INHBA	Mono_INHBA	IL6	IL6R_IL6ST	4,04059E-04	11006,5	3,11762E-02	1139	7,68918E-01	1407	1,34396E+00	2615	7,38880E-01	38663	0	11208,5
Responder	Mono_CD14	Macro_FOLR2+APOE+	S100A9	TLR4	4,04146E-04	5314,9	1,52754E-02	4112	1,01933E+00	538	1,79307E+00	1073	8,84448E-01	9643	0	11208,5
Responder	Mono_INHBA	Mono_INHBA	NAMPT	ITGA5_ITGB1	4,05405E-04	7448,5	1,11137E-02	7092	6,38680E-01	2409	8,47480E-01	6884	8,84434E-01	9649	0	11208,5
Responder	cDC_CLEC9A	pDC_LILRA4	SELPLG	SELL	4,05800E-04	4652,5	2,93143E-02	1289	9,98201E-01	587	2,82839E+00	97	8,82006E-01	10081	0	11208,5
Responder	Mono_INHBA	Mono_INHBA	CXCL8	SDC2	4,07195E-04	7580,7	2,71225E-02	1496	7,71138E-01	1395	1,45367E+00	2101	8,24807E-01	21703	0	11208,5

Responder	Macro_ISG15	Mono_CD16	HLA-B	LILRA1	4,07932E-04	6334,3	1,80589E-02	3024	5,60648E-01	3390	7,33296E-01	8512	9,06562E-01	5537	0	11208,5
Responder	Macro_ISG15	Mono_CD16	HLA-B	LILRB2	4,08143E-04	5474,7	1,03316E-02	8038	7,33714E-01	1623	8,85801E-01	6408	9,47684E-01	96	0	11208,5
Responder	Macro_ISG15	Mono_CD16	HLA-A	LILRA1	4,08777E-04	6471,5	1,88786E-02	2791	5,56100E-01	3463	7,22425E-01	8677	9,03004E-01	6218	0	11208,5
Responder	Macro_ISG15	Mono_CD16	HLA-A	LILRB2	4,08989E-04	5397,5	1,08005E-02	7454	7,29165E-01	1648	8,74930E-01	6537	9,45598E-01	140	0	11208,5
Responder	Macro_OLFML3	Mono_CD14	C1QB	CD33	4,10260E-04	8362,7	9,29219E-03	9672	4,12454E-01	6597	8,86325E-01	6398	8,93870E-01	7938	0	11208,5
Responder	cDC(CD1C)	Mast	VIM	CD44	4,10745E-04	7167,3	6,60782E-03	16921	5,55939E-01	3466	1,10942E+00	4096	9,45494E-01	145	0	11208,5
Responder	Macro_OLFML3	cDC_CLEC9A	CALM2	MYLK	4,11434E-04	9228,3	4,85595E-02	509	9,33193E-01	750	8,71560E-01	6570	7,98403E-01	27104	0	11208,5
Responder	Macro_FOLR2+APOE-	Macro_ISG15	CCL13	CCR2	4,13019E-04	13514,1	1,00416E-01	91	1,31746E+00	183	1,11964E+00	4016	6,54220E-01	52072	0	11208,5
Responder	Macro_ISG15	Mono_CD16	HLA-F	LILRB2	4,13024E-04	7075,7	1,15413E-02	6676	6,68431E-01	2121	7,84223E-01	7759	8,95615E-01	7614	0	11208,5
Responder	Macro_ISG15	pDC_LILRA4	CCL5	CXCR3	4,13324E-04	11166,1	2,92345E-02	1297	1,08232E+00	421	1,94222E+00	803	7,19621E-01	42101	0	11208,5
Responder	cDC(CD1C)	cDC_LAMP3	ENTPD1	ADORA2A	4,13521E-04	11671,5	3,28828E-02	1037	6,51280E-01	2298	1,10782E+00	4103	7,33113E-01	39711	0	11208,5
Responder	Macro_ISG15	Mono_CD16	B2M	LILRB2	4,13664E-04	5809,3	1,00289E-02	8451	7,05049E-01	1810	7,99201E-01	7552	9,53242E-01	25	0	11208,5
Responder	Mast	Macro_NLRP3	CSF1	CSF2RA	4,14271E-04	6918,3	3,50518E-02	933	8,87875E-01	877	1,69541E+00	1298	8,31823E-01	20275	0	11208,5
Responder	pDC_LILRA4	Macro_ISG15	LTB	CD40	4,14271E-04	6198,5	2,92339E-02	1298	1,05574E+00	458	2,69368E+00	135	8,43031E-01	17893	0	11208,5
Responder	Macro_LYVE1	pDC_LILRA4	MAML2	NOTCH4	4,15219E-04	11846,1	6,33001E-02	271	7,90510E-01	1299	1,74464E+00	1186	7,01612E-01	45266	0	11208,5
Responder	Mono_INHBA	Mono_INHBA	VEGFA	ITGB1	4,15513E-04	7254,5	1,71568E-02	3305	6,92299E-01	1916	1,13745E+00	3858	8,52125E-01	15985	0	11208,5
Responder	Macro_OLFML3	cDC_LAMP3	FN1	CD44	4,16447E-04	7605,3	1,48170E-02	4353	3,98243E-01	7026	6,61374E-01	9701	9,05406E-01	5738	0	11208,5
Responder	pDC_LILRA4	cDC(CD1C)	SPON2	ITGB1	4,17120E-04	9713,3	2,91924E-02	1301	8,38880E-01	1069	2,06912E+00	613	7,61983E-01	34375	0	11208,5
Responder	cDC(CD1C)	pDC_LILRA4	HLA-DPA1	CD4	4,18313E-04	5740,7	7,83059E-03	12897	5,15695E-01	4115	2,56914E+00	190	9,41874E-01	293	0	11208,5
Responder	pDC_LILRA4	Mono_CD16	SPON2	ITGA4	4,19026E-04	9025,5	4,54053E-02	577	9,91120E-01	598	1,69361E+00	1303	7,76662E-01	31441	0	11208,5
Responder	Macro_OLFML3	Macro_LYVE1	HLA-DRB5	CD4	4,19245E-04	6603,3	9,27011E-03	9714	6,10651E-01	2717	7,02525E-01	9012	9,40259E-01	365	0	11208,5
Responder	Mono_INHBA	Macro_NLRP3	HBEGF	CD44	4,19893E-04	7315,7	1,25819E-02	5763	6,05422E-01	2779	6,60455E-01	9717	8,98259E-01	7111	0	11208,5
Responder	Macro_FOLR2-APOE+	Mono_CD14	FN1	C5AR1	4,20109E-04	6475,5	1,91690E-02	2712	4,72259E-01	4987	1,15037E+00	3752	8,84069E-01	9718	0	11208,5
Responder	Macro_FOLR2-APOE+	pDC_LILRA4	C1QB	C1QBP	4,20974E-04	7586,1	9,26583E-03	9722	3,34190E-01	9368	1,23338E+00	3243	9,12771E-01	4389	0	11208,5
Responder	cDC_LAMP3	Macro_IER3	CCL19	CCRL2	4,22069E-04	7474,7	7,76495E-02	185	1,96449E+00	43	1,67741E+00	1346	8,11117E-01	24591	0	11208,5
Responder	Macro_OLFML3	Macro_FOLR2+APOE-	C1QB	LRP1	4,22492E-04	7498,7	9,26205E-03	9729	4,71784E-01	4997	7,67371E-01	7990	9,17240E-01	3569	0	11208,5
Responder	Macro_ISG15	Mono_CD16	RPS19	C5AR1	4,22709E-04	5866,7	1,14963E-02	6715	7,23163E-01	1685	7,38252E-01	8445	9,30629E-01	1280	0	11208,5
Responder	Macro_OLFML3	cDC_CLEC9A	C3	CD81	4,24450E-04	7450,9	1,11749E-02	7019	4,17187E-01	6448	1,30147E+00	2841	8,83959E-01	9738	0	11208,5
Responder	Macro_ISG15	Macro_LYVE1	CCL8	CCR1	4,24780E-04	9583,9	5,25002E-02	413	8,45999E-01	1032	1,69281E+00	1309	7,63893E-01	33957	0	11208,5
Responder	Macro_ISG15	Macro_ISG15	C3	IFITM1	4,25322E-04	6187,9	1,66397E-02	3498	5,52777E-01	3526	1,28100E+00	2965	8,83954E-01	9742	0	11208,5
Responder	cDC(CD1C)	pDC_LILRA4	HMGB1	CXCR4	4,26799E-04	6205,7	7,48128E-03	13904	5,14428E-01	4136	2,68688E+00	140	9,28136E-01	1640	0	11208,5
Responder	Mono_CD14	cDC(CD1C)	TGFB1	CXCR4	4,27510E-04	7183,9	9,24943E-03	9752	5,12966E-01	4163	1,60331E+00	1558	8,86802E-01	9238	0	11208,5
Responder	pDC_LILRA4	cDC_CLEC9A	SPON2	ITGB1	4,28645E-04	9753,7	2,90968E-02	1313	8,37663E-01	1072	1,98042E+00	749	7,61685E-01	34426	0	11208,5
Responder	Mono_CD14	Macro_IER3	VCAN	ITGA4	4,29266E-04	4994,1	2,38172E-02	1901	9,44019E-01	721	1,66462E+00	1380	8,83831E-01	9760	0	11208,5
Responder	Macro_FOLR2+APOE-	pDC_LILRA4	GNAI2	CXCR3	4,29615E-04	6390,9	2,90957E-02	1314	1,04849E+00	469	2,02270E+00	680	8,41213E-01	18283	0	11208,5
Responder	Macro_ISG15	Mast	SPP1	CD44	4,29706E-04	4527,1	1,68905E-02	3393	7,20116E-01	1704	1,48586E+00	1972	9,12934E-01	4358	0	11208,5
Responder	pDC_LILRA4	Macro_NLRP3	APP	CD74	4,31559E-04	3692,7	1,43849E-02	4600	7,86767E-01	1316	1,74015E+00	1202	9,45768E-01	137	0	11208,5
Responder	Mono_CD14	Mast	CD55	ADGRE2	4,33239E-04	4923,7	2,74002E-02	1465	9,28619E-01	762	1,65506E+00	1405	8,83757E-01	9778	0	11208,5
Responder	Macro_ISG15	Macro_LYVE1	TNFSF13B	TFRC	4,34126E-04	7329,3	1,23925E-02	5910	6,07394E-01	2755	8,40541E-01	6991	8,83697E-01	9782	0	11208,5
Responder	Mono_INHBA	Mono_INHBA	INHBA	ACTR2	4,35825E-04	5536,5	2,55882E-02	1667	8,57065E-01	992	1,82768E+00	1004	8,67635E-01	12811	0	11208,5
Responder	Macro_OLFML3	Mono_CD16	C3	LRP1	4,36350E-04	7497,9	1,41294E-02	4756	4,98414E-01	4431	8,16274E-01	7302	8,83620E-01	9792	0	11208,5
Responder	Mono_INHBA	Mono_CD14	TNF	TNFRSF1B	4,38311E-04	6590,7	1,54196E-02	4036	6,47416E-01	2331	1,43629E+00	2185	8,65669E-01	13193	0	11208,5
Responder	Macro_OLFML3	Macro_FOLR2-APOE+	FN1	ITGA5_ITGB1	4,38726E-04	6471,3	2,44076E-02	1818	5,92445E-01	2934	1,10128E+00	4165	8,70806E-01	12231	0	11208,5
Responder	Mono_CD14	Mono_CD16	HLA-C	LILRB2	4,39254E-04	7092,5	9,21744E-03	9805	4,69379E-01	5067	6,99179E-01	9054	9,41082E-01	328	0	11208,5
Responder	Mono_CD14	cDC(CD1C)	S100A9	CD68	4,41723E-04	4724,1	9,21115E-03	9816	7,82722E-01	1342	1,99787E+00	716	9,37666E-01	538	0	11208,5
Responder	Mono_INHBA	Mono_CD14	THBS1	CD36	4,43734E-04	7556,1	1,83570E-02	2932	5,69270E-01	3282	1,22410E+00	3297	8,46888E-01	17061	0	11208,5
Responder	Macro_ISG15	Macro_IER3	SPP1	CD44	4,43978E-04	7798,3	1,11071E-02	7097	4,19740E-01	6370	8,74443E-01	6543	8,94769E-01	7773	0	11208,5
Responder	Macro_OLFML3	Macro_ISG15	HLA-DRA	LAG3	4,45562E-04	7262,9	1,46440E-02	4441	5,96360E-01	2882	7,70851E-01	7950	8,83438E-01	9833	0	11208,5
Responder	Macro_OLFML3	Macro_OLFML3	C1QA	CD33	4,46242E-04	6693,7	1,42808E-02	4664	5,68576E-01	3292	6,52962E-01	9836	9,12319E-01	4468	0	11208,5
Responder	Macro_OLFML3	Macro_NLRP3	CXCL9	FCGR2A	4,47150E-04	8975,5	1,22075E-02	6060	3,23497E-01	9840	7,50446E-01	8252	8,85143E-01	9517	0	11208,5
Responder	Macro_OLFML3	pDC_LILRA4	C3	CD46	4,47939E-04	6473,1	1,51329E-02	4187	5,12931E-01	4164	2,11309E+00	567	8,70764E-01	12239	0	11208,5
Responder	Macro_FOLR2+APOE+	pDC_LILRA4	CCL5	CXCR3	4,48318E-04	9090,1	5,12066E-02	439	1,22741E+00	250	1,68110E+00	1333	7,72563E-01	32220	0	11208,5
Responder	Macro_ISG15	Mono_INHBA	S100A9	CD36	4,49427E-04	8075,1	1,23064E-02	5985	5,01776E-01	4372	7,04792E-01	8960	8,83346E-01	9850	0	11208,5
Responder	cDC_CLEC9A	cDC(CD1C)	HLA-DOB	CD4	4,50317E-04	6248,7	2,88352E-02	1335	1,05742E+00	453	1,75059E+00	1170	8,46832E-01	17077	0	11208,5
Responder	Mono_INHBA	Mono_INHBA	VEGFA	SIRPA	4,50900E-04	8882,7	1,84972E-02	2895	6,68301E-01	2122	1,09693E+00	4194	8,13926E-01	23994	0	11208,5
Responder	Macro_ISG15	Mast	ARPC5	ADRB2	4,51026E-04	5619,1	2,74879E-02	1450	7,23228E-01	1683	1,13281E+00	3897	8,83287E-01	9857	0	11208,5
Responder	pDC_LILRA4	cDC_CLEC9A	CXCL2	XCR1	4,51319E-04	7772,7	2,88274E-02	1336	2,35458E+00	17	2,11037E+00	573	8,05510E-01	25729	0	11208,5
Responder	Mono_INHBA	Mono_CD14	IL6	IL6R_IL6ST	4,52173E-04	10471,7	3,39625E-02	988	7,89893E-01	1303	1,54794E+00	1758	7,47053E-01	37101	0	11208,5
Responder	pDC_LILRA4	Macro_OLFML3	TNFSF9	HLA-DPA1	4,53449E-04	3549,5	2,77582E-02	1427	1,04037E+00	491	2,23536E+00	421	9,13748E-01	4200	0	11208,5
Responder	Mono_INHBA	Macro_FOLR2-APOE+	LGALS3	ENG	4,53548E-04	7295,1	1,33244E-02	5228	6,02861E-01	2806	8,12640E-01	7365	8,83247E-01	9868	0	11208,5
Responder	Macro_OLFML3	Mono_INHBA	C3	ITGAM	4,53875E-04	7392,3	2,05742E-02	2422	5,98878E-01	2848	1,09613E+00	4201	8,50819E-01	16282	0	11208,5
Responder	Mono_INHBA	Mono_CD14	CCL3	CCR1	4,54728E-04	5503,9	1,81167E-02	3010	8,26718E-01	1122	1,75423E+00	1161	8,77082E-01	11018	0	11208,5
Responder	Macro_ISG15	cDC_CLEC9A	TNFSF13B	CD40	4,55159E-04	6553,9	1,24340E-02	5867	5,54772E-01	3487	1,40304E+00	2332	8,83204E-01	9875	0	11208,5
Responder	Mono_INHBA	Mono_CD14	CCL4	CCR1	4,56437E-04	6500,7	1,56666E-02	3936	6,43976E-01	2364	1,60368E+00	1554	8,64463E-01	13441	0	11208,5
Responder	Macro_FOLR2+APOE+	pDC_LILRA4	TNF	PTPRS	4,57361E-04	11198,1	3,51114E-02	931	1,45417E+00	136	1,67859E+00	1342	7,18040E-01	42373	0	11208,5

Responder	Macro_OLFML3	Macro_IER3	FN1	C5AR1	4,58858E-04	7446,5	1,88243E-02	2808	4,82461E-01	4766	7,30464E-01	8559	8,83136E-01	9891	0	11208,5
Responder	Mono_INHBA	Mono_CD14	VCAN	SELL	4,59440E-04	5714,3	2,46095E-02	1787	6,99216E-01	1858	1,57805E+00	1644	8,71698E-01	12074	0	11208,5
Responder	Macro_NLRP3	Mono_INHBA	HBEGF	CD44	4,62457E-04	4563,5	1,80472E-02	3029	8,79979E-01	900	1,19474E+00	3459	9,13599E-01	4221	0	11208,5
Responder	Mono_INHBA	Mono_CD14	CCL3L1	CCR1	4,62890E-04	7306,9	1,95120E-02	2632	6,65146E-01	2145	1,58621E+00	1618	8,38124E-01	18931	0	11208,5
Responder	Macro_FOLR2+APOE-	pDC_LILRA4	CXCL12	CXCR4	4,66524E-04	4517,7	2,86954E-02	1351	8,77770E-01	911	2,83906E+00	91	8,88018E-01	9027	0	11208,5
Responder	cDC(CD1C)	pDC_LILRA4	HLA-DMB	CD4	4,68212E-04	6210,9	9,14520E-03	9931	4,71595E-01	5003	2,45098E+00	265	9,11400E-01	4647	0	11208,5
Responder	Mast	Macro_FOLR2+APOE+	CSF1	CSF2RA	4,69605E-04	5439,9	5,63896E-02	352	1,04799E+00	471	1,67389E+00	1354	8,62514E-01	13814	0	11208,5
Responder	Mono_INHBA	cDC_CLEC9A	CXCL2	DPP4	4,71667E-04	8861,3	5,97147E-02	309	1,04199E+00	485	1,67344E+00	1356	7,79186E-01	30948	0	11208,5
Responder	cDC(CD1C)	pDC_LILRA4	HLA-DQA2	CD4	4,71759E-04	6801,9	9,13818E-03	9946	3,92353E-01	7225	2,42403E+00	284	9,07591E-01	5346	0	11208,5
Responder	Macro_IER3	Mono_CD14	CCL3L1	CCR1	4,72700E-04	5639,7	2,85813E-02	1357	9,98184E-01	588	1,74023E+00	1199	8,62380E-01	13846	0	11208,5
Responder	Mono_CD14	Macro_FOLR2+APOE+	VCAN	CD44	4,73183E-04	7287,3	1,15908E-02	6626	6,75797E-01	2066	6,46870E-01	9952	9,01045E-01	6584	0	11208,5
Responder	Macro_LYVE1	pDC_LILRA4	HMGB1	TLR9	4,73589E-04	5419,3	7,39804E-02	196	1,47638E+00	128	1,22677E+00	3279	8,70421E-01	12285	0	11208,5
Responder	Mono_INHBA	Mono_CD14	INHBA	ACTR2	4,78208E-04	5697,5	2,39976E-02	1879	8,23116E-01	1136	2,00264E+00	711	8,63906E-01	13553	0	11208,5
Responder	Mast	Macro_OLFML3	CSF1	CSF3R	4,78929E-04	5417,9	7,37773E-02	197	1,09349E+00	398	1,67030E+00	1363	8,62005E-01	13923	0	11208,5
Responder	Macro_OLFML3	Mast	ARPC5	ADRB2	4,79157E-04	6759,9	2,81348E-02	1404	7,41135E-01	1575	6,46056E-01	9977	8,84480E-01	9635	0	11208,5
Responder	Macro_FOLR2-APOE+	pDC_LILRA4	CCL5	CXCR3	4,79972E-04	10690,9	3,38876E-02	992	1,11304E+00	373	1,67005E+00	1364	7,34277E-01	39517	0	11208,5
Responder	Macro_OLFML3	Mono_INHBA	FN1	ITGAV_ITGB1	4,79982E-04	6924,9	2,16338E-02	2249	5,06841E-01	4261	1,18501E+00	3528	8,64853E-01	13378	0	11208,5
Responder	cDC(CD1C)	Macro_ISG15	SPN	SIGLEC1	4,80426E-04	13440,3	1,76966E-02	3135	5,21070E-01	4025	1,08864E+00	4262	7,05597E-01	44571	0	11208,5
Responder	Macro_OLFML3	pDC_LILRA4	ALCAM	NRP1	4,82207E-04	12167,3	1,61958E-02	3667	5,31391E-01	3837	1,08851E+00	4266	7,43075E-01	37858	0	11208,5
Responder	Macro_FOLR2-APOE+	Mono_CD14	C3	ITGAX	4,85190E-04	8637,3	1,24001E-02	5901	3,31808E-01	9466	8,68942E-01	6609	8,82506E-01	10002	0	11208,5
Responder	Mono_INHBA	Macro_FOLR2+APOE+	INHBA	ENG	4,86264E-04	7458,9	4,25564E-02	652	9,79173E-01	631	1,66802E+00	1370	8,16637E-01	23433	0	11208,5
Responder	pDC_LILRA4	Mast	HSPA8	ADRB2	4,87318E-04	4004,9	2,98273E-02	1249	7,76542E-01	1371	2,46747E+00	251	9,04355E-01	5945	0	11208,5
Responder	Mast	Mono_INHBA	CSF1	CSF2RA	4,91548E-04	7278,7	3,16993E-02	1107	8,62720E-01	970	1,66629E+00	1375	8,24673E-01	21733	0	11208,5
Responder	pDC_LILRA4	Mast	SPON2	ITGAM	4,92610E-04	12998,7	3,23758E-02	1068	8,43208E-01	1044	1,66599E+00	1376	6,68442E-01	50297	0	11208,5
Responder	Macro_ISG15	Mast	APOE	ABCA1	4,93247E-04	8389,3	1,01112E-02	8337	3,18842E-01	10035	8,65240E-01	6657	9,05547E-01	5709	0	11208,5
Responder	Macro_OLFML3	Macro_LYVE1	FN1	SDC2	4,93899E-04	9247,7	3,27284E-02	1042	6,16666E-01	2648	1,08495E+00	4292	7,98622E-01	27048	0	11208,5
Responder	Macro_OLFML3	Macro_LYVE1	APOE	TREM2	4,94353E-04	6082,3	1,68616E-02	3406	5,05403E-01	4293	6,32818E-01	10201	9,30467E-01	1303	0	11208,5
Responder	Macro_NLRP3	Macro_OLFML3	VCAN	TLR2	4,95463E-04	6025,7	2,69162E-02	1519	7,89592E-01	1308	8,51939E-01	6824	8,86647E-01	9269	0	11208,5
Responder	Mono_INHBA	Macro_ISG15	TIMP1	CD63	4,96697E-04	5241,9	9,07301E-03	10049	6,70669E-01	2108	1,31579E+00	2765	9,48663E-01	79	0	11208,5
Responder	pDC_LILRA4	Macro_IFI27	SPON2	ITGAM	4,97940E-04	13270,1	2,99472E-02	1233	8,28080E-01	1116	1,66409E+00	1381	6,59745E-01	51412	0	11208,5
Responder	pDC_LILRA4	Mono_CD14	SPON2	ITGA4	4,97940E-04	10867,7	2,83692E-02	1381	8,24217E-01	1127	1,86674E+00	936	7,33246E-01	39686	0	11208,5
Responder	pDC_LILRA4	Mast	SPON2	ITGA4	5,00082E-04	10944,1	2,83572E-02	1383	8,24099E-01	1128	1,69318E+00	1306	7,33205E-01	39695	0	11208,5
Responder	Mono_INHBA	Macro_FOLR2-APOE+	IL6	IL6R_IL6ST	5,03496E-04	11979,5	2,65827E-02	1557	7,32956E-01	1626	1,11187E+00	4086	7,23214E-01	41420	0	11208,5
Responder	Macro_OLFML3	Mono_CD16	C3	CD46	5,05408E-04	6483,3	1,86229E-02	2859	5,80250E-01	3093	9,93916E-01	5172	8,81998E-01	10084	0	11208,5
Responder	Macro_NLRP3	Macro_OLFML3	CD52	SIGLEC10	5,05909E-04	6574,7	1,49556E-02	4278	6,94444E-01	1905	7,04315E-01	8970	9,01433E-01	6512	0	11208,5
Responder	cDC_LAMP3	Mono_CD16	CCL19	CCRL2	5,06546E-04	8847,7	5,17922E-02	430	1,85228E+00	54	1,66108E+00	1389	7,78130E-01	31157	0	11208,5
Responder	pDC_LILRA4	cDC_CLEC9A	SPON2	ITGA4	5,06546E-04	10829,9	2,82733E-02	1389	8,23277E-01	1134	2,03810E+00	659	7,32915E-01	39759	0	11208,5
Responder	Macro_ISG15	cDC_LAMP3	TNFSF13B	CD40	5,06662E-04	7549,5	1,21444E-02	6107	5,41807E-01	3670	8,64102E-01	6673	8,81984E-01	10089	0	11208,5
Responder	Mono_INHBA	Macro_FOLR2-APOE+	CXCL8	SDC2	5,07188E-04	8314,3	2,42590E-02	1837	7,24481E-01	1677	1,20506E+00	3404	8,16599E-01	23445	0	11208,5
Responder	Mono_CD14	Mono_INHBA	NAMPT	ADORA2A	5,07629E-04	7153,1	3,19128E-02	1097	9,57261E-01	679	1,66091E+00	1390	8,26325E-01	21391	0	11208,5
Responder	Macro_OLFML3	Macro_LYVE1	SERPINA1	LRP1	5,08114E-04	6388,7	1,48691E-02	4323	7,52094E-01	1511	5,01771E-01	12892	9,25801E-01	2009	0	11208,5
Responder	cDC(CD1C)	Macro_FOLR2+APOE-	HMGB1	CD163	5,11365E-04	6751,9	8,56980E-03	11132	5,16039E-01	4107	1,08109E+00	4330	9,20524E-01	2982	0	11208,5
Responder	Mono_INHBA	cDC_LAMP3	ADM	RAMP1	5,12909E-04	10223,1	8,54526E-02	139	1,28055E+00	204	1,33094E+00	2690	7,48212E-01	36874	0	11208,5
Responder	Mono_INHBA	pDC_LILRA4	HMGB1	THBD	5,12970E-04	7622,9	1,02110E-02	8203	3,87667E-01	7371	1,73443E+00	1218	8,81870E-01	10114	0	11208,5
Responder	Macro_FOLR2+APOE+	pDC_LILRA4	CXCL11	CXCR3	5,13065E-04	14039,3	3,98904E-02	736	1,07757E+00	433	1,65848E+00	1395	6,14568E-01	56424	0	11208,5
Responder	cDC(CD1C)	Macro_ISG15	HLA-DRA	LAG3	5,13732E-04	6378,7	1,42043E-02	4708	5,51795E-01	3538	1,40417E+00	2322	8,81859E-01	10117	0	11208,5
Responder	Macro_ISG15	Macro_OLFML3	TNFSF13B	CD40	5,16276E-04	7793,5	1,21064E-02	6146	5,40103E-01	3694	7,82212E-01	7792	8,81820E-01	10127	0	11208,5
Responder	cDC(CD1C)	Mast	HBEGF	CD82	5,16975E-04	9352,5	1,48350E-02	4342	5,38596E-01	3715	1,18340E+00	3540	8,14091E-01	23957	0	11208,5
Responder	Mono_INHBA	Macro_FOLR2-APOE+	VEGFA	NRP1	5,18385E-04	9160,7	2,35984E-02	1939	7,68808E-01	1409	1,19296E+00	3479	7,95223E-01	27768	0	11208,5
Responder	Mast	Macro_IFI27	CSF1	CSF3R	5,18539E-04	6590,5	4,98471E-02	475	9,56901E-01	680	1,65700E+00	1400	8,36990E-01	19189	0	11208,5
Responder	Macro_NLRP3	cDC(CD1C)	S100A8	ITGB2	5,18830E-04	5265,9	1,19671E-02	6288	5,39574E-01	3701	1,07605E+00	4369	9,35408E-01	763	0	11208,5
Responder	Mono_INHBA	Macro_FOLR2-APOE+	VEGFA	NRP2	5,18855E-04	6913,1	3,01251E-02	1217	9,02837E-01	834	1,25406E+00	3124	8,41661E-01	18182	0	11208,5
Responder	Macro_NLRP3	cDC(CD1C)	S100A9	ITGB2	5,19854E-04	5302,5	1,01122E-02	8335	5,59643E-01	3406	1,27439E+00	2998	9,37401E-01	565	0	11208,5
Responder	Mono_INHBA	Macro_FOLR2-APOE+	VEGFA	SIRPA	5,20268E-04	7409,5	2,55878E-02	1668	8,17651E-01	1164	1,13391E+00	3889	8,37259E-01	19118	0	11208,5
Responder	Mast	Macro_IFI27	CSF1	CSF2RA	5,21842E-04	6771,3	3,68243E-02	855	9,01176E-01	839	1,65607E+00	1403	8,35245E-01	19551	0	11208,5
Responder	Mono_CD14	Mono_CD16	HLA-B	LILRB2	5,22165E-04	7190,7	9,02391E-03	10150	4,68814E-01	5078	6,82201E-01	9327	9,44226E-01	190	0	11208,5
Responder	Mono_INHBA	Macro_FOLR2-APOE+	ADM	CALCRL	5,22630E-04	12150,3	2,45538E-02	1796	6,24144E-01	2555	1,13953E+00	3844	7,23620E-01	41348	0	11208,5
Responder	Macro_NLRP3	cDC(CD1C)	VCAN	CD44	5,22680E-04	5879,9	1,31774E-02	5327	6,06878E-01	2760	1,05492E+00	4576	9,06619E-01	5528	0	11208,5
Responder	Macro_NLRP3	cDC(CD1C)	HBEGF	CD44	5,26812E-04	7961,5	1,02158E-02	8197	4,71548E-01	5005	8,84215E-01	6426	8,88337E-01	8971	0	11208,5
Responder	Mono_INHBA	Mono_INHBA	TIMP1	CD63	5,27378E-04	6195,1	8,16342E-03	12034	5,72189E-01	3238	1,07713E+00	4364	9,46028E-01	131	0	11208,5
Responder	Macro_OLFML3	Mono_CD14	C1QA	CD33	5,30188E-04	8756,3	9,00849E-03	10181	4,00731E-01	6950	8,25901E-01	7176	8,92056E-01	8266	0	11208,5
Responder	Mono_INHBA	Macro_NLRP3	GNAI2	C5AR1	5,30709E-04	8260,1	9,65664E-03	9031	5,33183E-01	3816	6,34060E-01	10183	8,98444E-01	7062	0	11208,5
Responder	Macro_FOLR2-APOE+	Mono_CD14	MMP9	ITGAM	5,31835E-04	8142,9	3,32833E-02	1017	7,97092E-01	1266	1,65172E+00	1412	8,05029E-01	25811	0	11208,5
Responder	pDC_LILRA4	Mono_CD14	PROC	THBD	5,34072E-04	13172,3	5,73481E-02	338	7,83102E-01	1335	1,65064E+00	1414	6,58562E-01	51566	0	11208,5
Responder	Mono_INHBA	pDC_LILRA4	LGALS1	CD69	5,37256E-04	6788,9	8,99848E-03	10208	4,95713E-01	4489	1,67897E+00	1340	9,00443E-01	6699	0	11208,5

Responder	Macro_OLFML3	cDC(CD1C)	LGALS3BP	CD33	5,37452E-04	7995,9	1,89028E-02	2785	5,01042E-01	4385	1,07714E+00	4363	8,46080E-01	17238	0	11208,5
Responder	Mono_CD14	cDC(CD1C)	AGTRAP	RACK1	5,37519E-04	7777,9	8,99841E-03	10209	4,05071E-01	6820	1,67753E+00	1344	8,86342E-01	9308	0	11208,5
Responder	Macro_NLRP3	Mono_INHBA	THBS1	LRP1	5,38836E-04	5869,5	2,05919E-02	2419	7,36110E-01	1607	1,06191E+00	4498	8,84586E-01	9615	0	11208,5
Responder	Macro_OLFML3	pDC_LILRA4	FARP2	PLXNA4	5,39693E-04	16583,1	4,86378E-02	505	8,54935E-01	1006	8,07938E-01	7435	4,88763E-01	62761	0	11208,5
Responder	pDC_LILRA4	Macro_FOLR2+APOE-	SPON2	ITGB1	5,39693E-04	12317,7	2,79249E-02	1419	8,22747E-01	1141	1,82561E+00	1009	7,57934E-01	35093	0,001	22926,5
Responder	Macro_OLFML3	cDC_LAMP3	C3	CD81	5,40685E-04	9056,9	1,06083E-02	7671	4,01492E-01	6929	6,86923E-01	9255	8,81263E-01	10221	0	11208,5
Responder	Macro_ISG15	Mono_CD16	SPP1	CD44	5,41479E-04	8404,5	1,13051E-02	6889	4,30022E-01	6084	6,31408E-01	10224	8,95598E-01	7617	0	11208,5
Responder	Macro_OLFML3	pDC_LILRA4	HSP90B1	TLR9	5,41952E-04	6460,9	6,92342E-02	220	1,36504E+00	162	9,69839E-01	5421	8,55360E-01	15293	0	11208,5
Responder	pDC_LILRA4	Macro_ISG15	APP	CD74	5,41952E-04	3656,5	1,41721E-02	4723	7,67439E-01	1421	1,95841E+00	781	9,45384E-01	149	0	11208,5
Responder	Mono_CD14	cDC(CD1C)	VCAN	ITGA4	5,42274E-04	4965,3	2,26552E-02	2079	9,24354E-01	773	2,14223E+00	539	8,81239E-01	10227	0	11208,5
Responder	Macro_OLFML3	cDC_CLEC9A	CXCL11	CXCR3	5,42299E-04	13655,5	6,17482E-02	287	7,39605E-01	1586	1,07373E+00	4395	6,64858E-01	50801	0	11208,5
Responder	Macro_OLFML3	Macro_ISG15	C1QA	CD93	5,43335E-04	8467,1	8,98942E-03	10231	4,61581E-01	5256	8,09083E-01	7418	8,92234E-01	8222	0	11208,5
Responder	Mono_INHBA	Macro_FOLR2-APOE+	INHBA	ACTR2	5,44247E-04	5317,3	2,83971E-02	1378	9,17020E-01	792	1,64126E+00	1447	8,73502E-01	11761	0	11208,5
Responder	Macro_NLRP3	Mono_INHBA	S100A8	CD36	5,44664E-04	6192,7	2,05977E-02	2417	7,19621E-01	1707	7,47114E-01	8296	8,97003E-01	7335	0	11208,5
Responder	Macro_NLRP3	Mono_INHBA	S100A8	CD68	5,44930E-04	5660,3	1,33845E-02	5187	6,40142E-01	2398	6,89923E-01	9206	9,41588E-01	302	0	11208,5
Responder	Macro_NLRP3	Mono_INHBA	S100A9	CD36	5,45729E-04	5696,3	1,74051E-02	3226	7,39689E-01	1585	9,45454E-01	5684	9,00054E-01	6778	0	11208,5
Responder	Macro_NLRP3	Mono_INHBA	S100A9	CD68	5,45996E-04	5376,1	1,13099E-02	6884	6,60210E-01	2201	8,88264E-01	6367	9,43402E-01	220	0	11208,5
Responder	Macro_OLFML3	Macro_IFI27	C3	LRP1	5,46796E-04	7448,9	1,34709E-02	5143	4,82778E-01	4753	9,26628E-01	5896	8,81143E-01	10244	0	11208,5
Responder	Macro_OLFML3	pDC_LILRA4	HMGB1	TLR9	5,47627E-04	6349,3	6,34272E-02	267	1,33203E+00	176	9,13021E-01	6067	8,61491E-01	14028	0	11208,5
Responder	Macro_NLRP3	Mono_INHBA	VCAN	ITGB1	5,48132E-04	5955,1	1,82778E-02	2961	7,42431E-01	1563	1,07392E+00	4392	8,84422E-01	9651	0	11208,5
Responder	pDC_LILRA4	cDC_CLEC9A	TNFSF9	HLA-DPA1	5,53077E-04	3566,5	2,70015E-02	1510	1,00264E+00	572	2,73256E+00	125	9,12653E-01	4417	0	11208,5
Responder	Macro_OLFML3	cDC(CD1C)	B2M	CD1A	5,55638E-04	5596,5	5,66683E-02	349	7,65361E-01	1433	1,05661E+00	4552	8,80125E-01	10440	0	11208,5
Responder	cDC_CLEC9A	pDC_LILRA4	HSP90B1	TLR9	5,56789E-04	6086,9	5,96616E-02	310	1,26540E+00	212	1,64550E+00	1434	8,45909E-01	17270	0	11208,5
Responder	Macro_OLFML3	Macro_LYVE1	C1QA	CD33	5,57555E-04	7310,1	1,27212E-02	5652	5,18925E-01	4058	6,28042E-01	10284	9,07582E-01	5348	0	11208,5
Responder	cDC_LAMP3	Mast	LGALS1	CD69	5,61014E-04	4756,3	1,46571E-02	4433	7,36238E-01	1606	1,18666E+00	3513	9,20275E-01	3021	0	11208,5
Responder	Mono_INHBA	Macro_ISG15	IL6	IL6R_IL6ST	5,61513E-04	12794,9	2,19195E-02	2205	6,95630E-01	1893	1,15559E+00	3710	7,03499E-01	44958	0	11208,5
Responder	Macro_IER3	pDC_LILRA4	GNAI2	CXCR3	5,62566E-04	6209,3	3,20705E-02	1083	1,10396E+00	385	1,64274E+00	1439	8,47607E-01	16931	0	11208,5
Responder	Macro_NLRP3	Mono_INHBA	NAMPT	ITGA5_ITGB1	5,63271E-04	7266,1	1,15821E-02	6633	6,72552E-01	2088	8,30971E-01	7120	8,86528E-01	9281	0	11208,5
Responder	Macro_NLRP3	Mono_INHBA	VEGFA	CD44	5,63545E-04	6789,9	1,37393E-02	4990	6,94739E-01	1901	8,91813E-01	6331	8,85131E-01	9519	0	11208,5
Responder	Mono_INHBA	Macro_ISG15	CCL3	CCR5	5,65516E-04	7067,9	2,73535E-02	1475	9,08554E-01	816	1,60125E+00	1563	8,31799E-01	20277	0	11208,5
Responder	cDC(CD1C)	Macro_ISG15	HLA-DPA1	LAG3	5,65516E-04	6574,3	1,46436E-02	4442	5,45054E-01	3618	1,38630E+00	2423	8,76354E-01	11180	0	11208,5
Responder	Macro_FOLR2-APOE+	Macro_NLRP3	PLAU	PLAUR	5,66010E-04	6785,3	1,42958E-02	4656	7,16577E-01	1727	9,15772E-01	6020	8,80810E-01	10315	0	11208,5
Responder	Mono_INHBA	Macro_ISG15	CCL4	CCR1	5,68533E-04	5581,7	1,97320E-02	2584	7,35008E-01	1613	1,60367E+00	1555	8,77420E-01	10948	0	11208,5
Responder	Macro_NLRP3	Mono_CD14	THBS1	CD36	5,69034E-04	4947,7	3,32926E-02	1016	9,19038E-01	786	1,60061E+00	1568	8,81641E-01	10160	0	11208,5
Responder	Mono_INHBA	Macro_ISG15	CCL4	CCR5	5,69036E-04	8120,3	2,36542E-02	1930	7,25812E-01	1669	1,45070E+00	2111	8,15509E-01	23683	0	11208,5
Responder	Macro_FOLR2+APOE+	Macro_FOLR2-APOE+	APOE	LRP1	5,71561E-04	4236,3	1,73476E-02	3246	8,23107E-01	1137	1,06636E+00	4454	9,31812E-01	1136	0	11208,5
Responder	pDC_LILRA4	cDC_CLEC9A	SPON2	ITGB2	5,73064E-04	9547,6	3,04112E-02	1186	7,63487E-01	1448	2,02405E+00	676	8,39586E-01	18625	0,01	25803
Responder	Mast	Macro_ISG15	TIMP3	CD44	5,74238E-04	4377,3	4,91484E-02	492	1,02011E+00	534	1,64045E+00	1449	8,92351E-01	8203	0	11208,5
Responder	Macro_NLRP3	Mono_CD14	S100A8	ITGB2	5,75678E-04	6126,9	1,12393E-02	6958	4,88034E-01	4649	8,49613E-01	6858	9,33487E-01	961	0	11208,5
Responder	Macro_NLRP3	Mono_CD14	S100A8	CD36	5,75956E-04	4728,7	2,39734E-02	1882	7,95546E-01	1272	1,23598E+00	3232	9,03806E-01	6049	0	11208,5
Responder	Macro_NLRP3	Mono_CD14	S100A9	ITGB2	5,76791E-04	6029,9	9,49720E-03	9296	5,08103E-01	4243	1,04795E+00	4651	9,35535E-01	751	0	11208,5
Responder	Macro_NLRP3	Mono_CD14	S100A9	CD36	5,77069E-04	4517,1	2,02575E-02	2481	8,15614E-01	1180	1,43432E+00	2198	9,06676E-01	5518	0	11208,5
Responder	pDC_LILRA4	Macro_NLRP3	RARRES2	CCRL2	5,78951E-04	11045,5	3,94819E-02	754	7,62607E-01	1453	2,33387E+00	347	7,22952E-01	41465	0	11208,5
Responder	Macro_FOLR2+APOE-	cDC(CD1C)	CXCL12	CXCR4	5,78951E-04	4791,5	2,74649E-02	1453	8,41210E-01	1055	1,92186E+00	837	8,85821E-01	9404	0	11208,5
Responder	Mono_INHBA	Mono_CD16	CD99	PILRA	5,79023E-04	7880,3	1,08528E-02	7400	6,02477E-01	2809	6,23226E-01	10362	8,95526E-01	7622	0	11208,5
Responder	Mono_INHBA	Macro_ISG15	CCL3L1	CCR1	5,79694E-04	6378,9	2,45753E-02	1791	7,56178E-01	1497	1,58621E+00	1619	8,53171E-01	15779	0	11208,5
Responder	Macro_IFI27	pDC_LILRA4	CD14	TLR9	5,80124E-04	5840,5	6,98329E-02	217	1,31991E+00	181	1,24377E+00	3175	8,59588E-01	14421	0	11208,5
Responder	Macro_ISG15	Macro_OLFML3	CCL8	CCR5	5,80133E-04	13850,1	4,61844E-02	561	7,89782E-01	1305	1,63920E+00	1454	6,31208E-01	54722	0	11208,5
Responder	Mono_INHBA	Macro_ISG15	CCL3L1	CCR5	5,80205E-04	9319,9	2,94601E-02	1281	7,46983E-01	1536	1,43323E+00	2204	7,82056E-01	30370	0	11208,5
Responder	Mono_INHBA	Macro_ISG15	TNF	VSIR	5,81741E-04	7303,7	1,97158E-02	2592	6,53561E-01	2279	1,06446E+00	4474	8,52215E-01	15965	0	11208,5
Responder	Macro_NLRP3	Mono_CD14	HBEGF	CD44	5,82945E-04	5350,3	1,48511E-02	4336	7,13290E-01	1748	1,15000E+00	3758	9,05589E-01	5701	0	11208,5
Responder	Mono_CD14	Mono_INHBA	S100A8	CD69	5,82945E-04	5853,3	1,20660E-02	6184	1,08266E+00	420	1,78896E+00	1078	8,80392E-01	10376	0	11208,5
Responder	Macro_IFI27	Mono_CD14	FN1	PLAUR	5,83793E-04	5295,5	1,62362E-02	3656	5,25744E-01	3932	1,23949E+00	3203	9,12294E-01	4478	0	11208,5
Responder	Mono_CD14	cDC(CD1C)	VCAN	ITGB1	5,84069E-04	5348,5	1,69007E-02	3391	8,33574E-01	1085	2,02372E+00	678	8,80358E-01	10380	0	11208,5
Responder	Macro_OLFML3	Macro_LYVE1	MDK	SDC3	5,84306E-04	7557,7	5,21449E-02	423	7,73828E-01	1380	1,06366E+00	4479	8,31705E-01	20298	0	11208,5
Responder	Macro_NLRP3	Mono_CD14	GNAI2	C5AR1	5,84351E-04	7286,9	9,50071E-03	9284	5,15284E-01	4118	1,05062E+00	4622	8,97699E-01	7202	0	11208,5
Responder	Macro_FOLR2+APOE+	cDC_CLEC9A	APOE	SCARB1	5,84878E-04	4519,1	2,74363E-02	1458	9,09088E-01	815	1,77269E+00	1115	8,93527E-01	7999	0	11208,5
Responder	Macro_ISG15	Macro_LYVE1	C1QB	LRP1	5,87453E-04	7369,7	1,08174E-02	7432	4,59223E-01	5306	6,22222E-01	10392	9,22945E-01	2510	0	11208,5
Responder	pDC_LILRA4	Macro_FOLR2-APOE+	SPON2	ITGAM	5,89649E-04	11989,9	4,25780E-02	647	9,06758E-01	821	1,63524E+00	1462	6,98067E-01	45811	0	11208,5
Responder	Macro_OLFML3	Mono_CD14	HLA-A	LILRA1	5,90853E-04	8101,5	1,17761E-02	6454	5,24176E-01	3964	7,35919E-01	8477	8,80279E-01	10404	0	11208,5
Responder	Mono_INHBA	Macro_LYVE1	HMGB1	CD163	5,91705E-04	7410,7	9,13297E-03	9949	5,91424E-01	2949	6,21312E-01	10407	9,22821E-01	2540	0	11208,5
Responder	Macro_IER3	pDC_LILRA4	TNF	NOTCH1	5,92044E-04	10174,9	3,27397E-02	1041	7,61904E-01	1464	2,02942E+00	669	7,50144E-01	36492	0	11208,5
Responder	Macro_OLFML3	cDC(CD1C)	C3	CD46	5,93093E-04	7187,7	1,45728E-02	4496	5,02126E-01	4363	1,22551E+00	3288	8,68627E-01	12583	0	11208,5
Responder	Mono_CD14	Macro_FOLR2-APOE+	CXCL8	SDC2	5,95648E-04	7333,9	2,73965E-02	1467	8,07381E-01	1216	1,72439E+00	1235	8,25532E-01	21543	0	11208,5
Responder	Mono_INHBA	Macro_ISG15	INHBA	ACTR2	5,96218E-04	5878,1	2,36652E-02	1925	8,16021E-01	1176	1,67054E+00	1362	8,63084E-01	13719	0	11208,5

Responder	Macro_FOLR2-APOE+	Mast	ARPC5	ADRB2	5,96268E-04	6714,3	2,59091E-02	1636	6,79527E-01	2026	7,48540E-01	8278	8,80203E-01	10423	0	11208,5
Responder	cDC_CLEC9A	Mast	IL16	CD9	5,96852E-04	5043,1	2,97407E-02	1257	8,39580E-01	1065	1,63260E+00	1468	8,81282E-01	10217	0	11208,5
Responder	Mono_CD14	cDC_CLEC9A	VCAN	ITGB1	5,97699E-04	5392,1	1,68453E-02	3416	8,32357E-01	1090	1,93501E+00	818	8,80185E-01	10428	0	11208,5
Responder	cDC(CD1C)	Mast	CD55	ADGRE2	5,97785E-04	7735,7	1,56510E-02	3941	5,91376E-01	2950	1,06123E+00	4505	8,51763E-01	16074	0	11208,5
Responder	Mono_INHBA	Macro_LYVE1	INHBA	ENG	5,98059E-04	8938,1	2,87962E-02	1342	8,53404E-01	1012	1,63237E+00	1469	7,85571E-01	29659	0	11208,5
Responder	Macro_NLRP3	Macro_FOLR2-APOE+	THBS1	LRP1	5,98273E-04	6679,9	1,92546E-02	2690	7,01805E-01	1836	8,09089E-01	7417	8,81114E-01	10248	0	11208,5
Responder	Mono_INHBA	Macro_NLRP3	TNF	TNFRSF1B	5,99355E-04	6789,3	1,62097E-02	3662	6,81852E-01	1998	1,06459E+00	4473	8,68548E-01	12605	0	11208,5
Responder	pDC_LILRA4	Macro_LYVE1	HSP90B1	LRP1	6,00928E-04	4159,1	1,45515E-02	4511	8,26362E-01	1124	2,24036E+00	418	9,17421E-01	3534	0	11208,5
Responder	Macro_OLFML3	Mono_CD16	FN1	CD44	6,01978E-04	7285,5	1,77301E-02	3120	4,94715E-01	4513	4,88889E-01	13211	9,12818E-01	4375	0	11208,5
Responder	Mono_CD14	Macro_FOLR2+APOE-	CXCL8	SDC3	6,02899E-04	5436,3	2,73703E-02	1473	8,23082E-01	1138	1,86892E+00	933	8,69510E-01	12429	0	11208,5
Responder	Macro_OLFML3	Macro_FOLR2+APOE+	HLA-DPA1	LAG3	6,03164E-04	7304,1	1,74201E-02	3220	6,61595E-01	2185	6,19082E-01	10447	8,85457E-01	9460	0	11208,5
Responder	Mono_INHBA	Mono_INHBA	VEGFA	NRP1	6,04083E-04	11205,3	1,60977E-02	3726	6,54143E-01	2276	1,05919E+00	4517	7,62322E-01	34299	0	11208,5
Responder	Mono_CD14	Macro_OLFML3	VCAN	ITGB1	6,05767E-04	5862,9	1,67968E-02	3442	8,31291E-01	1092	1,25588E+00	3116	8,80033E-01	10456	0	11208,5
Responder	Mast	Mono_CD14	PLAT	ITGAM	6,08987E-04	9303,1	1,16742E-01	62	9,36569E-01	744	1,62971E+00	1478	7,68454E-01	33023	0	11208,5
Responder	pDC_LILRA4	cDC_CLEC9A	CALM1	MYLK	6,08987E-04	12214,2	3,70805E-02	843	7,60091E-01	1478	2,14794E+00	535	7,71632E-01	32412	0,01	25803
Responder	cDC(CD1C)	Macro_LYVE1	LGALS9	LRP1	6,09542E-04	7671,3	1,06594E-02	7618	5,24957E-01	3947	9,98434E-01	5114	8,79962E-01	10469	0	11208,5
Responder	cDC(CD1C)	pDC_LILRA4	HLA-DMA	CD4	6,09899E-04	5888,7	8,53198E-03	11201	4,93683E-01	4528	2,46939E+00	250	9,24446E-01	2256	0	11208,5
Responder	Macro_NLRP3	Macro_FOLR2-APOE+	VCAN	ITGB1	6,10124E-04	6360,5	1,84558E-02	2905	7,47495E-01	1535	8,69763E-01	6601	8,84916E-01	9553	0	11208,5
Responder	Mono_CD14	cDC_CLEC9A	ADAM10	CADM1	6,10209E-04	8667,1	2,74238E-02	1461	8,54554E-01	1007	1,62966E+00	1479	7,93148E-01	28180	0	11208,5
Responder	Mono_CD14	Macro_FOLR2+APOE+	S100A9	CD68	6,10416E-04	5208,5	8,86107E-03	10472	7,67407E-01	1422	1,40770E+00	2304	9,36524E-01	636	0	11208,5
Responder	Macro_NLRP3	Macro_FOLR2-APOE+	VCAN	CD44	6,10707E-04	5778,9	1,62499E-02	3649	7,31104E-01	1638	7,38206E-01	8446	9,15121E-01	3953	0	11208,5
Responder	Macro_IER3	pDC_LILRA4	ICAM1	SPN	6,12658E-04	5680,9	2,72767E-02	1481	9,28531E-01	764	1,97934E+00	751	8,60641E-01	14200	0	11208,5
Responder	Macro_NLRP3	Mono_CD14	S100A9	TLR4	6,13628E-04	6555,9	1,39910E-02	4841	6,06636E-01	2764	1,19153E+00	3483	8,79884E-01	10483	0	11208,5
Responder	Mono_INHBA	Macro_NLRP3	IL1B	IL1R2_IL1RAP	6,15757E-04	8847,1	1,57175E-02	3908	6,29185E-01	2508	1,11725E+00	4038	8,20659E-01	22573	0	11208,5
Responder	cDC(CD1C)	Mono_CD14	ANXA1	DYSF	6,17897E-04	8149,9	1,67929E-02	3444	4,92717E-01	4543	1,29948E+00	2860	8,39193E-01	18694	0	11208,5
Responder	cDC(CD1C)	cDC_CLEC9A	HLA-DQA1	LAG3	6,18433E-04	7342,5	1,44651E-02	4544	5,54188E-01	3497	1,54754E+00	1762	8,53476E-01	15701	0	11208,5
Responder	Mono_INHBA	Macro_NLRP3	IL6	IL6R_IL6ST	6,18969E-04	10592,7	3,48678E-02	943	7,98280E-01	1260	1,28504E+00	2940	7,49531E-01	36612	0	11208,5
Responder	Macro_NLRP3	Macro_FOLR2-APOE+	HBEGF	CD9	6,20390E-04	7072,7	2,08432E-02	2374	6,65609E-01	2139	6,24655E-01	10337	8,86345E-01	9305	0	11208,5
Responder	Macro_OLFML3	Mono_CD14	SERPING1	LRP1	6,23052E-04	7784,1	1,16372E-02	6581	4,71041E-01	5021	9,52934E-01	5595	8,79730E-01	10515	0	11208,5
Responder	cDC(CD1C)	Macro_FOLR2+APOE+	HLA-DRB5	LAG3	6,23272E-04	7276,7	1,61953E-02	3668	4,92395E-01	4553	1,06106E+00	4507	8,69348E-01	12447	0	11208,5
Responder	Macro_ISG15	Macro_FOLR2-APOE+	CCL8	CCR5	6,25002E-04	14357,3	3,83271E-02	793	7,57057E-01	1491	1,65489E+00	1407	6,09250E-01	56887	0	11208,5
Responder	pDC_LILRA4	Macro_ISG15	SPON2	ITGB1	6,25002E-04	10155,7	2,71976E-02	1491	8,13491E-01	1189	1,67317E+00	1357	7,55505E-01	35533	0	11208,5
Responder	pDC_LILRA4	cDC(CD1C)	TNFSF9	HLA-DPA1	6,25432E-04	3612,9	2,64824E-02	1567	9,76753E-01	634	2,82718E+00	98	9,11876E-01	4557	0	11208,5
Responder	Mono_CD14	Mono_CD14	CD55	CR1	6,26246E-04	7160,7	2,71932E-02	1492	8,11556E-01	1201	1,66535E+00	1378	8,30638E-01	20524	0	11208,5
Responder	Mono_INHBA	cDC(CD1C)	VEGFA	CD44	6,26317E-04	7029,9	1,23692E-02	5931	5,56746E-01	3455	1,11844E+00	4029	8,79682E-01	10526	0	11208,5
Responder	Macro_IFI27	Macro_NLRP3	MMP12	PLAUR	6,28737E-04	5408,3	2,71603E-02	1494	8,27978E-01	1117	1,68548E+00	1321	8,72688E-01	11901	0	11208,5
Responder	cDC(CD1C)	Macro_OLFML3	CD52	SIGLEC10	6,28999E-04	9346,7	9,54839E-03	9208	4,03224E-01	6873	7,07648E-01	8909	8,79625E-01	10535	0	11208,5
Responder	Macro_FOLR2+APOE-	cDC_CLEC9A	C1QB	C1QBP	6,29768E-04	4721,1	1,47819E-02	4364	6,76467E-01	2058	1,05575E+00	4565	9,29660E-01	1410	0	11208,5
Responder	Macro_OLFML3	Macro_IER3	C3	C3AR1	6,33190E-04	8661,3	1,27642E-02	5617	4,05054E-01	6821	6,95932E-01	9111	8,79513E-01	10549	0	11208,5
Responder	pDC_LILRA4	cDC(CD1C)	ASIP	MGRN1	6,33740E-04	13519,9	6,13278E-02	292	8,19733E-01	1151	1,62456E+00	1498	6,42561E-01	53450	0	11208,5
Responder	Mono_CD14	pDC_LILRA4	ADM	CALCRL	6,33740E-04	10120,9	3,19048E-02	1098	7,55824E-01	1498	2,89848E+00	82	7,49028E-01	36718	0	11208,5
Responder	cDC(CD1C)	pDC_LILRA4	HLA-DRB1	CD4	6,35219E-04	6065,1	7,44302E-03	14026	4,91551E-01	4575	2,52620E+00	215	9,41602E-01	301	0	11208,5
Responder	Mono_CD14	Mono_CD16	S100A8	CD36	6,35294E-04	5418,9	1,44622E-02	4547	1,14102E+00	347	2,22214E+00	436	8,79483E-01	10556	0	11208,5
Responder	Mono_CD14	Mono_CD16	GNAI2	C5AR1	6,36197E-04	8544,3	8,81311E-03	10559	4,62880E-01	5226	7,78438E-01	7843	8,94198E-01	7885	0	11208,5
Responder	cDC(CD1C)	Mono_CD14	ANXA1	FPR1	6,37403E-04	7872,7	8,81041E-03	10563	3,74354E-01	7828	1,30857E+00	2802	8,98987E-01	6962	0	11208,5
Responder	Mast	Macro_FOLR2-APOE+	TIMP3	CD44	6,37509E-04	3902,3	6,21060E-02	284	1,15718E+00	319	1,62380E+00	1501	9,03085E-01	6199	0	11208,5
Responder	Mast	Macro_IER3	CSF1	CSF2RA	6,38769E-04	6726,5	3,75570E-02	826	9,06673E-01	822	1,62342E+00	1502	8,36596E-01	19274	0	11208,5
Responder	Macro_OLFML3	Macro_OLFML3	C1QB	C1QBP	6,40123E-04	6669,9	1,21109E-02	6139	5,94928E-01	2897	6,11828E-01	10572	9,22859E-01	2533	0	11208,5
Responder	Mono_INHBA	Macro_NLRP3	INHBA	ENG	6,40156E-04	11386,1	1,69196E-02	3384	7,44851E-01	1549	1,51588E+00	1854	7,37409E-01	38935	0	11208,5
Responder	Macro_NLRP3	Macro_ISG15	S100A9	ITGB2	6,40426E-04	7668,5	8,81614E-03	10552	4,51027E-01	5525	6,41015E-01	10071	9,33254E-01	986	0	11208,5
Responder	Mono_INHBA	Macro_FOLR2-APOE+	CCL3L1	CCR5	6,41256E-04	13121,9	1,44014E-02	4586	5,80068E-01	3097	1,14716E+00	3779	7,15008E-01	42939	0	11208,5
Responder	Mono_INHBA	Macro_ISG15	VEGFA	SIRPA	6,43462E-04	8992,1	1,83890E-02	2926	6,66022E-01	2137	1,05395E+00	4590	8,13482E-01	24099	0	11208,5
Responder	Macro_OLFML3	Mono_INHBA	GRN	TNFRSF1B	6,43764E-04	7460,9	8,80277E-03	10584	5,78985E-01	3122	6,28281E-01	10277	9,25188E-01	2113	0	11208,5
Responder	Macro_NLRP3	Macro_ISG15	VCAN	CD44	6,44677E-04	6712,5	1,28596E-02	5551	5,94026E-01	2915	7,54862E-01	8186	9,05581E-01	5702	0	11208,5
Responder	pDC_LILRA4	Macro_LYVE1	SPON2	ITGB1	6,45092E-04	8538,1	4,07000E-02	713	9,85349E-01	615	1,62191E+00	1507	7,90798E-01	28647	0	11208,5
Responder	pDC_LILRA4	cDC_CLEC9A	COPA	P2RY6	6,45092E-04	6982,5	2,70272E-02	1507	7,98002E-01	1263	2,53968E+00	210	8,29714E-01	20724	0	11208,5
Responder	Mono_INHBA	Macro_NLRP3	INHBA	ACTR2	6,46361E-04	5438,7	2,70180E-02	1508	8,87582E-01	879	1,67578E+00	1350	8,70726E-01	12248	0	11208,5
Responder	cDC_CLEC9A	Mast	HSPA8	ADRB2	6,47633E-04	4308,9	2,91274E-02	1310	7,53016E-01	1509	1,66923E+00	1366	9,03323E-01	6151	0	11208,5
Responder	cDC(CD1C)	pDC_LILRA4	SPINT1	ST14	6,47890E-04	13639,7	1,83562E-02	2933	4,90451E-01	4598	1,90289E+00	865	6,80242E-01	48594	0	11208,5
Responder	pDC_LILRA4	pDC_LILRA4	SPON2	ITGA4	6,50180E-04	10927,1	2,70008E-02	1511	8,10810E-01	1204	2,56976E+00	189	7,28383E-01	40523	0	11208,5
Responder	pDC_LILRA4	Macro_IFI27	SPON2	ITGB2	6,52734E-04	6489,7	3,28880E-02	1036	8,18818E-01	1155	1,62005E+00	1513	8,44788E-01	17536	0	11208,5
Responder	pDC_LILRA4	cDC_LAMP3	SPON2	ITGA5	6,54013E-04	13677,7	2,69853E-02	1514	8,37898E-01	1071	1,70830E+00	1270	6,43662E-01	53325	0	11208,5
Responder	Macro_OLFML3	pDC_LILRA4	CXCL12	DPP4	6,56577E-04	14609,7	5,92343E-02	315	7,51215E-01	1516	1,00326E+00	5061	6,29270E-01	54948	0	11208,5
Responder	Mono_INHBA	cDC(CD1C)	MIF	CD44_CD74	6,56815E-04	7534,1	6,36263E-03	17922	4,89902E-01	4614	1,14278E+00	3818	9,47163E-01	108	0	11208,5
Responder	Macro_OLFML3	Mono_CD16	B2M	LILRB2	6,58495E-04	6161,3	1,04767E-02	7851	8,30795E-01	1096	6,08580E-01	10632	9,54206E-01	19	0	11208,5

Responder	cDC(CD1C)	Macro_ISG15	ANXA1	FPR1	6,62531E-04	8574,3	8,77115E-03	10645	3,71757E-01	7920	9,10939E-01	6094	8,98784E-01	7004	0	11208,5
Responder	Macro_FOLR2+APOE+	pDC_LILRA4	HSP90B1	TLR9	6,62784E-04	6547,7	6,72903E-02	232	1,34480E+00	167	9,66474E-01	5459	8,53589E-01	15672	0	11208,5
Responder	pDC_LILRA4	Mono_CD14	PROC	ITGAM	6,64308E-04	13273,5	7,48427E-02	193	8,16340E-01	1174	1,61523E+00	1522	6,52649E-01	52270	0	11208,5
Responder	Mono_INHBA	Macro_OLFML3	CCL3L1	CCR1	6,65264E-04	8529,5	1,70654E-02	3337	6,21160E-01	2592	1,05001E+00	4629	8,28829E-01	20881	0	11208,5
Responder	Macro_NLRP3	Macro_NLRP3	THBS1	LRP1	6,67212E-04	6291,3	1,98808E-02	2555	7,17869E-01	1716	9,16007E-01	6017	8,82780E-01	9960	0	11208,5
Responder	Macro_NLRP3	cDC_LAMP3	IL10	CD70	6,68489E-04	15544,1	1,16513E-01	63	1,24103E+00	233	7,37710E-01	8450	5,99402E-01	57766	0	11208,5
Responder	Macro_NLRP3	Macro_ISG15	CD52	SIGLEC10	6,69405E-04	9201,1	9,42285E-03	9425	4,77972E-01	4863	6,52795E-01	9842	8,78923E-01	10667	0	11208,5
Responder	cDC_CLEC9A	cDC_CLEC9A	CALM1	MYLK	6,69495E-04	8762,7	4,37678E-02	621	8,93433E-01	855	1,61432E+00	1526	7,85911E-01	29603	0	11208,5
Responder	Mono_CD14	Macro_IER3	VCAN	ITGB1	6,69719E-04	5749,1	1,64460E-02	3579	8,23578E-01	1132	1,44216E+00	2158	8,78914E-01	10668	0	11208,5
Responder	Macro_OLFML3	Macro_ISG15	HLA-DPA1	LAG3	6,71290E-04	7261,9	1,53491E-02	4070	6,08671E-01	2740	7,94485E-01	7618	8,78880E-01	10673	0	11208,5
Responder	Macro_NLRP3	Macro_FOLR2-APOE+	S100A9	CD68	6,71919E-04	6463,1	1,07365E-02	7519	6,18193E-01	2627	6,06020E-01	10675	9,41997E-01	286	0	11208,5
Responder	Mono_CD14	Macro_LYVE1	THBS1	LRP1	6,73179E-04	6426,3	1,84476E-02	2908	6,98373E-01	1864	9,65472E-01	5472	8,78853E-01	10679	0	11208,5
Responder	Mono_INHBA	cDC_CLEC9A	NECTIN2	CD226	6,73223E-04	13653,7	3,36898E-02	998	7,62461E-01	1457	1,06127E+00	4504	6,69866E-01	50101	0	11208,5
Responder	cDC_CLEC9A	Mono_INHBA	BTLA	TNFRSF14	6,73403E-04	7178,5	4,61406E-02	563	1,69469E+00	80	1,61312E+00	1529	8,20894E-01	22512	0	11208,5
Responder	Macro_OLFML3	Macro_ISG15	C3	NRP1	6,73794E-04	9010,1	1,43162E-02	4644	5,83780E-01	3046	1,18219E+00	3548	8,20517E-01	22604	0	11208,5
Responder	Mono_INHBA	cDC_CLEC9A	IL1B	SIGIRR	6,74938E-04	6934,9	1,43738E-02	4605	6,30745E-01	2487	1,34070E+00	2633	8,62938E-01	13741	0	11208,5
Responder	Mono_INHBA	cDC_CLEC9A	IL6	IL6R_IL6ST	6,75510E-04	11658,9	2,59186E-02	1634	7,26006E-01	1668	1,50896E+00	1884	7,20674E-01	41900	0	11208,5
Responder	Macro_NLRP3	Macro_NLRP3	S100A8	CD36	6,75704E-04	6831,1	1,90245E-02	2755	6,84235E-01	1979	6,34926E-01	10163	8,93275E-01	8050	0	11208,5
Responder	Macro_NLRP3	Macro_NLRP3	S100A9	CD36	6,76970E-04	6261,1	1,60756E-02	3731	7,04303E-01	1817	8,33266E-01	7086	8,96423E-01	7463	0	11208,5
Responder	Macro_OLFML3	Macro_IFI27	FN1	CD44	6,78237E-04	9156,1	1,19745E-02	6285	3,04108E-01	10695	6,43371E-01	10030	8,95883E-01	7562	0	11208,5
Responder	Macro_NLRP3	Macro_NLRP3	VCAN	ITGB1	6,79189E-04	6571,3	1,70349E-02	3349	7,07077E-01	1800	9,03412E-01	6179	8,80774E-01	10320	0	11208,5
Responder	Mono_INHBA	cDC_CLEC9A	CCL5	CXCR3	6,79526E-04	12592,3	2,49637E-02	1747	6,76252E-01	2060	1,27937E+00	2975	7,03416E-01	44971	0	11208,5
Responder	Macro_NLRP3	Macro_NLRP3	VCAN	CD44	6,80142E-04	4763,5	1,89743E-02	2764	8,41258E-01	1053	9,26669E-01	5895	9,20950E-01	2897	0	11208,5
Responder	Mono_INHBA	cDC(CD1C)	HBEGF	CD44	6,83007E-04	9586,9	8,73797E-03	10710	3,71042E-01	7939	7,88707E-01	7693	8,80348E-01	10384	0	11208,5
Responder	Macro_NLRP3	Macro_NLRP3	HBEGF	CD44	6,84922E-04	6271,9	1,47099E-02	4402	7,05928E-01	1807	7,55963E-01	8172	9,05180E-01	5770	0	11208,5
Responder	Mono_INHBA	Macro_IFI27	TIMP1	CD63	6,85881E-04	5753,5	8,73319E-03	10719	6,33878E-01	2456	1,08517E+00	4290	9,47725E-01	94	0	11208,5
Responder	Mono_INHBA	cDC_CLEC9A	ADM	RAMP1	6,86451E-04	12454,5	4,93832E-02	488	8,71497E-01	940	1,26580E+00	3058	6,93158E-01	46578	0	11208,5
Responder	Mono_INHBA	cDC_CLEC9A	ADM	CALCRL	6,87030E-04	11979,9	2,42803E-02	1834	6,20254E-01	2601	1,33080E+00	2691	7,22498E-01	41565	0	11208,5
Responder	Mono_INHBA	Macro_FOLR2-APOE+	CXCL8	SDC3	6,87030E-04	8652,1	1,54671E-02	4012	5,55731E-01	3471	1,04607E+00	4667	8,33586E-01	19902	0	11208,5
Responder	Mono_INHBA	cDC_CLEC9A	COPA	P2RY6	6,88190E-04	9824,3	1,63126E-02	3630	6,00452E-01	2830	1,29913E+00	2865	7,91031E-01	28588	0	11208,5
Responder	Macro_OLFML3	Macro_ISG15	FN1	C5AR1	6,88445E-04	6924,3	1,72605E-02	3270	4,51476E-01	5514	1,13198E+00	3902	8,78585E-01	10727	0	11208,5
Responder	Macro_NLRP3	Macro_NLRP3	GNAI2	C5AR1	6,88766E-04	7438,5	1,09201E-02	7311	6,18134E-01	2629	6,44389E-01	10014	9,03918E-01	6030	0	11208,5
Responder	Mono_INHBA	Macro_FOLR2-APOE+	VEGFA	ITGAV	6,90515E-04	8231,1	2,93913E-02	1285	7,74590E-01	1378	1,04559E+00	4673	8,20481E-01	22611	0	11208,5
Responder	Mono_INHBA	cDC_CLEC9A	CALM1	MYLK	6,92845E-04	8519,7	4,90076E-02	494	9,97912E-01	590	1,35788E+00	2550	7,95270E-01	27756	0	11208,5
Responder	Macro_NLRP3	pDC_LILRA4	CXCL2	DPP4	6,93167E-04	8374,3	6,92195E-02	221	1,07666E+00	435	1,60762E+00	1544	7,91631E-01	28463	0	11208,5
Responder	Macro_ISG15	Mono_CD14	HLA-B	LILRA1	6,93917E-04	9815,7	1,00274E-02	8456	3,19947E-01	9978	7,21240E-01	8692	8,78489E-01	10744	0	11208,5
Responder	Mono_INHBA	cDC_CLEC9A	ADAM10	CADM1	6,94012E-04	7787,3	3,51615E-02	928	9,40560E-01	733	1,52767E+00	1819	8,12795E-01	24248	0	11208,5
Responder	Macro_FOLR2+APOE-	pDC_LILRA4	CXCL12	DPP4	6,94498E-04	11381,1	1,24058E-01	51	1,09329E+00	400	1,60721E+00	1545	7,10684E-01	43701	0	11208,5
Responder	Macro_OLFML3	cDC_LAMP3	C3	ITGAX	6,95210E-04	7636,1	1,14840E-02	6731	4,80339E-01	4822	1,04591E+00	4671	8,78468E-01	10748	0	11208,5
Responder	Macro_FOLR2+APOE+	Macro_ISG15	APOE	TREM2	6,95766E-04	4685,1	1,60590E-02	3735	6,53513E-01	2280	1,04391E+00	4682	9,28873E-01	1520	0	11208,5
Responder	Mono_INHBA	cDC_CLEC9A	TNF	TRADD	6,96937E-04	11031,1	1,60236E-02	3754	5,33193E-01	3814	1,11720E+00	4039	7,71947E-01	32340	0	11208,5
Responder	Mono_CD16	pDC_LILRA4	SELPLG	SELL	6,97165E-04	4989,7	2,66517E-02	1547	9,42747E-01	723	2,22666E+00	432	8,76960E-01	11038	0	11208,5
Responder	Macro_FOLR2+APOE-	Macro_LYVE1	CCL13	CCR1	6,97377E-04	10581,3	6,66466E-02	238	1,28984E+00	198	1,02310E+00	4867	7,50657E-01	36395	0	11208,5
Responder	Mono_CD14	Macro_OLFML3	CD52	SIGLEC10	6,97477E-04	9547,9	9,74487E-03	8880	4,13806E-01	6567	6,00653E-01	10755	8,80700E-01	10329	0	11208,5
Responder	Macro_FOLR2+APOE+	Mono_CD14	APOE	SORL1	6,97523E-04	4702,1	1,42346E-02	4685	6,77426E-01	2050	1,43869E+00	2174	9,18129E-01	3393	0	11208,5
Responder	Macro_ISG15	Mono_CD14	FN1	PLAUR	6,97801E-04	9721,3	8,71816E-03	10756	3,45105E-01	8919	7,66422E-01	7995	8,84020E-01	9728	0	11208,5
Responder	Macro_ISG15	Macro_NLRP3	GNAI2	C5AR1	6,99099E-04	9078,1	8,71627E-03	10760	4,69957E-01	5052	6,21461E-01	10403	8,93674E-01	7967	0	11208,5
Responder	Mono_INHBA	cDC_CLEC9A	CCL5	DPP4	6,99283E-04	15924,3	2,72862E-02	1480	5,08704E-01	4235	1,08002E+00	4338	5,91836E-01	58360	0	11208,5
Responder	Macro_NLRP3	cDC(CD1C)	AGTRAP	RACK1	6,99749E-04	8426,3	8,71548E-03	10762	3,86695E-01	7397	1,24349E+00	3177	8,84722E-01	9587	0	11208,5
Responder	Mono_INHBA	cDC_CLEC9A	INHBA	ACTR2	6,99871E-04	5343,7	2,62001E-02	1606	8,70126E-01	944	2,21333E+00	443	8,68986E-01	12517	0	11208,5
Responder	Macro_FOLR2-APOE+	Mast	HSPA8	ADRB2	7,00400E-04	6824,7	2,46764E-02	1779	6,03387E-01	2801	6,00166E-01	10764	8,95836E-01	7571	0	11208,5
Responder	Macro_OLFML3	Macro_IER3	C1QB	LRP1	7,00725E-04	8107,9	9,18331E-03	9863	4,68360E-01	5087	6,00072E-01	10765	9,16916E-01	3616	0	11208,5
Responder	Mono_INHBA	Macro_ISG15	VEGFA	CD44	7,01051E-04	7811,9	1,20709E-02	6176	5,43894E-01	3637	8,18384E-01	7272	8,78383E-01	10766	0	11208,5
Responder	pDC_LILRA4	Macro_FOLR2+APOE+	SPON2	ITGB2	7,01178E-04	5597,5	4,27038E-02	643	1,03811E+00	495	1,60468E+00	1550	8,61151E-01	14091	0	11208,5
Responder	Mono_INHBA	pDC_LILRA4	TNF	NOTCH1	7,03403E-04	12269,9	2,26172E-02	2086	5,30892E-01	3844	1,78598E+00	1082	7,13909E-01	43129	0	11208,5
Responder	Macro_FOLR2-APOE+	Mono_CD14	C1QA	CD93	7,04640E-04	9193,3	1,03120E-02	8060	3,45905E-01	8896	5,99100E-01	10777	8,98658E-01	7025	0	11208,5
Responder	Mono_INHBA	pDC_LILRA4	APP	TNFRSF21	7,05174E-04	8474,9	2,47715E-02	1769	1,04213E+00	483	1,81591E+00	1029	7,94663E-01	27885	0	11208,5
Responder	Macro_OLFML3	Mast	C3	ITGAM	7,05765E-04	8854,1	1,53236E-02	4087	5,29776E-01	3860	1,04237E+00	4699	8,31139E-01	20416	0	11208,5
Responder	Macro_NLRP3	cDC_CLEC9A	S100A8	ITGB2	7,06603E-04	7738,5	9,59409E-03	9125	3,71529E-01	7924	7,11466E-01	8838	9,28402E-01	1597	0	11208,5
Responder	Mono_INHBA	pDC_LILRA4	ICAM1	SPN	7,06949E-04	6548,1	2,18225E-02	2216	7,82106E-01	1345	1,90184E+00	868	8,46717E-01	17103	0	11208,5
Responder	cDC_CLEC9A	pDC_LILRA4	FARP2	PLXNA4	7,09102E-04	12951,7	1,99961E-01	23	1,23551E+00	240	1,51388E+00	1863	6,59688E-01	51424	0	11208,5
Responder	Macro_FOLR2+APOE-	Mono_CD14	CCL13	CCR1	7,09102E-04	10095,9	6,59204E-02	240	1,28598E+00	201	1,42423E+00	2241	7,49630E-01	36589	0	11208,5
Responder	Mono_CD14	pDC_LILRA4	CXCL2	DPP4	7,09250E-04	10340,9	4,19698E-02	670	8,01419E-01	1242	1,60349E+00	1556	7,47367E-01	37028	0	11208,5
Responder	Macro_ISG15	Macro_FOLR2+APOE+	CCL8	CCR5	7,10601E-04	14236,9	4,06500E-02	714	7,66732E-01	1424	1,60346E+00	1557	6,16231E-01	56281	0	11208,5
Responder	Mono_INHBA	pDC_LILRA4	CD14	TLR9	7,11102E-04	6270,1	5,96232E-02	311	1,22619E+00	252	1,26016E+00	3091	8,49776E-01	16488	0	11208,5

Post_R_Myeloid_Myeloid_CellCel

Responder	Macro_NLRP3	cDC_CLEC9A	CD52	SIGLEC10	7,14502E-04	6487,7	1,20284E-02	6225	5,79914E-01	3100	1,18928E+00	3496	8,91324E-01	8409	0	11208,5
Responder	pDC_LILRA4	Macro_FOLR2-APOE+	CD24	SIGLEC10	7,14665E-04	14997,3	4,52229E-02	581	7,43320E-01	1560	1,78597E+00	1083	6,89164E-01	47237	0,004	24525,5
Responder	Macro_NLRP3	cDC_CLEC9A	LYZ	ITGAL	7,15825E-04	5836,5	1,30525E-02	5416	6,25171E-01	2537	1,19322E+00	3475	9,01274E-01	6546	0	11208,5
Responder	Macro_OLFML3	Macro_IFI27	C3	ITGAM	7,18864E-04	9262,1	1,41741E-02	4721	5,14648E-01	4129	1,04046E+00	4718	8,25596E-01	21534	0	11208,5
Responder	Macro_ISG15	Mono_INHBA	LGALS9	CD44	7,19142E-04	7300,7	8,69190E-03	10821	5,67090E-01	3317	8,75626E-01	6531	9,11482E-01	4626	0	11208,5
Responder	Macro_OLFML3	Macro_ISG15	HLA-DRB1	LAG3	7,20139E-04	7885,9	1,45031E-02	4533	5,80263E-01	3092	6,56821E-01	9772	8,78035E-01	10824	0	11208,5
Responder	Macro_ISG15	Mono_CD14	CXCL10	TLR4	7,21472E-04	5900,7	3,47243E-02	952	7,42107E-01	1565	2,04577E+00	650	8,56153E-01	15128	0	11208,5
Responder	Mono_INHBA	Mono_CD14	VEGFA	SIRPA	7,21866E-04	10118,3	1,41686E-02	4726	5,77128E-01	3151	1,22927E+00	3267	7,92889E-01	28239	0	11208,5
Responder	Mono_CD14	Macro_FOLR2+APOE-	VCAN	ITGB1	7,22471E-04	5596,9	1,61669E-02	3681	8,17441E-01	1165	1,78020E+00	1099	8,78000E-01	10831	0	11208,5
Responder	Mast	Macro_LYVE1	CSF1	CSF2RA	7,22839E-04	6663,5	3,83778E-02	790	9,12832E-01	807	1,60066E+00	1566	8,38069E-01	18946	0	11208,5
Responder	Macro_NLRP3	pDC_LILRA4	THBS1	CD47	7,23472E-04	4752,3	2,38673E-02	1896	8,33872E-01	1083	2,00607E+00	705	8,88916E-01	8869	0	11208,5
Responder	Macro_ISG15	pDC_LILRA4	S100A8	CD69	7,23806E-04	7873,3	1,15315E-02	6686	3,72654E-01	7895	1,31956E+00	2742	8,77986E-01	10835	0	11208,5
Responder	Mono_INHBA	pDC_LILRA4	S100A9	CD36	7,25143E-04	8202,1	1,11079E-02	7096	4,60812E-01	5276	8,70440E-01	6591	8,77963E-01	10839	0	11208,5
Responder	Mono_INHBA	pDC_LILRA4	MIF	CD74_CXCR4	7,26084E-04	7148,3	6,04388E-03	19439	5,10258E-01	4211	1,98622E+00	733	9,45355E-01	150	0	11208,5
Responder	Macro_OLFML3	Macro_FOLR2+APOE+	HLA-DRA	LAG3	7,29166E-04	7312,3	1,66199E-02	3502	6,49283E-01	2312	5,95448E-01	10851	8,89798E-01	8688	0	11208,5
Responder	Macro_NLRP3	pDC_LILRA4	S100A8	CD36	7,29502E-04	5476,7	1,81388E-02	3001	6,64315E-01	2158	1,35971E+00	2539	8,90981E-01	8477	0	11208,5
Responder	Macro_FOLR2-APOE+	Macro_IER3	MMP9	CD44	7,30174E-04	7231,7	1,61543E-02	3689	6,42098E-01	2382	7,64310E-01	8025	8,77871E-01	10854	0	11208,5
Responder	Mono_INHBA	pDC_LILRA4	COPA	P2RY6	7,30320E-04	9679,7	1,56846E-02	3925	5,70485E-01	3262	1,95865E+00	780	7,87767E-01	29223	0	11208,5
Responder	Macro_NLRP3	pDC_LILRA4	S100A9	CD36	7,30511E-04	5374,5	1,53273E-02	4084	6,84384E-01	1976	1,55805E+00	1715	8,94189E-01	7889	0	11208,5
Responder	Macro_NLRP3	cDC(CD1C)	CD52	SIGLEC10	7,30511E-04	8317,9	9,23798E-03	9767	4,70739E-01	5031	1,03822E+00	4728	8,77865E-01	10855	0	11208,5
Responder	cDC(CD1C)	cDC_CLEC9A	HLA-DQB2	LAG3	7,32142E-04	10956,5	2,25870E-02	2090	4,83415E-01	4743	1,61974E+00	1514	7,57221E-01	35227	0	11208,5
Responder	Macro_ISG15	cDC(CD1C)	B2M	CD1A	7,33207E-04	5601,7	5,42465E-02	380	6,39615E-01	2401	1,24724E+00	3156	8,77802E-01	10863	0	11208,5
Responder	Macro_FOLR2+APOE+	Macro_FOLR2+APOE-	APOE	LRP1	7,33358E-04	4793,9	1,41468E-02	4745	7,31971E-01	1629	1,08960E+00	4255	9,25040E-01	2132	0	11208,5
Responder	Macro_OLFML3	Mono_CD16	LGALS3BP	ITGB1	7,33544E-04	7595,5	1,71469E-02	3309	6,09991E-01	2725	6,51465E-01	9871	8,77801E-01	10864	0	11208,5
Responder	Mono_INHBA	Mono_CD14	ICAM1	ITGAX_ITGB2	7,34558E-04	7871,1	8,66960E-03	10867	4,54820E-01	5415	1,15968E+00	3679	8,92450E-01	8186	0	11208,5
Responder	cDC(CD1C)	pDC_LILRA4	MAML2	NOTCH4	7,35185E-04	16434,9	1,65268E-02	3544	4,83064E-01	4748	1,61614E+00	1520	5,45755E-01	61154	0	11208,5
Responder	Mono_INHBA	pDC_LILRA4	PSEN1	NOTCH4	7,35794E-04	13292,7	3,64477E-02	870	6,18389E-01	2623	1,82574E+00	1008	6,65248E-01	50754	0	11208,5
Responder	Macro_NLRP3	pDC_LILRA4	HLA-C	NOTCH4	7,36927E-04	5595,3	3,04649E-02	1180	5,83386E-01	3051	1,57446E+00	1663	8,77747E-01	10874	0	11208,5
Responder	Mono_CD16	pDC_LILRA4	HLA-C	NOTCH4	7,37983E-04	5044,9	3,36611E-02	1000	7,50384E-01	1520	1,59874E+00	1577	8,83000E-01	9919	0	11208,5
Responder	pDC_LILRA4	Macro_FOLR2-APOE+	SPON2	ITGB2	7,39370E-04	6226,3	3,54639E-02	916	8,76364E-01	916	1,59850E+00	1578	8,49668E-01	16513	0	11208,5
Responder	Macro_OLFML3	cDC_CLEC9A	TNFSF13B	CD40	7,39641E-04	8325,9	1,12032E-02	6995	4,67714E-01	5102	8,07474E-01	7442	8,77720E-01	10882	0	11208,5
Responder	Macro_NLRP3	pDC_LILRA4	HMGB1	THBD	7,40321E-04	6639,9	1,14477E-02	6763	4,77498E-01	4876	1,71049E+00	1260	8,87696E-01	9092	0	11208,5
Responder	Mono_INHBA	pDC_LILRA4	HSP90B1	TLR9	7,41298E-04	5956,5	6,54126E-02	246	1,32526E+00	179	1,45738E+00	2086	8,51812E-01	16063	0	11208,5
Responder	Macro_NLRP3	Macro_OLFML3	S100A9	ITGB2	7,41342E-04	6102,3	1,18977E-02	6349	7,09277E-01	1782	5,93469E-01	10887	9,42006E-01	285	0	11208,5
Responder	Mono_INHBA	Macro_LYVE1	CCL3	CCR1	7,43387E-04	5743,9	1,83163E-02	2949	8,30582E-01	1098	1,35310E+00	2571	8,77671E-01	10893	0	11208,5
Responder	Macro_NLRP3	pDC_LILRA4	AGTRAP	RACK1	7,43387E-04	6274,3	1,06635E-02	7614	5,14707E-01	4128	2,07711E+00	608	8,94617E-01	7813	0	11208,5
Responder	Mono_INHBA	pDC_LILRA4	HMGB1	TLR9	7,44369E-04	6197,1	5,42237E-02	381	1,20614E+00	272	1,26209E+00	3077	8,51870E-01	16047	0	11208,5
Responder	Macro_NLRP3	pDC_LILRA4	S100A8	CD69	7,44753E-04	5299,1	1,67786E-02	3450	5,96972E-01	2874	1,60518E+00	1549	8,96692E-01	7414	0	11208,5
Responder	cDC_CLEC9A	cDC_CLEC9A	ICAM3	ITGAL	7,44935E-04	8378,7	3,00295E-02	1229	8,07213E-01	1217	1,59736E+00	1582	8,00898E-01	26657	0	11208,5
Responder	Mono_INHBA	pDC_LILRA4	TNF	TRAF2	7,46833E-04	13909,7	2,06207E-02	2414	4,97761E-01	4445	1,52084E+00	1836	6,72862E-01	49645	0	11208,5
Responder	Macro_ISG15	Macro_FOLR2-APOE+	CCL8	CCR1	7,47728E-04	11076,1	3,68051E-02	857	7,39971E-01	1584	1,59752E+00	1580	7,30381E-01	40151	0	11208,5
Responder	Mono_INHBA	pDC_LILRA4	TNF	TRADD	7,48067E-04	10720,3	1,52142E-02	4141	5,13329E-01	4156	1,91310E+00	853	7,67353E-01	33243	0	11208,5
Responder	Macro_ISG15	Macro_NLRP3	S100A9	CD36	7,48863E-04	8941,5	1,13664E-02	6835	4,66391E-01	5139	5,92603E-01	10909	8,79190E-01	10616	0	11208,5
Responder	Mono_INHBA	pDC_LILRA4	CCL5	DPP4	7,50540E-04	14183,9	3,86749E-02	777	6,99122E-01	1859	1,35176E+00	2580	6,33199E-01	54495	0	11208,5
Responder	Mast	Mono_CD16	CSF1	CSF3R	7,51930E-04	7285,7	4,08083E-02	711	9,05308E-01	826	1,59562E+00	1587	8,22877E-01	22096	0	11208,5
Responder	Mono_INHBA	pDC_LILRA4	MAML2	NOTCH4	7,52398E-04	13717,7	3,81263E-02	800	6,25039E-01	2539	1,83740E+00	987	6,45998E-01	53054	0	11208,5
Responder	Mono_INHBA	pDC_LILRA4	INHBA	ACTR2	7,53019E-04	6617,7	1,83444E-02	2938	7,02451E-01	1829	2,68995E+00	136	8,47329E-01	16977	0	11208,5
Responder	cDC(CD1C)	Macro_OLFML3	HLA-DQA1	CD4	7,53639E-04	6954,3	7,72836E-03	13206	4,82010E-01	4778	1,09269E+00	4226	9,30124E-01	1353	0	11208,5
Responder	Mono_INHBA	Macro_IER3	VEGFA	CD44	7,54371E-04	7980,1	1,33929E-02	5178	6,00838E-01	2827	5,91740E-01	10925	8,83826E-01	9762	0	11208,5
Responder	Macro_NLRP3	Macro_IFI27	S100A9	CD68	7,54371E-04	5656,3	1,15736E-02	6642	6,79536E-01	2025	7,53079E-01	8212	9,44014E-01	194	0	11208,5
Responder	Mono_CD14	Macro_LYVE1	NAMPT	ITGA5_ITGB1	7,55061E-04	7344,3	9,73535E-03	8894	6,66240E-01	2135	1,17976E+00	3557	8,77493E-01	10927	0	11208,5
Responder	Macro_OLFML3	Macro_FOLR2+APOE+	HLA-DRB5	LAG3	7,57483E-04	7408,1	1,87621E-02	2827	6,60589E-01	2196	6,51002E-01	9875	8,77479E-01	10934	0	11208,5
Responder	Macro_FOLR2-APOE+	Macro_LYVE1	APOE	LRP1	7,61997E-04	5907,1	1,63576E-02	3615	6,41418E-01	2386	5,90507E-01	10947	9,29921E-01	1379	0	11208,5
Responder	Macro_NLRP3	Macro_IFI27	HBEGF	CD9	7,62693E-04	7716,7	1,80489E-02	3027	5,86379E-01	3014	6,06827E-01	10662	8,78890E-01	10672	0	11208,5
Responder	Mono_INHBA	cDC_LAMP3	VEGFA	NRP2	7,66050E-04	6291,1	3,29572E-02	1035	9,58053E-01	675	1,59183E+00	1597	8,47557E-01	16940	0	11208,5
Responder	Mono_CD14	Mono_CD16	TGFB1	CXCR4	7,67232E-04	9484,1	8,63110E-03	10962	4,58413E-01	5334	6,41876E-01	10057	8,83282E-01	9859	0	11208,5
Responder	Mono_INHBA	Macro_IFI27	CXCL8	SDC3	7,68019E-04	8140,7	1,62876E-02	3637	5,72989E-01	3226	1,19072E+00	3487	8,37140E-01	19145	0	11208,5
Responder	Mono_CD14	pDC_LILRA4	VCAN	CD44	7,70035E-04	7034,5	8,62701E-03	10970	5,83182E-01	3054	1,98221E+00	745	8,87078E-01	9195	0	11208,5
Responder	pDC_LILRA4	Macro_NLRP3	SPON2	ITGB2	7,70320E-04	6493,3	3,30059E-02	1031	8,21452E-01	1146	1,59100E+00	1600	8,45023E-01	17481	0	11208,5
Responder	Macro_ISG15	Mono_CD16	CD14	ITGA4	7,70386E-04	9814,1	8,63770E-03	10944	3,96906E-01	7069	5,88836E-01	10971	8,88883E-01	8878	0	11208,5
Responder	Macro_FOLR2-APOE+	Mono_INHBA	PKM	CD44	7,70738E-04	6742,3	8,62651E-03	10972	6,52327E-01	2290	7,60263E-01	8109	9,31851E-01	1132	0	11208,5
Responder	Macro_NLRP3	Macro_LYVE1	THBS1	CD36	7,71792E-04	4442,9	4,50899E-02	584	1,11011E+00	375	1,34571E+00	2606	8,96574E-01	7441	0	11208,5
Responder	Macro_NLRP3	Macro_LYVE1	THBS1	LRP1	7,72144E-04	4493,1	3,19836E-02	1088	1,02834E+00	515	1,13319E+00	3895	9,05232E-01	5759	0	11208,5
Responder	cDC(CD1C)	cDC_CLEC9A	HLA-DQB1	LAG3	7,73700E-04	7538,1	1,40384E-02	4810	5,35796E-01	3760	1,49355E+00	1946	8,52206E-01	15966	0	11208,5
Responder	cDC(CD1C)	Macro_OLFML3	HLA-DPB1	CD4	7,73700E-04	7230,5	6,95932E-03	15602	4,80664E-01	4810	1,12942E+00	3936	9,37024E-01	596	0	11208,5

Responder	Mono_INHBA	pDC_LILRA4	TNF	TRPM2	7,75601E-04	11161,7	1,79962E-02	3049	4,80642E-01	4813	1,28427E+00	2945	7,64717E-01	33793	0	11208,5
Responder	Mono_INHBA	Mono_INHBA	VEGFA	NRP2	7,76870E-04	10553,7	1,40302E-02	4815	5,89049E-01	2971	1,14535E+00	3794	7,83905E-01	29980	0	11208,5
Responder	cDC(CD1C)	pDC_LILRA4	VEGFA	NRP1	7,77505E-04	11750,5	1,40292E-02	4816	5,17615E-01	4074	1,46320E+00	2067	7,49639E-01	36587	0	11208,5
Responder	cDC_CLEC9A	Mono_CD14	BTLA	TNFRSF14	7,78906E-04	8161,5	3,48152E-02	949	1,59888E+00	93	1,58964E+00	1606	7,99247E-01	26951	0	11208,5
Responder	Mono_INHBA	Macro_NLRP3	VCAN	CD44	7,80268E-04	7561,5	1,22813E-02	6003	5,53777E-01	3503	5,87403E-01	10999	9,03595E-01	6094	0	11208,5
Responder	Macro_NLRP3	Macro_LYVE1	S100A8	CD36	7,82754E-04	4382,9	3,24685E-02	1058	9,86615E-01	610	9,81081E-01	5289	9,16208E-01	3749	0	11208,5
Responder	Macro_ISG15	Mono_CD16	HLA-C	LILRA3	7,82754E-04	6216,7	2,95683E-02	1270	6,80153E-01	2021	5,87023E-01	11006	9,06306E-01	5578	0	11208,5
Responder	Macro_NLRP3	Macro_LYVE1	S100A8	CD68	7,83109E-04	4483,5	1,77176E-02	3125	9,08480E-01	818	8,24626E-01	7192	9,48840E-01	74	0	11208,5
Responder	pDC_LILRA4	cDC_CLEC9A	ADAM10	CADM1	7,83223E-04	8649,5	2,61786E-02	1609	8,40714E-01	1059	2,20084E+00	453	7,89310E-01	28918	0	11208,5
Responder	Macro_NLRP3	Macro_LYVE1	S100A9	TLR4	7,83465E-04	7771,3	1,47863E-02	4363	6,28791E-01	2511	5,97174E-01	10813	8,82775E-01	9961	0	11208,5
Responder	Mono_CD14	cDC_LAMP3	S100A8	CD68	7,83821E-04	5473,7	8,61126E-03	11009	7,99567E-01	1250	1,41602E+00	2271	9,28211E-01	1630	0	11208,5
Responder	Macro_NLRP3	Macro_LYVE1	S100A9	CD68	7,84533E-04	4231,9	1,49714E-02	4267	9,28549E-01	763	1,02297E+00	4869	9,50440E-01	52	0	11208,5
Responder	cDC_CLEC9A	cDC_CLEC9A	CALM2	MYLK	7,86109E-04	8732,1	4,30645E-02	635	8,21789E-01	1144	1,58828E+00	1611	7,88565E-01	29062	0	11208,5
Responder	Mono_INHBA	pDC_LILRA4	IL10	IFNLR1	7,87079E-04	15426,9	1,01039E-01	89	7,71462E-01	1392	1,02754E+00	4831	5,73880E-01	59614	0	11208,5
Responder	Macro_OLFML3	Mono_CD14	FN1	ITGA5_ITGB1	7,87079E-04	7655,9	1,72542E-02	3274	4,79855E-01	4831	1,36544E+00	2518	8,50011E-01	16448	0	11208,5
Responder	Macro_NLRP3	Macro_LYVE1	VCAN	ITGB1	7,87387E-04	6802,1	1,82110E-02	2977	7,40532E-01	1580	7,30472E-01	8558	8,84235E-01	9687	0	11208,5
Responder	Macro_OLFML3	Macro_OLFML3	LGALS9	LRP1	7,87488E-04	25385,7	5,28480E-03	23816	1,36012E-01	22235	-5,00524E-01	50644	8,37707E-01	19025	0	11208,5
Responder	Macro_OLFML3	Macro_LYVE1	HLA-DPA1	CD4	7,87745E-04	6928,3	8,60702E-03	11020	6,11657E-01	2703	6,70605E-01	9526	9,44409E-01	184	0	11208,5
Responder	pDC_LILRA4	Macro_IER3	SPON2	ITGA5	7,89002E-04	13382,3	3,00711E-02	1226	8,57688E-01	988	1,58750E+00	1613	6,55980E-01	51876	0	11208,5
Responder	Mono_INHBA	Macro_IFI27	INHBA	ACTR2	7,90289E-04	7303,1	1,72472E-02	3278	6,79033E-01	2035	1,44303E+00	2149	8,43297E-01	17845	0	11208,5
Responder	Macro_IER3	Macro_ISG15	CCL3L1	CCR5	7,90452E-04	7623,3	4,31534E-02	631	1,08002E+00	428	1,58726E+00	1614	8,12837E-01	24235	0	11208,5
Responder	Mono_CD14	Mono_CD16	S100A12	CD36	7,93356E-04	7527,3	2,60966E-02	1616	1,00918E+00	558	1,84646E+00	973	8,17401E-01	23281	0	11208,5
Responder	Macro_FOLR2-APOE+	Mono_CD16	C3	IFITM1	7,94199E-04	7141,5	2,06244E-02	2413	5,73326E-01	3219	5,85217E-01	11038	8,94520E-01	7829	0	11208,5
Responder	Macro_NLRP3	pDC_LILRA4	THBS1	SCARB1	7,96267E-04	8196,9	2,60615E-02	1618	7,34386E-01	1617	1,68657E+00	1318	8,08082E-01	25223	0	11208,5
Responder	Macro_OLFML3	Macro_LYVE1	HLA-DPB1	CD4	7,98887E-04	7171,9	8,59611E-03	11051	6,08882E-01	2737	6,09120E-01	10622	9,42976E-01	241	0	11208,5
Responder	Macro_NLRP3	Macro_ISG15	HBEGF	CD44	8,00334E-04	9060,5	9,96941E-03	8532	4,58697E-01	5322	5,84156E-01	11055	8,87120E-01	9185	0	11208,5
Responder	Macro_NLRP3	Macro_LYVE1	HMGB1	CD163	8,07235E-04	6807,1	1,02391E-02	8164	6,81255E-01	2005	5,97372E-01	10808	9,26795E-01	1850	0	11208,5
Responder	Mono_INHBA	Mono_CD16	INHBA	ACTR2	8,07981E-04	5410,7	2,78124E-02	1425	9,04539E-01	828	1,58442E+00	1626	8,72348E-01	11966	0	11208,5
Responder	pDC_LILRA4	pDC_LILRA4	COPA	P2RY6	8,09453E-04	7111,9	2,59868E-02	1627	7,68035E-01	1416	3,19919E+00	43	8,26923E-01	21265	0	11208,5
Responder	cDC(CD1C)	cDC(CD1C)	HMGB1	CXCR4	8,11067E-04	6801,1	7,16047E-03	14932	4,77868E-01	4868	1,76968E+00	1126	9,26661E-01	1871	0	11208,5
Responder	Mono_INHBA	Mast	PTGS2	ALOX5	8,11253E-04	5990,1	2,10033E-02	2352	6,55442E-01	2262	1,26718E+00	3043	8,76730E-01	11085	0	11208,5
Responder	Macro_NLRP3	cDC_LAMP3	THBS1	ITGA4	8,13084E-04	6348,5	2,18755E-02	2207	7,66994E-01	1423	9,19281E-01	5976	8,77491E-01	10928	0	11208,5
Responder	Mono_INHBA	Macro_OLFML3	CCL4	CCR5	8,13692E-04	11011,3	1,39337E-02	4872	5,91623E-01	2948	1,14893E+00	3767	7,72345E-01	32261	0	11208,5
Responder	cDC_CLEC9A	pDC_LILRA4	CCL5	DPP4	8,15359E-04	13627,7	4,29784E-02	637	7,31789E-01	1631	1,60833E+00	1541	6,45363E-01	53121	0	11208,5
Responder	Mono_INHBA	Macro_IER3	SPP1	CD44	8,16388E-04	10740,1	8,60549E-03	11030	2,95759E-01	11099	6,27205E-01	10297	8,82137E-01	10066	0	11208,5
Responder	Mono_INHBA	Macro_IER3	INHBA	ENG	8,16840E-04	9188,3	2,73234E-02	1478	8,39942E-01	1063	1,58298E+00	1632	7,81116E-01	30560	0	11208,5
Responder	Macro_LYVE1	pDC_LILRA4	APP	TNFRSF21	8,18323E-04	8340,5	2,59355E-02	1633	1,05579E+00	457	1,69827E+00	1292	7,98384E-01	27112	0	11208,5
Responder	Macro_NLRP3	cDC_LAMP3	VCAN	ITGA4	8,18966E-04	6064,5	2,23415E-02	2126	7,90642E-01	1298	9,75804E-01	5338	8,80507E-01	10352	0	11208,5
Responder	Macro_ISG15	pDC_LILRA4	S100A9	CD36	8,19704E-04	7626,1	1,08373E-02	7417	4,46471E-01	5641	1,31738E+00	2756	8,76635E-01	11108	0	11208,5
Responder	Mono_INHBA	Macro_LYVE1	IL6	IL6R_IL6ST	8,20945E-04	11487,7	2,97917E-02	1252	7,59378E-01	1481	1,11915E+00	4023	7,34474E-01	39474	0	11208,5
Responder	cDC(CD1C)	Macro_ISG15	HLA-DRB1	LAG3	8,22268E-04	6802,7	1,39188E-02	4885	5,20910E-01	4026	1,34336E+00	2617	8,75816E-01	11277	0	11208,5
Responder	Mast	Macro_OLFML3	CSF1	SIRPA	8,22782E-04	7832,9	4,26068E-02	646	9,74591E-01	639	1,58111E+00	1636	8,09008E-01	25035	0	11208,5
Responder	Mono_INHBA	Macro_LYVE1	CXCL8	SDC3	8,25585E-04	5429,7	3,02565E-02	1205	8,66824E-01	956	1,40130E+00	2342	8,75092E-01	11437	0	11208,5
Responder	Mono_INHBA	Macro_LYVE1	CXCL8	SDC2	8,26249E-04	9840,3	1,83956E-02	2923	6,28943E-01	2510	1,03598E+00	4747	7,94968E-01	27813	0	11208,5
Responder	Mono_CD14	Mono_CD14	GNAI2	C5AR1	8,26367E-04	7673,9	8,57115E-03	11126	4,43446E-01	5717	1,43431E+00	2199	8,92874E-01	8119	0	11208,5
Responder	Macro_FOLR2+APOE+	cDC(CD1C)	C1QB	CD33	8,26914E-04	5452,3	1,38998E-02	4892	6,16094E-01	2652	1,13357E+00	3891	9,11513E-01	4618	0	11208,5
Responder	Mono_CD14	Macro_ISG15	VCAN	ITGB1	8,27110E-04	5786,1	1,57458E-02	3892	8,08185E-01	1213	1,62776E+00	1489	8,76580E-01	11128	0	11208,5
Responder	Macro_OLFML3	Macro_LYVE1	MDK	LRP1	8,30249E-04	5965,5	3,86313E-02	780	8,04729E-01	1228	1,00681E+00	5034	8,74402E-01	11577	0	11208,5
Responder	Mono_INHBA	Macro_LYVE1	CCL4	CCR1	8,31579E-04	6823,3	1,58392E-02	3845	6,47840E-01	2325	1,20255E+00	3417	8,65103E-01	13321	0	11208,5
Responder	Mono_CD14	cDC_LAMP3	S100A9	ITGB2	8,33821E-04	5457,3	8,56177E-03	11146	6,88919E-01	1944	1,50347E+00	1910	9,32337E-01	1078	0	11208,5
Responder	Macro_OLFML3	Macro_ISG15	APOE	SORL1	8,34195E-04	10027,7	8,56060E-03	11147	3,28580E-01	9618	5,98142E-01	10794	8,96871E-01	7371	0	11208,5
Responder	pDC_LILRA4	Macro_FOLR2+APOE+	APP	LRP10	8,34751E-04	6853,1	2,58263E-02	1644	1,01996E+00	535	2,15867E+00	521	8,31429E-01	20357	0	11208,5
Responder	Mono_INHBA	cDC_CLEC9A	ICAM1	SPN	8,34923E-04	9278,7	1,38852E-02	4904	5,52857E-01	3523	1,27916E+00	2979	8,15028E-01	23779	0	11208,5
Responder	pDC_LILRA4	Macro_OLFML3	TNFSF9	TRAF2	8,36255E-04	15207,1	2,62685E-02	1595	7,29845E-01	1645	1,70234E+00	1283	5,62658E-01	60304	0	11208,5
Responder	Mono_CD14	Macro_LYVE1	GNAI2	C5AR1	8,38695E-04	9420,1	8,55622E-03	11159	4,42247E-01	5743	5,95323E-01	10855	8,92790E-01	8135	0	11208,5
Responder	Macro_FOLR2+APOE+	Mono_CD14	APOE	LDLR	8,39268E-04	4366,9	3,83653E-02	792	9,75850E-01	637	1,57760E+00	1647	8,95947E-01	7550	0	11208,5
Responder	pDC_LILRA4	Mono_CD14	LTB	TNFRSF1A	8,39268E-04	6120,7	2,58003E-02	1647	1,00837E+00	559	2,98733E+00	73	8,46681E-01	17116	0	11208,5
Responder	Mono_CD14	Mono_CD16	GNAI2	FPR1	8,40199E-04	9447,5	8,55342E-03	11163	3,83635E-01	7505	7,24612E-01	8645	8,89680E-01	8716	0	11208,5
Responder	Mono_INHBA	Macro_LYVE1	THBS1	LRP1	8,40952E-04	7144,9	1,76353E-02	3158	6,78571E-01	2039	7,56682E-01	8154	8,76435E-01	11165	0	11208,5
Responder	Mono_INHBA	Macro_LYVE1	VEGFA	NRP1	8,41641E-04	6965,1	3,98391E-02	738	1,01708E+00	541	1,34205E+00	2628	8,34593E-01	19710	0	11208,5
Responder	Mono_INHBA	Macro_LYVE1	VEGFA	NRP2	8,42315E-04	8192,3	2,38088E-02	1907	7,79695E-01	1360	1,01881E+00	4900	8,25345E-01	21586	0	11208,5
Responder	Macro_IER3	pDC_LILRA4	TNF	TRAF2	8,43802E-04	11735,5	2,98497E-02	1244	7,28773E-01	1650	1,76429E+00	1139	7,12201E-01	43436	0	11208,5
Responder	Macro_NLRP3	Mono_CD14	THBS1	LRP1	8,43969E-04	6243,5	1,76260E-02	3159	6,60027E-01	2205	1,19334E+00	3472	8,76407E-01	11173	0	11208,5
Responder	Mono_CD14	Mast	HBEGF	CD82	8,45317E-04	5998,3	2,88223E-02	1339	7,94290E-01	1283	1,57713E+00	1651	8,59228E-01	14510	0	11208,5
Responder	Mono_INHBA	Macro_LYVE1	CCL3L1	CCR1	8,45691E-04	7638,5	1,97270E-02	2585	6,69011E-01	2120	1,18508E+00	3527	8,38866E-01	18752	0	11208,5

Post_R_Myeloid_Myeloid_CellCel

Responder	Macro_ISG15	Macro_NLRP3	CCL8	CCR1	8,48352E-04	11225,1	3,51117E-02	930	7,28531E-01	1653	1,66916E+00	1367	7,25718E-01	40967	0	11208,5
Responder	Macro_NLRP3	Mono_CD16	VCAN	ITGB1	8,48511E-04	6042,3	1,98574E-02	2558	7,87365E-01	1314	9,02531E-01	6192	8,88591E-01	8939	0	11208,5
Responder	Mono_INHBA	cDC_LAMP3	IL10	CD70	8,50908E-04	15653,7	1,05335E-01	80	1,21143E+00	263	7,65058E-01	8014	5,87235E-01	58703	0	11208,5
Responder	Macro_NLRP3	Mono_CD14	S100A8	TLR4	8,51170E-04	6824,5	1,65574E-02	3528	5,86568E-01	3013	9,93190E-01	5181	8,76302E-01	11192	0	11208,5
Responder	Macro_OLFML3	cDC_CLEC9A	CXCL12	CXCR3	8,51394E-04	13008,3	3,82344E-02	797	7,28345E-01	1655	9,30873E-01	5854	6,99883E-01	45527	0	11208,5
Responder	Macro_NLRP3	Macro_NLRP3	NAMPT	ITGA5_ITGB1	8,54979E-04	9203,7	9,51006E-03	9270	5,69087E-01	3286	5,84223E-01	11052	8,76229E-01	11202	0	11208,5
Responder	Mono_INHBA	Macro_FOLR2-APOE+	CCL3L1	CCR1	8,56561E-04	9576,3	1,38295E-02	4936	5,62982E-01	3367	1,08979E+00	4253	8,13395E-01	24117	0	11208,5
Responder	Macro_OLFML3	Macro_LYVE1	C1QA	CD93	8,56889E-04	8512,7	1,00539E-02	8428	4,96267E-01	4475	5,75659E-01	11207	8,97498E-01	7245	0	11208,5
Responder	Mono_INHBA	cDC(CD1C)	VCAN	CD44	8,56889E-04	10098,5	8,52921E-03	11207	3,19397E-01	10009	7,15654E-01	8783	8,86506E-01	9285	0	11208,5
Responder	cDC(CD1C)	Macro_FOLR2+APOE+	HLA-DQA2	LAG3	8,57244E-04	9166,5	1,93946E-02	2654	4,74635E-01	4937	1,06579E+00	4464	8,20676E-01	22569	0	11208,5
Responder	Macro_FOLR2-APOE+	Mono_INHBA	PLAU	PLAUR	8,59568E-04	7537,3	1,31057E-02	5369	6,32575E-01	2470	8,08555E-01	7425	8,76171E-01	11214	0	11208,5
Responder	Macro_NLRP3	Mono_CD16	LYZ	ITGAL	8,60719E-04	6125,5	1,47867E-02	4362	6,85931E-01	1967	7,97609E-01	7576	9,06687E-01	5514	0	11208,5
Responder	pDC_LILRA4	Macro_ISG15	APP	RPSA	8,61350E-04	3835,5	2,09454E-02	2360	1,10281E+00	389	2,43136E+00	277	9,09764E-01	4943	0	11208,5
Responder	Macro_FOLR2-APOE+	Mono_CD14	PLAU	PLAUR	8,61486E-04	6522,1	1,30982E-02	5373	6,32043E-01	2477	1,40302E+00	2333	8,76140E-01	11219	0	11208,5
Responder	Macro_FOLR2-APOE+	pDC_LILRA4	HSP90B1	TLR9	8,63844E-04	6439,3	6,35356E-02	265	1,30572E+00	187	1,11271E+00	4079	8,49965E-01	16457	0	11208,5
Responder	cDC(CD1C)	Mono_CD14	CD55	CR1	8,64095E-04	10763,5	1,55328E-02	3987	4,74312E-01	4947	1,07152E+00	4409	7,87538E-01	29266	0	11208,5
Responder	Mono_INHBA	cDC_CLEC9A	CALM3	MYLK	8,64782E-04	12729,5	4,10201E-02	706	8,05514E-01	1223	1,01458E+00	4948	6,99712E-01	45562	0	11208,5
Responder	Macro_NLRP3	Mast	CD55	ADGRE2	8,68033E-04	5748,3	2,49174E-02	1749	8,57355E-01	991	1,10897E+00	4097	8,78789E-01	10696	0	11208,5
Responder	Macro_OLFML3	Macro_IFI27	C1QB	CD33	8,69192E-04	9939,5	8,51026E-03	11239	3,88321E-01	7350	5,77764E-01	11172	8,89628E-01	8728	0	11208,5
Responder	Macro_FOLR2-APOE+	pDC_LILRA4	SEMA6B	PLXNA4	8,70349E-04	14076,7	2,21305E-01	18	1,20969E+00	266	1,23057E+00	3262	6,22713E-01	55629	0	11208,5
Responder	cDC_CLEC9A	pDC_LILRA4	HLA-DPA1	CD4	8,70349E-04	5573,1	7,98410E-03	12470	5,41582E-01	3676	2,47453E+00	245	9,42403E-01	266	0	11208,5
Responder	Mono_CD16	cDC_CLEC9A	ADAM10	CADM1	8,71342E-04	6593,9	4,91185E-02	493	1,09570E+00	395	1,57302E+00	1668	8,36911E-01	19205	0	11208,5
Responder	Macro_NLRP3	Mast	HBEGF	CD44	8,71903E-04	5199,5	1,68208E-02	3431	8,16016E-01	1177	9,68929E-01	5437	9,10780E-01	4744	0	11208,5
Responder	Macro_OLFML3	Macro_ISG15	HLA-DPB1	LAG3	8,71903E-04	7566,5	1,53296E-02	4083	6,05897E-01	2774	7,32999E-01	8521	8,75981E-01	11246	0	11208,5
Responder	Macro_NLRP3	Mast	HBEGF	CD9	8,73454E-04	4592,3	2,84852E-02	1369	8,82290E-01	892	1,28674E+00	2927	9,01154E-01	6565	0	11208,5
Responder	cDC(CD1C)	Mono_CD16	GNAI2	C5AR1	8,75397E-04	8427,9	8,50326E-03	11255	4,39592E-01	5823	9,46938E-01	5672	8,92493E-01	8181	0	11208,5
Responder	Macro_NLRP3	Mast	ARPC5	ADRB2	8,75786E-04	5775,3	2,82768E-02	1388	7,45067E-01	1546	9,95467E-01	5153	8,84737E-01	9581	0	11208,5
Responder	Macro_NLRP3	cDC(CD1C)	S100A8	CD69	8,75786E-04	8989,1	1,11037E-02	7111	4,72092E-01	4991	6,22639E-01	10379	8,75946E-01	11256	0	11208,5
Responder	Mono_INHBA	Macro_LYVE1	INHBA	ACTR2	8,77222E-04	5615,3	2,65157E-02	1561	8,76861E-01	915	1,48513E+00	1977	8,69666E-01	12415	0	11208,5
Responder	Mono_INHBA	cDC(CD1C)	LGALS1	PTPRC	8,77222E-04	7879,7	6,38911E-03	17818	4,73234E-01	4966	1,08825E+00	4267	9,31794E-01	1139	0	11208,5
Responder	Macro_FOLR2-APOE+	Mono_CD14	RPS19	C5AR1	8,77733E-04	7105,9	8,49859E-03	11261	5,14830E-01	4124	9,26290E-01	5899	9,20219E-01	3037	0	11208,5
Responder	Macro_ISG15	cDC(CD1C)	TNFSF13B	HLA-DPB1	8,78903E-04	5134,7	8,49681E-03	11264	6,61392E-01	2186	1,84741E+00	970	9,51202E-01	45	0	11208,5
Responder	Macro_NLRP3	Mast	HSPA8	ADRB2	8,80074E-04	6533,9	2,42599E-02	1836	5,89386E-01	2966	7,06369E-01	8929	8,95039E-01	7730	0	11208,5
Responder	Macro_ISG15	Macro_FOLR2+APOE+	CCL8	CCR1	8,80652E-04	9982,5	4,83955E-02	513	8,18270E-01	1159	1,57048E+00	1674	7,56473E-01	35358	0	11208,5
Responder	Macro_OLFML3	cDC(CD1C)	FN1	ITGA4_ITGB1	8,80701E-04	6741,9	1,82895E-02	2956	4,72916E-01	4971	1,44065E+00	2164	8,69678E-01	12410	0	11208,5
Responder	Macro_NLRP3	Mast	VEGFA	CD44	8,80855E-04	7817,3	1,28056E-02	5587	6,30777E-01	2486	6,65999E-01	9617	8,81504E-01	10188	0	11208,5
Responder	Macro_ISG15	Macro_OLFML3	CD14	ITGB2	8,83594E-04	7720,3	8,49044E-03	11276	4,86952E-01	4671	6,02944E-01	10726	9,35837E-01	720	0	11208,5
Responder	Macro_OLFML3	Macro_FOLR2+APOE-	MDK	SDC3	8,84890E-04	8571,7	4,17689E-02	678	6,47185E-01	2334	1,01196E+00	4977	8,15601E-01	23661	0	11208,5
Responder	Mast	pDC_LILRA4	TIMP3	CD44	8,85331E-04	6005,3	2,54829E-02	1677	7,69750E-01	1403	2,02177E+00	681	8,56506E-01	15057	0	11208,5
Responder	Macro_OLFML3	Macro_FOLR2-APOE+	C3	ITGAM	8,89093E-04	7633,9	2,01523E-02	2504	5,93326E-01	2922	1,01162E+00	4983	8,49500E-01	16552	0	11208,5
Responder	cDC(CD1C)	Macro_OLFML3	HMGB1	TLR2	8,89487E-04	10144,5	8,48417E-03	11291	3,13878E-01	10246	6,71640E-01	9503	8,90994E-01	8474	0	11208,5
Responder	Mono_INHBA	cDC_LAMP3	IL6	IL6R_IL6ST	8,91200E-04	9276,3	4,59024E-02	568	8,88557E-01	873	1,51087E+00	1876	7,74446E-01	31856	0	11208,5
Responder	Mono_CD14	Macro_FOLR2+APOE+	CD52	SIGLEC10	8,92643E-04	8477,9	1,14012E-02	6808	5,13265E-01	4159	5,70329E-01	11299	8,88703E-01	8915	0	11208,5
Responder	Macro_LYVE1	cDC_LAMP3	IL10	CD70	8,96608E-04	16681,9	1,04050E-01	82	1,20803E+00	270	4,95907E-01	13036	5,85747E-01	58813	0	11208,5
Responder	Macro_OLFML3	Macro_FOLR2-APOE+	FN1	ITGA6	9,01791E-04	11176,7	4,26339E-02	645	5,33268E-01	3812	1,01006E+00	5001	7,57293E-01	35217	0	11208,5
Responder	pDC_LILRA4	Mono_CD14	SELPLG	SELL	9,02628E-04	5095,9	2,53827E-02	1688	8,77936E-01	910	2,98896E+00	72	8,74304E-01	11601	0	11208,5
Responder	Macro_OLFML3	cDC(CD1C)	C1QA	CD93	9,05754E-04	8569,5	8,46963E-03	11332	4,44644E-01	5687	9,31015E-01	5850	8,89337E-01	8770	0	11208,5
Responder	Mono_INHBA	cDC_LAMP3	ADM	CALCRL	9,06053E-04	11356,9	2,96534E-02	1263	6,96678E-01	1882	1,07387E+00	4393	7,42087E-01	38038	0	11208,5
Responder	Mono_INHBA	Mono_CD14	ICAM1	ITGAM_ITGB2	9,06954E-04	8658,5	8,46902E-03	11335	4,30198E-01	6078	1,07563E+00	4373	8,80895E-01	10298	0	11208,5
Responder	Macro_NLRP3	Macro_NLRP3	VCAN	TLR2	9,07354E-04	6495,1	2,17721E-02	2226	6,82835E-01	1991	9,42246E-01	5714	8,75543E-01	11336	0	11208,5
Responder	Macro_ISG15	pDC_LILRA4	ADM	CALCRL	9,07384E-04	10575,3	2,90331E-02	1316	7,21908E-01	1691	2,50241E+00	232	7,40058E-01	38429	0	11208,5
Responder	Macro_OLFML3	Macro_FOLR2-APOE+	SERPING1	LRP1	9,08155E-04	8407,9	1,27124E-02	5656	5,12819E-01	4168	5,68679E-01	11338	8,84328E-01	9669	0	11208,5
Responder	Mono_INHBA	cDC_LAMP3	ICAM1	IL2RG	9,10159E-04	7350,3	1,33538E-02	5205	6,79231E-01	2029	8,41880E-01	6966	8,75488E-01	11343	0	11208,5
Responder	Macro_ISG15	Mono_INHBA	SERPING1	LRP1	9,10962E-04	8996,7	1,07520E-02	7497	3,77882E-01	7704	8,22117E-01	7229	8,75482E-01	11345	0	11208,5
Responder	Macro_FOLR2+APOE+	cDC(CD1C)	C1QA	CD33	9,11045E-04	5471,3	1,37038E-02	5014	6,23857E-01	2558	1,14438E+00	3804	9,10655E-01	4772	0	11208,5
Responder	Mono_INHBA	Macro_OLFML3	CCL4	CCR1	9,11760E-04	7714,5	1,37022E-02	5015	5,99990E-01	2833	1,06748E+00	4443	8,56421E-01	15073	0	11208,5
Responder	Macro_OLFML3	pDC_LILRA4	CXCL12	CXCR4	9,12475E-04	7548,9	1,37012E-02	5016	5,35698E-01	3761	2,23511E+00	423	8,45670E-01	17336	0	11208,5
Responder	Macro_NLRP3	Macro_FOLR2+APOE-	S100A9	CD68	9,14178E-04	6616,1	9,93507E-03	8577	5,59455E-01	3410	6,71924E-01	9495	9,39840E-01	390	0	11208,5
Responder	Macro_NLRP3	Mono_CD16	VCAN	ITGA4	9,17403E-04	6817,7	2,03164E-02	2469	7,46303E-01	1539	8,02169E-01	7511	8,75417E-01	11361	0	11208,5
Responder	Macro_OLFML3	cDC(CD1C)	B2M	CD1B	9,18544E-04	8146,3	3,64240E-02	872	7,21166E-01	1698	5,02067E-01	12882	8,61238E-01	14071	0	11208,5
Responder	Mono_INHBA	cDC_LAMP3	SPP1	S1PR1	9,18930E-04	11612,5	5,91027E-02	320	7,31265E-01	1636	1,08110E+00	4328	7,28111E-01	40570	0	11208,5
Responder	Macro_NLRP3	Macro_FOLR2-APOE+	HBEGF	CD44	9,19829E-04	7661,7	1,25978E-02	5748	5,95774E-01	2889	5,67500E-01	11367	8,98316E-01	7096	0	11208,5
Responder	Macro_FOLR2+APOE-	cDC_LAMP3	F13A1	ITGA4	9,20146E-04	5086,3	2,83497E-02	1384	1,08495E+00	414	1,56433E+00	1699	8,78589E-01	10726	0	11208,5
Responder	Mono_INHBA	cDC_LAMP3	INHBA	ACTR2	9,21089E-04	6670,1	2,05049E-02	2432	7,48566E-01	1528	1,33299E+00	2682	8,54392E-01	15500	0	11208,5
Responder	Macro_ISG15	Mast	IL1B	ADRB2	9,26320E-04	5265,5	3,62282E-02	877	8,42277E-01	1049	1,53059E+00	1810	8,75314E-01	11383	0	11208,5

Post_R_Myeloid_Myeloid_CellCel

Responder	Macro_NLRP3	Mono_CD16	S100A9	TLR4	9,27542E-04	8415,7	1,28454E-02	5564	5,74727E-01	3190	6,02664E-01	10730	8,75296E-01	11386	0	11208,5
Responder	cDC(CD1C)	cDC_CLEC9A	ICAM3	ITGAL	9,28314E-04	12199,9	1,54683E-02	4011	4,70531E-01	5038	1,30298E+00	2834	7,42733E-01	37908	0	11208,5
Responder	Macro_ISG15	Macro_OLFML3	TNFSF13B	HLA-DPB1	9,29988E-04	5970,3	8,44822E-03	11392	6,53593E-01	2278	1,01622E+00	4927	9,51069E-01	46	0	11208,5
Responder	cDC(CD1C)	Macro_OLFML3	ENTPD1	ADORA3	9,31216E-04	8930,7	1,66097E-02	3507	4,70341E-01	5042	1,05430E+00	4586	8,31633E-01	20310	0	11208,5
Responder	Macro_ISG15	Mono_CD16	CD14	ITGB1	9,34487E-04	8868,1	8,44256E-03	11403	4,37969E-01	5872	6,89198E-01	9220	9,00794E-01	6637	0	11208,5
Responder	Mast	cDC_CLEC9A	CSF1	CSF3R	9,36263E-04	8921,5	2,52323E-02	1709	8,16401E-01	1173	1,96061E+00	776	7,85091E-01	29741	0	11208,5
Responder	Macro_OLFML3	Macro_IER3	C1QA	CD93	9,37769E-04	9142,5	9,15915E-03	9904	4,67111E-01	5117	5,65167E-01	11411	8,93130E-01	8072	0	11208,5
Responder	Macro_OLFML3	cDC_CLEC9A	CALM1	MYLK	9,39509E-04	12403,7	3,49880E-02	936	7,18368E-01	1711	4,29735E-01	14733	7,66473E-01	33430	0	11208,5
Responder	pDC_LILRA4	cDC_LAMP3	SEMA7A	PLXNC1	9,41135E-04	11361,5	2,74494E-02	1456	7,18296E-01	1712	1,63204E+00	1471	7,25744E-01	40960	0	11208,5
Responder	cDC_CLEC9A	Macro_ISG15	BTLA	TNFRSF14	9,42762E-04	7371,9	4,41413E-02	611	1,67778E+00	84	1,55877E+00	1713	8,17614E-01	23243	0	11208,5
Responder	Macro_NLRP3	pDC_LILRA4	THBS1	CD36	9,42762E-04	5708,3	2,51899E-02	1713	7,87807E-01	1311	1,72433E+00	1236	8,66298E-01	13073	0	11208,5
Responder	Macro_NLRP3	Mast	HBEGF	CD82	9,43949E-04	5300,5	3,80058E-02	808	9,62167E-01	667	1,39219E+00	2393	8,75140E-01	11426	0	11208,5
Responder	Mast	Macro_FOLR2-APOE+	CSF1	CSF3R	9,44391E-04	6830,9	4,72232E-02	531	9,41924E-01	729	1,55840E+00	1714	8,33267E-01	19972	0	11208,5
Responder	Mono_CD14	Macro_FOLR2-APOE+	VCAN	TLR1	9,44391E-04	6682,9	2,51858E-02	1714	8,69883E-01	945	1,61584E+00	1521	8,42422E-01	18026	0	11208,5
Responder	Mono_INHBA	Mono_CD16	IL1B	SIGIRR	9,45090E-04	6349,5	1,75749E-02	3174	7,07860E-01	1790	1,12238E+00	3997	8,74400E-01	11578	0	11208,5
Responder	Macro_ISG15	Macro_OLFML3	CCL8	CCR1	9,47656E-04	10234,5	4,54170E-02	576	7,98149E-01	1261	1,55774E+00	1716	7,50575E-01	36411	0	11208,5
Responder	Macro_OLFML3	Macro_NLRP3	C3	CD81	9,47673E-04	9859,3	9,45232E-03	9385	3,69475E-01	8000	6,86223E-01	9268	8,75093E-01	11435	0	11208,5
Responder	Macro_ISG15	Mono_INHBA	CCL8	CCR1	9,49290E-04	11367,9	3,35297E-02	1008	7,17844E-01	1717	1,78557E+00	1085	7,21106E-01	41821	0	11208,5
Responder	Macro_OLFML3	Mono_CD16	HLA-B	LILRA1	9,49503E-04	6585,7	1,91651E-02	2714	6,88849E-01	1945	5,38422E-01	11994	9,09049E-01	5067	0	11208,5
Responder	pDC_LILRA4	Macro_LYVE1	APP	APLP2	9,55848E-04	3966,7	2,51499E-02	1721	1,15174E+00	330	2,39195E+00	311	9,02773E-01	6263	0	11208,5
Responder	Mono_CD14	Macro_OLFML3	VCAN	ITGA4	9,58496E-04	5732,9	2,01534E-02	2501	8,82018E-01	894	1,34737E+00	2600	8,74977E-01	11461	0	11208,5
Responder	Mono_CD14	cDC_LAMP3	AGTRAP	RACK1	9,58496E-04	10217,1	8,42335E-03	11461	3,68469E-01	8032	6,18427E-01	10459	8,82973E-01	9925	0	11208,5
Responder	Macro_OLFML3	cDC(CD1C)	FN1	ITGA5_ITGB1	9,59117E-04	7657,3	1,74603E-02	3213	4,68780E-01	5080	1,37204E+00	2491	8,50766E-01	16294	0	11208,5
Responder	Macro_OLFML3	Macro_LYVE1	HMGB1	CD163	9,59138E-04	8323,7	1,06831E-02	7592	7,17313E-01	1720	2,72240E-01	19469	9,28222E-01	1629	0	11208,5
Responder	Mono_CD14	Mono_CD16	VCAN	TLR2	9,59332E-04	5579,9	2,15452E-02	2264	8,16081E-01	1175	1,54013E+00	1789	8,74972E-01	11463	0	11208,5
Responder	Macro_NLRP3	Macro_FOLR2+APOE+	TIMP1	CD63	9,60169E-04	4961,5	1,15185E-02	6693	9,75458E-01	638	8,98229E-01	6248	9,54173E-01	20	0	11208,5
Responder	Macro_NLRP3	Macro_FOLR2+APOE+	CD52	SIGLEC10	9,63104E-04	6076,9	1,74977E-02	3196	7,93904E-01	1284	6,73990E-01	9473	9,08190E-01	5223	0	11208,5
Responder	Macro_IFI27	Macro_ISG15	C3	IFITM1	9,64087E-04	4346,1	2,64947E-02	1563	7,41334E-01	1572	1,55586E+00	1726	9,05766E-01	5661	0	11208,5
Responder	Macro_FOLR2-APOE+	Macro_LYVE1	MMP9	LRP1	9,67730E-04	5249,9	3,69834E-02	844	1,04311E+00	482	1,42580E+00	2232	8,74872E-01	11483	0	11208,5
Responder	Macro_OLFML3	Macro_FOLR2+APOE-	C3	C3AR1	9,68573E-04	8626,7	1,17079E-02	6522	3,79241E-01	7656	8,97217E-01	6262	8,74861E-01	11485	0	11208,5
Responder	Macro_NLRP3	Mono_CD14	VEGFA	CD44	9,68995E-04	8072,1	1,13061E-02	6888	5,28051E-01	3892	8,47075E-01	6886	8,74844E-01	11486	0	11208,5
Responder	cDC(CD1C)	Macro_FOLR2+APOE+	TIMP1	CD63	9,73298E-04	6433,5	7,64080E-03	13425	6,56319E-01	2248	9,99562E-01	5099	9,44314E-01	187	0	11208,5
Responder	cDC(CD1C)	cDC_LAMP3	GSTP1	TRAF2	9,74048E-04	10014,1	1,35420E-02	5100	4,81238E-01	4797	1,04854E+00	4648	8,12383E-01	24317	0	11208,5
Responder	Mono_CD16	Macro_OLFML3	CD52	SIGLEC10	9,74799E-04	4555,5	2,14702E-02	2276	1,04530E+00	475	9,99504E-01	5101	9,16371E-01	3717	0	11208,5
Responder	pDC_LILRA4	Mono_INHBA	RARRES2	CCRL2	9,75701E-04	11482,9	3,59236E-02	890	7,15521E-01	1733	2,31112E+00	365	7,13395E-01	43218	0	11208,5
Responder	cDC_CLEC9A	Macro_OLFML3	HLA-DQA2	CD4	9,75763E-04	8425,5	8,76701E-03	10656	3,72677E-01	7892	8,60504E-01	6723	9,05837E-01	5648	0	11208,5
Responder	Macro_OLFML3	Macro_OLFML3	FN1	ITGA6	9,79315E-04	10778,7	4,68311E-02	539	5,55531E-01	3474	9,99075E-01	5107	7,65818E-01	33565	0	11208,5
Responder	Macro_OLFML3	cDC_CLEC9A	PDCD1LG2	PDCD1	9,80706E-04	17821,1	8,12822E-02	162	7,15413E-01	1736	4,08373E-01	15308	5,55041E-01	60691	0	11208,5
Responder	Mono_CD14	Mono_CD14	VCAN	TLR2	9,82142E-04	5391,1	2,14313E-02	2287	8,14254E-01	1186	1,97388E+00	757	8,74681E-01	11517	0	11208,5
Responder	Mono_CD14	Macro_FOLR2+APOE+	VCAN	TLR2	9,82569E-04	5761,1	2,14305E-02	2288	8,14242E-01	1187	1,34665E+00	2604	8,74679E-01	11518	0	11208,5
Responder	Mono_CD14	Macro_FOLR2-APOE+	VCAN	TLR2	9,85558E-04	5638,7	2,14232E-02	2291	8,14125E-01	1188	1,48441E+00	1981	8,74661E-01	11525	0	11208,5
Responder	Macro_FOLR2-APOE+	Mono_CD14	C3	IFITM1	9,85986E-04	7915,9	1,39651E-02	4859	3,47504E-01	8832	1,24849E+00	3154	8,74660E-01	11526	0	11208,5
Responder	Mono_INHBA	Mono_CD16	IL6	FCGR3B	9,86117E-04	10938,7	1,25373E-01	49	9,98326E-01	586	1,46922E+00	2036	7,26600E-01	40814	0	11208,5
Responder	Mono_INHBA	Mono_CD14	GNAI2	C5AR1	9,86842E-04	8364,5	8,40147E-03	11528	4,30333E-01	6072	1,04029E+00	4720	8,91913E-01	8294	0	11208,5
Responder	cDC_CLEC9A	Macro_OLFML3	SELPLG	ITGB2	9,87270E-04	6636,5	1,09915E-02	7236	5,97586E-01	2862	9,05216E-01	6155	9,05498E-01	5721	0	11208,5
Responder	Macro_NLRP3	Macro_FOLR2+APOE+	THBS1	CD47	9,89983E-04	7657,9	1,81450E-02	2998	6,86367E-01	1960	6,10981E-01	10590	8,74644E-01	11533	0	11208,5
Responder	Macro_FOLR2+APOE+	Mono_CD14	MMP12	PLAUR	9,94135E-04	5679,7	2,49891E-02	1744	7,45054E-01	1547	1,75220E+00	1166	8,67988E-01	12733	0	11208,5
Responder	cDC_CLEC9A	Macro_OLFML3	SERPINF1	PLXDC2	9,94570E-04	5421,7	1,67102E-02	3466	6,54697E-01	2270	1,37474E+00	2474	8,95201E-01	7690	0	11208,5
Responder	Macro_OLFML3	Macro_IER3	APOE	LRP1	9,96294E-04	7935,1	1,07528E-02	7495	4,54190E-01	5441	5,58759E-01	11550	9,14957E-01	3981	0	11208,5
Responder	Macro_IER3	pDC_LILRA4	HSP90B1	TLR9	9,98526E-04	6500,5	6,20692E-02	285	1,29046E+00	197	1,11428E+00	4066	8,48470E-01	16746	0	11208,5
Responder	Macro_FOLR2-APOE+	Macro_LYVE1	A2M	LRP1	9,98884E-04	8056,1	1,63370E-02	3624	5,97462E-01	2863	5,58500E-01	11556	8,77009E-01	11029	0	11208,5
Responder	pDC_LILRA4	Macro_IFI27	FAM3D	FPR1	1,00089E-03	20123,5	2,49479E-02	1748	7,41577E-01	1570	1,73592E+00	1213	6,71099E-01	49908	0,988	46178,5
Responder	cDC_CLEC9A	Macro_OLFML3	CD52	SIGLEC10	1,00148E-03	8539,1	1,10364E-02	7190	4,83363E-01	4745	6,23424E-01	10358	8,87084E-01	9194	0	11208,5
Responder	Macro_OLFML3	cDC_CLEC9A	HLA-DRA	LAG3	1,00365E-03	8024,7	1,23720E-02	5928	5,35506E-01	3763	7,90935E-01	7657	8,74473E-01	11567	0	11208,5
Responder	Macro_OLFML3	cDC(CD1C)	CXCL9	FCGR2A	1,00408E-03	8535,3	1,14586E-02	6753	2,86678E-01	11568	1,26782E+00	3039	8,81885E-01	10108	0	11208,5
Responder	Macro_OLFML3	pDC_LILRA4	MMP2	PECAM1	1,00429E-03	15791,3	2,49120E-02	1750	3,98210E-01	7028	1,60424E+00	1552	6,03175E-01	57418	0	11208,5
Responder	Macro_OLFML3	Macro_FOLR2+APOE+	HLA-DPB1	LAG3	1,00495E-03	7639,3	1,73980E-02	3229	6,58820E-01	2218	5,57597E-01	11570	8,82695E-01	9971	0	11208,5
Responder	cDC(CD1C)	Macro_FOLR2+APOE+	ENTPD1	TMIGD3	1,00520E-03	10385,5	3,97192E-02	742	7,00195E-01	1851	9,96455E-01	5141	7,68619E-01	32985	0	11208,5
Responder	Macro_IFI27	Mono_INHBA	MMP12	PLAUR	1,00598E-03	5786,5	2,48993E-02	1751	7,43976E-01	1556	1,57826E+00	1642	8,67781E-01	12775	0	11208,5
Responder	cDC_CLEC9A	Macro_OLFML3	HMGB1	TLR2	1,00712E-03	7679,9	9,91156E-03	8605	4,70242E-01	5044	8,83270E-01	6447	8,98319E-01	7095	0	11208,5
Responder	Macro_FOLR2+APOE+	Macro_LYVE1	APOE	TREM2	1,00750E-03	4073,5	2,19951E-02	2187	7,79760E-01	1358	9,96360E-01	5144	9,38589E-01	470	0	11208,5
Responder	Macro_IFI27	Mono_CD14	MMP12	PLAUR	1,00768E-03	5558,5	2,48849E-02	1752	7,43444E-01	1559	2,17272E+00	490	8,67748E-01	12783	0	11208,5
Responder	Mono_INHBA	Mast	HBEGF	CD82	1,01059E-03	5836,5	3,25078E-02	1054	8,61661E-01	979	1,29669E+00	2872	8,66350E-01	13069	0	11208,5
Responder	Macro_OLFML3	Macro_FOLR2-APOE+	APOE	SDC2	1,01498E-03	6581,5	2,39075E-02	1889	7,02039E-01	1833	8,87535E-01	6384	8,74323E-01	11593	0	11208,5
Responder	Mono_INHBA	Mono_INHBA	SPP1	ITGA5_ITGB1	1,01522E-03	8702,5	1,34553E-02	5154	4,69769E-01	5055	1,29088E+00	2910	8,36998E-01	19185	0	11208,5

Post_R_Myeloid_Myeloid_CellCel

Responder	Macro_ISG15	Mono_INHBA	TNFSF13B	TFRC	1,01761E-03	7734,9	1,03860E-02	7970	5,25131E-01	3944	1,12808E+00	3953	8,74308E-01	11599	0	11208,5
Responder	cDC_CLEC9A	cDC(CD1C)	SELPLG	ITGB2	1,01761E-03	7019,9	9,34196E-03	9573	4,47952E-01	5601	1,58614E+00	1620	8,98307E-01	7097	0	11208,5
Responder	Macro_FOLR2+APOE+	Macro_LYVE1	C1QB	LRP1	1,01988E-03	3919,3	2,07408E-02	2396	9,89666E-01	600	9,95074E-01	5160	9,43134E-01	232	0	11208,5
Responder	cDC(CD1C)	Mono_CD16	PTPN6	CLEC12A	1,01988E-03	7982,9	1,34422E-02	5160	4,91787E-01	4565	1,02489E+00	4854	8,60998E-01	14127	0	11208,5
Responder	Macro_OLFML3	Mono_INHBA	MDK	SDC4	1,02299E-03	10309,5	4,98640E-02	473	6,69727E-01	2116	9,94729E-01	5164	7,70703E-01	32586	0	11208,5
Responder	Mono_INHBA	Mast	ACTR2	ADRB2	1,02533E-03	6638,7	2,74583E-02	1454	6,98141E-01	1866	1,12645E+00	3962	8,58241E-01	14703	0	11208,5
Responder	Macro_FOLR2+APOE-	Macro_LYVE1	CCL18	CCR1	1,02651E-03	7965,7	2,87406E-02	1350	7,11635E-01	1763	1,76301E+00	1140	8,12140E-01	24367	0	11208,5
Responder	Mast	Macro_LYVE1	PLAT	LRP1	1,02662E-03	6105,3	1,29720E-01	47	1,18259E+00	289	1,40379E+00	2325	8,48941E-01	16657	0	11208,5
Responder	Mono_INHBA	Macro_ISG15	TNF	TNFRSF1A	1,02767E-03	8978,1	1,56809E-02	3928	5,15935E-01	4111	9,94309E-01	5170	8,30855E-01	20473	0	11208,5
Responder	Macro_ISG15	Macro_LYVE1	RPS19	C5AR1	1,02774E-03	6620,7	1,11612E-02	7038	7,02530E-01	1827	5,55137E-01	11622	9,29668E-01	1408	0	11208,5
Responder	cDC_LAMP3	Macro_FOLR2-APOE+	CCL19	CCRL2	1,02823E-03	9288,3	4,67859E-02	541	1,83055E+00	58	1,54710E+00	1764	7,69231E-01	32870	0	11208,5
Responder	cDC_CLEC9A	cDC(CD1C)	HMGB1	CXCR4	1,03172E-03	5430,5	8,36516E-03	11606	6,34232E-01	2449	1,98131E+00	746	9,31773E-01	1143	0	11208,5
Responder	Macro_NLRP3	Macro_IFI27	S100A8	CD68	1,03217E-03	6069,9	1,36966E-02	5018	6,59467E-01	2215	5,54738E-01	11632	9,42219E-01	276	0	11208,5
Responder	Mono_CD14	Macro_ISG15	VCAN	SELL	1,03342E-03	5281,1	2,47739E-02	1767	9,16240E-01	795	2,06474E+00	620	8,72070E-01	12015	0	11208,5
Responder	Macro_FOLR2+APOE-	Macro_FOLR2+APOE+	CCL13	CCR1	1,03371E-03	11159,9	6,14358E-02	290	1,26211E+00	214	9,00773E-01	6213	7,42961E-01	37874	0	11208,5
Responder	Mono_INHBA	Macro_FOLR2-APOE+	IL1B	IL1R1_IL1RAP	1,03550E-03	11324,9	1,33912E-02	5180	5,96957E-01	2877	1,00794E+00	5023	7,71969E-01	32336	0	11208,5
Responder	Macro_IER3	pDC_LILRA4	PLAU	ST14	1,03861E-03	9213,7	2,50530E-02	1734	7,10849E-01	1770	1,81869E+00	1025	7,82220E-01	30331	0	11208,5
Responder	Macro_OLFML3	cDC(CD1C)	PSAP	CD1B	1,04035E-03	9308,3	4,18231E-02	675	7,10713E-01	1771	4,95971E-01	13034	8,33838E-01	19853	0	11208,5
Responder	pDC_LILRA4	cDC_LAMP3	FAM3D	FPR1	1,04035E-03	19317,2	2,47623E-02	1771	7,40296E-01	1581	1,66695E+00	1372	6,70274E-01	50039	0,884	41823
Responder	cDC_CLEC9A	Macro_FOLR2+APOE+	BTLA	TNFRSF14	1,04081E-03	6496,5	6,14285E-02	291	1,82402E+00	59	1,35120E+00	2585	8,40975E-01	18339	0	11208,5
Responder	Mono_INHBA	cDC(CD1C)	TNF	TNFRSF1A	1,04338E-03	9280,3	1,40994E-02	4773	4,64644E-01	5190	1,23730E+00	3218	8,23253E-01	22012	0	11208,5
Responder	Mono_INHBA	Macro_IER3	IL6	IL6R_IL6ST	1,04496E-03	11019,7	3,26642E-02	1045	7,82956E-01	1337	1,15629E+00	3702	7,43353E-01	37806	0	11208,5
Responder	Mono_INHBA	Macro_FOLR2-APOE+	CCL3	CCR5	1,04654E-03	10473,3	1,33716E-02	5194	7,41640E-01	1568	1,31518E+00	2772	7,75664E-01	31624	0	11208,5
Responder	Macro_ISG15	Mono_CD14	HLA-A	LILRA1	1,04689E-03	9951,7	1,04825E-02	7845	3,15399E-01	10180	7,10369E-01	8860	8,74010E-01	11665	0	11208,5
Responder	cDC_CLEC9A	Macro_LYVE1	HMGB1	CD163	1,04892E-03	3951,5	1,33698E-02	5197	9,35499E-01	747	1,52109E+00	1835	9,35346E-01	770	0	11208,5
Responder	pDC_LILRA4	Macro_FOLR2-APOE+	VEGFB	NRP1	1,04906E-03	10565,7	2,47236E-02	1776	7,18292E-01	1713	2,16556E+00	505	7,44278E-01	37626	0	11208,5
Responder	pDC_LILRA4	cDC(CD1C)	SPON2	ITGAM	1,05081E-03	13900,5	2,47167E-02	1777	7,95499E-01	1274	1,70553E+00	1273	6,37881E-01	53970	0	11208,5
Responder	Mono_INHBA	Macro_NLRP3	NAMPT	ITGA5_ITGB1	1,05139E-03	9474,7	9,12540E-03	9965	5,35216E-01	3773	6,00732E-01	10752	8,73973E-01	11675	0	11208,5
Responder	Mono_INHBA	Macro_FOLR2-APOE+	NAMPT	ITGA5_ITGB1	1,05184E-03	9072,7	9,95921E-03	8543	5,72681E-01	3229	5,53159E-01	11676	8,78710E-01	10707	0	11208,5
Responder	Macro_ISG15	cDC_CLEC9A	LGALS9	HAVCR2	1,05229E-03	8831,1	8,78773E-03	10617	3,74917E-01	7809	1,30129E+00	2844	8,73972E-01	11677	0	11208,5
Responder	Macro_FOLR2+APOE+	Macro_FOLR2-APOE+	APOE	ABCA1	1,05289E-03	4184,5	2,01684E-02	2499	9,08517E-01	817	9,90644E-01	5202	9,31226E-01	1196	0	11208,5
Responder	Macro_FOLR2-APOE+	Macro_IER3	SPP1	CD44	1,05409E-03	8288,1	1,20010E-02	6260	4,64043E-01	5207	5,52888E-01	11681	8,98358E-01	7084	0	11208,5
Responder	Mono_INHBA	Macro_IER3	SPP1	PTGER4	1,05448E-03	9091,9	1,51984E-02	4156	4,73930E-01	4955	1,11930E+00	4020	8,27650E-01	21120	0	11208,5
Responder	Macro_ISG15	Macro_IER3	CCL8	CCR1	1,06133E-03	11669,5	3,22429E-02	1077	7,09151E-01	1783	1,55135E+00	1745	7,17153E-01	42534	0	11208,5
Responder	Mono_CD14	cDC(CD1C)	NAMPT	ITGA5_ITGB1	1,06178E-03	7239,9	9,10581E-03	9996	6,72487E-01	2090	1,73818E+00	1207	8,73854E-01	11698	0	11208,5
Responder	cDC(CD1C)	Macro_OLFML3	HLA-DQB1	CD4	1,06247E-03	7287,7	7,50038E-03	13849	4,63618E-01	5214	1,03870E+00	4726	9,29464E-01	1441	0	11208,5
Responder	cDC_CLEC9A	Mono_INHBA	LGALS9	CD44	1,06360E-03	6960,7	8,31098E-03	11702	5,39681E-01	3699	1,23492E+00	3237	9,09657E-01	4957	0	11208,5
Responder	Macro_FOLR2+APOE-	pDC_LILRA4	CXCL12	CD4	1,06486E-03	5930,3	2,65895E-02	1555	7,08869E-01	1785	2,25389E+00	404	8,58254E-01	14699	0	11208,5
Responder	Mono_INHBA	Macro_IER3	VEGFA	NRP1	1,06487E-03	10240,3	1,97938E-02	2569	7,10646E-01	1772	1,01254E+00	4971	7,80537E-01	30681	0	11208,5
Responder	Macro_OLFML3	Macro_FOLR2-APOE+	LGALS3BP	ITGB1	1,06496E-03	8085,3	1,59366E-02	3795	5,70121E-01	3266	6,18697E-01	10452	8,73820E-01	11705	0	11208,5
Responder	Mono_INHBA	Macro_IER3	VEGFA	NRP2	1,06568E-03	7805,7	2,34344E-02	1962	7,72395E-01	1388	1,33904E+00	2641	8,24200E-01	21829	0	11208,5
Responder	pDC_LILRA4	Macro_OLFML3	SELPLG	ITGB2	1,06568E-03	4647,1	1,33319E-02	5218	7,27587E-01	1663	1,88793E+00	887	9,13440E-01	4259	0	11208,5
Responder	Mono_CD14	Mast	ARPC5	ADRB2	1,06815E-03	6191,5	2,30017E-02	2032	5,99046E-01	2845	1,24653E+00	3160	8,73784E-01	11712	0	11208,5
Responder	Macro_FOLR2+APOE+	pDC_LILRA4	GNAI2	CXCR3	1,06839E-03	5985,7	3,51831E-02	927	1,16200E+00	313	1,54095E+00	1787	8,53494E-01	15693	0	11208,5
Responder	Macro_OLFML3	pDC_LILRA4	HLA-G	CD4	1,06889E-03	7979,9	1,62505E-02	3648	4,63019E-01	5222	1,72593E+00	1233	8,39813E-01	18588	0	11208,5
Responder	Mono_CD16	pDC_LILRA4	HMGB1	TLR9	1,06949E-03	6173,3	6,10643E-02	295	1,29971E+00	190	1,04776E+00	4653	8,59211E-01	14520	0	11208,5
Responder	cDC_CLEC9A	cDC_LAMP3	BTLA	TNFRSF14	1,07016E-03	6818,7	5,25328E-02	412	1,74876E+00	71	1,54059E+00	1788	8,30234E-01	20614	0	11208,5
Responder	cDC_CLEC9A	Mono_CD14	SELPLG	ITGB2	1,07409E-03	7824,5	8,77380E-03	10640	3,96412E-01	7084	1,35970E+00	2540	8,95405E-01	7650	0	11208,5
Responder	Macro_NLRP3	Macro_OLFML3	THBS1	CD47	1,07638E-03	7596,7	1,78302E-02	3095	6,78252E-01	2042	6,49194E-01	9908	8,73682E-01	11730	0	11208,5
Responder	Mono_CD14	Macro_LYVE1	HP	CD163	1,07672E-03	6641,5	1,19428E-01	59	1,17428E+00	296	1,47821E+00	2003	8,34907E-01	19641	0	11208,5
Responder	Mono_INHBA	Mono_INHBA	ADM	GPR84	1,07696E-03	13005,9	2,59699E-02	1631	5,07946E-01	4246	9,87074E-01	5232	7,16199E-01	42712	0	11208,5
Responder	Macro_FOLR2+APOE-	Mono_CD14	CCL18	CCR1	1,07725E-03	7886,7	2,84275E-02	1374	7,07771E-01	1792	2,16413E+00	508	8,11303E-01	24551	0	11208,5
Responder	Mono_INHBA	cDC(CD1C)	VEGFA	SIRPA	1,07857E-03	10548,9	1,33206E-02	5234	5,59268E-01	3413	1,16131E+00	3669	7,87776E-01	29220	0	11208,5
Responder	pDC_LILRA4	Macro_NLRP3	CD24	SIGLEC10	1,08081E-03	15900	3,98494E-02	737	7,07471E-01	1794	1,82622E+00	1007	6,75455E-01	49271	0,018	26691
Responder	Macro_FOLR2+APOE+	Mast	LGALS1	CD69	1,08101E-03	4366,5	1,84572E-02	2904	8,88971E-01	870	9,86468E-01	5237	9,28333E-01	1613	0	11208,5
Responder	Macro_OLFML3	Macro_NLRP3	FN1	ITGAV_ITGB1	1,08182E-03	7772,7	1,93135E-02	2676	4,62246E-01	5238	1,00953E+00	5004	8,58085E-01	14737	0	11208,5
Responder	Mono_INHBA	Mono_INHBA	ADAM10	CD44	1,08282E-03	8796,3	9,45623E-03	9370	5,57420E-01	3442	7,52826E-01	8217	8,73601E-01	11744	0	11208,5
Responder	cDC_CLEC9A	Mono_CD14	GNAI2	C5AR1	1,08282E-03	7771,5	8,42705E-03	11444	4,32310E-01	6021	1,50132E+00	1919	8,92060E-01	8265	0	11208,5
Responder	cDC_CLEC9A	Macro_OLFML3	BTLA	TNFRSF14	1,08397E-03	6530,5	6,10451E-02	297	1,82077E+00	62	1,33776E+00	2649	8,40556E-01	18436	0	11208,5
Responder	Macro_ISG15	Macro_NLRP3	SERPING1	LRP1	1,08559E-03	9749,1	1,03807E-02	7977	3,59640E-01	8383	6,76214E-01	9427	8,73554E-01	11750	0	11208,5
Responder	Macro_FOLR2-APOE+	cDC(CD1C)	MMP9	CD44	1,08605E-03	6604,5	1,49196E-02	4295	5,98006E-01	2859	1,29101E+00	2909	8,73545E-01	11751	0	11208,5
Responder	Macro_OLFML3	Mono_CD16	HLA-C	LILRA1	1,08744E-03	6609,5	2,03884E-02	2452	6,82657E-01	1993	5,49516E-01	11754	9,05872E-01	5640	0	11208,5
Responder	pDC_LILRA4	Mast	APP	RPSA	1,08751E-03	3964,5	2,01037E-02	2514	1,07844E+00	429	2,23298E+00	426	9,08067E-01	5245	0	11208,5
Responder	cDC_CLEC9A	cDC_CLEC9A	CCL5	CXCR3	1,08795E-03	11855,7	2,77416E-02	1430	7,08920E-01	1784	1,53594E+00	1798	7,14302E-01	43058	0	11208,5
Responder	Mono_INHBA	Macro_IER3	INHBA	ACTR2	1,08832E-03	5722,5	2,55389E-02	1671	8,56012E-01	996	1,50430E+00	1900	8,67524E-01	12837	0	11208,5

Post_R_Myeloid_Myeloid_CellCel

Responder	pDC_LILRA4	Mono_CD16	APP	CD74	1,09077E-03	4350,9	1,32941E-02	5249	6,87728E-01	1952	1,25077E+00	3142	9,43710E-01	203	0	11208,5
Responder	Macro_NLRP3	Mono_INHBA	THBS1	CD36	1,09115E-03	5891,5	2,86047E-02	1355	8,43113E-01	1045	1,11174E+00	4087	8,73490E-01	11762	0	11208,5
Responder	cDC_CLEC9A	Mono_CD14	HSPA1A	TLR4	1,09208E-03	7870,9	8,74632E-03	10697	4,48786E-01	5578	1,54486E+00	1776	8,81956E-01	10095	0	11208,5
Responder	Macro_FOLR2-APOE+	pDC_LILRA4	LGALS1	CD69	1,09208E-03	7777,9	8,27902E-03	11764	4,16703E-01	6462	1,47074E+00	2031	8,96645E-01	7424	0	11208,5
Responder	Macro_OLFML3	Macro_FOLR2+APOE-	SERPING1	LRP1	1,09300E-03	9641,3	1,03668E-02	7997	4,21683E-01	6314	5,91917E-01	10921	8,73480E-01	11766	0	11208,5
Responder	Macro_FOLR2+APOE-	cDC_CLEC9A	CXCL12	CXCR3	1,09333E-03	9215,3	8,00768E-02	168	1,07042E+00	441	1,53482E+00	1801	7,71423E-01	32458	0	11208,5
Responder	Mono_CD14	Mono_CD16	NAMPT	INSR	1,09512E-03	6553,7	2,45012E-02	1802	8,75870E-01	917	1,55711E+00	1718	8,46648E-01	17123	0	11208,5
Responder	Mono_INHBA	Macro_FOLR2+APOE-	CXCL8	SDC3	1,09732E-03	6210,1	2,42359E-02	1841	7,40182E-01	1583	1,34960E+00	2591	8,62453E-01	13827	0	11208,5
Responder	pDC_LILRA4	Macro_IER3	SEMA7A	PLXNC1	1,09872E-03	11327,7	2,81368E-02	1403	7,25750E-01	1671	1,53418E+00	1804	7,28199E-01	40552	0	11208,5
Responder	cDC(CD1C)	Macro_LYVE1	SPN	SIGLEC1	1,10060E-03	11709,9	2,46556E-02	1784	7,32963E-01	1625	9,84000E-01	5261	7,38833E-01	38671	0	11208,5
Responder	Macro_OLFML3	cDC_CLEC9A	LGALS9	HAVCR2	1,10186E-03	9651,9	8,69505E-03	10814	3,68321E-01	8041	8,85269E-01	6411	8,73387E-01	11785	0	11208,5
Responder	Macro_OLFML3	Mono_CD14	CXCL9	FCGR2A	1,10233E-03	8559,9	1,13884E-02	6815	2,83227E-01	11786	1,30686E+00	2816	8,81565E-01	10174	0	11208,5
Responder	cDC(CD1C)	Mono_CD14	GNAI2	C5AR1	1,10279E-03	7870,1	8,26981E-03	11787	4,20158E-01	6350	1,60281E+00	1560	8,91150E-01	8445	0	11208,5
Responder	cDC_CLEC9A	Macro_FOLR2-APOE+	SELPLG	ITGB2	1,10373E-03	8885,7	8,73381E-03	10718	3,92784E-01	7207	7,95999E-01	7602	8,95191E-01	7693	0	11208,5
Responder	Mono_INHBA	Macro_FOLR2+APOE-	VEGFA	NRP1	1,10554E-03	10028,5	2,01485E-02	2507	7,16068E-01	1729	1,08112E+00	4327	7,82054E-01	30371	0	11208,5
Responder	Macro_NLRP3	pDC_LILRA4	HMGB1	TLR9	1,10588E-03	5895,5	6,07910E-02	300	1,29597E+00	194	1,23815E+00	3210	8,58939E-01	14565	0	11208,5
Responder	Mono_CD14	Mono_CD16	S100A9	CD36	1,10654E-03	6591,9	1,01984E-02	8215	9,86872E-01	608	1,76661E+00	1133	8,73312E-01	11795	0	11208,5
Responder	Macro_OLFML3	Mast	MDK	NCL	1,10701E-03	7230,3	2,13182E-02	2306	4,96472E-01	4472	8,88194E-01	6369	8,73306E-01	11796	0	11208,5
Responder	Macro_OLFML3	Mono_CD14	GRN	TNFRSF1B	1,10701E-03	7192,3	8,26403E-03	11796	5,35173E-01	3774	8,63894E-01	6678	9,22972E-01	2505	0	11208,5
Responder	Macro_OLFML3	Mono_INHBA	LGALS3BP	ITGB1	1,10748E-03	7486,7	1,57829E-02	3874	5,65057E-01	3335	8,22858E-01	7219	8,73285E-01	11797	0	11208,5
Responder	cDC_CLEC9A	Macro_FOLR2-APOE+	SERPINF1	PLXDC2	1,10889E-03	7586,5	1,20194E-02	6241	4,39721E-01	5821	1,12803E+00	3954	8,78709E-01	10708	0	11208,5
Responder	Mono_INHBA	Macro_FOLR2+APOE+	CCL3	CCR1	1,11077E-03	6180,3	1,68842E-02	3395	8,02853E-01	1236	1,23077E+00	3258	8,73233E-01	11804	0	11208,5
Responder	Mono_INHBA	Macro_FOLR2+APOE-	INHBA	ACTR2	1,11133E-03	6554,5	2,02058E-02	2493	7,42181E-01	1564	1,53190E+00	1807	8,53476E-01	15700	0	11208,5
Responder	Mono_INHBA	cDC_CLEC9A	MIF	CD74_CXCR4	1,11133E-03	7608,7	6,39180E-03	17804	4,60904E-01	5274	1,16489E+00	3644	9,46783E-01	113	0	11208,5
Responder	pDC_LILRA4	cDC(CD1C)	PROC	THBD	1,11137E-03	14042,9	4,44406E-02	605	7,04993E-01	1811	1,57778E+00	1645	6,29343E-01	54945	0	11208,5
Responder	Macro_ISG15	Macro_NLRP3	CXCL10	SDC4	1,11137E-03	9474,5	3,15432E-02	1119	7,09718E-01	1780	1,53055E+00	1811	7,76596E-01	31454	0	11208,5
Responder	Mono_CD14	Mono_CD14	NAMPT	ITGA5_ITGB1	1,11265E-03	7288,1	8,99833E-03	10212	6,83562E-01	1987	1,73158E+00	1225	8,73199E-01	11808	0	11208,5
Responder	Macro_OLFML3	Macro_ISG15	FN1	ITGA5_ITGB1	1,11381E-03	8158,9	1,68500E-02	3413	4,60674E-01	5277	1,10221E+00	4155	8,48493E-01	16741	0	11208,5
Responder	cDC(CD1C)	Macro_LYVE1	GNAI2	C5AR1	1,11548E-03	9180,9	8,25540E-03	11814	4,18959E-01	6389	7,63823E-01	8032	8,91065E-01	8461	0	11208,5
Responder	cDC(CD1C)	Macro_NLRP3	CIRBP	TREM1	1,11713E-03	7941,7	1,32475E-02	5281	4,78450E-01	4848	1,00811E+00	5019	8,65017E-01	13352	0	11208,5
Responder	cDC(CD1C)	Mono_CD16	GNAI2	FPR1	1,11737E-03	9349,3	8,25271E-03	11818	3,60348E-01	8348	8,93113E-01	6321	8,87912E-01	9051	0	11208,5
Responder	Macro_OLFML3	Mono_INHBA	FN1	ITGA4_ITGB1	1,11796E-03	7470,9	1,77265E-02	3121	4,60127E-01	5282	1,01091E+00	4991	8,67896E-01	12752	0	11208,5
Responder	Macro_NLRP3	Mono_CD16	HLA-C	LILRA3	1,11832E-03	6206,5	3,07493E-02	1161	7,41290E-01	1573	5,46664E-01	11820	9,07955E-01	5270	0	11208,5
Responder	Mono_INHBA	Mast	GNAS	ADRB2	1,11880E-03	7286,7	2,23379E-02	2127	6,47153E-01	2335	9,81710E-01	5283	8,54520E-01	15480	0	11208,5
Responder	pDC_LILRA4	Mono_CD14	SEMA7A	PLXNC1	1,12047E-03	11472,5	2,61711E-02	1610	7,04435E-01	1816	1,89721E+00	876	7,20973E-01	41852	0	11208,5
Responder	Macro_IER3	pDC_LILRA4	TNF	TRPM2	1,12412E-03	8788,5	2,60504E-02	1619	7,11653E-01	1762	1,52771E+00	1818	7,96353E-01	27535	0	11208,5
Responder	Mono_INHBA	pDC_LILRA4	HGF	NRP1	1,12714E-03	15304,3	1,56696E-02	3933	4,59875E-01	5293	1,18908E+00	3499	6,50143E-01	52588	0	11208,5
Responder	cDC(CD1C)	pDC_LILRA4	FAM3C	ADGRG5	1,12881E-03	17219,3	3,35556E-02	1007	4,59838E-01	5295	9,93444E-01	5178	4,13797E-01	63408	0	11208,5
Responder	cDC_CLEC9A	Macro_ISG15	HLA-DRA	LAG3	1,12924E-03	7880,3	1,29425E-02	5499	4,23928E-01	6247	9,70930E-01	5404	8,76926E-01	11043	0	11208,5
Responder	Macro_FOLR2-APOE+	pDC_LILRA4	HLA-DQA2	CD4	1,12924E-03	8173,1	8,24077E-03	11843	3,31949E-01	9458	1,43912E+00	2171	9,03164E-01	6185	0	11208,5
Responder	cDC_CLEC9A	Macro_ISG15	HLA-DPB1	LAG3	1,13402E-03	6880,1	1,45215E-02	4526	5,34417E-01	3790	1,26886E+00	3032	8,73010E-01	11844	0	11208,5
Responder	Macro_NLRP3	pDC_LILRA4	CD14	TLR9	1,13542E-03	6403,3	6,02639E-02	304	1,23207E+00	246	1,13131E+00	3909	8,50457E-01	16349	0	11208,5
Responder	cDC_CLEC9A	Macro_ISG15	HLA-DPA1	LAG3	1,13833E-03	6522,7	1,49307E-02	4292	5,70941E-01	3254	1,29169E+00	2905	8,77402E-01	10954	0	11208,5
Responder	Mono_INHBA	cDC(CD1C)	TNF	TRADD	1,13890E-03	12266,9	1,32119E-02	5307	4,64190E-01	5203	1,13073E+00	3917	7,54520E-01	35699	0	11208,5
Responder	cDC(CD1C)	pDC_LILRA4	CXCL8	CD79A	1,14227E-03	14275,1	2,28077E-02	2059	4,59105E-01	5311	1,16775E+00	3628	6,76088E-01	49169	0	11208,5
Responder	Mono_INHBA	cDC_CLEC9A	CD52	SIGLEC10	1,14313E-03	9916,5	8,42500E-03	11457	3,38616E-01	9182	9,29842E-01	5863	8,72840E-01	11872	0	11208,5
Responder	Mono_INHBA	Macro_NLRP3	CCL3L1	CCR1	1,14650E-03	9716,9	1,31932E-02	5316	5,51543E-01	3544	1,16143E+00	3665	8,09794E-01	24851	0	11208,5
Responder	cDC(CD1C)	cDC_CLEC9A	HLA-DRA	LAG3	1,14796E-03	7235,7	1,20005E-02	6261	4,90941E-01	4587	1,42425E+00	2240	8,72790E-01	11882	0	11208,5
Responder	Mono_CD14	cDC(CD1C)	VCAN	TLR1	1,14805E-03	6668,7	2,42905E-02	1831	8,60227E-01	983	1,96371E+00	770	8,40005E-01	18551	0	11208,5
Responder	Macro_ISG15	Mono_CD16	HLA-C	LILRB2	1,14892E-03	7189,9	9,69409E-03	8969	5,44640E-01	3626	5,43632E-01	11884	9,42464E-01	262	0	11208,5
Responder	Macro_ISG15	cDC(CD1C)	S100A8	ITGB2	1,14892E-03	8685,5	8,22471E-03	11884	3,15256E-01	10188	7,90430E-01	7668	9,23111E-01	2479	0	11208,5
Responder	pDC_LILRA4	Mono_INHBA	APP	LRP1	1,14990E-03	4925,3	2,42864E-02	1832	1,04383E+00	480	2,58572E+00	184	8,77507E-01	10922	0	11208,5
Responder	cDC_CLEC9A	Macro_OLFML3	HMGB1	HAVCR2	1,15183E-03	8036,3	8,22243E-03	11890	4,71537E-01	5006	8,92675E-01	6327	9,05310E-01	5750	0	11208,5
Responder	Macro_OLFML3	pDC_LILRA4	HLA-B	CANX	1,15329E-03	7837,9	5,89389E-03	20217	4,58638E-01	5324	1,45474E+00	2098	9,40779E-01	342	0	11208,5
Responder	cDC(CD1C)	Macro_LYVE1	SAA1	CD36	1,15500E-03	11898,5	1,44199E-02	4575	5,59543E-01	3409	9,77329E-01	5326	7,58577E-01	34974	0	11208,5
Responder	Mono_INHBA	Macro_FOLR2+APOE+	CCL3	CCR5	1,15585E-03	10230,1	1,41820E-02	4718	7,51314E-01	1515	1,26375E+00	3067	7,80742E-01	30642	0	11208,5
Responder	Macro_OLFML3	Macro_IFI27	APOE	SORL1	1,15668E-03	8041,7	1,18569E-02	6390	4,32989E-01	5999	5,42876E-01	11900	9,10992E-01	4711	0	11208,5
Responder	Macro_ISG15	cDC_LAMP3	IL10	CD70	1,15783E-03	16274,3	8,83477E-02	128	1,16645E+00	307	6,66846E-01	9604	5,65770E-01	60124	0	11208,5
Responder	Mono_CD16	cDC_CLEC9A	CD52	SIGLEC10	1,15841E-03	4508,3	1,72679E-02	3266	9,30772E-01	758	1,48447E+00	1979	9,07638E-01	5330	0	11208,5
Responder	Mono_INHBA	cDC(CD1C)	ICAM1	ITGAX_ITGB2	1,15862E-03	8442,7	8,21712E-03	11904	4,07621E-01	6738	1,15947E+00	3681	8,89851E-01	8682	0	11208,5
Responder	Mono_INHBA	Macro_FOLR2+APOE+	CCL4	CCR1	1,16012E-03	7379,5	1,46008E-02	4478	6,20111E-01	2605	1,08022E+00	4335	8,60282E-01	14271	0	11208,5
Responder	Mono_CD14	Macro_IER3	HBEGF	CD44	1,16203E-03	10859,7	8,38849E-03	11555	3,47763E-01	8822	5,42444E-01	11911	8,78182E-01	10802	0	11208,5
Responder	Mono_INHBA	pDC_LILRA4	THBS1	CD47	1,16354E-03	7584,3	1,31601E-02	5336	4,84104E-01	4731	1,62957E+00	1480	8,55951E-01	15166	0	11208,5
Responder	Macro_OLFML3	Macro_FOLR2+APOE+	APOE	SCARB1	1,16496E-03	7554,3	1,82750E-02	2962	5,86171E-01	3019	7,23080E-01	8665	8,72597E-01	11917	0	11208,5
Responder	Mono_INHBA	cDC(CD1C)	TNF	VSIR	1,16525E-03	9093,1	1,32638E-02	5269	4,58314E-01	5338	1,45629E+00	2090	8,25474E-01	21560	0	11208,5

Responder	Macro_OLFML3	Macro_LYVE1	HLA-DRA	CD4	1,16937E-03	7208,3	8,21166E-03	11926	5,99345E-01	2841	6,46971E-01	9950	9,46650E-01	116	0	11208,5
Responder	Macro_IER3	Mast	IL1B	ADRB2	1,17289E-03	4019,1	6,38995E-02	262	1,16467E+00	309	1,44773E+00	2124	9,03132E-01	6192	0	11208,5
Responder	Macro_OLFML3	Macro_FOLR2+APOE+	C3	CR1	1,17299E-03	11671,5	1,38834E-02	4907	5,46464E-01	3596	9,75136E-01	5347	7,67114E-01	33299	0	11208,5
Responder	Mono_INHBA	Macro_LYVE1	VEGFA	EPHB2	1,17644E-03	11838,3	4,00265E-02	729	7,50773E-01	1518	9,74603E-01	5351	7,29160E-01	40385	0	11208,5
Responder	Mono_INHBA	Macro_FOLR2+APOE+	CCL3L1	CCR1	1,17817E-03	8206,1	1,81846E-02	2982	6,41281E-01	2388	1,06275E+00	4484	8,33288E-01	19968	0	11208,5
Responder	Mono_INHBA	Macro_FOLR2+APOE+	CCL3L1	CCR5	1,17903E-03	12868,7	1,52742E-02	4113	5,89573E-01	2963	1,09573E+00	4205	7,20965E-01	41854	0	11208,5
Responder	Macro_ISG15	Mast	ADM	ADRB2	1,17984E-03	8319,3	5,20790E-02	424	8,79672E-01	901	1,51761E+00	1848	7,97859E-01	27215	0	11208,5
Responder	Macro_OLFML3	Macro_OLFML3	LGALS9	CD47	1,18120E-03	19435,3	6,72749E-03	16470	2,47085E-01	13781	-2,00126E-01	39181	8,49574E-01	16536	0	11208,5
Responder	cDC(CD1C)	Mono_INHBA	ENTPD1	ADORA2A	1,18249E-03	14196,7	2,18534E-02	2209	4,59310E-01	5304	9,74302E-01	5358	6,91294E-01	46904	0	11208,5
Responder	Macro_OLFML3	Macro_FOLR2+APOE+	HLA-DQB1	LAG3	1,18267E-03	7284,9	1,97375E-02	2582	7,03093E-01	1824	7,10599E-01	8857	8,72402E-01	11953	0	11208,5
Responder	cDC_CLEC9A	Macro_NLRP3	GNAI2	C5AR1	1,18366E-03	7042,9	9,68604E-03	8986	5,35161E-01	3775	1,09508E+00	4211	8,98583E-01	7034	0	11208,5
Responder	cDC_CLEC9A	Macro_NLRP3	HMGB1	THBD	1,18465E-03	8497,3	8,60534E-03	11031	4,66376E-01	5140	1,23997E+00	3200	8,72664E-01	11907	0	11208,5
Responder	Macro_OLFML3	Mono_CD14	SERPINA1	LRP1	1,18663E-03	9692,3	8,19428E-03	11961	3,83782E-01	7502	5,61928E-01	11485	9,02559E-01	6305	0	11208,5
Responder	Macro_OLFML3	cDC(CD1C)	CXCL12	CXCR4	1,18683E-03	8337,1	1,31137E-02	5363	4,99138E-01	4422	1,31791E+00	2752	8,42788E-01	17940	0	11208,5
Responder	Macro_OLFML3	Macro_FOLR2+APOE+	HLA-DQA1	LAG3	1,18713E-03	7549,5	1,99216E-02	2551	6,96852E-01	1880	6,36369E-01	10146	8,72379E-01	11962	0	11208,5
Responder	Macro_NLRP3	Mono_CD16	THBS1	ITGA4	1,19110E-03	7147,5	1,98927E-02	2553	7,22655E-01	1686	7,45646E-01	8320	8,72292E-01	11970	0	11208,5
Responder	Macro_ISG15	Macro_LYVE1	S100A8	CD68	1,19259E-03	6339,1	1,21769E-02	6082	6,84162E-01	1980	5,39004E-01	11973	9,38933E-01	452	0	11208,5
Responder	Macro_NLRP3	cDC_CLEC9A	THBS1	SCARB1	1,19310E-03	7664,1	3,04522E-02	1182	7,87786E-01	1312	1,51541E+00	1855	8,19867E-01	22763	0	11208,5
Responder	pDC_LILRA4	Macro_FOLR2+APOE+	RARRES2	CMKLR1	1,19500E-03	13082,9	3,93083E-02	759	6,99570E-01	1856	1,99523E+00	719	6,64283E-01	50872	0	11208,5
Responder	Macro_FOLR2-APOE+	Macro_ISG15	MMP9	CD44	1,20058E-03	7186,9	1,45597E-02	4505	5,85154E-01	3033	9,90954E-01	5199	8,72190E-01	11989	0	11208,5
Responder	pDC_LILRA4	Macro_LYVE1	SPON2	ITGAM	1,20072E-03	12686,3	3,62179E-02	878	8,67141E-01	955	1,51446E+00	1859	6,80749E-01	48531	0	11208,5
Responder	Macro_OLFML3	Mono_CD16	HLA-G	LILRB2	1,20263E-03	6938,7	2,61163E-02	1612	8,24046E-01	1129	8,35740E-01	7061	8,63301E-01	13683	0	11208,5
Responder	Macro_NLRP3	Macro_NLRP3	S100A9	TLR4	1,20309E-03	8511,9	1,21344E-02	6115	5,54920E-01	3480	6,57212E-01	9762	8,72155E-01	11994	0	11208,5
Responder	Mono_INHBA	cDC(CD1C)	PTGS2	ALOX5	1,20959E-03	7914,1	1,44018E-02	4585	4,55989E-01	5389	1,27921E+00	2978	8,54850E-01	15410	0	11208,5
Responder	cDC_CLEC9A	cDC_CLEC9A	HMGB1	HAVCR2	1,21013E-03	6555,3	8,66674E-03	10873	5,03787E-01	4326	1,82244E+00	1011	9,07542E-01	5358	0	11208,5
Responder	cDC_CLEC9A	cDC_CLEC9A	CD52	SIGLEC10	1,21214E-03	9013,5	8,87623E-03	10436	3,68833E-01	8020	1,10839E+00	4101	8,75707E-01	11302	0	11208,5
Responder	Mono_INHBA	Macro_FOLR2+APOE+	INHBA	ACTR2	1,21224E-03	6282,7	2,33095E-02	1978	8,08427E-01	1210	1,25337E+00	3129	8,62187E-01	13888	0	11208,5
Responder	Macro_OLFML3	Mono_CD14	HLA-C	LILRA1	1,21467E-03	9350,3	1,13209E-02	6872	4,41955E-01	5754	5,37459E-01	12017	8,77620E-01	10900	0	11208,5
Responder	Macro_OLFML3	pDC_LILRA4	CCL5	DPP4	1,21605E-03	15954,1	3,85212E-02	784	6,97955E-01	1867	5,67660E-01	11363	6,32736E-01	54548	0	11208,5
Responder	pDC_LILRA4	cDC_CLEC9A	CALM2	MYLK	1,21797E-03	13567,9	3,69550E-02	847	6,97928E-01	1868	2,10178E+00	588	7,75529E-01	31644	0,237	32892,5
Responder	Macro_OLFML3	Macro_NLRP3	APOE	LDLR	1,21872E-03	6874,5	2,40160E-02	1875	6,29636E-01	2501	8,57301E-01	6763	8,72000E-01	12025	0	11208,5
Responder	cDC_CLEC9A	Mast	ACTR2	ADRB2	1,21990E-03	6131,1	2,81149E-02	1405	7,11201E-01	1768	1,51210E+00	1869	8,59673E-01	14405	0	11208,5
Responder	Mono_CD14	Mono_CD16	CD99	PILRA	1,22024E-03	9522,9	8,16666E-03	12028	4,53501E-01	5464	7,18890E-01	8720	8,81456E-01	10194	0	11208,5
Responder	cDC_CLEC9A	pDC_LILRA4	HLA-DQA2	CD4	1,22328E-03	5739,1	1,03861E-02	7969	4,76350E-01	4903	2,47811E+00	242	9,12820E-01	4373	0	11208,5
Responder	cDC_CLEC9A	pDC_LILRA4	HLA-DQB1	CD4	1,22481E-03	5222,9	8,86022E-03	10474	5,64017E-01	3355	2,47425E+00	247	9,34734E-01	830	0	11208,5
Responder	pDC_LILRA4	Macro_ISG15	SPON2	ITGA4	1,22570E-03	11691,7	2,40463E-02	1872	7,81865E-01	1347	1,64546E+00	1435	7,16769E-01	42596	0	11208,5
Responder	Macro_NLRP3	Macro_FOLR2-APOE+	NAMPT	ITGA5_ITGB1	1,22736E-03	8858,9	1,03790E-02	7979	6,06553E-01	2766	5,36650E-01	12042	8,80893E-01	10299	0	11208,5
Responder	Mono_CD14	Macro_ISG15	NAMPT	ITGA5_ITGB1	1,22838E-03	7613,1	8,78754E-03	10618	6,64381E-01	2155	1,46835E+00	2040	8,71880E-01	12044	0	11208,5
Responder	Macro_OLFML3	cDC_LAMP3	FN1	ITGA4_ITGB1	1,23297E-03	7232,1	1,89983E-02	2760	4,88199E-01	4646	9,63123E-01	5493	8,71817E-01	12053	0	11208,5
Responder	pDC_LILRA4	Macro_NLRP3	SPON2	ITGA4	1,23734E-03	11567,1	2,51986E-02	1711	7,93154E-01	1289	1,50956E+00	1878	7,21496E-01	41749	0	11208,5
Responder	Macro_ISG15	Macro_FOLR2-APOE+	SERPING1	LRP1	1,23810E-03	10400,1	1,00537E-02	8429	3,43576E-01	8974	5,69296E-01	11326	8,71775E-01	12063	0	11208,5
Responder	cDC_CLEC9A	pDC_LILRA4	HMGB1	THBD	1,24169E-03	4801,1	1,49479E-02	4283	7,31741E-01	1632	2,63421E+00	165	9,00322E-01	6717	0	11208,5
Responder	Macro_NLRP3	Macro_ISG15	VCAN	TLR2	1,24221E-03	6716,1	2,03133E-02	2471	6,52561E-01	2285	9,57898E-01	5545	8,71715E-01	12071	0	11208,5
Responder	cDC_CLEC9A	Mono_CD16	HLA-C	LILRA3	1,24523E-03	4767,9	3,01383E-02	1213	7,09661E-01	1781	1,09547E+00	4208	9,07113E-01	5429	0	11208,5
Responder	Mono_INHBA	Macro_FOLR2+APOE+	TNF	VSIR	1,24736E-03	6457,5	2,73534E-02	1476	8,84684E-01	886	8,66974E-01	6636	8,71668E-01	12081	0	11208,5
Responder	cDC_CLEC9A	pDC_LILRA4	HMGB1	CXCR4	1,24736E-03	5020,5	8,73993E-03	10706	6,70793E-01	2106	2,89851E+00	81	9,33153E-01	1001	0	11208,5
Responder	Mono_CD14	Macro_FOLR2-APOE+	HBEGF	CD9	1,24839E-03	7801,3	1,58068E-02	3862	4,97731E-01	4447	8,09588E-01	7406	8,71652E-01	12083	0	11208,5
Responder	Mono_INHBA	Macro_LYVE1	THBS1	CD36	1,24884E-03	6619,9	2,48619E-02	1760	7,60338E-01	1476	9,69202E-01	5433	8,65537E-01	13222	0	11208,5
Responder	pDC_LILRA4	Mono_INHBA	SPON2	ITGB2	1,24906E-03	11499	2,74024E-02	1464	6,96269E-01	1884	1,51522E+00	1856	8,32445E-01	20162	0,19	32129
Responder	Mono_CD16	Macro_FOLR2+APOE+	CD52	SIGLEC10	1,24974E-03	4274,5	2,51196E-02	1725	1,14476E+00	343	9,69179E-01	5434	9,22193E-01	2662	0	11208,5
Responder	cDC_CLEC9A	Macro_ISG15	SELPLG	ITGB2	1,25253E-03	9272,1	8,14461E-03	12091	3,39337E-01	9160	9,52763E-01	5599	8,91869E-01	8302	0	11208,5
Responder	cDC(CD1C)	Mono_CD14	HSPA8	LDLR	1,25517E-03	7939,7	1,44642E-02	4545	4,54194E-01	5440	1,29917E+00	2864	8,53720E-01	15641	0	11208,5
Responder	cDC(CD1C)	cDC_CLEC9A	HLA-DPB1	LAG3	1,25517E-03	6952,3	1,30257E-02	5440	5,52842E-01	3524	1,58427E+00	1627	8,66861E-01	12962	0	11208,5
Responder	cDC_CLEC9A	Macro_ISG15	HLA-DRB1	LAG3	1,25876E-03	8213,9	1,28749E-02	5539	4,14860E-01	6536	9,45680E-01	5683	8,71514E-01	12103	0	11208,5
Responder	pDC_LILRA4	Macro_IFI27	TNFSF9	HLA-DPA1	1,26061E-03	4165,1	2,35533E-02	1943	8,30691E-01	1097	1,76739E+00	1131	9,07052E-01	5446	0	11208,5
Responder	Mono_CD14	Macro_OLFML3	CXCL8	SDC3	1,26152E-03	7917,1	1,58585E-02	3839	6,08663E-01	2741	1,41972E+00	2258	8,35312E-01	19539	0	11208,5
Responder	cDC_CLEC9A	cDC_CLEC9A	LGALS9	HAVCR2	1,26240E-03	9012,9	8,40262E-03	11524	3,47509E-01	8831	1,66058E+00	1391	8,71483E-01	12110	0	11208,5
Responder	Macro_OLFML3	Macro_FOLR2+APOE+	FN1	IL17RC	1,26425E-03	12523,3	3,55972E-02	908	5,13213E-01	4160	9,67174E-01	5450	7,26193E-01	40890	0	11208,5
Responder	Mono_CD14	Macro_OLFML3	VCAN	SELL	1,26516E-03	5991,1	2,25185E-02	2096	8,87612E-01	878	1,31551E+00	2768	8,66650E-01	13005	0	11208,5
Responder	Mono_INHBA	pDC_LILRA4	APP	RPSA	1,26606E-03	6971,9	9,46994E-03	9348	7,23224E-01	1684	2,16661E+00	502	8,71452E-01	12117	0	11208,5
Responder	Mono_INHBA	Macro_NLRP3	VEGFA	ITGB1	1,26790E-03	7919,1	1,59901E-02	3770	6,56944E-01	2240	9,66935E-01	5454	8,47633E-01	16923	0	11208,5
Responder	pDC_LILRA4	pDC_LILRA4	SPON2	ITGB1	1,26875E-03	10495,9	2,38741E-02	1894	7,71189E-01	1394	2,65694E+00	156	7,43268E-01	37827	0	11208,5
Responder	cDC_CLEC9A	Macro_LYVE1	LGALS9	LRP1	1,26919E-03	7010,9	1,16618E-02	6558	5,76360E-01	3166	1,05918E+00	4518	8,84628E-01	9604	0	11208,5
Responder	pDC_LILRA4	Macro_FOLR2+APOE+	TNFSF9	HLA-DPA1	1,27064E-03	4387,9	2,35194E-02	1947	8,29003E-01	1108	1,42921E+00	2219	9,06991E-01	5457	0	11208,5
Responder	cDC_CLEC9A	Macro_LYVE1	HLA-DQA2	CD4	1,27129E-03	6083,9	1,08912E-02	7347	5,08694E-01	4236	1,17139E+00	3604	9,14692E-01	4024	0	11208,5

Responder	Macro_ISG15	pDC_LILRA4	S100A8	CD36	1,27181E-03	7876,9	1,24664E-02	5842	4,39997E-01	5816	1,07408E+00	4390	8,71388E-01	12128	0	11208,5
Responder	cDC_CLEC9A	Macro_LYVE1	HLA-DQA1	CD4	1,27181E-03	6622,3	8,49068E-03	11275	4,85523E-01	4702	1,01650E+00	4922	9,33120E-01	1004	0	11208,5
Responder	cDC_CLEC9A	Macro_LYVE1	HLA-DQB1	CD4	1,27286E-03	5614,3	9,29114E-03	9676	5,96361E-01	2881	1,16753E+00	3629	9,36168E-01	677	0	11208,5
Responder	cDC_CLEC9A	Macro_NLRP3	SELPLG	ITGB2	1,27286E-03	9714,5	8,12847E-03	12130	3,37872E-01	9216	7,88503E-01	7698	8,91773E-01	8320	0	11208,5
Responder	Macro_FOLR2+APOE-	Macro_OLFML3	CCL13	CCR5	1,27313E-03	14729,7	5,86290E-02	322	1,23363E+00	243	9,69488E-01	5427	6,14289E-01	56448	0	11208,5
Responder	cDC_CLEC9A	Macro_LYVE1	HLA-DPB1	CD4	1,27391E-03	6228,3	8,14297E-03	12094	5,37402E-01	3736	1,14498E+00	3797	9,41503E-01	306	0	11208,5
Responder	Macro_OLFML3	Macro_LYVE1	HLA-DRB1	CD4	1,27496E-03	7741,1	8,13263E-03	12115	5,83248E-01	3053	5,32941E-01	12134	9,43991E-01	195	0	11208,5
Responder	Mono_INHBA	Mono_CD16	VEGFA	ITGB1	1,27522E-03	7204,9	1,86395E-02	2855	7,37233E-01	1599	9,66054E-01	5462	8,57272E-01	14900	0	11208,5
Responder	Macro_ISG15	cDC_CLEC9A	CXCL11	DPP4	1,27668E-03	14099,1	1,21237E-01	52	7,82762E-01	1339	1,50540E+00	1898	6,19123E-01	55998	0	11208,5
Responder	pDC_LILRA4	Mono_CD14	SPON2	ITGB1	1,27867E-03	10648,1	2,38311E-02	1899	7,70642E-01	1399	1,89328E+00	880	7,43096E-01	37854	0	11208,5
Responder	cDC_CLEC9A	Macro_LYVE1	HLA-DPA1	CD4	1,27917E-03	5967,9	8,37241E-03	11589	5,73926E-01	3209	1,16781E+00	3627	9,43679E-01	206	0	11208,5
Responder	Mono_CD14	Macro_ISG15	VCAN	TLR1	1,28066E-03	6779,7	2,38213E-02	1900	8,55166E-01	1004	1,83718E+00	988	8,38690E-01	18798	0	11208,5
Responder	Macro_FOLR2+APOE+	Macro_OLFML3	APOE	SORL1	1,28073E-03	4155,7	2,21226E-02	2167	8,68963E-01	947	9,65625E-01	5468	9,33246E-01	988	0	11208,5
Responder	cDC(CD1C)	Mono_CD14	VIM	CD44	1,28441E-03	8083,3	5,83405E-03	20547	4,53213E-01	5472	1,29050E+00	2912	9,42194E-01	277	0	11208,5
Responder	Macro_ISG15	Macro_LYVE1	CD14	TLR4	1,28656E-03	9711,5	1,05518E-02	7746	4,06466E-01	6781	6,06650E-01	10666	8,71169E-01	12156	0	11208,5
Responder	Macro_FOLR2-APOE+	Mono_CD14	TIMP2	CD44	1,28815E-03	9964,9	8,11254E-03	12159	4,14426E-01	6546	6,68022E-01	9583	8,80716E-01	10328	0	11208,5
Responder	cDC_CLEC9A	Macro_LYVE1	CALR	LRP1	1,29027E-03	7834,7	9,42349E-03	9424	4,67444E-01	5107	8,12462E-01	7368	9,03724E-01	6066	0	11208,5
Responder	Macro_ISG15	Macro_FOLR2+APOE+	APOE	TREM2	1,29133E-03	8623,5	1,29806E-02	5477	2,88080E-01	11489	5,31648E-01	12165	9,21514E-01	2778	0	11208,5
Responder	Macro_OLFML3	Macro_FOLR2+APOE-	LILRB4	LAIR1	1,29239E-03	9981,5	9,61519E-03	9094	3,63740E-01	8226	5,31596E-01	12167	8,86933E-01	9212	0	11208,5
Responder	Macro_OLFML3	Macro_NLRP3	FN1	ITGA4_ITGB1	1,29365E-03	7559,9	1,77642E-02	3113	4,61169E-01	5271	9,64470E-01	5482	8,68018E-01	12725	0	11208,5
Responder	Macro_NLRP3	cDC_CLEC9A	S100A9	ITGB2	1,29612E-03	7602,5	8,10699E-03	12174	3,91598E-01	7244	9,09807E-01	6102	9,30594E-01	1284	0	11208,5
Responder	Macro_FOLR2-APOE+	cDC_CLEC9A	LYZ	ITGAL	1,29718E-03	10220,9	9,74438E-03	8883	3,26577E-01	9707	5,31142E-01	12176	8,87486E-01	9130	0	11208,5
Responder	cDC_CLEC9A	Macro_LYVE1	GNAI2	C5AR1	1,29718E-03	9343,5	8,41237E-03	11488	4,31111E-01	6055	6,62327E-01	9685	8,91976E-01	8281	0	11208,5
Responder	Macro_OLFML3	pDC_LILRA4	HMGB1	CXCR4	1,30015E-03	7125,7	6,98364E-03	15503	4,52607E-01	5489	1,64966E+00	1420	9,25807E-01	2008	0	11208,5
Responder	cDC_CLEC9A	Macro_LYVE1	CXCL9	FCGR2A	1,30359E-03	10596,9	9,37525E-03	9513	3,60518E-01	8341	5,50698E-01	11734	8,71027E-01	12188	0	11208,5
Responder	Macro_ISG15	Mast	LGALS9	CD44	1,30412E-03	8554,3	8,10123E-03	12189	5,03128E-01	4339	6,49812E-01	9893	9,08601E-01	5142	0	11208,5
Responder	Mono_INHBA	Macro_NLRP3	ICAM1	ITGAX_ITGB2	1,30466E-03	9820,9	8,09966E-03	12190	4,10459E-01	6651	6,30065E-01	10250	8,89143E-01	8805	0	11208,5
Responder	pDC_LILRA4	Macro_IER3	SPON2	ITGB2	1,30470E-03	6502,9	3,34295E-02	1013	8,30916E-01	1094	1,50290E+00	1912	8,45856E-01	17287	0	11208,5
Responder	Macro_FOLR2-APOE+	Mast	FN1	CD44	1,30519E-03	7805,5	1,46145E-02	4468	4,59156E-01	5307	5,30504E-01	12191	9,04815E-01	5853	0	11208,5
Responder	cDC_CLEC9A	Macro_IFI27	SELPLG	ITGB2	1,30573E-03	9671,9	8,09944E-03	12192	3,35239E-01	9324	8,17552E-01	7279	8,91601E-01	8356	0	11208,5
Responder	pDC_LILRA4	Macro_IER3	GAS6	MERTK	1,30672E-03	8890,3	2,37786E-02	1913	9,14421E-01	799	2,27743E+00	388	7,83099E-01	30143	0	11208,5
Responder	Mono_INHBA	Mono_CD16	LGALS1	PTPRC	1,30787E-03	7123,3	8,09702E-03	12196	6,72257E-01	2097	6,63377E-01	9664	9,38948E-01	451	0	11208,5
Responder	cDC_CLEC9A	pDC_LILRA4	HLA-DQA1	CD4	1,30841E-03	6076,9	8,09688E-03	12197	4,53179E-01	5473	2,32322E+00	352	9,31623E-01	1154	0	11208,5
Responder	cDC_CLEC9A	Macro_LYVE1	HSP90B1	LRP1	1,30948E-03	8626,3	9,04069E-03	10115	4,47825E-01	5604	7,04677E-01	8962	8,97507E-01	7242	0	11208,5
Responder	cDC_CLEC9A	cDC(CD1C)	SERPINF1	PLXDC2	1,31002E-03	8191,5	1,04344E-02	7904	3,67079E-01	8099	1,60700E+00	1546	8,70968E-01	12200	0	11208,5
Responder	cDC_CLEC9A	Macro_LYVE1	HSPA1A	TLR4	1,31109E-03	8238,5	9,24352E-03	9758	4,70940E-01	5026	9,50507E-01	5628	8,84804E-01	9572	0	11208,5
Responder	Mono_CD14	Macro_FOLR2+APOE+	VCAN	ITGB1	1,31217E-03	6558,7	1,42180E-02	4699	7,74596E-01	1377	1,22333E+00	3305	8,70952E-01	12204	0	11208,5
Responder	Macro_OLFML3	cDC_CLEC9A	CALM3	MYLK	1,31480E-03	15632,7	3,07960E-02	1158	6,92290E-01	1917	4,71946E-01	13624	6,68761E-01	50256	0	11208,5
Responder	pDC_LILRA4	Mono_CD16	APP	NOTCH2	1,31886E-03	5715,1	2,36952E-02	1919	1,02600E+00	523	2,34484E+00	336	8,58795E-01	14589	0	11208,5
Responder	Mono_INHBA	Macro_LYVE1	MMP9	LRP1	1,32260E-03	9451,1	1,48662E-02	4325	6,18345E-01	2625	9,61392E-01	5513	8,15936E-01	23584	0	11208,5
Responder	Mono_INHBA	Macro_FOLR2-APOE+	HBEGF	CD9	1,32404E-03	8131,9	1,78279E-02	3096	5,65103E-01	3334	5,29147E-01	12226	8,78233E-01	10795	0	11208,5
Responder	Macro_OLFML3	Macro_ISG15	LILRB4	LAIR1	1,32620E-03	9652,1	1,00069E-02	8486	3,84601E-01	7469	5,28960E-01	12230	8,88919E-01	8867	0	11208,5
Responder	Mono_CD14	cDC(CD1C)	HP	ITGB2	1,32637E-03	7659,1	5,58302E-02	356	6,06144E-01	2771	1,48566E+00	1974	8,23430E-01	21986	0	11208,5
Responder	Macro_FOLR2+APOE+	Mono_INHBA	APOE	SDC2	1,32699E-03	4516,5	3,48672E-02	945	1,02305E+00	530	1,49969E+00	1923	8,93634E-01	7976	0	11208,5
Responder	Mast	pDC_LILRA4	HMGB1	TLR9	1,32879E-03	5934,9	5,81585E-02	329	1,25996E+00	216	1,30776E+00	2811	8,56236E-01	15110	0	11208,5
Responder	cDC_LAMP3	pDC_LILRA4	GNAI2	CXCR3	1,32903E-03	7278,5	2,44859E-02	1806	9,62542E-01	664	1,49968E+00	1924	8,29351E-01	20790	0	11208,5
Responder	cDC_CLEC9A	Mono_CD16	HLA-B	LILRA1	1,33055E-03	5966,9	1,73938E-02	3230	4,83562E-01	4739	1,02666E+00	4835	9,04960E-01	5822	0	11208,5
Responder	cDC_CLEC9A	Mono_CD16	HLA-B	LILRB2	1,33109E-03	5135,5	9,95105E-03	8551	6,56627E-01	2245	1,17917E+00	3559	9,46746E-01	114	0	11208,5
Responder	cDC_CLEC9A	Mono_CD16	HLA-A	LILRB2	1,33436E-03	5870,5	9,64269E-03	9052	5,47805E-01	3578	9,83642E-01	5262	9,42606E-01	252	0	11208,5
Responder	pDC_LILRA4	Macro_LYVE1	TNFSF9	HLA-DPA1	1,33489E-03	4375,5	2,33222E-02	1975	8,19171E-01	1153	1,47420E+00	2015	9,06635E-01	5526	0	11208,5
Responder	Macro_FOLR2-APOE+	pDC_LILRA4	HMGB1	TLR9	1,33684E-03	6348,9	5,80083E-02	330	1,25791E+00	220	1,02566E+00	4844	8,56077E-01	15142	0	11208,5
Responder	cDC_CLEC9A	Mono_CD16	HLA-C	LILRA1	1,33763E-03	6937,1	1,72713E-02	3265	4,01082E-01	6940	8,99571E-01	6233	8,98556E-01	7039	0	11208,5
Responder	cDC_CLEC9A	Mono_CD16	HLA-C	LILRB2	1,33817E-03	5583,7	9,88097E-03	8655	5,74147E-01	3205	1,05208E+00	4610	9,42980E-01	240	0	11208,5
Responder	Macro_FOLR2-APOE+	cDC(CD1C)	APP	RPSA	1,33817E-03	7937,1	9,33839E-03	9578	5,18511E-01	4061	1,35119E+00	2586	8,70667E-01	12252	0	11208,5
Responder	Macro_NLRP3	Macro_IER3	VCAN	CD44	1,33927E-03	7068,1	1,42680E-02	4669	6,50971E-01	2299	5,28217E-01	12254	9,09931E-01	4910	0	11208,5
Responder	Macro_NLRP3	Mast	AGTRAP	RACK1	1,33981E-03	7641,3	1,24705E-02	5837	6,33450E-01	2461	5,28207E-01	12255	9,01771E-01	6445	0	11208,5
Responder	Mono_CD14	cDC(CD1C)	VCAN	TLR2	1,34059E-03	5976,9	1,80703E-02	3021	7,60347E-01	1475	1,92321E+00	835	8,65030E-01	13345	0	11208,5
Responder	cDC_CLEC9A	Mono_CD16	HLA-F	LILRB2	1,34200E-03	5562,7	1,29624E-02	5489	7,36820E-01	1603	1,29203E+00	2902	9,00920E-01	6611	0	11208,5
Responder	Mono_CD14	cDC(CD1C)	VCAN	SELL	1,34344E-03	5617,7	2,22603E-02	2138	8,84335E-01	889	1,98777E+00	731	8,65982E-01	13122	0	11208,5
Responder	cDC_CLEC9A	Mono_CD16	B2M	LILRB2	1,34364E-03	6085,1	9,30880E-03	9641	5,02799E-01	4346	9,91978E-01	5192	9,51553E-01	38	0	11208,5
Responder	cDC_CLEC9A	Mono_CD14	HMGB1	THBD	1,34364E-03	8530,9	8,29465E-03	11729	4,53376E-01	5468	1,48236E+00	1987	8,70606E-01	12262	0	11208,5
Responder	Mono_INHBA	Macro_FOLR2-APOE+	SPP1	ITGAV_ITGB5	1,34439E-03	10986,1	1,95069E-02	2634	4,50590E-01	5536	1,04281E+00	4694	7,79638E-01	30858	0	11208,5
Responder	Macro_NLRP3	Mono_CD16	IL1B	SIGIRR	1,34474E-03	7320,7	1,64146E-02	3594	6,56056E-01	2251	8,17193E-01	7286	8,70601E-01	12264	0	11208,5
Responder	cDC(CD1C)	pDC_LILRA4	ICAM1	SPN	1,34535E-03	9251,3	1,28758E-02	5537	5,41926E-01	3664	1,89664E+00	878	8,09272E-01	24969	0	11208,5
Responder	Mono_CD14	Mono_CD16	B2M	LILRB2	1,34584E-03	8689,1	8,72559E-03	10740	3,39003E-01	9168	5,27629E-01	12266	9,50040E-01	63	0	11208,5

Responder	Mono_CD14	cDC(CD1C)	ADM	CALCRL	1,34821E-03	13971,1	1,62147E-02	3661	4,54039E-01	5445	1,85847E+00	950	6,80270E-01	48591	0	11208,5
Responder	Macro_NLRP3	Mono_INHBA	ADAM10	CD44	1,34913E-03	9377,3	8,95438E-03	10298	5,36679E-01	3750	6,80796E-01	9358	8,70560E-01	12272	0	11208,5
Responder	Macro_ISG15	Macro_NLRP3	CXCL10	TLR4	1,34954E-03	6649,9	3,01164E-02	1220	6,90391E-01	1934	1,51146E+00	1874	8,47162E-01	17013	0	11208,5
Responder	Mono_CD14	cDC(CD1C)	S100A12	TLR4	1,35012E-03	10348,9	1,55463E-02	3982	8,88665E-01	871	2,06575E+00	619	7,58142E-01	35064	0	11208,5
Responder	Mono_CD14	cDC(CD1C)	S100A12	CD36	1,35108E-03	8855,3	1,83345E-02	2941	9,60983E-01	671	2,10362E+00	584	7,89568E-01	28872	0	11208,5
Responder	Macro_OLFML3	Macro_ISG15	HLA-DRB5	LAG3	1,35133E-03	7389,3	1,65316E-02	3540	6,07666E-01	2749	8,26405E-01	7173	8,70511E-01	12276	0	11208,5
Responder	Macro_NLRP3	Macro_ISG15	S100A9	TLR4	1,35188E-03	8375,7	1,17831E-02	6446	5,45135E-01	3617	7,45210E-01	8330	8,70508E-01	12277	0	11208,5
Responder	cDC_CLEC9A	Mono_CD16	GNAI2	C5AR1	1,35519E-03	8506,7	8,66494E-03	10875	4,51744E-01	5508	8,45443E-01	6908	8,93393E-01	8034	0	11208,5
Responder	Macro_OLFML3	Macro_LYVE1	PSAP	LRP1	1,35573E-03	7298,3	1,11175E-02	7085	6,90061E-01	1937	4,00375E-01	15535	9,35748E-01	726	0	11208,5
Responder	cDC_CLEC9A	Mono_CD16	GNAI2	FPR1	1,35574E-03	9428,5	8,40962E-03	11499	3,72499E-01	7900	7,91617E-01	7646	8,88846E-01	8889	0	11208,5
Responder	Macro_OLFML3	Macro_IER3	FN1	ITGA4_ITGB1	1,35961E-03	7469,7	1,85186E-02	2887	4,77750E-01	4871	9,11065E-01	6091	8,70382E-01	12291	0	11208,5
Responder	Mono_CD14	cDC(CD1C)	NAMPT	INSR	1,35971E-03	7140,9	2,06903E-02	2404	8,19589E-01	1152	1,65427E+00	1408	8,35349E-01	19532	0	11208,5
Responder	cDC_CLEC9A	Macro_NLRP3	HMGB1	CD163	1,36016E-03	6885,5	8,05688E-03	12292	5,19262E-01	4050	1,18954E+00	3495	9,18237E-01	3382	0	11208,5
Responder	Macro_NLRP3	cDC(CD1C)	THBS1	CD47	1,36071E-03	6444,3	1,67992E-02	3441	6,51677E-01	2293	1,27754E+00	2986	8,70359E-01	12293	0	11208,5
Responder	Macro_FOLR2+APOE-	Macro_OLFML3	CCL13	CCR1	1,36113E-03	11437,9	5,76547E-02	333	1,24199E+00	232	8,88032E-01	6372	7,36849E-01	39044	0	11208,5
Responder	Macro_OLFML3	cDC_CLEC9A	TNFSF13B	HLA-DPB1	1,36645E-03	7231,1	7,21069E-03	14778	4,95055E-01	4504	9,56527E-01	5559	9,47249E-01	106	0	11208,5
Responder	cDC_CLEC9A	Macro_LYVE1	HMGB1	TLR4	1,36681E-03	9240,1	9,13907E-03	9944	4,36513E-01	5913	8,51314E-01	6831	8,70279E-01	12304	0	11208,5
Responder	Macro_OLFML3	Mono_CD14	HLA-B	LILRA1	1,36792E-03	9382,9	1,06416E-02	7641	4,48148E-01	5595	5,26365E-01	12306	8,81627E-01	10164	0	11208,5
Responder	Mono_CD14	cDC(CD1C)	RETN	CAP1	1,36838E-03	6607,3	3,04013E-02	1188	6,33397E-01	2462	1,77414E+00	1111	8,46868E-01	17067	0	11208,5
Responder	Mast	pDC_LILRA4	HSP90B1	TLR9	1,36927E-03	6511,7	5,75631E-02	334	1,24356E+00	231	1,27373E+00	3000	8,43561E-01	17785	0	11208,5
Responder	Macro_LYVE1	Mast	PLTP	ABCA1	1,37025E-03	4645,7	2,68607E-02	1524	1,04046E+00	490	1,49383E+00	1944	8,93194E-01	8062	0	11208,5
Responder	Mono_INHBA	Macro_FOLR2-APOE+	CCL3	CCR1	1,37418E-03	7290,1	1,28406E-02	5567	7,24554E-01	1676	1,25781E+00	3104	8,57291E-01	14895	0	11208,5
Responder	cDC_CLEC9A	Mast	ARPC5	ADRB2	1,37517E-03	5855,7	2,41159E-02	1862	6,29889E-01	2499	1,36168E+00	2531	8,76370E-01	11178	0	11208,5
Responder	Macro_FOLR2-APOE+	Macro_LYVE1	CXCL8	SDC3	1,37572E-03	6240,3	2,77085E-02	1434	8,12844E-01	1195	1,00519E+00	5044	8,70204E-01	12320	0	11208,5
Responder	Macro_OLFML3	Macro_NLRP3	C1QB	CD33	1,37628E-03	10251,7	8,04320E-03	12321	3,73906E-01	7842	6,07451E-01	10653	8,86826E-01	9234	0	11208,5
Responder	Mono_INHBA	Macro_FOLR2+APOE+	IL6	IL6R_IL6ST	1,38001E-03	13402,1	2,07431E-02	2395	6,85834E-01	1968	9,54974E-01	5573	6,97714E-01	45866	0	11208,5
Responder	Macro_FOLR2-APOE+	Mast	PKM	CD44	1,38020E-03	8009,3	8,04028E-03	12328	5,88365E-01	2986	5,34450E-01	12102	9,29583E-01	1422	0	11208,5
Responder	Macro_FOLR2-APOE+	Mono_CD14	APOE	LRP1	1,38076E-03	9777,9	9,01456E-03	10171	2,73105E-01	12329	6,50664E-01	9884	9,07841E-01	5297	0	11208,5
Responder	Mono_CD14	cDC_LAMP3	ADM	RAMP1	1,38563E-03	11218,5	6,31923E-02	273	1,14782E+00	336	1,47381E+00	2016	7,18738E-01	42259	0	11208,5
Responder	Macro_FOLR2+APOE+	pDC_LILRA4	APOE	SCARB1	1,38697E-03	4870,3	2,34804E-02	1952	8,55689E-01	999	1,94384E+00	800	8,85891E-01	9392	0	11208,5
Responder	cDC_CLEC9A	Macro_LYVE1	HLA-DOB	CD4	1,38805E-03	5304,5	4,22728E-02	665	1,27910E+00	205	1,45339E+00	2102	8,70032E-01	12342	0	11208,5
Responder	Macro_NLRP3	Macro_FOLR2+APOE-	THBS1	LRP1	1,39030E-03	7456,1	1,57019E-02	3914	6,10669E-01	2716	8,32326E-01	7096	8,70009E-01	12346	0	11208,5
Responder	cDC_CLEC9A	Macro_IER3	SELPLG	ITGB2	1,39030E-03	9830,1	8,23281E-03	11862	3,47337E-01	8844	7,00401E-01	9039	8,92387E-01	8197	0	11208,5
Responder	Mono_INHBA	cDC_LAMP3	TNF	TRADD	1,39073E-03	9333,3	2,33488E-02	1971	7,12960E-01	1750	9,53752E-01	5584	8,03384E-01	26153	0	11208,5
Responder	Macro_OLFML3	Macro_FOLR2-APOE+	FN1	ITGA4_ITGB1	1,39143E-03	7569,5	1,83925E-02	2925	4,73933E-01	4953	8,85192E-01	6413	8,69996E-01	12348	0	11208,5
Responder	Macro_OLFML3	Macro_OLFML3	HLA-DRA	CD4	1,39385E-03	10215,5	6,61005E-03	16911	4,63328E-01	5218	3,36086E-01	17403	9,40898E-01	337	0	11208,5
Responder	cDC_CLEC9A	Mono_CD16	HSPA1A	TLR4	1,39707E-03	9306,3	8,03020E-03	12358	4,16877E-01	6458	9,55997E-01	5564	8,77437E-01	10943	0	11208,5
Responder	cDC(CD1C)	Macro_OLFML3	SERPINF1	PLXDC2	1,39820E-03	8982,7	1,02479E-02	8143	3,87663E-01	7372	9,32459E-01	5830	8,69951E-01	12360	0	11208,5
Responder	Macro_FOLR2+APOE-	Mono_CD16	F13A1	ITGB1	1,39960E-03	4908,3	2,51977E-02	1712	1,08167E+00	423	1,49106E+00	1958	8,86786E-01	9240	0	11208,5
Responder	Macro_NLRP3	Macro_IER3	VCAN	ITGA4	1,39990E-03	7114,5	1,84076E-02	2916	7,04508E-01	1815	8,18589E-01	7270	8,69937E-01	12363	0	11208,5
Responder	Mono_CD14	Mono_INHBA	HP	CD163	1,40151E-03	9462,1	6,32758E-02	272	6,81793E-01	1999	1,08214E+00	4317	7,86374E-01	29514	0	11208,5
Responder	pDC_LILRA4	Macro_LYVE1	SEMA7A	PLXNC1	1,40382E-03	10640,1	3,31489E-02	1026	7,80098E-01	1356	1,49066E+00	1960	7,44116E-01	37650	0	11208,5
Responder	pDC_LILRA4	Macro_NLRP3	APP	LRP1	1,40382E-03	5058,5	2,34478E-02	1960	1,02559E+00	525	2,43981E+00	273	8,75606E-01	11326	0	11208,5
Responder	Mono_INHBA	pDC_LILRA4	S100A8	CD69	1,40500E-03	10041,5	9,95649E-03	8546	3,05320E-01	10631	8,07199E-01	7450	8,69899E-01	12372	0	11208,5
Responder	Macro_ISG15	pDC_LILRA4	TNFSF13B	TFRC	1,41069E-03	7789,3	9,58630E-03	9143	4,92344E-01	4554	1,57507E+00	1659	8,69839E-01	12382	0	11208,5
Responder	Mono_CD14	Mono_INHBA	TGFB1	SDC2	1,41235E-03	12273,9	1,60928E-02	3727	4,84783E-01	4714	9,52264E-01	5606	7,52299E-01	36114	0	11208,5
Responder	cDC_CLEC9A	Macro_FOLR2+APOE-	HMGB1	CD163	1,41468E-03	5330,3	1,00116E-02	8476	6,72403E-01	2093	1,29272E+00	2898	9,26029E-01	1976	0	11208,5
Responder	cDC_CLEC9A	Macro_NLRP3	HMGB1	TLR2	1,41525E-03	9241,1	8,01728E-03	12390	3,63485E-01	8236	9,73577E-01	5373	8,88215E-01	8998	0	11208,5
Responder	Mono_INHBA	Mono_INHBA	VEGFA	ITGAV	1,41532E-03	11564,5	1,52063E-02	4148	5,54194E-01	3496	9,52198E-01	5609	7,66762E-01	33361	0	11208,5
Responder	Mono_CD14	Mono_INHBA	VCAN	TLR2	1,41631E-03	6082,9	1,80931E-02	3014	7,60712E-01	1473	1,65794E+00	1397	8,65103E-01	13322	0	11208,5
Responder	pDC_LILRA4	Macro_IER3	SPON2	ITGB1	1,41868E-03	10096,1	2,84070E-02	1377	8,28884E-01	1109	1,48757E+00	1967	7,59501E-01	34819	0	11208,5
Responder	Mono_CD14	Macro_IFI27	S100A12	TLR4	1,41868E-03	8566,7	2,33908E-02	1967	9,39637E-01	737	1,92812E+00	828	7,93603E-01	28093	0	11208,5
Responder	cDC_CLEC9A	Macro_FOLR2+APOE+	HLA-DRA	LAG3	1,41926E-03	7583,1	1,46889E-02	4411	4,76851E-01	4889	7,95528E-01	7609	8,83595E-01	9798	0	11208,5
Responder	cDC(CD1C)	cDC_CLEC9A	LTB	CD40	1,42028E-03	10782,7	1,27664E-02	5614	4,49966E-01	5553	1,95119E+00	786	7,80178E-01	30752	0	11208,5
Responder	cDC_CLEC9A	Macro_FOLR2+APOE+	HLA-DRB1	LAG3	1,42269E-03	7896,7	1,46121E-02	4469	4,67783E-01	5098	7,70278E-01	7951	8,78435E-01	10757	0	11208,5
Responder	cDC_CLEC9A	Macro_FOLR2+APOE+	HLA-DPB1	LAG3	1,42499E-03	6496,7	1,64809E-02	3563	5,87340E-01	3002	1,09346E+00	4221	8,79862E-01	10489	0	11208,5
Responder	cDC_CLEC9A	Macro_FOLR2+APOE+	HLA-DQB1	LAG3	1,42556E-03	6564,3	1,88047E-02	2814	6,46299E-01	2340	1,11601E+00	4051	8,69684E-01	12408	0	11208,5
Responder	cDC_CLEC9A	Macro_FOLR2+APOE+	HLA-DPA1	LAG3	1,42959E-03	6181,7	1,69452E-02	3374	6,23864E-01	2557	1,11629E+00	4047	8,84048E-01	9722	0	11208,5
Responder	Mono_INHBA	pDC_LILRA4	ICAM1	IL2RG	1,43016E-03	6627,7	1,20257E-02	6229	6,15445E-01	2658	2,05835E+00	627	8,69665E-01	12416	0	11208,5
Responder	Mono_CD14	Mono_INHBA	RETN	TLR4	1,43023E-03	12824,7	2,78336E-02	1424	5,14495E-01	4134	1,10535E+00	4127	7,13333E-01	43230	0	11208,5
Responder	cDC_CLEC9A	Macro_FOLR2+APOE+	SELPLG	ITGB2	1,43074E-03	7220,1	1,05168E-02	7796	5,54526E-01	3491	8,02182E-01	7510	9,03592E-01	6095	0	11208,5
Responder	Mono_INHBA	Mast	ADAM10	CD44	1,43074E-03	10200,1	8,81361E-03	10556	4,93458E-01	4534	5,27013E-01	12285	8,69664E-01	12417	0	11208,5
Responder	Mono_INHBA	Macro_LYVE1	ADAM10	GPNMB	1,43223E-03	7101,1	1,71440E-02	3311	7,61768E-01	1467	9,50661E-01	5626	8,62156E-01	13893	0	11208,5
Responder	pDC_LILRA4	Macro_FOLR2+APOE+	SELPLG	ITGB2	1,43223E-03	4895,5	1,27561E-02	5626	6,84527E-01	1975	1,78489E+00	1086	9,11679E-01	4582	0	11208,5
Responder	Macro_OLFML3	cDC_CLEC9A	HLA-DPA1	LAG3	1,43362E-03	8004,1	1,29677E-02	5486	5,47817E-01	3577	8,14569E-01	7327	8,69617E-01	12422	0	11208,5

Post_R_Myeloid_Myeloid_CellCel

Responder	Macro_FOLR2-APOE+	Mono_CD14	GNAI2	C5AR1	1,43420E-03	9291,9	8,00347E-03	12423	3,99575E-01	6991	8,32132E-01	7100	8,89552E-01	8737	0	11208,5
Responder	cDC_CLEC9A	Macro_IFI27	SERPINF1	PLXDC2	1,43478E-03	8903,9	1,01839E-02	8238	3,55598E-01	8535	1,10699E+00	4114	8,69597E-01	12424	0	11208,5
Responder	Macro_ISG15	cDC_CLEC9A	TNFSF13B	HLA-DPB1	1,43478E-03	5701,9	8,00287E-03	12424	5,82113E-01	3073	1,55209E+00	1740	9,49793E-01	64	0	11208,5
Responder	Macro_FOLR2-APOE+	Mono_CD16	MMP9	CD44	1,43536E-03	8038,7	1,64422E-02	3583	6,52380E-01	2289	5,21275E-01	12425	8,78815E-01	10688	0	11208,5
Responder	Mono_CD14	Mono_INHBA	THBS1	SDC4	1,43623E-03	10652,7	2,38115E-02	1904	5,63371E-01	3362	9,53391E-01	5590	7,77895E-01	31199	0	11208,5
Responder	Mono_INHBA	cDC_LAMP3	VEGFA	CD44	1,43651E-03	9194,7	1,13919E-02	6814	5,14648E-01	4130	5,21190E-01	12427	8,75257E-01	11394	0	11208,5
Responder	Macro_NLRP3	Macro_ISG15	TIMP1	CD63	1,43651E-03	7337,5	8,00246E-03	12427	5,43851E-01	3638	6,85915E-01	9272	9,45518E-01	142	0	11208,5
Responder	Macro_NLRP3	Macro_IER3	THBS1	LRP1	1,43709E-03	8000,5	1,55684E-02	3974	6,07245E-01	2757	6,65027E-01	9635	8,69526E-01	12428	0	11208,5
Responder	cDC(CD1C)	Macro_LYVE1	HLA-DRB5	CD4	1,43767E-03	6831,1	8,00188E-03	12429	4,42458E-01	5738	1,11258E+00	4081	9,35991E-01	699	0	11208,5
Responder	Macro_OLFML3	Mast	POMC	ADRB2	1,43793E-03	13739,5	3,15705E-02	1117	7,15657E-01	1732	9,32120E-01	5836	6,78657E-01	48804	0	11208,5
Responder	cDC(CD1C)	Mono_INHBA	LGALS9	CD44	1,44023E-03	7725,3	7,59661E-03	13549	4,88279E-01	4645	1,17417E+00	3590	9,05896E-01	5634	0	11208,5
Responder	Mono_INHBA	Macro_LYVE1	S100A9	CD36	1,44224E-03	6776,3	1,98832E-02	2554	7,83111E-01	1334	4,91815E-01	13149	9,05884E-01	5636	0	11208,5
Responder	Macro_ISG15	Mono_INHBA	S100A9	CD68	1,44520E-03	8171,7	7,99673E-03	12442	4,22297E-01	6295	6,47601E-01	9941	9,33404E-01	972	0	11208,5
Responder	Macro_ISG15	Mono_CD16	LGALS9	PTPRC	1,44636E-03	9348,3	7,99521E-03	12444	4,72437E-01	4980	5,20912E-01	12432	9,05711E-01	5677	0	11208,5
Responder	Mono_CD14	pDC_LILRA4	VCAN	ITGB1	1,44753E-03	6038,9	1,38217E-02	4940	7,65883E-01	1429	2,61154E+00	171	8,69355E-01	12446	0	11208,5
Responder	Mono_CD14	Mono_INHBA	RETN	CAP1	1,44827E-03	8892,3	2,33304E-02	1974	4,52048E-01	5505	1,01764E+00	4911	8,28904E-01	20863	0	11208,5
Responder	cDC_CLEC9A	Macro_FOLR2+APOE+	CD52	SIGLEC10	1,44869E-03	7693,9	1,29123E-02	5517	5,82823E-01	3062	5,93099E-01	10895	8,94711E-01	7787	0	11208,5
Responder	Macro_OLFML3	Macro_NLRP3	LGALS3BP	ITGB1	1,44927E-03	8354,7	1,47097E-02	4403	5,29702E-01	3862	6,52346E-01	9851	8,69337E-01	12449	0	11208,5
Responder	Macro_FOLR2-APOE+	Macro_NLRP3	RPS19	C5AR1	1,44927E-03	7448,5	9,76828E-03	8834	6,17680E-01	2633	5,20058E-01	12449	9,25183E-01	2118	0	11208,5
Responder	Macro_FOLR2+APOE-	Macro_FOLR2+APOE+	CCL18	CCR1	1,45085E-03	8375,7	2,64935E-02	1564	6,83905E-01	1982	1,64068E+00	1448	8,05850E-01	25676	0	11208,5
Responder	cDC_CLEC9A	Macro_FOLR2+APOE+	HMGB1	CD163	1,45452E-03	7288,1	8,69926E-03	10799	5,69589E-01	3278	7,47677E-01	8289	9,21071E-01	2866	0	11208,5
Responder	cDC_CLEC9A	Macro_LYVE1	CD59	STAB1	1,45802E-03	5912,5	1,96512E-02	2603	8,82537E-01	891	1,39183E+00	2396	8,69253E-01	12464	0	11208,5
Responder	pDC_LILRA4	Macro_FOLR2+APOE-	TNFSF9	HLA-DPA1	1,45837E-03	6697,5	2,28716E-02	2049	7,96701E-01	1269	1,59333E+00	1591	9,05806E-01	5652	0,001	22926,5
Responder	Mono_CD14	Mono_CD14	VCAN	ITGB1	1,45861E-03	6206,7	1,37968E-02	4957	7,65336E-01	1434	1,84787E+00	969	8,69252E-01	12465	0	11208,5
Responder	Macro_FOLR2-APOE+	Mono_INHBA	APOE	LRP1	1,46037E-03	8866,5	1,05314E-02	7778	3,49189E-01	8758	5,19231E-01	12468	9,14144E-01	4120	0	11208,5
Responder	pDC_LILRA4	Macro_OLFML3	APP	RPSA	1,46271E-03	6143,9	1,12034E-02	6994	8,20791E-01	1148	1,81725E+00	1026	8,80578E-01	10343	0	11208,5
Responder	pDC_LILRA4	Macro_OLFML3	APP	APLP2	1,46388E-03	5463,9	1,59036E-02	3813	8,70837E-01	941	1,81589E+00	1030	8,80720E-01	10327	0	11208,5
Responder	Mono_CD14	Mono_CD14	THBS1	CD36	1,46445E-03	7114,1	1,92026E-02	2703	5,89072E-01	2970	1,43289E+00	2205	8,49785E-01	16484	0	11208,5
Responder	Macro_FOLR2+APOE-	Mast	LGALS1	CD69	1,46649E-03	5363,1	1,27102E-02	5660	6,57988E-01	2227	1,15272E+00	3728	9,14888E-01	3992	0	11208,5
Responder	Macro_OLFML3	Macro_IER3	APOE	LDLR	1,46741E-03	7193,9	2,28439E-02	2051	6,14025E-01	2675	7,99074E-01	7555	8,69182E-01	12480	0	11208,5
Responder	Mono_CD14	Mono_CD14	CD55	ADGRE5	1,46954E-03	6724,1	1,46193E-02	4462	6,75806E-01	2065	1,64820E+00	1426	8,59407E-01	14459	0	11208,5
Responder	pDC_LILRA4	Macro_OLFML3	CD99	PILRA	1,46976E-03	8104,7	8,64124E-03	10931	4,36161E-01	5923	1,30906E+00	2800	8,84376E-01	9661	0	11208,5
Responder	Mono_INHBA	Macro_ISG15	VEGFA	NRP1	1,47158E-03	12681,5	1,27040E-02	5665	6,02263E-01	2814	9,77900E-01	5321	7,40212E-01	38399	0	11208,5
Responder	Mast	Mono_CD14	HDC	HRH2	1,47257E-03	10050,9	1,28373E-01	48	8,48264E-01	1027	1,48134E+00	1992	7,53046E-01	35979	0	11208,5
Responder	Mono_CD14	Mono_INHBA	VIM	CD44	1,47361E-03	7386,9	6,72815E-03	16469	5,56496E-01	3458	9,47282E-01	5667	9,45957E-01	132	0	11208,5
Responder	Macro_OLFML3	Macro_ISG15	C3	CD46	1,47566E-03	8296,5	1,26981E-02	5669	4,65964E-01	5151	9,85833E-01	5243	8,60569E-01	14211	0	11208,5
Responder	pDC_LILRA4	Macro_OLFML3	LILRB4	LAIR1	1,47625E-03	6534,7	1,14522E-02	6759	4,49567E-01	5561	1,62502E+00	1496	8,95407E-01	7649	0	11208,5
Responder	Mono_CD14	Mono_CD14	HP	TLR4	1,48179E-03	12459,5	7,72452E-02	187	6,53137E-01	2283	1,40279E+00	2334	6,95239E-01	46285	0	11208,5
Responder	Mono_CD14	Mono_CD14	HP	ITGB2	1,48282E-03	8236,1	5,24347E-02	415	5,54604E-01	3490	1,25922E+00	3096	8,18822E-01	22971	0	11208,5
Responder	Macro_NLRP3	Macro_NLRP3	THBS1	CD36	1,48335E-03	6320,7	2,64198E-02	1574	8,07727E-01	1214	9,99551E-01	5100	8,69035E-01	12507	0	11208,5
Responder	Macro_ISG15	Macro_ISG15	CCL2	CCR1	1,48351E-03	8496,1	3,22595E-02	1074	6,81966E-01	1997	1,54610E+00	1768	8,01984E-01	26433	0	11208,5
Responder	Mono_CD14	Mono_CD14	HP	ASGR1	1,48384E-03	12653,1	8,68643E-02	136	6,72297E-01	2096	1,26029E+00	3090	6,92198E-01	46735	0	11208,5
Responder	Mono_CD14	Mono_CD14	HP	ASGR2	1,48487E-03	13961,7	1,71956E-01	28	7,85286E-01	1325	1,31993E+00	2740	6,33061E-01	54507	0	11208,5
Responder	Mono_CD14	Mono_INHBA	HP	ASGR1	1,48487E-03	13618,9	7,79962E-02	182	6,32160E-01	2475	9,46119E-01	5678	6,80609E-01	48551	0	11208,5
Responder	Mono_CD14	Mono_CD14	HP	ITGAM	1,48589E-03	12286,9	9,66962E-02	99	7,25393E-01	1674	1,40408E+00	2323	6,96162E-01	46130	0	11208,5
Responder	Macro_OLFML3	Macro_IFI27	C1QA	CD33	1,48750E-03	10474,9	8,25043E-03	11822	3,76598E-01	7748	5,17341E-01	12514	8,87750E-01	9082	0	11208,5
Responder	Macro_OLFML3	pDC_LILRA4	COPA	P2RY6	1,49000E-03	11180,5	1,26832E-02	5683	5,12931E-01	4165	1,47174E+00	2023	7,69470E-01	32823	0	11208,5
Responder	Macro_OLFML3	pDC_LILRA4	APP	RPSA	1,49009E-03	8040,5	8,11322E-03	12158	6,81578E-01	2000	1,81602E+00	1028	8,62540E-01	13808	0	11208,5
Responder	Macro_FOLR2-APOE+	cDC_LAMP3	MMP9	CD44	1,49286E-03	8266,1	1,37407E-02	4988	5,55908E-01	3468	6,93760E-01	9143	8,68928E-01	12523	0	11208,5
Responder	Macro_FOLR2+APOE-	Mono_INHBA	F13A1	ITGB1	1,49450E-03	5016,9	2,31932E-02	2002	1,03674E+00	499	1,66245E+00	1385	8,82558E-01	9990	0	11208,5
Responder	pDC_LILRA4	Macro_OLFML3	COPA	CD74	1,49465E-03	5894,9	7,96502E-03	12526	5,45830E-01	3605	1,75075E+00	1169	9,33462E-01	966	0	11208,5
Responder	Mono_INHBA	Macro_LYVE1	HSP90B1	LRP1	1,49584E-03	8611,5	9,91215E-03	8604	5,07685E-01	4251	5,16555E-01	12528	9,01663E-01	6466	0	11208,5
Responder	Macro_IER3	Mast	LGALS1	CD69	1,49618E-03	4712,1	1,68321E-02	3423	8,23653E-01	1130	9,44874E-01	5689	9,25205E-01	2110	0	11208,5
Responder	cDC_LAMP3	pDC_LILRA4	CXCL10	CXCR3	1,49891E-03	9637,3	3,56040E-02	907	1,05737E+00	454	1,47817E+00	2004	7,65638E-01	33613	0	11208,5
Responder	Mono_CD14	Mono_CD14	VCAN	ITGA4	1,49928E-03	6128,9	1,64241E-02	3590	8,18911E-01	1154	1,82133E+00	1017	8,63350E-01	13675	0	11208,5
Responder	pDC_LILRA4	cDC_LAMP3	APP	RPSA	1,50032E-03	4109,9	1,89757E-02	2763	1,04579E+00	474	2,24772E+00	411	9,05628E-01	5693	0	11208,5
Responder	Mono_CD14	Mono_CD16	IL1B	SIGIRR	1,50422E-03	6580,5	1,59117E-02	3808	6,33604E-01	2459	1,29462E+00	2885	8,68839E-01	12542	0	11208,5
Responder	Mono_CD14	Mono_INHBA	THBS1	CD36	1,50446E-03	8640,7	1,64987E-02	3556	5,13147E-01	4161	9,44025E-01	5697	8,39840E-01	18581	0	11208,5
Responder	cDC(CD1C)	cDC_LAMP3	VEGFA	NRP2	1,50549E-03	11418,9	1,26652E-02	5698	5,09528E-01	4220	1,09130E+00	4236	7,75110E-01	31732	0	11208,5
Responder	cDC_CLEC9A	Macro_FOLR2+APOE+	HLA-DRB5	LAG3	1,50722E-03	7863,7	1,60469E-02	3744	4,82673E-01	4757	8,35717E-01	7062	8,68825E-01	12547	0	11208,5
Responder	Mono_INHBA	Macro_LYVE1	SPP1	ITGAV_ITGB5	1,50757E-03	12382,1	1,52036E-02	4151	4,44021E-01	5700	9,46502E-01	5674	7,57484E-01	35177	0	11208,5
Responder	Mono_CD14	Mono_CD14	RETN	TLR4	1,50860E-03	10336,5	4,15236E-02	686	6,42982E-01	2374	1,68362E+00	1323	7,52434E-01	36091	0	11208,5
Responder	Mono_CD14	Macro_FOLR2+APOE+	AGTRAP	RACK1	1,50902E-03	7384,3	1,33081E-02	5241	6,79375E-01	2027	5,15606E-01	12550	9,04613E-01	5895	0	11208,5
Responder	pDC_LILRA4	Macro_OLFML3	HSP90B1	TLR2	1,51083E-03	5754,3	1,19907E-02	6270	5,63967E-01	3356	1,90259E+00	866	8,98406E-01	7071	0	11208,5
Responder	Macro_OLFML3	Macro_ISG15	C3	LRP1	1,51143E-03	8889,3	1,07486E-02	7499	4,18136E-01	6419	8,57073E-01	6766	8,68804E-01	12554	0	11208,5

Responder	Mono_CD14	Mono_INHBA	ADAM15	ITGA5	1,51276E-03	13953,9	1,96837E-02	2597	4,76906E-01	4887	9,43195E-01	5705	7,00955E-01	45372	0	11208,5
Responder	Macro_OLFML3	Macro_FOLR2-APOE+	C1QA	CD93	1,51565E-03	10273,1	7,95369E-03	12561	4,27832E-01	6136	5,33330E-01	12123	8,86206E-01	9337	0	11208,5
Responder	Macro_OLFML3	cDC_CLEC9A	ADAM10	CADM1	1,51692E-03	11391,5	1,79110E-02	3075	7,48819E-01	1526	9,42685E-01	5709	7,56025E-01	35439	0	11208,5
Responder	pDC_LILRA4	cDC(CD1C)	APP	APLP2	1,51746E-03	5900,1	1,37374E-02	4991	8,05027E-01	1227	2,56288E+00	195	8,72812E-01	11879	0	11208,5
Responder	Macro_OLFML3	cDC_CLEC9A	HLA-DRB1	LAG3	1,51988E-03	8654,5	1,22529E-02	6027	5,19409E-01	4049	6,76905E-01	9420	8,68716E-01	12568	0	11208,5
Responder	Mono_INHBA	Macro_FOLR2+APOE+	LGALS3	ENG	1,52109E-03	9758,3	1,01938E-02	8220	4,83405E-01	4744	5,36371E-01	12049	8,68714E-01	12570	0	11208,5
Responder	Macro_OLFML3	Macro_LYVE1	FN1	ITGA4_ITGB1	1,52290E-03	7941,3	1,79726E-02	3059	4,65256E-01	5175	7,88864E-01	7691	8,68684E-01	12573	0	11208,5
Responder	Macro_FOLR2+APOE+	Macro_LYVE1	APOE	ABCA1	1,52633E-03	4230,9	2,08450E-02	2372	9,24427E-01	772	9,41995E-01	5718	9,32275E-01	1084	0	11208,5
Responder	pDC_LILRA4	cDC(CD1C)	SELPLG	ITGB2	1,52654E-03	5380,1	1,13311E-02	6869	5,77953E-01	3138	2,56885E+00	191	9,06792E-01	5494	0	11208,5
Responder	Macro_ISG15	Macro_ISG15	LGALS9	HAVCR2	1,52836E-03	11018,1	7,98940E-03	12455	3,17769E-01	10088	7,16699E-01	8757	8,68632E-01	12582	0	11208,5
Responder	Macro_OLFML3	Mast	ACTR2	ADRB2	1,53002E-03	7265,7	3,05522E-02	1177	7,59680E-01	1480	6,99997E-01	9044	8,64613E-01	13419	0	11208,5
Responder	Macro_ISG15	Macro_ISG15	CXCL10	TLR4	1,53002E-03	6705,1	2,92446E-02	1295	6,80606E-01	2018	1,59945E+00	1574	8,45250E-01	17430	0	11208,5
Responder	Macro_ISG15	Macro_FOLR2+APOE+	TIMP1	CD63	1,53140E-03	6528,1	7,94223E-03	12587	6,81127E-01	2006	8,63257E-01	6687	9,45323E-01	152	0	11208,5
Responder	pDC_LILRA4	cDC(CD1C)	SERPINF1	PLXDC2	1,53201E-03	4844,7	1,63777E-02	3606	7,60374E-01	1474	3,01970E+00	62	8,94255E-01	7873	0	11208,5
Responder	Macro_ISG15	Macro_NLRP3	PLAU	PLAUR	1,53201E-03	7492,3	1,14383E-02	6777	6,37296E-01	2426	1,06591E+00	4462	8,68598E-01	12588	0	11208,5
Responder	Mast	Macro_LYVE1	CSF1	CSF3R	1,53450E-03	7531,5	3,90271E-02	767	8,95141E-01	854	1,47307E+00	2020	8,19601E-01	22808	0	11208,5
Responder	cDC_CLEC9A	Macro_FOLR2+APOE+	HMGB1	HAVCR2	1,53566E-03	8695,5	7,94072E-03	12594	4,51089E-01	5524	7,60674E-01	8101	9,03806E-01	6050	0	11208,5
Responder	Macro_FOLR2-APOE+	Mono_CD14	C1QA	CR1	1,53749E-03	8873,5	1,73143E-02	3259	4,62530E-01	5233	5,35561E-01	12070	8,68579E-01	12597	0	11208,5
Responder	Macro_ISG15	Macro_FOLR2+APOE+	TNFSF13B	CD40	1,53871E-03	10269,3	9,49715E-03	9297	4,23282E-01	6268	5,38951E-01	11974	8,68574E-01	12599	0	11208,5
Responder	Macro_OLFML3	Mono_INHBA	A2M	LRP1	1,54299E-03	8489,7	1,40258E-02	4820	4,69251E-01	5070	7,17306E-01	8744	8,68544E-01	12606	0	11208,5
Responder	Macro_IFI27	pDC_LILRA4	GNAI2	CXCR3	1,54574E-03	7391,5	2,30428E-02	2025	9,35633E-01	746	1,68713E+00	1317	8,25009E-01	21661	0	11208,5
Responder	cDC_CLEC9A	Macro_OLFML3	LGALS9	HAVCR2	1,54667E-03	11013,5	7,97185E-03	12504	3,15259E-01	10187	7,30812E-01	8556	8,68507E-01	12612	0	11208,5
Responder	pDC_LILRA4	cDC(CD1C)	APP	NCSTN	1,55025E-03	7559,1	2,30176E-02	2027	9,61471E-01	669	2,57867E+00	187	8,15429E-01	23704	0	11208,5
Responder	Macro_OLFML3	Mono_INHBA	TNFSF13B	TFRC	1,55035E-03	10276,3	9,35792E-03	9544	4,38073E-01	5867	5,32523E-01	12144	8,68468E-01	12618	0	11208,5
Responder	cDC_CLEC9A	Macro_LYVE1	HLA-DRB5	CD4	1,55650E-03	7392,3	7,92857E-03	12628	4,32735E-01	6009	8,87240E-01	6388	9,35715E-01	728	0	11208,5
Responder	Mono_INHBA	Mono_INHBA	CCL3L1	CCR1	1,55691E-03	9846,7	1,25988E-02	5747	5,40855E-01	3683	1,27784E+00	2983	8,06218E-01	25612	0	11208,5
Responder	pDC_LILRA4	Macro_LYVE1	HMGB1	CD163	1,55703E-03	4832,3	1,02140E-02	8202	6,79217E-01	2030	1,90999E+00	856	9,26712E-01	1865	0	11208,5
Responder	pDC_LILRA4	Mono_INHBA	APP	APLP2	1,55959E-03	4782,9	1,86562E-02	2848	9,54457E-01	684	2,42177E+00	287	8,88853E-01	8887	0	11208,5
Responder	Macro_ISG15	Macro_IFI27	S100A9	CD68	1,56268E-03	8499,5	8,18318E-03	11989	4,41623E-01	5770	5,12416E-01	12638	9,34117E-01	892	0	11208,5
Responder	Macro_NLRP3	Mono_CD16	CD52	SIGLEC10	1,56639E-03	9539,1	9,73376E-03	8896	4,90136E-01	4610	5,12244E-01	12644	8,80640E-01	10337	0	11208,5
Responder	pDC_LILRA4	Mono_INHBA	SELPLG	ITGB2	1,57135E-03	8347,7	8,18543E-03	11981	3,42690E-01	8996	1,69543E+00	1297	8,92110E-01	8256	0	11208,5
Responder	Mono_INHBA	cDC(CD1C)	TIMP1	CD63	1,57291E-03	7034,5	6,95946E-03	15601	4,41838E-01	5762	1,40744E+00	2305	9,41807E-01	296	0	11208,5
Responder	Macro_ISG15	Macro_FOLR2+APOE-	CCL8	CCR1	1,57293E-03	12261,1	2,77668E-02	1426	6,78913E-01	2037	1,65730E+00	1398	7,01753E-01	45236	0	11208,5
Responder	Macro_OLFML3	Macro_OLFML3	LGALS9	HAVCR2	1,57488E-03	15341,1	8,24928E-03	11827	3,36071E-01	9288	-4,44993E-02	32099	8,70448E-01	12283	0	11208,5
Responder	Mono_INHBA	Macro_IFI27	HBEGF	CD9	1,57882E-03	8977,5	1,54378E-02	4025	4,85873E-01	4693	5,11319E-01	12664	8,70325E-01	12297	0	11208,5
Responder	pDC_LILRA4	Mono_INHBA	SERPINF1	PLXDC2	1,58069E-03	6389,1	1,15365E-02	6681	6,19018E-01	2616	2,40759E+00	296	8,76506E-01	11144	0	11208,5
Responder	cDC(CD1C)	Mono_INHBA	PIGF	FLT1	1,58149E-03	14965,1	2,98396E-02	1246	5,78127E-01	3135	9,36994E-01	5770	6,42419E-01	53466	0	11208,5
Responder	Mono_INHBA	Macro_FOLR2+APOE+	ADAM10	GPNMB	1,58569E-03	6722,1	1,90304E-02	2751	8,64207E-01	963	9,16505E-01	6013	8,68242E-01	12675	0	11208,5
Responder	Macro_OLFML3	cDC(CD1C)	C3	CD81	1,58694E-03	9738,5	8,36124E-03	11616	3,39254E-01	9163	1,11853E+00	4028	8,68234E-01	12677	0	11208,5
Responder	Mono_CD14	Macro_FOLR2-APOE+	CXCL8	SDC3	1,58795E-03	7333,5	1,74675E-02	3211	6,38632E-01	2410	1,56540E+00	1691	8,41852E-01	18147	0	11208,5
Responder	Macro_NLRP3	Macro_LYVE1	SERPINA1	LRP1	1,58882E-03	6947,7	1,30402E-02	5429	6,22743E-01	2573	5,10857E-01	12680	9,21165E-01	2848	0	11208,5
Responder	Macro_OLFML3	cDC(CD1C)	MDK	NCL	1,59195E-03	7225,5	1,94617E-02	2639	4,38205E-01	5861	1,15243E+00	3734	8,68179E-01	12685	0	11208,5
Responder	Mono_CD14	Macro_FOLR2-APOE+	VCAN	ITGA4	1,59227E-03	7237,7	1,37086E-02	5011	7,72958E-01	1385	1,33924E+00	2636	8,52335E-01	15948	0	11208,5
Responder	pDC_LILRA4	Macro_NLRP3	APP	APLP2	1,59352E-03	4191,3	2,28781E-02	2046	1,08272E+00	419	2,48077E+00	240	8,98538E-01	7043	0	11208,5
Responder	Mono_CD14	Macro_LYVE1	THBS1	CD36	1,59572E-03	6088,5	2,60071E-02	1623	7,80141E-01	1355	1,17799E+00	3565	8,68136E-01	12691	0	11208,5
Responder	Macro_FOLR2-APOE+	Mono_INHBA	FN1	C5AR1	1,59698E-03	9292,3	1,64845E-02	3561	3,75849E-01	7775	5,10250E-01	12693	8,76110E-01	11224	0	11208,5
Responder	cDC_CLEC9A	Macro_FOLR2+APOE+	SERPINF1	PLXDC2	1,59761E-03	9848,9	9,92518E-03	8590	3,43743E-01	8968	7,82587E-01	7784	8,68131E-01	12694	0	11208,5
Responder	Macro_NLRP3	pDC_LILRA4	APP	TNFRSF21	1,59812E-03	8825,5	2,28729E-02	2048	1,01985E+00	536	1,75394E+00	1163	7,88081E-01	29172	0	11208,5
Responder	Macro_FOLR2-APOE+	Mono_INHBA	GRN	TNFRSF1B	1,59887E-03	8608,7	7,90029E-03	12696	4,70730E-01	5032	5,70649E-01	11294	9,21357E-01	2813	0	11208,5
Responder	Macro_FOLR2+APOE-	pDC_LILRA4	C1QB	C1QBP	1,59984E-03	4860,1	1,25434E-02	5787	5,70362E-01	3264	1,55592E+00	1725	9,24098E-01	2316	0	11208,5
Responder	Mono_CD14	Mono_INHBA	PKM	CD44	1,60093E-03	8304,9	6,66265E-03	16709	4,40929E-01	5788	9,74422E-01	5355	9,23177E-01	2464	0	11208,5
Responder	pDC_LILRA4	Mono_INHBA	HSP90B1	LRP1	1,60139E-03	6392,7	9,36866E-03	9525	5,34134E-01	3797	2,16909E+00	496	8,99135E-01	6937	0	11208,5
Responder	Macro_OLFML3	Mono_INHBA	MDK	LRP1	1,60310E-03	7946,3	2,48719E-02	1755	5,12501E-01	4173	9,35533E-01	5790	8,48167E-01	16805	0	11208,5
Responder	Mono_INHBA	Macro_ISG15	VEGFA	EPHB2	1,60418E-03	14104,1	2,41892E-02	1849	6,22111E-01	2579	9,35278E-01	5791	6,76678E-01	49093	0	11208,5
Responder	Mono_CD14	Macro_FOLR2-APOE+	ADM	CALCRL	1,60418E-03	13685,7	1,81576E-02	2993	4,91408E-01	4576	1,28241E+00	2957	6,92450E-01	46694	0	11208,5
Responder	pDC_LILRA4	Mono_CD14	APP	RPSA	1,60707E-03	6100,3	1,10783E-02	7138	8,17168E-01	1168	2,15768E+00	523	8,79986E-01	10464	0	11208,5
Responder	Mono_CD14	Macro_IER3	NAMPT	ITGA5_ITGB1	1,60833E-03	8324,1	8,21444E-03	11914	6,35638E-01	2439	1,21594E+00	3348	8,68066E-01	12711	0	11208,5
Responder	Mono_CD14	Macro_FOLR2-APOE+	S100A12	CD36	1,60853E-03	9280,1	1,70506E-02	3340	9,53012E-01	692	1,78389E+00	1088	7,83474E-01	30072	0	11208,5
Responder	pDC_LILRA4	Mono_CD14	APP	APLP2	1,60897E-03	4237,3	2,22116E-02	2148	1,06247E+00	446	2,89157E+00	85	8,97182E-01	7299	0	11208,5
Responder	pDC_LILRA4	Mono_CD14	APP	LRP1	1,60960E-03	5380,9	2,07884E-02	2389	9,67746E-01	652	2,71715E+00	128	8,68900E-01	12527	0	11208,5
Responder	Mono_INHBA	Mono_CD16	ICAM1	ITGAL_ITGB2	1,61023E-03	10555,7	7,89108E-03	12714	4,36718E-01	5905	5,43972E-01	11867	8,76731E-01	11084	0	11208,5
Responder	Mono_CD14	Macro_FOLR2-APOE+	RETN	TLR4	1,61071E-03	12782,1	2,97441E-02	1256	5,32425E-01	3829	9,51704E-01	5613	7,20072E-01	42004	0	11208,5
Responder	Mono_INHBA	Mono_CD16	HLA-C	LILRA3	1,61277E-03	6955,5	2,75021E-02	1448	5,73197E-01	3223	5,08993E-01	12718	9,03184E-01	6180	0	11208,5
Responder	cDC_CLEC9A	cDC_CLEC9A	HLA-DPA1	LAG3	1,61277E-03	7331,5	1,26142E-02	5731	5,10087E-01	4214	1,31177E+00	2786	8,68042E-01	12718	0	11208,5
Responder	Macro_ISG15	Mono_CD14	CD14	TLR4	1,61404E-03	8669,9	9,98421E-03	8514	3,84311E-01	7479	1,20101E+00	3428	8,68034E-01	12720	0	11208,5

Responder	pDC_LILRA4	Mono_INHBA	SEMA7A	PLXNC1	1,61429E-03	12115,1	2,36216E-02	1935	6,76789E-01	2055	1,57664E+00	1653	7,10548E-01	43724	0	11208,5
Responder	cDC(CD1C)	Macro_LYVE1	GAS6	MERTK	1,61507E-03	12517,1	1,43680E-02	4609	6,80978E-01	2007	9,34259E-01	5801	7,37290E-01	38960	0	11208,5
Responder	pDC_LILRA4	Mono_CD14	SELPLG	ITGB2	1,61912E-03	5820,3	1,06420E-02	7640	5,26413E-01	3920	2,34241E+00	342	9,04106E-01	5991	0	11208,5
Responder	Macro_FOLR2-APOE+	Mono_CD16	SPP1	ITGA4_ITGB1	1,61975E-03	7391,5	1,68689E-02	3403	5,79906E-01	3101	8,77021E-01	6516	8,68002E-01	12729	0	11208,5
Responder	Macro_OLFML3	cDC_LAMP3	APOE	SORL1	1,62294E-03	10867,9	8,08046E-03	12232	3,13372E-01	10268	5,07980E-01	12734	8,94171E-01	7897	0	11208,5
Responder	Macro_OLFML3	Macro_FOLR2-APOE+	APOE	SCARB1	1,62485E-03	7882,3	1,68415E-02	3417	5,60929E-01	3387	7,23207E-01	8662	8,67987E-01	12737	0	11208,5
Responder	cDC(CD1C)	pDC_LILRA4	APP	RPSA	1,62549E-03	7276,5	8,90611E-03	10382	7,05917E-01	1808	2,47439E+00	246	8,67975E-01	12738	0	11208,5
Responder	pDC_LILRA4	cDC_CLEC9A	SPON2	ITGA5	1,62591E-03	14278,9	2,28035E-02	2060	8,11081E-01	1203	1,64779E+00	1430	6,24128E-01	55493	0	11208,5
Responder	Macro_ISG15	Macro_OLFML3	CD14	TLR1	1,62613E-03	9120,3	1,24935E-02	5819	4,19421E-01	6373	6,74507E-01	9462	8,67969E-01	12739	0	11208,5
Responder	Macro_OLFML3	cDC_LAMP3	ENTPD1	ADORA2A	1,62824E-03	13373,5	3,47229E-02	953	6,76173E-01	2061	4,61053E-01	13900	7,38406E-01	38745	0	11208,5
Responder	cDC_CLEC9A	Macro_ISG15	HMGB1	HAVCR2	1,62932E-03	7784,5	7,87940E-03	12744	4,46638E-01	5635	1,23785E+00	3213	9,03468E-01	6122	0	11208,5
Responder	Mono_CD14	cDC_LAMP3	VCAN	ITGB1	1,63124E-03	6598,7	1,34787E-02	5138	7,58342E-01	1487	1,38906E+00	2413	8,67921E-01	12747	0	11208,5
Responder	Mono_INHBA	Macro_FOLR2-APOE+	VEGFA	ITGB1	1,63591E-03	7607,5	1,73239E-02	3254	6,97363E-01	1875	9,33285E-01	5820	8,52735E-01	15880	0	11208,5
Responder	Macro_ISG15	pDC_LILRA4	LGALS9	CD47	1,63636E-03	8463,7	9,10132E-03	10001	4,09302E-01	6685	1,57277E+00	1669	8,67884E-01	12755	0	11208,5
Responder	Macro_OLFML3	Mono_CD14	HLA-A	APLP2	1,63812E-03	7634,7	6,60273E-03	16935	5,34076E-01	3799	9,32946E-01	5822	9,39633E-01	409	0	11208,5
Responder	Mast	Mono_CD14	PLAT	LRP1	1,63992E-03	8037,5	7,14881E-02	208	8,14280E-01	1185	1,46395E+00	2066	8,06651E-01	25520	0	11208,5
Responder	Macro_FOLR2+APOE+	cDC(CD1C)	APOE	SORL1	1,64033E-03	5281,7	1,24900E-02	5824	6,35066E-01	2445	1,34687E+00	2602	9,13079E-01	4329	0	11208,5
Responder	cDC(CD1C)	Macro_ISG15	HLA-DRB5	LAG3	1,64254E-03	7771,9	1,42699E-02	4667	4,39472E-01	5826	1,23646E+00	3229	8,61991E-01	13929	0	11208,5
Responder	pDC_LILRA4	Mono_CD14	HSP90B1	LRP1	1,64536E-03	7523,7	8,01926E-03	12384	4,58050E-01	5345	2,30052E+00	375	8,91860E-01	8306	0	11208,5
Responder	Macro_ISG15	Mono_INHBA	HBEGF	CD44	1,64536E-03	9594,9	7,87383E-03	12769	4,88337E-01	4644	7,77997E-01	7852	8,74754E-01	11501	0	11208,5
Responder	Mono_CD14	Mono_CD14	RETN	CAP1	1,64696E-03	8562,5	2,28383E-02	2052	4,39429E-01	5830	1,35708E+00	2554	8,27387E-01	21168	0	11208,5
Responder	cDC_CLEC9A	pDC_LILRA4	HLA-DPB1	CD4	1,65149E-03	5847,5	7,76530E-03	13096	5,05058E-01	4302	2,45171E+00	264	9,40181E-01	367	0	11208,5
Responder	pDC_LILRA4	Macro_FOLR2-APOE+	APP	RPSA	1,65182E-03	4552,5	1,65780E-02	3518	9,76378E-01	636	2,12506E+00	558	8,99695E-01	6842	0	11208,5
Responder	pDC_LILRA4	Macro_FOLR2-APOE+	APP	APLP2	1,65376E-03	4668,9	1,96126E-02	2615	9,83513E-01	618	2,17607E+00	486	8,91299E-01	8417	0	11208,5
Responder	Mono_CD14	Mono_INHBA	SERPINA1	LRP1	1,65376E-03	10074,7	7,86967E-03	12782	2,72739E-01	12345	8,10638E-01	7395	9,00767E-01	6643	0	11208,5
Responder	Macro_OLFML3	cDC_CLEC9A	CCL5	CXCR3	1,65401E-03	14628,5	2,48645E-02	1757	6,75086E-01	2072	4,95270E-01	13051	7,03001E-01	45054	0	11208,5
Responder	pDC_LILRA4	Macro_FOLR2-APOE+	APP	LRP1	1,65440E-03	5177,1	2,27092E-02	2073	1,00952E+00	556	2,33290E+00	348	8,73852E-01	11700	0	11208,5
Responder	Mono_CD14	Macro_ISG15	CD55	ADGRE5	1,65918E-03	6296,3	1,66854E-02	3475	7,33934E-01	1620	1,41496E+00	2275	8,67205E-01	12903	0	11208,5
Responder	pDC_LILRA4	Macro_FOLR2-APOE+	CD99	CD81	1,66024E-03	7343,9	8,94326E-03	10321	5,03484E-01	4331	1,61130E+00	1531	8,86225E-01	9328	0	11208,5
Responder	Mono_CD14	Macro_ISG15	CD55	ADGRE2	1,66029E-03	8549,3	1,32252E-02	5295	5,94179E-01	2909	1,01393E+00	4958	8,40813E-01	18376	0	11208,5
Responder	Macro_NLRP3	Macro_IFI27	S100A9	ITGB2	1,66089E-03	8258,3	8,76724E-03	10654	4,46930E-01	5628	5,05804E-01	12793	9,33081E-01	1008	0	11208,5
Responder	cDC_LAMP3	Macro_FOLR2+APOE+	CCL19	CCRL2	1,66108E-03	8773,1	5,48507E-02	373	1,86555E+00	51	1,46095E+00	2075	7,83044E-01	30158	0	11208,5
Responder	Macro_LYVE1	pDC_LILRA4	CCL5	CXCR3	1,66344E-03	12370,5	2,26945E-02	2076	1,03913E+00	492	1,61094E+00	1532	6,93380E-01	46544	0	11208,5
Responder	pDC_LILRA4	Macro_FOLR2-APOE+	TNFSF9	HLA-DPA1	1,66348E-03	4629,1	2,17662E-02	2227	7,41576E-01	1571	1,46454E+00	2060	9,03671E-01	6079	0	11208,5
Responder	Mono_CD14	Macro_ISG15	HP	TLR4	1,66587E-03	13809,3	6,50552E-02	250	5,91636E-01	2947	9,56474E-01	5560	6,76745E-01	49081	0	11208,5
Responder	pDC_LILRA4	Macro_FOLR2-APOE+	SELPLG	ITGB2	1,66674E-03	6006,9	1,05935E-02	7702	5,22785E-01	3989	1,77871E+00	1104	9,03908E-01	6031	0	11208,5
Responder	pDC_LILRA4	Macro_FOLR2-APOE+	LILRB4	LAIR1	1,66804E-03	8925,5	8,91403E-03	10369	2,89471E-01	11418	1,55433E+00	1730	8,83080E-01	9902	0	11208,5
Responder	Mono_CD14	Macro_ISG15	HP	CD163	1,66922E-03	10056,5	5,83687E-02	325	6,38754E-01	2408	9,65866E-01	5463	7,79515E-01	30878	0	11208,5
Responder	cDC_CLEC9A	Macro_IER3	HMGB1	THBD	1,67195E-03	9598,7	7,87308E-03	12775	4,35739E-01	5934	9,83188E-01	5266	8,67640E-01	12810	0	11208,5
Responder	Mast	Macro_FOLR2+APOE-	CSF1	CSF3R	1,67292E-03	8986,5	2,64930E-02	1565	8,23596E-01	1131	1,45877E+00	2080	7,89175E-01	28948	0	11208,5
Responder	Macro_OLFML3	Macro_IFI27	C3	CD46	1,67652E-03	7866,9	1,43175E-02	4643	4,97202E-01	4459	9,01474E-01	6207	8,67615E-01	12817	0	11208,5
Responder	pDC_LILRA4	Macro_FOLR2-APOE+	SERPINF1	PLXDC2	1,67718E-03	4388,9	1,88656E-02	2795	8,33016E-01	1088	2,54073E+00	209	9,00757E-01	6644	0	11208,5
Responder	Macro_FOLR2+APOE-	Macro_FOLR2-APOE+	F13A1	ITGB1	1,67767E-03	5129,5	2,34191E-02	1965	1,04180E+00	486	1,45829E+00	2082	8,83060E-01	9906	0	11208,5
Responder	Macro_OLFML3	cDC_CLEC9A	MDK	NOTCH2	1,67819E-03	9250,7	2,10715E-02	2339	4,38278E-01	5858	1,24895E+00	3153	8,15477E-01	23695	0	11208,5
Responder	Mast	Macro_IER3	CSF1	CSF3R	1,68242E-03	7827,3	3,60593E-02	882	8,78201E-01	909	1,45770E+00	2084	8,13680E-01	24053	0	11208,5
Responder	Mono_CD14	Macro_ISG15	VCAN	ITGA4	1,68494E-03	6955,3	1,39214E-02	4884	7,76559E-01	1369	1,60006E+00	1569	8,53301E-01	15746	0	11208,5
Responder	cDC_CLEC9A	cDC_CLEC9A	HLA-DRA	LAG3	1,68701E-03	8956,7	1,09345E-02	7292	3,63074E-01	8252	9,91014E-01	5198	8,67535E-01	12833	0	11208,5
Responder	Mono_INHBA	pDC_LILRA4	THBS1	CD36	1,68945E-03	9127,7	1,38893E-02	4898	4,38039E-01	5868	1,34783E+00	2598	8,27920E-01	21066	0	11208,5
Responder	Macro_ISG15	Mono_CD16	CXCL10	TLR4	1,68958E-03	6490,9	3,18812E-02	1101	7,10198E-01	1774	1,45691E+00	2087	8,50812E-01	16284	0	11208,5
Responder	pDC_LILRA4	Macro_FOLR2-APOE+	RPS19	C5AR1	1,68964E-03	5642,5	8,57702E-03	11114	6,87364E-01	1955	1,85012E+00	960	9,20555E-01	2975	0	11208,5
Responder	Macro_NLRP3	pDC_LILRA4	LGALS1	CD69	1,69030E-03	8569,9	7,85403E-03	12838	3,70030E-01	7976	1,28558E+00	2934	8,94177E-01	7893	0	11208,5
Responder	Mono_INHBA	cDC_CLEC9A	THBS1	SCARB1	1,69171E-03	11355,5	1,67909E-02	3445	4,38018E-01	5870	1,13891E+00	3850	7,71674E-01	32404	0	11208,5
Responder	Mono_CD14	Macro_ISG15	S100A4	CCR5	1,69623E-03	7642,5	1,60354E-02	3751	6,60540E-01	2197	1,12382E+00	3988	8,46867E-01	17068	0	11208,5
Responder	Macro_ISG15	cDC_LAMP3	ADM	RAMP1	1,69653E-03	12045,1	5,75043E-02	336	1,11390E+00	372	1,07774E+00	4355	7,09107E-01	43954	0	11208,5
Responder	Mono_CD14	Mono_CD16	HLA-F	LILRB2	1,69756E-03	9553,5	7,84940E-03	12849	4,90769E-01	4592	7,74012E-01	7905	8,76173E-01	11213	0	11208,5
Responder	Mono_CD14	Macro_ISG15	S100A12	CD36	1,69963E-03	9247,3	1,71306E-02	3316	9,53508E-01	689	1,81362E+00	1035	7,83870E-01	29988	0	11208,5
Responder	Macro_NLRP3	Mono_CD16	HLA-C	LILRB2	1,70152E-03	7086,3	1,00813E-02	8379	6,05776E-01	2775	5,03273E-01	12855	9,43517E-01	214	0	11208,5
Responder	Mono_CD14	Macro_ISG15	RETN	TLR4	1,70190E-03	11525,3	3,49708E-02	938	5,81480E-01	3077	1,23729E+00	3219	7,36093E-01	39184	0	11208,5
Responder	Macro_ISG15	Macro_FOLR2-APOE+	CD14	ITGB1	1,70285E-03	9626,3	7,84665E-03	12857	3,98098E-01	7032	6,56430E-01	9783	8,97475E-01	7251	0	11208,5
Responder	Macro_NLRP3	cDC_CLEC9A	ADAM10	CADM1	1,70394E-03	8040,5	3,32954E-02	1015	9,19819E-01	785	1,45564E+00	2093	8,08611E-01	25101	0	11208,5
Responder	pDC_LILRA4	Macro_FOLR2-APOE+	HSP90B1	LRP1	1,70417E-03	6931,7	8,76022E-03	10666	4,99828E-01	4407	1,91626E+00	848	8,96049E-01	7529	0	11208,5
Responder	Macro_FOLR2+APOE+	pDC_LILRA4	APOE	SORL1	1,71100E-03	4951,3	1,24144E-02	5887	6,33229E-01	2465	1,92882E+00	827	9,12837E-01	4369	0	11208,5
Responder	pDC_LILRA4	Macro_ISG15	APP	APLP2	1,71147E-03	5467,7	1,53809E-02	4055	8,54956E-01	1005	2,24483E+00	413	8,78953E-01	10657	0	11208,5
Responder	Macro_IER3	pDC_LILRA4	CXCL2	DPP4	1,71472E-03	8397,1	7,26688E-02	202	1,11151E+00	374	1,36824E+00	2503	7,95613E-01	27698	0	11208,5
Responder	Mono_CD14	cDC_CLEC9A	CD52	SIGLEC10	1,71813E-03	10374,3	7,83751E-03	12880	2,99275E-01	10936	1,08562E+00	4286	8,68774E-01	12561	0	11208,5

Responder	cDC(CD1C)	cDC_LAMP3	LTB	CD40	1,71898E-03	11276,3	1,24691E-02	5840	4,37001E-01	5894	1,41225E+00	2285	7,78150E-01	31154	0	11208,5
Responder	Macro_OLFML3	Mast	GNAS	ADRB2	1,72081E-03	8731,9	2,35087E-02	1950	6,71882E-01	2100	4,73206E-01	13589	8,57667E-01	14812	0	11208,5
Responder	pDC_LILRA4	Macro_ISG15	SELPLG	ITGB2	1,72147E-03	6476,3	9,87882E-03	8656	4,69337E-01	5068	1,93547E+00	817	9,00831E-01	6632	0	11208,5
Responder	Macro_OLFML3	pDC_LILRA4	LGALS9	CD47	1,72214E-03	8974,1	9,00533E-03	10192	4,02705E-01	6886	1,15675E+00	3698	8,67275E-01	12886	0	11208,5
Responder	Mast	pDC_LILRA4	TNF	TNFRSF21	1,72322E-03	9324,5	2,24829E-02	2101	1,03082E+00	509	1,72895E+00	1228	7,75876E-01	31576	0	11208,5
Responder	Macro_ISG15	Mono_CD14	CCL2	CCR2	1,72564E-03	11730,7	4,67783E-02	542	6,71664E-01	2102	1,56448E+00	1696	7,14045E-01	43105	0	11208,5
Responder	pDC_LILRA4	Macro_ISG15	SERPINF1	PLXDC2	1,72883E-03	6479,3	1,13580E-02	6842	6,13807E-01	2677	2,31776E+00	358	8,75660E-01	11311	0	11208,5
Responder	pDC_LILRA4	Mono_CD14	HSP90B1	TLR4	1,72950E-03	7295,3	1,04614E-02	7875	5,08083E-01	4244	2,46499E+00	252	8,67236E-01	12897	0	11208,5
Responder	Macro_FOLR2-APOE+	pDC_LILRA4	CXCL8	CD79A	1,73535E-03	9524,3	5,83763E-02	324	8,16772E-01	1169	1,45186E+00	2106	7,69547E-01	32814	0	11208,5
Responder	Macro_ISG15	Macro_LYVE1	CXCL10	TLR4	1,73778E-03	6010,9	3,66983E-02	862	7,64261E-01	1444	1,45142E+00	2107	8,59524E-01	14433	0	11208,5
Responder	pDC_LILRA4	Macro_OLFML3	HSP90B1	TLR1	1,73824E-03	6791,5	1,30907E-02	5381	5,43193E-01	3646	1,93849E+00	812	8,67171E-01	12910	0	11208,5
Responder	Macro_ISG15	Macro_IER3	CD14	ITGA4	1,73958E-03	10621,9	7,82614E-03	12912	3,55111E-01	8550	6,05256E-01	10690	8,83916E-01	9749	0	11208,5
Responder	Mono_INHBA	cDC(CD1C)	ICAM1	ITGAM_ITGB2	1,74093E-03	10244,3	7,82513E-03	12914	3,28327E-01	9629	8,88080E-01	6371	8,76684E-01	11099	0	11208,5
Responder	pDC_LILRA4	Macro_IER3	SPON2	ITGAM	1,74265E-03	13736,7	2,73096E-02	1479	8,11650E-01	1200	1,45092E+00	2109	6,49322E-01	52687	0	11208,5
Responder	Mono_CD14	Macro_NLRP3	CD55	CR1	1,74427E-03	10705,5	1,43625E-02	4612	5,95240E-01	2894	1,09573E+00	4206	7,80911E-01	30607	0	11208,5
Responder	cDC_CLEC9A	Mono_CD14	HMGB1	TLR4	1,74498E-03	8746,3	8,64749E-03	10915	4,14358E-01	6551	1,44567E+00	2137	8,67126E-01	12920	0	11208,5
Responder	Macro_FOLR2-APOE+	Macro_NLRP3	MMP12	PLAUR	1,74508E-03	5971,9	2,38132E-02	1902	7,80542E-01	1352	1,45083E+00	2110	8,65202E-01	13287	0	11208,5
Responder	Mono_INHBA	Macro_OLFML3	IL6	IL6R_IL6ST	1,74543E-03	12920,3	2,34484E-02	1959	7,08598E-01	1787	9,24706E-01	5917	7,10483E-01	43730	0	11208,5
Responder	Macro_OLFML3	Mono_CD14	MDK	LRP1	1,74543E-03	8573,9	2,12895E-02	2309	4,36417E-01	5917	1,06697E+00	4450	8,37879E-01	18985	0	11208,5
Responder	Macro_NLRP3	cDC(CD1C)	VCAN	ITGA4	1,74768E-03	6433,9	1,75094E-02	3190	6,84843E-01	1973	1,29620E+00	2874	8,67081E-01	12924	0	11208,5
Responder	Macro_OLFML3	Mono_CD16	C1QB	CD33	1,74836E-03	10769,9	7,81989E-03	12925	3,67014E-01	8102	5,32485E-01	12146	8,85405E-01	9468	0	11208,5
Responder	Macro_OLFML3	cDC_CLEC9A	CALR	SCARF1	1,74997E-03	8485,1	2,62535E-02	1598	6,70234E-01	2112	7,11191E-01	8843	8,39354E-01	18664	0	11208,5
Responder	cDC(CD1C)	Macro_LYVE1	TNFSF13B	TFRC	1,75039E-03	9502,3	9,12957E-03	9955	3,75828E-01	7777	9,49535E-01	5643	8,67051E-01	12928	0	11208,5
Responder	pDC_LILRA4	Macro_ISG15	HSP90B1	TLR2	1,75106E-03	7544,7	9,04916E-03	10089	4,26936E-01	6156	2,00855E+00	700	8,84822E-01	9570	0	11208,5
Responder	Macro_LYVE1	Mono_INHBA	PLTP	ABCA1	1,75241E-03	4632,1	2,73804E-02	1472	1,05146E+00	462	1,45049E+00	2113	8,94104E-01	7905	0	11208,5
Responder	Macro_ISG15	Macro_FOLR2+APOE+	CD14	ITGB2	1,75445E-03	8567,5	8,12376E-03	12139	4,43892E-01	5703	4,99910E-01	12934	9,34499E-01	853	0	11208,5
Responder	Mono_CD14	Mono_INHBA	VCAN	ITGA4	1,75469E-03	7674,7	1,23761E-02	5925	7,50409E-01	1519	1,38651E+00	2419	8,45783E-01	17302	0	11208,5
Responder	cDC(CD1C)	cDC_CLEC9A	HLA-DPA1	LAG3	1,75934E-03	7427,1	1,23717E-02	5929	4,84199E-01	4729	1,40639E+00	2313	8,66926E-01	12956	0	11208,5
Responder	Macro_FOLR2+APOE+	Mono_INHBA	C1QB	LRP1	1,76516E-03	5115,1	1,33535E-02	5206	6,97437E-01	1873	9,23798E-01	5934	9,30108E-01	1354	0	11208,5
Responder	Macro_OLFML3	pDC_LILRA4	HGF	NRP1	1,76750E-03	16325,1	1,36211E-02	5062	4,35608E-01	5936	1,00827E+00	5017	6,34047E-01	54402	0	11208,5
Responder	pDC_LILRA4	Macro_NLRP3	CD99	PILRA	1,76806E-03	7539,5	8,92043E-03	10360	4,54440E-01	5428	1,68190E+00	1329	8,85992E-01	9372	0	11208,5
Responder	Mono_CD14	Macro_NLRP3	HP	ASGR1	1,76866E-03	11489,7	1,17162E-01	61	8,09423E-01	1207	1,18435E+00	3536	7,23127E-01	41436	0	11208,5
Responder	Mono_CD14	Macro_NLRP3	HP	ASGR2	1,76983E-03	15498,9	1,20595E-01	54	6,72441E-01	2092	9,43846E-01	5699	5,90968E-01	58441	0	11208,5
Responder	Macro_NLRP3	pDC_LILRA4	APP	RPSA	1,77011E-03	7458,1	8,74415E-03	10698	7,00945E-01	1846	2,10465E+00	581	8,66920E-01	12957	0	11208,5
Responder	Macro_OLFML3	cDC(CD1C)	HLA-DQA2	CD4	1,77216E-03	9084,5	7,80747E-03	12960	3,22612E-01	9882	1,03792E+00	4731	9,00776E-01	6641	0	11208,5
Responder	Mono_CD14	Macro_NLRP3	HP	CD163	1,77217E-03	8816,9	7,19695E-02	207	7,58041E-01	1489	1,14667E+00	3783	7,96988E-01	27397	0	11208,5
Responder	pDC_LILRA4	Macro_NLRP3	SELPLG	ITGB2	1,77284E-03	6550,1	9,85924E-03	8684	4,67873E-01	5094	1,77121E+00	1119	9,00743E-01	6645	0	11208,5
Responder	pDC_LILRA4	cDC_LAMP3	LCN2	FCGR2A	1,77451E-03	18800,6	2,23643E-02	2122	6,76023E-01	2063	1,68029E+00	1337	7,30896E-01	40077	0,998	48404
Responder	Mono_CD14	Macro_NLRP3	IL1B	IL1R2_IL1RAP	1,77568E-03	9288,3	1,42301E-02	4686	5,54929E-01	3479	1,28949E+00	2917	8,13227E-01	24151	0	11208,5
Responder	cDC_CLEC9A	Mono_CD16	HMGB1	CXCR4	1,77626E-03	6719,9	7,80594E-03	12966	5,79679E-01	3103	1,01988E+00	4894	9,29540E-01	1428	0	11208,5
Responder	cDC(CD1C)	Macro_OLFML3	LTB	CD40	1,77685E-03	11383,9	1,24300E-02	5870	4,35297E-01	5944	1,33036E+00	2695	7,77879E-01	31202	0	11208,5
Responder	Mono_INHBA	cDC(CD1C)	ICAM1	IL2RG	1,78175E-03	7303,3	1,14406E-02	6773	5,87347E-01	3001	1,35665E+00	2560	8,66812E-01	12974	0	11208,5
Responder	Macro_FOLR2+APOE+	Macro_IER3	APOE	LRP1	1,78389E-03	5165,5	1,40265E-02	4819	7,28547E-01	1652	9,22302E-01	5950	9,24743E-01	2198	0	11208,5
Responder	pDC_LILRA4	Macro_NLRP3	SERPINF1	PLXDC2	1,78450E-03	5523,3	1,39280E-02	4879	6,88847E-01	1946	2,43036E+00	279	8,86346E-01	9304	0	11208,5
Responder	Macro_ISG15	Mono_CD14	SERPING1	LRP1	1,78656E-03	10086,3	9,20333E-03	9831	3,01798E-01	10824	9,53550E-01	5587	8,66754E-01	12981	0	11208,5
Responder	cDC_CLEC9A	Macro_NLRP3	BTLA	TNFRSF14	1,79183E-03	7475,9	4,38224E-02	619	1,67508E+00	85	1,44676E+00	2129	8,17072E-01	23338	0	11208,5
Responder	Macro_IER3	Mono_CD14	TNF	TNFRSF1B	1,79183E-03	4998,9	2,23208E-02	2129	8,78427E-01	907	1,67973E+00	1338	8,85759E-01	9412	0	11208,5
Responder	Macro_OLFML3	Macro_FOLR2-APOE+	C1QB	C1QBP	1,79207E-03	9485,7	8,22982E-03	11871	4,30048E-01	6083	4,98024E-01	12989	9,07934E-01	5277	0	11208,5
Responder	Mono_CD14	Macro_NLRP3	VCAN	ITGA4	1,79213E-03	6837,1	1,45885E-02	4489	7,87848E-01	1310	1,46415E+00	2063	8,56207E-01	15115	0	11208,5
Responder	Mono_CD14	Macro_NLRP3	VCAN	SELL	1,79331E-03	6560,7	1,80145E-02	3036	8,30444E-01	1100	1,56548E+00	1690	8,53219E-01	15769	0	11208,5
Responder	Macro_OLFML3	Macro_NLRP3	C1QA	CD33	1,79346E-03	10770,5	7,79763E-03	12991	3,62183E-01	8288	5,47028E-01	11809	8,84907E-01	9556	0	11208,5
Responder	Macro_NLRP3	Macro_IER3	THBS1	ITGA4	1,79484E-03	7462,7	1,80237E-02	3031	6,80860E-01	2010	7,62066E-01	8071	8,66694E-01	12993	0	11208,5
Responder	Macro_NLRP3	Macro_ISG15	S100A8	TLR4	1,79622E-03	8965,7	1,39445E-02	4866	5,25067E-01	3945	5,46869E-01	11814	8,66689E-01	12995	0	11208,5
Responder	pDC_LILRA4	Macro_NLRP3	CIRBP	TREM1	1,79691E-03	6837,7	1,38127E-02	4947	5,07564E-01	4252	1,87190E+00	926	8,67438E-01	12855	0	11208,5
Responder	cDC_CLEC9A	Macro_LYVE1	SELPLG	ITGB2	1,80314E-03	11174,9	7,79543E-03	13005	3,07661E-01	10521	5,20797E-01	12436	8,89738E-01	8704	0	11208,5
Responder	Mono_CD14	Macro_NLRP3	RETN	TLR4	1,80395E-03	11491,9	3,60133E-02	883	5,91265E-01	2951	1,14930E+00	3765	7,38937E-01	38652	0	11208,5
Responder	pDC_LILRA4	Macro_NLRP3	HSP90B1	LRP1	1,80800E-03	6668,3	9,04513E-03	10105	5,15892E-01	4112	2,02318E+00	679	8,97530E-01	7237	0	11208,5
Responder	pDC_LILRA4	Macro_NLRP3	HSP90B1	TLR2	1,80869E-03	7046,9	9,69902E-03	8961	4,57210E-01	5366	1,99290E+00	723	8,88309E-01	8976	0	11208,5
Responder	pDC_LILRA4	Macro_NLRP3	HSP90B1	ASGR1	1,81008E-03	5537,7	1,58675E-02	3834	6,64369E-01	2156	2,24655E+00	412	8,82054E-01	10078	0	11208,5
Responder	pDC_LILRA4	cDC_CLEC9A	APP	RPSA	1,81287E-03	4436,9	1,66701E-02	3484	9,79046E-01	633	3,03960E+00	60	8,99945E-01	6799	0	11208,5
Responder	cDC(CD1C)	Mono_CD14	CIRBP	TREM1	1,81463E-03	7960,1	1,23144E-02	5976	4,37421E-01	5884	1,35914E+00	2543	8,60696E-01	14189	0	11208,5
Responder	pDC_LILRA4	pDC_LILRA4	SEMA4A	NRP1_PLXNA4	1,81676E-03	11236,9	2,22593E-02	2139	7,68169E-01	1413	2,11054E+00	572	7,26465E-01	40852	0	11208,5
Responder	Mono_CD14	Macro_NLRP3	NAMPT	INSR	1,81940E-03	8145,1	1,67149E-02	3463	7,60877E-01	1472	1,50923E+00	1880	8,20146E-01	22702	0	11208,5
Responder	Mono_CD14	Macro_LYVE1	VCAN	TLR2	1,82054E-03	6198,5	1,85438E-02	2876	7,67941E-01	1417	1,37856E+00	2461	8,66532E-01	13030	0	11208,5
Responder	Macro_OLFML3	cDC_CLEC9A	HLA-DPB1	LAG3	1,82124E-03	8313,3	1,29512E-02	5497	5,45043E-01	3619	7,53083E-01	8211	8,66530E-01	13031	0	11208,5

Responder	Macro_OLFML3	Macro_IFI27	APOE	LRP1	1,82194E-03	9738,7	8,61235E-03	11007	3,74691E-01	7814	4,96020E-01	13032	9,05914E-01	5632	0	11208,5
Responder	pDC_LILRA4	Macro_ISG15	LILRB4	LAIR1	1,82194E-03	9434,9	8,44929E-03	11388	2,60157E-01	13032	1,76228E+00	1145	8,80287E-01	10401	0	11208,5
Responder	Macro_OLFML3	Macro_NLRP3	A2M	LRP1	1,82263E-03	9229,3	1,35415E-02	5101	4,51009E-01	5526	5,71403E-01	11278	8,66524E-01	13033	0	11208,5
Responder	pDC_LILRA4	cDC_CLEC9A	SELPLG	ITGB2	1,82333E-03	7136,7	9,08419E-03	10027	4,09908E-01	6666	2,20427E+00	450	8,97022E-01	7332	0	11208,5
Responder	Mono_CD14	Macro_OLFML3	TIMP1	CD63	1,82333E-03	7389,7	7,78423E-03	13034	5,37486E-01	3733	7,13891E-01	8803	9,44801E-01	170	0	11208,5
Responder	cDC_LAMP3	pDC_LILRA4	HLA-DOB	CD4	1,82428E-03	7346,7	2,22486E-02	2142	7,23843E-01	1680	1,88186E+00	897	8,29248E-01	20806	0	11208,5
Responder	cDC(CD1C)	cDC_CLEC9A	TNFSF13B	CD40	1,82613E-03	9175,5	9,16017E-03	9903	3,23206E-01	9857	1,51203E+00	1871	8,66498E-01	13038	0	11208,5
Responder	pDC_LILRA4	cDC_CLEC9A	COPA	CD74	1,83104E-03	5486,7	8,15032E-03	12076	5,85707E-01	3026	2,48260E+00	239	9,34173E-01	884	0	11208,5
Responder	Mono_CD14	Macro_NLRP3	RETN	CAP1	1,83135E-03	9137,5	2,32086E-02	1995	4,48924E-01	5576	9,19450E-01	5973	8,28532E-01	20935	0	11208,5
Responder	Macro_IER3	Mono_INHBA	TNF	TNFRSF1B	1,83182E-03	4984,1	2,37759E-02	1916	9,22239E-01	779	1,44412E+00	2145	8,88915E-01	8872	0	11208,5
Responder	Macro_FOLR2+APOE-	cDC(CD1C)	F13A1	ITGA4	1,83434E-03	5647,5	2,22182E-02	2146	9,79148E-01	632	1,88472E+00	892	8,64980E-01	13359	0	11208,5
Responder	cDC_CLEC9A	pDC_LILRA4	MIF	CD74_CXCR4	1,83524E-03	8039,3	5,52653E-03	22325	4,31686E-01	6038	2,27762E+00	387	9,42997E-01	238	0	11208,5
Responder	pDC_LILRA4	Mono_CD16	SAA1	FPR2	1,83686E-03	11179,7	3,10551E-02	1144	6,65108E-01	2147	2,22993E+00	430	7,25715E-01	40969	0	11208,5
Responder	Macro_ISG15	Macro_FOLR2-APOE+	CXCL10	SDC4	1,83686E-03	10244,3	2,68644E-02	1523	6,76958E-01	2052	1,44385E+00	2147	7,62360E-01	34291	0	11208,5
Responder	Mono_CD14	cDC_CLEC9A	ITGB2	CD226	1,83734E-03	7301,9	2,84985E-02	1366	6,82823E-01	1992	1,25231E+00	3132	8,38632E-01	18811	0	11208,5
Responder	Macro_FOLR2-APOE+	Mono_CD14	APOE	LDLR	1,83807E-03	7890,7	2,17783E-02	2225	4,67626E-01	5105	7,77650E-01	7860	8,66442E-01	13055	0	11208,5
Responder	Mono_CD14	Macro_OLFML3	HBEGF	CD9	1,84089E-03	9298,1	1,44203E-02	4574	4,45894E-01	5651	5,38217E-01	11998	8,66428E-01	13059	0	11208,5
Responder	Macro_ISG15	Macro_LYVE1	SERPINA1	LRP1	1,84441E-03	7994,1	1,11185E-02	7084	4,86831E-01	4674	4,94950E-01	13064	9,15179E-01	3940	0	11208,5
Responder	pDC_LILRA4	Macro_FOLR2+APOE+	GAS6	MERTK	1,84444E-03	9221,5	2,22023E-02	2150	8,91936E-01	860	2,12009E+00	562	7,77218E-01	31327	0	11208,5
Responder	pDC_LILRA4	cDC(CD1C)	LTB	CD40	1,84697E-03	7198,9	2,21995E-02	2151	9,63406E-01	663	2,91277E+00	79	8,23948E-01	21893	0	11208,5
Responder	Mono_CD14	cDC_CLEC9A	THBS1	SCARB1	1,84697E-03	10792,7	1,75643E-02	3175	4,57820E-01	5351	1,34770E+00	2599	7,75617E-01	31630	0	11208,5
Responder	cDC(CD1C)	cDC_CLEC9A	HLA-DRB1	LAG3	1,84724E-03	7710,7	1,17593E-02	6469	4,60056E-01	5284	1,36344E+00	2524	8,66353E-01	13068	0	11208,5
Responder	Macro_FOLR2-APOE+	Mono_INHBA	C3	ITGAX	1,85148E-03	9982,5	1,23315E-02	5962	3,28618E-01	9615	4,94566E-01	13074	8,82218E-01	10053	0	11208,5
Responder	Mono_CD14	cDC_CLEC9A	ICAM3	ITGAL	1,85180E-03	9965,9	2,16555E-02	2246	6,13590E-01	2681	1,57063E+00	1673	7,73548E-01	32021	0	11208,5
Responder	Macro_FOLR2-APOE+	Mono_CD16	FN1	C5AR1	1,85219E-03	8179,5	1,97102E-02	2593	4,91693E-01	4568	4,94499E-01	13075	8,85488E-01	9453	0	11208,5
Responder	Mono_CD14	cDC_CLEC9A	IL1B	SIGIRR	1,85542E-03	7392,3	1,30136E-02	5455	5,56489E-01	3459	1,51293E+00	1865	8,56952E-01	14974	0	11208,5
Responder	Macro_ISG15	Mono_INHBA	CD14	ITGB1	1,85574E-03	9107,3	7,77096E-03	13080	3,93035E-01	7198	8,60591E-01	6720	8,97028E-01	7330	0	11208,5
Responder	pDC_LILRA4	Macro_NLRP3	APP	NOTCH2	1,85711E-03	5939,3	2,21866E-02	2155	9,98728E-01	584	2,37657E+00	320	8,54759E-01	15429	0	11208,5
Responder	Mono_INHBA	Macro_FOLR2+APOE+	CCL4	CCR5	1,85784E-03	11830,1	1,22640E-02	6012	5,68573E-01	3293	1,11320E+00	4075	7,60928E-01	34562	0	11208,5
Responder	Mono_CD14	cDC_CLEC9A	S100A8	CD68	1,85784E-03	8099,7	5,61647E-03	21763	7,07182E-01	1799	1,69447E+00	1301	9,12604E-01	4427	0	11208,5
Responder	Mono_CD14	cDC_CLEC9A	S100A9	CD68	1,86027E-03	11645,5	3,96060E-03	35023	5,53031E-01	3519	1,23893E+00	3206	9,07951E-01	5271	0	11208,5
Responder	Macro_OLFML3	Macro_OLFML3	FN1	ITGA4_ITGB1	1,86071E-03	8570,9	1,72155E-02	3290	4,50606E-01	5535	6,59297E-01	9734	8,66210E-01	13087	0	11208,5
Responder	Mono_CD14	cDC_CLEC9A	VCAN	ITGA4	1,86269E-03	6083,1	1,63686E-02	3611	8,17971E-01	1162	1,99269E+00	725	8,63150E-01	13709	0	11208,5
Responder	Mono_CD14	cDC_CLEC9A	VCAN	SELL	1,86390E-03	5924,1	2,04803E-02	2435	8,61742E-01	976	1,88342E+00	895	8,61072E-01	14106	0	11208,5
Responder	Mono_INHBA	cDC(CD1C)	VEGFA	ITGB1	1,86390E-03	8956,5	1,22609E-02	6017	5,43930E-01	3635	1,24121E+00	3191	8,29682E-01	20731	0	11208,5
Responder	pDC_LILRA4	pDC_LILRA4	TGFB1	CXCR4	1,86426E-03	8058,1	7,81457E-03	12940	4,60644E-01	5278	3,18832E+00	45	8,78061E-01	10819	0	11208,5
Responder	Macro_OLFML3	pDC_LILRA4	B2M	TFRC	1,86512E-03	8648,7	6,23873E-03	18478	4,32408E-01	6018	9,96380E-01	5142	9,23569E-01	2397	0	11208,5
Responder	Mono_CD14	cDC_CLEC9A	VCAN	TLR1	1,86633E-03	8364,7	1,60401E-02	3747	7,71237E-01	1393	2,02098E+00	685	8,10117E-01	24790	0	11208,5
Responder	Macro_OLFML3	Macro_LYVE1	FN1	ITGAV_ITGB1	1,86640E-03	7356,7	2,21249E-02	2166	5,18139E-01	4065	8,98013E-01	6249	8,66160E-01	13095	0	11208,5
Responder	pDC_LILRA4	Macro_IER3	APP	PTGER2	1,86984E-03	10404,1	2,21594E-02	2160	9,48141E-01	707	2,21486E+00	441	7,44953E-01	37504	0	11208,5
Responder	Mono_CD14	cDC_CLEC9A	ADM	RAMP1	1,87120E-03	13468,1	3,65189E-02	868	7,38762E-01	1590	1,40867E+00	2299	6,60166E-01	51375	0	11208,5
Responder	Mono_CD14	cDC_CLEC9A	ADM	CALCRL	1,87241E-03	13571,9	1,79553E-02	3064	4,87518E-01	4659	1,47367E+00	2017	6,91256E-01	46911	0	11208,5
Responder	Mono_CD14	cDC_CLEC9A	COPA	P2RY6	1,87485E-03	9683,7	1,61281E-02	3704	5,97049E-01	2871	1,51207E+00	1870	7,90089E-01	28765	0	11208,5
Responder	Macro_FOLR2+APOE-	Macro_OLFML3	CCL18	CCR1	1,87495E-03	8656,9	2,48630E-02	1758	6,63784E-01	2162	1,62794E+00	1486	8,00833E-01	26670	0	11208,5
Responder	Macro_NLRP3	cDC(CD1C)	VCAN	ITGB1	1,87497E-03	7240,3	1,30620E-02	5405	5,94063E-01	2913	1,17768E+00	3568	8,66110E-01	13107	0	11208,5
Responder	Mono_CD14	cDC_CLEC9A	CXCL2	DPP4	1,87607E-03	12231,7	2,96108E-02	1268	6,11001E-01	2713	1,33175E+00	2685	7,13043E-01	43284	0	11208,5
Responder	cDC_CLEC9A	Macro_FOLR2+APOE-	BTLA	TNFRSF14	1,87751E-03	8911,5	2,92432E-02	1296	1,55175E+00	104	1,44089E+00	2163	7,84889E-01	29786	0	11208,5
Responder	pDC_LILRA4	pDC_LILRA4	HMGB1	THBD	1,87998E-03	6419,7	1,14197E-02	6795	4,75459E-01	4924	3,02311E+00	61	8,87574E-01	9110	0	11208,5
Responder	Mono_CD14	cDC_CLEC9A	CALM3	MYLK	1,88095E-03	12519,5	3,89842E-02	770	7,82967E-01	1336	1,29484E+00	2882	6,94337E-01	46401	0	11208,5
Responder	Mono_INHBA	Macro_NLRP3	CCL3	CCR1	1,88095E-03	7438,1	1,22498E-02	6031	7,13114E-01	1749	1,32945E+00	2701	8,54385E-01	15501	0	11208,5
Responder	Mono_INHBA	Mono_CD14	ICAM1	ITGAL_ITGB2	1,88142E-03	10069,3	7,75761E-03	13116	3,55730E-01	8531	9,00792E-01	6212	8,75807E-01	11279	0	11208,5
Responder	Mono_INHBA	Mast	TIMP1	CD63	1,88429E-03	6946,7	7,75615E-03	13120	5,28095E-01	3891	8,90180E-01	6342	9,44707E-01	172	0	11208,5
Responder	Mono_CD14	cDC_CLEC9A	GNAI2	CXCR3	1,88462E-03	8610,3	1,99478E-02	2546	6,61786E-01	2180	1,23775E+00	3215	8,14353E-01	23902	0	11208,5
Responder	pDC_LILRA4	pDC_LILRA4	HSP90B1	TLR7	1,88572E-03	5692,5	2,14430E-02	2283	6,93503E-01	1911	3,07059E+00	56	8,66654E-01	13004	0	11208,5
Responder	Macro_OLFML3	Macro_FOLR2+APOE+	FN1	ITGA6	1,88585E-03	13705,9	2,59874E-02	1626	4,44972E-01	5676	9,14921E-01	6035	7,08968E-01	43984	0	11208,5
Responder	Macro_LYVE1	pDC_LILRA4	C1QB	C1QBP	1,88707E-03	5121,5	1,22440E-02	6036	5,48788E-01	3568	1,40112E+00	2347	9,23247E-01	2448	0	11208,5
Responder	Mast	Macro_NLRP3	ANXA1	FPR1	1,88830E-03	6011,5	1,22442E-02	6035	6,36984E-01	2429	9,14619E-01	6037	9,12980E-01	4348	0	11208,5
Responder	Mono_CD14	cDC_CLEC9A	RETN	CAP1	1,88952E-03	6681,5	2,99329E-02	1236	6,21383E-01	2588	1,78363E+00	1089	8,45858E-01	17286	0	11208,5
Responder	Mono_CD14	cDC_CLEC9A	TGFB1	LPP	1,89197E-03	7843,3	1,49751E-02	4264	6,23089E-01	2567	1,58497E+00	1623	8,35234E-01	19554	0	11208,5
Responder	Macro_NLRP3	cDC_CLEC9A	VCAN	ITGB1	1,89220E-03	7395,5	1,30192E-02	5449	5,92846E-01	2929	1,08898E+00	4260	8,65920E-01	13131	0	11208,5
Responder	Mono_CD14	pDC_LILRA4	APP	TNFRSF21	1,89443E-03	9707,7	1,83406E-02	2940	9,66666E-01	654	1,92888E+00	826	7,69054E-01	32910	0	11208,5
Responder	pDC_LILRA4	Macro_IFI27	APP	RPSA	1,89652E-03	6682,9	9,88921E-03	8641	7,82745E-01	1340	2,15384E+00	528	8,73859E-01	11697	0	11208,5
Responder	pDC_LILRA4	Macro_IFI27	APP	APLP2	1,89797E-03	6356,9	1,25549E-02	5779	7,69104E-01	1405	2,08190E+00	606	8,67732E-01	12786	0	11208,5
Responder	Mono_CD14	pDC_LILRA4	ICAM1	SPN	1,89812E-03	7928,3	1,61301E-02	3702	6,29289E-01	2505	1,96153E+00	774	8,26059E-01	21452	0	11208,5
Responder	Macro_OLFML3	Macro_NLRP3	GRN	TNFRSF1B	1,90014E-03	8133,1	8,68748E-03	10835	5,69609E-01	3277	4,92188E-01	13142	9,24730E-01	2203	0	11208,5

Responder	Macro_OLFML3	Mono_INHBA	B2M	LILRB2	1,90181E-03	11135,9	6,12731E-03	19012	4,25987E-01	6183	2,87698E-01	18941	9,40951E-01	335	0	11208,5
Responder	Mono_CD14	pDC_LILRA4	THBS1	CD36	1,90181E-03	8645,5	1,45291E-02	4522	4,57841E-01	5350	1,55662E+00	1722	8,31104E-01	20425	0	11208,5
Responder	Macro_OLFML3	cDC(CD1C)	HLA-DQB1	CD4	1,90181E-03	8116,3	6,65206E-03	16742	4,31474E-01	6048	1,05933E+00	4515	9,25427E-01	2068	0	11208,5
Responder	Mono_INHBA	Macro_FOLR2+APOE+	VEGFA	NRP1	1,90304E-03	11425,7	1,63276E-02	3628	6,57658E-01	2231	9,14004E-01	6049	7,63604E-01	34012	0	11208,5
Responder	Mono_CD14	pDC_LILRA4	THBS1	CD47	1,90304E-03	7220,9	1,37663E-02	4975	5,03906E-01	4323	1,83836E+00	985	8,58705E-01	14613	0	11208,5
Responder	Mono_CD14	cDC(CD1C)	HBEGF	CD44	1,90375E-03	10239,3	7,74733E-03	13147	3,03670E-01	10716	1,06915E+00	4429	8,73864E-01	11696	0	11208,5
Responder	pDC_LILRA4	Macro_IFI27	SELPLG	ITGB2	1,90520E-03	6571,1	9,82403E-03	8738	4,65240E-01	5176	1,80026E+00	1058	9,00583E-01	6675	0	11208,5
Responder	Mono_INHBA	cDC_CLEC9A	VEGFA	ITGB1	1,90551E-03	9083,3	1,22207E-02	6051	5,42714E-01	3650	1,15250E+00	3731	8,29450E-01	20776	0	11208,5
Responder	cDC_CLEC9A	Mast	LGALS9	CD44	1,90593E-03	7957,1	7,74620E-03	13150	4,75719E-01	4914	1,00910E+00	5008	9,06724E-01	5505	0	11208,5
Responder	pDC_LILRA4	Macro_IFI27	SERPINF1	PLXDC2	1,90883E-03	4961,7	1,59845E-02	3773	7,48893E-01	1524	2,51969E+00	220	8,93100E-01	8083	0	11208,5
Responder	cDC_CLEC9A	Macro_LYVE1	SERPINF1	PLXDC2	1,90883E-03	9850,5	9,53398E-03	9227	3,25814E-01	9737	9,24205E-01	5926	8,65812E-01	13154	0	11208,5
Responder	Macro_IFI27	cDC_CLEC9A	C1QB	C1QBP	1,91168E-03	5669,3	1,28746E-02	5540	5,59848E-01	3403	9,13671E-01	6056	9,25007E-01	2139	0	11208,5
Responder	Macro_ISG15	Macro_LYVE1	CD14	ITGB1	1,91319E-03	10303,9	7,74258E-03	13160	3,91136E-01	7255	5,17139E-01	12517	8,96859E-01	7379	0	11208,5
Responder	Mono_CD14	pDC_LILRA4	SELPLG	SELL	1,91416E-03	7301,1	1,45604E-02	4504	6,90917E-01	1930	2,26434E+00	400	8,40464E-01	18463	0	11208,5
Responder	Mono_INHBA	Macro_FOLR2-APOE+	SPP1	ITGAV_ITGB1	1,91664E-03	9325,9	1,32139E-02	5304	4,30897E-01	6060	1,02490E+00	4853	8,36911E-01	19204	0	11208,5
Responder	Macro_FOLR2-APOE+	Macro_LYVE1	THBS1	LRP1	1,91974E-03	7882,3	1,45839E-02	4491	6,04187E-01	2792	7,84355E-01	7751	8,65775E-01	13169	0	11208,5
Responder	Mono_CD14	pDC_LILRA4	S100A8	ITGB2	1,92035E-03	6281,3	7,26343E-03	14588	6,71000E-01	2105	2,59077E+00	181	9,18583E-01	3324	0	11208,5
Responder	pDC_LILRA4	Mono_CD16	SEMA7A	PLXNC1	1,92133E-03	11690,1	2,63895E-02	1580	7,06803E-01	1801	1,43690E+00	2180	7,21808E-01	41681	0	11208,5
Responder	Macro_IER3	pDC_LILRA4	TNF	TRADD	1,92133E-03	8499,7	2,20234E-02	2180	7,44340E-01	1554	2,15654E+00	525	7,98728E-01	27031	0	11208,5
Responder	Macro_NLRP3	Mono_CD14	GNAI2	FPR1	1,92265E-03	10310,5	7,73909E-03	13173	3,24268E-01	9800	7,83250E-01	7774	8,84674E-01	9597	0	11208,5
Responder	Mono_CD14	pDC_LILRA4	S100A9	ITGB2	1,92408E-03	8994,9	5,12200E-03	25018	5,16848E-01	4093	2,13524E+00	545	9,14218E-01	4110	0	11208,5
Responder	Macro_NLRP3	Macro_FOLR2+APOE+	S100A9	ITGB2	1,92704E-03	6736,5	1,13839E-02	6819	6,66217E-01	2136	4,90435E-01	13179	9,40788E-01	340	0	11208,5
Responder	pDC_LILRA4	Macro_IER3	LTB	TNFRSF1A	1,92914E-03	6758,9	2,20201E-02	2183	9,53749E-01	686	2,36460E+00	325	8,36114E-01	19392	0	11208,5
Responder	Mono_INHBA	Mono_CD16	TNF	TNFRSF1B	1,92923E-03	7623,3	1,54371E-02	4026	6,48176E-01	2320	8,11811E-01	7380	8,65734E-01	13182	0	11208,5
Responder	Mono_CD14	pDC_LILRA4	VCAN	ITGA4	1,93030E-03	6168,1	1,56319E-02	3948	8,05505E-01	1224	2,52436E+00	217	8,60407E-01	14243	0	11208,5
Responder	cDC_CLEC9A	Macro_LYVE1	IL16	CD4	1,93143E-03	6611,5	1,86671E-02	2846	6,75545E-01	2068	1,15045E+00	3750	8,65728E-01	13185	0	11208,5
Responder	pDC_LILRA4	Mono_CD16	CD99	PILRA	1,93279E-03	5054,5	1,30038E-02	5466	7,21768E-01	1693	1,92503E+00	832	9,03687E-01	6073	0	11208,5
Responder	Mono_CD14	pDC_LILRA4	VEGFA	NRP1	1,93653E-03	12455,3	1,27140E-02	5654	4,91369E-01	4577	1,38083E+00	2448	7,40288E-01	38389	0	11208,5
Responder	pDC_LILRA4	Macro_LYVE1	CD99	CD81	1,93729E-03	5836,5	1,11571E-02	7044	6,50493E-01	2302	1,70972E+00	1266	8,96908E-01	7362	0	11208,5
Responder	pDC_LILRA4	Macro_LYVE1	CD99	PILRA	1,93876E-03	6706,9	1,00870E-02	8372	5,30810E-01	3846	1,51943E+00	1841	8,92053E-01	8267	0	11208,5
Responder	Mono_CD14	pDC_LILRA4	COPA	P2RY6	1,93903E-03	9685,1	1,55072E-02	3995	5,67083E-01	3318	2,17159E+00	492	7,86815E-01	29412	0	11208,5
Responder	cDC_LAMP3	cDC_CLEC9A	CXCL2	XCR1	1,93957E-03	7911,1	3,03062E-02	1201	2,36149E+00	16	1,43603E+00	2187	8,09399E-01	24943	0	11208,5
Responder	Mono_INHBA	Mast	INHBA	ACTR2	1,94028E-03	9331,1	1,21801E-02	6079	5,70878E-01	3256	1,24499E+00	3167	8,18919E-01	22945	0	11208,5
Responder	pDC_LILRA4	Mono_INHBA	APP	TNFRSF21	1,94219E-03	8793,9	2,19824E-02	2188	1,02068E+00	532	2,50743E+00	227	7,84746E-01	29814	0	11208,5
Responder	pDC_LILRA4	Macro_LYVE1	SELPLG	ITGB2	1,94465E-03	7072,7	9,45529E-03	9373	4,37662E-01	5881	1,50351E+00	1909	8,98857E-01	6992	0	11208,5
Responder	pDC_LILRA4	pDC_LILRA4	NUCB2	ERAP1	1,94480E-03	10164,9	2,59846E-02	1628	6,61266E-01	2189	2,59138E+00	180	7,54948E-01	35619	0	11208,5
Responder	pDC_LILRA4	Macro_FOLR2+APOE+	APP	NCSTN	1,94480E-03	7806,5	2,19780E-02	2189	9,48485E-01	706	2,16094E+00	515	8,11926E-01	24414	0	11208,5
Responder	pDC_LILRA4	Macro_LYVE1	LILRB4	LAIR1	1,94612E-03	9081,5	8,86323E-03	10465	2,86266E-01	11597	1,43792E+00	2178	8,82785E-01	9959	0	11208,5
Responder	Mono_CD14	pDC_LILRA4	PSEN1	NOTCH4	1,94654E-03	14128,9	2,91220E-02	1312	5,67414E-01	3310	1,80887E+00	1045	6,39819E-01	53769	0	11208,5
Responder	Mono_CD14	pDC_LILRA4	NAMPT	INSR	1,94905E-03	7327,7	1,95380E-02	2628	8,02571E-01	1238	1,74223E+00	1191	8,31371E-01	20373	0	11208,5
Responder	Mono_INHBA	Macro_FOLR2+APOE+	CXCL8	SDC3	1,94905E-03	8659,3	1,62890E-02	3635	5,73020E-01	3225	9,11358E-01	6086	8,37146E-01	19142	0	11208,5
Responder	Macro_ISG15	Mono_CD14	S100A8	ITGB2	1,95276E-03	10321,1	7,72450E-03	13214	2,63716E-01	12825	5,63990E-01	11440	9,20855E-01	2918	0	11208,5
Responder	pDC_LILRA4	Mono_CD14	HSP90B1	ASGR1	1,95350E-03	7029,5	1,17642E-02	6463	5,27243E-01	3907	2,32249E+00	354	8,65579E-01	13215	0	11208,5
Responder	Mono_INHBA	Macro_ISG15	HBEGF	CD44	1,95572E-03	10944,9	8,52720E-03	11214	3,58190E-01	8441	4,88648E-01	13218	8,79056E-01	10643	0	11208,5
Responder	cDC(CD1C)	cDC(CD1C)	TNFSF13B	HLA-DPB1	1,95659E-03	7334,3	6,25963E-03	18378	4,29826E-01	6092	1,95640E+00	783	9,43601E-01	210	0	11208,5
Responder	pDC_LILRA4	Macro_LYVE1	CALR	LRP1	1,95720E-03	5681,5	1,07095E-02	7557	5,59656E-01	3405	1,74334E+00	1190	9,09148E-01	5047	0	11208,5
Responder	Macro_NLRP3	pDC_LILRA4	PSEN1	NOTCH4	1,95793E-03	12701,7	4,25677E-02	648	6,60974E-01	2194	1,75238E+00	1165	6,82303E-01	48293	0	11208,5
Responder	pDC_LILRA4	Macro_LYVE1	SERPINF1	PLXDC2	1,95794E-03	5241,3	1,49644E-02	4274	7,19109E-01	1708	2,33690E+00	345	8,89911E-01	8671	0	11208,5
Responder	cDC_LAMP3	cDC_LAMP3	CCL19	CCRL2	1,96056E-03	10801,1	3,22525E-02	1075	1,76748E+00	67	1,43452E+00	2195	7,34579E-01	39460	0	11208,5
Responder	cDC(CD1C)	Macro_ISG15	C3	IFITM1	1,96090E-03	7544,7	1,18797E-02	6368	4,61702E-01	5252	1,57267E+00	1670	8,65523E-01	13225	0	11208,5
Responder	pDC_LILRA4	cDC_LAMP3	SPON2	ITGB1	1,96319E-03	11023,1	2,32816E-02	1982	7,63648E-01	1447	1,43447E+00	2196	7,40863E-01	38282	0	11208,5
Responder	Macro_ISG15	Macro_LYVE1	LRPAP1	LRP1	1,96387E-03	9906,7	1,07289E-02	7528	4,95632E-01	4491	4,94494E-01	13077	8,65501E-01	13229	0	11208,5
Responder	cDC(CD1C)	Macro_OLFML3	HLA-DMB	CD4	1,96387E-03	9106,5	7,71954E-03	13229	3,67922E-01	8061	8,33372E-01	7084	9,04315E-01	5950	0	11208,5
Responder	Macro_OLFML3	Macro_OLFML3	LGALS9	MRC2	1,96853E-03	18915,9	1,24070E-02	5896	2,98903E-01	10951	-7,25205E-03	30475	7,52648E-01	36049	0	11208,5
Responder	Mono_INHBA	Macro_LYVE1	CALR	LRP1	1,96981E-03	9171,5	9,24697E-03	9755	4,54787E-01	5418	4,87839E-01	13237	9,02898E-01	6239	0	11208,5
Responder	Macro_FOLR2-APOE+	cDC_CLEC9A	LGALS9	HAVCR2	1,97056E-03	10656,3	7,71732E-03	13238	2,98737E-01	10957	1,02283E+00	4871	8,66643E-01	13007	0	11208,5
Responder	cDC(CD1C)	Macro_OLFML3	HLA-DQA2	CD4	1,97801E-03	10024,1	7,71362E-03	13248	2,88680E-01	11467	8,06424E-01	7458	9,00234E-01	6739	0	11208,5
Responder	Mono_CD14	cDC(CD1C)	S100A8	CD36	1,97932E-03	6914,1	1,01606E-02	8277	1,09283E+00	402	2,47931E+00	241	8,59486E-01	14442	0	11208,5
Responder	Mono_CD14	Macro_IFI27	S100A8	TLR4	1,98060E-03	6269,7	1,29627E-02	5488	1,07148E+00	440	2,30381E+00	374	8,62414E-01	13838	0	11208,5
Responder	Macro_OLFML3	cDC_CLEC9A	MDK	NCL	1,98474E-03	7660,3	1,85363E-02	2880	4,09162E-01	6693	1,08863E+00	4263	8,65367E-01	13257	0	11208,5
Responder	Mono_CD14	Macro_IFI27	CXCL8	SDC3	1,98822E-03	7015,5	1,83941E-02	2924	6,55890E-01	2254	1,71004E+00	1264	8,45262E-01	17427	0	11208,5
Responder	Macro_FOLR2-APOE+	Mast	TIMP2	CD44	1,98924E-03	9520,1	9,18851E-03	9853	5,17153E-01	4087	4,86946E-01	13263	8,87104E-01	9189	0	11208,5
Responder	Mono_CD14	Macro_IFI27	VCAN	TLR2	1,98950E-03	6984,1	1,52592E-02	4122	7,15258E-01	1737	1,38167E+00	2444	8,54851E-01	15409	0	11208,5
Responder	Macro_FOLR2+APOE+	pDC_LILRA4	PLAU	IGF2R	1,98965E-03	8867,3	2,18914E-02	2206	6,80063E-01	2022	1,66243E+00	1386	7,96442E-01	27514	0	11208,5
Responder	pDC_LILRA4	Macro_LYVE1	HSP90B1	TLR4	1,99149E-03	7090,7	1,10561E-02	7169	5,30237E-01	3855	1,87063E+00	931	8,70386E-01	12290	0	11208,5

Responder	Mono_CD14	cDC_CLEC9A	NAMPT	ITGA5_ITGB1	1,99449E-03	8394,9	7,83020E-03	12898	6,16724E-01	2646	1,49251E+00	1952	8,65299E-01	13270	0	11208,5
Responder	Mono_CD14	Macro_IFI27	VCAN	TLR1	1,99460E-03	8554,7	1,61817E-02	3676	7,72765E-01	1387	1,51799E+00	1844	8,10792E-01	24658	0	11208,5
Responder	cDC_CLEC9A	pDC_LILRA4	LGALS9	CD47	1,99599E-03	8732,9	8,70247E-03	10795	3,81893E-01	7568	1,93206E+00	821	8,65293E-01	13272	0	11208,5
Responder	cDC(CD1C)	Mono_CD16	ICAM3	ITGAL	1,99715E-03	12012,7	1,75234E-02	3187	5,31291E-01	3838	9,07371E-01	6124	7,54470E-01	35706	0	11208,5
Responder	Mono_CD14	Macro_FOLR2-APOE+	IL1B	IL1R1_IL1RAP	2,00098E-03	11800,9	1,21239E-02	6127	5,22701E-01	3991	1,18018E+00	3553	7,63101E-01	34125	0	11208,5
Responder	Macro_ISG15	cDC_LAMP3	CXCL10	SDC4	2,00564E-03	9309,5	3,34990E-02	1009	7,23412E-01	1682	1,43136E+00	2212	7,81771E-01	30436	0	11208,5
Responder	Macro_OLFML3	Macro_LYVE1	CALR	LRP1	2,00610E-03	7855,5	1,21206E-02	6131	6,60836E-01	2195	3,96564E-01	15620	9,14132E-01	4123	0	11208,5
Responder	cDC_CLEC9A	Macro_FOLR2+APOE+	LGALS9	HAVCR2	2,00730E-03	11894,5	7,69872E-03	13287	2,94811E-01	11157	5,98811E-01	10783	8,66503E-01	13037	0	11208,5
Responder	Macro_OLFML3	Macro_ISG15	HLA-DQB1	LAG3	2,00957E-03	7287,5	1,73909E-02	3231	6,50170E-01	2303	8,86002E-01	6405	8,65190E-01	13290	0	11208,5
Responder	Mono_INHBA	Macro_IER3	CCL3L1	CCR1	2,00995E-03	10412,7	1,21153E-02	6134	5,32162E-01	3832	1,04362E+00	4689	8,03143E-01	26200	0	11208,5
Responder	Mono_CD14	Macro_NLRP3	GNAI2	FPR1	2,01032E-03	10370,9	7,69714E-03	13291	3,12145E-01	10330	8,12375E-01	7369	8,84397E-01	9656	0	11208,5
Responder	Mono_CD14	Macro_IER3	CD55	ADGRE2	2,01184E-03	6487,3	1,95249E-02	2629	7,42812E-01	1561	1,15125E+00	3745	8,65189E-01	13293	0	11208,5
Responder	Macro_OLFML3	Mono_CD16	HLA-F	LILRB2	2,01486E-03	7609,9	1,30514E-02	5417	7,41103E-01	1576	4,85008E-01	13297	9,01224E-01	6551	0	11208,5
Responder	Mono_INHBA	Mono_CD16	VCAN	ITGB1	2,01486E-03	9184,9	1,28529E-02	5558	4,99885E-01	4403	5,63265E-01	11458	8,65172E-01	13297	0	11208,5
Responder	Macro_IFI27	Macro_OLFML3	APOE	TREM2	2,01634E-03	4974,9	2,31863E-02	2003	6,59181E-01	2216	6,98194E-01	9075	9,40091E-01	372	0	11208,5
Responder	Macro_OLFML3	Macro_ISG15	HLA-DQA1	LAG3	2,01866E-03	7486,7	1,75532E-02	3176	6,43928E-01	2366	8,11771E-01	7381	8,65165E-01	13302	0	11208,5
Responder	pDC_LILRA4	cDC_LAMP3	SERPINF1	PLXDC2	2,01866E-03	7188,7	9,98205E-03	8517	5,73631E-01	3214	2,28372E+00	381	8,68458E-01	12623	0	11208,5
Responder	Macro_FOLR2+APOE+	Mono_CD14	RPS19	C5AR1	2,01895E-03	5040,9	1,26570E-02	5704	8,07712E-01	1215	9,06458E-01	6141	9,33670E-01	936	0	11208,5
Responder	Macro_FOLR2+APOE+	Macro_IFI27	APOE	SORL1	2,02024E-03	5199,7	1,54668E-02	4013	7,07346E-01	1795	9,06418E-01	6142	9,21195E-01	2840	0	11208,5
Responder	Macro_OLFML3	Macro_FOLR2+APOE+	C1QB	CD33	2,02093E-03	8953,9	1,04963E-02	7819	4,49617E-01	5560	4,84680E-01	13305	8,99513E-01	6877	0	11208,5
Responder	Macro_FOLR2-APOE+	Mono_CD14	MMP12	PLAUR	2,02171E-03	6091,9	2,18182E-02	2218	6,96008E-01	1887	1,93808E+00	813	8,60018E-01	14333	0	11208,5
Responder	Macro_OLFML3	cDC_CLEC9A	HLA-DQB1	LAG3	2,02410E-03	8018,5	1,46928E-02	4410	5,89316E-01	2969	9,06086E-01	6145	8,55052E-01	15360	0	11208,5
Responder	Macro_NLRP3	Macro_NLRP3	S100A9	CD68	2,02473E-03	7554,1	9,63338E-03	9066	5,37345E-01	3737	4,84438E-01	13310	9,38963E-01	449	0	11208,5
Responder	cDC(CD1C)	cDC_LAMP3	TNFSF13B	CD40	2,02626E-03	10124,9	8,94684E-03	10314	3,10241E-01	10410	9,73096E-01	5380	8,65130E-01	13312	0	11208,5
Responder	Macro_NLRP3	Macro_FOLR2-APOE+	S100A9	ITGB2	2,02930E-03	7796,3	9,45391E-03	9379	5,04475E-01	4312	4,84252E-01	13316	9,35397E-01	766	0	11208,5
Responder	Mono_CD14	Macro_LYVE1	CD55	CR1	2,03315E-03	9591,3	1,79293E-02	3072	6,55373E-01	2264	1,06547E+00	4467	7,99294E-01	26945	0	11208,5
Responder	pDC_LILRA4	Mono_CD16	HLA-B	LILRA1	2,03540E-03	6146,9	1,63737E-02	3608	3,65342E-01	8163	1,65022E+00	1415	9,02329E-01	6340	0	11208,5
Responder	pDC_LILRA4	Mono_CD16	HLA-B	LILRB2	2,03617E-03	5134,1	9,36746E-03	9531	5,38407E-01	3720	1,80272E+00	1055	9,45201E-01	156	0	11208,5
Responder	pDC_LILRA4	Mono_CD16	HLA-A	LILRB2	2,03693E-03	7147,5	8,18900E-03	11974	3,20100E-01	9972	1,46059E+00	2078	9,38023E-01	505	0	11208,5
Responder	Macro_NLRP3	Macro_FOLR2+APOE+	ADAM10	GPNMB	2,03770E-03	7107,1	1,80204E-02	3035	8,43466E-01	1041	8,44475E-01	6924	8,65092E-01	13327	0	11208,5
Responder	cDC_CLEC9A	Macro_FOLR2-APOE+	BTLA	TNFRSF14	2,03786E-03	7068,1	4,98310E-02	476	1,72591E+00	73	1,42703E+00	2224	8,26480E-01	21359	0	11208,5
Responder	cDC(CD1C)	cDC_CLEC9A	LGALS9	HAVCR2	2,03999E-03	10050,9	7,68037E-03	13330	2,96106E-01	11081	1,59984E+00	1570	8,66365E-01	13065	0	11208,5
Responder	pDC_LILRA4	Mono_CD16	APP	APLP2	2,04152E-03	5527,5	1,53480E-02	4071	8,53956E-01	1011	2,03176E+00	665	8,78839E-01	10682	0	11208,5
Responder	cDC(CD1C)	cDC_CLEC9A	CD52	SIGLEC10	2,04305E-03	10461,3	7,67948E-03	13334	2,88693E-01	11466	1,19261E+00	3480	8,67608E-01	12818	0	11208,5
Responder	Macro_OLFML3	Mono_CD16	SAA1	FPR2	2,04327E-03	13677,5	3,03002E-02	1202	6,58085E-01	2226	5,24939E-01	12345	7,23259E-01	41406	0	11208,5
Responder	Macro_NLRP3	pDC_LILRA4	VEGFA	NRP1	2,04597E-03	9117,7	2,29540E-02	2036	6,95703E-01	1890	1,42657E+00	2227	7,92960E-01	28227	0	11208,5
Responder	pDC_LILRA4	Mono_CD16	HLA-F	LILRB2	2,04842E-03	5672,7	1,17603E-02	6468	6,78974E-01	2036	1,74017E+00	1201	8,96490E-01	7450	0	11208,5
Responder	Macro_FOLR2+APOE+	Macro_ISG15	C3	IFITM1	2,04872E-03	5100,7	2,50061E-02	1741	7,12853E-01	1751	1,04960E+00	4639	9,03269E-01	6164	0	11208,5
Responder	pDC_LILRA4	Mono_CD16	B2M	LILRB2	2,05073E-03	5447,7	9,21618E-03	9807	4,76786E-01	4893	1,69920E+00	1289	9,51322E-01	41	0	11208,5
Responder	pDC_LILRA4	Mono_CD16	SELPLG	ITGB2	2,05226E-03	7419,7	9,06683E-03	10064	4,08609E-01	6713	1,54764E+00	1761	8,96934E-01	7352	0	11208,5
Responder	pDC_LILRA4	Macro_IER3	LTB	CD40	2,05410E-03	7323,7	2,17492E-02	2230	9,57496E-01	678	2,39404E+00	309	8,22457E-01	22193	0	11208,5
Responder	Macro_FOLR2-APOE+	Mast	IL1B	ADRB2	2,05611E-03	7076,3	3,01897E-02	1211	7,71925E-01	1390	7,52461E-01	8221	8,65019E-01	13351	0	11208,5
Responder	Mast	Mono_CD16	TIMP3	CD44	2,05837E-03	4547,9	5,55031E-02	363	1,08733E+00	410	1,17077E+00	3609	8,98054E-01	7149	0	11208,5
Responder	Mono_INHBA	cDC_LAMP3	ICAM1	IL2RA	2,05915E-03	12494,5	1,40941E-02	4780	4,79615E-01	4834	9,04212E-01	6172	7,55769E-01	35478	0	11208,5
Responder	Macro_FOLR2+APOE-	Mono_CD14	C1QA	CD93	2,06046E-03	6013,9	1,37727E-02	4972	5,85770E-01	3024	9,04117E-01	6173	9,11096E-01	4692	0	11208,5
Responder	pDC_LILRA4	Mono_CD16	SERPINF1	PLXDC2	2,06151E-03	7312,3	9,84119E-03	8716	5,69518E-01	3280	2,13159E+00	548	8,67644E-01	12809	0	11208,5
Responder	pDC_LILRA4	Macro_FOLR2-APOE+	SEMA7A	PLXNC1	2,06225E-03	11628,5	2,67779E-02	1530	7,11014E-01	1769	1,42578E+00	2233	7,23273E-01	41402	0	11208,5
Responder	Macro_FOLR2-APOE+	cDC_LAMP3	SPP1	CD44	2,06460E-03	9802,9	1,02080E-02	8206	3,77852E-01	7705	4,82338E-01	13362	8,90729E-01	8533	0	11208,5
Responder	cDC(CD1C)	Macro_OLFML3	TNFSF13B	CD40	2,06691E-03	10350,5	8,91880E-03	10364	3,08537E-01	10479	8,91206E-01	6336	8,64946E-01	13365	0	11208,5
Responder	Mono_CD16	pDC_LILRA4	VCAN	SELL	2,07316E-03	6022,5	2,17117E-02	2237	7,54207E-01	1505	1,55372E+00	1732	8,64527E-01	13430	0	11208,5
Responder	Mono_CD14	Macro_LYVE1	CXCL8	SDC2	2,08013E-03	8591,9	2,07747E-02	2391	7,11843E-01	1761	1,55531E+00	1728	8,04703E-01	25871	0	11208,5
Responder	Macro_FOLR2-APOE+	Mast	GNAS	ADRB2	2,08398E-03	6952,3	2,64960E-02	1562	7,34980E-01	1614	8,40559E-01	6990	8,64813E-01	13387	0	11208,5
Responder	cDC(CD1C)	Mono_INHBA	CD1D	LILRB2	2,08408E-03	9613,7	1,53143E-02	4093	4,25647E-01	6191	1,12665E+00	3961	8,20470E-01	22615	0	11208,5
Responder	Mono_CD14	Macro_LYVE1	VCAN	ITGA4	2,08408E-03	7507,7	1,30945E-02	5378	7,62566E-01	1454	1,28588E+00	2929	8,49427E-01	16569	0	11208,5
Responder	Mono_INHBA	cDC_CLEC9A	ICAM1	ITGAL_ITGB2	2,08554E-03	9444,1	7,65497E-03	13389	4,06987E-01	6762	1,07009E+00	4422	8,75080E-01	11439	0	11208,5
Responder	Mono_CD14	Macro_LYVE1	VCAN	TLR1	2,08672E-03	7845,5	1,89220E-02	2777	8,02321E-01	1240	1,52795E+00	1817	8,22501E-01	22185	0	11208,5
Responder	Macro_OLFML3	Macro_FOLR2+APOE+	HLA-DRB1	LAG3	2,08710E-03	8048,1	1,64599E-02	3575	6,33186E-01	2466	4,81419E-01	13391	8,84651E-01	9600	0	11208,5
Responder	Macro_OLFML3	Macro_LYVE1	C1QA	CR1	2,09178E-03	8096,3	1,62016E-02	3665	6,05984E-01	2772	6,75678E-01	9439	8,64742E-01	13397	0	11208,5
Responder	Macro_IER3	Macro_NLRP3	PLAU	PLAUR	2,09333E-03	4905,5	2,27318E-02	2070	9,50628E-01	699	1,07786E+00	4352	9,03088E-01	6198	0	11208,5
Responder	pDC_LILRA4	Mast	LGALS1	CD69	2,09490E-03	7589,3	7,90810E-03	12682	4,64983E-01	5183	1,81170E+00	1039	8,94501E-01	7834	0	11208,5
Responder	Macro_IFI27	pDC_LILRA4	HMGB1	TLR9	2,09846E-03	6470,3	5,24564E-02	414	1,18196E+00	291	1,12854E+00	3948	8,49768E-01	16490	0	11208,5
Responder	Macro_FOLR2+APOE+	pDC_LILRA4	EFNA4	EPHA2	2,10057E-03	15732,3	5,91592E-02	318	7,66578E-01	1426	1,42305E+00	2247	3,90972E-01	63462	0	11208,5
Responder	Macro_OLFML3	Macro_OLFML3	LILRB4	LAIR1	2,10260E-03	8292,7	1,35633E-02	5086	5,74010E-01	3208	3,91698E-01	15756	9,03069E-01	6205	0	11208,5
Responder	Mono_CD14	Macro_LYVE1	RETN	TLR4	2,10525E-03	10678,5	4,38841E-02	618	6,65136E-01	2146	1,08926E+00	4257	7,57547E-01	35163	0	11208,5
Responder	Macro_OLFML3	cDC(CD1C)	HLA-DQA1	CD4	2,10658E-03	8250,5	6,71410E-03	16514	4,25232E-01	6208	9,85096E-01	5252	9,25412E-01	2070	0	11208,5

Responder	cDC_CLEC9A	Mono_CD16	LGALS9	PTPRC	2,10665E-03	8567,1	7,64483E-03	13416	4,45028E-01	5675	8,80204E-01	6480	9,03780E-01	6056	0	11208,5
Responder	Macro_LYVE1	cDC_CLEC9A	C1QB	C1QBP	2,11057E-03	5155,5	1,44290E-02	4565	6,54893E-01	2268	9,00949E-01	6211	9,28866E-01	1525	0	11208,5
Responder	pDC_LILRA4	Macro_IER3	APP	RPSA	2,11058E-03	4530,7	1,68957E-02	3392	9,85578E-01	614	1,97514E+00	755	9,00548E-01	6684	0	11208,5
Responder	Macro_OLFML3	Mono_CD14	MDK	NOTCH2	2,11190E-03	9772,9	2,03282E-02	2466	4,25160E-01	6212	1,03891E+00	4723	8,12759E-01	24255	0	11208,5
Responder	Macro_OLFML3	cDC(CD1C)	C3	C3AR1	2,11215E-03	9279,1	9,76314E-03	8846	3,31720E-01	9471	1,19687E+00	3447	8,64575E-01	13423	0	11208,5
Responder	pDC_LILRA4	Macro_IER3	APP	APLP2	2,11294E-03	5252,1	1,64796E-02	3564	8,88334E-01	876	2,06118E+00	623	8,82576E-01	9989	0	11208,5
Responder	Macro_FOLR2+APOE-	Macro_NLRP3	F13A1	ITGB1	2,11437E-03	5333,7	2,16161E-02	2252	1,00138E+00	577	1,49194E+00	1955	8,78859E-01	10676	0	11208,5
Responder	cDC_CLEC9A	Macro_ISG15	LGALS9	HAVCR2	2,11609E-03	10699,7	7,63927E-03	13428	2,90360E-01	11376	1,07599E+00	4370	8,66054E-01	13116	0	11208,5
Responder	Mono_INHBA	Macro_OLFML3	LGALS3	ENG	2,11688E-03	10357,9	9,48273E-03	9323	4,56272E-01	5387	5,20526E-01	12442	8,64536E-01	13429	0	11208,5
Responder	cDC(CD1C)	Macro_FOLR2+APOE+	ADAM10	GPNMB	2,11990E-03	8569,1	1,20320E-02	6218	7,20482E-01	1700	9,98404E-01	5115	8,39737E-01	18604	0	11208,5
Responder	Macro_NLRP3	Macro_IER3	VCAN	ITGB1	2,12003E-03	8836,5	1,27106E-02	5659	5,84067E-01	3043	5,96127E-01	10839	8,64521E-01	13433	0	11208,5
Responder	pDC_LILRA4	Macro_IER3	CD99	CD81	2,12003E-03	8807,7	7,71585E-03	13243	4,21980E-01	6300	1,35667E+00	2559	8,78567E-01	10728	0	11208,5
Responder	Mono_CD14	Macro_LYVE1	NAMPT	INSR	2,12123E-03	9817,3	1,22739E-02	6006	6,95290E-01	1894	1,38632E+00	2422	7,96235E-01	27556	0	11208,5
Responder	pDC_LILRA4	Macro_IER3	SELPLG	ITGB2	2,12240E-03	6493,5	9,98579E-03	8511	4,77337E-01	4882	1,68311E+00	1326	9,01311E-01	6540	0	11208,5
Responder	pDC_LILRA4	Macro_OLFML3	CD99	CD81	2,12240E-03	8990,9	7,63621E-03	13436	4,16691E-01	6463	1,27120E+00	3020	8,78013E-01	10827	0	11208,5
Responder	Mono_INHBA	Macro_OLFML3	CXCL8	SDC3	2,12257E-03	9492,7	1,40424E-02	4806	5,25762E-01	3931	9,00395E-01	6220	8,26775E-01	21298	0	11208,5
Responder	Macro_OLFML3	Macro_LYVE1	LGALS3BP	ITGB1	2,12635E-03	8749,3	1,57252E-02	3905	5,63158E-01	3363	4,79407E-01	13441	8,73082E-01	11829	0	11208,5
Responder	cDC(CD1C)	Macro_NLRP3	ANXA2	TLR2	2,12635E-03	10065,5	7,63425E-03	13441	3,08602E-01	10476	9,97179E-01	5131	8,82122E-01	10071	0	11208,5
Responder	Mono_INHBA	Mast	VEGFA	ITGAV	2,12658E-03	12188,1	1,39772E-02	4849	5,35097E-01	3776	9,00211E-01	6223	7,59141E-01	34884	0	11208,5
Responder	Mono_CD14	Macro_LYVE1	ADAM10	GPNMB	2,12658E-03	8036,7	1,33713E-02	5195	6,75762E-01	2067	1,05265E+00	4606	8,46712E-01	17107	0	11208,5
Responder	Macro_FOLR2-APOE+	Mast	ACTR2	ADRB2	2,12794E-03	6611,5	3,04725E-02	1179	7,58095E-01	1488	9,40031E-01	5739	8,64460E-01	13443	0	11208,5
Responder	Macro_OLFML3	Mast	LGALS1	CD69	2,12794E-03	7948,1	1,10772E-02	7141	5,92354E-01	2935	4,79328E-01	13443	9,09378E-01	5013	0	11208,5
Responder	cDC_CLEC9A	Macro_NLRP3	RPS19	C5AR1	2,12794E-03	7248,3	7,63415E-03	13443	4,86909E-01	4672	1,24602E+00	3162	9,16191E-01	3756	0	11208,5
Responder	pDC_LILRA4	Macro_IER3	SERPINF1	PLXDC2	2,12952E-03	5519,3	1,40229E-02	4821	6,91618E-01	1924	2,28056E+00	383	8,86687E-01	9260	0	11208,5
Responder	Macro_FOLR2-APOE+	Mono_INHBA	LGALS9	CD44	2,13110E-03	9132,9	7,63316E-03	13447	4,90909E-01	4588	5,97163E-01	10814	9,06100E-01	5607	0	11208,5
Responder	Macro_OLFML3	Macro_NLRP3	C3	CD46	2,13190E-03	8182,9	1,35564E-02	5089	4,82520E-01	4762	8,85927E-01	6407	8,64446E-01	13448	0	11208,5
Responder	cDC(CD1C)	pDC_LILRA4	HLA-DRB5	CD4	2,13586E-03	6491,7	7,63075E-03	13453	4,10113E-01	6663	2,41930E+00	290	9,34554E-01	844	0	11208,5
Responder	pDC_LILRA4	cDC_LAMP3	SEMA3C	NRP2_PLXNA1	2,13936E-03	13399,9	4,65730E-02	550	6,86444E-01	1959	1,41947E+00	2261	6,63143E-01	51021	0	11208,5
Responder	pDC_LILRA4	Macro_IER3	RPS19	C5AR1	2,13983E-03	5904,5	8,24125E-03	11842	6,72168E-01	2098	1,76794E+00	1128	9,19083E-01	3246	0	11208,5
Responder	Mono_INHBA	Macro_NLRP3	IL1B	SIGIRR	2,13999E-03	8306,1	1,20239E-02	6233	5,74134E-01	3206	1,02233E+00	4877	8,52035E-01	16006	0	11208,5
Responder	Mono_CD14	Macro_ISG15	RETN	CAP1	2,15076E-03	9198,9	2,22448E-02	2144	4,24206E-01	6241	1,02556E+00	4848	8,25498E-01	21553	0	11208,5
Responder	Macro_OLFML3	cDC_LAMP3	C1QB	C1QBP	2,15179E-03	10128,3	7,66935E-03	13349	4,06238E-01	6784	4,78037E-01	13473	9,04943E-01	5827	0	11208,5
Responder	Macro_OLFML3	Mono_CD14	C3	CD46	2,15211E-03	8317,7	1,20188E-02	6242	4,52862E-01	5483	1,15070E+00	3747	8,57238E-01	14908	0	11208,5
Responder	pDC_LILRA4	Macro_FOLR2+APOE-	APP	RPSA	2,15338E-03	6344,9	1,06021E-02	7682	8,03384E-01	1232	2,00580E+00	706	8,77646E-01	10896	0	11208,5
Responder	pDC_LILRA4	Macro_FOLR2+APOE-	APP	APLP2	2,15498E-03	5430,7	1,54901E-02	4002	8,58275E-01	987	2,30842E+00	368	8,79329E-01	10588	0	11208,5
Responder	pDC_LILRA4	Mono_CD14	ASIP	MGRN1	2,15892E-03	14368,3	4,82635E-02	516	7,65933E-01	1428	1,41687E+00	2268	6,14607E-01	56421	0	11208,5
Responder	cDC(CD1C)	Mast	LGALS9	CD44	2,16022E-03	8905,9	7,08037E-03	15184	4,24317E-01	6235	9,48360E-01	5654	9,02853E-01	6248	0	11208,5
Responder	Mono_CD14	cDC_LAMP3	S100A9	CD68	2,16293E-03	8128,3	6,07245E-03	19289	6,45416E-01	2349	9,60481E-01	5516	9,24321E-01	2279	0	11208,5
Responder	pDC_LILRA4	Macro_FOLR2+APOE-	HMGB1	CD163	2,16379E-03	7233,5	7,64849E-03	13407	4,16121E-01	6484	1,68162E+00	1330	9,16263E-01	3738	0	11208,5
Responder	Mono_CD14	Macro_FOLR2+APOE+	VCAN	TLR1	2,16734E-03	7382,3	2,14853E-02	2271	8,29970E-01	1101	1,47420E+00	2014	8,31586E-01	20317	0	11208,5
Responder	Mono_CD14	cDC_LAMP3	VCAN	TLR1	2,17107E-03	8680,1	1,57298E-02	3901	7,67890E-01	1418	1,54589E+00	1771	8,08610E-01	25102	0	11208,5
Responder	pDC_LILRA4	Macro_FOLR2+APOE+	APP	APLP2	2,17182E-03	4951,9	1,83249E-02	2945	9,44395E-01	720	1,91640E+00	847	8,87965E-01	9039	0	11208,5
Responder	Macro_NLRP3	Macro_NLRP3	S100A9	ITGB2	2,17343E-03	8371,5	8,79866E-03	10593	4,49563E-01	5563	4,76756E-01	13500	9,33192E-01	993	0	11208,5
Responder	Macro_OLFML3	Mono_CD16	APOE	SORL1	2,17504E-03	9265,1	1,02278E-02	8181	3,81388E-01	7580	4,76504E-01	13502	9,04815E-01	5854	0	11208,5
Responder	Mono_CD14	cDC_LAMP3	ADM	CALCRL	2,17787E-03	12710,5	2,19287E-02	2200	5,63942E-01	3358	1,21674E+00	3345	7,12171E-01	43441	0	11208,5
Responder	pDC_LILRA4	Macro_FOLR2+APOE+	CD99	CD81	2,18069E-03	5854,3	1,12582E-02	6943	6,57208E-01	2236	1,58877E+00	1608	8,97324E-01	7276	0	11208,5
Responder	Macro_OLFML3	Mono_CD14	CD14	TLR4	2,18554E-03	10863,9	9,32579E-03	9604	3,28831E-01	9604	6,22324E-01	10388	8,64077E-01	13515	0	11208,5
Responder	Mono_INHBA	cDC(CD1C)	SPP1	CD44	2,18634E-03	10371,7	7,94774E-03	12570	2,51666E-01	13516	1,15391E+00	3719	8,77940E-01	10845	0	11208,5
Responder	cDC(CD1C)	pDC_LILRA4	SELPLG	SELL	2,18741E-03	8314,1	1,19966E-02	6268	6,37520E-01	2424	2,21883E+00	439	8,27047E-01	21231	0	11208,5
Responder	pDC_LILRA4	Macro_FOLR2+APOE+	LILRB4	LAIR1	2,18877E-03	7182,5	1,06982E-02	7570	4,02008E-01	6909	1,48333E+00	1982	8,92175E-01	8243	0	11208,5
Responder	Macro_OLFML3	Mast	VEGFB	ADRB2	2,18990E-03	14250,1	2,14653E-02	2279	6,76865E-01	2054	7,87719E-01	7709	6,84263E-01	48000	0	11208,5
Responder	Mast	Macro_IER3	TIMP3	CD44	2,19557E-03	4318,3	5,45312E-02	378	1,07705E+00	434	1,41381E+00	2281	8,97242E-01	7290	0	11208,5
Responder	pDC_LILRA4	Macro_FOLR2+APOE+	SERPINF1	PLXDC2	2,19607E-03	5108,3	1,55785E-02	3969	7,37037E-01	1601	2,19528E+00	459	8,91865E-01	8304	0	11208,5
Responder	Macro_NLRP3	Macro_OLFML3	TIMP1	CD63	2,19769E-03	7449,5	8,97589E-03	10256	6,63342E-01	2169	4,75657E-01	13530	9,48400E-01	84	0	11208,5
Responder	cDC(CD1C)	Macro_NLRP3	VIM	CD44	2,20110E-03	8860,5	5,77859E-03	20872	4,45850E-01	5653	8,96459E-01	6278	9,41934E-01	291	0	11208,5
Responder	Mono_INHBA	cDC_CLEC9A	LGALS3	LAG3	2,20385E-03	9807,5	1,51253E-02	4190	5,45913E-01	3601	8,96278E-01	6280	8,15158E-01	23758	0	11208,5
Responder	Mono_CD14	Mono_CD16	ICAM1	SPN	2,20660E-03	10407,7	1,29729E-02	5481	5,05920E-01	4277	8,99842E-01	6229	8,09851E-01	24843	0	11208,5
Responder	cDC_CLEC9A	Macro_FOLR2-APOE+	IL16	CD9	2,20745E-03	6992,3	2,17618E-02	2228	6,22899E-01	2571	9,70525E-01	5412	8,63945E-01	13542	0	11208,5
Responder	cDC(CD1C)	cDC(CD1C)	CD48	CD2	2,20797E-03	12554,3	1,95474E-02	2626	4,22741E-01	6283	1,19936E+00	3435	7,35946E-01	39219	0	11208,5
Responder	pDC_LILRA4	cDC(CD1C)	COPA	CD74	2,20827E-03	6273,9	7,59908E-03	13543	4,67074E-01	5118	2,28174E+00	382	9,31986E-01	1118	0	11208,5
Responder	Mono_CD14	Macro_NLRP3	SERPINA1	LRP1	2,21072E-03	10936,1	7,59791E-03	13546	2,54497E-01	13357	6,64735E-01	9639	8,99185E-01	6930	0	11208,5
Responder	Macro_FOLR2-APOE+	Macro_LYVE1	C3	LRP1	2,21235E-03	7943,9	1,74999E-02	3191	5,28588E-01	3878	4,74926E-01	13548	8,94177E-01	7894	0	11208,5
Responder	cDC_CLEC9A	Macro_FOLR2+APOE+	HLA-DQA1	LAG3	2,21235E-03	7460,5	1,71846E-02	3301	5,35461E-01	3768	9,64974E-01	5477	8,63923E-01	13548	0	11208,5
Responder	cDC_CLEC9A	Mono_CD16	BTLA	TNFRSF14	2,21832E-03	7298,1	4,66641E-02	546	1,69912E+00	78	1,41139E+00	2289	8,21721E-01	22369	0	11208,5
Responder	Mono_INHBA	pDC_LILRA4	ADA	DPP4	2,22038E-03	16607,7	3,02238E-02	1209	6,59523E-01	2213	8,95510E-01	6292	5,17403E-01	62116	0	11208,5

Responder	Mono_CD14	Mono_CD16	CD1D	LILRB2	2,22591E-03	9401,7	1,48900E-02	4311	6,32852E-01	2468	9,21992E-01	5954	8,18392E-01	23067	0	11208,5
Responder	Macro_OLFML3	pDC_LILRA4	TNFSF13B	TFRC	2,22791E-03	9567,9	8,63738E-03	10947	4,05286E-01	6811	9,79506E-01	5306	8,63824E-01	13567	0	11208,5
Responder	Mono_CD14	Mono_CD16	ICAM3	ITGAL	2,22868E-03	9714,7	2,45326E-02	1799	6,74349E-01	2082	1,17502E+00	3583	7,84288E-01	29901	0	11208,5
Responder	cDC(CD1C)	Macro_OLFML3	TNFSF13B	HLA-DPB1	2,22868E-03	8047,5	6,22383E-03	18541	4,22027E-01	6298	1,12522E+00	3974	9,43449E-01	216	0	11208,5
Responder	cDC(CD1C)	Mono_CD14	PKM	CD44	2,23006E-03	7925,9	6,61227E-03	16899	4,22000E-01	6299	1,32785E+00	2707	9,22908E-01	2516	0	11208,5
Responder	Macro_ISG15	pDC_LILRA4	SEMA6B	PLXNA4	2,23140E-03	14702,9	1,58593E-01	32	1,08049E+00	427	1,30301E+00	2832	5,82849E-01	59015	0	11208,5
Responder	Macro_NLRP3	cDC(CD1C)	THBS1	ITGA4	2,23778E-03	6698,1	1,71443E-02	3310	6,61195E-01	2192	1,23967E+00	3201	8,63778E-01	13579	0	11208,5
Responder	cDC_CLEC9A	Macro_NLRP3	HSPA1A	TLR4	2,23778E-03	9687,7	7,58567E-03	13579	3,97069E-01	7066	1,01054E+00	4994	8,74341E-01	11591	0	11208,5
Responder	Mono_INHBA	Macro_OLFML3	PTGS2	ALOX5	2,23860E-03	9065,3	1,66936E-02	3471	5,25234E-01	3940	4,92874E-01	13127	8,63774E-01	13580	0	11208,5
Responder	Macro_NLRP3	Macro_FOLR2+APOE-	S100A8	CD68	2,24025E-03	7095,9	1,17575E-02	6471	5,39387E-01	3704	4,73583E-01	13582	9,37920E-01	514	0	11208,5
Responder	Macro_ISG15	Mono_CD14	GNAI2	C5AR1	2,24355E-03	9389,7	7,58332E-03	13586	3,67106E-01	8098	1,02769E+00	4830	8,86875E-01	9226	0	11208,5
Responder	cDC(CD1C)	Macro_ISG15	HLA-DQA2	LAG3	2,24534E-03	9723,7	1,70889E-02	3332	4,21712E-01	6310	1,24119E+00	3192	8,11173E-01	24576	0	11208,5
Responder	pDC_LILRA4	Mono_INHBA	SPON2	ITGA4	2,24698E-03	12391,1	2,13771E-02	2299	7,55715E-01	1500	1,43192E+00	2207	7,04676E-01	44741	0	11208,5
Responder	cDC_CLEC9A	Mono_INHBA	HLA-F	LILRB2	2,25016E-03	10253,3	7,58110E-03	13594	3,32012E-01	9456	9,71153E-01	5399	8,74274E-01	11609	0	11208,5
Responder	Macro_NLRP3	Macro_FOLR2+APOE-	S100A9	CD36	2,25099E-03	10115,5	8,61912E-03	10994	5,05830E-01	4282	6,16261E-01	10498	8,63709E-01	13595	0	11208,5
Responder	Macro_IER3	pDC_LILRA4	MMP9	TLR9	2,25221E-03	13459,5	5,17931E-02	429	1,30114E+00	189	9,88557E-01	5221	6,68786E-01	50250	0	11208,5
Responder	pDC_LILRA4	Macro_NLRP3	PROC	THBD	2,25274E-03	13233,3	5,94962E-02	312	7,96101E-01	1271	1,40824E+00	2301	6,62684E-01	51074	0	11208,5
Responder	Macro_FOLR2-APOE+	Mono_CD14	CD14	TLR4	2,25928E-03	10422,3	9,25862E-03	9736	3,23171E-01	9860	7,88070E-01	7702	8,63652E-01	13605	0	11208,5
Responder	Mono_CD14	Mono_CD16	VCAN	SELL	2,26070E-03	7642,7	1,43863E-02	4598	7,84392E-01	1329	1,42226E+00	2250	8,38570E-01	18828	0	11208,5
Responder	Macro_ISG15	Macro_NLRP3	TNFSF13B	TFRC	2,26344E-03	9912,5	8,60736E-03	11019	4,52209E-01	5501	7,52376E-01	8224	8,63619E-01	13610	0	11208,5
Responder	Macro_OLFML3	Macro_FOLR2+APOE+	FN1	SDC2	2,26771E-03	14682,5	1,24009E-02	5899	4,30501E-01	6069	8,92721E-01	6326	7,09399E-01	43910	0	11208,5
Responder	Macro_OLFML3	Mono_CD16	C1QA	CD33	2,27093E-03	11358,1	7,58114E-03	13593	3,55291E-01	8544	4,72062E-01	13619	8,83465E-01	9826	0	11208,5
Responder	Mono_CD14	Mono_CD16	RETN	TLR4	2,27192E-03	11307,7	3,81238E-02	801	6,11073E-01	2710	1,09475E+00	4214	7,44392E-01	37605	0	11208,5
Responder	Macro_FOLR2-APOE+	Mono_INHBA	APOE	SDC2	2,27260E-03	8114,7	1,97926E-02	2570	5,14829E-01	4125	6,99732E-01	9049	8,63573E-01	13621	0	11208,5
Responder	Mono_INHBA	Macro_FOLR2-APOE+	HBEGF	CD44	2,27260E-03	9058,5	1,07753E-02	7474	4,95268E-01	4502	4,71992E-01	13621	8,90954E-01	8487	0	11208,5
Responder	cDC_CLEC9A	Macro_IER3	BTLA	TNFRSF14	2,27297E-03	7386,5	4,55102E-02	574	1,68936E+00	81	1,40715E+00	2308	8,19880E-01	22761	0	11208,5
Responder	Macro_FOLR2+APOE+	cDC_LAMP3	IL10	CD70	2,27311E-03	18596,3	5,49707E-02	372	1,07807E+00	431	2,98629E-01	18571	5,06843E-01	62399	0	11208,5
Responder	Macro_FOLR2+APOE-	Macro_IER3	F13A1	ITGA4	2,27587E-03	5765,5	2,33579E-02	1970	9,98812E-01	582	1,40712E+00	2309	8,67874E-01	12758	0	11208,5
Responder	Macro_ISG15	Mono_INHBA	PLAU	PLAUR	2,27594E-03	8344,7	1,04861E-02	7842	5,53295E-01	3513	9,58692E-01	5535	8,63558E-01	13625	0	11208,5
Responder	pDC_LILRA4	Macro_OLFML3	APP	LRP10	2,27878E-03	7614,7	2,12755E-02	2310	9,51222E-01	698	2,10872E+00	577	8,17407E-01	23280	0	11208,5
Responder	Macro_ISG15	Mono_CD14	PLAU	PLAUR	2,28179E-03	7590,3	1,04800E-02	7849	5,52762E-01	3527	1,55315E+00	1735	8,63524E-01	13632	0	11208,5
Responder	Macro_NLRP3	Macro_FOLR2+APOE-	VCAN	ITGB1	2,28346E-03	7921,1	1,24949E-02	5817	5,77930E-01	3140	9,34170E-01	5806	8,63516E-01	13634	0	11208,5
Responder	Macro_IER3	pDC_LILRA4	HMGB1	TLR9	2,28360E-03	9463,5	5,17212E-02	432	1,17191E+00	301	8,40747E-01	6986	8,48864E-01	16672	0,001	22926,5
Responder	Mono_CD14	Macro_OLFML3	RETN	CAP1	2,28459E-03	7522,7	3,13151E-02	1135	6,56833E-01	2243	8,90924E-01	6338	8,48778E-01	16689	0	11208,5
Responder	Macro_ISG15	Mono_CD16	SPP1	ITGA4_ITGB1	2,28681E-03	7200,1	1,56124E-02	3953	5,35603E-01	3762	1,19858E+00	3439	8,63504E-01	13638	0	11208,5
Responder	Macro_OLFML3	Macro_LYVE1	C3	ITGAM	2,28741E-03	8623,9	1,71421E-02	3313	5,53708E-01	3505	8,90833E-01	6340	8,38863E-01	18753	0	11208,5
Responder	cDC_CLEC9A	Macro_NLRP3	GNAI2	FPR1	2,28765E-03	10402,9	7,56773E-03	13639	3,01009E-01	10864	8,79380E-01	6492	8,83527E-01	9811	0	11208,5
Responder	Macro_OLFML3	pDC_LILRA4	CD38	PECAM1	2,28882E-03	13039,3	1,67324E-02	3458	4,20410E-01	6341	1,39860E+00	2357	7,21059E-01	41832	0	11208,5
Responder	Macro_FOLR2+APOE-	Macro_FOLR2+APOE+	CCL13	CCR5	2,29411E-03	15103,1	5,16033E-02	433	1,21058E+00	265	9,33752E-01	5812	5,99061E-01	57797	0	11208,5
Responder	Mono_CD14	Mono_CD16	RETN	CAP1	2,29589E-03	7611,1	2,93106E-02	1290	6,05423E-01	2778	9,93619E-01	5175	8,44483E-01	17604	0	11208,5
Responder	Mono_CD14	Macro_LYVE1	TIMP1	CD63	2,29605E-03	7940,7	7,56359E-03	13649	5,06256E-01	4271	6,22523E-01	10382	9,44047E-01	193	0	11208,5
Responder	Macro_FOLR2-APOE+	cDC_CLEC9A	APOE	SCARB1	2,29773E-03	8232,3	1,55744E-02	3971	4,00865E-01	6947	9,72732E-01	5384	8,63441E-01	13651	0	11208,5
Responder	cDC_CLEC9A	pDC_LILRA4	HLA-DRB5	CD4	2,30532E-03	6635,5	7,56084E-03	13660	4,00391E-01	6971	2,19396E+00	464	9,34272E-01	874	0	11208,5
Responder	Mono_INHBA	Mono_CD14	LGALS1	PTPRC	2,30724E-03	9065,3	5,93270E-03	20026	4,20048E-01	6354	8,95878E-01	6288	9,29401E-01	1450	0	11208,5
Responder	Mono_CD14	Macro_ISG15	HBEGF	CD44	2,30785E-03	11226,3	7,56046E-03	13663	2,90819E-01	11358	7,69088E-01	7969	8,72513E-01	11933	0	11208,5
Responder	Mono_CD14	Mast	CD55	ADGRE5	2,31008E-03	7660,1	1,32899E-02	5254	6,38405E-01	2416	1,15179E+00	3741	8,53548E-01	15681	0	11208,5
Responder	Mono_CD14	Macro_IFI27	HBEGF	CD9	2,31039E-03	8789,9	1,36876E-02	5026	4,18502E-01	6406	7,91759E-01	7643	8,63382E-01	13666	0	11208,5
Responder	cDC_CLEC9A	cDC_CLEC9A	HLA-DPB1	LAG3	2,31377E-03	7753,1	1,22685E-02	6009	4,73563E-01	4960	1,28895E+00	2918	8,63367E-01	13670	0	11208,5
Responder	Mono_CD14	Mast	HP	CD22	2,31577E-03	13565,1	3,13875E-01	11	9,33078E-01	753	1,26988E+00	3028	6,48104E-01	52825	0	11208,5
Responder	pDC_LILRA4	Macro_ISG15	RPS19	C5AR1	2,31631E-03	6317,1	7,55660E-03	13673	6,41183E-01	2389	2,16946E+00	495	9,15799E-01	3820	0	11208,5
Responder	Macro_OLFML3	cDC(CD1C)	GRN	TNFRSF1A	2,31716E-03	10122,5	7,55646E-03	13674	3,52401E-01	8642	6,64904E-01	9637	8,96487E-01	7451	0	11208,5
Responder	Macro_IFI27	cDC(CD1C)	HLA-DQA2	CD4	2,31885E-03	9715,3	7,65447E-03	13391	3,08215E-01	10491	8,63951E-01	6677	8,99888E-01	6809	0	11208,5
Responder	Mono_CD14	Mast	VCAN	ITGA4	2,32055E-03	6212,9	1,64172E-02	3591	8,18793E-01	1156	1,64778E+00	1431	8,63325E-01	13678	0	11208,5
Responder	cDC_CLEC9A	Mono_CD14	SELPLG	SELL	2,32225E-03	5897,7	2,09268E-02	2365	7,47935E-01	1531	2,00625E+00	704	8,63308E-01	13680	0	11208,5
Responder	Macro_IER3	Macro_ISG15	CCL4	CCR1	2,32850E-03	4887,3	2,54931E-02	1674	9,61568E-01	668	1,40373E+00	2327	8,90543E-01	8559	0	11208,5
Responder	Mono_INHBA	Mono_INHBA	VIM	CD44	2,32904E-03	6937,5	7,55007E-03	13688	7,00675E-01	1849	7,76940E-01	7867	9,48829E-01	75	0	11208,5
Responder	Macro_IFI27	cDC(CD1C)	B2M	CD1A	2,32989E-03	6697,1	5,22670E-02	419	5,36836E-01	3747	8,51485E-01	6828	8,75795E-01	11283	0	11208,5
Responder	Mono_CD14	Mast	GNAS	ADRB2	2,33005E-03	8305,7	1,75882E-02	3171	5,46833E-01	3592	1,02722E+00	4832	8,39023E-01	18725	0	11208,5
Responder	Macro_FOLR2-APOE+	Macro_LYVE1	CD59	STAB1	2,33075E-03	7012,5	1,77186E-02	3124	8,24402E-01	1126	9,24817E-01	5914	8,63257E-01	13690	0	11208,5
Responder	Macro_ISG15	Macro_FOLR2-APOE+	TNFSF13B	CD40	2,33415E-03	10407,1	8,66214E-03	10885	3,85897E-01	7427	7,12917E-01	8821	8,63232E-01	13694	0	11208,5
Responder	pDC_LILRA4	Mono_CD14	CIRBP	TREM1	2,34012E-03	7209,7	1,28398E-02	5569	4,66536E-01	5135	2,22294E+00	435	8,63182E-01	13701	0	11208,5
Responder	pDC_LILRA4	Macro_FOLR2+APOE-	VEGFB	NRP1	2,34030E-03	11347,9	2,11092E-02	2331	6,65552E-01	2141	2,05372E+00	633	7,28950E-01	40426	0	11208,5
Responder	Mono_INHBA	Macro_IFI27	VEGFA	CD44	2,34525E-03	10787,5	9,20645E-03	9824	4,20513E-01	6340	5,03187E-01	12858	8,63157E-01	13707	0	11208,5
Responder	Mono_CD14	Mast	PTGS2	ALOX5	2,34584E-03	8435,9	1,41742E-02	4720	4,57375E-01	5363	9,82518E-01	5272	8,53859E-01	15616	0	11208,5
Responder	Mono_CD14	Mast	ACTR2	ADRB2	2,35016E-03	6675,7	2,57515E-02	1651	6,64191E-01	2159	1,30141E+00	2842	8,54292E-01	15518	0	11208,5

Responder	Macro_OLFML3	cDC_CLEC9A	FN1	ITGA4_ITGB1	2,35160E-03	7681,3	1,57010E-02	3916	4,19115E-01	6385	1,32152E+00	2735	8,60783E-01	14162	0	11208,5
Responder	Mono_CD14	Macro_NLRP3	RPS19	C5AR1	2,35296E-03	8952,3	7,54110E-03	13716	4,81207E-01	4799	5,75974E-01	11201	9,15719E-01	3837	0	11208,5
Responder	Macro_OLFML3	Mono_CD14	SAA1	CD36	2,35304E-03	12434,5	1,47740E-02	4369	4,47221E-01	5620	8,87449E-01	6386	7,60791E-01	34589	0	11208,5
Responder	pDC_LILRA4	cDC_LAMP3	CD24	SIGLEC10	2,35807E-03	19184,8	3,07875E-02	1159	6,47014E-01	2337	1,68040E+00	1336	6,46563E-01	53003	0,651	38089
Responder	Macro_ISG15	Mono_CD16	CD14	TLR4	2,35811E-03	10806,1	9,16674E-03	9894	3,52402E-01	8641	6,12140E-01	10565	8,63064E-01	13722	0	11208,5
Responder	Mono_CD14	Macro_FOLR2-APOE+	S100A8	CD69	2,35897E-03	7543,1	8,84438E-03	10500	1,04735E+00	472	1,53039E+00	1812	8,63048E-01	13723	0	11208,5
Responder	Macro_IFI27	cDC(CD1C)	C1QA	CD33	2,35983E-03	9559,7	1,00825E-02	8376	3,24176E-01	9807	5,79099E-01	11140	8,97360E-01	7267	0	11208,5
Responder	Macro_IFI27	cDC(CD1C)	C1QB	CD33	2,36069E-03	10067,5	9,86976E-03	8671	2,94652E-01	11165	5,43700E-01	11881	8,96697E-01	7412	0	11208,5
Responder	pDC_LILRA4	Mono_CD14	SERPINF1	PLXDC2	2,36069E-03	7755,5	9,09261E-03	10015	5,47661E-01	3580	2,46951E+00	249	8,63035E-01	13725	0	11208,5
Responder	Mono_CD14	Macro_IER3	CD55	ADGRE5	2,36170E-03	7420,3	1,42183E-02	4698	6,64525E-01	2154	1,09092E+00	4239	8,57719E-01	14802	0	11208,5
Responder	Macro_OLFML3	Macro_OLFML3	ADAM10	AXL	2,36217E-03	23889,7	7,34743E-03	14319	1,96256E-01	17204	-1,04051E-01	34811	7,20630E-01	41906	0	11208,5
Responder	Macro_IFI27	cDC(CD1C)	C1QB	C1QBP	2,36241E-03	10110,5	8,12875E-03	12127	3,01570E-01	10832	5,87189E-01	11005	9,07416E-01	5380	0	11208,5
Responder	Macro_ISG15	cDC(CD1C)	CCL2	CCR2	2,36402E-03	12098,9	4,28708E-02	640	6,46872E-01	2339	1,58294E+00	1633	7,05057E-01	44674	0	11208,5
Responder	cDC(CD1C)	Macro_OLFML3	HLA-DRA	CD4	2,36749E-03	8222,3	6,41156E-03	17702	4,18764E-01	6396	9,69403E-01	5429	9,40045E-01	376	0	11208,5
Responder	pDC_LILRA4	Macro_IFI27	CD24	SIGLEC10	2,36997E-03	19603	3,06715E-02	1166	6,46240E-01	2341	1,73417E+00	1219	6,46132E-01	53037	0,805	40252
Responder	cDC_CLEC9A	pDC_LILRA4	IL16	CD4	2,37276E-03	6136,7	1,78014E-02	3106	6,43201E-01	2372	2,45717E+00	258	8,62943E-01	13739	0	11208,5
Responder	Mono_INHBA	pDC_LILRA4	S100A8	CD36	2,37621E-03	10364,5	1,07636E-02	7486	3,72664E-01	7893	5,61727E-01	11492	8,62932E-01	13743	0	11208,5
Responder	pDC_LILRA4	Mono_CD16	HSP90B1	ASGR1	2,37967E-03	7396,1	1,12404E-02	6956	5,09741E-01	4218	1,91475E+00	851	8,62908E-01	13747	0	11208,5
Responder	Macro_OLFML3	Mono_INHBA	APOE	SORL1	2,38140E-03	11447,7	7,72590E-03	13212	3,02142E-01	10795	4,67331E-01	13749	8,92029E-01	8274	0	11208,5
Responder	Mono_INHBA	Macro_OLFML3	VCAN	TLR2	2,38400E-03	9034,9	1,74218E-02	3219	5,02111E-01	4364	5,12673E-01	12631	8,62882E-01	13752	0	11208,5
Responder	Macro_OLFML3	Mono_CD14	APOE	ABCA1	2,38400E-03	9888,5	7,53032E-03	13752	3,90879E-01	7263	7,04145E-01	8974	8,92169E-01	8245	0	11208,5
Responder	Macro_ISG15	Mono_INHBA	LGALS9	LRP1	2,38574E-03	11686,7	7,85231E-03	12842	3,11540E-01	10356	6,28610E-01	10273	8,62860E-01	13754	0	11208,5
Responder	Mono_INHBA	Mono_INHBA	TIMP2	CD44	2,39095E-03	10115,5	7,52719E-03	13760	4,65069E-01	5182	6,81790E-01	9338	8,76725E-01	11089	0	11208,5
Responder	Macro_IFI27	Mono_INHBA	FN1	CD44	2,39181E-03	7007,7	1,52633E-02	4118	5,12465E-01	4174	6,44113E-01	10017	9,06669E-01	5521	0	11208,5
Responder	Mono_CD14	Macro_IER3	VCAN	TLR2	2,39366E-03	6848,7	1,60212E-02	3757	7,27481E-01	1664	1,30187E+00	2839	8,57848E-01	14775	0	11208,5
Responder	pDC_LILRA4	Mast	ARPC5	ADRB2	2,39442E-03	6780,5	1,89723E-02	2765	4,87508E-01	4660	1,62267E+00	1505	8,62777E-01	13764	0	11208,5
Responder	Macro_IFI27	Mono_INHBA	FN1	PLAUR	2,39442E-03	6650,7	1,62455E-02	3653	5,26277E-01	3924	6,45031E-01	9999	9,12317E-01	4469	0	11208,5
Responder	Macro_IFI27	Mono_INHBA	APOE	SDC2	2,39616E-03	7710,9	2,25952E-02	2089	6,09316E-01	2731	6,22901E-01	10372	8,71188E-01	12154	0	11208,5
Responder	pDC_LILRA4	pDC_LILRA4	APP	LRP10	2,39989E-03	7553,3	2,10115E-02	2351	9,47233E-01	710	3,51393E+00	26	8,16473E-01	23471	0	11208,5
Responder	Mono_CD14	Macro_IER3	VCAN	TLR1	2,40097E-03	7538,7	2,00899E-02	2519	8,14919E-01	1181	1,62430E+00	1499	8,26831E-01	21286	0	11208,5
Responder	Macro_OLFML3	cDC_CLEC9A	HLA-DQA2	LAG3	2,40097E-03	10030,9	1,72448E-02	3280	4,80454E-01	4817	8,84677E-01	6419	8,11868E-01	24430	0	11208,5
Responder	cDC_CLEC9A	Mono_INHBA	VIM	CD44	2,40139E-03	5982,1	7,52258E-03	13772	6,95852E-01	1889	1,28154E+00	2964	9,48740E-01	77	0	11208,5
Responder	Mono_CD14	Macro_ISG15	TIMP1	CD63	2,40389E-03	7905,3	6,94004E-03	15672	4,17995E-01	6421	9,24149E-01	5928	9,41730E-01	297	0	11208,5
Responder	Macro_IFI27	Mono_INHBA	SPP1	CD44	2,40401E-03	8171,3	8,93790E-03	10330	5,05103E-01	4301	9,79619E-01	5302	8,84093E-01	9715	0	11208,5
Responder	Mono_INHBA	Mono_CD16	HLA-C	LILRB2	2,40837E-03	8278,7	9,01670E-03	10166	4,37683E-01	5880	4,65603E-01	13780	9,40469E-01	359	0	11208,5
Responder	Macro_LYVE1	pDC_LILRA4	MMP9	TLR9	2,41132E-03	13379,1	5,10356E-02	444	1,29893E+00	192	1,05689E+00	4547	6,67152E-01	50504	0	11208,5
Responder	Mono_CD14	Macro_IER3	RETN	TLR4	2,41269E-03	12581,1	2,88605E-02	1332	5,24132E-01	3966	1,13957E+00	3843	7,17023E-01	42556	0	11208,5
Responder	Macro_IFI27	Mono_INHBA	C3	ITGAX	2,41712E-03	8965,7	1,33940E-02	5177	3,68831E-01	8021	5,79569E-01	11130	8,86445E-01	9292	0	11208,5
Responder	Macro_IFI27	Mono_INHBA	MMP9	CD44	2,41888E-03	6150,3	1,53112E-02	4096	7,08771E-01	1786	1,43445E+00	2197	8,74969E-01	11464	0	11208,5
Responder	Macro_ISG15	Macro_ISG15	TNFSF13B	CD40	2,42151E-03	10278,5	8,57076E-03	11130	3,81806E-01	7571	7,88938E-01	7688	8,62604E-01	13795	0	11208,5
Responder	Macro_NLRP3	Mono_CD14	ANXA1	FPR1	2,42151E-03	11903,9	7,51465E-03	13795	2,59838E-01	13052	4,94171E-01	13087	8,91532E-01	8377	0	11208,5
Responder	Mono_INHBA	cDC_LAMP3	SPP1	PTGER4	2,42152E-03	10026,7	1,33701E-02	5196	4,17555E-01	6433	1,09491E+00	4212	8,18316E-01	23084	0	11208,5
Responder	Macro_IFI27	Mono_INHBA	C3	LRP1	2,42239E-03	10768,5	1,22377E-02	6040	2,76572E-01	12140	4,88653E-01	13217	8,76023E-01	11237	0	11208,5
Responder	cDC_CLEC9A	Macro_LYVE1	HLA-DMB	CD4	2,42502E-03	9965,7	7,51243E-03	13799	3,13816E-01	10247	7,42273E-01	8381	9,03132E-01	6193	0	11208,5
Responder	Mono_INHBA	Mono_INHBA	TNF	TNFRSF21	2,42594E-03	13696,1	1,31764E-02	5329	4,90841E-01	4590	8,83905E-01	6436	7,26042E-01	40917	0	11208,5
Responder	Macro_IFI27	Mono_INHBA	SERPING1	LRP1	2,43382E-03	12305,7	8,79525E-03	10601	2,61414E-01	12959	4,87210E-01	13252	8,64113E-01	13508	0	11208,5
Responder	Macro_IFI27	Mono_INHBA	APOE	ABCA1	2,43558E-03	6248,3	1,59603E-02	3788	5,99669E-01	2837	5,88530E-01	10974	9,23344E-01	2434	0	11208,5
Responder	Macro_OLFML3	Macro_FOLR2-APOE+	A2M	LRP1	2,43558E-03	9949,9	1,31150E-02	5361	4,34945E-01	5956	4,64485E-01	13811	8,64662E-01	13413	0	11208,5
Responder	Mono_INHBA	Mono_CD16	LGALS1	ITGB1	2,43735E-03	9026,5	7,50873E-03	13813	5,38967E-01	3709	5,55172E-01	11621	9,10635E-01	4781	0	11208,5
Responder	Mono_CD14	Macro_FOLR2+APOE-	HP	CD163	2,43775E-03	7853,3	8,94302E-02	122	9,11182E-01	811	1,24984E+00	3148	8,13995E-01	23977	0	11208,5
Responder	pDC_LILRA4	Macro_FOLR2+APOE-	FAM3D	FPR1	2,43916E-03	19951,3	2,09290E-02	2364	7,13842E-01	1746	1,84650E+00	972	6,51430E-01	52431	0,901	42243,5
Responder	Macro_FOLR2-APOE+	Macro_IFI27	SPP1	CD44	2,44000E-03	11815,9	8,24967E-03	11825	2,83718E-01	11757	4,64335E-01	13816	8,79924E-01	10473	0	11208,5
Responder	Mono_CD14	Macro_FOLR2+APOE-	S100A9	ITGB2	2,44368E-03	6313,5	7,35198E-03	14305	6,28400E-01	2514	1,53708E+00	1793	9,27370E-01	1747	0	11208,5
Responder	Macro_ISG15	Macro_IFI27	CCL8	CCR1	2,44827E-03	13234,9	2,25868E-02	2091	6,43919E-01	2367	1,52192E+00	1834	6,79706E-01	48674	0	11208,5
Responder	Mono_CD14	Macro_FOLR2+APOE-	VCAN	TLR2	2,44961E-03	7381,1	1,35751E-02	5081	6,88247E-01	1950	1,56092E+00	1707	8,47444E-01	16959	0	11208,5
Responder	Macro_OLFML3	Macro_ISG15	HLA-A	LILRB1	2,45150E-03	8190,5	7,50507E-03	13829	5,04918E-01	4305	6,94872E-01	9123	9,23076E-01	2487	0	11208,5
Responder	Mono_INHBA	Mono_CD14	ADAM10	CD44	2,45416E-03	10850,9	7,78154E-03	13046	3,90732E-01	7269	7,08088E-01	8899	8,62442E-01	13832	0	11208,5
Responder	Macro_ISG15	pDC_LILRA4	HSP90B1	TLR9	2,45465E-03	7101,5	5,08675E-02	448	1,17386E+00	298	1,12233E+00	3998	8,35227E-01	19555	0	11208,5
Responder	Macro_IFI27	Mono_CD14	FN1	CD44	2,46037E-03	8759,9	1,25602E-02	5776	3,45776E-01	8900	5,99375E-01	10773	8,98089E-01	7142	0	11208,5
Responder	Macro_IFI27	Mono_CD14	FN1	C5AR1	2,46126E-03	6802,3	1,86595E-02	2847	4,61606E-01	5254	1,03817E+00	4729	8,82681E-01	9973	0	11208,5
Responder	Macro_IFI27	Mono_CD14	APOE	LDLR	2,46304E-03	7412,1	2,48621E-02	1759	5,62113E-01	3378	7,00819E-01	9031	8,73920E-01	11684	0	11208,5
Responder	Macro_IFI27	Mono_CD14	APOE	LRP1	2,46393E-03	8580,7	1,02910E-02	8088	3,67592E-01	8075	5,73833E-01	11235	9,13233E-01	4297	0	11208,5
Responder	cDC_CLEC9A	Macro_ISG15	HLA-DQB1	LAG3	2,47463E-03	6882,7	1,65691E-02	3522	5,93376E-01	2921	1,29141E+00	2907	8,62342E-01	13855	0	11208,5
Responder	Macro_FOLR2-APOE+	Mono_CD14	ANXA1	FPR1	2,47642E-03	11743,9	7,49720E-03	13857	2,58296E-01	13141	5,33341E-01	12122	8,91419E-01	8391	0	11208,5
Responder	Macro_IFI27	Mono_CD14	APOE	SORL1	2,47820E-03	10406,9	9,22451E-03	9788	2,63690E-01	12826	5,61909E-01	11486	9,00275E-01	6726	0	11208,5

Responder	Macro_IFI27	Mono_CD14	C3	ITGAX	2,47999E-03	7820,3	1,34685E-02	5145	3,72021E-01	7915	9,53945E-01	5583	8,86724E-01	9250	0	11208,5
Responder	Macro_IFI27	Mono_CD14	MMP9	CD44	2,48268E-03	7314,9	1,25996E-02	5745	5,42082E-01	3662	1,38971E+00	2409	8,63911E-01	13550	0	11208,5
Responder	cDC(CD1C)	Mast	CALM1	KCNQ1	2,48545E-03	9204,9	1,62479E-02	3651	4,16348E-01	6476	1,12018E+00	4012	8,29949E-01	20677	0	11208,5
Responder	Macro_IFI27	Mono_CD14	C1QB	LRP1	2,48626E-03	10514,5	8,11616E-03	12150	2,99911E-01	10908	4,65358E-01	13789	9,12088E-01	4517	0	11208,5
Responder	Macro_IFI27	Mono_CD14	C1QA	CD93	2,48716E-03	7247,9	1,20384E-02	6212	4,65558E-01	5163	7,71886E-01	7931	9,05492E-01	5725	0	11208,5
Responder	Macro_IER3	Mono_CD14	PLAU	PLAUR	2,49113E-03	4634,1	2,08274E-02	2381	8,66094E-01	960	1,56510E+00	1693	8,99191E-01	6928	0	11208,5
Responder	pDC_LILRA4	Mono_CD16	HSP90B1	TLR4	2,49254E-03	8001,5	9,60488E-03	9106	4,76174E-01	4907	1,87612E+00	911	8,62240E-01	13875	0	11208,5
Responder	Macro_IFI27	Mono_CD14	C3	IFITM1	2,49434E-03	7212,1	1,51684E-02	4174	3,87717E-01	7370	1,33350E+00	2679	8,79121E-01	10629	0	11208,5
Responder	Mono_INHBA	cDC_LAMP3	NAMPT	ADORA2A	2,49447E-03	7660,3	3,75705E-02	823	9,25760E-01	769	8,80166E-01	6482	8,37726E-01	19019	0	11208,5
Responder	Macro_LYVE1	pDC_LILRA4	HLA-C	NOTCH4	2,49729E-03	5504,1	3,15857E-02	1116	6,41949E-01	2383	1,41223E+00	2286	8,79672E-01	10527	0	11208,5
Responder	Macro_ISG15	Macro_ISG15	CCL2	CCR5	2,50037E-03	11899,7	3,86718E-02	778	6,72771E-01	2086	1,39313E+00	2384	7,14377E-01	43042	0	11208,5
Responder	Macro_IFI27	Mono_CD14	C1QA	CR1	2,50334E-03	7333,9	2,02128E-02	2491	5,82183E-01	3072	7,08347E-01	8894	8,77165E-01	11004	0	11208,5
Responder	Macro_ISG15	Mono_INHBA	S100A8	CD36	2,50514E-03	9021,9	1,41563E-02	4734	4,95303E-01	4500	4,61491E-01	13889	8,78345E-01	10778	0	11208,5
Responder	pDC_LILRA4	Macro_FOLR2+APOE-	APP	LRP1	2,50604E-03	5820,5	1,85191E-02	2886	9,18388E-01	788	2,35613E+00	330	8,62175E-01	13890	0	11208,5
Responder	Macro_OLFML3	Macro_FOLR2+APOE+	ENTPD1	TMIGD3	2,50655E-03	12520,7	4,19419E-02	672	7,25088E-01	1675	3,49692E-01	16997	7,73425E-01	32051	0	11208,5
Responder	Macro_NLRP3	Macro_FOLR2-APOE+	TIMP1	CD63	2,50695E-03	7774,9	8,60279E-03	11036	6,17543E-01	2637	4,61456E-01	13891	9,47351E-01	102	0	11208,5
Responder	pDC_LILRA4	Macro_OLFML3	SPON2	ITGA4	2,51274E-03	10289,7	3,48107E-02	950	8,87324E-01	880	1,39278E+00	2388	7,52775E-01	36022	0	11208,5
Responder	cDC_CLEC9A	Mono_CD16	HMGB1	TLR4	2,51327E-03	10404,5	7,93946E-03	12598	3,82449E-01	7547	8,56804E-01	6771	8,62127E-01	13898	0	11208,5
Responder	Macro_NLRP3	Macro_LYVE1	THBS1	CD47	2,51598E-03	8579,1	1,45659E-02	4501	5,94106E-01	2911	6,22867E-01	10374	8,62096E-01	13901	0	11208,5
Responder	Mono_INHBA	Mono_INHBA	PKM	CD44	2,51689E-03	7742,7	7,48292E-03	13902	5,29226E-01	3871	7,69994E-01	7957	9,27195E-01	1775	0	11208,5
Responder	cDC_CLEC9A	Macro_ISG15	HMGB1	TLR2	2,52323E-03	9864,9	7,48010E-03	13909	3,33211E-01	9406	9,89229E-01	5216	8,84725E-01	9585	0	11208,5
Responder	pDC_LILRA4	cDC(CD1C)	TGFB1	CXCR4	2,52414E-03	8613,1	7,47948E-03	13910	4,24084E-01	6245	2,27112E+00	395	8,75695E-01	11307	0	11208,5
Responder	Mono_CD14	Macro_FOLR2+APOE+	CXCL8	SDC3	2,52470E-03	7204,5	1,83957E-02	2922	6,55921E-01	2253	1,43068E+00	2215	8,45268E-01	17424	0	11208,5
Responder	cDC_CLEC9A	Macro_OLFML3	HLA-DQB1	CD4	2,52505E-03	7726,1	7,47899E-03	13911	4,60344E-01	5281	8,56647E-01	6774	9,29370E-01	1456	0	11208,5
Responder	Mono_INHBA	Macro_ISG15	ADM	GPR84	2,52774E-03	15257,1	1,78482E-02	3093	4,15545E-01	6504	8,88018E-01	6373	6,76596E-01	49107	0	11208,5
Responder	Mono_INHBA	Macro_FOLR2+APOE-	VEGFA	ITGB1	2,52774E-03	9617,1	1,17285E-02	6504	5,27798E-01	3899	9,97693E-01	5127	8,26523E-01	21347	0	11208,5
Responder	cDC_CLEC9A	Macro_IER3	SERPINF1	PLXDC2	2,53960E-03	10614,1	8,93414E-03	10337	2,98323E-01	10976	8,67866E-01	6622	8,61992E-01	13927	0	11208,5
Responder	Macro_FOLR2+APOE+	Macro_LYVE1	SERPING1	LRP1	2,53992E-03	4771,9	2,54776E-02	1678	1,00648E+00	566	8,77446E-01	6512	9,15419E-01	3895	0	11208,5
Responder	cDC_CLEC9A	Mono_CD16	SELPLG	ITGB2	2,54051E-03	11536,1	7,47516E-03	13928	2,78609E-01	12026	5,64923E-01	11419	8,87663E-01	9099	0	11208,5
Responder	pDC_LILRA4	Macro_IER3	VEGFB	NRP1	2,54070E-03	11451,5	2,07376E-02	2397	6,60130E-01	2202	1,98514E+00	736	7,27192E-01	40714	0	11208,5
Responder	Macro_NLRP3	Mono_CD16	S100A9	CD68	2,54508E-03	7000,9	1,10195E-02	7206	6,38928E-01	2407	4,60000E-01	13933	9,42703E-01	250	0	11208,5
Responder	Mono_INHBA	Macro_NLRP3	ANXA2	TLR2	2,54599E-03	11807,3	7,64656E-03	13412	3,09916E-01	10426	4,59880E-01	13934	8,82206E-01	10056	0	11208,5
Responder	Macro_FOLR2+APOE-	Mono_CD16	F13A1	ITGA4	2,54694E-03	5504,5	2,57801E-02	1650	1,04061E+00	489	1,39070E+00	2399	8,73429E-01	11776	0	11208,5
Responder	cDC_CLEC9A	Macro_NLRP3	CIRBP	TREM1	2,54782E-03	8414,9	1,25789E-02	5767	4,44007E-01	5702	9,66174E-01	5461	8,61965E-01	13936	0	11208,5
Responder	Macro_NLRP3	Macro_ISG15	VCAN	ITGB1	2,54965E-03	8464,9	1,21695E-02	6088	5,68674E-01	3291	7,81727E-01	7799	8,61953E-01	13938	0	11208,5
Responder	Mast	Macro_FOLR2+APOE-	CSF1	CSF2RA	2,55007E-03	9035,1	2,07187E-02	2400	7,80326E-01	1354	1,54543E+00	1772	7,91783E-01	28441	0	11208,5
Responder	Mono_CD14	Macro_FOLR2+APOE+	ICAM1	IL2RA	2,55214E-03	11036,9	1,83272E-02	2944	5,49156E-01	3565	8,76566E-01	6520	7,79187E-01	30947	0	11208,5
Responder	Macro_OLFML3	Mono_CD14	GNAI2	C5AR1	2,55239E-03	11111,3	7,46982E-03	13941	3,58334E-01	8430	5,12711E-01	12628	8,86116E-01	9349	0	11208,5
Responder	pDC_LILRA4	cDC_CLEC9A	ASIP	MGRN1	2,55319E-03	14441,1	4,74158E-02	528	7,62442E-01	1458	1,39051E+00	2401	6,12506E-01	56610	0	11208,5
Responder	Macro_OLFML3	Mono_INHBA	LGALS9	CD44	2,55514E-03	8857,1	8,60022E-03	11042	5,60493E-01	3396	4,59606E-01	13944	9,11053E-01	4695	0	11208,5
Responder	Mono_INHBA	Macro_ISG15	LGALS3	LAG3	2,55827E-03	8942,1	1,79029E-02	3078	6,06767E-01	2762	8,76194E-01	6524	8,27523E-01	21138	0	11208,5
Responder	Macro_OLFML3	cDC(CD1C)	C3	ITGAM	2,56902E-03	10275,9	1,16985E-02	6531	4,82067E-01	4776	1,08191E+00	4320	8,11342E-01	24544	0	11208,5
Responder	Mono_CD14	cDC_LAMP3	CD14	ITGA4	2,57075E-03	11706,5	7,46175E-03	13961	2,60837E-01	12991	6,34400E-01	10176	8,81447E-01	10196	0	11208,5
Responder	Mono_CD14	Macro_FOLR2+APOE+	ADAM10	GPNMB	2,57210E-03	7510,5	1,48425E-02	4339	7,78202E-01	1366	1,01849E+00	4906	8,53364E-01	15733	0	11208,5
Responder	Mono_INHBA	Mono_INHBA	CCL3	CCR1	2,57210E-03	7571,1	1,16979E-02	6533	7,02427E-01	1830	1,44586E+00	2134	8,51494E-01	16150	0	11208,5
Responder	Mono_CD14	Macro_FOLR2-APOE+	TIMP1	CD63	2,57536E-03	7799,9	7,46067E-03	13966	4,91687E-01	4569	6,99690E-01	9051	9,43684E-01	205	0	11208,5
Responder	Mono_CD16	Macro_FOLR2+APOE+	TIMP1	CD63	2,57577E-03	5324,9	1,24805E-02	5833	1,05463E+00	459	6,95779E-01	9112	9,55895E-01	12	0	11208,5
Responder	Macro_NLRP3	Macro_NLRP3	S100A8	TLR4	2,57720E-03	9242,5	1,43602E-02	4614	5,34851E-01	3779	4,58872E-01	13968	8,68376E-01	12643	0	11208,5
Responder	Mono_INHBA	Mono_CD14	S100A9	TLR4	2,57813E-03	10768,1	1,01395E-02	8299	3,83065E-01	7527	5,03924E-01	12837	8,61803E-01	13969	0	11208,5
Responder	Macro_IFI27	Macro_FOLR2-APOE+	LGALS3	ENG	2,57997E-03	11005,9	9,81447E-03	8749	3,60359E-01	8347	4,69000E-01	13696	8,66537E-01	13029	0	11208,5
Responder	cDC_CLEC9A	cDC_CLEC9A	HLA-DRB1	LAG3	2,58090E-03	9322,1	1,08774E-02	7371	3,54006E-01	8593	9,65764E-01	5466	8,61775E-01	13972	0	11208,5
Responder	cDC(CD1C)	Macro_NLRP3	PKM	CD44	2,58598E-03	8657,7	6,54942E-03	17135	4,14637E-01	6542	9,33810E-01	5810	9,22567E-01	2593	0	11208,5
Responder	Macro_FOLR2-APOE+	Macro_ISG15	PLAU	PLAUR	2,58829E-03	9636,9	1,01719E-02	8254	4,25497E-01	6197	7,31375E-01	8545	8,61756E-01	13980	0	11208,5
Responder	Macro_OLFML3	cDC_CLEC9A	CXCL11	DPP4	2,59062E-03	16452,5	6,74927E-02	229	5,72057E-01	3240	8,74379E-01	6545	5,48091E-01	61040	0	11208,5
Responder	Mono_INHBA	Macro_FOLR2-APOE+	ICAM1	ITGAM_ITGB2	2,59942E-03	11539,7	7,82890E-03	12900	3,56372E-01	8505	4,57868E-01	13992	8,76710E-01	11093	0	11208,5
Responder	Mono_INHBA	Macro_NLRP3	ICAM1	ITGAM_ITGB2	2,59942E-03	11761,7	7,45252E-03	13992	3,39368E-01	9154	5,05996E-01	12791	8,74022E-01	11663	0	11208,5
Responder	pDC_LILRA4	Macro_IER3	APP	LRP1	2,60128E-03	5880,9	1,83617E-02	2929	9,14964E-01	798	2,18884E+00	475	8,61667E-01	13994	0	11208,5
Responder	Macro_NLRP3	cDC_LAMP3	VCAN	CD44	2,60221E-03	8172,7	1,21362E-02	6114	5,64780E-01	3343	4,57667E-01	13995	9,03076E-01	6203	0	11208,5
Responder	Macro_IFI27	Macro_ISG15	FN1	PLAUR	2,60314E-03	8957,5	1,26089E-02	5737	3,19198E-01	10018	5,67852E-01	11356	9,01638E-01	6468	0	11208,5
Responder	Macro_NLRP3	Macro_ISG15	CD55	ADGRE5	2,60779E-03	7632,3	1,51735E-02	4169	6,62670E-01	2173	8,68876E-01	6610	8,61640E-01	14001	0	11208,5
Responder	cDC_CLEC9A	Mono_CD14	HLA-B	LILRA1	2,61618E-03	10070,1	9,65807E-03	9028	2,42861E-01	14010	1,01461E+00	4947	8,76472E-01	11157	0	11208,5
Responder	Macro_OLFML3	Macro_IFI27	C1QB	C1QBP	2,61618E-03	8972,3	7,44890E-03	14010	3,96873E-01	7070	8,78949E-01	6496	9,03681E-01	6077	0	11208,5
Responder	pDC_LILRA4	cDC_CLEC9A	APP	NOTCH2	2,61626E-03	6160,5	2,05755E-02	2421	9,69607E-01	647	2,89913E+00	80	8,50017E-01	16446	0	11208,5
Responder	cDC_CLEC9A	Macro_NLRP3	SERPINF1	PLXDC2	2,61992E-03	10337,7	8,87366E-03	10443	2,95552E-01	11114	1,01766E+00	4909	8,61588E-01	14014	0	11208,5
Responder	cDC(CD1C)	Mono_INHBA	GRN	TNFRSF1B	2,62172E-03	7698,9	7,42595E-03	14075	4,13832E-01	6565	1,20538E+00	3401	9,19084E-01	3245	0	11208,5

Responder	cDC(CD1C)	Macro_FOLR2+APOE+	HMGB1	CD163	2,62179E-03	9559,3	7,44646E-03	14016	4,13224E-01	6581	5,36046E-01	12058	9,15230E-01	3933	0	11208,5
Responder	Mono_CD16	pDC_LILRA4	AGTRAP	RACK1	2,62328E-03	5452,1	1,22966E-02	5992	6,01397E-01	2822	2,02728E+00	672	9,01148E-01	6566	0	11208,5
Responder	Macro_ISG15	cDC(CD1C)	CD14	ITGA4	2,62834E-03	9814,9	7,44430E-03	14023	3,35446E-01	9320	1,08286E+00	4311	8,81325E-01	10212	0	11208,5
Responder	Macro_NLRP3	Mast	ACTR2	ADRB2	2,62928E-03	6711,3	2,89926E-02	1321	7,28658E-01	1651	9,74550E-01	5352	8,61517E-01	14024	0	11208,5
Responder	Macro_FOLR2-APOE+	cDC_CLEC9A	TNFSF13B	CD40	2,63022E-03	11432,1	8,41439E-03	11482	2,70455E-01	12457	7,67762E-01	7988	8,61510E-01	14025	0	11208,5
Responder	pDC_LILRA4	cDC_LAMP3	TNFSF9	HLA-DPA1	2,63110E-03	7950,7	2,05470E-02	2428	6,80780E-01	2013	1,40270E+00	2335	9,01133E-01	6571	0,015	26406,5
Responder	Macro_NLRP3	Macro_OLFML3	S100A9	CD68	2,63491E-03	7270,7	1,04329E-02	7907	5,95936E-01	2888	4,56165E-01	14030	9,41208E-01	320	0	11208,5
Responder	pDC_LILRA4	Macro_NLRP3	APP	RPSA	2,63679E-03	8156,7	7,96832E-03	12513	7,27138E-01	1665	1,67004E+00	1365	8,61468E-01	14032	0	11208,5
Responder	Mono_CD14	Macro_LYVE1	HBEGF	CD9	2,63867E-03	9773,5	1,32514E-02	5277	4,02192E-01	6905	5,63865E-01	11443	8,61461E-01	14034	0	11208,5
Responder	Mono_INHBA	Macro_LYVE1	VEGFA	SIRPA	2,64050E-03	9253,9	1,89187E-02	2779	6,77179E-01	2051	8,71307E-01	6577	8,15626E-01	23654	0	11208,5
Responder	pDC_LILRA4	Macro_LYVE1	LTB	TNFRSF1A	2,64177E-03	7030,9	2,05446E-02	2429	9,32428E-01	754	2,30452E+00	372	8,31306E-01	20391	0	11208,5
Responder	cDC_CLEC9A	Macro_ISG15	HLA-DRB5	LAG3	2,64337E-03	8215,9	1,41392E-02	4749	4,29750E-01	6093	1,01112E+00	4990	8,61442E-01	14039	0	11208,5
Responder	Mono_INHBA	cDC(CD1C)	IL1B	SIGIRR	2,64364E-03	7984,7	1,16374E-02	6579	5,64822E-01	3341	1,40257E+00	2337	8,49963E-01	16458	0	11208,5
Responder	pDC_LILRA4	Macro_FOLR2-APOE+	SPON2	ITGA4	2,64497E-03	11964,7	2,36787E-02	1921	7,78264E-01	1365	1,38465E+00	2430	7,15203E-01	42899	0	11208,5
Responder	Macro_OLFML3	Macro_FOLR2+APOE+	C1QB	C1QBP	2,64526E-03	8493,5	9,80073E-03	8776	4,96785E-01	4462	4,55834E-01	14041	9,14980E-01	3980	0	11208,5
Responder	pDC_LILRA4	Macro_LYVE1	SAA1	CD36	2,64817E-03	9049,7	2,05077E-02	2431	6,45313E-01	2351	2,33754E+00	344	7,89346E-01	28914	0	11208,5
Responder	cDC_LAMP3	cDC_CLEC9A	CD274	PDCD1	2,65435E-03	15003,5	1,32652E-01	45	1,04943E+00	466	7,84203E-01	7760	6,23585E-01	55538	0	11208,5
Responder	Macro_ISG15	cDC_CLEC9A	TNFSF10	TNFRSF10B	2,65458E-03	7835,9	2,07612E-02	2393	6,86716E-01	1957	1,38395E+00	2433	8,27293E-01	21188	0	11208,5
Responder	Mono_INHBA	Macro_IFI27	VEGFA	SIRPA	2,65623E-03	11174,9	1,32293E-02	5294	5,57343E-01	3444	8,70789E-01	6587	7,87200E-01	29341	0	11208,5
Responder	Mono_CD14	Macro_NLRP3	S100A8	CD69	2,65658E-03	7697,9	8,59271E-03	11066	1,04459E+00	476	1,56723E+00	1686	8,61333E-01	14053	0	11208,5
Responder	Macro_NLRP3	Macro_IFI27	TIMP1	CD63	2,65658E-03	8595,1	7,70274E-03	13276	5,07060E-01	4258	4,55299E-01	14053	9,44526E-01	180	0	11208,5
Responder	Mast	cDC_CLEC9A	ANXA1	DYSF	2,65779E-03	6013,3	2,52455E-02	1703	7,82863E-01	1338	1,38367E+00	2434	8,64839E-01	13383	0	11208,5
Responder	Macro_OLFML3	cDC_CLEC9A	FN1	ITGA5_ITGB1	2,65781E-03	8871,9	1,50143E-02	4251	4,13017E-01	6588	1,12637E+00	3963	8,40929E-01	18349	0	11208,5
Responder	Macro_FOLR2-APOE+	Mono_CD16	LGALS1	PTPRC	2,66036E-03	8566,9	7,44963E-03	14007	5,93246E-01	2925	4,55146E-01	14057	9,36516E-01	637	0	11208,5
Responder	Macro_NLRP3	Mast	ADAM10	CD44	2,66415E-03	10981,9	8,34587E-03	11636	4,72717E-01	4976	4,54983E-01	14061	8,66543E-01	13028	0	11208,5
Responder	cDC_CLEC9A	cDC_LAMP3	ENTPD1	ADORA2A	2,66422E-03	10912,1	3,59433E-02	889	6,92682E-01	1914	1,38333E+00	2436	7,41728E-01	38113	0	11208,5
Responder	Mono_INHBA	Mono_CD14	TNF	VSIR	2,66570E-03	10486,3	1,17536E-02	6478	4,12613E-01	6593	1,04190E+00	4706	8,16595E-01	23446	0	11208,5
Responder	Macro_NLRP3	Mono_CD16	HLA-F	LILRB2	2,66604E-03	10266,7	8,40920E-03	11504	5,17708E-01	4070	4,54938E-01	14063	8,79862E-01	10488	0	11208,5
Responder	Macro_IFI27	Macro_NLRP3	FN1	C5AR1	2,66889E-03	7152,7	2,14472E-02	2282	5,64457E-01	3348	6,31935E-01	10216	8,89701E-01	8709	0	11208,5
Responder	Macro_IFI27	Macro_NLRP3	FN1	PLAUR	2,66984E-03	5822,5	1,77207E-02	3123	6,10278E-01	2721	7,52249E-01	8227	9,15732E-01	3833	0	11208,5
Responder	cDC(CD1C)	Macro_LYVE1	HMGB1	TLR4	2,67174E-03	12046,1	7,82294E-03	12918	2,80149E-01	11946	6,39683E-01	10089	8,61246E-01	14069	0	11208,5
Responder	cDC_CLEC9A	cDC(CD1C)	HLA-DQA2	CD4	2,67269E-03	9181,1	7,42915E-03	14070	2,87013E-01	11545	1,46859E+00	2038	8,98534E-01	7044	0	11208,5
Responder	Macro_LYVE1	Mono_INHBA	TIMP2	CD44	2,67362E-03	5931,3	1,38836E-02	4906	7,81476E-01	1349	8,69984E-01	6598	9,06181E-01	5595	0	11208,5
Responder	Macro_OLFML3	cDC_LAMP3	C3	CD46	2,67520E-03	8992,9	1,16208E-02	6599	4,45183E-01	5670	9,05724E-01	6150	8,55164E-01	15337	0	11208,5
Responder	cDC(CD1C)	Macro_NLRP3	GNAI2	FPR1	2,67649E-03	10404,3	7,42653E-03	14074	2,88857E-01	11457	9,80875E-01	5291	8,82554E-01	9991	0	11208,5
Responder	cDC(CD1C)	Mono_CD16	SAA1	FPR2	2,67996E-03	14069,7	2,18362E-02	2212	5,79339E-01	3110	8,69720E-01	6602	6,89309E-01	47216	0	11208,5
Responder	Macro_NLRP3	cDC(CD1C)	NAMPT	ITGA5_ITGB1	2,68125E-03	10274,7	7,42478E-03	14079	4,82888E-01	4750	8,07406E-01	7445	8,62170E-01	13891	0	11208,5
Responder	Mast	Mast	HSPA8	ADRB2	2,68154E-03	5104,3	2,76101E-02	1439	7,02006E-01	1834	1,06772E+00	4437	9,00962E-01	6603	0	11208,5
Responder	cDC_LAMP3	pDC_LILRA4	ALCAM	NRP1	2,68356E-03	10282,3	2,11810E-02	2321	6,35285E-01	2442	1,41335E+00	2283	7,67846E-01	33157	0	11208,5
Responder	Macro_FOLR2+APOE-	pDC_LILRA4	HMGB1	TLR9	2,68838E-03	9962,9	4,99665E-02	469	1,14791E+00	335	1,11290E+00	4076	8,46637E-01	17125	0,033	27809,5
Responder	Mono_CD14	Macro_ISG15	CXCL8	SDC3	2,69267E-03	9550,3	1,16083E-02	6610	5,29498E-01	3864	1,53424E+00	1803	8,12717E-01	24266	0	11208,5
Responder	cDC(CD1C)	Macro_OLFML3	HLA-DPA1	CD4	2,69427E-03	8187,9	6,60987E-03	16912	4,12022E-01	6611	9,51537E-01	5615	9,37057E-01	593	0	11208,5
Responder	Macro_IFI27	cDC(CD1C)	CXCL12	CXCR4	2,69652E-03	6656,1	1,75472E-02	3181	6,04814E-01	2784	1,47428E+00	2012	8,61134E-01	14095	0	11208,5
Responder	Macro_IFI27	Macro_NLRP3	C3	ITGAX	2,69747E-03	9874,7	1,27638E-02	5619	3,41840E-01	9039	4,65915E-01	13774	8,83997E-01	9733	0	11208,5
Responder	Macro_NLRP3	Macro_FOLR2-APOE+	S100A9	TLR4	2,69939E-03	10438,3	1,00220E-02	8463	4,96080E-01	4480	4,59618E-01	13942	8,61107E-01	14098	0	11208,5
Responder	Macro_IFI27	Macro_NLRP3	MMP9	CD44	2,70130E-03	7930,5	1,24798E-02	5834	5,34720E-01	3783	9,95671E-01	5151	8,63349E-01	13676	0	11208,5
Responder	cDC(CD1C)	cDC_CLEC9A	CCL5	CXCR3	2,70224E-03	16753,9	1,20064E-02	6254	5,23874E-01	3973	8,68557E-01	6616	6,21901E-01	55718	0	11208,5
Responder	Macro_IER3	cDC(CD1C)	TNF	TNFRSF1A	2,70300E-03	6852,9	2,04097E-02	2448	6,95656E-01	1891	1,48074E+00	1994	8,48577E-01	16723	0	11208,5
Responder	cDC(CD1C)	cDC_CLEC9A	HMGB1	HAVCR2	2,70322E-03	8463,9	7,41863E-03	14102	3,47422E-01	8840	1,61081E+00	1534	9,00808E-01	6635	0	11208,5
Responder	Mono_CD14	Macro_NLRP3	HP	TLR4	2,70384E-03	13903,5	6,69946E-02	236	6,01421E-01	2821	8,68477E-01	6617	6,79950E-01	48635	0	11208,5
Responder	pDC_LILRA4	Mono_CD16	HSP90B1	TLR2	2,70609E-03	9359,7	7,41796E-03	14105	3,50945E-01	8696	1,74474E+00	1185	8,74300E-01	11604	0	11208,5
Responder	Macro_ISG15	Mono_INHBA	NAMPT	ITGA5_ITGB1	2,70897E-03	10914,5	7,41760E-03	14108	3,71445E-01	7925	8,08154E-01	7432	8,62113E-01	13899	0	11208,5
Responder	Macro_NLRP3	Macro_NLRP3	VEGFA	CD44	2,71186E-03	9588,5	1,11986E-02	7000	5,20688E-01	4029	4,53033E-01	14111	8,74320E-01	11594	0	11208,5
Responder	Macro_FOLR2-APOE+	Mono_CD14	GRN	TNFRSF1B	2,71186E-03	8437,9	7,41678E-03	14111	4,26918E-01	6158	8,06261E-01	7460	9,19038E-01	3252	0	11208,5
Responder	Mono_INHBA	Mono_CD16	SPP1	ITGA4_ITGB1	2,71344E-03	9299,9	1,20961E-02	6155	4,11622E-01	6623	9,51338E-01	5619	8,47756E-01	16894	0	11208,5
Responder	pDC_LILRA4	Macro_IER3	TNFSF9	HLA-DPA1	2,71664E-03	7733,3	2,04180E-02	2445	6,74350E-01	2081	1,27641E+00	2990	9,00852E-01	6625	0,004	24525,5
Responder	Macro_NLRP3	Mono_CD14	S100A12	CD36	2,71927E-03	7331,7	3,31204E-02	1027	6,56138E-01	2250	1,37988E+00	2453	8,34520E-01	19720	0	11208,5
Responder	Macro_OLFML3	Macro_FOLR2-APOE+	MDK	SDC2	2,71985E-03	11196,9	4,18088E-02	676	6,31484E-01	2481	8,67426E-01	6627	7,58502E-01	34992	0	11208,5
Responder	Mono_CD14	Macro_OLFML3	NAMPT	ITGA5_ITGB1	2,72148E-03	9469,3	7,41210E-03	14121	5,98602E-01	2852	9,84444E-01	5257	8,62069E-01	13908	0	11208,5
Responder	pDC_LILRA4	Mono_INHBA	PIGF	FLT1	2,72253E-03	12959,5	4,23833E-02	663	6,74454E-01	2080	1,37981E+00	2454	6,81644E-01	48392	0	11208,5
Responder	Macro_FOLR2-APOE+	Mono_CD14	MMP9	LRP1	2,72579E-03	7311,3	2,03813E-02	2455	6,74803E-01	2076	1,48596E+00	1970	8,38461E-01	18847	0	11208,5
Responder	Macro_ISG15	Macro_LYVE1	B2M	TFRC	2,73016E-03	9228,3	7,72030E-03	13228	4,21712E-01	6309	4,52475E-01	14130	9,30758E-01	1266	0	11208,5
Responder	Macro_ISG15	cDC(CD1C)	CCL8	CCR1	2,73232E-03	13369,9	2,10843E-02	2335	6,33769E-01	2457	1,77486E+00	1109	6,72166E-01	49740	0	11208,5
Responder	Macro_OLFML3	pDC_LILRA4	MDK	NOTCH2	2,73270E-03	9429,1	1,95387E-02	2627	4,11225E-01	6635	1,53336E+00	1805	8,09727E-01	24870	0	11208,5
Responder	cDC(CD1C)	Macro_LYVE1	TNFSF12	CD163	2,73270E-03	8807,3	1,15805E-02	6635	6,83426E-01	1989	1,07587E+00	4371	8,33932E-01	19833	0	11208,5

Responder	cDC(CD1C)	Mono_CD14	ARPC5	LDLR	2,73592E-03	9445,7	1,38169E-02	4946	4,11126E-01	6637	1,26880E+00	3033	8,26264E-01	21404	0	11208,5
Responder	Macro_OLFML3	Mono_CD16	HLA-A	LILRB1	2,73596E-03	9146,3	7,40716E-03	14136	4,98671E-01	4430	4,81995E-01	13372	9,22609E-01	2585	0	11208,5
Responder	Macro_OLFML3	Mono_CD14	CIRBP	TREM1	2,74371E-03	10129,5	1,23496E-02	5946	4,39373E-01	5833	4,76106E-01	13516	8,60867E-01	14144	0	11208,5
Responder	Macro_IFI27	cDC_CLEC9A	APOE	SCARB1	2,74468E-03	7457,3	1,77797E-02	3112	4,95351E-01	4498	8,95901E-01	6287	8,71062E-01	12181	0	11208,5
Responder	Mono_CD14	Macro_ISG15	S100A8	CD36	2,74557E-03	7339,5	9,49345E-03	9304	1,08535E+00	413	2,18930E+00	474	8,55335E-01	15298	0	11208,5
Responder	Mono_INHBA	Macro_FOLR2-APOE+	VCAN	ITGB1	2,74662E-03	9827,5	1,19457E-02	6304	4,60014E-01	5286	5,30496E-01	12192	8,60845E-01	14147	0	11208,5
Responder	Macro_FOLR2-APOE+	Mono_INHBA	ADAM10	CD44	2,74954E-03	11419,9	7,57308E-03	13620	4,79590E-01	4835	4,85529E-01	13286	8,60823E-01	14150	0	11208,5
Responder	Mono_CD14	Macro_IFI27	VCAN	ITGA4	2,75207E-03	8059,7	1,15693E-02	6647	7,36757E-01	1604	1,35062E+00	2587	8,41335E-01	18252	0	11208,5
Responder	Mono_CD14	Macro_NLRP3	ANXA1	FPR1	2,75343E-03	12040,5	7,40258E-03	14154	2,48855E-01	13669	5,11768E-01	12653	8,90803E-01	8518	0	11208,5
Responder	Macro_OLFML3	Macro_OLFML3	ADAM10	TREM2	2,75578E-03	16192,5	8,39238E-03	11546	3,25634E-01	9749	1,76614E-01	22827	8,06109E-01	25632	0	11208,5
Responder	Mono_INHBA	Mono_INHBA	CCL5	SDC4	2,76017E-03	15281,1	1,58914E-02	3818	4,10448E-01	6652	8,94062E-01	6309	6,81450E-01	48418	0	11208,5
Responder	Macro_IFI27	cDC_CLEC9A	C1QA	CD93	2,76122E-03	10903,1	7,97616E-03	12491	3,04643E-01	10661	5,95401E-01	10852	8,86348E-01	9303	0	11208,5
Responder	Macro_OLFML3	Macro_OLFML3	HLA-DQB1	CD4	2,76219E-03	8709,3	7,84997E-03	12848	5,17138E-01	4088	4,51237E-01	14163	9,30943E-01	1239	0	11208,5
Responder	Macro_ISG15	Macro_NLRP3	LGALS9	LRP1	2,76414E-03	12709,9	7,58115E-03	13592	2,93298E-01	11231	4,82707E-01	13353	8,60768E-01	14165	0	11208,5
Responder	Mono_INHBA	Macro_FOLR2-APOE+	CCL4	CCR5	2,76504E-03	12091,1	1,15632E-02	6655	5,58898E-01	3418	1,16463E+00	3647	7,55535E-01	35527	0	11208,5
Responder	pDC_LILRA4	pDC_LILRA4	CCL5	DPP4	2,76515E-03	14541,7	2,99571E-02	1232	6,32947E-01	2467	2,30506E+00	371	6,03065E-01	57430	0	11208,5
Responder	Macro_OLFML3	Macro_ISG15	FN1	ITGAV_ITGB1	2,76667E-03	8893,9	1,57441E-02	3894	4,10288E-01	6656	9,83452E-01	5263	8,45182E-01	17448	0	11208,5
Responder	Macro_IFI27	cDC_CLEC9A	LYZ	ITGAL	2,76805E-03	7826,9	1,09614E-02	7266	4,36425E-01	5916	8,63356E-01	6686	8,93229E-01	8058	0	11208,5
Responder	Macro_IFI27	pDC_LILRA4	HLA-DQA2	CD4	2,77489E-03	5658,3	1,07011E-02	7568	4,97552E-01	4450	1,87347E+00	917	9,14002E-01	4148	0	11208,5
Responder	pDC_LILRA4	Macro_FOLR2-APOE+	CALR	ITGAV	2,77489E-03	8950,7	1,02659E-02	8122	3,32391E-01	9448	1,53570E+00	1799	8,60733E-01	14176	0	11208,5
Responder	Macro_IFI27	pDC_LILRA4	HLA-DQA1	CD4	2,77587E-03	7313,9	7,44965E-03	14005	3,72178E-01	7907	1,49663E+00	1935	9,28921E-01	1514	0	11208,5
Responder	Macro_IFI27	pDC_LILRA4	HLA-DQB1	CD4	2,77783E-03	6905,3	7,60427E-03	13530	4,01714E-01	6921	1,62788E+00	1487	9,29914E-01	1380	0	11208,5
Responder	Macro_OLFML3	cDC_CLEC9A	HLA-DRB5	LAG3	2,78077E-03	8147,1	1,39667E-02	4856	5,46812E-01	3593	8,46489E-01	6896	8,60708E-01	14182	0	11208,5
Responder	Macro_OLFML3	Macro_ISG15	HLA-DQA2	LAG3	2,78132E-03	9157,3	2,04116E-02	2446	5,41309E-01	3681	8,64593E-01	6665	8,24406E-01	21786	0	11208,5
Responder	Macro_FOLR2-APOE+	Mono_INHBA	PLTP	ABCA1	2,78175E-03	8279,7	1,46647E-02	4430	5,80617E-01	3086	7,34917E-01	8491	8,60707E-01	14183	0	11208,5
Responder	Macro_FOLR2-APOE+	pDC_LILRA4	TGFB1	CXCR4	2,78175E-03	8747,7	7,39302E-03	14183	4,40383E-01	5808	1,78241E+00	1092	8,75061E-01	11447	0	11208,5
Responder	Macro_IFI27	pDC_LILRA4	HLA-C	NOTCH4	2,78371E-03	5906,9	2,95556E-02	1273	5,35876E-01	3758	1,46125E+00	2072	8,76112E-01	11223	0	11208,5
Responder	Macro_OLFML3	Macro_IFI27	CXCL9	FCGR2A	2,78960E-03	11000,5	1,05137E-02	7801	2,40218E-01	14191	5,96242E-01	10835	8,77328E-01	10967	0	11208,5
Responder	Macro_NLRP3	pDC_LILRA4	HSP90B1	TLR9	2,79175E-03	7351,9	4,97339E-02	478	1,16207E+00	312	1,02227E+00	4878	8,33671E-01	19883	0	11208,5
Responder	Macro_IFI27	pDC_LILRA4	HLA-DRB5	CD4	2,79354E-03	7092,9	7,42140E-03	14094	3,80998E-01	7595	1,58184E+00	1634	9,33698E-01	933	0	11208,5
Responder	Macro_IFI27	pDC_LILRA4	APOE	SCARB1	2,79748E-03	7891,9	1,52161E-02	4140	4,41952E-01	5755	1,06706E+00	4447	8,62063E-01	13909	0	11208,5
Responder	Mono_INHBA	Mono_INHBA	ICAM1	ITGAX_ITGB2	2,79945E-03	10651,3	7,38722E-03	14201	3,61363E-01	8314	6,48999E-01	9909	8,84523E-01	9624	0	11208,5
Responder	Macro_IFI27	pDC_LILRA4	CXCL12	CXCR4	2,80438E-03	6088,1	1,83334E-02	2942	6,41375E-01	2387	2,39148E+00	312	8,63733E-01	13591	0	11208,5
Responder	Macro_OLFML3	Macro_FOLR2-APOE+	C1QB	CD33	2,80635E-03	11546,9	7,38339E-03	14208	3,53543E-01	8604	4,68794E-01	13703	8,82459E-01	10011	0	11208,5
Responder	Macro_FOLR2-APOE+	Macro_OLFML3	MMP9	EPHB2	2,80750E-03	14821,7	2,42423E-02	1839	6,30156E-01	2493	9,92406E-01	5187	6,43173E-01	53381	0	11208,5
Responder	Mono_INHBA	Macro_NLRP3	HSP90B1	ASGR1	2,80833E-03	10831,1	1,08085E-02	7444	3,45692E-01	8903	5,22743E-01	12390	8,60574E-01	14210	0	11208,5
Responder	Mono_CD14	Macro_OLFML3	ADAM10	TREM2	2,80914E-03	9724,3	1,28497E-02	5561	4,31370E-01	6051	8,63593E-01	6682	8,37252E-01	19119	0	11208,5
Responder	Macro_IFI27	pDC_LILRA4	HMGB1	THBD	2,81426E-03	8020,7	9,87822E-03	8657	3,63493E-01	8234	1,60088E+00	1565	8,80133E-01	10439	0	11208,5
Responder	pDC_LILRA4	Mono_CD14	HSP90B1	TLR2	2,81822E-03	9267,9	7,37873E-03	14220	3,49118E-01	8760	2,17850E+00	485	8,74008E-01	11666	0	11208,5
Responder	Macro_IFI27	pDC_LILRA4	C1QB	C1QBP	2,82021E-03	5895,9	1,09250E-02	7303	4,53743E-01	5454	1,41385E+00	2280	9,19109E-01	3234	0	11208,5
Responder	pDC_LILRA4	Macro_FOLR2+APOE+	HSP90B1	TLR2	2,82120E-03	9521,3	7,37848E-03	14223	3,49106E-01	8761	1,55127E+00	1746	8,74006E-01	11668	0	11208,5
Responder	Macro_IER3	Mono_CD14	CCL4	CCR1	2,82488E-03	5532,1	2,02407E-02	2485	8,70536E-01	943	1,40373E+00	2326	8,78782E-01	10698	0	11208,5
Responder	pDC_LILRA4	Macro_FOLR2-APOE+	HSP90B1	TLR2	2,83212E-03	9439,7	7,37596E-03	14234	3,48989E-01	8769	1,68903E+00	1314	8,73987E-01	11673	0	11208,5
Responder	pDC_LILRA4	Macro_NLRP3	SEMA7A	PLXNC1	2,83492E-03	12976,7	2,02248E-02	2488	6,39956E-01	2399	1,39296E+00	2386	6,94326E-01	46402	0	11208,5
Responder	Macro_ISG15	Macro_IFI27	SPP1	CD44	2,83810E-03	11582,1	7,63518E-03	13438	2,39415E-01	14240	7,85890E-01	7736	8,75774E-01	11288	0	11208,5
Responder	pDC_LILRA4	Macro_OLFML3	APP	NCSTN	2,84162E-03	8142,3	2,02208E-02	2490	9,26536E-01	767	2,16257E+00	511	8,05481E-01	25735	0	11208,5
Responder	Mono_CD14	Macro_FOLR2+APOE+	VCAN	ITGA4	2,84878E-03	8285,9	1,15075E-02	6706	7,35710E-01	1611	1,17796E+00	3566	8,40977E-01	18338	0	11208,5
Responder	pDC_LILRA4	cDC(CD1C)	MIF	CD74_CXCR4	2,85001E-03	9768,1	4,69878E-03	28155	3,53158E-01	8615	2,28895E+00	379	9,38476E-01	483	0	11208,5
Responder	pDC_LILRA4	Macro_FOLR2+APOE-	LILRB4	LAIR1	2,85108E-03	9909,7	8,11856E-03	12145	2,39296E-01	14253	1,76492E+00	1137	8,78167E-01	10805	0	11208,5
Responder	Mono_CD14	Mono_INHBA	GNAI2	C5AR1	2,85108E-03	10281,3	7,37081E-03	14253	3,47036E-01	8858	7,94189E-01	7623	8,85441E-01	9464	0	11208,5
Responder	pDC_LILRA4	Macro_FOLR2+APOE-	COPA	CD74	2,85308E-03	7249,9	7,36909E-03	14255	4,17577E-01	6432	1,25340E+00	3128	9,31006E-01	1226	0	11208,5
Responder	Macro_ISG15	Mono_CD14	S100A9	TLR4	2,86009E-03	9551,3	9,89243E-03	8639	3,68724E-01	8024	9,50868E-01	5623	8,60327E-01	14262	0	11208,5
Responder	Macro_ISG15	Mono_INHBA	VEGFA	CD44	2,86109E-03	9686,5	8,77920E-03	10631	5,29383E-01	3869	7,37166E-01	8461	8,60327E-01	14263	0	11208,5
Responder	pDC_LILRA4	Mono_INHBA	GAS6	MERTK	2,86180E-03	9594,5	2,01853E-02	2496	8,63163E-01	966	2,30939E+00	367	7,68864E-01	32935	0	11208,5
Responder	Mono_INHBA	Macro_NLRP3	ADM	GPR84	2,86208E-03	15516,5	1,72342E-02	3284	4,08559E-01	6714	8,61492E-01	6711	6,72754E-01	49665	0	11208,5
Responder	cDC_CLEC9A	Macro_ISG15	HSPA1A	TLR4	2,86210E-03	9786,3	7,36608E-03	14264	3,87284E-01	7381	1,09854E+00	4184	8,72719E-01	11894	0	11208,5
Responder	cDC(CD1C)	Macro_NLRP3	HMGB1	THBD	2,86310E-03	10859,3	7,36607E-03	14265	3,10011E-01	10420	1,02834E+00	4821	8,63771E-01	13582	0	11208,5
Responder	Macro_ISG15	Macro_OLFML3	LILRB4	LAIR1	2,86812E-03	9845,9	1,04204E-02	7920	3,88748E-01	7336	4,46947E-01	14270	8,90903E-01	8495	0	11208,5
Responder	Macro_IFI27	Macro_LYVE1	LGALS9	LRP1	2,86812E-03	10440,3	8,92105E-03	10358	4,35814E-01	5933	5,22802E-01	12388	8,70237E-01	12314	0	11208,5
Responder	Macro_OLFML3	Macro_IER3	FN1	ITGAV_ITGB1	2,86875E-03	8648,5	1,72463E-02	3279	4,40644E-01	5802	8,60799E-01	6718	8,51051E-01	16235	0	11208,5
Responder	Macro_IFI27	Macro_LYVE1	HLA-DQA2	CD4	2,87214E-03	7447,7	1,12216E-02	6976	5,29897E-01	3858	5,66747E-01	11381	9,15850E-01	3815	0	11208,5
Responder	Mono_INHBA	Mono_INHBA	VCAN	ITGB1	2,87315E-03	9160,5	1,18305E-02	6410	4,54951E-01	5411	7,34658E-01	8498	8,60264E-01	14275	0	11208,5
Responder	Macro_FOLR2-APOE+	pDC_LILRA4	LGALS9	CD47	2,87516E-03	10047,3	7,99272E-03	12450	3,33121E-01	9413	1,29431E+00	2888	8,60258E-01	14277	0	11208,5
Responder	Mono_INHBA	Macro_ISG15	SPP1	CD44	2,87617E-03	11304,5	7,75604E-03	13121	2,38814E-01	14278	8,53850E-01	6803	8,76626E-01	11112	0	11208,5
Responder	Macro_NLRP3	cDC_CLEC9A	THBS1	CD47	2,87919E-03	7490,7	1,41166E-02	4763	5,82527E-01	3066	1,10379E+00	4135	8,60223E-01	14281	0	11208,5

Post_R_Myeloid_Myeloid_CellCel

Responder	pDC_LILRA4	Macro_LYVE1	LTBP3	ITGB5	2,88207E-03	14920,9	5,57989E-02	357	6,41183E-01	2390	1,36946E+00	2502	5,94336E-01	58147	0	11208,5
Responder	Macro_NLRP3	Macro_OLFML3	VCAN	ITGA4	2,88626E-03	8948,9	1,55759E-02	3970	6,42507E-01	2378	5,01335E-01	12900	8,60191E-01	14288	0	11208,5
Responder	cDC_CLEC9A	Mono_INHBA	LGALS9	LRP1	2,88828E-03	11253,7	7,50819E-03	13815	2,84131E-01	11730	9,87901E-01	5225	8,60187E-01	14290	0	11208,5
Responder	Macro_NLRP3	Mono_CD16	VCAN	TLR2	2,89131E-03	8258,5	1,66516E-02	3493	5,76570E-01	3161	6,94091E-01	9137	8,60185E-01	14293	0	11208,5
Responder	Macro_OLFML3	Macro_IFI27	C1QB	LRP1	2,89131E-03	10020,3	7,35527E-03	14293	3,88861E-01	7331	5,37333E-01	12021	9,08060E-01	5248	0	11208,5
Responder	Macro_IFI27	Mono_CD14	SPP1	CD44	2,89434E-03	10443,3	7,35502E-03	14296	3,38415E-01	9193	9,34881E-01	5796	8,73726E-01	11723	0	11208,5
Responder	Mono_INHBA	Macro_FOLR2+APOE+	VEGFA	NRP2	2,89554E-03	9212,5	2,04620E-02	2438	7,14444E-01	1739	8,59250E-01	6734	8,14157E-01	23943	0	11208,5
Responder	pDC_LILRA4	Mono_CD16	SPON2	ITGB2	2,89563E-03	7002,7	3,03531E-02	1194	7,62188E-01	1461	1,36742E+00	2506	8,39457E-01	18644	0	11208,5
Responder	Macro_OLFML3	Macro_OLFML3	HLA-DPA1	CD4	2,89705E-03	9807,5	6,92830E-03	15702	4,75640E-01	4918	3,59720E-01	16722	9,38431E-01	487	0	11208,5
Responder	Macro_OLFML3	pDC_LILRA4	HSP90B1	TLR7	2,89722E-03	9127,7	1,54598E-02	4017	4,14604E-01	6543	8,59240E-01	6735	8,46592E-01	17135	0	11208,5
Responder	Mono_CD14	cDC_LAMP3	VEGFA	NRP2	2,89722E-03	12227,7	1,14778E-02	6735	4,83282E-01	4747	1,00893E+00	5011	7,66414E-01	33437	0	11208,5
Responder	Mono_CD14	Macro_FOLR2-APOE+	SERPINA1	LRP1	2,89941E-03	11711,3	7,35858E-03	14286	2,38433E-01	14301	5,57817E-01	11568	8,97725E-01	7193	0	11208,5
Responder	Macro_FOLR2-APOE+	cDC(CD1C)	APP	CD74	2,90227E-03	8258,5	6,68672E-03	16616	4,09924E-01	6665	1,09854E+00	4185	9,22420E-01	2618	0	11208,5
Responder	Macro_IFI27	Macro_LYVE1	APOE	LRP1	2,90448E-03	5829,5	1,86738E-02	2839	7,35904E-01	1610	5,13676E-01	12597	9,34115E-01	893	0	11208,5
Responder	Mono_CD14	Macro_IER3	HP	TLR4	2,90732E-03	14815,7	5,36883E-02	390	5,34287E-01	3793	8,58750E-01	6741	6,55394E-01	51946	0	11208,5
Responder	Macro_OLFML3	Macro_OLFML3	CXCL9	FCGR2A	2,90753E-03	9630,7	1,30250E-02	5443	3,63693E-01	8228	4,45054E-01	14309	8,88397E-01	8965	0	11208,5
Responder	Macro_ISG15	pDC_LILRA4	CXCL8	CD79A	2,91605E-03	11251,5	3,96598E-02	746	6,28565E-01	2512	1,44437E+00	2143	7,33503E-01	39648	0	11208,5
Responder	Mono_INHBA	Macro_OLFML3	CD52	SIGLEC10	2,91770E-03	9699,7	1,04753E-02	7852	4,53147E-01	5474	4,44880E-01	14319	8,84445E-01	9645	0	11208,5
Responder	Macro_FOLR2-APOE+	Mono_INHBA	MMP12	PLAUR	2,92280E-03	6449,1	2,18308E-02	2214	6,96540E-01	1883	1,34361E+00	2616	8,60052E-01	14324	0	11208,5
Responder	Macro_IFI27	Macro_LYVE1	CXCL9	FCGR2A	2,93302E-03	10228,7	9,61847E-03	9090	3,67498E-01	8080	5,96862E-01	10821	8,72459E-01	11944	0	11208,5
Responder	Macro_FOLR2-APOE+	cDC(CD1C)	MRC1	PTPRC	2,93607E-03	9281,7	1,37750E-02	4971	4,87167E-01	4665	1,08397E+00	4303	8,26938E-01	21261	0	11208,5
Responder	pDC_LILRA4	Macro_LYVE1	LGALS9	LRP1	2,93814E-03	10094,7	7,48142E-03	13903	3,61991E-01	8293	1,32198E+00	2730	8,59972E-01	14339	0	11208,5
Responder	Macro_ISG15	Mast	HBEGF	CD44	2,93814E-03	11141,1	7,33875E-03	14339	4,24375E-01	6234	5,52184E-01	11698	8,70848E-01	12226	0	11208,5
Responder	cDC(CD1C)	pDC_LILRA4	LGALS9	CD47	2,93916E-03	9710,7	7,95444E-03	12559	3,30491E-01	9519	1,87132E+00	927	8,59969E-01	14340	0	11208,5
Responder	Mono_INHBA	Macro_FOLR2+APOE+	VEGFA	EPHB2	2,93947E-03	14825,9	2,13933E-02	2296	5,99397E-01	2840	8,57630E-01	6760	6,63098E-01	51025	0	11208,5
Responder	Macro_NLRP3	pDC_LILRA4	MAML2	NOTCH4	2,94000E-03	13755,3	3,84640E-02	786	6,27259E-01	2519	1,68793E+00	1315	6,47006E-01	52948	0	11208,5
Responder	Macro_FOLR2+APOE+	cDC(CD1C)	C1QB	C1QBP	2,94287E-03	5406,1	1,14479E-02	6762	6,23013E-01	2569	1,17706E+00	3571	9,20831E-01	2920	0	11208,5
Responder	cDC(CD1C)	cDC(CD1C)	HLA-DRA	CD4	2,94343E-03	9190,1	5,43315E-03	22900	3,33099E-01	9414	1,57749E+00	1648	9,35205E-01	780	0	11208,5
Responder	Macro_IFI27	Macro_LYVE1	A2M	LRP1	2,94737E-03	8170,7	1,57295E-02	3902	5,79173E-01	3116	5,78034E-01	11162	8,74951E-01	11465	0	11208,5
Responder	pDC_LILRA4	Macro_LYVE1	CD24	SIGLEC10	2,95030E-03	21113,9	2,77360E-02	1431	6,26656E-01	2522	1,55967E+00	1710	6,34548E-01	54342	0,982	45564,5
Responder	cDC(CD1C)	cDC(CD1C)	HLA-DQA1	CD4	2,95030E-03	7790,5	6,54900E-03	17136	3,96346E-01	7087	1,70078E+00	1287	9,24548E-01	2234	0	11208,5
Responder	Macro_ISG15	Macro_FOLR2+APOE-	SERPING1	LRP1	2,95045E-03	12379,9	8,19868E-03	11951	2,52440E-01	13476	5,92533E-01	10913	8,59937E-01	14351	0	11208,5
Responder	Macro_IFI27	Macro_LYVE1	C3	LRP1	2,95148E-03	7187,5	1,90078E-02	2759	5,68801E-01	3290	5,59929E-01	11527	8,98024E-01	7153	0	11208,5
Responder	Macro_OLFML3	Macro_LYVE1	APOE	SCARB1	2,95148E-03	8607,1	1,46838E-02	4417	5,22936E-01	3985	6,98338E-01	9073	8,59932E-01	14352	0	11208,5
Responder	cDC(CD1C)	cDC(CD1C)	HLA-DRB1	CD4	2,95374E-03	9710,3	5,32397E-03	23558	3,02213E-01	10787	1,51669E+00	1850	9,31679E-01	1148	0	11208,5
Responder	Macro_NLRP3	Mono_CD14	NAMPT	ITGA5_ITGB1	2,95457E-03	10330,1	7,33714E-03	14355	4,93963E-01	4523	8,00805E-01	7531	8,61463E-01	14033	0	11208,5
Responder	cDC(CD1C)	cDC(CD1C)	HLA-DQB1	CD4	2,95718E-03	8130,7	6,35581E-03	17961	3,77953E-01	7700	1,64679E+00	1433	9,23839E-01	2351	0	11208,5
Responder	Macro_IFI27	Macro_LYVE1	SERPING1	LRP1	2,96074E-03	8069,9	1,36609E-02	5037	5,53642E-01	3506	5,58486E-01	11558	8,87958E-01	9040	0	11208,5
Responder	cDC(CD1C)	Mono_CD16	APP	FPR2	2,96161E-03	12467,5	1,96032E-02	2618	5,87921E-01	2992	8,56655E-01	6773	7,38402E-01	38746	0	11208,5
Responder	Macro_NLRP3	Mono_CD14	VCAN	TLR2	2,96384E-03	7248,1	1,65635E-02	3525	5,74743E-01	3188	1,12785E+00	3955	8,59866E-01	14364	0	11208,5
Responder	cDC(CD1C)	cDC(CD1C)	HLA-DPB1	CD4	2,96407E-03	8172,1	5,89732E-03	20196	3,95000E-01	7127	1,73751E+00	1209	9,31958E-01	1120	0	11208,5
Responder	Macro_NLRP3	Macro_FOLR2+APOE+	VCAN	TLR2	2,96487E-03	9041,1	1,65630E-02	3526	5,74731E-01	3189	5,00614E-01	12917	8,59864E-01	14365	0	11208,5
Responder	Macro_ISG15	Mono_INHBA	GRN	TNFRSF1B	2,96693E-03	8767,5	7,33014E-03	14367	4,02339E-01	6902	7,63550E-01	8039	9,18600E-01	3321	0	11208,5
Responder	Macro_ISG15	Macro_FOLR2+APOE+	CXCL10	SDC4	2,96752E-03	10783,7	2,41149E-02	1863	6,57707E-01	2230	1,36284E+00	2527	7,52442E-01	36090	0	11208,5
Responder	Macro_IFI27	Macro_LYVE1	HMGB1	CD163	2,96797E-03	8206,7	8,83530E-03	10514	5,67250E-01	3312	4,87757E-01	13239	9,21633E-01	2760	0	11208,5
Responder	pDC_LILRA4	Macro_LYVE1	COPA	CD74	2,96797E-03	7418,9	7,32959E-03	14368	4,09078E-01	6698	1,17775E+00	3567	9,30833E-01	1253	0	11208,5
Responder	Macro_NLRP3	Macro_FOLR2-APOE+	VCAN	TLR2	2,96900E-03	8482,3	1,65573E-02	3529	5,74614E-01	3196	6,38378E-01	10109	8,59843E-01	14369	0	11208,5
Responder	Macro_FOLR2-APOE+	cDC(CD1C)	SPP1	ITGA4_ITGB1	2,97016E-03	8379,1	1,26004E-02	5743	4,52525E-01	5491	1,26161E+00	3081	8,50373E-01	16372	0	11208,5
Responder	Macro_FOLR2-APOE+	cDC(CD1C)	SPP1	ITGA5_ITGB1	2,97187E-03	9463,1	1,20291E-02	6224	4,48390E-01	5591	1,19300E+00	3478	8,29210E-01	20814	0	11208,5
Responder	cDC(CD1C)	Macro_NLRP3	GRN	TNFRSF1B	2,97314E-03	8034,3	7,32869E-03	14373	4,04456E-01	6839	1,06929E+00	4428	9,18593E-01	3323	0	11208,5
Responder	Mono_INHBA	cDC(CD1C)	S100A9	ITGB2	2,97520E-03	9528,3	7,32847E-03	14375	3,36071E-01	9287	5,86787E-01	11010	9,27262E-01	1761	0	11208,5
Responder	Macro_FOLR2-APOE+	cDC(CD1C)	SPP1	PTGER4	2,97530E-03	8297,3	1,61120E-02	3715	5,29820E-01	3859	1,38634E+00	2421	8,31774E-01	20283	0	11208,5
Responder	Macro_OLFML3	Macro_FOLR2+APOE+	LILRB4	LAIR1	2,97530E-03	9571,5	1,26704E-02	5693	5,26452E-01	3918	2,50013E-01	20257	9,00047E-01	6781	0	11208,5
Responder	Macro_FOLR2+APOE+	Mono_CD14	C1QB	LRP1	2,97873E-03	5463,9	1,14302E-02	6783	6,21353E-01	2589	1,05523E+00	4574	9,24881E-01	2165	0	11208,5
Responder	Macro_NLRP3	Macro_ISG15	S100A8	ITGB2	2,98246E-03	8152,1	1,04333E-02	7906	4,30959E-01	6058	4,42675E-01	14382	9,31139E-01	1206	0	11208,5
Responder	Macro_FOLR2-APOE+	cDC(CD1C)	MIF	CD74_CXCR4	2,98388E-03	8615,3	5,49285E-03	22531	4,55875E-01	5393	1,15661E+00	3700	9,42832E-01	244	0	11208,5
Responder	pDC_LILRA4	Macro_FOLR2+APOE+	SPON2	ITGAM	2,98481E-03	14799,5	2,03388E-02	2464	7,68229E-01	1412	1,36133E+00	2532	6,15077E-01	56381	0	11208,5
Responder	pDC_LILRA4	Mono_INHBA	LIN7C	ABCA1	2,98827E-03	9908,3	2,52518E-02	1701	6,25467E-01	2533	1,44796E+00	2123	7,73775E-01	31976	0	11208,5
Responder	Mono_CD14	Mono_INHBA	THBS1	LRP1	2,99076E-03	9280,5	1,18771E-02	6372	4,06144E-01	6790	8,94195E-01	6307	8,53391E-01	15725	0	11208,5
Responder	Macro_IER3	cDC_CLEC9A	TNF	TRADD	2,99174E-03	8690,5	2,31951E-02	2001	7,64204E-01	1445	1,36064E+00	2534	8,02862E-01	26264	0	11208,5
Responder	Macro_IFI27	Mono_INHBA	APOE	LRP1	2,99388E-03	8152,5	1,20227E-02	6236	4,43676E-01	5710	4,42400E-01	14393	9,19200E-01	3215	0	11208,5
Responder	Macro_OLFML3	Macro_LYVE1	SAA1	CD36	2,99521E-03	11138,1	2,00092E-02	2535	6,38290E-01	2417	6,32548E-01	10205	7,87293E-01	29325	0	11208,5
Responder	Mono_CD16	pDC_LILRA4	PSEN1	NOTCH4	2,99869E-03	13256,1	3,74598E-02	831	6,25431E-01	2536	1,66320E+00	1382	6,68290E-01	50323	0	11208,5
Responder	Mono_INHBA	Macro_NLRP3	VEGFA	SIRPA	2,99937E-03	11154,7	1,33689E-02	5198	5,60285E-01	3399	8,54591E-01	6795	7,88079E-01	29173	0	11208,5
Responder	Macro_ISG15	cDC_LAMP3	ICAM1	IL2RG	3,00429E-03	8943,3	1,01380E-02	8301	5,38151E-01	3726	8,34432E-01	7078	8,59678E-01	14403	0	11208,5

Responder	pDC_LILRA4	cDC_LAMP3	APP	APLP2	3,00534E-03	7116,7	1,09487E-02	7280	7,20305E-01	1701	1,83549E+00	990	8,59676E-01	14404	0	11208,5
Responder	pDC_LILRA4	Macro_IFI27	SPON2	ITGA4	3,00912E-03	12713,5	1,99836E-02	2539	7,42062E-01	1566	1,39602E+00	2371	6,97613E-01	45883	0	11208,5
Responder	Macro_ISG15	Macro_NLRP3	CD14	TLR4	3,00951E-03	11111,1	8,65929E-03	10895	3,32595E-01	9439	6,66688E-01	9605	8,59664E-01	14408	0	11208,5
Responder	cDC_CLEC9A	Macro_LYVE1	HLA-DMA	CD4	3,01160E-03	9375,9	7,31753E-03	14410	3,29624E-01	9557	7,39667E-01	8427	9,18906E-01	3277	0	11208,5
Responder	cDC(CD1C)	cDC(CD1C)	HLA-DPA1	CD4	3,01261E-03	9124,7	5,60120E-03	21874	3,26357E-01	9715	1,55963E+00	1711	9,31994E-01	1115	0	11208,5
Responder	Macro_IFI27	Mono_CD16	CD99	PILRA	3,01578E-03	11062,7	7,36672E-03	14261	4,09136E-01	6695	5,42942E-01	11898	8,75964E-01	11251	0	11208,5
Responder	Macro_OLFML3	pDC_LILRA4	PDCD1LG2	PDCD2	3,01665E-03	15153,3	2,00280E-02	2528	4,05513E-01	6805	1,29410E+00	2889	6,52114E-01	52336	0	11208,5
Responder	Macro_OLFML3	Macro_IFI27	MDK	SDC3	3,02011E-03	10850,5	2,80705E-02	1411	4,79992E-01	4828	8,53078E-01	6807	7,83827E-01	29998	0	11208,5
Responder	cDC(CD1C)	Macro_LYVE1	B2M	TFRC	3,02102E-03	9350,5	7,31456E-03	14419	2,73684E-01	12293	8,14590E-01	7326	9,28998E-01	1506	0	11208,5
Responder	Macro_FOLR2-APOE+	Mast	PLTP	ABCA1	3,02731E-03	8270,9	1,43863E-02	4597	5,69624E-01	3276	7,78250E-01	7848	8,59554E-01	14425	0	11208,5
Responder	Macro_NLRP3	Macro_IER3	CD55	ADGRE2	3,02941E-03	8309,3	1,77557E-02	3117	6,71548E-01	2103	6,05168E-01	10691	8,59553E-01	14427	0	11208,5
Responder	Macro_IER3	pDC_LILRA4	ADM	CALCRL	3,03006E-03	12270,1	2,07995E-02	2387	6,24666E-01	2545	1,92811E+00	829	7,06723E-01	44381	0	11208,5
Responder	Mast	Macro_FOLR2+APOE+	CSF1	CSF3R	3,03356E-03	7976,9	3,55263E-02	914	8,75158E-01	921	1,35884E+00	2546	8,12549E-01	24295	0	11208,5
Responder	cDC(CD1C)	cDC(CD1C)	HLA-DRB5	CD4	3,03356E-03	10820,1	5,45825E-03	22741	2,20776E-01	15449	1,40978E+00	2295	9,23530E-01	2407	0	11208,5
Responder	Mono_CD14	Mono_CD16	HP	ASGR1	3,03574E-03	13589,9	8,29973E-02	154	6,54795E-01	2269	8,52558E-01	6816	6,87326E-01	47502	0	11208,5
Responder	Macro_OLFML3	Macro_LYVE1	FN1	PLAUR	3,03748E-03	8786,3	1,52917E-02	4106	4,05149E-01	6817	3,54327E-01	16876	9,09867E-01	4924	0	11208,5
Responder	pDC_LILRA4	Macro_FOLR2+APOE+	CD52	SIGLEC10	3,04624E-03	11395,1	7,30596E-03	14443	3,24747E-01	9782	7,57656E-01	8141	8,64718E-01	13401	0	11208,5
Responder	Mono_CD14	Macro_IFI27	RETN	TLR4	3,04794E-03	16103,3	1,61940E-02	3671	4,05251E-01	6813	8,51984E-01	6823	6,54940E-01	52001	0	11208,5
Responder	Macro_IFI27	Mono_CD16	HLA-A	LILRB2	3,05046E-03	7540,3	9,84389E-03	8707	5,79321E-01	3111	4,40777E-01	14447	9,43162E-01	228	0	11208,5
Responder	Mono_CD14	Macro_ISG15	HP	ITGAM	3,05842E-03	15250,1	5,56463E-02	359	5,51659E-01	3542	8,51475E-01	6829	6,34787E-01	54312	0	11208,5
Responder	Macro_FOLR2-APOE+	Macro_ISG15	ANXA2	TLR2	3,05892E-03	11801,9	7,56995E-03	13629	3,29500E-01	9564	4,40596E-01	14455	8,81682E-01	10153	0	11208,5
Responder	Macro_FOLR2-APOE+	cDC(CD1C)	MMP9	ITGAM	3,06192E-03	13575,7	1,25215E-02	5799	5,41810E-01	3669	1,05018E+00	4624	7,16918E-01	42578	0	11208,5
Responder	Macro_OLFML3	Mono_CD14	A2M	LRP1	3,06210E-03	9197,5	1,20057E-02	6256	3,93167E-01	7195	8,48740E-01	6870	8,59408E-01	14458	0	11208,5
Responder	cDC(CD1C)	cDC(CD1C)	MIF	CD74_CXCR4	3,06518E-03	9043,3	5,08845E-03	25247	4,03564E-01	6865	1,60527E+00	1548	9,40736E-01	348	0	11208,5
Responder	Macro_IFI27	Mono_CD16	C3	IFITM1	3,06527E-03	6524,9	2,24014E-02	2116	6,13539E-01	2682	6,70220E-01	9533	8,98356E-01	7085	0	11208,5
Responder	Macro_IFI27	Mono_CD16	LYZ	ITGAL	3,06845E-03	8449,7	1,24177E-02	5885	4,97184E-01	4460	4,67745E-01	13738	8,99034E-01	6957	0	11208,5
Responder	Macro_FOLR2-APOE+	cDC(CD1C)	MMP9	IFNAR1	3,07244E-03	9370,9	1,74984E-02	3194	6,07943E-01	2746	1,43457E+00	2194	7,96454E-01	27512	0	11208,5
Responder	Mono_INHBA	Macro_ISG15	TNF	TNFRSF1B	3,07595E-03	9174,3	1,13591E-02	6839	4,70441E-01	5040	9,41737E-01	5722	8,46886E-01	17062	0	11208,5
Responder	Mono_CD14	pDC_LILRA4	THBS1	SCARB1	3,07771E-03	11707,9	1,50318E-02	4239	4,04421E-01	6840	1,51885E+00	1842	7,61778E-01	34410	0	11208,5
Responder	Macro_OLFML3	Macro_FOLR2-APOE+	HLA-DQA2	CD4	3,07908E-03	11559,5	7,41768E-03	14106	2,98863E-01	10952	4,40175E-01	14474	8,98464E-01	7057	0	11208,5
Responder	Macro_IFI27	Mast	SPP1	CD44	3,08120E-03	9444,7	8,33051E-03	11667	4,41141E-01	5783	7,53806E-01	8197	8,80438E-01	10368	0	11208,5
Responder	Macro_FOLR2-APOE+	cDC(CD1C)	INHBA	ACTR2	3,08474E-03	9682,5	1,32520E-02	5276	4,26011E-01	6181	1,10780E+00	4104	8,25090E-01	21643	0	11208,5
Responder	pDC_LILRA4	Mono_CD14	APP	NOTCH2	3,08637E-03	6298,7	1,98498E-02	2561	9,56488E-01	681	2,68910E+00	138	8,47713E-01	16905	0	11208,5
Responder	Macro_IFI27	Mast	MMP9	CD44	3,08973E-03	6758,9	1,42707E-02	4666	6,44809E-01	2359	1,20864E+00	3382	8,71068E-01	12179	0	11208,5
Responder	Macro_NLRP3	Macro_ISG15	THBS1	CD47	3,08973E-03	8156,9	1,39109E-02	4889	5,77222E-01	3148	8,36216E-01	7055	8,59338E-01	14484	0	11208,5
Responder	Macro_OLFML3	Macro_LYVE1	HLA-A	APLP2	3,09346E-03	8516,3	7,47618E-03	13923	6,23341E-01	2563	4,33326E-01	14651	9,43062E-01	236	0	11208,5
Responder	Macro_NLRP3	Mono_CD16	THBS1	LRP1	3,09400E-03	9575,1	1,30789E-02	5387	5,43381E-01	3644	4,91934E-01	13148	8,59319E-01	14488	0	11208,5
Responder	Macro_IFI27	Mast	APOE	ABCA1	3,09506E-03	6182,7	1,56574E-02	3938	5,88676E-01	2981	6,31862E-01	10218	9,22663E-01	2568	0	11208,5
Responder	Mono_INHBA	Macro_ISG15	TNF	TRADD	3,09532E-03	12565,9	1,38053E-02	4951	4,78753E-01	4844	8,50319E-01	6850	7,58566E-01	34976	0	11208,5
Responder	Macro_FOLR2-APOE+	Mono_INHBA	MMP9	LRP1	3,09700E-03	6778,5	2,38109E-02	1905	7,50886E-01	1517	1,35453E+00	2564	8,48718E-01	16698	0	11208,5
Responder	Macro_IFI27	Mast	ARPC5	ADRB2	3,09720E-03	6090,9	2,46599E-02	1782	6,44947E-01	2355	1,09646E+00	4197	8,77573E-01	10912	0	11208,5
Responder	Macro_NLRP3	Macro_IFI27	VCAN	CD44	3,09934E-03	9507,3	9,80800E-03	8762	4,70646E-01	5035	4,39664E-01	14493	8,93346E-01	8038	0	11208,5
Responder	Macro_ISG15	Macro_IER3	CXCL10	TLR4	3,10055E-03	7547,9	2,41347E-02	1859	6,23257E-01	2565	1,50173E+00	1917	8,32270E-01	20190	0	11208,5
Responder	Macro_FOLR2+APOE-	Macro_FOLR2-APOE+	CCL13	CCR5	3,10117E-03	15117,1	4,86545E-02	504	1,20090E+00	277	9,85180E-01	5251	5,91975E-01	58345	0	11208,5
Responder	Macro_NLRP3	Macro_IER3	S100A9	TLR4	3,10148E-03	9842,5	9,72426E-03	8914	4,87787E-01	4652	6,47486E-01	9943	8,59294E-01	14495	0	11208,5
Responder	Macro_OLFML3	Macro_IER3	FN1	ITGA5_ITGB1	3,10415E-03	9133,9	1,57511E-02	3888	4,31931E-01	6032	8,49798E-01	6855	8,44107E-01	17686	0	11208,5
Responder	Macro_IFI27	Mast	HSPA8	ADRB2	3,10469E-03	7148,9	2,24750E-02	2102	5,29382E-01	3870	6,34701E-01	10169	8,91395E-01	8395	0	11208,5
Responder	Macro_NLRP3	Mono_INHBA	SERPINA1	LRP1	3,10469E-03	10570,1	8,39562E-03	11538	3,30515E-01	9517	4,39581E-01	14498	9,03621E-01	6089	0	11208,5
Responder	Macro_FOLR2-APOE+	Mono_INHBA	VEGFA	CD44	3,10576E-03	10766,9	8,62854E-03	10967	5,24360E-01	3961	4,89458E-01	13199	8,59283E-01	14499	0	11208,5
Responder	pDC_LILRA4	Macro_IFI27	CD99	CD81	3,10897E-03	9134,1	7,29411E-03	14502	3,93974E-01	7166	1,63859E+00	1457	8,75537E-01	11337	0	11208,5
Responder	Mono_INHBA	Macro_FOLR2-APOE+	SPP1	ITGA5_ITGB1	3,10946E-03	10036,3	1,20576E-02	6193	4,03770E-01	6858	9,96562E-01	5138	8,29378E-01	20784	0	11208,5
Responder	Mono_CD14	Mono_INHBA	ADAM10	CD44	3,11541E-03	10350,9	7,37528E-03	14236	4,71415E-01	5009	8,54814E-01	6793	8,59230E-01	14508	0	11208,5
Responder	Macro_FOLR2-APOE+	Macro_OLFML3	MMP9	IFNAR1	3,12010E-03	9808,7	1,93371E-02	2665	6,42485E-01	2379	8,49199E-01	6864	8,04434E-01	25927	0	11208,5
Responder	cDC(CD1C)	Macro_OLFML3	LGALS9	HAVCR2	3,12293E-03	12352,9	7,28662E-03	14515	2,63856E-01	12818	6,70068E-01	9538	8,63289E-01	13685	0	11208,5
Responder	Macro_IFI27	Macro_NLRP3	SPP1	CD44	3,12832E-03	11793,3	7,28510E-03	14520	3,31052E-01	9499	5,40840E-01	11930	8,73198E-01	11809	0	11208,5
Responder	pDC_LILRA4	Mono_INHBA	HSP90B1	ASGR1	3,13371E-03	7767,5	1,05631E-02	7736	4,87106E-01	4667	2,00831E+00	701	8,59190E-01	14525	0	11208,5
Responder	Macro_NLRP3	Mono_CD14	ADAM10	CD44	3,13479E-03	11623,9	7,36858E-03	14257	3,69991E-01	7979	6,36058E-01	10149	8,59175E-01	14526	0	11208,5
Responder	Macro_IER3	Macro_ISG15	CCL3	CCR1	3,14336E-03	4881,9	2,44444E-02	1813	9,88369E-01	604	1,35227E+00	2577	8,92339E-01	8207	0	11208,5
Responder	Mono_INHBA	Mono_CD14	S100A8	CD36	3,14775E-03	9096,9	1,42259E-02	4692	5,03894E-01	4324	4,38003E-01	14538	8,78607E-01	10722	0	11208,5
Responder	Macro_OLFML3	Macro_OLFML3	TNFSF13	TNFRSF1A	3,14936E-03	14267,7	1,10022E-02	7226	3,34372E-01	9358	9,26094E-02	26192	8,45571E-01	17354	0	11208,5
Responder	cDC(CD1C)	Mono_CD16	LGALS1	PTPRC	3,15217E-03	8441,1	6,39516E-03	17786	4,64553E-01	5194	8,47566E-01	6882	9,31825E-01	1135	0	11208,5
Responder	cDC(CD1C)	Macro_NLRP3	CD1D	LILRB2	3,16112E-03	10077,5	1,46981E-02	4408	4,02699E-01	6887	1,05220E+00	4608	8,17425E-01	23276	0	11208,5
Responder	cDC_CLEC9A	pDC_LILRA4	SEMA4A	NRP1_PLXNA4	3,16130E-03	11805,3	2,14852E-02	2272	7,56365E-01	1495	1,35163E+00	2582	7,22934E-01	41469	0	11208,5
Responder	cDC_CLEC9A	Macro_NLRP3	HLA-F	LILRB2	3,16403E-03	10904,5	7,27604E-03	14553	3,09063E-01	10462	8,96700E-01	6273	8,71999E-01	12026	0	11208,5
Responder	Macro_OLFML3	Macro_FOLR2-APOE+	APOE	SORL1	3,16729E-03	8892,3	1,12192E-02	6982	4,12790E-01	6590	4,37207E-01	14556	9,08725E-01	5125	0	11208,5

Responder	cDC_CLEC9A	Mono_INHBA	ARF6	PLD1	3,17571E-03	12061,5	2,05736E-02	2423	6,21567E-01	2586	1,44100E+00	2161	7,20501E-01	41929	0	11208,5
Responder	pDC_LILRA4	Macro_FOLR2-APOE+	GAS6	MERTK	3,17571E-03	9720,7	1,97257E-02	2586	8,56607E-01	994	2,19070E+00	467	7,66811E-01	33348	0	11208,5
Responder	pDC_LILRA4	Macro_FOLR2-APOE+	GAS6	AXL	3,17932E-03	8963,7	1,97224E-02	2587	7,75234E-01	1374	2,16331E+00	510	7,88228E-01	29139	0	11208,5
Responder	Mono_CD14	Macro_LYVE1	TNFSF13B	TFRC	3,18474E-03	11817,9	7,95402E-03	12560	2,92400E-01	11277	6,74027E-01	9472	8,58903E-01	14572	0	11208,5
Responder	Macro_NLRP3	Macro_LYVE1	ADAM10	GPNMB	3,18583E-03	7503,7	1,62341E-02	3658	7,41027E-01	1577	8,78631E-01	6502	8,58883E-01	14573	0	11208,5
Responder	Macro_OLFML3	Macro_OLFML3	HLA-DRB1	CD4	3,18720E-03	11131,5	6,54643E-03	17148	4,47231E-01	5619	2,22056E-01	21171	9,37972E-01	511	0	11208,5
Responder	Mono_INHBA	cDC_CLEC9A	APP	CD74	3,18990E-03	10416,5	5,00026E-03	25859	4,02314E-01	6903	1,19498E+00	3457	9,11362E-01	4655	0	11208,5
Responder	Macro_FOLR2-APOE+	Mono_INHBA	CXCL8	SDC2	3,19170E-03	8461,7	2,48384E-02	1763	7,17157E-01	1723	1,05756E+00	4541	8,18360E-01	23073	0	11208,5
Responder	Macro_NLRP3	cDC_CLEC9A	IL1B	SIGIRR	3,19239E-03	7766,1	1,34249E-02	5166	5,78941E-01	3125	1,03551E+00	4752	8,58848E-01	14579	0	11208,5
Responder	pDC_LILRA4	Macro_IFI27	COPA	CD74	3,19239E-03	7291,7	7,26679E-03	14579	3,95563E-01	7107	1,41541E+00	2273	9,30556E-01	1291	0	11208,5
Responder	Macro_FOLR2-APOE+	Mono_INHBA	SPP1	ITGAV_ITGB1	3,19351E-03	8545,5	1,49043E-02	4304	4,86451E-01	4681	1,00597E+00	5039	8,44961E-01	17495	0	11208,5
Responder	Macro_FOLR2-APOE+	Mono_INHBA	SPP1	ITGA5_ITGB1	3,19532E-03	6892,7	1,87645E-02	2825	6,38053E-01	2421	1,21657E+00	3346	8,58436E-01	14663	0	11208,5
Responder	pDC_LILRA4	Macro_NLRP3	HSP90B1	TLR4	3,19787E-03	8409,7	9,07318E-03	10048	4,56367E-01	5385	1,93067E+00	823	8,58823E-01	14584	0	11208,5
Responder	Macro_FOLR2-APOE+	Mono_INHBA	SPP1	PTGER4	3,20074E-03	10812,5	1,19979E-02	6267	4,38855E-01	5846	9,21701E-01	5956	8,10127E-01	24785	0	11208,5
Responder	Mono_INHBA	Macro_ISG15	VEGFA	ITGB1	3,20255E-03	10142,5	1,14231E-02	6792	5,18541E-01	4060	8,45249E-01	6910	8,24623E-01	21742	0	11208,5
Responder	Macro_NLRP3	pDC_LILRA4	ADM	CALCRL	3,20465E-03	12322,7	2,04892E-02	2434	6,21001E-01	2594	1,99271E+00	724	7,05162E-01	44653	0	11208,5
Responder	Macro_ISG15	Macro_IER3	TNFSF13B	TFRC	3,20775E-03	10941,1	7,93810E-03	12601	4,24770E-01	6223	6,40251E-01	10080	8,58782E-01	14593	0	11208,5
Responder	Macro_OLFML3	cDC(CD1C)	LGALS3BP	ITGB1	3,21342E-03	9235,5	1,12790E-02	6916	4,16688E-01	6464	9,26619E-01	5897	8,53501E-01	15692	0	11208,5
Responder	Mono_INHBA	Macro_OLFML3	ICAM1	ITGAL_ITGB2	3,21876E-03	10260,7	9,23062E-03	9781	4,26480E-01	6167	4,35285E-01	14603	8,84957E-01	9544	0	11208,5
Responder	Macro_OLFML3	cDC(CD1C)	SERPINF1	PLXDC2	3,22206E-03	12633,7	8,46164E-03	11354	2,36534E-01	14423	5,57372E-01	11577	8,58728E-01	14606	0	11208,5
Responder	Mono_INHBA	Macro_ISG15	VEGFA	NRP2	3,22250E-03	12408,5	1,12738E-02	6921	5,35310E-01	3771	8,88240E-01	6368	7,64805E-01	33774	0	11208,5
Responder	pDC_LILRA4	Macro_LYVE1	FARP2	PLXNA1	3,22283E-03	14854,9	3,65574E-02	865	6,20518E-01	2599	1,47832E+00	2002	6,01355E-01	57600	0	11208,5
Responder	Macro_NLRP3	pDC_LILRA4	S100A8	CD68	3,22317E-03	9647,3	7,25822E-03	14607	2,60758E-01	12997	8,55372E-01	6786	9,22304E-01	2638	0	11208,5
Responder	Macro_NLRP3	Macro_ISG15	CCL3	CCR1	3,22537E-03	8250,1	1,31436E-02	5348	4,97697E-01	4448	9,50222E-01	5637	8,58712E-01	14609	0	11208,5
Responder	cDC_CLEC9A	Macro_LYVE1	HLA-DRA	CD4	3,22537E-03	7816,5	7,25756E-03	14609	4,26913E-01	6159	8,47051E-01	6888	9,43444E-01	218	0	11208,5
Responder	cDC_CLEC9A	Macro_NLRP3	HMGB1	TLR4	3,22648E-03	10805,5	7,49996E-03	13850	3,62642E-01	8272	9,11352E-01	6087	8,58708E-01	14610	0	11208,5
Responder	Macro_IER3	Mono_INHBA	PLAU	PLAUR	3,22796E-03	5375,1	2,08394E-02	2377	8,66626E-01	957	9,70643E-01	5409	8,99218E-01	6924	0	11208,5
Responder	Macro_FOLR2-APOE+	pDC_LILRA4	PSEN1	NOTCH4	3,23741E-03	13358,5	3,67014E-02	861	6,20154E-01	2603	1,63520E+00	1463	6,66020E-01	50657	0	11208,5
Responder	Macro_FOLR2-APOE+	Macro_NLRP3	GRN	TNFRSF1B	3,24640E-03	9401,3	7,79681E-03	12997	4,61354E-01	5262	4,34556E-01	14628	9,20878E-01	2911	0	11208,5
Responder	Mono_INHBA	Macro_ISG15	ICAM1	ITGAM_ITGB2	3,24640E-03	11764,5	7,25075E-03	14628	3,14793E-01	10208	5,95856E-01	10844	8,72503E-01	11934	0	11208,5
Responder	Macro_ISG15	cDC(CD1C)	B2M	CD1B	3,25084E-03	7767,7	3,48674E-02	944	5,95420E-01	2892	6,92688E-01	9162	8,58608E-01	14632	0	11208,5
Responder	Mono_INHBA	cDC_CLEC9A	GNAI2	CXCR3	3,25718E-03	9465,1	1,95529E-02	2625	6,48672E-01	2316	8,43729E-01	6940	8,12837E-01	24236	0	11208,5
Responder	pDC_LILRA4	Mono_CD14	SELPLG	ITGAM	3,25938E-03	7436,3	1,96252E-02	2609	6,97202E-01	1877	2,48727E+00	238	8,26984E-01	21249	0	11208,5
Responder	cDC_CLEC9A	Mono_INHBA	GNAI2	C5AR1	3,26420E-03	10296,5	7,24689E-03	14644	3,35900E-01	9299	8,61194E-01	6715	8,84578E-01	9616	0	11208,5
Responder	Macro_IFI27	Mono_CD16	HLA-C	LILRA3	3,26866E-03	6905,5	2,98315E-02	1247	6,93780E-01	1908	4,33455E-01	14648	9,06681E-01	5516	0	11208,5
Responder	Mono_INHBA	Macro_IER3	CCL3	CCR1	3,27002E-03	8015,1	1,12489E-02	6947	6,93734E-01	1909	1,21163E+00	3367	8,49002E-01	16644	0	11208,5
Responder	cDC_LAMP3	Macro_OLFML3	CCL19	CCRL2	3,27407E-03	10019,7	4,03013E-02	723	1,80241E+00	65	1,34424E+00	2613	7,55724E-01	35489	0	11208,5
Responder	Macro_ISG15	Macro_NLRP3	CD14	ITGB1	3,27424E-03	10297,5	7,24255E-03	14653	3,57680E-01	8465	6,90079E-01	9202	8,93730E-01	7959	0	11208,5
Responder	Macro_OLFML3	Macro_IER3	C3	CD46	3,27647E-03	8890,5	1,22634E-02	6014	4,57580E-01	5357	8,22876E-01	7218	8,58466E-01	14655	0	11208,5
Responder	cDC_CLEC9A	Macro_OLFML3	IL16	CD9	3,27759E-03	8145,9	1,98530E-02	2559	5,71061E-01	3250	6,99153E-01	9056	8,58459E-01	14656	0	11208,5
Responder	Mono_CD14	Macro_FOLR2-APOE+	S100A8	CD36	3,28695E-03	7378,9	9,44913E-03	9389	1,08486E+00	415	2,15958E+00	519	8,55045E-01	15363	0	11208,5
Responder	cDC(CD1C)	Macro_FOLR2-APOE+	CALR	ITGAV	3,28767E-03	10418,7	9,88127E-03	8654	3,03621E-01	10719	8,50627E-01	6847	8,58428E-01	14665	0	11208,5
Responder	Mast	cDC_LAMP3	TIMP3	CD44	3,29620E-03	4743,9	4,63838E-02	553	9,90859E-01	599	1,34326E+00	2619	8,89539E-01	8740	0	11208,5
Responder	Macro_FOLR2+APOE+	Macro_IFI27	APOE	LRP1	3,29767E-03	6176,7	1,12344E-02	6962	6,49047E-01	2314	8,59563E-01	6731	9,16646E-01	3668	0	11208,5
Responder	Macro_FOLR2-APOE+	Macro_FOLR2-APOE+	SPP1	ITGAV_ITGB1	3,29777E-03	7452,9	1,84278E-02	2912	5,99181E-01	2843	9,50587E-01	5627	8,58358E-01	14674	0	11208,5
Responder	pDC_LILRA4	Macro_LYVE1	SEMA3C	NRP1_NRP2_PLXND1	3,30359E-03	12303,9	4,50104E-02	585	7,65663E-01	1430	1,34303E+00	2621	6,98999E-01	45675	0	11208,5
Responder	Mono_CD14	cDC_CLEC9A	TNFSF13B	CD40	3,30677E-03	11162,1	7,98069E-03	12480	2,39778E-01	14213	1,23652E+00	3227	8,58322E-01	14682	0	11208,5
Responder	Macro_OLFML3	Macro_LYVE1	APOE	SDC2	3,30789E-03	8077,3	1,81290E-02	3004	6,06501E-01	2767	7,18452E-01	8724	8,58319E-01	14683	0	11208,5
Responder	Macro_IFI27	pDC_LILRA4	HSP90B1	TLR9	3,31212E-03	7500,1	4,80907E-02	521	1,14496E+00	342	1,00502E+00	5046	8,31328E-01	20383	0	11208,5
Responder	Macro_OLFML3	Macro_FOLR2+APOE+	C3	ITGAX	3,31692E-03	11840,1	8,06207E-03	12278	3,99721E-01	6989	4,56086E-01	14034	8,58284E-01	14691	0	11208,5
Responder	cDC(CD1C)	Macro_LYVE1	VEGFA	NRP1	3,31806E-03	12141,3	1,53098E-02	4097	5,68557E-01	3295	8,41525E-01	6973	7,57746E-01	35133	0	11208,5
Responder	Macro_ISG15	Macro_NLRP3	GRN	TNFRSF1B	3,32030E-03	9358,3	7,23413E-03	14694	3,92963E-01	7200	6,27457E-01	10292	9,18106E-01	3397	0	11208,5
Responder	Macro_FOLR2-APOE+	Mono_INHBA	FABP5	RXRA	3,32178E-03	11059,9	1,13796E-02	6825	4,85461E-01	4705	8,74878E-01	6539	8,03983E-01	26022	0	11208,5
Responder	cDC_CLEC9A	Macro_ISG15	SPN	SIGLEC1	3,32213E-03	11506,1	2,38752E-02	1892	6,24854E-01	2542	1,34219E+00	2626	7,35718E-01	39262	0	11208,5
Responder	Mono_INHBA	Macro_ISG15	VEGFA	ITGAV	3,32550E-03	12344,7	1,39609E-02	4860	5,34845E-01	3780	8,41287E-01	6977	7,59035E-01	34898	0	11208,5
Responder	Macro_FOLR2-APOE+	Mono_INHBA	MMP9	ITGAM	3,33108E-03	10539,7	2,20216E-02	2182	6,58620E-01	2220	1,06440E+00	4475	7,70566E-01	32613	0	11208,5
Responder	Macro_FOLR2-APOE+	Macro_FOLR2-APOE+	APOE	TREM2	3,33729E-03	8096,9	1,48744E-02	4318	3,61032E-01	8322	4,30799E-01	14709	9,26300E-01	1927	0	11208,5
Responder	Mono_INHBA	Mono_CD16	HLA-F	LILRB2	3,33729E-03	11491,1	7,23017E-03	14709	4,60971E-01	5273	4,50459E-01	14180	8,71646E-01	12085	0	11208,5
Responder	Macro_FOLR2-APOE+	Mono_INHBA	MMP9	IFNAR1	3,34041E-03	9863,9	1,72517E-02	3275	6,03310E-01	2803	1,08607E+00	4281	7,95301E-01	27752	0	11208,5
Responder	Macro_OLFML3	Mono_INHBA	SERPINA1	LRP1	3,34751E-03	9085,1	9,57312E-03	9165	4,59865E-01	5294	4,30495E-01	14718	9,09187E-01	5040	0	11208,5
Responder	Mono_CD14	Macro_FOLR2+APOE-	VCAN	ITGA4	3,35162E-03	8259,3	1,12082E-02	6991	7,30646E-01	1642	1,31661E+00	2762	8,39207E-01	18693	0	11208,5
Responder	cDC(CD1C)	Macro_LYVE1	LRPAP1	LRP1	3,35206E-03	9568,3	9,48326E-03	9322	4,40829E-01	5791	8,54426E-01	6798	8,58155E-01	14722	0	11208,5
Responder	Macro_ISG15	Macro_LYVE1	CALR	LRP1	3,35548E-03	9727,3	8,97069E-03	10266	4,34977E-01	5955	4,30182E-01	14725	9,01561E-01	6482	0	11208,5
Responder	Macro_OLFML3	cDC_CLEC9A	NECTIN2	CD226	3,35567E-03	16881,1	1,95008E-02	2635	6,36511E-01	2431	5,84577E-01	11046	6,06878E-01	57085	0	11208,5
Responder	Macro_OLFML3	Macro_OLFML3	HLA-DQA2	CD4	3,36118E-03	9549,7	9,21345E-03	9811	4,08277E-01	6723	4,29828E-01	14730	9,07934E-01	5276	0	11208,5

Responder	Mono_INHBA	Mono_CD16	ICAM1	SPN	3,36286E-03	8766,7	1,75510E-02	3178	6,58737E-01	2219	8,40157E-01	6997	8,32042E-01	20231	0	11208,5
Responder	Macro_FOLR2+APOE+	Mono_CD16	APOE	SORL1	3,36662E-03	5906,5	1,33417E-02	5215	6,55745E-01	2258	8,40047E-01	6999	9,15661E-01	3852	0	11208,5
Responder	Macro_IER3	Macro_LYVE1	CCL3L1	CCR1	3,36690E-03	5895,7	2,88962E-02	1330	1,00205E+00	575	1,33910E+00	2638	8,63029E-01	13727	0	11208,5
Responder	cDC_CLEC9A	Macro_NLRP3	LGALS9	LRP1	3,37603E-03	12050,1	7,24891E-03	14639	2,65890E-01	12696	8,41998E-01	6964	8,58060E-01	14743	0	11208,5
Responder	Mono_CD14	cDC_LAMP3	ICAM1	IL2RG	3,37833E-03	8949,5	9,87048E-03	8670	5,26415E-01	3919	9,01565E-01	6205	8,58058E-01	14745	0	11208,5
Responder	Macro_NLRP3	cDC_LAMP3	ICAM1	IL2RG	3,38062E-03	10398,1	9,86504E-03	8674	5,26177E-01	3925	4,79635E-01	13436	8,58024E-01	14747	0	11208,5
Responder	cDC_CLEC9A	Macro_LYVE1	HLA-DRB1	CD4	3,38406E-03	7992,5	7,21965E-03	14750	4,17845E-01	6426	8,21801E-01	7233	9,40759E-01	345	0	11208,5
Responder	Macro_ISG15	Mast	GNAS	ADRB2	3,39439E-03	7054,1	2,36157E-02	1936	6,74143E-01	2083	9,81578E-01	5284	8,57944E-01	14759	0	11208,5
Responder	Macro_ISG15	Macro_IFI27	TNFSF13B	HLA-DPB1	3,39439E-03	8507,1	7,21704E-03	14759	4,55985E-01	5390	5,82927E-01	11073	9,47271E-01	105	0	11208,5
Responder	Macro_ISG15	cDC(CD1C)	CD14	ITGB2	3,39554E-03	7883,3	7,21625E-03	14760	3,37318E-01	9238	1,28387E+00	2947	9,30779E-01	1263	0	11208,5
Responder	cDC(CD1C)	Mono_CD14	HMGB1	TLR4	3,40015E-03	11306,3	7,40215E-03	14156	2,57994E-01	13164	1,23404E+00	3239	8,57909E-01	14764	0	11208,5
Responder	Macro_OLFML3	Macro_ISG15	HLA-C	LILRB1	3,40015E-03	9622,1	7,21493E-03	14764	4,22698E-01	6286	4,96412E-01	13027	9,21283E-01	2825	0	11208,5
Responder	Macro_FOLR2-APOE+	Mono_CD14	THBS1	CD36	3,40243E-03	8561,7	1,51807E-02	4166	4,94885E-01	4508	1,25178E+00	3135	8,34161E-01	19791	0	11208,5
Responder	Macro_IER3	Mono_CD14	CCL3	CCR1	3,40451E-03	5521,1	1,94081E-02	2648	8,97337E-01	850	1,35228E+00	2576	8,80745E-01	10323	0	11208,5
Responder	Macro_NLRP3	Macro_NLRP3	GNAI2	FPR1	3,40706E-03	10681,1	8,53191E-03	11202	3,83983E-01	7489	4,28685E-01	14770	8,89557E-01	8736	0	11208,5
Responder	Macro_ISG15	Macro_ISG15	CD14	TLR4	3,40706E-03	11109,1	8,40862E-03	11506	3,22810E-01	9872	7,54685E-01	8189	8,57882E-01	14770	0	11208,5
Responder	cDC(CD1C)	Macro_FOLR2-APOE+	LGALS3	ENG	3,40822E-03	11861,7	8,48283E-03	11297	2,68356E-01	12558	6,73881E-01	9474	8,57877E-01	14771	0	11208,5
Responder	pDC_LILRA4	Macro_OLFML3	SPON2	ITGAM	3,41206E-03	14004,5	2,60164E-02	1622	8,03595E-01	1231	1,33774E+00	2650	6,43779E-01	53311	0	11208,5
Responder	cDC_CLEC9A	pDC_LILRA4	HLA-DRA	CD4	3,41375E-03	6977,1	6,92096E-03	15724	3,94569E-01	7146	2,15377E+00	529	9,42163E-01	278	0	11208,5
Responder	Macro_FOLR2-APOE+	Mono_CD14	MRC1	PTPRC	3,42139E-03	10304,5	1,27910E-02	5602	4,33981E-01	5986	8,91592E-01	6333	8,21570E-01	22393	0	11208,5
Responder	Macro_FOLR2+APOE+	Macro_ISG15	APOE	SORL1	3,42519E-03	6341,7	1,11669E-02	7030	6,02937E-01	2805	9,61684E-01	5510	9,08531E-01	5155	0	11208,5
Responder	Macro_FOLR2-APOE+	Mono_INHBA	CD14	ITGB1	3,42556E-03	11543,1	7,20621E-03	14786	3,31894E-01	9460	4,47655E-01	14253	8,93491E-01	8008	0	11208,5
Responder	Macro_OLFML3	cDC_CLEC9A	LGALS3BP	ITGB1	3,42709E-03	9489,3	1,12421E-02	6953	4,15472E-01	6507	8,37914E-01	7031	8,53296E-01	15747	0	11208,5
Responder	pDC_LILRA4	Macro_ISG15	CD99	PILRA	3,43251E-03	9625,3	7,20442E-03	14792	3,42095E-01	9030	1,59278E+00	1593	8,74748E-01	11503	0	11208,5
Responder	cDC_CLEC9A	Mono_INHBA	HMGB1	THBD	3,43599E-03	10268,7	7,20396E-03	14795	4,07744E-01	6735	1,03235E+00	4779	8,62456E-01	13826	0	11208,5
Responder	Mono_CD14	Mono_CD14	CD55	ADGRE2	3,43852E-03	9144,3	1,11622E-02	7037	5,45506E-01	3609	1,26876E+00	3034	8,29133E-01	20833	0	11208,5
Responder	Macro_LYVE1	Mono_INHBA	PDGFC	FLT1	3,43940E-03	12487,5	1,12426E-01	68	1,02111E+00	531	9,85987E-01	5241	7,00875E-01	45389	0	11208,5
Responder	cDC(CD1C)	Macro_OLFML3	HLA-DMA	CD4	3,44646E-03	8701,1	7,20192E-03	14804	3,90010E-01	7298	8,51785E-01	6825	9,18310E-01	3370	0	11208,5
Responder	Mono_INHBA	Mast	CALM1	KCNQ1	3,44998E-03	8470,3	1,92986E-02	2678	5,70822E-01	3257	8,36750E-01	7043	8,41749E-01	18165	0	11208,5
Responder	pDC_LILRA4	Macro_FOLR2+APOE-	SPON2	ITGA4	3,44999E-03	16035,6	1,93599E-02	2660	7,35952E-01	1609	1,36201E+00	2530	6,94258E-01	46412	0,021	26967
Responder	Macro_OLFML3	Mono_CD16	C1QB	LRP1	3,45228E-03	10196,9	7,71481E-03	13246	4,04497E-01	6837	4,26979E-01	14809	9,10033E-01	4884	0	11208,5
Responder	Macro_NLRP3	Mono_INHBA	TIMP1	CD63	3,45345E-03	9237,9	7,20020E-03	14810	4,45371E-01	5662	4,47256E-01	14262	9,42732E-01	247	0	11208,5
Responder	Macro_FOLR2-APOE+	Mono_CD14	APOC2	LDLR	3,45380E-03	15587,3	2,79619E-02	1416	4,47282E-01	5617	8,43898E-01	6936	6,48674E-01	52759	0	11208,5
Responder	Macro_FOLR2-APOE+	Mono_CD14	SPP1	ITGA5_ITGB1	3,46338E-03	9472,1	1,18871E-02	6359	4,59464E-01	5303	1,18640E+00	3517	8,28367E-01	20973	0	11208,5
Responder	cDC(CD1C)	Mono_INHBA	VEGFA	CD44	3,46512E-03	9508,1	8,39729E-03	11535	5,16651E-01	4096	9,28447E-01	5881	8,57633E-01	14820	0	11208,5
Responder	Mono_CD14	cDC(CD1C)	S100A8	TLR4	3,46514E-03	8468,9	8,61548E-03	11001	1,02051E+00	533	2,44143E+00	272	8,36338E-01	19330	0	11208,5
Responder	Macro_NLRP3	cDC_CLEC9A	CXCL2	DPP4	3,46524E-03	9945,9	4,88362E-02	497	8,86246E-01	882	1,33588E+00	2664	7,61401E-01	34478	0	11208,5
Responder	cDC_CLEC9A	pDC_LILRA4	TNF	TNFRSF21	3,46524E-03	10042,1	1,93404E-02	2664	1,00254E+00	573	1,61693E+00	1517	7,62514E-01	34248	0	11208,5
Responder	Macro_FOLR2-APOE+	pDC_LILRA4	MAML2	NOTCH4	3,46906E-03	13990,7	3,65415E-02	866	6,14622E-01	2665	1,59109E+00	1599	6,41129E-01	53615	0	11208,5
Responder	Macro_FOLR2+APOE-	cDC_CLEC9A	CXCL12	DPP4	3,47289E-03	12879,1	8,75264E-02	132	9,02869E-01	833	1,33547E+00	2666	6,73554E-01	49556	0	11208,5
Responder	Macro_FOLR2-APOE+	Macro_NLRP3	GNAI2	C5AR1	3,47800E-03	9546,1	9,19918E-03	9837	5,02426E-01	4355	4,25899E-01	14831	8,96209E-01	7499	0	11208,5
Responder	Mono_CD14	Macro_NLRP3	HP	ITGAM	3,47874E-03	14612,9	6,76056E-02	228	6,02274E-01	2812	8,36013E-01	7058	6,57043E-01	51758	0	11208,5
Responder	Mono_INHBA	cDC(CD1C)	VCAN	ITGA4	3,48259E-03	9850,1	1,13332E-02	6865	3,97362E-01	7060	9,56929E-01	5555	8,39954E-01	18562	0	11208,5
Responder	Macro_ISG15	Mast	HBEGF	CD9	3,48387E-03	8621,3	1,24279E-02	5873	4,90648E-01	4593	8,69990E-01	6596	8,57587E-01	14836	0	11208,5
Responder	pDC_LILRA4	Mast	LIN7C	ABCA1	3,48437E-03	9981,3	2,47725E-02	1768	6,14474E-01	2669	1,49130E+00	1957	7,72093E-01	32304	0	11208,5
Responder	cDC(CD1C)	Mono_CD16	CD99	PILRA	3,48739E-03	10498,3	7,19216E-03	14839	3,99454E-01	6993	7,72843E-01	7922	8,74655E-01	11529	0	11208,5
Responder	cDC_CLEC9A	Mono_CD14	CIRBP	TREM1	3,49092E-03	8444,5	1,16929E-02	6537	4,02978E-01	6877	1,31721E+00	2758	8,57562E-01	14842	0	11208,5
Responder	Mono_CD14	pDC_LILRA4	TGFB1	TGFBR1_TGFBR2	3,49222E-03	10454,9	1,17794E-02	6449	3,97087E-01	7065	1,72030E+00	1243	8,02608E-01	26309	0	11208,5
Responder	pDC_LILRA4	cDC(CD1C)	SEMA7A	PLXNC1	3,49588E-03	12983,9	1,93209E-02	2672	6,30155E-01	2492	1,67371E+00	1355	6,89452E-01	47192	0	11208,5
Responder	Macro_LYVE1	cDC(CD1C)	CD14	ITGA4	3,49916E-03	11350,7	7,38886E-03	14195	3,29182E-01	9585	5,62312E-01	11477	8,80933E-01	10288	0	11208,5
Responder	pDC_LILRA4	pDC_LILRA4	GNAI2	CXCR3	3,50356E-03	7895,9	1,93170E-02	2674	8,66164E-01	959	2,51556E+00	222	8,11911E-01	24416	0	11208,5
Responder	Macro_ISG15	Macro_IER3	SERPING1	LRP1	3,50387E-03	13260,1	8,12899E-03	12125	2,49016E-01	13659	4,25234E-01	14853	8,59422E-01	14455	0	11208,5
Responder	pDC_LILRA4	cDC(CD1C)	B2M	CD1A	3,50392E-03	11311,4	4,98503E-02	474	4,11352E-01	6629	2,14723E+00	536	8,73197E-01	11810	0,571	37108
Responder	Macro_FOLR2-APOE+	Macro_FOLR2-APOE+	LGALS3	ENG	3,50741E-03	9589,3	1,18211E-02	6415	4,98993E-01	4426	4,25060E-01	14856	8,76932E-01	11041	0	11208,5
Responder	Macro_NLRP3	Mono_CD14	SERPINA1	LRP1	3,50741E-03	11628,3	7,18637E-03	14856	2,54431E-01	13363	5,71014E-01	11286	8,96633E-01	7428	0	11208,5
Responder	Mono_INHBA	Mono_CD16	LYZ	ITGAL	3,50978E-03	8881,1	1,20944E-02	6158	4,71420E-01	5008	4,25024E-01	14858	8,97830E-01	7173	0	11208,5
Responder	Macro_LYVE1	cDC(CD1C)	B2M	CD1A	3,51214E-03	6811,1	5,29170E-02	406	5,70585E-01	3259	7,64312E-01	8024	8,76465E-01	11158	0	11208,5
Responder	cDC_CLEC9A	Mono_CD16	CD52	SIGLEC10	3,51332E-03	13272,5	7,18294E-03	14861	2,79055E-01	12003	4,31353E-01	14698	8,63722E-01	13592	0	11208,5
Responder	Macro_FOLR2-APOE+	Macro_NLRP3	ANXA2	TLR2	3,51569E-03	11221,3	8,11358E-03	12157	3,59774E-01	8377	4,24944E-01	14863	8,85251E-01	9501	0	11208,5
Responder	Mono_INHBA	Macro_LYVE1	LRPAP1	LRP1	3,51687E-03	11043,7	9,37515E-03	9514	4,36072E-01	5927	4,68730E-01	13705	8,57455E-01	14864	0	11208,5
Responder	cDC_CLEC9A	Macro_FOLR2+APOE+	MIF	TNFRSF14	3,51805E-03	10300,5	7,18176E-03	14865	4,06197E-01	6787	6,62778E-01	9674	8,88350E-01	8968	0	11208,5
Responder	cDC(CD1C)	Mast	HBEGF	CD9	3,52125E-03	8855,3	1,11187E-02	7080	4,58719E-01	5320	1,07794E+00	4350	8,50654E-01	16318	0	11208,5
Responder	Macro_OLFML3	Macro_IER3	SERPING1	LRP1	3,52279E-03	10488,7	1,02787E-02	8107	4,18259E-01	6414	4,24617E-01	14869	8,73007E-01	11845	0	11208,5
Responder	pDC_LILRA4	Macro_LYVE1	APP	NOTCH2	3,52282E-03	6484,1	1,92924E-02	2679	9,46413E-01	714	2,14978E+00	534	8,45866E-01	17285	0	11208,5
Responder	Macro_ISG15	Mono_INHBA	CXCL10	TLR4	3,52668E-03	7727,3	2,32761E-02	1985	6,13620E-01	2680	1,46751E+00	2043	8,29726E-01	20720	0	11208,5

Responder	Macro_FOLR2+APOE+	pDC_LILRA4	HMGB1	TLR9	3,52989E-03	8092,7	4,68938E-02	538	1,10588E+00	383	6,25552E-01	10322	8,42471E-01	18012	0	11208,5
Responder	pDC_LILRA4	Macro_IFI27	LTB	TNFRSF1A	3,53055E-03	7259,9	1,92834E-02	2681	9,14204E-01	800	2,37362E+00	321	8,26817E-01	21289	0	11208,5
Responder	Macro_OLFML3	Macro_FOLR2+APOE+	C1QA	CD33	3,53227E-03	9478,3	1,01759E-02	8248	4,37894E-01	5877	4,24257E-01	14877	8,97784E-01	7181	0	11208,5
Responder	Mast	Mono_INHBA	PLAT	LRP1	3,53828E-03	7591,3	8,35174E-02	149	8,90364E-01	867	1,33252E+00	2683	8,18491E-01	23049	0	11208,5
Responder	Mono_CD14	Mono_CD16	ANXA1	FPR1	3,53940E-03	11101,1	8,22609E-03	11879	3,20345E-01	9962	4,24006E-01	14883	8,95829E-01	7573	0	11208,5
Responder	Macro_LYVE1	pDC_LILRA4	PSEN1	NOTCH4	3,54215E-03	13487,9	3,57378E-02	900	6,13449E-01	2684	1,58664E+00	1615	6,63054E-01	51032	0	11208,5
Responder	Macro_OLFML3	Macro_OLFML3	TNFSF13	TNFRSF14	3,54292E-03	15274,1	9,78605E-03	8798	3,23975E-01	9819	-2,58946E-02	31262	8,55422E-01	15283	0	11208,5
Responder	cDC(CD1C)	Macro_NLRP3	HSP90B1	ASGR1	3,54535E-03	10273,5	1,02445E-02	8150	3,10160E-01	10414	8,61764E-01	6707	8,57327E-01	14888	0	11208,5
Responder	Macro_LYVE1	cDC(CD1C)	CXCL12	CXCR4	3,54773E-03	6476,7	1,83069E-02	2952	6,22921E-01	2570	1,46698E+00	2047	8,63648E-01	13606	0	11208,5
Responder	Macro_FOLR2-APOE+	cDC_CLEC9A	APP	CD74	3,55012E-03	7000,7	7,17177E-03	14892	5,28557E-01	3879	1,29940E+00	2861	9,24889E-01	2163	0	11208,5
Responder	cDC(CD1C)	Mono_CD14	ACTR2	LDLR	3,55240E-03	10704,1	1,46169E-02	4464	3,96089E-01	7096	1,23046E+00	3263	7,96554E-01	27489	0	11208,5
Responder	Mast	Mono_CD14	ANXA1	FPR1	3,55826E-03	5911,3	1,11064E-02	7099	5,77269E-01	3147	1,26918E+00	3029	9,09027E-01	5073	0	11208,5
Responder	pDC_LILRA4	Macro_LYVE1	SPON2	ITGA4	3,56155E-03	12225,9	2,26180E-02	2085	7,67872E-01	1419	1,33128E+00	2689	7,10513E-01	43728	0	11208,5
Responder	cDC_CLEC9A	Macro_OLFML3	HLA-DOB	CD4	3,56325E-03	6251,9	3,40279E-02	982	1,14308E+00	346	1,14250E+00	3820	8,57265E-01	14903	0	11208,5
Responder	Macro_FOLR2-APOE+	Mono_INHBA	SPP1	ITGA4_ITGB1	3,56609E-03	9390,3	1,22125E-02	6057	4,39736E-01	5820	8,31873E-01	7103	8,48373E-01	16763	0	11208,5
Responder	Macro_OLFML3	cDC_CLEC9A	HLA-DQA1	LAG3	3,56805E-03	8216,5	1,48298E-02	4347	5,83074E-01	3056	8,31855E-01	7104	8,55026E-01	15367	0	11208,5
Responder	Mono_CD14	Macro_NLRP3	THBS1	CD36	3,57001E-03	9411,5	1,52385E-02	4132	4,77761E-01	4870	8,31837E-01	7105	8,34423E-01	19742	0	11208,5
Responder	Macro_IFI27	Macro_LYVE1	C3	NRP1	3,57043E-03	8170,5	2,46972E-02	1778	5,84028E-01	3044	6,48746E-01	9913	8,57234E-01	14909	0	11208,5
Responder	Macro_FOLR2-APOE+	Mono_CD14	FABP5	RXRA	3,57393E-03	8414,7	1,59071E-02	3812	5,91716E-01	2944	1,22959E+00	3265	8,29041E-01	20844	0	11208,5
Responder	pDC_LILRA4	Mono_INHBA	CIRBP	TREM1	3,57522E-03	8039,7	1,16274E-02	6591	4,15408E-01	6511	1,84559E+00	975	8,57219E-01	14913	0	11208,5
Responder	Mono_INHBA	pDC_LILRA4	CD99	PILRB	3,57589E-03	12215,7	1,30047E-02	5464	3,95497E-01	7108	8,88798E-01	6362	7,79229E-01	30936	0	11208,5
Responder	Macro_OLFML3	Macro_ISG15	APOE	ABCA1	3,57642E-03	10273,5	8,39962E-03	11533	4,17545E-01	6435	4,23116E-01	14914	8,97312E-01	7277	0	11208,5
Responder	Mono_INHBA	Macro_FOLR2-APOE+	CCL4	CCR1	3,57982E-03	8799,1	1,11040E-02	7110	5,41812E-01	3668	1,10725E+00	4108	8,43004E-01	17901	0	11208,5
Responder	Macro_LYVE1	cDC(CD1C)	C1QA	CD33	3,58002E-03	8828,1	1,08785E-02	7366	3,90050E-01	7292	5,54500E-01	11638	9,00807E-01	6636	0	11208,5
Responder	Macro_LYVE1	cDC(CD1C)	C1QB	CD33	3,58122E-03	8856,1	1,10614E-02	7159	3,89696E-01	7305	5,30979E-01	12180	9,01858E-01	6428	0	11208,5
Responder	Macro_NLRP3	Macro_ISG15	NAMPT	ITGA5_ITGB1	3,58242E-03	11480,3	7,16526E-03	14919	4,74782E-01	4934	5,37582E-01	12013	8,60042E-01	14327	0	11208,5
Responder	Macro_LYVE1	cDC(CD1C)	C1QB	C1QBP	3,58482E-03	8803,1	9,11016E-03	9987	3,96614E-01	7077	5,74468E-01	11227	9,12094E-01	4516	0	11208,5
Responder	Macro_FOLR2-APOE+	Mono_CD14	PLAU	ITGAM	3,58572E-03	13101,3	1,55492E-02	3981	4,09419E-01	6679	8,57810E-01	6756	7,48166E-01	36882	0	11208,5
Responder	cDC_CLEC9A	pDC_LILRA4	HLA-DMB	CD4	3,58602E-03	9049,1	7,16401E-03	14922	2,81472E-01	11887	2,04899E+00	641	9,01035E-01	6587	0	11208,5
Responder	Macro_IER3	pDC_LILRA4	PSEN1	NOTCH4	3,58883E-03	13487,9	3,55748E-02	910	6,12315E-01	2696	1,61371E+00	1527	6,62543E-01	51098	0	11208,5
Responder	cDC(CD1C)	Macro_FOLR2+APOE+	TNF	VSIR	3,59162E-03	10053,5	1,10986E-02	7116	5,77955E-01	3137	1,06571E+00	4465	8,12263E-01	24341	0	11208,5
Responder	Macro_LYVE1	cDC(CD1C)	TIMP2	CD44	3,59203E-03	10815,7	7,85895E-03	12815	3,73045E-01	7875	5,59457E-01	11535	8,79037E-01	10645	0	11208,5
Responder	Macro_FOLR2-APOE+	Mono_CD14	MMP9	IFNAR1	3,59359E-03	9798,7	1,63700E-02	3610	5,86747E-01	3010	1,35400E+00	2567	7,90998E-01	28598	0	11208,5
Responder	Mono_INHBA	Macro_ISG15	LGALS3	ENG	3,59444E-03	10739,5	8,38441E-03	11567	4,14363E-01	6550	6,75498E-01	9443	8,57164E-01	14929	0	11208,5
Responder	Macro_LYVE1	cDC(CD1C)	CD14	ITGB2	3,59444E-03	8994,5	7,16251E-03	14929	3,31053E-01	9498	7,63317E-01	8044	9,30538E-01	1293	0	11208,5
Responder	Macro_OLFML3	Macro_LYVE1	HLA-DMB	CD4	3,59564E-03	8377,3	1,01224E-02	8322	5,52665E-01	3528	4,22631E-01	14930	9,15415E-01	3898	0	11208,5
Responder	Macro_FOLR2-APOE+	Macro_FOLR2+APOE-	CXCL8	SDC3	3,59805E-03	7169,1	2,21949E-02	2153	6,86201E-01	1964	9,53487E-01	5588	8,57151E-01	14932	0	11208,5
Responder	Macro_LYVE1	cDC(CD1C)	HMGB1	CXCR4	3,60046E-03	6741,3	7,79631E-03	12999	5,60398E-01	3398	1,04621E+00	4666	9,29500E-01	1435	0	11208,5
Responder	Macro_OLFML3	pDC_LILRA4	SELPLG	SELL	3,60056E-03	9200,5	1,07800E-02	7472	6,12181E-01	2699	1,54991E+00	1751	8,19265E-01	22872	0	11208,5
Responder	Macro_NLRP3	Mono_INHBA	S100A9	TLR4	3,61495E-03	10213,9	9,37829E-03	9505	4,78149E-01	4858	6,13267E-01	10552	8,57090E-01	14946	0	11208,5
Responder	Macro_OLFML3	Macro_ISG15	GRN	TNFRSF1A	3,61495E-03	10214,5	8,40407E-03	11519	4,03692E-01	6861	4,21912E-01	14946	9,01317E-01	6538	0	11208,5
Responder	Macro_IER3	cDC(CD1C)	TNF	VSIR	3,62017E-03	6694,9	1,92001E-02	2704	6,89325E-01	1941	1,69974E+00	1288	8,50538E-01	16333	0	11208,5
Responder	Macro_FOLR2+APOE-	Macro_FOLR2-APOE+	CCL13	CCR1	3,62155E-03	12136,1	4,67224E-02	545	1,18381E+00	287	9,27808E-01	5885	7,15964E-01	42755	0	11208,5
Responder	Macro_LYVE1	Mono_INHBA	ADAM10	CD44	3,62342E-03	10760,9	8,25349E-03	11816	5,07711E-01	4250	4,81357E-01	13393	8,65898E-01	13137	0	11208,5
Responder	Macro_ISG15	cDC_LAMP3	SPP1	S1PR1	3,62409E-03	10232,3	7,62838E-02	189	8,55246E-01	1002	1,32834E+00	2705	7,52623E-01	36057	0	11208,5
Responder	Macro_FOLR2-APOE+	Macro_LYVE1	CALR	LRP1	3,62464E-03	7974,5	1,15716E-02	6646	6,21470E-01	2587	4,21660E-01	14954	9,12295E-01	4477	0	11208,5
Responder	Macro_FOLR2-APOE+	Macro_LYVE1	LGALS9	LRP1	3,62585E-03	9607,5	1,07107E-02	7554	5,27587E-01	3903	4,21423E-01	14955	8,80215E-01	10417	0	11208,5
Responder	pDC_LILRA4	Macro_ISG15	HSP90B1	TLR4	3,62827E-03	8610,1	8,81053E-03	10562	4,46582E-01	5637	2,01867E+00	686	8,57033E-01	14957	0	11208,5
Responder	Macro_FOLR2+APOE+	pDC_LILRA4	HLA-DMB	CD4	3,62918E-03	4995,7	1,10817E-02	7135	6,57427E-01	2234	1,77158E+00	1118	9,18855E-01	3283	0	11208,5
Responder	Macro_ISG15	Mono_CD14	LGALS9	CD44	3,63313E-03	9283,9	7,15258E-03	14961	4,00401E-01	6969	8,30888E-01	7121	9,03297E-01	6160	0	11208,5
Responder	Mono_CD14	cDC_CLEC9A	ANXA1	DYSF	3,63712E-03	9572,9	1,52629E-02	4119	3,94734E-01	7139	9,80823E-01	5292	8,32642E-01	20106	0	11208,5
Responder	Macro_NLRP3	Macro_ISG15	VCAN	SELL	3,63799E-03	6856,3	1,91470E-02	2721	6,76728E-01	2057	1,21871E+00	3330	8,56996E-01	14965	0	11208,5
Responder	Macro_ISG15	cDC(CD1C)	S100A9	ITGB2	3,64529E-03	8552,9	7,14991E-03	14971	3,21730E-01	9914	1,03373E+00	4765	9,26426E-01	1906	0	11208,5
Responder	pDC_LILRA4	Macro_FOLR2-APOE+	SEMA3C	NRP1_NRP2_PLXND1	3,64773E-03	13305,7	3,58900E-02	893	6,49192E-01	2313	1,32667E+00	2711	6,74655E-01	49403	0	11208,5
Responder	Macro_FOLR2+APOE+	Macro_LYVE1	CP	SLC40A1	3,64794E-03	12680,9	6,21236E-02	283	1,01424E+00	547	1,08222E+00	4316	6,90394E-01	47050	0	11208,5
Responder	Mono_INHBA	Macro_FOLR2-APOE+	ICAM1	ITGAL_ITGB2	3,64894E-03	12584,3	7,34671E-03	14320	3,07218E-01	10541	4,20729E-01	14974	8,72817E-01	11878	0	11208,5
Responder	Mono_CD14	Mono_CD14	ICAM3	ITGAL	3,64906E-03	14611,3	1,12842E-02	6913	3,94571E-01	7145	1,09388E+00	4219	7,11469E-01	43571	0	11208,5
Responder	Macro_IFI27	Mono_CD16	CXCL12	CXCR4	3,65016E-03	9193,9	1,63742E-02	3607	5,50261E-01	3555	5,12851E-01	12624	8,56945E-01	14975	0	11208,5
Responder	cDC_CLEC9A	Macro_ISG15	HMGB1	TLR4	3,65870E-03	10888,9	7,28284E-03	14525	3,52857E-01	8625	9,99349E-01	5104	8,56916E-01	14982	0	11208,5
Responder	pDC_LILRA4	Macro_FOLR2+APOE-	HSP90B1	LRP1	3,65870E-03	8610,1	7,14387E-03	14982	4,08692E-01	6709	1,93950E+00	809	8,86159E-01	9342	0	11208,5
Responder	Mast	Macro_IFI27	TIMP3	CD44	3,65959E-03	5264,3	3,74855E-02	830	8,96724E-01	851	1,32525E+00	2714	8,78633E-01	10718	0	11208,5
Responder	cDC(CD1C)	cDC(CD1C)	ANXA1	FPR1	3,65992E-03	10206,9	7,14357E-03	14983	2,64074E-01	12805	1,23940E+00	3204	8,89059E-01	8834	0	11208,5
Responder	Mono_INHBA	pDC_LILRA4	NAMPT	INSR	3,66103E-03	9937,3	1,52867E-02	4108	5,79100E-01	3118	8,27972E-01	7151	8,13465E-01	24101	0	11208,5
Responder	cDC(CD1C)	Mast	CALM2	KCNQ1	3,67303E-03	8954,7	1,69495E-02	3371	3,94249E-01	7157	1,16024E+00	3676	8,36217E-01	19361	0	11208,5
Responder	Macro_FOLR2-APOE+	cDC(CD1C)	LGALS1	PTPRC	3,67503E-03	9327,7	5,87828E-03	20294	3,94223E-01	7158	8,80020E-01	6485	9,29098E-01	1493	0	11208,5

Post_R_Myeloid_Myeloid_CellCel

Responder	pDC_LILRA4	cDC_CLEC9A	PROC	THBD	3,67543E-03	13952,1	4,88396E-02	496	7,31613E-01	1633	1,32412E+00	2718	6,40283E-01	53705	0	11208,5
Responder	pDC_LILRA4	Macro_ISG15	TNFSF9	HLA-DPA1	3,67703E-03	9096,4	1,91488E-02	2720	6,11059E-01	2712	1,58390E+00	1629	8,97949E-01	7159	0,142	31262
Responder	Macro_OLFML3	Macro_IER3	C3	ITGAM	3,67904E-03	10254,9	1,29257E-02	5513	4,98218E-01	4435	8,27293E-01	7160	8,18858E-01	22958	0	11208,5
Responder	Macro_FOLR2-APOE+	Macro_FOLR2-APOE+	APOE	SDC2	3,68196E-03	9719,9	1,77030E-02	3133	4,68172E-01	5089	4,51124E-01	14168	8,56867E-01	15001	0	11208,5
Responder	cDC_CLEC9A	Macro_OLFML3	MIF	TNFRSF14	3,68810E-03	10403,1	7,13694E-03	15006	4,02954E-01	6878	6,49341E-01	9901	8,88039E-01	9022	0	11208,5
Responder	Mast	Mono_CD16	ANXA1	FPR1	3,69108E-03	5764,1	1,36063E-02	5066	7,08474E-01	1789	8,26857E-01	7166	9,17080E-01	3591	0	11208,5
Responder	pDC_LILRA4	Macro_LYVE1	SPON2	ITGB2	3,69133E-03	6879,5	3,16536E-02	1109	7,91241E-01	1295	1,32330E+00	2722	8,42264E-01	18063	0	11208,5
Responder	Mono_CD14	cDC_LAMP3	HBEGF	CD44	3,69425E-03	13052,5	7,13518E-03	15011	2,61573E-01	12952	4,71894E-01	13630	8,69258E-01	12461	0	11208,5
Responder	Mast	Macro_ISG15	ANXA1	FPR1	3,69510E-03	6650,7	1,10569E-02	7168	5,74671E-01	3194	8,71548E-01	6571	9,08843E-01	5112	0	11208,5
Responder	pDC_LILRA4	cDC_CLEC9A	CCL5	CXCR3	3,69531E-03	13238,3	1,93366E-02	2666	6,10077E-01	2723	2,23267E+00	427	6,76100E-01	49167	0	11208,5
Responder	Macro_NLRP3	Macro_OLFML3	THBS1	ITGA4	3,69671E-03	9457,5	1,52511E-02	4125	6,18859E-01	2620	4,44812E-01	14321	8,56746E-01	15013	0	11208,5
Responder	Mono_INHBA	Macro_ISG15	ANXA2	TLR2	3,69794E-03	12492,5	7,13422E-03	15014	2,79642E-01	11974	4,75532E-01	13534	8,78554E-01	10732	0	11208,5
Responder	Mono_CD14	Macro_OLFML3	VCAN	CD44	3,70657E-03	9044,7	9,95891E-03	8544	6,24802E-01	2543	4,19167E-01	15021	8,94072E-01	7907	0	11208,5
Responder	Macro_LYVE1	Mono_INHBA	GRN	TNFRSF1B	3,70903E-03	8822,9	8,11085E-03	12165	4,95987E-01	4482	4,71987E-01	13622	9,22305E-01	2637	0	11208,5
Responder	pDC_LILRA4	Macro_LYVE1	SEMA3C	NRP1_PLXNA1	3,71125E-03	13744,5	5,58389E-02	355	7,40728E-01	1579	1,32261E+00	2727	6,47806E-01	52853	0	11208,5
Responder	Macro_LYVE1	pDC_LILRA4	CXCL12	CXCR4	3,71924E-03	5911,1	1,91270E-02	2729	6,59481E-01	2214	2,38418E+00	316	8,66208E-01	13088	0	11208,5
Responder	cDC(CD1C)	cDC_LAMP3	GNAI2	S1PR1	3,72131E-03	12081,7	2,84754E-02	1370	5,42185E-01	3659	8,25419E-01	7181	7,47546E-01	36990	0	11208,5
Responder	Macro_LYVE1	Mono_INHBA	HBEGF	CD44	3,72140E-03	9721,3	9,22930E-03	9785	5,40518E-01	3687	4,55467E-01	14049	8,83200E-01	9877	0	11208,5
Responder	Macro_LYVE1	Mono_INHBA	PLAU	PLAUR	3,72263E-03	10160,9	9,66756E-03	9017	5,28523E-01	3880	5,35056E-01	12083	8,58699E-01	14616	0	11208,5
Responder	Macro_IER3	cDC(CD1C)	TNF	TRADD	3,72324E-03	9532,7	1,91249E-02	2730	6,95201E-01	1896	1,37418E+00	2480	7,87146E-01	29349	0	11208,5
Responder	Macro_ISG15	cDC_CLEC9A	NECTIN2	CD226	3,72770E-03	10931,1	6,16196E-02	288	1,01038E+00	553	1,30100E+00	2848	7,32917E-01	39758	0	11208,5
Responder	Mono_INHBA	Macro_NLRP3	GNAI2	FPR1	3,73007E-03	12148,9	7,54476E-03	13704	2,99032E-01	10946	4,18356E-01	15040	8,83370E-01	9846	0	11208,5
Responder	Mono_INHBA	cDC(CD1C)	NAMPT	ITGA5_ITGB1	3,73007E-03	10685,3	7,12446E-03	15040	4,49017E-01	5574	8,23915E-01	7206	8,59698E-01	14398	0	11208,5
Responder	Macro_LYVE1	Mono_INHBA	F13A1	ITGB1	3,73255E-03	7316,9	1,47944E-02	4359	6,30202E-01	2491	1,17154E+00	3603	8,57182E-01	14923	0	11208,5
Responder	Macro_OLFML3	Mono_CD14	C3	C3AR1	3,73255E-03	10680,3	8,54301E-03	11182	3,01904E-01	10820	9,95846E-01	5149	8,56567E-01	15042	0	11208,5
Responder	cDC(CD1C)	Macro_ISG15	ANXA2	TLR2	3,73255E-03	10800,9	7,12274E-03	15042	2,78328E-01	12037	1,01283E+00	4968	8,78468E-01	10749	0	11208,5
Responder	Macro_IFI27	Mast	FN1	CD44	3,73627E-03	8522,7	1,42260E-02	4690	4,48503E-01	5587	4,18300E-01	15045	9,03648E-01	6083	0	11208,5
Responder	Macro_LYVE1	Mono_INHBA	A2M	LRP1	3,73876E-03	8811,3	1,37275E-02	5000	4,55302E-01	5402	6,69061E-01	9566	8,67311E-01	12880	0	11208,5
Responder	cDC_CLEC9A	pDC_LILRA4	HLA-DRB1	CD4	3,74108E-03	7097,7	6,88481E-03	15871	3,85501E-01	7437	2,12852E+00	554	9,39422E-01	418	0	11208,5
Responder	Mono_CD14	cDC_LAMP3	TNFSF13B	CD40	3,74373E-03	12668,3	7,79483E-03	13007	2,26813E-01	15051	6,97588E-01	9083	8,56884E-01	14992	0	11208,5
Responder	cDC(CD1C)	Mono_INHBA	TNFSF13B	TFRC	3,74497E-03	10820,3	7,65140E-03	13401	2,93565E-01	11219	1,23708E+00	3221	8,56536E-01	15052	0	11208,5
Responder	Macro_LYVE1	Mono_INHBA	PSAP	LRP1	3,74497E-03	9361,9	7,42946E-03	14067	4,52270E-01	5498	4,79798E-01	13432	9,22514E-01	2604	0	11208,5
Responder	Mono_CD14	Mono_INHBA	HP	TLR4	3,74969E-03	15048,9	5,17782E-02	431	5,24650E-01	3956	8,24532E-01	7195	6,51291E-01	52454	0	11208,5
Responder	Macro_NLRP3	Macro_FOLR2+APOE-	S100A8	CD36	3,74995E-03	10712,9	1,02002E-02	8214	4,85761E-01	4696	4,17920E-01	15056	8,59721E-01	14390	0	11208,5
Responder	Mono_INHBA	Mono_CD14	PTGS2	ALOX5	3,76393E-03	9428,9	1,23155E-02	5974	3,92958E-01	7202	9,85684E-01	5245	8,44869E-01	17515	0	11208,5
Responder	Macro_FOLR2-APOE+	pDC_LILRA4	MMP9	IFNAR1	3,76742E-03	8657,1	1,90814E-02	2741	6,37682E-01	2423	1,97227E+00	761	8,03384E-01	26152	0	11208,5
Responder	cDC(CD1C)	Mono_INHBA	GNAI2	C5AR1	3,76992E-03	10280,5	7,11167E-03	15072	3,23748E-01	9828	9,62689E-01	5499	8,83613E-01	9795	0	11208,5
Responder	pDC_LILRA4	pDC_LILRA4	APP	NOTCH2	3,77145E-03	6436,3	1,90789E-02	2742	9,42554E-01	726	3,18354E+00	47	8,45139E-01	17458	0	11208,5
Responder	Macro_NLRP3	Macro_IFI27	THBS1	LRP1	3,77492E-03	9351,5	1,24693E-02	5839	5,27745E-01	3901	6,02288E-01	10733	8,56410E-01	15076	0	11208,5
Responder	Macro_FOLR2-APOE+	Macro_FOLR2-APOE+	SPP1	ITGA5_ITGB1	3,77822E-03	7988,7	1,68153E-02	3434	5,72054E-01	3241	9,22245E-01	5952	8,51640E-01	16108	0	11208,5
Responder	Macro_LYVE1	cDC_CLEC9A	CALM1	MYLK	3,78135E-03	8967,7	4,95783E-02	481	1,00929E+00	557	1,00705E+00	5032	7,96211E-01	27560	0	11208,5
Responder	Macro_FOLR2-APOE+	Macro_FOLR2-APOE+	SPP1	ITGAV_ITGB5	3,78231E-03	9245,3	2,72039E-02	1490	6,18874E-01	2618	9,68492E-01	5439	8,06878E-01	25471	0	11208,5
Responder	Macro_FOLR2+APOE-	Macro_LYVE1	F13A1	ITGB1	3,78357E-03	5299,9	2,31085E-02	2014	1,03484E+00	502	1,31900E+00	2745	8,82368E-01	10030	0	11208,5
Responder	cDC(CD1C)	pDC_LILRA4	LGALS1	CD69	3,79248E-03	9499,7	7,10715E-03	15090	2,88009E-01	11493	1,86316E+00	942	8,89356E-01	8765	0	11208,5
Responder	Macro_FOLR2-APOE+	Macro_IFI27	MMP9	CD44	3,79877E-03	9618,7	1,11047E-02	7105	4,61773E-01	5250	6,75757E-01	9435	8,56315E-01	15095	0	11208,5
Responder	pDC_LILRA4	Macro_FOLR2+APOE+	SEMA7A	PLXNC1	3,79976E-03	12242,9	2,40549E-02	1870	6,81487E-01	2001	1,31842E+00	2749	7,12413E-01	43386	0	11208,5
Responder	Macro_IFI27	cDC(CD1C)	C3	ITGAX	3,80129E-03	11096,7	1,00609E-02	8419	2,26085E-01	15097	7,27088E-01	8610	8,71228E-01	12149	0	11208,5
Responder	cDC_CLEC9A	Macro_ISG15	HLA-DQA1	LAG3	3,80255E-03	7817,3	1,51416E-02	4182	4,82538E-01	4761	1,14038E+00	3837	8,56310E-01	15098	0	11208,5
Responder	Mono_CD14	Macro_LYVE1	CD14	TLR4	3,80633E-03	13294,7	8,28904E-03	11741	2,26057E-01	15101	4,78578E-01	13459	8,57007E-01	14964	0	11208,5
Responder	Mono_INHBA	Macro_ISG15	VCAN	CD44	3,81011E-03	11612,9	8,32348E-03	11679	3,06546E-01	10577	4,15595E-01	15104	8,85272E-01	9496	0	11208,5
Responder	Macro_LYVE1	Mono_CD14	TIMP2	CD44	3,81515E-03	7014,5	1,14248E-02	6789	6,14788E-01	2663	8,25246E-01	7185	8,97561E-01	7227	0	11208,5
Responder	Macro_OLFML3	Mono_CD14	CXCL10	TLR4	3,81600E-03	9578,7	1,90266E-02	2753	3,78345E-01	7686	1,37716E+00	2464	8,15009E-01	23782	0	11208,5
Responder	cDC(CD1C)	Mono_CD14	HMGB1	THBD	3,81642E-03	10877,5	7,10012E-03	15109	2,97012E-01	11034	1,27073E+00	3024	8,61593E-01	14012	0	11208,5
Responder	Macro_OLFML3	cDC(CD1C)	MDK	ITGA4_ITGB1	3,81927E-03	9710,9	1,77168E-02	3126	3,92195E-01	7229	1,05404E+00	4588	8,21501E-01	22403	0	11208,5
Responder	Mono_CD16	Mono_CD14	SELPLG	SELL	3,82007E-03	6605,9	1,90261E-02	2754	6,92481E-01	1915	1,40452E+00	2320	8,57592E-01	14832	0	11208,5
Responder	Macro_OLFML3	Mono_CD14	B2M	LILRB2	3,82183E-03	11347,9	5,44658E-03	22817	3,62628E-01	8274	4,61373E-01	13893	9,37593E-01	547	0	11208,5
Responder	Macro_FOLR2-APOE+	Mono_CD14	PKM	CD44	3,82274E-03	8376,9	7,09877E-03	15114	4,85638E-01	4701	7,15525E-01	8786	9,25396E-01	2075	0	11208,5
Responder	Macro_OLFML3	Mono_INHBA	C3	IFITM1	3,82527E-03	9886,3	1,01674E-02	8268	5,03341E-01	4334	6,15796E-01	10505	8,56205E-01	15116	0	11208,5
Responder	Macro_LYVE1	Mono_CD14	CD14	TLR4	3,82527E-03	9940,1	9,90987E-03	8608	3,78047E-01	7698	6,80455E-01	9365	8,67605E-01	12821	0	11208,5
Responder	pDC_LILRA4	Macro_ISG15	ASIP	MGRN1	3,83229E-03	14079,5	5,53427E-02	365	7,95086E-01	1280	1,31723E+00	2757	6,30684E-01	54787	0	11208,5
Responder	Macro_OLFML3	Mono_INHBA	HLA-A	LILRB2	3,84048E-03	8442,7	7,09623E-03	15128	5,33134E-01	3818	5,79599E-01	11129	9,33727E-01	930	0	11208,5
Responder	Mono_INHBA	Macro_FOLR2+APOE+	CD52	SIGLEC10	3,85319E-03	8824,5	1,22559E-02	6022	5,52606E-01	3530	4,14555E-01	15138	8,92228E-01	8224	0	11208,5
Responder	Mono_INHBA	Macro_ISG15	ICAM1	ITGAX_ITGB2	3,85828E-03	11230,1	7,09219E-03	15142	3,19215E-01	10017	6,57174E-01	9763	8,82425E-01	10020	0	11208,5
Responder	Macro_FOLR2-APOE+	pDC_LILRA4	ALCAM	NRP1	3,86089E-03	10195,3	2,19623E-02	2193	6,51568E-01	2294	1,31586E+00	2764	7,71059E-01	32517	0	11208,5
Responder	pDC_LILRA4	Macro_OLFML3	NUCB2	ERAP1	3,86499E-03	11454,3	2,11302E-02	2328	6,06574E-01	2765	1,58174E+00	1635	7,35318E-01	39335	0	11208,5

Post_R_Myeloid_Myeloid_CellCel

Responder	cDC(CD1C)	Macro_ISG15	GRN	TNFRSF1A	3,86593E-03	10754,1	7,08961E-03	15148	2,38539E-01	14296	9,99012E-01	5109	8,93490E-01	8009	0	11208,5
Responder	Macro_FOLR2-APOE+	pDC_LILRA4	HLA-DRB5	CD4	3,86976E-03	7754,3	7,08928E-03	15151	3,34810E-01	9344	1,48331E+00	1983	9,32266E-01	1085	0	11208,5
Responder	Mono_INHBA	Macro_OLFML3	VEGFA	EPHB2	3,87311E-03	15164,7	2,00983E-02	2517	5,88876E-01	2975	8,19836E-01	7255	6,56088E-01	51868	0	11208,5
Responder	Macro_ISG15	Mast	VEGFA	CD44	3,87615E-03	11237,3	8,18260E-03	11991	4,65421E-01	5170	5,11353E-01	12661	8,56044E-01	15156	0	11208,5
Responder	Mono_INHBA	cDC(CD1C)	LGALS3	ENG	3,87999E-03	9894,7	8,23124E-03	11868	4,08518E-01	6715	1,05893E+00	4523	8,56032E-01	15159	0	11208,5
Responder	Macro_LYVE1	Mono_CD14	GRN	TNFRSF1B	3,88127E-03	8438,7	7,61446E-03	13500	4,52175E-01	5502	7,07599E-01	8910	9,20012E-01	3073	0	11208,5
Responder	Macro_IER3	Macro_NLRP3	CCL3L1	CCR1	3,88141E-03	7319,7	1,93255E-02	2670	8,84580E-01	887	1,31545E+00	2769	8,37472E-01	19064	0	11208,5
Responder	Mono_INHBA	Macro_NLRP3	VCAN	ITGB1	3,88255E-03	10274,3	1,10260E-02	7196	4,19596E-01	6371	5,64146E-01	11435	8,55977E-01	15161	0	11208,5
Responder	Mono_CD14	Macro_OLFML3	TNFSF13B	CD40	3,88255E-03	12996,3	7,77039E-03	13083	2,25109E-01	15161	6,15698E-01	10508	8,56691E-01	15021	0	11208,5
Responder	cDC(CD1C)	Mono_CD16	LGALS9	PTPRC	3,88562E-03	9592,1	6,98772E-03	15490	3,93626E-01	7177	8,19460E-01	7261	8,99801E-01	6824	0	11208,5
Responder	Macro_LYVE1	Mono_CD14	PLAU	PLAUR	3,88895E-03	8535,7	9,66200E-03	9023	5,27991E-01	3895	1,12952E+00	3933	8,58664E-01	14619	0	11208,5
Responder	cDC_LAMP3	pDC_LILRA4	HMGB1	TLR9	3,88976E-03	11156	4,59810E-02	565	1,09339E+00	399	8,94129E-01	6308	8,41162E-01	18292	0,098	30216
Responder	Macro_LYVE1	Mono_CD14	GNAI2	C5AR1	3,89537E-03	7600,3	9,63279E-03	9071	5,25491E-01	3935	8,62731E-01	6695	8,98331E-01	7092	0	11208,5
Responder	Macro_ISG15	Macro_NLRP3	LGALS9	CD44	3,89537E-03	10877,3	7,08459E-03	15171	3,93039E-01	7197	4,36846E-01	14566	9,02879E-01	6244	0	11208,5
Responder	Macro_LYVE1	Mono_CD14	GNAI2	FPR1	3,89665E-03	10748,7	7,84669E-03	12856	3,34475E-01	9354	5,95359E-01	10853	8,85377E-01	9472	0	11208,5
Responder	Macro_LYVE1	Mono_CD14	HMGB1	THBD	3,89794E-03	11373,7	7,73060E-03	13200	3,79542E-01	7644	5,47261E-01	11801	8,66588E-01	13015	0	11208,5
Responder	cDC_CLEC9A	Mono_INHBA	HMGB1	CD163	3,89794E-03	8075,5	7,08364E-03	15173	4,43014E-01	5725	1,12501E+00	3976	9,13272E-01	4295	0	11208,5
Responder	Macro_FOLR2-APOE+	Macro_FOLR2-APOE+	MMP9	LRP1	3,89815E-03	7372,1	2,22645E-02	2137	7,16581E-01	1726	1,10170E+00	4164	8,44357E-01	17625	0	11208,5
Responder	pDC_LILRA4	Macro_IER3	HSP90B1	LRP1	3,90179E-03	8746,3	7,08314E-03	15176	4,05268E-01	6812	1,77220E+00	1117	8,85727E-01	9418	0	11208,5
Responder	Macro_LYVE1	Mono_CD14	A2M	LRP1	3,90308E-03	9524,3	1,17503E-02	6484	3,79219E-01	7659	8,00495E-01	7535	8,58104E-01	14735	0	11208,5
Responder	Macro_LYVE1	cDC(CD1C)	LGALS3	ENG	3,90308E-03	10456,3	8,21351E-03	11918	4,06535E-01	6779	8,24214E-01	7199	8,55899E-01	15177	0	11208,5
Responder	Mono_INHBA	Macro_NLRP3	ICAM1	ITGAL_ITGB2	3,90694E-03	12567,1	7,08192E-03	15180	3,04780E-01	10655	4,74839E-01	13555	8,70765E-01	12237	0	11208,5
Responder	Macro_LYVE1	Mono_CD14	C1QB	LRP1	3,90822E-03	9231,9	9,09604E-03	10011	3,94955E-01	7133	4,52637E-01	14124	9,16551E-01	3683	0	11208,5
Responder	Macro_ISG15	Macro_FOLR2-APOE+	CXCL10	TLR4	3,91025E-03	7594,1	2,48737E-02	1754	6,31551E-01	2480	1,31386E+00	2776	8,34365E-01	19752	0	11208,5
Responder	Macro_FOLR2+APOE-	Macro_FOLR2-APOE+	CCL18	CCR1	3,91025E-03	9583,5	2,01485E-02	2506	6,05607E-01	2776	1,66771E+00	1371	7,83535E-01	30056	0	11208,5
Responder	Macro_LYVE1	Mono_CD14	C1QA	CD93	3,91080E-03	6787,9	1,29888E-02	5472	5,31432E-01	3835	7,47287E-01	8293	9,08694E-01	5131	0	11208,5
Responder	Macro_OLFML3	Macro_LYVE1	HLA-DMA	CD4	3,91595E-03	8204,7	9,31281E-03	9630	5,70129E-01	3265	4,12957E-01	15187	9,27448E-01	1733	0	11208,5
Responder	Macro_OLFML3	Mono_CD14	LGALS9	CD44	3,92111E-03	10990,1	7,07714E-03	15191	3,93805E-01	7170	4,14868E-01	15127	9,02833E-01	6254	0	11208,5
Responder	Macro_LYVE1	Mono_CD14	CIRBP	TREM1	3,92240E-03	10282,3	1,14897E-02	6723	3,91719E-01	7241	5,77560E-01	11177	8,56488E-01	15062	0	11208,5
Responder	Macro_LYVE1	Mono_CD14	HMGB1	TLR4	3,92628E-03	11801,7	8,05945E-03	12284	3,40524E-01	9099	5,10570E-01	12688	8,63016E-01	13729	0	11208,5
Responder	Macro_OLFML3	Macro_NLRP3	APOE	ABCA1	3,93016E-03	10107,7	8,65278E-03	10907	4,25311E-01	6203	4,12471E-01	15198	8,98672E-01	7022	0	11208,5
Responder	Macro_LYVE1	Mono_CD14	HSP90B1	TLR4	3,93404E-03	11360,3	8,69563E-03	10812	3,39370E-01	9153	6,15390E-01	10515	8,56227E-01	15113	0	11208,5
Responder	Macro_FOLR2-APOE+	cDC(CD1C)	TGFB1	CXCR4	3,93404E-03	10366,5	7,07600E-03	15201	4,03823E-01	6855	8,65207E-01	6658	8,72646E-01	11910	0	11208,5
Responder	Macro_OLFML3	Macro_OLFML3	TNF	TNFRSF1A	3,93645E-03	40675,9	2,39582E-03	51311	4,51703E-02	31835	-8,25076E-01	57334	6,57538E-01	51691	0	11208,5
Responder	Macro_OLFML3	Macro_OLFML3	LGALS3BP	CD33	3,93792E-03	8597,3	2,20232E-02	2181	5,65458E-01	3331	5,83797E-01	11062	8,55767E-01	15204	0	11208,5
Responder	Macro_FOLR2-APOE+	Macro_FOLR2-APOE+	APOE	ABCA1	3,93804E-03	10402,5	1,14487E-02	6761	4,00293E-01	6974	1,90690E-01	22314	9,10728E-01	4755	0	11208,5
Responder	Macro_FOLR2-APOE+	Mono_INHBA	VIM	CD44	3,94051E-03	7746,5	7,07297E-03	15206	6,16983E-01	2644	6,69060E-01	9567	9,47221E-01	107	0	11208,5
Responder	cDC_CLEC9A	Macro_IER3	HLA-DQA2	CD4	3,94181E-03	10797,5	7,07259E-03	15207	2,64181E-01	12800	8,17018E-01	7288	8,96270E-01	7484	0	11208,5
Responder	Mono_INHBA	Macro_FOLR2+APOE+	ICAM1	IL2RA	3,94648E-03	9619,5	2,47949E-02	1764	7,01972E-01	1835	8,16882E-01	7290	8,04091E-01	26000	0	11208,5
Responder	Macro_ISG15	Mono_INHBA	VCAN	CD44	3,94959E-03	10647,1	7,06957E-03	15213	4,47819E-01	5605	6,37868E-01	10118	8,76716E-01	11091	0	11208,5
Responder	cDC(CD1C)	cDC_CLEC9A	COPA	CD74	3,95281E-03	10081,7	4,95019E-03	26220	3,90050E-01	7293	1,45533E+00	2095	9,17079E-01	3592	0	11208,5
Responder	Mono_CD14	Mono_INHBA	HP	ITGAM	3,95493E-03	14857,9	6,39781E-02	261	5,86922E-01	3007	8,16768E-01	7294	6,50803E-01	52519	0	11208,5
Responder	Macro_LYVE1	Mono_CD14	C1QA	CR1	3,95608E-03	7057,3	2,18086E-02	2221	6,48057E-01	2323	6,83748E-01	9300	8,81200E-01	10234	0	11208,5
Responder	Macro_FOLR2-APOE+	Macro_FOLR2-APOE+	MMP9	ITGAM	3,95704E-03	10802,7	2,15701E-02	2260	6,53068E-01	2284	9,79897E-01	5299	7,68730E-01	32962	0	11208,5
Responder	Macro_LYVE1	Mono_CD14	HSPA1A	TLR4	3,95738E-03	11696,9	7,88437E-03	12730	3,56645E-01	8495	4,23856E-01	14888	8,76448E-01	11163	0	11208,5
Responder	Mono_INHBA	cDC_LAMP3	VCAN	ITGA4	3,96129E-03	9091,9	1,44607E-02	4548	5,03162E-01	4338	6,36538E-01	10143	8,55664E-01	15222	0	11208,5
Responder	pDC_LILRA4	Mast	CD55	ADGRE2	3,96519E-03	6729,9	1,66578E-02	3491	6,20274E-01	2600	1,76974E+00	1125	8,55656E-01	15225	0	11208,5
Responder	Macro_OLFML3	cDC(CD1C)	HLA-DPA1	CD4	3,96763E-03	9048,9	5,87104E-03	20328	3,89975E-01	7300	9,67810E-01	5445	9,33470E-01	963	0	11208,5
Responder	cDC_CLEC9A	cDC_CLEC9A	HMGB1	THBD	3,97171E-03	10223,3	7,06400E-03	15230	4,01888E-01	6912	1,15585E+00	3705	8,61288E-01	14061	0	11208,5
Responder	Macro_FOLR2-APOE+	Macro_FOLR2-APOE+	MMP9	IFNAR1	3,97399E-03	9482,9	1,91098E-02	2734	6,38216E-01	2418	1,01631E+00	4923	8,03502E-01	26131	0	11208,5
Responder	Mono_CD14	Mono_CD14	VIM	CD44	3,97399E-03	9462,5	5,53661E-03	22264	3,89808E-01	7303	9,02544E-01	6190	9,40753E-01	347	0	11208,5
Responder	Mono_CD14	Macro_FOLR2-APOE+	RETN	CAP1	3,98036E-03	9103,1	2,42990E-02	1829	4,76891E-01	4888	8,15983E-01	7306	8,31769E-01	20284	0	11208,5
Responder	Macro_LYVE1	pDC_LILRA4	ADM	CALCRL	3,98088E-03	12704,5	1,90647E-02	2746	6,04178E-01	2793	1,88411E+00	893	6,97617E-01	45882	0	11208,5
Responder	Macro_IFI27	Macro_IER3	CXCL9	FCGR2A	3,98346E-03	13429,3	8,05800E-03	12288	2,24017E-01	15239	4,37904E-01	14542	8,62281E-01	13869	0	11208,5
Responder	cDC(CD1C)	Mono_CD16	SEMA4A	LILRB2	3,98461E-03	9415,3	1,21375E-02	6112	6,49700E-01	2308	8,15718E-01	7308	8,32503E-01	20140	0	11208,5
Responder	cDC_CLEC9A	Macro_IFI27	BTLA	TNFRSF14	3,98925E-03	8912,9	3,01971E-02	1210	1,55982E+00	99	1,30982E+00	2795	7,87587E-01	29252	0	11208,5
Responder	cDC_CLEC9A	Macro_ISG15	HLA-F	LILRB1	3,99523E-03	10766,7	8,01788E-03	12387	3,03796E-01	10711	1,08643E+00	4279	8,55547E-01	15248	0	11208,5
Responder	Macro_OLFML3	cDC_LAMP3	FN1	ITGA5_ITGB1	3,99524E-03	10086,7	1,32366E-02	5290	3,89418E-01	7313	8,83653E-01	6443	8,32319E-01	20179	0	11208,5
Responder	Macro_OLFML3	Macro_IFI27	C3	ITGAX	3,99654E-03	11074,3	7,70900E-03	13260	3,91403E-01	7250	7,40776E-01	8404	8,55539E-01	15249	0	11208,5
Responder	Macro_LYVE1	Mono_CD14	HBEGF	CD44	3,99916E-03	11949,9	7,59481E-03	13558	3,73830E-01	7845	4,10729E-01	15251	8,72764E-01	11887	0	11208,5
Responder	Macro_LYVE1	pDC_LILRA4	HMGB1	THBD	3,99951E-03	5382,9	1,39315E-02	4873	6,57907E-01	2228	1,69911E+00	1290	8,97118E-01	7315	0	11208,5
Responder	cDC_CLEC9A	Macro_FOLR2-APOE+	HLA-DQA2	CD4	4,00047E-03	10679,3	7,05825E-03	15252	2,63264E-01	12846	8,70851E-01	6586	8,96175E-01	7504	0	11208,5
Responder	Macro_NLRP3	Mono_INHBA	GNAI2	C5AR1	4,00310E-03	10686,5	8,17019E-03	12020	4,18873E-01	6393	4,10500E-01	15254	8,90561E-01	8557	0	11208,5
Responder	Mono_INHBA	Macro_ISG15	VCAN	SELL	4,00590E-03	10385,3	1,23931E-02	5908	3,89248E-01	7318	8,79441E-01	6491	8,28219E-01	21001	0	11208,5
Responder	pDC_LILRA4	Macro_FOLR2-APOE+	COPA	CD74	4,00704E-03	8025,5	7,05547E-03	15257	3,50084E-01	8732	1,18700E+00	3511	9,29596E-01	1419	0	11208,5

Responder	Mono_CD14	Macro_IFI27	S100A9	TLR4	4,00835E-03	7632,5	9,14100E-03	9940	9,17330E-01	790	1,84827E+00	966	8,55513E-01	15258	0	11208,5
Responder	Mono_INHBA	Macro_IER3	ICAM1	ITGAL_ITGB2	4,01755E-03	12839,5	7,13861E-03	14999	3,06558E-01	10575	4,10053E-01	15265	8,71213E-01	12150	0	11208,5
Responder	cDC(CD1C)	Macro_OLFML3	HLA-DQB2	LAG3	4,02086E-03	13195,1	1,61528E-02	3693	3,89017E-01	7325	1,33073E+00	2692	7,25092E-01	41057	0	11208,5
Responder	Macro_NLRP3	Macro_OLFML3	VCAN	ITGB1	4,02282E-03	9614,1	1,29817E-02	5476	5,91780E-01	2943	4,09846E-01	15269	8,65753E-01	13174	0	11208,5
Responder	Macro_OLFML3	cDC(CD1C)	HLA-DRB5	CD4	4,02514E-03	8663,7	6,32334E-03	18115	3,88970E-01	7327	9,99730E-01	5095	9,28566E-01	1573	0	11208,5
Responder	Macro_IFI27	pDC_LILRA4	C3	IFITM1	4,02941E-03	10424,9	1,00451E-02	8440	2,27762E-01	14981	1,42897E+00	2221	8,55458E-01	15274	0	11208,5
Responder	cDC(CD1C)	Mono_CD16	C3	IFITM1	4,03073E-03	10711,9	1,00444E-02	8441	3,33908E-01	9382	6,87031E-01	9253	8,55454E-01	15275	0	11208,5
Responder	Macro_IER3	Macro_NLRP3	TNF	TNFRSF1B	4,04389E-03	5153,3	2,34645E-02	1955	9,12863E-01	806	1,30803E+00	2808	8,88263E-01	8989	0	11208,5
Responder	Macro_IER3	Macro_ISG15	TNF	VSIR	4,05234E-03	5585,7	2,85397E-02	1360	8,84572E-01	888	1,30790E+00	2810	8,74024E-01	11662	0	11208,5
Responder	Mono_CD14	Mono_CD16	HP	TLR4	4,05305E-03	13807,3	7,09206E-02	212	6,21228E-01	2591	8,13929E-01	7340	6,86114E-01	47685	0	11208,5
Responder	cDC(CD1C)	Mono_INHBA	HBEGF	CD44	4,05453E-03	9947,3	7,04443E-03	15293	4,56407E-01	5384	9,85952E-01	5242	8,68529E-01	12609	0	11208,5
Responder	Macro_OLFML3	Macro_LYVE1	FN1	ITGA5_ITGB1	4,05736E-03	8402,5	1,86674E-02	2845	4,62533E-01	5232	8,13619E-01	7342	8,54960E-01	15385	0	11208,5
Responder	Macro_OLFML3	cDC(CD1C)	FN1	ITGAV_ITGB1	4,05952E-03	8833,1	1,49902E-02	4257	3,88492E-01	7343	1,23639E+00	3230	8,41945E-01	18127	0	11208,5
Responder	Macro_ISG15	pDC_LILRA4	TNF	TNFRSF21	4,06080E-03	9994,7	1,88155E-02	2812	9,97822E-01	591	2,04593E+00	649	7,60014E-01	34713	0	11208,5
Responder	Macro_ISG15	Mono_INHBA	PKM	CD44	4,06382E-03	8443,9	7,04324E-03	15300	4,81898E-01	4780	7,13784E-01	8808	9,25124E-01	2123	0	11208,5
Responder	pDC_LILRA4	Mono_INHBA	ADAM10	CD44	4,07313E-03	9836,9	7,04040E-03	15307	4,57574E-01	5358	1,42599E+00	2231	8,56397E-01	15080	0	11208,5
Responder	Mono_INHBA	Mono_CD14	NAMPT	ITGA5_ITGB1	4,07446E-03	10727,7	7,04037E-03	15308	4,60091E-01	5283	8,17314E-01	7283	8,58981E-01	14556	0	11208,5
Responder	Macro_NLRP3	Mono_INHBA	HBEGF	CD9	4,07579E-03	10612,1	1,19703E-02	6287	4,14028E-01	6561	4,69087E-01	13695	8,55281E-01	15309	0	11208,5
Responder	Macro_OLFML3	Macro_FOLR2-APOE+	C1QA	CD33	4,07579E-03	12171,3	7,15797E-03	14940	3,41819E-01	9041	4,08371E-01	15309	8,80476E-01	10358	0	11208,5
Responder	cDC(CD1C)	Macro_OLFML3	HMGB1	HAVCR2	4,08112E-03	10626,7	7,03830E-03	15313	3,15172E-01	10192	6,81044E-01	9355	8,98432E-01	7065	0	11208,5
Responder	Macro_ISG15	Mono_CD14	SAA1	CD36	4,08200E-03	9195,5	2,29101E-02	2040	6,02468E-01	2810	1,30598E+00	2817	7,98409E-01	27102	0	11208,5
Responder	cDC_CLEC9A	Macro_IFI27	IL16	CD9	4,08245E-03	7712,7	1,88443E-02	2799	5,43669E-01	3641	9,52696E-01	5601	8,55262E-01	15314	0	11208,5
Responder	Mono_CD14	Macro_NLRP3	IL1B	SIGIRR	4,08327E-03	8752,9	1,08861E-02	7354	4,99878E-01	4404	1,19456E+00	3461	8,45658E-01	17337	0	11208,5
Responder	Macro_FOLR2+APOE+	pDC_LILRA4	CXCL10	CXCR3	4,08625E-03	9242,1	4,12635E-02	696	1,08348E+00	416	1,30577E+00	2818	7,78614E-01	31072	0	11208,5
Responder	pDC_LILRA4	Macro_OLFML3	APP	LRP1	4,08778E-03	6384,3	1,65196E-02	3549	8,74898E-01	923	1,87260E+00	923	8,55245E-01	15318	0	11208,5
Responder	Mono_INHBA	cDC_CLEC9A	COPA	CD74	4,08977E-03	10370,3	4,91923E-03	26470	3,88157E-01	7357	1,24205E+00	3185	9,16840E-01	3631	0	11208,5
Responder	Mono_INHBA	Mast	VIM	CD44	4,09045E-03	8158,1	7,03700E-03	15320	6,36713E-01	2430	5,51127E-01	11722	9,47093E-01	110	0	11208,5
Responder	pDC_LILRA4	Mono_INHBA	CD24	SIGLEC10	4,09050E-03	21663,7	2,39681E-02	1883	6,01519E-01	2819	1,65262E+00	1409	6,17458E-01	56155	0,987	46052,5
Responder	Mono_INHBA	Macro_LYVE1	CD99	CD81	4,09179E-03	9799,7	9,31164E-03	9633	5,31202E-01	3841	4,07917E-01	15321	8,88244E-01	8995	0	11208,5
Responder	cDC(CD1C)	Macro_FOLR2+APOE+	LGALS9	HAVCR2	4,09179E-03	13317,5	7,03697E-03	15321	2,43409E-01	13980	5,38067E-01	12002	8,61219E-01	14076	0	11208,5
Responder	cDC(CD1C)	Macro_FOLR2-APOE+	ALCAM	NRP1	4,09410E-03	14574,9	1,21416E-02	6110	3,87977E-01	7359	9,91518E-01	5194	7,14625E-01	43003	0	11208,5
Responder	pDC_LILRA4	Macro_FOLR2+APOE-	GAS6	MERTK	4,09476E-03	9942,1	1,87900E-02	2820	8,43259E-01	1043	2,30834E+00	369	7,62438E-01	34270	0	11208,5
Responder	Macro_ISG15	cDC(CD1C)	TNFSF10	TNFRSF10B	4,09901E-03	7789,9	2,14332E-02	2286	6,96918E-01	1879	1,30497E+00	2821	8,29557E-01	20755	0	11208,5
Responder	Macro_NLRP3	Macro_FOLR2-APOE+	S100A8	CD68	4,10248E-03	7085,3	1,27060E-02	5661	5,98124E-01	2858	4,07680E-01	15329	9,40141E-01	370	0	11208,5
Responder	Macro_NLRP3	Macro_IER3	S100A8	TLR4	4,10382E-03	10510,3	1,15080E-02	6705	4,67718E-01	5101	4,49146E-01	14207	8,55199E-01	15330	0	11208,5
Responder	cDC(CD1C)	Macro_OLFML3	HLA-DRB1	CD4	4,10713E-03	8719,5	6,28272E-03	18294	3,87878E-01	7365	9,08596E-01	6113	9,36765E-01	617	0	11208,5
Responder	cDC_CLEC9A	cDC_CLEC9A	GNAI2	CXCR3	4,10754E-03	8626,5	1,96125E-02	2616	6,50649E-01	2301	1,30475E+00	2823	8,13068E-01	24184	0	11208,5
Responder	Macro_LYVE1	Macro_FOLR2-APOE+	F13A1	ITGB1	4,10784E-03	7636,1	1,49385E-02	4288	6,35266E-01	2443	9,67382E-01	5449	8,57774E-01	14792	0	11208,5
Responder	Macro_LYVE1	Mono_CD14	CCL3	CCR1	4,11052E-03	9173,7	1,24077E-02	5895	5,14513E-01	4133	6,83903E-01	9297	8,55180E-01	15335	0	11208,5
Responder	pDC_LILRA4	Macro_FOLR2-APOE+	APP	LRP10	4,11181E-03	8117,5	1,87679E-02	2824	9,13344E-01	804	2,17567E+00	488	8,07861E-01	25263	0	11208,5
Responder	Mono_CD16	Macro_ISG15	CD52	SIGLEC10	4,11365E-03	6091,3	1,35274E-02	5112	8,28830E-01	1110	9,47984E-01	5658	8,96883E-01	7368	0	11208,5
Responder	Macro_LYVE1	Macro_FOLR2-APOE+	A2M	LRP1	4,11588E-03	10372,1	1,28360E-02	5570	4,20996E-01	6329	4,16240E-01	15093	8,63400E-01	13660	0	11208,5
Responder	Macro_OLFML3	cDC_CLEC9A	COPA	P2RY6	4,11801E-03	11939,5	1,31910E-02	5319	5,42897E-01	3648	8,12222E-01	7370	7,72933E-01	32152	0	11208,5
Responder	Macro_FOLR2+APOE+	Mono_INHBA	C1QA	CD93	4,12019E-03	6158,7	1,33311E-02	5219	6,76901E-01	2053	8,12192E-01	7371	9,09767E-01	4942	0	11208,5
Responder	pDC_LILRA4	Macro_IFI27	SPON2	ITGB1	4,12035E-03	12058,7	1,87625E-02	2826	7,06128E-01	1804	1,39898E+00	2353	7,19616E-01	42102	0	11208,5
Responder	Macro_FOLR2-APOE+	Macro_ISG15	SPP1	ITGA5_ITGB1	4,12455E-03	10177,3	1,16086E-02	6609	4,40283E-01	5812	9,23177E-01	5938	8,26676E-01	21319	0	11208,5
Responder	Macro_ISG15	Mono_CD16	LGALS3BP	ITGB1	4,12528E-03	10899,3	1,15771E-02	6637	3,51412E-01	8676	5,12693E-01	12629	8,55124E-01	15346	0	11208,5
Responder	Macro_LYVE1	Macro_FOLR2-APOE+	PLTP	ABCA1	4,13066E-03	5810,1	2,24217E-02	2108	9,46569E-01	713	9,75822E-01	5337	8,84267E-01	9684	0	11208,5
Responder	pDC_LILRA4	cDC_LAMP3	LTB	TNFRSF1A	4,13746E-03	7393,7	1,87255E-02	2830	9,06142E-01	824	2,28806E+00	380	8,24705E-01	21726	0	11208,5
Responder	Mono_CD14	Macro_IFI27	VCAN	ITGB1	4,14144E-03	7674,1	1,08624E-02	7387	7,00822E-01	1847	1,35358E+00	2570	8,55053E-01	15358	0	11208,5
Responder	cDC_CLEC9A	Mono_INHBA	PKM	CD44	4,14144E-03	7584,9	7,02535E-03	15358	4,79971E-01	4829	1,07359E+00	4396	9,25036E-01	2133	0	11208,5
Responder	cDC(CD1C)	Macro_FOLR2+APOE+	ICAM1	IL2RA	4,14420E-03	12633,3	1,46296E-02	4455	4,61793E-01	5249	8,11675E-01	7382	7,59195E-01	34872	0	11208,5
Responder	Macro_ISG15	Macro_FOLR2+APOE-	S100A9	CD68	4,14683E-03	10533,1	7,02467E-03	15362	3,21542E-01	9925	4,31261E-01	14699	9,29261E-01	1471	0	11208,5
Responder	Macro_OLFML3	cDC(CD1C)	HLA-DPB1	CD4	4,14858E-03	9248,9	5,86359E-03	20368	3,87200E-01	7384	9,06324E-01	6143	9,31776E-01	1141	0	11208,5
Responder	Macro_FOLR2-APOE+	Macro_FOLR2+APOE+	ADAM10	GPNMB	4,15899E-03	8387,7	1,52406E-02	4131	7,86377E-01	1321	6,49208E-01	9907	8,55013E-01	15371	0	11208,5
Responder	cDC(CD1C)	Macro_LYVE1	C3	LRP1	4,16305E-03	12085,5	8,52270E-03	11219	2,89170E-01	11437	5,76739E-01	11189	8,55004E-01	15374	0	11208,5
Responder	pDC_LILRA4	Macro_LYVE1	ASIP	ATRN	4,16323E-03	14747,5	1,04061E-01	81	8,99298E-01	846	1,30244E+00	2836	5,86368E-01	58766	0	11208,5
Responder	cDC_CLEC9A	Macro_FOLR2-APOE+	LGALS9	LRP1	4,16576E-03	12765,3	7,02058E-03	15376	2,49825E-01	13614	7,35080E-01	8489	8,56100E-01	15139	0	11208,5
Responder	Macro_FOLR2-APOE+	pDC_LILRA4	HLA-DQA1	CD4	4,17525E-03	8241,7	7,01784E-03	15383	3,18136E-01	10069	1,32363E+00	2719	9,26924E-01	1829	0	11208,5
Responder	Mono_INHBA	Macro_FOLR2+APOE-	TIMP1	CD63	4,17712E-03	8676,1	6,52139E-03	17246	3,94408E-01	7152	8,10571E-01	7397	9,40000E-01	377	0	11208,5
Responder	Macro_LYVE1	Macro_FOLR2-APOE+	LGALS3	ENG	4,18204E-03	8067,9	1,32957E-02	5246	6,00878E-01	2826	5,77922E-01	11169	8,83136E-01	9890	0	11208,5
Responder	Macro_IFI27	Macro_LYVE1	C1QB	LRP1	4,18340E-03	6820,9	1,47274E-02	4393	6,68223E-01	2123	4,05201E-01	15389	9,33225E-01	991	0	11208,5
Responder	Macro_FOLR2-APOE+	Macro_ISG15	LGALS9	HAVCR2	4,18748E-03	13866,7	7,01623E-03	15392	2,41588E-01	14088	4,38236E-01	14532	8,61043E-01	14113	0	11208,5
Responder	Mono_INHBA	Mast	TIMP2	CD44	4,19020E-03	11889,1	7,01567E-03	15394	4,01107E-01	6939	4,55976E-01	14037	8,72872E-01	11867	0	11208,5
Responder	Macro_OLFML3	Macro_FOLR2+APOE-	C3	ITGAX	4,19156E-03	11449,7	7,62977E-03	13457	3,89536E-01	7308	6,50792E-01	9880	8,54899E-01	15395	0	11208,5

Responder	Macro_OLFML3	Macro_OLFML3	HLA-DRA	LAG3	4,19429E-03	11153,7	8,84765E-03	10491	4,41108E-01	5784	5,01925E-01	12888	8,54887E-01	15397	0	11208,5
Responder	Mono_INHBA	Macro_LYVE1	S100A9	CD68	4,19475E-03	7624,9	1,08500E-02	7405	7,04977E-01	1812	3,35361E-01	17425	9,42283E-01	274	0	11208,5
Responder	Macro_FOLR2-APOE+	pDC_LILRA4	PLAU	IGF2R	4,19774E-03	9562,1	1,86694E-02	2844	6,11610E-01	2704	1,87095E+00	929	7,83232E-01	30125	0	11208,5
Responder	Macro_FOLR2-APOE+	Macro_ISG15	MMP9	EPHB2	4,20138E-03	13934,7	2,91767E-02	1304	6,63371E-01	2168	1,10785E+00	4102	6,64140E-01	50891	0	11208,5
Responder	pDC_LILRA4	Macro_OLFML3	SPON2	ITGB1	4,20207E-03	10184,7	2,90130E-02	1319	8,36597E-01	1075	1,30129E+00	2845	7,61424E-01	34476	0	11208,5
Responder	Macro_FOLR2-APOE+	Macro_ISG15	MMP9	LRP1	4,20359E-03	11521,3	1,15048E-02	6707	4,77879E-01	4866	8,25349E-01	7183	7,95904E-01	27642	0	11208,5
Responder	cDC_CLEC9A	Mast	VIM	CD44	4,20792E-03	6754,1	7,01137E-03	15407	6,31890E-01	2478	1,05573E+00	4566	9,47002E-01	111	0	11208,5
Responder	Macro_NLRP3	Mono_INHBA	TIMP2	CD44	4,20929E-03	11061,7	7,01137E-03	15408	4,39392E-01	5832	5,87933E-01	10986	8,72838E-01	11874	0	11208,5
Responder	Macro_FOLR2-APOE+	Macro_ISG15	MMP12	PLAUR	4,21023E-03	7989,5	1,69438E-02	3377	4,89461E-01	4622	1,26643E+00	3051	8,44095E-01	17689	0	11208,5
Responder	Macro_IER3	Macro_FOLR2-APOE+	CCL3L1	CCR5	4,21073E-03	10663,3	2,10953E-02	2334	9,13106E-01	805	1,30118E+00	2847	7,52258E-01	36122	0	11208,5
Responder	Macro_OLFML3	Macro_ISG15	FN1	ITGA4_ITGB1	4,21244E-03	8860,3	1,41149E-02	4764	3,86323E-01	7413	9,71580E-01	5395	8,54279E-01	15521	0	11208,5
Responder	cDC(CD1C)	Mono_INHBA	CIRBP	TREM1	4,21466E-03	9282,3	1,11516E-02	7054	3,86293E-01	7414	9,81790E-01	5280	8,54643E-01	15455	0	11208,5
Responder	Macro_FOLR2+APOE+	cDC_CLEC9A	C1QA	CD93	4,21466E-03	6339,5	1,08409E-02	7414	6,04323E-01	2790	1,16068E+00	3673	9,00913E-01	6612	0	11208,5
Responder	cDC(CD1C)	Macro_LYVE1	ADAM10	GPNMB	4,21687E-03	9229,5	1,08393E-02	7415	6,18043E-01	2630	1,03256E+00	4777	8,32588E-01	20117	0	11208,5
Responder	Macro_IFI27	cDC(CD1C)	APOE	SORL1	4,21749E-03	12074,3	8,09399E-03	12207	2,21329E-01	15414	4,70081E-01	13668	8,94250E-01	7874	0	11208,5
Responder	Mono_INHBA	Macro_IFI27	ICAM1	ITGAM_ITGB2	4,22160E-03	12742,9	7,00886E-03	15417	2,88261E-01	11481	4,83066E-01	13345	8,70604E-01	12263	0	11208,5
Responder	Macro_FOLR2-APOE+	Macro_OLFML3	APP	CD74	4,22297E-03	8988,9	7,00873E-03	15418	4,88680E-01	4635	5,67553E-01	11366	9,24087E-01	2317	0	11208,5
Responder	Macro_IER3	Mono_CD14	ICAM1	ITGAX_ITGB2	4,22352E-03	6190,1	1,08365E-02	7418	6,01245E-01	2823	1,23718E+00	3220	9,02698E-01	6281	0	11208,5
Responder	Macro_NLRP3	Mono_CD16	S100A8	TLR4	4,22571E-03	9272,7	1,52017E-02	4153	5,54659E-01	3489	4,04324E-01	15420	8,71597E-01	12093	0	11208,5
Responder	Macro_FOLR2-APOE+	Macro_FOLR2-APOE+	CXCL8	SDC2	4,22796E-03	9542,1	2,22161E-02	2147	6,70500E-01	2109	8,08953E-01	7420	8,09920E-01	24826	0	11208,5
Responder	Macro_OLFML3	Mono_CD14	FN1	ITGA4_ITGB1	4,22796E-03	8488,9	1,40879E-02	4786	3,86075E-01	7420	1,19227E+00	3481	8,54160E-01	15549	0	11208,5
Responder	pDC_LILRA4	cDC_CLEC9A	GAS6	AXL	4,22809E-03	9159,5	1,86490E-02	2851	7,59947E-01	1479	2,55847E+00	198	7,83520E-01	30061	0	11208,5
Responder	Macro_ISG15	Mono_INHBA	S100A8	CD68	4,22845E-03	8855,9	9,19883E-03	9840	4,15823E-01	6494	4,04301E-01	15422	9,30380E-01	1315	0	11208,5
Responder	Macro_ISG15	Macro_OLFML3	ICAM1	ITGAL_ITGB2	4,23668E-03	13078,5	7,00775E-03	15428	2,85400E-01	11645	4,27837E-01	14787	8,70172E-01	12324	0	11208,5
Responder	Macro_FOLR2-APOE+	Macro_ISG15	MMP9	ITGAM	4,24130E-03	11150,3	1,91537E-02	2718	6,23358E-01	2562	1,09911E+00	4178	7,58000E-01	35085	0	11208,5
Responder	Mono_CD14	Macro_LYVE1	CD99	CD81	4,24218E-03	11767,1	7,00693E-03	15432	3,82225E-01	7557	5,03581E-01	12847	8,73332E-01	11791	0	11208,5
Responder	Mono_CD14	Macro_LYVE1	HP	TLR4	4,24353E-03	13243,5	8,16363E-02	160	6,75292E-01	2070	8,08439E-01	7427	7,01065E-01	45352	0	11208,5
Responder	cDC_CLEC9A	Mono_CD16	HLA-F	LILRB1	4,24493E-03	11373,7	7,91328E-03	12664	2,97548E-01	11010	8,73549E-01	6552	8,54734E-01	15434	0	11208,5
Responder	Macro_FOLR2-APOE+	Macro_ISG15	MMP9	IFNAR1	4,25244E-03	10047,1	1,67734E-02	3452	5,94324E-01	2906	1,06622E+00	4456	7,93003E-01	28213	0	11208,5
Responder	cDC(CD1C)	Mono_CD16	HMGB1	CXCR4	4,25244E-03	8765,5	6,68179E-03	16634	4,23315E-01	6266	8,08247E-01	7431	9,24275E-01	2288	0	11208,5
Responder	cDC_CLEC9A	Macro_LYVE1	BTLA	TNFRSF14	4,25249E-03	7563,5	4,48787E-02	591	1,68402E+00	83	1,28001E+00	2972	8,18846E-01	22963	0	11208,5
Responder	Macro_IFI27	pDC_LILRA4	HLA-DPB1	CD4	4,25318E-03	7376,7	7,00279E-03	15440	3,78930E-01	7665	1,48254E+00	1985	9,37208E-01	585	0	11208,5
Responder	Macro_LYVE1	Macro_ISG15	CCL3	CCR1	4,25456E-03	7952,9	1,56275E-02	3950	6,05545E-01	2777	6,83899E-01	9298	8,68890E-01	12531	0	11208,5
Responder	Macro_LYVE1	Macro_ISG15	CCL4	CCR1	4,25732E-03	9542,9	1,33577E-02	5203	4,84332E-01	4724	5,77930E-01	11168	8,54848E-01	15411	0	11208,5
Responder	Macro_FOLR2-APOE+	Mono_INHBA	C3	LRP1	4,25869E-03	12026,7	1,12669E-02	6929	2,36359E-01	14438	4,03650E-01	15444	8,71464E-01	12114	0	11208,5
Responder	Macro_ISG15	Macro_ISG15	GRN	TNFRSF1A	4,26836E-03	12278,3	6,99814E-03	15451	2,27046E-01	15032	5,57180E-01	11579	8,92871E-01	8121	0	11208,5
Responder	Mono_CD14	Macro_OLFML3	RETN	TLR4	4,27031E-03	13277,5	2,89878E-02	1322	5,25327E-01	3937	8,07664E-01	7439	7,17469E-01	42481	0	11208,5
Responder	Macro_ISG15	Macro_IER3	SPP1	PTGER4	4,27169E-03	7331,7	1,96166E-02	2614	5,97911E-01	2861	1,36654E+00	2510	8,45097E-01	17465	0	11208,5
Responder	Macro_IFI27	Mono_CD16	B2M	LILRB2	4,27250E-03	7705,3	9,66298E-03	9020	6,02270E-01	2813	4,03450E-01	15454	9,52406E-01	31	0	11208,5
Responder	Macro_OLFML3	Macro_OLFML3	FN1	ITGAV_ITGB1	4,27665E-03	8762,1	1,82594E-02	2967	4,61644E-01	5253	7,06920E-01	8925	8,54633E-01	15457	0	11208,5
Responder	Mono_CD16	Mono_CD16	CD52	SIGLEC10	4,27926E-03	6328,1	1,39737E-02	4852	8,40995E-01	1057	8,07433E-01	7443	8,98374E-01	7080	0	11208,5
Responder	Mono_INHBA	Macro_NLRP3	CCL5	CCRL2	4,29496E-03	13466,3	1,21162E-02	6133	3,85160E-01	7450	9,14464E-01	6041	7,50101E-01	36499	0	11208,5
Responder	Macro_OLFML3	pDC_LILRA4	IGF1	IGF2R	4,29721E-03	15921,5	1,08015E-02	7451	4,41871E-01	5758	1,56424E+00	1700	6,42174E-01	53490	0	11208,5
Responder	cDC(CD1C)	cDC_CLEC9A	APP	CD74	4,30396E-03	10761,5	4,70255E-03	28123	3,85007E-01	7454	1,50276E+00	1913	9,08851E-01	5109	0	11208,5
Responder	pDC_LILRA4	Macro_LYVE1	APP	TNFRSF21	4,31119E-03	9555,1	1,85783E-02	2870	9,81253E-01	623	2,29258E+00	377	7,70196E-01	32697	0	11208,5
Responder	Macro_ISG15	Mono_CD14	S100A8	TLR4	4,31274E-03	10142,9	1,13795E-02	6826	3,62250E-01	8285	7,07568E-01	8912	8,54502E-01	15483	0	11208,5
Responder	Macro_LYVE1	Mono_CD14	HSP90B1	ASGR1	4,31971E-03	11506,3	9,77848E-03	8817	3,58530E-01	8422	4,72885E-01	13596	8,54456E-01	15488	0	11208,5
Responder	Macro_FOLR2+APOE+	Mono_INHBA	SERPING1	LRP1	4,32650E-03	6283,9	1,64032E-02	3597	7,14250E-01	1743	8,06170E-01	7464	8,96740E-01	7407	0	11208,5
Responder	Macro_NLRP3	Macro_LYVE1	S100A12	CD36	4,32668E-03	6459,7	4,48568E-02	592	8,47206E-01	1028	1,12498E+00	3977	8,54417E-01	15493	0	11208,5
Responder	Mono_INHBA	Macro_NLRP3	IL1A	IL1R2_IL1RAP	4,32876E-03	16171,5	3,68457E-02	852	5,16792E-01	4095	8,06109E-01	7465	6,05203E-01	57237	0	11208,5
Responder	Mono_CD14	cDC_CLEC9A	COPA	CD74	4,32876E-03	10272,1	4,86358E-03	26881	3,84754E-01	7465	1,45499E+00	2096	9,16406E-01	3710	0	11208,5
Responder	Macro_OLFML3	Macro_OLFML3	HLA-B	LILRA1	4,32996E-03	23068,1	3,97514E-03	34855	2,59888E-01	13049	-7,45797E-02	33468	8,19885E-01	22760	0	11208,5
Responder	Macro_LYVE1	pDC_LILRA4	HMGB1	CXCR4	4,33323E-03	5637,1	8,14560E-03	12089	5,96959E-01	2875	1,96341E+00	771	9,30922E-01	1242	0	11208,5
Responder	Mono_INHBA	pDC_LILRA4	THBS1	SCARB1	4,33328E-03	12244,7	1,43699E-02	4608	3,84618E-01	7467	1,31006E+00	2794	7,57668E-01	35146	0	11208,5
Responder	cDC(CD1C)	Macro_FOLR2-APOE+	RPS19	C5AR1	4,33554E-03	9371,3	6,15322E-03	18867	3,84607E-01	7468	1,12815E+00	3952	9,07532E-01	5361	0	11208,5
Responder	cDC(CD1C)	Macro_ISG15	LGALS9	HAVCR2	4,35047E-03	12018,5	6,98263E-03	15510	2,38958E-01	14270	1,01525E+00	4937	8,60755E-01	14167	0	11208,5
Responder	Macro_NLRP3	Macro_FOLR2+APOE+	THBS1	LRP1	4,35609E-03	9798,7	1,37310E-02	4995	5,60111E-01	3400	4,01101E-01	15514	8,62235E-01	13876	0	11208,5
Responder	Macro_LYVE1	Macro_ISG15	LGALS3	ENG	4,35749E-03	11762,5	8,36635E-03	11601	4,12380E-01	6599	4,40780E-01	14446	8,57032E-01	14958	0	11208,5
Responder	Mono_CD14	Mono_CD14	GNAI2	FPR1	4,35890E-03	10885,9	6,98189E-03	15516	2,52430E-01	13477	1,16694E+00	3636	8,79317E-01	10592	0	11208,5
Responder	Mono_CD14	Macro_OLFML3	HP	ITGB2	4,36274E-03	8012,1	6,56882E-02	244	7,55778E-01	1499	8,04733E-01	7480	8,34942E-01	19629	0	11208,5
Responder	Macro_NLRP3	Mono_INHBA	S100A9	ITGB2	4,36311E-03	10550,7	7,30490E-03	14446	3,24380E-01	9795	4,00971E-01	15519	9,27154E-01	1785	0	11208,5
Responder	Macro_FOLR2+APOE-	cDC(CD1C)	MRC1	PTPRC	4,36421E-03	7141,1	1,85249E-02	2882	6,45976E-01	2343	1,42027E+00	2255	8,47122E-01	17017	0	11208,5
Responder	pDC_LILRA4	Mono_CD16	TGFB1	CXCR4	4,36592E-03	9913,3	6,97947E-03	15521	3,69531E-01	7996	1,30969E+00	2796	8,71880E-01	12045	0	11208,5
Responder	Macro_IFI27	pDC_LILRA4	APOE	SORL1	4,36733E-03	10316,1	8,04497E-03	12317	2,19492E-01	15522	1,05203E+00	4612	8,93963E-01	7921	0	11208,5
Responder	cDC(CD1C)	Mono_CD16	GRN	TNFRSF1B	4,36733E-03	9126,1	6,97935E-03	15522	3,70780E-01	7951	8,16514E-01	7298	9,16748E-01	3651	0	11208,5

Responder	Macro_NLRP3	pDC_LILRA4	CXCL8	CD79A	4,36865E-03	11614,3	3,78368E-02	814	6,10233E-01	2722	1,29483E+00	2883	7,28879E-01	40444	0	11208,5
Responder	Macro_FOLR2-APOE+	Macro_NLRP3	APOE	LDLR	4,36874E-03	10383,7	1,77834E-02	3111	3,95769E-01	7103	4,20889E-01	14973	8,54275E-01	15523	0	11208,5
Responder	cDC_CLEC9A	Macro_LYVE1	B2M	TFRC	4,36874E-03	10651,3	7,16594E-03	14916	2,19462E-01	15523	6,45253E-01	9992	9,28318E-01	1617	0	11208,5
Responder	Macro_FOLR2-APOE+	Macro_ISG15	SPP1	ITGAV_ITGB1	4,36956E-03	11094,1	1,08467E-02	7407	3,89897E-01	7302	8,04416E-01	7483	8,22988E-01	22070	0	11208,5
Responder	Mono_INHBA	Macro_FOLR2-APOE+	LGALS1	ITGB1	4,37296E-03	9778,9	6,97873E-03	15526	4,99096E-01	4424	5,22404E-01	12399	9,07611E-01	5337	0	11208,5
Responder	cDC(CD1C)	Mono_INHBA	ARF6	PLD1	4,37639E-03	15383,3	1,38619E-02	4915	3,84024E-01	7486	1,05633E+00	4558	6,79073E-01	48749	0	11208,5
Responder	cDC_CLEC9A	pDC_LILRA4	HLA-DMA	CD4	4,38001E-03	8398,9	6,97815E-03	15531	2,97280E-01	11023	2,04639E+00	647	9,17118E-01	3585	0	11208,5
Responder	Mono_CD14	Mast	TGFB1	SMAD3	4,38095E-03	13840,1	1,97195E-02	2590	4,05736E-01	6801	8,04105E-01	7488	7,24790E-01	41113	0	11208,5
Responder	Mono_INHBA	Macro_FOLR2-APOE+	ADM	GPR84	4,38551E-03	14837,5	2,00997E-02	2516	4,41160E-01	5782	8,03955E-01	7490	6,89454E-01	47191	0	11208,5
Responder	Mono_INHBA	Mono_INHBA	GNAI2	C5AR1	4,39412E-03	12101,1	7,22489E-03	14728	3,33922E-01	9378	4,00170E-01	15541	8,84423E-01	9650	0	11208,5
Responder	Macro_FOLR2-APOE+	pDC_LILRA4	CXCL9	CXCR3	4,39534E-03	11991,1	1,85144E-02	2889	9,57944E-01	676	1,30259E+00	2835	7,18163E-01	42347	0	11208,5
Responder	Macro_FOLR2+APOE-	Macro_NLRP3	CCL13	CCR1	4,39623E-03	12167,3	4,45727E-02	601	1,17237E+00	300	9,99450E-01	5102	7,11151E-01	43625	0	11208,5
Responder	Macro_OLFML3	Macro_FOLR2-APOE+	C3	CD46	4,39837E-03	9651,1	1,14382E-02	6778	4,41662E-01	5767	7,04866E-01	8958	8,54181E-01	15544	0	11208,5
Responder	Mono_INHBA	Mast	PKM	CD44	4,40403E-03	9196,9	6,97440E-03	15548	4,65263E-01	5174	5,44180E-01	11864	9,24783E-01	2190	0	11208,5
Responder	Macro_FOLR2+APOE-	Mono_CD14	CXCL12	ITGA5	4,40872E-03	10143,7	2,92656E-02	1294	6,54587E-01	2272	1,29350E+00	2892	7,68296E-01	33052	0	11208,5
Responder	Macro_OLFML3	Mono_INHBA	APOE	LDLR	4,41065E-03	9343,7	1,40412E-02	4807	4,96781E-01	4463	8,02898E-01	7501	8,38945E-01	18739	0	11208,5
Responder	pDC_LILRA4	Macro_NLRP3	LTB	TNFRSF1A	4,41319E-03	7427,5	1,85027E-02	2893	9,02922E-01	832	2,40990E+00	295	8,23838E-01	21909	0	11208,5
Responder	cDC(CD1C)	Mono_CD14	GRN	TNFRSF1B	4,41537E-03	8112,9	6,97147E-03	15556	3,70020E-01	7978	1,44099E+00	2162	9,16705E-01	3660	0	11208,5
Responder	Mono_CD14	cDC(CD1C)	HP	ITGAM	4,41982E-03	16708,1	3,63781E-02	874	4,70111E-01	5048	8,02544E-01	7505	5,84259E-01	58905	0	11208,5
Responder	Macro_FOLR2-APOE+	Mono_CD16	HLA-A	LILRB2	4,42389E-03	7611,3	1,00655E-02	8410	6,14032E-01	2674	3,99098E-01	15562	9,43756E-01	202	0	11208,5
Responder	Macro_FOLR2-APOE+	Macro_NLRP3	SPP1	ITGAV_ITGB1	4,42441E-03	9685,3	1,33058E-02	5242	4,41855E-01	5759	8,30495E-01	7125	8,37384E-01	19092	0	11208,5
Responder	Mono_INHBA	cDC_CLEC9A	CALR	SCARF1	4,42671E-03	9822,9	2,00291E-02	2527	4,64185E-01	5204	8,02466E-01	7508	8,20262E-01	22667	0	11208,5
Responder	Macro_FOLR2-APOE+	Macro_NLRP3	SPP1	ITGA5_ITGB1	4,42671E-03	8341,7	1,54075E-02	4044	5,34589E-01	3786	9,69818E-01	5423	8,46031E-01	17247	0	11208,5
Responder	Macro_FOLR2-APOE+	Macro_LYVE1	THBS1	CD36	4,42674E-03	7259,3	2,05601E-02	2425	6,85954E-01	1966	9,96875E-01	5133	8,54093E-01	15564	0	11208,5
Responder	Macro_IFI27	Macro_ISG15	HLA-DRB5	LAG3	4,42958E-03	10519,1	1,38784E-02	4908	4,10357E-01	6653	3,99002E-01	15566	8,60328E-01	14260	0	11208,5
Responder	Mono_INHBA	Macro_FOLR2-APOE+	VCAN	CD44	4,43243E-03	9541,9	1,05179E-02	7791	4,43623E-01	5713	3,98939E-01	15568	8,96630E-01	7429	0	11208,5
Responder	Macro_OLFML3	Macro_ISG15	C1QB	CD33	4,43385E-03	11460,7	6,96735E-03	15569	3,40702E-01	9089	5,94583E-01	10868	8,79418E-01	10569	0	11208,5
Responder	Macro_NLRP3	Macro_LYVE1	S100A8	TLR4	4,43528E-03	8662,7	1,74986E-02	3193	6,08722E-01	2739	3,98834E-01	15570	8,79267E-01	10603	0	11208,5
Responder	Macro_FOLR2+APOE+	Macro_NLRP3	C1QA	CD93	4,43820E-03	6149,3	1,34603E-02	5152	6,80666E-01	2017	8,01927E-01	7513	9,10162E-01	4856	0	11208,5
Responder	Mono_INHBA	Mono_CD14	SDC2	PTPRJ	4,44051E-03	17196,5	1,35252E-02	5114	4,06639E-01	6774	8,01922E-01	7514	6,25337E-01	55372	0	11208,5
Responder	Macro_NLRP3	Macro_IER3	CD52	SIGLEC10	4,44240E-03	10199,3	9,63041E-03	9075	4,86092E-01	4688	3,98646E-01	15575	8,80077E-01	10450	0	11208,5
Responder	pDC_LILRA4	Macro_ISG15	RARRES2	CMKLR1	4,44454E-03	14145,9	2,93308E-02	1288	5,94811E-01	2900	2,10995E+00	575	6,30891E-01	54758	0	11208,5
Responder	Mono_INHBA	Macro_LYVE1	SERPINA1	LRP1	4,44742E-03	8929,1	1,07384E-02	7517	4,59946E-01	5290	3,68769E-01	16445	9,13819E-01	4185	0	11208,5
Responder	Macro_IFI27	pDC_LILRA4	HLA-DPA1	CD4	4,45097E-03	7427,1	6,96448E-03	15581	3,69632E-01	7995	1,50935E+00	1879	9,38581E-01	472	0	11208,5
Responder	Macro_NLRP3	pDC_LILRA4	VCAN	ITGB1	4,45239E-03	7888,3	1,06824E-02	7593	5,26371E-01	3922	1,76550E+00	1136	8,54014E-01	15582	0	11208,5
Responder	pDC_LILRA4	Macro_FOLR2+APOE+	APP	LRP1	4,45382E-03	6449,1	1,61946E-02	3669	8,67830E-01	952	1,92491E+00	833	8,54011E-01	15583	0	11208,5
Responder	Macro_FOLR2-APOE+	pDC_LILRA4	APOE	SCARB1	4,45525E-03	8931,5	1,33288E-02	5221	3,47465E-01	8836	1,14389E+00	3808	8,53999E-01	15584	0	11208,5
Responder	Macro_IFI27	Mono_INHBA	FN1	C5AR1	4,45811E-03	10048,7	1,60463E-02	3745	3,65196E-01	8169	3,98045E-01	15586	8,74641E-01	11535	0	11208,5
Responder	Macro_LYVE1	cDC(CD1C)	GRN	TNFRSF1A	4,46097E-03	12036,7	6,96251E-03	15588	2,69404E-01	12505	5,08609E-01	12724	8,92626E-01	8158	0	11208,5
Responder	Macro_IER3	Mono_INHBA	CCL3L1	CCR1	4,47153E-03	7401,5	1,84548E-02	2906	8,73893E-01	928	1,43186E+00	2208	8,34310E-01	19757	0	11208,5
Responder	Macro_IFI27	Macro_FOLR2-APOE+	MMP9	CD44	4,47243E-03	9613,1	1,06879E-02	7583	4,24566E-01	6229	8,07207E-01	7449	8,53946E-01	15596	0	11208,5
Responder	Macro_OLFML3	Macro_FOLR2+APOE+	HLA-DQA2	LAG3	4,47604E-03	9058,3	2,31657E-02	2008	5,94232E-01	2907	6,89191E-01	9221	8,33380E-01	19947	0	11208,5
Responder	Macro_FOLR2+APOE+	Macro_FOLR2-APOE+	APOE	SORL1	4,48209E-03	5674,5	1,46349E-02	4453	6,87147E-01	1956	8,00750E-01	7532	9,19165E-01	3223	0	11208,5
Responder	pDC_LILRA4	Macro_FOLR2-APOE+	APP	PTGER2	4,48507E-03	11211,9	1,84464E-02	2909	9,16211E-01	796	2,23482E+00	424	7,27143E-01	40722	0	11208,5
Responder	Macro_LYVE1	Macro_NLRP3	PLAU	PLAUR	4,48535E-03	9052,1	1,05454E-02	7754	6,12525E-01	2692	6,42273E-01	10050	8,63890E-01	13556	0	11208,5
Responder	Macro_FOLR2+APOE-	Macro_NLRP3	CCL18	CCR1	4,48959E-03	9779,9	1,92215E-02	2697	5,94167E-01	2910	1,73935E+00	1204	7,79514E-01	30880	0	11208,5
Responder	Macro_LYVE1	pDC_LILRA4	TNF	TNFRSF21	4,48959E-03	10463,9	1,84328E-02	2910	9,94379E-01	595	1,35961E+00	2541	7,58135E-01	35065	0	11208,5
Responder	Macro_NLRP3	Mono_CD14	VCAN	ITGB1	4,48966E-03	8687,1	1,06631E-02	7615	5,25825E-01	3930	1,00184E+00	5074	8,53902E-01	15608	0	11208,5
Responder	cDC_CLEC9A	Macro_FOLR2+APOE+	HLA-DQA2	CD4	4,49686E-03	11317,3	6,95624E-03	15613	2,56731E-01	13234	7,07808E-01	8904	8,95496E-01	7627	0	11208,5
Responder	Macro_LYVE1	Macro_NLRP3	GNAI2	C5AR1	4,49830E-03	8159,9	1,10719E-02	7146	6,28342E-01	2515	4,56499E-01	14019	9,04516E-01	5911	0	11208,5
Responder	Mono_CD14	Macro_FOLR2+APOE+	VCAN	SELL	4,50298E-03	9527,9	1,07183E-02	7541	7,37835E-01	1594	1,11557E+00	4055	8,17643E-01	23241	0	11208,5
Responder	Macro_LYVE1	Macro_NLRP3	A2M	LRP1	4,50695E-03	9606,5	1,32535E-02	5275	4,37061E-01	5892	5,23158E-01	12381	8,65276E-01	13276	0	11208,5
Responder	Macro_OLFML3	Macro_OLFML3	HLA-DPB1	CD4	4,51290E-03	10220,7	6,91952E-03	15729	4,72865E-01	4974	2,98234E-01	18583	9,36855E-01	609	0	11208,5
Responder	cDC_CLEC9A	Macro_ISG15	CD52	SIGLEC10	4,51561E-03	12940,3	6,95351E-03	15626	2,66890E-01	12628	5,71904E-01	11269	8,61800E-01	13970	0	11208,5
Responder	Mono_CD16	cDC(CD1C)	CD52	SIGLEC10	4,51695E-03	5570,1	1,32620E-02	5270	8,21597E-01	1145	1,33341E+00	2680	8,95963E-01	7547	0	11208,5
Responder	Macro_OLFML3	cDC(CD1C)	CD14	ITGA4	4,51706E-03	12502,1	6,95337E-03	15627	2,79966E-01	11960	5,04181E-01	12830	8,77710E-01	10885	0	11208,5
Responder	Macro_OLFML3	Mono_CD16	CD1D	LILRB2	4,52129E-03	9316,9	1,84068E-02	2917	6,94379E-01	1906	6,07236E-01	10657	8,33620E-01	19896	0	11208,5
Responder	Macro_FOLR2-APOE+	Macro_IFI27	APOE	TREM2	4,52574E-03	8577,1	1,42666E-02	4672	3,38262E-01	9204	3,96295E-01	15633	9,24863E-01	2168	0	11208,5
Responder	Mono_CD14	Macro_ISG15	GNAI2	FPR1	4,53008E-03	11812,1	6,95078E-03	15636	2,49833E-01	13613	7,69304E-01	7965	8,79080E-01	10638	0	11208,5
Responder	cDC(CD1C)	Macro_NLRP3	HSPA8	LDLR	4,53096E-03	9863,3	1,18110E-02	6423	3,82337E-01	7553	9,42405E-01	5712	8,40608E-01	18420	0	11208,5
Responder	Mono_CD14	pDC_LILRA4	HMGB1	TLR9	4,53492E-03	12861,2	3,97075E-02	744	1,00758E+00	561	1,28822E+00	2920	8,31116E-01	20422	0,767	39659
Responder	Macro_FOLR2-APOE+	Macro_NLRP3	MMP9	LRP1	4,53563E-03	7082,3	2,29886E-02	2033	7,32645E-01	1627	1,20862E+00	3383	8,46448E-01	17160	0	11208,5
Responder	Macro_LYVE1	Macro_NLRP3	TIMP2	CD44	4,54458E-03	8571,5	1,13162E-02	6878	6,07425E-01	2754	4,31205E-01	14703	8,97121E-01	7314	0	11208,5
Responder	Macro_ISG15	Mono_CD16	C3	IFITM1	4,55622E-03	9864,3	1,40690E-02	4796	4,24983E-01	6218	3,95365E-01	15654	8,75066E-01	11445	0	11208,5
Responder	Macro_ISG15	Mono_INHBA	SPP1	ITGA5_ITGB1	4,55767E-03	6962,3	1,73668E-02	3238	5,93750E-01	2918	1,53812E+00	1792	8,53667E-01	15655	0	11208,5

Post_R_Myeloid_Myeloid_CellCel

Responder	Mono_CD14	pDC_LILRA4	APP	RPSA	4,56204E-03	8996,5	7,01147E-03	15406	6,47758E-01	2326	2,27959E+00	384	8,53657E-01	15658	0	11208,5
Responder	Macro_NLRP3	Macro_OLFML3	S100A8	ITGB2	4,56641E-03	6793,9	1,40802E-02	4790	6,89209E-01	1942	3,95129E-01	15661	9,40150E-01	368	0	11208,5
Responder	cDC(CD1C)	Macro_ISG15	ANXA1	FPR2_FPR3	4,56933E-03	9428,7	1,02546E-02	8138	4,07922E-01	6732	9,70989E-01	5402	8,53637E-01	15663	0	11208,5
Responder	Macro_IFI27	Macro_ISG15	CCL18	CCR1	4,57141E-03	8638,3	2,52347E-02	1707	5,93105E-01	2928	1,87329E+00	920	8,02014E-01	26428	0	11208,5
Responder	Macro_LYVE1	Macro_NLRP3	HSP90B1	ASGR1	4,57371E-03	9727,3	1,31892E-02	5320	4,95656E-01	4490	3,96949E-01	15609	8,72093E-01	12009	0	11208,5
Responder	Macro_ISG15	Macro_FOLR2-APOE+	SPP1	ITGAV_ITGB1	4,58393E-03	7342,9	1,70552E-02	3339	5,54878E-01	3482	1,27214E+00	3012	8,53587E-01	15673	0	11208,5
Responder	Macro_NLRP3	Mono_CD16	GNAI2	C5AR1	4,58540E-03	9293,3	9,76891E-03	8833	5,34718E-01	3784	3,94749E-01	15674	8,98970E-01	6967	0	11208,5
Responder	Macro_NLRP3	Mono_CD14	CD55	ADGRE5	4,58979E-03	7815,5	1,32946E-02	5248	6,04542E-01	2787	1,10211E+00	4157	8,53570E-01	15677	0	11208,5
Responder	Macro_FOLR2-APOE+	Macro_NLRP3	FABP5	RXRA	4,59672E-03	9432,5	1,47224E-02	4394	5,63911E-01	3359	9,00303E-01	6221	8,23486E-01	21980	0	11208,5
Responder	Macro_IER3	Macro_OLFML3	CCL3L1	CCR5	4,60348E-03	9882,5	2,54200E-02	1683	9,45831E-01	715	1,28549E+00	2935	7,69224E-01	32871	0	11208,5
Responder	Mono_CD14	Macro_OLFML3	CD14	TLR1	4,60885E-03	12348,1	9,81436E-03	8750	2,39012E-01	14267	5,46436E-01	11825	8,53515E-01	15690	0	11208,5
Responder	Mono_INHBA	Macro_FOLR2-APOE+	ICAM1	ITGAX_ITGB2	4,60885E-03	11924,3	7,41735E-03	14109	3,42073E-01	9031	3,94180E-01	15690	8,84731E-01	9583	0	11208,5
Responder	Macro_FOLR2-APOE+	Macro_NLRP3	MMP9	ITGAM	4,61091E-03	10252,3	2,32702E-02	1987	6,73973E-01	2084	1,08365E+00	4306	7,75405E-01	31676	0	11208,5
Responder	pDC_LILRA4	Macro_FOLR2+APOE+	COPA	CD74	4,61472E-03	8882,3	6,93211E-03	15694	3,23536E-01	9839	9,04392E-01	6168	9,29017E-01	1502	0	11208,5
Responder	Mono_CD14	Macro_FOLR2+APOE+	RETN	TLR4	4,61564E-03	13658,7	2,70616E-02	1503	5,07248E-01	4257	7,96782E-01	7589	7,10448E-01	43736	0	11208,5
Responder	Mono_INHBA	Macro_OLFML3	ADAM10	TREM2	4,61767E-03	8526,5	1,64752E-02	3568	5,17376E-01	4079	7,61605E-01	8081	8,53484E-01	15696	0	11208,5
Responder	Macro_IFI27	Macro_NLRP3	RPS19	C5AR1	4,62797E-03	10311,5	6,92830E-03	15703	4,43657E-01	5712	4,39989E-01	14480	9,12391E-01	4454	0	11208,5
Responder	Macro_FOLR2-APOE+	Macro_NLRP3	MMP9	IFNAR1	4,62987E-03	10895,1	1,49794E-02	4262	5,60624E-01	3391	9,55984E-01	5565	7,83565E-01	30049	0	11208,5
Responder	Mono_INHBA	cDC_LAMP3	VEGFA	SIRPA	4,62987E-03	12091,3	1,17864E-02	6443	5,26952E-01	3911	7,96237E-01	7595	7,77367E-01	31299	0	11208,5
Responder	Macro_LYVE1	cDC_CLEC9A	CALM3	MYLK	4,63107E-03	11222,5	5,71626E-02	341	9,84278E-01	617	1,08574E+00	4285	7,33381E-01	39661	0	11208,5
Responder	Macro_ISG15	Mast	ACTR2	ADRB2	4,64125E-03	7255,1	2,53948E-02	1685	6,57096E-01	2239	9,69316E-01	5431	8,53422E-01	15712	0	11208,5
Responder	Macro_OLFML3	cDC_CLEC9A	HMGB1	HAVCR2	4,64125E-03	11399,5	6,92516E-03	15712	2,85601E-01	11634	5,73596E-01	11238	8,97690E-01	7205	0	11208,5
Responder	Mono_INHBA	Mono_INHBA	MMP9	CD44	4,65456E-03	8313,1	1,05946E-02	7698	5,81666E-01	3076	1,13713E+00	3862	8,53398E-01	15721	0	11208,5
Responder	Macro_LYVE1	cDC_CLEC9A	HMGB1	HAVCR2	4,65752E-03	8366,1	8,07739E-03	12242	4,29952E-01	6086	8,87343E-01	6387	9,04545E-01	5907	0	11208,5
Responder	cDC(CD1C)	Macro_OLFML3	ANXA1	FPR1	4,66196E-03	12313,7	7,39361E-03	14182	2,80617E-01	11922	3,92930E-01	15726	8,90744E-01	8530	0	11208,5
Responder	Macro_LYVE1	cDC_CLEC9A	C1QA	CD93	4,66938E-03	10024,5	8,60587E-03	11028	3,70517E-01	7959	5,70802E-01	11291	8,90120E-01	8636	0	11208,5
Responder	Mono_INHBA	Mono_CD14	TNF	TNFRSF1A	4,67992E-03	10942,7	1,14958E-02	6716	3,80208E-01	7616	1,13090E+00	3913	8,07907E-01	25260	0	11208,5
Responder	pDC_LILRA4	Macro_FOLR2+APOE+	CD99	PILRA	4,68871E-03	10495,7	6,91634E-03	15744	3,23235E-01	9854	1,15232E+00	3736	8,72495E-01	11936	0	11208,5
Responder	Macro_OLFML3	Mono_INHBA	VIM	CD44	4,69072E-03	20884,3	4,80064E-03	27307	2,18376E-01	15586	-4,69804E-01	49699	9,36651E-01	621	0	11208,5
Responder	Macro_ISG15	Mono_CD14	CCL2	CCR1	4,69129E-03	9551,3	2,56130E-02	1665	5,90935E-01	2954	1,54610E+00	1767	7,83026E-01	30162	0	11208,5
Responder	pDC_LILRA4	Macro_FOLR2-APOE+	CD99	PILRA	4,69168E-03	10300,5	6,91586E-03	15746	3,23204E-01	9858	1,31828E+00	2750	8,72492E-01	11940	0	11208,5
Responder	pDC_LILRA4	Mast	CD24	SIGLEC10	4,70060E-03	24010,3	2,23438E-02	2125	5,90682E-01	2956	1,61943E+00	1515	6,09137E-01	56892	1	56563,5
Responder	cDC(CD1C)	Mast	VEGFA	CD44	4,70063E-03	10872,9	7,82664E-03	12909	4,52689E-01	5486	7,02634E-01	9009	8,53282E-01	15752	0	11208,5
Responder	Mono_INHBA	Macro_LYVE1	VEGFA	ITGB1	4,70149E-03	8027,3	1,70941E-02	3331	6,90400E-01	1933	7,93995E-01	7625	8,51894E-01	16039	0	11208,5
Responder	cDC_CLEC9A	Macro_IER3	CXCL9	FCGR2A	4,70362E-03	13927,3	7,85423E-03	12832	2,17037E-01	15674	3,91740E-01	15754	8,60753E-01	14168	0	11208,5
Responder	Macro_OLFML3	Macro_OLFML3	HLA-DRB5	CD4	4,70810E-03	9356,1	7,46206E-03	13959	4,74634E-01	4938	3,91640E-01	15757	9,33867E-01	918	0	11208,5
Responder	cDC_CLEC9A	Macro_LYVE1	IL16	CD9	4,70959E-03	8496,9	1,82437E-02	2971	5,27359E-01	3905	7,24801E-01	8642	8,53245E-01	15758	0	11208,5
Responder	cDC_CLEC9A	Macro_ISG15	HLA-F	LILRB2	4,70959E-03	11120,9	6,91346E-03	15758	2,81788E-01	11877	1,08808E+00	4268	8,69119E-01	12493	0	11208,5
Responder	Macro_LYVE1	pDC_LILRA4	HLA-DQA2	CD4	4,71258E-03	9104,1	7,78091E-03	13050	3,00996E-01	10866	1,15263E+00	3729	9,00623E-01	6667	0	11208,5
Responder	Macro_ISG15	pDC_LILRA4	ICAM1	IL2RG	4,71557E-03	8501,1	9,12971E-03	9954	4,74365E-01	4944	2,05090E+00	637	8,53240E-01	15762	0	11208,5
Responder	Macro_LYVE1	pDC_LILRA4	HLA-DRB1	CD4	4,71557E-03	7295,3	6,98629E-03	15495	4,04780E-01	6827	1,35692E+00	2555	9,39837E-01	391	0	11208,5
Responder	Mono_INHBA	Mono_INHBA	LGALS1	ITGB1	4,72006E-03	9105,1	6,91142E-03	15765	4,94033E-01	4522	7,26565E-01	8617	9,07204E-01	5413	0	11208,5
Responder	Mono_INHBA	Macro_LYVE1	VCAN	ITGB1	4,72156E-03	10643,7	1,17873E-02	6442	4,53052E-01	5476	3,91206E-01	15766	8,60044E-01	14326	0	11208,5
Responder	Macro_LYVE1	pDC_LILRA4	APP	RPSA	4,72156E-03	6745,1	9,91494E-03	8603	7,36884E-01	1602	2,04898E+00	642	8,74003E-01	11670	0	11208,5
Responder	Macro_OLFML3	Macro_OLFML3	HLA-B	LILRB2	4,72344E-03	19919,5	4,57726E-03	29223	2,93885E-01	11205	-3,50737E-01	45534	9,23414E-01	2427	0	11208,5
Responder	Macro_FOLR2+APOE-	Mono_INHBA	CXCL12	ITGA5	4,72391E-03	8700,7	4,11235E-02	704	7,95158E-01	1277	1,28175E+00	2961	7,97186E-01	27353	0	11208,5
Responder	Macro_FOLR2-APOE+	cDC_CLEC9A	NECTIN2	CD226	4,72554E-03	13770,3	3,52765E-02	921	7,76545E-01	1370	9,17693E-01	5994	6,74935E-01	49358	0	11208,5
Responder	Macro_ISG15	Mono_CD16	HLA-C	LILRA1	4,72605E-03	9096,1	1,69446E-02	3375	3,71574E-01	7922	3,91127E-01	15769	8,97682E-01	7206	0	11208,5
Responder	Mono_CD14	Macro_ISG15	HP	ASGR1	4,73278E-03	15405,9	5,40115E-02	385	5,23608E-01	3976	7,92827E-01	7638	6,39419E-01	53822	0	11208,5
Responder	Macro_OLFML3	pDC_LILRA4	FN1	ITGA4_ITGB1	4,73278E-03	8103,3	1,37788E-02	4969	3,79645E-01	7638	1,92562E+00	831	8,52773E-01	15870	0	11208,5
Responder	pDC_LILRA4	Macro_FOLR2+APOE-	SERPINF1	PLXDC2	4,73505E-03	8955,9	7,73470E-03	13192	5,08012E-01	4245	2,31768E+00	359	8,53191E-01	15775	0	11208,5
Responder	Macro_ISG15	Mono_CD16	SEMA4A	LILRB2	4,73805E-03	7771,5	1,65923E-02	3514	7,47900E-01	1532	8,51712E-01	6826	8,53184E-01	15777	0	11208,5
Responder	Macro_LYVE1	pDC_LILRA4	HLA-DMA	CD4	4,74406E-03	6599,3	8,19995E-03	11945	4,51715E-01	5509	1,51853E+00	1843	9,23048E-01	2491	0	11208,5
Responder	Mono_CD14	cDC_CLEC9A	VCAN	CD44	4,74406E-03	9815,9	6,90777E-03	15781	5,29459E-01	3867	8,48324E-01	6874	8,75459E-01	11349	0	11208,5
Responder	Macro_LYVE1	pDC_LILRA4	HLA-DMB	CD4	4,74556E-03	8799,3	7,54187E-03	13714	3,17734E-01	10091	1,30473E+00	2824	9,03303E-01	6159	0	11208,5
Responder	Macro_FOLR2-APOE+	cDC_CLEC9A	SPP1	ITGA4_ITGB1	4,75211E-03	9590,5	1,08170E-02	7433	3,98725E-01	7016	1,14249E+00	3821	8,40403E-01	18474	0	11208,5
Responder	Macro_FOLR2-APOE+	Mono_CD16	HLA-B	LILRB2	4,75760E-03	7755,1	9,72021E-03	8924	6,09864E-01	2726	3,90375E-01	15790	9,46151E-01	127	0	11208,5
Responder	Macro_LYVE1	pDC_LILRA4	HLA-DRB5	CD4	4,76062E-03	7307,9	7,44455E-03	14022	3,84217E-01	7482	1,29202E+00	2903	9,33794E-01	924	0	11208,5
Responder	Macro_OLFML3	cDC(CD1C)	HLA-DMB	CD4	4,76062E-03	10420,9	6,90473E-03	15792	3,30984E-01	9502	7,19835E-01	8707	8,99379E-01	6895	0	11208,5
Responder	cDC_CLEC9A	Mono_INHBA	HLA-DOB	CD4	4,76137E-03	7524,9	2,25051E-02	2099	9,52988E-01	693	1,28046E+00	2969	8,30058E-01	20655	0	11208,5
Responder	Macro_FOLR2-APOE+	cDC(CD1C)	CD14	ITGA4	4,76665E-03	11954,9	6,90328E-03	15796	2,74306E-01	12262	6,69926E-01	9539	8,77322E-01	10969	0	11208,5
Responder	Mono_CD14	cDC_LAMP3	NAMPT	ITGA5_ITGB1	4,76816E-03	9574,3	6,90309E-03	15797	5,93126E-01	2927	1,24979E+00	3150	8,57784E-01	14789	0	11208,5
Responder	Macro_IFI27	Mono_CD16	HLA-C	LILRB2	4,77269E-03	7899,1	9,78040E-03	8811	5,58266E-01	3427	3,90065E-01	15800	9,42704E-01	249	0	11208,5
Responder	Macro_FOLR2-APOE+	cDC_CLEC9A	COPA	CD74	4,78364E-03	9693,3	5,39495E-03	23114	4,17242E-01	6443	1,04390E+00	4683	9,20293E-01	3018	0	11208,5
Responder	Macro_FOLR2-APOE+	cDC_CLEC9A	COPA	P2RY6	4,78607E-03	9605,1	1,78902E-02	3084	6,29538E-01	2502	1,10098E+00	4167	7,98558E-01	27064	0	11208,5

Responder	Macro_ISG15	cDC_LAMP3	TNFSF13B	HLA-DPB1	4,78630E-03	9518,7	6,89904E-03	15809	4,04945E-01	6822	4,71910E-01	13626	9,46134E-01	128	0	11208,5
Responder	Macro_FOLR2-APOE+	cDC_CLEC9A	CCL20	CXCR3	4,78851E-03	14268,1	3,42827E-02	969	7,15516E-01	1734	9,94697E-01	5165	6,52670E-01	52264	0	11208,5
Responder	Mono_INHBA	cDC_CLEC9A	ICAM1	ITGAX_ITGB2	4,79387E-03	10731,7	6,89715E-03	15814	3,08850E-01	10470	9,27651E-01	5886	8,80971E-01	10280	0	11208,5
Responder	Macro_OLFML3	Macro_FOLR2+APOE+	CXCL9	FCGR2A	4,79539E-03	11260,5	1,12705E-02	6923	2,77430E-01	12086	3,89370E-01	15815	8,81020E-01	10270	0	11208,5
Responder	cDC(CD1C)	Macro_NLRP3	HMGB1	CD163	4,79842E-03	9019,5	6,89660E-03	15817	3,62898E-01	8259	9,77911E-01	5320	9,12207E-01	4493	0	11208,5
Responder	Macro_IFI27	Macro_ISG15	HLA-DQB1	LAG3	4,79994E-03	10417,5	1,42204E-02	4695	4,31073E-01	6056	4,45039E-01	14310	8,53015E-01	15818	0	11208,5
Responder	Macro_LYVE1	pDC_LILRA4	HLA-DPA1	CD4	4,80145E-03	7862,3	6,89617E-03	15819	3,58113E-01	8444	1,21616E+00	3347	9,38296E-01	493	0	11208,5
Responder	Macro_LYVE1	pDC_LILRA4	LGALS1	CD69	4,80601E-03	7017,1	8,84722E-03	10493	4,79102E-01	4842	1,56445E+00	1697	8,99680E-01	6845	0	11208,5
Responder	pDC_LILRA4	Mono_INHBA	CALR	LRP1	4,80905E-03	9956,7	6,89507E-03	15824	2,67428E-01	12603	1,67206E+00	1359	8,89250E-01	8789	0	11208,5
Responder	cDC(CD1C)	Macro_OLFML3	HLA-DRA	LAG3	4,81209E-03	9817,9	8,58197E-03	11094	3,96543E-01	7080	1,13524E+00	3881	8,52985E-01	15826	0	11208,5
Responder	Macro_FOLR2-APOE+	cDC_CLEC9A	CALM1	MYLK	4,81533E-03	10337,1	3,87656E-02	773	7,93691E-01	1285	8,56709E-01	6772	7,75524E-01	31647	0	11208,5
Responder	pDC_LILRA4	Macro_LYVE1	APP	NCSTN	4,81793E-03	8576,1	1,81867E-02	2981	9,01128E-01	840	2,19356E+00	465	7,97040E-01	27386	0	11208,5
Responder	Macro_NLRP3	Macro_IER3	S100A9	ITGB2	4,81817E-03	8733,9	8,91160E-03	10371	4,59027E-01	5313	3,88654E-01	15830	9,33589E-01	947	0	11208,5
Responder	Macro_FOLR2-APOE+	cDC_CLEC9A	ADAM10	CADM1	4,82511E-03	8883,1	2,81593E-02	1401	8,62730E-01	969	1,26038E+00	3089	7,95311E-01	27748	0	11208,5
Responder	Macro_NLRP3	Mono_INHBA	S100A8	TLR4	4,82578E-03	10925,7	1,10986E-02	7117	4,58081E-01	5343	4,14927E-01	15125	8,52941E-01	15835	0	11208,5
Responder	Macro_ISG15	Macro_FOLR2+APOE+	TNFSF13B	HLA-DPB1	4,82883E-03	9233,3	7,37522E-03	14239	4,81373E-01	4790	3,88363E-01	15837	9,47810E-01	92	0	11208,5
Responder	cDC(CD1C)	cDC_CLEC9A	HLA-DRB5	LAG3	4,83001E-03	8858,5	1,20559E-02	6194	3,78618E-01	7678	1,25654E+00	3110	8,51653E-01	16102	0	11208,5
Responder	Macro_OLFML3	Macro_NLRP3	MDK	LRP1	4,83246E-03	8511,3	2,40130E-02	1876	4,94259E-01	4518	7,89630E-01	7679	8,45890E-01	17275	0	11208,5
Responder	Macro_IFI27	cDC(CD1C)	C3	IFITM1	4,83493E-03	12904,9	9,64214E-03	9055	2,15181E-01	15788	5,12651E-01	12632	8,52909E-01	15841	0	11208,5
Responder	Macro_LYVE1	pDC_LILRA4	HSP90B1	TLR7	4,83646E-03	7360,7	1,78236E-02	3097	5,24790E-01	3950	1,22099E+00	3313	8,55605E-01	15235	0	11208,5
Responder	Mono_CD14	pDC_LILRA4	CD55	ADGRE5	4,83736E-03	8270,7	1,06034E-02	7681	5,62824E-01	3371	2,34433E+00	339	8,38863E-01	18754	0	11208,5
Responder	Macro_IFI27	Macro_OLFML3	ANXA2	TLR2	4,83799E-03	13359,7	7,30182E-03	14463	2,30891E-01	14783	3,88204E-01	15843	8,79788E-01	10501	0	11208,5
Responder	Macro_IFI27	pDC_LILRA4	HLA-DRA	CD4	4,83951E-03	7309,5	6,89249E-03	15844	3,89174E-01	7320	1,50792E+00	1891	9,42051E-01	284	0	11208,5
Responder	Macro_LYVE1	Mono_CD14	FABP5	RXRA	4,84162E-03	7470,7	1,81787E-02	2986	6,95130E-01	1899	1,39727E+00	2363	8,38294E-01	18897	0	11208,5
Responder	Macro_NLRP3	cDC(CD1C)	VEGFA	CD44	4,84410E-03	12561,1	7,77727E-03	13062	2,86308E-01	11589	5,81285E-01	11099	8,52886E-01	15847	0	11208,5
Responder	Mono_INHBA	Macro_IER3	VEGFA	SIRPA	4,84717E-03	10639,7	1,50722E-02	4214	5,96160E-01	2885	7,89024E-01	7685	7,97920E-01	27206	0	11208,5
Responder	Mono_CD14	Mono_CD14	RPS19	C5AR1	4,84717E-03	9223,1	6,56090E-03	17094	3,78356E-01	7685	9,82206E-01	5274	9,10188E-01	4854	0	11208,5
Responder	Macro_FOLR2-APOE+	cDC_CLEC9A	TGFB1	LPP	4,85700E-03	10515,1	1,14562E-02	6756	5,13945E-01	4142	8,46875E-01	6890	8,15967E-01	23579	0	11208,5
Responder	Macro_LYVE1	Macro_NLRP3	F13A1	ITGB1	4,85940E-03	8001,1	1,37884E-02	4963	5,94848E-01	2899	1,00103E+00	5078	8,52817E-01	15857	0	11208,5
Responder	Mast	pDC_LILRA4	HDC	HRH2	4,86539E-03	13000,3	5,78200E-02	331	5,88109E-01	2991	2,03954E+00	656	6,71752E-01	49815	0	11208,5
Responder	Macro_FOLR2-APOE+	pDC_LILRA4	TNF	TNFRSF21	4,86931E-03	11837,5	1,41019E-02	4772	9,55410E-01	682	1,32180E+00	2731	7,32740E-01	39794	0	11208,5
Responder	Macro_LYVE1	pDC_LILRA4	HGF	NRP1	4,87015E-03	11729,9	3,16349E-02	1111	6,49002E-01	2315	1,27570E+00	2992	7,25307E-01	41023	0	11208,5
Responder	Macro_FOLR2-APOE+	cDC_LAMP3	SPP1	ITGA4_ITGB1	4,87628E-03	9063,3	1,30886E-02	5382	4,67809E-01	5097	7,84087E-01	7761	8,52776E-01	15868	0	11208,5
Responder	pDC_LILRA4	Macro_OLFML3	HMGB1	TLR2	4,88089E-03	10638,9	7,57207E-03	13622	2,13960E-01	15871	1,27217E+00	3011	8,85347E-01	9482	0	11208,5
Responder	Macro_FOLR2+APOE+	pDC_LILRA4	MDK	NCL	4,88923E-03	5477,5	2,42983E-02	1830	5,87542E-01	2996	1,84526E+00	976	8,80370E-01	10377	0	11208,5
Responder	Mono_INHBA	Macro_NLRP3	CCL4	CCR1	4,89151E-03	8979,5	1,05931E-02	7703	5,30372E-01	3850	1,17890E+00	3561	8,39862E-01	18575	0	11208,5
Responder	Macro_NLRP3	Mono_CD16	THBS1	CD47	4,89166E-03	9521,1	1,24993E-02	5813	5,40835E-01	3684	5,85920E-01	11022	8,52746E-01	15878	0	11208,5
Responder	Macro_FOLR2-APOE+	cDC_CLEC9A	CALM3	MYLK	4,89646E-03	13425,7	3,95957E-02	749	7,89740E-01	1306	7,87935E-01	7705	6,95986E-01	46160	0	11208,5
Responder	Macro_FOLR2-APOE+	pDC_LILRA4	APP	NCSTN	4,89893E-03	11388,7	1,44384E-02	4560	4,53607E-01	5460	1,06259E+00	4487	7,77732E-01	31228	0	11208,5
Responder	Macro_FOLR2-APOE+	pDC_LILRA4	ICAM1	SPN	4,90141E-03	8903,9	1,45619E-02	4503	5,87190E-01	3003	1,31207E+00	2784	8,18589E-01	23021	0	11208,5
Responder	Macro_FOLR2-APOE+	pDC_LILRA4	THBS1	CD47	4,90884E-03	8872,1	1,08830E-02	7358	4,09720E-01	6670	1,65724E+00	1399	8,43838E-01	17725	0	11208,5
Responder	Macro_ISG15	Mono_CD14	GRN	TNFRSF1B	4,91018E-03	8875,5	6,88153E-03	15890	3,58527E-01	8423	9,99162E-01	5106	9,16207E-01	3750	0	11208,5
Responder	Mono_CD16	pDC_LILRA4	HGF	NRP1	4,91315E-03	12359,9	2,78644E-02	1422	6,04335E-01	2789	1,27364E+00	3001	7,12485E-01	43379	0	11208,5
Responder	cDC(CD1C)	Macro_LYVE1	HSPA1A	TLR4	4,91327E-03	12489,7	6,88145E-03	15892	2,32020E-01	14713	7,60583E-01	8105	8,68891E-01	12530	0	11208,5
Responder	Macro_OLFML3	cDC(CD1C)	HLA-DRA	CD4	4,91380E-03	9434,1	5,60135E-03	21872	3,77664E-01	7712	9,44175E-01	5693	9,36122E-01	685	0	11208,5
Responder	Macro_FOLR2+APOE+	Mono_CD14	C1QA	CR1	4,91795E-03	4939,7	2,74725E-02	1452	8,81864E-01	895	1,27362E+00	3002	8,92763E-01	8141	0	11208,5
Responder	pDC_LILRA4	Mono_INHBA	HLA-F	LILRB2	4,92100E-03	10836,9	6,87807E-03	15897	2,74165E-01	12271	1,41929E+00	2262	8,68827E-01	12546	0	11208,5
Responder	Macro_FOLR2-APOE+	Macro_IER3	PLTP	ABCA1	4,92255E-03	10329,9	1,28571E-02	5553	5,09228E-01	4227	4,28903E-01	14763	8,52633E-01	15898	0	11208,5
Responder	Mono_CD14	cDC_CLEC9A	S100A8	CD69	4,92410E-03	8565,7	7,45503E-03	13985	1,03212E+00	507	1,72870E+00	1229	8,52632E-01	15899	0	11208,5
Responder	Macro_OLFML3	cDC_LAMP3	PDGFB	S1PR1	4,92754E-03	19449,1	5,92003E-02	317	5,87135E-01	3004	2,57611E-01	19980	4,90704E-01	62736	0	11208,5
Responder	cDC_CLEC9A	Macro_OLFML3	IL16	CD4	4,93650E-03	8414,1	1,50263E-02	4246	5,39528E-01	3702	8,39568E-01	7007	8,52610E-01	15907	0	11208,5
Responder	pDC_LILRA4	Macro_IFI27	CD99	PILRA	4,93650E-03	10275,3	6,87594E-03	15907	3,20590E-01	9954	1,40559E+00	2316	8,72169E-01	11991	0	11208,5
Responder	Macro_FOLR2+APOE+	pDC_LILRA4	SIRPA	CD47	4,93864E-03	8359,9	1,33526E-02	5208,5	5,35472E-01	3766,5	1,54604E+00	1769,5	8,33875E-01	19846,5	0	11208,5
Responder	Mono_INHBA	Macro_ISG15	NAMPT	ITGA5_ITGB1	4,94271E-03	11882,3	6,87544E-03	15911	4,40910E-01	5790	5,54091E-01	11652	8,57539E-01	14850	0	11208,5
Responder	Macro_IFI27	Macro_ISG15	MMP12	PLAUR	4,94426E-03	7091,3	1,93254E-02	2671	5,36897E-01	3745	1,50108E+00	1920	8,52554E-01	15912	0	11208,5
Responder	Macro_FOLR2+APOE+	Macro_ISG15	APOE	ABCA1	4,94612E-03	6643,3	1,09569E-02	7271	6,91901E-01	1922	7,86659E-01	7725	9,08927E-01	5090	0	11208,5
Responder	cDC(CD1C)	Mono_INHBA	ADAM15	ITGA5	4,94861E-03	16080,5	1,46853E-02	4415	4,02694E-01	6888	7,86605E-01	7726	6,69379E-01	50165	0	11208,5
Responder	Mono_CD14	Mast	ADAM10	CD44	4,94892E-03	11904,5	6,87408E-03	15915	4,07453E-01	6742	6,29001E-01	10266	8,54920E-01	15391	0	11208,5
Responder	Macro_OLFML3	Macro_IER3	HLA-DQA2	CD4	4,95048E-03	11827,3	7,43275E-03	14057	2,99781E-01	10915	3,86342E-01	15916	8,98556E-01	7040	0	11208,5
Responder	Macro_FOLR2-APOE+	pDC_LILRA4	SELPLG	SELL	4,95110E-03	8573,7	1,17539E-02	6476	6,32465E-01	2472	1,74925E+00	1177	8,25581E-01	21535	0	11208,5
Responder	Macro_FOLR2-APOE+	pDC_LILRA4	SEMA4A	NRP1_PLXNA4	4,95609E-03	14475,7	1,47341E-02	4387	6,53410E-01	2281	8,86121E-01	6404	6,83621E-01	48098	0	11208,5
Responder	cDC(CD1C)	cDC(CD1C)	LGALS1	PTPRC	4,95640E-03	10966,5	5,04623E-03	25550	2,65530E-01	12720	1,27244E+00	3010	9,23904E-01	2344	0	11208,5
Responder	Macro_FOLR2-APOE+	cDC(CD1C)	TNFSF13B	HLA-DPB1	4,96109E-03	8727,9	5,75000E-03	21025	3,77075E-01	7731	1,21214E+00	3363	9,41299E-01	312	0	11208,5
Responder	Macro_FOLR2-APOE+	pDC_LILRA4	FN1	CD79A	4,96358E-03	12103,3	6,25689E-02	281	5,32823E-01	3824	1,08410E+00	4300	7,26103E-01	40903	0	11208,5
Responder	pDC_LILRA4	Mast	TXLNA	STX3	4,96604E-03	12257,5	3,35771E-02	1006	5,86742E-01	3012	1,39305E+00	2385	7,10834E-01	43676	0	11208,5

Responder	Macro_OLFML3	Macro_ISG15	APOE	LRP1	4,96605E-03	11988,7	6,87190E-03	15926	3,10049E-01	10419	4,26466E-01	14820	8,95842E-01	7570	0	11208,5
Responder	Mono_CD14	cDC(CD1C)	S100A9	CD36	4,96761E-03	8694,5	7,16502E-03	14921	9,38676E-01	739	2,02377E+00	677	8,52463E-01	15927	0	11208,5
Responder	cDC_CLEC9A	Macro_FOLR2+APOE+	ENTPD1	TMIGD3	4,97087E-03	9578,7	4,34161E-02	625	7,41597E-01	1569	1,27197E+00	3013	7,76438E-01	31478	0	11208,5
Responder	Macro_FOLR2-APOE+	pDC_LILRA4	CXCL10	CXCR3	4,97359E-03	13813,5	1,58106E-02	3860	9,66059E-01	657	9,62083E-01	5506	6,85239E-01	47836	0	11208,5
Responder	Macro_NLRP3	cDC_LAMP3	VCAN	ITGB1	4,97854E-03	10204,9	1,04173E-02	7928	5,18830E-01	4059	5,43028E-01	11895	8,52441E-01	15934	0	11208,5
Responder	Macro_OLFML3	Mono_CD16	APOE	LRP1	4,98322E-03	9967,1	9,03334E-03	10130	3,90327E-01	7281	3,85666E-01	15937	9,07928E-01	5279	0	11208,5
Responder	Macro_NLRP3	Macro_OLFML3	VCAN	TLR1	4,98479E-03	7243,9	2,93855E-02	1286	7,68818E-01	1408	8,87840E-01	6379	8,52390E-01	15938	0	11208,5
Responder	Macro_NLRP3	Macro_ISG15	GNAI2	FPR1	4,98635E-03	11996,3	7,70461E-03	13272	3,21670E-01	9918	3,85614E-01	15939	8,84446E-01	9644	0	11208,5
Responder	Macro_FOLR2-APOE+	Macro_NLRP3	SPP1	ITGA4_ITGB1	4,98862E-03	9504,1	1,22385E-02	6038	4,40779E-01	5796	7,85435E-01	7742	8,48510E-01	16736	0	11208,5
Responder	Macro_FOLR2-APOE+	pDC_LILRA4	SPP1	PTGER4	4,98862E-03	9709,7	1,20984E-02	6151	4,41077E-01	5785	1,98243E+00	743	8,10767E-01	24661	0	11208,5
Responder	Macro_FOLR2-APOE+	pDC_LILRA4	HGF	NRP1	5,00117E-03	16056,5	1,41408E-02	4748	4,41764E-01	5764	1,04964E+00	4638	6,38380E-01	53924	0	11208,5
Responder	Macro_FOLR2-APOE+	pDC_LILRA4	VEGFA	NRP1	5,00368E-03	12144,7	1,44156E-02	4579	5,25324E-01	3939	1,02421E+00	4860	7,52179E-01	36137	0	11208,5
Responder	Macro_FOLR2-APOE+	pDC_LILRA4	ADM	CALCRL	5,00871E-03	14151,5	1,44376E-02	4561	5,49529E-01	3562	1,83187E+00	998	6,67516E-01	50428	0	11208,5
Responder	pDC_LILRA4	Macro_LYVE1	RARRES2	CCRL2	5,00959E-03	13078,9	2,61380E-02	1611	5,86029E-01	3021	1,87988E+00	902	6,79816E-01	48652	0	11208,5
Responder	cDC_CLEC9A	Mono_CD14	GNAI2	FPR1	5,01143E-03	11053,1	6,86451E-03	15955	2,41294E-01	14104	1,23394E+00	3240	8,78414E-01	10758	0	11208,5
Responder	Macro_FOLR2-APOE+	Macro_LYVE1	HMGB1	CD163	5,01457E-03	8095,7	9,77040E-03	8828	6,43191E-01	2373	3,84879E-01	15957	9,25190E-01	2112	0	11208,5
Responder	Macro_OLFML3	Macro_FOLR2+APOE-	A2M	LRP1	5,01771E-03	11389,1	1,06951E-02	7575	3,43810E-01	8963	4,87722E-01	13240	8,52278E-01	15959	0	11208,5
Responder	Macro_FOLR2-APOE+	pDC_LILRA4	VEGFB	NRP1	5,01878E-03	15696,9	1,16291E-02	6589	4,61883E-01	5245	1,02883E+00	4814	6,66235E-01	50628	0	11208,5
Responder	cDC(CD1C)	Mono_INHBA	LGALS9	LRP1	5,02086E-03	12635,1	6,86282E-03	15961	2,32729E-01	14673	9,27157E-01	5890	8,54695E-01	15443	0	11208,5
Responder	Macro_FOLR2-APOE+	pDC_LILRA4	COPA	P2RY6	5,02129E-03	9245,7	1,72015E-02	3296	5,99571E-01	2839	1,76050E+00	1151	7,95382E-01	27734	0	11208,5
Responder	Macro_LYVE1	Macro_FOLR2-APOE+	LGALS1	ITGB1	5,02715E-03	10547,7	6,86142E-03	15965	4,82485E-01	4764	4,07888E-01	15322	9,06898E-01	5479	0	11208,5
Responder	Macro_FOLR2-APOE+	cDC(CD1C)	NRG1	HLA-DPB1	5,02886E-03	9329,3	1,05414E-02	7758	5,02262E-01	4359	1,34439E+00	2610	8,29753E-01	20711	0	11208,5
Responder	Mono_INHBA	Mono_CD16	GNAI2	C5AR1	5,03030E-03	10345,7	8,63863E-03	10943	4,49767E-01	5556	3,84419E-01	15967	8,93248E-01	8054	0	11208,5
Responder	Macro_FOLR2+APOE+	cDC_LAMP3	APOE	SORL1	5,03138E-03	6827,9	1,05406E-02	7759	5,87729E-01	2995	8,71522E-01	6572	9,06104E-01	5605	0	11208,5
Responder	Mono_CD14	Macro_FOLR2+APOE-	THBS1	PTPRJ	5,03391E-03	13221,3	1,19493E-02	6301	3,76159E-01	7760	8,09200E-01	7414	7,66512E-01	33423	0	11208,5
Responder	Macro_FOLR2-APOE+	pDC_LILRA4	FAM3C	ADGRG5	5,03643E-03	17374,5	4,27179E-02	642	5,05115E-01	4300	8,07015E-01	7452	4,43349E-01	63270	0	11208,5
Responder	cDC(CD1C)	cDC_CLEC9A	ENTPD1	ADORA2B	5,03643E-03	16407,9	2,41367E-02	1858	3,76152E-01	7761	1,00917E+00	5007	6,16979E-01	56205	0	11208,5
Responder	Macro_NLRP3	Macro_LYVE1	TIMP1	CD63	5,03976E-03	8100,1	8,72147E-03	10748	6,32111E-01	2476	3,84289E-01	15973	9,47692E-01	95	0	11208,5
Responder	Macro_FOLR2-APOE+	pDC_LILRA4	CXCL2	DPP4	5,04148E-03	10630,1	4,62450E-02	557	8,44602E-01	1038	1,01217E+00	4975	7,56414E-01	35372	0	11208,5
Responder	pDC_LILRA4	Mono_CD16	TNFSF9	HLA-DPA1	5,05160E-03	12221,9	1,78963E-02	3079	5,48603E-01	3571	8,76101E-01	6526	8,94808E-01	7767	0,799	40166,5
Responder	Mono_INHBA	Mono_CD16	SPP1	CD44	5,05240E-03	11673,7	8,75887E-03	10670	3,06041E-01	10601	3,84171E-01	15981	8,83052E-01	9908	0	11208,5
Responder	Mono_CD14	cDC(CD1C)	IL1B	SIGIRR	5,06173E-03	8607,1	1,05361E-02	7771	4,90566E-01	4596	1,57480E+00	1662	8,43513E-01	17798	0	11208,5
Responder	Macro_FOLR2-APOE+	Macro_LYVE1	C3	NRP1	5,06505E-03	8870,9	2,27381E-02	2068	5,43815E-01	3639	5,63743E-01	11450	8,52101E-01	15989	0	11208,5
Responder	Macro_FOLR2-APOE+	pDC_LILRA4	PLAU	ST14	5,06934E-03	11960,1	1,57556E-02	3887	4,76798E-01	4892	1,65660E+00	1402	7,40151E-01	38411	0	11208,5
Responder	Mast	Mono_INHBA	ADAM12	SDC4	5,07198E-03	12354,9	1,20850E-01	53	9,70040E-01	646	1,07146E+00	4411	7,00360E-01	45456	0	11208,5
Responder	Mono_INHBA	pDC_LILRA4	ALCAM	NRP1	5,07442E-03	14933,1	1,05333E-02	7776	4,13380E-01	6577	1,17505E+00	3582	6,99917E-01	45522	0	11208,5
Responder	Macro_FOLR2-APOE+	pDC_LILRA4	C3	NRP1	5,07696E-03	7760,1	2,08361E-02	2379	4,92872E-01	4541	1,18542E+00	3522	8,46512E-01	17150	0	11208,5
Responder	pDC_LILRA4	Macro_FOLR2+APOE+	SPON2	ITGB1	5,07782E-03	10947,1	2,45587E-02	1795	7,79902E-01	1357	1,26874E+00	3035	7,45956E-01	37340	0	11208,5
Responder	cDC_CLEC9A	cDC(CD1C)	MIF	CD74_CXCR4	5,08272E-03	6523,3	6,48862E-03	17364	5,84683E-01	3036	1,88170E+00	899	9,47160E-01	109	0	11208,5
Responder	Macro_OLFML3	Macro_LYVE1	CXCL10	TLR4	5,08713E-03	10273,7	2,01082E-02	2512	4,00499E-01	6962	7,82802E-01	7781	8,19141E-01	22905	0	11208,5
Responder	Macro_FOLR2-APOE+	pDC_LILRA4	MMP2	PECAM1	5,08967E-03	15728,7	2,53632E-02	1690	4,03729E-01	6860	1,57498E+00	1661	6,05321E-01	57224	0	11208,5
Responder	cDC_CLEC9A	cDC_CLEC9A	HLA-DQB1	LAG3	5,09522E-03	7733,3	1,39984E-02	4835	5,32522E-01	3827	1,31150E+00	2788	8,52026E-01	16008	0	11208,5
Responder	cDC(CD1C)	Mono_INHBA	HLA-DPB1	CD4	5,10725E-03	11400,3	4,60270E-03	29000	2,90571E-01	11369	1,26738E+00	3041	9,23666E-01	2383	0	11208,5
Responder	Macro_FOLR2-APOE+	pDC_LILRA4	HMGB1	CXCR4	5,11006E-03	8084,1	6,38700E-03	17822	3,78485E-01	7683	1,76230E+00	1143	9,22681E-01	2564	0	11208,5
Responder	Macro_FOLR2-APOE+	pDC_LILRA4	FN1	DPP4	5,11261E-03	11103,5	6,91155E-02	222	7,13468E-01	1747	1,02620E+00	4840	7,44976E-01	37500	0	11208,5
Responder	cDC(CD1C)	Macro_ISG15	CD1D	LILRB2	5,11261E-03	10221,7	1,39656E-02	4858	3,75424E-01	7791	1,24358E+00	3176	8,13580E-01	24075	0	11208,5
Responder	Mono_INHBA	Macro_LYVE1	LGALS1	ITGB1	5,11275E-03	10619,9	6,88617E-03	15867	4,92134E-01	4558	3,83113E-01	16019	9,07050E-01	5447	0	11208,5
Responder	Mono_INHBA	Mono_CD14	GNAI2	FPR1	5,11275E-03	12038,7	6,84367E-03	16019	2,39317E-01	14251	7,72920E-01	7921	8,78252E-01	10794	0	11208,5
Responder	Mono_INHBA	Macro_IER3	TIMP1	CD63	5,11435E-03	9053,7	6,84277E-03	16020	4,29204E-01	6106	5,54934E-01	11626	9,41342E-01	308	0	11208,5
Responder	Macro_OLFML3	Macro_OLFML3	HLA-B	LILRB1	5,11690E-03	14272,7	6,25015E-03	18427	3,91334E-01	7251	-3,40116E-02	31608	9,21065E-01	2869	0	11208,5
Responder	cDC_LAMP3	pDC_LILRA4	FARP2	PLXNA4	5,11871E-03	15334,5	9,39140E-02	109	9,68804E-01	649	1,02199E+00	4881	5,70535E-01	59825	0	11208,5
Responder	Macro_FOLR2+APOE-	Mono_INHBA	CCL13	CCR1	5,11871E-03	12131,5	4,25644E-02	649	1,16169E+00	314	1,11586E+00	4054	7,06392E-01	44432	0	11208,5
Responder	Macro_ISG15	Mono_CD16	PTPN6	CLEC12A	5,12073E-03	10763,5	1,16034E-02	6618	4,17674E-01	6430	4,75343E-01	13537	8,51960E-01	16024	0	11208,5
Responder	cDC_CLEC9A	Mast	VEGFB	ADRB2	5,12200E-03	12912,3	2,35350E-02	1945	6,91005E-01	1929	1,26717E+00	3044	6,94121E-01	46435	0	11208,5
Responder	Mono_CD14	Mono_INHBA	ADM	GPR84	5,12795E-03	14715,7	1,92048E-02	2702	3,75210E-01	7797	1,12995E+00	3928	6,84558E-01	47943	0	11208,5
Responder	Macro_ISG15	Mono_INHBA	CXCL9	FCGR2A	5,12873E-03	13114,1	8,69764E-03	10805	2,34588E-01	14546	3,82690E-01	16029	8,66754E-01	12982	0	11208,5
Responder	Macro_FOLR2-APOE+	pDC_LILRA4	ADA	DPP4	5,13051E-03	16113,7	3,97496E-02	741	7,14352E-01	1742	9,17394E-01	5998	5,51474E-01	60879	0	11208,5
Responder	Macro_LYVE1	Macro_LYVE1	F13A1	ITGB1	5,13353E-03	8045,7	1,47404E-02	4383	6,28303E-01	2516	8,28092E-01	7148	8,56958E-01	14973	0	11208,5
Responder	cDC_CLEC9A	Mono_CD14	LGALS9	CD44	5,13353E-03	9028,5	6,83912E-03	16032	3,72993E-01	7876	1,19018E+00	3491	9,01322E-01	6535	0	11208,5
Responder	Mono_INHBA	Macro_FOLR2+APOE-	HMGB1	CD163	5,13513E-03	11430,5	6,83898E-03	16033	3,28329E-01	9627	3,92942E-01	15725	9,11870E-01	4559	0	11208,5
Responder	Mono_INHBA	Macro_IER3	VEGFA	ITGAV	5,13563E-03	11412,1	1,68778E-02	3398	5,80164E-01	3095	7,81581E-01	7800	7,75957E-01	31559	0	11208,5
Responder	Mono_INHBA	Macro_LYVE1	S100A8	CD68	5,13563E-03	10301,9	1,05137E-02	7800	6,16829E-01	2645	2,66471E-02	29014	9,34584E-01	842	0	11208,5
Responder	Mono_CD14	Macro_FOLR2+APOE+	CD99	CD81	5,13673E-03	12296,9	7,07044E-03	15211	3,88940E-01	7328	3,82627E-01	16034	8,73830E-01	11703	0	11208,5
Responder	Macro_FOLR2-APOE+	pDC_LILRA4	PAM	DPP4	5,14076E-03	16390,7	5,56376E-02	360	7,63695E-01	1446	9,05383E-01	6153	4,86530E-01	62786	0	11208,5
Responder	Macro_LYVE1	Macro_LYVE1	A2M	LRP1	5,14314E-03	6400,9	2,13218E-02	2305	7,47531E-01	1534	7,40338E-01	8415	8,90665E-01	8542	0	11208,5

Post_R_Myeloid_Myeloid_CellCel

Responder	Macro_IFI27	Mono_CD16	FN1	C5AR1	5,14635E-03	8893,5	1,91862E-02	2709	4,81040E-01	4800	3,82294E-01	16040	8,84115E-01	9710	0	11208,5
Responder	Macro_FOLR2+APOE-	Mono_INHBA	CCL18	CCR1	5,14665E-03	9957,9	1,83555E-02	2934	5,83480E-01	3049	1,85576E+00	952	7,75526E-01	31646	0	11208,5
Responder	Macro_LYVE1	Macro_LYVE1	C1QB	LRP1	5,14956E-03	6513,9	1,65054E-02	3554	7,63267E-01	1449	3,92479E-01	15738	9,36690E-01	620	0	11208,5
Responder	Macro_LYVE1	Macro_LYVE1	PSAP	LRP1	5,15117E-03	6353,7	1,15395E-02	6678	7,44499E-01	1550	5,51074E-01	11724	9,36859E-01	608	0	11208,5
Responder	cDC_CLEC9A	Macro_OLFML3	HLA-DQA1	CD4	5,15759E-03	9386,1	6,83465E-03	16047	3,49506E-01	8750	7,05612E-01	8948	9,26024E-01	1977	0	11208,5
Responder	Macro_LYVE1	Macro_LYVE1	PLTP	ABCA1	5,16081E-03	5829,3	2,31739E-02	2005	9,62480E-01	665	9,27173E-01	5889	8,85944E-01	9379	0	11208,5
Responder	cDC_CLEC9A	Macro_ISG15	GNAI2	FPR1	5,16081E-03	11879,5	6,83392E-03	16049	2,38696E-01	14284	8,36309E-01	7052	8,78175E-01	10804	0	11208,5
Responder	Mono_INHBA	pDC_LILRA4	HSP90B1	TLR7	5,16387E-03	8804,3	1,46065E-02	4474	3,74826E-01	7811	1,34678E+00	2603	8,42868E-01	17925	0	11208,5
Responder	cDC_CLEC9A	Mono_CD16	HLA-A	LILRA1	5,16902E-03	9685,3	1,68548E-02	3409	3,74740E-01	7813	8,31137E-01	7115	8,97923E-01	7163	0,001	22926,5
Responder	Macro_NLRP3	Macro_IER3	CD55	ADGRE5	5,17852E-03	9509,9	1,29300E-02	5508	5,93260E-01	2924	5,44832E-01	11849	8,51824E-01	16060	0	11208,5
Responder	cDC_CLEC9A	Mono_CD16	RPS19	C5AR1	5,18335E-03	8769,5	6,82936E-03	16063	4,03493E-01	6868	9,96379E-01	5143	9,11814E-01	4565	0	11208,5
Responder	Macro_LYVE1	Macro_LYVE1	ADAM10	GPNMB	5,18497E-03	8448,3	1,49634E-02	4275	7,12059E-01	1760	6,79192E-01	9385	8,53871E-01	15613	0	11208,5
Responder	Macro_IFI27	pDC_LILRA4	C3	NRP1	5,18820E-03	7240,1	2,26314E-02	2081	5,33086E-01	3819	1,27043E+00	3026	8,51805E-01	16066	0	11208,5
Responder	Macro_OLFML3	Mono_INHBA	LILRB4	LAIR1	5,19466E-03	11372,9	8,98621E-03	10236	3,30241E-01	9530	3,81283E-01	16070	8,83496E-01	9820	0	11208,5
Responder	pDC_LILRA4	Macro_NLRP3	TNFSF9	HLA-DPA1	5,19480E-03	12236	1,77985E-02	3107	5,43729E-01	3640	1,22104E+00	3312	8,94550E-01	7823	0,938	43298
Responder	Mono_CD16	pDC_LILRA4	HSP90B1	TLR9	5,19706E-03	12159,6	4,25298E-02	654	1,08708E+00	411	7,49415E-01	8264	8,22537E-01	22174	0,0659999999999999	29295
Responder	Macro_FOLR2-APOE+	cDC_CLEC9A	QDPR	DYSF	5,19997E-03	16227,1	1,85241E-02	2883	4,25535E-01	6196	7,79931E-01	7825	6,46361E-01	53023	0	11208,5
Responder	Macro_FOLR2+APOE+	Macro_NLRP3	MMP12	PLAUR	5,20116E-03	5742,5	2,72739E-02	1482	8,29588E-01	1103	1,26495E+00	3060	8,72920E-01	11859	0	11208,5
Responder	Macro_LYVE1	Macro_LYVE1	HMGB1	CD163	5,20274E-03	6010,7	1,24606E-02	5851	8,61665E-01	978	5,85987E-01	11021	9,33184E-01	995	0	11208,5
Responder	Macro_NLRP3	Macro_FOLR2-APOE+	THBS1	CD47	5,20274E-03	9692,7	1,23053E-02	5987	5,35834E-01	3759	5,64273E-01	11434	8,51761E-01	16075	0	11208,5
Responder	Mono_CD14	Mono_CD14	LYZ	ITGAL	5,20274E-03	9892,9	6,82479E-03	16075	4,10204E-01	6660	1,29302E+00	2895	8,68442E-01	12626	0	11208,5
Responder	Macro_LYVE1	Macro_LYVE1	HSP90B1	LRP1	5,21084E-03	8048,1	1,20954E-02	6157	6,57649E-01	2232	3,90761E-01	15782	9,10142E-01	4861	0	11208,5
Responder	Mono_INHBA	Macro_IER3	ADM	GPR84	5,21550E-03	14815,7	2,04317E-02	2443	4,44938E-01	5678	7,79677E-01	7831	6,91206E-01	46918	0	11208,5
Responder	cDC(CD1C)	cDC(CD1C)	CD58	CD2	5,21809E-03	15595,3	2,17228E-02	2234	3,74191E-01	7832	1,08447E+00	4297	6,51595E-01	52405	0	11208,5
Responder	Macro_OLFML3	Macro_LYVE1	HSP90B1	LRP1	5,21809E-03	11505,7	1,04913E-02	7832	5,47463E-01	3585	2,90114E-02	28923	9,04152E-01	5980	0	11208,5
Responder	Macro_FOLR2-APOE+	Macro_NLRP3	C3	ITGAX	5,21895E-03	11025,1	1,17513E-02	6481	3,01627E-01	10830	3,80912E-01	16085	8,79691E-01	10521	0	11208,5
Responder	Mono_CD14	Macro_OLFML3	ICAM1	ITGAL_ITGB2	5,22057E-03	13046,3	6,82283E-03	16086	2,73663E-01	12298	4,94970E-01	13062	8,68654E-01	12577	0	11208,5
Responder	Macro_FOLR2-APOE+	Macro_IFI27	CXCL8	SDC3	5,22069E-03	9523,1	1,49159E-02	4297	5,19009E-01	4057	7,94605E-01	7616	8,31054E-01	20437	0	11208,5
Responder	pDC_LILRA4	Macro_FOLR2+APOE-	CD24	SIGLEC10	5,22606E-03	21370,4	2,11424E-02	2326	5,82667E-01	3065	1,54319E+00	1782	6,02538E-01	57489	0,899	42190
Responder	cDC(CD1C)	Mast	TXLNA	STX3	5,22847E-03	15867,1	1,99567E-02	2543	4,43657E-01	5711	7,78873E-01	7836	6,54595E-01	52037	0	11208,5
Responder	Macro_LYVE1	Macro_LYVE1	CD59	STAB1	5,22869E-03	5723,9	2,40519E-02	1871	1,01492E+00	545	1,05328E+00	4602	8,80314E-01	10393	0	11208,5
Responder	Macro_OLFML3	Mono_INHBA	HLA-C	LILRB2	5,22869E-03	10000,1	6,82190E-03	16091	4,50913E-01	5527	3,81139E-01	16076	9,32163E-01	1098	0	11208,5
Responder	pDC_LILRA4	Macro_OLFML3	SEMA7A	PLXNC1	5,24104E-03	12633,9	2,24471E-02	2104	6,64054E-01	2160	1,26372E+00	3068	7,05275E-01	44629	0	11208,5
Responder	cDC(CD1C)	Macro_LYVE1	LGALS9	COLEC12	5,24406E-03	13667,3	2,69074E-02	1520	4,82653E-01	4759	7,78530E-01	7842	7,14591E-01	43007	0	11208,5
Responder	cDC_CLEC9A	cDC(CD1C)	CD52	SIGLEC10	5,25637E-03	12026,9	6,81709E-03	16108	2,59658E-01	13061	9,57333E-01	5552	8,60616E-01	14205	0	11208,5
Responder	Macro_FOLR2-APOE+	Macro_IFI27	MMP9	LRP1	5,25708E-03	9960,9	1,44186E-02	4577	5,42521E-01	3656	8,94904E-01	6300	8,13630E-01	24063	0	11208,5
Responder	Macro_LYVE1	cDC(CD1C)	APP	RPSA	5,25800E-03	10820,1	6,81680E-03	16109	4,05929E-01	6799	1,12914E+00	3940	8,51889E-01	16044	0	11208,5
Responder	Macro_FOLR2+APOE+	Mono_CD16	SAA1	FPR2	5,26017E-03	10274,3	6,33398E-02	270	9,65477E-01	658	6,11771E-01	10577	7,90735E-01	28658	0	11208,5
Responder	Macro_IFI27	cDC_CLEC9A	CALM3	MYLK	5,26105E-03	12865,1	3,61064E-02	879	7,51696E-01	1512	1,26285E+00	3072	6,86300E-01	47654	0	11208,5
Responder	Macro_ISG15	Macro_NLRP3	ANXA2	TLR2	5,26126E-03	13960,5	6,81632E-03	16111	2,21283E-01	15420	3,88322E-01	15838	8,76101E-01	11225	0	11208,5
Responder	Mono_INHBA	Macro_NLRP3	CD99	PILRA	5,27270E-03	12363,3	7,44493E-03	14021	3,35149E-01	9331	3,80095E-01	16118	8,76536E-01	11138	0	11208,5
Responder	Macro_FOLR2+APOE+	Macro_NLRP3	C1QB	LRP1	5,27535E-03	5626,1	1,28924E-02	5527	6,79196E-01	2031	7,77895E-01	7854	9,28957E-01	1510	0	11208,5
Responder	Macro_LYVE1	cDC_LAMP3	F13A1	ITGA4	5,27598E-03	7316,9	1,80836E-02	3018	6,78413E-01	2040	1,07342E+00	4398	8,52498E-01	15920	0	11208,5
Responder	Macro_FOLR2-APOE+	Macro_IFI27	MMP9	ITGAM	5,27796E-03	12560,3	1,51713E-02	4172	5,74391E-01	3201	1,00874E+00	5015	7,35984E-01	39205	0	11208,5
Responder	Mono_INHBA	cDC_LAMP3	TNF	TRAF2	5,28058E-03	13645,5	2,77578E-02	1428	5,87367E-01	3000	7,77853E-01	7856	7,04698E-01	44735	0	11208,5
Responder	cDC(CD1C)	Macro_IER3	HLA-DQA1	CD4	5,28058E-03	8948,9	6,23468E-03	18498	3,73515E-01	7856	1,04920E+00	4641	9,22814E-01	2541	0	11208,5
Responder	Macro_FOLR2-APOE+	Macro_IFI27	MMP9	IFNAR1	5,28319E-03	12227,9	1,19043E-02	6343	5,02858E-01	4345	9,91552E-01	5193	7,63448E-01	34050	0	11208,5
Responder	Mast	Mono_CD14	CSF1	CSF2RA	5,28613E-03	9666,1	1,79042E-02	3077	7,59208E-01	1482	1,56885E+00	1678	7,79492E-01	30885	0	11208,5
Responder	Macro_NLRP3	Macro_ISG15	AGTRAP	RACK1	5,28744E-03	10376,9	9,37740E-03	9507	4,30193E-01	6079	3,79561E-01	16127	8,88403E-01	8963	0	11208,5
Responder	Mono_CD14	pDC_LILRA4	ICAM1	IL2RG	5,29072E-03	8708,5	8,88879E-03	10411	4,62629E-01	5230	2,11804E+00	564	8,51558E-01	16129	0	11208,5
Responder	Macro_OLFML3	Macro_NLRP3	HLA-A	LILRB2	5,29564E-03	9086,3	6,81068E-03	16132	5,10186E-01	4213	5,05146E-01	12809	9,32444E-01	1069	0	11208,5
Responder	Macro_ISG15	Macro_NLRP3	S100A9	TLR4	5,29728E-03	12728,7	8,57969E-03	11105	3,17007E-01	10110	4,16550E-01	15087	8,51552E-01	16133	0	11208,5
Responder	cDC(CD1C)	pDC_LILRA4	TNFSF13B	TFRC	5,30057E-03	11378,7	7,06226E-03	15234	2,60778E-01	12994	1,68406E+00	1322	8,51543E-01	16135	0	11208,5
Responder	Macro_ISG15	Macro_NLRP3	GNAI2	FPR1	5,30057E-03	13606,3	6,81004E-03	16135	2,35805E-01	14476	4,05757E-01	15379	8,77988E-01	10833	0	11208,5
Responder	Macro_OLFML3	Mono_INHBA	SPP1	CD44	5,30714E-03	11535,9	7,55041E-03	13687	4,62956E-01	5223	3,79100E-01	16139	8,75165E-01	11422	0	11208,5
Responder	Macro_NLRP3	pDC_LILRA4	ICAM1	IL2RG	5,31043E-03	8860,7	8,88390E-03	10425	4,62390E-01	5234	1,69611E+00	1295	8,51523E-01	16141	0	11208,5
Responder	Mono_CD14	Mono_INHBA	VEGFA	CD44	5,31537E-03	10473,3	7,61002E-03	13513	4,90406E-01	4599	8,46071E-01	6902	8,51517E-01	16144	0	11208,5
Responder	Macro_NLRP3	Macro_LYVE1	HBEGF	CD9	5,32031E-03	8967,3	1,74736E-02	3207	5,70069E-01	3268	3,78932E-01	16147	8,77156E-01	11006	0	11208,5
Responder	Macro_FOLR2-APOE+	pDC_LILRA4	HLA-DMA	CD4	5,32690E-03	9194,7	6,80775E-03	16151	2,75741E-01	12177	1,33415E+00	2677	9,16174E-01	3760	0	11208,5
Responder	Macro_FOLR2+APOE+	Macro_NLRP3	APOE	ABCA1	5,33306E-03	6541,1	1,12871E-02	6908	6,99667E-01	1855	7,76014E-01	7876	9,10148E-01	4858	0	11208,5
Responder	Macro_IFI27	Mono_CD16	HLA-B	LILRB2	5,34176E-03	8109,7	9,41218E-03	9445	5,47467E-01	3584	3,78576E-01	16160	9,45325E-01	151	0	11208,5
Responder	cDC_CLEC9A	Mono_INHBA	CIRBP	TREM1	5,34837E-03	9895,3	1,05888E-02	7708	3,51850E-01	8655	9,39857E-01	5741	8,51396E-01	16164	0	11208,5
Responder	Macro_LYVE1	Mono_CD16	HLA-C	LILRA3	5,35168E-03	6902,5	3,18806E-02	1102	7,99853E-01	1249	3,84433E-01	15966	9,09454E-01	4987	0	11208,5
Responder	Macro_OLFML3	Macro_NLRP3	MDK	NCL	5,35334E-03	11557,3	1,52466E-02	4128	3,05915E-01	10607	3,78348E-01	16167	8,53574E-01	15676	0	11208,5
Responder	Macro_LYVE1	Mono_CD16	CD99	PILRA	5,35665E-03	6706,3	1,43957E-02	4589	7,98964E-01	1252	5,74470E-01	11225	9,08023E-01	5257	0	11208,5

Responder	Mast	Mono_CD14	ANXA1	DYSF	5,36185E-03	6811,3	2,11692E-02	2323	6,95632E-01	1892	1,26009E+00	3092	8,54212E-01	15541	0	11208,5
Responder	Macro_LYVE1	Mono_INHBA	TIMP2	ITGB1	5,36328E-03	10021,7	1,09008E-02	7337	5,08599E-01	4239	5,78460E-01	11151	8,51358E-01	16173	0	11208,5
Responder	cDC(CD1C)	Macro_OLFML3	HSP90B1	TLR2	5,36660E-03	12832,1	7,74148E-03	13164	2,09757E-01	16175	5,17806E-01	12502	8,76627E-01	11111	0	11208,5
Responder	cDC(CD1C)	cDC_CLEC9A	QDPR	DYSF	5,36739E-03	17019,7	1,40672E-02	4797	3,72721E-01	7889	1,03470E+00	4758	6,14312E-01	56446	0	11208,5
Responder	Macro_ISG15	cDC_CLEC9A	ADAM10	CADM1	5,37200E-03	10879,7	1,78398E-02	3094	7,48027E-01	1530	1,26409E+00	3064	7,55658E-01	35502	0	11208,5
Responder	Macro_NLRP3	Mono_CD14	LYZ	ITGAL	5,37324E-03	11122,7	6,80139E-03	16179	4,06153E-01	6789	7,16471E-01	8764	8,68246E-01	12673	0	11208,5
Responder	Macro_OLFML3	Macro_NLRP3	HLA-A	APLP2	5,37988E-03	8729,3	6,80086E-03	16183	5,54324E-01	3493	5,22144E-01	12402	9,40466E-01	360	0	11208,5
Responder	Macro_FOLR2-APOE+	Macro_FOLR2-APOE+	SPP1	PTGER4	5,38593E-03	10731,3	1,28800E-02	5536	4,58360E-01	5337	7,74786E-01	7896	8,15523E-01	23679	0	11208,5
Responder	cDC(CD1C)	Macro_FOLR2-APOE+	HLA-DQA1	CD4	5,38859E-03	8870,9	6,22205E-03	18553	3,72597E-01	7897	1,10304E+00	4144	9,22742E-01	2552	0	11208,5
Responder	Macro_NLRP3	Macro_FOLR2+APOE+	VCAN	ITGB1	5,39153E-03	10723,1	1,09887E-02	7237	5,35085E-01	3777	3,77301E-01	16190	8,55768E-01	15203	0	11208,5
Responder	Macro_LYVE1	Mono_CD16	LGALS1	ITGB1	5,39486E-03	9755,5	7,38250E-03	14213	5,22355E-01	3997	4,40656E-01	14454	9,09943E-01	4905	0	11208,5
Responder	Macro_LYVE1	Mono_CD16	LGALS1	PTPRC	5,39652E-03	7652,7	7,96091E-03	12540	6,55645E-01	2259	5,48860E-01	11772	9,38460E-01	484	0	11208,5
Responder	Macro_IFI27	Macro_LYVE1	MMP9	LRP1	5,39742E-03	7248,1	2,14844E-02	2273	7,45449E-01	1545	1,25871E+00	3099	8,41998E-01	18115	0	11208,5
Responder	cDC_CLEC9A	Mono_CD14	HLA-DOB	CD4	5,39742E-03	8418,3	1,78154E-02	3099	8,75622E-01	918	1,33754E+00	2652	8,12936E-01	24214	0	11208,5
Responder	Macro_FOLR2-APOE+	Mono_INHBA	TNFSF13B	TFRC	5,39986E-03	14006,3	7,02846E-03	15347	2,40814E-01	14151	4,92811E-01	13130	8,51239E-01	16195	0	11208,5
Responder	cDC(CD1C)	Macro_FOLR2+APOE+	HMGB1	HAVCR2	5,39986E-03	11543,3	6,79716E-03	16195	2,94725E-01	11161	5,49043E-01	11765	8,96831E-01	7387	0	11208,5
Responder	Macro_OLFML3	Macro_NLRP3	C1QA	CR1	5,40153E-03	9085,9	1,29785E-02	5480	5,45851E-01	3603	7,05939E-01	8942	8,51237E-01	16196	0	11208,5
Responder	Mono_INHBA	Mono_CD14	MMP9	ITGAM	5,40453E-03	13832,5	1,33789E-02	5189	3,72322E-01	7903	1,18730E+00	3508	7,23590E-01	41354	0	11208,5
Responder	Macro_FOLR2-APOE+	Mono_INHBA	A2M	LRP1	5,40486E-03	11816,5	1,05182E-02	7790	3,05233E-01	10635	4,87224E-01	13251	8,51225E-01	16198	0	11208,5
Responder	Macro_OLFML3	Macro_OLFML3	HLA-DQA1	CD4	5,40820E-03	9097,7	7,92319E-03	12641	5,10896E-01	4198	3,77006E-01	16200	9,30929E-01	1241	0	11208,5
Responder	Mono_INHBA	Macro_FOLR2+APOE-	CCL3L1	CCR1	5,40985E-03	11165,7	1,04334E-02	7905	5,01924E-01	4368	1,14957E+00	3762	7,91060E-01	28585	0	11208,5
Responder	cDC(CD1C)	Mono_CD16	HMGB1	TLR4	5,41154E-03	13680,9	6,79608E-03	16202	2,26085E-01	15096	6,45173E-01	9995	8,52623E-01	15903	0	11208,5
Responder	Macro_LYVE1	Mono_CD16	CXCL12	CXCR4	5,41488E-03	9015,3	1,70830E-02	3333	5,68367E-01	3297	5,05546E-01	12803	8,59523E-01	14435	0	11208,5
Responder	Macro_LYVE1	Mono_INHBA	LGALS1	ITGB1	5,41488E-03	9680,5	6,79524E-03	16204	4,77421E-01	4877	6,12049E-01	10567	9,06488E-01	5546	0	11208,5
Responder	Macro_ISG15	pDC_LILRA4	SEMA4A	NRP1_PLXNA4	5,41782E-03	11413,5	2,39837E-02	1881	7,94467E-01	1282	1,25840E+00	3103	7,33816E-01	39593	0	11208,5
Responder	Macro_FOLR2+APOE+	Mono_INHBA	SPP1	CD44	5,41784E-03	6530,1	1,35326E-02	5105	6,44672E-01	2360	7,73562E-01	7908	9,03712E-01	6069	0	11208,5
Responder	cDC(CD1C)	Macro_IER3	HLA-DPB1	CD4	5,41784E-03	9297,5	5,61427E-03	21774	3,72168E-01	7908	1,08594E+00	4284	9,30382E-01	1313	0	11208,5
Responder	Mono_CD14	Macro_NLRP3	THBS1	LRP1	5,41822E-03	9961,3	1,14669E-02	6746	3,87903E-01	7363	7,48292E-01	8283	8,51179E-01	16206	0	11208,5
Responder	pDC_LILRA4	pDC_LILRA4	HLA-B	CANX	5,41955E-03	15155,6	5,03543E-03	25620	1,35131E-01	22331	2,56654E+00	192	9,36238E-01	668	0,021	26967
Responder	Macro_NLRP3	Macro_NLRP3	CD52	SIGLEC10	5,41989E-03	13037,3	6,79493E-03	16207	3,75153E-01	7799	3,92744E-01	15731	8,60421E-01	14241	0	11208,5
Responder	Macro_LYVE1	Mono_CD16	TIMP2	ITGB1	5,42324E-03	10302,9	1,18429E-02	6397	5,53533E-01	3508	4,07067E-01	15347	8,56527E-01	15054	0	11208,5
Responder	Macro_FOLR2-APOE+	Macro_IER3	SPP1	ITGA4_ITGB1	5,42491E-03	9388,3	1,27582E-02	5623	4,57360E-01	5364	7,32029E-01	8536	8,51163E-01	16210	0	11208,5
Responder	Macro_LYVE1	Mono_CD16	F13A1	ITGB1	5,42658E-03	7197,9	1,60730E-02	3732	6,75136E-01	2071	1,00015E+00	5089	8,62181E-01	13889	0	11208,5
Responder	Macro_FOLR2-APOE+	pDC_LILRA4	HLA-DRB1	CD4	5,42993E-03	7626,3	6,79362E-03	16213	3,68176E-01	8045	1,42895E+00	2222	9,39041E-01	443	0	11208,5
Responder	Macro_ISG15	Macro_FOLR2-APOE+	LILRB4	LAIR1	5,43663E-03	13065,1	8,11091E-03	12164	2,28652E-01	14927	3,76261E-01	16217	8,78117E-01	10809	0	11208,5
Responder	Macro_OLFML3	Mono_CD14	MMP2	PECAM1	5,43919E-03	16162,1	2,98825E-02	1239	4,71254E-01	5014	7,73312E-01	7916	6,24730E-01	55433	0	11208,5
Responder	Macro_LYVE1	Mono_CD14	ADAM10	CD44	5,44167E-03	13324,3	6,79181E-03	16220	3,41023E-01	9076	4,36619E-01	14570	8,54172E-01	15547	0	11208,5
Responder	Mast	pDC_LILRA4	ADAM12	ITGB1	5,44339E-03	10971,3	3,06186E-02	1170	5,79427E-01	3108	1,65933E+00	1394	7,42406E-01	37976	0	11208,5
Responder	Macro_LYVE1	Macro_NLRP3	PLTP	ABCA1	5,44670E-03	8580,1	1,25482E-02	5784	7,37720E-01	1595	7,61192E-01	8090	8,51098E-01	16223	0	11208,5
Responder	cDC_CLEC9A	cDC_CLEC9A	HLA-DRB5	LAG3	5,45510E-03	9309,5	1,19455E-02	6305	3,68896E-01	8019	1,03120E+00	4787	8,51071E-01	16228	0	11208,5
Responder	Macro_LYVE1	pDC_LILRA4	LGALS9	CD47	5,45678E-03	12220,9	6,88666E-03	15862	2,57115E-01	13210	1,05372E+00	4595	8,51062E-01	16229	0	11208,5
Responder	Macro_LYVE1	cDC_CLEC9A	ADAM10	CADM1	5,46390E-03	8546,7	3,06893E-02	1164	8,90851E-01	864	1,25621E+00	3112	8,02225E-01	26385	0	11208,5
Responder	Macro_ISG15	pDC_LILRA4	HMGB1	TLR9	5,46782E-03	12089,4	4,19669E-02	671	1,03848E+00	494	1,00462E+00	5048	8,34964E-01	19625	0,364	34609
Responder	Macro_NLRP3	Macro_OLFML3	VCAN	SELL	5,46856E-03	9335,9	1,74038E-02	3227	6,48100E-01	2322	4,69474E-01	13686	8,51047E-01	16236	0	11208,5
Responder	Macro_ISG15	Macro_FOLR2-APOE+	LGALS9	LRP1	5,46856E-03	13692,1	7,34235E-03	14330	2,77234E-01	12105	3,75789E-01	16236	8,58839E-01	14581	0	11208,5
Responder	Macro_FOLR2-APOE+	Mono_INHBA	THBS1	SDC4	5,47133E-03	12459,3	1,88243E-02	2807	4,69184E-01	5072	7,72274E-01	7928	7,56932E-01	35281	0	11208,5
Responder	cDC(CD1C)	Macro_OLFML3	ADAM10	TREM2	5,47670E-03	11227,7	1,04165E-02	7930	3,73651E-01	7854	8,43503E-01	6947	8,22439E-01	22199	0	11208,5
Responder	cDC_CLEC9A	Macro_ISG15	IL16	CCR5	5,47931E-03	12077,3	2,81956E-02	1397	6,12185E-01	2698	1,25598E+00	3115	7,20260E-01	41968	0	11208,5
Responder	cDC(CD1C)	Macro_FOLR2-APOE+	HLA-DPB1	CD4	5,48476E-03	9232,7	5,60289E-03	21858	3,71251E-01	7933	1,13977E+00	3840	9,30316E-01	1324	0	11208,5
Responder	Mono_INHBA	Macro_LYVE1	S100A8	CD36	5,49014E-03	9266,7	1,92670E-02	2686	6,94963E-01	1900	1,83102E-01	22604	8,93877E-01	7935	0	11208,5
Responder	Mono_INHBA	Macro_NLRP3	VEGFA	ITGAV	5,49014E-03	13583,7	1,17413E-02	6489	5,00357E-01	4395	7,71759E-01	7935	7,42847E-01	37891	0	11208,5
Responder	pDC_LILRA4	Mono_CD16	APP	LRP1	5,49725E-03	6640,9	1,54255E-02	4035	8,51100E-01	1017	2,01574E+00	691	8,50951E-01	16253	0	11208,5
Responder	Mono_INHBA	Macro_ISG15	GNAI2	FPR1	5,49725E-03	13763,3	6,81317E-03	16122	2,36719E-01	14407	3,75285E-01	16253	8,78013E-01	10826	0	11208,5
Responder	Macro_FOLR2-APOE+	Macro_FOLR2+APOE+	APOE	TREM2	5,50571E-03	7396,1	1,76075E-02	3168	4,63428E-01	5216	3,75101E-01	16258	9,31855E-01	1130	0	11208,5
Responder	Mono_CD14	Mono_CD16	CD14	ITGA4	5,50571E-03	13651,1	6,78541E-03	16258	2,16497E-01	15707	4,60764E-01	13906	8,76392E-01	11176	0	11208,5
Responder	Macro_NLRP3	Macro_LYVE1	VCAN	TLR2	5,50910E-03	9625,5	1,43319E-02	4631	5,28429E-01	3885	5,32526E-01	12143	8,50918E-01	16260	0	11208,5
Responder	Mono_INHBA	Macro_LYVE1	PSAP	LRP1	5,51418E-03	9613,1	8,63950E-03	10939	3,70398E-01	7964	3,75010E-01	16263	9,27738E-01	1691	0	11208,5
Responder	Macro_OLFML3	Macro_ISG15	TNFSF13	TNFRSF1A	5,52096E-03	10848,9	1,19477E-02	6303	3,74614E-01	7819	5,12094E-01	12647	8,50877E-01	16267	0	11208,5
Responder	Macro_FOLR2-APOE+	Macro_LYVE1	SPP1	ITGAV_ITGB5	5,52249E-03	10444,9	2,12025E-02	2319	6,12305E-01	2697	8,72185E-01	6563	7,86714E-01	29437	0	11208,5
Responder	Macro_OLFML3	Macro_ISG15	HLA-B	LILRB1	5,52436E-03	9842,9	6,78205E-03	16269	4,28890E-01	6114	4,85318E-01	13291	9,23983E-01	2332	0	11208,5
Responder	Macro_FOLR2-APOE+	cDC(CD1C)	GRN	TNFRSF1A	5,52776E-03	12095,3	6,78175E-03	16271	2,44146E-01	13940	6,07272E-01	10655	8,91359E-01	8402	0	11208,5
Responder	Macro_LYVE1	Mast	GNAS	ADRB2	5,53115E-03	6271,3	3,15203E-02	1120	8,41099E-01	1056	8,83804E-01	6440	8,74646E-01	11532	0	11208,5
Responder	Macro_LYVE1	Mast	HBEGF	CD9	5,53285E-03	8818,5	1,45673E-02	4500	5,42829E-01	3649	5,47460E-01	11798	8,67014E-01	12937	0	11208,5
Responder	Macro_OLFML3	Mono_INHBA	MDK	NCL	5,53625E-03	11466,1	1,45978E-02	4482	2,85551E-01	11637	4,68039E-01	13727	8,50836E-01	16276	0	11208,5
Responder	Macro_ISG15	Mono_CD16	GRN	TNFRSF1B	5,53625E-03	11094,7	6,88931E-03	15850	3,59287E-01	8398	3,74682E-01	16276	9,16251E-01	3741	0	11208,5

Responder	Macro_ISG15	Mono_CD14	CD14	ITGB2	5,54476E-03	9040,9	6,77737E-03	16281	2,85778E-01	11628	1,05743E+00	4542	9,28730E-01	1545	0	11208,5
Responder	cDC(CD1C)	cDC_LAMP3	NAMPT	ADORA2A	5,54685E-03	10395,1	2,15872E-02	2257	5,83913E-01	3045	7,70006E-01	7956	7,96465E-01	27509	0	11208,5
Responder	Macro_FOLR2-APOE+	cDC(CD1C)	INHBA	ENG	5,54685E-03	14014,7	1,49822E-02	4261	3,70637E-01	7956	9,49151E-01	5646	7,25466E-01	41002	0	11208,5
Responder	Macro_LYVE1	Mast	TIMP2	CD44	5,54987E-03	6926,5	1,29401E-02	5500	7,17514E-01	1719	6,44171E-01	10016	9,03146E-01	6189	0	11208,5
Responder	Macro_LYVE1	Mast	ARPC5	ADRB2	5,55158E-03	5795,7	3,08672E-02	1155	8,16771E-01	1170	8,67061E-01	6634	8,89132E-01	8811	0	11208,5
Responder	Macro_LYVE1	Macro_FOLR2-APOE+	TIMP2	ITGB1	5,55499E-03	10976,3	1,10070E-02	7221	5,13663E-01	4145	3,74299E-01	16287	8,51970E-01	16020	0	11208,5
Responder	Macro_IFI27	Macro_FOLR2-APOE+	APOE	SDC2	5,55669E-03	9350,7	2,02097E-02	2492	5,62659E-01	3374	3,74293E-01	16288	8,64797E-01	13391	0	11208,5
Responder	Mono_CD14	Mono_CD14	SELPLG	SELL	5,56042E-03	10116,5	1,03944E-02	7961	4,40650E-01	5801	1,44220E+00	2156	8,16552E-01	23456	0	11208,5
Responder	cDC_CLEC9A	Macro_NLRP3	LGALS9	CD44	5,57035E-03	9974,1	6,77411E-03	16296	3,65630E-01	8153	7,96137E-01	7597	9,00896E-01	6616	0	11208,5
Responder	Macro_LYVE1	Mast	HSPA8	ADRB2	5,57206E-03	5974,9	2,90862E-02	1315	7,51629E-01	1514	6,62866E-01	9670	9,03261E-01	6167	0	11208,5
Responder	Macro_LYVE1	Mast	ACTR2	ADRB2	5,57377E-03	7261,5	2,84535E-02	1373	7,17936E-01	1715	7,83900E-01	7764	8,60393E-01	14247	0	11208,5
Responder	Macro_ISG15	cDC(CD1C)	LGALS9	HAVCR2	5,57377E-03	12555,7	6,77367E-03	16298	2,30742E-01	14794	9,25224E-01	5909	8,58924E-01	14569	0	11208,5
Responder	Macro_FOLR2-APOE+	Macro_FOLR2-APOE+	SPP1	ITGA4_ITGB1	5,58746E-03	9520,7	1,26713E-02	5691	4,53543E-01	5462	7,06157E-01	8936	8,50730E-01	16306	0	11208,5
Responder	Macro_OLFML3	Macro_FOLR2+APOE-	LGALS3BP	ITGB1	5,59604E-03	10251,1	1,07893E-02	7465	4,00556E-01	6958	6,83104E-01	9313	8,50704E-01	16311	0	11208,5
Responder	Macro_FOLR2+APOE+	Macro_FOLR2-APOE+	APOE	SDC2	5,60365E-03	5023,9	3,11861E-02	1138	9,76396E-01	635	1,25108E+00	3139	8,88214E-01	8999	0	11208,5
Responder	pDC_LILRA4	Macro_IFI27	SEMA7A	PLXNC1	5,60365E-03	13620,9	1,76887E-02	3139	6,12456E-01	2693	1,38537E+00	2427	6,79925E-01	48637	0	11208,5
Responder	Macro_FOLR2-APOE+	Macro_NLRP3	APOE	LRP1	5,61149E-03	9937,7	1,01678E-02	8267	3,30948E-01	9503	3,73328E-01	16320	9,12755E-01	4390	0	11208,5
Responder	Mono_CD14	Macro_NLRP3	TGFB1	CXCR4	5,62009E-03	12382,5	6,76582E-03	16325	2,93846E-01	11209	5,95948E-01	10840	8,70134E-01	12330	0	11208,5
Responder	Macro_IER3	Macro_ISG15	CCL4	CCR5	5,62978E-03	7207,3	3,05604E-02	1175	9,52372E-01	694	1,25076E+00	3144	8,34007E-01	19815	0	11208,5
Responder	Macro_OLFML3	Macro_LYVE1	ALCAM	NRP1	5,62978E-03	13495,3	1,76742E-02	3144	5,82334E-01	3067	4,66835E-01	13759	7,51325E-01	36298	0	11208,5
Responder	Mono_INHBA	Mono_INHBA	THBS1	LRP1	5,63043E-03	10215,1	1,13540E-02	6845	3,86342E-01	7412	6,85406E-01	9279	8,50552E-01	16331	0	11208,5
Responder	Macro_ISG15	Macro_FOLR2-APOE+	LGALS3BP	ITGB1	5,63560E-03	11762,9	1,07599E-02	7488	3,11541E-01	10355	4,79925E-01	13429	8,50531E-01	16334	0	11208,5
Responder	Mono_INHBA	Macro_FOLR2+APOE-	VEGFA	NRP2	5,64509E-03	11498,5	1,37905E-02	4962	5,84376E-01	3038	7,67103E-01	7992	7,82442E-01	30292	0	11208,5
Responder	Macro_OLFML3	Mono_INHBA	B2M	TFRC	5,65460E-03	9281,7	6,75917E-03	16345	4,65195E-01	5178	5,49397E-01	11757	9,26349E-01	1920	0	11208,5
Responder	cDC(CD1C)	Macro_NLRP3	HMGB1	TLR2	5,65980E-03	12400,7	6,86269E-03	15962	2,07121E-01	16348	7,61947E-01	8076	8,80259E-01	10409	0	11208,5
Responder	cDC_LAMP3	pDC_LILRA4	MAML2	NOTCH4	5,66124E-03	14536,5	3,08376E-02	1156	5,77130E-01	3150	1,65999E+00	1393	6,21380E-01	55775	0	11208,5
Responder	Macro_IER3	Macro_FOLR2+APOE+	CCL3L1	CCR5	5,66649E-03	10478,7	2,23738E-02	2121	9,22780E-01	777	1,24975E+00	3151	7,57700E-01	35136	0	11208,5
Responder	pDC_LILRA4	Macro_FOLR2+APOE+	ADAM10	GPNMB	5,66846E-03	7066,7	1,41686E-02	4725	7,64361E-01	1442	1,58967E+00	1605	8,50433E-01	16353	0	11208,5
Responder	cDC_CLEC9A	Macro_OLFML3	HLA-F	LILRB1	5,67019E-03	13160,9	7,38906E-03	14194	2,66239E-01	12673	5,67096E-01	11375	8,50427E-01	16354	0	11208,5
Responder	pDC_LILRA4	Mast	PROC	PROCR	5,67175E-03	15270,7	1,41729E-01	42	8,41507E-01	1052	1,24959E+00	3152	5,51151E-01	60899	0	11208,5
Responder	pDC_LILRA4	Macro_IER3	RARRES2	CCRL2	5,67175E-03	13197,5	2,54644E-02	1679	5,77115E-01	3152	1,88221E+00	896	6,76968E-01	49052	0	11208,5
Responder	cDC(CD1C)	Macro_IER3	RPS19	C5AR1	5,67260E-03	9929,7	5,91233E-03	20123	3,69411E-01	8002	1,04597E+00	4668	9,05842E-01	5647	0	11208,5
Responder	Macro_ISG15	Macro_LYVE1	PSAP	LRP1	5,67366E-03	9080,3	9,04784E-03	10094	4,23073E-01	6274	3,71937E-01	16356	9,29270E-01	1469	0	11208,5
Responder	Macro_OLFML3	Macro_ISG15	C1QA	CD33	5,68060E-03	12044,9	6,75463E-03	16360	3,28979E-01	9595	5,34160E-01	12106	8,77390E-01	10955	0	11208,5
Responder	cDC_CLEC9A	Macro_FOLR2+APOE-	CD59	STAB1	5,68233E-03	7559,3	1,43678E-02	4610	6,62425E-01	2174	1,19742E+00	3443	8,50407E-01	16361	0	11208,5
Responder	cDC(CD1C)	Mono_CD16	ICAM1	SPN	5,68363E-03	12221,7	1,03556E-02	8006	4,18557E-01	6403	8,34951E-01	7072	7,91893E-01	28419	0	11208,5
Responder	Macro_ISG15	Mono_CD16	GNAI2	C5AR1	5,68581E-03	11390,7	7,79740E-03	12993	3,86540E-01	7403	3,71820E-01	16363	8,88264E-01	8986	0	11208,5
Responder	Macro_FOLR2-APOE+	Macro_LYVE1	MMP9	EPHB2	5,68639E-03	11840,3	4,82794E-02	515	7,92033E-01	1291	1,14717E+00	3778	7,17808E-01	42409	0	11208,5
Responder	pDC_LILRA4	Macro_LYVE1	SERPING1	LRP1	5,68928E-03	12085,7	7,02649E-03	15354	2,99401E-01	10934	8,71736E-01	6567	8,50385E-01	16365	0	11208,5
Responder	pDC_LILRA4	Macro_ISG15	APP	LRP10	5,69282E-03	8369,7	1,76467E-02	3156	8,96407E-01	852	2,24934E+00	409	8,03035E-01	26223	0	11208,5
Responder	Macro_LYVE1	Macro_IER3	PLTP	ABCA1	5,69450E-03	5321,1	2,40055E-02	1878	9,80069E-01	626	1,14448E+00	3803	8,87713E-01	9090	0	11208,5
Responder	Macro_ISG15	Macro_OLFML3	LGALS9	HAVCR2	5,69450E-03	12082,3	8,33722E-03	11653	3,42667E-01	8998	3,71521E-01	16368	8,71045E-01	12184	0	11208,5
Responder	Mono_INHBA	Macro_FOLR2+APOE+	MIF	TNFRSF14	5,69972E-03	10655,1	7,85406E-03	12836	4,84769E-01	4715	3,71380E-01	16371	8,92711E-01	8145	0	11208,5
Responder	Macro_ISG15	Mono_INHBA	TIMP2	CD44	5,69972E-03	10924,9	6,75292E-03	16371	4,26527E-01	6166	7,25364E-01	8633	8,70739E-01	12246	0	11208,5
Responder	Macro_FOLR2-APOE+	Mast	LGALS9	CD44	5,70146E-03	11000,7	7,11444E-03	15062	4,26947E-01	6155	3,71349E-01	16372	9,03063E-01	6206	0	11208,5
Responder	Macro_FOLR2-APOE+	Macro_LYVE1	C1QB	LRP1	5,70574E-03	8667,1	1,24908E-02	5821	5,48670E-01	3570	2,24735E-01	21068	9,27906E-01	1668	0	11208,5
Responder	Macro_OLFML3	Macro_FOLR2+APOE+	C3	CD46	5,70843E-03	10385,7	1,07638E-02	7485	4,28653E-01	6119	6,01991E-01	10740	8,50355E-01	16376	0	11208,5
Responder	Mono_CD14	Macro_LYVE1	RETN	CAP1	5,70851E-03	9238,9	2,43265E-02	1828	4,77596E-01	4872	7,64951E-01	8015	8,31848E-01	20271	0	11208,5
Responder	cDC(CD1C)	Macro_OLFML3	HLA-DQA1	LAG3	5,72514E-03	9606,1	1,03445E-02	8021	4,59790E-01	5298	1,25853E+00	3102	8,31246E-01	20401	0	11208,5
Responder	Macro_NLRP3	cDC(CD1C)	VCAN	SELL	5,72938E-03	7415,1	1,72043E-02	3294	6,44823E-01	2358	1,14174E+00	3827	8,50314E-01	16388	0	11208,5
Responder	Mono_INHBA	Macro_FOLR2+APOE+	VEGFA	SIRPA	5,73069E-03	9694,1	1,83328E-02	2943	6,64839E-01	2150	7,64315E-01	8023	8,13249E-01	24146	0	11208,5
Responder	Macro_ISG15	Mono_INHBA	HLA-F	LILRB2	5,73287E-03	13409,7	6,74994E-03	16390	2,63622E-01	12830	4,63341E-01	13838	8,67752E-01	12782	0	11208,5
Responder	Macro_FOLR2-APOE+	cDC_CLEC9A	SPP1	ITGA5_ITGB1	5,73347E-03	11041,3	1,03439E-02	8024	3,92627E-01	7213	9,47337E-01	5666	8,18256E-01	23095	0	11208,5
Responder	Macro_LYVE1	Macro_IER3	CD59	STAB1	5,73462E-03	7659,1	1,57254E-02	3904	7,31500E-01	1634	8,86274E-01	6401	8,56060E-01	15148	0	11208,5
Responder	Mast	Mono_CD16	CSF1	CSF2RA	5,74039E-03	9875,3	1,76114E-02	3165	7,57010E-01	1492	1,40133E+00	2341	7,78071E-01	31170	0	11208,5
Responder	Macro_OLFML3	Macro_FOLR2+APOE-	FN1	CD44	5,75214E-03	12703,3	9,02422E-03	10148	2,06405E-01	16401	3,95246E-01	15659	8,81932E-01	10100	0	11208,5
Responder	Macro_FOLR2-APOE+	Macro_LYVE1	C3	CD81	5,75294E-03	10114,9	1,35472E-02	5096	3,93754E-01	7171	2,83823E-01	19086	8,93474E-01	8013	0	11208,5
Responder	cDC(CD1C)	Mono_CD14	SAA1	CD36	5,75294E-03	14076,5	1,06470E-02	7631	3,68474E-01	8031	1,23223E+00	3250	7,29725E-01	40262	0	11208,5
Responder	pDC_LILRA4	cDC_LAMP3	ICAM1	IL2RG	5,75389E-03	9457,9	8,70547E-03	10792	4,75305E-01	4927	1,12679E+00	3960	8,50236E-01	16402	0	11208,5
Responder	Macro_ISG15	Macro_FOLR2-APOE+	CD14	ITGB2	5,75389E-03	10826,5	6,74648E-03	16402	2,82150E-01	11851	4,93727E-01	13100	9,28579E-01	1571	0	11208,5
Responder	Macro_OLFML3	Macro_FOLR2+APOE-	FN1	ITGA4_ITGB1	5,75564E-03	9420,5	1,32365E-02	5291	3,67995E-01	8054	9,06077E-01	6146	8,50234E-01	16403	0	11208,5
Responder	Macro_FOLR2-APOE+	Mono_CD14	PLAU	ITGA5	5,75851E-03	14752,5	1,28037E-02	5589	3,70414E-01	7961	7,63810E-01	8033	7,25702E-01	40971	0	11208,5
Responder	Macro_FOLR2-APOE+	Macro_LYVE1	APOE	ABCA1	5,75851E-03	10561,5	1,18328E-02	6407	4,16204E-01	6482	1,42041E-01	24190	9,12060E-01	4520	0	11208,5
Responder	Mono_INHBA	Macro_ISG15	TNF	NOTCH1	5,76130E-03	15553,1	1,59405E-02	3794	4,45331E-01	5664	7,63702E-01	8034	6,76891E-01	49065	0	11208,5
Responder	Mono_CD14	Macro_OLFML3	ICAM1	ITGAM_ITGB2	5,76442E-03	13703,3	6,74554E-03	16408	2,54375E-01	13366	4,23410E-01	14905	8,68432E-01	12629	0	11208,5

Responder	Macro_LYVE1	Macro_FOLR2+APOE-	A2M	LRP1	5,76618E-03	11876,5	1,04676E-02	7864	3,29861E-01	9550	4,39477E-01	14501	8,50920E-01	16259	0	11208,5
Responder	cDC(CD1C)	Macro_ISG15	HMGB1	HAVCR2	5,76969E-03	10259,1	6,74467E-03	16411	2,90274E-01	11382	1,02622E+00	4839	8,96471E-01	7455	0	11208,5
Responder	Macro_LYVE1	Macro_FOLR2+APOE-	CD59	STAB1	5,77849E-03	7231,7	1,75853E-02	3172	7,94807E-01	1281	8,58870E-01	6739	8,62811E-01	13758	0	11208,5
Responder	Macro_IER3	Macro_FOLR2-APOE+	CCL3L1	CCR1	5,78290E-03	7225,3	2,02576E-02	2480	8,96020E-01	853	1,24381E+00	3173	8,40652E-01	18412	0	11208,5
Responder	Mono_INHBA	Macro_OLFML3	ANXA2	TLR2	5,78377E-03	10321,9	9,45325E-03	9381	4,16673E-01	6465	3,69573E-01	16419	8,92788E-01	8136	0	11208,5
Responder	Mono_CD16	Macro_LYVE1	SERPINA1	LRP1	5,78643E-03	5043,5	1,65102E-02	3553	8,68159E-01	950	7,63394E-01	8043	9,29318E-01	1463	0	11208,5
Responder	Macro_NLRP3	Mono_INHBA	PSEN1	CD44	5,78906E-03	10249,1	9,27770E-03	9702	5,16405E-01	4102	6,54828E-01	9811	8,50126E-01	16422	0	11208,5
Responder	Macro_FOLR2-APOE+	Macro_LYVE1	MMP9	ITGAM	5,78922E-03	11869,3	1,83481E-02	2937	6,13451E-01	2683	8,59109E-01	6736	7,54037E-01	35782	0	11208,5
Responder	Macro_OLFML3	cDC(CD1C)	CD14	ITGB2	5,79435E-03	10007,5	6,74036E-03	16425	2,81837E-01	11873	7,05186E-01	8954	9,28549E-01	1577	0	11208,5
Responder	Macro_FOLR2-APOE+	cDC(CD1C)	SPP1	ITGAV_ITGB1	5,79762E-03	10938,3	1,03273E-02	8043	3,68102E-01	8047	1,05736E+00	4543	8,19385E-01	22850	0	11208,5
Responder	Macro_FOLR2-APOE+	Macro_IER3	SPP1	PTGER4	5,79788E-03	7402,5	2,11953E-02	2320	6,42214E-01	2381	1,04499E+00	4676	8,50096E-01	16427	0	11208,5
Responder	Macro_IER3	pDC_LILRA4	MAML2	NOTCH4	5,79889E-03	14619,9	3,06217E-02	1168	5,75711E-01	3176	1,56575E+00	1688	6,20553E-01	55859	0	11208,5
Responder	Macro_ISG15	cDC(CD1C)	ICAM1	IL2RG	5,79964E-03	9342,7	8,68556E-03	10839	4,46267E-01	5644	1,34920E+00	2594	8,50090E-01	16428	0	11208,5
Responder	cDC_CLEC9A	Macro_OLFML3	HLA-DPA1	CD4	5,79964E-03	8165,1	6,73945E-03	16428	4,37909E-01	5876	8,56924E-01	6770	9,37628E-01	543	0	11208,5
Responder	Macro_FOLR2+APOE-	Mono_INHBA	GRN	TNFRSF1B	5,80042E-03	5847,3	1,03207E-02	8048	7,61063E-01	1470	8,23378E-01	7214	9,30510E-01	1296	0	11208,5
Responder	cDC(CD1C)	Macro_IER3	HMGB1	THBD	5,80317E-03	12445,7	6,73927E-03	16430	2,79375E-01	11992	7,71558E-01	7939	8,58453E-01	14659	0	11208,5
Responder	cDC_CLEC9A	Mono_CD14	HLA-F	LILRB2	5,80494E-03	11357,5	6,73886E-03	16431	2,68654E-01	12543	1,14483E+00	3800	8,67657E-01	12805	0	11208,5
Responder	Macro_FOLR2-APOE+	Macro_LYVE1	MMP9	IFNAR1	5,80603E-03	9339,7	2,02588E-02	2479	6,59799E-01	2210	9,54683E-01	5576	8,08070E-01	25225	0	11208,5
Responder	Mono_CD14	Mast	HP	ITGAM	5,81164E-03	16018,5	4,76507E-02	524	5,17820E-01	4068	7,63004E-01	8052	6,16626E-01	56240	0	11208,5
Responder	Macro_LYVE1	pDC_LILRA4	AGTRAP	RACK1	5,81378E-03	10443,9	6,73764E-03	16436	3,06317E-01	10588	1,54371E+00	1779	8,70933E-01	12208	0	11208,5
Responder	Macro_FOLR2-APOE+	Macro_LYVE1	NRG1	MS4A4A	5,81725E-03	10940,5	2,22896E-02	2134	8,58982E-01	984	8,96774E-01	6270	7,63167E-01	34106	0	11208,5
Responder	Macro_NLRP3	Macro_FOLR2+APOE-	HMGB1	CD163	5,81908E-03	10227,9	7,66728E-03	13356	4,18160E-01	6418	3,69002E-01	16439	9,16357E-01	3718	0	11208,5
Responder	Macro_NLRP3	Macro_OLFML3	ADAM10	TREM2	5,82085E-03	9057,7	1,56009E-02	3957	4,96634E-01	4468	6,89575E-01	9215	8,50042E-01	16440	0	11208,5
Responder	cDC(CD1C)	Mono_CD14	GNAI2	FPR1	5,82085E-03	11230,9	6,73643E-03	16440	2,29143E-01	14887	1,33544E+00	2667	8,77405E-01	10952	0	11208,5
Responder	Mono_CD14	Macro_IER3	AGTRAP	RACK1	5,82439E-03	11096,5	8,69778E-03	10804	3,85936E-01	7423	3,68856E-01	16442	8,84619E-01	9605	0	11208,5
Responder	Macro_FOLR2-APOE+	Mono_INHBA	THBS1	CD36	5,82568E-03	10613,5	1,30431E-02	5425	4,18960E-01	6388	7,62908E-01	8057	8,23396E-01	21989	0	11208,5
Responder	Macro_IFI27	Macro_LYVE1	C3	CD81	5,82794E-03	9062,1	1,47145E-02	4398	4,33967E-01	5988	3,68826E-01	16444	8,97343E-01	7272	0	11208,5
Responder	pDC_LILRA4	Mono_INHBA	LILRB4	LAIR1	5,82794E-03	10862,9	7,58748E-03	13575	2,05797E-01	16444	1,61461E+00	1524	8,74502E-01	11563	0	11208,5
Responder	Mono_INHBA	Mono_CD14	ANXA1	FPR1	5,82971E-03	12737,7	6,90273E-03	15800	2,05757E-01	16445	5,82096E-01	11085	8,87356E-01	9150	0	11208,5
Responder	pDC_LILRA4	Mono_INHBA	GRN	TNFRSF1B	5,83858E-03	8442,3	6,73374E-03	16450	3,30800E-01	9509	1,76267E+00	1142	9,15370E-01	3902	0	11208,5
Responder	cDC(CD1C)	cDC_CLEC9A	TNFSF13B	HLA-DPB1	5,84168E-03	8359,9	5,89574E-03	20203	3,50547E-01	8712	1,66108E+00	1388	9,41986E-01	288	0	11208,5
Responder	Mono_INHBA	Mono_CD14	HSPA1A	TLR4	5,85279E-03	12558,5	6,73045E-03	16458	2,33293E-01	14642	7,90225E-01	7670	8,67622E-01	12814	0	11208,5
Responder	Macro_FOLR2+APOE-	Macro_IER3	CCL18	CCR1	5,85778E-03	10271,9	1,76510E-02	3152	5,74786E-01	3187	1,62154E+00	1509	7,72101E-01	32303	0	11208,5
Responder	Macro_OLFML3	Macro_FOLR2-APOE+	HLA-A	APLP2	5,86149E-03	11841,7	5,83013E-03	20569	4,55118E-01	5407	2,17444E-01	21329	9,36006E-01	695	0	11208,5
Responder	Macro_ISG15	Mono_INHBA	LGALS3BP	ITGB1	5,86525E-03	11034,5	1,06561E-02	7623	3,06478E-01	10581	6,84086E-01	9295	8,49914E-01	16465	0	11208,5
Responder	Macro_FOLR2-APOE+	pDC_LILRA4	HLA-DQB1	CD4	5,86703E-03	8858,3	6,72903E-03	16466	2,88611E-01	11470	1,25099E+00	3140	9,25823E-01	2007	0	11208,5
Responder	Macro_ISG15	Macro_ISG15	CCL2	CCR2	5,86852E-03	12807,7	3,90162E-02	768	6,22414E-01	2577	1,24151E+00	3189	6,95167E-01	46296	0	11208,5
Responder	Macro_IFI27	cDC_CLEC9A	HLA-DRB5	LAG3	5,87059E-03	11592,1	1,17252E-02	6510	3,49503E-01	8751	4,19086E-01	15023	8,49887E-01	16468	0	11208,5
Responder	Macro_IFI27	Macro_IER3	MMP12	PLAUR	5,87237E-03	8330,3	1,85280E-02	2881	5,07272E-01	4256	8,51036E-01	6837	8,49886E-01	16469	0	11208,5
Responder	Macro_LYVE1	Mono_CD14	CD14	ITGB2	5,87951E-03	10656,1	6,72690E-03	16473	2,79513E-01	11981	5,36878E-01	12034	9,28482E-01	1584	0	11208,5
Responder	Mono_INHBA	Macro_NLRP3	VCAN	TLR2	5,88308E-03	10061,1	1,40922E-02	4782	3,95354E-01	7116	6,02980E-01	10724	8,49845E-01	16475	0	11208,5
Responder	Macro_IFI27	pDC_LILRA4	HLA-DMA	CD4	5,88486E-03	9272,1	6,72568E-03	16476	2,65368E-01	12732	1,45210E+00	2104	9,15707E-01	3840	0	11208,5
Responder	Mono_INHBA	Mono_INHBA	LGALS3	ENG	5,90096E-03	11492,3	7,45071E-03	14000	3,78735E-01	7675	7,61007E-01	8093	8,49783E-01	16485	0	11208,5
Responder	Macro_FOLR2+APOE-	cDC_CLEC9A	CXCL2	XCR1	5,90374E-03	8241,3	3,19422E-02	1096	2,36914E+00	15	1,03270E+00	4775	8,13421E-01	24112	0	11208,5
Responder	Macro_FOLR2-APOE+	Mono_CD16	CD99	PILRA	5,90454E-03	10089,1	9,30845E-03	9642	5,16825E-01	4094	3,66998E-01	16487	8,88124E-01	9014	0	11208,5
Responder	Macro_IFI27	pDC_LILRA4	HLA-DRB1	CD4	5,90633E-03	7746,7	6,72204E-03	16488	3,54578E-01	8568	1,47531E+00	2010	9,38737E-01	459	0	11208,5
Responder	Macro_ISG15	Mono_CD14	LGALS9	LRP1	5,90991E-03	13205,3	6,72132E-03	16490	2,35456E-01	14492	7,60044E-01	8113	8,53396E-01	15723	0	11208,5
Responder	Mono_INHBA	cDC(CD1C)	ICAM1	SPN	5,91044E-03	11647,7	1,02918E-02	8087	4,49073E-01	5571	1,02562E+00	4847	7,91384E-01	28525	0	11208,5
Responder	Macro_ISG15	Macro_FOLR2-APOE+	SPP1	ITGAV_ITGB5	5,91164E-03	9142,3	2,51776E-02	1716	5,74571E-01	3197	1,29005E+00	2914	8,00776E-01	26676	0	11208,5
Responder	Macro_FOLR2-APOE+	cDC_LAMP3	SPP1	PTGER4	5,91613E-03	8028,1	1,86456E-02	2853	5,85839E-01	3023	1,02059E+00	4888	8,41746E-01	18168	0	11208,5
Responder	Macro_FOLR2-APOE+	Mono_CD14	CIRBP	TREM1	5,91708E-03	11590,3	1,03187E-02	8051	3,26821E-01	9694	5,17749E-01	12504	8,49754E-01	16494	0	11208,5
Responder	pDC_LILRA4	Macro_ISG15	CD99	CD81	5,92067E-03	10080,7	6,71991E-03	16496	3,55845E-01	8526	1,48442E+00	1980	8,71000E-01	12193	0	11208,5
Responder	Mono_CD14	Macro_IFI27	HP	ITGAM	5,92181E-03	16261,9	4,40763E-02	613	5,02692E-01	4349	7,61104E-01	8091	6,07368E-01	57048	0	11208,5
Responder	Mono_INHBA	cDC_LAMP3	MIF	TNFRSF14	5,93864E-03	11088,5	6,71668E-03	16506	4,09516E-01	6676	5,60773E-01	11511	8,84987E-01	9541	0	11208,5
Responder	Mono_INHBA	Mono_CD16	VCAN	ITGA4	5,94044E-03	10444,7	1,31500E-02	5343	4,58822E-01	5317	4,62903E-01	13848	8,49697E-01	16507	0	11208,5
Responder	Macro_ISG15	Macro_ISG15	S100A9	TLR4	5,94404E-03	12549,1	8,33132E-03	11665	3,07222E-01	10540	5,04547E-01	12823	8,49685E-01	16509	0	11208,5
Responder	Macro_ISG15	Mono_CD14	S100A9	ITGB2	5,94404E-03	9986,5	6,71507E-03	16509	2,70190E-01	12476	8,07291E-01	7447	9,24259E-01	2292	0	11208,5
Responder	Macro_IER3	cDC_LAMP3	ADM	RAMP1	5,94519E-03	14971,7	4,11966E-02	700	1,01666E+00	542	5,03443E-01	12851	6,73553E-01	49557	0	11208,5
Responder	Macro_OLFML3	Mono_CD16	LYZ	ITGAL	5,94745E-03	14865,3	1,09131E-02	7321	3,77304E-01	7722	-2,19223E-01	39975	8,93019E-01	8100	0	11208,5
Responder	Macro_FOLR2-APOE+	cDC_CLEC9A	LGALS9	MRC2	5,95602E-03	12495,3	1,60217E-02	3756	3,66939E-01	8103	7,82490E-01	7788	7,75673E-01	31621	0	11208,5
Responder	Macro_LYVE1	Mono_INHBA	LGALS3	ENG	5,96026E-03	12365,9	7,43466E-03	14050	3,76752E-01	7744	5,26289E-01	12309	8,49646E-01	16518	0	11208,5
Responder	pDC_LILRA4	Macro_FOLR2-APOE+	APP	NOTCH2	5,96039E-03	6885,5	1,74744E-02	3206	9,13552E-01	803	2,14335E+00	538	8,39303E-01	18672	0	11208,5
Responder	Macro_FOLR2+APOE-	cDC_CLEC9A	NECTIN2	CD226	5,96200E-03	12159,1	5,47877E-02	374	9,49738E-01	701	8,60153E-01	6725	7,21259E-01	41787	0	11208,5
Responder	Macro_OLFML3	cDC(CD1C)	C3	LRP1	5,96206E-03	11047,1	7,82692E-03	12908	3,48760E-01	8781	9,33317E-01	5819	8,49645E-01	16519	0	11208,5
Responder	Mono_INHBA	Macro_OLFML3	ADM	GPR84	5,96459E-03	15546,5	1,80216E-02	3033	4,17518E-01	6437	7,60376E-01	8106	6,77653E-01	48948	0	11208,5

Responder	Mono_INHBA	Macro_ISG15	CXCL8	SDC3	5,96459E-03	11212,1	1,02789E-02	8106	4,46598E-01	5636	1,01491E+00	4941	8,03284E-01	26169	0	11208,5
Responder	Macro_IFI27	Mono_INHBA	CXCL9	FCGR2A	5,96567E-03	12707,7	7,84789E-03	12853	2,04698E-01	16521	7,15979E-01	8772	8,60705E-01	14184	0	11208,5
Responder	Macro_NLRP3	Mast	GNAS	ADRB2	5,97109E-03	8344,1	2,06662E-02	2408	6,11845E-01	2702	7,09400E-01	8878	8,49618E-01	16524	0	11208,5
Responder	Macro_OLFML3	pDC_LILRA4	CXCL12	CD4	5,97604E-03	10366,5	1,26957E-02	5671	3,66797E-01	8110	1,64994E+00	1418	8,07094E-01	25425	0	11208,5
Responder	Mono_CD14	Mono_CD16	HLA-A	LILRB2	5,97832E-03	11180,5	7,68188E-03	13325	2,40665E-01	14161	3,65778E-01	16528	9,36138E-01	680	0	11208,5
Responder	cDC(CD1C)	Macro_IER3	HSPA8	LDLR	5,97890E-03	10366,7	1,12345E-02	6961	3,66725E-01	8111	8,84178E-01	6428	8,37227E-01	19125	0	11208,5
Responder	Macro_FOLR2-APOE+	cDC(CD1C)	C1QA	CD33	5,98556E-03	12542,1	8,63668E-03	10950	2,04523E-01	16532	4,06313E-01	15366	8,90009E-01	8654	0	11208,5
Responder	Macro_OLFML3	pDC_LILRA4	IRAK4	TLR7	5,98750E-03	15248,7	1,61623E-02	3686	3,66642E-01	8114	1,06192E+00	4497	6,79132E-01	48738	0	11208,5
Responder	Macro_OLFML3	cDC_LAMP3	MDK	NCL	5,99280E-03	11538,3	1,54106E-02	4042	3,11061E-01	10374	3,65626E-01	16536	8,54241E-01	15531	0	11208,5
Responder	Macro_FOLR2-APOE+	cDC_LAMP3	SPP1	S1PR1	5,99324E-03	10402,7	8,24233E-02	157	8,99549E-01	845	1,00678E+00	5035	7,59758E-01	34768	0	11208,5
Responder	Macro_IER3	cDC(CD1C)	ICAM1	ITGAX_ITGB2	5,99898E-03	6553,5	1,02709E-02	8118	5,54046E-01	3499	1,23697E+00	3223	9,00318E-01	6719	0	11208,5
Responder	Macro_LYVE1	Macro_FOLR2+APOE+	PLTP	ABCA1	6,00005E-03	9456,7	1,24814E-02	5830	7,36307E-01	1605	5,25166E-01	12340	8,50760E-01	16300	0	11208,5
Responder	Macro_NLRP3	Mono_CD14	CIRBP	TREM1	6,00005E-03	11002,1	1,02889E-02	8090	3,25172E-01	9772	6,78152E-01	9400	8,49570E-01	16540	0	11208,5
Responder	Macro_IER3	Macro_ISG15	TNF	TNFRSF1A	6,00395E-03	6668,5	2,26990E-02	2075	7,46946E-01	1537	1,23775E+00	3214	8,55281E-01	15308	0	11208,5
Responder	cDC(CD1C)	Macro_ISG15	GNAI2	FPR1	6,00550E-03	11917,9	6,70641E-03	16543	2,26545E-01	15073	9,37804E-01	5760	8,77164E-01	11005	0	11208,5
Responder	Macro_ISG15	Macro_FOLR2-APOE+	S100A9	CD68	6,00731E-03	10014,7	7,59136E-03	13565	3,80280E-01	7612	3,65358E-01	16544	9,31769E-01	1144	0	11208,5
Responder	Macro_FOLR2-APOE+	Mono_CD16	HLA-B	LILRA1	6,01048E-03	9428,7	1,69903E-02	3358	4,36799E-01	5901	2,37870E-01	20654	9,03946E-01	6022	0	11208,5
Responder	Mono_CD14	Macro_LYVE1	VEGFA	NRP1	6,01048E-03	12937,9	1,38745E-02	4910	5,42312E-01	3658	7,59149E-01	8122	7,48596E-01	36791	0	11208,5
Responder	Macro_ISG15	Mono_CD14	TNFSF13B	TFRC	6,01276E-03	11531,1	6,84042E-03	16028	3,79766E-01	7633	8,99132E-01	6239	8,49514E-01	16547	0	11208,5
Responder	Macro_LYVE1	Macro_FOLR2+APOE+	ADAM10	GPNMB	6,01458E-03	8033,9	1,66099E-02	3506	8,14498E-01	1183	6,45036E-01	9998	8,60264E-01	14274	0	11208,5
Responder	Mono_INHBA	Macro_NLRP3	SPP1	ITGA5_ITGB1	6,01623E-03	10646,7	1,10481E-02	7174	3,66305E-01	8124	1,04414E+00	4681	8,23102E-01	22046	0	11208,5
Responder	Mono_CD14	cDC_CLEC9A	ICAM1	SPN	6,01623E-03	11504,1	1,02632E-02	8124	4,00040E-01	6979	1,33885E+00	2643	7,91154E-01	28566	0	11208,5
Responder	cDC_CLEC9A	Macro_LYVE1	SPN	SIGLEC1	6,02034E-03	9980,7	3,32639E-02	1018	8,36747E-01	1074	1,23755E+00	3217	7,66678E-01	33386	0	11208,5
Responder	Macro_FOLR2-APOE+	Mono_CD16	HLA-A	LILRA1	6,02487E-03	9466,5	1,75938E-02	3170	4,40967E-01	5786	2,46593E-01	20356	8,99873E-01	6812	0	11208,5
Responder	Macro_OLFML3	cDC(CD1C)	LGALS9	HAVCR2	6,02549E-03	14080,5	6,70223E-03	16554	2,24145E-01	15233	5,09204E-01	12714	8,58281E-01	14693	0	11208,5
Responder	cDC(CD1C)	pDC_LILRA4	IL16	CD4	6,04217E-03	9864,9	1,02581E-02	8133	3,67573E-01	8076	2,03897E+00	657	8,26977E-01	21250	0	11208,5
Responder	cDC(CD1C)	Macro_FOLR2+APOE+	HLA-DQA1	CD4	6,04506E-03	9342,1	6,13211E-03	18975	3,66065E-01	8134	9,39995E-01	5740	9,22222E-01	2653	0	11208,5
Responder	pDC_LILRA4	Macro_ISG15	HLA-F	LILRB1	6,05101E-03	11595,1	7,27434E-03	14562	2,45949E-01	13835	1,53456E+00	1802	8,49429E-01	16568	0	11208,5
Responder	Macro_OLFML3	Macro_OLFML3	HLA-DPA1	LAG3	6,06198E-03	11057,3	9,27364E-03	9708	4,53419E-01	5466	5,25559E-01	12330	8,49404E-01	16574	0	11208,5
Responder	cDC(CD1C)	Macro_FOLR2+APOE+	GRN	TNFRSF1A	6,06381E-03	12838,3	6,69707E-03	16575	2,10381E-01	16129	5,49628E-01	11750	8,90749E-01	8529	0	11208,5
Responder	Macro_FOLR2-APOE+	Macro_ISG15	GRN	TNFRSF1A	6,06746E-03	12016,3	7,54246E-03	13711	2,95436E-01	11119	3,64280E-01	16577	8,96401E-01	7466	0	11208,5
Responder	Macro_OLFML3	pDC_LILRA4	C3	CD81	6,07479E-03	11128,9	6,69460E-03	16581	2,93091E-01	11240	1,72313E+00	1238	8,54990E-01	15377	0	11208,5
Responder	Macro_NLRP3	Mono_INHBA	VCAN	TLR2	6,08029E-03	8807,1	1,39836E-02	4845	5,21200E-01	4020	8,11907E-01	7378	8,49350E-01	16584	0	11208,5
Responder	Macro_FOLR2+APOE-	Macro_IER3	CCL13	CCR1	6,08032E-03	12772,7	4,09308E-02	708	1,15299E+00	325	8,81637E-01	6466	7,02318E-01	45156	0	11208,5
Responder	cDC(CD1C)	pDC_LILRA4	CD14	TLR9	6,08066E-03	17509,1	3,14402E-02	1128	9,67481E-01	653	1,23651E+00	3228	8,04218E-01	25973	1	56563,5
Responder	Mono_CD14	Mono_CD14	CD14	TLR4	6,08212E-03	12146,3	7,84317E-03	12865	2,03903E-01	16585	1,07293E+00	4399	8,53586E-01	15674	0	11208,5
Responder	Macro_ISG15	Macro_LYVE1	HMGB1	CD163	6,08395E-03	10713,7	7,06854E-03	15216	4,23770E-01	6252	3,63841E-01	16586	9,13188E-01	4306	0	11208,5
Responder	Macro_NLRP3	pDC_LILRA4	HMGB1	CXCR4	6,08762E-03	7459,3	6,69339E-03	16588	4,16549E-01	6470	1,97479E+00	756	9,24336E-01	2274	0	11208,5
Responder	Macro_FOLR2-APOE+	Mono_CD16	HLA-C	LILRA3	6,08849E-03	7778,5	2,91265E-02	1311	6,57283E-01	2235	3,02869E-01	18450	9,05664E-01	5688	0	11208,5
Responder	Macro_IFI27	cDC(CD1C)	C1QA	CD93	6,08946E-03	13343,7	6,96684E-03	15572	2,64660E-01	12767	3,63806E-01	16589	8,79354E-01	10582	0	11208,5
Responder	Macro_OLFML3	Mono_CD14	LGALS3BP	CD33	6,09720E-03	10596,1	1,38925E-02	4895	3,97613E-01	7049	7,56737E-01	8152	8,24942E-01	21676	0	11208,5
Responder	Macro_OLFML3	Macro_OLFML3	HLA-B	CANX	6,09731E-03	17175,5	4,98684E-03	25956	3,51824E-01	8660	-2,04522E-01	39344	9,35948E-01	709	0	11208,5
Responder	Mono_INHBA	Macro_OLFML3	ICAM1	ITGAM_ITGB2	6,10048E-03	10819,1	9,12606E-03	9964	4,07192E-01	6752	3,63726E-01	16595	8,84759E-01	9576	0	11208,5
Responder	Mono_CD14	pDC_LILRA4	SIRPB2	CD47	6,10302E-03	12956,5	1,31809E-02	5322	3,65608E-01	8154	1,55720E+00	1717	7,40328E-01	38381	0	11208,5
Responder	Macro_NLRP3	cDC(CD1C)	VCAN	TLR2	6,10415E-03	8210,3	1,39660E-02	4857	5,20835E-01	4027	1,07717E+00	4362	8,49270E-01	16597	0	11208,5
Responder	Macro_FOLR2-APOE+	cDC(CD1C)	CD14	ITGB2	6,10783E-03	9633,1	6,69181E-03	16599	2,76177E-01	12157	8,70932E-01	6582	9,28309E-01	1619	0	11208,5
Responder	pDC_LILRA4	Mono_INHBA	ASIP	MGRN1	6,10822E-03	14858,5	4,29391E-02	639	7,44006E-01	1555	1,23561E+00	3233	6,00673E-01	57657	0	11208,5
Responder	Macro_FOLR2+APOE-	cDC(CD1C)	CXCL12	ITGA4	6,10822E-03	9043,7	2,11692E-02	2324	5,72471E-01	3233	1,26808E+00	3038	8,07156E-01	25415	0	11208,5
Responder	Macro_OLFML3	cDC_CLEC9A	GNAI2	CXCR3	6,10822E-03	12342,1	1,73847E-02	3233	5,76673E-01	3159	3,16148E-01	18032	8,03731E-01	26078	0	11208,5
Responder	Macro_FOLR2-APOE+	Macro_LYVE1	SPP1	ITGA4_ITGB1	6,10967E-03	10002,9	1,23820E-02	5917	4,44866E-01	5682	6,09828E-01	10607	8,49258E-01	16600	0	11208,5
Responder	Mono_INHBA	Macro_IER3	ICAM1	ITGAX_ITGB2	6,12257E-03	12843,7	6,95768E-03	15608	3,06080E-01	10597	3,63264E-01	16607	8,81429E-01	10198	0	11208,5
Responder	cDC(CD1C)	Macro_ISG15	LTB	TNFRSF1A	6,12632E-03	11972,7	1,05937E-02	7701	3,65359E-01	8162	1,49415E+00	1942	7,79670E-01	30850	0	11208,5
Responder	Macro_LYVE1	Macro_ISG15	PLTP	ABCA1	6,12994E-03	8694,5	1,21811E-02	6078	7,29954E-01	1643	7,71837E-01	7932	8,49207E-01	16611	0	11208,5
Responder	Mono_CD14	Macro_LYVE1	HMGB1	CD163	6,12994E-03	9936,3	6,68799E-03	16611	3,92865E-01	7204	6,47440E-01	9944	9,10969E-01	4714	0	11208,5
Responder	cDC(CD1C)	pDC_LILRA4	MIF	CD74_CXCR4	6,14844E-03	11526,3	4,33397E-03	31444	2,50568E-01	13580	2,00118E+00	712	9,36101E-01	687	0	11208,5
Responder	Mono_CD14	Macro_FOLR2-APOE+	THBS1	LRP1	6,15027E-03	10582,9	1,11057E-02	7100	3,71839E-01	7919	6,41374E-01	10065	8,49141E-01	16622	0	11208,5
Responder	Macro_IER3	cDC_CLEC9A	ICAM1	SPN	6,15247E-03	7852,7	1,73556E-02	3241	6,99282E-01	1857	1,35667E+00	2558	8,31258E-01	20399	0	11208,5
Responder	Macro_OLFML3	cDC(CD1C)	HMGB1	CXCR4	6,16138E-03	9026,7	6,68418E-03	16628	4,16047E-01	6486	7,32460E-01	8528	9,24287E-01	2283	0	11208,5
Responder	Macro_FOLR2-APOE+	Mono_CD16	SPP1	CD44	6,16431E-03	9471,1	1,22149E-02	6055	4,74325E-01	4945	3,09853E-01	18214	8,99162E-01	6933	0	11208,5
Responder	Macro_FOLR2-APOE+	Macro_IER3	APOE	LDLR	6,17436E-03	11012,7	1,69155E-02	3386	3,80158E-01	7617	3,62662E-01	16635	8,51133E-01	16217	0	11208,5
Responder	Macro_OLFML3	Macro_ISG15	LGALS3BP	ITGB1	6,17807E-03	11018,9	1,05083E-02	7808	3,91299E-01	7252	5,30661E-01	12189	8,49021E-01	16637	0	11208,5
Responder	cDC_CLEC9A	Macro_OLFML3	ANXA2	TLR2	6,17807E-03	13768,7	6,98299E-03	15507	2,03358E-01	16637	4,37962E-01	14540	8,77406E-01	10951	0	11208,5
Responder	Macro_ISG15	Macro_ISG15	HLA-A	LILRB1	6,17992E-03	10353,7	6,68064E-03	16638	2,96141E-01	11078	6,69322E-01	9559	9,18842E-01	3285	0	11208,5
Responder	cDC_CLEC9A	cDC(CD1C)	HMGB1	HAVCR2	6,18364E-03	9179,7	6,68041E-03	16640	3,59611E-01	8386	1,44638E+00	2131	8,96026E-01	7533	0	11208,5
Responder	Mono_CD14	Macro_IFI27	TIMP1	CD63	6,18922E-03	8987,9	6,68011E-03	16643	3,81204E-01	7589	6,93532E-01	9149	9,40674E-01	350	0	11208,5

Post_R_Myeloid_Myeloid_CellCel

Responder	Macro_OLFML3	Mono_CD14	CALR	LRP1	6,19294E-03	12446,9	6,67959E-03	16645	2,92524E-01	11271	4,56721E-01	14014	8,87677E-01	9096	0	11208,5
Responder	Macro_NLRP3	cDC_LAMP3	ADM	RAMP1	6,19977E-03	14724,1	4,05819E-02	715	1,01299E+00	550	5,68033E-01	11352	6,71898E-01	49795	0	11208,5
Responder	Macro_FOLR2-APOE+	Macro_LYVE1	PSAP	LRP1	6,20248E-03	8128,3	1,02226E-02	8188	5,74619E-01	3195	3,48192E-01	17052	9,33179E-01	998	0	11208,5
Responder	cDC(CD1C)	Macro_FOLR2+APOE+	HLA-DPB1	CD4	6,20248E-03	9693,9	5,52191E-03	22353	3,64718E-01	8188	9,76726E-01	5329	9,29843E-01	1391	0	11208,5
Responder	cDC_CLEC9A	Macro_ISG15	SERPINF1	PLXDC2	6,20597E-03	12833,7	7,23628E-03	14684	2,20512E-01	15467	9,05060E-01	6157	8,48970E-01	16652	0	11208,5
Responder	Macro_FOLR2-APOE+	cDC_CLEC9A	FN1	DPP4	6,21132E-03	13523,3	4,87629E-02	500	5,23050E-01	3983	7,54461E-01	8191	7,10454E-01	43734	0	11208,5
Responder	Macro_ISG15	Mono_CD16	S100A9	TLR4	6,21156E-03	12500,1	9,08247E-03	10031	3,36815E-01	9258	3,62002E-01	16655	8,55115E-01	15348	0	11208,5
Responder	pDC_LILRA4	pDC_LILRA4	HMGB1	CXCR4	6,21156E-03	7348,3	6,67699E-03	16655	4,14511E-01	6545	3,28741E+00	40	9,24250E-01	2293	0	11208,5
Responder	Macro_LYVE1	pDC_LILRA4	HLA-DRA	CD4	6,21343E-03	8098,1	6,67689E-03	16656	3,48313E-01	8802	1,18823E+00	3503	9,41177E-01	321	0	11208,5
Responder	cDC(CD1C)	cDC_CLEC9A	CALM3	MYLK	6,21365E-03	18748,4	2,75350E-02	1446	6,56177E-01	2249	1,23209E+00	3252	6,56249E-01	51848	0,391	34947
Responder	Macro_FOLR2+APOE+	Macro_LYVE1	SAA1	CD36	6,21693E-03	7859,1	4,18273E-02	674	9,45681E-01	716	7,19380E-01	8712	8,42555E-01	17985	0	11208,5
Responder	Mono_INHBA	Mast	MMP9	CD44	6,21716E-03	9337,7	9,87466E-03	8663	5,17704E-01	4071	9,11317E-01	6088	8,48941E-01	16658	0	11208,5
Responder	cDC_LAMP3	Mono_INHBA	CXCL9	FCGR2A	6,21902E-03	11102,7	1,01311E-02	8310	2,85007E-01	11673	4,99744E-01	12940	8,75320E-01	11382	0	11208,5
Responder	pDC_LILRA4	cDC_LAMP3	FARP2	PLXNA1	6,21923E-03	15461,5	3,00433E-02	1227	5,70978E-01	3253	1,42226E+00	2249	5,77616E-01	59370	0	11208,5
Responder	Macro_OLFML3	Macro_IFI27	SERPING1	LRP1	6,22836E-03	12641,1	8,23262E-03	11863	3,38760E-01	9178	3,61878E-01	16664	8,60185E-01	14292	0	11208,5
Responder	Macro_FOLR2+APOE+	Mono_CD14	C1QB	CD33	6,23492E-03	7606,1	1,02156E-02	8199	5,12666E-01	4170	8,13168E-01	7350	8,98281E-01	7103	0	11208,5
Responder	cDC_LAMP3	Mono_INHBA	CIRBP	TREM1	6,23771E-03	10574,5	1,21128E-02	6138	4,45115E-01	5672	4,03300E-01	15457	8,59703E-01	14397	0	11208,5
Responder	Mono_CD16	Mast	ARPC5	ADRB2	6,24083E-03	5350,9	3,29842E-02	1034	8,75371E-01	919	9,71859E-01	5392	8,92359E-01	8201	0	11208,5
Responder	cDC(CD1C)	cDC_CLEC9A	LGALS9	MRC2	6,24083E-03	11489,7	1,59450E-02	3792	3,64309E-01	8201	1,35950E+00	2542	7,75255E-01	31705	0	11208,5
Responder	cDC(CD1C)	Macro_FOLR2-APOE+	VEGFA	NRP2	6,24379E-03	12953,1	1,15768E-02	6639	4,54312E-01	5431	7,53539E-01	8202	7,67182E-01	33285	0	11208,5
Responder	Macro_FOLR2-APOE+	Mono_CD16	MMP9	LRP1	6,24674E-03	9977,3	1,51234E-02	4192	5,58157E-01	3428	7,84550E-01	7748	8,17221E-01	23310	0	11208,5
Responder	pDC_LILRA4	Macro_OLFML3	SPON2	ITGA5	6,24719E-03	15406,5	1,73179E-02	3258	7,75901E-01	1373	1,31079E+00	2789	5,91343E-01	58404	0	11208,5
Responder	Macro_IFI27	Mono_INHBA	A2M	LRP1	6,26020E-03	12106,3	1,01271E-02	8315	2,86944E-01	11553	5,06757E-01	12774	8,48810E-01	16681	0	11208,5
Responder	Macro_OLFML3	Mono_CD14	HLA-A	LILRB2	6,26155E-03	8804,1	6,30785E-03	18180	4,69776E-01	5054	7,53274E-01	8208	9,29988E-01	1370	0	11208,5
Responder	Mono_CD14	Macro_OLFML3	CD14	ITGB2	6,26207E-03	10731,3	6,66973E-03	16682	3,06543E-01	10578	4,74872E-01	13554	9,28199E-01	1634	0	11208,5
Responder	Macro_OLFML3	Macro_NLRP3	MDK	SORL1	6,26451E-03	9454,9	2,43621E-02	1824	4,28129E-01	6128	7,53183E-01	8209	8,33572E-01	19905	0	11208,5
Responder	cDC_LAMP3	Mono_CD14	HLA-F	LILRB2	6,26583E-03	13168,5	6,69177E-03	16600	2,64295E-01	12789	5,24450E-01	12353	8,67254E-01	12892	0	11208,5
Responder	Macro_NLRP3	pDC_LILRA4	VCAN	CD44	6,27898E-03	10499,3	6,66754E-03	16691	3,43671E-01	8969	1,13618E+00	3871	8,73516E-01	11757	0	11208,5
Responder	Macro_IFI27	pDC_LILRA4	CXCL9	DPP4	6,28084E-03	12822,9	4,24213E-02	661	6,45462E-01	2348	1,23044E+00	3264	6,92830E-01	46633	0	11208,5
Responder	pDC_LILRA4	Mono_CD14	HSPA8	LDLR	6,29028E-03	8332,1	1,33670E-02	5199	3,71894E-01	7916	2,04993E+00	640	8,48725E-01	16697	0	11208,5
Responder	cDC_LAMP3	Mono_CD14	RPS19	C5AR1	6,29028E-03	6687,9	8,85775E-03	10478	5,40126E-01	3693	9,78708E-01	5314	9,21725E-01	2746	0	11208,5
Responder	Macro_FOLR2-APOE+	Mono_CD16	LYZ	ITGAL	6,29718E-03	11639,1	1,10390E-02	7185	3,87337E-01	7379	1,35531E-01	24432	8,93566E-01	7991	0	11208,5
Responder	cDC_LAMP3	Mono_CD14	CIRBP	TREM1	6,29782E-03	8381,1	1,33758E-02	5191	4,96242E-01	4476	7,80653E-01	7814	8,65579E-01	13216	0	11208,5
Responder	Macro_LYVE1	Mono_CD14	HSP90B1	LRP1	6,29782E-03	12697,9	6,66568E-03	16701	2,89337E-01	11427	4,50918E-01	14171	8,82617E-01	9982	0	11208,5
Responder	Macro_ISG15	pDC_LILRA4	HLA-DMB	CD4	6,29970E-03	10235,3	6,66562E-03	16702	2,33645E-01	14615	1,63383E+00	1466	8,97773E-01	7185	0	11208,5
Responder	Macro_FOLR2-APOE+	Mono_CD16	FABP5	RXRA	6,30314E-03	9003,3	1,60039E-02	3764	5,93987E-01	2916	8,89205E-01	6359	8,29471E-01	20769	0	11208,5
Responder	pDC_LILRA4	Mono_CD14	CD24	SIGLEC10	6,31461E-03	24416,1	1,92413E-02	2695	5,69984E-01	3270	1,76491E+00	1138	5,91203E-01	58414	1	56563,5
Responder	Mono_INHBA	Mono_CD14	S100A9	ITGB2	6,31480E-03	11526,3	6,88277E-03	15884	2,84531E-01	11704	3,60347E-01	16710	9,25118E-01	2125	0	11208,5
Responder	Macro_FOLR2-APOE+	pDC_LILRA4	THBS1	CD36	6,32699E-03	10524,7	1,14861E-02	6727	3,63655E-01	8230	1,37550E+00	2471	8,13962E-01	23987	0	11208,5
Responder	Macro_FOLR2-APOE+	Mono_INHBA	PLAU	ITGA5	6,32998E-03	12344,7	1,79915E-02	3051	5,10985E-01	4195	7,52054E-01	8231	7,58232E-01	35038	0	11208,5
Responder	Macro_FOLR2-APOE+	Mono_CD16	MMP9	IFNAR1	6,32998E-03	10659,3	1,59911E-02	3768	5,79629E-01	3106	8,98948E-01	6243	7,89055E-01	28971	0	11208,5
Responder	Macro_FOLR2-APOE+	cDC_CLEC9A	THBS1	SCARB1	6,32998E-03	12736,1	1,38856E-02	4903	3,63634E-01	8231	1,16658E+00	3637	7,54507E-01	35701	0	11208,5
Responder	Macro_NLRP3	Mono_INHBA	THBS1	CD47	6,33183E-03	9228,7	1,17073E-02	6523	5,20419E-01	4034	7,90789E-01	7659	8,48588E-01	16719	0	11208,5
Responder	pDC_LILRA4	Mono_CD16	HLA-F	LILRB1	6,33372E-03	11950,9	7,17944E-03	14872	2,39702E-01	14221	1,32169E+00	2733	8,48587E-01	16720	0	11208,5
Responder	Macro_IFI27	pDC_LILRA4	LGALS9	CD47	6,34320E-03	12214,1	6,65720E-03	16725	2,41348E-01	14100	1,39569E+00	2372	8,48902E-01	16665	0	11208,5
Responder	pDC_LILRA4	Macro_NLRP3	CALR	LRP1	6,34699E-03	10506,3	6,65696E-03	16727	2,49186E-01	13648	1,52616E+00	1822	8,87508E-01	9126	0	11208,5
Responder	pDC_LILRA4	Mono_INHBA	APP	NOTCH2	6,34850E-03	6892,1	1,72511E-02	3276	9,09516E-01	814	2,39460E+00	308	8,38434E-01	18854	0	11208,5
Responder	Macro_FOLR2-APOE+	Macro_IER3	APOE	ABCA1	6,35268E-03	8840,3	1,22574E-02	6021	4,33793E-01	5989	3,59346E-01	16730	9,13464E-01	4253	0	11208,5
Responder	Mono_CD14	cDC_LAMP3	THBS1	ITGA4	6,35391E-03	9724,9	1,26174E-02	5728	4,37028E-01	5893	7,51567E-01	8239	8,44715E-01	17556	0	11208,5
Responder	cDC_CLEC9A	cDC_CLEC9A	CALM3	MYLK	6,35416E-03	16998,5	3,23866E-02	1065	7,09905E-01	1776	1,22743E+00	3277	6,74315E-01	49454	0,07	29420,5
Responder	Macro_FOLR2-APOE+	pDC_LILRA4	CXCL12	DPP4	6,35691E-03	16963,7	2,95201E-02	1277	5,94415E-01	2905	7,51313E-01	8240	5,45095E-01	61188	0	11208,5
Responder	Macro_LYVE1	Macro_LYVE1	CD99	CD81	6,35838E-03	8407,3	1,23514E-02	5945	7,27689E-01	1660	3,59161E-01	16733	9,01515E-01	6490	0	11208,5
Responder	Macro_ISG15	Macro_LYVE1	HLA-A	APLP2	6,36028E-03	10039,9	6,65492E-03	16734	4,14563E-01	6544	4,07775E-01	15324	9,39856E-01	389	0	11208,5
Responder	cDC(CD1C)	pDC_LILRA4	HLA-B	CANX	6,36549E-03	11337,3	5,13090E-03	24941	1,71108E-01	19179	1,98232E+00	744	9,36797E-01	614	0	11208,5
Responder	Macro_FOLR2-APOE+	cDC(CD1C)	LGALS3	ENG	6,36979E-03	12514,5	7,30252E-03	14459	3,04650E-01	10660	6,71351E-01	9506	8,48497E-01	16739	0	11208,5
Responder	Mono_CD16	Macro_LYVE1	SPN	SIGLEC1	6,37239E-03	9907,9	4,20460E-02	669	9,42627E-01	725	7,98541E-01	7565	7,86976E-01	29372	0	11208,5
Responder	Macro_OLFML3	Macro_LYVE1	LGALS3BP	CD33	6,37550E-03	9244,5	1,96180E-02	2613	5,15807E-01	4114	5,58877E-01	11545	8,48482E-01	16742	0	11208,5
Responder	cDC_LAMP3	Macro_ISG15	HLA-F	LILRB2	6,38121E-03	13110,7	6,86515E-03	15952	2,77429E-01	12087	4,67705E-01	13739	8,68720E-01	12567	0	11208,5
Responder	cDC_LAMP3	Macro_ISG15	HLA-F	LILRB1	6,38312E-03	12758,9	7,96185E-03	12535	2,99437E-01	10930	4,66049E-01	13772	8,55113E-01	15349	0	11208,5
Responder	Macro_FOLR2+APOE-	Mast	PLTP	ABCA1	6,38819E-03	6065,5	1,88435E-02	2800	7,37858E-01	1593	1,22652E+00	3283	8,75069E-01	11443	0	11208,5
Responder	Macro_LYVE1	cDC_CLEC9A	LGALS9	HAVCR2	6,39075E-03	13171,7	6,64937E-03	16750	2,22731E-01	15323	7,82240E-01	7791	8,57799E-01	14786	0	11208,5
Responder	pDC_LILRA4	Mono_CD16	SEMA4A	LILRB2	6,39265E-03	6996,1	1,53993E-02	4047	7,21602E-01	1695	1,70385E+00	1279	8,48449E-01	16751	0	11208,5
Responder	Macro_FOLR2-APOE+	Mast	ADM	ADRB2	6,39595E-03	12159,5	2,58979E-02	1638	7,07294E-01	1798	8,47070E-01	6887	7,35686E-01	39266	0	11208,5
Responder	Macro_FOLR2-APOE+	Mast	VEGFB	ADRB2	6,39896E-03	12578,7	2,90169E-02	1317	7,28455E-01	1654	9,23015E-01	5939	7,15888E-01	42775	0	11208,5
Responder	Macro_FOLR2-APOE+	Macro_LYVE1	ADAM10	GPNMB	6,40029E-03	8849,7	1,37299E-02	4997	6,83938E-01	1981	6,83364E-01	9307	8,48422E-01	16755	0	11208,5

Responder	cDC_LAMP3	Macro_ISG15	CXCL9	FCGR2A	6,40029E-03	12177,9	9,31981E-03	9616	2,27225E-01	15018	5,05628E-01	12797	8,70693E-01	12250	0	11208,5
Responder	Macro_IER3	Mono_CD14	ICAM1	ITGAM_ITGB2	6,40197E-03	6812,1	1,05858E-02	7711	5,76623E-01	3160	1,15313E+00	3726	8,92110E-01	8255	0	11208,5
Responder	Macro_LYVE1	Macro_LYVE1	LRPAP1	LRP1	6,40602E-03	9649,7	1,26542E-02	5705	5,80339E-01	3087	3,58162E-01	16758	8,74821E-01	11490	0	11208,5
Responder	Macro_OLFML3	Macro_OLFML3	HLA-DRB1	LAG3	6,41176E-03	12140,7	8,76250E-03	10660	4,25011E-01	6217	3,87895E-01	15857	8,48388E-01	16761	0	11208,5
Responder	Macro_ISG15	Macro_FOLR2-APOE+	NAMPT	ITGA5_ITGB1	6,41176E-03	13291,9	6,64709E-03	16761	3,05446E-01	10624	5,13833E-01	12594	8,55463E-01	15272	0	11208,5
Responder	Mono_CD14	cDC(CD1C)	ICAM1	IL2RG	6,41367E-03	9516,1	8,45636E-03	11372	4,34531E-01	5969	1,41634E+00	2269	8,48378E-01	16762	0	11208,5
Responder	pDC_LILRA4	cDC_CLEC9A	CALR	SCARF1	6,41664E-03	7519,9	2,31970E-02	2000	5,69054E-01	3288	2,05797E+00	628	8,30833E-01	20475	0	11208,5
Responder	cDC_LAMP3	Macro_ISG15	C3	IFITM1	6,41750E-03	9530,7	1,02150E-02	8200	4,29852E-01	6089	8,32362E-01	7095	8,56492E-01	15061	0	11208,5
Responder	Mono_INHBA	Macro_OLFML3	MIF	TNFRSF14	6,42708E-03	10785,7	7,80504E-03	12969	4,81525E-01	4787	3,57944E-01	16769	8,92411E-01	8195	0	11208,5
Responder	pDC_LILRA4	Macro_FOLR2+APOE+	HMGB1	CD163	6,42899E-03	9376,1	6,64591E-03	16770	3,13307E-01	10273	1,13658E+00	3868	9,10713E-01	4761	0	11208,5
Responder	pDC_LILRA4	Macro_NLRP3	GRN	TNFRSF1B	6,43474E-03	8679,5	6,64555E-03	16773	3,21424E-01	9929	1,62658E+00	1492	9,14858E-01	3995	0	11208,5
Responder	Macro_FOLR2-APOE+	Mast	INHBA	SMAD3	6,43517E-03	14319,9	3,83732E-02	791	5,64638E-01	3346	8,18020E-01	7275	6,77403E-01	48979	0	11208,5
Responder	Macro_NLRP3	cDC(CD1C)	ICAM1	IL2RG	6,43666E-03	10101,7	8,45171E-03	11383	4,34292E-01	5975	9,94406E-01	5168	8,48343E-01	16774	0	11208,5
Responder	Macro_LYVE1	Macro_FOLR2+APOE-	HMGB1	CD163	6,44242E-03	8565,9	9,33079E-03	9594	5,98569E-01	2854	3,57617E-01	16777	9,23581E-01	2396	0	11208,5
Responder	Macro_FOLR2+APOE+	Mono_CD16	APOE	LRP1	6,44727E-03	6285,3	1,17835E-02	6444	6,64683E-01	2153	7,49209E-01	8270	9,18451E-01	3351	0	11208,5
Responder	Macro_NLRP3	Macro_IER3	HBEGF	CD44	6,44818E-03	9498,3	1,10613E-02	7160	5,15641E-01	4116	3,57511E-01	16780	8,92220E-01	8227	0	11208,5
Responder	Mono_INHBA	Macro_NLRP3	THBS1	LRP1	6,44818E-03	11052,1	1,09620E-02	7264	3,68101E-01	8048	5,39503E-01	11960	8,48304E-01	16780	0	11208,5
Responder	Macro_FOLR2+APOE-	Mono_CD14	MRC1	PTPRC	6,45660E-03	7754,3	1,72015E-02	3295	5,92790E-01	2930	1,22789E+00	3274	8,42261E-01	18064	0	11208,5
Responder	Macro_LYVE1	Mono_CD14	ANXA1	FPR1	6,46164E-03	11956,1	7,90538E-03	12685	2,94370E-01	11178	3,57238E-01	16787	8,93958E-01	7922	0	11208,5
Responder	cDC_CLEC9A	Mono_CD14	RPS19	C5AR1	6,46357E-03	8331,5	6,64187E-03	16788	3,84059E-01	7485	1,65225E+00	1410	9,10688E-01	4766	0	11208,5
Responder	Macro_FOLR2-APOE+	Mast	MMP9	ITGAM	6,46849E-03	12109,9	1,64016E-02	3598	5,89519E-01	2965	1,01064E+00	4992	7,43490E-01	37786	0	11208,5
Responder	Mono_CD14	Mast	PKM	CD44	6,46849E-03	9759,5	6,20988E-03	18620	3,76967E-01	7733	7,48609E-01	8277	9,20644E-01	2959	0	11208,5
Responder	cDC_LAMP3	Macro_NLRP3	RPS19	C5AR1	6,47127E-03	6991,5	1,01811E-02	8240	6,42976E-01	2375	5,72476E-01	11256	9,26603E-01	1878	0	11208,5
Responder	Macro_ISG15	Macro_ISG15	HLA-F	LILRB1	6,47706E-03	13728,9	7,13883E-03	14997	2,35406E-01	14495	5,78614E-01	11149	8,48222E-01	16795	0	11208,5
Responder	cDC_LAMP3	Macro_NLRP3	CIRBP	TREM1	6,48284E-03	9334,3	1,43894E-02	4594	5,37271E-01	3738	4,29617E-01	14738	8,69772E-01	12393	0	11208,5
Responder	Macro_OLFML3	cDC_CLEC9A	QDPR	DYSF	6,48368E-03	16034,1	1,96338E-02	2608	4,38685E-01	5850	7,48357E-01	8282	6,52982E-01	52222	0	11208,5
Responder	Macro_ISG15	Mono_CD14	VEGFA	CD44	6,48477E-03	12034,5	7,22442E-03	14731	3,62695E-01	8266	6,92428E-01	9168	8,48199E-01	16799	0	11208,5
Responder	cDC(CD1C)	Mono_CD14	CD1D	LILRB2	6,48976E-03	10377,9	1,36129E-02	5064	3,62289E-01	8284	1,30032E+00	2853	8,11632E-01	24480	0	11208,5
Responder	Mast	Macro_LYVE1	TIMP3	CD44	6,49459E-03	5800,7	4,16446E-02	682	9,40723E-01	732	8,64081E-01	6675	8,84132E-01	9706	0	11208,5
Responder	Macro_ISG15	Macro_LYVE1	S100A9	TLR4	6,50216E-03	11354,7	1,04548E-02	7882	3,90878E-01	7264	3,56512E-01	16808	8,63616E-01	13611	0	11208,5
Responder	Macro_NLRP3	cDC_CLEC9A	HMGB1	HAVCR2	6,50216E-03	11095,9	6,63734E-03	16808	2,49543E-01	13628	8,98728E-01	6244	8,95725E-01	7591	0	11208,5
Responder	cDC(CD1C)	Mono_INHBA	CALR	LRP1	6,50603E-03	11337,3	6,63673E-03	16810	2,38657E-01	14286	9,86990E-01	5233	8,87356E-01	9149	0	11208,5
Responder	pDC_LILRA4	Macro_FOLR2+APOE+	SPON2	ITGA4	6,50821E-03	12921,5	1,98768E-02	2556	7,41016E-01	1578	1,22336E+00	3304	6,97048E-01	45961	0	11208,5
Responder	Macro_FOLR2-APOE+	cDC_CLEC9A	LGALS9	SLC1A5	6,51108E-03	11124,9	1,05378E-02	7769	3,62072E-01	8291	8,38227E-01	7027	8,26609E-01	21329	0	11208,5
Responder	Macro_ISG15	Macro_OLFML3	ICAM1	ITGAM_ITGB2	6,51184E-03	13751,9	6,92836E-03	15701	2,66112E-01	12678	3,56278E-01	16813	8,69952E-01	12359	0	11208,5
Responder	Mono_CD14	Macro_ISG15	S100A9	CD36	6,51184E-03	9316,3	6,69455E-03	16582	9,31201E-01	757	1,73377E+00	1221	8,48141E-01	16813	0	11208,5
Responder	Macro_FOLR2-APOE+	pDC_LILRA4	HLA-DRA	CD4	6,51959E-03	8020,3	6,63513E-03	16817	3,40400E-01	9106	1,33916E+00	2637	9,41003E-01	333	0	11208,5
Responder	Macro_ISG15	pDC_LILRA4	PSEN1	NOTCH4	6,51972E-03	14180,5	2,91649E-02	1307	5,67712E-01	3306	1,67918E+00	1339	6,39988E-01	53742	0	11208,5
Responder	pDC_LILRA4	Mono_INHBA	CD99	PILRA	6,53317E-03	10572,7	6,63347E-03	16824	3,04716E-01	10658	1,51784E+00	1846	8,70155E-01	12327	0	11208,5
Responder	Macro_FOLR2+APOE-	cDC_CLEC9A	CALM3	MYLK	6,53700E-03	15621,7	3,96159E-02	748	7,89964E-01	1302	1,22197E+00	3309	6,96040E-01	46154	0,017	26595,5
Responder	pDC_LILRA4	pDC_LILRA4	RARRES2	CMKLR1	6,53700E-03	14415,5	2,67320E-02	1539	5,67524E-01	3309	2,76830E+00	111	6,20025E-01	55910	0	11208,5
Responder	cDC_LAMP3	pDC_LILRA4	HLA-DQA2	CD4	6,54483E-03	8384,3	8,05462E-03	12298	3,19419E-01	10006	1,46992E+00	2033	9,02160E-01	6376	0	11208,5
Responder	cDC_LAMP3	pDC_LILRA4	HLA-DQA1	CD4	6,54677E-03	8079,5	7,09312E-03	15138	3,27558E-01	9662	1,34123E+00	2631	9,27285E-01	1758	0	11208,5
Responder	Mono_CD14	Mono_CD16	CD14	ITGB1	6,54677E-03	12293,7	6,63211E-03	16831	2,57560E-01	13182	5,61126E-01	11501	8,89476E-01	8746	0	11208,5
Responder	Macro_OLFML3	Macro_FOLR2-APOE+	TNFSF13B	HLA-DPB1	6,54731E-03	17058,1	5,99778E-03	19685	2,78992E-01	12006	-2,68505E-01	42127	9,42453E-01	264	0	11208,5
Responder	Mono_INHBA	Mono_INHBA	NAMPT	ADORA2A	6,54775E-03	9623,3	2,49688E-02	1746	7,33790E-01	1621	7,46653E-01	8303	8,08007E-01	25238	0	11208,5
Responder	cDC_LAMP3	pDC_LILRA4	HLA-DQB1	CD4	6,54872E-03	8237,5	6,96841E-03	15566	3,19545E-01	10000	1,34621E+00	2605	9,27014E-01	1808	0	11208,5
Responder	cDC_LAMP3	pDC_LILRA4	HLA-DPB1	CD4	6,55066E-03	8130,1	6,69423E-03	16583	3,27890E-01	9649	1,37153E+00	2494	9,35869E-01	716	0	11208,5
Responder	Macro_OLFML3	cDC(CD1C)	HLA-DRB1	CD4	6,55388E-03	9968,9	5,54744E-03	22190	3,61567E-01	8305	8,30146E-01	7128	9,32976E-01	1013	0	11208,5
Responder	cDC_CLEC9A	Macro_LYVE1	RPS19	C5AR1	6,55455E-03	9541,1	6,63030E-03	16835	3,82859E-01	7532	8,13264E-01	7345	9,10617E-01	4785	0	11208,5
Responder	Mono_INHBA	cDC(CD1C)	SPP1	PTGER4	6,56001E-03	10731,5	1,15533E-02	6667	3,61536E-01	8307	1,46065E+00	2077	8,07206E-01	25398	0	11208,5
Responder	Macro_FOLR2+APOE+	Macro_NLRP3	APOE	LDLR	6,56588E-03	5167,3	3,13278E-02	1134	9,03993E-01	830	1,22084E+00	3314	8,86114E-01	9350	0	11208,5
Responder	Macro_OLFML3	Macro_OLFML3	C3	ITGAX	6,57014E-03	11477,3	9,14022E-03	9942	4,25121E-01	6215	3,55407E-01	16843	8,65747E-01	13178	0	11208,5
Responder	cDC(CD1C)	Macro_NLRP3	LGALS9	LRP1	6,57990E-03	13523,7	6,62583E-03	16848	2,14487E-01	15837	7,81254E-01	7806	8,52499E-01	15919	0	11208,5
Responder	Macro_ISG15	Macro_ISG15	PLAU	PLAUR	6,58576E-03	11103,1	8,13871E-03	12103	3,46216E-01	8885	8,81512E-01	6468	8,47930E-01	16851	0	11208,5
Responder	pDC_LILRA4	Macro_IFI27	APP	LRP1	6,59945E-03	6821,3	1,47066E-02	4406	8,35464E-01	1077	2,12610E+00	557	8,47899E-01	16858	0	11208,5
Responder	Macro_LYVE1	pDC_LILRA4	HLA-DPB1	CD4	6,59945E-03	8641,5	6,62347E-03	16858	3,16185E-01	10152	1,09073E+00	4241	9,35549E-01	748	0	11208,5
Responder	cDC_CLEC9A	Macro_ISG15	HLA-DOB	CD4	6,60063E-03	7428,7	2,35037E-02	1951	9,69462E-01	648	1,22005E+00	3320	8,33098E-01	20016	0	11208,5
Responder	Macro_OLFML3	Macro_FOLR2+APOE+	HLA-DQB2	LAG3	6,61805E-03	11810,9	2,88186E-02	1340	5,66504E-01	3323	5,31175E-01	12174	7,78910E-01	31009	0	11208,5
Responder	pDC_LILRA4	cDC_CLEC9A	HMGB1	HAVCR2	6,62101E-03	9979,7	6,62108E-03	16869	2,47505E-01	13759	2,21135E+00	446	8,95610E-01	7616	0	11208,5
Responder	cDC(CD1C)	cDC_CLEC9A	HLA-DQA2	LAG3	6,62155E-03	10876,9	1,44376E-02	4562	3,60858E-01	8327	1,26127E+00	3083	7,97922E-01	27204	0	11208,5
Responder	Macro_IFI27	Mono_CD14	C3	LRP1	6,62297E-03	11846,5	1,04751E-02	7853	2,00489E-01	16870	6,20086E-01	10426	8,67328E-01	12875	0	11208,5
Responder	Mono_CD14	Mono_INHBA	TNFSF13B	TFRC	6,62297E-03	13285,7	6,66619E-03	16695	2,10137E-01	16143	9,61570E-01	5512	8,47857E-01	16870	0	11208,5
Responder	pDC_LILRA4	Macro_FOLR2+APOE+	VEGFB	NRP1	6,62387E-03	12479,1	1,71061E-02	3324	6,07142E-01	2759	1,88661E+00	890	7,07685E-01	44214	0	11208,5
Responder	Macro_FOLR2-APOE+	Mono_CD14	TFPI	LRP1	6,62773E-03	13819,9	2,17223E-02	2235	4,83548E-01	4740	7,45219E-01	8329	7,16853E-01	42587	0	11208,5

Responder	Macro_ISG15	cDC_LAMP3	SPP1	ITGA4_ITGB1	6,62886E-03	8919,9	1,21137E-02	6136	4,23506E-01	6259	1,10564E+00	4123	8,47850E-01	16873	0	11208,5
Responder	Mono_INHBA	Mono_INHBA	CCL4	CCR1	6,63082E-03	9140,7	1,01158E-02	8330	5,19685E-01	4044	1,29530E+00	2877	8,36737E-01	19244	0	11208,5
Responder	Mono_CD14	Macro_FOLR2-APOE+	S100A9	CD36	6,63279E-03	9365,5	6,66330E-03	16708	9,30704E-01	759	1,70404E+00	1277	8,47840E-01	16875	0	11208,5
Responder	Macro_FOLR2-APOE+	Macro_OLFML3	APOE	SORL1	6,64319E-03	10572,7	1,25581E-02	5777	3,60740E-01	8334	1,65671E-01	23254	9,13294E-01	4290	0	11208,5
Responder	Macro_IFI27	Macro_FOLR2-APOE+	APOE	TREM2	6,64656E-03	7624,7	1,69806E-02	3361	4,55519E-01	5397	3,53968E-01	16882	9,30695E-01	1275	0	11208,5
Responder	Macro_OLFML3	cDC_CLEC9A	APP	CD74	6,64938E-03	12844,5	4,28389E-03	31921	3,60668E-01	8336	8,44382E-01	6927	9,04915E-01	5830	0	11208,5
Responder	cDC_CLEC9A	Macro_FOLR2+APOE+	LGALS9	CD47	6,65247E-03	14284,1	6,61602E-03	16885	2,34388E-01	14559	5,36973E-01	12030	8,48504E-01	16738	0	11208,5
Responder	Mono_INHBA	Mono_INHBA	THBS1	SDC4	6,65248E-03	11438,9	2,27630E-02	2066	5,43568E-01	3642	7,44602E-01	8337	7,73980E-01	31941	0	11208,5
Responder	Macro_OLFML3	Mono_INHBA	MDK	NOTCH2	6,66178E-03	11368,9	1,76669E-02	3146	3,78188E-01	7694	7,44417E-01	8340	8,01848E-01	26456	0	11208,5
Responder	Macro_NLRP3	cDC_CLEC9A	COPA	P2RY6	6,66466E-03	9506,5	1,76503E-02	3153	6,25114E-01	2538	1,21863E+00	3331	7,97470E-01	27302	0	11208,5
Responder	Macro_OLFML3	Macro_NLRP3	HLA-DQA2	CD4	6,67219E-03	12531,1	6,61338E-03	16895	2,49858E-01	13610	5,03050E-01	12862	8,93107E-01	8080	0	11208,5
Responder	Mono_INHBA	Mono_CD14	IL1B	SIGIRR	6,67730E-03	9011,3	1,01041E-02	8345	5,27885E-01	3896	1,23803E+00	3212	8,40730E-01	18395	0	11208,5
Responder	Macro_FOLR2-APOE+	Mono_CD14	MMP2	PECAM1	6,68351E-03	16172,1	3,04238E-02	1185	4,76773E-01	4894	7,44049E-01	8347	6,26832E-01	55226	0	11208,5
Responder	Macro_OLFML3	Macro_IER3	APOE	SORL1	6,68405E-03	10817,3	9,04095E-03	10114	3,43795E-01	8965	3,53295E-01	16901	8,99369E-01	6898	0	11208,5
Responder	Macro_NLRP3	Macro_OLFML3	HBEGF	CD9	6,68603E-03	8741,5	1,90149E-02	2758	6,13771E-01	2678	3,53284E-01	16902	8,81638E-01	10161	0	11208,5
Responder	Mono_INHBA	Macro_IFI27	LGALS3	ENG	6,68800E-03	12296,1	7,21579E-03	14761	3,69771E-01	7989	6,09201E-01	10619	8,47727E-01	16903	0	11208,5
Responder	Macro_ISG15	Macro_FOLR2-APOE+	CD14	TLR4	6,68998E-03	13812,1	7,15188E-03	14965	2,73755E-01	12289	4,69094E-01	13694	8,47725E-01	16904	0	11208,5
Responder	Macro_OLFML3	Macro_OLFML3	HLA-DQA2	LAG3	6,69049E-03	13043,5	1,23323E-02	5961	3,86056E-01	7421	5,95667E-01	10847	7,84916E-01	29780	0	11208,5
Responder	Mono_CD16	Mast	S100A8	CD69	6,70218E-03	5145,1	2,63355E-02	1584	9,31351E-01	756	7,43713E-01	8353	9,15785E-01	3824	0	11208,5
Responder	Macro_OLFML3	Mono_INHBA	C3	CD46	6,70218E-03	9979,7	1,01000E-02	8353	4,15848E-01	6493	8,66324E-01	6643	8,46260E-01	17201	0	11208,5
Responder	Macro_NLRP3	Macro_NLRP3	IL1B	SIGIRR	6,70583E-03	9566,5	1,12301E-02	6968	5,22330E-01	3999	7,17135E-01	8745	8,47678E-01	16912	0	11208,5
Responder	cDC_LAMP3	Macro_LYVE1	CXCL9	FCGR2A	6,70781E-03	9633,3	1,24168E-02	5886	4,47807E-01	5606	3,80628E-01	16098	8,86004E-01	9368	0	11208,5
Responder	Macro_FOLR2-APOE+	Macro_IER3	MMP9	LRP1	6,71465E-03	8614,9	1,80021E-02	3044	6,22021E-01	2583	9,57643E-01	5550	8,29877E-01	20689	0	11208,5
Responder	Macro_ISG15	Macro_OLFML3	S100A9	ITGB2	6,71575E-03	9152,7	8,41238E-03	11487	4,71364E-01	5011	3,52806E-01	16917	9,31779E-01	1140	0	11208,5
Responder	Macro_NLRP3	Macro_FOLR2-APOE+	CD52	SIGLEC10	6,73164E-03	12158,7	7,71120E-03	13254	4,11003E-01	6638	3,52491E-01	16925	8,67845E-01	12768	0	11208,5
Responder	cDC_CLEC9A	Macro_OLFML3	HLA-DPA1	LAG3	6,73761E-03	9933,3	9,02085E-03	10157	4,15689E-01	6500	1,02276E+00	4873	8,47627E-01	16928	0	11208,5
Responder	Macro_IER3	Macro_FOLR2+APOE+	CCL3L1	CCR1	6,74085E-03	6293,3	2,66369E-02	1550	9,74318E-01	640	1,21677E+00	3344	8,58145E-01	14724	0	11208,5
Responder	cDC(CD1C)	Mono_CD16	CD48	CD244	6,74590E-03	13716,5	2,02673E-02	2477	3,59949E-01	8367	8,52564E-01	6815	7,33091E-01	39715	0	11208,5
Responder	Macro_LYVE1	Macro_IFI27	LGALS3	ENG	6,74956E-03	13460,5	7,20024E-03	14808	3,67788E-01	8070	3,74483E-01	16282	8,47588E-01	16934	0	11208,5
Responder	Mono_INHBA	Macro_NLRP3	PTGS2	ALOX5	6,75953E-03	10046,7	1,28353E-02	5572	4,08662E-01	6710	6,55105E-01	9804	8,47559E-01	16939	0	11208,5
Responder	Macro_OLFML3	Macro_NLRP3	HLA-A	LILRA1	6,75953E-03	13068,1	7,27044E-03	14571	4,09195E-01	6690	3,52171E-01	16939	8,52450E-01	15932	0	11208,5
Responder	pDC_LILRA4	Macro_NLRP3	HLA-F	LILRB2	6,76751E-03	11470,3	6,60130E-03	16943	2,51217E-01	13544	1,34484E+00	2608	8,66469E-01	13048	0	11208,5
Responder	Macro_FOLR2-APOE+	Mono_CD14	C3	CR1	6,76783E-03	10552,3	2,35113E-02	1949	4,13023E-01	6587	7,42673E-01	8374	8,10840E-01	24643	0	11208,5
Responder	Mono_INHBA	Macro_FOLR2+APOE+	ICAM1	ITGAL_ITGB2	6,76951E-03	11496,7	8,57847E-03	11110	3,69935E-01	7984	3,52027E-01	16944	8,81174E-01	10237	0	11208,5
Responder	cDC_CLEC9A	Macro_NLRP3	HLA-DOB	CD4	6,77030E-03	7290,7	2,44251E-02	1816	9,84662E-01	616	1,21573E+00	3349	8,35755E-01	19464	0	11208,5
Responder	Macro_OLFML3	Macro_FOLR2-APOE+	LILRB4	LAIR1	6,77097E-03	10352,5	1,05573E-02	7741	4,13915E-01	6564	3,21012E-01	17874	8,91535E-01	8375	0	11208,5
Responder	Macro_FOLR2-APOE+	Macro_IER3	MMP9	ITGAM	6,77411E-03	13587,7	1,38351E-02	4932	5,57961E-01	3432	7,95569E-01	7607	7,26930E-01	40759	0	11208,5
Responder	Macro_NLRP3	Mono_CD14	S100A9	CD68	6,77551E-03	10619,1	6,60071E-03	16947	3,15090E-01	10193	4,98551E-01	12970	9,27187E-01	1777	0	11208,5
Responder	pDC_LILRA4	Macro_IER3	APP	TNFRSF21	6,77817E-03	10778,7	1,45640E-02	4502	9,34759E-01	748	2,15872E+00	520	7,47948E-01	36915	0	11208,5
Responder	Macro_NLRP3	Mast	CD55	ADGRE5	6,77950E-03	9663,7	1,20856E-02	6166	5,67140E-01	3315	6,05707E-01	10680	8,47511E-01	16949	0	11208,5
Responder	Macro_NLRP3	cDC_CLEC9A	TNFSF13B	CD40	6,78551E-03	13490,5	7,41062E-03	14126	1,99456E-01	16952	6,70235E-01	9532	8,53756E-01	15634	0	11208,5
Responder	Mono_INHBA	Macro_ISG15	CD52	SIGLEC10	6,78551E-03	14580,7	6,60002E-03	16952	2,36674E-01	14412	3,93360E-01	15711	8,58664E-01	14620	0	11208,5
Responder	Macro_FOLR2-APOE+	Macro_IER3	MMP9	IFNAR1	6,78668E-03	11371,9	1,45055E-02	4532	5,51723E-01	3541	8,42778E-01	6956	7,80827E-01	30622	0	11208,5
Responder	Macro_IER3	Mono_CD14	TNF	VSIR	6,78801E-03	7564,1	1,70140E-02	3352	6,43625E-01	2369	1,28534E+00	2938	8,42691E-01	17953	0	11208,5
Responder	Macro_OLFML3	cDC_LAMP3	CXCL10	SDC4	6,79297E-03	14295,7	1,83551E-02	2935	3,59650E-01	8382	7,62744E-01	8060	7,26158E-01	40893	0	11208,5
Responder	Macro_FOLR2-APOE+	Macro_IER3	CD59	STAB1	6,79926E-03	9804,9	1,15847E-02	6631	5,40982E-01	3682	7,57807E-01	8136	8,36189E-01	19367	0	11208,5
Responder	cDC_LAMP3	Mono_CD16	HLA-F	LILRB2	6,79953E-03	6910,5	1,28718E-02	5542	7,32461E-01	1628	6,71657E-01	9502	9,00606E-01	6672	0	11208,5
Responder	pDC_LILRA4	cDC_CLEC9A	NUCB2	ERAP1	6,79983E-03	12249,5	1,73538E-02	3244	5,64028E-01	3354	2,05132E+00	636	7,15720E-01	42805	0	11208,5
Responder	Macro_NLRP3	Mono_CD14	VCAN	ITGA4	6,80153E-03	8458,9	1,26937E-02	5673	5,79400E-01	3109	9,75300E-01	5344	8,47428E-01	16960	0	11208,5
Responder	Macro_ISG15	Macro_ISG15	LILRB4	LAIR1	6,80554E-03	12787,3	7,68805E-03	13312	1,99338E-01	16962	5,84210E-01	11053	8,75223E-01	11401	0	11208,5
Responder	Macro_FOLR2+APOE+	Mono_INHBA	APOE	SORL1	6,80556E-03	7171,1	1,00781E-02	8386	5,76498E-01	3164	8,30874E-01	7122	9,04178E-01	5975	0	11208,5
Responder	Macro_NLRP3	Mast	VCAN	ITGA4	6,80755E-03	8896,5	1,26883E-02	5679	5,79282E-01	3113	8,01744E-01	7519	8,47401E-01	16963	0	11208,5
Responder	Macro_IER3	Macro_FOLR2+APOE+	TNF	VSIR	6,81404E-03	4979,3	3,95956E-02	750	1,11570E+00	371	1,11042E+00	4088	8,90974E-01	8479	0	11208,5
Responder	cDC_LAMP3	Mono_INHBA	HLA-F	LILRB2	6,81960E-03	12656,9	7,52813E-03	13754	3,27653E-01	9661	3,50775E-01	16969	8,73888E-01	11692	0	11208,5
Responder	cDC(CD1C)	cDC_CLEC9A	LGALS9	SLC1A5	6,82133E-03	10223,5	1,04873E-02	7841	3,59441E-01	8391	1,41524E+00	2274	8,26265E-01	21403	0	11208,5
Responder	Macro_NLRP3	Mono_CD16	HLA-C	LILRA1	6,82161E-03	8846,5	1,76214E-02	3160	4,32711E-01	6010	3,50768E-01	16970	8,99467E-01	6884	0	11208,5
Responder	Macro_ISG15	Mono_CD16	HLA-F	LILRB1	6,82161E-03	14975,5	7,04569E-03	15286	2,29159E-01	14884	3,65737E-01	16529	8,47375E-01	16970	0	11208,5
Responder	Macro_ISG15	Mono_CD14	C3	IFITM1	6,82362E-03	11584,1	9,52637E-03	9238	1,99160E-01	16971	1,05864E+00	4524	8,52149E-01	15979	0	11208,5
Responder	Macro_ISG15	Mono_CD16	HLA-A	LILRB1	6,83569E-03	11393,7	6,59348E-03	16977	2,89894E-01	11397	4,56444E-01	14022	9,18351E-01	3364	0	11208,5
Responder	Macro_FOLR2+APOE+	Mono_CD14	C1QA	CD33	6,83712E-03	7624,1	1,00715E-02	8396	5,20428E-01	4033	8,23970E-01	7205	8,97310E-01	7278	0	11208,5
Responder	Macro_OLFML3	Mono_CD16	APP	FPR2	6,84132E-03	16009,1	1,78580E-02	3090	5,63583E-01	3361	1,98282E-01	22031	7,29297E-01	40355	0	11208,5
Responder	Macro_FOLR2-APOE+	Mono_INHBA	CALR	LRP1	6,84375E-03	11975,7	7,45009E-03	14004	3,29241E-01	9582	3,50384E-01	16981	8,93006E-01	8103	0	11208,5
Responder	Macro_FOLR2-APOE+	Mast	VIM	CD44	6,84576E-03	9246,1	6,59232E-03	16982	5,53021E-01	3520	4,43247E-01	14372	9,45433E-01	148	0	11208,5
Responder	cDC_CLEC9A	pDC_LILRA4	PAM	DPP4	6,84726E-03	15598,7	7,12274E-02	211	8,30848E-01	1095	1,21219E+00	3362	5,17397E-01	62117	0	11208,5
Responder	Macro_FOLR2-APOE+	Macro_FOLR2+APOE-	MMP9	LRP1	6,84977E-03	8254,7	1,81565E-02	2994	6,25445E-01	2535	1,12494E+00	3978	8,30479E-01	20558	0	11208,5

Responder	Macro_FOLR2-APOE+	Mono_INHBA	LGALS9	LRP1	6,84979E-03	14777,9	6,89584E-03	15823	2,35359E-01	14499	3,50147E-01	16984	8,54992E-01	15375	0	11208,5
Responder	cDC_LAMP3	Mast	GNAS	ADRB2	6,85786E-03	6557,1	2,69415E-02	1517	7,44390E-01	1552	9,75428E-01	5343	8,65785E-01	13165	0	11208,5
Responder	Macro_NLRP3	pDC_LILRA4	COPA	P2RY6	6,86510E-03	9266,7	1,69708E-02	3365	5,95148E-01	2896	1,87815E+00	907	7,94281E-01	27957	0	11208,5
Responder	cDC_LAMP3	Mast	ARPC5	ADRB2	6,86594E-03	7112,1	2,36727E-02	1922	6,17620E-01	2636	7,40024E-01	8420	8,75361E-01	11374	0	11208,5
Responder	Macro_NLRP3	cDC_LAMP3	THBS1	CD47	6,86594E-03	9884,7	1,14679E-02	6745	5,14249E-01	4139	6,24430E-01	10339	8,47256E-01	16992	0	11208,5
Responder	Macro_ISG15	Mast	VCAN	CD44	6,86796E-03	12555,7	6,58915E-03	16993	3,83857E-01	7494	4,12054E-01	15215	8,72862E-01	11868	0	11208,5
Responder	cDC_LAMP3	Mast	HSPA8	ADRB2	6,87403E-03	6155,3	2,62222E-02	1601	6,55350E-01	2265	7,22497E-01	8674	8,98637E-01	7028	0	11208,5
Responder	pDC_LILRA4	Mono_INHBA	TNFSF9	HLA-DPA1	6,88148E-03	13570,2	1,66360E-02	3500	4,85758E-01	4697	1,30148E+00	2840	8,91321E-01	8410	0,998	48404
Responder	Macro_NLRP3	cDC_CLEC9A	VCAN	ITGA4	6,88414E-03	8166,1	1,26507E-02	5706	5,78459E-01	3131	1,14666E+00	3784	8,47209E-01	17001	0	11208,5
Responder	cDC_CLEC9A	Mono_CD14	HSPA8	LDLR	6,89022E-03	9111,7	1,30534E-02	5414	3,48368E-01	8796	1,25170E+00	3136	8,47195E-01	17004	0	11208,5
Responder	Macro_FOLR2-APOE+	cDC_CLEC9A	CXCL2	DPP4	6,89101E-03	12885,7	3,26271E-02	1048	6,54184E-01	2275	7,40431E-01	8413	7,22864E-01	41484	0	11208,5
Responder	Macro_FOLR2-APOE+	cDC_LAMP3	CD14	ITGA4	6,89225E-03	11016,9	8,80836E-03	10567	3,80105E-01	7621	3,49535E-01	17005	8,89845E-01	8683	0	11208,5
Responder	Macro_ISG15	Macro_NLRP3	S100A8	CD36	6,90441E-03	10112,7	1,30750E-02	5394	4,59917E-01	5291	3,49303E-01	17011	8,74036E-01	11659	0	11208,5
Responder	pDC_LILRA4	cDC_CLEC9A	QDPR	DYSF	6,90685E-03	12508,7	3,01090E-02	1222	5,62818E-01	3372	1,74503E+00	1182	6,99719E-01	45559	0	11208,5
Responder	cDC(CD1C)	pDC_LILRA4	GSTP1	TRAF2	6,91329E-03	11396,3	1,00601E-02	8420	3,91632E-01	7242	1,79153E+00	1074	7,88676E-01	29037	0	11208,5
Responder	Macro_ISG15	Mono_CD14	ICAM1	ITGAX_ITGB2	6,91863E-03	10591,7	6,58182E-03	17018	3,13740E-01	10254	1,15223E+00	3737	8,78496E-01	10741	0	11208,5
Responder	Mono_INHBA	cDC(CD1C)	NAMPT	INSR	6,91967E-03	9879,9	1,61883E-02	3672	5,96118E-01	2887	7,40007E-01	8422	8,17774E-01	23210	0	11208,5
Responder	Macro_OLFML3	Macro_LYVE1	LILRB4	LAIR1	6,91967E-03	11175,5	1,04971E-02	7818	4,10710E-01	6644	2,04602E-01	21785	8,91259E-01	8422	0	11208,5
Responder	Macro_FOLR2-APOE+	Mono_CD14	VEGFA	CD44	6,93084E-03	13225,7	7,10044E-03	15105	3,57672E-01	8467	4,44720E-01	14324	8,47081E-01	17024	0	11208,5
Responder	Macro_NLRP3	Macro_OLFML3	THBS1	LRP1	6,93287E-03	10007,3	1,40066E-02	4831	5,67180E-01	3314	3,48796E-01	17025	8,63411E-01	13658	0	11208,5
Responder	Macro_OLFML3	cDC_CLEC9A	FAM3C	PDCD1	6,93677E-03	20244,9	3,82347E-02	796	5,62148E-01	3377	1,76709E-01	22823	4,70710E-01	63020	0	11208,5
Responder	Mono_INHBA	Mono_CD16	VEGFA	CD44	6,93695E-03	9116,9	1,36316E-02	5056	6,11120E-01	2709	3,48705E-01	17027	8,84730E-01	9584	0	11208,5
Responder	cDC(CD1C)	pDC_LILRA4	YBX1	NOTCH1	6,93882E-03	10675,9	1,05785E-02	7715	3,58368E-01	8428	1,93932E+00	810	8,08097E-01	25218	0	11208,5
Responder	cDC_CLEC9A	Macro_OLFML3	HLA-DRA	LAG3	6,94306E-03	12544,7	7,81966E-03	12928	2,68675E-01	12541	7,02004E-01	9016	8,47056E-01	17030	0	11208,5
Responder	Macro_IER3	pDC_LILRA4	CCL5	DPP4	6,94876E-03	14793,1	3,37020E-02	997	6,61374E-01	2187	1,20946E+00	3379	6,17074E-01	56194	0	11208,5
Responder	Macro_LYVE1	Mono_INHBA	LGALS9	CD44	6,95122E-03	11689,7	6,57686E-03	17034	4,14903E-01	6534	3,56577E-01	16806	8,99569E-01	6866	0	11208,5
Responder	Mono_CD16	cDC(CD1C)	AGTRAP	RACK1	6,95801E-03	7277,9	1,00503E-02	8434	4,73385E-01	4963	1,19366E+00	3469	8,91792E-01	8315	0	11208,5
Responder	Macro_FOLR2+APOE+	Macro_ISG15	C1QA	CD93	6,96121E-03	7484,7	1,00502E-02	8435	5,81278E-01	3081	8,07152E-01	7451	8,97481E-01	7248	0	11208,5
Responder	Macro_OLFML3	Mono_CD16	CXCL9	FCGR2A	6,96347E-03	12468,1	9,66269E-03	9022	1,98380E-01	17040	4,90617E-01	13174	8,72714E-01	11896	0	11208,5
Responder	Mast	pDC_LILRA4	TGFB1	CXCR4	6,96442E-03	6319,1	1,01118E-02	8336	5,71060E-01	3251	2,31200E+00	364	8,91200E-01	8436	0	11208,5
Responder	Mono_CD14	Mast	VEGFA	CD44	6,96551E-03	11995,7	7,09287E-03	15139	4,26444E-01	6168	6,20258E-01	10422	8,47012E-01	17041	0	11208,5
Responder	pDC_LILRA4	Macro_NLRP3	HMGB1	THBD	6,96755E-03	12171,1	6,57417E-03	17042	2,10094E-01	16145	1,62887E+00	1484	8,56940E-01	14976	0	11208,5
Responder	cDC(CD1C)	Macro_OLFML3	HLA-DQB1	LAG3	6,99008E-03	9906,3	1,00394E-02	8444	4,41398E-01	5775	1,20454E+00	3407	8,29822E-01	20697	0	11208,5
Responder	Macro_FOLR2+APOE-	Macro_LYVE1	C1QB	LRP1	7,00289E-03	5657,5	1,69090E-02	3388	7,84842E-01	1327	5,47279E-01	11800	9,37403E-01	564	0	11208,5
Responder	Macro_FOLR2+APOE+	Macro_OLFML3	ANXA2	TLR2	7,00616E-03	9971,1	1,00306E-02	8449	4,66531E-01	5136	3,34637E-01	17443	8,95592E-01	7619	0	11208,5
Responder	Macro_OLFML3	cDC_CLEC9A	CXCL12	DPP4	7,00892E-03	16497,3	4,17914E-02	677	5,60797E-01	3389	7,31526E-01	8544	5,87752E-01	58668	0	11208,5
Responder	Mono_CD16	Macro_OLFML3	ADAM10	TREM2	7,01059E-03	6918,3	2,30149E-02	2028	6,72511E-01	2089	8,06949E-01	7453	8,73176E-01	11813	0	11208,5
Responder	cDC(CD1C)	Macro_ISG15	HLA-DPB1	CD4	7,01495E-03	10906,3	4,80693E-03	27268	3,07045E-01	10548	1,20696E+00	3390	9,25183E-01	2117	0	11208,5
Responder	Mono_INHBA	Mono_CD14	ARPC5	LDLR	7,01582E-03	10958,1	1,32335E-02	5293	3,75088E-01	7803	7,37696E-01	8452	8,23146E-01	22034	0	11208,5
Responder	Macro_LYVE1	Macro_NLRP3	TIMP2	ITGB1	7,03116E-03	11368,5	1,01596E-02	8278	4,73244E-01	4965	4,07948E-01	15318	8,46847E-01	17073	0	11208,5
Responder	Mono_INHBA	pDC_LILRA4	VEGFA	ITGB1	7,03194E-03	9871,3	1,00272E-02	8457	4,76239E-01	4905	1,82903E+00	1002	8,14998E-01	23784	0	11208,5
Responder	cDC(CD1C)	Mast	HBEGF	CD44	7,03940E-03	11412,1	6,56571E-03	17077	3,92445E-01	7221	7,60139E-01	8110	8,64458E-01	13444	0	11208,5
Responder	pDC_LILRA4	Macro_FOLR2-APOE+	HSP90B1	TLR4	7,04765E-03	10086,5	7,49372E-03	13867	3,97527E-01	7054	1,73308E+00	1222	8,46826E-01	17081	0	11208,5
Responder	Macro_NLRP3	cDC_LAMP3	NAMPT	ADORA2A	7,04940E-03	7555,1	3,91542E-02	763	9,59632E-01	674	8,63657E-01	6680	8,40513E-01	18450	0	11208,5
Responder	Macro_ISG15	Mast	PKM	CD44	7,04971E-03	10103,3	6,56461E-03	17082	4,17935E-01	6423	4,87971E-01	13232	9,22650E-01	2571	0	11208,5
Responder	Mono_CD16	Macro_OLFML3	ICAM2	ITGAL_ITGB2	7,05384E-03	9458,7	2,00110E-02	2534	6,44003E-01	2363	3,78290E-01	16169	8,56707E-01	15019	0	11208,5
Responder	Macro_IFI27	Mono_CD16	MMP9	CD44	7,05590E-03	12587,5	9,55161E-03	9201	3,54715E-01	8566	3,54184E-01	16877	8,46796E-01	17085	0	11208,5
Responder	pDC_LILRA4	Mast	ADAM10	CD44	7,06830E-03	10984,3	6,56196E-03	17091	3,93612E-01	7178	1,20018E+00	3431	8,52015E-01	16013	0	11208,5
Responder	Macro_OLFML3	Macro_OLFML3	MDK	SORL1	7,07037E-03	8923,5	2,96581E-02	1262	5,24051E-01	3968	5,81973E-01	11087	8,46773E-01	17092	0	11208,5
Responder	Macro_ISG15	Macro_LYVE1	CXCL8	SDC3	7,07244E-03	7755,9	1,88247E-02	2806	6,24637E-01	2546	9,97702E-01	5126	8,46769E-01	17093	0	11208,5
Responder	Macro_FOLR2-APOE+	Mono_CD16	LGALS1	ITGB1	7,07657E-03	10957,7	6,90837E-03	15779	4,59956E-01	5289	3,46941E-01	17095	9,07186E-01	5417	0	11208,5
Responder	Mono_INHBA	pDC_LILRA4	CALM1	INSR	7,08046E-03	11475,1	1,09454E-02	7283	4,20539E-01	6338	7,36297E-01	8472	8,13586E-01	24074	0	11208,5
Responder	Macro_FOLR2-APOE+	cDC_CLEC9A	CALR	SCARF1	7,08371E-03	8651,7	2,50643E-02	1731	6,30868E-01	2483	7,36287E-01	8473	8,36204E-01	19363	0	11208,5
Responder	Mono_CD16	cDC_CLEC9A	CXCL2	XCR1	7,08382E-03	8702,5	2,71577E-02	1495	2,34678E+00	18	1,10690E+00	4117	8,00793E-01	26674	0	11208,5
Responder	cDC_CLEC9A	Mono_INHBA	CXCL9	FCGR2A	7,09108E-03	13157,3	7,64944E-03	13404	1,97718E-01	17102	6,69814E-01	9542	8,59162E-01	14530	0	11208,5
Responder	Mono_INHBA	cDC(CD1C)	VCAN	SELL	7,09345E-03	11374,3	1,11356E-02	7068	3,57343E-01	8476	8,02471E-01	7507	8,20474E-01	22612	0	11208,5
Responder	cDC_CLEC9A	pDC_LILRA4	PSEN1	NOTCH4	7,09978E-03	14314,3	2,80284E-02	1414	5,59804E-01	3404	1,69554E+00	1296	6,35396E-01	54249	0	11208,5
Responder	pDC_LILRA4	Mono_INHBA	VEGFB	NRP1	7,09978E-03	12510,3	1,68652E-02	3404	6,03627E-01	2800	2,03179E+00	664	7,06215E-01	44475	0	11208,5
Responder	Macro_IFI27	cDC_CLEC9A	HLA-DPA1	LAG3	7,10560E-03	11816,7	1,10033E-02	7225	3,38137E-01	9209	3,46597E-01	17109	8,60018E-01	14332	0	11208,5
Responder	Mono_INHBA	Mono_CD14	VEGFA	ITGB1	7,10644E-03	10575,9	1,00091E-02	8480	4,75693E-01	4916	1,06536E+00	4468	8,14862E-01	23807	0	11208,5
Responder	cDC_CLEC9A	Macro_OLFML3	HLA-DPB1	CD4	7,11391E-03	8622,1	6,55475E-03	17113	4,01385E-01	6932	8,34099E-01	7080	9,35234E-01	777	0	11208,5
Responder	Macro_ISG15	Macro_FOLR2-APOE+	SPP1	ITGA5_ITGB1	7,11807E-03	7874,7	1,55628E-02	3976	5,27751E-01	3900	1,24380E+00	3174	8,46683E-01	17115	0	11208,5
Responder	Mono_INHBA	Macro_IER3	TNF	TRPM2	7,12272E-03	13104,3	1,57467E-02	3891	4,38432E-01	5856	7,35344E-01	8485	7,52492E-01	36081	0	11208,5
Responder	Macro_IER3	Macro_OLFML3	CCL3L1	CCR1	7,12414E-03	6516,1	2,49976E-02	1743	9,54197E-01	685	1,20403E+00	3408	8,54235E-01	15536	0	11208,5
Responder	Mono_INHBA	Macro_OLFML3	MIF	CD74_CXCR4	7,12431E-03	9258,7	6,55298E-03	17118	5,10873E-01	4199	4,70103E-01	13667	9,47406E-01	101	0	11208,5

Responder	Mono_INHBA	Mono_INHBA	THBS1	CD36	7,12923E-03	9468,7	1,57722E-02	3879	4,93344E-01	4538	7,35235E-01	8487	8,36788E-01	19231	0	11208,5
Responder	Mono_CD16	Mono_INHBA	ADAM10	CD44	7,13902E-03	6866,5	1,32098E-02	5310	7,12556E-01	1755	7,98171E-01	7569	8,90935E-01	8490	0	11208,5
Responder	Macro_FOLR2-APOE+	Macro_NLRP3	SPP1	PTGER4	7,13902E-03	12336,5	1,03512E-02	8014	4,02447E-01	6893	7,35059E-01	8490	7,98512E-01	27077	0	11208,5
Responder	Macro_FOLR2+APOE+	pDC_LILRA4	HLA-DQA1	CD4	7,14855E-03	5456,3	8,94534E-03	10319	5,59363E-01	3412	1,62087E+00	1511	9,34730E-01	831	0	11208,5
Responder	cDC(CD1C)	pDC_LILRA4	HLA-DOB	CD4	7,14881E-03	12447,7	1,00015E-02	8493	3,69312E-01	8004	1,93776E+00	814	7,65044E-01	33719	0	11208,5
Responder	Mono_INHBA	Macro_FOLR2-APOE+	TNF	TRPM2	7,15534E-03	13911,1	1,39843E-02	4842	4,05362E-01	6809	7,34756E-01	8495	7,41274E-01	38201	0	11208,5
Responder	cDC(CD1C)	Macro_NLRP3	HLA-DPB1	CD4	7,16690E-03	10461,9	4,99537E-03	25898	3,22246E-01	9890	1,20264E+00	3415	9,26503E-01	1898	0	11208,5
Responder	cDC_CLEC9A	Mono_CD16	PTPN6	CLEC12A	7,16812E-03	10483,3	1,06679E-02	7610	3,79967E-01	7627	7,12349E-01	8832	8,46580E-01	17139	0	11208,5
Responder	cDC_CLEC9A	Mast	PKM	CD44	7,16812E-03	8861,9	6,54793E-03	17139	4,16009E-01	6488	8,47777E-01	6879	9,22559E-01	2595	0	11208,5
Responder	Macro_IFI27	Macro_NLRP3	A2M	LRP1	7,18277E-03	13281,1	9,77735E-03	8821	2,68703E-01	12538	3,60854E-01	16692	8,46541E-01	17146	0	11208,5
Responder	Macro_IFI27	cDC_CLEC9A	HLA-DRA	LAG3	7,18905E-03	11410,7	1,08896E-02	7349	3,57679E-01	8466	3,45166E-01	17149	8,67298E-01	12881	0	11208,5
Responder	Macro_OLFML3	Macro_IER3	LGALS3BP	ITGB1	7,19324E-03	11690,7	1,09756E-02	7251	4,06693E-01	6772	3,45062E-01	17151	8,51788E-01	16071	0	11208,5
Responder	Macro_FOLR2-APOE+	Macro_OLFML3	SPP1	ITGA4_ITGB1	7,19534E-03	10848,9	1,18604E-02	6387	4,30216E-01	6077	4,80262E-01	13420	8,46482E-01	17152	0	11208,5
Responder	Macro_ISG15	Mono_CD14	FN1	C5AR1	7,19744E-03	12030,7	1,00194E-02	8469	2,80967E-01	11909	5,65097E-01	11414	8,46466E-01	17153	0	11208,5
Responder	cDC_CLEC9A	Mast	POMC	ADRB2	7,19754E-03	13885,5	2,68054E-02	1527	6,88826E-01	1947	1,20187E+00	3420	6,60561E-01	51325	0	11208,5
Responder	cDC_CLEC9A	Mono_CD16	ICAM3	ITGAL	7,20982E-03	8249,1	3,40193E-02	983	8,67973E-01	951	1,20175E+00	3422	8,10658E-01	24681	0	11208,5
Responder	Mono_CD16	cDC(CD1C)	ADAM10	CD44	7,21424E-03	12676,7	7,47757E-03	13918	3,04125E-01	10694	4,87643E-01	13244	8,60061E-01	14319	0	11208,5
Responder	Macro_FOLR2-APOE+	Macro_LYVE1	SPP1	ITGAV_ITGB1	7,22265E-03	9133,5	1,52427E-02	4130	4,97748E-01	4446	7,18977E-01	8718	8,46426E-01	17165	0	11208,5
Responder	Macro_LYVE1	Mono_CD16	F13A1	ITGA4	7,22475E-03	8127,7	1,64445E-02	3581	6,34074E-01	2453	8,99788E-01	6230	8,46424E-01	17166	0	11208,5
Responder	cDC(CD1C)	cDC(CD1C)	HLA-DMB	CD4	7,22475E-03	9949,1	6,54153E-03	17166	2,82258E-01	11849	1,44146E+00	2159	8,96907E-01	7363	0	11208,5
Responder	cDC_LAMP3	pDC_LILRA4	PDCD1LG2	PDCD2	7,22827E-03	13027,7	2,85690E-02	1358	5,58458E-01	3425	1,42568E+00	2234	6,91244E-01	46913	0	11208,5
Responder	Macro_OLFML3	Macro_IER3	C1QB	CD33	7,23739E-03	12899,1	6,54033E-03	17172	3,27523E-01	9663	4,12037E-01	15216	8,76024E-01	11236	0	11208,5
Responder	Mono_CD16	cDC(CD1C)	ICAM2	ITGAM_ITGB2	7,24582E-03	8478,9	1,69641E-02	3366	5,45850E-01	3604	8,31084E-01	7117	8,46754E-01	17099	0	11208,5
Responder	Mono_CD14	Mast	CALM3	KCNQ1	7,24728E-03	13405,5	1,53515E-02	4069	3,55877E-01	8523	7,73708E-01	7906	7,56721E-01	35321	0	11208,5
Responder	Macro_IFI27	Macro_NLRP3	APOE	LDLR	7,24793E-03	9864,9	2,03015E-02	2474	4,90256E-01	4605	3,44058E-01	17177	8,62326E-01	13860	0	11208,5
Responder	Mono_CD14	Mono_CD14	SERPINA1	LRP1	7,24793E-03	11705,5	6,73618E-03	16442	1,96655E-01	17177	9,42072E-01	5716	8,93596E-01	7984	0	11208,5
Responder	cDC(CD1C)	Macro_OLFML3	HLA-DPA1	LAG3	7,25004E-03	10043,5	8,84741E-03	10492	3,89801E-01	7304	1,11738E+00	4035	8,46370E-01	17178	0	11208,5
Responder	Mono_CD16	cDC(CD1C)	B2M	CD1A	7,25215E-03	6671,9	5,33129E-02	394	5,91142E-01	2952	7,84408E-01	7749	8,76868E-01	11056	0	11208,5
Responder	Macro_OLFML3	Mono_CD14	LGALS9	LRP1	7,25215E-03	15185,1	6,65043E-03	16747	2,28859E-01	14910	3,44024E-01	17179	8,52732E-01	15881	0	11208,5
Responder	Macro_IFI27	cDC_CLEC9A	C3	ITGAX	7,25849E-03	12220,3	9,37212E-03	9521	1,96587E-01	17182	6,28036E-01	10285	8,67197E-01	12905	0	11208,5
Responder	pDC_LILRA4	Mono_INHBA	PROC	THBD	7,25908E-03	14032,7	4,98073E-02	477	7,37469E-01	1597	1,20063E+00	3430	6,42539E-01	53451	0	11208,5
Responder	Macro_FOLR2-APOE+	Macro_FOLR2+APOE+	MMP9	EPHB2	7,26049E-03	14522,7	2,58043E-02	1646	6,40657E-01	2394	1,03020E+00	4794	6,50305E-01	52571	0	11208,5
Responder	Macro_ISG15	pDC_LILRA4	VCAN	SELL	7,26271E-03	6977,1	1,61744E-02	3679	6,69474E-01	2118	2,01187E+00	696	8,46343E-01	17184	0	11208,5
Responder	Mono_CD16	cDC(CD1C)	SELPLG	ITGB2	7,26271E-03	8577,7	8,49345E-03	11268	3,92498E-01	7218	9,84409E-01	5258	8,93875E-01	7936	0	11208,5
Responder	Macro_FOLR2-APOE+	pDC_LILRA4	HLA-DMB	CD4	7,26694E-03	10813,7	6,53718E-03	17186	2,21320E-01	15415	1,29409E+00	2890	8,96877E-01	7369	0	11208,5
Responder	Mono_CD14	Macro_FOLR2-APOE+	HP	ITGAM	7,27371E-03	15176,9	6,26663E-02	279	5,81370E-01	3078	7,32261E-01	8531	6,48445E-01	52788	0	11208,5
Responder	cDC(CD1C)	Macro_LYVE1	IL16	CD4	7,27702E-03	10959,7	1,07570E-02	7490	3,99917E-01	6983	7,32247E-01	8532	8,30348E-01	20585	0	11208,5
Responder	Macro_IFI27	Macro_FOLR2+APOE+	APOE	TREM2	7,27761E-03	7289,9	2,01006E-02	2515	5,57914E-01	3433	2,98270E-01	18582	9,35941E-01	711	0	11208,5
Responder	cDC(CD1C)	cDC(CD1C)	HLA-DQA2	CD4	7,27963E-03	11100,5	6,53651E-03	17192	2,03016E-01	16655	1,41451E+00	2276	8,92548E-01	8171	0	11208,5
Responder	Mono_INHBA	Mono_CD14	VIM	CD44	7,28033E-03	8468,9	6,21297E-03	18604	5,33987E-01	3801	7,32202E-01	8533	9,43884E-01	198	0	11208,5
Responder	cDC(CD1C)	Macro_OLFML3	HLA-DPB1	LAG3	7,28175E-03	9247,5	9,31515E-03	9626	4,58444E-01	5331	1,29526E+00	2879	8,46296E-01	17193	0	11208,5
Responder	Mono_INHBA	Macro_LYVE1	TNF	TRPM2	7,28364E-03	13326,1	1,52331E-02	4134	4,28796E-01	6115	7,32148E-01	8534	7,49392E-01	36639	0	11208,5
Responder	Macro_ISG15	Macro_ISG15	CXCL11	CCR5	7,28379E-03	13908,5	5,83358E-02	327	5,57746E-01	3434	1,22263E+00	3307	6,61095E-01	51266	0	11208,5
Responder	Macro_NLRP3	Mast	TIMP2	CD44	7,29235E-03	13076,3	6,53490E-03	17198	3,75430E-01	7788	3,62120E-01	16653	8,68880E-01	12534	0	11208,5
Responder	cDC_CLEC9A	Macro_ISG15	HMGB1	CD163	7,29659E-03	9057,9	6,53430E-03	17200	3,99975E-01	6980	1,00874E+00	5014	9,10022E-01	4887	0	11208,5
Responder	Macro_IER3	Macro_ISG15	CCL3	CCR5	7,30236E-03	7169,7	2,93032E-02	1291	9,79173E-01	630	1,19930E+00	3437	8,36561E-01	19282	0	11208,5
Responder	Macro_NLRP3	Mono_INHBA	VIM	CD44	7,30295E-03	9624,3	6,53367E-03	17203	5,22380E-01	3996	3,99271E-01	15559	9,45203E-01	155	0	11208,5
Responder	Macro_FOLR2+APOE-	Mast	ARPC5	ADRB2	7,30855E-03	5829,3	2,53388E-02	1691	6,63738E-01	2163	1,19888E+00	3438	8,79024E-01	10646	0	11208,5
Responder	Mono_INHBA	Macro_FOLR2-APOE+	THBS1	LRP1	7,30932E-03	11879,7	1,06167E-02	7665	3,52036E-01	8648	4,32585E-01	14671	8,46234E-01	17206	0	11208,5
Responder	Mono_INHBA	Mono_CD16	PTGS2	ALOX5	7,31145E-03	10614,5	1,25746E-02	5770	4,00784E-01	6949	5,40462E-01	11938	8,46228E-01	17207	0	11208,5
Responder	pDC_LILRA4	Macro_OLFML3	HSP90B1	TLR7	7,31357E-03	7357,5	1,53721E-02	4060	5,47623E-01	3581	1,98802E+00	730	8,46222E-01	17208	0	11208,5
Responder	Macro_FOLR2-APOE+	pDC_LILRA4	HSP90B1	TLR7	7,32013E-03	9577,5	1,41873E-02	4716	3,55289E-01	8545	1,00211E+00	5070	8,40930E-01	18348	0	11208,5
Responder	Macro_FOLR2-APOE+	Macro_FOLR2+APOE+	C3	CD81	7,32345E-03	10899,5	1,36700E-02	5034	4,00469E-01	6966	1,62869E-01	23356	8,93902E-01	7933	0	11208,5
Responder	Mono_INHBA	cDC(CD1C)	SDC2	PTPRJ	7,32345E-03	18746,9	1,07054E-02	7560	3,56169E-01	8516	7,31301E-01	8546	5,97573E-01	57904	0	11208,5
Responder	Macro_ISG15	Macro_IER3	SPP1	ITGA4_ITGB1	7,32846E-03	9206,9	1,18078E-02	6426	4,13057E-01	6586	1,05358E+00	4599	8,46193E-01	17215	0	11208,5
Responder	cDC(CD1C)	Macro_IER3	HLA-DQB1	CD4	7,33010E-03	9399,7	6,05076E-03	19400	3,55122E-01	8548	9,95211E-01	5158	9,22091E-01	2684	0	11208,5
Responder	Mono_CD16	cDC(CD1C)	TIMP1	CD63	7,33272E-03	9336,5	6,65094E-03	16745	3,94191E-01	7160	5,75115E-01	11216	9,40552E-01	353	0	11208,5
Responder	Macro_IER3	Macro_IER3	CCL3L1	CCR1	7,33337E-03	7809,3	1,77465E-02	3118	8,65199E-01	962	1,19764E+00	3442	8,31587E-01	20316	0	11208,5
Responder	cDC(CD1C)	Macro_OLFML3	GRN	TNFRSF1A	7,33911E-03	13071,3	6,52860E-03	17220	1,98297E-01	17051	5,79527E-01	11133	8,89504E-01	8744	0	11208,5
Responder	pDC_LILRA4	Macro_IER3	SEMA3C	NRP1_NRP2_PLXND1	7,33959E-03	14425,9	2,81020E-02	1407	5,57407E-01	3443	1,24839E+00	3155	6,47258E-01	52916	0	11208,5
Responder	Mono_INHBA	Mono_INHBA	TGM2	SDC4	7,34009E-03	15207,9	2,97606E-02	1253	5,78887E-01	3126	7,31169E-01	8551	6,55753E-01	51901	0	11208,5
Responder	cDC_CLEC9A	Mono_CD16	SEMA4A	LILRB2	7,34124E-03	8044,5	1,48638E-02	4329	7,09798E-01	1777	9,44944E-01	5687	8,46159E-01	17221	0	11208,5
Responder	Macro_IFI27	Macro_NLRP3	C3	LRP1	7,34337E-03	11926,1	1,18151E-02	6420	2,58331E-01	13136	3,42750E-01	17222	8,74102E-01	11644	0	11208,5
Responder	Macro_IER3	cDC_LAMP3	TNF	TRADD	7,34580E-03	7363,5	3,37988E-02	993	9,43972E-01	722	1,19719E+00	3444	8,30970E-01	20450	0	11208,5
Responder	cDC(CD1C)	pDC_LILRA4	PLAU	IGF2R	7,35675E-03	13575,9	9,94760E-03	8556	4,26317E-01	6171	1,89141E+00	884	7,25084E-01	41060	0	11208,5

Post_R_Myeloid_Myeloid_CellCel

Responder	Macro_FOLR2-APOE+	Macro_FOLR2+APOE+	MMP9	IFNAR1	7,36009E-03	9725,1	1,98118E-02	2568	6,51401E-01	2296	8,42050E-01	6963	8,06334E-01	25590	0	11208,5
Responder	Macro_FOLR2-APOE+	Macro_FOLR2+APOE-	CD59	STAB1	7,37010E-03	9161,5	1,29548E-02	5494	6,04289E-01	2791	7,30403E-01	8560	8,43702E-01	17754	0	11208,5
Responder	cDC_CLEC9A	cDC(CD1C)	CD59	CD2	7,37070E-03	12905,1	2,48660E-02	1756	5,70909E-01	3255	1,19681E+00	3448	7,04032E-01	44858	0	11208,5
Responder	Macro_IFI27	Mono_INHBA	GRN	TNFRSF1B	7,37969E-03	11258,5	6,52284E-03	17239	3,05502E-01	10622	4,93776E-01	13098	9,14130E-01	4125	0	11208,5
Responder	Macro_FOLR2-APOE+	cDC(CD1C)	CD59	CD2	7,39016E-03	14526,7	2,24206E-02	2109	5,12774E-01	4169	7,29794E-01	8566	6,93134E-01	46581	0	11208,5
Responder	Macro_ISG15	Mono_INHBA	GNAI2	C5AR1	7,39683E-03	13479,7	6,52133E-03	17247	2,70696E-01	12440	3,87571E-01	15866	8,79083E-01	10637	0	11208,5
Responder	Macro_IER3	cDC_CLEC9A	CXCL2	DPP4	7,40040E-03	10039,9	5,12698E-02	437	9,21087E-01	782	1,09650E+00	4196	7,65790E-01	33576	0	11208,5
Responder	Macro_FOLR2-APOE+	Macro_ISG15	CD14	TLR4	7,40755E-03	14033,7	7,79753E-03	12992	2,61670E-01	12948	3,41749E-01	17252	8,53221E-01	15768	0	11208,5
Responder	Macro_FOLR2-APOE+	Mono_CD14	GNAI2	FPR1	7,41615E-03	13492,1	6,51947E-03	17256	2,08560E-01	16253	5,64760E-01	11425	8,75633E-01	11318	0	11208,5
Responder	Mono_INHBA	pDC_LILRA4	SDC2	PTPRJ	7,42033E-03	17439,7	1,06074E-02	7672	3,54416E-01	8575	1,54862E+00	1756	5,96466E-01	57987	0	11208,5
Responder	Macro_FOLR2-APOE+	Macro_NLRP3	A2M	LRP1	7,42044E-03	12988,5	1,01550E-02	8282	2,86992E-01	11546	3,41320E-01	17258	8,48986E-01	16648	0	11208,5
Responder	Macro_IFI27	Macro_NLRP3	SERPING1	LRP1	7,42259E-03	13530,3	8,49152E-03	11272	2,43172E-01	13993	3,41307E-01	17259	8,62037E-01	13919	0	11208,5
Responder	Macro_OLFML3	pDC_LILRA4	FAM3C	ADGRG5	7,43376E-03	17779,5	3,69657E-02	846	4,76690E-01	4897	7,28952E-01	8579	4,25583E-01	63367	0	11208,5
Responder	Macro_LYVE1	Mono_CD16	HLA-C	LILRB2	7,43766E-03	7740,3	1,04522E-02	7887	6,64340E-01	2157	3,41042E-01	17266	9,44472E-01	183	0	11208,5
Responder	Macro_OLFML3	Macro_OLFML3	HLA-DPB1	LAG3	7,43766E-03	11513,7	9,26188E-03	9732	4,50645E-01	5534	4,64073E-01	13828	8,45923E-01	17266	0	11208,5
Responder	Macro_FOLR2+APOE+	Mono_CD14	SPP1	CD44	7,44049E-03	7893,5	1,11360E-02	7066	4,77983E-01	4862	7,28824E-01	8581	8,94892E-01	7750	0	11208,5
Responder	Mono_CD14	cDC(CD1C)	RETN	TLR4	7,44049E-03	17905,1	1,07631E-02	7487	3,54279E-01	8581	9,89608E-01	5211	6,07440E-01	57038	0	11208,5
Responder	Macro_NLRP3	Mono_CD16	GNAI2	FPR1	7,44843E-03	10202,7	9,48106E-03	9329	4,55473E-01	5398	3,40923E-01	17271	8,94633E-01	7807	0	11208,5
Responder	Mono_CD16	Mono_INHBA	TNFSF13B	TFRC	7,44843E-03	14570,1	7,01234E-03	15402	2,39450E-01	14235	3,90897E-01	15780	8,51094E-01	16225	0	11208,5
Responder	Macro_FOLR2-APOE+	cDC_CLEC9A	NRG1	HLA-DPB1	7,45058E-03	10453,7	9,92860E-03	8584	4,22983E-01	6276	1,04907E+00	4642	8,25481E-01	21558	0	11208,5
Responder	cDC(CD1C)	Macro_FOLR2-APOE+	HLA-DQB1	CD4	7,46406E-03	9320,5	6,03850E-03	19469	3,54204E-01	8588	1,04904E+00	4644	9,22018E-01	2693	0	11208,5
Responder	Mono_INHBA	Macro_NLRP3	CIRBP	TREM1	7,46786E-03	13094,9	9,76748E-03	8837	2,99176E-01	10939	3,40799E-01	17280	8,46216E-01	17210	0	11208,5
Responder	Macro_ISG15	Macro_FOLR2+APOE+	ICAM1	ITGAL_ITGB2	7,47218E-03	14737,7	6,51264E-03	17282	2,28855E-01	14911	3,44579E-01	17163	8,65976E-01	13124	0	11208,5
Responder	Macro_ISG15	Macro_LYVE1	LGALS3BP	ITGB1	7,47435E-03	12666,3	1,06172E-02	7664	3,04579E-01	10666	3,40634E-01	17283	8,49680E-01	16510	0	11208,5
Responder	Macro_OLFML3	Macro_OLFML3	HLA-DQA1	LAG3	7,47713E-03	11093,3	1,06053E-02	7678	4,88676E-01	4636	5,42845E-01	11901	8,32985E-01	20043	0	11208,5
Responder	cDC(CD1C)	Mono_CD14	HSPA1A	TLR4	7,48300E-03	12082,3	6,51130E-03	17287	2,09866E-01	16165	1,35494E+00	2563	8,65709E-01	13188	0	11208,5
Responder	Macro_ISG15	Macro_LYVE1	SPP1	ITGAV_ITGB5	7,48343E-03	10268,7	1,96232E-02	2610	5,68002E-01	3299	1,19374E+00	3466	7,80148E-01	30760	0	11208,5
Responder	Macro_OLFML3	cDC(CD1C)	IL15RA	AXL	7,48430E-03	14645,7	2,19461E-02	2194	3,53982E-01	8594	7,99338E-01	7550	7,10805E-01	43682	0	11208,5
Responder	Mono_CD16	Mono_INHBA	HBEGF	CD44	7,48733E-03	11069,9	7,78474E-03	13032	4,84907E-01	4713	4,28970E-01	14758	8,74130E-01	11638	0	11208,5
Responder	Mono_INHBA	cDC(CD1C)	APP	RPSA	7,48733E-03	11107,3	6,51085E-03	17289	3,92269E-01	7228	1,24677E+00	3158	8,48969E-01	16653	0	11208,5
Responder	Mono_CD16	Mono_INHBA	SERPINA1	LRP1	7,49166E-03	7092,5	1,06297E-02	7652	5,75930E-01	3171	6,92118E-01	9169	9,13417E-01	4262	0	11208,5
Responder	Macro_LYVE1	Mono_INHBA	CD14	ITGB1	7,50684E-03	11313,5	7,71310E-03	13250	3,86770E-01	7395	3,40041E-01	17298	8,96683E-01	7416	0	11208,5
Responder	Mono_CD16	Mono_INHBA	CIRBP	TREM1	7,50901E-03	10232,5	1,21871E-02	6073	4,49661E-01	5558	4,57212E-01	14007	8,60072E-01	14316	0	11208,5
Responder	Macro_OLFML3	Macro_FOLR2+APOE+	FN1	PLAUR	7,51136E-03	12305,5	1,36084E-02	5065	3,53673E-01	8602	-1,60934E-02	30832	9,04969E-01	5820	0	11208,5
Responder	Macro_IFI27	Mono_CD16	HLA-F	LILRB2	7,51769E-03	11830,9	7,47780E-03	13915	4,72887E-01	4972	3,39760E-01	17303	8,73518E-01	11756	0	11208,5
Responder	Macro_ISG15	Macro_IER3	CD14	TLR4	7,52856E-03	13336,9	6,93941E-03	15674	2,65462E-01	12726	6,56961E-01	9768	8,45769E-01	17308	0	11208,5
Responder	Macro_OLFML3	cDC_CLEC9A	C1QB	CD33	7,53074E-03	11454,1	6,50650E-03	17309	3,26479E-01	9712	7,85279E-01	7745	8,75742E-01	11296	0	11208,5
Responder	pDC_LILRA4	cDC_CLEC9A	GNAS	ADCY7	7,53727E-03	8383,1	9,89736E-03	8627	5,01264E-01	4377	2,27368E+00	391	8,45751E-01	17312	0	11208,5
Responder	Macro_ISG15	Macro_FOLR2-APOE+	SPP1	ITGA4_ITGB1	7,53944E-03	9309,3	1,17274E-02	6507	4,09240E-01	6689	1,02771E+00	4829	8,45748E-01	17313	0	11208,5
Responder	Macro_IFI27	Macro_FOLR2-APOE+	MMP12	PLAUR	7,54380E-03	8650,3	1,73743E-02	3236	4,64409E-01	5197	8,95194E-01	6295	8,45738E-01	17315	0	11208,5
Responder	Macro_LYVE1	cDC(CD1C)	C1QA	CD93	7,54598E-03	12336,3	7,51686E-03	13788	3,30534E-01	9516	3,39207E-01	17316	8,83327E-01	9853	0	11208,5
Responder	Macro_ISG15	Macro_OLFML3	CXCL11	CCR5	7,54868E-03	16430,5	3,43633E-02	966	4,23557E-01	6258	9,20858E-01	5961	5,99544E-01	57759	0	11208,5
Responder	Mono_CD16	Mono_CD14	ADAM10	CD44	7,55034E-03	8128,9	1,08704E-02	7376	5,45867E-01	3602	7,53433E-01	8205	8,81098E-01	10253	0	11208,5
Responder	cDC(CD1C)	Macro_OLFML3	HLA-DRB1	LAG3	7,55252E-03	10512,9	8,40951E-03	11500	3,65658E-01	8152	1,07443E+00	4385	8,45724E-01	17319	0	11208,5
Responder	cDC_CLEC9A	Macro_FOLR2+APOE-	HLA-DOB	CD4	7,55287E-03	8127,9	1,97695E-02	2575	9,07859E-01	820	1,19304E+00	3477	8,20721E-01	22559	0	11208,5
Responder	Macro_OLFML3	cDC_CLEC9A	HLA-DQB2	LAG3	7,55548E-03	12735,7	2,14528E-02	2281	4,52727E-01	5485	7,26661E-01	8615	7,52454E-01	36089	0	11208,5
Responder	cDC_CLEC9A	Macro_OLFML3	LGALS9	CD47	7,55906E-03	14359,9	6,50123E-03	17322	2,26273E-01	15086	5,75185E-01	11214	8,47375E-01	16969	0	11208,5
Responder	Macro_OLFML3	Macro_NLRP3	MDK	NOTCH2	7,57591E-03	9896,5	2,27214E-02	2071	4,67399E-01	5108	7,26386E-01	8621	8,21081E-01	22474	0	11208,5
Responder	Mono_CD16	Mono_CD14	CD55	ADGRE5	7,57872E-03	7187,5	1,51583E-02	4177	7,04803E-01	1814	1,03945E+00	4722	8,61580E-01	14016	0	11208,5
Responder	Mono_CD16	Mono_CD14	HLA-C	LILRA1	7,58309E-03	11021,7	1,08110E-02	7439	3,59008E-01	8412	3,62994E-01	16620	8,75124E-01	11429	0	11208,5
Responder	cDC_LAMP3	Mono_INHBA	RPS19	C5AR1	7,58747E-03	10302,3	7,61728E-03	13488	4,43715E-01	5708	3,38586E-01	17335	9,16106E-01	3772	0	11208,5
Responder	Mono_CD16	Mono_CD14	ICAM2	ITGAM_ITGB2	7,58966E-03	7486,1	1,83600E-02	2931	6,47721E-01	2328	1,01863E+00	4901	8,51814E-01	16062	0	11208,5
Responder	Mono_CD16	Mono_CD14	ICAM2	ITGAL_ITGB2	7,59184E-03	8424,9	1,68177E-02	3433	5,73254E-01	3222	8,43796E-01	6937	8,45701E-01	17324	0	11208,5
Responder	Macro_OLFML3	Macro_LYVE1	LGALS9	COLEC12	7,59727E-03	16803,1	3,04622E-02	1181	5,54867E-01	3484	6,39629E-02	27406	7,27075E-01	40736	0	11208,5
Responder	Macro_IFI27	Macro_IER3	MMP9	CD44	7,59842E-03	11560,7	9,38435E-03	9496	3,44433E-01	8947	5,97219E-01	10812	8,45646E-01	17340	0	11208,5
Responder	cDC_CLEC9A	cDC_CLEC9A	HLA-DQA1	LAG3	7,60280E-03	8827,9	1,27924E-02	5601	4,21684E-01	6313	1,16046E+00	3675	8,45624E-01	17342	0	11208,5
Responder	Mono_CD16	Mono_CD14	SELPLG	ITGB2	7,60499E-03	9883,9	7,97689E-03	12489	3,40958E-01	9079	7,57969E-01	8134	8,90862E-01	8509	0	11208,5
Responder	Macro_ISG15	Macro_OLFML3	SPP1	ITGAV_ITGB1	7,60663E-03	10548,7	1,16426E-02	6574	3,96951E-01	7068	8,49440E-01	6860	8,28086E-01	21033	0	11208,5
Responder	Macro_ISG15	Macro_OLFML3	SPP1	ITGA4_ITGB1	7,61346E-03	10328,3	1,09770E-02	7250	3,85913E-01	7424	8,01817E-01	7517	8,41385E-01	18242	0	11208,5
Responder	Macro_NLRP3	Macro_FOLR2+APOE-	THBS1	CD47	7,61596E-03	9864,5	1,11773E-02	7017	5,06758E-01	4262	6,73010E-01	9487	8,45588E-01	17348	0	11208,5
Responder	Macro_ISG15	Macro_OLFML3	SPP1	PTGER4	7,61689E-03	12058,9	1,04736E-02	7854	3,79487E-01	7647	8,64656E-01	6663	7,99456E-01	26922	0	11208,5
Responder	Macro_NLRP3	Mast	PSEN1	CD44	7,61816E-03	11944,3	8,64722E-03	10916	4,52442E-01	5493	4,29015E-01	14755	8,45587E-01	17349	0	11208,5
Responder	Macro_ISG15	Macro_OLFML3	CCL2	CCR1	7,62031E-03	10874,3	2,24015E-02	2115	5,46948E-01	3589	1,00990E+00	5002	7,71429E-01	32457	0	11208,5
Responder	Macro_IER3	cDC(CD1C)	NRG1	HLA-DPB1	7,62272E-03	6754,5	1,66618E-02	3488	6,32357E-01	2474	1,43370E+00	2203	8,59698E-01	14399	0	11208,5
Responder	Macro_ISG15	Macro_OLFML3	CCL2	CCR5	7,62373E-03	14583,7	2,27800E-02	2062	5,38582E-01	3716	1,09136E+00	4234	6,57489E-01	51698	0	11208,5

Responder	Mono_INHBA	Mono_CD14	PKM	CD44	7,62373E-03	9994,1	6,15770E-03	18841	3,62537E-01	8276	7,25256E-01	8635	9,20336E-01	3010	0	11208,5
Responder	pDC_LILRA4	cDC(CD1C)	APP	LRP10	7,62909E-03	8575,3	1,66600E-02	3489	8,81503E-01	896	2,56422E+00	194	7,98445E-01	27089	0	11208,5
Responder	Mono_CD16	Mono_CD14	S100A9	CD36	7,63134E-03	8589,9	1,40750E-02	4794	5,67749E-01	3304	4,19952E-01	15002	8,90089E-01	8641	0	11208,5
Responder	Macro_OLFML3	Macro_FOLR2+APOE+	FN1	ITGA4_ITGB1	7,63134E-03	10973,1	1,23127E-02	5979	3,49105E-01	8762	5,58318E-01	11561	8,45569E-01	17355	0	11208,5
Responder	Macro_OLFML3	Mono_CD14	HLA-F	LILRB2	7,63354E-03	13974,9	6,78512E-03	16260	2,72936E-01	12333	3,37801E-01	17356	8,68050E-01	12717	0	11208,5
Responder	Macro_FOLR2-APOE+	Macro_OLFML3	LILRB4	LAIR1	7,63744E-03	11747,3	1,02491E-02	8141	3,78651E-01	7677	1,70195E-01	23071	8,90095E-01	8639	0	11208,5
Responder	Macro_NLRP3	cDC(CD1C)	IL1B	SIGIRR	7,63794E-03	8859,5	1,08691E-02	7377	5,13018E-01	4162	1,09738E+00	4192	8,45556E-01	17358	0	11208,5
Responder	Mono_INHBA	Macro_OLFML3	IL1B	SIGIRR	7,64772E-03	10147,5	9,88884E-03	8642	5,22699E-01	3992	7,53002E-01	8215	8,39283E-01	18680	0	11208,5
Responder	Mono_CD16	Mono_CD14	SERPINA1	LRP1	7,65335E-03	7654,9	9,09866E-03	10006	4,99847E-01	4406	8,23551E-01	7213	9,07067E-01	5441	0	11208,5
Responder	Mono_CD16	Mono_CD14	GNAI2	C5AR1	7,65996E-03	8173,3	8,99700E-03	10215	4,76356E-01	4902	8,50868E-01	6842	8,95171E-01	7699	0	11208,5
Responder	Mono_CD16	Mono_CD14	GNAI2	FPR1	7,66217E-03	11682,7	7,32878E-03	14372	2,85340E-01	11653	5,83496E-01	11065	8,81866E-01	10115	0	11208,5
Responder	Macro_ISG15	cDC(CD1C)	TNFSF13B	CD40	7,66437E-03	12463,7	6,50842E-03	17301	2,89470E-01	11419	1,00803E+00	5020	8,45465E-01	17370	0	11208,5
Responder	cDC(CD1C)	Mast	ST6GAL1	CD22	7,66834E-03	16215,3	3,39633E-02	987	4,62081E-01	5240	7,24381E-01	8648	6,28793E-01	54993	0	11208,5
Responder	Macro_FOLR2-APOE+	pDC_LILRA4	TNFSF13B	TFRC	7,66879E-03	13569,9	6,48728E-03	17372	2,08027E-01	16294	9,39794E-01	5743	8,46094E-01	17232	0	11208,5
Responder	Macro_ISG15	cDC(CD1C)	S100A8	CD69	7,67320E-03	14268,3	7,63127E-03	13452	2,47773E-01	13744	3,37016E-01	17374	8,54094E-01	15563	0	11208,5
Responder	Macro_OLFML3	pDC_LILRA4	MDK	SORL1	7,67378E-03	10740,1	1,66430E-02	3496	2,88317E-01	11479	1,54516E+00	1774	8,05438E-01	25743	0	11208,5
Responder	Mono_CD16	Mono_CD14	CIRBP	TREM1	7,67983E-03	8190,7	1,34579E-02	5153	5,00789E-01	4388	8,34565E-01	7076	8,65934E-01	13128	0	11208,5
Responder	Macro_IER3	Mono_CD14	TNF	TNFRSF1A	7,68018E-03	7900,7	1,66409E-02	3497	6,11219E-01	2706	1,37434E+00	2478	8,34989E-01	19614	0	11208,5
Responder	Macro_FOLR2-APOE+	Macro_NLRP3	APOE	SORL1	7,68646E-03	11033,1	1,03156E-02	8056	2,64817E-01	12753	3,36881E-01	17380	9,05182E-01	5768	0	11208,5
Responder	pDC_LILRA4	Macro_IER3	LILRB4	LAIR1	7,69310E-03	11350,1	7,39954E-03	14166	1,93943E-01	17383	1,43974E+00	2169	8,73119E-01	11824	0	11208,5
Responder	Macro_NLRP3	Mono_CD16	HLA-B	LILRB2	7,70860E-03	8501,3	9,27742E-03	9704	5,20167E-01	4041	3,36651E-01	17390	9,44951E-01	163	0	11208,5
Responder	cDC_LAMP3	Mono_INHBA	VIM	CD44	7,71525E-03	9142,9	6,48320E-03	17393	5,13527E-01	4150	5,05584E-01	12801	9,45001E-01	162	0	11208,5
Responder	Macro_ISG15	cDC(CD1C)	SPP1	ITGA4_ITGB1	7,71969E-03	8703,7	1,16618E-02	6559	4,08222E-01	6725	1,58317E+00	1631	8,45381E-01	17395	0	11208,5
Responder	Macro_ISG15	Macro_NLRP3	LGALS3BP	ITGB1	7,72635E-03	12440,5	9,93155E-03	8580	2,71123E-01	12413	5,13574E-01	12603	8,45367E-01	17398	0	11208,5
Responder	Mono_CD16	Macro_FOLR2-APOE+	ADAM10	TREM2	7,72857E-03	9788,3	1,68551E-02	3407	4,68849E-01	5077	4,62724E-01	13855	8,54904E-01	15394	0	11208,5
Responder	Mono_INHBA	Macro_ISG15	VCAN	TLR2	7,72857E-03	10516,5	1,31480E-02	5346	3,65080E-01	8174	6,18632E-01	10455	8,45366E-01	17399	0	11208,5
Responder	Macro_OLFML3	Macro_FOLR2+APOE+	C1QB	LRP1	7,73301E-03	10331,7	8,09949E-03	12191	4,21226E-01	6326	3,36146E-01	17401	9,12005E-01	4532	0	11208,5
Responder	Macro_OLFML3	Macro_ISG15	B2M	LILRB1	7,73746E-03	10459,9	6,48033E-03	17403	3,97771E-01	7046	4,02971E-01	15464	9,31376E-01	1178	0	11208,5
Responder	pDC_LILRA4	cDC_LAMP3	ASIP	MGRN1	7,73792E-03	13788,9	6,40872E-02	259	8,31097E-01	1093	1,18791E+00	3506	6,47599E-01	52878	0	11208,5
Responder	Mono_INHBA	Mono_INHBA	HBEGF	CD9	7,73968E-03	12669,7	1,02386E-02	8165	3,13522E-01	10263	3,73579E-01	16308	8,45340E-01	17404	0	11208,5
Responder	Macro_ISG15	Mono_CD14	HBEGF	CD44	7,74413E-03	12129,9	6,47939E-03	17406	3,21649E-01	9919	7,33259E-01	8513	8,63681E-01	13603	0	11208,5
Responder	Macro_LYVE1	Macro_NLRP3	GRN	TNFRSF1B	7,74635E-03	9688,1	8,00462E-03	12421	4,86611E-01	4677	3,35894E-01	17407	9,21831E-01	2727	0	11208,5
Responder	cDC(CD1C)	Macro_FOLR2+APOE+	ENTPD1	ADORA3	7,75465E-03	11295,3	1,37068E-02	5012	3,51518E-01	8673	7,43058E-01	8367	8,17753E-01	23216	0	11208,5
Responder	Macro_ISG15	Macro_NLRP3	HLA-F	LILRB2	7,75526E-03	14371,9	6,47832E-03	17411	2,40673E-01	14159	3,88888E-01	15826	8,65377E-01	13255	0	11208,5
Responder	cDC(CD1C)	Mono_CD14	VEGFA	CD44	7,75971E-03	11914,1	6,91015E-03	15773	3,49963E-01	8735	8,83709E-01	6441	8,45314E-01	17413	0	11208,5
Responder	Mono_CD16	Macro_FOLR2-APOE+	ICAM2	ITGAM_ITGB2	7,76417E-03	10078,1	1,69722E-02	3364	5,73896E-01	3210	4,00872E-01	15520	8,46785E-01	17088	0	11208,5
Responder	cDC_CLEC9A	cDC(CD1C)	LGALS9	HAVCR2	7,76640E-03	12664,9	6,47682E-03	17416	2,03333E-01	16638	1,28452E+00	2944	8,56187E-01	15118	0	11208,5
Responder	cDC_LAMP3	pDC_LILRA4	PSEN1	NOTCH4	7,77657E-03	14545,1	2,70955E-02	1499	5,53312E-01	3512	1,52822E+00	1815	6,31467E-01	54691	0	11208,5
Responder	cDC_CLEC9A	pDC_LILRA4	HLA-B	CANX	7,77884E-03	9870,7	5,34914E-03	23410	2,53351E-01	13427	1,94298E+00	802	9,38019E-01	506	0	11208,5
Responder	Macro_IFI27	Macro_ISG15	C3	C3AR1	7,77979E-03	12557,3	1,15911E-02	6624	1,93459E-01	17422	3,85763E-01	15934	8,74311E-01	11598	0	11208,5
Responder	pDC_LILRA4	Macro_LYVE1	B2M	TFRC	7,78425E-03	9599,3	7,09464E-03	15134	1,93449E-01	17424	1,35247E+00	2574	9,27985E-01	1656	0	11208,5
Responder	Mono_INHBA	Macro_LYVE1	VEGFA	ITGAV	7,78590E-03	11625,1	1,67828E-02	3447	5,78688E-01	3129	7,21663E-01	8682	7,75466E-01	31659	0	11208,5
Responder	Mast	Macro_NLRP3	PLAT	LRP1	7,78948E-03	7884,9	8,06333E-02	165	8,72122E-01	938	1,18661E+00	3514	8,15866E-01	23599	0	11208,5
Responder	Macro_ISG15	Macro_OLFML3	CXCL10	TLR4	7,79285E-03	7866,5	2,42412E-02	1840	6,24452E-01	2549	1,16982E+00	3616	8,32577E-01	20119	0	11208,5
Responder	Mono_CD14	Mast	VIM	CD44	7,79981E-03	8594,7	6,27093E-03	18339	4,92534E-01	4549	7,21468E-01	8686	9,44130E-01	191	0	11208,5
Responder	Macro_OLFML3	Macro_ISG15	HLA-A	LILRB2	7,81109E-03	8751,1	6,47129E-03	17436	4,82910E-01	4749	6,96528E-01	9103	9,30817E-01	1259	0	11208,5
Responder	Macro_OLFML3	pDC_LILRA4	FLT3LG	FLT3	7,81375E-03	18792,9	1,34728E-02	5140	3,51118E-01	8690	8,64165E-01	6672	5,12157E-01	62254	0	11208,5
Responder	Mono_INHBA	cDC(CD1C)	CD52	SIGLEC10	7,82006E-03	13242,3	6,47054E-03	17440	2,29441E-01	14863	7,78789E-01	7839	8,57457E-01	14861	0	11208,5
Responder	Macro_FOLR2+APOE-	cDC(CD1C)	F13A1	ITGB1	7,82181E-03	6053,9	1,65747E-02	3519	8,88367E-01	875	1,76621E+00	1134	8,63997E-01	13533	0	11208,5
Responder	Macro_ISG15	Mono_INHBA	B2M	TFRC	7,82454E-03	9678,7	6,47031E-03	17442	3,39449E-01	9148	7,40018E-01	8421	9,24845E-01	2174	0	11208,5
Responder	cDC(CD1C)	Macro_LYVE1	CXCL8	SDC3	7,82770E-03	11619,9	1,08258E-02	7423	4,55178E-01	5406	7,21080E-01	8694	8,07347E-01	25368	0	11208,5
Responder	pDC_LILRA4	cDC(CD1C)	NUCB2	ERAP1	7,82829E-03	12575,1	1,65727E-02	3520	5,55227E-01	3476	1,82021E+00	1023	7,11012E-01	43648	0	11208,5
Responder	Mono_CD14	pDC_LILRA4	LGALS1	CD69	7,83352E-03	11123,5	6,46966E-03	17446	2,18001E-01	15608	1,54939E+00	1753	8,84646E-01	9602	0	11208,5
Responder	Macro_OLFML3	Macro_FOLR2+APOE+	TOR2A	ATP5F1B	7,83477E-03	12958,9	1,65713E-02	3521	6,42965E-01	2376	1,63530E-01	23324	8,12166E-01	24365	0	11208,5
Responder	Macro_LYVE1	Macro_FOLR2-APOE+	CD59	STAB1	7,84250E-03	9002,3	1,32487E-02	5279	6,47197E-01	2333	7,17514E-01	8741	8,45175E-01	17450	0	11208,5
Responder	Macro_ISG15	pDC_LILRA4	ICAM1	SPN	7,84774E-03	7811,7	1,65673E-02	3523	6,41026E-01	2392	1,89439E+00	879	8,27972E-01	21056	0	11208,5
Responder	pDC_LILRA4	Macro_OLFML3	HSP90B1	TLR4	7,85825E-03	10400,3	7,30316E-03	14452	3,90428E-01	7277	1,58904E+00	1607	8,45148E-01	17457	0	11208,5
Responder	Mono_CD16	Macro_FOLR2-APOE+	SERPINA1	LRP1	7,86951E-03	8543,1	9,93936E-03	8569	5,41625E-01	3675	4,39296E-01	14506	9,10725E-01	4757	0	11208,5
Responder	pDC_LILRA4	Macro_FOLR2+APOE+	SPN	SIGLEC1	7,87373E-03	13390,5	1,65618E-02	3527	5,53095E-01	3517	1,27927E+00	2976	6,98667E-01	45724	0	11208,5
Responder	Macro_IFI27	Mono_INHBA	C1QB	LRP1	7,87401E-03	9831,7	9,48186E-03	9325	3,75994E-01	7767	3,33925E-01	17464	9,18126E-01	3394	0	11208,5
Responder	Macro_LYVE1	Macro_NLRP3	PSAP	LRP1	7,87627E-03	10475,5	7,17290E-03	14889	4,34029E-01	5983	3,33895E-01	17465	9,21248E-01	2832	0	11208,5
Responder	Macro_LYVE1	pDC_LILRA4	HLA-DQB1	CD4	7,87627E-03	9786,7	6,46524E-03	17465	2,54523E-01	13356	1,04864E+00	4646	9,24438E-01	2258	0	11208,5
Responder	Macro_ISG15	cDC(CD1C)	NRG1	HLA-DPB1	7,88019E-03	8198,5	1,24370E-02	5866	5,42555E-01	3654	1,48999E+00	1961	8,41117E-01	18303	0	11208,5
Responder	Macro_ISG15	cDC(CD1C)	SECTM1	CD7	7,88370E-03	16684,1	1,77371E-02	3119	3,64465E-01	8196	1,04152E+00	4712	6,17160E-01	56185	0	11208,5
Responder	Macro_OLFML3	Macro_LYVE1	PDGFB	LRP1	7,88675E-03	14487,7	1,97338E-02	2583	5,52645E-01	3529	4,65210E-01	13793	7,23701E-01	41325	0	11208,5

Post_R_Myeloid_Myeloid_CellCel

Responder	Macro_ISG15	cDC(CD1C)	CD274	CD80	7,89072E-03	17986,1	1,66780E-02	3479	4,13084E-01	6585	7,44602E-01	8338	5,62405E-01	60320	0	11208,5
Responder	Mono_CD16	Macro_FOLR2-APOE+	CD52	SIGLEC10	7,89207E-03	7784,9	1,10702E-02	7150	7,61861E-01	1466	6,47680E-01	9938	8,87237E-01	9162	0	11208,5
Responder	Mono_CD16	Macro_FOLR2-APOE+	ADAM10	GPNMB	7,90110E-03	9047,1	1,50015E-02	4255	5,69074E-01	3287	5,92585E-01	10910	8,54030E-01	15575	0	11208,5
Responder	Macro_FOLR2-APOE+	cDC(CD1C)	PLAU	PLAUR	7,90110E-03	13022,7	7,78625E-03	13030	2,57105E-01	13212	6,33821E-01	10187	8,45053E-01	17476	0	11208,5
Responder	Macro_ISG15	Macro_FOLR2-APOE+	TNFSF13B	TFRC	7,90789E-03	12717,3	6,46247E-03	17479	3,64271E-01	8204	6,77857E-01	9404	8,45845E-01	17291	0	11208,5
Responder	Macro_FOLR2-APOE+	pDC_LILRA4	HLA-DPB1	CD4	7,92827E-03	8850,9	6,45909E-03	17488	2,88995E-01	11451	1,22667E+00	3282	9,34787E-01	825	0	11208,5
Responder	Macro_ISG15	Macro_OLFML3	TNFSF10	RIPK1	7,92941E-03	14143,1	1,13045E-02	6890	5,09455E-01	4222	7,18478E-01	8723	7,33309E-01	39672	0	11208,5
Responder	Macro_FOLR2-APOE+	pDC_LILRA4	ICAM1	IL2RG	7,93280E-03	9888,5	8,02462E-03	12366	4,20530E-01	6339	1,46858E+00	2039	8,44977E-01	17490	0	11208,5
Responder	cDC(CD1C)	Macro_LYVE1	HEBP1	FPR3	7,94352E-03	12855,5	1,14354E-02	6780	3,50186E-01	8727	8,03846E-01	7494	7,83485E-01	30068	0	11208,5
Responder	cDC_CLEC9A	pDC_LILRA4	CXCL9	DPP4	7,94548E-03	15580	4,13486E-02	691	6,38482E-01	2415	1,18427E+00	3538	6,90098E-01	47092	0,003	24164
Responder	cDC(CD1C)	Macro_OLFML3	COPA	CD74	7,94705E-03	11878,7	4,83765E-03	27043	3,50173E-01	8728	7,23483E-01	8661	9,16201E-01	3753	0	11208,5
Responder	cDC_CLEC9A	pDC_LILRA4	HLA-C	NOTCH4	7,95203E-03	8029,3	2,98595E-02	1242	5,51756E-01	3539	2,12326E+00	561	8,76666E-01	11102	0,002	23702,5
Responder	Macro_ISG15	cDC(CD1C)	SPP1	ITGA5_ITGB1	7,95411E-03	9786,5	1,11331E-02	7071	4,04087E-01	6849	1,51456E+00	1858	8,23659E-01	21946	0	11208,5
Responder	Macro_NLRP3	cDC_CLEC9A	VCAN	SELL	7,95778E-03	7974,5	1,58286E-02	3848	6,22230E-01	2578	1,03739E+00	4737	8,44933E-01	17501	0	11208,5
Responder	Mono_CD16	Macro_ISG15	CD55	ADGRE5	7,96005E-03	7167,9	1,73006E-02	3260	7,62931E-01	1451	8,06216E-01	7462	8,69276E-01	12458	0	11208,5
Responder	Macro_ISG15	cDC(CD1C)	SPP1	PTGER4	7,96117E-03	8576,5	1,49119E-02	4301	4,85517E-01	4703	1,70789E+00	1271	8,26288E-01	21399	0	11208,5
Responder	Mono_CD16	Mono_INHBA	LGALS9	CD44	7,96460E-03	11580,9	6,45458E-03	17504	4,06104E-01	6793	4,05343E-01	15386	8,98718E-01	7013	0	11208,5
Responder	Macro_ISG15	cDC(CD1C)	CCL2	CCR1	7,96471E-03	15194,3	1,03996E-02	7953	3,82568E-01	7543	1,22702E+00	3278	6,96930E-01	45989	0	11208,5
Responder	Macro_FOLR2+APOE+	Macro_NLRP3	RPS19	C5AR1	7,97151E-03	5995,1	1,45480E-02	4516	9,10563E-01	812	5,00225E-01	12923	9,37853E-01	516	0	11208,5
Responder	Macro_FOLR2+APOE-	Mono_INHBA	PLTP	ABCA1	7,97824E-03	6039,5	1,92081E-02	2699	7,48852E-01	1525	1,18318E+00	3543	8,76112E-01	11222	0	11208,5
Responder	Macro_FOLR2+APOE+	Mono_CD14	APOE	ABCA1	7,98594E-03	6496,1	9,82294E-03	8739	6,65235E-01	2143	1,06769E+00	4439	9,04303E-01	5951	0	11208,5
Responder	Mono_CD16	Macro_ISG15	SELPLG	ITGB2	7,98738E-03	12647,1	7,40485E-03	14144	2,83882E-01	11746	3,51032E-01	16967	8,87191E-01	9170	0	11208,5
Responder	Macro_ISG15	cDC(CD1C)	ADM	CALCRL	7,98948E-03	14819,3	1,47552E-02	4378	4,20123E-01	6351	1,46240E+00	2071	6,69927E-01	50088	0	11208,5
Responder	pDC_LILRA4	Macro_IER3	HSP90B1	TLR4	7,99650E-03	10290,7	7,27109E-03	14570	3,89233E-01	7319	1,92095E+00	838	8,44860E-01	17518	0	11208,5
Responder	Mast	pDC_LILRA4	SEMA4A	NRP1_PLXNA4	7,99793E-03	13216,3	1,65240E-02	3546	6,80705E-01	2014	1,25145E+00	3138	6,95887E-01	46175	0	11208,5
Responder	Macro_NLRP3	Mono_CD14	HLA-C	LILRA1	7,99879E-03	13457,9	9,78448E-03	8805	1,92010E-01	17519	3,38712E-01	17331	8,69570E-01	12426	0	11208,5
Responder	Macro_NLRP3	Macro_LYVE1	CCL3	CCR1	8,00335E-03	10978,3	1,05505E-02	7749	4,10530E-01	6650	5,49096E-01	11763	8,44848E-01	17521	0	11208,5
Responder	Mono_CD14	Macro_IFI27	HP	ITGB2	8,01076E-03	9848,5	4,84045E-02	512	4,93430E-01	4536	7,17069E-01	8746	8,12814E-01	24240	0	11208,5
Responder	Macro_FOLR2+APOE-	cDC_CLEC9A	F13A1	ITGB1	8,01108E-03	6110,7	1,65205E-02	3548	8,87150E-01	881	1,67751E+00	1345	8,63804E-01	13571	0	11208,5
Responder	cDC(CD1C)	Macro_FOLR2-APOE+	GRN	TNFRSF1A	8,01707E-03	12906,3	6,44924E-03	17527	1,92604E-01	17472	6,75139E-01	9449	8,88901E-01	8875	0	11208,5
Responder	Macro_FOLR2+APOE+	Mono_CD16	HLA-A	LILRB2	8,02497E-03	5444,5	1,19089E-02	6339	9,02779E-01	835	7,16923E-01	8750	9,48056E-01	90	0	11208,5
Responder	Mono_INHBA	Macro_FOLR2-APOE+	IL1B	IL1R2_IL1RAP	8,02853E-03	11939,5	9,81387E-03	8751	5,36925E-01	3744	9,26988E-01	5892	7,83356E-01	30102	0	11208,5
Responder	Mono_CD16	Macro_ISG15	S100A4	CCR5	8,03080E-03	7296,1	1,84265E-02	2913	8,29324E-01	1106	9,15131E-01	6030	8,55664E-01	15223	0	11208,5
Responder	Mono_INHBA	Macro_LYVE1	LGALS3	ENG	8,03080E-03	13188,1	6,89775E-03	15813	3,57636E-01	8470	5,00722E-01	12916	8,44795E-01	17533	0	11208,5
Responder	Macro_ISG15	pDC_LILRA4	LGALS1	CD69	8,03080E-03	11418,5	6,44750E-03	17533	2,15568E-01	15766	1,28408E+00	2946	8,84471E-01	9639	0	11208,5
Responder	Mono_INHBA	cDC_CLEC9A	MIF	CD44_CD74	8,03564E-03	9296,1	6,10375E-03	19121	3,90753E-01	7268	7,16891E-01	8753	9,46114E-01	130	0	11208,5
Responder	Macro_LYVE1	Macro_NLRP3	CD99	PILRA	8,03767E-03	9942,1	9,87526E-03	8662	5,31636E-01	3834	3,31339E-01	17536	8,91027E-01	8470	0	11208,5
Responder	cDC(CD1C)	Macro_FOLR2+APOE+	TNFSF13B	CD40	8,03767E-03	14109,7	6,99658E-03	15454	1,91716E-01	17536	6,47944E-01	9929	8,50130E-01	16421	0	11208,5
Responder	pDC_LILRA4	Macro_FOLR2-APOE+	CALR	LRP1	8,03767E-03	11009,9	6,44727E-03	17536	2,33122E-01	14654	1,41924E+00	2263	8,85900E-01	9388	0	11208,5
Responder	Macro_FOLR2+APOE+	Macro_IER3	APOE	SORL1	8,04276E-03	6754,9	1,17935E-02	6437	6,18152E-01	2628	7,16837E-01	8755	9,10774E-01	4746	0	11208,5
Responder	Macro_LYVE1	Macro_OLFML3	ADAM10	TREM2	8,04684E-03	10328,5	1,43797E-02	4603	4,67667E-01	5103	4,90135E-01	13188	8,44772E-01	17540	0	11208,5
Responder	Mono_CD16	Macro_ISG15	TIMP1	CD63	8,04914E-03	7615,5	8,67079E-03	10862	6,23023E-01	2568	4,83465E-01	13340	9,47547E-01	99	0	11208,5
Responder	Macro_LYVE1	cDC_LAMP3	ADM	RAMP1	8,04922E-03	15497,1	3,77605E-02	816	9,96170E-01	592	4,59438E-01	13950	6,63907E-01	50919	0	11208,5
Responder	cDC(CD1C)	cDC_CLEC9A	MIF	CD74_CXCR4	8,06380E-03	12254,5	4,58346E-03	29168	2,01214E-01	16811	1,17985E+00	3556	9,37754E-01	529	0	11208,5
Responder	cDC_LAMP3	pDC_LILRA4	SPINT1	ST14	8,07041E-03	12870,5	2,14258E-02	2290	5,49916E-01	3557	1,70121E+00	1286	6,96820E-01	46011	0	11208,5
Responder	Macro_FOLR2-APOE+	Macro_NLRP3	FN1	CD44	8,07843E-03	10110,3	1,27805E-02	5604	3,49067E-01	8765	3,17538E-01	17989	8,98882E-01	6985	0	11208,5
Responder	Macro_FOLR2-APOE+	Macro_LYVE1	APP	LRP1	8,08131E-03	9574,9	1,40040E-02	4832	6,29908E-01	2497	5,48389E-01	11782	8,44716E-01	17555	0	11208,5
Responder	Mono_CD14	Macro_LYVE1	ICAM1	IL2RA	8,08200E-03	15022,1	1,12091E-02	6990	3,49048E-01	8766	7,28170E-01	8591	7,34018E-01	39555	0	11208,5
Responder	Mono_INHBA	Mono_CD16	GNAI2	FPR1	8,09052E-03	11442,3	8,38409E-03	11568	3,70522E-01	7958	3,30594E-01	17559	8,88695E-01	8918	0	11208,5
Responder	Macro_OLFML3	Macro_NLRP3	MDK	SDC4	8,09273E-03	14359,1	2,52286E-02	1710	4,54061E-01	5444	7,16211E-01	8769	7,05083E-01	44664	0	11208,5
Responder	Mono_INHBA	Macro_NLRP3	LGALS1	ITGB1	8,09513E-03	10333,1	6,44145E-03	17561	4,58678E-01	5323	5,56053E-01	11602	9,04197E-01	5971	0	11208,5
Responder	cDC(CD1C)	Macro_NLRP3	VEGFA	CD44	8,09743E-03	13397,9	6,84446E-03	16018	3,42600E-01	9004	4,89668E-01	13197	8,44688E-01	17562	0	11208,5
Responder	cDC(CD1C)	Macro_OLFML3	HLA-DRB5	CD4	8,09974E-03	9672,3	6,44118E-03	17563	3,06440E-01	10583	8,01693E-01	7521	9,29176E-01	1486	0	11208,5
Responder	cDC_CLEC9A	pDC_LILRA4	ADA	DPP4	8,10349E-03	15965,9	3,22838E-02	1072	6,71380E-01	2104	1,17874E+00	3562	5,25630E-01	61883	0	11208,5
Responder	Mono_CD16	Macro_NLRP3	ADAM10	CD44	8,10435E-03	9895,5	1,07670E-02	7482	5,38505E-01	3718	3,59391E-01	16729	8,80596E-01	10340	0	11208,5
Responder	Macro_NLRP3	Mast	PTGS2	ALOX5	8,10666E-03	11059,5	1,22794E-02	6005	4,02422E-01	6895	4,71967E-01	13623	8,44677E-01	17566	0	11208,5
Responder	Macro_FOLR2+APOE+	Mono_CD14	SERPING1	LRP1	8,11421E-03	6595,1	1,40406E-02	4808	6,38167E-01	2419	9,37603E-01	5765	8,89314E-01	8775	0	11208,5
Responder	Macro_OLFML3	Mono_INHBA	PIGF	FLT1	8,11675E-03	16598,9	2,61057E-02	1614	5,49453E-01	3564	5,66014E-01	11391	6,26923E-01	55217	0	11208,5
Responder	Macro_IFI27	Macro_NLRP3	GRN	TNFRSF1B	8,12745E-03	12171,7	6,43740E-03	17575	2,96126E-01	11079	3,57683E-01	16776	9,13611E-01	4220	0	11208,5
Responder	Macro_OLFML3	pDC_LILRA4	PLAU	IGF2R	8,13932E-03	14105,3	9,79533E-03	8782	4,23082E-01	6273	1,29205E+00	2901	7,23544E-01	41362	0	11208,5
Responder	Macro_FOLR2-APOE+	Mono_CD16	HLA-C	LILRA1	8,14292E-03	11280,1	1,66914E-02	3473	3,48704E-01	8783	1,06974E-01	25598	8,96989E-01	7338	0	11208,5
Responder	pDC_LILRA4	Mono_CD14	APP	CD74	8,14292E-03	15236,7	9,79443E-03	8783	3,69966E-01	7983	1,46606E+00	2054	9,35023E-01	800	1	56563,5
Responder	Mono_CD16	Macro_ISG15	AGTRAP	RACK1	8,14365E-03	9598,1	1,08136E-02	7437	5,16883E-01	4092	3,29726E-01	17582	8,95274E-01	7671	0	11208,5
Responder	Macro_FOLR2-APOE+	pDC_LILRA4	HLA-DPA1	CD4	8,15292E-03	8955,7	6,43605E-03	17586	2,80518E-01	11928	1,20651E+00	3395	9,36267E-01	661	0	11208,5
Responder	Mono_CD16	cDC(CD1C)	HMGB1	CXCR4	8,15987E-03	9088,1	6,43517E-03	17589	3,83726E-01	7503	8,67198E-01	6632	9,22948E-01	2508	0	11208,5

Responder	Mono_INHBA	Macro_LYVE1	MIF	CD74_CXCR4	8,16915E-03	9896,7	6,43432E-03	17593	5,21804E-01	4010	3,64730E-01	16560	9,46949E-01	112	0	11208,5
Responder	Macro_OLFML3	Mono_INHBA	PSAP	LRP1	8,17148E-03	10726,7	7,15776E-03	14941	3,97833E-01	7043	3,29098E-01	17594	9,21171E-01	2847	0	11208,5
Responder	Macro_ISG15	Macro_LYVE1	TNFSF12	CD163	8,17380E-03	8768,5	1,35487E-02	5093	7,27816E-01	1659	7,47741E-01	8287	8,44518E-01	17595	0	11208,5
Responder	Macro_IFI27	Macro_NLRP3	HLA-DQA2	CD4	8,17380E-03	13786,1	6,48378E-03	17389	2,35461E-01	14491	3,29083E-01	17595	8,92159E-01	8247	0	11208,5
Responder	Mono_INHBA	cDC_CLEC9A	ICAM1	IL2RG	8,17612E-03	10805,5	7,96867E-03	12511	4,20597E-01	6337	8,87909E-01	6375	8,44518E-01	17596	0	11208,5
Responder	Macro_FOLR2+APOE+	pDC_LILRA4	ADM	CALCRL	8,18325E-03	13483,7	1,64611E-02	3574	5,73427E-01	3217	1,80140E+00	1057	6,81908E-01	48362	0	11208,5
Responder	pDC_LILRA4	Macro_FOLR2-APOE+	APP	TNFRSF21	8,18609E-03	11764,9	1,21101E-02	6140	9,06338E-01	823	2,18676E+00	477	7,30161E-01	40176	0	11208,5
Responder	Macro_ISG15	Mono_INHBA	CD274	CD80	8,19694E-03	17072,5	2,12265E-02	2315	4,71670E-01	5001	7,36079E-01	8476	5,91823E-01	58362	0	11208,5
Responder	Mono_INHBA	Macro_IFI27	TNF	TRPM2	8,19694E-03	15626,5	1,10868E-02	7130	3,50994E-01	8693	7,14367E-01	8798	7,18395E-01	42303	0	11208,5
Responder	Macro_OLFML3	Macro_OLFML3	C3	ITGAM	8,20778E-03	10867,7	1,23136E-02	5978	4,90163E-01	4609	7,14120E-01	8801	8,15233E-01	23742	0	11208,5
Responder	cDC(CD1C)	Macro_NLRP3	FN1	PLAUR	8,21103E-03	11473,5	6,43046E-03	17611	3,61727E-01	8301	8,10038E-01	7401	8,67482E-01	12846	0	11208,5
Responder	Macro_ISG15	Mono_CD14	ICAM1	ITGAM_ITGB2	8,22269E-03	11578,3	6,42955E-03	17616	2,89118E-01	11440	1,06818E+00	4433	8,65667E-01	13194	0	11208,5
Responder	Macro_OLFML3	pDC_LILRA4	LILRB4	LAIR1	8,23671E-03	12630,1	6,42808E-03	17622	1,94000E-01	17380	1,16853E+00	3623	8,65116E-01	13317	0	11208,5
Responder	Macro_IER3	Macro_ISG15	TNF	TNFRSF1B	8,23671E-03	6520,1	1,64430E-02	3582	7,01453E-01	1841	1,18518E+00	3524	8,69361E-01	12445	0	11208,5
Responder	Mono_INHBA	Mono_CD16	B2M	LILRB2	8,23904E-03	9719,1	8,73909E-03	10708	3,42796E-01	8994	3,27957E-01	17623	9,50076E-01	62	0	11208,5
Responder	Macro_OLFML3	cDC_LAMP3	C3	IFITM1	8,24138E-03	12521,7	8,44037E-03	11408	4,73679E-01	4958	3,35864E-01	17410	8,44361E-01	17624	0	11208,5
Responder	Macro_IFI27	cDC_CLEC9A	LGALS9	HAVCR2	8,24372E-03	12878,9	6,42783E-03	17625	2,06963E-01	16362	1,12420E+00	3985	8,55719E-01	15214	0	11208,5
Responder	Mono_CD16	Macro_NLRP3	SERPINA1	LRP1	8,24840E-03	7825,9	1,02626E-02	8125	5,57689E-01	3436	5,46214E-01	11830	9,12018E-01	4530	0	11208,5
Responder	cDC_CLEC9A	cDC(CD1C)	HMGB1	THBD	8,24840E-03	10834,3	6,42774E-03	17627	3,75268E-01	7794	1,40951E+00	2296	8,55553E-01	15246	0	11208,5
Responder	Macro_OLFML3	Macro_LYVE1	FN1	CD44	8,25123E-03	10909,1	1,33031E-02	5243	3,48107E-01	8813	1,82197E-01	22626	9,00689E-01	6655	0	11208,5
Responder	Mono_INHBA	Macro_FOLR2+APOE+	ADAM10	TREM2	8,25776E-03	11331,1	1,42827E-02	4662	4,16109E-01	6485	3,61681E-01	16669	8,44328E-01	17631	0	11208,5
Responder	Mono_CD16	Macro_NLRP3	GNAI2	C5AR1	8,25776E-03	8633,1	1,03411E-02	8027	5,79207E-01	3114	4,44635E-01	14327	9,01526E-01	6489	0	11208,5
Responder	Macro_OLFML3	Macro_LYVE1	B2M	TFRC	8,26352E-03	9594,9	8,06497E-03	12267	5,47458E-01	3586	2,61854E-01	19814	9,32152E-01	1099	0	11208,5
Responder	Macro_FOLR2+APOE+	cDC_CLEC9A	CALM3	MYLK	8,26481E-03	12614,9	4,99710E-02	468	9,04638E-01	827	7,30873E-01	8555	7,20031E-01	42016	0	11208,5
Responder	Macro_OLFML3	Macro_FOLR2-APOE+	HLA-B	CANX	8,26481E-03	15317,3	5,07034E-03	25373	3,61656E-01	8302	-2,14785E-02	31061	9,36444E-01	642	0	11208,5
Responder	Macro_ISG15	Mono_INHBA	SAA1	CD36	8,26576E-03	10921,5	1,96841E-02	2596	5,26543E-01	3917	8,17116E-01	7287	7,85918E-01	29599	0	11208,5
Responder	Macro_OLFML3	Mast	CALM2	KCNQ1	8,26948E-03	10565,1	1,91221E-02	2731	5,06103E-01	4273	3,50426E-01	16977	8,44310E-01	17636	0	11208,5
Responder	Macro_ISG15	Mono_INHBA	CXCL8	SDC2	8,27302E-03	10462,9	1,68748E-02	3400	5,28951E-01	3876	1,05008E+00	4627	7,87846E-01	29203	0	11208,5
Responder	cDC_CLEC9A	Mono_CD14	LGALS9	LRP1	8,27417E-03	13097,1	6,42676E-03	17638	2,08048E-01	16293	1,11933E+00	4019	8,50571E-01	16327	0	11208,5
Responder	Mono_CD16	Macro_NLRP3	CD52	SIGLEC10	8,27651E-03	8259,5	9,75477E-03	8860	7,26012E-01	1667	6,87933E-01	9240	8,80753E-01	10322	0	11208,5
Responder	Mono_INHBA	Macro_FOLR2+APOE+	ADM	GPR84	8,27666E-03	16463,1	1,57014E-02	3915	3,91121E-01	7256	7,13045E-01	8820	6,62419E-01	51116	0	11208,5
Responder	Mono_INHBA	pDC_LILRA4	SEMA4A	NRP1_PLXNA4	8,27666E-03	16831,5	9,77819E-03	8820	5,77831E-01	3142	8,40523E-01	6994	6,37715E-01	53993	0	11208,5
Responder	Mono_CD16	Macro_NLRP3	CIRBP	TREM1	8,27886E-03	9016,5	1,44776E-02	4541	5,41818E-01	3667	4,83528E-01	13334	8,70118E-01	12332	0	11208,5
Responder	Macro_ISG15	Mono_INHBA	SPP1	ITGAV_ITGB1	8,28030E-03	8641,3	1,37942E-02	4959	4,42148E-01	5746	1,32753E+00	2708	8,39823E-01	18585	0	11208,5
Responder	Macro_ISG15	Mono_INHBA	SPP1	ITGA4_ITGB1	8,28757E-03	9354,7	1,13028E-02	6891	3,95433E-01	7111	1,15343E+00	3722	8,43327E-01	17841	0	11208,5
Responder	cDC(CD1C)	Macro_FOLR2+APOE+	HLA-DQB1	CD4	8,29121E-03	9830,7	5,95122E-03	19933	3,47672E-01	8824	8,86001E-01	6406	9,21493E-01	2782	0	11208,5
Responder	Macro_ISG15	Mono_INHBA	SPP1	PTGER4	8,29485E-03	10928,1	1,11042E-02	7108	3,94552E-01	7147	1,24326E+00	3180	8,04102E-01	25997	0	11208,5
Responder	Macro_ISG15	Mono_INHBA	CCL2	CCR1	8,29850E-03	12173,9	1,65382E-02	3538	4,66644E-01	5133	1,23773E+00	3216	7,43583E-01	37774	0	11208,5
Responder	Macro_ISG15	Macro_LYVE1	SPP1	ITGA4_ITGB1	8,31412E-03	9683,9	1,14597E-02	6751	4,00563E-01	6957	9,31383E-01	5848	8,44236E-01	17655	0	11208,5
Responder	Macro_NLRP3	Macro_NLRP3	CIRBP	TREM1	8,31412E-03	11938,1	1,10685E-02	7152	3,66200E-01	8130	3,27116E-01	17655	8,54178E-01	15545	0	11208,5
Responder	Macro_ISG15	Macro_LYVE1	HLA-DMB	CD4	8,31647E-03	12766,7	6,98981E-03	15482	2,65989E-01	12684	3,27111E-01	17656	8,99932E-01	6803	0	11208,5
Responder	cDC_LAMP3	Mast	ACTR2	ADRB2	8,32354E-03	8847,5	2,20035E-02	2185	5,89642E-01	2964	6,31761E-01	10221	8,44228E-01	17659	0	11208,5
Responder	Mono_CD16	cDC_CLEC9A	LGALS9	HAVCR2	8,32590E-03	13291,9	6,52574E-03	17229	2,13932E-01	15874	8,31007E-01	7119	8,56650E-01	15029	0	11208,5
Responder	Macro_ISG15	Macro_FOLR2-APOE+	TNFSF13B	HLA-DPB1	8,32826E-03	10777,7	6,65671E-03	16730	3,66050E-01	8135	3,27056E-01	17661	9,45216E-01	154	0	11208,5
Responder	Macro_NLRP3	pDC_LILRA4	VCAN	ITGA4	8,33297E-03	7941,9	1,20814E-02	6168	5,65993E-01	3327	1,67832E+00	1343	8,44205E-01	17663	0	11208,5
Responder	Macro_ISG15	Mono_INHBA	MDK	SDC2	8,33864E-03	14456,5	2,50541E-02	1733	4,01062E-01	6941	7,44020E-01	8348	7,08570E-01	44052	0	11208,5
Responder	Macro_FOLR2+APOE+	Mast	POMC	ADRB2	8,34390E-03	10684,9	6,49345E-02	252	9,03524E-01	831	1,01670E+00	4919	7,51790E-01	36214	0	11208,5
Responder	cDC(CD1C)	Macro_NLRP3	HMGB1	TLR4	8,34477E-03	14195,7	6,41987E-03	17668	2,06278E-01	16411	6,99721E-01	9050	8,49009E-01	16641	0	11208,5
Responder	Macro_FOLR2+APOE-	cDC(CD1C)	C1QA	CD33	8,34962E-03	7711,9	1,15351E-02	6683	4,44388E-01	5691	7,11330E-01	8840	9,03395E-01	6137	0	11208,5
Responder	Mono_CD16	cDC_CLEC9A	ICAM2	ITGAL_ITGB2	8,35186E-03	7952,1	1,65952E-02	3513	6,24511E-01	2548	1,01310E+00	4964	8,44830E-01	17527	0	11208,5
Responder	Macro_LYVE1	Macro_NLRP3	CXCL12	CXCR4	8,35422E-03	10971,7	1,33912E-02	5179	4,03800E-01	6856	4,59618E-01	13943	8,44170E-01	17672	0	11208,5
Responder	Mono_INHBA	Mono_INHBA	SDC2	PTPRJ	8,36427E-03	17975,9	1,23371E-02	5956	3,85374E-01	7442	7,11190E-01	8844	6,14506E-01	56429	0	11208,5
Responder	Macro_ISG15	Macro_NLRP3	HLA-A	LILRA1	8,36605E-03	16137,3	6,47178E-03	17434	2,00417E-01	16879	3,26620E-01	17677	8,44981E-01	17488	0	11208,5
Responder	pDC_LILRA4	Macro_OLFML3	HLA-F	LILRB1	8,36842E-03	13328,1	6,70384E-03	16552	2,08393E-01	16264	1,01523E+00	4938	8,44131E-01	17678	0	11208,5
Responder	Macro_ISG15	Macro_NLRP3	HBEGF	CD44	8,36842E-03	14028,3	6,41780E-03	17678	3,14286E-01	10227	3,39218E-01	17315	8,63118E-01	13713	0	11208,5
Responder	Macro_NLRP3	Mono_CD14	CCL3	CCR1	8,37078E-03	9839,7	1,04356E-02	7903	4,06665E-01	6773	9,50225E-01	5635	8,44129E-01	17679	0	11208,5
Responder	Mono_CD16	cDC_CLEC9A	TNFSF13B	CD40	8,37078E-03	11787,9	8,39510E-03	11540	2,69090E-01	12523	6,65849E-01	9620	8,61373E-01	14048	0	11208,5
Responder	Macro_IFI27	Macro_ISG15	HLA-DPA1	LAG3	8,37789E-03	10743,5	1,30239E-02	5444	3,98991E-01	7006	3,26513E-01	17682	8,69862E-01	12377	0	11208,5
Responder	cDC(CD1C)	Macro_FOLR2-APOE+	LGALS9	LRP1	8,37789E-03	14347,7	6,41712E-03	17682	1,98423E-01	17034	6,74336E-01	9466	8,50475E-01	16348	0	11208,5
Responder	Macro_NLRP3	Macro_LYVE1	LRPAP1	LRP1	8,38737E-03	11960,5	9,23604E-03	9771	4,29952E-01	6087	3,26428E-01	17686	8,56539E-01	15050	0	11208,5
Responder	Mono_CD14	Mono_CD16	RPS19	C5AR1	8,39448E-03	11401,5	6,74611E-03	16406	3,97790E-01	7044	3,26334E-01	17689	9,11320E-01	4660	0	11208,5
Responder	Mono_CD16	cDC_CLEC9A	HMGB1	HAVCR2	8,39923E-03	11556,1	6,66718E-03	16692	2,53280E-01	13431	7,08335E-01	8895	8,95934E-01	7554	0	11208,5
Responder	Macro_OLFML3	cDC(CD1C)	TNFSF13	TNFRSF1A	8,41586E-03	10890,1	1,07427E-02	7510	3,23323E-01	9850	7,55086E-01	8184	8,44006E-01	17698	0	11208,5
Responder	Macro_OLFML3	Mono_INHBA	HLA-B	LILRB2	8,41586E-03	10306,9	6,41260E-03	17698	4,57106E-01	5367	3,70045E-01	16410	9,34517E-01	851	0	11208,5
Responder	Macro_NLRP3	Macro_ISG15	S100A4	CCR5	8,42062E-03	9176,3	1,53476E-02	4073	6,11983E-01	2701	6,33057E-01	10199	8,44003E-01	17700	0	11208,5

Post_R_Myeloid_Myeloid_CellCel

Responder	Mono_CD16	cDC_CLEC9A	AGTRAP	RACK1	8,42538E-03	11459,9	7,09727E-03	15123	3,05104E-01	10641	7,26151E-01	8623	8,73828E-01	11704	0	11208,5
Responder	Mono_INHBA	Macro_IER3	IL1B	SIGIRR	8,42673E-03	9892,9	9,75464E-03	8861	5,19466E-01	4047	8,81217E-01	6471	8,38360E-01	18877	0	11208,5
Responder	Macro_NLRP3	Mono_INHBA	PKM	CD44	8,43490E-03	10488,9	6,40970E-03	17706	4,13700E-01	6569	4,48409E-01	14228	9,21794E-01	2733	0	11208,5
Responder	Mono_INHBA	Mono_INHBA	COL6A3	CD44	8,43779E-03	12114,3	1,08637E-02	7385	5,06175E-01	4272	7,10233E-01	8864	7,89718E-01	28842	0	11208,5
Responder	Macro_OLFML3	cDC(CD1C)	HLA-DMA	CD4	8,44148E-03	10221,1	6,35247E-03	17990	3,48447E-01	8792	7,10162E-01	8865	9,13478E-01	4250	0	11208,5
Responder	Mono_CD16	cDC_CLEC9A	CALM1	MYLK	8,44327E-03	9581,3	4,42086E-02	609	9,02221E-01	836	9,32744E-01	5826	7,86753E-01	29427	0	11208,5
Responder	cDC(CD1C)	cDC_CLEC9A	FAM3C	PDCD1	8,44597E-03	18672,1	3,47075E-02	954	5,45296E-01	3613	4,41200E-01	14427	4,58672E-01	63158	0	11208,5
Responder	cDC(CD1C)	Macro_ISG15	HLA-DQA1	CD4	8,44597E-03	10473,1	5,33812E-03	23470	3,08392E-01	10484	1,17023E+00	3613	9,17101E-01	3590	0	11208,5
Responder	Mono_CD16	pDC_LILRA4	LGALS9	CD47	8,44682E-03	12377,3	6,75861E-03	16346	2,48316E-01	13707	1,10249E+00	4152	8,49869E-01	16473	0	11208,5
Responder	Macro_FOLR2-APOE+	Macro_ISG15	CCL18	CCR1	8,44886E-03	14543,9	1,25761E-02	5769	3,46751E-01	8867	7,27190E-01	8608	7,40913E-01	38267	0	11208,5
Responder	Mono_CD14	Mono_CD14	ICAM1	ITGAX_ITGB2	8,45159E-03	10815,9	6,40814E-03	17713	3,02003E-01	10810	1,21936E+00	3327	8,77061E-01	11021	0	11208,5
Responder	Mono_INHBA	cDC(CD1C)	TGM2	ITGA4	8,45624E-03	14840,1	1,23591E-02	5940	3,46746E-01	8869	8,49735E-01	6856	7,23697E-01	41327	0	11208,5
Responder	Macro_OLFML3	Macro_LYVE1	MDK	NCL	8,45636E-03	10357,5	1,85195E-02	2885	4,08636E-01	6712	3,25826E-01	17715	8,65314E-01	13267	0	11208,5
Responder	Macro_FOLR2+APOE+	pDC_LILRA4	PLAU	ST14	8,45959E-03	11086,7	1,84748E-02	2900	5,45251E-01	3615	1,44808E+00	2122	7,55166E-01	35588	0	11208,5
Responder	cDC(CD1C)	Macro_NLRP3	CALR	LRP1	8,46113E-03	12163,9	6,40755E-03	17717	2,20415E-01	15472	8,41087E-01	6981	8,85588E-01	9441	0	11208,5
Responder	Macro_LYVE1	pDC_LILRA4	HLA-DQA1	CD4	8,47308E-03	10057,3	6,40710E-03	17722	2,41703E-01	14082	1,01706E+00	4914	9,23780E-01	2360	0	11208,5
Responder	Macro_ISG15	cDC_LAMP3	TNFSF13B	TFRC	8,47786E-03	11995,5	6,40696E-03	17724	3,61995E-01	8292	9,75991E-01	5334	8,45282E-01	17419	0	11208,5
Responder	Macro_OLFML3	Mono_CD16	CXCL10	TLR4	8,48583E-03	11224,3	1,74687E-02	3210	3,46436E-01	8877	7,88292E-01	7700	8,08483E-01	25126	0	11208,5
Responder	Macro_ISG15	Mono_INHBA	PLAU	ITGA5	8,48583E-03	13401,9	1,43952E-02	4590	4,31705E-01	6036	9,02192E-01	6196	7,37208E-01	38979	0	11208,5
Responder	Macro_NLRP3	cDC(CD1C)	HMGB1	CXCR4	8,48743E-03	8729,7	6,40638E-03	17728	3,79989E-01	7626	1,05759E+00	4540	9,22789E-01	2546	0	11208,5
Responder	Mono_CD14	cDC(CD1C)	TNFSF13B	HLA-DPB1	8,48954E-03	8915,5	5,45362E-03	22764	3,46398E-01	8878	1,68090E+00	1334	9,39819E-01	393	0	11208,5
Responder	Mono_CD16	Mono_CD14	HBEGF	CD44	8,49701E-03	13742,3	6,40608E-03	17732	3,18219E-01	10063	3,84232E-01	15977	8,63010E-01	13731	0	11208,5
Responder	Macro_OLFML3	Mono_INHBA	CALR	LRP1	8,49941E-03	11526,9	7,80356E-03	12976	3,68608E-01	8026	3,25288E-01	17733	8,95200E-01	7691	0	11208,5
Responder	Macro_ISG15	cDC(CD1C)	LGALS9	CD47	8,49941E-03	13613,5	6,40606E-03	17733	2,27106E-01	15026	8,44239E-01	6932	8,46419E-01	17168	0	11208,5
Responder	Macro_OLFML3	Macro_FOLR2-APOE+	MDK	LRP1	8,50180E-03	9020,9	2,32566E-02	1989	4,78195E-01	4855	6,82712E-01	9318	8,43792E-01	17734	0	11208,5
Responder	Mono_CD16	pDC_LILRA4	TNFSF13B	TFRC	8,50180E-03	13862,3	6,47241E-03	17429	2,06663E-01	16380	8,37881E-01	7032	8,45945E-01	17262	0	11208,5
Responder	Mono_CD16	pDC_LILRA4	S100A8	CD36	8,50900E-03	11772,5	1,04197E-02	7922	3,59063E-01	8409	3,43503E-01	17197	8,61000E-01	14126	0	11208,5
Responder	Mono_CD16	pDC_LILRA4	S100A9	CD36	8,51140E-03	9588,1	1,06494E-02	7629	4,36519E-01	5912	5,43676E-01	11883	8,75687E-01	11308	0	11208,5
Responder	Macro_NLRP3	Mono_CD14	ICAM1	ITGAX_ITGB2	8,51140E-03	11674,7	6,40462E-03	17738	3,01765E-01	10825	7,97433E-01	7578	8,77031E-01	11024	0	11208,5
Responder	cDC(CD1C)	cDC_LAMP3	ICAM1	IL2RG	8,51380E-03	10915,9	7,87910E-03	12746	4,39052E-01	5841	8,36673E-01	7045	8,43774E-01	17739	0	11208,5
Responder	Mono_CD14	Mono_CD14	ANXA1	FPR1	8,51620E-03	12295,5	6,71470E-03	16512	1,89140E-01	17740	8,66332E-01	6642	8,85968E-01	9375	0	11208,5
Responder	Macro_OLFML3	Macro_OLFML3	SERPINF1	PLXDC2	8,52100E-03	9510,5	1,35509E-02	5092	5,24152E-01	3965	3,25113E-01	17742	8,84956E-01	9545	0	11208,5
Responder	Macro_IFI27	Macro_ISG15	HLA-DRA	LAG3	8,52340E-03	10396,3	1,28893E-02	5530	4,18533E-01	6405	3,25082E-01	17743	8,76704E-01	11095	0	11208,5
Responder	Macro_ISG15	Macro_OLFML3	CD14	TLR4	8,52820E-03	14881,1	6,97002E-03	15562	2,66656E-01	12647	3,25054E-01	17745	8,46055E-01	17243	0	11208,5
Responder	Macro_NLRP3	Mono_CD14	THBS1	ITGA4	8,53542E-03	8856,3	1,24290E-02	5872	5,55771E-01	3470	9,18777E-01	5983	8,43726E-01	17748	0	11208,5
Responder	Macro_OLFML3	Macro_FOLR2-APOE+	MDK	SDC3	8,53780E-03	11554,7	2,66565E-02	1546	4,62734E-01	5228	7,08432E-01	8891	7,79416E-01	30900	0	11208,5
Responder	Mono_CD16	pDC_LILRA4	LGALS1	CD69	8,53782E-03	8491,7	8,02476E-03	12365	3,88780E-01	7334	1,13755E+00	3857	8,95190E-01	7694	0	11208,5
Responder	Macro_LYVE1	cDC_CLEC9A	ANXA1	DYSF	8,54263E-03	10011,1	1,79694E-02	3061	4,99964E-01	4401	4,71729E-01	13634	8,43709E-01	17751	0	11208,5
Responder	Macro_OLFML3	Macro_NLRP3	CXCL10	SDC4	8,54524E-03	14411,1	1,72835E-02	3262	3,45956E-01	8893	8,61930E-01	6702	7,20136E-01	41990	0	11208,5
Responder	Mono_CD16	pDC_LILRA4	HMGB1	THBD	8,55226E-03	6720,5	1,14992E-02	6710	4,81235E-01	4798	1,52010E+00	1838	8,87920E-01	9048	0	11208,5
Responder	Macro_ISG15	Macro_LYVE1	SPP1	CD44	8,55226E-03	12448,3	8,48231E-03	11300	2,83413E-01	11774	3,24716E-01	17755	8,81385E-01	10204	0	11208,5
Responder	Macro_NLRP3	Mast	THBS1	ITGA4	8,55467E-03	9328,1	1,24237E-02	5876	5,55633E-01	3472	7,45221E-01	8328	8,43698E-01	17756	0	11208,5
Responder	Mono_CD14	Macro_IER3	TGFB1	CXCR4	8,55467E-03	14337,9	6,48149E-03	17399	2,68760E-01	12533	3,24699E-01	17756	8,67689E-01	12793	0	11208,5
Responder	Macro_NLRP3	Macro_LYVE1	CXCL8	SDC3	8,55949E-03	8334,9	1,79593E-02	3063	6,06305E-01	2770	8,48163E-01	6875	8,43691E-01	17758	0	11208,5
Responder	pDC_LILRA4	Macro_LYVE1	ADAM10	GPNMB	8,56672E-03	7653,1	1,27641E-02	5618	6,61922E-01	2178	1,62383E+00	1500	8,43672E-01	17761	0	11208,5
Responder	Mono_CD16	pDC_LILRA4	HMGB1	CXCR4	8,57396E-03	7473,3	6,72348E-03	16482	4,20286E-01	6344	1,78440E+00	1087	9,24492E-01	2245	0	11208,5
Responder	Macro_OLFML3	Macro_NLRP3	C3	IFITM1	8,57637E-03	12349,3	8,34970E-03	11630	4,72122E-01	4990	3,78739E-01	16153	8,43650E-01	17765	0	11208,5
Responder	Mono_CD16	pDC_LILRA4	S100A8	CD69	8,57879E-03	11052,5	9,63835E-03	9059	2,91719E-01	11311	5,88975E-01	10968	8,68050E-01	12716	0	11208,5
Responder	Macro_FOLR2+APOE+	Macro_NLRP3	APP	APLP2	8,57879E-03	12869,1	8,49330E-03	11270	3,76570E-01	7749	3,72162E-01	16352	8,43649E-01	17766	0	11208,5
Responder	Macro_OLFML3	Macro_FOLR2-APOE+	HLA-DRA	CD4	8,58335E-03	12266,7	5,32170E-03	23570	3,53915E-01	8598	3,46433E-01	17114	9,34574E-01	843	0	11208,5
Responder	Mono_INHBA	Macro_IER3	CCL4	CCR1	8,58626E-03	9724,3	9,72760E-03	8904	5,10992E-01	4194	1,06108E+00	4506	8,34046E-01	19809	0	11208,5
Responder	Mono_INHBA	cDC(CD1C)	LGALS1	ITGB1	8,58626E-03	12361,9	4,93916E-03	26307	3,45664E-01	8904	8,30326E-01	7126	8,92062E-01	8264	0	11208,5
Responder	Macro_ISG15	pDC_LILRA4	AGTRAP	RACK1	8,58845E-03	10836,7	6,39900E-03	17770	2,88341E-01	11477	1,83026E+00	1000	8,68007E-01	12728	0	11208,5
Responder	Mono_CD16	Macro_IFI27	ADAM10	TREM2	8,59570E-03	10254,7	1,61664E-02	3682	4,46078E-01	5647	4,28219E-01	14781	8,52297E-01	15955	0	11208,5
Responder	Macro_ISG15	Mono_CD14	TNFSF10	TNFRSF10B	8,59747E-03	10044,3	1,41196E-02	4759	5,85880E-01	3022	1,11635E+00	4046	7,97993E-01	27186	0	11208,5
Responder	Macro_IER3	Mast	HSPA8	ADRB2	8,60121E-03	6007,7	2,75470E-02	1444	6,99887E-01	1854	7,07652E-01	8908	9,00860E-01	6624	0	11208,5
Responder	cDC_CLEC9A	Macro_IER3	HLA-DOB	CD4	8,60296E-03	7024,3	2,74512E-02	1455	1,03458E+00	503	1,09902E+00	4179	8,43615E-01	17776	0	11208,5
Responder	cDC(CD1C)	Macro_NLRP3	PLAU	PLAUR	8,60538E-03	10418,1	7,61724E-03	13489	5,31283E-01	3839	9,36224E-01	5777	8,43611E-01	17777	0	11208,5
Responder	Macro_IER3	Macro_NLRP3	ICAM1	ITGAX_ITGB2	8,61243E-03	7747,9	1,01241E-02	8320	5,56884E-01	3453	7,07568E-01	8911	8,99670E-01	6847	0	11208,5
Responder	Macro_FOLR2-APOE+	Macro_ISG15	SPP1	ITGA4_ITGB1	8,61992E-03	11180,9	9,72431E-03	8913	3,65933E-01	8138	7,92545E-01	7639	8,33131E-01	20006	0	11208,5
Responder	Mono_CD16	Macro_NLRP3	ICAM2	ITGAM_ITGB2	8,62233E-03	10068,7	1,61563E-02	3688	5,56892E-01	3452	4,49001E-01	14211	8,43561E-01	17784	0	11208,5
Responder	Macro_FOLR2+APOE+	Mono_INHBA	APOE	LDLR	8,62411E-03	6866,3	1,83161E-02	2950	7,71138E-01	1396	1,16644E+00	3639	8,56103E-01	15138	0	11208,5
Responder	Mono_CD16	Mono_CD14	VCAN	SELL	8,62718E-03	9171,7	1,54995E-02	4000	5,03940E-01	4321	7,31580E-01	8543	8,43551E-01	17786	0	11208,5
Responder	Mono_INHBA	Macro_FOLR2+APOE+	ICAM1	ITGAM_ITGB2	8,62961E-03	11639,3	8,61190E-03	11008	3,67979E-01	8058	3,24001E-01	17787	8,81770E-01	10135	0	11208,5
Responder	cDC(CD1C)	Macro_NLRP3	HLA-DQA1	CD4	8,63101E-03	10040,9	5,54738E-03	22191	3,23592E-01	9836	1,16591E+00	3640	9,18551E-01	3329	0	11208,5

Responder	Macro_ISG15	Mono_INHBA	NAMPT	ADORA2A	8,63117E-03	12112,7	1,66650E-02	3487	4,66555E-01	5134	7,07327E-01	8916	7,74684E-01	31818	0	11208,5
Responder	Macro_LYVE1	cDC(CD1C)	CD99	PILRA	8,63203E-03	13018,3	6,39461E-03	17788	3,25795E-01	9738	4,71139E-01	13647	8,68069E-01	12710	0	11208,5
Responder	Macro_FOLR2+APOE+	Macro_NLRP3	PLAU	PLAUR	8,63867E-03	6754,9	1,67631E-02	3454	7,85029E-01	1326	7,07253E-01	8918	8,88917E-01	8868	0	11208,5
Responder	cDC(CD1C)	Macro_OLFML3	APP	CD74	8,63867E-03	12484,9	4,59564E-03	29063	3,45130E-01	8918	7,70908E-01	7948	9,07894E-01	5287	0	11208,5
Responder	pDC_LILRA4	Mast	ACTR2	ADRB2	8,65172E-03	7482,5	1,96851E-02	2595	5,43527E-01	3643	1,98873E+00	728	8,36765E-01	19238	0	11208,5
Responder	Macro_FOLR2+APOE+	Mono_CD16	B2M	LILRB2	8,65692E-03	6825,9	1,02466E-02	8145	7,66193E-01	1427	4,83804E-01	13327	9,53718E-01	22	0	11208,5
Responder	Mono_CD16	Macro_IFI27	CD52	SIGLEC10	8,65876E-03	10218,1	7,50811E-03	13816	6,64782E-01	2151	5,95883E-01	10843	8,66307E-01	13072	0	11208,5
Responder	Macro_NLRP3	cDC_CLEC9A	THBS1	ITGA4	8,66362E-03	8530,9	1,23869E-02	5911	5,54811E-01	3485	1,09013E+00	4249	8,43502E-01	17801	0	11208,5
Responder	Macro_OLFML3	Macro_IER3	HLA-DQA1	CD4	8,66849E-03	11152,1	6,39186E-03	17803	4,02401E-01	6898	3,33520E-01	17477	9,23696E-01	2374	0	11208,5
Responder	Mono_CD14	Macro_OLFML3	COPA	CD74	8,67249E-03	12073,1	4,75301E-03	27695	3,44877E-01	8927	7,23144E-01	8663	9,15521E-01	3872	0	11208,5
Responder	cDC_CLEC9A	Macro_FOLR2-APOE+	HLA-DOB	CD4	8,67580E-03	6942,7	2,73956E-02	1468	1,03367E+00	504	1,15285E+00	3727	8,43481E-01	17806	0	11208,5
Responder	Macro_OLFML3	Macro_ISG15	HLA-DQB2	LAG3	8,67625E-03	11826,3	2,53924E-02	1686	5,13581E-01	4147	7,06577E-01	8928	7,67820E-01	33162	0	11208,5
Responder	Macro_ISG15	Mono_CD14	SAA1	FPR1	8,68378E-03	9596,9	1,56199E-02	3951	4,33216E-01	5996	1,14098E+00	3829	8,18692E-01	23000	0	11208,5
Responder	pDC_LILRA4	cDC(CD1C)	HMGB1	CXCR4	8,68555E-03	7920,5	6,39067E-03	17810	3,77951E-01	7702	2,37021E+00	322	9,22701E-01	2560	0	11208,5
Responder	cDC(CD1C)	Mono_CD14	HLA-DRA	CD4	8,68633E-03	16042,9	3,35680E-03	41228	1,51305E-01	20869	1,16444E+00	3648	9,18995E-01	3261	0	11208,5
Responder	Macro_ISG15	Mono_CD14	SAA1	FPR2	8,68755E-03	13776,1	2,41671E-02	1856	4,82342E-01	4769	9,57743E-01	5549	7,00064E-01	45498	0	11208,5
Responder	Macro_ISG15	Macro_ISG15	HLA-B	LILRB1	8,68799E-03	10397,5	6,39059E-03	17811	3,00689E-01	10877	6,80192E-01	9372	9,21869E-01	2719	0	11208,5
Responder	Macro_ISG15	Macro_NLRP3	SPP1	ITGA4_ITGB1	8,69042E-03	9417,7	1,13269E-02	6871	3,96476E-01	7082	1,10699E+00	4115	8,43467E-01	17812	0	11208,5
Responder	Mono_INHBA	Mono_INHBA	PIGF	FLT1	8,69508E-03	16347,1	2,43929E-02	1821	5,36299E-01	3753	7,06246E-01	8933	6,18953E-01	56020	0	11208,5
Responder	Macro_ISG15	Mono_CD14	SPP1	ITGA5_ITGB1	8,69508E-03	9792,9	1,10016E-02	7227	4,15161E-01	6523	1,50796E+00	1889	8,22795E-01	22117	0	11208,5
Responder	Macro_IFI27	Macro_NLRP3	GNAI2	C5AR1	8,69530E-03	13356,1	6,39009E-03	17814	3,13554E-01	10261	3,61874E-01	16665	8,77998E-01	10832	0	11208,5
Responder	pDC_LILRA4	pDC_LILRA4	ICAM1	IL2RG	8,69775E-03	9772,9	7,83965E-03	12873	4,11519E-01	6627	2,34326E+00	341	8,43443E-01	17815	0	11208,5
Responder	cDC_LAMP3	Mono_CD16	RPS19	C5AR1	8,70019E-03	8996,1	9,10780E-03	9991	5,59560E-01	3408	3,22835E-01	17816	9,22723E-01	2557	0	11208,5
Responder	Macro_FOLR2-APOE+	Macro_ISG15	APP	RPSA	8,70751E-03	12700,7	7,77579E-03	13066	3,96662E-01	7075	3,22759E-01	17819	8,60002E-01	14335	0	11208,5
Responder	Macro_ISG15	Mono_CD14	VCAN	SELL	8,71395E-03	9958,9	1,15465E-02	6672	4,19208E-01	6383	1,18973E+00	3494	8,23127E-01	22037	0	11208,5
Responder	Macro_FOLR2-APOE+	Macro_FOLR2+APOE+	MMP9	ITGAM	8,71395E-03	15675,9	1,03037E-02	8070	5,14539E-01	4132	7,05982E-01	8938	6,96724E-01	46031	0	11208,5
Responder	Macro_OLFML3	Macro_FOLR2+APOE-	MDK	LRP1	8,72150E-03	10199,3	1,89655E-02	2767	3,87059E-01	7390	7,05949E-01	8940	8,29874E-01	20691	0	11208,5
Responder	cDC(CD1C)	Macro_FOLR2-APOE+	RTN4	GJB2	8,72150E-03	14829,1	1,85084E-02	2891	3,44588E-01	8940	8,10023E-01	7402	7,10671E-01	43704	0	11208,5
Responder	Mono_CD16	pDC_LILRA4	THBS1	CD47	8,72708E-03	9729,9	1,08101E-02	7442	4,07338E-01	6744	9,69454E-01	5428	8,43394E-01	17827	0	11208,5
Responder	Mono_CD14	Mono_CD14	THBS1	LRP1	8,73198E-03	10338,7	1,01664E-02	8269	3,30061E-01	9541	1,02563E+00	4846	8,43393E-01	17829	0	11208,5
Responder	Macro_FOLR2-APOE+	Mono_CD14	SPP1	ITGA4_ITGB1	8,73284E-03	10660,3	9,70570E-03	8943	3,65684E-01	8149	1,01324E+00	4963	8,32998E-01	20038	0	11208,5
Responder	Macro_ISG15	Mono_INHBA	CD14	TLR4	8,73442E-03	13859,3	6,69251E-03	16597	2,55824E-01	13285	6,22743E-01	10376	8,43391E-01	17830	0	11208,5
Responder	Macro_FOLR2+APOE-	Macro_LYVE1	CD59	STAB1	8,73492E-03	6166,9	1,96387E-02	2606	8,82161E-01	893	1,16279E+00	3655	8,69217E-01	12472	0	11208,5
Responder	cDC(CD1C)	Mono_INHBA	NAMPT	ITGA5_ITGB1	8,73687E-03	12874,5	6,38566E-03	17831	2,96833E-01	11043	7,37320E-01	8459	8,52965E-01	15831	0	11208,5
Responder	cDC(CD1C)	Mono_INHBA	CXCL8	SDC2	8,74041E-03	15056,5	9,70442E-03	8945	3,59491E-01	8390	7,73454E-01	7914	7,37956E-01	38825	0	11208,5
Responder	Mono_INHBA	cDC_CLEC9A	LGALS1	ITGB1	8,74420E-03	12654,9	4,92298E-03	26433	3,44447E-01	8946	7,41621E-01	8392	8,91904E-01	8295	0	11208,5
Responder	Mono_CD16	Macro_ISG15	HLA-C	LILRB1	8,74668E-03	11434,3	6,88999E-03	15848	3,39751E-01	9131	3,21947E-01	17835	9,19595E-01	3149	0	11208,5
Responder	Macro_NLRP3	cDC_CLEC9A	NAMPT	ITGA5_ITGB1	8,74668E-03	12503,9	6,38466E-03	17835	4,27125E-01	6152	5,61742E-01	11491	8,52955E-01	15833	0	11208,5
Responder	pDC_LILRA4	Macro_LYVE1	HSP90B1	TLR2	8,75158E-03	10915,5	6,38457E-03	17837	3,02804E-01	10752	1,58318E+00	1630	8,65822E-01	13150	0	11208,5
Responder	Macro_FOLR2+APOE+	Macro_IER3	APOE	LDLR	8,75580E-03	5359,5	2,97988E-02	1251	8,88381E-01	874	1,16262E+00	3658	8,83565E-01	9806	0	11208,5
Responder	Macro_NLRP3	cDC_CLEC9A	CCL20	CXCR3	8,76974E-03	12963,7	4,54604E-02	575	7,86433E-01	1320	1,16255E+00	3660	6,83931E-01	48055	0	11208,5
Responder	Macro_ISG15	Mono_CD14	CD38	PECAM1	8,77452E-03	14460,7	1,61528E-02	3692	4,08109E-01	6729	7,53625E-01	8199	7,17500E-01	42475	0	11208,5
Responder	Mono_INHBA	Macro_IER3	VCAN	ITGA4	8,77614E-03	11057,7	1,19145E-02	6335	4,17027E-01	6454	4,79323E-01	13444	8,43288E-01	17847	0	11208,5
Responder	Macro_FOLR2-APOE+	Macro_NLRP3	PKM	CD44	8,78844E-03	10276,7	7,03129E-03	15341	4,78276E-01	4852	3,21484E-01	17852	9,25065E-01	2130	0	11208,5
Responder	cDC_CLEC9A	Macro_OLFML3	HLA-DRB5	CD4	8,79091E-03	10564,3	6,38217E-03	17853	2,96718E-01	11045	5,76354E-01	11194	9,28873E-01	1521	0	11208,5
Responder	Mono_CD14	Macro_FOLR2+APOE+	HP	CD163	8,79351E-03	9553,7	7,77076E-02	184	8,08367E-01	1211	7,04802E-01	8959	8,03123E-01	26206	0	11208,5
Responder	Mono_INHBA	Mono_INHBA	HSP90B1	LRP1	8,79583E-03	13905,9	6,38170E-03	17855	2,15457E-01	15774	4,45279E-01	14307	8,80343E-01	10385	0	11208,5
Responder	Mono_CD14	Macro_FOLR2+APOE+	CD14	ITGB2	8,79829E-03	12025,1	6,38168E-03	17856	2,63483E-01	12835	3,71838E-01	16362	9,26713E-01	1864	0	11208,5
Responder	Macro_FOLR2-APOE+	Mono_CD16	MRC1	PTPRC	8,80322E-03	8796,5	1,74573E-02	3215	6,86190E-01	1965	6,59091E-01	9736	8,43239E-01	17858	0	11208,5
Responder	Macro_FOLR2-APOE+	pDC_LILRA4	C3	IFITM1	8,80322E-03	11565,7	9,24825E-03	9753	1,87549E-01	17858	1,34397E+00	2614	8,50273E-01	16395	0	11208,5
Responder	Mono_CD16	Macro_ISG15	CD55	ADGRE2	8,81062E-03	10406,9	1,37128E-02	5009	6,23175E-01	2566	4,05182E-01	15390	8,43221E-01	17861	0	11208,5
Responder	Mono_CD16	Mono_CD14	HMGB1	THBD	8,81062E-03	15509,1	6,38093E-03	17861	2,02870E-01	16666	3,68253E-01	16460	8,55101E-01	15350	0	11208,5
Responder	Macro_LYVE1	Mono_INHBA	C1QB	LRP1	8,81309E-03	8876,5	1,06266E-02	7656	4,71039E-01	5022	3,21203E-01	17862	9,22309E-01	2634	0	11208,5
Responder	Macro_IFI27	Macro_LYVE1	HLA-DQB1	CD4	8,81802E-03	9743,9	7,97410E-03	12496	4,34058E-01	5982	3,21160E-01	17864	9,31445E-01	1169	0	11208,5
Responder	Macro_OLFML3	Mono_INHBA	HLA-A	APLP2	8,82610E-03	10856,9	5,54581E-03	22200	4,26061E-01	6179	4,63152E-01	13842	9,34492E-01	855	0	11208,5
Responder	Macro_OLFML3	Macro_FOLR2-APOE+	HLA-DQA1	CD4	8,82790E-03	10853,9	6,37890E-03	17868	4,01483E-01	6930	3,87354E-01	15872	9,23625E-01	2391	0	11208,5
Responder	cDC_CLEC9A	Macro_IER3	CD59	STAB1	8,83284E-03	8155,1	1,28482E-02	5562	5,99118E-01	2844	1,22482E+00	3291	8,43156E-01	17870	0	11208,5
Responder	Mono_INHBA	Mono_CD16	HLA-B	LILRB2	8,83778E-03	9403,1	8,69603E-03	10810	4,02394E-01	6900	3,21026E-01	17872	9,43243E-01	225	0	11208,5
Responder	Macro_ISG15	Mono_INHBA	RARRES2	CCRL2	8,83921E-03	16992,5	1,67067E-02	3468	4,19792E-01	6368	7,04292E-01	8971	6,29282E-01	54947	0	11208,5
Responder	Macro_FOLR2+APOE+	Macro_OLFML3	C1QB	CD33	8,83964E-03	6100,3	1,61944E-02	3670	6,80511E-01	2019	6,40228E-01	10081	9,17484E-01	3523	0	11208,5
Responder	Macro_LYVE1	cDC_CLEC9A	CALM2	MYLK	8,84647E-03	9386,1	4,65947E-02	548	8,93360E-01	856	8,76388E-01	6522	7,95058E-01	27796	0	11208,5
Responder	pDC_LILRA4	Macro_LYVE1	SEMA3C	NRP2_PLXNA1	8,84665E-03	14513,3	3,65360E-02	867	6,22035E-01	2582	1,16099E+00	3671	6,35520E-01	54238	0	11208,5
Responder	Mono_CD16	Macro_LYVE1	CD52	SIGLEC10	8,84768E-03	11800,1	6,78952E-03	16231	6,45198E-01	2353	4,21381E-01	14957	8,60373E-01	14251	0	11208,5
Responder	Macro_FOLR2-APOE+	Mono_CD14	CALR	LRP1	8,84768E-03	13076,1	6,37704E-03	17876	2,53158E-01	13435	4,81817E-01	13377	8,85346E-01	9484	0	11208,5
Responder	Macro_ISG15	Macro_IER3	TNFSF13B	CD40	8,85263E-03	14347,7	6,37642E-03	17878	2,83560E-01	11767	4,89297E-01	13204	8,44121E-01	17681	0	11208,5

Responder	cDC(CD1C)	pDC_LILRA4	VCAN	SELL	8,85367E-03	11487,9	7,82622E-03	12911	5,41729E-01	3672	1,64228E+00	1440	7,93020E-01	28208	0	11208,5
Responder	Mono_INHBA	Mono_CD14	FABP5	RXRA	8,86595E-03	12395,5	1,04537E-02	7883	3,43450E-01	8978	8,72975E-01	6556	7,97209E-01	27352	0	11208,5
Responder	Macro_NLRP3	Mono_INHBA	THBS1	SDC4	8,86687E-03	7823,9	4,12833E-02	695	8,93336E-01	857	1,12111E+00	4004	8,21799E-01	22355	0	11208,5
Responder	Mono_CD16	Macro_LYVE1	ADAM10	GPNMB	8,86997E-03	5868,7	2,39491E-02	1885	9,16903E-01	794	9,96006E-01	5147	8,80845E-01	10309	0	11208,5
Responder	cDC(CD1C)	cDC(CD1C)	GRN	TNFRSF1A	8,86997E-03	11826,7	6,37457E-03	17885	1,87248E-01	17881	1,24200E+00	3186	8,88325E-01	8973	0	11208,5
Responder	Macro_FOLR2-APOE+	Macro_LYVE1	SPP1	PTGER4	8,88126E-03	10786,3	1,32118E-02	5308	4,65695E-01	5158	7,03970E-01	8982	8,17429E-01	23275	0	11208,5
Responder	Mono_CD16	Macro_LYVE1	HMGB1	CD163	8,88238E-03	7690,1	1,02851E-02	8094	6,84992E-01	1970	4,06979E-01	15353	9,26947E-01	1825	0	11208,5
Responder	Macro_FOLR2+APOE+	Mono_CD14	GRN	TNFRSF1B	8,88509E-03	5604,9	9,68906E-03	8983	7,17251E-01	1721	1,05899E+00	4520	9,28441E-01	1592	0	11208,5
Responder	Macro_FOLR2-APOE+	cDC_CLEC9A	HLA-DRB5	LAG3	8,88734E-03	12776,9	1,12005E-02	6998	3,03315E-01	10735	3,20552E-01	17892	8,46943E-01	17051	0	11208,5
Responder	Macro_ISG15	cDC_LAMP3	SPP1	PTGER4	8,88879E-03	8015,3	1,72568E-02	3273	5,41536E-01	3677	1,34215E+00	2627	8,36521E-01	19291	0	11208,5
Responder	cDC_CLEC9A	Mono_CD14	SELPLG	ITGAM	8,88879E-03	8871,7	1,61800E-02	3677	5,67202E-01	3313	1,50456E+00	1899	8,12736E-01	24261	0	11208,5
Responder	Mono_INHBA	cDC_LAMP3	CD274	CD80	8,88892E-03	16303,5	2,60990E-02	1615	5,56821E-01	3454	7,03896E-01	8984	6,16527E-01	56256	0	11208,5
Responder	pDC_LILRA4	Macro_OLFML3	HSP90B1	LRP1	8,88983E-03	9953,9	6,37256E-03	17893	3,65203E-01	8168	1,45597E+00	2092	8,80267E-01	10408	0	11208,5
Responder	Macro_IER3	Mono_CD14	HSPA1A	TLR4	8,89275E-03	7916,7	9,90229E-03	8620	5,72357E-01	3237	8,00676E-01	7533	8,88266E-01	8985	0	11208,5
Responder	Mono_INHBA	Macro_FOLR2+APOE-	CCL3	CCR1	8,89275E-03	8762,7	9,68730E-03	8985	6,63496E-01	2166	1,31758E+00	2753	8,39171E-01	18701	0	11208,5
Responder	Macro_OLFML3	Macro_IER3	A2M	LRP1	8,89728E-03	12393,9	1,06042E-02	7679	3,40385E-01	9108	3,20423E-01	17896	8,51740E-01	16078	0	11208,5
Responder	Mono_INHBA	Macro_FOLR2-APOE+	MIF	TNFRSF14	8,89728E-03	12163,5	6,37124E-03	17896	3,86660E-01	7399	4,47206E-01	14265	8,82272E-01	10049	0	11208,5
Responder	Macro_IFI27	pDC_LILRA4	CXCL12	DPP4	8,90991E-03	13430,7	7,92603E-02	176	8,56891E-01	993	1,15963E+00	3680	6,62556E-01	51096	0	11208,5
Responder	cDC_CLEC9A	cDC_CLEC9A	CCL5	DPP4	8,90991E-03	15210,3	3,03224E-02	1200	5,41372E-01	3680	1,33659E+00	2661	6,04515E-01	57302	0	11208,5
Responder	Mono_CD14	Macro_FOLR2-APOE+	TGFB1	SDC2	8,91577E-03	13751,1	1,43938E-02	4591	4,38126E-01	5863	7,03656E-01	8991	7,41758E-01	38102	0	11208,5
Responder	Macro_OLFML3	Macro_LYVE1	HLA-G	CD4	8,91968E-03	10395,5	1,70409E-02	3347	4,95363E-01	4497	4,19211E-01	15020	8,42981E-01	17905	0	11208,5
Responder	Macro_IER3	cDC(CD1C)	ICAM1	ITGAM_ITGB2	8,92729E-03	7883,3	9,78094E-03	8810	4,74752E-01	4935	9,65583E-01	5469	8,88245E-01	8994	0	11208,5
Responder	Mast	cDC(CD1C)	TGFB1	CXCR4	8,93883E-03	7041,3	9,67824E-03	8997	5,34500E-01	3788	1,39480E+00	2377	8,89057E-01	8836	0	11208,5
Responder	Mono_CD14	Macro_FOLR2+APOE+	ICAM1	ITGAM_ITGB2	8,94461E-03	14848,5	6,36550E-03	17915	2,15162E-01	15789	3,83686E-01	15998	8,65083E-01	13332	0	11208,5
Responder	cDC_LAMP3	cDC_LAMP3	CD274	CD80	8,94870E-03	12573,9	5,66935E-02	348	8,91689E-01	861	9,67145E-01	5451	7,03227E-01	45001	0	11208,5
Responder	Macro_IFI27	cDC_CLEC9A	HLA-DPB1	LAG3	8,94961E-03	12010,7	1,10638E-02	7158	3,47434E-01	8839	3,19787E-01	17917	8,57155E-01	14931	0	11208,5
Responder	Macro_ISG15	Macro_OLFML3	S100A8	ITGB2	8,95037E-03	10485,3	9,67696E-03	9000	4,64890E-01	5187	1,09506E-01	25477	9,28687E-01	1554	0	11208,5
Responder	Macro_OLFML3	Macro_ISG15	HLA-DQA2	CD4	8,95710E-03	12970,3	6,36390E-03	17920	2,34658E-01	14544	5,07370E-01	12755	8,91258E-01	8424	0	11208,5
Responder	Macro_ISG15	Macro_FOLR2+APOE+	CXCL10	TLR4	8,95930E-03	8170,5	2,26304E-02	2082	6,06374E-01	2769	1,15894E+00	3687	8,27730E-01	21106	0	11208,5
Responder	Mono_CD16	cDC_LAMP3	CD52	SIGLEC10	8,96210E-03	10405,1	7,53651E-03	13728	6,65556E-01	2140	5,42120E-01	11917	8,66525E-01	13032	0	11208,5
Responder	Macro_FOLR2-APOE+	Macro_FOLR2-APOE+	INHBA	ENG	8,96579E-03	11592,9	2,42526E-02	1838	5,64979E-01	3338	7,02860E-01	9004	7,70754E-01	32576	0	11208,5
Responder	Macro_LYVE1	Mono_INHBA	HSP90B1	LRP1	8,96961E-03	11779,1	7,78731E-03	13025	3,65421E-01	8156	3,19485E-01	17925	8,90437E-01	8581	0	11208,5
Responder	Macro_LYVE1	Mono_CD14	CD99	PILRA	8,96961E-03	13738,1	6,36240E-03	17925	3,23891E-01	9822	3,51343E-01	16959	8,67779E-01	12776	0	11208,5
Responder	Macro_OLFML3	Macro_OLFML3	APOE	LRP1	8,96964E-03	10403,7	9,67410E-03	9005	4,14125E-01	6558	2,42528E-01	20497	9,10753E-01	4750	0	11208,5
Responder	Macro_LYVE1	cDC(CD1C)	GNAI2	FPR1	8,97211E-03	13656,3	6,36217E-03	17926	2,24195E-01	15229	5,26188E-01	12313	8,74297E-01	11605	0	11208,5
Responder	Macro_IFI27	Macro_IFI27	APOE	TREM2	8,97461E-03	8048,1	1,62868E-02	3638	4,32748E-01	6007	3,19464E-01	17927	9,29337E-01	1460	0	11208,5
Responder	Macro_OLFML3	Macro_LYVE1	TNFSF12	CD163	8,97711E-03	9417,3	1,32108E-02	5309	7,20195E-01	1702	5,90906E-01	10939	8,42853E-01	17928	0	11208,5
Responder	Macro_IFI27	cDC_CLEC9A	CXCL9	CXCR3	8,98761E-03	10876,5	2,73820E-02	1471	6,22593E-01	2576	1,15805E+00	3691	7,56030E-01	35436	0	11208,5
Responder	cDC(CD1C)	cDC(CD1C)	MIF	CD44_CD74	8,99469E-03	11912,5	4,56254E-03	29338	2,30212E-01	14828	1,15774E+00	3692	9,38195E-01	496	0	11208,5
Responder	Macro_FOLR2+APOE+	Mono_INHBA	MMP12	PLAUR	9,00178E-03	6182,1	2,50034E-02	1742	7,45587E-01	1543	1,15773E+00	3693	8,68021E-01	12724	0	11208,5
Responder	Mono_CD14	Macro_ISG15	ANXA1	FPR1	9,00469E-03	13778,5	6,68478E-03	16624	1,86542E-01	17939	4,68697E-01	13707	8,85743E-01	9414	0	11208,5
Responder	Macro_ISG15	Macro_ISG15	ANXA2	TLR2	9,00469E-03	14830,1	6,35961E-03	17939	1,91009E-01	17602	4,03974E-01	15430	8,72288E-01	11971	0	11208,5
Responder	Macro_ISG15	Macro_IER3	CD14	ITGB2	9,00720E-03	12204,1	6,35948E-03	17940	2,36702E-01	14409	3,98129E-01	15582	9,26595E-01	1881	0	11208,5
Responder	Macro_OLFML3	cDC(CD1C)	MDK	NOTCH2	9,00827E-03	11753,3	1,56367E-02	3946	3,42353E-01	9015	9,02045E-01	6199	7,91971E-01	28398	0	11208,5
Responder	Macro_LYVE1	Mono_CD14	PSAP	LRP1	9,01222E-03	10227,7	6,35937E-03	17942	3,76187E-01	7758	6,11232E-01	10586	9,16769E-01	3644	0	11208,5
Responder	Macro_IER3	pDC_LILRA4	APP	TNFRSF21	9,01597E-03	10531,1	1,61520E-02	3695	9,40984E-01	731	1,51314E+00	1864	7,57577E-01	35157	0	11208,5
Responder	Mono_CD16	Mono_CD16	HLA-C	LILRA1	9,01976E-03	7795,5	1,94701E-02	2637	5,99710E-01	2836	3,75051E-01	16261	9,03889E-01	6035	0	11208,5
Responder	Mono_CD16	Mono_CD16	HLA-C	LILRB2	9,02227E-03	6411,7	1,11390E-02	7065	7,72775E-01	1386	5,27556E-01	12270	9,46117E-01	129	0	11208,5
Responder	cDC(CD1C)	Macro_FOLR2+APOE-	HLA-DPB1	CD4	9,02307E-03	13218,1	4,04323E-03	34205	2,45443E-01	13858	1,17995E+00	3555	9,18970E-01	3264	0	11208,5
Responder	Mono_CD16	Mono_CD16	HLA-C	LILRA3	9,02479E-03	5765,3	3,39753E-02	986	9,08289E-01	819	5,70946E-01	11288	9,12040E-01	4525	0	11208,5
Responder	Macro_FOLR2-APOE+	Mono_INHBA	LGALS1	ITGB1	9,02479E-03	10851,7	6,35882E-03	17947	4,15022E-01	6530	5,18334E-01	12488	9,03636E-01	6085	0	11208,5
Responder	Mono_CD14	Macro_FOLR2+APOE+	HP	ITGB2	9,02763E-03	8504,3	6,28514E-02	278	7,12718E-01	1753	7,01699E-01	9020	8,31877E-01	20262	0	11208,5
Responder	Mono_CD16	Mono_CD16	ICAM2	ITGAL_ITGB2	9,02982E-03	9429,9	1,71070E-02	3323	6,54241E-01	2274	4,86977E-01	13260	8,46811E-01	17084	0	11208,5
Responder	Mast	Macro_LYVE1	HSPG2	LRP1	9,03090E-03	10330,7	8,21881E-02	158	8,90630E-01	865	7,68550E-01	7975	7,76625E-01	31447	0	11208,5
Responder	Macro_ISG15	Macro_FOLR2-APOE+	CXCL11	CCR5	9,03151E-03	17006,5	2,85171E-02	1364	3,90832E-01	7265	9,36550E-01	5772	5,76964E-01	59423	0	11208,5
Responder	Macro_IFI27	Mono_INHBA	LGALS9	CD44	9,03233E-03	10475,5	6,35773E-03	17950	3,99136E-01	7001	6,98541E-01	9067	8,98028E-01	7151	0	11208,5
Responder	Mono_CD16	Mono_CD16	B2M	LILRB2	9,04492E-03	7913,3	9,85634E-03	8688	6,56576E-01	2246	3,36373E-01	17396	9,52853E-01	28	0	11208,5
Responder	Macro_OLFML3	Macro_OLFML3	HLA-DQB1	LAG3	9,05013E-03	10808,1	1,05073E-02	7811	4,94918E-01	4507	6,17076E-01	10481	8,33015E-01	20033	0	11208,5
Responder	Macro_FOLR2-APOE+	Mono_CD16	SPP1	PTGER4	9,05090E-03	11520,1	1,18367E-02	6406	4,35291E-01	5945	7,01270E-01	9026	8,09084E-01	25015	0	11208,5
Responder	Mono_INHBA	Macro_IER3	TNF	TNFRSF1B	9,07266E-03	11311,7	1,06443E-02	7633	4,39286E-01	5835	4,60437E-01	13916	8,42625E-01	17966	0	11208,5
Responder	Macro_OLFML3	pDC_LILRA4	AGTRAP	RACK1	9,07266E-03	11150,5	6,35552E-03	17966	2,86033E-01	11614	1,44328E+00	2148	8,67616E-01	12816	0	11208,5
Responder	Macro_FOLR2+APOE-	Macro_IER3	F13A1	ITGB1	9,07287E-03	6639,1	1,61288E-02	3703	8,78371E-01	908	1,18466E+00	3531	8,62387E-01	13845	0	11208,5
Responder	Mono_INHBA	Macro_FOLR2+APOE+	LGALS3	LAG3	9,07422E-03	8847,9	2,03185E-02	2468	6,59691E-01	2211	7,00792E-01	9032	8,36369E-01	19320	0	11208,5
Responder	Macro_LYVE1	Macro_ISG15	PLAU	PLAUR	9,07771E-03	13386,9	7,50342E-03	13839	3,21444E-01	9928	4,57876E-01	13991	8,42615E-01	17968	0	11208,5
Responder	Macro_FOLR2-APOE+	cDC(CD1C)	HMGB1	CXCR4	9,08200E-03	9820,9	6,11312E-03	19090	3,41925E-01	9034	8,45098E-01	6912	9,21103E-01	2860	0	11208,5

Responder	Macro_OLFML3	cDC_CLEC9A	LGALS9	SLC1A5	9,08589E-03	10454,5	1,18728E-02	6375	4,31656E-01	6041	7,00670E-01	9035	8,34992E-01	19613	0	11208,5
Responder	Macro_ISG15	Mono_CD16	GNAI2	FPR1	9,09288E-03	12633,7	7,56764E-03	13640	3,07295E-01	10534	3,17995E-01	17974	8,83526E-01	9812	0	11208,5
Responder	Macro_ISG15	Macro_FOLR2-APOE+	CXCL8	SDC2	9,09368E-03	11764,9	1,50932E-02	4206	4,82294E-01	4774	8,01467E-01	7524	7,78372E-01	31112	0	11208,5
Responder	Macro_OLFML3	Macro_NLRP3	PLAU	PLAUR	9,09541E-03	12861,1	7,50064E-03	13848	5,28048E-01	3893	3,36866E-01	17381	8,42591E-01	17975	0	11208,5
Responder	Mono_INHBA	pDC_LILRA4	SIRPA	CD47	9,09758E-03	10863,7	9,65247E-03	9038,5	3,86121E-01	7417,5	1,50906E+00	1882,5	8,10167E-01	24771,5	0	11208,5
Responder	Mono_INHBA	Macro_IER3	ICAM1	ITGAM_ITGB2	9,10047E-03	13662,9	7,04175E-03	15304	2,86095E-01	11603	3,17905E-01	17977	8,70868E-01	12222	0	11208,5
Responder	Macro_NLRP3	pDC_LILRA4	ICAM1	SPN	9,10141E-03	8134,7	1,61212E-02	3707	6,29051E-01	2509	1,53960E+00	1790	8,26020E-01	21459	0	11208,5
Responder	cDC_CLEC9A	Macro_IER3	CD52	SIGLEC10	9,11566E-03	14044,5	7,10668E-03	15091	2,75011E-01	12223	3,17755E-01	17983	8,63093E-01	13717	0	11208,5
Responder	Macro_IFI27	Mono_INHBA	TIMP2	CD44	9,12327E-03	11892,1	6,35275E-03	17986	4,06608E-01	6776	6,10255E-01	10599	8,67262E-01	12891	0	11208,5
Responder	Macro_ISG15	Macro_FOLR2-APOE+	SPP1	PTGER4	9,12489E-03	10637,7	1,19206E-02	6328	4,14057E-01	6559	1,09634E+00	4199	8,09629E-01	24894	0	11208,5
Responder	Mono_INHBA	pDC_LILRA4	HBEGF	CD82	9,12489E-03	12372,1	1,00335E-02	8447	3,41717E-01	9045	1,28779E+00	2923	7,82669E-01	30237	0	11208,5
Responder	Macro_ISG15	Macro_FOLR2-APOE+	CCL2	CCR1	9,12879E-03	11915,7	1,81537E-02	2997	4,88771E-01	4632	1,04968E+00	4636	7,52367E-01	36105	0	11208,5
Responder	Macro_ISG15	Macro_FOLR2-APOE+	CCL2	CCR5	9,13270E-03	15309,7	1,89045E-02	2784	5,05857E-01	4279	1,10705E+00	4112	6,36194E-01	54165	0	11208,5
Responder	Macro_FOLR2-APOE+	cDC(CD1C)	C3	ITGAX	9,13342E-03	12403,1	9,26280E-03	9727	1,85871E-01	17990	6,42085E-01	10056	8,66520E-01	13034	0	11208,5
Responder	Macro_NLRP3	Mono_CD14	HMGB1	THBD	9,13596E-03	14628,9	6,35238E-03	17991	1,99133E-01	16974	5,58646E-01	11553	8,54823E-01	15418	0	11208,5
Responder	Macro_ISG15	Macro_FOLR2-APOE+	CD14	TLR1	9,14358E-03	12895,3	8,27583E-03	11769	2,80975E-01	11908	5,56474E-01	11597	8,42532E-01	17994	0	11208,5
Responder	pDC_LILRA4	Mast	ST6GAL1	CD22	9,14432E-03	13715,9	4,52437E-02	580	5,38766E-01	3713	1,51013E+00	1877	6,61600E-01	51201	0	11208,5
Responder	Macro_OLFML3	cDC_LAMP3	FN1	ITGAV_ITGB1	9,14443E-03	10924,1	1,22071E-02	6061	3,41505E-01	9050	8,23787E-01	7210	8,27795E-01	21091	0	11208,5
Responder	Macro_LYVE1	Mast	CALM1	KCNQ1	9,15120E-03	9636,3	1,95233E-02	2630	5,82200E-01	3070	4,85917E-01	13276	8,42519E-01	17997	0	11208,5
Responder	cDC_CLEC9A	Macro_FOLR2+APOE+	HLA-DOB	CD4	9,15374E-03	7288,9	2,69996E-02	1512	1,02713E+00	517	9,89809E-01	5209	8,42517E-01	17998	0	11208,5
Responder	Mono_CD16	Mono_CD16	PTPN6	CLEC12A	9,16901E-03	9121,9	1,53258E-02	4085	5,67709E-01	3307	4,41041E-01	14434	8,68661E-01	12575	0	11208,5
Responder	Mono_CD16	Mast	ADAM10	CD44	9,17920E-03	7979,3	1,23121E-02	5981	6,48593E-01	2317	5,72358E-01	11258	8,87468E-01	9132	0	11208,5
Responder	Mono_CD16	Mast	CD55	ADGRE5	9,18685E-03	9079,3	1,37798E-02	4968	6,67401E-01	2130	5,43047E-01	11893	8,55797E-01	15197	0	11208,5
Responder	Macro_ISG15	Mono_INHBA	ADM	GPR84	9,18753E-03	16287,3	1,74761E-02	3205	3,41294E-01	9061	7,33872E-01	8505	6,74286E-01	49457	0	11208,5
Responder	Mono_CD16	Mast	CD55	ADGRE2	9,18940E-03	5472,5	2,84104E-02	1376	9,57616E-01	677	1,04631E+00	4664	8,85604E-01	9437	0	11208,5
Responder	pDC_LILRA4	Mono_INHBA	HSP90B1	TLR4	9,19195E-03	10630,7	7,01239E-03	15401	3,79596E-01	7642	1,88673E+00	889	8,42471E-01	18013	0	11208,5
Responder	cDC_LAMP3	Macro_NLRP3	FN1	PLAUR	9,19195E-03	13465,9	6,34563E-03	18013	3,59859E-01	8371	3,58562E-01	16745	8,66717E-01	12992	0	11208,5
Responder	Macro_ISG15	Macro_FOLR2+APOE+	ICAM1	ITGAM_ITGB2	9,19451E-03	14891,1	6,53802E-03	17183	2,26899E-01	15042	3,16553E-01	18014	8,66636E-01	13008	0	11208,5
Responder	Macro_FOLR2+APOE-	Mono_CD14	F13A1	ITGA4	9,19455E-03	7006,5	1,61074E-02	3720	8,73704E-01	929	1,56383E+00	1703	8,45073E-01	17472	0	11208,5
Responder	cDC_CLEC9A	Macro_IFI27	HLA-DOB	CD4	9,20174E-03	7557,3	2,32055E-02	1996	9,64543E-01	662	1,15348E+00	3721	8,32209E-01	20199	0	11208,5
Responder	Macro_ISG15	Macro_FOLR2-APOE+	ADM	CALCRL	9,20324E-03	14960,3	1,65232E-02	3547	4,57492E-01	5359	8,86330E-01	6397	6,82316E-01	48290	0	11208,5
Responder	Mono_CD16	Mast	IL1B	ADRB2	9,20472E-03	9184,5	2,46673E-02	1781	7,07585E-01	1793	4,09536E-01	15275	8,52784E-01	15865	0	11208,5
Responder	Macro_OLFML3	Mono_INHBA	TNFSF13	SDC2	9,20894E-03	11462,1	2,08456E-02	2371	5,38310E-01	3722	6,60483E-01	9715	7,82431E-01	30294	0	11208,5
Responder	pDC_LILRA4	Macro_IFI27	GAS6	AXL	9,20894E-03	9939,3	1,61048E-02	3722	7,23713E-01	1681	2,15657E+00	524	7,70822E-01	32561	0	11208,5
Responder	Macro_LYVE1	Mono_CD16	B2M	LILRB2	9,22261E-03	8101,1	9,78315E-03	8807	6,36020E-01	2435	3,16278E-01	18025	9,52686E-01	30	0	11208,5
Responder	Macro_IFI27	Mono_CD14	SERPING1	LRP1	9,22261E-03	13775,5	7,52844E-03	13753	1,85330E-01	18025	6,18643E-01	10454	8,54721E-01	15437	0	11208,5
Responder	Macro_FOLR2+APOE-	Mast	F13A1	ITGA4	9,22334E-03	7149,5	1,61005E-02	3724	8,73586E-01	932	1,39027E+00	2405	8,45045E-01	17478	0	11208,5
Responder	Macro_NLRP3	Macro_NLRP3	HMGB1	THBD	9,22517E-03	15432,9	6,59032E-03	16990	2,12132E-01	15995	3,16255E-01	18026	8,57090E-01	14945	0	11208,5
Responder	Mono_CD16	Mast	HBEGF	CD9	9,22773E-03	9857,7	1,22872E-02	5997	4,87218E-01	4664	5,20963E-01	12430	8,56891E-01	14989	0	11208,5
Responder	Macro_OLFML3	Macro_LYVE1	MDK	SDC2	9,23079E-03	13000,7	3,17035E-02	1106	5,35946E-01	3756	6,98342E-01	9072	7,32265E-01	39861	0	11208,5
Responder	Mono_CD14	Mono_CD16	CD52	SIGLEC10	9,23285E-03	15168,9	6,34237E-03	18029	2,09497E-01	16192	4,08582E-01	15303	8,56230E-01	15112	0	11208,5
Responder	Mono_CD16	Mast	AGTRAP	RACK1	9,24310E-03	7252,1	1,43804E-02	4601	7,20140E-01	1703	4,78372E-01	13463	9,07904E-01	5285	0	11208,5
Responder	Macro_FOLR2-APOE+	Macro_LYVE1	LRPAP1	LRP1	9,24310E-03	10745,1	1,09779E-02	7248	5,06586E-01	4267	3,16087E-01	18033	8,66831E-01	12969	0	11208,5
Responder	cDC(CD1C)	Macro_NLRP3	TNFSF13B	TFRC	9,24310E-03	13799,3	6,34106E-03	18033	2,20642E-01	15459	8,61370E-01	6714	8,44604E-01	17582	0	11208,5
Responder	Macro_ISG15	pDC_LILRA4	MDK	NCL	9,24822E-03	9351,5	1,28177E-02	5581	3,00872E-01	10871	1,79835E+00	1062	8,42394E-01	18035	0	11208,5
Responder	Mono_CD14	Macro_FOLR2+APOE+	ICAM1	ITGAL_ITGB2	9,24822E-03	14719,1	6,34079E-03	18035	2,17118E-01	15668	4,11712E-01	15227	8,64417E-01	13457	0	11208,5
Responder	Macro_OLFML3	Macro_IER3	C1QA	CD33	9,25079E-03	13608,3	6,34064E-03	18036	3,15800E-01	10166	3,51614E-01	16951	8,73947E-01	11680	0	11208,5
Responder	Mono_CD16	Mast	HSPA8	ADRB2	9,26105E-03	6000,5	2,79325E-02	1418	7,12847E-01	1752	6,94662E-01	9127	9,01479E-01	6497	0	11208,5
Responder	Mono_CD16	Mast	ACTR2	ADRB2	9,26362E-03	6827,3	2,98451E-02	1245	7,45615E-01	1542	8,83197E-01	6448	8,63236E-01	13693	0	11208,5
Responder	Macro_FOLR2-APOE+	Macro_LYVE1	HSP90B1	LRP1	9,26628E-03	10932,3	9,62772E-03	9081	4,88148E-01	4647	1,71880E-01	23015	9,00365E-01	6710	0	11208,5
Responder	cDC_CLEC9A	Macro_OLFML3	HLA-DPB1	LAG3	9,26875E-03	10529,1	8,77364E-03	10641	3,79165E-01	7660	9,99938E-01	5093	8,42360E-01	18043	0	11208,5
Responder	Macro_OLFML3	Macro_FOLR2+APOE+	C3	ITGAM	9,27023E-03	12357,1	9,62641E-03	9082	4,54797E-01	5417	7,37706E-01	8451	7,95967E-01	27627	0	11208,5
Responder	Macro_NLRP3	Macro_OLFML3	S100A9	TLR4	9,27903E-03	11429,7	9,76715E-03	8838	4,88981E-01	4631	3,15579E-01	18047	8,59560E-01	14424	0	11208,5
Responder	Macro_OLFML3	cDC(CD1C)	LGALS9	CD47	9,28675E-03	15365,1	6,33849E-03	18050	2,20509E-01	15468	4,28219E-01	14782	8,45729E-01	17317	0	11208,5
Responder	Mono_CD16	Macro_IER3	CD55	ADGRE5	9,29189E-03	9044,3	1,47425E-02	4382	6,93521E-01	1910	4,82172E-01	13366	8,59913E-01	14355	0	11208,5
Responder	Mono_CD16	Macro_IER3	CD55	ADGRE2	9,29447E-03	7975,1	2,02447E-02	2483	7,71809E-01	1391	5,42508E-01	11908	8,67286E-01	12885	0	11208,5
Responder	Macro_FOLR2-APOE+	cDC_CLEC9A	C1QA	CD93	9,29447E-03	14205,3	6,83236E-03	16053	1,84989E-01	18053	4,22615E-01	14931	8,78316E-01	10781	0	11208,5
Responder	Macro_LYVE1	pDC_LILRA4	CXCL12	DPP4	9,30279E-03	13298,7	8,26916E-02	156	8,74997E-01	922	1,15233E+00	3735	6,67277E-01	50472	0	11208,5
Responder	cDC_CLEC9A	cDC(CD1C)	HLA-DQB1	CD4	9,30477E-03	8300,7	6,33769E-03	18057	3,74680E-01	7815	1,46474E+00	2059	9,23739E-01	2364	0	11208,5
Responder	Macro_IFI27	Mono_CD16	MMP12	PLAUR	9,31250E-03	9618,3	1,64826E-02	3562	4,31282E-01	6052	6,89875E-01	9209	8,42270E-01	18060	0	11208,5
Responder	pDC_LILRA4	Mono_CD14	HMGB1	THBD	9,31250E-03	12557,3	6,33681E-03	18060	1,97095E-01	17141	1,87126E+00	928	8,54671E-01	15449	0	11208,5
Responder	Macro_ISG15	Macro_FOLR2-APOE+	TNFSF10	RIPK1	9,31773E-03	11622,9	1,63910E-02	3602	5,74953E-01	3183	8,40490E-01	6995	7,68034E-01	33126	0	11208,5
Responder	Macro_FOLR2+APOE-	cDC_CLEC9A	F13A1	ITGA4	9,32453E-03	6923,5	1,60529E-02	3738	8,72764E-01	935	1,73518E+00	1216	8,44851E-01	17520	0	11208,5
Responder	Mono_CD16	Mono_INHBA	NAMPT	ITGA5_ITGB1	9,33056E-03	11127,1	9,07388E-03	10045	4,91198E-01	4581	3,14768E-01	18067	8,73661E-01	11734	0	11208,5
Responder	Mono_INHBA	Macro_FOLR2-APOE+	IL1A	IL1R1_IL1RAP	9,34155E-03	17574,1	3,13923E-02	1132	4,84564E-01	4719	6,96800E-01	9100	5,31417E-01	61711	0	11208,5

Post_R_Myeloid_Myeloid_CellCel

Responder	Mono_INHBA	cDC_CLEC9A	ANXA1	DYSF	9,34553E-03	10115,5	1,56903E-02	3924	4,11351E-01	6630	6,96587E-01	9101	8,34557E-01	19714	0	11208,5
Responder	Mono_INHBA	Macro_ISG15	PTGS2	ALOX5	9,34950E-03	10555,3	1,17344E-02	6494	3,75401E-01	7792	6,96569E-01	9102	8,41676E-01	18180	0	11208,5
Responder	Macro_FOLR2-APOE+	cDC_CLEC9A	HMGB1	HAVCR2	9,36159E-03	12520,1	6,33351E-03	18079	2,11479E-01	16045	6,86234E-01	9267	8,93516E-01	8001	0	11208,5
Responder	pDC_LILRA4	Macro_IER3	PROC	THBD	9,36812E-03	13804,9	5,44335E-02	379	7,65464E-01	1431	1,15146E+00	3744	6,52674E-01	52262	0	11208,5
Responder	Macro_LYVE1	Macro_NLRP3	LGALS1	ITGB1	9,36936E-03	11116,5	6,33317E-03	18082	4,42066E-01	5748	4,41537E-01	14419	9,03460E-01	6125	0	11208,5
Responder	Macro_OLFML3	Macro_IER3	HLA-DQB1	CD4	9,37195E-03	10739,7	6,33279E-03	18083	4,08642E-01	6711	4,07751E-01	15326	9,23712E-01	2370	0	11208,5
Responder	Mono_CD16	Macro_IER3	CD52	SIGLEC10	9,37454E-03	6704,9	1,38254E-02	4938	8,36951E-01	1073	6,93835E-01	9140	8,97886E-01	7165	0	11208,5
Responder	Macro_IFI27	cDC_CLEC9A	HLA-DQB1	LAG3	9,37454E-03	11461,5	1,20141E-02	6247	3,70219E-01	7971	4,65123E-01	13797	8,42128E-01	18084	0	11208,5
Responder	Mono_CD16	Macro_FOLR2-APOE+	ICAM2	ITGAL_ITGB2	9,38751E-03	10728,3	1,59269E-02	3798	5,24742E-01	3952	3,63733E-01	16594	8,42117E-01	18089	0	11208,5
Responder	Macro_FOLR2-APOE+	Macro_FOLR2-APOE+	LGALS1	ITGB1	9,38751E-03	11863,3	6,42075E-03	17665	4,20085E-01	6353	3,14173E-01	18089	9,04058E-01	6001	0	11208,5
Responder	Macro_OLFML3	Mono_CD14	CD14	ITGB2	9,38751E-03	11895,9	6,33043E-03	18089	2,30297E-01	14821	4,78747E-01	13456	9,26439E-01	1905	0	11208,5
Responder	pDC_LILRA4	Mono_INHBA	RARRES2	CMKLR1	9,39725E-03	14948,9	2,37986E-02	1910	5,36724E-01	3748	1,98369E+00	740	6,06240E-01	57138	0	11208,5
Responder	Mono_CD14	Macro_FOLR2-APOE+	HP	ITGB2	9,39730E-03	9468,7	5,21957E-02	422	5,50976E-01	3548	6,95516E-01	9114	8,18483E-01	23051	0	11208,5
Responder	Macro_OLFML3	Mast	IL16	CD9	9,40049E-03	9543,5	1,53477E-02	4072	5,24792E-01	3949	6,22145E-01	10394	8,42089E-01	18094	0	11208,5
Responder	pDC_LILRA4	Mono_CD16	GRN	TNFRSF1B	9,40309E-03	9529,7	6,32878E-03	18095	2,87748E-01	11506	1,37380E+00	2482	9,12937E-01	4357	0	11208,5
Responder	Mono_INHBA	Macro_NLRP3	ADAM10	CD44	9,40569E-03	12807,7	7,70757E-03	13263	3,83370E-01	7517	3,14047E-01	18096	8,61875E-01	13954	0	11208,5
Responder	Macro_LYVE1	Mono_CD14	PLTP	ABCA1	9,40829E-03	8608,5	1,09204E-02	7309	7,03288E-01	1823	1,05287E+00	4605	8,42078E-01	18097	0	11208,5
Responder	Mono_INHBA	Mono_INHBA	ICAM1	ITGAM_ITGB2	9,41089E-03	13841,1	6,32788E-03	18098	2,69101E-01	12521	4,58481E-01	13978	8,64737E-01	13400	0	11208,5
Responder	cDC_LAMP3	pDC_LILRA4	APP	TNFRSF21	9,41184E-03	10558,5	1,60380E-02	3750	9,39645E-01	736	1,52888E+00	1814	7,56926E-01	35284	0	11208,5
Responder	Macro_OLFML3	Macro_IFI27	FN1	ITGAV_ITGB1	9,41327E-03	11387,7	1,16317E-02	6586	3,40104E-01	9118	7,52343E-01	8225	8,24326E-01	21801	0	11208,5
Responder	Mono_CD14	Mono_CD14	AGTRAP	RACK1	9,41609E-03	13027,7	6,32733E-03	18100	2,35062E-01	14520	7,38377E-01	8444	8,67361E-01	12866	0	11208,5
Responder	Macro_IFI27	Macro_ISG15	HLA-DQA1	LAG3	9,42389E-03	11466,7	1,39312E-02	4874	4,01536E-01	6927	3,13789E-01	18103	8,51108E-01	16221	0	11208,5
Responder	Mono_CD16	Macro_FOLR2+APOE-	SERPINA1	LRP1	9,42389E-03	9835,1	8,10544E-03	12177	4,50489E-01	5540	4,62534E-01	13862	9,02079E-01	6388	0	11208,5
Responder	Macro_OLFML3	Mono_CD14	MMP2	CCR2	9,42630E-03	18546,9	3,60118E-02	884	3,54274E-01	8582	5,66803E-01	11379	5,55288E-01	60681	0	11208,5
Responder	Macro_OLFML3	Macro_OLFML3	HLA-A	LILRA1	9,44331E-03	19792,7	4,39892E-03	30832	3,35916E-01	9297	1,34974E-01	24462	8,17980E-01	23164	0	11208,5
Responder	Mono_CD14	Macro_LYVE1	CD99	PILRA	9,44734E-03	14622,9	6,33484E-03	18072	2,62542E-01	12887	3,13285E-01	18112	8,67530E-01	12835	0	11208,5
Responder	Macro_FOLR2+APOE+	cDC_CLEC9A	CALM2	MYLK	9,44734E-03	9769,3	4,62015E-02	559	8,85387E-01	885	7,50424E-01	8253	7,94367E-01	27941	0	11208,5
Responder	Mono_CD16	Macro_FOLR2+APOE+	ADAM10	TREM2	9,44995E-03	9138,1	1,99521E-02	2545	5,71244E-01	3248	4,07026E-01	15351	8,65056E-01	13338	0	11208,5
Responder	Mono_CD14	Mono_INHBA	TIMP2	CD44	9,45256E-03	11295,1	6,32352E-03	18114	4,05152E-01	6816	8,10682E-01	7394	8,66996E-01	12943	0	11208,5
Responder	Macro_OLFML3	Mono_CD16	MDK	NOTCH2	9,45329E-03	9634,7	2,42664E-02	1835	4,94669E-01	4514	6,94656E-01	9128	8,25862E-01	21488	0	11208,5
Responder	Mono_CD16	Mono_CD14	HSPA1A	TLR4	9,45517E-03	14170,3	6,92781E-03	15704	2,54390E-01	13365	3,13249E-01	18115	8,69273E-01	12459	0	11208,5
Responder	Macro_OLFML3	Mono_CD16	GRN	TNFRSF1B	9,46301E-03	9967,1	8,27338E-03	11776	5,35933E-01	3757	2,39413E-01	20598	9,23013E-01	2496	0	11208,5
Responder	Macro_FOLR2-APOE+	Mono_INHBA	LGALS3	ENG	9,46561E-03	14956,5	6,61005E-03	16910	2,74868E-01	12231	3,73427E-01	16314	8,41981E-01	18119	0	11208,5
Responder	Macro_FOLR2+APOE+	Macro_FOLR2+APOE-	C1QB	LRP1	9,47334E-03	6952,1	1,01824E-02	8239	5,71996E-01	3242	6,94214E-01	9133	9,20766E-01	2938	0	11208,5
Responder	Macro_NLRP3	Macro_ISG15	THBS1	LRP1	9,47345E-03	11048,7	9,94944E-03	8554	4,63103E-01	5221	5,32733E-01	12138	8,41963E-01	18122	0	11208,5
Responder	Mono_INHBA	Mast	CD55	ADGRE2	9,47606E-03	10226,9	1,34550E-02	5155	5,28342E-01	3888	5,07223E-01	12760	8,41962E-01	18123	0	11208,5
Responder	pDC_LILRA4	Mono_CD14	GRN	TNFRSF1B	9,47606E-03	9191,3	6,32163E-03	18123	2,86988E-01	11547	1,99828E+00	715	9,12892E-01	4363	0	11208,5
Responder	Macro_FOLR2-APOE+	Macro_LYVE1	ALCAM	NRP1	9,47736E-03	11023,7	2,39671E-02	1884	7,02511E-01	1828	6,94179E-01	9134	7,78678E-01	31064	0	11208,5
Responder	Macro_IER3	Mono_CD14	RPS19	C5AR1	9,47736E-03	6418,5	9,58997E-03	9134	5,91696E-01	2946	8,71736E-01	6566	9,24543E-01	2238	0	11208,5
Responder	Macro_IFI27	Macro_LYVE1	CD99	CD81	9,48653E-03	13805,9	6,32059E-03	18127	3,37860E-01	9217	3,27633E-01	17636	8,67519E-01	12841	0	11208,5
Responder	Macro_IER3	Mono_CD14	ICAM1	ITGAL_ITGB2	9,48941E-03	7797,3	9,69654E-03	8964	5,02156E-01	4361	9,78295E-01	5316	8,87440E-01	9137	0	11208,5
Responder	Macro_OLFML3	Macro_FOLR2-APOE+	HLA-DQB1	CD4	9,49176E-03	10469,9	6,31995E-03	18129	4,07724E-01	6736	4,61584E-01	13887	9,23640E-01	2389	0	11208,5
Responder	Macro_FOLR2+APOE-	Mono_INHBA	CXCL12	SDC4	9,51437E-03	10535,5	5,09752E-02	445	8,04612E-01	1229	1,14951E+00	3764	7,52723E-01	36031	0	11208,5
Responder	Macro_IFI27	cDC_CLEC9A	HLA-DRB1	LAG3	9,51535E-03	12225,7	1,06203E-02	7662	3,23083E-01	9863	3,12552E-01	18138	8,60344E-01	14257	0	11208,5
Responder	Macro_OLFML3	Mono_CD16	MDK	ITGA4_ITGB1	9,52322E-03	8973,9	2,37187E-02	1918	5,19576E-01	4045	6,69450E-01	9557	8,41899E-01	18141	0	11208,5
Responder	Macro_FOLR2-APOE+	Macro_FOLR2+APOE+	MMP9	LRP1	9,52563E-03	9980,3	1,58775E-02	3828	5,74887E-01	3184	6,93717E-01	9146	8,20827E-01	22535	0	11208,5
Responder	cDC_CLEC9A	cDC(CD1C)	IL16	CD4	9,52584E-03	8513,7	1,27333E-02	5644	4,53864E-01	5449	1,44766E+00	2125	8,41899E-01	18142	0	11208,5
Responder	Mono_INHBA	Mono_CD16	ICAM1	ITGAX_ITGB2	9,52584E-03	13007,7	6,99472E-03	15456	3,17828E-01	10086	3,12191E-01	18142	8,81706E-01	10146	0	11208,5
Responder	Macro_IFI27	Mono_CD14	CIRBP	TREM1	9,53109E-03	12568,1	9,14418E-03	9934	2,61732E-01	12944	6,09736E-01	10610	8,41876E-01	18144	0	11208,5
Responder	Macro_ISG15	Mono_INHBA	HLA-A	LILRB2	9,53372E-03	10433,3	6,31671E-03	18145	3,24357E-01	9796	5,54048E-01	11653	9,30034E-01	1364	0	11208,5
Responder	Macro_ISG15	Macro_ISG15	SAA1	FPR1	9,54176E-03	10538,3	1,55503E-02	3980	4,30618E-01	6065	7,43349E-01	8364	8,18360E-01	23074	0	11208,5
Responder	Macro_ISG15	Macro_ISG15	SAA1	FPR2	9,54580E-03	13308,3	2,81885E-02	1398	5,40671E-01	3686	8,03457E-01	7497	7,15971E-01	42752	0	11208,5
Responder	Macro_ISG15	Macro_ISG15	CCL3	CCR1	9,55388E-03	10789,7	9,78407E-03	8806	3,51830E-01	8658	8,62700E-01	6697	8,39840E-01	18579	0	11208,5
Responder	Macro_ISG15	Macro_ISG15	CCL3	CCR5	9,55791E-03	13901,9	1,17289E-02	6503	3,42635E-01	9001	7,09729E-01	8874	7,64054E-01	33923	0	11208,5
Responder	Macro_ISG15	Macro_ISG15	SPP1	ITGAV_ITGB1	9,56196E-03	11165,3	1,00388E-02	8445	3,45594E-01	8907	1,12597E+00	3967	8,17279E-01	23299	0	11208,5
Responder	Mono_CD16	Mono_INHBA	PSEN1	CD44	9,56529E-03	11521,1	8,16442E-03	12032	4,80862E-01	4805	5,65640E-01	11403	8,41799E-01	18157	0	11208,5
Responder	cDC(CD1C)	Mono_CD16	CALM1	INSR	9,56600E-03	11467,1	1,15560E-02	6663	3,39365E-01	9156	8,34602E-01	7075	8,17668E-01	23233	0	11208,5
Responder	Macro_ISG15	Macro_ISG15	SPP1	ITGA5_ITGB1	9,56600E-03	10291,9	1,07439E-02	7505	3,95980E-01	7098	1,24473E+00	3169	8,21060E-01	22479	0	11208,5
Responder	Macro_IFI27	Macro_LYVE1	CD59	STAB1	9,57583E-03	8751,3	1,25853E-02	5759	6,69980E-01	2115	8,77332E-01	6513	8,41784E-01	18161	0	11208,5
Responder	cDC(CD1C)	Mono_INHBA	ADAM17	ITGA5	9,57813E-03	15856,5	9,76439E-03	8843	3,39353E-01	9159	7,89642E-01	7678	7,17933E-01	42394	0	11208,5
Responder	cDC(CD1C)	pDC_LILRA4	HSP90B1	TLR7	9,58623E-03	9059,1	1,38442E-02	4928	3,39293E-01	9161	1,68580E+00	1320	8,39286E-01	18678	0	11208,5
Responder	cDC(CD1C)	Macro_NLRP3	ARPC5	LDLR	9,59028E-03	11585,7	1,12824E-02	6914	3,39269E-01	9162	9,12043E-01	6081	8,11235E-01	24563	0	11208,5
Responder	Macro_FOLR2-APOE+	Macro_LYVE1	FN1	C5AR1	9,59694E-03	9371,1	1,91356E-02	2725	4,71060E-01	5019	3,11383E-01	18169	8,83979E-01	9734	0	11208,5
Responder	Mono_CD16	Macro_ISG15	ICAM2	ITGAM_ITGB2	9,59958E-03	9818,9	1,57189E-02	3907	5,32317E-01	3831	5,38861E-01	11978	8,41742E-01	18170	0	11208,5
Responder	Mono_INHBA	cDC_LAMP3	THBS1	ITGA4	9,60222E-03	10782,9	1,20618E-02	6189	4,17226E-01	6444	5,42777E-01	11902	8,41739E-01	18171	0	11208,5

Responder	Macro_LYVE1	Mono_CD14	CCL18	CCR1	9,60281E-03	9092,9	2,53902E-02	1687	6,33322E-01	2463	1,14723E+00	3776	8,02501E-01	26330	0	11208,5
Responder	Macro_LYVE1	Macro_ISG15	CCL18	CCR1	9,61021E-03	8090,9	3,19789E-02	1090	7,24354E-01	1678	1,14723E+00	3777	8,20149E-01	22701	0	11208,5
Responder	Macro_FOLR2-APOE+	Mono_CD14	APP	APLP2	9,61544E-03	11502,1	8,24586E-03	11836	3,56322E-01	8511	7,82963E-01	7779	8,41689E-01	18176	0	11208,5
Responder	Macro_ISG15	cDC(CD1C)	LGALS9	PTPRC	9,61544E-03	11019,3	6,30878E-03	18176	2,73414E-01	12314	9,45787E-01	5682	8,95098E-01	7716	0	11208,5
Responder	Mono_CD16	Macro_FOLR2+APOE+	ADAM10	GPNMB	9,61808E-03	5628,9	2,65843E-02	1556	1,01934E+00	537	9,61850E-01	5509	8,86215E-01	9334	0	11208,5
Responder	Macro_NLRP3	cDC(CD1C)	S100A8	CD68	9,61808E-03	13184,9	6,50686E-03	17308	2,14229E-01	15858	3,11161E-01	18177	9,18297E-01	3373	0	11208,5
Responder	Macro_FOLR2-APOE+	cDC(CD1C)	ICAM1	IL2RG	9,62073E-03	11608,7	7,63423E-03	13442	3,92432E-01	7222	7,66876E-01	7993	8,41682E-01	18178	0	11208,5
Responder	Macro_ISG15	Macro_ISG15	CCL3L1	CCR1	9,62272E-03	11403,3	1,16593E-02	6563	3,79603E-01	7641	1,02968E+00	4804	8,00093E-01	26800	0	11208,5
Responder	Macro_ISG15	Macro_ISG15	CCL3L1	CCR5	9,62678E-03	14804,9	1,39768E-02	4851	3,70408E-01	7962	8,76710E-01	6519	7,11949E-01	43484	0	11208,5
Responder	Macro_OLFML3	cDC_CLEC9A	C1QA	CD33	9,62867E-03	11994,5	6,30785E-03	18181	3,14756E-01	10210	7,24855E-01	8640	8,73661E-01	11733	0	11208,5
Responder	Macro_ISG15	Mono_CD16	HLA-B	LILRB1	9,63397E-03	11422,9	6,30722E-03	18183	2,94442E-01	11174	4,67315E-01	13750	9,21395E-01	2799	0	11208,5
Responder	Mono_CD16	Macro_IFI27	ADAM10	GPNMB	9,63927E-03	10627,1	1,23814E-02	5920	4,67223E-01	5111	5,09403E-01	12711	8,41654E-01	18185	0	11208,5
Responder	Macro_NLRP3	Macro_NLRP3	THBS1	CD47	9,64192E-03	10634,1	1,05289E-02	7780	4,90043E-01	4611	5,66423E-01	11385	8,41646E-01	18186	0	11208,5
Responder	pDC_LILRA4	Mono_CD16	PTPN6	CLEC12A	9,65253E-03	9698,3	9,89613E-03	8631	3,48858E-01	8773	1,56555E+00	1689	8,41639E-01	18190	0	11208,5
Responder	cDC_LAMP3	Mono_CD16	HLA-B	LILRB2	9,65518E-03	8772,7	9,17604E-03	9881	4,99632E-01	4409	3,10839E-01	18191	9,44664E-01	174	0	11208,5
Responder	Mono_CD16	Macro_FOLR2+APOE-	ADAM10	GPNMB	9,65784E-03	10822,3	1,23785E-02	5924	4,67112E-01	5116	4,69982E-01	13671	8,41638E-01	18192	0	11208,5
Responder	Mono_INHBA	Mono_CD14	HSP90B1	ASGR1	9,66049E-03	13769,1	8,01346E-03	12406	2,08566E-01	16252	5,98679E-01	10786	8,41637E-01	18193	0	11208,5
Responder	Mast	Macro_OLFML3	ANXA1	FPR1	9,66315E-03	9790,1	9,32038E-03	9614	4,83531E-01	4742	3,53539E-01	16895	9,01514E-01	6491	0	11208,5
Responder	Macro_IER3	cDC_LAMP3	ICAM1	IL2RG	9,66339E-03	6191,9	1,66914E-02	3472	8,25657E-01	1125	9,19383E-01	5974	8,87147E-01	9180	0	11208,5
Responder	Macro_IER3	Macro_ISG15	PLAU	PLAUR	9,66746E-03	6519,3	1,61744E-02	3680	6,59547E-01	2212	8,93464E-01	6315	8,87140E-01	9181	0	11208,5
Responder	Macro_IER3	Mono_INHBA	HBEGF	CD44	9,66746E-03	7877,9	1,06954E-02	7574	5,96957E-01	2876	6,91589E-01	9181	8,90592E-01	8550	0	11208,5
Responder	Macro_FOLR2+APOE+	Macro_OLFML3	C1QA	CD33	9,66950E-03	6095,9	1,59660E-02	3785	6,88273E-01	1949	6,51030E-01	9874	9,16679E-01	3663	0	11208,5
Responder	pDC_LILRA4	Macro_FOLR2-APOE+	HSP90B1	TLR1	9,67643E-03	9623,7	8,67139E-03	10860	4,04747E-01	6829	1,82046E+00	1022	8,41607E-01	18199	0	11208,5
Responder	Mono_CD16	pDC_LILRA4	TNF	TNFRSF21	9,67692E-03	11157,3	1,59634E-02	3786	9,72160E-01	643	1,34822E+00	2596	7,44704E-01	37553	0	11208,5
Responder	pDC_LILRA4	Macro_LYVE1	HSP90B1	ASGR1	9,67909E-03	9997,5	8,00987E-03	12412	4,01780E-01	6918	1,71897E+00	1249	8,41607E-01	18200	0	11208,5
Responder	Macro_LYVE1	Mono_INHBA	HGF	CD44	9,68707E-03	8582,5	1,89353E-02	2774	6,48038E-01	2324	7,40948E-01	8403	8,41579E-01	18203	0	11208,5
Responder	Mono_CD14	Macro_OLFML3	TNFSF13B	HLA-DPB1	9,68785E-03	10124,9	5,42243E-03	22967	3,38599E-01	9186	8,49709E-01	6857	9,39657E-01	406	0	11208,5
Responder	Macro_LYVE1	Mast	CALM2	KCNQ1	9,68973E-03	10868,1	1,83484E-02	2936	4,66269E-01	5143	3,55254E-01	16849	8,41576E-01	18204	0	11208,5
Responder	cDC(CD1C)	Macro_ISG15	RPS19	C5AR1	9,71235E-03	10378,1	5,42116E-03	22974	3,38426E-01	9192	1,44749E+00	2127	9,02078E-01	6389	0	11208,5
Responder	Macro_ISG15	Macro_FOLR2+APOE+	CD14	TLR4	9,71904E-03	15701,5	6,50687E-03	17307	2,48578E-01	13687	3,14172E-01	18090	8,41524E-01	18215	0	11208,5
Responder	Macro_OLFML3	cDC_CLEC9A	MDK	ITGA4_ITGB1	9,72872E-03	11030,5	1,52093E-02	4145	3,38395E-01	9196	9,34918E-01	5795	8,10037E-01	24808	0	11208,5
Responder	Macro_NLRP3	Mono_CD16	B2M	LILRB2	9,73773E-03	8908,7	9,17400E-03	9885	4,64942E-01	5185	3,09655E-01	18222	9,51216E-01	43	0	11208,5
Responder	Mono_CD14	Macro_LYVE1	TGFB1	ITGB5	9,74100E-03	13903,7	2,52697E-02	1700	5,29029E-01	3875	6,90217E-01	9199	7,11702E-01	43536	0	11208,5
Responder	Mono_INHBA	Macro_FOLR2-APOE+	CD99	CD81	9,74842E-03	12389,1	7,46398E-03	13951	3,84193E-01	7483	3,09489E-01	18226	8,76786E-01	11077	0	11208,5
Responder	cDC_CLEC9A	Mast	ST6GAL1	CD22	9,75140E-03	14095,1	4,53228E-02	578	5,39304E-01	3707	1,14509E+00	3796	6,61796E-01	51186	0	11208,5
Responder	Macro_OLFML3	Macro_IFI27	APOE	SCARB1	9,76448E-03	10484,1	1,09662E-02	7257	4,57474E-01	5362	6,23230E-01	10361	8,41411E-01	18232	0	11208,5
Responder	Macro_OLFML3	cDC_LAMP3	SPP1	S1PR1	9,76634E-03	19506,7	3,17832E-02	1104	5,34125E-01	3798	-4,23752E-03	30336	6,62597E-01	51087	0	11208,5
Responder	Macro_ISG15	Macro_LYVE1	CCL2	CCR1	9,76634E-03	9903,5	2,58952E-02	1639	5,94799E-01	2901	1,14497E+00	3798	7,83955E-01	29971	0	11208,5
Responder	Macro_FOLR2-APOE+	Mono_CD16	HLA-C	LILRB2	9,76971E-03	8923,5	9,54925E-03	9206	5,21769E-01	4012	2,59479E-01	19908	9,42055E-01	283	0	11208,5
Responder	Mast	Macro_OLFML3	TIMP3	CD44	9,76984E-03	8197,3	2,94171E-02	1283	8,11369E-01	1202	4,58723E-01	13973	8,65105E-01	13320	0	11208,5
Responder	Mono_INHBA	Mono_CD14	CIRBP	TREM1	9,77252E-03	12361,3	9,07950E-03	10037	2,58148E-01	13150	6,91836E-01	9176	8,41403E-01	18235	0	11208,5
Responder	Macro_NLRP3	Macro_IER3	VCAN	TLR2	9,77520E-03	10810,1	1,23823E-02	5916	4,87969E-01	4650	4,55836E-01	14040	8,41403E-01	18236	0	11208,5
Responder	Macro_ISG15	Mast	TIMP2	CD44	9,77788E-03	12731,7	6,29401E-03	18237	3,62565E-01	8275	4,99551E-01	12949	8,66726E-01	12989	0	11208,5
Responder	Macro_FOLR2-APOE+	Mono_INHBA	FN1	SDC2	9,79025E-03	11689,9	2,66223E-02	1552	4,33209E-01	5997	6,89804E-01	9211	7,81505E-01	30481	0	11208,5
Responder	Mono_INHBA	Mono_INHBA	CCL5	CCRL2	9,79025E-03	14436,3	1,10242E-02	7199	3,38073E-01	9211	8,91706E-01	6332	7,41145E-01	38231	0	11208,5
Responder	Mono_CD16	Macro_LYVE1	THBS1	CD36	9,79666E-03	9906,3	2,04223E-02	2444	6,83572E-01	1986	3,09090E-01	18244	8,53673E-01	15649	0	11208,5
Responder	cDC_CLEC9A	Macro_NLRP3	HLA-DQA2	CD4	9,79934E-03	11931,7	6,29292E-03	18245	2,14259E-01	15855	9,33725E-01	5814	8,90713E-01	8536	0	11208,5
Responder	Macro_IFI27	Macro_NLRP3	CXCL12	CXCR4	9,80471E-03	11243,5	1,28355E-02	5571	3,85694E-01	7433	4,66922E-01	13758	8,41362E-01	18247	0	11208,5
Responder	pDC_LILRA4	Macro_OLFML3	APP	NOTCH2	9,80849E-03	7441,7	1,55927E-02	3961	8,79540E-01	902	1,94701E+00	792	8,31470E-01	20345	0	11208,5
Responder	Mast	cDC(CD1C)	ANXA1	FPR1	9,81278E-03	7349,1	9,00517E-03	10193	4,66988E-01	5122	1,20001E+00	3432	8,99976E-01	6790	0	11208,5
Responder	Macro_ISG15	Macro_LYVE1	SPP1	ITGAV_ITGB1	9,82623E-03	8882,7	1,41073E-02	4768	4,53445E-01	5465	1,04053E+00	4717	8,41327E-01	18255	0	11208,5
Responder	Macro_ISG15	Macro_ISG15	CD14	ITGB2	9,83161E-03	11242,7	6,29135E-03	18257	2,28702E-01	14921	6,50491E-01	9886	9,26228E-01	1941	0	11208,5
Responder	Macro_OLFML3	Macro_OLFML3	HLA-A	APLP2	9,83648E-03	17230,9	4,72759E-03	27917	3,42441E-01	9009	-1,42729E-01	36574	9,29433E-01	1446	0	11208,5
Responder	Macro_ISG15	Macro_NLRP3	TNFSF10	TNFRSF10B	9,83969E-03	11442,5	1,18611E-02	6386	5,51590E-01	3543	9,15237E-01	6028	7,83579E-01	30047	0	11208,5
Responder	cDC(CD1C)	Macro_LYVE1	CD59	STAB1	9,83969E-03	10051,5	9,53853E-03	9223	5,78327E-01	3132	1,06195E+00	4496	8,22441E-01	22198	0	11208,5
Responder	Mono_INHBA	Macro_IER3	LGALS3	ENG	9,83969E-03	13947,7	6,54494E-03	17152	3,44174E-01	8956	4,51333E-01	14162	8,41322E-01	18260	0	11208,5
Responder	Mono_CD14	Macro_FOLR2-APOE+	TGFB1	ENG	9,84382E-03	11649,5	1,24631E-02	5849	4,06077E-01	6794	6,88944E-01	9224	8,08309E-01	25172	0	11208,5
Responder	Macro_FOLR2-APOE+	Macro_IER3	MMP12	PLAUR	9,84778E-03	9783,1	1,62447E-02	3654	4,59836E-01	5296	6,16388E-01	10494	8,41302E-01	18263	0	11208,5
Responder	Macro_FOLR2-APOE+	cDC(CD1C)	C1QB	C1QBP	9,84778E-03	13479,3	6,89427E-03	15834	1,82017E-01	18263	4,06723E-01	15360	9,00261E-01	6731	0	11208,5
Responder	pDC_LILRA4	Mono_INHBA	LTB	CD40	9,85141E-03	8697,9	1,57455E-02	3893	8,78689E-01	904	2,51580E+00	221	7,97634E-01	27263	0	11208,5
Responder	Mono_CD16	pDC_LILRA4	CD55	ADGRE5	9,85857E-03	8172,7	1,09943E-02	7231	5,91820E-01	2942	1,73558E+00	1215	8,41295E-01	18267	0	11208,5
Responder	Macro_FOLR2-APOE+	cDC_CLEC9A	ITGB2	CD226	9,87275E-03	8548,7	2,83686E-02	1382	6,79195E-01	2032	6,88605E-01	9231	8,38323E-01	18890	0	11208,5
Responder	pDC_LILRA4	cDC_LAMP3	APP	NOTCH2	9,87290E-03	7407,1	1,55356E-02	3985	8,78507E-01	905	2,15239E+00	531	8,31213E-01	20406	0	11208,5
Responder	Mono_CD14	Macro_FOLR2+APOE+	THBS1	CD47	9,88017E-03	12044,7	1,04657E-02	7865	3,56401E-01	8504	4,43267E-01	14371	8,41245E-01	18275	0	11208,5
Responder	Macro_OLFML3	Mono_CD14	HLA-G	LILRB2	9,88102E-03	11358,3	1,35772E-02	5079	3,55879E-01	8522	6,88532E-01	9233	8,19934E-01	22749	0	11208,5

Responder	cDC(CD1C)	Macro_ISG15	CD55	ADGRE5	9,88516E-03	11017,3	9,53074E-03	9234	3,96691E-01	7074	8,21134E-01	7240	8,31524E-01	20330	0	11208,5
Responder	Mast	cDC(CD1C)	PTGS2	ALOX5	9,88558E-03	8080,5	1,47565E-02	4377	4,70993E-01	5024	1,04193E+00	4705	8,56353E-01	15088	0	11208,5
Responder	Mono_CD16	cDC_CLEC9A	CALM2	MYLK	9,89442E-03	9294,5	4,58620E-02	570	8,78505E-01	906	9,41437E-01	5725	7,93764E-01	28063	0	11208,5
Responder	pDC_LILRA4	Mono_CD16	HLA-C	LILRA3	9,89759E-03	15662,5	2,29457E-02	2037	3,37329E-01	9237	1,32130E+00	2737	8,94971E-01	7738	1	56563,5
Responder	Mono_CD14	Macro_NLRP3	HP	ITGB2	9,90588E-03	9923,3	4,85780E-02	508	4,96064E-01	4481	6,88021E-01	9239	8,13086E-01	24180	0	11208,5
Responder	Macro_FOLR2-APOE+	Mono_CD14	CD14	ITGB2	9,90994E-03	11329,5	6,28483E-03	18286	2,24637E-01	15194	6,44492E-01	10012	9,26192E-01	1947	0	11208,5
Responder	cDC(CD1C)	Macro_FOLR2+APOE+	LTB	TNFRSF1A	9,91833E-03	13083,1	1,00071E-02	8484	3,37202E-01	9242	1,04476E+00	4679	7,74738E-01	31802	0	11208,5
Responder	Mast	Mono_INHBA	ANXA1	FPR1	9,92079E-03	11447,3	7,06161E-03	15238	3,64982E-01	8182	4,70758E-01	13653	8,88488E-01	8955	0	11208,5
Responder	Macro_IER3	cDC_CLEC9A	ICAM1	ITGAL_ITGB2	9,92248E-03	7381,9	9,56825E-03	9175	5,53412E-01	3510	1,14760E+00	3773	8,86773E-01	9243	0	11208,5
Responder	Macro_OLFML3	Macro_IFI27	HLA-DQA2	CD4	9,92621E-03	13467,1	6,28317E-03	18292	2,29739E-01	14849	4,40802E-01	14442	8,90638E-01	8544	0	11208,5
Responder	Macro_FOLR2-APOE+	Mono_CD14	LGALS1	PTPRC	9,92664E-03	10827,7	5,45836E-03	22739	3,41037E-01	9072	6,87647E-01	9244	9,26618E-01	1875	0	11208,5
Responder	Macro_NLRP3	Macro_IFI27	S100A8	ITGB2	9,93435E-03	8976,9	1,03755E-02	7985	4,26861E-01	6160	3,07464E-01	18295	9,30961E-01	1236	0	11208,5
Responder	Macro_OLFML3	pDC_LILRA4	FN1	PLAUR	9,93435E-03	11791,5	7,98376E-03	12471	1,81672E-01	18295	8,85028E-01	6416	8,79432E-01	10567	0	11208,5
Responder	cDC_LAMP3	Mono_INHBA	PKM	CD44	9,93707E-03	11094,7	6,28205E-03	18296	3,99959E-01	6981	3,80062E-01	16120	9,21065E-01	2868	0	11208,5
Responder	Macro_FOLR2-APOE+	Macro_ISG15	LILRB4	LAIR1	9,93979E-03	14497,5	7,56167E-03	13656	1,89241E-01	17730	3,07458E-01	18297	8,74315E-01	11596	0	11208,5
Responder	Macro_LYVE1	cDC(CD1C)	LGALS1	PTPRC	9,93979E-03	8289,3	6,28171E-03	18297	4,56622E-01	5377	9,73735E-01	5369	9,31254E-01	1195	0	11208,5
Responder	pDC_LILRA4	Macro_OLFML3	HMGB1	HAVCR2	9,94522E-03	11265,9	6,28164E-03	18299	2,15255E-01	15784	1,28158E+00	2963	8,93124E-01	8075	0	11208,5
Responder	Macro_FOLR2+APOE+	Macro_NLRP3	GRN	TNFRSF1B	9,94742E-03	6312,5	1,01855E-02	8233	7,51687E-01	1513	6,87285E-01	9249	9,30083E-01	1359	0	11208,5
Responder	Macro_FOLR2-APOE+	Mono_CD14	LGALS9	CD44	9,95337E-03	11651,7	6,28134E-03	18302	3,24221E-01	9804	5,52425E-01	11692	8,97473E-01	7252	0	11208,5
Responder	Mono_INHBA	cDC(CD1C)	VEGFA	ITGAV	9,95575E-03	14565,5	9,52128E-03	9251	4,65865E-01	5153	9,51211E-01	5620	7,22326E-01	41595	0	11208,5
Responder	Macro_FOLR2-APOE+	Mono_CD14	A2M	LRP1	9,95881E-03	13009,5	9,00322E-03	10197	2,29150E-01	14885	6,18657E-01	10453	8,41106E-01	18304	0	11208,5
Responder	pDC_LILRA4	Macro_IER3	LIN7C	ABCA1	9,96190E-03	11010,3	2,21392E-02	2163	5,54078E-01	3498	1,14195E+00	3824	7,62055E-01	34358	0	11208,5
[truncated: 1,975 more chars]
