# Supplementary material for: A single-cell pan-cancer analysis to show the variability of tumor-infiltrating myeloid cells in immune checkpoint blockade
Source: Nat Commun. 2024 Jul 21;15:6142. doi: 10.1038/s41467-024-50478-8 (PMC11271490; doi:10.1038/s41467-024-50478-8)
Supplement: Supplementary file 9 — Supplementary Data 6 [file 41467_2024_50478_MOESM9_ESM.pdf]

Myeloid_vs_CD4_Post_NR

Response	source	target	ligand.complex	receptor.complex	aggregate_rank	mean_rank	natmi.edge_specificity	natmi.rank	connectome.weight_sc	connectome.rank	logfc.logfc_comb	logfc.rank	sca.LRscore	sca.rank	cellphonedb.pvalue	cellphonedb.rank
Myeloid_vs_CD4_Non-responder	CD4(TNF+ T)	cDC_CLEC9A	XCL1	XCR1	2,21039E-07	30218,3	3,88304E-01	6	6,71871E+00	10	2,39482E+00	18226	8,09985E-01	78429	0	54420,5
Myeloid_vs_CD4_Non-responder	Mono_CD14	CD4(TGFB1+ Th17)	S100A8	CD69	5,46320E-07	11534,7	2,27940E-02	1833	3,57775E+00	440	4,32384E+00	910	9,64104E-01	70	0	54420,5
Myeloid_vs_CD4_Non-responder	Mono_CD14	CD4(IL26+ Th17)	S100A8	CD69	1,09898E-06	11666,7	2,08242E-02	2184	3,49313E+00	496	4,22452E+00	1093	9,62507E-01	140	0	54420,5
Myeloid_vs_CD4_Non-responder	Mono_CD14	CD4(AREG+ Tm)	S100A8	CD69	2,15884E-06	11861,5	1,90314E-02	2587	3,41611E+00	561	4,07494E+00	1444	9,60848E-01	295	0	54420,5
Myeloid_vs_CD4_Non-responder	CD4(CRTAM- T)	cDC(CD1C)	B2M	CD1C	3,38924E-06	18862,5	1,88478E-02	2649	2,45564E+00	2757	1,81064E+00	33605	9,57121E-01	881	0	54420,5
Myeloid_vs_CD4_Non-responder	Mono_CD14	CD4(CRTAM- T)	S100A8	CD69	3,40792E-06	12123,3	1,79708E-02	2901	3,37055E+00	609	3,84086E+00	2259	9,59755E-01	427	0	54420,5
Myeloid_vs_CD4_Non-responder	CD4(Tn)	cDC(CD1C)	B2M	CD1C	4,65374E-06	19268,3	1,84840E-02	2763	2,38469E+00	3137	1,76786E+00	35060	9,56719E-01	961	0	54420,5
Myeloid_vs_CD4_Non-responder	Mono_CD14	CD4(TNF+ T)	S100A8	CD69	7,58970E-06	12153,7	1,61248E-02	3547	3,29124E+00	674	4,10913E+00	1358	9,57609E-01	769	0	54420,5
Myeloid_vs_CD4_Non-responder	Macro_NLRP3	CD4(TGFB1+ Th17)	S100A8	CD69	8,65505E-06	12529,7	1,80174E-02	2892	2,89738E+00	1251	3,55967E+00	3666	9,59805E-01	419	0	54420,5
Myeloid_vs_CD4_Non-responder	cDC_LAMP3	CD4(Tn)	CCL19	CCR7	1,05154E-05	15809,1	1,09021E-01	69	5,49383E+00	25	3,06894E+00	7763	9,22012E-01	16768	0	54420,5
Myeloid_vs_CD4_Non-responder	CD4(CRTAM- T)	Mono_CD16	ANXA1	FPR1	1,43898E-05	17707,9	8,13966E-03	11723	2,00961E+00	5991	2,78063E+00	11467	9,46018E-01	4938	0	54420,5
Myeloid_vs_CD4_Non-responder	cDC_LAMP3	CD4(TNFRSF9+ Treg)	CCL17	CCR8	1,48484E-05	18445,9	3,68340E-01	7	4,51960E+00	82	3,68798E+00	2942	8,89134E-01	34778	0	54420,5
Myeloid_vs_CD4_Non-responder	Mono_CD14	CD4(Tn)	S100A8	CD69	1,57156E-05	12663,5	1,46043E-02	4259	3,22593E+00	717	3,75126E+00	2635	9,55553E-01	1286	0	54420,5
Myeloid_vs_CD4_Non-responder	CD4(TGFB1+ Th17)	pDC_LILRA4	TNF	PTPRS	1,67133E-05	16035,3	1,04883E-01	74	4,49357E+00	87	2,89054E+00	9982	9,24319E-01	15613	0	54420,5
Myeloid_vs_CD4_Non-responder	Macro_NLRP3	CD4(IL26+ Th17)	S100A8	CD69	1,68298E-05	12861,9	1,64604E-02	3401	2,81276E+00	1463	3,46035E+00	4333	9,58025E-01	692	0	54420,5
Myeloid_vs_CD4_Non-responder	CD4(ISG+ Treg)	pDC_LILRA4	BST2	LILRA4	1,72865E-05	13975,3	3,34659E-02	853	4,09714E+00	172	3,83832E+00	2270	9,31389E-01	12161	0	54420,5
Myeloid_vs_CD4_Non-responder	pDC_LILRA4	CD4(TGFB1+ Th17)	SCT	ADRB2	1,78851E-05	25351,1	9,90804E-02	90	4,87460E+00	57	3,24478E+00	6045	8,32449E-01	66143	0	54420,5
Myeloid_vs_CD4_Non-responder	CD4(CRTAM- T)	Mono_CD14	ANXA1	FPR1	2,12367E-05	17522,9	8,52655E-03	10867	2,08224E+00	5282	2,70007E+00	12672	9,47192E-01	4373	0	54420,5
Myeloid_vs_CD4_Non-responder	CD4(TNF+ T)	pDC_LILRA4	TNF	PTPRS	2,43388E-05	15433,7	9,27474E-02	105	4,41531E+00	95	3,40391E+00	4701	9,19905E-01	17847	0	54420,5
Myeloid_vs_CD4_Non-responder	cDC_LAMP3	CD4(ISG+ Treg)	CCL17	CCR8	2,48042E-05	21928,9	2,34189E-01	10	4,30958E+00	106	3,14064E+00	7002	8,64770E-01	48106	0	54420,5
Myeloid_vs_CD4_Non-responder	cDC_LAMP3	CD4(ISG+ Treg)	CCL17	CCR4	2,62271E-05	20055,7	1,06679E-01	73	4,29859E+00	109	3,10103E+00	7419	8,82655E-01	38257	0	54420,5
Myeloid_vs_CD4_Non-responder	cDC_LAMP3	CD4(TNFRSF9+ Treg)	CCL17	CCR4	3,17831E-05	20702,9	9,51118E-02	99	4,25334E+00	120	3,11312E+00	7298	8,76580E-01	41577	0	54420,5
Myeloid_vs_CD4_Non-responder	Mono_CD14	CD4(GZMK+ Teff)	S100A8	CD69	3,27200E-05	13157,1	1,31951E-02	5122	3,16539E+00	774	3,58440E+00	3515	9,53347E-01	1954	0	54420,5
Myeloid_vs_CD4_Non-responder	CD4(Tn)	cDC_CLEC9A	FLT3LG	FLT3	3,52880E-05	31322,1	3,06971E-02	1006	3,21408E+00	723	1,64909E+00	39230	8,41302E-01	61231	0	54420,5
Myeloid_vs_CD4_Non-responder	cDC_LAMP3	CD4(CXCL13+ Tfh)	CCL17	CCR4	3,55959E-05	21456,3	8,71594E-02	117	4,22223E+00	127	3,01048E+00	8411	8,71779E-01	44206	0	54420,5
Myeloid_vs_CD4_Non-responder	CD4(TNFRSF9+ Treg)	cDC(CD1C)	B2M	CD1C	3,71666E-05	16803,1	2,01854E-02	2319	2,71648E+00	1730	2,10426E+00	24940	9,58506E-01	606	0	54420,5
Myeloid_vs_CD4_Non-responder	cDC_LAMP3	CD4(TGFB1+ Th17)	CCL19	CXCR3	3,96243E-05	19485,9	8,27375E-02	134	5,19858E+00	33	2,77130E+00	11602	8,95498E-01	31240	0	54420,5
Myeloid_vs_CD4_Non-responder	CD4(TNF+ T)	Macro_NLRP3	HSPA1A	TLR4	4,00601E-05	19739,7	1,04791E-02	7703	1,74484E+00	9541	2,59304E+00	14387	9,30382E-01	12647	0	54420,5
Myeloid_vs_CD4_Non-responder	cDC_LAMP3	CD4(Tn)	CCL17	CCR4	4,14167E-05	21836,9	8,24409E-02	137	4,20377E+00	136	3,00218E+00	8524	8,68636E-01	45967	0	54420,5
Myeloid_vs_CD4_Non-responder	Macro_NLRP3	CD4(AREG+ Tm)	S100A8	CD69	4,22946E-05	13340,5	1,50433E-02	4038	2,73574E+00	1667	3,31076E+00	5464	9,56178E-01	1113	0	54420,5
Myeloid_vs_CD4_Non-responder	cDC_LAMP3	CD4(IL26+ Th17)	CCL22	DPP4	4,32488E-05	21203,5	1,13955E-01	60	4,19149E+00	140	3,29740E+00	5572	8,68910E-01	45825	0	54420,5
Myeloid_vs_CD4_Non-responder	Macro_NLRP3	CD4(TNF+ T)	S100A8	CD69	4,36945E-05	13885,1	1,27457E-02	5509	2,61088E+00	2086	3,34495E+00	5175	9,52571E-01	2235	0	54420,5
Myeloid_vs_CD4_Non-responder	CD4(TNFRSF9+ Treg)	pDC_LILRA4	BST2	LILRA4	4,80005E-05	17844,1	1,67178E-02	3311	3,70796E+00	374	3,28916E+00	5641	9,05612E-01	25474	0	54420,5
Myeloid_vs_CD4_Non-responder	cDC_LAMP3	CD4(GZMK+ Teff)	CCL19	CCR7	5,30022E-05	18149,7	7,71865E-02	155	5,25874E+00	31	2,71878E+00	12385	9,08657E-01	23757	0	54420,5
Myeloid_vs_CD4_Non-responder	cDC_LAMP3	CD4(NME1+ T)	CCL17	CCR4	5,36876E-05	22513,7	7,70824E-02	156	4,18281E+00	144	2,90695E+00	9730	8,64754E-01	48118	0	54420,5
Myeloid_vs_CD4_Non-responder	CD4(CRTAM- T)	Macro_ISG15	ANXA1	FPR1	5,52602E-05	19635,3	6,80553E-03	15343	1,75916E+00	9276	2,74811E+00	11924	9,41261E-01	7213	0	54420,5
Myeloid_vs_CD4_Non-responder	cDC_LAMP3	CD4(ISG+ Treg)	CCL22	CCR4	5,64731E-05	18791,5	1,12214E-01	61	4,12664E+00	160	3,20224E+00	6416	8,92427E-01	32900	0	54420,5
Myeloid_vs_CD4_Non-responder	cDC_LAMP3	CD4(CRTAM- T)	CCL19	CXCR3	6,29975E-05	20147,5	7,56361E-02	169	5,15592E+00	36	2,71008E+00	12519	8,91224E-01	33593	0	54420,5
Myeloid_vs_CD4_Non-responder	cDC_LAMP3	CD4(CRTAM- T)	CCL19	CCR7	6,44957E-05	18280,1	7,55012E-02	171	5,24629E+00	32	2,71034E+00	12507	9,07737E-01	24270	0	54420,5
Myeloid_vs_CD4_Non-responder	CD4(CRTAM- T)	pDC_LILRA4	COPA	P2RY6	6,83695E-05	21645,9	1,22580E-02	5871	3,51026E+00	483	3,22894E+00	6167	8,77112E-01	41288	0	54420,5
Myeloid_vs_CD4_Non-responder	CD4(ISG+ Treg)	Mono_CD16	HLA-F	LILRB2	6,92423E-05	20004,9	6,60205E-03	16051	2,49185E+00	2579	2,67019E+00	13133	9,27915E-01	13841	0	54420,5
Myeloid_vs_CD4_Non-responder	cDC_LAMP3	CD4(TNFRSF9+ Treg)	CCL22	CCR4	7,14548E-05	19405,5	1,00047E-01	85	4,08139E+00	180	3,21433E+00	6299	8,86792E-01	36043	0	54420,5
Myeloid_vs_CD4_Non-responder	cDC_LAMP3	CD4(TGFB1+ Th17)	CCL22	DPP4	7,22500E-05	23626,1	8,26404E-02	136	4,07984E+00	181	3,16477E+00	6763	8,49503E-01	56630	0	54420,5
Myeloid_vs_CD4_Non-responder	cDC_LAMP3	CD4(NME1+ T)	CCL17	CCR8	7,71133E-05	31141,5	7,82885E-02	151	4,06551E+00	187	2,83391E+00	10753	7,87115E-01	90196	0	54420,5
Myeloid_vs_CD4_Non-responder	cDC_LAMP3	CD4(AREG+ Tm)	CCL22	DPP4	7,87695E-05	24009,5	7,80757E-02	152	4,06356E+00	189	3,18082E+00	6620	8,45834E-01	58666	0	54420,5
Myeloid_vs_CD4_Non-responder	cDC_LAMP3	CD4(CXCL13+ Tfh)	CCL22	CCR4	8,21346E-05	20091,9	9,16819E-02	109	4,05028E+00	193	3,11168E+00	7312	8,82335E-01	38425	0	54420,5
Myeloid_vs_CD4_Non-responder	cDC_LAMP3	CD4(Tn)	CCL22	CCR4	8,64396E-05	20434,1	8,67186E-02	119	4,03182E+00	198	3,10339E+00	7388	8,79415E-01	40045	0	54420,5
Myeloid_vs_CD4_Non-responder	cDC_LAMP3	CD4(NME1+ T)	CCL22	CCR4	9,35558E-05	21045,7	8,10821E-02	142	4,01086E+00	206	3,00815E+00	8448	8,75806E-01	42012	0	54420,5
Myeloid_vs_CD4_Non-responder	CD4(CRTAM- T)	Macro_OLFML3	ANXA1	FPR1	1,03808E-04	20337,3	7,03641E-03	14573	1,80250E+00	8554	2,43543E+00	17405	9,42176E-01	6734	0	54420,5
Myeloid_vs_CD4_Non-responder	CD4(Tn)	pDC_LILRA4	HMGB1	TLR9	1,05747E-04	27382,9	3,24264E-02	910	2,64864E+00	1943	1,36922E+00	50734	8,99588E-01	28907	0	54420,5
Myeloid_vs_CD4_Non-responder	cDC_LAMP3	CD4(AREG+ Tm)	CCL19	CCR7	1,06684E-04	19053,1	6,64433E-02	220	5,17940E+00	35	2,66646E+00	13192	9,02244E-01	27398	0	54420,5
Myeloid_vs_CD4_Non-responder	Mono_CD14	CD4(IFNG+ Tfh/Th1)	S100A8	CD69	1,07140E-04	14062,9	1,11714E-02	6907	3,07845E+00	890	3,40051E+00	4733	9,49502E-01	3364	0	54420,5
Myeloid_vs_CD4_Non-responder	cDC_LAMP3	CD4(IL26+ Th17)	CCL22	CCR4	1,13571E-04	22149,7	6,86722E-02	208	3,96471E+00	227	2,98832E+00	8717	8,66486E-01	47176	0	54420,5
Myeloid_vs_CD4_Non-responder	CD4(TNFRSF9+ Treg)	Mono_CD16	HLA-F	LILRB2	1,13708E-04	20625,9	6,16341E-03	17725	2,44875E+00	2783	2,66544E+00	13208	9,25582E-01	14993	0	54420,5
Myeloid_vs_CD4_Non-responder	pDC_LILRA4	CD4(TNF+ T)	SCT	ADRB2	1,14572E-04	28636,5	6,54367E-02	228	4,73953E+00	67	3,30256E+00	5525	8,01495E-01	82942	0	54420,5
Myeloid_vs_CD4_Non-responder	cDC_LAMP3	CD4(GZMK+ Teff)	CCL19	CXCR3	1,15578E-04	21246,1	6,53125E-02	229	5,09389E+00	40	2,62069E+00	13933	8,83904E-01	37608	0	54420,5
Myeloid_vs_CD4_Non-responder	cDC_LAMP3	CD4(IL26+ Th17)	CCL17	CCR4	1,16588E-04	23709,1	6,52846E-02	230	4,13666E+00	157	2,88711E+00	10019	8,54742E-01	53719	0	54420,5
Myeloid_vs_CD4_Non-responder	CD4(TNF+ T)	Macro_FOLR2+APOE+	HSPA1A	TLR4	1,18602E-04	20644,7	1,02605E-02	7979	1,72074E+00	9984	2,41222E+00	17875	9,29696E-01	12965	0	54420,5
Myeloid_vs_CD4_Non-responder	CD4(ISG+ Treg)	Mono_CD16	HLA-B	LILRA1	1,20973E-04	21111,5	1,11086E-02	6975	2,42548E+00	2908	1,79228E+00	34232	9,41596E-01	7022	0	54420,5
Myeloid_vs_CD4_Non-responder	CD4(Tn)	pDC_LILRA4	HSP90B1	TLR9	1,23344E-04	31854,9	2,22391E-02	1914	2,62012E+00	2046	1,34393E+00	51854	8,63069E-01	49040	0	54420,5
Myeloid_vs_CD4_Non-responder	Mono_CD14	CD4(TGFB1+ Th17)	VCAN	CD44	1,24991E-04	13486,1	1,61628E-02	3532	3,50546E+00	486	3,12237E+00	7181	9,53795E-01	1811	0	54420,5
Myeloid_vs_CD4_Non-responder	CD4(NME1+ T)	pDC_LILRA4	HMGB1	THBD	1,28575E-04	23376,1	6,17129E-03	17689	1,73968E+00	9619	2,45589E+00	16986	9,19284E-01	18166	0	54420,5
Myeloid_vs_CD4_Non-responder	cDC_LAMP3	CD4(TNF+ T)	CCL22	DPP4	1,32264E-04	25607,7	6,33387E-02	245	4,01101E+00	205	3,18285E+00	6590	8,31697E-01	66578	0	54420,5
Myeloid_vs_CD4_Non-responder	Mono_CD14	CD4(NME1+ T)	S100A8	CD69	1,33903E-04	14095,7	1,08116E-02	7307	3,06300E+00	918	3,48687E+00	4157	9,48712E-01	3676	0	54420,5
Myeloid_vs_CD4_Non-responder	CD4(TNF+ T)	Macro_ISG15	HSPA1A	TLR4	1,43021E-04	23803,1	7,91266E-03	12262	1,46191E+00	16358	2,38025E+00	18557	9,20715E-01	17418	0	54420,5
Myeloid_vs_CD4_Non-responder	CD4(IFNG+ Tfh/Th1)	pDC_LILRA4	CXCL13	CXCR3	1,57943E-04	18059,7	6,04424E-02	264	1,39680E+00	18419	3,16379E+00	6775	9,34906E-01	10420	0	54420,5
Myeloid_vs_CD4_Non-responder	CD4(ISG+ Treg)	cDC(CD1C)	B2M	CD1C	1,58134E-04	16316,9	2,06347E-02	2224	2,80410E+00	1489	2,18403E+00	22917	9,58942E-01	534	0	54420,5
Myeloid_vs_CD4_Non-responder	CD4(IFNG+ Tfh/Th1)	cDC(CD1C)	B2M	CD1C	1,58558E-04	16332,9	2,06244E-02	2226	2,80208E+00	1494	2,18050E+00	22988	9,58932E-01	536	0	54420,5
Myeloid_vs_CD4_Non-responder	CD4(CRTAM- T)	Macro_ISG15	ANXA1	FPR2_FPR3	1,59787E-04	19680,9	1,22998E-02	5831	2,09725E+00	5147	2,35981E+00	18973	9,27457E-01	14033	0	54420,5
Myeloid_vs_CD4_Non-responder	Macro_NLRP3	CD4(CRTAM- T)	S100A8	CD69	1,63325E-04	13974,7	1,42049E-02	4497	2,69018E+00	1807	3,07669E+00	7683	9,54960E-01	1466	0	54420,5

Myeloid_vs_CD4_Post_NR

Myeloid_vs_CD4_Non-responder	cDC_LAMP3	CD4(ISG+ Treg)	CCL19	CXCR3	1,64163E-04	22114,1	5,93955E-02	273	5,05834E+00	42	2,53070E+00	15542	8,78942E-01	40293	0	54420,5
Myeloid_vs_CD4_Non-responder	CD4(IFNG+ Tfh/Th1)	pDC_LILRA4	HMGB1	THBD	1,64473E-04	24214,7	5,97684E-03	18532	1,70452E+00	10309	2,35421E+00	19083	9,18088E-01	18729	0	54420,5
Myeloid_vs_CD4_Non-responder	Mono_CD14	CD4(ISG+ Treg)	S100A8	CD69	1,70941E-04	14276,3	1,04294E-02	7772	3,04658E+00	944	3,48578E+00	4164	9,47829E-01	4081	0	54420,5
Myeloid_vs_CD4_Non-responder	CD4(TNF+ T)	Mono_CD14	HSPA1A	TLR4	1,74129E-04	21380,7	9,82976E-03	8596	1,67326E+00	10909	2,34313E+00	19302	9,28281E-01	13676	0	54420,5
Myeloid_vs_CD4_Non-responder	CD4(TNFRSF9+ Treg)	Mono_CD16	HLA-B	LILRA1	1,78010E-04	22845,3	1,04919E-02	7677	2,25846E+00	3869	1,61877E+00	40408	9,40005E-01	7852	0	54420,5
Myeloid_vs_CD4_Non-responder	Macro_NLRP3	CD4(TGFB1+ Th17)	IL1B	ADRB2	1,80719E-04	18329,9	2,01294E-02	2326	3,08769E+00	880	3,83670E+00	2278	8,94592E-01	31745	0	54420,5
Myeloid_vs_CD4_Non-responder	Mono_CD14	CD4(CXCL13+ Tfh)	S100A8	CD69	1,81443E-04	14525,3	1,03362E-02	7890	3,04257E+00	948	3,34408E+00	5183	9,47607E-01	4185	0	54420,5
Myeloid_vs_CD4_Non-responder	cDC_LAMP3	CD4(GZMK+ Teff)	CCL22	CCR4	1,83930E-04	23512,5	5,73373E-02	289	3,92256E+00	254	2,93052E+00	9446	8,55702E-01	53153	0	54420,5
Myeloid_vs_CD4_Non-responder	Macro_NLRP3	CD4(TGFB1+ Th17)	VCAN	CD44	1,84832E-04	14166,1	1,39965E-02	4618	3,08753E+00	881	3,05300E+00	7927	9,50519E-01	2984	0	54420,5
Myeloid_vs_CD4_Non-responder	cDC_LAMP3	CD4(TNFRSF9+ Treg)	CCL19	CXCR3	1,87761E-04	22401,5	5,72802E-02	292	5,04563E+00	43	2,51118E+00	15911	8,77000E-01	41341	0	54420,5
Myeloid_vs_CD4_Non-responder	Mono_CD14	CD4(CRTAM- T)	VCAN	CD44	1,87802E-04	13770,7	1,55149E-02	3822	3,45713E+00	530	3,05053E+00	7959	9,52886E-01	2122	0	54420,5
Myeloid_vs_CD4_Non-responder	pDC_LILRA4	CD4(Tn)	APP	RPSA	1,88082E-04	14330,7	1,42323E-02	4478	2,42163E+00	2928	3,05038E+00	7962	9,53602E-01	1865	0	54420,5
Myeloid_vs_CD4_Non-responder	Macro_FOLR2+APOE+	CD4(IFNG+ Tfh/Th1)	HLA-DRA	LAG3	1,91563E-04	15225,3	1,22893E-02	5848	1,83903E+00	7999	4,01912E+00	1613	9,43276E-01	6246	0	54420,5
Myeloid_vs_CD4_Non-responder	CD4(TNFRSF9+ Treg)	Mono_CD16	HLA-A	LILRA1	1,93560E-04	21536,7	1,14209E-02	6661	2,36936E+00	3229	1,75974E+00	35353	9,39649E-01	8020	0	54420,5
Myeloid_vs_CD4_Non-responder	CD4(ISG+ Treg)	Mono_CD16	HLA-A	LILRA1	1,94996E-04	21762,5	1,14078E-02	6672	2,36626E+00	3250	1,72724E+00	36435	9,39616E-01	8035	0	54420,5
Myeloid_vs_CD4_Non-responder	CD4(IFNG+ Tfh/Th1)	Mono_CD16	HLA-F	LILRB2	1,95495E-04	21569,3	5,72600E-03	19754	2,40576E+00	3023	2,59230E+00	14401	9,23007E-01	16248	0	54420,5
Myeloid_vs_CD4_Non-responder	cDC_LAMP3	CD4(CXCL13+ Tfh)	CCL19	CCR7	1,96853E-04	20829,3	5,64328E-02	299	5,10548E+00	39	2,41719E+00	17770	8,94802E-01	31618	0	54420,5
Myeloid_vs_CD4_Non-responder	CD4(IL26+ Th17)	cDC(CD1C)	B2M	CD1C	1,97391E-04	17386,3	1,97608E-02	2396	2,63367E+00	2011	2,01186E+00	27429	9,58081E-01	675	0	54420,5
Myeloid_vs_CD4_Non-responder	cDC_LAMP3	CD4(AREG+ Tm)	CCL22	CCR4	1,99489E-04	23690,9	5,63856E-02	301	3,91902E+00	257	2,90763E+00	9725	8,54666E-01	53751	0	54420,5
Myeloid_vs_CD4_Non-responder	pDC_LILRA4	CD4(ISG+ Treg)	GZMB	IGF2R	2,03834E-04	21549,9	1,96580E-02	2422	3,78408E+00	338	4,44129E+00	740	8,61657E-01	49829	0	54420,5
Myeloid_vs_CD4_Non-responder	cDC_CLEC9A	CD4(IFNG+ Tfh/Th1)	HLA-DPB1	LAG3	2,04076E-04	15487,5	1,29303E-02	5336	1,82997E+00	8128	3,94006E+00	1868	9,40340E-01	7685	0	54420,5
Myeloid_vs_CD4_Non-responder	CD4(GZMK+ Teff)	pDC_LILRA4	HMGB1	THBD	2,07196E-04	24942,3	5,68033E-03	19985	1,65090E+00	11391	2,34839E+00	19205	9,16155E-01	19710	0	54420,5
Myeloid_vs_CD4_Non-responder	cDC(CD1C)	CD4(IFNG+ Tfh/Th1)	HLA-DRA	LAG3	2,10810E-04	15694,9	1,21894E-02	5934	1,82565E+00	8195	3,57380E+00	3587	9,43058E-01	6338	0	54420,5
Myeloid_vs_CD4_Non-responder	CD4(CRTAM- T)	Macro_NLRP3	ANXA1	FPR1	2,11745E-04	23532,1	5,66377E-03	20072	1,54482E+00	13990	2,34415E+00	19280	9,35973E-01	9898	0	54420,5
Myeloid_vs_CD4_Non-responder	pDC_LILRA4	CD4(AREG+ Tm)	APP	RPSA	2,15115E-04	14454,7	1,40203E-02	4603	2,40277E+00	3040	3,02567E+00	8237	9,53269E-01	1973	0	54420,5
Myeloid_vs_CD4_Non-responder	CD4(GZMK+ Teff)	cDC(CD1C)	B2M	CD1C	2,18441E-04	17789,7	1,94893E-02	2479	2,58073E+00	2200	1,95564E+00	29114	9,57802E-01	735	0	54420,5
Myeloid_vs_CD4_Non-responder	cDC_LAMP3	CD4(CRTAM- T)	CCL22	DPP4	2,28232E-04	27052,1	5,45489E-02	322	3,97967E+00	218	3,06587E+00	7795	8,20981E-01	72505	0	54420,5
Myeloid_vs_CD4_Non-responder	Macro_ISG15	CD4(TGFB1+ Th17)	S100A8	CD69	2,30589E-04	16347,7	1,04084E-02	7794	1,81357E+00	8383	3,13746E+00	7032	9,47779E-01	4109	0	54420,5
Myeloid_vs_CD4_Non-responder	cDC_LAMP3	CD4(GZMK+ Teff)	CCL17	CCR4	2,32496E-04	25185,9	5,45090E-02	325	4,09451E+00	174	2,82931E+00	10811	8,43181E-01	60199	0	54420,5
Myeloid_vs_CD4_Non-responder	CD4(TNFRSF9+ Treg)	Mono_CD16	HLA-A	LILRB2	2,32619E-04	17947,9	5,58536E-03	20453	2,59641E+00	2132	2,72045E+00	12362	9,60114E-01	372	0	54420,5
Myeloid_vs_CD4_Non-responder	CD4(ISG+ Treg)	Mono_CD16	HLA-A	LILRB2	2,34559E-04	18056,5	5,57898E-03	20487	2,59332E+00	2140	2,68794E+00	12859	9,60092E-01	376	0	54420,5
Myeloid_vs_CD4_Non-responder	cDC_LAMP3	CD4(NME1+ T)	CCL19	CXCR3	2,38241E-04	22973,9	5,44024E-02	329	5,02834E+00	46	2,44526E+00	17196	8,74192E-01	42878	0	54420,5
Myeloid_vs_CD4_Non-responder	CD4(NME1+ T)	cDC(CD1C)	B2M	CD1C	2,40084E-04	18067,3	1,91605E-02	2559	2,51661E+00	2480	1,92338E+00	30077	9,57457E-01	800	0	54420,5
Myeloid_vs_CD4_Non-responder	cDC(CD1C)	CD4(IFNG+ Tfh/Th1)	HLA-DPB1	LAG3	2,45621E-04	16074,5	1,27408E-02	5512	1,80490E+00	8518	3,50129E+00	4039	9,39924E-01	7883	0	54420,5
Myeloid_vs_CD4_Non-responder	CD4(TNF+ T)	Mono_CD16	HSPA1A	TLR4	2,48204E-04	24497,7	7,75283E-03	12634	1,44428E+00	16900	2,27813E+00	20720	9,19967E-01	17814	0	54420,5
Myeloid_vs_CD4_Non-responder	Mono_CD14	CD4(TNFRSF9+ Treg)	S100A8	CD69	2,49519E-04	14809,9	9,85753E-03	8552	3,02201E+00	991	3,32419E+00	5345	9,46417E-01	4741	0	54420,5
Myeloid_vs_CD4_Non-responder	cDC_LAMP3	CD4(AREG+ Tm)	CCL17	CCR4	2,51424E-04	25375,1	5,36042E-02	338	4,09097E+00	177	2,80643E+00	11129	8,42071E-01	60811	0	54420,5
Myeloid_vs_CD4_Non-responder	cDC_CLEC9A	CD4(IFNG+ Tfh/Th1)	HLA-DRA	LAG3	2,52530E-04	15526,3	1,20063E-02	6101	1,80115E+00	8578	3,90321E+00	2014	9,42650E-01	6518	0	54420,5
Myeloid_vs_CD4_Non-responder	pDC_LILRA4	CD4(TNFRSF9+ Treg)	GZMB	IGF2R	2,56923E-04	21832,1	1,89414E-02	2618	3,77760E+00	342	4,45977E+00	712	8,59428E-01	51068	0	54420,5
Myeloid_vs_CD4_Non-responder	CD4(TNF+ T)	pDC_LILRA4	TNF	TNFRSF21	2,57937E-04	16543,3	2,85351E-02	1170	2,95012E+00	1137	3,35281E+00	5109	9,13916E-01	20880	0	54420,5
Myeloid_vs_CD4_Non-responder	CD4(TNFRSF9+ Treg)	Macro_FOLR2+APOE+	LTB	CD40	2,59795E-04	20960,3	1,29874E-02	5283	1,77779E+00	8947	2,26924E+00	20910	9,25057E-01	15241	0	54420,5
Myeloid_vs_CD4_Non-responder	CD4(GZMK+ Teff)	Mono_CD16	HLA-F	LILRB2	2,71364E-04	22041,9	5,46918E-03	21093	2,38053E+00	3162	2,59130E+00	14421	9,21360E-01	17113	0	54420,5
Myeloid_vs_CD4_Non-responder	Macro_NLRP3	CD4(Tn)	S100A8	CD69	2,71663E-04	15027,7	1,15439E-02	6549	2,54556E+00	2354	2,98708E+00	8738	9,50283E-01	3077	0	54420,5
Myeloid_vs_CD4_Non-responder	CD4(ISG+ Treg)	Macro_ISG15	HLA-F	LILRB2	2,71686E-04	23017,1	5,46870E-03	21098	2,07704E+00	5320	2,44965E+00	17131	9,21357E-01	17116	0	54420,5
Myeloid_vs_CD4_Non-responder	Macro_NLRP3	CD4(CRTAM- T)	VCAN	CD44	2,79735E-04	14494,3	1,34354E-02	4949	3,03920E+00	955	2,98116E+00	8803	9,49548E-01	3344	0	54420,5
Myeloid_vs_CD4_Non-responder	CD4(CXCL13+ Tfh)	pDC_LILRA4	CXCL13	CXCR3	2,80028E-04	17010,7	6,65983E-02	219	1,49028E+00	15486	3,25483E+00	5954	9,37796E-01	8974	0	54420,5
Myeloid_vs_CD4_Non-responder	CD4(Tn)	Mono_CD16	HLA-B	LILRA1	2,80238E-04	25027,7	9,81568E-03	8616	2,07536E+00	5341	1,43391E+00	47954	9,38099E-01	8807	0	54420,5
Myeloid_vs_CD4_Non-responder	CD4(CRTAM- T)	Macro_FOLR2+APOE+	TGFB1	ENG	2,83675E-04	22630,1	9,30115E-03	9411	1,76896E+00	9098	2,25257E+00	21281	9,17633E-01	18940	0	54420,5
Myeloid_vs_CD4_Non-responder	CD4(ISG+ Treg)	Mono_CD16	HLA-B	LILRB2	2,86552E-04	17954,1	5,43265E-03	21324	2,65253E+00	1919	2,75298E+00	11875	9,61428E-01	232	0	54420,5
Myeloid_vs_CD4_Non-responder	CD4(CXCL13+ Tfh)	cDC(CD1C)	B2M	CD1C	2,89417E-04	18659,3	1,88762E-02	2636	2,46116E+00	2725	1,84070E+00	32643	9,57152E-01	872	0	54420,5
Myeloid_vs_CD4_Non-responder	Mono_CD14	CD4(IFNG+ Tfh/Th1)	S100A8	ITGB2	2,93222E-04	14318,3	7,81648E-03	12482	3,33452E+00	640	4,12578E+00	1312	9,51243E-01	2737	0	54420,5
Myeloid_vs_CD4_Non-responder	CD4(IFNG+ Tfh/Th1)	pDC_LILRA4	BST2	LILRA4	2,93677E-04	16591,7	1,99623E-02	2360	3,78335E+00	339	3,44606E+00	4410	9,12925E-01	21429	0	54420,5
Myeloid_vs_CD4_Non-responder	CD4(CRTAM- T)	Mono_CD16	HLA-B	LILRA1	2,94975E-04	25726,3	9,74168E-03	8720	2,05532E+00	5523	1,36259E+00	51046	9,37879E-01	8922	0	54420,5
Myeloid_vs_CD4_Non-responder	CD4(CXCL13+ Tfh)	pDC_LILRA4	HMGB1	THBD	2,96912E-04	25830,1	5,40849E-03	21476	1,60174E+00	12551	2,31016E+00	20012	9,14252E-01	20691	0	54420,5
Myeloid_vs_CD4_Non-responder	cDC_CLEC9A	CD4(IFNG+ Tfh/Th1)	HLA-DPA1	LAG3	2,97858E-04	15548,7	1,24024E-02	5752	1,77789E+00	8944	3,92201E+00	1938	9,42280E-01	6689	0	54420,5
Myeloid_vs_CD4_Non-responder	CD4(IFNG+ Tfh/Th1)	Mono_CD16	HLA-A	LILRB2	3,02343E-04	18766,7	5,39420E-03	21554	2,50367E+00	2535	2,56774E+00	14858	9,59441E-01	466	0	54420,5
Myeloid_vs_CD4_Non-responder	Macro_ISG15	CD4(IL26+ Th17)	CXCL10	DPP4	3,04508E-04	22869,3	3,71921E-02	701	2,62514E+00	2034	3,72436E+00	2772	8,53474E-01	54419	0	54420,5
Myeloid_vs_CD4_Non-responder	Macro_OLFML3	CD4(IL26+ Th17)	CXCL10	DPP4	3,15074E-04	21582,7	4,33564E-02	522	3,02286E+00	988	3,71587E+00	2804	8,62806E-01	49179	0	54420,5
Myeloid_vs_CD4_Non-responder	CD4(IFNG+ Tfh/Th1)	Macro_FOLR2+APOE+	CD99	CD81	3,15108E-04	24302,3	5,67376E-03	20020	1,56126E+00	13566	2,23334E+00	21733	9,32178E-01	11772	0	54420,5
Myeloid_vs_CD4_Non-responder	CD4(CRTAM- T)	Mono_CD16	HLA-C	LILRB2	3,17082E-04	19472,1	5,05500E-03	23738	2,44450E+00	2810	2,52299E+00	15684	9,57938E-01	708	0	54420,5
Myeloid_vs_CD4_Non-responder	cDC_LAMP3	CD4(TNF+ T)	CCL19	CXCR3	3,19282E-04	23232,3	5,07324E-02	381	5,00629E+00	47	2,49202E+00	16294	8,70301E-01	45019	0	54420,5
Myeloid_vs_CD4_Non-responder	CD4(CRTAM- T)	Macro_FOLR2+APOE+	CD99	CD81	3,22203E-04	24194,9	5,72794E-03	19741	1,56952E+00	13349	2,22897E+00	21830	9,32478E-01	11634	0	54420,5
Myeloid_vs_CD4_Non-responder	pDC_LILRA4	CD4(NME1+ T)	APP	RPSA	3,22245E-04	14843,5	1,34159E-02	4969	2,34902E+00	3351	2,95507E+00	9124	9,52278E-01	2353	0	54420,5
Myeloid_vs_CD4_Non-responder	cDC(CD1C)	CD4(IFNG+ Tfh/Th1)	HLA-DRB5	LAG3	3,24763E-04	16395,5	1,28895E-02	5378	1,81037E+00	8438	3,41793E+00	4599	9,37485E-01	9142	0	54420,5
Myeloid_vs_CD4_Non-responder	CD4(CRTAM- T)	cDC(CD1C)	ANXA1	FPR1	3,26431E-04	21226,1	7,04067E-03	14555	1,80330E+00	8539	2,22663E+00	21887	9,42193E-01	6729	0	54420,5
Myeloid_vs_CD4_Non-responder	CD4(TGFB1+ Th17)	cDC(CD1C)	B2M	CD1C	3,31718E-04	18894,5	1,87422E-02	2678	2,43503E+00	2853	1,81007E+00	33625	9,57005E-01	896	0	54420,5
Myeloid_vs_CD4_Non-responder	CD4(AREG+ Tm)	cDC(CD1C)	B2M	CD1C	3,41132E-04	18981,9	1,87179E-02	2684	2,43030E+00	2880	1,79865E+00	34021	9,56979E-01	904	0	54420,5
Myeloid_vs_CD4_Non-responder	CD4(TNF+ T)	Mono_CD16	HLA-B	LILRA1	3,50307E-04	26229,1	9,50837E-03	9067	1,99215E+00	6169	1,33652E+00	52177	9,37169E-01	9312	0	54420,5
Myeloid_vs_CD4_Non-responder	CD4(TNF+ T)	Mono_CD16	IL23A	LILRB2	3,62025E-04	23794,9	3,23110E-02	921	2,52110E+00	2460	3,07458E+00	7705	8,55169E-01	53468	0	54420,5
Myeloid_vs_CD4_Non-responder	CD4(IL26+ Th17)	Macro_FOLR2+APOE+	CD99	CD81	3,65869E-04	23477,7	5,94169E-03	18675	1,60211E+00	12539	2,27769E+00	20729	9,33622E-01	11025	0	54420,5
Myeloid_vs_CD4_Non-responder	CD4(IL26+ Th17)	Macro_FOLR2+APOE+	CD99	PILRA	3,66114E-04	23148,5	5,60363E-03	20360	1,88962E+00	7311	2,22983E+00	21809	9,32019E-01	11842	0	54420,5

Myeloid_vs_CD4_Post_NR

Myeloid_vs_CD4_Non-responder	Macro_OLFML3	CD4(IFNG+ Tfh/Th1)	HLA-DRA	LAG3	3,67839E-04	16163,3	1,16313E-02	6469	1,75094E+00	9435	3,57701E+00	3567	9,41786E-01	6925	0	54420,5
Myeloid_vs_CD4_Non-responder	cDC(CD1C)	CD4(IFNG+ Tfh/Th1)	HLA-DPA1	LAG3	3,78886E-04	16204,9	1,21691E-02	5959	1,74619E+00	9506	3,48018E+00	4199	9,41761E-01	6940	0	54420,5
Myeloid_vs_CD4_Non-responder	CD4(NME1+ T)	Macro_FOLR2+APOE+	HMGB1	CD163	3,78960E-04	21208,7	5,22978E-03	22550	1,86884E+00	7594	2,60252E+00	14230	9,41175E-01	7249	0	54420,5
Myeloid_vs_CD4_Non-responder	CD4(Tn)	Macro_ISG15	RPS19	C5AR1	3,79717E-04	22107,7	5,42411E-03	21375	1,74584E+00	9515	2,19797E+00	22559	9,51384E-01	2669	0	54420,5
Myeloid_vs_CD4_Non-responder	Macro_ISG15	CD4(TGFB1+ Th17)	CXCL10	CXCR3	3,80929E-04	17845,7	2,56012E-02	1487	2,57645E+00	2212	3,67854E+00	2989	9,00997E-01	28120	0	54420,5
Myeloid_vs_CD4_Non-responder	CD4(CRTAM- T)	cDC_LAMP3	THBS1	TNFRSF11B	3,83204E-04	50534,5	1,76733E-02	2995	2,82738E+00	1422	1,10261E+00	63059	6,80440E-01	130776	0	54420,5
Myeloid_vs_CD4_Non-responder	CD4(IL26+ Th17)	Mono_CD16	CD99	PILRA	3,87609E-04	23306,9	5,55222E-03	20662	1,87322E+00	7539	2,22484E+00	21930	9,31727E-01	11983	0	54420,5
Myeloid_vs_CD4_Non-responder	CD4(NME1+ T)	Macro_FOLR2+APOE+	CD99	CD81	3,87865E-04	25148,9	5,40501E-03	21494	1,52028E+00	14642	2,19424E+00	22655	9,30628E-01	12533	0	54420,5
Myeloid_vs_CD4_Non-responder	CD4(AREG+ Tm)	Macro_ISG15	RPS19	C5AR1	3,88551E-04	22142,1	5,41975E-03	21403	1,74466E+00	9545	2,19400E+00	22663	9,51366E-01	2679	0	54420,5
Myeloid_vs_CD4_Non-responder	Mono_CD14	CD4(IFNG+ Tfh/Th1)	S100A9	ITGB2	3,92010E-04	15688,7	6,15347E-03	17762	3,03410E+00	968	3,83705E+00	2275	9,50440E-01	3018	0	54420,5
Myeloid_vs_CD4_Non-responder	CD4(CRTAM- T)	Macro_ISG15	RPS19	C5AR1	3,93115E-04	21665,3	5,62817E-03	20240	1,80107E+00	8582	2,19180E+00	22716	9,52231E-01	2368	0	54420,5
Myeloid_vs_CD4_Non-responder	Macro_OLFML3	CD4(TGFB1+ Th17)	CXCL10	CXCR3	3,98993E-04	16786,1	2,98445E-02	1060	2,97417E+00	1084	3,67005E+00	3036	9,07630E-01	24330	0	54420,5
Myeloid_vs_CD4_Non-responder	CD4(GZMK+ Teff)	Mono_CD16	HLA-A	LILRB2	3,99473E-04	19522,7	5,19138E-03	22789	2,40526E+00	3026	2,46567E+00	16801	9,58689E-01	577	0	54420,5
Myeloid_vs_CD4_Non-responder	CD4(Tn)	Mono_CD16	RPS19	C5AR1	4,00350E-04	22180,9	5,41534E-03	21428	1,74313E+00	9569	2,18886E+00	22799	9,51347E-01	2688	0	54420,5
Myeloid_vs_CD4_Non-responder	CD4(CRTAM- T)	Mono_CD16	HLA-C	LILRA1	4,03198E-04	23750,9	1,03364E-02	7889	2,21744E+00	4128	1,56229E+00	42660	9,36431E-01	9657	0	54420,5
Myeloid_vs_CD4_Non-responder	CD4(IFNG+ Tfh/Th1)	Macro_FOLR2+APOE+	CD99	PILRA	4,08047E-04	23905,5	5,35094E-03	21794	1,84877E+00	7847	2,18548E+00	22886	9,30543E-01	12580	0	54420,5
Myeloid_vs_CD4_Non-responder	CD4(AREG+ Tm)	Mono_CD16	RPS19	C5AR1	4,09297E-04	22212,5	5,41099E-03	21458	1,74195E+00	9589	2,18489E+00	22900	9,51328E-01	2695	0	54420,5
Myeloid_vs_CD4_Non-responder	CD4(CRTAM- T)	Macro_FOLR2+APOE+	ANXA1	FPR1	4,09386E-04	25290,9	5,17589E-03	22901	1,45323E+00	16638	2,25376E+00	21254	9,33221E-01	11241	0	54420,5
Myeloid_vs_CD4_Non-responder	CD4(CRTAM- T)	Mono_CD16	RPS19	C5AR1	4,14055E-04	21732,5	5,61907E-03	20285	1,79836E+00	8623	2,18269E+00	22953	9,52194E-01	2381	0	54420,5
Myeloid_vs_CD4_Non-responder	Mono_INHBA	CD4(TGFB1+ Th17)	SPP1	CD44	4,14181E-04	16694,7	1,46226E-02	4252	1,95482E+00	6572	2,90785E+00	9723	9,38675E-01	8506	0	54420,5
Myeloid_vs_CD4_Non-responder	CD4(ISG+ Treg)	Mono_CD16	B2M	LILRB2	4,15953E-04	18629,9	5,16648E-03	22974	2,58497E+00	2176	2,64426E+00	13527	9,64583E-01	52	0	54420,5
Myeloid_vs_CD4_Non-responder	CD4(CRTAM- T)	Macro_FOLR2+APOE+	CD99	PILRA	4,16043E-04	23816,1	5,40203E-03	21514	1,85703E+00	7744	2,18111E+00	22975	9,30849E-01	12427	0	54420,5
Myeloid_vs_CD4_Non-responder	CD4(IFNG+ Tfh/Th1)	Mono_CD16	B2M	LILRB2	4,17222E-04	18646,5	5,16388E-03	22988	2,58295E+00	2186	2,64073E+00	13585	9,64574E-01	53	0	54420,5
Myeloid_vs_CD4_Non-responder	CD4(IFNG+ Tfh/Th1)	Mono_CD16	CD99	PILRA	4,17312E-04	24065,7	5,30185E-03	22096	1,83236E+00	8102	2,18049E+00	22989	9,30245E-01	12721	0	54420,5
Myeloid_vs_CD4_Non-responder	Macro_FOLR2+APOE+	CD4(IFNG+ Tfh/Th1)	HLA-DRB5	LAG3	4,17721E-04	16439,3	1,23740E-02	5773	1,74557E+00	9522	3,73152E+00	2737	9,36278E-01	9744	0	54420,5
Myeloid_vs_CD4_Non-responder	CD4(ISG+ Treg)	Mono_CD16	HLA-C	LILRA1	4,21795E-04	23859,7	1,02696E-02	7971	2,20142E+00	4260	1,55595E+00	42879	9,36237E-01	9768	0	54420,5
Myeloid_vs_CD4_Non-responder	CD4(CRTAM- T)	pDC_LILRA4	TNF	PTPRS	4,23268E-04	24982,7	3,64531E-02	727	4,05225E+00	192	1,96459E+00	28819	8,78054E-01	40755	0	54420,5
Myeloid_vs_CD4_Non-responder	Macro_LYVE1	CD4(IFNG+ Tfh/Th1)	HLA-DRA	LAG3	4,25384E-04	17103,3	1,14823E-02	6603	1,73099E+00	9789	3,08611E+00	7579	9,41432E-01	7125	0	54420,5
Myeloid_vs_CD4_Non-responder	Macro_OLFML3	CD4(TNFRSF9+ Treg)	CXCL10	SDC4	4,26114E-04	23936,3	1,99331E-02	2368	2,87719E+00	1293	3,65583E+00	3104	8,46131E-01	58496	0	54420,5
Myeloid_vs_CD4_Non-responder	CD4(CRTAM- T)	Mono_CD16	CD99	PILRA	4,28133E-04	23972,5	5,35248E-03	21788	1,84062E+00	7973	2,17612E+00	23107	9,30552E-01	12574	0	54420,5
Myeloid_vs_CD4_Non-responder	CD4(TNF+ T)	cDC(CD1C)	B2M	CD1C	4,34020E-04	19360,9	1,81355E-02	2863	2,31672E+00	3525	1,77142E+00	34934	9,56323E-01	1062	0	54420,5
Myeloid_vs_CD4_Non-responder	Macro_ISG15	CD4(IL26+ Th17)	S100A8	CD69	4,34020E-04	17322,1	9,50894E-03	9065	1,72895E+00	9839	3,03814E+00	8095	9,45497E-01	5191	0	54420,5
Myeloid_vs_CD4_Non-responder	CD4(TNFRSF9+ Treg)	cDC_LAMP3	LTB	CD40	4,34470E-04	19088,7	1,71242E-02	3171	2,26067E+00	3852	2,17383E+00	23175	9,34096E-01	10825	0	54420,5
Myeloid_vs_CD4_Non-responder	CD4(TNFRSF9+ Treg)	Mono_CD16	HLA-B	LILRB2	4,36818E-04	19040,9	5,13102E-03	23200	2,48552E+00	2607	2,57947E+00	14634	9,60354E-01	343	0	54420,5
Myeloid_vs_CD4_Non-responder	CD4(ISG+ Treg)	Macro_ISG15	HLA-B	LILRB1	4,39082E-04	21802,3	5,15447E-03	23047	2,07605E+00	5331	2,17206E+00	23224	9,50502E-01	2989	0	54420,5
Myeloid_vs_CD4_Non-responder	CD4(CRTAM- T)	Mono_CD16	HLA-A	LILRA1	4,39615E-04	25966,3	1,00845E-02	8220	2,05226E+00	5553	1,34601E+00	51767	9,36022E-01	9871	0	54420,5
Myeloid_vs_CD4_Non-responder	CD4(GZMK+ Teff)	Macro_FOLR2+APOE+	CD99	CD81	4,41261E-04	25688,7	5,24376E-03	22458	1,49569E+00	15320	2,17110E+00	23247	9,29644E-01	12998	0	54420,5
Myeloid_vs_CD4_Non-responder	CD4(CRTAM- T)	pDC_LILRA4	HSP90B1	TLR9	4,47655E-04	29279,3	2,74214E-02	1280	2,72037E+00	1724	1,46769E+00	46521	8,74983E-01	42451	0	54420,5
Myeloid_vs_CD4_Non-responder	CD4(CRTAM- T)	pDC_LILRA4	HMGB1	TLR9	4,49763E-04	29283,1	3,02418E-02	1032	2,58959E+00	2154	1,20677E+00	58078	8,96394E-01	30731	0	54420,5
Myeloid_vs_CD4_Non-responder	CD4(CRTAM- T)	pDC_LILRA4	HRAS	TLR9	4,53575E-04	40762,3	7,69843E-02	158	2,80540E+00	1485	1,71625E+00	36791	7,41388E-01	110957	0	54420,5
Myeloid_vs_CD4_Non-responder	CD4(TNFRSF9+ Treg)	Macro_ISG15	HLA-F	LILRB2	4,53835E-04	23819,7	5,10536E-03	23378	2,03393E+00	5731	2,44490E+00	17207	9,18830E-01	18362	0	54420,5
Myeloid_vs_CD4_Non-responder	pDC_LILRA4	CD4(GZMK+ Teff)	APP	RPSA	4,59257E-04	14912,1	1,36880E-02	4787	2,37322E+00	3204	2,89058E+00	9981	9,52732E-01	2168	0	54420,5
Myeloid_vs_CD4_Non-responder	Macro_ISG15	CD4(TNFRSF9+ Treg)	CXCL10	SDC4	4,59974E-04	25502,9	1,70991E-02	3185	2,47947E+00	2643	3,66432E+00	3063	8,35881E-01	64203	0	54420,5
Myeloid_vs_CD4_Non-responder	CD4(IFNG+ Tfh/Th1)	Mono_CD16	HLA-C	LILRB2	4,66495E-04	19331,5	5,08455E-03	23507	2,45898E+00	2740	2,54375E+00	15309	9,58055E-01	681	0	54420,5
Myeloid_vs_CD4_Non-responder	CD4(TNFRSF9+ Treg)	Mono_CD16	HLA-C	LILRA1	4,66926E-04	24507,9	1,01006E-02	8200	2,16091E+00	4584	1,49630E+00	45312	9,35740E-01	10023	0	54420,5
Myeloid_vs_CD4_Non-responder	CD4(TNF+ T)	pDC_LILRA4	BST2	LILRA4	4,75807E-04	19415,7	1,33246E-02	5033	3,62911E+00	416	3,25552E+00	5941	8,95460E-01	31268	0	54420,5
Myeloid_vs_CD4_Non-responder	CD4(IFNG+ Tfh/Th1)	Macro_FOLR2+APOE+	HMGB1	CD163	4,81875E-04	22000,5	5,06500E-03	23660	1,83367E+00	8084	2,50085E+00	16126	9,40282E-01	7712	0	54420,5
Myeloid_vs_CD4_Non-responder	CD4(TNFRSF9+ Treg)	Mono_CD16	B2M	LILRB2	4,90593E-04	19141,3	5,05398E-03	23745	2,49735E+00	2560	2,56449E+00	14914	9,64205E-01	67	0	54420,5
Myeloid_vs_CD4_Non-responder	cDC_CLEC9A	CD4(IFNG+ Tfh/Th1)	HLA-DQB1	LAG3	4,91839E-04	15487,9	1,47726E-02	4190	1,94080E+00	6712	3,91797E+00	1961	9,35479E-01	10156	0	54420,5
Myeloid_vs_CD4_Non-responder	CD4(NME1+ T)	Macro_FOLR2+APOE+	CD99	PILRA	5,04488E-04	24704,3	5,09748E-03	23430	1,80779E+00	8477	2,14639E+00	23878	9,28958E-01	13316	0	54420,5
Myeloid_vs_CD4_Non-responder	CD4(IL26+ Th17)	Macro_ISG15	CD99	PILRA	5,05651E-04	24252,3	5,28985E-03	22167	1,78950E+00	8778	2,17469E+00	23144	9,30171E-01	12752	0	54420,5
Myeloid_vs_CD4_Non-responder	Macro_FOLR2+APOE+	CD4(ISG+ Treg)	C3	IFITM1	5,11015E-04	17255,7	1,09867E-02	7116	1,78298E+00	8873	2,86965E+00	10255	9,44723E-01	5614	0	54420,5
Myeloid_vs_CD4_Non-responder	CD4(IL26+ Th17)	Mono_CD16	HLA-A	LILRB2	5,13853E-04	20266,3	5,02261E-03	23966	2,32338E+00	3484	2,36956E+00	18772	9,58030E-01	689	0	54420,5
Myeloid_vs_CD4_Non-responder	CD4(ISG+ Treg)	Mono_CD16	HLA-C	LILRB2	5,14067E-04	19562,9	5,02233E-03	23968	2,42848E+00	2888	2,51665E+00	15805	9,57807E-01	733	0	54420,5
Myeloid_vs_CD4_Non-responder	Macro_ISG15	CD4(CRTAM- T)	CXCL10	CXCR3	5,16093E-04	18468,9	2,34039E-02	1760	2,53378E+00	2403	3,61732E+00	3311	8,96922E-01	30450	0	54420,5
Myeloid_vs_CD4_Non-responder	Macro_LYVE1	CD4(IFNG+ Tfh/Th1)	HLA-DRB5	LAG3	5,16141E-04	17966,1	1,20555E-02	6061	1,70554E+00	10281	2,97092E+00	8922	9,35496E-01	10146	0	54420,5
Myeloid_vs_CD4_Non-responder	CD4(Tn)	Macro_FOLR2+APOE+	RPS19	C5AR1	5,18371E-04	22967,7	5,21385E-03	22656	1,68095E+00	10749	2,14169E+00	24008	9,50462E-01	3005	0	54420,5
Myeloid_vs_CD4_Non-responder	CD4(NME1+ T)	Mono_CD16	CD99	PILRA	5,19235E-04	24884,7	5,05071E-03	23781	1,79138E+00	8733	2,14139E+00	24016	9,28654E-01	13473	0	54420,5
Myeloid_vs_CD4_Non-responder	CD4(CRTAM- T)	cDC_CLEC9A	FLT3LG	FLT3	5,19801E-04	29923,7	3,57254E-02	758	3,26480E+00	693	1,68266E+00	38034	8,51169E-01	55713	0	54420,5
Myeloid_vs_CD4_Non-responder	CD4(NME1+ T)	Mono_CD16	HLA-A	LILRB2	5,23574E-04	20157,5	5,00945E-03	24056	2,31699E+00	3524	2,40194E+00	18089	9,57977E-01	698	0	54420,5
Myeloid_vs_CD4_Non-responder	CD4(TNFRSF9+ Treg)	Macro_ISG15	HLA-A	LILRB1	5,23683E-04	22013,3	5,29937E-03	22106	2,01993E+00	5865	2,13952E+00	24057	9,48836E-01	3618	0	54420,5
Myeloid_vs_CD4_Non-responder	CD4(AREG+ Tm)	Macro_FOLR2+APOE+	RPS19	C5AR1	5,28160E-04	22997,1	5,20966E-03	22681	1,67977E+00	10770	2,13772E+00	24098	9,50443E-01	3016	0	54420,5
Myeloid_vs_CD4_Non-responder	CD4(ISG+ Treg)	pDC_LILRA4	HMGB1	THBD	5,29147E-04	27888,1	5,00135E-03	24107	1,52812E+00	14429	2,13834E+00	24079	9,11134E-01	22405	0	54420,5
Myeloid_vs_CD4_Non-responder	CD4(NME1+ T)	pDC_LILRA4	BST2	LILRA4	5,31787E-04	17501,9	1,76865E-02	2990	3,73047E+00	364	3,29379E+00	5604	9,07992E-01	24131	0	54420,5
Myeloid_vs_CD4_Non-responder	CD4(CRTAM- T)	Macro_FOLR2+APOE+	RPS19	C5AR1	5,33994E-04	22484,3	5,41001E-03	21464	1,73618E+00	9688	2,13552E+00	24151	9,51324E-01	2698	0	54420,5
Myeloid_vs_CD4_Non-responder	Macro_ISG15	CD4(TNF+ T)	CXCL10	DPP4	5,37653E-04	27947,1	2,06723E-02	2215	2,44466E+00	2808	3,60981E+00	3357	8,12823E-01	76935	0	54420,5
Myeloid_vs_CD4_Non-responder	Macro_OLFML3	CD4(CRTAM- T)	CXCL10	CXCR3	5,40509E-04	17351,5	2,72829E-02	1292	2,93150E+00	1183	3,60883E+00	3363	9,03799E-01	26499	0	54420,5
Myeloid_vs_CD4_Non-responder	Macro_ISG15	CD4(AREG+ Tm)	CXCL10	DPP4	5,44331E-04	26082,9	2,54821E-02	1498	2,49721E+00	2561	3,60778E+00	3371	8,28218E-01	68564	0	54420,5
Myeloid_vs_CD4_Non-responder	cDC_LAMP3	CD4(IFNG+ Tfh/Th1)	CCL22	CCR4	5,46818E-04	25639,9	4,43410E-02	499	3,87422E+00	284	2,84914E+00	10531	8,39097E-01	62465	0	54420,5
Myeloid_vs_CD4_Non-responder	CD4(CXCL13+ Tfh)	Mono_CD16	HLA-A	LILRB2	5,47280E-04	20448,5	4,98037E-03	24270	2,30288E+00	3612	2,34768E+00	19218	9,57860E-01	722	0	54420,5
Myeloid_vs_CD4_Non-responder	CD4(IFNG+ Tfh/Th1)	Macro_ISG15	CD99	PILRA	5,47393E-04	25080,7	5,05131E-03	23777	1,74864E+00	9467	2,13034E+00	24271	9,28658E-01	13468	0	54420,5

Myeloid_vs_CD4_Post_NR

Myeloid_vs_CD4_Non-responder	CD4(IL26+ Th17)	Mono_CD16	HLA-B	LILRB2	5,48521E-04	19773,3	4,97895E-03	24281	2,40132E+00	3045	2,47177E+00	16697	9,59778E-01	423	0	54420,5
Myeloid_vs_CD4_Non-responder	Macro_FOLR2+APOE+	CD4(IFNG+ Tfh/Th1)	HLA-DPB1	LAG3	5,48949E-04	16567,1	1,19275E-02	6172	1,69724E+00	10443	3,68270E+00	2964	9,38035E-01	8836	0	54420,5
Myeloid_vs_CD4_Non-responder	CD4(CRTAM- T)	Macro_IER3	ANXA1	FPR1	5,50784E-04	21797,1	6,98784E-03	14734	1,79338E+00	8702	2,12937E+00	24301	9,41987E-01	6828	0	54420,5
Myeloid_vs_CD4_Non-responder	CD4(IL26+ Th17)	pDC_LILRA4	HMGB1	THBD	5,50898E-04	27972,7	4,97570E-03	24302	1,52348E+00	14561	2,13841E+00	24072	9,10926E-01	22508	0	54420,5
Myeloid_vs_CD4_Non-responder	pDC_LILRA4	CD4(IL26+ Th17)	APP	RPSA	5,59171E-04	16059,1	1,12447E-02	6827	2,15591E+00	4622	2,85257E+00	10492	9,48102E-01	3934	0	54420,5
Myeloid_vs_CD4_Non-responder	CD4(IL26+ Th17)	Mono_CD16	HLA-C	LILRB2	5,59566E-04	19807,5	4,96672E-03	24378	2,40122E+00	3046	2,48584E+00	16419	9,57582E-01	774	0	54420,5
Myeloid_vs_CD4_Non-responder	CD4(CRTAM- T)	Macro_ISG15	CD99	PILRA	5,61635E-04	24971,5	5,09954E-03	23415	1,75690E+00	9321	2,12597E+00	24396	9,28972E-01	13305	0	54420,5
Myeloid_vs_CD4_Non-responder	CD4(TNF+ T)	Macro_ISG15	IL23A	LILRB2	5,62539E-04	26305,3	2,67643E-02	1342	2,10628E+00	5056	2,85404E+00	10469	8,43111E-01	60239	0	54420,5
Myeloid_vs_CD4_Non-responder	CD4(AREG+ Tm)	Mono_CD16	HLA-F	LILRB2	5,63825E-04	23447,1	4,96163E-03	24415	2,33065E+00	3441	2,50228E+00	16087	9,17758E-01	18872	0	54420,5
Myeloid_vs_CD4_Non-responder	CD4(GZMK+ Teff)	Mono_CD16	HLA-B	LILRB2	5,65675E-04	19621,5	4,95941E-03	24431	2,39050E+00	3108	2,52120E+00	15715	9,59702E-01	433	0	54420,5
Myeloid_vs_CD4_Non-responder	Macro_OLFML3	CD4(TNF+ T)	CXCL10	DPP4	5,67148E-04	26327,7	2,40985E-02	1665	2,84238E+00	1383	3,60132E+00	3418	8,24210E-01	70752	0	54420,5
Myeloid_vs_CD4_Non-responder	CD4(TNFRSF9+ Treg)	cDC_CLEC9A	LTB	CD40	5,70204E-04	22643,1	1,19104E-02	6190	1,65207E+00	11363	2,12324E+00	24470	9,22001E-01	16772	0	54420,5
Myeloid_vs_CD4_Non-responder	Macro_FOLR2+APOE+	CD4(IFNG+ Tfh/Th1)	HLA-DPA1	LAG3	5,71024E-04	16242,7	1,17669E-02	6327	1,69157E+00	10548	3,77949E+00	2495	9,40833E-01	7423	0	54420,5
Myeloid_vs_CD4_Non-responder	Macro_OLFML3	CD4(AREG+ Tm)	CXCL10	DPP4	5,71587E-04	24560,1	2,97055E-02	1080	2,89493E+00	1258	3,59929E+00	3427	8,38854E-01	62615	0	54420,5
Myeloid_vs_CD4_Non-responder	CD4(IL26+ Th17)	Mono_CD16	B2M	LILRB2	5,75584E-04	19735,5	4,94766E-03	24516	2,41454E+00	2971	2,47208E+00	16693	9,63836E-01	77	0	54420,5
Myeloid_vs_CD4_Non-responder	pDC_LILRA4	CD4(CXCL13+ Tfh)	APP	RPSA	5,76591E-04	15126,9	1,34322E-02	4954	2,35047E+00	3341	2,84561E+00	10574	9,52305E-01	2345	0	54420,5
Myeloid_vs_CD4_Non-responder	CD4(GZMK+ Teff)	Macro_FOLR2+APOE+	CD99	PILRA	5,77229E-04	25221,9	4,94540E-03	24530	1,78320E+00	8871	2,12324E+00	24469	9,27952E-01	13819	0	54420,5
Myeloid_vs_CD4_Non-responder	CD4(TNFRSF9+ Treg)	Mono_CD16	HLA-C	LILRB2	5,81359E-04	19973,9	4,93969E-03	24565	2,38796E+00	3119	2,45700E+00	16971	9,57471E-01	794	0	54420,5
Myeloid_vs_CD4_Non-responder	CD4(Tn)	Mono_CD16	HLA-B	LILRB2	5,86839E-04	20473,5	4,80034E-03	25585	2,30242E+00	3614	2,39462E+00	18231	9,59067E-01	517	0	54420,5
Myeloid_vs_CD4_Non-responder	CD4(CRTAM- T)	Mono_CD16	HLA-A	LILRB2	5,89690E-04	20730,5	4,93180E-03	24635	2,27932E+00	3750	2,30672E+00	20088	9,57661E-01	759	0	54420,5
Myeloid_vs_CD4_Non-responder	CD4(Tn)	Mono_CD16	B2M	LILRB2	5,91372E-04	21585,9	4,62799E-03	26974	2,16556E+00	4554	2,22809E+00	21850	9,62654E-01	131	0	54420,5
Myeloid_vs_CD4_Non-responder	Macro_ISG15	CD4(TGFB1+ Th17)	CXCL10	DPP4	5,95145E-04	25593,5	2,69719E-02	1315	2,51348E+00	2494	3,59173E+00	3474	8,32222E-01	66264	0	54420,5
Myeloid_vs_CD4_Non-responder	CD4(TNF+ T)	Macro_OLFML3	HSPA1A	TLR4	5,95216E-04	26580,5	7,02775E-03	14603	1,36435E+00	19552	2,11503E+00	24681	9,16277E-01	19646	0	54420,5
Myeloid_vs_CD4_Non-responder	Macro_FOLR2+APOE+	CD4(IFNG+ Tfh/Th1)	HLA-DRB1	LAG3	5,95290E-04	16222,7	1,17446E-02	6345	1,68523E+00	10660	3,81944E+00	2335	9,40966E-01	7353	0	54420,5
Myeloid_vs_CD4_Non-responder	cDC_CLEC9A	CD4(IFNG+ Tfh/Th1)	HLA-DRB1	LAG3	6,01031E-04	16250,7	1,17323E-02	6364	1,68357E+00	10686	3,80090E+00	2410	9,40937E-01	7373	0	54420,5
Myeloid_vs_CD4_Non-responder	CD4(TGFB1+ Th17)	Macro_FOLR2+APOE+	CD99	CD81	6,05658E-04	21576,9	6,65047E-03	15862	1,71019E+00	10176	2,40398E+00	18052	9,37030E-01	9374	0	54420,5
Myeloid_vs_CD4_Non-responder	CD4(TGFB1+ Th17)	Macro_FOLR2+APOE+	CD99	PILRA	6,06025E-04	21396,3	6,27208E-03	17261	1,99770E+00	6116	2,35612E+00	19047	9,35504E-01	10137	0	54420,5
Myeloid_vs_CD4_Non-responder	cDC_CLEC9A	CD4(IFNG+ Tfh/Th1)	HLA-DRB5	LAG3	6,06813E-04	16956,5	1,18724E-02	6229	1,68252E+00	10712	3,66807E+00	3044	9,35033E-01	10377	0	54420,5
Myeloid_vs_CD4_Non-responder	Mono_INHBA	CD4(CRTAM- T)	SPP1	CD44	6,08824E-04	17195,9	1,40364E-02	4592	1,90648E+00	7106	2,83601E+00	10721	9,37487E-01	9140	0	54420,5
Myeloid_vs_CD4_Non-responder	CD4(TGFB1+ Th17)	Macro_FOLR2+APOE+	TGFB1	ENG	6,13895E-04	20831,7	1,05238E-02	7646	1,89149E+00	7281	2,39891E+00	18139	9,22182E-01	16672	0	54420,5
Myeloid_vs_CD4_Non-responder	CD4(IFNG+ Tfh/Th1)	Mono_CD16	HLA-B	LILRB2	6,15256E-04	20060,9	4,90301E-03	24845	2,35927E+00	3293	2,44160E+00	17285	9,59480E-01	461	0	54420,5
Myeloid_vs_CD4_Non-responder	CD4(GZMK+ Teff)	Mono_CD16	CD99	PILRA	6,18109E-04	25398,1	4,90003E-03	24868	1,76680E+00	9146	2,11825E+00	24602	9,27644E-01	13954	0	54420,5
Myeloid_vs_CD4_Non-responder	CD4(ISG+ Treg)	Macro_ISG15	HLA-A	LILRB1	6,18233E-04	22193,7	5,29331E-03	22145	2,01683E+00	5903	2,10702E+00	24869	9,48808E-01	3631	0	54420,5
Myeloid_vs_CD4_Non-responder	Macro_OLFML3	CD4(TGFB1+ Th17)	CXCL10	DPP4	6,19333E-04	24120,5	3,14422E-02	963	2,91120E+00	1220	3,58324E+00	3521	8,42658E-01	60478	0	54420,5
Myeloid_vs_CD4_Non-responder	CD4(ISG+ Treg)	Macro_FOLR2+APOE+	HLA-F	LILRB2	6,21347E-04	25529,9	4,89701E-03	24894	1,86779E+00	7606	2,23851E+00	21603	9,17262E-01	19126	0	54420,5
Myeloid_vs_CD4_Non-responder	CD4(TGFB1+ Th17)	Macro_NLRP3	ANXA1	FPR1	6,23722E-04	25599,1	5,31274E-03	22021	1,47814E+00	15853	2,10817E+00	24845	9,34029E-01	10856	0	54420,5
Myeloid_vs_CD4_Non-responder	CD4(GZMK+ Teff)	Mono_CD16	B2M	LILRB2	6,36977E-04	20119,7	4,87968E-03	25018	2,36160E+00	3283	2,41587E+00	17791	9,63594E-01	86	0	54420,5
Myeloid_vs_CD4_Non-responder	CD4(TGFB1+ Th17)	Mono_CD16	ANXA1	FPR1	6,38506E-04	19029,9	7,63518E-03	12962	1,94293E+00	6692	2,54465E+00	15288	9,44361E-01	5787	0	54420,5
Myeloid_vs_CD4_Non-responder	CD4(TGFB1+ Th17)	Mono_CD16	HLA-A	LILRB2	6,38889E-04	19713,7	5,16097E-03	23008	2,39051E+00	3107	2,43408E+00	17435	9,58573E-01	598	0	54420,5
Myeloid_vs_CD4_Non-responder	CD4(TGFB1+ Th17)	Mono_CD16	HLA-C	LILRB2	6,40805E-04	18428,1	5,34130E-03	21844	2,58487E+00	2177	2,66759E+00	13176	9,59034E-01	523	0	54420,5
Myeloid_vs_CD4_Non-responder	CD4(TGFB1+ Th17)	Mono_CD16	CD99	PILRA	6,41573E-04	21526,7	6,21454E-03	17502	1,98129E+00	6281	2,35113E+00	19149	9,35225E-01	10281	0	54420,5
Myeloid_vs_CD4_Non-responder	CD4(TGFB1+ Th17)	Mono_CD16	HLA-B	LILRB2	6,42598E-04	20325,5	4,87362E-03	25062	2,34300E+00	3378	2,39202E+00	18291	9,59363E-01	476	0	54420,5
Myeloid_vs_CD4_Non-responder	CD4(Tn)	Mono_CD16	HLA-A	LILRA1	6,44240E-04	28024,9	9,42960E-03	9194	1,89687E+00	7221	1,19966E+00	58413	9,33982E-01	10876	0	54420,5
Myeloid_vs_CD4_Non-responder	cDC(CD1C)	CD4(IFNG+ Tfh/Th1)	HLA-DQB1	LAG3	6,47044E-04	16551,7	1,40526E-02	4579	1,85499E+00	7773	3,35402E+00	5098	9,33955E-01	10888	0	54420,5
Myeloid_vs_CD4_Non-responder	CD4(TGFB1+ Th17)	Macro_OLFML3	ANXA1	FPR1	6,54740E-04	22069,3	6,60031E-03	16054	1,73582E+00	9695	2,19944E+00	22526	9,40409E-01	7651	0	54420,5
Myeloid_vs_CD4_Non-responder	Macro_NLRP3	CD4(GZMK+ Teff)	S100A8	CD69	6,58825E-04	15963,5	1,04300E-02	7771	2,48502E+00	2610	2,82023E+00	10938	9,47830E-01	4078	0	54420,5
Myeloid_vs_CD4_Non-responder	CD4(Tn)	pDC_LILRA4	HMGB1	THBD	6,68122E-04	28292,1	4,84784E-03	25258	1,50036E+00	15169	2,16083E+00	23539	9,09864E-01	23074	0	54420,5
Myeloid_vs_CD4_Non-responder	CD4(IL26+ Th17)	pDC_LILRA4	TNF	PTPRS	6,68672E-04	22620,5	4,23779E-02	552	4,09046E+00	178	2,24723E+00	21416	8,85890E-01	36536	0	54420,5
Myeloid_vs_CD4_Non-responder	CD4(TNFRSF9+ Treg)	Macro_FOLR2+APOE+	ADAM10	GPNMB	6,74378E-04	24027,3	9,55216E-03	8998	2,10890E+00	5027	2,81567E+00	11003	8,78186E-01	40688	0	54420,5
Myeloid_vs_CD4_Non-responder	Macro_FOLR2+APOE+	CD4(ISG+ Treg)	HLA-DRA	LAG3	6,77037E-04	17995,9	8,84970E-03	10222	1,66832E+00	11014	3,60659E+00	3379	9,33826E-01	10944	0	54420,5
Myeloid_vs_CD4_Non-responder	CD4(ISG+ Treg)	Macro_ISG15	HLA-F	LILRB1	6,81184E-04	25596,9	6,26400E-03	17299	1,91537E+00	7002	2,08926E+00	25356	9,08402E-01	23907	0	54420,5
Myeloid_vs_CD4_Non-responder	pDC_LILRA4	CD4(CRTAM- T)	APP	RPSA	6,85793E-04	15200,3	1,34858E-02	4918	2,35524E+00	3311	2,81219E+00	11050	9,52396E-01	2302	0	54420,5
Myeloid_vs_CD4_Non-responder	CD4(ISG+ Treg)	pDC_LILRA4	COPA	P2RY6	6,90447E-04	25371,7	8,43663E-03	11069	3,40834E+00	570	3,08925E+00	7543	8,55519E-01	53256	0	54420,5
Myeloid_vs_CD4_Non-responder	cDC_LAMP3	CD4(IFNG+ Tfh/Th1)	CCL17	CCR4	6,90572E-04	27439,5	4,21537E-02	561	4,04617E+00	195	2,74793E+00	11928	8,25429E-01	70093	0	54420,5
Myeloid_vs_CD4_Non-responder	CD4(AREG+ Tm)	pDC_LILRA4	HMGB1	THBD	6,91182E-04	28716,1	4,82299E-03	25430	1,49587E+00	15317	2,09426E+00	25219	9,09653E-01	23194	0	54420,5
Myeloid_vs_CD4_Non-responder	CD4(GZMK+ Teff)	Macro_FOLR2+APOE+	HMGB1	CD163	6,99512E-04	22700,3	4,81372E-03	25491	1,78006E+00	8914	2,49502E+00	16245	9,38837E-01	8431	0	54420,5
Myeloid_vs_CD4_Non-responder	CD4(AREG+ Tm)	Mono_CD16	ANXA1	FPR1	7,00748E-04	24136,5	5,65645E-03	20107	1,68139E+00	10741	2,08417E+00	25500	9,35935E-01	9914	0	54420,5
Myeloid_vs_CD4_Non-responder	CD4(NME1+ T)	Macro_ISG15	CD99	PILRA	7,01572E-04	25942,1	4,81204E-03	25506	1,70766E+00	10227	2,09124E+00	25302	9,27033E-01	14255	0	54420,5
Myeloid_vs_CD4_Non-responder	Macro_FOLR2+APOE+	CD4(IFNG+ Tfh/Th1)	HLA-DQB1	LAG3	7,03058E-04	16195,3	1,38250E-02	4712	1,82787E+00	8168	3,76861E+00	2556	9,33449E-01	11120	0	54420,5
Myeloid_vs_CD4_Non-responder	cDC_CLEC9A	CD4(IFNG+ Tfh/Th1)	HLA-DQA1	LAG3	7,05550E-04	15500,5	1,53596E-02	3887	1,99495E+00	6139	3,92573E+00	1926	9,33420E-01	11130	0	54420,5
Myeloid_vs_CD4_Non-responder	Mono_CD14	CD4(Tn)	VCAN	SELL	7,06299E-04	14848,5	2,29626E-02	1808	3,36740E+00	612	3,21726E+00	6269	9,33418E-01	11133	0	54420,5
Myeloid_vs_CD4_Non-responder	CD4(TNFRSF9+ Treg)	Macro_ISG15	HLA-F	LILRB1	7,06538E-04	26433,5	5,84782E-03	19160	1,87226E+00	7555	2,08452E+00	25490	9,05502E-01	25542	0	54420,5
Myeloid_vs_CD4_Non-responder	CD4(NME1+ T)	Mono_CD16	B2M	LILRB2	7,16972E-04	20455,3	4,79737E-03	25617	2,29748E+00	3651	2,38361E+00	18491	9,63294E-01	97	0	54420,5
Myeloid_vs_CD4_Non-responder	CD4(GZMK+ Teff)	Mono_CD16	HLA-B	LILRA1	7,22166E-04	23637,7	1,01410E-02	8141	2,16344E+00	4573	1,56050E+00	42730	9,39039E-01	8324	0	54420,5
Myeloid_vs_CD4_Non-responder	CD4(GZMK+ Teff)	Mono_CD16	HLA-A	LILRA1	7,23182E-04	24094,1	1,06153E-02	7524	2,17820E+00	4450	1,50496E+00	44961	9,37541E-01	9115	0	54420,5
Myeloid_vs_CD4_Non-responder	CD4(GZMK+ Teff)	Mono_CD16	HLA-C	LILRA1	7,24963E-04	25478,9	9,73477E-03	8731	2,07318E+00	5366	1,42538E+00	48315	9,34622E-01	10562	0	54420,5
Myeloid_vs_CD4_Non-responder	Macro_FOLR2+APOE+	CD4(IFNG+ Tfh/Th1)	HLA-DQA1	LAG3	7,26237E-04	15513,1	1,52964E-02	3918	1,98765E+00	6214	3,95752E+00	1801	9,33292E-01	11212	0	54420,5
Myeloid_vs_CD4_Non-responder	CD4(CRTAM- T)	Mono_CD16	HLA-B	LILRB2	7,32988E-04	20860,7	4,76415E-03	25878	2,28238E+00	3727	2,32329E+00	19738	9,58918E-01	540	0	54420,5
Myeloid_vs_CD4_Non-responder	CD4(Tn)	pDC_LILRA4	COPA	P2RY6	7,33659E-04	25406,9	8,35066E-03	11241	3,40605E+00	577	3,12478E+00	7160	8,54885E-01	53636	0	54420,5
Myeloid_vs_CD4_Non-responder	Macro_OLFML3	CD4(IFNG+ Tfh/Th1)	HLA-DRB5	LAG3	7,34173E-04	17562,9	1,16732E-02	6427	1,65748E+00	11243	3,35258E+00	5111	9,34517E-01	10613	0	54420,5
Myeloid_vs_CD4_Non-responder	CD4(CRTAM- T)	Macro_ISG15	TGFB1	ENG	7,41655E-04	27379,7	7,03309E-03	14584	1,43684E+00	17114	2,07228E+00	25791	9,06434E-01	24989	0	54420,5

Myeloid_vs_CD4_Post_NR

Myeloid_vs_CD4_Non-responder	CD4(TNF+ T)	Macro_ISG15	RPS19	C5AR1	7,46556E-04	23442,9	5,16229E-03	22997	1,67498E+00	10877	2,07109E+00	25825	9,50227E-01	3095	0	54420,5
Myeloid_vs_CD4_Non-responder	cDC(CD1C)	CD4(ISG+ Treg)	HLA-DRA	LAG3	7,50239E-04	18785,3	8,77775E-03	10348	1,65495E+00	11305	3,16127E+00	6795	9,33573E-01	11058	0	54420,5
Myeloid_vs_CD4_Non-responder	CD4(NME1+ T)	Mono_CD16	HLA-B	LILRB2	7,53229E-04	20739,7	4,76501E-03	25871	2,28286E+00	3723	2,35134E+00	19145	9,58921E-01	539	0	54420,5
Myeloid_vs_CD4_Non-responder	CD4(TNF+ T)	Mono_CD16	HLA-A	LILRA1	7,56790E-04	28756,3	9,15527E-03	9662	1,83178E+00	8108	1,16095E+00	60261	9,33066E-01	11330	0	54420,5
Myeloid_vs_CD4_Non-responder	CD4(NME1+ T)	Mono_CD16	HLA-F	LILRB2	7,57606E-04	24361,3	4,76168E-03	25901	2,31101E+00	3562	2,39460E+00	18233	9,16193E-01	19690	0	54420,5
Myeloid_vs_CD4_Non-responder	CD4(GZMK+ Teff)	Mono_CD16	HLA-C	LILRB2	7,58484E-04	20670,7	4,76077E-03	25907	2,30024E+00	3633	2,38608E+00	18431	9,56713E-01	962	0	54420,5
Myeloid_vs_CD4_Non-responder	CD4(TGFB1+ Th17)	pDC_LILRA4	TNF	TNFRSF21	7,63033E-04	17086,9	3,22688E-02	922	3,02839E+00	976	2,83944E+00	10666	9,18632E-01	18450	0	54420,5
Myeloid_vs_CD4_Non-responder	CD4(TNF+ T)	Mono_INHBA	HSPA1A	TLR4	7,64947E-04	25513,3	7,77103E-03	12587	1,44629E+00	16836	2,06623E+00	25951	9,20054E-01	17772	0	54420,5
Myeloid_vs_CD4_Non-responder	Macro_ISG15	CD4(AREG+ Tm)	S100A8	CD69	7,66564E-04	18527,3	8,69027E-03	10537	1,65193E+00	11367	2,88856E+00	10001	9,43130E-01	6311	0	54420,5
Myeloid_vs_CD4_Non-responder	CD4(TGFB1+ Th17)	pDC_LILRA4	TGFB1	TGFBR1_TGFBR2	7,68047E-04	25855,3	8,43862E-03	11062	1,30277E+00	21747	2,39537E+00	18217	9,08509E-01	23830	0	54420,5
Myeloid_vs_CD4_Non-responder	CD4(TGFB1+ Th17)	pDC_LILRA4	HMGB1	THBD	7,71305E-04	27757,1	5,05865E-03	23717	1,53848E+00	14152	2,12895E+00	24318	9,11594E-01	22178	0	54420,5
Myeloid_vs_CD4_Non-responder	CD4(ISG+ Treg)	Macro_ISG15	B2M	LILRB1	7,74575E-04	22577,1	4,90193E-03	24855	2,00849E+00	6006	2,06333E+00	26016	9,54509E-01	1588	0	54420,5
Myeloid_vs_CD4_Non-responder	CD4(IFNG+ Tfh/Th1)	Macro_ISG15	HLA-F	LILRB2	7,75617E-04	25021,9	4,74304E-03	26023	1,99095E+00	6186	2,37176E+00	18725	9,16042E-01	19755	0	54420,5
Myeloid_vs_CD4_Non-responder	CD4(IL26+ Th17)	cDC_CLEC9A	FLT3LG	FLT3	7,79064E-04	26620,3	4,80257E-02	434	3,38886E+00	596	1,86546E+00	31853	8,68953E-01	45798	0	54420,5
Myeloid_vs_CD4_Non-responder	CD4(TNF+ T)	Mono_CD16	RPS19	C5AR1	7,79350E-04	23513,5	5,15394E-03	23054	1,67227E+00	10935	2,06198E+00	26048	9,50189E-01	3110	0	54420,5
Myeloid_vs_CD4_Non-responder	cDC(CD1C)	CD4(IFNG+ Tfh/Th1)	HLA-DRB1	LAG3	7,80458E-04	17169,1	1,14800E-02	6607	1,64938E+00	11419	3,28186E+00	5710	9,40330E-01	7689	0	54420,5
Myeloid_vs_CD4_Non-responder	CD4(Tn)	Mono_CD16	ANXA1	FPR1	7,81747E-04	25133,3	5,26273E-03	22337	1,62935E+00	11863	2,06143E+00	26064	9,33737E-01	10982	0	54420,5
Myeloid_vs_CD4_Non-responder	CD4(TNF+ T)	Macro_FOLR2+APOE+	CD52	SIGLEC10	7,84901E-04	23314,7	7,62995E-03	12982	1,73447E+00	9727	2,06058E+00	26085	9,28873E-01	13359	0	54420,5
Myeloid_vs_CD4_Non-responder	CD4(IFNG+ Tfh/Th1)	Macro_ISG15	B2M	LILRB1	7,87311E-04	22602,9	4,89947E-03	24875	2,00647E+00	6028	2,05980E+00	26101	9,54498E-01	1590	0	54420,5
Myeloid_vs_CD4_Non-responder	CD4(TNFRSF9+ Treg)	pDC_LILRA4	COPA	P2RY6	7,91272E-04	25645,3	8,24585E-03	11459	3,40325E+00	581	3,07546E+00	7699	8,54100E-01	54067	0	54420,5
Myeloid_vs_CD4_Non-responder	CD4(TNFRSF9+ Treg)	pDC_LILRA4	HMGB1	THBD	7,94274E-04	28839,9	4,84015E-03	25304	1,49897E+00	15219	2,05772E+00	26147	9,09799E-01	23109	0	54420,5
Myeloid_vs_CD4_Non-responder	CD4(Tn)	Macro_NLRP3	RPS19	C5AR1	7,95641E-04	22903,5	5,39593E-03	21545	1,73714E+00	9668	2,05740E+00	26156	9,51264E-01	2728	0	54420,5
Myeloid_vs_CD4_Non-responder	CD4(CXCL13+ Tfh)	Mono_CD16	B2M	LILRB2	7,97164E-04	20982,1	4,72618E-03	26166	2,24203E+00	3982	2,30093E+00	20232	9,63029E-01	110	0	54420,5
Myeloid_vs_CD4_Non-responder	CD4(TNF+ T)	Macro_FOLR2+APOE+	TNF	TNFRSF1A	8,00673E-04	27352,5	8,14129E-03	11720	1,20616E+00	25780	2,37461E+00	18653	9,04354E-01	26189	0	54420,5
Myeloid_vs_CD4_Non-responder	CD4(CXCL13+ Tfh)	Mono_CD16	HLA-B	LILRB2	8,00673E-04	20852,7	4,72369E-03	26189	2,25998E+00	3861	2,34715E+00	19227	9,58749E-01	566	0	54420,5
Myeloid_vs_CD4_Non-responder	CD4(CRTAM- T)	Mono_CD16	B2M	LILRB2	8,07730E-04	21131,5	4,71908E-03	26235	2,23651E+00	4015	2,27087E+00	20875	9,63002E-01	112	0	54420,5
Myeloid_vs_CD4_Non-responder	CD4(TNF+ T)	Macro_FOLR2+APOE+	CD99	CD81	8,12204E-04	27603,5	4,71625E-03	26264	1,41526E+00	17794	2,10967E+00	24808	9,26097E-01	14731	0	54420,5
Myeloid_vs_CD4_Non-responder	CD4(AREG+ Tm)	Macro_NLRP3	RPS19	C5AR1	8,12359E-04	22936,9	5,39160E-03	21570	1,73596E+00	9693	2,05343E+00	26265	9,51245E-01	2736	0	54420,5
Myeloid_vs_CD4_Non-responder	CD4(TGFB1+ Th17)	Mono_CD14	ANXA1	FPR1	8,16698E-04	18875,5	7,99809E-03	12045	2,01556E+00	5921	2,46409E+00	16834	9,45569E-01	5157	0	54420,5
Myeloid_vs_CD4_Non-responder	Macro_OLFML3	CD4(TGFB1+ Th17)	CXCL10	SDC4	8,18821E-04	25663,5	1,66350E-02	3343	2,83150E+00	1412	3,52899E+00	3869	8,33985E-01	65273	0	54420,5
Myeloid_vs_CD4_Non-responder	CD4(NME1+ T)	Macro_NLRP3	HMGB1	THBD	8,19186E-04	25084,7	6,23879E-03	17418	1,75694E+00	9320	2,05228E+00	26309	9,19687E-01	17956	0	54420,5
Myeloid_vs_CD4_Non-responder	Macro_IER3	CD4(IFNG+ Tfh/Th1)	HLA-DRA	LAG3	8,19350E-04	17832,3	1,08213E-02	7297	1,64250E+00	11561	3,05287E+00	7930	9,39776E-01	7953	0	54420,5
Myeloid_vs_CD4_Non-responder	CD4(AREG+ Tm)	Macro_ISG15	ANXA1	FPR1	8,21524E-04	27367,3	4,72934E-03	26142	1,43094E+00	17287	2,05164E+00	26324	9,30354E-01	12663	0	54420,5
Myeloid_vs_CD4_Non-responder	Macro_ISG15	CD4(GZMK+ Teff)	CXCL10	CXCR3	8,21958E-04	19521,1	2,02095E-02	2312	2,47175E+00	2680	3,52793E+00	3874	8,89938E-01	34319	0	54420,5
Myeloid_vs_CD4_Non-responder	CD4(CRTAM- T)	Macro_NLRP3	RPS19	C5AR1	8,23242E-04	22453,3	5,59894E-03	20384	1,79237E+00	8713	2,05123E+00	26335	9,52113E-01	2414	0	54420,5
Myeloid_vs_CD4_Non-responder	CD4(CRTAM- T)	cDC_CLEC9A	ANXA1	DYSF	8,34584E-04	24771,9	1,82034E-02	2845	2,25459E+00	3894	1,74632E+00	35778	9,03036E-01	26922	0	54420,5
Myeloid_vs_CD4_Non-responder	CD4(TGFB1+ Th17)	Macro_ISG15	ANXA1	FPR2_FPR3	8,35031E-04	21263,7	1,15375E-02	6557	2,03057E+00	5759	2,12383E+00	24452	9,25275E-01	15130	0	54420,5
Myeloid_vs_CD4_Non-responder	CD4(TGFB1+ Th17)	Macro_ISG15	ANXA1	FPR1	8,35189E-04	21158,5	6,38374E-03	16828	1,69248E+00	10532	2,51213E+00	15892	9,39467E-01	8120	0	54420,5
Myeloid_vs_CD4_Non-responder	CD4(TGFB1+ Th17)	Macro_ISG15	HLA-C	LILRB1	8,38039E-04	22745,3	5,06780E-03	23626	2,00839E+00	6008	2,08666E+00	25424	9,47468E-01	4248	0	54420,5
Myeloid_vs_CD4_Non-responder	CD4(TGFB1+ Th17)	Macro_ISG15	CD99	PILRA	8,38832E-04	22324,9	5,92087E-03	18787	1,89757E+00	7212	2,30098E+00	20231	9,33743E-01	10974	0	54420,5
Myeloid_vs_CD4_Non-responder	CD4(TGFB1+ Th17)	Mono_CD16	B2M	LILRB2	8,39467E-04	21200,7	4,69263E-03	26438	2,21590E+00	4143	2,27030E+00	20887	9,62902E-01	115	0	54420,5
Myeloid_vs_CD4_Non-responder	CD4(AREG+ Tm)	Macro_FOLR2+APOE+	CD99	CD81	8,43444E-04	27848,1	4,74061E-03	26045	1,41897E+00	17661	2,04738E+00	26463	9,26273E-01	14651	0	54420,5
Myeloid_vs_CD4_Non-responder	cDC_LAMP3	CD4(CRTAM- T)	CCL22	CCR4	8,45511E-04	26716,3	3,94772E-02	621	3,85614E+00	293	2,78928E+00	11337	8,31098E-01	66910	0	54420,5
Myeloid_vs_CD4_Non-responder	CD4(AREG+ Tm)	Mono_CD16	B2M	LILRB2	8,47116E-04	21268,7	4,68655E-03	26486	2,21117E+00	4187	2,25888E+00	21131	9,62879E-01	119	0	54420,5
Myeloid_vs_CD4_Non-responder	CD4(TGFB1+ Th17)	Macro_ISG15	TGFB1	ENG	8,48075E-04	24889,9	7,95761E-03	12137	1,55937E+00	13608	2,21862E+00	22079	9,11542E-01	22205	0	54420,5
Myeloid_vs_CD4_Non-responder	Macro_OLFML3	CD4(GZMK+ Teff)	CXCL10	CXCR3	8,48620E-04	18333,3	2,35591E-02	1738	2,86948E+00	1315	3,51943E+00	3916	8,97227E-01	30277	0	54420,5
Myeloid_vs_CD4_Non-responder	Macro_LYVE1	CD4(IFNG+ Tfh/Th1)	HLA-DPA1	LAG3	8,53321E-04	17791,3	1,13763E-02	6703	1,63850E+00	11681	3,02476E+00	8253	9,39886E-01	7899	0	54420,5
Myeloid_vs_CD4_Non-responder	CD4(AREG+ Tm)	Mono_CD16	HLA-A	LILRB2	8,55625E-04	21990,9	4,68038E-03	26539	2,15734E+00	4609	2,16613E+00	23398	9,56588E-01	988	0	54420,5
Myeloid_vs_CD4_Non-responder	Macro_ISG15	CD4(IFNG+ Tfh/Th1)	HLA-DRA	LAG3	8,56198E-04	17091,1	1,07873E-02	7331	1,63797E+00	11691	3,50456E+00	4014	9,39687E-01	7999	0	54420,5
Myeloid_vs_CD4_Non-responder	CD4(TNFRSF9+ Treg)	Macro_OLFML3	LTB	CD40	8,56593E-04	22091,5	1,29841E-02	5286	1,77740E+00	8957	2,04418E+00	26545	9,25048E-01	15249	0	54420,5
Myeloid_vs_CD4_Non-responder	CD4(TGFB1+ Th17)	Macro_ISG15	RPS19	C5AR1	8,63066E-04	23391,1	5,23738E-03	22503	1,69530E+00	10478	2,04247E+00	26585	9,50568E-01	2969	0	54420,5
Myeloid_vs_CD4_Non-responder	CD4(GZMK+ Teff)	Macro_ISG15	CD99	PILRA	8,71049E-04	26486,5	4,66848E-03	26634	1,68307E+00	10699	2,06810E+00	25899	9,26002E-01	14780	0	54420,5
Myeloid_vs_CD4_Non-responder	CD4(TNFRSF9+ Treg)	Macro_FOLR2+APOE+	LTB	TNFRSF1A	8,75473E-04	25743,5	9,00679E-03	9939	1,23962E+00	24357	2,03966E+00	26661	9,28914E-01	13340	0	54420,5
Myeloid_vs_CD4_Non-responder	Macro_FOLR2+APOE+	CD4(Tn)	APOE	LSR	8,82226E-04	18585,9	2,01563E-02	2324	1,89234E+00	7273	3,77826E+00	2503	9,03960E-01	26409	0	54420,5
Myeloid_vs_CD4_Non-responder	Macro_FOLR2+APOE+	CD4(Tn)	MMP9	CD44	8,88023E-04	24519,5	9,48745E-03	9099	1,49895E+00	15220	2,43873E+00	17338	9,03763E-01	26520	0	54420,5
Myeloid_vs_CD4_Non-responder	CD4(TNF+ T)	Macro_ISG15	CCL5	CCR1	8,88687E-04	22555,9	2,52327E-02	1533	2,01345E+00	5944	2,13605E+00	24141	9,03345E-01	26741	0	54420,5
Myeloid_vs_CD4_Non-responder	Macro_FOLR2+APOE+	CD4(Tn)	C3	IFITM1	8,89352E-04	23402,5	7,69123E-03	12818	1,39598E+00	18453	2,27577E+00	20767	9,34639E-01	10554	0	54420,5
Myeloid_vs_CD4_Non-responder	Macro_FOLR2+APOE+	CD4(CRTAM- T)	LGALS9	CD44	8,93350E-04	23128,7	5,20370E-03	22713	1,65135E+00	11380	2,29233E+00	20432	9,42259E-01	6698	0	54420,5
Myeloid_vs_CD4_Non-responder	CD4(TNF+ T)	Mono_CD16	HLA-B	LILRB2	8,96692E-04	21253,1	4,65005E-03	26789	2,21920E+00	4121	2,29722E+00	20318	9,58438E-01	617	0	54420,5
Myeloid_vs_CD4_Non-responder	cDC_CLEC9A	CD4(ISG+ Treg)	HLA-DRA	LAG3	8,96941E-04	18458,9	8,64593E-03	10613	1,63044E+00	11830	3,49068E+00	4120	9,33102E-01	11311	0	54420,5
Myeloid_vs_CD4_Non-responder	Macro_FOLR2+APOE+	CD4(CRTAM- T)	CD14	ITGB1	8,98870E-04	19475,5	6,19052E-03	17611	1,97491E+00	6358	3,06495E+00	7808	9,33348E-01	11180	0	54420,5
Myeloid_vs_CD4_Non-responder	Macro_FOLR2+APOE+	CD4(CRTAM- T)	CD14	ITGA4	8,99037E-04	22577,3	4,92931E-03	24653	1,87405E+00	7523	2,91060E+00	9691	9,22324E-01	16599	0	54420,5
Myeloid_vs_CD4_Non-responder	CD4(NME1+ T)	Mono_CD16	HLA-C	LILRB2	9,02733E-04	21110,9	4,64668E-03	26825	2,24430E+00	3967	2,34644E+00	19239	9,56208E-01	1103	0	54420,5
Myeloid_vs_CD4_Non-responder	CD4(TGFB1+ Th17)	Mono_CD16	RPS19	C5AR1	9,05935E-04	23466,1	5,22891E-03	22558	1,69259E+00	10529	2,03336E+00	26844	9,50530E-01	2979	0	54420,5
Myeloid_vs_CD4_Non-responder	Macro_FOLR2+APOE+	CD4(CRTAM- T)	SPP1	CD44	9,10500E-04	19394,3	1,00486E-02	8278	1,46312E+00	16318	3,56175E+00	3657	9,26947E-01	14298	0	54420,5
Myeloid_vs_CD4_Non-responder	CD4(TNF+ T)	Mono_CD16	HLA-C	LILRA1	9,10736E-04	28103,9	8,93600E-03	10059	1,88165E+00	7425	1,23473E+00	56739	9,31957E-01	11876	0	54420,5
Myeloid_vs_CD4_Non-responder	Macro_FOLR2+APOE+	CD4(CRTAM- T)	MMP9	CD44	9,17467E-04	18822,1	1,40045E-02	4613	1,87225E+00	7556	2,93255E+00	9424	9,19418E-01	18097	0	54420,5
Myeloid_vs_CD4_Non-responder	CD4(GZMK+ Teff)	pDC_LILRA4	COPA	P2RY6	9,22553E-04	25781,3	8,07132E-03	11886	3,39860E+00	584	3,12005E+00	7207	8,52762E-01	54809	0	54420,5
Myeloid_vs_CD4_Non-responder	CD4(CRTAM- T)	pDC_LILRA4	TGFB1	TGFBR1_TGFBR2	9,27741E-04	28600,5	7,45822E-03	13435	1,18023E+00	26972	2,24903E+00	21376	9,03245E-01	26799	0	54420,5
Myeloid_vs_CD4_Non-responder	Macro_FOLR2+APOE+	CD4(TNFRSF9+ Treg)	HLA-DRA	LAG3	9,28085E-04	22079,9	5,82126E-03	19288	1,51802E+00	14715	3,50589E+00	4006	9,19647E-01	17970	0	54420,5
Myeloid_vs_CD4_Non-responder	Macro_FOLR2+APOE+	CD4(TNFRSF9+ Treg)	HLA-DQA1	LAG3	9,28773E-04	21834,3	7,24569E-03	13992	1,66664E+00	11046	3,44429E+00	4421	9,05919E-01	25292	0	54420,5

Myeloid_vs_CD4_Post_NR

Myeloid_vs_CD4_Non-responder	CD4(AREG+ Tm)	pDC_LILRA4	TNF	PTPRS	9,28805E-04	23247,5	3,84962E-02	651	4,06542E+00	188	2,23125E+00	21775	8,80943E-01	39203	0	54420,5
Myeloid_vs_CD4_Non-responder	Macro_FOLR2+APOE+	CD4(TNFRSF9+ Treg)	HLA-DRB1	LAG3	9,29117E-04	23921,1	5,56323E-03	20588	1,36423E+00	19557	3,30621E+00	5500	9,16460E-01	19540	0	54420,5
Myeloid_vs_CD4_Non-responder	Macro_FOLR2+APOE+	CD4(TNFRSF9+ Treg)	HLA-DQB1	LAG3	9,29462E-04	23353,9	6,54872E-03	16249	1,50686E+00	15000	3,25538E+00	5943	9,06134E-01	25157	0	54420,5
Myeloid_vs_CD4_Non-responder	Macro_FOLR2+APOE+	CD4(TNFRSF9+ Treg)	HLA-DPB1	LAG3	9,30323E-04	24423,5	5,64989E-03	20142	1,37623E+00	19129	3,16947E+00	6726	9,12425E-01	21700	0	54420,5
Myeloid_vs_CD4_Non-responder	CD4(TNFRSF9+ Treg)	Macro_ISG15	HLA-A	LILRB2	9,30323E-04	20606,1	4,62654E-03	26987	2,18160E+00	4416	2,49991E+00	16150	9,56347E-01	1057	0	54420,5
Myeloid_vs_CD4_Non-responder	Macro_FOLR2+APOE+	CD4(TNFRSF9+ Treg)	HLA-DPA1	LAG3	9,34294E-04	23954,1	5,57383E-03	20528	1,37056E+00	19322	3,26627E+00	5852	9,16276E-01	19648	0	54420,5
Myeloid_vs_CD4_Non-responder	Macro_FOLR2+APOE+	CD4(TNFRSF9+ Treg)	CD86	CTLA4	9,35852E-04	19540,7	1,55355E-02	3813	1,95213E+00	6597	2,55861E+00	15036	9,19925E-01	17837	0	54420,5
Myeloid_vs_CD4_Non-responder	Mast	CD4(TGFB1+ Th17)	TIMP3	CD44	9,37347E-04	21291,1	5,24671E-02	352	3,31601E+00	654	1,74343E+00	35873	9,25213E-01	15156	0	54420,5
Myeloid_vs_CD4_Non-responder	CD4(ISG+ Treg)	Macro_ISG15	HLA-A	LILRB2	9,38453E-04	20746,9	4,62126E-03	27034	2,17850E+00	4445	2,46740E+00	16771	9,56323E-01	1064	0	54420,5
Myeloid_vs_CD4_Non-responder	Macro_FOLR2+APOE+	CD4(TNFRSF9+ Treg)	HLA-DRB5	LAG3	9,38626E-04	24045,1	5,86139E-03	19085	1,42457E+00	17470	3,21829E+00	6261	9,10012E-01	22989	0	54420,5
Myeloid_vs_CD4_Non-responder	CD4(GZMK+ Teff)	Macro_ISG15	RPS19	C5AR1	9,42103E-04	23582,3	5,19946E-03	22738	1,68504E+00	10663	2,02616E+00	27055	9,50397E-01	3035	0	54420,5
Myeloid_vs_CD4_Non-responder	Macro_FOLR2+APOE+	CD4(TNFRSF9+ Treg)	CXCL16	CXCR6	9,49438E-04	23643,7	1,05003E-02	7669	1,55549E+00	13699	2,48792E+00	16381	9,04598E-01	26049	0	54420,5
Myeloid_vs_CD4_Non-responder	CD4(Tn)	Mono_CD16	HLA-A	LILRB2	9,50841E-04	22212,7	4,61152E-03	27105	2,12393E+00	4900	2,16036E+00	23559	9,56279E-01	1079	0	54420,5
Myeloid_vs_CD4_Non-responder	CD4(IL26+ Th17)	Mono_CD16	HLA-F	LILRB2	9,55938E-04	24566,7	4,60867E-03	27134	2,29597E+00	3656	2,43982E+00	17319	9,14930E-01	20304	0	54420,5
Myeloid_vs_CD4_Non-responder	CD4(TNF+ T)	cDC_CLEC9A	FLT3LG	FLT3	9,57431E-04	28079,7	3,98814E-02	608	3,30671E+00	661	1,83315E+00	32871	8,58005E-01	51838	0	54420,5
Myeloid_vs_CD4_Non-responder	Macro_FOLR2+APOE+	CD4(ISG+ Treg)	HLA-DQA1	LAG3	9,62828E-04	18040,9	1,10152E-02	7084	1,81694E+00	8327	3,54498E+00	3771	9,22315E-01	16602	0	54420,5
Myeloid_vs_CD4_Non-responder	Macro_FOLR2+APOE+	CD4(ISG+ Treg)	HLA-DRB1	LAG3	9,63182E-04	19442,1	8,45744E-03	11017	1,51453E+00	14795	3,40691E+00	4675	9,31159E-01	12303	0	54420,5
Myeloid_vs_CD4_Non-responder	Macro_FOLR2+APOE+	CD4(ISG+ Treg)	HLA-DQB1	LAG3	9,63537E-04	19136,7	9,95561E-03	8412	1,65716E+00	11260	3,35607E+00	5076	9,22496E-01	16515	0	54420,5
Myeloid_vs_CD4_Non-responder	Macro_FOLR2+APOE+	CD4(ISG+ Treg)	HLA-DPB1	LAG3	9,64424E-04	19870,9	8,58917E-03	10743	1,52653E+00	14479	3,27017E+00	5815	9,27777E-01	13897	0	54420,5
Myeloid_vs_CD4_Non-responder	pDC_LILRA4	CD4(ISG+ Treg)	APP	RPSA	9,64970E-04	15882,9	1,22564E-02	5872	2,24590E+00	3958	2,74064E+00	12052	9,50182E-01	3112	0	54420,5
Myeloid_vs_CD4_Non-responder	Macro_FOLR2+APOE+	CD4(ISG+ Treg)	HLA-DPA1	LAG3	9,68689E-04	19475,7	8,47355E-03	10978	1,52086E+00	14628	3,36696E+00	4992	9,31005E-01	12360	0	54420,5
Myeloid_vs_CD4_Non-responder	Macro_ISG15	CD4(CRTAM- T)	CXCL10	DPP4	9,70028E-04	29511,7	1,78035E-02	2943	2,41332E+00	2979	3,49283E+00	4097	8,01192E-01	83119	0	54420,5
Myeloid_vs_CD4_Non-responder	Macro_FOLR2+APOE+	CD4(ISG+ Treg)	CD86	CTLA4	9,70292E-04	20067,5	1,62577E-02	3477	1,98511E+00	6246	2,34871E+00	19198	9,21582E-01	16996	0	54420,5
Myeloid_vs_CD4_Non-responder	Macro_FOLR2+APOE+	CD4(ISG+ Treg)	HLA-DRB5	LAG3	9,72612E-04	19605,3	8,91071E-03	10117	1,57487E+00	13192	3,31899E+00	5388	9,25754E-01	14909	0	54420,5
Myeloid_vs_CD4_Non-responder	cDC(CD1C)	CD4(IFNG+ Tfh/Th1)	HLA-DQA1	LAG3	9,76034E-04	16720,1	1,44696E-02	4331	1,89211E+00	7275	3,30828E+00	5487	9,31541E-01	12087	0	54420,5
Myeloid_vs_CD4_Non-responder	CD4(NME1+ T)	Macro_LYVE1	HMGB1	CD163	9,76189E-04	22916,7	5,59705E-03	20396	1,99299E+00	6163	2,01918E+00	27248	9,43025E-01	6356	0	54420,5
Myeloid_vs_CD4_Non-responder	CD4(Tn)	pDC_LILRA4	TNF	PTPRS	9,79162E-04	34006,7	1,49278E-02	4110	3,91343E+00	263	1,65284E+00	39098	8,21674E-01	72142	0	54420,5
Myeloid_vs_CD4_Non-responder	Macro_FOLR2+APOE+	CD4(ISG+ Treg)	LGALS3	LAG3	9,79957E-04	24603,7	9,61768E-03	8907	1,23503E+00	24553	2,73497E+00	12145	9,10000E-01	22993	0	54420,5
Myeloid_vs_CD4_Non-responder	CD4(TGFB1+ Th17)	Macro_FOLR2+APOE+	ANXA1	FPR1	9,82115E-04	27594,1	4,85510E-03	25200	1,38655E+00	18790	2,01778E+00	27281	9,31199E-01	12279	0	54420,5
Myeloid_vs_CD4_Non-responder	Macro_FOLR2+APOE+	CD4(ISG+ Treg)	CXCL16	CXCR6	9,84096E-04	22194,7	1,22547E-02	5878	1,62649E+00	11927	2,49128E+00	16311	9,11060E-01	22437	0	54420,5
Myeloid_vs_CD4_Non-responder	CD4(GZMK+ Teff)	Mono_CD16	RPS19	C5AR1	9,85720E-04	23656,3	5,19105E-03	22792	1,68233E+00	10717	2,01705E+00	27301	9,50358E-01	3051	0	54420,5
Myeloid_vs_CD4_Non-responder	CD4(AREG+ Tm)	cDC_CLEC9A	FLT3LG	FLT3	9,86488E-04	28870,7	3,86939E-02	644	3,29474E+00	671	1,74775E+00	35725	8,56154E-01	52893	0	54420,5
Myeloid_vs_CD4_Non-responder	CD4(CXCL13+ Tfh)	Macro_FOLR2+APOE+	HMGB1	CD163	9,91329E-04	23539,7	4,58335E-03	27332	1,73090E+00	9790	2,45680E+00	16976	9,37414E-01	9180	0	54420,5
Myeloid_vs_CD4_Non-responder	cDC_LAMP3	CD4(IFNG+ Tfh/Th1)	CCL19	CXCR3	9,92351E-04	26014,1	3,78108E-02	673	4,92866E+00	51	2,30459E+00	20135	8,52788E-01	54791	0	54420,5
Myeloid_vs_CD4_Non-responder	Macro_OLFML3	CD4(IFNG+ Tfh/Th1)	HLA-DPB1	LAG3	9,94576E-04	17674,5	1,13322E-02	6753	1,61844E+00	12145	3,31418E+00	5433	9,36530E-01	9621	0	54420,5
Myeloid_vs_CD4_Non-responder	CD4(TNF+ T)	Macro_FOLR2+APOE+	RPS19	C5AR1	9,96781E-04	24392,5	4,96218E-03	24411	1,61009E+00	12332	2,01481E+00	27362	9,49284E-01	3437	0	54420,5
Myeloid_vs_CD4_Non-responder	CD4(AREG+ Tm)	Mono_CD16	HLA-C	LILRB2	9,97693E-04	21636,9	4,57988E-03	27367	2,21155E+00	4184	2,26473E+00	21026	9,55904E-01	1187	0	54420,5
Myeloid_vs_CD4_Non-responder	CD4(TNF+ T)	pDC_LILRA4	HMGB1	THBD	1,00061E-03	29453,7	4,57857E-03	27383	1,45167E+00	16694	2,12707E+00	24367	9,07493E-01	24404	0	54420,5
Myeloid_vs_CD4_Non-responder	Macro_IFI27	CD4(IFNG+ Tfh/Th1)	HLA-DRA	LAG3	1,00492E-03	17967,3	1,15376E-02	6555	1,73840E+00	9644	2,73307E+00	12177	9,41564E-01	7040	0	54420,5
Myeloid_vs_CD4_Non-responder	Macro_FOLR2+APOE+	CD4(TNF+ T)	APOE	LDLR	1,00886E-03	19029,5	1,38051E-02	4724	1,87916E+00	7462	3,94048E+00	1864	9,03472E-01	26677	0	54420,5
Myeloid_vs_CD4_Non-responder	CD4(TNFRSF9+ Treg)	Macro_FOLR2+APOE+	HLA-F	LILRB2	1,01089E-03	26456,1	4,57166E-03	27439	1,82469E+00	8207	2,23377E+00	21724	9,14616E-01	20490	0	54420,5
Myeloid_vs_CD4_Non-responder	CD4(IFNG+ Tfh/Th1)	Macro_ISG15	HLA-F	LILRB1	1,01181E-03	27737,7	5,43280E-03	21323	1,82928E+00	8140	2,01138E+00	27444	9,02305E-01	27361	0	54420,5
Myeloid_vs_CD4_Non-responder	Macro_FOLR2+APOE+	CD4(CRTAM- T)	TIMP2	CD44	1,01218E-03	25464,1	6,78999E-03	15397	1,59412E+00	12721	2,01126E+00	27446	9,20882E-01	17336	0	54420,5
Myeloid_vs_CD4_Non-responder	Mono_INHBA	CD4(TGFB1+ Th17)	IL1B	ADRB2	1,02350E-03	20748,7	1,56349E-02	3761	2,44126E+00	2826	3,48455E+00	4172	8,82071E-01	38564	0	54420,5
Myeloid_vs_CD4_Non-responder	Macro_OLFML3	CD4(CRTAM- T)	CXCL10	DPP4	1,02641E-03	27806,9	2,07543E-02	2201	2,81104E+00	1468	3,48433E+00	4176	8,13124E-01	76769	0	54420,5
Myeloid_vs_CD4_Non-responder	CD4(Tn)	Mono_CD16	HLA-C	LILRA1	1,02847E-03	28865,3	8,74270E-03	10420	1,83530E+00	8054	1,18391E+00	59183	9,31261E-01	12249	0	54420,5
Myeloid_vs_CD4_Non-responder	CD4(CXCL13+ Tfh)	Macro_ISG15	RPS19	C5AR1	1,03000E-03	23725,5	5,18333E-03	22846	1,68067E+00	10756	2,00832E+00	27542	9,50323E-01	3063	0	54420,5
Myeloid_vs_CD4_Non-responder	cDC_LAMP3	CD4(CRTAM- T)	CCL17	CCR4	1,03387E-03	28574,9	3,75299E-02	687	4,02809E+00	200	2,68808E+00	12856	8,16899E-01	74711	0	54420,5
Myeloid_vs_CD4_Non-responder	CD4(NME1+ T)	Macro_ISG15	RPS19	C5AR1	1,03506E-03	24344,5	4,97424E-03	24314	1,62408E+00	11996	2,00736E+00	27569	9,49342E-01	3423	0	54420,5
Myeloid_vs_CD4_Non-responder	Macro_FOLR2+APOE+	CD4(GZMK+ Teff)	SPP1	CD44	1,04259E-03	24108,9	7,58779E-03	13094	1,17969E+00	27008	3,17501E+00	6675	9,16848E-01	19347	0	54420,5
Myeloid_vs_CD4_Non-responder	Mono_CD14	CD4(CRTAM- T)	VCAN	ITGB1	1,04474E-03	16282,7	1,52440E-02	3944	3,24074E+00	707	2,88523E+00	10044	9,31169E-01	12298	0	54420,5
Myeloid_vs_CD4_Non-responder	Mast	CD4(CRTAM- T)	TIMP3	CD44	1,04589E-03	21963,5	5,03639E-02	386	3,26767E+00	691	1,67159E+00	38455	9,23785E-01	15865	0	54420,5
Myeloid_vs_CD4_Non-responder	Macro_FOLR2+APOE+	CD4(GZMK+ Teff)	MMP9	CD44	1,04922E-03	22810,7	1,05750E-02	7575	1,58883E+00	12858	2,54582E+00	15271	9,08380E-01	23929	0	54420,5
Myeloid_vs_CD4_Non-responder	Macro_FOLR2+APOE+	CD4(GZMK+ Teff)	C3	IFITM1	1,05112E-03	22839,1	7,95547E-03	12141	1,42701E+00	17397	2,30333E+00	20174	9,35663E-01	10063	0	54420,5
Myeloid_vs_CD4_Non-responder	Macro_NLRP3	CD4(NME1+ T)	S100A8	CD69	1,05412E-03	17444,5	8,54599E-03	10819	2,38263E+00	3151	2,72269E+00	12326	9,42679E-01	6506	0	54420,5
Myeloid_vs_CD4_Non-responder	CD4(AREG+ Tm)	Mono_CD14	ANXA1	FPR1	1,05740E-03	23893,7	5,92531E-03	18757	1,75402E+00	9364	2,00361E+00	27687	9,37313E-01	9240	0	54420,5
Myeloid_vs_CD4_Non-responder	Macro_NLRP3	CD4(ISG+ Treg)	S100A8	CD69	1,06018E-03	17691,7	8,24386E-03	11465	2,36621E+00	3251	2,72160E+00	12344	9,41699E-01	6978	0	54420,5
Myeloid_vs_CD4_Non-responder	Macro_FOLR2+APOE+	CD4(CRTAM- T)	LGALS9	PTPRC	1,06104E-03	26499,3	4,57211E-03	27433	1,46244E+00	16344	2,00253E+00	27706	9,42496E-01	6593	0	54420,5
Myeloid_vs_CD4_Non-responder	CD4(TNF+ T)	Mono_CD16	B2M	LILRB2	1,06276E-03	21840,9	4,54073E-03	27715	2,09759E+00	5145	2,23165E+00	21768	9,62310E-01	156	0	54420,5
Myeloid_vs_CD4_Non-responder	CD4(TGFB1+ Th17)	cDC_CLEC9A	FLT3LG	FLT3	1,06709E-03	29519,5	3,72693E-02	698	3,28037E+00	682	1,69436E+00	37577	8,53829E-01	54220	0	54420,5
Myeloid_vs_CD4_Non-responder	Mono_INHBA	CD4(CXCL13+ Tfh)	IL6	IL6R_IL6ST	1,07014E-03	24710,1	3,80503E-02	664	3,25958E+00	699	2,17955E+00	23022	8,70809E-01	44745	0	54420,5
Myeloid_vs_CD4_Non-responder	Macro_FOLR2+APOE+	CD4(AREG+ Tm)	APOE	SORL1	1,07838E-03	22285,9	5,06329E-03	23669	1,78651E+00	8821	3,65215E+00	3135	9,12991E-01	21384	0	54420,5
Myeloid_vs_CD4_Non-responder	CD4(GZMK+ Teff)	Macro_ISG15	HLA-F	LILRB2	1,07838E-03	25619,5	4,53031E-03	27796	1,96572E+00	6450	2,37076E+00	18745	9,14261E-01	20686	0	54420,5
Myeloid_vs_CD4_Non-responder	Macro_FOLR2+APOE+	CD4(AREG+ Tm)	MMP9	CD44	1,07974E-03	23728,5	1,00379E-02	8292	1,54444E+00	13998	2,46533E+00	16809	9,06188E-01	25123	0	54420,5
Myeloid_vs_CD4_Non-responder	CD4(CXCL13+ Tfh)	Mono_CD16	RPS19	C5AR1	1,08013E-03	23801,5	5,17495E-03	22906	1,67796E+00	10800	1,99921E+00	27805	9,50285E-01	3076	0	54420,5
Myeloid_vs_CD4_Non-responder	cDC_CLEC9A	CD4(ISG+ Treg)	HLA-DPA1	LAG3	1,08057E-03	18480,5	8,93115E-03	10066	1,60718E+00	12404	3,50948E+00	3984	9,32675E-01	11528	0	54420,5
Myeloid_vs_CD4_Non-responder	Macro_FOLR2+APOE+	CD4(AREG+ Tm)	C3	IFITM1	1,08168E-03	23090,9	7,85232E-03	12404	1,41490E+00	17808	2,28639E+00	20559	9,35270E-01	10263	0	54420,5
Myeloid_vs_CD4_Non-responder	CD4(TNFRSF9+ Treg)	Macro_ISG15	HLA-B	LILRB1	1,08441E-03	23587,9	4,86829E-03	25111	1,90904E+00	7068	1,99855E+00	27827	9,49141E-01	3513	0	54420,5
Myeloid_vs_CD4_Non-responder	Mono_CD14	CD4(ISG+ Treg)	VCAN	SELL	1,08640E-03	15869,9	2,11849E-02	2109	3,31803E+00	651	2,90605E+00	9748	9,30870E-01	12421	0	54420,5
Myeloid_vs_CD4_Non-responder	CD4(NME1+ T)	Mono_CD16	RPS19	C5AR1	1,08675E-03	24427,5	4,96620E-03	24383	1,62137E+00	12062	1,99825E+00	27839	9,49303E-01	3433	0	54420,5
Myeloid_vs_CD4_Non-responder	CD4(CRTAM- T)	pDC_LILRA4	HMGB1	THBD	1,09497E-03	30360,7	4,52124E-03	27881	1,44130E+00	16988	1,99838E+00	27834	9,06963E-01	24680	0	54420,5

Myeloid_vs_CD4_Post_NR

Myeloid_vs_CD4_Non-responder	Macro_FOLR2+APOE+	CD4(NME1+ T)	CD14	ITGB1	1,09615E-03	21332,5	5,34191E-03	21839	1,88962E+00	7312	2,91921E+00	9592	9,28613E-01	13499	0	54420,5
Myeloid_vs_CD4_Non-responder	Macro_FOLR2+APOE+	CD4(NME1+ T)	CD14	ITGA4	1,09635E-03	22185,7	5,07955E-03	23549	1,88811E+00	7340	2,92187E+00	9561	9,23392E-01	16058	0	54420,5
Myeloid_vs_CD4_Non-responder	CD4(TNF+ T)	Macro_ISG15	TNF	TNFRSF1A	1,09989E-03	28420,9	7,78532E-03	12553	1,15962E+00	27906	2,31422E+00	19929	9,02403E-01	27296	0	54420,5
Myeloid_vs_CD4_Non-responder	Macro_FOLR2+APOE+	CD4(CRTAM- T)	C3	IFITM1	1,10542E-03	26514,5	6,77982E-03	15422	1,28895E+00	22279	1,99510E+00	27934	9,30679E-01	12517	0	54420,5
Myeloid_vs_CD4_Non-responder	CD4(TNF+ T)	Macro_LYVE1	HSPA1A	TLR4	1,10779E-03	26481,5	7,43411E-03	13493	1,40915E+00	17999	1,99483E+00	27946	9,18408E-01	18549	0	54420,5
Myeloid_vs_CD4_Non-responder	Macro_FOLR2+APOE+	CD4(NME1+ T)	C1QB	C1QBP	1,12114E-03	16917,1	6,53967E-03	16294	2,32428E+00	3477	4,04091E+00	1543	9,37998E-01	8851	0	54420,5
Myeloid_vs_CD4_Non-responder	Macro_LYVE1	CD4(IFNG+ Tfh/Th1)	HLA-DPB1	LAG3	1,12118E-03	18837,9	1,12138E-02	6864	1,60277E+00	12521	2,84344E+00	10605	9,36217E-01	9779	0	54420,5
Myeloid_vs_CD4_Non-responder	Macro_FOLR2+APOE+	CD4(NME1+ T)	C3	IFITM1	1,12174E-03	27349,1	6,28414E-03	17226	1,23074E+00	24728	2,03975E+00	26658	9,28190E-01	13713	0	54420,5
Myeloid_vs_CD4_Non-responder	Mono_CD14	CD4(IFNG+ Tfh/Th1)	VCAN	ITGB1	1,12364E-03	16309,5	1,49938E-02	4065	3,23053E+00	716	2,90168E+00	9818	9,30637E-01	12528	0	54420,5
Myeloid_vs_CD4_Non-responder	CD4(TGFB1+ Th17)	cDC(CD1C)	ANXA1	FPR1	1,13259E-03	23169,9	6,60430E-03	16042	1,73662E+00	9677	1,99065E+00	28070	9,40426E-01	7640	0	54420,5
Myeloid_vs_CD4_Non-responder	CD4(TNFRSF9+ Treg)	pDC_LILRA4	SIRPG	CD47	1,13441E-03	25266,1	1,84182E-02	2779,5	1,15603E+00	28079,5	2,57999E+00	14618,5	9,03917E-01	26432,5	0	54420,5
Myeloid_vs_CD4_Non-responder	CD4(ISG+ Treg)	Macro_ISG15	HLA-B	LILRB2	1,13623E-03	20552,9	4,50004E-03	28088	2,23772E+00	4007	2,53244E+00	15507	9,57780E-01	742	0	54420,5
Myeloid_vs_CD4_Non-responder	Macro_FOLR2+APOE+	CD4(IFNG+ Tfh/Th1)	CD14	ITGB1	1,13683E-03	19603,7	6,08893E-03	18041	1,96470E+00	6460	3,08140E+00	7636	9,32832E-01	11461	0	54420,5
Myeloid_vs_CD4_Non-responder	Macro_FOLR2+APOE+	CD4(IFNG+ Tfh/Th1)	CD14	ITGA4	1,13704E-03	21150,5	5,44841E-03	21218	1,92263E+00	6923	3,01808E+00	8331	9,25835E-01	14860	0	54420,5
Myeloid_vs_CD4_Non-responder	Macro_FOLR2+APOE+	CD4(Tn)	APOE	SORL1	1,13886E-03	23863,9	4,49829E-03	28101	1,75111E+00	9432	3,61343E+00	3334	9,08175E-01	24032	0	54420,5
Myeloid_vs_CD4_Non-responder	Macro_FOLR2+APOE+	CD4(IFNG+ Tfh/Th1)	HLA-DMB	CD4	1,13926E-03	22177,3	4,98738E-03	24211	1,96623E+00	6447	2,88059E+00	10108	9,24150E-01	15700	0	54420,5
Myeloid_vs_CD4_Non-responder	Macro_FOLR2+APOE+	CD4(IFNG+ Tfh/Th1)	CD86	CTLA4	1,14028E-03	19051,3	1,76575E-02	2998	2,04906E+00	5583	2,46920E+00	16740	9,24515E-01	15515	0	54420,5
Myeloid_vs_CD4_Non-responder	Macro_FOLR2+APOE+	CD4(CRTAM- T)	HBEGF	CD44	1,15005E-03	29251,1	4,83111E-03	25377	1,27592E+00	22795	1,98786E+00	28156	9,24525E-01	15507	0	54420,5
Myeloid_vs_CD4_Non-responder	Macro_FOLR2+APOE+	CD4(IFNG+ Tfh/Th1)	LGALS3	LAG3	1,15086E-03	20185,9	1,33558E-02	5006	1,40574E+00	18101	3,14750E+00	6939	9,22571E-01	16463	0	54420,5
Myeloid_vs_CD4_Non-responder	Macro_FOLR2+APOE+	CD4(IFNG+ Tfh/Th1)	CXCL16	CXCR6	1,15496E-03	17792,7	1,81049E-02	2875	1,86326E+00	7666	2,96230E+00	9043	9,25655E-01	14959	0	54420,5
Myeloid_vs_CD4_Non-responder	CD4(IFNG+ Tfh/Th1)	Macro_ISG15	HLA-A	LILRB1	1,15598E-03	23355,3	5,11800E-03	23289	1,92719E+00	6884	1,98682E+00	28185	9,47984E-01	3998	0	54420,5
Myeloid_vs_CD4_Non-responder	cDC_CLEC9A	CD4(ISG+ Treg)	HLA-DPB1	LAG3	1,15893E-03	18306,1	9,31127E-03	9395	1,65927E+00	11212	3,52753E+00	3876	9,30436E-01	12627	0	54420,5
Myeloid_vs_CD4_Non-responder	CD4(TGFB1+ Th17)	Macro_FOLR2+APOE+	RPS19	C5AR1	1,15947E-03	24330,7	5,03436E-03	23880	1,63041E+00	11831	1,98620E+00	28202	9,49630E-01	3320	0	54420,5
Myeloid_vs_CD4_Non-responder	Macro_FOLR2+APOE+	CD4(IFNG+ Tfh/Th1)	C3	IFITM1	1,15988E-03	22093,9	8,22766E-03	11506	1,45898E+00	16440	2,38060E+00	18553	9,36669E-01	9550	0	54420,5
Myeloid_vs_CD4_Non-responder	Macro_FOLR2+APOE+	CD4(CRTAM- T)	C1QB	C1QBP	1,16009E-03	20876,5	4,48584E-03	28205	2,15716E+00	4611	3,80047E+00	2411	9,26088E-01	14735	0	54420,5
Myeloid_vs_CD4_Non-responder	pDC_LILRA4	CD4(IFNG+ Tfh/Th1)	APP	RPSA	1,16109E-03	15820,5	1,27091E-02	5542	2,28616E+00	3705	2,70333E+00	12633	9,51033E-01	2802	0	54420,5
Myeloid_vs_CD4_Non-responder	Macro_OLFML3	CD4(IFNG+ Tfh/Th1)	HLA-DPA1	LAG3	1,16181E-03	17474,9	1,10764E-02	7019	1,59776E+00	12635	3,36522E+00	5004	9,39127E-01	8296	0	54420,5
Myeloid_vs_CD4_Non-responder	Macro_OLFML3	CD4(IFNG+ Tfh/Th1)	HLA-DQB1	LAG3	1,16724E-03	17899,9	1,25464E-02	5638	1,67549E+00	10865	3,25705E+00	5926	9,30371E-01	12650	0	54420,5
Myeloid_vs_CD4_Non-responder	Macro_FOLR2+APOE+	CD4(IFNG+ Tfh/Th1)	LGALS3BP	ITGB1	1,17435E-03	27395,7	8,41683E-03	11110	1,42935E+00	17338	1,98398E+00	28274	9,04983E-01	25836	0	54420,5
Myeloid_vs_CD4_Non-responder	CD4(TNF+ T)	Mono_CD16	HLA-A	LILRB2	1,17497E-03	22790,9	4,47736E-03	28277	2,05884E+00	5495	2,12165E+00	24514	9,55658E-01	1248	0	54420,5
Myeloid_vs_CD4_Non-responder	Macro_FOLR2+APOE+	CD4(CXCL13+ Tfh)	CD86	CTLA4	1,17663E-03	24186,9	1,15963E-02	6502	1,77217E+00	9052	2,02402E+00	27103	9,08471E-01	23857	0	54420,5
Myeloid_vs_CD4_Non-responder	CD4(TNFRSF9+ Treg)	Macro_ISG15	B2M	LILRB1	1,17726E-03	23405,3	4,79519E-03	25636	1,92086E+00	6946	1,98357E+00	28288	9,54028E-01	1736	0	54420,5
Myeloid_vs_CD4_Non-responder	Macro_IER3	CD4(IFNG+ Tfh/Th1)	HLA-DRB5	LAG3	1,18730E-03	18772,1	1,11741E-02	6903	1,59474E+00	12705	2,99954E+00	8561	9,33167E-01	11271	0	54420,5
Myeloid_vs_CD4_Non-responder	CD4(AREG+ Tm)	Macro_FOLR2+APOE+	CD99	PILRA	1,18833E-03	27265,5	4,47089E-03	28341	1,70648E+00	10261	1,99952E+00	27785	9,24507E-01	15520	0	54420,5
Myeloid_vs_CD4_Non-responder	CD4(Tn)	Mono_CD14	ANXA1	FPR1	1,19421E-03	24866,1	5,51288E-03	20873	1,70198E+00	10358	1,98087E+00	28369	9,35160E-01	10310	0	54420,5
Myeloid_vs_CD4_Non-responder	CD4(IFNG+ Tfh/Th1)	Macro_ISG15	HLA-A	LILRB2	1,19590E-03	21702,7	4,46820E-03	28377	2,08885E+00	5228	2,34721E+00	19224	9,55614E-01	1264	0	54420,5
Myeloid_vs_CD4_Non-responder	Macro_FOLR2+APOE+	CD4(CXCL13+ Tfh)	C1QB	C1QBP	1,19800E-03	20300,7	4,70983E-03	26318	2,17538E+00	4476	3,80929E+00	2377	9,27738E-01	13912	0	54420,5
Myeloid_vs_CD4_Non-responder	Macro_FOLR2+APOE+	CD4(CXCL13+ Tfh)	C3	IFITM1	1,19843E-03	20009,5	9,09859E-03	9766	1,56125E+00	13567	2,60170E+00	14244	9,39588E-01	8050	0	54420,5
Myeloid_vs_CD4_Non-responder	CD4(TNFRSF9+ Treg)	Macro_ISG15	LTB	TNFRSF1A	1,20371E-03	26791,3	8,61298E-03	10692	1,19307E+00	26378	1,97927E+00	28414	9,27424E-01	14052	0	54420,5
Myeloid_vs_CD4_Non-responder	Macro_FOLR2+APOE+	CD4(IL26+ Th17)	HLA-DRA	LAG3	1,20732E-03	23037,1	5,53183E-03	20786	1,50366E+00	15098	3,24991E+00	6001	9,17743E-01	18880	0	54420,5
Myeloid_vs_CD4_Non-responder	Macro_FOLR2+APOE+	CD4(IL26+ Th17)	HLA-DQA1	LAG3	1,20817E-03	22781,3	6,88544E-03	15059	1,65228E+00	11358	3,18831E+00	6531	9,03723E-01	26538	0	54420,5
Myeloid_vs_CD4_Non-responder	Macro_FOLR2+APOE+	CD4(IL26+ Th17)	HLA-DRB1	LAG3	1,20859E-03	25032,5	5,28663E-03	22184	1,34987E+00	20044	3,05023E+00	7964	9,14487E-01	20550	0	54420,5
Myeloid_vs_CD4_Non-responder	Macro_FOLR2+APOE+	CD4(IL26+ Th17)	HLA-DQB1	LAG3	1,20902E-03	24458,7	6,22312E-03	17473	1,49250E+00	15413	2,99940E+00	8563	9,03943E-01	26424	0	54420,5
Myeloid_vs_CD4_Non-responder	Macro_FOLR2+APOE+	CD4(IL26+ Th17)	HLA-DPB1	LAG3	1,20987E-03	25640,5	5,36898E-03	21693	1,36187E+00	19639	2,91349E+00	9654	9,10366E-01	22796	0	54420,5
Myeloid_vs_CD4_Non-responder	Macro_FOLR2+APOE+	CD4(IL26+ Th17)	HLA-DPA1	LAG3	1,21455E-03	25090,5	5,29671E-03	22125	1,35620E+00	19829	3,01028E+00	8416	9,14299E-01	20662	0	54420,5
Myeloid_vs_CD4_Non-responder	CD4(TNFRSF9+ Treg)	pDC_LILRA4	TNF	PTPRS	1,21487E-03	23695,5	3,61010E-02	745	4,04998E+00	194	2,21922E+00	22068	8,77533E-01	41050	0	54420,5
Myeloid_vs_CD4_Non-responder	CD4(ISG+ Treg)	Mono_CD16	HLA-B	LILRB1	1,21861E-03	24245,1	4,62769E-03	26978	1,89010E+00	7302	1,97677E+00	28484	9,47904E-01	4041	0	54420,5
Myeloid_vs_CD4_Non-responder	Macro_FOLR2+APOE+	CD4(IL26+ Th17)	HLA-DRB5	LAG3	1,21904E-03	25230,1	5,56997E-03	20554	1,41021E+00	17956	2,96231E+00	9040	9,07902E-01	24180	0	54420,5
Myeloid_vs_CD4_Non-responder	CD4(GZMK+ Teff)	Macro_ISG15	HLA-F	LILRB1	1,22892E-03	28352,9	5,18914E-03	22803	1,80405E+00	8531	2,01037E+00	27478	9,00263E-01	28532	0	54420,5
Myeloid_vs_CD4_Non-responder	Macro_FOLR2+APOE+	CD4(IL26+ Th17)	CXCL16	CXCR6	1,23064E-03	21189,5	1,32525E-02	5077	1,66687E+00	11041	2,57721E+00	14677	9,14181E-01	20732	0	54420,5
Myeloid_vs_CD4_Non-responder	Macro_FOLR2+APOE+	CD4(IL26+ Th17)	MMP9	CD44	1,23323E-03	25492,1	9,00358E-03	9944	1,45896E+00	16441	2,36761E+00	18800	9,01462E-01	27855	0	54420,5
Myeloid_vs_CD4_Non-responder	Macro_FOLR2+APOE+	CD4(IL26+ Th17)	C1QB	C1QBP	1,23517E-03	20184,5	4,74228E-03	26031	2,17802E+00	4453	3,85121E+00	2202	9,27968E-01	13816	0	54420,5
Myeloid_vs_CD4_Non-responder	Macro_FOLR2+APOE+	CD4(IL26+ Th17)	C3	IFITM1	1,23561E-03	24556,1	7,21922E-03	14053	1,34055E+00	20385	2,20453E+00	22397	9,32678E-01	11525	0	54420,5
Myeloid_vs_CD4_Non-responder	CD4(TNF+ T)	Macro_FOLR2+APOE+	CD99	PILRA	1,23625E-03	26996,5	4,44791E-03	28566	1,70276E+00	10342	2,06181E+00	26050	9,24327E-01	15604	0	54420,5
Myeloid_vs_CD4_Non-responder	cDC(CD1C)	CD4(ISG+ Treg)	HLA-DPB1	LAG3	1,24027E-03	19239,3	9,17488E-03	9628	1,63419E+00	11751	3,08876E+00	7550	9,29957E-01	12847	0	54420,5
Myeloid_vs_CD4_Non-responder	Macro_ISG15	CD4(TGFB1+ Th17)	CXCL10	SDC4	1,24100E-03	27382,3	1,42698E-02	4453	2,43378E+00	2863	3,53749E+00	3814	8,23095E-01	71361	0	54420,5
Myeloid_vs_CD4_Non-responder	Macro_FOLR2+APOE+	CD4(TGFB1+ Th17)	LGALS9	PTPRC	1,24124E-03	25931,1	4,66328E-03	26671	1,48744E+00	15569	2,04026E+00	26643	9,43028E-01	6352	0	54420,5
Myeloid_vs_CD4_Non-responder	Macro_FOLR2+APOE+	CD4(TGFB1+ Th17)	LGALS9	CD44	1,24146E-03	22256,9	5,42100E-03	21394	1,69968E+00	10392	2,36417E+00	18870	9,43362E-01	6208	0	54420,5
Myeloid_vs_CD4_Non-responder	CD4(IFNG+ Tfh/Th1)	Macro_ISG15	RPS19	C5AR1	1,24363E-03	24005,5	5,15923E-03	23017	1,67415E+00	10890	1,97228E+00	28600	9,50213E-01	3100	0	54420,5
Myeloid_vs_CD4_Non-responder	Macro_ISG15	CD4(ISG+ Treg)	CXCL10	CXCR3	1,24677E-03	20285,3	1,83786E-02	2791	2,43620E+00	2846	3,43793E+00	4460	8,85201E-01	36909	0	54420,5
Myeloid_vs_CD4_Non-responder	CD4(GZMK+ Teff)	Macro_FOLR2+APOE+	RPS19	C5AR1	1,25826E-03	24539,3	4,99791E-03	24138	1,62015E+00	12092	1,96988E+00	28667	9,49456E-01	3379	0	54420,5
Myeloid_vs_CD4_Non-responder	CD4(ISG+ Treg)	Macro_FOLR2+APOE+	ADAM10	GPNMB	1,25972E-03	26122,5	8,05855E-03	11918	2,05783E+00	5501	2,68472E+00	12898	8,68795E-01	45875	0	54420,5
Myeloid_vs_CD4_Non-responder	Macro_FOLR2+APOE+	CD4(ISG+ Treg)	ICAM1	IL2RG	1,26222E-03	30590,1	5,44148E-03	21263	1,14886E+00	28428	1,96911E+00	28685	9,15227E-01	20154	0	54420,5
Myeloid_vs_CD4_Non-responder	CD4(Tn)	Mono_CD16	HLA-F	LILRB2	1,26222E-03	25055,1	4,43811E-03	28685	2,27921E+00	3751	2,44121E+00	17293	9,13451E-01	21126	0	54420,5
Myeloid_vs_CD4_Non-responder	Macro_IFI27	CD4(IFNG+ Tfh/Th1)	HLA-DPA1	LAG3	1,26279E-03	18609,7	1,14833E-02	6602	1,65304E+00	11340	2,68400E+00	12906	9,40150E-01	7780	0	54420,5
Myeloid_vs_CD4_Non-responder	Macro_FOLR2+APOE+	CD4(TGFB1+ Th17)	SPP1	CD44	1,26332E-03	18781,1	1,04682E-02	7718	1,51146E+00	14875	3,63359E+00	3237	9,28320E-01	13655	0	54420,5
Myeloid_vs_CD4_Non-responder	Macro_FOLR2+APOE+	CD4(TGFB1+ Th17)	CXCL9	CXCR3	1,26729E-03	17996,1	2,83316E-02	1194	3,10397E+00	854	3,32820E+00	5317	9,00856E-01	28195	0	54420,5
Myeloid_vs_CD4_Non-responder	Macro_FOLR2+APOE+	CD4(TGFB1+ Th17)	HBEGF	CD44	1,26817E-03	28044,1	5,03286E-03	23889	1,32425E+00	20990	2,05970E+00	26104	9,25940E-01	14817	0	54420,5
Myeloid_vs_CD4_Non-responder	CD4(ISG+ Treg)	Macro_ISG15	RPS19	C5AR1	1,26906E-03	24572,9	4,97455E-03	24311	1,62416E+00	11995	1,96784E+00	28716	9,49344E-01	3422	0	54420,5
Myeloid_vs_CD4_Non-responder	Macro_FOLR2+APOE+	CD4(TGFB1+ Th17)	LGALS1	CD69	1,27038E-03	23687,5	5,61033E-03	20323	1,20909E+00	25661	2,47524E+00	16629	9,55137E-01	1404	0	54420,5
Myeloid_vs_CD4_Non-responder	Macro_FOLR2+APOE+	CD4(CRTAM- T)	LGALS3BP	ITGB1	1,27237E-03	27281,7	8,55726E-03	10796	1,43956E+00	17031	1,96753E+00	28731	9,05692E-01	25430	0	54420,5

Myeloid_vs_CD4_Post_NR

Myeloid_vs_CD4_Non-responder	Macro_FOLR2+APOE+	CD4(TGFB1+ Th17)	MMP9	CD44	1,27370E-03	18287,9	1,45894E-02	4270	1,92059E+00	6950	3,00439E+00	8490	9,20920E-01	17309	0	54420,5
Myeloid_vs_CD4_Non-responder	Macro_FOLR2+APOE+	CD4(TGFB1+ Th17)	TIMP2	CD44	1,27814E-03	24512,1	7,07354E-03	14463	1,64245E+00	11564	2,08310E+00	25528	9,22360E-01	16585	0	54420,5
Myeloid_vs_CD4_Non-responder	CD4(AREG+ Tm)	Mono_CD16	CD99	PILRA	1,27925E-03	27478,7	4,42987E-03	28762	1,69008E+00	10568	1,99453E+00	27962	9,24185E-01	15681	0	54420,5
Myeloid_vs_CD4_Non-responder	cDC_LAMP3	CD4(NME1+ T)	CCL19	CCR7	1,28397E-03	24734,7	3,54012E-02	766	4,95017E+00	49	2,15569E+00	23666	8,70750E-01	44772	0	54420,5
Myeloid_vs_CD4_Non-responder	CD4(TGFB1+ Th17)	Macro_ISG15	HLA-C	LILRB2	1,28996E-03	21262,1	4,42438E-03	28810	2,17005E+00	4521	2,44705E+00	17167	9,55170E-01	1392	0	54420,5
Myeloid_vs_CD4_Non-responder	Macro_FOLR2+APOE+	CD4(TNFRSF9+ Treg)	CD14	ITGB1	1,29602E-03	24036,3	4,42156E-03	28837	1,79711E+00	8643	2,77475E+00	11550	9,22086E-01	16731	0	54420,5
Myeloid_vs_CD4_Non-responder	Macro_LYVE1	CD4(IFNG+ Tfh/Th1)	HLA-DRB1	LAG3	1,29615E-03	18481,7	1,09936E-02	7111	1,58345E+00	12992	2,92000E+00	9582	9,39104E-01	8303	0	54420,5
Myeloid_vs_CD4_Non-responder	Macro_OLFML3	CD4(ISG+ Treg)	CXCL10	CXCR3	1,29782E-03	19027,1	2,14247E-02	2072	2,83393E+00	1406	3,42944E+00	4521	8,92766E-01	32716	0	54420,5
Myeloid_vs_CD4_Non-responder	CD4(IL26+ Th17)	pDC_LILRA4	BST2	LILRA4	1,29984E-03	18804,7	1,46051E-02	4258	3,65887E+00	396	3,23922E+00	6095	8,99678E-01	28854	0	54420,5
Myeloid_vs_CD4_Non-responder	CD4(AREG+ Tm)	Mono_CD16	HLA-B	LILRA1	1,30046E-03	29197,7	8,82935E-03	10258	1,80828E+00	8470	1,11447E+00	62438	9,34952E-01	10402	0	54420,5
Myeloid_vs_CD4_Non-responder	CD4(IFNG+ Tfh/Th1)	Mono_CD16	RPS19	C5AR1	1,30187E-03	24087,7	5,15089E-03	23078	1,67144E+00	10962	1,96317E+00	28863	9,50175E-01	3115	0	54420,5
Myeloid_vs_CD4_Non-responder	CD4(AREG+ Tm)	Mono_CD16	HLA-A	LILRA1	1,30203E-03	27806,7	9,57040E-03	8970	1,93028E+00	6841	1,20543E+00	58144	9,34437E-01	10658	0	54420,5
Myeloid_vs_CD4_Non-responder	CD4(AREG+ Tm)	Mono_CD16	HLA-C	LILRA1	1,30518E-03	26948,9	9,36490E-03	9309	1,98449E+00	6252	1,30403E+00	53637	9,33429E-01	11126	0	54420,5
Myeloid_vs_CD4_Non-responder	CD4(IFNG+ Tfh/Th1)	Macro_ISG15	HLA-C	LILRB1	1,30684E-03	24195,3	4,82420E-03	25422	1,88250E+00	7413	1,96282E+00	28885	9,46228E-01	4836	0	54420,5
Myeloid_vs_CD4_Non-responder	CD4(TNFRSF9+ Treg)	Macro_LYVE1	CD59	STAB1	1,32344E-03	22884,1	7,89089E-03	12304	2,55596E+00	2309	2,48546E+00	16429	8,99489E-01	28958	0	54420,5
Myeloid_vs_CD4_Non-responder	CD4(TNF+ T)	cDC_CLEC9A	TNFSF9	HLA-DPA1	1,32572E-03	23980,5	6,15717E-03	17749	1,50933E+00	14922	3,53340E+00	3843	8,99472E-01	28968	0	54420,5
Myeloid_vs_CD4_Non-responder	Macro_OLFML3	CD4(ISG+ Treg)	HLA-DRA	LAG3	1,32577E-03	19452,1	8,37585E-03	11199	1,58024E+00	13067	3,16448E+00	6767	9,32105E-01	11807	0	54420,5
Myeloid_vs_CD4_Non-responder	CD4(CXCL13+ Tfh)	Mono_CD16	HLA-C	LILRB2	1,32893E-03	22473,3	4,40750E-03	28982	2,12703E+00	4872	2,19382E+00	22669	9,55088E-01	1423	0	54420,5
Myeloid_vs_CD4_Non-responder	CD4(TNF+ T)	Mono_CD16	CD99	PILRA	1,33031E-03	27194,7	4,40711E-03	28988	1,68636E+00	10630	2,05682E+00	26175	9,24004E-01	15760	0	54420,5
Myeloid_vs_CD4_Non-responder	Macro_FOLR2+APOE+	CD4(AREG+ Tm)	SPP1	CD44	1,33329E-03	25073,7	7,20244E-03	14105	1,13531E+00	29001	3,09453E+00	7480	9,14839E-01	20362	0	54420,5
Myeloid_vs_CD4_Non-responder	Macro_FOLR2+APOE+	CD4(CXCL13+ Tfh)	LGALS9	PTPRC	1,33467E-03	27422,9	4,40519E-03	29007	1,41668E+00	17736	1,96339E+00	28855	9,41479E-01	7096	0	54420,5
Myeloid_vs_CD4_Non-responder	CD4(ISG+ Treg)	Mono_CD16	RPS19	C5AR1	1,33582E-03	24660,7	4,96650E-03	24380	1,62146E+00	12059	1,95873E+00	29012	9,49305E-01	3432	0	54420,5
Myeloid_vs_CD4_Non-responder	CD4(Tn)	Macro_ISG15	ANXA1	FPR1	1,34436E-03	28656,9	4,40015E-03	29049	1,37890E+00	19032	2,02890E+00	26975	9,27980E-01	13808	0	54420,5
Myeloid_vs_CD4_Non-responder	CD4(Tn)	Mono_CD14	RPS19	C5AR1	1,34784E-03	24029,3	5,19626E-03	22758	1,67552E+00	10862	1,95726E+00	29064	9,50382E-01	3042	0	54420,5
Myeloid_vs_CD4_Non-responder	Macro_FOLR2+APOE+	CD4(IFNG+ Tfh/Th1)	HLA-DQA2	LAG3	1,34807E-03	22851,5	1,15852E-02	6510	1,45588E+00	16561	3,07476E+00	7701	8,99285E-01	29065	0	54420,5
Myeloid_vs_CD4_Non-responder	cDC_LAMP3	CD4(ISG+ Treg)	CCL19	CCR7	1,34811E-03	24238,5	3,47110E-02	785	4,94507E+00	50	2,28731E+00	20537	8,69638E-01	45400	0	54420,5
Myeloid_vs_CD4_Non-responder	CD4(ISG+ Treg)	Macro_FOLR2+APOE+	CD28	CD86	1,35341E-03	24225,7	1,27926E-02	5465,5	1,82309E+00	8231,5	2,14498E+00	23922,5	8,99225E-01	29088,5	0	54420,5
Myeloid_vs_CD4_Non-responder	Macro_FOLR2+APOE+	CD4(ISG+ Treg)	CD86	CD28	1,35364E-03	24225,7	1,27926E-02	5465,5	1,82309E+00	8231,5	2,14498E+00	23922,5	8,99225E-01	29088,5	0	54420,5
Myeloid_vs_CD4_Non-responder	Macro_FOLR2+APOE+	CD4(IL26+ Th17)	CXCL9	DPP4	1,35838E-03	23051,3	4,11586E-02	578	3,15267E+00	788	3,37402E+00	4931	8,53275E-01	54539	0	54420,5
Myeloid_vs_CD4_Non-responder	Macro_FOLR2+APOE+	CD4(AREG+ Tm)	CD14	ITGB1	1,36134E-03	23986,5	4,39226E-03	29122	1,79416E+00	8694	2,82720E+00	10838	9,21847E-01	16858	0	54420,5
Myeloid_vs_CD4_Non-responder	Macro_ISG15	CD4(TNFRSF9+ Treg)	CXCL10	CXCR3	1,36157E-03	20567,1	1,77240E-02	2979	2,42350E+00	2914	3,41842E+00	4595	8,83345E-01	37927	0	54420,5
Myeloid_vs_CD4_Non-responder	Macro_OLFML3	CD4(IFNG+ Tfh/Th1)	HLA-DRB1	LAG3	1,36401E-03	17667,5	1,09384E-02	7170	1,57597E+00	13162	3,33950E+00	5221	9,38960E-01	8364	0	54420,5
Myeloid_vs_CD4_Non-responder	CD4(TNF+ T)	Macro_FOLR2+APOE+	IFNG	IFNGR1_IFNGR2	1,36719E-03	23116,9	2,17944E-02	1993	1,38720E+00	18765	2,79578E+00	11259	8,99140E-01	29147	0	54420,5
Myeloid_vs_CD4_Non-responder	cDC(CD1C)	CD4(ISG+ Treg)	HLA-DPA1	LAG3	1,36971E-03	19513,3	8,76313E-03	10379	1,57549E+00	13176	3,06765E+00	7776	9,32076E-01	11815	0	54420,5
Myeloid_vs_CD4_Non-responder	CD4(AREG+ Tm)	Mono_CD14	RPS19	C5AR1	1,37754E-03	24066,5	5,19209E-03	22783	1,67434E+00	10888	1,95329E+00	29191	9,50363E-01	3050	0	54420,5
Myeloid_vs_CD4_Non-responder	CD4(CXCL13+ Tfh)	Macro_FOLR2+APOE+	RPS19	C5AR1	1,38582E-03	24702,7	4,98241E-03	24252	1,61578E+00	12210	1,95204E+00	29226	9,49382E-01	3405	0	54420,5
Myeloid_vs_CD4_Non-responder	CD4(ISG+ Treg)	Macro_ISG15	CD28	CD86	1,38748E-03	24160,5	1,27317E-02	5518,5	1,81500E+00	8354,5	2,17039E+00	23275,5	8,99008E-01	29233,5	0	54420,5
Myeloid_vs_CD4_Non-responder	CD4(CRTAM- T)	Mono_CD14	RPS19	C5AR1	1,39366E-03	23555,1	5,39175E-03	21568	1,73075E+00	9794	1,95109E+00	29259	9,51246E-01	2734	0	54420,5
Myeloid_vs_CD4_Non-responder	CD4(NME1+ T)	Macro_FOLR2+APOE+	RPS19	C5AR1	1,39390E-03	25371,9	4,78142E-03	25755	1,55919E+00	13613	1,95108E+00	29260	9,48383E-01	3811	0	54420,5
Myeloid_vs_CD4_Non-responder	Mono_CD14	CD4(GZMK+ Teff)	VCAN	CD44	1,39394E-03	15932,9	1,17155E-02	6380	3,17370E+00	765	2,66380E+00	13235	9,46164E-01	4864	0	54420,5
Myeloid_vs_CD4_Non-responder	CD4(IFNG+ Tfh/Th1)	Macro_NLRP3	HMGB1	THBD	1,39843E-03	26085,7	6,04221E-03	18248	1,72178E+00	9968	1,95060E+00	29279	9,18496E-01	18513	0	54420,5
Myeloid_vs_CD4_Non-responder	Macro_OLFML3	CD4(TNFRSF9+ Treg)	CXCL10	CXCR3	1,41026E-03	19285,3	2,06617E-02	2216	2,82122E+00	1442	3,40993E+00	4650	8,91017E-01	33698	0	54420,5
Myeloid_vs_CD4_Non-responder	CD4(TNF+ T)	Mono_CD16	HLA-C	LILRB2	1,41595E-03	22579,9	4,37013E-03	29352	2,10871E+00	5028	2,19543E+00	22623	9,54905E-01	1476	0	54420,5
Myeloid_vs_CD4_Non-responder	CD4(CXCL13+ Tfh)	Mono_CD16	HLA-F	LILRB2	1,41716E-03	25528,5	4,36987E-03	29357	2,27251E+00	3788	2,37774E+00	18598	9,12837E-01	21479	0	54420,5
Myeloid_vs_CD4_Non-responder	Macro_FOLR2-APOE+	CD4(IFNG+ Tfh/Th1)	HLA-DRA	LAG3	1,41722E-03	18631,5	1,10832E-02	7010	1,67757E+00	10809	2,66021E+00	13291	9,40449E-01	7627	0	54420,5
Myeloid_vs_CD4_Non-responder	CD4(TNFRSF9+ Treg)	Macro_FOLR2+APOE+	LTA_LTB	LTBR	1,42224E-03	27050,1	1,18165E-02	6271	1,47679E+00	15902	1,94719E+00	29378	8,98944E-01	29279	0	54420,5
Myeloid_vs_CD4_Non-responder	Macro_FOLR2+APOE+	CD4(NME1+ T)	CD86	CTLA4	1,44048E-03	25243,1	1,08516E-02	7257	1,73815E+00	9646	1,94490E+00	29453	9,05673E-01	25439	0	54420,5
Myeloid_vs_CD4_Non-responder	CD4(GZMK+ Teff)	Macro_NLRP3	HMGB1	THBD	1,44195E-03	26810,1	5,74246E-03	19672	1,66816E+00	11017	1,94478E+00	29459	9,16571E-01	19482	0	54420,5
Myeloid_vs_CD4_Non-responder	Macro_FOLR2+APOE+	CD4(ISG+ Treg)	TNFSF13	TNFRSF14	1,44195E-03	28420,9	4,67867E-03	26553	1,59496E+00	12697	2,35973E+00	18975	8,98643E-01	29459	0	54420,5
Myeloid_vs_CD4_Non-responder	CD4(GZMK+ Teff)	pDC_LILRA4	BST2	LILRA4	1,44207E-03	24995,5	7,48685E-03	13350	3,49346E+00	495	2,97419E+00	8889	8,65243E-01	47823	0	54420,5
Myeloid_vs_CD4_Non-responder	CD4(TNFRSF9+ Treg)	Mono_CD16	HLA-A	LILRB1	1,44636E-03	24553,3	4,75778E-03	25922	1,83398E+00	8074	1,94424E+00	29477	9,46155E-01	4873	0	54420,5
Myeloid_vs_CD4_Non-responder	CD4(TNF+ T)	cDC(CD1C)	TNFSF9	HLA-DPA1	1,44734E-03	25108,7	6,04134E-03	18251	1,47764E+00	15873	3,09158E+00	7518	8,98611E-01	29481	0	54420,5
Myeloid_vs_CD4_Non-responder	Macro_ISG15	CD4(CRTAM- T)	S100A8	CD69	1,45355E-03	19762,3	8,20595E-03	11555	1,60637E+00	12425	2,65448E+00	13377	9,41572E-01	7034	0	54420,5
Myeloid_vs_CD4_Non-responder	Macro_FOLR2+APOE+	CD4(IL26+ Th17)	LGALS1	CD69	1,45472E-03	25563,5	5,12552E-03	23239	1,12447E+00	29511	2,37592E+00	18628	9,53160E-01	2019	0	54420,5
Myeloid_vs_CD4_Non-responder	Macro_NLRP3	CD4(Tn)	VCAN	SELL	1,45952E-03	15652,3	1,98849E-02	2379	2,94947E+00	1138	3,14789E+00	6933	9,28804E-01	13391	0	54420,5
Myeloid_vs_CD4_Non-responder	CD4(CRTAM- T)	Macro_ISG15	HLA-C	LILRB1	1,46015E-03	24418,3	4,79616E-03	25630	1,86801E+00	7602	1,94207E+00	29533	9,46080E-01	4906	0	54420,5
Myeloid_vs_CD4_Non-responder	CD4(TNFRSF9+ Treg)	Macro_ISG15	LTB	CD40	1,46114E-03	25298,1	1,04731E-02	7714	1,48430E+00	15685	1,94192E+00	29537	9,17249E-01	19134	0	54420,5
Myeloid_vs_CD4_Non-responder	CD4(TGFB1+ Th17)	Macro_ISG15	CCL4	CCR1	1,47106E-03	22612,9	1,33249E-02	5032	2,16710E+00	4542	2,33326E+00	19493	8,98462E-01	29577	0	54420,5
Myeloid_vs_CD4_Non-responder	CD4(GZMK+ Teff)	Macro_ISG15	HLA-B	LILRB1	1,47256E-03	24514,1	4,70547E-03	26346	1,81402E+00	8374	1,94027E+00	29583	9,48314E-01	3847	0	54420,5
Myeloid_vs_CD4_Non-responder	Macro_LYVE1	CD4(IFNG+ Tfh/Th1)	HLA-DQB1	LAG3	1,47929E-03	19775,3	1,19312E-02	6168	1,60216E+00	12537	2,72351E+00	12314	9,28725E-01	13437	0	54420,5
Myeloid_vs_CD4_Non-responder	Macro_ISG15	CD4(TNF+ T)	CXCL10	CXCR3	1,48866E-03	21477,5	1,56980E-02	3724	2,38416E+00	3141	3,39926E+00	4736	8,76944E-01	41366	0	54420,5
Myeloid_vs_CD4_Non-responder	CD4(TNF+ T)	Macro_IER3	HSPA1A	TLR4	1,48981E-03	27103,7	7,27774E-03	13908	1,39191E+00	18587	1,93780E+00	29652	9,17608E-01	18951	0	54420,5
Myeloid_vs_CD4_Non-responder	Macro_FOLR2+APOE+	CD4(TNF+ T)	CD14	ITGA4	1,49560E-03	23997,1	4,33820E-03	29675	1,81874E+00	8303	2,99330E+00	8642	9,17623E-01	18945	0	54420,5
Myeloid_vs_CD4_Non-responder	Macro_FOLR2+APOE+	CD4(IFNG+ Tfh/Th1)	B2M	CD3D	1,49592E-03	31453,7	4,52790E-03	27818	9,81690E-01	36883	1,68001E+00	38138	9,67513E-01	9	0	54420,5
Myeloid_vs_CD4_Non-responder	Macro_FOLR2+APOE+	CD4(TNF+ T)	C3	IFITM1	1,50115E-03	28919,3	5,89194E-03	18941	1,18468E+00	26762	1,93617E+00	29697	9,26012E-01	14776	0	54420,5
Myeloid_vs_CD4_Non-responder	CD4(ISG+ Treg)	Macro_ISG15	HLA-C	LILRB1	1,50494E-03	24561,9	4,76517E-03	25869	1,85200E+00	7810	1,93573E+00	29712	9,45914E-01	4998	0	54420,5
Myeloid_vs_CD4_Non-responder	Macro_NLRP3	CD4(IFNG+ Tfh/Th1)	S100A9	ITGB2	1,51010E-03	17690,9	5,21300E-03	22661	2,58132E+00	2196	3,44493E+00	4418	9,46384E-01	4759	0	54420,5
Myeloid_vs_CD4_Non-responder	CD4(TGFB1+ Th17)	pDC_LILRA4	CCL20	CXCR3	1,52352E-03	24540,9	3,15445E-02	955	1,11901E+00	29785	2,69917E+00	12686	9,06669E-01	24858	0	54420,5
Myeloid_vs_CD4_Non-responder	CD4(TNF+ T)	Macro_FOLR2+APOE+	IL23A	LILRB2	1,52737E-03	28238,7	2,39664E-02	1681	1,89704E+00	7217	2,64290E+00	13547	8,35670E-01	64328	0	54420,5
Myeloid_vs_CD4_Non-responder	CD4(GZMK+ Teff)	Macro_ISG15	CCL5	CCR1	1,53044E-03	23405,1	2,63198E-02	1399	2,04115E+00	5659	1,93246E+00	29812	9,05171E-01	25735	0	54420,5
Myeloid_vs_CD4_Non-responder	Macro_FOLR2+APOE+	CD4(AREG+ Tm)	SPP1	PTGER4	1,53532E-03	22998,9	1,24583E-02	5709	1,32371E+00	21009	3,50324E+00	4025	8,98007E-01	29831	0	54420,5

Myeloid_vs_CD4_Post_NR

Myeloid_vs_CD4_Non-responder	CD4(AREG+ Tm)	Mono_CD16	HLA-B	LILRB2	1,53996E-03	23322,1	4,31797E-03	29849	2,03533E+00	5722	2,07517E+00	25703	9,56937E-01	916	0	54420,5
Myeloid_vs_CD4_Non-responder	Macro_OLFML3	CD4(TNF+ T)	CXCL10	CXCR3	1,54311E-03	20120,9	1,82998E-02	2816	2,78188E+00	1549	3,39077E+00	4794	8,84982E-01	37025	0	54420,5
Myeloid_vs_CD4_Non-responder	CD4(TNF+ T)	Macro_NLRP3	RPS19	C5AR1	1,54332E-03	24330,1	5,13547E-03	23174	1,66628E+00	11051	1,93052E+00	29862	9,50104E-01	3143	0	54420,5
Myeloid_vs_CD4_Non-responder	Macro_LYVE1	CD4(ISG+ Treg)	HLA-DRA	LAG3	1,54603E-03	20897,9	8,26855E-03	11411	1,56029E+00	13589	2,67357E+00	13062	9,31696E-01	12007	0	54420,5
Myeloid_vs_CD4_Non-responder	Macro_FOLR2+APOE+	CD4(TGFB1+ Th17)	FN1	CD44	1,54823E-03	31516,7	7,22427E-03	14040	1,12322E+00	29573	1,92978E+00	29881	8,98287E-01	29669	0	54420,5
Myeloid_vs_CD4_Non-responder	Macro_FOLR2+APOE+	CD4(IFNG+ Tfh/Th1)	LGALS9	PTPRC	1,56097E-03	27890,1	4,34917E-03	29565	1,40132E+00	18264	1,92837E+00	29930	9,41126E-01	7271	0	54420,5
Myeloid_vs_CD4_Non-responder	Macro_NLRP3	CD4(IFNG+ Tfh/Th1)	S100A8	CD69	1,57523E-03	17504,1	8,83038E-03	10252	2,39809E+00	3066	2,63633E+00	13654	9,43557E-01	6128	0	54420,5
Myeloid_vs_CD4_Non-responder	Macro_FOLR2+APOE+	CD4(TGFB1+ Th17)	C1QB	C1QBP	1,57747E-03	21431,5	4,30269E-03	29993	2,14225E+00	4755	3,77234E+00	2540	9,24649E-01	15449	0	54420,5
Myeloid_vs_CD4_Non-responder	CD4(TNF+ T)	Mono_CD16	ANXA1	FPR1	1,58010E-03	28598,5	4,34453E-03	29614	1,50798E+00	14963	1,92565E+00	30003	9,27554E-01	13992	0	54420,5
Myeloid_vs_CD4_Non-responder	CD4(GZMK+ Teff)	Macro_ISG15	HLA-A	LILRB2	1,58352E-03	22719,5	4,30019E-03	30016	1,99045E+00	6188	2,24513E+00	21466	9,54794E-01	1507	0	54420,5
Myeloid_vs_CD4_Non-responder	Macro_FOLR2+APOE+	CD4(ISG+ Treg)	MMP9	CD44	1,58537E-03	26997,7	8,28130E-03	11388	1,39927E+00	18328	2,27310E+00	20829	8,97686E-01	30023	0	54420,5
Myeloid_vs_CD4_Non-responder	CD4(IL26+ Th17)	Macro_ISG15	RPS19	C5AR1	1,58696E-03	26441,5	4,29895E-03	30029	1,44131E+00	16987	2,07590E+00	25689	9,45717E-01	5082	0	54420,5
Myeloid_vs_CD4_Non-responder	CD4(TNF+ T)	Macro_FOLR2+APOE-	TNFSF9	HLA-DPA1	1,59145E-03	28565,9	5,91468E-03	18819	1,44298E+00	16940	2,19626E+00	22604	8,97641E-01	30046	0	54420,5
Myeloid_vs_CD4_Non-responder	Macro_IER3	CD4(IFNG+ Tfh/Th1)	HLA-DQB1	LAG3	1,59386E-03	19692,5	1,17549E-02	6336	1,58115E+00	13042	2,81757E+00	10969	9,28230E-01	13695	0	54420,5
Myeloid_vs_CD4_Non-responder	Macro_FOLR2+APOE+	CD4(CXCL13+ Tfh)	MMP9	CD44	1,59463E-03	27146,9	8,27102E-03	11404	1,39842E+00	18359	2,24389E+00	21493	8,97628E-01	30058	0	54420,5
Myeloid_vs_CD4_Non-responder	Macro_FOLR2+APOE+	CD4(NME1+ T)	MMP9	CD44	1,59809E-03	27141,1	8,26282E-03	11422	1,39774E+00	18385	2,24773E+00	21407	8,97583E-01	30071	0	54420,5
Myeloid_vs_CD4_Non-responder	Macro_FOLR2+APOE+	CD4(IFNG+ Tfh/Th1)	LYZ	ITGAL	1,60527E-03	24351,3	4,29362E-03	30098	1,69572E+00	10466	3,40719E+00	4674	9,11705E-01	22098	0	54420,5
Myeloid_vs_CD4_Non-responder	CD4(TNF+ T)	Mono_CD16	CD52	SIGLEC10	1,60714E-03	26882,5	6,32818E-03	17040	1,46357E+00	16306	1,92264E+00	30105	9,22440E-01	16541	0	54420,5
Myeloid_vs_CD4_Non-responder	CD4(IL26+ Th17)	Mono_CD16	RPS19	C5AR1	1,61035E-03	26527,9	4,29200E-03	30117	1,43860E+00	17052	2,06679E+00	25940	9,45675E-01	5110	0	54420,5
Myeloid_vs_CD4_Non-responder	CD4(AREG+ Tm)	pDC_LILRA4	BST2	LILRA4	1,61266E-03	24444,9	7,98352E-03	12074	3,50500E+00	487	2,93123E+00	9438	8,68944E-01	45805	0	54420,5
Myeloid_vs_CD4_Non-responder	CD4(ISG+ Treg)	cDC_LAMP3	LTB	CD40	1,61450E-03	23360,3	1,42127E-02	4490	2,10323E+00	5085	1,65345E+00	39066	9,28123E-01	13740	0	54420,5
Myeloid_vs_CD4_Non-responder	Macro_FOLR2+APOE+	CD4(TGFB1+ Th17)	C3	IFITM1	1,62188E-03	27799,7	6,39156E-03	16800	1,24335E+00	24196	1,92126E+00	30160	9,28753E-01	13422	0	54420,5
Myeloid_vs_CD4_Non-responder	CD4(TGFB1+ Th17)	Macro_FOLR2+APOE+	HMGB1	CD163	1,62457E-03	25315,5	4,28689E-03	30170	1,66764E+00	11031	2,27558E+00	20772	9,35424E-01	10184	0	54420,5
Myeloid_vs_CD4_Non-responder	Macro_FOLR2-APOE+	CD4(ISG+ Treg)	C3	IFITM1	1,63608E-03	19262,7	1,38474E-02	4696	2,12474E+00	4890	1,94968E+00	29303	9,50464E-01	3004	0	54420,5
Myeloid_vs_CD4_Non-responder	CD4(ISG+ Treg)	Macro_ISG15	B2M	LILRB2	1,64812E-03	21415,5	4,27957E-03	30257	2,17016E+00	4520	2,42372E+00	17629	9,61221E-01	251	0	54420,5
Myeloid_vs_CD4_Non-responder	CD4(IFNG+ Tfh/Th1)	Macro_ISG15	B2M	LILRB2	1,65522E-03	21438,9	4,27742E-03	30283	2,16814E+00	4536	2,42019E+00	17702	9,61211E-01	253	0	54420,5
Myeloid_vs_CD4_Non-responder	CD4(IFNG+ Tfh/Th1)	Macro_LYVE1	HMGB1	CD163	1,65549E-03	23877,3	5,42069E-03	21396	1,95783E+00	6542	1,91750E+00	30284	9,42159E-01	6744	0	54420,5
Myeloid_vs_CD4_Non-responder	Macro_OLFML3	CD4(IFNG+ Tfh/Th1)	HLA-DQA1	LAG3	1,65636E-03	17866,7	1,29560E-02	5311	1,71722E+00	10047	3,27930E+00	5725	9,27933E-01	13830	0	54420,5
Myeloid_vs_CD4_Non-responder	CD4(IL26+ Th17)	Macro_NLRP3	RPS19	C5AR1	1,65795E-03	27366,9	4,27662E-03	30293	1,43261E+00	17236	1,93532E+00	29731	9,45583E-01	5154	0	54420,5
Myeloid_vs_CD4_Non-responder	CD4(Tn)	Mono_CD16	HLA-C	LILRB2	1,65960E-03	23144,3	4,27560E-03	30299	2,06236E+00	5465	2,14461E+00	23931	9,54432E-01	1606	0	54420,5
Myeloid_vs_CD4_Non-responder	CD4(CXCL13+ Tfh)	Macro_FOLR2+APOE+	CD99	CD81	1,66097E-03	30056,3	4,27531E-03	30304	1,34802E+00	20114	1,95821E+00	29028	9,22667E-01	16415	0	54420,5
Myeloid_vs_CD4_Non-responder	CD4(TGFB1+ Th17)	Macro_ISG15	HLA-A	LILRB2	1,66206E-03	22959,9	4,27501E-03	30308	1,97569E+00	6341	2,21354E+00	22187	9,54667E-01	1543	0	54420,5
Myeloid_vs_CD4_Non-responder	pDC_LILRA4	CD4(IFNG+ Tfh/Th1)	B2M	CD3D	1,66211E-03	29740,7	4,50598E-03	28030	9,60835E-01	38082	1,98769E+00	28161	9,67437E-01	10	0	54420,5
Myeloid_vs_CD4_Non-responder	CD4(IFNG+ Tfh/Th1)	Macro_FOLR2+APOE+	RPS19	C5AR1	1,66618E-03	24995,5	4,95924E-03	24433	1,60926E+00	12356	1,91600E+00	30323	9,49270E-01	3445	0	54420,5
Myeloid_vs_CD4_Non-responder	Mono_INHBA	CD4(IFNG+ Tfh/Th1)	IL6	IL6R_IL6ST	1,66914E-03	26139,1	3,30062E-02	874	3,18569E+00	751	2,08955E+00	25346	8,62597E-01	49304	0	54420,5
Myeloid_vs_CD4_Non-responder	CD4(TNF+ T)	Macro_FOLR2+APOE+	TNFSF9	HLA-DPA1	1,67637E-03	25256,1	5,84170E-03	19185	1,42301E+00	17523	3,39089E+00	4792	8,97070E-01	30360	0	54420,5
Myeloid_vs_CD4_Non-responder	Macro_FOLR2+APOE+	CD4(ISG+ Treg)	C1QB	C1QBP	1,68051E-03	21541,5	4,26908E-03	30375	2,13952E+00	4779	3,76699E+00	2564	9,24375E-01	15569	0	54420,5
Myeloid_vs_CD4_Non-responder	CD4(CXCL13+ Tfh)	Macro_FOLR2+APOE+	CD28	CD86	1,68966E-03	24650,7	1,21824E-02	5942,5	1,79613E+00	8658,5	2,14902E+00	23823,5	8,96989E-01	30408,5	0	54420,5
Myeloid_vs_CD4_Non-responder	Macro_FOLR2+APOE+	CD4(CXCL13+ Tfh)	CD86	CD28	1,68994E-03	24650,7	1,21824E-02	5942,5	1,79613E+00	8658,5	2,14902E+00	23823,5	8,96989E-01	30408,5	0	54420,5
Myeloid_vs_CD4_Non-responder	Macro_FOLR2+APOE+	CD4(TNFRSF9+ Treg)	GRN	TNFRSF1B	1,70081E-03	20769,7	4,26280E-03	30448	1,81140E+00	8414	3,32902E+00	5306	9,45360E-01	5260	0	54420,5
Myeloid_vs_CD4_Non-responder	CD4(ISG+ Treg)	Mono_CD16	HLA-A	LILRB1	1,70332E-03	24769,1	4,75235E-03	25968	1,83088E+00	8114	1,91173E+00	30457	9,46126E-01	4886	0	54420,5
Myeloid_vs_CD4_Non-responder	CD4(GZMK+ Teff)	Macro_LYVE1	HMGB1	CD163	1,70388E-03	24508,1	5,15177E-03	23073	1,90421E+00	7127	1,91168E+00	30459	9,40757E-01	7461	0	54420,5
Myeloid_vs_CD4_Non-responder	CD4(ISG+ Treg)	Macro_FOLR2+APOE+	RPS19	C5AR1	1,70528E-03	25611,1	4,78172E-03	25752	1,55927E+00	13611	1,91156E+00	30464	9,48385E-01	3808	0	54420,5
Myeloid_vs_CD4_Non-responder	Macro_FOLR2+APOE+	CD4(CRTAM- T)	CXCL9	CXCR3	1,72101E-03	18629,1	2,58999E-02	1443	3,06131E+00	920	3,26698E+00	5842	8,96775E-01	30520	0	54420,5
Myeloid_vs_CD4_Non-responder	CD4(CXCL13+ Tfh)	Macro_ISG15	CD28	CD86	1,72270E-03	24579,9	1,21244E-02	6001,5	1,78804E+00	8795,5	2,17443E+00	23155,5	8,96768E-01	30526,5	0	54420,5
Myeloid_vs_CD4_Non-responder	CD4(TNFRSF9+ Treg)	Macro_ISG15	HLA-B	LILRB2	1,73118E-03	22068,7	4,25020E-03	30556	2,07071E+00	5388	2,35893E+00	18997	9,56609E-01	982	0	54420,5
Myeloid_vs_CD4_Non-responder	CD4(NME1+ T)	pDC_LILRA4	TNF	PTPRS	1,73432E-03	24485,3	3,26725E-02	891	4,02787E+00	201	2,18607E+00	22867	8,72069E-01	44047	0	54420,5
Myeloid_vs_CD4_Non-responder	CD4(IFNG+ Tfh/Th1)	Macro_FOLR2+APOE+	HLA-F	LILRB2	1,74055E-03	27910,9	4,24721E-03	30589	1,78171E+00	8895	2,16063E+00	23547	9,11697E-01	22103	0	54420,5
Myeloid_vs_CD4_Non-responder	CD4(CRTAM- T)	Macro_FOLR2+APOE+	ANXA1	FPR2_FPR3	1,74141E-03	23493,7	1,09235E-02	7187	1,76417E+00	9200	1,90723E+00	30592	9,23363E-01	16069	0	54420,5
Myeloid_vs_CD4_Non-responder	CD4(CXCL13+ Tfh)	Macro_NLRP3	HMGB1	THBD	1,74825E-03	27744,9	5,46764E-03	21107	1,61900E+00	12124	1,90655E+00	30616	9,14677E-01	20457	0	54420,5
Myeloid_vs_CD4_Non-responder	Macro_FOLR2+APOE+	CD4(TNFRSF9+ Treg)	MMP9	CD44	1,76027E-03	27451,9	8,07421E-03	11881	1,38215E+00	18921	2,24897E+00	21379	8,96517E-01	30658	0	54420,5
Myeloid_vs_CD4_Non-responder	CD4(IL26+ Th17)	Macro_ISG15	HLA-C	LILRB1	1,76085E-03	24941,1	4,71241E-03	26291	1,82473E+00	8206	1,90492E+00	30660	9,45629E-01	5128	0	54420,5
Myeloid_vs_CD4_Non-responder	CD4(ISG+ Treg)	Macro_FOLR2+APOE+	HMGB1	CD163	1,76487E-03	25457,5	4,23833E-03	30674	1,65728E+00	11253	2,28498E+00	20587	9,35079E-01	10353	0	54420,5
Myeloid_vs_CD4_Non-responder	pDC_LILRA4	CD4(TGFB1+ Th17)	APP	RPSA	1,78131E-03	16456,9	1,18830E-02	6215	2,21268E+00	4175	2,61187E+00	14089	9,49444E-01	3385	0	54420,5
Myeloid_vs_CD4_Non-responder	Macro_FOLR2+APOE+	CD4(CRTAM- T)	SPP1	PTGER4	1,78162E-03	23576,1	1,20290E-02	6084	1,30115E+00	21820	3,38727E+00	4824	8,96391E-01	30732	0	54420,5
Myeloid_vs_CD4_Non-responder	Macro_FOLR2+APOE+	CD4(ISG+ Treg)	TNFSF13	FAS	1,78191E-03	25350,3	9,49759E-03	9083	1,58867E+00	12862	2,32680E+00	19653	8,96388E-01	30733	0	54420,5
Myeloid_vs_CD4_Non-responder	CD4(TNF+ T)	pDC_LILRA4	COPA	P2RY6	1,78776E-03	27006,7	7,20323E-03	14102	3,37545E+00	606	3,13674E+00	7042	8,45474E-01	58863	0	54420,5
Myeloid_vs_CD4_Non-responder	CD4(TGFB1+ Th17)	Macro_NLRP3	RPS19	C5AR1	1,78859E-03	24299,1	5,21017E-03	22678	1,68660E+00	10628	1,90190E+00	30756	9,50445E-01	3013	0	54420,5
Myeloid_vs_CD4_Non-responder	Macro_NLRP3	CD4(TNF+ T)	IL1B	ADRB2	1,79905E-03	21199,1	1,32942E-02	5050	2,95262E+00	1131	3,89449E+00	2052	8,73372E-01	43342	0	54420,5
Myeloid_vs_CD4_Non-responder	CD4(AREG+ Tm)	Macro_ISG15	CD99	PILRA	1,81874E-03	28740,1	4,22054E-03	30859	1,60635E+00	12426	1,94438E+00	29472	9,22471E-01	16523	0	54420,5
Myeloid_vs_CD4_Non-responder	Macro_FOLR2+APOE+	CD4(ISG+ Treg)	GRN	TNFRSF1B	1,82937E-03	21200,3	4,21682E-03	30895	1,80392E+00	8533	3,16945E+00	6727	9,45079E-01	5426	0	54420,5
Myeloid_vs_CD4_Non-responder	CD4(IL26+ Th17)	Macro_FOLR2+APOE+	HMGB1	CD163	1,82996E-03	25533,1	4,21660E-03	30897	1,65264E+00	11352	2,28505E+00	20582	9,34922E-01	10414	0	54420,5
Myeloid_vs_CD4_Non-responder	cDC(CD1C)	CD4(ISG+ Treg)	HLA-DRB5	LAG3	1,83036E-03	19631,9	9,28192E-03	9437	1,63967E+00	11639	3,00539E+00	8476	9,27144E-01	14187	0	54420,5
Myeloid_vs_CD4_Non-responder	CD4(IFNG+ Tfh/Th1)	Macro_ISG15	HLA-C	LILRB2	1,84691E-03	22490,3	4,21170E-03	30954	2,04417E+00	5629	2,32321E+00	19741	9,54103E-01	1707	0	54420,5
Myeloid_vs_CD4_Non-responder	CD4(TNF+ T)	Macro_IFI27	TNFSF9	HLA-DPA1	1,85617E-03	28898,3	5,70090E-03	19892	1,38448E+00	18839	2,29539E+00	20355	8,95938E-01	30985	0	54420,5
Myeloid_vs_CD4_Non-responder	CD4(ISG+ Treg)	Mono_CD16	HLA-F	LILRB1	1,85917E-03	28398,7	5,62383E-03	20264	1,72942E+00	9827	1,89398E+00	30995	9,03817E-01	26487	0	54420,5
Myeloid_vs_CD4_Non-responder	Macro_FOLR2+APOE+	CD4(TNF+ T)	MMP9	CD44	1,86037E-03	27382,1	7,97048E-03	12114	1,37358E+00	19221	2,30410E+00	20156	8,95915E-01	30999	0	54420,5
Myeloid_vs_CD4_Non-responder	Macro_FOLR2+APOE+	CD4(Tn)	C1QB	C1QBP	1,86187E-03	21690,9	4,20735E-03	31004	2,13449E+00	4816	3,80795E+00	2381	9,23864E-01	15833	0	54420,5
Myeloid_vs_CD4_Non-responder	CD4(TGFB1+ Th17)	Macro_IER3	ANXA1	FPR1	1,86337E-03	23856,1	6,55474E-03	16229	1,72670E+00	9874	1,89339E+00	31009	9,40214E-01	7748	0	54420,5
Myeloid_vs_CD4_Non-responder	CD4(Tn)	Macro_FOLR2+APOE+	B2M	LILRB2	1,86434E-03	29047,7	3,43277E-03	41741	1,54150E+00	14060	1,79642E+00	34093	9,56896E-01	924	0	54420,5
Myeloid_vs_CD4_Non-responder	Macro_ISG15	CD4(NME1+ T)	CXCL10	CXCR3	1,86495E-03	21040,7	1,68336E-02	3276	2,40621E+00	3021	3,35250E+00	5112	8,80663E-01	39374	0	54420,5

Myeloid_vs_CD4_Post_NR

Myeloid_vs_CD4_Non-responder	CD4(TNFRSF9+ Treg)	cDC_LAMP3	TNFSF10	TNFRSF11B	1,86925E-03	51172,9	1,61147E-02	3553	2,72533E+00	1701	9,42783E-01	70978	7,00422E-01	125212	0	54420,5
Myeloid_vs_CD4_Non-responder	CD4(AREG+ Tm)	Macro_ISG15	HLA-F	LILRB1	1,87784E-03	30265,5	4,70757E-03	26333	1,75417E+00	9363	1,92136E+00	30154	8,95805E-01	31057	0	54420,5
Myeloid_vs_CD4_Non-responder	CD4(TGFB1+ Th17)	Mono_CD16	HLA-C	LILRB1	1,88268E-03	25400,5	4,54988E-03	27639	1,82243E+00	8251	1,89138E+00	31073	9,44719E-01	5619	0	54420,5
Myeloid_vs_CD4_Non-responder	CD4(IL26+ Th17)	Macro_ISG15	B2M	LILRB1	1,88480E-03	24364,3	4,69432E-03	26428	1,83806E+00	8012	1,89116E+00	31080	9,53560E-01	1881	0	54420,5
Myeloid_vs_CD4_Non-responder	CD4(IL26+ Th17)	Macro_ISG15	HLA-B	LILRB1	1,88814E-03	24739,1	4,72401E-03	26185	1,82484E+00	8203	1,89084E+00	31091	9,48410E-01	3796	0	54420,5
Myeloid_vs_CD4_Non-responder	Macro_FOLR2+APOE-	CD4(IFNG+ Tfh/Th1)	HLA-DRA	LAG3	1,88867E-03	18211,5	1,17368E-02	6353	1,76506E+00	9180	2,59867E+00	14301	9,42033E-01	6803	0	54420,5
Myeloid_vs_CD4_Non-responder	pDC_LILRA4	CD4(TNFRSF9+ Treg)	APP	RPSA	1,88970E-03	16494,1	1,18933E-02	6206	2,21360E+00	4165	2,59845E+00	14303	9,49465E-01	3376	0	54420,5
Myeloid_vs_CD4_Non-responder	CD4(TNF+ T)	Macro_ISG15	CD99	PILRA	1,89057E-03	28450,9	4,19885E-03	31099	1,60264E+00	12526	2,00667E+00	27588	9,22287E-01	16621	0	54420,5
Myeloid_vs_CD4_Non-responder	CD4(ISG+ Treg)	Macro_OLFML3	HLA-F	LILRB2	1,89209E-03	29631,3	4,19858E-03	31104	1,61216E+00	12282	1,99309E+00	27993	9,11233E-01	22357	0	54420,5
Myeloid_vs_CD4_Non-responder	CD4(TNFRSF9+ Treg)	Mono_CD16	HLA-F	LILRB1	1,90184E-03	29368,7	5,25018E-03	22417	1,68631E+00	10632	1,88924E+00	31136	9,00787E-01	28238	0	54420,5
Myeloid_vs_CD4_Non-responder	Macro_FOLR2+APOE+	CD4(TGFB1+ Th17)	TNFSF13	FAS	1,91593E-03	25634,1	9,33772E-03	9352	1,58018E+00	13069	2,30434E+00	20147	8,95597E-01	31182	0	54420,5
Myeloid_vs_CD4_Non-responder	CD4(IFNG+ Tfh/Th1)	pDC_LILRA4	SIRPG	CD47	1,91870E-03	27429,3	1,62285E-02	3498,5	1,09056E+00	31191,5	2,38939E+00	18360,5	8,98278E-01	29675,5	0	54420,5
Myeloid_vs_CD4_Non-responder	Macro_NLRP3	CD4(GZMK+ Teff)	VCAN	CD44	1,91990E-03	17033,5	1,01453E-02	8135	2,75577E+00	1609	2,59443E+00	14361	9,42379E-01	6642	0	54420,5
Myeloid_vs_CD4_Non-responder	CD4(CRTAM- T)	Macro_ISG15	HLA-C	LILRB2	1,93011E-03	22673,5	4,18723E-03	31228	2,02968E+00	5769	2,30246E+00	20199	9,53976E-01	1751	0	54420,5
Myeloid_vs_CD4_Non-responder	Macro_FOLR2+APOE+	CD4(Tn)	SPP1	CD44	1,93227E-03	26075,7	6,80744E-03	15337	1,08981E+00	31235	3,06793E+00	7774	9,12616E-01	21612	0	54420,5
Myeloid_vs_CD4_Non-responder	CD4(TNFRSF9+ Treg)	Macro_ISG15	B2M	LILRB2	1,93258E-03	22104,5	4,18638E-03	31236	2,08253E+00	5276	2,34395E+00	19288	9,60808E-01	302	0	54420,5
Myeloid_vs_CD4_Non-responder	CD4(TNF+ T)	Macro_LYVE1	TNFSF9	HLA-DPA1	1,93289E-03	27763,3	5,64777E-03	20155	1,36995E+00	19346	2,63616E+00	13658	8,95500E-01	31237	0	54420,5
Myeloid_vs_CD4_Non-responder	Macro_LYVE1	CD4(IFNG+ Tfh/Th1)	HLA-DQA1	LAG3	1,93461E-03	19391,3	1,25193E-02	5663	1,66676E+00	11043	2,78222E+00	11441	9,26778E-01	14389	0	54420,5
Myeloid_vs_CD4_Non-responder	Macro_FOLR2+APOE+	CD4(IL26+ Th17)	CD14	ITGA4	1,93568E-03	24883,3	4,18560E-03	31246	1,80446E+00	8523	2,84589E+00	10572	9,16259E-01	19655	0	54420,5
Myeloid_vs_CD4_Non-responder	CD4(GZMK+ Teff)	Macro_NLRP3	RPS19	C5AR1	1,94064E-03	24505,7	5,17245E-03	22922	1,67634E+00	10843	1,88558E+00	31262	9,50274E-01	3081	0	54420,5
Myeloid_vs_CD4_Non-responder	Macro_OLFML3	CD4(NME1+ T)	CXCL10	CXCR3	1,94343E-03	19727,5	1,96236E-02	2436	2,80393E+00	1490	3,34401E+00	5184	8,88489E-01	35107	0	54420,5
Myeloid_vs_CD4_Non-responder	CD4(TNF+ T)	Macro_FOLR2+APOE+	CD40LG	CD9	1,94841E-03	23227,7	1,68431E-02	3273	1,49795E+00	15250	2,74974E+00	11908	8,95424E-01	31287	0	54420,5
Myeloid_vs_CD4_Non-responder	CD4(GZMK+ Teff)	Macro_ISG15	HLA-A	LILRB1	1,95028E-03	24602,5	4,92556E-03	24689	1,82878E+00	8154	1,88474E+00	31293	9,47031E-01	4456	0	54420,5
Myeloid_vs_CD4_Non-responder	Macro_NLRP3	CD4(IFNG+ Tfh/Th1)	S100A8	ITGB2	1,95450E-03	16843,9	6,17850E-03	17656	2,65416E+00	1914	3,36160E+00	5035	9,45491E-01	5194	0	54420,5
Myeloid_vs_CD4_Non-responder	Macro_FOLR2+APOE+	CD4(IL26+ Th17)	ICAM1	IL2RG	1,96152E-03	32480,3	5,00702E-03	24069	1,08807E+00	31329	1,90627E+00	30624	9,11942E-01	21959	0	54420,5
Myeloid_vs_CD4_Non-responder	CD4(AREG+ Tm)	Macro_FOLR2+APOE+	CD52	SIGLEC10	1,97120E-03	25699,1	7,14399E-03	14280	1,66753E+00	11034	1,79030E+00	34303	9,26668E-01	14458	0	54420,5
Myeloid_vs_CD4_Non-responder	Macro_ISG15	CD4(TNF+ T)	S100A8	CD69	1,97280E-03	20136,3	7,36302E-03	13662	1,52707E+00	14461	2,92275E+00	9545	9,38519E-01	8593	0	54420,5
Myeloid_vs_CD4_Non-responder	CD4(ISG+ Treg)	cDC(CD1C)	B2M	CD1A	1,97975E-03	25976,5	2,90521E-02	1119	1,87445E+00	7516	1,33255E+00	52353	9,26617E-01	14474	0	54420,5
Myeloid_vs_CD4_Non-responder	Macro_NLRP3	CD4(TGFB1+ Th17)	HBEGF	CD44	1,98458E-03	17365,9	9,71263E-03	8763	2,23915E+00	4000	2,58757E+00	14483	9,45559E-01	5163	0	54420,5
Myeloid_vs_CD4_Non-responder	Macro_FOLR2+APOE+	CD4(GZMK+ Teff)	LGALS9	PTPRC	2,00033E-03	28972,1	4,18140E-03	31295	1,35533E+00	19854	1,87873E+00	31452	9,40026E-01	7839	0	54420,5
Myeloid_vs_CD4_Non-responder	Mono_CD16	CD4(TGFB1+ Th17)	S100A8	CD69	2,00127E-03	19993,3	8,38338E-03	11185	1,52514E+00	14514	2,67196E+00	13102	9,42158E-01	6745	0	54420,5
Myeloid_vs_CD4_Non-responder	Macro_FOLR2+APOE-	CD4(IFNG+ Tfh/Th1)	HLA-DPA1	LAG3	2,01209E-03	18507,3	1,19140E-02	6185	1,71154E+00	10151	2,58487E+00	14534	9,41178E-01	7246	0	54420,5
Myeloid_vs_CD4_Non-responder	CD4(TNFRSF9+ Treg)	Macro_ISG15	HLA-C	LILRB1	2,02397E-03	25207,5	4,68676E-03	26484	1,81148E+00	8412	1,87608E+00	31526	9,45488E-01	5195	0	54420,5
Myeloid_vs_CD4_Non-responder	Mono_CD14	CD4(AREG+ Tm)	VCAN	CD44	2,02840E-03	16463,7	1,11205E-02	6964	3,12932E+00	817	2,58331E+00	14564	9,44821E-01	5553	0	54420,5
Myeloid_vs_CD4_Non-responder	CD4(IL26+ Th17)	Macro_ISG15	HLA-A	LILRB2	2,02847E-03	23715,7	4,16039E-03	31540	1,90856E+00	7081	2,14902E+00	23822	9,54076E-01	1715	0	54420,5
Myeloid_vs_CD4_Non-responder	CD4(ISG+ Treg)	Macro_ISG15	HLA-C	LILRB2	2,02912E-03	22807,9	4,16017E-03	31542	2,01366E+00	5942	2,29612E+00	20338	9,53833E-01	1797	0	54420,5
Myeloid_vs_CD4_Non-responder	CD4(ISG+ Treg)	Macro_OLFML3	HLA-B	LILRB1	2,03330E-03	26383,3	4,19922E-03	31094	1,73885E+00	9633	1,87517E+00	31555	9,45452E-01	5214	0	54420,5
Myeloid_vs_CD4_Non-responder	Macro_NLRP3	CD4(CRTAM- T)	VCAN	ITGB1	2,03769E-03	17310,7	1,32008E-02	5117	2,82281E+00	1436	2,81586E+00	10999	9,26412E-01	14581	0	54420,5
Myeloid_vs_CD4_Non-responder	Mono_CD14	CD4(TNFRSF9+ Treg)	VCAN	SELL	2,04153E-03	16638,5	1,85093E-02	2747	3,24372E+00	706	2,83526E+00	10731	9,26397E-01	14588	0	54420,5
Myeloid_vs_CD4_Non-responder	CD4(CRTAM- T)	Mono_CD16	HLA-C	LILRA3	2,05086E-03	24487,5	1,22403E-02	5898	1,96511E+00	6453	1,60176E+00	41061	9,26365E-01	14605	0	54420,5
Myeloid_vs_CD4_Non-responder	CD4(CXCL13+ Tfh)	Macro_LYVE1	HMGB1	CD163	2,05108E-03	25360,1	4,90522E-03	24827	1,85506E+00	7771	1,87345E+00	31610	9,39376E-01	8172	0	54420,5
Myeloid_vs_CD4_Non-responder	Macro_NLRP3	CD4(TNFRSF9+ Treg)	CXCL8	CD79A	2,05369E-03	25747,5	4,93992E-02	411	2,53436E+00	2401	3,33212E+00	5282	8,32293E-01	66223	0	54420,5
Myeloid_vs_CD4_Non-responder	Macro_NLRP3	CD4(CXCL13+ Tfh)	S100A8	CD69	2,06132E-03	18214,9	8,17021E-03	11642	2,36221E+00	3277	2,57991E+00	14624	9,41452E-01	7111	0	54420,5
Myeloid_vs_CD4_Non-responder	CD4(NME1+ T)	Macro_ISG15	HLA-A	LILRB2	2,06670E-03	23588,9	4,14950E-03	31658	1,90218E+00	7155	2,18140E+00	22972	9,54018E-01	1739	0	54420,5
Myeloid_vs_CD4_Non-responder	Mono_CD14	CD4(NME1+ T)	VCAN	ITGB1	2,07183E-03	17416,1	1,31543E-02	5159	3,15544E+00	784	2,73949E+00	12074	9,26291E-01	14643	0	54420,5
Myeloid_vs_CD4_Non-responder	Macro_FOLR2+APOE+	CD4(IFNG+ Tfh/Th1)	MMP9	CD44	2,08209E-03	28251,3	7,76052E-03	12610	1,35623E+00	19827	2,19262E+00	22694	8,94664E-01	31705	0	54420,5
Myeloid_vs_CD4_Non-responder	CD4(TNFRSF9+ Treg)	Macro_FOLR2+APOE+	HLA-A	LILRB2	2,09196E-03	22956,9	4,14289E-03	31735	1,97235E+00	6383	2,28877E+00	20496	9,53983E-01	1750	0	54420,5
Myeloid_vs_CD4_Non-responder	CD4(ISG+ Treg)	Mono_CD16	B2M	LILRB1	2,10087E-03	25176,7	4,40096E-03	29039	1,82254E+00	8246	1,86805E+00	31762	9,52110E-01	2416	0	54420,5
Myeloid_vs_CD4_Non-responder	CD4(CXCL13+ Tfh)	Macro_NLRP3	RPS19	C5AR1	2,10418E-03	24654,3	5,15641E-03	23033	1,67198E+00	10943	1,86775E+00	31772	9,50200E-01	3103	0	54420,5
Myeloid_vs_CD4_Non-responder	CD4(ISG+ Treg)	Mono_CD16	HLA-C	LILRA3	2,10639E-03	24610,3	1,21612E-02	5963	1,94910E+00	6626	1,59542E+00	41337	9,26144E-01	14705	0	54420,5
Myeloid_vs_CD4_Non-responder	Macro_ISG15	CD4(IFNG+ Tfh/Th1)	HLA-DRB1	LAG3	2,10639E-03	18052,5	1,05124E-02	7658	1,51822E+00	14705	3,43044E+00	4516	9,37811E-01	8963	0	54420,5
Myeloid_vs_CD4_Non-responder	CD4(ISG+ Treg)	Macro_FOLR2+APOE+	HLA-A	LILRB2	2,11081E-03	23113,5	4,13816E-03	31792	1,96926E+00	6408	2,25627E+00	21193	9,53958E-01	1754	0	54420,5
Myeloid_vs_CD4_Non-responder	cDC_LAMP3	CD4(AREG+ Tm)	CCL19	CXCR3	2,11263E-03	27799,9	3,10254E-02	984	4,88789E+00	55	2,24125E+00	21541	8,39935E-01	61999	0	54420,5
Myeloid_vs_CD4_Non-responder	CD4(NME1+ T)	Macro_NLRP3	RPS19	C5AR1	2,11380E-03	25283,7	4,94840E-03	24509	1,61538E+00	12221	1,86679E+00	31801	9,49217E-01	3467	0	54420,5
Myeloid_vs_CD4_Non-responder	Macro_FOLR2+APOE+	CD4(TNFRSF9+ Treg)	CD14	ITGA4	2,12113E-03	25031,1	4,13549E-03	31823	1,79977E+00	8602	2,85633E+00	10438	9,15796E-01	19872	0	54420,5
Myeloid_vs_CD4_Non-responder	Macro_NLRP3	CD4(ISG+ Treg)	VCAN	SELL	2,12157E-03	16781,3	1,83454E-02	2801	2,90010E+00	1244	2,83668E+00	10709	9,26094E-01	14732	0	54420,5
Myeloid_vs_CD4_Non-responder	CD4(IFNG+ Tfh/Th1)	Macro_FOLR2+APOE+	CD52	SIGLEC10	2,12880E-03	24916,7	7,32437E-03	13769	1,69238E+00	10537	1,86560E+00	31846	9,27511E-01	14011	0	54420,5
Myeloid_vs_CD4_Non-responder	CD4(IL26+ Th17)	Macro_FOLR2+APOE+	RPS19	C5AR1	2,13516E-03	27655,1	4,13230E-03	31865	1,37642E+00	19121	2,01962E+00	27236	9,44693E-01	5633	0	54420,5
Myeloid_vs_CD4_Non-responder	Macro_FOLR2+APOE+	CD4(TNFRSF9+ Treg)	TNFSF13	FAS	2,13818E-03	25501,1	9,08886E-03	9778	1,56698E+00	13420	2,40587E+00	18013	8,94328E-01	31874	0	54420,5
Myeloid_vs_CD4_Non-responder	CD4(IFNG+ Tfh/Th1)	Mono_CD16	B2M	LILRB1	2,14086E-03	25213,9	4,39875E-03	29065	1,82052E+00	8280	1,86452E+00	31882	9,52098E-01	2422	0	54420,5
Myeloid_vs_CD4_Non-responder	Macro_FOLR2+APOE+	CD4(TNFRSF9+ Treg)	HLA-DMB	CD4	2,14153E-03	25023,5	4,13073E-03	31884	1,86940E+00	7582	2,73659E+00	12117	9,17275E-01	19114	0	54420,5
Myeloid_vs_CD4_Non-responder	CD4(TNF+ T)	Macro_OLFML3	TNFSF9	HLA-DPA1	2,15095E-03	27389,9	5,49887E-03	20948	1,32921E+00	20816	2,97661E+00	8853	8,94244E-01	31912	0	54420,5
Myeloid_vs_CD4_Non-responder	CD4(IFNG+ Tfh/Th1)	Macro_ISG15	CCL5	CCR1	2,15264E-03	25039,5	2,06526E-02	2220	1,89674E+00	7225	1,94593E+00	29415	8,94240E-01	31917	0	54420,5
Myeloid_vs_CD4_Non-responder	CD4(CXCL13+ Tfh)	Macro_ISG15	HLA-A	LILRB2	2,16244E-03	23970,5	4,12541E-03	31946	1,88807E+00	7343	2,12714E+00	24362	9,53890E-01	1781	0	54420,5
Myeloid_vs_CD4_Non-responder	CD4(IL26+ Th17)	Macro_ISG15	HLA-B	LILRB2	2,16684E-03	23015,7	4,12423E-03	31959	1,98651E+00	6229	2,25123E+00	21305	9,55981E-01	1165	0	54420,5
Myeloid_vs_CD4_Non-responder	CD4(TGFB1+ Th17)	Macro_FOLR2+APOE+	TNF	TNFRSF1A	2,17533E-03	28344,7	9,20654E-03	9575	1,28443E+00	22481	1,86125E+00	31984	9,09542E-01	23263	0	54420,5
Myeloid_vs_CD4_Non-responder	cDC_CLEC9A	CD4(ISG+ Treg)	HLA-DRB1	LAG3	2,17908E-03	19484,9	8,44860E-03	11041	1,51286E+00	14833	3,38837E+00	4813	9,31125E-01	12317	0	54420,5
Myeloid_vs_CD4_Non-responder	CD4(IFNG+ Tfh/Th1)	Macro_ISG15	HLA-B	LILRB1	2,18214E-03	25207,5	4,65196E-03	26772	1,78279E+00	8875	1,86068E+00	32004	9,48033E-01	3966	0	54420,5
Myeloid_vs_CD4_Non-responder	CD4(TNFRSF9+ Treg)	cDC(CD1C)	B2M	CD1A	2,18484E-03	27025,9	2,84194E-02	1184	1,78683E+00	8814	1,25278E+00	55868	9,25865E-01	14843	0	54420,5
Myeloid_vs_CD4_Non-responder	Macro_NLRP3	CD4(IFNG+ Tfh/Th1)	VCAN	ITGB1	2,19292E-03	17359,7	1,29842E-02	5285	2,81260E+00	1465	2,83232E+00	10771	9,25846E-01	14857	0	54420,5
Myeloid_vs_CD4_Non-responder	CD4(TGFB1+ Th17)	pDC_LILRA4	TGFB1	ACVR1_TGFBR1_TGFBR2	2,19478E-03	30311,1	8,50002E-03	10916	1,16075E+00	27862	2,05200E+00	26316	8,94009E-01	32041	0	54420,5

Myeloid_vs_CD4_Post_NR

Myeloid_vs_CD4_Non-responder	CD4(TNF+ T)	Macro_FOLR2-APOE+	TNFSF9	HLA-DPA1	2,19821E-03	30388,3	5,46860E-03	21099	1,32092E+00	21107	2,17068E+00	23264	8,93982E-01	32051	0	54420,5
Myeloid_vs_CD4_Non-responder	Macro_FOLR2+APOE+	CD4(TNFRSF9+ Treg)	ICAM1	IL2RG	2,20164E-03	32747,9	4,90594E-03	24822	1,07392E+00	32061	1,92482E+00	30022	9,11120E-01	22414	0	54420,5
Myeloid_vs_CD4_Non-responder	CD4(IL26+ Th17)	Macro_ISG15	HLA-C	LILRB2	2,20164E-03	23120,1	4,11411E-03	32061	1,98640E+00	6230	2,26531E+00	21019	9,53587E-01	1870	0	54420,5
Myeloid_vs_CD4_Non-responder	Macro_FOLR2+APOE+	CD4(CRTAM- T)	FN1	CD44	2,21472E-03	32829,9	6,93467E-03	14895	1,07489E+00	32010	1,85793E+00	32099	8,96403E-01	30725	0	54420,5
Myeloid_vs_CD4_Non-responder	CD4(AREG+ Tm)	Macro_ISG15	HLA-F	LILRB2	2,21851E-03	27394,3	4,10988E-03	32110	1,91584E+00	6997	2,28174E+00	20647	9,10365E-01	22797	0	54420,5
Myeloid_vs_CD4_Non-responder	CD4(Tn)	Macro_FOLR2+APOE+	HMGB1	CD163	2,22439E-03	25855,9	4,10824E-03	32127	1,62952E+00	11854	2,30747E+00	20068	9,34126E-01	10810	0	54420,5
Myeloid_vs_CD4_Non-responder	CD4(GZMK+ Teff)	Macro_ISG15	HLA-B	LILRB2	2,22578E-03	22863,9	4,10805E-03	32131	1,97569E+00	6342	2,30066E+00	20237	9,55898E-01	1189	0	54420,5
Myeloid_vs_CD4_Non-responder	Macro_FOLR2+APOE+	CD4(TNFRSF9+ Treg)	CCL4	CCR8	2,24838E-03	23798,1	4,88809E-02	422	1,20836E+00	25689	3,21824E+00	6263	8,93697E-01	32196	0	54420,5
Myeloid_vs_CD4_Non-responder	CD4(TNFRSF9+ Treg)	Macro_FOLR2+APOE+	HMGB1	CD163	2,24943E-03	26347,9	4,10172E-03	32199	1,62813E+00	11888	2,20435E+00	22400	9,34077E-01	10832	0	54420,5
Myeloid_vs_CD4_Non-responder	Macro_IFI27	CD4(IFNG+ Tfh/Th1)	HLA-DRB5	LAG3	2,25774E-03	19472,9	1,17338E-02	6360	1,66509E+00	11075	2,56173E+00	14968	9,34675E-01	10541	0	54420,5
Myeloid_vs_CD4_Non-responder	Macro_FOLR2+APOE+	CD4(TNFRSF9+ Treg)	LGALS9	PTPRC	2,26274E-03	29453,5	4,09916E-03	32237	1,33278E+00	20701	1,86728E+00	31787	9,39464E-01	8122	0	54420,5
Myeloid_vs_CD4_Non-responder	CD4(IL26+ Th17)	Macro_ISG15	B2M	LILRB2	2,26695E-03	22880,1	4,09831E-03	32249	1,99972E+00	6096	2,25155E+00	21299	9,60406E-01	336	0	54420,5
Myeloid_vs_CD4_Non-responder	CD4(TGFB1+ Th17)	Macro_ISG15	HLA-A	LILRB1	2,26765E-03	24892,1	4,89671E-03	24898	1,81402E+00	8373	1,85315E+00	32251	9,46883E-01	4518	0	54420,5
Myeloid_vs_CD4_Non-responder	CD4(TNFRSF9+ Treg)	Mono_CD16	HLA-C	LILRA3	2,27491E-03	25266,1	1,19611E-02	6142	1,90858E+00	7080	1,53577E+00	43691	9,25574E-01	14997	0	54420,5
Myeloid_vs_CD4_Non-responder	Macro_NLRP3	CD4(TNFRSF9+ Treg)	S100A8	CD69	2,28085E-03	18627,7	7,79183E-03	12539	2,34164E+00	3382	2,56001E+00	15007	9,40131E-01	7790	0	54420,5
Myeloid_vs_CD4_Non-responder	CD4(TNFRSF9+ Treg)	Macro_ISG15	HLA-C	LILRB2	2,28918E-03	23337,7	4,09171E-03	32312	1,97315E+00	6378	2,23647E+00	21662	9,53466E-01	1916	0	54420,5
Myeloid_vs_CD4_Non-responder	Macro_FOLR2-APOE+	CD4(IFNG+ Tfh/Th1)	HLA-DPA1	LAG3	2,28919E-03	19544,5	1,10154E-02	7083	1,58948E+00	12841	2,55929E+00	15021	9,38969E-01	8357	0	54420,5
Myeloid_vs_CD4_Non-responder	CD4(NME1+ T)	Macro_FOLR2+APOE+	ADAM10	GPNMB	2,29038E-03	27418,7	7,25327E-03	13973	2,03030E+00	5762	2,63425E+00	13687	8,62678E-01	49251	0	54420,5
Myeloid_vs_CD4_Non-responder	CD4(AREG+ Tm)	Macro_FOLR2+APOE+	HMGB1	CD163	2,30481E-03	26237,1	4,08718E-03	32356	1,62502E+00	11974	2,24090E+00	21555	9,33968E-01	10880	0	54420,5
Myeloid_vs_CD4_Non-responder	CD4(CRTAM- T)	Macro_ISG15	HLA-A	LILRB2	2,31337E-03	24345,1	4,08518E-03	32380	1,86450E+00	7648	2,08618E+00	25434	9,53674E-01	1843	0	54420,5
Myeloid_vs_CD4_Non-responder	Mono_CD14	CD4(Tn)	VCAN	CD44	2,32035E-03	16853,3	1,05107E-02	7663	3,08382E+00	885	2,55671E+00	15073	9,43332E-01	6225	0	54420,5
Myeloid_vs_CD4_Non-responder	CD4(CXCL13+ Tfh)	cDC_LAMP3	CD28	CD86	2,32375E-03	26029,5	1,25137E-02	5667,5	1,84232E+00	7946,5	1,84835E+00	32409,5	8,98222E-01	29703,5	0	54420,5
Myeloid_vs_CD4_Non-responder	Macro_OLFML3	CD4(IL26+ Th17)	CXCL9	DPP4	2,33571E-03	23814,1	3,89412E-02	635	2,99510E+00	1035	3,19301E+00	6494	8,49774E-01	56486	0	54420,5
Myeloid_vs_CD4_Non-responder	Macro_IFI27	CD4(IFNG+ Tfh/Th1)	HLA-DPB1	LAG3	2,33848E-03	19429,1	1,14945E-02	6589	1,63993E+00	11628	2,55499E+00	15103	9,36951E-01	9405	0	54420,5
Myeloid_vs_CD4_Non-responder	CD4(TGFB1+ Th17)	pDC_LILRA4	TGFB1	CXCR4	2,35257E-03	25240,9	5,53876E-03	20735	1,06581E+00	32489	2,55585E+00	15092	9,49217E-01	3468	0	54420,5
Myeloid_vs_CD4_Non-responder	CD4(NME1+ T)	Macro_LYVE1	CD59	STAB1	2,35692E-03	24753,3	6,88476E-03	15061	2,50709E+00	2517	2,34475E+00	19267	8,93154E-01	32501	0	54420,5
Myeloid_vs_CD4_Non-responder	CD4(TNF+ T)	Mono_CD14	ANXA1	FPR1	2,36272E-03	28171,3	4,55103E-03	27622	1,58061E+00	13055	1,84509E+00	32517	9,29098E-01	13242	0	54420,5
Myeloid_vs_CD4_Non-responder	CD4(ISG+ Treg)	cDC_LAMP3	CD28	CD86	2,37036E-03	25627,9	1,31405E-02	5175,5	1,86928E+00	7585,5	1,84431E+00	32538,5	9,00434E-01	28419,5	0	54420,5
Myeloid_vs_CD4_Non-responder	CD4(TNFRSF9+ Treg)	Macro_OLFML3	HLA-A	LILRB1	2,38753E-03	26733,1	4,31726E-03	29857	1,68273E+00	10708	1,84263E+00	32585	9,43626E-01	6095	0	54420,5
Myeloid_vs_CD4_Non-responder	CD4(ISG+ Treg)	pDC_LILRA4	LGALS9	CD47	2,38863E-03	30196,9	4,06755E-03	32588	1,27994E+00	22644	2,68294E+00	12926	9,00460E-01	28406	0	54420,5
Myeloid_vs_CD4_Non-responder	Macro_FOLR2+APOE+	CD4(IFNG+ Tfh/Th1)	ICAM1	IL2RG	2,39157E-03	33512,3	4,83308E-03	25357	1,06373E+00	32596	1,84679E+00	32462	9,10512E-01	22726	0	54420,5
Myeloid_vs_CD4_Non-responder	CD4(NME1+ T)	Mono_CD16	HLA-B	LILRA1	2,40891E-03	25477,7	9,74345E-03	8718	2,05580E+00	5517	1,39064E+00	49816	9,37884E-01	8917	0	54420,5
Myeloid_vs_CD4_Non-responder	CD4(NME1+ T)	Mono_CD16	HLA-A	LILRA1	2,41201E-03	24976,7	1,02433E-02	7996	2,08994E+00	5216	1,44124E+00	47613	9,36488E-01	9638	0	54420,5
Myeloid_vs_CD4_Non-responder	CD4(IFNG+ Tfh/Th1)	Macro_ISG15	HLA-B	LILRB2	2,41292E-03	23404,1	4,06133E-03	32654	1,94446E+00	6676	2,22106E+00	22021	9,55656E-01	1249	0	54420,5
Myeloid_vs_CD4_Non-responder	CD4(TGFB1+ Th17)	cDC_CLEC9A	TGFB1	LPP	2,41477E-03	30074,3	8,55603E-03	10798	1,19908E+00	26124	1,84031E+00	32659	9,04023E-01	26370	0	54420,5
Myeloid_vs_CD4_Non-responder	CD4(NME1+ T)	Mono_CD16	HLA-C	LILRA1	2,41946E-03	26069,9	9,50149E-03	9081	2,01724E+00	5902	1,38573E+00	50030	9,33877E-01	10916	0	54420,5
Myeloid_vs_CD4_Non-responder	CD4(GZMK+ Teff)	Macro_FOLR2+APOE+	HLA-F	LILRB2	2,42811E-03	28623,3	4,05672E-03	32695	1,75647E+00	9333	2,15962E+00	23573	9,09833E-01	23095	0	54420,5
Myeloid_vs_CD4_Non-responder	CD4(NME1+ T)	Macro_FOLR2+APOE+	CD52	SIGLEC10	2,43690E-03	26758,9	6,82926E-03	15263	1,62418E+00	11994	1,71293E+00	36916	9,25123E-01	15201	0	54420,5
Myeloid_vs_CD4_Non-responder	CD4(TNFRSF9+ Treg)	Macro_FOLR2+APOE+	CD28	CD86	2,44113E-03	25574,9	1,11312E-02	6953,5	1,74968E+00	9454,5	2,12897E+00	24315,5	8,92744E-01	32730,5	0	54420,5
Myeloid_vs_CD4_Non-responder	Macro_FOLR2+APOE+	CD4(TNFRSF9+ Treg)	CD86	CD28	2,44150E-03	25574,9	1,11312E-02	6953,5	1,74968E+00	9454,5	2,12897E+00	24315,5	8,92744E-01	32730,5	0	54420,5
Myeloid_vs_CD4_Non-responder	Mast	CD4(GZMK+ Teff)	TIMP3	CD44	2,44451E-03	26364,7	3,80304E-02	665	2,98425E+00	1059	1,28485E+00	54448	9,13289E-01	21231	0	54420,5
Myeloid_vs_CD4_Non-responder	Macro_FOLR2+APOE+	CD4(TGFB1+ Th17)	CXCL9	DPP4	2,44451E-03	25778,1	2,98484E-02	1059	3,04101E+00	952	3,24139E+00	6076	8,32001E-01	66383	0	54420,5
Myeloid_vs_CD4_Non-responder	CD4(TNFRSF9+ Treg)	pDC_LILRA4	HSP90B1	TLR9	2,45485E-03	32853,9	2,14437E-02	2064	2,60473E+00	2107	1,26299E+00	55444	8,60902E-01	50234	0	54420,5
Myeloid_vs_CD4_Non-responder	CD4(TNFRSF9+ Treg)	pDC_LILRA4	HMGB1	TLR9	2,46001E-03	28306,3	3,23749E-02	916	2,64725E+00	1954	1,26611E+00	55302	8,99516E-01	28939	0	54420,5
Myeloid_vs_CD4_Non-responder	CD4(TNFRSF9+ Treg)	pDC_LILRA4	CD70	TNFRSF17	2,46516E-03	41861,1	1,56947E-01	27	2,14952E+00	4681	1,77193E+00	34916	7,30143E-01	115261	0	54420,5
Myeloid_vs_CD4_Non-responder	CD4(IL26+ Th17)	Mono_CD14	RPS19	C5AR1	2,46735E-03	28843,5	4,11836E-03	32016	1,37099E+00	19309	1,83519E+00	32800	9,44605E-01	5672	0	54420,5
Myeloid_vs_CD4_Non-responder	CD4(GZMK+ Teff)	Macro_ISG15	B2M	LILRB1	2,47036E-03	24999,7	4,62983E-03	26948	1,78511E+00	8844	1,83494E+00	32808	9,53252E-01	1978	0	54420,5
Myeloid_vs_CD4_Non-responder	CD4(TNFRSF9+ Treg)	Macro_ISG15	RPS19	C5AR1	2,47299E-03	25734,7	4,86683E-03	25117	1,59501E+00	12695	1,83481E+00	32815	9,48815E-01	3626	0	54420,5
Myeloid_vs_CD4_Non-responder	Macro_FOLR2+APOE+	CD4(ISG+ Treg)	CCL3	CCR4	2,48016E-03	21286,9	1,68736E-02	3256	1,73524E+00	9706	3,22293E+00	6218	8,92580E-01	32834	0	54420,5
Myeloid_vs_CD4_Non-responder	Macro_LYVE1	CD4(ISG+ Treg)	HLA-DRB5	LAG3	2,48281E-03	21920,3	8,68134E-03	10556	1,53483E+00	14247	2,55839E+00	15042	9,24852E-01	15336	0	54420,5
Myeloid_vs_CD4_Non-responder	cDC_CLEC9A	CD4(ISG+ Treg)	HLA-DQB1	LAG3	2,48914E-03	18071,7	1,06380E-02	7503	1,77010E+00	9082	3,50544E+00	4007	9,24833E-01	15346	0	54420,5
Myeloid_vs_CD4_Non-responder	CD4(TNFRSF9+ Treg)	Macro_ISG15	CD28	CD86	2,49113E-03	25517,5	1,10782E-02	7014,5	1,74160E+00	9591,5	2,15439E+00	23697,5	8,92516E-01	32863,5	0	54420,5
Myeloid_vs_CD4_Non-responder	Macro_IER3	CD4(IFNG+ Tfh/Th1)	HLA-DPB1	LAG3	2,49232E-03	19802,5	1,03989E-02	7804	1,49490E+00	15351	2,84901E+00	10533	9,33927E-01	10904	0	54420,5
Myeloid_vs_CD4_Non-responder	CD4(GZMK+ Teff)	Macro_ISG15	B2M	LILRB2	2,49265E-03	23385,5	4,04201E-03	32867	1,94678E+00	6647	2,19533E+00	22624	9,60142E-01	369	0	54420,5
Myeloid_vs_CD4_Non-responder	Macro_ISG15	CD4(IFNG+ Tfh/Th1)	B2M	CD3D	2,49300E-03	36818,3	4,30007E-03	30017	7,64936E-01	50511	1,40673E+00	49128	9,66692E-01	15	0	54420,5
Myeloid_vs_CD4_Non-responder	CD4(CXCL13+ Tfh)	Macro_ISG15	CIRBP	TREM1	2,49303E-03	28386,5	6,44297E-03	16619	1,49601E+00	15309	1,83323E+00	32868	9,10520E-01	22716	0	54420,5
Myeloid_vs_CD4_Non-responder	Macro_ISG15	CD4(IFNG+ Tfh/Th1)	HLA-DRB5	LAG3	2,50058E-03	19254,3	1,03760E-02	7829	1,49442E+00	15364	3,22365E+00	6212	9,30819E-01	12446	0	54420,5
Myeloid_vs_CD4_Non-responder	pDC_LILRA4	CD4(TNF+ T)	APP	RPSA	2,50377E-03	17322,7	1,07616E-02	7368	2,11295E+00	4992	2,53998E+00	15369	9,47011E-01	4464	0	54420,5
Myeloid_vs_CD4_Non-responder	Macro_FOLR2+APOE+	CD4(AREG+ Tm)	C1QB	C1QBP	2,50405E-03	22263,5	4,03975E-03	32897	2,12086E+00	4926	3,77511E+00	2522	9,22422E-01	16552	0	54420,5
Myeloid_vs_CD4_Non-responder	CD4(Tn)	Macro_FOLR2+APOE+	CD99	CD81	2,50557E-03	31237,5	4,03916E-03	32901	1,31201E+00	21406	1,92642E+00	29986	9,20615E-01	17474	0	54420,5
Myeloid_vs_CD4_Non-responder	CD4(IFNG+ Tfh/Th1)	Macro_NLRP3	RPS19	C5AR1	2,51396E-03	24949,5	5,13243E-03	23190	1,66545E+00	11068	1,83171E+00	32923	9,50090E-01	3146	0	54420,5
Myeloid_vs_CD4_Non-responder	CD4(TGFB1+ Th17)	Macro_ISG15	HLA-B	LILRB2	2,51434E-03	23747,1	4,03699E-03	32924	1,92819E+00	6865	2,17148E+00	23238	9,55529E-01	1288	0	54420,5
Myeloid_vs_CD4_Non-responder	CD4(ISG+ Treg)	Macro_NLRP3	HLA-F	LILRB2	2,52275E-03	30984,9	4,03462E-03	32946	1,55215E+00	13792	1,90880E+00	30545	9,09608E-01	23221	0	54420,5
Myeloid_vs_CD4_Non-responder	CD4(TNF+ T)	Mono_CD14	RPS19	C5AR1	2,52965E-03	25572,3	4,94544E-03	24529	1,60466E+00	12471	1,83038E+00	32964	9,49203E-01	3477	0	54420,5
Myeloid_vs_CD4_Non-responder	CD4(IL26+ Th17)	Macro_ISG15	HLA-F	LILRB1	2,53118E-03	31756,7	4,37269E-03	29318	1,71949E+00	10007	1,85890E+00	32070	8,92310E-01	32968	0	54420,5
Myeloid_vs_CD4_Non-responder	CD4(TNFRSF9+ Treg)	Macro_ISG15	TIGIT	NECTIN2	2,53541E-03	21442,5	4,22320E-02	557,5	2,55461E+00	2317,5	2,45868E+00	16937,5	8,92298E-01	32979,5	0	54420,5
Myeloid_vs_CD4_Non-responder	CD4(CXCL13+ Tfh)	Macro_FOLR2+APOE+	CD99	PILRA	2,53810E-03	29403,1	4,03206E-03	32986	1,63553E+00	11725	1,91036E+00	30516	9,20822E-01	17368	0	54420,5
Myeloid_vs_CD4_Non-responder	CD4(ISG+ Treg)	Macro_FOLR2+APOE+	HLA-B	LILRB2	2,55276E-03	22859,5	4,02961E-03	33024	2,02848E+00	5776	2,32131E+00	19778	9,55490E-01	1299	0	54420,5
Myeloid_vs_CD4_Non-responder	CD4(GZMK+ Teff)	cDC(CD1C)	B2M	CD1A	2,55383E-03	29100,1	2,74394E-02	1278	1,65107E+00	11384	1,10416E+00	62971	9,24652E-01	15447	0	54420,5
Myeloid_vs_CD4_Non-responder	CD4(TNFRSF9+ Treg)	cDC_LAMP3	CD28	CD86	2,55391E-03	26958,3	1,14339E-02	6646,5	1,79587E+00	8663,5	1,82830E+00	33027,5	8,94022E-01	32033,5	0	54420,5
Myeloid_vs_CD4_Non-responder	Macro_FOLR2-APOE+	CD4(IFNG+ Tfh/Th1)	HLA-DRB5	LAG3	2,56289E-03	19533,3	1,17770E-02	6318	1,67052E+00	10977	2,53505E+00	15461	9,34787E-01	10490	0	54420,5

Myeloid_vs_CD4_Post_NR

Myeloid_vs_CD4_Non-responder	cDC_LAMP3	CD4(TGFB1+ Th17)	CCL19	CCR7	2,56512E-03	26605,5	2,96519E-02	1085	4,90771E+00	53	2,02876E+00	26976	8,60446E-01	50493	0	54420,5
Myeloid_vs_CD4_Non-responder	CD4(ISG+ Treg)	Macro_NLRP3	RPS19	C5AR1	2,56942E-03	25534,3	4,94870E-03	24502	1,61547E+00	12217	1,82727E+00	33067	9,49218E-01	3465	0	54420,5
Myeloid_vs_CD4_Non-responder	CD4(ISG+ Treg)	pDC_LILRA4	SIRPG	CD47	2,58383E-03	28755,9	1,49967E-02	4060,5	1,05372E+00	33104,5	2,29068E+00	20461,5	8,94615E-01	31732,5	0	54420,5
Myeloid_vs_CD4_Non-responder	Macro_FOLR2-APOE+	CD4(TNF+ T)	APOE	LDLR	2,59111E-03	18867,9	1,50579E-02	4028	2,04543E+00	5615	3,28113E+00	5716	9,07194E-01	24560	0	54420,5
Myeloid_vs_CD4_Non-responder	CD4(TNFRSF9+ Treg)	Mono_CD16	RPS19	C5AR1	2,59242E-03	25825,5	4,85896E-03	25172	1,59230E+00	12764	1,82570E+00	33126	9,48776E-01	3645	0	54420,5
Myeloid_vs_CD4_Non-responder	Mono_CD14	CD4(NME1+ T)	S100A8	ITGB2	2,59245E-03	16374,9	5,95014E-03	18636	3,17508E+00	762	3,81809E+00	2339	9,44512E-01	5717	0	54420,5
Myeloid_vs_CD4_Non-responder	CD4(TNFRSF9+ Treg)	Macro_ISG15	CIRBP	TREM1	2,59516E-03	28823,1	6,27763E-03	17242	1,47279E+00	16007	1,82549E+00	33133	9,09455E-01	23313	0	54420,5
Myeloid_vs_CD4_Non-responder	Macro_FOLR2+APOE+	CD4(NME1+ T)	LGALS3BP	ITGB1	2,64846E-03	30058,5	7,38421E-03	13602	1,35426E+00	19903	1,82178E+00	33268	8,99204E-01	29099	0	54420,5
Myeloid_vs_CD4_Non-responder	CD4(NME1+ T)	Macro_ISG15	HLA-A	LILRB1	2,65763E-03	25633,7	4,75294E-03	25962	1,74051E+00	9611	1,82102E+00	33291	9,46129E-01	4884	0	54420,5
Myeloid_vs_CD4_Non-responder	cDC_CLEC9A	CD4(ISG+ Treg)	HLA-DRB5	LAG3	2,66349E-03	20330,7	8,54948E-03	10813	1,51181E+00	14866	3,25554E+00	5940	9,24319E-01	15614	0	54420,5
Myeloid_vs_CD4_Non-responder	CD4(CXCL13+ Tfh)	pDC_LILRA4	BST2	LILRA4	2,66523E-03	20254,9	1,23273E-02	5809	3,60594E+00	425	3,11189E+00	7310	8,91762E-01	33310	0	54420,5
Myeloid_vs_CD4_Non-responder	Macro_FOLR2+APOE+	CD4(IL26+ Th17)	SPP1	CD44	2,66963E-03	27124,1	6,46026E-03	16556	1,04983E+00	33321	2,99681E+00	8595	9,10506E-01	22728	0	54420,5
Myeloid_vs_CD4_Non-responder	CD4(GZMK+ Teff)	Mono_CD16	HLA-C	LILRA3	2,67485E-03	26304,7	1,15279E-02	6566	1,82085E+00	8274	1,46485E+00	46632	9,24294E-01	15631	0	54420,5
Myeloid_vs_CD4_Non-responder	Macro_OLFML3	CD4(ISG+ Treg)	CXCL10	SDC4	2,68555E-03	28657,3	1,23559E-02	5786	2,77222E+00	1572	3,45899E+00	4341	8,12365E-01	77167	0	54420,5
Myeloid_vs_CD4_Non-responder	CD4(IFNG+ Tfh/Th1)	Macro_FOLR2+APOE+	HLA-A	LILRB2	2,68891E-03	24275,7	4,00110E-03	33369	1,87961E+00	7455	2,13607E+00	24140	9,53213E-01	1994	0	54420,5
Myeloid_vs_CD4_Non-responder	CD4(TNFRSF9+ Treg)	cDC_CLEC9A	FLT3LG	FLT3	2,71294E-03	33747,7	2,59570E-02	1435	3,16627E+00	771	1,51790E+00	44435	8,29783E-01	67677	0	54420,5
Myeloid_vs_CD4_Non-responder	Macro_FOLR2+APOE+	CD4(AREG+ Tm)	LGALS1	CD69	2,71764E-03	27807,9	4,68424E-03	26507	1,04745E+00	33440	2,22633E+00	21897	9,51109E-01	2775	0	54420,5
Myeloid_vs_CD4_Non-responder	CD4(CXCL13+ Tfh)	Mono_CD16	CD99	PILRA	2,71805E-03	29632,9	3,99507E-03	33441	1,61913E+00	12120	1,90536E+00	30650	9,20485E-01	17533	0	54420,5
Myeloid_vs_CD4_Non-responder	CD4(IFNG+ Tfh/Th1)	Mono_CD16	HLA-F	LILRB1	2,72415E-03	30922,3	4,87758E-03	25037	1,64333E+00	11532	1,81610E+00	33456	8,97449E-01	30166	0	54420,5
Myeloid_vs_CD4_Non-responder	CD4(GZMK+ Teff)	Mono_CD16	HLA-F	LILRB1	2,73434E-03	31619,5	4,65882E-03	26705	1,61809E+00	12153	1,81509E+00	33481	8,95318E-01	31338	0	54420,5
Myeloid_vs_CD4_Non-responder	CD4(Tn)	Macro_ISG15	HLA-B	LILRB1	2,75277E-03	25929,7	4,55454E-03	27584	1,72594E+00	9885	1,81369E+00	33526	9,47509E-01	4233	0	54420,5
Myeloid_vs_CD4_Non-responder	CD4(NME1+ T)	Macro_ISG15	HLA-F	LILRB1	2,75359E-03	31540,3	4,51786E-03	27921	1,73452E+00	9725	1,81367E+00	33528	8,93869E-01	32107	0	54420,5
Myeloid_vs_CD4_Non-responder	Macro_FOLR2+APOE+	CD4(TNFRSF9+ Treg)	LGALS3	LAG3	2,76222E-03	30042,1	6,32643E-03	17050	1,08473E+00	31505	2,63427E+00	13686	8,91311E-01	33549	0	54420,5
Myeloid_vs_CD4_Non-responder	CD4(TGFB1+ Th17)	Macro_ISG15	HLA-B	LILRB1	2,78038E-03	25646,7	4,62408E-03	27012	1,76652E+00	9156	1,81110E+00	33593	9,47884E-01	4052	0	54420,5
Myeloid_vs_CD4_Non-responder	Macro_OLFML3	CD4(NME1+ T)	CXCL10	SDC4	2,78920E-03	28734,5	1,22807E-02	5861	2,77117E+00	1576	3,44349E+00	4426	8,11899E-01	77389	0	54420,5
Myeloid_vs_CD4_Non-responder	CD4(ISG+ Treg)	Macro_OLFML3	HLA-A	LILRB1	2,79199E-03	26966,1	4,31233E-03	29904	1,67963E+00	10773	1,81013E+00	33621	9,43595E-01	6112	0	54420,5
Myeloid_vs_CD4_Non-responder	CD4(AREG+ Tm)	cDC_LAMP3	LTB	CD40	2,79313E-03	25343,7	1,25702E-02	5626	2,01441E+00	5932	1,50579E+00	44935	9,23917E-01	15805	0	54420,5
Myeloid_vs_CD4_Non-responder	Macro_NLRP3	CD4(Tn)	IL1B	SIGIRR	2,79656E-03	19481,5	7,79864E-03	12524	2,79986E+00	1503	3,45126E+00	4380	9,07153E-01	24580	0	54420,5
Myeloid_vs_CD4_Non-responder	Macro_NLRP3	CD4(Tn)	VCAN	ITGB1	2,80030E-03	22027,9	7,89572E-03	12290	2,57275E+00	2227	2,48378E+00	16462	9,06858E-01	24740	0	54420,5
Myeloid_vs_CD4_Non-responder	Macro_NLRP3	CD4(Tn)	VCAN	CD44	2,80155E-03	18126,3	9,10190E-03	9758	2,66589E+00	1877	2,48734E+00	16394	9,39361E-01	8182	0	54420,5
Myeloid_vs_CD4_Non-responder	Macro_FOLR2+APOE+	CD4(NME1+ T)	HLA-DRB5	LAG3	2,80239E-03	30519,7	3,97928E-03	33646	1,33180E+00	20731	2,80645E+00	11128	8,92845E-01	32673	0	54420,5
Myeloid_vs_CD4_Non-responder	Macro_NLRP3	CD4(CRTAM- T)	HBEGF	CD44	2,80281E-03	17937,3	9,32329E-03	9375	2,19081E+00	4350	2,51573E+00	15819	9,44496E-01	5722	0	54420,5
Myeloid_vs_CD4_Non-responder	Macro_NLRP3	CD4(Tn)	HBEGF	CD44	2,80405E-03	23634,3	6,31611E-03	17093	1,81751E+00	8318	2,02191E+00	27168	9,33360E-01	11172	0	54420,5
Myeloid_vs_CD4_Non-responder	Macro_NLRP3	CD4(Tn)	VEGFA	CD44	2,81365E-03	23338,7	8,88102E-03	10176	2,31469E+00	3536	1,87554E+00	31540	9,21529E-01	17021	0	54420,5
Myeloid_vs_CD4_Non-responder	Macro_FOLR2+APOE+	CD4(ISG+ Treg)	LGALS9	PTPRC	2,81490E-03	30385,7	3,97702E-03	33676	1,29930E+00	21902	1,81808E+00	33384	9,38598E-01	8546	0	54420,5
Myeloid_vs_CD4_Non-responder	CD4(Tn)	Macro_ISG15	HLA-B	LILRB2	2,81699E-03	23999,5	3,97628E-03	33681	1,88761E+00	7348	2,17408E+00	23167	9,55206E-01	1381	0	54420,5
Myeloid_vs_CD4_Non-responder	cDC(CD1C)	CD4(ISG+ Treg)	HLA-DRB1	LAG3	2,81877E-03	20913,5	8,26693E-03	11413	1,47867E+00	15842	2,86932E+00	10259	9,30425E-01	12633	0	54420,5
Myeloid_vs_CD4_Non-responder	Macro_NLRP3	CD4(CRTAM- T)	THBS1	ITGA4	2,82537E-03	22740,1	9,03032E-03	9886	1,83687E+00	8026	2,47937E+00	16550	9,06749E-01	24818	0	54420,5
Myeloid_vs_CD4_Non-responder	Macro_NLRP3	CD4(CRTAM- T)	CD14	ITGB1	2,82746E-03	27868,9	4,34503E-03	29611	1,40157E+00	18262	2,31127E+00	19984	9,21457E-01	17067	0	54420,5
Myeloid_vs_CD4_Non-responder	CD4(NME1+ T)	Macro_ISG15	B2M	LILRB2	2,83124E-03	23887,3	3,97382E-03	33715	1,88267E+00	7408	2,16307E+00	23477	9,59815E-01	416	0	54420,5
Myeloid_vs_CD4_Non-responder	Macro_NLRP3	CD4(AREG+ Tm)	VCAN	CD44	2,83200E-03	17654,3	9,63003E-03	8886	2,71139E+00	1743	2,51394E+00	15861	9,40947E-01	7361	0	54420,5
Myeloid_vs_CD4_Non-responder	CD4(NME1+ T)	cDC(CD1C)	B2M	CD1A	2,83410E-03	29785,1	2,69765E-02	1314	1,58696E+00	12913	1,07190E+00	64547	9,24057E-01	15731	0	54420,5
Myeloid_vs_CD4_Non-responder	Macro_NLRP3	CD4(CRTAM- T)	IL1B	SIGIRR	2,84091E-03	20845,9	6,84296E-03	15212	2,76371E+00	1590	3,34554E+00	5168	9,01499E-01	27839	0	54420,5
Myeloid_vs_CD4_Non-responder	pDC_LILRA4	CD4(AREG+ Tm)	APP	PTGER2	2,84128E-03	21659,3	2,11969E-02	2107	2,40696E+00	3016	3,26010E+00	5898	8,74236E-01	42855	0	54420,5
Myeloid_vs_CD4_Non-responder	Macro_NLRP3	CD4(CRTAM- T)	VCAN	ITGA4	2,84470E-03	19539,7	1,05114E-02	7661	2,72195E+00	1714	2,66152E+00	13272	9,14345E-01	20631	0	54420,5
Myeloid_vs_CD4_Non-responder	Macro_NLRP3	CD4(CRTAM- T)	VEGFA	ITGB1	2,84597E-03	22126,3	1,28805E-02	5385	2,47161E+00	2681	2,20407E+00	22403	9,05157E-01	25742	0	54420,5
Myeloid_vs_CD4_Non-responder	CD4(TGFB1+ Th17)	pDC_LILRA4	COPA	P2RY6	2,84695E-03	21119,3	1,30241E-02	5262	3,53069E+00	470	3,25985E+00	5902	8,80342E-01	39542	0	54420,5
Myeloid_vs_CD4_Non-responder	CD4(TNF+ T)	Macro_OLFML3	IFNG	IFNGR1_IFNGR2	2,84850E-03	26991,7	1,82868E-02	2822	1,20101E+00	26027	2,40936E+00	17933	8,90900E-01	33756	0	54420,5
Myeloid_vs_CD4_Non-responder	Macro_FOLR2+APOE+	CD4(TNF+ T)	CXCL9	DPP4	2,85262E-03	28059,1	2,28770E-02	1820	2,97219E+00	1088	3,25947E+00	5906	8,12581E-01	77061	0	54420,5
Myeloid_vs_CD4_Non-responder	CD4(AREG+ Tm)	pDC_LILRA4	COPA	P2RY6	2,85580E-03	28226,3	6,64269E-03	15895	3,36050E+00	616	3,02196E+00	8282	8,40108E-01	61918	0	54420,5
Myeloid_vs_CD4_Non-responder	Macro_OLFML3	CD4(ISG+ Treg)	HLA-DRB5	LAG3	2,85721E-03	21272,9	8,40607E-03	11137	1,48678E+00	15589	2,94005E+00	9321	9,23725E-01	15897	0	54420,5
Myeloid_vs_CD4_Non-responder	Macro_NLRP3	CD4(CRTAM- T)	NAMPT	ITGA5_ITGB1	2,86329E-03	28876,3	5,07803E-03	23558	1,31677E+00	21273	2,15912E+00	23591	9,12746E-01	21539	0	54420,5
Myeloid_vs_CD4_Non-responder	Macro_NLRP3	CD4(CRTAM- T)	VEGFA	CD44	2,86372E-03	18165,1	1,31094E-02	5203	2,68800E+00	1810	2,36937E+00	18775	9,34503E-01	10617	0	54420,5
Myeloid_vs_CD4_Non-responder	CD4(TGFB1+ Th17)	Macro_LYVE1	CD59	STAB1	2,86457E-03	25207,9	6,56174E-03	16204	2,49140E+00	2582	2,35649E+00	19039	8,90839E-01	33794	0	54420,5
Myeloid_vs_CD4_Non-responder	CD4(GZMK+ Teff)	Macro_ISG15	HLA-C	LILRB1	2,86584E-03	26356,7	4,51700E-03	27932	1,72375E+00	9931	1,80515E+00	33797	9,44530E-01	5703	0	54420,5
Myeloid_vs_CD4_Non-responder	CD4(CRTAM- T)	Mono_INHBA	ANXA1	FPR1	2,86881E-03	30892,1	4,31167E-03	29908	1,29099E+00	22203	1,80501E+00	33804	9,27298E-01	14125	0	54420,5
Myeloid_vs_CD4_Non-responder	Macro_NLRP3	CD4(TNFRSF9+ Treg)	ICAM1	IL2RA	2,87815E-03	21112,7	2,14812E-02	2058	1,61703E+00	12184	2,94673E+00	9232	9,01771E-01	27669	0	54420,5
Myeloid_vs_CD4_Non-responder	Macro_FOLR2+APOE+	CD4(AREG+ Tm)	CXCL9	DPP4	2,87825E-03	26243,7	2,81997E-02	1203	3,02474E+00	983	3,25744E+00	5924	8,27992E-01	68688	0	54420,5
Myeloid_vs_CD4_Non-responder	Macro_NLRP3	CD4(TNFRSF9+ Treg)	ICAM1	IL2RG	2,87900E-03	26016,9	6,36632E-03	16895	1,37945E+00	19013	2,19855E+00	22546	9,21121E-01	17210	0	54420,5
Myeloid_vs_CD4_Non-responder	Macro_NLRP3	CD4(TNFRSF9+ Treg)	THBS1	ITGA4	2,87943E-03	24711,7	7,57608E-03	13114	1,76259E+00	9226	2,42510E+00	17600	8,99056E-01	29198	0	54420,5
Myeloid_vs_CD4_Non-responder	Macro_FOLR2+APOE+	CD4(TNFRSF9+ Treg)	C3	IFITM1	2,89009E-03	32089,5	5,08076E-03	23535	1,08942E+00	31253	1,80377E+00	33854	9,20775E-01	17385	0	54420,5
Myeloid_vs_CD4_Non-responder	CD4(TNF+ T)	Macro_OLFML3	TNF	TNFRSF1A	2,89350E-03	32364,7	6,86805E-03	15116	1,03968E+00	33862	1,99667E+00	27886	8,96742E-01	30539	0	54420,5
Myeloid_vs_CD4_Non-responder	CD4(TNFRSF9+ Treg)	Mono_CD16	HLA-B	LILRB1	2,89350E-03	26455,5	4,37076E-03	29340	1,72309E+00	9944	1,80326E+00	33862	9,46475E-01	4711	0	54420,5
Myeloid_vs_CD4_Non-responder	Macro_NLRP3	CD4(TNFRSF9+ Treg)	IL1B	SIGIRR	2,89607E-03	19509,5	7,82297E-03	12469	2,80078E+00	1499	3,40970E+00	4656	9,07284E-01	24503	0	54420,5
Myeloid_vs_CD4_Non-responder	Macro_NLRP3	CD4(TNFRSF9+ Treg)	S100A8	ITGB2	2,89649E-03	20946,3	4,37412E-03	29298	2,45914E+00	2738	3,01818E+00	8329	9,35875E-01	9946	0	54420,5
Myeloid_vs_CD4_Non-responder	CD4(TGFB1+ Th17)	Macro_FOLR2+APOE+	HLA-C	LILRB2	2,89863E-03	23728,5	3,96186E-03	33874	1,96081E+00	6510	2,23592E+00	21674	9,52746E-01	2164	0	54420,5
Myeloid_vs_CD4_Non-responder	Macro_NLRP3	CD4(TNFRSF9+ Treg)	CCL3	CCR4	2,89949E-03	21187,9	1,66535E-02	3335	1,86536E+00	7636	3,10691E+00	7353	8,91949E-01	33195	0	54420,5
Myeloid_vs_CD4_Non-responder	Macro_NLRP3	CD4(TNFRSF9+ Treg)	CCL4	CCR8	2,89992E-03	24009,3	5,10032E-02	373	1,25030E+00	23890	2,87201E+00	10233	8,95699E-01	31130	0	54420,5
Myeloid_vs_CD4_Non-responder	Macro_NLRP3	CD4(TNFRSF9+ Treg)	VCAN	ITGB1	2,90077E-03	20402,3	9,42866E-03	9197	2,64501E+00	1964	2,52567E+00	15640	9,14086E-01	20790	0	54420,5
Myeloid_vs_CD4_Non-responder	Macro_NLRP3	CD4(TNFRSF9+ Treg)	VCAN	ITGA4	2,90120E-03	21068,5	8,81864E-03	10271	2,64767E+00	1951	2,60725E+00	14159	9,07214E-01	24541	0	54420,5
Myeloid_vs_CD4_Non-responder	Macro_NLRP3	CD4(TNFRSF9+ Treg)	VCAN	SELL	2,90163E-03	17650,9	1,60285E-02	3587	2,82579E+00	1426	2,76589E+00	11698	9,21338E-01	17123	0	54420,5

Myeloid_vs_CD4_Post_NR

Myeloid_vs_CD4_Non-responder	Macro_NLRP3	CD4(TNFRSF9+ Treg)	VCAN	CD44	2,90206E-03	20058,1	7,74609E-03	12653	2,54910E+00	2338	2,29758E+00	20304	9,34601E-01	10575	0	54420,5
Myeloid_vs_CD4_Non-responder	CD4(NME1+ T)	Macro_ISG15	B2M	LILRB1	2,90249E-03	25605,5	4,55172E-03	27614	1,72100E+00	9982	1,80268E+00	33883	9,52872E-01	2128	0	54420,5
Myeloid_vs_CD4_Non-responder	Macro_NLRP3	CD4(TNFRSF9+ Treg)	HBEGF	CD44	2,90720E-03	26618,3	5,37526E-03	21660	1,70071E+00	10381	1,83214E+00	32909	9,28165E-01	13721	0	54420,5
Myeloid_vs_CD4_Non-responder	Macro_NLRP3	CD4(TNFRSF9+ Treg)	HBEGF	CD82	2,91235E-03	22241,3	1,10522E-02	7043	1,93488E+00	6778	2,36184E+00	18922	9,08157E-01	24043	0	54420,5
Myeloid_vs_CD4_Non-responder	CD4(TGFB1+ Th17)	Mono_CD14	RPS19	C5AR1	2,91536E-03	25530,5	5,01737E-03	24001	1,62498E+00	11975	1,80177E+00	33913	9,49550E-01	3343	0	54420,5
Myeloid_vs_CD4_Non-responder	Macro_IFI27	CD4(IFNG+ Tfh/Th1)	HLA-DRB1	LAG3	2,92736E-03	19880,5	1,08926E-02	7216	1,56976E+00	13340	2,50721E+00	15996	9,38840E-01	8430	0	54420,5
Myeloid_vs_CD4_Non-responder	CD4(TGFB1+ Th17)	Macro_ISG15	TNF	TNFRSF1A	2,92828E-03	29478,7	8,80400E-03	10297	1,23789E+00	24442	1,80086E+00	33943	9,07686E-01	24291	0	54420,5
Myeloid_vs_CD4_Non-responder	Macro_NLRP3	CD4(ISG+ Treg)	HLA-DRA	LAG3	2,93216E-03	28739,1	5,86725E-03	19062	1,11389E+00	30017	2,20560E+00	22368	9,19938E-01	17828	0	54420,5
Myeloid_vs_CD4_Non-responder	CD4(CRTAM- T)	cDC(CD1C)	B2M	CD1A	2,93380E-03	31283,3	2,65363E-02	1373	1,52599E+00	14491	9,59162E-01	70127	9,23478E-01	16005	0	54420,5
Myeloid_vs_CD4_Non-responder	Mast	CD4(AREG+ Tm)	TIMP3	CD44	2,93408E-03	27374,9	3,60990E-02	746	2,93986E+00	1161	1,20437E+00	58174	9,11203E-01	22373	0	54420,5
Myeloid_vs_CD4_Non-responder	Macro_NLRP3	CD4(ISG+ Treg)	ICAM1	IL2RA	2,93994E-03	22511,3	2,08233E-02	2185	1,59763E+00	12636	2,56680E+00	14868	9,00385E-01	28447	0	54420,5
Myeloid_vs_CD4_Non-responder	Macro_NLRP3	CD4(ISG+ Treg)	ICAM1	IL2RG	2,94081E-03	24479,9	7,06128E-03	14497	1,45438E+00	16606	2,24283E+00	21513	9,24804E-01	15363	0	54420,5
Myeloid_vs_CD4_Non-responder	Macro_NLRP3	CD4(ISG+ Treg)	THBS1	CD47	2,94167E-03	24198,7	7,66676E-03	12882	1,84221E+00	7948	2,52551E+00	15641	8,97549E-01	30102	0	54420,5
Myeloid_vs_CD4_Non-responder	CD4(CRTAM- T)	Macro_LYVE1	CD59	STAB1	2,94514E-03	25299,1	6,51796E-03	16364	2,48927E+00	2589	2,35161E+00	19140	8,90513E-01	33982	0	54420,5
Myeloid_vs_CD4_Non-responder	Macro_FOLR2+APOE+	CD4(NME1+ T)	HLA-DRA	LAG3	2,94730E-03	27929,3	3,95203E-03	33987	1,42525E+00	17439	3,09406E+00	7486	9,04125E-01	26314	0	54420,5
Myeloid_vs_CD4_Non-responder	CD4(NME1+ T)	Mono_CD16	HLA-C	LILRA3	2,94815E-03	26955,1	1,12516E-02	6819	1,76492E+00	9187	1,42520E+00	48324	9,23441E-01	16025	0	54420,5
Myeloid_vs_CD4_Non-responder	CD4(Tn)	Macro_ISG15	HLA-F	LILRB1	2,95034E-03	32349,9	4,21086E-03	30973	1,70273E+00	10345	1,86029E+00	32017	8,90485E-01	33994	0	54420,5
Myeloid_vs_CD4_Non-responder	Mono_CD14	CD4(IFNG+ Tfh/Th1)	VCAN	ITGA4	2,95751E-03	17370,9	1,34166E-02	4968	3,18845E+00	749	2,83837E+00	10679	9,23430E-01	16038	0	54420,5
Myeloid_vs_CD4_Non-responder	Macro_IER3	CD4(ISG+ Treg)	HLA-DRA	LAG3	2,95751E-03	21902,7	7,79255E-03	12536	1,47180E+00	16038	2,64034E+00	13589	9,29785E-01	12930	0	54420,5
Myeloid_vs_CD4_Non-responder	Macro_NLRP3	CD4(ISG+ Treg)	IL1B	SIGIRR	2,95816E-03	18213,9	9,02989E-03	9888	2,84645E+00	1367	3,49359E+00	4093	9,13145E-01	21301	0	54420,5
Myeloid_vs_CD4_Non-responder	Macro_NLRP3	CD4(ISG+ Treg)	S100A8	ITGB2	2,95859E-03	21587,7	4,17810E-03	31337	2,43795E+00	2840	2,98852E+00	8713	9,34486E-01	10628	0	54420,5
Myeloid_vs_CD4_Non-responder	Macro_NLRP3	CD4(ISG+ Treg)	CCL3	CCR4	2,96121E-03	20371,5	1,86788E-02	2697	1,91061E+00	7052	3,09482E+00	7479	8,97356E-01	30209	0	54420,5
Myeloid_vs_CD4_Non-responder	Macro_NLRP3	CD4(ISG+ Treg)	VCAN	ITGB1	2,96251E-03	21731,9	8,21420E-03	11530	2,58776E+00	2163	2,47002E+00	16720	9,08514E-01	23826	0	54420,5
Myeloid_vs_CD4_Non-responder	Macro_NLRP3	CD4(ISG+ Treg)	VCAN	CD44	2,96382E-03	19764,7	7,94476E-03	12163	2,56621E+00	2256	2,32171E+00	19766	9,35370E-01	10218	0	54420,5
Myeloid_vs_CD4_Non-responder	CD4(NME1+ T)	Macro_ISG15	HLA-B	LILRB2	2,96818E-03	24351,1	3,94702E-03	34035	1,86805E+00	7601	2,13080E+00	24258	9,55047E-01	1441	0	54420,5
Myeloid_vs_CD4_Non-responder	CD4(Tn)	Macro_IER3	RPS19	C5AR1	2,96905E-03	24117,5	5,53933E-03	20731	1,78140E+00	8898	1,79826E+00	34037	9,51868E-01	2501	0	54420,5
Myeloid_vs_CD4_Non-responder	Macro_NLRP3	CD4(ISG+ Treg)	HBEGF	CD44	2,96948E-03	26155,1	5,51313E-03	20871	1,71783E+00	10039	1,85628E+00	32157	9,29005E-01	13288	0	54420,5
Myeloid_vs_CD4_Non-responder	CD4(CRTAM- T)	Macro_ISG15	HLA-B	LILRB2	2,97341E-03	24500,7	3,94630E-03	34047	1,86757E+00	7610	2,10275E+00	24984	9,55043E-01	1442	0	54420,5
Myeloid_vs_CD4_Non-responder	Macro_NLRP3	CD4(ISG+ Treg)	HBEGF	CD82	2,97428E-03	23696,7	9,94284E-03	8429	1,88397E+00	7390	2,23607E+00	21670	9,03649E-01	26574	0	54420,5
Myeloid_vs_CD4_Non-responder	CD4(ISG+ Treg)	Macro_ISG15	CIRBP	TREM1	2,97603E-03	28984,1	6,28793E-03	17208	1,47424E+00	15968	1,79758E+00	34053	9,09523E-01	23271	0	54420,5
Myeloid_vs_CD4_Non-responder	Macro_FOLR2+APOE+	CD4(AREG+ Tm)	APOE	LSR	2,98172E-03	20850,7	1,50094E-02	4051	1,80173E+00	8571	3,65115E+00	3145	8,90377E-01	34066	0	54420,5
Myeloid_vs_CD4_Non-responder	CD4(Tn)	pDC_LILRA4	BST2	LILRA4	2,98445E-03	26782,3	6,46782E-03	16531	3,46978E+00	521	2,90651E+00	9737	8,56483E-01	52702	0	54420,5
Myeloid_vs_CD4_Non-responder	CD4(NME1+ T)	Macro_ISG15	HLA-F	LILRB2	2,98478E-03	28527,7	3,94426E-03	34073	1,89619E+00	7233	2,17406E+00	23169	9,08673E-01	23743	0	54420,5
Myeloid_vs_CD4_Non-responder	CD4(GZMK+ Teff)	Macro_ISG15	HLA-C	LILRB2	2,98653E-03	24295,3	3,94350E-03	34077	1,88542E+00	7371	2,16554E+00	23409	9,52641E-01	2199	0	54420,5
Myeloid_vs_CD4_Non-responder	CD4(TNF+ T)	Mast	IL23A	SIGLEC6	2,99148E-03	40221,1	1,77281E-01	18	2,83084E+00	1414	1,38068E+00	50241	7,77345E-01	95012	0	54420,5
Myeloid_vs_CD4_Non-responder	Macro_NLRP3	CD4(TNF+ T)	THBS1	ITGA4	2,99707E-03	23687,1	7,94744E-03	12157	1,78156E+00	8896	2,56206E+00	14957	9,01207E-01	28005	0	54420,5
Myeloid_vs_CD4_Non-responder	Macro_NLRP3	CD4(TNF+ T)	IL1B	SIGIRR	3,00895E-03	17872,9	9,27946E-03	9443	2,85589E+00	1349	3,59727E+00	3442	9,14220E-01	20710	0	54420,5
Myeloid_vs_CD4_Non-responder	cDC_LAMP3	CD4(Tn)	CCL19	CXCR3	3,00979E-03	28515,3	2,84807E-02	1176	4,87260E+00	58	2,23423E+00	21713	8,34098E-01	65209	0	54420,5
Myeloid_vs_CD4_Non-responder	Macro_NLRP3	CD4(TNF+ T)	VCAN	ITGB1	3,01115E-03	21576,3	8,13624E-03	11735	2,58409E+00	2183	2,53468E+00	15471	9,08117E-01	24072	0	54420,5
Myeloid_vs_CD4_Non-responder	Macro_NLRP3	CD4(TNF+ T)	VCAN	ITGA4	3,01160E-03	20244,7	9,25090E-03	9486	2,66664E+00	1874	2,74421E+00	11988	9,09209E-01	23455	0	54420,5
Myeloid_vs_CD4_Non-responder	Macro_NLRP3	CD4(TNF+ T)	VCAN	CD44	3,01248E-03	19923,5	7,64657E-03	12932	2,54053E+00	2374	2,35271E+00	19119	9,34204E-01	10772	0	54420,5
Myeloid_vs_CD4_Non-responder	Macro_FOLR2+APOE+	CD4(CXCL13+ Tfh)	HLA-DMB	CD4	3,01380E-03	25853,1	3,93912E-03	34139	1,84775E+00	7858	2,69112E+00	12804	9,15455E-01	20044	0	54420,5
Myeloid_vs_CD4_Non-responder	Macro_NLRP3	CD4(TNF+ T)	HBEGF	CD44	3,01557E-03	26430,7	5,30621E-03	22072	1,69214E+00	10543	1,88728E+00	31203	9,27733E-01	13915	0	54420,5
Myeloid_vs_CD4_Non-responder	Macro_NLRP3	CD4(TNF+ T)	HBEGF	CD82	3,02131E-03	25898,5	8,09117E-03	11845	1,79901E+00	8612	2,19105E+00	22733	8,94297E-01	31882	0	54420,5
Myeloid_vs_CD4_Non-responder	CD4(NME1+ T)	Macro_ISG15	HMGB1	HAVCR2	3,02220E-03	31737,1	4,45825E-03	28473	1,17580E+00	27178	1,79436E+00	34158	9,26671E-01	14456	0	54420,5
Myeloid_vs_CD4_Non-responder	CD4(AREG+ Tm)	cDC(CD1C)	B2M	CD1A	3,02360E-03	31567,9	2,63533E-02	1393	1,50065E+00	15165	9,47175E-01	70732	9,23233E-01	16129	0	54420,5
Myeloid_vs_CD4_Non-responder	CD4(AREG+ Tm)	Macro_IER3	RPS19	C5AR1	3,02397E-03	24152,5	5,53488E-03	20763	1,78022E+00	8912	1,79428E+00	34162	9,51850E-01	2505	0	54420,5
Myeloid_vs_CD4_Non-responder	Macro_FOLR2+APOE+	CD4(NME1+ T)	LGALS9	PTPRC	3,02485E-03	30696,1	3,94691E-03	34038	1,29104E+00	22201	1,79416E+00	34164	9,38379E-01	8657	0	54420,5
Myeloid_vs_CD4_Non-responder	CD4(TNFRSF9+ Treg)	Macro_FOLR2+APOE+	HLA-A	LILRB1	3,03816E-03	28645,9	3,93414E-03	34194	1,55118E+00	13819	1,81408E+00	33514	9,41102E-01	7282	0	54420,5
Myeloid_vs_CD4_Non-responder	cDC_LAMP3	CD4(IFNG+ Tfh/Th1)	HLA-DQA1	LAG3	3,04342E-03	20832,1	1,12846E-02	6793	1,52410E+00	14543	2,72797E+00	12248	9,23176E-01	16156	0	54420,5
Myeloid_vs_CD4_Non-responder	Macro_NLRP3	CD4(GZMK+ Teff)	THBS1	CD47	3,04483E-03	25479,3	6,82261E-03	15286	1,79492E+00	8683	2,51327E+00	15874	8,92060E-01	33133	0	54420,5
Myeloid_vs_CD4_Non-responder	Macro_LYVE1	CD4(ISG+ Treg)	HLA-DPA1	LAG3	3,05300E-03	21826,1	8,19225E-03	11590	1,46780E+00	16169	2,61223E+00	14082	9,29913E-01	12869	0	54420,5
Myeloid_vs_CD4_Non-responder	CD4(ISG+ Treg)	Macro_OLFML3	HLA-F	LILRB1	3,05374E-03	30819,1	5,10313E-03	23392	1,57817E+00	13112	1,79237E+00	34229	8,99510E-01	28942	0	54420,5
Myeloid_vs_CD4_Non-responder	CD4(CRTAM- T)	Macro_IER3	RPS19	C5AR1	3,05686E-03	23700,9	5,74773E-03	19640	1,83663E+00	8035	1,79209E+00	34236	9,52707E-01	2173	0	54420,5
Myeloid_vs_CD4_Non-responder	Macro_NLRP3	CD4(GZMK+ Teff)	IL1B	SIGIRR	3,05776E-03	17851,5	9,40586E-03	9236	2,86067E+00	1337	3,53095E+00	3854	9,14749E-01	20410	0	54420,5
Myeloid_vs_CD4_Non-responder	Macro_NLRP3	CD4(GZMK+ Teff)	S100A8	ITGB2	3,05820E-03	20899,9	4,34386E-03	29624	2,45587E+00	2756	3,08124E+00	7638	9,35667E-01	10061	0	54420,5
Myeloid_vs_CD4_Non-responder	Macro_NLRP3	CD4(GZMK+ Teff)	VCAN	ITGB1	3,06222E-03	23801,1	6,73527E-03	15562	2,51805E+00	2479	2,41439E+00	17829	8,99923E-01	28715	0	54420,5
Myeloid_vs_CD4_Non-responder	Macro_NLRP3	CD4(GZMK+ Teff)	VCAN	ITGA4	3,06267E-03	24576,9	6,45950E-03	16560	2,54415E+00	2358	2,45061E+00	17111	8,93255E-01	32435	0	54420,5
Myeloid_vs_CD4_Non-responder	Macro_NLRP3	CD4(GZMK+ Teff)	VCAN	SELL	3,06312E-03	21304,9	1,01387E-02	8144	2,63691E+00	1999	2,55817E+00	15047	9,03057E-01	26914	0	54420,5
Myeloid_vs_CD4_Non-responder	Macro_FOLR2+APOE+	CD4(GZMK+ Teff)	LGALS9	CD44	3,06312E-03	29909,7	3,92937E-03	34250	1,36792E+00	19424	1,90559E+00	30645	9,34126E-01	10809	0	54420,5
Myeloid_vs_CD4_Non-responder	Macro_ISG15	CD4(ISG+ Treg)	HLA-DRA	LAG3	3,06408E-03	20738,1	7,76813E-03	12593	1,46726E+00	16184	3,09203E+00	7513	9,29682E-01	12980	0	54420,5
Myeloid_vs_CD4_Non-responder	Macro_NLRP3	CD4(GZMK+ Teff)	HBEGF	CD44	3,06714E-03	21987,9	7,04013E-03	14557	1,90739E+00	7094	2,12900E+00	24310	9,36657E-01	9558	0	54420,5
Myeloid_vs_CD4_Non-responder	CD4(IFNG+ Tfh/Th1)	Mono_CD16	HLA-A	LILRB1	3,06938E-03	26168,3	4,59495E-03	27236	1,74123E+00	9601	1,79154E+00	34264	9,45261E-01	5320	0	54420,5
Myeloid_vs_CD4_Non-responder	CD4(Tn)	pDC_LILRA4	HLA-B	CANX	3,07616E-03	39282,5	2,54016E-03	61713	8,09765E-01	47395	1,86825E+00	31754	9,56092E-01	1130	0	54420,5
Myeloid_vs_CD4_Non-responder	Macro_NLRP3	CD4(GZMK+ Teff)	VEGFA	CD44	3,07835E-03	21872,3	9,89906E-03	8498	2,40457E+00	3030	1,98263E+00	28320	9,25365E-01	15093	0	54420,5
Myeloid_vs_CD4_Non-responder	CD4(IFNG+ Tfh/Th1)	Macro_ISG15	CIRBP	TREM1	3,07925E-03	29106,5	6,25614E-03	17335	1,46978E+00	16104	1,79078E+00	34286	9,09314E-01	23387	0	54420,5
Myeloid_vs_CD4_Non-responder	cDC(CD1C)	CD4(ISG+ Treg)	HLA-DQB1	LAG3	3,08558E-03	19755,9	1,01195E-02	8171	1,68428E+00	10677	2,94149E+00	9298	9,23078E-01	16213	0	54420,5
Myeloid_vs_CD4_Non-responder	Macro_FOLR2+APOE+	CD4(TNFRSF9+ Treg)	TNFSF13	TNFRSF14	3,08599E-03	31524,1	3,92597E-03	34301	1,50872E+00	14939	2,31593E+00	19892	8,90371E-01	34068	0	54420,5
Myeloid_vs_CD4_Non-responder	Macro_NLRP3	CD4(AREG+ Tm)	THBS1	ITGA4	3,08959E-03	26071,5	6,92539E-03	14930	1,72935E+00	9828	2,32772E+00	19629	8,94907E-01	31550	0	54420,5
Myeloid_vs_CD4_Non-responder	Macro_NLRP3	CD4(AREG+ Tm)	THBS1	CD47	3,09049E-03	25101,3	7,04629E-03	14542	1,80745E+00	8486	2,51630E+00	15809	8,93603E-01	32249	0	54420,5
Myeloid_vs_CD4_Non-responder	CD4(TGFB1+ Th17)	Macro_OLFML3	HLA-C	LILRB1	3,09770E-03	27679,3	4,12861E-03	31920	1,67118E+00	10969	1,78978E+00	34327	9,42127E-01	6760	0	54420,5

Myeloid_vs_CD4_Post_NR

Myeloid_vs_CD4_Non-responder	Macro_NLRP3	CD4(AREG+ Tm)	IL1B	SIGIRR	3,10448E-03	18666,1	8,56867E-03	10783	2,82900E+00	1419	3,47198E+00	4262	9,11043E-01	22446	0	54420,5
Myeloid_vs_CD4_Non-responder	Macro_NLRP3	CD4(AREG+ Tm)	VCAN	ITGB1	3,10855E-03	20256,1	9,36617E-03	9303	2,64206E+00	1975	2,57812E+00	14657	9,13824E-01	20925	0	54420,5
Myeloid_vs_CD4_Non-responder	Macro_NLRP3	CD4(AREG+ Tm)	VCAN	ITGA4	3,10900E-03	22212,1	8,06123E-03	11912	2,61443E+00	2067	2,50987E+00	15937	9,03365E-01	26724	0	54420,5
Myeloid_vs_CD4_Non-responder	Macro_NLRP3	CD4(AREG+ Tm)	VCAN	SELL	3,10945E-03	19369,5	1,20363E-02	6076	2,69777E+00	1787	2,76275E+00	11736	9,10312E-01	22828	0	54420,5
Myeloid_vs_CD4_Non-responder	Macro_ISG15	CD4(IFNG+ Tfh/Th1)	CD86	CTLA4	3,11167E-03	18988,9	1,75734E-02	3024	2,04098E+00	5662	2,49461E+00	16248	9,24349E-01	15590	0	54420,5
Myeloid_vs_CD4_Non-responder	CD4(IL26+ Th17)	Macro_ISG15	HLA-A	LILRB1	3,11217E-03	25796,5	4,76543E-03	25863	1,74689E+00	9490	1,78864E+00	34359	9,46196E-01	4850	0	54420,5
Myeloid_vs_CD4_Non-responder	Macro_NLRP3	CD4(AREG+ Tm)	HBEGF	CD44	3,11262E-03	22925,5	6,68259E-03	15758	1,86300E+00	7673	2,04851E+00	26429	9,35093E-01	10347	0	54420,5
Myeloid_vs_CD4_Non-responder	CD4(TNFRSF9+ Treg)	Mono_CD16	B2M	LILRB1	3,11806E-03	26212,9	4,30513E-03	29965	1,73491E+00	9714	1,78828E+00	34372	9,51605E-01	2593	0	54420,5
Myeloid_vs_CD4_Non-responder	Macro_NLRP3	CD4(AREG+ Tm)	VEGFA	CD44	3,12533E-03	22738,3	9,39633E-03	9255	2,36019E+00	3287	1,90215E+00	30751	9,23545E-01	15978	0	54420,5
Myeloid_vs_CD4_Non-responder	CD4(TNFRSF9+ Treg)	Macro_OLFML3	HLA-F	LILRB2	3,12533E-03	30842,3	3,91963E-03	34388	1,56906E+00	13363	1,98835E+00	28139	9,08413E-01	23901	0	54420,5
Myeloid_vs_CD4_Non-responder	CD4(TNFRSF9+ Treg)	Macro_OLFML3	HLA-F	LILRB1	3,13078E-03	31936,7	4,76408E-03	25880	1,53506E+00	14235	1,78763E+00	34400	8,96360E-01	30748	0	54420,5
Myeloid_vs_CD4_Non-responder	Macro_FOLR2+APOE+	CD4(GZMK+ Teff)	CXCL9	CXCR3	3,13898E-03	19681,9	2,23648E-02	1893	2,99928E+00	1026	3,17758E+00	6652	8,89783E-01	34418	0	54420,5
Myeloid_vs_CD4_Non-responder	Macro_FOLR2+APOE+	CD4(GZMK+ Teff)	C1QB	C1QBP	3,13989E-03	22698,3	3,91649E-03	34420	2,11083E+00	5011	3,77781E+00	2507	9,21306E-01	17133	0	54420,5
Myeloid_vs_CD4_Non-responder	CD4(AREG+ Tm)	Mono_CD16	HLA-C	LILRA3	3,14019E-03	27870,3	1,10899E-02	7005	1,73216E+00	9767	1,34350E+00	51873	9,22927E-01	16286	0	54420,5
Myeloid_vs_CD4_Non-responder	CD4(TGFB1+ Th17)	Macro_ISG15	CD99	CD81	3,14263E-03	34593,9	3,93922E-03	34137	1,07442E+00	32035	1,78687E+00	34426	9,19695E-01	17951	0	54420,5
Myeloid_vs_CD4_Non-responder	Macro_NLRP3	CD4(NME1+ T)	ICAM1	IL2RG	3,14309E-03	28785,9	5,63060E-03	20227	1,30013E+00	21864	1,99552E+00	27923	9,16543E-01	19495	0	54420,5
Myeloid_vs_CD4_Non-responder	CD4(ISG+ Treg)	cDC_CLEC9A	FLT3LG	FLT3	3,14325E-03	32467,1	2,82325E-02	1202	3,18922E+00	748	1,58875E+00	41615	8,35635E-01	64350	0	54420,5
Myeloid_vs_CD4_Non-responder	Macro_NLRP3	CD4(NME1+ T)	THBS1	ITGA4	3,14355E-03	22418,7	9,30556E-03	9403	1,85093E+00	7823	2,49063E+00	16325	9,08011E-01	24122	0	54420,5
Myeloid_vs_CD4_Non-responder	CD4(NME1+ T)	pDC_LILRA4	COPA	P2RY6	3,14396E-03	23600,9	1,00218E-02	8323	3,45062E+00	538	3,12006E+00	7206	8,65838E-01	47517	0	54420,5
Myeloid_vs_CD4_Non-responder	Macro_NLRP3	CD4(NME1+ T)	THBS1	CD47	3,14400E-03	25672,1	6,89284E-03	15042	1,79885E+00	8617	2,43401E+00	17437	8,92552E-01	32844	0	54420,5
Myeloid_vs_CD4_Non-responder	Mono_CD14	CD4(NME1+ T)	S100A9	ITGB2	3,14589E-03	18440,1	4,68421E-03	26508	2,87466E+00	1300	3,52936E+00	3866	9,43605E-01	6106	0	54420,5
Myeloid_vs_CD4_Non-responder	CD4(CXCL13+ Tfh)	Macro_ISG15	B2M	LILRB2	3,14903E-03	24615,9	3,91485E-03	34440	1,82722E+00	8175	2,08039E+00	25588	9,59526E-01	456	0	54420,5
Myeloid_vs_CD4_Non-responder	CD4(CXCL13+ Tfh)	Macro_ISG15	HLA-F	LILRB1	3,15543E-03	33022,5	4,14612E-03	31697	1,69602E+00	10464	1,79681E+00	34077	8,89727E-01	34454	0	54420,5
Myeloid_vs_CD4_Non-responder	CD4(CXCL13+ Tfh)	Macro_ISG15	HLA-B	LILRB2	3,15681E-03	24529,7	3,91279E-03	34457	1,84517E+00	7901	2,12661E+00	24382	9,54860E-01	1488	0	54420,5
Myeloid_vs_CD4_Non-responder	Macro_NLRP3	CD4(NME1+ T)	IL1B	SIGIRR	3,16047E-03	17766,3	9,61867E-03	8906	2,86872E+00	1316	3,47664E+00	4231	9,15617E-01	19958	0	54420,5
Myeloid_vs_CD4_Non-responder	CD4(GZMK+ Teff)	Mono_CD14	RPS19	C5AR1	3,16047E-03	25756,7	4,98105E-03	24260	1,61472E+00	12230	1,78545E+00	34465	9,49375E-01	3408	0	54420,5
Myeloid_vs_CD4_Non-responder	Macro_NLRP3	CD4(NME1+ T)	S100A8	ITGB2	3,16093E-03	20023,5	4,70326E-03	26362	2,49471E+00	2568	3,05391E+00	7923	9,38018E-01	8844	0	54420,5
Myeloid_vs_CD4_Non-responder	Macro_NLRP3	CD4(NME1+ T)	S100A9	ITGB2	3,16231E-03	21298,5	3,96829E-03	33782	2,42187E+00	2924	3,13724E+00	7035	9,39026E-01	8331	0	54420,5
Myeloid_vs_CD4_Non-responder	Macro_NLRP3	CD4(TNFRSF9+ Treg)	VEGFA	ITGB1	3,16414E-03	26509,1	9,19985E-03	9587	2,29381E+00	3670	1,91387E+00	30395	8,89695E-01	34473	0	54420,5
Myeloid_vs_CD4_Non-responder	Macro_NLRP3	CD4(NME1+ T)	VCAN	ITGB1	3,16506E-03	18613,7	1,13912E-02	6689	2,73751E+00	1659	2,67012E+00	13135	9,21226E-01	17165	0	54420,5
Myeloid_vs_CD4_Non-responder	Macro_NLRP3	CD4(NME1+ T)	VCAN	ITGA4	3,16552E-03	19292,7	1,08318E-02	7281	2,73601E+00	1666	2,67279E+00	13076	9,15513E-01	20020	0	54420,5
Myeloid_vs_CD4_Non-responder	Macro_NLRP3	CD4(NME1+ T)	VCAN	SELL	3,16598E-03	20215,1	1,17901E-02	6305	2,68987E+00	1809	2,54811E+00	15234	9,09465E-01	23307	0	54420,5
Myeloid_vs_CD4_Non-responder	Macro_NLRP3	CD4(NME1+ T)	VCAN	CD44	3,16644E-03	19897,5	7,92703E-03	12220	2,56469E+00	2265	2,29634E+00	20331	9,35303E-01	10251	0	54420,5
Myeloid_vs_CD4_Non-responder	Macro_NLRP3	CD4(NME1+ T)	VEGFA	ITGB1	3,16736E-03	24023,7	1,11148E-02	6971	2,38631E+00	3129	2,05833E+00	26135	8,98637E-01	29463	0	54420,5
Myeloid_vs_CD4_Non-responder	Macro_NLRP3	CD4(NME1+ T)	HBEGF	CD44	3,17103E-03	26339,9	5,50083E-03	20932	1,71630E+00	10065	1,83091E+00	32949	9,28931E-01	13333	0	54420,5
Myeloid_vs_CD4_Non-responder	Macro_NLRP3	CD4(NME1+ T)	HBEGF	CD82	3,17794E-03	23532,7	1,02670E-02	7972	1,89885E+00	7198	2,20971E+00	22268	9,05037E-01	25805	0	54420,5
Myeloid_vs_CD4_Non-responder	CD4(CRTAM- T)	Macro_ISG15	B2M	LILRB2	3,17932E-03	24801,9	3,90898E-03	34506	1,82169E+00	8259	2,05033E+00	26365	9,59497E-01	459	0	54420,5
Myeloid_vs_CD4_Non-responder	Macro_FOLR2+APOE+	CD4(NME1+ T)	LYZ	ITGAL	3,17978E-03	25894,3	3,90883E-03	34507	1,66558E+00	11064	3,33337E+00	5273	9,07852E-01	24207	0	54420,5
Myeloid_vs_CD4_Non-responder	Macro_FOLR2+APOE+	CD4(TNFRSF9+ Treg)	ICAM1	IL2RA	3,18300E-03	25360,5	1,65536E-02	3370	1,31150E+00	21426	2,67300E+00	13072	8,89611E-01	34514	0	54420,5
Myeloid_vs_CD4_Non-responder	CD4(TNF+ T)	Macro_IER3	TNFSF9	HLA-DPA1	3,18485E-03	31299,5	4,99418E-03	24171	1,19111E+00	26475	2,45991E+00	16913	8,89604E-01	34518	0	54420,5
Myeloid_vs_CD4_Non-responder	cDC_LAMP3	CD4(IFNG+ Tfh/Th1)	HLA-DRA	LAG3	3,18637E-03	20520,1	9,47511E-03	9126	1,46230E+00	16347	2,69308E+00	12780	9,35905E-01	9927	0	54420,5
Myeloid_vs_CD4_Non-responder	Macro_NLRP3	CD4(IFNG+ Tfh/Th1)	HLA-DRA	LAG3	3,20010E-03	22967,1	8,14766E-03	11704	1,28460E+00	22473	2,61813E+00	13972	9,31226E-01	12266	0	54420,5
Myeloid_vs_CD4_Non-responder	Macro_ISG15	CD4(Tn)	S100A8	CD69	3,20238E-03	22317,1	6,66875E-03	15799	1,46175E+00	16368	2,56488E+00	14904	9,35598E-01	10094	0	54420,5
Myeloid_vs_CD4_Non-responder	Macro_NLRP3	CD4(IFNG+ Tfh/Th1)	HLA-DRB1	LAG3	3,20288E-03	25831,9	7,53870E-03	13222	1,11517E+00	29955	2,43182E+00	17479	9,27380E-01	14083	0	54420,5
Myeloid_vs_CD4_Non-responder	Macro_NLRP3	CD4(IFNG+ Tfh/Th1)	ICAM1	IL2RG	3,20891E-03	26619,3	6,27178E-03	17262	1,36926E+00	19374	2,12051E+00	24546	9,20576E-01	17494	0	54420,5
Myeloid_vs_CD4_Non-responder	Macro_NLRP3	CD4(IFNG+ Tfh/Th1)	THBS1	ITGA4	3,20938E-03	21437,9	9,98130E-03	8375	1,88545E+00	7370	2,58685E+00	14497	9,10897E-01	22527	0	54420,5
Myeloid_vs_CD4_Non-responder	Macro_NLRP3	CD4(IFNG+ Tfh/Th1)	THBS1	CD47	3,20984E-03	26020,3	6,62446E-03	15961	1,78382E+00	8865	2,45842E+00	16941	8,90633E-01	33914	0	54420,5
Myeloid_vs_CD4_Non-responder	Macro_NLRP3	CD4(IFNG+ Tfh/Th1)	CD14	ITGB1	3,21170E-03	28064,1	4,27373E-03	30322	1,39136E+00	18607	2,32772E+00	19628	9,20856E-01	17343	0	54420,5
Myeloid_vs_CD4_Non-responder	Mono_INHBA	CD4(TGFB1+ Th17)	S100A8	CD69	3,21615E-03	17189,5	1,18017E-02	6294	2,01204E+00	5964	2,48779E+00	16386	9,50802E-01	2883	0	54420,5
Myeloid_vs_CD4_Non-responder	CD4(TNF+ T)	Macro_ISG15	IFNG	IFNGR1_IFNGR2	3,21634E-03	26869,5	1,77674E-02	2955	1,16637E+00	27627	2,57269E+00	14759	8,89492E-01	34586	0	54420,5
Myeloid_vs_CD4_Non-responder	CD4(ISG+ Treg)	Macro_FOLR2+APOE+	HLA-A	LILRB1	3,22612E-03	28892,1	3,92965E-03	34244	1,54809E+00	13891	1,78158E+00	34607	9,41071E-01	7298	0	54420,5
Myeloid_vs_CD4_Non-responder	CD4(TNF+ T)	cDC_LAMP3	LTB	CD40	3,22766E-03	25049,5	1,21430E-02	5983	1,99131E+00	6181	1,57180E+00	42262	9,22693E-01	16401	0	54420,5
Myeloid_vs_CD4_Non-responder	Macro_NLRP3	CD4(IFNG+ Tfh/Th1)	IL1B	SIGIRR	3,22845E-03	18279,9	8,98360E-03	9977	2,84470E+00	1374	3,47915E+00	4212	9,12941E-01	21416	0	54420,5
Myeloid_vs_CD4_Non-responder	Macro_NLRP3	CD4(IFNG+ Tfh/Th1)	VCAN	ITGA4	3,23358E-03	18554,7	1,16183E-02	6483	2,77053E+00	1579	2,76900E+00	11637	9,18185E-01	18654	0	54420,5
Myeloid_vs_CD4_Non-responder	Macro_NLRP3	CD4(IFNG+ Tfh/Th1)	VCAN	CD44	3,23452E-03	20607,9	7,44515E-03	13465	2,52317E+00	2453	2,24123E+00	21543	9,33379E-01	11158	0	54420,5
Myeloid_vs_CD4_Non-responder	Macro_NLRP3	CD4(IFNG+ Tfh/Th1)	VEGFA	ITGB1	3,23592E-03	22174,3	1,26691E-02	5562	2,46140E+00	2723	2,22052E+00	22031	9,04445E-01	26135	0	54420,5
Myeloid_vs_CD4_Non-responder	CD4(ISG+ Treg)	Macro_LYVE1	CD59	STAB1	3,24013E-03	25623,5	6,37191E-03	16869	2,48218E+00	2625	2,33002E+00	19566	8,89403E-01	34637	0	54420,5
Myeloid_vs_CD4_Non-responder	Macro_NLRP3	CD4(IFNG+ Tfh/Th1)	CXCL16	CXCR6	3,24060E-03	30297,1	9,13783E-03	9688	1,08956E+00	31244	2,04446E+00	26537	8,98430E-01	29596	0	54420,5
Myeloid_vs_CD4_Non-responder	Mono_CD14	CD4(IL26+ Th17)	VCAN	CD44	3,24537E-03	17406,7	9,97460E-03	8383	3,04383E+00	946	2,48559E+00	16424	9,41917E-01	6860	0	54420,5
Myeloid_vs_CD4_Non-responder	Macro_NLRP3	CD4(IFNG+ Tfh/Th1)	HBEGF	CD82	3,24574E-03	21472,7	1,20565E-02	6059	1,98096E+00	6284	2,38295E+00	18510	9,11720E-01	22090	0	54420,5
Myeloid_vs_CD4_Non-responder	Macro_FOLR2+APOE+	CD4(IFNG+ Tfh/Th1)	ITGB2	CD226	3,24715E-03	32769,3	6,73061E-03	15572	1,02668E+00	34557	2,11654E+00	24645	8,89383E-01	34652	0	54420,5
Myeloid_vs_CD4_Non-responder	Macro_IER3	CD4(IFNG+ Tfh/Th1)	HLA-DPA1	LAG3	3,24846E-03	19873,5	1,00598E-02	8255	1,45966E+00	16428	2,84852E+00	10539	9,36316E-01	9725	0	54420,5
Myeloid_vs_CD4_Non-responder	Macro_NLRP3	CD4(IFNG+ Tfh/Th1)	LYZ	ITGAL	3,24856E-03	26262,7	4,13675E-03	31807	1,63180E+00	11804	2,85950E+00	10390	9,10196E-01	22892	0	54420,5
Myeloid_vs_CD4_Non-responder	Macro_NLRP3	CD4(AREG+ Tm)	VEGFA	ITGB1	3,25137E-03	26241,7	9,13888E-03	9684	2,29086E+00	3685	1,96633E+00	28758	8,89368E-01	34661	0	54420,5
Myeloid_vs_CD4_Non-responder	CD4(IL26+ Th17)	Macro_FOLR2+APOE+	LTB	CD40	3,25841E-03	26057,3	1,01223E-02	8168	1,57351E+00	13228	1,77957E+00	34676	9,15947E-01	19794	0	54420,5
Myeloid_vs_CD4_Non-responder	Macro_NLRP3	CD4(CXCL13+ Tfh)	THBS1	ITGA4	3,27112E-03	26981,7	6,50182E-03	16420	1,70772E+00	10225	2,28283E+00	20621	8,91902E-01	33222	0	54420,5
Myeloid_vs_CD4_Non-responder	CD4(TNFRSF9+ Treg)	Macro_FOLR2+APOE+	RPS19	C5AR1	3,27112E-03	26826,3	4,67817E-03	26558	1,53012E+00	14382	1,77853E+00	34703	9,47846E-01	4068	0	54420,5
Myeloid_vs_CD4_Non-responder	Macro_NLRP3	CD4(CXCL13+ Tfh)	IL1B	SIGIRR	3,28859E-03	19171,1	8,12889E-03	11752	2,81236E+00	1466	3,42129E+00	4585	9,08885E-01	23632	0	54420,5
Myeloid_vs_CD4_Non-responder	Macro_NLRP3	CD4(CXCL13+ Tfh)	S100A8	ITGB2	3,28906E-03	21532,7	4,17597E-03	31359	2,43772E+00	2842	3,01108E+00	8405	9,34470E-01	10637	0	54420,5
Myeloid_vs_CD4_Non-responder	Macro_NLRP3	CD4(CXCL13+ Tfh)	VCAN	ITGB1	3,29285E-03	23008,5	7,39731E-03	13576	2,54926E+00	2336	2,38893E+00	18368	9,04067E-01	26342	0	54420,5
Myeloid_vs_CD4_Non-responder	Macro_NLRP3	CD4(CXCL13+ Tfh)	VCAN	ITGA4	3,29333E-03	22971,7	7,56818E-03	13129	2,59280E+00	2142	2,46499E+00	16816	9,00575E-01	28351	0	54420,5

Myeloid_vs_CD4_Post_NR

Myeloid_vs_CD4_Non-responder	Macro_NLRP3	CD4(CXCL13+ Tfh)	VCAN	SELL	3,29380E-03	20353,1	1,14509E-02	6629	2,67899E+00	1835	2,56574E+00	14888	9,08256E-01	23993	0	54420,5
Myeloid_vs_CD4_Non-responder	Macro_NLRP3	CD4(CXCL13+ Tfh)	VCAN	CD44	3,29427E-03	19906,7	7,93490E-03	12190	2,56536E+00	2262	2,29250E+00	20427	9,35333E-01	10234	0	54420,5
Myeloid_vs_CD4_Non-responder	Macro_NLRP3	CD4(CXCL13+ Tfh)	HBEGF	CD44	3,29902E-03	26353,1	5,50629E-03	20906	1,71698E+00	10055	1,82707E+00	33075	9,28964E-01	13309	0	54420,5
Myeloid_vs_CD4_Non-responder	CD4(TNF+ T)	Mono_CD16	HLA-F	LILRB2	3,29902E-03	27093,1	3,89147E-03	34762	2,22550E+00	4094	2,40084E+00	18114	9,08112E-01	24075	0	54420,5
Myeloid_vs_CD4_Non-responder	Macro_NLRP3	CD4(CXCL13+ Tfh)	HBEGF	CD82	3,30376E-03	23632,3	9,98306E-03	8374	1,88582E+00	7367	2,24245E+00	21518	9,03825E-01	26482	0	54420,5
Myeloid_vs_CD4_Non-responder	Macro_NLRP3	CD4(IFNG+ Tfh/Th1)	HBEGF	CD44	3,30947E-03	27482,1	5,16643E-03	22975	1,67479E+00	10882	1,77580E+00	34784	9,26833E-01	14349	0	54420,5
Myeloid_vs_CD4_Non-responder	Macro_FOLR2+APOE+	CD4(TNFRSF9+ Treg)	LYZ	ITGAL	3,31756E-03	25898,5	3,88927E-03	34801	1,66405E+00	11102	3,38416E+00	4847	9,07642E-01	24322	0	54420,5
Myeloid_vs_CD4_Non-responder	cDC_CLEC9A	CD4(ISG+ Treg)	HLA-DQA1	LAG3	3,32555E-03	18031,3	1,10607E-02	7035	1,82425E+00	8213	3,51320E+00	3961	9,22462E-01	16527	0	54420,5
Myeloid_vs_CD4_Non-responder	CD4(NME1+ T)	cDC_CLEC9A	HMGB1	THBD	3,32663E-03	29670,1	5,23437E-03	22524	1,50014E+00	15181	1,77498E+00	34820	9,12960E-01	21405	0	54420,5
Myeloid_vs_CD4_Non-responder	CD4(TGFB1+ Th17)	Macro_ISG15	B2M	LILRB2	3,33189E-03	24938,3	3,88706E-03	34831	1,80109E+00	8581	2,04976E+00	26387	9,59388E-01	472	0	54420,5
Myeloid_vs_CD4_Non-responder	Macro_NLRP3	CD4(IL26+ Th17)	ICAM1	IL2RG	3,33332E-03	25849,9	6,49749E-03	16434	1,39359E+00	18533	2,17999E+00	23010	9,21859E-01	16852	0	54420,5
Myeloid_vs_CD4_Non-responder	Macro_NLRP3	CD4(IL26+ Th17)	THBS1	ITGA4	3,33380E-03	24629,7	7,66787E-03	12876	1,76728E+00	9131	2,41466E+00	17821	8,99601E-01	28900	0	54420,5
Myeloid_vs_CD4_Non-responder	Macro_NLRP3	CD4(IL26+ Th17)	THBS1	CD47	3,33428E-03	25725,7	6,75082E-03	15509	1,79090E+00	8744	2,48042E+00	16537	8,91550E-01	33418	0	54420,5
Myeloid_vs_CD4_Non-responder	Macro_FOLR2+APOE+	CD4(TGFB1+ Th17)	APOE	SORL1	3,33572E-03	26198,5	3,88615E-03	34839	1,71275E+00	10131	3,50752E+00	3996	9,01891E-01	27606	0	54420,5
Myeloid_vs_CD4_Non-responder	Macro_NLRP3	CD4(IL26+ Th17)	IL1B	SIGIRR	3,34962E-03	18223,3	9,03044E-03	9885	2,84647E+00	1366	3,48803E+00	4146	9,13147E-01	21299	0	54420,5
Myeloid_vs_CD4_Non-responder	Macro_NLRP3	CD4(IL26+ Th17)	VCAN	ITGB1	3,35395E-03	22067,3	7,86176E-03	12378	2,57115E+00	2235	2,48427E+00	16451	9,06675E-01	24852	0	54420,5
Myeloid_vs_CD4_Non-responder	Macro_NLRP3	CD4(IL26+ Th17)	VCAN	ITGA4	3,35443E-03	21006,3	8,92548E-03	10083	2,65236E+00	1920	2,59681E+00	14328	9,07720E-01	24280	0	54420,5
Myeloid_vs_CD4_Non-responder	Macro_NLRP3	CD4(IL26+ Th17)	VCAN	CD44	3,35539E-03	18759,9	8,63769E-03	10632	2,62591E+00	2029	2,41622E+00	17783	9,37852E-01	8935	0	54420,5
Myeloid_vs_CD4_Non-responder	Macro_FOLR2+APOE+	CD4(IFNG+ Tfh/Th1)	SPP1	ITGA4_ITGB1	3,35876E-03	25817,9	9,22635E-03	9535	1,21548E+00	25375	3,38124E+00	4872	8,88928E-01	34887	0	54420,5
Myeloid_vs_CD4_Non-responder	Macro_NLRP3	CD4(IL26+ Th17)	HBEGF	CD44	3,35924E-03	24614,9	5,99398E-03	18440	1,77752E+00	8953	1,95079E+00	29269	9,31714E-01	11992	0	54420,5
Myeloid_vs_CD4_Non-responder	Macro_NLRP3	CD4(IL26+ Th17)	LGALS1	CD69	3,36117E-03	29805,3	4,75567E-03	25937	1,04124E+00	33773	1,85314E+00	32252	9,51460E-01	2644	0	54420,5
Myeloid_vs_CD4_Non-responder	CD4(AREG+ Tm)	Macro_ISG15	B2M	LILRB2	3,36213E-03	25029,9	3,88203E-03	34894	1,79636E+00	8654	2,03834E+00	26704	9,59362E-01	477	0	54420,5
Myeloid_vs_CD4_Non-responder	Macro_NLRP3	CD4(IL26+ Th17)	HBEGF	CD82	3,36502E-03	26483,7	7,91061E-03	12266	1,79073E+00	8750	2,12129E+00	24527	8,93226E-01	32455	0	54420,5
Myeloid_vs_CD4_Non-responder	CD4(Tn)	cDC(CD1C)	B2M	CD1A	3,37134E-03	32223,7	2,60241E-02	1431	1,45504E+00	16585	9,16384E-01	72326	9,22786E-01	16356	0	54420,5
Myeloid_vs_CD4_Non-responder	Macro_NLRP3	CD4(IL26+ Th17)	VEGFA	CD44	3,37419E-03	24218,5	8,42808E-03	11085	2,27471E+00	3773	1,80443E+00	33826	9,19616E-01	17988	0	54420,5
Myeloid_vs_CD4_Non-responder	CD4(TNF+ T)	Macro_FOLR2+APOE+	HMGB1	CD163	3,37612E-03	26981,9	3,88006E-03	34923	1,58083E+00	13052	2,27371E+00	20815	9,32346E-01	11699	0	54420,5
Myeloid_vs_CD4_Non-responder	Macro_ISG15	CD4(IFNG+ Tfh/Th1)	HLA-DQB1	LAG3	3,38406E-03	20200,3	1,06925E-02	7437	1,45455E+00	16601	3,11505E+00	7275	9,25011E-01	15268	0	54420,5
Myeloid_vs_CD4_Non-responder	Macro_FOLR2+APOE+	CD4(IL26+ Th17)	LGALS3	LAG3	3,38532E-03	31709,3	6,01189E-03	18364	1,07037E+00	32231	2,37829E+00	18589	8,88816E-01	34942	0	54420,5
Myeloid_vs_CD4_Non-responder	CD4(NME1+ T)	cDC_CLEC9A	FLT3LG	FLT3	3,38636E-03	33049,5	2,77701E-02	1248	3,18456E+00	753	1,53201E+00	43850	8,34498E-01	64976	0	54420,5
Myeloid_vs_CD4_Non-responder	CD4(TNF+ T)	Mast	CD40LG	CD9	3,39017E-03	28115,1	1,65253E-02	3380	1,47169E+00	16041	1,77105E+00	34952	8,94529E-01	31782	0	54420,5
Myeloid_vs_CD4_Non-responder	Macro_NLRP3	CD4(TGFB1+ Th17)	THBS1	CD47	3,39114E-03	24766,5	7,31257E-03	13794	1,82237E+00	8253	2,50486E+00	16043	8,95354E-01	31322	0	54420,5
Myeloid_vs_CD4_Non-responder	Macro_NLRP3	CD4(TGFB1+ Th17)	CD55	ADGRE5	3,39211E-03	27501,3	8,59789E-03	10722	1,17810E+00	27074	1,83249E+00	32896	9,30930E-01	12394	0	54420,5
Myeloid_vs_CD4_Non-responder	CD4(AREG+ Tm)	Macro_ISG15	HLA-A	LILRB2	3,39550E-03	26127,1	3,87692E-03	34963	1,74252E+00	9580	1,94559E+00	29420	9,52505E-01	2252	0	54420,5
Myeloid_vs_CD4_Non-responder	CD4(IFNG+ Tfh/Th1)	Macro_LYVE1	CD59	STAB1	3,40085E-03	25660,3	6,29102E-03	17197	2,47825E+00	2651	2,35531E+00	19059	8,88773E-01	34974	0	54420,5
Myeloid_vs_CD4_Non-responder	CD4(NME1+ T)	Macro_ISG15	HLA-B	LILRB1	3,40085E-03	26369,7	4,52103E-03	27883	1,70638E+00	10263	1,77042E+00	34974	9,47325E-01	4308	0	54420,5
Myeloid_vs_CD4_Non-responder	CD4(IL26+ Th17)	Macro_FOLR2+APOE+	CD40LG	CD9	3,40425E-03	25499,5	1,46631E-02	4232	1,43662E+00	17121	2,46901E+00	16743	8,88754E-01	34981	0	54420,5
Myeloid_vs_CD4_Non-responder	Macro_NLRP3	CD4(IFNG+ Tfh/Th1)	ICAM1	ITGAL_ITGB2	3,40571E-03	32074,9	3,87572E-03	34984	1,31896E+00	21189	2,15095E+00	23766	9,04649E-01	26015	0	54420,5
Myeloid_vs_CD4_Non-responder	Macro_NLRP3	CD4(TGFB1+ Th17)	IL1B	SIGIRR	3,40620E-03	19830,9	7,56865E-03	13127	2,79116E+00	1527	3,39375E+00	4770	9,05885E-01	25310	0	54420,5
Myeloid_vs_CD4_Non-responder	Macro_IER3	CD4(IFNG+ Tfh/Th1)	HLA-DQA1	LAG3	3,40799E-03	21304,9	1,09988E-02	7105	1,49107E+00	15456	2,68382E+00	12912	9,22262E-01	16631	0	54420,5
Myeloid_vs_CD4_Non-responder	Macro_NLRP3	CD4(TGFB1+ Th17)	VCAN	ITGB1	3,40912E-03	24474,5	6,55427E-03	16230	2,50952E+00	2507	2,32101E+00	19784	8,98690E-01	29431	0	54420,5
Myeloid_vs_CD4_Non-responder	Macro_NLRP3	CD4(TGFB1+ Th17)	VCAN	SELL	3,41010E-03	21577,3	1,05253E-02	7641	2,64931E+00	1937	2,41138E+00	17890	9,04683E-01	25998	0	54420,5
Myeloid_vs_CD4_Non-responder	Macro_NLRP3	CD4(TNFRSF9+ Treg)	CD86	CTLA4	3,41253E-03	33747,5	7,50737E-03	13307	1,07443E+00	32030	1,79988E+00	33982	8,88717E-01	34998	0	54420,5
Myeloid_vs_CD4_Non-responder	Macro_NLRP3	CD4(TGFB1+ Th17)	LGALS1	CD69	3,41790E-03	27537,1	5,20550E-03	22698	1,12586E+00	29448	1,95246E+00	29216	9,53505E-01	1903	0	54420,5
Myeloid_vs_CD4_Non-responder	Macro_NLRP3	CD4(TGFB1+ Th17)	NAMPT	ITGA5_ITGB1	3,43649E-03	30993,7	4,27035E-03	30359	1,35361E+00	19927	2,11088E+00	24777	9,05597E-01	25485	0	54420,5
Myeloid_vs_CD4_Non-responder	Macro_NLRP3	CD4(TGFB1+ Th17)	VEGFA	CD44	3,43698E-03	17641,7	1,36568E-02	4808	2,73633E+00	1665	2,44121E+00	17295	9,35744E-01	10020	0	54420,5
Myeloid_vs_CD4_Non-responder	CD4(CXCL13+ Tfh)	Mono_CD14	RPS19	C5AR1	3,44876E-03	25927,3	4,96560E-03	24386	1,61035E+00	12324	1,76762E+00	35072	9,49301E-01	3434	0	54420,5
Myeloid_vs_CD4_Non-responder	CD4(IFNG+ Tfh/Th1)	Mono_CD16	HLA-C	LILRB1	3,45073E-03	27172,3	4,33117E-03	29731	1,69655E+00	10454	1,76754E+00	35076	9,43419E-01	6180	0	54420,5
Myeloid_vs_CD4_Non-responder	Mast	CD4(Tn)	TIMP3	CD44	3,45124E-03	27920,9	3,41193E-02	808	2,89437E+00	1260	1,17777E+00	59488	9,08895E-01	23628	0	54420,5
Myeloid_vs_CD4_Non-responder	CD4(NME1+ T)	Macro_ISG15	HMGB1	THBD	3,46206E-03	36273,9	3,87299E-03	35020	1,15209E+00	28274	1,76678E+00	35099	9,00224E-01	28556	0	54420,5
Myeloid_vs_CD4_Non-responder	CD4(CXCL13+ Tfh)	Macro_ISG15	HLA-A	LILRB1	3,46255E-03	26107,5	4,72535E-03	26178	1,72640E+00	9880	1,76675E+00	35100	9,45981E-01	4959	0	54420,5
Myeloid_vs_CD4_Non-responder	CD4(NME1+ T)	Mono_CD14	RPS19	C5AR1	3,46601E-03	26597,1	4,76529E-03	25866	1,55376E+00	13740	1,76665E+00	35107	9,48300E-01	3852	0	54420,5
Myeloid_vs_CD4_Non-responder	CD4(ISG+ Treg)	Macro_OLFML3	B2M	LILRB1	3,46996E-03	27443,9	3,99349E-03	33465	1,67129E+00	10966	1,76645E+00	35115	9,49845E-01	3253	0	54420,5
Myeloid_vs_CD4_Non-responder	CD4(CXCL13+ Tfh)	Macro_ISG15	HLA-B	LILRB1	3,47639E-03	26579,3	4,48182E-03	28239	1,68350E+00	10689	1,76623E+00	35128	9,47107E-01	4420	0	54420,5
Myeloid_vs_CD4_Non-responder	CD4(CXCL13+ Tfh)	Macro_LYVE1	CD59	STAB1	3,48035E-03	25826,3	6,24792E-03	17364	2,47616E+00	2664	2,33080E+00	19547	8,88433E-01	35136	0	54420,5
Myeloid_vs_CD4_Non-responder	Macro_FOLR2+APOE+	CD4(Tn)	LGALS9	PTPRC	3,48927E-03	31215,9	3,86348E-03	35154	1,26817E+00	23137	1,78815E+00	34378	9,37758E-01	8990	0	54420,5
Myeloid_vs_CD4_Non-responder	CD4(NME1+ T)	Macro_ISG15	HLA-C	LILRB1	3,49126E-03	27111,7	4,40875E-03	28966	1,66782E+00	11025	1,76551E+00	35158	9,43891E-01	5989	0	54420,5
Myeloid_vs_CD4_Non-responder	Macro_FOLR2+APOE+	CD4(NME1+ T)	HLA-DQB1	LAG3	3,51465E-03	29330,7	4,44590E-03	28594	1,41410E+00	17831	2,84354E+00	10603	8,88318E-01	35205	0	54420,5
Myeloid_vs_CD4_Non-responder	CD4(ISG+ Treg)	Macro_FOLR2+APOE+	HLA-F	LILRB1	3,51665E-03	32925,3	4,65026E-03	26788	1,44662E+00	16823	1,76382E+00	35209	8,95232E-01	31386	0	54420,5
Myeloid_vs_CD4_Non-responder	CD4(Tn)	pDC_LILRA4	MIF	CD74_CXCR4	3,52770E-03	42897,7	1,98949E-03	78920	6,91158E-01	56150	2,15321E+00	23724	9,55583E-01	1274	0	54420,5
Myeloid_vs_CD4_Non-responder	CD4(IFNG+ Tfh/Th1)	Macro_OLFML3	B2M	LILRB1	3,53316E-03	27481,9	3,99148E-03	33492	1,66927E+00	10997	1,76292E+00	35242	9,49833E-01	3258	0	54420,5
Myeloid_vs_CD4_Non-responder	Macro_IER3	CD4(IFNG+ Tfh/Th1)	HLA-DRB1	LAG3	3,53938E-03	20188,5	9,99367E-03	8362	1,44792E+00	16793	2,76864E+00	11644	9,36319E-01	9723	0	54420,5
Myeloid_vs_CD4_Non-responder	CD4(NME1+ T)	pDC_LILRA4	HMGB1	TLR9	3,54419E-03	23597,9	4,12787E-02	575	2,88796E+00	1277	1,66428E+00	38714	9,09976E-01	23003	0	54420,5
Myeloid_vs_CD4_Non-responder	Macro_OLFML3	CD4(ISG+ Treg)	HLA-DPB1	LAG3	3,54761E-03	21493,5	8,16046E-03	11668	1,44773E+00	16803	2,90165E+00	9819	9,26043E-01	14757	0	54420,5
Myeloid_vs_CD4_Non-responder	CD4(TNF+ T)	Macro_FOLR2+APOE+	CD40LG	ITGAM_ITGB2	3,54923E-03	32932,7	1,00618E-02	8251	1,01219E+00	35274	1,84950E+00	32366	8,89890E-01	34352	0	54420,5
Myeloid_vs_CD4_Non-responder	Macro_FOLR2+APOE+	CD4(ISG+ Treg)	CD14	ITGB1	3,55578E-03	26191,9	3,85204E-03	35287	1,73986E+00	9617	2,71911E+00	12376	9,16987E-01	19259	0	54420,5
Myeloid_vs_CD4_Non-responder	CD4(TNF+ T)	Macro_ISG15	HLA-B	LILRB2	3,55830E-03	25098,7	3,85179E-03	35292	1,80439E+00	8525	2,07668E+00	25669	9,54520E-01	1587	0	54420,5
Myeloid_vs_CD4_Non-responder	CD4(GZMK+ Teff)	Macro_FOLR2+APOE+	HLA-A	LILRB2	3,56940E-03	25556,9	3,85066E-03	35314	1,78120E+00	8900	2,03399E+00	26826	9,52351E-01	2324	0	54420,5
Myeloid_vs_CD4_Non-responder	Macro_OLFML3	CD4(TGFB1+ Th17)	CXCL9	DPP4	3,57729E-03	26679,3	2,82403E-02	1201	2,88344E+00	1283	3,06038E+00	7855	8,28094E-01	68637	0	54420,5
Myeloid_vs_CD4_Non-responder	CD4(TNF+ T)	cDC(CD1C)	IFNG	IFNGR1_IFNGR2	3,57749E-03	29386,9	1,72720E-02	3128	1,11181E+00	30152	2,14552E+00	23904	8,88094E-01	35330	0	54420,5
Myeloid_vs_CD4_Non-responder	CD4(NME1+ T)	Macro_ISG15	HLA-C	LILRB2	3,58307E-03	24943,7	3,84901E-03	35341	1,82949E+00	8134	2,12590E+00	24399	9,52091E-01	2424	0	54420,5
Myeloid_vs_CD4_Non-responder	Macro_FOLR2+APOE+	CD4(ISG+ Treg)	ICAM1	IL2RA	3,58408E-03	27182,3	1,60466E-02	3579	1,29210E+00	22158	2,29307E+00	20411	8,88075E-01	35343	0	54420,5

Myeloid_vs_CD4_Post_NR

Myeloid_vs_CD4_Non-responder	Macro_FOLR2+APOE+	CD4(NME1+ T)	HLA-DQA1	LAG3	3,58712E-03	27172,9	4,91907E-03	24728	1,57388E+00	13213	3,03245E+00	8154	8,88067E-01	35349	0	54420,5
Myeloid_vs_CD4_Non-responder	CD4(TNFRSF9+ Treg)	Macro_FOLR2+APOE+	HLA-F	LILRB1	3,60135E-03	34163,9	4,34130E-03	29650	1,40352E+00	18183	1,75908E+00	35377	8,91964E-01	33189	0	54420,5
Myeloid_vs_CD4_Non-responder	Macro_ISG15	CD4(IFNG+ Tfh/Th1)	CXCL10	CXCR3	3,60499E-03	24276,1	1,16997E-02	6396	2,30652E+00	3587	3,21182E+00	6327	8,60184E-01	50650	0	54420,5
Myeloid_vs_CD4_Non-responder	CD4(CRTAM- T)	pDC_LILRA4	TGFB1	ACVR1_TGFBR1_TGFBR2	3,60594E-03	33533,7	7,51249E-03	13293	1,03822E+00	33926	1,90567E+00	30643	8,88013E-01	35386	0	54420,5
Myeloid_vs_CD4_Non-responder	Macro_OLFML3	CD4(IFNG+ Tfh/Th1)	CXCL10	CXCR3	3,62156E-03	22619,9	1,36388E-02	4823	2,70424E+00	1772	3,20333E+00	6406	8,69154E-01	45678	0	54420,5
Myeloid_vs_CD4_Non-responder	Macro_FOLR2+APOE+	CD4(CXCL13+ Tfh)	TNFSF13	FAS	3,62227E-03	27697,3	7,97106E-03	12112	1,50767E+00	14969	2,24017E+00	21567	8,87964E-01	35418	0	54420,5
Myeloid_vs_CD4_Non-responder	CD4(Tn)	Macro_NLRP3	HMGB1	THBD	3,62841E-03	30453,7	4,90086E-03	24865	1,51762E+00	14723	1,75722E+00	35430	9,10309E-01	22830	0	54420,5
Myeloid_vs_CD4_Non-responder	CD4(TGFB1+ Th17)	Macro_OLFML3	TGFB1	ENG	3,63712E-03	31525,1	6,09905E-03	17999	1,31883E+00	21195	1,75675E+00	35447	9,00214E-01	28564	0	54420,5
Myeloid_vs_CD4_Non-responder	CD4(IL26+ Th17)	Macro_FOLR2+APOE+	CD52	SIGLEC10	3,63764E-03	25973,9	7,11296E-03	14357	1,66326E+00	11122	1,75665E+00	35448	9,26520E-01	14522	0	54420,5
Myeloid_vs_CD4_Non-responder	CD4(TGFB1+ Th17)	Macro_OLFML3	CD99	PILRA	3,63815E-03	30491,1	4,33597E-03	29692	1,44574E+00	16857	1,75664E+00	35449	9,23431E-01	16037	0	54420,5
Myeloid_vs_CD4_Non-responder	Macro_NLRP3	CD4(TGFB1+ Th17)	HBEGF	CD82	3,63866E-03	28112,3	7,09277E-03	14415	1,75320E+00	9383	2,03175E+00	26893	8,87909E-01	35450	0	54420,5
Myeloid_vs_CD4_Non-responder	cDC_LAMP3	CD4(CXCL13+ Tfh)	CCL19	CXCR3	3,64393E-03	29291,3	2,72539E-02	1295	4,86523E+00	60	2,15302E+00	23727	8,31029E-01	66954	0	54420,5
Myeloid_vs_CD4_Non-responder	CD4(CRTAM- T)	pDC_LILRA4	HLA-C	NOTCH4	3,66686E-03	28187,5	1,48799E-02	4133	1,68228E+00	10720	1,27889E+00	54718	9,21681E-01	16946	0	54420,5
Myeloid_vs_CD4_Non-responder	Macro_NLRP3	CD4(Tn)	THBS1	CD47	3,66749E-03	26590,7	6,25730E-03	17331	1,76325E+00	9217	2,48304E+00	16479	8,87825E-01	35506	0	54420,5
Myeloid_vs_CD4_Non-responder	Macro_FOLR2+APOE+	CD4(IFNG+ Tfh/Th1)	HLA-DQA1	CD4	3,67059E-03	22581,7	3,83603E-03	35512	1,73679E+00	9673	3,35633E+00	5072	9,39255E-01	8231	0	54420,5
Myeloid_vs_CD4_Non-responder	CD4(IL26+ Th17)	Macro_FOLR2-APOE+	ADAM10	TREM2	3,67111E-03	28974,5	7,44353E-03	13471	1,87917E+00	7461	1,79925E+00	34007	8,87809E-01	35513	0	54420,5
Myeloid_vs_CD4_Non-responder	Macro_FOLR2+APOE+	CD4(NME1+ T)	HLA-DPB1	LAG3	3,67266E-03	31082,1	3,83569E-03	35516	1,28347E+00	22522	2,75763E+00	11803	8,95665E-01	31149	0	54420,5
Myeloid_vs_CD4_Non-responder	CD4(Tn)	Macro_ISG15	B2M	LILRB2	3,69131E-03	25496,1	3,83352E-03	35552	1,75074E+00	9437	2,00755E+00	27560	9,59117E-01	511	0	54420,5
Myeloid_vs_CD4_Non-responder	CD4(ISG+ Treg)	Macro_FOLR2+APOE+	B2M	LILRB2	3,70275E-03	23843,1	3,83219E-03	35574	1,96091E+00	6507	2,21259E+00	22202	9,59110E-01	512	0	54420,5
Myeloid_vs_CD4_Non-responder	Macro_NLRP3	CD4(CXCL13+ Tfh)	CCL3	CCR4	3,70744E-03	22100,3	1,52611E-02	3936	1,83425E+00	8069	3,00426E+00	8493	8,87669E-01	35583	0	54420,5
Myeloid_vs_CD4_Non-responder	CD4(CRTAM- T)	Macro_FOLR2+APOE+	HMGB1	CD163	3,70796E-03	27823,9	3,83147E-03	35584	1,57046E+00	13309	2,14502E+00	23920	9,31947E-01	11886	0	54420,5
Myeloid_vs_CD4_Non-responder	Macro_NLRP3	CD4(IFNG+ Tfh/Th1)	HLA-DRB5	LAG3	3,70900E-03	29374,1	6,49387E-03	16449	1,00642E+00	35586	2,32711E+00	19644	9,14121E-01	20771	0	54420,5
Myeloid_vs_CD4_Non-responder	CD4(ISG+ Treg)	cDC_LAMP3	TNFSF10	TNFRSF11B	3,71118E-03	44142,5	2,88907E-02	1140	2,87272E+00	1307	1,16913E+00	59861	7,57900E-01	103984	0	54420,5
Myeloid_vs_CD4_Non-responder	Macro_FOLR2+APOE+	CD4(IL26+ Th17)	LGALS9	PTPRC	3,71526E-03	31521,1	3,83091E-03	35598	1,25924E+00	23520	1,77141E+00	34935	9,37511E-01	9132	0	54420,5
Myeloid_vs_CD4_Non-responder	Mono_CD16	CD4(IL26+ Th17)	S100A8	CD69	3,71947E-03	21423,3	7,65893E-03	12906	1,44052E+00	17008	2,57264E+00	14760	9,39646E-01	8022	0	54420,5
Myeloid_vs_CD4_Non-responder	CD4(IFNG+ Tfh/Th1)	Macro_FOLR2+APOE+	B2M	LILRB2	3,72518E-03	23873,3	3,83026E-03	35617	1,95889E+00	6529	2,20906E+00	22286	9,59100E-01	514	0	54420,5
Myeloid_vs_CD4_Non-responder	CD4(TGFB1+ Th17)	Macro_FOLR2+APOE+	HLA-A	LILRB2	3,74037E-03	25861,7	3,82810E-03	35646	1,76645E+00	9159	2,00241E+00	27712	9,52217E-01	2371	0	54420,5
Myeloid_vs_CD4_Non-responder	CD4(ISG+ Treg)	Macro_ISG15	TIGIT	NECTIN2	3,74877E-03	23795,5	3,82841E-02	653,5	2,48099E+00	2632,5	2,07949E+00	25608,5	8,87490E-01	35662,5	0	54420,5
Myeloid_vs_CD4_Non-responder	CD4(ISG+ Treg)	Macro_FOLR2+APOE+	HLA-B	LILRB1	3,75140E-03	28264,5	3,82657E-03	35667	1,60730E+00	12402	1,84662E+00	32469	9,43005E-01	6364	0	54420,5
Myeloid_vs_CD4_Non-responder	CD4(ISG+ Treg)	Macro_FOLR2+APOE+	LTB	CD40	3,76088E-03	25622,7	1,07793E-02	7343	1,62035E+00	12084	1,74885E+00	35685	9,18336E-01	18581	0	54420,5
Myeloid_vs_CD4_Non-responder	CD4(IL26+ Th17)	Macro_ISG15	CIRBP	TREM1	3,76141E-03	30104,3	5,96367E-03	18585	1,42870E+00	17350	1,74883E+00	35686	9,07321E-01	24480	0	54420,5
Myeloid_vs_CD4_Non-responder	Macro_NLRP3	CD4(IFNG+ Tfh/Th1)	CD14	ITGA4	3,77566E-03	30558,7	3,82416E-03	35713	1,34929E+00	20073	2,26440E+00	21037	9,12728E-01	21550	0	54420,5
Myeloid_vs_CD4_Non-responder	CD4(ISG+ Treg)	pDC_LILRA4	HLA-C	NOTCH4	3,77608E-03	28346,5	1,47837E-02	4183	1,66627E+00	11052	1,27255E+00	55003	9,21446E-01	17074	0	54420,5
Myeloid_vs_CD4_Non-responder	Macro_FOLR2+APOE-	CD4(IFNG+ Tfh/Th1)	HLA-DRB5	LAG3	3,78385E-03	19492,3	1,21683E-02	5960	1,71972E+00	10002	2,45173E+00	17083	9,35777E-01	9996	0	54420,5
Myeloid_vs_CD4_Non-responder	cDC_LAMP3	CD4(TNF+ T)	CCL19	CCR7	3,79041E-03	26915,9	2,68983E-02	1321	4,88737E+00	56	2,10478E+00	24923	8,54490E-01	53859	0	54420,5
Myeloid_vs_CD4_Non-responder	CD4(Tn)	Macro_NLRP3	VIM	CD44	3,79610E-03	58956,9	1,79227E-03	85877	4,28992E-01	79691	8,93254E-01	73474	9,55413E-01	1322	0	54420,5
Myeloid_vs_CD4_Non-responder	CD4(CRTAM- T)	Mono_CD16	HLA-C	LILRB1	3,80110E-03	27422,7	4,30600E-03	29959	1,68206E+00	10724	1,74679E+00	35761	9,43263E-01	6249	0	54420,5
Myeloid_vs_CD4_Non-responder	CD4(Tn)	Macro_ISG15	HLA-A	LILRB2	3,80535E-03	26475,7	3,81988E-03	35769	1,70911E+00	10198	1,93982E+00	29601	9,52168E-01	2390	0	54420,5
Myeloid_vs_CD4_Non-responder	CD4(TGFB1+ Th17)	Mono_CD16	HLA-C	LILRA3	3,80854E-03	22857,3	1,29336E-02	5331	2,10548E+00	5062	1,74636E+00	35775	9,28222E-01	13698	0	54420,5
Myeloid_vs_CD4_Non-responder	CD4(CRTAM- T)	Mast	CALR	ITGA2B	3,81561E-03	47215,5	2,79009E-02	1217	1,95979E+00	6521	2,23672E-01	108988	8,34587E-01	64931	0	54420,5
Myeloid_vs_CD4_Non-responder	Macro_FOLR2+APOE+	CD4(CRTAM- T)	SPP1	ITGA4_ITGB1	3,81867E-03	26389,1	8,91865E-03	10099	1,19630E+00	26246	3,31928E+00	5386	8,87243E-01	35794	0	54420,5
Myeloid_vs_CD4_Non-responder	CD4(IL26+ Th17)	Macro_ISG15	HLA-F	LILRB2	3,81920E-03	28840,3	3,81752E-03	35795	1,88116E+00	7436	2,21928E+00	22065	9,07309E-01	24485	0	54420,5
Myeloid_vs_CD4_Non-responder	Macro_OLFML3	CD4(IFNG+ Tfh/Th1)	B2M	CD3D	3,82220E-03	42266,1	4,15767E-03	31570	6,29465E-01	61298	1,08318E+00	64019	9,66146E-01	23	0	54420,5
Myeloid_vs_CD4_Non-responder	Macro_FOLR2+APOE+	CD4(CRTAM- T)	LGALS1	CD69	3,82401E-03	30069,1	4,42318E-03	28820	1,00189E+00	35804	1,99226E+00	28021	9,49758E-01	3280	0	54420,5
Myeloid_vs_CD4_Non-responder	Mono_INHBA	CD4(GZMK+ Teff)	SPP1	CD44	3,83333E-03	20912,7	1,05991E-02	7540	1,62306E+00	12028	2,44927E+00	17140	9,28733E-01	13435	0	54420,5
Myeloid_vs_CD4_Non-responder	Macro_FOLR2+APOE+	CD4(TNF+ T)	CD14	ITGB1	3,83363E-03	26159,9	3,81548E-03	35822	1,73619E+00	9686	2,78377E+00	11414	9,16623E-01	19457	0	54420,5
Myeloid_vs_CD4_Non-responder	CD4(GZMK+ Teff)	Mono_CD16	HLA-B	LILRB1	3,83523E-03	27620,3	4,22458E-03	30821	1,62807E+00	11891	1,74499E+00	35825	9,45607E-01	5144	0	54420,5
Myeloid_vs_CD4_Non-responder	CD4(ISG+ Treg)	Macro_FOLR2+APOE+	CD99	CD81	3,84488E-03	32537,3	3,81432E-03	35843	1,27773E+00	22727	1,88797E+00	31184	9,18497E-01	18512	0	54420,5
Myeloid_vs_CD4_Non-responder	CD4(TNF+ T)	Mono_CD16	HLA-C	LILRA3	3,84730E-03	29187,1	1,05820E-02	7563	1,62932E+00	11864	1,27420E+00	54932	9,21243E-01	17156	0	54420,5
Myeloid_vs_CD4_Non-responder	Macro_OLFML3	CD4(TGFB1+ Th17)	CXCL9	CXCR3	3,86470E-03	18695,9	2,68052E-02	1334	2,94640E+00	1148	3,14719E+00	6944	8,98355E-01	29633	0	54420,5
Myeloid_vs_CD4_Non-responder	Mono_CD14	CD4(TNFRSF9+ Treg)	S100A8	ITGB2	3,86559E-03	17006,5	5,53375E-03	20771	3,13950E+00	807	3,78236E+00	2484	9,42580E-01	6550	0	54420,5
Myeloid_vs_CD4_Non-responder	CD4(Tn)	Macro_FOLR2+APOE+	CD99	PILRA	3,87825E-03	30557,9	3,80935E-03	35905	1,59952E+00	12601	1,87856E+00	31454	9,18725E-01	18409	0	54420,5
Myeloid_vs_CD4_Non-responder	CD4(CRTAM- T)	Macro_ISG15	HLA-B	LILRB1	3,87879E-03	26560,1	4,52020E-03	27888	1,70590E+00	10275	1,74237E+00	35906	9,47320E-01	4311	0	54420,5
Myeloid_vs_CD4_Non-responder	CD4(CXCL13+ Tfh)	Macro_ISG15	CD99	PILRA	3,90098E-03	31039,1	3,80628E-03	35947	1,53540E+00	14224	1,85521E+00	32183	9,18695E-01	18421	0	54420,5
Myeloid_vs_CD4_Non-responder	CD4(TNF+ T)	Macro_LYVE1	TNF	TNFRSF1A	3,90478E-03	33472,1	7,05093E-03	14525	1,06359E+00	32602	1,74148E+00	35954	8,97952E-01	29859	0	54420,5
Myeloid_vs_CD4_Non-responder	Macro_FOLR2+APOE+	CD4(TNFRSF9+ Treg)	CCL3	CCR4	3,90533E-03	22220,3	1,50440E-02	4036	1,68999E+00	10570	3,23501E+00	6120	8,86953E-01	35955	0	54420,5
Myeloid_vs_CD4_Non-responder	CD4(TNFRSF9+ Treg)	Macro_FOLR2+APOE+	HLA-B	LILRB2	3,90696E-03	24715,1	3,80589E-03	35958	1,86146E+00	7688	2,14780E+00	23847	9,54259E-01	1662	0	54420,5
Myeloid_vs_CD4_Non-responder	CD4(TNF+ T)	Macro_ISG15	TNFSF9	HLA-DPA1	3,90859E-03	31664,5	4,73325E-03	26115	1,11971E+00	29748	2,73926E+00	12078	8,86942E-01	35961	0	54420,5
Myeloid_vs_CD4_Non-responder	CD4(GZMK+ Teff)	pDC_LILRA4	CCL5	CXCR3	3,90967E-03	26324,5	2,49088E-02	1568	9,99222E-01	35963	2,16521E+00	23421	9,22998E-01	16250	0	54420,5
Myeloid_vs_CD4_Non-responder	Macro_NLRP3	CD4(TNF+ T)	VEGFA	CD44	3,91348E-03	25687,9	7,46101E-03	13427	2,18933E+00	4359	1,74092E+00	35970	9,14994E-01	20263	0	54420,5
Myeloid_vs_CD4_Non-responder	CD4(ISG+ Treg)	Mono_CD16	HLA-C	LILRB1	3,92165E-03	27612,5	4,27817E-03	30270	1,66604E+00	11057	1,74045E+00	35985	9,43089E-01	6330	0	54420,5
Myeloid_vs_CD4_Non-responder	pDC_LILRA4	CD4(TNFRSF9+ Treg)	APP	CD74	3,92216E-03	18664,9	6,52093E-03	16350	2,29750E+00	3650	2,44330E+00	17241	9,54255E-01	1663	0	54420,5
Myeloid_vs_CD4_Non-responder	CD4(AREG+ Tm)	Macro_FOLR2+APOE+	ADAM10	GPNMB	3,92571E-03	29142,5	6,27679E-03	17245	1,99691E+00	6122	2,63182E+00	13747	8,53886E-01	54178	0	54420,5
Myeloid_vs_CD4_Non-responder	CD4(AREG+ Tm)	Macro_OLFML3	ANXA1	FPR1	3,94897E-03	28701,7	4,88978E-03	24946	1,47428E+00	15964	1,73896E+00	36035	9,31427E-01	12143	0	54420,5
Myeloid_vs_CD4_Non-responder	Mast	CD4(IL26+ Th17)	TIMP3	CD44	3,96293E-03	28875,5	3,23791E-02	915	2,85438E+00	1351	1,10665E+00	62850	9,06704E-01	24841	0	54420,5
Myeloid_vs_CD4_Non-responder	CD4(IFNG+ Tfh/Th1)	pDC_LILRA4	HMGB1	TLR9	3,98039E-03	24556,7	3,99781E-02	605	2,85280E+00	1354	1,56260E+00	42646	9,08656E-01	23758	0	54420,5
Myeloid_vs_CD4_Non-responder	Mono_CD14	CD4(NME1+ T)	VCAN	ITGA4	3,98461E-03	18043,7	1,25083E-02	5671	3,15394E+00	786	2,74215E+00	12030	9,20915E-01	17311	0	54420,5
Myeloid_vs_CD4_Non-responder	CD4(AREG+ Tm)	Macro_ISG15	HLA-C	LILRB2	3,99576E-03	25652,9	3,79367E-03	36120	1,79673E+00	8645	2,04419E+00	26544	9,51760E-01	2535	0	54420,5
Myeloid_vs_CD4_Non-responder	Macro_OLFML3	CD4(AREG+ Tm)	CXCL9	DPP4	4,00373E-03	27127,1	2,66805E-02	1358	2,86717E+00	1321	3,07643E+00	7685	8,24012E-01	70851	0	54420,5
Myeloid_vs_CD4_Non-responder	CD4(TNFRSF9+ Treg)	pDC_LILRA4	HLA-C	NOTCH4	4,01882E-03	29160,1	1,45405E-02	4303	1,62575E+00	11952	1,21290E+00	57776	9,20844E-01	17349	0	54420,5
Myeloid_vs_CD4_Non-responder	Mono_CD14	CD4(GZMK+ Teff)	S100A8	ITGB2	4,01985E-03	17014,1	5,49547E-03	20961	3,13623E+00	813	3,84541E+00	2238	9,42392E-01	6638	0	54420,5

Myeloid_vs_CD4_Post_NR

Myeloid_vs_CD4_Non-responder	CD4(IL26+ Th17)	Macro_NLRP3	HMGB1	THBD	4,02795E-03	30173,3	5,03013E-03	23904	1,54074E+00	14077	1,73480E+00	36178	9,11366E-01	22287	0	54420,5
Myeloid_vs_CD4_Non-responder	CD4(ISG+ Treg)	Macro_NLRP3	HMGB1	THBD	4,02850E-03	30099,9	5,05605E-03	23732	1,54538E+00	13975	1,73473E+00	36179	9,11573E-01	22193	0	54420,5
Myeloid_vs_CD4_Non-responder	cDC_LAMP3	CD4(IFNG+ Tfh/Th1)	HLA-DPB1	LAG3	4,04144E-03	21042,7	9,89206E-03	8507	1,42781E+00	17374	2,66484E+00	13221	9,32368E-01	11691	0	54420,5
Myeloid_vs_CD4_Non-responder	Macro_NLRP3	CD4(TGFB1+ Th17)	ICAM1	IL2RG	4,04411E-03	36506,9	3,92727E-03	34278	1,11647E+00	29903	1,73386E+00	36207	9,01689E-01	27726	0	54420,5
Myeloid_vs_CD4_Non-responder	Macro_OLFML3	CD4(ISG+ Treg)	HLA-DPA1	LAG3	4,06052E-03	21268,7	7,97628E-03	12097	1,42706E+00	17395	2,95269E+00	9158	9,29037E-01	13273	0	54420,5
Myeloid_vs_CD4_Non-responder	Macro_FOLR2+APOE+	CD4(NME1+ T)	HLA-DPA1	LAG3	4,06594E-03	30488,3	3,78405E-03	36246	1,27779E+00	22724	2,85443E+00	10464	9,00173E-01	28587	0	54420,5
Myeloid_vs_CD4_Non-responder	CD4(TNF+ T)	Macro_FOLR2-APOE+	CD40LG	CD9	4,06987E-03	26831,3	1,84536E-02	2773	1,63105E+00	11819	1,73235E+00	36253	8,99623E-01	28891	0	54420,5
Myeloid_vs_CD4_Non-responder	CD4(IFNG+ Tfh/Th1)	Mono_CD14	RPS19	C5AR1	4,08111E-03	26244,5	4,94251E-03	24549	1,60383E+00	12495	1,73157E+00	36273	9,49188E-01	3485	0	54420,5
Myeloid_vs_CD4_Non-responder	Macro_FOLR2-APOE+	CD4(IFNG+ Tfh/Th1)	HLA-DRB1	LAG3	4,08240E-03	20458,5	1,06475E-02	7491	1,53653E+00	14201	2,43477E+00	17419	9,38183E-01	8761	0	54420,5
Myeloid_vs_CD4_Non-responder	Macro_FOLR2+APOE+	CD4(AREG+ Tm)	CD14	ITGA4	4,09068E-03	26687,7	3,78031E-03	36290	1,76653E+00	9154	2,75896E+00	11782	9,12268E-01	21792	0	54420,5
Myeloid_vs_CD4_Non-responder	Macro_ISG15	CD4(TNFRSF9+ Treg)	CCL18	CCR8	4,10746E-03	28235,5	1,44732E-01	33	1,94339E+00	6687	3,18559E+00	6558	8,19170E-01	73479	0	54420,5
Myeloid_vs_CD4_Non-responder	Macro_FOLR2+APOE+	CD4(AREG+ Tm)	LGALS3BP	ITGB1	4,11667E-03	33342,3	6,07150E-03	18117	1,25881E+00	23543	1,72978E+00	36336	8,89981E-01	34295	0	54420,5
Myeloid_vs_CD4_Non-responder	Macro_FOLR2+APOE+	CD4(NME1+ T)	HLA-DRB1	LAG3	4,12007E-03	30430,3	3,77686E-03	36342	1,27146E+00	23003	2,89438E+00	9941	9,00388E-01	28445	0	54420,5
Myeloid_vs_CD4_Non-responder	CD4(TNFRSF9+ Treg)	Macro_FOLR2-APOE+	ADAM10	TREM2	4,12801E-03	29238,1	7,20958E-03	14082	1,86857E+00	7596	1,80694E+00	33736	8,86209E-01	36356	0	54420,5
Myeloid_vs_CD4_Non-responder	CD4(Tn)	Mono_CD16	CD99	PILRA	4,13938E-03	30793,3	3,77440E-03	36376	1,58312E+00	12999	1,87357E+00	31608	9,18381E-01	18563	0	54420,5
Myeloid_vs_CD4_Non-responder	Macro_IER3	CD4(ISG+ Treg)	HLA-DRB5	LAG3	4,14210E-03	23004,9	8,04664E-03	11945	1,42404E+00	17484	2,58700E+00	14493	9,22171E-01	16682	0	54420,5
Myeloid_vs_CD4_Non-responder	Macro_FOLR2+APOE+	CD4(Tn)	SPP1	PTGER4	4,14223E-03	26424,3	9,74129E-03	8721	1,18088E+00	26942	3,28769E+00	5657	8,86177E-01	36381	0	54420,5
Myeloid_vs_CD4_Non-responder	Macro_FOLR2+APOE+	CD4(CRTAM- T)	TIMP2	ITGB1	4,14280E-03	31633,3	6,67143E-03	15789	1,37773E+00	19081	1,84597E+00	32494	8,86177E-01	36382	0	54420,5
Myeloid_vs_CD4_Non-responder	Macro_ISG15	CD4(IFNG+ Tfh/Th1)	CXCL16	CXCR6	4,15504E-03	19612,7	1,56845E-02	3736	1,65442E+00	11315	2,80868E+00	11094	9,20564E-01	17498	0	54420,5
Myeloid_vs_CD4_Non-responder	Macro_LYVE1	CD4(ISG+ Treg)	HLA-DPB1	LAG3	4,15967E-03	23197,9	8,07521E-03	11874	1,43206E+00	17248	2,43091E+00	17503	9,25683E-01	14944	0	54420,5
Myeloid_vs_CD4_Non-responder	CD4(IFNG+ Tfh/Th1)	Mono_CD16	CD52	SIGLEC10	4,16333E-03	28759,5	6,07474E-03	18100	1,42148E+00	17575	1,72766E+00	36418	9,20965E-01	17284	0	54420,5
Myeloid_vs_CD4_Non-responder	CD4(IFNG+ Tfh/Th1)	Macro_FOLR2+APOE+	HLA-C	LILRB2	4,16963E-03	25247,9	3,77142E-03	36429	1,83493E+00	8059	2,11207E+00	24743	9,51624E-01	2588	0	54420,5
Myeloid_vs_CD4_Non-responder	CD4(ISG+ Treg)	Mono_CD14	RPS19	C5AR1	4,17363E-03	26860,5	4,76558E-03	25861	1,55384E+00	13734	1,72713E+00	36436	9,48302E-01	3851	0	54420,5
Myeloid_vs_CD4_Non-responder	Macro_IFI27	CD4(ISG+ Treg)	C3	IFITM1	4,17989E-03	20514,1	1,22987E-02	5833	1,93972E+00	6727	1,88083E+00	31396	9,47596E-01	4194	0	54420,5
Myeloid_vs_CD4_Non-responder	Macro_FOLR2+APOE+	CD4(ISG+ Treg)	SPP1	CD44	4,18510E-03	28800,5	5,94200E-03	18674	9,90134E-01	36456	2,90230E+00	9811	9,07040E-01	24641	0	54420,5
Myeloid_vs_CD4_Non-responder	CD4(TNF+ T)	Macro_FOLR2+APOE+	CCL5	CCRL2	4,19199E-03	28746,7	1,99326E-02	2369	1,47223E+00	16018	1,72625E+00	36468	8,89722E-01	34458	0	54420,5
Myeloid_vs_CD4_Non-responder	CD4(AREG+ Tm)	Mono_CD16	HLA-F	LILRB1	4,19314E-03	33794,7	4,22647E-03	30805	1,56822E+00	13388	1,72608E+00	36470	8,90665E-01	33890	0	54420,5
Myeloid_vs_CD4_Non-responder	CD4(CRTAM- T)	Macro_ISG15	HLA-A	LILRB1	4,19947E-03	26574,1	4,67927E-03	26548	1,70284E+00	10341	1,72579E+00	36481	9,45730E-01	5080	0	54420,5
Myeloid_vs_CD4_Non-responder	CD4(TNFRSF9+ Treg)	Macro_NLRP3	HLA-F	LILRB2	4,20696E-03	32270,5	3,76656E-03	36494	1,50905E+00	14928	1,90406E+00	30690	9,06742E-01	24820	0	54420,5
Myeloid_vs_CD4_Non-responder	CD4(Tn)	Mono_CD16	HLA-C	LILRA3	4,20710E-03	30031,9	1,03531E-02	7871	1,58297E+00	13005	1,22338E+00	57309	9,20446E-01	17554	0	54420,5
Myeloid_vs_CD4_Non-responder	CD4(TGFB1+ Th17)	Macro_NLRP3	HMGB1	THBD	4,20753E-03	29969,7	5,11398E-03	23320	1,55574E+00	13695	1,72534E+00	36495	9,12031E-01	21918	0	54420,5
Myeloid_vs_CD4_Non-responder	Macro_FOLR2+APOE+	CD4(CXCL13+ Tfh)	SPP1	CD44	4,21099E-03	28903,7	5,93463E-03	18712	9,89285E-01	36501	2,87308E+00	10216	9,06988E-01	24669	0	54420,5
Myeloid_vs_CD4_Non-responder	Macro_NLRP3	CD4(NME1+ T)	LYZ	ITGAL	4,21099E-03	27987,3	3,76602E-03	36501	1,60166E+00	12553	2,78568E+00	11384	9,06284E-01	25078	0	54420,5
Myeloid_vs_CD4_Non-responder	Macro_FOLR2+APOE+	CD4(ISG+ Treg)	HLA-DMB	CD4	4,21734E-03	26835,9	3,76506E-03	36512	1,82807E+00	8164	2,61214E+00	14084	9,13689E-01	20999	0	54420,5
Myeloid_vs_CD4_Non-responder	Macro_FOLR2+APOE+	CD4(NME1+ T)	SPP1	CD44	4,23354E-03	28910,3	5,92875E-03	18742	9,88607E-01	36540	2,87693E+00	10156	9,06946E-01	24693	0	54420,5
Myeloid_vs_CD4_Non-responder	CD4(Tn)	Macro_LYVE1	HMGB1	CD163	4,23528E-03	27841,7	4,39674E-03	29079	1,75367E+00	9372	1,72412E+00	36543	9,36184E-01	9794	0	54420,5
Myeloid_vs_CD4_Non-responder	Macro_NLRP3	CD4(AREG+ Tm)	ICAM1	IL2RG	4,23817E-03	37044,7	3,83505E-03	35525	1,10653E+00	30402	1,72401E+00	36548	9,00631E-01	28328	0	54420,5
Myeloid_vs_CD4_Non-responder	CD4(TGFB1+ Th17)	Macro_FOLR2+APOE+	HLA-C	LILRB1	4,24049E-03	29694,3	3,76223E-03	36552	1,53964E+00	14117	1,76122E+00	35302	9,39541E-01	8080	0	54420,5
Myeloid_vs_CD4_Non-responder	CD4(TNF+ T)	Macro_NLRP3	HMGB1	THBD	4,24572E-03	31647,3	4,62865E-03	26966	1,46893E+00	16133	1,72346E+00	36561	9,07948E-01	24156	0	54420,5
Myeloid_vs_CD4_Non-responder	CD4(TNF+ T)	Macro_ISG15	B2M	LILRB2	4,25269E-03	25943,7	3,76124E-03	36573	1,68278E+00	10706	2,01111E+00	27451	9,58742E-01	568	0	54420,5
Myeloid_vs_CD4_Non-responder	CD4(TNFRSF9+ Treg)	Macro_FOLR2+APOE-	CD59	STAB1	4,27191E-03	30655,5	5,92298E-03	18777	1,91824E+00	6980	1,72534E+00	36494	8,85759E-01	36606	0	54420,5
Myeloid_vs_CD4_Non-responder	Macro_FOLR2+APOE+	CD4(NME1+ T)	ICAM1	IL2RG	4,27366E-03	36425,9	4,33899E-03	29666	9,94597E-01	36207	1,72179E+00	36609	9,06021E-01	25227	0	54420,5
Myeloid_vs_CD4_Non-responder	CD4(TNF+ T)	Macro_FOLR2+APOE+	CSF1	SIRPA	4,27380E-03	28391,3	1,87111E-02	2685	1,90561E+00	7114	2,42392E+00	17625	8,43321E-01	60112	0	54420,5
Myeloid_vs_CD4_Non-responder	cDC(CD1C)	CD4(ISG+ Treg)	HLA-DQA1	LAG3	4,27380E-03	19945,3	1,04198E-02	7782	1,72141E+00	9975	2,89575E+00	9924	9,20301E-01	17625	0	54420,5
Myeloid_vs_CD4_Non-responder	CD4(TNF+ T)	Macro_FOLR2+APOE+	CCL5	CCR1	4,27425E-03	29005,3	1,73648E-02	3095	1,46286E+00	16326	1,78275E+00	34575	8,85756E-01	36610	0	54420,5
Myeloid_vs_CD4_Non-responder	CD4(Tn)	cDC_LAMP3	CD40LG	CD40	4,29564E-03	33743,5	1,28359E-02	5431	1,93383E+00	6790	1,60859E+00	40777	8,41183E-01	61299	0	54420,5
Myeloid_vs_CD4_Non-responder	Mono_CD14	CD4(TNF+ T)	VCAN	CD44	4,31077E-03	18411,3	8,83008E-03	10254	2,95845E+00	1115	2,42208E+00	17664	9,38491E-01	8603	0	54420,5
Myeloid_vs_CD4_Non-responder	CD4(CXCL13+ Tfh)	Macro_ISG15	B2M	LILRB1	4,31291E-03	26523,9	4,48418E-03	28216	1,66555E+00	11066	1,72000E+00	36676	9,52535E-01	2241	0	54420,5
Myeloid_vs_CD4_Non-responder	CD4(NME1+ T)	Macro_FOLR2+APOE+	CD28	CD86	4,31526E-03	28029,7	9,63242E-03	8881,5	1,68346E+00	10690,5	1,94425E+00	29475,5	8,85621E-01	36680,5	0	54420,5
Myeloid_vs_CD4_Non-responder	Macro_FOLR2+APOE+	CD4(NME1+ T)	CD86	CD28	4,31585E-03	28029,7	9,63242E-03	8881,5	1,68346E+00	10690,5	1,94425E+00	29475,5	8,85621E-01	36680,5	0	54420,5
Myeloid_vs_CD4_Non-responder	cDC_LAMP3	CD4(IFNG+ Tfh/Th1)	B2M	CD3D	4,32057E-03	46963,1	4,09901E-03	32242	5,73654E-01	66169	7,31186E-01	81958	9,65912E-01	26	0	54420,5
Myeloid_vs_CD4_Non-responder	Macro_FOLR2+APOE+	CD4(CRTAM- T)	PLAU	ITGB1	4,33353E-03	26757,7	1,14553E-02	6625	1,85380E+00	7787	1,98489E+00	28245	8,85570E-01	36711	0	54420,5
Myeloid_vs_CD4_Non-responder	Macro_FOLR2+APOE-	CD4(IFNG+ Tfh/Th1)	HLA-DPB1	LAG3	4,34988E-03	19851,7	1,15943E-02	6503	1,65314E+00	11336	2,42017E+00	17705	9,37206E-01	9294	0	54420,5
Myeloid_vs_CD4_Non-responder	CD4(CRTAM- T)	Macro_FOLR2+APOE+	HLA-C	LILRB2	4,35127E-03	25474,9	3,74950E-03	36741	1,82044E+00	8283	2,09132E+00	25299	9,51490E-01	2631	0	54420,5
Myeloid_vs_CD4_Non-responder	Macro_FOLR2+APOE-	CD4(IFNG+ Tfh/Th1)	HLA-DRB1	LAG3	4,35276E-03	19825,7	1,12397E-02	6835	1,61681E+00	12188	2,42006E+00	17708	9,39734E-01	7977	0	54420,5
Myeloid_vs_CD4_Non-responder	Macro_NLRP3	CD4(NME1+ T)	CD14	ITGB1	4,35304E-03	31130,5	3,74940E-03	36744	1,31627E+00	21286	2,16552E+00	23411	9,15953E-01	19791	0	54420,5
Myeloid_vs_CD4_Non-responder	CD4(TNFRSF9+ Treg)	Macro_FOLR2+APOE+	B2M	LILRB2	4,36431E-03	24702,1	3,74874E-03	36763	1,87329E+00	7537	2,13282E+00	24211	9,58676E-01	579	0	54420,5
Myeloid_vs_CD4_Non-responder	pDC_LILRA4	CD4(IFNG+ Tfh/Th1)	HLA-DRA	LAG3	4,36906E-03	19419,7	9,13656E-03	9691	1,41698E+00	17725	3,39165E+00	4787	9,34805E-01	10475	0	54420,5
Myeloid_vs_CD4_Non-responder	CD4(TNF+ T)	Macro_ISG15	CCL5	CCRL2	4,37441E-03	29441,3	1,82929E-02	2817	1,37120E+00	19298	1,80241E+00	33891	8,85440E-01	36780	0	54420,5
Myeloid_vs_CD4_Non-responder	Macro_NLRP3	CD4(TNFRSF9+ Treg)	LYZ	ITGAL	4,37738E-03	27939,9	3,74718E-03	36785	1,60013E+00	12583	2,83647E+00	10713	9,06071E-01	25198	0	54420,5
Myeloid_vs_CD4_Non-responder	CD4(TNFRSF9+ Treg)	Macro_ISG15	LTA_LTB	LTBR	4,37917E-03	32637,1	8,91860E-03	10100	1,15859E+00	27959	1,80155E+00	33918	8,85428E-01	36788	0	54420,5
Myeloid_vs_CD4_Non-responder	CD4(TNF+ T)	Macro_ISG15	HLA-B	LILRB1	4,37917E-03	27256,9	4,41195E-03	28931	1,64272E+00	11556	1,71630E+00	36788	9,46712E-01	4589	0	54420,5
Myeloid_vs_CD4_Non-responder	CD4(Tn)	Macro_OLFML3	ANXA1	FPR1	4,38215E-03	29930,7	4,54942E-03	27645	1,42224E+00	17548	1,71622E+00	36793	9,29087E-01	13247	0	54420,5
Myeloid_vs_CD4_Non-responder	Macro_FOLR2+APOE+	CD4(TGFB1+ Th17)	SPP1	PTGER4	4,38572E-03	26764,7	9,59499E-03	8933	1,17319E+00	27298	3,20642E+00	6373	8,85412E-01	36799	0	54420,5
Myeloid_vs_CD4_Non-responder	CD4(NME1+ T)	Macro_ISG15	CD28	CD86	4,39885E-03	27945,5	9,58655E-03	8943,5	1,67537E+00	10871,5	1,96967E+00	28670,5	8,85379E-01	36821,5	0	54420,5
Myeloid_vs_CD4_Non-responder	Macro_FOLR2-APOE+	CD4(IFNG+ Tfh/Th1)	HLA-DPB1	LAG3	4,40953E-03	20586,3	1,09428E-02	7166	1,56689E+00	13423	2,41734E+00	17767	9,35483E-01	10155	0	54420,5
Myeloid_vs_CD4_Non-responder	Macro_FOLR2+APOE+	CD4(IFNG+ Tfh/Th1)	TIMP2	ITGB1	4,41200E-03	31768,5	6,56194E-03	16202	1,36752E+00	19441	1,86242E+00	31936	8,85340E-01	36843	0	54420,5
Myeloid_vs_CD4_Non-responder	CD4(IFNG+ Tfh/Th1)	Macro_OLFML3	HLA-F	LILRB1	4,41979E-03	33629,7	4,42597E-03	28793	1,49208E+00	15430	1,71449E+00	36856	8,92891E-01	32649	0	54420,5
Myeloid_vs_CD4_Non-responder	CD4(CRTAM- T)	pDC_LILRA4	TNF	TNFRSF21	4,42668E-03	27879,7	1,12153E-02	6860	2,58706E+00	2165	1,91349E+00	30407	8,69380E-01	45546	0	54420,5
Myeloid_vs_CD4_Non-responder	Macro_FOLR2+APOE+	CD4(ISG+ Treg)	APOE	LSR	4,43300E-03	21705,7	1,35428E-02	4884	1,77591E+00	8988	3,60931E+00	3358	8,85258E-01	36878	0	54420,5
Myeloid_vs_CD4_Non-responder	Mast	CD4(TGFB1+ Th17)	ADCYAP1	ADRB2	4,43527E-03	41958,5	9,93026E-02	87	2,82526E+00	1430	1,24349E+00	56315	7,72191E-01	97540	0	54420,5

Myeloid_vs_CD4_Non-responder	Macro_ISG15	CD4(ISG+ Treg)	CCL2	CCR4	4,44371E-03	25693,1	3,51389E-02	775	1,92798E+00	6869	3,25464E+00	5956	8,42722E-01	60445	0	54420,5
Myeloid_vs_CD4_Non-responder	CD4(GZMK+ Teff)	Macro_OLFML3	HLA-F	LILRB1	4,44563E-03	34437,9	4,22747E-03	30792	1,46685E+00	16199	1,71349E+00	36899	8,90677E-01	33879	0	54420,5
Myeloid_vs_CD4_Non-responder	Mono_INHBA	CD4(TNFRSF9+ Treg)	SPP1	CCR8	4,45129E-03	21688,1	1,19477E-01	53	1,92778E+00	6873	3,34412E+00	5182	8,76002E-01	41912	0	54420,5
Myeloid_vs_CD4_Non-responder	CD4(IFNG+ Tfh/Th1)	Macro_FOLR2+APOE+	ADAM10	GPNMB	4,46684E-03	26306,1	7,85498E-03	12396	2,05087E+00	5559	2,71416E+00	12455	8,67330E-01	46700	0	54420,5
Myeloid_vs_CD4_Non-responder	CD4(CRTAM- T)	Mono_INHBA	TGFB1	SDC2	4,50271E-03	30765,9	1,53193E-02	3902	1,92547E+00	6900	1,47158E+00	46350	8,75339E-01	42257	0	54420,5
Myeloid_vs_CD4_Non-responder	Macro_FOLR2+APOE+	CD4(ISG+ Treg)	CXCL9	CXCR3	4,50438E-03	20473,5	2,03387E-02	2280	2,96373E+00	1105	3,08759E+00	7566	8,85039E-01	36996	0	54420,5
Myeloid_vs_CD4_Non-responder	Macro_NLRP3	CD4(IFNG+ Tfh/Th1)	CD86	CTLA4	4,51108E-03	32243,9	8,53277E-03	10854	1,17137E+00	27376	1,71047E+00	37007	8,94892E-01	31562	0	54420,5
Myeloid_vs_CD4_Non-responder	Macro_LYVE1	CD4(ISG+ Treg)	HLA-DRB1	LAG3	4,51978E-03	22765,1	7,91664E-03	12254	1,41274E+00	17880	2,50746E+00	15987	9,29010E-01	13284	0	54420,5
Myeloid_vs_CD4_Non-responder	Macro_NLRP3	CD4(ISG+ Treg)	VEGFA	CD44	4,52450E-03	25554,9	7,75196E-03	12637	2,21501E+00	4151	1,70992E+00	37029	9,16470E-01	19537	0	54420,5
Myeloid_vs_CD4_Non-responder	Mono_CD14	CD4(CRTAM- T)	VCAN	ITGA4	4,53161E-03	18263,9	1,21383E-02	5986	3,13988E+00	806	2,73089E+00	12215	9,19815E-01	17892	0	54420,5
Myeloid_vs_CD4_Non-responder	CD4(IL26+ Th17)	Mono_CD16	HLA-C	LILRB1	4,53184E-03	28065,5	4,23081E-03	30747	1,63878E+00	11670	1,70964E+00	37041	9,42790E-01	6449	0	54420,5
Myeloid_vs_CD4_Non-responder	Mast	CD4(IL26+ Th17)	ADCYAP1	DPP4	4,53410E-03	41925,5	1,44622E-01	34	2,81989E+00	1446	1,23969E+00	56500	7,72824E-01	97227	0	54420,5
Myeloid_vs_CD4_Non-responder	Macro_FOLR2+APOE+	CD4(TGFB1+ Th17)	GRN	TNFRSF1B	4,53551E-03	23375,9	3,72989E-03	37047	1,72472E+00	9910	2,99740E+00	8586	9,41806E-01	6916	0	54420,5
Myeloid_vs_CD4_Non-responder	Macro_FOLR2+APOE+	CD4(AREG+ Tm)	LGALS9	CD44	4,53673E-03	31449,7	3,72982E-03	37049	1,32353E+00	21017	1,82511E+00	33144	9,32504E-01	11618	0	54420,5
Myeloid_vs_CD4_Non-responder	CD4(GZMK+ Teff)	Macro_ISG15	CIRBP	TREM1	4,55759E-03	31453,3	5,57978E-03	20485	1,37480E+00	19175	1,70859E+00	37083	9,04485E-01	26103	0	54420,5
Myeloid_vs_CD4_Non-responder	Macro_NLRP3	CD4(Tn)	CCL3	CCR4	4,56005E-03	22560,1	1,44349E-02	4346	1,81579E+00	8339	2,99597E+00	8608	8,84864E-01	37087	0	54420,5
Myeloid_vs_CD4_Non-responder	Macro_ISG15	CD4(TNFRSF9+ Treg)	CD86	CTLA4	4,56127E-03	19483,3	1,54616E-02	3843	1,94404E+00	6682	2,58403E+00	14549	9,19749E-01	17922	0	54420,5
Myeloid_vs_CD4_Non-responder	CD4(IL26+ Th17)	Macro_FOLR2+APOE+	HLA-A	LILRB2	4,57914E-03	26837,5	3,72547E-03	37118	1,69932E+00	10400	1,93789E+00	29650	9,51595E-01	2599	0	54420,5
Myeloid_vs_CD4_Non-responder	CD4(ISG+ Treg)	Macro_FOLR2+APOE+	HLA-C	LILRB2	4,58160E-03	25645,9	3,72527E-03	37122	1,80442E+00	8524	2,08498E+00	25473	9,51340E-01	2690	0	54420,5
Myeloid_vs_CD4_Non-responder	CD4(TGFB1+ Th17)	Mono_CD16	HLA-C	LILRA1	4,58840E-03	22174,3	1,09218E-02	7189	2,35781E+00	3302	1,70689E+00	37133	9,38051E-01	8827	0	54420,5
Myeloid_vs_CD4_Non-responder	Macro_FOLR2+APOE+	CD4(IFNG+ Tfh/Th1)	PLAU	ITGB1	4,60201E-03	26810,1	1,12673E-02	6807	1,84359E+00	7924	2,00134E+00	27744	8,84729E-01	37155	0	54420,5
Myeloid_vs_CD4_Non-responder	Macro_FOLR2+APOE+	CD4(TNFRSF9+ Treg)	SPP1	CCR8	4,60267E-03	24850,9	8,55326E-02	123	1,48442E+00	15678	4,06986E+00	1457	8,56681E-01	52576	0	54420,5
Myeloid_vs_CD4_Non-responder	CD4(IFNG+ Tfh/Th1)	Mono_CD16	HLA-B	LILRA1	4,62504E-03	24407,7	1,00256E-02	8316	2,13221E+00	4835	1,48090E+00	45980	9,38711E-01	8487	0	54420,5
Myeloid_vs_CD4_Non-responder	Macro_FOLR2+APOE+	CD4(NME1+ T)	TNFSF13	FAS	4,62683E-03	28769,5	7,46692E-03	13405	1,48092E+00	15782	2,17882E+00	23045	8,84673E-01	37195	0	54420,5
Myeloid_vs_CD4_Non-responder	Macro_NLRP3	CD4(IL26+ Th17)	CXCL2	DPP4	4,62806E-03	22508,9	2,73374E-02	1284	2,52102E+00	2464	3,14583E+00	6965	8,66021E-01	47411	0	54420,5
Myeloid_vs_CD4_Non-responder	CD4(IFNG+ Tfh/Th1)	Mono_CD16	HLA-A	LILRA1	4,62904E-03	22923,3	1,10300E-02	7070	2,27661E+00	3764	1,60704E+00	40846	9,38654E-01	8516	0	54420,5
Myeloid_vs_CD4_Non-responder	CD4(IFNG+ Tfh/Th1)	Mono_CD16	HLA-C	LILRA1	4,63606E-03	23533,9	1,03968E-02	7807	2,23193E+00	4041	1,58305E+00	41819	9,36604E-01	9582	0	54420,5
Myeloid_vs_CD4_Non-responder	CD4(IFNG+ Tfh/Th1)	Mono_CD16	HLA-C	LILRA3	4,63807E-03	24263,9	1,23119E-02	5825	1,97960E+00	6297	1,62252E+00	40274	9,26564E-01	14503	0	54420,5
Myeloid_vs_CD4_Non-responder	Macro_FOLR2+APOE+	CD4(ISG+ Treg)	LYZ	ITGAL	4,64490E-03	26766,3	3,71745E-03	37224	1,65059E+00	11398	3,31969E+00	5379	9,05731E-01	25410	0	54420,5
Myeloid_vs_CD4_Non-responder	Mono_CD14	CD4(TNFRSF9+ Treg)	VCAN	ITGB1	4,64710E-03	18984,3	1,08880E-02	7221	3,06293E+00	919	2,59504E+00	14353	9,19570E-01	18008	0	54420,5
Myeloid_vs_CD4_Non-responder	CD4(TNFRSF9+ Treg)	pDC_LILRA4	TNF	TNFRSF21	4,65730E-03	26557,5	1,11070E-02	6980	2,58479E+00	2178	2,16812E+00	23347	8,68828E-01	45862	0	54420,5
Myeloid_vs_CD4_Non-responder	Macro_FOLR2+APOE+	CD4(CRTAM- T)	CXCL9	DPP4	4,66708E-03	29646,7	1,97022E-02	2415	2,94085E+00	1158	3,14248E+00	6985	8,00939E-01	83255	0	54420,5
Myeloid_vs_CD4_Non-responder	CD4(NME1+ T)	Macro_FOLR2+APOE+	HLA-A	LILRB2	4,66865E-03	26695,9	3,71571E-03	37262	1,69293E+00	10521	1,97027E+00	28658	9,51535E-01	2618	0	54420,5
Myeloid_vs_CD4_Non-responder	CD4(CRTAM- T)	Macro_ISG15	CCL5	CCR1	4,68275E-03	26765,5	2,14307E-02	2070	1,91657E+00	6993	1,64539E+00	39384	8,95977E-01	30960	0	54420,5
Myeloid_vs_CD4_Non-responder	Macro_NLRP3	CD4(ISG+ Treg)	VCAN	ITGA4	4,68873E-03	26980,1	5,40655E-03	21483	2,49794E+00	2559	2,35137E+00	19144	8,84470E-01	37294	0	54420,5
Myeloid_vs_CD4_Non-responder	CD4(GZMK+ Teff)	pDC_LILRA4	HLA-C	NOTCH4	4,69249E-03	30472,7	1,40138E-02	4607	1,53802E+00	14163	1,14198E+00	61120	9,19489E-01	18053	0	54420,5
Myeloid_vs_CD4_Non-responder	Mono_CD14	CD4(TNFRSF9+ Treg)	S100A9	ITGB2	4,69845E-03	19281,3	4,35641E-03	29501	2,83908E+00	1392	3,49363E+00	4092	9,41643E-01	7001	0	54420,5
Myeloid_vs_CD4_Non-responder	CD4(IL26+ Th17)	Macro_LYVE1	HMGB1	CD163	4,70006E-03	27616,1	4,51271E-03	27974	1,77680E+00	8972	1,70171E+00	37312	9,36957E-01	9402	0	54420,5
Myeloid_vs_CD4_Non-responder	CD4(TNFRSF9+ Treg)	Macro_OLFML3	HLA-B	LILRB1	4,70132E-03	28955,3	3,96608E-03	33808	1,57184E+00	13274	1,70166E+00	37314	9,43960E-01	5960	0	54420,5
Myeloid_vs_CD4_Non-responder	CD4(ISG+ Treg)	Macro_LYVE1	HMGB1	CD163	4,70384E-03	27543,3	4,53596E-03	27744	1,78143E+00	8897	1,70163E+00	37318	9,37109E-01	9337	0	54420,5
Myeloid_vs_CD4_Non-responder	CD4(TNFRSF9+ Treg)	Macro_FOLR2+APOE+	CD52	SIGLEC10	4,70667E-03	29754,5	6,08247E-03	18067	1,52132E+00	14616	1,51872E+00	44405	9,21012E-01	17264	0	54420,5
Myeloid_vs_CD4_Non-responder	Macro_OLFML3	CD4(AREG+ Tm)	CXCL10	CXCR3	4,71419E-03	24469,5	1,11913E-02	6890	2,66348E+00	1893	3,13999E+00	7009	8,57490E-01	52135	0	54420,5
Myeloid_vs_CD4_Non-responder	CD4(GZMK+ Teff)	Macro_FOLR2+APOE+	CD28	CD86	4,72341E-03	27995,3	9,39931E-03	9247,5	1,67316E+00	10912,5	1,99141E+00	28046,5	8,84375E-01	37349,5	0	54420,5
Myeloid_vs_CD4_Non-responder	Mono_INHBA	CD4(TNFRSF9+ Treg)	CXCL8	CD79A	4,72404E-03	27420,1	4,35411E-02	517	2,25327E+00	3901	3,13945E+00	7014	8,23298E-01	71248	0	54420,5
Myeloid_vs_CD4_Non-responder	Macro_FOLR2+APOE+	CD4(GZMK+ Teff)	CD86	CD28	4,72404E-03	27995,3	9,39931E-03	9247,5	1,67316E+00	10912,5	1,99141E+00	28046,5	8,84375E-01	37349,5	0	54420,5
Myeloid_vs_CD4_Non-responder	CD4(TNF+ T)	Macro_ISG15	HLA-A	LILRB2	4,73037E-03	27341,1	3,70875E-03	37360	1,64402E+00	11518	1,90111E+00	30777	9,51491E-01	2630	0	54420,5
Myeloid_vs_CD4_Non-responder	CD4(TNFRSF9+ Treg)	Mast	CALR	ITGA2B	4,73983E-03	48291,9	2,60570E-02	1424	1,91363E+00	7022	1,86697E-01	110817	8,29585E-01	67776	0	54420,5
Myeloid_vs_CD4_Non-responder	Macro_FOLR2+APOE+	CD4(TNFRSF9+ Treg)	SPP1	CD44	4,75638E-03	29325,9	5,79341E-03	19409	9,73020E-01	37401	2,87816E+00	10138	9,05967E-01	25261	0	54420,5
Myeloid_vs_CD4_Non-responder	CD4(ISG+ Treg)	Mono_CD14	HLA-F	LILRB2	4,75893E-03	34001,9	3,70568E-03	37405	1,43176E+00	17263	1,74823E+00	35712	9,06051E-01	25209	0	54420,5
Myeloid_vs_CD4_Non-responder	Macro_OLFML3	CD4(ISG+ Treg)	HLA-DRB1	LAG3	4,75964E-03	21548,3	7,87693E-03	12333	1,40527E+00	18119	2,92697E+00	9494	9,28844E-01	13375	0	54420,5
Myeloid_vs_CD4_Non-responder	Mono_CD14	CD4(AREG+ Tm)	VCAN	ITGB1	4,79039E-03	18854,3	1,08158E-02	7304	3,05999E+00	922	2,64749E+00	13476	9,19324E-01	18149	0	54420,5
Myeloid_vs_CD4_Non-responder	Macro_FOLR2+APOE+	CD4(Tn)	CD14	ITGB1	4,79082E-03	26799,9	3,70269E-03	37455	1,72485E+00	9906	2,73286E+00	12182	9,15469E-01	20036	0	54420,5
Myeloid_vs_CD4_Non-responder	CD4(GZMK+ Teff)	Macro_FOLR2+APOE+	CD52	SIGLEC10	4,79656E-03	29294,5	6,06222E-03	18155	1,51853E+00	14699	1,58201E+00	41866	9,20890E-01	17332	0	54420,5
Myeloid_vs_CD4_Non-responder	CD4(Tn)	Mono_CD16	TNFSF8	TNFRSF8	4,80501E-03	58389,5	2,55645E-02	1489	7,83064E-01	49272	8,48731E-01	75748	7,41230E-01	111018	0	54420,5
Myeloid_vs_CD4_Non-responder	CD4(TNF+ T)	pDC_LILRA4	CCL5	CXCR3	4,80619E-03	25885,5	2,38799E-02	1695	9,71521E-01	37479	2,36880E+00	18785	9,21485E-01	17048	0	54420,5
Myeloid_vs_CD4_Non-responder	CD4(TGFB1+ Th17)	pDC_LILRA4	CCL5	CXCR3	4,81004E-03	28237,7	2,38768E-02	1696	9,71437E-01	37485	1,90933E+00	30534	9,21480E-01	17053	0	54420,5
Myeloid_vs_CD4_Non-responder	CD4(GZMK+ Teff)	Macro_ISG15	CD28	CD86	4,81132E-03	27923,3	9,35455E-03	9322,5	1,66507E+00	11077,5	2,01682E+00	27308,5	8,84131E-01	37487,5	0	54420,5
Myeloid_vs_CD4_Non-responder	CD4(TNF+ T)	Macro_OLFML3	IL23A	LILRB2	4,81304E-03	31368,5	2,05482E-02	2244	1,64141E+00	11586	2,39748E+00	18171	8,24829E-01	70421	0	54420,5
Myeloid_vs_CD4_Non-responder	CD4(IL26+ Th17)	Mono_CD16	B2M	LILRB1	4,83189E-03	27400,1	4,21457E-03	30926	1,65210E+00	11361	1,69588E+00	37519	9,51113E-01	2774	0	54420,5
Myeloid_vs_CD4_Non-responder	CD4(IL26+ Th17)	Mono_CD16	HLA-B	LILRB1	4,84156E-03	27870,5	4,24122E-03	30645	1,63889E+00	11663	1,69556E+00	37534	9,45708E-01	5090	0	54420,5
Myeloid_vs_CD4_Non-responder	Macro_NLRP3	CD4(GZMK+ Teff)	THBS1	ITGA4	4,84349E-03	28957,1	5,54935E-03	20678	1,65907E+00	11214	2,26846E+00	20936	8,84026E-01	37537	0	54420,5
Myeloid_vs_CD4_Non-responder	CD4(CXCL13+ Tfh)	Macro_FOLR2+APOE+	HLA-A	LILRB2	4,86094E-03	27151,1	3,69414E-03	37564	1,67883E+00	10786	1,91601E+00	30322	9,51400E-01	2663	0	54420,5
Myeloid_vs_CD4_Non-responder	CD4(CRTAM- T)	Macro_OLFML3	ANXA1	FPR2_FPR3	4,86611E-03	28863,7	7,68416E-03	12834	1,45817E+00	16479	1,69450E+00	37572	9,09953E-01	23013	0	54420,5
Myeloid_vs_CD4_Non-responder	CD4(IL26+ Th17)	Macro_FOLR2+APOE+	HLA-B	LILRB2	4,86935E-03	25894,5	3,69309E-03	37577	1,77726E+00	8960	2,04010E+00	26648	9,53598E-01	1867	0	54420,5
Myeloid_vs_CD4_Non-responder	CD4(TNFRSF9+ Treg)	Macro_FOLR2+APOE+	ADAM10	TREM2	4,87104E-03	28195,9	6,04671E-03	18227	1,56099E+00	13573	2,65054E+00	13431	8,77034E-01	41328	0	54420,5
Myeloid_vs_CD4_Non-responder	CD4(TNFRSF9+ Treg)	Macro_NLRP3	RPS19	C5AR1	4,87324E-03	26781,9	4,84155E-03	25294	1,58631E+00	12924	1,69424E+00	37583	9,48688E-01	3688	0	54420,5
Myeloid_vs_CD4_Non-responder	CD4(CRTAM- T)	cDC_CLEC9A	TGFB1	LPP	4,87973E-03	33327,3	7,56199E-03	13151	1,07655E+00	31930	1,69397E+00	37593	8,98529E-01	29542	0	54420,5
Myeloid_vs_CD4_Non-responder	CD4(TNF+ T)	Macro_ISG15	HLA-F	LILRB1	4,88622E-03	34875,5	3,69222E-03	37599	1,64901E+00	11430	1,81992E+00	33325	8,83909E-01	37603	0	54420,5
Myeloid_vs_CD4_Non-responder	CD4(TGFB1+ Th17)	pDC_LILRA4	TGFB1	ACVR1_TGFBR2	4,89793E-03	34173,5	6,85533E-03	15168	9,68830E-01	37621	1,89367E+00	31004	8,92876E-01	32654	0	54420,5
Myeloid_vs_CD4_Non-responder	Macro_NLRP3	CD4(TNFRSF9+ Treg)	S100A9	ITGB2	4,89923E-03	22401,5	3,69059E-03	37623	2,38630E+00	3130	3,10151E+00	7411	9,36916E-01	9423	0	54420,5
Myeloid_vs_CD4_Non-responder	CD4(TNF+ T)	Mono_CD14	CD40LG	ITGAM_ITGB2	4,90705E-03	34839,7	9,32759E-03	9368	9,92099E-01	36364	1,69296E+00	37635	8,86123E-01	36411	0	54420,5

Myeloid_vs_CD4_Post_NR

Myeloid_vs_CD4_Non-responder	CD4(IFNG+ Tfh/Th1)	Macro_ISG15	HMGB1	HAVCR2	4,91357E-03	33134,7	4,31777E-03	29852	1,14064E+00	28760	1,69268E+00	37645	9,25575E-01	14996	0	54420,5
Myeloid_vs_CD4_Non-responder	CD4(GZMK+ Teff)	pDC_LILRA4	HMGB1	TLR9	4,91424E-03	24878,5	3,79948E-02	666	2,79918E+00	1506	1,55677E+00	42850	9,06522E-01	24950	0	54420,5
Myeloid_vs_CD4_Non-responder	Mono_INHBA	CD4(TNFRSF9+ Treg)	CCL3	CCR4	4,91986E-03	20515,9	1,76478E-02	3005	1,97369E+00	6370	3,12971E+00	7112	8,94712E-01	31672	0	54420,5
Myeloid_vs_CD4_Non-responder	Mono_CD14	CD4(GZMK+ Teff)	S100A9	ITGB2	4,91986E-03	19277,5	4,32627E-03	29768	2,83581E+00	1401	3,55669E+00	3686	9,41452E-01	7112	0	54420,5
Myeloid_vs_CD4_Non-responder	CD4(TGFB1+ Th17)	Macro_LYVE1	HMGB1	CD163	4,92010E-03	27452,1	4,58793E-03	27293	1,79180E+00	8725	1,69224E+00	37655	9,37443E-01	9167	0	54420,5
Myeloid_vs_CD4_Non-responder	CD4(CRTAM- T)	pDC_LILRA4	BST2	LILRA4	4,92221E-03	27828,5	6,03533E-03	18276	3,45973E+00	527	2,83207E+00	10778	8,52177E-01	55141	0	54420,5
Myeloid_vs_CD4_Non-responder	CD4(AREG+ Tm)	Macro_ISG15	CIRBP	TREM1	4,94039E-03	31577,5	5,57893E-03	20489	1,37468E+00	19183	1,69121E+00	37686	9,04478E-01	26109	0	54420,5
Myeloid_vs_CD4_Non-responder	Macro_FOLR2+APOE+	CD4(IL26+ Th17)	CD14	ITGB1	4,94039E-03	26866,7	3,68677E-03	37686	1,72325E+00	9939	2,73336E+00	12171	9,15302E-01	20117	0	54420,5
Myeloid_vs_CD4_Non-responder	Macro_NLRP3	CD4(CXCL13+ Tfh)	ICAM1	IL2RG	4,94104E-03	37918,5	3,71260E-03	37307	1,09332E+00	31055	1,69119E+00	37687	8,99170E-01	29123	0	54420,5
Myeloid_vs_CD4_Non-responder	CD4(GZMK+ Teff)	cDC_LAMP3	CD28	CD86	4,94432E-03	29519,3	9,65491E-03	8854,5	1,71935E+00	10012,5	1,69074E+00	37692,5	8,85739E-01	36616,5	0	54420,5
Myeloid_vs_CD4_Non-responder	CD4(AREG+ Tm)	Macro_NLRP3	HMGB1	THBD	4,94891E-03	30985,5	4,87574E-03	25045	1,51313E+00	14830	1,69065E+00	37699	9,10099E-01	22933	0	54420,5
Myeloid_vs_CD4_Non-responder	Mono_INHBA	CD4(IFNG+ Tfh/Th1)	HLA-DRA	LAG3	4,95162E-03	21842,5	9,00913E-03	9935	1,39992E+00	18304	2,51389E+00	15862	9,34375E-01	10691	0	54420,5
Myeloid_vs_CD4_Non-responder	Macro_OLFML3	CD4(ISG+ Treg)	HLA-DQB1	LAG3	4,95478E-03	21652,9	9,03486E-03	9881	1,50478E+00	15067	2,84452E+00	10589	9,18955E-01	18307	0	54420,5
Myeloid_vs_CD4_Non-responder	Macro_FOLR2+APOE+	CD4(NME1+ T)	SPP1	ITGA4_ITGB1	4,95482E-03	27456,5	8,32112E-03	11317	1,16068E+00	27864	3,25204E+00	5973	8,83727E-01	37708	0	54420,5
Myeloid_vs_CD4_Non-responder	CD4(TNF+ T)	Macro_LYVE1	HMGB1	CD163	4,96008E-03	28941,1	4,15253E-03	31629	1,70498E+00	10295	1,69036E+00	37716	9,34455E-01	10645	0	54420,5
Myeloid_vs_CD4_Non-responder	CD4(IL26+ Th17)	Macro_FOLR2+APOE+	HLA-C	LILRB2	4,96140E-03	26028,3	3,68402E-03	37718	1,77716E+00	8963	2,05417E+00	26252	9,51082E-01	2788	0	54420,5
Myeloid_vs_CD4_Non-responder	Mono_CD14	CD4(ISG+ Treg)	VCAN	CD44	4,96216E-03	18295,5	9,17442E-03	9630	2,98414E+00	1060	2,39108E+00	18314	9,39586E-01	8053	0	54420,5
Myeloid_vs_CD4_Non-responder	Macro_NLRP3	CD4(AREG+ Tm)	S100A8	ITGB2	4,96798E-03	23475,9	3,68318E-03	37728	2,38446E+00	3139	2,92567E+00	9500	9,30519E-01	12592	0	54420,5
Myeloid_vs_CD4_Non-responder	Macro_ISG15	CD4(GZMK+ Teff)	S100A8	CD69	4,96849E-03	24160,3	6,02523E-03	18320	1,40121E+00	18265	2,39802E+00	18159	9,32473E-01	11637	0	54420,5
Myeloid_vs_CD4_Non-responder	CD4(CRTAM- T)	Macro_ISG15	B2M	LILRB1	4,96995E-03	26774,1	4,47745E-03	28275	1,66003E+00	11188	1,68994E+00	37731	9,52501E-01	2256	0	54420,5
Myeloid_vs_CD4_Non-responder	CD4(IFNG+ Tfh/Th1)	Macro_OLFML3	HLA-A	LILRB1	4,97061E-03	28584,3	4,16951E-03	31438	1,58998E+00	12831	1,68993E+00	37732	9,42692E-01	6500	0	54420,5
Myeloid_vs_CD4_Non-responder	CD4(GZMK+ Teff)	Mono_CD16	HLA-A	LILRB1	4,98248E-03	27678,7	4,42217E-03	28828	1,64283E+00	11553	1,68946E+00	37750	9,44261E-01	5842	0	54420,5
Myeloid_vs_CD4_Non-responder	Mast	CD4(ISG+ Treg)	TIMP3	CD44	4,98556E-03	30278,3	2,97816E-02	1065	2,79469E+00	1517	1,01214E+00	67509	9,03107E-01	26880	0	54420,5
Myeloid_vs_CD4_Non-responder	Mono_INHBA	CD4(TNFRSF9+ Treg)	IL6	IL6R_IL6ST	4,98556E-03	28630,9	2,53503E-02	1517	3,11452E+00	837	1,99595E+00	27912	8,46197E-01	58468	0	54420,5
Myeloid_vs_CD4_Non-responder	CD4(TGFB1+ Th17)	Macro_ISG15	B2M	LILRB1	4,98710E-03	26931,9	4,45235E-03	28519	1,63942E+00	11650	1,68938E+00	37757	9,52374E-01	2313	0	54420,5
Myeloid_vs_CD4_Non-responder	Macro_FOLR2-APOE+	CD4(CXCL13+ Tfh)	C3	IFITM1	4,99105E-03	22255,5	1,14677E-02	6614	1,90301E+00	7147	1,68174E+00	38072	9,45831E-01	5024	0	54420,5
Myeloid_vs_CD4_Non-responder	Mast	CD4(CXCL13+ Tfh)	TIMP3	CD44	4,99858E-03	30575,1	2,97447E-02	1072	2,79384E+00	1519	9,82925E-01	68948	9,03052E-01	26916	0	54420,5
Myeloid_vs_CD4_Non-responder	CD4(AREG+ Tm)	Macro_FOLR2+APOE+	HLA-F	LILRB2	5,00165E-03	30738,3	3,68024E-03	37779	1,70660E+00	10257	2,07061E+00	25841	9,05757E-01	25394	0	54420,5
Myeloid_vs_CD4_Non-responder	Macro_FOLR2+APOE+	CD4(IFNG+ Tfh/Th1)	TNFSF13	FAS	5,01490E-03	28937,7	7,30966E-03	13808	1,47257E+00	16010	2,19436E+00	22651	8,83583E-01	37799	0	54420,5
Myeloid_vs_CD4_Non-responder	Mast	CD4(NME1+ T)	TIMP3	CD44	5,01814E-03	30542,1	2,97152E-02	1077	2,79316E+00	1522	9,86767E-01	68760	9,03009E-01	26931	0	54420,5
Myeloid_vs_CD4_Non-responder	CD4(GZMK+ Teff)	Macro_FOLR2+APOE+	HLA-B	LILRB2	5,02021E-03	25727,5	3,67860E-03	37807	1,76644E+00	9161	2,08952E+00	25348	9,53511E-01	1901	0	54420,5
Myeloid_vs_CD4_Non-responder	CD4(GZMK+ Teff)	Mono_CD16	ANXA1	FPR1	5,02619E-03	32578,3	3,67798E-03	37816	1,41988E+00	17627	1,73686E+00	36115	9,21754E-01	16913	0	54420,5
Myeloid_vs_CD4_Non-responder	CD4(TNF+ T)	Macro_FOLR2+APOE+	TNFSF14	LTBR	5,02999E-03	26941,5	2,76413E-02	1263	1,59139E+00	12796	2,38854E+00	18378	8,65193E-01	47850	0	54420,5
Myeloid_vs_CD4_Non-responder	Mono_INHBA	CD4(IL26+ Th17)	S100A8	CD69	5,03319E-03	18145,5	1,07819E-02	7339	1,92742E+00	6881	2,38847E+00	18381	9,48645E-01	3706	0	54420,5
Myeloid_vs_CD4_Non-responder	CD4(Tn)	Macro_ISG15	HLA-F	LILRB2	5,04616E-03	29473,1	3,67623E-03	37846	1,86440E+00	7649	2,22067E+00	22029	9,05711E-01	25421	0	54420,5
Myeloid_vs_CD4_Non-responder	CD4(GZMK+ Teff)	Macro_ISG15	HMGB1	HAVCR2	5,05349E-03	34338,3	4,10357E-03	32179	1,08702E+00	31379	1,68686E+00	37857	9,23804E-01	15856	0	54420,5
Myeloid_vs_CD4_Non-responder	CD4(TNFRSF9+ Treg)	Macro_OLFML3	B2M	LILRB1	5,05616E-03	28647,1	3,90653E-03	34539	1,58366E+00	12985	1,68668E+00	37861	9,49318E-01	3430	0	54420,5
Myeloid_vs_CD4_Non-responder	Mono_CD14	CD4(ISG+ Treg)	S100A8	ITGB2	5,05672E-03	17450,3	5,28577E-03	22192	3,11832E+00	831	3,75270E+00	2629	9,41327E-01	7179	0	54420,5
Myeloid_vs_CD4_Non-responder	Macro_FOLR2+APOE+	CD4(TNF+ T)	SPP1	CD44	5,05817E-03	29415,9	5,71898E-03	19786	9,64447E-01	37864	2,93330E+00	9414	9,05415E-01	25595	0	54420,5
Myeloid_vs_CD4_Non-responder	Macro_NLRP3	CD4(AREG+ Tm)	LGALS1	CD69	5,06686E-03	32514,1	4,34623E-03	29596	9,64220E-01	37877	1,70355E+00	37251	9,49338E-01	3426	0	54420,5
Myeloid_vs_CD4_Non-responder	Mono_CD14	CD4(CXCL13+ Tfh)	S100A8	ITGB2	5,06703E-03	17434,3	5,28306E-03	22214	3,11809E+00	832	3,77526E+00	2521	9,41313E-01	7184	0	54420,5
Myeloid_vs_CD4_Non-responder	CD4(GZMK+ Teff)	pDC_LILRA4	TNF	PTPRS	5,07705E-03	27741,7	2,52409E-02	1531	3,97994E+00	217	1,92235E+00	30117	8,56970E-01	52423	0	54420,5
Myeloid_vs_CD4_Non-responder	Macro_FOLR2+APOE+	CD4(ISG+ Treg)	HLA-DQA2	LAG3	5,07757E-03	27854,3	8,34269E-03	11257	1,28518E+00	22443	2,66223E+00	13258	8,83411E-01	37893	0	54420,5
Myeloid_vs_CD4_Non-responder	CD4(IFNG+ Tfh/Th1)	Macro_FOLR2+APOE+	HLA-F	LILRB1	5,07757E-03	36027,7	4,03320E-03	32971	1,36054E+00	19688	1,68594E+00	37893	8,88365E-01	35166	0	54420,5
Myeloid_vs_CD4_Non-responder	Macro_NLRP3	CD4(TNFRSF9+ Treg)	VEGFA	CD44	5,08494E-03	25961,3	7,55811E-03	13164	2,19790E+00	4294	1,68578E+00	37904	9,15496E-01	20024	0	54420,5
Myeloid_vs_CD4_Non-responder	Macro_NLRP3	CD4(TNFRSF9+ Treg)	HLA-DRA	LAG3	5,09031E-03	35869,7	3,85943E-03	35203	9,63591E-01	37912	2,10490E+00	24921	9,03092E-01	26892	0	54420,5
Myeloid_vs_CD4_Non-responder	CD4(GZMK+ Teff)	Macro_FOLR2+APOE+	HLA-F	LILRB1	5,10375E-03	36934,5	3,85231E-03	35284	1,33530E+00	20596	1,68493E+00	37932	8,86070E-01	36440	0	54420,5
Myeloid_vs_CD4_Non-responder	CD4(IL26+ Th17)	Macro_FOLR2+APOE+	B2M	LILRB2	5,10712E-03	25678,5	3,66988E-03	37937	1,79048E+00	8755	2,04041E+00	26640	9,58253E-01	640	0	54420,5
Myeloid_vs_CD4_Non-responder	Macro_NLRP3	CD4(NME1+ T)	VEGFA	CD44	5,11924E-03	25761,9	7,73466E-03	12680	2,21349E+00	4166	1,68455E+00	37955	9,16385E-01	19588	0	54420,5
Myeloid_vs_CD4_Non-responder	CD4(TGFB1+ Th17)	Macro_LYVE1	TGFB1	ENG	5,12127E-03	28271,5	7,83433E-03	12443	1,54342E+00	14019	1,68446E+00	37958	9,10910E-01	22517	0	54420,5
Myeloid_vs_CD4_Non-responder	CD4(IL26+ Th17)	cDC_LAMP3	LTB	CD40	5,12869E-03	23540,3	1,33464E-02	5016	2,05639E+00	5511	1,68417E+00	37969	9,25997E-01	14785	0	54420,5
Myeloid_vs_CD4_Non-responder	CD4(NME1+ T)	pDC_LILRA4	HLA-C	NOTCH4	5,13733E-03	31302,7	1,36780E-02	4794	1,48209E+00	15745	1,10233E+00	63076	9,18586E-01	18478	0	54420,5
Myeloid_vs_CD4_Non-responder	CD4(AREG+ Tm)	Macro_ISG15	HLA-C	LILRB1	5,14018E-03	27979,5	4,34537E-03	29609	1,63506E+00	11732	1,68381E+00	37986	9,43506E-01	6150	0	54420,5
Myeloid_vs_CD4_Non-responder	Macro_FOLR2-APOE+	CD4(Tn)	APOE	LSR	5,14376E-03	18680,9	2,19855E-02	1963	2,05861E+00	5497	3,11891E+00	7221	9,07665E-01	24303	0	54420,5
Myeloid_vs_CD4_Non-responder	Macro_FOLR2+APOE+	CD4(TNFRSF9+ Treg)	CXCL9	CXCR3	5,14966E-03	20753,3	1,96143E-02	2438	2,95102E+00	1136	3,06808E+00	7772	8,83181E-01	38000	0	54420,5
Myeloid_vs_CD4_Non-responder	CD4(IFNG+ Tfh/Th1)	Macro_FOLR2+APOE+	IFNG	IFNGR1_IFNGR2	5,15916E-03	29879,7	1,56656E-02	3746	1,23853E+00	24406	1,96474E+00	28812	8,83151E-01	38014	0	54420,5
Myeloid_vs_CD4_Non-responder	Macro_NLRP3	CD4(GZMK+ Teff)	S100A9	ITGB2	5,16255E-03	22377,7	3,66506E-03	38019	2,38303E+00	3147	3,16456E+00	6766	9,36710E-01	9536	0	54420,5
Myeloid_vs_CD4_Non-responder	CD4(IL26+ Th17)	pDC_LILRA4	CCL20	CXCR3	5,17070E-03	29262,1	2,38004E-02	1708	9,61724E-01	38031	2,30475E+00	20132	8,94048E-01	32019	0	54420,5
Myeloid_vs_CD4_Non-responder	CD4(TNFRSF9+ Treg)	Macro_FOLR2+APOE+	HLA-C	LILRB2	5,17274E-03	26312,7	3,66397E-03	38034	1,76390E+00	9206	2,02533E+00	27072	9,50955E-01	2831	0	54420,5
Myeloid_vs_CD4_Non-responder	Mono_INHBA	CD4(ISG+ Treg)	CCL3	CCR4	5,17718E-03	19721,5	1,97940E-02	2393	2,01894E+00	5881	3,11762E+00	7237	8,99996E-01	28676	0	54420,5
Myeloid_vs_CD4_Non-responder	Macro_LYVE1	CD4(IFNG+ Tfh/Th1)	F13A1	ITGB1	5,18074E-03	19391,7	1,29831E-02	5288	2,74069E+00	1650	2,38260E+00	18518	9,21420E-01	17082	0	54420,5
Myeloid_vs_CD4_Non-responder	Macro_FOLR2+APOE+	CD4(TNFRSF9+ Treg)	C1QB	C1QBP	5,21709E-03	23817,9	3,66029E-03	38099	2,08998E+00	5214	3,67838E+00	2991	9,18819E-01	18365	0	54420,5
Myeloid_vs_CD4_Non-responder	CD4(TNFRSF9+ Treg)	Mono_CD16	HLA-C	LILRB1	5,22463E-03	28401,9	4,20778E-03	30998	1,62553E+00	11961	1,68080E+00	38110	9,42642E-01	6520	0	54420,5
Myeloid_vs_CD4_Non-responder	Macro_NLRP3	CD4(CXCL13+ Tfh)	VEGFA	CD44	5,22737E-03	25784,1	7,74234E-03	12663	2,21416E+00	4159	1,68070E+00	38114	9,16423E-01	19564	0	54420,5
Myeloid_vs_CD4_Non-responder	CD4(IFNG+ Tfh/Th1)	cDC(CD1C)	B2M	CD1A	5,22880E-03	26015,5	2,90375E-02	1122	1,87243E+00	7552	1,32902E+00	52495	9,26600E-01	14488	0	54420,5
Myeloid_vs_CD4_Non-responder	Macro_FOLR2+APOE+	CD4(AREG+ Tm)	LGALS9	PTPRC	5,23217E-03	33052,9	3,68177E-03	37755	1,21835E+00	25262	1,68043E+00	38121	9,36337E-01	9706	0	54420,5
Myeloid_vs_CD4_Non-responder	CD4(CRTAM- T)	Macro_FOLR2+APOE+	HLA-A	LILRB2	5,23835E-03	27631,7	3,65812E-03	38130	1,65526E+00	11294	1,87504E+00	31560	9,51173E-01	2754	0	54420,5
Myeloid_vs_CD4_Non-responder	CD4(NME1+ T)	Macro_ISG15	HMGB1	TLR2	5,24729E-03	33489,3	4,13122E-03	31876	1,27779E+00	22723	1,67980E+00	38143	9,14960E-01	20284	0	54420,5
Myeloid_vs_CD4_Non-responder	Macro_FOLR2+APOE+	CD4(TNF+ T)	LGALS3BP	ITGB1	5,25211E-03	35748,7	5,27421E-03	22268	1,20084E+00	26033	1,68635E+00	37872	8,82897E-01	38150	0	54420,5
Myeloid_vs_CD4_Non-responder	CD4(ISG+ Treg)	Macro_OLFML3	LTB	CD40	5,25295E-03	27328,1	1,07765E-02	7344	1,61996E+00	12095	1,52379E+00	44197	9,18327E-01	18584	0	54420,5
Myeloid_vs_CD4_Non-responder	Macro_FOLR2+APOE+	CD4(TNFRSF9+ Treg)	CCL18	CCR8	5,26344E-03	28277,3	1,39899E-01	39	1,89186E+00	7278	3,39086E+00	4793	8,16641E-01	74856	0	54420,5

Myeloid_vs_CD4_Post_NR

Myeloid_vs_CD4_Non-responder	Mast	CD4(TNFRSF9+ Treg)	TIMP3	CD44	5,27586E-03	30670,5	2,90369E-02	1123	2,77757E+00	1561	9,88002E-01	68700	9,01993E-01	27548	0	54420,5
Myeloid_vs_CD4_Non-responder	CD4(AREG+ Tm)	Macro_ISG15	B2M	LILRB1	5,29007E-03	27054,7	4,44658E-03	28584	1,63469E+00	11738	1,67796E+00	38205	9,52344E-01	2326	0	54420,5
Myeloid_vs_CD4_Non-responder	Macro_FOLR2+APOE+	CD4(TGFB1+ Th17)	CXCL16	CXCR6	5,29839E-03	30418,9	6,62102E-03	15971	1,39849E+00	18355	2,09773E+00	25131	8,82759E-01	38217	0	54420,5
Myeloid_vs_CD4_Non-responder	Macro_NLRP3	CD4(ISG+ Treg)	VEGFA	ITGB1	5,30047E-03	28151,9	8,01486E-03	12012	2,23656E+00	4013	1,85823E+00	32094	8,82745E-01	38220	0	54420,5
Myeloid_vs_CD4_Non-responder	CD4(TGFB1+ Th17)	pDC_LILRA4	HRAS	TLR9	5,30265E-03	41965,1	6,83393E-02	212	2,77519E+00	1565	1,67690E+00	38245	7,29806E-01	115383	0	54420,5
Myeloid_vs_CD4_Non-responder	Macro_FOLR2+APOE+	CD4(TNFRSF9+ Treg)	LGALS3BP	ITGB1	5,30394E-03	33618,5	6,11201E-03	17944	1,26176E+00	23400	1,67733E+00	38225	8,90306E-01	34103	0	54420,5
Myeloid_vs_CD4_Non-responder	CD4(CXCL13+ Tfh)	Macro_ISG15	HLA-C	LILRB2	5,31644E-03	26846,9	3,65088E-03	38243	1,71221E+00	10139	1,97328E+00	28576	9,50871E-01	2856	0	54420,5
Myeloid_vs_CD4_Non-responder	CD4(TGFB1+ Th17)	Macro_ISG15	CCL5	CCR1	5,32409E-03	25380,1	2,52294E-02	1535	2,01337E+00	5945	1,67658E+00	38254	9,03339E-01	26746	0	54420,5
Myeloid_vs_CD4_Non-responder	CD4(IL26+ Th17)	Macro_IER3	RPS19	C5AR1	5,33174E-03	28510,3	4,39027E-03	29148	1,47687E+00	15896	1,67618E+00	38265	9,46254E-01	4822	0	54420,5
Myeloid_vs_CD4_Non-responder	cDC_CLEC9A	CD4(IFNG+ Tfh/Th1)	CXCL16	CXCR6	5,33924E-03	20595,9	1,47046E-02	4215	1,56988E+00	13334	2,72133E+00	12348	9,18173E-01	18662	0	54420,5
Myeloid_vs_CD4_Non-responder	Macro_ISG15	CD4(ISG+ Treg)	CD86	CTLA4	5,34480E-03	20004,5	1,61802E-02	3520	1,97703E+00	6327	2,37412E+00	18667	9,21410E-01	17088	0	54420,5
Myeloid_vs_CD4_Non-responder	cDC(CD1C)	CD4(IFNG+ Tfh/Th1)	HLA-DQA2	LAG3	5,34814E-03	17628,1	1,82903E-02	2820	2,12973E+00	4852	3,10420E+00	7378	9,18162E-01	18670	0	54420,5
Myeloid_vs_CD4_Non-responder	Mono_INHBA	CD4(ISG+ Treg)	IL6	IL6R_IL6ST	5,34969E-03	29061,9	2,48572E-02	1572	3,11066E+00	846	1,94979E+00	29296	8,44914E-01	59175	0	54420,5
Myeloid_vs_CD4_Non-responder	Macro_FOLR2+APOE+	CD4(CXCL13+ Tfh)	CCL3	CCR4	5,37999E-03	23158,9	1,37862E-02	4736	1,65888E+00	11217	3,13237E+00	7087	8,82501E-01	38334	0	54420,5
Myeloid_vs_CD4_Non-responder	Macro_NLRP3	CD4(TNF+ T)	ICAM1	IL2RG	5,39896E-03	38013,9	3,64403E-03	38361	1,08593E+00	31446	1,73419E+00	36196	8,98321E-01	29646	0	54420,5
Myeloid_vs_CD4_Non-responder	Macro_IFI27	CD4(IFNG+ Tfh/Th1)	HLA-DQB1	LAG3	5,40407E-03	21116,9	1,18734E-02	6225	1,59529E+00	12691	2,37203E+00	18720	9,28564E-01	13528	0	54420,5
Myeloid_vs_CD4_Non-responder	Macro_ISG15	CD4(TGFB1+ Th17)	VCAN	CD44	5,41081E-03	23537,3	5,93227E-03	18726	1,53178E+00	14325	2,53731E+00	15412	9,25959E-01	14803	0	54420,5
Myeloid_vs_CD4_Non-responder	Macro_ISG15	CD4(IFNG+ Tfh/Th1)	HLA-DPA1	LAG3	5,41193E-03	19967,1	9,53419E-03	9027	1,38827E+00	18727	3,12787E+00	7128	9,34698E-01	10533	0	54420,5
Myeloid_vs_CD4_Non-responder	Macro_ISG15	CD4(IFNG+ Tfh/Th1)	HLA-DQA1	LAG3	5,41306E-03	21360,9	1,01090E-02	8185	1,38826E+00	18728	3,11633E+00	7257	9,19183E-01	18214	0	54420,5
Myeloid_vs_CD4_Non-responder	Mast	CD4(TNF+ T)	TIMP3	CD44	5,41724E-03	30210,5	2,86639E-02	1157	2,76900E+00	1582	1,04314E+00	66005	9,01420E-01	27888	0	54420,5
Myeloid_vs_CD4_Non-responder	CD4(IFNG+ Tfh/Th1)	Macro_OLFML3	HLA-F	LILRB2	5,41940E-03	32660,9	3,64146E-03	38390	1,52608E+00	14490	1,91521E+00	30348	9,05304E-01	25656	0	54420,5
Myeloid_vs_CD4_Non-responder	CD4(IFNG+ Tfh/Th1)	cDC_CLEC9A	HMGB1	THBD	5,42152E-03	30962,7	5,06944E-03	23617	1,46498E+00	16268	1,67330E+00	38393	9,11680E-01	22115	0	54420,5
Myeloid_vs_CD4_Non-responder	Macro_FOLR2+APOE+	CD4(CRTAM- T)	APOE	LSR	5,42364E-03	22206,1	1,28046E-02	5456	1,76292E+00	9218	3,58066E+00	3540	8,82380E-01	38396	0	54420,5
Myeloid_vs_CD4_Non-responder	CD4(AREG+ Tm)	pDC_LILRA4	HLA-C	NOTCH4	5,43783E-03	32391,9	1,34814E-02	4921	1,44934E+00	16757	1,02063E+00	67111	9,18043E-01	18750	0	54420,5
Myeloid_vs_CD4_Non-responder	Macro_FOLR2+APOE+	CD4(TNFRSF9+ Treg)	SECTM1	CD7	5,44061E-03	29136,3	1,38064E-02	4722	1,40186E+00	18252	1,67266E+00	38420	8,97939E-01	29867	0	54420,5
Myeloid_vs_CD4_Non-responder	CD4(CXCL13+ Tfh)	pDC_LILRA4	SIRPG	CD47	5,44132E-03	32237,1	1,17030E-02	6393,5	9,55239E-01	38374,5	2,15958E+00	23575,5	8,82340E-01	38421,5	0	54420,5
Myeloid_vs_CD4_Non-responder	CD4(ISG+ Treg)	Macro_FOLR2+APOE+	B2M	LILRB1	5,44203E-03	29445,9	3,63909E-03	38422	1,53974E+00	14113	1,73789E+00	36075	9,47585E-01	4199	0	54420,5
Myeloid_vs_CD4_Non-responder	CD4(AREG+ Tm)	Mono_CD16	CD52	SIGLEC10	5,44912E-03	29698,5	5,92513E-03	18760	1,39664E+00	18427	1,65236E+00	39112	9,20053E-01	17773	0	54420,5
Myeloid_vs_CD4_Non-responder	Macro_FOLR2+APOE+	CD4(IFNG+ Tfh/Th1)	HLA-DQB2	LAG3	5,45408E-03	25843,7	1,52876E-02	3922	1,43543E+00	17155	2,54496E+00	15282	8,82305E-01	38439	0	54420,5
Myeloid_vs_CD4_Non-responder	CD4(NME1+ T)	Macro_FOLR2+APOE+	CALR	LRP1	5,45479E-03	34311,1	3,63766E-03	38440	1,38139E+00	18950	1,69994E+00	37377	9,11216E-01	22368	0	54420,5
Myeloid_vs_CD4_Non-responder	CD4(TNF+ T)	cDC(CD1C)	B2M	CD1A	5,45929E-03	32707,9	2,55333E-02	1493	1,38707E+00	18769	9,19943E-01	72136	9,22105E-01	16721	0	54420,5
Myeloid_vs_CD4_Non-responder	CD4(IFNG+ Tfh/Th1)	Macro_FOLR2+APOE+	B2M	LILRB1	5,46188E-03	29487,9	3,63727E-03	38450	1,53772E+00	14172	1,73436E+00	36190	9,47573E-01	4207	0	54420,5
Myeloid_vs_CD4_Non-responder	CD4(IFNG+ Tfh/Th1)	Macro_FOLR2+APOE+	HLA-B	LILRB2	5,46544E-03	26410,3	3,63676E-03	38455	1,73521E+00	9707	2,00993E+00	27493	9,53257E-01	1976	0	54420,5
Myeloid_vs_CD4_Non-responder	CD4(TNF+ T)	Macro_IER3	IFNG	IFNGR1_IFNGR2	5,46686E-03	31834,9	1,54029E-02	3868	1,03686E+00	33987	1,97828E+00	28442	8,82275E-01	38457	0	54420,5
Myeloid_vs_CD4_Non-responder	CD4(TNF+ T)	Macro_IER3	RPS19	C5AR1	5,47326E-03	25650,3	5,27195E-03	22278	1,71053E+00	10169	1,67138E+00	38466	9,50722E-01	2918	0	54420,5
Myeloid_vs_CD4_Non-responder	CD4(TGFB1+ Th17)	Macro_FOLR2+APOE+	ANXA1	FPR2_FPR3	5,47682E-03	25711,3	1,02465E-02	7989	1,69749E+00	10439	1,67125E+00	38471	9,21068E-01	17237	0	54420,5
Myeloid_vs_CD4_Non-responder	Macro_NLRP3	CD4(TNF+ T)	VEGFA	ITGB1	5,47753E-03	27839,9	7,93879E-03	12179	2,23289E+00	4036	1,92289E+00	30092	8,82250E-01	38472	0	54420,5
Myeloid_vs_CD4_Non-responder	Mono_INHBA	CD4(AREG+ Tm)	SPP1	CD44	5,47968E-03	21766,7	1,00608E-02	8253	1,57867E+00	13093	2,36879E+00	18787	9,26988E-01	14280	0	54420,5
Myeloid_vs_CD4_Non-responder	Mono_CD14	CD4(TNFRSF9+ Treg)	VCAN	CD44	5,50809E-03	18558,3	8,94500E-03	10040	2,96703E+00	1100	2,36694E+00	18812	9,38864E-01	8419	0	54420,5
Myeloid_vs_CD4_Non-responder	CD4(TNF+ T)	Macro_ISG15	ANXA1	FPR1	5,51321E-03	32941,1	3,63244E-03	38522	1,25753E+00	23605	1,89313E+00	31023	9,21304E-01	17135	0	54420,5
Myeloid_vs_CD4_Non-responder	Macro_NLRP3	CD4(GZMK+ Teff)	ICAM1	IL2RG	5,51751E-03	37949,7	3,63181E-03	38528	1,08461E+00	31509	1,75353E+00	35555	8,98168E-01	29736	0	54420,5
Myeloid_vs_CD4_Non-responder	CD4(ISG+ Treg)	pDC_LILRA4	HLA-F	LILRB1	5,51823E-03	38894,1	3,63173E-03	38529	1,15077E+00	28337	1,76641E+00	35117	8,83059E-01	38067	0	54420,5
Myeloid_vs_CD4_Non-responder	Macro_LYVE1	CD4(CRTAM- T)	F13A1	ITGB1	5,52633E-03	19351,7	1,31997E-02	5119	2,75090E+00	1625	2,36615E+00	18828	9,22017E-01	16766	0	54420,5
Myeloid_vs_CD4_Non-responder	Mono_CD14	CD4(NME1+ T)	VCAN	CD44	5,54004E-03	18415,3	9,15395E-03	9665	2,98261E+00	1065	2,36571E+00	18840	9,39523E-01	8086	0	54420,5
Myeloid_vs_CD4_Non-responder	Macro_ISG15	CD4(TNFRSF9+ Treg)	CCL2	CCR4	5,54092E-03	26672,3	3,13289E-02	968	1,88273E+00	7407	3,26673E+00	5846	8,34966E-01	64720	0	54420,5
Myeloid_vs_CD4_Non-responder	CD4(CRTAM- T)	Macro_ISG15	TGFB1	SDC2	5,54748E-03	28300,3	1,48610E-02	4139	1,88261E+00	7410	1,84979E+00	32358	8,73672E-01	43174	0	54420,5
Myeloid_vs_CD4_Non-responder	CD4(IFNG+ Tfh/Th1)	Macro_ISG15	CCL4	CCR1	5,55989E-03	25668,9	9,51412E-03	9056	1,97661E+00	6330	2,31293E+00	19951	8,82033E-01	38587	0	54420,5
Myeloid_vs_CD4_Non-responder	CD4(Tn)	cDC(CD1C)	MIF	CD74_CXCR4	5,56046E-03	53807,9	1,88641E-03	82502	5,68694E-01	66586	1,08497E+00	63928	9,54441E-01	1603	0	54420,5
Myeloid_vs_CD4_Non-responder	CD4(ISG+ Treg)	Macro_FOLR2+APOE+	B2M	TFRC	5,56061E-03	33981,7	3,78818E-03	36186	9,51498E-01	38588	1,69989E+00	37381	9,49578E-01	3333	0	54420,5
Myeloid_vs_CD4_Non-responder	Macro_FOLR2+APOE+	CD4(IL26+ Th17)	CD86	CTLA4	5,56781E-03	32080,5	6,57935E-03	16135	1,54298E+00	14030	1,70431E+00	37219	8,82023E-01	38598	0	54420,5
Myeloid_vs_CD4_Non-responder	CD4(GZMK+ Teff)	cDC_CLEC9A	HMGB1	THBD	5,56854E-03	31923,7	4,81795E-03	25465	1,41136E+00	17914	1,66748E+00	38599	9,09610E-01	23220	0	54420,5
Myeloid_vs_CD4_Non-responder	CD4(CXCL13+ Tfh)	Macro_FOLR2+APOE+	CALR	LRP1	5,57287E-03	34041,9	3,62768E-03	38605	1,37943E+00	19014	1,74715E+00	35747	9,11105E-01	22423	0	54420,5
Myeloid_vs_CD4_Non-responder	CD4(TNF+ T)	Macro_FOLR2+APOE+	LTB	CD40	5,57720E-03	27870,3	9,20958E-03	9571	1,50843E+00	14946	1,66720E+00	38611	9,12237E-01	21803	0	54420,5
Myeloid_vs_CD4_Non-responder	Macro_FOLR2+APOE+	CD4(ISG+ Treg)	SECTM1	CD7	5,60325E-03	27950,1	1,52324E-02	3954	1,49274E+00	15404	1,66602E+00	38647	9,02356E-01	27325	0	54420,5
Myeloid_vs_CD4_Non-responder	CD4(IFNG+ Tfh/Th1)	Macro_OLFML3	HLA-C	LILRB1	5,60542E-03	29746,7	3,93016E-03	34234	1,54530E+00	13977	1,66594E+00	38650	9,40769E-01	7452	0	54420,5
Myeloid_vs_CD4_Non-responder	CD4(IFNG+ Tfh/Th1)	Macro_FOLR2+APOE+	CD40LG	CD9	5,60666E-03	27005,7	1,27546E-02	5501	1,38292E+00	18898	2,43507E+00	17412	8,81672E-01	38797	0	54420,5
Myeloid_vs_CD4_Non-responder	CD4(IFNG+ Tfh/Th1)	pDC_LILRA4	HLA-C	NOTCH4	5,61358E-03	27921,1	1,49669E-02	4078	1,69677E+00	10450	1,29964E+00	53827	9,21891E-01	16830	0	54420,5
Myeloid_vs_CD4_Non-responder	CD4(IFNG+ Tfh/Th1)	Mono_CD16	HLA-B	LILRB1	5,61631E-03	28476,5	4,17653E-03	31353	1,59684E+00	12658	1,66539E+00	38665	9,45312E-01	5286	0	54420,5
Myeloid_vs_CD4_Non-responder	CD4(IFNG+ Tfh/Th1)	Macro_ISG15	HMGB1	THBD	5,62794E-03	37815,1	3,75096E-03	36723	1,11692E+00	29876	1,66510E+00	38681	8,98777E-01	29375	0	54420,5
Myeloid_vs_CD4_Non-responder	Mono_CD16	CD4(Tn)	VCAN	SELL	5,63012E-03	30297,5	7,50582E-03	13312	1,26849E+00	23121	2,07101E+00	25827	8,89075E-01	34807	0	54420,5
Myeloid_vs_CD4_Non-responder	CD4(Tn)	Mono_CD16	HLA-F	LILRB1	5,63158E-03	36218,5	3,78052E-03	36287	1,51678E+00	14742	1,66500E+00	38686	8,85116E-01	36957	0	54420,5
Myeloid_vs_CD4_Non-responder	Mono_CD14	CD4(CXCL13+ Tfh)	VCAN	CD44	5,63324E-03	18425,5	9,16304E-03	9649	2,98329E+00	1063	2,36187E+00	18921	9,39551E-01	8074	0	54420,5
Myeloid_vs_CD4_Non-responder	CD4(IFNG+ Tfh/Th1)	Macro_FOLR2+APOE+	B2M	TFRC	5,64104E-03	34033,5	3,78627E-03	36207	9,49477E-01	38699	1,69636E+00	37505	9,49566E-01	3336	0	54420,5
Myeloid_vs_CD4_Non-responder	Mono_CD16	CD4(Tn)	S100A8	CD69	5,64542E-03	28378,9	5,37132E-03	21681	1,17332E+00	27289	2,09938E+00	25089	9,28765E-01	13415	0	54420,5
Myeloid_vs_CD4_Non-responder	Macro_FOLR2+APOE+	CD4(IFNG+ Tfh/Th1)	TNFSF13	TNFRSF14	5,64761E-03	33442,9	3,62185E-03	38708	1,47388E+00	15980	2,22910E+00	21826	8,86374E-01	36280	0	54420,5
Myeloid_vs_CD4_Non-responder	cDC_CLEC9A	CD4(IFNG+ Tfh/Th1)	B2M	CD3D	5,64938E-03	44200,9	4,00580E-03	33312	4,84979E-01	74273	1,18814E+00	58965	9,65532E-01	34	0	54420,5
Myeloid_vs_CD4_Non-responder	Mono_CD16	CD4(CRTAM- T)	LGALS9	CD44	5,64980E-03	30618,9	3,88907E-03	34807	1,29220E+00	22152	1,90148E+00	30765	9,33808E-01	10950	0	54420,5
Myeloid_vs_CD4_Non-responder	Macro_NLRP3	CD4(IL26+ Th17)	HLA-DRA	LAG3	5,64980E-03	38341,3	3,66754E-03	37976	9,49227E-01	38711	1,84892E+00	32389	9,00838E-01	28210	0	54420,5
Myeloid_vs_CD4_Non-responder	CD4(IFNG+ Tfh/Th1)	pDC_LILRA4	COPA	P2RY6	5,65991E-03	24805,9	8,85790E-03	10209	3,41958E+00	559	3,11271E+00	7300	8,58505E-01	51541	0	54420,5
Myeloid_vs_CD4_Non-responder	Mono_CD16	CD4(CRTAM- T)	CD55	ADGRE5	5,66514E-03	29861,3	7,63413E-03	12967	1,07207E+00	32142	1,75489E+00	35512	9,27008E-01	14265	0	54420,5
Myeloid_vs_CD4_Non-responder	CD4(IL26+ Th17)	Mono_CD16	HLA-F	LILRB1	5,66953E-03	35527,7	3,92581E-03	34303	1,53354E+00	14268	1,66361E+00	38738	8,87020E-01	35909	0	54420,5

Myeloid_vs_CD4_Post_NR

Myeloid_vs_CD4_Non-responder	CD4(TNF+ T)	Macro_ISG15	HLA-C	LILRB2	5,67026E-03	27026,7	3,61993E-03	38739	1,69389E+00	10503	1,97489E+00	28534	9,50672E-01	2937	0	54420,5
Myeloid_vs_CD4_Non-responder	Mast	CD4(IFNG+ Tfh/Th1)	TIMP3	CD44	5,67080E-03	31467,3	2,79088E-02	1222	2,75165E+00	1619	9,31655E-01	71524	9,00227E-01	28551	0	54420,5
Myeloid_vs_CD4_Non-responder	Macro_OLFML3	CD4(CRTAM- T)	CXCL9	CXCR3	5,67080E-03	19360,9	2,45045E-02	1619	2,90374E+00	1237	3,08597E+00	7581	8,94184E-01	31947	0	54420,5
Myeloid_vs_CD4_Non-responder	CD4(CXCL13+ Tfh)	Macro_ISG15	HLA-F	LILRB2	5,67099E-03	30065,7	3,61971E-03	38740	1,85769E+00	7740	2,15720E+00	23628	9,05047E-01	25800	0	54420,5
Myeloid_vs_CD4_Non-responder	CD4(AREG+ Tm)	Macro_ISG15	ANXA1	FPR2_FPR3	5,67392E-03	26757,3	8,54744E-03	10817	1,76903E+00	9097	1,66335E+00	38744	9,14221E-01	20708	0	54420,5
Myeloid_vs_CD4_Non-responder	CD4(GZMK+ Teff)	Macro_FOLR2+APOE+	B2M	LILRB2	5,67685E-03	26357,7	3,61946E-03	38748	1,73754E+00	9654	1,98420E+00	28267	9,57975E-01	699	0	54420,5
Myeloid_vs_CD4_Non-responder	Mono_CD16	CD4(CRTAM- T)	VCAN	ITGB1	5,68931E-03	36030,5	4,98282E-03	24246	1,14183E+00	28709	1,73898E+00	36034	8,85512E-01	36743	0	54420,5
Myeloid_vs_CD4_Non-responder	Mono_CD16	CD4(CRTAM- T)	VCAN	CD44	5,69151E-03	29207,9	5,07138E-03	23601	1,35822E+00	19763	1,90428E+00	30684	9,20402E-01	17571	0	54420,5
Myeloid_vs_CD4_Non-responder	Mono_CD16	CD4(CRTAM- T)	HBEGF	CD44	5,69445E-03	36194,5	3,74919E-03	36747	1,05557E+00	33018	1,72177E+00	36613	9,15189E-01	20174	0	54420,5
Myeloid_vs_CD4_Non-responder	cDC_LAMP3	CD4(IL26+ Th17)	CCL19	CCR7	5,69855E-03	27694,1	2,44885E-02	1623	4,86958E+00	59	2,09518E+00	25195	8,48558E-01	57173	0	54420,5
Myeloid_vs_CD4_Non-responder	Mono_CD16	CD4(CRTAM- T)	S100A8	CD69	5,70989E-03	24940,3	6,60945E-03	16021	1,31794E+00	21226	2,18898E+00	22797	9,35329E-01	10237	0	54420,5
Myeloid_vs_CD4_Non-responder	CD4(TGFB1+ Th17)	cDC(CD1C)	TGFB1	ENG	5,71062E-03	30585,3	6,74992E-03	15510	1,40307E+00	18202	1,66178E+00	38794	9,04677E-01	26000	0	54420,5
Myeloid_vs_CD4_Non-responder	CD4(TNFRSF9+ Treg)	Macro_OLFML3	LTB	TNFRSF1A	5,71210E-03	30918,3	7,59819E-03	13067	1,07313E+00	32104	1,66172E+00	38796	9,23091E-01	16204	0	54420,5
Myeloid_vs_CD4_Non-responder	Macro_FOLR2+APOE+	CD4(IFNG+ Tfh/Th1)	SPP1	CD44	5,71799E-03	30186,5	5,56834E-03	20560	9,47095E-01	38804	2,82181E+00	10910	9,04266E-01	26238	0	54420,5
Myeloid_vs_CD4_Non-responder	CD4(IFNG+ Tfh/Th1)	Macro_FOLR2+APOE+	HLA-A	LILRB1	5,71799E-03	30703,5	3,79950E-03	36038	1,45844E+00	16463	1,66138E+00	38804	9,40130E-01	7792	0	54420,5
Myeloid_vs_CD4_Non-responder	Mono_CD16	CD4(TNFRSF9+ Treg)	HLA-DRA	LAG3	5,72610E-03	34955,7	3,95074E-03	33998	9,89397E-01	36494	2,16063E+00	23548	9,04111E-01	26318	0	54420,5
Myeloid_vs_CD4_Non-responder	CD4(CXCL13+ Tfh)	pDC_LILRA4	HMGB1	TLR9	5,73333E-03	25469,7	3,61765E-02	741	2,75002E+00	1628	1,51855E+00	44411	9,04424E-01	26148	0	54420,5
Myeloid_vs_CD4_Non-responder	CD4(TGFB1+ Th17)	Macro_FOLR2+APOE+	HLA-B	LILRB2	5,73348E-03	26853,5	3,61497E-03	38825	1,71894E+00	10021	1,96035E+00	28963	9,53123E-01	2038	0	54420,5
Myeloid_vs_CD4_Non-responder	CD4(NME1+ T)	pDC_LILRA4	HRAS	TLR9	5,74030E-03	42991,7	6,10117E-02	262	2,74959E+00	1629	1,65021E+00	39187	7,18479E-01	119460	0	54420,5
Myeloid_vs_CD4_Non-responder	CD4(TNFRSF9+ Treg)	Macro_FOLR2+APOE+	HLA-B	LILRB1	5,74308E-03	31156,9	3,61412E-03	38838	1,44029E+00	17015	1,67311E+00	38398	9,41451E-01	7113	0	54420,5
Myeloid_vs_CD4_Non-responder	Macro_FOLR2+APOE+	CD4(IFNG+ Tfh/Th1)	HLA-C	CD3D	5,74826E-03	31101,7	4,78757E-03	25697	9,46285E-01	38845	1,72757E+00	36423	9,62811E-01	123	0	54420,5
Myeloid_vs_CD4_Non-responder	CD4(TNF+ T)	cDC_LAMP3	TNFSF9	HLA-DPA1	5,75122E-03	37706,7	4,26182E-03	30456	9,90717E-01	36433	1,98058E+00	28375	8,81574E-01	38849	0	54420,5
Myeloid_vs_CD4_Non-responder	cDC_CLEC9A	CD4(TNFRSF9+ Treg)	HLA-DPA1	LAG3	5,75222E-03	22649,5	5,87483E-03	19023	1,45688E+00	16529	3,40878E+00	4661	9,18272E-01	18614	0	54420,5
Myeloid_vs_CD4_Non-responder	Mono_CD16	CD4(TNFRSF9+ Treg)	CD86	CTLA4	5,75418E-03	31238,5	8,38544E-03	11182	1,17043E+00	27429	1,88883E+00	31154	8,94070E-01	32007	0	54420,5
Myeloid_vs_CD4_Non-responder	Mono_CD16	CD4(TNFRSF9+ Treg)	SECTM1	CD7	5,76752E-03	26535,1	1,59689E-02	3608	1,57651E+00	13152	1,76008E+00	35339	9,04416E-01	26156	0	54420,5
Myeloid_vs_CD4_Non-responder	Macro_FOLR2+APOE+	CD4(IL26+ Th17)	APOE	SORL1	5,77049E-03	27448,5	3,61235E-03	38875	1,69559E+00	10470	3,50727E+00	3997	8,98611E-01	29480	0	54420,5
Myeloid_vs_CD4_Non-responder	CD4(Tn)	cDC_CLEC9A	ANXA1	DYSF	5,78489E-03	34710,5	1,17695E-02	6324	1,87433E+00	7517	1,02712E+00	66797	8,82194E-01	38494	0	54420,5
Myeloid_vs_CD4_Non-responder	CD4(ISG+ Treg)	pDC_LILRA4	SELPLG	SELL	5,79354E-03	31748,3	6,45648E-03	16574	9,45289E-01	38906	2,06477E+00	25976	9,10245E-01	22865	0	54420,5
Myeloid_vs_CD4_Non-responder	pDC_LILRA4	CD4(ISG+ Treg)	APP	CD74	5,79583E-03	19577,7	6,05750E-03	18173	2,23373E+00	4030	2,35530E+00	19060	9,52619E-01	2205	0	54420,5
Myeloid_vs_CD4_Non-responder	Macro_ISG15	CD4(TGFB1+ Th17)	HBEGF	CD44	5,79702E-03	22460,1	6,56241E-03	16198	1,62328E+00	12024	2,35526E+00	19061	9,34540E-01	10597	0	54420,5
Myeloid_vs_CD4_Non-responder	Mono_CD16	CD4(TNFRSF9+ Treg)	S100A8	CD69	5,80173E-03	37916,3	3,62549E-03	38642	9,69399E-01	37593	1,67231E+00	38435	9,14614E-01	20491	0	54420,5
Myeloid_vs_CD4_Non-responder	CD4(TGFB1+ Th17)	Mono_CD16	HLA-A	LILRB1	5,80919E-03	28045,7	4,39627E-03	29085	1,62807E+00	11890	1,65787E+00	38927	9,44106E-01	5906	0	54420,5
Myeloid_vs_CD4_Non-responder	CD4(AREG+ Tm)	Macro_LYVE1	HMGB1	CD163	5,81292E-03	28394,9	4,37420E-03	29296	1,74918E+00	9458	1,65756E+00	38932	9,36030E-01	9868	0	54420,5
Myeloid_vs_CD4_Non-responder	Mono_CD16	CD4(ISG+ Treg)	HLA-DRA	LAG3	5,81516E-03	28016,5	6,00607E-03	18393	1,13970E+00	28801	2,26132E+00	21089	9,20795E-01	17379	0	54420,5
Myeloid_vs_CD4_Non-responder	Macro_FOLR2+APOE+	CD4(IFNG+ Tfh/Th1)	HBEGF	CD82	5,81815E-03	35077,9	6,24742E-03	17368	1,06607E+00	32475	1,85507E+00	32187	8,81436E-01	38939	0	54420,5
Myeloid_vs_CD4_Non-responder	Macro_FOLR2+APOE+	CD4(GZMK+ Teff)	CD86	CTLA4	5,82413E-03	32307,3	6,50231E-03	16417	1,53946E+00	14122	1,69304E+00	37630	8,81409E-01	38947	0	54420,5
Myeloid_vs_CD4_Non-responder	Macro_NLRP3	CD4(NME1+ T)	CCL3	CCR4	5,83161E-03	23361,9	1,34967E-02	4911	1,79483E+00	8684	2,90073E+00	9837	8,81396E-01	38957	0	54420,5
Myeloid_vs_CD4_Non-responder	Macro_ISG15	CD4(ISG+ Treg)	CXCL10	SDC4	5,83224E-03	30591,9	1,05992E-02	7538	2,37450E+00	3198	3,46748E+00	4288	8,00396E-01	83515	0	54420,5
Myeloid_vs_CD4_Non-responder	Macro_NLRP3	CD4(ISG+ Treg)	HLA-DRB1	LAG3	5,83610E-03	32398,1	5,42873E-03	21345	9,44466E-01	38963	2,01929E+00	27245	9,15518E-01	20017	0	54420,5
Myeloid_vs_CD4_Non-responder	Mono_CD16	CD4(ISG+ Treg)	HLA-DPA1	LAG3	5,83985E-03	30218,3	5,84445E-03	19173	1,02491E+00	34637	2,13648E+00	24126	9,18077E-01	18735	0	54420,5
Myeloid_vs_CD4_Non-responder	Mono_CD16	CD4(ISG+ Treg)	CD86	CTLA4	5,84359E-03	31942,1	8,77521E-03	10358	1,20342E+00	25918	1,67892E+00	38178	8,96203E-01	30836	0	54420,5
Myeloid_vs_CD4_Non-responder	CD4(GZMK+ Teff)	Mono_CD14	ANXA1	FPR1	5,84359E-03	32025,7	3,85280E-03	35274	1,49251E+00	15412	1,65630E+00	38973	9,23413E-01	16049	0	54420,5
Myeloid_vs_CD4_Non-responder	Mono_CD16	CD4(ISG+ Treg)	HLA-DRB5	LAG3	5,85335E-03	29154,9	6,12046E-03	17923	1,08780E+00	31341	2,30954E+00	20026	9,11768E-01	22064	0	54420,5
Myeloid_vs_CD4_Non-responder	Mono_CD16	CD4(ISG+ Treg)	SECTM1	CD7	5,85410E-03	25565,1	1,76183E-02	3012	1,66739E+00	11035	1,75344E+00	35558	9,08581E-01	23800	0	54420,5
Myeloid_vs_CD4_Non-responder	Macro_FOLR2+APOE-	CD4(ISG+ Treg)	C3	IFITM1	5,86168E-03	21305,5	1,17356E-02	6356	1,87245E+00	7551	1,81588E+00	33461	9,46420E-01	4739	0	54420,5
Myeloid_vs_CD4_Non-responder	Mono_CD16	CD4(ISG+ Treg)	HLA-DPB1	LAG3	5,86311E-03	32735,9	5,41973E-03	21404	9,43947E-01	38999	2,05428E+00	26248	9,10749E-01	22608	0	54420,5
Myeloid_vs_CD4_Non-responder	Mono_CD16	CD4(ISG+ Treg)	VCAN	SELL	5,86537E-03	33386,7	6,92472E-03	14933	1,21912E+00	25232	1,75980E+00	35351	8,85038E-01	36997	0	54420,5
Myeloid_vs_CD4_Non-responder	CD4(TNF+ T)	Macro_FOLR2+APOE+	TNFSF14	TNFRSF14	5,86762E-03	31299,7	2,07919E-02	2192	9,43768E-01	39005	2,08398E+00	25506	8,88027E-01	35375	0	54420,5
Myeloid_vs_CD4_Non-responder	Mono_CD16	CD4(ISG+ Treg)	CXCL16	CXCR6	5,87364E-03	35089,9	6,98696E-03	14737	9,55006E-01	38385	1,88859E+00	31166	8,85514E-01	36741	0	54420,5
Myeloid_vs_CD4_Non-responder	CD4(TNF+ T)	Mono_CD14	CSF1	CSF3R	5,87423E-03	28562,1	1,82549E-02	2830	1,71511E+00	10091	2,35232E+00	19126	8,50048E-01	56343	0	54420,5
Myeloid_vs_CD4_Non-responder	CD4(TNFRSF9+ Treg)	pDC_LILRA4	IRAK4	TLR7	5,87757E-03	39502,9	1,06613E-02	7478	1,87192E+00	7558	1,86828E+00	31753	7,74745E-01	96305	0	54420,5
Myeloid_vs_CD4_Non-responder	CD4(IL26+ Th17)	pDC_LILRA4	HSP90B1	TLR9	5,88055E-03	28430,7	2,84763E-02	1178	2,74077E+00	1649	1,53864E+00	43576	8,77033E-01	41330	0	54420,5
Myeloid_vs_CD4_Non-responder	CD4(TNF+ T)	Macro_LYVE1	IFNG	IFNGR1_IFNGR2	5,88494E-03	32719,1	1,51056E-02	4003	1,00496E+00	35660	1,91119E+00	30484	8,81259E-01	39028	0	54420,5
Myeloid_vs_CD4_Non-responder	Macro_FOLR2+APOE+	CD4(CRTAM- T)	TNFSF13	FAS	5,88570E-03	29749,5	6,98948E-03	14728	1,45558E+00	16570	2,14222E+00	24000	8,81259E-01	39029	0	54420,5
Myeloid_vs_CD4_Non-responder	CD4(CRTAM- T)	pDC_LILRA4	TGFB1	CXCR4	5,88947E-03	28231,9	4,89527E-03	24906	9,43278E-01	39034	2,40951E+00	17928	9,46156E-01	4871	0	54420,5
Myeloid_vs_CD4_Non-responder	Mono_CD16	CD4(ISG+ Treg)	S100A8	CD69	5,89249E-03	35680,1	3,83582E-03	35514	9,93967E-01	36247	1,83390E+00	32847	9,16791E-01	19372	0	54420,5
Myeloid_vs_CD4_Non-responder	CD4(TNFRSF9+ Treg)	Macro_NLRP3	HMGB1	THBD	5,89702E-03	31201,7	4,89309E-03	24921	1,51623E+00	14755	1,65411E+00	39044	9,10244E-01	22868	0	54420,5
Myeloid_vs_CD4_Non-responder	CD4(TNF+ T)	Macro_ISG15	CD40LG	CD9	5,93563E-03	29132,7	1,26263E-02	5593	1,14946E+00	28398	2,39813E+00	18157	8,81143E-01	39095	0	54420,5
Myeloid_vs_CD4_Non-responder	Macro_ISG15	CD4(IL26+ Th17)	TNFSF10	CCR6	5,94016E-03	20368,3	2,36165E-02	1733	1,61886E+00	12127	2,59352E+00	14380	9,17137E-01	19181	0	54420,5
Myeloid_vs_CD4_Non-responder	CD4(ISG+ Treg)	Macro_FOLR2+APOE+	CD99	PILRA	5,94627E-03	31828,3	3,59730E-03	39109	1,56524E+00	13461	1,84012E+00	32665	9,16561E-01	19486	0	54420,5
Myeloid_vs_CD4_Non-responder	pDC_LILRA4	CD4(NME1+ T)	APP	CD74	5,95582E-03	19823,3	5,83864E-03	19194	2,20361E+00	4245	2,37125E+00	18732	9,51781E-01	2525	0	54420,5
Myeloid_vs_CD4_Non-responder	pDC_LILRA4	CD4(Tn)	APP	PTGER2	5,95745E-03	25306,7	1,41378E-02	4536	2,27979E+00	3747	3,08488E+00	7593	8,50234E-01	56237	0	54420,5
Myeloid_vs_CD4_Non-responder	CD4(TNFRSF9+ Treg)	Macro_FOLR2+APOE+	CD59	STAB1	5,95844E-03	28233,3	5,40887E-03	21471	1,75163E+00	9419	2,46957E+00	16731	8,81083E-01	39125	0	54420,5
Myeloid_vs_CD4_Non-responder	CD4(Tn)	Macro_ISG15	CD99	PILRA	5,95844E-03	32288,9	3,59604E-03	39125	1,49940E+00	15202	1,82342E+00	33205	9,16548E-01	19492	0	54420,5
Myeloid_vs_CD4_Non-responder	Mono_CD16	CD4(TNF+ T)	S100A8	CD69	5,96225E-03	25276,7	5,93052E-03	18733	1,23863E+00	24401	2,45725E+00	16965	9,31972E-01	11864	0	54420,5
Myeloid_vs_CD4_Non-responder	Macro_NLRP3	CD4(TGFB1+ Th17)	VCAN	ITGA4	5,97674E-03	28176,5	5,05886E-03	23713	2,48268E+00	2623	2,26703E+00	20977	8,81030E-01	39149	0	54420,5
Myeloid_vs_CD4_Non-responder	CD4(NME1+ T)	pDC_LILRA4	SIRPG	CD47	5,98896E-03	32626,9	1,14077E-02	6673,5	9,46409E-01	38835,5	2,14020E+00	24039,5	8,81007E-01	39165,5	0	54420,5
Myeloid_vs_CD4_Non-responder	CD4(TNF+ T)	Macro_ISG15	B2M	LILRB1	5,99431E-03	28148,3	4,30822E-03	29938	1,52111E+00	14622	1,65072E+00	39172	9,51622E-01	2589	0	54420,5
Myeloid_vs_CD4_Non-responder	CD4(NME1+ T)	Macro_FOLR2+APOE+	HMGB1	HAVCR2	6,00503E-03	34271,3	4,16352E-03	31505	1,10194E+00	30630	1,65024E+00	39186	9,24313E-01	15615	0	54420,5
Myeloid_vs_CD4_Non-responder	CD4(ISG+ Treg)	Macro_FOLR2+APOE+	CD52	SIGLEC10	6,03702E-03	30393,1	5,82582E-03	19261	1,48597E+00	15616	1,51444E+00	44581	9,19429E-01	18087	0	54420,5
Myeloid_vs_CD4_Non-responder	Mono_CD16	CD4(GZMK+ Teff)	S100A8	CD69	6,03805E-03	30928,5	4,85300E-03	25216	1,11278E+00	30089	1,93252E+00	29811	9,25334E-01	15106	0	54420,5

Myeloid_vs_CD4_Post_NR

Myeloid_vs_CD4_Non-responder	Macro_FOLR2+APOE+	CD4(IFNG+ Tfh/Th1)	C1QB	C1QBP	6,04113E-03	24114,3	3,58967E-03	39233	2,08423E+00	5263	3,69151E+00	2927	9,18089E-01	18728	0	54420,5
Myeloid_vs_CD4_Non-responder	Macro_ISG15	CD4(NME1+ T)	CXCL10	SDC4	6,04265E-03	30677,3	1,05346E-02	7630	2,37345E+00	3203	3,45198E+00	4374	7,99907E-01	83759	0	54420,5
Myeloid_vs_CD4_Non-responder	cDC(CD1C)	CD4(IFNG+ Tfh/Th1)	CXCL16	CXCR6	6,04677E-03	21674,1	1,51090E-02	3999	1,60477E+00	12469	2,34465E+00	19269	9,19186E-01	18213	0	54420,5
Myeloid_vs_CD4_Non-responder	CD4(CXCL13+ Tfh)	Macro_ISG15	HMGB1	HAVCR2	6,05577E-03	35777,7	3,90718E-03	34529	1,03786E+00	33945	1,64863E+00	39252	9,22060E-01	16742	0	54420,5
Myeloid_vs_CD4_Non-responder	CD4(TNF+ T)	cDC_LAMP3	TNFSF10	TNFRSF11B	6,05818E-03	45163,9	2,40085E-02	1674	2,81640E+00	1455	1,22984E+00	56988	7,40515E-01	111282	0	54420,5
Myeloid_vs_CD4_Non-responder	CD4(Tn)	cDC_LAMP3	LTB	CD40	6,05897E-03	28996,3	1,03943E-02	7810	1,89675E+00	7224	1,24455E+00	56248	9,16962E-01	19279	0	54420,5
Myeloid_vs_CD4_Non-responder	CD4(Tn)	Mast	CALR	ITGA2B	6,06534E-03	49628,1	2,39930E-02	1675	1,86436E+00	7650	1,34173E-01	113358	8,23672E-01	71037	0	54420,5
Myeloid_vs_CD4_Non-responder	CD4(TNF+ T)	cDC(CD1C)	TNF	TNFRSF1A	6,07276E-03	36137,5	6,09768E-03	18008	9,38950E-01	39274	1,76043E+00	35328	8,91102E-01	33657	0	54420,5
Myeloid_vs_CD4_Non-responder	Macro_FOLR2+APOE+	CD4(IL26+ Th17)	TNFSF10	CCR6	6,07354E-03	34920,7	1,14972E-02	6587	9,38919E-01	39275	1,69720E+00	37482	8,85355E-01	36839	0	54420,5
Myeloid_vs_CD4_Non-responder	Macro_OLFML3	CD4(ISG+ Treg)	C3	IFITM1	6,08956E-03	22268,9	8,23775E-03	11481	1,45458E+00	16600	2,34305E+00	19304	9,36705E-01	9539	0	54420,5
Myeloid_vs_CD4_Non-responder	CD4(CXCL13+ Tfh)	Macro_FOLR2-APOE+	ADAM10	TREM2	6,09057E-03	30980,5	6,48821E-03	16466	1,83589E+00	8046	1,72013E+00	36673	8,80784E-01	39297	0	54420,5
Myeloid_vs_CD4_Non-responder	CD4(AREG+ Tm)	Macro_NLRP3	ANXA1	FPR1	6,09212E-03	33782,9	3,93589E-03	34171	1,21659E+00	25334	1,64768E+00	39299	9,24164E-01	15690	0	54420,5
Myeloid_vs_CD4_Non-responder	Macro_NLRP3	CD4(Tn)	ICAM1	IL2RG	6,10298E-03	38433,7	3,58485E-03	39313	1,07955E+00	31776	1,72290E+00	36577	8,97571E-01	30082	0	54420,5
Myeloid_vs_CD4_Non-responder	Mono_CD16	CD4(AREG+ Tm)	S100A8	CD69	6,10919E-03	23142,9	6,99954E-03	14697	1,36350E+00	19584	2,42306E+00	17647	9,37042E-01	9366	0	54420,5
Myeloid_vs_CD4_Non-responder	CD4(Tn)	Macro_ISG15	B2M	LILRB1	6,11152E-03	27634,9	4,39102E-03	29142	1,58908E+00	12851	1,64717E+00	39324	9,52058E-01	2437	0	54420,5
Myeloid_vs_CD4_Non-responder	Mono_INHBA	CD4(Tn)	SPP1	CD44	6,11411E-03	22466,9	9,50905E-03	9064	1,53318E+00	14284	2,34219E+00	19324	9,25057E-01	15242	0	54420,5
Myeloid_vs_CD4_Non-responder	Macro_OLFML3	CD4(TNF+ T)	CXCL9	DPP4	6,11698E-03	28973,1	2,16444E-02	2025	2,81462E+00	1458	3,07846E+00	7662	8,08328E-01	79300	0	54420,5
Myeloid_vs_CD4_Non-responder	Mono_CD14	CD4(ISG+ Treg)	S100A9	ITGB2	6,13331E-03	19876,1	4,16118E-03	31531	2,81790E+00	1449	3,46397E+00	4311	9,40371E-01	7669	0	54420,5
Myeloid_vs_CD4_Non-responder	Macro_NLRP3	CD4(Tn)	VEGFA	ITGB1	6,13643E-03	28460,3	7,70411E-03	12773	2,22155E+00	4110	1,87198E+00	31642	8,80683E-01	39356	0	54420,5
Myeloid_vs_CD4_Non-responder	Mono_CD16	CD4(TNFRSF9+ Treg)	HLA-DRB5	LAG3	6,13955E-03	36298,5	4,02598E-03	33062	9,37503E-01	39360	2,20885E+00	22293	8,93402E-01	32357	0	54420,5
Myeloid_vs_CD4_Non-responder	Macro_IFI27	CD4(IFNG+ Tfh/Th1)	HLA-DQA1	LAG3	6,13997E-03	21368,9	1,21129E-02	6013	1,61980E+00	12104	2,34131E+00	19345	9,25651E-01	14962	0	54420,5
Myeloid_vs_CD4_Non-responder	Macro_NLRP3	CD4(ISG+ Treg)	LYZ	ITGAL	6,14579E-03	28921,1	3,58164E-03	39368	1,58668E+00	12917	2,77200E+00	11593	9,04130E-01	26307	0	54420,5
Myeloid_vs_CD4_Non-responder	Mono_CD14	CD4(CXCL13+ Tfh)	S100A9	ITGB2	6,14734E-03	19850,7	4,15906E-03	31549	2,81767E+00	1450	3,48653E+00	4159	9,40356E-01	7675	0	54420,5
Myeloid_vs_CD4_Non-responder	CD4(CRTAM- T)	Macro_OLFML3	HLA-C	LILRB1	6,16376E-03	30049,1	3,90732E-03	34523	1,53081E+00	14361	1,64518E+00	39391	9,40606E-01	7550	0	54420,5
Myeloid_vs_CD4_Non-responder	Macro_NLRP3	CD4(CRTAM- T)	THBS1	CD47	6,17786E-03	28913,1	5,43359E-03	21318	1,71710E+00	10049	2,34026E+00	19369	8,80601E-01	39409	0	54420,5
Myeloid_vs_CD4_Non-responder	Macro_NLRP3	CD4(CRTAM- T)	ICAM1	IL2RG	6,20219E-03	38928,7	3,59865E-03	39091	1,08104E+00	31701	1,64410E+00	39440	8,97748E-01	29991	0	54420,5
Myeloid_vs_CD4_Non-responder	CD4(AREG+ Tm)	Macro_ISG15	HLA-B	LILRB2	6,20298E-03	28053,3	3,57672E-03	39441	1,62052E+00	12080	1,85463E+00	32202	9,52885E-01	2123	0	54420,5
Myeloid_vs_CD4_Non-responder	Mono_CD16	CD4(NME1+ T)	S100A8	CD69	6,20691E-03	34990,9	3,97640E-03	33679	1,01039E+00	35373	1,83499E+00	32807	9,18154E-01	18675	0	54420,5
Myeloid_vs_CD4_Non-responder	Macro_OLFML3	CD4(IFNG+ Tfh/Th1)	CXCL16	CXCR6	6,21926E-03	21996,9	1,41476E-02	4527	1,52181E+00	14598	2,45372E+00	17030	9,16710E-01	19409	0	54420,5
Myeloid_vs_CD4_Non-responder	Mono_CD16	CD4(IFNG+ Tfh/Th1)	HLA-DRA	LAG3	6,22266E-03	22412,7	8,34043E-03	11264	1,31040E+00	21454	2,67386E+00	13059	9,31971E-01	11866	0	54420,5
Myeloid_vs_CD4_Non-responder	Macro_ISG15	CD4(IFNG+ Tfh/Th1)	HLA-DPB1	LAG3	6,22300E-03	20625,7	9,44277E-03	9171	1,36834E+00	19412	3,07348E+00	7714	9,30888E-01	12411	0	54420,5
Myeloid_vs_CD4_Non-responder	CD4(Tn)	Macro_FOLR2+APOE+	MIF	CD44_CD74	6,22388E-03	49564,3	1,87398E-03	82902	5,71855E-01	66336	1,56645E+00	42466	9,54128E-01	1697	0	54420,5
Myeloid_vs_CD4_Non-responder	Macro_FOLR2+APOE+	CD4(NME1+ T)	CXCL9	CXCR3	6,22424E-03	21260,1	1,86289E-02	2712	2,93373E+00	1175	3,00216E+00	8525	8,80496E-01	39468	0	54420,5
Myeloid_vs_CD4_Non-responder	CD4(NME1+ T)	cDC_LAMP3	CD28	CD86	6,22424E-03	29631,7	9,89436E-03	8504,5	1,72965E+00	9820,5	1,64359E+00	39468,5	8,86973E-01	35944,5	0	54420,5
Myeloid_vs_CD4_Non-responder	Mono_CD16	CD4(IFNG+ Tfh/Th1)	HLA-DQA2	LAG3	6,22424E-03	28094,5	8,75903E-03	10387	1,17186E+00	27359	2,75910E+00	11776	8,85896E-01	36530	0	54420,5
Myeloid_vs_CD4_Non-responder	Mono_CD16	CD4(IFNG+ Tfh/Th1)	HLA-DRB1	LAG3	6,22740E-03	26306,1	7,40355E-03	13561	1,09685E+00	30875	2,39269E+00	18277	9,26769E-01	14397	0	54420,5
Myeloid_vs_CD4_Non-responder	CD4(GZMK+ Teff)	Macro_OLFML3	HLA-B	LILRB1	6,22897E-03	30337,7	3,83343E-03	35553	1,47682E+00	15901	1,64339E+00	39474	9,43054E-01	6340	0	54420,5
Myeloid_vs_CD4_Non-responder	Mono_CD16	CD4(IFNG+ Tfh/Th1)	HLA-DPB1	LAG3	6,23213E-03	26114,5	7,52621E-03	13259	1,11465E+00	29981	2,46681E+00	16782	9,23224E-01	16130	0	54420,5
Myeloid_vs_CD4_Non-responder	Mono_CD16	CD4(AREG+ Tm)	LGALS1	CD69	6,23213E-03	32840,1	4,22983E-03	30761	9,35558E-01	39478	1,74419E+00	35850	9,48681E-01	3691	0	54420,5
Myeloid_vs_CD4_Non-responder	Macro_NLRP3	CD4(IL26+ Th17)	VEGFA	ITGB1	6,23608E-03	28503,7	7,67098E-03	12868	2,21995E+00	4117	1,87248E+00	31630	8,80456E-01	39483	0	54420,5
Myeloid_vs_CD4_Non-responder	CD4(AREG+ Tm)	Macro_FOLR2+APOE+	TIMP1	CD63	6,23795E-03	41073,9	1,54982E-03	95389	1,44293E+00	16941	2,34879E+00	19195	9,16687E-01	19424	0	54420,5
Myeloid_vs_CD4_Non-responder	CD4(TGFB1+ Th17)	Macro_IER3	RPS19	C5AR1	6,24635E-03	25661,9	5,34863E-03	21807	1,73086E+00	9792	1,64276E+00	39496	9,51059E-01	2794	0	54420,5
Myeloid_vs_CD4_Non-responder	Mono_CD16	CD4(IFNG+ Tfh/Th1)	HLA-DPA1	LAG3	6,24872E-03	24143,3	8,11599E-03	11790	1,19562E+00	26269	2,54901E+00	15217	9,29607E-01	13020	0	54420,5
Myeloid_vs_CD4_Non-responder	CD4(TNFRSF9+ Treg)	Macro_NLRP3	LTB	TNFRSF1A	6,25031E-03	33731,1	6,48442E-03	16482	9,41494E-01	39135	1,64263E+00	39501	9,17272E-01	19117	0	54420,5
Myeloid_vs_CD4_Non-responder	Mono_CD16	CD4(IFNG+ Tfh/Th1)	CD86	CTLA4	6,25268E-03	29861,7	9,53078E-03	9034	1,26737E+00	23164	1,79941E+00	34003	8,99982E-01	28687	0	54420,5
Myeloid_vs_CD4_Non-responder	Mono_CD16	CD4(IFNG+ Tfh/Th1)	HLA-DRB5	LAG3	6,26535E-03	23391,5	8,49928E-03	10918	1,25851E+00	23564	2,72208E+00	12338	9,24113E-01	15717	0	54420,5
Myeloid_vs_CD4_Non-responder	Mono_CD16	CD4(IFNG+ Tfh/Th1)	VCAN	ITGB1	6,27566E-03	36230,1	4,90105E-03	24863	1,13162E+00	29180	1,75543E+00	35491	8,84670E-01	37196	0	54420,5
Myeloid_vs_CD4_Non-responder	CD4(NME1+ T)	pDC_LILRA4	HSP90B1	TLR9	6,28203E-03	29145,5	2,76313E-02	1264	2,72443E+00	1705	1,47783E+00	46113	8,75399E-01	42225	0	54420,5
Myeloid_vs_CD4_Non-responder	Mono_CD16	CD4(IFNG+ Tfh/Th1)	CXCL16	CXCR6	6,28281E-03	26843,3	1,03224E-02	7913	1,19177E+00	26436	2,35961E+00	18979	9,03859E-01	26468	0	54420,5
Myeloid_vs_CD4_Non-responder	CD4(TNF+ T)	Macro_ISG15	CD52	SIGLEC10	6,28519E-03	34384,9	4,71611E-03	26266	1,12810E+00	29348	1,64165E+00	39545	9,11247E-01	22345	0	54420,5
Myeloid_vs_CD4_Non-responder	Macro_FOLR2+APOE+	CD4(IL26+ Th17)	HLA-DMB	CD4	6,28599E-03	27601,9	3,56966E-03	39546	1,80598E+00	8506	2,65687E+00	13340	9,11565E-01	22197	0	54420,5
Myeloid_vs_CD4_Non-responder	CD4(TGFB1+ Th17)	Macro_ISG15	TGFB1	SDC2	6,28678E-03	26238,7	1,68146E-02	3281	2,00514E+00	6040	1,99612E+00	27905	8,80332E-01	39547	0	54420,5
Myeloid_vs_CD4_Non-responder	Mono_CD16	CD4(IFNG+ Tfh/Th1)	S100A8	CD69	6,29951E-03	34989,9	4,10872E-03	32121	1,02584E+00	34591	1,74863E+00	35690	9,19375E-01	18127	0	54420,5
Myeloid_vs_CD4_Non-responder	Macro_FOLR2+APOE+	CD4(NME1+ T)	APOE	LDLR	6,31545E-03	23571,7	8,51900E-03	10891	1,75214E+00	9410	3,57925E+00	3554	8,80275E-01	39583	0	54420,5
Myeloid_vs_CD4_Non-responder	CD4(Tn)	Macro_ISG15	ANXA1	FPR2_FPR3	6,31784E-03	27700,9	7,95249E-03	12148	1,71699E+00	10054	1,64061E+00	39586	9,11349E-01	22296	0	54420,5
Myeloid_vs_CD4_Non-responder	cDC(CD1C)	CD4(TNFRSF9+ Treg)	HLA-DRA	LAG3	6,33456E-03	22994,7	5,77393E-03	19501	1,50465E+00	15069	3,06057E+00	7848	9,19345E-01	18135	0	54420,5
Myeloid_vs_CD4_Non-responder	Macro_NLRP3	CD4(NME1+ T)	CD14	ITGA4	6,34262E-03	32351,9	3,56526E-03	39617	1,31477E+00	21318	2,16819E+00	23345	9,09896E-01	23059	0	54420,5
Myeloid_vs_CD4_Non-responder	CD4(GZMK+ Teff)	Mono_CD16	B2M	LILRB1	6,34422E-03	28225,3	4,15666E-03	31580	1,59916E+00	12612	1,63966E+00	39619	9,50791E-01	2895	0	54420,5
Myeloid_vs_CD4_Non-responder	Macro_FOLR2+APOE+	CD4(TGFB1+ Th17)	LGALS3	MCAM	6,34823E-03	25998,3	1,98485E-02	2384	1,33373E+00	20663	2,68451E+00	12900	8,80217E-01	39624	0	54420,5
Myeloid_vs_CD4_Non-responder	CD4(GZMK+ Teff)	Macro_ISG15	HMGB1	THBD	6,34983E-03	39250,7	3,56487E-03	39626	1,06331E+00	32616	1,65928E+00	38881	8,96439E-01	30710	0	54420,5
Myeloid_vs_CD4_Non-responder	Macro_IER3	CD4(ISG+ Treg)	HLA-DQB1	LAG3	6,35604E-03	24180,9	8,46486E-03	10996	1,41045E+00	17944	2,40504E+00	18026	9,16495E-01	19518	0	54420,5
Myeloid_vs_CD4_Non-responder	CD4(ISG+ Treg)	Mono_CD16	CD99	PILRA	6,35624E-03	32082,7	3,56430E-03	39634	1,54883E+00	13875	1,83512E+00	32802	9,16208E-01	19682	0	54420,5
Myeloid_vs_CD4_Non-responder	Macro_FOLR2+APOE+	CD4(IFNG+ Tfh/Th1)	HLA-DMA	CD4	6,36186E-03	26071,5	3,56391E-03	39641	1,54301E+00	14029	2,84891E+00	10535	9,32263E-01	11732	0	54420,5
Myeloid_vs_CD4_Non-responder	CD4(ISG+ Treg)	Macro_OLFML3	HLA-C	LILRB1	6,37229E-03	30279,5	3,88206E-03	34893	1,51480E+00	14789	1,63884E+00	39654	9,40425E-01	7641	0	54420,5
Myeloid_vs_CD4_Non-responder	Mono_CD16	CD4(CXCL13+ Tfh)	S100A8	CD69	6,38757E-03	36820,5	3,80155E-03	36015	9,89964E-01	36465	1,69220E+00	37656	9,16448E-01	19546	0	54420,5
Myeloid_vs_CD4_Non-responder	CD4(TGFB1+ Th17)	Macro_FOLR2+APOE+	CCL4	CCR1	6,39241E-03	28865,9	9,17000E-03	9636	1,61651E+00	12197	1,97996E+00	28397	8,80103E-01	39679	0	54420,5
Myeloid_vs_CD4_Non-responder	CD4(Tn)	Macro_FOLR2+APOE+	HLA-B	LILRB2	6,39885E-03	27185,5	3,56061E-03	39687	1,67836E+00	10796	1,96294E+00	28872	9,52783E-01	2152	0	54420,5
Myeloid_vs_CD4_Non-responder	cDC(CD1C)	CD4(TNFRSF9+ Treg)	HLA-DPA1	LAG3	6,39915E-03	23871,1	5,76432E-03	19552	1,42519E+00	17441	2,96695E+00	8966	9,17556E-01	18976	0	54420,5
Myeloid_vs_CD4_Non-responder	Mono_CD16	CD4(IL26+ Th17)	HLA-DRA	LAG3	6,40047E-03	37328,3	3,75431E-03	36676	9,75033E-01	37263	1,90465E+00	30672	9,01877E-01	27610	0	54420,5
Myeloid_vs_CD4_Non-responder	CD4(TNFRSF9+ Treg)	Macro_FOLR2+APOE+	B2M	LILRB1	6,41257E-03	30836,1	3,55985E-03	39704	1,45212E+00	16681	1,65813E+00	38922	9,47036E-01	4453	0	54420,5
Myeloid_vs_CD4_Non-responder	CD4(IFNG+ Tfh/Th1)	Macro_FOLR2+APOE+	HLA-C	LILRB1	6,41903E-03	32028,1	3,58138E-03	39371	1,41376E+00	17842	1,63738E+00	39712	9,38126E-01	8795	0	54420,5

Myeloid_vs_CD4_Post_NR

Myeloid_vs_CD4_Non-responder	CD4(NME1+ T)	Macro_FOLR2+APOE+	B2M	LILRB2	6,43197E-03	27009,7	3,55840E-03	39728	1,67342E+00	10908	1,95194E+00	29228	9,57631E-01	764	0	54420,5
Myeloid_vs_CD4_Non-responder	CD4(AREG+ Tm)	Macro_FOLR2+APOE+	CD40LG	CD9	6,43609E-03	27710,3	1,20684E-02	6050	1,36361E+00	19581	2,40092E+00	18112	8,78757E-01	40388	0	54420,5
Myeloid_vs_CD4_Non-responder	cDC_CLEC9A	CD4(IFNG+ Tfh/Th1)	HLA-DOB	CD4	6,45067E-03	23504,3	2,36606E-02	1728	3,79190E+00	330	2,00708E+00	27573	8,91454E-01	33470	0	54420,5
Myeloid_vs_CD4_Non-responder	Macro_FOLR2+APOE+	CD4(IL26+ Th17)	LGALS3BP	ITGB1	6,45630E-03	36670,3	5,09628E-03	23440	1,18790E+00	26622	1,63593E+00	39758	8,81112E-01	39111	0	54420,5
Myeloid_vs_CD4_Non-responder	CD4(TNF+ T)	cDC_LAMP3	CD40LG	CD40	6,45873E-03	25129,7	2,45806E-02	1609	2,16025E+00	4590	2,09253E+00	25268	8,79945E-01	39761	0	54420,5
Myeloid_vs_CD4_Non-responder	Mono_CD16	CD4(IL26+ Th17)	CXCL16	CXCR6	6,46117E-03	33385,1	7,55583E-03	13168	9,95386E-01	36167	1,97452E+00	28544	8,89422E-01	34626	0	54420,5
Myeloid_vs_CD4_Non-responder	Mono_CD16	CD4(IL26+ Th17)	LGALS1	CD69	6,46198E-03	30102,1	4,62831E-03	26970	1,01258E+00	35250	1,89378E+00	30999	9,50829E-01	2871	0	54420,5
Myeloid_vs_CD4_Non-responder	Macro_FOLR2+APOE+	CD4(Tn)	LGALS3BP	ITGB1	6,46767E-03	36602,1	5,11829E-03	23287	1,18950E+00	26546	1,63544E+00	39772	8,81337E-01	38985	0	54420,5
Myeloid_vs_CD4_Non-responder	Macro_ISG15	CD4(CXCL13+ Tfh)	CCL2	CCR4	6,47810E-03	27671,1	2,87094E-02	1153	1,85163E+00	7814	3,16409E+00	6771	8,28861E-01	68197	0	54420,5
Myeloid_vs_CD4_Non-responder	CD4(IL26+ Th17)	Macro_FOLR2+APOE+	ADAM10	GPNMB	6,47988E-03	23758,3	9,86212E-03	8544	2,11950E+00	4937	2,80798E+00	11103	8,79884E-01	39787	0	54420,5
Myeloid_vs_CD4_Non-responder	Mono_CD16	CD4(IL26+ Th17)	TNFSF10	CCR6	6,48151E-03	28649,5	1,55671E-02	3800	1,16726E+00	27576	1,96838E+00	28702	8,99861E-01	28749	0	54420,5
Myeloid_vs_CD4_Non-responder	Mono_CD16	CD4(TGFB1+ Th17)	LGALS9	CD44	6,48395E-03	29300,9	4,05148E-03	32760	1,34053E+00	20387	1,97332E+00	28574	9,35061E-01	10363	0	54420,5
Myeloid_vs_CD4_Non-responder	Macro_ISG15	CD4(CRTAM- T)	CD14	ITGB1	6,48730E-03	20753,3	5,75043E-03	19621	1,83819E+00	8010	2,93753E+00	9363	9,31018E-01	12352	0	54420,5
Myeloid_vs_CD4_Non-responder	Mono_CD16	CD4(TGFB1+ Th17)	CD55	ADGRE5	6,50353E-03	25552,9	9,22460E-03	9541	1,24212E+00	24245	1,98369E+00	28281	9,33158E-01	11277	0	54420,5
Myeloid_vs_CD4_Non-responder	CD4(IL26+ Th17)	cDC_CLEC9A	LTB	CD40	6,51906E-03	28427,7	9,28282E-03	9433	1,44778E+00	16802	1,63358E+00	39835	9,12554E-01	21648	0	54420,5
Myeloid_vs_CD4_Non-responder	CD4(TNFRSF9+ Treg)	Macro_OLFML3	HLA-A	LILRB2	6,51988E-03	26774,3	3,55202E-03	39836	1,71672E+00	10060	2,04335E+00	26561	9,50485E-01	2994	0	54420,5
Myeloid_vs_CD4_Non-responder	Mono_CD16	CD4(TGFB1+ Th17)	VCAN	CD44	6,53298E-03	28007,5	5,28316E-03	22211	1,40655E+00	18073	1,97612E+00	28502	9,21888E-01	16831	0	54420,5
Myeloid_vs_CD4_Non-responder	Mono_CD16	CD4(TGFB1+ Th17)	HBEGF	CD44	6,53790E-03	34614,3	3,90575E-03	34549	1,10391E+00	30533	1,79361E+00	34184	9,16763E-01	19385	0	54420,5
Myeloid_vs_CD4_Non-responder	Mono_CD16	CD4(TGFB1+ Th17)	LGALS1	CD69	6,54118E-03	27806,3	5,06609E-03	23645	1,09719E+00	30858	1,99310E+00	27992	9,52899E-01	2116	0	54420,5
Myeloid_vs_CD4_Non-responder	Macro_FOLR2+APOE+	CD4(CXCL13+ Tfh)	CD14	ITGA4	6,55925E-03	27891,7	3,54909E-03	39884	1,74490E+00	9538	2,71407E+00	12456	9,09709E-01	23160	0	54420,5
Myeloid_vs_CD4_Non-responder	Mono_CD16	CD4(TGFB1+ Th17)	TNFSF10	CCR6	6,57077E-03	32744,7	1,26568E-02	5575	1,02443E+00	34662	1,77329E+00	34870	8,90143E-01	34196	0	54420,5
Myeloid_vs_CD4_Non-responder	CD4(ISG+ Treg)	Macro_OLFML3	HLA-A	LILRB2	6,57077E-03	26980,9	3,54796E-03	39898	1,71363E+00	10116	2,01084E+00	27463	9,50458E-01	3007	0	54420,5
Myeloid_vs_CD4_Non-responder	CD4(TNF+ T)	Mono_CD16	IFNG	IFNGR1_IFNGR2	6,58066E-03	30674,3	1,46569E-02	4238	1,00652E+00	35580	2,34742E+00	19223	8,79672E-01	39910	0	54420,5
Myeloid_vs_CD4_Non-responder	Macro_OLFML3	CD4(ISG+ Treg)	HLA-DQA1	LAG3	6,60362E-03	21545,5	9,32979E-03	9364	1,54651E+00	13938	2,86677E+00	10294	9,16153E-01	19711	0	54420,5
Myeloid_vs_CD4_Non-responder	CD4(IL26+ Th17)	Macro_OLFML3	CD99	PILRA	6,60874E-03	33604,5	3,87386E-03	35012	1,33766E+00	20514	1,63035E+00	39944	9,19350E-01	18132	0	54420,5
Myeloid_vs_CD4_Non-responder	Macro_FOLR2+APOE+	CD4(Tn)	CCL3	CCR4	6,61288E-03	23678,7	1,30399E-02	5247	1,64042E+00	11612	3,12408E+00	7165	8,79585E-01	39949	0	54420,5
Myeloid_vs_CD4_Non-responder	Macro_FOLR2+APOE+	CD4(NME1+ T)	HLA-DMB	CD4	6,62447E-03	27982,1	3,54345E-03	39963	1,80302E+00	8541	2,57850E+00	14650	9,11267E-01	22336	0	54420,5
Myeloid_vs_CD4_Non-responder	CD4(CXCL13+ Tfh)	Mono_CD14	CIRBP	TREM1	6,62530E-03	29420,9	6,59564E-03	16069	1,52921E+00	14406	1,63001E+00	39964	9,11470E-01	22245	0	54420,5
Myeloid_vs_CD4_Non-responder	Macro_NLRP3	CD4(IFNG+ Tfh/Th1)	VEGFA	CD44	6,64356E-03	26743,7	7,26447E-03	13947	2,17197E+00	4505	1,62943E+00	39986	9,13950E-01	20860	0	54420,5
Myeloid_vs_CD4_Non-responder	Mono_CD16	CD4(IFNG+ Tfh/Th1)	B2M	CD3D	6,64580E-03	48161,9	3,92307E-03	34337	4,06272E-01	81897	9,59406E-01	70115	9,65183E-01	40	0	54420,5
Myeloid_vs_CD4_Non-responder	CD4(Tn)	Macro_ISG15	HLA-C	LILRB2	6,64688E-03	27807,7	3,54162E-03	39990	1,64754E+00	11458	1,92408E+00	30049	9,50157E-01	3121	0	54420,5
Myeloid_vs_CD4_Non-responder	CD4(CXCL13+ Tfh)	cDC_CLEC9A	HMGB1	THBD	6,65270E-03	33141,5	4,58737E-03	27297	1,36221E+00	19631	1,62925E+00	39997	9,07573E-01	24362	0	54420,5
Myeloid_vs_CD4_Non-responder	Mono_CD14	CD4(AREG+ Tm)	VCAN	SELL	6,67152E-03	18092,3	1,38993E-02	4667	3,11569E+00	834	2,83212E+00	10777	9,16016E-01	19763	0	54420,5
Myeloid_vs_CD4_Non-responder	CD4(Tn)	Macro_FOLR2+APOE+	SEMA4D	CD72	6,68593E-03	33815,1	1,38950E-02	4669	1,84531E+00	7899	1,67048E+00	38496	8,37066E-01	63591	0	54420,5
Myeloid_vs_CD4_Non-responder	Mono_CD16	CD4(ISG+ Treg)	HLA-DRB1	LAG3	6,69439E-03	33025,9	5,33140E-03	21901	9,26147E-01	40047	1,98015E+00	28385	9,14816E-01	20376	0	54420,5
Myeloid_vs_CD4_Non-responder	Mast	CD4(TGFB1+ Th17)	ADCYAP1	DPP4	6,69645E-03	45845,3	1,04880E-01	75	2,70823E+00	1761	1,10706E+00	62831	7,43393E-01	110139	0	54420,5
Myeloid_vs_CD4_Non-responder	Mono_INHBA	CD4(TNF+ T)	IL1B	ADRB2	6,70818E-03	24224,3	1,03259E-02	7908	2,30620E+00	3591	3,54234E+00	3787	8,58728E-01	51415	0	54420,5
Myeloid_vs_CD4_Non-responder	Macro_IFI27	CD4(ISG+ Treg)	HLA-DRA	LAG3	6,71092E-03	22177,3	8,30840E-03	11332	1,56769E+00	13407	2,32054E+00	19793	9,31849E-01	11934	0	54420,5
Myeloid_vs_CD4_Non-responder	CD4(Tn)	Macro_FOLR2+APOE+	HLA-B	CANX	6,72654E-03	46972,7	2,29703E-03	68832	6,98819E-01	55559	1,28828E+00	54287	9,53931E-01	1765	0	54420,5
Myeloid_vs_CD4_Non-responder	CD4(NME1+ T)	Macro_FOLR2+APOE+	HLA-B	LILRB2	6,74553E-03	27631,5	3,53440E-03	40108	1,65880E+00	11220	1,91967E+00	30203	9,52617E-01	2206	0	54420,5
Myeloid_vs_CD4_Non-responder	CD4(GZMK+ Teff)	Macro_IER3	RPS19	C5AR1	6,74637E-03	25882,9	5,30991E-03	22050	1,72060E+00	9987	1,62644E+00	40109	9,50890E-01	2848	0	54420,5
Myeloid_vs_CD4_Non-responder	Macro_NLRP3	CD4(IFNG+ Tfh/Th1)	HLA-DPB1	LAG3	6,75226E-03	30835,9	6,09122E-03	18033	9,24707E-01	40116	2,24208E+00	21530	9,15383E-01	20080	0	54420,5
Myeloid_vs_CD4_Non-responder	CD4(CRTAM- T)	Macro_FOLR2+APOE+	HLA-B	LILRB2	6,75394E-03	27808,5	3,53376E-03	40118	1,65832E+00	11229	1,89162E+00	31066	9,52613E-01	2209	0	54420,5
Myeloid_vs_CD4_Non-responder	CD4(NME1+ T)	Mono_CD16	HLA-A	LILRB1	6,77332E-03	28983,7	4,26720E-03	30400	1,55456E+00	13724	1,62573E+00	40141	9,43315E-01	6233	0	54420,5
Myeloid_vs_CD4_Non-responder	CD4(NME1+ T)	Macro_FOLR2+APOE+	HLA-F	LILRB2	6,77501E-03	32090,5	3,53193E-03	40143	1,68695E+00	10623	1,96292E+00	28874	9,03987E-01	26392	0	54420,5
Myeloid_vs_CD4_Non-responder	CD4(GZMK+ Teff)	Macro_FOLR2+APOE+	HLA-C	LILRB2	6,78345E-03	27545,1	3,53126E-03	40153	1,67618E+00	10846	1,95441E+00	29158	9,50087E-01	3148	0	54420,5
Myeloid_vs_CD4_Non-responder	CD4(NME1+ T)	cDC_LAMP3	LTB	CD40	6,79821E-03	28651,9	1,00896E-02	8216	1,88027E+00	7445	1,31097E+00	53319	9,15822E-01	19859	0	54420,5
Myeloid_vs_CD4_Non-responder	CD4(Tn)	Macro_NLRP3	ANXA1	FPR1	6,79952E-03	35470,1	3,66193E-03	38069	1,16455E+00	27704	1,62495E+00	40172	9,21597E-01	16985	0	54420,5
Myeloid_vs_CD4_Non-responder	CD4(TNF+ T)	Macro_IFI27	CD40LG	CD9	6,80629E-03	29987,7	1,56393E-02	3758	1,39847E+00	18356	1,62483E+00	40180	8,91901E-01	33224	0	54420,5
Myeloid_vs_CD4_Non-responder	Macro_IFI27	CD4(IFNG+ Tfh/Th1)	B2M	CD3D	6,81185E-03	58093,1	3,91192E-03	34467	3,95663E-01	82979	2,53183E-02	118558	9,65135E-01	41	0	54420,5
Myeloid_vs_CD4_Non-responder	Macro_FOLR2+APOE+	CD4(GZMK+ Teff)	TIMP2	CD44	6,81816E-03	32453,5	5,12720E-03	23228	1,31069E+00	21444	1,62453E+00	40194	9,10026E-01	22981	0	54420,5
Myeloid_vs_CD4_Non-responder	CD4(AREG+ Tm)	Macro_OLFML3	HLA-F	LILRB1	6,82155E-03	36885,1	3,83514E-03	35521	1,41697E+00	17726	1,62447E+00	40198	8,85844E-01	36560	0	54420,5
Myeloid_vs_CD4_Non-responder	Macro_NLRP3	CD4(IFNG+ Tfh/Th1)	HLA-DPA1	LAG3	6,82494E-03	30531,5	6,11144E-03	17947	9,23317E-01	40202	2,21457E+00	22164	9,19741E-01	17924	0	54420,5
Myeloid_vs_CD4_Non-responder	Mono_CD16	CD4(IL26+ Th17)	HLA-DRB5	LAG3	6,83599E-03	38650,9	3,82582E-03	35681	9,23139E-01	40215	1,95286E+00	29201	8,90950E-01	33737	0	54420,5
Myeloid_vs_CD4_Non-responder	Macro_FOLR2+APOE+	CD4(Tn)	LGALS9	CD44	6,83854E-03	32777,1	3,52527E-03	40218	1,27804E+00	22712	1,79850E+00	34029	9,30708E-01	12506	0	54420,5
Myeloid_vs_CD4_Non-responder	Macro_NLRP3	CD4(ISG+ Treg)	S100A9	ITGB2	6,84024E-03	23149,5	3,52520E-03	40220	2,36511E+00	3259	3,07185E+00	7729	9,35547E-01	10119	0	54420,5
Myeloid_vs_CD4_Non-responder	Macro_OLFML3	CD4(Tn)	CXCL10	CXCR3	6,85275E-03	25309,3	1,02733E-02	7966	2,64819E+00	1947	3,13297E+00	7077	8,52181E-01	55136	0	54420,5
Myeloid_vs_CD4_Non-responder	Macro_FOLR2+APOE+	CD4(TNF+ T)	LGALS1	CD69	6,86237E-03	30792,5	3,96882E-03	33778	9,22589E-01	40246	2,26053E+00	21100	9,47109E-01	4418	0	54420,5
Myeloid_vs_CD4_Non-responder	Macro_NLRP3	CD4(CXCL13+ Tfh)	S100A9	ITGB2	6,87005E-03	23108,9	3,52340E-03	40255	2,36488E+00	3262	3,09441E+00	7481	9,35532E-01	10126	0	54420,5
Myeloid_vs_CD4_Non-responder	Macro_FOLR2+APOE+	CD4(TNF+ T)	CXCL9	CXCR3	6,87285E-03	21633,1	1,73722E-02	3088	2,91168E+00	1219	3,04892E+00	7974	8,76772E-01	41664	0	54420,5
Myeloid_vs_CD4_Non-responder	CD4(ISG+ Treg)	pDC_LILRA4	TNF	PTPRS	6,87536E-03	30212,1	1,92904E-02	2526	3,94156E+00	243	1,86921E+00	31729	8,39689E-01	62142	0	54420,5
Myeloid_vs_CD4_Non-responder	Macro_ISG15	CD4(CRTAM- T)	VCAN	CD44	6,87829E-03	24469,5	5,69446E-03	19919	1,48345E+00	15707	2,46547E+00	16806	9,24545E-01	15495	0	54420,5
Myeloid_vs_CD4_Non-responder	pDC_LILRA4	CD4(TGFB1+ Th17)	APP	PTGER2	6,88543E-03	25607,5	1,37925E-02	4734	2,27357E+00	3780	3,04849E+00	7979	8,48653E-01	57124	0	54420,5
Myeloid_vs_CD4_Non-responder	CD4(TNFRSF9+ Treg)	Mono_CD14	CIRBP	TREM1	6,89227E-03	29837,9	6,42638E-03	16693	1,50599E+00	15025	1,62228E+00	40281	9,10415E-01	22770	0	54420,5
Myeloid_vs_CD4_Non-responder	Macro_FOLR2+APOE+	CD4(ISG+ Treg)	LGALS3BP	ITGB1	6,91368E-03	36087,3	5,32475E-03	21944	1,20451E+00	25859	1,62169E+00	40306	8,83389E-01	37907	0	54420,5
Myeloid_vs_CD4_Non-responder	CD4(TNF+ T)	Macro_NLRP3	IL23A	LILRB2	6,91589E-03	32359,5	1,97458E-02	2402	1,58140E+00	13037	2,31319E+00	19947	8,21933E-01	71991	0	54420,5
Myeloid_vs_CD4_Non-responder	cDC_CLEC9A	CD4(TNFRSF9+ Treg)	HLA-DRA	LAG3	6,91724E-03	22670,3	5,68722E-03	19948	1,48014E+00	15801	3,38998E+00	4801	9,18782E-01	18381	0	54420,5
Myeloid_vs_CD4_Non-responder	CD4(CXCL13+ Tfh)	Macro_ISG15	TIGIT	NECTIN2	6,92484E-03	25388,5	3,24031E-02	911,5	2,37131E+00	3215,5	1,99038E+00	28075,5	8,78891E-01	40319,5	0	54420,5
Myeloid_vs_CD4_Non-responder	CD4(IL26+ Th17)	Macro_ISG15	CD99	CD81	6,92570E-03	38275,3	3,51940E-03	40320	9,66346E-01	37754	1,66059E+00	38831	9,15433E-01	20051	0	54420,5
Myeloid_vs_CD4_Non-responder	CD4(TNF+ T)	Macro_OLFML3	CD52	SIGLEC10	6,93610E-03	30586,5	5,79546E-03	19402	1,35271E+00	19962	1,60486E+00	40950	9,19235E-01	18198	0	54420,5
Myeloid_vs_CD4_Non-responder	CD4(TNFRSF9+ Treg)	Macro_LYVE1	HMGB1	CD163	6,93859E-03	28628,9	4,38977E-03	29153	1,75228E+00	9406	1,62101E+00	40335	9,36136E-01	9830	0	54420,5

Myeloid_vs_CD4_Post_NR

Myeloid_vs_CD4_Non-responder	Macro_FOLR2+APOE+	CD4(CXCL13+ Tfh)	SPP1	PTGER4	6,94289E-03	28531,1	8,45610E-03	11020	1,11332E+00	30054	3,15893E+00	6821	8,78844E-01	40340	0	54420,5
Myeloid_vs_CD4_Non-responder	Macro_FOLR2+APOE+	CD4(GZMK+ Teff)	CXCL16	CXCR6	6,94719E-03	31201,1	6,14452E-03	17797	1,37920E+00	19022	2,12493E+00	24421	8,78838E-01	40345	0	54420,5
Myeloid_vs_CD4_Non-responder	cDC_CLEC9A	CD4(TNFRSF9+ Treg)	HLA-DPB1	LAG3	6,94825E-03	22352,1	6,12488E-03	17896	1,50897E+00	14931	3,42683E+00	4542	9,15597E-01	19971	0	54420,5
Myeloid_vs_CD4_Non-responder	CD4(ISG+ Treg)	pDC_LILRA4	TNFSF13B	TNFRSF17	6,96376E-03	45724,3	1,54133E-02	3863	2,02313E+00	5833	1,52378E+00	44198	7,16075E-01	120307	0	54420,5
Myeloid_vs_CD4_Non-responder	Mast	CD4(AREG+ Tm)	ADCYAP1	DPP4	6,96968E-03	46113,9	9,90872E-02	89	2,69195E+00	1797	1,12311E+00	62013	7,37936E-01	112250	0	54420,5
Myeloid_vs_CD4_Non-responder	Macro_LYVE1	CD4(ISG+ Treg)	HLA-DQB1	LAG3	6,97258E-03	24328,7	8,59180E-03	10736	1,43146E+00	17274	2,31098E+00	19989	9,17063E-01	19224	0	54420,5
Myeloid_vs_CD4_Non-responder	Macro_FOLR2-APOE+	CD4(IFNG+ Tfh/Th1)	B2M	CD3D	6,97790E-03	59647,1	3,89999E-03	34641	3,84317E-01	84096	-1,25135E-01	125036	9,65083E-01	42	0	54420,5
Myeloid_vs_CD4_Non-responder	Mono_CD14	CD4(IFNG+ Tfh/Th1)	VCAN	CD44	6,98884E-03	19063,5	8,59748E-03	10723	2,94110E+00	1157	2,31060E+00	20001	9,37716E-01	9016	0	54420,5
Myeloid_vs_CD4_Non-responder	CD4(AREG+ Tm)	Macro_ISG15	CCL5	CCR1	6,98948E-03	28277,1	1,80513E-02	2885	1,83046E+00	8121	1,61923E+00	40394	8,87704E-01	35565	0	54420,5
Myeloid_vs_CD4_Non-responder	CD4(IL26+ Th17)	Macro_FOLR2+APOE+	ADAM10	TREM2	6,99035E-03	27808,7	6,24293E-03	17397	1,57159E+00	13281	2,64285E+00	13550	8,78746E-01	40395	0	54420,5
Myeloid_vs_CD4_Non-responder	Macro_NLRP3	CD4(CXCL13+ Tfh)	THBS1	CD47	6,99641E-03	29434,1	5,24475E-03	22452	1,70653E+00	10259	2,32744E+00	19637	8,78729E-01	40402	0	54420,5
Myeloid_vs_CD4_Non-responder	CD4(IL26+ Th17)	Mono_CD16	CD52	SIGLEC10	7,00594E-03	30031,3	5,89940E-03	18894	1,39236E+00	18576	1,61870E+00	40413	9,19893E-01	17853	0	54420,5
Myeloid_vs_CD4_Non-responder	CD4(Tn)	Mono_CD16	HLA-B	LILRB1	7,01287E-03	29372,9	4,08907E-03	32336	1,53999E+00	14102	1,61841E+00	40421	9,44762E-01	5585	0	54420,5
Myeloid_vs_CD4_Non-responder	CD4(ISG+ Treg)	Macro_ISG15	LGALS9	HAVCR2	7,01287E-03	34322,7	3,85503E-03	35250	1,37069E+00	19319	2,21248E+00	22203	8,78697E-01	40421	0	54420,5
Myeloid_vs_CD4_Non-responder	CD4(NME1+ T)	Mono_CD16	HLA-F	LILRB1	7,01374E-03	35291,1	4,05615E-03	32703	1,54857E+00	13882	1,61839E+00	40422	8,88646E-01	35028	0	54420,5
Myeloid_vs_CD4_Non-responder	Macro_FOLR2+APOE+	CD4(TNFRSF9+ Treg)	APOE	LSR	7,01895E-03	22799,7	1,19353E-02	6166	1,74762E+00	9479	3,58557E+00	3505	8,78682E-01	40428	0	54420,5
Myeloid_vs_CD4_Non-responder	CD4(TGFB1+ Th17)	Mono_INHBA	TGFB1	SDC2	7,02676E-03	28444,5	1,73331E-02	3105	2,04800E+00	5592	1,61791E+00	40437	8,81923E-01	38668	0	54420,5
Myeloid_vs_CD4_Non-responder	Mast	CD4(TNF+ T)	ADCYAP1	ADRB2	7,03882E-03	44822,7	6,55834E-02	226	2,69019E+00	1806	1,30127E+00	53753	7,33665E-01	113908	0	54420,5
Myeloid_vs_CD4_Non-responder	Macro_FOLR2+APOE+	CD4(IFNG+ Tfh/Th1)	CD14	ITGB2	7,04503E-03	23409,3	3,51103E-03	40458	1,96281E+00	6484	3,10591E+00	7364	9,39043E-01	8320	0	54420,5
Myeloid_vs_CD4_Non-responder	CD4(TNF+ T)	Mono_CD16	TNFSF9	HLA-DPA1	7,05026E-03	38291,9	4,02918E-03	33026	9,27061E-01	39992	2,16041E+00	23557	8,78612E-01	40464	0	54420,5
Myeloid_vs_CD4_Non-responder	CD4(IL26+ Th17)	pDC_LILRA4	HSP90B1	TLR7	7,05636E-03	29317,9	8,18738E-03	11601	1,91153E+00	7042	1,82755E+00	33055	8,78598E-01	40471	0	54420,5
Myeloid_vs_CD4_Non-responder	CD4(CRTAM- T)	Macro_FOLR2+APOE+	HLA-C	LILRB1	7,06508E-03	32357,3	3,56057E-03	39690	1,39927E+00	18327	1,61663E+00	40481	9,37957E-01	8868	0	54420,5
Myeloid_vs_CD4_Non-responder	CD4(TGFB1+ Th17)	pDC_LILRA4	HMGB1	TLR9	7,07737E-03	27407,7	3,38364E-02	823	2,68676E+00	1811	1,33734E+00	52140	9,01494E-01	27844	0	54420,5
Myeloid_vs_CD4_Non-responder	Macro_LYVE1	CD4(NME1+ T)	C1QB	C1QBP	7,07740E-03	20447,9	5,66502E-03	20066	2,02196E+00	5846	2,82209E+00	10905	9,33689E-01	11002	0	54420,5
Myeloid_vs_CD4_Non-responder	CD4(ISG+ Treg)	pDC_LILRA4	HSP90B1	TLR9	7,07848E-03	33103,3	2,06034E-02	2233	2,58847E+00	2159	1,26902E+00	55158	8,58491E-01	51546	0	54420,5
Myeloid_vs_CD4_Non-responder	CD4(NME1+ T)	Mono_CD16	CD52	SIGLEC10	7,08424E-03	31029,9	5,66410E-03	20071	1,35329E+00	19941	1,57498E+00	42153	9,18380E-01	18564	0	54420,5
Myeloid_vs_CD4_Non-responder	CD4(ISG+ Treg)	pDC_LILRA4	HMGB1	TLR9	7,08874E-03	27395,1	3,34532E-02	854	2,67640E+00	1846	1,34673E+00	51731	9,00987E-01	28124	0	54420,5
Myeloid_vs_CD4_Non-responder	Macro_FOLR2+APOE+	CD4(NME1+ T)	TIMP2	ITGB1	7,09130E-03	34805,1	5,75689E-03	19589	1,29243E+00	22137	1,70022E+00	37368	8,78527E-01	40511	0	54420,5
Myeloid_vs_CD4_Non-responder	CD4(TGFB1+ Th17)	Mono_CD16	HLA-B	LILRB1	7,09742E-03	29003,5	4,15150E-03	31638	1,58057E+00	13059	1,61581E+00	40518	9,45157E-01	5382	0	54420,5
Myeloid_vs_CD4_Non-responder	CD4(ISG+ Treg)	pDC_LILRA4	CD70	TNFRSF17	7,10157E-03	47382,1	9,87986E-02	91	2,03421E+00	5728	1,47218E+00	46320	6,82208E-01	130351	0	54420,5
Myeloid_vs_CD4_Non-responder	CD4(ISG+ Treg)	pDC_LILRA4	IRAK4	TLR7	7,11185E-03	37730,1	1,23704E-02	5774	1,90940E+00	7064	1,88236E+00	31358	7,87453E-01	90034	0	54420,5
Myeloid_vs_CD4_Non-responder	cDC_LAMP3	CD4(IFNG+ Tfh/Th1)	HLA-DRB5	LAG3	7,12404E-03	22737,5	9,21559E-03	9561	1,34855E+00	20100	2,54484E+00	15284	9,26902E-01	14322	0	54420,5
Myeloid_vs_CD4_Non-responder	CD4(NME1+ T)	Macro_FOLR2+APOE+	HMGB1	TLR2	7,12813E-03	32779,3	4,36451E-03	29409	1,34311E+00	20294	1,61494E+00	40553	9,17073E-01	19220	0	54420,5
Myeloid_vs_CD4_Non-responder	CD4(IFNG+ Tfh/Th1)	cDC_LAMP3	CD28	CD86	7,13604E-03	31304,9	8,59023E-03	10740,5	1,67355E+00	10904,5	1,61481E+00	40562,5	8,79692E-01	39896,5	0	54420,5
Myeloid_vs_CD4_Non-responder	CD4(CXCL13+ Tfh)	Macro_FOLR2+APOE+	B2M	LILRB2	7,13956E-03	27939,5	3,50560E-03	40566	1,61798E+00	12156	1,86926E+00	31726	9,57327E-01	829	0	54420,5
Myeloid_vs_CD4_Non-responder	Macro_ISG15	CD4(IFNG+ Tfh/Th1)	CD14	ITGB1	7,14056E-03	20890,3	5,65606E-03	20112	1,82798E+00	8166	2,95398E+00	9142	9,30484E-01	12611	0	54420,5
Myeloid_vs_CD4_Non-responder	Macro_FOLR2+APOE-	CD4(IFNG+ Tfh/Th1)	B2M	CD3D	7,14395E-03	60062,1	3,88816E-03	34817	3,73066E-01	85244	-1,43547E-01	125786	9,65032E-01	43	0	54420,5
Myeloid_vs_CD4_Non-responder	CD4(TNF+ T)	Macro_ISG15	HLA-C	LILRB1	7,14837E-03	29542,5	4,14636E-03	31695	1,53222E+00	14317	1,61451E+00	40576	9,42244E-01	6704	0	54420,5
Myeloid_vs_CD4_Non-responder	Macro_ISG15	CD4(Tn)	CCL2	CCR4	7,17114E-03	28210,9	2,71552E-02	1301	1,83317E+00	8091	3,15579E+00	6855	8,24878E-01	70387	0	54420,5
Myeloid_vs_CD4_Non-responder	CD4(CXCL13+ Tfh)	Macro_FOLR2+APOE+	HLA-B	LILRB2	7,17130E-03	27872,7	3,50375E-03	40602	1,63592E+00	11719	1,91548E+00	30333	9,52420E-01	2289	0	54420,5
Myeloid_vs_CD4_Non-responder	Macro_ISG15	CD4(ISG+ Treg)	HLA-DRB1	LAG3	7,17230E-03	22003,5	7,57011E-03	13123	1,34752E+00	20135	3,01791E+00	8333	9,27520E-01	14006	0	54420,5
Myeloid_vs_CD4_Non-responder	pDC_LILRA4	CD4(TNFRSF9+ Treg)	APP	LRP10	7,17890E-03	20863,1	1,19671E-02	6133	2,27139E+00	3796	3,03817E+00	8094	8,94330E-01	31872	0	54420,5
Myeloid_vs_CD4_Non-responder	Macro_NLRP3	CD4(TNFRSF9+ Treg)	THBS1	CD47	7,18721E-03	29591,9	5,20586E-03	22696	1,70435E+00	10314	2,31520E+00	19909	8,78332E-01	40620	0	54420,5
Myeloid_vs_CD4_Non-responder	CD4(CXCL13+ Tfh)	Macro_ISG15	HLA-C	LILRB1	7,19518E-03	29355,1	4,18182E-03	31290	1,55054E+00	13832	1,61289E+00	40629	9,42475E-01	6604	0	54420,5
Myeloid_vs_CD4_Non-responder	Mono_CD14	CD4(NME1+ T)	VCAN	SELL	7,20691E-03	18851,1	1,36149E-02	4840	3,10780E+00	851	2,61748E+00	13984	9,15217E-01	20160	0	54420,5
Myeloid_vs_CD4_Non-responder	CD4(IFNG+ Tfh/Th1)	Macro_FOLR2+APOE+	CD28	CD86	7,21645E-03	29706,9	8,36282E-03	11217,5	1,62736E+00	11908,5	1,91548E+00	30334,5	8,78265E-01	40653,5	0	54420,5
Myeloid_vs_CD4_Non-responder	Macro_FOLR2+APOE+	CD4(IFNG+ Tfh/Th1)	CD86	CD28	7,21734E-03	29706,9	8,36282E-03	11217,5	1,62736E+00	11908,5	1,91548E+00	30334,5	8,78265E-01	40653,5	0	54420,5
Myeloid_vs_CD4_Non-responder	CD4(CRTAM- T)	Macro_FOLR2+APOE+	B2M	LILRB2	7,22000E-03	28174,5	3,50034E-03	40657	1,61245E+00	12274	1,83920E+00	32686	9,57296E-01	835	0	54420,5
Myeloid_vs_CD4_Non-responder	CD4(TNF+ T)	pDC_LILRA4	HLA-C	NOTCH4	7,22774E-03	34033,3	1,28639E-02	5404	1,34650E+00	20175	9,51329E-01	70514	9,16262E-01	19653	0	54420,5
Myeloid_vs_CD4_Non-responder	CD4(IFNG+ Tfh/Th1)	Macro_NLRP3	HLA-F	LILRB2	7,23778E-03	34176,5	3,49925E-03	40677	1,46607E+00	16224	1,83092E+00	32948	9,03583E-01	26613	0	54420,5
Myeloid_vs_CD4_Non-responder	CD4(CXCL13+ Tfh)	Macro_FOLR2+APOE+	ADAM10	GPNMB	7,24583E-03	25313,7	8,59640E-03	10726	2,07622E+00	5327	2,72886E+00	12238	8,72434E-01	43857	0	54420,5
Myeloid_vs_CD4_Non-responder	CD4(TGFB1+ Th17)	Mono_CD14	CD99	PILRA	7,26540E-03	35601,9	3,60572E-03	38984	1,23756E+00	24453	1,61075E+00	40708	9,16651E-01	19444	0	54420,5
Myeloid_vs_CD4_Non-responder	CD4(CRTAM- T)	Macro_OLFML3	TGFB1	ENG	7,27522E-03	34949,3	5,39046E-03	21579	1,19630E+00	26245	1,61041E+00	40719	8,94529E-01	31783	0	54420,5
Myeloid_vs_CD4_Non-responder	CD4(ISG+ Treg)	Macro_FOLR2+APOE+	HLA-C	LILRB1	7,28058E-03	32614,1	3,53756E-03	40048	1,38325E+00	18888	1,61029E+00	40725	9,37768E-01	8989	0	54420,5
Myeloid_vs_CD4_Non-responder	CD4(GZMK+ Teff)	Mono_CD16	HLA-C	LILRB1	7,29490E-03	29808,9	4,05537E-03	32711	1,53780E+00	14168	1,60987E+00	40741	9,41637E-01	7004	0	54420,5
Myeloid_vs_CD4_Non-responder	Macro_LYVE1	CD4(IFNG+ Tfh/Th1)	B2M	CD3D	7,30999E-03	55439,5	3,87717E-03	34958	3,62604E-01	86323	3,68770E-01	101452	9,64984E-01	44	0	54420,5
Myeloid_vs_CD4_Non-responder	pDC_LILRA4	CD4(ISG+ Treg)	APP	LRP10	7,31682E-03	20214,3	1,30333E-02	5252	2,30610E+00	3593	3,03318E+00	8147	8,98296E-01	29659	0	54420,5
Myeloid_vs_CD4_Non-responder	CD4(GZMK+ Teff)	Macro_FOLR2+APOE+	HLA-B	LILRB1	7,32090E-03	32713,7	3,49324E-03	40770	1,34527E+00	20219	1,61483E+00	40558	9,40506E-01	7601	0	54420,5
Myeloid_vs_CD4_Non-responder	CD4(CXCL13+ Tfh)	Macro_IER3	RPS19	C5AR1	7,32449E-03	26056,3	5,29344E-03	22144	1,71623E+00	10067	1,60861E+00	40774	9,50817E-01	2876	0	54420,5
Myeloid_vs_CD4_Non-responder	CD4(ISG+ Treg)	cDC_CLEC9A	LTB	CD40	7,32692E-03	27911,3	9,88537E-03	8511	1,49463E+00	15359	1,60286E+00	41020	9,15031E-01	20246	0	54420,5
Myeloid_vs_CD4_Non-responder	cDC(CD1C)	CD4(TNFRSF9+ Treg)	HLA-DPB1	LAG3	7,33113E-03	23472,9	6,03516E-03	18278	1,48389E+00	15692	2,98806E+00	8725	9,15025E-01	20249	0	54420,5
Myeloid_vs_CD4_Non-responder	CD4(IFNG+ Tfh/Th1)	Macro_ISG15	CD28	CD86	7,33258E-03	29639,7	8,32299E-03	11312,5	1,61927E+00	12117,5	1,94089E+00	29564,5	8,78010E-01	40783,5	0	54420,5
Myeloid_vs_CD4_Non-responder	Mono_CD16	CD4(TNFRSF9+ Treg)	VCAN	SELL	7,34337E-03	35957,5	6,05015E-03	18213	1,14481E+00	28584	1,68901E+00	37775	8,77989E-01	40795	0	54420,5
Myeloid_vs_CD4_Non-responder	CD4(IL26+ Th17)	Macro_OLFML3	HLA-C	LILRB1	7,34787E-03	30810,9	3,83908E-03	35472	1,48753E+00	15564	1,60803E+00	40800	9,40112E-01	7798	0	54420,5
Myeloid_vs_CD4_Non-responder	CD4(NME1+ T)	Macro_IER3	RPS19	C5AR1	7,35688E-03	26646,1	5,07991E-03	23546	1,65964E+00	11202	1,60764E+00	40810	9,49845E-01	3252	0	54420,5
Myeloid_vs_CD4_Non-responder	Mono_CD14	CD4(TNF+ T)	VCAN	ITGA4	7,36913E-03	18811,5	1,06827E-02	7450	3,08457E+00	883	2,81358E+00	11028	9,14976E-01	20276	0	54420,5
Myeloid_vs_CD4_Non-responder	pDC_LILRA4	CD4(IFNG+ Tfh/Th1)	HLA-DRB5	LAG3	7,37195E-03	20724,1	9,17636E-03	9625	1,34362E+00	20278	3,37838E+00	4895	9,26757E-01	14402	0	54420,5
Myeloid_vs_CD4_Non-responder	CD4(NME1+ T)	Mono_CD16	B2M	LILRB1	7,37312E-03	28977,3	4,08654E-03	32363	1,53505E+00	14237	1,60740E+00	40828	9,50391E-01	3038	0	54420,5
Myeloid_vs_CD4_Non-responder	Macro_NLRP3	CD4(TNF+ T)	CXCL2	DPP4	7,37984E-03	27709,7	1,51948E-02	3967	2,34054E+00	3386	3,03128E+00	8171	8,28150E-01	68604	0	54420,5
Myeloid_vs_CD4_Non-responder	Macro_FOLR2+APOE+	CD4(NME1+ T)	PLAU	ITGB1	7,38939E-03	29114,7	9,88501E-03	8512	1,76850E+00	9106	1,83915E+00	32689	8,77885E-01	40846	0	54420,5
Myeloid_vs_CD4_Non-responder	CD4(CXCL13+ Tfh)	Macro_NLRP3	CIRBP	TREM1	7,41203E-03	31245,1	6,01075E-03	18372	1,40203E+00	18246	1,60649E+00	40871	9,07651E-01	24316	0	54420,5

Myeloid_vs_CD4_Post_NR

Myeloid_vs_CD4_Non-responder	Mono_CD16	CD4(TGFB1+ Th17)	LGALS9	PTPRC	7,41203E-03	34878,3	3,48518E-03	40871	1,12829E+00	29341	1,64942E+00	39220	9,34682E-01	10539	0	54420,5
Myeloid_vs_CD4_Non-responder	CD4(TNF+ T)	pDC_LILRA4	TNFSF9	HLA-DPA1	7,41566E-03	36860,7	3,97410E-03	33707	9,11989E-01	40875	2,58964E+00	14450	8,77876E-01	40851	0	54420,5
Myeloid_vs_CD4_Non-responder	CD4(Tn)	Macro_ISG15	CIRBP	TREM1	7,42201E-03	34102,3	4,99419E-03	24170	1,29256E+00	22131	1,60620E+00	40882	8,99587E-01	28908	0	54420,5
Myeloid_vs_CD4_Non-responder	Macro_NLRP3	CD4(Tn)	VCAN	ITGA4	7,43109E-03	28786,7	4,76044E-03	25908	2,46959E+00	2691	2,30969E+00	20022	8,77806E-01	40892	0	54420,5
Myeloid_vs_CD4_Non-responder	CD4(ISG+ Treg)	Mast	CALR	ITGA2B	7,43792E-03	49114,7	2,48540E-02	1573	1,88492E+00	7375	1,51416E-01	112538	8,26218E-01	69667	0	54420,5
Myeloid_vs_CD4_Non-responder	Macro_NLRP3	CD4(AREG+ Tm)	CXCL2	DPP4	7,44056E-03	25786,7	1,87301E-02	2680	2,39309E+00	3094	3,02925E+00	8194	8,42529E-01	60545	0	54420,5
Myeloid_vs_CD4_Non-responder	pDC_LILRA4	CD4(IFNG+ Tfh/Th1)	HLA-DQA1	LAG3	7,46118E-03	21455,1	9,70830E-03	8776	1,34195E+00	20341	3,38776E+00	4819	9,17668E-01	18919	0	54420,5
Myeloid_vs_CD4_Non-responder	CD4(CXCL13+ Tfh)	Mono_CD16	HLA-B	LILRA1	7,46261E-03	25613,7	9,65895E-03	8851	2,03292E+00	5738	1,38645E+00	49990	9,37630E-01	9069	0	54420,5
Myeloid_vs_CD4_Non-responder	CD4(CXCL13+ Tfh)	Mono_CD16	HLA-A	LILRA1	7,46830E-03	25506,1	1,01838E-02	8084	2,07583E+00	5334	1,38698E+00	49966	9,36315E-01	9726	0	54420,5
Myeloid_vs_CD4_Non-responder	CD4(IL26+ Th17)	pDC_LILRA4	HMGB1	TLR9	7,47650E-03	27424,3	3,32817E-02	863	2,67177E+00	1862	1,34680E+00	51724	9,00758E-01	28252	0	54420,5
Myeloid_vs_CD4_Non-responder	CD4(Tn)	Macro_OLFML3	B2M	LILRB2	7,48443E-03	34684,3	2,94318E-03	51649	1,28587E+00	22409	1,55099E+00	43080	9,53609E-01	1863	0	54420,5
Myeloid_vs_CD4_Non-responder	CD4(CXCL13+ Tfh)	Mono_CD16	HLA-C	LILRA1	7,48682E-03	28021,9	9,01241E-03	9931	1,89997E+00	7184	1,23312E+00	56827	9,32227E-01	11747	0	54420,5
Myeloid_vs_CD4_Non-responder	CD4(CXCL13+ Tfh)	Mono_CD16	HLA-C	LILRA3	7,48968E-03	29070,5	1,06725E-02	7464	1,64764E+00	11456	1,27259E+00	55002	9,21551E-01	17010	0	54420,5
Myeloid_vs_CD4_Non-responder	CD4(IL26+ Th17)	pDC_LILRA4	TNF	TNFRSF21	7,49125E-03	25053,3	1,30382E-02	5248	2,62527E+00	2033	2,19613E+00	22607	8,77696E-01	40958	0	54420,5
Myeloid_vs_CD4_Non-responder	CD4(TGFB1+ Th17)	Macro_FOLR2+APOE+	B2M	LILRB2	7,49308E-03	28345,1	3,48071E-03	40960	1,59184E+00	12783	1,83863E+00	32698	9,57181E-01	864	0	54420,5
Myeloid_vs_CD4_Non-responder	Macro_LYVE1	CD4(ISG+ Treg)	HLA-DQA1	LAG3	7,50395E-03	23758,5	9,01533E-03	9923	1,49605E+00	15307	2,36969E+00	18771	9,14827E-01	20371	0	54420,5
Myeloid_vs_CD4_Non-responder	Mono_INHBA	CD4(CXCL13+ Tfh)	CCL3	CCR4	7,50959E-03	21366,5	1,61722E-02	3525	1,94258E+00	6694	3,02706E+00	8220	8,90528E-01	33973	0	54420,5
Myeloid_vs_CD4_Non-responder	CD4(NME1+ T)	Macro_ISG15	HMGB1	CD163	7,51231E-03	31469,5	3,47974E-03	40981	1,27722E+00	22745	2,07120E+00	25823	9,28830E-01	13378	0	54420,5
Myeloid_vs_CD4_Non-responder	Macro_NLRP3	CD4(CRTAM- T)	CD55	ADGRE5	7,52515E-03	32147,7	7,11548E-03	14349	1,00806E+00	35498	1,60368E+00	40995	9,24592E-01	15476	0	54420,5
Myeloid_vs_CD4_Non-responder	CD4(TGFB1+ Th17)	Macro_NLRP3	TGFB1	ENG	7,52882E-03	39048,7	4,28117E-03	30245	1,08356E+00	31568	1,60354E+00	40999	8,83155E-01	38011	0	54420,5
Myeloid_vs_CD4_Non-responder	Macro_NLRP3	CD4(TNF+ T)	THBS1	CD47	7,52974E-03	29391,3	5,13751E-03	23158	1,70052E+00	10383	2,40679E+00	17995	8,77624E-01	41000	0	54420,5
Myeloid_vs_CD4_Non-responder	CD4(GZMK+ Teff)	Macro_OLFML3	HLA-F	LILRB2	7,53801E-03	33546,9	3,47814E-03	41009	1,50084E+00	15159	1,91420E+00	30387	9,03319E-01	26759	0	54420,5
Myeloid_vs_CD4_Non-responder	CD4(AREG+ Tm)	Macro_FOLR2+APOE+	B2M	LILRB2	7,56931E-03	28462,1	3,47621E-03	41043	1,58711E+00	12906	1,82721E+00	33071	9,57155E-01	870	0	54420,5
Myeloid_vs_CD4_Non-responder	CD4(CXCL13+ Tfh)	Macro_FOLR2+APOE+	CD40LG	CD9	7,57996E-03	28609,7	1,12212E-02	6854	1,33978E+00	20424	2,36664E+00	18817	8,74826E-01	42533	0	54420,5
Myeloid_vs_CD4_Non-responder	CD4(ISG+ Treg)	Macro_FOLR2+APOE+	LTA_LTB	LTBR	7,59516E-03	31526,5	9,81564E-03	8617	1,36964E+00	19358	1,60157E+00	41071	8,90200E-01	34166	0	54420,5
Myeloid_vs_CD4_Non-responder	CD4(CXCL13+ Tfh)	Mono_CD16	HLA-F	LILRB1	7,59886E-03	36989,5	3,72239E-03	37166	1,51007E+00	14911	1,60153E+00	41075	8,84326E-01	37375	0	54420,5
Myeloid_vs_CD4_Non-responder	CD4(AREG+ Tm)	Macro_FOLR2+APOE+	LTB	CD40	7,60904E-03	27983,9	9,53353E-03	9029	1,53153E+00	14338	1,60120E+00	41086	9,13611E-01	21046	0	54420,5
Myeloid_vs_CD4_Non-responder	Macro_NLRP3	CD4(Tn)	GNAI2	S1PR1	7,61275E-03	37051,9	8,01354E-03	12015	9,18951E-01	40474	1,70332E+00	37260	8,77437E-01	41090	0	54420,5
Myeloid_vs_CD4_Non-responder	Macro_FOLR2+APOE+	CD4(GZMK+ Teff)	HBEGF	CD44	7,61460E-03	38185,1	3,64803E-03	38297	9,92493E-01	36346	1,60112E+00	41092	9,14122E-01	20770	0	54420,5
Myeloid_vs_CD4_Non-responder	CD4(TNF+ T)	Macro_NLRP3	TNF	TNFRSF1A	7,62294E-03	35563,5	5,86131E-03	19086	9,08043E-01	41101	1,97759E+00	28460	8,89169E-01	34750	0	54420,5
Myeloid_vs_CD4_Non-responder	CD4(AREG+ Tm)	Macro_FOLR2+APOE+	HLA-A	LILRB2	7,63965E-03	29841,9	3,47163E-03	41119	1,53328E+00	14277	1,73446E+00	36186	9,49944E-01	3207	0	54420,5
Myeloid_vs_CD4_Non-responder	cDC_CLEC9A	CD4(IL26+ Th17)	HLA-DPA1	LAG3	7,64348E-03	23666,5	5,58274E-03	20468	1,44252E+00	16950	3,15280E+00	6881	9,16337E-01	19613	0	54420,5
Myeloid_vs_CD4_Non-responder	CD4(GZMK+ Teff)	Macro_FOLR2+APOE+	TGFB1	ENG	7,66197E-03	37047,5	4,36791E-03	29375	1,27456E+00	22848	1,60008E+00	41143	8,84186E-01	37451	0	54420,5
Myeloid_vs_CD4_Non-responder	CD4(ISG+ Treg)	Macro_FOLR2-APOE+	ADAM10	TREM2	7,66663E-03	32046,5	6,08226E-03	18069	1,81750E+00	8319	1,67599E+00	38276	8,77350E-01	41148	0	54420,5
Myeloid_vs_CD4_Non-responder	CD4(IL26+ Th17)	Mono_CD16	HLA-B	LILRA1	7,67046E-03	23999,9	1,01809E-02	8086	2,17426E+00	4487	1,51107E+00	44726	9,39151E-01	8280	0	54420,5
Myeloid_vs_CD4_Non-responder	Macro_FOLR2+APOE+	CD4(CXCL13+ Tfh)	CD14	ITGB1	7,67408E-03	28201,1	3,46896E-03	41156	1,70136E+00	10369	2,63802E+00	13622	9,12912E-01	21438	0	54420,5
Myeloid_vs_CD4_Non-responder	CD4(CXCL13+ Tfh)	Macro_OLFML3	CD28	CD86	7,67781E-03	34238,7	8,32776E-03	11295,5	1,25872E+00	23551,5	1,59957E+00	41160,5	8,78041E-01	40765,5	0	54420,5
Myeloid_vs_CD4_Non-responder	CD4(TNF+ T)	cDC(CD1C)	CD40LG	CD9	7,69462E-03	33278,9	1,17451E-02	6344	1,07664E+00	31922	1,84454E+00	32530	8,77303E-01	41178	0	54420,5
Myeloid_vs_CD4_Non-responder	Macro_FOLR2+APOE+	CD4(IFNG+ Tfh/Th1)	HLA-DQB1	CD4	7,70022E-03	24727,9	3,46704E-03	41184	1,57701E+00	13138	3,16742E+00	6741	9,39399E-01	8156	0	54420,5
Myeloid_vs_CD4_Non-responder	CD4(GZMK+ Teff)	Macro_ISG15	CCL5	CCRL2	7,71051E-03	30431,7	1,90810E-02	2572	1,39891E+00	18340	1,59882E+00	41195	8,87562E-01	35631	0	54420,5
Myeloid_vs_CD4_Non-responder	CD4(TNFRSF9+ Treg)	Macro_NLRP3	CIRBP	TREM1	7,71426E-03	31740,3	5,85650E-03	19117	1,37881E+00	19035	1,59875E+00	41199	9,06555E-01	24930	0	54420,5
Myeloid_vs_CD4_Non-responder	Macro_FOLR2+APOE+	CD4(TGFB1+ Th17)	MRC1	PTPRC	7,71894E-03	34034,9	6,73979E-03	15547	1,09864E+00	30788	1,59865E+00	41204	9,00827E-01	28215	0	54420,5
Myeloid_vs_CD4_Non-responder	CD4(IL26+ Th17)	cDC_LAMP3	CD28	CD86	7,73394E-03	31688,7	8,39101E-03	11167,5	1,66498E+00	11081,5	1,59840E+00	41220,5	8,78445E-01	40553,5	0	54420,5
Myeloid_vs_CD4_Non-responder	Macro_NLRP3	CD4(CXCL13+ Tfh)	VEGFA	ITGB1	7,74520E-03	29749,3	7,21780E-03	14057	2,19806E+00	4293	1,77714E+00	34744	8,77214E-01	41232	0	54420,5
Myeloid_vs_CD4_Non-responder	Macro_FOLR2+APOE+	CD4(GZMK+ Teff)	CD72	CD5	7,77154E-03	28130,1	2,33780E-02	1763	1,93255E+00	6811	1,72834E+00	36396	8,77163E-01	41260	0	54420,5
Myeloid_vs_CD4_Non-responder	Mono_INHBA	CD4(Tn)	CCL3	CCR4	7,77621E-03	21818,9	1,52967E-02	3917	1,92412E+00	6912	3,01877E+00	8319	8,87785E-01	35526	0	54420,5
Myeloid_vs_CD4_Non-responder	CD4(IL26+ Th17)	Mono_CD16	ANXA1	FPR1	7,78096E-03	34538,9	3,49705E-03	40709	1,39597E+00	18454	1,59716E+00	41270	9,19916E-01	17841	0	54420,5
Myeloid_vs_CD4_Non-responder	CD4(IFNG+ Tfh/Th1)	Macro_FOLR2+APOE+	CALR	LRP1	7,78096E-03	35477,5	3,46275E-03	41270	1,34704E+00	20156	1,68154E+00	38080	9,09202E-01	23461	0	54420,5
Myeloid_vs_CD4_Non-responder	Macro_NLRP3	CD4(CRTAM- T)	CD14	ITGA4	7,81779E-03	32992,1	3,45980E-03	41309	1,30071E+00	21839	2,15692E+00	23636	9,08657E-01	23756	0	54420,5
Myeloid_vs_CD4_Non-responder	CD4(AREG+ Tm)	Macro_FOLR2+APOE+	HLA-F	LILRB1	7,81969E-03	39609,9	3,49480E-03	40752	1,28543E+00	22434	1,59592E+00	41311	8,81060E-01	39132	0	54420,5
Myeloid_vs_CD4_Non-responder	CD4(TNFRSF9+ Treg)	Mono_CD14	HLA-F	LILRB2	7,82442E-03	35443,9	3,45948E-03	41316	1,38865E+00	18706	1,74349E+00	35872	9,03084E-01	26905	0	54420,5
Myeloid_vs_CD4_Non-responder	CD4(ISG+ Treg)	Macro_OLFML3	CD28	CD86	7,83579E-03	33595,3	8,74488E-03	10413,5	1,28569E+00	22420,5	1,59552E+00	41328,5	8,80633E-01	39393,5	0	54420,5
Myeloid_vs_CD4_Non-responder	CD4(IL26+ Th17)	Macro_FOLR2+APOE+	CD28	CD86	7,84432E-03	30077,1	8,16887E-03	11646,5	1,61879E+00	12129,5	1,89907E+00	30851,5	8,77006E-01	41337,5	0	54420,5
Myeloid_vs_CD4_Non-responder	Macro_FOLR2+APOE+	CD4(IL26+ Th17)	CD86	CD28	7,84527E-03	30077,1	8,16887E-03	11646,5	1,61879E+00	12129,5	1,89907E+00	30851,5	8,77006E-01	41337,5	0	54420,5
Myeloid_vs_CD4_Non-responder	Macro_ISG15	CD4(CRTAM- T)	HBEGF	CD44	7,85115E-03	23321,5	6,29935E-03	17162	1,57495E+00	13191	2,28342E+00	20610	9,33278E-01	11224	0	54420,5
Myeloid_vs_CD4_Non-responder	CD4(TNF+ T)	Macro_NLRP3	IFNG	IFNGR1_IFNGR2	7,85382E-03	32118,5	1,39402E-02	4639	9,73217E-01	37394	2,18919E+00	22792	8,76994E-01	41347	0	54420,5
Myeloid_vs_CD4_Non-responder	CD4(NME1+ T)	pDC_LILRA4	HSP90B1	TLR7	7,86047E-03	30056,9	7,94442E-03	12164	1,89519E+00	7243	1,76674E+00	35103	8,76982E-01	41354	0	54420,5
Myeloid_vs_CD4_Non-responder	CD4(CRTAM- T)	Macro_NLRP3	HMGB1	THBD	7,86998E-03	32825,5	4,57070E-03	27450	1,45856E+00	16458	1,59477E+00	41364	9,07420E-01	24435	0	54420,5
Myeloid_vs_CD4_Non-responder	cDC_CLEC9A	CD4(TNFRSF9+ Treg)	HLA-DRB1	LAG3	7,87332E-03	23976,9	5,55742E-03	20625	1,36256E+00	19613	3,28767E+00	5659	9,16420E-01	19567	0	54420,5
Myeloid_vs_CD4_Non-responder	Macro_FOLR2+APOE+	CD4(ISG+ Treg)	LGALS9	CD47	7,87664E-03	35224,9	3,45644E-03	41371	1,33943E+00	20447	2,01882E+00	27260	8,92922E-01	32626	0	54420,5
Myeloid_vs_CD4_Non-responder	CD4(IFNG+ Tfh/Th1)	pDC_LILRA4	HSP90B1	TLR9	7,87815E-03	31185,3	2,40218E-02	1672	2,65460E+00	1912	1,35574E+00	51362	8,67562E-01	46560	0	54420,5
Myeloid_vs_CD4_Non-responder	CD4(CRTAM- T)	Macro_FOLR2+APOE+	CD52	SIGLEC10	7,88072E-03	32550,7	5,55623E-03	20630	1,44884E+00	16768	1,34048E+00	52008	9,17657E-01	18927	0	54420,5
Myeloid_vs_CD4_Non-responder	CD4(ISG+ Treg)	Mono_CD14	CIRBP	TREM1	7,88426E-03	30032,7	6,43693E-03	16650	1,50744E+00	14978	1,59437E+00	41379	9,10482E-01	22736	0	54420,5
Myeloid_vs_CD4_Non-responder	CD4(IL26+ Th17)	Macro_OLFML3	B2M	LILRB1	7,88711E-03	30060,1	3,82435E-03	35707	1,50085E+00	15158	1,59427E+00	41382	9,48804E-01	3633	0	54420,5
Myeloid_vs_CD4_Non-responder	CD4(ISG+ Treg)	Macro_OLFML3	HLA-B	LILRB2	7,89379E-03	26591,1	3,45490E-03	41389	1,77285E+00	9036	2,07589E+00	25690	9,52100E-01	2420	0	54420,5
Myeloid_vs_CD4_Non-responder	CD4(TNFRSF9+ Treg)	Mono_CD14	RPS19	C5AR1	7,89474E-03	28228,3	4,66239E-03	26682	1,52469E+00	14528	1,59410E+00	41390	9,47763E-01	4121	0	54420,5
Myeloid_vs_CD4_Non-responder	Macro_NLRP3	CD4(TNF+ T)	S100A8	ITGB2	7,89570E-03	24385,5	3,45474E-03	41391	2,35977E+00	3290	2,94755E+00	9221	9,28420E-01	13605	0	54420,5
Myeloid_vs_CD4_Non-responder	CD4(IL26+ Th17)	Macro_OLFML3	HLA-B	LILRB1	7,90047E-03	30603,7	3,84853E-03	35344	1,48764E+00	15559	1,59396E+00	41396	9,43159E-01	6299	0	54420,5
Myeloid_vs_CD4_Non-responder	Mono_CD14	CD4(ISG+ Treg)	VCAN	ITGB1	7,90741E-03	20112,3	9,48556E-03	9104	3,00569E+00	1013	2,53939E+00	15376	9,14321E-01	20648	0	54420,5
Myeloid_vs_CD4_Non-responder	pDC_LILRA4	CD4(IL26+ Th17)	APP	PTGER2	7,91317E-03	27644,1	1,12714E-02	6802	2,22815E+00	4068	3,01435E+00	8369	8,35229E-01	64561	0	54420,5
Myeloid_vs_CD4_Non-responder	CD4(IL26+ Th17)	Mono_CD16	HLA-A	LILRB1	7,92531E-03	29176,3	4,27841E-03	30267	1,56094E+00	13576	1,59335E+00	41422	9,43385E-01	6196	0	54420,5

Myeloid_vs_CD4_Post_NR

Myeloid_vs_CD4_Non-responder	Mono_CD16	CD4(NME1+ T)	VCAN	ITGB1	7,92818E-03	39944,3	4,29976E-03	30021	1,05653E+00	32972	1,59324E+00	41425	8,77823E-01	40883	0	54420,5
Myeloid_vs_CD4_Non-responder	CD4(Tn)	cDC_CLEC9A	MIF	CD74_CXCR4	7,93519E-03	53275,9	1,80382E-03	85459	4,86446E-01	74134	1,37572E+00	50447	9,53457E-01	1919	0	54420,5
Myeloid_vs_CD4_Non-responder	Macro_FOLR2+APOE+	CD4(TNFRSF9+ Treg)	HBEGF	CD82	7,93967E-03	36663,5	5,72701E-03	19745	1,01999E+00	34872	1,83397E+00	32843	8,76816E-01	41437	0	54420,5
Myeloid_vs_CD4_Non-responder	Macro_NLRP3	CD4(TGFB1+ Th17)	CXCL2	DPP4	7,95179E-03	25324,3	1,98252E-02	2388	2,40937E+00	2998	3,01320E+00	8383	8,46262E-01	58432	0	54420,5
Myeloid_vs_CD4_Non-responder	CD4(TNF+ T)	Macro_FOLR2+APOE+	HLA-B	LILRB2	7,96653E-03	28572,7	3,44913E-03	41465	1,59515E+00	12694	1,86555E+00	31850	9,52062E-01	2434	0	54420,5
Myeloid_vs_CD4_Non-responder	CD4(ISG+ Treg)	Macro_FOLR2+APOE+	LGALS9	LRP1	7,96749E-03	31857,7	4,02238E-03	33117	1,70622E+00	10265	2,30979E+00	20020	8,76769E-01	41466	0	54420,5
Myeloid_vs_CD4_Non-responder	CD4(IL26+ Th17)	Macro_ISG15	CD28	CD86	7,97518E-03	29998,7	8,12997E-03	11748,5	1,61071E+00	12318,5	1,92448E+00	30031,5	8,76748E-01	41474,5	0	54420,5
Myeloid_vs_CD4_Non-responder	CD4(TGFB1+ Th17)	pDC_LILRA4	TGFB1	LPP	7,98383E-03	39330,3	4,98991E-03	24198	9,02012E-01	41483	1,74734E+00	35741	8,77947E-01	40809	0	54420,5
Myeloid_vs_CD4_Non-responder	Macro_ISG15	CD4(NME1+ T)	CCL2	CCR4	8,00440E-03	29041,9	2,53902E-02	1513	1,81221E+00	8402	3,06056E+00	7849	8,19971E-01	73025	0	54420,5
Myeloid_vs_CD4_Non-responder	CD4(NME1+ T)	Macro_FOLR2+APOE+	HLA-C	LILRB2	8,00503E-03	28348,5	3,44663E-03	41505	1,62024E+00	12088	1,91476E+00	30369	9,49509E-01	3360	0	54420,5
Myeloid_vs_CD4_Non-responder	Macro_ISG15	CD4(TGFB1+ Th17)	LGALS9	CD44	8,00884E-03	22183,1	5,54149E-03	20716	1,73128E+00	9782	2,30937E+00	20031	9,43946E-01	5966	0	54420,5
Myeloid_vs_CD4_Non-responder	CD4(IFNG+ Tfh/Th1)	pDC_LILRA4	TNF	PTPRS	8,01704E-03	29145,1	2,21322E-02	1929	3,95989E+00	229	1,85907E+00	32061	8,48724E-01	57086	0	54420,5
Myeloid_vs_CD4_Non-responder	Macro_NLRP3	CD4(ISG+ Treg)	CD86	CTLA4	8,06403E-03	34494,9	7,85632E-03	12394	1,10742E+00	30355	1,58997E+00	41566	8,90944E-01	33739	0	54420,5
Myeloid_vs_CD4_Non-responder	CD4(TNF+ T)	Macro_NLRP3	CD40LG	ITGAM_ITGB2	8,08053E-03	37274,3	8,41290E-03	11122	9,00742E-01	41583	1,62985E+00	39969	8,80811E-01	39277	0	54420,5
Myeloid_vs_CD4_Non-responder	CD4(CRTAM- T)	pDC_LILRA4	HSP90B1	TLR7	8,08247E-03	30211,5	7,88409E-03	12314	1,89113E+00	7287	1,75660E+00	35451	8,76571E-01	41585	0	54420,5
Myeloid_vs_CD4_Non-responder	CD4(TNF+ T)	cDC_CLEC9A	CD52	SIGLEC10	8,10290E-03	34335,9	4,82254E-03	25433	1,15025E+00	28363	1,58898E+00	41606	9,12145E-01	21857	0	54420,5
Myeloid_vs_CD4_Non-responder	Mono_CD14	CD4(CXCL13+ Tfh)	VCAN	SELL	8,12938E-03	18969,9	1,32233E-02	5093	3,09692E+00	864	2,63511E+00	13676	9,14078E-01	20796	0	54420,5
Myeloid_vs_CD4_Non-responder	CD4(CXCL13+ Tfh)	cDC_CLEC9A	CXCL13	CXCR3	8,13802E-03	31673,5	2,37971E-02	1709	8,99840E-01	41642	1,86128E+00	31983	9,00120E-01	28613	0	54420,5
Myeloid_vs_CD4_Non-responder	CD4(TNF+ T)	pDC_LILRA4	CD70	TNFRSF17	8,14013E-03	43948,7	1,26651E-01	49	2,08944E+00	5220	1,70145E+00	37327	7,08501E-01	122727	0	54420,5
Myeloid_vs_CD4_Non-responder	CD4(GZMK+ Teff)	Macro_OLFML3	HLA-A	LILRB1	8,14095E-03	30347,3	4,01273E-03	33229	1,49158E+00	15445	1,58785E+00	41645	9,41648E-01	6997	0	54420,5
Myeloid_vs_CD4_Non-responder	CD4(IFNG+ Tfh/Th1)	Mono_CD14	CIRBP	TREM1	8,15366E-03	30159,9	6,40438E-03	16759	1,50297E+00	15114	1,58757E+00	41658	9,10275E-01	22848	0	54420,5
Myeloid_vs_CD4_Non-responder	Macro_FOLR2-APOE+	CD4(AREG+ Tm)	APOE	SORL1	8,17340E-03	22017,3	5,52279E-03	20825	1,95278E+00	6593	2,99280E+00	8653	9,16380E-01	19595	0	54420,5
Myeloid_vs_CD4_Non-responder	CD4(NME1+ T)	cDC_CLEC9A	HMGB1	HAVCR2	8,17424E-03	36828,3	3,81840E-03	35781	1,01545E+00	35099	1,58694E+00	41679	9,21230E-01	17162	0	54420,5
Myeloid_vs_CD4_Non-responder	Mono_INHBA	CD4(TNF+ T)	S100A8	CD69	8,18101E-03	20636,9	8,34871E-03	11246	1,72554E+00	9893	2,27307E+00	20830	9,42045E-01	6795	0	54420,5
Myeloid_vs_CD4_Non-responder	CD4(IFNG+ Tfh/Th1)	Macro_OLFML3	CD99	PILRA	8,20960E-03	34914,9	3,69918E-03	37498	1,29681E+00	21997	1,58600E+00	41715	9,17623E-01	18944	0	54420,5
Myeloid_vs_CD4_Non-responder	Macro_IFI27	CD4(ISG+ Treg)	HLA-DPA1	LAG3	8,22982E-03	23032,9	8,26932E-03	11408	1,48233E+00	15740	2,27147E+00	20862	9,30217E-01	12734	0	54420,5
Myeloid_vs_CD4_Non-responder	CD4(AREG+ Tm)	Macro_ISG15	HLA-A	LILRB1	8,23324E-03	28728,9	4,44073E-03	28653	1,58085E+00	13051	1,58521E+00	41739	9,44371E-01	5781	0	54420,5
Myeloid_vs_CD4_Non-responder	Mono_CD14	CD4(TNF+ T)	VCAN	ITGB1	8,23441E-03	19952,1	9,39554E-03	9258	3,00202E+00	1019	2,60405E+00	14198	9,13947E-01	20865	0	54420,5
Myeloid_vs_CD4_Non-responder	CD4(TGFB1+ Th17)	pDC_LILRA4	HSP90B1	TLR9	8,24006E-03	31468,5	2,36150E-02	1734	2,64673E+00	1956	1,33839E+00	52101	8,66577E-01	47131	0	54420,5
Myeloid_vs_CD4_Non-responder	Mono_INHBA	CD4(IL26+ Th17)	SPP1	CD44	8,24359E-03	23342,5	9,02408E-03	9896	1,49319E+00	15391	2,27107E+00	20871	9,23222E-01	16134	0	54420,5
Myeloid_vs_CD4_Non-responder	CD4(IL26+ Th17)	Macro_FOLR2+APOE+	TGFB1	ENG	8,24607E-03	35878,5	4,71033E-03	26313	1,30888E+00	21510	1,58499E+00	41752	8,87995E-01	35397	0	54420,5
Myeloid_vs_CD4_Non-responder	CD4(CXCL13+ Tfh)	cDC(CD1C)	B2M	CD1A	8,25125E-03	30948,5	2,65762E-02	1367	1,53151E+00	14339	9,89223E-01	68636	9,23531E-01	15980	0	54420,5
Myeloid_vs_CD4_Non-responder	CD4(NME1+ T)	Macro_FOLR2+APOE-	CD59	STAB1	8,25595E-03	33431,9	5,16777E-03	22963	1,86936E+00	7584	1,58463E+00	41762	8,78674E-01	40430	0	54420,5
Myeloid_vs_CD4_Non-responder	Mono_CD16	CD4(IFNG+ Tfh/Th1)	ITGB2	CD226	8,25892E-03	36752,1	6,06009E-03	18163	8,97874E-01	41765	1,86345E+00	31910	8,84115E-01	37502	0	54420,5
Myeloid_vs_CD4_Non-responder	Macro_OLFML3	CD4(TNFRSF9+ Treg)	HLA-DRA	LAG3	8,26657E-03	23881,3	5,50957E-03	20886	1,42994E+00	17322	3,06378E+00	7818	9,17590E-01	18960	0	54420,5
Myeloid_vs_CD4_Non-responder	CD4(IFNG+ Tfh/Th1)	Macro_OLFML3	HLA-A	LILRB2	8,26683E-03	28521,3	3,43045E-03	41773	1,62398E+00	12003	1,89065E+00	31096	9,49659E-01	3314	0	54420,5
Myeloid_vs_CD4_Non-responder	CD4(Tn)	Mono_INHBA	VIM	CD44	8,27335E-03	67357,3	1,62692E-03	92309	3,22334E-01	90542	4,40791E-01	97555	9,53305E-01	1960	0	54420,5
Myeloid_vs_CD4_Non-responder	CD4(Tn)	pDC_LILRA4	APP	TNFRSF21	8,28168E-03	36113,1	5,49935E-03	20943	2,64523E+00	1961	2,57115E+00	14791	7,90438E-01	88450	0	54420,5
Myeloid_vs_CD4_Non-responder	CD4(Tn)	Macro_NLRP3	CD55	ADGRE2	8,28466E-03	28153,5	8,39469E-03	11163	1,88891E+00	7322	2,06120E+00	26071	8,76221E-01	41791	0	54420,5
Myeloid_vs_CD4_Non-responder	Macro_FOLR2+APOE+	CD4(IL26+ Th17)	TNFSF13	FAS	8,29457E-03	30990,7	6,35615E-03	16933	1,42198E+00	17556	2,13141E+00	24243	8,76199E-01	41801	0	54420,5
Myeloid_vs_CD4_Non-responder	Macro_ISG15	CD4(IFNG+ Tfh/Th1)	TGM2	ADGRG1	8,30614E-03	31366,5	1,26167E-01	50	2,20602E+00	4225	2,19195E+00	22710	8,15576E-01	75427	0	54420,5
Myeloid_vs_CD4_Non-responder	cDC(CD1C)	CD4(IFNG+ Tfh/Th1)	B2M	CD3D	8,30614E-03	55763,9	3,80469E-03	35978	2,93655E-01	93621	4,93626E-01	94750	9,64664E-01	50	0	54420,5
Myeloid_vs_CD4_Non-responder	Macro_NLRP3	CD4(CRTAM- T)	VCAN	SELL	8,31543E-03	28100,9	5,85020E-03	19147	2,49938E+00	2549	2,19787E+00	22566	8,76178E-01	41822	0	54420,5
Myeloid_vs_CD4_Non-responder	CD4(AREG+ Tm)	pDC_LILRA4	HMGB1	TLR9	8,32341E-03	28005,9	3,22601E-02	923	2,64415E+00	1966	1,30265E+00	53688	8,99355E-01	29032	0	54420,5
Myeloid_vs_CD4_Non-responder	CD4(CRTAM- T)	Macro_FOLR2+APOE+	SEMA4D	CD72	8,35615E-03	36136,7	1,22546E-02	5879	1,80422E+00	8527	1,54395E+00	43364	8,28317E-01	68493	0	54420,5
Myeloid_vs_CD4_Non-responder	CD4(TGFB1+ Th17)	Macro_FOLR2+APOE-	CD59	STAB1	8,35727E-03	34011,9	4,92531E-03	24693	1,85367E+00	7790	1,59637E+00	41292	8,76089E-01	41864	0	54420,5
Myeloid_vs_CD4_Non-responder	CD4(CRTAM- T)	Macro_OLFML3	CD99	PILRA	8,38626E-03	34746,1	3,73450E-03	36977	1,30507E+00	21657	1,58163E+00	41893	9,17981E-01	18783	0	54420,5
Myeloid_vs_CD4_Non-responder	CD4(Tn)	cDC_LAMP3	CD28	CD86	8,39126E-03	32154,9	8,02109E-03	11994,5	1,64907E+00	11427,5	1,60257E+00	41033,5	8,76018E-01	41898,5	0	54420,5
Myeloid_vs_CD4_Non-responder	Mono_CD16	CD4(IFNG+ Tfh/Th1)	LGALS3	LAG3	8,39427E-03	31646,7	8,35726E-03	11231	8,95078E-01	41901	2,12785E+00	24347	9,04080E-01	26334	0	54420,5
Myeloid_vs_CD4_Non-responder	CD4(TNF+ T)	Macro_ISG15	CSF1	SIRPA	8,41322E-03	30937,9	1,63527E-02	3438	1,68252E+00	10711	2,26685E+00	20981	8,34213E-01	65139	0	54420,5
Myeloid_vs_CD4_Non-responder	Macro_FOLR2+APOE+	CD4(GZMK+ Teff)	APOE	LSR	8,41432E-03	23237,1	1,13530E-02	6729	1,73736E+00	9659	3,59429E+00	3456	8,75991E-01	41921	0	54420,5
Myeloid_vs_CD4_Non-responder	Macro_FOLR2+APOE+	CD4(NME1+ T)	CCL3	CCR4	8,41833E-03	24517,5	1,21923E-02	5931	1,61946E+00	12111	3,02884E+00	8200	8,75980E-01	41925	0	54420,5
Myeloid_vs_CD4_Non-responder	CD4(TNF+ T)	Macro_OLFML3	ANXA1	FPR1	8,42235E-03	34267,7	3,75567E-03	36651	1,30087E+00	21829	1,58045E+00	41929	9,22505E-01	16509	0	54420,5
Myeloid_vs_CD4_Non-responder	CD4(IFNG+ Tfh/Th1)	Macro_FOLR2-APOE+	ADAM10	TREM2	8,42637E-03	32142,1	5,92861E-03	18745	1,81054E+00	8432	1,70543E+00	37180	8,75966E-01	41933	0	54420,5
Myeloid_vs_CD4_Non-responder	Macro_FOLR2+APOE+	CD4(CXCL13+ Tfh)	HLA-DQB1	LAG3	8,42737E-03	33692,3	3,50488E-03	40584	1,37258E+00	19256	2,72657E+00	12267	8,75965E-01	41934	0	54420,5
Myeloid_vs_CD4_Non-responder	Macro_IER3	CD4(ISG+ Treg)	HLA-DPB1	LAG3	8,42876E-03	24473,5	7,48837E-03	13344	1,32419E+00	20991	2,43648E+00	17385	9,23045E-01	16227	0	54420,5
Myeloid_vs_CD4_Non-responder	CD4(Tn)	Macro_FOLR2+APOE+	HLA-A	LILRB2	8,43944E-03	30253,3	3,42055E-03	41946	1,49987E+00	15188	1,72869E+00	36383	9,49590E-01	3329	0	54420,5
Myeloid_vs_CD4_Non-responder	cDC(CD1C)	CD4(IL26+ Th17)	HLA-DRA	LAG3	8,44122E-03	24217,1	5,48685E-03	20999	1,49028E+00	15485	2,80459E+00	11150	9,17434E-01	19031	0	54420,5
Myeloid_vs_CD4_Non-responder	Mono_INHBA	CD4(AREG+ Tm)	S100A8	CD69	8,44489E-03	19430,1	9,85363E-03	8558	1,85040E+00	7829	2,23888E+00	21595	9,46407E-01	4748	0	54420,5
Myeloid_vs_CD4_Non-responder	Macro_ISG15	CD4(ISG+ Treg)	HLA-DRB5	LAG3	8,45524E-03	23588,1	7,47190E-03	13385	1,32371E+00	21008	2,81112E+00	11065	9,19470E-01	18062	0	54420,5
Myeloid_vs_CD4_Non-responder	CD4(TNFRSF9+ Treg)	Macro_FOLR2+APOE+	TIGIT	NECTIN2	8,45927E-03	26200,9	2,73201E-02	1287,5	1,80200E+00	8563,5	2,25361E+00	21257,5	8,69512E-01	45475,5	0	54420,5
Myeloid_vs_CD4_Non-responder	CD4(IL26+ Th17)	Macro_FOLR2+APOE+	HLA-F	LILRB2	8,46159E-03	32416,1	3,41844E-03	41968	1,67191E+00	10946	2,00815E+00	27547	9,02560E-01	27199	0	54420,5
Myeloid_vs_CD4_Non-responder	CD4(IL26+ Th17)	Macro_FOLR2+APOE+	HLA-C	LILRB1	8,46563E-03	33218,1	3,49839E-03	40696	1,35599E+00	19834	1,57948E+00	41972	9,37442E-01	9168	0	54420,5
Myeloid_vs_CD4_Non-responder	CD4(Tn)	Macro_ISG15	HLA-A	LILRB1	8,46663E-03	29106,1	4,37539E-03	29277	1,54745E+00	13908	1,57944E+00	41973	9,43980E-01	5952	0	54420,5
Myeloid_vs_CD4_Non-responder	Macro_NLRP3	CD4(IL26+ Th17)	S100A8	ITGB2	8,47067E-03	24637,5	3,41793E-03	41977	2,35579E+00	3309	2,90926E+00	9711	9,28064E-01	13770	0	54420,5
Myeloid_vs_CD4_Non-responder	Macro_FOLR2+APOE+	CD4(IL26+ Th17)	SPP1	PTGER4	8,47773E-03	29356,5	7,99797E-03	12046	1,08924E+00	31260	3,13330E+00	7072	8,75847E-01	41984	0	54420,5
Myeloid_vs_CD4_Non-responder	CD4(TNFRSF9+ Treg)	Macro_OLFML3	HLA-C	LILRB1	8,47874E-03	31205,3	3,81819E-03	35786	1,47428E+00	15965	1,57919E+00	41985	9,39959E-01	7870	0	54420,5
Myeloid_vs_CD4_Non-responder	CD4(GZMK+ Teff)	Macro_FOLR2+APOE+	CCL5	CCR1	8,48076E-03	30045,5	1,81129E-02	2872	1,49056E+00	15475	1,57916E+00	41987	8,87873E-01	35473	0	54420,5
Myeloid_vs_CD4_Non-responder	Mast	CD4(TNF+ T)	ADCYAP1	DPP4	8,48291E-03	47679,9	8,03843E-02	144	2,63940E+00	1985	1,12514E+00	61922	7,17212E-01	119928	0	54420,5
Myeloid_vs_CD4_Non-responder	Mono_CD16	CD4(CRTAM- T)	LGALS9	PTPRC	8,48784E-03	35698,1	3,41704E-03	41994	1,10329E+00	30567	1,61168E+00	40676	9,34077E-01	10833	0	54420,5
Myeloid_vs_CD4_Non-responder	Macro_FOLR2+APOE-	CD4(IFNG+ Tfh/Th1)	HLA-DQA1	LAG3	8,48803E-03	21243,9	1,25924E-02	5612	1,67521E+00	10873	2,26466E+00	21029	9,26976E-01	14285	0	54420,5

Myeloid_vs_CD4_Post_NR

Myeloid_vs_CD4_Non-responder	CD4(TGFB1+ Th17)	Mono_CD16	HLA-B	LILRA1	8,49670E-03	24882,1	9,96554E-03	8391	2,11594E+00	4964	1,43132E+00	48059	9,38537E-01	8576	0	54420,5
Myeloid_vs_CD4_Non-responder	cDC(CD1C)	CD4(IL26+ Th17)	HLA-DPA1	LAG3	8,51151E-03	25170,9	5,47772E-03	21044	1,41082E+00	17932	2,71097E+00	12496	9,15607E-01	19962	0	54420,5
Myeloid_vs_CD4_Non-responder	cDC_CLEC9A	CD4(IL26+ Th17)	HLA-DPB1	LAG3	8,51778E-03	23366,7	5,82035E-03	19294	1,49460E+00	15362	3,17085E+00	6709	9,13605E-01	21048	0	54420,5
Myeloid_vs_CD4_Non-responder	CD4(AREG+ Tm)	Macro_OLFML3	LTB	CD40	8,52248E-03	29858,3	9,53105E-03	9033	1,53113E+00	14350	1,37614E+00	50437	9,13601E-01	21051	0	54420,5
Myeloid_vs_CD4_Non-responder	Macro_NLRP3	CD4(IL26+ Th17)	CXCL16	CXCR6	8,52327E-03	37802,3	6,68872E-03	15734	8,93177E-01	42029	1,65937E+00	38875	8,83284E-01	37953	0	54420,5
Myeloid_vs_CD4_Non-responder	Macro_FOLR2-APOE+	CD4(IFNG+ Tfh/Th1)	C3	IFITM1	8,52847E-03	24783,5	1,03700E-02	7841	1,80073E+00	8587	1,46063E+00	46786	9,43195E-01	6283	0	54420,5
Myeloid_vs_CD4_Non-responder	CD4(IFNG+ Tfh/Th1)	Macro_ISG15	HMGB1	TLR2	8,53138E-03	35009,3	4,00104E-03	33371	1,24263E+00	24226	1,57812E+00	42037	9,13706E-01	20992	0	54420,5
Myeloid_vs_CD4_Non-responder	Macro_OLFML3	CD4(CXCL13+ Tfh)	CXCL10	CXCR3	8,54004E-03	25927,7	9,83083E-03	8591	2,64082E+00	1981	3,05176E+00	7943	8,49386E-01	56703	0	54420,5
Myeloid_vs_CD4_Non-responder	Mono_CD14	CD4(IL26+ Th17)	VCAN	ITGA4	8,54603E-03	19502,9	1,03069E-02	7930	3,07029E+00	904	2,66618E+00	13194	9,13573E-01	21066	0	54420,5
Myeloid_vs_CD4_Non-responder	CD4(CRTAM- T)	Macro_FOLR2+APOE-	CD59	STAB1	8,55068E-03	34141,9	4,89245E-03	24926	1,85155E+00	7815	1,59149E+00	41492	8,75725E-01	42056	0	54420,5
Myeloid_vs_CD4_Non-responder	CD4(TNFRSF9+ Treg)	Macro_NLRP3	HLA-A	LILRB2	8,55068E-03	28018,1	3,41330E-03	42056	1,65671E+00	11265	1,95906E+00	29000	9,49539E-01	3349	0	54420,5
Myeloid_vs_CD4_Non-responder	Macro_FOLR2+APOE+	CD4(IFNG+ Tfh/Th1)	SPP1	PTGER4	8,56085E-03	29456,1	7,97726E-03	12094	1,08815E+00	31321	3,10396E+00	7379	8,75706E-01	42066	0	54420,5
Myeloid_vs_CD4_Non-responder	Macro_OLFML3	CD4(GZMK+ Teff)	CXCL9	CXCR3	8,56320E-03	20483,3	2,11599E-02	2115	2,84171E+00	1386	2,99657E+00	8599	8,87038E-01	35896	0	54420,5
Myeloid_vs_CD4_Non-responder	Mono_INHBA	CD4(TGFB1+ Th17)	INHBA	TGFBR3	8,56745E-03	32003,3	7,24898E-02	185	2,63746E+00	1995	2,08352E+00	25518	8,10974E-01	77898	0	54420,5
Myeloid_vs_CD4_Non-responder	Macro_FOLR2+APOE+	CD4(CXCL13+ Tfh)	HLA-DQA1	LAG3	8,57510E-03	31079,9	3,87790E-03	34949	1,53236E+00	14313	2,91548E+00	9637	8,75690E-01	42080	0	54420,5
Myeloid_vs_CD4_Non-responder	Macro_FOLR2+APOE+	CD4(NME1+ T)	CD72	CD5	8,58224E-03	29184,3	2,27466E-02	1843	1,91825E+00	6979	1,61397E+00	40592	8,75680E-01	42087	0	54420,5
Myeloid_vs_CD4_Non-responder	CD4(TGFB1+ Th17)	Macro_OLFML3	CD99	CD81	8,60265E-03	37601,9	3,70096E-03	37477	1,01855E+00	34942	1,57577E+00	42107	9,17360E-01	19063	0	54420,5
Myeloid_vs_CD4_Non-responder	CD4(CRTAM- T)	Macro_LYVE1	ANXA1	FPR1	8,60367E-03	34549,1	3,89013E-03	34786	1,21185E+00	25552	1,57575E+00	42108	9,23753E-01	15879	0	54420,5
Myeloid_vs_CD4_Non-responder	CD4(ISG+ Treg)	Macro_NLRP3	HLA-A	LILRB2	8,60776E-03	28239,5	3,40940E-03	42112	1,65362E+00	11327	1,92656E+00	29979	9,49512E-01	3359	0	54420,5
Myeloid_vs_CD4_Non-responder	CD4(NME1+ T)	Mono_CD16	HLA-B	LILRB1	8,63744E-03	29913,7	4,05898E-03	32682	1,52043E+00	14638	1,57513E+00	42141	9,44569E-01	5687	0	54420,5
Myeloid_vs_CD4_Non-responder	cDC_LAMP3	CD4(TNFRSF9+ Treg)	CD86	CTLA4	8,69469E-03	20525,7	1,59580E-02	3618	1,99831E+00	6110	2,25795E+00	21160	9,20908E-01	17320	0	54420,5
Myeloid_vs_CD4_Non-responder	CD4(CRTAM- T)	Macro_FOLR2+APOE+	CALR	LRP1	8,72593E-03	36138,5	3,40183E-03	42227	1,33508E+00	20609	1,64075E+00	39577	9,08467E-01	23859	0	54420,5
Myeloid_vs_CD4_Non-responder	CD4(IFNG+ Tfh/Th1)	Macro_IER3	RPS19	C5AR1	8,73317E-03	26411,1	5,26882E-03	22292	1,70971E+00	10187	1,57256E+00	42234	9,50708E-01	2922	0	54420,5
Myeloid_vs_CD4_Non-responder	CD4(TNF+ T)	cDC_CLEC9A	IFNG	IFNGR1_IFNGR2	8,73627E-03	32855,9	1,35312E-02	4890	9,15627E-01	40655	2,21875E+00	22077	8,75378E-01	42237	0	54420,5
Myeloid_vs_CD4_Non-responder	Macro_LYVE1	CD4(IFNG+ Tfh/Th1)	F13A1	ITGA4	8,74573E-03	20739,7	1,16173E-02	6484	2,69862E+00	1785	2,31929E+00	19817	9,13345E-01	21192	0	54420,5
Myeloid_vs_CD4_Non-responder	CD4(GZMK+ Teff)	Macro_ISG15	HMGB1	TLR2	8,74765E-03	36271,3	3,80255E-03	35999	1,18901E+00	26572	1,57230E+00	42248	9,11679E-01	22117	0	54420,5
Myeloid_vs_CD4_Non-responder	CD4(CXCL13+ Tfh)	pDC_LILRA4	APP	TNFRSF21	8,75852E-03	31683,1	7,51978E-03	13275	2,70817E+00	1762	2,65743E+00	13333	8,15179E-01	75625	0	54420,5
Myeloid_vs_CD4_Non-responder	CD4(CXCL13+ Tfh)	pDC_LILRA4	HLA-C	NOTCH4	8,76973E-03	33861,1	1,29739E-02	5293	1,36482E+00	19532	9,49717E-01	70588	9,16588E-01	19472	0	54420,5
Myeloid_vs_CD4_Non-responder	CD4(TGFB1+ Th17)	Mono_INHBA	TGFB1	ENG	8,77979E-03	34150,7	5,65551E-03	20114	1,26143E+00	23417	1,57148E+00	42279	8,96772E-01	30523	0	54420,5
Myeloid_vs_CD4_Non-responder	CD4(TNFRSF9+ Treg)	Macro_IFI27	ADAM10	TREM2	8,78083E-03	33894,7	6,06885E-03	18134	1,56685E+00	13425	1,57147E+00	42280	8,77231E-01	41214	0	54420,5
Myeloid_vs_CD4_Non-responder	CD4(CXCL13+ Tfh)	Mono_CD16	HLA-A	LILRB1	8,78187E-03	29545,1	4,24243E-03	30636	1,54045E+00	14088	1,57147E+00	42281	9,43159E-01	6300	0	54420,5
Myeloid_vs_CD4_Non-responder	Macro_FOLR2-APOE+	CD4(AREG+ Tm)	APOE	LSR	8,78238E-03	20946,3	1,63715E-02	3435	1,96800E+00	6427	2,99180E+00	8674	8,94545E-01	31775	0	54420,5
Myeloid_vs_CD4_Non-responder	CD4(TNF+ T)	cDC_LAMP3	IFNG	IFNGR1_IFNGR2	8,78499E-03	34362,7	1,35103E-02	4905	9,60389E-01	38103	1,85789E+00	32101	8,75294E-01	42284	0	54420,5
Myeloid_vs_CD4_Non-responder	CD4(AREG+ Tm)	Macro_FOLR2+APOE+	HLA-C	LILRB2	8,80266E-03	29197,1	3,39708E-03	42301	1,58749E+00	12895	1,83306E+00	32874	9,49161E-01	3495	0	54420,5
Myeloid_vs_CD4_Non-responder	CD4(CXCL13+ Tfh)	Mono_CD16	HLA-B	LILRB1	8,80370E-03	30174,1	4,02378E-03	33090	1,49755E+00	15259	1,57095E+00	42302	9,44341E-01	5799	0	54420,5
Myeloid_vs_CD4_Non-responder	CD4(ISG+ Treg)	Macro_NLRP3	CIRBP	TREM1	8,80578E-03	31931,1	5,86611E-03	19066	1,38026E+00	18984	1,57084E+00	42304	9,06625E-01	24881	0	54420,5
Myeloid_vs_CD4_Non-responder	CD4(TGFB1+ Th17)	Macro_OLFML3	HLA-C	LILRB2	8,80682E-03	27718,1	3,39680E-03	42305	1,70518E+00	10292	1,99049E+00	28074	9,49159E-01	3499	0	54420,5
Myeloid_vs_CD4_Non-responder	CD4(CXCL13+ Tfh)	pDC_LILRA4	COPA	P2RY6	8,81787E-03	23982,7	9,59818E-03	8929	3,43932E+00	544	3,12814E+00	7125	8,63310E-01	48895	0	54420,5
Myeloid_vs_CD4_Non-responder	CD4(IFNG+ Tfh/Th1)	Macro_FOLR2+APOE+	CCL5	CCR1	8,82036E-03	32573,7	1,42128E-02	4489	1,34615E+00	20196	1,59263E+00	41445	8,75224E-01	42318	0	54420,5
Myeloid_vs_CD4_Non-responder	CD4(CXCL13+ Tfh)	pDC_LILRA4	IGFL2	IGFLR1	8,82109E-03	28034,3	7,70399E-02	157	1,52577E+00	14495	2,67633E+00	13035	8,46857E-01	58064	0	54420,5
Myeloid_vs_CD4_Non-responder	CD4(ISG+ Treg)	Macro_ISG15	CD99	PILRA	8,82557E-03	33627,9	3,39587E-03	42323	1,46511E+00	16264	1,78497E+00	34493	9,14331E-01	20639	0	54420,5
Myeloid_vs_CD4_Non-responder	CD4(NME1+ T)	Mono_CD16	HLA-C	LILRB1	8,82662E-03	30754,5	3,95819E-03	33926	1,48187E+00	15750	1,57023E+00	42324	9,40967E-01	7352	0	54420,5
Myeloid_vs_CD4_Non-responder	pDC_LILRA4	CD4(IL26+ Th17)	APP	LRP10	8,82665E-03	21360,5	1,14127E-02	6668	2,25335E+00	3900	2,99052E+00	8689	8,92068E-01	33125	0	54420,5
Myeloid_vs_CD4_Non-responder	Macro_NLRP3	CD4(IFNG+ Tfh/Th1)	HLA-DQB1	LAG3	8,82870E-03	33717,1	5,93897E-03	18689	8,88037E-01	42326	2,08238E+00	25546	9,01894E-01	27604	0	54420,5
Myeloid_vs_CD4_Non-responder	Macro_FOLR2+APOE+	CD4(ISG+ Treg)	CD72	CD5	8,82975E-03	29032,5	2,25497E-02	1870	1,91379E+00	7019	1,64194E+00	39526	8,75206E-01	42327	0	54420,5
Myeloid_vs_CD4_Non-responder	CD4(CXCL13+ Tfh)	Macro_FOLR2+APOE+	ADAM10	TREM2	8,85653E-03	29910,3	5,44169E-03	21261	1,52831E+00	14425	2,56373E+00	14926	8,71235E-01	44519	0	54420,5
Myeloid_vs_CD4_Non-responder	CD4(CXCL13+ Tfh)	Macro_ISG15	HMGB1	THBD	8,86527E-03	40851,7	3,39427E-03	42361	1,01415E+00	35171	1,62106E+00	40333	8,94140E-01	31973	0	54420,5
Myeloid_vs_CD4_Non-responder	CD4(ISG+ Treg)	Macro_FOLR2+APOE+	CSF1	SIRPA	8,87105E-03	34557,7	1,32181E-02	5104	1,79312E+00	8704	1,89423E+00	30987	8,18969E-01	73573	0	54420,5
Myeloid_vs_CD4_Non-responder	CD4(TGFB1+ Th17)	Mono_INHBA	ANXA1	FPR1	8,87888E-03	33969,7	4,04444E-03	32836	1,22431E+00	25011	1,56903E+00	42374	9,25112E-01	15207	0	54420,5
Myeloid_vs_CD4_Non-responder	CD4(IL26+ Th17)	Macro_FOLR2+APOE+	CD40LG	ITGAM_ITGB2	8,88517E-03	36789,3	8,75948E-03	10386	9,50858E-01	38624	1,56876E+00	42380	8,82914E-01	38136	0	54420,5
Myeloid_vs_CD4_Non-responder	Macro_LYVE1	CD4(TNFRSF9+ Treg)	HLA-DRA	LAG3	8,89045E-03	25525,7	5,43898E-03	21282	1,40999E+00	17971	2,57288E+00	14755	9,17102E-01	19200	0	54420,5
Myeloid_vs_CD4_Non-responder	cDC(CD1C)	CD4(TNFRSF9+ Treg)	HLA-DRB1	LAG3	8,89368E-03	25635,9	5,43792E-03	21284	1,32837E+00	20845	2,76863E+00	11647	9,15584E-01	19983	0	54420,5
Myeloid_vs_CD4_Non-responder	CD4(CRTAM- T)	Macro_ISG15	CD99	CD81	8,89460E-03	39580,1	3,39279E-03	42389	9,33753E-01	39582	1,61187E+00	40671	9,14004E-01	20838	0	54420,5
Myeloid_vs_CD4_Non-responder	Macro_FOLR2+APOE+	CD4(CRTAM- T)	LGALS1	ITGB1	8,91245E-03	33328,1	3,39213E-03	42406	9,77973E-01	37103	2,23621E+00	21666	9,33590E-01	11045	0	54420,5
Myeloid_vs_CD4_Non-responder	CD4(ISG+ Treg)	Macro_IER3	RPS19	C5AR1	8,91561E-03	26964,1	5,08022E-03	23540	1,65972E+00	11200	1,56812E+00	42409	9,49847E-01	3251	0	54420,5
Myeloid_vs_CD4_Non-responder	Macro_FOLR2+APOE+	CD4(IFNG+ Tfh/Th1)	B2M	CD247	8,93033E-03	32866,5	4,83321E-03	25356	8,86459E-01	42423	1,59569E+00	41319	9,57389E-01	814	0	54420,5
Myeloid_vs_CD4_Non-responder	CD4(TGFB1+ Th17)	Macro_FOLR2+APOE+	LGALS3	ENG	8,93981E-03	36846,1	3,39076E-03	42432	1,34213E+00	20334	1,73057E+00	36305	8,96375E-01	30739	0	54420,5
Myeloid_vs_CD4_Non-responder	CD4(TNFRSF9+ Treg)	pDC_LILRA4	HLA-F	LILRB1	8,94402E-03	40501,1	3,39044E-03	42436	1,10766E+00	30342	1,76167E+00	35278	8,79463E-01	40029	0	54420,5
Myeloid_vs_CD4_Non-responder	CD4(TNF+ T)	Macro_NLRP3	CCL5	CCRL2	8,94613E-03	33320,5	1,50076E-02	4054	1,16878E+00	27516	1,67902E+00	38174	8,75011E-01	42438	0	54420,5
Myeloid_vs_CD4_Non-responder	CD4(ISG+ Treg)	Macro_FOLR2+APOE+	LGALS9	HAVCR2	8,94929E-03	36763,3	3,60017E-03	39065	1,29682E+00	21996	2,06836E+00	25894	8,75005E-01	42441	0	54420,5
Myeloid_vs_CD4_Non-responder	Mast	CD4(IFNG+ Tfh/Th1)	B2M	CD3D	8,97016E-03	61927,5	3,77860E-03	36318	2,68826E-01	96468	-6,01854E-02	122377	9,64547E-01	54	0	54420,5
Myeloid_vs_CD4_Non-responder	Mono_CD14	CD4(TNFRSF9+ Treg)	VCAN	ITGA4	8,97160E-03	19555,1	1,01836E-02	8085	3,06560E+00	911	2,67662E+00	13027	9,13096E-01	21332	0	54420,5
Myeloid_vs_CD4_Non-responder	CD4(ISG+ Treg)	Macro_LYVE1	LGALS9	LRP1	8,97568E-03	35010,7	3,88924E-03	34803	1,65988E+00	11196	1,85589E+00	32168	8,74939E-01	42466	0	54420,5
Myeloid_vs_CD4_Non-responder	CD4(TGFB1+ Th17)	Macro_FOLR2+APOE+	CALR	LRP1	8,99366E-03	36300,7	3,38745E-03	42483	1,33225E+00	20718	1,63154E+00	39909	9,08291E-01	23973	0	54420,5
Myeloid_vs_CD4_Non-responder	CD4(IL26+ Th17)	Macro_FOLR2+APOE+	B2M	LILRB1	9,00214E-03	32371,3	3,48496E-03	40875	1,36931E+00	19370	1,56572E+00	42491	9,46500E-01	4700	0	54420,5
Myeloid_vs_CD4_Non-responder	CD4(IFNG+ Tfh/Th1)	Macro_ISG15	CCL5	CCRL2	9,01167E-03	33075,1	1,49724E-02	4073	1,25449E+00	23722	1,61229E+00	40660	8,74882E-01	42500	0	54420,5
Myeloid_vs_CD4_Non-responder	CD4(IL26+ Th17)	Macro_FOLR2+APOE+	HLA-B	LILRB1	9,02440E-03	32969,1	3,50701E-03	40537	1,35609E+00	19831	1,56540E+00	42512	9,40616E-01	7545	0	54420,5
Myeloid_vs_CD4_Non-responder	CD4(TNFRSF9+ Treg)	Macro_OLFML3	LTA_LTB	LTBR	9,03183E-03	35000,7	8,60077E-03	10713	1,12369E+00	29548	1,56529E+00	42519	8,83575E-01	37803	0	54420,5
Myeloid_vs_CD4_Non-responder	cDC(CD1C)	CD4(IL26+ Th17)	HLA-DPB1	LAG3	9,03528E-03	24760,1	5,73509E-03	19704	1,46953E+00	16115	2,73208E+00	12190	9,13021E-01	21371	0	54420,5
Myeloid_vs_CD4_Non-responder	CD4(NME1+ T)	Macro_IER3	HMGB1	CD163	9,04033E-03	35412,1	3,39444E-03	42353	1,24838E+00	23963	1,56509E+00	42527	9,28005E-01	13797	0	54420,5
Myeloid_vs_CD4_Non-responder	CD4(IL26+ Th17)	Mono_CD16	HLA-C	LILRA3	9,06268E-03	24978,1	1,20266E-02	6088	1,92183E+00	6932	1,56461E+00	42548	9,25762E-01	14902	0	54420,5

Myeloid_vs_CD4_Post_NR

Myeloid_vs_CD4_Non-responder	Macro_FOLR2+APOE+	CD4(TGFB1+ Th17)	CXCL10	CXCR3	9,07013E-03	24378,5	1,50909E-02	4009	1,59130E+00	12797	3,03637E+00	8111	8,74800E-01	42555	0	54420,5
Myeloid_vs_CD4_Non-responder	CD4(CXCL13+ Tfh)	pDC_LILRA4	HSP90B1	TLR9	9,07452E-03	32236,7	2,20988E-02	1935	2,61740E+00	2054	1,30635E+00	53533	8,62694E-01	49241	0	54420,5
Myeloid_vs_CD4_Non-responder	CD4(IFNG+ Tfh/Th1)	Macro_NLRP3	CIRBP	TREM1	9,09040E-03	32071,1	5,83645E-03	19212	1,37580E+00	19145	1,56404E+00	42574	9,06410E-01	25004	0	54420,5
Myeloid_vs_CD4_Non-responder	cDC_LAMP3	CD4(TNFRSF9+ Treg)	CCL19	CCR7	9,09196E-03	29858,1	2,14991E-02	2056	4,84750E+00	61	1,90101E+00	30784	8,40001E-01	61969	0	54420,5
Myeloid_vs_CD4_Non-responder	Macro_FOLR2-APOE+	CD4(ISG+ Treg)	HLA-DRA	LAG3	9,09928E-03	23095,5	7,98116E-03	12079	1,50686E+00	15001	2,24768E+00	21410	9,30562E-01	12567	0	54420,5
Myeloid_vs_CD4_Non-responder	CD4(IFNG+ Tfh/Th1)	Macro_OLFML3	HLA-B	LILRB1	9,10429E-03	31303,3	3,78984E-03	36166	1,44559E+00	16864	1,56379E+00	42587	9,42746E-01	6479	0	54420,5
Myeloid_vs_CD4_Non-responder	Mono_CD16	CD4(TNFRSF9+ Treg)	CXCL16	CXCR6	9,10536E-03	37569,1	5,98670E-03	18474	8,84003E-01	42588	1,88523E+00	31278	8,77446E-01	41085	0	54420,5
Myeloid_vs_CD4_Non-responder	CD4(IL26+ Th17)	Macro_IFI27	ADAM10	TREM2	9,10643E-03	33543,9	6,26578E-03	17291	1,57744E+00	13123	1,56378E+00	42589	8,78940E-01	40296	0	54420,5
Myeloid_vs_CD4_Non-responder	CD4(Tn)	Macro_ISG15	HLA-C	LILRB1	9,11177E-03	30466,5	4,05667E-03	32696	1,48587E+00	15623	1,56369E+00	42594	9,41646E-01	6999	0	54420,5
Myeloid_vs_CD4_Non-responder	Macro_FOLR2+APOE+	CD4(IL26+ Th17)	TNFSF13	TNFRSF14	9,11712E-03	34891,3	3,38031E-03	42599	1,44620E+00	16843	2,20314E+00	22426	8,82852E-01	38168	0	54420,5
Myeloid_vs_CD4_Non-responder	CD4(Tn)	Macro_OLFML3	HLA-F	LILRB1	9,12890E-03	39599,7	3,43048E-03	41771	1,36553E+00	19509	1,56340E+00	42610	8,80083E-01	39688	0	54420,5
Myeloid_vs_CD4_Non-responder	CD4(TGFB1+ Th17)	Mono_CD16	HLA-F	LILRB2	9,13854E-03	30094,7	3,37919E-03	42619	2,17516E+00	4479	2,24618E+00	21445	9,02051E-01	27510	0	54420,5
Myeloid_vs_CD4_Non-responder	CD4(IFNG+ Tfh/Th1)	Macro_OLFML3	CD52	SIGLEC10	9,15865E-03	32866,1	5,56335E-03	20587	1,31062E+00	21446	1,40988E+00	48978	9,17705E-01	18899	0	54420,5
Myeloid_vs_CD4_Non-responder	CD4(IFNG+ Tfh/Th1)	cDC_LAMP3	TNFSF10	TNFRSF11B	9,17062E-03	47653,7	2,14402E-02	2065	2,78677E+00	1537	1,06859E+00	64745	7,29497E-01	115501	0	54420,5
Myeloid_vs_CD4_Non-responder	Macro_NLRP3	CD4(ISG+ Treg)	THBS1	ITGA4	9,17828E-03	31898,5	4,64476E-03	26835	1,61286E+00	12266	2,16922E+00	23315	8,74588E-01	42656	0	54420,5
Myeloid_vs_CD4_Non-responder	CD4(TGFB1+ Th17)	pDC_LILRA4	TNF	TNFRSF1A	9,19766E-03	35242,3	5,73148E-03	19727	8,82620E-01	42674	2,14035E+00	24037	8,88061E-01	35353	0	54420,5
Myeloid_vs_CD4_Non-responder	CD4(IL26+ Th17)	Macro_OLFML3	HLA-F	LILRB1	9,19874E-03	38849,7	3,56232E-03	39666	1,38229E+00	18916	1,56201E+00	42675	8,82059E-01	38571	0	54420,5
Myeloid_vs_CD4_Non-responder	CD4(Tn)	Macro_FOLR2+APOE+	CD28	CD86	9,20413E-03	30567,7	7,80875E-03	12503,5	1,60288E+00	12518,5	1,90324E+00	30715,5	8,74553E-01	42680,5	0	54420,5
Myeloid_vs_CD4_Non-responder	Macro_FOLR2+APOE+	CD4(Tn)	CD86	CD28	9,20521E-03	30567,7	7,80875E-03	12503,5	1,60288E+00	12518,5	1,90324E+00	30715,5	8,74553E-01	42680,5	0	54420,5
Myeloid_vs_CD4_Non-responder	CD4(CRTAM- T)	Macro_LYVE1	HMGB1	CD163	9,21600E-03	30132,3	4,10054E-03	32220	1,69462E+00	10493	1,56168E+00	42691	9,34068E-01	10837	0	54420,5
Myeloid_vs_CD4_Non-responder	CD4(GZMK+ Teff)	pDC_LILRA4	SELPLG	SELL	9,23653E-03	34162,1	5,62776E-03	20243	8,81986E-01	42710	2,01606E+00	27326	9,04473E-01	26111	0	54420,5
Myeloid_vs_CD4_Non-responder	Macro_FOLR2+APOE+	CD4(CRTAM- T)	MRC1	PTPRC	9,24302E-03	34788,7	6,60802E-03	16027	1,07364E+00	32072	1,56092E+00	42716	8,99941E-01	28708	0	54420,5
Myeloid_vs_CD4_Non-responder	CD4(ISG+ Treg)	Macro_FOLR2+APOE-	CD59	STAB1	9,24302E-03	34624,1	4,78282E-03	25739	1,84445E+00	7909	1,56990E+00	42336	8,74487E-01	42716	0	54420,5
Myeloid_vs_CD4_Non-responder	cDC_CLEC9A	CD4(IL26+ Th17)	HLA-DRA	LAG3	9,24325E-03	23713,5	5,40446E-03	21497	1,46578E+00	16241	3,13400E+00	7066	9,16859E-01	19343	0	54420,5
Myeloid_vs_CD4_Non-responder	CD4(Tn)	Macro_ISG15	CD55	ADGRE2	9,25276E-03	28486,9	8,12958E-03	11750	1,84013E+00	7982	2,08202E+00	25557	8,74470E-01	42725	0	54420,5
Myeloid_vs_CD4_Non-responder	CD4(AREG+ Tm)	cDC_LAMP3	CD28	CD86	9,26251E-03	32621,7	7,92732E-03	12217,5	1,64503E+00	11496,5	1,56029E+00	42734,5	8,75378E-01	42239,5	0	54420,5
Myeloid_vs_CD4_Non-responder	CD4(GZMK+ Teff)	Macro_FOLR2+APOE+	HLA-A	LILRB1	9,28963E-03	32670,5	3,65663E-03	38145	1,36003E+00	19707	1,55930E+00	42759	9,39042E-01	8321	0	54420,5
Myeloid_vs_CD4_Non-responder	Macro_FOLR2+APOE+	CD4(IL26+ Th17)	B2M	CD3D	9,30214E-03	38004,1	3,74746E-03	36779	7,92779E-01	48555	1,38139E+00	50210	9,64405E-01	56	0	54420,5
Myeloid_vs_CD4_Non-responder	Mono_CD14	CD4(Tn)	VCAN	ITGB1	9,30499E-03	20372,9	9,11779E-03	9729	2,99068E+00	1042	2,55314E+00	15139	9,12760E-01	21534	0	54420,5
Myeloid_vs_CD4_Non-responder	CD4(IFNG+ Tfh/Th1)	Macro_ISG15	HMGB1	CD163	9,30702E-03	32804,7	3,37009E-03	42775	1,24205E+00	24251	1,96952E+00	28674	9,27764E-01	13903	0	54420,5
Myeloid_vs_CD4_Non-responder	Macro_FOLR2+APOE+	CD4(TNF+ T)	CXCL16	CXCR6	9,31029E-03	32347,1	5,65888E-03	20094	1,35955E+00	19722	2,11267E+00	24721	8,74386E-01	42778	0	54420,5
Myeloid_vs_CD4_Non-responder	Macro_NLRP3	CD4(GZMK+ Teff)	HBEGF	CD82	9,32444E-03	31537,3	5,47374E-03	21068	1,67891E+00	10783	1,97132E+00	28624	8,74352E-01	42791	0	54420,5
Myeloid_vs_CD4_Non-responder	CD4(Tn)	Macro_ISG15	CD28	CD86	9,34844E-03	30488,1	7,77156E-03	12585,5	1,59479E+00	12701,5	1,92865E+00	29919,5	8,74291E-01	42813,5	0	54420,5
Myeloid_vs_CD4_Non-responder	CD4(TNF+ T)	Macro_FOLR2+APOE+	B2M	LILRB2	9,35608E-03	29644,5	3,36804E-03	42820	1,47353E+00	15992	1,79998E+00	33977	9,56502E-01	1013	0	54420,5
Myeloid_vs_CD4_Non-responder	CD4(AREG+ Tm)	Macro_FOLR2+APOE+	ANXA1	FPR1	9,37358E-03	36630,9	3,59685E-03	39118	1,12501E+00	29483	1,55729E+00	42836	9,20946E-01	17297	0	54420,5
Myeloid_vs_CD4_Non-responder	Macro_LYVE1	CD4(TNFRSF9+ Treg)	HLA-DPA1	LAG3	9,39899E-03	26688,3	5,38879E-03	21590	1,31750E+00	21248	2,51154E+00	15904	9,14972E-01	20279	0	54420,5
Myeloid_vs_CD4_Non-responder	CD4(TGFB1+ Th17)	Macro_OLFML3	HLA-A	LILRB1	9,40974E-03	30759,7	3,98923E-03	33517	1,47682E+00	15900	1,55627E+00	42869	9,41487E-01	7092	0	54420,5
Myeloid_vs_CD4_Non-responder	Macro_LYVE1	CD4(IFNG+ Tfh/Th1)	CD14	ITGB1	9,42936E-03	23404,3	5,64590E-03	20158	1,82477E+00	8205	2,23829E+00	21608	9,30426E-01	12630	0	54420,5
Myeloid_vs_CD4_Non-responder	Mast	CD4(CRTAM- T)	ADCYAP1	DPP4	9,43523E-03	49850,1	6,92289E-02	203	2,60806E+00	2095	1,00815E+00	67740	7,01819E-01	124792	0	54420,5
Myeloid_vs_CD4_Non-responder	Mono_CD14	CD4(IL26+ Th17)	VCAN	ITGB1	9,45641E-03	20401,7	9,07858E-03	9791	2,98908E+00	1047	2,55364E+00	15126	9,12588E-01	21624	0	54420,5
Myeloid_vs_CD4_Non-responder	CD4(AREG+ Tm)	pDC_LILRA4	HSP90B1	TLR9	9,47968E-03	32509,1	2,15593E-02	2043	2,60697E+00	2100	1,29689E+00	53936	8,61224E-01	50046	0	54420,5
Myeloid_vs_CD4_Non-responder	CD4(TNF+ T)	Macro_FOLR2+APOE-	HSPA1A	TLR4	9,48350E-03	33552,9	5,62167E-03	20277	1,20933E+00	25645	1,55458E+00	42936	9,07307E-01	24486	0	54420,5
Myeloid_vs_CD4_Non-responder	CD4(IL26+ Th17)	Macro_OLFML3	LTB	CD40	9,48902E-03	27714,3	1,01196E-02	8170	1,57311E+00	13240	1,55452E+00	42941	9,15937E-01	19800	0	54420,5
Myeloid_vs_CD4_Non-responder	CD4(IFNG+ Tfh/Th1)	Macro_ISG15	CD99	CD81	9,49787E-03	39796,7	3,36070E-03	42949	9,25492E-01	40082	1,61623E+00	40495	9,13630E-01	21037	0	54420,5
Myeloid_vs_CD4_Non-responder	Macro_FOLR2+APOE+	CD4(GZMK+ Teff)	HLA-DQB1	LAG3	9,50782E-03	34163,3	3,38418E-03	42544	1,36726E+00	19456	2,78254E+00	11438	8,74049E-01	42958	0	54420,5
Myeloid_vs_CD4_Non-responder	Macro_ISG15	CD4(AREG+ Tm)	CXCL10	CXCR3	9,53591E-03	26325,3	9,60012E-03	8923	2,26576E+00	3821	3,14848E+00	6924	8,47861E-01	57538	0	54420,5
Myeloid_vs_CD4_Non-responder	CD4(AREG+ Tm)	Macro_FOLR2+APOE+	CD28	CD86	9,58777E-03	30957,3	7,71746E-03	12724,5	1,59885E+00	12616,5	1,86096E+00	31994,5	8,73907E-01	43030,5	0	54420,5
Myeloid_vs_CD4_Non-responder	Macro_FOLR2+APOE+	CD4(AREG+ Tm)	CD86	CD28	9,58888E-03	30957,3	7,71746E-03	12724,5	1,59885E+00	12616,5	1,86096E+00	31994,5	8,73907E-01	43030,5	0	54420,5
Myeloid_vs_CD4_Non-responder	Macro_FOLR2+APOE-	CD4(IFNG+ Tfh/Th1)	HLA-DQB1	LAG3	9,60106E-03	21523,7	1,20575E-02	6057	1,61723E+00	12179	2,23435E+00	21709	9,29073E-01	13253	0	54420,5
Myeloid_vs_CD4_Non-responder	CD4(Tn)	Macro_FOLR2+APOE+	MIF	CD74_CXCR4	9,60470E-03	54273,1	1,76024E-03	87089	4,58856E-01	76783	1,36463E+00	50959	9,52912E-01	2114	0	54420,5
Myeloid_vs_CD4_Non-responder	Macro_FOLR2+APOE+	CD4(NME1+ T)	TNFSF13	TNFRSF14	9,64136E-03	35383,9	3,35271E-03	43078	1,44304E+00	16936	2,13699E+00	24112	8,82427E-01	38373	0	54420,5
Myeloid_vs_CD4_Non-responder	CD4(TNFRSF9+ Treg)	Macro_FOLR2+APOE+	HLA-C	LILRB1	9,65592E-03	33611,7	3,47935E-03	40990	1,34273E+00	20304	1,55064E+00	43091	9,37282E-01	9253	0	54420,5
Myeloid_vs_CD4_Non-responder	CD4(IFNG+ Tfh/Th1)	Macro_OLFML3	IFNG	IFNGR1_IFNGR2	9,65816E-03	35578,5	1,31444E-02	5170	1,05234E+00	33180	1,57832E+00	42029	8,73788E-01	43093	0	54420,5
Myeloid_vs_CD4_Non-responder	CD4(IFNG+ Tfh/Th1)	Macro_FOLR2+APOE-	CD59	STAB1	9,66152E-03	34609,9	4,72210E-03	26209	1,84052E+00	7975	1,59520E+00	41349	8,73784E-01	43096	0	54420,5
Myeloid_vs_CD4_Non-responder	Macro_FOLR2+APOE+	CD4(GZMK+ Teff)	HLA-DQA1	LAG3	9,67274E-03	31546,1	3,74435E-03	36827	1,52704E+00	14463	2,97145E+00	8914	8,73770E-01	43106	0	54420,5
Myeloid_vs_CD4_Non-responder	CD4(TNF+ T)	pDC_LILRA4	HMGB1	TLR9	9,67649E-03	28038,9	3,06253E-02	1012	2,59995E+00	2122	1,33546E+00	52223	8,96977E-01	30417	0	54420,5
Myeloid_vs_CD4_Non-responder	CD4(IL26+ Th17)	Macro_FOLR2+APOE+	LTB	TNFRSF1A	9,68059E-03	32782,3	7,01979E-03	14633	1,03533E+00	34084	1,54999E+00	43113	9,20232E-01	17661	0	54420,5
Myeloid_vs_CD4_Non-responder	CD4(NME1+ T)	Macro_FOLR2+APOE+	CD59	STAB1	9,68846E-03	30741,9	4,71921E-03	26234	1,70276E+00	10343	2,32886E+00	19592	8,73750E-01	43120	0	54420,5
Myeloid_vs_CD4_Non-responder	pDC_LILRA4	CD4(IFNG+ Tfh/Th1)	APP	LRP10	9,69521E-03	21706,9	1,10199E-02	7077	2,24056E+00	3994	2,96623E+00	8974	8,90370E-01	34069	0	54420,5
Myeloid_vs_CD4_Non-responder	Mono_INHBA	CD4(AREG+ Tm)	SPP1	PTGER4	9,69725E-03	19982,7	1,74024E-02	3076	1,76707E+00	9138	2,77750E+00	11514	9,12327E-01	21765	0	54420,5
Myeloid_vs_CD4_Non-responder	Macro_FOLR2+APOE+	CD4(CXCL13+ Tfh)	ITGB2	CD226	9,70082E-03	38109,7	4,98571E-03	24223	9,24505E-01	40128	1,97054E+00	28646	8,73737E-01	43131	0	54420,5
Myeloid_vs_CD4_Non-responder	Macro_OLFML3	CD4(Tn)	C3	IFITM1	9,70194E-03	31431,9	5,76683E-03	19541	1,06758E+00	32388	1,74917E+00	35679	9,25274E-01	15131	0	54420,5
Myeloid_vs_CD4_Non-responder	Macro_OLFML3	CD4(CRTAM- T)	LGALS9	PTPRC	9,72220E-03	32650,7	3,84414E-03	35411	1,23609E+00	24510	1,63363E+00	39834	9,37612E-01	9078	0	54420,5
Myeloid_vs_CD4_Non-responder	Macro_OLFML3	CD4(CRTAM- T)	LGALS9	CD44	9,72333E-03	28088,5	4,37516E-03	29283	1,42500E+00	17448	1,92343E+00	30073	9,37356E-01	9218	0	54420,5
Myeloid_vs_CD4_Non-responder	CD4(IL26+ Th17)	Macro_ISG15	CD40LG	CD9	9,73348E-03	32128,7	1,09920E-02	7112	1,08812E+00	31323	2,11739E+00	24628	8,73691E-01	43160	0	54420,5
Myeloid_vs_CD4_Non-responder	Macro_NLRP3	CD4(TNF+ T)	NAMPT	ITGA5_ITGB1	9,73799E-03	36330,1	3,34845E-03	43164	1,18372E+00	26814	2,08187E+00	25558	8,94676E-01	31694	0	54420,5
Myeloid_vs_CD4_Non-responder	CD4(CRTAM- T)	pDC_LILRA4	CCL5	CXCR3	9,74250E-03	30288,5	2,02818E-02	2297	8,74640E-01	43168	1,87814E+00	31467	9,15370E-01	20090	0	54420,5
Myeloid_vs_CD4_Non-responder	Mono_CD16	CD4(TNFRSF9+ Treg)	HLA-DPA1	LAG3	9,74589E-03	37590,9	3,84443E-03	35406	8,74612E-01	43171	2,03578E+00	26774	9,00882E-01	28183	0	54420,5
Myeloid_vs_CD4_Non-responder	CD4(IFNG+ Tfh/Th1)	Macro_FOLR2+APOE+	HMGB1	HAVCR2	9,75266E-03	35833,1	4,03232E-03	32981	1,06677E+00	32437	1,54856E+00	43177	9,23185E-01	16150	0	54420,5
Myeloid_vs_CD4_Non-responder	Macro_OLFML3	CD4(CRTAM- T)	CD14	ITGB1	9,75492E-03	28466,9	4,22799E-03	30781	1,36521E+00	19517	2,30752E+00	20067	9,20463E-01	17549	0	54420,5
Myeloid_vs_CD4_Non-responder	Macro_OLFML3	CD4(CRTAM- T)	CD14	ITGA4	9,75605E-03	33731,5	3,36661E-03	42838	1,26435E+00	23286	2,15318E+00	23725	9,07518E-01	24388	0	54420,5

Myeloid_vs_CD4_Post_NR

Myeloid_vs_CD4_Non-responder	CD4(AREG+ Tm)	Macro_ISG15	CD28	CD86	9,76735E-03	30899,5	7,68071E-03	12839,5	1,59076E+00	12812,5	1,88637E+00	31234,5	8,73643E-01	43190,5	0	54420,5
Myeloid_vs_CD4_Non-responder	CD4(TNF+ T)	Macro_OLFML3	LTB	CD40	9,77158E-03	29667,3	9,20718E-03	9572	1,50804E+00	14961	1,44214E+00	47575	9,12227E-01	21808	0	54420,5
Myeloid_vs_CD4_Non-responder	Mono_CD14	CD4(AREG+ Tm)	S100A8	ITGB2	9,77392E-03	18794,1	4,65964E-03	26702	3,06483E+00	913	3,68984E+00	2936	9,37747E-01	8999	0	54420,5
Myeloid_vs_CD4_Non-responder	pDC_LILRA4	CD4(CXCL13+ Tfh)	APP	LRP10	9,78655E-03	21778,3	1,09339E-02	7173	2,23777E+00	4006	2,96428E+00	9003	8,89987E-01	34289	0	54420,5
Myeloid_vs_CD4_Non-responder	pDC_LILRA4	CD4(IL26+ Th17)	B2M	CD3D	9,80008E-03	35866,1	3,72931E-03	37062	7,71924E-01	50017	1,68907E+00	37772	9,64322E-01	59	0	54420,5
Myeloid_vs_CD4_Non-responder	Macro_OLFML3	CD4(CRTAM- T)	FN1	CD44	9,80132E-03	32242,3	7,35471E-03	13679	1,11886E+00	29791	1,79440E+00	34153	8,99102E-01	29168	0	54420,5
Myeloid_vs_CD4_Non-responder	Macro_FOLR2+APOE+	CD4(IL26+ Th17)	LGALS9	CD44	9,81039E-03	34359,9	3,34548E-03	43228	1,23805E+00	24431	1,72739E+00	36430	9,29000E-01	13290	0	54420,5
Myeloid_vs_CD4_Non-responder	Macro_FOLR2+APOE+	CD4(CXCL13+ Tfh)	LYZ	ITGAL	9,81720E-03	28676,9	3,34536E-03	43234	1,62144E+00	12060	3,29181E+00	5628	9,01131E-01	28042	0	54420,5
Myeloid_vs_CD4_Non-responder	CD4(CRTAM- T)	Mono_CD16	HLA-B	LILRB1	9,82175E-03	30136,7	4,05825E-03	32684	1,51995E+00	14651	1,54708E+00	43238	9,44565E-01	5690	0	54420,5
Myeloid_vs_CD4_Non-responder	CD4(NME1+ T)	Macro_OLFML3	CD99	PILRA	9,83197E-03	36292,5	3,52395E-03	40244	1,25583E+00	23664	1,54690E+00	43247	9,15770E-01	19887	0	54420,5
Myeloid_vs_CD4_Non-responder	Macro_OLFML3	CD4(CRTAM- T)	HBEGF	CD44	9,84562E-03	32731,1	4,24271E-03	30632	1,15609E+00	28076	1,84027E+00	32661	9,19867E-01	17866	0	54420,5
Myeloid_vs_CD4_Non-responder	CD4(Tn)	pDC_LILRA4	HLA-C	NOTCH4	9,86726E-03	35013,7	1,25857E-02	5618	1,30015E+00	21863	9,00513E-01	73110	9,15419E-01	20057	0	54420,5
Myeloid_vs_CD4_Non-responder	CD4(IL26+ Th17)	pDC_LILRA4	SIRPG	CD47	9,87068E-03	34386,3	9,91832E-03	8469,5	9,01875E-01	41498,5	2,13065E+00	24261,5	8,73476E-01	43281,5	0	54420,5
Myeloid_vs_CD4_Non-responder	Mono_CD16	CD4(Tn)	GNAI2	S1PR1	9,87296E-03	37633,1	7,62151E-03	13008	8,72586E-01	43283	1,77410E+00	34845	8,74714E-01	42609	0	54420,5
Myeloid_vs_CD4_Non-responder	CD4(GZMK+ Teff)	Macro_NLRP3	HLA-F	LILRB2	9,88095E-03	35099,1	3,34231E-03	43290	1,44083E+00	17000	1,82991E+00	32983	9,01565E-01	27802	0	54420,5
Myeloid_vs_CD4_Non-responder	CD4(IL26+ Th17)	Mono_CD14	CIRBP	TREM1	9,88666E-03	31204,1	6,10498E-03	17981	1,46190E+00	16359	1,54561E+00	43295	9,08301E-01	23965	0	54420,5
Myeloid_vs_CD4_Non-responder	Macro_OLFML3	CD4(CRTAM- T)	S100A8	CD69	9,89351E-03	33146,1	4,61258E-03	27094	9,57167E-01	38262	1,92645E+00	29983	9,23560E-01	15971	0	54420,5
Myeloid_vs_CD4_Non-responder	Macro_OLFML3	CD4(TNFRSF9+ Treg)	TNFSF13	FAS	9,91638E-03	30791,1	7,84562E-03	12419	1,35815E+00	19766	1,87652E+00	31517	8,87173E-01	35833	0	54420,5
Myeloid_vs_CD4_Non-responder	CD4(CXCL13+ Tfh)	Macro_FOLR2+APOE-	CD59	STAB1	9,91638E-03	34903,5	4,68975E-03	26459	1,83843E+00	8009	1,57069E+00	42308	8,73404E-01	43321	0	54420,5
Myeloid_vs_CD4_Non-responder	Macro_OLFML3	CD4(TNFRSF9+ Treg)	TNFSF13	TNFRSF14	9,91752E-03	38258,3	3,38895E-03	42453	1,29989E+00	21875	1,78658E+00	34437	8,82984E-01	38106	0	54420,5
Myeloid_vs_CD4_Non-responder	Macro_OLFML3	CD4(TNFRSF9+ Treg)	HLA-DQA1	LAG3	9,92898E-03	26375,9	6,13706E-03	17832	1,39621E+00	18443	2,76607E+00	11693	8,98599E-01	29491	0	54420,5
Myeloid_vs_CD4_Non-responder	Macro_OLFML3	CD4(TNFRSF9+ Treg)	HLA-DRB1	LAG3	9,93127E-03	26565,3	5,18138E-03	22864	1,25497E+00	23698	2,82627E+00	10848	9,13697E-01	20996	0	54420,5
Myeloid_vs_CD4_Non-responder	Macro_OLFML3	CD4(TNFRSF9+ Treg)	HLA-DQB1	LAG3	9,93356E-03	26511,3	5,94306E-03	18666	1,35448E+00	19889	2,74382E+00	11998	9,01925E-01	27583	0	54420,5
Myeloid_vs_CD4_Non-responder	Macro_OLFML3	CD4(TNFRSF9+ Treg)	HLA-DPB1	LAG3	9,93929E-03	26415,5	5,36788E-03	21703	1,29743E+00	21966	2,80095E+00	11186	9,10357E-01	22802	0	54420,5
Myeloid_vs_CD4_Non-responder	Macro_OLFML3	CD4(CRTAM- T)	CXCL9	DPP4	9,94534E-03	30639,5	1,86407E-02	2707	2,78328E+00	1547	2,96147E+00	9053	7,96487E-01	85470	0	54420,5
Myeloid_vs_CD4_Non-responder	Mono_CD16	CD4(CRTAM- T)	NAMPT	ITGA5_ITGB1	9,94617E-03	40600,3	3,55366E-03	39817	8,71392E-01	43347	1,76271E+00	35249	8,97446E-01	30168	0	54420,5
Myeloid_vs_CD4_Non-responder	Macro_OLFML3	CD4(TNFRSF9+ Treg)	ICAM1	IL2RA	9,94732E-03	31476,9	1,21765E-02	5948	1,04011E+00	33840	2,31221E+00	19963	8,73606E-01	43213	0	54420,5
Myeloid_vs_CD4_Non-responder	Macro_OLFML3	CD4(CRTAM- T)	TNFSF13	FAS	9,95650E-03	36147,1	6,03341E-03	18286	1,24675E+00	24039	1,61287E+00	40634	8,73345E-01	43356	0	54420,5
Myeloid_vs_CD4_Non-responder	Macro_FOLR2+APOE+	CD4(AREG+ Tm)	TIMP2	CD44	9,96110E-03	34061,5	4,86682E-03	25118	1,26631E+00	23215	1,54405E+00	43360	9,07869E-01	24194	0	54420,5
Myeloid_vs_CD4_Non-responder	Mono_CD16	CD4(IFNG+ Tfh/Th1)	VCAN	ITGA4	9,96224E-03	39175,3	4,38549E-03	29192	1,08955E+00	31245	1,69212E+00	37658	8,73338E-01	43361	0	54420,5
Myeloid_vs_CD4_Non-responder	Macro_OLFML3	CD4(TNFRSF9+ Treg)	HLA-DPA1	LAG3	9,96569E-03	26200,3	5,24673E-03	22439	1,27676E+00	22768	2,85199E+00	10498	9,13927E-01	20876	0	54420,5
Myeloid_vs_CD4_Non-responder	Macro_OLFML3	CD4(TNFRSF9+ Treg)	CD86	CTLA4	9,97374E-03	26646,7	1,06199E-02	7519	1,41472E+00	17810	2,00916E+00	27521	9,04748E-01	25963	0	54420,5
Myeloid_vs_CD4_Non-responder	Macro_OLFML3	CD4(TNFRSF9+ Treg)	HLA-DRB5	LAG3	9,99330E-03	26159,7	5,52944E-03	20799	1,33648E+00	20558	2,83936E+00	10668	9,07596E-01	24353	0	54420,5

Myeloid_vs_CD4_Post_R

Response	source	target	ligand.complex	receptor.complex	aggregate_rank	mean_rank	natmi.edge_specificity	natmi.rank	connectome.weight_sc	connectome.rank	logfc.logfc_comb	logfc.rank	sca.LRscore	sca.rank	cellphonedb.pvalue	cellphonedb.rank
Myeloid_vs_CD4_Responder	Mono_CD14	CD4(TGFB1+ Th17)	S100A8	CD69	2,09601E-08	8832,4	3,19480E-02	708	4,30815E+00	191	4,94554E+00	48	9,65423E-01	3	0	43212
Myeloid_vs_CD4_Responder	Mono_CD14	CD4(AREG+ Tm)	S100A8	CD69	2,16262E-07	8981,4	2,41872E-02	1270	4,05445E+00	241	4,46608E+00	110	9,60466E-01	74	0	43212
Myeloid_vs_CD4_Responder	Mono_CD14	CD4(CRTAM- T)	S100A8	CD69	3,77091E-07	9041,4	2,27348E-02	1466	4,00697E+00	258	4,29340E+00	150	9,59273E-01	121	0	43212
Myeloid_vs_CD4_Responder	Mono_CD14	CD4(TNF+ T)	S100A8	CD69	3,90385E-07	9031,4	2,26363E-02	1480	4,00374E+00	261	4,74184E+00	73	9,59188E-01	131	0	43212
Myeloid_vs_CD4_Responder	Mono_CD14	CD4(IL26+ Th17)	S100A8	CD69	4,13230E-07	9077	2,20287E-02	1568	3,98388E+00	266	4,23951E+00	171	9,58652E-01	168	0	43212
Myeloid_vs_CD4_Responder	Mono_CD14	CD4(Tn)	S100A8	CD69	4,97533E-07	9146,4	2,03841E-02	1791	3,93012E+00	283	4,23228E+00	172	9,57087E-01	274	0	43212
Myeloid_vs_CD4_Responder	Mono_CD14	CD4(GZMK+ Teff)	S100A8	CD69	1,43575E-06	9235,2	1,90135E-02	2041	3,88531E+00	290	4,10891E+00	224	9,55634E-01	409	0	43212
Myeloid_vs_CD4_Responder	CD4(ISG+ Treg)	pDC_LILRA4	BST2	LILRA4	1,60074E-06	10752,2	4,99359E-02	289	5,93572E+00	40	3,79199E+00	418	9,27063E-01	9802	0	43212
Myeloid_vs_CD4_Responder	Mono_CD14	CD4(ISG+ Treg)	S100A8	CD69	1,60242E-06	9243,2	1,87868E-02	2098	3,87790E+00	292	4,21068E+00	180	9,55379E-01	434	0	43212
Myeloid_vs_CD4_Responder	CD4(Tn)	cDC(CD1C)	B2M	CD1C	1,96452E-06	12530,6	2,29699E-02	1430	3,18373E+00	725	1,75679E+00	15078	9,46626E-01	2208	0	43212
Myeloid_vs_CD4_Responder	CD4(AREG+ Tm)	cDC(CD1C)	B2M	CD1C	2,09162E-06	12813,6	2,28106E-02	1456	3,15686E+00	756	1,69945E+00	16401	9,46450E-01	2243	0	43212
Myeloid_vs_CD4_Responder	Mono_CD14	CD4(CXCL13+ Tfh)	S100A8	CD69	3,60324E-06	9423	1,69066E-02	2571	3,81644E+00	305	3,98940E+00	286	9,53077E-01	741	0	43212
Myeloid_vs_CD4_Responder	cDC_LAMP3	CD4(ISG+ Treg)	CCL19	CXCR3	3,96029E-06	15930,8	1,14547E-01	37	6,11079E+00	26	2,12554E+00	8631	8,84885E-01	27748	0	43212
Myeloid_vs_CD4_Responder	Macro_NLRP3	CD4(TGFB1+ Th17)	S100A8	CD69	5,78108E-06	10235,6	1,59147E-02	2895	2,35831E+00	2700	3,13898E+00	1382	9,51706E-01	989	0	43212
Myeloid_vs_CD4_Responder	CD4(TNFRSF9+ Treg)	cDC(CD1C)	B2M	CD1C	7,72658E-06	11113,8	2,50704E-02	1196	3,53790E+00	431	2,09451E+00	9047	9,48794E-01	1683	0	43212
Myeloid_vs_CD4_Responder	cDC_LAMP3	CD4(Tn)	CCL19	CCR7	7,82043E-06	14482,4	1,01913E-01	52	6,08757E+00	28	2,27566E+00	6867	8,97608E-01	22253	0	43212
Myeloid_vs_CD4_Responder	CD4(CRTAM- T)	Mono_CD14	ANXA1	FPR1	8,83576E-06	14698	8,55330E-03	8956	1,88430E+00	6239	2,22440E+00	7438	9,31795E-01	7645	0	43212
Myeloid_vs_CD4_Responder	cDC_LAMP3	CD4(CRTAM- T)	CCL19	CCR7	1,22170E-05	15108,4	9,47317E-02	65	6,04187E+00	32	2,13623E+00	8500	8,94201E-01	23733	0	43212
Myeloid_vs_CD4_Responder	CD4(Tn)	pDC_LILRA4	LYPD3	AGR2	1,24306E-05	33638,8	8,13341E-02	95	2,44529E+00	2338	2,66830E+00	3509	6,44171E-01	119040	0	43212
Myeloid_vs_CD4_Responder	Mono_CD14	CD4(CRTAM- T)	VCAN	CD44	1,35817E-05	10856,4	1,48323E-02	3337	3,11324E+00	815	2,65817E+00	3588	9,42501E-01	3330	0	43212
Myeloid_vs_CD4_Responder	Mono_CD14	CD4(NME1+ T)	S100A8	CD69	1,36421E-05	9820,6	1,42498E-02	3592	3,72959E+00	338	3,87816E+00	355	9,49103E-01	1606	0	43212
Myeloid_vs_CD4_Responder	CD4(IFNG+ Tfh/Th1)	pDC_LILRA4	BST2	LILRA4	1,39187E-05	12694,8	2,92072E-02	861	5,55105E+00	79	3,43832E+00	765	9,06723E-01	18557	0	43212
Myeloid_vs_CD4_Responder	CD4(AREG+ Tm)	Mono_CD14	ANXA1	FPR1	1,51634E-05	15587,4	7,83348E-03	10353	1,80016E+00	7257	2,16572E+00	8116	9,28948E-01	8999	0	43212
Myeloid_vs_CD4_Responder	CD4(IL26+ Th17)	cDC(CD1C)	B2M	CD1C	1,64833E-05	12118,4	2,37137E-02	1324	3,30914E+00	590	1,83563E+00	13463	9,47425E-01	2003	0	43212
Myeloid_vs_CD4_Responder	Mono_CD14	CD4(GZMK+ Teff)	VCAN	CD44	1,69226E-05	11056	1,40768E-02	3657	3,06223E+00	881	2,63436E+00	3738	9,41068E-01	3792	0	43212
Myeloid_vs_CD4_Responder	Mono_CD14	CD4(IFNG+ Tfh/Th1)	S100A8	CD69	1,81058E-05	9927,8	1,37216E-02	3857	3,71232E+00	344	3,82060E+00	399	9,48183E-01	1827	0	43212
Myeloid_vs_CD4_Responder	CD4(AREG+ Tm)	cDC_CLEC9A	FLT3LG	FLT3	2,22966E-05	23589,2	3,60384E-02	554	4,09588E+00	229	1,60150E+00	18926	8,25319E-01	55025	0	43212
Myeloid_vs_CD4_Responder	Macro_OLFML3	CD4(IFNG+ Tfh/Th1)	HLA-DQA1	LAG3	2,26168E-05	10962,6	1,72576E-02	2466	2,12014E+00	4079	3,29719E+00	1010	9,40259E-01	4046	0	43212
Myeloid_vs_CD4_Responder	Macro_OLFML3	CD4(ISG+ Treg)	C3	IFITM1	2,28824E-05	10137,2	2,34375E-02	1361	2,67650E+00	1633	2,58678E+00	4091	9,55923E-01	389	0	43212
Myeloid_vs_CD4_Responder	cDC_LAMP3	CD4(CRTAM- T)	CCL19	CXCR3	2,39359E-05	17831	8,21931E-02	91	5,93540E+00	41	2,02269E+00	10118	8,66871E-01	35693	0	43212
Myeloid_vs_CD4_Responder	Mono_CD14	CD4(AREG+ Tm)	VCAN	CD44	2,44552E-05	11242,8	1,36878E-02	3880	3,03596E+00	925	2,57859E+00	4160	9,40286E-01	4037	0	43212
Myeloid_vs_CD4_Responder	CD4(AREG+ Tm)	pDC_LILRA4	TNF	PTPRS	2,46202E-05	19131,6	5,91606E-02	203	4,60788E+00	137	2,50154E+00	4754	8,41418E-01	47352	0	43212
Myeloid_vs_CD4_Responder	cDC_LAMP3	CD4(NME1+ T)	CCL19	CXCR3	2,71938E-05	18331,4	8,03784E-02	97	5,92557E+00	42	1,91097E+00	12032	8,65577E-01	36274	0	43212
Myeloid_vs_CD4_Responder	CD4(AREG+ Tm)	pDC_LILRA4	HSP90B1	TLR9	2,73255E-05	23174	3,10357E-02	751	3,20252E+00	705	1,30001E+00	28418	8,51239E-01	42784	0	43212
Myeloid_vs_CD4_Responder	CD4(AREG+ Tm)	pDC_LILRA4	HMGB1	TLR9	2,75531E-05	22743,2	3,11700E-02	742	3,09908E+00	834	1,10891E+00	35674	8,72175E-01	33254	0	43212
Myeloid_vs_CD4_Responder	Macro_OLFML3	CD4(IFNG+ Tfh/Th1)	HLA-DRB5	LAG3	2,81254E-05	10847,2	1,61131E-02	2821	2,09308E+00	4309	3,51089E+00	662	9,42824E-01	3232	0	43212
Myeloid_vs_CD4_Responder	Mono_CD14	CD4(TNFRSF9+ Treg)	S100A8	CD69	2,81254E-05	10106,4	1,28997E-02	4309	3,68545E+00	357	3,75735E+00	448	9,46644E-01	2206	0	43212
Myeloid_vs_CD4_Responder	Macro_OLFML3	CD4(IFNG+ Tfh/Th1)	HLA-DRA	LAG3	2,85426E-05	10635,4	1,43710E-02	3530	2,09103E+00	4325	3,73731E+00	467	9,48955E-01	1643	0	43212
Myeloid_vs_CD4_Responder	CD4(Tn)	Mono_CD14	ANXA1	FPR1	2,96886E-05	16201,2	7,23183E-03	11842	1,72983E+00	8191	2,20901E+00	7610	9,26265E-01	10151	0	43212
Myeloid_vs_CD4_Responder	cDC(CD1C)	CD4(IFNG+ Tfh/Th1)	HLA-DQA1	LAG3	3,15091E-05	10964,6	1,68333E-02	2594	2,07561E+00	4434	3,95445E+00	307	9,39556E-01	4276	0	43212
Myeloid_vs_CD4_Responder	Mono_CD14	CD4(Tn)	VCAN	CD44	3,53413E-05	11450,8	1,29833E-02	4249	2,98839E+00	991	2,56854E+00	4238	9,38786E-01	4564	0	43212
Myeloid_vs_CD4_Responder	CD4(ISG+ Treg)	cDC(CD1C)	B2M	CD1C	3,81492E-05	10889,8	2,56898E-02	1144	3,64235E+00	377	2,16046E+00	8189	9,49384E-01	1527	0	43212
Myeloid_vs_CD4_Responder	CD4(CRTAM- T)	Macro_NLRP3	ANXA1	FPR1	4,14931E-05	14919,4	9,42953E-03	7577	2,02832E+00	4861	1,87717E+00	12662	9,34830E-01	6285	0	43212
Myeloid_vs_CD4_Responder	CD4(CRTAM- T)	cDC(CD1C)	ANXA1	FPR1	4,17559E-05	16948,4	6,93510E-03	12678	1,61833E+00	9904	2,16304E+00	8147	9,24822E-01	10801	0	43212
Myeloid_vs_CD4_Responder	cDC(CD1C)	CD4(IFNG+ Tfh/Th1)	HLA-DRA	LAG3	4,25349E-05	10747	1,39395E-02	3731	2,03693E+00	4782	4,18231E+00	188	9,48212E-01	1822	0	43212
Myeloid_vs_CD4_Responder	CD4(CRTAM- T)	Macro_ISG15	ANXA1	FPR1	4,28206E-05	15803	8,51519E-03	9025	1,87804E+00	6308	1,87214E+00	12742	9,31653E-01	7728	0	43212
Myeloid_vs_CD4_Responder	Macro_FOLR2+APOE+	CD4(IFNG+ Tfh/Th1)	HLA-DQA1	LAG3	4,32098E-05	11469,8	1,64466E-02	2714	2,03504E+00	4801	2,93102E+00	2106	9,38893E-01	4516	0	43212
Myeloid_vs_CD4_Responder	Macro_NLRP3	CD4(AREG+ Tm)	S100A8	CD69	4,50609E-05	11697,8	1,20488E-02	4852	2,10460E+00	4209	2,65951E+00	3570	9,44894E-01	2646	0	43212
Myeloid_vs_CD4_Responder	CD4(Tn)	Macro_NLRP3	ANXA1	FPR1	4,66651E-05	16262,6	7,97268E-03	10043	1,87385E+00	6360	1,86178E+00	12963	9,29527E-01	8735	0	43212
Myeloid_vs_CD4_Responder	cDC_LAMP3	CD4(GZMK+ Teff)	CCL19	CXCR3	4,73306E-05	18640,4	7,29561E-02	128	5,88533E+00	46	1,97484E+00	10913	8,59840E-01	38903	0	43212
Myeloid_vs_CD4_Responder	Macro_OLFML3	CD4(IFNG+ Tfh/Th1)	HLA-DQB1	LAG3	4,75051E-05	11204,6	1,65827E-02	2670	2,02259E+00	4917	3,21876E+00	1184	9,40271E-01	4040	0	43212
Myeloid_vs_CD4_Responder	CD4(NME1+ T)	pDC_LILRA4	BST2	LILRA4	4,76684E-05	13606,2	2,39042E-02	1300	5,45265E+00	84	3,17531E+00	1295	8,97898E-01	22140	0	43212
Myeloid_vs_CD4_Responder	cDC_LAMP3	CD4(GZMK+ Teff)	CCL19	CCR7	4,80723E-05	17000,2	7,27992E-02	129	5,90228E+00	45	1,89630E+00	12307	8,81083E-01	29308	0	43212
Myeloid_vs_CD4_Responder	CD4(Tn)	Macro_ISG15	ANXA1	FPR1	4,85673E-05	17337,2	7,19960E-03	11916	1,72356E+00	8267	1,85675E+00	13067	9,26113E-01	10224	0	43212
Myeloid_vs_CD4_Responder	CD4(TGFB1+ Th17)	cDC(CD1C)	B2M	CD1C	4,99962E-05	12062,2	2,36573E-02	1333	3,29964E+00	600	1,85306E+00	13143	9,47366E-01	2023	0	43212
Myeloid_vs_CD4_Responder	CD4(AREG+ Tm)	Mono_CD14	RPS19	C5AR1	5,02249E-05	15064,4	6,75655E-03	13155	1,92106E+00	5844	1,97405E+00	10930	9,46707E-01	2181	0	43212
Myeloid_vs_CD4_Responder	CD4(CRTAM- T)	cDC(CD1C)	B2M	CD1C	5,47922E-05	12120,2	2,35553E-02	1343	3,28244E+00	616	1,84025E+00	13386	9,47258E-01	2044	0	43212
Myeloid_vs_CD4_Responder	cDC_LAMP3	CD4(TGFB1+ Th17)	CCL19	CCR7	5,50065E-05	17410,8	6,95154E-02	138	5,88138E+00	47	1,84603E+00	13270	8,78643E-01	30387	0	43212
Myeloid_vs_CD4_Responder	cDC(CD1C)	CD4(IFNG+ Tfh/Th1)	HLA-DPB1	LAG3	5,80601E-05	10918	1,43960E-02	3520	1,99142E+00	5172	4,13524E+00	213	9,45579E-01	2473	0	43212
Myeloid_vs_CD4_Responder	CD4(TNF+ T)	Macro_LYVE1	HSPA1A	TLR4	5,81460E-05	14115,6	1,04147E-02	6338	2,15589E+00	3801	2,64388E+00	3681	9,18287E-01	13546	0	43212
Myeloid_vs_CD4_Responder	CD4(TNF+ T)	pDC_LILRA4	TNF	PTPRS	5,90602E-05	17776,8	6,87051E-02	143	4,66805E+00	128	2,82665E+00	2579	8,51144E-01	42822	0	43212
Myeloid_vs_CD4_Responder	cDC_LAMP3	CD4(TNFRSF9+ Treg)	CCL19	CXCR3	6,24066E-05	19380,8	6,76349E-02	147	5,85649E+00	48	1,88543E+00	12505	8,55214E-01	40992	0	43212
Myeloid_vs_CD4_Responder	Macro_OLFML3	CD4(IFNG+ Tfh/Th1)	HLA-DPB1	LAG3	6,24546E-05	11065,6	1,43136E-02	3554	1,98129E+00	5268	3,42738E+00	787	9,45431E-01	2507	0	43212
Myeloid_vs_CD4_Responder	CD4(AREG+ Tm)	Macro_NLRP3	ANXA1	FPR1	6,41069E-05	15792,4	8,63597E-03	8802	1,94418E+00	5620	1,81849E+00	13813	9,32100E-01	7515	0	43212
Myeloid_vs_CD4_Responder	cDC_LAMP3	CD4(IFNG+ Tfh/Th1)	CCL19	CXCR3	6,41144E-05	19437,4	6,73738E-02	149	5,85507E+00	49	1,87621E+00	12674	8,54974E-01	41103	0	43212
Myeloid_vs_CD4_Responder	CD4(TGFB1+ Th17)	Mono_CD14	RPS19	C5AR1	6,51579E-05	15353,6	6,54193E-03	13858	1,87306E+00	6373	1,97289E+00	10946	9,45887E-01	2379	0	43212
Myeloid_vs_CD4_Responder	CD4(AREG+ Tm)	Macro_ISG15	ANXA1	FPR1	6,63419E-05	16791,4	7,79857E-03	10439	1,79389E+00	7338	1,81345E+00	13908	9,28801E-01	9060	0	43212
Myeloid_vs_CD4_Responder	CD4(IL26+ Th17)	Mono_CD14	RPS19	C5AR1	6,69163E-05	15955,6	6,52412E-03	13932	1,86907E+00	6420	1,81832E+00	13817	9,45817E-01	2397	0	43212

Myeloid_vs_CD4_Post_R

Myeloid_vs_CD4_Responder	CD4(CRTAM- T)	Mono_CD14	RPS19	C5AR1	6,96000E-05	15570,8	6,49108E-03	14042	1,86168E+00	6512	1,93267E+00	11650	9,45687E-01	2438	0	43212
Myeloid_vs_CD4_Responder	CD4(TGFB1+ Th17)	pDC_LILRA4	TNF	PTPRS	7,02725E-05	18335,6	6,63218E-02	156	4,65302E+00	130	2,56094E+00	4297	8,48893E-01	43883	0	43212
Myeloid_vs_CD4_Responder	CD4(CRTAM- T)	Mono_CD16	ANXA1	FPR1	7,11754E-05	14402	1,04785E-02	6265	2,20074E+00	3517	1,80337E+00	14105	9,37970E-01	4911	0	43212
Myeloid_vs_CD4_Responder	CD4(AREG+ Tm)	pDC_LILRA4	TNF	TNFRSF21	7,14729E-05	19310,2	2,25293E-02	1489	3,36286E+00	540	2,30013E+00	6625	8,47237E-01	44685	0	43212
Myeloid_vs_CD4_Responder	Macro_NLRP3	CD4(CRTAM- T)	S100A8	CD69	7,19303E-05	12255,4	1,13252E-02	5459	2,05712E+00	4608	2,48683E+00	4882	9,43259E-01	3116	0	43212
Myeloid_vs_CD4_Responder	Mono_CD14	CD4(TGFB1+ Th17)	VCAN	CD44	7,35107E-05	11941,8	1,22632E-02	4700	2,93976E+00	1067	2,41598E+00	5489	9,37125E-01	5241	0	43212
Myeloid_vs_CD4_Responder	Macro_NLRP3	CD4(TNF+ T)	S100A8	CD69	7,45249E-05	11715,6	1,12762E-02	5508	2,05390E+00	4633	2,93527E+00	2081	9,43143E-01	3144	0	43212
Myeloid_vs_CD4_Responder	Mono_CD14	CD4(IFNG+ Tfh/Th1)	S100A8	ITGB2	7,63020E-05	10205,4	1,08840E-02	5878	3,93175E+00	282	4,36961E+00	133	9,49403E-01	1522	0	43212
Myeloid_vs_CD4_Responder	Macro_OLFML3	CD4(IFNG+ Tfh/Th1)	HLA-DPA1	LAG3	7,67489E-05	11047,8	1,41110E-02	3643	1,95239E+00	5549	3,49305E+00	680	9,46805E-01	2155	0	43212
Myeloid_vs_CD4_Responder	CD4(IL26+ Th17)	pDC_LILRA4	BST2	LILRA4	7,69685E-05	18265	1,12466E-02	5553	5,21776E+00	97	2,81254E+00	2653	8,57794E-01	39810	0	43212
Myeloid_vs_CD4_Responder	CD4(TNF+ T)	Mast	HSPA8	ADRB2	7,78732E-05	15006,4	1,77619E-02	2331	1,81679E+00	7056	1,79189E+00	14361	9,30937E-01	8072	0	43212
Myeloid_vs_CD4_Responder	cDC_LAMP3	CD4(CXCL13+ Tfh)	CCL19	CCR7	7,86040E-05	18065,8	6,48301E-02	165	5,85156E+00	50	1,76628E+00	14875	8,74874E-01	32027	0	43212
Myeloid_vs_CD4_Responder	CD4(TNF+ T)	Mono_CD14	HSPA1A	TLR4	7,91833E-05	14035,6	9,85447E-03	7015	2,09510E+00	4296	3,19317E+00	1246	9,16189E-01	14409	0	43212
Myeloid_vs_CD4_Responder	CD4(Tn)	Mono_CD16	ANXA1	FPR1	8,01219E-05	15596,4	8,85962E-03	8450	2,04626E+00	4699	1,78798E+00	14443	9,32905E-01	7178	0	43212
Myeloid_vs_CD4_Responder	cDC(CD1C)	CD4(IFNG+ Tfh/Th1)	HLA-DQB1	LAG3	8,04894E-05	11328	1,58442E-02	2925	1,94454E+00	5616	3,80054E+00	411	9,38979E-01	4476	0	43212
Myeloid_vs_CD4_Responder	CD4(AREG+ Tm)	cDC(CD1C)	ANXA1	FPR1	8,25359E-05	18046,4	6,35146E-03	14529	1,53419E+00	11463	2,10436E+00	8913	9,21708E-01	12115	0	43212
Myeloid_vs_CD4_Responder	cDC_CLEC9A	CD4(IFNG+ Tfh/Th1)	HLA-DQB1	LAG3	8,31350E-05	11370,2	1,57990E-02	2949	1,93977E+00	5662	3,65733E+00	514	9,38897E-01	4514	0	43212
Myeloid_vs_CD4_Responder	Macro_OLFML3	CD4(ISG+ Treg)	HLA-DRA	LAG3	8,35432E-05	11709,6	1,12637E-02	5523	1,93912E+00	5669	3,36289E+00	883	9,42722E-01	3261	0	43212
Myeloid_vs_CD4_Responder	pDC_LILRA4	CD4(ISG+ Treg)	GZMB	IGF2R	8,48228E-05	18357,8	2,19839E-02	1577	2,88465E+00	1172	3,87868E+00	354	8,45486E-01	45474	0	43212
Myeloid_vs_CD4_Responder	Mono_CD14	CD4(TNFRSF9+ Treg)	VCAN	CD44	8,54283E-05	12115,8	1,18205E-02	5035	2,90987E+00	1117	2,41345E+00	5514	9,36033E-01	5701	0	43212
Myeloid_vs_CD4_Responder	CD4(IFNG+ Tfh/Th1)	cDC(CD1C)	B2M	CD1C	8,67619E-05	10953,8	2,54532E-02	1163	3,60246E+00	398	2,14353E+00	8407	9,49161E-01	1589	0	43212
Myeloid_vs_CD4_Responder	Macro_OLFML3	CD4(ISG+ Treg)	HLA-DRB5	LAG3	8,89256E-05	12095	1,26292E-02	4460	1,94116E+00	5650	3,13647E+00	1394	9,35892E-01	5759	0	43212
Myeloid_vs_CD4_Responder	Macro_NLRP3	CD4(IL26+ Th17)	S100A8	CD69	9,18998E-05	12505,6	1,09735E-02	5807	2,03404E+00	4811	2,43294E+00	5344	9,42409E-01	3354	0	43212
Myeloid_vs_CD4_Responder	CD4(CXCL13+ Tfh)	Mono_CD14	RPS19	C5AR1	9,40981E-05	16583,6	6,25015E-03	14915	1,80780E+00	7167	1,76509E+00	14908	9,44707E-01	2716	0	43212
Myeloid_vs_CD4_Responder	CD4(Tn)	Mono_CD14	RPS19	C5AR1	9,79774E-05	16060,8	6,22401E-03	15036	1,80195E+00	7233	1,90868E+00	12074	9,44598E-01	2749	0	43212
Myeloid_vs_CD4_Responder	CD4(CXCL13+ Tfh)	cDC(CD1C)	B2M	CD1C	1,01246E-04	12508,2	2,32246E-02	1393	3,22666E+00	669	1,75428E+00	15135	9,46904E-01	2132	0	43212
Myeloid_vs_CD4_Responder	cDC_CLEC9A	CD4(IFNG+ Tfh/Th1)	HLA-DPA1	LAG3	1,05513E-04	11150	1,37264E-02	3854	1,90434E+00	6013	3,90951E+00	338	9,46105E-01	2333	0	43212
Myeloid_vs_CD4_Responder	Mono_CD14	CD4(IL26+ Th17)	VCAN	CD44	1,07756E-04	12147,6	1,20169E-02	4877	2,92313E+00	1094	2,35688E+00	6045	9,36525E-01	5510	0	43212
Myeloid_vs_CD4_Responder	CD4(AREG+ Tm)	Mono_CD16	ANXA1	FPR1	1,08185E-04	15204,4	9,59669E-03	7341	2,11659E+00	4100	1,74468E+00	15337	9,35363E-01	6032	0	43212
Myeloid_vs_CD4_Responder	CD4(TGFB1+ Th17)	pDC_LILRA4	TNF	TNFRSF21	1,09961E-04	18448,8	2,52565E-02	1179	3,40801E+00	507	2,35953E+00	6024	8,54486E-01	41322	0	43212
Myeloid_vs_CD4_Responder	Macro_FOLR2+APOE+	CD4(IFNG+ Tfh/Th1)	HLA-DRA	LAG3	1,11402E-04	11602,8	1,28223E-02	4350	1,89690E+00	6096	2,94887E+00	2029	9,46122E-01	2327	0	43212
Myeloid_vs_CD4_Responder	CD4(TGFB1+ Th17)	pDC_LILRA4	BST2	LILRA4	1,11475E-04	16137,4	1,50365E-02	3238	5,28809E+00	92	2,95959E+00	1990	8,74604E-01	32155	0	43212
Myeloid_vs_CD4_Responder	CD4(TGFB1+ Th17)	Mono_CD14	ANXA1	FPR1	1,15685E-04	18972,8	6,39217E-03	14393	1,63168E+00	9686	1,73604E+00	15544	9,21938E-01	12029	0	43212
Myeloid_vs_CD4_Responder	cDC_CLEC9A	CD4(IFNG+ Tfh/Th1)	HLA-DPB1	LAG3	1,18591E-04	11320,6	1,35591E-02	3944	1,88841E+00	6193	3,87355E+00	361	9,44017E-01	2893	0	43212
Myeloid_vs_CD4_Responder	Macro_OLFML3	CD4(IFNG+ Tfh/Th1)	HLA-DRB1	LAG3	1,20269E-04	11303	1,35760E-02	3936	1,88665E+00	6215	3,35510E+00	900	9,46405E-01	2252	0	43212
Myeloid_vs_CD4_Responder	cDC(CD1C)	CD4(ISG+ Treg)	HLA-DRA	LAG3	1,21422E-04	11839,4	1,09255E-02	5848	1,88502E+00	6230	3,80789E+00	402	9,41893E-01	3505	0	43212
Myeloid_vs_CD4_Responder	cDC_CLEC9A	CD4(IFNG+ Tfh/Th1)	HLA-DRA	LAG3	1,24147E-04	11342,2	1,27013E-02	4418	1,88172E+00	6265	3,76995E+00	434	9,45880E-01	2382	0	43212
Myeloid_vs_CD4_Responder	CD4(TNFRSF9+ Treg)	Mono_CD16	HLA-A	LILRB2	1,26727E-04	15012,6	6,62141E-03	13571	2,59335E+00	1864	1,72388E+00	15830	9,54206E-01	586	0	43212
Myeloid_vs_CD4_Responder	CD4(TNF+ T)	Mono_CD16	HSPA1A	TLR4	1,28579E-04	15197	9,04762E-03	8166	2,00754E+00	5041	2,64303E+00	3690	9,12850E-01	15876	0	43212
Myeloid_vs_CD4_Responder	Macro_IFI27	CD4(IFNG+ Tfh/Th1)	HLA-DRA	LAG3	1,30544E-04	11553,4	1,26490E-02	4449	1,87517E+00	6345	3,15478E+00	1350	9,45774E-01	2411	0	43212
Myeloid_vs_CD4_Responder	cDC(CD1C)	CD4(IFNG+ Tfh/Th1)	HLA-DPA1	LAG3	1,34165E-04	11274,2	1,34625E-02	4011	1,87138E+00	6389	3,97840E+00	297	9,45608E-01	2462	0	43212
Myeloid_vs_CD4_Responder	cDC_CLEC9A	CD4(IFNG+ Tfh/Th1)	HLA-DQA1	LAG3	1,34249E-04	11838,2	1,48867E-02	3309	1,87135E+00	6390	3,60880E+00	554	9,35971E-01	5726	0	43212
Myeloid_vs_CD4_Responder	Macro_FOLR2+APOE+	CD4(Tn)	APOE	LSR	1,45033E-04	13106,6	2,11443E-02	1684	2,58583E+00	1888	3,20741E+00	1209	9,09033E-01	17540	0	43212
Myeloid_vs_CD4_Responder	CD4(GZMK+ Teff)	cDC(CD1C)	B2M	CD1C	1,45301E-04	11772	2,41051E-02	1286	3,37513E+00	530	1,91866E+00	11908	9,47832E-01	1924	0	43212
Myeloid_vs_CD4_Responder	Macro_FOLR2+APOE+	CD4(CRTAM- T)	APOE	LSR	1,48028E-04	13213,6	2,08309E-02	1729	2,58148E+00	1901	3,12911E+00	1410	9,08413E-01	17816	0	43212
Myeloid_vs_CD4_Responder	CD4(Tn)	cDC(CD1C)	ANXA1	FPR1	1,52078E-04	18884	5,86363E-03	16418	1,46386E+00	13098	2,14765E+00	8350	9,18776E-01	13342	0	43212
Myeloid_vs_CD4_Responder	CD4(CXCL13+ Tfh)	Macro_LYVE1	HMGB1	CD163	1,57336E-04	16414,6	6,17251E-03	15229	2,51315E+00	2110	1,69360E+00	16530	9,37756E-01	4992	0	43212
Myeloid_vs_CD4_Responder	CD4(GZMK+ Teff)	Mono_CD14	RPS19	C5AR1	1,57479E-04	17267,6	5,83425E-03	16533	1,71478E+00	8404	1,76210E+00	14970	9,42881E-01	3219	0	43212
Myeloid_vs_CD4_Responder	CD4(NME1+ T)	cDC(CD1C)	B2M	CD1C	1,57739E-04	11749,4	2,39772E-02	1298	3,35357E+00	549	1,92682E+00	11746	9,47700E-01	1942	0	43212
Myeloid_vs_CD4_Responder	CD4(TGFB1+ Th17)	cDC_CLEC9A	FLT3LG	FLT3	1,57926E-04	20075,6	5,48849E-02	234	4,27413E+00	194	1,76124E+00	14995	8,53602E-01	41743	0	43212
Myeloid_vs_CD4_Responder	Macro_NLRP3	CD4(Tn)	S100A8	CD69	1,58147E-04	12917,4	1,01542E-02	6660	1,98028E+00	5273	2,42572E+00	5400	9,40267E-01	4042	0	43212
Myeloid_vs_CD4_Responder	cDC(CD1C)	CD4(IFNG+ Tfh/Th1)	HLA-DRB5	LAG3	1,58900E-04	11723	1,39087E-02	3753	1,84768E+00	6668	3,82934E+00	392	9,38727E-01	4590	0	43212
Myeloid_vs_CD4_Responder	CD4(ISG+ Treg)	Mono_CD16	HLA-A	LILRB2	1,62202E-04	15094,2	6,69876E-03	13311	2,62522E+00	1771	1,68957E+00	16631	9,54459E-01	546	0	43212
Myeloid_vs_CD4_Responder	cDC(CD1C)	CD4(ISG+ Treg)	HLA-DPB1	LAG3	1,67951E-04	12081,2	1,12833E-02	5503	1,83950E+00	6762	3,76082E+00	443	9,38959E-01	4486	0	43212
Myeloid_vs_CD4_Responder	CD4(TNF+ T)	cDC(CD1C)	B2M	CD1C	1,68843E-04	13112	2,12218E-02	1669	2,88895E+00	1163	1,68433E+00	16765	9,44591E-01	2751	0	43212
Myeloid_vs_CD4_Responder	Macro_FOLR2+APOE-	CD4(ISG+ Treg)	CCL13	CXCR3	1,70284E-04	23621,6	8,74094E-02	78	4,04970E+00	243	1,38926E+00	25247	8,37288E-01	49328	0	43212
Myeloid_vs_CD4_Responder	CD4(TNF+ T)	pDC_LILRA4	BST2	LILRA4	1,71160E-04	14586	1,92430E-02	1996	5,36615E+00	88	3,29411E+00	1012	8,87517E-01	26622	0	43212
Myeloid_vs_CD4_Responder	CD4(Tn)	Macro_LYVE1	HMGB1	CD163	1,74610E-04	16904,4	5,84098E-03	16509	2,46608E+00	2258	1,67891E+00	16878	9,36126E-01	5665	0	43212
Myeloid_vs_CD4_Responder	CD4(TNF+ T)	Macro_NLRP3	HSPA1A	TLR4	1,74869E-04	15588	8,54677E-03	8966	1,95319E+00	5542	2,69647E+00	3337	9,10558E-01	16883	0	43212
Myeloid_vs_CD4_Responder	Macro_ISG15	CD4(ISG+ Treg)	CXCL10	CXCR3	1,75536E-04	13126,6	4,42555E-02	377	2,54313E+00	2013	2,95982E+00	1988	9,07901E-01	18043	0	43212
Myeloid_vs_CD4_Responder	cDC_CLEC9A	CD4(IFNG+ Tfh/Th1)	HLA-DRB5	LAG3	1,76155E-04	11819,4	1,37812E-02	3820	1,83349E+00	6844	3,62978E+00	533	9,38462E-01	4688	0	43212
Myeloid_vs_CD4_Responder	cDC_LAMP3	CD4(AREG+ Tm)	CCL19	CCR7	1,77350E-04	19547,6	5,35409E-02	248	5,77971E+00	53	1,66489E+00	17258	8,64021E-01	36967	0	43212
Myeloid_vs_CD4_Responder	CD4(GZMK+ Teff)	Macro_LYVE1	HMGB1	CD163	1,77946E-04	16037,8	6,29892E-03	14732	2,53110E+00	2047	1,73954E+00	15457	9,38345E-01	4741	0	43212
Myeloid_vs_CD4_Responder	Macro_OLFML3	CD4(ISG+ Treg)	HLA-DPB1	LAG3	1,82659E-04	12383,8	1,12188E-02	5587	1,82937E+00	6907	3,05296E+00	1655	9,38795E-01	4558	0	43212
Myeloid_vs_CD4_Responder	Macro_FOLR2+APOE-	CD4(IFNG+ Tfh/Th1)	HLA-DRA	LAG3	1,83077E-04	11882,2	1,22805E-02	4682	1,82898E+00	6911	2,96126E+00	1984	9,45011E-01	2622	0	43212
Myeloid_vs_CD4_Responder	pDC_LILRA4	CD4(TGFB1+ Th17)	SCT	ADRB2	1,83106E-04	29053,8	5,33113E-02	252	5,60711E+00	73	2,56373E+00	4269	7,24235E-01	97463	0	43212
Myeloid_vs_CD4_Responder	Macro_FOLR2+APOE-	CD4(IFNG+ Tfh/Th1)	HLA-DRB5	LAG3	1,83707E-04	12210,6	1,37351E-02	3848	1,82836E+00	6917	2,87922E+00	2346	9,38365E-01	4730	0	43212
Myeloid_vs_CD4_Responder	Macro_LYVE1	CD4(IFNG+ Tfh/Th1)	HLA-DRA	LAG3	1,87732E-04	11987	1,22533E-02	4707	1,82557E+00	6955	2,86020E+00	2429	9,44953E-01	2632	0	43212
Myeloid_vs_CD4_Responder	Mono_CD14	CD4(Tn)	VCAN	SELL	1,90200E-04	12359,6	2,22460E-02	1532	3,09091E+00	845	2,93923E+00	2068	9,16826E-01	14141	0	43212

Myeloid_vs_CD4_Post_R

Myeloid_vs_CD4_Responder	CD4(ISG+ Treg)	Mono_CD16	HLA-F	LILRB2	1,95285E-04	16662,4	8,99837E-03	8238	2,55409E+00	1986	1,66487E+00	17260	9,20531E-01	12616	0	43212
Myeloid_vs_CD4_Responder	cDC(CD1C)	CD4(IFNG+ Tfh/Th1)	HLA-DRB1	LAG3	1,95758E-04	11465,4	1,30291E-02	4224	1,81897E+00	7029	3,91181E+00	334	9,45353E-01	2528	0	43212
Myeloid_vs_CD4_Responder	CD4(ISG+ Treg)	Mono_CD16	HLA-B	LILRB2	1,98187E-04	15437,8	6,36562E-03	14487	2,62123E+00	1778	1,66210E+00	17311	9,55751E-01	401	0	43212
Myeloid_vs_CD4_Responder	Macro_FOLR2-APOE+	CD4(IFNG+ Tfh/Th1)	HLA-DRA	LAG3	1,99530E-04	11922	1,21767E-02	4760	1,81597E+00	7063	2,98636E+00	1882	9,44790E-01	2693	0	43212
Myeloid_vs_CD4_Responder	CD4(TNF+ T)	Macro_ISG15	HSPA1A	TLR4	2,03042E-04	15717,6	8,29935E-03	9415	1,92634E+00	5795	2,79156E+00	2771	9,09355E-01	17395	0	43212
Myeloid_vs_CD4_Responder	Macro_OLFML3	CD4(ISG+ Treg)	HLA-DQB1	LAG3	2,04264E-04	12690,4	1,29972E-02	4241	1,87068E+00	6399	2,84434E+00	2495	9,33052E-01	7105	0	43212
Myeloid_vs_CD4_Responder	Macro_OLFML3	CD4(ISG+ Treg)	HLA-DQA1	LAG3	2,04605E-04	12366,4	1,35262E-02	3971	1,96823E+00	5401	2,92277E+00	2140	9,33038E-01	7108	0	43212
Myeloid_vs_CD4_Responder	Macro_LYVE1	CD4(IFNG+ Tfh/Th1)	HLA-DRB5	LAG3	2,09081E-04	12411,4	1,35693E-02	3938	1,80989E+00	7147	2,77558E+00	2871	9,38013E-01	4889	0	43212
Myeloid_vs_CD4_Responder	pDC_LILRA4	CD4(TNFRSF9+ Treg)	GZMB	IGF2R	2,10884E-04	19556,8	1,85610E-02	2141	2,85033E+00	1248	3,90243E+00	343	8,34105E-01	50840	0	43212
Myeloid_vs_CD4_Responder	CD4(TNFRSF9+ Treg)	Mono_CD14	RPS19	C5AR1	2,14620E-04	17877,6	5,78871E-03	16751	1,70459E+00	8558	1,65047E+00	17589	9,42670E-01	3278	0	43212
Myeloid_vs_CD4_Responder	Macro_IFI27	CD4(IFNG+ Tfh/Th1)	HLA-DRB5	LAG3	2,15043E-04	12199,6	1,35271E-02	3969	1,80520E+00	7198	3,04327E+00	1684	9,37922E-01	4935	0	43212
Myeloid_vs_CD4_Responder	Mono_CD14	CD4(CXCL13+ Tfh)	VCAN	CD44	2,15635E-04	12987	1,06425E-02	6104	2,83032E+00	1287	2,25195E+00	7129	9,32817E-01	7203	0	43212
Myeloid_vs_CD4_Responder	Macro_FOLR2+APOE+	CD4(TGFB1+ Th17)	APOE	LSR	2,16203E-04	13776,4	1,84884E-02	2159	2,54897E+00	2001	3,07166E+00	1586	9,03329E-01	19924	0	43212
Myeloid_vs_CD4_Responder	Macro_OLFML3	CD4(IL26+ Th17)	HLA-DRA	LAG3	2,19567E-04	12678	9,68501E-03	7236	1,86193E+00	6507	3,02073E+00	1765	9,38506E-01	4670	0	43212
Myeloid_vs_CD4_Responder	Macro_OLFML3	CD4(ISG+ Treg)	HLA-DPA1	LAG3	2,21856E-04	12332,2	1,10600E-02	5736	1,80047E+00	7255	3,11863E+00	1439	9,40326E-01	4019	0	43212
Myeloid_vs_CD4_Responder	CD4(ISG+ Treg)	Macro_LYVE1	HMGB1	CD163	2,26338E-04	16563,2	6,24932E-03	14920	2,52406E+00	2071	1,64322E+00	17777	9,38116E-01	4836	0	43212
Myeloid_vs_CD4_Responder	Mono_CD14	CD4(IFNG+ Tfh/Th1)	S100A9	ITGB2	2,32377E-04	11401,6	7,66958E-03	10744	3,47996E+00	463	3,85480E+00	377	9,46595E-01	2212	0	43212
Myeloid_vs_CD4_Responder	CD4(ISG+ Treg)	cDC_CLEC9A	LTB	CD40	2,35260E-04	17809,6	1,36124E-02	3915	1,59108E+00	10365	1,82770E+00	13641	9,08200E-01	17915	0	43212
Myeloid_vs_CD4_Responder	cDC(CD1C)	CD4(ISG+ Treg)	HLA-DQA1	LAG3	2,45989E-04	12239,6	1,31936E-02	4135	1,92370E+00	5819	3,58003E+00	585	9,32256E-01	7447	0	43212
Myeloid_vs_CD4_Responder	Macro_FOLR2+APOE-	CD4(CRTAM- T)	CCL13	CXCR3	2,49068E-04	26607	6,27203E-02	183	3,87432E+00	294	1,28641E+00	28925	8,13396E-01	60421	0	43212
Myeloid_vs_CD4_Responder	Macro_FOLR2+APOE-	CD4(NME1+ T)	CCL13	CXCR3	2,50762E-04	27601,2	6,13356E-02	190	3,86448E+00	295	1,17469E+00	33085	8,11696E-01	61224	0	43212
Myeloid_vs_CD4_Responder	Mono_CD14	CD4(IL26+ Th17)	VCAN	ITGA4	2,51300E-04	11551,2	2,38781E-02	1306	3,25305E+00	639	2,89806E+00	2271	9,25916E-01	10328	0	43212
Myeloid_vs_CD4_Responder	Macro_FOLR2+APOE+	CD4(TNFRSF9+ Treg)	APOE	LSR	2,54937E-04	13883,2	1,79636E-02	2282	2,54168E+00	2015	3,11590E+00	1450	9,02064E-01	20457	0	43212
Myeloid_vs_CD4_Responder	Macro_NLRP3	CD4(GZMK+ Teff)	S100A8	CD69	2,55255E-04	13559,8	9,47146E-03	7517	1,93547E+00	5707	2,30235E+00	6600	9,38282E-01	4763	0	43212
Myeloid_vs_CD4_Responder	Mono_CD14	CD4(NME1+ T)	S100A8	ITGB2	2,55876E-04	10822	9,22382E-03	7861	3,83913E+00	298	4,17986E+00	192	9,45277E-01	2547	0	43212
Myeloid_vs_CD4_Responder	Mono_CD14	CD4(ISG+ Treg)	S100A8	ITGB2	2,57592E-04	10884,6	9,10294E-03	8072	3,83239E+00	299	4,14618E+00	206	9,44935E-01	2634	0	43212
Myeloid_vs_CD4_Responder	CD4(IFNG+ Tfh/Th1)	Mono_CD14	RPS19	C5AR1	2,63370E-04	16662	6,00510E-03	15845	1,75299E+00	7866	1,83990E+00	13395	9,43653E-01	2992	0	43212
Myeloid_vs_CD4_Responder	Macro_FOLR2+APOE-	CD4(GZMK+ Teff)	CCL13	CXCR3	2,64514E-04	27811	5,56717E-02	227	3,82425E+00	303	1,23856E+00	30641	8,04180E-01	64672	0	43212
Myeloid_vs_CD4_Responder	cDC(CD1C)	CD4(IL26+ Th17)	HLA-DRA	LAG3	2,68939E-04	12752,4	9,39418E-03	7617	1,80784E+00	7166	3,46573E+00	719	9,37620E-01	5048	0	43212
Myeloid_vs_CD4_Responder	Mono_INHBA	CD4(IL26+ Th17)	CCL3	CCR4	2,74341E-04	13096,2	2,76750E-02	983	2,44664E+00	2335	2,88046E+00	2339	9,11183E-01	16612	0	43212
Myeloid_vs_CD4_Responder	Macro_FOLR2+APOE-	CD4(TNFRSF9+ Treg)	CCL18	CCR8	2,76089E-04	16935,6	1,25716E-01	28	2,44370E+00	2344	3,51276E+00	654	8,60878E-01	38440	0	43212
Myeloid_vs_CD4_Responder	Macro_NLRP3	CD4(ISG+ Treg)	S100A8	CD69	2,76125E-04	13436	9,35856E-03	7668	1,92806E+00	5775	2,40411E+00	5597	9,37934E-01	4928	0	43212
Myeloid_vs_CD4_Responder	CD4(ISG+ Treg)	Mono_CD16	B2M	LILRB2	2,76786E-04	15933	5,97488E-03	15974	2,59749E+00	1849	1,61678E+00	18507	9,59262E-01	123	0	43212
Myeloid_vs_CD4_Responder	Mono_CD14	CD4(GZMK+ Teff)	S100A8	ITGB2	2,80423E-04	11120,6	8,61068E-03	8853	3,80492E+00	312	4,21828E+00	178	9,43471E-01	3048	0	43212
Myeloid_vs_CD4_Responder	Mono_CD14	CD4(TNFRSF9+ Treg)	S100A8	ITGB2	2,82219E-04	11158,6	8,55126E-03	8960	3,80161E+00	313	4,15384E+00	201	9,43286E-01	3107	0	43212
Myeloid_vs_CD4_Responder	pDC_LILRA4	CD4(TGFB1+ Th17)	GZMB	IGF2R	2,84577E-04	19976	1,76030E-02	2368	2,84073E+00	1259	3,84170E+00	386	8,30406E-01	52655	0	43212
Myeloid_vs_CD4_Responder	cDC(CD1C)	CD4(IL26+ Th17)	HLA-DPB1	LAG3	2,86511E-04	13081,4	9,70182E-03	7209	1,76232E+00	7740	3,41866E+00	797	9,34486E-01	6449	0	43212
Myeloid_vs_CD4_Responder	Mono_CD14	CD4(IL26+ Th17)	S100A8	ITGB2	2,87642E-04	11200	8,47629E-03	9086	3,79742E+00	316	4,11249E+00	223	9,43050E-01	3163	0	43212
Myeloid_vs_CD4_Responder	cDC(CD1C)	CD4(ISG+ Treg)	HLA-DQB1	LAG3	2,87683E-04	12739,6	1,24184E-02	4595	1,79263E+00	7354	3,42612E+00	789	9,31615E-01	7748	0	43212
Myeloid_vs_CD4_Responder	Macro_FOLR2+APOE-	CD4(TNFRSF9+ Treg)	CCL13	CXCR3	2,89461E-04	29060	5,16113E-02	269	3,79540E+00	317	1,14915E+00	34041	7,98148E-01	67461	0	43212
Myeloid_vs_CD4_Responder	Macro_IER3	CD4(IFNG+ Tfh/Th1)	HLA-DRA	LAG3	2,90923E-04	12434	1,17326E-02	5099	1,76030E+00	7770	2,72733E+00	3142	9,43813E-01	2947	0	43212
Myeloid_vs_CD4_Responder	Macro_FOLR2+APOE-	CD4(IFNG+ Tfh/Th1)	CCL13	CXCR3	2,91286E-04	29163,2	5,14119E-02	272	3,79399E+00	318	1,13994E+00	34401	7,97836E-01	67613	0	43212
Myeloid_vs_CD4_Responder	cDC_CLEC9A	CD4(ISG+ Treg)	HLA-DQB1	LAG3	2,93744E-04	12815,8	1,23830E-02	4627	1,78785E+00	7414	3,28291E+00	1037	9,31524E-01	7789	0	43212
Myeloid_vs_CD4_Responder	Macro_FOLR2+APOE+	CD4(ISG+ Treg)	HLA-DQA1	LAG3	2,94340E-04	13180	1,28906E-02	4316	1,88312E+00	6254	2,55660E+00	4325	9,31519E-01	7793	0	43212
Myeloid_vs_CD4_Responder	Mono_CD14	CD4(CXCL13+ Tfh)	S100A8	ITGB2	2,94952E-04	11249,4	8,37912E-03	9250	3,79200E+00	320	4,13694E+00	210	9,42739E-01	3255	0	43212
Myeloid_vs_CD4_Responder	Macro_FOLR2+APOE+	CD4(IFNG+ Tfh/Th1)	HLA-DPB1	LAG3	2,97035E-04	12418,8	1,24957E-02	4532	1,75752E+00	7811	2,74869E+00	3014	9,41819E-01	3525	0	43212
Myeloid_vs_CD4_Responder	CD4(IFNG+ Tfh/Th1)	pDC_LILRA4	HMGB1	THBD	3,00917E-04	16778,2	7,62326E-03	10862	1,89923E+00	6070	2,38136E+00	5795	9,08121E-01	17952	0	43212
Myeloid_vs_CD4_Responder	CD4(ISG+ Treg)	cDC_CLEC9A	FLT3LG	FLT3	3,02354E-04	21158,2	4,77682E-02	324	4,20682E+00	203	1,70776E+00	16211	8,44710E-01	45841	0	43212
Myeloid_vs_CD4_Responder	Macro_ISG15	CD4(TGFB1+ Th17)	S100A8	CD69	3,05229E-04	12576,8	1,09378E-02	5839	1,75305E+00	7865	2,82565E+00	2586	9,42321E-01	3382	0	43212
Myeloid_vs_CD4_Responder	cDC_CLEC9A	CD4(ISG+ Treg)	HLA-DPA1	LAG3	3,06457E-04	12396,8	1,07585E-02	5984	1,75243E+00	7873	3,53510E+00	633	9,39546E-01	4282	0	43212
Myeloid_vs_CD4_Responder	Macro_OLFML3	CD4(IL26+ Th17)	HLA-DPB1	LAG3	3,07072E-04	13625,4	9,64634E-03	7281	1,75219E+00	7877	2,71080E+00	3240	9,34310E-01	6517	0	43212
Myeloid_vs_CD4_Responder	CD4(CRTAM- T)	cDC_CLEC9A	FLT3LG	FLT3	3,11734E-04	21215,8	4,70419E-02	329	4,19995E+00	209	1,71574E+00	16013	8,43702E-01	46316	0	43212
Myeloid_vs_CD4_Responder	Macro_OLFML3	CD4(IL26+ Th17)	HLA-DRB5	LAG3	3,16572E-04	13256,6	1,08590E-02	5896	1,86398E+00	6488	2,79431E+00	2749	9,31210E-01	7938	0	43212
Myeloid_vs_CD4_Responder	CD4(IFNG+ Tfh/Th1)	Macro_LYVE1	HMGB1	CD163	3,18086E-04	15188	6,81526E-03	12996	2,60441E+00	1831	1,81057E+00	13960	9,40585E-01	3941	0	43212
Myeloid_vs_CD4_Responder	Macro_IFI27	CD4(IFNG+ Tfh/Th1)	HLA-DQA1	LAG3	3,21005E-04	12929,4	1,36967E-02	3874	1,74648E+00	7966	2,80891E+00	2671	9,33428E-01	6924	0	43212
Myeloid_vs_CD4_Responder	Macro_FOLR2+APOE+	CD4(ISG+ Treg)	HLA-DRA	LAG3	3,23879E-04	13291,6	1,00499E-02	6801	1,74498E+00	7984	2,57445E+00	4189	9,39564E-01	4272	0	43212
Myeloid_vs_CD4_Responder	CD4(Tn)	cDC_CLEC9A	FLT3LG	FLT3	3,25301E-04	24411	3,21383E-02	699	4,05899E+00	239	1,59711E+00	19032	8,16908E-01	58873	0	43212
Myeloid_vs_CD4_Responder	CD4(IFNG+ Tfh/Th1)	Mono_CD16	B2M	LILRB2	3,26617E-04	16095,6	5,91986E-03	16192	2,55760E+00	1972	1,59985E+00	18961	9,59081E-01	141	0	43212
Myeloid_vs_CD4_Responder	Macro_OLFML3	CD4(CXCL13+ Tfh)	C3	IFITM1	3,30008E-04	11513,8	1,78815E-02	2296	2,37249E+00	2639	2,17467E+00	8022	9,49858E-01	1400	0	43212
Myeloid_vs_CD4_Responder	CD4(TNFRSF9+ Treg)	pDC_LILRA4	BST2	LILRA4	3,36224E-04	15350,2	1,70870E-02	2516	5,32614E+00	89	3,01319E+00	1789	8,81448E-01	29145	0	43212
Myeloid_vs_CD4_Responder	Mono_CD14	CD4(ISG+ Treg)	VCAN	SELL	3,37547E-04	11533	2,84818E-02	908	3,23335E+00	661	2,84080E+00	2508	9,25776E-01	10376	0	43212
Myeloid_vs_CD4_Responder	CD4(Tn)	pDC_LILRA4	TNF	PTPRS	3,37547E-04	26225	2,68407E-02	1038	4,40411E+00	172	1,92645E+00	11756	7,81366E-01	74947	0	43212
Myeloid_vs_CD4_Responder	Macro_FOLR2-APOE+	CD4(IFNG+ Tfh/Th1)	HLA-DRB5	LAG3	3,39033E-04	12623,6	1,29217E-02	4296	1,73781E+00	8077	2,94558E+00	2042	9,36576E-01	5491	0	43212
Myeloid_vs_CD4_Responder	CD4(GZMK+ Teff)	Mono_CD14	ANXA1	FPR1	3,40860E-04	21092,8	5,27027E-03	19294	1,50053E+00	12222	1,73092E+00	15660	9,14705E-01	15076	0	43212
Myeloid_vs_CD4_Responder	cDC_CLEC9A	CD4(ISG+ Treg)	HLA-DPB1	LAG3	3,41859E-04	12661,4	1,06274E-02	6120	1,73650E+00	8094	3,49913E+00	676	9,37220E-01	5205	0	43212
Myeloid_vs_CD4_Responder	Macro_ISG15	CD4(TGFB1+ Th17)	CXCL10	DPP4	3,44390E-04	17930	5,82942E-02	212	2,51190E+00	2116	2,83696E+00	2525	8,53936E-01	41585	0	43212
Myeloid_vs_CD4_Responder	CD4(GZMK+ Teff)	pDC_LILRA4	HMGB1	THBD	3,44497E-04	17674	7,04570E-03	12372	1,82593E+00	6946	2,31033E+00	6505	9,04781E-01	19335	0	43212
Myeloid_vs_CD4_Responder	cDC_LAMP3	CD4(AREG+ Tm)	CCL19	CXCR3	3,44693E-04	22341,8	4,60048E-02	346	5,73924E+00	59	1,75518E+00	15115	8,29686E-01	52977	0	43212
Myeloid_vs_CD4_Responder	Macro_OLFML3	CD4(ISG+ Treg)	HLA-DRB1	LAG3	3,47395E-04	12703	1,06406E-02	6105	1,73474E+00	8127	2,98068E+00	1905	9,39880E-01	4166	0	43212

Myeloid_vs_CD4_Responder	Mono_CD14	CD4(TGFB1+ Th17)	S100A8	ITGB2	3,50680E-04	12580,6	6,66694E-03	13434	3,69648E+00	349	3,98176E+00	294	9,36248E-01	5614	0	43212
Myeloid_vs_CD4_Responder	CD4(Tn)	pDC_LILRA4	HSP90B1	TLR9	3,53793E-04	24019,8	2,79594E-02	953	3,14728E+00	771	1,27720E+00	29229	8,44508E-01	45934	0	43212
Myeloid_vs_CD4_Responder	CD4(Tn)	pDC_LILRA4	HMGB1	TLR9	3,54619E-04	21184,4	3,32654E-02	648	3,15131E+00	762	1,26596E+00	29660	8,75759E-01	31640	0	43212
Myeloid_vs_CD4_Responder	CD4(ISG+ Treg)	Mono_CD14	RPS19	C5AR1	3,55869E-04	19250,2	5,23798E-03	19461	1,58141E+00	10549	1,60350E+00	18870	9,39908E-01	4159	0	43212
Myeloid_vs_CD4_Responder	CD4(ISG+ Treg)	pDC_LILRA4	HMGB1	THBD	3,56235E-04	17957,2	6,99023E-03	12516	1,81888E+00	7031	2,21402E+00	7562	9,04440E-01	19465	0	43212
Myeloid_vs_CD4_Responder	cDC_CLEC9A	CD4(ISG+ Treg)	HLA-DRA	LAG3	3,58494E-04	12700,4	9,95503E-03	6906	1,72981E+00	8192	3,39553E+00	835	9,39294E-01	4357	0	43212
Myeloid_vs_CD4_Responder	Mono_INHBA	CD4(CXCL13+ Tfh)	CCL3	CCR4	3,60016E-04	13643	2,43266E-02	1258	2,39224E+00	2563	2,90048E+00	2255	9,05824E-01	18927	0	43212
Myeloid_vs_CD4_Responder	CD4(CRTAM- T)	Macro_LYVE1	HMGB1	CD163	3,60649E-04	17767	5,61835E-03	17495	2,43447E+00	2391	1,57951E+00	19513	9,34954E-01	6224	0	43212
Myeloid_vs_CD4_Responder	Macro_FOLR2+APOE+	CD4(AREG+ Tm)	APOE	LSR	3,64625E-04	14206,6	1,69002E-02	2574	2,52692E+00	2059	3,06815E+00	1600	8,99335E-01	21588	0	43212
Myeloid_vs_CD4_Responder	CD4(IFNG+ Tfh/Th1)	Mono_CD16	HLA-A	LILRB2	3,65573E-04	15968,8	6,42422E-03	14278	2,51210E+00	2115	1,57718E+00	19566	9,53541E-01	673	0	43212
Myeloid_vs_CD4_Responder	Macro_IFI27	CD4(IFNG+ Tfh/Th1)	HLA-DPB1	LAG3	3,70031E-04	12402,6	1,22277E-02	4726	1,72453E+00	8258	2,93452E+00	2086	9,41223E-01	3731	0	43212
Myeloid_vs_CD4_Responder	Macro_OLFML3	CD4(IL26+ Th17)	HLA-DPA1	LAG3	3,72513E-04	13510,2	9,50980E-03	7464	1,72329E+00	8272	2,77647E+00	2866	9,35945E-01	5737	0	43212
Myeloid_vs_CD4_Responder	Macro_IFI27	CD4(ISG+ Treg)	HLA-DRA	LAG3	3,72691E-04	13136	9,91408E-03	6959	1,72326E+00	8273	2,78036E+00	2837	9,39177E-01	4399	0	43212
Myeloid_vs_CD4_Responder	CD4(TNFRSF9+ Treg)	Mono_CD16	HLA-F	LILRB2	3,74535E-04	18747,8	7,15969E-03	12018	2,40668E+00	2489	1,57377E+00	19661	9,11758E-01	16359	0	43212
Myeloid_vs_CD4_Responder	CD4(CXCL13+ Tfh)	pDC_LILRA4	HMGB1	THBD	3,76253E-04	17958,6	6,90430E-03	12755	1,80798E+00	7163	2,26440E+00	6984	9,03904E-01	19679	0	43212
Myeloid_vs_CD4_Responder	cDC_LAMP3	CD4(TGFB1+ Th17)	CCL19	CXCR3	3,77217E-04	22677,2	4,49918E-02	362	5,73374E+00	60	1,71627E+00	15997	8,28107E-01	53755	0	43212
Myeloid_vs_CD4_Responder	Mono_CD14	CD4(AREG+ Tm)	S100A8	ITGB2	3,77217E-04	12983,4	6,32158E-03	14645	3,67721E+00	362	3,93622E+00	318	9,34642E-01	6380	0	43212
Myeloid_vs_CD4_Responder	CD4(AREG+ Tm)	Macro_NLRP3	RPS19	C5AR1	3,80478E-04	15680,8	7,76598E-03	10522	2,18821E+00	3602	1,57096E+00	19723	9,50113E-01	1345	0	43212
Myeloid_vs_CD4_Responder	Macro_LYVE1	CD4(IFNG+ Tfh/Th1)	HLA-DRB1	LAG3	3,83110E-04	12377,6	1,22296E-02	4724	1,72003E+00	8331	2,81518E+00	2637	9,43694E-01	2984	0	43212
Myeloid_vs_CD4_Responder	CD4(TGFB1+ Th17)	Macro_NLRP3	RPS19	C5AR1	3,83769E-04	15906,2	7,51929E-03	11099	2,14021E+00	3924	1,56980E+00	19757	9,49342E-01	1539	0	43212
Myeloid_vs_CD4_Responder	CD4(TGFB1+ Th17)	cDC(CD1C)	ANXA1	FPR1	3,84254E-04	22225,8	5,18283E-03	19762	1,36571E+00	15800	1,67468E+00	16989	9,14050E-01	15366	0	43212
Myeloid_vs_CD4_Responder	cDC(CD1C)	CD4(ISG+ Treg)	HLA-DPA1	LAG3	3,85657E-04	12556	1,05517E-02	6193	1,71946E+00	8345	3,60398E+00	558	9,38992E-01	4472	0	43212
Myeloid_vs_CD4_Responder	Macro_FOLR2+APOE+	CD4(IFNG+ Tfh/Th1)	HLA-DRB1	LAG3	3,88767E-04	12466,8	1,22113E-02	4737	1,71777E+00	8362	2,74517E+00	3032	9,43654E-01	2991	0	43212
Myeloid_vs_CD4_Responder	Mono_CD14	CD4(IFNG+ Tfh/Th1)	VCAN	CD44	3,96530E-04	13503,6	9,80994E-03	7078	2,77410E+00	1401	2,22566E+00	7423	9,30220E-01	8404	0	43212
Myeloid_vs_CD4_Responder	Mono_CD14	CD4(NME1+ T)	VCAN	CD44	3,99330E-04	13544,8	9,80181E-03	7091	2,77355E+00	1404	2,21037E+00	7598	9,30193E-01	8419	0	43212
Myeloid_vs_CD4_Responder	Macro_ISG15	CD4(CRTAM- T)	CXCL10	CXCR3	4,00214E-04	14651,4	3,17554E-02	719	2,36775E+00	2656	2,85697E+00	2438	8,93053E-01	24232	0	43212
Myeloid_vs_CD4_Responder	Mono_CD14	CD4(CRTAM- T)	S100A8	ITGB2	4,00423E-04	13471	5,92632E-03	16167	3,65516E+00	373	3,92680E+00	326	9,32642E-01	7277	0	43212
Myeloid_vs_CD4_Responder	Mono_CD14	CD4(Tn)	S100A8	ITGB2	4,04716E-04	13622	5,80963E-03	16658	3,64865E+00	375	3,94602E+00	314	9,32015E-01	7551	0	43212
Myeloid_vs_CD4_Responder	Macro_ISG15	CD4(IL26+ Th17)	TNFSF10	CCR6	4,06869E-04	13023	3,26273E-02	670	1,86281E+00	6500	2,33340E+00	6274	9,30112E-01	8459	0	43212
Myeloid_vs_CD4_Responder	CD4(AREG+ Tm)	pDC_LILRA4	BST2	LILRA4	4,09151E-04	20348,2	8,84761E-03	8471	5,17324E+00	101	2,73394E+00	3098	8,42524E-01	46859	0	43212
Myeloid_vs_CD4_Responder	Macro_FOLR2+APOE-	CD4(ISG+ Treg)	CCL13	CCR5	4,15549E-04	38403,6	4,38713E-02	380	3,81496E+00	307	1,13312E+00	34670	6,70029E-01	113449	0	43212
Myeloid_vs_CD4_Responder	Macro_FOLR2+APOE+	CD4(ISG+ Treg)	APOE	LSR	4,20222E-04	14347,2	1,64894E-02	2700	2,52122E+00	2079	3,02893E+00	1737	8,98216E-01	22008	0	43212
Myeloid_vs_CD4_Responder	pDC_LILRA4	CD4(NME1+ T)	GZMB	IGF2R	4,20684E-04	20483	1,64841E-02	2701	2,82951E+00	1288	3,85716E+00	373	8,25731E-01	54841	0	43212
Myeloid_vs_CD4_Responder	Mono_INHBA	CD4(TNFRSF9+ Treg)	CCL3	CCR4	4,21146E-04	14075,2	2,22286E-02	1534	2,35816E+00	2702	2,86276E+00	2411	9,01906E-01	20517	0	43212
Myeloid_vs_CD4_Responder	CD4(TNFRSF9+ Treg)	cDC_CLEC9A	LTB	CD40	4,25171E-04	19044,6	1,19923E-02	4894	1,48142E+00	12691	1,79726E+00	14260	9,02779E-01	20166	0	43212
Myeloid_vs_CD4_Responder	Macro_IFI27	CD4(IFNG+ Tfh/Th1)	HLA-DQB1	LAG3	4,28935E-04	12953,4	1,35595E-02	3943	1,70307E+00	8573	2,83470E+00	2541	9,34362E-01	6498	0	43212
Myeloid_vs_CD4_Responder	CD4(IFNG+ Tfh/Th1)	Macro_FOLR2+APOE-	HMGB1	CD163	4,30255E-04	18764,6	5,10343E-03	20214	1,96051E+00	5466	1,66006E+00	17363	9,31969E-01	7568	0	43212
Myeloid_vs_CD4_Responder	CD4(TNFRSF9+ Treg)	Mono_CD16	B2M	LILRB2	4,34958E-04	16472	5,83082E-03	16559	2,49304E+00	2171	1,55083E+00	20258	9,58782E-01	160	0	43212
Myeloid_vs_CD4_Responder	CD4(TNF+ T)	Macro_FOLR2-APOE+	HSPA1A	TLR4	4,37648E-04	17566	7,05895E-03	12329	1,79174E+00	7364	2,51535E+00	4642	9,02458E-01	20283	0	43212
Myeloid_vs_CD4_Responder	cDC_CLEC9A	CD4(IFNG+ Tfh/Th1)	HLA-DRB1	LAG3	4,43322E-04	12086,4	1,20520E-02	4850	1,69805E+00	8645	3,54292E+00	623	9,43304E-01	3102	0	43212
Myeloid_vs_CD4_Responder	CD4(TNFRSF9+ Treg)	Mono_CD16	HLA-C	LILRB2	4,44378E-04	16457,4	6,13377E-03	15381	2,44253E+00	2353	1,54758E+00	20345	9,51655E-01	996	0	43212
Myeloid_vs_CD4_Responder	cDC(CD1C)	CD4(ISG+ Treg)	HLA-DRB5	LAG3	4,49626E-04	13276,8	1,09014E-02	5871	1,69576E+00	8676	3,45492E+00	736	9,31335E-01	7889	0	43212
Myeloid_vs_CD4_Responder	Macro_FOLR2+APOE+	CD4(IFNG+ Tfh/Th1)	HLA-DQB1	LAG3	4,51263E-04	13531,6	1,34868E-02	3994	1,69539E+00	8684	2,44709E+00	5198	9,34197E-01	6570	0	43212
Myeloid_vs_CD4_Responder	Macro_OLFML3	CD4(IFNG+ Tfh/Th1)	C3	IFITM1	4,54365E-04	11924,2	1,73574E-02	2441	2,34381E+00	2772	2,05606E+00	9605	9,49145E-01	1591	0	43212
Myeloid_vs_CD4_Responder	CD4(TNF+ T)	Macro_LYVE1	HMGB1	CD163	4,56522E-04	18144,2	5,05965E-03	20455	2,35515E+00	2716	1,68928E+00	16639	9,31695E-01	7699	0	43212
Myeloid_vs_CD4_Responder	CD4(AREG+ Tm)	Mono_CD16	HLA-C	LILRA3	4,57778E-04	25576,6	1,67614E-02	2613	2,34230E+00	2779	5,21487E-01	65064	9,16663E-01	14215	0	43212
Myeloid_vs_CD4_Responder	cDC_LAMP3	CD4(IFNG+ Tfh/Th1)	HLA-DRA	LAG3	4,58065E-04	12695,4	1,12011E-02	5607	1,69368E+00	8717	2,81552E+00	2634	9,42571E-01	3307	0	43212
Myeloid_vs_CD4_Responder	CD4(Tn)	Mono_CD16	HLA-C	LILRA3	4,59247E-04	25152,4	1,69240E-02	2566	2,36227E+00	2687	5,50242E-01	63243	9,17031E-01	14054	0	43212
Myeloid_vs_CD4_Responder	CD4(NME1+ T)	Mono_CD14	RPS19	C5AR1	4,62467E-04	18779	5,23155E-03	19505	1,57998E+00	10577	1,69791E+00	16429	9,39873E-01	4172	0	43212
Myeloid_vs_CD4_Responder	CD4(GZMK+ Teff)	Mono_CD16	HLA-C	LILRB2	4,66654E-04	16415,4	6,19841E-03	15124	2,46677E+00	2256	1,53966E+00	20545	9,51896E-01	940	0	43212
Myeloid_vs_CD4_Responder	cDC_LAMP3	CD4(Tn)	CCL19	CXCR3	4,67210E-04	23071,2	4,22781E-02	403	5,71903E+00	62	1,72295E+00	15849	8,23634E-01	55830	0	43212
Myeloid_vs_CD4_Responder	Mono_CD14	CD4(ISG+ Treg)	VCAN	CD44	4,72319E-04	13779	9,56959E-03	7381	2,75787E+00	1444	2,17025E+00	8073	9,29410E-01	8785	0	43212
Myeloid_vs_CD4_Responder	Macro_ISG15	CD4(AREG+ Tm)	CXCL10	DPP4	4,79624E-04	20501,6	4,00341E-02	446	2,36587E+00	2665	2,78371E+00	2823	8,28910E-01	53362	0	43212
Myeloid_vs_CD4_Responder	Macro_IFI27	CD4(IFNG+ Tfh/Th1)	HLA-DPA1	LAG3	4,81287E-04	12439,4	1,19734E-02	4904	1,68539E+00	8827	2,97431E+00	1929	9,42514E-01	3325	0	43212
Myeloid_vs_CD4_Responder	Mono_CD14	CD4(TNF+ T)	S100A8	ITGB2	4,83530E-04	15395,8	4,74468E-03	22477	3,58923E+00	410	3,98723E+00	288	9,25312E-01	10592	0	43212
Myeloid_vs_CD4_Responder	CD4(Tn)	pDC_LILRA4	HMGB1	THBD	4,84408E-04	18542,6	6,53347E-03	13890	1,76091E+00	7759	2,24970E+00	7153	9,01480E-01	20699	0	43212
Myeloid_vs_CD4_Responder	cDC_LAMP3	CD4(TNFRSF9+ Treg)	CCL17	CCR8	4,85885E-04	20773,2	3,12910E-01	7	3,58895E+00	411	2,26793E+00	6947	8,29049E-01	53289	0	43212
Myeloid_vs_CD4_Responder	Macro_FOLR2+APOE+	CD4(IFNG+ Tfh/Th1)	HLA-DPA1	LAG3	4,86901E-04	12796,2	1,19562E-02	4918	1,68324E+00	8853	2,64760E+00	3662	9,42475E-01	3336	0	43212
Myeloid_vs_CD4_Responder	cDC_CLEC9A	CD4(ISG+ Treg)	HLA-DRB5	LAG3	4,93001E-04	13431,6	1,08015E-02	5944	1,68158E+00	8881	3,25536E+00	1101	9,31040E-01	8020	0	43212
Myeloid_vs_CD4_Responder	CD4(TNF+ T)	Macro_OLFML3	HSPA1A	TLR4	4,94317E-04	17937	6,87944E-03	12824	1,77226E+00	7621	2,44204E+00	5245	9,01319E-01	20783	0	43212
Myeloid_vs_CD4_Responder	CD4(NME1+ T)	Macro_LYVE1	HMGB1	CD163	4,98275E-04	12550	8,58434E-03	8905	2,85558E+00	1234	2,24370E+00	7220	9,46715E-01	2179	0	43212
Myeloid_vs_CD4_Responder	CD4(CRTAM- T)	Macro_NLRP3	RPS19	C5AR1	4,99932E-04	16176,2	7,46084E-03	11228	2,12884E+00	4021	1,52958E+00	20830	9,49154E-01	1590	0	43212
Myeloid_vs_CD4_Responder	Macro_FOLR2-APOE+	CD4(IFNG+ Tfh/Th1)	HLA-DRB1	LAG3	5,02036E-04	12535,6	1,18923E-02	4969	1,67829E+00	8922	2,86954E+00	2383	9,42946E-01	3192	0	43212
Myeloid_vs_CD4_Responder	cDC_LAMP3	CD4(IFNG+ Tfh/Th1)	HLA-DQA1	LAG3	5,04258E-04	13533,8	1,30412E-02	4215	1,67770E+00	8932	2,64066E+00	3702	9,31888E-01	7608	0	43212
Myeloid_vs_CD4_Responder	Macro_FOLR2+APOE-	CD4(ISG+ Treg)	HLA-DRA	LAG3	5,06041E-04	13659,6	9,62524E-03	7307	1,67706E+00	8940	2,58684E+00	4090	9,38327E-01	4749	0	43212
Myeloid_vs_CD4_Responder	CD4(TNF+ T)	Macro_IER3	HSPA1A	TLR4	5,06324E-04	17604	6,84923E-03	12906	1,76898E+00	7658	2,69380E+00	3361	9,01123E-01	20883	0	43212
Myeloid_vs_CD4_Responder	CD4(NME1+ T)	Macro_NLRP3	HMGB1	CD163	5,08024E-04	17695,6	5,17309E-03	19812	1,83688E+00	6801	1,95477E+00	11276	9,32398E-01	7377	0	43212
Myeloid_vs_CD4_Responder	Macro_FOLR2+APOE-	CD4(ISG+ Treg)	HLA-DRB5	LAG3	5,08500E-04	14189,6	1,07654E-02	5978	1,67644E+00	8951	2,50480E+00	4732	9,30932E-01	8075	0	43212
Myeloid_vs_CD4_Responder	CD4(TNF+ T)	Mono_CD14	RPS19	C5AR1	5,10582E-04	19470,6	4,98539E-03	20918	1,52492E+00	11674	1,67901E+00	16876	9,38497E-01	4673	0	43212

Myeloid_vs_CD4_Responder	cDC_CLEC9A	CD4(IL26+ Th17)	HLA-DPA1	LAG3	5,13445E-04	13482,4	9,25058E-03	7823	1,67524E+00	8973	3,19293E+00	1248	9,35112E-01	6156	0	43212
Myeloid_vs_CD4_Responder	CD4(AREG+ Tm)	Mono_CD16	HLA-B	LILRB2	5,15776E-04	17894,8	5,74859E-03	16937	2,32063E+00	2893	1,37454E+00	25761	9,53544E-01	671	0	43212
Myeloid_vs_CD4_Responder	Macro_LYVE1	CD4(ISG+ Treg)	HLA-DRA	LAG3	5,18880E-04	13839,8	9,60396E-03	7331	1,67366E+00	8997	2,48578E+00	4886	9,38263E-01	4773	0	43212
Myeloid_vs_CD4_Responder	Macro_ISG15	CD4(GZMK+ Teff)	CXCL10	CXCR3	5,23750E-04	15292,4	2,81867E-02	934	2,31768E+00	2908	2,80912E+00	2670	8,87225E-01	26738	0	43212
Myeloid_vs_CD4_Responder	CD4(NME1+ T)	pDC_LILRA4	HMGB1	THBD	5,25396E-04	14197	9,60207E-03	7336	2,15040E+00	3852	2,81450E+00	2639	9,17306E-01	13946	0	43212
Myeloid_vs_CD4_Responder	Macro_FOLR2+APOE-	CD4(IFNG+ Tfh/Th1)	HLA-DPB1	LAG3	5,28955E-04	12846,6	1,17925E-02	5060	1,67097E+00	9041	2,77735E+00	2861	9,40212E-01	4059	0	43212
Myeloid_vs_CD4_Responder	CD4(AREG+ Tm)	Macro_LYVE1	HMGB1	CD163	5,29027E-04	18308	5,47305E-03	18210	2,41384E+00	2460	1,52186E+00	21067	9,34153E-01	6591	0	43212
Myeloid_vs_CD4_Responder	Macro_LYVE1	CD4(IFNG+ Tfh/Th1)	HLA-DPA1	LAG3	5,30341E-04	12788,6	1,18560E-02	4997	1,67072E+00	9047	2,70374E+00	3286	9,42246E-01	3401	0	43212
Myeloid_vs_CD4_Responder	CD4(NME1+ T)	pDC_LILRA4	HMGB1	TLR9	5,31693E-04	16161,6	4,88894E-02	305	3,54081E+00	430	1,83075E+00	13572	8,95237E-01	23289	0	43212
Myeloid_vs_CD4_Responder	CD4(IL26+ Th17)	pDC_LILRA4	HMGB1	THBD	5,43112E-04	19250,8	6,36757E-03	14480	1,73985E+00	8054	2,07435E+00	9330	9,00332E-01	21178	0	43212
Myeloid_vs_CD4_Responder	CD4(NME1+ T)	cDC_CLEC9A	HMGB1	HAVCR2	5,44909E-04	20703,8	4,93934E-03	21192	1,26670E+00	18916	2,11136E+00	8813	9,23401E-01	11386	0	43212
Myeloid_vs_CD4_Responder	Macro_FOLR2+APOE+	CD4(IL26+ Th17)	HLA-DRA	LAG3	5,45518E-04	14924,2	8,64132E-03	8795	1,66780E+00	9112	2,23229E+00	7356	9,35132E-01	6146	0	43212
Myeloid_vs_CD4_Responder	cDC(CD1C)	CD4(ISG+ Treg)	HLA-DRB1	LAG3	5,48592E-04	12831	1,02120E-02	6590	1,66706E+00	9125	3,53739E+00	629	9,38707E-01	4599	0	43212
Myeloid_vs_CD4_Responder	cDC_LAMP3	CD4(NME1+ T)	CCL19	CCR7	5,51593E-04	22321,6	4,03931E-02	438	5,69603E+00	64	1,46273E+00	22923	8,46603E-01	44971	0	43212
Myeloid_vs_CD4_Responder	CD4(IFNG+ Tfh/Th1)	Mono_CD16	HLA-B	LILRB2	5,53057E-04	16607,2	6,02953E-03	15748	2,45750E+00	2290	1,51533E+00	21255	9,54589E-01	531	0	43212
Myeloid_vs_CD4_Responder	Macro_FOLR2-APOE+	CD4(ISG+ Treg)	HLA-DRA	LAG3	5,58371E-04	13709	9,54391E-03	7420	1,66406E+00	9166	2,61194E+00	3893	9,38081E-01	4854	0	43212
Myeloid_vs_CD4_Responder	Macro_NLRP3	CD4(CXCL13+ Tfh)	S100A8	CD69	5,59332E-04	14612	8,42194E-03	9170	1,86660E+00	6452	2,18284E+00	7922	9,34793E-01	6304	0	43212
Myeloid_vs_CD4_Responder	Macro_FOLR2-APOE+	CD4(IFNG+ Tfh/Th1)	HLA-DQA1	LAG3	5,61016E-04	13637,4	1,29028E-02	4304	1,66317E+00	9177	2,63878E+00	3714	9,31549E-01	7780	0	43212
Myeloid_vs_CD4_Responder	Macro_IFI27	CD4(IFNG+ Tfh/Th1)	HLA-DRB1	LAG3	5,62705E-04	12586,4	1,17670E-02	5076	1,66279E+00	9184	2,91629E+00	2178	9,42660E-01	3282	0	43212
Myeloid_vs_CD4_Responder	Mast	CD4(CRTAM- T)	TIMP3	CD44	5,64216E-04	13589	4,37239E-02	383	3,50929E+00	443	1,83443E+00	13489	9,25677E-01	10418	0	43212
Myeloid_vs_CD4_Responder	Macro_IFI27	CD4(ISG+ Treg)	C3	IFITM1	5,70959E-04	13876,2	1,28933E-02	4312	1,67351E+00	8999	2,08290E+00	9218	9,41471E-01	3640	0	43212
Myeloid_vs_CD4_Responder	CD4(CRTAM- T)	pDC_LILRA4	HMGB1	THBD	5,74725E-04	19188,4	6,28445E-03	14788	1,72930E+00	8200	2,15031E+00	8323	8,99741E-01	21419	0	43212
Myeloid_vs_CD4_Responder	cDC_CLEC9A	CD4(IL26+ Th17)	HLA-DPB1	LAG3	5,75856E-04	13816,4	9,13783E-03	8006	1,65931E+00	9238	3,15697E+00	1342	9,32628E-01	7284	0	43212
Myeloid_vs_CD4_Responder	cDC_LAMP3	CD4(IFNG+ Tfh/Th1)	HLA-DPB1	LAG3	5,79055E-04	12884,2	1,16889E-02	5129	1,65822E+00	9251	2,80696E+00	2680	9,39964E-01	4149	0	43212
Myeloid_vs_CD4_Responder	Macro_LYVE1	CD4(ISG+ Treg)	HLA-DRB5	LAG3	5,80537E-04	14493,2	1,06354E-02	6113	1,65798E+00	9257	2,40116E+00	5630	9,30541E-01	8254	0	43212
Myeloid_vs_CD4_Responder	Macro_OLFML3	CD4(IL26+ Th17)	HLA-DRB1	LAG3	5,83260E-04	14031,8	9,14924E-03	7984	1,65755E+00	9268	2,63852E+00	3718	9,35469E-01	5977	0	43212
Myeloid_vs_CD4_Responder	cDC_CLEC9A	CD4(ISG+ Treg)	HLA-DQA1	LAG3	5,86241E-04	13427,6	1,16679E-02	5149	1,71944E+00	8348	3,23438E+00	1149	9,28271E-01	9280	0	43212
Myeloid_vs_CD4_Responder	CD4(Tn)	Macro_NLRP3	RPS19	C5AR1	5,89636E-04	16623,6	7,15387E-03	12043	2,06911E+00	4486	1,50559E+00	21529	9,48130E-01	1848	0	43212
Myeloid_vs_CD4_Responder	CD4(TNF+ T)	Mono_INHBA	HSPA1A	TLR4	5,90595E-04	17987,8	6,60555E-03	13628	1,74254E+00	8017	2,66286E+00	3546	8,99497E-01	21536	0	43212
Myeloid_vs_CD4_Responder	Macro_ISG15	CD4(NME1+ T)	CXCL10	CXCR3	5,92214E-04	14876	3,10543E-02	750	2,35791E+00	2703	2,74525E+00	3031	8,91982E-01	24684	0	43212
Myeloid_vs_CD4_Responder	Macro_FOLR2+APOE-	CD4(ISG+ Treg)	CCL18	CCR8	5,95697E-04	19029,6	9,39047E-02	66	2,28995E+00	3037	2,96971E+00	1948	8,42471E-01	46885	0	43212
Myeloid_vs_CD4_Responder	Macro_IFI27	CD4(ISG+ Treg)	HLA-DRB5	LAG3	5,97269E-04	14099,6	1,06023E-02	6146	1,65328E+00	9324	2,66885E+00	3507	9,30440E-01	8309	0	43212
Myeloid_vs_CD4_Responder	Macro_OLFML3	CD4(IL26+ Th17)	HLA-DQB1	LAG3	5,97521E-04	14052,8	1,11755E-02	5633	1,79349E+00	7343	2,50218E+00	4751	9,28178E-01	9325	0	43212
Myeloid_vs_CD4_Responder	Macro_OLFML3	CD4(IL26+ Th17)	HLA-DQA1	LAG3	5,99290E-04	13607,4	1,16304E-02	5187	1,89104E+00	6166	2,58061E+00	4140	9,28164E-01	9332	0	43212
Myeloid_vs_CD4_Responder	CD4(IL26+ Th17)	Macro_LYVE1	HMGB1	CD163	5,99839E-04	18071,6	5,69266E-03	17164	2,44502E+00	2340	1,50355E+00	21603	9,35352E-01	6039	0	43212
Myeloid_vs_CD4_Responder	cDC_CLEC9A	CD4(IL26+ Th17)	HLA-DRA	LAG3	6,00303E-04	13884,8	8,55972E-03	8946	1,65262E+00	9336	3,05337E+00	1652	9,34843E-01	6278	0	43212
Myeloid_vs_CD4_Responder	CD4(NME1+ T)	Macro_FOLR2+APOE-	HMGB1	CD163	6,03039E-04	14899,8	6,42815E-03	14260	2,21168E+00	3455	2,09320E+00	9069	9,38930E-01	4503	0	43212
Myeloid_vs_CD4_Responder	CD4(NME1+ T)	Macro_FOLR2+APOE+	HMGB1	CD163	6,19244E-04	18520,8	5,58554E-03	17656	1,96005E+00	5474	1,56215E+00	19953	9,34776E-01	6309	0	43212
Myeloid_vs_CD4_Responder	Macro_ISG15	CD4(IFNG+ Tfh/Th1)	HLA-DRA	LAG3	6,19786E-04	12735,4	1,08387E-02	5915	1,64824E+00	9412	3,08041E+00	1563	9,41674E-01	3575	0	43212
Myeloid_vs_CD4_Responder	Macro_OLFML3	CD4(TGFB1+ Th17)	C3	IFITM1	6,21050E-04	12440,6	1,62319E-02	2774	2,28222E+00	3080	1,96173E+00	11150	9,47502E-01	1987	0	43212
Myeloid_vs_CD4_Responder	Macro_IFI27	CD4(IL26+ Th17)	HLA-DRA	LAG3	6,28390E-04	14660	8,52451E-03	9014	1,64607E+00	9445	2,43820E+00	5285	9,34718E-01	6344	0	43212
Myeloid_vs_CD4_Responder	Macro_OLFML3	CD4(AREG+ Tm)	FN1	CD44	6,29566E-04	14534,8	1,28078E-02	4355	1,96198E+00	5457	1,85380E+00	13126	9,34300E-01	6524	0	43212
Myeloid_vs_CD4_Responder	Macro_FOLR2+APOE-	CD4(IFNG+ Tfh/Th1)	HLA-DRB1	LAG3	6,30489E-04	12796,6	1,16253E-02	5194	1,64525E+00	9453	2,79436E+00	2748	9,42332E-01	3376	0	43212
Myeloid_vs_CD4_Responder	Macro_OLFML3	CD4(AREG+ Tm)	APOE	SORL1	6,34779E-04	18532,2	5,08252E-03	20333	1,91379E+00	5915	2,82656E+00	2581	9,01664E-01	20620	0	43212
Myeloid_vs_CD4_Responder	Macro_OLFML3	CD4(AREG+ Tm)	C3	IFITM1	6,36524E-04	15093,2	1,26726E-02	4432	2,08747E+00	4348	1,57379E+00	19660	9,40994E-01	3814	0	43212
Myeloid_vs_CD4_Responder	CD4(IL26+ Th17)	pDC_LILRA4	TNF	PTPRS	6,37348E-04	22974,2	3,86745E-02	484	4,47872E+00	151	2,06430E+00	9479	8,10963E-01	61545	0	43212
Myeloid_vs_CD4_Responder	CD4(AREG+ Tm)	pDC_LILRA4	HMGB1	THBD	6,38710E-04	19615,4	6,12191E-03	15424	1,70867E+00	8489	2,09265E+00	9076	8,98553E-01	21876	0	43212
Myeloid_vs_CD4_Responder	Macro_LYVE1	CD4(IFNG+ Tfh/Th1)	HLA-DPB1	LAG3	6,38938E-04	13285,4	1,15653E-02	5248	1,64301E+00	9485	2,56784E+00	4242	9,39663E-01	4240	0	43212
Myeloid_vs_CD4_Responder	Mast	CD4(GZMK+ Teff)	TIMP3	CD44	6,40221E-04	13850,2	4,14968E-02	419	3,45827E+00	472	1,81062E+00	13959	9,23859E-01	11189	0	43212
Myeloid_vs_CD4_Responder	cDC(CD1C)	CD4(IL26+ Th17)	HLA-DPA1	LAG3	6,41862E-04	13669	9,07272E-03	8121	1,64228E+00	9496	3,26182E+00	1085	9,34520E-01	6431	0	43212
Myeloid_vs_CD4_Responder	Macro_FOLR2+APOE-	CD4(IFNG+ Tfh/Th1)	HLA-DPA1	LAG3	6,42661E-04	12858,6	1,16269E-02	5190	1,64211E+00	9499	2,78114E+00	2829	9,41713E-01	3563	0	43212
Myeloid_vs_CD4_Responder	CD4(ISG+ Treg)	Mono_CD16	HLA-C	LILRB2	6,45896E-04	16774,2	6,13360E-03	15383	2,44247E+00	2354	1,49349E+00	21925	9,51654E-01	997	0	43212
Myeloid_vs_CD4_Responder	Macro_OLFML3	CD4(Tn)	FN1	CD44	6,47075E-04	14897,2	1,21486E-02	4782	1,91441E+00	5911	1,84375E+00	13309	9,32660E-01	7272	0	43212
Myeloid_vs_CD4_Responder	Macro_OLFML3	CD4(Tn)	APOE	SORL1	6,51661E-04	17727	5,45597E-03	18293	1,93396E+00	5726	2,94154E+00	2061	9,04763E-01	19343	0	43212
Myeloid_vs_CD4_Responder	Macro_OLFML3	CD4(Tn)	C3	IFITM1	6,53146E-04	13147,8	1,46557E-02	3412	2,19598E+00	3544	1,86353E+00	12928	9,44903E-01	2643	0	43212
Myeloid_vs_CD4_Responder	Macro_OLFML3	CD4(IL26+ Th17)	HLA-DQA2	CD4	6,57616E-04	20151	4,99320E-03	20876	1,63043E+00	9704	1,93976E+00	11530	9,13891E-01	15433	0	43212
Myeloid_vs_CD4_Responder	Macro_OLFML3	CD4(IL26+ Th17)	HLA-DQA2	LAG3	6,57766E-04	16608	1,38226E-02	3797	1,77329E+00	7609	2,10387E+00	8919	9,04343E-01	19503	0	43212
Myeloid_vs_CD4_Responder	CD4(TNF+ T)	Macro_FOLR2+APOE+	HSPA1A	TLR4	6,57766E-04	18745,2	6,42232E-03	14285	1,72266E+00	8284	2,36926E+00	5940	8,98218E-01	22005	0	43212
Myeloid_vs_CD4_Responder	Macro_OLFML3	CD4(IL26+ Th17)	CD14	ITGA4	6,60760E-04	16981,2	7,32479E-03	11619	1,81435E+00	7087	1,93063E+00	11677	9,23600E-01	11311	0	43212
Myeloid_vs_CD4_Responder	CD4(TGFB1+ Th17)	pDC_LILRA4	HMGB1	THBD	6,64971E-04	19830,2	6,06394E-03	15624	1,70131E+00	8598	2,05209E+00	9664	8,98118E-01	22053	0	43212
Myeloid_vs_CD4_Responder	Macro_OLFML3	CD4(IL26+ Th17)	FN1	ITGA4_ITGB1	6,65876E-04	16039,8	1,59192E-02	2894	2,00370E+00	5071	1,92982E+00	11696	9,09528E-01	17326	0	43212
Myeloid_vs_CD4_Responder	Macro_OLFML3	CD4(IL26+ Th17)	FN1	CD44	6,66027E-04	16382,2	1,12443E-02	5556	1,84914E+00	6654	1,63209E+00	18069	9,30190E-01	8420	0	43212
Myeloid_vs_CD4_Responder	Macro_OLFML3	CD4(IL26+ Th17)	CXCL16	CXCR6	6,71631E-04	18191,6	1,66646E-02	2643	1,58022E+00	10568	1,60989E+00	18685	9,12929E-01	15850	0	43212
Myeloid_vs_CD4_Responder	CD4(IFNG+ Tfh/Th1)	Mono_CD16	HLA-F	LILRB2	6,71935E-04	20151,6	6,40463E-03	14350	2,34614E+00	2755	1,48817E+00	22099	9,07171E-01	18342	0	43212
Myeloid_vs_CD4_Responder	Macro_OLFML3	CD4(IL26+ Th17)	C3	IFITM1	6,74829E-04	13259	1,52550E-02	3156	2,22877E+00	3355	1,79943E+00	14203	9,45937E-01	2369	0	43212
Myeloid_vs_CD4_Responder	cDC_LAMP3	CD4(ISG+ Treg)	CCL17	CCR8	6,75839E-04	24550,8	2,33731E-01	10	3,43520E+00	485	1,72488E+00	15807	8,07373E-01	63240	0	43212
Myeloid_vs_CD4_Responder	Mast	CD4(AREG+ Tm)	TIMP3	CD44	6,78618E-04	14179,4	4,03502E-02	442	3,43201E+00	486	1,75484E+00	15120	9,22867E-01	11637	0	43212
Myeloid_vs_CD4_Responder	CD4(TNFRSF9+ Treg)	Macro_LYVE1	HMGB1	CD163	6,82337E-04	18718,6	5,36379E-03	18816	2,39833E+00	2531	1,48597E+00	22167	9,33530E-01	6867	0	43212
Myeloid_vs_CD4_Responder	Macro_ISG15	CD4(TNFRSF9+ Treg)	CXCL10	SDC4	6,83370E-04	22570,2	2,04388E-02	1785	2,25909E+00	3181	2,72461E+00	3158	8,11026E-01	61515	0	43212

Myeloid_vs_CD4_Post_R

Myeloid_vs_CD4_Responder	Macro_ISG15	CD4(TNFRSF9+ Treg)	CXCL10	CXCR3	6,88475E-04	15768,4	2,61309E-02	1099	2,28883E+00	3045	2,71971E+00	3189	8,83381E-01	28297	0	43212
Myeloid_vs_CD4_Responder	Macro_OLFML3	CD4(TGFB1+ Th17)	FN1	CD44	6,88671E-04	15942,4	1,14748E-02	5324	1,86578E+00	6468	1,69120E+00	16597	9,30846E-01	8111	0	43212
Myeloid_vs_CD4_Responder	CD4(Tn)	pDC_LILRA4	BST2	LILRA4	6,93605E-04	21661	7,73054E-03	10603	5,15251E+00	102	2,71829E+00	3197	8,33362E-01	51191	0	43212
Myeloid_vs_CD4_Responder	Macro_IER3	CD4(ISG+ Treg)	ICAM1	IL2RG	6,93660E-04	14510,4	1,20480E-02	4855	1,87332E+00	6367	2,05093E+00	9685	9,30168E-01	8433	0	43212
Myeloid_vs_CD4_Responder	CD4(TNFRSF9+ Treg)	pDC_LILRA4	HMGB1	THBD	6,93959E-04	19928	5,99970E-03	15869	1,69316E+00	8728	2,05676E+00	9589	8,97630E-01	22242	0	43212
Myeloid_vs_CD4_Responder	CD4(CRTAM- T)	pDC_LILRA4	BST2	LILRA4	6,94788E-04	19693	9,46199E-03	7532	5,18464E+00	99	2,78914E+00	2794	8,46926E-01	44828	0	43212
Myeloid_vs_CD4_Responder	CD4(CRTAM- T)	Mono_INHBA	ANXA1	FPR1	6,94895E-04	22794,8	5,43832E-03	18404	1,37232E+00	15594	1,48324E+00	22248	9,15921E-01	14516	0	43212
Myeloid_vs_CD4_Responder	Macro_OLFML3	CD4(TGFB1+ Th17)	APOE	SORL1	6,95051E-04	16371,8	6,38258E-03	14422	1,98400E+00	5239	2,86134E+00	2422	9,11310E-01	16564	0	43212
Myeloid_vs_CD4_Responder	cDC(CD1C)	CD4(IL26+ Th17)	HLA-DQA1	LAG3	6,95918E-04	13235,2	1,13444E-02	5446	1,84651E+00	6686	3,23787E+00	1139	9,27329E-01	9693	0	43212
Myeloid_vs_CD4_Responder	CD4(CRTAM- T)	pDC_LILRA4	COPA	P2RY6	6,97616E-04	25047,6	9,77645E-03	7120	2,11460E+00	4119	2,12116E+00	8679	8,09785E-01	62108	0	43212
Myeloid_vs_CD4_Responder	CD4(TGFB1+ Th17)	Macro_LYVE1	HMGB1	CD163	7,05264E-04	18646,2	5,42122E-03	18493	2,40648E+00	2492	1,48129E+00	22314	9,33859E-01	6720	0	43212
Myeloid_vs_CD4_Responder	Macro_OLFML3	CD4(Tn)	APOE	LSR	7,07162E-04	15125,2	1,62093E-02	2782	1,97889E+00	5288	2,95245E+00	2018	8,97430E-01	22326	0	43212
Myeloid_vs_CD4_Responder	CD4(CRTAM- T)	cDC_CLEC9A	ANXA1	DYSF	7,08271E-04	17760	1,94148E-02	1961	2,08934E+00	4336	1,67570E+00	16958	8,97418E-01	22333	0	43212
Myeloid_vs_CD4_Responder	Macro_OLFML3	CD4(TNFRSF9+ Treg)	FN1	CD44	7,13201E-04	16220,2	1,10605E-02	5735	1,83588E+00	6819	1,68867E+00	16664	9,29653E-01	8671	0	43212
Myeloid_vs_CD4_Responder	Macro_OLFML3	CD4(TNFRSF9+ Treg)	GRN	TNFRSF1B	7,16556E-04	18126,2	4,83665E-03	21859	1,65963E+00	9235	1,97683E+00	10872	9,36637E-01	5453	0	43212
Myeloid_vs_CD4_Responder	Macro_OLFML3	CD4(TNFRSF9+ Treg)	C3	IFITM1	7,22819E-04	15264	1,21355E-02	4796	2,05808E+00	4598	1,57958E+00	19511	9,39780E-01	4203	0	43212
Myeloid_vs_CD4_Responder	Macro_ISG15	CD4(IFNG+ Tfh/Th1)	CXCL10	CXCR3	7,24914E-04	15800,2	2,60300E-02	1112	2,28742E+00	3057	2,71049E+00	3245	8,83181E-01	28375	0	43212
Myeloid_vs_CD4_Responder	Macro_OLFML3	CD4(CRTAM- T)	FN1	ITGA4_ITGB1	7,35479E-04	18202,4	1,21414E-02	4789	1,87048E+00	6402	1,78939E+00	14418	8,97746E-01	22191	0	43212
Myeloid_vs_CD4_Responder	Macro_OLFML3	CD4(CRTAM- T)	FN1	CD44	7,35805E-04	13761	1,38787E-02	3775	2,03926E+00	4767	1,93338E+00	11635	9,36722E-01	5416	0	43212
Myeloid_vs_CD4_Responder	CD4(CRTAM- T)	pDC_LILRA4	TNF	PTPRS	7,41199E-04	23250,8	3,78860E-02	508	4,47375E+00	154	2,02525E+00	10077	8,09379E-01	62303	0	43212
Myeloid_vs_CD4_Responder	Macro_OLFML3	CD4(CRTAM- T)	C3	IFITM1	7,44181E-04	13038,2	1,52422E-02	3161	2,22807E+00	3362	1,85529E+00	13085	9,45916E-01	2371	0	43212
Myeloid_vs_CD4_Responder	Macro_OLFML3	CD4(TNF+ T)	FN1	CD44	7,55133E-04	19040,2	7,97157E-03	10047	1,61296E+00	10003	1,62226E+00	18335	9,18161E-01	13604	0	43212
Myeloid_vs_CD4_Responder	Macro_OLFML3	CD4(TNF+ T)	C3	IFITM1	7,60489E-04	17718,2	8,83712E-03	8490	1,87760E+00	6318	1,48720E+00	22133	9,30154E-01	8438	0	43212
Myeloid_vs_CD4_Responder	Macro_OLFML3	CD4(ISG+ Treg)	B2M	CD3D	7,60803E-04	38036,4	3,97213E-03	29041	6,92013E-01	47324	4,34243E-01	70601	9,63900E-01	4	0	43212
Myeloid_vs_CD4_Responder	Macro_OLFML3	CD4(CRTAM- T)	APOE	LSR	7,61497E-04	15289,2	1,59691E-02	2877	1,97454E+00	5332	2,87415E+00	2366	8,96740E-01	22659	0	43212
Myeloid_vs_CD4_Responder	CD4(GZMK+ Teff)	Macro_FOLR2+APOE-	HMGB1	CD163	7,64358E-04	20022,4	4,71678E-03	22676	1,88720E+00	6207	1,58903E+00	19244	9,29429E-01	8773	0	43212
Myeloid_vs_CD4_Responder	Macro_OLFML3	CD4(ISG+ Treg)	HLA-DQA2	LAG3	7,65538E-04	14944,2	1,60758E-02	2830	1,85047E+00	6636	2,44603E+00	5210	9,10678E-01	16833	0	43212
Myeloid_vs_CD4_Responder	Mono_CD14	CD4(NME1+ T)	S100A9	ITGB2	7,70550E-04	12330,2	6,49972E-03	14014	3,38734E+00	518	3,66504E+00	509	9,42254E-01	3398	0	43212
Myeloid_vs_CD4_Responder	CD4(IFNG+ Tfh/Th1)	Mono_CD16	HLA-C	LILRB2	7,78620E-04	17153,8	5,95836E-03	16034	2,37676E+00	2620	1,46779E+00	22760	9,50983E-01	1143	0	43212
Myeloid_vs_CD4_Responder	Macro_OLFML3	CD4(ISG+ Treg)	GRN	TNFRSF1B	7,80504E-04	18579,8	4,83246E-03	21880	1,65901E+00	9242	1,85455E+00	13101	9,36611E-01	5464	0	43212
Myeloid_vs_CD4_Responder	Mast	CD4(Tn)	TIMP3	CD44	7,82448E-04	14396,2	3,82734E-02	496	3,38444E+00	522	1,74480E+00	15333	9,20965E-01	12418	0	43212
Myeloid_vs_CD4_Responder	Macro_OLFML3	CD4(TGFB1+ Th17)	C1QB	C1QBP	7,85486E-04	17701,6	4,69740E-03	22800	1,96199E+00	5456	2,74321E+00	3049	9,17196E-01	13991	0	43212
Myeloid_vs_CD4_Responder	cDC(CD1C)	CD4(IL26+ Th17)	HLA-DQB1	LAG3	7,86487E-04	13843,6	1,06778E-02	6064	1,71544E+00	8393	3,08396E+00	1550	9,26645E-01	9999	0	43212
Myeloid_vs_CD4_Responder	Macro_OLFML3	CD4(ISG+ Treg)	C1QB	C1QBP	7,87728E-04	16999	5,02742E-03	20652	1,98606E+00	5221	2,76022E+00	2959	9,19738E-01	12951	0	43212
Myeloid_vs_CD4_Responder	Macro_ISG15	CD4(IL26+ Th17)	CXCL10	DPP4	7,90292E-04	22113,6	3,24584E-02	676	2,30528E+00	2960	2,69630E+00	3341	8,13519E-01	60379	0	43212
Myeloid_vs_CD4_Responder	cDC_CLEC9A	CD4(IL26+ Th17)	HLA-DQB1	LAG3	7,98001E-04	13974,8	1,06474E-02	6100	1,71067E+00	8462	2,94075E+00	2064	9,26548E-01	10036	0	43212
Myeloid_vs_CD4_Responder	Macro_FOLR2+APOE+	CD4(IL26+ Th17)	HLA-DQA1	LAG3	7,99880E-04	14741,4	1,10838E-02	5717	1,80594E+00	7183	2,21444E+00	7553	9,26542E-01	10042	0	43212
Myeloid_vs_CD4_Responder	Macro_IER3	CD4(IFNG+ Tfh/Th1)	HLA-DRB1	LAG3	8,00821E-04	13224,8	1,13406E-02	5450	1,61001E+00	10045	2,62157E+00	3831	9,41655E-01	3586	0	43212
Myeloid_vs_CD4_Responder	Macro_FOLR2-APOE+	CD4(CRTAM- T)	SPP1	CD44	8,03962E-04	15099,8	9,43014E-03	7575	1,79108E+00	7379	2,23857E+00	7278	9,26510E-01	10055	0	43212
Myeloid_vs_CD4_Responder	Macro_OLFML3	CD4(CXCL13+ Tfh)	FN1	CD44	8,04794E-04	17811,8	9,95822E-03	6900	1,75633E+00	7827	1,52716E+00	20909	9,26141E-01	10211	0	43212
Myeloid_vs_CD4_Responder	CD4(ISG+ Treg)	Macro_FOLR2+APOE-	HMGB1	CD163	8,06552E-04	20653,6	4,67964E-03	22921	1,88016E+00	6285	1,49272E+00	21951	9,29169E-01	8899	0	43212
Myeloid_vs_CD4_Responder	Macro_IER3	CD4(ISG+ Treg)	HLA-DRA	LAG3	8,11223E-04	14514,2	9,19580E-03	7900	1,60838E+00	10078	2,35291E+00	6078	9,36993E-01	5303	0	43212
Myeloid_vs_CD4_Responder	Mono_CD14	CD4(ISG+ Treg)	S100A9	ITGB2	8,12590E-04	12417,4	6,41454E-03	14316	3,38060E+00	524	3,63136E+00	532	9,41894E-01	3503	0	43212
Myeloid_vs_CD4_Responder	CD4(IFNG+ Tfh/Th1)	cDC_CLEC9A	CD52	SIGLEC10	8,13083E-04	25495,2	5,00092E-03	20835	1,20142E+00	21305	1,46119E+00	22958	9,05227E-01	19166	0	43212
Myeloid_vs_CD4_Responder	Macro_FOLR2-APOE+	CD4(IFNG+ Tfh/Th1)	HLA-DPB1	LAG3	8,13442E-04	13344,8	1,12783E-02	5506	1,60768E+00	10085	2,68176E+00	3427	9,38947E-01	4494	0	43212
Myeloid_vs_CD4_Responder	Macro_OLFML3	CD4(IL26+ Th17)	C1QB	C1QBP	8,15032E-04	17778,8	4,67346E-03	22969	1,96025E+00	5471	2,72188E+00	3177	9,17002E-01	14065	0	43212
Myeloid_vs_CD4_Responder	cDC(CD1C)	CD4(IL26+ Th17)	HLA-DRB5	LAG3	8,22364E-04	14465,2	9,37343E-03	7640	1,61858E+00	9899	3,11276E+00	1462	9,26346E-01	10113	0	43212
Myeloid_vs_CD4_Responder	Macro_FOLR2+APOE+	CD4(ISG+ Treg)	HLA-DPB1	LAG3	8,22684E-04	14524,4	9,79389E-03	7101	1,60561E+00	10114	2,37427E+00	5884	9,34774E-01	6311	0	43212
Myeloid_vs_CD4_Responder	Macro_OLFML3	CD4(GZMK+ Teff)	FN1	CD44	8,24121E-04	14150,4	1,31718E-02	4143	1,98824E+00	5202	1,90957E+00	12060	9,35155E-01	6135	0	43212
Myeloid_vs_CD4_Responder	Macro_OLFML3	CD4(CXCL13+ Tfh)	C1QB	C1QBP	8,24837E-04	17778,4	4,66503E-03	23024	1,95963E+00	5476	2,73629E+00	3085	9,16933E-01	14095	0	43212
Myeloid_vs_CD4_Responder	Macro_OLFML3	CD4(GZMK+ Teff)	C3	IFITM1	8,32027E-04	12968,8	1,51180E-02	3208	2,22127E+00	3407	1,88106E+00	12583	9,45706E-01	2434	0	43212
Myeloid_vs_CD4_Responder	CD4(Tn)	Mono_CD16	HLA-C	LILRB2	8,32333E-04	19124,8	5,54543E-03	17866	2,22193E+00	3400	1,26798E+00	29592	9,49282E-01	1554	0	43212
Myeloid_vs_CD4_Responder	Macro_OLFML3	CD4(IFNG+ Tfh/Th1)	HLA-DQA2	CD4	8,37090E-04	19943,2	4,99683E-03	20865	1,63082E+00	9695	1,99814E+00	10523	9,13919E-01	15421	0	43212
Myeloid_vs_CD4_Responder	Macro_OLFML3	CD4(IFNG+ Tfh/Th1)	HLA-DQA2	LAG3	8,37271E-04	13092,8	2,05106E-02	1772	2,00238E+00	5082	2,82045E+00	2607	9,20104E-01	12791	0	43212
Myeloid_vs_CD4_Responder	CD4(GZMK+ Teff)	Mono_CD16	HLA-B	LILRB2	8,42177E-04	17182,2	5,87537E-03	16377	2,38240E+00	2596	1,45548E+00	23120	9,54025E-01	606	0	43212
Myeloid_vs_CD4_Responder	Macro_OLFML3	CD4(IFNG+ Tfh/Th1)	FN1	ITGA4_ITGB1	8,48022E-04	17040,4	1,36974E-02	3872	1,92474E+00	5808	1,89661E+00	12300	9,03150E-01	20010	0	43212
Myeloid_vs_CD4_Responder	Macro_OLFML3	CD4(IFNG+ Tfh/Th1)	FN1	CD44	8,48388E-04	18576	9,17923E-03	7927	1,70012E+00	8612	1,50088E+00	21694	9,23307E-01	11435	0	43212
Myeloid_vs_CD4_Responder	CD4(TNFRSF9+ Treg)	Mono_CD16	HLA-B	LILRB2	8,49121E-04	17163,8	5,89632E-03	16292	2,39260E+00	2558	1,45443E+00	23158	9,54103E-01	599	0	43212
Myeloid_vs_CD4_Responder	CD4(ISG+ Treg)	pDC_LILRA4	TNF	PTPRS	8,49507E-04	23200,2	3,62815E-02	544	4,46363E+00	157	2,15502E+00	8261	8,06018E-01	63827	0	43212
Myeloid_vs_CD4_Responder	Macro_FOLR2+APOE-	CD4(IL26+ Th17)	HLA-DRA	LAG3	8,59771E-04	15371,4	8,27615E-03	9463	1,59988E+00	10228	2,24468E+00	7209	9,33810E-01	6745	0	43212
Myeloid_vs_CD4_Responder	Mono_CD14	CD4(TNFRSF9+ Treg)	VCAN	SELL	8,60174E-04	12904,2	2,08421E-02	1727	3,05884E+00	886	2,67990E+00	3438	9,14307E-01	15258	0	43212
Myeloid_vs_CD4_Responder	Macro_OLFML3	CD4(NME1+ T)	HLA-DRA	LAG3	8,62032E-04	18009	5,11354E-03	20162	1,63843E+00	9570	2,72562E+00	3151	9,17284E-01	13950	0	43212
Myeloid_vs_CD4_Responder	Macro_OLFML3	CD4(NME1+ T)	HLA-DQA1	LAG3	8,62775E-04	18838,6	6,14068E-03	15348	1,66754E+00	9116	2,28550E+00	6772	9,03739E-01	19745	0	43212
Myeloid_vs_CD4_Responder	Macro_OLFML3	CD4(NME1+ T)	HLA-DRB1	LAG3	8,63146E-04	20166,2	4,83067E-03	21894	1,43405E+00	13866	2,34340E+00	6174	9,13296E-01	15685	0	43212
Myeloid_vs_CD4_Responder	Macro_OLFML3	CD4(NME1+ T)	HLA-DQB1	LAG3	8,63518E-04	19526	5,90051E-03	16277	1,56999E+00	10769	2,20707E+00	7634	9,03758E-01	19738	0	43212
Myeloid_vs_CD4_Responder	Macro_OLFML3	CD4(NME1+ T)	HLA-DPB1	LAG3	8,64447E-04	19381,8	5,09313E-03	20265	1,52869E+00	11589	2,41569E+00	5491	9,11776E-01	16352	0	43212
Myeloid_vs_CD4_Responder	Mono_CD14	CD4(GZMK+ Teff)	S100A9	ITGB2	8,68270E-04	12772,2	6,06766E-03	15607	3,35313E+00	550	3,70346E+00	486	9,40354E-01	4006	0	43212
Myeloid_vs_CD4_Responder	Macro_OLFML3	CD4(NME1+ T)	HLA-DPA1	LAG3	8,68733E-04	19298,4	5,02103E-03	20698	1,49979E+00	12237	2,48136E+00	4925	9,13921E-01	15420	0	43212
Myeloid_vs_CD4_Responder	cDC_CLEC9A	CD4(IL26+ Th17)	HLA-DRB5	LAG3	8,69400E-04	14712,2	9,28756E-03	7767	1,60439E+00	10134	2,91320E+00	2191	9,26032E-01	10257	0	43212

Myeloid_vs_CD4_Responder	CD4(TNF+ T)	Mono_CD16	HLA-F	LILRB2	8,71163E-04	22604,6	4,89930E-03	21464	2,22546E+00	3384	1,50116E+00	21686	8,95258E-01	23277	0	43212
Myeloid_vs_CD4_Responder	Macro_OLFML3	CD4(NME1+ T)	HLA-DRB5	LAG3	8,71912E-04	18523,2	5,73342E-03	16992	1,64048E+00	9526	2,49920E+00	4775	9,07718E-01	18111	0	43212
Myeloid_vs_CD4_Responder	Macro_OLFML3	CD4(NME1+ T)	FN1	ITGA4_ITGB1	8,73598E-04	17488,6	1,31946E-02	4134	1,90712E+00	5988	1,83842E+00	13423	9,01502E-01	20686	0	43212
Myeloid_vs_CD4_Responder	Macro_OLFML3	CD4(NME1+ T)	FN1	CD44	8,73974E-04	18681,2	9,17163E-03	7943	1,69957E+00	8622	1,48558E+00	22178	9,23277E-01	11451	0	43212
Myeloid_vs_CD4_Responder	Macro_LYVE1	CD4(IL26+ Th17)	HLA-DRA	LAG3	8,78772E-04	15632,4	8,25786E-03	9488	1,59648E+00	10285	2,14362E+00	8404	9,33741E-01	6773	0	43212
Myeloid_vs_CD4_Responder	CD4(CXCL13+ Tfh)	Macro_FOLR2+APOE-	HMGB1	CD163	8,82260E-04	20499,6	4,62212E-03	23336	1,86925E+00	6417	1,54310E+00	20461	9,28761E-01	9072	0	43212
Myeloid_vs_CD4_Responder	Mono_CD14	CD4(TNFRSF9+ Treg)	S100A9	ITGB2	8,84061E-04	12825	6,02578E-03	15759	3,34982E+00	555	3,63903E+00	526	9,40159E-01	4073	0	43212
Myeloid_vs_CD4_Responder	Macro_OLFML3	CD4(NME1+ T)	C1QB	C1QBP	8,84152E-04	14686,8	6,42464E-03	14274	2,08796E+00	4343	2,87745E+00	2356	9,28337E-01	9249	0	43212
Myeloid_vs_CD4_Responder	Macro_OLFML3	CD4(NME1+ T)	C3	IFITM1	8,84531E-04	14215,4	1,28472E-02	4337	2,09702E+00	4280	1,73466E+00	15578	9,41372E-01	3670	0	43212
Myeloid_vs_CD4_Responder	Macro_FOLR2+APOE-	CD4(IL26+ Th17)	HLA-DRB5	LAG3	8,92971E-04	15950,2	9,25647E-03	7819	1,59926E+00	10239	2,16264E+00	8154	9,25917E-01	10327	0	43212
Myeloid_vs_CD4_Responder	Macro_ISG15	CD4(IL26+ Th17)	CD14	ITGA4	8,93992E-04	15009,6	7,84194E-03	10330	1,92581E+00	5800	2,42493E+00	5408	9,25972E-01	10298	0	43212
Myeloid_vs_CD4_Responder	CD4(TNF+ T)	pDC_LILRA4	HMGB1	THBD	8,98445E-04	20070,4	5,65951E-03	17307	1,64998E+00	9380	2,26008E+00	7032	8,94917E-01	23421	0	43212
Myeloid_vs_CD4_Responder	Mono_CD14	CD4(CXCL13+ Tfh)	S100A9	ITGB2	8,99993E-04	12967,4	5,90449E-03	16260	3,34021E+00	560	3,62213E+00	543	9,39585E-01	4262	0	43212
Myeloid_vs_CD4_Responder	Mono_CD14	CD4(IFNG+ Tfh/Th1)	VCAN	ITGB1	9,00779E-04	13178,6	1,45614E-02	3440	3,01300E+00	953	2,67200E+00	3492	9,15329E-01	14796	0	43212
Myeloid_vs_CD4_Responder	Macro_OLFML3	CD4(ISG+ Treg)	FN1	CD44	9,02095E-04	19126	8,95434E-03	8305	1,68389E+00	8844	1,44547E+00	23440	9,22424E-01	11829	0	43212
Myeloid_vs_CD4_Responder	CD4(TGFB1+ Th17)	Mono_CD16	ANXA1	FPR1	9,02874E-04	19204,2	7,83096E-03	10356	1,94811E+00	5589	1,31500E+00	27858	9,28938E-01	9006	0	43212
Myeloid_vs_CD4_Responder	CD4(GZMK+ Teff)	pDC_LILRA4	HLA-C	NOTCH4	9,11758E-04	18259	1,80420E-02	2262	1,72143E+00	8308	1,80716E+00	14023	8,94794E-01	23490	0	43212
Myeloid_vs_CD4_Responder	cDC(CD1C)	CD4(IL26+ Th17)	HLA-DRB1	LAG3	9,11821E-04	13997,4	8,78068E-03	8591	1,58987E+00	10382	3,19523E+00	1241	9,34217E-01	6561	0	43212
Myeloid_vs_CD4_Responder	Macro_ISG15	CD4(CRTAM- T)	CXCL10	DPP4	9,13048E-04	23263	2,81131E-02	941	2,27053E+00	3129	2,66860E+00	3508	8,02371E-01	65525	0	43212
Myeloid_vs_CD4_Responder	CD4(AREG+ Tm)	Mono_CD16	HLA-C	LILRB2	9,14590E-04	19414,4	5,49217E-03	18124	2,20195E+00	3510	1,23923E+00	30606	9,49049E-01	1620	0	43212
Myeloid_vs_CD4_Responder	CD4(Tn)	Mono_INHBA	ANXA1	FPR1	9,15646E-04	25519,4	4,59810E-03	23510	1,21784E+00	20675	1,46785E+00	22759	9,09230E-01	17441	0	43212
Myeloid_vs_CD4_Responder	Mast	CD4(TGFB1+ Th17)	TIMP3	CD44	9,16066E-04	15361,2	3,61506E-02	548	3,33581E+00	565	1,59224E+00	19170	9,18864E-01	13311	0	43212
Myeloid_vs_CD4_Responder	CD4(IL26+ Th17)	pDC_LILRA4	COPA	P2RY6	9,20834E-04	27768,4	7,80981E-03	10408	2,05041E+00	4664	2,01347E+00	10247	7,91883E-01	70311	0	43212
Myeloid_vs_CD4_Responder	CD4(IL26+ Th17)	Mono_CD16	HLA-B	LILRB2	9,21699E-04	17124,6	5,98438E-03	15931	2,43550E+00	2386	1,44219E+00	23541	9,54426E-01	553	0	43212
Myeloid_vs_CD4_Responder	CD4(CXCL13+ Tfh)	pDC_LILRA4	BST2	LILRA4	9,24649E-04	16471,4	1,43864E-02	3523	5,27602E+00	94	2,89872E+00	2266	8,72160E-01	33262	0	43212
Myeloid_vs_CD4_Responder	CD4(CRTAM- T)	Macro_OLFML3	ANXA1	FPR1	9,28571E-04	19656,2	7,17785E-03	11972	1,65823E+00	9250	1,44104E+00	23576	9,26009E-01	10271	0	43212
Myeloid_vs_CD4_Responder	Mono_CD14	CD4(IL26+ Th17)	S100A9	ITGB2	9,29026E-04	12896	5,97296E-03	15983	3,34563E+00	558	3,59767E+00	569	9,39911E-01	4158	0	43212
Myeloid_vs_CD4_Responder	Macro_FOLR2-APOE+	CD4(IL26+ Th17)	HLA-DRA	LAG3	9,32368E-04	15404,8	8,20622E-03	9582	1,58687E+00	10441	2,26978E+00	6926	9,33547E-01	6863	0	43212
Myeloid_vs_CD4_Responder	Macro_FOLR2+APOE+	CD4(IL26+ Th17)	CD14	ITGA4	9,33071E-04	14971,2	8,78709E-03	8579	2,12950E+00	4014	2,00307E+00	10443	9,29779E-01	8608	0	43212
Myeloid_vs_CD4_Responder	Macro_FOLR2-APOE+	CD4(ISG+ Treg)	HLA-DRB5	LAG3	9,40117E-04	14717,6	1,01278E-02	6698	1,58589E+00	10463	2,57116E+00	4215	9,28944E-01	9000	0	43212
Myeloid_vs_CD4_Responder	Macro_FOLR2+APOE-	CD4(IFNG+ Tfh/Th1)	HLA-DQA1	LAG3	9,47203E-04	14207,6	1,21587E-02	4776	1,58510E+00	10483	2,61272E+00	3883	9,29631E-01	8684	0	43212
Myeloid_vs_CD4_Responder	CD4(TGFB1+ Th17)	cDC_CLEC9A	CD52	SIGLEC10	9,48031E-04	24242,4	5,45389E-03	18306	1,28247E+00	18408	1,43824E+00	23674	9,08881E-01	17612	0	43212
Myeloid_vs_CD4_Responder	CD4(CXCL13+ Tfh)	pDC_LILRA4	LYPD3	AGR2	9,48142E-04	33793,8	7,84658E-02	108	2,43733E+00	2372	2,66208E+00	3553	6,40045E-01	119724	0	43212
Myeloid_vs_CD4_Responder	CD4(TNFRSF9+ Treg)	pDC_LILRA4	HLA-C	NOTCH4	9,49835E-04	18344,6	1,78539E-02	2302	1,69720E+00	8654	1,81508E+00	13872	8,94300E-01	23683	0	43212
Myeloid_vs_CD4_Responder	CD4(ISG+ Treg)	pDC_LILRA4	HLA-C	NOTCH4	9,50637E-04	18571,8	1,78534E-02	2303	1,69713E+00	8656	1,76099E+00	15001	8,94298E-01	23687	0	43212
Myeloid_vs_CD4_Responder	Macro_OLFML3	CD4(IFNG+ Tfh/Th1)	B2M	CD3D	9,50990E-04	36897,8	3,94641E-03	29316	6,85677E-01	47724	5,34845E-01	64232	9,63787E-01	5	0	43212
Myeloid_vs_CD4_Responder	CD4(GZMK+ Teff)	Mono_CD16	HLA-F	LILRB2	9,51440E-04	21445	5,73342E-03	16993	2,29233E+00	3026	1,43751E+00	23691	9,02403E-01	20303	0	43212
Myeloid_vs_CD4_Responder	CD4(ISG+ Treg)	Macro_ISG15	HLA-F	LILRB2	9,52444E-04	24727,6	4,79925E-03	22092	1,44079E+00	13682	1,52551E+00	20956	8,94286E-01	23696	0	43212
Myeloid_vs_CD4_Responder	Mono_CD14	CD4(TNF+ T)	VCAN	CD44	9,54328E-04	14097	8,51929E-03	9021	2,68695E+00	1606	2,34704E+00	6143	9,25500E-01	10503	0	43212
Myeloid_vs_CD4_Responder	cDC_LAMP3	CD4(IL26+ Th17)	CCL17	CCR4	9,55217E-04	23171,4	1,11053E-01	41	3,32820E+00	577	1,61078E+00	18662	8,28904E-01	53365	0	43212
Myeloid_vs_CD4_Responder	Macro_FOLR2+APOE+	CD4(TNF+ T)	APOE	LDLR	9,56059E-04	14790,8	1,43069E-02	3563	2,62558E+00	1767	3,37673E+00	865	8,92324E-01	24547	0	43212
Myeloid_vs_CD4_Responder	CD4(IFNG+ Tfh/Th1)	Macro_NLRP3	RPS19	C5AR1	9,57076E-04	17335	6,90225E-03	12765	2,02015E+00	4933	1,43681E+00	23719	9,47243E-01	2046	0	43212
Myeloid_vs_CD4_Responder	Mono_INHBA	CD4(IFNG+ Tfh/Th1)	HLA-DRA	LAG3	9,58263E-04	13412,6	1,03217E-02	6460	1,58343E+00	10514	2,77818E+00	2854	9,40317E-01	4023	0	43212
Myeloid_vs_CD4_Responder	cDC_LAMP3	CD4(IFNG+ Tfh/Th1)	HLA-DQB1	LAG3	9,59339E-04	14092,8	1,24256E-02	4591	1,58324E+00	10517	2,54608E+00	4409	9,31633E-01	7735	0	43212
Myeloid_vs_CD4_Responder	Macro_IFI27	CD4(ISG+ Treg)	HLA-DQA1	LAG3	9,64729E-04	15078	1,07352E-02	6009	1,59457E+00	10313	2,43449E+00	5324	9,25448E-01	10532	0	43212
Myeloid_vs_CD4_Responder	CD4(IFNG+ Tfh/Th1)	Mono_CD16	CD99	PILRA	9,64767E-04	18983,2	7,27014E-03	11754	2,03646E+00	4789	1,43585E+00	23757	9,23370E-01	11404	0	43212
Myeloid_vs_CD4_Responder	CD4(GZMK+ Teff)	Mono_CD16	HLA-A	LILRB2	9,66799E-04	17180	6,10271E-03	15465	2,38149E+00	2598	1,43558E+00	23767	9,52407E-01	858	0	43212
Myeloid_vs_CD4_Responder	Macro_FOLR2+APOE-	CD4(IFNG+ Tfh/Th1)	HLA-DQB1	LAG3	9,69781E-04	14037	1,24104E-02	4603	1,58163E+00	10546	2,58917E+00	4070	9,31594E-01	7754	0	43212
Myeloid_vs_CD4_Responder	Mast	CD4(IL26+ Th17)	TIMP3	CD44	9,71769E-04	15743,8	3,54244E-02	572	3,31918E+00	582	1,53314E+00	20728	9,18104E-01	13625	0	43212
Myeloid_vs_CD4_Responder	Macro_FOLR2+APOE-	CD4(AREG+ Tm)	CCL13	CXCR3	9,71769E-04	33027,2	3,51056E-02	582	3,67815E+00	360	1,01890E+00	39328	7,65320E-01	81654	0	43212
Myeloid_vs_CD4_Responder	Macro_LYVE1	CD4(IL26+ Th17)	HLA-DRB5	LAG3	9,74126E-04	16365,4	9,14470E-03	7995	1,58080E+00	10558	2,05900E+00	9558	9,25499E-01	10504	0	43212
Myeloid_vs_CD4_Responder	Macro_FOLR2+APOE+	CD4(IFNG+ Tfh/Th1)	HLA-DRB5	LAG3	9,88349E-04	14438,8	1,14967E-02	5304	1,57918E+00	10597	2,36566E+00	5967	9,33016E-01	7114	0	43212
Myeloid_vs_CD4_Responder	Mono_INHBA	CD4(Tn)	CCL3	CCR4	9,93851E-04	16055	1,58394E-02	2929	2,25436E+00	3210	2,65565E+00	3610	8,85861E-01	27314	0	43212
Myeloid_vs_CD4_Responder	CD4(TGFB1+ Th17)	cDC_CLEC9A	LTB	CD40	9,98113E-04	22406	1,03660E-02	6397	1,37134E+00	15624	1,43083E+00	23919	8,96193E-01	22878	0	43212
Myeloid_vs_CD4_Responder	CD4(NME1+ T)	Mono_INHBA	HMGB1	CD163	9,98321E-04	19523,2	4,54820E-03	23920	1,65027E+00	9370	1,92332E+00	11808	9,28226E-01	9306	0	43212
Myeloid_vs_CD4_Responder	Macro_OLFML3	CD4(CRTAM- T)	C1QB	C1QBP	1,00125E-03	18051,8	4,54585E-03	23934	1,95094E+00	5563	2,74410E+00	3041	9,15942E-01	14509	0	43212
Myeloid_vs_CD4_Responder	Macro_FOLR2-APOE+	CD4(ISG+ Treg)	C3	IFITM1	1,00272E-03	14743,2	1,18705E-02	4986	1,57623E+00	10636	2,00097E+00	10470	9,39152E-01	4412	0	43212
Myeloid_vs_CD4_Responder	Macro_IFI27	CD4(IL26+ Th17)	HLA-DRB5	LAG3	1,00384E-03	15758,8	9,11627E-03	8054	1,57610E+00	10639	2,32669E+00	6333	9,25392E-01	10556	0	43212
Myeloid_vs_CD4_Responder	Macro_NLRP3	CD4(IFNG+ Tfh/Th1)	HLA-DRA	LAG3	1,00978E-03	13574	1,02562E-02	6540	1,57522E+00	10655	2,69118E+00	3379	9,40138E-01	4084	0	43212
Myeloid_vs_CD4_Responder	Mast	CD4(TNFRSF9+ Treg)	TIMP3	CD44	1,01207E-03	15501	3,48454E-02	593	3,30591E+00	594	1,58971E+00	19228	9,17482E-01	13878	0	43212
Myeloid_vs_CD4_Responder	Macro_OLFML3	CD4(TNFRSF9+ Treg)	CD86	CTLA4	1,01979E-03	19895,6	1,29323E-02	4289	1,60936E+00	10060	1,42766E+00	24022	9,08238E-01	17895	0	43212
Myeloid_vs_CD4_Responder	CD4(Tn)	Macro_OLFML3	ANXA1	FPR1	1,03045E-03	21572	6,06888E-03	15602	1,50376E+00	12155	1,42565E+00	24072	9,20050E-01	12819	0	43212
Myeloid_vs_CD4_Responder	Macro_IFI27	CD4(ISG+ Treg)	HLA-DPB1	LAG3	1,03080E-03	14437,6	9,58383E-03	7357	1,57262E+00	10711	2,56010E+00	4302	9,34110E-01	6606	0	43212
Myeloid_vs_CD4_Responder	CD4(TNF+ T)	Macro_OLFML3	TNFSF9	HLA-DPA1	1,03516E-03	18426,4	6,58845E-03	13683	1,67041E+00	9055	2,93427E+00	2088	8,93350E-01	24094	0	43212
Myeloid_vs_CD4_Responder	CD4(AREG+ Tm)	Mono_INHBA	ANXA1	FPR1	1,03667E-03	24501,6	4,98064E-03	20943	1,28818E+00	18209	1,42455E+00	24101	9,12474E-01	16043	0	43212
Myeloid_vs_CD4_Responder	Macro_FOLR2-APOE+	CD4(IFNG+ Tfh/Th1)	HLA-DPA1	LAG3	1,03801E-03	13432,4	1,10649E-02	5732	1,57192E+00	10730	2,67524E+00	3473	9,40338E-01	4015	0	43212
Myeloid_vs_CD4_Responder	CD4(ISG+ Treg)	cDC_CLEC9A	CD52	SIGLEC10	1,03990E-03	25456,4	5,08388E-03	20322	1,21627E+00	20737	1,42423E+00	24116	9,05930E-01	18895	0	43212
Myeloid_vs_CD4_Responder	Mono_INHBA	CD4(TGFB1+ Th17)	S100A8	CD69	1,04029E-03	14532,4	9,44388E-03	7561	1,57137E+00	10736	2,32479E+00	6348	9,38198E-01	4805	0	43212
Myeloid_vs_CD4_Responder	CD4(AREG+ Tm)	cDC_CLEC9A	ANXA1	DYSF	1,04033E-03	18642,2	1,77809E-02	2326	2,00519E+00	5060	1,61701E+00	18495	8,93301E-01	24118	0	43212

Myeloid_vs_CD4_Responder	Macro_ISG15	CD4(ISG+ Treg)	CXCL10	SDC4	1,04182E-03	23329,8	1,88529E-02	2074	2,24139E+00	3284	2,64575E+00	3668	8,04759E-01	64411	0	43212
Myeloid_vs_CD4_Responder	CD4(NME1+ T)	Mono_CD16	HLA-F	LILRB2	1,04465E-03	20333,6	6,57598E-03	13734	2,35988E+00	2695	1,42329E+00	24138	9,08277E-01	17889	0	43212
Myeloid_vs_CD4_Responder	Macro_OLFML3	CD4(IFNG+ Tfh/Th1)	CD14	ITGA4	1,04487E-03	23362	4,51949E-03	24139	1,54574E+00	11231	1,60337E+00	18874	9,04724E-01	19354	0	43212
Myeloid_vs_CD4_Responder	CD4(Tn)	pDC_LILRA4	APP	TNFRSF21	1,04908E-03	30026,8	9,20104E-03	7888	3,21454E+00	688	1,98346E+00	10759	7,50406E-01	87587	0	43212
Myeloid_vs_CD4_Responder	CD4(IFNG+ Tfh/Th1)	pDC_LILRA4	HMGB1	TLR9	1,06011E-03	19474	3,88142E-02	477	3,28964E+00	608	1,39762E+00	24973	8,83911E-01	28100	0	43212
Myeloid_vs_CD4_Responder	Macro_FOLR2+APOE-	CD4(TGFB1+ Th17)	CCL13	CXCR3	1,06359E-03	33522,2	3,43326E-02	609	3,67266E+00	365	9,79992E-01	40947	7,63314E-01	82478	0	43212
Myeloid_vs_CD4_Responder	CD4(ISG+ Treg)	Mono_CD14	HLA-F	LILRB2	1,06383E-03	24960,8	4,67805E-03	22926	1,40865E+00	14570	1,56552E+00	19870	8,93071E-01	24226	0	43212
Myeloid_vs_CD4_Responder	Macro_LYVE1	CD4(ISG+ Treg)	HLA-DRB1	LAG3	1,06488E-03	14397	9,58536E-03	7354	1,56812E+00	10800	2,44076E+00	5258	9,36860E-01	5361	0	43212
Myeloid_vs_CD4_Responder	CD4(NME1+ T)	Macro_NLRP3	HMGB1	THBD	1,07264E-03	24925,8	5,52780E-03	17950	1,38311E+00	15295	1,41956E+00	24266	8,93804E-01	23906	0	43212
Myeloid_vs_CD4_Responder	Macro_OLFML3	CD4(TGFB1+ Th17)	FN1	ITGA4_ITGB1	1,07375E-03	19341,2	1,09588E-02	5824	1,83016E+00	6890	1,69442E+00	16509	8,92946E-01	24271	0	43212
Myeloid_vs_CD4_Responder	CD4(IL26+ Th17)	pDC_LILRA4	HLA-C	NOTCH4	1,07530E-03	19410,8	1,73494E-02	2445	1,63221E+00	9675	1,65672E+00	17444	8,92937E-01	24278	0	43212
Myeloid_vs_CD4_Responder	Macro_OLFML3	CD4(NME1+ T)	CD14	ITGB1	1,07641E-03	22363,8	4,50255E-03	24283	1,57841E+00	10609	1,63808E+00	17914	9,13030E-01	15801	0	43212
Myeloid_vs_CD4_Responder	CD4(IFNG+ Tfh/Th1)	pDC_LILRA4	HLA-C	NOTCH4	1,07707E-03	19039,2	1,73433E-02	2447	1,63143E+00	9687	1,73529E+00	15564	8,92920E-01	24286	0	43212
Myeloid_vs_CD4_Responder	Macro_FOLR2+APOE+	CD4(ISG+ Treg)	HLA-DRB1	LAG3	1,08046E-03	14545,6	9,57103E-03	7379	1,56585E+00	10840	2,37075E+00	5919	9,36816E-01	5378	0	43212
Myeloid_vs_CD4_Responder	Macro_FOLR2-APOE+	CD4(GZMK+ Teff)	SPP1	CD44	1,08399E-03	15593,8	8,94981E-03	8316	1,74006E+00	8046	2,21476E+00	7546	9,24710E-01	10849	0	43212
Myeloid_vs_CD4_Responder	Mono_INHBA	CD4(TGFB1+ Th17)	CXCL2	DPP4	1,09737E-03	17484	3,31729E-02	652	2,64296E+00	1723	2,63525E+00	3733	8,61645E-01	38100	0	43212
Myeloid_vs_CD4_Responder	CD4(NME1+ T)	Macro_ISG15	HMGB1	HAVCR2	1,09853E-03	24692,6	4,49062E-03	24382	1,17303E+00	22396	1,53739E+00	20616	9,19964E-01	12857	0	43212
Myeloid_vs_CD4_Responder	CD4(AREG+ Tm)	Mono_INHBA	VIM	CD44	1,10217E-03	38551,8	2,34188E-03	59562	6,11451E-01	52729	1,08107E+00	36780	9,55060E-01	476	0	43212
Myeloid_vs_CD4_Responder	CD4(IL26+ Th17)	Macro_NLRP3	RPS19	C5AR1	1,10395E-03	16855	7,49882E-03	11139	2,13623E+00	3961	1,41523E+00	24406	9,49276E-01	1557	0	43212
Myeloid_vs_CD4_Responder	CD4(IFNG+ Tfh/Th1)	pDC_LILRA4	TNF	PTPRS	1,10571E-03	23949,2	3,40530E-02	621	4,44958E+00	160	2,05620E+00	9604	8,01014E-01	66149	0	43212
Myeloid_vs_CD4_Responder	cDC_LAMP3	CD4(CXCL13+ Tfh)	CCL17	CCR4	1,10926E-03	23934,2	9,76172E-02	60	3,27381E+00	622	1,63079E+00	18102	8,19565E-01	57675	0	43212
Myeloid_vs_CD4_Responder	Macro_OLFML3	CD4(IL26+ Th17)	TNFSF10	CCR6	1,10984E-03	19590,2	2,22384E-02	1533	1,41033E+00	14523	1,41461E+00	24432	9,16579E-01	14251	0	43212
Myeloid_vs_CD4_Responder	Macro_ISG15	CD4(NME1+ T)	CXCL10	SDC4	1,12629E-03	23506	1,85136E-02	2151	2,23760E+00	3303	2,62996E+00	3766	8,03329E-01	65098	0	43212
Myeloid_vs_CD4_Responder	CD4(NME1+ T)	Mono_CD16	HLA-A	LILRB2	1,12675E-03	17381	6,06305E-03	15626	2,36330E+00	2681	1,41194E+00	24506	9,52242E-01	880	0	43212
Myeloid_vs_CD4_Responder	Macro_IER3	CD4(IFNG+ Tfh/Th1)	HLA-DQA1	LAG3	1,12903E-03	14659,6	1,19178E-02	4947	1,55981E+00	10962	2,44770E+00	5191	9,28973E-01	8986	0	43212
Myeloid_vs_CD4_Responder	Macro_OLFML3	CD4(IFNG+ Tfh/Th1)	CD14	ITGB1	1,13459E-03	22130,4	4,46682E-03	24540	1,57431E+00	10669	1,70457E+00	16292	9,12713E-01	15939	0	43212
Myeloid_vs_CD4_Responder	Macro_OLFML3	CD4(IFNG+ Tfh/Th1)	CXCL16	CXCR6	1,13713E-03	21589,4	1,26010E-02	4477	1,39841E+00	14843	1,41063E+00	24551	9,01160E-01	20864	0	43212
Myeloid_vs_CD4_Responder	Macro_OLFML3	CD4(ISG+ Treg)	HLA-DQA2	CD4	1,13898E-03	21915,6	4,46400E-03	24559	1,57502E+00	10662	1,82081E+00	13763	9,09379E-01	17382	0	43212
Myeloid_vs_CD4_Responder	CD4(TGFB1+ Th17)	Mono_CD16	HLA-F	LILRB2	1,14061E-03	20958	6,18505E-03	15175	2,32854E+00	2846	1,41019E+00	24566	9,05692E-01	18991	0	43212
Myeloid_vs_CD4_Responder	CD4(GZMK+ Teff)	Macro_NLRP3	CCL5	CCRL2	1,14247E-03	19589	1,92471E-02	1995	1,69593E+00	8673	1,58028E+00	19491	8,92242E-01	24574	0	43212
Myeloid_vs_CD4_Responder	CD4(AREG+ Tm)	Mono_INHBA	RPS19	C5AR1	1,14573E-03	19348,2	5,81034E-03	16646	1,67063E+00	9051	1,40944E+00	24588	9,42770E-01	3244	0	43212
Myeloid_vs_CD4_Responder	CD4(ISG+ Treg)	Mono_INHBA	HLA-F	LILRB2	1,14619E-03	23976,6	5,26272E-03	19336	1,56367E+00	10875	1,40940E+00	24590	8,98565E-01	21870	0	43212
Myeloid_vs_CD4_Responder	CD4(AREG+ Tm)	Mono_CD16	HLA-A	LILRB2	1,14764E-03	20017,6	5,56577E-03	17756	2,15842E+00	3790	1,14996E+00	34014	9,50258E-01	1316	0	43212
Myeloid_vs_CD4_Responder	CD4(TNF+ T)	pDC_LILRA4	TNF	TNFRSF21	1,15227E-03	17787,8	2,61641E-02	1091	3,42304E+00	496	2,62524E+00	3801	8,56667E-01	40339	0	43212
Myeloid_vs_CD4_Responder	CD4(TGFB1+ Th17)	Mono_INHBA	RPS19	C5AR1	1,15297E-03	19726	5,62578E-03	17459	1,62263E+00	9836	1,40828E+00	24619	9,41893E-01	3504	0	43212
Myeloid_vs_CD4_Responder	CD4(TNF+ T)	pDC_LILRA4	COPA	P2RY6	1,16009E-03	27093,4	7,82159E-03	10376	2,05080E+00	4658	2,26776E+00	6949	7,92007E-01	70272	0	43212
Myeloid_vs_CD4_Responder	CD4(NME1+ T)	Mono_CD14	HMGB1	THBD	1,16166E-03	24051,2	5,32821E-03	18987	1,34552E+00	16414	1,67474E+00	16987	8,92046E-01	24656	0	43212
Myeloid_vs_CD4_Responder	Macro_IER3	CD4(ISG+ Treg)	CCL3L1	CCR5	1,16202E-03	22521,6	1,84472E-02	2171	2,88409E+00	1173	2,62458E+00	3806	8,09517E-01	62246	0	43212
Myeloid_vs_CD4_Responder	CD4(TNF+ T)	cDC_CLEC9A	TNFSF9	HLA-DPA1	1,16354E-03	18591,8	6,40886E-03	14338	1,62236E+00	9840	3,35073E+00	905	8,92026E-01	24664	0	43212
Myeloid_vs_CD4_Responder	Macro_OLFML3	CD4(IFNG+ Tfh/Th1)	HLA-DMB	CD4	1,16756E-03	21842,6	4,44940E-03	24681	1,71984E+00	8337	1,67299E+00	17035	9,12690E-01	15948	0	43212
Myeloid_vs_CD4_Responder	Macro_OLFML3	CD4(IL26+ Th17)	HLA-DMB	CD4	1,17420E-03	22159,2	4,44616E-03	24709	1,71946E+00	8347	1,61460E+00	18567	9,12661E-01	15961	0	43212
Myeloid_vs_CD4_Responder	CD4(IL26+ Th17)	pDC_LILRA4	HSP90B1	TLR9	1,17772E-03	22486,6	3,36790E-02	632	3,24999E+00	641	1,32561E+00	27483	8,56340E-01	40465	0	43212
Myeloid_vs_CD4_Responder	Macro_IFI27	CD4(ISG+ Treg)	HLA-DQB1	LAG3	1,19094E-03	15121,4	1,06277E-02	6119	1,55116E+00	11112	2,46028E+00	5098	9,26485E-01	10066	0	43212
Myeloid_vs_CD4_Responder	CD4(NME1+ T)	pDC_LILRA4	HRAS	TLR9	1,19239E-03	37549,6	6,83459E-02	145	3,24631E+00	645	1,36827E+00	25959	6,50680E-01	117787	0	43212
Myeloid_vs_CD4_Responder	CD4(GZMK+ Teff)	pDC_LILRA4	TNF	PTPRS	1,20715E-03	24211	3,32541E-02	649	4,44455E+00	162	2,02964E+00	10008	7,99115E-01	67024	0	43212
Myeloid_vs_CD4_Responder	Macro_OLFML3	CD4(IL26+ Th17)	C3	CD46	1,21442E-03	18117,8	9,98322E-03	6878	2,17585E+00	3672	1,91618E+00	11951	8,91516E-01	24876	0	43212
Myeloid_vs_CD4_Responder	cDC_LAMP3	CD4(TNFRSF9+ Treg)	CCL17	CCR4	1,21828E-03	24753,8	8,91983E-02	73	3,23972E+00	652	1,59307E+00	19145	8,12800E-01	60687	0	43212
Myeloid_vs_CD4_Responder	Mono_CD14	CD4(CRTAM- T)	VCAN	ITGB1	1,22072E-03	13360,2	1,42610E-02	3588	3,00243E+00	972	2,61471E+00	3870	9,14517E-01	15159	0	43212
Myeloid_vs_CD4_Responder	Macro_ISG15	CD4(CRTAM- T)	SPP1	CD44	1,23013E-03	15281,6	8,72772E-03	8665	1,66982E+00	9070	2,56559E+00	4257	9,23830E-01	11204	0	43212
Myeloid_vs_CD4_Responder	CD4(ISG+ Treg)	pDC_LILRA4	HRAS	TLR9	1,23695E-03	37834,6	6,45617E-02	168	3,23510E+00	657	1,36507E+00	26097	6,44179E-01	119039	0	43212
Myeloid_vs_CD4_Responder	cDC_CLEC9A	CD4(ISG+ Treg)	HLA-DRB1	LAG3	1,23748E-03	13771,8	9,44612E-03	7559	1,54613E+00	11221	3,16850E+00	1315	9,36426E-01	5552	0	43212
Myeloid_vs_CD4_Responder	cDC_LAMP3	CD4(ISG+ Treg)	CCL19	CCR7	1,24070E-03	23597,4	3,30606E-02	658	5,64937E+00	67	1,46802E+00	22753	8,33139E-01	51297	0	43212
Myeloid_vs_CD4_Responder	CD4(GZMK+ Teff)	Mono_CD16	CD99	PILRA	1,24175E-03	19516,8	7,02625E-03	12430	2,00918E+00	5026	1,39716E+00	24987	9,22154E-01	11929	0	43212
Myeloid_vs_CD4_Responder	Macro_OLFML3	CD4(TNFRSF9+ Treg)	CXCL16	CXCR6	1,24548E-03	22340,6	1,16544E-02	5159	1,35605E+00	16086	1,39673E+00	25002	8,97627E-01	22244	0	43212
Myeloid_vs_CD4_Responder	Macro_LYVE1	CD4(IFNG+ Tfh/Th1)	HLA-DQA1	LAG3	1,24616E-03	14942,8	1,17799E-02	5068	1,54535E+00	11241	2,35652E+00	6049	9,28588E-01	9144	0	43212
Myeloid_vs_CD4_Responder	Macro_FOLR2-APOE+	CD4(AREG+ Tm)	SPP1	CD44	1,25096E-03	15959,4	8,70251E-03	8703	1,71380E+00	8422	2,15899E+00	8208	9,23729E-01	11252	0	43212
Myeloid_vs_CD4_Responder	CD4(TNF+ T)	cDC(CD1C)	TNFSF9	HLA-DPA1	1,25598E-03	18846,2	6,28564E-03	14782	1,58940E+00	10397	3,41962E+00	796	8,91087E-01	25044	0	43212
Myeloid_vs_CD4_Responder	Macro_OLFML3	CD4(TGFB1+ Th17)	APOE	LSR	1,25648E-03	16027,4	1,41734E-02	3616	1,94202E+00	5639	2,81670E+00	2624	8,91085E-01	25046	0	43212
Myeloid_vs_CD4_Responder	Macro_FOLR2+APOE-	CD4(IL26+ Th17)	CD14	ITGA4	1,25839E-03	16301	7,44720E-03	11269	1,84073E+00	6748	2,08332E+00	9207	9,24182E-01	11069	0	43212
Myeloid_vs_CD4_Responder	Macro_FOLR2+APOE+	CD4(ISG+ Treg)	HLA-DQB1	LAG3	1,25971E-03	16030	1,05707E-02	6179	1,54348E+00	11272	2,07267E+00	9348	9,26301E-01	10139	0	43212
Myeloid_vs_CD4_Responder	CD4(CRTAM- T)	Macro_ISG15	ANXA1	FPR2_FPR3	1,26756E-03	21067,8	9,95247E-03	6907	1,70046E+00	8609	1,39380E+00	25090	8,99528E-01	21521	0	43212
Myeloid_vs_CD4_Responder	CD4(Tn)	Mono_CD16	HLA-A	LILRB2	1,26887E-03	20050,2	5,52345E-03	17979	2,14099E+00	3921	1,15629E+00	33786	9,50077E-01	1353	0	43212
Myeloid_vs_CD4_Responder	Macro_IER3	CD4(IFNG+ Tfh/Th1)	HLA-DPB1	LAG3	1,27337E-03	14025	1,07458E-02	5999	1,54214E+00	11303	2,53153E+00	4532	9,37546E-01	5079	0	43212
Myeloid_vs_CD4_Responder	cDC_LAMP3	CD4(ISG+ Treg)	HLA-DRA	LAG3	1,27647E-03	14854,2	8,77927E-03	8594	1,54176E+00	11310	2,44110E+00	5252	9,35611E-01	5903	0	43212
Myeloid_vs_CD4_Responder	CD4(NME1+ T)	pDC_LILRA4	HSP90B1	TLR9	1,28231E-03	22794,2	3,26882E-02	669	3,23220E+00	662	1,30790E+00	28111	8,54494E-01	41317	0	43212
Myeloid_vs_CD4_Responder	CD4(Tn)	Mono_CD16	B2M	LILRB2	1,28519E-03	19595,4	5,34231E-03	18915	2,13887E+00	3938	1,21311E+00	31628	9,57018E-01	284	0	43212
Myeloid_vs_CD4_Responder	Mono_CD14	CD4(NME1+ T)	VCAN	ITGB1	1,28905E-03	13228,2	1,46779E-02	3401	3,01710E+00	946	2,60552E+00	3942	9,15637E-01	14640	0	43212
Myeloid_vs_CD4_Responder	CD4(AREG+ Tm)	cDC_CLEC9A	CALM1	MYLK	1,30165E-03	28503,2	2,83438E-02	919	2,13686E+00	3955	9,66480E-01	41539	8,29912E-01	52891	0	43212
Myeloid_vs_CD4_Responder	CD4(CRTAM- T)	Mono_CD16	HLA-B	LILRB2	1,30616E-03	17658,8	5,83569E-03	16528	2,36307E+00	2683	1,38936E+00	25241	9,53876E-01	630	0	43212

Myeloid_vs_CD4_Post_R

Myeloid_vs_CD4_Responder	CD4(IL26+ Th17)	Mono_CD16	HLA-C	LILRB2	1,30823E-03	17648,2	5,96046E-03	16024	2,37754E+00	2614	1,38922E+00	25249	9,50991E-01	1142	0	43212
Myeloid_vs_CD4_Responder	Macro_FOLR2-APOE+	CD4(IFNG+ Tfh/Th1)	HLA-DQB1	LAG3	1,30912E-03	14559,4	1,19988E-02	4890	1,53813E+00	11383	2,46711E+00	5044	9,30512E-01	8268	0	43212
Myeloid_vs_CD4_Responder	CD4(TGFB1+ Th17)	Macro_NLRP3	ANXA1	FPR1	1,31161E-03	19793,8	7,04700E-03	12366	1,77569E+00	7568	1,38881E+00	25262	9,25376E-01	10561	0	43212
Myeloid_vs_CD4_Responder	Macro_OLFML3	CD4(CRTAM- T)	CD14	ITGB1	1,31524E-03	22667,2	4,37468E-03	25276	1,56373E+00	10872	1,64728E+00	17672	9,11879E-01	16304	0	43212
Myeloid_vs_CD4_Responder	CD4(Tn)	Macro_FOLR2+APOE-	HMGB1	CD163	1,31707E-03	21246,2	4,37386E-03	25283	1,82218E+00	6990	1,52840E+00	20865	9,26913E-01	9881	0	43212
Myeloid_vs_CD4_Responder	Macro_FOLR2+APOE+	CD4(ISG+ Treg)	B2M	CD3D	1,33135E-03	41459,6	3,88491E-03	29972	5,95218E-01	53908	2,90809E-01	80199	9,63512E-01	7	0	43212
Myeloid_vs_CD4_Responder	CD4(TGFB1+ Th17)	Macro_FOLR2+APOE+	CD52	SIGLEC10	1,34192E-03	23009,6	7,93379E-03	10126	1,80869E+00	7157	9,31022E-01	43098	9,23258E-01	11455	0	43212
Myeloid_vs_CD4_Responder	Mono_CD16	CD4(TGFB1+ Th17)	S100A8	CD69	1,34330E-03	15313,8	9,14211E-03	7996	1,53468E+00	11458	2,11925E+00	8709	9,37249E-01	5194	0	43212
Myeloid_vs_CD4_Responder	Macro_OLFML3	CD4(IFNG+ Tfh/Th1)	HLA-DQB2	LAG3	1,34782E-03	17389,8	2,57023E-02	1141	1,87069E+00	6398	1,98152E+00	10798	8,90251E-01	25400	0	43212
Myeloid_vs_CD4_Responder	CD4(GZMK+ Teff)	pDC_LILRA4	HMGB1	TLR9	1,34796E-03	20359,4	3,58735E-02	561	3,21633E+00	686	1,32659E+00	27454	8,79807E-01	29884	0	43212
Myeloid_vs_CD4_Responder	CD4(ISG+ Treg)	Macro_ISG15	LTB	TNFRSF1A	1,35074E-03	23067,6	1,01325E-02	6692	1,18248E+00	22004	1,38476E+00	25411	9,07953E-01	18019	0	43212
Myeloid_vs_CD4_Responder	Macro_IFI27	CD4(ISG+ Treg)	HLA-DPA1	LAG3	1,35345E-03	14447,8	9,38457E-03	7627	1,53348E+00	11480	2,59989E+00	3989	9,35547E-01	5931	0	43212
Myeloid_vs_CD4_Responder	CD4(NME1+ T)	pDC_LILRA4	HLA-C	NOTCH4	1,35527E-03	20611,6	1,63877E-02	2734	1,50832E+00	12067	1,57520E+00	19617	8,90181E-01	25428	0	43212
Myeloid_vs_CD4_Responder	CD4(TGFB1+ Th17)	Macro_ISG15	ANXA1	FPR1	1,36007E-03	21007,6	6,36368E-03	14497	1,62541E+00	9792	1,38377E+00	25446	9,21777E-01	12091	0	43212
Myeloid_vs_CD4_Responder	CD4(GZMK+ Teff)	Macro_NLRP3	ANXA1	FPR1	1,36221E-03	21653	5,81017E-03	16650	1,64455E+00	9467	1,38368E+00	25454	9,18433E-01	13482	0	43212
Myeloid_vs_CD4_Responder	CD4(AREG+ Tm)	pDC_LILRA4	COPA	P2RY6	1,36227E-03	28465,4	7,36287E-03	11499	2,03582E+00	4796	2,01029E+00	10302	7,86985E-01	72518	0	43212
Myeloid_vs_CD4_Responder	CD4(TNF+ T)	pDC_LILRA4	MAML2	NOTCH4	1,36320E-03	35556,4	2,11893E-02	1674	1,57989E+00	10582	1,94153E+00	11501	6,80568E-01	110813	0	43212
Myeloid_vs_CD4_Responder	CD4(NME1+ T)	Mono_CD16	B2M	LILRB2	1,36650E-03	17909,2	5,57656E-03	17700	2,30871E+00	2944	1,38314E+00	25470	9,57892E-01	220	0	43212
Myeloid_vs_CD4_Responder	CD4(AREG+ Tm)	Macro_OLFML3	ANXA1	FPR1	1,37403E-03	20947,8	6,57378E-03	13746	1,57409E+00	10675	1,38235E+00	25498	9,22940E-01	11608	0	43212
Myeloid_vs_CD4_Responder	Macro_FOLR2+APOE-	CD4(Tn)	CCL13	CXCR3	1,37546E-03	33954	3,22618E-02	693	3,65795E+00	372	9,86669E-01	40694	7,57648E-01	84799	0	43212
Myeloid_vs_CD4_Responder	Macro_FOLR2+APOE+	CD4(ISG+ Treg)	HLA-DPA1	LAG3	1,37627E-03	15047,4	9,37108E-03	7646	1,53133E+00	11529	2,27318E+00	6896	9,35503E-01	5954	0	43212
Myeloid_vs_CD4_Responder	Macro_IER3	CD4(IL26+ Th17)	HLA-DRA	LAG3	1,37814E-03	16522,2	7,90691E-03	10193	1,53120E+00	11533	2,01075E+00	10290	9,32385E-01	7383	0	43212
Myeloid_vs_CD4_Responder	cDC_CLEC9A	CD4(IL26+ Th17)	HLA-DQA1	LAG3	1,38425E-03	14674,2	1,00325E-02	6826	1,64225E+00	9497	2,89222E+00	2290	9,23078E-01	11546	0	43212
Myeloid_vs_CD4_Responder	CD4(ISG+ Treg)	pDC_LILRA4	HMGB1	TLR9	1,38733E-03	21106,2	3,55910E-02	569	3,20929E+00	696	1,23027E+00	30970	8,79388E-01	30084	0	43212
Myeloid_vs_CD4_Responder	Macro_OLFML3	CD4(IFNG+ Tfh/Th1)	C1QB	C1QBP	1,39817E-03	18593,2	4,33839E-03	25587	1,93581E+00	5704	2,72958E+00	3128	9,14127E-01	15335	0	43212
Myeloid_vs_CD4_Responder	Macro_FOLR2+APOE+	CD4(IL26+ Th17)	HLA-DPB1	LAG3	1,40602E-03	16490,2	8,42116E-03	9171	1,52842E+00	11592	2,03211E+00	9972	9,30017E-01	8504	0	43212
Myeloid_vs_CD4_Responder	CD4(GZMK+ Teff)	Macro_ISG15	ANXA1	FPR1	1,40721E-03	23156,8	5,24678E-03	19422	1,49427E+00	12379	1,37865E+00	25620	9,14530E-01	15151	0	43212
Myeloid_vs_CD4_Responder	CD4(Tn)	Macro_ISG15	ANXA1	FPR2_FPR3	1,40941E-03	22810,2	8,41482E-03	9181	1,54598E+00	11222	1,37841E+00	25628	8,91686E-01	24808	0	43212
Myeloid_vs_CD4_Responder	Macro_OLFML3	CD4(CRTAM- T)	APOE	SORL1	1,41024E-03	20375,4	4,33302E-03	25631	1,87330E+00	6368	2,74921E+00	3010	8,94361E-01	23656	0	43212
Myeloid_vs_CD4_Responder	Macro_ISG15	CD4(AREG+ Tm)	CXCL10	CXCR3	1,41149E-03	18242,6	1,77740E-02	2330	2,17158E+00	3697	2,58946E+00	4065	8,62018E-01	37909	0	43212
Myeloid_vs_CD4_Responder	Macro_OLFML3	CD4(TNFRSF9+ Treg)	APOE	LSR	1,41189E-03	16163,8	1,37710E-02	3826	1,93474E+00	5719	2,86094E+00	2425	8,89680E-01	25637	0	43212
Myeloid_vs_CD4_Responder	Macro_OLFML3	CD4(CXCL13+ Tfh)	FN1	ITGA4_ITGB1	1,41823E-03	19963,6	1,02283E-02	6570	1,80541E+00	7190	1,66742E+00	17186	8,89604E-01	25660	0	43212
Myeloid_vs_CD4_Responder	CD4(IL26+ Th17)	cDC_CLEC9A	FLT3LG	FLT3	1,42727E-03	24936,8	3,19918E-02	706	4,05761E+00	240	1,50671E+00	21499	8,16566E-01	59027	0	43212
Myeloid_vs_CD4_Responder	Macro_FOLR2+APOE+	CD4(CRTAM- T)	APOE	LDLR	1,42796E-03	15286	1,33039E-02	4081	2,60632E+00	1821	3,18065E+00	1277	8,88782E-01	26039	0	43212
Myeloid_vs_CD4_Responder	Macro_FOLR2-APOE+	CD4(ISG+ Treg)	HLA-DRB1	LAG3	1,42852E-03	14616	9,32101E-03	7721	1,52638E+00	11639	2,49512E+00	4803	9,36028E-01	5705	0	43212
Myeloid_vs_CD4_Responder	CD4(TGFB1+ Th17)	pDC_LILRA4	HLA-C	NOTCH4	1,43183E-03	21198	1,61620E-02	2797	1,47926E+00	12750	1,50596E+00	21522	8,89501E-01	25709	0	43212
Myeloid_vs_CD4_Responder	cDC_LAMP3	CD4(ISG+ Treg)	HLA-DQA1	LAG3	1,43528E-03	15930,2	1,02215E-02	6579	1,52578E+00	11653	2,26624E+00	6959	9,23738E-01	11248	0	43212
Myeloid_vs_CD4_Responder	CD4(ISG+ Treg)	cDC(CD1C)	LTB	TNFRSF1A	1,43768E-03	23169,6	9,11053E-03	8064	1,09299E+00	25730	1,60008E+00	18953	9,03413E-01	19889	0	43212
Myeloid_vs_CD4_Responder	CD4(Tn)	pDC_LILRA4	HLA-C	NOTCH4	1,44020E-03	21048,6	1,61414E-02	2808	1,47659E+00	12810	1,53548E+00	20674	8,89438E-01	25739	0	43212
Myeloid_vs_CD4_Responder	CD4(Tn)	cDC_CLEC9A	ANXA1	DYSF	1,44076E-03	18949,8	1,64153E-02	2721	1,93486E+00	5717	1,66031E+00	17358	8,89432E-01	25741	0	43212
Myeloid_vs_CD4_Responder	Mono_INHBA	CD4(TGFB1+ Th17)	LGALS1	CD69	1,44108E-03	14050,2	7,30775E-03	11665	1,63198E+00	9678	2,46902E+00	5028	9,53563E-01	668	0	43212
Myeloid_vs_CD4_Responder	CD4(GZMK+ Teff)	Mono_CD16	B2M	LILRB2	1,44272E-03	17913,6	5,60631E-03	17556	2,33027E+00	2837	1,37498E+00	25748	9,57999E-01	215	0	43212
Myeloid_vs_CD4_Responder	Macro_OLFML3	CD4(TNFRSF9+ Treg)	HLA-DQB1	LAG3	1,44552E-03	22957,8	4,32548E-03	25698	1,50326E+00	12169	2,18065E+00	7952	8,89381E-01	25758	0	43212
Myeloid_vs_CD4_Responder	CD4(CXCL13+ Tfh)	pDC_LILRA4	HMGB1	TLR9	1,44744E-03	20796,6	3,51536E-02	581	3,19838E+00	711	1,28065E+00	29128	8,78731E-01	30351	0	43212
Myeloid_vs_CD4_Responder	Macro_OLFML3	CD4(TNFRSF9+ Treg)	HLA-DQA1	LAG3	1,44946E-03	22102	4,50154E-03	24289	1,60081E+00	10198	2,25908E+00	7039	8,89360E-01	25772	0	43212
Myeloid_vs_CD4_Responder	Macro_FOLR2+APOE+	CD4(GZMK+ Teff)	APOE	LSR	1,46547E-03	15560,4	1,32298E-02	4117	2,47597E+00	2229	3,03472E+00	1711	8,87697E-01	26533	0	43212
Myeloid_vs_CD4_Responder	CD4(NME1+ T)	Mono_CD16	HLA-B	LILRB2	1,46840E-03	17842,4	5,79244E-03	16726	2,34200E+00	2781	1,37229E+00	25839	9,53712E-01	654	0	43212
Myeloid_vs_CD4_Responder	CD4(Tn)	Mono_CD16	HLA-F	LILRB2	1,47067E-03	23016,2	5,09747E-03	20241	2,24134E+00	3285	1,37202E+00	25847	8,97102E-01	22496	0	43212
Myeloid_vs_CD4_Responder	Mono_INHBA	CD4(ISG+ Treg)	CCL3	CCR4	1,47073E-03	16733	1,43496E-02	3538	2,23016E+00	3348	2,58256E+00	4122	8,80771E-01	29445	0	43212
Myeloid_vs_CD4_Responder	Mono_CD14	CD4(TGFB1+ Th17)	S100A9	ITGB2	1,47184E-03	14919	4,69797E-03	22796	3,24469E+00	646	3,46695E+00	717	9,32762E-01	7224	0	43212
Myeloid_vs_CD4_Responder	Mono_INHBA	CD4(AREG+ Tm)	CXCL2	DPP4	1,47706E-03	20020,4	2,27819E-02	1460	2,49693E+00	2158	2,58200E+00	4128	8,37689E-01	49144	0	43212
Myeloid_vs_CD4_Responder	Macro_FOLR2-APOE+	CD4(IL26+ Th17)	CD14	ITGA4	1,48124E-03	16738,2	7,27203E-03	11747	1,80298E+00	7220	2,02413E+00	10097	9,23344E-01	11415	0	43212
Myeloid_vs_CD4_Responder	CD4(NME1+ T)	Mono_CD14	HMGB1	TLR4	1,48208E-03	24339	5,54949E-03	17845	1,38989E+00	15103	1,57412E+00	19648	8,89069E-01	25887	0	43212
Myeloid_vs_CD4_Responder	CD4(CRTAM- T)	Mono_CD14	ANXA1	DYSF	1,48782E-03	20025,8	1,62800E-02	2763	1,83833E+00	6781	1,50784E+00	21466	8,89024E-01	25907	0	43212
Myeloid_vs_CD4_Responder	Mono_INHBA	CD4(ISG+ Treg)	ICAM1	IL2RG	1,49115E-03	16708,8	9,63887E-03	7294	1,58802E+00	10421	1,97878E+00	10850	9,22566E-01	11767	0	43212
Myeloid_vs_CD4_Responder	CD4(AREG+ Tm)	Mono_CD16	RPS19	C5AR1	1,49415E-03	17832,6	6,94729E-03	12646	1,97154E+00	5363	1,36904E+00	25929	9,47405E-01	2013	0	43212
Myeloid_vs_CD4_Responder	CD4(CRTAM- T)	Mono_CD16	HLA-F	LILRB2	1,49645E-03	21933	5,71011E-03	17091	2,29046E+00	3034	1,36880E+00	25937	9,02224E-01	20391	0	43212
Myeloid_vs_CD4_Responder	CD4(AREG+ Tm)	pDC_LILRA4	HLA-C	NOTCH4	1,49703E-03	21357,8	1,59863E-02	2868	1,45662E+00	13273	1,50673E+00	21497	8,88963E-01	25939	0	43212
Myeloid_vs_CD4_Responder	CD4(AREG+ Tm)	Mono_CD16	B2M	LILRB2	1,49828E-03	20113,2	5,30525E-03	19106	2,11200E+00	4148	1,15577E+00	33805	9,56875E-01	295	0	43212
Myeloid_vs_CD4_Responder	CD4(CRTAM- T)	Mono_INHBA	RPS19	C5AR1	1,50571E-03	20091,8	5,58204E-03	17676	1,61125E+00	10029	1,36806E+00	25969	9,41680E-01	3573	0	43212
Myeloid_vs_CD4_Responder	CD4(TGFB1+ Th17)	Mono_CD16	RPS19	C5AR1	1,50861E-03	18094,2	6,72661E-03	13246	1,92354E+00	5821	1,36788E+00	25979	9,46595E-01	2213	0	43212
Myeloid_vs_CD4_Responder	CD4(CRTAM- T)	pDC_LILRA4	HLA-C	NOTCH4	1,50890E-03	21449,2	1,59603E-02	2880	1,45326E+00	13357	1,49679E+00	21817	8,88882E-01	25980	0	43212
Myeloid_vs_CD4_Responder	Macro_FOLR2+APOE-	CD4(ISG+ Treg)	HLA-DPB1	LAG3	1,50963E-03	15118,4	9,24276E-03	7832	1,51906E+00	11804	2,40293E+00	5609	9,32986E-01	7135	0	43212
Myeloid_vs_CD4_Responder	Macro_LYVE1	CD4(IL26+ Th17)	CD14	ITGA4	1,51064E-03	16365,6	7,78355E-03	10483	1,91322E+00	5924	1,92370E+00	11806	9,25715E-01	10403	0	43212
Myeloid_vs_CD4_Responder	Macro_LYVE1	CD4(ISG+ Treg)	HLA-DPA1	LAG3	1,51114E-03	15034,6	9,29252E-03	7762	1,51881E+00	11807	2,32932E+00	6305	9,35249E-01	6087	0	43212
Myeloid_vs_CD4_Responder	cDC_LAMP3	CD4(TNF+ T)	CCL19	CCR7	1,51295E-03	23497,2	3,15515E-02	727	5,63976E+00	69	1,53898E+00	20570	8,29866E-01	52908	0	43212
Myeloid_vs_CD4_Responder	CD4(AREG+ Tm)	Mast	HSPA8	ADRB2	1,52070E-03	20649	1,45977E-02	3432	1,51822E+00	11826	1,15606E+00	33795	9,24357E-01	10980	0	43212
Myeloid_vs_CD4_Responder	CD4(NME1+ T)	cDC_CLEC9A	CD52	SIGLEC10	1,52347E-03	26478	4,88668E-03	21543	1,18098E+00	22058	1,36688E+00	26030	9,04231E-01	19547	0	43212
Myeloid_vs_CD4_Responder	CD4(TNF+ T)	cDC(CD1C)	CSF1	CSF2RA	1,52878E-03	28399,2	8,80946E-03	8537	1,75506E+00	7846	1,92197E+00	11842	7,91320E-01	70559	0	43212

Myeloid_vs_CD4_Responder	pDC_LILRA4	CD4(TGFB1+ Th17)	APP	RPSA	1,53639E-03	13922	1,17469E-02	5087	1,51729E+00	11857	2,46662E+00	5049	9,39162E-01	4405	0	43212
Myeloid_vs_CD4_Responder	Mast	CD4(CXCL13+ Tfh)	TIMP3	CD44	1,55043E-03	16853	3,13727E-02	736	3,22636E+00	671	1,42821E+00	24010	9,13420E-01	15636	0	43212
Myeloid_vs_CD4_Responder	CD4(GZMK+ Teff)	cDC(CD1C)	ANXA1	FPR1	1,55089E-03	25071,4	4,27318E-03	26123	1,23456E+00	20102	1,66955E+00	17121	9,06160E-01	18799	0	43212
Myeloid_vs_CD4_Responder	Macro_OLFML3	CD4(AREG+ Tm)	FN1	ITGA4_ITGB1	1,56578E-03	20104,2	1,00012E-02	6858	1,79735E+00	7292	1,67475E+00	16986	8,88496E-01	26173	0	43212
Myeloid_vs_CD4_Responder	CD4(IL26+ Th17)	Mono_CD16	CD99	PILRA	1,57279E-03	19947,4	7,19403E-03	11928	2,02794E+00	4866	1,30660E+00	28156	9,22997E-01	11575	0	43212
Myeloid_vs_CD4_Responder	Mono_CD14	CD4(IFNG+ Tfh/Th1)	VCAN	ITGA4	1,57303E-03	13995,6	1,47331E-02	3377	2,98444E+00	996	2,57080E+00	4217	9,07555E-01	18176	0	43212
Myeloid_vs_CD4_Responder	CD4(CXCL13+ Tfh)	Macro_NLRP3	RPS19	C5AR1	1,57538E-03	17527,2	7,18392E-03	11960	2,07495E+00	4441	1,36200E+00	26205	9,48233E-01	1818	0	43212
Myeloid_vs_CD4_Responder	CD4(Tn)	pDC_LILRA4	COPA	P2RY6	1,57642E-03	28607,6	7,19263E-03	11935	2,03027E+00	4846	2,05377E+00	9641	7,85018E-01	73404	0	43212
Myeloid_vs_CD4_Responder	CD4(TNFRSF9+ Treg)	pDC_LILRA4	COPA	P2RY6	1,58056E-03	28739	7,19073E-03	11943	2,03021E+00	4847	2,01132E+00	10278	7,84996E-01	73415	0	43212
Myeloid_vs_CD4_Responder	CD4(Tn)	Mono_CD16	HLA-B	LILRB2	1,58186E-03	20308,8	5,30168E-03	19124	2,10292E+00	4225	1,15040E+00	33998	9,51718E-01	985	0	43212
Myeloid_vs_CD4_Responder	Macro_OLFML3	CD4(Tn)	FN1	ITGA4_ITGB1	1,58321E-03	19787,2	9,97499E-03	6885	1,79550E+00	7313	1,74631E+00	15295	8,88366E-01	26231	0	43212
Myeloid_vs_CD4_Responder	Macro_LYVE1	CD4(TGFB1+ Th17)	LGALS1	CD69	1,58628E-03	14427,6	7,18491E-03	11954	1,60851E+00	10074	2,34410E+00	6167	9,53186E-01	731	0	43212
Myeloid_vs_CD4_Responder	CD4(Tn)	cDC_CLEC9A	CALM1	MYLK	1,59182E-03	28562,6	2,75749E-02	989	2,10272E+00	4234	9,89356E-01	40567	8,27962E-01	53811	0	43212
Myeloid_vs_CD4_Responder	Macro_NLRP3	CD4(CRTAM- T)	VCAN	CD44	1,59619E-03	13805	1,14634E-02	5332	2,44258E+00	2351	1,91474E+00	11973	9,35109E-01	6157	0	43212
Myeloid_vs_CD4_Responder	pDC_LILRA4	CD4(CRTAM- T)	APP	RPSA	1,60615E-03	13943,2	1,16906E-02	5126	1,51133E+00	11992	2,48006E+00	4935	9,39024E-01	4451	0	43212
Myeloid_vs_CD4_Responder	CD4(GZMK+ Teff)	Macro_NLRP3	RPS19	C5AR1	1,60811E-03	18060,6	6,70588E-03	13293	1,98193E+00	5258	1,35901E+00	26313	9,46517E-01	2227	0	43212
Myeloid_vs_CD4_Responder	Macro_FOLR2-APOE+	CD4(ISG+ Treg)	HLA-DQA1	LAG3	1,60825E-03	16063,6	1,01130E-02	6716	1,51126E+00	11996	2,26436E+00	6985	9,23361E-01	11409	0	43212
Myeloid_vs_CD4_Responder	Macro_ISG15	CD4(GZMK+ Teff)	SPP1	CD44	1,61351E-03	15803	8,28317E-03	9449	1,61881E+00	9893	2,54178E+00	4455	9,21971E-01	12006	0	43212
Myeloid_vs_CD4_Responder	Macro_IFI27	CD4(ISG+ Treg)	HLA-DRB1	LAG3	1,61404E-03	14677,8	9,22281E-03	7862	1,51087E+00	12007	2,54187E+00	4452	9,35710E-01	5856	0	43212
Myeloid_vs_CD4_Responder	pDC_LILRA4	CD4(AREG+ Tm)	APP	RPSA	1,61773E-03	14014,4	1,16841E-02	5134	1,51064E+00	12014	2,44106E+00	5253	9,39009E-01	4459	0	43212
Myeloid_vs_CD4_Responder	CD4(CXCL13+ Tfh)	pDC_LILRA4	HLA-C	NOTCH4	1,62499E-03	22190,8	1,57014E-02	2993	1,41992E+00	14230	1,42292E+00	24151	8,88072E-01	26368	0	43212
Myeloid_vs_CD4_Responder	Macro_OLFML3	CD4(TNFRSF9+ Treg)	FN1	ITGA4_ITGB1	1,63611E-03	20169,8	9,90220E-03	6970	1,79347E+00	7344	1,67713E+00	16919	8,88003E-01	26404	0	43212
Myeloid_vs_CD4_Responder	Macro_FOLR2-APOE+	CD4(IL26+ Th17)	HLA-DRB5	LAG3	1,64002E-03	16515,4	8,70830E-03	8694	1,50871E+00	12056	2,22900E+00	7394	9,23796E-01	11221	0	43212
Myeloid_vs_CD4_Responder	Macro_FOLR2-APOE+	CD4(Tn)	SPP1	CD44	1,64483E-03	16448,8	8,25460E-03	9495	1,66622E+00	9137	2,14894E+00	8335	9,21846E-01	12065	0	43212
Myeloid_vs_CD4_Responder	CD4(CRTAM- T)	Mono_CD16	CD99	PILRA	1,66419E-03	19837,8	7,00929E-03	12470	2,00728E+00	5045	1,35315E+00	26494	9,22067E-01	11968	0	43212
Myeloid_vs_CD4_Responder	CD4(IL26+ Th17)	Macro_FOLR2+APOE-	HMGB1	CD163	1,66544E-03	22682,8	4,26280E-03	26206	1,80112E+00	7243	1,35305E+00	26498	9,26037E-01	10255	0	43212
Myeloid_vs_CD4_Responder	cDC_LAMP3	CD4(ISG+ Treg)	HLA-DPB1	LAG3	1,66631E-03	15174,4	9,16155E-03	7959	1,50631E+00	12105	2,43254E+00	5350	9,32710E-01	7246	0	43212
Myeloid_vs_CD4_Responder	CD4(IL26+ Th17)	cDC_CLEC9A	CALM1	MYLK	1,67415E-03	29533,2	2,76856E-02	982	2,10764E+00	4186	8,74398E-01	45595	8,28247E-01	53691	0	43212
Myeloid_vs_CD4_Responder	Macro_IER3	CD4(IFNG+ Tfh/Th1)	HLA-DQB1	LAG3	1,68201E-03	15104,6	1,16846E-02	5133	1,50492E+00	12134	2,32315E+00	6369	9,29649E-01	8675	0	43212
Myeloid_vs_CD4_Responder	Macro_ISG15	CD4(Tn)	CXCL10	CXCR3	1,68910E-03	18871,2	1,63342E-02	2748	2,15138E+00	3839	2,55723E+00	4320	8,56916E-01	40237	0	43212
Myeloid_vs_CD4_Responder	CD4(GZMK+ Teff)	Mono_INHBA	CCL5	CCRL2	1,68946E-03	20279,6	1,75125E-02	2401	1,58987E+00	10384	1,60506E+00	18827	8,87617E-01	26574	0	43212
Myeloid_vs_CD4_Responder	CD4(IL26+ Th17)	pDC_LILRA4	TNF	TNFRSF21	1,69026E-03	23744,8	1,47279E-02	3382	3,23371E+00	659	1,86289E+00	12942	8,17658E-01	58529	0	43212
Myeloid_vs_CD4_Responder	CD4(TNFRSF9+ Treg)	Macro_ISG15	LTB	TNFRSF1A	1,69902E-03	24974,8	8,92652E-03	8346	1,07282E+00	26604	1,35431E+00	26455	9,02519E-01	20257	0	43212
Myeloid_vs_CD4_Responder	Macro_NLRP3	CD4(NME1+ T)	S100A8	CD69	1,72480E-03	16218,2	7,09848E-03	12212	1,77974E+00	7509	2,07160E+00	9360	9,29385E-01	8798	0	43212
Myeloid_vs_CD4_Responder	CD4(CRTAM- T)	Macro_FOLR2+APOE-	HMGB1	CD163	1,72731E-03	22346,6	4,20715E-03	26692	1,79057E+00	7387	1,42901E+00	23986	9,25585E-01	10456	0	43212
Myeloid_vs_CD4_Responder	Macro_FOLR2+APOE+	CD4(TGFB1+ Th17)	APOE	SORL1	1,72812E-03	13620,6	8,32576E-03	9355	2,59094E+00	1872	3,11630E+00	1446	9,21480E-01	12218	0	43212
Myeloid_vs_CD4_Responder	Macro_OLFML3	CD4(IFNG+ Tfh/Th1)	HLA-DQA1	CD4	1,73509E-03	17758	4,20435E-03	26716	1,74857E+00	7937	2,47488E+00	4983	9,35526E-01	5942	0	43212
Myeloid_vs_CD4_Responder	Mono_CD14	CD4(Tn)	S100A9	ITGB2	1,73575E-03	16348,6	4,09386E-03	27774	3,19686E+00	714	3,43120E+00	779	9,28315E-01	9264	0	43212
Myeloid_vs_CD4_Responder	Macro_OLFML3	CD4(TNFRSF9+ Treg)	HLA-DRB5	LAG3	1,73996E-03	21901,6	4,20299E-03	26731	1,57375E+00	10679	2,47278E+00	5001	8,93864E-01	23885	0	43212
Myeloid_vs_CD4_Responder	Mono_CD14	CD4(TNF+ T)	S100A9	ITGB2	1,74462E-03	18862	3,34342E-03	37306	3,13744E+00	781	3,47241E+00	708	9,21278E-01	12303	0	43212
Myeloid_vs_CD4_Responder	Macro_ISG15	CD4(AREG+ Tm)	S100A8	CD69	1,74481E-03	15519,6	8,28081E-03	9455	1,49935E+00	12248	2,34619E+00	6149	9,34276E-01	6534	0	43212
Myeloid_vs_CD4_Responder	CD4(IL26+ Th17)	Macro_FOLR2+APOE+	CD52	SIGLEC10	1,74537E-03	24541,8	7,53270E-03	11066	1,75936E+00	7778	8,16199E-01	48404	9,21399E-01	12249	0	43212
Myeloid_vs_CD4_Responder	Macro_OLFML3	CD4(IL26+ Th17)	HLA-DQA1	CD4	1,74746E-03	17868,6	4,20129E-03	26754	1,74819E+00	7941	2,41650E+00	5483	9,35504E-01	5953	0	43212
Myeloid_vs_CD4_Responder	Macro_ISG15	CD4(IFNG+ Tfh/Th1)	HLA-DRB1	LAG3	1,75096E-03	13638	1,04398E-02	6305	1,49854E+00	12259	2,93837E+00	2071	9,39339E-01	4343	0	43212
Myeloid_vs_CD4_Responder	Macro_ISG15	CD4(TGFB1+ Th17)	CXCL10	CXCR3	1,75686E-03	18455,8	1,73826E-02	2429	2,16609E+00	3728	2,55055E+00	4378	8,60688E-01	38532	0	43212
Myeloid_vs_CD4_Responder	cDC_LAMP3	CD4(Tn)	CCL17	CCR4	1,75797E-03	28531,8	6,35600E-02	172	3,13593E+00	784	1,38596E+00	25376	7,85644E-01	73115	0	43212
Myeloid_vs_CD4_Responder	CD4(Tn)	Mono_INHBA	RPS19	C5AR1	1,75827E-03	20788,2	5,35237E-03	18874	1,55152E+00	11105	1,34407E+00	26787	9,40515E-01	3963	0	43212
Myeloid_vs_CD4_Responder	CD4(IL26+ Th17)	pDC_LILRA4	HMGB1	TLR9	1,75923E-03	22674,2	3,24208E-02	680	3,13026E+00	790	1,09061E+00	36404	8,74353E-01	32285	0	43212
Myeloid_vs_CD4_Responder	cDC(CD1C)	CD4(AREG+ Tm)	LGALS1	CD69	1,75925E-03	21369,2	4,36971E-03	25319	1,08485E+00	26076	2,14765E+00	8351	9,40754E-01	3888	0	43212
Myeloid_vs_CD4_Responder	CD4(NME1+ T)	Macro_ISG15	HMGB1	CD163	1,76221E-03	21137,4	4,19548E-03	26799	1,54494E+00	11250	1,81288E+00	13918	9,25490E-01	10508	0	43212
Myeloid_vs_CD4_Responder	Macro_OLFML3	CD4(IFNG+ Tfh/Th1)	C3	CD46	1,76418E-03	18770,6	9,11796E-03	8050	2,14683E+00	3873	1,91852E+00	11913	8,87053E-01	26805	0	43212
Myeloid_vs_CD4_Responder	Macro_FOLR2+APOE+	CD4(NME1+ T)	C1QB	C1QBP	1,78365E-03	13876,6	7,06306E-03	12317	2,29231E+00	3027	2,75505E+00	2986	9,31425E-01	7841	0	43212
Myeloid_vs_CD4_Responder	Macro_ISG15	CD4(ISG+ Treg)	HLA-DRA	LAG3	1,78763E-03	14851	8,49516E-03	9055	1,49633E+00	12324	2,70599E+00	3271	9,34613E-01	6393	0	43212
Myeloid_vs_CD4_Responder	Mono_CD14	CD4(AREG+ Tm)	S100A9	ITGB2	1,79831E-03	15462	4,45460E-03	24631	3,22542E+00	672	3,42140E+00	793	9,31074E-01	8002	0	43212
Myeloid_vs_CD4_Responder	Macro_IFI27	CD4(IL26+ Th17)	HLA-DPB1	LAG3	1,80017E-03	16285,6	8,24055E-03	9523	1,49544E+00	12346	2,21794E+00	7510	9,29308E-01	8837	0	43212
Myeloid_vs_CD4_Responder	cDC(CD1C)	CD4(IL26+ Th17)	HLA-DQA2	CD4	1,80503E-03	22057,4	4,18037E-03	26928	1,36754E+00	15738	2,37898E+00	5821	9,06638E-01	18588	0	43212
Myeloid_vs_CD4_Responder	cDC(CD1C)	CD4(IL26+ Th17)	HLA-DQA2	LAG3	1,80537E-03	17542,6	1,15725E-02	5242	1,51039E+00	12023	2,54309E+00	4438	8,96377E-01	22798	0	43212
Myeloid_vs_CD4_Responder	cDC_LAMP3	CD4(IFNG+ Tfh/Th1)	HLA-DPA1	LAG3	1,81392E-03	14103,4	1,04452E-02	6303	1,49451E+00	12370	2,59457E+00	4027	9,38701E-01	4605	0	43212
Myeloid_vs_CD4_Responder	cDC(CD1C)	CD4(IL26+ Th17)	HLA-DMB	CD4	1,81511E-03	20634,4	4,21229E-03	26635	1,62881E+00	9727	2,29424E+00	6690	9,10483E-01	16908	0	43212
Myeloid_vs_CD4_Responder	Mono_CD14	CD4(NME1+ T)	VCAN	SELL	1,81548E-03	13916,8	1,73509E-02	2444	2,97909E+00	1005	2,54385E+00	4427	9,06847E-01	18496	0	43212
Myeloid_vs_CD4_Responder	Macro_IER3	CD4(CXCL13+ Tfh)	CCL3	CCR4	1,81668E-03	13678,2	2,60606E-02	1106	2,55952E+00	1964	2,54384E+00	4428	9,08720E-01	17681	0	43212
Myeloid_vs_CD4_Responder	Macro_OLFML3	CD4(AREG+ Tm)	APOE	LSR	1,82084E-03	16592,6	1,29558E-02	4275	1,91998E+00	5857	2,81319E+00	2644	8,86650E-01	26975	0	43212
Myeloid_vs_CD4_Responder	Macro_OLFML3	CD4(CXCL13+ Tfh)	C3	CD46	1,82185E-03	18887,8	9,04356E-03	8179	2,14433E+00	3896	1,90299E+00	12174	8,86642E-01	26978	0	43212
Myeloid_vs_CD4_Responder	Macro_NLRP3	CD4(GZMK+ Teff)	VCAN	CD44	1,83122E-03	14188	1,08795E-02	5880	2,39156E+00	2567	1,89093E+00	12400	9,33504E-01	6881	0	43212
Myeloid_vs_CD4_Responder	cDC(CD1C)	CD4(IL26+ Th17)	CXCL16	CXCR6	1,83234E-03	16456,6	1,54881E-02	3071	1,49106E+00	12458	2,31762E+00	6421	9,09975E-01	17121	0	43212
Myeloid_vs_CD4_Responder	Macro_FOLR2+APOE-	CD4(ISG+ Treg)	HLA-DRB1	LAG3	1,83411E-03	15034,6	9,11172E-03	8063	1,49334E+00	12405	2,41994E+00	5451	9,35345E-01	6042	0	43212
Myeloid_vs_CD4_Responder	Macro_ISG15	CD4(AREG+ Tm)	SPP1	CD44	1,84456E-03	16151,2	8,05429E-03	9898	1,59254E+00	10340	2,48600E+00	4883	9,20957E-01	12423	0	43212
Myeloid_vs_CD4_Responder	CD4(ISG+ Treg)	Macro_LYVE1	LGALS9	LRP1	1,84527E-03	19461,8	8,17603E-03	9645	2,56336E+00	1943	1,73938E+00	15462	8,86510E-01	27047	0	43212
Myeloid_vs_CD4_Responder	CD4(CRTAM- T)	pDC_LILRA4	HMGB1	TLR9	1,84824E-03	22142,2	3,19975E-02	705	3,11971E+00	804	1,16656E+00	33378	8,73629E-01	32612	0	43212

Myeloid_vs_CD4_Post_R

Myeloid_vs_CD4_Responder	Macro_OLFML3	CD4(TNFRSF9+ Treg)	APOE	SORL1	1,85176E-03	20814,2	4,16544E-03	27066	1,86425E+00	6482	2,78071E+00	2833	8,92483E-01	24478	0	43212
Myeloid_vs_CD4_Responder	CD4(NME1+ T)	pDC_LILRA4	TNF	PTPRS	1,85281E-03	25141,4	3,01769E-02	805	4,42515E+00	167	1,97460E+00	10920	7,91208E-01	70603	0	43212
Myeloid_vs_CD4_Responder	CD4(ISG+ Treg)	cDC(CD1C)	CSF1	CSF2RA	1,86266E-03	22014	1,71385E-02	2500	2,01682E+00	4962	1,92206E+00	11840	8,40996E-01	47556	0	43212
Myeloid_vs_CD4_Responder	Macro_LYVE1	CD4(ISG+ Treg)	HLA-DPB1	LAG3	1,86384E-03	15799	9,06471E-03	8133	1,49110E+00	12456	2,19342E+00	7804	9,32376E-01	7390	0	43212
Myeloid_vs_CD4_Responder	Macro_LYVE1	CD4(IL26+ Th17)	HLA-DRB1	LAG3	1,86677E-03	16326,8	8,24186E-03	9522	1,49093E+00	12461	2,09860E+00	8985	9,32244E-01	7454	0	43212
Myeloid_vs_CD4_Responder	cDC(CD1C)	CD4(TGFB1+ Th17)	LGALS1	CD69	1,87721E-03	16462,2	5,77178E-03	16830	1,33856E+00	16615	2,62712E+00	3789	9,48050E-01	1865	0	43212
Myeloid_vs_CD4_Responder	CD4(AREG+ Tm)	Macro_ISG15	ANXA1	FPR2_FPR3	1,87791E-03	22305,8	9,11489E-03	8056	1,61631E+00	9938	1,33511E+00	27142	8,95485E-01	23181	0	43212
Myeloid_vs_CD4_Responder	Macro_FOLR2+APOE-	CD4(ISG+ Treg)	HLA-DPA1	LAG3	1,87972E-03	15139,6	9,11298E-03	8059	1,49020E+00	12483	2,40672E+00	5570	9,34656E-01	6374	0	43212
Myeloid_vs_CD4_Responder	CD4(TNFRSF9+ Treg)	Mono_CD16	HLA-C	LILRA3	1,88621E-03	21489,6	1,87195E-02	2111	2,58287E+00	1895	8,29841E-01	47736	9,20787E-01	12494	0	43212
Myeloid_vs_CD4_Responder	Macro_OLFML3	CD4(CXCL13+ Tfh)	HLA-DQA2	CD4	1,88692E-03	22628	4,15494E-03	27168	1,54266E+00	11286	1,87043E+00	12770	9,06379E-01	18704	0	43212
Myeloid_vs_CD4_Responder	CD4(IL26+ Th17)	Macro_LYVE1	SPN	SIGLEC1	1,88773E-03	36766,2	1,29339E-02	4288	2,19554E+00	3548	6,89669E-01	55058	7,74665E-01	77725	0	43212
Myeloid_vs_CD4_Responder	pDC_LILRA4	CD4(IL26+ Th17)	APP	RPSA	1,88858E-03	14431,4	1,14827E-02	5317	1,48929E+00	12498	2,31453E+00	6462	9,38509E-01	4668	0	43212
Myeloid_vs_CD4_Responder	Macro_FOLR2+APOE+	CD4(IL26+ Th17)	HLA-DRB1	LAG3	1,89391E-03	16551	8,22954E-03	9540	1,48867E+00	12507	2,02859E+00	10024	9,32196E-01	7472	0	43212
Myeloid_vs_CD4_Responder	cDC(CD1C)	CD4(TNFRSF9+ Treg)	HLA-DQA1	LAG3	1,89701E-03	21578,6	4,39085E-03	25140	1,55628E+00	11028	2,91634E+00	2177	8,88129E-01	26336	0	43212
Myeloid_vs_CD4_Responder	Macro_LYVE1	CD4(IFNG+ Tfh/Th1)	HLA-DQB1	LAG3	1,89807E-03	15329,6	1,15284E-02	5277	1,48842E+00	12514	2,28590E+00	6765	9,29208E-01	8880	0	43212
Myeloid_vs_CD4_Responder	Mono_CD14	CD4(CRTAM- T)	S100A9	ITGB2	1,89884E-03	16137	4,17608E-03	26970	3,20337E+00	703	3,41198E+00	815	9,28974E-01	8985	0	43212
Myeloid_vs_CD4_Responder	cDC(CD1C)	CD4(TNFRSF9+ Treg)	CD86	CTLA4	1,90925E-03	14666	1,55820E-02	3041	1,87900E+00	6295	2,34138E+00	6190	9,15715E-01	14592	0	43212
Myeloid_vs_CD4_Responder	cDC_LAMP3	CD4(ISG+ Treg)	CCL17	CCR4	1,91741E-03	29767,4	5,75816E-02	216	3,11172E+00	819	1,31287E+00	27926	7,77209E-01	76664	0	43212
Myeloid_vs_CD4_Responder	CD4(GZMK+ Teff)	Macro_ISG15	CCL5	CCR1	1,91943E-03	20079,8	1,74957E-02	2403	1,62052E+00	9868	1,64799E+00	17655	8,86010E-01	27261	0	43212
Myeloid_vs_CD4_Responder	cDC(CD1C)	CD4(TNFRSF9+ Treg)	CXCL16	CXCR6	1,92825E-03	20136,6	1,08317E-02	5924	1,26689E+00	18910	2,10446E+00	8910	8,94214E-01	23727	0	43212
Myeloid_vs_CD4_Responder	Macro_IER3	CD4(IFNG+ Tfh/Th1)	HLA-DPA1	LAG3	1,93517E-03	14364,4	1,03796E-02	6385	1,48632E+00	12576	2,47472E+00	4985	9,38519E-01	4664	0	43212
Myeloid_vs_CD4_Responder	CD4(TNF+ T)	Mono_CD14	CSF1	CSF3R	1,94423E-03	29910,8	9,92744E-03	6945	1,58579E+00	10468	1,88072E+00	12591	7,77961E-01	76338	0	43212
Myeloid_vs_CD4_Responder	Mono_CD14	CD4(Tn)	VCAN	ITGB1	1,95676E-03	14051,4	1,24845E-02	4541	2,93988E+00	1066	2,60301E+00	3965	9,09171E-01	17473	0	43212
Myeloid_vs_CD4_Responder	cDC(CD1C)	CD4(TNFRSF9+ Treg)	HLA-DQB1	LAG3	1,95883E-03	22886,8	4,13285E-03	27372	1,42521E+00	14119	2,76242E+00	2949	8,87120E-01	26782	0	43212
Myeloid_vs_CD4_Responder	Macro_OLFML3	CD4(TNFRSF9+ Treg)	HLA-DQA2	CD4	1,96134E-03	22783,8	4,13247E-03	27379	1,54031E+00	11337	1,85035E+00	13189	9,06149E-01	18802	0	43212
Myeloid_vs_CD4_Responder	CD4(NME1+ T)	Macro_ISG15	HMGB1	TLR2	1,96672E-03	25911	4,76992E-03	22298	1,32407E+00	17042	1,32834E+00	27394	9,04083E-01	19609	0	43212
Myeloid_vs_CD4_Responder	CD4(CRTAM- T)	Mono_CD16	RPS19	C5AR1	1,97571E-03	18444,2	6,67432E-03	13402	1,91216E+00	5935	1,32765E+00	27419	9,46398E-01	2253	0	43212
Myeloid_vs_CD4_Responder	CD4(TNFRSF9+ Treg)	cDC(CD1C)	CD28	CD86	1,97643E-03	19839	1,11536E-02	5654,5	1,63185E+00	9680,5	1,84825E+00	13226,5	8,85603E-01	27421,5	0	43212
Myeloid_vs_CD4_Responder	CD4(TGFB1+ Th17)	Mono_CD16	HLA-B	LILRB2	1,97643E-03	18306	5,69340E-03	17161	2,29375E+00	3023	1,32764E+00	27421	9,53330E-01	713	0	43212
Myeloid_vs_CD4_Responder	cDC(CD1C)	CD4(TNFRSF9+ Treg)	CD86	CD28	1,97679E-03	19839	1,11536E-02	5654,5	1,63185E+00	9680,5	1,84825E+00	13226,5	8,85603E-01	27421,5	0	43212
Myeloid_vs_CD4_Responder	Macro_OLFML3	CD4(NME1+ T)	C3	CD46	1,97931E-03	19160,6	8,85759E-03	8453	2,13810E+00	3947	1,87097E+00	12762	8,85594E-01	27429	0	43212
Myeloid_vs_CD4_Responder	Macro_OLFML3	CD4(TNF+ T)	FN1	ITGA4_ITGB1	1,98328E-03	19764,2	9,43232E-03	7571	1,77777E+00	7539	1,85702E+00	13059	8,85562E-01	27440	0	43212
Myeloid_vs_CD4_Responder	CD4(ISG+ Treg)	Mono_CD14	CSF1	CSF3R	1,99676E-03	23449,2	1,93135E-02	1981	1,84755E+00	6673	1,88081E+00	12590	8,30133E-01	52790	0	43212
Myeloid_vs_CD4_Responder	Macro_OLFML3	CD4(ISG+ Treg)	APOE	LSR	2,00944E-03	16794	1,26409E-02	4455	1,91428E+00	5913	2,77397E+00	2878	8,85407E-01	27512	0	43212
Myeloid_vs_CD4_Responder	cDC(CD1C)	CD4(ISG+ Treg)	HLA-DQA2	LAG3	2,01310E-03	15992,2	1,34589E-02	4012	1,58758E+00	10427	2,88525E+00	2315	9,03183E-01	19995	0	43212
Myeloid_vs_CD4_Responder	CD4(TNFRSF9+ Treg)	cDC_CLEC9A	CD52	SIGLEC10	2,01456E-03	27432,2	4,70124E-03	22783	1,14781E+00	23392	1,32406E+00	27526	9,02543E-01	20248	0	43212
Myeloid_vs_CD4_Responder	CD4(Tn)	Mast	CD55	ADGRE2	2,01492E-03	19673	1,29782E-02	4256	2,03656E+00	4786	1,61400E+00	18584	8,85361E-01	27527	0	43212
Myeloid_vs_CD4_Responder	Macro_IER3	CD4(IL26+ Th17)	CCL3	CCR4	2,01834E-03	13172	2,96476E-02	843	2,61391E+00	1800	2,52383E+00	4589	9,13929E-01	15416	0	43212
Myeloid_vs_CD4_Responder	CD4(ISG+ Treg)	Mono_CD14	CIRBP	TREM1	2,01859E-03	21913,4	9,31381E-03	7727	1,68301E+00	8857	1,32361E+00	27537	8,97655E-01	22234	0	43212
Myeloid_vs_CD4_Responder	cDC(CD1C)	CD4(ISG+ Treg)	ICAM1	IL2RG	2,01969E-03	23369,6	5,68719E-03	17181	1,12004E+00	24551	1,95862E+00	11216	9,01494E-01	20688	0	43212
Myeloid_vs_CD4_Responder	CD4(IL26+ Th17)	cDC_CLEC9A	CD52	SIGLEC10	2,02115E-03	25849,2	5,17817E-03	19783	1,23314E+00	20145	1,32341E+00	27544	9,06710E-01	18562	0	43212
Myeloid_vs_CD4_Responder	cDC(CD1C)	CD4(ISG+ Treg)	CD86	CTLA4	2,02703E-03	15654,2	1,48376E-02	3334	1,84093E+00	6744	2,06209E+00	9511	9,13807E-01	15470	0	43212
Myeloid_vs_CD4_Responder	CD4(TNF+ T)	Macro_IFI27	TNFSF9	HLA-DPA1	2,02850E-03	21723	5,59041E-03	17623	1,40341E+00	14722	2,41553E+00	5494	8,85268E-01	27564	0	43212
Myeloid_vs_CD4_Responder	CD4(TGFB1+ Th17)	pDC_LILRA4	HMGB1	TLR9	2,03069E-03	23122,4	3,08748E-02	762	3,09172E+00	843	1,06834E+00	37316	8,71644E-01	33479	0	43212
Myeloid_vs_CD4_Responder	CD4(ISG+ Treg)	Macro_NLRP3	HLA-F	LILRB2	2,03403E-03	25213,8	5,05095E-03	20510	1,50752E+00	12081	1,32235E+00	27579	8,96678E-01	22687	0	43212
Myeloid_vs_CD4_Responder	CD4(TNF+ T)	Macro_FOLR2+APOE+	TNFSF9	HLA-DPA1	2,04104E-03	22477,8	5,58237E-03	17674	1,40126E+00	14778	2,08882E+00	9127	8,85195E-01	27598	0	43212
Myeloid_vs_CD4_Responder	Macro_OLFML3	CD4(ISG+ Treg)	CXCL9	CXCR3	2,04178E-03	18422	2,35242E-02	1350	1,71464E+00	8408	1,93914E+00	11540	8,85191E-01	27600	0	43212
Myeloid_vs_CD4_Responder	Mono_CD14	CD4(NME1+ T)	VCAN	ITGA4	2,04698E-03	14556,4	1,33938E-02	4045	2,94510E+00	1058	2,52089E+00	4611	9,03479E-01	19856	0	43212
Myeloid_vs_CD4_Responder	cDC(CD1C)	CD4(ISG+ Treg)	CXCL16	CXCR6	2,04845E-03	19131	1,23860E-02	4623	1,34172E+00	16524	2,02108E+00	10138	9,00391E-01	21158	0	43212
Myeloid_vs_CD4_Responder	Macro_OLFML3	CD4(NME1+ T)	CD14	ITGA4	2,05402E-03	24848,2	4,10865E-03	27633	1,50641E+00	12103	1,55345E+00	20189	9,00537E-01	21104	0	43212
Myeloid_vs_CD4_Responder	cDC(CD1C)	CD4(ISG+ Treg)	LYZ	ITGAL	2,05848E-03	20436,8	4,52527E-03	24091	1,56623E+00	10831	2,90357E+00	2241	8,98711E-01	21809	0	43212
Myeloid_vs_CD4_Responder	Macro_OLFML3	CD4(ISG+ Treg)	FN1	ITGA4_ITGB1	2,05997E-03	20847	9,34356E-03	7688	1,77432E+00	7592	1,63101E+00	18094	8,85082E-01	27649	0	43212
Myeloid_vs_CD4_Responder	CD4(IFNG+ Tfh/Th1)	Macro_NLRP3	HMGB1	CD163	2,06109E-03	22649	4,10701E-03	27652	1,58571E+00	10470	1,52164E+00	21076	9,24751E-01	10835	0	43212
Myeloid_vs_CD4_Responder	cDC_LAMP3	CD4(IFNG+ Tfh/Th1)	HLA-DRB5	LAG3	2,06484E-03	15080,4	1,05864E-02	6165	1,47783E+00	12786	2,48308E+00	4911	9,30391E-01	8328	0	43212
Myeloid_vs_CD4_Responder	CD4(IL26+ Th17)	Mono_CD16	HLA-A	LILRB2	2,07588E-03	20980,2	5,40283E-03	18609	2,09129E+00	4323	1,06923E+00	37278	9,49551E-01	1479	0	43212
Myeloid_vs_CD4_Responder	Macro_IFI27	CD4(IL26+ Th17)	HLA-DQA1	LAG3	2,07879E-03	16960,8	9,23057E-03	7852	1,51738E+00	11852	2,09233E+00	9080	9,20068E-01	12808	0	43212
Myeloid_vs_CD4_Responder	cDC_LAMP3	CD4(CXCL13+ Tfh)	CCL19	CXCR3	2,07883E-03	27211	2,95181E-02	853	5,64987E+00	65	1,44373E+00	23492	7,96009E-01	68433	0	43212
Myeloid_vs_CD4_Responder	cDC(CD1C)	CD4(CXCL13+ Tfh)	CD86	CTLA4	2,08129E-03	22842,4	8,01312E-03	9968	1,49192E+00	12436	1,50847E+00	21439	8,86249E-01	27157	0	43212
Myeloid_vs_CD4_Responder	CD4(IL26+ Th17)	Mono_CD16	HLA-C	LILRA3	2,08778E-03	23305	1,81906E-02	2230	2,51789E+00	2091	6,71479E-01	56040	9,19735E-01	12952	0	43212
Myeloid_vs_CD4_Responder	CD4(TNFRSF9+ Treg)	Mono_INHBA	HLA-F	LILRB2	2,09070E-03	27739,4	4,18737E-03	26869	1,41626E+00	14339	1,31830E+00	27731	8,87664E-01	26546	0	43212
Myeloid_vs_CD4_Responder	Macro_FOLR2+APOE+	CD4(IFNG+ Tfh/Th1)	B2M	CD3D	2,09199E-03	40259,8	3,85977E-03	30279	5,88882E-01	54343	3,91411E-01	73454	9,63398E-01	11	0	43212
Myeloid_vs_CD4_Responder	CD4(AREG+ Tm)	Macro_FOLR2+APOE-	HMGB1	CD163	2,09598E-03	23067,6	4,09834E-03	27745	1,76994E+00	7647	1,37135E+00	25871	9,24678E-01	10863	0	43212
Myeloid_vs_CD4_Responder	cDC(CD1C)	CD4(IL26+ Th17)	HLA-DQA1	CD4	2,09787E-03	17488,2	4,09798E-03	27750	1,70366E+00	8570	3,07376E+00	1580	9,34748E-01	6329	0	43212
Myeloid_vs_CD4_Responder	CD4(TNFRSF9+ Treg)	pDC_LILRA4	HMGB1	TLR9	2,09825E-03	23146,4	3,05477E-02	780	3,08356E+00	857	1,07302E+00	37127	8,71047E-01	33756	0	43212
Myeloid_vs_CD4_Responder	CD4(IL26+ Th17)	Mono_CD16	B2M	LILRB2	2,09973E-03	18667,2	5,51529E-03	18012	2,26429E+00	3160	1,29195E+00	28713	9,57669E-01	239	0	43212
Myeloid_vs_CD4_Responder	CD4(TNFRSF9+ Treg)	Macro_ISG15	HLA-A	LILRB1	2,10279E-03	23750,8	4,09543E-03	27763	1,45432E+00	13330	1,35009E+00	26586	9,31376E-01	7863	0	43212
Myeloid_vs_CD4_Responder	CD4(TNF+ T)	Macro_LYVE1	TNFSF9	HLA-DPA1	2,11266E-03	22486	5,53558E-03	17911	1,38874E+00	15132	2,14495E+00	8386	8,84766E-01	27789	0	43212
Myeloid_vs_CD4_Responder	Macro_IFI27	CD4(IL26+ Th17)	HLA-DQB1	LAG3	2,11589E-03	17029	9,13809E-03	8005	1,47397E+00	12866	2,11812E+00	8726	9,21173E-01	12336	0	43212
Myeloid_vs_CD4_Responder	CD4(CRTAM- T)	Mast	HSPA8	ADRB2	2,11911E-03	21168,6	1,41262E-02	3636	1,47373E+00	12871	1,13399E+00	34642	9,23201E-01	11482	0	43212

Myeloid_vs_CD4_Responder	CD4(Tn)	pDC_LILRA4	MAML2	NOTCH4	2,12234E-03	34745,8	2,45794E-02	1237	1,61626E+00	9940	1,86549E+00	12876	6,96480E-01	106464	0	43212
Myeloid_vs_CD4_Responder	CD4(TGFB1+ Th17)	Mono_CD16	CD99	PILRA	2,12298E-03	20483,2	6,86429E-03	12877	1,99106E+00	5176	1,28803E+00	28865	9,21313E-01	12286	0	43212
Myeloid_vs_CD4_Responder	CD4(ISG+ Treg)	Macro_ISG15	HLA-A	LILRB1	2,12485E-03	23712,4	4,14328E-03	27272	1,48619E+00	12582	1,31578E+00	27821	9,31746E-01	7675	0	43212
Myeloid_vs_CD4_Responder	cDC_LAMP3	CD4(TNF+ T)	CCL19	CXCR3	2,13244E-03	26457	2,91614E-02	864	5,64793E+00	68	1,58887E+00	19248	7,95020E-01	68893	0	43212
Myeloid_vs_CD4_Responder	Macro_OLFML3	CD4(NME1+ T)	HLA-DQA2	CD4	2,13633E-03	23300,8	4,08621E-03	27851	1,53547E+00	11439	1,76087E+00	15003	9,05669E-01	18999	0	43212
Myeloid_vs_CD4_Responder	CD4(AREG+ Tm)	Mono_CD14	ANXA1	DYSF	2,13710E-03	21110	1,49099E-02	3299	1,75419E+00	7855	1,44916E+00	23331	8,84613E-01	27853	0	43212
Myeloid_vs_CD4_Responder	cDC(CD1C)	CD4(IFNG+ Tfh/Th1)	HLA-DQA2	CD4	2,14671E-03	21941,8	4,18341E-03	26899	1,36792E+00	15725	2,43736E+00	5296	9,06669E-01	18577	0	43212
Myeloid_vs_CD4_Responder	cDC(CD1C)	CD4(IFNG+ Tfh/Th1)	HLA-DQA2	LAG3	2,14709E-03	14105,6	1,71717E-02	2492	1,73949E+00	8060	3,25967E+00	1087	9,13324E-01	15677	0	43212
Myeloid_vs_CD4_Responder	cDC_LAMP3	CD4(NME1+ T)	CCL17	CCR4	2,14717E-03	31659,6	4,89270E-02	303	3,07669E+00	867	1,22288E+00	31237	7,62790E-01	82679	0	43212
Myeloid_vs_CD4_Responder	cDC(CD1C)	CD4(IFNG+ Tfh/Th1)	HLA-DQA1	CD4	2,14748E-03	17442,4	4,10097E-03	27716	1,70404E+00	8566	3,13214E+00	1405	9,34771E-01	6313	0	43212
Myeloid_vs_CD4_Responder	Macro_OLFML3	CD4(ISG+ Treg)	CXCL16	CXCR6	2,16022E-03	21786,2	1,33269E-02	4073	1,43088E+00	13943	1,31335E+00	27913	9,03626E-01	19790	0	43212
Myeloid_vs_CD4_Responder	cDC(CD1C)	CD4(IFNG+ Tfh/Th1)	HLA-DMB	CD4	2,16022E-03	20502,4	4,21536E-03	26602	1,62919E+00	9718	2,35262E+00	6081	9,10513E-01	16899	0	43212
Myeloid_vs_CD4_Responder	cDC(CD1C)	CD4(IFNG+ Tfh/Th1)	CD86	CTLA4	2,16138E-03	16424,8	1,34553E-02	4014	1,77024E+00	7645	2,02416E+00	10095	9,09877E-01	17158	0	43212
Myeloid_vs_CD4_Responder	Macro_FOLR2-APOE+	CD4(TGFB1+ Th17)	SPP1	CD44	2,16197E-03	17412	7,79677E-03	10442	1,61760E+00	9920	1,99639E+00	10549	9,19766E-01	12937	0	43212
Myeloid_vs_CD4_Responder	cDC(CD1C)	CD4(TNFRSF9+ Treg)	GRN	TNFRSF1B	2,16447E-03	19738,4	4,08016E-03	27924	1,40902E+00	14564	2,45457E+00	5140	9,31398E-01	7852	0	43212
Myeloid_vs_CD4_Responder	Macro_NLRP3	CD4(IFNG+ Tfh/Th1)	S100A8	CD69	2,16590E-03	16696,4	6,83534E-03	12943	1,76248E+00	7737	2,01404E+00	10240	9,28135E-01	9350	0	43212
Myeloid_vs_CD4_Responder	cDC(CD1C)	CD4(IFNG+ Tfh/Th1)	HLA-DQB2	LAG3	2,17146E-03	15278,2	2,70612E-02	1025	1,94735E+00	5596	2,91035E+00	2204	8,92742E-01	24354	0	43212
Myeloid_vs_CD4_Responder	cDC(CD1C)	CD4(IFNG+ Tfh/Th1)	CXCL16	CXCR6	2,17885E-03	19344,6	1,17114E-02	5115	1,30924E+00	17522	2,11836E+00	8721	8,97851E-01	22153	0	43212
Myeloid_vs_CD4_Responder	cDC(CD1C)	CD4(ISG+ Treg)	GRN	TNFRSF1B	2,17885E-03	19980,6	4,07662E-03	27961	1,40839E+00	14579	2,33229E+00	6283	9,31371E-01	7868	0	43212
Myeloid_vs_CD4_Responder	Mast	CD4(IFNG+ Tfh/Th1)	TIMP3	CD44	2,18175E-03	17341,6	2,89186E-02	874	3,17015E+00	742	1,40192E+00	24836	9,10145E-01	17044	0	43212
Myeloid_vs_CD4_Responder	pDC_LILRA4	CD4(CXCL13+ Tfh)	APP	RPSA	2,18232E-03	14503,4	1,12948E-02	5490	1,46938E+00	12968	2,36564E+00	5968	9,38031E-01	4879	0	43212
Myeloid_vs_CD4_Responder	CD4(TNFRSF9+ Treg)	Mono_CD14	CIRBP	TREM1	2,18470E-03	22723,8	8,66301E-03	8765	1,61510E+00	9967	1,31161E+00	27976	8,94279E-01	23699	0	43212
Myeloid_vs_CD4_Responder	cDC(CD1C)	CD4(IFNG+ Tfh/Th1)	LYZ	ITGAL	2,18588E-03	21740,6	4,08144E-03	27908	1,53299E+00	11490	2,90653E+00	2229	8,93915E-01	23864	0	43212
Myeloid_vs_CD4_Responder	cDC_CLEC9A	CD4(IL26+ Th17)	HLA-DRB1	LAG3	2,18958E-03	15237	8,12214E-03	9755	1,46895E+00	12979	2,82634E+00	2583	9,31780E-01	7656	0	43212
Myeloid_vs_CD4_Responder	Mast	CD4(NME1+ T)	TIMP3	CD44	2,19168E-03	17448,8	2,88946E-02	876	3,16960E+00	743	1,38663E+00	25351	9,10111E-01	17062	0	43212
Myeloid_vs_CD4_Responder	cDC(CD1C)	CD4(NME1+ T)	HLA-DRA	LAG3	2,19292E-03	18104,2	4,95999E-03	21066	1,58434E+00	10496	3,17062E+00	1310	9,16120E-01	14437	0	43212
Myeloid_vs_CD4_Responder	CD4(NME1+ T)	Macro_NLRP3	GNAI2	C5AR1	2,19370E-03	24125,2	4,28157E-03	26054	2,08474E+00	4367	1,59838E+00	18994	8,84135E-01	27999	0	43212
Myeloid_vs_CD4_Responder	cDC(CD1C)	CD4(NME1+ T)	HLA-DQA1	LAG3	2,19448E-03	18242	5,98968E-03	15907	1,62301E+00	9830	2,94276E+00	2057	9,02651E-01	20204	0	43212
Myeloid_vs_CD4_Responder	cDC(CD1C)	CD4(NME1+ T)	HLA-DRB1	LAG3	2,19527E-03	20179,4	4,63607E-03	23239	1,36638E+00	15774	2,90012E+00	2259	9,11654E-01	16413	0	43212
Myeloid_vs_CD4_Responder	cDC(CD1C)	CD4(NME1+ T)	HLA-DQB1	LAG3	2,19605E-03	19287,4	5,63774E-03	17409	1,49194E+00	12434	2,78885E+00	2798	9,01758E-01	20584	0	43212
Myeloid_vs_CD4_Responder	cDC(CD1C)	CD4(NME1+ T)	HLA-DPB1	LAG3	2,19683E-03	18470,8	5,12242E-03	20107	1,53882E+00	11369	3,12355E+00	1425	9,12007E-01	16241	0	43212
Myeloid_vs_CD4_Responder	Macro_IER3	CD4(TNFRSF9+ Treg)	CCL3	CCR4	2,19828E-03	14121,8	2,38130E-02	1312	2,52543E+00	2064	2,50612E+00	4724	9,04910E-01	19297	0	43212
Myeloid_vs_CD4_Responder	cDC(CD1C)	CD4(NME1+ T)	HLA-DPA1	LAG3	2,20587E-03	19562	4,79026E-03	22148	1,41878E+00	14264	2,96671E+00	1963	9,12052E-01	16223	0	43212
Myeloid_vs_CD4_Responder	CD4(NME1+ T)	cDC_CLEC9A	HMGB1	THBD	2,20666E-03	28372,4	4,53769E-03	24009	1,19664E+00	21472	1,39264E+00	25137	8,84067E-01	28032	0	43212
Myeloid_vs_CD4_Responder	cDC(CD1C)	CD4(NME1+ T)	CD86	CTLA4	2,20784E-03	19188,4	1,07730E-02	5974	1,63307E+00	9660	1,71965E+00	15919	9,00337E-01	21177	0	43212
Myeloid_vs_CD4_Responder	CD4(GZMK+ Teff)	Mono_CD16	ANXA1	FPR1	2,20784E-03	20864,4	6,45654E-03	14156	1,81697E+00	7051	1,30988E+00	28035	9,22298E-01	11868	0	43212
Myeloid_vs_CD4_Responder	cDC(CD1C)	CD4(NME1+ T)	HLA-DRB5	LAG3	2,21296E-03	20534,2	4,94903E-03	21131	1,39508E+00	14960	2,81765E+00	2621	9,01369E-01	20747	0	43212
Myeloid_vs_CD4_Responder	CD4(TGFB1+ Th17)	Mono_CD16	B2M	LILRB2	2,21651E-03	18558	5,50218E-03	18071	2,25478E+00	3207	1,30938E+00	28057	9,57621E-01	243	0	43212
Myeloid_vs_CD4_Responder	Macro_OLFML3	CD4(AREG+ Tm)	C3	CD46	2,22443E-03	19357	8,57832E-03	8912	2,12873E+00	4023	1,88201E+00	12561	8,83961E-01	28077	0	43212
Myeloid_vs_CD4_Responder	cDC(CD1C)	CD4(NME1+ T)	LYZ	ITGAL	2,23634E-03	21075,2	4,28786E-03	26004	1,54845E+00	11168	2,92301E+00	2137	8,96232E-01	22855	0	43212
Myeloid_vs_CD4_Responder	CD4(TGFB1+ Th17)	Macro_FOLR2+APOE-	HMGB1	CD163	2,23952E-03	23467,2	4,05953E-03	28115	1,76258E+00	7736	1,33079E+00	27291	9,24346E-01	10982	0	43212
Myeloid_vs_CD4_Responder	CD4(NME1+ T)	Mono_CD16	HLA-C	LILRB2	2,24311E-03	18690	5,63006E-03	17445	2,25366E+00	3216	1,30770E+00	28124	9,49645E-01	1453	0	43212
Myeloid_vs_CD4_Responder	cDC_LAMP3	CD4(IL26+ Th17)	HLA-DRA	LAG3	2,24758E-03	16876,6	7,54876E-03	11040	1,46458E+00	13066	2,09894E+00	8978	9,30909E-01	8087	0	43212
Myeloid_vs_CD4_Responder	CD4(TNF+ T)	Macro_FOLR2+APOE-	TNFSF9	HLA-DPA1	2,25590E-03	22647,6	5,42862E-03	18456	1,36013E+00	15955	2,22236E+00	7459	8,83768E-01	28156	0	43212
Myeloid_vs_CD4_Responder	CD4(TNF+ T)	Mast	CD40LG	CD9	2,25972E-03	19511,6	2,02354E-02	1815	1,91865E+00	5867	1,85542E+00	13084	8,71408E-01	33580	0	43212
Myeloid_vs_CD4_Responder	Macro_ISG15	CD4(IFNG+ Tfh/Th1)	HLA-DPB1	LAG3	2,26717E-03	14266,6	1,01103E-02	6720	1,46392E+00	13095	2,85228E+00	2462	9,35737E-01	5844	0	43212
Myeloid_vs_CD4_Responder	Macro_IFI27	CD4(IFNG+ Tfh/Th1)	HLA-DQA2	LAG3	2,27531E-03	13306,2	2,01086E-02	1839	1,97074E+00	5376	2,75296E+00	2997	9,19373E-01	13107	0	43212
Myeloid_vs_CD4_Responder	cDC_LAMP3	CD4(TGFB1+ Th17)	CCL17	CCR4	2,27698E-03	32968,8	4,31394E-02	389	3,05325E+00	893	1,17591E+00	33044	7,51213E-01	87306	0	43212
Myeloid_vs_CD4_Responder	Macro_OLFML3	CD4(GZMK+ Teff)	FN1	ITGA4_ITGB1	2,28004E-03	20857,2	9,07977E-03	8108	1,76610E+00	7704	1,67258E+00	17046	8,83618E-01	28216	0	43212
Myeloid_vs_CD4_Responder	CD4(ISG+ Treg)	pDC_LILRA4	TNF	TNFRSF21	2,28756E-03	23933	1,38166E-02	3801	3,21862E+00	679	1,95361E+00	11309	8,12848E-01	60664	0	43212
Myeloid_vs_CD4_Responder	CD4(Tn)	Mono_CD16	RPS19	C5AR1	2,30194E-03	18999	6,39971E-03	14368	1,85243E+00	6611	1,30366E+00	28270	9,45322E-01	2534	0	43212
Myeloid_vs_CD4_Responder	CD4(ISG+ Treg)	pDC_LILRA4	COPA	P2RY6	2,31703E-03	29296,2	6,96942E-03	12567	2,02298E+00	4913	1,95743E+00	11235	7,82346E-01	74554	0	43212
Myeloid_vs_CD4_Responder	Mono_INHBA	CD4(IL26+ Th17)	CXCL2	DPP4	2,31820E-03	21746,4	1,84708E-02	2161	2,43635E+00	2378	2,49460E+00	4810	8,22919E-01	56171	0	43212
Myeloid_vs_CD4_Responder	cDC(CD1C)	CD4(CRTAM- T)	LGALS1	CD69	2,31991E-03	22910	4,10731E-03	27651	1,03737E+00	28314	1,97497E+00	10909	9,39005E-01	4464	0	43212
Myeloid_vs_CD4_Responder	Macro_OLFML3	CD4(IFNG+ Tfh/Th1)	HLA-DQB1	CD4	2,32155E-03	18498,6	4,03992E-03	28318	1,65102E+00	9357	2,39645E+00	5671	9,35538E-01	5935	0	43212
Myeloid_vs_CD4_Responder	Macro_OLFML3	CD4(IL26+ Th17)	HLA-DQB1	CD4	2,33181E-03	18618,4	4,03698E-03	28343	1,65064E+00	9361	2,33807E+00	6230	9,35517E-01	5946	0	43212
Myeloid_vs_CD4_Responder	CD4(AREG+ Tm)	Macro_LYVE1	RPS19	C5AR1	2,33221E-03	19227,4	6,74478E-03	13190	1,91794E+00	5875	1,21234E+00	31661	9,46663E-01	2199	0	43212
Myeloid_vs_CD4_Responder	cDC_LAMP3	CD4(CRTAM- T)	CCL17	CCR4	2,34843E-03	33019,6	4,15275E-02	417	3,04673E+00	907	1,20703E+00	31867	7,47637E-01	88695	0	43212
Myeloid_vs_CD4_Responder	Mono_INHBA	CD4(NME1+ T)	CCL3	CCR4	2,35241E-03	17937,4	1,21928E-02	4753	2,19512E+00	3555	2,49257E+00	4834	8,71951E-01	33333	0	43212
Myeloid_vs_CD4_Responder	Macro_IER3	CD4(ISG+ Treg)	HLA-DRB1	LAG3	2,36420E-03	15687,8	8,88854E-03	8407	1,45810E+00	13236	2,24715E+00	7182	9,34591E-01	6402	0	43212
Myeloid_vs_CD4_Responder	cDC_LAMP3	CD4(AREG+ Tm)	CCL17	CCR4	2,37421E-03	33537,8	4,05023E-02	435	3,04258E+00	912	1,16422E+00	33481	7,45272E-01	89649	0	43212
Myeloid_vs_CD4_Responder	Macro_FOLR2-APOE+	CD4(TGFB1+ Th17)	LGALS1	CD69	2,37680E-03	15236,6	6,72347E-03	13254	1,52036E+00	11777	2,26785E+00	6948	9,51683E-01	992	0	43212
Myeloid_vs_CD4_Responder	Macro_IER3	CD4(ISG+ Treg)	CCL3	CCR5	2,37685E-03	21128,6	1,24871E-02	4539	2,56108E+00	1960	2,49003E+00	4851	8,33576E-01	51081	0	43212
Myeloid_vs_CD4_Responder	Macro_FOLR2-APOE+	CD4(IL26+ Th17)	SPP1	CD44	2,37750E-03	17810,8	7,64015E-03	10823	1,60096E+00	10196	1,93728E+00	11568	9,19014E-01	13255	0	43212
Myeloid_vs_CD4_Responder	Macro_ISG15	CD4(Tn)	SPP1	CD44	2,37891E-03	16703	7,63974E-03	10824	1,54497E+00	11248	2,47596E+00	4974	9,19012E-01	13257	0	43212
Myeloid_vs_CD4_Responder	cDC(CD1C)	CD4(TNF+ T)	LGALS1	CD69	2,38997E-03	21891	4,08951E-03	27816	1,03415E+00	28483	2,42341E+00	5424	9,38880E-01	4520	0	43212
Myeloid_vs_CD4_Responder	CD4(TNFRSF9+ Treg)	pDC_LILRA4	TNF	PTPRS	2,39298E-03	27949,4	2,24057E-02	1513	4,37615E+00	181	1,84557E+00	13277	7,65549E-01	81564	0	43212
Myeloid_vs_CD4_Responder	CD4(NME1+ T)	Macro_NLRP3	HMGB1	TLR2	2,39333E-03	25026,4	5,11247E-03	20168	1,39806E+00	14853	1,29778E+00	28491	9,07048E-01	18408	0	43212
Myeloid_vs_CD4_Responder	CD4(ISG+ Treg)	cDC_LAMP3	LTB	CD40	2,39796E-03	21007,6	1,32954E-02	4088	1,56195E+00	10909	1,29743E+00	28502	9,07213E-01	18327	0	43212

Myeloid_vs_CD4_Post_R

Myeloid_vs_CD4_Responder	Macro_IFI27	CD4(IL26+ Th17)	HLA-DPA1	LAG3	2,39933E-03	16306,2	8,06922E-03	9860	1,45629E+00	13286	2,25773E+00	7058	9,30841E-01	8115	0	43212
Myeloid_vs_CD4_Responder	CD4(TNF+ T)	pDC_LILRA4	HMGB1	TLR9	2,40014E-03	21908,4	2,88157E-02	881	3,04038E+00	917	1,27633E+00	29264	8,67733E-01	35268	0	43212
Myeloid_vs_CD4_Responder	CD4(IL26+ Th17)	Mono_CD16	RPS19	C5AR1	2,40145E-03	19241,2	6,70829E-03	13289	1,91955E+00	5860	1,21331E+00	31621	9,46526E-01	2224	0	43212
Myeloid_vs_CD4_Responder	CD4(TGFB1+ Th17)	pDC_LILRA4	COPA	P2RY6	2,40782E-03	29667,2	6,70450E-03	13298	2,01434E+00	4986	1,97456E+00	10922	7,79029E-01	75918	0	43212
Myeloid_vs_CD4_Responder	Macro_FOLR2-APOE+	CD4(ISG+ Treg)	HLA-DPB1	LAG3	2,40994E-03	15860	8,83975E-03	8484	1,45577E+00	13301	2,30734E+00	6546	9,31579E-01	7757	0	43212
Myeloid_vs_CD4_Responder	CD4(CRTAM- T)	Mono_CD16	B2M	LILRB2	2,41314E-03	18696,2	5,47846E-03	18182	2,23758E+00	3304	1,29657E+00	28538	9,57533E-01	245	0	43212
Myeloid_vs_CD4_Responder	CD4(ISG+ Treg)	Macro_LYVE1	TNFSF12	CD163	2,41420E-03	22964	7,66337E-03	10758	2,62603E+00	1766	1,86776E+00	12826	8,43831E-01	46258	0	43212
Myeloid_vs_CD4_Responder	CD4(TNFRSF9+ Treg)	Macro_FOLR2+APOE-	HMGB1	CD163	2,42373E-03	23580,8	4,01653E-03	28563	1,75443E+00	7853	1,33546E+00	27130	9,23973E-01	11146	0	43212
Myeloid_vs_CD4_Responder	Macro_FOLR2+APOE+	CD4(IL26+ Th17)	HLA-DPA1	LAG3	2,43413E-03	17247,8	8,05762E-03	9889	1,45414E+00	13335	1,93102E+00	11671	9,30795E-01	8132	0	43212
Myeloid_vs_CD4_Responder	CD4(NME1+ T)	Macro_NLRP3	RPS19	C5AR1	2,43819E-03	19455,8	6,01314E-03	15809	1,84713E+00	6675	1,29482E+00	28597	9,43689E-01	2986	0	43212
Myeloid_vs_CD4_Responder	CD4(CXCL13+ Tfh)	Mono_CD14	CIRBP	TREM1	2,44373E-03	22888,8	8,62776E-03	8817	1,61143E+00	10018	1,29463E+00	28610	8,94087E-01	23787	0	43212
Myeloid_vs_CD4_Responder	Macro_FOLR2-APOE+	CD4(TNFRSF9+ Treg)	SPP1	CCR8	2,45114E-03	16455,2	8,43675E-02	83	2,02448E+00	4902	2,91872E+00	2167	8,75172E-01	31912	0	43212
Myeloid_vs_CD4_Responder	Macro_ISG15	CD4(CRTAM- T)	S100A8	CD69	2,47436E-03	16504,8	7,78356E-03	10482	1,45187E+00	13391	2,17351E+00	8038	9,32349E-01	7401	0	43212
Myeloid_vs_CD4_Responder	pDC_LILRA4	CD4(Tn)	APP	RPSA	2,48232E-03	14479,4	1,11237E-02	5679	1,45124E+00	13402	2,46830E+00	5039	9,37586E-01	5065	0	43212
Myeloid_vs_CD4_Responder	Mast	CD4(ISG+ Treg)	TIMP3	CD44	2,48402E-03	17819	2,82101E-02	933	3,15392E+00	760	1,34651E+00	26693	9,09125E-01	17497	0	43212
Myeloid_vs_CD4_Responder	CD4(ISG+ Treg)	Macro_ISG15	HLA-F	LILRB1	2,50281E-03	26513,8	5,56561E-03	17759	1,41507E+00	14377	1,29108E+00	28747	8,82973E-01	28474	0	43212
Myeloid_vs_CD4_Responder	CD4(CRTAM- T)	Macro_FOLR2+APOE+	ANXA1	FPR1	2,50631E-03	21731,4	6,66603E-03	13435	1,57411E+00	10672	1,25712E+00	29964	9,23435E-01	11374	0	43212
Myeloid_vs_CD4_Responder	Macro_OLFML3	CD4(ISG+ Treg)	LYZ	ITGAL	2,51939E-03	25469,2	3,99500E-03	28785	1,37898E+00	15411	1,73160E+00	15645	8,92896E-01	24293	0	43212
Myeloid_vs_CD4_Responder	Macro_FOLR2-APOE+	CD4(IL26+ Th17)	HLA-DRB1	LAG3	2,52020E-03	16559,4	8,01456E-03	9963	1,44919E+00	13454	2,15296E+00	8288	9,31355E-01	7880	0	43212
Myeloid_vs_CD4_Responder	Macro_OLFML3	CD4(TNFRSF9+ Treg)	C3	CD46	2,52114E-03	19667,2	8,30035E-03	9414	2,11941E+00	4086	1,86756E+00	12835	8,82261E-01	28789	0	43212
Myeloid_vs_CD4_Responder	CD4(TNFRSF9+ Treg)	Macro_NLRP3	RPS19	C5AR1	2,53488E-03	18923,8	6,65354E-03	13474	1,97175E+00	5359	1,24738E+00	30299	9,46318E-01	2275	0	43212
Myeloid_vs_CD4_Responder	Macro_NLRP3	CD4(AREG+ Tm)	VCAN	CD44	2,53709E-03	14561,8	1,05789E-02	6170	2,36530E+00	2668	1,83515E+00	13477	9,32629E-01	7282	0	43212
Myeloid_vs_CD4_Responder	Macro_ISG15	CD4(TNF+ T)	S100A8	CD69	2,53783E-03	15708	7,74982E-03	10561	1,44865E+00	13478	2,62195E+00	3825	9,32212E-01	7464	0	43212
Myeloid_vs_CD4_Responder	CD4(CRTAM- T)	pDC_LILRA4	HSP90B1	TLR9	2,54251E-03	24402,8	2,80615E-02	944	3,14911E+00	765	1,22219E+00	31271	8,44747E-01	45822	0	43212
Myeloid_vs_CD4_Responder	Macro_FOLR2-APOE+	CD4(TNFRSF9+ Treg)	SPP1	CD44	2,55185E-03	17765,4	7,51527E-03	11105	1,58770E+00	10425	1,99386E+00	10588	9,18398E-01	13497	0	43212
Myeloid_vs_CD4_Responder	Macro_IER3	CD4(IL26+ Th17)	CD14	ITGA4	2,55629E-03	17664,2	7,11270E-03	12171	1,76864E+00	7662	1,83383E+00	13503	9,22556E-01	11773	0	43212
Myeloid_vs_CD4_Responder	CD4(ISG+ Treg)	Mono_CD16	HLA-B	LILRA1	2,56295E-03	23354,4	1,11307E-02	5669	1,89410E+00	6129	8,02143E-01	49114	9,20437E-01	12648	0	43212
Myeloid_vs_CD4_Responder	Macro_OLFML3	CD4(TNFRSF9+ Treg)	C1QB	C1QBP	2,56790E-03	19677,6	3,98558E-03	28895	1,91008E+00	5954	2,66805E+00	3510	9,10738E-01	16817	0	43212
Myeloid_vs_CD4_Responder	Mono_CD14	CD4(GZMK+ Teff)	VCAN	ITGA4	2,56918E-03	15264,8	1,18819E-02	4981	2,90069E+00	1140	2,47927E+00	4942	8,98129E-01	22049	0	43212
Myeloid_vs_CD4_Responder	CD4(ISG+ Treg)	Mono_CD16	HLA-C	LILRA3	2,57410E-03	22034,4	1,87190E-02	2112	2,58281E+00	1896	7,75752E-01	50457	9,20786E-01	12495	0	43212
Myeloid_vs_CD4_Responder	CD4(IL26+ Th17)	cDC_CLEC9A	LTB	CD40	2,58483E-03	24570,6	9,29065E-03	7763	1,29855E+00	17858	1,28627E+00	28933	8,90987E-01	25087	0	43212
Myeloid_vs_CD4_Responder	CD4(AREG+ Tm)	cDC_CLEC9A	CD52	SIGLEC10	2,59154E-03	27512,8	4,75692E-03	22387	1,15777E+00	22968	1,28589E+00	28948	9,03059E-01	20049	0	43212
Myeloid_vs_CD4_Responder	CD4(AREG+ Tm)	Macro_FOLR2+APOE+	CD52	SIGLEC10	2,59275E-03	25728	6,91990E-03	12713	1,68399E+00	8843	7,78676E-01	50320	9,18271E-01	13552	0	43212
Myeloid_vs_CD4_Responder	cDC_CLEC9A	CD4(IFNG+ Tfh/Th1)	HLA-DQA2	LAG3	2,59574E-03	13086,2	1,95167E-02	1943	1,92413E+00	5814	3,34940E+00	906	9,18259E-01	13556	0	43212
Myeloid_vs_CD4_Responder	cDC_LAMP3	CD4(IL26+ Th17)	HLA-DQA1	LAG3	2,60174E-03	18125,6	8,78881E-03	8572	1,44860E+00	13480	1,92408E+00	11800	9,18246E-01	13564	0	43212
Myeloid_vs_CD4_Responder	CD4(ISG+ Treg)	cDC(CD1C)	CD28	CD86	2,60814E-03	21195,6	1,03654E-02	6398,5	1,59521E+00	10305,5	1,67118E+00	17076,5	8,81838E-01	28985,5	0	43212
Myeloid_vs_CD4_Responder	cDC(CD1C)	CD4(ISG+ Treg)	CD86	CD28	2,60859E-03	21195,6	1,03654E-02	6398,5	1,59521E+00	10305,5	1,67118E+00	17076,5	8,81838E-01	28985,5	0	43212
Myeloid_vs_CD4_Responder	Macro_OLFML3	CD4(ISG+ Treg)	HLA-DMB	CD4	2,61941E-03	24241,8	3,97494E-03	29010	1,66405E+00	9167	1,49565E+00	21856	9,08091E-01	17964	0	43212
Myeloid_vs_CD4_Responder	CD4(ISG+ Treg)	Mono_CD16	HLA-A	LILRA1	2,62129E-03	23148	1,17132E-02	5112	1,89809E+00	6082	8,29617E-01	47744	9,18202E-01	13590	0	43212
Myeloid_vs_CD4_Responder	Macro_OLFML3	CD4(TGFB1+ Th17)	C3	CD46	2,63117E-03	19828,4	8,21358E-03	9570	2,11650E+00	4101	1,84830E+00	13223	8,81714E-01	29036	0	43212
Myeloid_vs_CD4_Responder	CD4(TNFRSF9+ Treg)	cDC_CLEC9A	FLT3LG	FLT3	2,63422E-03	26036,2	2,79175E-02	961	4,01907E+00	254	1,49167E+00	21986	8,06143E-01	63768	0	43212
Myeloid_vs_CD4_Responder	CD4(IL26+ Th17)	Mono_CD16	HLA-B	LILRA1	2,63565E-03	26576,6	1,04641E-02	6281	1,70837E+00	8495	5,82237E-01	61286	9,18146E-01	13609	0	43212
Myeloid_vs_CD4_Responder	CD4(ISG+ Treg)	Mast	HSPA8	ADRB2	2,65539E-03	21735,6	1,40297E-02	3683	1,46462E+00	13065	1,07314E+00	37120	9,22958E-01	11598	0	43212
Myeloid_vs_CD4_Responder	Macro_ISG15	CD4(ISG+ Treg)	B2M	CD3D	2,66241E-03	39010,2	3,80237E-03	30919	5,03605E-01	60610	5,97718E-01	60296	9,63133E-01	14	0	43212
Myeloid_vs_CD4_Responder	CD4(AREG+ Tm)	cDC_CLEC9A	CALM2	MYLK	2,66438E-03	33137	2,05651E-02	1763	2,00739E+00	5043	8,69557E-01	45823	7,92952E-01	69844	0	43212
Myeloid_vs_CD4_Responder	Mono_INHBA	CD4(CRTAM- T)	CXCL2	DPP4	2,66905E-03	22926,2	1,59981E-02	2864	2,40160E+00	2520	2,46690E+00	5046	8,12203E-01	60989	0	43212
Myeloid_vs_CD4_Responder	Macro_FOLR2+APOE-	CD4(IL26+ Th17)	HLA-DPB1	LAG3	2,67601E-03	17171,4	7,94728E-03	10094	1,44187E+00	13662	2,06077E+00	9532	9,28108E-01	9357	0	43212
Myeloid_vs_CD4_Responder	Macro_LYVE1	CD4(IL26+ Th17)	HLA-DPA1	LAG3	2,68137E-03	17169,8	7,99007E-03	10013	1,44163E+00	13669	1,98716E+00	10694	9,30523E-01	8261	0	43212
Myeloid_vs_CD4_Responder	Macro_ISG15	CD4(IFNG+ Tfh/Th1)	HLA-DRB5	LAG3	2,69520E-03	15139,8	1,02523E-02	6543	1,44065E+00	13687	2,68028E+00	3436	9,29346E-01	8821	0	43212
Myeloid_vs_CD4_Responder	Macro_LYVE1	CD4(TNFRSF9+ Treg)	CCL18	CCR8	2,69558E-03	18648,2	1,12284E-01	39	2,22800E+00	3363	2,46433E+00	5063	8,53972E-01	41564	0	43212
Myeloid_vs_CD4_Responder	Macro_NLRP3	CD4(Tn)	VCAN	CD44	2,70214E-03	14940,6	1,00344E-02	6823	2,31773E+00	2907	1,82511E+00	13696	9,30950E-01	8065	0	43212
Myeloid_vs_CD4_Responder	CD4(CRTAM- T)	cDC_CLEC9A	LTB	CD40	2,70632E-03	25615,8	8,51116E-03	9034	1,24579E+00	19665	1,27796E+00	29200	8,86658E-01	26968	0	43212
Myeloid_vs_CD4_Responder	cDC_LAMP3	CD4(GZMK+ Teff)	CCL17	CCR4	2,71095E-03	36400,4	2,92249E-02	860	2,99692E+00	975	1,10999E+00	35627	7,13079E-01	101328	0	43212
Myeloid_vs_CD4_Responder	CD4(CRTAM- T)	pDC_LILRA4	TNF	TNFRSF21	2,71140E-03	24066	1,44276E-02	3508	3,22873E+00	666	1,82384E+00	13708	8,16117E-01	59236	0	43212
Myeloid_vs_CD4_Responder	cDC_LAMP3	CD4(IFNG+ Tfh/Th1)	HLA-DRB1	LAG3	2,71295E-03	14804,2	9,96394E-03	6896	1,43965E+00	13710	2,43603E+00	5306	9,37996E-01	4897	0	43212
Myeloid_vs_CD4_Responder	Mono_CD14	CD4(TGFB1+ Th17)	VCAN	ITGB1	2,71441E-03	14578,4	1,18227E-02	5033	2,91659E+00	1103	2,46276E+00	5075	9,06898E-01	18469	0	43212
Myeloid_vs_CD4_Responder	CD4(TNFRSF9+ Treg)	Macro_FOLR2+APOE+	CD52	SIGLEC10	2,73156E-03	25448,6	6,83891E-03	12934	1,67403E+00	8993	8,16844E-01	48370	9,17829E-01	13734	0	43212
Myeloid_vs_CD4_Responder	CD4(TNF+ T)	Macro_FOLR2-APOE+	TNFSF9	HLA-DPA1	2,73190E-03	23844,8	5,16624E-03	19852	1,28994E+00	18156	2,11646E+00	8749	8,81199E-01	29255	0	43212
Myeloid_vs_CD4_Responder	CD4(CXCL13+ Tfh)	cDC(CD1C)	CD28	CD86	2,73470E-03	20868	1,02366E-02	6561,5	1,58921E+00	10400,5	1,76531E+00	14904,5	8,81185E-01	29261,5	0	43212
Myeloid_vs_CD4_Responder	cDC(CD1C)	CD4(CXCL13+ Tfh)	CD86	CD28	2,73517E-03	20868	1,02366E-02	6561,5	1,58921E+00	10400,5	1,76531E+00	14904,5	8,81185E-01	29261,5	0	43212
Myeloid_vs_CD4_Responder	CD4(TNF+ T)	Macro_NLRP3	RPS19	C5AR1	2,74359E-03	20043	5,73020E-03	17007	1,79208E+00	7360	1,27592E+00	29280	9,42395E-01	3356	0	43212
Myeloid_vs_CD4_Responder	CD4(TNFRSF9+ Treg)	Mono_CD16	HLA-A	LILRA1	2,74714E-03	22951	1,15779E-02	5240	1,86622E+00	6460	8,63927E-01	46089	9,17765E-01	13754	0	43212
Myeloid_vs_CD4_Responder	CD4(NME1+ T)	Mono_CD16	CD99	PILRA	2,74875E-03	20882,2	6,61266E-03	13600	1,96292E+00	5443	1,27576E+00	29291	9,19949E-01	12865	0	43212
Myeloid_vs_CD4_Responder	CD4(IFNG+ Tfh/Th1)	Mono_INHBA	RPS19	C5AR1	2,76003E-03	21775,8	5,16412E-03	19868	1,50256E+00	12187	1,27529E+00	29315	9,39506E-01	4297	0	43212
Myeloid_vs_CD4_Responder	Mono_INHBA	CD4(IFNG+ Tfh/Th1)	LGALS3	LAG3	2,78875E-03	15405,4	1,44138E-02	3511	1,43639E+00	13807	2,55075E+00	4375	9,21683E-01	12122	0	43212
Myeloid_vs_CD4_Responder	CD4(TNFRSF9+ Treg)	Mono_CD16	HLA-B	LILRA1	2,80140E-03	26637,8	1,03101E-02	6476	1,66548E+00	9148	5,94480E-01	60530	9,17587E-01	13823	0	43212
Myeloid_vs_CD4_Responder	Macro_OLFML3	CD4(CRTAM- T)	FN1	ITGA5_ITGB1	2,80600E-03	18986,8	1,26734E-02	4430	1,93283E+00	5734	1,90459E+00	12146	8,80855E-01	29412	0	43212
Myeloid_vs_CD4_Responder	Mono_INHBA	CD4(ISG+ Treg)	CCL3L1	CCR5	2,80663E-03	26784,8	1,25936E-02	4483	1,99626E+00	5133	2,48405E+00	4902	7,78339E-01	76194	0	43212
Myeloid_vs_CD4_Responder	CD4(ISG+ Treg)	Macro_ISG15	HLA-B	LILRB1	2,81268E-03	24195,6	3,93722E-03	29426	1,48220E+00	12671	1,28831E+00	28854	9,33638E-01	6815	0	43212

Myeloid_vs_CD4_Post_R

Myeloid_vs_CD4_Responder	CD4(CRTAM- T)	cDC_CLEC9A	CD52	SIGLEC10	2,82273E-03	28392,4	4,55093E-03	23898	1,12091E+00	24515	1,27175E+00	29447	9,01104E-01	20890	0	43212
Myeloid_vs_CD4_Responder	cDC(CD1C)	CD4(IL26+ Th17)	LGALS1	CD69	2,82609E-03	23667	3,97974E-03	28961	1,01429E+00	29454	1,92108E+00	11861	9,38095E-01	4847	0	43212
Myeloid_vs_CD4_Responder	Mono_INHBA	CD4(IFNG+ Tfh/Th1)	HLA-DRB1	LAG3	2,82922E-03	14485,4	9,92205E-03	6950	1,43447E+00	13858	2,67737E+00	3455	9,37874E-01	4952	0	43212
Myeloid_vs_CD4_Responder	Macro_FOLR2-APOE+	CD4(IL26+ Th17)	HLA-DQA1	LAG3	2,83481E-03	18272,4	8,69553E-03	8721	1,43407E+00	13865	1,92220E+00	11835	9,17844E-01	13729	0	43212
Myeloid_vs_CD4_Responder	CD4(TGFB1+ Th17)	Macro_OLFML3	CD52	SIGLEC10	2,83801E-03	24656,8	6,78117E-03	13094	1,56411E+00	10866	9,50557E-01	42243	9,17508E-01	13869	0	43212
Myeloid_vs_CD4_Responder	Macro_LYVE1	CD4(IFNG+ Tfh/Th1)	LGALS3	LAG3	2,83961E-03	15861,2	1,43827E-02	3525	1,43373E+00	13871	2,30787E+00	6541	9,21605E-01	12157	0	43212
Myeloid_vs_CD4_Responder	Macro_IFI27	CD4(IL26+ Th17)	HLA-DRB1	LAG3	2,84041E-03	16594,6	7,93013E-03	10146	1,43369E+00	13872	2,19971E+00	7712	9,31016E-01	8031	0	43212
Myeloid_vs_CD4_Responder	Mono_INHBA	CD4(ISG+ Treg)	CCL3	CCR5	2,84376E-03	21369,4	1,16563E-02	5156	2,39381E+00	2552	2,84667E+00	2482	8,28745E-01	53445	0	43212
Myeloid_vs_CD4_Responder	CD4(AREG+ Tm)	Mono_CD16	CD99	PILRA	2,84761E-03	21053,6	6,53626E-03	13881	1,95437E+00	5526	1,26756E+00	29606	9,19520E-01	13043	0	43212
Myeloid_vs_CD4_Responder	CD4(Tn)	cDC_CLEC9A	CALM2	MYLK	2,84863E-03	32883	2,01725E-02	1826	1,99318E+00	5159	9,17382E-01	43683	7,91366E-01	70535	0	43212
Myeloid_vs_CD4_Responder	CD4(ISG+ Treg)	Macro_FOLR2+APOE+	CD52	SIGLEC10	2,85243E-03	23775	7,39554E-03	11421	1,74249E+00	8020	9,17014E-01	43698	9,20731E-01	12524	0	43212
Myeloid_vs_CD4_Responder	Macro_FOLR2+APOE-	CD4(ISG+ Treg)	HLA-DQA1	LAG3	2,85403E-03	16825,8	9,52982E-03	7434	1,43318E+00	13889	2,23830E+00	7282	9,21233E-01	12312	0	43212
Myeloid_vs_CD4_Responder	CD4(NME1+ T)	Mono_INHBA	HMGB1	THBD	2,86468E-03	28903,8	4,62759E-03	23299	1,21357E+00	20816	1,26960E+00	29534	8,85068E-01	27658	0	43212
Myeloid_vs_CD4_Responder	CD4(TGFB1+ Th17)	Macro_LYVE1	RPS19	C5AR1	2,86529E-03	19524,6	6,53054E-03	13903	1,86994E+00	6408	1,21118E+00	31709	9,45842E-01	2391	0	43212
Myeloid_vs_CD4_Responder	Macro_OLFML3	CD4(ISG+ Treg)	C3	CD46	2,86565E-03	20171,8	8,03153E-03	9931	2,11039E+00	4167	1,80765E+00	14013	8,80540E-01	29536	0	43212
Myeloid_vs_CD4_Responder	cDC(CD1C)	CD4(ISG+ Treg)	HLA-DQB2	LAG3	2,86905E-03	17248,8	2,12101E-02	1672	1,79544E+00	7314	2,53594E+00	4503	8,80508E-01	29543	0	43212
Myeloid_vs_CD4_Responder	CD4(GZMK+ Teff)	pDC_LILRA4	HSP90B1	TLR9	2,87334E-03	24301,2	2,73684E-02	1004	3,13667E+00	783	1,25831E+00	29910	8,43100E-01	46597	0	43212
Myeloid_vs_CD4_Responder	Macro_OLFML3	CD4(IFNG+ Tfh/Th1)	HLA-DRB5	CD4	2,87780E-03	17848,6	3,92551E-03	29561	1,72151E+00	8306	2,68858E+00	3398	9,38281E-01	4766	0	43212
Myeloid_vs_CD4_Responder	CD4(Tn)	Mono_CD14	ANXA1	DYSF	2,87926E-03	21481,8	1,37647E-02	3831	1,68386E+00	8847	1,49245E+00	21955	8,80471E-01	29564	0	43212
Myeloid_vs_CD4_Responder	Macro_OLFML3	CD4(TGFB1+ Th17)	HLA-DQA1	LAG3	2,88267E-03	24193,4	3,92479E-03	29571	1,57733E+00	10624	2,10877E+00	8850	8,82432E-01	28710	0	43212
Myeloid_vs_CD4_Responder	Mono_INHBA	CD4(ISG+ Treg)	HLA-DRA	LAG3	2,88629E-03	15927,2	8,08994E-03	9816	1,43152E+00	13929	2,40376E+00	5599	9,33103E-01	7080	0	43212
Myeloid_vs_CD4_Responder	Macro_OLFML3	CD4(IL26+ Th17)	HLA-DRB5	CD4	2,88852E-03	17929	3,92266E-03	29583	1,72113E+00	8314	2,63020E+00	3760	9,38260E-01	4776	0	43212
Myeloid_vs_CD4_Responder	cDC_LAMP3	CD4(ISG+ Treg)	HLA-DQB1	LAG3	2,89196E-03	16748	9,73901E-03	7163	1,43133E+00	13936	2,17166E+00	8061	9,23455E-01	11368	0	43212
Myeloid_vs_CD4_Responder	CD4(IFNG+ Tfh/Th1)	cDC_CLEC9A	HMGB1	HAVCR2	2,89292E-03	26823	3,92143E-03	29592	1,01554E+00	29401	1,67822E+00	16893	9,14830E-01	15017	0	43212
Myeloid_vs_CD4_Responder	CD4(TGFB1+ Th17)	Mast	CD40LG	CD9	2,90124E-03	19058,4	2,38666E-02	1308	1,98505E+00	5227	1,71895E+00	15936	8,80375E-01	29609	0	43212
Myeloid_vs_CD4_Responder	Mono_CD16	CD4(TGFB1+ Th17)	LGALS1	CD69	2,90414E-03	16509,6	6,51698E-03	13951	1,48091E+00	12705	1,94010E+00	11525	9,50960E-01	1155	0	43212
Myeloid_vs_CD4_Responder	CD4(ISG+ Treg)	Mono_INHBA	HLA-A	LILRB2	2,90810E-03	21997,2	3,91779E-03	29623	1,63479E+00	9624	1,43410E+00	23820	9,41273E-01	3707	0	43212
Myeloid_vs_CD4_Responder	CD4(TNFRSF9+ Treg)	cDC_LAMP3	LTB	CD40	2,91154E-03	22383,6	1,17130E-02	5113	1,45229E+00	13373	1,26698E+00	29630	9,01740E-01	20590	0	43212
Myeloid_vs_CD4_Responder	CD4(IL26+ Th17)	Macro_LYVE1	RPS19	C5AR1	2,91228E-03	20768,4	6,51275E-03	13961	1,86596E+00	6465	1,05661E+00	37792	9,45773E-01	2412	0	43212
Myeloid_vs_CD4_Responder	Macro_FOLR2+APOE-	CD4(ISG+ Treg)	HLA-DQB1	LAG3	2,93517E-03	16662,6	9,72708E-03	7181	1,42972E+00	13989	2,21475E+00	7548	9,23411E-01	11383	0	43212
Myeloid_vs_CD4_Responder	CD4(CRTAM- T)	Mono_CD16	HLA-B	LILRA1	2,93763E-03	27595,6	1,02041E-02	6606	1,63594E+00	9609	5,29410E-01	64559	9,17196E-01	13992	0	43212
Myeloid_vs_CD4_Responder	cDC_LAMP3	CD4(IL26+ Th17)	HLA-DPB1	LAG3	2,94420E-03	17213	7,87745E-03	10259	1,42912E+00	14000	2,09038E+00	9111	9,27813E-01	9483	0	43212
Myeloid_vs_CD4_Responder	Macro_ISG15	CD4(IL26+ Th17)	S100A8	CD69	2,94995E-03	16971,6	7,54180E-03	11052	1,42878E+00	14007	2,11962E+00	8703	9,31347E-01	7884	0	43212
Myeloid_vs_CD4_Responder	Macro_OLFML3	CD4(IFNG+ Tfh/Th1)	HLA-DMA	CD4	2,95603E-03	21537,2	3,90851E-03	29720	1,55238E+00	11086	1,86110E+00	12982	9,25079E-01	10686	0	43212
Myeloid_vs_CD4_Responder	CD4(TGFB1+ Th17)	cDC(CD1C)	CD52	SIGLEC10	2,96399E-03	30328,4	4,18868E-03	26858	1,01400E+00	29468	1,26385E+00	29736	8,97346E-01	22368	0	43212
Myeloid_vs_CD4_Responder	Macro_OLFML3	CD4(IL26+ Th17)	HLA-DMA	CD4	2,96998E-03	21773,4	3,90566E-03	29748	1,55200E+00	11093	1,80271E+00	14116	9,25053E-01	10698	0	43212
Myeloid_vs_CD4_Responder	CD4(TGFB1+ Th17)	Mono_CD16	HLA-C	LILRA3	2,97802E-03	25520,8	1,69456E-02	2561	2,36493E+00	2673	5,20720E-01	65117	9,17079E-01	14041	0	43212
Myeloid_vs_CD4_Responder	Macro_FOLR2+APOE+	CD4(ISG+ Treg)	HLA-DRB5	LAG3	2,98881E-03	17370,6	9,01095E-03	8224	1,42726E+00	14054	1,99124E+00	10640	9,24989E-01	10723	0	43212
Myeloid_vs_CD4_Responder	CD4(CRTAM- T)	Macro_LYVE1	RPS19	C5AR1	3,01214E-03	19904,4	6,47977E-03	14082	1,85857E+00	6544	1,17096E+00	33233	9,45642E-01	2451	0	43212
Myeloid_vs_CD4_Responder	CD4(TNFRSF9+ Treg)	Mast	HSPA8	ADRB2	3,03476E-03	22677,4	1,36136E-02	3912	1,42536E+00	14109	9,99867E-01	40102	9,21881E-01	12052	0	43212
Myeloid_vs_CD4_Responder	Macro_ISG15	CD4(IFNG+ Tfh/Th1)	B2M	CD3D	3,04266E-03	38026,8	3,77776E-03	31210	4,97268E-01	61090	6,98320E-01	54606	9,63017E-01	16	0	43212
Myeloid_vs_CD4_Responder	CD4(TGFB1+ Th17)	Mono_CD16	HLA-A	LILRB2	3,05275E-03	18919,4	5,73859E-03	16972	2,22962E+00	3353	1,25825E+00	29912	9,50976E-01	1148	0	43212
Myeloid_vs_CD4_Responder	Macro_ISG15	CD4(TGFB1+ Th17)	SPP1	CD44	3,05413E-03	17581,2	7,21601E-03	11874	1,49634E+00	12321	2,32340E+00	6367	9,16863E-01	14132	0	43212
Myeloid_vs_CD4_Responder	CD4(NME1+ T)	Mono_CD14	HMGB1	TLR2	3,05837E-03	28563,2	3,88942E-03	29923	1,13388E+00	23940	1,48192E+00	22289	8,94863E-01	23452	0	43212
Myeloid_vs_CD4_Responder	Mono_CD14	CD4(CXCL13+ Tfh)	VCAN	ITGA4	3,06306E-03	15216,4	1,21416E-02	4788	2,90832E+00	1119	2,43795E+00	5288	8,99114E-01	21675	0	43212
Myeloid_vs_CD4_Responder	Mono_CD14	CD4(CXCL13+ Tfh)	VCAN	SELL	3,06646E-03	14984	1,44251E-02	3509	2,91226E+00	1108	2,43783E+00	5290	8,98748E-01	21801	0	43212
Myeloid_vs_CD4_Responder	Macro_NLRP3	CD4(ISG+ Treg)	HLA-DRA	LAG3	3,07529E-03	16181	8,03860E-03	9920	1,42331E+00	14157	2,31676E+00	6437	9,32904E-01	7179	0	43212
Myeloid_vs_CD4_Responder	CD4(TGFB1+ Th17)	pDC_LILRA4	HSP90B1	TLR9	3,09317E-03	25133	2,68321E-02	1042	3,12704E+00	794	1,16541E+00	33423	8,41787E-01	47194	0	43212
Myeloid_vs_CD4_Responder	Macro_OLFML3	CD4(ISG+ Treg)	CXCL10	CXCR3	3,12539E-03	18894	2,42490E-02	1266	1,53653E+00	11414	2,13416E+00	8525	8,79475E-01	30053	0	43212
Myeloid_vs_CD4_Responder	Macro_FOLR2-APOE+	CD4(ISG+ Treg)	HLA-DPA1	LAG3	3,13338E-03	15975,4	8,67252E-03	8755	1,42001E+00	14225	2,30082E+00	6617	9,33126E-01	7068	0	43212
Myeloid_vs_CD4_Responder	CD4(AREG+ Tm)	Mono_CD16	HLA-B	LILRA1	3,13596E-03	28011,4	1,00518E-02	6798	1,59350E+00	10327	5,14584E-01	65492	9,16623E-01	14228	0	43212
Myeloid_vs_CD4_Responder	CD4(IL26+ Th17)	Mono_INHBA	RPS19	C5AR1	3,13788E-03	20849,8	5,61045E-03	17538	1,61864E+00	9896	1,25371E+00	30077	9,41819E-01	3526	0	43212
Myeloid_vs_CD4_Responder	CD4(CRTAM- T)	Mono_CD16	HLA-C	LILRA3	3,14458E-03	25709,2	1,67341E-02	2620	2,33894E+00	2787	5,11549E-01	65689	9,16600E-01	14238	0	43212
Myeloid_vs_CD4_Responder	CD4(CRTAM- T)	Macro_FOLR2+APOE+	CD52	SIGLEC10	3,14716E-03	26307,2	6,62025E-03	13575	1,64713E+00	9429	7,64537E-01	51079	9,16595E-01	14241	0	43212
Myeloid_vs_CD4_Responder	CD4(GZMK+ Teff)	cDC_CLEC9A	CD52	SIGLEC10	3,14938E-03	29366,2	4,33607E-03	25607	1,08247E+00	26185	1,25296E+00	30099	8,98928E-01	21728	0	43212
Myeloid_vs_CD4_Responder	CD4(AREG+ Tm)	pDC_LILRA4	CD96	NECTIN1	3,15234E-03	41969,8	3,87096E-02	481,5	6,41795E-01	50696,5	1,48429E+00	22215,5	7,35820E-01	93243,5	0	43212
Myeloid_vs_CD4_Responder	Macro_NLRP3	CD4(IL26+ Th17)	VCAN	ITGA4	3,15494E-03	13958,2	1,84546E-02	2168	2,58238E+00	1897	2,15463E+00	8264	9,16579E-01	14250	0	43212
Myeloid_vs_CD4_Responder	Macro_ISG15	CD4(IL26+ Th17)	HLA-DRA	LAG3	3,15926E-03	16742,8	7,30447E-03	11676	1,41914E+00	14255	2,36383E+00	5989	9,29844E-01	8582	0	43212
Myeloid_vs_CD4_Responder	CD4(TNFRSF9+ Treg)	Mono_INHBA	HLA-A	LILRB2	3,15986E-03	22012,4	3,87255E-03	30119	1,60292E+00	10161	1,46841E+00	22740	9,40951E-01	3830	0	43212
Myeloid_vs_CD4_Responder	Macro_NLRP3	CD4(TNFRSF9+ Treg)	S100A8	CD69	3,17227E-03	17439,8	6,42590E-03	14270	1,73561E+00	8110	1,95078E+00	11359	9,26048E-01	10248	0	43212
Myeloid_vs_CD4_Responder	CD4(AREG+ Tm)	Mono_CD16	HLA-F	LILRB2	3,18195E-03	24953,4	4,61582E-03	23385	2,20273E+00	3506	1,25109E+00	30161	8,92430E-01	24503	0	43212
Myeloid_vs_CD4_Responder	CD4(NME1+ T)	cDC_CLEC9A	FLT3LG	FLT3	3,18213E-03	26969,8	2,66787E-02	1057	4,00736E+00	256	1,40001E+00	24903	8,02572E-01	65421	0	43212
Myeloid_vs_CD4_Responder	Macro_FOLR2+APOE+	CD4(CXCL13+ Tfh)	APOE	LSR	3,18370E-03	16525,6	1,14246E-02	5358	2,45091E+00	2321	2,95044E+00	2023	8,80174E-01	29714	0	43212
Myeloid_vs_CD4_Responder	CD4(NME1+ T)	cDC(CD1C)	HMGB1	THBD	3,18723E-03	28776	4,12897E-03	27414	1,11967E+00	24573	1,61664E+00	18510	8,79141E-01	30171	0	43212
Myeloid_vs_CD4_Responder	CD4(AREG+ Tm)	cDC(CD1C)	MIF	CD74_CXCR4	3,18811E-03	39645,2	2,01099E-03	69497	4,90685E-01	61566	1,46387E+00	22893	9,51318E-01	1058	0	43212
Myeloid_vs_CD4_Responder	Macro_NLRP3	CD4(ISG+ Treg)	VCAN	SELL	3,20450E-03	14010,6	2,20127E-02	1571	2,56269E+00	1949	2,09736E+00	9014	9,16423E-01	14307	0	43212
Myeloid_vs_CD4_Responder	cDC(CD1C)	CD4(ISG+ Treg)	C3	IFITM1	3,21851E-03	22910,4	5,78108E-03	16786	9,96995E-01	30230	2,06879E+00	9401	9,15046E-01	14923	0	43212
Myeloid_vs_CD4_Responder	CD4(IL26+ Th17)	cDC_CLEC9A	CALM2	MYLK	3,23105E-03	34702,6	1,95335E-02	1939	1,97005E+00	5385	7,61354E-01	51246	7,88696E-01	71731	0	43212
Myeloid_vs_CD4_Responder	Macro_FOLR2+APOE-	CD4(IL26+ Th17)	HLA-DRB1	LAG3	3,23434E-03	17080	7,83461E-03	10350	1,41615E+00	14341	2,07778E+00	9277	9,30626E-01	8220	0	43212

Myeloid_vs_CD4_Post_R

Myeloid_vs_CD4_Responder	CD4(CXCL13+ Tfh)	Mono_CD16	HLA-F	LILRB2	3,24147E-03	24240,2	4,93531E-03	21219	2,22834E+00	3359	1,24803E+00	30273	8,95600E-01	23138	0	43212
Myeloid_vs_CD4_Responder	cDC(CD1C)	CD4(IFNG+ Tfh/Th1)	HLA-DQB1	CD4	3,24147E-03	18539,2	3,86000E-03	30273	1,57297E+00	10705	2,97823E+00	1914	9,34151E-01	6592	0	43212
Myeloid_vs_CD4_Responder	Macro_OLFML3	CD4(TNF+ T)	APOE	LDLR	3,24683E-03	17137,8	1,09677E-02	5815	2,01864E+00	4948	3,12177E+00	1431	8,78874E-01	30283	0	43212
Myeloid_vs_CD4_Responder	cDC(CD1C)	CD4(IL26+ Th17)	HLA-DQB1	CD4	3,25756E-03	18597,2	3,85719E-03	30303	1,57259E+00	10714	2,91984E+00	2159	9,34129E-01	6598	0	43212
Myeloid_vs_CD4_Responder	CD4(TGFB1+ Th17)	Mono_CD16	HLA-B	LILRA1	3,26793E-03	28767	9,95525E-03	6905	1,56662E+00	10823	4,67685E-01	68516	9,16254E-01	14379	0	43212
Myeloid_vs_CD4_Responder	CD4(CXCL13+ Tfh)	Mono_CD16	HLA-B	LILRB2	3,28074E-03	19096	5,56181E-03	17784	2,22964E+00	3352	1,24622E+00	30346	9,52807E-01	786	0	43212
Myeloid_vs_CD4_Responder	CD4(ISG+ Treg)	Macro_NLRP3	GNAI2	C5AR1	3,28669E-03	25964,6	3,85996E-03	30274	2,04237E+00	4734	1,51580E+00	21246	8,78719E-01	30357	0	43212
Myeloid_vs_CD4_Responder	CD4(CRTAM- T)	cDC_CLEC9A	TGFB1	LPP	3,28886E-03	25675,2	8,93920E-03	8329	9,94532E-01	30361	1,59337E+00	19137	8,85819E-01	27337	0	43212
Myeloid_vs_CD4_Responder	Macro_LYVE1	CD4(IL26+ Th17)	HLA-DPB1	LAG3	3,30088E-03	18176,6	7,79419E-03	10457	1,41391E+00	14416	1,85126E+00	13173	9,27457E-01	9625	0	43212
Myeloid_vs_CD4_Responder	CD4(CRTAM- T)	Mono_CD16	HLA-A	LILRB2	3,31439E-03	19053	5,71645E-03	17064	2,22050E+00	3413	1,24438E+00	30408	9,50885E-01	1168	0	43212
Myeloid_vs_CD4_Responder	Macro_NLRP3	CD4(TGFB1+ Th17)	LGALS1	CD69	3,31879E-03	16249,4	6,37832E-03	14436	1,45443E+00	13328	2,09765E+00	9006	9,50457E-01	1265	0	43212
Myeloid_vs_CD4_Responder	Macro_FOLR2+APOE-	CD4(IL26+ Th17)	HLA-DPA1	LAG3	3,32868E-03	17206,6	7,83569E-03	10345	1,41301E+00	14447	2,06456E+00	9472	9,29890E-01	8557	0	43212
Myeloid_vs_CD4_Responder	Macro_ISG15	CD4(IL26+ Th17)	SPP1	CD44	3,32958E-03	17935,8	7,07106E-03	12294	1,47971E+00	12738	2,26429E+00	6987	9,16086E-01	14448	0	43212
Myeloid_vs_CD4_Responder	Mono_INHBA	CD4(GZMK+ Teff)	INHBA	TGFBR3	3,35765E-03	24035	5,85678E-02	208	2,92789E+00	1086	2,22139E+00	7471	7,96535E-01	68198	0	43212
Myeloid_vs_CD4_Responder	CD4(Tn)	Macro_FOLR2+APOE+	ANXA1	FPR1	3,37422E-03	23867	5,63613E-03	17418	1,41963E+00	14240	1,24173E+00	30517	9,17287E-01	13948	0	43212
Myeloid_vs_CD4_Responder	CD4(IFNG+ Tfh/Th1)	Mono_CD14	HMGB1	THBD	3,37643E-03	31090,8	4,23017E-03	26476	1,09435E+00	25663	1,24161E+00	30521	8,80422E-01	29582	0	43212
Myeloid_vs_CD4_Responder	Macro_OLFML3	CD4(CRTAM- T)	C3	CD46	3,38973E-03	20495,6	7,69505E-03	10684	2,09911E+00	4268	1,82042E+00	13769	8,78271E-01	30545	0	43212
Myeloid_vs_CD4_Responder	CD4(NME1+ T)	Mono_CD16	GNAI2	C5AR1	3,41477E-03	27153,8	3,83021E-03	30590	1,86806E+00	6433	1,39645E+00	25005	8,78306E-01	30529	0	43212
Myeloid_vs_CD4_Responder	Macro_OLFML3	CD4(Tn)	CD14	ITGB1	3,41756E-03	24540,6	3,82971E-03	30595	1,50119E+00	12216	1,63558E+00	17980	9,06385E-01	18700	0	43212
Myeloid_vs_CD4_Responder	CD4(TNF+ T)	cDC_LAMP3	TNFSF9	HLA-DPA1	3,42036E-03	25238,8	4,87686E-03	21606	1,21253E+00	20850	2,03579E+00	9926	8,78149E-01	30600	0	43212
Myeloid_vs_CD4_Responder	Mono_CD16	CD4(ISG+ Treg)	B2M	CD3D	3,42289E-03	46427,8	3,73694E-03	31723	4,30976E-01	66226	1,38679E-01	90960	9,62823E-01	18	0	43212
Myeloid_vs_CD4_Responder	cDC(CD1C)	CD4(TGFB1+ Th17)	HLA-DQA1	LAG3	3,42819E-03	23507	3,82828E-03	30614	1,53280E+00	11494	2,76602E+00	2927	8,81134E-01	29288	0	43212
Myeloid_vs_CD4_Responder	CD4(GZMK+ Teff)	Macro_OLFML3	TNFSF9	HLA-DPA1	3,44221E-03	21911,6	4,86969E-03	21649	1,58405E+00	10500	2,66160E+00	3558	8,78070E-01	30639	0	43212
Myeloid_vs_CD4_Responder	CD4(TGFB1+ Th17)	Mono_CD16	HLA-C	LILRB2	3,44502E-03	19324,6	5,55254E-03	17832	2,22459E+00	3389	1,23846E+00	30644	9,49313E-01	1546	0	43212
Myeloid_vs_CD4_Responder	cDC_LAMP3	CD4(TNFRSF9+ Treg)	CCL19	CCR7	3,45025E-03	26481,4	2,61206E-02	1101	5,60520E+00	75	1,29036E+00	28782	8,16113E-01	59237	0	43212
Myeloid_vs_CD4_Responder	Mono_CD14	CD4(CRTAM- T)	VCAN	ITGA4	3,45992E-03	15628	1,13986E-02	5382	2,88650E+00	1167	2,41364E+00	5512	8,96213E-01	22867	0	43212
Myeloid_vs_CD4_Responder	Macro_IER3	CD4(ISG+ Treg)	HLA-DQA1	LAG3	3,46376E-03	17493,8	9,34094E-03	7695	1,40790E+00	14595	2,07328E+00	9339	9,20504E-01	12628	0	43212
Myeloid_vs_CD4_Responder	CD4(AREG+ Tm)	Mast	CD55	ADGRE2	3,47322E-03	22551,8	1,12606E-02	5530	1,94953E+00	5576	1,31796E+00	27747	8,77957E-01	30694	0	43212
Myeloid_vs_CD4_Responder	Macro_ISG15	CD4(TNF+ T)	CXCL10	CXCR3	3,47468E-03	22027,2	1,12666E-02	5520	2,08028E+00	4399	2,42315E+00	5426	8,32605E-01	51579	0	43212
Myeloid_vs_CD4_Responder	CD4(TNFRSF9+ Treg)	Macro_ISG15	HLA-F	LILRB2	3,48398E-03	28848,6	3,81860E-03	30713	1,29338E+00	18043	1,43441E+00	23809	8,82985E-01	28466	0	43212
Myeloid_vs_CD4_Responder	cDC(CD1C)	CD4(IFNG+ Tfh/Th1)	LGALS3	LAG3	3,50217E-03	21447,4	9,17633E-03	7934	9,87059E-01	30745	2,40308E+00	5607	9,03755E-01	19739	0	43212
Myeloid_vs_CD4_Responder	Mono_CD14	CD4(IL26+ Th17)	VCAN	ITGB1	3,50618E-03	14867,2	1,14172E-02	5368	2,90231E+00	1136	2,41115E+00	5537	9,05414E-01	19083	0	43212
Myeloid_vs_CD4_Responder	Macro_ISG15	CD4(IFNG+ Tfh/Th1)	HLA-DQA1	LAG3	3,50656E-03	15642,8	1,04561E-02	6293	1,40643E+00	14641	2,72591E+00	3148	9,24533E-01	10920	0	43212
Myeloid_vs_CD4_Responder	CD4(TNF+ T)	Macro_IER3	TNFSF9	HLA-DPA1	3,50673E-03	25780	4,84625E-03	21790	1,20434E+00	21188	1,91593E+00	11957	8,77811E-01	30753	0	43212
Myeloid_vs_CD4_Responder	Mono_CD14	CD4(TNFRSF9+ Treg)	VCAN	ITGB1	3,50804E-03	14900,4	1,12545E-02	5538	2,89658E+00	1146	2,43899E+00	5277	9,04797E-01	19329	0	43212
Myeloid_vs_CD4_Responder	Macro_FOLR2+APOE+	CD4(Tn)	APOE	SORL1	3,51591E-03	14656,6	7,11705E-03	12158	2,54090E+00	2023	3,19650E+00	1239	9,15614E-01	14651	0	43212
Myeloid_vs_CD4_Responder	Macro_OLFML3	CD4(ISG+ Treg)	HLA-DQB2	LAG3	3,51700E-03	20590,4	2,01450E-02	1834	1,71878E+00	8353	1,60710E+00	18782	8,77771E-01	30771	0	43212
Myeloid_vs_CD4_Responder	CD4(IFNG+ Tfh/Th1)	Mono_CD16	RPS19	C5AR1	3,52329E-03	19846,6	6,17462E-03	15220	1,80347E+00	7217	1,23489E+00	30782	9,44389E-01	2802	0	43212
Myeloid_vs_CD4_Responder	Macro_ISG15	CD4(IFNG+ Tfh/Th1)	HLA-DPA1	LAG3	3,52435E-03	14664,8	9,73438E-03	7171	1,40573E+00	14660	2,78108E+00	2830	9,36641E-01	5451	0	43212
Myeloid_vs_CD4_Responder	CD4(CXCL13+ Tfh)	pDC_LILRA4	HSP90B1	TLR9	3,53151E-03	25419	2,60226E-02	1114	3,11250E+00	817	1,15594E+00	33801	8,39736E-01	48151	0	43212
Myeloid_vs_CD4_Responder	Mono_CD14	CD4(TNF+ T)	VCAN	SELL	3,54722E-03	16377,6	1,12408E-02	5559	2,83952E+00	1264	2,47793E+00	4956	8,86822E-01	26897	0	43212
Myeloid_vs_CD4_Responder	Macro_NLRP3	CD4(IFNG+ Tfh/Th1)	HLA-DRB1	LAG3	3,55352E-03	14921,2	9,67962E-03	7246	1,40447E+00	14691	2,57034E+00	4225	9,37149E-01	5232	0	43212
Myeloid_vs_CD4_Responder	CD4(NME1+ T)	cDC(CD1C)	HMGB1	HAVCR2	3,56354E-03	26847,6	3,80729E-03	30852	1,03037E+00	28662	1,71725E+00	15980	9,13672E-01	15532	0	43212
Myeloid_vs_CD4_Responder	CD4(IL26+ Th17)	Macro_OLFML3	CD52	SIGLEC10	3,56864E-03	26292,6	6,43835E-03	14204	1,51478E+00	11916	8,35734E-01	47424	9,15524E-01	14707	0	43212
Myeloid_vs_CD4_Responder	CD4(NME1+ T)	Mast	HSPA8	ADRB2	3,58379E-03	19648,8	1,52295E-02	3165	1,57784E+00	10617	1,23209E+00	30887	9,25825E-01	10363	0	43212
Myeloid_vs_CD4_Responder	Macro_ISG15	CD4(TNFRSF9+ Treg)	SPP1	CCR8	3,58831E-03	16861,6	7,80832E-02	109	1,90323E+00	6024	3,24574E+00	1123	8,70882E-01	33840	0	43212
Myeloid_vs_CD4_Responder	CD4(ISG+ Treg)	cDC(CD1C)	LTB	CD40	3,58902E-03	28174,4	7,12525E-03	12135	9,94932E-01	30342	1,41884E+00	24287	8,77416E-01	30896	0	43212
Myeloid_vs_CD4_Responder	CD4(IL26+ Th17)	cDC_CLEC9A	CALM3	MYLK	3,59424E-03	41783,2	1,70972E-02	2510	1,94866E+00	5584	7,72416E-01	50658	6,94973E-01	106952	0	43212
Myeloid_vs_CD4_Responder	Macro_ISG15	CD4(TNFRSF9+ Treg)	SPP1	CD44	3,59808E-03	17997,6	6,95548E-03	12624	1,46644E+00	13027	2,32087E+00	6387	9,15451E-01	14738	0	43212
Myeloid_vs_CD4_Responder	CD4(Tn)	Mono_CD16	CD99	PILRA	3,60240E-03	22490,2	5,76790E-03	16849	1,86843E+00	6428	1,23147E+00	30919	9,14769E-01	15043	0	43212
Myeloid_vs_CD4_Responder	CD4(IFNG+ Tfh/Th1)	Mono_CD14	CIRBP	TREM1	3,60706E-03	24069	8,04902E-03	9906	1,55104E+00	11115	1,23132E+00	30927	8,90754E-01	25185	0	43212
Myeloid_vs_CD4_Responder	CD4(TNFRSF9+ Treg)	Macro_NLRP3	HLA-F	LILRB2	3,60881E-03	29212,4	4,01887E-03	28536	1,36011E+00	15957	1,23126E+00	30930	8,85600E-01	27427	0	43212
Myeloid_vs_CD4_Responder	CD4(IL26+ Th17)	cDC(CD1C)	CD28	CD86	3,61173E-03	22076,4	9,52059E-03	7449,5	1,55592E+00	11034,5	1,64421E+00	17750,5	8,77337E-01	30935,5	0	43212
Myeloid_vs_CD4_Responder	cDC(CD1C)	CD4(IL26+ Th17)	CD86	CD28	3,61231E-03	22076,4	9,52059E-03	7449,5	1,55592E+00	11034,5	1,64421E+00	17750,5	8,77337E-01	30935,5	0	43212
Myeloid_vs_CD4_Responder	Mono_CD16	CD4(IFNG+ Tfh/Th1)	B2M	CD3D	3,61299E-03	45142	3,71275E-03	32035	4,24639E-01	66723	2,39281E-01	83721	9,62707E-01	19	0	43212
Myeloid_vs_CD4_Responder	CD4(TNFRSF9+ Treg)	cDC(CD1C)	LTB	TNFRSF1A	3,61698E-03	25209,8	8,02622E-03	9939	9,83329E-01	30944	1,56964E+00	19760	8,97742E-01	22194	0	43212
Myeloid_vs_CD4_Responder	CD4(IFNG+ Tfh/Th1)	pDC_LILRA4	HSP90B1	TLR9	3,62642E-03	25407,8	2,58222E-02	1129	3,10890E+00	822	1,16453E+00	33463	8,39215E-01	48413	0	43212
Myeloid_vs_CD4_Responder	CD4(GZMK+ Teff)	Macro_NLRP3	HMGB1	CD163	3,64336E-03	24293,2	3,79585E-03	30989	1,51240E+00	11967	1,45061E+00	23286	9,21964E-01	12012	0	43212
Myeloid_vs_CD4_Responder	Mast	CD4(IL26+ Th17)	TNFSF10	CCR6	3,64498E-03	17375,8	2,29359E-02	1436	1,44071E+00	13685	1,76988E+00	14787	9,17752E-01	13759	0	43212
Myeloid_vs_CD4_Responder	CD4(CRTAM- T)	Mono_CD16	HLA-C	LILRB2	3,65395E-03	19508	5,48321E-03	18158	2,19860E+00	3529	1,22929E+00	31007	9,49010E-01	1634	0	43212
Myeloid_vs_CD4_Responder	Mono_CD14	CD4(AREG+ Tm)	VCAN	ITGA4	3,66839E-03	15735	1,12583E-02	5532	2,88238E+00	1177	2,40160E+00	5623	8,95636E-01	23131	0	43212
Myeloid_vs_CD4_Responder	CD4(IL26+ Th17)	Macro_FOLR2+APOE+	ADAM10	GPNMB	3,68292E-03	20007	1,01337E-02	6688	2,70406E+00	1571	1,65404E+00	17508	8,77075E-01	31056	0	43212
Myeloid_vs_CD4_Responder	Macro_NLRP3	CD4(IFNG+ Tfh/Th1)	HLA-DRB5	LAG3	3,69329E-03	15790,4	9,87504E-03	7003	1,39864E+00	14837	2,53087E+00	4539	9,28105E-01	9361	0	43212
Myeloid_vs_CD4_Responder	CD4(TNF+ T)	Macro_FOLR2+APOE-	HMGB1	CD163	3,69539E-03	23072,4	3,78879E-03	31077	1,71125E+00	8452	1,53877E+00	20576	9,21897E-01	12045	0	43212
Myeloid_vs_CD4_Responder	Macro_FOLR2+APOE-	CD4(IL26+ Th17)	F13A1	ITGA4	3,71275E-03	13256,4	2,34438E-02	1359	3,41074E+00	505	2,32464E+00	6349	9,15185E-01	14857	0	43212
Myeloid_vs_CD4_Responder	Macro_OLFML3	CD4(NME1+ T)	LYZ	ITGAL	3,71744E-03	26164,2	3,78541E-03	31114	1,36120E+00	15931	1,75103E+00	15187	8,90292E-01	25377	0	43212
Myeloid_vs_CD4_Responder	CD4(NME1+ T)	Mast	ARPC5	ADRB2	3,73599E-03	22972	1,10181E-02	5775	1,61335E+00	9996	1,40482E+00	24732	8,76893E-01	31145	0	43212
Myeloid_vs_CD4_Responder	CD4(ISG+ Treg)	Macro_ISG15	LTB	CD40	3,73839E-03	25630,2	9,38304E-03	7631	1,20242E+00	21272	1,22549E+00	31149	8,91467E-01	24887	0	43212
Myeloid_vs_CD4_Responder	CD4(ISG+ Treg)	Mast	IL16	CD9	3,74139E-03	20461	1,07395E-02	6004	1,94132E+00	5645	1,70460E+00	16290	8,76862E-01	31154	0	43212

Myeloid_vs_CD4_Post_R

Myeloid_vs_CD4_Responder	CD4(IFNG+ Tfh/Th1)	Macro_NLRP3	CD99	PILRA	3,76910E-03	25406,4	4,98723E-03	20909	1,42521E+00	14117	1,22399E+00	31200	9,08926E-01	17594	0	43212
Myeloid_vs_CD4_Responder	CD4(NME1+ T)	Macro_OLFML3	HMGB1	TLR2	3,77575E-03	22594	6,32042E-03	14652	1,65897E+00	9243	1,22376E+00	31211	9,15612E-01	14652	0	43212
Myeloid_vs_CD4_Responder	Macro_LYVE1	CD4(ISG+ Treg)	B2M	CD3D	3,80309E-03	47046,8	3,70918E-03	32084	4,00175E-01	68705	1,35068E-01	91213	9,62690E-01	20	0	43212
Myeloid_vs_CD4_Responder	Macro_OLFML3	CD4(TNF+ T)	C3	CD46	3,81889E-03	20011,4	7,45865E-03	11234	2,09118E+00	4324	2,02979E+00	10005	8,76593E-01	31282	0	43212
Myeloid_vs_CD4_Responder	Macro_OLFML3	CD4(TGFB1+ Th17)	HLA-DQB1	LAG3	3,82133E-03	25187,2	3,77129E-03	31286	1,47978E+00	12734	2,03034E+00	9998	8,82454E-01	28706	0	43212
Myeloid_vs_CD4_Responder	CD4(GZMK+ Teff)	cDC_CLEC9A	TNFSF9	HLA-DPA1	3,82255E-03	22007,2	4,73695E-03	22545	1,53601E+00	11424	3,07806E+00	1567	8,76583E-01	31288	0	43212
Myeloid_vs_CD4_Responder	Mono_CD14	CD4(GZMK+ Teff)	VCAN	SELL	3,82360E-03	16423,2	1,14189E-02	5363	2,84358E+00	1257	2,39259E+00	5703	8,87608E-01	26581	0	43212
Myeloid_vs_CD4_Responder	CD4(ISG+ Treg)	pDC_LILRA4	PSEN1	NOTCH4	3,83308E-03	37002,2	1,88889E-02	2066	1,62360E+00	9821	1,76170E+00	14979	6,63744E-01	114933	0	43212
Myeloid_vs_CD4_Responder	CD4(NME1+ T)	Macro_IER3	HMGB1	THBD	3,83907E-03	27742	5,05742E-03	20466	1,29452E+00	18015	1,22092E+00	31315	8,89509E-01	25702	0	43212
Myeloid_vs_CD4_Responder	Mono_INHBA	CD4(AREG+ Tm)	IL1B	SIGIRR	3,84951E-03	18862,8	6,62140E-03	13572	2,02613E+00	4882	2,59486E+00	4024	8,82628E-01	28624	0	43212
Myeloid_vs_CD4_Responder	CD4(ISG+ Treg)	Macro_NLRP3	HMGB1	CD163	3,85258E-03	25051,4	3,76597E-03	31337	1,50536E+00	12125	1,35429E+00	26456	9,21679E-01	12127	0	43212
Myeloid_vs_CD4_Responder	cDC(CD1C)	CD4(ISG+ Treg)	HLA-DMB	CD4	3,85381E-03	22436,8	3,76585E-03	31339	1,57340E+00	10690	2,17529E+00	8008	9,05811E-01	18935	0	43212
Myeloid_vs_CD4_Responder	Mono_INHBA	CD4(AREG+ Tm)	SPP1	CD44	3,85442E-03	20288,6	6,24024E-03	14961	1,25321E+00	19388	2,24034E+00	7259	9,11155E-01	16623	0	43212
Myeloid_vs_CD4_Responder	Mono_INHBA	CD4(AREG+ Tm)	SPP1	PTGER4	3,85504E-03	18408,2	1,16871E-02	5130	1,37697E+00	15455	2,44357E+00	5231	8,95903E-01	23013	0	43212
Myeloid_vs_CD4_Responder	CD4(CRTAM- T)	Mast	CD55	ADGRE2	3,85750E-03	23119	1,09484E-02	5834	1,93371E+00	5729	1,27102E+00	29475	8,76443E-01	31345	0	43212
Myeloid_vs_CD4_Responder	Mono_INHBA	CD4(AREG+ Tm)	VCAN	CD44	3,85811E-03	19896	6,84729E-03	12908	1,56031E+00	10950	1,61343E+00	18596	9,17609E-01	13814	0	43212
Myeloid_vs_CD4_Responder	Macro_LYVE1	CD4(ISG+ Treg)	HLA-DQA1	LAG3	3,85909E-03	17929,8	9,23288E-03	7843	1,39343E+00	15005	1,98210E+00	10786	9,20077E-01	12803	0	43212
Myeloid_vs_CD4_Responder	Mono_INHBA	CD4(AREG+ Tm)	HBEGF	CD44	3,86242E-03	23395,2	6,62969E-03	13541	1,40728E+00	14620	1,26233E+00	29775	9,12973E-01	15828	0	43212
Myeloid_vs_CD4_Responder	Mono_INHBA	CD4(AREG+ Tm)	LGALS1	CD69	3,86550E-03	17865	5,53256E-03	17925	1,37827E+00	15424	1,98955E+00	10659	9,46998E-01	2105	0	43212
Myeloid_vs_CD4_Responder	CD4(ISG+ Treg)	Macro_OLFML3	CD52	SIGLEC10	3,87818E-03	25603,2	6,32111E-03	14650	1,49791E+00	12280	9,36549E-01	42850	9,14810E-01	15024	0	43212
Myeloid_vs_CD4_Responder	Mono_INHBA	CD4(AREG+ Tm)	S100A8	CD69	3,88032E-03	18896,8	7,14978E-03	12053	1,31767E+00	17243	1,84532E+00	13283	9,29621E-01	8693	0	43212
Myeloid_vs_CD4_Responder	Mono_INHBA	CD4(AREG+ Tm)	VEGFA	CD44	3,88403E-03	18625	9,81134E-03	7074	2,07342E+00	4454	1,48070E+00	22340	9,12470E-01	16045	0	43212
Myeloid_vs_CD4_Responder	Mono_INHBA	CD4(AREG+ Tm)	INHBA	TGFBR3	3,88556E-03	25334,6	5,03700E-02	282	2,88533E+00	1169	2,16044E+00	8190	7,84043E-01	73820	0	43212
Myeloid_vs_CD4_Responder	CD4(ISG+ Treg)	Macro_NLRP3	HLA-A	LILRB2	3,89890E-03	23197,8	3,76014E-03	31412	1,57865E+00	10603	1,34705E+00	26676	9,40127E-01	4086	0	43212
Myeloid_vs_CD4_Responder	CD4(IFNG+ Tfh/Th1)	Macro_OLFML3	IFNG	IFNGR1_IFNGR2	3,91071E-03	22206,2	2,30433E-02	1419	1,43941E+00	13721	1,51574E+00	21248	8,76217E-01	31431	0	43212
Myeloid_vs_CD4_Responder	Mono_INHBA	CD4(Tn)	IL1B	SIGIRR	3,91444E-03	19017,2	6,47418E-03	14098	2,01973E+00	4936	2,64103E+00	3700	8,81459E-01	29140	0	43212
Myeloid_vs_CD4_Responder	CD4(TNFRSF9+ Treg)	pDC_LILRA4	PSEN1	NOTCH4	3,91758E-03	38744	1,43410E-02	3543	1,56591E+00	10838	1,75765E+00	15063	6,32347E-01	121064	0	43212
Myeloid_vs_CD4_Responder	Mono_INHBA	CD4(Tn)	SPP1	CD44	3,92005E-03	21096,4	5,91907E-03	16197	1,20564E+00	21139	2,23030E+00	7375	9,08993E-01	17559	0	43212
Myeloid_vs_CD4_Responder	Mono_INHBA	CD4(Tn)	SPP1	PTGER4	3,92067E-03	21067,8	9,35113E-03	7680	1,24022E+00	19876	2,27384E+00	6892	8,85036E-01	27679	0	43212
Myeloid_vs_CD4_Responder	Mono_INHBA	CD4(Tn)	VCAN	ITGB1	3,92192E-03	24117	6,24531E-03	14941	1,46423E+00	13083	1,63786E+00	17924	8,76233E-01	31425	0	43212
Myeloid_vs_CD4_Responder	Mono_CD14	CD4(CRTAM- T)	VCAN	SELL	3,92274E-03	15909,6	1,24249E-02	4592	2,86657E+00	1216	2,38605E+00	5753	8,91751E-01	24775	0	43212
Myeloid_vs_CD4_Responder	Mono_INHBA	CD4(Tn)	VCAN	SELL	3,92317E-03	19380,8	1,11285E-02	5674	1,61526E+00	9961	1,97408E+00	10929	8,86317E-01	27128	0	43212
Myeloid_vs_CD4_Responder	CD4(CXCL13+ Tfh)	pDC_LILRA4	TNF	PTPRS	3,92367E-03	28090,6	2,24377E-02	1507	4,37635E+00	180	1,80645E+00	14043	7,65677E-01	81511	0	43212
Myeloid_vs_CD4_Responder	Mono_INHBA	CD4(Tn)	VCAN	CD44	3,92379E-03	20548,6	6,49487E-03	14028	1,51274E+00	11963	1,60339E+00	18873	9,15589E-01	14667	0	43212
Myeloid_vs_CD4_Responder	CD4(IFNG+ Tfh/Th1)	Mono_INHBA	HLA-A	LILRB2	3,92629E-03	23624,2	3,75722E-03	31456	1,52168E+00	11752	1,32171E+00	27606	9,40105E-01	4095	0	43212
Myeloid_vs_CD4_Responder	CD4(CXCL13+ Tfh)	pDC_LILRA4	APP	TNFRSF21	3,92671E-03	26054	1,35458E-02	3953	3,29946E+00	602	2,09660E+00	9022	7,84850E-01	73481	0	43212
Myeloid_vs_CD4_Responder	Mono_INHBA	CD4(Tn)	HBEGF	CD44	3,92816E-03	24168,4	6,28847E-03	14772	1,35970E+00	15971	1,25229E+00	30122	9,10851E-01	16765	0	43212
Myeloid_vs_CD4_Responder	Mono_INHBA	CD4(Tn)	LGALS1	CD69	3,93003E-03	20806,8	4,66263E-03	23034	1,25394E+00	19367	1,75576E+00	15103	9,42537E-01	3318	0	43212
Myeloid_vs_CD4_Responder	CD4(Tn)	Macro_LYVE1	RPS19	C5AR1	3,93078E-03	20489,8	6,21317E-03	15076	1,79883E+00	7276	1,14697E+00	34122	9,44552E-01	2763	0	43212
Myeloid_vs_CD4_Responder	Mono_INHBA	CD4(TGFB1+ Th17)	INHBA	SMAD3	3,93182E-03	27372,4	3,08502E-02	766	2,88292E+00	1176	2,11979E+00	8699	7,61989E-01	83009	0	43212
Myeloid_vs_CD4_Responder	Macro_OLFML3	CD4(ISG+ Treg)	HLA-DQA1	CD4	3,93753E-03	19523,2	3,75602E-03	31474	1,69278E+00	8733	2,29755E+00	6661	9,32040E-01	7536	0	43212
Myeloid_vs_CD4_Responder	Mono_INHBA	CD4(Tn)	S100A8	CD69	3,94191E-03	22086,4	6,02556E-03	15763	1,19334E+00	21600	1,61153E+00	18645	9,23815E-01	11212	0	43212
Myeloid_vs_CD4_Responder	Mono_INHBA	CD4(Tn)	VEGFA	CD44	3,94504E-03	19094,2	9,30637E-03	7742	2,02585E+00	4885	1,47066E+00	22667	9,10337E-01	16965	0	43212
Myeloid_vs_CD4_Responder	Macro_IER3	CD4(ISG+ Treg)	HLA-DPB1	LAG3	3,94809E-03	16841	8,42236E-03	9166	1,39022E+00	15093	2,15711E+00	8231	9,30021E-01	8503	0	43212
Myeloid_vs_CD4_Responder	cDC(CD1C)	CD4(TNFRSF9+ Treg)	HLA-DPB1	LAG3	3,94818E-03	22182,4	3,75509E-03	31491	1,47209E+00	12900	3,09713E+00	1503	8,98724E-01	21806	0	43212
Myeloid_vs_CD4_Responder	cDC(CD1C)	CD4(IFNG+ Tfh/Th1)	HLA-DMA	CD4	3,94880E-03	20583,8	3,75495E-03	31492	1,48824E+00	12520	2,54410E+00	4422	9,23678E-01	11273	0	43212
Myeloid_vs_CD4_Responder	CD4(CXCL13+ Tfh)	pDC_LILRA4	COPA	P2RY6	3,95319E-03	27550	7,94302E-03	10106	2,05476E+00	4624	2,02245E+00	10123	7,93273E-01	69685	0	43212
Myeloid_vs_CD4_Responder	Mono_INHBA	CD4(IL26+ Th17)	HLA-DRA	LAG3	3,95445E-03	18159,4	6,95604E-03	12620	1,35433E+00	16145	2,06160E+00	9519	9,28233E-01	9301	0	43212
Myeloid_vs_CD4_Responder	Mono_INHBA	CD4(IL26+ Th17)	HLA-DRB1	LAG3	3,95822E-03	19898,4	6,68674E-03	13370	1,20537E+00	21155	1,96079E+00	11170	9,25334E-01	10585	0	43212
Myeloid_vs_CD4_Responder	Mono_INHBA	CD4(IL26+ Th17)	HLA-DPB1	LAG3	3,96073E-03	23920,8	5,65729E-03	17318	1,02363E+00	28993	1,73542E+00	15559	9,15911E-01	14522	0	43212
Myeloid_vs_CD4_Responder	Mono_INHBA	CD4(IL26+ Th17)	ICAM1	IL2RG	3,96450E-03	28806,8	4,92225E-03	21319	1,08184E+00	26216	1,26010E+00	29852	8,94892E-01	23435	0	43212
Myeloid_vs_CD4_Responder	Mono_INHBA	CD4(IL26+ Th17)	THBS1	ITGA4	3,96513E-03	22808,4	8,93778E-03	8330	1,18644E+00	21857	1,79762E+00	14256	8,88026E-01	26387	0	43212
Myeloid_vs_CD4_Responder	cDC(CD1C)	CD4(IL26+ Th17)	HLA-DMA	CD4	3,96639E-03	20687,4	3,75222E-03	31520	1,48786E+00	12531	2,48572E+00	4889	9,23652E-01	11285	0	43212
Myeloid_vs_CD4_Responder	Mono_INHBA	CD4(IL26+ Th17)	CD14	ITGA4	3,96828E-03	22358,4	5,10169E-03	20223	1,33524E+00	16711	1,78716E+00	14462	9,09820E-01	17184	0	43212
Myeloid_vs_CD4_Responder	Mono_INHBA	CD4(IL26+ Th17)	HLA-DPA1	LAG3	3,97143E-03	23477,6	5,69940E-03	17137	1,01708E+00	29331	1,79181E+00	14367	9,18777E-01	13341	0	43212
Myeloid_vs_CD4_Responder	Mono_INHBA	CD4(IL26+ Th17)	HLA-DRB5	LAG3	3,97899E-03	22231,4	6,33962E-03	14582	1,11744E+00	24682	1,89299E+00	12361	9,11842E-01	16320	0	43212
Myeloid_vs_CD4_Responder	Mono_INHBA	CD4(IL26+ Th17)	IL1B	SIGIRR	3,98530E-03	17628,2	7,59648E-03	10924	2,06852E+00	4495	2,62146E+00	3833	8,89559E-01	25677	0	43212
Myeloid_vs_CD4_Responder	CD4(CXCL13+ Tfh)	pDC_LILRA4	MAML2	NOTCH4	3,98698E-03	34823	2,52716E-02	1177	1,62368E+00	9820	1,79432E+00	14317	6,99407E-01	105589	0	43212
Myeloid_vs_CD4_Responder	Mono_INHBA	CD4(IL26+ Th17)	LGALS1	PTPRC	3,98909E-03	21841,4	3,82529E-03	30652	1,28587E+00	18295	1,71033E+00	16148	9,52126E-01	900	0	43212
Myeloid_vs_CD4_Responder	Mono_INHBA	CD4(IL26+ Th17)	SPP1	ITGA4_ITGB1	3,99036E-03	21702,4	7,75618E-03	10543	1,29493E+00	17998	2,31636E+00	6443	8,78788E-01	30316	0	43212
Myeloid_vs_CD4_Responder	Mono_INHBA	CD4(IL26+ Th17)	SPP1	CD44	3,99099E-03	22848,2	5,47847E-03	18181	1,14038E+00	23697	2,01864E+00	10180	9,05743E-01	18971	0	43212
Myeloid_vs_CD4_Responder	Mono_INHBA	CD4(IL26+ Th17)	SPP1	PTGER4	3,99162E-03	22119	8,79770E-03	8553	1,20782E+00	21056	2,11131E+00	8814	8,81896E-01	28960	0	43212
Myeloid_vs_CD4_Responder	Macro_ISG15	CD4(IL26+ Th17)	DEFB1	CCR6	3,99318E-03	21087,6	1,40270E-01	21	2,84000E+00	1263	1,60340E+00	18872	8,52916E-01	42070	0	43212
Myeloid_vs_CD4_Responder	Macro_LYVE1	CD4(IFNG+ Tfh/Th1)	B2M	CD3D	3,99318E-03	45766	3,68518E-03	32392	3,93839E-01	69228	2,35670E-01	83977	9,62573E-01	21	0	43212
Myeloid_vs_CD4_Responder	Mono_INHBA	CD4(IL26+ Th17)	VCAN	ITGA4	3,99352E-03	17854	1,19449E-02	4928	1,77740E+00	7545	1,93290E+00	11646	8,98371E-01	21939	0	43212
Myeloid_vs_CD4_Responder	Mono_INHBA	CD4(IL26+ Th17)	VCAN	CD44	3,99415E-03	22743,6	6,01141E-03	15814	1,44748E+00	13514	1,39172E+00	25173	9,12552E-01	16005	0	43212
Myeloid_vs_CD4_Responder	Mono_INHBA	CD4(IL26+ Th17)	LGALS3	LAG3	3,99732E-03	20752,8	9,71382E-03	7199	1,20729E+00	21075	1,83417E+00	13496	9,06202E-01	18782	0	43212
Myeloid_vs_CD4_Responder	Mono_INHBA	CD4(IL26+ Th17)	CCL20	CCR6	3,99858E-03	19058,4	4,57517E-02	350	1,23486E+00	20081	2,15911E+00	8205	8,94873E-01	23444	0	43212
Myeloid_vs_CD4_Responder	Mono_INHBA	CD4(IL26+ Th17)	CXCL16	CXCR6	4,00048E-03	21825,8	1,19169E-02	4949	1,22039E+00	20585	1,61509E+00	18551	8,98646E-01	21832	0	43212

Myeloid_vs_CD4_Post_R

Myeloid_vs_CD4_Responder	Macro_OLFML3	CD4(TNFRSF9+ Treg)	HLA-DRA	LAG3	4,00048E-03	21639,4	3,74858E-03	31574	1,57170E+00	10734	2,69920E+00	3316	9,04714E-01	19361	0	43212
Myeloid_vs_CD4_Responder	Mono_INHBA	CD4(IL26+ Th17)	LGALS1	CD69	4,00238E-03	19813,2	5,03881E-03	20583	1,30771E+00	17571	1,76298E+00	14955	9,44602E-01	2745	0	43212
Myeloid_vs_CD4_Responder	cDC(CD1C)	CD4(IL26+ Th17)	CD86	CTLA4	4,01444E-03	26074,6	6,56983E-03	13756	1,41811E+00	14285	1,32411E+00	27524	8,75848E-01	31596	0	43212
Myeloid_vs_CD4_Responder	Mono_INHBA	CD4(IL26+ Th17)	S100A8	CD69	4,01635E-03	21057	6,51171E-03	13965	1,24711E+00	19608	1,61876E+00	18441	9,26501E-01	10059	0	43212
Myeloid_vs_CD4_Responder	Mono_INHBA	CD4(IL26+ Th17)	VEGFA	CD44	4,02016E-03	21155,4	8,61363E-03	8844	1,96059E+00	5465	1,25899E+00	29893	9,07130E-01	18363	0	43212
Myeloid_vs_CD4_Responder	CD4(IFNG+ Tfh/Th1)	Mono_INHBA	HLA-F	LILRB2	4,02207E-03	30141	3,74577E-03	31608	1,35572E+00	16100	1,23270E+00	30865	8,81986E-01	28920	0	43212
Myeloid_vs_CD4_Responder	Mast	CD4(TNF+ T)	TIMP3	CD44	4,03854E-03	17169,6	2,51139E-02	1192	3,08299E+00	858	1,52330E+00	21030	9,04207E-01	19556	0	43212
Myeloid_vs_CD4_Responder	Mono_INHBA	CD4(TGFB1+ Th17)	ICAM1	IL2RG	4,04247E-03	22809,4	6,66935E-03	13425	1,26934E+00	18838	1,53412E+00	20712	9,08345E-01	17860	0	43212
Myeloid_vs_CD4_Responder	Macro_FOLR2-APOE+	CD4(ISG+ Treg)	HLA-DQB1	LAG3	4,06041E-03	17399,8	9,40446E-03	7604	1,38621E+00	15202	2,09269E+00	9075	9,22210E-01	11906	0	43212
Myeloid_vs_CD4_Responder	Mono_INHBA	CD4(TGFB1+ Th17)	IL1B	SIGIRR	4,06167E-03	15630,4	9,69295E-03	7224	2,15966E+00	3777	2,75193E+00	3002	9,00975E-01	20937	0	43212
Myeloid_vs_CD4_Responder	Macro_NLRP3	CD4(IL26+ Th17)	THBS1	ITGA4	4,06666E-03	15271,2	1,62097E-02	2781	1,93916E+00	5667	2,06406E+00	9488	9,14385E-01	15208	0	43212
Myeloid_vs_CD4_Responder	Mono_INHBA	CD4(TGFB1+ Th17)	SPP1	CD44	4,06809E-03	22342,6	5,59077E-03	17619	1,15702E+00	23004	2,07774E+00	9278	9,06605E-01	18600	0	43212
Myeloid_vs_CD4_Responder	Mono_INHBA	CD4(TGFB1+ Th17)	SPP1	PTGER4	4,06873E-03	20087,8	1,02298E-02	6569	1,29165E+00	18095	2,27552E+00	6870	8,89526E-01	25693	0	43212
Myeloid_vs_CD4_Responder	Macro_FOLR2-APOE+	CD4(CXCL13+ Tfh)	SPP1	CD44	4,07083E-03	19431,2	6,76630E-03	13130	1,50815E+00	12068	1,83235E+00	13534	9,14377E-01	15212	0	43212
Myeloid_vs_CD4_Responder	Mono_INHBA	CD4(TGFB1+ Th17)	VCAN	CD44	4,07130E-03	22124,2	6,13464E-03	15379	1,46411E+00	13090	1,45083E+00	23278	9,13358E-01	15662	0	43212
Myeloid_vs_CD4_Responder	cDC(CD1C)	CD4(ISG+ Treg)	HLA-DQA2	CD4	4,09190E-03	23992,2	3,73732E-03	31717	1,31213E+00	17431	2,26003E+00	7033	9,01787E-01	20568	0	43212
Myeloid_vs_CD4_Responder	Mono_INHBA	CD4(TGFB1+ Th17)	VEGFA	CD44	4,10611E-03	20567,8	8,79020E-03	8568	1,97723E+00	5305	1,31810E+00	27745	9,07981E-01	18009	0	43212
Myeloid_vs_CD4_Responder	CD4(CXCL13+ Tfh)	Mono_CD16	B2M	LILRB2	4,10805E-03	19494	5,40152E-03	18612	2,18180E+00	3638	1,21060E+00	31742	9,57244E-01	266	0	43212
Myeloid_vs_CD4_Responder	CD4(GZMK+ Teff)	cDC(CD1C)	TNFSF9	HLA-DPA1	4,10870E-03	22332,6	4,64587E-03	23168	1,50304E+00	12175	3,14694E+00	1365	8,75529E-01	31743	0	43212
Myeloid_vs_CD4_Responder	Macro_OLFML3	CD4(TNFRSF9+ Treg)	HLA-DPB1	LAG3	4,11777E-03	23148,6	3,73361E-03	31757	1,46195E+00	13140	2,38927E+00	5721	8,98463E-01	21913	0	43212
Myeloid_vs_CD4_Responder	CD4(CXCL13+ Tfh)	Macro_LYVE1	RPS19	C5AR1	4,12218E-03	21613,6	6,23926E-03	14963	1,80468E+00	7206	1,00338E+00	39959	9,44662E-01	2728	0	43212
Myeloid_vs_CD4_Responder	Mono_INHBA	CD4(TNFRSF9+ Treg)	ICAM1	IL2RA	4,12685E-03	19304,2	1,80061E-02	2269	1,44294E+00	13635	2,20743E+00	7631	8,80044E-01	29774	0	43212
Myeloid_vs_CD4_Responder	Macro_FOLR2-APOE+	CD4(CRTAM- T)	SPP1	S1PR1	4,12808E-03	18706	3,21116E-02	700	1,92020E+00	5854	2,47279E+00	5000	8,60148E-01	38764	0	43212
Myeloid_vs_CD4_Responder	Mono_INHBA	CD4(TNFRSF9+ Treg)	ICAM1	IL2RG	4,12815E-03	23321,8	6,15763E-03	15290	1,21442E+00	20799	1,63324E+00	18034	9,04967E-01	19274	0	43212
Myeloid_vs_CD4_Responder	CD4(CRTAM- T)	pDC_LILRA4	MAML2	NOTCH4	4,13061E-03	35484,4	2,37611E-02	1318	1,60748E+00	10088	1,74739E+00	15269	6,92889E-01	107535	0	43212
Myeloid_vs_CD4_Responder	Mono_INHBA	CD4(TNFRSF9+ Treg)	CD86	CTLA4	4,13986E-03	21013,2	1,06904E-02	6042	1,38122E+00	15345	1,59303E+00	19147	8,99991E-01	21320	0	43212
Myeloid_vs_CD4_Responder	Mono_CD14	CD4(IL26+ Th17)	CD14	ITGA4	4,14011E-03	18002,6	6,16030E-03	15278	1,56339E+00	10880	2,29465E+00	6686	9,17263E-01	13957	0	43212
Myeloid_vs_CD4_Responder	Mono_INHBA	CD4(TNFRSF9+ Treg)	IL1B	SIGIRR	4,15094E-03	17708,8	7,49468E-03	11149	2,06409E+00	4547	2,64778E+00	3660	8,88895E-01	25976	0	43212
Myeloid_vs_CD4_Responder	Mono_CD14	CD4(TGFB1+ Th17)	VCAN	ITGA4	4,15709E-03	15609,4	1,15757E-02	5241	2,89170E+00	1156	2,37565E+00	5868	8,96928E-01	22570	0	43212
Myeloid_vs_CD4_Responder	Mono_INHBA	CD4(TNFRSF9+ Treg)	SPP1	CD44	4,15747E-03	22936	5,38892E-03	18682	1,12712E+00	24224	2,07521E+00	9320	9,05037E-01	19242	0	43212
Myeloid_vs_CD4_Responder	Mono_INHBA	CD4(TNFRSF9+ Treg)	CCL4	CCR8	4,15943E-03	15728	5,52684E-02	228	1,48299E+00	12652	2,96497E+00	1967	9,01763E-01	20581	0	43212
Myeloid_vs_CD4_Responder	Mono_INHBA	CD4(TNFRSF9+ Treg)	VCAN	SELL	4,16204E-03	20910,4	1,04262E-02	6322	1,58319E+00	10518	1,71475E+00	16039	8,82991E-01	28461	0	43212
Myeloid_vs_CD4_Responder	Mono_INHBA	CD4(TNFRSF9+ Treg)	VCAN	CD44	4,16270E-03	22589,8	5,91315E-03	16217	1,43422E+00	13862	1,44830E+00	23361	9,11892E-01	16297	0	43212
Myeloid_vs_CD4_Responder	Mono_INHBA	CD4(TNFRSF9+ Treg)	CXCL16	CXCR6	4,17252E-03	27387,4	8,33412E-03	9343	9,96223E-01	30272	1,40194E+00	24835	8,81161E-01	29275	0	43212
Myeloid_vs_CD4_Responder	CD4(AREG+ Tm)	Mono_CD14	VIM	CD44	4,18075E-03	44395,6	1,92714E-03	72144	4,26582E-01	66570	1,03063E+00	38839	9,50686E-01	1213	0	43212
Myeloid_vs_CD4_Responder	Mono_INHBA	CD4(TNFRSF9+ Treg)	VEGFA	CD44	4,19418E-03	20880,8	8,47284E-03	9091	1,94733E+00	5597	1,31557E+00	27829	9,06433E-01	18675	0	43212
Myeloid_vs_CD4_Responder	CD4(NME1+ T)	Mono_CD14	GNAI2	C5AR1	4,19418E-03	24755,2	3,72505E-03	31874	1,81758E+00	7046	2,00146E+00	10463	8,76811E-01	31181	0	43212
Myeloid_vs_CD4_Responder	Macro_OLFML3	CD4(CXCL13+ Tfh)	CD14	ITGA4	4,20011E-03	26733,4	3,72453E-03	31883	1,46963E+00	12964	1,47052E+00	22672	8,96054E-01	22936	0	43212
Myeloid_vs_CD4_Responder	Mono_INHBA	CD4(CRTAM- T)	ICAM1	IL2RG	4,21198E-03	28769,6	4,77468E-03	22270	1,06600E+00	26921	1,32807E+00	27404	8,93452E-01	24041	0	43212
Myeloid_vs_CD4_Responder	CD4(ISG+ Treg)	Mono_INHBA	HLA-B	LILRB2	4,21264E-03	22538,2	3,72295E-03	31902	1,63080E+00	9696	1,40663E+00	24674	9,42916E-01	3207	0	43212
Myeloid_vs_CD4_Responder	Macro_IER3	CD4(IL26+ Th17)	HLA-DRB1	LAG3	4,21881E-03	18022,2	7,64271E-03	10816	1,38091E+00	15352	1,90499E+00	12137	9,29821E-01	8594	0	43212
Myeloid_vs_CD4_Responder	CD4(TNFRSF9+ Treg)	Mono_CD14	HLA-F	LILRB2	4,22057E-03	29168,8	3,72216E-03	31914	1,26124E+00	19113	1,47443E+00	22539	8,81657E-01	29066	0	43212
Myeloid_vs_CD4_Responder	Mono_INHBA	CD4(CRTAM- T)	IL1B	SIGIRR	4,22983E-03	18421	6,95908E-03	12603	2,04081E+00	4747	2,60577E+00	3938	8,85180E-01	27605	0	43212
Myeloid_vs_CD4_Responder	cDC(CD1C)	CD4(TNFRSF9+ Treg)	LYZ	ITGAL	4,23248E-03	23066,8	3,72085E-03	31932	1,50598E+00	12111	2,87983E+00	2344	8,89449E-01	25735	0	43212
Myeloid_vs_CD4_Responder	Macro_OLFML3	CD4(AREG+ Tm)	C1QB	C1QBP	4,23381E-03	20604,6	3,72072E-03	31934	1,89076E+00	6170	2,64579E+00	3667	9,07904E-01	18040	0	43212
Myeloid_vs_CD4_Responder	CD4(CXCL13+ Tfh)	Mono_CD16	HLA-B	LILRA1	4,23489E-03	30232,6	9,72516E-03	7184	1,50251E+00	12188	3,86264E-01	73792	9,15352E-01	14787	0	43212
Myeloid_vs_CD4_Responder	Mono_INHBA	CD4(CRTAM- T)	SPP1	CD44	4,23712E-03	18967	6,76200E-03	13143	1,33050E+00	16851	2,31993E+00	6399	9,14352E-01	15230	0	43212
Myeloid_vs_CD4_Responder	Mono_INHBA	CD4(CRTAM- T)	SPP1	PTGER4	4,23779E-03	20645	9,79464E-03	7099	1,26618E+00	18938	2,23723E+00	7294	8,87372E-01	26682	0	43212
Myeloid_vs_CD4_Responder	Mono_INHBA	CD4(CRTAM- T)	VCAN	ITGB1	4,23911E-03	22580,4	7,13403E-03	12103	1,52678E+00	11629	1,64956E+00	17614	8,83269E-01	28344	0	43212
Myeloid_vs_CD4_Responder	Mono_INHBA	CD4(CRTAM- T)	VCAN	CD44	4,24110E-03	18656,4	7,41980E-03	11353	1,63759E+00	9585	1,69301E+00	16547	9,20594E-01	12585	0	43212
Myeloid_vs_CD4_Responder	Mono_INHBA	CD4(CRTAM- T)	VEGFA	ITGB1	4,24310E-03	21434,2	1,02222E-02	6578	2,03989E+00	4759	1,51683E+00	21215	8,76277E-01	31407	0	43212
Myeloid_vs_CD4_Responder	Mono_INHBA	CD4(CRTAM- T)	HBEGF	CD44	4,24575E-03	21822,4	7,18401E-03	11959	1,48456E+00	12620	1,34192E+00	26880	9,16111E-01	14441	0	43212
Myeloid_vs_CD4_Responder	CD4(CXCL13+ Tfh)	Macro_NLRP3	HMGB1	CD163	4,24708E-03	24918,2	3,71967E-03	31954	1,49445E+00	12373	1,40467E+00	24739	9,21232E-01	12313	0	43212
Myeloid_vs_CD4_Responder	Mono_INHBA	CD4(CRTAM- T)	LGALS1	CD69	4,24841E-03	19213,6	5,20034E-03	19671	1,33079E+00	16834	1,81687E+00	13841	9,45422E-01	2510	0	43212
Myeloid_vs_CD4_Responder	CD4(CXCL13+ Tfh)	Mono_CD16	HLA-C	LILRA3	4,25318E-03	26753	1,64627E-02	2711	2,30560E+00	2958	4,37677E-01	70387	9,15973E-01	14497	0	43212
Myeloid_vs_CD4_Responder	CD4(NME1+ T)	Macro_LYVE1	GNAI2	C5AR1	4,25573E-03	28817,6	3,71856E-03	31967	1,81447E+00	7085	1,23975E+00	30591	8,76716E-01	31233	0	43212
Myeloid_vs_CD4_Responder	cDC(CD1C)	CD4(IL26+ Th17)	TNFSF10	CCR6	4,26039E-03	24476	1,19666E-02	4911	9,62958E-01	31974	1,68960E+00	16630	8,89623E-01	25653	0	43212
Myeloid_vs_CD4_Responder	Mono_INHBA	CD4(CRTAM- T)	S100A8	CD69	4,26372E-03	20384,6	6,72045E-03	13259	1,27019E+00	18817	1,67265E+00	17044	9,27568E-01	9591	0	43212
Myeloid_vs_CD4_Responder	Mono_INHBA	CD4(CRTAM- T)	VEGFA	CD44	4,26972E-03	17567,8	1,06317E-02	6116	2,15071E+00	3847	1,56029E+00	20017	9,15624E-01	14647	0	43212
Myeloid_vs_CD4_Responder	CD4(TNFRSF9+ Treg)	Macro_NLRP3	HLA-A	LILRB2	4,26972E-03	23227,2	3,71672E-03	31988	1,54678E+00	11202	1,38137E+00	25536	9,39799E-01	4198	0	43212
Myeloid_vs_CD4_Responder	Macro_OLFML3	CD4(CRTAM- T)	APOE	LDLR	4,27707E-03	17810,6	1,01988E-02	6612	1,99938E+00	5107	2,92569E+00	2123	8,74952E-01	31999	0	43212
Myeloid_vs_CD4_Responder	CD4(CXCL13+ Tfh)	Mono_CD16	RPS19	C5AR1	4,27801E-03	20033,6	6,42659E-03	14266	1,85828E+00	6547	1,16007E+00	33635	9,45430E-01	2508	0	43212
Myeloid_vs_CD4_Responder	CD4(IFNG+ Tfh/Th1)	Macro_ISG15	HLA-A	LILRB1	4,27908E-03	25627,6	3,97347E-03	29027	1,37308E+00	15575	1,20339E+00	32002	9,30404E-01	8322	0	43212
Myeloid_vs_CD4_Responder	Mono_INHBA	CD4(TNF+ T)	IL1B	SIGIRR	4,29246E-03	19221	6,27352E-03	14821	2,01101E+00	5010	2,72304E+00	3172	8,79804E-01	29890	0	43212
Myeloid_vs_CD4_Responder	Macro_FOLR2-APOE+	CD4(IL26+ Th17)	HLA-DPB1	LAG3	4,29318E-03	18129	7,60076E-03	10917	1,37858E+00	15421	1,96518E+00	11081	9,26607E-01	10014	0	43212
Myeloid_vs_CD4_Responder	CD4(AREG+ Tm)	Macro_NLRP3	CCL5	CCRL2	4,29448E-03	24733,4	1,37248E-02	3855	1,43931E+00	13725	1,23306E+00	30850	8,74875E-01	32025	0	43212
Myeloid_vs_CD4_Responder	Mono_INHBA	CD4(Tn)	INHBA	TGFBR3	4,29764E-03	26184,2	4,50213E-02	361	2,85757E+00	1230	2,15371E+00	8278	7,74388E-01	77840	0	43212
Myeloid_vs_CD4_Responder	Mono_INHBA	CD4(TNF+ T)	VCAN	CD44	4,30051E-03	27603	4,26174E-03	26214	1,21130E+00	20902	1,38189E+00	25519	8,97818E-01	22168	0	43212
Myeloid_vs_CD4_Responder	Mono_INHBA	CD4(TNF+ T)	LGALS1	CD69	4,30521E-03	17889,4	5,17780E-03	19784	1,32757E+00	16944	2,26531E+00	6971	9,45310E-01	2536	0	43212

Myeloid_vs_CD4_Post_R

Myeloid_vs_CD4_Responder	Mono_INHBA	CD4(TNF+ T)	S100A8	CD69	4,31597E-03	18759,2	6,69132E-03	13350	1,26697E+00	18908	2,12109E+00	8681	9,27422E-01	9645	0	43212
Myeloid_vs_CD4_Responder	Mono_INHBA	CD4(TNF+ T)	VEGFA	CD44	4,31732E-03	24404,6	6,10657E-03	15470	1,72441E+00	8260	1,24916E+00	30231	8,91591E-01	24850	0	43212
Myeloid_vs_CD4_Responder	CD4(NME1+ T)	Macro_OLFML3	HMGB1	HAVCR2	4,32001E-03	26233	4,68612E-03	22879	1,21384E+00	20812	1,20180E+00	32063	9,21518E-01	12199	0	43212
Myeloid_vs_CD4_Responder	Mono_CD14	CD4(TNF+ T)	VCAN	ITGA4	4,32110E-03	15584	1,07976E-02	5946	2,86885E+00	1209	2,65671E+00	3602	8,93667E-01	23951	0	43212
Myeloid_vs_CD4_Responder	Mono_INHBA	CD4(ISG+ Treg)	HLA-DRB1	LAG3	4,33215E-03	17408,2	7,77673E-03	10498	1,28255E+00	18405	2,30295E+00	6595	9,30386E-01	8331	0	43212
Myeloid_vs_CD4_Responder	Mono_INHBA	CD4(ISG+ Treg)	HLA-DPB1	LAG3	4,33486E-03	20754	6,57948E-03	13719	1,10082E+00	25378	2,07758E+00	9280	9,21547E-01	12181	0	43212
Myeloid_vs_CD4_Responder	Mono_INHBA	CD4(ISG+ Treg)	ICAM1	IL2RA	4,33959E-03	17673,8	2,31157E-02	1408	1,61830E+00	9905	2,06733E+00	9431	8,92616E-01	24413	0	43212
Myeloid_vs_CD4_Responder	Mono_INHBA	CD4(ISG+ Treg)	HLA-DPA1	LAG3	4,34906E-03	20399,8	6,62845E-03	13545	1,09426E+00	25667	2,13397E+00	8530	9,24236E-01	11045	0	43212
Myeloid_vs_CD4_Responder	CD4(AREG+ Tm)	Macro_FOLR2+APOE+	ANXA1	FPR1	4,35205E-03	23216,2	6,10503E-03	15475	1,48996E+00	12488	1,19843E+00	32198	9,20268E-01	12708	0	43212
Myeloid_vs_CD4_Responder	CD4(CXCL13+ Tfh)	Mono_INHBA	RPS19	C5AR1	4,35313E-03	21803	5,37485E-03	18769	1,55737E+00	11003	1,20048E+00	32112	9,40632E-01	3919	0	43212
Myeloid_vs_CD4_Responder	Mono_INHBA	CD4(ISG+ Treg)	CD86	CTLA4	4,35380E-03	23281	1,01797E-02	6634	1,34315E+00	16472	1,31374E+00	27900	8,97766E-01	22187	0	43212
Myeloid_vs_CD4_Responder	CD4(ISG+ Treg)	Macro_NLRP3	RPS19	C5AR1	4,35652E-03	20149,8	6,02053E-03	15783	1,84857E+00	6660	1,20041E+00	32117	9,43722E-01	2977	0	43212
Myeloid_vs_CD4_Responder	Mono_INHBA	CD4(ISG+ Treg)	HLA-DRB5	LAG3	4,36059E-03	19465,8	7,37303E-03	11473	1,19463E+00	21557	2,23515E+00	7317	9,17726E-01	13770	0	43212
Myeloid_vs_CD4_Responder	CD4(Tn)	cDC_CLEC9A	CD52	SIGLEC10	4,36330E-03	30309,8	4,20675E-03	26698	1,05933E+00	27229	1,20029E+00	32127	8,97544E-01	22283	0	43212
Myeloid_vs_CD4_Responder	Mono_INHBA	CD4(ISG+ Treg)	IL1B	SIGIRR	4,36602E-03	15453	9,99272E-03	6870	2,17269E+00	3687	2,72489E+00	3155	9,02325E-01	20341	0	43212
Myeloid_vs_CD4_Responder	Mono_INHBA	CD4(ISG+ Treg)	SPP1	CD44	4,37282E-03	27325,4	4,36275E-03	25385	9,75122E-01	31339	1,83201E+00	13541	8,95563E-01	23150	0	43212
Myeloid_vs_CD4_Responder	Macro_IFI27	CD4(ISG+ Treg)	B2M	CD3D	4,37335E-03	46871	3,66362E-03	32719	3,49607E-01	72984	2,15353E-01	85417	9,62467E-01	23	0	43212
Myeloid_vs_CD4_Responder	Mono_INHBA	CD4(ISG+ Treg)	CCL4	CCR8	4,37554E-03	18466,6	4,12833E-02	422	1,32923E+00	16896	2,42192E+00	5432	8,88062E-01	26371	0	43212
Myeloid_vs_CD4_Responder	Mono_INHBA	CD4(ISG+ Treg)	VCAN	SELL	4,37962E-03	17864,2	1,42479E-02	3594	1,75770E+00	7808	1,87564E+00	12682	8,98184E-01	22025	0	43212
Myeloid_vs_CD4_Responder	Mono_INHBA	CD4(ISG+ Treg)	VCAN	CD44	4,38031E-03	27162,4	4,78715E-03	22171	1,28222E+00	18417	1,20510E+00	31946	9,03029E-01	20066	0	43212
Myeloid_vs_CD4_Responder	Mono_INHBA	CD4(ISG+ Treg)	LGALS3	LAG3	4,38508E-03	18219,8	1,12973E-02	5487	1,28448E+00	18337	2,17633E+00	7993	9,12426E-01	16070	0	43212
Myeloid_vs_CD4_Responder	Mono_INHBA	CD4(ISG+ Treg)	CXCL16	CXCR6	4,39122E-03	26290,2	9,53010E-03	7433	1,07105E+00	26684	1,31856E+00	27721	8,88004E-01	26401	0	43212
Myeloid_vs_CD4_Responder	Mono_INHBA	CD4(ISG+ Treg)	LGALS1	CD69	4,39258E-03	22010	4,29727E-03	25920	1,20173E+00	21291	1,73415E+00	15591	9,40287E-01	4036	0	43212
Myeloid_vs_CD4_Responder	Macro_ISG15	CD4(Tn)	S100A8	CD69	4,39493E-03	17824,2	6,97875E-03	12547	1,37502E+00	15514	2,11239E+00	8796	9,28824E-01	9052	0	43212
Myeloid_vs_CD4_Responder	Mono_INHBA	CD4(ISG+ Treg)	LYZ	ITGAL	4,40488E-03	21988	4,42743E-03	24846	1,53168E+00	11520	2,16260E+00	8156	8,97712E-01	22206	0	43212
Myeloid_vs_CD4_Responder	Macro_OLFML3	CD4(Tn)	C1QB	C1QBP	4,40762E-03	20618,2	3,70195E-03	32192	1,88939E+00	6184	2,69025E+00	3383	9,07692E-01	18120	0	43212
Myeloid_vs_CD4_Responder	Mono_INHBA	CD4(ISG+ Treg)	S100A8	CD69	4,41241E-03	23273,6	5,55341E-03	17827	1,14113E+00	23660	1,58992E+00	19222	9,20893E-01	12447	0	43212
Myeloid_vs_CD4_Responder	Mono_INHBA	CD4(TGFB1+ Th17)	CCL3	CCR4	4,41977E-03	18948,4	1,07505E-02	5992	2,17169E+00	3694	2,44559E+00	5217	8,64757E-01	36627	0	43212
Myeloid_vs_CD4_Responder	Mono_CD16	CD4(IFNG+ Tfh/Th1)	HLA-DRA	LAG3	4,42037E-03	16804,4	8,65327E-03	8777	1,37429E+00	15537	2,00689E+00	10369	9,35174E-01	6127	0	43212
Myeloid_vs_CD4_Responder	Macro_OLFML3	CD4(CXCL13+ Tfh)	HLA-DMB	CD4	4,42408E-03	24950,8	3,69974E-03	32216	1,63169E+00	9685	1,54528E+00	20403	9,05053E-01	19238	0	43212
Myeloid_vs_CD4_Responder	CD4(GZMK+ Teff)	Mono_INHBA	RPS19	C5AR1	4,43576E-03	22771	5,01720E-03	20722	1,46435E+00	13078	1,19749E+00	32233	9,38680E-01	4610	0	43212
Myeloid_vs_CD4_Responder	CD4(GZMK+ Teff)	Macro_LYVE1	CCL5	CCR1	4,44678E-03	24772,8	1,40440E-02	3678	1,40462E+00	14686	1,25501E+00	30039	8,74433E-01	32249	0	43212
Myeloid_vs_CD4_Responder	CD4(ISG+ Treg)	Macro_ISG15	B2M	LILRB1	4,45713E-03	24745,4	3,69555E-03	32264	1,45847E+00	13230	1,24299E+00	30460	9,38791E-01	4561	0	43212
Myeloid_vs_CD4_Responder	Mono_INHBA	CD4(CXCL13+ Tfh)	IL1B	SIGIRR	4,46266E-03	18424,2	6,96561E-03	12583	2,04109E+00	4745	2,59778E+00	4000	8,85228E-01	27581	0	43212
Myeloid_vs_CD4_Responder	Mono_INHBA	CD4(CXCL13+ Tfh)	LGALS1	PTPRC	4,46681E-03	21947	3,73128E-03	31796	1,25606E+00	19299	1,78941E+00	14417	9,51555E-01	1011	0	43212
Myeloid_vs_CD4_Responder	Mono_CD14	CD4(TGFB1+ Th17)	VCAN	SELL	4,46747E-03	16241,8	1,18471E-02	5004	2,85337E+00	1237	2,36129E+00	6014	8,89432E-01	25742	0	43212
Myeloid_vs_CD4_Responder	Mono_INHBA	CD4(CXCL13+ Tfh)	SPP1	CD44	4,46889E-03	25179,8	4,85186E-03	21759	1,04757E+00	27803	1,91371E+00	11987	9,00429E-01	21138	0	43212
Myeloid_vs_CD4_Responder	Mono_INHBA	CD4(CXCL13+ Tfh)	SPP1	PTGER4	4,46958E-03	20848,8	9,46001E-03	7537	1,24659E+00	19632	2,31544E+00	6454	8,85624E-01	27409	0	43212
Myeloid_vs_CD4_Responder	Mono_INHBA	CD4(CXCL13+ Tfh)	VCAN	CD44	4,47304E-03	25086	5,32385E-03	19007	1,35467E+00	16133	1,28679E+00	28911	9,07582E-01	18167	0	43212
Myeloid_vs_CD4_Responder	CD4(Tn)	Macro_FOLR2+APOE+	CD52	SIGLEC10	4,47716E-03	27921	6,11958E-03	15435	1,58555E+00	10475	6,93071E-01	54895	9,13539E-01	15588	0	43212
Myeloid_vs_CD4_Responder	Mono_INHBA	CD4(CXCL13+ Tfh)	LGALS1	CD69	4,48136E-03	24721,2	3,86720E-03	30190	1,14026E+00	23705	1,51287E+00	21308	9,37257E-01	5191	0	43212
Myeloid_vs_CD4_Responder	CD4(TNF+ T)	Macro_ISG15	TNFSF9	HLA-DPA1	4,48483E-03	26258,2	4,54500E-03	23942	1,12375E+00	24372	2,22230E+00	7461	8,74328E-01	32304	0	43212
Myeloid_vs_CD4_Responder	Mono_INHBA	CD4(CXCL13+ Tfh)	S100A8	CD69	4,49595E-03	26082,6	4,99762E-03	20859	1,07966E+00	26318	1,36865E+00	25940	9,16966E-01	14084	0	43212
Myeloid_vs_CD4_Responder	CD4(TNFRSF9+ Treg)	Macro_ISG15	LTB	CD40	4,49803E-03	27669,8	8,26630E-03	9470	1,09275E+00	25740	1,19504E+00	32323	8,85183E-01	27604	0	43212
Myeloid_vs_CD4_Responder	CD4(NME1+ T)	Mast	GNAS	ADRB2	4,50918E-03	24762,4	1,23887E-02	4618	1,49636E+00	12320	1,19468E+00	32339	8,76493E-01	31323	0	43212
Myeloid_vs_CD4_Responder	CD4(Tn)	Mono_CD16	HLA-B	LILRA1	4,51194E-03	32467,4	9,27030E-03	7793	1,37579E+00	15493	2,90448E-01	80220	9,13478E-01	15619	0	43212
Myeloid_vs_CD4_Responder	CD4(TNF+ T)	Macro_FOLR2+APOE+	ADAM10	GPNMB	4,51870E-03	23551	6,48094E-03	14077	2,58327E+00	1894	1,73283E+00	15625	8,50880E-01	42947	0	43212
Myeloid_vs_CD4_Responder	CD4(AREG+ Tm)	Macro_NLRP3	VIM	CD44	4,52906E-03	48323,4	1,90882E-03	72743	4,18416E-01	67214	6,50831E-01	57185	9,50462E-01	1263	0	43212
Myeloid_vs_CD4_Responder	Mono_INHBA	CD4(GZMK+ Teff)	IL1B	SIGIRR	4,53363E-03	17254,4	7,83448E-03	10351	2,07887E+00	4409	2,71054E+00	3242	8,91066E-01	25058	0	43212
Myeloid_vs_CD4_Responder	CD4(TGFB1+ Th17)	Mast	HSPA8	ADRB2	4,53675E-03	23471,2	1,30367E-02	4219	1,37092E+00	15641	9,65145E-01	41597	9,20307E-01	12687	0	43212
Myeloid_vs_CD4_Responder	Mono_INHBA	CD4(GZMK+ Teff)	SPP1	CD44	4,53924E-03	19757,4	6,41758E-03	14300	1,27948E+00	18483	2,29612E+00	6668	9,12283E-01	16124	0	43212
Myeloid_vs_CD4_Responder	Mono_INHBA	CD4(GZMK+ Teff)	VCAN	CD44	4,54274E-03	19315,2	7,04187E-03	12386	1,58658E+00	10449	1,66920E+00	17133	9,18662E-01	13396	0	43212
Myeloid_vs_CD4_Responder	Mono_INHBA	CD4(GZMK+ Teff)	HBEGF	CD44	4,54835E-03	22634	6,81808E-03	12986	1,43354E+00	13876	1,31811E+00	27744	9,14080E-01	15352	0	43212
Myeloid_vs_CD4_Responder	CD4(TNFRSF9+ Treg)	Mono_CD16	HLA-C	LILRA1	4,54919E-03	25689,6	1,07253E-02	6019	1,71540E+00	8394	6,87628E-01	55171	9,13369E-01	15652	0	43212
Myeloid_vs_CD4_Responder	CD4(ISG+ Treg)	Mono_CD16	HLA-C	LILRA1	4,55032E-03	26293,6	1,07250E-02	6020	1,71534E+00	8395	6,33538E-01	58188	9,13368E-01	15653	0	43212
Myeloid_vs_CD4_Responder	Mono_INHBA	CD4(GZMK+ Teff)	LGALS1	CD69	4,55046E-03	22335,6	4,34911E-03	25494	1,20914E+00	20986	1,63239E+00	18060	9,40623E-01	3926	0	43212
Myeloid_vs_CD4_Responder	CD4(ISG+ Treg)	cDC(CD1C)	B2M	CD1A	4,55538E-03	21185,6	3,23366E-02	689	1,99777E+00	5122	1,19287E+00	32405	8,92439E-01	24500	0	43212
Myeloid_vs_CD4_Responder	Mono_INHBA	CD4(GZMK+ Teff)	S100A8	CD69	4,56452E-03	23685,6	5,62040E-03	17484	1,14854E+00	23355	1,48816E+00	22100	9,21329E-01	12277	0	43212
Myeloid_vs_CD4_Responder	Mono_INHBA	CD4(GZMK+ Teff)	VEGFA	CD44	4,56805E-03	18087,4	1,00902E-02	6748	2,09969E+00	4265	1,53648E+00	20639	9,13583E-01	15573	0	43212
Myeloid_vs_CD4_Responder	Macro_FOLR2+APOE+	CD4(IL26+ Th17)	HLA-DQB1	LAG3	4,57073E-03	18484,2	9,08913E-03	8096	1,46630E+00	13031	1,73051E+00	15671	9,20978E-01	12411	0	43212
Myeloid_vs_CD4_Responder	CD4(TGFB1+ Th17)	cDC_CLEC9A	CALM1	MYLK	4,57492E-03	30440	2,61219E-02	1100	2,03820E+00	4774	8,33982E-01	47516	8,24072E-01	55598	0	43212
Myeloid_vs_CD4_Responder	cDC(CD1C)	CD4(Tn)	LGALS1	CD69	4,57792E-03	25105,2	3,68262E-03	32437	9,60526E-01	32093	1,91386E+00	11984	9,35803E-01	5800	0	43212
Myeloid_vs_CD4_Responder	Mono_INHBA	CD4(IFNG+ Tfh/Th1)	HLA-DQA2	LAG3	4,57933E-03	23545	8,78943E-03	8571	1,07949E+00	26326	1,96365E+00	11117	8,82887E-01	28499	0	43212
Myeloid_vs_CD4_Responder	Mono_INHBA	CD4(IFNG+ Tfh/Th1)	HLA-DQA1	LAG3	4,58074E-03	22017,6	7,02506E-03	12433	1,04640E+00	27874	2,08331E+00	9208	9,09435E-01	17361	0	43212
Myeloid_vs_CD4_Responder	Mono_INHBA	CD4(IFNG+ Tfh/Th1)	HLA-DQB1	LAG3	4,58356E-03	20683	7,79901E-03	10437	1,09426E+00	25669	2,07953E+00	9257	9,15225E-01	14840	0	43212
Myeloid_vs_CD4_Responder	Mono_INHBA	CD4(IFNG+ Tfh/Th1)	HLA-DPB1	LAG3	4,58498E-03	17108,6	8,39452E-03	9213	1,25273E+00	19412	2,45200E+00	5161	9,29914E-01	8545	0	43212
Myeloid_vs_CD4_Responder	Mono_INHBA	CD4(IFNG+ Tfh/Th1)	ICAM1	IL2RG	4,58992E-03	23912,4	6,03188E-03	15740	1,20092E+00	21320	1,57287E+00	19677	9,04076E-01	19613	0	43212
Myeloid_vs_CD4_Responder	Macro_OLFML3	CD4(TNFRSF9+ Treg)	HLA-DPA1	LAG3	4,59558E-03	23136	3,68077E-03	32462	1,43305E+00	13892	2,45494E+00	5138	9,00896E-01	20976	0	43212
Myeloid_vs_CD4_Responder	Mono_INHBA	CD4(IFNG+ Tfh/Th1)	HLA-DPA1	LAG3	4,59913E-03	16817,2	8,45700E-03	9111	1,24618E+00	19654	2,50839E+00	4703	9,32337E-01	7406	0	43212

Myeloid_vs_CD4_Post_R

Myeloid_vs_CD4_Responder	Mono_INHBA	CD4(IFNG+ Tfh/Th1)	CD86	CTLA4	4,60338E-03	24650,2	9,23137E-03	7847	1,27246E+00	18732	1,27581E+00	29286	8,93190E-01	24174	0	43212
Myeloid_vs_CD4_Responder	Mono_INHBA	CD4(IFNG+ Tfh/Th1)	HLA-DRB5	LAG3	4,60905E-03	16235	9,40698E-03	7601	1,34654E+00	16380	2,60957E+00	3909	9,26468E-01	10073	0	43212
Myeloid_vs_CD4_Responder	Macro_OLFML3	CD4(TNFRSF9+ Treg)	HLA-DMB	CD4	4,61118E-03	25139,2	3,67973E-03	32484	1,62933E+00	9715	1,52520E+00	20963	9,04820E-01	19322	0	43212
Myeloid_vs_CD4_Responder	Mono_INHBA	CD4(IFNG+ Tfh/Th1)	IL1B	SIGIRR	4,61615E-03	18075,6	7,21432E-03	11877	2,05191E+00	4645	2,62315E+00	3820	8,86998E-01	26824	0	43212
Myeloid_vs_CD4_Responder	Mono_INHBA	CD4(IFNG+ Tfh/Th1)	SPP1	CD44	4,62255E-03	26678,4	4,47232E-03	24499	9,91352E-01	30537	1,88742E+00	12470	8,96717E-01	22674	0	43212
Myeloid_vs_CD4_Responder	Mono_INHBA	CD4(IFNG+ Tfh/Th1)	VCAN	ITGB1	4,62397E-03	22098	7,28430E-03	11713	1,53735E+00	11397	1,70684E+00	16236	8,84339E-01	27932	0	43212
Myeloid_vs_CD4_Responder	Mono_INHBA	CD4(IFNG+ Tfh/Th1)	VCAN	ITGA4	4,62468E-03	23591,4	7,37018E-03	11484	1,50879E+00	12054	1,60564E+00	18816	8,74112E-01	32391	0	43212
Myeloid_vs_CD4_Responder	Mono_INHBA	CD4(IFNG+ Tfh/Th1)	VCAN	CD44	4,62539E-03	26384,4	4,90738E-03	21404	1,29845E+00	17861	1,26051E+00	29844	9,04109E-01	19601	0	43212
Myeloid_vs_CD4_Responder	Macro_OLFML3	CD4(TGFB1+ Th17)	FN1	ITGA5_ITGB1	4,62824E-03	20149,2	1,11295E-02	5671	1,90917E+00	5964	1,84011E+00	13391	8,73868E-01	32508	0	43212
Myeloid_vs_CD4_Responder	Mono_INHBA	CD4(IFNG+ Tfh/Th1)	VEGFA	ITGB1	4,62895E-03	20947,4	1,04375E-02	6309	2,05047E+00	4661	1,57412E+00	19649	8,77402E-01	30906	0	43212
Myeloid_vs_CD4_Responder	Mono_INHBA	CD4(IFNG+ Tfh/Th1)	CXCL16	CXCR6	4,63251E-03	26336	9,01103E-03	8223	1,03858E+00	28257	1,41583E+00	24386	8,85189E-01	27602	0	43212
Myeloid_vs_CD4_Responder	CD4(GZMK+ Teff)	Mono_CD14	CCL5	CCR1	4,64178E-03	22513,8	1,38910E-02	3764	1,39504E+00	14962	1,63068E+00	18104	8,73830E-01	32527	0	43212
Myeloid_vs_CD4_Responder	Mono_INHBA	CD4(IFNG+ Tfh/Th1)	LYZ	ITGAL	4,64463E-03	23340,6	3,99319E-03	28804	1,49844E+00	12264	2,16556E+00	8118	8,92874E-01	24305	0	43212
Myeloid_vs_CD4_Responder	CD4(IFNG+ Tfh/Th1)	Macro_NLRP3	CCL5	CCRL2	4,64535E-03	23616,8	1,34626E-02	4010	1,42713E+00	14060	1,41935E+00	24270	8,73815E-01	32532	0	43212
Myeloid_vs_CD4_Responder	Mono_INHBA	CD4(IFNG+ Tfh/Th1)	S100A8	CD69	4,65035E-03	30506,4	4,05612E-03	28153	9,75541E-01	31320	1,19985E+00	32145	9,08666E-01	17702	0	43212
Myeloid_vs_CD4_Responder	CD4(TGFB1+ Th17)	cDC(CD1C)	TNFSF13B	HLA-DPB1	4,65536E-03	31285,8	1,53114E-03	86041	1,58416E+00	10499	3,33413E+00	932	9,13162E-01	15745	0	43212
Myeloid_vs_CD4_Responder	Macro_OLFML3	CD4(GZMK+ Teff)	APOE	LSR	4,66681E-03	18341,2	1,01420E-02	6671	1,86903E+00	6421	2,77976E+00	2840	8,73751E-01	32562	0	43212
Myeloid_vs_CD4_Responder	Mono_INHBA	CD4(NME1+ T)	ICAM1	IL2RG	4,67900E-03	25557,4	5,68563E-03	17188	1,16376E+00	22730	1,42963E+00	23960	9,01482E-01	20697	0	43212
Myeloid_vs_CD4_Responder	Mono_INHBA	CD4(TNFRSF9+ Treg)	LGALS1	PTPRC	4,68259E-03	22512,6	3,67280E-03	32584	1,23752E+00	19974	1,72921E+00	15703	9,51190E-01	1090	0	43212
Myeloid_vs_CD4_Responder	Mono_INHBA	CD4(NME1+ T)	HLA-DRA	LAG3	4,68403E-03	26887,6	3,67269E-03	32586	1,13083E+00	24061	1,76649E+00	14869	9,03829E-01	19710	0	43212
Myeloid_vs_CD4_Responder	CD4(AREG+ Tm)	cDC_LAMP3	CD96	PVR	4,70109E-03	47682,4	2,41050E-02	1287	1,10803E+00	25102	2,56624E-01	82514	7,53830E-01	86297	0	43212
Myeloid_vs_CD4_Responder	Mono_INHBA	CD4(NME1+ T)	IL1B	SIGIRR	4,70347E-03	19438,2	6,30617E-03	14703	2,01243E+00	5001	2,53515E+00	4513	8,80078E-01	29762	0	43212
Myeloid_vs_CD4_Responder	Mono_INHBA	CD4(GZMK+ Teff)	LGALS1	PTPRC	4,70419E-03	22438,2	3,67060E-03	32614	1,23682E+00	20001	1,74761E+00	15266	9,51176E-01	1098	0	43212
Myeloid_vs_CD4_Responder	CD4(TGFB1+ Th17)	cDC_CLEC9A	ANXA1	DYSF	4,70996E-03	22892,2	1,45093E-02	3460	1,83671E+00	6804	1,18733E+00	32622	8,83216E-01	28363	0	43212
Myeloid_vs_CD4_Responder	Mono_INHBA	CD4(NME1+ T)	LGALS1	ITGB1	4,70996E-03	23943,2	3,67021E-03	32622	1,26025E+00	19151	1,82627E+00	13665	9,24187E-01	11066	0	43212
Myeloid_vs_CD4_Responder	Mono_INHBA	CD4(NME1+ T)	SPP1	CD44	4,71068E-03	26745,2	4,46862E-03	24527	9,90803E-01	30558	1,87213E+00	12743	8,96679E-01	22686	0	43212
Myeloid_vs_CD4_Responder	Mono_INHBA	CD4(NME1+ T)	VCAN	ITGB1	4,71285E-03	22349,2	7,34256E-03	11564	1,54145E+00	11319	1,64036E+00	17852	8,84746E-01	27799	0	43212
Myeloid_vs_CD4_Responder	Mono_INHBA	CD4(NME1+ T)	VCAN	CD44	4,71502E-03	26502,4	4,90332E-03	21434	1,29790E+00	17872	1,24521E+00	30380	9,04073E-01	19614	0	43212
Myeloid_vs_CD4_Responder	Mono_INHBA	CD4(NME1+ T)	VEGFA	ITGB1	4,71935E-03	21256,8	1,05210E-02	6224	2,05457E+00	4627	1,50763E+00	21475	8,77830E-01	30746	0	43212
Myeloid_vs_CD4_Responder	Mono_INHBA	CD4(NME1+ T)	LGALS3	LAG3	4,72008E-03	29320,8	5,12875E-03	20068	9,83791E-01	30921	1,53906E+00	20567	8,75314E-01	31836	0	43212
Myeloid_vs_CD4_Responder	Mono_INHBA	CD4(CRTAM- T)	INHBA	TGFBR3	4,72282E-03	27382	3,95091E-02	460	2,82896E+00	1290	2,07948E+00	9259	7,62775E-01	82689	0	43212
Myeloid_vs_CD4_Responder	CD4(Tn)	pDC_LILRA4	TNF	TNFRSF21	4,72365E-03	27601,6	1,02214E-02	6580	3,15910E+00	753	1,72504E+00	15804	7,88837E-01	71659	0	43212
Myeloid_vs_CD4_Responder	CD4(ISG+ Treg)	cDC_CLEC9A	LGALS9	HAVCR2	4,72731E-03	22953,8	5,24323E-03	19439	1,51415E+00	11929	2,21516E+00	7543	8,73549E-01	32646	0	43212
Myeloid_vs_CD4_Responder	Macro_OLFML3	CD4(NME1+ T)	FN1	ITGA4_ITGB7	4,73528E-03	19855,8	1,35428E-02	3954	1,89595E+00	6109	1,84216E+00	13347	8,73526E-01	32657	0	43212
Myeloid_vs_CD4_Responder	Mono_INHBA	CD4(NME1+ T)	LYZ	ITGAL	4,73963E-03	22635,8	4,19515E-03	26800	1,51390E+00	11935	2,18204E+00	7930	8,95211E-01	23302	0	43212
Myeloid_vs_CD4_Responder	Mono_INHBA	CD4(NME1+ T)	S100A8	CD69	4,74399E-03	29453,8	4,21227E-03	26638	9,92810E-01	30453	1,25741E+00	29951	9,10221E-01	17015	0	43212
Myeloid_vs_CD4_Responder	CD4(CXCL13+ Tfh)	Mast	HSPA8	ADRB2	4,74580E-03	24199	1,29722E-02	4260	1,36483E+00	15823	8,89011E-01	44924	9,20125E-01	12776	0	43212
Myeloid_vs_CD4_Responder	Mono_INHBA	CD4(NME1+ T)	INHBA	ACTR2	4,75271E-03	17841,8	1,47700E-02	3357	2,86980E+00	1205	2,09114E+00	9099	8,74253E-01	32336	0	43212
Myeloid_vs_CD4_Responder	CD4(GZMK+ Teff)	Macro_NLRP3	CD99	PILRA	4,75634E-03	26185,6	4,81993E-03	21971	1,39794E+00	14857	1,18531E+00	32686	9,07504E-01	18202	0	43212
Myeloid_vs_CD4_Responder	pDC_LILRA4	CD4(TNFRSF9+ Treg)	APP	RPSA	4,76217E-03	15816,6	1,03060E-02	6484	1,36457E+00	15837	2,21987E+00	7490	9,35314E-01	6060	0	43212
Myeloid_vs_CD4_Responder	Macro_OLFML3	CD4(TGFB1+ Th17)	HLA-DRB5	LAG3	4,76727E-03	24035,8	3,66449E-03	32701	1,55026E+00	11134	2,32246E+00	6371	8,87182E-01	26761	0	43212
Myeloid_vs_CD4_Responder	CD4(TGFB1+ Th17)	Macro_OLFML3	TNFSF13B	HLA-DPB1	4,77154E-03	31981,8	1,52238E-03	86383	1,57403E+00	10676	2,62627E+00	3793	9,12935E-01	15845	0	43212
Myeloid_vs_CD4_Responder	Macro_OLFML3	CD4(IFNG+ Tfh/Th1)	FN1	ITGA4_ITGB7	4,77748E-03	19950	1,35113E-02	3978	1,87564E+00	6338	1,83357E+00	13507	8,73397E-01	32715	0	43212
Myeloid_vs_CD4_Responder	cDC(CD1C)	CD4(ISG+ Treg)	HLA-DQA1	CD4	4,77967E-03	19054,2	3,66366E-03	32718	1,64825E+00	9411	2,95481E+00	2009	9,31247E-01	7921	0	43212
Myeloid_vs_CD4_Responder	CD4(TNFRSF9+ Treg)	Macro_ISG15	CD28	CD86	4,78333E-03	26487	8,85372E-03	8462,5	1,30489E+00	17652,5	1,24508E+00	30384,5	8,73375E-01	32723,5	0	43212
Myeloid_vs_CD4_Responder	Mono_INHBA	CD4(TGFB1+ Th17)	INHBA	TGFBR3	4,78829E-03	27584,4	3,87903E-02	479	2,82522E+00	1299	2,05736E+00	9582	7,61109E-01	83350	0	43212
Myeloid_vs_CD4_Responder	CD4(ISG+ Treg)	cDC(CD1C)	CD52	SIGLEC10	4,79723E-03	31944,4	3,90450E-03	29760	9,47793E-01	32742	1,24984E+00	30209	8,94065E-01	23799	0	43212
Myeloid_vs_CD4_Responder	CD4(IFNG+ Tfh/Th1)	Macro_ISG15	B2M	LILRB1	4,80236E-03	25204,8	3,66151E-03	32749	1,41858E+00	14275	1,22606E+00	31127	9,38525E-01	4661	0	43212
Myeloid_vs_CD4_Responder	CD4(TNF+ T)	cDC_CLEC9A	FLT3LG	FLT3	4,81021E-03	27216,2	2,38946E-02	1302	3,98102E+00	270	1,49720E+00	21795	7,93695E-01	69502	0	43212
Myeloid_vs_CD4_Responder	CD4(AREG+ Tm)	Macro_LYVE1	SPN	SIGLEC1	4,81078E-03	39354,4	1,05803E-02	6168	2,14455E+00	3895	6,31050E-01	58341	7,56654E-01	85156	0	43212
Myeloid_vs_CD4_Responder	Macro_FOLR2+APOE+	CD4(AREG+ Tm)	APOE	SORL1	4,81743E-03	15254,4	6,62990E-03	13539	2,52073E+00	2081	3,08152E+00	1556	9,12834E-01	15884	0	43212
Myeloid_vs_CD4_Responder	Mono_INHBA	CD4(TGFB1+ Th17)	VCAN	ITGB1	4,81925E-03	25532,6	5,91428E-03	16214	1,44094E+00	13679	1,49761E+00	21786	8,73250E-01	32772	0	43212
Myeloid_vs_CD4_Responder	CD4(NME1+ T)	Mono_CD16	GNAI2	FPR1	4,83471E-03	29384,2	3,71991E-03	31950	1,73609E+00	8103	1,23282E+00	30863	8,73207E-01	32793	0	43212
Myeloid_vs_CD4_Responder	Mono_INHBA	CD4(NME1+ T)	VCAN	SELL	4,84577E-03	23291,2	8,67972E-03	8739	1,50344E+00	12164	1,57869E+00	19533	8,73183E-01	32808	0	43212
Myeloid_vs_CD4_Responder	pDC_LILRA4	CD4(GZMK+ Teff)	APP	RPSA	4,85532E-03	15631,8	1,02799E-02	6516	1,36180E+00	15916	2,31755E+00	6422	9,35237E-01	6093	0	43212
Myeloid_vs_CD4_Responder	Mono_INHBA	CD4(IL26+ Th17)	INHBA	ACTR2	4,86056E-03	18012,4	1,44719E-02	3486	2,86001E+00	1226	2,07573E+00	9310	8,73128E-01	32828	0	43212
Myeloid_vs_CD4_Responder	CD4(ISG+ Treg)	Mono_CD14	LTB	TNFRSF1A	4,87019E-03	26592,8	7,42819E-03	11325	9,45661E-01	32841	1,49682E+00	21814	8,94132E-01	23772	0	43212
Myeloid_vs_CD4_Responder	cDC(CD1C)	CD4(TNF+ T)	CD86	CTLA4	4,88207E-03	24138,6	6,24395E-03	14947	1,40144E+00	14774	1,76532E+00	14903	8,73056E-01	32857	0	43212
Myeloid_vs_CD4_Responder	Mono_INHBA	CD4(IFNG+ Tfh/Th1)	INHBA	TGFBR3	4,88364E-03	27751,4	3,74535E-02	516	2,81829E+00	1312	2,09589E+00	9031	7,57907E-01	84686	0	43212
Myeloid_vs_CD4_Responder	Mono_INHBA	CD4(IFNG+ Tfh/Th1)	ICAM1	ITGAL_ITGB2	4,89546E-03	31074,2	3,65244E-03	32875	1,11205E+00	24918	1,45009E+00	23308	8,77069E-01	31058	0	43212
Myeloid_vs_CD4_Responder	Macro_OLFML3	CD4(NME1+ T)	HLA-DQA2	LAG3	4,91709E-03	22586,4	7,29814E-03	11689	1,54979E+00	11142	1,80876E+00	13985	8,72928E-01	32904	0	43212
Myeloid_vs_CD4_Responder	Macro_IFI27	CD4(IL26+ Th17)	CD14	ITGA4	4,92096E-03	20009	5,97529E-03	15971	1,52351E+00	11711	1,77431E+00	14706	9,16098E-01	14445	0	43212
Myeloid_vs_CD4_Responder	CD4(GZMK+ Teff)	cDC_CLEC9A	ANXA1	DYSF	4,93280E-03	24479,4	1,19628E-02	4915	1,70556E+00	8537	1,18221E+00	32808	8,72889E-01	32925	0	43212
Myeloid_vs_CD4_Responder	Macro_IFI27	CD4(IFNG+ Tfh/Th1)	B2M	CD3D	4,94357E-03	45665,2	3,63991E-03	33036	3,43271E-01	73556	3,15955E-01	78496	9,62350E-01	26	0	43212
Myeloid_vs_CD4_Responder	Macro_OLFML3	CD4(GZMK+ Teff)	CD14	ITGA4	4,96058E-03	26800,8	3,64485E-03	32962	1,46200E+00	13137	1,51183E+00	21330	8,95042E-01	23363	0	43212
Myeloid_vs_CD4_Responder	CD4(TGFB1+ Th17)	Macro_LYVE1	SPN	SIGLEC1	4,96068E-03	35400,4	1,47330E-02	3378	2,23451E+00	3321	7,07994E-01	54056	7,85829E-01	73035	0	43212
Myeloid_vs_CD4_Responder	Macro_IER3	CD4(TNFRSF9+ Treg)	CCL4	CCR8	4,96910E-03	13799,2	7,14049E-02	132	1,83146E+00	6875	2,79234E+00	2766	9,12540E-01	16011	0	43212
Myeloid_vs_CD4_Responder	Macro_OLFML3	CD4(Tn)	C3	CD46	4,97640E-03	21289,2	6,95745E-03	12612	2,07437E+00	4446	1,85013E+00	13193	8,72781E-01	32983	0	43212
Myeloid_vs_CD4_Responder	Mono_INHBA	CD4(IFNG+ Tfh/Th1)	LGALS1	ITGB1	5,00059E-03	23810,6	3,64109E-03	33015	1,25615E+00	19296	1,89276E+00	12367	9,23907E-01	11163	0	43212

Myeloid_vs_CD4_Post_R

Myeloid_vs_CD4_Responder	Mono_INHBA	CD4(ISG+ Treg)	HLA-DQB1	LAG3	5,00437E-03	25419,6	6,11273E-03	15449	9,42343E-01	33020	1,70511E+00	16275	9,05283E-01	19142	0	43212
Myeloid_vs_CD4_Responder	Macro_OLFML3	CD4(GZMK+ Teff)	C1QB	C1QBP	5,00968E-03	20858	3,64040E-03	33027	1,88490E+00	6231	2,68976E+00	3390	9,06987E-01	18430	0	43212
Myeloid_vs_CD4_Responder	Mono_INHBA	CD4(TNFRSF9+ Treg)	LYZ	ITGAL	5,01044E-03	24770,2	3,64040E-03	33028	1,47143E+00	12915	2,13886E+00	8467	8,88369E-01	26229	0	43212
Myeloid_vs_CD4_Responder	CD4(IFNG+ Tfh/Th1)	cDC(CD1C)	B2M	CD1A	5,02183E-03	21426,8	3,20388E-02	704	1,95788E+00	5494	1,17594E+00	33043	8,91994E-01	24681	0	43212
Myeloid_vs_CD4_Responder	Macro_OLFML3	CD4(NME1+ T)	HLA-DMB	CD4	5,03019E-03	25868	3,63854E-03	33054	1,62449E+00	9804	1,43571E+00	23763	9,04334E-01	19507	0	43212
Myeloid_vs_CD4_Responder	CD4(TNFRSF9+ Treg)	Mono_CD16	RPS19	C5AR1	5,03099E-03	21680,6	5,95212E-03	16062	1,75507E+00	7845	1,04546E+00	38220	9,43417E-01	3064	0	43212
Myeloid_vs_CD4_Responder	CD4(CRTAM- T)	Macro_FOLR2+APOE+	TIMP1	CD63	5,04930E-03	32220,4	1,74848E-03	78228	1,68786E+00	8799	1,76991E+00	14786	9,12403E-01	16077	0	43212
Myeloid_vs_CD4_Responder	cDC(CD1C)	CD4(TNFRSF9+ Treg)	HLA-DRA	LAG3	5,05230E-03	21881,4	3,63602E-03	33083	1,51760E+00	11846	3,14420E+00	1370	9,03392E-01	19896	0	43212
Myeloid_vs_CD4_Responder	Macro_FOLR2+APOE-	CD4(IL26+ Th17)	HLA-DQA1	LAG3	5,06398E-03	19180,6	8,19410E-03	9614	1,35600E+00	16089	1,89614E+00	12311	9,15577E-01	14677	0	43212
Myeloid_vs_CD4_Responder	Macro_FOLR2-APOE+	CD4(Tn)	SPP1	S1PR1	5,07093E-03	19138,6	2,96438E-02	844	1,88080E+00	6280	2,54857E+00	4390	8,55269E-01	40967	0	43212
Myeloid_vs_CD4_Responder	CD4(TNFRSF9+ Treg)	Macro_ISG15	HLA-C	LILRB1	5,08215E-03	26920,6	3,79382E-03	31019	1,30350E+00	17692	1,17379E+00	33122	9,27650E-01	9558	0	43212
Myeloid_vs_CD4_Responder	CD4(NME1+ T)	Macro_ISG15	HMGB1	TLR4	5,12139E-03	29909,8	4,67373E-03	22963	1,22113E+00	20558	1,17251E+00	33173	8,80313E-01	29643	0	43212
Myeloid_vs_CD4_Responder	cDC(CD1C)	CD4(IL26+ Th17)	HLA-DQB2	LAG3	5,12217E-03	18952,4	1,82372E-02	2221	1,71825E+00	8357	2,19377E+00	7798	8,72333E-01	33174	0	43212
Myeloid_vs_CD4_Responder	CD4(ISG+ Treg)	Macro_OLFML3	LTB	CD40	5,12680E-03	21975,8	1,32537E-02	4110	1,55812E+00	10987	1,17234E+00	33180	9,07081E-01	18390	0	43212
Myeloid_vs_CD4_Responder	cDC(CD1C)	CD4(TNFRSF9+ Treg)	HLA-DRB5	LAG3	5,12680E-03	24609,6	3,62798E-03	33180	1,32835E+00	16920	2,79123E+00	2775	8,86680E-01	26961	0	43212
Myeloid_vs_CD4_Responder	CD4(IL26+ Th17)	Mono_CD16	HLA-C	LILRA1	5,13167E-03	27924,4	1,04222E-02	6331	1,65042E+00	9367	5,29266E-01	64568	9,12228E-01	16144	0	43212
Myeloid_vs_CD4_Responder	Macro_OLFML3	CD4(TGFB1+ Th17)	CD14	ITGB1	5,13840E-03	26135,2	3,62671E-03	33195	1,47789E+00	12784	1,49533E+00	21864	9,04049E-01	19621	0	43212
Myeloid_vs_CD4_Responder	cDC_LAMP3	CD4(IL26+ Th17)	HLA-DQB1	LAG3	5,14281E-03	19185,2	8,37398E-03	9263	1,35414E+00	16153	1,82950E+00	13607	9,17944E-01	13691	0	43212
Myeloid_vs_CD4_Responder	CD4(TGFB1+ Th17)	Mono_CD16	HLA-A	LILRA1	5,14901E-03	30271,8	1,00343E-02	6824	1,50249E+00	12189	3,98300E-01	72976	9,12201E-01	16158	0	43212
Myeloid_vs_CD4_Responder	CD4(GZMK+ Teff)	Mono_INHBA	HLA-C	LILRB2	5,15235E-03	24623,2	3,62516E-03	33213	1,47634E+00	12813	1,28419E+00	28991	9,38016E-01	4887	0	43212
Myeloid_vs_CD4_Responder	CD4(TNF+ T)	Macro_IFI27	HSPA1A	TLR4	5,15778E-03	25195	3,84320E-03	30452	1,44278E+00	13641	2,41997E+00	5450	8,72233E-01	33220	0	43212
Myeloid_vs_CD4_Responder	CD4(GZMK+ Teff)	cDC_CLEC9A	HMGB1	HAVCR2	5,16088E-03	28922,6	3,62433E-03	33224	9,42226E-01	33022	1,60719E+00	18776	9,11710E-01	16379	0	43212
Myeloid_vs_CD4_Responder	CD4(GZMK+ Teff)	Mono_CD14	HMGB1	THBD	5,18189E-03	33346,8	3,90968E-03	29705	1,02104E+00	29133	1,17058E+00	33251	8,76212E-01	31433	0	43212
Myeloid_vs_CD4_Responder	Macro_IER3	CD4(ISG+ Treg)	HLA-DQB1	LAG3	5,18506E-03	18215	9,15816E-03	7967	1,35300E+00	16187	1,94873E+00	11398	9,21253E-01	12311	0	43212
Myeloid_vs_CD4_Responder	CD4(CXCL13+ Tfh)	Mono_CD16	HLA-A	LILRB2	5,18734E-03	19750,8	5,63462E-03	17426	2,18679E+00	3613	1,17026E+00	33258	9,50548E-01	1245	0	43212
Myeloid_vs_CD4_Responder	CD4(ISG+ Treg)	Mast	ARPC5	ADRB2	5,19437E-03	24725	1,01057E-02	6725	1,56462E+00	10859	1,26904E+00	29562	8,72151E-01	33267	0	43212
Myeloid_vs_CD4_Responder	Macro_OLFML3	CD4(CXCL13+ Tfh)	HLA-DQA1	LAG3	5,20062E-03	25623,6	3,61981E-03	33275	1,56491E+00	10853	2,01776E+00	10188	8,78171E-01	30590	0	43212
Myeloid_vs_CD4_Responder	Macro_FOLR2+APOE-	CD4(IL26+ Th17)	HLA-DQB1	LAG3	5,20628E-03	19028,2	8,36372E-03	9283	1,35253E+00	16204	1,87259E+00	12732	9,17898E-01	13710	0	43212
Myeloid_vs_CD4_Responder	CD4(AREG+ Tm)	Macro_OLFML3	CD52	SIGLEC10	5,21503E-03	27727,8	5,91458E-03	16211	1,43941E+00	13720	7,98212E-01	49330	9,12184E-01	16166	0	43212
Myeloid_vs_CD4_Responder	CD4(IL26+ Th17)	Mono_CD14	CIRBP	TREM1	5,22175E-03	24422,8	8,14161E-03	9709	1,56070E+00	10937	1,16881E+00	33302	8,91309E-01	24954	0	43212
Myeloid_vs_CD4_Responder	CD4(TNFRSF9+ Treg)	pDC_LILRA4	HSP90B1	TLR9	5,22841E-03	26852	2,34454E-02	1358	3,06622E+00	878	1,07051E+00	37225	8,32593E-01	51587	0	43212
Myeloid_vs_CD4_Responder	CD4(CRTAM- T)	Mono_CD16	HLA-A	LILRA1	5,23383E-03	30525	9,99556E-03	6866	1,49337E+00	12403	3,84427E-01	73918	9,12046E-01	16226	0	43212
Myeloid_vs_CD4_Responder	CD4(TGFB1+ Th17)	Macro_OLFML3	TNFSF9	HLA-DPA1	5,23745E-03	23400,2	4,35751E-03	25433	1,55831E+00	10984	2,59121E+00	4050	8,71995E-01	33322	0	43212
Myeloid_vs_CD4_Responder	CD4(NME1+ T)	Mono_INHBA	HLA-F	LILRB2	5,24846E-03	30193,2	3,84598E-03	30426	1,36946E+00	15682	1,16782E+00	33336	8,83353E-01	28310	0	43212
Myeloid_vs_CD4_Responder	CD4(Tn)	cDC_CLEC9A	LTB	CD40	5,25870E-03	27960	7,48065E-03	11180	1,17604E+00	22269	1,16751E+00	33349	8,80010E-01	29790	0	43212
Myeloid_vs_CD4_Responder	CD4(CXCL13+ Tfh)	Mono_CD16	CD99	PILRA	5,26028E-03	23085,6	5,70425E-03	17114	1,86131E+00	6513	1,16743E+00	33351	9,14336E-01	15238	0	43212
Myeloid_vs_CD4_Responder	Macro_IER3	CD4(TNFRSF9+ Treg)	ICAM1	IL2RG	5,28294E-03	19545,2	7,69666E-03	10680	1,49972E+00	12239	1,70539E+00	16265	9,14137E-01	15330	0	43212
Myeloid_vs_CD4_Responder	Macro_OLFML3	CD4(ISG+ Treg)	APOE	SORL1	5,28319E-03	22835,4	3,61180E-03	33380	1,83435E+00	6837	2,70795E+00	3262	8,85446E-01	27486	0	43212
Myeloid_vs_CD4_Responder	CD4(IFNG+ Tfh/Th1)	Mono_INHBA	HMGB1	CD163	5,29031E-03	25242,2	3,61090E-03	33389	1,39910E+00	14824	1,49019E+00	22026	9,20148E-01	12760	0	43212
Myeloid_vs_CD4_Responder	CD4(GZMK+ Teff)	Macro_ISG15	HLA-C	LILRB1	5,29666E-03	26701,6	3,83380E-03	30546	1,32774E+00	16939	1,16587E+00	33397	9,28001E-01	9414	0	43212
Myeloid_vs_CD4_Responder	Macro_OLFML3	CD4(ISG+ Treg)	HLA-DQB1	CD4	5,30856E-03	20390,8	3,60912E-03	33412	1,59523E+00	10304	2,21912E+00	7499	9,32054E-01	7527	0	43212
Myeloid_vs_CD4_Responder	pDC_LILRA4	CD4(NME1+ T)	APP	RPSA	5,31333E-03	15592,4	1,01674E-02	6645	1,34988E+00	16289	2,40683E+00	5569	9,34903E-01	6247	0	43212
Myeloid_vs_CD4_Responder	CD4(TNFRSF9+ Treg)	Macro_ISG15	B2M	LILRB1	5,34281E-03	26135,4	3,60644E-03	33455	1,35402E+00	16157	1,17703E+00	33001	9,38086E-01	4852	0	43212
Myeloid_vs_CD4_Responder	Macro_ISG15	CD4(IFNG+ Tfh/Th1)	HLA-DQB1	LAG3	5,34512E-03	16328,2	1,02096E-02	6598	1,34903E+00	16314	2,49139E+00	4838	9,25106E-01	10679	0	43212
Myeloid_vs_CD4_Responder	CD4(IFNG+ Tfh/Th1)	Macro_NLRP3	HLA-A	LILRB2	5,34920E-03	25002,8	3,60603E-03	33463	1,46553E+00	13050	1,23467E+00	30789	9,38939E-01	4500	0	43212
Myeloid_vs_CD4_Responder	Macro_FOLR2-APOE+	CD4(ISG+ Treg)	SPP1	CCR8	5,34982E-03	19045	6,30191E-02	182	1,87073E+00	6396	2,37568E+00	5867	8,58345E-01	39568	0	43212
Myeloid_vs_CD4_Responder	Mono_INHBA	CD4(NME1+ T)	INHBA	TGFBR3	5,35103E-03	29170	3,15824E-02	724	2,78781E+00	1374	2,05639E+00	9598	7,41923E-01	90942	0	43212
Myeloid_vs_CD4_Responder	CD4(AREG+ Tm)	Mast	VIM	CD44	5,36645E-03	43730,6	2,18273E-03	64253	5,40512E-01	57839	7,33702E-01	52673	9,53525E-01	676	0	43212
Myeloid_vs_CD4_Responder	cDC(CD1C)	CD4(TGFB1+ Th17)	HLA-DQB1	LAG3	5,37723E-03	25026,4	3,60334E-03	33498	1,40173E+00	14764	2,61211E+00	3891	8,80071E-01	29767	0	43212
Myeloid_vs_CD4_Responder	Macro_OLFML3	CD4(IFNG+ Tfh/Th1)	LYZ	ITGAL	5,37964E-03	27032	3,60318E-03	33501	1,34574E+00	16405	1,73456E+00	15582	8,87859E-01	26460	0	43212
Myeloid_vs_CD4_Responder	CD4(IFNG+ Tfh/Th1)	cDC(CD1C)	CD52	SIGLEC10	5,38366E-03	32046,6	3,84078E-03	30479	9,32949E-01	33506	1,28681E+00	28910	8,93283E-01	24126	0	43212
Myeloid_vs_CD4_Responder	CD4(ISG+ Treg)	Macro_FOLR2+APOE+	ADAM10	GPNMB	5,40458E-03	21011,2	9,16066E-03	7961	2,67188E+00	1649	1,60938E+00	18702	8,71529E-01	33532	0	43212
Myeloid_vs_CD4_Responder	Mono_INHBA	CD4(CRTAM- T)	CCL3	CCR4	5,41859E-03	19202,4	1,03488E-02	6424	2,16516E+00	3734	2,47672E+00	4972	8,62514E-01	37670	0	43212
Myeloid_vs_CD4_Responder	Macro_ISG15	CD4(ISG+ Treg)	HLA-DRB1	LAG3	5,42585E-03	16208,6	8,18254E-03	9638	1,34663E+00	16377	2,56395E+00	4266	9,32015E-01	7550	0	43212
Myeloid_vs_CD4_Responder	Mono_CD14	CD4(ISG+ Treg)	VCAN	ITGB1	5,43835E-03	15522,6	1,03411E-02	6432	2,86443E+00	1220	2,37681E+00	5853	9,01089E-01	20896	0	43212
Myeloid_vs_CD4_Responder	CD4(NME1+ T)	Macro_LYVE1	CALR	LRP1	5,43932E-03	22374,6	5,94829E-03	16082	2,04053E+00	4751	1,16145E+00	33575	9,16567E-01	14253	0	43212
Myeloid_vs_CD4_Responder	Macro_NLRP3	CD4(IL26+ Th17)	HLA-DRA	LAG3	5,44650E-03	18533,6	6,91190E-03	12739	1,34612E+00	16393	1,97460E+00	10921	9,28021E-01	9403	0	43212
Myeloid_vs_CD4_Responder	CD4(ISG+ Treg)	cDC_CLEC9A	HMGB1	HAVCR2	5,45635E-03	29613,4	3,59580E-03	33596	9,35184E-01	33377	1,51087E+00	21361	9,11391E-01	16521	0	43212
Myeloid_vs_CD4_Responder	CD4(IL26+ Th17)	Macro_LYVE1	ADAM10	GPNMB	5,47343E-03	20953,2	9,12918E-03	8028	2,43640E+00	2377	1,65289E+00	17532	8,71336E-01	33617	0	43212
Myeloid_vs_CD4_Responder	Mono_INHBA	CD4(IL26+ Th17)	VCAN	ITGB1	5,48565E-03	26283,8	5,71143E-03	17085	1,42666E+00	14067	1,44599E+00	23423	8,71305E-01	33632	0	43212
Myeloid_vs_CD4_Responder	CD4(GZMK+ Teff)	Mono_CD14	CIRBP	TREM1	5,54709E-03	25707,8	7,33199E-03	11600	1,47622E+00	12816	1,15817E+00	33707	8,86131E-01	27204	0	43212
Myeloid_vs_CD4_Responder	CD4(TNFRSF9+ Treg)	Mono_INHBA	HLA-C	LILRB2	5,55450E-03	24803,8	3,58735E-03	33716	1,45211E+00	13382	1,29211E+00	28701	9,37710E-01	5008	0	43212
Myeloid_vs_CD4_Responder	CD4(ISG+ Treg)	Mono_INHBA	HLA-C	LILRB2	5,55532E-03	25197,2	3,58726E-03	33717	1,45204E+00	13387	1,23380E+00	30660	9,37709E-01	5010	0	43212
Myeloid_vs_CD4_Responder	pDC_LILRA4	CD4(IFNG+ Tfh/Th1)	APP	RPSA	5,55846E-03	15975,4	1,01029E-02	6729	1,34305E+00	16479	2,25407E+00	7110	9,34709E-01	6347	0	43212
Myeloid_vs_CD4_Responder	CD4(CXCL13+ Tfh)	Mast	CD40LG	CD9	5,56240E-03	20721,6	1,87214E-02	2110	1,89097E+00	6167	1,69550E+00	16482	8,66987E-01	35637	0	43212
Myeloid_vs_CD4_Responder	CD4(CXCL13+ Tfh)	Mono_CD16	HLA-A	LILRA1	5,56634E-03	31755,4	9,85248E-03	7020	1,45966E+00	13203	3,10310E-01	78857	9,11466E-01	16485	0	43212
Myeloid_vs_CD4_Responder	Macro_FOLR2-APOE+	CD4(IL26+ Th17)	HLA-DPA1	LAG3	5,57159E-03	18288,2	7,45697E-03	11240	1,34282E+00	16489	1,95866E+00	11214	9,28258E-01	9286	0	43212
Myeloid_vs_CD4_Responder	CD4(TNFRSF9+ Treg)	Macro_OLFML3	CD52	SIGLEC10	5,57816E-03	27492,2	5,84536E-03	16494	1,42945E+00	13993	8,36379E-01	47386	9,11711E-01	16376	0	43212
Myeloid_vs_CD4_Responder	cDC_LAMP3	CD4(ISG+ Treg)	HLA-DPA1	LAG3	5,58079E-03	16943,2	8,18674E-03	9625	1,34259E+00	16496	2,22015E+00	7485	9,31305E-01	7898	0	43212

Myeloid_vs_CD4_Post_R

Myeloid_vs_CD4_Responder	CD4(GZMK+ Teff)	Mono_CD16	RPS19	C5AR1	5,58090E-03	20710,6	5,99895E-03	15872	1,76526E+00	7717	1,15708E+00	33748	9,43626E-01	3004	0	43212
Myeloid_vs_CD4_Responder	CD4(TNF+ T)	cDC_CLEC9A	LTB	CD40	5,58504E-03	29552,8	6,34605E-03	14557	1,09924E+00	25441	1,23435E+00	30801	8,71050E-01	33753	0	43212
Myeloid_vs_CD4_Responder	Mono_INHBA	CD4(ISG+ Treg)	HBEGF	CD82	5,60161E-03	24217,6	1,06155E-02	6135	1,47920E+00	12752	1,39025E+00	25216	8,71011E-01	33773	0	43212
Myeloid_vs_CD4_Responder	CD4(IL26+ Th17)	Mast	HSPA8	ADRB2	5,60187E-03	25371	1,27315E-02	4397	1,34212E+00	16512	7,92542E-01	49655	9,19434E-01	13079	0	43212
Myeloid_vs_CD4_Responder	CD4(TNF+ T)	Macro_LYVE1	ADAM10	GPNMB	5,60715E-03	24883	5,83851E-03	16516	2,31561E+00	2917	1,73168E+00	15642	8,44135E-01	46128	0	43212
Myeloid_vs_CD4_Responder	CD4(ISG+ Treg)	cDC(CD1C)	TNFSF13B	HLA-DPB1	5,63094E-03	32010,4	1,46377E-03	88570	1,57073E+00	10752	3,30927E+00	984	9,11362E-01	16534	0	43212
Myeloid_vs_CD4_Responder	CD4(CXCL13+ Tfh)	Mono_CD16	HLA-C	LILRB2	5,64653E-03	20230,2	5,39429E-03	18647	2,16525E+00	3733	1,15542E+00	33827	9,48613E-01	1732	0	43212
Myeloid_vs_CD4_Responder	CD4(NME1+ T)	cDC_CLEC9A	LTB	CD40	5,66659E-03	28693,4	7,10920E-03	12183	1,15090E+00	23264	1,15480E+00	33851	8,77295E-01	30957	0	43212
Myeloid_vs_CD4_Responder	Macro_OLFML3	CD4(GZMK+ Teff)	C3	CD46	5,66659E-03	21708,8	6,71906E-03	13263	2,06637E+00	4521	1,82501E+00	13697	8,70833E-01	33851	0	43212
Myeloid_vs_CD4_Responder	CD4(TGFB1+ Th17)	Mono_INHBA	HLA-F	LILRB2	5,67245E-03	31345,6	3,61735E-03	33307	1,33812E+00	16629	1,15472E+00	33858	8,80158E-01	29722	0	43212
Myeloid_vs_CD4_Responder	Macro_ISG15	CD4(CXCL13+ Tfh)	SPP1	CD44	5,67876E-03	19607,4	6,26230E-03	14868	1,38690E+00	15184	2,15937E+00	8203	9,11298E-01	16570	0	43212
Myeloid_vs_CD4_Responder	CD4(GZMK+ Teff)	Macro_LYVE1	CCL5	CCRL2	5,69343E-03	26344,6	1,27421E-02	4391	1,29820E+00	17866	1,19386E+00	32371	8,70752E-01	33883	0	43212
Myeloid_vs_CD4_Responder	Mono_INHBA	CD4(CXCL13+ Tfh)	VEGFA	CD44	5,69427E-03	22982,4	7,62843E-03	10848	1,86778E+00	6440	1,15406E+00	33884	9,01885E-01	20528	0	43212
Myeloid_vs_CD4_Responder	CD4(ISG+ Treg)	Macro_NLRP3	HLA-B	LILRB2	5,70015E-03	23800	3,57314E-03	33891	1,57466E+00	10666	1,31958E+00	27696	9,41801E-01	3535	0	43212
Myeloid_vs_CD4_Responder	cDC(CD1C)	CD4(ISG+ Treg)	B2M	CD3D	5,70377E-03	39837	3,60254E-03	33512	2,81810E-01	79150	9,26914E-01	43281	9,62162E-01	30	0	43212
Myeloid_vs_CD4_Responder	CD4(ISG+ Treg)	Macro_ISG15	HLA-A	LILRB2	5,70604E-03	22797,2	3,57276E-03	33898	1,51191E+00	11982	1,55021E+00	20281	9,38672E-01	4613	0	43212
Myeloid_vs_CD4_Responder	CD4(GZMK+ Teff)	Mono_INHBA	HLA-A	LILRB2	5,71530E-03	25937,6	3,57182E-03	33909	1,39107E+00	15070	1,18011E+00	32882	9,38665E-01	4615	0	43212
Myeloid_vs_CD4_Responder	CD4(IFNG+ Tfh/Th1)	Macro_ISG15	CD99	PILRA	5,74232E-03	29916,6	4,02784E-03	28434	1,16834E+00	22555	1,15237E+00	33941	8,99689E-01	21441	0	43212
Myeloid_vs_CD4_Responder	CD4(TNFRSF9+ Treg)	Macro_ISG15	HLA-F	LILRB1	5,74655E-03	30604,6	4,42837E-03	24835	1,26765E+00	18888	1,19998E+00	32142	8,70636E-01	33946	0	43212
Myeloid_vs_CD4_Responder	CD4(ISG+ Treg)	Macro_OLFML3	LGALS9	HAVCR2	5,75502E-03	27900,4	4,97443E-03	20984	1,46129E+00	13153	1,30561E+00	28197	8,70613E-01	33956	0	43212
Myeloid_vs_CD4_Responder	CD4(AREG+ Tm)	Mast	CD40LG	CD9	5,76594E-03	21656,6	1,68562E-02	2583	1,85686E+00	6562	1,65597E+00	17462	8,60818E-01	38464	0	43212
Myeloid_vs_CD4_Responder	Macro_FOLR2-APOE+	CD4(IFNG+ Tfh/Th1)	SPP1	CD44	5,77125E-03	20453	6,23700E-03	14976	1,45193E+00	13390	1,80607E+00	14048	9,11134E-01	16639	0	43212
Myeloid_vs_CD4_Responder	Mono_INHBA	CD4(CRTAM- T)	LGALS1	ITGB1	5,77284E-03	24365,8	3,56598E-03	33977	1,24557E+00	19672	1,83547E+00	13469	9,23171E-01	11499	0	43212
Myeloid_vs_CD4_Responder	CD4(TNF+ T)	Mono_CD16	TNFSF9	HLA-DPA1	5,77794E-03	30421,2	4,24771E-03	26331	1,04422E+00	27990	1,53813E+00	20590	8,70564E-01	33983	0	43212
Myeloid_vs_CD4_Responder	CD4(ISG+ Treg)	Macro_OLFML3	TNFSF13B	HLA-DPB1	5,77934E-03	32736,4	1,45540E-03	88907	1,56060E+00	10941	2,60141E+00	3977	9,11130E-01	16645	0	43212
Myeloid_vs_CD4_Responder	Mono_INHBA	CD4(IFNG+ Tfh/Th1)	SPP1	ITGA4_ITGB1	5,78559E-03	23631	6,67366E-03	13408	1,21597E+00	20750	2,28316E+00	6793	8,70551E-01	33992	0	43212
Myeloid_vs_CD4_Responder	CD4(NME1+ T)	Mono_CD14	HSPA1A	TLR4	5,79155E-03	30400,4	3,72855E-03	31838	1,19931E+00	21385	1,50439E+00	21568	8,70536E-01	33999	0	43212
Myeloid_vs_CD4_Responder	Macro_FOLR2-APOE+	CD4(NME1+ T)	SPP1	CD44	5,79420E-03	20532	6,23183E-03	15000	1,45138E+00	13401	1,79077E+00	14391	9,11101E-01	16656	0	43212
Myeloid_vs_CD4_Responder	CD4(Tn)	Mono_CD14	CIRBP	TREM1	5,79667E-03	25910,4	7,23638E-03	11825	1,46625E+00	13033	1,15028E+00	34005	8,85467E-01	27477	0	43212
Myeloid_vs_CD4_Responder	Mono_CD14	CD4(AREG+ Tm)	VCAN	ITGB1	5,79938E-03	15552,4	1,02238E-02	6575	2,86030E+00	1225	2,39748E+00	5661	9,00579E-01	21089	0	43212
Myeloid_vs_CD4_Responder	Mono_INHBA	CD4(TNFRSF9+ Treg)	VCAN	ITGB1	5,80605E-03	26287,2	5,63000E-03	17446	1,42093E+00	14202	1,47384E+00	22560	8,70498E-01	34016	0	43212
Myeloid_vs_CD4_Responder	Macro_LYVE1	CD4(ISG+ Treg)	HLA-DQB1	LAG3	5,82537E-03	18520,8	9,03579E-03	8188	1,33650E+00	16679	1,91148E+00	12022	9,20764E-01	12503	0	43212
Myeloid_vs_CD4_Responder	Mono_INHBA	CD4(TNF+ T)	ICAM1	IL2RG	5,82570E-03	33667,6	3,56219E-03	34039	9,35883E-01	33355	1,32928E+00	27362	8,78683E-01	30370	0	43212
Myeloid_vs_CD4_Responder	CD4(TGFB1+ Th17)	cDC_CLEC9A	TNFSF9	HLA-DPA1	5,82742E-03	23498,4	4,23873E-03	26401	1,51027E+00	12029	3,00767E+00	1809	8,70445E-01	34041	0	43212
Myeloid_vs_CD4_Responder	CD4(IL26+ Th17)	cDC(CD1C)	CD52	SIGLEC10	5,83255E-03	32308,8	3,97692E-03	28992	9,64663E-01	31881	1,14902E+00	34047	8,94932E-01	23412	0	43212
Myeloid_vs_CD4_Responder	Macro_OLFML3	CD4(ISG+ Treg)	CD86	CTLA4	5,84970E-03	22295	1,23145E-02	4666	1,57129E+00	10738	1,14837E+00	34067	9,06178E-01	18792	0	43212
Myeloid_vs_CD4_Responder	Macro_FOLR2-APOE+	CD4(IL26+ Th17)	CXCL16	CXCR6	5,85938E-03	18186,4	1,59918E-02	2865	1,52923E+00	11569	1,68683E+00	16704	9,11277E-01	16582	0	43212
Myeloid_vs_CD4_Responder	CD4(ISG+ Treg)	Macro_FOLR2-APOE+	LTB	CD40	5,86087E-03	26072,6	9,48309E-03	7497	1,21161E+00	20888	1,14807E+00	34080	8,91979E-01	24686	0	43212
Myeloid_vs_CD4_Responder	CD4(AREG+ Tm)	Mono_CD16	HLA-A	LILRA1	5,87849E-03	32258	9,73208E-03	7175	1,43129E+00	13937	2,90011E-01	80248	9,10969E-01	16718	0	43212
Myeloid_vs_CD4_Responder	CD4(TNF+ T)	Mono_CD14	ANXA1	FPR1	5,88326E-03	28232,8	3,55829E-03	34106	1,30041E+00	17792	1,42908E+00	23984	8,98081E-01	22070	0	43212
Myeloid_vs_CD4_Responder	CD4(GZMK+ Teff)	cDC_CLEC9A	CRTAM	CADM1	5,88532E-03	27828	6,30579E-02	180,5	1,48731E+00	12547,5	1,85186E+00	13162,5	7,92548E-01	70037,5	0	43212
Myeloid_vs_CD4_Responder	Macro_NLRP3	CD4(IFNG+ Tfh/Th1)	HLA-DPB1	LAG3	5,88532E-03	16045	9,06157E-03	8140	1,33484E+00	16723	2,50116E+00	4758	9,32365E-01	7392	0	43212
Myeloid_vs_CD4_Responder	CD4(TNF+ T)	Macro_NLRP3	TNFSF9	HLA-DPA1	5,90572E-03	28799	4,22452E-03	26525	1,03802E+00	28280	1,92161E+00	11846	8,70255E-01	34132	0	43212
Myeloid_vs_CD4_Responder	CD4(GZMK+ Teff)	pDC_LILRA4	TNF	TNFRSF21	5,90587E-03	25151,2	1,26637E-02	4436	3,19953E+00	709	1,82823E+00	13626	8,06130E-01	63773	0	43212
Myeloid_vs_CD4_Responder	Macro_IER3	CD4(ISG+ Treg)	HLA-DPA1	LAG3	5,90587E-03	17324	8,13536E-03	9720	1,33440E+00	16738	2,10030E+00	8962	9,31104E-01	7988	0	43212
Myeloid_vs_CD4_Responder	CD4(IFNG+ Tfh/Th1)	Macro_NLRP3	HLA-F	LILRB2	5,93433E-03	31734,8	3,59504E-03	33609	1,29957E+00	17814	1,14565E+00	34165	8,79832E-01	29874	0	43212
Myeloid_vs_CD4_Responder	CD4(IL26+ Th17)	Mast	CD40LG	CD9	5,93966E-03	22218,4	1,65183E-02	2685	1,85068E+00	6629	1,57731E+00	19565	8,59601E-01	39001	0	43212
Myeloid_vs_CD4_Responder	CD4(GZMK+ Teff)	pDC_LILRA4	BST2	LILRA4	5,94160E-03	19100,8	1,00775E-02	6763	5,19606E+00	98	2,83667E+00	2527	8,50967E-01	42904	0	43212
Myeloid_vs_CD4_Responder	CD4(GZMK+ Teff)	pDC_LILRA4	COPA	P2RY6	5,95401E-03	28843,2	7,07055E-03	12299	2,02628E+00	4881	2,04396E+00	9805	7,83570E-01	74019	0	43212
Myeloid_vs_CD4_Responder	CD4(CXCL13+ Tfh)	cDC_CLEC9A	HMGB1	HAVCR2	5,95694E-03	29615	3,55160E-03	34191	9,24278E-01	33935	1,56125E+00	19987	9,10890E-01	16750	0	43212
Myeloid_vs_CD4_Responder	Macro_OLFML3	CD4(TGFB1+ Th17)	CD14	ITGA4	5,96915E-03	27860,6	3,55095E-03	34205	1,45301E+00	13360	1,40822E+00	24621	8,93810E-01	23905	0	43212
Myeloid_vs_CD4_Responder	Macro_IER3	CD4(Tn)	CCL3	CCR4	5,97375E-03	16128,4	1,69684E-02	2552	2,42164E+00	2436	2,29901E+00	6642	8,89296E-01	25800	0	43212
Myeloid_vs_CD4_Responder	CD4(TNFRSF9+ Treg)	Macro_LYVE1	RPS19	C5AR1	5,98857E-03	23366,8	5,77862E-03	16798	1,70148E+00	8594	8,88761E-01	44934	9,42623E-01	3296	0	43212
Myeloid_vs_CD4_Responder	CD4(TGFB1+ Th17)	cDC_CLEC9A	TNFSF13B	HLA-DPB1	5,99966E-03	32737,8	1,44213E-03	89389	1,48116E+00	12698	3,07244E+00	1584	9,10758E-01	16806	0	43212
Myeloid_vs_CD4_Responder	CD4(TNF+ T)	Mono_CD16	HLA-B	LILRB2	6,00676E-03	20957	5,02536E-03	20667	1,96831E+00	5400	1,14332E+00	34248	9,50473E-01	1258	0	43212
Myeloid_vs_CD4_Responder	CD4(NME1+ T)	Mono_INHBA	HLA-A	LILRB2	6,02784E-03	26306,8	3,54600E-03	34272	1,37288E+00	15581	1,15647E+00	33777	9,38455E-01	4692	0	43212
Myeloid_vs_CD4_Responder	Macro_IFI27	CD4(TNFRSF9+ Treg)	CCL18	CCR8	6,03967E-03	20036,6	8,86040E-02	75	1,84773E+00	6667	3,10985E+00	1467	8,38577E-01	48762	0	43212
Myeloid_vs_CD4_Responder	Macro_IER3	CD4(IL26+ Th17)	HLA-DQA1	LAG3	6,04418E-03	20132,6	8,03170E-03	9929	1,33071E+00	16838	1,73112E+00	15655	9,14800E-01	15029	0	43212
Myeloid_vs_CD4_Responder	CD4(Tn)	Mono_CD16	HLA-A	LILRA1	6,04976E-03	32314,4	9,65809E-03	7268	1,41386E+00	14417	2,96332E-01	79833	9,10659E-01	16842	0	43212
Myeloid_vs_CD4_Responder	CD4(GZMK+ Teff)	Macro_FOLR2+APOE+	CCL5	CCR1	6,05074E-03	26553	1,29460E-02	4283	1,33593E+00	16696	1,14247E+00	34298	8,69895E-01	34276	0	43212
Myeloid_vs_CD4_Responder	Macro_ISG15	CD4(GZMK+ Teff)	S100A8	CD69	6,07492E-03	18955,4	6,50950E-03	13976	1,33022E+00	16860	1,98902E+00	10666	9,26489E-01	10063	0	43212
Myeloid_vs_CD4_Responder	CD4(TNFRSF9+ Treg)	Macro_OLFML3	LTB	CD40	6,07548E-03	23361,8	1,16763E-02	5142	1,44846E+00	13486	1,14190E+00	34326	9,01601E-01	20643	0	43212
Myeloid_vs_CD4_Responder	CD4(IFNG+ Tfh/Th1)	Macro_ISG15	HLA-B	LILRB1	6,08167E-03	26835,4	3,72935E-03	31825	1,31847E+00	17226	1,14154E+00	34333	9,31938E-01	7581	0	43212
Myeloid_vs_CD4_Responder	Macro_OLFML3	CD4(TNFRSF9+ Treg)	HLA-DRB1	LAG3	6,08522E-03	24192,2	3,54121E-03	34337	1,36732E+00	15743	2,31698E+00	6433	9,00186E-01	21236	0	43212
Myeloid_vs_CD4_Responder	CD4(CRTAM- T)	Macro_NLRP3	CD99	PILRA	6,08965E-03	26552,2	4,80829E-03	22044	1,39604E+00	14919	1,14130E+00	34342	9,07403E-01	18244	0	43212
Myeloid_vs_CD4_Responder	Mono_INHBA	CD4(ISG+ Treg)	HLA-DQA2	LAG3	6,10563E-03	28673	6,88901E-03	12799	9,27580E-01	33753	1,58923E+00	19241	8,69693E-01	34360	0	43212
Myeloid_vs_CD4_Responder	CD4(IFNG+ Tfh/Th1)	Mono_CD14	HMGB1	TLR4	6,10651E-03	31475,8	4,40584E-03	25024	1,13872E+00	23762	1,14098E+00	34361	8,77168E-01	31020	0	43212
Myeloid_vs_CD4_Responder	CD4(TNF+ T)	Mono_CD16	B2M	LILRB2	6,11452E-03	21189,8	4,93573E-03	21216	1,84410E+00	6712	1,14065E+00	34370	9,55360E-01	439	0	43212
Myeloid_vs_CD4_Responder	Macro_FOLR2+APOE-	CD4(NME1+ T)	C1QB	C1QBP	6,13111E-03	16798,6	5,75818E-03	16900	1,87464E+00	6350	2,29952E+00	6635	9,24607E-01	10896	0	43212

Myeloid_vs_CD4_Post_R

Myeloid_vs_CD4_Responder	CD4(AREG+ Tm)	Mono_INHBA	CCL5	CCRL2	6,13946E-03	25767,8	1,24878E-02	4537	1,33325E+00	16763	1,25784E+00	29929	8,69613E-01	34398	0	43212
Myeloid_vs_CD4_Responder	CD4(GZMK+ Teff)	Macro_LYVE1	RPS19	C5AR1	6,14946E-03	22311	5,82408E-03	16587	1,71166E+00	8449	1,00039E+00	40078	9,42834E-01	3229	0	43212
Myeloid_vs_CD4_Responder	CD4(TNFRSF9+ Treg)	Macro_ISG15	TIGIT	NECTIN2	6,15823E-03	21379,8	3,14104E-02	733,5	1,74254E+00	8018,5	1,54071E+00	20515,5	8,69556E-01	34419,5	0	43212
Myeloid_vs_CD4_Responder	CD4(TNF+ T)	Mast	IL16	CD9	6,16926E-03	24900,2	6,72874E-03	13238	1,78463E+00	7450	1,67668E+00	16927	8,49320E-01	43674	0	43212
Myeloid_vs_CD4_Responder	Macro_FOLR2-APOE+	CD4(AREG+ Tm)	SPP1	PTGER4	6,17634E-03	15139,6	1,62986E-02	2761	1,83755E+00	6789	2,36222E+00	6004	9,10422E-01	16932	0	43212
Myeloid_vs_CD4_Responder	Mono_INHBA	CD4(CRTAM- T)	NAMPT	ITGA5_ITGB1	6,18332E-03	31918,6	3,91783E-03	29622	9,13889E-01	34447	1,36714E+00	26011	8,88223E-01	26301	0	43212
Myeloid_vs_CD4_Responder	CD4(TNFRSF9+ Treg)	Macro_ISG15	HLA-A	LILRB2	6,20669E-03	22903,4	3,53151E-03	34473	1,48004E+00	12725	1,58452E+00	19359	9,38337E-01	4748	0	43212
Myeloid_vs_CD4_Responder	Macro_OLFML3	CD4(IL26+ Th17)	HLA-DQB2	LAG3	6,20939E-03	23868,4	1,73215E-02	2454	1,64159E+00	9504	1,26494E+00	29696	8,69437E-01	34476	0	43212
Myeloid_vs_CD4_Responder	cDC(CD1C)	CD4(CXCL13+ Tfh)	HLA-DQA1	LAG3	6,21119E-03	24822,6	3,53080E-03	34478	1,52038E+00	11775	2,67502E+00	3475	8,76833E-01	31173	0	43212
Myeloid_vs_CD4_Responder	Mono_INHBA	CD4(NME1+ T)	HLA-DRB1	LAG3	6,21479E-03	29447	3,53050E-03	34482	9,81869E-01	31012	1,66568E+00	17232	9,00050E-01	21297	0	43212
Myeloid_vs_CD4_Responder	CD4(TGFB1+ Th17)	cDC(CD1C)	TNFSF9	HLA-DPA1	6,23735E-03	23846,8	4,15724E-03	27149	1,47731E+00	12796	3,07656E+00	1570	8,69346E-01	34507	0	43212
Myeloid_vs_CD4_Responder	CD4(ISG+ Treg)	Macro_FOLR2+APOE-	TNFSF12	CD163	6,23750E-03	27430,8	5,73851E-03	16975	1,98213E+00	5254	1,71725E+00	15979	8,23812E-01	55734	0	43212
Myeloid_vs_CD4_Responder	Mono_INHBA	CD4(AREG+ Tm)	CCL3	CCR4	6,24567E-03	19486,8	1,00933E-02	6744	2,16101E+00	3771	2,43391E+00	5329	8,61026E-01	38378	0	43212
Myeloid_vs_CD4_Responder	Macro_OLFML3	CD4(TGFB1+ Th17)	FN1	ITGA4_ITGB7	6,25092E-03	20661,6	1,25648E-02	4503	1,88451E+00	6237	1,76806E+00	14834	8,69327E-01	34522	0	43212
Myeloid_vs_CD4_Responder	Mono_CD16	CD4(IFNG+ Tfh/Th1)	HLA-DPA1	LAG3	6,25465E-03	16739	9,09767E-03	8082	1,32620E+00	16987	2,09691E+00	9018	9,34604E-01	6396	0	43212
Myeloid_vs_CD4_Responder	CD4(TNFRSF9+ Treg)	Macro_FOLR2+APOE+	ADAM10	GPNMB	6,25636E-03	21192,6	8,80656E-03	8542	2,66017E+00	1675	1,63438E+00	18006	8,69306E-01	34528	0	43212
Myeloid_vs_CD4_Responder	CD4(NME1+ T)	cDC(CD1C)	CD52	SIGLEC10	6,25817E-03	33254,8	3,75305E-03	31510	9,12510E-01	34530	1,19249E+00	32420	8,92177E-01	24602	0	43212
Myeloid_vs_CD4_Responder	cDC_LAMP3	CD4(ISG+ Treg)	HLA-DRB5	LAG3	6,26753E-03	18088,2	8,29743E-03	9422	1,32592E+00	16996	2,10866E+00	8852	9,22077E-01	11959	0	43212
Myeloid_vs_CD4_Responder	Mono_INHBA	CD4(TNFRSF9+ Treg)	S100A8	CD69	6,26905E-03	32014,4	3,81316E-03	30778	9,48672E-01	32701	1,13660E+00	34542	9,06070E-01	18839	0	43212
Myeloid_vs_CD4_Responder	CD4(GZMK+ Teff)	Macro_IER3	CCL5	CCRL2	6,27631E-03	26654,6	1,24137E-02	4597	1,27812E+00	18517	1,19307E+00	32397	8,69275E-01	34550	0	43212
Myeloid_vs_CD4_Responder	CD4(GZMK+ Teff)	Mono_CD16	HLA-B	LILRA1	6,27756E-03	26673,8	1,02734E-02	6523	1,65527E+00	9298	5,95524E-01	60444	9,17453E-01	13892	0	43212
Myeloid_vs_CD4_Responder	CD4(IFNG+ Tfh/Th1)	Mono_INHBA	HLA-B	LILRB2	6,27904E-03	24858,4	3,52639E-03	34553	1,46707E+00	13017	1,25986E+00	29857	9,41439E-01	3653	0	43212
Myeloid_vs_CD4_Responder	CD4(GZMK+ Teff)	Mono_CD16	HLA-A	LILRA1	6,28330E-03	27074	1,06788E-02	6061	1,65436E+00	9307	5,75629E-01	61695	9,14663E-01	15095	0	43212
Myeloid_vs_CD4_Responder	CD4(GZMK+ Teff)	Mono_CD16	HLA-C	LILRA1	6,29192E-03	25648	1,08383E-02	5916	1,73964E+00	8057	6,79707E-01	55574	9,13783E-01	15481	0	43212
Myeloid_vs_CD4_Responder	CD4(GZMK+ Teff)	Mono_CD16	HLA-C	LILRA3	6,29479E-03	21510,2	1,89168E-02	2058	2,60711E+00	1819	8,21921E-01	48123	9,21168E-01	12339	0	43212
Myeloid_vs_CD4_Responder	Macro_FOLR2+APOE-	CD4(CXCL13+ Tfh)	CCL13	CXCR3	6,29821E-03	39410,8	2,25248E-02	1492	3,58878E+00	412	7,07455E-01	54081	7,23161E-01	97857	0	43212
Myeloid_vs_CD4_Responder	CD4(IFNG+ Tfh/Th1)	Mast	GNAS	ADRB2	6,30088E-03	26051,8	1,13116E-02	5478	1,44955E+00	13442	1,13581E+00	34577	8,71485E-01	33550	0	43212
Myeloid_vs_CD4_Responder	CD4(IFNG+ Tfh/Th1)	Macro_NLRP3	GNAI2	C5AR1	6,30452E-03	27155,6	3,52464E-03	34581	2,00868E+00	5031	1,54487E+00	20411	8,73793E-01	32543	0	43212
Myeloid_vs_CD4_Responder	Macro_NLRP3	CD4(TGFB1+ Th17)	VCAN	CD44	6,34379E-03	15966,4	9,47786E-03	7507	2,26910E+00	3137	1,67255E+00	17049	9,29094E-01	8927	0	43212
Myeloid_vs_CD4_Responder	Macro_OLFML3	CD4(IFNG+ Tfh/Th1)	LGALS3BP	ITGB1	6,34840E-03	24806,2	8,36583E-03	9276	1,44055E+00	13691	1,13442E+00	34629	8,95389E-01	23223	0	43212
Myeloid_vs_CD4_Responder	Macro_FOLR2-APOE+	CD4(ISG+ Treg)	SPP1	CD44	6,35102E-03	20965,4	6,08419E-03	15542	1,43570E+00	13828	1,75066E+00	15191	9,10125E-01	17054	0	43212
Myeloid_vs_CD4_Responder	Macro_OLFML3	CD4(NME1+ T)	GRN	TNFRSF1B	6,35849E-03	24390	3,52032E-03	34640	1,46235E+00	13127	1,52650E+00	20926	9,26531E-01	10045	0	43212
Myeloid_vs_CD4_Responder	CD4(Tn)	Macro_NLRP3	HMGB1	CD163	6,36308E-03	25956,4	3,51989E-03	34645	1,44738E+00	13518	1,38998E+00	25226	9,19205E-01	13181	0	43212
Myeloid_vs_CD4_Responder	CD4(ISG+ Treg)	pDC_LILRA4	HSP90B1	TLR9	6,37355E-03	27624,8	2,24698E-02	1501	3,04870E+00	902	1,01509E+00	39490	8,29610E-01	53019	0	43212
Myeloid_vs_CD4_Responder	CD4(NME1+ T)	Mono_INHBA	RPS19	C5AR1	6,38147E-03	25010,2	4,49890E-03	24310	1,32955E+00	16881	1,13330E+00	34665	9,35466E-01	5983	0	43212
Myeloid_vs_CD4_Responder	Macro_FOLR2+APOE-	CD4(IFNG+ Tfh/Th1)	HLA-DQA2	LAG3	6,38290E-03	15413,8	1,58435E-02	2926	1,63491E+00	9623	2,56983E+00	4232	9,10084E-01	17076	0	43212
Myeloid_vs_CD4_Responder	CD4(GZMK+ Teff)	Mast	HSPA8	ADRB2	6,39452E-03	24239,2	1,27799E-02	4370	1,34669E+00	16376	9,04534E-01	44217	9,19575E-01	13021	0	43212
Myeloid_vs_CD4_Responder	Macro_ISG15	CD4(ISG+ Treg)	S100A8	CD69	6,39452E-03	18777,6	6,43190E-03	14244	1,32281E+00	17084	2,09079E+00	9107	9,26080E-01	10241	0	43212
Myeloid_vs_CD4_Responder	pDC_LILRA4	CD4(ISG+ Treg)	APP	CD74	6,39743E-03	16330,2	5,71110E-03	17086	1,55603E+00	11033	2,32611E+00	6338	9,40418E-01	3982	0	43212
Myeloid_vs_CD4_Responder	Macro_IFI27	CD4(CXCL13+ Tfh)	C3	IFITM1	6,40471E-03	17975,6	9,83683E-03	7039	1,36950E+00	15680	1,67079E+00	17091	9,33556E-01	6856	0	43212
Myeloid_vs_CD4_Responder	CD4(GZMK+ Teff)	Macro_IFI27	TNFSF9	HLA-DPA1	6,41005E-03	26194,2	4,13201E-03	27382	1,31706E+00	17265	2,14285E+00	8416	8,69000E-01	34696	0	43212
Myeloid_vs_CD4_Responder	Macro_NLRP3	CD4(TNFRSF9+ Treg)	VCAN	CD44	6,43386E-03	16218,6	9,13567E-03	8015	2,23920E+00	3292	1,67002E+00	17111	9,27873E-01	9463	0	43212
Myeloid_vs_CD4_Responder	CD4(GZMK+ Teff)	Macro_FOLR2+APOE+	TNFSF9	HLA-DPA1	6,44152E-03	27317,4	4,12607E-03	27447	1,31491E+00	17344	1,81615E+00	13854	8,68918E-01	34730	0	43212
Myeloid_vs_CD4_Responder	CD4(AREG+ Tm)	Macro_OLFML3	MIF	CD74_CXCR4	6,45777E-03	51811,2	1,85709E-03	74463	3,44811E-01	73402	4,99940E-01	66468	9,49441E-01	1511	0	43212
Myeloid_vs_CD4_Responder	Mono_INHBA	CD4(Tn)	VEGFA	ITGB1	6,46474E-03	22627,6	8,94878E-03	8319	1,97735E+00	5302	1,50513E+00	21550	8,68883E-01	34755	0	43212
Myeloid_vs_CD4_Responder	cDC(CD1C)	CD4(TNFRSF9+ Treg)	HLA-DPA1	LAG3	6,47218E-03	23610,4	3,51159E-03	34763	1,35205E+00	16219	2,94029E+00	2065	8,98776E-01	21793	0	43212
Myeloid_vs_CD4_Responder	CD4(IL26+ Th17)	Macro_LYVE1	HSP90B1	LRP1	6,48709E-03	24260	5,30588E-03	19102	2,02509E+00	4894	1,13042E+00	34779	9,04839E-01	19313	0	43212
Myeloid_vs_CD4_Responder	Mono_INHBA	CD4(NME1+ T)	VCAN	ITGA4	6,50950E-03	24884,8	6,70020E-03	13309	1,46945E+00	12967	1,55573E+00	20133	8,68774E-01	34803	0	43212
Myeloid_vs_CD4_Responder	Macro_IFI27	CD4(ISG+ Treg)	HLA-DQA2	LAG3	6,51013E-03	15239,4	1,57608E-02	2963	1,81882E+00	7032	2,37854E+00	5827	9,09870E-01	17163	0	43212
Myeloid_vs_CD4_Responder	Macro_ISG15	CD4(CXCL13+ Tfh)	CXCL10	CXCR3	6,51992E-03	22191,8	1,14043E-02	5376	2,08221E+00	4383	2,27801E+00	6844	8,33450E-01	51144	0	43212
Myeloid_vs_CD4_Responder	Macro_NLRP3	CD4(IFNG+ Tfh/Th1)	HLA-DPA1	LAG3	6,52635E-03	15991,2	9,04798E-03	8165	1,31999E+00	17174	2,48039E+00	4934	9,34437E-01	6471	0	43212
Myeloid_vs_CD4_Responder	CD4(NME1+ T)	Macro_ISG15	HLA-F	LILRB2	6,54042E-03	31424,8	3,50728E-03	34836	1,24658E+00	19634	1,28393E+00	29000	8,78519E-01	30442	0	43212
Myeloid_vs_CD4_Responder	cDC(CD1C)	CD4(IFNG+ Tfh/Th1)	HLA-DPB1	CD4	6,54136E-03	18524,2	3,50718E-03	34837	1,61985E+00	9875	3,31293E+00	977	9,41241E-01	3720	0	43212
Myeloid_vs_CD4_Responder	CD4(IFNG+ Tfh/Th1)	Macro_FOLR2+APOE+	HMGB1	CD163	6,54700E-03	24198	4,43446E-03	24781	1,70888E+00	8486	1,12901E+00	34843	9,27377E-01	9668	0	43212
Myeloid_vs_CD4_Responder	Macro_OLFML3	CD4(ISG+ Treg)	HLA-DRB5	CD4	6,54700E-03	19621,8	3,50692E-03	34843	1,66572E+00	9144	2,51125E+00	4680	9,34934E-01	6230	0	43212
Myeloid_vs_CD4_Responder	CD4(ISG+ Treg)	Macro_FOLR2+APOE+	LGALS9	HAVCR2	6,56581E-03	29419,8	4,80400E-03	22066	1,42777E+00	14039	1,17921E+00	32919	8,68637E-01	34863	0	43212
Myeloid_vs_CD4_Responder	cDC(CD1C)	CD4(CXCL13+ Tfh)	HLA-DMB	CD4	6,57146E-03	23406,2	3,50513E-03	34869	1,54104E+00	11330	2,22491E+00	7432	9,02706E-01	20188	0	43212
Myeloid_vs_CD4_Responder	CD4(TNF+ T)	Mono_CD16	HLA-A	LILRB2	6,57240E-03	21321,8	5,00559E-03	20800	1,92763E+00	5783	1,12817E+00	34870	9,47690E-01	1944	0	43212
Myeloid_vs_CD4_Responder	cDC(CD1C)	CD4(IL26+ Th17)	HLA-DPB1	CD4	6,57995E-03	18561,4	3,50463E-03	34878	1,61947E+00	9881	3,25454E+00	1104	9,41221E-01	3732	0	43212
Myeloid_vs_CD4_Responder	CD4(IL26+ Th17)	Mono_CD16	HLA-A	LILRA1	6,58706E-03	33933,2	9,44717E-03	7558	1,36416E+00	15846	2,09278E-01	85835	9,09757E-01	17215	0	43212
Myeloid_vs_CD4_Responder	Mono_INHBA	CD4(IFNG+ Tfh/Th1)	VEGFA	CD44	6,58750E-03	23929,4	7,03169E-03	12419	1,81156E+00	7127	1,12778E+00	34886	8,98222E-01	22003	0	43212
Myeloid_vs_CD4_Responder	CD4(CXCL13+ Tfh)	Macro_ISG15	CD28	CD86	6,59883E-03	28096,2	8,12580E-03	9741,5	1,26225E+00	19073,5	1,16214E+00	33555,5	8,68555E-01	34898,5	0	43212
Myeloid_vs_CD4_Responder	Macro_FOLR2+APOE-	CD4(TNF+ T)	CCL13	CXCR3	6,61072E-03	38009,6	2,22527E-02	1529	3,58685E+00	413	8,52590E-01	46615	7,21943E-01	98279	0	43212
Myeloid_vs_CD4_Responder	Macro_OLFML3	CD4(IL26+ Th17)	CD14	ITGB1	6,61397E-03	26995,6	3,50232E-03	34914	1,46362E+00	13104	1,44371E+00	23493	9,02524E-01	20255	0	43212
Myeloid_vs_CD4_Responder	CD4(TNFRSF9+ Treg)	cDC(CD1C)	B2M	CD1A	6,61682E-03	21992,6	3,15569E-02	726	1,89332E+00	6137	1,12692E+00	34917	8,91262E-01	24971	0	43212
Myeloid_vs_CD4_Responder	CD4(IFNG+ Tfh/Th1)	Mono_INHBA	CCL5	CCRL2	6,61682E-03	24691	1,22493E-02	4709	1,32107E+00	17142	1,44413E+00	23475	8,68516E-01	34917	0	43212
Myeloid_vs_CD4_Responder	CD4(IFNG+ Tfh/Th1)	pDC_LILRA4	CXCL13	CXCR3	6,62819E-03	19716,4	3,35969E-02	635	9,04391E-01	34929	2,57351E+00	4198	9,13506E-01	15608	0	43212
Myeloid_vs_CD4_Responder	Macro_OLFML3	CD4(IFNG+ Tfh/Th1)	HLA-DRA	CD4	6,62819E-03	18266,2	3,50110E-03	34929	1,71946E+00	8346	2,91500E+00	2184	9,44870E-01	2660	0	43212
Myeloid_vs_CD4_Responder	Mono_INHBA	CD4(TNF+ T)	SPP1	CD44	6,63674E-03	28794,2	3,88392E-03	29984	9,04199E-01	34938	2,00880E+00	10328	8,90000E-01	25509	0	43212

Myeloid_vs_CD4_Post_R

Myeloid_vs_CD4_Responder	CD4(IL26+ Th17)	Mono_INHBA	HLA-B	LILRB2	6,64909E-03	25621,2	3,49999E-03	34951	1,44508E+00	13571	1,18672E+00	32646	9,41231E-01	3726	0	43212
Myeloid_vs_CD4_Responder	Macro_FOLR2+APOE+	CD4(TNFRSF9+ Treg)	GRN	TNFRSF1B	6,65117E-03	15756,4	5,67067E-03	17258	1,93593E+00	5702	2,10746E+00	8870	9,41197E-01	3740	0	43212
Myeloid_vs_CD4_Responder	CD4(GZMK+ Teff)	Macro_FOLR2+APOE+	CD52	SIGLEC10	6,65267E-03	27013,8	6,30769E-03	14695	1,60869E+00	10069	7,45748E-01	52031	9,14727E-01	15062	0	43212
Myeloid_vs_CD4_Responder	cDC(CD1C)	CD4(IFNG+ Tfh/Th1)	B2M	CD3D	6,65389E-03	39157,6	3,57922E-03	33824	2,75473E-01	79743	1,02752E+00	38974	9,62044E-01	35	0	43212
Myeloid_vs_CD4_Responder	CD4(GZMK+ Teff)	Macro_LYVE1	TNFSF9	HLA-DPA1	6,66147E-03	27287,8	4,09148E-03	27796	1,30239E+00	17729	1,87228E+00	12738	8,68438E-01	34964	0	43212
Myeloid_vs_CD4_Responder	Mono_INHBA	CD4(NME1+ T)	SPP1	ITGA4_ITGB1	6,66528E-03	24255,2	6,42872E-03	14257	1,19835E+00	21409	2,22496E+00	7430	8,68430E-01	34968	0	43212
Myeloid_vs_CD4_Responder	Macro_OLFML3	CD4(IL26+ Th17)	HLA-DRA	CD4	6,66719E-03	18328,4	3,49855E-03	34970	1,71908E+00	8351	2,85661E+00	2441	9,44852E-01	2668	0	43212
Myeloid_vs_CD4_Responder	Macro_FOLR2+APOE+	CD4(ISG+ Treg)	GRN	TNFRSF1B	6,68265E-03	16136,2	5,66575E-03	17279	1,93530E+00	5709	1,98519E+00	10730	9,41173E-01	3751	0	43212
Myeloid_vs_CD4_Responder	Macro_OLFML3	CD4(CRTAM- T)	CD14	ITGA4	6,69296E-03	27871,2	3,49661E-03	34997	1,44780E+00	13506	1,44621E+00	23417	8,93076E-01	24224	0	43212
Myeloid_vs_CD4_Responder	CD4(IFNG+ Tfh/Th1)	Mast	HSPA8	ADRB2	6,70253E-03	21314,2	1,40291E-02	3684	1,46457E+00	13067	1,12481E+00	35007	9,22956E-01	11601	0	43212
Myeloid_vs_CD4_Responder	Macro_OLFML3	CD4(CXCL13+ Tfh)	HLA-DQA1	CD4	6,70349E-03	20444	3,49598E-03	35008	1,66042E+00	9220	2,34717E+00	6139	9,29732E-01	8641	0	43212
Myeloid_vs_CD4_Responder	CD4(CXCL13+ Tfh)	Mono_CD14	HMGB1	THBD	6,71019E-03	34140,4	3,83121E-03	30582	1,00309E+00	29958	1,12464E+00	35015	8,75108E-01	31935	0	43212
Myeloid_vs_CD4_Responder	CD4(ISG+ Treg)	Mono_INHBA	B2M	LILRB2	6,72362E-03	23316,4	3,49443E-03	35029	1,60707E+00	10095	1,36131E+00	26226	9,47390E-01	2020	0	43212
Myeloid_vs_CD4_Responder	CD4(CRTAM- T)	Macro_OLFML3	CD52	SIGLEC10	6,73083E-03	28453,4	5,65846E-03	17311	1,40255E+00	14741	7,84073E-01	50058	9,10395E-01	16945	0	43212
Myeloid_vs_CD4_Responder	Mono_CD14	CD4(CXCL13+ Tfh)	VCAN	ITGB1	6,74200E-03	15824,2	9,94445E-03	6923	2,85046E+00	1247	2,34646E+00	6147	8,99332E-01	21592	0	43212
Myeloid_vs_CD4_Responder	Mono_INHBA	CD4(IFNG+ Tfh/Th1)	HBEGF	CD82	6,74861E-03	24389,2	1,01031E-02	6727	1,45400E+00	13339	1,44017E+00	23613	8,68206E-01	35055	0	43212
Myeloid_vs_CD4_Responder	CD4(TNFRSF9+ Treg)	Mono_CD16	HLA-A	LILRB1	6,74861E-03	25675,4	4,04200E-03	28289	1,43821E+00	13759	1,12369E+00	35055	9,30955E-01	8062	0	43212
Myeloid_vs_CD4_Responder	CD4(ISG+ Treg)	Macro_ISG15	LGALS9	HAVCR2	6,75920E-03	26526,4	4,76690E-03	22313	1,42047E+00	14211	1,64119E+00	17830	8,68194E-01	35066	0	43212
Myeloid_vs_CD4_Responder	Mono_INHBA	CD4(TGFB1+ Th17)	LGALS1	PTPRC	6,76306E-03	24151,4	3,49197E-03	35070	1,18019E+00	22098	1,59794E+00	19005	9,50004E-01	1372	0	43212
Myeloid_vs_CD4_Responder	CD4(IL26+ Th17)	Mono_CD16	HLA-F	LILRB2	6,76499E-03	27848,8	3,94963E-03	29288	2,14932E+00	3859	1,12326E+00	35072	8,84716E-01	27813	0	43212
Myeloid_vs_CD4_Responder	Macro_OLFML3	CD4(ISG+ Treg)	HLA-DMA	CD4	6,76692E-03	23949,2	3,49172E-03	35074	1,49659E+00	12311	1,68376E+00	16774	9,21076E-01	12375	0	43212
Myeloid_vs_CD4_Responder	CD4(NME1+ T)	Mono_INHBA	CALR	LRP1	6,80365E-03	29625,4	3,82967E-03	30597	1,32067E+00	17150	1,12231E+00	35112	8,98113E-01	22056	0	43212
Myeloid_vs_CD4_Responder	Macro_OLFML3	CD4(GZMK+ Teff)	APOE	SORL1	6,82111E-03	23344,4	3,48747E-03	35130	1,82764E+00	6924	2,71010E+00	3251	8,83658E-01	28205	0	43212
Myeloid_vs_CD4_Responder	Macro_OLFML3	CD4(IFNG+ Tfh/Th1)	HLA-DPB1	CD4	6,82694E-03	19226,6	3,48712E-03	35136	1,60972E+00	10051	2,60507E+00	3948	9,41082E-01	3786	0	43212
Myeloid_vs_CD4_Responder	cDC(CD1C)	CD4(TNFRSF9+ Treg)	HLA-DMB	CD4	6,84055E-03	23533,4	3,48618E-03	35150	1,53869E+00	11370	2,20484E+00	7657	9,02467E-01	20278	0	43212
Myeloid_vs_CD4_Responder	CD4(IL26+ Th17)	Mono_INHBA	HLA-C	LILRB2	6,84444E-03	26709,8	3,48599E-03	35154	1,38712E+00	15178	1,13375E+00	34648	9,36868E-01	5357	0	43212
Myeloid_vs_CD4_Responder	CD4(TGFB1+ Th17)	pDC_LILRA4	MAML2	NOTCH4	6,84779E-03	37575,2	1,87797E-02	2099	1,55405E+00	11066	1,65899E+00	17388	6,67306E-01	114111	0	43212
Myeloid_vs_CD4_Responder	CD4(TGFB1+ Th17)	Mono_CD16	HLA-C	LILRA1	6,86309E-03	30882,8	9,70895E-03	7202	1,49746E+00	12290	3,78507E-01	74312	9,09349E-01	17398	0	43212
Myeloid_vs_CD4_Responder	CD4(IFNG+ Tfh/Th1)	Mono_INHBA	HLA-C	LILRB2	6,86686E-03	26122,4	3,48477E-03	35177	1,38634E+00	15198	1,21232E+00	31663	9,36858E-01	5362	0	43212
Myeloid_vs_CD4_Responder	Macro_ISG15	CD4(ISG+ Treg)	ICAM1	IL2RG	6,86769E-03	19851,4	7,31768E-03	11635	1,31313E+00	17401	1,98018E+00	10824	9,12134E-01	16185	0	43212
Myeloid_vs_CD4_Responder	Macro_OLFML3	CD4(IL26+ Th17)	HLA-DPB1	CD4	6,86979E-03	19331	3,48459E-03	35180	1,60934E+00	10061	2,54668E+00	4403	9,41062E-01	3799	0	43212
Myeloid_vs_CD4_Responder	Macro_IER3	CD4(IL26+ Th17)	HLA-DPB1	LAG3	6,87228E-03	19409,6	7,24187E-03	11813	1,31304E+00	17404	1,81495E+00	13876	9,24945E-01	10743	0	43212
Myeloid_vs_CD4_Responder	pDC_LILRA4	CD4(TNFRSF9+ Treg)	APP	CD74	6,87382E-03	16461	5,63864E-03	17405	1,54474E+00	11254	2,32785E+00	6319	9,40059E-01	4115	0	43212
Myeloid_vs_CD4_Responder	CD4(Tn)	Mono_CD16	HLA-C	LILRA1	6,88762E-03	30502,6	9,69652E-03	7215	1,49480E+00	12357	4,08028E-01	72315	9,09296E-01	17414	0	43212
Myeloid_vs_CD4_Responder	CD4(ISG+ Treg)	Mono_CD14	HLA-A	LILRB2	6,89913E-03	23060,4	3,48253E-03	35210	1,47978E+00	12735	1,59023E+00	19216	9,37932E-01	4929	0	43212
Myeloid_vs_CD4_Responder	CD4(ISG+ Treg)	Macro_ISG15	HLA-C	LILRB1	6,91777E-03	27343,8	3,79372E-03	31023	1,30344E+00	17696	1,11970E+00	35229	9,27650E-01	9559	0	43212
Myeloid_vs_CD4_Responder	Macro_ISG15	CD4(ISG+ Treg)	HLA-DPB1	LAG3	6,92146E-03	17034,2	7,92427E-03	10157	1,31200E+00	17436	2,47786E+00	4957	9,28011E-01	9409	0	43212
Myeloid_vs_CD4_Responder	CD4(AREG+ Tm)	Macro_ISG15	CCL5	CCR1	6,93251E-03	25449	1,24758E-02	4549	1,36390E+00	15855	1,30077E+00	28385	8,67787E-01	35244	0	43212
Myeloid_vs_CD4_Responder	pDC_LILRA4	CD4(IFNG+ Tfh/Th1)	HLA-DRA	LAG3	6,95696E-03	15612,2	8,15027E-03	9692	1,31124E+00	17459	3,45864E+00	728	9,33335E-01	6970	0	43212
Myeloid_vs_CD4_Responder	CD4(GZMK+ Teff)	Macro_NLRP3	HLA-C	LILRB2	6,96009E-03	26065,4	3,47928E-03	35272	1,42020E+00	14219	1,19715E+00	32244	9,36811E-01	5380	0	43212
Myeloid_vs_CD4_Responder	CD4(IFNG+ Tfh/Th1)	Macro_LYVE1	CALR	LRP1	6,96700E-03	23495,8	5,49471E-03	18108	1,98156E+00	5265	1,11852E+00	35279	9,13484E-01	15615	0	43212
Myeloid_vs_CD4_Responder	cDC(CD1C)	CD4(CXCL13+ Tfh)	HLA-DQA2	CD4	6,96897E-03	25070,4	3,47857E-03	35281	1,27977E+00	18471	2,30965E+00	6517	8,98564E-01	21871	0	43212
Myeloid_vs_CD4_Responder	Macro_OLFML3	CD4(CXCL13+ Tfh)	HLA-DQB1	LAG3	6,97490E-03	26725	3,47824E-03	35287	1,46736E+00	13009	1,93933E+00	11537	8,78194E-01	30580	0	43212
Myeloid_vs_CD4_Responder	Macro_OLFML3	CD4(TNFRSF9+ Treg)	HLA-DQA1	CD4	6,98974E-03	20564	3,47707E-03	35302	1,65807E+00	9256	2,32709E+00	6329	9,29555E-01	8721	0	43212
Myeloid_vs_CD4_Responder	Mono_CD16	CD4(IFNG+ Tfh/Th1)	HLA-DRB5	LAG3	6,99260E-03	17923,2	9,08341E-03	8103	1,31052E+00	17482	2,01633E+00	10207	9,25267E-01	10612	0	43212
Myeloid_vs_CD4_Responder	Macro_LYVE1	CD4(NME1+ T)	C1QB	C1QBP	6,99415E-03	17345,4	5,62074E-03	17483	1,83064E+00	6884	2,18351E+00	7914	9,23761E-01	11234	0	43212
Myeloid_vs_CD4_Responder	Macro_FOLR2+APOE+	CD4(IFNG+ Tfh/Th1)	HLA-DQA2	LAG3	6,99570E-03	16019	1,54854E-02	3072	1,60671E+00	10099	2,33828E+00	6228	9,09144E-01	17484	0	43212
Myeloid_vs_CD4_Responder	CD4(TNFRSF9+ Treg)	Macro_FOLR2-APOE+	LTB	CD40	7,00758E-03	28107,2	8,35444E-03	9301	1,10195E+00	25327	1,11763E+00	35320	8,85721E-01	27376	0	43212
Myeloid_vs_CD4_Responder	CD4(AREG+ Tm)	cDC(CD1C)	MIF	CD44_CD74	7,01833E-03	41923,4	1,89225E-03	73299	4,34003E-01	65981	1,38083E+00	25549	9,49206E-01	1576	0	43212
Myeloid_vs_CD4_Responder	Macro_FOLR2-APOE+	CD4(IL26+ Th17)	HLA-DQB1	LAG3	7,06271E-03	19996,4	8,08632E-03	9819	1,30903E+00	17527	1,75053E+00	15195	9,16618E-01	14229	0	43212
Myeloid_vs_CD4_Responder	CD4(CXCL13+ Tfh)	Macro_FOLR2+APOE+	CD52	SIGLEC10	7,08463E-03	30898,4	5,60975E-03	17541	1,52285E+00	11732	5,23952E-01	64917	9,10041E-01	17090	0	43212
Myeloid_vs_CD4_Responder	Mono_INHBA	CD4(GZMK+ Teff)	ICAM1	IL2RG	7,09731E-03	35613,8	3,46747E-03	35410	9,25717E-01	33859	1,13518E+00	34605	8,77239E-01	30983	0	43212
Myeloid_vs_CD4_Responder	CD4(TNFRSF9+ Treg)	Macro_LYVE1	HLA-A	APLP2	7,10433E-03	27453	3,50676E-03	34845	1,17639E+00	22250	1,11515E+00	35417	9,49324E-01	1541	0	43212
Myeloid_vs_CD4_Responder	Mono_INHBA	CD4(ISG+ Treg)	HLA-DQA1	LAG3	7,11035E-03	26921,6	5,50612E-03	18052	8,94491E-01	35423	1,70889E+00	16177	8,98889E-01	21744	0	43212
Myeloid_vs_CD4_Responder	CD4(TNF+ T)	Mono_INHBA	RPS19	C5AR1	7,12441E-03	25994,4	4,28721E-03	26008	1,27449E+00	18655	1,11440E+00	35437	9,33996E-01	6660	0	43212
Myeloid_vs_CD4_Responder	CD4(GZMK+ Teff)	Macro_ISG15	CD99	PILRA	7,16169E-03	30859	3,89272E-03	29897	1,14106E+00	23668	1,11368E+00	35474	8,98139E-01	22044	0	43212
Myeloid_vs_CD4_Responder	CD4(GZMK+ Teff)	Macro_FOLR2+APOE-	TNFSF9	HLA-DPA1	7,16673E-03	27470,8	4,01243E-03	28605	1,27377E+00	18683	1,94969E+00	11375	8,67319E-01	35479	0	43212
Myeloid_vs_CD4_Responder	CD4(IFNG+ Tfh/Th1)	Mono_INHBA	B2M	LILRB2	7,17179E-03	23670,8	3,46225E-03	35484	1,56718E+00	10815	1,34438E+00	26773	9,47159E-01	2070	0	43212
Myeloid_vs_CD4_Responder	CD4(ISG+ Treg)	cDC_CLEC9A	TNFSF13B	HLA-DPB1	7,17754E-03	33439,8	1,37868E-03	91712	1,46772E+00	13003	3,04758E+00	1672	9,08912E-01	17600	0	43212
Myeloid_vs_CD4_Responder	CD4(AREG+ Tm)	Mono_CD16	HLA-C	LILRA1	7,18229E-03	31050,4	9,60339E-03	7332	1,47482E+00	12841	3,79273E-01	74264	9,08897E-01	17603	0	43212
Myeloid_vs_CD4_Responder	Mono_INHBA	CD4(GZMK+ Teff)	SPP1	PTGER4	7,18392E-03	25596,4	6,73726E-03	13217	1,08720E+00	25989	2,02609E+00	10068	8,67276E-01	35496	0	43212
Myeloid_vs_CD4_Responder	CD4(IL26+ Th17)	cDC_CLEC9A	TGFB1	LPP	7,18999E-03	28970,8	7,63465E-03	10832	8,93227E-01	35502	1,41282E+00	24477	8,77596E-01	30831	0	43212
Myeloid_vs_CD4_Responder	CD4(ISG+ Treg)	Macro_ISG15	TIGIT	NECTIN2	7,19101E-03	23747,4	3,01781E-02	803,5	1,71277E+00	8437,5	1,23489E+00	30780,5	8,67270E-01	35503,5	0	43212
Myeloid_vs_CD4_Responder	cDC(CD1C)	CD4(TNFRSF9+ Treg)	HLA-DQA2	CD4	7,20114E-03	25191,6	3,45976E-03	35513	1,27742E+00	18538	2,28958E+00	6731	8,98316E-01	21964	0	43212
Myeloid_vs_CD4_Responder	CD4(NME1+ T)	Macro_LYVE1	HSP90B1	LRP1	7,20215E-03	24713,6	5,14978E-03	19962	2,00729E+00	5044	1,11272E+00	35514	9,03545E-01	19836	0	43212
Myeloid_vs_CD4_Responder	Mono_INHBA	CD4(NME1+ T)	VEGFA	CD44	7,20722E-03	24064,8	7,02587E-03	12431	1,81101E+00	7136	1,11248E+00	35519	8,98184E-01	22026	0	43212
Myeloid_vs_CD4_Responder	Macro_OLFML3	CD4(CRTAM- T)	CXCL9	CXCR3	7,21433E-03	21224,6	1,68797E-02	2579	1,53925E+00	11357	1,83628E+00	13449	8,67217E-01	35526	0	43212
Myeloid_vs_CD4_Responder	Macro_FOLR2+APOE+	CD4(IL26+ Th17)	HLA-DRB5	LAG3	7,21877E-03	20142,8	7,74796E-03	10573	1,35008E+00	16284	1,64908E+00	17626	9,19579E-01	13019	0	43212
Myeloid_vs_CD4_Responder	cDC_CLEC9A	CD4(IFNG+ Tfh/Th1)	TGM2	ADGRG1	7,22389E-03	28218	1,13709E-01	38	1,99099E+00	5177	1,61366E+00	18594	7,83460E-01	74069	0	43212

Myeloid_vs_CD4_Post_R

Myeloid_vs_CD4_Responder	CD4(CRTAM- T)	Mono_CD16	HLA-C	LILRA1	7,23149E-03	31201,2	9,58773E-03	7349	1,47147E+00	12914	3,69335E-01	74897	9,08829E-01	17634	0	43212
Myeloid_vs_CD4_Responder	Mono_INHBA	CD4(NME1+ T)	HBEGF	CD82	7,24179E-03	25115,6	9,92164E-03	6951	1,44507E+00	13572	1,35956E+00	26290	8,67166E-01	35553	0	43212
Myeloid_vs_CD4_Responder	CD4(IL26+ Th17)	Mono_CD14	THBS1	CD36	7,26975E-03	30897,8	6,08863E-03	15526	1,71281E+00	8436	1,64794E+00	17658	7,93336E-01	69657	0	43212
Myeloid_vs_CD4_Responder	Macro_OLFML3	CD4(AREG+ Tm)	CD14	ITGA4	7,28057E-03	28143,8	3,45357E-03	35591	1,44368E+00	13621	1,43417E+00	23818	8,92483E-01	24477	0	43212
Myeloid_vs_CD4_Responder	CD4(ISG+ Treg)	Mono_CD16	GNAI2	C5AR1	7,29183E-03	29338,4	3,45305E-03	35602	1,82570E+00	6953	1,31387E+00	27894	8,72657E-01	33031	0	43212
Myeloid_vs_CD4_Responder	Macro_OLFML3	CD4(TNFRSF9+ Treg)	CD14	ITGB1	7,29900E-03	27044,6	3,45239E-03	35609	1,45788E+00	13242	1,47156E+00	22633	9,01891E-01	20527	0	43212
Myeloid_vs_CD4_Responder	Macro_OLFML3	CD4(IFNG+ Tfh/Th1)	CD86	CTLA4	7,30002E-03	23437,2	1,11673E-02	5641	1,50060E+00	12221	1,11045E+00	35610	9,01937E-01	20502	0	43212
Myeloid_vs_CD4_Responder	CD4(TNF+ T)	cDC_CLEC9A	CD52	SIGLEC10	7,30105E-03	34030,2	3,45235E-03	35611	9,24352E-01	33929	1,22848E+00	31045	8,88094E-01	26354	0	43212
Myeloid_vs_CD4_Responder	Macro_NLRP3	CD4(TGFB1+ Th17)	CXCL2	DPP4	7,30499E-03	19704,4	2,71297E-02	1020	2,21243E+00	3449	2,25354E+00	7116	8,49216E-01	43725	0	43212
Myeloid_vs_CD4_Responder	CD4(Tn)	Mono_CD14	HMGB1	THBD	7,31849E-03	35528,8	3,62544E-03	33207	9,56023E-01	32310	1,10995E+00	35628	8,72060E-01	33287	0	43212
Myeloid_vs_CD4_Responder	CD4(IL26+ Th17)	Macro_LYVE1	CD99	PILRA	7,31938E-03	25823,8	5,58037E-03	17689	1,59132E+00	10361	9,50642E-01	42236	9,13472E-01	15621	0	43212
Myeloid_vs_CD4_Responder	CD4(Tn)	Macro_IER3	CD55	ADGRE2	7,31952E-03	25189,2	9,24807E-03	7826	1,52349E+00	11712	1,32267E+00	27567	8,67010E-01	35629	0	43212
Myeloid_vs_CD4_Responder	Mono_CD14	CD4(AREG+ Tm)	IL1B	SIGIRR	7,32877E-03	20015,8	5,99480E-03	15887	1,82621E+00	6941	2,72983E+00	3126	8,77380E-01	30913	0	43212
Myeloid_vs_CD4_Responder	Mono_CD14	CD4(AREG+ Tm)	HBEGF	CD44	7,34215E-03	23733	5,87807E-03	16369	1,25413E+00	19359	1,49838E+00	21757	9,08073E-01	17968	0	43212
Myeloid_vs_CD4_Responder	Mono_CD14	CD4(AREG+ Tm)	LGALS1	CD69	7,34524E-03	24142,8	3,97775E-03	28982	9,85954E-01	30803	1,86604E+00	12862	9,38080E-01	4855	0	43212
Myeloid_vs_CD4_Responder	CD4(TNFRSF9+ Treg)	Mono_INHBA	HLA-B	LILRB2	7,35348E-03	25934,2	3,44848E-03	35662	1,40218E+00	14749	1,19896E+00	32179	9,40820E-01	3869	0	43212
Myeloid_vs_CD4_Responder	cDC(CD1C)	CD4(ISG+ Treg)	HLA-DQB1	CD4	7,35554E-03	20337,6	3,44839E-03	35664	1,51718E+00	11863	2,80089E+00	2716	9,30597E-01	8233	0	43212
Myeloid_vs_CD4_Responder	CD4(GZMK+ Teff)	pDC_LILRA4	MAML2	NOTCH4	7,35637E-03	38948,2	1,54036E-02	3100	1,51783E+00	11840	1,64600E+00	17712	6,44956E-01	118877	0	43212
Myeloid_vs_CD4_Responder	Mono_CD14	CD4(TNFRSF9+ Treg)	VCAN	ITGA4	7,36200E-03	16729,2	9,75830E-03	7135	2,83832E+00	1268	2,36484E+00	5980	8,88762E-01	26051	0	43212
Myeloid_vs_CD4_Responder	cDC_CLEC9A	CD4(ISG+ Treg)	HLA-DQA2	LAG3	7,36604E-03	14723,2	1,52969E-02	3139	1,77222E+00	7622	2,97498E+00	1925	9,08637E-01	17718	0	43212
Myeloid_vs_CD4_Responder	cDC(CD1C)	CD4(NME1+ T)	HLA-DMB	CD4	7,36689E-03	23922	3,44715E-03	35675	1,53384E+00	11471	2,11535E+00	8765	9,01971E-01	20487	0	43212
Myeloid_vs_CD4_Responder	Macro_IER3	CD4(IFNG+ Tfh/Th1)	ICAM1	IL2RG	7,39350E-03	20052,4	7,53949E-03	11056	1,48623E+00	12581	1,64502E+00	17735	9,13324E-01	15678	0	43212
Myeloid_vs_CD4_Responder	CD4(IFNG+ Tfh/Th1)	Macro_FOLR2+APOE+	IFNG	IFNGR1_IFNGR2	7,39585E-03	25561,2	1,94901E-02	1951	1,30850E+00	17551	1,27333E+00	29389	8,66845E-01	35703	0	43212
Myeloid_vs_CD4_Responder	Mono_CD14	CD4(Tn)	IL1B	SIGIRR	7,40103E-03	20196,4	5,86151E-03	16427	1,81981E+00	7021	2,77601E+00	2867	8,76166E-01	31455	0	43212
Myeloid_vs_CD4_Responder	Mono_CD14	CD4(Tn)	VCAN	ITGA4	7,40829E-03	17835	8,42249E-03	9164	2,79908E+00	1356	2,33918E+00	6219	8,81274E-01	29224	0	43212
Myeloid_vs_CD4_Responder	CD4(AREG+ Tm)	cDC(CD1C)	CD52	SIGLEC10	7,40829E-03	34496	3,65339E-03	32856	8,89293E-01	35715	1,11150E+00	35562	8,90876E-01	25135	0	43212
Myeloid_vs_CD4_Responder	Mono_CD14	CD4(Tn)	HBEGF	CD44	7,41451E-03	24607	5,57553E-03	17704	1,20656E+00	21109	1,48834E+00	22093	9,05843E-01	18917	0	43212
Myeloid_vs_CD4_Responder	CD4(TNFRSF9+ Treg)	pDC_LILRA4	TNF	TNFRSF21	7,42265E-03	29799,2	8,53249E-03	9001	3,13114E+00	789	1,64416E+00	17753	7,73403E-01	78241	0	43212
Myeloid_vs_CD4_Responder	CD4(TNFRSF9+ Treg)	Macro_NLRP3	HLA-C	LILRB2	7,43425E-03	26269,4	3,44300E-03	35740	1,39596E+00	14924	1,20507E+00	31949	9,36500E-01	5522	0	43212
Myeloid_vs_CD4_Responder	CD4(ISG+ Treg)	Macro_NLRP3	HLA-C	LILRB2	7,43529E-03	26677	3,44290E-03	35741	1,39590E+00	14926	1,15098E+00	33983	9,36499E-01	5523	0	43212
Myeloid_vs_CD4_Responder	CD4(TNFRSF9+ Treg)	Mono_CD14	HLA-A	LILRB2	7,44257E-03	23159	3,44232E-03	35748	1,44791E+00	13503	1,62454E+00	18274	9,37593E-01	5058	0	43212
Myeloid_vs_CD4_Responder	Mono_CD14	CD4(IL26+ Th17)	THBS1	ITGA4	7,46029E-03	21466,6	9,34949E-03	7681	1,22905E+00	20291	1,98435E+00	10748	8,90246E-01	25401	0	43212
Myeloid_vs_CD4_Responder	CD4(IFNG+ Tfh/Th1)	Macro_ISG15	CCL5	CCR1	7,46968E-03	24414	1,22375E-02	4718	1,35172E+00	16228	1,48706E+00	22138	8,66677E-01	35774	0	43212
Myeloid_vs_CD4_Responder	Mono_INHBA	CD4(TGFB1+ Th17)	NAMPT	ITGA5_ITGB1	7,46968E-03	34409,4	3,44056E-03	35774	8,90237E-01	35657	1,30266E+00	28317	8,81610E-01	29087	0	43212
Myeloid_vs_CD4_Responder	Mono_CD14	CD4(IL26+ Th17)	IL1B	SIGIRR	7,48640E-03	18662,4	6,87761E-03	12832	1,86860E+00	6425	2,75644E+00	2980	8,84581E-01	27863	0	43212
Myeloid_vs_CD4_Responder	CD4(TNF+ T)	cDC_CLEC9A	COPA	P2RY6	7,49262E-03	28699,4	8,13475E-03	9722	2,13521E+00	3968	1,64245E+00	17796	7,95222E-01	68799	0	43212
Myeloid_vs_CD4_Responder	Mono_CD14	CD4(IL26+ Th17)	HBEGF	CD44	7,50000E-03	27254,4	5,16051E-03	19884	1,14130E+00	23654	1,27667E+00	29256	9,02493E-01	20266	0	43212
Myeloid_vs_CD4_Responder	Mono_CD14	CD4(IL26+ Th17)	LGALS1	CD69	7,50419E-03	26956	3,62277E-03	33246	9,15390E-01	34379	1,63947E+00	17882	9,35309E-01	6061	0	43212
Myeloid_vs_CD4_Responder	Macro_OLFML3	CD4(NME1+ T)	HLA-DQA1	CD4	7,50839E-03	20905	3,43815E-03	35811	1,65322E+00	9325	2,23761E+00	7287	9,29186E-01	8890	0	43212
Myeloid_vs_CD4_Responder	Macro_OLFML3	CD4(IFNG+ Tfh/Th1)	HLA-DPA1	CD4	7,51258E-03	19278,6	3,43776E-03	35815	1,58082E+00	10557	2,67074E+00	3500	9,42559E-01	3309	0	43212
Myeloid_vs_CD4_Responder	Mono_CD14	CD4(IL26+ Th17)	LYZ	ITGAL	7,51468E-03	22763,4	3,55591E-03	34132	1,76923E+00	7654	2,93881E+00	2069	8,87200E-01	26750	0	43212
Myeloid_vs_CD4_Responder	Mono_CD14	CD4(IL26+ Th17)	AGTRAP	RACK1	7,51678E-03	28193,6	5,53372E-03	17917	9,44948E-01	32877	1,11387E+00	35464	9,23175E-01	11498	0	43212
Myeloid_vs_CD4_Responder	CD4(GZMK+ Teff)	Macro_LYVE1	CALR	LRP1	7,52098E-03	24183,4	5,19954E-03	19674	1,94318E+00	5627	1,10484E+00	35823	9,11277E-01	16581	0	43212
Myeloid_vs_CD4_Responder	CD4(AREG+ Tm)	Macro_LYVE1	HSP90B1	LRP1	7,52203E-03	25335	4,88944E-03	21527	1,97762E+00	5301	1,10483E+00	35824	9,01261E-01	20811	0	43212
Myeloid_vs_CD4_Responder	CD4(GZMK+ Teff)	Mono_INHBA	HLA-B	LILRB2	7,53674E-03	26027,4	3,43623E-03	35838	1,39197E+00	15051	1,20001E+00	32141	9,40721E-01	3895	0	43212
Myeloid_vs_CD4_Responder	CD4(AREG+ Tm)	Macro_LYVE1	MIF	CD74_CXCR4	7,54683E-03	54447,8	1,82347E-03	75675	3,48819E-01	73049	3,12961E-01	78668	9,49000E-01	1635	0	43212
Myeloid_vs_CD4_Responder	Mono_CD14	CD4(TGFB1+ Th17)	ICAM1	IL2RG	7,54936E-03	27792,6	4,92966E-03	21265	9,71579E-01	31538	1,57787E+00	19552	8,94963E-01	23396	0	43212
Myeloid_vs_CD4_Responder	CD4(IFNG+ Tfh/Th1)	Macro_ISG15	HMGB1	HAVCR2	7,54936E-03	32755,4	3,56518E-03	33993	9,21857E-01	34041	1,10425E+00	35850	9,11045E-01	16681	0	43212
Myeloid_vs_CD4_Responder	Macro_OLFML3	CD4(IL26+ Th17)	HLA-DPA1	CD4	7,55147E-03	19366	3,43526E-03	35852	1,58044E+00	10563	2,61236E+00	3888	9,42539E-01	3315	0	43212
Myeloid_vs_CD4_Responder	cDC(CD1C)	CD4(GZMK+ Teff)	LGALS1	CD69	7,55779E-03	26926,6	3,43500E-03	35858	9,15719E-01	34359	1,79049E+00	14401	9,33680E-01	6803	0	43212
Myeloid_vs_CD4_Responder	Macro_OLFML3	CD4(IFNG+ Tfh/Th1)	GRN	TNFRSF1B	7,57150E-03	24636	3,43437E-03	35871	1,44947E+00	13444	1,55148E+00	20239	9,25685E-01	10414	0	43212
Myeloid_vs_CD4_Responder	Mono_CD14	CD4(TGFB1+ Th17)	IL1B	SIGIRR	7,57467E-03	16472	8,77569E-03	8599	1,95974E+00	5475	2,88691E+00	2303	8,96451E-01	22771	0	43212
Myeloid_vs_CD4_Responder	Macro_IER3	CD4(IFNG+ Tfh/Th1)	HLA-DRB5	LAG3	7,58448E-03	17405,4	8,97665E-03	8276	1,29863E+00	17852	2,27305E+00	6899	9,24857E-01	10788	0	43212
Myeloid_vs_CD4_Responder	Mono_CD14	CD4(TGFB1+ Th17)	HBEGF	CD44	7,59157E-03	26500,6	5,26629E-03	19316	1,15793E+00	22960	1,33578E+00	27115	9,03382E-01	19900	0	43212
Myeloid_vs_CD4_Responder	Mono_CD14	CD4(TGFB1+ Th17)	LGALS1	CD69	7,59475E-03	18218	5,25407E-03	19389	1,23966E+00	19894	2,34550E+00	6156	9,45687E-01	2439	0	43212
Myeloid_vs_CD4_Responder	CD4(TNF+ T)	Mono_INHBA	TNFSF9	HLA-DPA1	7,59475E-03	30338,8	3,94858E-03	29297	9,64199E-01	31913	1,94960E+00	11379	8,66394E-01	35893	0	43212
Myeloid_vs_CD4_Responder	Macro_LYVE1	CD4(IL26+ Th17)	HLA-DQA1	LAG3	7,60262E-03	20744,8	7,93879E-03	10113	1,31625E+00	17302	1,63994E+00	17863	9,14346E-01	15234	0	43212
Myeloid_vs_CD4_Responder	Macro_FOLR2+APOE-	CD4(ISG+ Treg)	B2M	CD3D	7,60387E-03	52342,4	3,53687E-03	34399	2,08920E-01	86219	4,09222E-02	97842	9,61826E-01	40	0	43212
Myeloid_vs_CD4_Responder	Mono_CD14	CD4(TGFB1+ Th17)	LYZ	ITGAL	7,60427E-03	20799,2	4,08975E-03	27814	1,80254E+00	7226	2,97568E+00	1921	8,94011E-01	23823	0	43212
Myeloid_vs_CD4_Responder	CD4(NME1+ T)	Macro_LYVE1	LRPAP1	LRP1	7,60851E-03	27706	5,39053E-03	18676	1,97584E+00	5316	1,11506E+00	35420	8,66360E-01	35906	0	43212
Myeloid_vs_CD4_Responder	Mono_CD14	CD4(TGFB1+ Th17)	NAMPT	ITGA5_ITGB1	7,61805E-03	24586,8	4,39740E-03	25096	1,20248E+00	21269	2,06489E+00	9463	8,93828E-01	23894	0	43212
Myeloid_vs_CD4_Responder	CD4(IL26+ Th17)	Macro_NLRP3	HMGB1	CD163	7,63503E-03	27668,6	3,43051E-03	35931	1,42632E+00	14075	1,21462E+00	31559	9,18245E-01	13566	0	43212
Myeloid_vs_CD4_Responder	CD4(TNFRSF9+ Treg)	cDC(CD1C)	LTB	CD40	7,63928E-03	30657,2	6,27723E-03	14810	8,85271E-01	35935	1,38840E+00	25285	8,70437E-01	34044	0	43212
Myeloid_vs_CD4_Responder	CD4(CXCL13+ Tfh)	Macro_LYVE1	CALR	LRP1	7,64779E-03	23647,6	5,48199E-03	18164	1,97990E+00	5277	1,10157E+00	35943	9,13392E-01	15642	0	43212
Myeloid_vs_CD4_Responder	Mono_CD14	CD4(TNFRSF9+ Treg)	ICAM1	IL2RG	7,64992E-03	28669	4,55142E-03	23891	9,16662E-01	34309	1,67700E+00	16923	8,91151E-01	25010	0	43212
Myeloid_vs_CD4_Responder	CD4(GZMK+ Teff)	Macro_OLFML3	CCL5	CCR1	7,66483E-03	27171,4	1,21492E-02	4781	1,28609E+00	18284	1,16047E+00	33621	8,66258E-01	35959	0	43212
Myeloid_vs_CD4_Responder	Macro_FOLR2-APOE+	CD4(IFNG+ Tfh/Th1)	LGALS3	LAG3	7,67716E-03	17478,6	1,27875E-02	4365	1,29687E+00	17908	2,18120E+00	7945	9,17252E-01	13963	0	43212
Myeloid_vs_CD4_Responder	Mono_CD14	CD4(AREG+ Tm)	AGTRAP	RACK1	7,67763E-03	29094	5,31958E-03	19039	8,99845E-01	35153	1,10110E+00	35971	9,21764E-01	12095	0	43212
Myeloid_vs_CD4_Responder	CD4(AREG+ Tm)	pDC_LILRA4	MAML2	NOTCH4	7,67882E-03	38815,2	1,58362E-02	2932	1,52247E+00	11737	1,63832E+00	17909	6,48120E-01	118286	0	43212

Myeloid_vs_CD4_Post_R

Myeloid_vs_CD4_Responder	Mono_CD14	CD4(TNFRSF9+ Treg)	IL1B	SIGIRR	7,68296E-03	18741,6	6,78544E-03	13078	1,86417E+00	6485	2,78276E+00	2826	8,83890E-01	28107	0	43212
Myeloid_vs_CD4_Responder	cDC_LAMP3	CD4(IFNG+ Tfh/Th1)	HLA-DQA2	LAG3	7,69046E-03	16189,6	1,51356E-02	3200	1,57917E+00	10598	2,36015E+00	6022	9,08196E-01	17916	0	43212
Myeloid_vs_CD4_Responder	Mono_CD14	CD4(TNFRSF9+ Treg)	HBEGF	CD44	7,70006E-03	27110,2	5,07616E-03	20366	1,12803E+00	24187	1,33325E+00	27207	9,01765E-01	20579	0	43212
Myeloid_vs_CD4_Responder	Mono_CD14	CD4(TNFRSF9+ Treg)	LYZ	ITGAL	7,71505E-03	19678,4	4,46609E-03	24545	1,82603E+00	6943	3,06061E+00	1634	8,98110E-01	22058	0	43212
Myeloid_vs_CD4_Responder	Mono_INHBA	CD4(ISG+ Treg)	INHBA	ACTR2	7,71719E-03	19191,8	1,27966E-02	4357	2,80500E+00	1346	1,96780E+00	11036	8,66156E-01	36008	0	43212
Myeloid_vs_CD4_Responder	CD4(CXCL13+ Tfh)	Mono_CD16	HLA-C	LILRA1	7,72381E-03	32473,8	9,43224E-03	7572	1,43813E+00	13762	2,95464E-01	79887	9,08150E-01	17936	0	43212
Myeloid_vs_CD4_Responder	CD4(IFNG+ Tfh/Th1)	Macro_ISG15	HLA-A	LILRB2	7,72470E-03	24570,8	3,42634E-03	36015	1,39880E+00	14833	1,43782E+00	23681	9,37457E-01	5113	0	43212
Myeloid_vs_CD4_Responder	Mono_CD14	CD4(CRTAM- T)	CD14	ITGB1	7,74832E-03	24554,2	3,67919E-03	32491	1,31276E+00	17408	2,01131E+00	10280	9,04670E-01	19380	0	43212
Myeloid_vs_CD4_Responder	Mono_INHBA	CD4(TGFB1+ Th17)	HBEGF	CD44	7,75047E-03	26122,6	5,93968E-03	16126	1,31108E+00	17464	1,09973E+00	36039	9,08507E-01	17772	0	43212
Myeloid_vs_CD4_Responder	Macro_FOLR2+APOE+	CD4(ISG+ Treg)	C1QB	C1QBP	7,76228E-03	15996,8	5,52700E-03	17959	2,19041E+00	3588	2,63782E+00	3721	9,23166E-01	11504	0	43212
Myeloid_vs_CD4_Responder	Mono_CD14	CD4(CRTAM- T)	IL1B	SIGIRR	7,76769E-03	19505,8	6,30053E-03	14727	1,84089E+00	6745	2,74075E+00	3064	8,80031E-01	29781	0	43212
Myeloid_vs_CD4_Responder	Mono_CD14	CD4(CRTAM- T)	HBEGF	CD44	7,78170E-03	22115,2	6,36955E-03	14473	1,33141E+00	16813	1,57796E+00	19548	9,11370E-01	16530	0	43212
Myeloid_vs_CD4_Responder	Mono_CD14	CD4(CRTAM- T)	LGALS1	CD69	7,78386E-03	26058,2	3,73890E-03	31700	9,38474E-01	33232	1,69336E+00	16537	9,36257E-01	5610	0	43212
Myeloid_vs_CD4_Responder	Macro_ISG15	CD4(CRTAM- T)	SPP1	S1PR1	7,78473E-03	18983	2,97197E-02	837	1,79895E+00	7273	2,79980E+00	2727	8,55427E-01	40866	0	43212
Myeloid_vs_CD4_Responder	cDC_CLEC9A	CD4(ISG+ Treg)	B2M	CD3D	7,79385E-03	42961,4	3,52934E-03	34496	2,00568E-01	87097	7,86142E-01	49961	9,61787E-01	41	0	43212
Myeloid_vs_CD4_Responder	CD4(CRTAM- T)	Macro_FOLR2-APOE+	ANXA1	FPR1	7,80222E-03	31637,4	3,72762E-03	31850	1,09115E+00	25803	1,09828E+00	36087	9,00189E-01	21235	0	43212
Myeloid_vs_CD4_Responder	Mono_CD14	CD4(CRTAM- T)	NAMPT	ITGA5_ITGB1	7,80547E-03	22870,2	5,00739E-03	20792	1,22614E+00	20380	2,12938E+00	8584	8,99836E-01	21383	0	43212
Myeloid_vs_CD4_Responder	CD4(CXCL13+ Tfh)	Macro_FOLR2+APOE+	ADAM10	GPNMB	7,82062E-03	21817,6	8,30522E-03	9396	2,64359E+00	1722	1,61094E+00	18654	8,65940E-01	36104	0	43212
Myeloid_vs_CD4_Responder	CD4(CRTAM- T)	cDC_CLEC9A	COPA	CD74	7,82447E-03	26144,4	2,40500E-03	57804	1,29499E+00	17996	3,02660E+00	1744	9,26713E-01	9966	0	43212
Myeloid_vs_CD4_Responder	Macro_OLFML3	CD4(NME1+ T)	CXCL9	CXCR3	7,83037E-03	21879	1,65071E-02	2690	1,52942E+00	11566	1,72457E+00	15814	8,65927E-01	36113	0	43212
Myeloid_vs_CD4_Responder	Mono_CD14	CD4(TNF+ T)	IL1B	SIGIRR	7,83037E-03	20446,6	5,67985E-03	17210	1,81109E+00	7133	2,85802E+00	2437	8,74447E-01	32241	0	43212
Myeloid_vs_CD4_Responder	cDC(CD1C)	CD4(NME1+ T)	HLA-DQA2	CD4	7,83037E-03	25588	3,42103E-03	36113	1,27257E+00	18729	2,20009E+00	7710	8,97801E-01	22176	0	43212
Myeloid_vs_CD4_Responder	Mono_CD14	CD4(TNF+ T)	VCAN	ITGB1	7,83579E-03	15826	9,49580E-03	7486	2,83467E+00	1278	2,50691E+00	4718	8,97223E-01	22436	0	43212
Myeloid_vs_CD4_Responder	Mono_CD14	CD4(TNF+ T)	HBEGF	CD44	7,84230E-03	33531,6	3,65850E-03	32788	9,05114E-01	34880	1,26684E+00	29632	8,86275E-01	27146	0	43212
Myeloid_vs_CD4_Responder	Mono_INHBA	CD4(IFNG+ Tfh/Th1)	LGALS1	PTPRC	7,84230E-03	24431,2	3,42010E-03	36124	1,15740E+00	22989	1,62202E+00	18341	9,49508E-01	1490	0	43212
Myeloid_vs_CD4_Responder	Mono_CD14	CD4(TNF+ T)	LGALS1	CD69	7,84447E-03	24517,6	3,72269E-03	31906	9,35253E-01	33375	2,14180E+00	8433	9,36128E-01	5662	0	43212
Myeloid_vs_CD4_Responder	Mono_INHBA	CD4(TNFRSF9+ Treg)	HBEGF	CD44	7,84990E-03	26648	5,72524E-03	17019	1,28118E+00	18437	1,09720E+00	36131	9,06967E-01	18441	0	43212
Myeloid_vs_CD4_Responder	Mono_CD14	CD4(TNF+ T)	LYZ	ITGAL	7,84990E-03	22362,8	3,61653E-03	33320	1,77301E+00	7612	3,17798E+00	1289	8,88043E-01	26381	0	43212
Myeloid_vs_CD4_Responder	CD4(NME1+ T)	Mono_CD14	HLA-F	LILRB2	7,85969E-03	31742,4	3,41870E-03	36140	1,21444E+00	20798	1,32395E+00	27529	8,77147E-01	31033	0	43212
Myeloid_vs_CD4_Responder	Macro_ISG15	CD4(IFNG+ Tfh/Th1)	SPP1	CD44	7,86669E-03	20688,4	5,77243E-03	16825	1,33068E+00	16839	2,13308E+00	8545	9,07950E-01	18021	0	43212
Myeloid_vs_CD4_Responder	CD4(NME1+ T)	cDC(CD1C)	CD28	CD86	7,86948E-03	24698,4	7,75088E-03	10558,5	1,47364E+00	12875,5	1,53455E+00	20696,5	8,65835E-01	36149,5	0	43212
Myeloid_vs_CD4_Responder	cDC(CD1C)	CD4(NME1+ T)	CD86	CD28	7,87057E-03	24698,4	7,75088E-03	10558,5	1,47364E+00	12875,5	1,53455E+00	20696,5	8,65835E-01	36149,5	0	43212
Myeloid_vs_CD4_Responder	CD4(IFNG+ Tfh/Th1)	Macro_OLFML3	CD52	SIGLEC10	7,87685E-03	25494	6,21796E-03	15058	1,48307E+00	12648	9,73515E-01	41230	9,14167E-01	15322	0	43212
Myeloid_vs_CD4_Responder	Mono_CD14	CD4(ISG+ Treg)	ICAM1	IL2RA	7,88582E-03	20535,8	1,70860E-02	2517	1,32054E+00	17153	2,11108E+00	8817	8,77248E-01	30980	0	43212
Myeloid_vs_CD4_Responder	Mono_CD14	CD4(ISG+ Treg)	ICAM1	IL2RG	7,88800E-03	20058,6	7,12459E-03	12139	1,29026E+00	18144	2,02254E+00	10121	9,11057E-01	16677	0	43212
Myeloid_vs_CD4_Responder	Macro_ISG15	CD4(NME1+ T)	SPP1	CD44	7,88872E-03	20739,6	5,76764E-03	16851	1,33013E+00	16866	2,11779E+00	8735	9,07916E-01	18034	0	43212
Myeloid_vs_CD4_Responder	CD4(TNFRSF9+ Treg)	Macro_LYVE1	ADAM10	GPNMB	7,89041E-03	22248,6	7,93361E-03	10127	2,39251E+00	2560	1,63323E+00	18035	8,63261E-01	37309	0	43212
Myeloid_vs_CD4_Responder	CD4(IFNG+ Tfh/Th1)	Macro_ISG15	HLA-F	LILRB2	7,89673E-03	31442,8	3,41589E-03	36174	1,23284E+00	20157	1,34881E+00	26625	8,77103E-01	31046	0	43212
Myeloid_vs_CD4_Responder	CD4(AREG+ Tm)	cDC_CLEC9A	MIF	CD74_CXCR4	7,91576E-03	47077,2	1,81142E-03	76053	2,97668E-01	77700	1,08194E+00	36746	9,48840E-01	1675	0	43212
Myeloid_vs_CD4_Responder	Mono_INHBA	CD4(TGFB1+ Th17)	VEGFA	ITGB1	7,91639E-03	24027	8,47446E-03	9089	1,95405E+00	5533	1,36488E+00	26109	8,65749E-01	36192	0	43212
Myeloid_vs_CD4_Responder	Mono_CD14	CD4(ISG+ Treg)	SECTM1	CD7	7,92077E-03	30084,8	1,16093E-02	5209	9,51545E-01	32535	1,13532E+00	34596	8,68609E-01	34872	0	43212
Myeloid_vs_CD4_Responder	Mono_CD14	CD4(ISG+ Treg)	IL1B	SIGIRR	7,92624E-03	16261,4	9,04709E-03	8169	1,97277E+00	5345	2,85987E+00	2430	8,97856E-01	22151	0	43212
Myeloid_vs_CD4_Responder	Mono_CD14	CD4(ISG+ Treg)	VCAN	ITGA4	7,93719E-03	16940,8	9,54935E-03	7409	2,83218E+00	1284	2,33479E+00	6261	8,87687E-01	26538	0	43212
Myeloid_vs_CD4_Responder	CD4(CRTAM- T)	Mono_INHBA	HLA-B	LILRB2	7,94268E-03	26724	3,41302E-03	36216	1,37264E+00	15587	1,13389E+00	34646	9,40532E-01	3959	0	43212
Myeloid_vs_CD4_Responder	cDC(CD1C)	CD4(CXCL13+ Tfh)	LYZ	ITGAL	7,96243E-03	24465,8	3,41185E-03	36234	1,48284E+00	12658	2,82430E+00	2593	8,85114E-01	27632	0	43212
Myeloid_vs_CD4_Responder	Mono_CD14	CD4(ISG+ Treg)	LYZ	ITGAL	7,96463E-03	17595,4	5,43163E-03	18440	1,88628E+00	6222	3,08435E+00	1547	9,06723E-01	18556	0	43212
Myeloid_vs_CD4_Responder	Mono_INHBA	CD4(ISG+ Treg)	VCAN	ITGB1	7,96573E-03	27781,2	5,17310E-03	19811	1,38878E+00	15130	1,41166E+00	24516	8,65652E-01	36237	0	43212
Myeloid_vs_CD4_Responder	CD4(IL26+ Th17)	Macro_NLRP3	CD99	PILRA	7,96683E-03	26556	4,93502E-03	21222	1,41670E+00	14327	1,09474E+00	36238	9,08490E-01	17781	0	43212
Myeloid_vs_CD4_Responder	CD4(TNFRSF9+ Treg)	Mono_INHBA	B2M	LILRB2	7,99214E-03	24480,4	3,41017E-03	36261	1,50262E+00	12185	1,29536E+00	28581	9,46778E-01	2163	0	43212
Myeloid_vs_CD4_Responder	CD4(TNFRSF9+ Treg)	cDC(CD1C)	CD52	SIGLEC10	7,99545E-03	34454,2	3,61063E-03	33394	8,79332E-01	36264	1,14967E+00	34031	8,90302E-01	25370	0	43212
Myeloid_vs_CD4_Responder	cDC(CD1C)	CD4(CXCL13+ Tfh)	HLA-DQA1	CD4	7,99545E-03	20052,2	3,41002E-03	36264	1,61589E+00	9949	3,00443E+00	1819	9,28915E-01	9017	0	43212
Myeloid_vs_CD4_Responder	CD4(ISG+ Treg)	Mono_CD16	CD99	PILRA	7,99951E-03	24106	5,49677E-03	18099	1,83811E+00	6785	1,08615E+00	36567	9,12874E-01	15867	0	43212
Myeloid_vs_CD4_Responder	CD4(TNFRSF9+ Treg)	Macro_OLFML3	ENTPD1	ADORA3	8,00185E-03	27663	9,95795E-03	6901	1,84774E+00	6665	1,19278E+00	32407	8,37742E-01	49130	0	43212
Myeloid_vs_CD4_Responder	CD4(IFNG+ Tfh/Th1)	Macro_ISG15	HLA-C	LILRB1	8,00427E-03	28364,2	3,68533E-03	32388	1,23773E+00	19965	1,09400E+00	36272	9,26671E-01	9984	0	43212
Myeloid_vs_CD4_Responder	CD4(ISG+ Treg)	Macro_LYVE1	ADAM10	GPNMB	8,01090E-03	22048	8,25261E-03	9501	2,40422E+00	2508	1,60823E+00	18741	8,65571E-01	36278	0	43212
Myeloid_vs_CD4_Responder	cDC(CD1C)	CD4(TGFB1+ Th17)	LYZ	ITGAL	8,02968E-03	24518	3,40731E-03	36295	1,48250E+00	12664	2,79490E+00	2747	8,85046E-01	27672	0	43212
Myeloid_vs_CD4_Responder	Mono_CD14	CD4(CXCL13+ Tfh)	IL1B	SIGIRR	8,03522E-03	19504,6	6,30644E-03	14702	1,84117E+00	6741	2,73275E+00	3109	8,80080E-01	29759	0	43212
Myeloid_vs_CD4_Responder	CD4(GZMK+ Teff)	Macro_NLRP3	HLA-A	LILRB2	8,04518E-03	27463,8	3,42809E-03	35974	1,33492E+00	16718	1,09307E+00	36309	9,37472E-01	5106	0	43212
Myeloid_vs_CD4_Responder	CD4(NME1+ T)	Mono_CD16	RPS19	C5AR1	8,05072E-03	22376,2	5,37923E-03	18742	1,63046E+00	9703	1,09290E+00	36314	9,40655E-01	3910	0	43212
Myeloid_vs_CD4_Responder	Mono_CD14	CD4(CXCL13+ Tfh)	HBEGF	CD44	8,05072E-03	30090,2	4,57027E-03	23728	1,04848E+00	27771	1,17174E+00	33201	8,97016E-01	22539	0	43212
Myeloid_vs_CD4_Responder	Mono_CD16	CD4(IL26+ Th17)	CXCL16	CXCR6	8,05964E-03	19221,2	1,48812E-02	3312	1,44505E+00	13573	1,62961E+00	18134	9,08324E-01	17875	0	43212
Myeloid_vs_CD4_Responder	Mono_CD14	CD4(CXCL13+ Tfh)	LYZ	ITGAL	8,06070E-03	20761,6	4,09521E-03	27766	1,80288E+00	7221	3,00508E+00	1815	8,94075E-01	23794	0	43212
Myeloid_vs_CD4_Responder	cDC(CD1C)	CD4(IL26+ Th17)	LGALS9	PTPRC	8,06958E-03	30951	3,40478E-03	36331	9,18180E-01	34231	1,29926E+00	28445	9,20714E-01	12536	0	43212
Myeloid_vs_CD4_Responder	Macro_OLFML3	CD4(CXCL13+ Tfh)	APOE	LSR	8,07403E-03	19646	8,75814E-03	8621	1,84397E+00	6714	2,69547E+00	3348	8,65435E-01	36335	0	43212
Myeloid_vs_CD4_Responder	Macro_OLFML3	CD4(IL26+ Th17)	GRN	TNFRSF1B	8,07625E-03	25390,6	3,40441E-03	36337	1,44498E+00	13578	1,45114E+00	23266	9,25383E-01	10560	0	43212
Myeloid_vs_CD4_Responder	CD4(Tn)	Mast	CD40LG	CD9	8,07827E-03	23997	1,32869E-02	4095	1,79159E+00	7366	1,55959E+00	20035	8,45944E-01	45277	0	43212
Myeloid_vs_CD4_Responder	Mono_CD14	CD4(GZMK+ Teff)	IL1B	SIGIRR	8,10630E-03	18283,6	7,09309E-03	12228	1,87895E+00	6296	2,84551E+00	2489	8,86146E-01	27193	0	43212
Myeloid_vs_CD4_Responder	Mono_CD14	CD4(TNFRSF9+ Treg)	SECTM1	CD7	8,10964E-03	30787,2	1,09724E-02	5809	9,01964E-01	35060	1,16409E+00	33488	8,65356E-01	36367	0	43212
Myeloid_vs_CD4_Responder	Mono_CD14	CD4(GZMK+ Teff)	VCAN	ITGB1	8,11299E-03	17466,6	7,92818E-03	10150	2,77948E+00	1394	2,31548E+00	6453	8,88601E-01	26124	0	43212

Myeloid_vs_CD4_Post_R

Myeloid_vs_CD4_Responder	cDC_LAMP3	CD4(TGFB1+ Th17)	CCL22	DPP4	8,11666E-03	24301,6	1,03331E-01	49	2,57640E+00	1912	2,22994E+00	7378	7,94877E-01	68957	0	43212
Myeloid_vs_CD4_Responder	Mono_CD14	CD4(GZMK+ Teff)	HBEGF	CD44	8,12080E-03	22994,8	6,04511E-03	15694	1,28039E+00	18456	1,55416E+00	20175	9,09236E-01	17437	0	43212
Myeloid_vs_CD4_Responder	CD4(IL26+ Th17)	Mono_INHBA	HSP90B1	LRP1	8,12415E-03	32281	3,41607E-03	36170	1,30522E+00	17640	1,09128E+00	36380	8,84118E-01	28003	0	43212
Myeloid_vs_CD4_Responder	CD4(IFNG+ Tfh/Th1)	Macro_LYVE1	CD99	CD81	8,13085E-03	26506,8	5,43084E-03	18443	1,21537E+00	20767	1,09109E+00	36386	9,17851E-01	13726	0	43212
Myeloid_vs_CD4_Responder	CD4(TNFRSF9+ Treg)	cDC_LAMP3	CD28	CD86	8,14091E-03	29200,8	8,08303E-03	9831,5	1,19532E+00	21527,5	1,09078E+00	36395,5	8,68253E-01	35037,5	0	43212
Myeloid_vs_CD4_Responder	CD4(ISG+ Treg)	pDC_LILRA4	MAML2	NOTCH4	8,15132E-03	38789	1,60321E-02	2845	1,52457E+00	11685	1,62731E+00	18187	6,49521E-01	118016	0	43212
Myeloid_vs_CD4_Responder	Mono_CD14	CD4(IFNG+ Tfh/Th1)	HLA-DRA	LAG3	8,15322E-03	21846,2	5,71064E-03	17089	1,00543E+00	29848	2,27980E+00	6827	9,21378E-01	12255	0	43212
Myeloid_vs_CD4_Responder	Macro_ISG15	CD4(ISG+ Treg)	HLA-DRB5	LAG3	8,15653E-03	18066	8,03562E-03	9926	1,28873E+00	18190	2,30586E+00	6560	9,20917E-01	12442	0	43212
Myeloid_vs_CD4_Responder	CD4(NME1+ T)	Mast	ACTR2	ADRB2	8,15994E-03	26183,4	1,21480E-02	4784	1,48451E+00	12621	1,15392E+00	33888	8,65256E-01	36412	0	43212
Myeloid_vs_CD4_Responder	CD4(CRTAM- T)	Macro_OLFML3	ANXA2	TLR2	8,16218E-03	29886,8	4,10184E-03	27708	1,57002E+00	10767	1,09033E+00	36414	8,76475E-01	31333	0	43212
Myeloid_vs_CD4_Responder	CD4(AREG+ Tm)	Macro_FOLR2-APOE+	MIF	CD44_CD74	8,16963E-03	47730,2	1,85470E-03	74560	4,39644E-01	65515	7,15539E-01	53662	9,48721E-01	1702	0	43212
Myeloid_vs_CD4_Responder	Mono_CD14	CD4(IFNG+ Tfh/Th1)	ICAM1	IL2RG	8,17228E-03	29351,8	4,45847E-03	24602	9,03167E-01	34992	1,61662E+00	18512	8,90146E-01	25441	0	43212
Myeloid_vs_CD4_Responder	Macro_FOLR2-APOE+	CD4(ISG+ Treg)	B2M	CD3D	8,17378E-03	52061,2	3,52268E-03	34607	1,93180E-01	87852	8,76239E-02	94592	9,61752E-01	43	0	43212
Myeloid_vs_CD4_Responder	Mono_CD14	CD4(ISG+ Treg)	HBEGF	CD44	8,17676E-03	32647,4	4,10954E-03	27622	9,76037E-01	31303	1,09005E+00	36427	8,92003E-01	24673	0	43212
Myeloid_vs_CD4_Responder	Mono_CD14	CD4(IFNG+ Tfh/Th1)	CD14	ITGB1	8,17901E-03	24035,4	3,75669E-03	31464	1,32334E+00	17067	2,06859E+00	9406	9,05565E-01	19028	0	43212
Myeloid_vs_CD4_Responder	Mono_CD14	CD4(IFNG+ Tfh/Th1)	CD14	ITGA4	8,18013E-03	25147,8	3,80098E-03	30934	1,29478E+00	18007	1,96740E+00	11041	8,96996E-01	22545	0	43212
Myeloid_vs_CD4_Responder	Macro_FOLR2+APOE+	CD4(CRTAM- T)	SPP1	CD44	8,18437E-03	21681,4	6,51744E-03	13950	1,28828E+00	18206	1,66758E+00	17179	9,12899E-01	15860	0	43212
Myeloid_vs_CD4_Responder	cDC(CD1C)	CD4(TNFRSF9+ Treg)	HLA-DRB1	LAG3	8,20036E-03	24360	3,39856E-03	36448	1,29964E+00	17811	2,87370E+00	2370	8,98324E-01	21959	0	43212
Myeloid_vs_CD4_Responder	CD4(ISG+ Treg)	Mono_CD16	HLA-A	LILRB1	8,20261E-03	25663,8	4,08922E-03	27820	1,47008E+00	12945	1,08938E+00	36450	9,31328E-01	7892	0	43212
Myeloid_vs_CD4_Responder	Mono_CD14	CD4(IFNG+ Tfh/Th1)	IL1B	SIGIRR	8,20824E-03	19129	6,53161E-03	13899	1,85199E+00	6614	2,75813E+00	2972	8,81919E-01	28948	0	43212
Myeloid_vs_CD4_Responder	cDC_LAMP3	CD4(ISG+ Treg)	HLA-DRB1	LAG3	8,21403E-03	17925,2	7,80956E-03	10410	1,28774E+00	18223	2,06162E+00	9518	9,30522E-01	8263	0	43212
Myeloid_vs_CD4_Responder	cDC(CD1C)	CD4(ISG+ Treg)	IL1B	SIGIRR	8,22176E-03	26843,4	4,81707E-03	21991	1,07849E+00	26358	2,34144E+00	6189	8,65121E-01	36467	0	43212
Myeloid_vs_CD4_Responder	Mono_CD14	CD4(IFNG+ Tfh/Th1)	HBEGF	CD44	8,22176E-03	31733,6	4,21276E-03	26631	9,92267E-01	30480	1,14546E+00	34173	8,93192E-01	24172	0	43212
Myeloid_vs_CD4_Responder	Mono_INHBA	CD4(TNFRSF9+ Treg)	INHBA	ACTR2	8,23078E-03	19065	1,25656E-02	4502	2,79741E+00	1360	2,04614E+00	9776	8,65097E-01	36475	0	43212
Myeloid_vs_CD4_Responder	Mono_CD14	CD4(IFNG+ Tfh/Th1)	LYZ	ITGAL	8,23529E-03	18636	4,89891E-03	21468	1,85303E+00	6597	3,08731E+00	1536	9,02265E-01	20367	0	43212
Myeloid_vs_CD4_Responder	CD4(TNFRSF9+ Treg)	Mono_INHBA	CD28	CD86	8,24094E-03	30090,4	7,65219E-03	10791,5	1,13407E+00	23930,5	1,09990E+00	36033,5	8,65089E-01	36484,5	0	43212
Myeloid_vs_CD4_Responder	cDC(CD1C)	CD4(IFNG+ Tfh/Th1)	HLA-DRA	CD4	8,24094E-03	18523,2	3,39597E-03	36484	1,66536E+00	9150	3,36000E+00	891	9,44071E-01	2879	0	43212
Myeloid_vs_CD4_Responder	Mono_INHBA	CD4(TNFRSF9+ Treg)	CD86	CD28	8,24207E-03	30090,4	7,65219E-03	10791,5	1,13407E+00	23930,5	1,09990E+00	36033,5	8,65089E-01	36484,5	0	43212
Myeloid_vs_CD4_Responder	Mono_CD14	CD4(IFNG+ Tfh/Th1)	LGALS3	LAG3	8,25789E-03	24632,2	7,87171E-03	10275	8,75133E-01	36499	1,99435E+00	10582	8,96876E-01	22593	0	43212
Myeloid_vs_CD4_Responder	Macro_IER3	CD4(ISG+ Treg)	CCL3	CCR4	8,25843E-03	16843	1,53724E-02	3115	2,39744E+00	2535	2,22592E+00	7422	8,84340E-01	27931	0	43212
Myeloid_vs_CD4_Responder	CD4(ISG+ Treg)	Macro_ISG15	HLA-B	LILRB2	8,26016E-03	23365,4	3,39508E-03	36501	1,50792E+00	12074	1,52274E+00	21044	9,40384E-01	3996	0	43212
Myeloid_vs_CD4_Responder	CD4(TGFB1+ Th17)	Macro_FOLR2+APOE+	TNFSF13B	HLA-DPB1	8,27006E-03	36555	1,32903E-03	93613	1,35026E+00	16277	1,94758E+00	11418	9,07383E-01	18255	0	43212
Myeloid_vs_CD4_Responder	cDC(CD1C)	CD4(ISG+ Treg)	LGALS1	CD69	8,27601E-03	26761,6	3,39406E-03	36515	9,08310E-01	34726	1,89225E+00	12374	9,33308E-01	6981	0	43212
Myeloid_vs_CD4_Responder	Mono_CD14	CD4(NME1+ T)	CD14	ITGB1	8,27941E-03	24123	3,78673E-03	31100	1,32744E+00	16948	2,00211E+00	10453	9,05905E-01	18902	0	43212
Myeloid_vs_CD4_Responder	Mono_CD14	CD4(NME1+ T)	CD14	ITGA4	8,28054E-03	26898,6	3,45546E-03	35565	1,25544E+00	19321	1,91748E+00	11930	8,92510E-01	24465	0	43212
Myeloid_vs_CD4_Responder	cDC(CD1C)	CD4(IL26+ Th17)	HLA-DRA	CD4	8,28394E-03	18555,2	3,39350E-03	36522	1,66498E+00	9157	3,30161E+00	1000	9,44052E-01	2885	0	43212
Myeloid_vs_CD4_Responder	Mono_INHBA	CD4(AREG+ Tm)	VCAN	ITGB1	8,29983E-03	27806,6	5,11441E-03	20155	1,38465E+00	15250	1,43232E+00	23880	8,64987E-01	36536	0	43212
Myeloid_vs_CD4_Responder	CD4(TGFB1+ Th17)	Macro_FOLR2+APOE+	TIMP1	CD63	8,30170E-03	34712,2	1,54517E-03	85546	1,65040E+00	9368	1,66831E+00	17162	9,07335E-01	18273	0	43212
Myeloid_vs_CD4_Responder	Mono_CD14	CD4(NME1+ T)	IL1B	SIGIRR	8,30665E-03	20604,4	5,70941E-03	17095	1,81251E+00	7114	2,67013E+00	3502	8,74732E-01	32099	0	43212
Myeloid_vs_CD4_Responder	cDC(CD1C)	CD4(TNFRSF9+ Treg)	HLA-DQA1	CD4	8,30665E-03	20144,2	3,39157E-03	36542	1,61354E+00	9992	2,98435E+00	1893	9,28736E-01	9082	0	43212
Myeloid_vs_CD4_Responder	CD4(TNFRSF9+ Treg)	cDC(CD1C)	B2M	CD1B	8,30712E-03	29284,8	2,02835E-02	1805	1,78596E+00	7437	5,79185E-01	61472	8,73878E-01	32498	0	43212
Myeloid_vs_CD4_Responder	Mono_CD14	CD4(NME1+ T)	HBEGF	CD44	8,32485E-03	31875,6	4,20927E-03	26669	9,91718E-01	30513	1,13016E+00	34793	8,93153E-01	24191	0	43212
Myeloid_vs_CD4_Responder	Macro_OLFML3	CD4(AREG+ Tm)	FN1	ITGA4_ITGB7	8,33283E-03	21208,6	1,16341E-02	5180	1,85345E+00	6590	1,78554E+00	14496	8,64894E-01	36565	0	43212
Myeloid_vs_CD4_Responder	Mono_CD14	CD4(NME1+ T)	LYZ	ITGAL	8,33966E-03	18115,6	5,14667E-03	19978	1,86849E+00	6427	3,10379E+00	1485	9,04419E-01	19476	0	43212
Myeloid_vs_CD4_Responder	CD4(AREG+ Tm)	Mono_INHBA	MIF	CD44_CD74	8,34105E-03	44826,2	1,85070E-03	74708	4,98633E-01	60984	9,21689E-01	43507	9,48668E-01	1720	0	43212
Myeloid_vs_CD4_Responder	CD4(TNFRSF9+ Treg)	Mono_INHBA	RPS19	C5AR1	8,34879E-03	23756,8	4,97804E-03	20965	1,45416E+00	13334	1,08586E+00	36579	9,38454E-01	4694	0	43212
Myeloid_vs_CD4_Responder	cDC(CD1C)	CD4(IFNG+ Tfh/Th1)	HLA-DRB5	CD4	8,35792E-03	20227,6	3,38847E-03	36587	1,47611E+00	12819	3,00703E+00	1811	9,33881E-01	6709	0	43212
Myeloid_vs_CD4_Responder	Macro_FOLR2+APOE-	CD4(IFNG+ Tfh/Th1)	B2M	CD3D	8,36375E-03	51123,2	3,51397E-03	34731	2,02583E-01	86878	1,41524E-01	90751	9,61707E-01	44	0	43212
Myeloid_vs_CD4_Responder	CD4(NME1+ T)	Mono_INHBA	HLA-B	LILRB2	8,37050E-03	27085,6	3,38773E-03	36598	1,35157E+00	16239	1,11682E+00	35359	9,40323E-01	4020	0	43212
Myeloid_vs_CD4_Responder	Mono_INHBA	CD4(CXCL13+ Tfh)	ICAM1	IL2RG	8,38079E-03	34324	3,80284E-03	30913	9,61708E-01	32035	1,08516E+00	36607	8,82125E-01	28853	0	43212
Myeloid_vs_CD4_Responder	CD4(TGFB1+ Th17)	cDC(CD1C)	LTB	TNFRSF1A	8,38308E-03	29929	6,93774E-03	12671	8,73245E-01	36609	1,20321E+00	32010	8,90856E-01	25143	0	43212
Myeloid_vs_CD4_Responder	Macro_ISG15	CD4(AREG+ Tm)	SPP1	PTGER4	8,39362E-03	15307,4	1,50846E-02	3221	1,71629E+00	8385	2,68923E+00	3394	9,07215E-01	18325	0	43212
Myeloid_vs_CD4_Responder	Macro_NLRP3	CD4(ISG+ Treg)	ICAM1	IL2RG	8,39539E-03	21706,8	7,12066E-03	12149	1,28980E+00	18160	1,62252E+00	18326	9,11034E-01	16687	0	43212
Myeloid_vs_CD4_Responder	cDC(CD1C)	CD4(IL26+ Th17)	HLA-DRB5	CD4	8,39683E-03	20282,6	3,38600E-03	36621	1,47573E+00	12827	2,94865E+00	2031	9,33858E-01	6722	0	43212
Myeloid_vs_CD4_Responder	CD4(GZMK+ Teff)	Macro_LYVE1	CD99	PILRA	8,40249E-03	25330,6	5,45023E-03	18330	1,57255E+00	10716	1,04121E+00	38382	9,12535E-01	16013	0	43212
Myeloid_vs_CD4_Responder	CD4(CRTAM- T)	Macro_NLRP3	HMGB1	CD163	8,40371E-03	27344,4	3,38573E-03	36627	1,41577E+00	14355	1,29058E+00	28768	9,17750E-01	13760	0	43212
Myeloid_vs_CD4_Responder	CD4(IFNG+ Tfh/Th1)	Macro_NLRP3	HLA-B	LILRB2	8,43358E-03	26312,2	3,38449E-03	36653	1,41093E+00	14505	1,17281E+00	33159	9,40296E-01	4032	0	43212
Myeloid_vs_CD4_Responder	Macro_FOLR2+APOE+	CD4(TGFB1+ Th17)	LGALS1	CD69	8,45055E-03	19865	5,48582E-03	18152	1,28393E+00	18357	1,65672E+00	17443	9,46785E-01	2161	0	43212
Myeloid_vs_CD4_Responder	CD4(CRTAM- T)	Macro_IER3	ANXA1	FPR1	8,46123E-03	30364	4,02764E-03	28438	1,14046E+00	23691	1,08341E+00	36677	9,03613E-01	19802	0	43212
Myeloid_vs_CD4_Responder	Mono_CD14	CD4(IFNG+ Tfh/Th1)	HLA-DRB1	LAG3	8,47970E-03	24008,2	5,37546E-03	18762	8,71820E-01	36693	2,22098E+00	7475	9,17434E-01	13899	0	43212
Myeloid_vs_CD4_Responder	cDC(CD1C)	CD4(ISG+ Treg)	ICAM1	IL2RA	8,48201E-03	23367,8	1,36389E-02	3898	1,15032E+00	23286	2,04717E+00	9748	8,64591E-01	36695	0	43212
Myeloid_vs_CD4_Responder	Macro_OLFML3	CD4(CXCL13+ Tfh)	HLA-DRB5	LAG3	8,49820E-03	25419,4	3,37974E-03	36709	1,53785E+00	11387	2,23146E+00	7366	8,83070E-01	28423	0	43212
Myeloid_vs_CD4_Responder	cDC(CD1C)	CD4(CRTAM- T)	VEGFA	CD44	8,50399E-03	35091,4	4,08567E-03	27857	9,28892E-01	33699	1,08265E+00	36714	8,70586E-01	33975	0	43212
Myeloid_vs_CD4_Responder	CD4(AREG+ Tm)	cDC_CLEC9A	NELL2	ROBO3	8,50453E-03	53839,4	2,07313E-02	1737	8,09152E-01	40150	5,66840E-01	62269	6,27410E-01	121829	0	43212
Myeloid_vs_CD4_Responder	CD4(TGFB1+ Th17)	Mast	IL16	CD9	8,51672E-03	22770,6	8,59624E-03	8882	1,85759E+00	6552	1,62052E+00	18394	8,64332E-01	36813	0	43212
Myeloid_vs_CD4_Responder	Macro_LYVE1	CD4(AREG+ Tm)	LGALS1	CD69	8,52211E-03	18569,6	5,43956E-03	18397	1,35480E+00	16126	1,86464E+00	12896	9,46571E-01	2217	0	43212
Myeloid_vs_CD4_Responder	CD4(Tn)	Macro_LYVE1	HSP90B1	LRP1	8,53415E-03	26712	4,40480E-03	25035	1,92238E+00	5830	1,08202E+00	36740	8,96518E-01	22743	0	43212
Myeloid_vs_CD4_Responder	CD4(TNF+ T)	Macro_NLRP3	ANXA1	FPR1	8,54344E-03	28684,8	3,92281E-03	29582	1,44443E+00	13600	1,08185E+00	36748	9,02459E-01	20282	0	43212
Myeloid_vs_CD4_Responder	CD4(CRTAM- T)	Macro_LYVE1	CD99	PILRA	8,54905E-03	25732,4	5,43707E-03	18412	1,57066E+00	10754	9,97197E-01	40219	9,12438E-01	16065	0	43212

Myeloid_vs_CD4_Responder	CD4(GZMK+ Teff)	Macro_ISG15	HLA-B	LILRB1	8,54926E-03	28161,2	3,63400E-03	33107	1,24337E+00	19755	1,08169E+00	36753	9,31112E-01	7979	0	43212
Myeloid_vs_CD4_Responder	cDC_CLEC9A	CD4(IFNG+ Tfh/Th1)	B2M	CD3D	8,55370E-03	42177,2	3,50650E-03	34849	1,94231E-01	87753	8,86744E-01	45027	9,61667E-01	45	0	43212
Myeloid_vs_CD4_Responder	CD4(GZMK+ Teff)	Macro_FOLR2-APOE+	TNFSF9	HLA-DPA1	8,55973E-03	29044	3,81849E-03	30715	1,20358E+00	21223	1,84379E+00	13308	8,64443E-01	36762	0	43212
Myeloid_vs_CD4_Responder	CD4(ISG+ Treg)	Mono_CD14	HLA-F	LILRB1	8,57021E-03	33314,2	3,97439E-03	29019	1,06195E+00	27126	1,24344E+00	30443	8,64422E-01	36771	0	43212
Myeloid_vs_CD4_Responder	Mono_INHBA	CD4(CRTAM- T)	LGALS1	PTPRC	8,57954E-03	24988,6	3,37573E-03	36779	1,14334E+00	23574	1,56806E+00	19800	9,49194E-01	1578	0	43212
Myeloid_vs_CD4_Responder	Macro_LYVE1	CD4(ISG+ Treg)	LGALS3	LAG3	8,57967E-03	18979,2	1,12729E-02	5513	1,28181E+00	18429	1,93345E+00	11634	9,12340E-01	16108	0	43212
Myeloid_vs_CD4_Responder	Mono_CD14	CD4(Tn)	AGTRAP	RACK1	8,58771E-03	28898,4	5,17832E-03	19781	8,70093E-01	36786	1,19788E+00	32220	9,20788E-01	12493	0	43212
Myeloid_vs_CD4_Responder	CD4(ISG+ Treg)	Macro_LYVE1	HLA-A	APLP2	8,59004E-03	27347,8	3,54772E-03	34244	1,20826E+00	21032	1,08084E+00	36788	9,49603E-01	1463	0	43212
Myeloid_vs_CD4_Responder	CD4(NME1+ T)	Macro_NLRP3	HLA-F	LILRB2	8,59355E-03	31792,8	3,69122E-03	32321	1,31331E+00	17394	1,08078E+00	36791	8,81220E-01	29246	0	43212
Myeloid_vs_CD4_Responder	Mono_CD16	CD4(AREG+ Tm)	S100A8	CD69	8,60133E-03	20277,4	6,92132E-03	12708	1,28097E+00	18441	1,63978E+00	17867	9,28551E-01	9159	0	43212
Myeloid_vs_CD4_Responder	CD4(TNFRSF9+ Treg)	Macro_ISG15	HLA-B	LILRB1	8,60172E-03	28051,4	3,64696E-03	32933	1,25358E+00	19381	1,08064E+00	36798	9,31226E-01	7933	0	43212
Myeloid_vs_CD4_Responder	Mono_CD14	CD4(IFNG+ Tfh/Th1)	THBS1	ITGA4	8,61342E-03	29278,6	5,76875E-03	16844	9,60442E-01	32096	1,65709E+00	17433	8,64341E-01	36808	0	43212
Myeloid_vs_CD4_Responder	CD4(GZMK+ Teff)	cDC(CD1C)	CD28	CD86	8,62747E-03	24541	7,55205E-03	11033,5	1,46439E+00	13073,5	1,61464E+00	18565,5	8,64318E-01	36820,5	0	43212
Myeloid_vs_CD4_Responder	cDC(CD1C)	CD4(GZMK+ Teff)	CD86	CD28	8,62864E-03	24541	7,55205E-03	11033,5	1,46439E+00	13073,5	1,61464E+00	18565,5	8,64318E-01	36820,5	0	43212
Myeloid_vs_CD4_Responder	CD4(IFNG+ Tfh/Th1)	Mono_INHBA	CD99	PILRA	8,63098E-03	32260,8	3,70864E-03	32090	1,08287E+00	26165	1,08002E+00	36823	8,95901E-01	23014	0	43212
Myeloid_vs_CD4_Responder	CD4(IFNG+ Tfh/Th1)	Macro_LYVE1	CD99	PILRA	8,63684E-03	24621	5,63941E-03	17402	1,59983E+00	10229	1,07989E+00	36828	9,13887E-01	15434	0	43212
Myeloid_vs_CD4_Responder	Macro_ISG15	CD4(ISG+ Treg)	SPP1	CD44	8,64840E-03	21152	5,63100E-03	17440	1,31445E+00	17362	2,07767E+00	9279	9,06908E-01	18467	0	43212
Myeloid_vs_CD4_Responder	CD4(IFNG+ Tfh/Th1)	Mono_INHBA	CALR	LRP1	8,66032E-03	31436,2	3,53764E-03	34388	1,26169E+00	19101	1,07938E+00	36848	8,94426E-01	23632	0	43212
Myeloid_vs_CD4_Responder	Macro_OLFML3	CD4(CRTAM- T)	FN1	ITGA4_ITGB7	8,66620E-03	21380,6	1,15090E-02	5291	1,84666E+00	6681	1,76661E+00	14866	8,64261E-01	36853	0	43212
Myeloid_vs_CD4_Responder	CD4(IFNG+ Tfh/Th1)	Macro_ISG15	HLA-F	LILRB1	8,68385E-03	33150,4	3,96135E-03	29153	1,20712E+00	21081	1,11438E+00	35438	8,64230E-01	36868	0	43212
Myeloid_vs_CD4_Responder	Macro_NLRP3	CD4(IFNG+ Tfh/Th1)	S100A8	ITGB2	8,68656E-03	15968,2	5,42180E-03	18488	1,98191E+00	5260	2,56305E+00	4277	9,29793E-01	8604	0	43212
Myeloid_vs_CD4_Responder	Macro_FOLR2+APOE+	CD4(IFNG+ Tfh/Th1)	CD14	ITGA4	8,68838E-03	20258,4	5,42174E-03	18489	1,86089E+00	6518	1,67581E+00	16952	9,12286E-01	16121	0	43212
Myeloid_vs_CD4_Responder	CD4(IFNG+ Tfh/Th1)	Macro_LYVE1	RPS19	C5AR1	8,72988E-03	21387,6	5,99464E-03	15890	1,74987E+00	7918	1,07819E+00	36907	9,43607E-01	3011	0	43212
Myeloid_vs_CD4_Responder	Mono_CD14	CD4(ISG+ Treg)	HBEGF	CD82	8,74882E-03	24588,4	9,41199E-03	7597	1,32605E+00	16993	1,62630E+00	18217	8,64099E-01	36923	0	43212
Myeloid_vs_CD4_Responder	CD4(IFNG+ Tfh/Th1)	Macro_ISG15	CCL4	CCR1	8,75408E-03	23886,8	7,69175E-03	10694	1,45555E+00	13305	1,99167E+00	10634	8,53928E-01	41589	0	43212
Myeloid_vs_CD4_Responder	CD4(NME1+ T)	Macro_NLRP3	HMGB1	TLR4	8,76423E-03	30144,6	4,81306E-03	22014	1,24798E+00	19580	1,07742E+00	36936	8,81852E-01	28981	0	43212
Myeloid_vs_CD4_Responder	CD4(IL26+ Th17)	Mast	ACTR2	ADRB2	8,76898E-03	26481,2	1,19029E-02	4957	1,47472E+00	12845	1,13851E+00	34452	8,64063E-01	36940	0	43212
Myeloid_vs_CD4_Responder	CD4(TNF+ T)	Mono_CD14	CIRBP	TREM1	8,77016E-03	29835,6	5,59532E-03	17605	1,29502E+00	17994	1,07735E+00	36941	8,71765E-01	33426	0	43212
Myeloid_vs_CD4_Responder	Macro_NLRP3	CD4(IFNG+ Tfh/Th1)	S100A9	ITGB2	8,77054E-03	16577,8	4,57810E-03	23668	2,08941E+00	4334	2,58522E+00	4098	9,31947E-01	7577	0	43212
Myeloid_vs_CD4_Responder	Macro_OLFML3	CD4(CRTAM- T)	LGALS3BP	ITGB1	8,78679E-03	25479,2	8,19325E-03	9615	1,42997E+00	13975	1,07713E+00	36955	8,94409E-01	23639	0	43212
Myeloid_vs_CD4_Responder	CD4(TGFB1+ Th17)	Macro_LYVE1	TNFSF12	CD163	8,79074E-03	27195,8	5,41326E-03	18545	2,53621E+00	2035	1,78532E+00	14502	8,19537E-01	57685	0	43212
Myeloid_vs_CD4_Responder	Macro_LYVE1	CD4(IFNG+ Tfh/Th1)	HLA-DQA2	LAG3	8,79625E-03	17181,2	1,46212E-02	3429	1,53867E+00	11371	2,07269E+00	9346	9,06745E-01	18548	0	43212
Myeloid_vs_CD4_Responder	CD4(TNF+ T)	Macro_ISG15	ANXA1	FPR1	8,80583E-03	30935,2	3,54243E-03	34323	1,29414E+00	18025	1,07681E+00	36971	8,97876E-01	22145	0	43212
Myeloid_vs_CD4_Responder	CD4(TNFRSF9+ Treg)	cDC(CD1C)	CSF1	CSF2RA	8,81647E-03	33421	6,42539E-03	14272	1,68013E+00	8896	1,61486E+00	18559	7,64069E-01	82166	0	43212
Myeloid_vs_CD4_Responder	CD4(AREG+ Tm)	Macro_OLFML3	MIF	CD44_CD74	8,83612E-03	51269,2	1,83440E-03	75290	3,53703E-01	72626	5,47308E-01	63447	9,48452E-01	1771	0	43212
Myeloid_vs_CD4_Responder	CD4(TGFB1+ Th17)	Macro_NLRP3	CD99	PILRA	8,83684E-03	27393,6	4,70883E-03	22735	1,37982E+00	15384	1,07617E+00	36997	9,06521E-01	18640	0	43212
Myeloid_vs_CD4_Responder	CD4(AREG+ Tm)	Mono_INHBA	HLA-B	LILRB2	8,84042E-03	27283,6	3,36208E-03	37000	1,33021E+00	16862	1,11907E+00	35251	9,40110E-01	4093	0	43212
Myeloid_vs_CD4_Responder	Mono_CD14	CD4(NME1+ T)	ICAM1	IL2RG	8,84401E-03	31253,6	4,20254E-03	26736	8,66008E-01	37003	1,47338E+00	22576	8,87222E-01	26741	0	43212
Myeloid_vs_CD4_Responder	CD4(Tn)	cDC_CLEC9A	HMGB1	HAVCR2	8,85955E-03	30938,8	3,36084E-03	37016	8,77208E-01	36382	1,54656E+00	20361	9,08624E-01	17723	0	43212
Myeloid_vs_CD4_Responder	CD4(TGFB1+ Th17)	cDC_LAMP3	FLT3LG	FLT3	8,87196E-03	36821,8	2,06360E-02	1754	1,77337E+00	7607	6,61194E-01	56630	7,81433E-01	74906	0	43212
Myeloid_vs_CD4_Responder	Macro_NLRP3	CD4(IL26+ Th17)	VCAN	CD44	8,88288E-03	16402	9,28748E-03	7768	2,25246E+00	3222	1,61344E+00	18595	9,28422E-01	9213	0	43212
Myeloid_vs_CD4_Responder	CD4(NME1+ T)	Macro_FOLR2+APOE+	HMGB1	HAVCR2	8,88831E-03	27830,4	4,52556E-03	24089	1,18032E+00	22093	1,07540E+00	37040	9,20249E-01	12718	0	43212
Myeloid_vs_CD4_Responder	Macro_OLFML3	CD4(CXCL13+ Tfh)	HLA-DQB1	CD4	8,89311E-03	21343,8	3,35925E-03	37044	1,56287E+00	10892	2,26874E+00	6938	9,29746E-01	8633	0	43212
Myeloid_vs_CD4_Responder	CD4(IL26+ Th17)	Macro_NLRP3	HLA-B	LILRB2	8,89671E-03	27106,2	3,35915E-03	37047	1,38893E+00	15126	1,09968E+00	36043	9,40085E-01	4103	0	43212
Myeloid_vs_CD4_Responder	Mono_INHBA	CD4(IL26+ Th17)	HLA-DQB1	LAG3	8,90032E-03	29530,6	5,25596E-03	19377	8,65158E-01	37050	1,36295E+00	26170	8,98608E-01	21844	0	43212
Myeloid_vs_CD4_Responder	CD4(NME1+ T)	Macro_IER3	HMGB1	TLR4	8,90993E-03	34381,6	3,85711E-03	30306	1,06377E+00	27021	1,07475E+00	37058	8,69821E-01	34311	0	43212
Myeloid_vs_CD4_Responder	CD4(ISG+ Treg)	Mono_CD14	GNAI2	C5AR1	8,91233E-03	26694,2	3,35824E-03	37060	1,77522E+00	7574	1,91889E+00	11900	8,71102E-01	33725	0	43212
Myeloid_vs_CD4_Responder	Macro_NLRP3	CD4(ISG+ Treg)	LYZ	ITGAL	8,91437E-03	18205,8	5,41302E-03	18546	1,87970E+00	6287	2,55116E+00	4372	9,06578E-01	18612	0	43212
Myeloid_vs_CD4_Responder	Macro_FOLR2+APOE+	CD4(NME1+ T)	CD14	ITGB1	8,91808E-03	19389,6	5,40142E-03	18614	1,89356E+00	6135	1,71052E+00	16142	9,19990E-01	12845	0	43212
Myeloid_vs_CD4_Responder	CD4(TNFRSF9+ Treg)	Macro_LYVE1	LRPAP1	LRP1	8,92918E-03	28012,8	5,15708E-03	19912	1,95944E+00	5481	1,14021E+00	34385	8,63777E-01	37074	0	43212
Myeloid_vs_CD4_Responder	CD4(ISG+ Treg)	Mono_CD14	HMGB1	THBD	8,92918E-03	34282	3,87889E-03	30035	1,01400E+00	29467	1,07426E+00	37074	8,75782E-01	31622	0	43212
Myeloid_vs_CD4_Responder	Macro_FOLR2-APOE+	CD4(IFNG+ Tfh/Th1)	B2M	CD3D	8,93359E-03	50808,2	3,49988E-03	34956	1,86843E-01	88479	1,88226E-01	87347	9,61633E-01	47	0	43212
Myeloid_vs_CD4_Responder	CD4(TNF+ T)	Mono_CD16	RPS19	C5AR1	8,94123E-03	23078	5,12612E-03	20085	1,57540E+00	10653	1,07399E+00	37084	9,39295E-01	4356	0	43212
Myeloid_vs_CD4_Responder	CD4(AREG+ Tm)	Macro_ISG15	RPS19	C5AR1	8,94847E-03	30153,6	3,35647E-03	37090	1,02118E+00	29125	1,22713E+00	31088	9,26039E-01	10253	0	43212
Myeloid_vs_CD4_Responder	Mono_INHBA	CD4(IL26+ Th17)	VEGFA	ITGB1	8,96054E-03	24704,6	8,18379E-03	9635	1,93977E+00	5661	1,31326E+00	27915	8,63708E-01	37100	0	43212
Myeloid_vs_CD4_Responder	cDC(CD1C)	CD4(ISG+ Treg)	HLA-DMA	CD4	8,99198E-03	22643,2	3,35454E-03	37126	1,43245E+00	13907	2,36677E+00	5962	9,19607E-01	13009	0	43212
Myeloid_vs_CD4_Responder	CD4(CXCL13+ Tfh)	cDC_CLEC9A	APP	CD74	8,99811E-03	29031,6	2,15335E-03	65087	1,33104E+00	16824	3,14030E+00	1378	9,06470E-01	18657	0	43212
Myeloid_vs_CD4_Responder	CD4(TGFB1+ Th17)	Macro_IFI27	TNFSF13B	HLA-DPB1	8,99998E-03	36476,8	1,30052E-03	94723	1,31728E+00	17256	2,13342E+00	8535	9,06468E-01	18658	0	43212
Myeloid_vs_CD4_Responder	CD4(ISG+ Treg)	Macro_NLRP3	B2M	LILRB2	9,00894E-03	24618,4	3,35381E-03	37140	1,55092E+00	11119	1,27426E+00	29356	9,46357E-01	2265	0	43212
Myeloid_vs_CD4_Responder	Macro_NLRP3	CD4(Tn)	VCAN	SELL	9,00932E-03	14912,6	1,71932E-02	2481	2,42024E+00	2441	2,19580E+00	7766	9,06460E-01	18663	0	43212
Myeloid_vs_CD4_Responder	Macro_OLFML3	CD4(TNFRSF9+ Treg)	TNFSF13	FAS	9,01016E-03	26896,2	1,18903E-02	4973	1,48117E+00	12695	1,07264E+00	37141	8,65135E-01	36460	0	43212
Myeloid_vs_CD4_Responder	CD4(GZMK+ Teff)	Macro_OLFML3	CD52	SIGLEC10	9,01119E-03	29320,4	5,39131E-03	18664	1,36411E+00	15848	7,65284E-01	51051	9,08402E-01	17827	0	43212
Myeloid_vs_CD4_Responder	Mono_INHBA	CD4(CRTAM- T)	SPP1	ITGA4_ITGB1	9,01258E-03	25473,4	5,91555E-03	16206	1,16172E+00	22806	2,17594E+00	8000	8,63604E-01	37143	0	43212
Myeloid_vs_CD4_Responder	CD4(ISG+ Treg)	Mono_CD16	GNAI2	FPR1	9,01622E-03	31708,6	3,35361E-03	37146	1,69373E+00	8715	1,15024E+00	34006	8,67356E-01	35464	0	43212
Myeloid_vs_CD4_Responder	cDC(CD1C)	CD4(NME1+ T)	HLA-DQA1	CD4	9,01744E-03	20389,8	3,35360E-03	37147	1,60869E+00	10068	2,89487E+00	2284	9,28362E-01	9238	0	43212
Myeloid_vs_CD4_Responder	Mono_INHBA	CD4(ISG+ Treg)	VEGFA	CD44	9,02715E-03	24613,8	6,85941E-03	12884	1,79533E+00	7316	1,07237E+00	37155	8,97082E-01	22502	0	43212
Myeloid_vs_CD4_Responder	CD4(GZMK+ Teff)	Mono_INHBA	HLA-F	LILRB2	9,02837E-03	32484	3,35321E-03	37156	1,30191E+00	17749	1,18204E+00	32813	8,76101E-01	31490	0	43212
Myeloid_vs_CD4_Responder	CD4(ISG+ Treg)	Macro_LYVE1	GNAI2	C5AR1	9,04296E-03	31105	3,35239E-03	37168	1,77210E+00	7624	1,15718E+00	33742	8,71004E-01	33779	0	43212
Myeloid_vs_CD4_Responder	CD4(CXCL13+ Tfh)	Macro_LYVE1	ADAM10	GPNMB	9,05801E-03	22928,6	7,48196E-03	11178	2,37593E+00	2624	1,60979E+00	18689	8,59764E-01	38940	0	43212

Myeloid_vs_CD4_Post_R

Myeloid_vs_CD4_Responder	CD4(ISG+ Treg)	Mono_CD16	RPS19	C5AR1	9,08243E-03	23130,4	5,38584E-03	18702	1,63189E+00	9679	9,98481E-01	40158	9,40689E-01	3901	0	43212
Myeloid_vs_CD4_Responder	Macro_ISG15	CD4(TNFRSF9+ Treg)	CD86	CTLA4	9,10125E-03	18627,6	1,23690E-02	4635	1,55204E+00	11092	1,73820E+00	15487	9,06366E-01	18712	0	43212
Myeloid_vs_CD4_Responder	Mono_CD14	CD4(Tn)	LGALS1	CD69	9,11373E-03	28568,2	3,35230E-03	37169	8,61626E-01	37226	1,63225E+00	18063	9,32922E-01	7171	0	43212
Myeloid_vs_CD4_Responder	Macro_OLFML3	CD4(TNFRSF9+ Treg)	B2M	CD3D	9,12353E-03	41930,2	3,49693E-03	34994	5,74900E-01	55365	3,52218E-01	76032	9,61617E-01	48	0	43212
Myeloid_vs_CD4_Responder	CD4(NME1+ T)	Macro_NLRP3	GNAI2	FPR1	9,12721E-03	30997,8	3,34751E-03	37237	1,56368E+00	10874	1,30663E+00	28154	8,67251E-01	35512	0	43212
Myeloid_vs_CD4_Responder	Mono_INHBA	CD4(NME1+ T)	HLA-DRB5	LAG3	9,13456E-03	32713,2	3,34723E-03	37243	8,93941E-01	35455	1,59788E+00	19008	8,82570E-01	28648	0	43212
Myeloid_vs_CD4_Responder	CD4(GZMK+ Teff)	Mono_CD14	HMGB1	TLR4	9,14192E-03	33671,8	4,07204E-03	28004	1,06541E+00	26950	1,06995E+00	37249	8,72860E-01	32944	0	43212
Myeloid_vs_CD4_Responder	CD4(TNF+ T)	Mono_CD16	HLA-C	LILRB2	9,14315E-03	22517,6	4,64268E-03	23194	1,88343E+00	6251	1,06995E+00	37250	9,44830E-01	2681	0	43212
Myeloid_vs_CD4_Responder	CD4(CRTAM- T)	Macro_LYVE1	ANXA1	FPR1	9,14929E-03	31917,8	3,71831E-03	31969	1,08962E+00	25869	1,06981E+00	37255	9,00077E-01	21284	0	43212
Myeloid_vs_CD4_Responder	CD4(CRTAM- T)	Macro_ISG15	CD99	PILRA	9,15297E-03	31259,4	3,88333E-03	29996	1,13916E+00	23745	1,06967E+00	37258	8,98028E-01	22086	0	43212
Myeloid_vs_CD4_Responder	Mono_INHBA	CD4(CXCL13+ Tfh)	VCAN	ITGB1	9,15789E-03	28503,2	4,97467E-03	20981	1,37481E+00	15522	1,38130E+00	25539	8,63362E-01	37262	0	43212
Myeloid_vs_CD4_Responder	CD4(NME1+ T)	Macro_NLRP3	HLA-A	LILRB2	9,16772E-03	27861,4	3,40330E-03	36357	1,31673E+00	17278	1,06943E+00	37270	9,37259E-01	5190	0	43212
Myeloid_vs_CD4_Responder	cDC(CD1C)	CD4(TGFB1+ Th17)	IL1B	SIGIRR	9,16895E-03	27270,6	4,67256E-03	22975	1,06546E+00	26948	2,36848E+00	5947	8,63334E-01	37271	0	43212
Myeloid_vs_CD4_Responder	CD4(ISG+ Treg)	Macro_FOLR2-APOE+	LTB	TNFRSF1A	9,17264E-03	26667	9,21723E-03	7865	1,10233E+00	25314	1,06926E+00	37274	9,03920E-01	19670	0	43212
Myeloid_vs_CD4_Responder	Macro_FOLR2+APOE+	CD4(CRTAM- T)	APOE	SORL1	9,18248E-03	16669,4	5,65222E-03	17347	2,48025E+00	2212	3,00417E+00	1821	9,06274E-01	18755	0	43212
Myeloid_vs_CD4_Responder	CD4(IFNG+ Tfh/Th1)	Macro_NLRP3	HLA-C	LILRB2	9,19481E-03	27650,2	3,34454E-03	37292	1,33019E+00	16863	1,12528E+00	34990	9,35632E-01	5894	0	43212
Myeloid_vs_CD4_Responder	CD4(IFNG+ Tfh/Th1)	pDC_LILRA4	TNF	TNFRSF21	9,19576E-03	24845	1,29680E-02	4263	3,20457E+00	702	1,85479E+00	13098	8,07978E-01	62950	0	43212
Myeloid_vs_CD4_Responder	CD4(NME1+ T)	Mono_CD14	CIRBP	TREM1	9,20591E-03	26659,8	7,18284E-03	11964	1,46066E+00	13177	1,06866E+00	37301	8,85090E-01	27645	0	43212
Myeloid_vs_CD4_Responder	Macro_NLRP3	CD4(AREG+ Tm)	CXCL2	DPP4	9,20855E-03	22688,8	1,86316E-02	2127	2,06640E+00	4520	2,20029E+00	7705	8,23549E-01	55880	0	43212
Myeloid_vs_CD4_Responder	Macro_NLRP3	CD4(ISG+ Treg)	IL1B	SIGIRR	9,21202E-03	16595,2	9,33301E-03	7706	2,03322E+00	4822	2,40170E+00	5621	8,99274E-01	21615	0	43212
Myeloid_vs_CD4_Responder	CD4(IL26+ Th17)	Macro_ISG15	HLA-B	LILRB1	9,22073E-03	27670,2	3,70142E-03	32199	1,29648E+00	17931	1,06840E+00	37313	9,31699E-01	7696	0	43212
Myeloid_vs_CD4_Responder	CD4(Tn)	Macro_IER3	ANXA1	FPR1	9,24175E-03	34121	3,40537E-03	36325	9,85983E-01	30799	1,06802E+00	37330	8,96053E-01	22939	0	43212
Myeloid_vs_CD4_Responder	CD4(ISG+ Treg)	Macro_ISG15	CD28	CD86	9,24423E-03	28702,6	8,22808E-03	9543,5	1,26824E+00	18869,5	1,06801E+00	37332,5	8,69267E-01	34555,5	0	43212
Myeloid_vs_CD4_Responder	Macro_OLFML3	CD4(NME1+ T)	LGALS3BP	ITGB1	9,25290E-03	25274	8,43274E-03	9147	1,44465E+00	13592	1,06794E+00	37339	8,95762E-01	23080	0	43212
Myeloid_vs_CD4_Responder	Macro_OLFML3	CD4(TNFRSF9+ Treg)	HLA-DQB1	CD4	9,25786E-03	21477,2	3,34108E-03	37343	1,56052E+00	10945	2,24867E+00	7169	9,29569E-01	8717	0	43212
Myeloid_vs_CD4_Responder	Macro_IER3	CD4(IL26+ Th17)	HLA-DQB1	LAG3	9,25852E-03	21105,6	7,87454E-03	10268	1,27582E+00	18591	1,60657E+00	18795	9,15598E-01	14662	0	43212
Myeloid_vs_CD4_Responder	CD4(TGFB1+ Th17)	Macro_NLRP3	HLA-F	LILRB2	9,26530E-03	33005,8	3,47178E-03	35359	1,28197E+00	18423	1,06768E+00	37349	8,77975E-01	30686	0	43212
Myeloid_vs_CD4_Responder	Macro_IER3	CD4(TGFB1+ Th17)	ICAM1	IL2RG	9,26997E-03	19278,2	8,33628E-03	9338	1,55464E+00	11060	1,60627E+00	18801	9,17218E-01	13980	0	43212
Myeloid_vs_CD4_Responder	CD4(IFNG+ Tfh/Th1)	pDC_LILRA4	COPA	P2RY6	9,27570E-03	28046,8	7,58663E-03	10955	2,04313E+00	4729	2,03231E+00	9967	7,89484E-01	71371	0	43212
Myeloid_vs_CD4_Responder	Mono_CD14	CD4(TNFRSF9+ Treg)	ICAM1	IL2RA	9,27646E-03	23057,6	1,33093E-02	4080	1,14518E+00	23504	2,25119E+00	7134	8,63153E-01	37358	0	43212
Myeloid_vs_CD4_Responder	CD4(IL26+ Th17)	Macro_OLFML3	ADAM10	TREM2	9,28019E-03	24202,2	8,77254E-03	8604	1,85993E+00	6527	1,38780E+00	25307	8,63150E-01	37361	0	43212
Myeloid_vs_CD4_Responder	CD4(IFNG+ Tfh/Th1)	Mono_CD14	HLA-A	LILRB2	9,28764E-03	24840,6	3,33980E-03	37367	1,36667E+00	15767	1,47784E+00	22429	9,36703E-01	5428	0	43212
Myeloid_vs_CD4_Responder	CD4(AREG+ Tm)	cDC_CLEC9A	LTB	CD40	9,29013E-03	29527,6	7,03964E-03	12392	1,14619E+00	23457	1,06723E+00	37369	8,76764E-01	31208	0	43212
Myeloid_vs_CD4_Responder	Macro_OLFML3	CD4(GZMK+ Teff)	GRN	TNFRSF1B	9,29137E-03	25126,8	3,33970E-03	37370	1,43528E+00	13836	1,54642E+00	20369	9,24718E-01	10847	0	43212
Myeloid_vs_CD4_Responder	CD4(TGFB1+ Th17)	Macro_OLFML3	ANXA1	FPR1	9,29289E-03	26718,6	5,36424E-03	18813	1,40560E+00	14663	9,52672E-01	42143	9,15392E-01	14762	0	43212
Myeloid_vs_CD4_Responder	CD4(CRTAM- T)	Mono_INHBA	HLA-F	LILRB2	9,29635E-03	33092,8	3,33958E-03	37374	1,30004E+00	17800	1,11333E+00	35489	8,75880E-01	31589	0	43212
Myeloid_vs_CD4_Responder	CD4(NME1+ T)	Mono_CD16	HMGB1	TLR2	9,29883E-03	31480,6	3,91010E-03	29701	1,13835E+00	23778	1,06704E+00	37376	8,95112E-01	23336	0	43212
Myeloid_vs_CD4_Responder	CD4(IFNG+ Tfh/Th1)	pDC_LILRA4	PSEN1	NOTCH4	9,30246E-03	39168,8	1,35498E-02	3951	1,55588E+00	11038	1,73544E+00	15558	6,25725E-01	122085	0	43212
Myeloid_vs_CD4_Responder	pDC_LILRA4	CD4(ISG+ Treg)	B2M	CD3D	9,31346E-03	38433,6	3,49422E-03	35033	1,61590E-01	91033	1,46527E+00	22841	9,61603E-01	49	0	43212
Myeloid_vs_CD4_Responder	CD4(CXCL13+ Tfh)	pDC_LILRA4	TNF	TNFRSF21	9,32352E-03	29999,8	8,54465E-03	8973	3,13134E+00	788	1,60504E+00	18829	7,73528E-01	78197	0	43212
Myeloid_vs_CD4_Responder	Mono_INHBA	CD4(CXCL13+ Tfh)	LYZ	ITGAL	9,32748E-03	26273,8	3,33808E-03	37399	1,44829E+00	13492	2,08333E+00	9206	8,83998E-01	28060	0	43212
Myeloid_vs_CD4_Responder	Mono_INHBA	CD4(CXCL13+ Tfh)	VCAN	ITGA4	9,32998E-03	26537	6,07379E-03	15581	1,43267E+00	13900	1,47280E+00	22591	8,63077E-01	37401	0	43212
Myeloid_vs_CD4_Responder	Macro_ISG15	CD4(IL26+ Th17)	HLA-DRB1	LAG3	9,33503E-03	18342,2	7,03566E-03	12407	1,26944E+00	18835	2,22179E+00	7465	9,27072E-01	9792	0	43212
Myeloid_vs_CD4_Responder	CD4(GZMK+ Teff)	Mono_INHBA	HMGB1	CD163	9,34495E-03	27177,6	3,33733E-03	37413	1,32579E+00	17003	1,41916E+00	24274	9,17205E-01	13986	0	43212
Myeloid_vs_CD4_Responder	Macro_FOLR2+APOE+	CD4(IFNG+ Tfh/Th1)	CD14	ITGB1	9,35422E-03	19171,4	5,35857E-03	18845	1,88946E+00	6181	1,77701E+00	14654	9,19696E-01	12965	0	43212
Myeloid_vs_CD4_Responder	CD4(GZMK+ Teff)	Mono_INHBA	CALR	LRP1	9,35620E-03	32621,6	3,34760E-03	37236	1,22331E+00	20481	1,06570E+00	37422	8,91791E-01	24757	0	43212
Myeloid_vs_CD4_Responder	CD4(ISG+ Treg)	Mono_INHBA	HLA-F	LILRB1	9,35995E-03	32571,6	4,40018E-03	25069	1,15644E+00	23028	1,06566E+00	37425	8,70276E-01	34124	0	43212
Myeloid_vs_CD4_Responder	Macro_OLFML3	CD4(Tn)	LGALS3BP	ITGB1	9,37371E-03	26959,2	7,17258E-03	11987	1,36743E+00	15740	1,06543E+00	37436	8,87960E-01	26421	0	43212
Myeloid_vs_CD4_Responder	Mono_CD14	CD4(IFNG+ Tfh/Th1)	SECTM1	CD7	9,38123E-03	31841,4	1,05404E-02	6203	8,68336E-01	36863	1,11339E+00	35487	8,62999E-01	37442	0	43212
Myeloid_vs_CD4_Responder	CD4(AREG+ Tm)	cDC_CLEC9A	MIF	CD44_CD74	9,38555E-03	47487,2	1,81526E-03	75931	3,22389E-01	75422	9,77742E-01	41045	9,48195E-01	1826	0	43212
Myeloid_vs_CD4_Responder	Macro_FOLR2-APOE+	CD4(TGFB1+ Th17)	SPP1	S1PR1	9,38692E-03	21949,6	2,21402E-02	1551	1,76100E+00	7756	2,22792E+00	7400	8,36253E-01	49829	0	43212
Myeloid_vs_CD4_Responder	Macro_NLRP3	CD4(TNF+ T)	VCAN	CD44	9,39656E-03	19036,4	6,58429E-03	13698	2,01628E+00	4967	1,60361E+00	18867	9,16116E-01	14438	0	43212
Myeloid_vs_CD4_Responder	CD4(ISG+ Treg)	Mono_CD16	HLA-F	LILRB1	9,41636E-03	28480,6	5,49300E-03	18117	1,39895E+00	14825	1,06468E+00	37470	8,82292E-01	28779	0	43212
Myeloid_vs_CD4_Responder	Mono_INHBA	CD4(TGFB1+ Th17)	LYZ	ITGAL	9,41887E-03	26381,6	3,33363E-03	37472	1,44795E+00	13500	2,05393E+00	9635	8,83929E-01	28089	0	43212
Myeloid_vs_CD4_Responder	CD4(TGFB1+ Th17)	Macro_FOLR2-APOE+	CD40LG	CD9	9,42893E-03	25044,2	1,74636E-02	2413	1,48505E+00	12613	1,27017E+00	29503	8,62926E-01	37480	0	43212
Myeloid_vs_CD4_Responder	Mono_INHBA	CD4(TNFRSF9+ Treg)	SPP1	CCR8	9,44647E-03	19356,6	6,04968E-02	196	1,56390E+00	10870	3,00008E+00	1832	8,55844E-01	40673	0	43212
Myeloid_vs_CD4_Responder	CD4(NME1+ T)	Macro_NLRP3	CD99	PILRA	9,45159E-03	28050,4	4,53621E-03	24017	1,35168E+00	16233	1,06390E+00	37498	9,04926E-01	19292	0	43212
Myeloid_vs_CD4_Responder	Macro_ISG15	CD4(Tn)	SPP1	S1PR1	9,45747E-03	19506,2	2,74357E-02	1000	1,75955E+00	7776	2,87559E+00	2363	8,50412E-01	43180	0	43212
Myeloid_vs_CD4_Responder	Mono_INHBA	CD4(TNFRSF9+ Treg)	VEGFA	ITGB1	9,46168E-03	24643,8	8,06712E-03	9864	1,93404E+00	5724	1,34111E+00	26913	8,62860E-01	37506	0	43212
Myeloid_vs_CD4_Responder	Macro_FOLR2+APOE+	CD4(IFNG+ Tfh/Th1)	LGALS3	LAG3	9,47389E-03	18368	1,24392E-02	4577	1,26699E+00	18907	1,98483E+00	10739	9,16198E-01	14405	0	43212
Myeloid_vs_CD4_Responder	CD4(GZMK+ Teff)	Macro_LYVE1	HSP90B1	LRP1	9,47556E-03	27126	4,31169E-03	25797	1,91176E+00	5938	1,06313E+00	37517	8,95523E-01	23166	0	43212
Myeloid_vs_CD4_Responder	CD4(IFNG+ Tfh/Th1)	Macro_ISG15	HMGB1	CD163	9,47935E-03	27673	3,33087E-03	37520	1,29377E+00	18032	1,37974E+00	25585	9,17132E-01	14016	0	43212
Myeloid_vs_CD4_Responder	CD4(TGFB1+ Th17)	Mono_CD14	CIRBP	TREM1	9,48693E-03	26786,6	7,13509E-03	12099	1,45568E+00	13302	1,06280E+00	37526	8,84750E-01	27794	0	43212
Myeloid_vs_CD4_Responder	CD4(TGFB1+ Th17)	Mono_INHBA	HLA-B	LILRB2	9,50591E-03	27959,4	3,32980E-03	37541	1,30332E+00	17698	1,07217E+00	37162	9,39838E-01	4184	0	43212
Myeloid_vs_CD4_Responder	CD4(CXCL13+ Tfh)	Mono_INHBA	CALR	LRP1	9,50717E-03	31617,4	3,52945E-03	34491	1,26004E+00	19167	1,06243E+00	37542	8,94317E-01	23675	0	43212
Myeloid_vs_CD4_Responder	CD4(IFNG+ Tfh/Th1)	Mono_CD14	HLA-F	LILRB2	9,50971E-03	31801,6	3,32962E-03	37544	1,20070E+00	21327	1,38882E+00	25261	8,75718E-01	31664	0	43212
Myeloid_vs_CD4_Responder	CD4(TNFRSF9+ Treg)	Macro_FOLR2+APOE+	TIGIT	NECTIN2	9,51858E-03	25538,8	2,79279E-02	959,5	1,59050E+00	10373,5	1,11067E+00	35597,5	8,62746E-01	37551,5	0	43212
Myeloid_vs_CD4_Responder	Mono_INHBA	CD4(ISG+ Treg)	LGALS1	PTPRC	9,51984E-03	25390,4	3,32937E-03	37552	1,12864E+00	24163	1,54672E+00	20357	9,48860E-01	1668	0	43212
Myeloid_vs_CD4_Responder	cDC(CD1C)	CD4(NME1+ T)	HLA-DQA2	LAG3	9,52491E-03	24331,6	6,11010E-03	15459	1,28689E+00	18255	2,24798E+00	7176	8,62742E-01	37556	0	43212

Myeloid_vs_CD4_Post_R

Myeloid_vs_CD4_Responder	CD4(ISG+ Treg)	Mono_CD16	HLA-B	LILRB1	9,53126E-03	26160	3,88586E-03	29964	1,46609E+00	13041	1,06191E+00	37561	9,33230E-01	7022	0	43212
Myeloid_vs_CD4_Responder	CD4(GZMK+ Teff)	Macro_ISG15	HLA-A	LILRB1	9,53760E-03	28172,6	3,77739E-03	31217	1,24247E+00	19790	1,06179E+00	37566	9,28747E-01	9078	0	43212
Myeloid_vs_CD4_Responder	CD4(TNF+ T)	cDC_CLEC9A	CD40LG	CD40	9,53999E-03	28069,2	1,48641E-02	3321	1,31878E+00	17212	1,60046E+00	18941	8,19603E-01	57660	0	43212
Myeloid_vs_CD4_Responder	Mono_INHBA	CD4(IFNG+ Tfh/Th1)	INHBA	ACTR2	9,55666E-03	19428,6	1,20624E-02	4843	2,78089E+00	1392	2,02284E+00	10115	8,62694E-01	37581	0	43212
Myeloid_vs_CD4_Responder	cDC_LAMP3	CD4(IL26+ Th17)	HLA-DPA1	LAG3	9,58099E-03	19468,8	7,03927E-03	12393	1,26541E+00	18962	1,87799E+00	12645	9,26315E-01	10132	0	43212
Myeloid_vs_CD4_Responder	CD4(TGFB1+ Th17)	Macro_FOLR2+APOE+	ADAM10	GPNMB	9,58594E-03	22635,6	7,85107E-03	10310	2,62858E+00	1758	1,54986E+00	20294	8,62642E-01	37604	0	43212
Myeloid_vs_CD4_Responder	Mono_INHBA	CD4(CXCL13+ Tfh)	VCAN	SELL	9,61018E-03	25820,6	7,21613E-03	11873	1,43661E+00	13800	1,47267E+00	22595	8,62601E-01	37623	0	43212
Myeloid_vs_CD4_Responder	Macro_FOLR2+APOE+	CD4(ISG+ Treg)	C3	IFITM1	9,64174E-03	16256,2	1,21689E-02	4767	1,60461E+00	10132	1,59842E+00	18993	9,39858E-01	4177	0	43212
Myeloid_vs_CD4_Responder	cDC(CD1C)	CD4(CXCL13+ Tfh)	HLA-DQB1	LAG3	9,64856E-03	26448,4	3,32333E-03	37653	1,38931E+00	15118	2,52110E+00	4608	8,75736E-01	31651	0	43212
Myeloid_vs_CD4_Responder	Macro_FOLR2+APOE+	CD4(ISG+ Treg)	APOE	LDLR	9,64966E-03	17835,4	9,24442E-03	7830	2,52839E+00	2056	3,06457E+00	1618	8,69477E-01	34461	0	43212
Myeloid_vs_CD4_Responder	CD4(TGFB1+ Th17)	Macro_IFI27	TNFSF9	HLA-DPA1	9,65240E-03	28113	3,69742E-03	32236	1,29132E+00	18109	2,07247E+00	9352	8,62544E-01	37656	0	43212
Myeloid_vs_CD4_Responder	CD4(IFNG+ Tfh/Th1)	Macro_NLRP3	B2M	LILRB2	9,65753E-03	25031,2	3,32293E-03	37660	1,51103E+00	12001	1,25733E+00	29955	9,46122E-01	2328	0	43212
Myeloid_vs_CD4_Responder	CD4(TGFB1+ Th17)	Macro_LYVE1	CD99	PILRA	9,65943E-03	26551	5,32460E-03	19002	1,55444E+00	11063	9,32075E-01	43043	9,11600E-01	16435	0	43212
Myeloid_vs_CD4_Responder	Mono_CD14	CD4(ISG+ Treg)	HLA-DRA	LAG3	9,66009E-03	26738,2	4,47591E-03	24477	8,53513E-01	37662	1,90538E+00	12130	9,12089E-01	16210	0	43212
Myeloid_vs_CD4_Responder	Macro_FOLR2-APOE+	CD4(CRTAM- T)	MMP9	CD44	9,66140E-03	18018,6	1,23022E-02	4669	1,85509E+00	6572	1,59798E+00	19003	9,11135E-01	16637	0	43212
Myeloid_vs_CD4_Responder	Macro_IER3	CD4(ISG+ Treg)	IL1B	SIGIRR	9,67117E-03	16884,4	9,48059E-03	7502	2,06442E+00	4545	2,19035E+00	7836	8,99982E-01	21327	0	43212
Myeloid_vs_CD4_Responder	Macro_FOLR2-APOE+	CD4(IL26+ Th17)	SPP1	ITGA4_ITGB1	9,67476E-03	17502	1,08166E-02	5934	1,75551E+00	7837	2,23501E+00	7319	8,95416E-01	23208	0	43212
Myeloid_vs_CD4_Responder	cDC(CD1C)	CD4(GZMK+ Teff)	VEGFA	CD44	9,69091E-03	36523,6	3,87756E-03	30058	8,77876E-01	36342	1,05884E+00	37686	8,67613E-01	35320	0	43212
Myeloid_vs_CD4_Responder	cDC(CD1C)	CD4(CXCL13+ Tfh)	LGALS9	PTPRC	9,69477E-03	31042	3,32111E-03	37689	8,88375E-01	35759	1,37833E+00	25632	9,19801E-01	12918	0	43212
Myeloid_vs_CD4_Responder	CD4(TGFB1+ Th17)	Macro_FOLR2+APOE+	TNFSF9	HLA-DPA1	9,70635E-03	29342,4	3,69210E-03	32313	1,28917E+00	18182	1,74576E+00	15307	8,62459E-01	37698	0	43212
Myeloid_vs_CD4_Responder	CD4(ISG+ Treg)	Mono_INHBA	LGALS9	LRP1	9,73084E-03	24673	5,26394E-03	19331	1,84349E+00	6723	1,70024E+00	16382	8,62406E-01	37717	0	43212
Myeloid_vs_CD4_Responder	CD4(CRTAM- T)	cDC(CD1C)	CD52	SIGLEC10	9,73987E-03	35633,6	3,49519E-03	35019	8,52438E-01	37724	1,09736E+00	36126	8,88705E-01	26087	0	43212
Myeloid_vs_CD4_Responder	CD4(GZMK+ Teff)	Macro_FOLR2+APOE+	HMGB1	CD163	9,74116E-03	25831,4	4,09849E-03	27744	1,63557E+00	9615	1,05798E+00	37725	9,24679E-01	10861	0	43212
Myeloid_vs_CD4_Responder	CD4(CXCL13+ Tfh)	Macro_OLFML3	APP	CD74	9,75616E-03	30398	2,10440E-03	66605	1,28792E+00	18219	2,48366E+00	4903	9,05490E-01	19051	0	43212
Myeloid_vs_CD4_Responder	CD4(CRTAM- T)	Macro_NLRP3	ANXA2	TLR2	9,76830E-03	33737,8	3,31790E-03	37746	1,30910E+00	17526	1,16435E+00	33477	8,64528E-01	36728	0	43212
Myeloid_vs_CD4_Responder	Mono_INHBA	CD4(IFNG+ Tfh/Th1)	S100A9	ITGB2	9,76960E-03	23403,4	3,31782E-03	37747	1,52253E+00	11736	1,91816E+00	11920	9,20999E-01	12402	0	43212
Myeloid_vs_CD4_Responder	CD4(ISG+ Treg)	Macro_FOLR2+APOE+	TNFSF13B	HLA-DPB1	9,77003E-03	37331,2	1,27055E-03	95896	1,33683E+00	16668	1,92272E+00	11822	9,05475E-01	19058	0	43212
Myeloid_vs_CD4_Responder	CD4(NME1+ T)	Mono_INHBA	HSP90B1	LRP1	9,80978E-03	32992,2	3,31556E-03	37778	1,28743E+00	18233	1,07358E+00	37093	8,82580E-01	28645	0	43212
Myeloid_vs_CD4_Responder	Macro_FOLR2-APOE+	CD4(TGFB1+ Th17)	SPP1	PTGER4	9,81174E-03	16353,8	1,42662E-02	3579	1,75223E+00	7875	2,19416E+00	7792	9,04841E-01	19311	0	43212
Myeloid_vs_CD4_Responder	CD4(CXCL13+ Tfh)	Mast	IL16	CD9	9,81175E-03	24752	7,12202E-03	12145	1,80000E+00	7258	1,59557E+00	19079	8,52918E-01	42066	0	43212
Myeloid_vs_CD4_Responder	CD4(ISG+ Treg)	Macro_LYVE1	LRPAP1	LRP1	9,81887E-03	28948	5,04889E-03	20524	1,95183E+00	5556	1,05668E+00	37785	8,62524E-01	37663	0	43212
Myeloid_vs_CD4_Responder	CD4(ISG+ Treg)	pDC_LILRA4	LGALS9	CD47	9,86443E-03	33427,8	3,31242E-03	37820	8,69200E-01	36823	1,81547E+00	13867	8,67443E-01	35417	0	43212
Myeloid_vs_CD4_Responder	Macro_OLFML3	CD4(TNF+ T)	CD14	ITGA4	9,86834E-03	27390	3,31226E-03	37823	1,43015E+00	13968	1,68928E+00	16640	8,90462E-01	25307	0	43212
Myeloid_vs_CD4_Responder	CD4(AREG+ Tm)	Macro_NLRP3	CD99	PILRA	9,87356E-03	28282,6	4,48380E-03	24426	1,34313E+00	16475	1,05570E+00	37827	9,04425E-01	19473	0	43212
Myeloid_vs_CD4_Responder	CD4(IFNG+ Tfh/Th1)	Mono_CD16	HLA-B	LILRA1	9,87356E-03	25605,2	1,05430E-02	6200	1,73037E+00	8180	6,55373E-01	56949	9,18428E-01	13485	0	43212
Myeloid_vs_CD4_Responder	Macro_ISG15	CD4(CXCL13+ Tfh)	S100A8	CD69	9,87356E-03	20740,4	5,78819E-03	16753	1,26134E+00	19110	1,86951E+00	12789	9,22388E-01	11838	0	43212
Myeloid_vs_CD4_Responder	CD4(IFNG+ Tfh/Th1)	Mono_CD16	HLA-A	LILRA1	9,88156E-03	24809,6	1,12331E-02	5568	1,78497E+00	7446	7,17228E-01	53592	9,16617E-01	14230	0	43212
Myeloid_vs_CD4_Responder	Macro_NLRP3	CD4(ISG+ Treg)	B2M	CD3D	9,88322E-03	52478,8	3,47823E-03	35288	1,43844E-01	92954	1,39801E-01	90888	9,61518E-01	52	0	43212
Myeloid_vs_CD4_Responder	CD4(IL26+ Th17)	Macro_LYVE1	LRPAP1	LRP1	9,88662E-03	28880,6	5,01764E-03	20721	1,94964E+00	5574	1,07470E+00	37059	8,62156E-01	37837	0	43212
Myeloid_vs_CD4_Responder	CD4(ISG+ Treg)	Macro_IER3	HLA-F	LILRB2	9,88793E-03	31443,4	4,01549E-03	28576	1,23299E+00	20150	1,05534E+00	37838	8,85557E-01	27441	0	43212
Myeloid_vs_CD4_Responder	CD4(ISG+ Treg)	Mono_INHBA	HMGB1	CD163	9,88793E-03	27987,4	3,31105E-03	37838	1,31875E+00	17215	1,32284E+00	27558	9,16905E-01	14114	0	43212
Myeloid_vs_CD4_Responder	CD4(IFNG+ Tfh/Th1)	Mono_CD16	HLA-C	LILRA1	9,89156E-03	26954,6	1,04186E-02	6333	1,64963E+00	9388	6,07837E-01	59691	9,12214E-01	16149	0	43212
Myeloid_vs_CD4_Responder	CD4(IFNG+ Tfh/Th1)	Mono_CD16	HLA-C	LILRA3	9,89557E-03	22461,2	1,81842E-02	2232	2,51710E+00	2094	7,50050E-01	51811	9,19722E-01	12957	0	43212
Myeloid_vs_CD4_Responder	Macro_IER3	CD4(TGFB1+ Th17)	IL1B	SIGIRR	9,89889E-03	17025,6	9,19619E-03	7899	2,05139E+00	4652	2,21739E+00	7518	8,98603E-01	21847	0	43212
Myeloid_vs_CD4_Responder	Macro_OLFML3	CD4(ISG+ Treg)	TNFSF13	TNFRSF14	9,92457E-03	27457,4	6,54038E-03	13865	1,54527E+00	11243	1,05474E+00	37866	8,76979E-01	31101	0	43212
Myeloid_vs_CD4_Responder	CD4(TNFRSF9+ Treg)	Macro_NLRP3	HLA-B	LILRB2	9,92457E-03	27449,8	3,30972E-03	37866	1,34603E+00	16396	1,11192E+00	35537	9,39666E-01	4238	0	43212
Myeloid_vs_CD4_Responder	CD4(ISG+ Treg)	Mono_CD14	HLA-B	LILRB2	9,93506E-03	23617,4	3,30934E-03	37874	1,47579E+00	12823	1,56275E+00	19937	9,39663E-01	4241	0	43212
Myeloid_vs_CD4_Responder	Macro_FOLR2-APOE+	CD4(TNF+ T)	SPP1	CD44	9,96783E-03	21691	5,41643E-03	18524	1,36478E+00	15825	1,92745E+00	11737	9,05255E-01	19157	0	43212
Myeloid_vs_CD4_Responder	Macro_OLFML3	CD4(IFNG+ Tfh/Th1)	HLA-DRB1	CD4	9,97841E-03	20196,8	3,30742E-03	37907	1,51508E+00	11905	2,53278E+00	4525	9,42129E-01	3435	0	43212
Myeloid_vs_CD4_Responder	Macro_ISG15	CD4(ISG+ Treg)	SPP1	CCR8	9,97921E-03	19278,2	5,83250E-02	209	1,74948E+00	7921	2,70269E+00	3294	8,53574E-01	41755	0	43212

Myeloid_vs_CD8_Post_NR

Response	source	target	ligand.complex	receptor.complex	aggregate_rank	mean_rank	natmi.edge_specificity	natmi.rank	connectome.weight_sc	connectome.rank	logfc.logfc_comb	logfc.rank	sca.LRscore	sca.rank	cellphonedb.pvalue	cellphonedb.rank
Myeloid_vs_CD8_Non-responder	CD8(EOMES+ NK-like)	cDC_CLEC9A	XCL1	XCR1	2,29150E-07	22202	2,02454E-01	12	5,73037E+00	7	2,43925E+00	12650	9,00391E-01	36441	0	61900
Myeloid_vs_CD8_Non-responder	CD8(EOMES+ NK-like)	cDC_CLEC9A	XCL2	XCR1	5,74422E-07	21948	1,74171E-01	19	5,70266E+00	9	2,41011E+00	13137	9,02987E-01	34675	0	61900
Myeloid_vs_CD8_Non-responder	Mono_CD14	CD8(ZNF683+KLRB1+ T)	S100A8	CD69	7,94600E-07	13107,8	1,88986E-02	2373	2,94977E+00	363	4,03354E+00	849	9,64066E-01	54	0	61900
Myeloid_vs_CD8_Non-responder	Mono_CD14	CD8(Tc17)	S100A8	CD69	1,04222E-06	13146,6	1,82922E-02	2540	2,91535E+00	385	4,05281E+00	810	9,63496E-01	98	0	61900
Myeloid_vs_CD8_Non-responder	CD8(ZNF683+KLRB1+ T)	cDC_CLEC9A	XCL2	XCR1	2,41966E-06	24806,4	1,38252E-01	39	5,53396E+00	11	2,07332E+00	20155	8,92389E-01	41927	0	61900
Myeloid_vs_CD8_Non-responder	cDC_LAMP3	CD8(Tc17)	CCL22	DPP4	2,94133E-06	23587,4	1,59537E-01	24	4,44229E+00	43	3,46237E+00	2573	8,75113E-01	53397	0	61900
Myeloid_vs_CD8_Non-responder	CD8(IL7R+ZNF683+ Tm)	cDC_CLEC9A	XCL1	XCR1	2,94133E-06	26099,6	1,33258E-01	43	5,44746E+00	12	2,14833E+00	18377	8,80004E-01	50166	0	61900
Myeloid_vs_CD8_Non-responder	CD8(NME1+ T)	cDC(CD1C)	B2M	CD1C	3,88354E-06	19939,6	1,69392E-02	2958	1,85177E+00	3533	1,76335E+00	29006	9,54924E-01	2301	0	61900
Myeloid_vs_CD8_Non-responder	cDC_LAMP3	CD8(GZMK+ Early Tem)	CCL22	CCR4	4,46796E-06	29505,6	1,64320E-01	21	4,21939E+00	53	3,28242E+00	3552	8,30969E-01	82002	0	61900
Myeloid_vs_CD8_Non-responder	cDC_LAMP3	CD8(Tn)	CCL19	CCR7	4,46796E-06	20494,4	1,22927E-01	53	4,49787E+00	40	3,31846E+00	3346	8,99342E-01	37133	0	61900
Myeloid_vs_CD8_Non-responder	cDC_LAMP3	CD8(ISG+ T)	CCL22	CCR4	4,63810E-06	30083,2	1,54382E-01	27	4,20001E+00	54	3,26235E+00	3694	8,26542E-01	84741	0	61900
Myeloid_vs_CD8_Non-responder	cDC_LAMP3	CD8(Tn)	CCL22	CCR4	5,53646E-06	31015	1,39785E-01	37	4,17154E+00	59	3,25307E+00	3756	8,19306E-01	89323	0	61900
Myeloid_vs_CD8_Non-responder	Macro_NLRP3	CD8(Tc17)	S100A8	CD69	7,07991E-06	14388,2	1,44589E-02	4108	2,37208E+00	1137	3,20326E+00	4074	9,59128E-01	722	0	61900
Myeloid_vs_CD8_Non-responder	Mono_CD14	CD8(IL7R+ZNF683+ Tm)	S100A8	CD69	7,45773E-06	13858	1,43739E-02	4162	2,69297E+00	586	3,62809E+00	1886	9,59012E-01	756	0	61900
Myeloid_vs_CD8_Non-responder	CD8(NME1+ T)	pDC_LILRA4	BST2	LILRA4	7,70322E-06	19568,4	1,94736E-02	2228	2,92739E+00	377	3,18627E+00	4196	9,11365E-01	29141	0	61900
Myeloid_vs_CD8_Non-responder	Macro_NLRP3	CD8(ZNF683+KLRB1+ T)	S100A8	CD69	7,81342E-06	14311,8	1,49383E-02	3842	2,40649E+00	1044	3,18399E+00	4211	9,59762E-01	562	0	61900
Myeloid_vs_CD8_Non-responder	CD8(ZNF683+KLRB1+ T)	cDC_CLEC9A	XCL1	XCR1	8,47432E-06	29578	1,07751E-01	73	5,34317E+00	15	1,79537E+00	28011	8,68325E-01	57891	0	61900
Myeloid_vs_CD8_Non-responder	CD8(ID2+CXCR4+ T)	cDC_CLEC9A	XCL1	XCR1	8,70799E-06	29130	1,07387E-01	74	5,34168E+00	16	1,87320E+00	25618	8,68132E-01	58042	0	61900
Myeloid_vs_CD8_Non-responder	Mono_CD14	CD8(EOMES+ NK-like)	S100A8	CD69	1,01939E-05	14045,4	1,38012E-02	4502	2,66047E+00	634	3,55579E+00	2154	9,58205E-01	1037	0	61900
Myeloid_vs_CD8_Non-responder	CD8(ID2+CXCR4+ T)	cDC_CLEC9A	XCL2	XCR1	1,17596E-05	28237,8	9,88662E-02	86	5,34897E+00	14	1,86489E+00	25858	8,75198E-01	53331	0	61900
Myeloid_vs_CD8_Non-responder	pDC_LILRA4	CD8(Temra)	SCT	ADRB2	1,34568E-05	27712,2	9,54328E-02	92	3,88930E+00	87	3,28364E+00	3545	8,45245E-01	72937	0	61900
Myeloid_vs_CD8_Non-responder	Mono_CD14	CD8(Temra)	S100A8	CD69	1,38439E-05	14268,8	1,32950E-02	4862	2,63174E+00	669	3,45632E+00	2608	9,57451E-01	1305	0	61900
Myeloid_vs_CD8_Non-responder	Mono_CD14	CD8(Tn)	S100A8	CD69	1,56594E-05	14260	1,30727E-02	5015	2,61913E+00	690	3,53155E+00	2265	9,57106E-01	1430	0	61900
Myeloid_vs_CD8_Non-responder	CD8(ISG+ T)	cDC(CD1C)	B2M	CD1C	1,64693E-05	18212	1,79923E-02	2615	2,10304E+00	2038	1,97329E+00	22754	9,56204E-01	1753	0	61900
Myeloid_vs_CD8_Non-responder	Mono_CD14	CD8(GZMK+ Early Tem)	S100A8	CD69	1,89010E-05	14344,2	1,27675E-02	5258	2,60180E+00	718	3,53419E+00	2253	9,56619E-01	1592	0	61900
Myeloid_vs_CD8_Non-responder	CD8(ISG+ T)	pDC_LILRA4	BST2	LILRA4	1,98324E-05	17641,2	2,59228E-02	1291	3,07947E+00	273	3,50212E+00	2404	9,22260E-01	22338	0	61900
Myeloid_vs_CD8_Non-responder	Mono_CD14	CD8(ID2+CXCR4+ T)	S100A8	CD69	2,02956E-05	14398,2	1,26666E-02	5353	2,59608E+00	723	3,51220E+00	2356	9,56454E-01	1659	0	61900
Myeloid_vs_CD8_Non-responder	pDC_LILRA4	CD8(NME1+ T)	APP	RPSA	2,07209E-05	15647,4	1,29342E-02	5121	2,46923E+00	913	3,03632E+00	5381	9,49398E-01	4922	0	61900
Myeloid_vs_CD8_Non-responder	Macro_OLFML3	CD8(Tc17)	CXCL10	DPP4	2,57243E-05	24289,8	5,88243E-02	261	2,70576E+00	578	3,76962E+00	1426	8,69254E-01	57284	0	61900
Myeloid_vs_CD8_Non-responder	CD8(Tn)	Mono_CD14	ANXA1	FPR1	2,75603E-05	21316,6	6,45582E-03	15734	1,55573E+00	6688	2,46064E+00	12286	9,42602E-01	9975	0	61900
Myeloid_vs_CD8_Non-responder	Mono_CD14	CD8(LAYN+ T)	S100A8	CD69	2,85312E-05	14898,8	1,21290E-02	5832	2,56556E+00	767	3,22464E+00	3948	9,55541E-01	2047	0	61900
Myeloid_vs_CD8_Non-responder	Macro_ISG15	CD8(Tc17)	CXCL10	DPP4	3,02806E-05	25617,4	5,04608E-02	348	2,37557E+00	1126	3,73963E+00	1506	8,60290E-01	63207	0	61900
Myeloid_vs_CD8_Non-responder	Mono_CD14	CD8(ISG+ T)	S100A8	CD69	3,12673E-05	14680	1,19694E-02	5968	2,55650E+00	783	3,46169E+00	2581	9,55259E-01	2168	0	61900
Myeloid_vs_CD8_Non-responder	cDC_LAMP3	CD8(GZMK+ Early Tem)	CCL17	CCR4	3,15911E-05	31531,2	1,50771E-01	29	3,43590E+00	141	3,11609E+00	4726	8,16762E-01	90860	0	61900
Myeloid_vs_CD8_Non-responder	CD8(Terminal Tex)	cDC_CLEC9A	XCL1	XCR1	3,20404E-05	32389,8	7,88164E-02	142	5,22487E+00	20	1,74453E+00	29630	8,49398E-01	70257	0	61900
Myeloid_vs_CD8_Non-responder	cDC_LAMP3	CD8(ISG+ T)	CCL17	CCR4	3,24929E-05	32117,6	1,41652E-01	35	3,41652E+00	143	3,09603E+00	4891	8,12047E-01	93619	0	61900
Myeloid_vs_CD8_Non-responder	Mono_CD14	CD8(Terminal Tex)	S100A8	CD69	3,29007E-05	14816	1,18844E-02	6045	2,55168E+00	787	3,36105E+00	3118	9,55107E-01	2230	0	61900
Myeloid_vs_CD8_Non-responder	CD8(IL7R+ZNF683+ Tm)	cDC_CLEC9A	XCL2	XCR1	3,29485E-05	31459	7,83718E-02	144	5,25271E+00	18	1,64487E+00	33138	8,61949E-01	62095	0	61900
Myeloid_vs_CD8_Non-responder	cDC_LAMP3	CD8(Tn)	CCL17	CCR4	3,52744E-05	33073,8	1,28259E-01	46	3,38806E+00	149	3,08675E+00	4961	8,04349E-01	98313	0	61900
Myeloid_vs_CD8_Non-responder	pDC_LILRA4	CD8(Tc17)	SCT	ADRB2	3,52744E-05	29628,6	7,76109E-02	149	3,80530E+00	94	3,18921E+00	4178	8,31238E-01	81822	0	61900
Myeloid_vs_CD8_Non-responder	CD8(Tn)	Mono_CD16	ANXA1	FPR1	3,88113E-05	21693,6	6,16289E-03	16849	1,49751E+00	7663	2,53017E+00	11167	9,41332E-01	10889	0	61900
Myeloid_vs_CD8_Non-responder	Mono_CD14	CD8(GZMK+ Tex)	S100A8	CD69	4,01289E-05	15010	1,14998E-02	6355	2,52985E+00	818	3,29516E+00	3476	9,54396E-01	2501	0	61900
Myeloid_vs_CD8_Non-responder	pDC_LILRA4	CD8(GZMK+ Early Tem)	APP	RPSA	4,16286E-05	15983	1,26449E-02	5366	2,44516E+00	969	2,92122E+00	6414	9,48852E-01	5266	0	61900
Myeloid_vs_CD8_Non-responder	CD8(GZMK+ Early Tem)	cDC_CLEC9A	TNFSF9	HLA-DPA1	4,28760E-05	22870,8	6,33524E-03	16195	1,27349E+00	13186	2,98004E+00	5885	9,31225E-01	17188	0	61900
Myeloid_vs_CD8_Non-responder	CD8(GZMK+ Early Tem)	cDC(CD1C)	TNFSF9	HLA-DPA1	4,69516E-05	24260,8	6,21606E-03	16659	1,24418E+00	14109	2,53269E+00	11133	9,30614E-01	17503	0	61900
Myeloid_vs_CD8_Non-responder	CD8(Terminal Tex)	cDC_CLEC9A	XCL2	XCR1	4,97618E-05	31719	7,13027E-02	177	5,21951E+00	21	1,71043E+00	30751	8,56228E-01	65746	0	61900
Myeloid_vs_CD8_Non-responder	pDC_LILRA4	CD8(ISG+ T)	APP	RPSA	5,27422E-05	16175	1,24021E-02	5579	2,42496E+00	1005	2,88170E+00	6808	9,48379E-01	5583	0	61900
Myeloid_vs_CD8_Non-responder	Mono_CD14	CD8(GZMK+ Tem)	S100A8	CD69	5,46425E-05	15380,4	1,09824E-02	6869	2,50049E+00	870	3,16563E+00	4357	9,53384E-01	2906	0	61900
Myeloid_vs_CD8_Non-responder	CD8(GZMK+ Early Tem)	Macro_FOLR2+APOE+	TNFSF9	HLA-DPA1	5,57698E-05	24143	6,01065E-03	17534	1,19366E+00	15935	2,84023E+00	7230	9,29522E-01	18116	0	61900
Myeloid_vs_CD8_Non-responder	CD8(Temra)	cDC_CLEC9A	XCL2	XCR1	5,73315E-05	32061,8	6,98303E-02	190	5,21260E+00	22	1,68633E+00	31597	8,54939E-01	66600	0	61900
Myeloid_vs_CD8_Non-responder	cDC_LAMP3	CD8(GZMK+ Tem)	CCL19	CXCR3	5,79359E-05	22457,4	6,97767E-02	191	4,17609E+00	58	2,67202E+00	9268	8,93853E-01	40870	0	61900
Myeloid_vs_CD8_Non-responder	pDC_LILRA4	CD8(Tn)	APP	RPSA	5,90628E-05	16403,6	1,20217E-02	5927	2,39331E+00	1070	2,86116E+00	7005	9,47611E-01	6116	0	61900
Myeloid_vs_CD8_Non-responder	CD8(NME1+ T)	Macro_FOLR2+APOE+	HMGB1	CD163	5,94011E-05	20274,2	5,82651E-03	18346	1,72286E+00	4637	2,72192E+00	8601	9,45330E-01	7887	0	61900
Myeloid_vs_CD8_Non-responder	CD8(NME1+ T)	Macro_LYVE1	HMGB1	CD163	6,05759E-05	21436,2	6,23568E-03	16591	1,82278E+00	3765	2,14641E+00	18418	9,47058E-01	6507	0	61900
Myeloid_vs_CD8_Non-responder	pDC_LILRA4	CD8(IL7R+ZNF683+ Tm)	APP	RPSA	7,00931E-05	16793,4	1,13004E-02	6518	2,33331E+00	1227	2,86077E+00	7008	9,46054E-01	7314	0	61900
Myeloid_vs_CD8_Non-responder	pDC_LILRA4	CD8(Temra)	APP	RPSA	7,23235E-05	16412,6	1,21500E-02	5814	2,40399E+00	1050	2,82665E+00	7372	9,47874E-01	5927	0	61900
Myeloid_vs_CD8_Non-responder	cDC_LAMP3	CD8(Terminal Tex)	CCL22	DPP4	7,33905E-05	31411,6	6,51228E-02	215	4,11137E+00	62	3,15897E+00	4391	8,17417E-01	90490	0	61900
Myeloid_vs_CD8_Non-responder	CD8(GZMK+ Early Tem)	cDC(CD1C)	B2M	CD1C	7,34976E-05	19216,2	1,74759E-02	2764	1,97985E+00	2657	1,83557E+00	26738	9,55590E-01	2022	0	61900
Myeloid_vs_CD8_Non-responder	CD8(ITM2C+ T)	pDC_LILRA4	TNF	PTPRS	7,47606E-05	20800,8	6,39107E-02	217	3,49549E+00	127	2,89091E+00	6718	9,02478E-01	35042	0	61900
Myeloid_vs_CD8_Non-responder	CD8(NME1+ T)	cDC_LAMP3	TNFSF10	TNFRSF11B	7,65760E-05	53094,4	2,30119E-02	1622	2,25600E+00	1466	9,67756E-01	65809	7,34065E-01	134675	0	61900
Myeloid_vs_CD8_Non-responder	CD8(GZMK+ Early Tem)	Macro_OLFML3	TNFSF9	HLA-DPA1	7,71342E-05	26507,4	5,65791E-03	19148	1,10690E+00	19330	2,42324E+00	12901	9,27515E-01	19258	0	61900
Myeloid_vs_CD8_Non-responder	cDC_LAMP3	CD8(Tn)	CCL22	DPP4	9,21870E-05	31933,2	6,12915E-02	241	4,09794E+00	64	3,17348E+00	4289	8,12849E-01	93172	0	61900
Myeloid_vs_CD8_Non-responder	CD8(GZMK+ Early Tem)	Macro_LYVE1	TNFSF9	HLA-DPA1	9,21954E-05	27367,8	5,81111E-03	18413	1,14458E+00	17744	2,07869E+00	20032	9,28408E-01	18750	0	61900
Myeloid_vs_CD8_Non-responder	Macro_NLRP3	CD8(IL7R+ZNF683+ Tm)	S100A8	CD69	9,42862E-05	16140,8	1,13618E-02	6473	2,14969E+00	1844	2,77854E+00	7882	9,54133E-01	2605	0	61900
Myeloid_vs_CD8_Non-responder	cDC_LAMP3	CD8(IL7R+ZNF683+ Tm)	CCL19	CXCR3	9,52683E-05	23370,4	6,09329E-02	245	4,10925E+00	63	2,67253E+00	9262	8,87250E-01	45382	0	61900
Myeloid_vs_CD8_Non-responder	Macro_NLRP3	CD8(Temra)	IL1B	ADRB2	9,73151E-05	20316,2	1,94756E-02	2227	2,49549E+00	874	3,58370E+00	2041	9,03194E-01	34539	0	61900
Myeloid_vs_CD8_Non-responder	pDC_LILRA4	CD8(Tc17)	APP	RPSA	1,03925E-04	16933,8	1,08826E-02	6986	2,29855E+00	1328	2,92433E+00	6377	9,45084E-01	8078	0	61900
Myeloid_vs_CD8_Non-responder	CD8(Temra)	pDC_LILRA4	TNF	PTPRS	1,04815E-04	21567,6	5,93895E-02	257	3,45981E+00	137	2,74438E+00	8306	8,99201E-01	37238	0	61900
Myeloid_vs_CD8_Non-responder	CD8(NME1+ T)	Macro_NLRP3	HMGB1	THBD	1,05131E-04	23505,2	6,95522E-03	14088	1,64564E+00	5435	2,28340E+00	15538	9,25240E-01	20565	0	61900
Myeloid_vs_CD8_Non-responder	CD8(GZMK+ Early Tem)	pDC_LILRA4	TNF	PTPRS	1,05359E-04	22338	5,23881E-02	321	3,40456E+00	146	2,76141E+00	8106	8,93372E-01	41217	0	61900
Myeloid_vs_CD8_Non-responder	CD8(NME1+ T)	cDC_LAMP3	CD200R1	CD200	1,08529E-04	49074	3,33098E-02	789,5	2,04545E+00	2310,5	1,41168E+00	42770,5	7,27224E-01	137599,5	0	61900
Myeloid_vs_CD8_Non-responder	CD8(NME1+ T)	pDC_LILRA4	HMGB1	THBD	1,10929E-04	22333,8	6,87997E-03	14311	1,63195E+00	5624	2,68868E+00	9047	9,24863E-01	20787	0	61900
Myeloid_vs_CD8_Non-responder	pDC_LILRA4	CD8(GZMK+ Tex)	APP	RPSA	1,11299E-04	16952,8	1,14464E-02	6399	2,34545E+00	1204	2,75166E+00	8219	9,46380E-01	7042	0	61900
Myeloid_vs_CD8_Non-responder	CD8(Tn)	Macro_OLFML3	ANXA1	FPR1	1,16065E-04	25141	5,32757E-03	20976	1,33151E+00	11453	2,18213E+00	17637	9,37179E-01	13739	0	61900
Myeloid_vs_CD8_Non-responder	CD8(GZMK+ Tem)	cDC_CLEC9A	XCL2	XCR1	1,18251E-04	35412,6	5,74959E-02	273	5,15467E+00	23	1,47171E+00	40118	8,42467E-01	74749	0	61900
Myeloid_vs_CD8_Non-responder	CD8(GZMK+ Tex)	cDC_CLEC9A	XCL2	XCR1	1,19117E-04	34132	5,71880E-02	274	5,15322E+00	24	1,63603E+00	33476	8,42110E-01	74986	0	61900

Myeloid_vs_CD8_Post_NR

Myeloid_vs_CD8_Non-responder	pDC_LILRA4	CD8(ITM2C+ T)	SCT	ADRB2	1,19987E-04	32562,2	5,71565E-02	275	3,70889E+00	102	3,10465E+00	4823	8,08683E-01	95711	0	61900
Myeloid_vs_CD8_Non-responder	CD8(ZNF683+KLRB1+ T)	cDC(CD1C)	B2M	CD1C	1,21471E-04	17179,6	1,88282E-02	2399	2,30250E+00	1315	2,12643E+00	18870	9,57145E-01	1414	0	61900
Myeloid_vs_CD8_Non-responder	CD8(ZNF683+KLRB1+ T)	Macro_FOLR2+APOE+	CD99	CD81	1,27562E-04	24977,6	5,48817E-03	20084	1,34972E+00	10927	2,15684E+00	18206	9,37122E-01	13771	0	61900
Myeloid_vs_CD8_Non-responder	CD8(ZNF683+KLRB1+ T)	Macro_FOLR2+APOE+	CD99	PILRA	1,27651E-04	23872,2	5,66955E-03	19099	1,58102E+00	6324	2,19798E+00	17291	9,35597E-01	14747	0	61900
Myeloid_vs_CD8_Non-responder	CD8(Tc17)	pDC_LILRA4	TNF	PTPRS	1,27956E-04	21930	5,55989E-02	284	3,42990E+00	142	2,76759E+00	8020	8,96172E-01	39304	0	61900
Myeloid_vs_CD8_Non-responder	Macro_OLFML3	CD8(IL7R+ZNF683+ Tm)	CXCL10	CXCR3	1,28230E-04	20725	2,21032E-02	1770	2,44818E+00	962	3,49368E+00	2443	9,00232E-01	36550	0	61900
Myeloid_vs_CD8_Non-responder	Macro_OLFML3	CD8(GZMK+ Tem)	CXCL10	CXCR3	1,28386E-04	19822,4	2,53113E-02	1352	2,51502E+00	849	3,49316E+00	2444	9,06155E-01	32567	0	61900
Myeloid_vs_CD8_Non-responder	cDC_LAMP3	CD8(ISG+ T)	CCL19	CCR7	1,29762E-04	27280,4	5,55231E-02	286	4,09480E+00	65	2,68867E+00	9048	8,57238E-01	65103	0	61900
Myeloid_vs_CD8_Non-responder	pDC_LILRA4	CD8(ID2+CXCR4+ T)	APP	RPSA	1,31409E-04	17311,6	1,09784E-02	6878	2,30652E+00	1308	2,72344E+00	8571	9,45311E-01	7901	0	61900
Myeloid_vs_CD8_Non-responder	Mono_CD14	CD8(Tn)	VCAN	CD44	1,32628E-04	17186,6	1,14921E-02	6357	2,61204E+00	704	2,72250E+00	8591	9,44646E-01	8381	0	61900
Myeloid_vs_CD8_Non-responder	CD8(ZNF683+KLRB1+ T)	Mono_CD16	CD99	PILRA	1,34262E-04	23995,6	5,61753E-03	19377	1,56785E+00	6541	2,20034E+00	17245	9,35319E-01	14915	0	61900
Myeloid_vs_CD8_Non-responder	CD8(ZNF683+KLRB1+ T)	Mono_CD16	HLA-F	LILRB2	1,34418E-04	22904,2	5,74870E-03	18738	1,95908E+00	2768	2,62196E+00	9901	9,24067E-01	21214	0	61900
Myeloid_vs_CD8_Non-responder	Macro_OLFML3	CD8(Tn)	CXCL10	DPP4	1,35882E-04	33065,4	2,25993E-02	1690	2,36142E+00	1165	3,48073E+00	2491	8,04720E-01	98081	0	61900
Myeloid_vs_CD8_Non-responder	CD8(ZNF683+KLRB1+ T)	Mono_CD16	HLA-C	LILRB2	1,40757E-04	19002,4	5,19083E-03	21801	2,25831E+00	1458	2,68326E+00	9111	9,59058E-01	742	0	61900
Myeloid_vs_CD8_Non-responder	CD8(NME1+ T)	pDC_LILRA4	TNF	PTPRS	1,44506E-04	27182,8	3,14313E-02	872	3,23919E+00	203	2,37459E+00	13768	8,66484E-01	59171	0	61900
Myeloid_vs_CD8_Non-responder	pDC_LILRA4	CD8(ITM2C+ T)	APP	RPSA	1,45804E-04	17220,6	1,11894E-02	6634	2,32408E+00	1253	2,70648E+00	8799	9,45801E-01	7517	0	61900
Myeloid_vs_CD8_Non-responder	Macro_OLFML3	CD8(Terminal Tex)	CXCL10	DPP4	1,45864E-04	32465,2	2,40120E-02	1506	2,37485E+00	1127	3,46623E+00	2551	8,09440E-01	95242	0	61900
Myeloid_vs_CD8_Non-responder	Macro_NLRP3	CD8(EOMES+ NK-like)	S100A8	CD69	1,46197E-04	16521,2	1,09090E-02	6955	2,11719E+00	1969	2,70624E+00	8805	9,53235E-01	2977	0	61900
Myeloid_vs_CD8_Non-responder	CD8(Tn)	Macro_ISG15	ANXA1	FPR1	1,47400E-04	24511	5,15276E-03	22003	1,29677E+00	12510	2,49230E+00	11755	9,36190E-01	14387	0	61900
Myeloid_vs_CD8_Non-responder	Macro_ISG15	CD8(IL7R+ZNF683+ Tm)	CXCL10	CXCR3	1,48433E-04	22036,4	1,89606E-02	2357	2,11799E+00	1964	3,46369E+00	2566	8,93131E-01	41395	0	61900
Myeloid_vs_CD8_Non-responder	Macro_ISG15	CD8(GZMK+ Tem)	CXCL10	CXCR3	1,48951E-04	21014,8	2,17126E-02	1816	2,18483E+00	1713	3,46317E+00	2569	8,99428E-01	37076	0	61900
Myeloid_vs_CD8_Non-responder	CD8(ZNF683+KLRB1+ T)	Macro_FOLR2+APOE+	CD52	SIGLEC10	1,49760E-04	24362	7,14697E-03	13554	1,54286E+00	6874	1,99766E+00	22073	9,30823E-01	17409	0	61900
Myeloid_vs_CD8_Non-responder	CD8(ID2+CXCR4+ T)	cDC(CD1C)	B2M	CD1C	1,52606E-04	18102,6	1,81321E-02	2590	2,13642E+00	1900	1,98544E+00	22426	9,56366E-01	1697	0	61900
Myeloid_vs_CD8_Non-responder	cDC_LAMP3	CD8(NME1+ T)	CCL19	CXCR3	1,55371E-04	24973,4	5,32343E-02	313	4,05106E+00	68	2,44099E+00	12628	8,80315E-01	49958	0	61900
Myeloid_vs_CD8_Non-responder	pDC_LILRA4	CD8(Terminal Tex)	APP	RPSA	1,56860E-04	16841,4	1,19334E-02	6009	2,38597E+00	1083	2,69406E+00	8963	9,47428E-01	6252	0	61900
Myeloid_vs_CD8_Non-responder	cDC_LAMP3	CD8(ITM2C+ T)	CCL19	CXCR3	1,58359E-04	24619,4	5,29785E-02	316	4,04913E+00	70	2,56565E+00	10676	8,80061E-01	50135	0	61900
Myeloid_vs_CD8_Non-responder	CD8(NME1+ T)	pDC_LILRA4	HSP90B1	TLR9	1,60094E-04	32438	2,86910E-02	1051	2,15976E+00	1800	1,33528E+00	46268	8,78449E-01	51171	0	61900
Myeloid_vs_CD8_Non-responder	pDC_LILRA4	CD8(GZMK+ Tem)	APP	RPSA	1,60212E-04	17479,2	1,08559E-02	7021	2,29633E+00	1335	2,69074E+00	9011	9,45021E-01	8129	0	61900
Myeloid_vs_CD8_Non-responder	CD8(Tn)	cDC(CD1C)	ANXA1	FPR1	1,60575E-04	26078,4	5,33079E-03	20958	1,33215E+00	11428	1,98682E+00	22383	9,37197E-01	13723	0	61900
Myeloid_vs_CD8_Non-responder	Macro_ISG15	CD8(Tn)	CXCL10	DPP4	1,60638E-04	34911,4	1,93862E-02	2244	2,03122E+00	2380	3,45074E+00	2635	7,92388E-01	105398	0	61900
Myeloid_vs_CD8_Non-responder	CD8(GZMK+ Tex)	cDC(CD1C)	B2M	CD1C	1,60638E-04	18387,6	1,79196E-02	2635	2,08570E+00	2116	1,94646E+00	23498	9,56119E-01	1789	0	61900
Myeloid_vs_CD8_Non-responder	CD8(NME1+ T)	pDC_LILRA4	HMGB1	TLR9	1,61001E-04	22515,2	4,60080E-02	405	2,57161E+00	756	1,95274E+00	23320	9,16140E-01	26195	0	61900
Myeloid_vs_CD8_Non-responder	CD8(NME1+ T)	pDC_LILRA4	HRAS	TLR9	1,63376E-04	47389	6,31132E-02	226	2,23073E+00	1546	1,65229E+00	32860	7,20318E-01	140413	0	61900
Myeloid_vs_CD8_Non-responder	CD8(ITM2C+ T)	cDC(CD1C)	B2M	CD1C	1,64664E-04	18527,2	1,78298E-02	2657	2,06428E+00	2207	1,92793E+00	24035	9,56014E-01	1837	0	61900
Myeloid_vs_CD8_Non-responder	Macro_NLRP3	CD8(GZMK+ Early Tem)	S100A8	CD69	1,66566E-04	16985	1,00920E-02	7868	2,05852E+00	2243	2,68464E+00	9100	9,51468E-01	3814	0	61900
Myeloid_vs_CD8_Non-responder	CD8(Terminal Tex)	cDC(CD1C)	B2M	CD1C	1,68194E-04	18792,8	1,77792E-02	2676	2,05221E+00	2279	1,88581E+00	25252	9,55954E-01	1857	0	61900
Myeloid_vs_CD8_Non-responder	cDC_LAMP3	CD8(ISG+ T)	CCL19	CXCR3	1,68521E-04	24902,4	5,20247E-02	326	4,04192E+00	71	2,51138E+00	11473	8,79099E-01	50742	0	61900
Myeloid_vs_CD8_Non-responder	CD8(ZNF683+KLRB1+ T)	Mono_CD16	HLA-A	LILRB2	1,68698E-04	19723,8	5,06584E-03	22605	2,13968E+00	1886	2,51773E+00	11356	9,58646E-01	872	0	61900
Myeloid_vs_CD8_Non-responder	Macro_NLRP3	CD8(Tn)	S100A8	CD69	1,68822E-04	16868	1,03333E-02	7597	2,07585E+00	2154	2,68200E+00	9131	9,52011E-01	3558	0	61900
Myeloid_vs_CD8_Non-responder	Macro_FOLR2+APOE+	CD8(Temra)	CXCL9	KIR2DL3	1,71017E-04	30375	1,28565E-01	45	2,71936E+00	566	3,43944E+00	2691	8,23482E-01	86673	0	61900
Myeloid_vs_CD8_Non-responder	CD8(GZMK+ Early Tem)	pDC_LILRA4	TNF	TNFRSF21	1,71176E-04	24463,8	1,61739E-02	3250	2,22755E+00	1554	2,67971E+00	9163	8,85647E-01	46452	0	61900
Myeloid_vs_CD8_Non-responder	CD8(ZNF683+KLRB1+ T)	Macro_ISG15	CD99	PILRA	1,71705E-04	24918,8	5,35207E-03	20847	1,50063E+00	7600	2,14295E+00	18499	9,33839E-01	15748	0	61900
Myeloid_vs_CD8_Non-responder	Macro_ISG15	CD8(Terminal Tex)	CXCL10	DPP4	1,74636E-04	34280	2,05980E-02	1990	2,04465E+00	2317	3,43623E+00	2710	7,97331E-01	102483	0	61900
Myeloid_vs_CD8_Non-responder	CD8(IL7R+ZNF683+ Tm)	cDC(CD1C)	B2M	CD1C	1,75983E-04	18944,2	1,76312E-02	2717	2,01691E+00	2457	1,87004E+00	25718	9,55778E-01	1929	0	61900
Myeloid_vs_CD8_Non-responder	cDC_LAMP3	CD8(NME1+ T)	CCL22	DPP4	1,78998E-04	33772,6	5,11742E-02	336	4,06248E+00	67	3,08902E+00	4946	7,98738E-01	101614	0	61900
Myeloid_vs_CD8_Non-responder	Mono_CD14	CD8(ITM2C+ T)	S100A8	CD69	1,80295E-04	16236,4	9,13640E-03	9284	2,39572E+00	1060	3,24048E+00	3846	9,49119E-01	5092	0	61900
Myeloid_vs_CD8_Non-responder	CD8(Temra)	cDC(CD1C)	B2M	CD1C	1,81239E-04	19018,2	1,75570E-02	2744	1,99919E+00	2554	1,86257E+00	25924	9,55688E-01	1969	0	61900
Myeloid_vs_CD8_Non-responder	CD8(Tn)	Macro_ISG15	ANXA1	FPR2_FPR3	1,81971E-04	23452,8	9,31822E-03	8980	1,58216E+00	6304	2,20500E+00	17130	9,21294E-01	22950	0	61900
Myeloid_vs_CD8_Non-responder	CD8(Tc17)	cDC(CD1C)	B2M	CD1C	1,83015E-04	19074,2	1,75017E-02	2753	1,98599E+00	2621	1,85710E+00	26092	9,55622E-01	2005	0	61900
Myeloid_vs_CD8_Non-responder	CD8(GZMK+ Early Tem)	cDC_CLEC9A	XCL2	XCR1	1,84355E-04	36142,6	5,07526E-02	341	5,12299E+00	25	1,51277E+00	38400	8,34011E-01	80047	0	61900
Myeloid_vs_CD8_Non-responder	CD8(GZMK+ Tem)	cDC(CD1C)	B2M	CD1C	1,86800E-04	19144,6	1,74521E-02	2772	1,97417E+00	2699	1,84907E+00	26317	9,55561E-01	2035	0	61900
Myeloid_vs_CD8_Non-responder	Macro_FOLR2+APOE+	CD8(Tc17)	CXCL9	DPP4	1,87000E-04	25773,2	5,55402E-02	285	2,69153E+00	590	3,42476E+00	2773	8,60099E-01	63318	0	61900
Myeloid_vs_CD8_Non-responder	CD8(GZMK+ Tex)	pDC_LILRA4	CXCL13	CXCR3	1,87589E-04	22221,4	3,06459E-02	917	1,16333E+00	17040	2,75677E+00	8160	9,21079E-01	23090	0	61900
Myeloid_vs_CD8_Non-responder	Macro_NLRP3	CD8(ID2+CXCR4+ T)	S100A8	CD69	1,87942E-04	17085,2	1,00123E-02	7960	2,05280E+00	2272	2,66265E+00	9382	9,51285E-01	3912	0	61900
Myeloid_vs_CD8_Non-responder	CD8(EOMES+ NK-like)	cDC(CD1C)	B2M	CD1C	1,88409E-04	19178,4	1,74383E-02	2780	1,97088E+00	2715	1,84489E+00	26451	9,55545E-01	2046	0	61900
Myeloid_vs_CD8_Non-responder	Macro_FOLR2+APOE+	CD8(EOMES+ NK-like)	CXCL9	KIR2DL3	1,89015E-04	31277,6	1,16927E-01	61	2,67162E+00	622	3,42255E+00	2783	8,16480E-01	91022	0	61900
Myeloid_vs_CD8_Non-responder	CD8(LAYN+ T)	cDC(CD1C)	B2M	CD1C	1,89622E-04	19201,6	1,74152E-02	2786	1,96535E+00	2741	1,84243E+00	26523	9,55516E-01	2058	0	61900
Myeloid_vs_CD8_Non-responder	pDC_LILRA4	CD8(LAYN+ T)	APP	RPSA	2,01861E-04	17320,4	1,12697E-02	6548	2,33076E+00	1233	2,64930E+00	9553	9,45984E-01	7368	0	61900
Myeloid_vs_CD8_Non-responder	CD8(NME1+ T)	Mono_CD16	CD99	PILRA	2,07166E-04	26994	4,94583E-03	23397	1,42836E+00	9006	1,94461E+00	23553	9,31359E-01	17114	0	61900
Myeloid_vs_CD8_Non-responder	CD8(NME1+ T)	cDC_CLEC9A	FLT3LG	FLT3	2,08007E-04	40082,6	2,32066E-02	1595	2,54686E+00	794	1,58689E+00	35358	8,00179E-01	100766	0	61900
Myeloid_vs_CD8_Non-responder	CD8(NME1+ T)	Macro_FOLR2+APOE+	CD99	PILRA	2,10263E-04	26858,2	4,99162E-03	23093	1,44153E+00	8730	1,94225E+00	23623	9,31653E-01	16945	0	61900
Myeloid_vs_CD8_Non-responder	CD8(GZMK+ Early Tem)	Macro_ISG15	CCL4	CCR1	2,11557E-04	24122,2	1,16636E-02	6216	1,54216E+00	6889	1,94100E+00	23652	9,22898E-01	21954	0	61900
Myeloid_vs_CD8_Non-responder	CD8(NME1+ T)	cDC_CLEC9A	XCL1	XCR1	2,11689E-04	43614,2	4,07867E-02	516	5,06938E+00	30	1,14143E+00	56115	8,02263E-01	99510	0	61900
Myeloid_vs_CD8_Non-responder	CD8(GZMK+ Early Tem)	Mono_CD16	HLA-F	LILRB2	2,12857E-04	24641,6	5,14346E-03	22061	1,89239E+00	3212	2,45615E+00	12354	9,20070E-01	23681	0	61900
Myeloid_vs_CD8_Non-responder	CD8(NME1+ T)	cDC_CLEC9A	XCL2	XCR1	2,12999E-04	39733	4,46087E-02	428	5,09414E+00	28	1,24896E+00	50515	8,24886E-01	85794	0	61900
Myeloid_vs_CD8_Non-responder	CD8(IL7R+ZNF683+ Tm)	Mono_CD16	ANXA1	FPR1	2,13262E-04	26695,4	5,17333E-03	21895	1,32470E+00	11674	1,93980E+00	23690	9,36309E-01	14318	0	61900
Myeloid_vs_CD8_Non-responder	Macro_FOLR2+APOE+	CD8(ISG+ T)	C3	IFITM1	2,13380E-04	18714,6	1,03810E-02	7536	1,55517E+00	6696	2,79049E+00	7753	9,42994E-01	9688	0	61900
Myeloid_vs_CD8_Non-responder	CD8(ISG+ T)	Mono_CD16	HLA-F	LILRB2	2,14751E-04	24819,2	5,13320E-03	22131	1,89126E+00	3223	2,41120E+00	13119	9,19997E-01	23723	0	61900
Myeloid_vs_CD8_Non-responder	CD8(EOMES+ NK-like)	Macro_FOLR2+APOE+	CD99	PILRA	2,14797E-04	26825,6	4,92503E-03	23543	1,42782E+00	9020	1,98368E+00	22476	9,31224E-01	17189	0	61900
Myeloid_vs_CD8_Non-responder	CD8(GZMK+ Tem)	Mono_CD16	HLA-A	LILRB2	2,14978E-04	20402	4,89734E-03	23728	2,03862E+00	2355	2,42315E+00	12905	9,57970E-01	1122	0	61900
Myeloid_vs_CD8_Non-responder	CD8(NME1+ T)	pDC_LILRA4	COPA	P2RY6	2,15916E-04	28362,4	8,88295E-03	9717	2,67637E+00	612	3,01329E+00	5587	8,58984E-01	63996	0	61900
Myeloid_vs_CD8_Non-responder	CD8(Temra)	Mono_CD16	HLA-C	LILRB2	2,16204E-04	20116	4,89373E-03	23755	2,08687E+00	2112	2,49775E+00	11658	9,57885E-01	1155	0	61900
Myeloid_vs_CD8_Non-responder	Macro_OLFML3	CD8(NME1+ T)	CXCL10	DPP4	2,16739E-04	35023	1,88689E-02	2384	2,32596E+00	1244	3,39628E+00	2914	7,90155E-01	106673	0	61900
Myeloid_vs_CD8_Non-responder	CD8(Tn)	Macro_ISG15	RPS19	C5AR1	2,24332E-04	24625,4	4,87045E-03	23931	1,42110E+00	9166	1,97777E+00	22633	9,48501E-01	5497	0	61900
Myeloid_vs_CD8_Non-responder	CD8(EOMES+ NK-like)	Mono_CD16	CD99	PILRA	2,26450E-04	26968	4,87985E-03	23866	1,41465E+00	9319	1,98605E+00	22406	9,30928E-01	17349	0	61900
Myeloid_vs_CD8_Non-responder	CD8(Tn)	Mono_CD16	RPS19	C5AR1	2,26875E-04	24663,8	4,86257E-03	23985	1,41893E+00	9222	1,97538E+00	22688	9,48462E-01	5524	0	61900
Myeloid_vs_CD8_Non-responder	CD8(GZMK+ Tex)	Mono_CD16	HLA-F	LILRB2	2,28012E-04	24964,2	5,06851E-03	22583	1,88413E+00	3291	2,41557E+00	13038	9,19529E-01	24009	0	61900

Myeloid_vs_CD8_Non-responder	Macro_OLFML3	CD8(ITM2C+ T)	CXCL10	CXCR3	2,29596E-04	21834,8	1,92178E-02	2283	2,38806E+00	1078	3,38680E+00	2971	8,93772E-01	40942	0	61900
Myeloid_vs_CD8_Non-responder	CD8(ITM2C+ T)	cDC_CLEC9A	XCL2	XCR1	2,30038E-04	37749,6	4,80965E-02	381	5,11052E+00	26	1,38473E+00	44001	8,30256E-01	82440	0	61900
Myeloid_vs_CD8_Non-responder	CD8(ISG+ T)	Macro_ISG15	RPS19	C5AR1	2,40777E-04	24993,8	4,82749E-03	24272	1,41046E+00	9408	1,93695E+00	23757	9,48284E-01	5632	0	61900
Myeloid_vs_CD8_Non-responder	CD8(ISG+ T)	Mono_CD16	RPS19	C5AR1	2,42768E-04	25031,2	4,81968E-03	24312	1,40829E+00	9453	1,93456E+00	23837	9,48245E-01	5654	0	61900
Myeloid_vs_CD8_Non-responder	Macro_NLRP3	CD8(ISG+ T)	S100A8	CD69	2,42920E-04	17549,2	9,46111E-03	8762	2,01323E+00	2474	2,61214E+00	10011	9,49956E-01	4599	0	61900
Myeloid_vs_CD8_Non-responder	CD8(GZMK+ Early Tem)	pDC_LILRA4	BST2	LILRA4	2,44555E-04	27012,8	8,70179E-03	10028	2,67339E+00	620	2,79417E+00	7709	8,72990E-01	54807	0	61900
Myeloid_vs_CD8_Non-responder	CD8(NME1+ T)	cDC_CLEC9A	HMGB1	THBD	2,44721E-04	27087	5,83546E-03	18297	1,44193E+00	8722	1,99454E+00	22165	9,18938E-01	24351	0	61900
Myeloid_vs_CD8_Non-responder	CD8(GZMK+ Tex)	cDC_CLEC9A	XCL1	XCR1	2,44723E-04	39003,2	4,69320E-02	393	5,09451E+00	27	1,48170E+00	39697	8,13160E-01	92999	0	61900
Myeloid_vs_CD8_Non-responder	CD8(IL7R+ZNF683+ Tm)	Macro_FOLR2+APOE+	CD99	PILRA	2,45879E-04	27394,4	4,81228E-03	24374	1,40462E+00	9538	1,94314E+00	23594	9,30478E-01	17566	0	61900
Myeloid_vs_CD8_Non-responder	CD8(GZMK+ Tem)	cDC_CLEC9A	TNFSF9	HLA-DPA1	2,47903E-04	27080	4,80794E-03	24414	1,16134E+00	17108	2,66226E+00	9385	9,21849E-01	22593	0	61900
Myeloid_vs_CD8_Non-responder	Macro_NLRP3	CD8(Temra)	S100A8	CD69	2,49508E-04	16973,2	1,05089E-02	7405	2,08846E+00	2105	2,60678E+00	10079	9,52395E-01	3377	0	61900
Myeloid_vs_CD8_Non-responder	CD8(Temra)	pDC_LILRA4	TNF	TNFRSF21	2,54733E-04	23495	1,83355E-02	2524	2,28280E+00	1383	2,66268E+00	9381	8,91847E-01	42287	0	61900
Myeloid_vs_CD8_Non-responder	CD8(Temra)	pDC_LILRA4	BST2	LILRA4	2,57627E-04	25543,8	9,90840E-03	8094	2,70184E+00	580	2,86314E+00	6988	8,80016E-01	50157	0	61900
Myeloid_vs_CD8_Non-responder	Mono_CD14	CD8(IL7R+ZNF683+ Tm)	VCAN	CD44	2,57727E-04	18054,8	1,05828E-02	7317	2,53919E+00	807	2,60068E+00	10162	9,42451E-01	10088	0	61900
Myeloid_vs_CD8_Non-responder	Macro_ISG15	CD8(ZNF683+KLRB1+ T)	S100A8	CD69	2,57928E-04	18652,6	8,62962E-03	10164	1,54106E+00	6909	2,74856E+00	8258	9,47724E-01	6032	0	61900
Myeloid_vs_CD8_Non-responder	CD8(Temra)	pDC_LILRA4	COPA	P2RY6	2,59234E-04	27824	9,22690E-03	9115	2,68606E+00	598	3,08593E+00	4968	8,61269E-01	62539	0	61900
Myeloid_vs_CD8_Non-responder	CD8(GZMK+ Tem)	Mono_CD16	HLA-C	LILRB2	2,59434E-04	20707	4,77834E-03	24637	2,02028E+00	2439	2,40415E+00	13235	9,57401E-01	1324	0	61900
Myeloid_vs_CD8_Non-responder	cDC_CLEC9A	CD8(GZMK+ Tem)	HLA-DQB1	LAG3	2,60949E-04	17711,8	8,61630E-03	10194	1,56362E+00	6584	3,02190E+00	5503	9,50387E-01	4378	0	61900
Myeloid_vs_CD8_Non-responder	cDC_LAMP3	CD8(GZMK+ Early Tem)	CCL19	CXCR3	2,61143E-04	25959	4,59840E-02	406	3,99627E+00	74	2,46287E+00	12249	8,72385E-01	55166	0	61900
Myeloid_vs_CD8_Non-responder	CD8(IL7R+ZNF683+ Tm)	Mono_CD16	CD99	PILRA	2,63087E-04	27544,4	4,76813E-03	24706	1,39145E+00	9870	1,94550E+00	23526	9,30180E-01	17720	0	61900
Myeloid_vs_CD8_Non-responder	CD8(EOMES+ NK-like)	Macro_FOLR2+APOE+	CD99	CD81	2,63460E-04	28474,2	4,76748E-03	24713	1,19653E+00	15833	1,94254E+00	23613	9,32844E-01	16312	0	61900
Myeloid_vs_CD8_Non-responder	CD8(GZMK+ Tex)	Mono_CD16	HLA-A	LILRB2	2,63514E-04	21027	4,76733E-03	24714	1,96066E+00	2763	2,33795E+00	14447	9,57425E-01	1311	0	61900
Myeloid_vs_CD8_Non-responder	CD8(EOMES+ NK-like)	pDC_LILRA4	CCL5	CXCR3	2,66836E-04	24551,4	1,10791E-02	6766	9,91610E-01	24643	1,96202E+00	23068	9,47227E-01	6380	0	61900
Myeloid_vs_CD8_Non-responder	CD8(GZMK+ Early Tem)	Mono_CD16	HLA-A	LILRB2	2,68509E-04	21061	4,75507E-03	24807	1,95331E+00	2807	2,33727E+00	14453	9,57373E-01	1338	0	61900
Myeloid_vs_CD8_Non-responder	CD8(NME1+ T)	Macro_FOLR2+APOE+	CD99	CD81	2,69810E-04	28467	4,83194E-03	24234	1,21023E+00	15296	1,90111E+00	24831	9,33263E-01	16074	0	61900
Myeloid_vs_CD8_Non-responder	Macro_ISG15	CD8(ITM2C+ T)	CXCL10	CXCR3	2,70143E-04	23291,8	1,64855E-02	3124	2,05787E+00	2246	3,35681E+00	3138	8,86269E-01	46051	0	61900
Myeloid_vs_CD8_Non-responder	CD8(ZNF683+KLRB1+ T)	Macro_ISG15	HLA-C	LILRB1	2,74240E-04	23510,2	4,74394E-03	24912	1,69565E+00	4904	2,09367E+00	19632	9,47498E-01	6203	0	61900
Myeloid_vs_CD8_Non-responder	cDC_CLEC9A	CD8(GZMK+ Tem)	HLA-DQA1	LAG3	2,75505E-04	18278,2	8,53281E-03	10335	1,51136E+00	7395	2,91864E+00	6448	9,48778E-01	5313	0	61900
Myeloid_vs_CD8_Non-responder	CD8(Temra)	Macro_ISG15	RPS19	C5AR1	2,81246E-04	25369,4	4,72719E-03	25038	1,38560E+00	10009	1,93236E+00	23899	9,47767E-01	6001	0	61900
Myeloid_vs_CD8_Non-responder	Macro_FOLR2+APOE+	CD8(GZMK+ Tem)	HLA-DQA1	LAG3	2,82844E-04	18260,4	8,49771E-03	10404	1,50539E+00	7507	2,95414E+00	6109	9,48677E-01	5382	0	61900
Myeloid_vs_CD8_Non-responder	CD8(Temra)	Mono_CD16	RPS19	C5AR1	2,85029E-04	25413,6	4,71955E-03	25105	1,38343E+00	10062	1,92997E+00	23972	9,47727E-01	6029	0	61900
Myeloid_vs_CD8_Non-responder	CD8(GZMK+ Tem)	cDC(CD1C)	TNFSF9	HLA-DPA1	2,86166E-04	29052,2	4,71749E-03	25125	1,13203E+00	18264	2,21491E+00	16935	9,21162E-01	23037	0	61900
Myeloid_vs_CD8_Non-responder	CD8(ISG+ T)	Mono_CD16	HLA-A	LILRB2	2,87993E-04	21229	4,71391E-03	25157	1,92862E+00	2961	2,32403E+00	14725	9,57195E-01	1402	0	61900
Myeloid_vs_CD8_Non-responder	CD8(GZMK+ Tex)	Macro_ISG15	RPS19	C5AR1	2,90232E-04	25541,6	4,70928E-03	25196	1,38116E+00	10127	1,91438E+00	24420	9,47673E-01	6065	0	61900
Myeloid_vs_CD8_Non-responder	CD8(ZNF683+KLRB1+ T)	Mono_CD16	B2M	LILRB2	2,90405E-04	20255,6	4,70924E-03	25199	2,12112E+00	1949	2,47198E+00	12095	9,63024E-01	135	0	61900
Myeloid_vs_CD8_Non-responder	CD8(NME1+ T)	Macro_ISG15	CD99	PILRA	2,91155E-04	28178	4,71211E-03	25180	1,36114E+00	10643	1,88722E+00	25212	9,29795E-01	17955	0	61900
Myeloid_vs_CD8_Non-responder	CD8(ISG+ T)	Mono_CD16	HLA-B	LILRB2	2,92775E-04	20735,2	4,70302E-03	25240	1,99764E+00	2562	2,40379E+00	13241	9,59098E-01	733	0	61900
Myeloid_vs_CD8_Non-responder	CD8(GZMK+ Tex)	Mono_CD16	RPS19	C5AR1	2,93414E-04	25580,2	4,70167E-03	25251	1,37899E+00	10163	1,91200E+00	24485	9,47633E-01	6102	0	61900
Myeloid_vs_CD8_Non-responder	CD8(ITM2C+ T)	cDC_CLEC9A	TNFSF9	HLA-DPA1	2,95398E-04	18870,4	9,05889E-03	9414	1,47351E+00	8081	3,15362E+00	4438	9,41831E-01	10519	0	61900
Myeloid_vs_CD8_Non-responder	CD8(GZMK+ Early Tem)	Macro_IER3	TNFSF9	HLA-DPA1	2,96038E-04	31034,6	5,13861E-03	22090	9,79182E-01	25296	1,90370E+00	24757	9,24211E-01	21130	0	61900
Myeloid_vs_CD8_Non-responder	Macro_ISG15	CD8(NME1+ T)	CXCL10	DPP4	2,98183E-04	36917,4	1,61862E-02	3244	1,99576E+00	2568	3,36628E+00	3090	7,77158E-01	113785	0	61900
Myeloid_vs_CD8_Non-responder	pDC_LILRA4	CD8(EOMES+ NK-like)	APP	RPSA	2,98740E-04	18248	9,61575E-03	8514	2,19315E+00	1682	2,72224E+00	8595	9,41783E-01	10549	0	61900
Myeloid_vs_CD8_Non-responder	CD8(Tn)	Macro_FOLR2+APOE+	RPS19	C5AR1	3,03425E-04	25627,6	4,68165E-03	25421	1,36908E+00	10414	1,92111E+00	24220	9,47527E-01	6183	0	61900
Myeloid_vs_CD8_Non-responder	Macro_OLFML3	CD8(ISG+ T)	CXCL10	CXCR3	3,03680E-04	22042	1,88718E-02	2381	2,38086E+00	1105	3,33253E+00	3264	8,92906E-01	41560	0	61900
Myeloid_vs_CD8_Non-responder	Mono_CD14	CD8(GZMK+ Tem)	VCAN	CD44	3,06191E-04	18050	1,07385E-02	7166	2,55166E+00	788	2,56950E+00	10615	9,42845E-01	9781	0	61900
Myeloid_vs_CD8_Non-responder	CD8(ZNF683+KLRB1+ T)	Mono_CD16	HLA-C	LILRA3	3,07927E-04	22972,4	1,25661E-02	5426	1,89405E+00	3205	1,87824E+00	25496	9,28262E-01	18835	0	61900
Myeloid_vs_CD8_Non-responder	CD8(ZNF683+KLRB1+ T)	Macro_ISG15	HLA-F	LILRB2	3,08895E-04	26288,2	4,76184E-03	24753	1,62671E+00	5696	2,38626E+00	13580	9,17189E-01	25512	0	61900
Myeloid_vs_CD8_Non-responder	CD8(IL7R+ZNF683+ Tm)	Macro_FOLR2+APOE+	CD99	CD81	3,14259E-04	29132,6	4,65833E-03	25600	1,17333E+00	16660	1,90200E+00	24805	9,32115E-01	16698	0	61900
Myeloid_vs_CD8_Non-responder	CD8(Tn)	Macro_IER3	ANXA1	FPR1	3,14750E-04	26852,4	5,29079E-03	21195	1,32420E+00	11695	1,87361E+00	25608	9,36975E-01	13864	0	61900
Myeloid_vs_CD8_Non-responder	CD8(GZMK+ Early Tem)	Mono_CD16	HLA-B	LILRB2	3,15181E-04	20995,2	4,65658E-03	25615	1,96544E+00	2740	2,36622E+00	13926	9,58903E-01	795	0	61900
Myeloid_vs_CD8_Non-responder	Macro_ISG15	CD8(Tc17)	S100A8	CD69	3,15872E-04	18942,8	8,35272E-03	10699	1,50665E+00	7480	2,76783E+00	8017	9,46910E-01	6618	0	61900
Myeloid_vs_CD8_Non-responder	CD8(EOMES+ NK-like)	Macro_ISG15	CD99	PILRA	3,18517E-04	28157,8	4,64925E-03	25669	1,34744E+00	10997	1,92866E+00	24014	9,29355E-01	18209	0	61900
Myeloid_vs_CD8_Non-responder	CD8(IL7R+ZNF683+ Tm)	Mono_CD14	ANXA1	FPR1	3,20882E-04	26321,8	5,41922E-03	20484	1,38291E+00	10076	1,87028E+00	25707	9,37679E-01	13442	0	61900
Myeloid_vs_CD8_Non-responder	Mono_CD14	CD8(LAYN+ T)	VCAN	CD44	3,21624E-04	17972,6	1,09072E-02	6957	2,56518E+00	768	2,56041E+00	10748	9,43264E-01	9490	0	61900
Myeloid_vs_CD8_Non-responder	CD8(GZMK+ Tex)	pDC_LILRA4	CCL5	CXCR3	3,22132E-04	25308,8	1,08647E-02	7008	9,70352E-01	25727	1,88876E+00	25165	9,46737E-01	6744	0	61900
Myeloid_vs_CD8_Non-responder	CD8(ISG+ T)	Macro_FOLR2+APOE+	RPS19	C5AR1	3,22758E-04	26022,2	4,64036E-03	25737	1,35844E+00	10708	1,88029E+00	25430	9,47306E-01	6336	0	61900
Myeloid_vs_CD8_Non-responder	Mono_CD14	CD8(GZMK+ Early Tem)	VCAN	CD44	3,23638E-04	18189,2	1,05594E-02	7351	2,53731E+00	811	2,55950E+00	10765	9,42390E-01	10119	0	61900
Myeloid_vs_CD8_Non-responder	CD8(EOMES+ NK-like)	Mono_CD16	HLA-A	LILRB2	3,24644E-04	21676	4,63596E-03	25767	1,88187E+00	3311	2,26572E+00	15889	9,56852E-01	1513	0	61900
Myeloid_vs_CD8_Non-responder	CD8(ZNF683+KLRB1+ T)	Macro_ISG15	HLA-A	LILRB1	3,27806E-04	24939,2	4,62971E-03	25817	1,57702E+00	6384	1,92814E+00	24030	9,46976E-01	6565	0	61900
Myeloid_vs_CD8_Non-responder	CD8(ZNF683+KLRB1+ T)	Mono_CD16	HLA-B	LILRB2	3,28250E-04	21194,8	4,62859E-03	25824	1,94603E+00	2865	2,33237E+00	14561	9,58784E-01	824	0	61900
Myeloid_vs_CD8_Non-responder	CD8(GZMK+ Early Tem)	cDC_CLEC9A	XCL1	XCR1	3,29239E-04	40934,6	4,34310E-02	456	5,08019E+00	29	1,34744E+00	45723	8,07199E-01	96565	0	61900
Myeloid_vs_CD8_Non-responder	CD8(ZNF683+KLRB1+ T)	Macro_FOLR2+APOE+	HMGB1	CD163	3,33045E-04	24949,2	4,62195E-03	25899	1,42952E+00	8983	2,28700E+00	15463	9,39028E-01	12501	0	61900
Myeloid_vs_CD8_Non-responder	CD8(ZNF683+KLRB1+ T)	Mono_CD16	CD52	SIGLEC10	3,33302E-04	27651,8	5,92760E-03	17900	1,32685E+00	11616	1,86326E+00	25903	9,24552E-01	20940	0	61900
Myeloid_vs_CD8_Non-responder	cDC_LAMP3	CD8(Tn)	CCL19	CXCR3	3,33574E-04	26492,2	4,33030E-02	459	3,97601E+00	76	2,44340E+00	12589	8,69003E-01	57437	0	61900
Myeloid_vs_CD8_Non-responder	CD8(NME1+ T)	Macro_ISG15	HMGB1	TLR2	3,34075E-04	29869,6	4,62020E-03	25915	1,27071E+00	13268	1,89412E+00	25018	9,20812E-01	23247	0	61900
Myeloid_vs_CD8_Non-responder	Mono_CD14	CD8(NME1+ T)	S100A8	CD69	3,34333E-04	17347,2	8,26928E-03	10854	2,34651E+00	1202	2,97168E+00	5960	9,46657E-01	6820	0	61900
Myeloid_vs_CD8_Non-responder	cDC_LAMP3	CD8(Terminal Tex)	CCL19	CXCR3	3,35025E-04	26885,8	4,32402E-02	460	3,97553E+00	77	2,33517E+00	14506	8,68921E-01	57486	0	61900
Myeloid_vs_CD8_Non-responder	CD8(ITM2C+ T)	cDC(CD1C)	TNFSF9	HLA-DPA1	3,40455E-04	19997,2	8,88848E-03	9705	1,44419E+00	8673	2,70627E+00	8804	9,41309E-01	10904	0	61900
Myeloid_vs_CD8_Non-responder	CD8(ZNF683+KLRB1+ T)	pDC_LILRA4	HMGB1	THBD	3,41029E-04	27112,6	5,45762E-03	20264	1,33861E+00	11233	2,25376E+00	16144	9,16409E-01	26022	0	61900
Myeloid_vs_CD8_Non-responder	Macro_NLRP3	CD8(Tc17)	IL1B	ADRB2	3,42545E-04	21947,2	1,58386E-02	3399	2,41149E+00	1035	3,48928E+00	2461	8,93773E-01	40941	0	61900
Myeloid_vs_CD8_Non-responder	CD8(GZMK+ Tex)	Mono_CD16	HLA-B	LILRB2	3,42604E-04	21296,6	4,60247E-03	26046	1,92793E+00	2965	2,32470E+00	14710	9,58672E-01	862	0	61900
Myeloid_vs_CD8_Non-responder	CD8(IL7R+ZNF683+ Tm)	Mono_CD16	HLA-A	LILRB2	3,43526E-04	21891,6	4,60084E-03	26060	1,86081E+00	3466	2,23783E+00	16473	9,56695E-01	1559	0	61900
Myeloid_vs_CD8_Non-responder	pDC_LILRA4	CD8(IL7R+ZNF683+ Tm)	APP	PTGER2	3,46150E-04	25826	1,94346E-02	2235	2,46132E+00	931	3,30548E+00	3411	8,64204E-01	60653	0	61900
Myeloid_vs_CD8_Non-responder	CD8(GZMK+ Tem)	Mono_CD16	HLA-B	LILRB2	3,47765E-04	21461,2	4,59272E-03	26124	1,92117E+00	3012	2,29114E+00	15390	9,58630E-01	880	0	61900
Myeloid_vs_CD8_Non-responder	Macro_ISG15	CD8(ISG+ T)	CXCL10	CXCR3	3,51300E-04	23501,4	1,61887E-02	3242	2,05066E+00	2288	3,30253E+00	3428	8,85350E-01	46649	0	61900
Myeloid_vs_CD8_Non-responder	CD8(ID2+CXCR4+ T)	Mono_CD16	HLA-B	LILRB2	3,56912E-04	21261,6	4,62623E-03	25852	1,94440E+00	2876	2,31768E+00	14851	9,58773E-01	829	0	61900
Myeloid_vs_CD8_Non-responder	CD8(ID2+CXCR4+ T)	Mono_CD16	HLA-A	LILRB2	3,57320E-04	21021,6	4,75721E-03	24789	1,95459E+00	2798	2,34595E+00	14287	9,57382E-01	1334	0	61900

Myeloid_vs_CD8_Non-responder	CD8(ID2+CXCR4+ T)	Mono_CD16	HLA-C	LILRB2	3,57660E-04	21612,2	4,58192E-03	26218	1,90694E+00	3116	2,30082E+00	15204	9,56537E-01	1623	0	61900
Myeloid_vs_CD8_Non-responder	CD8(GZMK+ Tex)	pDC_LILRA4	HMGB1	THBD	3,61489E-04	27566,2	5,39218E-03	20627	1,32512E+00	11664	2,19664E+00	17313	9,15946E-01	26327	0	61900
Myeloid_vs_CD8_Non-responder	CD8(ZNF683+KLRB1+ T)	Macro_NLRP3	HMGB1	THBD	3,62382E-04	28953,6	5,51731E-03	19924	1,35230E+00	10858	1,84848E+00	26340	9,16825E-01	25746	0	61900
Myeloid_vs_CD8_Non-responder	CD8(GZMK+ Tex)	Macro_FOLR2+APOE+	HMGB1	CD163	3,63691E-04	25387	4,56654E-03	26359	1,41603E+00	9285	2,22988E+00	16629	9,38682E-01	12762	0	61900
Myeloid_vs_CD8_Non-responder	CD8(GZMK+ Tem)	Macro_FOLR2+APOE+	TNFSF9	HLA-DPA1	3,67084E-04	28762	4,56160E-03	26408	1,08150E+00	20454	2,52246E+00	11280	9,19933E-01	23768	0	61900
Myeloid_vs_CD8_Non-responder	CD8(Tn)	Macro_NLRP3	RPS19	C5AR1	3,70152E-04	25481	4,84515E-03	24139	1,41413E+00	9329	1,84488E+00	26452	9,48374E-01	5585	0	61900
Myeloid_vs_CD8_Non-responder	CD8(Temra)	Macro_FOLR2+APOE+	RPS19	C5AR1	3,79552E-04	26425,8	4,54395E-03	26585	1,33358E+00	11384	1,87570E+00	25555	9,46780E-01	6705	0	61900
Myeloid_vs_CD8_Non-responder	CD8(IL7R+ZNF683+ Tm)	Macro_ISG15	CD99	PILRA	3,80266E-04	28805	4,54281E-03	26595	1,32424E+00	11693	1,88811E+00	25188	9,28591E-01	18649	0	61900
Myeloid_vs_CD8_Non-responder	CD8(GZMK+ Tex)	pDC_LILRA4	GZMB	IGF2R	3,81411E-04	24435	1,38084E-02	4494	1,21489E+00	15142	2,36001E+00	14028	9,15456E-01	26611	0	61900
Myeloid_vs_CD8_Non-responder	CD8(GZMK+ Tem)	pDC_LILRA4	CCL5	CXCR3	3,82057E-04	25415,6	1,09808E-02	6873	9,81861E-01	25143	1,83901E+00	26620	9,47004E-01	6542	0	61900
Myeloid_vs_CD8_Non-responder	CD8(IL7R+ZNF683+ Tm)	Mono_CD16	HLA-C	LILRB2	3,83710E-04	21787,8	4,53789E-03	26643	1,88153E+00	3314	2,29200E+00	15369	9,56335E-01	1713	0	61900
Myeloid_vs_CD8_Non-responder	CD8(Tc17)	cDC_CLEC9A	FLT3LG	FLT3	3,84674E-04	31365,4	4,54048E-02	415	2,78921E+00	493	1,95831E+00	23179	8,48515E-01	70840	0	61900
Myeloid_vs_CD8_Non-responder	CD8(ID2+CXCR4+ T)	Mono_CD16	B2M	LILRB2	3,84864E-04	21227,6	4,53514E-03	26659	1,95503E+00	2796	2,33099E+00	14598	9,62347E-01	185	0	61900
Myeloid_vs_CD8_Non-responder	Macro_OLFML3	CD8(GZMK+ Early Tem)	CXCL10	CXCR3	3,87147E-04	23076,2	1,66806E-02	3052	2,33520E+00	1226	3,28401E+00	3542	8,86860E-01	45661	0	61900
Myeloid_vs_CD8_Non-responder	CD8(ID2+CXCR4+ T)	Macro_FOLR2+APOE+	CD99	PILRA	3,87179E-04	28921,6	4,53231E-03	26691	1,34702E+00	11003	1,84901E+00	26319	9,28514E-01	18695	0	61900
Myeloid_vs_CD8_Non-responder	Macro_NLRP3	CD8(Tn)	VCAN	CD44	3,87301E-04	18501,8	9,95179E-03	8031	2,28004E+00	1391	2,62049E+00	9921	9,40760E-01	11266	0	61900
Myeloid_vs_CD8_Non-responder	pDC_LILRA4	CD8(Temra)	APP	PTGER2	3,87472E-04	25277,4	2,07659E-02	1957	2,48634E+00	884	3,28388E+00	3543	8,68045E-01	58103	0	61900
Myeloid_vs_CD8_Non-responder	CD8(Temra)	Mono_CD16	HLA-A	LILRB2	3,87832E-04	22249	4,53150E-03	26700	1,81923E+00	3794	2,20398E+00	17160	9,56379E-01	1691	0	61900
Myeloid_vs_CD8_Non-responder	CD8(Temra)	Macro_FOLR2+APOE+	CD99	PILRA	3,88559E-04	29006	4,53031E-03	26710	1,34660E+00	11013	1,83644E+00	26703	9,28500E-01	18704	0	61900
Myeloid_vs_CD8_Non-responder	CD8(GZMK+ Tex)	Macro_FOLR2+APOE+	RPS19	C5AR1	3,90454E-04	26606,6	4,52673E-03	26736	1,32914E+00	11539	1,85773E+00	26063	9,46684E-01	6795	0	61900
Myeloid_vs_CD8_Non-responder	Macro_OLFML3	CD8(Temra)	CXCL9	KIR2DL3	3,90729E-04	31092	1,21638E-01	54	2,59480E+00	726	3,28242E+00	3553	8,19421E-01	89227	0	61900
Myeloid_vs_CD8_Non-responder	Macro_FOLR2+APOE+	CD8(GZMK+ Tem)	HLA-DQB1	LAG3	3,91250E-04	18713,8	8,06360E-03	11295	1,46721E+00	8207	2,87549E+00	6867	9,48801E-01	5300	0	61900
Myeloid_vs_CD8_Non-responder	CD8(IL7R+ZNF683+ Tm)	Mono_CD16	HLA-B	LILRB2	3,91917E-04	21820,4	4,52474E-03	26756	1,87404E+00	3375	2,25640E+00	16083	9,58333E-01	988	0	61900
Myeloid_vs_CD8_Non-responder	CD8(GZMK+ Tem)	Macro_FOLR2+APOE+	CD99	PILRA	3,94855E-04	29050	4,52029E-03	26796	1,34454E+00	11071	1,83546E+00	26744	9,28426E-01	18739	0	61900
Myeloid_vs_CD8_Non-responder	CD8(NME1+ T)	Macro_ISG15	TIGIT	NECTIN2	3,99943E-04	34998,8	1,54662E-02	3577,5	1,84460E+00	3581,5	1,66437E+00	32384,5	8,44341E-01	73550,5	0	61900
Myeloid_vs_CD8_Non-responder	CD8(GZMK+ Early Tem)	Mono_CD16	HLA-C	LILRB2	4,02128E-04	22056,2	4,51127E-03	26894	1,86617E+00	3432	2,24626E+00	16306	9,56212E-01	1749	0	61900
Myeloid_vs_CD8_Non-responder	CD8(Temra)	Mono_CD16	HLA-B	LILRB2	4,05053E-04	21954,2	4,50718E-03	26933	1,86186E+00	3460	2,23851E+00	16460	9,58255E-01	1018	0	61900
Myeloid_vs_CD8_Non-responder	CD8(NME1+ T)	Macro_FOLR2+APOE+	HMGB1	TLR2	4,05880E-04	29287,2	4,88111E-03	23856	1,32378E+00	11711	1,82926E+00	26944	9,22792E-01	22025	0	61900
Myeloid_vs_CD8_Non-responder	CD8(ZNF683+KLRB1+ T)	Mono_CD16	HLA-C	LILRA1	4,06333E-04	22281,6	1,06167E-02	7287	2,08768E+00	2110	1,82917E+00	26950	9,38086E-01	13161	0	61900
Myeloid_vs_CD8_Non-responder	CD8(ZNF683+KLRB1+ T)	pDC_LILRA4	BST2	LILRA4	4,07283E-04	20453,2	1,67171E-02	3026	2,86239E+00	426	3,27562E+00	3603	9,05004E-01	33311	0	61900
Myeloid_vs_CD8_Non-responder	CD8(ISG+ T)	Mono_CD16	B2M	LILRB2	4,09585E-04	21384,6	4,50016E-03	26993	1,92166E+00	3010	2,31884E+00	14825	9,62206E-01	195	0	61900
Myeloid_vs_CD8_Non-responder	CD8(ID2+CXCR4+ T)	Mono_CD16	RPS19	C5AR1	4,14693E-04	26680,6	4,49101E-03	27060	1,32670E+00	11621	1,86467E+00	25866	9,46484E-01	6956	0	61900
Myeloid_vs_CD8_Non-responder	CD8(ID2+CXCR4+ T)	Mono_CD16	CD99	PILRA	4,14923E-04	29094	4,49073E-03	27063	1,33385E+00	11375	1,85137E+00	26258	9,28208E-01	18874	0	61900
Myeloid_vs_CD8_Non-responder	Macro_NLRP3	CD8(Terminal Tex)	S100A8	CD69	4,16007E-04	17888,2	9,39393E-03	8869	2,00840E+00	2500	2,51150E+00	11472	9,49786E-01	4700	0	61900
Myeloid_vs_CD8_Non-responder	CD8(Temra)	Mono_CD16	CD99	PILRA	4,17228E-04	29178,8	4,48874E-03	27093	1,33343E+00	11391	1,83880E+00	26627	9,28193E-01	18883	0	61900
Myeloid_vs_CD8_Non-responder	CD8(EOMES+ NK-like)	Mono_CD16	HLA-F	LILRB2	4,20394E-04	26795,8	4,48402E-03	27134	1,81973E+00	3791	2,35016E+00	14201	9,14878E-01	26953	0	61900
Myeloid_vs_CD8_Non-responder	CD8(GZMK+ Tex)	Mono_CD16	B2M	LILRB2	4,21868E-04	21552,2	4,48198E-03	27153	1,90432E+00	3139	2,29201E+00	15368	9,62133E-01	201	0	61900
Myeloid_vs_CD8_Non-responder	CD8(ID2+CXCR4+ T)	pDC_LILRA4	HMGB1	THBD	4,23035E-04	28436	5,19907E-03	21745	1,28529E+00	12832	2,14110E+00	18535	9,14532E-01	27168	0	61900
Myeloid_vs_CD8_Non-responder	CD8(GZMK+ Tem)	Mono_CD16	CD99	PILRA	4,24438E-04	29223,8	4,47882E-03	27186	1,33137E+00	11457	1,83783E+00	26651	9,28120E-01	18925	0	61900
Myeloid_vs_CD8_Non-responder	Macro_OLFML3	CD8(Tc17)	CXCL9	DPP4	4,24983E-04	26410,4	5,25479E-02	320	2,56697E+00	766	3,26774E+00	3655	8,56733E-01	65411	0	61900
Myeloid_vs_CD8_Non-responder	CD8(EOMES+ NK-like)	Mono_CD16	HLA-C	LILRB2	4,25689E-04	22116,8	4,47708E-03	27202	1,84644E+00	3567	2,25551E+00	16099	9,56053E-01	1816	0	61900
Myeloid_vs_CD8_Non-responder	CD8(GZMK+ Tem)	Macro_ISG15	HLA-A	LILRB1	4,26158E-04	26246,2	4,47571E-03	27208	1,47597E+00	8046	1,83356E+00	26814	9,46121E-01	7263	0	61900
Myeloid_vs_CD8_Non-responder	CD8(IL7R+ZNF683+ Tm)	Mono_CD16	HLA-F	LILRB2	4,28356E-04	26804,8	4,47395E-03	27236	1,81862E+00	3798	2,35683E+00	14083	9,14790E-01	27007	0	61900
Myeloid_vs_CD8_Non-responder	CD8(ITM2C+ T)	Macro_FOLR2+APOE+	TNFSF9	HLA-DPA1	4,28593E-04	19816,4	8,59476E-03	10239	1,39367E+00	9804	3,01381E+00	5580	9,40373E-01	11559	0	61900
Myeloid_vs_CD8_Non-responder	CD8(Temra)	Macro_ISG15	HLA-C	LILRB1	4,29221E-04	25656,8	4,47242E-03	27247	1,52421E+00	7188	1,90816E+00	24608	9,46012E-01	7341	0	61900
Myeloid_vs_CD8_Non-responder	CD8(GZMK+ Tex)	Macro_FOLR2+APOE+	CD99	PILRA	4,29773E-04	29199	4,47209E-03	27254	1,33463E+00	11357	1,84217E+00	26534	9,28069E-01	18950	0	61900
Myeloid_vs_CD8_Non-responder	Macro_OLFML3	CD8(EOMES+ NK-like)	CXCL9	KIR2DL3	4,31924E-04	31981,6	1,10627E-01	69	2,54707E+00	793	3,26553E+00	3675	8,12294E-01	93471	0	61900
Myeloid_vs_CD8_Non-responder	CD8(ID2+CXCR4+ T)	Macro_ISG15	RPS19	C5AR1	4,33253E-04	26638,2	4,49829E-03	27008	1,32887E+00	11554	1,86706E+00	25806	9,46525E-01	6923	0	61900
Myeloid_vs_CD8_Non-responder	Macro_OLFML3	CD8(Tn)	CXCL10	CXCR3	4,34721E-04	23599,2	1,57080E-02	3459	2,31494E+00	1287	3,26454E+00	3683	8,83812E-01	47667	0	61900
Myeloid_vs_CD8_Non-responder	CD8(GZMK+ Tex)	Mono_CD16	HLA-C	LILRB2	4,38677E-04	22348,4	4,45925E-03	27366	1,83615E+00	3646	2,21205E+00	16980	9,55969E-01	1850	0	61900
Myeloid_vs_CD8_Non-responder	Macro_OLFML3	CD8(NME1+ T)	CXCL10	CXCR3	4,39291E-04	21938,2	1,93106E-02	2260	2,39000E+00	1076	3,26214E+00	3696	8,94000E-01	40759	0	61900
Myeloid_vs_CD8_Non-responder	cDC_LAMP3	CD8(EOMES+ NK-like)	CCL19	CXCR3	4,42725E-04	27215,6	4,02240E-02	529	3,95273E+00	80	2,40303E+00	13257	8,64748E-01	60312	0	61900
Myeloid_vs_CD8_Non-responder	CD8(GZMK+ Early Tem)	Macro_ISG15	RPS19	C5AR1	4,50422E-04	26680,4	4,56749E-03	26346	1,34602E+00	11026	1,81085E+00	27511	9,46910E-01	6619	0	61900
Myeloid_vs_CD8_Non-responder	CD8(GZMK+ Tem)	Macro_ISG15	RPS19	C5AR1	4,55686E-04	26972,2	4,43744E-03	27575	1,31379E+00	11992	1,85391E+00	26178	9,46179E-01	7216	0	61900
Myeloid_vs_CD8_Non-responder	CD8(GZMK+ Early Tem)	Mono_CD16	RPS19	C5AR1	4,56513E-04	26728,6	4,56010E-03	26424	1,34385E+00	11092	1,80847E+00	27585	9,46869E-01	6642	0	61900
Myeloid_vs_CD8_Non-responder	CD8(EOMES+ NK-like)	Mono_CD16	HLA-B	LILRB2	4,58336E-04	22277	4,43362E-03	27607	1,81086E+00	3865	2,21819E+00	16873	9,57925E-01	1140	0	61900
Myeloid_vs_CD8_Non-responder	CD8(GZMK+ Tex)	Mono_CD16	CD99	PILRA	4,59666E-04	29377,2	4,43107E-03	27623	1,32146E+00	11764	1,84454E+00	26463	9,27761E-01	19136	0	61900
Myeloid_vs_CD8_Non-responder	CD8(GZMK+ Tem)	Mono_CD16	RPS19	C5AR1	4,60248E-04	27018,4	4,43026E-03	27630	1,31162E+00	12065	1,85152E+00	26249	9,46138E-01	7248	0	61900
Myeloid_vs_CD8_Non-responder	Mono_CD14	CD8(ISG+ T)	VCAN	CD44	4,60909E-04	18877,8	9,80262E-03	8242	2,47669E+00	896	2,49111E+00	11774	9,40338E-01	11577	0	61900
Myeloid_vs_CD8_Non-responder	CD8(ISG+ T)	Mono_CD16	HLA-C	LILRB2	4,65772E-04	22492,4	4,42331E-03	27696	1,81540E+00	3827	2,20548E+00	17119	9,55798E-01	1920	0	61900
Myeloid_vs_CD8_Non-responder	CD8(ISG+ T)	Macro_NLRP3	RPS19	C5AR1	4,67709E-04	25876,4	4,80241E-03	24468	1,40349E+00	9570	1,80406E+00	27719	9,48156E-01	5725	0	61900
Myeloid_vs_CD8_Non-responder	CD8(ITM2C+ T)	cDC_CLEC9A	XCL1	XCR1	4,69826E-04	42173	3,94164E-02	545	5,06378E+00	31	1,31509E+00	47263	7,99539E-01	101126	0	61900
Myeloid_vs_CD8_Non-responder	CD8(Tn)	cDC(CD1C)	B2M	CD1C	4,74802E-04	20651,2	1,66876E-02	3048	1,79176E+00	4041	1,68024E+00	31831	9,54601E-01	2436	0	61900
Myeloid_vs_CD8_Non-responder	CD8(IL7R+ZNF683+ Tm)	Mono_CD16	B2M	LILRB2	4,77063E-04	22107,6	4,40986E-03	27829	1,83553E+00	3657	2,21559E+00	16921	9,61836E-01	231	0	61900
Myeloid_vs_CD8_Non-responder	CD8(Temra)	Macro_NLRP3	RPS19	C5AR1	4,80501E-04	26256,8	4,70263E-03	25243	1,37863E+00	10175	1,79946E+00	27869	9,47638E-01	6097	0	61900
Myeloid_vs_CD8_Non-responder	CD8(ID2+CXCR4+ T)	Macro_FOLR2+APOE+	HMGB1	CD163	4,82747E-04	26265,4	4,40299E-03	27895	1,37620E+00	10241	2,17434E+00	17814	9,37624E-01	13477	0	61900
Myeloid_vs_CD8_Non-responder	CD8(Temra)	Macro_FOLR2+APOE+	ADAM10	GPNMB	4,87876E-04	30522	7,75361E-03	11945	1,64629E+00	5428	2,64148E+00	9663	8,59526E-01	63674	0	61900
Myeloid_vs_CD8_Non-responder	CD8(Temra)	Mono_CD16	B2M	LILRB2	4,92956E-04	22204	4,39129E-03	28012	1,81781E+00	3804	2,20812E+00	17062	9,61759E-01	242	0	61900
Myeloid_vs_CD8_Non-responder	CD8(ID2+CXCR4+ T)	Macro_FOLR2+APOE+	CD99	CD81	4,96574E-04	30848	4,38732E-03	28053	1,11572E+00	18972	1,80787E+00	27602	9,30194E-01	17713	0	61900
Myeloid_vs_CD8_Non-responder	CD8(Temra)	Macro_FOLR2+APOE+	CD99	CD81	4,98702E-04	30940	4,38538E-03	28077	1,11531E+00	18989	1,79530E+00	28013	9,30179E-01	17721	0	61900
Myeloid_vs_CD8_Non-responder	CD8(GZMK+ Tex)	Macro_NLRP3	HMGB1	THBD	5,03337E-04	29522	5,45116E-03	20308	1,33881E+00	11225	1,79136E+00	28129	9,16364E-01	26048	0	61900
Myeloid_vs_CD8_Non-responder	CD8(Tn)	pDC_LILRA4	BST2	LILRA4	5,05997E-04	23487,4	1,21478E-02	5815	2,75465E+00	535	2,97797E+00	5904	8,90364E-01	43283	0	61900
Myeloid_vs_CD8_Non-responder	CD8(GZMK+ Tem)	Macro_FOLR2+APOE+	CD99	CD81	5,07106E-04	30987,6	4,37569E-03	28171	1,11325E+00	19055	1,79432E+00	28040	9,30108E-01	17772	0	61900
Myeloid_vs_CD8_Non-responder	CD8(Tn)	pDC_LILRA4	COPA	P2RY6	5,07819E-04	26695,2	1,01736E-02	7773	2,71275E+00	572	3,15462E+00	4426	8,67002E-01	58805	0	61900
Myeloid_vs_CD8_Non-responder	CD8(GZMK+ Early Tem)	Mono_CD16	B2M	LILRB2	5,10988E-04	22398,4	4,37101E-03	28214	1,79846E+00	3967	2,18112E+00	17662	9,61673E-01	249	0	61900
Myeloid_vs_CD8_Non-responder	Mono_CD14	CD8(Tc17)	VCAN	CD44	5,13316E-04	19128,2	9,55330E-03	8611	2,45671E+00	942	2,47157E+00	12100	9,39611E-01	12088	0	61900
Myeloid_vs_CD8_Non-responder	Macro_ISG15	CD8(NME1+ T)	CXCL10	CXCR3	5,13650E-04	23403,8	1,65650E-02	3095	2,05980E+00	2233	3,23214E+00	3896	8,86511E-01	45895	0	61900

Myeloid_vs_CD8_Non-responder	CD8(GZMK+ Tem)	Macro_ISG15	HLA-C	LILRB1	5,14075E-04	26756,4	4,36696E-03	28248	1,45762E+00	8403	1,81456E+00	27400	9,45400E-01	7831	0	61900
Myeloid_vs_CD8_Non-responder	CD8(GZMK+ Tem)	Mono_CD16	B2M	LILRB2	5,15624E-04	22361,6	4,36506E-03	28265	1,79279E+00	4027	2,19462E+00	17362	9,61648E-01	254	0	61900
Myeloid_vs_CD8_Non-responder	CD8(EOMES+ NK-like)	Mono_CD16	B2M	LILRB2	5,19191E-04	22394	4,36162E-03	28304	1,78950E+00	4059	2,19045E+00	17450	9,61634E-01	257	0	61900
Myeloid_vs_CD8_Non-responder	CD8(Tc17)	Macro_FOLR2+APOE+	LTB	TNFRSF1A	5,20200E-04	25483,6	9,75956E-03	8297	1,35932E+00	10686	2,09308E+00	19645	9,14979E-01	26890	0	61900
Myeloid_vs_CD8_Non-responder	CD8(NME1+ T)	pDC_LILRA4	CD70	TNFRSF17	5,22302E-04	46571,6	1,17080E-01	60	1,80382E+00	3918	1,68559E+00	31631	7,32550E-01	135349	0	61900
Myeloid_vs_CD8_Non-responder	CD8(EOMES+ NK-like)	pDC_LILRA4	HMGB1	THBD	5,24532E-04	29589,6	4,94875E-03	23378	1,23367E+00	14454	2,08502E+00	19854	9,12583E-01	28362	0	61900
Myeloid_vs_CD8_Non-responder	Macro_FOLR2+APOE+	CD8(GZMK+ Tem)	LGALS3	LAG3	5,26485E-04	21461,2	8,28631E-03	10823	1,30800E+00	12178	2,55910E+00	10771	9,40271E-01	11634	0	61900
Myeloid_vs_CD8_Non-responder	pDC_LILRA4	CD8(Tc17)	APP	PTGER2	5,31449E-04	27072,8	1,70787E-02	2906	2,41704E+00	1021	3,22554E+00	3941	8,56441E-01	65596	0	61900
Myeloid_vs_CD8_Non-responder	CD8(GZMK+ Tex)	Macro_NLRP3	RPS19	C5AR1	5,34314E-04	26443,6	4,68482E-03	25393	1,37419E+00	10290	1,78149E+00	28467	9,47544E-01	6168	0	61900
Myeloid_vs_CD8_Non-responder	CD8(Tc17)	Mono_CD16	HLA-B	LILRB2	5,34971E-04	22713,8	4,36112E-03	28311	1,76060E+00	4311	2,17543E+00	17789	9,57591E-01	1258	0	61900
Myeloid_vs_CD8_Non-responder	CD8(Tc17)	Mono_CD16	HLA-A	LILRB2	5,35441E-04	21982,6	4,58180E-03	26221	1,84939E+00	3547	2,22884E+00	16649	9,56609E-01	1596	0	61900
Myeloid_vs_CD8_Non-responder	CD8(Tc17)	Mono_CD16	HLA-C	LILRB2	5,36005E-04	21556,6	4,57993E-03	26237	1,90579E+00	3125	2,31606E+00	14889	9,56527E-01	1632	0	61900
Myeloid_vs_CD8_Non-responder	CD8(Tc17)	Mono_CD16	HLA-F	LILRB2	5,37041E-04	25130,6	4,98475E-03	23136	1,87490E+00	3366	2,42515E+00	12870	9,18910E-01	24381	0	61900
Myeloid_vs_CD8_Non-responder	CD8(Tc17)	Mono_CD16	B2M	LILRB2	5,37418E-04	22281,2	4,37745E-03	28150	1,80461E+00	3915	2,20265E+00	17194	9,61701E-01	247	0	61900
Myeloid_vs_CD8_Non-responder	cDC(CD1C)	CD8(GZMK+ Tem)	HLA-DQB1	LAG3	5,38515E-04	19593,2	8,19633E-03	10990	1,49036E+00	7799	2,46294E+00	12248	9,49196E-01	5029	0	61900
Myeloid_vs_CD8_Non-responder	CD8(ISG+ T)	pDC_LILRA4	TNF	PTPRS	5,39729E-04	25945,4	3,53585E-02	690	3,27018E+00	191	2,46251E+00	12255	8,73149E-01	54691	0	61900
Myeloid_vs_CD8_Non-responder	cDC_CLEC9A	CD8(Terminal Tex)	HLA-DQB1	LAG3	5,39902E-04	18935,6	7,61912E-03	12256	1,47067E+00	8143	2,95456E+00	6107	9,47406E-01	6272	0	61900
Myeloid_vs_CD8_Non-responder	CD8(ZNF683+KLRB1+ T)	pDC_LILRA4	TNF	PTPRS	5,41078E-04	25219,8	3,79149E-02	585	3,29035E+00	179	2,52302E+00	11273	8,76964E-01	52162	0	61900
Myeloid_vs_CD8_Non-responder	CD8(ISG+ T)	pDC_LILRA4	COPA	P2RY6	5,44081E-04	30149,2	7,60820E-03	12280	2,64044E+00	655	3,00998E+00	5613	8,49338E-01	70298	0	61900
Myeloid_vs_CD8_Non-responder	CD8(NME1+ T)	Macro_ISG15	RPS19	C5AR1	5,47200E-04	27414,2	4,44794E-03	27475	1,31639E+00	11929	1,77754E+00	28603	9,46239E-01	7164	0	61900
Myeloid_vs_CD8_Non-responder	CD8(GZMK+ Tex)	Macro_FOLR2+APOE+	CD99	CD81	5,49307E-04	31166,2	4,32903E-03	28625	1,10333E+00	19504	1,80103E+00	27825	9,29758E-01	17977	0	61900
Myeloid_vs_CD8_Non-responder	CD8(ISG+ T)	Macro_FOLR2+APOE+	CD99	PILRA	5,50171E-04	30049	4,32812E-03	28634	1,30500E+00	12255	1,79915E+00	27884	9,26969E-01	19572	0	61900
Myeloid_vs_CD8_Non-responder	Macro_NLRP3	CD8(LAYN+ T)	VCAN	CD44	5,50923E-04	19371	9,44526E-03	8786	2,23318E+00	1537	2,45840E+00	12313	9,39288E-01	12319	0	61900
Myeloid_vs_CD8_Non-responder	CD8(IL7R+ZNF683+ Tm)	Macro_ISG15	ANXA1	FPR1	5,52288E-04	30287,8	4,32540E-03	28656	1,12396E+00	18631	1,90194E+00	24806	9,30758E-01	17446	0	61900
Myeloid_vs_CD8_Non-responder	CD8(NME1+ T)	Mono_CD16	RPS19	C5AR1	5,53059E-04	27460	4,44074E-03	27555	1,31422E+00	11984	1,77515E+00	28664	9,46198E-01	7197	0	61900
Myeloid_vs_CD8_Non-responder	CD8(ID2+CXCR4+ T)	Macro_FOLR2+APOE+	RPS19	C5AR1	5,53542E-04	27779,4	4,32392E-03	28669	1,27685E+00	13073	1,81040E+00	27523	9,45515E-01	7732	0	61900
Myeloid_vs_CD8_Non-responder	Mono_INHBA	CD8(Temra)	IL1B	ADRB2	5,54983E-04	22954,6	1,51271E-02	3753	1,98018E+00	2650	3,21746E+00	3999	8,91571E-01	42471	0	61900
Myeloid_vs_CD8_Non-responder	CD8(Temra)	pDC_LILRA4	SELPLG	SELL	5,57651E-04	26408,2	7,57504E-03	12357	1,50336E+00	7546	2,45927E+00	12303	8,98136E-01	37935	0	61900
Myeloid_vs_CD8_Non-responder	cDC_CLEC9A	CD8(GZMK+ Tex)	HLA-DQB1	LAG3	5,62827E-04	18961,6	7,56374E-03	12386	1,46551E+00	8245	2,97923E+00	5893	9,47224E-01	6384	0	61900
Myeloid_vs_CD8_Non-responder	CD8(IL7R+ZNF683+ Tm)	Macro_ISG15	RPS19	C5AR1	5,63264E-04	27162,6	4,31437E-03	28769	1,28329E+00	12895	1,91206E+00	24482	9,45458E-01	7767	0	61900
Myeloid_vs_CD8_Non-responder	CD8(GZMK+ Tem)	pDC_LILRA4	HMGB1	THBD	5,65028E-04	30201,2	4,86932E-03	23938	1,21729E+00	15061	2,02681E+00	21320	9,11936E-01	28787	0	61900
Myeloid_vs_CD8_Non-responder	CD8(NME1+ T)	Mono_CD16	HLA-A	LILRB2	5,65420E-04	23561,4	4,31247E-03	28791	1,68787E+00	4967	2,07933E+00	20015	9,55334E-01	2134	0	61900
Myeloid_vs_CD8_Non-responder	Macro_OLFML3	CD8(EOMES+ NK-like)	CXCL10	CXCR3	5,67421E-04	24284,8	1,45911E-02	4029	2,29167E+00	1355	3,22417E+00	3952	8,79971E-01	50188	0	61900
Myeloid_vs_CD8_Non-responder	cDC_CLEC9A	CD8(Terminal Tex)	HLA-DQA1	LAG3	5,68398E-04	19645,6	7,54530E-03	12417	1,41841E+00	9232	2,85130E+00	7102	9,45705E-01	7577	0	61900
Myeloid_vs_CD8_Non-responder	CD8(IL7R+ZNF683+ Tm)	Mono_CD16	RPS19	C5AR1	5,70644E-04	27211,2	4,30739E-03	28844	1,28112E+00	12941	1,90968E+00	24559	9,45417E-01	7812	0	61900
Myeloid_vs_CD8_Non-responder	CD8(ZNF683+KLRB1+ T)	Macro_ISG15	B2M	LILRB1	5,73916E-04	25221	4,30381E-03	28877	1,55846E+00	6651	1,88239E+00	25362	9,52528E-01	3315	0	61900
Myeloid_vs_CD8_Non-responder	CD8(Tc17)	cDC_LAMP3	LTB	CD40	5,74811E-04	20616	2,01549E-02	2067	2,25130E+00	1482	2,33299E+00	14548	9,21091E-01	23083	0	61900
Myeloid_vs_CD8_Non-responder	CD8(ISG+ T)	pDC_LILRA4	HMGB1	THBD	5,77802E-04	30123	4,84674E-03	24124	1,21263E+00	15224	2,06168E+00	20451	9,11749E-01	28916	0	61900
Myeloid_vs_CD8_Non-responder	CD8(ZNF683+KLRB1+ T)	Macro_ISG15	HLA-C	LILRB2	5,78202E-04	21702,4	4,29974E-03	28920	1,92594E+00	2980	2,44757E+00	12516	9,55196E-01	2196	0	61900
Myeloid_vs_CD8_Non-responder	CD8(ISG+ T)	Macro_ISG15	HLA-B	LILRB1	5,79602E-04	26654,6	4,29813E-03	28934	1,43499E+00	8864	1,81420E+00	27412	9,47548E-01	6163	0	61900
Myeloid_vs_CD8_Non-responder	CD8(EOMES+ NK-like)	pDC_LILRA4	BST2	LILRA4	5,80041E-04	20812,4	1,61097E-02	3273	2,84807E+00	442	3,20608E+00	4059	9,03401E-01	34388	0	61900
Myeloid_vs_CD8_Non-responder	CD8(GZMK+ Tem)	Macro_OLFML3	TNFSF9	HLA-DPA1	5,84426E-04	31991,6	4,29390E-03	28982	9,94747E-01	24495	2,10546E+00	19370	9,17677E-01	25211	0	61900
Myeloid_vs_CD8_Non-responder	Macro_FOLR2+APOE+	CD8(Terminal Tex)	HLA-DQA1	LAG3	5,85359E-04	19639,4	7,51426E-03	12510	1,41245E+00	9370	2,88681E+00	6756	9,45599E-01	7661	0	61900
Myeloid_vs_CD8_Non-responder	CD8(IL7R+ZNF683+ Tm)	Macro_NLRP3	RPS19	C5AR1	5,86445E-04	28088	4,29196E-03	29002	1,27632E+00	13093	1,77917E+00	28551	9,45324E-01	7894	0	61900
Myeloid_vs_CD8_Non-responder	CD8(GZMK+ Early Tem)	Macro_ISG15	TNFSF9	HLA-DPA1	5,88774E-04	30941,4	4,87014E-03	23933	9,13151E-01	29025	2,18660E+00	17548	9,22310E-01	22301	0	61900
Myeloid_vs_CD8_Non-responder	CD8(ISG+ T)	Mono_CD16	CD99	PILRA	5,91009E-04	30232,8	4,28841E-03	29047	1,29183E+00	12653	1,80152E+00	27812	9,26657E-01	19752	0	61900
Myeloid_vs_CD8_Non-responder	CD8(Tn)	Macro_NLRP3	ANXA1	FPR1	5,91213E-04	29359,6	4,28828E-03	29049	1,12498E+00	18583	2,09062E+00	19701	9,30480E-01	17565	0	61900
Myeloid_vs_CD8_Non-responder	Macro_NLRP3	CD8(GZMK+ Tex)	S100A8	CD69	5,91652E-04	18323,2	9,08995E-03	9359	1,98658E+00	2618	2,44562E+00	12544	9,48996E-01	5195	0	61900
Myeloid_vs_CD8_Non-responder	CD8(GZMK+ Tem)	Macro_LYVE1	TNFSF9	HLA-DPA1	5,94987E-04	33212,2	4,41017E-03	27826	1,03243E+00	22710	1,76091E+00	29086	9,18681E-01	24539	0	61900
Myeloid_vs_CD8_Non-responder	cDC_CLEC9A	CD8(GZMK+ Tex)	HLA-DQA1	LAG3	5,96124E-04	19680,6	7,49046E-03	12568	1,41325E+00	9346	2,87597E+00	6858	9,45518E-01	7731	0	61900
Myeloid_vs_CD8_Non-responder	CD8(ID2+CXCR4+ T)	Macro_ISG15	CD99	PILRA	6,01150E-04	30455,4	4,27852E-03	29146	1,26663E+00	13390	1,79398E+00	28053	9,26578E-01	19788	0	61900
Myeloid_vs_CD8_Non-responder	CD8(Temra)	Macro_ISG15	CD99	PILRA	6,03836E-04	30548,4	4,27663E-03	29172	1,26622E+00	13400	1,78141E+00	28471	9,26563E-01	19799	0	61900
Myeloid_vs_CD8_Non-responder	pDC_LILRA4	CD8(EOMES+ NK-like)	APP	PTGER2	6,07579E-04	28651,6	1,46261E-02	4012	2,37096E+00	1139	3,19695E+00	4123	8,46645E-01	72084	0	61900
Myeloid_vs_CD8_Non-responder	Macro_NLRP3	CD8(GZMK+ Tem)	VCAN	CD44	6,08743E-04	19458,6	9,29917E-03	9007	2,21966E+00	1586	2,46750E+00	12165	9,38842E-01	12635	0	61900
Myeloid_vs_CD8_Non-responder	CD8(ID2+CXCR4+ T)	Macro_ISG15	HLA-A	LILRB1	6,08820E-04	27457,2	4,34765E-03	28456	1,39193E+00	9853	1,75636E+00	29220	9,45376E-01	7857	0	61900
Myeloid_vs_CD8_Non-responder	CD8(ISG+ T)	pDC_LILRA4	SELPLG	SELL	6,11214E-04	26530,2	7,54129E-03	12428	1,50163E+00	7586	2,43940E+00	12648	8,97931E-01	38089	0	61900
Myeloid_vs_CD8_Non-responder	CD8(GZMK+ Early Tem)	Macro_FOLR2+APOE+	CD99	PILRA	6,14361E-04	30453	4,26759E-03	29273	1,29255E+00	12634	1,77707E+00	28620	9,26491E-01	19838	0	61900
Myeloid_vs_CD8_Non-responder	Macro_FOLR2+APOE+	CD8(GZMK+ Tex)	HLA-DQA1	LAG3	6,14457E-04	19674,2	7,45964E-03	12665	1,40728E+00	9479	2,91147E+00	6508	9,45411E-01	7819	0	61900
Myeloid_vs_CD8_Non-responder	CD8(GZMK+ Tem)	Macro_ISG15	CD99	PILRA	6,14886E-04	30592,8	4,26718E-03	29278	1,26416E+00	13449	1,78043E+00	28497	9,26488E-01	19840	0	61900
Myeloid_vs_CD8_Non-responder	CD8(GZMK+ Tem)	Macro_FOLR2+APOE+	RPS19	C5AR1	6,16147E-04	28135,2	4,26543E-03	29290	1,26177E+00	13528	1,79725E+00	27950	9,45163E-01	8008	0	61900
Myeloid_vs_CD8_Non-responder	CD8(GZMK+ Early Tem)	Macro_FOLR2+APOE+	RPS19	C5AR1	6,16568E-04	27848	4,39044E-03	28024	1,29400E+00	12590	1,75419E+00	29294	9,45907E-01	7432	0	61900
Myeloid_vs_CD8_Non-responder	CD8(ZNF683+KLRB1+ T)	Macro_FOLR2+APOE+	HLA-F	LILRB2	6,18254E-04	29115,8	4,26405E-03	29310	1,45906E+00	8379	2,17365E+00	17825	9,12898E-01	28165	0	61900
Myeloid_vs_CD8_Non-responder	CD8(GZMK+ Early Tem)	Macro_ISG15	HLA-F	LILRB2	6,24076E-04	28578,2	4,26050E-03	29365	1,56002E+00	6622	2,22045E+00	16812	9,12865E-01	28192	0	61900
Myeloid_vs_CD8_Non-responder	CD8(Tc17)	Macro_FOLR2+APOE+	CD99	PILRA	6,24183E-04	30319,6	4,26049E-03	29366	1,29109E+00	12669	1,80213E+00	27791	9,26435E-01	19872	0	61900
Myeloid_vs_CD8_Non-responder	CD8(Tn)	Mono_CD14	RPS19	C5AR1	6,24289E-04	26720,2	4,66585E-03	25543	1,36473E+00	10550	1,75233E+00	29367	9,47443E-01	6241	0	61900
Myeloid_vs_CD8_Non-responder	CD8(ZNF683+KLRB1+ T)	Mono_CD16	HLA-C	LILRB1	6,25992E-04	26218,2	4,25912E-03	29383	1,55658E+00	6679	1,90109E+00	24832	9,44751E-01	8297	0	61900
Myeloid_vs_CD8_Non-responder	CD8(Tn)	Macro_FOLR2+APOE+	ANXA1	FPR2_FPR3	6,27805E-04	27935	8,27554E-03	10842	1,31952E+00	11827	1,75152E+00	29400	9,16883E-01	25706	0	61900
Myeloid_vs_CD8_Non-responder	CD8(GZMK+ Early Tem)	Macro_ISG15	HLA-B	LILRB1	6,29622E-04	27175	4,25568E-03	29417	1,40278E+00	9586	1,77663E+00	28634	9,47301E-01	6338	0	61900
Myeloid_vs_CD8_Non-responder	CD8(ISG+ T)	Macro_ISG15	HLA-F	LILRB2	6,33806E-04	28805,4	4,25200E-03	29456	1,55889E+00	6648	2,17550E+00	17788	9,12786E-01	28235	0	61900
Myeloid_vs_CD8_Non-responder	cDC(CD1C)	CD8(GZMK+ Tem)	HLA-DRB5	LAG3	6,34573E-04	19723	7,42063E-03	12769	1,45602E+00	8438	2,48115E+00	11925	9,51953E-01	3583	0	61900
Myeloid_vs_CD8_Non-responder	CD8(ITM2C+ T)	Macro_OLFML3	TNFSF9	HLA-DPA1	6,36926E-04	21671,2	8,09037E-03	11240	1,30692E+00	12208	2,59682E+00	10227	9,38655E-01	12781	0	61900
Myeloid_vs_CD8_Non-responder	CD8(GZMK+ Tex)	Macro_ISG15	HLA-A	LILRB1	6,39421E-04	27450,4	4,35690E-03	28352	1,39800E+00	9694	1,74836E+00	29508	9,45431E-01	7798	0	61900
Myeloid_vs_CD8_Non-responder	CD8(GZMK+ Early Tem)	Macro_ISG15	HLA-A	LILRB1	6,41808E-04	27531	4,34570E-03	28471	1,39065E+00	9886	1,74768E+00	29530	9,45364E-01	7868	0	61900
Myeloid_vs_CD8_Non-responder	CD8(Tn)	Macro_FOLR2+APOE+	CD40LG	CD9	6,43032E-04	29305,2	3,03448E-02	942	1,28593E+00	12812	2,59047E+00	10312	8,64347E-01	60560	0	61900
Myeloid_vs_CD8_Non-responder	Macro_ISG15	CD8(GZMK+ Early Tem)	CXCL10	CXCR3	6,43194E-04	24643,8	1,43090E-02	4203	2,00501E+00	2519	3,25402E+00	3751	8,78935E-01	50846	0	61900
Myeloid_vs_CD8_Non-responder	CD8(Tn)	Mono_CD16	HLA-A	LILRB2	6,50219E-04	24082,4	4,23718E-03	29607	1,64272E+00	5477	2,03409E+00	21142	9,54957E-01	2286	0	61900
Myeloid_vs_CD8_Non-responder	CD8(Tc17)	Macro_ISG15	LTB	TNFRSF1A	6,50878E-04	26372,6	9,33284E-03	8967	1,31951E+00	11828	2,03163E+00	21207	9,13224E-01	27961	0	61900

Myeloid_vs_CD8_Post_NR

Myeloid_vs_CD8_Non-responder	CD8(NME1+ T)	Mono_CD16	B2M	LILRB2	6,50988E-04	23251,2	4,23676E-03	29614	1,67039E+00	5147	2,10890E+00	19268	9,61094E-01	327		0	61900
Myeloid_vs_CD8_Non-responder	CD8(Tc17)	Macro_FOLR2+APOE+	LTB	CD40	6,58830E-04	22367	1,52860E-02	3661	1,84288E+00	3598	2,41827E+00	12991	9,10439E-01	29685		0	61900
Myeloid_vs_CD8_Non-responder	CD8(ZNF683+KLRB1+ T)	Macro_ISG15	HLA-B	LILRB1	6,59163E-04	27557	4,23010E-03	29688	1,38338E+00	10066	1,74278E+00	29685	9,47151E-01	6446		0	61900
Myeloid_vs_CD8_Non-responder	CD8(Tc17)	Macro_OLFML3	LTB	CD40	6,60051E-04	23235,4	1,52820E-02	3664	1,84255E+00	3600	2,19650E+00	17317	9,10428E-01	29696		0	61900
Myeloid_vs_CD8_Non-responder	CD8(GZMK+ Early Tem)	Mono_CD16	CD99	PILRA	6,60718E-04	30629,4	4,22844E-03	29702	1,27938E+00	12995	1,77943E+00	28536	9,26177E-01	20014		0	61900
Myeloid_vs_CD8_Non-responder	CD8(GZMK+ Tex)	Macro_ISG15	CD99	PILRA	6,68990E-04	30751,2	4,22168E-03	29776	1,25424E+00	13770	1,78715E+00	28269	9,26122E-01	20041		0	61900
Myeloid_vs_CD8_Non-responder	CD8(Tc17)	Mono_CD16	CD99	PILRA	6,69215E-04	30494,6	4,22141E-03	29778	1,27792E+00	13044	1,80450E+00	27707	9,26120E-01	20044		0	61900
Myeloid_vs_CD8_Non-responder	Macro_NLRP3	CD8(IL7R+ZNF683+ Tm)	VCAN	CD44	6,71356E-04	19472,8	9,16442E-03	9238	2,20720E+00	1630	2,49868E+00	11643	9,38422E-01	12953		0	61900
Myeloid_vs_CD8_Non-responder	CD8(NME1+ T)	Mono_CD16	HLA-B	LILRB2	6,74513E-04	23864,6	4,21688E-03	29825	1,66060E+00	5264	2,04634E+00	20837	9,56903E-01	1497		0	61900
Myeloid_vs_CD8_Non-responder	CD8(ID2+CXCR4+ T)	pDC_LILRA4	CCL5	CXCR3	6,76664E-04	27436,2	1,01915E-02	7751	9,03620E-01	29621	1,73798E+00	29844	9,45101E-01	8065		0	61900
Myeloid_vs_CD8_Non-responder	CD8(Tn)	Mono_CD16	HLA-B	LILRB2	6,77458E-04	23836,8	4,21391E-03	29851	1,65854E+00	5289	2,05396E+00	20640	9,56889E-01	1504		0	61900
Myeloid_vs_CD8_Non-responder	Macro_NLRP3	CD8(GZMK+ Early Tem)	VCAN	CD44	6,78117E-04	19626	9,14408E-03	9271	2,20532E+00	1637	2,45749E+00	12336	9,38357E-01	12986		0	61900
Myeloid_vs_CD8_Non-responder	CD8(GZMK+ Early Tem)	Macro_IFI27	TNFSF9	HLA-DPA1	6,82922E-04	29154,4	5,86577E-03	18168	1,15803E+00	17236	1,73616E+00	29899	9,28718E-01	18569		0	61900
Myeloid_vs_CD8_Non-responder	CD8(ID2+CXCR4+ T)	Macro_NLRP3	HMGB1	THBD	6,84408E-04	30513,8	5,25594E-03	21416	1,29898E+00	12441	1,73582E+00	29912	9,14956E-01	26900		0	61900
Myeloid_vs_CD8_Non-responder	CD8(GZMK+ Tex)	Macro_ISG15	HLA-B	LILRB1	6,86929E-04	27765,2	4,20623E-03	29925	1,36527E+00	10526	1,73511E+00	29934	9,47009E-01	6541		0	61900
Myeloid_vs_CD8_Non-responder	CD8(EOMES+ NK-like)	Macro_ISG15	CCL5	CCR1	6,87732E-04	23530,6	1,39606E-02	4417	1,59753E+00	6083	1,73490E+00	29941	9,34613E-01	15312		0	61900
Myeloid_vs_CD8_Non-responder	CD8(ISG+ T)	Macro_ISG15	HLA-A	LILRB1	6,89226E-04	27846	4,30808E-03	28840	1,36596E+00	10507	1,73444E+00	29954	9,45139E-01	8029		0	61900
Myeloid_vs_CD8_Non-responder	CD8(ID2+CXCR4+ T)	Macro_NLRP3	RPS19	C5AR1	6,90608E-04	27574,8	4,47492E-03	27222	1,32190E+00	11757	1,73417E+00	29966	9,46393E-01	7029		0	61900
Myeloid_vs_CD8_Non-responder	CD8(Temra)	Macro_FOLR2+APOE+	TGFB1	ENG	6,97317E-04	28527,8	6,83556E-03	14447	1,30802E+00	12176	1,92588E+00	24092	9,09951E-01	30024		0	61900
Myeloid_vs_CD8_Non-responder	CD8(GZMK+ Tex)	Macro_ISG15	HLA-F	LILRB2	6,97666E-04	28987,6	4,19842E-03	30027	1,55176E+00	6744	2,17988E+00	17694	9,12280E-01	28573		0	61900
Myeloid_vs_CD8_Non-responder	CD8(NME1+ T)	Mono_CD16	HLA-F	LILRB2	7,01041E-04	28458,6	4,19684E-03	30056	1,78808E+00	4074	2,18028E+00	17682	9,12265E-01	28581		0	61900
Myeloid_vs_CD8_Non-responder	CD8(ZNF683+KLRB1+ T)	Macro_ISG15	HLA-A	LILRB2	7,01858E-04	22761,2	4,19621E-03	30063	1,80731E+00	3895	2,28203E+00	15574	9,54747E-01	2374		0	61900
Myeloid_vs_CD8_Non-responder	CD8(ISG+ T)	Mono_CD16	HLA-B	LILRA1	7,05876E-04	24827,8	9,61904E-03	8509	1,82701E+00	3732	1,54969E+00	36879	9,38145E-01	13119		0	61900
Myeloid_vs_CD8_Non-responder	CD8(EOMES+ NK-like)	Macro_FOLR2+APOE+	HMGB1	CD163	7,07832E-04	27428,4	4,19100E-03	30114	1,32458E+00	11683	2,11826E+00	19044	9,36165E-01	14401		0	61900
Myeloid_vs_CD8_Non-responder	CD8(ISG+ T)	Macro_FOLR2+APOE+	CD99	CD81	7,09125E-04	32119,4	4,18966E-03	30125	1,07371E+00	20817	1,75801E+00	29166	9,28682E-01	18589		0	61900
Myeloid_vs_CD8_Non-responder	CD8(IL7R+ZNF683+ Tm)	pDC_LILRA4	HMGB1	THBD	7,12545E-04	31474,2	4,61404E-03	25970	1,16464E+00	16985	1,98746E+00	22362	9,09749E-01	30154		0	61900
Myeloid_vs_CD8_Non-responder	CD8(ID2+CXCR4+ T)	Macro_ISG15	HLA-B	LILRB1	7,14319E-04	27668,2	4,22794E-03	29710	1,38174E+00	10110	1,72808E+00	30169	9,47138E-01	6452		0	61900
Myeloid_vs_CD8_Non-responder	pDC_LILRA4	CD8(GZMK+ Early Tem)	SCT	ADRB2	7,17515E-04	37373,6	3,57206E-02	674	3,60785E+00	109	2,91594E+00	6476	7,69668E-01	117709		0	61900
Myeloid_vs_CD8_Non-responder	CD8(LAYN+ T)	pDC_LILRA4	COPA	P2RY6	7,18003E-04	23566,6	1,40456E-02	4362	2,82189E+00	461	3,23422E+00	3884	8,84521E-01	47226		0	61900
Myeloid_vs_CD8_Non-responder	CD8(Tc17)	Macro_ISG15	HLA-C	LILRB1	7,21212E-04	28416	4,18563E-03	30169	1,34313E+00	11112	1,72647E+00	30227	9,44295E-01	8672		0	61900
Myeloid_vs_CD8_Non-responder	pDC_LILRA4	CD8(IL7R+ZNF683+ Tm)	SCT	ADRB2	7,21763E-04	37355	3,56765E-02	676	3,60764E+00	110	2,93120E+00	6323	7,69559E-01	117766		0	61900
Myeloid_vs_CD8_Non-responder	CD8(Tn)	Mono_CD16	B2M	LILRB2	7,30084E-04	23964,2	4,17385E-03	30301	1,61038E+00	5906	2,02579E+00	21343	9,60814E-01	371		0	61900
Myeloid_vs_CD8_Non-responder	CD8(GZMK+ Tem)	Macro_NLRP3	RPS19	C5AR1	7,42699E-04	27924,6	4,41439E-03	27791	1,30682E+00	12209	1,72102E+00	30405	9,46046E-01	7318		0	61900
Myeloid_vs_CD8_Non-responder	CD8(NME1+ T)	Macro_FOLR2+APOE+	RPS19	C5AR1	7,43065E-04	28578,4	4,27552E-03	29179	1,26437E+00	13439	1,72088E+00	30408	9,45225E-01	7966		0	61900
Myeloid_vs_CD8_Non-responder	Macro_OLFML3	CD8(Terminal Tex)	CXCL10	CXCR3	7,44155E-04	23758,6	1,56853E-02	3471	2,31446E+00	1288	3,15632E+00	4415	8,83737E-01	47719		0	61900
Myeloid_vs_CD8_Non-responder	CD8(GZMK+ Early Tem)	Mono_CD16	HLA-B	LILRA1	7,47205E-04	25262,2	9,52404E-03	8667	1,79481E+00	4005	1,51212E+00	38429	9,37856E-01	13310		0	61900
Myeloid_vs_CD8_Non-responder	CD8(ZNF683+KLRB1+ T)	Mono_CD16	HLA-A	LILRB1	7,52151E-04	27966,4	4,15656E-03	30482	1,43795E+00	8799	1,73555E+00	29919	9,44204E-01	8732		0	61900
Myeloid_vs_CD8_Non-responder	CD8(Temra)	Mono_CD16	HLA-F	LILRB2	7,55241E-04	28183,4	4,15481E-03	30507	1,78345E+00	4116	2,28294E+00	15552	9,11861E-01	28842		0	61900
Myeloid_vs_CD8_Non-responder	CD8(Tn)	cDC_LAMP3	LTB	CD40	7,56727E-04	23688	1,48323E-02	3895	1,99653E+00	2565	2,09682E+00	19561	9,09203E-01	30519		0	61900
Myeloid_vs_CD8_Non-responder	CD8(GZMK+ Tem)	Mono_CD16	HLA-F	LILRB2	7,60827E-04	28370,2	4,15123E-03	30552	1,78306E+00	4117	2,24057E+00	16419	9,11826E-01	28863		0	61900
Myeloid_vs_CD8_Non-responder	CD8(LAYN+ T)	Macro_FOLR2+APOE+	CD99	CD81	7,61699E-04	31604,6	4,27891E-03	29138	1,09268E+00	19975	1,76980E+00	28817	9,29377E-01	18193		0	61900
Myeloid_vs_CD8_Non-responder	CD8(LAYN+ T)	Macro_FOLR2+APOE+	CD99	PILRA	7,62073E-04	29601,6	4,42032E-03	27726	1,32397E+00	11705	1,81094E+00	27507	9,27680E-01	19170		0	61900
Myeloid_vs_CD8_Non-responder	cDC(CD1C)	CD8(GZMK+ Tex)	HLA-DQB1	LAG3	7,63016E-04	21093	7,19508E-03	13381	1,39225E+00	9845	2,42027E+00	12945	9,45961E-01	7394		0	61900
Myeloid_vs_CD8_Non-responder	CD8(IL7R+ZNF683+ Tm)	Macro_FOLR2+APOE+	RPS19	C5AR1	7,66823E-04	28346,4	4,14713E-03	30600	1,23127E+00	14533	1,85541E+00	26141	9,44430E-01	8558		0	61900
Myeloid_vs_CD8_Non-responder	cDC(CD1C)	CD8(Terminal Tex)	HLA-DQB1	LAG3	7,68417E-04	21098,4	7,24776E-03	13233	1,39742E+00	9714	2,39560E+00	13405	9,46147E-01	7240		0	61900
Myeloid_vs_CD8_Non-responder	CD8(LAYN+ T)	Macro_FOLR2+APOE+	RPS19	C5AR1	7,69081E-04	26856,8	4,57917E-03	26246	1,34266E+00	11122	1,78205E+00	28448	9,46974E-01	6568		0	61900
Myeloid_vs_CD8_Non-responder	CD8(ID2+CXCR4+ T)	Macro_ISG15	B2M	LILRB1	7,69583E-04	27158,4	4,14470E-03	30622	1,39237E+00	9839	1,74140E+00	29727	9,51669E-01	3704		0	61900
Myeloid_vs_CD8_Non-responder	cDC_CLEC9A	CD8(GZMK+ Tem)	HLA-DPB1	LAG3	7,74299E-04	18293,2	7,18249E-03	13431	1,49377E+00	7732	2,98787E+00	5815	9,54179E-01	2588		0	61900
Myeloid_vs_CD8_Non-responder	CD8(EOMES+ NK-like)	Macro_FOLR2+APOE+	CD52	SIGLEC10	7,77152E-04	28929,6	5,77180E-03	18609	1,31502E+00	11963	1,71222E+00	30682	9,23617E-01	21494		0	61900
Myeloid_vs_CD8_Non-responder	Macro_FOLR2+APOE+	CD8(IL7R+ZNF683+ Tm)	CXCL9	CXCR3	7,79130E-04	22163,4	2,08692E-02	1939	2,43395E+00	992	3,14882E+00	4484	8,92979E-01	41502		0	61900
Myeloid_vs_CD8_Non-responder	Macro_FOLR2+APOE+	CD8(GZMK+ Tem)	CXCL9	CXCR3	7,80674E-04	21189,4	2,38982E-02	1515	2,50079E+00	868	3,14830E+00	4487	8,99285E-01	37177		0	61900
Myeloid_vs_CD8_Non-responder	CD8(ISG+ T)	Mono_CD14	RPS19	C5AR1	7,81341E-04	27139,6	4,62470E-03	25871	1,35408E+00	10827	1,71151E+00	30715	9,47222E-01	6385		0	61900
Myeloid_vs_CD8_Non-responder	CD8(ZNF683+KLRB1+ T)	Macro_LYVE1	HMGB1	CD163	7,81722E-04	26853	4,94653E-03	23392	1,52944E+00	7106	1,71149E+00	30718	9,40942E-01	11149		0	61900
Myeloid_vs_CD8_Non-responder	CD8(ID2+CXCR4+ T)	Macro_ISG15	HLA-C	LILRB1	7,83250E-04	28502,4	4,18745E-03	30139	1,34428E+00	11079	1,71123E+00	30730	9,44306E-01	8664		0	61900
Myeloid_vs_CD8_Non-responder	CD8(GZMK+ Early Tem)	Macro_FOLR2+APOE+	CD99	CD81	7,89387E-04	32567,6	4,13107E-03	30778	1,06125E+00	21385	1,73593E+00	29908	9,28214E-01	18867		0	61900
Myeloid_vs_CD8_Non-responder	Macro_NLRP3	CD8(ITM2C+ T)	S100A8	CD69	7,89841E-04	20362	7,22182E-03	13300	1,85244E+00	3527	2,39094E+00	13499	9,43132E-01	9584		0	61900
Myeloid_vs_CD8_Non-responder	CD8(GZMK+ Tex)	pDC_LILRA4	BST2	LILRA4	7,89985E-04	20933,8	1,60442E-02	3304	2,84652E+00	444	3,14562E+00	4505	9,03223E-01	34516		0	61900
Myeloid_vs_CD8_Non-responder	CD8(Tc17)	Macro_ISG15	HLA-F	LILRB2	7,91441E-04	29207,4	4,12904E-03	30794	1,54253E+00	6878	2,18946E+00	17469	9,11611E-01	28996		0	61900
Myeloid_vs_CD8_Non-responder	CD8(LAYN+ T)	Mono_CD16	HLA-B	LILRB2	7,92469E-04	22018,8	4,50883E-03	26919	1,86300E+00	3452	2,22055E+00	16810	9,58262E-01	1013		0	61900
Myeloid_vs_CD8_Non-responder	CD8(LAYN+ T)	Mono_CD16	ANXA1	FPR1	7,92726E-04	25899,8	5,27522E-03	21289	1,34249E+00	11129	2,02916E+00	21273	9,36888E-01	13908		0	61900
Myeloid_vs_CD8_Non-responder	CD8(LAYN+ T)	Mono_CD16	HLA-A	LILRB2	7,93113E-04	21584,2	4,66558E-03	25547	1,89964E+00	3173	2,26899E+00	15824	9,56983E-01	1477		0	61900
Myeloid_vs_CD8_Non-responder	CD8(LAYN+ T)	Mono_CD16	HLA-C	LILRB2	7,93885E-04	21083,2	4,69462E-03	25302	1,97197E+00	2708	2,35847E+00	14049	9,57039E-01	1457		0	61900
Myeloid_vs_CD8_Non-responder	CD8(LAYN+ T)	Mono_CD16	CD99	PILRA	7,94530E-04	29778	4,37977E-03	28131	1,31080E+00	12097	1,81330E+00	27431	9,27370E-01	19331		0	61900
Myeloid_vs_CD8_Non-responder	CD8(LAYN+ T)	Mono_CD16	B2M	LILRB2	7,95690E-04	22425,6	4,35582E-03	28364	1,78397E+00	4106	2,18798E+00	17499	9,61609E-01	259		0	61900
Myeloid_vs_CD8_Non-responder	CD8(Tc17)	Macro_FOLR2+APOE+	CD99	CD81	7,97499E-04	32434,2	4,12420E-03	30841	1,05979E+00	21441	1,76099E+00	29085	9,28159E-01	18904		0	61900
Myeloid_vs_CD8_Non-responder	CD8(LAYN+ T)	Mono_CD16	RPS19	C5AR1	7,98534E-04	25827	4,75613E-03	24796	1,39251E+00	9834	1,83633E+00	26708	9,47918E-01	5897		0	61900
Myeloid_vs_CD8_Non-responder	CD8(GZMK+ Tem)	Macro_FOLR2+APOE+	HMGB1	CD163	7,98534E-04	28019,4	4,12373E-03	30849	1,30820E+00	12169	2,06006E+00	20478	9,35680E-01	14701		0	61900
Myeloid_vs_CD8_Non-responder	CD8(Terminal Tex)	pDC_LILRA4	BST2	LILRA4	7,98845E-04	20147,4	1,79571E-02	2623	2,89163E+00	403	3,14327E+00	4522	9,08036E-01	31289		0	61900
Myeloid_vs_CD8_Non-responder	CD8(Temra)	Mono_CD14	RPS19	C5AR1	8,00607E-04	27560,2	4,52861E-03	26723	1,32923E+00	11530	1,70692E+00	30865	9,46695E-01	6783		0	61900
Myeloid_vs_CD8_Non-responder	CD8(LAYN+ T)	Macro_NLRP3	RPS19	C5AR1	8,04245E-04	26730,8	4,73909E-03	24945	1,38771E+00	9954	1,70582E+00	30893	9,47829E-01	5962		0	61900
Myeloid_vs_CD8_Non-responder	Mono_CD14	CD8(ID2+CXCR4+ T)	VCAN	CD44	8,13351E-04	19936	8,85480E-03	9778	2,40076E+00	1055	2,39849E+00	13347	9,37421E-01	13600		0	61900
Myeloid_vs_CD8_Non-responder	CD8(ZNF683+KLRB1+ T)	pDC_LILRA4	CCL5	CXCR3	8,14581E-04	27423,8	1,03214E-02	7607	9,16496E-01	28840	1,70364E+00	30972	9,45429E-01	7800		0	61900
Myeloid_vs_CD8_Non-responder	Macro_FOLR2+APOE+	CD8(Terminal Tex)	HLA-DQB1	LAG3	8,15707E-04	20182,8	7,13039E-03	13610	1,37426E+00	10286	2,80815E+00	7554	9,45729E-01	7564		0	61900
Myeloid_vs_CD8_Non-responder	CD8(GZMK+ Tex)	Macro_FOLR2+APOE+	CD52	SIGLEC10	8,16292E-04	28399,6	6,00242E-03	17566	1,35323E+00	10840	1,70340E+00	30985	9,24988E-01	20707		0	61900
Myeloid_vs_CD8_Non-responder	CD8(ISG+ T)	Macro_ISG15	B2M	LILRB1	8,16950E-04	27504,6	4,11273E-03	30990	1,35900E+00	10693	1,72925E+00	30136	9,51491E-01	3804		0	61900
Myeloid_vs_CD8_Non-responder	Macro_FOLR2+APOE+	CD8(GZMK+ Tem)	HLA-DRB5	LAG3	8,19486E-04	19378,4	7,12387E-03	13626	1,39967E+00	9653	2,79926E+00	7661	9,51011E-01	4052		0	61900
Myeloid_vs_CD8_Non-responder	CD8(IL7R+ZNF683+ Tm)	Macro_ISG15	HLA-C	LILRB1	8,20383E-04	28844,4	4,14721E-03	30597	1,31887E+00	11847	1,70241E+00	31016	9,44052E-01	8862		0	61900

Myeloid_vs_CD8_Post_NR

Myeloid_vs_CD8_Non-responder	CD8(GZMK+ Tem)	Macro_ISG15	HLA-B	LILRB1	8,24624E-04	28057,2	4,19732E-03	30047	1,35851E+00	10707	1,70155E+00	31048	9,46956E-01	6584	0	61900
Myeloid_vs_CD8_Non-responder	CD8(ISG+ T)	Macro_FOLR2+APOE+	HMGB1	CD163	8,28482E-04	27932,8	4,10461E-03	31077	1,30354E+00	12290	2,09493E+00	19610	9,35540E-01	14787	0	61900
Myeloid_vs_CD8_Non-responder	CD8(ISG+ T)	pDC_LILRA4	TNF	TNFRSF21	8,29708E-04	29053,8	1,09163E-02	6946	2,09317E+00	2085	2,38080E+00	13669	8,64180E-01	60669	0	61900
Myeloid_vs_CD8_Non-responder	Macro_FOLR2+APOE+	CD8(Tn)	CXCL9	DPP4	8,32248E-04	35016,6	2,13376E-02	1872	2,34718E+00	1200	3,13587E+00	4585	7,92127E-01	105526	0	61900
Myeloid_vs_CD8_Non-responder	CD8(GZMK+ Tem)	Macro_FOLR2+APOE+	IFNG	IFNGR1_IFNGR2	8,34766E-04	29959,6	7,91615E-03	11619	1,12802E+00	18439	1,69923E+00	31124	9,15266E-01	26716	0	61900
Myeloid_vs_CD8_Non-responder	CD8(GZMK+ Tex)	Macro_ISG15	B2M	LILRB1	8,40279E-04	27815,8	4,09611E-03	31165	1,34166E+00	11151	1,70242E+00	31014	9,51397E-01	3849	0	61900
Myeloid_vs_CD8_Non-responder	Macro_FOLR2+APOE+	CD8(GZMK+ Tem)	HLA-DRA	LAG3	8,41712E-04	18278	7,08276E-03	13719	1,44172E+00	8725	3,03495E+00	5389	9,56466E-01	1657	0	61900
Myeloid_vs_CD8_Non-responder	Macro_FOLR2+APOE+	CD8(GZMK+ Tex)	HLA-DQB1	LAG3	8,44612E-04	20212	7,07857E-03	13731	1,36910E+00	10413	2,83282E+00	7305	9,45542E-01	7711	0	61900
Myeloid_vs_CD8_Non-responder	cDC(CD1C)	CD8(GZMK+ Tem)	HLA-DPB1	LAG3	8,45338E-04	19492,2	7,07728E-03	13734	1,47074E+00	8142	2,54410E+00	10978	9,53856E-01	2707	0	61900
Myeloid_vs_CD8_Non-responder	CD8(Tc17)	Macro_OLFML3	LTB	TNFRSF1A	8,47041E-04	29940,2	8,23324E-03	10925	1,21693E+00	15076	1,71523E+00	30585	9,08127E-01	31215	0	61900
Myeloid_vs_CD8_Non-responder	Macro_NLRP3	CD8(LAYN+ T)	S100A8	CD69	8,51895E-04	18215	9,58728E-03	8554	2,02228E+00	2425	2,37510E+00	13761	9,50270E-01	4435	0	61900
Myeloid_vs_CD8_Non-responder	CD8(ISG+ T)	Macro_ISG15	CD99	PILRA	8,53983E-04	31658,2	4,08576E-03	31266	1,22462E+00	14781	1,74413E+00	29644	9,24995E-01	20700	0	61900
Myeloid_vs_CD8_Non-responder	Mono_CD14	CD8(Tn)	VCAN	SELL	8,62700E-04	22153	1,51453E-02	3739	2,61617E+00	695	3,12824E+00	4641	8,95429E-01	39790	0	61900
Myeloid_vs_CD8_Non-responder	CD8(Tc17)	pDC_LILRA4	HMGB1	THBD	8,65655E-04	32579,8	4,41736E-03	27761	1,12408E+00	18626	1,95517E+00	23261	9,07945E-01	31351	0	61900
Myeloid_vs_CD8_Non-responder	CD8(Temra)	Mono_CD16	HLA-C	LILRA3	8,67589E-04	24784,8	1,18469E-02	6070	1,72261E+00	4641	1,69273E+00	31365	9,26275E-01	19948	0	61900
Myeloid_vs_CD8_Non-responder	CD8(IL7R+ZNF683+ Tm)	Mono_CD16	HLA-B	LILRA1	8,69810E-04	26563,6	9,25440E-03	9068	1,70341E+00	4822	1,40230E+00	43194	9,37014E-01	13834	0	61900
Myeloid_vs_CD8_Non-responder	CD8(GZMK+ Early Tem)	pDC_LILRA4	HMGB1	THBD	8,78710E-04	32820,8	4,40475E-03	27876	1,12148E+00	18737	1,92408E+00	24146	9,07825E-01	31445	0	61900
Myeloid_vs_CD8_Non-responder	cDC_LAMP3	CD8(IL7R+ZNF683+ Tm)	CCL19	CCR7	8,80631E-04	32481	3,41398E-02	747	3,96692E+00	79	2,37054E+00	13844	8,24822E-01	85835	0	61900
Myeloid_vs_CD8_Non-responder	CD8(GZMK+ Early Tem)	pDC_LILRA4	CCL5	CXCR3	8,80808E-04	27352	1,04155E-02	7505	9,25822E-01	28285	1,69019E+00	31460	9,45662E-01	7610	0	61900
Myeloid_vs_CD8_Non-responder	cDC(CD1C)	CD8(GZMK+ Tem)	HLA-DRA	LAG3	8,81242E-04	19400,4	7,02517E-03	13880	1,43017E+00	8972	2,57464E+00	10525	9,56296E-01	1725	0	61900
Myeloid_vs_CD8_Non-responder	CD8(NME1+ T)	Mono_CD16	HLA-C	LILRB2	8,86985E-04	24758,6	4,06551E-03	31504	1,60894E+00	5916	2,00773E+00	21815	9,53982E-01	2658	0	61900
Myeloid_vs_CD8_Non-responder	CD8(Temra)	Mono_CD16	HLA-B	LILRA1	8,86999E-04	26780	9,21848E-03	9136	1,69123E+00	4939	1,38441E+00	44022	9,36899E-01	13903	0	61900
Myeloid_vs_CD8_Non-responder	CD8(GZMK+ Tex)	Mono_CD14	RPS19	C5AR1	8,87126E-04	27765,4	4,51146E-03	26888	1,32479E+00	11672	1,68895E+00	31505	9,46599E-01	6862	0	61900
Myeloid_vs_CD8_Non-responder	Macro_FOLR2+APOE+	CD8(Terminal Tex)	CXCL9	DPP4	8,87695E-04	34417,6	2,26714E-02	1682	2,36061E+00	1170	3,12136E+00	4686	7,97075E-01	102650	0	61900
Myeloid_vs_CD8_Non-responder	CD8(IL7R+ZNF683+ Tm)	Mono_CD14	RPS19	C5AR1	8,98304E-04	29513,2	4,13314E-03	30758	1,22692E+00	14687	1,68663E+00	31584	9,44341E-01	8637	0	61900
Myeloid_vs_CD8_Non-responder	cDC(CD1C)	CD8(GZMK+ Tem)	HLA-DQA2	LAG3	9,02150E-04	19865,6	1,15690E-02	6298	1,72737E+00	4598	2,44426E+00	12569	9,36801E-01	13963	0	61900
Myeloid_vs_CD8_Non-responder	CD8(GZMK+ Tem)	Macro_ISG15	HLA-A	LILRB2	9,02150E-04	23695,6	4,05663E-03	31611	1,70626E+00	4795	2,18745E+00	17523	9,54011E-01	2649	0	61900
Myeloid_vs_CD8_Non-responder	CD8(LAYN+ T)	pDC_LILRA4	HMGB1	THBD	9,03578E-04	31218,6	4,70410E-03	25232	1,18321E+00	16282	1,96264E+00	23049	9,10540E-01	29630	0	61900
Myeloid_vs_CD8_Non-responder	CD8(Temra)	Macro_ISG15	HLA-C	LILRB2	9,06439E-04	23307,4	4,05364E-03	31641	1,75450E+00	4351	2,26206E+00	15963	9,53918E-01	2682	0	61900
Myeloid_vs_CD8_Non-responder	Macro_NLRP3	CD8(Tc17)	CXCL2	DPP4	9,07460E-04	24822,2	3,68736E-02	630	2,19593E+00	1674	3,11646E+00	4721	8,72341E-01	55186	0	61900
Myeloid_vs_CD8_Non-responder	Macro_OLFML3	CD8(GZMK+ Tem)	HLA-DQB1	LAG3	9,08774E-04	21451,4	7,31784E-03	13050	1,33713E+00	11286	2,36229E+00	13989	9,46391E-01	7032	0	61900
Myeloid_vs_CD8_Non-responder	Macro_ISG15	CD8(Tn)	CXCL10	CXCR3	9,14303E-04	25225,4	1,34747E-02	4733	1,98474E+00	2627	3,23455E+00	3882	8,75703E-01	52985	0	61900
Myeloid_vs_CD8_Non-responder	pDC_LILRA4	CD8(GZMK+ Tem)	APP	CD74	9,19809E-04	18669,8	6,97955E-03	14032	2,27737E+00	1405	2,37639E+00	13746	9,55027E-01	2266	0	61900
Myeloid_vs_CD8_Non-responder	Mono_CD14	CD8(ITM2C+ T)	VCAN	CD44	9,23940E-04	20147,8	8,63279E-03	10159	2,38297E+00	1094	2,38873E+00	13538	9,36672E-01	14048	0	61900
Myeloid_vs_CD8_Non-responder	Macro_ISG15	CD8(Terminal Tex)	CXCL10	CXCR3	9,25207E-04	25395,2	1,34552E-02	4752	1,98427E+00	2631	3,12632E+00	4653	8,75624E-01	53040	0	61900
Myeloid_vs_CD8_Non-responder	Mono_CD14	CD8(GZMK+ Tex)	VCAN	CD44	9,25234E-04	20227,2	8,63076E-03	10162	2,38281E+00	1096	2,36634E+00	13925	9,36665E-01	14053	0	61900
Myeloid_vs_CD8_Non-responder	CD8(LAYN+ T)	Macro_OLFML3	ANXA1	FPR1	9,29152E-04	30634,8	4,56022E-03	26422	1,17649E+00	16533	1,68112E+00	31798	9,32442E-01	16521	0	61900
Myeloid_vs_CD8_Non-responder	CD8(EOMES+ NK-like)	Macro_NLRP3	HMGB1	THBD	9,37068E-04	31770	5,00287E-03	23011	1,24736E+00	13998	1,67974E+00	31852	9,13016E-01	28089	0	61900
Myeloid_vs_CD8_Non-responder	CD8(LAYN+ T)	Mono_CD14	ANXA1	FPR1	9,40752E-04	25521	5,52596E-03	19882	1,40071E+00	9622	1,95963E+00	23137	9,38247E-01	13064	0	61900
Myeloid_vs_CD8_Non-responder	CD8(GZMK+ Early Tem)	Macro_NLRP3	RPS19	C5AR1	9,46521E-04	27665,4	4,54376E-03	26587	1,33905E+00	11218	1,67796E+00	31916	9,46779E-01	6706	0	61900
Myeloid_vs_CD8_Non-responder	CD8(Temra)	cDC_CLEC9A	XCL1	XCR1	9,47586E-04	45261,8	3,35824E-02	775	5,03993E+00	32	1,16457E+00	54926	7,86394E-01	108676	0	61900
Myeloid_vs_CD8_Non-responder	CD8(ZNF683+KLRB1+ T)	pDC_LILRA4	SELPLG	SELL	9,47707E-04	23684,2	9,29518E-03	9012	1,59155E+00	6178	2,66068E+00	9407	9,07123E-01	31924	0	61900
Myeloid_vs_CD8_Non-responder	CD8(GZMK+ Early Tem)	Macro_ISG15	CD99	PILRA	9,48598E-04	32080,4	4,02862E-03	31930	1,21216E+00	15238	1,72204E+00	30368	9,24505E-01	20966	0	61900
Myeloid_vs_CD8_Non-responder	CD8(IL7R+ZNF683+ Tm)	pDC_LILRA4	BST2	LILRA4	9,49017E-04	26222,2	9,25855E-03	9059	2,68652E+00	597	2,86122E+00	7003	8,76388E-01	52552	0	61900
Myeloid_vs_CD8_Non-responder	Mono_CD14	CD8(Temra)	VCAN	CD44	9,49280E-04	20249,8	8,65355E-03	10114	2,38463E+00	1084	2,35376E+00	14145	9,36743E-01	14006	0	61900
Myeloid_vs_CD8_Non-responder	CD8(IL7R+ZNF683+ Tm)	pDC_LILRA4	COPA	P2RY6	9,53245E-04	30251,4	7,53155E-03	12458	2,63828E+00	659	3,02057E+00	5515	8,48689E-01	70725	0	61900
Myeloid_vs_CD8_Non-responder	CD8(EOMES+ NK-like)	Macro_ISG15	HLA-A	LILRB1	9,55302E-04	28730,4	4,23684E-03	29613	1,31921E+00	11837	1,67613E+00	31975	9,44705E-01	8327	0	61900
Myeloid_vs_CD8_Non-responder	cDC_CLEC9A	CD8(GZMK+ Tem)	HLA-DPA1	LAG3	9,56425E-04	18485,4	6,92843E-03	14172	1,45783E+00	8397	2,96896E+00	5991	9,55691E-01	1967	0	61900
Myeloid_vs_CD8_Non-responder	CD8(LAYN+ T)	Macro_ISG15	ANXA1	FPR2_FPR3	9,56946E-04	27995,4	7,97608E-03	11474	1,42714E+00	9042	1,70399E+00	30955	9,15468E-01	26606	0	61900
Myeloid_vs_CD8_Non-responder	CD8(LAYN+ T)	Macro_ISG15	ANXA1	FPR1	9,57096E-04	29389,6	4,41059E-03	27823	1,14175E+00	17868	1,99129E+00	22252	9,31384E-01	17105	0	61900
Myeloid_vs_CD8_Non-responder	CD8(LAYN+ T)	Macro_ISG15	HLA-A	LILRB1	9,57694E-04	28517,2	4,26391E-03	29311	1,33698E+00	11291	1,67940E+00	31861	9,44872E-01	8223	0	61900
Myeloid_vs_CD8_Non-responder	CD8(LAYN+ T)	Macro_ISG15	HLA-C	LILRB1	9,58593E-04	27473,2	4,29045E-03	29018	1,40931E+00	9428	1,76888E+00	28845	9,44942E-01	8175	0	61900
Myeloid_vs_CD8_Non-responder	CD8(LAYN+ T)	Macro_ISG15	CD99	PILRA	9,59342E-04	31173	4,17280E-03	30317	1,24359E+00	14131	1,75591E+00	29237	9,25723E-01	20280	0	61900
Myeloid_vs_CD8_Non-responder	CD8(Tc17)	Macro_ISG15	CD99	PILRA	9,60842E-04	31948,6	4,02192E-03	32012	1,21070E+00	15281	1,74711E+00	29555	9,24447E-01	20995	0	61900
Myeloid_vs_CD8_Non-responder	CD8(NME1+ T)	Macro_ISG15	HMGB1	THBD	9,61292E-04	32388,8	4,31775E-03	28737	1,16582E+00	16936	1,98754E+00	22356	9,06987E-01	32015	0	61900
Myeloid_vs_CD8_Non-responder	cDC_CLEC9A	CD8(GZMK+ Tem)	HLA-DRA	LAG3	9,63075E-04	18770	6,91967E-03	14197	1,40902E+00	9435	2,91647E+00	6472	9,55979E-01	1846	0	61900
Myeloid_vs_CD8_Non-responder	cDC_LAMP3	CD8(ZNF683+KLRB1+ T)	CCL19	CXCR3	9,67162E-04	28897,2	3,34542E-02	783	3,90157E+00	85	2,34872E+00	14232	8,53604E-01	67486	0	61900
Myeloid_vs_CD8_Non-responder	CD8(LAYN+ T)	Macro_ISG15	RPS19	C5AR1	9,67312E-04	25782	4,76383E-03	24740	1,39468E+00	9780	1,83871E+00	26628	9,47958E-01	5862	0	61900
Myeloid_vs_CD8_Non-responder	CD8(ZNF683+KLRB1+ T)	cDC_CLEC9A	FLT3LG	FLT3	9,74555E-04	34499,8	3,34314E-02	786	2,65849E+00	638	1,88768E+00	25202	8,27775E-01	83973	0	61900
Myeloid_vs_CD8_Non-responder	Macro_FOLR2+APOE+	CD8(LAYN+ T)	CD14	ITGA4	9,74594E-04	20071,6	6,90487E-03	14240	1,76499E+00	4284	2,97794E+00	5905	9,36704E-01	14029	0	61900
Myeloid_vs_CD8_Non-responder	CD8(Tc17)	cDC_CLEC9A	LTB	CD40	9,74728E-04	23737,8	1,40183E-02	4385	1,73655E+00	4519	2,27118E+00	15781	9,06846E-01	32104	0	61900
Myeloid_vs_CD8_Non-responder	CD8(Temra)	Mono_CD16	HLA-C	LILRB1	9,74880E-04	28826,4	4,01534E-03	32105	1,38514E+00	10017	1,71558E+00	30575	9,43192E-01	9535	0	61900
Myeloid_vs_CD8_Non-responder	CD8(ID2+CXCR4+ T)	Macro_FOLR2+APOE+	CD52	SIGLEC10	9,82495E-04	28183	6,19290E-03	16752	1,38479E+00	10030	1,67092E+00	32155	9,26065E-01	20078	0	61900
Myeloid_vs_CD8_Non-responder	CD8(Temra)	Mono_CD16	HLA-C	LILRA1	9,84316E-04	24074,2	1,00091E-02	7964	1,91624E+00	3050	1,64366E+00	33181	9,36352E-01	14276	0	61900
Myeloid_vs_CD8_Non-responder	CD8(Tn)	Mono_CD16	HLA-C	LILRB2	9,86627E-04	25192,4	4,00788E-03	32182	1,57568E+00	6408	1,97555E+00	22684	9,53667E-01	2788	0	61900
Myeloid_vs_CD8_Non-responder	Macro_FOLR2+APOE+	CD8(Terminal Tex)	B2M	CD3D	9,87250E-04	35888,4	2,94225E-03	50522	9,67957E-01	25862	1,44818E+00	41151	9,66143E-01	7	0	61900
Myeloid_vs_CD8_Non-responder	cDC_LAMP3	CD8(GZMK+ Tex)	CCL19	CXCR3	9,94404E-04	29183,4	3,32321E-02	794	3,89989E+00	86	2,29179E+00	15381	8,53187E-01	67756	0	61900
Myeloid_vs_CD8_Non-responder	CD8(IL7R+ZNF683+ Tm)	Macro_ISG15	HLA-B	LILRB1	1,00500E-03	28776,8	4,13519E-03	30731	1,31138E+00	12077	1,66681E+00	32301	9,46580E-01	6875	0	61900
Myeloid_vs_CD8_Non-responder	CD8(EOMES+ NK-like)	Macro_ISG15	HLA-C	LILRB1	1,01015E-03	29497	4,09164E-03	31214	1,28378E+00	12880	1,66592E+00	32334	9,43694E-01	9157	0	61900
Myeloid_vs_CD8_Non-responder	Macro_NLRP3	CD8(ISG+ T)	VCAN	CD44	1,01835E-03	20421	8,48876E-03	10417	2,14469E+00	1861	2,38910E+00	13527	9,36171E-01	14400	0	61900
Myeloid_vs_CD8_Non-responder	CD8(ZNF683+KLRB1+ T)	Mono_CD16	HLA-A	LILRA1	1,02302E-03	23631,8	1,03611E-02	7564	1,96905E+00	2722	1,66363E+00	32416	9,37477E-01	13557	0	61900
Myeloid_vs_CD8_Non-responder	CD8(LAYN+ T)	Macro_FOLR2+APOE+	HMGB1	CD163	1,03125E-03	28990,6	3,98381E-03	32468	1,27412E+00	13163	1,99589E+00	22124	9,34633E-01	15298	0	61900
Myeloid_vs_CD8_Non-responder	cDC_CLEC9A	CD8(GZMK+ Tem)	HLA-DRB5	LAG3	1,03204E-03	20085,8	6,83507E-03	14449	1,34483E+00	11063	2,73293E+00	8460	9,50038E-01	4557	0	61900
Myeloid_vs_CD8_Non-responder	CD8(GZMK+ Tex)	Macro_ISG15	CCL5	CCR1	1,03364E-03	24201,6	1,36904E-02	4574	1,57627E+00	6398	1,66164E+00	32483	9,34013E-01	15653	0	61900
Myeloid_vs_CD8_Non-responder	CD8(Tn)	cDC_CLEC9A	FLT3LG	FLT3	1,03470E-03	35030,6	3,28878E-02	810	2,65255E+00	646	1,82418E+00	27093	8,26603E-01	84704	0	61900
Myeloid_vs_CD8_Non-responder	CD8(ISG+ T)	cDC_LAMP3	TNFSF10	TNFRSF11B	1,03773E-03	46723	3,35397E-02	776	2,37927E+00	1109	1,22244E+00	51875	7,69183E-01	117955	0	61900
Myeloid_vs_CD8_Non-responder	CD8(ITM2C+ T)	pDC_LILRA4	BST2	LILRA4	1,04084E-03	22554,4	1,31697E-02	4945	2,77874E+00	509	3,10574E+00	4814	8,94245E-01	40604	0	61900

Myeloid_vs_CD8_Post_NR

Myeloid_vs_CD8_Non-responder	CD8(NME1+ T)	pDC_LILRA4	SELPLG	SELL	1,04105E-03	27321,6	7,27541E-03	13157	1,48799E+00	7837	2,33624E+00	14481	8,96274E-01	39233	0	61900
Myeloid_vs_CD8_Non-responder	CD8(ISG+ T)	cDC_LAMP3	CD200R1	CD200	1,04209E-03	47468,4	3,78275E-02	588,5	2,06956E+00	2186,5	1,46462E+00	40430,5	7,39655E-01	132236,5	0	61900
Myeloid_vs_CD8_Non-responder	CD8(GZMK+ Tem)	cDC_CLEC9A	XCL1	XCR1	1,04745E-03	46034,6	3,27441E-02	815	5,03650E+00	33	1,11468E+00	57595	7,84263E-01	109830	0	61900
Myeloid_vs_CD8_Non-responder	Mono_CD14	CD8(ITM2C+ T)	S100A8	ITGB2	1,05668E-03	16966,6	6,80451E-03	14536	2,54957E+00	789	3,67711E+00	1721	9,47928E-01	5887	0	61900
Myeloid_vs_CD8_Non-responder	CD8(Temra)	Macro_FOLR2+APOE+	SPON2	ITGB2	1,06097E-03	28414,4	7,67861E-03	12126	1,12888E+00	18406	2,21169E+00	16987	9,06043E-01	32653	0	61900
Myeloid_vs_CD8_Non-responder	Mast	CD8(Tn)	TIMP3	CD44	1,06286E-03	26758,2	3,73624E-02	606	2,52896E+00	821	1,44982E+00	41076	9,10931E-01	29388	0	61900
Myeloid_vs_CD8_Non-responder	CD8(GZMK+ Early Tem)	Macro_ISG15	HLA-C	LILRB1	1,06471E-03	29344,6	4,12289E-03	30860	1,30351E+00	12292	1,65667E+00	32676	9,43896E-01	8995	0	61900
Myeloid_vs_CD8_Non-responder	cDC(CD1C)	CD8(GZMK+ Tem)	HLA-DPA1	LAG3	1,06499E-03	19785,2	6,79809E-03	14565	1,42852E+00	9004	2,52161E+00	11298	9,55287E-01	2159	0	61900
Myeloid_vs_CD8_Non-responder	CD8(ISG+ T)	Macro_NLRP3	HMGB1	THBD	1,06618E-03	32329,8	4,89975E-03	23711	1,22632E+00	14720	1,65641E+00	32685	9,12186E-01	28633	0	61900
Myeloid_vs_CD8_Non-responder	CD8(Temra)	pDC_LILRA4	HMGB1	THBD	1,07075E-03	34169,6	4,21400E-03	29850	1,08214E+00	20437	1,86181E+00	25948	9,05956E-01	32713	0	61900
Myeloid_vs_CD8_Non-responder	CD8(GZMK+ Tex)	Macro_LYVE1	HMGB1	CD163	1,08011E-03	27438	4,88722E-03	23816	1,51595E+00	7335	1,65437E+00	32770	9,40606E-01	11369	0	61900
Myeloid_vs_CD8_Non-responder	CD8(GZMK+ Tem)	Macro_ISG15	HLA-C	LILRB2	1,08722E-03	24106,4	3,95806E-03	32813	1,68791E+00	4966	2,16845E+00	17949	9,53390E-01	2904	0	61900
Myeloid_vs_CD8_Non-responder	CD8(GZMK+ Tex)	Macro_ISG15	HLA-A	LILRB2	1,10288E-03	24561,2	3,94894E-03	32907	1,62829E+00	5670	2,10225E+00	19439	9,53417E-01	2890	0	61900
Myeloid_vs_CD8_Non-responder	CD8(NME1+ T)	Macro_ISG15	HMGB1	HAVCR2	1,10691E-03	32136	3,94675E-03	32931	1,07285E+00	20853	1,79181E+00	28119	9,31777E-01	16877	0	61900
Myeloid_vs_CD8_Non-responder	CD8(Temra)	Macro_ISG15	HLA-B	LILRB1	1,11551E-03	29037,8	4,11914E-03	30919	1,29920E+00	12430	1,64892E+00	32982	9,46481E-01	6958	0	61900
Myeloid_vs_CD8_Non-responder	CD8(ID2+CXCR4+ T)	Macro_ISG15	HLA-A	LILRB2	1,11771E-03	24560,8	3,94056E-03	32995	1,62222E+00	5752	2,11025E+00	19245	9,53370E-01	2912	0	61900
Myeloid_vs_CD8_Non-responder	CD8(GZMK+ Early Tem)	Mono_CD16	HLA-A	LILRA1	1,11854E-03	25751	9,72549E-03	8349	1,78268E+00	4121	1,48317E+00	39637	9,35596E-01	14748	0	61900
Myeloid_vs_CD8_Non-responder	CD8(IL7R+ZNF683+ Tm)	Macro_ISG15	HLA-A	LILRB1	1,12025E-03	29163,4	4,20474E-03	29945	1,29816E+00	12469	1,64824E+00	33010	9,44507E-01	8493	0	61900
Myeloid_vs_CD8_Non-responder	CD8(GZMK+ Early Tem)	Macro_ISG15	HLA-A	LILRB2	1,12212E-03	24612,4	3,93879E-03	33021	1,62094E+00	5770	2,10157E+00	19453	9,53360E-01	2918	0	61900
Myeloid_vs_CD8_Non-responder	pDC_LILRA4	CD8(Terminal Tex)	B2M	CD3D	1,12827E-03	33771,8	2,92800E-03	50832	9,44970E-01	27132	1,76396E+00	28987	9,66063E-01	8	0	61900
Myeloid_vs_CD8_Non-responder	Macro_OLFML3	CD8(GZMK+ Tem)	HLA-DRB5	LAG3	1,13411E-03	21244,2	6,72042E-03	14800	1,32305E+00	11724	2,41798E+00	13002	9,49635E-01	4795	0	61900
Myeloid_vs_CD8_Non-responder	CD8(NME1+ T)	Macro_NLRP3	RPS19	C5AR1	1,14404E-03	28426,8	4,42483E-03	27678	1,30942E+00	12128	1,64465E+00	33149	9,46106E-01	7279	0	61900
Myeloid_vs_CD8_Non-responder	Mono_CD14	CD8(Temra)	S100A8	ITGB2	1,14802E-03	17128,6	6,70593E-03	14846	2,54085E+00	803	3,61346E+00	1949	9,47567E-01	6145	0	61900
Myeloid_vs_CD8_Non-responder	CD8(NME1+ T)	Macro_FOLR2+APOE-	HMGB1	CD163	1,14818E-03	25426,4	5,71383E-03	18905	1,69534E+00	4906	1,64397E+00	33173	9,44824E-01	8248	0	61900
Myeloid_vs_CD8_Non-responder	Macro_OLFML3	CD8(GZMK+ Tem)	HLA-DRA	LAG3	1,14924E-03	19948,4	6,70352E-03	14850	1,36568E+00	10516	2,58907E+00	10327	9,55306E-01	2149	0	61900
Myeloid_vs_CD8_Non-responder	CD8(Tc17)	Macro_FOLR2+APOE+	CD52	SIGLEC10	1,15148E-03	29341	5,80494E-03	18442	1,32051E+00	11794	1,64336E+00	33192	9,23819E-01	21377	0	61900
Myeloid_vs_CD8_Non-responder	Macro_NLRP3	CD8(Tc17)	VCAN	CD44	1,15472E-03	20681,8	8,27286E-03	10846	2,12472E+00	1936	2,36957E+00	13859	9,35397E-01	14868	0	61900
Myeloid_vs_CD8_Non-responder	CD8(LAYN+ T)	Macro_FOLR2+APOE+	CD52	SIGLEC10	1,16018E-03	28630,4	6,09252E-03	17162	1,36816E+00	10439	1,64234E+00	33242	9,25503E-01	20409	0	61900
Myeloid_vs_CD8_Non-responder	Macro_NLRP3	CD8(GZMK+ Tem)	S100A8	CD69	1,16083E-03	19111,8	8,68099E-03	10061	1,95721E+00	2780	2,31608E+00	14888	9,47871E-01	5930	0	61900
Myeloid_vs_CD8_Non-responder	CD8(Tn)	Macro_FOLR2+APOE+	ANXA1	FPR1	1,16227E-03	31668	3,91889E-03	33254	1,05157E+00	21837	1,99727E+00	22089	9,27509E-01	19260	0	61900
Myeloid_vs_CD8_Non-responder	CD8(GZMK+ Early Tem)	pDC_LILRA4	COPA	P2RY6	1,16574E-03	31852,8	6,68898E-03	14904	2,61452E+00	697	2,96596E+00	6025	8,40913E-01	75738	0	61900
Myeloid_vs_CD8_Non-responder	CD8(ID2+CXCR4+ T)	Mono_CD14	RPS19	C5AR1	1,16630E-03	29004,4	4,30933E-03	28827	1,27250E+00	13217	1,64162E+00	33277	9,45428E-01	7801	0	61900
Myeloid_vs_CD8_Non-responder	CD8(ISG+ T)	Mono_CD16	HLA-A	LILRA1	1,16728E-03	25963	9,64130E-03	8479	1,75799E+00	4326	1,46994E+00	40201	9,35334E-01	14909	0	61900
Myeloid_vs_CD8_Non-responder	CD8(Terminal Tex)	Macro_FOLR2+APOE+	CD99	CD81	1,16752E-03	30386,6	4,51881E-03	26817	1,14367E+00	17786	1,78954E+00	28196	9,31146E-01	17234	0	61900
Myeloid_vs_CD8_Non-responder	CD8(Terminal Tex)	Macro_FOLR2+APOE+	CD99	PILRA	1,16805E-03	28543,6	4,66814E-03	25524	1,37497E+00	10266	1,83068E+00	26897	9,29488E-01	18131	0	61900
Myeloid_vs_CD8_Non-responder	CD8(GZMK+ Tem)	Mono_CD16	HLA-A	LILRB1	1,17103E-03	29602,8	4,01830E-03	32068	1,33690E+00	11294	1,64097E+00	33304	9,43306E-01	9448	0	61900
Myeloid_vs_CD8_Non-responder	CD8(Tc17)	Macro_ISG15	RPS19	C5AR1	1,17174E-03	29397,8	3,91384E-03	33308	1,18403E+00	16252	1,86811E+00	25770	9,42891E-01	9759	0	61900
Myeloid_vs_CD8_Non-responder	CD8(Terminal Tex)	Macro_FOLR2+APOE+	IFNG	IFNGR1_IFNGR2	1,17332E-03	24816,4	9,47957E-03	8730	1,24235E+00	14172	2,22233E+00	16771	9,21999E-01	22509	0	61900
Myeloid_vs_CD8_Non-responder	CD8(Terminal Tex)	Macro_FOLR2+APOE+	RPS19	C5AR1	1,17808E-03	25783,2	4,74654E-03	24887	1,38581E+00	10002	1,85327E+00	26196	9,47868E-01	5931	0	61900
Myeloid_vs_CD8_Non-responder	CD8(GZMK+ Early Tem)	Macro_FOLR2+APOE-	TNFSF9	HLA-DPA1	1,17844E-03	29113,8	6,08574E-03	17199	1,21213E+00	15239	1,64002E+00	33346	9,29927E-01	17885	0	61900
Myeloid_vs_CD8_Non-responder	CD8(ZNF683+KLRB1+ T)	Mono_CD14	CIRBP	TREM1	1,17985E-03	31736,8	5,95564E-03	17775	1,24855E+00	13956	1,68374E+00	31699	9,04941E-01	33354	0	61900
Myeloid_vs_CD8_Non-responder	CD8(Terminal Tex)	Macro_FOLR2+APOE+	CD52	SIGLEC10	1,18215E-03	26706,4	6,54940E-03	15405	1,44386E+00	8680	1,77984E+00	28526	9,27958E-01	19021	0	61900
Myeloid_vs_CD8_Non-responder	CD8(Tc17)	Macro_ISG15	HLA-A	LILRB1	1,18322E-03	29356,8	4,18734E-03	30141	1,28674E+00	12787	1,63925E+00	33373	9,44398E-01	8583	0	61900
Myeloid_vs_CD8_Non-responder	CD8(ID2+CXCR4+ T)	Mono_CD16	HLA-F	LILRB2	1,18481E-03	29309	3,90825E-03	33382	1,75628E+00	4339	2,23502E+00	16523	9,09372E-01	30401	0	61900
Myeloid_vs_CD8_Non-responder	CD8(Terminal Tex)	Macro_FOLR2+APOE+	HMGB1	CD163	1,18606E-03	26814	4,35809E-03	28339	1,36527E+00	10527	2,09252E+00	19656	9,37323E-01	13648	0	61900
Myeloid_vs_CD8_Non-responder	CD8(IL7R+ZNF683+ Tm)	Macro_FOLR2+APOE+	HMGB1	CD163	1,18712E-03	29230,6	3,90754E-03	33395	1,25555E+00	13734	2,02070E+00	21490	9,34040E-01	15634	0	61900
Myeloid_vs_CD8_Non-responder	CD8(Tc17)	Mono_CD16	RPS19	C5AR1	1,18730E-03	29451,8	3,90751E-03	33396	1,18186E+00	16342	1,86573E+00	25842	9,42847E-01	9779	0	61900
Myeloid_vs_CD8_Non-responder	CD8(Terminal Tex)	Macro_FOLR2+APOE+	HSPA1A	TLR4	1,18872E-03	29205,2	6,60837E-03	15186	1,23981E+00	14249	1,75092E+00	29423	9,17572E-01	25268	0	61900
Myeloid_vs_CD8_Non-responder	CD8(ISG+ T)	Macro_ISG15	HLA-A	LILRB2	1,19300E-03	24842,6	3,90469E-03	33428	1,59625E+00	6102	2,08834E+00	19772	9,53166E-01	3011	0	61900
Myeloid_vs_CD8_Non-responder	CD8(NME1+ T)	pDC_LILRA4	CXCL13	CXCR3	1,19872E-03	28612	2,03311E-02	2037	9,12616E-01	29066	2,23118E+00	16597	9,04817E-01	33460	0	61900
Myeloid_vs_CD8_Non-responder	CD8(ZNF683+KLRB1+ T)	Macro_ISG15	B2M	LILRB2	1,20374E-03	23314,6	3,90082E-03	33488	1,78875E+00	4067	2,23628E+00	16500	9,59520E-01	618	0	61900
Myeloid_vs_CD8_Non-responder	CD8(Terminal Tex)	Macro_NLRP3	RPS19	C5AR1	1,20410E-03	25693,2	4,91231E-03	23629	1,43086E+00	8961	1,77704E+00	28622	9,48710E-01	5354	0	61900
Myeloid_vs_CD8_Non-responder	CD8(Terminal Tex)	Macro_NLRP3	HMGB1	THBD	1,20590E-03	31264,6	5,20233E-03	21727	1,28805E+00	12753	1,65400E+00	32785	9,14556E-01	27158	0	61900
Myeloid_vs_CD8_Non-responder	CD8(Terminal Tex)	Macro_NLRP3	HSPA1A	TLR4	1,21113E-03	27755,6	6,74917E-03	14703	1,25893E+00	13621	1,93577E+00	23802	9,18366E-01	24752	0	61900
Myeloid_vs_CD8_Non-responder	CD8(Terminal Tex)	Mono_CD16	HLA-B	LILRB2	1,21420E-03	22756,8	4,42258E-03	27702	1,80321E+00	3925	2,11591E+00	19098	9,57875E-01	1159	0	61900
Myeloid_vs_CD8_Non-responder	CD8(ISG+ T)	Macro_ISG15	HLA-B	LILRB2	1,21457E-03	24157,8	3,89567E-03	33548	1,66528E+00	5207	2,16809E+00	17957	9,55239E-01	2177	0	61900
Myeloid_vs_CD8_Non-responder	CD8(Terminal Tex)	Mono_CD16	HLA-A	LILRB2	1,21583E-03	22346,2	4,55936E-03	26430	1,83594E+00	3650	2,16080E+00	18110	9,56507E-01	1641	0	61900
Myeloid_vs_CD8_Non-responder	CD8(Terminal Tex)	Mono_CD16	HLA-C	LILRB2	1,21801E-03	24500	4,17665E-03	30273	1,67307E+00	5111	1,97257E+00	22772	9,54570E-01	2444	0	61900
Myeloid_vs_CD8_Non-responder	CD8(Terminal Tex)	Mono_CD16	CD99	PILRA	1,21873E-03	28705,8	4,62532E-03	25862	1,36180E+00	10626	1,83305E+00	26827	9,29186E-01	18314	0	61900
Myeloid_vs_CD8_Non-responder	CD8(Terminal Tex)	Mono_CD16	HLA-F	LILRB2	1,21964E-03	27909,8	4,30933E-03	28829	1,80048E+00	3947	2,21204E+00	16981	9,13317E-01	27892	0	61900
Myeloid_vs_CD8_Non-responder	CD8(Terminal Tex)	Mono_CD16	B2M	LILRB2	1,22110E-03	21918,8	4,44687E-03	27484	1,87083E+00	3398	2,23136E+00	16593	9,61989E-01	219	0	61900
Myeloid_vs_CD8_Non-responder	CD8(Tc17)	Macro_NLRP3	RPS19	C5AR1	1,22110E-03	30357,8	3,89351E-03	33584	1,17706E+00	16515	1,73522E+00	29926	9,42751E-01	9864	0	61900
Myeloid_vs_CD8_Non-responder	CD8(Terminal Tex)	Mono_CD16	IFNG	IFNGR1_IFNGR2	1,22273E-03	33739,6	6,37507E-03	16029	9,01815E-01	29745	1,77416E+00	28685	9,06485E-01	32339	0	61900
Myeloid_vs_CD8_Non-responder	CD8(ID2+CXCR4+ T)	Mono_CD16	ANXA1	FPR1	1,22474E-03	33333	3,89136E-03	33604	1,10081E+00	19623	1,67080E+00	32158	9,27272E-01	19380	0	61900
Myeloid_vs_CD8_Non-responder	CD8(Terminal Tex)	Mono_CD16	RPS19	C5AR1	1,22528E-03	24836,4	4,92997E-03	23499	1,43567E+00	8848	1,90754E+00	24631	9,48797E-01	5304	0	61900
Myeloid_vs_CD8_Non-responder	CD8(Terminal Tex)	Mono_CD16	CD52	SIGLEC10	1,22747E-03	30582,4	5,43199E-03	20408	1,22784E+00	14648	1,64544E+00	33118	9,21449E-01	22838	0	61900
Myeloid_vs_CD8_Non-responder	CD8(LAYN+ T)	Macro_ISG15	HLA-B	LILRB1	1,23021E-03	29153,4	4,12065E-03	30894	1,30034E+00	12391	1,63096E+00	33634	9,46491E-01	6948	0	61900
Myeloid_vs_CD8_Non-responder	CD8(LAYN+ T)	Macro_ISG15	HLA-C	LILRB2	1,23131E-03	24620,4	3,88871E-03	33640	1,63960E+00	5520	2,12277E+00	18952	9,52996E-01	3090	0	61900
Myeloid_vs_CD8_Non-responder	Macro_FOLR2-APOE+	CD8(ISG+ T)	C3	IFITM1	1,23391E-03	19911,2	1,30840E-02	5011	1,82922E+00	3714	1,93977E+00	23693	9,48904E-01	5238	0	61900
Myeloid_vs_CD8_Non-responder	Macro_FOLR2+APOE+	CD8(GZMK+ Tem)	HLA-DPB1	LAG3	1,23608E-03	19842,4	6,62549E-03	15128	1,37184E+00	10350	2,73340E+00	8450	9,52382E-01	3384	0	61900
Myeloid_vs_CD8_Non-responder	CD8(Temra)	pDC_LILRA4	GZMB	IGF2R	1,23644E-03	30885,8	1,05637E-02	7346	1,01078E+00	23668	1,80009E+00	27847	9,04498E-01	33668	0	61900
Myeloid_vs_CD8_Non-responder	CD8(Tn)	Macro_ISG15	CD28	CD86	1,23672E-03	28993	1,57381E-02	3444,5	1,53894E+00	6954,5	2,30478E+00	15130,5	8,68832E-01	57535,5	0	61900
Myeloid_vs_CD8_Non-responder	Macro_FOLR2+APOE+	CD8(NME1+ T)	CXCL9	DPP4	1,23809E-03	37006,2	1,78155E-02	2660	2,31172E+00	1296	3,05141E+00	5244	7,76883E-01	113931	0	61900
Myeloid_vs_CD8_Non-responder	Macro_FOLR2+APOE+	CD8(Terminal Tex)	LGALS3	LAG3	1,23832E-03	23168,4	7,32733E-03	13028	1,21506E+00	15135	2,49176E+00	11762	9,36722E-01	14017	0	61900
Myeloid_vs_CD8_Non-responder	CD8(NME1+ T)	Mono_CD16	HLA-B	LILRA1	1,24315E-03	29700	8,62474E-03	10169	1,48997E+00	7807	1,19225E+00	53474	9,34903E-01	15150	0	61900
Myeloid_vs_CD8_Non-responder	CD8(Terminal Tex)	Macro_OLFML3	IFNG	IFNGR1_IFNGR2	1,24473E-03	29487,2	7,95391E-03	11524	1,07569E+00	20721	1,83726E+00	26677	9,15451E-01	26614	0	61900
Myeloid_vs_CD8_Non-responder	CD8(Tn)	Mono_CD16	HLA-B	LILRA1	1,24638E-03	29632	8,61866E-03	10188	1,48791E+00	7840	1,19987E+00	53072	9,34881E-01	15160	0	61900

Myeloid_vs_CD8_Post_NR

Myeloid_vs_CD8_Non-responder	CD8(EOMES+ NK-like)	Macro_ISG15	HLA-B	LILRB1	1,24658E-03	29712,4	4,05191E-03	31662	1,24820E+00	13972	1,62860E+00	33723	9,46063E-01	7305	0	61900
Myeloid_vs_CD8_Non-responder	CD8(GZMK+ Tem)	Mono_CD14	RPS19	C5AR1	1,24732E-03	29372,2	4,25104E-03	29465	1,25742E+00	13682	1,62847E+00	33727	9,45076E-01	8087	0	61900
Myeloid_vs_CD8_Non-responder	Mast	CD8(LAYN+ T)	TIMP3	CD44	1,24804E-03	28561,8	3,54607E-02	687	2,48210E+00	890	1,28773E+00	48570	9,08789E-01	30762	0	61900
Myeloid_vs_CD8_Non-responder	CD8(Tn)	Macro_FOLR2+APOE+	CD40LG	CD40	1,25220E-03	33742,4	3,69306E-02	626	1,60266E+00	6000	2,30214E+00	15178	8,26088E-01	85008	0	61900
Myeloid_vs_CD8_Non-responder	CD8(NME1+ T)	Macro_ISG15	HMGB1	CD163	1,25640E-03	28592	3,87678E-03	33776	1,24673E+00	14016	2,18839E+00	17492	9,33796E-01	15776	0	61900
Myeloid_vs_CD8_Non-responder	Mono_CD16	CD8(Tc17)	S100A8	CD69	1,25739E-03	23188,4	6,72766E-03	14776	1,27633E+00	13092	2,30130E+00	15194	9,41201E-01	10980	0	61900
Myeloid_vs_CD8_Non-responder	CD8(IL7R+ZNF683+ Tm)	Macro_ISG15	B2M	LILRB1	1,26367E-03	28974,2	4,03021E-03	31907	1,27287E+00	13205	1,62600E+00	33815	9,51021E-01	4044	0	61900
Myeloid_vs_CD8_Non-responder	Macro_FOLR2+APOE+	CD8(ZNF683+KLRB1+ T)	B2M	CD3D	1,26929E-03	35774,8	2,92037E-03	51017	9,60309E-01	26272	1,48219E+00	39676	9,66021E-01	9	0	61900
Myeloid_vs_CD8_Non-responder	CD8(ZNF683+KLRB1+ T)	Macro_OLFML3	HLA-C	LILRB1	1,28644E-03	28537,2	3,86477E-03	33936	1,44346E+00	8689	1,79950E+00	27867	9,42159E-01	10294	0	61900
Myeloid_vs_CD8_Non-responder	CD8(LAYN+ T)	Macro_ISG15	HLA-A	LILRB2	1,28701E-03	25332,8	3,86466E-03	33939	1,56727E+00	6545	2,03329E+00	21166	9,52936E-01	3114	0	61900
Myeloid_vs_CD8_Non-responder	CD8(ZNF683+KLRB1+ T)	Mono_CD16	B2M	LILRB1	1,28948E-03	28220,8	3,86397E-03	33952	1,41939E+00	9210	1,68980E+00	31479	9,50030E-01	4563	0	61900
Myeloid_vs_CD8_Non-responder	Mono_CD14	CD8(LAYN+ T)	VCAN	ITGA4	1,29284E-03	17346,4	1,70006E-02	2935	2,77539E+00	513	2,95894E+00	6082	9,34627E-01	15302	0	61900
Myeloid_vs_CD8_Non-responder	CD8(GZMK+ Tex)	Macro_ISG15	HLA-C	LILRB1	1,29347E-03	29938,6	4,07534E-03	31387	1,27349E+00	13187	1,62246E+00	33973	9,43588E-01	9246	0	61900
Myeloid_vs_CD8_Non-responder	Macro_FOLR2+APOE+	CD8(GZMK+ Tex)	LGALS3	LAG3	1,29583E-03	23184,2	7,27407E-03	13163	1,20990E+00	15311	2,51643E+00	11376	9,36506E-01	14171	0	61900
Myeloid_vs_CD8_Non-responder	Macro_FOLR2+APOE+	CD8(GZMK+ Tem)	HLA-DPA1	LAG3	1,29583E-03	19437,2	6,57345E-03	15311	1,37800E+00	10196	2,82916E+00	7334	9,54564E-01	2445	0	61900
Myeloid_vs_CD8_Non-responder	CD8(GZMK+ Tem)	Mono_CD16	HLA-C	LILRB1	1,29786E-03	30201,8	3,92067E-03	33235	1,31855E+00	11864	1,62197E+00	33996	9,42549E-01	10014	0	61900
Myeloid_vs_CD8_Non-responder	Macro_FOLR2+APOE+	CD8(ITM2C+ T)	CXCL9	CXCR3	1,29832E-03	23419,6	1,81449E-02	2585	2,37383E+00	1129	3,04194E+00	5329	8,86109E-01	46155	0	61900
Myeloid_vs_CD8_Non-responder	CD8(ISG+ T)	Mono_CD16	HLA-B	LILRB1	1,30034E-03	30143,6	3,85887E-03	34009	1,29592E+00	12533	1,62161E+00	34009	9,44804E-01	8267	0	61900
Myeloid_vs_CD8_Non-responder	CD8(GZMK+ Tem)	Macro_NLRP3	HMGB1	THBD	1,30053E-03	32504,2	4,92258E-03	23557	1,23098E+00	14547	1,62154E+00	34010	9,12372E-01	28507	0	61900
Myeloid_vs_CD8_Non-responder	CD8(IL7R+ZNF683+ Tm)	Mono_CD16	HLA-A	LILRA1	1,30182E-03	27016,2	9,41005E-03	8848	1,69018E+00	4948	1,38373E+00	44056	9,34595E-01	15329	0	61900
Myeloid_vs_CD8_Non-responder	CD8(GZMK+ Early Tem)	Macro_ISG15	HLA-B	LILRB2	1,30474E-03	24517	3,85720E-03	34032	1,63307E+00	5607	2,13052E+00	18779	9,55027E-01	2267	0	61900
Myeloid_vs_CD8_Non-responder	CD8(NME1+ T)	pDC_LILRA4	TNF	TNFRSF21	1,30817E-03	30603,6	9,70387E-03	8377	2,06218E+00	2218	2,29288E+00	15348	8,57122E-01	65175	0	61900
Myeloid_vs_CD8_Non-responder	CD8(GZMK+ Tem)	Mono_CD14	ANXA1	FPR1	1,30916E-03	33066,8	3,97697E-03	32547	1,14246E+00	17838	1,62042E+00	34055	9,28002E-01	18994	0	61900
Myeloid_vs_CD8_Non-responder	CD8(ZNF683+KLRB1+ T)	Macro_ISG15	CIRBP	TREM1	1,30974E-03	30953,6	5,81778E-03	18381	1,22216E+00	14871	1,87557E+00	25558	9,03929E-01	34058	0	61900
Myeloid_vs_CD8_Non-responder	cDC(CD1C)	CD8(GZMK+ Tem)	HLA-DQA1	LAG3	1,31085E-03	20793,8	8,03838E-03	11347	1,42737E+00	9032	2,29261E+00	15356	9,47308E-01	6334	0	61900
Myeloid_vs_CD8_Non-responder	Macro_ISG15	CD8(IL7R+ZNF683+ Tm)	S100A8	CD69	1,31185E-03	23182	6,56352E-03	15359	1,28426E+00	12863	2,34311E+00	14344	9,40514E-01	11444	0	61900
Myeloid_vs_CD8_Non-responder	cDC(CD1C)	CD8(Terminal Tex)	HLA-DRB5	LAG3	1,31353E-03	21208,8	6,56184E-03	15364	1,36308E+00	10582	2,41382E+00	13070	9,49061E-01	5128	0	61900
Myeloid_vs_CD8_Non-responder	CD8(ZNF683+KLRB1+ T)	Macro_FOLR2+APOE+	HLA-C	LILRB2	1,31860E-03	24010,4	3,85025E-03	34104	1,75828E+00	4324	2,23495E+00	16528	9,52773E-01	3196	0	61900
Myeloid_vs_CD8_Non-responder	CD8(ISG+ T)	pDC_LILRA4	HSP90B1	TLR9	1,32222E-03	37767,6	1,98873E-02	2123	1,97694E+00	2677	1,12124E+00	57208	8,57487E-01	64930	0	61900
Myeloid_vs_CD8_Non-responder	CD8(Temra)	Macro_ISG15	B2M	LILRB1	1,32228E-03	29197,8	4,01323E-03	32124	1,25515E+00	13744	1,61853E+00	34123	9,50922E-01	4098	0	61900
Myeloid_vs_CD8_Non-responder	CD8(ISG+ T)	pDC_LILRA4	HMGB1	TLR9	1,32440E-03	29365,6	3,24112E-02	829	2,15229E+00	1833	1,32574E+00	46709	9,01665E-01	35557	0	61900
Myeloid_vs_CD8_Non-responder	Mast	CD8(GZMK+ Tem)	TIMP3	CD44	1,32451E-03	28574,2	3,49122E-02	705	2,46858E+00	917	1,29682E+00	48147	9,08141E-01	31202	0	61900
Myeloid_vs_CD8_Non-responder	CD8(Terminal Tex)	Mono_CD16	HSPA1A	TLR4	1,32985E-03	34957,2	4,99329E-03	23068	1,02041E+00	23216	1,61771E+00	34162	9,06335E-01	32440	0	61900
Myeloid_vs_CD8_Non-responder	Macro_FOLR2+APOE+	CD8(LAYN+ T)	CD14	ITGB1	1,33310E-03	20637,2	6,60533E-03	15198	1,73018E+00	4576	2,95727E+00	6090	9,34414E-01	15422	0	61900
Myeloid_vs_CD8_Non-responder	CD8(NME1+ T)	Macro_OLFML3	HMGB1	TLR2	1,33805E-03	31715,2	4,61809E-03	25934	1,27028E+00	13279	1,61662E+00	34204	9,20795E-01	23259	0	61900
Myeloid_vs_CD8_Non-responder	CD8(Terminal Tex)	Macro_NLRP3	IFNG	IFNGR1_IFNGR2	1,34099E-03	35781	6,06334E-03	17301	8,71414E-01	31711	1,61632E+00	34219	9,04338E-01	33774	0	61900
Myeloid_vs_CD8_Non-responder	CD8(ISG+ T)	Macro_ISG15	HLA-C	LILRB1	1,34373E-03	30225,8	4,04249E-03	31770	1,25275E+00	13830	1,61588E+00	34233	9,43372E-01	9396	0	61900
Myeloid_vs_CD8_Non-responder	CD8(EOMES+ NK-like)	Macro_ISG15	HLA-A	LILRB2	1,34629E-03	25473	3,84012E-03	34246	1,54951E+00	6774	2,03002E+00	21257	9,52793E-01	3188	0	61900
Myeloid_vs_CD8_Non-responder	CD8(ZNF683+KLRB1+ T)	Macro_ISG15	HLA-F	LILRB1	1,34746E-03	29701,2	5,25378E-03	21429	1,39642E+00	9740	2,03237E+00	21185	9,03620E-01	34252	0	61900
Myeloid_vs_CD8_Non-responder	CD8(IL7R+ZNF683+ Tm)	Macro_ISG15	ANXA1	FPR2_FPR3	1,35417E-03	28896,2	7,82201E-03	11801	1,40934E+00	9427	1,61464E+00	34286	9,14710E-01	27067	0	61900
Myeloid_vs_CD8_Non-responder	CD8(Temra)	Macro_ISG15	HLA-A	LILRB1	1,35634E-03	29875	4,14137E-03	30658	1,25657E+00	13701	1,61439E+00	34297	9,44107E-01	8819	0	61900
Myeloid_vs_CD8_Non-responder	CD8(IL7R+ZNF683+ Tm)	pDC_LILRA4	TNF	PTPRS	1,35922E-03	27805,4	3,05183E-02	929	3,23199E+00	206	2,27528E+00	15691	8,64770E-01	60301	0	61900
Myeloid_vs_CD8_Non-responder	Mono_CD14	CD8(GZMK+ Tem)	S100A8	ITGB2	1,36148E-03	17435,4	6,52218E-03	15505	2,52459E+00	830	3,52441E+00	2301	9,46872E-01	6641	0	61900
Myeloid_vs_CD8_Non-responder	CD8(ZNF683+KLRB1+ T)	Macro_ISG15	HLA-B	LILRB2	1,36189E-03	24794,8	3,83401E-03	34325	1,61367E+00	5870	2,09668E+00	19567	9,54897E-01	2312	0	61900
Myeloid_vs_CD8_Non-responder	cDC_LAMP3	CD8(Temra)	CCL19	CXCR3	1,36213E-03	30037	3,05073E-02	930	3,87930E+00	88	2,26471E+00	15913	8,47748E-01	71354	0	61900
Myeloid_vs_CD8_Non-responder	CD8(LAYN+ T)	Mono_CD14	RPS19	C5AR1	1,36427E-03	28098,6	4,56372E-03	26380	1,33831E+00	11243	1,61327E+00	34337	9,46889E-01	6633	0	61900
Myeloid_vs_CD8_Non-responder	CD8(Tc17)	Macro_ISG15	B2M	LILRB1	1,36546E-03	29365,2	4,00059E-03	32257	1,24195E+00	14188	1,61306E+00	34343	9,50849E-01	4138	0	61900
Myeloid_vs_CD8_Non-responder	CD8(ISG+ T)	pDC_LILRA4	CD70	TNFRSF17	1,36788E-03	51947,4	7,01552E-02	186	1,64668E+00	5424	1,52261E+00	37966	6,79511E-01	154261	0	61900
Myeloid_vs_CD8_Non-responder	CD8(ID2+CXCR4+ T)	Macro_ISG15	HLA-B	LILRB2	1,36844E-03	24881	3,83206E-03	34358	1,61203E+00	5894	2,08198E+00	19936	9,54886E-01	2317	0	61900
Myeloid_vs_CD8_Non-responder	cDC(CD1C)	CD8(GZMK+ Tex)	HLA-DRB5	LAG3	1,37047E-03	21212,8	6,51414E-03	15531	1,35792E+00	10722	2,43848E+00	12666	9,48884E-01	5245	0	61900
Myeloid_vs_CD8_Non-responder	CD8(GZMK+ Tem)	Macro_ISG15	CCL5	CCR1	1,37363E-03	24492,8	1,38366E-02	4476	1,58778E+00	6227	1,61189E+00	34384	9,34340E-01	15477	0	61900
Myeloid_vs_CD8_Non-responder	Mono_CD16	CD8(ZNF683+KLRB1+ T)	S100A8	CD69	1,38507E-03	22802,4	6,95069E-03	14105	1,31074E+00	12102	2,28203E+00	15573	9,42097E-01	10332	0	61900
Myeloid_vs_CD8_Non-responder	CD8(Temra)	Mono_CD16	HLA-A	LILRA1	1,39136E-03	27512,2	9,26822E-03	9047	1,64860E+00	5410	1,34989E+00	45613	9,34130E-01	15591	0	61900
Myeloid_vs_CD8_Non-responder	Macro_NLRP3	CD8(Tn)	VCAN	SELL	1,39562E-03	23652	1,31154E-02	4980	2,28418E+00	1378	3,02623E+00	5461	8,88498E-01	44541	0	61900
Myeloid_vs_CD8_Non-responder	CD8(Terminal Tex)	pDC_LILRA4	CXCL13	CXCR3	1,39716E-03	25216,2	2,63491E-02	1240	1,05889E+00	21493	2,31909E+00	14818	9,15411E-01	26630	0	61900
Myeloid_vs_CD8_Non-responder	CD8(Terminal Tex)	pDC_LILRA4	CCL5	CXCR3	1,39878E-03	23434,2	1,16329E-02	6238	1,04651E+00	22076	2,02329E+00	21415	9,48433E-01	5542	0	61900
Myeloid_vs_CD8_Non-responder	CD8(IL7R+ZNF683+ Tm)	Mono_CD16	HLA-C	LILRA1	1,40190E-03	26580,4	9,28130E-03	9032	1,71090E+00	4751	1,43791E+00	41598	9,34065E-01	15621	0	61900
Myeloid_vs_CD8_Non-responder	CD8(Terminal Tex)	pDC_LILRA4	GZMB	IGF2R	1,40203E-03	24999,2	1,32591E-02	4885	1,18034E+00	16388	2,34721E+00	14268	9,13872E-01	27555	0	61900
Myeloid_vs_CD8_Non-responder	CD8(Tn)	Macro_FOLR2+APOE+	CD28	CD86	1,40225E-03	29027,2	1,58134E-02	3410,5	1,54541E+00	6831,5	2,27908E+00	15622,5	8,69104E-01	57371,5	0	61900
Myeloid_vs_CD8_Non-responder	CD8(Terminal Tex)	pDC_LILRA4	HMGB1	THBD	1,40284E-03	29000,2	5,14605E-03	22047	1,27436E+00	13157	2,05928E+00	20496	9,14130E-01	27401	0	61900
Myeloid_vs_CD8_Non-responder	Mast	CD8(IL7R+ZNF683+ Tm)	TIMP3	CD44	1,40323E-03	28367,6	3,44063E-02	734	2,45611E+00	944	1,32800E+00	46607	9,07530E-01	31653	0	61900
Myeloid_vs_CD8_Non-responder	Macro_ISG15	CD8(EOMES+ NK-like)	CXCL10	CXCR3	1,40849E-03	25990,4	1,25166E-02	5478	1,96147E+00	2757	3,19418E+00	4140	8,71633E-01	55677	0	61900
Myeloid_vs_CD8_Non-responder	CD8(GZMK+ Early Tem)	Macro_FOLR2+APOE+	HLA-F	LILRB2	1,40956E-03	31832,8	3,81511E-03	34562	1,39237E+00	9840	2,00784E+00	21809	9,08373E-01	31053	0	61900
Myeloid_vs_CD8_Non-responder	pDC_LILRA4	CD8(ZNF683+KLRB1+ T)	B2M	CD3D	1,41031E-03	33752,8	2,90623E-03	51360	9,37322E-01	27571	1,79797E+00	27923	9,65941E-01	10	0	61900
Myeloid_vs_CD8_Non-responder	CD8(Terminal Tex)	pDC_LILRA4	TNF	PTPRS	1,41508E-03	27599,6	3,02827E-02	948	3,23013E+00	208	2,34219E+00	14360	8,64316E-01	60582	0	61900
Myeloid_vs_CD8_Non-responder	CD8(GZMK+ Tex)	Macro_ISG15	HLA-B	LILRB2	1,41691E-03	24944,2	3,81238E-03	34598	1,59556E+00	6114	2,08900E+00	19749	9,54775E-01	2360	0	61900
Myeloid_vs_CD8_Non-responder	CD8(IL7R+ZNF683+ Tm)	Macro_ISG15	HLA-A	LILRB2	1,41999E-03	25770,2	3,81103E-03	34613	1,52845E+00	7123	2,00213E+00	21952	9,52621E-01	3263	0	61900
Myeloid_vs_CD8_Non-responder	CD8(ZNF683+KLRB1+ T)	cDC_LAMP3	LTB	CD40	1,42265E-03	25639,4	1,28358E-02	5199	1,90097E+00	3165	1,95324E+00	23307	9,03056E-01	34626	0	61900
Myeloid_vs_CD8_Non-responder	Macro_OLFML3	CD8(Terminal Tex)	HLA-DQB1	LAG3	1,42279E-03	23307	6,47094E-03	15680	1,24418E+00	14108	2,29495E+00	15305	9,43184E-01	9542	0	61900
Myeloid_vs_CD8_Non-responder	Mast	CD8(GZMK+ Early Tem)	TIMP3	CD44	1,42401E-03	28786,6	3,43300E-02	737	2,45423E+00	951	1,28682E+00	48624	9,07437E-01	31721	0	61900
Myeloid_vs_CD8_Non-responder	CD8(ISG+ T)	Macro_FOLR2+APOE+	HLA-F	LILRB2	1,42903E-03	32116,6	3,80750E-03	34657	1,39124E+00	9874	1,96289E+00	23046	9,08290E-01	31106	0	61900
Myeloid_vs_CD8_Non-responder	CD8(GZMK+ Tem)	Macro_ISG15	B2M	LILRB1	1,43027E-03	29542,6	3,98926E-03	32398	1,23013E+00	14578	1,60503E+00	34663	9,50782E-01	4174	0	61900
Myeloid_vs_CD8_Non-responder	CD8(GZMK+ Early Tem)	Mono_CD16	HLA-C	LILRA1	1,43852E-03	27063,6	9,22685E-03	9116	1,69554E+00	4905	1,39217E+00	43673	9,33884E-01	15724	0	61900
Myeloid_vs_CD8_Non-responder	CD8(GZMK+ Tem)	Macro_ISG15	HLA-B	LILRB2	1,44020E-03	25160,4	3,80430E-03	34711	1,58880E+00	6217	2,05545E+00	20597	9,54729E-01	2377	0	61900
Myeloid_vs_CD8_Non-responder	CD8(ISG+ T)	cDC_CLEC9A	FLT3LG	FLT3	1,44833E-03	36831	2,87396E-02	1044	2,60726E+00	711	1,74498E+00	29618	8,16727E-01	90882	0	61900
Myeloid_vs_CD8_Non-responder	Macro_OLFML3	CD8(GZMK+ Tem)	HLA-DQA1	LAG3	1,45040E-03	22479,4	7,19751E-03	13374	1,28453E+00	12854	2,27251E+00	15757	9,44481E-01	8512	0	61900
Myeloid_vs_CD8_Non-responder	CD8(ID2+CXCR4+ T)	Mono_CD14	ANXA1	FPR1	1,45897E-03	32757,2	4,07632E-03	31371	1,15903E+00	17198	1,60127E+00	34801	9,28822E-01	18516	0	61900

Myeloid_vs_CD8_Post_NR

Myeloid_vs_CD8_Non-responder	CD8(EOMES+ NK-like)	Macro_ISG15	B2M	LILRB1	1,46317E-03	29608,4	3,98611E-03	32441	1,22684E+00	14695	1,60085E+00	34821	9,50764E-01	4185	0	61900
Myeloid_vs_CD8_Non-responder	CD8(NME1+ T)	Macro_NLRP3	HMGB1	TLR4	1,46548E-03	32216,6	5,42052E-03	20476	1,28636E+00	12797	1,70086E+00	31078	9,02763E-01	34832	0	61900
Myeloid_vs_CD8_Non-responder	CD8(GZMK+ Tem)	Mono_CD16	ANXA1	FPR1	1,46548E-03	33681,6	3,79652E-03	34832	1,08425E+00	20334	1,68995E+00	31471	9,26435E-01	19871	0	61900
Myeloid_vs_CD8_Non-responder	CD8(ID2+CXCR4+ T)	Macro_ISG15	HLA-C	LILRB2	1,46843E-03	25376	3,79536E-03	34846	1,57457E+00	6427	2,06512E+00	20362	9,52449E-01	3345	0	61900
Myeloid_vs_CD8_Non-responder	CD8(Tc17)	Macro_ISG15	HLA-A	LILRB2	1,46864E-03	25918	3,79526E-03	34847	1,51703E+00	7320	1,99314E+00	22207	9,52528E-01	3316	0	61900
Myeloid_vs_CD8_Non-responder	Mono_CD14	CD8(EOMES+ NK-like)	VCAN	CD44	1,46891E-03	21195,4	7,83704E-03	11775	2,31922E+00	1268	2,29956E+00	15226	9,33743E-01	15808	0	61900
Myeloid_vs_CD8_Non-responder	CD8(ZNF683+KLRB1+ T)	Macro_OLFML3	CD99	PILRA	1,47244E-03	33919	3,91943E-03	33247	1,13788E+00	18035	1,59970E+00	34865	9,23540E-01	21548	0	61900
Myeloid_vs_CD8_Non-responder	CD8(Tc17)	Macro_ISG15	HLA-C	LILRB2	1,47349E-03	25311,4	3,79371E-03	34870	1,57342E+00	6449	2,08036E+00	19986	9,52439E-01	3352	0	61900
Myeloid_vs_CD8_Non-responder	CD8(NME1+ T)	Macro_FOLR2+APOE+	CD52	SIGLEC10	1,47539E-03	29846	5,74585E-03	18750	1,31072E+00	12103	1,59940E+00	34879	9,23458E-01	21598	0	61900
Myeloid_vs_CD8_Non-responder	CD8(Terminal Tex)	Mono_CD14	RPS19	C5AR1	1,47687E-03	26941,6	4,73053E-03	25021	1,38146E+00	10118	1,68449E+00	31675	9,47784E-01	5994	0	61900
Myeloid_vs_CD8_Non-responder	CD8(GZMK+ Tem)	Mono_CD16	HLA-C	LILRA3	1,47793E-03	25769,4	1,15675E-02	6302	1,65602E+00	5322	1,59912E+00	34891	9,25456E-01	20432	0	61900
Myeloid_vs_CD8_Non-responder	CD8(ID2+CXCR4+ T)	Macro_LYVE1	HMGB1	CD163	1,47963E-03	28425,4	4,71219E-03	25179	1,47612E+00	8042	1,59883E+00	34899	9,39579E-01	12107	0	61900
Myeloid_vs_CD8_Non-responder	Macro_OLFML3	CD8(GZMK+ Tex)	HLA-DQB1	LAG3	1,48097E-03	23302,8	6,42390E-03	15841	1,23902E+00	14270	2,31962E+00	14811	9,42989E-01	9692	0	61900
Myeloid_vs_CD8_Non-responder	CD8(ZNF683+KLRB1+ T)	Macro_FOLR2+APOE+	SPON2	ITGB2	1,48196E-03	29849	7,10161E-03	13682	1,08377E+00	20357	2,14740E+00	18396	9,02664E-01	34910	0	61900
Myeloid_vs_CD8_Non-responder	CD8(Terminal Tex)	cDC_CLEC9A	IFNG	IFNGR1_IFNGR2	1,48260E-03	36550,2	5,88544E-03	18080	8,24338E-01	34913	1,64272E+00	33225	9,03042E-01	34633	0	61900
Myeloid_vs_CD8_Non-responder	CD8(LAYN+ T)	Macro_ISG15	B2M	LILRB1	1,48409E-03	29684,8	3,98081E-03	32506	1,22131E+00	14894	1,59839E+00	34920	9,50733E-01	4204	0	61900
Myeloid_vs_CD8_Non-responder	CD8(Terminal Tex)	Mono_CD14	HSPA1A	TLR4	1,48472E-03	30321,6	6,33096E-03	16209	1,20212E+00	15607	1,68702E+00	31562	9,15936E-01	26330	0	61900
Myeloid_vs_CD8_Non-responder	CD8(Terminal Tex)	Macro_ISG15	CD99	PILRA	1,49495E-03	30041,4	4,40675E-03	27857	1,29458E+00	12568	1,77565E+00	28654	9,27576E-01	19228	0	61900
Myeloid_vs_CD8_Non-responder	Macro_ISG15	CD8(LAYN+ T)	CD14	ITGA4	1,49533E-03	21127	6,41400E-03	15880	1,65411E+00	5350	2,85026E+00	7121	9,34482E-01	15384	0	61900
Myeloid_vs_CD8_Non-responder	CD8(Terminal Tex)	Macro_ISG15	B2M	LILRB1	1,49838E-03	28565	4,06403E-03	31533	1,30817E+00	12172	1,64177E+00	33263	9,51215E-01	3957	0	61900
Myeloid_vs_CD8_Non-responder	CD8(Terminal Tex)	Macro_ISG15	IFNG	IFNGR1_IFNGR2	1,50095E-03	29072	7,72803E-03	11998	1,04556E+00	22126	1,99839E+00	22053	9,14329E-01	27283	0	61900
Myeloid_vs_CD8_Non-responder	CD8(Terminal Tex)	Macro_ISG15	CCL5	CCR1	1,50224E-03	22736,6	1,46583E-02	3993	1,65243E+00	5364	1,79617E+00	27982	9,36087E-01	14444	0	61900
Myeloid_vs_CD8_Non-responder	Macro_OLFML3	CD8(ISG+ T)	C3	IFITM1	1,50347E-03	23516,8	7,78358E-03	11882	1,29182E+00	12654	2,26522E+00	15902	9,34742E-01	15246	0	61900
Myeloid_vs_CD8_Non-responder	CD8(LAYN+ T)	pDC_LILRA4	BST2	LILRA4	1,50549E-03	22449,8	1,35846E-02	4653	2,78853E+00	496	3,01156E+00	5603	8,95702E-01	39597	0	61900
Myeloid_vs_CD8_Non-responder	CD8(Terminal Tex)	Macro_ISG15	RPS19	C5AR1	1,50675E-03	24796,2	4,93796E-03	23447	1,43784E+00	8803	1,90993E+00	24551	9,48836E-01	5280	0	61900
Myeloid_vs_CD8_Non-responder	CD8(NME1+ T)	Macro_IER3	HMGB1	CD163	1,50739E-03	31945,6	3,78175E-03	35029	1,22352E+00	14826	1,68236E+00	31754	9,33025E-01	16219	0	61900
Myeloid_vs_CD8_Non-responder	CD8(Terminal Tex)	Macro_ISG15	HSPA1A	TLR4	1,51710E-03	33842,2	5,09623E-03	22357	1,03440E+00	22606	1,71878E+00	30473	9,07198E-01	31875	0	61900
Myeloid_vs_CD8_Non-responder	Macro_NLRP3	CD8(Tn)	HBEGF	CD44	1,52060E-03	22084,2	7,05004E-03	13805	1,84100E+00	3613	2,26308E+00	15948	9,34889E-01	15155	0	61900
Myeloid_vs_CD8_Non-responder	CD8(Terminal Tex)	Macro_ISG15	HLA-A	LILRB2	1,52360E-03	26405,8	3,77667E-03	35104	1,50357E+00	7543	1,92511E+00	24113	9,52417E-01	3369	0	61900
Myeloid_vs_CD8_Non-responder	Mast	CD8(Tc17)	ADCYAP1	DPP4	1,52706E-03	43639,4	1,95177E-01	14	2,43777E+00	985	1,37416E+00	44497	7,82435E-01	110801	0	61900
Myeloid_vs_CD8_Non-responder	CD8(GZMK+ Tem)	Mono_CD16	HLA-B	LILRA1	1,52997E-03	26072,6	9,39343E-03	8870	1,75054E+00	4376	1,43705E+00	41639	9,37453E-01	13578	0	61900
Myeloid_vs_CD8_Non-responder	CD8(IL7R+ZNF683+ Tm)	Macro_OLFML3	ANXA1	FPR1	1,53186E-03	31672	4,47214E-03	27253	1,15870E+00	17207	1,59177E+00	35142	9,31825E-01	16858	0	61900
Myeloid_vs_CD8_Non-responder	CD8(GZMK+ Tem)	Mono_CD16	HLA-A	LILRA1	1,53336E-03	24708,8	1,00165E-02	7955	1,86799E+00	3418	1,56906E+00	36078	9,36479E-01	14193	0	61900
Myeloid_vs_CD8_Non-responder	CD8(GZMK+ Early Tem)	Macro_ISG15	B2M	LILRB1	1,53448E-03	29581	3,99470E-03	32318	1,23580E+00	14375	1,59153E+00	35154	9,50814E-01	4158	0	61900
Myeloid_vs_CD8_Non-responder	CD8(GZMK+ Tem)	Mono_CD16	HLA-C	LILRA1	1,53637E-03	25060,8	9,77309E-03	8277	1,84965E+00	3545	1,55006E+00	36861	9,35637E-01	14721	0	61900
Myeloid_vs_CD8_Non-responder	CD8(Tn)	Macro_FOLR2+APOE+	LTB	TNFRSF1A	1,53973E-03	31210,8	7,18220E-03	13434	1,10455E+00	19443	1,85692E+00	26099	9,02268E-01	35178	0	61900
Myeloid_vs_CD8_Non-responder	CD8(ZNF683+KLRB1+ T)	Macro_OLFML3	HLA-A	LILRB1	1,53973E-03	30599	3,77171E-03	35178	1,32483E+00	11670	1,63396E+00	33537	9,41589E-01	10710	0	61900
Myeloid_vs_CD8_Non-responder	Mono_INHBA	CD8(Tc17)	IL1B	ADRB2	1,56330E-03	24973,2	1,23021E-02	5675	1,89617E+00	3191	3,12304E+00	4678	8,81168E-01	49422	0	61900
Myeloid_vs_CD8_Non-responder	CD8(Tc17)	Macro_FOLR2+APOE+	RPS19	C5AR1	1,56572E-03	30696,4	3,76213E-03	35296	1,13201E+00	18265	1,81146E+00	27491	9,41817E-01	10530	0	61900
Myeloid_vs_CD8_Non-responder	CD8(ISG+ T)	Mono_CD16	HLA-C	LILRA1	1,56674E-03	27682	9,04693E-03	9439	1,64478E+00	5452	1,35138E+00	45549	9,33273E-01	16070	0	61900
Myeloid_vs_CD8_Non-responder	CD8(GZMK+ Tex)	Macro_FOLR2+APOE+	HLA-F	LILRB2	1,57306E-03	32338,4	3,75952E-03	35329	1,38411E+00	10047	1,96726E+00	22920	9,07760E-01	31496	0	61900
Myeloid_vs_CD8_Non-responder	CD8(IL7R+ZNF683+ Tm)	Macro_ISG15	HLA-C	LILRB2	1,57506E-03	25609,6	3,75889E-03	35338	1,54916E+00	6777	2,05630E+00	20573	9,52230E-01	3460	0	61900
Myeloid_vs_CD8_Non-responder	CD8(GZMK+ Early Tem)	Macro_FOLR2+APOE+	CCL4	CCR1	1,57796E-03	31645,4	8,02674E-03	11371	1,12354E+00	18648	1,58705E+00	35351	9,08507E-01	30957	0	61900
Myeloid_vs_CD8_Non-responder	CD8(ZNF683+KLRB1+ T)	Macro_FOLR2+APOE+	HLA-A	LILRB2	1,58019E-03	25289,8	3,75754E-03	35361	1,63966E+00	5518	2,06941E+00	20247	9,52301E-01	3423	0	61900
Myeloid_vs_CD8_Non-responder	CD8(ID2+CXCR4+ T)	Macro_ISG15	B2M	LILRB2	1,58355E-03	24689,2	3,75661E-03	35376	1,62267E+00	5745	2,09529E+00	19600	9,58782E-01	825	0	61900
Myeloid_vs_CD8_Non-responder	CD8(GZMK+ Tem)	Macro_IER3	TNFSF9	HLA-DPA1	1,58624E-03	38055,4	3,89980E-03	33497	8,67025E-01	31989	1,58592E+00	35388	9,13967E-01	27503	0	61900
Myeloid_vs_CD8_Non-responder	CD8(Tc17)	Macro_ISG15	HLA-B	LILRB1	1,58713E-03	30627,6	3,98566E-03	32446	1,19794E+00	15775	1,58584E+00	35392	9,45641E-01	7625	0	61900
Myeloid_vs_CD8_Non-responder	cDC_CLEC9A	CD8(Terminal Tex)	HLA-DPB1	LAG3	1,59135E-03	19584,2	6,35126E-03	16134	1,40082E+00	9620	2,92054E+00	6431	9,51414E-01	3836	0	61900
Myeloid_vs_CD8_Non-responder	CD8(GZMK+ Early Tem)	Mono_CD14	RPS19	C5AR1	1,59297E-03	29138,8	4,37562E-03	28173	1,28965E+00	12698	1,58542E+00	35418	9,45821E-01	7505	0	61900
Myeloid_vs_CD8_Non-responder	CD8(Temra)	Macro_ISG15	HLA-A	LILRB2	1,59297E-03	26300,8	3,75359E-03	35418	1,48686E+00	7856	1,96829E+00	22895	9,52277E-01	3435	0	61900
Myeloid_vs_CD8_Non-responder	CD8(GZMK+ Early Tem)	Macro_FOLR2-APOE+	TNFSF9	HLA-DPA1	1,59838E-03	31144,8	5,62675E-03	19326	1,09924E+00	19700	1,58479E+00	35442	9,27329E-01	19356	0	61900
Myeloid_vs_CD8_Non-responder	CD8(IL7R+ZNF683+ Tm)	pDC_LILRA4	SELPLG	SELL	1,59949E-03	28906	6,34548E-03	16155	1,44032E+00	8747	2,35992E+00	14030	8,89743E-01	43698	0	61900
Myeloid_vs_CD8_Non-responder	CD8(GZMK+ Tem)	Macro_FOLR2+APOE+	TGFB1	ENG	1,60131E-03	33273,6	5,64916E-03	19202	1,16479E+00	16976	1,65297E+00	32835	9,01830E-01	35455	0	61900
Myeloid_vs_CD8_Non-responder	CD8(Tc17)	Mono_CD14	RPS19	C5AR1	1,60447E-03	31931,4	3,74943E-03	35469	1,12766E+00	18455	1,64268E+00	33228	9,41725E-01	10605	0	61900
Myeloid_vs_CD8_Non-responder	CD8(GZMK+ Early Tem)	Mono_CD16	HLA-B	LILRB1	1,60538E-03	30758,4	3,82076E-03	34492	1,26371E+00	13468	1,58404E+00	35473	9,44545E-01	8459	0	61900
Myeloid_vs_CD8_Non-responder	CD8(ITM2C+ T)	Macro_LYVE1	TNFSF9	HLA-DPA1	1,60805E-03	22428,4	8,30943E-03	10776	1,34459E+00	11069	2,25227E+00	16177	9,39420E-01	12220	0	61900
Myeloid_vs_CD8_Non-responder	CD8(IL7R+ZNF683+ Tm)	Macro_ISG15	HLA-B	LILRB2	1,60810E-03	25653	3,74800E-03	35485	1,54167E+00	6898	2,02070E+00	21489	9,54406E-01	2493	0	61900
Myeloid_vs_CD8_Non-responder	pDC_LILRA4	CD8(Tn)	APP	PTGER2	1,60926E-03	30603,4	1,22366E-02	5731	2,32605E+00	1243	3,14076E+00	4540	8,34704E-01	79603	0	61900
Myeloid_vs_CD8_Non-responder	Macro_NLRP3	CD8(ID2+CXCR4+ T)	VCAN	CD44	1,61195E-03	21540,2	7,66798E-03	12149	2,06876E+00	2189	2,29649E+00	15276	9,33065E-01	16187	0	61900
Myeloid_vs_CD8_Non-responder	cDC(CD1C)	CD8(Terminal Tex)	HLA-DQA2	LAG3	1,61351E-03	21024,2	1,02301E-02	7708	1,63442E+00	5587	2,37692E+00	13735	9,33060E-01	16191	0	61900
Myeloid_vs_CD8_Non-responder	CD8(ISG+ T)	Macro_ISG15	TIGIT	NECTIN2	1,61756E-03	35053,6	1,47602E-02	3938,5	1,82427E+00	3757,5	1,72892E+00	30143,5	8,41245E-01	75528,5	0	61900
Myeloid_vs_CD8_Non-responder	pDC_LILRA4	CD8(Terminal Tex)	APP	CD74	1,61821E-03	19305	6,81997E-03	14496	2,25398E+00	1474	2,25107E+00	16203	9,54528E-01	2452	0	61900
Myeloid_vs_CD8_Non-responder	CD8(IL7R+ZNF683+ Tm)	Macro_NLRP3	HMGB1	THBD	1,62082E-03	33864,2	4,66450E-03	25555	1,17833E+00	16468	1,58218E+00	35541	9,10195E-01	29857	0	61900
Myeloid_vs_CD8_Non-responder	CD8(Tn)	Macro_IER3	RPS19	C5AR1	1,62425E-03	26882,6	4,97390E-03	23210	1,44961E+00	8567	1,58155E+00	35556	9,49012E-01	5180	0	61900
Myeloid_vs_CD8_Non-responder	cDC(CD1C)	CD8(GZMK+ Tex)	HLA-DQA1	LAG3	1,62841E-03	22475,6	7,05643E-03	13786	1,32926E+00	11528	2,24994E+00	16229	9,43960E-01	8935	0	61900
Myeloid_vs_CD8_Non-responder	CD8(Tc17)	Macro_FOLR2+APOE+	HMGB1	CD163	1,63295E-03	30271,4	3,74098E-03	35594	1,21499E+00	15136	1,98841E+00	22334	9,32685E-01	16393	0	61900
Myeloid_vs_CD8_Non-responder	CD8(Terminal Tex)	cDC(CD1C)	IFNG	IFNGR1_IFNGR2	1,63318E-03	32440	7,51252E-03	12512	1,00025E+00	24222	1,58075E+00	35595	9,13215E-01	27971	0	61900
Myeloid_vs_CD8_Non-responder	CD8(Tc17)	pDC_LILRA4	SELPLG	SELL	1,63639E-03	25441,6	8,17977E-03	11029	1,53436E+00	7025	2,49846E+00	11645	9,01596E-01	35609	0	61900
Myeloid_vs_CD8_Non-responder	Macro_ISG15	CD8(GZMK+ Tem)	LGALS3	LAG3	1,64183E-03	24058,8	7,48894E-03	12575	1,18383E+00	16263	2,26400E+00	15928	9,37366E-01	13628	0	61900
Myeloid_vs_CD8_Non-responder	CD8(IL7R+ZNF683+ Tm)	cDC_CLEC9A	FLT3LG	FLT3	1,64325E-03	36566,4	2,90940E-02	1022	2,61113E+00	707	1,76906E+00	28839	8,17643E-01	90364	0	61900
Myeloid_vs_CD8_Non-responder	CD8(GZMK+ Early Tem)	Macro_ISG15	HLA-C	LILRB2	1,64445E-03	25966,4	3,73684E-03	35644	1,53380E+00	7035	2,01057E+00	21735	9,52096E-01	3518	0	61900
Myeloid_vs_CD8_Non-responder	CD8(LAYN+ T)	Macro_ISG15	HLA-B	LILRB2	1,64907E-03	25924,2	3,73481E-03	35664	1,53064E+00	7086	1,98485E+00	22440	9,54329E-01	2531	0	61900
Myeloid_vs_CD8_Non-responder	Macro_OLFML3	CD8(IL7R+ZNF683+ Tm)	CXCL9	CXCR3	1,65188E-03	22888,6	1,97449E-02	2146	2,30939E+00	1299	2,99180E+00	5782	8,90304E-01	43316	0	61900
Myeloid_vs_CD8_Non-responder	CD8(Temra)	Macro_ISG15	HLA-B	LILRB2	1,65370E-03	25830,8	3,73345E-03	35684	1,52949E+00	7104	2,00281E+00	21930	9,54321E-01	2536	0	61900
Myeloid_vs_CD8_Non-responder	cDC_CLEC9A	CD8(GZMK+ Tex)	HLA-DPB1	LAG3	1,65493E-03	19618	6,30509E-03	16296	1,39566E+00	9757	2,94520E+00	6200	9,51245E-01	3937	0	61900
Myeloid_vs_CD8_Non-responder	CD8(Temra)	Mono_CD16	SPON2	ITGB2	1,65555E-03	31756,2	6,91365E-03	14216	1,00847E+00	23783	1,95793E+00	23190	9,01480E-01	35692	0	61900
Myeloid_vs_CD8_Non-responder	Macro_OLFML3	CD8(GZMK+ Tem)	CXCL9	CXCR3	1,65694E-03	21881,8	2,26107E-02	1687	2,37623E+00	1123	2,99128E+00	5788	8,96749E-01	38911	0	61900

Myeloid_vs_CD8_Post_NR

Myeloid_vs_CD8_Non-responder	Macro_OLFML3	CD8(NME1+ T)	CXCL10	SDC4	1,65694E-03	35289,4	1,21756E-02	5788	2,34818E+00	1198	3,44510E+00	2663	7,93218E-01	104898	0	61900
Myeloid_vs_CD8_Non-responder	CD8(EOMES+ NK-like)	Mono_CD16	CD52	SIGLEC10	1,66159E-03	33540,8	4,78706E-03	24578	1,09901E+00	19709	1,57782E+00	35718	9,16752E-01	25799	0	61900
Myeloid_vs_CD8_Non-responder	Macro_ISG15	CD8(EOMES+ NK-like)	S100A8	CD69	1,66211E-03	24022,2	6,30199E-03	16314	1,25176E+00	13858	2,27081E+00	15788	9,39366E-01	12251	0	61900
Myeloid_vs_CD8_Non-responder	CD8(ZNF683+KLRB1+ T)	Macro_ISG15	CD52	SIGLEC10	1,66275E-03	34906,8	4,41757E-03	27756	1,05935E+00	21467	1,57773E+00	35723	9,13635E-01	27688	0	61900
Myeloid_vs_CD8_Non-responder	CD8(GZMK+ Early Tem)	Macro_FOLR2+APOE+	HMGB1	CD163	1,66392E-03	30502,6	3,73030E-03	35728	1,21239E+00	15233	1,95732E+00	23212	9,32596E-01	16440	0	61900
Myeloid_vs_CD8_Non-responder	cDC(CD1C)	CD8(GZMK+ Tex)	HLA-DQA2	LAG3	1,66411E-03	20990,8	1,01558E-02	7792	1,62926E+00	5655	2,40159E+00	13288	9,32832E-01	16319	0	61900
Myeloid_vs_CD8_Non-responder	Macro_FOLR2+APOE+	CD8(Terminal Tex)	HLA-DRB5	LAG3	1,66570E-03	20939,6	6,29941E-03	16323	1,30672E+00	12213	2,73192E+00	8472	9,48065E-01	5790	0	61900
Myeloid_vs_CD8_Non-responder	pDC_LILRA4	CD8(NME1+ T)	APP	CD74	1,66811E-03	19628,4	6,53078E-03	15468	2,21159E+00	1619	2,24455E+00	16329	9,53578E-01	2826	0	61900
Myeloid_vs_CD8_Non-responder	Macro_OLFML3	CD8(GZMK+ Tem)	HLA-DPB1	LAG3	1,67292E-03	21723,4	6,29478E-03	16341	1,29944E+00	12421	2,36219E+00	13993	9,51207E-01	3962	0	61900
Myeloid_vs_CD8_Non-responder	CD8(ISG+ T)	Macro_ISG15	B2M	LILRB2	1,67512E-03	24933,6	3,72763E-03	35776	1,58930E+00	6209	2,08314E+00	19902	9,58629E-01	881	0	61900
Myeloid_vs_CD8_Non-responder	CD8(LAYN+ T)	Mono_CD16	HLA-C	LILRB1	1,67559E-03	31079,8	3,85197E-03	34082	1,27024E+00	13282	1,57629E+00	35778	9,42069E-01	10357	0	61900
Myeloid_vs_CD8_Non-responder	Macro_FOLR2+APOE+	CD8(ISG+ T)	CXCL9	CXCR3	1,68325E-03	23655,2	1,78182E-02	2658	2,36662E+00	1149	2,98766E+00	5819	8,85189E-01	46750	0	61900
Myeloid_vs_CD8_Non-responder	CD8(Tc17)	Macro_NLRP3	LTB	TNFRSF1A	1,68686E-03	32466,4	7,02638E-03	13876	1,10435E+00	19460	1,69535E+00	31270	9,01297E-01	35826	0	61900
Myeloid_vs_CD8_Non-responder	Macro_OLFML3	CD8(ZNF683+KLRB1+ T)	CXCL10	CXCR3	1,68838E-03	26086,2	1,21354E-02	5825	2,24050E+00	1513	3,16987E+00	4321	8,69892E-01	56872	0	61900
Myeloid_vs_CD8_Non-responder	CD8(GZMK+ Early Tem)	Macro_ISG15	CCL5	CCR1	1,69105E-03	26166,4	1,31243E-02	4973	1,53174E+00	7065	1,46307E+00	40508	9,32700E-01	16386	0	61900
Myeloid_vs_CD8_Non-responder	CD8(Tc17)	Macro_ISG15	LTB	CD40	1,69134E-03	25846,8	1,23266E-02	5646	1,59464E+00	6139	2,09046E+00	19704	9,01270E-01	35845	0	61900
Myeloid_vs_CD8_Non-responder	Macro_FOLR2+APOE+	CD8(IL7R+ZNF683+ Tm)	APOE	LSR	1,69866E-03	21416,2	2,35737E-02	1550	1,61651E+00	5837	3,40825E+00	2842	9,02616E-01	34952	0	61900
Myeloid_vs_CD8_Non-responder	CD8(NME1+ T)	Mast	GPI	NTRK1	1,70812E-03	49259	3,36937E-02	771	1,61547E+00	5848	5,25575E-01	93177	8,26783E-01	84599	0	61900
Myeloid_vs_CD8_Non-responder	CD8(ITM2C+ T)	Macro_FOLR2+APOE+	HLA-F	LILRB2	1,71006E-03	31121	3,89456E-03	33572	1,40417E+00	9554	2,07682E+00	20076	9,09227E-01	30503	0	61900
Myeloid_vs_CD8_Non-responder	CD8(Tc17)	Macro_ISG15	CIRBP	TREM1	1,71196E-03	32900,4	5,45762E-03	20263	1,16842E+00	16832	1,74642E+00	29575	9,01118E-01	35932	0	61900
Myeloid_vs_CD8_Non-responder	CD8(ITM2C+ T)	Macro_FOLR2+APOE+	IFNG	IFNGR1_IFNGR2	1,71411E-03	28754	7,41794E-03	12774	1,09158E+00	20028	2,04794E+00	20796	9,12711E-01	28272	0	61900
Myeloid_vs_CD8_Non-responder	CD8(NME1+ T)	Mono_CD16	HLA-A	LILRA1	1,71504E-03	29445,4	8,82024E-03	9828	1,51724E+00	7317	1,22523E+00	51737	9,32589E-01	16445	0	61900
Myeloid_vs_CD8_Non-responder	CD8(ITM2C+ T)	Macro_FOLR2+APOE+	RPS19	C5AR1	1,71841E-03	27808,6	4,38096E-03	28119	1,29156E+00	12658	1,76739E+00	28889	9,45852E-01	7477	0	61900
Myeloid_vs_CD8_Non-responder	CD8(EOMES+ NK-like)	Macro_ISG15	HLA-F	LILRB2	1,72032E-03	31338,2	3,71426E-03	35967	1,48736E+00	7846	2,11447E+00	19135	9,07252E-01	31843	0	61900
Myeloid_vs_CD8_Non-responder	CD8(ITM2C+ T)	Macro_FOLR2+APOE+	CD52	SIGLEC10	1,72128E-03	30181,8	5,51981E-03	19914	1,27327E+00	13193	1,63802E+00	33414	9,22028E-01	22488	0	61900
Myeloid_vs_CD8_Non-responder	Macro_FOLR2+APOE+	CD8(GZMK+ Tem)	HLA-DRB1	LAG3	1,72406E-03	20088,4	6,26446E-03	16467	1,32272E+00	11736	2,77377E+00	7936	9,54668E-01	2403	0	61900
Myeloid_vs_CD8_Non-responder	CD8(GZMK+ Tex)	Macro_ISG15	B2M	LILRB2	1,72655E-03	25167,6	3,71257E-03	35993	1,57195E+00	6471	2,05632E+00	20572	9,58548E-01	902	0	61900
Myeloid_vs_CD8_Non-responder	CD8(ITM2C+ T)	Macro_FOLR2+APOE+	HSPA1A	TLR4	1,72679E-03	25807,6	8,11606E-03	11178	1,46808E+00	8192	1,82432E+00	27085	9,25018E-01	20683	0	61900
Myeloid_vs_CD8_Non-responder	CD8(Terminal Tex)	Macro_ISG15	HLA-A	LILRB1	1,72775E-03	30035	4,16683E-03	30387	1,27328E+00	13192	1,57121E+00	35998	9,44269E-01	8698	0	61900
Myeloid_vs_CD8_Non-responder	Macro_FOLR2+APOE+	CD8(Terminal Tex)	HLA-DRA	LAG3	1,72816E-03	19609,8	6,26306E-03	16477	1,34877E+00	10957	2,96762E+00	6000	9,53833E-01	2715	0	61900
Myeloid_vs_CD8_Non-responder	CD8(LAYN+ T)	Macro_FOLR2+APOE+	TGFB1	ENG	1,73159E-03	33924,4	5,64441E-03	19226	1,16422E+00	17004	1,57063E+00	36014	9,01793E-01	35478	0	61900
Myeloid_vs_CD8_Non-responder	CD8(Temra)	Mono_CD14	SPON2	ITGB2	1,73447E-03	32988,4	6,83693E-03	14443	9,96393E-01	24413	1,79059E+00	28160	9,00983E-01	36026	0	61900
Myeloid_vs_CD8_Non-responder	cDC(CD1C)	CD8(Terminal Tex)	HLA-DPB1	LAG3	1,73640E-03	20923,6	6,25822E-03	16497	1,37779E+00	10205	2,47677E+00	11993	9,51072E-01	4023	0	61900
Myeloid_vs_CD8_Non-responder	cDC_CLEC9A	CD8(GZMK+ Tem)	HLA-DRB1	LAG3	1,73764E-03	20159,8	6,25791E-03	16500	1,32117E+00	11773	2,75238E+00	8211	9,54645E-01	2415	0	61900
Myeloid_vs_CD8_Non-responder	CD8(ITM2C+ T)	Macro_NLRP3	RPS19	C5AR1	1,74074E-03	27609,8	4,53395E-03	26674	1,33661E+00	11303	1,69116E+00	31419	9,46724E-01	6753	0	61900
Myeloid_vs_CD8_Non-responder	CD8(EOMES+ NK-like)	Macro_ISG15	HLA-C	LILRB2	1,74195E-03	26086,8	3,70852E-03	36057	1,51407E+00	7365	2,01981E+00	21517	9,51922E-01	3595	0	61900
Myeloid_vs_CD8_Non-responder	Macro_FOLR2+APOE+	CD8(GZMK+ Tex)	HLA-DRB5	LAG3	1,74425E-03	20968	6,25363E-03	16516	1,30156E+00	12347	2,75659E+00	8162	9,47885E-01	5915	0	61900
Myeloid_vs_CD8_Non-responder	CD8(ID2+CXCR4+ T)	pDC_LILRA4	BST2	LILRA4	1,74455E-03	23513,2	1,20564E-02	5890	2,75249E+00	539	2,99790E+00	5720	8,89995E-01	43517	0	61900
Myeloid_vs_CD8_Non-responder	Macro_OLFML3	CD8(GZMK+ Tex)	CXCL10	CXCR3	1,74630E-03	26234,6	1,20548E-02	5892	2,23882E+00	1518	3,11294E+00	4760	8,69515E-01	57103	0	61900
Myeloid_vs_CD8_Non-responder	CD8(ITM2C+ T)	Macro_NLRP3	HSPA1A	TLR4	1,74727E-03	24519,6	8,28897E-03	10814	1,48721E+00	7847	2,00917E+00	21767	9,25746E-01	20270	0	61900
Myeloid_vs_CD8_Non-responder	CD8(GZMK+ Tex)	Mono_CD16	CD52	SIGLEC10	1,74800E-03	32810,2	4,97833E-03	23175	1,13722E+00	18058	1,56900E+00	36082	9,18235E-01	24836	0	61900
Myeloid_vs_CD8_Non-responder	Macro_OLFML3	CD8(Tn)	CXCL9	DPP4	1,74892E-03	35894,2	2,01880E-02	2058	2,22263E+00	1575	2,97885E+00	5895	7,87530E-01	108043	0	61900
Myeloid_vs_CD8_Non-responder	CD8(Tn)	Macro_LYVE1	CD59	STAB1	1,75005E-03	29659,4	6,25041E-03	16530	2,00642E+00	2515	2,36852E+00	13882	8,75010E-01	53470	0	61900
Myeloid_vs_CD8_Non-responder	CD8(ITM2C+ T)	Mono_CD16	HLA-B	LILRA1	1,75018E-03	24328,8	9,84242E-03	8183	1,90274E+00	3148	1,57702E+00	35749	9,38808E-01	12664	0	61900
Myeloid_vs_CD8_Non-responder	CD8(IL7R+ZNF683+ Tm)	Macro_ISG15	HLA-F	LILRB2	1,75018E-03	31349,6	3,70593E-03	36091	1,48625E+00	7867	2,12113E+00	18991	9,07157E-01	31899	0	61900
Myeloid_vs_CD8_Non-responder	CD8(ITM2C+ T)	Mono_CD16	HLA-B	LILRB2	1,75042E-03	20367	4,81224E-03	24375	2,07336E+00	2168	2,43112E+00	12779	9,59546E-01	613	0	61900
Myeloid_vs_CD8_Non-responder	CD8(ITM2C+ T)	Mono_CD16	HLA-B	LILRB1	1,75066E-03	29196	3,94848E-03	32912	1,37164E+00	10354	1,64894E+00	32980	9,45399E-01	7834	0	61900
Myeloid_vs_CD8_Non-responder	CD8(ITM2C+ T)	Mono_CD16	HLA-A	LILRB2	1,75212E-03	20875,4	4,79896E-03	24493	1,97962E+00	2660	2,35826E+00	14053	9,57560E-01	1271	0	61900
Myeloid_vs_CD8_Non-responder	CD8(ITM2C+ T)	Mono_CD16	HLA-A	LILRB1	1,75236E-03	30724	3,93758E-03	33038	1,27790E+00	13045	1,57609E+00	35782	9,42761E-01	9855	0	61900
Myeloid_vs_CD8_Non-responder	CD8(ZNF683+KLRB1+ T)	Macro_NLRP3	CIRBP	TREM1	1,75358E-03	33777,2	5,42750E-03	20435	1,14742E+00	17619	1,65310E+00	32827	9,00871E-01	36105	0	61900
Myeloid_vs_CD8_Non-responder	CD8(ITM2C+ T)	Mono_CD16	HLA-C	LILRB2	1,75382E-03	23651,6	4,24143E-03	29565	1,71046E+00	4755	2,08958E+00	19731	9,54903E-01	2307	0	61900
Myeloid_vs_CD8_Non-responder	CD8(ITM2C+ T)	Mono_CD16	HLA-F	LILRB2	1,75576E-03	24191,6	5,25056E-03	21443	1,90419E+00	3140	2,52513E+00	11241	9,20825E-01	23234	0	61900
Myeloid_vs_CD8_Non-responder	CD8(ITM2C+ T)	Mono_CD16	TNFSF9	HLA-DPA1	1,75649E-03	30895,6	5,92804E-03	17896	9,34988E-01	27736	1,77887E+00	28563	9,29067E-01	18383	0	61900
Myeloid_vs_CD8_Non-responder	CD8(Tn)	pDC_LILRA4	TNF	PTPRS	1,75703E-03	28695,6	2,86324E-02	1057	3,21710E+00	212	2,18460E+00	17586	8,60996E-01	62723	0	61900
Myeloid_vs_CD8_Non-responder	CD8(ITM2C+ T)	Mono_CD16	B2M	LILRB2	1,75746E-03	21702,2	4,45952E-03	27364	1,88290E+00	3303	2,27348E+00	15731	9,62041E-01	213	0	61900
Myeloid_vs_CD8_Non-responder	CD8(ITM2C+ T)	Mono_CD16	RPS19	C5AR1	1,76136E-03	26689,4	4,55026E-03	26523	1,34141E+00	11158	1,82166E+00	27181	9,46815E-01	6685	0	61900
Myeloid_vs_CD8_Non-responder	CD8(EOMES+ NK-like)	Macro_ISG15	RPS19	C5AR1	1,76209E-03	31101,6	3,70184E-03	36140	1,13149E+00	18290	1,78583E+00	28315	9,41373E-01	10863	0	61900
Myeloid_vs_CD8_Non-responder	CD8(ITM2C+ T)	Mono_CD16	HSPA1A	TLR4	1,76600E-03	30267,6	6,13250E-03	16988	1,24869E+00	13951	1,69111E+00	31424	9,14702E-01	27075	0	61900
Myeloid_vs_CD8_Non-responder	Macro_NLRP3	CD8(Temra)	VCAN	CD44	1,76838E-03	21899,6	7,49370E-03	12560	2,05264E+00	2275	2,25176E+00	16189	9,32343E-01	16574	0	61900
Myeloid_vs_CD8_Non-responder	CD8(Tc17)	Macro_FOLR2+APOE+	HLA-F	LILRB2	1,77579E-03	32595,4	3,69739E-03	36196	1,37488E+00	10269	1,97684E+00	22649	9,07060E-01	31963	0	61900
Myeloid_vs_CD8_Non-responder	CD8(ITM2C+ T)	Macro_OLFML3	IFNG	IFNGR1_IFNGR2	1,77873E-03	34465,2	6,22409E-03	16635	9,24911E-01	28337	1,66288E+00	32444	9,05464E-01	33010	0	61900
Myeloid_vs_CD8_Non-responder	CD8(EOMES+ NK-like)	Mono_CD16	RPS19	C5AR1	1,78193E-03	31160,4	3,69585E-03	36221	1,12932E+00	18392	1,78344E+00	28398	9,41328E-01	10891	0	61900
Myeloid_vs_CD8_Non-responder	Macro_NLRP3	CD8(ITM2C+ T)	VCAN	CD44	1,78727E-03	21778,6	7,47573E-03	12622	2,05097E+00	2285	2,28673E+00	15467	9,32268E-01	16619	0	61900
Myeloid_vs_CD8_Non-responder	Mono_CD14	CD8(LAYN+ T)	VCAN	ITGB1	1,78769E-03	17708,8	1,62631E-02	3211	2,74057E+00	553	2,93828E+00	6260	9,32267E-01	16620	0	61900
Myeloid_vs_CD8_Non-responder	CD8(GZMK+ Tex)	Macro_ISG15	HLA-C	LILRB2	1,78907E-03	26399,2	3,69375E-03	36250	1,50378E+00	7541	1,97636E+00	22664	9,51830E-01	3641	0	61900
Myeloid_vs_CD8_Non-responder	Macro_NLRP3	CD8(GZMK+ Tex)	VCAN	CD44	1,78938E-03	21871,4	7,47397E-03	12627	2,05081E+00	2287	2,26433E+00	15919	9,32260E-01	16624	0	61900
Myeloid_vs_CD8_Non-responder	Macro_OLFML3	CD8(GZMK+ Tex)	HLA-DQA1	LAG3	1,79191E-03	24418	6,31827E-03	16257	1,18642E+00	16167	2,22984E+00	16630	9,40964E-01	11136	0	61900
Myeloid_vs_CD8_Non-responder	Mast	CD8(ISG+ T)	TIMP3	CD44	1,79691E-03	29943,4	3,18697E-02	849	2,39361E+00	1069	1,21843E+00	52078	9,04266E-01	33821	0	61900
Myeloid_vs_CD8_Non-responder	CD8(ITM2C+ T)	Macro_IFI27	TNFSF9	HLA-DPA1	1,79698E-03	23971,4	8,38760E-03	10635	1,35804E+00	10717	1,90973E+00	24556	9,39686E-01	12049	0	61900
Myeloid_vs_CD8_Non-responder	CD8(ID2+CXCR4+ T)	Mono_CD16	HLA-A	LILRB1	1,79723E-03	31098,2	3,90333E-03	33449	1,25286E+00	13827	1,56378E+00	36283	9,42525E-01	10032	0	61900
Myeloid_vs_CD8_Non-responder	CD8(Terminal Tex)	Macro_FOLR2+APOE+	LGALS3	ENG	1,79971E-03	37823,6	3,80248E-03	34747	1,09296E+00	19964	1,56349E+00	36293	9,00734E-01	36214	0	61900
Myeloid_vs_CD8_Non-responder	Macro_FOLR2+APOE+	CD8(GZMK+ Tex)	HLA-DRA	LAG3	1,80249E-03	19643,4	6,21754E-03	16655	1,34361E+00	11098	2,99228E+00	5780	9,53672E-01	2784	0	61900
Myeloid_vs_CD8_Non-responder	Macro_ISG15	CD8(GZMK+ Tem)	HLA-DRA	LAG3	1,80292E-03	21204,4	6,21714E-03	16656	1,26815E+00	13336	2,51841E+00	11345	9,53670E-01	2785	0	61900
Myeloid_vs_CD8_Non-responder	cDC(CD1C)	CD8(GZMK+ Tex)	HLA-DPB1	LAG3	1,81142E-03	20925,4	6,21273E-03	16676	1,37263E+00	10329	2,50143E+00	11611	9,50902E-01	4111	0	61900
Myeloid_vs_CD8_Non-responder	cDC(CD1C)	CD8(Terminal Tex)	HLA-DRA	LAG3	1,81227E-03	20835,6	6,21214E-03	16678	1,33723E+00	11283	2,50730E+00	11523	9,53653E-01	2794	0	61900
Myeloid_vs_CD8_Non-responder	CD8(ITM2C+ T)	Macro_IER3	TNFSF9	HLA-DPA1	1,81389E-03	25196	7,34781E-03	12965	1,17919E+00	16438	2,07728E+00	20066	9,35824E-01	14611	0	61900
Myeloid_vs_CD8_Non-responder	CD8(NME1+ T)	Macro_FOLR2+APOE+	HMGB1	HAVCR2	1,82339E-03	34542	3,68583E-03	36388	1,01689E+00	23376	1,64961E+00	32954	9,29571E-01	18092	0	61900

Myeloid_vs_CD8_Post_NR

Myeloid_vs_CD8_Non-responder	cDC(CD1C)	CD8(Terminal Tex)	HLA-DQA1	LAG3	1,82722E-03	22482,6	7,10809E-03	13663	1,33442E+00	11362	2,22527E+00	16713	9,44152E-01	8775	0	61900
Myeloid_vs_CD8_Non-responder	CD8(ITM2C+ T)	cDC_LAMP3	TNFSF9	HLA-DPA1	1,83142E-03	31042,8	6,27032E-03	16446	9,93861E-01	24536	1,59734E+00	34962	9,30895E-01	17370	0	61900
Myeloid_vs_CD8_Non-responder	Macro_FOLR2+APOE+	CD8(GZMK+ Tex)	B2M	CD3D	1,83334E-03	36867,6	2,84989E-03	52834	9,35674E-01	27698	1,43100E+00	41893	9,65617E-01	13	0	61900
Myeloid_vs_CD8_Non-responder	CD8(Terminal Tex)	Macro_ISG15	B2M	LILRB2	1,83419E-03	25678,8	3,68349E-03	36431	1,53846E+00	6970	1,99566E+00	22131	9,58392E-01	962	0	61900
Myeloid_vs_CD8_Non-responder	CD8(EOMES+ NK-like)	Macro_NLRP3	RPS19	C5AR1	1,83771E-03	32147,4	3,68261E-03	36445	1,12452E+00	18598	1,65294E+00	32838	9,41229E-01	10956	0	61900
Myeloid_vs_CD8_Non-responder	CD8(Tn)	Mono_CD16	HLA-A	LILRA1	1,83881E-03	30188,8	8,66625E-03	10093	1,47209E+00	8107	1,17999E+00	54104	9,32033E-01	16740	0	61900
Myeloid_vs_CD8_Non-responder	CD8(IL7R+ZNF683+ Tm)	pDC_LILRA4	CCL5	CXCR3	1,83897E-03	29208,2	9,95058E-03	8036	8,79732E-01	31138	1,55992E+00	36450	9,44477E-01	8517	0	61900
Myeloid_vs_CD8_Non-responder	CD8(ZNF683+KLRB1+ T)	cDC_CLEC9A	HMGB1	THBD	1,84124E-03	34361	4,62905E-03	25820	1,14859E+00	17561	1,55962E+00	36459	9,09882E-01	30065	0	61900
Myeloid_vs_CD8_Non-responder	Macro_OLFML3	CD8(GZMK+ Tem)	HLA-DPA1	LAG3	1,85262E-03	21484	6,18768E-03	16772	1,29124E+00	12666	2,41216E+00	13103	9,53234E-01	2979	0	61900
Myeloid_vs_CD8_Non-responder	CD8(Tn)	Macro_ISG15	LTB	TNFRSF1A	1,85619E-03	32397,4	6,86817E-03	14342	1,06474E+00	21220	1,79546E+00	28007	9,00279E-01	36518	0	61900
Myeloid_vs_CD8_Non-responder	CD8(Temra)	Macro_ISG15	CCL4	CCR1	1,85950E-03	27254,6	1,09822E-02	6870	1,49734E+00	7668	1,55806E+00	36531	9,20729E-01	23304	0	61900
Myeloid_vs_CD8_Non-responder	CD8(LAYN+ T)	Macro_NLRP3	HMGB1	THBD	1,86612E-03	33686,4	4,75556E-03	24803	1,19690E+00	15817	1,55737E+00	36557	9,10982E-01	29355	0	61900
Myeloid_vs_CD8_Non-responder	CD8(ZNF683+KLRB1+ T)	Macro_FOLR2+APOE+	IFNG	IFNGR1_IFNGR2	1,87021E-03	35553,6	5,78937E-03	18522	9,72475E-01	25621	1,55693E+00	36573	9,02319E-01	35152	0	61900
Myeloid_vs_CD8_Non-responder	CD8(Terminal Tex)	Macro_ISG15	CCL4	CCR1	1,87123E-03	27727,6	1,04528E-02	7469	1,46251E+00	8307	1,55692E+00	36577	9,18907E-01	24385	0	61900
Myeloid_vs_CD8_Non-responder	CD8(EOMES+ NK-like)	Macro_ISG15	HLA-B	LILRB2	1,87175E-03	26329,8	3,67251E-03	36579	1,47849E+00	8001	1,98250E+00	22502	9,53961E-01	2667	0	61900
Myeloid_vs_CD8_Non-responder	CD8(GZMK+ Early Tem)	pDC_LILRA4	SELPLG	SELL	1,87304E-03	29344,8	6,17369E-03	16819	1,43151E+00	8943	2,33856E+00	14435	8,88390E-01	44627	0	61900
Myeloid_vs_CD8_Non-responder	CD8(Terminal Tex)	cDC_CLEC9A	TNFSF9	HLA-DPA1	1,87584E-03	31688,4	3,67163E-03	36595	1,07789E+00	20614	2,59074E+00	10310	9,11567E-01	29023	0	61900
Myeloid_vs_CD8_Non-responder	Macro_OLFML3	CD8(Terminal Tex)	CXCL9	DPP4	1,87606E-03	35324	2,14500E-02	1848	2,23606E+00	1531	2,96434E+00	6037	7,92559E-01	105304	0	61900
Myeloid_vs_CD8_Non-responder	CD8(LAYN+ T)	Macro_NLRP3	ANXA1	FPR1	1,87815E-03	36007	3,67063E-03	36604	9,69956E-01	25748	1,58961E+00	35244	9,25278E-01	20539	0	61900
Myeloid_vs_CD8_Non-responder	cDC(CD1C)	CD8(GZMK+ Tex)	HLA-DRA	LAG3	1,88091E-03	20834,2	6,16699E-03	16837	1,33207E+00	11431	2,53197E+00	11145	9,53491E-01	2858	0	61900
Myeloid_vs_CD8_Non-responder	CD8(GZMK+ Tex)	Mono_CD16	HLA-A	LILRB1	1,88432E-03	31095,2	3,91163E-03	33336	1,25893E+00	13620	1,55577E+00	36628	9,42582E-01	9992	0	61900
Myeloid_vs_CD8_Non-responder	CD8(GZMK+ Tem)	Macro_ISG15	TNFSF9	HLA-DPA1	1,88432E-03	37871,2	3,69605E-03	36216	8,00994E-01	36628	1,86882E+00	25756	9,11834E-01	28856	0	61900
Myeloid_vs_CD8_Non-responder	CD8(GZMK+ Early Tem)	Mono_CD16	HLA-A	LILRB1	1,88972E-03	31184,8	3,90157E-03	33473	1,25158E+00	13862	1,55509E+00	36649	9,42512E-01	10040	0	61900
Myeloid_vs_CD8_Non-responder	CD8(ISG+ T)	Macro_FOLR2+APOE+	CD52	SIGLEC10	1,89179E-03	31251	5,37856E-03	20697	1,24987E+00	13919	1,55482E+00	36657	9,21091E-01	23082	0	61900
Myeloid_vs_CD8_Non-responder	CD8(Tc17)	Mono_CD14	CIRBP	TREM1	1,89437E-03	33849	5,58694E-03	19544	1,19481E+00	15890	1,55459E+00	36667	9,02157E-01	35244	0	61900
Myeloid_vs_CD8_Non-responder	CD8(ZNF683+KLRB1+ T)	pDC_LILRA4	HLA-C	NOTCH4	1,89592E-03	25727,6	1,50178E-02	3803	1,70452E+00	4811	1,55436E+00	36673	9,23689E-01	21451	0	61900
Myeloid_vs_CD8_Non-responder	CD8(ITM2C+ T)	pDC_LILRA4	TNFSF9	HLA-DPA1	1,90265E-03	28847,6	5,84700E-03	18250	9,21048E-01	28580	2,21829E+00	16872	9,28612E-01	18636	0	61900
Myeloid_vs_CD8_Non-responder	CD8(ISG+ T)	Macro_ISG15	HLA-C	LILRB2	1,90524E-03	26617,8	3,66397E-03	36709	1,48304E+00	7917	1,96978E+00	22848	9,51645E-01	3715	0	61900
Myeloid_vs_CD8_Non-responder	CD8(ITM2C+ T)	pDC_LILRA4	CCL5	CXCR3	1,90654E-03	29258	9,94043E-03	8052	8,78727E-01	31207	1,55671E+00	36588	9,44450E-01	8543	0	61900
Myeloid_vs_CD8_Non-responder	CD8(LAYN+ T)	Mono_CD16	HLA-C	LILRA3	1,90758E-03	26364,8	1,13649E-02	6469	1,60771E+00	5935	1,55344E+00	36718	9,24844E-01	20802	0	61900
Myeloid_vs_CD8_Non-responder	CD8(Tn)	cDC_LAMP3	CD40LG	CD40	1,90819E-03	30945,6	4,86937E-02	374	2,01108E+00	2484	2,21686E+00	16899	8,45065E-01	73071	0	61900
Myeloid_vs_CD8_Non-responder	CD8(Terminal Tex)	Macro_ISG15	HLA-B	LILRB2	1,90836E-03	26977	3,66338E-03	36721	1,47084E+00	8141	1,88021E+00	25437	9,53907E-01	2686	0	61900
Myeloid_vs_CD8_Non-responder	CD8(ITM2C+ T)	pDC_LILRA4	HMGB1	THBD	1,90888E-03	35205,8	3,97901E-03	32527	1,03368E+00	22647	1,90781E+00	24622	9,03483E-01	34333	0	61900
Myeloid_vs_CD8_Non-responder	CD8(Temra)	Macro_OLFML3	SPON2	ITGB2	1,90992E-03	33724,2	6,68214E-03	14926	9,72028E-01	25651	1,75108E+00	29417	8,99957E-01	36727	0	61900
Myeloid_vs_CD8_Non-responder	cDC_LAMP3	CD8(ITM2C+ T)	CCL19	CCR7	1,91574E-03	34377,4	2,80414E-02	1104	3,93045E+00	81	2,36285E+00	13985	8,10148E-01	94817	0	61900
Myeloid_vs_CD8_Non-responder	CD8(ITM2C+ T)	Macro_FOLR2-APOE+	TNFSF9	HLA-DPA1	1,92296E-03	25544,2	8,04582E-03	11334	1,29925E+00	12429	1,75837E+00	29156	9,38496E-01	12902	0	61900
Myeloid_vs_CD8_Non-responder	CD8(GZMK+ Tem)	pDC_LILRA4	BST2	LILRA4	1,92327E-03	23545	1,21359E-02	5824	2,75437E+00	536	2,95014E+00	6153	8,90316E-01	43312	0	61900
Myeloid_vs_CD8_Non-responder	CD8(GZMK+ Tem)	pDC_LILRA4	SELPLG	SELL	1,92372E-03	28315,2	6,68061E-03	14932	1,45750E+00	8406	2,34449E+00	14315	8,92242E-01	42023	0	61900
Myeloid_vs_CD8_Non-responder	CD8(NME1+ T)	Mono_CD14	RPS19	C5AR1	1,92531E-03	29929,8	4,26109E-03	29356	1,26002E+00	13577	1,55210E+00	36786	9,45137E-01	8030	0	61900
Myeloid_vs_CD8_Non-responder	CD8(GZMK+ Tem)	pDC_LILRA4	COPA	P2RY6	1,93353E-03	25934,6	1,10288E-02	6813	2,73686E+00	554	3,12023E+00	4693	8,71587E-01	55713	0	61900
Myeloid_vs_CD8_Non-responder	CD8(ITM2C+ T)	Macro_FOLR2+APOE+	CD99	PILRA	1,93449E-03	33922,6	3,65756E-03	36821	1,16703E+00	16880	1,70523E+00	30914	9,21063E-01	23098	0	61900
Myeloid_vs_CD8_Non-responder	Macro_ISG15	CD8(LAYN+ T)	CD14	ITGB1	1,94203E-03	21738,4	6,13576E-03	16975	1,61930E+00	5794	2,82959E+00	7328	9,32118E-01	16695	0	61900
Myeloid_vs_CD8_Non-responder	CD8(ZNF683+KLRB1+ T)	Macro_OLFML3	HLA-F	LILRB2	1,94264E-03	33743	3,65589E-03	36852	1,25424E+00	13773	1,93205E+00	23908	9,06583E-01	32282	0	61900
Myeloid_vs_CD8_Non-responder	CD8(ZNF683+KLRB1+ T)	Mono_CD16	HLA-B	LILRB1	1,94423E-03	31253,2	3,79779E-03	34813	1,24431E+00	14104	1,55020E+00	36858	9,44386E-01	8591	0	61900
Myeloid_vs_CD8_Non-responder	CD8(Tc17)	Macro_NLRP3	HMGB1	THBD	1,94686E-03	35028,2	4,46568E-03	27303	1,13777E+00	18040	1,54989E+00	36868	9,08399E-01	31030	0	61900
Myeloid_vs_CD8_Non-responder	cDC_CLEC9A	CD8(Terminal Tex)	HLA-DPA1	LAG3	1,95686E-03	19825,2	6,12660E-03	17008	1,36488E+00	10537	2,90163E+00	6602	9,53012E-01	3079	0	61900
Myeloid_vs_CD8_Non-responder	CD8(ID2+CXCR4+ T)	Mono_CD16	B2M	LILRB1	1,95877E-03	30714,2	3,72111E-03	35862	1,25331E+00	13809	1,54881E+00	36913	9,49128E-01	5087	0	61900
Myeloid_vs_CD8_Non-responder	CD8(IL7R+ZNF683+ Tm)	Macro_ISG15	B2M	LILRB2	1,95931E-03	25995	3,65284E-03	36915	1,50316E+00	7551	1,97990E+00	22579	9,58225E-01	1030	0	61900
Myeloid_vs_CD8_Non-responder	cDC_LAMP3	CD8(Tc17)	CCL19	CCR7	1,96433E-03	34663,2	2,78715E-02	1118	3,92944E+00	82	2,30726E+00	15088	8,09681E-01	95128	0	61900
Myeloid_vs_CD8_Non-responder	cDC(CD1C)	CD8(GZMK+ Tem)	HLA-DRB1	LAG3	1,96453E-03	22223,4	6,12335E-03	17025	1,28916E+00	12717	2,21757E+00	16885	9,54172E-01	2590	0	61900
Myeloid_vs_CD8_Non-responder	CD8(ITM2C+ T)	Mono_CD14	RPS19	C5AR1	1,96728E-03	29075,8	4,36618E-03	28258	1,28720E+00	12775	1,59861E+00	34912	9,45765E-01	7534	0	61900
Myeloid_vs_CD8_Non-responder	CD8(ITM2C+ T)	Mono_CD14	HSPA1A	TLR4	1,97368E-03	26685,6	7,77535E-03	11897	1,43040E+00	8969	1,76042E+00	29100	9,23517E-01	21562	0	61900
Myeloid_vs_CD8_Non-responder	cDC_CLEC9A	CD8(Terminal Tex)	HLA-DRA	LAG3	1,97631E-03	20193,8	6,11885E-03	17051	1,31607E+00	11940	2,84913E+00	7134	9,53317E-01	2944	0	61900
Myeloid_vs_CD8_Non-responder	CD8(ITM2C+ T)	Macro_ISG15	HLA-B	LILRB2	1,97662E-03	23620,8	3,98614E-03	32440	1,74100E+00	4467	2,19542E+00	17344	9,55728E-01	1953	0	61900
Myeloid_vs_CD8_Non-responder	CD8(ITM2C+ T)	Macro_ISG15	HLA-B	LILRB1	1,97688E-03	25910,2	4,39794E-03	27944	1,51071E+00	7403	1,84153E+00	26551	9,48116E-01	5753	0	61900
Myeloid_vs_CD8_Non-responder	CD8(ITM2C+ T)	Macro_ISG15	HLA-A	LILRB2	1,97876E-03	24335,2	3,97514E-03	32564	1,64726E+00	5417	2,12257E+00	18957	9,53564E-01	2838	0	61900
Myeloid_vs_CD8_Non-responder	CD8(ITM2C+ T)	Macro_ISG15	HLA-A	LILRB1	1,97902E-03	27148,8	4,38580E-03	28071	1,41697E+00	9263	1,76867E+00	28852	9,45601E-01	7658	0	61900
Myeloid_vs_CD8_Non-responder	CD8(ID2+CXCR4+ T)	pDC_LILRA4	COPA	P2RY6	1,98145E-03	25214,8	1,17409E-02	6150	2,75693E+00	531	3,20824E+00	4051	8,75048E-01	53442	0	61900
Myeloid_vs_CD8_Non-responder	CD8(ITM2C+ T)	Macro_ISG15	HLA-F	LILRB2	1,98331E-03	27981,2	4,34922E-03	28437	1,57182E+00	6475	2,28944E+00	15426	9,13682E-01	27668	0	61900
Myeloid_vs_CD8_Non-responder	CD8(ITM2C+ T)	Macro_ISG15	HLA-F	LILRB1	1,98357E-03	31662,4	4,79853E-03	24494	1,34153E+00	11154	1,93554E+00	23808	8,99600E-01	36956	0	61900
Myeloid_vs_CD8_Non-responder	CD8(ITM2C+ T)	Macro_ISG15	TNFSF9	HLA-DPA1	1,98384E-03	24921,4	6,96392E-03	14066	1,11316E+00	19060	2,36018E+00	14023	9,34194E-01	15558	0	61900
Myeloid_vs_CD8_Non-responder	CD8(ITM2C+ T)	Macro_ISG15	B2M	LILRB2	1,98465E-03	25379,4	3,69397E-03	36248	1,55053E+00	6761	2,03778E+00	21045	9,58449E-01	943	0	61900
Myeloid_vs_CD8_Non-responder	CD8(ITM2C+ T)	Macro_ISG15	B2M	LILRB1	1,98491E-03	28139,2	4,07559E-03	31382	1,32024E+00	11805	1,68389E+00	31694	9,51281E-01	3915	0	61900
Myeloid_vs_CD8_Non-responder	CD8(ITM2C+ T)	Macro_ISG15	IFNG	IFNGR1_IFNGR2	1,98679E-03	34080	6,04733E-03	17372	8,94789E-01	30189	1,82401E+00	27097	9,04223E-01	33842	0	61900
Myeloid_vs_CD8_Non-responder	CD8(ITM2C+ T)	Macro_ISG15	RPS19	C5AR1	1,99136E-03	26639,6	4,55763E-03	26450	1,34358E+00	11099	1,82405E+00	27095	9,46855E-01	6654	0	61900
Myeloid_vs_CD8_Non-responder	Macro_ISG15	CD8(ITM2C+ T)	CCL2	CCR5	1,99191E-03	34958	2,12497E-02	1881	1,59271E+00	6161	3,31436E+00	3369	7,98933E-01	101479	0	61900
Myeloid_vs_CD8_Non-responder	CD8(ITM2C+ T)	Macro_ISG15	HSPA1A	TLR4	1,99809E-03	29317,2	6,25892E-03	16493	1,26267E+00	13500	1,79218E+00	28105	9,15494E-01	26588	0	61900
Myeloid_vs_CD8_Non-responder	CD8(Temra)	Macro_OLFML3	HLA-C	LILRB1	2,00133E-03	31590,2	3,64357E-03	37072	1,27202E+00	13234	1,61399E+00	34314	9,40532E-01	11431	0	61900
Myeloid_vs_CD8_Non-responder	CD8(ITM2C+ T)	Macro_FOLR2+APOE-	TNFSF9	HLA-DPA1	2,00646E-03	24004,8	8,70213E-03	10027	1,41214E+00	9378	1,81360E+00	27427	9,40721E-01	11292	0	61900
Myeloid_vs_CD8_Non-responder	Mast	CD8(Tc17)	TIMP3	CD44	2,00998E-03	30326	3,10591E-02	890	2,37363E+00	1131	1,19889E+00	53133	9,03145E-01	34576	0	61900
Myeloid_vs_CD8_Non-responder	Macro_OLFML3	CD8(Terminal Tex)	HLA-DQA1	LAG3	2,01011E-03	24420,4	6,36453E-03	16075	1,19159E+00	15999	2,20517E+00	17125	9,41167E-01	11003	0	61900
Myeloid_vs_CD8_Non-responder	CD8(ZNF683+KLRB1+ T)	Macro_OLFML3	CD52	SIGLEC10	2,01079E-03	31317	5,42860E-03	20429	1,23845E+00	14290	1,54342E+00	37107	9,21427E-01	22859	0	61900
Myeloid_vs_CD8_Non-responder	CD8(EOMES+ NK-like)	Macro_LYVE1	HMGB1	CD163	2,01866E-03	29673,8	4,48531E-03	27124	1,42450E+00	9101	1,54275E+00	37136	9,38163E-01	13108	0	61900
Myeloid_vs_CD8_Non-responder	CD8(GZMK+ Tex)	pDC_LILRA4	SELPLG	SELL	2,01975E-03	26060,2	7,77711E-03	11893	1,51372E+00	7370	2,47656E+00	11998	8,99333E-01	37140	0	61900
Myeloid_vs_CD8_Non-responder	CD8(GZMK+ Tex)	Mono_CD16	HLA-B	LILRB1	2,02111E-03	31519,6	3,77636E-03	35110	1,22620E+00	14726	1,54252E+00	37145	9,44238E-01	8717	0	61900
Myeloid_vs_CD8_Non-responder	Macro_FOLR2+APOE+	CD8(NME1+ T)	HLA-DRA	LAG3	2,02247E-03	25843	4,27014E-03	29244	1,12279E+00	18677	2,54167E+00	11001	9,44627E-01	8393	0	61900
Myeloid_vs_CD8_Non-responder	Macro_FOLR2+APOE+	CD8(NME1+ T)	HLA-DQA2	LAG3	2,02301E-03	36447,6	4,41793E-03	27751	8,68718E-01	31874	1,89137E+00	25088	9,01575E-01	35625	0	61900

Myeloid_vs_CD8_Non-responder	CD8(Tc17)	pDC_LILRA4	BST2	LILRA4	2,02352E-03	23801,4	1,16861E-02	6194	2,74376E+00	550	2,99239E+00	5779	8,88458E-01	44584	0	61900
Myeloid_vs_CD8_Non-responder	Macro_FOLR2+APOE+	CD8(NME1+ T)	HLA-DQA1	LAG3	2,02356E-03	25544,2	5,12320E-03	22200	1,18646E+00	16166	2,46086E+00	12281	9,34864E-01	15174	0	61900
Myeloid_vs_CD8_Non-responder	Macro_FOLR2+APOE+	CD8(NME1+ T)	HLA-DRB1	LAG3	2,02410E-03	29354,6	3,77679E-03	35101	1,00380E+00	24038	2,28049E+00	15595	9,42369E-01	10139	0	61900
Myeloid_vs_CD8_Non-responder	Macro_FOLR2+APOE+	CD8(NME1+ T)	HLA-DQB1	LAG3	2,02465E-03	26442,6	4,86148E-03	23994	1,14828E+00	17576	2,38220E+00	13647	9,35018E-01	15096	0	61900
Myeloid_vs_CD8_Non-responder	Macro_FOLR2+APOE+	CD8(NME1+ T)	HLA-DPB1	LAG3	2,02656E-03	28916,4	3,99445E-03	32325	1,05291E+00	21767	2,24011E+00	16426	9,39502E-01	12164	0	61900
Myeloid_vs_CD8_Non-responder	CD8(Temra)	Macro_ISG15	B2M	LILRB2	2,02683E-03	26154,4	3,63745E-03	37166	1,48544E+00	7877	1,97242E+00	22773	9,58140E-01	1056	0	61900
Myeloid_vs_CD8_Non-responder	CD8(ISG+ T)	Mono_CD16	HLA-A	LILRB1	2,02819E-03	31572,8	3,86780E-03	33890	1,22689E+00	14691	1,54186E+00	37171	9,42276E-01	10212	0	61900
Myeloid_vs_CD8_Non-responder	Macro_FOLR2+APOE+	CD8(NME1+ T)	ICAM1	IL2RG	2,02956E-03	39759,4	3,66518E-03	36695	9,17885E-01	28748	1,54559E+00	37021	9,03347E-01	34433	0	61900
Myeloid_vs_CD8_Non-responder	CD8(IL7R+ZNF683+ Tm)	Macro_ISG15	CCL5	CCR1	2,03041E-03	27755,4	1,25385E-02	5454	1,48565E+00	7874	1,33280E+00	46380	9,31252E-01	17169	0	61900
Myeloid_vs_CD8_Non-responder	Macro_FOLR2+APOE+	CD8(NME1+ T)	CD14	ITGB1	2,03092E-03	27217,8	4,15798E-03	30471	1,45538E+00	8453	2,55991E+00	10754	9,18724E-01	24511	0	61900
Myeloid_vs_CD8_Non-responder	Macro_FOLR2+APOE+	CD8(NME1+ T)	CD14	ITGA4	2,03119E-03	28998,8	3,76899E-03	35211	1,40537E+00	9521	2,46535E+00	12203	9,16203E-01	26159	0	61900
Myeloid_vs_CD8_Non-responder	Macro_FOLR2+APOE+	CD8(NME1+ T)	HLA-DPA1	LAG3	2,03639E-03	28169,8	3,96308E-03	32735	1,05907E+00	21480	2,33587E+00	14494	9,42238E-01	10240	0	61900
Myeloid_vs_CD8_Non-responder	Macro_NLRP3	CD8(ITM2C+ T)	IL1B	ADRB2	2,04283E-03	24701,6	1,16643E-02	6214	2,31507E+00	1286	3,40472E+00	2865	8,78352E-01	51243	0	61900
Myeloid_vs_CD8_Non-responder	CD8(ISG+ T)	Macro_IER3	RPS19	C5AR1	2,04489E-03	27341,4	4,93003E-03	23498	1,43896E+00	8774	1,54073E+00	37232	9,48797E-01	5303	0	61900
Myeloid_vs_CD8_Non-responder	Macro_FOLR2+APOE+	CD8(NME1+ T)	HLA-DRB5	LAG3	2,04489E-03	27966,4	4,29492E-03	28971	1,08074E+00	20484	2,30597E+00	15114	9,37785E-01	13363	0	61900
Myeloid_vs_CD8_Non-responder	CD8(GZMK+ Tem)	Macro_FOLR2+APOE+	HLA-A	LILRB2	2,04874E-03	26519,8	3,63256E-03	37246	1,53860E+00	6963	1,97484E+00	22708	9,51527E-01	3782	0	61900
Myeloid_vs_CD8_Non-responder	cDC_CLEC9A	CD8(GZMK+ Tex)	HLA-DPA1	LAG3	2,05273E-03	19861,2	6,08207E-03	17217	1,35972E+00	10674	2,92629E+00	6358	9,52849E-01	3157	0	61900
Myeloid_vs_CD8_Non-responder	CD8(Tn)	Macro_OLFML3	ANXA1	FPR2_FPR3	2,05507E-03	34662,4	5,82145E-03	18368	1,07571E+00	20720	1,53979E+00	37269	9,02459E-01	35055	0	61900
Myeloid_vs_CD8_Non-responder	Macro_FOLR2+APOE+	CD8(NME1+ T)	LGALS3	LAG3	2,05562E-03	30223	4,99575E-03	23055	9,89076E-01	24771	2,06581E+00	20350	9,24376E-01	21039	0	61900
Myeloid_vs_CD8_Non-responder	CD8(Tn)	Macro_NLRP3	HSPA1A	TLR4	2,05617E-03	31316,6	6,39988E-03	15941	1,20715E+00	15412	1,53973E+00	37273	9,16352E-01	26057	0	61900
Myeloid_vs_CD8_Non-responder	CD8(GZMK+ Tem)	Macro_OLFML3	HLA-A	LILRB1	2,05976E-03	32475,4	3,64626E-03	37036	1,22378E+00	14813	1,53939E+00	37286	9,40651E-01	11342	0	61900
Myeloid_vs_CD8_Non-responder	CD8(NME1+ T)	Macro_IER3	HMGB1	THBD	2,06031E-03	37152,2	3,97063E-03	32628	1,10267E+00	19546	1,53936E+00	37288	9,03391E-01	34399	0	61900
Myeloid_vs_CD8_Non-responder	CD8(Temra)	Macro_FOLR2+APOE+	HLA-C	LILRB2	2,06169E-03	26005,2	3,62988E-03	37293	1,58684E+00	6241	2,04944E+00	20764	9,51429E-01	3828	0	61900
Myeloid_vs_CD8_Non-responder	Macro_FOLR2+APOE+	CD8(NME1+ T)	CXCL16	CXCR6	2,06308E-03	32030,4	5,46870E-03	20189	1,18319E+00	16283	1,91165E+00	24496	8,99117E-01	37284	0	61900
Myeloid_vs_CD8_Non-responder	Mono_CD14	CD8(ISG+ T)	VCAN	SELL	2,06713E-03	23851,2	1,28704E-02	5174	2,55734E+00	780	2,94037E+00	6239	8,87560E-01	45163	0	61900
Myeloid_vs_CD8_Non-responder	cDC_CLEC9A	CD8(GZMK+ Tex)	HLA-DRA	LAG3	2,07241E-03	20230,8	6,07438E-03	17259	1,31091E+00	12089	2,87380E+00	6887	9,53155E-01	3019	0	61900
Myeloid_vs_CD8_Non-responder	Macro_FOLR2+APOE+	CD8(NME1+ T)	C1QB	C1QBP	2,07250E-03	20515	5,88662E-03	18075	1,80797E+00	3891	3,53769E+00	2229	9,32521E-01	16480	0	61900
Myeloid_vs_CD8_Non-responder	Macro_FOLR2+APOE+	CD8(NME1+ T)	C3	IFITM1	2,07361E-03	35456,8	4,86348E-03	23978	8,64049E-01	32192	1,60174E+00	34787	9,18848E-01	24427	0	61900
Myeloid_vs_CD8_Non-responder	CD8(Temra)	Macro_FOLR2+APOE+	IFNG	IFNGR1_IFNGR2	2,07416E-03	35211	5,97543E-03	17685	9,86083E-01	24937	1,53808E+00	37338	9,03704E-01	34195	0	61900
Myeloid_vs_CD8_Non-responder	Macro_FOLR2+APOE+	CD8(NME1+ T)	LYZ	ITGAL	2,07500E-03	28201,6	3,94985E-03	32897	1,32594E+00	11644	2,87321E+00	6897	9,13679E-01	27670	0	61900
Myeloid_vs_CD8_Non-responder	Macro_FOLR2+APOE+	CD8(GZMK+ Early Tem)	CXCL9	CXCR3	2,07789E-03	24763,2	1,57493E-02	3436	2,32097E+00	1261	2,93915E+00	6250	8,78766E-01	50969	0	61900
Myeloid_vs_CD8_Non-responder	CD8(Tc17)	Macro_ISG15	B2M	LILRB2	2,08390E-03	26276,6	3,62599E-03	37373	1,47224E+00	8102	1,96695E+00	22928	9,58077E-01	1080	0	61900
Myeloid_vs_CD8_Non-responder	CD8(NME1+ T)	Macro_FOLR2+APOE+	IFNG	IFNGR1_IFNGR2	2,08725E-03	35075,4	6,02945E-03	17441	9,90034E-01	24719	1,53681E+00	37385	9,04095E-01	33932	0	61900
Myeloid_vs_CD8_Non-responder	Macro_FOLR2+APOE+	CD8(ISG+ T)	HLA-DRA	LAG3	2,08809E-03	23230,6	4,84046E-03	24180	1,18746E+00	16138	2,77133E+00	7965	9,47816E-01	5970	0	61900
Myeloid_vs_CD8_Non-responder	CD8(ISG+ T)	Mono_CD16	B2M	LILRB1	2,08837E-03	31146	3,69241E-03	36270	1,21993E+00	14950	1,53666E+00	37389	9,48941E-01	5221	0	61900
Myeloid_vs_CD8_Non-responder	Macro_FOLR2+APOE+	CD8(ISG+ T)	HLA-DQA2	LAG3	2,08865E-03	32740,4	5,00799E-03	22969	9,33388E-01	27833	2,12104E+00	18993	9,06998E-01	32007	0	61900
Myeloid_vs_CD8_Non-responder	Macro_FOLR2+APOE+	CD8(ISG+ T)	HLA-DQA1	LAG3	2,08921E-03	23213	5,80745E-03	18429	1,25113E+00	13880	2,69053E+00	9017	9,38579E-01	12839	0	61900
Myeloid_vs_CD8_Non-responder	Macro_FOLR2+APOE+	CD8(ISG+ T)	HLA-DRB1	LAG3	2,08976E-03	26228	4,28122E-03	29118	1,06847E+00	21040	2,51015E+00	11489	9,45680E-01	7593	0	61900
Myeloid_vs_CD8_Non-responder	CD8(ITM2C+ T)	Mono_CD16	CD99	PILRA	2,08976E-03	34166,4	3,62400E-03	37394	1,15386E+00	17387	1,70760E+00	30845	9,20727E-01	23306	0	61900
Myeloid_vs_CD8_Non-responder	Macro_FOLR2+APOE+	CD8(ISG+ T)	HLA-DQB1	LAG3	2,09032E-03	23962,4	5,51078E-03	19960	1,21295E+00	15210	2,61187E+00	10018	9,38724E-01	12724	0	61900
Myeloid_vs_CD8_Non-responder	CD8(ID2+CXCR4+ T)	Mono_CD16	CD52	SIGLEC10	2,09116E-03	32459,6	5,13631E-03	22111	1,16878E+00	16817	1,53652E+00	37399	9,19400E-01	24071	0	61900
Myeloid_vs_CD8_Non-responder	Macro_FOLR2+APOE+	CD8(ISG+ T)	HLA-DPB1	LAG3	2,09228E-03	25871,8	4,52795E-03	26727	1,11758E+00	18897	2,46978E+00	12134	9,42968E-01	9701	0	61900
Myeloid_vs_CD8_Non-responder	CD8(Temra)	Macro_IER3	RPS19	C5AR1	2,09592E-03	27709,6	4,82760E-03	24271	1,41411E+00	9330	1,53614E+00	37416	9,48285E-01	5631	0	61900
Myeloid_vs_CD8_Non-responder	Macro_FOLR2+APOE+	CD8(ISG+ T)	HLA-DPA1	LAG3	2,10012E-03	25191	4,49239E-03	27044	1,12374E+00	18643	2,56554E+00	10678	9,45557E-01	7690	0	61900
Myeloid_vs_CD8_Non-responder	CD8(ID2+CXCR4+ T)	Mono_CD16	HLA-B	LILRB1	2,10321E-03	31388	3,79585E-03	34842	1,24267E+00	14158	1,53550E+00	37442	9,44373E-01	8598	0	61900
Myeloid_vs_CD8_Non-responder	CD8(LAYN+ T)	pDC_LILRA4	CXCL13	CXCR3	2,10687E-03	32413,6	1,77664E-02	2681	8,50278E-01	33092	1,82931E+00	26940	8,98850E-01	37455	0	61900
Myeloid_vs_CD8_Non-responder	CD8(GZMK+ Early Tem)	Macro_ISG15	B2M	LILRB2	2,10715E-03	26442,2	3,62066E-03	37456	1,46610E+00	8233	1,94542E+00	23527	9,58048E-01	1095	0	61900
Myeloid_vs_CD8_Non-responder	Macro_FOLR2+APOE+	CD8(ISG+ T)	HLA-DRB5	LAG3	2,10743E-03	25106	4,86855E-03	23944	1,14541E+00	17711	2,53564E+00	11094	9,41343E-01	10881	0	61900
Myeloid_vs_CD8_Non-responder	CD8(ZNF683+KLRB1+ T)	Mono_CD16	HLA-F	LILRB1	2,11052E-03	32956,4	4,71685E-03	25130	1,25735E+00	13685	1,83978E+00	26599	8,98822E-01	37468	0	61900
Myeloid_vs_CD8_Non-responder	Macro_FOLR2+APOE+	CD8(ISG+ T)	SPP1	CD44	2,11616E-03	29412,4	6,26160E-03	16480	8,90253E-01	30482	2,86065E+00	7010	9,08158E-01	31190	0	61900
Myeloid_vs_CD8_Non-responder	CD8(Tc17)	Mono_CD16	HLA-C	LILRB1	2,11616E-03	32226	3,75787E-03	35354	1,20406E+00	15536	1,53388E+00	37488	9,41390E-01	10852	0	61900
Myeloid_vs_CD8_Non-responder	Macro_FOLR2+APOE+	CD8(ISG+ T)	LGALS3	LAG3	2,11927E-03	27335,2	5,66298E-03	19132	1,05375E+00	21733	2,29548E+00	15296	9,28643E-01	18615	0	61900
Myeloid_vs_CD8_Non-responder	Macro_FOLR2+APOE+	CD8(ISG+ T)	MMP9	CD44	2,12804E-03	25857,2	9,02220E-03	9479	1,47789E+00	8012	2,45023E+00	12468	8,98903E-01	37427	0	61900
Myeloid_vs_CD8_Non-responder	CD8(GZMK+ Tem)	Macro_ISG15	B2M	LILRB2	2,12946E-03	26411	3,61573E-03	37535	1,46042E+00	8353	1,95892E+00	23163	9,58020E-01	1104	0	61900
Myeloid_vs_CD8_Non-responder	Macro_FOLR2+APOE+	CD8(ISG+ T)	C1QB	C1QBP	2,13088E-03	26576,6	3,71130E-03	36011	1,63032E+00	5638	3,31535E+00	3362	9,16478E-01	25972	0	61900
Myeloid_vs_CD8_Non-responder	CD8(IL7R+ZNF683+ Tm)	pDC_LILRA4	TNF	TNFRSF21	2,13276E-03	31343,4	9,42199E-03	8823	2,05497E+00	2262	2,19357E+00	17386	8,55307E-01	66346	0	61900
Myeloid_vs_CD8_Non-responder	cDC_CLEC9A	CD8(Terminal Tex)	HLA-DRB5	LAG3	2,13372E-03	21804	6,04404E-03	17388	1,25188E+00	13855	2,66559E+00	9359	9,47037E-01	6518	0	61900
Myeloid_vs_CD8_Non-responder	CD8(GZMK+ Tex)	Macro_FOLR2+APOE+	IFNG	IFNGR1_IFNGR2	2,13656E-03	34579	6,24198E-03	16557	1,00558E+00	23936	1,53225E+00	37560	9,05586E-01	32942	0	61900
Myeloid_vs_CD8_Non-responder	Macro_FOLR2+APOE+	CD8(GZMK+ Early Tem)	LGALS9	PTPRC	2,13941E-03	30335	3,85594E-03	34046	1,21838E+00	15014	1,79056E+00	28163	9,38953E-01	12552	0	61900
Myeloid_vs_CD8_Non-responder	CD8(EOMES+ NK-like)	Macro_ISG15	B2M	LILRB2	2,14055E-03	26452,4	3,61287E-03	37574	1,45713E+00	8411	1,95475E+00	23269	9,58004E-01	1108	0	61900
Myeloid_vs_CD8_Non-responder	CD8(Tc17)	Macro_ISG15	HLA-B	LILRB2	2,14254E-03	27000,4	3,61246E-03	37581	1,42823E+00	9011	1,93973E+00	23695	9,53598E-01	2815	0	61900
Myeloid_vs_CD8_Non-responder	CD8(ID2+CXCR4+ T)	pDC_LILRA4	GZMB	IGF2R	2,14368E-03	33049,2	9,26295E-03	9055	9,28951E-01	28098	1,77739E+00	28608	8,98670E-01	37585	0	61900
Myeloid_vs_CD8_Non-responder	Macro_FOLR2+APOE+	CD8(GZMK+ Early Tem)	HLA-DQA1	LAG3	2,14397E-03	30476,6	3,86919E-03	33871	1,06795E+00	21061	2,29551E+00	15295	9,25777E-01	20256	0	61900
Myeloid_vs_CD8_Non-responder	Macro_FOLR2+APOE+	CD8(GZMK+ Early Tem)	HLA-DQB1	LAG3	2,14454E-03	31673,2	3,67153E-03	36598	1,02976E+00	22826	2,21685E+00	16900	9,25951E-01	20142	0	61900
Myeloid_vs_CD8_Non-responder	CD8(GZMK+ Early Tem)	Macro_ISG15	HLA-F	LILRB1	2,14482E-03	32416,8	4,70065E-03	25263	1,32973E+00	11514	1,86656E+00	25818	8,98666E-01	37589	0	61900
Myeloid_vs_CD8_Non-responder	CD8(LAYN+ T)	Macro_ISG15	B2M	LILRB2	2,15913E-03	26502,4	3,60807E-03	37639	1,45160E+00	8526	1,95228E+00	23331	9,57978E-01	1116	0	61900
Myeloid_vs_CD8_Non-responder	CD8(Temra)	cDC_CLEC9A	SPON2	ITGB2	2,16142E-03	33767,4	6,48159E-03	15647	9,40461E-01	27403	1,85180E+00	26240	8,98577E-01	37647	0	61900
Myeloid_vs_CD8_Non-responder	CD8(ISG+ T)	Macro_ISG15	HLA-F	LILRB1	2,16171E-03	32726	4,69127E-03	25332	1,32860E+00	11567	1,82161E+00	27183	8,98575E-01	37648	0	61900
Myeloid_vs_CD8_Non-responder	pDC_LILRA4	CD8(ITM2C+ T)	APP	CD74	2,16463E-03	20155,6	6,27338E-03	16431	2,17386E+00	1755	2,19033E+00	17452	9,52680E-01	3240	0	61900
Myeloid_vs_CD8_Non-responder	Macro_FOLR2+APOE+	CD8(GZMK+ Early Tem)	SPP1	CD44	2,16659E-03	27798,4	6,74498E-03	14725	9,50878E-01	26814	2,92904E+00	6330	9,11212E-01	29223	0	61900
Myeloid_vs_CD8_Non-responder	Macro_FOLR2+APOE+	CD8(GZMK+ Early Tem)	LGALS3	LAG3	2,16918E-03	36234,4	3,77293E-03	35158	8,70558E-01	31763	1,90047E+00	24846	9,13960E-01	27505	0	61900
Myeloid_vs_CD8_Non-responder	Macro_FOLR2+APOE+	CD8(GZMK+ Early Tem)	MMP9	CD44	2,17783E-03	24752	9,71869E-03	8359	1,53851E+00	6968	2,51862E+00	11341	9,02232E-01	35192	0	61900
Myeloid_vs_CD8_Non-responder	CD8(Terminal Tex)	cDC(CD1C)	TNFSF9	HLA-DPA1	2,17986E-03	33911	3,60256E-03	37711	1,04858E+00	21987	2,14339E+00	18487	9,10798E-01	29470	0	61900
Myeloid_vs_CD8_Non-responder	Macro_FOLR2+APOE+	CD8(GZMK+ Early Tem)	C3	IFITM1	2,18072E-03	26152	7,07593E-03	13735	1,14118E+00	17892	2,06566E+00	20354	9,31774E-01	16879	0	61900
Myeloid_vs_CD8_Non-responder	Macro_FOLR2+APOE+	CD8(GZMK+ Early Tem)	TIMP2	CD44	2,18217E-03	35430	4,61575E-03	25951	1,11205E+00	19107	1,56688E+00	36167	9,03977E-01	34025	0	61900
Myeloid_vs_CD8_Non-responder	Macro_FOLR2+APOE+	CD8(ISG+ T)	LGALS9	PTPRC	2,18391E-03	32580,2	3,60177E-03	37725	1,13966E+00	17945	1,69002E+00	31466	9,36969E-01	13865	0	61900
Myeloid_vs_CD8_Non-responder	Macro_FOLR2+APOE+	CD8(Temra)	LGALS9	PTPRC	2,18767E-03	29437,6	3,96695E-03	32677	1,25277E+00	13829	1,83403E+00	26793	9,39761E-01	11989	0	61900

Myeloid_vs_CD8_Post_NR

Myeloid_vs_CD8_Non-responder	CD8(ITM2C+ T)	Macro_OLFML3	HSPA1A	TLR4	2,18883E-03	33007,6	5,55896E-03	19706	1,18525E+00	16216	1,52782E+00	37742	9,10792E-01	29474	0	61900
Myeloid_vs_CD8_Non-responder	CD8(NME1+ T)	Mono_CD16	HLA-C	LILRA1	2,19292E-03	30893,2	8,31513E-03	10764	1,43831E+00	8791	1,15363E+00	55501	9,30598E-01	17510	0	61900
Myeloid_vs_CD8_Non-responder	CD8(Temra)	Macro_ISG15	CCL5	CCR1	2,19980E-03	28994,4	1,22743E-02	5700	1,46487E+00	8261	1,22817E+00	51587	9,30568E-01	17524	0	61900
Myeloid_vs_CD8_Non-responder	Macro_FOLR2+APOE+	CD8(Temra)	CD14	ITGB1	2,20075E-03	23784	5,08904E-03	22409	1,55992E+00	6624	2,78176E+00	7849	9,25956E-01	20138	0	61900
Myeloid_vs_CD8_Non-responder	Macro_FOLR2+APOE+	CD8(Temra)	CD14	ITGA4	2,20104E-03	24229,2	4,91741E-03	23597	1,53707E+00	6980	2,73250E+00	8467	9,25864E-01	20202	0	61900
Myeloid_vs_CD8_Non-responder	cDC(CD1C)	CD8(Terminal Tex)	HLA-DPA1	LAG3	2,20373E-03	21289	6,01134E-03	17532	1,33557E+00	11325	2,45428E+00	12397	9,52585E-01	3291	0	61900
Myeloid_vs_CD8_Non-responder	CD8(Terminal Tex)	Macro_ISG15	HLA-B	LILRB1	2,20950E-03	30612,2	4,04183E-03	31776	1,24055E+00	14222	1,52632E+00	37813	9,45999E-01	7350	0	61900
Myeloid_vs_CD8_Non-responder	Macro_FOLR2+APOE+	CD8(Temra)	B2M	KLRD1	2,21037E-03	27917,4	5,17447E-03	21892	1,02452E+00	23038	1,73644E+00	29890	9,53479E-01	2867	0	61900
Myeloid_vs_CD8_Non-responder	Macro_ISG15	CD8(GZMK+ Tem)	HLA-DQB1	LAG3	2,21902E-03	24634,2	6,23653E-03	16586	1,14852E+00	17563	2,21943E+00	16843	9,42188E-01	10279	0	61900
Myeloid_vs_CD8_Non-responder	Macro_FOLR2+APOE+	CD8(Temra)	SPP1	CD44	2,22326E-03	32395,4	5,52760E-03	19870	7,98198E-01	36845	2,72330E+00	8572	9,02824E-01	34790	0	61900
Myeloid_vs_CD8_Non-responder	Macro_FOLR2+APOE+	CD8(Temra)	GRN	TNFRSF1B	2,22620E-03	25174,2	3,65149E-03	36935	1,45959E+00	8368	2,80658E+00	7576	9,41048E-01	11092	0	61900
Myeloid_vs_CD8_Non-responder	cDC_CLEC9A	CD8(GZMK+ Tex)	HLA-DRB5	LAG3	2,22694E-03	21834,8	6,00011E-03	17579	1,24672E+00	14017	2,69026E+00	9022	9,46854E-01	6656	0	61900
Myeloid_vs_CD8_Non-responder	Macro_FOLR2+APOE+	CD8(Temra)	APOE	SORL1	2,23238E-03	27283,6	4,64119E-03	25731	1,38121E+00	10126	3,18368E+00	4214	9,03328E-01	34447	0	61900
Myeloid_vs_CD8_Non-responder	CD8(GZMK+ Tem)	Macro_FOLR2+APOE+	CALR	LRP1	2,23680E-03	37261	3,59028E-03	37906	1,07270E+00	20860	1,55233E+00	36779	9,11830E-01	28860	0	61900
Myeloid_vs_CD8_Non-responder	Macro_FOLR2+APOE+	CD8(Temra)	C3	IFITM1	2,23946E-03	26347,8	7,11896E-03	13636	1,14657E+00	17649	2,00904E+00	21773	9,31967E-01	16781	0	61900
Myeloid_vs_CD8_Non-responder	CD8(ZNF683+KLRB1+ T)	cDC_CLEC9A	CD52	SIGLEC10	2,23946E-03	34897,2	4,51726E-03	26829	1,07701E+00	20660	1,52403E+00	37915	9,14511E-01	27182	0	61900
Myeloid_vs_CD8_Non-responder	Macro_FOLR2+APOE+	CD8(Temra)	LYZ	ITGAL	2,24034E-03	27052,8	4,19078E-03	30117	1,34801E+00	10981	2,97014E+00	5977	9,15985E-01	26289	0	61900
Myeloid_vs_CD8_Non-responder	Macro_FOLR2+APOE+	CD8(Tn)	LGALS9	CD44	2,24922E-03	29700,2	3,88244E-03	33705	1,26702E+00	13373	1,96737E+00	22916	9,32293E-01	16607	0	61900
Myeloid_vs_CD8_Non-responder	CD8(Tn)	Macro_FOLR2+APOE+	LTB	CD40	2,25634E-03	26195,4	1,12492E-02	6576	1,58811E+00	6223	2,18210E+00	17638	8,97125E-01	38640	0	61900
Myeloid_vs_CD8_Non-responder	CD8(ITM2C+ T)	pDC_LILRA4	CD70	TNFRSF17	2,25634E-03	40608,8	1,78099E-01	16	2,00816E+00	2503	2,00178E+00	21961	7,71595E-01	116664	0	61900
Myeloid_vs_CD8_Non-responder	pDC_LILRA4	CD8(GZMK+ Tex)	B2M	CD3D	2,25634E-03	34745,8	2,83610E-03	53191	9,12686E-01	29059	1,74678E+00	29563	9,65537E-01	16	0	61900
Myeloid_vs_CD8_Non-responder	Macro_FOLR2+APOE+	CD8(Tn)	CD14	ITGB1	2,25961E-03	25630,6	4,49306E-03	27042	1,49301E+00	7746	2,71344E+00	8708	9,21571E-01	22757	0	61900
Myeloid_vs_CD8_Non-responder	CD8(Terminal Tex)	Mono_CD16	ANXA1	FPR1	2,26438E-03	34885,2	3,81619E-03	34552	1,08768E+00	20202	1,52184E+00	37999	9,26611E-01	19773	0	61900
Myeloid_vs_CD8_Non-responder	CD8(NME1+ T)	Mast	CALR	ITGA2B	2,26739E-03	53052	2,78459E-02	1119	1,57416E+00	6438	1,46031E-01	117721	8,37186E-01	78082	0	61900
Myeloid_vs_CD8_Non-responder	Macro_FOLR2+APOE+	CD8(Tn)	CXCL9	CXCR3	2,26843E-03	25335,2	1,48311E-02	3897	2,30070E+00	1322	2,91968E+00	6439	8,75530E-01	53118	0	61900
Myeloid_vs_CD8_Non-responder	CD8(ITM2C+ T)	Macro_OLFML3	HLA-B	LILRB1	2,27243E-03	32061,4	3,58290E-03	38026	1,25852E+00	13635	1,54735E+00	36960	9,42837E-01	9786	0	61900
Myeloid_vs_CD8_Non-responder	Macro_FOLR2+APOE+	CD8(Tn)	APOE	LDLR	2,27482E-03	23026,6	1,25567E-02	5439	1,49557E+00	7699	3,32551E+00	3299	8,99862E-01	36796	0	61900
Myeloid_vs_CD8_Non-responder	CD8(GZMK+ Tex)	Macro_ISG15	HLA-F	LILRB1	2,27572E-03	32906,8	4,63215E-03	25796	1,32147E+00	11762	1,82598E+00	27039	8,97995E-01	38037	0	61900
Myeloid_vs_CD8_Non-responder	Macro_FOLR2+APOE+	CD8(Tn)	SPP1	CD44	2,27782E-03	25989,2	7,34077E-03	12988	1,02560E+00	22990	3,09204E+00	4920	9,14577E-01	27148	0	61900
Myeloid_vs_CD8_Non-responder	CD8(Tc17)	Macro_NLRP3	CIRBP	TREM1	2,27931E-03	36040,2	5,09150E-03	22394	1,09369E+00	19940	1,52395E+00	37918	8,97981E-01	38049	0	61900
Myeloid_vs_CD8_Non-responder	Macro_FOLR2+APOE+	CD8(Tn)	HBEGF	CD44	2,28351E-03	36446,8	3,65316E-03	36904	1,00452E+00	24003	1,71639E+00	30536	9,11784E-01	28891	0	61900
Myeloid_vs_CD8_Non-responder	CD8(ID2+CXCR4+ T)	Macro_FOLR2+APOE+	TGFB1	ENG	2,28441E-03	35470,8	5,18424E-03	21831	1,10866E+00	19265	1,56352E+00	36292	8,97962E-01	38066	0	61900
Myeloid_vs_CD8_Non-responder	Macro_ISG15	CD8(GZMK+ Tem)	HLA-DRB5	LAG3	2,28450E-03	23623,8	5,97358E-03	17694	1,18122E+00	16360	2,28916E+00	15432	9,46742E-01	6733	0	61900
Myeloid_vs_CD8_Non-responder	CD8(ZNF683+KLRB1+ T)	Mono_CD16	SPON2	ITGB2	2,28591E-03	33410,8	6,39414E-03	15955	9,63363E-01	26098	1,89364E+00	25030	8,97956E-01	38071	0	61900
Myeloid_vs_CD8_Non-responder	Macro_FOLR2+APOE+	CD8(Tn)	MMP9	CD44	2,28861E-03	23394,6	1,05772E-02	7323	1,61323E+00	5877	2,68162E+00	9137	9,05902E-01	32736	0	61900
Myeloid_vs_CD8_Non-responder	CD8(EOMES+ NK-like)	Macro_ISG15	CIRBP	TREM1	2,29012E-03	34640,8	5,08663E-03	22441	1,11306E+00	19065	1,68326E+00	31713	8,97937E-01	38085	0	61900
Myeloid_vs_CD8_Non-responder	CD8(LAYN+ T)	pDC_LILRA4	CCL5	CXCR3	2,29132E-03	30776	9,35422E-03	8932	8,20612E-01	35172	1,51964E+00	38089	9,42834E-01	9787	0	61900
Myeloid_vs_CD8_Non-responder	Macro_FOLR2+APOE+	CD8(Tn)	C3	IFITM1	2,29192E-03	30200,2	5,92083E-03	17922	9,96492E-01	24407	1,84014E+00	26584	9,25887E-01	20188	0	61900
Myeloid_vs_CD8_Non-responder	Macro_FOLR2+APOE+	CD8(Tn)	TIMP2	CD44	2,29282E-03	32531,6	5,02347E-03	22874	1,18677E+00	16156	1,72988E+00	30118	9,07588E-01	31610	0	61900
Myeloid_vs_CD8_Non-responder	Macro_FOLR2+APOE+	CD8(NME1+ T)	CXCL9	CXCR3	2,29341E-03	23607,6	1,82325E-02	2557	2,37576E+00	1124	2,91727E+00	6463	8,86352E-01	45994	0	61900
Myeloid_vs_CD8_Non-responder	CD8(ISG+ T)	Macro_LYVE1	HMGB1	CD163	2,29493E-03	30218	4,39285E-03	27999	1,40346E+00	9571	1,51941E+00	38101	9,37556E-01	13519	0	61900
Myeloid_vs_CD8_Non-responder	Macro_ISG15	CD8(Tn)	S100A8	CD69	2,29513E-03	24912	5,96937E-03	17715	1,21042E+00	15292	2,24657E+00	16302	9,37804E-01	13351	0	61900
Myeloid_vs_CD8_Non-responder	CD8(NME1+ T)	Macro_ISG15	B2M	LILRB1	2,29614E-03	31532	3,87201E-03	33834	1,10773E+00	19295	1,51931E+00	38105	9,50080E-01	4526	0	61900
Myeloid_vs_CD8_Non-responder	cDC(CD1C)	CD8(GZMK+ Tex)	HLA-DPA1	LAG3	2,29817E-03	21287,4	5,96765E-03	17721	1,33041E+00	11492	2,47894E+00	11958	9,52420E-01	3366	0	61900
Myeloid_vs_CD8_Non-responder	Macro_FOLR2+APOE+	CD8(IL7R+ZNF683+ Tm)	LGALS9	PTPRC	2,30096E-03	31533	3,69847E-03	36178	1,16961E+00	16789	1,75132E+00	29409	9,37747E-01	13389	0	61900
Myeloid_vs_CD8_Non-responder	CD8(GZMK+ Early Tem)	Macro_NLRP3	HMGB1	THBD	2,30307E-03	35338,2	4,45293E-03	27415	1,13517E+00	18137	1,51880E+00	38128	9,08279E-01	31111	0	61900
Myeloid_vs_CD8_Non-responder	CD8(ID2+CXCR4+ T)	Mono_CD16	HLA-C	LILRB1	2,30549E-03	32339,4	3,75950E-03	35330	1,20521E+00	15491	1,51864E+00	38136	9,41402E-01	10840	0	61900
Myeloid_vs_CD8_Non-responder	cDC_CLEC9A	CD8(ID2+CXCR4+ T)	HLA-DQB1	LAG3	2,30783E-03	22366,6	5,96429E-03	17740	1,31642E+00	11928	2,68226E+00	9123	9,40960E-01	11142	0	61900
Myeloid_vs_CD8_Non-responder	Macro_FOLR2+APOE+	CD8(IL7R+ZNF683+ Tm)	LGALS9	CD44	2,31063E-03	32088,8	3,57527E-03	38153	1,19418E+00	15913	1,84556E+00	26434	9,29644E-01	18044	0	61900
Myeloid_vs_CD8_Non-responder	CD8(GZMK+ Tex)	Macro_IER3	RPS19	C5AR1	2,31154E-03	27914,8	4,80932E-03	24398	1,40967E+00	9423	1,51816E+00	38156	9,48192E-01	5697	0	61900
Myeloid_vs_CD8_Non-responder	Macro_FOLR2+APOE+	CD8(IL7R+ZNF683+ Tm)	CD14	ITGA4	2,31245E-03	29680,4	3,59075E-03	37900	1,38493E+00	10024	2,52187E+00	11292	9,14324E-01	27286	0	61900
Myeloid_vs_CD8_Non-responder	CD8(Temra)	Macro_ISG15	TGFB1	ENG	2,31366E-03	34819,2	5,16873E-03	21912	1,03693E+00	22497	1,74475E+00	29624	8,97825E-01	38163	0	61900
Myeloid_vs_CD8_Non-responder	CD8(Tn)	Mono_CD16	HLA-C	LILRA1	2,31394E-03	31472,6	8,19726E-03	10987	1,40505E+00	9527	1,12146E+00	57197	9,30136E-01	17752	0	61900
Myeloid_vs_CD8_Non-responder	CD8(ZNF683+KLRB1+ T)	pDC_LILRA4	HMGB1	TLR9	2,31700E-03	26849,2	3,64963E-02	643	2,27828E+00	1400	1,51782E+00	38174	9,06803E-01	32129	0	61900
Myeloid_vs_CD8_Non-responder	Macro_FOLR2+APOE+	CD8(IL7R+ZNF683+ Tm)	B2M	KLRC1	2,32064E-03	32782,2	5,35465E-03	20832	8,97601E-01	30015	1,68027E+00	31828	9,27364E-01	19336	0	61900
Myeloid_vs_CD8_Non-responder	Macro_FOLR2+APOE+	CD8(Temra)	HLA-B	KLRD1	2,32156E-03	32510,8	5,15558E-03	21989	8,22886E-01	34997	1,51749E+00	38189	9,48537E-01	5479	0	61900
Myeloid_vs_CD8_Non-responder	CD8(NME1+ T)	Macro_ISG15	HLA-A	LILRB2	2,32521E-03	28273,4	3,57216E-03	38201	1,35551E+00	10791	1,84363E+00	26490	9,51139E-01	3985	0	61900
Myeloid_vs_CD8_Non-responder	Mono_CD14	CD8(ITM2C+ T)	S100A9	ITGB2	2,32594E-03	18692,4	5,35924E-03	20809	2,31793E+00	1272	3,38458E+00	2987	9,47073E-01	6494	0	61900
Myeloid_vs_CD8_Non-responder	CD8(Terminal Tex)	Macro_LYVE1	HMGB1	CD163	2,32977E-03	29250	4,66413E-03	25560	1,46519E+00	8253	1,51701E+00	38216	9,39287E-01	12321	0	61900
Myeloid_vs_CD8_Non-responder	Macro_NLRP3	CD8(LAYN+ T)	VCAN	ITGA4	2,33082E-03	18333	1,47220E-02	3965	2,44339E+00	973	2,85694E+00	7042	9,30090E-01	17785	0	61900
Myeloid_vs_CD8_Non-responder	Macro_FOLR2+APOE+	CD8(IL7R+ZNF683+ Tm)	SPP1	CD44	2,33160E-03	27684,2	6,75998E-03	14669	9,52759E-01	26698	2,97023E+00	5976	9,11302E-01	29178	0	61900
Myeloid_vs_CD8_Non-responder	CD8(Terminal Tex)	Macro_ISG15	HLA-F	LILRB2	2,33955E-03	32791,2	3,56956E-03	38248	1,46811E+00	8190	1,97634E+00	22665	9,05566E-01	32953	0	61900
Myeloid_vs_CD8_Non-responder	cDC_LAMP3	CD8(LAYN+ T)	CCL19	CXCR3	2,34021E-03	31648	2,65402E-02	1221	3,84931E+00	90	2,17560E+00	17786	8,38539E-01	77243	0	61900
Myeloid_vs_CD8_Non-responder	CD8(ITM2C+ T)	Macro_FOLR2+APOE+	HLA-B	LILRB2	2,34077E-03	26407	3,56943E-03	38252	1,57334E+00	6450	1,98280E+00	22494	9,53332E-01	2939	0	61900
Myeloid_vs_CD8_Non-responder	CD8(NME1+ T)	Macro_FOLR2+APOE+	HMGB1	TLR4	2,34169E-03	34015,6	5,30745E-03	21107	1,26724E+00	13363	1,51601E+00	38255	9,01834E-01	35453	0	61900
Myeloid_vs_CD8_Non-responder	Macro_FOLR2+APOE+	CD8(IL7R+ZNF683+ Tm)	MMP9	CD44	2,34291E-03	24610,2	9,74030E-03	8330	1,54039E+00	6920	2,55981E+00	10759	9,02330E-01	35142	0	61900
Myeloid_vs_CD8_Non-responder	CD8(Tn)	Macro_FOLR2+APOE+	CD99	PILRA	2,34291E-03	35104,8	3,56922E-03	38259	1,14885E+00	17551	1,61658E+00	34208	9,20169E-01	23606	0	61900
Myeloid_vs_CD8_Non-responder	CD8(Temra)	Macro_FOLR2+APOE+	HMGB1	CD163	2,34414E-03	31806	3,56876E-03	38263	1,17305E+00	16675	1,89506E+00	24990	9,31191E-01	17202	0	61900
Myeloid_vs_CD8_Non-responder	CD8(IL7R+ZNF683+ Tm)	Macro_IER3	RPS19	C5AR1	2,34444E-03	29488,2	4,40601E-03	27865	1,31180E+00	12059	1,51585E+00	38264	9,45998E-01	7353	0	61900
Myeloid_vs_CD8_Non-responder	Macro_FOLR2+APOE+	CD8(IL7R+ZNF683+ Tm)	C3	IFITM1	2,34597E-03	27273,2	6,68925E-03	14903	1,09274E+00	19969	2,01074E+00	21730	9,29966E-01	17864	0	61900
Myeloid_vs_CD8_Non-responder	Macro_FOLR2+APOE+	CD8(IL7R+ZNF683+ Tm)	TIMP2	CD44	2,34720E-03	35056,8	4,62602E-03	25857	1,11393E+00	19032	1,60807E+00	34538	9,04073E-01	33957	0	61900
Myeloid_vs_CD8_Non-responder	Macro_FOLR2+APOE+	CD8(GZMK+ Early Tem)	LGALS9	CD44	2,35273E-03	32395,2	3,56734E-03	38291	1,19230E+00	15984	1,80437E+00	27710	9,29572E-01	18091	0	61900
Myeloid_vs_CD8_Non-responder	Macro_FOLR2+APOE+	CD8(GZMK+ Tem)	LGALS9	PTPRC	2,35334E-03	29190,6	3,96849E-03	32663	1,25325E+00	13813	1,87407E+00	25592	9,39772E-01	11985	0	61900
Myeloid_vs_CD8_Non-responder	Macro_FOLR2+APOE+	CD8(GZMK+ Tem)	LGALS9	CD44	2,35365E-03	31969,2	3,62784E-03	37341	1,20664E+00	15435	1,81437E+00	27408	9,30120E-01	17762	0	61900
Myeloid_vs_CD8_Non-responder	Macro_FOLR2+APOE+	CD8(Temra)	C1QB	C1QBP	2,35488E-03	27292	3,56681E-03	38298	1,61852E+00	5804	3,28316E+00	3549	9,14946E-01	26909	0	61900
Myeloid_vs_CD8_Non-responder	CD8(LAYN+ T)	cDC_CLEC9A	XCL2	XCR1	2,35546E-03	47312,6	2,64898E-02	1225	5,00904E+00	34	1,00966E+00	63436	7,84015E-01	109968	0	61900
Myeloid_vs_CD8_Non-responder	Macro_OLFML3	CD8(Terminal Tex)	HLA-DRB5	LAG3	2,35812E-03	23073	5,94266E-03	17838	1,23011E+00	14580	2,35064E+00	14195	9,46611E-01	6852	0	61900

Myeloid_vs_CD8_Non-responder	Macro_FOLR2+APOE+	CD8(GZMK+ Tem)	HLA-DQA2	LAG3	2,36011E-03	25447,8	7,32790E-03	13024	1,18765E+00	16128	2,38466E+00	13599	9,21857E-01	22588	0	61900
Myeloid_vs_CD8_Non-responder	Macro_FOLR2+APOE+	CD8(GZMK+ Tem)	CD14	ITGB1	2,36689E-03	23905,4	5,08794E-03	22423	1,55980E+00	6628	2,73447E+00	8432	9,25948E-01	20144	0	61900
Myeloid_vs_CD8_Non-responder	Macro_FOLR2+APOE+	CD8(GZMK+ Tem)	CD14	ITGA4	2,36720E-03	23008	5,39468E-03	20611	1,59180E+00	6172	2,77412E+00	7930	9,28981E-01	18427	0	61900
Myeloid_vs_CD8_Non-responder	CD8(Terminal Tex)	Macro_IER3	RPS19	C5AR1	2,37524E-03	27239,8	5,04285E-03	22756	1,46634E+00	8228	1,51371E+00	38364	9,49344E-01	4951	0	61900
Myeloid_vs_CD8_Non-responder	Macro_ISG15	CD8(Temra)	S100A8	CD69	2,37887E-03	24985,4	6,07085E-03	17272	1,22303E+00	14850	2,17135E+00	17878	9,38293E-01	13027	0	61900
Myeloid_vs_CD8_Non-responder	pDC_LILRA4	CD8(GZMK+ Tex)	APP	CD74	2,38356E-03	20185,4	6,31934E-03	16251	2,18059E+00	1726	2,17088E+00	17887	9,52844E-01	3163	0	61900
Myeloid_vs_CD8_Non-responder	CD8(IL7R+ZNF683+ Tm)	Macro_FOLR2+APOE+	CD52	SIGLEC10	2,38485E-03	31395,4	5,44391E-03	20343	1,26070E+00	13561	1,51285E+00	38395	9,21529E-01	22778	0	61900
Myeloid_vs_CD8_Non-responder	CD8(Tn)	pDC_LILRA4	HMGB1	THBD	2,38796E-03	39226	3,55993E-03	38405	9,47248E-01	27025	1,70013E+00	31101	8,98521E-01	37699	0	61900
Myeloid_vs_CD8_Non-responder	Macro_FOLR2+APOE+	CD8(GZMK+ Tem)	HLA-DQB2	LAG3	2,38889E-03	27814	9,82481E-03	8209	1,19707E+00	15809	1,99842E+00	22051	9,08295E-01	31101	0	61900
Myeloid_vs_CD8_Non-responder	Macro_OLFML3	CD8(Terminal Tex)	HLA-DRA	LAG3	2,38930E-03	21515,6	5,92771E-03	17898	1,27273E+00	13210	2,52173E+00	11295	9,52606E-01	3275	0	61900
Myeloid_vs_CD8_Non-responder	CD8(ITM2C+ T)	Macro_FOLR2+APOE+	HLA-A	LILRB2	2,39076E-03	27375,6	3,55958E-03	38414	1,47960E+00	7983	1,90995E+00	24550	9,51057E-01	4031	0	61900
Myeloid_vs_CD8_Non-responder	Macro_FOLR2+APOE+	CD8(GZMK+ Tem)	SPP1	CD44	2,39107E-03	27470,8	6,85938E-03	14372	9,65225E-01	26006	2,93904E+00	6251	9,11890E-01	28825	0	61900
Myeloid_vs_CD8_Non-responder	Macro_FOLR2+APOE+	CD8(GZMK+ Tem)	GRN	TNFRSF1B	2,39574E-03	24396,4	3,81677E-03	34544	1,49017E+00	7803	2,81254E+00	7513	9,42264E-01	10222	0	61900
Myeloid_vs_CD8_Non-responder	CD8(ZNF683+KLRB1+ T)	Mono_CD14	SPON2	ITGB2	2,39636E-03	34719,8	6,32318E-03	16240	9,51286E-01	26791	1,72630E+00	30236	8,97443E-01	38432	0	61900
Myeloid_vs_CD8_Non-responder	CD8(EOMES+ NK-like)	Macro_FOLR2+APOE+	RPS19	C5AR1	2,39668E-03	32529	3,55834E-03	38433	1,07947E+00	20541	1,72917E+00	30138	9,40272E-01	11633	0	61900
Myeloid_vs_CD8_Non-responder	CD8(GZMK+ Tem)	Macro_OLFML3	HLA-C	LILRB1	2,39948E-03	33160,4	3,55766E-03	38442	1,20544E+00	15485	1,52039E+00	38056	9,39862E-01	11919	0	61900
Myeloid_vs_CD8_Non-responder	Macro_FOLR2+APOE+	CD8(GZMK+ Tem)	MMP9	CD44	2,40511E-03	24526,4	9,88352E-03	8134	1,55286E+00	6726	2,52863E+00	11188	9,02971E-01	34684	0	61900
Myeloid_vs_CD8_Non-responder	Macro_FOLR2+APOE+	CD8(GZMK+ Tem)	C1QB	C1QBP	2,40855E-03	26814,6	3,66560E-03	36682	1,62659E+00	5698	3,28738E+00	3520	9,16003E-01	26273	0	61900
Myeloid_vs_CD8_Non-responder	Macro_FOLR2+APOE+	CD8(GZMK+ Tem)	C3	IFITM1	2,40980E-03	31904,2	5,55092E-03	19756	9,50157E-01	26844	1,74744E+00	29544	9,23642E-01	21477	0	61900
Myeloid_vs_CD8_Non-responder	CD8(Tc17)	Mono_CD16	HLA-C	LILRA3	2,41137E-03	27061,2	1,10872E-02	6756	1,54153E+00	6902	1,51103E+00	38480	9,23980E-01	21268	0	61900
Myeloid_vs_CD8_Non-responder	Macro_FOLR2+APOE+	CD8(GZMK+ Tem)	LYZ	ITGAL	2,41168E-03	28179,2	3,94398E-03	32953	1,32540E+00	11658	2,89372E+00	6691	9,13620E-01	27694	0	61900
Myeloid_vs_CD8_Non-responder	Macro_FOLR2+APOE+	CD8(GZMK+ Tem)	TIMP2	CD44	2,41199E-03	35004,4	4,69404E-03	25306	1,12639E+00	18515	1,57689E+00	35757	9,04704E-01	33544	0	61900
Myeloid_vs_CD8_Non-responder	CD8(ID2+CXCR4+ T)	Macro_ISG15	CCL5	CCR1	2,41419E-03	25950,2	1,28421E-02	5191	1,50954E+00	7420	1,51086E+00	38489	9,32014E-01	16751	0	61900
Myeloid_vs_CD8_Non-responder	Macro_FOLR2+APOE+	CD8(GZMK+ Tex)	LGALS9	PTPRC	2,41890E-03	29438,6	3,92432E-03	33185	1,23957E+00	14257	1,87225E+00	25650	9,39454E-01	12201	0	61900
Myeloid_vs_CD8_Non-responder	Mono_CD14	CD8(ZNF683+KLRB1+ T)	VCAN	CD44	2,42237E-03	22439	6,91962E-03	14198	2,24572E+00	1500	2,22965E+00	16636	9,29786E-01	17961	0	61900
Myeloid_vs_CD8_Non-responder	Macro_FOLR2+APOE+	CD8(GZMK+ Tex)	HLA-DQA2	LAG3	2,42581E-03	27562,6	6,43273E-03	15809	1,08954E+00	20129	2,34199E+00	14365	9,17034E-01	25610	0	61900
Myeloid_vs_CD8_Non-responder	Macro_FOLR2+APOE+	CD8(GZMK+ Tex)	HLA-DRB1	LAG3	2,42644E-03	21772,6	5,49920E-03	20025	1,22462E+00	14782	2,73110E+00	8484	9,51763E-01	3672	0	61900
Myeloid_vs_CD8_Non-responder	CD8(GZMK+ Tex)	Mono_CD16	B2M	LILRB1	2,42707E-03	31560,4	3,67749E-03	36510	1,20259E+00	15588	1,50984E+00	38530	9,48843E-01	5274	0	61900
Myeloid_vs_CD8_Non-responder	CD8(IL7R+ZNF683+ Tm)	Mono_CD16	HLA-C	LILRB1	2,42770E-03	32740	3,72338E-03	35831	1,17980E+00	16414	1,50982E+00	38532	9,41136E-01	11023	0	61900
Myeloid_vs_CD8_Non-responder	Macro_FOLR2+APOE+	CD8(GZMK+ Tex)	HLA-DPB1	LAG3	2,42865E-03	21487,6	5,81613E-03	18392	1,27373E+00	13178	2,69073E+00	9012	9,49339E-01	4956	0	61900
Myeloid_vs_CD8_Non-responder	Macro_FOLR2+APOE+	CD8(GZMK+ Tex)	ICAM1	IL2RG	2,43085E-03	37316,2	3,87873E-03	33754	9,54091E-01	26609	1,68870E+00	31515	9,05792E-01	32803	0	61900
Myeloid_vs_CD8_Non-responder	Macro_FOLR2+APOE+	CD8(GZMK+ Tex)	CD14	ITGB1	2,43275E-03	26565,4	4,25952E-03	29378	1,46678E+00	8214	2,66351E+00	9377	9,19620E-01	23958	0	61900
Myeloid_vs_CD8_Non-responder	cDC_CLEC9A	CD8(ID2+CXCR4+ T)	HLA-DQA1	LAG3	2,43294E-03	23254,4	5,90650E-03	17981	1,26416E+00	13448	2,57900E+00	10463	9,39064E-01	12480	0	61900
Myeloid_vs_CD8_Non-responder	Macro_FOLR2+APOE+	CD8(GZMK+ Tex)	CD14	ITGA4	2,43306E-03	27614,4	3,99613E-03	32301	1,43142E+00	8947	2,59803E+00	10205	9,18422E-01	24719	0	61900
Myeloid_vs_CD8_Non-responder	CD8(Tc17)	Macro_ISG15	HLA-F	LILRB1	2,43527E-03	33143	4,55560E-03	26471	1,31224E+00	12049	1,83556E+00	26739	8,97230E-01	38556	0	61900
Myeloid_vs_CD8_Non-responder	CD8(GZMK+ Tem)	Mono_CD16	HLA-B	LILRB1	2,43717E-03	31881	3,76836E-03	35220	1,21944E+00	14976	1,50897E+00	38562	9,44182E-01	8747	0	61900
Myeloid_vs_CD8_Non-responder	CD8(Tc17)	Mono_CD16	CD52	SIGLEC10	2,43748E-03	33983,4	4,81454E-03	24355	1,10450E+00	19447	1,50896E+00	38563	9,16970E-01	25652	0	61900
Myeloid_vs_CD8_Non-responder	CD8(EOMES+ NK-like)	pDC_LILRA4	SELPLG	SELL	2,43780E-03	26645,2	7,42546E-03	12755	1,49569E+00	7698	2,45896E+00	12309	8,97220E-01	38564	0	61900
Myeloid_vs_CD8_Non-responder	Macro_ISG15	CD8(GZMK+ Early Tem)	CCL2	CCR4	2,43837E-03	39052	4,96343E-02	358	1,56214E+00	6599	3,21545E+00	4016	7,60489E-01	122387	0	61900
Myeloid_vs_CD8_Non-responder	Macro_FOLR2+APOE+	CD8(GZMK+ Tex)	HLA-DPA1	LAG3	2,43907E-03	20999,6	5,77045E-03	18620	1,27989E+00	12978	2,78649E+00	7788	9,51653E-01	3712	0	61900
Myeloid_vs_CD8_Non-responder	CD8(GZMK+ Tex)	Macro_ISG15	CCL4	CCR1	2,44033E-03	29034,6	9,56689E-03	8589	1,40424E+00	9551	1,50876E+00	38572	9,15546E-01	26561	0	61900
Myeloid_vs_CD8_Non-responder	Macro_FOLR2+APOE+	CD8(GZMK+ Tex)	CD86	CTLA4	2,44191E-03	22868,8	1,45198E-02	4074	1,70644E+00	4794	2,45599E+00	12358	9,08124E-01	31218	0	61900
Myeloid_vs_CD8_Non-responder	Macro_FOLR2+APOE+	CD8(GZMK+ Tex)	B2M	KLRC1	2,44350E-03	34009,6	4,91790E-03	23593	8,60975E-01	32402	1,69850E+00	31157	9,24446E-01	20996	0	61900
Myeloid_vs_CD8_Non-responder	Macro_FOLR2+APOE+	CD8(GZMK+ Tex)	B2M	KLRC2	2,44381E-03	36912	5,54226E-03	19794	8,14809E-01	35582	1,65001E+00	32944	9,03471E-01	34340	0	61900
Myeloid_vs_CD8_Non-responder	Macro_FOLR2+APOE+	CD8(GZMK+ Tex)	SECTM1	CD7	2,44761E-03	30810,8	1,06308E-02	7266	1,33651E+00	11305	1,54865E+00	36922	9,00087E-01	36661	0	61900
Myeloid_vs_CD8_Non-responder	Macro_OLFML3	CD8(GZMK+ Tex)	HLA-DRB5	LAG3	2,44938E-03	23089,4	5,89946E-03	18012	1,22494E+00	14771	2,37531E+00	13757	9,46427E-01	7007	0	61900
Myeloid_vs_CD8_Non-responder	CD8(LAYN+ T)	Mono_CD16	CD52	SIGLEC10	2,45110E-03	33021,2	5,05306E-03	22689	1,15215E+00	17435	1,50794E+00	38606	9,18792E-01	24476	0	61900
Myeloid_vs_CD8_Non-responder	Macro_FOLR2+APOE+	CD8(GZMK+ Tex)	HLA-DQB2	LAG3	2,45492E-03	29978,6	8,62463E-03	10170	1,09896E+00	19712	1,95575E+00	23245	9,02722E-01	34866	0	61900
Myeloid_vs_CD8_Non-responder	Macro_FOLR2+APOE+	CD8(GZMK+ Tex)	SPP1	CD44	2,45682E-03	32420,4	5,51305E-03	19946	7,96372E-01	36963	2,73588E+00	8416	9,02709E-01	34877	0	61900
Myeloid_vs_CD8_Non-responder	CD8(EOMES+ NK-like)	Mono_CD14	RPS19	C5AR1	2,45682E-03	33882,2	3,54633E-03	38624	1,07511E+00	20751	1,56039E+00	36433	9,40177E-01	11703	0	61900
Myeloid_vs_CD8_Non-responder	Macro_FOLR2+APOE+	CD8(GZMK+ Tex)	CD59	CD2	2,45778E-03	32039,4	7,82284E-03	11798	1,00013E+00	24227	1,57895E+00	35667	9,15468E-01	26605	0	61900
Myeloid_vs_CD8_Non-responder	CD8(Tn)	Macro_OLFML3	LTB	CD40	2,46510E-03	27294,4	1,12462E-02	6579	1,58778E+00	6228	1,96033E+00	23115	8,97113E-01	38650	0	61900
Myeloid_vs_CD8_Non-responder	Macro_FOLR2+APOE+	CD8(GZMK+ Tex)	CXCL16	CXCR6	2,46542E-03	21286,8	1,07407E-02	7164	1,54674E+00	6819	2,58754E+00	10352	9,25873E-01	20199	0	61900
Myeloid_vs_CD8_Non-responder	CD8(GZMK+ Tem)	Macro_FOLR2+APOE+	HLA-C	LILRB2	2,46638E-03	27035,6	3,54429E-03	38654	1,52026E+00	7258	1,95584E+00	23241	9,50875E-01	4125	0	61900
Myeloid_vs_CD8_Non-responder	Macro_FOLR2+APOE+	CD8(GZMK+ Tex)	C3	IFITM1	2,47469E-03	29093,2	6,10797E-03	17089	1,01993E+00	23247	1,94128E+00	23645	9,26947E-01	19585	0	61900
Myeloid_vs_CD8_Non-responder	cDC_CLEC9A	CD8(ISG+ T)	HLA-DQB1	LAG3	2,47770E-03	22322	5,88850E-03	18065	1,30936E+00	12131	2,75828E+00	8143	9,40604E-01	11371	0	61900
Myeloid_vs_CD8_Non-responder	cDC_LAMP3	CD8(ID2+CXCR4+ T)	CCL19	CXCR3	2,47923E-03	31681	2,61861E-02	1257	3,84664E+00	91	2,19504E+00	17351	8,37627E-01	77806	0	61900
Myeloid_vs_CD8_Non-responder	CD8(LAYN+ T)	Macro_FOLR2+APOE+	IFNG	IFNGR1_IFNGR2	2,48590E-03	33389,6	6,87918E-03	14315	1,05218E+00	21811	1,50535E+00	38715	9,09661E-01	30207	0	61900
Myeloid_vs_CD8_Non-responder	CD8(ITM2C+ T)	Macro_FOLR2+APOE+	CD99	CD81	2,48590E-03	36531	3,54055E-03	38715	9,35733E-01	27692	1,66409E+00	32396	9,22902E-01	21952	0	61900
Myeloid_vs_CD8_Non-responder	Macro_OLFML3	CD8(GZMK+ Tex)	HLA-DRA	LAG3	2,48737E-03	21527,4	5,88463E-03	18083	1,26757E+00	13355	2,54640E+00	10951	9,52441E-01	3348	0	61900
Myeloid_vs_CD8_Non-responder	Macro_FOLR2+APOE+	CD8(ZNF683+KLRB1+ T)	HLA-A	CD8B	2,48911E-03	32707,8	4,08007E-03	31323	8,64076E-01	32189	1,56188E+00	36362	9,56181E-01	1765	0	61900
Myeloid_vs_CD8_Non-responder	Macro_FOLR2+APOE+	CD8(ZNF683+KLRB1+ T)	ICAM1	IL2RG	2,49104E-03	30660	4,86729E-03	23951	1,12170E+00	18727	2,00522E+00	21875	9,15042E-01	26847	0	61900
Myeloid_vs_CD8_Non-responder	Macro_FOLR2+APOE+	CD8(ID2+CXCR4+ T)	HLA-DQA1	LAG3	2,49329E-03	23237,6	5,88220E-03	18094	1,25820E+00	13646	2,61450E+00	9985	9,38946E-01	12563	0	61900
Myeloid_vs_CD8_Non-responder	Macro_FOLR2+APOE+	CD8(ZNF683+KLRB1+ T)	HLA-C	CD8B	2,49426E-03	31903,6	4,04787E-03	31702	8,84793E-01	30804	1,64226E+00	33246	9,55940E-01	1866	0	61900
Myeloid_vs_CD8_Non-responder	Macro_FOLR2+APOE+	CD8(ZNF683+KLRB1+ T)	HLA-E	CD8B	2,49555E-03	33708,2	4,27134E-03	29227	8,25804E-01	34812	1,52430E+00	37900	9,49784E-01	4702	0	61900
Myeloid_vs_CD8_Non-responder	CD8(LAYN+ T)	Mono_CD16	HLA-C	LILRA1	2,49813E-03	25636,6	9,60185E-03	8533	1,80134E+00	3937	1,50437E+00	38753	9,35103E-01	15060	0	61900
Myeloid_vs_CD8_Non-responder	CD8(ITM2C+ T)	Mono_CD16	HLA-A	LILRA1	2,50167E-03	25470,4	9,81525E-03	8225	1,80900E+00	3878	1,50417E+00	38764	9,35872E-01	14585	0	61900
Myeloid_vs_CD8_Non-responder	CD8(GZMK+ Early Tem)	Macro_FOLR2+APOE+	IFNG	IFNGR1_IFNGR2	2,50329E-03	37901,4	5,13986E-03	22084	9,24974E-01	28331	1,51223E+00	38423	8,96948E-01	38769	0	61900
Myeloid_vs_CD8_Non-responder	CD8(ISG+ T)	cDC_LAMP3	CD28	CD80	2,50419E-03	48095,2	2,45129E-02	1444,5	1,55818E+00	6659,5	1,09505E+00	58657,5	7,80652E-01	111814,5	0	61900
Myeloid_vs_CD8_Non-responder	CD8(Tn)	Mono_CD16	CD99	PILRA	2,50619E-03	35349	3,53648E-03	38778	1,13568E+00	18120	1,61895E+00	34109	9,19830E-01	23838	0	61900
Myeloid_vs_CD8_Non-responder	Macro_FOLR2+APOE+	CD8(GZMK+ Tex)	B2M	KLRD1	2,50652E-03	34481,4	3,96654E-03	32682	8,56842E-01	32653	1,50387E+00	38779	9,47215E-01	6393	0	61900
Myeloid_vs_CD8_Non-responder	CD8(GZMK+ Tex)	Macro_FOLR2+APOE+	HLA-A	LILRB2	2,50846E-03	27656,6	3,53613E-03	38785	1,46064E+00	8348	1,88964E+00	25141	9,50903E-01	4109	0	61900
Myeloid_vs_CD8_Non-responder	CD8(ITM2C+ T)	Mono_CD16	CD52	SIGLEC10	2,50943E-03	35075,6	4,57806E-03	26260	1,05726E+00	21577	1,50362E+00	38788	9,15032E-01	26853	0	61900
Myeloid_vs_CD8_Non-responder	Macro_FOLR2+APOE+	CD8(ZNF683+KLRB1+ T)	CD59	CD2	2,51040E-03	31899	7,78734E-03	11871	9,96172E-01	24423	1,60650E+00	34603	9,15292E-01	26698	0	61900
Myeloid_vs_CD8_Non-responder	CD8(ISG+ T)	cDC_CLEC9A	TNFSF9	HLA-DPA1	2,51137E-03	32539,8	3,53575E-03	38794	1,06791E+00	21069	2,54501E+00	10967	9,10035E-01	29969	0	61900
Myeloid_vs_CD8_Non-responder	Macro_FOLR2+APOE+	CD8(Tc17)	CXCL10	DPP4	2,51194E-03	32003	2,97446E-02	984	1,55769E+00	6666	3,08140E+00	5013	8,25408E-01	85452	0	61900
Myeloid_vs_CD8_Non-responder	Macro_FOLR2+APOE+	CD8(ZNF683+KLRB1+ T)	CXCL16	CXCR6	2,51526E-03	22276,4	9,81966E-03	8217	1,48322E+00	7912	2,52200E+00	11290	9,22737E-01	22063	0	61900

Myeloid_vs_CD8_Post_NR

Myeloid_vs_CD8_Non-responder	Macro_FOLR2+APOE+	CD8(ZNF683+KLRB1+ T)	LGALS1	CD69	2,51590E-03	25996,4	4,23594E-03	29624	1,11985E+00	18807	2,19201E+00	17414	9,55089E-01	2237	0	61900
Myeloid_vs_CD8_Non-responder	CD8(NME1+ T)	Macro_FOLR2+APOE+	ITGB3BP	ITGB5	2,51855E-03	57495	3,00693E-02	963	1,24767E+00	13993	1,15497E+00	55421	6,76276E-01	155198	0	61900
Myeloid_vs_CD8_Non-responder	CD8(Tc17)	Mono_CD14	LTB	TNFRSF1A	2,52597E-03	34565,6	6,36980E-03	16051	1,04310E+00	22227	1,62617E+00	33811	8,96846E-01	38839	0	61900
Myeloid_vs_CD8_Non-responder	CD8(ITM2C+ T)	Macro_NLRP3	HMGB1	THBD	2,52792E-03	37765,4	4,02254E-03	32002	1,04737E+00	22045	1,50253E+00	38845	9,03957E-01	34035	0	61900
Myeloid_vs_CD8_Non-responder	CD8(GZMK+ Tex)	cDC_CLEC9A	HMGB1	THBD	2,52890E-03	35115,4	4,57355E-03	26295	1,13510E+00	18139	1,50249E+00	38848	9,09387E-01	30395	0	61900
Myeloid_vs_CD8_Non-responder	Macro_FOLR2+APOE+	CD8(EOMES+ NK-like)	HLA-B	KIR3DL1	2,53150E-03	31771,6	1,83465E-02	2521	1,00966E+00	23721	1,62055E+00	34054	9,00086E-01	36662	0	61900
Myeloid_vs_CD8_Non-responder	Macro_FOLR2+APOE+	CD8(EOMES+ NK-like)	HLA-B	KIR3DL2	2,53183E-03	31423,4	1,65860E-02	3089	1,00853E+00	23779	1,64280E+00	33222	9,02356E-01	35127	0	61900
Myeloid_vs_CD8_Non-responder	Macro_FOLR2+APOE+	CD8(EOMES+ NK-like)	HLA-B	KLRD1	2,53215E-03	29139,8	5,97499E-03	17688	9,37046E-01	27597	1,59728E+00	34964	9,52020E-01	3550	0	61900
Myeloid_vs_CD8_Non-responder	Macro_FOLR2+APOE+	CD8(EOMES+ NK-like)	HLA-A	KIR3DL1	2,53574E-03	31332,4	1,91113E-02	2309	1,03412E+00	22620	1,69033E+00	31457	8,97522E-01	38376	0	61900
Myeloid_vs_CD8_Non-responder	Macro_FOLR2+APOE+	CD8(EOMES+ NK-like)	HLA-A	KIR3DL2	2,53607E-03	30976	1,72775E-02	2826	1,03299E+00	22675	1,71258E+00	30674	8,99844E-01	36805	0	61900
Myeloid_vs_CD8_Non-responder	Macro_OLFML3	CD8(NME1+ T)	CXCL9	DPP4	2,53641E-03	37945,2	1,68556E-02	2985	2,18717E+00	1703	2,89439E+00	6688	7,72046E-01	116450	0	61900
Myeloid_vs_CD8_Non-responder	Macro_ISG15	CD8(Terminal Tex)	B2M	CD3D	2,53833E-03	42594,2	2,79420E-03	54267	7,29041E-01	42338	1,17326E+00	54448	9,65288E-01	18	0	61900
Myeloid_vs_CD8_Non-responder	Macro_FOLR2+APOE+	CD8(EOMES+ NK-like)	ICAM1	IL2RG	2,53900E-03	38888,4	3,64092E-03	37107	9,13771E-01	28989	1,68050E+00	31821	9,03057E-01	34625	0	61900
Myeloid_vs_CD8_Non-responder	Macro_FOLR2+APOE+	CD8(EOMES+ NK-like)	CD14	ITGB1	2,54031E-03	28619,6	3,80043E-03	34772	1,41523E+00	9305	2,58093E+00	10435	9,15303E-01	26686	0	61900
Myeloid_vs_CD8_Non-responder	Macro_FOLR2+APOE+	CD8(EOMES+ NK-like)	HLA-C	KIR3DL1	2,54292E-03	30694,6	1,89605E-02	2358	1,05484E+00	21690	1,77071E+00	28791	8,96995E-01	38734	0	61900
Myeloid_vs_CD8_Non-responder	Macro_FOLR2+APOE+	CD8(EOMES+ NK-like)	HLA-E	KLRC1_KLRD1	2,54358E-03	31782,6	6,61376E-03	15168	8,43481E-01	33581	1,67114E+00	32146	9,33191E-01	16118	0	61900
Myeloid_vs_CD8_Non-responder	Macro_FOLR2+APOE+	CD8(EOMES+ NK-like)	HLA-E	KLRC2_KLRD1	2,54390E-03	30341,2	7,39339E-03	12831	9,26954E-01	28213	1,67103E+00	32149	9,32278E-01	16613	0	61900
Myeloid_vs_CD8_Non-responder	Macro_FOLR2+APOE+	CD8(EOMES+ NK-like)	HLA-E	KLRC3_KLRD1	2,54423E-03	29038,8	7,46711E-03	12647	9,86538E-01	24909	1,70045E+00	31092	9,35762E-01	14646	0	61900
Myeloid_vs_CD8_Non-responder	Macro_FOLR2+APOE+	CD8(Terminal Tex)	HLA-DPB1	LAG3	2,54980E-03	21464,4	5,85871E-03	18198	1,27889E+00	13010	2,66606E+00	9353	9,49514E-01	4861	0	61900
Myeloid_vs_CD8_Non-responder	CD8(ID2+CXCR4+ T)	Macro_FOLR2+APOE+	HLA-A	LILRB2	2,55078E-03	27666,4	3,52862E-03	38915	1,45457E+00	8467	1,89764E+00	24915	9,50853E-01	4135	0	61900
Myeloid_vs_CD8_Non-responder	Macro_FOLR2+APOE+	CD8(EOMES+ NK-like)	B2M	KLRC1	2,55111E-03	28927,2	6,32728E-03	16224	9,79166E-01	25298	1,89953E+00	24869	9,32789E-01	16345	0	61900
Myeloid_vs_CD8_Non-responder	Macro_FOLR2+APOE+	CD8(EOMES+ NK-like)	B2M	KLRC2	2,55144E-03	25911,8	1,09527E-02	6907	1,14611E+00	17673	1,89932E+00	24875	9,29366E-01	18204	0	61900
Myeloid_vs_CD8_Non-responder	Macro_FOLR2+APOE+	CD8(EOMES+ NK-like)	B2M	KIR3DL1	2,55209E-03	27353,8	1,84137E-02	2499	1,21129E+00	15268	1,83949E+00	26608	9,09235E-01	30494	0	61900
Myeloid_vs_CD8_Non-responder	Macro_FOLR2+APOE+	CD8(EOMES+ NK-like)	B2M	KLRD1	2,55242E-03	25285,2	5,99688E-03	17596	1,13868E+00	17993	1,81623E+00	27355	9,56643E-01	1582	0	61900
Myeloid_vs_CD8_Non-responder	Mast	CD8(ID2+CXCR4+ T)	TIMP3	CD44	2,55420E-03	31602,2	2,87882E-02	1042	2,31768E+00	1276	1,12581E+00	56952	8,99773E-01	36841	0	61900
Myeloid_vs_CD8_Non-responder	Macro_FOLR2+APOE+	CD8(EOMES+ NK-like)	SECTM1	CD7	2,55570E-03	27813,6	1,18245E-02	6090	1,44698E+00	8618	1,76481E+00	28966	9,04771E-01	33494	0	61900
Myeloid_vs_CD8_Non-responder	CD8(IL7R+ZNF683+ Tm)	Macro_NLRP3	ANXA1	FPR1	2,56063E-03	37248,8	3,59972E-03	37754	9,52160E-01	26737	1,50026E+00	38945	9,24601E-01	20908	0	61900
Myeloid_vs_CD8_Non-responder	CD8(GZMK+ Early Tem)	Macro_FOLR2+APOE+	HLA-A	LILRB2	2,56128E-03	27728	3,52703E-03	38947	1,45328E+00	8493	1,88896E+00	25158	9,50843E-01	4142	0	61900
Myeloid_vs_CD8_Non-responder	Macro_FOLR2+APOE+	CD8(Tn)	C1QB	C1QBP	2,56457E-03	27466,4	3,52572E-03	38957	1,61517E+00	5851	3,30105E+00	3435	9,14494E-01	27189	0	61900
Myeloid_vs_CD8_Non-responder	CD8(ITM2C+ T)	Macro_ISG15	HLA-C	LILRB1	2,56490E-03	32494,2	3,87628E-03	33781	1,14780E+00	17595	1,49999E+00	38958	9,42240E-01	10237	0	61900
Myeloid_vs_CD8_Non-responder	CD8(Terminal Tex)	Mono_CD14	IFNG	IFNGR1_IFNGR2	2,56754E-03	39180,6	5,43262E-03	20404	7,88524E-01	37586	1,49986E+00	38966	8,99480E-01	37047	0	61900
Myeloid_vs_CD8_Non-responder	CD8(Tn)	cDC_CLEC9A	CD40LG	CD40	2,57455E-03	35516,6	3,38679E-02	759	1,49632E+00	7686	2,15506E+00	18243	8,19781E-01	88995	0	61900
Myeloid_vs_CD8_Non-responder	Macro_FOLR2+APOE+	CD8(EOMES+ NK-like)	C1QB	C1QBP	2,57777E-03	26219,8	3,78849E-03	34935	1,63662E+00	5558	3,33938E+00	3234	9,17263E-01	25472	0	61900
Myeloid_vs_CD8_Non-responder	Macro_FOLR2+APOE+	CD8(EOMES+ NK-like)	C3	IFITM1	2,57810E-03	26027,6	6,99701E-03	13977	1,13129E+00	18299	2,12546E+00	18888	9,31417E-01	17074	0	61900
Myeloid_vs_CD8_Non-responder	Macro_FOLR2+APOE+	CD8(GZMK+ Tem)	CD86	CTLA4	2,58174E-03	26686,4	1,11767E-02	6652	1,53289E+00	7046	2,12856E+00	18825	8,96609E-01	39009	0	61900
Myeloid_vs_CD8_Non-responder	Macro_FOLR2+APOE+	CD8(ISG+ T)	TIMP2	CD44	2,58240E-03	37617	4,28496E-03	29082	1,05142E+00	21846	1,49849E+00	39011	9,00700E-01	36246	0	61900
Myeloid_vs_CD8_Non-responder	Macro_FOLR2+APOE+	CD8(ID2+CXCR4+ T)	LGALS9	PTPRC	2,58405E-03	28731,2	4,03575E-03	31841	1,27408E+00	13165	1,89105E+00	25102	9,40246E-01	11648	0	61900
Myeloid_vs_CD8_Non-responder	Macro_FOLR2+APOE+	CD8(Temra)	B2M	KIR3DL1	2,58472E-03	31582,4	1,37949E-02	4506	1,03961E+00	22374	1,72998E+00	30114	8,96594E-01	39018	0	61900
Myeloid_vs_CD8_Non-responder	Mono_INHBA	CD8(Temra)	INHBA	TGFBR3	2,58609E-03	32257,6	8,29936E-02	124	2,31575E+00	1284	2,27511E+00	15696	8,30518E-01	82284	0	61900
Myeloid_vs_CD8_Non-responder	Macro_FOLR2+APOE+	CD8(ID2+CXCR4+ T)	HLA-DRA	LAG3	2,59002E-03	23237,6	4,90276E-03	23694	1,19453E+00	15900	2,69531E+00	8952	9,48131E-01	5742	0	61900
Myeloid_vs_CD8_Non-responder	Macro_FOLR2+APOE+	CD8(IL7R+ZNF683+ Tm)	C1QB	C1QBP	2,59002E-03	27484,6	3,52187E-03	39034	1,61485E+00	5858	3,30563E+00	3410	9,14451E-01	27221	0	61900
Myeloid_vs_CD8_Non-responder	Macro_FOLR2+APOE+	CD8(ID2+CXCR4+ T)	HLA-DQA2	LAG3	2,59035E-03	32875	5,07245E-03	22559	9,40452E-01	27405	2,04502E+00	20864	9,07536E-01	31647	0	61900
Myeloid_vs_CD8_Non-responder	Macro_FOLR2+APOE+	CD8(ID2+CXCR4+ T)	HLA-DRB1	LAG3	2,59102E-03	26256,4	4,33633E-03	28568	1,07553E+00	20730	2,43413E+00	12740	9,46008E-01	7344	0	61900
Myeloid_vs_CD8_Non-responder	CD8(ZNF683+KLRB1+ T)	Macro_FOLR2+APOE+	HLA-C	LILRB1	2,59102E-03	30597	3,52180E-03	39037	1,34508E+00	11057	1,76764E+00	28879	9,39575E-01	12112	0	61900
Myeloid_vs_CD8_Non-responder	Macro_FOLR2+APOE+	CD8(ID2+CXCR4+ T)	HLA-DQB1	LAG3	2,59135E-03	23991	5,58171E-03	19566	1,22002E+00	14944	2,53584E+00	11086	9,39091E-01	12459	0	61900
Myeloid_vs_CD8_Non-responder	Macro_FOLR2+APOE+	CD8(ID2+CXCR4+ T)	HLA-DPB1	LAG3	2,59334E-03	25909	4,58623E-03	26174	1,12464E+00	18592	2,39376E+00	13437	9,43311E-01	9442	0	61900
Myeloid_vs_CD8_Non-responder	Macro_FOLR2+APOE+	CD8(ID2+CXCR4+ T)	ICAM1	IL2RG	2,59566E-03	38987,8	3,66352E-03	36719	9,17604E-01	28767	1,64590E+00	33105	9,03328E-01	34448	0	61900
Myeloid_vs_CD8_Non-responder	Macro_FOLR2+APOE+	CD8(ID2+CXCR4+ T)	CD14	ITGB1	2,59733E-03	27764	3,93845E-03	33023	1,43073E+00	8964	2,68538E+00	9087	9,16676E-01	25846	0	61900
Myeloid_vs_CD8_Non-responder	Macro_FOLR2+APOE+	CD8(ID2+CXCR4+ T)	CD14	ITGA4	2,59766E-03	26874,2	4,17280E-03	30316	1,45168E+00	8523	2,62028E+00	9922	9,20028E-01	23710	0	61900
Myeloid_vs_CD8_Non-responder	CD8(NME1+ T)	Macro_FOLR2+APOE+	MIF	CD44_CD74	2,59810E-03	43810,2	2,00821E-03	82386	7,51158E-01	40464	1,64813E+00	33014	9,57508E-01	1287	0	61900
Myeloid_vs_CD8_Non-responder	Macro_FOLR2+APOE+	CD8(ID2+CXCR4+ T)	HLA-DPA1	LAG3	2,60365E-03	25199,8	4,55021E-03	26525	1,13080E+00	18319	2,48952E+00	11798	9,45885E-01	7457	0	61900
Myeloid_vs_CD8_Non-responder	Macro_FOLR2+APOE+	CD8(ID2+CXCR4+ T)	CD86	CTLA4	2,60565E-03	26602,8	1,12326E-02	6596	1,53580E+00	6998	2,13438E+00	18678	8,96840E-01	38842	0	61900
Myeloid_vs_CD8_Non-responder	Macro_FOLR2+APOE+	CD8(ID2+CXCR4+ T)	B2M	KLRC1	2,60698E-03	38813	4,03512E-03	31854	7,86944E-01	37717	1,54347E+00	37105	9,17240E-01	25489	0	61900
Myeloid_vs_CD8_Non-responder	Macro_OLFML3	CD8(GZMK+ Tem)	HLA-DRB1	LAG3	2,60726E-03	22719,6	5,83448E-03	18302	1,22044E+00	14929	2,28939E+00	15427	9,53104E-01	3040	0	61900
Myeloid_vs_CD8_Non-responder	Macro_FOLR2+APOE+	CD8(ID2+CXCR4+ T)	B2M	KLRC2	2,60732E-03	40133,4	4,84139E-03	24168	7,71893E-01	38837	1,53875E+00	37309	8,97412E-01	38453	0	61900
Myeloid_vs_CD8_Non-responder	Macro_FOLR2+APOE+	CD8(ID2+CXCR4+ T)	HLA-DRB5	LAG3	2,61065E-03	25147,8	4,93121E-03	23488	1,15247E+00	17427	2,45962E+00	12301	9,41695E-01	10623	0	61900
Myeloid_vs_CD8_Non-responder	Macro_FOLR2+APOE+	CD8(ID2+CXCR4+ T)	SECTM1	CD7	2,61099E-03	29713	1,13622E-02	6472	1,40420E+00	9553	1,57108E+00	36003	9,03039E-01	34637	0	61900
Myeloid_vs_CD8_Non-responder	cDC_CLEC9A	CD8(ISG+ T)	HLA-DQA1	LAG3	2,61507E-03	23226	5,83144E-03	18316	1,25710E+00	13689	2,65502E+00	9477	9,38697E-01	12748	0	61900
Myeloid_vs_CD8_Non-responder	Macro_FOLR2+APOE+	CD8(ID2+CXCR4+ T)	SPP1	CD44	2,61834E-03	31759,6	5,65616E-03	19157	8,14320E-01	35620	2,76804E+00	8012	9,03828E-01	34109	0	61900
Myeloid_vs_CD8_Non-responder	Macro_FOLR2+APOE+	CD8(ID2+CXCR4+ T)	CD59	CD2	2,61901E-03	33436	7,42082E-03	12767	9,55276E-01	26561	1,51884E+00	38127	9,13404E-01	27825	0	61900
Myeloid_vs_CD8_Non-responder	Macro_FOLR2+APOE+	CD8(GZMK+ Early Tem)	CD14	ITGA4	2,62035E-03	30184,8	3,51603E-03	39125	1,37636E+00	10236	2,48352E+00	11888	9,13497E-01	27775	0	61900
Myeloid_vs_CD8_Non-responder	Macro_FOLR2+APOE+	CD8(ID2+CXCR4+ T)	LGALS3	LAG3	2,62102E-03	27465	5,73587E-03	18798	1,06081E+00	21402	2,21946E+00	16841	9,29065E-01	18384	0	61900
Myeloid_vs_CD8_Non-responder	Macro_ISG15	CD8(GZMK+ Early Tem)	S100A8	CD69	2,62233E-03	25242,2	5,83000E-03	18329	1,19309E+00	15957	2,24921E+00	16245	9,37111E-01	13780	0	61900
Myeloid_vs_CD8_Non-responder	CD8(ID2+CXCR4+ T)	Mono_CD16	HLA-C	LILRA3	2,62303E-03	27182,6	1,10920E-02	6747	1,54268E+00	6877	1,49579E+00	39133	9,23995E-01	21256	0	61900
Myeloid_vs_CD8_Non-responder	Macro_FOLR2+APOE+	CD8(ID2+CXCR4+ T)	CXCL16	CXCR6	2,62605E-03	21451,2	1,07240E-02	7180	1,54559E+00	6829	2,53406E+00	11119	9,25819E-01	20228	0	61900
Myeloid_vs_CD8_Non-responder	Macro_NLRP3	CD8(EOMES+ NK-like)	VCAN	CD44	2,62793E-03	22946,8	6,78663E-03	14589	1,98722E+00	2611	2,19755E+00	17295	9,29150E-01	18339	0	61900
Myeloid_vs_CD8_Non-responder	CD8(ZNF683+KLRB1+ T)	Macro_OLFML3	SPON2	ITGB2	2,62907E-03	35528,6	6,18002E-03	16800	9,26921E-01	28216	1,68679E+00	31576	8,96385E-01	39151	0	61900
Myeloid_vs_CD8_Non-responder	Mono_CD14	CD8(Temra)	S100A9	ITGB2	2,63012E-03	18909	5,28159E-03	21246	2,30921E+00	1300	3,32094E+00	3328	9,46706E-01	6771	0	61900
Myeloid_vs_CD8_Non-responder	Macro_FOLR2+APOE+	CD8(ID2+CXCR4+ T)	C3	IFITM1	2,63310E-03	31487,4	5,56511E-03	19671	9,51934E-01	26749	1,80507E+00	27690	9,23732E-01	21427	0	61900
Myeloid_vs_CD8_Non-responder	Macro_OLFML3	CD8(Temra)	CXCL10	CXCR3	2,63355E-03	27132,6	1,10664E-02	6774	2,21823E+00	1594	3,08585E+00	4969	8,64585E-01	60426	0	61900
Myeloid_vs_CD8_Non-responder	Macro_OLFML3	CD8(ITM2C+ T)	CXCL9	CXCR3	2,63812E-03	24210,8	1,71673E-02	2872	2,24927E+00	1490	2,88492E+00	6778	8,83284E-01	48014	0	61900
Myeloid_vs_CD8_Non-responder	CD8(ITM2C+ T)	Macro_ISG15	HLA-C	LILRB2	2,63848E-03	28337,6	3,51332E-03	39179	1,37809E+00	10192	1,85389E+00	26179	9,50669E-01	4238	0	61900
Myeloid_vs_CD8_Non-responder	CD8(ZNF683+KLRB1+ T)	Macro_NLRP3	HLA-F	LILRB2	2,63949E-03	35259,8	3,51312E-03	39182	1,20615E+00	15459	1,84822E+00	26353	9,04883E-01	33405	0	61900
Myeloid_vs_CD8_Non-responder	CD8(Temra)	Macro_ISG15	SPON2	ITGB2	2,64928E-03	34918	6,17025E-03	16829	8,91453E-01	30414	1,85191E+00	26236	8,96311E-01	39211	0	61900
Myeloid_vs_CD8_Non-responder	Macro_FOLR2+APOE+	CD8(Tc17)	ICAM1	IL2RG	2,65097E-03	37810,6	3,79377E-03	34869	9,39687E-01	27446	1,69165E+00	31406	9,04842E-01	33432	0	61900
Myeloid_vs_CD8_Non-responder	CD8(Tn)	Macro_ISG15	HLA-A	LILRB2	2,65604E-03	29071,8	3,50980E-03	39231	1,31036E+00	12115	1,79839E+00	27906	9,50728E-01	4207	0	61900
Myeloid_vs_CD8_Non-responder	CD8(NME1+ T)	Macro_ISG15	B2M	LILRB2	2,65841E-03	27865,8	3,50945E-03	39238	1,33802E+00	11259	1,87320E+00	25616	9,57416E-01	1316	0	61900

Myeloid_vs_CD8_Post_NR

Myeloid_vs_CD8_Non-responder	CD8(GZMK+ Tex)	Macro_FOLR2+APOE+	GZMB	IGF2R	2,65977E-03	35350,2	9,24103E-03	9094	9,16187E-01	28855	1,49289E+00	39242	8,98562E-01	37660	0	61900
Myeloid_vs_CD8_Non-responder	Macro_FOLR2+APOE+	CD8(Terminal Tex)	HLA-DPA1	LAG3	2,66510E-03	20978,4	5,81270E-03	18405	1,28505E+00	12838	2,76182E+00	8101	9,51821E-01	3648	0	61900
Myeloid_vs_CD8_Non-responder	Macro_FOLR2+APOE+	CD8(Tc17)	SPP1	CD44	2,66893E-03	29994,4	6,10234E-03	17113	8,70279E-01	31783	2,84111E+00	7226	9,07078E-01	31950	0	61900
Myeloid_vs_CD8_Non-responder	Macro_FOLR2+APOE+	CD8(ID2+CXCR4+ T)	MRC1	PTPRC	2,67063E-03	35358,4	5,91349E-03	17948	1,07896E+00	20569	1,54353E+00	37101	8,96210E-01	39274	0	61900
Myeloid_vs_CD8_Non-responder	Macro_FOLR2+APOE+	CD8(ZNF683+KLRB1+ T)	HLA-B	CD8B	2,67369E-03	33926,8	3,91678E-03	33277	8,39613E-01	33825	1,49210E+00	39283	9,57346E-01	1349	0	61900
Myeloid_vs_CD8_Non-responder	Macro_FOLR2+APOE+	CD8(Tc17)	CXCL16	CXCR6	2,67641E-03	28024,6	6,68528E-03	14915	1,26708E+00	13369	2,14112E+00	18533	9,07869E-01	31406	0	61900
Myeloid_vs_CD8_Non-responder	CD8(ID2+CXCR4+ T)	Mono_CD16	HLA-A	LILRA1	2,67675E-03	25677	9,72987E-03	8346	1,78396E+00	4107	1,49186E+00	39292	9,35610E-01	14740	0	61900
Myeloid_vs_CD8_Non-responder	Macro_FOLR2+APOE+	CD8(Tc17)	LGALS1	CD69	2,67709E-03	26560,8	4,10002E-03	31125	1,08544E+00	20273	2,21128E+00	16999	9,54384E-01	2507	0	61900
Myeloid_vs_CD8_Non-responder	Macro_FOLR2+APOE+	CD8(Tc17)	APOE	SORL1	2,67778E-03	24921	5,46260E-03	20231	1,43279E+00	8908	3,25764E+00	3728	9,10213E-01	29838	0	61900
Myeloid_vs_CD8_Non-responder	CD8(ZNF683+KLRB1+ T)	Macro_OLFML3	B2M	LILRB1	2,67880E-03	30957,6	3,50621E-03	39298	1,30628E+00	12223	1,58822E+00	35301	9,47673E-01	6066	0	61900
Myeloid_vs_CD8_Non-responder	Macro_FOLR2+APOE+	CD8(Tc17)	MMP9	CD44	2,68050E-03	26235,2	8,79272E-03	9867	1,45791E+00	8396	2,43070E+00	12788	8,97726E-01	38225	0	61900
Myeloid_vs_CD8_Non-responder	cDC_LAMP3	CD8(GZMK+ Early Tem)	CCL19	CCR7	2,68294E-03	35658	2,57345E-02	1308	3,91666E+00	83	2,25267E+00	16169	8,03458E-01	98830	0	61900
Myeloid_vs_CD8_Non-responder	Macro_FOLR2+APOE+	CD8(Tc17)	C3	IFITM1	2,68357E-03	30453	5,82981E-03	18331	9,85090E-01	24981	1,84079E+00	26564	9,25353E-01	20489	0	61900
Myeloid_vs_CD8_Non-responder	CD8(EOMES+ NK-like)	Mono_CD14	CIRBP	TREM1	2,68357E-03	35646,2	5,20716E-03	21697	1,13946E+00	17962	1,49144E+00	39312	8,99005E-01	37360	0	61900
Myeloid_vs_CD8_Non-responder	CD8(ITM2C+ T)	Mono_CD16	B2M	LILRB1	2,68494E-03	31944,2	3,65907E-03	36797	1,18117E+00	16361	1,49130E+00	39316	9,48721E-01	5347	0	61900
Myeloid_vs_CD8_Non-responder	Macro_FOLR2+APOE+	CD8(LAYN+ T)	LGALS9	PTPRC	2,69417E-03	27973,8	4,15417E-03	30513	1,31076E+00	12099	1,91963E+00	24266	9,41053E-01	11091	0	61900
Myeloid_vs_CD8_Non-responder	Macro_FOLR2+APOE+	CD8(LAYN+ T)	LGALS9	CD44	2,69451E-03	31686,2	3,68484E-03	36410	1,22016E+00	14940	1,80528E+00	27683	9,30625E-01	17498	0	61900
Myeloid_vs_CD8_Non-responder	CD8(ISG+ T)	Macro_OLFML3	HLA-B	LILRB1	2,69725E-03	33175,6	3,50158E-03	39352	1,18280E+00	16299	1,52003E+00	38072	9,42215E-01	10255	0	61900
Myeloid_vs_CD8_Non-responder	Macro_FOLR2+APOE+	CD8(LAYN+ T)	HLA-DRA	LAG3	2,70171E-03	24193,8	4,69487E-03	25296	1,17095E+00	16742	2,57481E+00	10522	9,47056E-01	6509	0	61900
Myeloid_vs_CD8_Non-responder	Macro_FOLR2+APOE+	CD8(LAYN+ T)	HLA-DQA2	LAG3	2,70206E-03	34347,4	4,85736E-03	24021	9,16879E-01	28823	1,92452E+00	24130	9,05702E-01	32863	0	61900
Myeloid_vs_CD8_Non-responder	Macro_FOLR2+APOE+	CD8(LAYN+ T)	HLA-DQA1	LAG3	2,70240E-03	24152,4	5,63278E-03	19295	1,23463E+00	14423	2,49400E+00	11718	9,37693E-01	13426	0	61900
Myeloid_vs_CD8_Non-responder	Macro_FOLR2+APOE+	CD8(EOMES+ NK-like)	CXCL9	CXCR3	2,70274E-03	26095,8	1,37765E-02	4523	2,27743E+00	1404	2,87931E+00	6834	8,71455E-01	55818	0	61900
Myeloid_vs_CD8_Non-responder	Macro_FOLR2+APOE+	CD8(LAYN+ T)	HLA-DRB1	LAG3	2,70274E-03	27483,2	4,15245E-03	30534	1,05196E+00	21824	2,31363E+00	14947	9,44890E-01	8211	0	61900
Myeloid_vs_CD8_Non-responder	Macro_FOLR2+APOE+	CD8(LAYN+ T)	HLA-DQB1	LAG3	2,70309E-03	24998,6	5,34503E-03	20885	1,19644E+00	15839	2,41534E+00	13045	9,37840E-01	13324	0	61900
Myeloid_vs_CD8_Non-responder	Macro_FOLR2+APOE+	CD8(LAYN+ T)	HLA-DPB1	LAG3	2,70515E-03	27113,2	4,39176E-03	28009	1,10107E+00	19612	2,27326E+00	15740	9,42142E-01	10305	0	61900
Myeloid_vs_CD8_Non-responder	CD8(NME1+ T)	Macro_ISG15	HLA-A	LILRB1	2,70583E-03	32531,6	3,94120E-03	32983	1,12522E+00	18572	1,48974E+00	39377	9,42786E-01	9826	0	61900
Myeloid_vs_CD8_Non-responder	Macro_FOLR2+APOE+	CD8(LAYN+ T)	HLA-DPA1	LAG3	2,71547E-03	26343,8	4,35727E-03	28348	1,10723E+00	19314	2,36901E+00	13872	9,44765E-01	8285	0	61900
Myeloid_vs_CD8_Non-responder	CD8(NME1+ T)	Macro_FOLR2+APOE+	MFGE8	ITGB5	2,71561E-03	63249,6	2,60934E-02	1273	1,09767E+00	19766	1,04874E+00	61287	5,82931E-01	172022	0	61900
Myeloid_vs_CD8_Non-responder	Macro_FOLR2+APOE+	CD8(LAYN+ T)	MRC1	PTPRC	2,71581E-03	34476,6	6,08701E-03	17191	1,11564E+00	18977	1,57211E+00	35955	8,97548E-01	38360	0	61900
Myeloid_vs_CD8_Non-responder	Macro_FOLR2+APOE+	CD8(LAYN+ T)	CD86	CTLA4	2,71857E-03	22335,2	1,60327E-02	3308	1,78497E+00	4098	2,37711E+00	13731	9,12176E-01	28639	0	61900
Myeloid_vs_CD8_Non-responder	Macro_FOLR2+APOE+	CD8(Temra)	B2M	KIR2DL3	2,71926E-03	31225,4	1,61909E-02	3240	1,05624E+00	21627	1,73466E+00	29944	8,96004E-01	39416	0	61900
Myeloid_vs_CD8_Non-responder	Mast	CD8(Temra)	TIMP3	CD44	2,71971E-03	32259,8	2,81339E-02	1092	2,30155E+00	1317	1,08108E+00	59467	8,98732E-01	37523	0	61900
Myeloid_vs_CD8_Non-responder	Macro_FOLR2+APOE+	CD8(LAYN+ T)	HLA-DRB5	LAG3	2,72478E-03	26253,4	4,72212E-03	25081	1,12890E+00	18404	2,33911E+00	14424	9,40495E-01	11458	0	61900
Myeloid_vs_CD8_Non-responder	CD8(ISG+ T)	Macro_FOLR2+APOE+	HLA-A	LILRB2	2,72651E-03	28028,6	3,49650E-03	39437	1,42860E+00	9001	1,87572E+00	25553	9,50639E-01	4252	0	61900
Myeloid_vs_CD8_Non-responder	CD8(Temra)	Macro_NLRP3	SPON2	ITGB2	2,73447E-03	36320,2	6,12042E-03	17043	8,83609E-01	30871	1,66618E+00	32327	8,95934E-01	39460	0	61900
Myeloid_vs_CD8_Non-responder	Macro_FOLR2+APOE+	CD8(LAYN+ T)	SPP1	ITGA4_ITGB1	2,73620E-03	25456,2	1,06276E-02	7271	1,17155E+00	16728	3,31815E+00	3348	8,98000E-01	38034	0	61900
Myeloid_vs_CD8_Non-responder	Macro_FOLR2+APOE+	CD8(LAYN+ T)	SPP1	CD44	2,73655E-03	27202	6,96714E-03	14054	9,78741E-01	25323	2,92995E+00	6327	9,12515E-01	28406	0	61900
Myeloid_vs_CD8_Non-responder	Macro_NLRP3	CD8(IL7R+ZNF683+ Tm)	HBEGF	CD44	2,73713E-03	23375,8	6,49225E-03	15611	1,76816E+00	4254	2,14127E+00	18531	9,32335E-01	16583	0	61900
Myeloid_vs_CD8_Non-responder	Macro_FOLR2+APOE+	CD8(LAYN+ T)	CD59	CD2	2,73724E-03	30087,2	8,50023E-03	10400	1,07572E+00	20719	1,65263E+00	32847	9,18626E-01	24570	0	61900
Myeloid_vs_CD8_Non-responder	Macro_FOLR2+APOE+	CD8(LAYN+ T)	LGALS3	LAG3	2,73932E-03	28631	5,49265E-03	20058	1,03724E+00	22483	2,09896E+00	19512	9,27624E-01	19202	0	61900
Myeloid_vs_CD8_Non-responder	Macro_FOLR2+APOE+	CD8(LAYN+ T)	GRN	TNFRSF1B	2,74071E-03	25847,8	3,56271E-03	38360	1,44316E+00	8699	2,71305E+00	8716	9,40362E-01	11564	0	61900
Myeloid_vs_CD8_Non-responder	CD8(ZNF683+KLRB1+ T)	Macro_FOLR2+APOE+	B2M	LILRB2	2,74280E-03	25983,2	3,49303E-03	39484	1,62110E+00	5768	2,02367E+00	21406	9,57320E-01	1358	0	61900
Myeloid_vs_CD8_Non-responder	Macro_FOLR2+APOE+	CD8(LAYN+ T)	LGALS3BP	ITGB1	2,74314E-03	25100,2	9,98434E-03	7993	1,62533E+00	5714	2,16638E+00	17993	9,07156E-01	31901	0	61900
Myeloid_vs_CD8_Non-responder	CD8(NME1+ T)	Macro_ISG15	HLA-B	LILRB2	2,74349E-03	28728	3,49299E-03	39486	1,32823E+00	11581	1,81065E+00	27515	9,52848E-01	3158	0	61900
Myeloid_vs_CD8_Non-responder	Macro_ISG15	CD8(ID2+CXCR4+ T)	S100A8	CD69	2,74522E-03	25435	5,78394E-03	18545	1,18737E+00	16140	2,22722E+00	16674	9,36877E-01	13916	0	61900
Myeloid_vs_CD8_Non-responder	Macro_FOLR2+APOE+	CD8(LAYN+ T)	CXCL16	CXCR6	2,74523E-03	24169,4	8,86748E-03	9748	1,41756E+00	9248	2,27744E+00	15650	9,19022E-01	24301	0	61900
Myeloid_vs_CD8_Non-responder	CD8(IL7R+ZNF683+ Tm)	Mono_CD16	HLA-C	LILRA3	2,74592E-03	27408	1,09855E-02	6864	1,51727E+00	7314	1,48698E+00	39493	9,23655E-01	21469	0	61900
Myeloid_vs_CD8_Non-responder	CD8(LAYN+ T)	Mono_CD16	HLA-A	LILRB1	2,74731E-03	32399,4	3,82814E-03	34415	1,19791E+00	15777	1,48681E+00	39497	9,41996E-01	10408	0	61900
Myeloid_vs_CD8_Non-responder	Mast	CD8(ITM2C+ T)	TIMP3	CD44	2,74848E-03	31891,4	2,80664E-02	1102	2,29989E+00	1324	1,11605E+00	57513	8,98623E-01	37618	0	61900
Myeloid_vs_CD8_Non-responder	Macro_FOLR2+APOE+	CD8(LAYN+ T)	MMP9	CD44	2,74940E-03	24389	1,00388E-02	7927	1,56637E+00	6557	2,51954E+00	11327	9,03652E-01	34234	0	61900
Myeloid_vs_CD8_Non-responder	Macro_ISG15	CD8(ISG+ T)	CCL2	CCR4	2,75187E-03	39711	4,66323E-02	396	1,54276E+00	6876	3,19539E+00	4131	7,54761E-01	125252	0	61900
Myeloid_vs_CD8_Non-responder	Mast	CD8(GZMK+ Tex)	TIMP3	CD44	2,75260E-03	32138,2	2,80598E-02	1103	2,29973E+00	1325	1,09366E+00	58736	8,98612E-01	37627	0	61900
Myeloid_vs_CD8_Non-responder	Macro_FOLR2+APOE+	CD8(LAYN+ T)	C1QB	C1QBP	2,75288E-03	24594,6	4,22519E-03	29745	1,67229E+00	5121	3,33217E+00	3267	9,21309E-01	22940	0	61900
Myeloid_vs_CD8_Non-responder	Macro_FOLR2+APOE+	CD8(LAYN+ T)	C3	IFITM1	2,75358E-03	36723,2	4,64472E-03	25704	8,36648E-01	34013	1,56009E+00	36446	9,17115E-01	25553	0	61900
Myeloid_vs_CD8_Non-responder	Macro_FOLR2+APOE+	CD8(LAYN+ T)	LYZ	ITGAL	2,75497E-03	30255,4	3,52969E-03	38897	1,28745E+00	12771	2,84758E+00	7151	9,09139E-01	30558	0	61900
Myeloid_vs_CD8_Non-responder	Macro_FOLR2+APOE+	CD8(LAYN+ T)	TIMP2	CD44	2,75532E-03	34750,6	4,76778E-03	24710	1,13991E+00	17936	1,56779E+00	36133	9,05374E-01	33074	0	61900
Myeloid_vs_CD8_Non-responder	CD8(Tn)	Macro_ISG15	HLA-B	LILRB2	2,75776E-03	28706,4	3,49053E-03	39527	1,32617E+00	11640	1,81826E+00	27293	9,52832E-01	3172	0	61900
Myeloid_vs_CD8_Non-responder	CD8(LAYN+ T)	cDC(CD1C)	ANXA1	FPR1	2,75846E-03	32169,6	4,56298E-03	26394	1,17713E+00	16513	1,48581E+00	39529	9,32461E-01	16512	0	61900
Myeloid_vs_CD8_Non-responder	CD8(ISG+ T)	Mono_CD16	ANXA1	FPR1	2,75916E-03	36891	3,49006E-03	39531	1,03073E+00	22780	1,50623E+00	38680	9,23515E-01	21564	0	61900
Myeloid_vs_CD8_Non-responder	Macro_FOLR2+APOE+	CD8(Terminal Tex)	LGALS9	PTPRC	2,76230E-03	31822,4	3,73089E-03	35722	1,17965E+00	16424	1,67997E+00	31845	9,38001E-01	13221	0	61900
Myeloid_vs_CD8_Non-responder	Macro_LYVE1	CD8(LAYN+ T)	CD14	ITGA4	2,76495E-03	23439,2	6,40247E-03	15929	1,65151E+00	5377	2,13908E+00	18579	9,34427E-01	15411	0	61900
Myeloid_vs_CD8_Non-responder	CD8(ISG+ T)	Macro_FOLR2+APOE+	HLA-B	LILRB2	2,76755E-03	27109	3,48842E-03	39555	1,49762E+00	7659	1,95547E+00	23254	9,52819E-01	3177	0	61900
Myeloid_vs_CD8_Non-responder	Macro_FOLR2+APOE+	CD8(Terminal Tex)	HLA-DQA2	LAG3	2,76964E-03	27550,6	6,47983E-03	15655	1,09470E+00	19897	2,31732E+00	14860	9,17311E-01	25441	0	61900
Myeloid_vs_CD8_Non-responder	Macro_FOLR2+APOE+	CD8(Terminal Tex)	HLA-DRB1	LAG3	2,77035E-03	21739	5,53947E-03	19812	1,22978E+00	14590	2,70643E+00	8800	9,51930E-01	3593	0	61900
Myeloid_vs_CD8_Non-responder	CD8(GZMK+ Tem)	Macro_LYVE1	HMGB1	CD163	2,77806E-03	30434,6	4,41332E-03	27801	1,40812E+00	9460	1,48454E+00	39585	9,37692E-01	13427	0	61900
Myeloid_vs_CD8_Non-responder	CD8(ZNF683+KLRB1+ T)	Macro_FOLR2+APOE+	LTB	TNFRSF1A	2,78051E-03	34513,6	6,21546E-03	16663	1,00899E+00	23768	1,71333E+00	30645	8,95706E-01	39592	0	61900
Myeloid_vs_CD8_Non-responder	CD8(Tc17)	cDC(CD1C)	LTB	TNFRSF1A	2,78262E-03	33505,4	7,30974E-03	13071	1,13078E+00	18323	1,48400E+00	39598	9,03041E-01	34635	0	61900
Myeloid_vs_CD8_Non-responder	CD8(GZMK+ Tex)	Mono_CD16	HLA-A	LILRA1	2,78473E-03	25715	9,75057E-03	8310	1,79003E+00	4054	1,48385E+00	39604	9,35674E-01	14707	0	61900
Myeloid_vs_CD8_Non-responder	Mast	CD8(Temra)	ADCYAP1	ADRB2	2,78569E-03	43939,4	9,60874E-02	91	2,29791E+00	1333	1,28343E+00	48788	7,88419E-01	107585	0	61900
Myeloid_vs_CD8_Non-responder	CD8(Terminal Tex)	Macro_FOLR2+APOE+	TNFSF9	HLA-DPA1	2,78825E-03	33729,2	3,48351E-03	39614	9,98059E-01	24316	2,45093E+00	12454	9,09424E-01	30362	0	61900
Myeloid_vs_CD8_Non-responder	CD8(EOMES+ NK-like)	Mono_CD16	HLA-A	LILRB1	2,79106E-03	32633,8	3,80384E-03	34723	1,18015E+00	16397	1,48354E+00	39622	9,41821E-01	10527	0	61900
Myeloid_vs_CD8_Non-responder	CD8(LAYN+ T)	Macro_FOLR2+APOE+	HLA-C	LILRB2	2,79459E-03	27704,8	3,48219E-03	39632	1,47194E+00	8115	1,91016E+00	24543	9,50460E-01	4334	0	61900
Myeloid_vs_CD8_Non-responder	Macro_FOLR2+APOE+	CD8(Terminal Tex)	HLA-DQB2	LAG3	2,79812E-03	29998,2	8,68778E-03	10053	1,10413E+00	19471	1,93109E+00	23933	9,03042E-01	34634	0	61900
Myeloid_vs_CD8_Non-responder	CD8(GZMK+ Tem)	cDC_LAMP3	CD200R1	CD200	2,79815E-03	38608,6	8,03090E-02	133,5	2,29631E+00	1336,5	1,67467E+00	32014,5	8,05432E-01	97658,5	0	61900
Myeloid_vs_CD8_Non-responder	Macro_FOLR2+APOE+	CD8(Terminal Tex)	CD59	CD2	2,80059E-03	30813	8,25345E-03	10885	1,04818E+00	22001	1,62232E+00	33979	9,17518E-01	25300	0	61900
Myeloid_vs_CD8_Non-responder	cDC_LAMP3	CD8(GZMK+ Tem)	CD200	CD200R1	2,80231E-03	38608,6	8,03090E-02	133,5	2,29631E+00	1336,5	1,67467E+00	32014,5	8,05432E-01	97658,5	0	61900
Myeloid_vs_CD8_Non-responder	Macro_FOLR2+APOE+	CD8(Terminal Tex)	CXCL16	CXCR6	2,81084E-03	24806,4	8,26563E-03	10862	1,37606E+00	10243	2,31243E+00	14981	9,16368E-01	26046	0	61900

Myeloid_vs_CD8_Post_NR

Myeloid_vs_CD8_Non-responder	pDC_LILRA4	CD8(EOMES+ NK-like)	B2M	KLRC2	2,81413E-03	24551,2	1,08997E-02	6963	1,12312E+00	18663	2,21510E+00	16931	9,29206E-01	18299	0	61900
Myeloid_vs_CD8_Non-responder	Macro_FOLR2+APOE+	CD8(Temra)	LGALS3BP	ITGB1	2,81793E-03	28882,6	7,69237E-03	12090	1,45507E+00	8455	1,99086E+00	22270	8,95575E-01	39698	0	61900
Myeloid_vs_CD8_Non-responder	CD8(LAYN+ T)	pDC_LILRA4	HRAS	TLR9	2,81897E-03	44829,8	7,96515E-02	141	2,29552E+00	1341	1,73002E+00	30111	7,43150E-01	130656	0	61900
Myeloid_vs_CD8_Non-responder	Macro_FOLR2+APOE+	CD8(Terminal Tex)	C3	IFITM1	2,81935E-03	31256,4	5,75549E-03	18698	9,75781E-01	25469	1,74996E+00	29459	9,24909E-01	20756	0	61900
Myeloid_vs_CD8_Non-responder	Macro_ISG15	CD8(ZNF683+KLRB1+ T)	B2M	CD3D	2,82030E-03	42476	2,77342E-03	54816	7,21392E-01	42961	1,20726E+00	52683	9,65163E-01	20	0	61900
Myeloid_vs_CD8_Non-responder	Macro_FOLR2+APOE+	CD8(Terminal Tex)	LYZ	ITGAL	2,82078E-03	29822,2	3,60976E-03	37622	1,29479E+00	12559	2,85363E+00	7082	9,10061E-01	29948	0	61900
Myeloid_vs_CD8_Non-responder	Macro_FOLR2+APOE+	CD8(GZMK+ Tem)	LGALS3BP	ITGB1	2,82113E-03	29149,4	7,69071E-03	12095	1,45495E+00	8460	1,94358E+00	23585	8,95565E-01	39707	0	61900
Myeloid_vs_CD8_Non-responder	CD8(NME1+ T)	Macro_ISG15	HLA-F	LILRB2	2,82397E-03	33465,6	3,47638E-03	39715	1,45571E+00	8444	1,94458E+00	23555	9,04429E-01	33714	0	61900
Myeloid_vs_CD8_Non-responder	Macro_FOLR2+APOE+	CD8(ITM2C+ T)	LGALS9	PTPRC	2,82789E-03	32092,2	3,62105E-03	37446	1,14563E+00	17698	1,74393E+00	29649	9,37126E-01	13768	0	61900
Myeloid_vs_CD8_Non-responder	CD8(Terminal Tex)	Macro_FOLR2+APOE+	GZMB	IGF2R	2,83680E-03	36268,2	8,87342E-03	9736	8,81631E-01	31006	1,48008E+00	39751	8,96697E-01	38948	0	61900
Myeloid_vs_CD8_Non-responder	Macro_OLFML3	CD8(GZMK+ Tem)	HLA-DQA2	LAG3	2,83777E-03	22930	8,87955E-03	9726	1,38511E+00	10018	2,34497E+00	14303	9,28501E-01	18703	0	61900
Myeloid_vs_CD8_Non-responder	CD8(ITM2C+ T)	Mono_CD14	ANXA1	FPR1	2,84859E-03	36663,6	3,51835E-03	39093	1,06600E+00	21154	1,47916E+00	39784	9,23800E-01	21387	0	61900
Myeloid_vs_CD8_Non-responder	CD8(ITM2C+ T)	Mono_INHBA	HSPA1A	TLR4	2,85146E-03	31906,2	6,14690E-03	16926	1,25028E+00	13907	1,47898E+00	39792	9,14793E-01	27006	0	61900
Myeloid_vs_CD8_Non-responder	Macro_FOLR2+APOE+	CD8(GZMK+ Tem)	MRC1	PTPRC	2,85146E-03	35884,8	5,81494E-03	18397	1,05812E+00	21537	1,52655E+00	37798	8,95426E-01	39792	0	61900
Myeloid_vs_CD8_Non-responder	Macro_FOLR2+APOE+	CD8(Tc17)	TIMP2	CD44	2,85289E-03	38345,8	4,17598E-03	30278	1,03145E+00	22753	1,47896E+00	39796	8,99542E-01	37002	0	61900
Myeloid_vs_CD8_Non-responder	Macro_FOLR2+APOE+	CD8(Temra)	MRC1	PTPRC	2,85683E-03	36235	5,81269E-03	18406	1,05765E+00	21553	1,48651E+00	39509	8,95408E-01	39807	0	61900
Myeloid_vs_CD8_Non-responder	Macro_FOLR2+APOE+	CD8(ITM2C+ T)	SPP1	CD44	2,85899E-03	32359,6	5,51435E-03	19937	7,96535E-01	36950	2,75827E+00	8144	9,02719E-01	34867	0	61900
Myeloid_vs_CD8_Non-responder	CD8(ZNF683+KLRB1+ T)	Mono_CD16	HLA-B	LILRA1	2,86654E-03	25621,6	9,46679E-03	8749	1,77541E+00	4186	1,47828E+00	39834	9,37681E-01	13439	0	61900
Myeloid_vs_CD8_Non-responder	CD8(ISG+ T)	cDC(CD1C)	TNFSF9	HLA-DPA1	2,86942E-03	34836,4	3,46924E-03	39842	1,03860E+00	22417	2,09766E+00	19545	9,09255E-01	30478	0	61900
Myeloid_vs_CD8_Non-responder	Macro_FOLR2+APOE+	CD8(ITM2C+ T)	C3	IFITM1	2,87554E-03	29423,6	6,06488E-03	17294	1,01453E+00	23499	1,90535E+00	24704	9,26707E-01	19721	0	61900
Myeloid_vs_CD8_Non-responder	CD8(ITM2C+ T)	pDC_LILRA4	TNF	TNFRSF21	2,87590E-03	22544,6	1,97313E-02	2151	2,31848E+00	1270	2,80920E+00	7542	8,95335E-01	39860	0	61900
Myeloid_vs_CD8_Non-responder	Macro_FOLR2+APOE+	CD8(LAYN+ T)	HBEGF	CD44	2,88060E-03	39015,6	3,46723E-03	39873	9,57664E-01	26417	1,55430E+00	36679	9,09660E-01	30209	0	61900
Myeloid_vs_CD8_Non-responder	CD8(GZMK+ Early Tem)	Macro_OLFML3	HLA-B	LILRB1	2,88276E-03	33877,8	3,46700E-03	39879	1,15060E+00	17500	1,48246E+00	39665	9,41944E-01	10445	0	61900
Myeloid_vs_CD8_Non-responder	CD8(ZNF683+KLRB1+ T)	Macro_ISG15	CCL5	CCR1	2,89399E-03	26128	1,30058E-02	5061	1,52242E+00	7225	1,47652E+00	39910	9,32415E-01	16544	0	61900
Myeloid_vs_CD8_Non-responder	CD8(GZMK+ Tem)	Macro_ISG15	IFNG	IFNGR1_IFNGR2	2,91325E-03	35513,6	6,45348E-03	15742	9,31225E-01	27958	1,47530E+00	39963	9,07001E-01	32005	0	61900
Myeloid_vs_CD8_Non-responder	CD8(LAYN+ T)	Macro_FOLR2+APOE+	HLA-A	LILRB2	2,91471E-03	28622,8	3,46065E-03	39967	1,39962E+00	9656	1,82067E+00	27220	9,50397E-01	4371	0	61900
Myeloid_vs_CD8_Non-responder	CD8(LAYN+ T)	Macro_OLFML3	HLA-C	LILRB1	2,92055E-03	34170,6	3,49532E-03	39455	1,15712E+00	17263	1,47471E+00	39983	9,39360E-01	12252	0	61900
Myeloid_vs_CD8_Non-responder	CD8(Terminal Tex)	Macro_ISG15	HLA-C	LILRB2	2,92201E-03	29469	3,45966E-03	39987	1,34071E+00	11175	1,73687E+00	29879	9,50307E-01	4404	0	61900
Myeloid_vs_CD8_Non-responder	CD8(ITM2C+ T)	Macro_OLFML3	HLA-A	LILRB1	2,92420E-03	33764	3,57301E-03	38184	1,16478E+00	16978	1,47450E+00	39993	9,40082E-01	11765	0	61900
Myeloid_vs_CD8_Non-responder	CD8(IL7R+ZNF683+ Tm)	Mono_CD16	HLA-B	LILRB1	2,92823E-03	32736,4	3,71258E-03	35992	1,17231E+00	16703	1,47422E+00	40004	9,43788E-01	9083	0	61900
Myeloid_vs_CD8_Non-responder	CD8(EOMES+ NK-like)	Macro_FOLR2+APOE+	TGFB1	ENG	2,93482E-03	36691,2	4,87295E-03	23911	1,07108E+00	20918	1,55376E+00	36705	8,95090E-01	40022	0	61900
Myeloid_vs_CD8_Non-responder	CD8(Tn)	Macro_ISG15	B2M	LILRB2	2,93556E-03	28913,2	3,45734E-03	40024	1,27801E+00	13039	1,79010E+00	28176	9,57110E-01	1427	0	61900
Myeloid_vs_CD8_Non-responder	CD8(EOMES+ NK-like)	Mono_CD16	HLA-C	LILRB1	2,94290E-03	33503	3,67348E-03	36564	1,14471E+00	17742	1,47333E+00	40044	9,40761E-01	11265	0	61900
Myeloid_vs_CD8_Non-responder	CD8(GZMK+ Tex)	Mono_CD16	HLA-B	LILRA1	2,94904E-03	25756,8	9,41338E-03	8839	1,75730E+00	4333	1,47060E+00	40174	9,37515E-01	13538	0	61900
Myeloid_vs_CD8_Non-responder	CD8(Tn)	cDC_CLEC9A	ANXA1	DYSF	2,95173E-03	29199,8	1,35835E-02	4655	1,66341E+00	5235	1,61806E+00	34141	8,95033E-01	40068	0	61900
Myeloid_vs_CD8_Non-responder	CD8(Tn)	Macro_FOLR2+APOE+	CD99	CD81	2,95173E-03	37806,2	3,45504E-03	40068	9,17557E-01	28772	1,57544E+00	35805	9,22028E-01	22486	0	61900
Myeloid_vs_CD8_Non-responder	CD8(Temra)	cDC(CD1C)	SPON2	ITGB2	2,95209E-03	36203,6	6,57743E-03	15297	9,55547E-01	26540	1,47277E+00	40069	8,99243E-01	37212	0	61900
Myeloid_vs_CD8_Non-responder	CD8(GZMK+ Early Tem)	Macro_FOLR2+APOE+	HLA-B	LILRB2	2,95762E-03	27569	3,45397E-03	40084	1,46542E+00	8250	1,91791E+00	24326	9,52595E-01	3285	0	61900
Myeloid_vs_CD8_Non-responder	CD8(GZMK+ Tex)	Mono_CD16	HLA-C	LILRA1	2,95820E-03	27521,6	9,12045E-03	9317	1,66552E+00	5200	1,35796E+00	45244	9,33525E-01	15947	0	61900
Myeloid_vs_CD8_Non-responder	Macro_FOLR2+APOE+	CD8(ID2+CXCR4+ T)	B2M	CD3D	2,96128E-03	38588,4	2,77130E-03	54869	9,08201E-01	29354	1,32393E+00	46798	9,65150E-01	21	0	61900
Myeloid_vs_CD8_Non-responder	cDC_CLEC9A	CD8(LAYN+ T)	HLA-DQB1	LAG3	2,96431E-03	23235,4	5,71139E-03	18913	1,29285E+00	12626	2,56175E+00	10730	9,39745E-01	12008	0	61900
Myeloid_vs_CD8_Non-responder	CD8(ITM2C+ T)	Macro_ISG15	CD99	PILRA	2,96575E-03	35904,8	3,45275E-03	40106	1,08664E+00	20238	1,65020E+00	32934	9,18942E-01	24346	0	61900
Myeloid_vs_CD8_Non-responder	CD8(Tc17)	Macro_IER3	RPS19	C5AR1	2,96723E-03	31771,2	3,99698E-03	32293	1,21254E+00	15229	1,47190E+00	40110	9,43454E-01	9324	0	61900
Myeloid_vs_CD8_Non-responder	CD8(ZNF683+KLRB1+ T)	cDC_CLEC9A	SPON2	ITGB2	2,96871E-03	35608,6	5,99455E-03	17607	8,95355E-01	30164	1,78751E+00	28258	8,94961E-01	40114	0	61900
Myeloid_vs_CD8_Non-responder	CD8(GZMK+ Early Tem)	Macro_ISG15	CIRBP	TREM1	2,97463E-03	36498	4,76788E-03	24708	1,06550E+00	21181	1,60732E+00	34571	8,94933E-01	40130	0	61900
Myeloid_vs_CD8_Non-responder	Mono_CD14	CD8(GZMK+ Early Tem)	S100A8	ITGB2	2,97474E-03	18771	5,70632E-03	18930	2,45242E+00	955	3,43746E+00	2703	9,43409E-01	9367	0	61900
Myeloid_vs_CD8_Non-responder	Macro_FOLR2+APOE+	CD8(GZMK+ Tex)	MRC1	PTPRC	2,98428E-03	36167,6	5,75022E-03	18731	1,04444E+00	22170	1,52472E+00	37881	8,94901E-01	40156	0	61900
Myeloid_vs_CD8_Non-responder	CD8(ID2+CXCR4+ T)	Macro_IER3	RPS19	C5AR1	2,98428E-03	29083	4,59384E-03	26113	1,35738E+00	10736	1,47084E+00	40156	9,47054E-01	6510	0	61900
Myeloid_vs_CD8_Non-responder	Macro_FOLR2+APOE+	CD8(Terminal Tex)	CD14	ITGA4	2,98540E-03	30814,2	3,44920E-03	40159	1,36870E+00	10422	2,39962E+00	13327	9,12735E-01	28263	0	61900
Myeloid_vs_CD8_Non-responder	CD8(NME1+ T)	Macro_FOLR2+APOE+	HMGB1	THBD	2,99209E-03	39751,8	3,44810E-03	40177	1,00761E+00	23837	1,61806E+00	34142	8,97056E-01	38703	0	61900
Myeloid_vs_CD8_Non-responder	Macro_NLRP3	CD8(NME1+ T)	S100A8	CD69	2,99813E-03	22354,8	6,53640E-03	15448	1,80323E+00	3924	2,12213E+00	18968	9,40398E-01	11534	0	61900
Myeloid_vs_CD8_Non-responder	pDC_LILRA4	CD8(EOMES+ NK-like)	B2M	KLRD1	3,00184E-03	23791,4	5,96785E-03	17720	1,11569E+00	18974	2,13201E+00	18743	9,56543E-01	1620	0	61900
Myeloid_vs_CD8_Non-responder	CD8(NME1+ T)	Macro_OLFML3	HMGB1	CD163	3,00777E-03	32833,8	3,44504E-03	40219	1,14130E+00	17889	1,84844E+00	26344	9,30052E-01	17817	0	61900
Myeloid_vs_CD8_Non-responder	CD8(NME1+ T)	Macro_FOLR2+APOE+	ADAM10	GPNMB	3,01112E-03	35136	5,69173E-03	18989	1,56973E+00	6515	2,48603E+00	11848	8,39806E-01	76428	0	61900
Myeloid_vs_CD8_Non-responder	CD8(Temra)	Macro_ISG15	HLA-F	LILRB2	3,03253E-03	33110,2	3,44157E-03	40285	1,45108E+00	8540	2,04725E+00	20810	9,03993E-01	34016	0	61900
Myeloid_vs_CD8_Non-responder	Macro_FOLR2+APOE+	CD8(Tn)	CD14	ITGA4	3,03290E-03	30504,2	3,44156E-03	40286	1,36782E+00	10448	2,50405E+00	11567	9,12647E-01	28320	0	61900
Myeloid_vs_CD8_Non-responder	Macro_LYVE1	CD8(LAYN+ T)	CD14	ITGB1	3,04346E-03	24103,6	6,12473E-03	17017	1,61669E+00	5832	2,11841E+00	19041	9,32061E-01	16728	0	61900
Myeloid_vs_CD8_Non-responder	CD8(Tc17)	pDC_LILRA4	LTB	TNFRSF1A	3,04497E-03	31317	6,07576E-03	17253	1,01567E+00	23444	2,38066E+00	13670	8,94640E-01	40318	0	61900
Myeloid_vs_CD8_Non-responder	CD8(ID2+CXCR4+ T)	Macro_FOLR2+APOE+	CALR	LRP1	3,04610E-03	38308,2	3,43963E-03	40321	1,03974E+00	22363	1,54548E+00	37025	9,10092E-01	29932	0	61900
Myeloid_vs_CD8_Non-responder	CD8(ITM2C+ T)	Mono_CD16	HLA-F	LILRB1	3,04950E-03	35267,8	4,30813E-03	28839	1,20246E+00	15592	1,74296E+00	29678	8,94626E-01	40330	0	61900
Myeloid_vs_CD8_Non-responder	CD8(EOMES+ NK-like)	Macro_FOLR2+APOE+	HLA-A	LILRB2	3,05253E-03	28821,4	3,43868E-03	40338	1,38185E+00	10106	1,81740E+00	27319	9,50246E-01	4444	0	61900
Myeloid_vs_CD8_Non-responder	CD8(GZMK+ Tex)	Macro_ISG15	CIRBP	TREM1	3,05442E-03	36760,2	4,73415E-03	24996	1,06047E+00	21416	1,59166E+00	35146	8,94599E-01	40343	0	61900
Myeloid_vs_CD8_Non-responder	CD8(GZMK+ Tem)	Macro_ISG15	HLA-F	LILRB2	3,05442E-03	33343	3,43860E-03	40343	1,45069E+00	8550	2,00487E+00	21886	9,03956E-01	34036	0	61900
Myeloid_vs_CD8_Non-responder	CD8(ZNF683+KLRB1+ T)	Macro_FOLR2+APOE+	HLA-A	LILRB1	3,06541E-03	32858,4	3,43700E-03	40372	1,22646E+00	14712	1,60211E+00	34773	9,38980E-01	12535	0	61900
Myeloid_vs_CD8_Non-responder	Mono_INHBA	CD8(Tc17)	S100A8	CD69	3,06788E-03	19903	9,47089E-03	8740	1,66512E+00	5210	2,11647E+00	19080	9,49981E-01	4585	0	61900
Myeloid_vs_CD8_Non-responder	cDC(CD1C)	CD8(ID2+CXCR4+ T)	HLA-DQB1	LAG3	3,06788E-03	25238,2	5,67359E-03	19080	1,24317E+00	14145	2,12330E+00	18937	9,39557E-01	12129	0	61900
Myeloid_vs_CD8_Non-responder	CD8(Tn)	Macro_OLFML3	LTB	TNFRSF1A	3,07606E-03	37116	6,05896E-03	17326	9,62162E-01	26168	1,47906E+00	39786	8,94509E-01	40400	0	61900
Myeloid_vs_CD8_Non-responder	CD8(NME1+ T)	Mono_CD16	CD52	SIGLEC10	3,08482E-03	34569,4	4,76553E-03	24728	1,09471E+00	19896	1,46499E+00	40423	9,16580E-01	25900	0	61900
Myeloid_vs_CD8_Non-responder	CD8(LAYN+ T)	pDC_LILRA4	GZMB	IGF2R	3,08711E-03	36094,2	8,46025E-03	10474	8,78455E-01	31224	1,56013E+00	36444	8,94467E-01	40429	0	61900
Myeloid_vs_CD8_Non-responder	CD8(Tn)	Macro_ISG15	HLA-B	LILRB1	3,09170E-03	32915	3,85112E-03	34095	1,09588E+00	19843	1,46437E+00	40441	9,44751E-01	8296	0	61900
Myeloid_vs_CD8_Non-responder	CD8(ZNF683+KLRB1+ T)	Macro_FOLR2+APOE+	HLA-B	LILRB2	3,09246E-03	27926,6	3,43321E-03	40443	1,44601E+00	8636	1,88406E+00	25310	9,52459E-01	3344	0	61900
Myeloid_vs_CD8_Non-responder	CD8(GZMK+ Early Tem)	Mono_CD16	HLA-C	LILRB1	3,09629E-03	33326	3,70153E-03	36146	1,16444E+00	16996	1,46409E+00	40453	9,40972E-01	11135	0	61900
Myeloid_vs_CD8_Non-responder	CD8(ID2+CXCR4+ T)	Macro_FOLR2+APOE+	HLA-B	LILRB2	3,10510E-03	28025,8	3,43146E-03	40476	1,44438E+00	8669	1,86936E+00	25738	9,52447E-01	3346	0	61900
Myeloid_vs_CD8_Non-responder	CD8(ITM2C+ T)	Mono_CD16	IFNG	IFNGR1_IFNGR2	3,10587E-03	40012	4,98861E-03	23112	7,51040E-01	40478	1,59977E+00	34861	8,95559E-01	39709	0	61900
Myeloid_vs_CD8_Non-responder	CD8(ID2+CXCR4+ T)	Mono_CD16	HLA-B	LILRA1	3,10702E-03	25758,2	9,46197E-03	8759	1,77377E+00	4199	1,46358E+00	40481	9,37666E-01	13452	0	61900
Myeloid_vs_CD8_Non-responder	CD8(IL7R+ZNF683+ Tm)	Macro_FOLR2+APOE+	TGFB1	ENG	3,11201E-03	36641	4,80037E-03	24484	1,06232E+00	21330	1,59604E+00	34997	8,94383E-01	40494	0	61900
Myeloid_vs_CD8_Non-responder	CD8(Temra)	pDC_LILRA4	TGFB1	TGFBR1_TGFBR2	3,11509E-03	35431	5,04548E-03	22730	9,08949E-01	29309	1,97462E+00	22714	8,94376E-01	40502	0	61900

Myeloid_vs_CD8_Post_NR

Myeloid_vs_CD8_Non-responder	cDC_CLEC9A	CD8(LAYN+ T)	HLA-DQA1	LAG3	3,11842E-03	24186,8	5,65605E-03	19160	1,24059E+00	14220	2,45849E+00	12311	9,37813E-01	13343	0	61900
Myeloid_vs_CD8_Non-responder	CD8(ZNF683+KLRB1+ T)	Macro_OLFML3	HLA-F	LILRB1	3,13243E-03	35834,6	4,28013E-03	29126	1,14424E+00	17762	1,73820E+00	29838	8,94318E-01	40547	0	61900
Myeloid_vs_CD8_Non-responder	CD8(ID2+CXCR4+ T)	Macro_OLFML3	HLA-A	LILRB1	3,13243E-03	34204,2	3,54193E-03	38696	1,13975E+00	17940	1,46219E+00	40547	9,39836E-01	11938	0	61900
Myeloid_vs_CD8_Non-responder	CD8(Tc17)	Mono_CD16	HLA-C	LILRA1	3,13707E-03	26274	9,36728E-03	8914	1,73516E+00	4530	1,46196E+00	40559	9,34348E-01	15467	0	61900
Myeloid_vs_CD8_Non-responder	CD8(ZNF683+KLRB1+ T)	Macro_ISG15	HMGB1	THBD	3,15218E-03	41963,8	3,42510E-03	40598	8,72485E-01	31644	1,55262E+00	36765	8,96747E-01	38912	0	61900
Myeloid_vs_CD8_Non-responder	CD8(EOMES+ NK-like)	Macro_NLRP3	CIRBP	TREM1	3,15606E-03	38021,6	4,74540E-03	24898	1,03833E+00	22432	1,46080E+00	40608	8,94711E-01	40270	0	61900
Myeloid_vs_CD8_Non-responder	CD8(Terminal Tex)	Macro_ISG15	CCL5	CCRL2	3,15840E-03	29511,4	1,01971E-02	7744	1,19515E+00	15875	1,46075E+00	40614	9,23736E-01	21424	0	61900
Myeloid_vs_CD8_Non-responder	CD8(GZMK+ Tex)	pDC_LILRA4	HMGB1	TLR9	3,15917E-03	27418,2	3,60588E-02	659	2,26478E+00	1441	1,46070E+00	40616	9,06292E-01	32475	0	61900
Myeloid_vs_CD8_Non-responder	Macro_NLRP3	CD8(LAYN+ T)	VCAN	ITGB1	3,15930E-03	18756,2	1,40834E-02	4344	2,40858E+00	1041	2,83627E+00	7272	9,27580E-01	19224	0	61900
Myeloid_vs_CD8_Non-responder	Macro_FOLR2+APOE+	CD8(ID2+CXCR4+ T)	MMP9	CD44	3,16618E-03	27458,6	8,14983E-03	11089	1,40195E+00	9601	2,35762E+00	14069	8,94188E-01	40634	0	61900
Myeloid_vs_CD8_Non-responder	CD8(Temra)	Mono_CD16	ANXA1	FPR1	3,17398E-03	35460,8	3,80648E-03	34676	1,08599E+00	20260	1,45973E+00	40654	9,26525E-01	19814	0	61900
Myeloid_vs_CD8_Non-responder	Macro_NLRP3	CD8(GZMK+ Tem)	HBEGF	CD44	3,17537E-03	23379,8	6,58771E-03	15263	1,78062E+00	4145	2,11009E+00	19249	9,32794E-01	16342	0	61900
Myeloid_vs_CD8_Non-responder	CD8(ZNF683+KLRB1+ T)	Macro_ISG15	HMGB1	TLR2	3,18219E-03	38687,8	3,66503E-03	36698	9,77370E-01	25388	1,45920E+00	40675	9,11946E-01	28778	0	61900
Myeloid_vs_CD8_Non-responder	CD8(Tc17)	Macro_LYVE1	LTB	TNFRSF1A	3,19394E-03	31585	8,45247E-03	10489	1,23738E+00	14321	1,45867E+00	40705	9,09217E-01	30510	0	61900
Myeloid_vs_CD8_Non-responder	CD8(GZMK+ Tem)	Macro_IER3	RPS19	C5AR1	3,21084E-03	29449,6	4,53170E-03	26699	1,34230E+00	11137	1,45769E+00	40748	9,46712E-01	6764	0	61900
Myeloid_vs_CD8_Non-responder	Macro_FOLR2+APOE+	CD8(Terminal Tex)	B2M	KLRD1	3,21360E-03	34397,6	4,06170E-03	31560	8,70052E-01	31793	1,45749E+00	40755	9,47805E-01	5980	0	61900
Myeloid_vs_CD8_Non-responder	Macro_FOLR2+APOE+	CD8(ZNF683+KLRB1+ T)	HLA-C	CD8A	3,21834E-03	34517,4	3,41500E-03	40767	8,77383E-01	31298	1,51228E+00	38418	9,62082E-01	204	0	61900
Myeloid_vs_CD8_Non-responder	Macro_NLRP3	CD8(ISG+ T)	VCAN	SELL	3,22311E-03	25488,2	1,11453E-02	6677	2,22534E+00	1560	2,83837E+00	7256	8,80177E-01	50048	0	61900
Myeloid_vs_CD8_Non-responder	CD8(GZMK+ Early Tem)	Macro_ISG15	CD28	CD86	3,22396E-03	33141,4	1,17757E-02	6123,5	1,40835E+00	9451,5	2,10736E+00	19324,5	8,51404E-01	68907,5	0	61900
Myeloid_vs_CD8_Non-responder	CD8(NME1+ T)	Macro_ISG15	HLA-B	LILRB1	3,22743E-03	32957,4	3,85384E-03	34066	1,09794E+00	19750	1,45675E+00	40790	9,44770E-01	8281	0	61900
Myeloid_vs_CD8_Non-responder	Macro_FOLR2+APOE+	CD8(Tc17)	C1QB	C1QBP	3,22901E-03	28014,4	3,41394E-03	40794	1,60604E+00	5951	3,29639E+00	3468	9,13226E-01	27959	0	61900
Myeloid_vs_CD8_Non-responder	CD8(GZMK+ Tex)	Macro_FOLR2+APOE+	HLA-B	LILRB2	3,23020E-03	28132,2	3,41384E-03	40797	1,42791E+00	9018	1,87638E+00	25541	9,52331E-01	3405	0	61900
Myeloid_vs_CD8_Non-responder	Macro_FOLR2+APOE+	CD8(GZMK+ Tem)	HBEGF	CD44	3,23138E-03	39361,8	3,41360E-03	40800	9,44149E-01	27192	1,56339E+00	36300	9,09018E-01	30617	0	61900
Myeloid_vs_CD8_Non-responder	CD8(Temra)	Macro_NLRP3	HMGB1	THBD	3,23218E-03	36861,2	4,26010E-03	29371	1,09583E+00	19847	1,45654E+00	40802	9,06419E-01	32386	0	61900
Myeloid_vs_CD8_Non-responder	CD8(GZMK+ Early Tem)	cDC_LAMP3	CD28	CD80	3,23356E-03	46223	2,76101E-02	1142,5	1,60171E+00	6020,5	1,15000E+00	55675,5	7,90669E-01	106376,5	0	61900
Myeloid_vs_CD8_Non-responder	CD8(IL7R+ZNF683+ Tm)	Macro_FOLR2+APOE+	HLA-A	LILRB2	3,23535E-03	29219,6	3,41263E-03	40810	1,36079E+00	10652	1,78951E+00	28197	9,50066E-01	4539	0	61900
Myeloid_vs_CD8_Non-responder	CD8(Temra)	Mono_CD16	HLA-B	LILRB1	3,23574E-03	33044,6	3,69817E-03	36183	1,16013E+00	17166	1,45633E+00	40811	9,43684E-01	9163	0	61900
Myeloid_vs_CD8_Non-responder	pDC_LILRA4	CD8(ID2+CXCR4+ T)	B2M	CD3D	3,24324E-03	36256,8	2,75788E-03	55220	8,85213E-01	30783	1,63971E+00	33358	9,65068E-01	23	0	61900
Myeloid_vs_CD8_Non-responder	CD8(GZMK+ Early Tem)	cDC_LAMP3	TNFSF10	TNFRSF11B	3,24403E-03	61417,6	1,10581E-02	6782	2,11602E+00	1976	7,91761E-01	76275	6,56767E-01	160155	0	61900
Myeloid_vs_CD8_Non-responder	CD8(IL7R+ZNF683+ Tm)	Mono_CD16	HLA-A	LILRB1	3,24527E-03	33151,8	3,77503E-03	35129	1,15909E+00	17197	1,45565E+00	40835	9,41613E-01	10698	0	61900
Myeloid_vs_CD8_Non-responder	Macro_FOLR2+APOE+	CD8(ISG+ T)	CXCL16	CXCR6	3,24924E-03	33717,4	4,88523E-03	23831	1,14295E+00	17817	1,92195E+00	24194	8,93884E-01	40845	0	61900
Myeloid_vs_CD8_Non-responder	CD8(GZMK+ Early Tem)	cDC_LAMP3	CD200R1	CD200	3,25189E-03	45627,6	4,33755E-02	457,5	2,09918E+00	2055,5	1,53375E+00	37491,5	7,52614E-01	126233,5	0	61900
Myeloid_vs_CD8_Non-responder	CD8(Temra)	pDC_LILRA4	CCL5	CXCR3	3,25362E-03	30518,2	9,74090E-03	8329	8,58946E-01	32535	1,45529E+00	40856	9,43916E-01	8971	0	61900
Myeloid_vs_CD8_Non-responder	CD8(ZNF683+KLRB1+ T)	pDC_LILRA4	SIRPG	CD47	3,25800E-03	27955	1,14834E-02	6368,5	1,12432E+00	18611,5	2,47534E+00	12027,5	8,93854E-01	40867,5	0	61900
Myeloid_vs_CD8_Non-responder	CD8(Terminal Tex)	Macro_OLFML3	HSPA1A	TLR4	3,27198E-03	38256,2	4,52630E-03	26742	9,56979E-01	26452	1,45442E+00	40902	9,02084E-01	35285	0	61900
Myeloid_vs_CD8_Non-responder	CD8(GZMK+ Tex)	Macro_OLFML3	HLA-A	LILRB1	3,27518E-03	34196	3,54946E-03	38575	1,14582E+00	17692	1,45419E+00	40910	9,39896E-01	11903	0	61900
Myeloid_vs_CD8_Non-responder	CD8(NME1+ T)	Macro_FOLR2+APOE+	CALR	LRP1	3,27918E-03	39125,4	3,43451E-03	40414	1,03862E+00	22416	1,45392E+00	40920	9,10031E-01	29977	0	61900
Myeloid_vs_CD8_Non-responder	CD8(GZMK+ Early Tem)	Macro_OLFML3	HLA-A	LILRB1	3,28560E-03	34301,2	3,54033E-03	38723	1,13846E+00	18003	1,45351E+00	40936	9,39823E-01	11944	0	61900
Myeloid_vs_CD8_Non-responder	CD8(ID2+CXCR4+ T)	pDC_LILRA4	SELPLG	SELL	3,28640E-03	27737	6,89861E-03	14260	1,46868E+00	8177	2,39517E+00	13410	8,93776E-01	40938	0	61900
Myeloid_vs_CD8_Non-responder	CD8(GZMK+ Tem)	Macro_FOLR2+APOE+	HLA-B	LILRB2	3,28640E-03	28388,8	3,40661E-03	40938	1,42114E+00	9164	1,84283E+00	26511	9,52282E-01	3431	0	61900
Myeloid_vs_CD8_Non-responder	CD8(Tn)	pDC_LILRA4	TNF	TNFRSF21	3,28958E-03	32486,8	8,83974E-03	9797	2,04009E+00	2349	2,10289E+00	19424	8,51315E-01	68964	0	61900
Myeloid_vs_CD8_Non-responder	Macro_ISG15	CD8(GZMK+ Tem)	HLA-DRB1	LAG3	3,29819E-03	23068,8	5,60722E-03	19437	1,16639E+00	16904	2,38254E+00	13636	9,52208E-01	3467	0	61900
Myeloid_vs_CD8_Non-responder	CD8(Terminal Tex)	Mono_CD14	ANXA1	FPR1	3,30370E-03	34351	3,99758E-03	32288	1,14590E+00	17690	1,45231E+00	40981	9,28175E-01	18896	0	61900
Myeloid_vs_CD8_Non-responder	CD8(NME1+ T)	Mono_CD14	HMGB1	TLR4	3,30814E-03	35341,8	5,08464E-03	22456	1,22955E+00	14600	1,45212E+00	40992	8,99919E-01	36761	0	61900
Myeloid_vs_CD8_Non-responder	Macro_FOLR2+APOE+	CD8(EOMES+ NK-like)	LGALS9	PTPRC	3,30935E-03	34272,4	3,40296E-03	40995	1,07807E+00	20606	1,65126E+00	32907	9,35271E-01	14954	0	61900
Myeloid_vs_CD8_Non-responder	Macro_OLFML3	CD8(ISG+ T)	CXCL9	CXCR3	3,31262E-03	24470,8	1,68583E-02	2982	2,24207E+00	1509	2,83064E+00	7324	8,82344E-01	48639	0	61900
Myeloid_vs_CD8_Non-responder	Macro_NLRP3	CD8(LAYN+ T)	HBEGF	CD44	3,31943E-03	23267,4	6,69120E-03	14893	1,79414E+00	4012	2,10099E+00	19469	9,33281E-01	16063	0	61900
Myeloid_vs_CD8_Non-responder	CD8(NME1+ T)	Mono_CD14	HMGB1	THBD	3,31945E-03	40713,8	3,40129E-03	41020	9,99096E-01	24271	1,54034E+00	37253	8,96423E-01	39125	0	61900
Myeloid_vs_CD8_Non-responder	pDC_LILRA4	CD8(GZMK+ Early Tem)	APP	CD74	3,32143E-03	20551,4	6,27987E-03	16402	2,17481E+00	1750	2,10079E+00	19472	9,52703E-01	3233	0	61900
Myeloid_vs_CD8_Non-responder	CD8(GZMK+ Early Tem)	Mono_CD16	HLA-F	LILRB1	3,32147E-03	36158,2	4,22025E-03	29789	1,19066E+00	16033	1,67397E+00	32044	8,93650E-01	41025	0	61900
Myeloid_vs_CD8_Non-responder	cDC(CD1C)	CD8(ISG+ T)	HLA-DQB1	LAG3	3,32609E-03	25078,2	5,60149E-03	19479	1,23610E+00	14369	2,19932E+00	17264	9,39192E-01	12379	0	61900
Myeloid_vs_CD8_Non-responder	CD8(EOMES+ NK-like)	Mono_CD16	HLA-C	LILRA3	3,32836E-03	27939,4	1,08382E-02	7043	1,48218E+00	7935	1,45049E+00	41042	9,23178E-01	21777	0	61900
Myeloid_vs_CD8_Non-responder	Macro_NLRP3	CD8(GZMK+ Early Tem)	HBEGF	CD44	3,33010E-03	23589	6,47784E-03	15665	1,76627E+00	4274	2,10008E+00	19485	9,32265E-01	16621	0	61900
Myeloid_vs_CD8_Non-responder	CD8(ZNF683+KLRB1+ T)	Macro_ISG15	LTB	TNFRSF1A	3,33810E-03	35896,2	5,94370E-03	17834	9,69180E-01	25798	1,65188E+00	32883	8,93600E-01	41066	0	61900
Myeloid_vs_CD8_Non-responder	CD8(ID2+CXCR4+ T)	Macro_FOLR2+APOE+	HLA-C	LILRB2	3,33892E-03	28661,2	3,39860E-03	41068	1,40691E+00	9483	1,85250E+00	26218	9,49885E-01	4637	0	61900
Myeloid_vs_CD8_Non-responder	CD8(Tc17)	Macro_FOLR2+APOE+	HLA-A	LILRB2	3,33932E-03	29399,2	3,39851E-03	41069	1,34937E+00	10938	1,78053E+00	28494	9,49968E-01	4595	0	61900
Myeloid_vs_CD8_Non-responder	Mono_CD14	CD8(GZMK+ Tem)	S100A9	ITGB2	3,34325E-03	19320,8	5,13687E-03	22105	2,29295E+00	1352	3,23189E+00	3900	9,46001E-01	7347	0	61900
Myeloid_vs_CD8_Non-responder	CD8(Tc17)	Macro_FOLR2+APOE+	HLA-C	LILRB2	3,34991E-03	28586	3,39712E-03	41095	1,40576E+00	9509	1,86775E+00	25783	9,49875E-01	4643	0	61900
Myeloid_vs_CD8_Non-responder	CD8(ISG+ T)	Mono_CD16	HLA-F	LILRB1	3,35031E-03	36527	4,21183E-03	29869	1,18953E+00	16069	1,62902E+00	33701	8,93555E-01	41096	0	61900
Myeloid_vs_CD8_Non-responder	CD8(Terminal Tex)	Mono_CD16	B2M	LILRB1	3,35276E-03	32439,4	3,64869E-03	36990	1,16910E+00	16803	1,44919E+00	41102	9,48652E-01	5402	0	61900
Myeloid_vs_CD8_Non-responder	Macro_NLRP3	CD8(Tn)	CXCL2	DPP4	3,35395E-03	34520,8	1,41662E-02	4297	1,85159E+00	3535	2,82757E+00	7355	8,08996E-01	95517	0	61900
Myeloid_vs_CD8_Non-responder	Mono_CD14	CD8(NME1+ T)	VCAN	CD44	3,35954E-03	22963	6,96766E-03	14053	2,24957E+00	1489	2,09820E+00	19529	9,30011E-01	17844	0	61900
Myeloid_vs_CD8_Non-responder	Macro_FOLR2+APOE+	CD8(ITM2C+ T)	APOE	LDLR	3,36052E-03	24418,8	1,09516E-02	6908	1,45111E+00	8538	3,27193E+00	3627	8,93529E-01	41121	0	61900
Myeloid_vs_CD8_Non-responder	Macro_ISG15	CD8(Tn)	CCL2	CCR4	3,36333E-03	40694,2	4,22231E-02	486	1,51429E+00	7362	3,18611E+00	4198	7,45452E-01	129525	0	61900
Myeloid_vs_CD8_Non-responder	CD8(ZNF683+KLRB1+ T)	Macro_OLFML3	HLA-B	LILRB1	3,36379E-03	34418,4	3,44616E-03	40201	1,13119E+00	18306	1,44861E+00	41129	9,41779E-01	10556	0	61900
Myeloid_vs_CD8_Non-responder	Mono_INHBA	CD8(ZNF683+KLRB1+ T)	S100A8	CD69	3,37502E-03	19755,2	9,78486E-03	8267	1,69954E+00	4860	2,09720E+00	19552	9,50750E-01	4197	0	61900
Myeloid_vs_CD8_Non-responder	CD8(ID2+CXCR4+ T)	cDC_CLEC9A	HMGB1	THBD	3,39704E-03	36441,6	4,40975E-03	27830	1,09527E+00	19869	1,44695E+00	41210	9,07873E-01	31399	0	61900
Myeloid_vs_CD8_Non-responder	Macro_FOLR2+APOE+	CD8(GZMK+ Tex)	LGALS9	HAVCR2	3,39869E-03	30975,2	4,56596E-03	26366	1,33441E+00	11363	2,35965E+00	14033	8,93376E-01	41214	0	61900
Myeloid_vs_CD8_Non-responder	CD8(ID2+CXCR4+ T)	Mono_CD16	HLA-C	LILRA1	3,40282E-03	26402,8	9,37135E-03	8910	1,73631E+00	4522	1,44672E+00	41224	9,34362E-01	15458	0	61900
Myeloid_vs_CD8_Non-responder	CD8(Tc17)	Mono_CD16	HLA-A	LILRB1	3,40323E-03	33365,4	3,75940E-03	35332	1,14767E+00	17602	1,44666E+00	41225	9,41499E-01	10768	0	61900
Myeloid_vs_CD8_Non-responder	Macro_FOLR2+APOE+	CD8(ZNF683+KLRB1+ T)	CXCL9	CXCR3	3,41320E-03	28025,2	1,14579E-02	6392	2,22627E+00	1558	2,82500E+00	7399	8,60775E-01	62877	0	61900
Myeloid_vs_CD8_Non-responder	Macro_IER3	CD8(LAYN+ T)	CD14	ITGA4	3,41494E-03	25287,2	5,57419E-03	19611	1,46441E+00	8271	2,12808E+00	18838	9,30053E-01	17816	0	61900
Myeloid_vs_CD8_Non-responder	Macro_IFI27	CD8(ISG+ T)	C3	IFITM1	3,41997E-03	21671,2	1,16206E-02	6259	1,68085E+00	5038	1,80317E+00	27755	9,45951E-01	7404	0	61900
Myeloid_vs_CD8_Non-responder	Macro_LYVE1	CD8(GZMK+ Tem)	HLA-DRA	LAG3	3,42446E-03	21983	6,61764E-03	15155	1,34846E+00	10970	2,09385E+00	19625	9,55030E-01	2265	0	61900
Myeloid_vs_CD8_Non-responder	CD8(NME1+ T)	pDC_LILRA4	GZMB	IGF2R	3,42641E-03	34908,8	8,25392E-03	10884	8,65476E-01	32094	1,78393E+00	28385	8,93296E-01	41281	0	61900
Myeloid_vs_CD8_Non-responder	CD8(IL7R+ZNF683+ Tm)	Macro_LYVE1	HMGB1	CD163	3,42641E-03	31724,8	4,18194E-03	30211	1,35547E+00	10794	1,44519E+00	41281	9,36100E-01	14438	0	61900
Myeloid_vs_CD8_Non-responder	CD8(GZMK+ Tex)	Macro_ISG15	HMGB1	THBD	3,43264E-03	42833	3,38404E-03	41296	8,58991E-01	32533	1,49549E+00	39144	8,96187E-01	39292	0	61900

Myeloid_vs_CD8_Post_NR

Myeloid_vs_CD8_Non-responder	CD8(Tn)	Macro_ISG15	HLA-A	LILRB1	3,43846E-03	33548,4	3,87239E-03	33828	1,08007E+00	20515	1,44450E+00	41310	9,42309E-01	10189	0	61900
Myeloid_vs_CD8_Non-responder	CD8(IL7R+ZNF683+ Tm)	Macro_ISG15	CIRBP	TREM1	3,44262E-03	37349,4	4,60071E-03	26062	1,04056E+00	22321	1,59169E+00	35144	8,93244E-01	41320	0	61900
Myeloid_vs_CD8_Non-responder	Macro_FOLR2+APOE+	CD8(GZMK+ Tem)	CXCL16	CXCR6	3,44346E-03	33959,4	4,81971E-03	24310	1,13844E+00	18005	1,91972E+00	24260	8,93242E-01	41322	0	61900
Myeloid_vs_CD8_Non-responder	CD8(Terminal Tex)	Macro_FOLR2+APOE+	HLA-A	LILRB2	3,45221E-03	29978,2	3,38186E-03	41343	1,33592E+00	11317	1,71249E+00	30677	9,49851E-01	4654	0	61900
Myeloid_vs_CD8_Non-responder	Macro_OLFML3	CD8(Terminal Tex)	HLA-DPB1	LAG3	3,45247E-03	23590,6	5,56628E-03	19666	1,20650E+00	15440	2,29485E+00	15307	9,48273E-01	5640	0	61900
Myeloid_vs_CD8_Non-responder	Macro_FOLR2+APOE+	CD8(GZMK+ Early Tem)	MRC1	PTPRC	3,46225E-03	37253,6	5,65003E-03	19197	1,02326E+00	23091	1,44304E+00	41367	8,94071E-01	40713	0	61900
Myeloid_vs_CD8_Non-responder	CD8(NME1+ T)	cDC_CLEC9A	HMGB1	HAVCR2	3,46434E-03	37059,8	3,38031E-03	41372	9,51363E-01	26781	1,58282E+00	35515	9,26685E-01	19731	0	61900
Myeloid_vs_CD8_Non-responder	Mono_INHBA	CD8(Tn)	SPP1	CD44	3,46757E-03	22659,6	1,02540E-02	7685	1,37578E+00	10253	2,37434E+00	13772	9,26761E-01	19688	0	61900
Myeloid_vs_CD8_Non-responder	CD8(LAYN+ T)	Macro_IER3	RPS19	C5AR1	3,46937E-03	28377,6	4,86503E-03	23967	1,42319E+00	9122	1,44249E+00	41384	9,48474E-01	5515	0	61900
Myeloid_vs_CD8_Non-responder	CD8(Terminal Tex)	Macro_FOLR2+APOE+	CCL5	CCR1	3,47482E-03	29380	1,00877E-02	7871	1,23381E+00	14451	1,44222E+00	41397	9,23955E-01	21281	0	61900
Myeloid_vs_CD8_Non-responder	Macro_FOLR2+APOE+	CD8(Temra)	MMP9	CD44	3,48322E-03	27957,2	7,96460E-03	11503	1,38583E+00	10000	2,31289E+00	14966	8,93095E-01	41417	0	61900
Myeloid_vs_CD8_Non-responder	CD8(LAYN+ T)	Macro_ISG15	TIGIT	NECTIN2	3,48823E-03	26072	2,94649E-02	1002,5	2,24769E+00	1493,5	2,20093E+00	17225,5	8,82171E-01	48738,5	0	61900
Myeloid_vs_CD8_Non-responder	Macro_ISG15	CD8(LAYN+ T)	NECTIN2	TIGIT	3,49286E-03	26072	2,94649E-02	1002,5	2,24769E+00	1493,5	2,20093E+00	17225,5	8,82171E-01	48738,5	0	61900
Myeloid_vs_CD8_Non-responder	CD8(Tn)	cDC_CLEC9A	LTB	CD40	3,49375E-03	28002,4	1,03163E-02	7614	1,48177E+00	7943	2,03502E+00	21113	8,93061E-01	41442	0	61900
Myeloid_vs_CD8_Non-responder	CD8(GZMK+ Early Tem)	Mono_CD16	HLA-C	LILRA3	3,49628E-03	27894,4	1,09210E-02	6941	1,50191E+00	7575	1,44124E+00	41448	9,23448E-01	21608	0	61900
Myeloid_vs_CD8_Non-responder	cDC_CLEC9A	CD8(GZMK+ Tem)	HLA-DQA2	LAG3	3,49791E-03	22703,6	8,41202E-03	10583	1,32561E+00	11649	2,64245E+00	9654	9,26684E-01	19732	0	61900
Myeloid_vs_CD8_Non-responder	CD8(ID2+CXCR4+ T)	Macro_OLFML3	B2M	LILRB1	3,49839E-03	33844,8	3,37658E-03	41453	1,14019E+00	17927	1,44723E+00	41196	9,46731E-01	6748	0	61900
Myeloid_vs_CD8_Non-responder	CD8(GZMK+ Tex)	Macro_OLFML3	HLA-B	LILRB1	3,50261E-03	34735,2	3,42672E-03	40564	1,11309E+00	19064	1,44093E+00	41463	9,41624E-01	10685	0	61900
Myeloid_vs_CD8_Non-responder	Mono_CD14	CD8(GZMK+ Tem)	VCAN	ITGA4	3,50483E-03	19080,8	1,32823E-02	4867	2,60220E+00	717	2,75513E+00	8178	9,26670E-01	19742	0	61900
Myeloid_vs_CD8_Non-responder	Macro_FOLR2+APOE+	CD8(ZNF683+KLRB1+ T)	LGALS9	PTPRC	3,51487E-03	34428,2	3,37457E-03	41492	1,06928E+00	20999	1,65720E+00	32652	9,35017E-01	15098	0	61900
Myeloid_vs_CD8_Non-responder	CD8(ISG+ T)	Macro_OLFML3	HLA-A	LILRB1	3,51742E-03	34755	3,50969E-03	39233	1,11378E+00	19035	1,44027E+00	41498	9,39577E-01	12109	0	61900
Myeloid_vs_CD8_Non-responder	Macro_FOLR2+APOE+	CD8(ITM2C+ T)	MMP9	CD44	3,51869E-03	27847,8	7,94550E-03	11544	1,38417E+00	10042	2,34786E+00	14252	8,92980E-01	41501	0	61900
Myeloid_vs_CD8_Non-responder	Macro_ISG15	CD8(NME1+ T)	CXCL10	SDC4	3,51989E-03	37328	1,04445E-02	7477	2,01798E+00	2449	3,41511E+00	2813	7,80358E-01	112001	0	61900
Myeloid_vs_CD8_Non-responder	Macro_FOLR2+APOE+	CD8(ISG+ T)	CD59	CD2	3,52166E-03	35333,4	6,95054E-03	14106	9,02801E-01	29668	1,44005E+00	41508	9,10779E-01	29485	0	61900
Myeloid_vs_CD8_Non-responder	Macro_FOLR2+APOE+	CD8(GZMK+ Tex)	MMP9	CD44	3,52250E-03	27939,8	7,94363E-03	11550	1,38400E+00	10051	2,32547E+00	14688	8,92969E-01	41510	0	61900
Myeloid_vs_CD8_Non-responder	CD8(GZMK+ Tex)	Mono_CD16	HLA-F	LILRB1	3,52802E-03	36751,2	4,15875E-03	30461	1,18240E+00	16311	1,63340E+00	33561	8,92951E-01	41523	0	61900
Myeloid_vs_CD8_Non-responder	CD8(ITM2C+ T)	Macro_FOLR2+APOE+	HMGB1	CD163	3,54633E-03	32786,8	3,36975E-03	41566	1,12459E+00	18595	1,94105E+00	23650	9,29330E-01	18223	0	61900
Myeloid_vs_CD8_Non-responder	CD8(Tn)	Macro_ISG15	CD99	PILRA	3,54804E-03	37174,8	3,36936E-03	41570	1,06847E+00	21039	1,56156E+00	36375	9,18027E-01	24990	0	61900
Myeloid_vs_CD8_Non-responder	CD8(LAYN+ T)	Mono_CD16	HLA-B	LILRB1	3,55102E-03	33182,6	3,69953E-03	36167	1,16128E+00	17111	1,43837E+00	41577	9,43694E-01	9158	0	61900
Myeloid_vs_CD8_Non-responder	CD8(GZMK+ Tex)	pDC_LILRA4	COPA	P2RY6	3,55287E-03	27717,8	9,28377E-03	9028	2,68767E+00	595	3,11164E+00	4770	8,61636E-01	62296	0	61900
Myeloid_vs_CD8_Non-responder	CD8(Terminal Tex)	Macro_LYVE1	TNFSF9	HLA-DPA1	3,55957E-03	38642,8	3,36786E-03	41597	9,48982E-01	26928	1,68939E+00	31487	9,08024E-01	31302	0	61900
Myeloid_vs_CD8_Non-responder	CD8(NME1+ T)	Macro_ISG15	HLA-C	LILRB2	3,56171E-03	30021	3,36760E-03	41602	1,27657E+00	13082	1,77203E+00	28750	9,49666E-01	4771	0	61900
Myeloid_vs_CD8_Non-responder	CD8(ISG+ T)	Macro_ISG15	CIRBP	TREM1	3,56257E-03	37792,8	4,56303E-03	26392	1,03493E+00	22582	1,55897E+00	36486	8,92851E-01	41604	0	61900
Myeloid_vs_CD8_Non-responder	Macro_NLRP3	CD8(Terminal Tex)	CXCL2	DPP4	3,56289E-03	33873,6	1,50517E-02	3788	1,86502E+00	3443	2,81306E+00	7508	8,13636E-01	92729	0	61900
Myeloid_vs_CD8_Non-responder	Macro_ISG15	CD8(ZNF683+KLRB1+ T)	CXCL10	CXCR3	3,56428E-03	27956,8	1,04100E-02	7509	1,91031E+00	3085	3,13987E+00	4546	8,60965E-01	62744	0	61900
Myeloid_vs_CD8_Non-responder	cDC_CLEC9A	CD8(Terminal Tex)	HLA-DRB1	LAG3	3,57391E-03	21814,2	5,53368E-03	19841	1,22822E+00	14635	2,68504E+00	9092	9,51907E-01	3603	0	61900
Myeloid_vs_CD8_Non-responder	Macro_FOLR2+APOE+	CD8(Terminal Tex)	CXCL9	CXCR3	3,57823E-03	25565,2	1,48096E-02	3909	2,30023E+00	1323	2,81145E+00	7519	8,75451E-01	53175	0	61900
Myeloid_vs_CD8_Non-responder	CD8(IL7R+ZNF683+ Tm)	Macro_FOLR2+APOE+	HLA-C	LILRB2	3,57930E-03	28985,6	3,36594E-03	41643	1,38151E+00	10116	1,84369E+00	26487	9,49655E-01	4782	0	61900
Myeloid_vs_CD8_Non-responder	CD8(Terminal Tex)	cDC_LAMP3	TNFSF10	TNFRSF11B	3,58150E-03	52051,2	2,39107E-02	1513	2,26652E+00	1435	1,02949E+00	62384	7,37789E-01	133024	0	61900
Myeloid_vs_CD8_Non-responder	CD8(ISG+ T)	Mono_CD14	ANXA1	FPR1	3,58532E-03	36236,2	3,65594E-03	36849	1,08894E+00	20159	1,43671E+00	41657	9,25139E-01	20616	0	61900
Myeloid_vs_CD8_Non-responder	CD8(Tn)	Macro_ISG15	B2M	LILRB1	3,59005E-03	32979,8	3,81452E-03	34573	1,04772E+00	22025	1,43620E+00	41668	9,49724E-01	4733	0	61900
Myeloid_vs_CD8_Non-responder	CD8(EOMES+ NK-like)	Mono_CD16	HLA-B	LILRB1	3,59264E-03	33895,8	3,63781E-03	37155	1,10913E+00	19243	1,43602E+00	41674	9,43245E-01	9507	0	61900
Myeloid_vs_CD8_Non-responder	Macro_FOLR2+APOE+	CD8(IL7R+ZNF683+ Tm)	HBEGF	CD44	3,59436E-03	39515,4	3,36413E-03	41678	9,31683E-01	27936	1,59458E+00	35047	9,08412E-01	31016	0	61900
Myeloid_vs_CD8_Non-responder	CD8(ID2+CXCR4+ T)	Macro_FOLR2+APOE+	B2M	LILRB2	3,59609E-03	27801,6	3,36390E-03	41682	1,45501E+00	8457	1,88268E+00	25352	9,56544E-01	1617	0	61900
Myeloid_vs_CD8_Non-responder	Macro_OLFML3	CD8(GZMK+ Tex)	HLA-DPB1	LAG3	3,60352E-03	23600,8	5,52582E-03	19883	1,20134E+00	15640	2,31952E+00	14813	9,48094E-01	5768	0	61900
Myeloid_vs_CD8_Non-responder	CD8(Temra)	Macro_FOLR2+APOE+	HLA-A	LILRB2	3,62031E-03	29892,6	3,36120E-03	41738	1,31921E+00	11838	1,75567E+00	29243	9,49705E-01	4744	0	61900
Myeloid_vs_CD8_Non-responder	CD8(GZMK+ Tex)	pDC_LILRA4	SIRPG	CD47	3,62193E-03	29117	1,08357E-02	7046,5	1,09430E+00	19909,5	2,36738E+00	13905,5	8,91068E-01	42823,5	0	61900
Myeloid_vs_CD8_Non-responder	CD8(ID2+CXCR4+ T)	Macro_OLFML3	HLA-B	LILRB1	3,63030E-03	34567,2	3,44440E-03	40232	1,12956E+00	18376	1,43391E+00	41761	9,41765E-01	10567	0	61900
Myeloid_vs_CD8_Non-responder	CD8(ZNF683+KLRB1+ T)	Macro_ISG15	SPON2	ITGB2	3,63204E-03	36845,6	5,70660E-03	18928	8,46347E-01	33385	1,78762E+00	28250	8,92625E-01	41765	0	61900
Myeloid_vs_CD8_Non-responder	CD8(IL7R+ZNF683+ Tm)	Mono_CD16	B2M	LILRB1	3,63595E-03	32978,4	3,61833E-03	37491	1,13380E+00	18195	1,43342E+00	41774	9,48448E-01	5532	0	61900
Myeloid_vs_CD8_Non-responder	CD8(ITM2C+ T)	Mono_CD16	ANXA1	FPR1	3,64554E-03	37369,2	3,35871E-03	41796	1,00779E+00	23828	1,54869E+00	36920	9,22149E-01	22402	0	61900
Myeloid_vs_CD8_Non-responder	CD8(GZMK+ Early Tem)	Macro_FOLR2+APOE+	CD28	CD86	3,64755E-03	33191,4	1,18321E-02	6083,5	1,41482E+00	9316,5	2,08166E+00	19945,5	8,51706E-01	68711,5	0	61900
Myeloid_vs_CD8_Non-responder	Mono_CD14	CD8(GZMK+ Tex)	S100A8	ITGB2	3,65255E-03	19134	5,51263E-03	19952	2,43528E+00	989	3,42659E+00	2764	9,42480E-01	10065	0	61900
Myeloid_vs_CD8_Non-responder	CD8(Tc17)	Macro_OLFML3	HLA-C	LILRB1	3,65645E-03	35485,4	3,40993E-03	40859	1,09094E+00	20062	1,43230E+00	41821	9,38652E-01	12785	0	61900
Myeloid_vs_CD8_Non-responder	Mast	CD8(EOMES+ NK-like)	TIMP3	CD44	3,66173E-03	33589,6	2,54793E-02	1335	2,23614E+00	1530	1,02688E+00	62512	8,94132E-01	40671	0	61900
Myeloid_vs_CD8_Non-responder	Macro_FOLR2+APOE+	CD8(GZMK+ Early Tem)	HBEGF	CD44	3,66477E-03	39919,4	3,35667E-03	41840	9,29801E-01	28044	1,55339E+00	36722	9,08320E-01	31091	0	61900
Myeloid_vs_CD8_Non-responder	Macro_FOLR2+APOE+	CD8(LAYN+ T)	B2M	CD3D	3,66614E-03	39492,6	2,73048E-03	56046	8,93932E-01	30246	1,27427E+00	49245	9,64900E-01	26	0	61900
Myeloid_vs_CD8_Non-responder	CD8(GZMK+ Early Tem)	Mono_CD16	TNFSF9	HLA-DPA1	3,66652E-03	39013,4	4,14571E-03	30614	7,34976E-01	41844	1,60529E+00	34648	9,16341E-01	26061	0	61900
Myeloid_vs_CD8_Non-responder	Macro_FOLR2+APOE+	CD8(ZNF683+KLRB1+ T)	HLA-A	CD8A	3,66783E-03	35375	3,44217E-03	40272	8,56667E-01	32665	1,43190E+00	41847	9,62290E-01	191	0	61900
Myeloid_vs_CD8_Non-responder	CD8(IL7R+ZNF683+ Tm)	Macro_FOLR2+APOE+	HLA-B	LILRB2	3,67002E-03	29045,6	3,35619E-03	41852	1,37402E+00	10296	1,80808E+00	27593	9,51942E-01	3587	0	61900
Myeloid_vs_CD8_Non-responder	Macro_ISG15	CD8(GZMK+ Tex)	CXCL10	CXCR3	3,67259E-03	28117,8	1,03409E-02	7586	1,90863E+00	3099	3,08294E+00	4992	8,60566E-01	63012	0	61900
Myeloid_vs_CD8_Non-responder	CD8(ZNF683+KLRB1+ T)	Macro_FOLR2+APOE+	B2M	TFRC	3,67309E-03	33640,8	3,35583E-03	41859	9,88309E-01	24804	1,63964E+00	33363	9,47394E-01	6278	0	61900
Myeloid_vs_CD8_Non-responder	CD8(Tn)	Macro_OLFML3	CD40LG	CD40	3,67617E-03	34708,2	3,69209E-02	627	1,60232E+00	6007	2,08038E+00	19985	8,26069E-01	85022	0	61900
Myeloid_vs_CD8_Non-responder	CD8(ISG+ T)	Macro_FOLR2+APOE+	TNFSF9	HLA-DPA1	3,68320E-03	34645	3,35460E-03	41882	9,88081E-01	24813	2,40520E+00	13216	9,07859E-01	31414	0	61900
Myeloid_vs_CD8_Non-responder	CD8(LAYN+ T)	Macro_FOLR2+APOE+	ANXA1	FPR1	3,68452E-03	39084	3,35444E-03	41885	8,96547E-01	30084	1,49626E+00	39115	9,22104E-01	22436	0	61900
Myeloid_vs_CD8_Non-responder	Macro_ISG15	CD8(Terminal Tex)	HLA-DRA	LAG3	3,71001E-03	23019,2	5,49762E-03	20032	1,17520E+00	16591	2,45107E+00	12450	9,50876E-01	4123	0	61900
Myeloid_vs_CD8_Non-responder	CD8(GZMK+ Tex)	Mono_CD16	HLA-C	LILRB1	3,71098E-03	34032,2	3,65885E-03	36802	1,13442E+00	18171	1,42988E+00	41945	9,40649E-01	11343	0	61900
Myeloid_vs_CD8_Non-responder	CD8(ISG+ T)	Macro_OLFML3	B2M	LILRB1	3,71364E-03	34361,6	3,35054E-03	41951	1,10682E+00	19335	1,43508E+00	41708	9,46535E-01	6914	0	61900
Myeloid_vs_CD8_Non-responder	cDC_CLEC9A	CD8(GZMK+ Tex)	HLA-DRB1	LAG3	3,72665E-03	21847,4	5,49345E-03	20055	1,22306E+00	14847	2,70971E+00	8755	9,51739E-01	3680	0	61900
Myeloid_vs_CD8_Non-responder	Macro_ISG15	CD8(Terminal Tex)	LGALS3	LAG3	3,73391E-03	26056,2	6,62224E-03	15140	1,09088E+00	20065	2,19666E+00	17312	9,33657E-01	15864	0	61900
Myeloid_vs_CD8_Non-responder	CD8(EOMES+ NK-like)	Macro_ISG15	HLA-F	LILRB1	3,74339E-03	35570	4,09798E-03	31144	1,25707E+00	13690	1,76057E+00	29098	8,92245E-01	42018	0	61900
Myeloid_vs_CD8_Non-responder	CD8(ITM2C+ T)	Macro_IER3	RPS19	C5AR1	3,74695E-03	29238,4	4,65444E-03	25637	1,37208E+00	10342	1,42783E+00	42026	9,47382E-01	6287	0	61900
Myeloid_vs_CD8_Non-responder	CD8(ZNF683+KLRB1+ T)	Macro_NLRP3	SPON2	ITGB2	3,74829E-03	38348,8	5,66051E-03	19143	8,38502E-01	33891	1,60189E+00	34781	8,92235E-01	42029	0	61900
Myeloid_vs_CD8_Non-responder	CD8(GZMK+ Early Tem)	Macro_FOLR2+APOE+	HLA-C	LILRB2	3,74873E-03	29443,8	3,34620E-03	42030	1,36615E+00	10502	1,79795E+00	27925	9,49514E-01	4862	0	61900
Myeloid_vs_CD8_Non-responder	Macro_FOLR2+APOE+	CD8(EOMES+ NK-like)	SPP1	CD44	3,75186E-03	34801,4	5,00605E-03	22988	7,32785E-01	42037	2,66910E+00	9308	8,98389E-01	37774	0	61900
Myeloid_vs_CD8_Non-responder	Macro_FOLR2-APOE+	CD8(IL7R+ZNF683+ Tm)	APOE	LSR	3,75990E-03	21531	2,57130E-02	1310	1,75051E+00	4377	2,80054E+00	7647	9,06368E-01	32421	0	61900
Myeloid_vs_CD8_Non-responder	CD8(LAYN+ T)	Macro_FOLR2+APOE+	HLA-B	LILRB2	3,76303E-03	29383,2	3,34438E-03	42062	1,36298E+00	10589	1,77224E+00	28743	9,51862E-01	3622	0	61900

Myeloid_vs_CD8_Post_NR

Myeloid_vs_CD8_Non-responder	CD8(GZMK+ Early Tem)	pDC_LILRA4	HSP90B1	TLR9	3,76711E-03	37870	1,99175E-02	2116	1,97757E+00	2670	1,11085E+00	57788	8,57580E-01	64876	0	61900
Myeloid_vs_CD8_Non-responder	CD8(Tc17)	Mono_CD16	HLA-F	LILRB1	3,77064E-03	37017,8	4,09003E-03	31230	1,17317E+00	16667	1,64298E+00	33213	8,92152E-01	42079	0	61900
Myeloid_vs_CD8_Non-responder	CD8(GZMK+ Early Tem)	pDC_LILRA4	HMGB1	TLR9	3,77145E-03	31463,4	2,94556E-02	1004	2,06114E+00	2225	1,18814E+00	53691	8,97343E-01	38497	0	61900
Myeloid_vs_CD8_Non-responder	CD8(IL7R+ZNF683+ Tm)	Macro_ISG15	HLA-F	LILRB1	3,77333E-03	35569,6	4,08878E-03	31244	1,25596E+00	13721	1,76724E+00	28898	8,92137E-01	42085	0	61900
Myeloid_vs_CD8_Non-responder	CD8(Temra)	Macro_FOLR2+APOE+	HLA-B	LILRB2	3,77333E-03	29281	3,34316E-03	42085	1,36184E+00	10624	1,79019E+00	28172	9,51853E-01	3624	0	61900
Myeloid_vs_CD8_Non-responder	Macro_FOLR2+APOE+	CD8(ITM2C+ T)	LYZ	ITGAL	3,78140E-03	31181	3,34215E-03	42103	1,27028E+00	13280	2,90929E+00	6529	9,06859E-01	32093	0	61900
Myeloid_vs_CD8_Non-responder	CD8(EOMES+ NK-like)	pDC_LILRA4	GZMB	IGF2R	3,78769E-03	35022,4	8,04829E-03	11330	8,52540E-01	32942	1,83338E+00	26823	8,92088E-01	42117	0	61900
Myeloid_vs_CD8_Non-responder	CD8(Temra)	Mono_CD16	B2M	LILRB1	3,78859E-03	33258,2	3,60308E-03	37705	1,11608E+00	18959	1,42594E+00	42119	9,48345E-01	5608	0	61900
Myeloid_vs_CD8_Non-responder	CD8(Tc17)	Mono_CD16	LTB	TNFRSF1A	3,80030E-03	36483,8	5,75507E-03	18705	9,85748E-01	24952	1,60362E+00	34717	8,92057E-01	42145	0	61900
Myeloid_vs_CD8_Non-responder	CD8(ITM2C+ T)	Macro_OLFML3	HLA-F	LILRB2	3,80752E-03	36288,2	3,33910E-03	42161	1,19935E+00	15723	1,83522E+00	26754	9,02674E-01	34903	0	61900
Myeloid_vs_CD8_Non-responder	Macro_OLFML3	CD8(Terminal Tex)	HLA-DPA1	LAG3	3,80996E-03	23300,2	5,47158E-03	20169	1,19829E+00	15767	2,34482E+00	14305	9,50415E-01	4360	0	61900
Myeloid_vs_CD8_Non-responder	CD8(ID2+CXCR4+ T)	pDC_LILRA4	HMGB1	TLR9	3,81028E-03	28152,8	3,47674E-02	713	2,22496E+00	1561	1,40516E+00	43071	9,04732E-01	33519	0	61900
Myeloid_vs_CD8_Non-responder	CD8(ISG+ T)	Macro_FOLR2+APOE+	B2M	LILRB2	3,81701E-03	28124,8	3,33795E-03	42182	1,42164E+00	9152	1,87052E+00	25702	9,56383E-01	1688	0	61900
Myeloid_vs_CD8_Non-responder	Macro_LYVE1	CD8(LAYN+ T)	F13A1	ITGA4	3,82102E-03	19833,6	1,47068E-02	3973	2,38158E+00	1104	2,47619E+00	12007	9,25898E-01	20184	0	61900
Myeloid_vs_CD8_Non-responder	CD8(GZMK+ Early Tem)	cDC_LAMP3	TNFSF9	HLA-DPA1	3,83651E-03	38812,2	4,38508E-03	28083	7,93849E-01	37165	1,42376E+00	42225	9,18467E-01	24688	0	61900
Myeloid_vs_CD8_Non-responder	Macro_ISG15	CD8(ISG+ T)	S100A8	CD69	3,84099E-03	26497,4	5,46554E-03	20211	1,14780E+00	17597	2,17671E+00	17767	9,35182E-01	15012	0	61900
Myeloid_vs_CD8_Non-responder	CD8(ISG+ T)	Mono_CD16	HLA-C	LILRB1	3,84469E-03	34401,2	3,62936E-03	37305	1,11368E+00	19041	1,42330E+00	42243	9,40423E-01	11517	0	61900
Myeloid_vs_CD8_Non-responder	Macro_FOLR2+APOE+	CD8(Tc17)	APOE	LSR	3,85587E-03	24250,6	1,71432E-02	2883	1,49485E+00	7713	3,26478E+00	3680	8,87692E-01	45077	0	61900
Myeloid_vs_CD8_Non-responder	Macro_FOLR2+APOE+	CD8(ZNF683+KLRB1+ T)	B2M	KLRC1	3,85745E-03	42929,4	3,35663E-03	41843	7,30046E-01	42271	1,50753E+00	38629	9,09978E-01	30004	0	61900
Myeloid_vs_CD8_Non-responder	CD8(ISG+ T)	Macro_FOLR2+APOE+	IFNG	IFNGR1_IFNGR2	3,86979E-03	40148,6	4,61225E-03	25981	8,86388E-01	30712	1,47770E+00	39852	8,91834E-01	42298	0	61900
Myeloid_vs_CD8_Non-responder	CD8(Temra)	Mono_CD16	HLA-A	LILRB1	3,87574E-03	34002,2	3,71813E-03	35915	1,11750E+00	18902	1,42181E+00	42311	9,41194E-01	10983	0	61900
Myeloid_vs_CD8_Non-responder	Macro_ISG15	CD8(GZMK+ Tex)	HLA-DRA	LAG3	3,87892E-03	23035	5,45766E-03	20262	1,17004E+00	16776	2,47574E+00	12019	9,50706E-01	4218	0	61900
Myeloid_vs_CD8_Non-responder	CD8(GZMK+ Tex)	Macro_ISG15	TIGIT	NECTIN2	3,88340E-03	28065,6	2,40604E-02	1500,5	2,09207E+00	2092,5	2,12578E+00	18883,5	8,71226E-01	55951,5	0	61900
Myeloid_vs_CD8_Non-responder	Macro_ISG15	CD8(GZMK+ Tex)	LGALS3	LAG3	3,88340E-03	26053,2	6,57411E-03	15309	1,08572E+00	20268	2,22133E+00	16794	9,33430E-01	15995	0	61900
Myeloid_vs_CD8_Non-responder	Mono_CD14	CD8(ZNF683+KLRB1+ T)	S100A8	ITGB2	3,88565E-03	19151	5,45614E-03	20271	2,43029E+00	996	3,52039E+00	2317	9,42200E-01	10271	0	61900
Myeloid_vs_CD8_Non-responder	Macro_FOLR2+APOE+	CD8(ID2+CXCR4+ T)	HLA-DQB2	LAG3	3,88582E-03	35648,6	6,80084E-03	14556	9,49879E-01	26868	1,65878E+00	32586	8,91780E-01	42333	0	61900
Myeloid_vs_CD8_Non-responder	CD8(Tc17)	Mono_CD16	B2M	LILRB1	3,90237E-03	33466	3,59173E-03	37885	1,10288E+00	19532	1,42047E+00	42369	9,48267E-01	5644	0	61900
Myeloid_vs_CD8_Non-responder	CD8(ISG+ T)	Mono_CD16	CD52	SIGLEC10	3,90283E-03	36347,2	4,46090E-03	27354	1,03386E+00	22632	1,42042E+00	42370	9,14019E-01	27480	0	61900
Myeloid_vs_CD8_Non-responder	CD8(LAYN+ T)	Macro_LYVE1	HMGB1	CD163	3,90329E-03	31587	4,26358E-03	29323	1,37404E+00	10295	1,42037E+00	42371	9,36676E-01	14046	0	61900
Myeloid_vs_CD8_Non-responder	Macro_LYVE1	CD8(GZMK+ Tem)	HLA-DPA1	LAG3	3,91112E-03	22518,4	6,35523E-03	16117	1,32892E+00	11551	2,06761E+00	20305	9,53826E-01	2719	0	61900
Myeloid_vs_CD8_Non-responder	Macro_FOLR2+APOE+	CD8(ID2+CXCR4+ T)	C1QB	C1QBP	3,91298E-03	28498,4	3,32783E-03	42392	1,59900E+00	6065	3,28807E+00	3516	9,12208E-01	28619	0	61900
Myeloid_vs_CD8_Non-responder	CD8(EOMES+ NK-like)	Macro_FOLR2+APOE+	HLA-F	LILRB2	3,92823E-03	35196	3,32598E-03	42425	1,31970E+00	11820	1,90185E+00	24810	9,02501E-01	35025	0	61900
Myeloid_vs_CD8_Non-responder	CD8(GZMK+ Tem)	pDC_LILRA4	TNF	PTPRS	3,93710E-03	31123,2	2,33026E-02	1587	3,17505E+00	226	2,04507E+00	20863	8,48206E-01	71040	0	61900
Myeloid_vs_CD8_Non-responder	CD8(GZMK+ Tex)	Macro_FOLR2+APOE+	B2M	LILRB2	3,94307E-03	28423	3,32446E-03	42457	1,40430E+00	9549	1,84370E+00	26485	9,56298E-01	1724	0	61900
Myeloid_vs_CD8_Non-responder	CD8(GZMK+ Early Tem)	Mast	CALR	ITGA2B	3,94449E-03	52608,2	2,68927E-02	1189	1,54844E+00	6791	2,09459E-01	113614	8,34798E-01	79547	0	61900
Myeloid_vs_CD8_Non-responder	CD8(GZMK+ Tem)	Macro_IFI27	TNFSF9	HLA-DPA1	3,94771E-03	35641	4,45166E-03	27431	1,04587E+00	22114	1,41838E+00	42467	9,19030E-01	24293	0	61900
Myeloid_vs_CD8_Non-responder	pDC_LILRA4	CD8(LAYN+ T)	B2M	CD3D	3,94807E-03	37061,8	2,71726E-03	56415	8,70945E-01	31742	1,59005E+00	35224	9,64817E-01	28	0	61900
Myeloid_vs_CD8_Non-responder	CD8(NME1+ T)	Macro_ISG15	HLA-C	LILRB1	3,95190E-03	34697,6	3,71550E-03	35947	1,04628E+00	22089	1,41813E+00	42476	9,41077E-01	11076	0	61900
Myeloid_vs_CD8_Non-responder	CD8(NME1+ T)	Mast	AIMP1	NTRK1	3,95194E-03	60979,6	2,38681E-02	1517	1,49121E+00	7778	4,67771E-01	96880	7,29104E-01	136823	0	61900
Myeloid_vs_CD8_Non-responder	CD8(GZMK+ Tem)	Macro_FOLR2+APOE+	HLA-A	LILRB1	3,96261E-03	34962,4	3,32268E-03	42499	1,12540E+00	18564	1,50753E+00	38630	9,38004E-01	13219	0	61900
Myeloid_vs_CD8_Non-responder	Macro_FOLR2+APOE+	CD8(EOMES+ NK-like)	HLA-DQA1	LAG3	3,96308E-03	33460,8	3,32263E-03	42500	1,01629E+00	23411	2,26033E+00	16001	9,20372E-01	23492	0	61900
Myeloid_vs_CD8_Non-responder	Macro_FOLR2+APOE+	CD8(EOMES+ NK-like)	B2M	KIR2DL3	3,97148E-03	32532	1,47252E-02	3963	1,00850E+00	23780	1,71777E+00	30499	8,91499E-01	42518	0	61900
Myeloid_vs_CD8_Non-responder	CD8(ID2+CXCR4+ T)	Macro_OLFML3	HLA-C	LILRB1	3,97148E-03	35605,2	3,41142E-03	40837	1,09209E+00	20000	1,41706E+00	42518	9,38664E-01	12771	0	61900
Myeloid_vs_CD8_Non-responder	CD8(EOMES+ NK-like)	Macro_FOLR2+APOE+	HLA-C	LILRB2	3,98317E-03	29611,4	3,32083E-03	42543	1,34641E+00	11018	1,80720E+00	27635	9,49331E-01	4961	0	61900
Myeloid_vs_CD8_Non-responder	CD8(Temra)	Macro_FOLR2+APOE+	HLA-C	LILRB1	3,99019E-03	33989,2	3,32023E-03	42558	1,17364E+00	16649	1,58213E+00	35543	9,37880E-01	13296	0	61900
Myeloid_vs_CD8_Non-responder	Macro_OLFML3	CD8(GZMK+ Tex)	HLA-DPA1	LAG3	3,99209E-03	23314,8	5,43180E-03	20412	1,19313E+00	15952	2,36949E+00	13861	9,50243E-01	4449	0	61900
Myeloid_vs_CD8_Non-responder	CD8(Tn)	Macro_ISG15	HLA-C	LILRB2	3,99441E-03	30670,2	3,31986E-03	42567	1,24332E+00	14142	1,73986E+00	29777	9,49324E-01	4965	0	61900
Myeloid_vs_CD8_Non-responder	Macro_IER3	CD8(GZMK+ Tem)	HLA-DRA	LAG3	4,00201E-03	22978,2	6,23668E-03	16583	1,27207E+00	13231	2,06249E+00	20425	9,53740E-01	2752	0	61900
Myeloid_vs_CD8_Non-responder	CD8(GZMK+ Early Tem)	Mono_CD14	CIRBP	TREM1	4,00428E-03	37552,4	4,88086E-03	23858	1,09190E+00	20012	1,41549E+00	42588	8,96029E-01	39404	0	61900
Myeloid_vs_CD8_Non-responder	CD8(IL7R+ZNF683+ Tm)	Macro_FOLR2+APOE+	HLA-F	LILRB2	4,00757E-03	35209,4	3,31851E-03	42595	1,31859E+00	11861	1,90852E+00	24600	9,02402E-01	35091	0	61900
Myeloid_vs_CD8_Non-responder	Macro_FOLR2+APOE+	CD8(Tn)	CD59	CD2	4,01133E-03	36875,2	6,49679E-03	15594	8,52171E-01	32965	1,41523E+00	42603	9,07998E-01	31314	0	61900
Myeloid_vs_CD8_Non-responder	Macro_ISG15	CD8(Terminal Tex)	S100A8	CD69	4,01272E-03	27074,8	5,42673E-03	20439	1,14297E+00	17816	2,07607E+00	20097	9,34965E-01	15122	0	61900
Myeloid_vs_CD8_Non-responder	Macro_FOLR2+APOE+	CD8(ZNF683+KLRB1+ T)	HLA-C	CD3G	4,01416E-03	37643	3,31777E-03	42609	7,67207E-01	39196	1,46054E+00	40620	9,51326E-01	3890	0	61900
Myeloid_vs_CD8_Non-responder	Macro_FOLR2+APOE+	CD8(GZMK+ Tem)	ICAM1	IL2RG	4,01699E-03	42546,8	3,31761E-03	42615	8,58955E-01	32534	1,51592E+00	38260	8,98910E-01	37425	0	61900
Myeloid_vs_CD8_Non-responder	CD8(LAYN+ T)	Mono_CD16	HLA-A	LILRA1	4,01793E-03	26566,2	9,54246E-03	8635	1,72901E+00	4586	1,41489E+00	42617	9,35021E-01	15093	0	61900
Myeloid_vs_CD8_Non-responder	CD8(ID2+CXCR4+ T)	Macro_ISG15	CIRBP	TREM1	4,02264E-03	38663	4,42391E-03	27687	1,01418E+00	23516	1,53164E+00	37585	8,91361E-01	42627	0	61900
Myeloid_vs_CD8_Non-responder	CD8(GZMK+ Early Tem)	cDC_CLEC9A	CRTAM	CADM1	4,02387E-03	27926,2	3,39104E-02	756,5	1,72098E+00	4656,5	1,88196E+00	25378,5	8,84913E-01	46939,5	0	61900
Myeloid_vs_CD8_Non-responder	Macro_IER3	CD8(GZMK+ Tem)	HLA-DRB5	LAG3	4,02421E-03	23388,4	6,43306E-03	15808	1,26848E+00	13325	2,06164E+00	20454	9,48580E-01	5455	0	61900
Myeloid_vs_CD8_Non-responder	CD8(Terminal Tex)	pDC_LILRA4	SELPLG	SELL	4,02548E-03	28748,8	6,55894E-03	15375	1,45126E+00	8533	2,29510E+00	15303	8,91356E-01	42633	0	61900
Myeloid_vs_CD8_Non-responder	CD8(GZMK+ Early Tem)	Macro_IER3	RPS19	C5AR1	4,02595E-03	29322,8	4,66451E-03	25554	1,37453E+00	10280	1,41463E+00	42634	9,47436E-01	6246	0	61900
Myeloid_vs_CD8_Non-responder	CD8(GZMK+ Early Tem)	Macro_FOLR2+APOE+	CALR	LRP1	4,02642E-03	39439,8	3,31695E-03	42635	1,01290E+00	23569	1,51734E+00	38198	9,08595E-01	30897	0	61900
Myeloid_vs_CD8_Non-responder	CD8(Tc17)	Mono_CD16	ANXA1	FPR1	4,03587E-03	36600	3,67191E-03	36590	1,06249E+00	21323	1,41410E+00	42655	9,25290E-01	20532	0	61900
Myeloid_vs_CD8_Non-responder	CD8(Terminal Tex)	pDC_LILRA4	HMGB1	TLR9	4,03604E-03	28975,6	3,44128E-02	732	2,21402E+00	1607	1,32334E+00	46829	9,04289E-01	33810	0	61900
Myeloid_vs_CD8_Non-responder	Mast	CD8(Tc17)	ADCYAP1	ADRB2	4,04601E-03	46898	7,81433E-02	146	2,21390E+00	1609	1,18900E+00	53656	7,70664E-01	117179	0	61900
Myeloid_vs_CD8_Non-responder	Macro_OLFML3	CD8(GZMK+ Early Tem)	CXCL9	CXCR3	4,04803E-03	25643	1,49008E-02	3860	2,19641E+00	1673	2,78213E+00	7842	8,75785E-01	52940	0	61900
Myeloid_vs_CD8_Non-responder	Macro_FOLR2+APOE+	CD8(ID2+CXCR4+ T)	GRN	TNFRSF1B	4,06291E-03	27229	3,31297E-03	42712	1,39695E+00	9726	2,70815E+00	8777	9,38291E-01	13030	0	61900
Myeloid_vs_CD8_Non-responder	CD8(Tc17)	Macro_LYVE1	HMGB1	CD163	4,06577E-03	32804	4,00369E-03	32217	1,31491E+00	11967	1,41290E+00	42718	9,34785E-01	15218	0	61900
Myeloid_vs_CD8_Non-responder	cDC(CD1C)	CD8(Terminal Tex)	HLA-DRB1	LAG3	4,06578E-03	24087	5,41469E-03	20508	1,19621E+00	15846	2,15023E+00	18337	9,51407E-01	3844	0	61900
Myeloid_vs_CD8_Non-responder	CD8(GZMK+ Tem)	Mono_CD16	B2M	LILRB1	4,07434E-03	33686	3,58157E-03	38048	1,09106E+00	20053	1,41244E+00	42736	9,48198E-01	5693	0	61900
Myeloid_vs_CD8_Non-responder	Macro_FOLR2+APOE+	CD8(ISG+ T)	LGALS9	CD44	4,07625E-03	34464	3,31168E-03	42740	1,13167E+00	18281	1,73598E+00	29905	9,27098E-01	19494	0	61900
Myeloid_vs_CD8_Non-responder	Macro_ISG15	CD8(Tc17)	TNFSF10	CCR6	4,07989E-03	22410,8	3,60977E-02	657	1,84341E+00	3593	2,78016E+00	7863	8,97993E-01	38041	0	61900
Myeloid_vs_CD8_Non-responder	Macro_FOLR2+APOE+	CD8(ISG+ T)	HLA-DQB2	LAG3	4,08484E-03	35335,6	6,71441E-03	14818	9,42814E-01	27259	1,73480E+00	29943	8,91161E-01	42758	0	61900
Myeloid_vs_CD8_Non-responder	CD8(ITM2C+ T)	Mono_CD16	TNF	TNFRSF1B	4,08627E-03	41926,4	3,93979E-03	33002	7,23774E-01	42761	1,48467E+00	39578	9,06416E-01	32391	0	61900
Myeloid_vs_CD8_Non-responder	Macro_ISG15	CD8(GZMK+ Tex)	B2M	CD3D	4,08902E-03	43839,8	2,70649E-03	56753	6,96757E-01	45137	1,15607E+00	55380	9,64750E-01	29	0	61900
Myeloid_vs_CD8_Non-responder	CD8(Temra)	Macro_ISG15	CIRBP	TREM1	4,09440E-03	38993,8	4,40304E-03	27894	1,01106E+00	23655	1,50467E+00	38742	8,91131E-01	42778	0	61900
Myeloid_vs_CD8_Non-responder	CD8(EOMES+ NK-like)	Mono_CD16	HLA-A	LILRA1	4,09536E-03	26670,2	9,48187E-03	8729	1,71124E+00	4747	1,41162E+00	42780	9,34827E-01	15195	0	61900
Myeloid_vs_CD8_Non-responder	Mono_CD14	CD8(Terminal Tex)	VCAN	CD44	4,10144E-03	23136,6	6,99332E-03	13994	2,25163E+00	1479	2,05705E+00	20554	9,30131E-01	17756	0	61900

Myeloid_vs_CD8_Post_NR

Myeloid_vs_CD8_Non-responder	Macro_FOLR2+APOE+	CD8(NME1+ T)	CD59	CD2	4,10686E-03	34800,6	7,25096E-03	13225	9,36322E-01	27648	1,41110E+00	42804	9,12484E-01	28426	0	61900
Myeloid_vs_CD8_Non-responder	CD8(ITM2C+ T)	Macro_FOLR2+APOE+	B2M	LILRB1	4,11742E-03	28723,4	3,30781E-03	42826	1,38288E+00	10078	1,82517E+00	27056	9,56193E-01	1757	0	61900
Myeloid_vs_CD8_Non-responder	CD8(GZMK+ Tex)	Macro_FOLR2+APOE+	HLA-C	LILRB2	4,11934E-03	30009,6	3,30761E-03	42830	1,33613E+00	11315	1,76374E+00	28993	9,49235E-01	5010	0	61900
Myeloid_vs_CD8_Non-responder	Macro_FOLR2+APOE+	CD8(ISG+ T)	CD14	ITGB1	4,14103E-03	31474,6	3,30513E-03	42875	1,35962E+00	10677	2,49212E+00	11759	9,09731E-01	30162	0	61900
Myeloid_vs_CD8_Non-responder	Macro_IER3	CD8(GZMK+ Tem)	HLA-DQA2	LAG3	4,14282E-03	24832,4	8,04481E-03	11336	1,27888E+00	13011	2,19700E+00	17308	9,25154E-01	20607	0	61900
Myeloid_vs_CD8_Non-responder	CD8(ZNF683+KLRB1+ T)	cDC(CD1C)	SPON2	ITGB2	4,16523E-03	38178,8	6,08318E-03	17210	9,10440E-01	29216	1,40848E+00	42925	8,95649E-01	39643	0	61900
Myeloid_vs_CD8_Non-responder	CD8(EOMES+ NK-like)	Mono_CD16	B2M	LILRB1	4,17154E-03	33768	3,57874E-03	38098	1,08777E+00	20195	1,40827E+00	42938	9,48178E-01	5709	0	61900
Myeloid_vs_CD8_Non-responder	CD8(ITM2C+ T)	Macro_LYVE1	HSPA1A	TLR4	4,17203E-03	33185	5,88039E-03	18102	1,22081E+00	14917	1,40826E+00	42939	9,13050E-01	28067	0	61900
Myeloid_vs_CD8_Non-responder	CD8(GZMK+ Tex)	Macro_OLFML3	B2M	LILRB1	4,17252E-03	34833	3,33700E-03	42193	1,08948E+00	20135	1,40825E+00	42940	9,46433E-01	6997	0	61900
Myeloid_vs_CD8_Non-responder	CD8(ZNF683+KLRB1+ T)	Macro_OLFML3	HLA-C	LILRB2	4,17252E-03	27757,2	3,30112E-03	42940	1,55346E+00	6714	1,99335E+00	22199	9,49188E-01	5033	0	61900
Myeloid_vs_CD8_Non-responder	CD8(IL7R+ZNF683+ Tm)	Macro_OLFML3	HLA-C	LILRB1	4,17397E-03	36072,4	3,37863E-03	41414	1,06669E+00	21130	1,40824E+00	42943	9,38386E-01	12975	0	61900
Myeloid_vs_CD8_Non-responder	Macro_FOLR2+APOE+	CD8(ITM2C+ T)	CD59	CD2	4,17738E-03	36889	6,51495E-03	15529	8,54197E-01	32845	1,40809E+00	42950	9,08115E-01	31221	0	61900
Myeloid_vs_CD8_Non-responder	CD8(NME1+ T)	cDC_LAMP3	CD28	CD80	4,18263E-03	52566,4	1,91248E-02	2305,5	1,48244E+00	7930,5	9,40661E-01	67400,5	7,58664E-01	123295,5	0	61900
Myeloid_vs_CD8_Non-responder	CD8(GZMK+ Tem)	Macro_OLFML3	HLA-B	LILRB1	4,19345E-03	35131,4	3,41945E-03	40687	1,10632E+00	19359	1,40738E+00	42983	9,41566E-01	10728	0	61900
Myeloid_vs_CD8_Non-responder	CD8(ISG+ T)	Macro_ISG15	CD28	CD86	4,19952E-03	34910,4	1,04547E-02	7463,5	1,36481E+00	10545,5	2,05241E+00	20679,5	8,43719E-01	73963,5	0	61900
Myeloid_vs_CD8_Non-responder	CD8(Terminal Tex)	Macro_FOLR2+APOE+	B2M	LILRB2	4,20175E-03	29094,4	3,29843E-03	43000	1,37081E+00	10374	1,78305E+00	28414	9,56134E-01	1784	0	61900
Myeloid_vs_CD8_Non-responder	CD8(GZMK+ Tex)	Mono_CD16	HLA-C	LILRA3	4,20224E-03	28397	1,07951E-02	7103	1,47189E+00	8117	1,40703E+00	43001	9,23037E-01	21864	0	61900
Myeloid_vs_CD8_Non-responder	CD8(GZMK+ Early Tem)	pDC_LILRA4	TNFSF9	HLA-DPA1	4,20468E-03	36683	4,08903E-03	31240	7,21037E-01	43006	2,04472E+00	20872	9,15812E-01	26397	0	61900
Myeloid_vs_CD8_Non-responder	Macro_NLRP3	CD8(ITM2C+ T)	S100A8	ITGB2	4,21299E-03	20601	5,37859E-03	20696	2,00629E+00	2517	2,82756E+00	7356	9,41809E-01	10536	0	61900
Myeloid_vs_CD8_Non-responder	CD8(ZNF683+KLRB1+ T)	pDC_LILRA4	CD99	PILRB	4,21447E-03	40832,6	5,32191E-03	21018	8,03990E-01	36387	1,40640E+00	43026	8,92518E-01	41832	0	61900
Myeloid_vs_CD8_Non-responder	cDC(CD1C)	CD8(ID2+CXCR4+ T)	HLA-DQA2	LAG3	4,21537E-03	24274,2	8,00821E-03	11412	1,48018E+00	7971	2,10462E+00	19389	9,24996E-01	20699	0	61900
Myeloid_vs_CD8_Non-responder	CD8(ITM2C+ T)	cDC(CD1C)	IFNG	IFNGR1_IFNGR2	4,21545E-03	38173,6	5,87869E-03	18113	8,49474E-01	33155	1,40636E+00	43028	9,02992E-01	34672	0	61900
Myeloid_vs_CD8_Non-responder	Macro_FOLR2+APOE+	CD8(ID2+CXCR4+ T)	TIMP2	CD44	4,22133E-03	40731	3,87064E-03	33850	9,75487E-01	25480	1,40588E+00	43040	8,96059E-01	39385	0	61900
Myeloid_vs_CD8_Non-responder	CD8(ITM2C+ T)	Macro_NLRP3	IFNG	IFNGR1_IFNGR2	4,22231E-03	42522,2	4,74467E-03	24904	7,20639E-01	43042	1,44194E+00	41406	8,93191E-01	41359	0	61900
Myeloid_vs_CD8_Non-responder	CD8(LAYN+ T)	Mono_CD16	B2M	LILRB1	4,22329E-03	33856	3,57398E-03	38172	1,08224E+00	20433	1,40580E+00	43044	9,48146E-01	5731	0	61900
Myeloid_vs_CD8_Non-responder	Macro_LYVE1	CD8(GZMK+ Tex)	HLA-DRA	LAG3	4,22808E-03	23688,2	5,80924E-03	18424	1,25035E+00	13905	2,05118E+00	20715	9,52148E-01	3497	0	61900
Myeloid_vs_CD8_Non-responder	CD8(Terminal Tex)	Mono_INHBA	HSPA1A	TLR4	4,22820E-03	36694,2	5,00501E-03	22992	1,02201E+00	23149	1,40558E+00	43054	9,06435E-01	32376	0	61900
Myeloid_vs_CD8_Non-responder	Macro_OLFML3	CD8(Terminal Tex)	B2M	CD3D	4,22998E-03	49748	2,70167E-03	56896	5,79718E-01	57163	8,50024E-01	72751	9,64720E-01	30	0	61900
Myeloid_vs_CD8_Non-responder	cDC(CD1C)	CD8(GZMK+ Tex)	HLA-DRB1	LAG3	4,23047E-03	24075,8	5,37533E-03	20718	1,19105E+00	16017	2,17490E+00	17803	9,51238E-01	3941	0	61900
Myeloid_vs_CD8_Non-responder	Macro_FOLR2+APOE+	CD8(LAYN+ T)	LGALS1	ITGB1	4,23213E-03	32796,2	3,29571E-03	43062	9,97237E-01	24367	2,10581E+00	19361	9,34652E-01	15291	0	61900
Myeloid_vs_CD8_Non-responder	Mono_INHBA	CD8(LAYN+ T)	SPP1	CD44	4,23365E-03	23899,2	9,73212E-03	8344	1,32892E+00	11552	2,21225E+00	16978	9,24968E-01	20722	0	61900
Myeloid_vs_CD8_Non-responder	CD8(ITM2C+ T)	Mono_INHBA	TNFSF9	HLA-DPA1	4,24049E-03	36087,6	5,34190E-03	20901	8,34169E-01	34176	1,40499E+00	43079	9,25559E-01	20382	0	61900
Myeloid_vs_CD8_Non-responder	CD8(GZMK+ Tem)	pDC_LILRA4	HRAS	TLR9	4,24265E-03	48357,4	5,58955E-02	282	2,20245E+00	1648	1,65274E+00	32844	7,07923E-01	145113	0	61900
Myeloid_vs_CD8_Non-responder	Macro_NLRP3	CD8(ZNF683+KLRB1+ T)	VCAN	CD44	4,24481E-03	24432,8	5,99218E-03	17618	1,91373E+00	3063	2,12765E+00	18847	9,24941E-01	20736	0	61900
Myeloid_vs_CD8_Non-responder	Macro_FOLR2+APOE+	CD8(NME1+ T)	LGALS9	PTPRC	4,25034E-03	36040,6	3,29333E-03	43099	1,04412E+00	22180	1,53348E+00	37508	9,34273E-01	15516	0	61900
Myeloid_vs_CD8_Non-responder	CD8(GZMK+ Early Tem)	Macro_ISG15	TIGIT	NECTIN2	4,26351E-03	37724,6	1,25586E-02	5436,5	1,76087E+00	4307,5	1,60907E+00	34486,5	8,30159E-01	82492,5	0	61900
Myeloid_vs_CD8_Non-responder	pDC_LILRA4	CD8(ZNF683+KLRB1+ T)	APP	RPSA	4,26719E-03	23970,6	5,57257E-03	19622	1,85678E+00	3495	2,35735E+00	14072	9,24897E-01	20764	0	61900
Myeloid_vs_CD8_Non-responder	Macro_FOLR2+APOE+	CD8(IL7R+ZNF683+ Tm)	MRC1	PTPRC	4,26763E-03	38632,6	5,41930E-03	20483	9,74488E-01	25522	1,40380E+00	43134	8,92081E-01	42124	0	61900
Myeloid_vs_CD8_Non-responder	Macro_OLFML3	CD8(Tc17)	CXCL11	DPP4	4,27330E-03	37773,2	8,42638E-02	121	2,20130E+00	1654	2,15204E+00	18309	7,89758E-01	106882	0	61900
Myeloid_vs_CD8_Non-responder	Macro_FOLR2+APOE+	CD8(Terminal Tex)	ICAM1	IL2RG	4,27357E-03	43324,4	3,29077E-03	43146	8,54405E-01	32828	1,44999E+00	41071	8,98540E-01	37677	0	61900
Myeloid_vs_CD8_Non-responder	CD8(Terminal Tex)	Macro_IER3	IFNG	IFNGR1_IFNGR2	4,27505E-03	35771,2	6,69957E-03	14865	9,30128E-01	28028	1,40343E+00	43149	9,08568E-01	30914	0	61900
Myeloid_vs_CD8_Non-responder	Macro_FOLR2+APOE+	CD8(LAYN+ T)	ICAM1	IL2RG	4,28050E-03	43135,4	3,29010E-03	43160	8,54290E-01	32840	1,47223E+00	40091	8,98531E-01	37686	0	61900
Myeloid_vs_CD8_Non-responder	CD8(IL7R+ZNF683+ Tm)	Macro_FOLR2+APOE+	ANXA1	FPR1	4,28348E-03	40430,6	3,28964E-03	43166	8,78751E-01	31205	1,40691E+00	43003	9,21400E-01	22879	0	61900
Myeloid_vs_CD8_Non-responder	CD8(EOMES+ NK-like)	Macro_FOLR2+APOE+	HLA-B	LILRB2	4,29093E-03	29959,4	3,28859E-03	43181	1,31084E+00	12094	1,76988E+00	28812	9,51475E-01	3810	0	61900
Myeloid_vs_CD8_Non-responder	CD8(GZMK+ Tex)	Macro_ISG15	HMGB1	TLR2	4,30237E-03	39540	3,62109E-03	37445	9,63875E-01	26076	1,40207E+00	43204	9,11460E-01	29075	0	61900
Myeloid_vs_CD8_Non-responder	Macro_FOLR2+APOE+	CD8(ITM2C+ T)	HLA-C	CD8A	4,30735E-03	36338,6	3,28675E-03	43214	8,41377E-01	33723	1,41597E+00	42567	9,61378E-01	289	0	61900
Myeloid_vs_CD8_Non-responder	Macro_FOLR2+APOE+	CD8(GZMK+ Tex)	CXCL9	CXCR3	4,30906E-03	28215,8	1,13818E-02	6459	2,22459E+00	1564	2,76807E+00	8011	8,60375E-01	63145	0	61900
Myeloid_vs_CD8_Non-responder	CD8(EOMES+ NK-like)	Mono_CD16	HLA-C	LILRA1	4,31833E-03	27069,8	9,15692E-03	9254	1,67581E+00	5085	1,40142E+00	43236	9,33648E-01	15874	0	61900
Myeloid_vs_CD8_Non-responder	CD8(GZMK+ Tem)	Macro_FOLR2+APOE+	CCL5	CCRL2	4,34214E-03	31707,4	1,04883E-02	7424	1,20984E+00	15314	1,20049E+00	53042	9,24722E-01	20857	0	61900
Myeloid_vs_CD8_Non-responder	CD8(ISG+ T)	Mono_CD16	HLA-C	LILRA3	4,34486E-03	28595,8	1,07081E-02	7196	1,45115E+00	8536	1,40045E+00	43289	9,22749E-01	22058	0	61900
Myeloid_vs_CD8_Non-responder	CD8(Terminal Tex)	Macro_ISG15	HLA-F	LILRB1	4,35490E-03	37303,4	3,93833E-03	33024	1,23782E+00	14310	1,62245E+00	33974	8,90320E-01	43309	0	61900
Myeloid_vs_CD8_Non-responder	CD8(IL7R+ZNF683+ Tm)	Mono_CD14	CIRBP	TREM1	4,36044E-03	38408,4	4,70973E-03	25193	1,06695E+00	21112	1,39986E+00	43320	8,94355E-01	40517	0	61900
Myeloid_vs_CD8_Non-responder	CD8(GZMK+ Tex)	Mono_CD14	CIRBP	TREM1	4,36094E-03	37836,6	4,84632E-03	24129	1,08686E+00	20233	1,39983E+00	43321	8,95698E-01	39600	0	61900
Myeloid_vs_CD8_Non-responder	CD8(ISG+ T)	Macro_FOLR2+APOE+	HLA-C	LILRB2	4,36346E-03	30305	3,28095E-03	43326	1,31538E+00	11953	1,75716E+00	29198	9,49040E-01	5148	0	61900
Myeloid_vs_CD8_Non-responder	CD8(EOMES+ NK-like)	Macro_ISG15	CCL5	CCRL2	4,36749E-03	30807	9,71174E-03	8368	1,14025E+00	17925	1,39947E+00	43334	9,22000E-01	22508	0	61900
Myeloid_vs_CD8_Non-responder	CD8(Terminal Tex)	Macro_FOLR2+APOE+	HLA-B	LILRB2	4,36799E-03	30728,4	3,28041E-03	43335	1,30319E+00	12303	1,66759E+00	32270	9,51417E-01	3834	0	61900
Myeloid_vs_CD8_Non-responder	Macro_NLRP3	CD8(ZNF683+KLRB1+ T)	ICAM1	IL2RG	4,37467E-03	24806	6,31616E-03	16261	1,40436E+00	9547	2,28945E+00	15425	9,24638E-01	20897	0	61900
Myeloid_vs_CD8_Non-responder	CD8(ZNF683+KLRB1+ T)	Macro_FOLR2+APOE+	LTB	CD40	4,37606E-03	28474,4	9,73501E-03	8338	1,49255E+00	7757	2,03852E+00	21026	8,90260E-01	43351	0	61900
Myeloid_vs_CD8_Non-responder	CD8(Terminal Tex)	Macro_OLFML3	TNFSF9	HLA-DPA1	4,37808E-03	37524,4	3,27908E-03	43355	9,11302E-01	29157	2,03394E+00	21146	9,06902E-01	32064	0	61900
Myeloid_vs_CD8_Non-responder	CD8(Temra)	Macro_FOLR2+APOE+	CD52	SIGLEC10	4,38061E-03	34180,8	4,90352E-03	23685	1,17116E+00	16739	1,39910E+00	43360	9,17664E-01	25220	0	61900
Myeloid_vs_CD8_Non-responder	CD8(ZNF683+KLRB1+ T)	Macro_OLFML3	LTB	CD40	4,38111E-03	29740,8	9,73248E-03	8341	1,49221E+00	7761	1,81675E+00	27341	8,90247E-01	43361	0	61900
Myeloid_vs_CD8_Non-responder	Macro_IER3	CD8(Tc17)	CXCL2	DPP4	4,38196E-03	27519,2	2,99917E-02	971	1,85154E+00	3536	2,76480E+00	8057	8,60389E-01	63132	0	61900
Myeloid_vs_CD8_Non-responder	Macro_FOLR2+APOE+	CD8(GZMK+ Tem)	LGALS9	HAVCR2	4,38313E-03	32656,4	4,27879E-03	29142	1,30078E+00	12372	2,23610E+00	16503	8,90243E-01	43365	0	61900
Myeloid_vs_CD8_Non-responder	Macro_LYVE1	CD8(ISG+ T)	C3	IFITM1	4,38610E-03	22796,4	9,02299E-03	9477	1,41748E+00	9250	2,04309E+00	20911	9,39107E-01	12444	0	61900
Myeloid_vs_CD8_Non-responder	CD8(GZMK+ Early Tem)	Mono_CD16	B2M	LILRB1	4,38819E-03	33741,8	3,58645E-03	37962	1,09674E+00	19810	1,39894E+00	43375	9,48231E-01	5662	0	61900
Myeloid_vs_CD8_Non-responder	Macro_NLRP3	CD8(ITM2C+ T)	IL1B	SIGIRR	4,38833E-03	19923,8	9,93301E-03	8061	2,31762E+00	1278	3,38423E+00	2990	9,17385E-01	25390	0	61900
Myeloid_vs_CD8_Non-responder	cDC(CD1C)	CD8(ISG+ T)	HLA-DQA2	LAG3	4,41231E-03	24047,4	7,90644E-03	11634	1,47311E+00	8089	2,18064E+00	17671	9,24551E-01	20943	0	61900
Myeloid_vs_CD8_Non-responder	Macro_IER3	CD8(LAYN+ T)	CD14	ITGB1	4,41559E-03	26079,2	5,33237E-03	20947	1,42960E+00	8981	2,10742E+00	19321	9,27541E-01	19247	0	61900
Myeloid_vs_CD8_Non-responder	Mono_CD14	CD8(Temra)	VCAN	ITGB1	4,42992E-03	19549,8	1,25298E-02	5468	2,57032E+00	758	2,76276E+00	8087	9,23554E-01	21536	0	61900
Myeloid_vs_CD8_Non-responder	Macro_OLFML3	CD8(Tn)	CXCL9	CXCR3	4,43153E-03	26243,6	1,40320E-02	4374	2,17615E+00	1742	2,76266E+00	8088	8,72481E-01	55114	0	61900
Myeloid_vs_CD8_Non-responder	CD8(IL7R+ZNF683+ Tm)	cDC(CD1C)	ANXA1	FPR1	4,44718E-03	33329,6	4,47484E-03	27223	1,15934E+00	17188	1,39646E+00	43491	9,31844E-01	16846	0	61900
Myeloid_vs_CD8_Non-responder	Macro_FOLR2+APOE+	CD8(ITM2C+ T)	MRC1	PTPRC	4,44820E-03	39233,8	5,30585E-03	21119	9,50506E-01	26829	1,39641E+00	43493	8,91058E-01	42828	0	61900
Myeloid_vs_CD8_Non-responder	Macro_ISG15	CD8(GZMK+ Tem)	HLA-DPA1	LAG3	4,44935E-03	25031	5,32615E-03	20988	1,09749E+00	19774	2,17552E+00	17787	9,49776E-01	4706	0	61900
Myeloid_vs_CD8_Non-responder	Macro_FOLR2+APOE+	CD8(ITM2C+ T)	TIMP2	CD44	4,45281E-03	41427	3,77360E-03	35150	9,57702E-01	26415	1,39611E+00	43502	8,94871E-01	40168	0	61900
Myeloid_vs_CD8_Non-responder	CD8(GZMK+ Early Tem)	Macro_OLFML3	HLA-F	LILRB2	4,45281E-03	37189,8	3,27099E-03	43502	1,18755E+00	16134	1,76624E+00	28922	9,01765E-01	35491	0	61900
Myeloid_vs_CD8_Non-responder	CD8(IL7R+ZNF683+ Tm)	Macro_FOLR2+APOE+	B2M	LILRB2	4,45383E-03	29497,2	3,27097E-03	43504	1,33550E+00	11329	1,76728E+00	28897	9,55958E-01	1856	0	61900
Myeloid_vs_CD8_Non-responder	Mono_INHBA	CD8(GZMK+ Tem)	SPP1	CD44	4,46755E-03	24042,8	9,58159E-03	8560	1,31540E+00	11952	2,22134E+00	16792	9,24425E-01	21010	0	61900

Myeloid_vs_CD8_Post_NR

Myeloid_vs_CD8_Non-responder	CD8(NME1+ T)	Macro_FOLR2+APOE+	MIF	CD74_CXCR4	4,47522E-03	50908,2	1,84436E-03	89575	5,93570E-01	55515	1,34467E+00	45858	9,56377E-01	1693	0	61900
Myeloid_vs_CD8_Non-responder	CD8(ITM2C+ T)	Macro_OLFML3	HLA-F	LILRB1	4,47537E-03	38448,4	3,90924E-03	33366	1,08935E+00	20138	1,64137E+00	33292	8,89958E-01	43546	0	61900
Myeloid_vs_CD8_Non-responder	CD8(ZNF683+KLRB1+ T)	Macro_FOLR2+APOE+	HMGB1	TLR2	4,48463E-03	37899,6	3,87199E-03	33835	1,03044E+00	22792	1,39434E+00	43564	9,14127E-01	27407	0	61900
Myeloid_vs_CD8_Non-responder	Macro_FOLR2+APOE+	CD8(ZNF683+KLRB1+ T)	HLA-E	CD8A	4,48514E-03	36008	3,60353E-03	37699	8,18395E-01	35333	1,39433E+00	43565	9,56746E-01	1543	0	61900
Myeloid_vs_CD8_Non-responder	Macro_OLFML3	CD8(NME1+ T)	CXCL9	CXCR3	4,49117E-03	24439,8	1,72502E-02	2847	2,25121E+00	1484	2,76025E+00	8125	8,83532E-01	47843	0	61900
Myeloid_vs_CD8_Non-responder	CD8(GZMK+ Tex)	Macro_FOLR2+APOE+	CCL5	CCRL2	4,50827E-03	31343,6	1,03775E-02	7541	1,19833E+00	15766	1,25024E+00	50452	9,24351E-01	21059	0	61900
Myeloid_vs_CD8_Non-responder	CD8(ZNF683+KLRB1+ T)	Macro_FOLR2+APOE+	HLA-F	LILRB1	4,51611E-03	38401,2	3,90030E-03	33491	1,04586E+00	22115	1,70634E+00	30875	8,89845E-01	43625	0	61900
Myeloid_vs_CD8_Non-responder	Mono_CD14	CD8(NME1+ T)	S100A8	ITGB2	4,51661E-03	19615	5,31550E-03	21069	2,41785E+00	1019	3,32505E+00	3306	9,41485E-01	10781	0	61900
Myeloid_vs_CD8_Non-responder	CD8(Tc17)	Mono_CD16	HLA-B	LILRB1	4,51663E-03	34986,8	3,57833E-03	38103	1,05887E+00	21495	1,39326E+00	43626	9,42802E-01	9810	0	61900
Myeloid_vs_CD8_Non-responder	CD8(ITM2C+ T)	Macro_FOLR2+APOE+	HLA-B	LILRB1	4,51922E-03	34519,6	3,26494E-03	43631	1,16014E+00	17165	1,51550E+00	38278	9,40280E-01	11624	0	61900
Myeloid_vs_CD8_Non-responder	CD8(Tn)	Mono_INHBA	ANXA1	FPR1	4,52596E-03	38827,2	3,26455E-03	43644	9,21534E-01	28558	1,54710E+00	36964	9,21123E-01	23070	0	61900
Myeloid_vs_CD8_Non-responder	CD8(ISG+ T)	Macro_OLFML3	HLA-F	LILRB2	4,52700E-03	37531,8	3,26447E-03	43646	1,18642E+00	16168	1,72129E+00	30394	9,01676E-01	35551	0	61900
Myeloid_vs_CD8_Non-responder	Macro_OLFML3	CD8(Terminal Tex)	HLA-DQA2	LAG3	4,52915E-03	24603,4	7,85191E-03	11743	1,29216E+00	12642	2,27763E+00	15648	9,24309E-01	21084	0	61900
Myeloid_vs_CD8_Non-responder	CD8(ID2+CXCR4+ T)	Macro_ISG15	HMGB1	THBD	4,54466E-03	44557	3,26285E-03	43680	8,19165E-01	35271	1,43995E+00	41513	8,94478E-01	40421	0	61900
Myeloid_vs_CD8_Non-responder	CD8(Tc17)	Macro_ISG15	CCL4	CCR1	4,55872E-03	33477,6	7,16095E-03	13505	1,24598E+00	14041	1,39112E+00	43707	9,03652E-01	34235	0	61900
Myeloid_vs_CD8_Non-responder	CD8(NME1+ T)	Macro_ISG15	HMGB1	TLR4	4,56028E-03	39552,6	4,09298E-03	31201	1,06183E+00	21350	1,48388E+00	39602	8,89717E-01	43710	0	61900
Myeloid_vs_CD8_Non-responder	CD8(EOMES+ NK-like)	cDC_CLEC9A	HMGB1	THBD	4,56341E-03	38133,2	4,19743E-03	30045	1,04365E+00	22200	1,39088E+00	43716	9,05788E-01	32805	0	61900
Myeloid_vs_CD8_Non-responder	Macro_FOLR2+APOE+	CD8(LAYN+ T)	HLA-DQB2	LAG3	4,57700E-03	37352,4	6,51246E-03	15536	9,26305E-01	28256	1,53828E+00	37328	8,89672E-01	43742	0	61900
Myeloid_vs_CD8_Non-responder	CD8(Temra)	Mono_CD14	ANXA1	FPR1	4,58014E-03	34954	3,98741E-03	32419	1,14420E+00	17763	1,39020E+00	43748	9,28090E-01	18940	0	61900
Myeloid_vs_CD8_Non-responder	CD8(ZNF683+KLRB1+ T)	Mono_CD14	CD99	PILRA	4,58014E-03	39584,2	3,25934E-03	43748	9,70737E-01	25712	1,45704E+00	40775	9,16769E-01	25786	0	61900
Myeloid_vs_CD8_Non-responder	Macro_NLRP3	CD8(Temra)	S100A8	ITGB2	4,58040E-03	20903,4	5,30067E-03	21145	1,99757E+00	2564	2,76392E+00	8075	9,41408E-01	10833	0	61900
Myeloid_vs_CD8_Non-responder	CD8(ITM2C+ T)	Macro_OLFML3	B2M	LILRB1	4,59272E-03	35277,8	3,32028E-03	42557	1,06806E+00	21053	1,38972E+00	43772	9,46305E-01	7107	0	61900
Myeloid_vs_CD8_Non-responder	CD8(NME1+ T)	Macro_LYVE1	CD59	STAB1	4,59391E-03	32378,8	5,29860E-03	21161	1,95879E+00	2770	2,24208E+00	16381	8,65693E-01	59682	0	61900
Myeloid_vs_CD8_Non-responder	CD8(EOMES+ NK-like)	Macro_IER3	RPS19	C5AR1	4,59482E-03	33666,8	3,78047E-03	35048	1,15999E+00	17169	1,38961E+00	43776	9,41950E-01	10441	0	61900
Myeloid_vs_CD8_Non-responder	CD8(Temra)	Macro_FOLR2+APOE+	B2M	LILRB2	4,59744E-03	29717,2	3,25720E-03	43781	1,31778E+00	11890	1,75980E+00	29116	9,55869E-01	1899	0	61900
Myeloid_vs_CD8_Non-responder	Macro_FOLR2+APOE+	CD8(GZMK+ Tex)	GRN	TNFRSF1B	4,60112E-03	27593,6	3,25663E-03	43788	1,38653E+00	9978	2,69560E+00	8946	9,37793E-01	13356	0	61900
Myeloid_vs_CD8_Non-responder	Macro_FOLR2+APOE+	CD8(Tn)	LGALS3BP	ITGB1	4,60217E-03	30876,8	6,79151E-03	14576	1,38815E+00	9940	1,92255E+00	24178	8,89606E-01	43790	0	61900
Myeloid_vs_CD8_Non-responder	Macro_FOLR2+APOE+	CD8(ISG+ T)	CD14	ITGA4	4,60217E-03	31711,6	3,25649E-03	43790	1,34660E+00	11014	2,46879E+00	12151	9,10418E-01	29703	0	61900
Myeloid_vs_CD8_Non-responder	CD8(ITM2C+ T)	Macro_FOLR2+APOE+	HLA-A	LILRB1	4,60743E-03	36364,8	3,25593E-03	43800	1,06640E+00	21138	1,44264E+00	41380	9,37411E-01	13606	0	61900
Myeloid_vs_CD8_Non-responder	Macro_NLRP3	CD8(ITM2C+ T)	CCL3L1	CCR5	4,61037E-03	29091,2	1,50987E-02	3766	1,46779E+00	8198	2,91052E+00	6517	8,57284E-01	65075	0	61900
Myeloid_vs_CD8_Non-responder	Macro_LYVE1	CD8(GZMK+ Tem)	HLA-DRB5	LAG3	4,61593E-03	22425,2	6,94049E-03	14130	1,36484E+00	10540	2,03229E+00	21187	9,50400E-01	4369	0	61900
Myeloid_vs_CD8_Non-responder	CD8(LAYN+ T)	Macro_FOLR2+APOE+	CALR	LRP1	4,62112E-03	40721	3,25491E-03	43826	9,99330E-01	24263	1,42486E+00	42161	9,07808E-01	31455	0	61900
Myeloid_vs_CD8_Non-responder	Macro_NLRP3	CD8(ISG+ T)	HBEGF	CD44	4,63207E-03	24667,4	6,01360E-03	17518	1,70565E+00	4799	2,03169E+00	21206	9,29879E-01	17914	0	61900
Myeloid_vs_CD8_Non-responder	CD8(ID2+CXCR4+ T)	Macro_ISG15	ANXA1	FPR1	4,63590E-03	38469,6	3,25355E-03	43854	9,00073E-01	29881	1,63293E+00	33580	9,21000E-01	23133	0	61900
Myeloid_vs_CD8_Non-responder	Macro_OLFML3	CD8(GZMK+ Tex)	HLA-DQA2	LAG3	4,64655E-03	24586	7,79484E-03	11854	1,28700E+00	12779	2,30230E+00	15174	9,24053E-01	21223	0	61900
Myeloid_vs_CD8_Non-responder	Macro_OLFML3	CD8(ZNF683+KLRB1+ T)	B2M	CD3D	4,65282E-03	49636,4	2,68158E-03	57497	5,72070E-01	58015	8,84051E-01	70737	9,64592E-01	33	0	61900
Myeloid_vs_CD8_Non-responder	Macro_ISG15	CD8(Terminal Tex)	HLA-DRB5	LAG3	4,66191E-03	25874,4	5,28225E-03	21241	1,08828E+00	20181	2,22182E+00	16784	9,43555E-01	9266	0	61900
Myeloid_vs_CD8_Non-responder	CD8(ZNF683+KLRB1+ T)	Macro_ISG15	CD99	CD81	4,66345E-03	40364,4	3,25077E-03	43906	8,26049E-01	34798	1,53728E+00	37366	9,19809E-01	23852	0	61900
Myeloid_vs_CD8_Non-responder	CD8(Tn)	Macro_ISG15	HLA-C	LILRB1	4,67940E-03	35490,8	3,66283E-03	36729	1,01302E+00	23565	1,38596E+00	43936	9,40680E-01	11324	0	61900
Myeloid_vs_CD8_Non-responder	cDC(CD1C)	CD8(ISG+ T)	HLA-DQA1	LAG3	4,69362E-03	26755,2	5,49354E-03	20054	1,17311E+00	16672	2,02899E+00	21278	9,36957E-01	13872	0	61900
Myeloid_vs_CD8_Non-responder	CD8(EOMES+ NK-like)	Macro_OLFML3	CD99	PILRA	4,69700E-03	39311,6	3,40474E-03	40965	9,84686E-01	25004	1,38540E+00	43969	9,18419E-01	24720	0	61900
Myeloid_vs_CD8_Non-responder	CD8(LAYN+ T)	Macro_OLFML3	HLA-A	LILRB1	4,70074E-03	35655,6	3,47370E-03	39771	1,08480E+00	20307	1,38523E+00	43976	9,39283E-01	12324	0	61900
Myeloid_vs_CD8_Non-responder	Macro_FOLR2+APOE+	CD8(LAYN+ T)	TNFSF13	FAS	4,70181E-03	27727,8	9,81284E-03	8228	1,37581E+00	10252	2,34640E+00	14281	8,89311E-01	43978	0	61900
Myeloid_vs_CD8_Non-responder	Macro_FOLR2+APOE+	CD8(IL7R+ZNF683+ Tm)	SPP1	PTGER4	4,70609E-03	28367	9,84943E-03	8171	1,01600E+00	23428	3,16645E+00	4350	8,89303E-01	43986	0	61900
Myeloid_vs_CD8_Non-responder	CD8(GZMK+ Early Tem)	Macro_NLRP3	CIRBP	TREM1	4,70983E-03	40097	4,44803E-03	27474	9,90769E-01	24683	1,38486E+00	43993	8,91624E-01	42435	0	61900
Myeloid_vs_CD8_Non-responder	CD8(Tc17)	Macro_FOLR2+APOE+	B2M	LILRB2	4,70983E-03	29872,6	3,24693E-03	43993	1,30458E+00	12266	1,75434E+00	29286	9,55803E-01	1918	0	61900
Myeloid_vs_CD8_Non-responder	CD8(Terminal Tex)	Macro_FOLR2+APOE+	CCL5	CCRL2	4,71198E-03	29099,2	1,11112E-02	6726	1,27448E+00	13151	1,38477E+00	43997	9,26705E-01	19722	0	61900
Myeloid_vs_CD8_Non-responder	CD8(Temra)	cDC_CLEC9A	TGFB1	LPP	4,71251E-03	38548,4	5,70226E-03	18947	9,21338E-01	28566	1,49107E+00	39331	8,89281E-01	43998	0	61900
Myeloid_vs_CD8_Non-responder	CD8(ZNF683+KLRB1+ T)	Macro_ISG15	CCL4	CCR1	4,71626E-03	33340	7,26997E-03	13178	1,25315E+00	13815	1,38467E+00	44005	9,04308E-01	33802	0	61900
Myeloid_vs_CD8_Non-responder	Mono_INHBA	CD8(IL7R+ZNF683+ Tm)	SPP1	CD44	4,71943E-03	24096,4	9,44275E-03	8790	1,30294E+00	12310	2,25252E+00	16174	9,23914E-01	21308	0	61900
Myeloid_vs_CD8_Non-responder	CD8(GZMK+ Tem)	Macro_ISG15	CIRBP	TREM1	4,72109E-03	40217	4,23701E-03	29609	9,86289E-01	24926	1,46019E+00	40636	8,89253E-01	44014	0	61900
Myeloid_vs_CD8_Non-responder	CD8(ISG+ T)	Macro_FOLR2+APOE+	CD28	CD86	4,73498E-03	34954,2	1,05048E-02	7411,5	1,37129E+00	10363,5	2,02671E+00	21326,5	8,44033E-01	73769,5	0	61900
Myeloid_vs_CD8_Non-responder	Macro_FOLR2+APOE+	CD8(GZMK+ Early Tem)	HLA-DRB5	LAG3	4,73935E-03	33807,4	3,24365E-03	44048	9,62222E-01	26162	2,14063E+00	18548	9,29074E-01	18379	0	61900
Myeloid_vs_CD8_Non-responder	CD8(ISG+ T)	Macro_LYVE1	TNFSF9	HLA-DPA1	4,74150E-03	39799,6	3,24323E-03	44052	9,39004E-01	27491	1,64366E+00	33182	9,06437E-01	32373	0	61900
Myeloid_vs_CD8_Non-responder	Macro_LYVE1	CD8(Terminal Tex)	HLA-DRA	LAG3	4,74276E-03	23722,6	5,85177E-03	18225	1,25551E+00	13735	2,02651E+00	21335	9,52314E-01	3418	0	61900
Myeloid_vs_CD8_Non-responder	CD8(GZMK+ Early Tem)	Macro_FOLR2+APOE+	B2M	LILRB2	4,75120E-03	30072,8	3,24216E-03	44070	1,29844E+00	12457	1,73280E+00	30005	9,55772E-01	1932	0	61900
Myeloid_vs_CD8_Non-responder	CD8(GZMK+ Tem)	Macro_FOLR2+APOE+	HLA-C	LILRB1	4,75443E-03	35691,2	3,24194E-03	44076	1,10706E+00	19324	1,48853E+00	39419	9,37181E-01	13737	0	61900
Myeloid_vs_CD8_Non-responder	CD8(Terminal Tex)	Macro_ISG15	HLA-C	LILRB1	4,75929E-03	34046	3,81707E-03	34540	1,11042E+00	19176	1,38298E+00	44085	9,41820E-01	10529	0	61900
Myeloid_vs_CD8_Non-responder	Mono_CD14	CD8(ID2+CXCR4+ T)	S100A8	ITGB2	4,76096E-03	19611,4	5,26627E-03	21356	2,41349E+00	1030	3,41521E+00	2812	9,41228E-01	10959	0	61900
Myeloid_vs_CD8_Non-responder	Macro_FOLR2+APOE+	CD8(Tn)	APOE	LSR	4,76350E-03	25188,2	1,54883E-02	3564	1,46354E+00	8290	3,26670E+00	3666	8,82531E-01	48521	0	61900
Myeloid_vs_CD8_Non-responder	pDC_LILRA4	CD8(GZMK+ Tem)	HLA-DRA	LAG3	4,76444E-03	24329	5,26573E-03	21360	1,07738E+00	20649	2,41288E+00	13087	9,49860E-01	4649	0	61900
Myeloid_vs_CD8_Non-responder	Mono_INHBA	CD8(GZMK+ Early Tem)	SPP1	CD44	4,76965E-03	24290,6	9,42180E-03	8824	1,30106E+00	12368	2,21134E+00	16995	9,23836E-01	21366	0	61900
Myeloid_vs_CD8_Non-responder	Macro_LYVE1	CD8(GZMK+ Tex)	HLA-DPA1	LAG3	4,77486E-03	24307	5,57888E-03	19585	1,23081E+00	14552	2,02494E+00	21372	9,50871E-01	4126	0	61900
Myeloid_vs_CD8_Non-responder	Macro_FOLR2+APOE+	CD8(EOMES+ NK-like)	LGALS3	LAG3	4,77984E-03	39729,2	3,23997E-03	44123	8,18902E-01	35291	1,86528E+00	25853	9,07780E-01	31479	0	61900
Myeloid_vs_CD8_Non-responder	CD8(EOMES+ NK-like)	Macro_OLFML3	HLA-A	LILRB1	4,78254E-03	35941,8	3,45165E-03	40124	1,06703E+00	21108	1,38195E+00	44128	9,39102E-01	12449	0	61900
Myeloid_vs_CD8_Non-responder	CD8(GZMK+ Early Tem)	Macro_LYVE1	HMGB1	CD163	4,78471E-03	33139,4	3,99226E-03	32357	1,31231E+00	12047	1,38181E+00	44132	9,34698E-01	15261	0	61900
Myeloid_vs_CD8_Non-responder	CD8(NME1+ T)	Macro_IER3	RPS19	C5AR1	4,79719E-03	30085	4,54242E-03	26598	1,34490E+00	11061	1,38132E+00	44155	9,46771E-01	6711	0	61900
Myeloid_vs_CD8_Non-responder	Macro_FOLR2+APOE-	CD8(ISG+ T)	C3	IFITM1	4,79723E-03	22533	1,10886E-02	6752	1,62691E+00	5692	1,73261E+00	30011	9,44741E-01	8310	0	61900
Myeloid_vs_CD8_Non-responder	Macro_NLRP3	CD8(LAYN+ T)	THBS1	ITGA4	4,80101E-03	20534,8	1,20721E-02	5882	1,80602E+00	3902	2,64693E+00	9588	9,23775E-01	21402	0	61900
Myeloid_vs_CD8_Non-responder	CD8(EOMES+ NK-like)	Macro_FOLR2+APOE+	CCL5	CCR1	4,80208E-03	30679,8	9,60746E-03	8528	1,17891E+00	16451	1,38095E+00	44164	9,22224E-01	22356	0	61900
Myeloid_vs_CD8_Non-responder	CD8(GZMK+ Tem)	Macro_FOLR2+APOE+	B2M	LILRB2	4,80426E-03	30044,8	3,23774E-03	44168	1,29276E+00	12629	1,74630E+00	29583	9,55743E-01	1944	0	61900
Myeloid_vs_CD8_Non-responder	CD8(NME1+ T)	Macro_ISG15	HLA-F	LILRB1	4,80480E-03	38062	3,83552E-03	34300	1,22542E+00	14753	1,59068E+00	35188	8,89022E-01	44169	0	61900
Myeloid_vs_CD8_Non-responder	CD8(NME1+ T)	Macro_ISG15	CCL5	CCR1	4,80625E-03	31788,8	1,00320E-02	7940	1,28846E+00	12738	1,16379E+00	54958	9,23761E-01	21408	0	61900
Myeloid_vs_CD8_Non-responder	CD8(ID2+CXCR4+ T)	Macro_ISG15	HLA-F	LILRB2	4,80807E-03	34606,8	3,23734E-03	44175	1,42392E+00	9112	1,99932E+00	22035	9,01306E-01	35812	0	61900
Myeloid_vs_CD8_Non-responder	CD8(EOMES+ NK-like)	pDC_LILRA4	HMGB1	TLR9	4,81105E-03	29015,4	3,30934E-02	802	2,17333E+00	1756	1,34908E+00	45646	9,02584E-01	34973	0	61900
Myeloid_vs_CD8_Non-responder	Macro_FOLR2+APOE+	CD8(ZNF683+KLRB1+ T)	HLA-A	CD3G	4,81460E-03	38544,4	3,34415E-03	42063	7,46490E-01	40827	1,38016E+00	44187	9,51590E-01	3745	0	61900
Myeloid_vs_CD8_Non-responder	Macro_FOLR2+APOE+	CD8(GZMK+ Tem)	CD59	CD2	4,81678E-03	36350,8	6,77620E-03	14621	8,83347E-01	30884	1,38007E+00	44191	9,09742E-01	30158	0	61900

Myeloid_vs_CD8_Post_NR

Myeloid_vs_CD8_Non-responder	CD8(GZMK+ Early Tem)	Macro_OLFML3	HLA-F	LILRB1	4,82878E-03	39418,2	3,82950E-03	34396	1,07755E+00	20639	1,57239E+00	35943	8,88944E-01	44213	0	61900
Myeloid_vs_CD8_Non-responder	CD8(NME1+ T)	Macro_FOLR2+APOE+	HSP90B1	LRP1	4,82878E-03	41468,6	3,23549E-03	44213	1,03192E+00	22733	1,45273E+00	40969	8,98727E-01	37528	0	61900
Myeloid_vs_CD8_Non-responder	CD8(EOMES+ NK-like)	Macro_FOLR2+APOE+	B2M	LILRB2	4,83151E-03	30097,4	3,23519E-03	44218	1,28948E+00	12703	1,74213E+00	29712	9,55726E-01	1954	0	61900
Myeloid_vs_CD8_Non-responder	CD8(Tc17)	Macro_FOLR2+APOE+	HLA-B	LILRB2	4,83370E-03	30779,8	3,23482E-03	44222	1,26058E+00	13566	1,72712E+00	30201	9,51093E-01	4010	0	61900
Myeloid_vs_CD8_Non-responder	Macro_NLRP3	CD8(NME1+ T)	CXCL2	DPP4	4,83450E-03	36838,2	1,18278E-02	6087	1,81613E+00	3818	2,74311E+00	8332	7,94668E-01	104054	0	61900
Myeloid_vs_CD8_Non-responder	CD8(GZMK+ Tex)	Macro_FOLR2+APOE+	HLA-A	LILRB1	4,83479E-03	36831	3,23447E-03	44224	1,04744E+00	22040	1,42233E+00	42282	9,37217E-01	13709	0	61900
Myeloid_vs_CD8_Non-responder	CD8(Terminal Tex)	Mono_CD16	HLA-A	LILRB1	4,85341E-03	34160,8	3,74099E-03	35593	1,13421E+00	18183	1,37863E+00	44258	9,41363E-01	10870	0	61900
Myeloid_vs_CD8_Non-responder	CD8(ID2+CXCR4+ T)	Macro_FOLR2+APOE+	B2M	TFRC	4,85725E-03	37440,8	3,23177E-03	44265	8,22222E-01	35045	1,49865E+00	39006	9,46448E-01	6988	0	61900
Myeloid_vs_CD8_Non-responder	CD8(IL7R+ZNF683+ Tm)	Mono_CD16	CD52	SIGLEC10	4,85999E-03	36474,8	4,51511E-03	26851	1,04468E+00	22162	1,37844E+00	44270	9,14493E-01	27191	0	61900
Myeloid_vs_CD8_Non-responder	CD8(Temra)	cDC_CLEC9A	TNFSF9	HLA-DPA1	4,85999E-03	34596,6	3,23133E-03	44270	1,04556E+00	22127	2,46539E+00	12201	9,06281E-01	32485	0	61900
Myeloid_vs_CD8_Non-responder	CD8(ZNF683+KLRB1+ T)	Macro_FOLR2+APOE+	ADAM10	GPNMB	4,86155E-03	36115,8	5,24570E-03	21471	1,55317E+00	6720	2,57102E+00	10591	8,34241E-01	79897	0	61900
Myeloid_vs_CD8_Non-responder	cDC(CD1C)	CD8(GZMK+ Tem)	HLA-DQB2	LAG3	4,86419E-03	22067,6	1,46575E-02	3995	1,63762E+00	5549	2,19179E+00	17420	9,23650E-01	21474	0	61900
Myeloid_vs_CD8_Non-responder	CD8(LAYN+ T)	Macro_FOLR2+APOE+	B2M	LILRB2	4,86493E-03	30160	3,23089E-03	44279	1,28394E+00	12873	1,73967E+00	29784	9,55698E-01	1964	0	61900
Myeloid_vs_CD8_Non-responder	Macro_ISG15	CD8(GZMK+ Tem)	HLA-DPB1	LAG3	4,86596E-03	26004,8	5,24527E-03	21476	1,06970E+00	20981	2,12207E+00	18969	9,46796E-01	6698	0	61900
Myeloid_vs_CD8_Non-responder	CD8(ISG+ T)	Macro_OLFML3	HLA-F	LILRB1	4,87043E-03	39822	3,82186E-03	34477	1,07642E+00	20686	1,52744E+00	37758	8,88846E-01	44289	0	61900
Myeloid_vs_CD8_Non-responder	Macro_ISG15	CD8(GZMK+ Tex)	HLA-DRB5	LAG3	4,87125E-03	25894	5,24385E-03	21482	1,08312E+00	20381	2,24649E+00	16303	9,43360E-01	9404	0	61900
Myeloid_vs_CD8_Non-responder	Macro_FOLR2+APOE+	CD8(Temra)	CXCL9	CXCR3	4,87537E-03	29163,2	1,04486E-02	7473	2,20399E+00	1645	2,74099E+00	8356	8,55157E-01	66442	0	61900
Myeloid_vs_CD8_Non-responder	CD8(GZMK+ Tex)	Macro_FOLR2+APOE+	ADAM10	GPNMB	4,87567E-03	36308,6	5,24347E-03	21487	1,55309E+00	6721	2,50780E+00	11519	8,34211E-01	79916	0	61900
Myeloid_vs_CD8_Non-responder	Macro_FOLR2+APOE+	CD8(Temra)	HLA-E	KLRC3_KLRD1	4,88584E-03	37496	5,40540E-03	20555	7,05935E-01	44317	1,46966E+00	40210	9,25340E-01	20498	0	61900
Myeloid_vs_CD8_Non-responder	CD8(ID2+CXCR4+ T)	Macro_FOLR2+APOE+	HLA-A	LILRB1	4,90075E-03	36841	3,22761E-03	44344	1,04137E+00	22284	1,43033E+00	41924	9,37154E-01	13753	0	61900
Myeloid_vs_CD8_Non-responder	Macro_IER3	CD8(GZMK+ Tex)	HLA-DRA	LAG3	4,90133E-03	24875	5,47481E-03	20149	1,17396E+00	16634	2,01982E+00	21516	9,50779E-01	4176	0	61900
Myeloid_vs_CD8_Non-responder	Macro_FOLR2+APOE+	CD8(Tc17)	LGALS9	CD44	4,90296E-03	35176,6	3,22745E-03	44348	1,11170E+00	19118	1,71644E+00	30532	9,26223E-01	19985	0	61900
Myeloid_vs_CD8_Non-responder	Mono_CD14	CD8(LAYN+ T)	CD14	ITGA4	4,90754E-03	25881,2	5,23731E-03	21523	1,38831E+00	9935	2,20874E+00	17052	9,27998E-01	18996	0	61900
Myeloid_vs_CD8_Non-responder	Mast	CD8(Terminal Tex)	TIMP3	CD44	4,90918E-03	37302,6	2,27363E-02	1672	2,16855E+00	1774	7,84369E-01	76707	8,88618E-01	44460	0	61900
Myeloid_vs_CD8_Non-responder	CD8(ZNF683+KLRB1+ T)	Mono_CD14	HLA-F	LILRB2	4,91291E-03	38558,8	3,22670E-03	44366	1,10969E+00	19210	1,69143E+00	31412	9,01159E-01	35906	0	61900
Myeloid_vs_CD8_Non-responder	Macro_IER3	CD8(GZMK+ Tex)	HLA-DRB5	LAG3	4,91553E-03	25462,4	5,64721E-03	19210	1,17037E+00	16764	2,01897E+00	21532	9,45308E-01	7906	0	61900
Myeloid_vs_CD8_Non-responder	CD8(GZMK+ Tem)	Macro_ISG15	TGFB1	ENG	4,91568E-03	41175	4,27163E-03	29224	8,93699E-01	30267	1,47183E+00	40113	8,88743E-01	44371	0	61900
Myeloid_vs_CD8_Non-responder	CD8(GZMK+ Early Tem)	Macro_FOLR2+APOE+	HLA-A	LILRB1	4,91956E-03	36939,2	3,22615E-03	44378	1,04008E+00	22339	1,42165E+00	42319	9,37141E-01	13760	0	61900
Myeloid_vs_CD8_Non-responder	Mono_CD14	CD8(GZMK+ Tem)	VCAN	ITGB1	4,92354E-03	19669,6	1,25271E-02	5469	2,57020E+00	759	2,71548E+00	8679	9,23547E-01	21541	0	61900
Myeloid_vs_CD8_Non-responder	Mono_CD16	CD8(EOMES+ NK-like)	CFP	NCR1	4,92563E-03	30335	6,30704E-02	227	2,16803E+00	1777	2,68012E+00	9155	8,36299E-01	78616	0	61900
Myeloid_vs_CD8_Non-responder	CD8(NME1+ T)	Mono_CD16	HMGB1	TLR4	4,92733E-03	40912	4,01031E-03	32158	1,04785E+00	22020	1,38280E+00	44090	8,88712E-01	44392	0	61900
Myeloid_vs_CD8_Non-responder	CD8(LAYN+ T)	Macro_ISG15	TGFB1	ENG	4,93121E-03	41930,8	4,26804E-03	29268	8,93126E-01	30305	1,38949E+00	43782	8,88702E-01	44399	0	61900
Myeloid_vs_CD8_Non-responder	Macro_FOLR2+APOE+	CD8(GZMK+ Early Tem)	HLA-DRA	LAG3	4,93566E-03	31604,2	3,22493E-03	44407	1,00428E+00	24016	2,37632E+00	13748	9,36810E-01	13950	0	61900
Myeloid_vs_CD8_Non-responder	Mast	CD8(NME1+ T)	TIMP3	CD44	4,95311E-03	36837,8	2,26529E-02	1685	2,16649E+00	1782	8,25516E-01	74213	8,88436E-01	44609	0	61900
Myeloid_vs_CD8_Non-responder	Macro_FOLR2+APOE+	CD8(IL7R+ZNF683+ Tm)	APOE	SORL1	4,95737E-03	33013,6	3,38453E-03	41293	1,30230E+00	12330	3,06913E+00	5099	8,88636E-01	44446	0	61900
Myeloid_vs_CD8_Non-responder	CD8(GZMK+ Tex)	Macro_OLFML3	HLA-F	LILRB2	4,95737E-03	37794	3,22333E-03	44446	1,17929E+00	16435	1,72567E+00	30254	9,01113E-01	35935	0	61900
Myeloid_vs_CD8_Non-responder	CD8(Tc17)	pDC_LILRA4	TNF	TNFRSF21	4,96072E-03	23956,6	1,71652E-02	2873	2,25289E+00	1477	2,68588E+00	9081	8,88624E-01	44452	0	61900
Myeloid_vs_CD8_Non-responder	pDC_LILRA4	CD8(LAYN+ T)	COL24A1	ITGA2_ITGB1	4,96412E-03	40552,4	6,51411E-02	214	2,16557E+00	1784	1,52564E+00	37840	7,99703E-01	101024	0	61900
Myeloid_vs_CD8_Non-responder	CD8(Tc17)	Mono_CD16	HLA-A	LILRA1	4,96630E-03	27144,4	9,37110E-03	8911	1,67877E+00	5059	1,37475E+00	44462	9,34469E-01	15390	0	61900
Myeloid_vs_CD8_Non-responder	Macro_FOLR2+APOE+	CD8(LAYN+ T)	SECTM1	CD7	4,97133E-03	32777,6	1,02903E-02	7639	1,30501E+00	12254	1,37462E+00	44471	8,98614E-01	37624	0	61900
Myeloid_vs_CD8_Non-responder	Macro_FOLR2+APOE+	CD8(ZNF683+KLRB1+ T)	B2M	CD3G	4,97133E-03	34756,2	3,22208E-03	44471	9,23659E-01	28415	1,52932E+00	37680	9,57416E-01	1315	0	61900
Myeloid_vs_CD8_Non-responder	Mono_CD14	CD8(Temra)	VCAN	ITGA4	4,97264E-03	19770,2	1,21073E-02	5856	2,54747E+00	792	2,71350E+00	8707	9,23460E-01	21596	0	61900
Myeloid_vs_CD8_Non-responder	Macro_FOLR2+APOE+	CD8(IL7R+ZNF683+ Tm)	LGALS1	CD69	4,97356E-03	34439	3,22177E-03	44475	8,63055E-01	32251	1,78656E+00	28293	9,48840E-01	5276	0	61900
Myeloid_vs_CD8_Non-responder	CD8(ZNF683+KLRB1+ T)	Mono_CD16	HLA-G	LILRB2	4,97444E-03	32280,6	5,80605E-03	18433	1,96836E+00	2724	2,84465E+00	7183	8,48032E-01	71163	0	61900
Myeloid_vs_CD8_Non-responder	CD8(ZNF683+KLRB1+ T)	Macro_OLFML3	HLA-A	LILRB2	4,97636E-03	29522,4	3,22163E-03	44480	1,43483E+00	8866	1,82782E+00	26988	9,48682E-01	5378	0	61900
Myeloid_vs_CD8_Non-responder	CD8(Temra)	Macro_ISG15	HLA-F	LILRB1	4,99765E-03	37499,8	3,79711E-03	34823	1,22079E+00	14918	1,69335E+00	31340	8,88524E-01	44518	0	61900
Myeloid_vs_CD8_Non-responder	Macro_FOLR2+APOE+	CD8(GZMK+ Tex)	TIMP2	CD44	4,99934E-03	41637	3,77271E-03	35163	9,57539E-01	26422	1,37372E+00	44521	8,94860E-01	40179	0	61900
Myeloid_vs_CD8_Non-responder	CD8(ITM2C+ T)	Macro_NLRP3	TNF	TNFRSF1B	5,00439E-03	43232,2	3,85385E-03	34065	7,03417E-01	44530	1,41393E+00	42666	9,05476E-01	33000	0	61900
Myeloid_vs_CD8_Non-responder	Mast	CD8(ZNF683+KLRB1+ T)	TIMP3	CD44	5,01936E-03	35331	2,24967E-02	1704	2,16264E+00	1794	9,56970E-01	66442	8,88093E-01	44815	0	61900
Myeloid_vs_CD8_Non-responder	CD8(GZMK+ Tem)	Macro_ISG15	HLA-F	LILRB1	5,02014E-03	37834,6	3,79384E-03	34868	1,22040E+00	14930	1,65098E+00	32917	8,88482E-01	44558	0	61900
Myeloid_vs_CD8_Non-responder	CD8(Temra)	pDC_LILRA4	TGFB1	CXCR4	5,02071E-03	36557,6	3,21716E-03	44559	7,08227E-01	44120	2,03691E+00	21072	9,40964E-01	11137	0	61900
Myeloid_vs_CD8_Non-responder	Macro_ISG15	CD8(Terminal Tex)	HLA-DQB1	LAG3	5,02211E-03	26900,4	5,51477E-03	19935	1,05557E+00	21651	2,15209E+00	18308	9,38745E-01	12708	0	61900
Myeloid_vs_CD8_Non-responder	CD8(IL7R+ZNF683+ Tm)	Macro_OLFML3	HLA-B	LILRB1	5,02578E-03	36106,6	3,36884E-03	41576	1,05920E+00	21472	1,37264E+00	44568	9,41154E-01	11017	0	61900
Myeloid_vs_CD8_Non-responder	CD8(LAYN+ T)	Macro_IER3	ANXA1	FPR1	5,02860E-03	33326,8	4,52874E-03	26720	1,16918E+00	16800	1,37260E+00	44573	9,32224E-01	16641	0	61900
Myeloid_vs_CD8_Non-responder	Macro_FOLR2+APOE+	CD8(Temra)	ICAM1	IL2RG	5,02916E-03	43470,2	3,21611E-03	44574	8,41746E-01	33699	1,50381E+00	38782	8,97489E-01	38396	0	61900
Myeloid_vs_CD8_Non-responder	CD8(EOMES+ NK-like)	Macro_OLFML3	HLA-C	LILRB1	5,04611E-03	36950,4	3,33336E-03	42277	1,03159E+00	22745	1,37175E+00	44604	9,37995E-01	13226	0	61900
Myeloid_vs_CD8_Non-responder	Macro_FOLR2+APOE+	CD8(IL7R+ZNF683+ Tm)	HLA-DQA1	LAG3	5,05007E-03	34513,8	3,21423E-03	44611	1,00605E+00	23915	2,16906E+00	17924	9,19148E-01	24219	0	61900
Myeloid_vs_CD8_Non-responder	Macro_FOLR2+APOE+	CD8(GZMK+ Early Tem)	HLA-DQA2	LAG3	5,05687E-03	43904,4	3,33655E-03	42205	7,50200E-01	40548	1,72603E+00	30246	8,88398E-01	44623	0	61900
Myeloid_vs_CD8_Non-responder	Macro_NLRP3	CD8(Tn)	VEGFA	CD44	5,05923E-03	22863,2	9,61472E-03	8515	2,02319E+00	2421	2,08765E+00	19788	9,23307E-01	21692	0	61900
Myeloid_vs_CD8_Non-responder	Macro_NLRP3	CD8(Tc17)	HBEGF	CD44	5,06013E-03	25032,8	5,86065E-03	18186	1,68568E+00	4988	2,01216E+00	21693	9,29034E-01	18397	0	61900
Myeloid_vs_CD8_Non-responder	cDC_LAMP3	CD8(Terminal Tex)	B2M	CD3D	5,07563E-03	55938,4	2,66355E-03	58050	5,18201E-01	64596	4,95306E-01	95110	9,64477E-01	36	0	61900
Myeloid_vs_CD8_Non-responder	Macro_NLRP3	CD8(NME1+ T)	ICAM1	IL2RG	5,08356E-03	31338,2	4,75622E-03	24795	1,20054E+00	15669	1,82982E+00	26928	9,14141E-01	27399	0	61900
Myeloid_vs_CD8_Non-responder	Macro_NLRP3	CD8(NME1+ T)	THBS1	ITGA4	5,08412E-03	28294,8	6,58952E-03	15258	1,44640E+00	8627	2,13435E+00	18681	8,99535E-01	37008	0	61900
Myeloid_vs_CD8_Non-responder	Macro_NLRP3	CD8(NME1+ T)	THBS1	CD47	5,08469E-03	28859,2	6,62731E-03	15120	1,48594E+00	7871	2,23006E+00	16624	8,91125E-01	42781	0	61900
Myeloid_vs_CD8_Non-responder	Macro_LYVE1	CD8(LAYN+ T)	F13A1	ITGB1	5,09289E-03	20310,6	1,40688E-02	4356	2,34677E+00	1201	2,45553E+00	12367	9,23249E-01	21729	0	61900
Myeloid_vs_CD8_Non-responder	CD8(Terminal Tex)	Macro_FOLR2+APOE+	CALR	LRP1	5,10236E-03	41377	3,20892E-03	44703	9,89269E-01	24762	1,39256E+00	43650	9,07210E-01	31870	0	61900
Myeloid_vs_CD8_Non-responder	Macro_ISG15	CD8(GZMK+ Tex)	S100A8	CD69	5,10293E-03	27901,8	5,25113E-03	21437	1,12115E+00	18748	2,01019E+00	21740	9,33958E-01	15684	0	61900
Myeloid_vs_CD8_Non-responder	CD8(IL7R+ZNF683+ Tm)	Macro_NLRP3	CIRBP	TREM1	5,10407E-03	41035	4,29208E-03	28999	9,65826E-01	25972	1,36923E+00	44706	8,89887E-01	43598	0	61900
Myeloid_vs_CD8_Non-responder	Macro_FOLR2+APOE+	CD8(EOMES+ NK-like)	MMP9	CD44	5,10636E-03	29554	7,21310E-03	13325	1,32042E+00	11800	2,25868E+00	16035	8,88271E-01	44710	0	61900
Myeloid_vs_CD8_Non-responder	CD8(GZMK+ Tex)	Macro_NLRP3	CIRBP	TREM1	5,10693E-03	40405,4	4,41656E-03	27771	9,85735E-01	24957	1,36920E+00	44711	8,91280E-01	42688	0	61900
Myeloid_vs_CD8_Non-responder	CD8(ITM2C+ T)	Macro_NLRP3	HLA-F	LILRB2	5,10864E-03	37911,8	3,20870E-03	44714	1,15126E+00	17461	1,75140E+00	29406	9,00910E-01	36078	0	61900
Myeloid_vs_CD8_Non-responder	Macro_NLRP3	CD8(NME1+ T)	IL1B	SIGIRR	5,10921E-03	22617,2	7,60772E-03	12284	2,21831E+00	1593	3,06871E+00	5105	9,06700E-01	32204	0	61900
Myeloid_vs_CD8_Non-responder	Macro_NLRP3	CD8(NME1+ T)	S100A8	ITGB2	5,10978E-03	24514,4	4,20161E-03	29992	1,87457E+00	3370	2,47550E+00	12026	9,34661E-01	15284	0	61900
Myeloid_vs_CD8_Non-responder	Macro_NLRP3	CD8(NME1+ T)	S100A9	ITGB2	5,11093E-03	25870,8	3,54664E-03	38619	1,82235E+00	3768	2,58479E+00	10395	9,35719E-01	14672	0	61900
Myeloid_vs_CD8_Non-responder	CD8(Temra)	pDC_LILRA4	HLA-C	NOTCH4	5,11493E-03	28143,6	1,41583E-02	4303	1,53308E+00	7044	1,36885E+00	44725	9,21585E-01	22746	0	61900
Myeloid_vs_CD8_Non-responder	Macro_NLRP3	CD8(NME1+ T)	VCAN	ITGB1	5,11493E-03	23184,2	8,86531E-03	9751	2,13378E+00	1906	2,43891E+00	12659	9,10411E-01	29705	0	61900
Myeloid_vs_CD8_Non-responder	Macro_NLRP3	CD8(NME1+ T)	VCAN	ITGA4	5,11550E-03	24249,8	8,03594E-03	11352	2,08377E+00	2119	2,34435E+00	14320	9,07658E-01	31558	0	61900

Myeloid_vs_CD8_Post_NR

Myeloid_vs_CD8_Non-responder	Macro_NLRP3	CD8(NME1+ T)	VCAN	CD44	5,11664E-03	25014,2	6,03378E-03	17422	1,91757E+00	3042	1,99619E+00	22114	9,25181E-01	20593	0	61900
Myeloid_vs_CD8_Non-responder	Macro_NLRP3	CD8(NME1+ T)	HBEGF	CD44	5,12466E-03	31507,4	4,27444E-03	29193	1,47853E+00	7998	1,63878E+00	33384	9,17899E-01	25062	0	61900
Myeloid_vs_CD8_Non-responder	Macro_OLFML3	CD8(LAYN+ T)	CXCL10	CXCR3	5,12533E-03	28715,8	9,62740E-03	8500	2,18825E+00	1698	2,99675E+00	5729	8,56221E-01	65752	0	61900
Myeloid_vs_CD8_Non-responder	CD8(GZMK+ Tex)	Macro_OLFML3	HLA-F	LILRB1	5,12695E-03	40073,4	3,77370E-03	35145	1,06929E+00	20997	1,53181E+00	37579	8,88218E-01	44746	0	61900
Myeloid_vs_CD8_Non-responder	CD8(ISG+ T)	Macro_FOLR2+APOE+	B2M	TFRC	5,12981E-03	38176,8	3,20684E-03	44751	7,88851E-01	37568	1,48650E+00	39510	9,46251E-01	7155	0	61900
Myeloid_vs_CD8_Non-responder	cDC_CLEC9A	CD8(NME1+ T)	HLA-DQB1	LAG3	5,13497E-03	24558,6	5,19470E-03	21775	1,24469E+00	14084	2,52861E+00	11191	9,37003E-01	13843	0	61900
Myeloid_vs_CD8_Non-responder	Macro_NLRP3	CD8(NME1+ T)	HBEGF	CD82	5,13555E-03	24569	1,07632E-02	7139	1,84644E+00	3566	2,30042E+00	15214	9,02500E-01	35026	0	61900
Myeloid_vs_CD8_Non-responder	Macro_NLRP3	CD8(NME1+ T)	LYZ	ITGAL	5,14129E-03	30619,8	3,80554E-03	34691	1,27372E+00	13180	2,32490E+00	14703	9,12200E-01	28625	0	61900
Myeloid_vs_CD8_Non-responder	CD8(ISG+ T)	Macro_ISG15	CCL5	CCR1	5,14691E-03	32204,6	9,86427E-03	8157	1,27526E+00	13128	1,14261E+00	56050	9,23165E-01	21788	0	61900
Myeloid_vs_CD8_Non-responder	CD8(GZMK+ Tem)	pDC_LILRA4	HMGB1	TLR9	5,14754E-03	29677,8	3,25623E-02	821	2,15695E+00	1817	1,29087E+00	48429	9,01871E-01	35422	0	61900
Myeloid_vs_CD8_Non-responder	CD8(ISG+ T)	cDC_CLEC9A	HMGB1	THBD	5,15105E-03	38844,4	4,11091E-03	31012	1,02261E+00	23126	1,36754E+00	44788	9,04896E-01	33396	0	61900
Myeloid_vs_CD8_Non-responder	Macro_NLRP3	CD8(NME1+ T)	VEGFA	CD44	5,15220E-03	32050,4	5,82942E-03	18333	1,66073E+00	5263	1,46334E+00	40495	9,03607E-01	34261	0	61900
Myeloid_vs_CD8_Non-responder	CD8(GZMK+ Early Tem)	cDC_LAMP3	CD28	CD86	5,15714E-03	34424,6	1,21538E-02	5812,5	1,45179E+00	8518,5	1,78703E+00	28273,5	8,53392E-01	67618,5	0	61900
Myeloid_vs_CD8_Non-responder	CD8(ISG+ T)	Mono_CD14	CIRBP	TREM1	5,16141E-03	38874,6	4,67115E-03	25498	1,06133E+00	21381	1,36714E+00	44806	8,93966E-01	40788	0	61900
Myeloid_vs_CD8_Non-responder	Macro_NLRP3	CD8(ISG+ T)	ICAM1	IL2RG	5,17179E-03	34073	4,14259E-03	30646	1,12037E+00	18787	1,79195E+00	28111	9,08562E-01	30921	0	61900
Myeloid_vs_CD8_Non-responder	Macro_NLRP3	CD8(ISG+ T)	THBS1	ITGA4	5,17237E-03	30227,2	5,69347E-03	18981	1,38762E+00	9957	2,13778E+00	18601	8,92735E-01	41697	0	61900
Myeloid_vs_CD8_Non-responder	Macro_NLRP3	CD8(ISG+ T)	THBS1	CD47	5,17294E-03	28672	6,54152E-03	15427	1,48072E+00	7953	2,31586E+00	14895	8,90492E-01	43185	0	61900
Myeloid_vs_CD8_Non-responder	CD8(NME1+ T)	Macro_FOLR2-APOE+	HMGB1	CD163	5,17352E-03	31094,2	4,55070E-03	26515	1,41130E+00	9393	1,36673E+00	44827	9,38581E-01	12836	0	61900
Myeloid_vs_CD8_Non-responder	Macro_FOLR2+APOE+	CD8(GZMK+ Tem)	TNFSF13	FAS	5,18160E-03	28179	9,56436E-03	8599	1,36145E+00	10633	2,31439E+00	14922	8,88042E-01	44841	0	61900
Myeloid_vs_CD8_Non-responder	CD8(LAYN+ T)	Mono_CD16	HLA-B	LILRA1	5,18160E-03	26939,6	9,22185E-03	9129	1,69237E+00	4931	1,36646E+00	44841	9,36910E-01	13897	0	61900
Myeloid_vs_CD8_Non-responder	Macro_NLRP3	CD8(EOMES+ NK-like)	TIMP1	CD63	5,19087E-03	23069,2	3,67663E-03	36524	1,80541E+00	3908	3,14942E+00	4477	9,44457E-01	8537	0	61900
Myeloid_vs_CD8_Non-responder	Macro_NLRP3	CD8(ISG+ T)	IL1B	SIGIRR	5,19433E-03	22210	7,82459E-03	11794	2,22758E+00	1553	3,15650E+00	4412	9,07882E-01	31391	0	61900
Myeloid_vs_CD8_Non-responder	Macro_NLRP3	CD8(ISG+ T)	S100A8	ITGB2	5,19491E-03	27372	3,50524E-03	39311	1,79663E+00	3985	2,40643E+00	13197	9,28905E-01	18467	0	61900
Myeloid_vs_CD8_Non-responder	Macro_NLRP3	CD8(ISG+ T)	VCAN	ITGB1	5,20128E-03	25643,2	7,04692E-03	13815	2,03802E+00	2359	2,37112E+00	13832	9,00598E-01	36310	0	61900
Myeloid_vs_CD8_Non-responder	Macro_NLRP3	CD8(ISG+ T)	VCAN	ITGA4	5,20186E-03	25695,2	6,94321E-03	14121	2,02499E+00	2414	2,34779E+00	14255	9,01348E-01	35786	0	61900
Myeloid_vs_CD8_Non-responder	Macro_NLRP3	CD8(ISG+ T)	HLA-DRA	LAG3	5,20302E-03	41917,8	3,20917E-03	44697	7,08841E-01	44060	1,36579E+00	44878	9,36665E-01	14054	0	61900
Myeloid_vs_CD8_Non-responder	CD8(NME1+ T)	Macro_FOLR2+APOE+	HLA-A	LILRB2	5,20998E-03	32407,6	3,19873E-03	44890	1,18785E+00	16123	1,63101E+00	33631	9,48508E-01	5494	0	61900
Myeloid_vs_CD8_Non-responder	CD8(ITM2C+ T)	Macro_LYVE1	HMGB1	CD163	5,21056E-03	35253	3,60639E-03	37664	1,22450E+00	14789	1,36554E+00	44891	9,31526E-01	17021	0	61900
Myeloid_vs_CD8_Non-responder	CD8(ISG+ T)	Macro_FOLR2+APOE+	HLA-A	LILRB1	5,21811E-03	37420	3,19823E-03	44904	1,01540E+00	23456	1,40841E+00	42928	9,36884E-01	13912	0	61900
Myeloid_vs_CD8_Non-responder	CD8(Terminal Tex)	cDC_CLEC9A	HMGB1	THBD	5,22334E-03	37425,8	4,36478E-03	28268	1,08434E+00	20332	1,36514E+00	44913	9,07443E-01	31716	0	61900
Myeloid_vs_CD8_Non-responder	CD8(Tc17)	Macro_FOLR2+APOE+	ADAM10	GPNMB	5,22831E-03	27818,4	9,58426E-03	8558	1,71426E+00	4717	2,73886E+00	8375	8,71842E-01	55542	0	61900
Myeloid_vs_CD8_Non-responder	Macro_NLRP3	CD8(ISG+ T)	VEGFA	CD44	5,22857E-03	25515,2	8,20125E-03	10981	1,88784E+00	3256	1,85626E+00	26119	9,17485E-01	25320	0	61900
Myeloid_vs_CD8_Non-responder	CD8(GZMK+ Tem)	Macro_ISG15	ANXA1	FPR2_FPR3	5,23614E-03	35572,8	5,74029E-03	18773	1,16889E+00	16810	1,36478E+00	44935	9,01839E-01	35446	0	61900
Myeloid_vs_CD8_Non-responder	Macro_FOLR2+APOE+	CD8(LAYN+ T)	TIMP2	ITGB1	5,23673E-03	31194	7,10900E-03	13659	1,31531E+00	11956	1,94566E+00	23519	8,87906E-01	44936	0	61900
Myeloid_vs_CD8_Non-responder	CD8(Terminal Tex)	Macro_FOLR2+APOE+	HLA-F	LILRB2	5,23731E-03	36885	3,19640E-03	44937	1,30045E+00	12388	1,76372E+00	28994	9,00738E-01	36206	0	61900
Myeloid_vs_CD8_Non-responder	Macro_ISG15	CD8(GZMK+ Tex)	HLA-DQB1	LAG3	5,24036E-03	26915,4	5,47469E-03	20150	1,05041E+00	21889	2,17676E+00	17766	9,38535E-01	12872	0	61900
Myeloid_vs_CD8_Non-responder	Macro_NLRP3	CD8(GZMK+ Early Tem)	ICAM1	IL2RG	5,24314E-03	39337,2	3,37843E-03	41419	1,02053E+00	23212	1,64143E+00	33288	8,99732E-01	36867	0	61900
Myeloid_vs_CD8_Non-responder	Macro_NLRP3	CD8(GZMK+ Early Tem)	THBS1	ITGA4	5,24372E-03	29111,6	6,14725E-03	16924	1,41739E+00	9253	2,15252E+00	18299	8,96351E-01	39182	0	61900
Myeloid_vs_CD8_Non-responder	Macro_FOLR2+APOE+	CD8(ZNF683+KLRB1+ T)	SPP1	PTGER4	5,24722E-03	28777,6	9,56995E-03	8582	9,98779E-01	24282	3,18980E+00	4170	8,87878E-01	44954	0	61900
Myeloid_vs_CD8_Non-responder	CD8(ZNF683+KLRB1+ T)	Macro_FOLR2+APOE+	B2M	LILRB1	5,25131E-03	33349,2	3,19506E-03	44961	1,20790E+00	15383	1,55636E+00	36607	9,45320E-01	7895	0	61900
Myeloid_vs_CD8_Non-responder	CD8(EOMES+ NK-like)	Mono_CD16	HLA-B	LILRA1	5,25481E-03	27202,8	9,06802E-03	9398	1,64023E+00	5511	1,36410E+00	44967	9,36411E-01	14238	0	61900
Myeloid_vs_CD8_Non-responder	CD8(GZMK+ Tex)	Macro_FOLR2+APOE+	B2M	TFRC	5,26124E-03	38727	3,19389E-03	44978	7,71508E-01	38859	1,45967E+00	40660	9,46148E-01	7238	0	61900
Myeloid_vs_CD8_Non-responder	pDC_LILRA4	CD8(GZMK+ Tem)	HLA-DRB5	LAG3	5,26181E-03	25347,8	5,28294E-03	21235	1,05007E+00	21912	2,45262E+00	12427	9,43558E-01	9265	0	61900
Myeloid_vs_CD8_Non-responder	Macro_NLRP3	CD8(GZMK+ Early Tem)	IL1B	SIGIRR	5,26183E-03	21700,4	8,21623E-03	10951	2,24430E+00	1504	3,20161E+00	4093	9,09904E-01	30054	0	61900
Myeloid_vs_CD8_Non-responder	Macro_NLRP3	CD8(GZMK+ Early Tem)	S100A8	ITGB2	5,26241E-03	23241,8	4,51053E-03	26903	1,90914E+00	3094	2,58791E+00	10341	9,36794E-01	13971	0	61900
Myeloid_vs_CD8_Non-responder	Macro_NLRP3	CD8(GZMK+ Early Tem)	S100A9	ITGB2	5,26358E-03	24463,2	3,80741E-03	34659	1,85692E+00	3494	2,69720E+00	8925	9,37820E-01	13338	0	61900
Myeloid_vs_CD8_Non-responder	Macro_NLRP3	CD8(GZMK+ Early Tem)	VCAN	ITGA4	5,26944E-03	24848,6	7,49659E-03	12550	2,05476E+00	2264	2,36252E+00	13986	9,04705E-01	33543	0	61900
Myeloid_vs_CD8_Non-responder	cDC(CD1C)	CD8(LAYN+ T)	HLA-DQB1	LAG3	5,27958E-03	26443	5,43301E-03	20401	1,21959E+00	14967	2,00280E+00	21931	9,38314E-01	13016	0	61900
Myeloid_vs_CD8_Non-responder	Macro_FOLR2+APOE+	CD8(ISG+ T)	ICAM1	IL2RG	5,28351E-03	43622,4	3,19231E-03	45016	8,37711E-01	33954	1,50772E+00	38619	8,97147E-01	38623	0	61900
Myeloid_vs_CD8_Non-responder	Macro_NLRP3	CD8(GZMK+ Early Tem)	VEGFA	CD44	5,29232E-03	24448,6	8,83437E-03	9809	1,94847E+00	2849	1,92465E+00	24127	9,20256E-01	23558	0	61900
Myeloid_vs_CD8_Non-responder	Macro_OLFML3	CD8(EOMES+ NK-like)	CXCL9	CXCR3	5,29290E-03	27053,2	1,30343E-02	5047	2,15288E+00	1831	2,72229E+00	8594	8,68321E-01	57894	0	61900
Myeloid_vs_CD8_Non-responder	CD8(ISG+ T)	Macro_FOLR2+APOE+	HLA-B	LILRB1	5,30349E-03	35757,2	3,19084E-03	45050	1,08442E+00	20325	1,48817E+00	39434	9,39633E-01	12077	0	61900
Myeloid_vs_CD8_Non-responder	CD8(Terminal Tex)	Macro_ISG15	ANXA1	FPR1	5,30467E-03	40154,8	3,19070E-03	45052	8,86945E-01	30682	1,48397E+00	39601	9,20287E-01	23539	0	61900
Myeloid_vs_CD8_Non-responder	CD8(GZMK+ Early Tem)	Macro_OLFML3	HLA-C	LILRB1	5,30585E-03	36736,2	3,35882E-03	41793	1,05132E+00	21852	1,36250E+00	45054	9,38216E-01	13082	0	61900
Myeloid_vs_CD8_Non-responder	Macro_NLRP3	CD8(Temra)	ICAM1	ITGAL_ITGB2	5,30761E-03	35803,4	3,39750E-03	41086	1,16204E+00	17086	1,96974E+00	22849	9,00881E-01	36096	0	61900
Myeloid_vs_CD8_Non-responder	Macro_NLRP3	CD8(Temra)	ICAM1	IL2RG	5,30879E-03	33953,2	4,17347E-03	30311	1,12440E+00	18606	1,78803E+00	28237	9,08870E-01	30712	0	61900
Myeloid_vs_CD8_Non-responder	Macro_NLRP3	CD8(Temra)	THBS1	ITGA4	5,30938E-03	24238,2	8,59736E-03	10235	1,57810E+00	6374	2,40149E+00	13292	9,10931E-01	29390	0	61900
Myeloid_vs_CD8_Non-responder	CD8(LAYN+ T)	pDC_LILRA4	SELPLG	SELL	5,30997E-03	29676,8	6,09036E-03	17173	1,42724E+00	9038	2,30045E+00	15212	8,87714E-01	45061	0	61900
Myeloid_vs_CD8_Non-responder	Macro_NLRP3	CD8(Temra)	CD14	ITGB1	5,31233E-03	33888,8	3,57192E-03	38205	1,09495E+00	19882	2,02935E+00	21269	9,12868E-01	28188	0	61900
Myeloid_vs_CD8_Non-responder	Macro_NLRP3	CD8(Temra)	CD14	ITGA4	5,31291E-03	34745,8	3,45146E-03	40128	1,07210E+00	20881	1,98009E+00	22574	9,12762E-01	28246	0	61900
Myeloid_vs_CD8_Non-responder	Macro_FOLR2+APOE+	CD8(ZNF683+KLRB1+ T)	HLA-B	CD8A	5,31586E-03	36857,8	3,30441E-03	42889	8,32204E-01	34314	1,36212E+00	45071	9,63300E-01	115	0	61900
Myeloid_vs_CD8_Non-responder	Macro_OLFML3	CD8(Terminal Tex)	HLA-DRB1	LAG3	5,31808E-03	24709,4	5,15925E-03	21972	1,12750E+00	18468	2,22205E+00	16776	9,50277E-01	4431	0	61900
Myeloid_vs_CD8_Non-responder	Macro_FOLR2+APOE+	CD8(Tc17)	CXCL2	DPP4	5,31996E-03	30950,2	2,19192E-02	1795	1,44756E+00	8609	2,91928E+00	6445	8,40473E-01	76002	0	61900
Myeloid_vs_CD8_Non-responder	CD8(GZMK+ Early Tem)	Macro_FOLR2+APOE+	CCL5	CCRL2	5,32091E-03	34084,4	9,94841E-03	8042	1,15380E+00	17389	1,05167E+00	61116	9,22861E-01	21975	0	61900
Myeloid_vs_CD8_Non-responder	Macro_NLRP3	CD8(Temra)	IL1B	SIGIRR	5,33062E-03	20653	9,23984E-03	9095	2,28802E+00	1364	3,25011E+00	3776	9,14602E-01	27130	0	61900
Myeloid_vs_CD8_Non-responder	Macro_NLRP3	CD8(Temra)	S100A9	ITGB2	5,33180E-03	21808,8	4,47437E-03	27230	1,94535E+00	2871	2,87320E+00	6898	9,42364E-01	10145	0	61900
Myeloid_vs_CD8_Non-responder	Macro_NLRP3	CD8(Temra)	VCAN	ITGB1	5,33417E-03	20926,4	1,08504E-02	7028	2,23832E+00	1520	2,66076E+00	9405	9,18317E-01	24779	0	61900
Myeloid_vs_CD8_Non-responder	Macro_NLRP3	CD8(Temra)	VCAN	ITGA4	5,33476E-03	21160,6	1,04845E-02	7429	2,21547E+00	1600	2,61150E+00	10024	9,18216E-01	24850	0	61900
Myeloid_vs_CD8_Non-responder	Macro_NLRP3	CD8(GZMK+ Tem)	S100A8	ITGB2	5,33505E-03	21423,6	5,15542E-03	21990	1,98131E+00	2646	2,67486E+00	9230	9,40637E-01	11352	0	61900
Myeloid_vs_CD8_Non-responder	Macro_NLRP3	CD8(Temra)	VEGFA	ITGB1	5,33654E-03	26182	1,04829E-02	7432	1,98147E+00	2643	2,12791E+00	18842	8,94990E-01	40093	0	61900
Myeloid_vs_CD8_Non-responder	Macro_NLRP3	CD8(Temra)	HBEGF	CD44	5,34068E-03	26834,8	5,30868E-03	21101	1,61359E+00	5873	1,89435E+00	25011	9,25703E-01	20289	0	61900
Myeloid_vs_CD8_Non-responder	Macro_FOLR2+APOE+	CD8(Temra)	TIMP2	CD44	5,34778E-03	41692,6	3,78267E-03	35019	9,59364E-01	26322	1,36115E+00	45125	8,94984E-01	40097	0	61900
Myeloid_vs_CD8_Non-responder	Macro_LYVE1	CD8(Terminal Tex)	HLA-DPA1	LAG3	5,35111E-03	24336,6	5,61973E-03	19365	1,23597E+00	14371	2,00027E+00	22007	9,51041E-01	4040	0	61900
Myeloid_vs_CD8_Non-responder	Macro_NLRP3	CD8(Temra)	LYZ	ITGAL	5,35134E-03	29269	4,03767E-03	31821	1,29579E+00	12536	2,42183E+00	12926	9,14542E-01	27162	0	61900
Myeloid_vs_CD8_Non-responder	CD8(NME1+ T)	Macro_ISG15	CIRBP	TREM1	5,35787E-03	41775,6	4,09763E-03	31148	9,65491E-01	25989	1,36932E+00	44699	8,87595E-01	45142	0	61900
Myeloid_vs_CD8_Non-responder	Macro_NLRP3	CD8(Temra)	NAMPT	ITGA5_ITGB1	5,36261E-03	29944,6	4,58980E-03	26147	1,28917E+00	12716	2,14755E+00	18393	9,09116E-01	30567	0	61900
Myeloid_vs_CD8_Non-responder	Macro_NLRP3	CD8(Temra)	VEGFA	CD44	5,36321E-03	27586,2	7,23989E-03	13250	1,79579E+00	3993	1,71891E+00	30465	9,12640E-01	28323	0	61900

Myeloid_vs_CD8_Post_NR

Myeloid_vs_CD8_Non-responder	CD8(ISG+ T)	Mast	CALR	ITGA2B	5,37436E-03	56786,2	2,30897E-02	1611	1,44583E+00	8639	2,74637E-02	125446	8,24015E-01	86335	0	61900
Myeloid_vs_CD8_Non-responder	CD8(Terminal Tex)	Macro_ISG15	CD52	SIGLEC10	5,37569E-03	38989,6	4,04822E-03	31697	9,60342E-01	26269	1,35991E+00	45172	9,10127E-01	29910	0	61900
Myeloid_vs_CD8_Non-responder	Macro_NLRP3	CD8(Tn)	THBS1	ITGA4	5,37867E-03	29303,6	6,01705E-03	17499	1,40885E+00	9441	2,17304E+00	17837	8,95353E-01	39841	0	61900
Myeloid_vs_CD8_Non-responder	CD8(LAYN+ T)	Macro_FOLR2+APOE+	HLA-C	LILRB1	5,37926E-03	36803,4	3,18514E-03	45178	1,05874E+00	21503	1,44285E+00	41379	9,36659E-01	14057	0	61900
Myeloid_vs_CD8_Non-responder	CD8(ZNF683+KLRB1+ T)	cDC(CD1C)	B2M	CD1A	5,38619E-03	27960,4	2,64603E-02	1228	1,58291E+00	6293	1,28287E+00	48820	9,23520E-01	21561	0	61900
Myeloid_vs_CD8_Non-responder	Macro_NLRP3	CD8(Tn)	IL1B	SIGIRR	5,39655E-03	23854,6	6,70008E-03	14862	2,17955E+00	1730	3,09673E+00	4888	9,01186E-01	35893	0	61900
Myeloid_vs_CD8_Non-responder	cDC_CLEC9A	CD8(NME1+ T)	HLA-DQA1	LAG3	5,39760E-03	25583,2	5,14436E-03	22056	1,19243E+00	15980	2,42535E+00	12868	9,34990E-01	15112	0	61900
Myeloid_vs_CD8_Non-responder	Macro_NLRP3	CD8(Tn)	VCAN	ITGB1	5,40073E-03	22052	9,57973E-03	8565	2,17140E+00	1761	2,59244E+00	10283	9,13522E-01	27751	0	61900
Myeloid_vs_CD8_Non-responder	Macro_NLRP3	CD8(Tn)	VCAN	ITGA4	5,40133E-03	24994,2	7,33781E-03	12995	2,04622E+00	2307	2,38305E+00	13623	9,03778E-01	34146	0	61900
Myeloid_vs_CD8_Non-responder	Macro_FOLR2-APOE+	CD8(Tn)	APOE	LDLR	5,40169E-03	22993,2	1,36962E-02	4570	1,62957E+00	5651	2,71780E+00	8654	9,03708E-01	34191	0	61900
Myeloid_vs_CD8_Non-responder	Macro_NLRP3	CD8(Tn)	VEGFA	ITGB1	5,40372E-03	27740	9,25527E-03	9066	1,91455E+00	3058	2,05960E+00	20489	8,88991E-01	44187	0	61900
Myeloid_vs_CD8_Non-responder	Macro_FOLR2+APOE+	CD8(IL7R+ZNF683+ Tm)	HLA-C	CD8B	5,40491E-03	39492,2	3,27542E-03	43429	7,21181E-01	42984	1,35849E+00	45221	9,51260E-01	3927	0	61900
Myeloid_vs_CD8_Non-responder	CD8(Temra)	Macro_ISG15	ANXA1	FPR1	5,41149E-03	40762,6	3,18258E-03	45232	8,85250E-01	30780	1,42186E+00	42308	9,20194E-01	23593	0	61900
Myeloid_vs_CD8_Non-responder	CD8(Terminal Tex)	Macro_ISG15	HMGB1	THBD	5,41388E-03	45657,8	3,22957E-03	44303	8,08229E-01	36084	1,35814E+00	45236	8,93994E-01	40766	0	61900
Myeloid_vs_CD8_Non-responder	Macro_NLRP3	CD8(Tn)	NAMPT	ITGA5_ITGB1	5,42526E-03	35065,6	3,62985E-03	37295	1,16344E+00	17036	2,01202E+00	21695	8,98946E-01	37402	0	61900
Myeloid_vs_CD8_Non-responder	Macro_FOLR2+APOE+	CD8(Tc17)	LGALS9	PTPRC	5,42646E-03	36864	3,18143E-03	45257	1,00945E+00	23735	1,53856E+00	37316	9,33203E-01	16112	0	61900
Myeloid_vs_CD8_Non-responder	CD8(Tc17)	Macro_OLFML3	HLA-F	LILRB1	5,43786E-03	40364,2	3,71134E-03	36010	1,06006E+00	21433	1,54139E+00	37202	8,87388E-01	45276	0	61900
Myeloid_vs_CD8_Non-responder	pDC_LILRA4	CD8(GZMK+ Tem)	APP	LRP10	5,44011E-03	26103,8	9,51788E-03	8675	2,27487E+00	1411	2,97259E+00	5949	8,76331E-01	52584	0	61900
Myeloid_vs_CD8_Non-responder	Macro_NLRP3	CD8(IL7R+ZNF683+ Tm)	ICAM1	IL2RG	5,44267E-03	35311,2	3,90936E-03	33363	1,08990E+00	20110	1,77801E+00	28589	9,06127E-01	32594	0	61900
Myeloid_vs_CD8_Non-responder	Macro_NLRP3	CD8(IL7R+ZNF683+ Tm)	THBS1	ITGA4	5,44327E-03	28667,6	6,27789E-03	16410	1,42596E+00	9072	2,19087E+00	17442	8,97324E-01	38514	0	61900
Myeloid_vs_CD8_Non-responder	cDC(CD1C)	CD8(ID2+CXCR4+ T)	HLA-DRB5	LAG3	5,44727E-03	25539,6	5,13664E-03	22108	1,20883E+00	15355	2,14151E+00	18529	9,42806E-01	9806	0	61900
Myeloid_vs_CD8_Non-responder	Macro_NLRP3	CD8(IL7R+ZNF683+ Tm)	IL1B	SIGIRR	5,46614E-03	22623,4	7,48866E-03	12577	2,21323E+00	1614	3,16440E+00	4364	9,06030E-01	32662	0	61900
Myeloid_vs_CD8_Non-responder	CD8(ITM2C+ T)	Macro_FOLR2+APOE+	B2M	TFRC	5,46735E-03	39314,4	3,17788E-03	45325	7,50088E-01	40556	1,44114E+00	41453	9,46020E-01	7338	0	61900
Myeloid_vs_CD8_Non-responder	Macro_NLRP3	CD8(IL7R+ZNF683+ Tm)	VCAN	ITGB1	5,46976E-03	26458,8	6,59564E-03	15231	2,01425E+00	2469	2,34176E+00	14373	8,97596E-01	38321	0	61900
Myeloid_vs_CD8_Non-responder	Macro_FOLR2+APOE+	CD8(LAYN+ T)	PLAU	ITGB1	5,47037E-03	27537,8	1,22177E-02	5752	1,65842E+00	5290	2,10332E+00	19417	8,87307E-01	45330	0	61900
Myeloid_vs_CD8_Non-responder	Macro_NLRP3	CD8(IL7R+ZNF683+ Tm)	VCAN	ITGA4	5,47037E-03	24504,8	7,65591E-03	12183	2,06333E+00	2215	2,40087E+00	13305	9,05608E-01	32921	0	61900
Myeloid_vs_CD8_Non-responder	Macro_IER3	CD8(Terminal Tex)	HLA-DRA	LAG3	5,47992E-03	24900,6	5,51490E-03	19933	1,17912E+00	16443	1,99515E+00	22142	9,50949E-01	4085	0	61900
Myeloid_vs_CD8_Non-responder	CD8(GZMK+ Tex)	Macro_FOLR2+APOE+	CALR	LRP1	5,48547E-03	41027,2	3,17657E-03	45355	9,82192E-01	25120	1,46076E+00	40611	9,06783E-01	32150	0	61900
Myeloid_vs_CD8_Non-responder	Macro_NLRP3	CD8(IL7R+ZNF683+ Tm)	VEGFA	CD44	5,49454E-03	24197	8,85402E-03	9779	1,95035E+00	2834	1,96583E+00	22960	9,20338E-01	23512	0	61900
Myeloid_vs_CD8_Non-responder	Macro_OLFML3	CD8(ID2+CXCR4+ T)	CXCL10	CXCR3	5,49530E-03	28831,8	9,49893E-03	8705	2,18557E+00	1709	3,01619E+00	5560	8,55392E-01	66285	0	61900
Myeloid_vs_CD8_Non-responder	Macro_ISG15	CD8(Temra)	CXCL10	CXCR3	5,50638E-03	29077,6	9,49304E-03	8711	1,88804E+00	3251	3,05586E+00	5217	8,55354E-01	66309	0	61900
Myeloid_vs_CD8_Non-responder	Macro_NLRP3	CD8(GZMK+ Tem)	HLA-DRA	LAG3	5,50667E-03	30699,2	4,69579E-03	25291	9,63100E-01	26115	1,62941E+00	33686	9,47061E-01	6504	0	61900
Myeloid_vs_CD8_Non-responder	Macro_NLRP3	CD8(GZMK+ Tem)	HLA-DRB1	LAG3	5,50849E-03	36896,2	4,02109E-03	32027	7,89094E-01	37538	1,38146E+00	44148	9,44047E-01	8868	0	61900
Myeloid_vs_CD8_Non-responder	Macro_IER3	CD8(Terminal Tex)	HLA-DRB5	LAG3	5,50885E-03	25480,2	5,68856E-03	19006	1,17554E+00	16574	1,99430E+00	22172	9,45496E-01	7749	0	61900
Myeloid_vs_CD8_Non-responder	CD8(ZNF683+KLRB1+ T)	Macro_FOLR2+APOE+	CCL5	CCRL2	5,50981E-03	34070,8	9,85855E-03	8164	1,14447E+00	17748	1,06512E+00	60369	9,22538E-01	22173	0	61900
Myeloid_vs_CD8_Non-responder	Macro_NLRP3	CD8(GZMK+ Tem)	ICAM1	ITGAL_ITGB2	5,51273E-03	37125	3,27420E-03	43450	1,14261E+00	17829	1,88700E+00	25218	8,99218E-01	37228	0	61900
Myeloid_vs_CD8_Non-responder	Macro_NLRP3	CD8(GZMK+ Tem)	ICAM1	IL2RG	5,51395E-03	33274,8	4,30518E-03	28868	1,14161E+00	17875	1,80015E+00	27846	9,10149E-01	29885	0	61900
Myeloid_vs_CD8_Non-responder	Macro_NLRP3	CD8(GZMK+ Tem)	THBS1	ITGA4	5,51456E-03	23206,2	9,43178E-03	8806	1,63283E+00	5609	2,44312E+00	12593	9,14618E-01	27123	0	61900
Myeloid_vs_CD8_Non-responder	CD8(Tn)	Macro_FOLR2+APOE+	HSPA1A	TLR4	5,51516E-03	33289,6	6,26638E-03	16462	1,18802E+00	16115	1,35489E+00	45404	9,15540E-01	26567	0	61900
Myeloid_vs_CD8_Non-responder	CD8(GZMK+ Tem)	Macro_ISG15	ANXA1	FPR1	5,51699E-03	38942,2	3,17425E-03	45407	8,83510E-01	30876	1,65208E+00	32871	9,20097E-01	23657	0	61900
Myeloid_vs_CD8_Non-responder	Macro_NLRP3	CD8(GZMK+ Tem)	CD14	ITGB1	5,51759E-03	34145	3,57115E-03	38218	1,09483E+00	19893	1,98206E+00	22516	9,12860E-01	28198	0	61900
Myeloid_vs_CD8_Non-responder	Macro_NLRP3	CD8(GZMK+ Tem)	CD14	ITGA4	5,51820E-03	32572	3,78644E-03	34965	1,12683E+00	18494	2,02171E+00	21461	9,16380E-01	26040	0	61900
Myeloid_vs_CD8_Non-responder	CD8(Temra)	Macro_OLFML3	HLA-B	LILRB1	5,51820E-03	36465,2	3,35577E-03	41863	1,04702E+00	22061	1,35475E+00	45409	9,41046E-01	11093	0	61900
Myeloid_vs_CD8_Non-responder	Macro_NLRP3	CD8(GZMK+ Tem)	HLA-DRB5	LAG3	5,53463E-03	39432,6	3,73860E-03	35620	7,56794E-01	39996	1,39012E+00	43752	9,33613E-01	15895	0	61900
Myeloid_vs_CD8_Non-responder	CD8(IL7R+ZNF683+ Tm)	Macro_OLFML3	HLA-A	LILRB1	5,53523E-03	36527,2	3,42550E-03	40585	1,04597E+00	22108	1,35407E+00	45437	9,38884E-01	12606	0	61900
Myeloid_vs_CD8_Non-responder	CD8(ZNF683+KLRB1+ T)	Macro_NLRP3	HLA-C	LILRB2	5,53584E-03	29016,6	3,17220E-03	45438	1,50538E+00	7508	1,90953E+00	24566	9,48218E-01	5671	0	61900
Myeloid_vs_CD8_Non-responder	Macro_OLFML3	CD8(GZMK+ Tex)	HLA-DRB1	LAG3	5,53983E-03	24723	5,12175E-03	22204	1,12234E+00	18699	2,24672E+00	16296	9,50105E-01	4516	0	61900
Myeloid_vs_CD8_Non-responder	Macro_NLRP3	CD8(GZMK+ Tem)	IL1B	SIGIRR	5,54011E-03	22196,8	7,88609E-03	11678	2,23020E+00	1550	3,11864E+00	4705	9,08209E-01	31151	0	61900
Myeloid_vs_CD8_Non-responder	Macro_NLRP3	CD8(GZMK+ Tem)	S100A9	ITGB2	5,54194E-03	22354,8	4,35177E-03	28402	1,92910E+00	2958	2,78415E+00	7812	9,41605E-01	10702	0	61900
Myeloid_vs_CD8_Non-responder	Macro_NLRP3	CD8(GZMK+ Tem)	VCAN	ITGB1	5,54560E-03	21047	1,08481E-02	7030	2,23820E+00	1521	2,61347E+00	10000	9,18309E-01	24784	0	61900
Myeloid_vs_CD8_Non-responder	Macro_NLRP3	CD8(GZMK+ Tem)	VCAN	ITGA4	5,54621E-03	20379,8	1,15021E-02	6352	2,27020E+00	1423	2,65312E+00	9502	9,21628E-01	22722	0	61900
Myeloid_vs_CD8_Non-responder	Macro_NLRP3	CD8(GZMK+ Tem)	VEGFA	ITGB1	5,54926E-03	26410,6	1,04807E-02	7435	1,98135E+00	2645	2,08062E+00	19974	8,94979E-01	40099	0	61900
Myeloid_vs_CD8_Non-responder	Macro_FOLR2+APOE+	CD8(Terminal Tex)	CD86	CTLA4	5,55048E-03	29139,4	9,18192E-03	9209	1,42934E+00	8986	2,07396E+00	20140	8,87135E-01	45462	0	61900
Myeloid_vs_CD8_Non-responder	Macro_NLRP3	CD8(GZMK+ Tem)	LGALS3	LAG3	5,55109E-03	37999,6	4,91402E-03	23620	7,82830E-01	38046	1,36282E+00	45042	9,23797E-01	21390	0	61900
Myeloid_vs_CD8_Non-responder	CD8(Temra)	cDC(CD1C)	TNFSF9	HLA-DPA1	5,56148E-03	37071	3,17055E-03	45480	1,01625E+00	23413	2,01804E+00	21560	9,05471E-01	33002	0	61900
Myeloid_vs_CD8_Non-responder	Macro_NLRP3	CD8(GZMK+ Tem)	HBEGF	CD82	5,56209E-03	26860,4	8,77764E-03	9894	1,74762E+00	4401	2,22447E+00	16728	8,93152E-01	41379	0	61900
Myeloid_vs_CD8_Non-responder	Macro_NLRP3	CD8(GZMK+ Tem)	LYZ	ITGAL	5,56820E-03	30566,2	3,79988E-03	34779	1,27318E+00	13197	2,34542E+00	14296	9,12140E-01	28659	0	61900
Myeloid_vs_CD8_Non-responder	CD8(Tc17)	Macro_OLFML3	HLA-F	LILRB2	5,56820E-03	38109,2	3,17006E-03	45491	1,17006E+00	16775	1,73524E+00	29924	9,00368E-01	36456	0	61900
Myeloid_vs_CD8_Non-responder	Macro_NLRP3	CD8(GZMK+ Tem)	GNAI2	CXCR3	5,57004E-03	43008,8	4,57922E-03	26245	7,61405E-01	39627	1,35370E+00	45452	8,92540E-01	41820	0	61900
Myeloid_vs_CD8_Non-responder	CD8(Terminal Tex)	pDC_LILRA4	HSP90B1	TLR9	5,57069E-03	32862	2,76398E-02	1137	2,13793E+00	1891	1,32256E+00	46866	8,76442E-01	52516	0	61900
Myeloid_vs_CD8_Non-responder	Macro_NLRP3	CD8(GZMK+ Tem)	VEGFA	CD44	5,57739E-03	24244,2	8,98420E-03	9533	1,96281E+00	2752	1,93465E+00	23831	9,20871E-01	23205	0	61900
Myeloid_vs_CD8_Non-responder	CD8(NME1+ T)	Macro_OLFML3	MIF	CD44_CD74	5,58235E-03	51010,4	1,85744E-03	88959	6,41493E-01	50466	1,22314E+00	51834	9,55891E-01	1893	0	61900
Myeloid_vs_CD8_Non-responder	CD8(ISG+ T)	Mast	AIMP1	NTRK1	5,58249E-03	62916,6	1,93207E-02	2257	1,44007E+00	8752	4,73793E-01	96495	7,07733E-01	145179	0	61900
Myeloid_vs_CD8_Non-responder	CD8(Terminal Tex)	Macro_FOLR2+APOE+	B2M	TFRC	5,58475E-03	39954,2	3,16887E-03	45518	7,38019E-01	41584	1,39902E+00	43364	9,45947E-01	7405	0	61900
Myeloid_vs_CD8_Non-responder	cDC_CLEC9A	CD8(Terminal Tex)	HLA-DQA2	LAG3	5,59143E-03	24377,4	7,43849E-03	12728	1,23266E+00	14484	2,57511E+00	10518	9,22395E-01	22257	0	61900
Myeloid_vs_CD8_Non-responder	Macro_NLRP3	CD8(GZMK+ Tex)	HLA-DRA	LAG3	5,59211E-03	33884,6	4,12216E-03	30872	8,64991E-01	32135	1,58674E+00	35362	9,43698E-01	9154	0	61900
Myeloid_vs_CD8_Non-responder	Macro_NLRP3	CD8(GZMK+ Tex)	ICAM1	IL2RG	5,60010E-03	29577,8	5,03333E-03	22811	1,23675E+00	14344	1,97293E+00	22766	9,16337E-01	26068	0	61900
Myeloid_vs_CD8_Non-responder	Macro_NLRP3	CD8(GZMK+ Tex)	THBS1	ITGA4	5,60072E-03	27023,4	6,98663E-03	14009	1,47245E+00	8099	2,26703E+00	15861	9,02148E-01	35248	0	61900
Myeloid_vs_CD8_Non-responder	Macro_NLRP3	CD8(GZMK+ Tex)	SECTM1	CD7	5,62412E-03	33644,4	9,48798E-03	8720	1,23064E+00	14558	1,40975E+00	42863	8,94856E-01	40181	0	61900
Myeloid_vs_CD8_Non-responder	CD8(LAYN+ T)	Macro_FOLR2+APOE+	HLA-A	LILRB1	5,62412E-03	38395,4	3,16544E-03	45582	9,86417E-01	24919	1,35337E+00	45464	9,36579E-01	14112	0	61900
Myeloid_vs_CD8_Non-responder	CD8(ZNF683+KLRB1+ T)	pDC_LILRA4	TNF	TNFRSF21	5,62471E-03	28132,8	1,17056E-02	6183	2,11334E+00	1992	2,44131E+00	12624	8,68225E-01	57965	0	61900
Myeloid_vs_CD8_Non-responder	CD8(ISG+ T)	cDC_LAMP3	LTB	CD40	5,62474E-03	30454,8	9,10672E-03	9331	1,72247E+00	4644	1,70844E+00	30816	8,86958E-01	45583	0	61900
Myeloid_vs_CD8_Non-responder	Macro_FOLR2+APOE+	CD8(GZMK+ Tex)	LGALS3BP	ITGB1	5,62659E-03	31906,8	6,43851E-03	15795	1,36193E+00	10620	1,87262E+00	25633	8,86957E-01	45586	0	61900
Myeloid_vs_CD8_Non-responder	CD8(EOMES+ NK-like)	Mono_CD16	HLA-F	LILRB1	5,62906E-03	39794	3,67917E-03	36478	1,11800E+00	18878	1,56799E+00	36124	8,86952E-01	45590	0	61900
Myeloid_vs_CD8_Non-responder	Macro_NLRP3	CD8(GZMK+ Tex)	IL1B	SIGIRR	5,63029E-03	23623,4	6,84930E-03	14406	2,18592E+00	1708	3,09917E+00	4863	9,02162E-01	35240	0	61900
Myeloid_vs_CD8_Non-responder	Macro_NLRP3	CD8(GZMK+ Tex)	S100A8	ITGB2	5,63091E-03	23719,4	4,35743E-03	28345	1,89200E+00	3216	2,57704E+00	10492	9,35764E-01	14644	0	61900
Myeloid_vs_CD8_Non-responder	Macro_NLRP3	CD8(GZMK+ Tex)	S100A9	ITGB2	5,63215E-03	25009,2	3,67817E-03	36496	1,83979E+00	3621	2,68633E+00	9073	9,36806E-01	13956	0	61900

Myeloid_vs_CD8_Post_NR

Myeloid_vs_CD8_Non-responder	CD8(Temra)	cDC_CLEC9A	FLT3LG	FLT3	5,63498E-03	40833,8	2,11101E-02	1902	2,52397E+00	832	1,61699E+00	34193	7,92501E-01	105342	0	61900
Myeloid_vs_CD8_Non-responder	Macro_NLRP3	CD8(GZMK+ Tex)	VCAN	ITGB1	5,63647E-03	22648	9,08180E-03	9374	2,14518E+00	1858	2,54251E+00	10990	9,11390E-01	29118	0	61900
Myeloid_vs_CD8_Non-responder	Macro_NLRP3	CD8(GZMK+ Tex)	VCAN	ITGA4	5,63709E-03	23237,8	8,52021E-03	10360	2,10982E+00	2004	2,47703E+00	11989	9,10081E-01	29936	0	61900
Myeloid_vs_CD8_Non-responder	Macro_LYVE1	CD8(GZMK+ Tex)	HLA-DRB5	LAG3	5,63748E-03	24224,2	6,09265E-03	17161	1,26674E+00	13383	1,98962E+00	22304	9,47238E-01	6373	0	61900
Myeloid_vs_CD8_Non-responder	CD8(ITM2C+ T)	Macro_IER3	HSPA1A	TLR4	5,64389E-03	34048	5,75670E-03	18690	1,20713E+00	15413	1,34987E+00	45614	9,12202E-01	28623	0	61900
Myeloid_vs_CD8_Non-responder	Macro_NLRP3	CD8(GZMK+ Tex)	HBEGF	CD44	5,64513E-03	26793,6	5,29470E-03	21175	1,61177E+00	5896	1,90692E+00	24653	9,25613E-01	20344	0	61900
Myeloid_vs_CD8_Non-responder	Macro_NLRP3	CD8(GZMK+ Tex)	CXCL16	CXCR6	5,64637E-03	36462,4	5,42099E-03	20473	8,95578E-01	30148	1,66781E+00	32255	8,98719E-01	37536	0	61900
Myeloid_vs_CD8_Non-responder	Macro_NLRP3	CD8(GZMK+ Tex)	HBEGF	CD82	5,65318E-03	25126,8	9,94129E-03	8051	1,80553E+00	3904	2,34125E+00	14380	8,98949E-01	37399	0	61900
Myeloid_vs_CD8_Non-responder	CD8(GZMK+ Early Tem)	Mono_CD16	ANXA1	FPR1	5,65751E-03	39588,4	3,29207E-03	43124	9,96153E-01	24424	1,34933E+00	45636	9,21427E-01	22858	0	61900
Myeloid_vs_CD8_Non-responder	Macro_NLRP3	CD8(GZMK+ Tex)	VEGFA	CD44	5,66682E-03	27532,6	7,22083E-03	13303	1,79396E+00	4017	1,73149E+00	30051	9,12535E-01	28392	0	61900
Myeloid_vs_CD8_Non-responder	Macro_LYVE1	CD8(NME1+ T)	C1QB	C1QBP	5,67096E-03	24946	5,09931E-03	22338	1,56325E+00	6588	2,31887E+00	14824	9,27861E-01	19080	0	61900
Myeloid_vs_CD8_Non-responder	CD8(ZNF683+KLRB1+ T)	pDC_LILRA4	COPA	P2RY6	5,67195E-03	33407	5,94393E-03	17833	2,59352E+00	727	2,99144E+00	5786	8,32854E-01	80789	0	61900
Myeloid_vs_CD8_Non-responder	CD8(GZMK+ Early Tem)	Macro_FOLR2+APOE+	HLA-B	LILRB1	5,67924E-03	36536,2	3,15933E-03	45671	1,05222E+00	21809	1,45060E+00	41039	9,39351E-01	12262	0	61900
Myeloid_vs_CD8_Non-responder	CD8(IL7R+ZNF683+ Tm)	Mono_CD16	HLA-F	LILRB1	5,68546E-03	39791,2	3,67091E-03	36602	1,11689E+00	18935	1,57465E+00	35838	8,86839E-01	45681	0	61900
Myeloid_vs_CD8_Non-responder	CD8(ISG+ T)	Macro_OLFML3	TNFSF9	HLA-DPA1	5,69917E-03	38572	3,15773E-03	45703	9,01324E-01	29792	1,98821E+00	22337	9,05298E-01	33128	0	61900
Myeloid_vs_CD8_Non-responder	CD8(NME1+ T)	pDC_LILRA4	GNAI2	CXCR3	5,70478E-03	42662,2	4,07344E-03	31412	7,13953E-01	43630	1,71315E+00	30657	8,86797E-01	45712	0	61900
Myeloid_vs_CD8_Non-responder	CD8(Terminal Tex)	Macro_OLFML3	B2M	LILRB1	5,70478E-03	35834	3,31086E-03	42752	1,05599E+00	21635	1,34760E+00	45712	9,46233E-01	7171	0	61900
Myeloid_vs_CD8_Non-responder	CD8(Tn)	Macro_NLRP3	LTB	TNFRSF1A	5,70603E-03	40668,2	5,17081E-03	21904	8,49576E-01	33147	1,45918E+00	40676	8,86793E-01	45714	0	61900
Myeloid_vs_CD8_Non-responder	Macro_FOLR2+APOE+	CD8(GZMK+ Tem)	GNAI2	CXCR3	5,70790E-03	44057,8	4,21511E-03	29842	6,90714E-01	45717	1,51534E+00	38291	8,88501E-01	44539	0	61900
Myeloid_vs_CD8_Non-responder	Macro_NLRP3	CD8(ZNF683+KLRB1+ T)	IL1B	SIGIRR	5,70915E-03	22341	7,63024E-03	12234	2,21928E+00	1589	3,23647E+00	3870	9,06825E-01	32112	0	61900
Myeloid_vs_CD8_Non-responder	Macro_NLRP3	CD8(ZNF683+KLRB1+ T)	S100A8	ITGB2	5,70977E-03	23615,6	4,31277E-03	28788	1,88701E+00	3262	2,67084E+00	9289	9,35454E-01	14839	0	61900
Myeloid_vs_CD8_Non-responder	Macro_NLRP3	CD8(ZNF683+KLRB1+ T)	S100A9	ITGB2	5,71040E-03	24943,6	3,64048E-03	37117	1,83479E+00	3662	2,78013E+00	7864	9,36500E-01	14175	0	61900
Myeloid_vs_CD8_Non-responder	Macro_NLRP3	CD8(ZNF683+KLRB1+ T)	VCAN	ITGA4	5,71290E-03	27406,4	6,02132E-03	17480	1,97541E+00	2688	2,32153E+00	14768	8,94831E-01	40196	0	61900
Myeloid_vs_CD8_Non-responder	CD8(Tn)	Macro_FOLR2+APOE+	TGFB1	ENG	5,71727E-03	41484,8	4,10545E-03	31070	9,78422E-01	25342	1,39880E+00	43380	8,86766E-01	45732	0	61900
Myeloid_vs_CD8_Non-responder	CD8(Tn)	Macro_ISG15	LTB	CD40	5,71915E-03	30879,4	9,07135E-03	9396	1,33987E+00	11199	1,85429E+00	26167	8,86763E-01	45735	0	61900
Myeloid_vs_CD8_Non-responder	Macro_NLRP3	CD8(ZNF683+KLRB1+ T)	HBEGF	CD44	5,71977E-03	30704,6	4,24497E-03	29518	1,47468E+00	8067	1,77024E+00	28804	9,17638E-01	25234	0	61900
Myeloid_vs_CD8_Non-responder	Macro_NLRP3	CD8(ZNF683+KLRB1+ T)	CXCL16	CXCR6	5,72102E-03	38935,2	4,95614E-03	23320	8,32067E-01	34324	1,60227E+00	34767	8,94565E-01	40365	0	61900
Myeloid_vs_CD8_Non-responder	Macro_NLRP3	CD8(ZNF683+KLRB1+ T)	LGALS1	CD69	5,72165E-03	30488,6	3,93028E-03	33124	1,04169E+00	22271	1,66757E+00	32273	9,53455E-01	2875	0	61900
Myeloid_vs_CD8_Non-responder	Macro_NLRP3	CD8(ZNF683+KLRB1+ T)	HBEGF	CD82	5,72540E-03	27831,2	7,95660E-03	11520	1,70676E+00	4790	2,24559E+00	16314	8,88375E-01	44632	0	61900
Myeloid_vs_CD8_Non-responder	CD8(Terminal Tex)	Macro_IFI27	TNFSF9	HLA-DPA1	5,73104E-03	41173,2	3,39955E-03	41049	9,62427E-01	26150	1,34686E+00	45754	9,08414E-01	31013	0	61900
Myeloid_vs_CD8_Non-responder	Macro_NLRP3	CD8(ZNF683+KLRB1+ T)	VEGFA	CD44	5,73479E-03	31045,6	5,78922E-03	18525	1,65688E+00	5309	1,59480E+00	35037	9,03306E-01	34457	0	61900
Myeloid_vs_CD8_Non-responder	CD8(ID2+CXCR4+ T)	Macro_ISG15	HMGB1	TLR2	5,73918E-03	41113,6	3,49141E-03	39512	9,24049E-01	28384	1,34653E+00	45767	9,09978E-01	30005	0	61900
Myeloid_vs_CD8_Non-responder	Mono_INHBA	CD8(Temra)	SPP1	S1PR1	5,74435E-03	28792,4	3,84557E-02	564	1,53754E+00	6978	2,70391E+00	8838	8,56322E-01	65682	0	61900
Myeloid_vs_CD8_Non-responder	cDC_CLEC9A	CD8(GZMK+ Tex)	HLA-DQA2	LAG3	5,74534E-03	24402	7,38442E-03	12863	1,22750E+00	14657	2,59978E+00	10177	9,22134E-01	22413	0	61900
Myeloid_vs_CD8_Non-responder	Macro_NLRP3	CD8(EOMES+ NK-like)	ICAM1	IL2RG	5,75048E-03	30667	4,72474E-03	25056	1,19643E+00	15840	1,96473E+00	22986	9,13880E-01	27553	0	61900
Myeloid_vs_CD8_Non-responder	Macro_NLRP3	CD8(EOMES+ NK-like)	THBS1	ITGA4	5,75111E-03	31140,2	5,38843E-03	20654	1,36761E+00	10455	2,11070E+00	19225	8,90070E-01	43467	0	61900
Myeloid_vs_CD8_Non-responder	Macro_FOLR2+APOE+	CD8(GZMK+ Early Tem)	C1QB	C1QBP	5,76242E-03	29542	3,15365E-03	45804	1,58478E+00	6266	3,25162E+00	3766	9,10032E-01	29974	0	61900
Myeloid_vs_CD8_Non-responder	Macro_NLRP3	CD8(Tn)	CD14	ITGB1	5,76305E-03	37035,2	3,15361E-03	45805	1,02803E+00	22898	1,96104E+00	23098	9,07786E-01	31475	0	61900
Myeloid_vs_CD8_Non-responder	CD8(Terminal Tex)	Macro_ISG15	CIRBP	TREM1	5,76871E-03	42431,2	4,01982E-03	32042	9,53882E-01	26622	1,34637E+00	45778	8,86635E-01	45814	0	61900
Myeloid_vs_CD8_Non-responder	Macro_NLRP3	CD8(EOMES+ NK-like)	SECTM1	CD7	5,76934E-03	30218,2	1,05534E-02	7355	1,34111E+00	11166	1,62591E+00	33819	8,99758E-01	36851	0	61900
Myeloid_vs_CD8_Non-responder	CD8(ID2+CXCR4+ T)	Macro_ISG15	ANXA1	FPR2_FPR3	5,77060E-03	35344,6	5,88369E-03	18086	1,18546E+00	16209	1,34563E+00	45817	9,02926E-01	34711	0	61900
Myeloid_vs_CD8_Non-responder	Macro_NLRP3	CD8(EOMES+ NK-like)	IL1B	SIGIRR	5,77312E-03	22440,4	7,63546E-03	12223	2,21950E+00	1588	3,15883E+00	4393	9,06854E-01	32098	0	61900
Myeloid_vs_CD8_Non-responder	Macro_FOLR2+APOE+	CD8(EOMES+ NK-like)	HLA-DQB1	LAG3	5,77312E-03	34824	3,15289E-03	45821	9,78109E-01	25352	2,18167E+00	17644	9,20558E-01	23403	0	61900
Myeloid_vs_CD8_Non-responder	Macro_NLRP3	CD8(EOMES+ NK-like)	S100A8	ITGB2	5,77375E-03	25364,2	3,87025E-03	33855	1,83748E+00	3637	2,56199E+00	10726	9,32107E-01	16703	0	61900
Myeloid_vs_CD8_Non-responder	Macro_NLRP3	CD8(EOMES+ NK-like)	S100A9	ITGB2	5,77438E-03	26993,6	3,26693E-03	43580	1,78527E+00	4095	2,67128E+00	9282	9,33204E-01	16111	0	61900
Myeloid_vs_CD8_Non-responder	Macro_FOLR2+APOE+	CD8(GZMK+ Tex)	LYZ	ITGAL	5,77501E-03	32507,8	3,15278E-03	45824	1,25293E+00	13820	2,83928E+00	7242	9,04366E-01	33753	0	61900
Myeloid_vs_CD8_Non-responder	Macro_NLRP3	CD8(EOMES+ NK-like)	VCAN	ITGB1	5,77627E-03	23940,2	8,10297E-03	11208	2,09363E+00	2080	2,45993E+00	12297	9,06676E-01	32216	0	61900
Myeloid_vs_CD8_Non-responder	Macro_NLRP3	CD8(EOMES+ NK-like)	VCAN	ITGA4	5,77690E-03	26394,8	6,57121E-03	15319	2,00499E+00	2520	2,32071E+00	14788	8,98872E-01	37447	0	61900
Myeloid_vs_CD8_Non-responder	cDC_LAMP3	CD8(ZNF683+KLRB1+ T)	B2M	CD3D	5,78025E-03	55827,2	2,64375E-03	58684	5,10553E-01	65601	5,29314E-01	92910	9,64349E-01	41	0	61900
Myeloid_vs_CD8_Non-responder	Macro_NLRP3	CD8(EOMES+ NK-like)	HBEGF	CD44	5,78321E-03	28411,2	4,80778E-03	24417	1,54818E+00	6799	1,84014E+00	26583	9,22222E-01	22357	0	61900
Myeloid_vs_CD8_Non-responder	CD8(Tc17)	Macro_OLFML3	HLA-A	LILRB1	5,78510E-03	36774	3,41133E-03	40839	1,03455E+00	22599	1,34508E+00	45840	9,38765E-01	12692	0	61900
Myeloid_vs_CD8_Non-responder	CD8(Tn)	cDC_LAMP3	CD28	CD86	5,78531E-03	30045,8	1,62434E-02	3219,5	1,58237E+00	6301,5	1,98445E+00	22453,5	8,70623E-01	56354,5	0	61900
Myeloid_vs_CD8_Non-responder	Macro_NLRP3	CD8(GZMK+ Tem)	CD55	ADGRE5	5,79015E-03	37730,2	4,83941E-03	24187	8,34079E-01	34185	1,34489E+00	45848	9,21956E-01	22531	0	61900
Myeloid_vs_CD8_Non-responder	CD8(IL7R+ZNF683+ Tm)	Macro_OLFML3	CD99	PILRA	5,79141E-03	40328,8	3,32679E-03	42407	9,61486E-01	26201	1,34486E+00	45850	9,17547E-01	25286	0	61900
Myeloid_vs_CD8_Non-responder	cDC(CD1C)	CD8(LAYN+ T)	HLA-DQA2	LAG3	5,79433E-03	25304,4	7,66864E-03	12148	1,45660E+00	8427	1,98411E+00	22462	9,23479E-01	21585	0	61900
Myeloid_vs_CD8_Non-responder	CD8(Tc17)	Mono_CD14	ANXA1	FPR1	5,80026E-03	36063,8	3,84645E-03	34160	1,12070E+00	18768	1,34457E+00	45864	9,26879E-01	19627	0	61900
Myeloid_vs_CD8_Non-responder	Macro_NLRP3	CD8(EOMES+ NK-like)	VEGFA	CD44	5,80089E-03	29023,6	6,55677E-03	15381	1,73037E+00	4575	1,66471E+00	32375	9,08608E-01	30887	0	61900
Myeloid_vs_CD8_Non-responder	CD8(LAYN+ T)	Macro_ISG15	CIRBP	TREM1	5,80342E-03	41988	4,01229E-03	32136	9,52758E-01	26700	1,39945E+00	43335	8,86541E-01	45869	0	61900
Myeloid_vs_CD8_Non-responder	CD8(NME1+ T)	Macro_OLFML3	CD99	PILRA	5,81672E-03	39318,6	3,45078E-03	40136	9,98388E-01	24299	1,34397E+00	45890	9,18921E-01	24368	0	61900
Myeloid_vs_CD8_Non-responder	Macro_NLRP3	CD8(ID2+CXCR4+ T)	ICAM1	IL2RG	5,81735E-03	30756	4,75407E-03	24818	1,20026E+00	15683	1,93012E+00	23967	9,14123E-01	27412	0	61900
Myeloid_vs_CD8_Non-responder	Macro_NLRP3	CD8(ID2+CXCR4+ T)	THBS1	ITGA4	5,81799E-03	26436	7,29552E-03	13112	1,49271E+00	7754	2,28928E+00	15428	9,04041E-01	33986	0	61900
Myeloid_vs_CD8_Non-responder	Macro_FOLR2+APOE+	CD8(Tn)	LGALS9	PTPRC	5,81862E-03	37230,8	3,14946E-03	45893	9,99551E-01	24254	1,52620E+00	37820	9,32888E-01	16287	0	61900
Myeloid_vs_CD8_Non-responder	CD8(Temra)	Macro_FOLR2+APOE+	CALR	LRP1	5,82433E-03	41489,4	3,14928E-03	45902	9,76223E-01	25441	1,43244E+00	41816	9,06418E-01	32388	0	61900
Myeloid_vs_CD8_Non-responder	Macro_NLRP3	CD8(ID2+CXCR4+ T)	SECTM1	CD7	5,83830E-03	32414,8	1,01408E-02	7811	1,29832E+00	12461	1,43218E+00	41824	8,97945E-01	38078	0	61900
Myeloid_vs_CD8_Non-responder	Macro_NLRP3	CD8(ID2+CXCR4+ T)	IL1B	SIGIRR	5,84339E-03	24226,8	6,50221E-03	15579	2,17110E+00	1763	3,07113E+00	5086	8,99843E-01	36806	0	61900
Myeloid_vs_CD8_Non-responder	Macro_NLRP3	CD8(ID2+CXCR4+ T)	S100A8	ITGB2	5,84402E-03	24371,8	4,16269E-03	30432	1,87021E+00	3405	2,56567E+00	10675	9,34376E-01	15447	0	61900
Myeloid_vs_CD8_Non-responder	Macro_NLRP3	CD8(ID2+CXCR4+ T)	S100A9	ITGB2	5,84529E-03	25788,2	3,51379E-03	39167	1,81799E+00	3800	2,67495E+00	9229	9,35439E-01	14845	0	61900
Myeloid_vs_CD8_Non-responder	Macro_NLRP3	CD8(ID2+CXCR4+ T)	VCAN	ITGB1	5,84784E-03	23278,2	8,39724E-03	10612	2,10913E+00	2011	2,56438E+00	10691	9,08174E-01	31177	0	61900
Myeloid_vs_CD8_Non-responder	Macro_NLRP3	CD8(ID2+CXCR4+ T)	VCAN	ITGA4	5,84848E-03	22799,8	8,89691E-03	9691	2,13008E+00	1915	2,49928E+00	11639	9,11836E-01	28854	0	61900
Myeloid_vs_CD8_Non-responder	Macro_FOLR2+APOE+	CD8(GZMK+ Tex)	HLA-C	CD8A	5,85166E-03	37767,4	3,14607E-03	45945	8,01879E-01	36557	1,38431E+00	44024	9,60558E-01	411	0	61900
Myeloid_vs_CD8_Non-responder	CD8(ITM2C+ T)	Macro_FOLR2+APOE+	HLA-C	LILRB2	5,85357E-03	32449	3,14604E-03	45948	1,21043E+00	15288	1,64127E+00	33295	9,48015E-01	5814	0	61900
Myeloid_vs_CD8_Non-responder	Macro_NLRP3	CD8(ID2+CXCR4+ T)	HBEGF	CD44	5,85421E-03	26301,4	5,43214E-03	20407	1,62972E+00	5649	1,93908E+00	23712	9,26490E-01	19839	0	61900
Myeloid_vs_CD8_Non-responder	Macro_NLRP3	CD8(ID2+CXCR4+ T)	CXCL16	CXCR6	5,85548E-03	36906	5,41257E-03	20518	8,94429E-01	30206	1,61433E+00	34301	8,98648E-01	37605	0	61900
Myeloid_vs_CD8_Non-responder	Macro_NLRP3	CD8(ID2+CXCR4+ T)	HBEGF	CD82	5,86122E-03	27942,4	8,02112E-03	11378	1,70997E+00	4761	2,19607E+00	17331	8,88775E-01	44342	0	61900
Myeloid_vs_CD8_Non-responder	Macro_FOLR2+APOE+	CD8(ISG+ T)	MRC1	PTPRC	5,86122E-03	39856,8	5,27760E-03	21271	9,44533E-01	27159	1,34250E+00	45960	8,90799E-01	42994	0	61900
Myeloid_vs_CD8_Non-responder	CD8(EOMES+ NK-like)	Macro_FOLR2+APOE+	HLA-A	LILRB1	5,86441E-03	38708,8	3,14534E-03	45965	9,68652E-01	25824	1,35010E+00	45601	9,36389E-01	14254	0	61900
Myeloid_vs_CD8_Non-responder	Macro_NLRP3	CD8(ID2+CXCR4+ T)	VEGFA	CD44	5,87207E-03	27058,4	7,40826E-03	12804	1,81191E+00	3855	1,76364E+00	28997	9,13553E-01	27736	0	61900

Myeloid_vs_CD8_Post_NR

Myeloid_vs_CD8_Non-responder	CD8(GZMK+ Tem)	pDC_LILRA4	GZMB	IGF2R	5,88101E-03	38993,4	7,16484E-03	13496	7,96965E-01	36922	1,55478E+00	36658	8,86362E-01	45991	0	61900
Myeloid_vs_CD8_Non-responder	CD8(GZMK+ Tem)	Macro_OLFML3	ANXA1	FPR1	5,88165E-03	40572,8	3,28194E-03	43307	9,18249E-01	28733	1,34191E+00	45992	9,21315E-01	22932	0	61900
Myeloid_vs_CD8_Non-responder	CD8(LAYN+ T)	pDC_LILRA4	HMGB1	TLR9	5,88379E-03	30570,6	3,14574E-02	871	2,12288E+00	1944	1,22671E+00	51658	9,00333E-01	36480	0	61900
Myeloid_vs_CD8_Non-responder	Macro_NLRP3	CD8(Tc17)	ICAM1	IL2RG	5,88805E-03	29917	4,92309E-03	23554	1,22235E+00	14864	1,97588E+00	22676	9,15484E-01	26591	0	61900
Myeloid_vs_CD8_Non-responder	Macro_NLRP3	CD8(GZMK+ Tex)	VEGFA	ITGB1	5,89189E-03	28562,4	8,77420E-03	9900	1,88833E+00	3247	2,00967E+00	21757	8,86330E-01	46008	0	61900
Myeloid_vs_CD8_Non-responder	Macro_FOLR2+APOE+	CD8(Temra)	HLA-B	KIR3DL1	5,89317E-03	36973,4	1,37445E-02	4543	8,37979E-01	33933	1,51103E+00	38481	8,86329E-01	46010	0	61900
Myeloid_vs_CD8_Non-responder	CD8(NME1+ T)	Macro_FOLR2+APOE+	MIF	TNFRSF14	5,89445E-03	39808,2	3,46108E-03	39959	8,56770E-01	32659	1,34141E+00	46012	9,28836E-01	18511	0	61900
Myeloid_vs_CD8_Non-responder	CD8(GZMK+ Tem)	Macro_FOLR2+APOE+	CCL5	CCR1	5,89930E-03	32000,6	9,52218E-03	8670	1,16916E+00	16802	1,25794E+00	50065	9,21904E-01	22566	0	61900
Myeloid_vs_CD8_Non-responder	cDC(CD1C)	CD8(ISG+ T)	HLA-DRB5	LAG3	5,89930E-03	25410,4	5,07136E-03	22566	1,20177E+00	15620	2,21753E+00	16886	9,42460E-01	10080	0	61900
Myeloid_vs_CD8_Non-responder	CD8(ISG+ T)	cDC(CD1C)	B2M	CD1A	5,90133E-03	30520,6	2,52855E-02	1355	1,38345E+00	10061	1,12973E+00	56719	9,21900E-01	22568	0	61900
Myeloid_vs_CD8_Non-responder	CD8(GZMK+ Early Tem)	Macro_NLRP3	HLA-F	LILRB2	5,90278E-03	38869,8	3,14325E-03	46025	1,13946E+00	17961	1,68242E+00	31749	8,99986E-01	36714	0	61900
Myeloid_vs_CD8_Non-responder	Macro_NLRP3	CD8(Tc17)	IL1B	SIGIRR	5,90407E-03	23120,2	7,15333E-03	13529	2,19891E+00	1663	3,14068E+00	4541	9,04062E-01	33968	0	61900
Myeloid_vs_CD8_Non-responder	CD8(Tn)	Macro_FOLR2+APOE+	HLA-A	LILRB2	5,91177E-03	33388,8	3,14289E-03	46039	1,14270E+00	17827	1,58577E+00	35398	9,48077E-01	5780	0	61900
Myeloid_vs_CD8_Non-responder	Macro_NLRP3	CD8(Tc17)	LGALS1	CD69	5,91690E-03	31053,4	3,80417E-03	34716	1,00728E+00	23857	1,68684E+00	31574	9,52726E-01	3220	0	61900
Myeloid_vs_CD8_Non-responder	CD8(NME1+ T)	Macro_FOLR2+APOE+	B2M	LILRB2	5,91690E-03	31892,4	3,14258E-03	46047	1,17037E+00	16765	1,66059E+00	32521	9,55108E-01	2229	0	61900
Myeloid_vs_CD8_Non-responder	CD8(IL7R+ZNF683+ Tm)	Macro_FOLR2+APOE+	B2M	TFRC	5,91819E-03	40836,2	3,14250E-03	46049	7,02714E-01	44596	1,38325E+00	44073	9,45733E-01	7563	0	61900
Myeloid_vs_CD8_Non-responder	Macro_FOLR2+APOE+	CD8(NME1+ T)	LGALS9	CD47	5,92783E-03	40248,2	3,15300E-03	45817	1,11029E+00	19181	1,78695E+00	28279	8,86245E-01	46064	0	61900
Myeloid_vs_CD8_Non-responder	Macro_NLRP3	CD8(Tc17)	VEGFA	CD44	5,93298E-03	25880,8	7,99266E-03	11435	1,86787E+00	3419	1,83672E+00	26695	9,16504E-01	25955	0	61900
Myeloid_vs_CD8_Non-responder	Macro_OLFML3	CD8(ID2+CXCR4+ T)	HLA-DQB1	LAG3	5,94005E-03	28081,4	5,06548E-03	22606	1,08993E+00	20109	2,02265E+00	21438	9,36256E-01	14354	0	61900
Myeloid_vs_CD8_Non-responder	CD8(ZNF683+KLRB1+ T)	Macro_FOLR2+APOE+	HLA-B	LILRB1	5,94136E-03	37117,8	3,14034E-03	46085	1,03281E+00	22685	1,41675E+00	42530	9,39179E-01	12389	0	61900
Myeloid_vs_CD8_Non-responder	CD8(ID2+CXCR4+ T)	Mono_CD14	CIRBP	TREM1	5,94394E-03	39776	4,52874E-03	26721	1,04057E+00	22320	1,33982E+00	46089	8,92489E-01	41850	0	61900
Myeloid_vs_CD8_Non-responder	Macro_NLRP3	CD8(LAYN+ T)	TNF	ICOS	5,94458E-03	34724,8	1,16262E-02	6250	9,43180E-01	27242	1,52021E+00	38065	8,94874E-01	40167	0	61900
Myeloid_vs_CD8_Non-responder	Macro_FOLR2+APOE+	CD8(GZMK+ Tex)	C1QB	C1QBP	5,95168E-03	29653,6	3,13983E-03	46101	1,58365E+00	6282	3,23204E+00	3897	9,09852E-01	30088	0	61900
Myeloid_vs_CD8_Non-responder	CD8(Terminal Tex)	Macro_NLRP3	CCL5	CCRL2	5,95362E-03	33438	8,36580E-03	10680	1,03619E+00	22520	1,33949E+00	46104	9,16464E-01	25986	0	61900
Myeloid_vs_CD8_Non-responder	Macro_NLRP3	CD8(LAYN+ T)	ICAM1	IL2RG	5,95426E-03	33711,6	4,26948E-03	29254	1,13695E+00	18071	1,75646E+00	29217	9,09808E-01	30116	0	61900
Myeloid_vs_CD8_Non-responder	CD8(GZMK+ Tem)	pDC_LILRA4	HSP90B1	TLR9	5,95584E-03	32983,8	2,67679E-02	1199	2,11982E+00	1956	1,33738E+00	46185	8,74696E-01	53679	0	61900
Myeloid_vs_CD8_Non-responder	Macro_NLRP3	CD8(LAYN+ T)	CD55	ADGRE5	5,95685E-03	36142,8	5,17901E-03	21864	8,88609E-01	30587	1,35672E+00	45310	9,24362E-01	21053	0	61900
Myeloid_vs_CD8_Non-responder	Macro_NLRP3	CD8(LAYN+ T)	CD14	ITGB1	5,95814E-03	28061	4,63618E-03	25763	1,26520E+00	13421	2,20487E+00	17134	9,22697E-01	22087	0	61900
Myeloid_vs_CD8_Non-responder	Macro_NLRP3	CD8(LAYN+ T)	CD14	ITGA4	5,95878E-03	27123,2	4,84642E-03	24127	1,30002E+00	12404	2,22553E+00	16708	9,25363E-01	20477	0	61900
Myeloid_vs_CD8_Non-responder	CD8(GZMK+ Tem)	Macro_ISG15	CD28	CD86	5,96050E-03	36329,2	9,64028E-03	8480,5	1,33797E+00	11261,5	1,97799E+00	22626,5	8,38297E-01	77377,5	0	61900
Myeloid_vs_CD8_Non-responder	CD8(ID2+CXCR4+ T)	Macro_FOLR2+APOE+	HLA-B	LILRB1	5,96460E-03	37276,8	3,13874E-03	46121	1,03118E+00	22760	1,40205E+00	43205	9,39164E-01	12398	0	61900
Myeloid_vs_CD8_Non-responder	pDC_LILRA4	CD8(Terminal Tex)	APP	LRP10	5,96549E-03	26350,8	9,34086E-03	8953	2,26896E+00	1427	2,94178E+00	6229	8,75310E-01	53245	0	61900
Myeloid_vs_CD8_Non-responder	Macro_NLRP3	CD8(GZMK+ Tex)	HLA-DRB1	LAG3	5,96783E-03	40811	3,52988E-03	38892	6,90986E-01	45688	1,33879E+00	46126	9,40505E-01	11449	0	61900
Myeloid_vs_CD8_Non-responder	Mono_CD14	CD8(Tn)	VCAN	ITGB1	5,97913E-03	20557,8	1,10624E-02	6779	2,50340E+00	866	2,69445E+00	8960	9,19040E-01	24284	0	61900
Myeloid_vs_CD8_Non-responder	Macro_NLRP3	CD8(LAYN+ T)	IL1B	SIGIRR	5,98273E-03	27052,6	5,21888E-03	21637	2,11629E+00	1973	2,97987E+00	5887	8,89491E-01	43866	0	61900
Myeloid_vs_CD8_Non-responder	Macro_NLRP3	CD8(LAYN+ T)	S100A8	ITGB2	5,98338E-03	26107,4	3,79718E-03	34821	1,82930E+00	3712	2,41374E+00	13072	9,31502E-01	17032	0	61900
Myeloid_vs_CD8_Non-responder	Macro_NLRP3	CD8(LAYN+ T)	S100A9	ITGB2	5,98467E-03	27711,8	3,20526E-03	44780	1,77709E+00	4172	2,52302E+00	11272	9,32608E-01	16435	0	61900
Myeloid_vs_CD8_Non-responder	CD8(NME1+ T)	Macro_IER3	HMGB1	TLR2	5,98597E-03	36684,4	4,05103E-03	31671	1,15493E+00	17343	1,33826E+00	46154	9,15884E-01	26354	0	61900
Myeloid_vs_CD8_Non-responder	CD8(GZMK+ Tex)	cDC(CD1C)	B2M	CD1A	5,98921E-03	30936	2,51834E-02	1367	1,36611E+00	10503	1,10291E+00	58256	9,21754E-01	22654	0	61900
Myeloid_vs_CD8_Non-responder	CD8(Temra)	Mono_CD16	TNF	TNFRSF1B	5,98986E-03	45058,2	3,66108E-03	36768	6,88097E-01	45969	1,33814E+00	46160	9,03258E-01	34494	0	61900
Myeloid_vs_CD8_Non-responder	CD8(ISG+ T)	Macro_NLRP3	HLA-F	LILRB2	5,99051E-03	39254,6	3,13698E-03	46161	1,13833E+00	18009	1,63747E+00	33429	8,99896E-01	36774	0	61900
Myeloid_vs_CD8_Non-responder	Macro_NLRP3	CD8(LAYN+ T)	VEGFA	ITGB1	5,99116E-03	23155,6	1,36063E-02	4632	2,15173E+00	1835	2,30342E+00	15151	9,06628E-01	32260	0	61900
Myeloid_vs_CD8_Non-responder	CD8(ITM2C+ T)	cDC_CLEC9A	FLT3LG	FLT3	5,99203E-03	40602,2	2,07340E-02	1962	2,51986E+00	841	1,67087E+00	32156	7,91019E-01	106152	0	61900
Myeloid_vs_CD8_Non-responder	Macro_NLRP3	CD8(LAYN+ T)	CXCL16	CXCR6	5,99635E-03	43473,8	4,47555E-03	27212	7,66406E-01	39251	1,35771E+00	45254	8,89658E-01	43752	0	61900
Myeloid_vs_CD8_Non-responder	CD8(IL7R+ZNF683+ Tm)	cDC_LAMP3	LTB	CD40	5,99895E-03	31944,8	8,94967E-03	9585	1,71496E+00	4708	1,53739E+00	37357	8,86083E-01	46174	0	61900
Myeloid_vs_CD8_Non-responder	Macro_NLRP3	CD8(LAYN+ T)	HBEGF	CD82	6,00220E-03	27310	8,57561E-03	10268	1,73757E+00	4508	2,17926E+00	17709	8,92036E-01	42165	0	61900
Myeloid_vs_CD8_Non-responder	Macro_NLRP3	CD8(LAYN+ T)	LYZ	ITGAL	6,00545E-03	32834,4	3,40073E-03	41028	1,23523E+00	14406	2,29927E+00	15230	9,07590E-01	31608	0	61900
Myeloid_vs_CD8_Non-responder	CD8(GZMK+ Tex)	Macro_FOLR2+APOE+	HMGB1	TLR2	6,01000E-03	38717,4	3,82557E-03	34444	1,01695E+00	23372	1,33722E+00	46191	9,13652E-01	27680	0	61900
Myeloid_vs_CD8_Non-responder	CD8(Terminal Tex)	Macro_LYVE1	IFNG	IFNGR1_IFNGR2	6,01260E-03	36908	6,57025E-03	15323	9,03588E-01	29623	1,33708E+00	46195	9,07755E-01	31499	0	61900
Myeloid_vs_CD8_Non-responder	Macro_NLRP3	CD8(LAYN+ T)	VEGFA	CD44	6,01521E-03	24166,8	9,12535E-03	9302	1,97633E+00	2680	1,92556E+00	24106	9,21437E-01	22846	0	61900
Myeloid_vs_CD8_Non-responder	CD8(LAYN+ T)	Macro_OLFML3	HLA-B	LILRB1	6,01977E-03	36605,4	3,35699E-03	41830	1,04816E+00	22002	1,33679E+00	46206	9,41056E-01	11089	0	61900
Myeloid_vs_CD8_Non-responder	Mast	CD8(ITM2C+ T)	ADCYAP1	ADRB2	6,02226E-03	50647,8	5,75486E-02	272	2,11749E+00	1967	1,10444E+00	58161	7,42520E-01	130939	0	61900
Myeloid_vs_CD8_Non-responder	Macro_FOLR2+APOE+	CD8(IL7R+ZNF683+ Tm)	LGALS3	LAG3	6,02433E-03	41051,4	3,13427E-03	46213	8,08657E-01	36042	1,77402E+00	28690	9,06383E-01	32412	0	61900
Myeloid_vs_CD8_Non-responder	CD8(ZNF683+KLRB1+ T)	Macro_ISG15	HLA-G	LILRB1	6,02525E-03	40544,6	5,30619E-03	21115	1,40570E+00	9515	2,25506E+00	16113	8,11293E-01	94080	0	61900
Myeloid_vs_CD8_Non-responder	CD8(ISG+ T)	Macro_NLRP3	CIRBP	TREM1	6,02628E-03	41533,8	4,25692E-03	29404	9,60203E-01	26280	1,33650E+00	46216	8,89484E-01	43869	0	61900
Myeloid_vs_CD8_Non-responder	Macro_NLRP3	CD8(Terminal Tex)	HLA-DRA	LAG3	6,02954E-03	33915	4,15234E-03	30535	8,70153E-01	31788	1,56207E+00	36351	9,43891E-01	9001	0	61900
Myeloid_vs_CD8_Non-responder	CD8(GZMK+ Tem)	Macro_ISG15	CCL5	CCRL2	6,03454E-03	32110,6	9,62554E-03	8503	1,13050E+00	18339	1,27646E+00	49113	9,21679E-01	22698	0	61900
Myeloid_vs_CD8_Non-responder	Macro_NLRP3	CD8(Terminal Tex)	ICAM1	IL2RG	6,03803E-03	33855,4	4,27036E-03	29238	1,13706E+00	18065	1,73422E+00	29963	9,09816E-01	30111	0	61900
Myeloid_vs_CD8_Non-responder	Macro_NLRP3	CD8(Terminal Tex)	THBS1	ITGA4	6,03868E-03	29760	6,03041E-03	17436	1,40972E+00	9422	2,06861E+00	20269	8,95457E-01	39773	0	61900
Myeloid_vs_CD8_Non-responder	Macro_NLRP3	CD8(Terminal Tex)	THBS1	CD47	6,03933E-03	30014,6	6,09434E-03	17148	1,45355E+00	8488	2,21236E+00	16975	8,86991E-01	45562	0	61900
Myeloid_vs_CD8_Non-responder	CD8(ZNF683+KLRB1+ T)	cDC_CLEC9A	LTB	CD40	6,04587E-03	30568,8	8,92769E-03	9626	1,38621E+00	9987	1,89143E+00	25085	8,85959E-01	46246	0	61900
Myeloid_vs_CD8_Non-responder	Macro_FOLR2+APOE+	CD8(ITM2C+ T)	HLA-A	CD8A	6,05306E-03	37259,8	3,31289E-03	42713	8,20660E-01	35167	1,33559E+00	46257	9,61590E-01	262	0	61900
Myeloid_vs_CD8_Non-responder	CD8(ZNF683+KLRB1+ T)	Macro_OLFML3	LTB	TNFRSF1A	6,05699E-03	41377,2	5,24341E-03	21488	8,66600E-01	32022	1,33548E+00	46263	8,87491E-01	45213	0	61900
Myeloid_vs_CD8_Non-responder	CD8(Terminal Tex)	pDC_LILRA4	COPA	P2RY6	6,05941E-03	27806	9,30353E-03	9001	2,68822E+00	594	3,04205E+00	5328	8,61762E-01	62207	0	61900
Myeloid_vs_CD8_Non-responder	CD8(Tn)	Macro_ISG15	CD40LG	CD40	6,06249E-03	38255,8	2,97809E-02	980	1,35442E+00	10819	1,97434E+00	22725	8,10085E-01	94855	0	61900
Myeloid_vs_CD8_Non-responder	Macro_NLRP3	CD8(Terminal Tex)	IL1B	SIGIRR	6,06747E-03	23402,8	7,05131E-03	13801	2,19455E+00	1678	3,04802E+00	5275	9,03437E-01	34360	0	61900
Myeloid_vs_CD8_Non-responder	Macro_NLRP3	CD8(Temra)	THBS1	CD47	6,06813E-03	30035,2	5,96652E-03	17728	1,44578E+00	8642	2,27906E+00	15626	8,85924E-01	46280	0	61900
Myeloid_vs_CD8_Non-responder	Macro_NLRP3	CD8(Terminal Tex)	S100A8	ITGB2	6,06813E-03	27919	3,44619E-03	40200	1,79002E+00	4055	2,32733E+00	14651	9,28342E-01	18789	0	61900
Myeloid_vs_CD8_Non-responder	Macro_NLRP3	CD8(Terminal Tex)	VCAN	ITGB1	6,07140E-03	27808,6	6,16058E-03	16860	1,99134E+00	2593	2,20133E+00	17220	8,94418E-01	40470	0	61900
Myeloid_vs_CD8_Non-responder	CD8(Terminal Tex)	Macro_LYVE1	HSPA1A	TLR4	6,07140E-03	38199,4	4,78801E-03	24571	9,92530E-01	24594	1,33486E+00	46285	9,04538E-01	33647	0	61900
Myeloid_vs_CD8_Non-responder	Macro_NLRP3	CD8(Terminal Tex)	VCAN	ITGA4	6,07206E-03	25374,4	7,35410E-03	12958	2,04710E+00	2302	2,27862E+00	15631	9,03875E-01	34081	0	61900
Myeloid_vs_CD8_Non-responder	Macro_NLRP3	CD8(Terminal Tex)	VCAN	CD44	6,07272E-03	25207,6	6,05600E-03	17337	1,91963E+00	3023	1,95504E+00	23262	9,25308E-01	20516	0	61900
Myeloid_vs_CD8_Non-responder	Macro_FOLR2+APOE+	CD8(Tn)	APOE	SORL1	6,07272E-03	34169,6	3,20535E-03	44777	1,29105E+00	12670	3,05611E+00	5214	8,85916E-01	46287	0	61900
Myeloid_vs_CD8_Non-responder	CD8(ZNF683+KLRB1+ T)	Macro_ISG15	HMGB1	HAVCR2	6,07862E-03	42601,2	3,13080E-03	46296	7,79514E-01	38279	1,35689E+00	45295	9,24037E-01	21236	0	61900
Myeloid_vs_CD8_Non-responder	Macro_NLRP3	CD8(Terminal Tex)	HBEGF	CD44	6,08125E-03	31761,4	4,29019E-03	29022	1,48059E+00	7955	1,59764E+00	34950	9,18038E-01	24980	0	61900
Myeloid_vs_CD8_Non-responder	CD8(EOMES+ NK-like)	Macro_OLFML3	HLA-B	LILRB1	6,08256E-03	37392	3,30100E-03	42943	9,96017E-01	24432	1,33443E+00	46302	9,40588E-01	11383	0	61900
Myeloid_vs_CD8_Non-responder	Macro_NLRP3	CD8(Terminal Tex)	CXCL16	CXCR6	6,08322E-03	44931,8	4,17179E-03	30331	7,24903E-01	42662	1,39270E+00	43646	8,86160E-01	46120	0	61900

Myeloid_vs_CD8_Post_NR

Myeloid_vs_CD8_Non-responder	Macro_FOLR2+APOE+	CD8(Terminal Tex)	HLA-C	CD8B	6,08519E-03	39680,8	3,27852E-03	43368	7,21838E-01	42916	1,33440E+00	46306	9,51282E-01	3914	0	61900
Myeloid_vs_CD8_Non-responder	Macro_NLRP3	CD8(Terminal Tex)	HBEGF	CD82	6,08913E-03	25371,2	9,86569E-03	8156	1,80177E+00	3933	2,29927E+00	15231	8,98601E-01	37636	0	61900
Myeloid_vs_CD8_Non-responder	Macro_NLRP3	CD8(IL7R+ZNF683+ Tm)	GNAI2	CXCR3	6,08979E-03	46253,8	3,99883E-03	32274	6,94565E-01	45351	1,35422E+00	45431	8,85865E-01	46313	0	61900
Myeloid_vs_CD8_Non-responder	CD8(ZNF683+KLRB1+ T)	pDC_LILRA4	IRAK4	TLR7	6,09156E-03	42322,8	1,14301E-02	6416	1,51078E+00	7401	1,97336E+00	22753	7,78307E-01	113144	0	61900
Myeloid_vs_CD8_Non-responder	Macro_NLRP3	CD8(Terminal Tex)	LYZ	ITGAL	6,09308E-03	32364,4	3,47788E-03	39694	1,24257E+00	14162	2,30532E+00	15123	9,08527E-01	30943	0	61900
Myeloid_vs_CD8_Non-responder	CD8(Temra)	Macro_FOLR2+APOE+	B2M	TFRC	6,09703E-03	41303,4	3,12926E-03	46324	6,84994E-01	46249	1,37578E+00	44404	9,45625E-01	7640	0	61900
Myeloid_vs_CD8_Non-responder	Macro_NLRP3	CD8(Terminal Tex)	VEGFA	CD44	6,10163E-03	32363,4	5,85088E-03	18230	1,66278E+00	5240	1,42220E+00	42290	9,03767E-01	34157	0	61900
Myeloid_vs_CD8_Non-responder	CD8(Terminal Tex)	Mono_CD16	HLA-B	LILRB1	6,10427E-03	34940,2	3,62876E-03	37322	1,10148E+00	19596	1,33373E+00	46335	9,43179E-01	9548	0	61900
Myeloid_vs_CD8_Non-responder	Macro_NLRP3	CD8(ITM2C+ T)	ICAM1	ITGAL_ITGB2	6,11482E-03	36968,4	3,23912E-03	44140	1,12753E+00	18465	1,97113E+00	22811	8,98729E-01	37526	0	61900
Myeloid_vs_CD8_Non-responder	Macro_FOLR2+APOE+	CD8(GZMK+ Tem)	SPP1	ITGA4_ITGB1	6,11877E-03	29644	8,24694E-03	10900	9,99762E-01	24241	3,10484E+00	4822	8,85785E-01	46357	0	61900
Myeloid_vs_CD8_Non-responder	CD8(NME1+ T)	Macro_FOLR2+APOE+	HLA-B	LILRB2	6,11877E-03	32947,8	3,12783E-03	46357	1,16057E+00	17143	1,59803E+00	34932	9,50305E-01	4407	0	61900
Myeloid_vs_CD8_Non-responder	CD8(GZMK+ Tex)	Macro_FOLR2+APOE+	CCL5	CCR1	6,12805E-03	31685	9,42151E-03	8825	1,15766E+00	17248	1,30769E+00	47664	9,21520E-01	22788	0	61900
Myeloid_vs_CD8_Non-responder	CD8(ZNF683+KLRB1+ T)	Macro_ISG15	IFNG	IFNGR1_IFNGR2	6,13000E-03	42692,4	4,71966E-03	25104	7,75685E-01	38554	1,33299E+00	46374	8,92939E-01	41530	0	61900
Myeloid_vs_CD8_Non-responder	Macro_NLRP3	CD8(ITM2C+ T)	S100A9	ITGB2	6,13661E-03	21489,2	4,54015E-03	26619	1,95407E+00	2801	2,93685E+00	6269	9,42759E-01	9857	0	61900
Myeloid_vs_CD8_Non-responder	CD8(GZMK+ Tem)	cDC_CLEC9A	HMGB1	THBD	6,13860E-03	39049,6	4,13006E-03	30786	1,02727E+00	22930	1,33267E+00	46387	9,05096E-01	33245	0	61900
Myeloid_vs_CD8_Non-responder	Macro_NLRP3	CD8(ITM2C+ T)	VCAN	ITGA4	6,14125E-03	28839,6	5,51341E-03	19943	1,94809E+00	2854	2,24200E+00	16383	8,90611E-01	43118	0	61900
Myeloid_vs_CD8_Non-responder	Macro_FOLR2+APOE+	CD8(Terminal Tex)	MRC1	PTPRC	6,14522E-03	39070,4	5,46680E-03	20201	9,84529E-01	25011	1,33245E+00	46397	8,92500E-01	41843	0	61900
Myeloid_vs_CD8_Non-responder	Macro_FOLR2+APOE+	CD8(NME1+ T)	LGALS3BP	ITGB1	6,14787E-03	32883,4	6,28503E-03	16377	1,35053E+00	10899	1,76902E+00	28840	8,85742E-01	46401	0	61900
Myeloid_vs_CD8_Non-responder	Macro_NLRP3	CD8(ITM2C+ T)	HBEGF	CD44	6,14853E-03	26659,4	5,29595E-03	21170	1,61193E+00	5895	1,92932E+00	23995	9,25621E-01	20337	0	61900
Myeloid_vs_CD8_Non-responder	CD8(Tn)	Macro_FOLR2+APOE+	HLA-B	LILRB2	6,15052E-03	32914,6	3,12563E-03	46405	1,15851E+00	17215	1,60565E+00	34635	9,50288E-01	4418	0	61900
Myeloid_vs_CD8_Non-responder	Macro_NLRP3	CD8(ZNF683+KLRB1+ T)	THBS1	ITGA4	6,15184E-03	32443,2	4,93752E-03	23448	1,33804E+00	11257	2,11152E+00	19204	8,85721E-01	46407	0	61900
Myeloid_vs_CD8_Non-responder	Macro_NLRP3	CD8(ITM2C+ T)	LYZ	ITGAL	6,15715E-03	33720,4	3,22005E-03	44512	1,21805E+00	15031	2,36098E+00	14015	9,05275E-01	33144	0	61900
Myeloid_vs_CD8_Non-responder	CD8(IL7R+ZNF683+ Tm)	Macro_OLFML3	B2M	LILRB1	6,16445E-03	36430,2	3,28331E-03	43283	1,02068E+00	23205	1,33183E+00	46426	9,46020E-01	7337	0	61900
Myeloid_vs_CD8_Non-responder	Macro_FOLR2+APOE+	CD8(Temra)	HLA-B	KIR2DL3	6,16511E-03	36534,6	1,61317E-02	3265	8,54605E-01	32813	1,51571E+00	38268	8,85688E-01	46427	0	61900
Myeloid_vs_CD8_Non-responder	Macro_NLRP3	CD8(ITM2C+ T)	VEGFA	CD44	6,16777E-03	27379,6	7,22252E-03	13297	1,79412E+00	4013	1,75388E+00	29307	9,12545E-01	28381	0	61900
Myeloid_vs_CD8_Non-responder	CD8(ITM2C+ T)	Macro_NLRP3	TNFSF9	HLA-DPA1	6,16910E-03	40239,8	4,46389E-03	27323	6,83147E-01	46433	1,44441E+00	41315	9,19132E-01	24228	0	61900
Myeloid_vs_CD8_Non-responder	CD8(GZMK+ Tex)	Macro_FOLR2+APOE+	HLA-B	LILRB1	6,19172E-03	37452	3,12262E-03	46467	1,01471E+00	23489	1,40908E+00	42898	9,39017E-01	12506	0	61900
Myeloid_vs_CD8_Non-responder	CD8(NME1+ T)	pDC_LILRA4	CCL5	CXCR3	6,20772E-03	35323,4	7,96143E-03	11506	6,82538E-01	46491	1,39091E+00	43715	9,38331E-01	13005	0	61900
Myeloid_vs_CD8_Non-responder	CD8(ITM2C+ T)	Macro_ISG15	CIRBP	TREM1	6,22242E-03	41473,2	3,93474E-03	33073	9,41187E-01	27362	1,51014E+00	38518	8,85556E-01	46513	0	61900
Myeloid_vs_CD8_Non-responder	CD8(ITM2C+ T)	Macro_ISG15	CCL5	CCR1	6,22912E-03	27795,6	1,25257E-02	5471	1,48465E+00	7891	1,32959E+00	46523	9,31220E-01	17193	0	61900
Myeloid_vs_CD8_Non-responder	pDC_LILRA4	CD8(ISG+ T)	APP	CD74	6,23319E-03	22294,2	5,54166E-03	19800	2,06660E+00	2198	1,96844E+00	22888	9,49805E-01	4685	0	61900
Myeloid_vs_CD8_Non-responder	pDC_LILRA4	CD8(LAYN+ T)	COL24A1	ITGA1_ITGB1	6,23601E-03	38806,4	6,88152E-02	194	2,10999E+00	2002	1,48781E+00	39449	8,17422E-01	90487	0	61900
Myeloid_vs_CD8_Non-responder	Macro_NLRP3	CD8(Tc17)	VCAN	ITGA4	6,23782E-03	30498,2	4,97649E-03	23189	1,91921E+00	3028	2,17304E+00	17838	8,85519E-01	46536	0	61900
Myeloid_vs_CD8_Non-responder	CD8(NME1+ T)	Macro_IFI27	HMGB1	CD163	6,24386E-03	33427,8	4,08194E-03	31303	1,29683E+00	12507	1,32915E+00	46545	9,35372E-01	14884	0	61900
Myeloid_vs_CD8_Non-responder	CD8(Terminal Tex)	Macro_FOLR2+APOE+	HSP90B1	LRP1	6,25191E-03	42477,8	3,11694E-03	46557	1,01009E+00	23699	1,44001E+00	41509	8,97015E-01	38724	0	61900
Myeloid_vs_CD8_Non-responder	pDC_LILRA4	CD8(NME1+ T)	APP	LRP10	6,25611E-03	26525	9,23754E-03	9100	2,26551E+00	1438	2,91130E+00	6509	8,74702E-01	53678	0	61900
Myeloid_vs_CD8_Non-responder	Macro_FOLR2+APOE+	CD8(ISG+ T)	HBEGF	CD44	6,26333E-03	42615,8	3,11611E-03	46574	8,69176E-01	31847	1,48500E+00	39562	9,05176E-01	33196	0	61900
Myeloid_vs_CD8_Non-responder	CD8(GZMK+ Tem)	Macro_FOLR2+APOE+	HLA-B	LILRB1	6,26535E-03	37853	3,11600E-03	46577	1,00795E+00	23816	1,37552E+00	44422	9,38956E-01	12550	0	61900
Myeloid_vs_CD8_Non-responder	CD8(ID2+CXCR4+ T)	Macro_ISG15	HLA-F	LILRB1	6,26602E-03	39148,6	3,57178E-03	38209	1,19362E+00	15937	1,64543E+00	33119	8,85458E-01	46578	0	61900
Myeloid_vs_CD8_Non-responder	CD8(GZMK+ Tex)	Macro_ISG15	GZMB	IGF2R	6,27275E-03	41242,8	7,03584E-03	13844	7,71967E-01	38832	1,36260E+00	45050	8,85444E-01	46588	0	61900
Myeloid_vs_CD8_Non-responder	CD8(GZMK+ Tex)	Macro_OLFML3	HLA-C	LILRB1	6,27612E-03	37508	3,32008E-03	42564	1,02131E+00	23184	1,32829E+00	46593	9,37879E-01	13299	0	61900
Myeloid_vs_CD8_Non-responder	CD8(GZMK+ Tem)	Macro_OLFML3	HLA-A	LILRB2	6,28421E-03	31163,8	3,11447E-03	46605	1,33378E+00	11377	1,73324E+00	29995	9,47853E-01	5942	0	61900
Myeloid_vs_CD8_Non-responder	Macro_ISG15	CD8(GZMK+ Tem)	CD14	ITGA4	6,29584E-03	24443,2	5,01116E-03	22947	1,48092E+00	7950	2,64644E+00	9592	9,26509E-01	19827	0	61900
Myeloid_vs_CD8_Non-responder	CD8(GZMK+ Tex)	Macro_ISG15	CCL5	CCRL2	6,30010E-03	31809,2	9,52378E-03	8668	1,11899E+00	18840	1,32622E+00	46687	9,21294E-01	22951	0	61900
Myeloid_vs_CD8_Non-responder	Mono_CD14	CD8(LAYN+ T)	CD14	ITGB1	6,30330E-03	26726,4	5,01011E-03	22954	1,35350E+00	10832	2,18808E+00	17497	9,25418E-01	20449	0	61900
Myeloid_vs_CD8_Non-responder	CD8(NME1+ T)	Macro_FOLR2+APOE+	HLA-F	LILRB2	6,31596E-03	37666,2	3,11296E-03	46652	1,28806E+00	12752	1,73196E+00	30035	8,99549E-01	36992	0	61900
Myeloid_vs_CD8_Non-responder	CD8(NME1+ T)	Mono_CD16	B2M	LILRB1	6,31866E-03	36064,6	3,47629E-03	39717	9,68664E-01	25821	1,32672E+00	46656	9,47460E-01	6229	0	61900
Myeloid_vs_CD8_Non-responder	CD8(ITM2C+ T)	Macro_FOLR2+APOE+	HLA-F	LILRB1	6,32137E-03	41214	3,56233E-03	38368	9,90969E-01	24671	1,60951E+00	34471	8,85324E-01	46660	0	61900
Myeloid_vs_CD8_Non-responder	CD8(GZMK+ Tem)	Macro_FOLR2+APOE+	ADAM10	GPNMB	6,32146E-03	37128,2	5,00774E-03	22971	1,54433E+00	6849	2,47976E+00	11941	8,31006E-01	81980	0	61900
Myeloid_vs_CD8_Non-responder	CD8(ID2+CXCR4+ T)	pDC_LILRA4	HSP90B1	TLR9	6,32253E-03	32982,4	2,62102E-02	1256	2,10824E+00	2016	1,35679E+00	45301	8,73537E-01	54439	0	61900
Myeloid_vs_CD8_Non-responder	Macro_FOLR2+APOE+	CD8(ISG+ T)	LGALS9	CD47	6,32815E-03	40023,4	3,11219E-03	46670	1,10508E+00	19423	1,87275E+00	25630	8,85586E-01	46494	0	61900
Myeloid_vs_CD8_Non-responder	CD8(Temra)	Macro_OLFML3	HLA-C	LILRB2	6,32883E-03	30457,8	3,11218E-03	46671	1,38202E+00	10099	1,80784E+00	27603	9,47747E-01	6016	0	61900
Myeloid_vs_CD8_Non-responder	Macro_LYVE1	CD8(Terminal Tex)	HLA-DRB5	LAG3	6,33215E-03	24269,2	6,13726E-03	16970	1,27190E+00	13237	1,96495E+00	22981	9,47420E-01	6258	0	61900
Myeloid_vs_CD8_Non-responder	Macro_ISG15	CD8(ID2+CXCR4+ T)	B2M	CD3D	6,34389E-03	45982,2	2,63185E-03	59016	6,69284E-01	47684	1,04901E+00	61266	9,64271E-01	45	0	61900
Myeloid_vs_CD8_Non-responder	CD8(IL7R+ZNF683+ Tm)	Macro_FOLR2+APOE+	CCL5	CCRL2	6,35037E-03	36285	9,50434E-03	8695	1,10771E+00	19296	9,21405E-01	68536	9,21220E-01	22998	0	61900
Myeloid_vs_CD8_Non-responder	Macro_FOLR2+APOE+	CD8(Terminal Tex)	SECTM1	CD7	6,35600E-03	34072,2	9,70291E-03	8379	1,25064E+00	13894	1,32568E+00	46711	8,95905E-01	39477	0	61900
Myeloid_vs_CD8_Non-responder	CD8(Terminal Tex)	Macro_OLFML3	CD52	SIGLEC10	6,36076E-03	34928,8	4,97471E-03	23205	1,13945E+00	17963	1,32559E+00	46718	9,18207E-01	24858	0	61900
Myeloid_vs_CD8_Non-responder	Mast	CD8(Terminal Tex)	ADCYAP1	DPP4	6,36600E-03	54591,8	7,96712E-02	140	2,10685E+00	2023	1,07076E+00	60044	6,96760E-01	148852	0	61900
Myeloid_vs_CD8_Non-responder	Macro_OLFML3	CD8(ISG+ T)	HLA-DQB1	LAG3	6,37722E-03	27883,4	5,00111E-03	23023	1,08287E+00	20397	2,09867E+00	19514	9,35873E-01	14583	0	61900
Myeloid_vs_CD8_Non-responder	Mono_INHBA	CD8(ISG+ T)	SPP1	CD44	6,38045E-03	25518,8	8,74658E-03	9944	1,24043E+00	14224	2,14295E+00	18500	9,21178E-01	23026	0	61900
Myeloid_vs_CD8_Non-responder	CD8(GZMK+ Tem)	pDC_LILRA4	TNF	TNFRSF21	6,38260E-03	35710,4	7,19427E-03	13385	1,99803E+00	2560	1,96336E+00	23028	8,37802E-01	77679	0	61900
Myeloid_vs_CD8_Non-responder	CD8(IL7R+ZNF683+ Tm)	cDC(CD1C)	B2M	CD1A	6,39014E-03	32275,2	2,47782E-02	1410	1,29731E+00	12490	1,02649E+00	62541	9,21167E-01	23035	0	61900
Myeloid_vs_CD8_Non-responder	CD8(ID2+CXCR4+ T)	Macro_FOLR2+APOE+	HLA-C	LILRB1	6,39214E-03	38344,6	3,10868E-03	46764	9,93714E-01	24543	1,38520E+00	43977	9,35934E-01	14539	0	61900
Myeloid_vs_CD8_Non-responder	Macro_NLRP3	CD8(ITM2C+ T)	S100A4	CCR5	6,39419E-03	45038	4,03418E-03	31862	6,96091E-01	45206	1,48762E+00	39455	8,85159E-01	46767	0	61900
Myeloid_vs_CD8_Non-responder	CD8(Temra)	Macro_OLFML3	B2M	LILRB1	6,40103E-03	36743	3,26948E-03	43533	1,00296E+00	24078	1,32436E+00	46777	9,45912E-01	7427	0	61900
Myeloid_vs_CD8_Non-responder	CD8(Tc17)	Macro_FOLR2+APOE+	HLA-C	LILRB1	6,41472E-03	38226,4	3,10733E-03	46797	9,92564E-01	24593	1,40044E+00	43290	9,35921E-01	14552	0	61900
Myeloid_vs_CD8_Non-responder	Macro_NLRP3	CD8(NME1+ T)	VEGFA	ITGB1	6,41678E-03	29402,2	8,56504E-03	10289	1,87693E+00	3346	1,90607E+00	24676	8,85109E-01	46800	0	61900
Myeloid_vs_CD8_Non-responder	CD8(IL7R+ZNF683+ Tm)	pDC_LILRA4	HMGB1	TLR9	6,42212E-03	30456,2	3,08551E-02	906	2,10430E+00	2032	1,25152E+00	50386	8,99462E-01	37057	0	61900
Myeloid_vs_CD8_Non-responder	Macro_ISG15	CD8(ISG+ T)	CCL2	CCR5	6,42622E-03	42348,6	1,15596E-02	6310	1,42037E+00	9184	3,09651E+00	4889	7,45589E-01	129460	0	61900
Myeloid_vs_CD8_Non-responder	CD8(EOMES+ NK-like)	Macro_FOLR2+APOE+	CCL5	CCRL2	6,43256E-03	30337,6	1,05823E-02	7318	1,21959E+00	14969	1,32350E+00	46823	9,25031E-01	20678	0	61900
Myeloid_vs_CD8_Non-responder	Mono_CD14	CD8(Temra)	VCAN	SELL	6,43440E-03	27574,6	9,25351E-03	9070	2,46379E+00	926	2,67814E+00	9188	8,70016E-01	56789	0	61900
Myeloid_vs_CD8_Non-responder	CD8(ZNF683+KLRB1+ T)	Mono_INHBA	CIRBP	TREM1	6,43943E-03	37318	5,23746E-03	21521	1,11104E+00	19145	1,32323E+00	46833	8,99268E-01	37191	0	61900
Myeloid_vs_CD8_Non-responder	CD8(EOMES+ NK-like)	Macro_ISG15	HMGB1	THBD	6,43943E-03	46808,8	3,10574E-03	46833	7,67539E-01	39169	1,38388E+00	44047	8,92127E-01	42095	0	61900
Myeloid_vs_CD8_Non-responder	CD8(ISG+ T)	pDC_LILRA4	IRAK4	TLR7	6,44053E-03	45932,6	9,19167E-03	9191	1,45705E+00	8412	1,82758E+00	26995	7,58936E-01	123165	0	61900
Myeloid_vs_CD8_Non-responder	CD8(NME1+ T)	Mast	COPA	NTRK1	6,44462E-03	61885,8	1,92616E-02	2271	1,42008E+00	9193	3,23712E-01	106123	7,44575E-01	129942	0	61900
Myeloid_vs_CD8_Non-responder	CD8(GZMK+ Tem)	pDC_LILRA4	TGFB1	TGFBR1_TGFBR2	6,45251E-03	41891,6	4,16977E-03	30354	7,65719E-01	39307	1,70171E+00	31045	8,85028E-01	46852	0	61900
Myeloid_vs_CD8_Non-responder	Macro_FOLR2+APOE+	CD8(EOMES+ NK-like)	CXCL16	CXCR6	6,45388E-03	37179,4	4,07933E-03	31333	1,08738E+00	20215	1,87404E+00	25595	8,85025E-01	46854	0	61900

Myeloid_vs_CD8_Non-responder	CD8(ID2+CXCR4+ T)	Macro_OLFML3	ANXA1	FPR1	6,45388E-03	40108,8	3,36392E-03	41681	9,34812E-01	27743	1,32276E+00	46854	9,22205E-01	22366	0	61900
Myeloid_vs_CD8_Non-responder	CD8(Tn)	Macro_ISG15	HSPA1A	TLR4	6,45526E-03	38288,6	4,83249E-03	24229	9,82617E-01	25099	1,32275E+00	46856	9,04937E-01	33359	0	61900
Myeloid_vs_CD8_Non-responder	cDC_LAMP3	CD8(Tc17)	CCL19	CXCR3	6,45966E-03	34387,2	2,03250E-02	2038	3,80234E+00	95	2,12826E+00	18831	8,19652E-01	89072	0	61900
Myeloid_vs_CD8_Non-responder	CD8(GZMK+ Tem)	Macro_FOLR2+APOE-	TNFSF9	HLA-DPA1	6,47181E-03	35572,2	4,61859E-03	25929	1,09997E+00	19670	1,32225E+00	46880	9,20389E-01	23482	0	61900
Myeloid_vs_CD8_Non-responder	CD8(IL7R+ZNF683+ Tm)	Macro_FOLR2+APOE+	HLA-A	LILRB1	6,47319E-03	39335,6	3,12151E-03	46487	9,47593E-01	27006	1,32221E+00	46882	9,36163E-01	14403	0	61900
Myeloid_vs_CD8_Non-responder	CD8(LAYN+ T)	pDC_LILRA4	TGFB1	TGFBR1_TGFBR2	6,47595E-03	42523,2	4,16626E-03	30395	7,65146E-01	39343	1,61937E+00	34092	8,84985E-01	46886	0	61900
Myeloid_vs_CD8_Non-responder	CD8(GZMK+ Tem)	cDC_LAMP3	TNFSF10	TNFRSF11B	6,47847E-03	54224,4	2,02992E-02	2041	2,22423E+00	1567	9,68953E-01	65728	7,21644E-01	139886	0	61900
Myeloid_vs_CD8_Non-responder	CD8(ISG+ T)	Macro_OLFML3	HLA-C	LILRB1	6,48563E-03	37913,6	3,29332E-03	43100	1,00056E+00	24206	1,32171E+00	46900	9,37642E-01	13462	0	61900
Myeloid_vs_CD8_Non-responder	CD8(Temra)	cDC(CD1C)	B2M	CD1A	6,48656E-03	32471,2	2,46738E-02	1424	1,27959E+00	12991	1,01901E+00	62917	9,21014E-01	23124	0	61900
Myeloid_vs_CD8_Non-responder	CD8(Terminal Tex)	Mono_CD16	HLA-F	LILRB1	6,49393E-03	41854,4	3,53583E-03	38793	1,09875E+00	19721	1,42986E+00	41946	8,84945E-01	46912	0	61900
Myeloid_vs_CD8_Non-responder	Macro_FOLR2+APOE+	CD8(NME1+ T)	HLA-DQB2	LAG3	6,50085E-03	39342	5,92330E-03	17912	8,78145E-01	31247	1,50514E+00	38729	8,84931E-01	46922	0	61900
Myeloid_vs_CD8_Non-responder	CD8(Tc17)	Mono_CD16	HLA-B	LILRA1	6,50640E-03	27845,4	8,91975E-03	9640	1,58997E+00	6202	1,32134E+00	46930	9,35919E-01	14555	0	61900
Myeloid_vs_CD8_Non-responder	Macro_ISG15	CD8(NME1+ T)	CCL2	CCR5	6,51239E-03	42534,2	1,14672E-02	6385	1,41872E+00	9226	3,04101E+00	5338	7,44827E-01	129822	0	61900
Myeloid_vs_CD8_Non-responder	Macro_NLRP3	CD8(ZNF683+KLRB1+ T)	VCAN	ITGB1	6,52514E-03	29999,6	5,07430E-03	22544	1,93413E+00	2932	2,27625E+00	15665	8,84902E-01	46957	0	61900
Myeloid_vs_CD8_Non-responder	CD8(Temra)	Macro_OLFML3	HLA-A	LILRB1	6,54461E-03	37465,4	3,37387E-03	41499	1,00438E+00	24009	1,32022E+00	46985	9,38447E-01	12934	0	61900
Myeloid_vs_CD8_Non-responder	Macro_NLRP3	CD8(GZMK+ Tex)	LGALS3	LAG3	6,54810E-03	41639,8	4,31374E-03	28776	6,84721E-01	46282	1,32015E+00	46990	9,19083E-01	24251	0	61900
Myeloid_vs_CD8_Non-responder	CD8(Terminal Tex)	Macro_FOLR2+APOE+	HLA-C	LILRB2	6,54810E-03	33913,4	3,09799E-03	46990	1,17305E+00	16674	1,52425E+00	37903	9,47634E-01	6100	0	61900
Myeloid_vs_CD8_Non-responder	CD8(GZMK+ Tex)	Macro_NLRP3	HLA-F	LILRB2	6,55716E-03	39522	3,09745E-03	47003	1,13120E+00	18305	1,64184E+00	33259	8,99323E-01	37143	0	61900
Myeloid_vs_CD8_Non-responder	CD8(Temra)	Macro_LYVE1	HMGB1	CD163	6,56972E-03	34532,6	3,81937E-03	34505	1,27297E+00	13201	1,31954E+00	47021	9,33334E-01	16036	0	61900
Myeloid_vs_CD8_Non-responder	cDC_LAMP3	CD8(GZMK+ Early Tem)	CD200	CD200R1	6,57293E-03	45627,6	4,33755E-02	457,5	2,09918E+00	2055,5	1,53375E+00	37491,5	7,52614E-01	126233,5	0	61900
Myeloid_vs_CD8_Non-responder	CD8(Tn)	Macro_FOLR2+APOE+	B2M	LILRB2	6,58371E-03	33241,8	3,09592E-03	47041	1,11036E+00	19178	1,57748E+00	35732	9,54786E-01	2358	0	61900
Myeloid_vs_CD8_Non-responder	CD8(ZNF683+KLRB1+ T)	Macro_NLRP3	HLA-A	LILRB2	6,58651E-03	30921,4	3,09582E-03	47045	1,38675E+00	9973	1,74399E+00	29646	9,47704E-01	6043	0	61900
Myeloid_vs_CD8_Non-responder	CD8(Tc17)	Macro_OLFML3	B2M	LILRB1	6,58721E-03	36984	3,25918E-03	43751	9,89763E-01	24729	1,31889E+00	47046	9,45832E-01	7494	0	61900
Myeloid_vs_CD8_Non-responder	cDC_CLEC9A	CD8(ID2+CXCR4+ T)	HLA-DPB1	LAG3	6,58846E-03	23301,6	4,97180E-03	23217	1,24658E+00	14024	2,64823E+00	9570	9,45431E-01	7797	0	61900
Myeloid_vs_CD8_Non-responder	CD8(GZMK+ Early Tem)	cDC(CD1C)	B2M	CD1A	6,59397E-03	32901,8	2,45599E-02	1436	1,26025E+00	13571	9,92012E-01	64380	9,20846E-01	23222	0	61900
Myeloid_vs_CD8_Non-responder	CD8(ITM2C+ T)	Mono_CD14	CIRBP	TREM1	6,61175E-03	42510	4,02798E-03	31938	9,67583E-01	25878	1,31831E+00	47081	8,86737E-01	45753	0	61900
Myeloid_vs_CD8_Non-responder	CD8(NME1+ T)	Macro_FOLR2+APOE+	LGALS3	ENG	6,61947E-03	43790	3,09371E-03	47092	9,82718E-01	25089	1,42673E+00	42085	8,91123E-01	42784	0	61900
Myeloid_vs_CD8_Non-responder	Macro_FOLR2+APOE+	CD8(IL7R+ZNF683+ Tm)	CD14	ITGB1	6,62510E-03	32918,2	3,09347E-03	47100	1,33585E+00	11319	2,46276E+00	12252	9,06976E-01	32020	0	61900
Myeloid_vs_CD8_Non-responder	Macro_FOLR2+APOE+	CD8(EOMES+ NK-like)	LGALS1	CD69	6,62650E-03	36002,4	3,09340E-03	47102	8,30550E-01	34446	1,71426E+00	30617	9,47844E-01	5947	0	61900
Myeloid_vs_CD8_Non-responder	CD8(GZMK+ Tem)	cDC(CD1C)	B2M	CD1A	6,63375E-03	32803,6	2,45264E-02	1443	1,25457E+00	13762	1,00551E+00	63655	9,20796E-01	23258	0	61900
Myeloid_vs_CD8_Non-responder	Macro_ISG15	CD8(GZMK+ Tex)	CXCL16	CXCR6	6,63596E-03	23457,2	9,30476E-03	8997	1,37097E+00	10370	2,43297E+00	12759	9,20795E-01	23260	0	61900
Myeloid_vs_CD8_Non-responder	CD8(Temra)	Macro_IER3	SPON2	ITGB2	6,64270E-03	39720,2	6,01195E-03	17529	8,66536E-01	32029	1,31766E+00	47125	8,95097E-01	40018	0	61900
Myeloid_vs_CD8_Non-responder	Macro_IER3	CD8(Terminal Tex)	HLA-DQA2	LAG3	6,64594E-03	26762,2	7,11378E-03	13651	1,18593E+00	16191	2,12966E+00	18800	9,20782E-01	23269	0	61900
Myeloid_vs_CD8_Non-responder	Macro_ISG15	CD8(ID2+CXCR4+ T)	CXCL16	CXCR6	6,68149E-03	23661,2	9,29032E-03	9015	1,36982E+00	10402	2,37949E+00	13688	9,20738E-01	23301	0	61900
Myeloid_vs_CD8_Non-responder	Macro_ISG15	CD8(Terminal Tex)	HLA-DRB1	LAG3	6,68706E-03	25179,8	4,95829E-03	23306	1,07344E+00	20828	2,31520E+00	14903	9,49330E-01	4962	0	61900
Myeloid_vs_CD8_Non-responder	CD8(GZMK+ Tem)	pDC_LILRA4	HLA-C	NOTCH4	6,69486E-03	29420,2	1,38245E-02	4485	1,46650E+00	8222	1,27524E+00	49181	9,20719E-01	23313	0	61900
Myeloid_vs_CD8_Non-responder	cDC(CD1C)	CD8(ID2+CXCR4+ T)	HLA-DQA1	LAG3	6,69486E-03	26985,8	5,56425E-03	19679	1,18017E+00	16394	1,95297E+00	23313	9,37334E-01	13643	0	61900
Myeloid_vs_CD8_Non-responder	CD8(GZMK+ Tem)	Macro_FOLR2+APOE+	CD28	CD86	6,71272E-03	36378,2	9,68640E-03	8407,5	1,34444E+00	11077,5	1,95229E+00	23329,5	8,38620E-01	77176,5	0	61900
Myeloid_vs_CD8_Non-responder	Macro_OLFML3	CD8(ZNF683+KLRB1+ T)	CXCL9	CXCR3	6,72055E-03	29058,4	1,08406E-02	7040	2,10171E+00	2041	2,66798E+00	9326	8,57423E-01	64985	0	61900
Myeloid_vs_CD8_Non-responder	pDC_LILRA4	CD8(EOMES+ NK-like)	APP	LRP10	6,73739E-03	26522,8	9,10478E-03	9334	2,26108E+00	1449	2,99564E+00	5740	8,73906E-01	54191	0	61900
Myeloid_vs_CD8_Non-responder	Mast	CD8(Tn)	ADCYAP1	DPP4	6,74463E-03	54860,2	7,49839E-02	161	2,09342E+00	2083	1,08527E+00	59228	6,90316E-01	150929	0	61900
Myeloid_vs_CD8_Non-responder	CD8(ID2+CXCR4+ T)	Macro_ISG15	TGFB1	ENG	6,75981E-03	44100,8	3,92007E-03	33241	8,37571E-01	33962	1,38238E+00	44111	8,84426E-01	47290	0	61900
Myeloid_vs_CD8_Non-responder	Mono_INHBA	CD8(ITM2C+ T)	CCL2	CCR5	6,76058E-03	37325,4	1,96321E-02	2185	1,48697E+00	7854	2,66673E+00	9345	7,92498E-01	105343	0	61900
Myeloid_vs_CD8_Non-responder	Mono_CD14	CD8(ID2+CXCR4+ T)	VCAN	ITGB1	6,76903E-03	21620,2	9,69693E-03	8387	2,44113E+00	978	2,66638E+00	9349	9,14001E-01	27487	0	61900
Myeloid_vs_CD8_Non-responder	Macro_ISG15	CD8(GZMK+ Tem)	CCL2	CCR5	6,77114E-03	42851,8	1,11533E-02	6673	1,41314E+00	9350	3,05098E+00	5248	7,42181E-01	131088	0	61900
Myeloid_vs_CD8_Non-responder	CD8(ITM2C+ T)	cDC_CLEC9A	IFNG	IFNGR1_IFNGR2	6,77125E-03	43569,4	4,60547E-03	26022	6,73562E-01	47306	1,46833E+00	40276	8,91762E-01	42343	0	61900
Myeloid_vs_CD8_Non-responder	CD8(NME1+ T)	Macro_OLFML3	MIF	CD74_CXCR4	6,77666E-03	56400,8	1,76517E-03	93263	5,47556E-01	60942	1,00226E+00	63811	9,55452E-01	2088	0	61900
Myeloid_vs_CD8_Non-responder	CD8(GZMK+ Tem)	Macro_OLFML3	IFNG	IFNGR1_IFNGR2	6,78414E-03	36330,8	6,64211E-03	15068	9,61348E-01	26212	1,31417E+00	47324	9,08209E-01	31150	0	61900
Myeloid_vs_CD8_Non-responder	CD8(Temra)	Macro_ISG15	IFNG	IFNGR1_IFNGR2	6,78558E-03	42224	4,87135E-03	23929	7,89292E-01	37518	1,31414E+00	47326	8,94442E-01	40447	0	61900
Myeloid_vs_CD8_Non-responder	Macro_NLRP3	CD8(Terminal Tex)	HLA-DRB1	LAG3	6,78916E-03	40840,4	3,55572E-03	38468	6,96148E-01	45203	1,31413E+00	47331	9,40709E-01	11300	0	61900
Myeloid_vs_CD8_Non-responder	CD8(GZMK+ Tex)	pDC_LILRA4	HSP90B1	TLR9	6,78949E-03	33593,6	2,54372E-02	1339	2,09219E+00	2090	1,31778E+00	47119	8,71875E-01	55520	0	61900
Myeloid_vs_CD8_Non-responder	CD8(ITM2C+ T)	Macro_FOLR2+APOE+	IL16	CD9	6,79849E-03	33309	5,45332E-03	20288	1,05913E+00	21477	2,28360E+00	15536	8,84336E-01	47344	0	61900
Myeloid_vs_CD8_Non-responder	Macro_FOLR2+APOE+	CD8(EOMES+ NK-like)	CD14	ITGA4	6,80352E-03	32938	3,08201E-03	47351	1,32659E+00	11626	2,44171E+00	12615	9,08147E-01	31198	0	61900
Myeloid_vs_CD8_Non-responder	CD8(Temra)	Macro_FOLR2+APOE+	HLA-F	LILRB2	6,80639E-03	37242,2	3,08179E-03	47355	1,28343E+00	12892	1,83463E+00	26769	8,99094E-01	37295	0	61900
Myeloid_vs_CD8_Non-responder	Macro_FOLR2-APOE+	CD8(ITM2C+ T)	APOE	LDLR	6,80712E-03	24370,6	1,19455E-02	5998	1,58512E+00	6262	2,66422E+00	9367	8,97591E-01	38326	0	61900
Myeloid_vs_CD8_Non-responder	Macro_ISG15	CD8(GZMK+ Tex)	NECTIN2	TIGIT	6,80877E-03	28065,6	2,40604E-02	1500,5	2,09207E+00	2092,5	2,12578E+00	18883,5	8,71226E-01	55951,5	0	61900
Myeloid_vs_CD8_Non-responder	CD8(ID2+CXCR4+ T)	Macro_FOLR2+APOE+	IFNG	IFNGR1_IFNGR2	6,80999E-03	42662,8	4,37459E-03	28183	8,69007E-01	31859	1,31363E+00	47360	8,89255E-01	44012	0	61900
Myeloid_vs_CD8_Non-responder	Macro_IER3	CD8(GZMK+ Tex)	HLA-DQA2	LAG3	6,81946E-03	26746,6	7,06207E-03	13772	1,18077E+00	16374	2,15433E+00	18263	9,20516E-01	23424	0	61900
Myeloid_vs_CD8_Non-responder	cDC(CD1C)	CD8(NME1+ T)	HLA-DQB1	LAG3	6,82059E-03	27944,4	4,94150E-03	23425	1,17143E+00	16733	1,96965E+00	22854	9,35513E-01	14810	0	61900
Myeloid_vs_CD8_Non-responder	CD8(Tc17)	Macro_FOLR2+APOE+	HLA-A	LILRB1	6,82294E-03	39636,6	3,10860E-03	46766	9,36174E-01	27662	1,31322E+00	47378	9,36039E-01	14477	0	61900
Myeloid_vs_CD8_Non-responder	CD8(GZMK+ Early Tem)	Macro_FOLR2+APOE+	HLA-F	LILRB1	6,82510E-03	42272,2	3,48966E-03	39538	9,79167E-01	25297	1,54053E+00	37245	8,84274E-01	47381	0	61900
Myeloid_vs_CD8_Non-responder	Macro_ISG15	CD8(GZMK+ Tem)	HLA-DQA1	LAG3	6,83190E-03	27487	5,61594E-03	19384	1,01588E+00	23435	2,11074E+00	19224	9,37605E-01	13492	0	61900
Myeloid_vs_CD8_Non-responder	CD8(NME1+ T)	Macro_ISG15	IFNG	IFNGR1_IFNGR2	6,83447E-03	42058,8	4,91539E-03	23612	7,93243E-01	37218	1,31288E+00	47394	8,94866E-01	40170	0	61900
Myeloid_vs_CD8_Non-responder	CD8(Temra)	Mono_CD14	CIRBP	TREM1	6,83519E-03	40139,4	4,50737E-03	26930	1,03746E+00	22468	1,31285E+00	47395	8,92262E-01	42004	0	61900
Myeloid_vs_CD8_Non-responder	CD8(Tn)	Macro_ISG15	CD40LG	CD9	6,84322E-03	35133,8	2,27477E-02	1670	1,01564E+00	23445	2,23641E+00	16497	8,46550E-01	72157	0	61900
Myeloid_vs_CD8_Non-responder	CD8(GZMK+ Tem)	Macro_FOLR2+APOE+	HLA-F	LILRB2	6,84457E-03	37527,8	3,07914E-03	47408	1,28303E+00	12905	1,79226E+00	28102	8,99055E-01	37324	0	61900
Myeloid_vs_CD8_Non-responder	CD8(GZMK+ Tex)	cDC_LAMP3	TNFSF10	TNFRSF11B	6,84740E-03	54254,8	1,99893E-02	2099	2,22060E+00	1583	9,78370E-01	65185	7,20097E-01	140507	0	61900
Myeloid_vs_CD8_Non-responder	pDC_LILRA4	CD8(ZNF683+KLRB1+ T)	APP	LRP10	6,84962E-03	26445,8	9,07568E-03	9387	2,26011E+00	1450	3,05958E+00	5191	8,73730E-01	54301	0	61900
Myeloid_vs_CD8_Non-responder	CD8(IL7R+ZNF683+ Tm)	Macro_FOLR2+APOE+	HLA-C	LILRB1	6,85251E-03	38852,6	3,07881E-03	47419	9,68307E-01	25846	1,37638E+00	44380	9,35644E-01	14718	0	61900
Myeloid_vs_CD8_Non-responder	CD8(ISG+ T)	Macro_FOLR2+APOE+	HLA-F	LILRB1	6,85902E-03	42690	3,48270E-03	39624	9,78037E-01	25357	1,49558E+00	39141	8,84171E-01	47428	0	61900
Myeloid_vs_CD8_Non-responder	CD8(Temra)	Macro_FOLR2+APOE+	CCL5	CCRL2	6,85910E-03	37865	9,30407E-03	8998	1,08692E+00	20230	8,16774E-01	74738	9,20444E-01	23459	0	61900
Myeloid_vs_CD8_Non-responder	CD8(ID2+CXCR4+ T)	Macro_FOLR2+APOE+	B2M	LILRB1	6,87784E-03	36571,2	3,07694E-03	47454	1,04181E+00	22268	1,41537E+00	42593	9,44338E-01	8641	0	61900
Myeloid_vs_CD8_Non-responder	CD8(NME1+ T)	Macro_IER3	MIF	CD74_CXCR4	6,87967E-03	59541,4	1,76215E-03	93404	5,59531E-01	59512	7,18234E-01	80787	9,55416E-01	2104	0	61900
Myeloid_vs_CD8_Non-responder	CD8(Tc17)	Macro_FOLR2+APOE+	B2M	TFRC	6,88146E-03	41648,6	3,11940E-03	46519	6,71794E-01	47459	1,37031E+00	44659	9,45544E-01	7706	0	61900
Myeloid_vs_CD8_Non-responder	CD8(NME1+ T)	Mono_CD14	GNAI2	FPR1	6,89597E-03	39361,2	3,07554E-03	47479	1,20896E+00	15348	1,78381E+00	28387	8,89755E-01	43692	0	61900
Myeloid_vs_CD8_Non-responder	CD8(GZMK+ Tem)	Macro_OLFML3	B2M	LILRB1	6,89743E-03	37242,2	3,24995E-03	43929	9,77943E-01	25363	1,31085E+00	47481	9,45759E-01	7538	0	61900
Myeloid_vs_CD8_Non-responder	CD8(ZNF683+KLRB1+ T)	Macro_ISG15	HMGB1	CD163	6,89961E-03	37064,8	3,07530E-03	47484	9,53392E-01	26656	1,75347E+00	29322	9,26268E-01	19962	0	61900

Myeloid_vs_CD8_Post_NR

Myeloid_vs_CD8_Non-responder	Mono_INHBA	CD8(ITM2C+ T)	IL1B	ADRB2	6,90511E-03	28257,4	9,05991E-03	9413	1,79976E+00	3955	3,03848E+00	5361	8,64195E-01	60658	0	61900
Myeloid_vs_CD8_Non-responder	Macro_OLFML3	CD8(GZMK+ Tex)	B2M	CD3D	6,90748E-03	51234,2	2,61687E-03	59488	5,47435E-01	60953	8,32861E-01	73781	9,64173E-01	49	0	61900
Myeloid_vs_CD8_Non-responder	Macro_FOLR2+APOE+	CD8(ZNF683+KLRB1+ T)	HLA-B	CD3G	6,91779E-03	40028,2	3,21032E-03	44682	7,22027E-01	42905	1,31038E+00	47509	9,52872E-01	3145	0	61900
Myeloid_vs_CD8_Non-responder	Macro_FOLR2-APOE+	CD8(Tn)	APOE	LSR	6,94370E-03	25193,2	1,68939E-02	2971	1,59754E+00	6082	2,65900E+00	9431	8,86959E-01	45582	0	61900
Myeloid_vs_CD8_Non-responder	Macro_FOLR2+APOE+	CD8(ZNF683+KLRB1+ T)	MRC1	PTPRC	6,94842E-03	41900,6	4,94468E-03	23403	8,74156E-01	31504	1,30968E+00	47551	8,87589E-01	45145	0	61900
Myeloid_vs_CD8_Non-responder	CD8(Tn)	Macro_NLRP3	ANXA1	FPR2_FPR3	6,95646E-03	42629,4	3,94855E-03	32911	9,56570E-01	26478	1,37788E+00	44296	8,83988E-01	47562	0	61900
Myeloid_vs_CD8_Non-responder	CD8(ITM2C+ T)	Macro_FOLR2+APOE+	TNF	TNFRSF1A	6,96158E-03	37683	5,22127E-03	21623	9,49225E-01	26914	1,72086E+00	30409	8,83970E-01	47569	0	61900
Myeloid_vs_CD8_Non-responder	CD8(Tc17)	Macro_ISG15	ANXA1	FPR1	6,96817E-03	42125,4	3,07007E-03	47578	8,61749E-01	32344	1,37623E+00	44386	9,18862E-01	24419	0	61900
Myeloid_vs_CD8_Non-responder	CD8(IL7R+ZNF683+ Tm)	Macro_FOLR2+APOE+	HLA-B	LILRB1	6,97183E-03	38931	3,06988E-03	47583	9,60817E-01	26245	1,34078E+00	46047	9,38527E-01	12880	0	61900
Myeloid_vs_CD8_Non-responder	CD8(ID2+CXCR4+ T)	Macro_NLRP3	CIRBP	TREM1	6,97256E-03	42532,8	4,12714E-03	30814	9,39446E-01	27463	1,30918E+00	47584	8,87953E-01	44903	0	61900
Myeloid_vs_CD8_Non-responder	Macro_ISG15	CD8(GZMK+ Tex)	HLA-DRB1	LAG3	6,97328E-03	25196	4,92225E-03	23559	1,06828E+00	21045	2,33987E+00	14405	9,49154E-01	5071	0	61900
Myeloid_vs_CD8_Non-responder	Macro_FOLR2-APOE+	CD8(Tc17)	APOE	LSR	6,98674E-03	24331,4	1,86990E-02	2424	1,62885E+00	5662	2,65708E+00	9451	8,91949E-01	42220	0	61900
Myeloid_vs_CD8_Non-responder	Mono_INHBA	CD8(Tc17)	SPP1	CD44	6,98938E-03	25937,4	8,52411E-03	10351	1,22046E+00	14927	2,12341E+00	18936	9,20238E-01	23573	0	61900
Myeloid_vs_CD8_Non-responder	CD8(ISG+ T)	cDC_LAMP3	CD28	CD86	6,99321E-03	36220,4	1,07904E-02	7110,5	1,40825E+00	9454,5	1,73209E+00	30029,5	8,45791E-01	72607,5	0	61900
Myeloid_vs_CD8_Non-responder	Macro_OLFML3	CD8(ISG+ T)	HLA-DQA1	LAG3	7,00435E-03	29192,2	4,91888E-03	23586	1,03027E+00	22805	2,00889E+00	21776	9,33614E-01	15894	0	61900
Myeloid_vs_CD8_Non-responder	cDC(CD1C)	CD8(LAYN+ T)	HLA-DRB5	LAG3	7,00550E-03	26772,2	4,91883E-03	23587	1,18526E+00	16215	2,02101E+00	21478	9,41626E-01	10681	0	61900
Myeloid_vs_CD8_Non-responder	CD8(GZMK+ Tex)	Macro_ISG15	IFNG	IFNGR1_IFNGR2	7,00560E-03	41413	5,08865E-03	22418	8,08786E-01	36028	1,30832E+00	47629	8,96484E-01	39090	0	61900
Myeloid_vs_CD8_Non-responder	CD8(ITM2C+ T)	Mono_CD16	HLA-C	LILRB1	7,03802E-03	37071,8	3,48013E-03	39659	1,00873E+00	23771	1,30741E+00	47673	9,39236E-01	12356	0	61900
Myeloid_vs_CD8_Non-responder	CD8(Temra)	Macro_FOLR2+APOE+	TNFSF9	HLA-DPA1	7,03875E-03	36847,4	3,06577E-03	47674	9,65726E-01	25979	2,32558E+00	14686	9,04023E-01	33998	0	61900
Myeloid_vs_CD8_Non-responder	CD8(ISG+ T)	pDC_LILRA4	CCL5	CXCR3	7,04245E-03	35874,2	7,82830E-03	11784	6,69340E-01	47679	1,36973E+00	44685	9,37841E-01	13323	0	61900
Myeloid_vs_CD8_Non-responder	Macro_OLFML3	CD8(Terminal Tex)	CXCL9	CXCR3	7,05597E-03	26535,2	1,40117E-02	4389	2,17567E+00	1745	2,65444E+00	9483	8,72400E-01	55159	0	61900
Myeloid_vs_CD8_Non-responder	Macro_FOLR2+APOE+	CD8(EOMES+ NK-like)	TIMP2	CD44	7,05649E-03	44758,4	3,42576E-03	40579	8,93952E-01	30244	1,30694E+00	47698	8,90234E-01	43371	0	61900
Myeloid_vs_CD8_Non-responder	CD8(Terminal Tex)	Mono_CD16	HLA-A	LILRA1	7,06463E-03	27855,2	9,32520E-03	8972	1,66531E+00	5206	1,30671E+00	47709	9,34318E-01	15489	0	61900
Myeloid_vs_CD8_Non-responder	CD8(EOMES+ NK-like)	Macro_OLFML3	B2M	LILRB1	7,06685E-03	37333,2	3,24739E-03	43981	9,74656E-01	25516	1,30668E+00	47712	9,45739E-01	7557	0	61900
Myeloid_vs_CD8_Non-responder	cDC_CLEC9A	CD8(ISG+ T)	HLA-DPB1	LAG3	7,07375E-03	23286,6	4,90862E-03	23646	1,23951E+00	14259	2,72425E+00	8562	9,45101E-01	8066	0	61900
Myeloid_vs_CD8_Non-responder	CD8(LAYN+ T)	pDC_LILRA4	HSP90B1	TLR9	7,07484E-03	34415,2	2,48645E-02	1403	2,08030E+00	2134	1,25388E+00	50263	8,70597E-01	56376	0	61900
Myeloid_vs_CD8_Non-responder	Macro_FOLR2+APOE+	CD8(Terminal Tex)	HLA-C	CD3G	7,07797E-03	40838,6	3,11262E-03	46659	7,18510E-01	43233	1,30631E+00	47727	9,49826E-01	4674	0	61900
Myeloid_vs_CD8_Non-responder	CD8(Terminal Tex)	cDC_CLEC9A	CD52	SIGLEC10	7,08316E-03	38998,2	4,13957E-03	30673	9,78002E-01	25360	1,30620E+00	47734	9,11036E-01	29324	0	61900
Myeloid_vs_CD8_Non-responder	Macro_ISG15	CD8(ZNF683+KLRB1+ T)	NECTIN2	CD96	7,09451E-03	25297,6	1,98128E-02	2137	2,12441E+00	1937	2,09275E+00	19649	8,93856E-01	40865	0	61900
Myeloid_vs_CD8_Non-responder	Macro_FOLR2+APOE+	CD8(EOMES+ NK-like)	APOE	LSR	7,09510E-03	27513,8	1,24546E-02	5530	1,40614E+00	9501	3,16362E+00	4366	8,70752E-01	56272	0	61900
Myeloid_vs_CD8_Non-responder	CD8(Temra)	Macro_FOLR2+APOE+	ADAM10	TREM2	7,09932E-03	35803,2	4,90602E-03	23668	1,20105E+00	15648	2,40070E+00	13309	8,58226E-01	64491	0	61900
Myeloid_vs_CD8_Non-responder	cDC(CD1C)	CD8(NME1+ T)	HLA-DQA2	LAG3	7,12730E-03	26488,6	6,97488E-03	14036	1,40844E+00	9449	1,95097E+00	23366	9,20060E-01	23692	0	61900
Myeloid_vs_CD8_Non-responder	CD8(GZMK+ Early Tem)	Macro_FOLR2+APOE+	HLA-C	LILRB1	7,12928E-03	39536,8	3,06075E-03	47796	9,52946E-01	26686	1,33064E+00	46469	9,35467E-01	14833	0	61900
Myeloid_vs_CD8_Non-responder	CD8(Tc17)	Macro_FOLR2+APOE+	TGFB1	ENG	7,13301E-03	43121	3,85799E-03	34020	9,48548E-01	26945	1,36469E+00	44939	8,83607E-01	47801	0	61900
Myeloid_vs_CD8_Non-responder	Macro_ISG15	CD8(LAYN+ T)	S100A8	CD69	7,13313E-03	27488	5,53843E-03	19816	1,15685E+00	17272	1,93967E+00	23697	9,35582E-01	14755	0	61900
Myeloid_vs_CD8_Non-responder	Macro_FOLR2+APOE+	CD8(EOMES+ NK-like)	HLA-E	KIR3DL1	7,13375E-03	33816,6	2,00072E-02	2096	9,95849E-01	24442	1,65275E+00	32843	8,83606E-01	47802	0	61900
Myeloid_vs_CD8_Non-responder	Macro_FOLR2+APOE+	CD8(LAYN+ T)	CXCL9	CXCR3	7,13437E-03	30964,8	9,08991E-03	9360	2,17401E+00	1754	2,65188E+00	9519	8,46315E-01	72291	0	61900
Myeloid_vs_CD8_Non-responder	CD8(ITM2C+ T)	Macro_OLFML3	HLA-B	LILRB2	7,13823E-03	30913,2	3,06035E-03	47808	1,36852E+00	10428	1,74121E+00	29732	9,49787E-01	4698	0	61900
Myeloid_vs_CD8_Non-responder	CD8(NME1+ T)	Mono_CD16	HLA-F	LILRB1	7,14197E-03	42727,6	3,44354E-03	40249	1,08635E+00	20251	1,39810E+00	43425	8,83591E-01	47813	0	61900
Myeloid_vs_CD8_Non-responder	CD8(LAYN+ T)	Macro_OLFML3	B2M	LILRB1	7,15168E-03	37432,2	3,24307E-03	44057	9,69124E-01	25800	1,30422E+00	47826	9,45704E-01	7578	0	61900
Myeloid_vs_CD8_Non-responder	CD8(NME1+ T)	Macro_IFI27	MIF	CD74_CXCR4	7,15366E-03	67736,2	1,75336E-03	93858	5,28474E-01	63302	1,49861E-01	117475	9,55309E-01	2146	0	61900
Myeloid_vs_CD8_Non-responder	cDC(CD1C)	CD8(ID2+CXCR4+ T)	HLA-DPB1	LAG3	7,15418E-03	25138,6	4,89897E-03	23715	1,22355E+00	14825	2,20446E+00	17146	9,45050E-01	8107	0	61900
Myeloid_vs_CD8_Non-responder	CD8(LAYN+ T)	Macro_FOLR2+APOE+	HLA-B	LILRB1	7,16215E-03	39472,8	3,05908E-03	47840	9,49782E-01	26877	1,30493E+00	47799	9,38426E-01	12948	0	61900
Myeloid_vs_CD8_Non-responder	Macro_FOLR2+APOE+	CD8(GZMK+ Tex)	HLA-A	CD8A	7,16365E-03	38747	3,17109E-03	45464	7,81162E-01	38152	1,30393E+00	47842	9,60774E-01	377	0	61900
Myeloid_vs_CD8_Non-responder	Macro_FOLR2+APOE+	CD8(EOMES+ NK-like)	MRC1	PTPRC	7,17114E-03	41731,2	4,98628E-03	23128	8,82951E-01	30913	1,30374E+00	47852	8,88006E-01	44863	0	61900
Myeloid_vs_CD8_Non-responder	CD8(GZMK+ Tex)	Macro_FOLR2+APOE+	HLA-F	LILRB1	7,17264E-03	42950,6	3,43881E-03	40335	9,70909E-01	25703	1,49995E+00	38961	8,83520E-01	47854	0	61900
Myeloid_vs_CD8_Non-responder	CD8(GZMK+ Early Tem)	Macro_FOLR2+APOE+	CCL5	CCR1	7,17997E-03	34410,4	9,03199E-03	9467	1,11313E+00	19062	1,10912E+00	57886	9,19980E-01	23737	0	61900
Myeloid_vs_CD8_Non-responder	CD8(NME1+ T)	cDC(CD1C)	B2M	CD1A	7,18003E-03	34850,6	2,38056E-02	1523	1,13218E+00	18257	9,19796E-01	68652	9,19701E-01	23921	0	61900
Myeloid_vs_CD8_Non-responder	CD8(Temra)	Macro_FOLR2+APOE+	HLA-B	LILRB1	7,18089E-03	39302,6	3,05797E-03	47865	9,48639E-01	26942	1,32289E+00	46849	9,38415E-01	12957	0	61900
Myeloid_vs_CD8_Non-responder	Macro_NLRP3	CD8(LAYN+ T)	LGALS1	ITGB1	7,18239E-03	38122,4	3,05790E-03	47867	9,19079E-01	28691	1,58137E+00	35565	9,32327E-01	16589	0	61900
Myeloid_vs_CD8_Non-responder	Mono_CD14	CD8(EOMES+ NK-like)	S100A8	ITGB2	7,18584E-03	20392,4	4,89629E-03	23742	2,38076E+00	1106	3,41154E+00	2827	9,39180E-01	12387	0	61900
Myeloid_vs_CD8_Non-responder	Macro_FOLR2+APOE+	CD8(Temra)	HLA-A	KIR3DL1	7,19440E-03	36373,6	1,43175E-02	4200	8,62442E-01	32295	1,58081E+00	35590	8,83458E-01	47883	0	61900
Myeloid_vs_CD8_Non-responder	Macro_FOLR2+APOE+	CD8(ITM2C+ T)	CD14	ITGB2	7,19966E-03	28496,6	3,05677E-03	47890	1,45259E+00	8501	2,68704E+00	9064	9,34955E-01	15128	0	61900
Myeloid_vs_CD8_Non-responder	CD8(NME1+ T)	Macro_IER3	GNAI2	C5AR1	7,21621E-03	39715	3,05623E-03	47912	1,30615E+00	12227	1,62211E+00	33991	8,91466E-01	42545	0	61900
Myeloid_vs_CD8_Non-responder	Macro_ISG15	CD8(Tc17)	CXCL2	DPP4	7,21774E-03	32099,2	2,10474E-02	1910	1,40393E+00	9557	2,66147E+00	9395	8,37733E-01	77734	0	61900
Myeloid_vs_CD8_Non-responder	CD8(GZMK+ Tem)	Macro_ISG15	HMGB1	THBD	7,22224E-03	47927,6	3,05590E-03	47920	7,51159E-01	40463	1,32567E+00	46712	8,91346E-01	42643	0	61900
Myeloid_vs_CD8_Non-responder	CD8(EOMES+ NK-like)	pDC_LILRA4	HSP90B1	TLR9	7,22628E-03	33815	2,46258E-02	1430	2,07534E+00	2157	1,32352E+00	46822	8,70053E-01	56766	0	61900
Myeloid_vs_CD8_Non-responder	CD8(NME1+ T)	Macro_NLRP3	VIM	CD44	7,22628E-03	58028,2	1,68277E-03	97126	5,37910E-01	62132	9,50807E-01	66826	9,55289E-01	2157	0	61900
Myeloid_vs_CD8_Non-responder	Macro_FOLR2+APOE+	CD8(ID2+CXCR4+ T)	CXCL9	CXCR3	7,22656E-03	31064,6	8,96861E-03	9561	2,17134E+00	1762	2,67132E+00	9280	8,45439E-01	72820	0	61900
Myeloid_vs_CD8_Non-responder	Macro_FOLR2+APOE+	CD8(ZNF683+KLRB1+ T)	HLA-DQA1	LAG3	7,22676E-03	35308,2	3,05568E-03	47926	9,91061E-01	24665	2,23260E+00	16570	9,17249E-01	25480	0	61900
Myeloid_vs_CD8_Non-responder	Macro_FOLR2+APOE+	CD8(Tc17)	SPP1	PTGER4	7,23204E-03	30385	8,75880E-03	9923	9,48806E-01	26936	3,05349E+00	5233	8,83394E-01	47933	0	61900
Myeloid_vs_CD8_Non-responder	Macro_FOLR2+APOE+	CD8(Temra)	SPP1	ITGA4_ITGB1	7,24336E-03	30396	7,86655E-03	11712	9,72458E-01	25623	3,10767E+00	4797	8,83374E-01	47948	0	61900
Myeloid_vs_CD8_Non-responder	CD8(Terminal Tex)	Macro_ISG15	GZMB	IGF2R	7,24714E-03	42358,2	6,75595E-03	14679	7,37412E-01	41640	1,34980E+00	45619	8,83369E-01	47953	0	61900
Myeloid_vs_CD8_Non-responder	CD8(ISG+ T)	Macro_IFI27	TNFSF9	HLA-DPA1	7,24714E-03	42427,4	3,27374E-03	43456	9,52449E-01	26720	1,30112E+00	47953	9,06833E-01	32108	0	61900
Myeloid_vs_CD8_Non-responder	CD8(ZNF683+KLRB1+ T)	Macro_OLFML3	CD99	CD81	7,25243E-03	44008,2	3,05415E-03	47960	7,80029E-01	38238	1,32777E+00	46617	9,17478E-01	25326	0	61900
Myeloid_vs_CD8_Non-responder	CD8(ITM2C+ T)	Mast	IL16	CD9	7,25394E-03	40120,2	5,35042E-03	20855	1,03876E+00	22408	1,31097E+00	47476	8,83358E-01	47962	0	61900
Myeloid_vs_CD8_Non-responder	pDC_LILRA4	CD8(GZMK+ Tex)	APP	LRP10	7,26188E-03	26754,2	8,95738E-03	9577	2,25616E+00	1465	2,96574E+00	6027	8,73004E-01	54802	0	61900
Myeloid_vs_CD8_Non-responder	CD8(ISG+ T)	Macro_FOLR2+APOE+	B2M	LILRB1	7,26757E-03	37122,8	3,05320E-03	47980	1,00844E+00	23785	1,40322E+00	43157	9,44135E-01	8792	0	61900
Myeloid_vs_CD8_Non-responder	CD8(EOMES+ NK-like)	Macro_FOLR2+APOE+	ADAM10	GPNMB	7,26838E-03	34947	5,68124E-03	19043	1,56934E+00	6519	2,55988E+00	10756	8,39682E-01	76517	0	61900
Myeloid_vs_CD8_Non-responder	Macro_LYVE1	CD8(GZMK+ Tem)	CD14	ITGA4	7,27075E-03	27318,8	5,00216E-03	23015	1,47832E+00	8004	1,93526E+00	23814	9,26448E-01	19861	0	61900
Myeloid_vs_CD8_Non-responder	CD8(NME1+ T)	Macro_LYVE1	HMGB1	TLR2	7,27363E-03	37997,8	3,87180E-03	33838	1,11847E+00	18854	1,30031E+00	47988	9,14125E-01	27409	0	61900
Myeloid_vs_CD8_Non-responder	CD8(GZMK+ Tex)	Macro_ISG15	HMGB1	HAVCR2	7,29563E-03	43560,8	3,09327E-03	47106	7,66020E-01	39285	1,29977E+00	48017	9,23613E-01	21496	0	61900
Myeloid_vs_CD8_Non-responder	CD8(NME1+ T)	cDC(CD1C)	MIF	CD44_CD74	7,29926E-03	54889,6	1,85549E-03	89045	6,06247E-01	54177	9,40209E-01	67426	9,55869E-01	1900	0	61900
Myeloid_vs_CD8_Non-responder	Mono_CD14	CD8(Temra)	IL1B	ADRB2	7,29952E-03	26783,2	1,19419E-02	6000	1,60271E+00	5999	2,64630E+00	9594	8,79603E-01	50423	0	61900
Myeloid_vs_CD8_Non-responder	CD8(ITM2C+ T)	Macro_OLFML3	HLA-A	LILRB2	7,30171E-03	32323,4	3,05190E-03	48025	1,27478E+00	13141	1,66836E+00	32243	9,47349E-01	6308	0	61900
Myeloid_vs_CD8_Non-responder	CD8(NME1+ T)	cDC(CD1C)	MIF	CD74_CXCR4	7,30591E-03	50471,2	1,97656E-03	83790	6,98052E-01	45019	1,06328E+00	60462	9,57798E-01	1185	0	61900
Myeloid_vs_CD8_Non-responder	CD8(GZMK+ Early Tem)	Macro_FOLR2+APOE+	B2M	TFRC	7,31160E-03	41985,8	3,11481E-03	46596	6,65651E-01	48038	1,34878E+00	45653	9,45506E-01	7742	0	61900

Myeloid_vs_CD8_Post_NR

Myeloid_vs_CD8_Non-responder	Macro_FOLR2+APOE+	CD8(Terminal Tex)	HLA-A	CD3D	7,32150E-03	39121	3,05372E-03	47972	7,90789E-01	37402	1,29901E+00	48051	9,61462E-01	280	0	61900
Myeloid_vs_CD8_Non-responder	Macro_FOLR2+APOE+	CD8(IL7R+ZNF683+ Tm)	HLA-DQB1	LAG3	7,33598E-03	35930,2	3,05003E-03	48070	9,67864E-01	25863	2,09040E+00	19708	9,19336E-01	24110	0	61900
Myeloid_vs_CD8_Non-responder	Macro_FOLR2+APOE+	CD8(Terminal Tex)	SPP1	CD44	7,35049E-03	38312,4	4,46711E-03	27294	6,65193E-01	48089	2,42659E+00	12845	8,93071E-01	41434	0	61900
Myeloid_vs_CD8_Non-responder	Macro_FOLR2+APOE+	CD8(ITM2C+ T)	HLA-E	CD8A	7,35279E-03	37956,4	3,46820E-03	39857	7,82388E-01	38070	1,29802E+00	48092	9,55947E-01	1863	0	61900
Myeloid_vs_CD8_Non-responder	CD8(GZMK+ Early Tem)	Macro_ISG15	CCL5	CCRL2	7,35401E-03	34543,2	9,13003E-03	9295	1,07446E+00	20785	1,12764E+00	56852	9,19750E-01	23884	0	61900
Myeloid_vs_CD8_Non-responder	Macro_OLFML3	CD8(ID2+CXCR4+ T)	HLA-DQA1	LAG3	7,35879E-03	29415,2	4,98219E-03	23155	1,03734E+00	22478	1,93287E+00	23888	9,34009E-01	15655	0	61900
Myeloid_vs_CD8_Non-responder	Mono_CD14	CD8(GZMK+ Tex)	VCAN	ITGB1	7,36404E-03	21091,2	1,04874E-02	7426	2,47718E+00	895	2,64452E+00	9623	9,17032E-01	25612	0	61900
Myeloid_vs_CD8_Non-responder	Macro_FOLR2+APOE+	CD8(ZNF683+KLRB1+ T)	C3	IFITM1	7,36732E-03	45064,8	3,27425E-03	43448	6,64983E-01	48111	1,54462E+00	37070	9,02820E-01	34795	0	61900
Myeloid_vs_CD8_Non-responder	CD8(GZMK+ Early Tem)	Macro_OLFML3	B2M	LILRB1	7,37652E-03	37286	3,25438E-03	43837	9,83620E-01	25051	1,29736E+00	48123	9,45794E-01	7519	0	61900
Myeloid_vs_CD8_Non-responder	CD8(Tc17)	Macro_NLRP3	HLA-F	LILRB2	7,38035E-03	39859,4	3,04626E-03	48128	1,12197E+00	18714	1,65142E+00	32899	8,98567E-01	37656	0	61900
Myeloid_vs_CD8_Non-responder	CD8(NME1+ T)	Mono_CD16	HLA-A	LILRB1	7,38572E-03	37136,6	3,53841E-03	38749	9,86147E-01	24935	1,29715E+00	48135	9,39808E-01	11964	0	61900
Myeloid_vs_CD8_Non-responder	CD8(GZMK+ Tem)	pDC_LILRA4	SIRPG	CD47	7,38751E-03	32399,6	8,93195E-03	9615,5	1,00607E+00	23912,5	2,19990E+00	17255,5	8,81331E-01	49314,5	0	61900
Myeloid_vs_CD8_Non-responder	CD8(NME1+ T)	Mono_CD16	HLA-C	LILRA3	7,40311E-03	32201,2	9,84189E-03	8184	1,24468E+00	14086	1,20270E+00	52911	9,19688E-01	23925	0	61900
Myeloid_vs_CD8_Non-responder	CD8(Temra)	Mono_CD16	HLA-F	LILRB1	7,40492E-03	42063,2	3,40905E-03	40882	1,08172E+00	20449	1,50077E+00	38925	8,83072E-01	48160	0	61900
Myeloid_vs_CD8_Non-responder	CD8(GZMK+ Tem)	Mono_CD16	HLA-F	LILRB1	7,42108E-03	42441,4	3,40612E-03	40947	1,08133E+00	20458	1,45840E+00	40721	8,83028E-01	48181	0	61900
Myeloid_vs_CD8_Non-responder	Macro_ISG15	CD8(GZMK+ Early Tem)	CCL2	CCR5	7,42219E-03	43682	1,04013E-02	7520	1,39976E+00	9649	3,04931E+00	5261	7,35446E-01	134080	0	61900
Myeloid_vs_CD8_Non-responder	cDC_LAMP3	CD8(ISG+ T)	CD200	CD200R1	7,42616E-03	47468,4	3,78275E-02	588,5	2,06956E+00	2186,5	1,46462E+00	40430,5	7,39655E-01	132236,5	0	61900
Myeloid_vs_CD8_Non-responder	CD8(ZNF683+KLRB1+ T)	Macro_FOLR2+APOE+	CCL5	CCR1	7,43316E-03	34412	8,95041E-03	9584	1,10380E+00	19482	1,12257E+00	57144	9,19645E-01	23950	0	61900
Myeloid_vs_CD8_Non-responder	Macro_FOLR2+APOE+	CD8(GZMK+ Tem)	APOE	LDLR	7,43572E-03	26837,6	8,85677E-03	9773	1,39309E+00	9820	3,14729E+00	4495	8,83001E-01	48200	0	61900
Myeloid_vs_CD8_Non-responder	Macro_FOLR2+APOE+	CD8(IL7R+ZNF683+ Tm)	CD59	CD2	7,44035E-03	39256,8	6,10805E-03	17088	8,08795E-01	36027	1,29575E+00	48206	9,05388E-01	33063	0	61900
Myeloid_vs_CD8_Non-responder	Macro_NLRP3	CD8(Terminal Tex)	LGALS3	LAG3	7,45348E-03	41699,6	4,34532E-03	28477	6,89883E-01	45796	1,29549E+00	48223	9,19354E-01	24102	0	61900
Myeloid_vs_CD8_Non-responder	Macro_NLRP3	CD8(Terminal Tex)	HLA-DRB5	LAG3	7,45425E-03	43568,6	3,30593E-03	42856	6,63848E-01	48224	1,32278E+00	46853	9,29699E-01	18010	0	61900
Myeloid_vs_CD8_Non-responder	Macro_FOLR2+APOE+	CD8(ID2+CXCR4+ T)	LGALS3BP	ITGB1	7,45502E-03	32914,2	5,95319E-03	17792	1,32588E+00	11645	1,89448E+00	25009	8,82968E-01	48225	0	61900
Myeloid_vs_CD8_Non-responder	CD8(ISG+ T)	Macro_ISG15	HMGB1	THBD	7,46585E-03	47780,8	3,04173E-03	48239	7,46502E-01	40825	1,36054E+00	45155	8,91120E-01	42785	0	61900
Myeloid_vs_CD8_Non-responder	Macro_FOLR2+APOE+	CD8(NME1+ T)	CD86	CTLA4	7,46972E-03	30843	8,45418E-03	10486	1,39157E+00	9864	1,93850E+00	23721	8,82934E-01	48244	0	61900
Myeloid_vs_CD8_Non-responder	cDC_CLEC9A	CD8(Terminal Tex)	B2M	CD3D	7,47102E-03	53347,8	2,60299E-03	59935	4,20459E-01	78200	9,53680E-01	66651	9,64081E-01	53	0	61900
Myeloid_vs_CD8_Non-responder	Macro_ISG15	CD8(GZMK+ Tex)	CCL2	CCR5	7,47163E-03	43713,4	1,03513E-02	7575	1,39888E+00	9671	3,06512E+00	5141	7,34978E-01	134280	0	61900
Myeloid_vs_CD8_Non-responder	cDC(CD1C)	CD8(ID2+CXCR4+ T)	HLA-DRA	LAG3	7,47175E-03	24915,6	4,86290E-03	23982	1,18298E+00	16291	2,23500E+00	16524	9,47930E-01	5881	0	61900
Myeloid_vs_CD8_Non-responder	CD8(Tn)	Macro_NLRP3	HMGB1	THBD	7,47437E-03	42301,6	3,59887E-03	37767	9,60939E-01	26236	1,29486E+00	48250	8,99016E-01	37355	0	61900
Myeloid_vs_CD8_Non-responder	CD8(GZMK+ Tex)	Macro_FOLR2+APOE+	B2M	LILRB1	7,48134E-03	37615,6	3,04087E-03	48259	9,91098E-01	24661	1,37639E+00	44378	9,44028E-01	8880	0	61900
Myeloid_vs_CD8_Non-responder	Macro_FOLR2+APOE+	CD8(Temra)	HLA-C	KIR3DL1	7,50385E-03	35572	1,42046E-02	4272	8,83159E-01	30901	1,66119E+00	32499	8,82868E-01	48288	0	61900
Myeloid_vs_CD8_Non-responder	Mono_INHBA	CD8(Tn)	SPP1	S1PR1	7,50546E-03	31478	3,02361E-02	952	1,42453E+00	9100	2,63931E+00	9686	8,40886E-01	75752	0	61900
Myeloid_vs_CD8_Non-responder	Macro_FOLR2+APOE+	CD8(NME1+ T)	SPP1	CD44	7,51706E-03	38276	4,45072E-03	27445	6,63137E-01	48305	2,46774E+00	12164	8,92895E-01	41566	0	61900
Myeloid_vs_CD8_Non-responder	CD8(IL7R+ZNF683+ Tm)	cDC_CLEC9A	HMGB1	THBD	7,52173E-03	40774,4	3,91354E-03	33310	9,74618E-01	25518	1,29331E+00	48311	9,02757E-01	34833	0	61900
Myeloid_vs_CD8_Non-responder	CD8(GZMK+ Tem)	Macro_OLFML3	HLA-C	LILRB2	7,52563E-03	31845,2	3,03879E-03	48316	1,31544E+00	11950	1,71424E+00	30618	9,47153E-01	6442	0	61900
Myeloid_vs_CD8_Non-responder	CD8(GZMK+ Tex)	Macro_ISG15	HMGB1	CD163	7,53186E-03	37817,8	3,03843E-03	48324	9,39898E-01	27427	1,69635E+00	31232	9,25855E-01	20206	0	61900
Myeloid_vs_CD8_Non-responder	Macro_FOLR2+APOE+	CD8(Temra)	HLA-A	KIR2DL3	7,54122E-03	35966,8	1,68043E-02	2998	8,79068E-01	31187	1,58550E+00	35413	8,82803E-01	48336	0	61900
Myeloid_vs_CD8_Non-responder	Macro_NLRP3	CD8(GZMK+ Tex)	LYZ	ITGAL	7,54122E-03	35227,8	3,03759E-03	48336	1,20070E+00	15662	2,29097E+00	15395	9,02744E-01	34846	0	61900
Myeloid_vs_CD8_Non-responder	CD8(EOMES+ NK-like)	Macro_FOLR2+APOE+	HLA-C	LILRB1	7,54200E-03	39829,2	3,03755E-03	48337	9,33215E-01	27844	1,33989E+00	46086	9,35237E-01	14979	0	61900
Myeloid_vs_CD8_Non-responder	Macro_FOLR2+APOE+	CD8(Tc17)	HBEGF	CD44	7,55058E-03	43557,6	3,03685E-03	48348	8,49203E-01	33174	1,46546E+00	40400	9,04065E-01	33966	0	61900
Myeloid_vs_CD8_Non-responder	CD8(LAYN+ T)	Macro_ISG15	CCL5	CCR1	7,55214E-03	28739,4	1,17871E-02	6111	1,42653E+00	9055	1,29252E+00	48350	9,29248E-01	18281	0	61900
Myeloid_vs_CD8_Non-responder	CD8(EOMES+ NK-like)	Macro_ISG15	CD52	SIGLEC10	7,56152E-03	43273,8	3,56758E-03	38284	8,31506E-01	34368	1,29229E+00	48362	9,04822E-01	33455	0	61900
Myeloid_vs_CD8_Non-responder	CD8(EOMES+ NK-like)	Macro_FOLR2+APOE+	CALR	LRP1	7,56230E-03	42398,6	3,03610E-03	48363	9,51462E-01	26775	1,43936E+00	41530	9,04854E-01	33425	0	61900
Myeloid_vs_CD8_Non-responder	cDC(CD1C)	CD8(GZMK+ Tex)	CXCL16	CXCR6	7,57132E-03	25898,2	8,96338E-03	9566	1,32918E+00	11534	1,98536E+00	22427	9,19421E-01	24064	0	61900
Myeloid_vs_CD8_Non-responder	CD8(ZNF683+KLRB1+ T)	Macro_ISG15	CCL5	CCRL2	7,57377E-03	34548	9,04756E-03	9438	1,06514E+00	21205	1,14110E+00	56131	9,19414E-01	24066	0	61900
Myeloid_vs_CD8_Non-responder	CD8(NME1+ T)	pDC_LILRA4	SIRPG	CD47	7,57621E-03	32582	8,86911E-03	9743,5	1,00316E+00	24068,5	2,18126E+00	17656,5	8,80961E-01	49541,5	0	61900
Myeloid_vs_CD8_Non-responder	CD8(ITM2C+ T)	Macro_ISG15	CCL4	CCR1	7,58814E-03	30286	1,02515E-02	7687	1,44927E+00	8570	1,29168E+00	48396	9,18180E-01	24877	0	61900
Myeloid_vs_CD8_Non-responder	CD8(Tc17)	Macro_OLFML3	HLA-B	LILRB1	7,58892E-03	38626,2	3,24702E-03	43990	9,45756E-01	27100	1,29167E+00	48397	9,40126E-01	11744	0	61900
Myeloid_vs_CD8_Non-responder	Macro_OLFML3	CD8(ZNF683+KLRB1+ T)	S100A8	CD69	7,58964E-03	29528,4	4,85072E-03	24079	1,02267E+00	23120	2,02060E+00	21494	9,31469E-01	17049	0	61900
Myeloid_vs_CD8_Non-responder	Macro_NLRP3	CD8(Tc17)	THBS1	CD47	7,59127E-03	30983,8	5,60300E-03	19465	1,42369E+00	9113	2,25833E+00	16041	8,82709E-01	48400	0	61900
Myeloid_vs_CD8_Non-responder	Macro_FOLR2+APOE+	CD8(Temra)	B2M	CD247	7,59519E-03	38151,8	3,03383E-03	48405	8,47322E-01	33319	1,42214E+00	42293	9,49534E-01	4842	0	61900
Myeloid_vs_CD8_Non-responder	CD8(Tn)	Mono_CD14	HSPA1A	TLR4	7,60776E-03	34589,6	6,00332E-03	17563	1,15034E+00	17507	1,29099E+00	48421	9,13867E-01	27557	0	61900
Myeloid_vs_CD8_Non-responder	CD8(Tc17)	pDC_LILRA4	HMGB1	TLR9	7,60832E-03	31112,4	2,95399E-02	997	2,06374E+00	2214	1,21923E+00	52043	8,97475E-01	38408	0	61900
Myeloid_vs_CD8_Non-responder	cDC(CD1C)	CD8(ID2+CXCR4+ T)	CXCL16	CXCR6	7,61289E-03	26216,8	8,94947E-03	9586	1,32804E+00	11588	1,93188E+00	23912	9,19364E-01	24098	0	61900
Myeloid_vs_CD8_Non-responder	pDC_LILRA4	CD8(ISG+ T)	APP	LRP10	7,61666E-03	26861,6	8,87381E-03	9735	2,25338E+00	1475	2,95766E+00	6086	8,72484E-01	55112	0	61900
Myeloid_vs_CD8_Non-responder	CD8(Tc17)	Macro_FOLR2+APOE+	HLA-F	LILRB1	7,62584E-03	43283,2	3,38199E-03	41339	9,61679E-01	26192	1,50953E+00	38541	8,82660E-01	48444	0	61900
Myeloid_vs_CD8_Non-responder	CD8(EOMES+ NK-like)	Macro_ISG15	HMGB1	TLR2	7,63056E-03	43168	3,32330E-03	42481	8,72423E-01	31651	1,29046E+00	48450	9,07936E-01	31358	0	61900
Myeloid_vs_CD8_Non-responder	Macro_NLRP3	CD8(ITM2C+ T)	VCAN	ITGB1	7,63608E-03	30809,2	4,85578E-03	24039	1,92263E+00	3006	2,22930E+00	16644	8,82641E-01	48457	0	61900
Myeloid_vs_CD8_Non-responder	CD8(GZMK+ Tex)	Macro_OLFML3	HLA-A	LILRB2	7,63687E-03	32703,4	3,03179E-03	48458	1,25582E+00	13727	1,64804E+00	33018	9,47184E-01	6414	0	61900
Myeloid_vs_CD8_Non-responder	CD8(GZMK+ Tem)	Macro_FOLR2+APOE+	CD52	SIGLEC10	7,63923E-03	35901	4,72107E-03	25091	1,14093E+00	17904	1,29016E+00	48461	9,16220E-01	26149	0	61900
Myeloid_vs_CD8_Non-responder	Macro_FOLR2+APOE+	CD8(ZNF683+KLRB1+ T)	HLA-A	CD3D	7,64712E-03	39012,2	3,03101E-03	48471	7,83141E-01	38024	1,33302E+00	46370	9,61324E-01	296	0	61900
Myeloid_vs_CD8_Non-responder	Macro_NLRP3	CD8(ID2+CXCR4+ T)	HLA-DRA	LAG3	7,65580E-03	42311,8	3,25047E-03	43913	7,15906E-01	43443	1,28977E+00	48482	9,37043E-01	13821	0	61900
Myeloid_vs_CD8_Non-responder	CD8(NME1+ T)	cDC_LAMP3	TIGIT	PVR	7,66952E-03	56890,2	1,94869E-02	2223	1,10268E+00	19544	5,68297E-01	90435	7,83250E-01	110349	0	61900
Myeloid_vs_CD8_Non-responder	Macro_FOLR2+APOE+	CD8(Terminal Tex)	HLA-C	CD3D	7,67397E-03	38150,2	3,02962E-03	48505	8,11506E-01	35818	1,37939E+00	44222	9,61250E-01	306	0	61900
Myeloid_vs_CD8_Non-responder	pDC_LILRA4	CD8(Temra)	B2M	KLRD1	7,68419E-03	26336,2	5,14942E-03	22024	1,00153E+00	24156	2,05222E+00	20690	9,53372E-01	2911	0	61900
Myeloid_vs_CD8_Non-responder	CD8(Temra)	pDC_LILRA4	HSP90B1	TLR9	7,69679E-03	35001,4	2,39366E-02	1511	2,06103E+00	2227	1,22904E+00	51554	8,68440E-01	57815	0	61900
Myeloid_vs_CD8_Non-responder	Macro_FOLR2+APOE+	CD8(Terminal Tex)	MMP9	CD44	7,70329E-03	32332,2	6,43656E-03	15799	1,25282E+00	13828	2,01618E+00	21592	8,82492E-01	48542	0	61900
Myeloid_vs_CD8_Non-responder	CD8(Temra)	Macro_FOLR2+APOE+	HLA-A	LILRB1	7,70329E-03	40420,8	3,07447E-03	47505	9,06006E-01	29478	1,28836E+00	48542	9,35707E-01	14679	0	61900
Myeloid_vs_CD8_Non-responder	CD8(GZMK+ Early Tem)	Macro_ISG15	IFNG	IFNGR1_IFNGR2	7,70567E-03	45688,8	4,19016E-03	30122	7,28183E-01	42402	1,28830E+00	48545	8,87116E-01	45475	0	61900
Myeloid_vs_CD8_Non-responder	Macro_NLRP3	CD8(GZMK+ Tem)	HLA-DPB1	LAG3	7,71281E-03	43096,4	3,38355E-03	41309	6,62150E-01	48402	1,28814E+00	48554	9,34610E-01	15317	0	61900
Myeloid_vs_CD8_Non-responder	cDC(CD1C)	CD8(Terminal Tex)	HLA-DQB2	LAG3	7,71631E-03	23386	1,29612E-02	5094	1,54467E+00	6842	2,12445E+00	18912	9,19199E-01	24182	0	61900
Myeloid_vs_CD8_Non-responder	CD8(GZMK+ Early Tem)	Macro_FOLR2+APOE+	TGFB1	ENG	7,73667E-03	43684,4	3,77174E-03	35176	9,38135E-01	27528	1,35816E+00	45234	8,82440E-01	48584	0	61900
Myeloid_vs_CD8_Non-responder	CD8(ITM2C+ T)	Macro_FOLR2+APOE+	B2M	LILRB1	7,73986E-03	38100	3,02563E-03	48588	9,69678E-01	25765	1,35786E+00	45250	9,43895E-01	8997	0	61900
Myeloid_vs_CD8_Non-responder	CD8(GZMK+ Tex)	Macro_FOLR2+APOE+	HLA-C	LILRB1	7,74384E-03	40441,2	3,02545E-03	48593	9,22926E-01	28478	1,29643E+00	48178	9,35116E-01	15057	0	61900
Myeloid_vs_CD8_Non-responder	cDC(CD1C)	CD8(ISG+ T)	HLA-DPB1	LAG3	7,74605E-03	25022,2	4,83671E-03	24206	1,21648E+00	15090	2,28048E+00	15596	9,44717E-01	8319	0	61900
Myeloid_vs_CD8_Non-responder	CD8(ID2+CXCR4+ T)	Macro_OLFML3	HLA-A	LILRB2	7,74623E-03	32714,6	3,02536E-03	48596	1,24975E+00	13923	1,65604E+00	32700	9,47130E-01	6454	0	61900
Myeloid_vs_CD8_Non-responder	CD8(NME1+ T)	pDC_LILRA4	IRAK4	TLR7	7,74735E-03	46770,6	8,84210E-03	9792	1,44866E+00	8580	1,77550E+00	28657	7,55371E-01	124924	0	61900

Myeloid_vs_CD8_Post_NR

Myeloid_vs_CD8_Non-responder	CD8(Tn)	Mono_CD16	HLA-C	LILRA3	7,74853E-03	32868,8	9,70238E-03	8381	1,21142E+00	15263	1,17053E+00	54592	9,19160E-01	24208	0	61900
Myeloid_vs_CD8_Non-responder	CD8(GZMK+ Tem)	Macro_FOLR2+APOE+	B2M	TFRC	7,75341E-03	42009,8	3,11057E-03	46719	6,59974E-01	48605	1,36227E+00	45064	9,45471E-01	7761	0	61900
Myeloid_vs_CD8_Non-responder	Macro_FOLR2+APOE+	CD8(EOMES+ NK-like)	B2M	CD247	7,76458E-03	37553,4	3,02442E-03	48619	8,45358E-01	33456	1,50118E+00	38902	9,49460E-01	4890	0	61900
Myeloid_vs_CD8_Non-responder	CD8(Tn)	cDC(CD1C)	B2M	CD1A	7,76468E-03	36420,2	2,34521E-02	1566	1,07217E+00	20877	8,36687E-01	73537	9,19147E-01	24221	0	61900
Myeloid_vs_CD8_Non-responder	Macro_IER3	CD8(ISG+ T)	C3	IFITM1	7,76965E-03	26552,2	6,95811E-03	14083	1,20813E+00	15373	1,92094E+00	24225	9,31238E-01	17180	0	61900
Myeloid_vs_CD8_Non-responder	CD8(GZMK+ Early Tem)	Macro_OLFML3	HLA-A	LILRB2	7,77736E-03	32802,6	3,02399E-03	48635	1,24846E+00	13961	1,64736E+00	33055	9,47119E-01	6462	0	61900
Myeloid_vs_CD8_Non-responder	Macro_NLRP3	CD8(ID2+CXCR4+ T)	VEGFA	ITGB1	7,79177E-03	29296,2	8,11282E-03	11186	1,85228E+00	3530	2,03153E+00	21212	8,82322E-01	48653	0	61900
Myeloid_vs_CD8_Non-responder	Macro_FOLR2+APOE+	CD8(NME1+ T)	MMP9	CD44	7,79898E-03	32176,2	6,41294E-03	15886	1,25077E+00	13891	2,05732E+00	20542	8,82302E-01	48662	0	61900
Myeloid_vs_CD8_Non-responder	Macro_FOLR2+APOE+	CD8(Terminal Tex)	B2M	CD3G	7,79978E-03	37651	3,02285E-03	48663	8,74961E-01	31452	1,37510E+00	44442	9,56096E-01	1798	0	61900
Myeloid_vs_CD8_Non-responder	CD8(LAYN+ T)	Mono_CD16	HLA-F	LILRB2	7,80138E-03	34887,8	3,02278E-03	48665	1,65871E+00	5285	2,05155E+00	20706	8,98213E-01	37883	0	61900
Myeloid_vs_CD8_Non-responder	Macro_FOLR2+APOE+	CD8(ZNF683+KLRB1+ T)	SPP1	CD44	7,80779E-03	38058	4,42003E-03	27729	6,59288E-01	48673	2,59919E+00	10188	8,92564E-01	41800	0	61900
Myeloid_vs_CD8_Non-responder	Mast	CD8(NME1+ T)	ADCYAP1	DPP4	7,82012E-03	56997,8	6,26065E-02	232	2,05796E+00	2245	1,00081E+00	63900	6,70709E-01	156712	0	61900
Myeloid_vs_CD8_Non-responder	CD8(GZMK+ Tex)	Macro_FOLR2+APOE+	TIGIT	NECTIN2	7,83201E-03	34210,8	1,55648E-02	3524,5	1,47438E+00	8070,5	1,91952E+00	24275,5	8,44758E-01	73283,5	0	61900
Myeloid_vs_CD8_Non-responder	Macro_FOLR2+APOE+	CD8(Temra)	HLA-C	KIR2DL3	7,83591E-03	35181,4	1,66717E-02	3060	8,99785E-01	29898	1,66587E+00	32341	8,82210E-01	48708	0	61900
Myeloid_vs_CD8_Non-responder	Macro_IER3	CD8(GZMK+ Tem)	HLA-DQB1	LAG3	7,84954E-03	24519	6,85616E-03	14384	1,25660E+00	13700	1,91908E+00	24289	9,44714E-01	8322	0	61900
Myeloid_vs_CD8_Non-responder	CD8(Tn)	Macro_ISG15	CIRBP	TREM1	7,84959E-03	43975,8	3,68518E-03	36401	9,03950E-01	29601	1,40119E+00	43252	8,82193E-01	48725	0	61900
Myeloid_vs_CD8_Non-responder	CD8(ITM2C+ T)	Mono_CD16	HLA-C	LILRA3	7,85765E-03	30462,2	1,02678E-02	7669	1,34620E+00	11023	1,28456E+00	48735	9,21239E-01	22984	0	61900
Myeloid_vs_CD8_Non-responder	Macro_NLRP3	CD8(GZMK+ Tex)	HLA-DRB5	LAG3	7,85846E-03	43562	3,28190E-03	43309	6,58686E-01	48736	1,34745E+00	45721	9,29460E-01	18144	0	61900
Myeloid_vs_CD8_Non-responder	CD8(NME1+ T)	Mono_CD14	HMGB1	TLR2	7,85846E-03	42673	3,01982E-03	48736	9,45167E-01	27123	1,44000E+00	41510	9,03855E-01	34096	0	61900
Myeloid_vs_CD8_Non-responder	Macro_NLRP3	CD8(IL7R+ZNF683+ Tm)	S100A8	ITGB2	7,87621E-03	30211,2	3,01928E-03	48758	1,74224E+00	4455	2,33235E+00	14563	9,23816E-01	21380	0	61900
Myeloid_vs_CD8_Non-responder	CD8(Tc17)	Macro_IER3	LTB	TNFRSF1A	7,88995E-03	39020,2	5,84389E-03	18265	9,94035E-01	24525	1,28378E+00	48775	8,92792E-01	41636	0	61900
Myeloid_vs_CD8_Non-responder	CD8(GZMK+ Tem)	Macro_FOLR2+APOE+	HSP90B1	LRP1	7,89238E-03	43182	3,01861E-03	48778	9,91986E-01	24620	1,45483E+00	40883	8,95525E-01	39729	0	61900
Myeloid_vs_CD8_Non-responder	CD8(Temra)	Macro_OLFML3	TGFB1	ENG	7,89318E-03	44817,6	3,96153E-03	32750	8,40594E-01	33760	1,28365E+00	48779	8,84962E-01	46899	0	61900
Myeloid_vs_CD8_Non-responder	CD8(GZMK+ Tex)	Macro_ISG15	CD52	SIGLEC10	7,89885E-03	42172,8	3,71012E-03	36030	8,69716E-01	31812	1,28347E+00	48786	9,06496E-01	32336	0	61900
Myeloid_vs_CD8_Non-responder	CD8(Tc17)	Mono_CD16	TNF	TNFRSF1B	7,90371E-03	46566,2	3,42740E-03	40548	6,58185E-01	48792	1,36135E+00	45115	9,00337E-01	36476	0	61900
Myeloid_vs_CD8_Non-responder	CD8(Terminal Tex)	cDC_LAMP3	IFNG	IFNGR1_IFNGR2	7,91019E-03	39212	5,87635E-03	18127	8,58685E-01	32551	1,28328E+00	48800	9,02974E-01	34682	0	61900
Myeloid_vs_CD8_Non-responder	CD8(IL7R+ZNF683+ Tm)	Macro_IER3	ANXA1	FPR1	7,91262E-03	34537,8	4,44126E-03	27548	1,15139E+00	17458	1,28325E+00	48803	9,31605E-01	16980	0	61900
Myeloid_vs_CD8_Non-responder	CD8(EOMES+ NK-like)	pDC_LILRA4	SIRPG	CD47	7,92885E-03	31773	9,05633E-03	9419,5	1,01184E+00	23617,5	2,30631E+00	15104,5	8,82052E-01	48823,5	0	61900
Myeloid_vs_CD8_Non-responder	CD8(Terminal Tex)	Macro_FOLR2+APOE+	B2M	LILRB1	7,92966E-03	38686,6	3,01705E-03	48824	9,57609E-01	26418	1,31574E+00	47231	9,43820E-01	9060	0	61900
Myeloid_vs_CD8_Non-responder	CD8(ITM2C+ T)	Macro_FOLR2-APOE+	IL16	CD9	7,93210E-03	37943,8	5,97475E-03	17691	1,16236E+00	17069	1,28269E+00	48827	8,88925E-01	44232	0	61900
Myeloid_vs_CD8_Non-responder	Macro_FOLR2+APOE+	CD8(GZMK+ Early Tem)	HLA-DPB1	LAG3	7,93210E-03	35183,6	3,01672E-03	48827	9,34392E-01	27767	2,07477E+00	20124	9,31015E-01	17300	0	61900
Myeloid_vs_CD8_Non-responder	cDC(CD1C)	CD8(GZMK+ Tex)	HLA-DQB2	LAG3	7,94139E-03	23350	1,28670E-02	5179	1,53951E+00	6943	2,14912E+00	18366	9,18928E-01	24362	0	61900
Myeloid_vs_CD8_Non-responder	Macro_FOLR2+APOE+	CD8(GZMK+ Tex)	HLA-C	CD3G	7,95405E-03	41652,4	3,01575E-03	48854	6,95514E-01	45263	1,31771E+00	47123	9,49068E-01	5122	0	61900
Myeloid_vs_CD8_Non-responder	CD8(Temra)	Macro_NLRP3	CIRBP	TREM1	7,95487E-03	42900,6	4,10766E-03	31045	9,36331E-01	27646	1,28221E+00	48855	8,87717E-01	45057	0	61900
Myeloid_vs_CD8_Non-responder	CD8(ITM2C+ T)	Macro_NLRP3	CIRBP	TREM1	7,95568E-03	45594	3,67078E-03	36603	8,66456E-01	32037	1,28767E+00	48574	8,81989E-01	48856	0	61900
Myeloid_vs_CD8_Non-responder	CD8(EOMES+ NK-like)	Macro_OLFML3	HLA-F	LILRB1	7,95649E-03	43454,6	3,33852E-03	42171	1,00488E+00	23976	1,46640E+00	40369	8,81989E-01	48857	0	61900
Myeloid_vs_CD8_Non-responder	CD8(NME1+ T)	Macro_FOLR2+APOE+	HLA-C	LILRB2	7,95649E-03	34612	3,01555E-03	48857	1,10892E+00	19257	1,55941E+00	36464	9,46961E-01	6582	0	61900
Myeloid_vs_CD8_Non-responder	Macro_NLRP3	CD8(ISG+ T)	HBEGF	CD82	7,95975E-03	29856	7,01584E-03	13907	1,65994E+00	5269	2,10658E+00	19343	8,81982E-01	48861	0	61900
Myeloid_vs_CD8_Non-responder	CD8(Tn)	Macro_FOLR2+APOE+	HMGB1	CD163	7,96953E-03	36713	3,01484E-03	48873	1,03816E+00	22441	1,73338E+00	29986	9,25587E-01	20365	0	61900
Myeloid_vs_CD8_Non-responder	CD8(ID2+CXCR4+ T)	Macro_FOLR2+APOE+	HMGB1	TLR2	7,97279E-03	40226,8	3,68857E-03	36339	9,77120E-01	25396	1,28168E+00	48877	9,12203E-01	28622	0	61900
Myeloid_vs_CD8_Non-responder	Macro_FOLR2+APOE+	CD8(ZNF683+KLRB1+ T)	MMP9	CD44	7,98259E-03	31667,4	6,36872E-03	16058	1,24692E+00	14009	2,18878E+00	17481	8,81942E-01	48889	0	61900
Myeloid_vs_CD8_Non-responder	CD8(LAYN+ T)	Macro_ISG15	IFNG	IFNGR1_IFNGR2	7,98259E-03	39812,2	5,60811E-03	19432	8,55387E-01	32760	1,28141E+00	48889	9,00908E-01	36080	0	61900
Myeloid_vs_CD8_Non-responder	CD8(ZNF683+KLRB1+ T)	Macro_ISG15	HLA-G	LILRB2	7,98571E-03	36208,6	4,80935E-03	24397	1,63599E+00	5568	2,60895E+00	10052	8,35495E-01	79126	0	61900
Myeloid_vs_CD8_Non-responder	Macro_FOLR2+APOE+	CD8(LAYN+ T)	LGALS9	HAVCR2	7,99402E-03	36469	3,62794E-03	37338	1,22455E+00	14785	2,10307E+00	19419	8,81918E-01	48903	0	61900
Myeloid_vs_CD8_Non-responder	CD8(GZMK+ Tex)	Macro_FOLR2+APOE+	TGFB1	ENG	7,99566E-03	43911,2	3,73393E-03	35674	9,33571E-01	27825	1,35773E+00	45252	8,81916E-01	48905	0	61900
Myeloid_vs_CD8_Non-responder	Macro_FOLR2+APOE+	CD8(Terminal Tex)	C1QB	C1QBP	7,99729E-03	30566,6	3,01338E-03	48907	1,57333E+00	6451	3,16002E+00	4382	9,08152E-01	31193	0	61900
Myeloid_vs_CD8_Non-responder	Macro_FOLR2+APOE+	CD8(IL7R+ZNF683+ Tm)	ICAM1	IL2RG	8,01366E-03	45327	3,01258E-03	48927	8,07238E-01	36151	1,49378E+00	39211	8,94442E-01	40446	0	61900
Myeloid_vs_CD8_Non-responder	CD8(Terminal Tex)	cDC_LAMP3	CD200R1	CD200	8,01385E-03	48473,6	3,46694E-02	720,5	2,05271E+00	2273,5	1,43875E+00	41553,5	7,31174E-01	135920,5	0	61900
Myeloid_vs_CD8_Non-responder	CD8(EOMES+ NK-like)	Macro_FOLR2+APOE+	B2M	TFRC	8,01611E-03	42124,6	3,10812E-03	46775	6,56688E-01	48930	1,35810E+00	45240	9,45450E-01	7778	0	61900
Myeloid_vs_CD8_Non-responder	Macro_FOLR2+APOE+	CD8(Temra)	CD14	ITGB2	8,01611E-03	28952,4	3,01249E-03	48930	1,44387E+00	8679	2,62339E+00	9883	9,34510E-01	15370	0	61900
Myeloid_vs_CD8_Non-responder	Macro_FOLR2+APOE+	CD8(Terminal Tex)	APOE	LDLR	8,01775E-03	27095,8	8,66683E-03	10091	1,38783E+00	9949	3,13263E+00	4607	8,81876E-01	48932	0	61900
Myeloid_vs_CD8_Non-responder	cDC_LAMP3	CD8(Terminal Tex)	CD200	CD200R1	8,02082E-03	48473,6	3,46694E-02	720,5	2,05271E+00	2273,5	1,43875E+00	41553,5	7,31174E-01	135920,5	0	61900
Myeloid_vs_CD8_Non-responder	CD8(IL7R+ZNF683+ Tm)	Macro_OLFML3	HLA-F	LILRB1	8,02185E-03	43451,8	3,33103E-03	42323	1,00377E+00	24041	1,47307E+00	40058	8,81872E-01	48937	0	61900
Myeloid_vs_CD8_Non-responder	Macro_FOLR2+APOE+	CD8(IL7R+ZNF683+ Tm)	CXCL16	CXCR6	8,03005E-03	38750,4	3,83532E-03	34302	1,07055E+00	20943	1,80613E+00	27660	8,81849E-01	48947	0	61900
Myeloid_vs_CD8_Non-responder	CD8(GZMK+ Early Tem)	Mono_CD14	ANXA1	FPR1	8,03825E-03	38914,4	3,44855E-03	40171	1,05437E+00	21718	1,27981E+00	48957	9,23092E-01	21826	0	61900
Myeloid_vs_CD8_Non-responder	CD8(IL7R+ZNF683+ Tm)	pDC_LILRA4	HLA-C	NOTCH4	8,04806E-03	31581,2	1,31288E-02	4970	1,32775E+00	11591	1,16310E+00	54999	9,18814E-01	24446	0	61900
Myeloid_vs_CD8_Non-responder	CD8(EOMES+ NK-like)	cDC(CD1C)	B2M	CD1A	8,06082E-03	32873,4	2,45071E-02	1446	1,25129E+00	13874	1,00134E+00	63867	9,20767E-01	23280	0	61900
Myeloid_vs_CD8_Non-responder	Mono_CD14	CD8(LAYN+ T)	S100A8	ITGB2	8,06082E-03	20795,4	4,80386E-03	24456	2,37258E+00	1133	3,26328E+00	3691	9,38634E-01	12797	0	61900
Myeloid_vs_CD8_Non-responder	CD8(Tn)	Mono_CD14	LTB	TNFRSF1A	8,07939E-03	43526,8	4,68763E-03	25368	7,88325E-01	37599	1,39001E+00	43760	8,81775E-01	49007	0	61900
Myeloid_vs_CD8_Non-responder	CD8(ITM2C+ T)	Macro_FOLR2+APOE+	IL16	CD4	8,08598E-03	32978,2	5,53155E-03	19851	1,21923E+00	14983	2,11401E+00	19142	8,81766E-01	49015	0	61900
Myeloid_vs_CD8_Non-responder	cDC(CD1C)	CD8(ISG+ T)	HLA-DRA	LAG3	8,09409E-03	24812	4,80110E-03	24482	1,17592E+00	16557	2,31102E+00	15007	9,47614E-01	6114	0	61900
Myeloid_vs_CD8_Non-responder	CD8(EOMES+ NK-like)	Macro_NLRP3	CCL5	CCRL2	8,09423E-03	34949,6	7,96758E-03	11494	9,81292E-01	25183	1,27821E+00	49025	9,14578E-01	27146	0	61900
Myeloid_vs_CD8_Non-responder	Macro_FOLR2+APOE+	CD8(IL7R+ZNF683+ Tm)	HLA-A	CD8B	8,09919E-03	40491,8	3,30147E-03	42929	7,00464E-01	44811	1,27811E+00	49031	9,51524E-01	3788	0	61900
Myeloid_vs_CD8_Non-responder	CD8(EOMES+ NK-like)	Macro_FOLR2+APOE+	HLA-B	LILRB1	8,10167E-03	40420,6	3,00806E-03	49034	8,97638E-01	30014	1,30257E+00	47890	9,37938E-01	13265	0	61900
Myeloid_vs_CD8_Non-responder	Macro_FOLR2+APOE+	CD8(ZNF683+KLRB1+ T)	HLA-C	CD3D	8,11738E-03	38074,4	3,00709E-03	49053	8,03858E-01	36400	1,41340E+00	42694	9,61111E-01	325	0	61900
Myeloid_vs_CD8_Non-responder	cDC_CLEC9A	CD8(ID2+CXCR4+ T)	HLA-DPA1	LAG3	8,13903E-03	23580,6	4,79593E-03	24517	1,21064E+00	15283	2,62932E+00	9813	9,47215E-01	6390	0	61900
Myeloid_vs_CD8_Non-responder	Macro_FOLR2+APOE+	CD8(GZMK+ Tex)	HLA-B	KLRD1	8,13974E-03	40646,2	3,95206E-03	32876	6,55211E-01	49080	1,28492E+00	48716	9,41648E-01	10659	0	61900
Myeloid_vs_CD8_Non-responder	CD8(Terminal Tex)	Macro_OLFML3	HLA-A	LILRB1	8,14306E-03	37621,8	3,39462E-03	41126	1,02109E+00	23192	1,27704E+00	49084	9,38623E-01	12807	0	61900
Myeloid_vs_CD8_Non-responder	CD8(ITM2C+ T)	Macro_ISG15	TNF	TNFRSF1A	8,14555E-03	39181	4,99298E-03	23074	9,09417E-01	29277	1,65940E+00	32567	8,81658E-01	49087	0	61900
Myeloid_vs_CD8_Non-responder	Macro_NLRP3	CD8(GZMK+ Tex)	THBS1	CD47	8,14804E-03	31443,6	5,49020E-03	20073	1,41683E+00	9267	2,21731E+00	16888	8,81652E-01	49090	0	61900
Myeloid_vs_CD8_Non-responder	CD8(Terminal Tex)	Macro_IER3	HSPA1A	TLR4	8,16631E-03	39190,8	4,68731E-03	25373	9,78850E-01	25315	1,27647E+00	49112	9,03616E-01	34254	0	61900
Myeloid_vs_CD8_Non-responder	cDC_CLEC9A	CD8(ID2+CXCR4+ T)	HLA-DRA	LAG3	8,18932E-03	24040,6	4,78987E-03	24556	1,16183E+00	17095	2,57682E+00	10495	9,47556E-01	6157	0	61900
Myeloid_vs_CD8_Non-responder	CD8(Tn)	Macro_FOLR2+APOE+	CD52	SIGLEC10	8,21381E-03	36952	4,50128E-03	26987	1,10452E+00	19446	1,27543E+00	49169	9,14373E-01	27258	0	61900
Myeloid_vs_CD8_Non-responder	CD8(ISG+ T)	Macro_FOLR2+APOE+	HLA-C	LILRB1	8,21381E-03	40886,4	3,00106E-03	49169	9,02184E-01	29719	1,28985E+00	48476	9,34870E-01	15168	0	61900
Myeloid_vs_CD8_Non-responder	pDC_LILRA4	CD8(ID2+CXCR4+ T)	APP	CD74	8,22038E-03	22663	5,52406E-03	19894	2,06402E+00	2210	1,90910E+00	24580	9,49729E-01	4731	0	61900
Myeloid_vs_CD8_Non-responder	CD8(GZMK+ Tex)	pDC_LILRA4	TNF	PTPRS	8,22686E-03	37374,4	1,34333E-02	4770	3,09717E+00	263	1,90895E+00	24585	8,09257E-01	95354	0	61900
Myeloid_vs_CD8_Non-responder	CD8(ID2+CXCR4+ T)	Macro_ISG15	TIGIT	NECTIN2	8,23104E-03	28888,6	2,24826E-02	1711,5	2,04663E+00	2304,5	2,08147E+00	19952,5	8,67373E-01	58574,5	0	61900

Myeloid_vs_CD8_Post_NR

Myeloid_vs_CD8_Non-responder	CD8(GZMK+ Early Tem)	pDC_LILRA4	HLA-C	NOTCH4	8,23724E-03	32199,8	1,30518E-02	5030	1,31239E+00	12042	1,11736E+00	57434	9,18594E-01	24593	0	61900
Myeloid_vs_CD8_Non-responder	Macro_ISG15	CD8(ID2+CXCR4+ T)	NECTIN2	TIGIT	8,23809E-03	28888,6	2,24826E-02	1711,5	2,04663E+00	2304,5	2,08147E+00	19952,5	8,67373E-01	58574,5	0	61900
Myeloid_vs_CD8_Non-responder	CD8(Terminal Tex)	pDC_LILRA4	SIRPG	CD47	8,24057E-03	32197	8,96229E-03	9567,5	1,00748E+00	23849,5	2,23831E+00	16466,5	8,81508E-01	49201,5	0	61900
Myeloid_vs_CD8_Non-responder	Macro_NLRP3	CD8(Temra)	ICAM1	SPN	8,24979E-03	32404,2	6,15588E-03	16884	1,23521E+00	14409	2,09458E+00	19616	8,81502E-01	49212	0	61900
Myeloid_vs_CD8_Non-responder	pDC_LILRA4	CD8(ZNF683+KLRB1+ T)	APP	CD74	8,26063E-03	22927	5,37957E-03	20689	2,04284E+00	2329	1,90813E+00	24611	9,49092E-01	5106	0	61900
Myeloid_vs_CD8_Non-responder	CD8(ISG+ T)	Macro_OLFML3	HLA-A	LILRB2	8,27077E-03	33221,4	2,99782E-03	49237	1,22378E+00	14814	1,63413E+00	33530	9,46901E-01	6626	0	61900
Myeloid_vs_CD8_Non-responder	cDC_LAMP3	CD8(NME1+ T)	CD200	CD200R1	8,28047E-03	49074	3,33098E-02	789,5	2,04545E+00	2310,5	1,41168E+00	42770,5	7,27224E-01	137599,5	0	61900
Myeloid_vs_CD8_Non-responder	CD8(GZMK+ Early Tem)	Macro_OLFML3	CCL4	CCR1	8,28758E-03	40355,6	5,83040E-03	18324	8,70733E-01	31755	1,27403E+00	49257	8,94325E-01	40542	0	61900
Myeloid_vs_CD8_Non-responder	Macro_FOLR2+APOE+	CD8(Tn)	SPP1	PTGER4	8,29936E-03	31023,6	8,43207E-03	10534	9,28677E-01	28113	3,04469E+00	5300	8,81421E-01	49271	0	61900
Myeloid_vs_CD8_Non-responder	Macro_OLFML3	CD8(GZMK+ Tex)	CXCL9	CXCR3	8,31108E-03	29272,4	1,07686E-02	7133	2,10003E+00	2050	2,61106E+00	10031	8,57015E-01	65248	0	61900
Myeloid_vs_CD8_Non-responder	CD8(GZMK+ Tem)	cDC_CLEC9A	COPA	CD74	8,31277E-03	43957,4	1,59212E-03	101460	9,91347E-01	24651	2,46694E+00	12175	9,26918E-01	19601	0	61900
Myeloid_vs_CD8_Non-responder	CD8(ZNF683+KLRB1+ T)	Macro_OLFML3	B2M	LILRB2	8,32044E-03	30310	2,99485E-03	49296	1,41628E+00	9278	1,78207E+00	28446	9,54064E-01	2630	0	61900
Myeloid_vs_CD8_Non-responder	CD8(ISG+ T)	Mast	COPA	NTRK1	8,34006E-03	63600	1,64974E-02	3119	1,38414E+00	10043	3,20401E-01	106321	7,29568E-01	136617	0	61900
Myeloid_vs_CD8_Non-responder	CD8(NME1+ T)	Macro_IFI27	MIF	CD44_CD74	8,35134E-03	64795,2	1,77115E-03	93000	5,42359E-01	61584	3,38254E-01	105171	9,54878E-01	2321	0	61900
Myeloid_vs_CD8_Non-responder	Macro_FOLR2+APOE+	CD8(ZNF683+KLRB1+ T)	C1QB	C1QBP	8,35510E-03	30512,4	2,99320E-03	49337	1,57168E+00	6481	3,29981E+00	3443	9,07871E-01	31401	0	61900
Myeloid_vs_CD8_Non-responder	Macro_FOLR2+APOE+	CD8(GZMK+ Early Tem)	HLA-DPA1	LAG3	8,35679E-03	34426,4	2,99303E-03	49339	9,40552E-01	27399	2,17053E+00	17893	9,34108E-01	15601	0	61900
Myeloid_vs_CD8_Non-responder	CD8(GZMK+ Tem)	Macro_NLRP3	HLA-A	LILRB2	8,35933E-03	32734,8	2,99284E-03	49342	1,28570E+00	12821	1,64942E+00	32960	9,46859E-01	6651	0	61900
Myeloid_vs_CD8_Non-responder	CD8(NME1+ T)	cDC_CLEC9A	CRTAM	CADM1	8,36426E-03	42714,2	1,36533E-02	4598,5	1,38394E+00	10053,5	1,17485E+00	54356,5	8,29901E-01	82662,5	0	61900
Myeloid_vs_CD8_Non-responder	CD8(NME1+ T)	Macro_ISG15	GNAI2	C5AR1	8,36441E-03	37814,4	2,99266E-03	49348	1,27765E+00	13055	2,01833E+00	21551	8,90445E-01	43218	0	61900
Myeloid_vs_CD8_Non-responder	CD8(NME1+ T)	Macro_LYVE1	MIF	CD74_CXCR4	8,37266E-03	62657,4	1,76565E-03	93237	5,38289E-01	62089	5,13201E-01	93974	9,55458E-01	2087	0	61900
Myeloid_vs_CD8_Non-responder	CD8(Tn)	Mono_CD16	HLA-B	LILRB1	8,37883E-03	37646,8	3,45755E-03	40019	9,56808E-01	26462	1,27178E+00	49365	9,41870E-01	10488	0	61900
Myeloid_vs_CD8_Non-responder	CD8(IL7R+ZNF683+ Tm)	Macro_FOLR2+APOE+	B2M	LILRB1	8,37883E-03	39404,4	2,99194E-03	49365	9,22304E-01	28508	1,29997E+00	48007	9,43598E-01	9242	0	61900
Myeloid_vs_CD8_Non-responder	CD8(NME1+ T)	cDC_LAMP3	MIF	CD74_CXCR4	8,37977E-03	62448,2	1,71770E-03	95533	5,34799E-01	62521	5,75432E-01	89962	9,54868E-01	2325	0	61900
Myeloid_vs_CD8_Non-responder	Mono_CD14	CD8(GZMK+ Early Tem)	S100A9	ITGB2	8,38123E-03	21016,4	4,49430E-03	27030	2,22078E+00	1582	3,14494E+00	4510	9,42484E-01	10060	0	61900
Myeloid_vs_CD8_Non-responder	Macro_FOLR2+APOE+	CD8(ID2+CXCR4+ T)	LGALS9	CD44	8,38987E-03	37525	2,99147E-03	49378	1,05574E+00	21645	1,64337E+00	33191	9,23586E-01	21511	0	61900
Myeloid_vs_CD8_Non-responder	Macro_FOLR2+APOE+	CD8(Tc17)	CXCL12	CXCR4	8,39327E-03	33963,6	9,63594E-03	8486	1,25927E+00	13608	1,56019E+00	36442	8,81228E-01	49382	0	61900
Myeloid_vs_CD8_Non-responder	CD8(EOMES+ NK-like)	Macro_ISG15	TGFB1	ENG	8,39752E-03	45795,8	3,68470E-03	36414	7,99991E-01	36706	1,37262E+00	44572	8,81223E-01	49387	0	61900
Myeloid_vs_CD8_Non-responder	CD8(ISG+ T)	Macro_OLFML3	HLA-B	LILRB2	8,40262E-03	31910,8	2,99089E-03	49393	1,29280E+00	12627	1,71388E+00	30626	9,49237E-01	5008	0	61900
Myeloid_vs_CD8_Non-responder	CD8(Temra)	Macro_NLRP3	HLA-C	LILRB2	8,40858E-03	31941,4	2,99064E-03	49400	1,33394E+00	11373	1,72402E+00	30307	9,46752E-01	6727	0	61900
Myeloid_vs_CD8_Non-responder	CD8(Temra)	Mono_INHBA	TGFB1	SDC2	8,41280E-03	37682,4	1,16819E-02	6197	1,44730E+00	8613	1,23794E+00	51107	8,64296E-01	60595	0	61900
Myeloid_vs_CD8_Non-responder	cDC_CLEC9A	CD8(GZMK+ Tex)	CXCL16	CXCR6	8,41383E-03	24671	8,72348E-03	9980	1,29982E+00	12410	2,34356E+00	14337	9,18410E-01	24728	0	61900
Myeloid_vs_CD8_Non-responder	Macro_NLRP3	CD8(ITM2C+ T)	THBS1	ITGA4	8,41709E-03	34280,6	4,52103E-03	26791	1,31072E+00	12104	2,03200E+00	21198	8,81184E-01	49410	0	61900
Myeloid_vs_CD8_Non-responder	Macro_NLRP3	CD8(GZMK+ Tex)	CD14	ITGB1	8,42561E-03	38589	2,98969E-03	49420	1,00181E+00	24142	1,91111E+00	24511	9,05527E-01	32972	0	61900
Myeloid_vs_CD8_Non-responder	CD8(ZNF683+KLRB1+ T)	Mono_CD16	HLA-G	LILRB1	8,42834E-03	43921,4	4,76391E-03	24739	1,26663E+00	13389	2,06247E+00	20426	8,02902E-01	99153	0	61900
Myeloid_vs_CD8_Non-responder	CD8(EOMES+ NK-like)	pDC_LILRA4	TNF	PTPRS	8,43098E-03	36254,4	1,46220E-02	4017	3,10655E+00	257	1,94239E+00	23621	8,15715E-01	91477	0	61900
Myeloid_vs_CD8_Non-responder	Macro_ISG15	CD8(Tn)	HBEGF	CD44	8,43363E-03	28784,2	4,76341E-03	24743	1,27792E+00	13043	2,01358E+00	21656	9,21889E-01	22579	0	61900
Myeloid_vs_CD8_Non-responder	CD8(GZMK+ Early Tem)	Mast	AIMP1	NTRK1	8,43470E-03	66803,8	1,42284E-02	4253	1,38281E+00	10082	3,83536E-01	102227	6,75119E-01	155557	0	61900
Myeloid_vs_CD8_Non-responder	cDC_CLEC9A	CD8(LAYN+ T)	HLA-DPB1	LAG3	8,45081E-03	24276,4	4,76098E-03	24756	1,22300E+00	14851	2,52773E+00	11208	9,44303E-01	8667	0	61900
Myeloid_vs_CD8_Non-responder	cDC_CLEC9A	CD8(ID2+CXCR4+ T)	CXCL16	CXCR6	8,45610E-03	24906,6	8,70994E-03	10008	1,29867E+00	12448	2,29008E+00	15417	9,18352E-01	24760	0	61900
Myeloid_vs_CD8_Non-responder	Macro_ISG15	CD8(LAYN+ T)	B2M	CD3D	8,45709E-03	47057,2	2,59309E-03	60255	6,55016E-01	49098	9,99345E-01	63973	9,64015E-01	60	0	61900
Myeloid_vs_CD8_Non-responder	Macro_FOLR2+APOE+	CD8(EOMES+ NK-like)	LGALS3BP	ITGB1	8,45977E-03	34082	5,74457E-03	18759	1,31038E+00	12113	1,79004E+00	28178	8,81113E-01	49460	0	61900
Myeloid_vs_CD8_Non-responder	CD8(NME1+ T)	Mono_CD16	GNAI2	C5AR1	8,46233E-03	37872,8	2,98782E-03	49463	1,27548E+00	13122	2,01594E+00	21599	8,90366E-01	43280	0	61900
Myeloid_vs_CD8_Non-responder	CD8(EOMES+ NK-like)	pDC_LILRA4	COPA	P2RY6	8,48524E-03	29739,8	7,82698E-03	11788	2,64660E+00	650	3,04645E+00	5290	8,51143E-01	69071	0	61900
Myeloid_vs_CD8_Non-responder	CD8(LAYN+ T)	Macro_FOLR2+APOE+	B2M	TFRC	8,48631E-03	42287,6	3,10398E-03	46871	6,51155E-01	49491	1,35564E+00	45362	9,45416E-01	7814	0	61900
Myeloid_vs_CD8_Non-responder	CD8(EOMES+ NK-like)	pDC_LILRA4	HLA-C	NOTCH4	8,48657E-03	32264,2	1,29529E-02	5100	1,29265E+00	12631	1,12661E+00	56907	9,18309E-01	24783	0	61900
Myeloid_vs_CD8_Non-responder	CD8(IL7R+ZNF683+ Tm)	pDC_LILRA4	HSP90B1	TLR9	8,48680E-03	35301	2,29715E-02	1629	2,04099E+00	2340	1,23521E+00	51234	8,66071E-01	59402	0	61900
Myeloid_vs_CD8_Non-responder	CD8(ZNF683+KLRB1+ T)	Mono_CD16	ICAM3	ITGAL	8,49231E-03	44760,6	5,66804E-03	19105	7,00039E-01	44846	1,26908E+00	49498	8,82647E-01	48454	0	61900
Myeloid_vs_CD8_Non-responder	CD8(LAYN+ T)	Macro_OLFML3	HLA-C	LILRB2	8,50261E-03	32755,4	2,98555E-03	49510	1,26712E+00	13368	1,66856E+00	32230	9,46709E-01	6769	0	61900
Myeloid_vs_CD8_Non-responder	CD8(IL7R+ZNF683+ Tm)	Macro_FOLR2+APOE+	CCL5	CCR1	8,50516E-03	36624,6	8,62883E-03	10165	1,06704E+00	21107	9,78852E-01	65154	9,18283E-01	24797	0	61900
Myeloid_vs_CD8_Non-responder	Macro_OLFML3	CD8(LAYN+ T)	HLA-DQB1	LAG3	8,50516E-03	29409,6	4,85069E-03	24080	1,06636E+00	21140	1,90214E+00	24797	9,34951E-01	15131	0	61900
Myeloid_vs_CD8_Non-responder	CD8(LAYN+ T)	cDC_CLEC9A	HMGB1	THBD	8,51378E-03	40527,6	3,98993E-03	32388	9,93193E-01	24564	1,26850E+00	49523	9,03603E-01	34263	0	61900
Myeloid_vs_CD8_Non-responder	CD8(GZMK+ Tem)	Mono_CD14	CIRBP	TREM1	8,52324E-03	41364,8	4,33741E-03	28557	1,01269E+00	23577	1,26836E+00	49534	8,90401E-01	43256	0	61900
Myeloid_vs_CD8_Non-responder	CD8(NME1+ T)	Macro_NLRP3	MIF	CD44_CD74	8,55133E-03	51362,2	1,76569E-03	93232	6,87259E-01	46046	1,19616E+00	53284	9,54811E-01	2349	0	61900
Myeloid_vs_CD8_Non-responder	CD8(Temra)	Macro_NLRP3	TNF	TNFRSF1B	8,57239E-03	46511,2	3,58122E-03	38057	6,67740E-01	47842	1,26741E+00	49591	9,02290E-01	35166	0	61900
Myeloid_vs_CD8_Non-responder	CD8(GZMK+ Early Tem)	Mast	GPI	NTRK1	8,57919E-03	58964,8	1,92313E-02	2279	1,38061E+00	10141	2,64375E-01	109969	7,82894E-01	110535	0	61900
Myeloid_vs_CD8_Non-responder	CD8(ISG+ T)	Macro_ISG15	HMGB1	TLR2	8,58623E-03	44061,6	3,25480E-03	43830	8,51386E-01	33012	1,26712E+00	49607	9,07062E-01	31959	0	61900
Myeloid_vs_CD8_Non-responder	CD8(GZMK+ Tem)	Macro_FOLR2-APOE+	TNFSF9	HLA-DPA1	8,59143E-03	38191,2	4,27026E-03	29241	9,87085E-01	24870	1,26701E+00	49613	9,17468E-01	25332	0	61900
Myeloid_vs_CD8_Non-responder	CD8(ISG+ T)	Macro_ISG15	CCL4	CCR1	8,60095E-03	35190,2	6,87524E-03	14324	1,22719E+00	14677	1,26670E+00	49624	9,01865E-01	35426	0	61900
Myeloid_vs_CD8_Non-responder	CD8(Terminal Tex)	Macro_FOLR2+APOE+	HLA-DRB1	CD4	8,60442E-03	34680,6	2,98037E-03	49628	1,18213E+00	16331	1,60581E+00	34627	9,41287E-01	10917	0	61900
Myeloid_vs_CD8_Non-responder	CD8(GZMK+ Tem)	pDC_LILRA4	HLA-DRB1	CD4	8,60658E-03	36613	2,60290E-03	59938	1,03119E+00	22759	1,89937E+00	24873	9,37431E-01	13595	0	61900
Myeloid_vs_CD8_Non-responder	Macro_FOLR2+APOE+	CD8(GZMK+ Tex)	HLA-E	CD8A	8,60962E-03	39506,4	3,31974E-03	42571	7,42890E-01	41158	1,26636E+00	49634	9,55016E-01	2269	0	61900
Myeloid_vs_CD8_Non-responder	Macro_FOLR2+APOE+	CD8(Temra)	LGALS1	CD69	8,61223E-03	37799	2,97994E-03	49637	8,01821E-01	36563	1,61479E+00	34278	9,46913E-01	6617	0	61900
Myeloid_vs_CD8_Non-responder	Mono_CD14	CD8(ID2+CXCR4+ T)	VCAN	ITGA4	8,61617E-03	21197	1,02739E-02	7661	2,46207E+00	928	2,60129E+00	10156	9,17451E-01	25340	0	61900
Myeloid_vs_CD8_Non-responder	Macro_NLRP3	CD8(ID2+CXCR4+ T)	THBS1	CD47	8,61743E-03	31681,4	5,40452E-03	20561	1,41162E+00	9388	2,21581E+00	16915	8,80829E-01	49643	0	61900
Myeloid_vs_CD8_Non-responder	Macro_FOLR2+APOE+	CD8(ZNF683+KLRB1+ T)	LGALS3	LAG3	8,61917E-03	41846,6	2,97967E-03	49645	7,93673E-01	37179	1,83756E+00	26662	9,04214E-01	33847	0	61900
Myeloid_vs_CD8_Non-responder	CD8(Temra)	Macro_FOLR2+APOE+	B2M	LILRB1	8,62698E-03	39753,4	2,97934E-03	49654	9,04584E-01	29554	1,29250E+00	48351	9,43485E-01	9308	0	61900
Myeloid_vs_CD8_Non-responder	CD8(ZNF683+KLRB1+ T)	Macro_NLRP3	HMGB1	TLR4	8,62959E-03	41429,8	4,29989E-03	28919	9,93027E-01	24572	1,26594E+00	49657	8,92113E-01	42101	0	61900
Myeloid_vs_CD8_Non-responder	Macro_FOLR2+APOE+	CD8(EOMES+ NK-like)	HLA-B	KIR2DL3	8,63394E-03	38147,4	1,46714E-02	3987	8,06872E-01	36188	1,49883E+00	39000	8,80795E-01	49662	0	61900
Myeloid_vs_CD8_Non-responder	CD8(GZMK+ Tex)	pDC_LILRA4	HLA-C	NOTCH4	8,63610E-03	32843,2	1,29013E-02	5158	1,28237E+00	12915	1,08315E+00	59348	9,18159E-01	24895	0	61900
Myeloid_vs_CD8_Non-responder	Macro_FOLR2+APOE+	CD8(ITM2C+ T)	HLA-B	CD8A	8,64002E-03	38799,2	3,18031E-03	45284	7,96197E-01	36981	1,26581E+00	49669	9,62617E-01	162	0	61900
Myeloid_vs_CD8_Non-responder	Mono_INHBA	CD8(LAYN+ T)	SPP1	ITGA4_ITGB1	8,64089E-03	22345,6	1,48453E-02	3892	1,52172E+00	7232	2,60045E+00	10166	9,12321E-01	28538	0	61900
Myeloid_vs_CD8_Non-responder	CD8(NME1+ T)	Macro_ISG15	CD28	CD86	8,65224E-03	39056	8,15674E-03	11070,5	1,28908E+00	12720,5	1,89802E+00	24907,5	8,26648E-01	84681,5	0	61900
Myeloid_vs_CD8_Non-responder	CD8(Terminal Tex)	Macro_IER3	TNFSF9	HLA-DPA1	8,65308E-03	44546,8	2,97811E-03	49684	7,83580E-01	37983	1,51440E+00	38333	9,02757E-01	34834	0	61900
Myeloid_vs_CD8_Non-responder	Macro_FOLR2+APOE+	CD8(ID2+CXCR4+ T)	SPP1	PTGER4	8,66876E-03	31314,2	8,32229E-03	10749	9,21914E-01	28541	3,00227E+00	5679	8,80735E-01	49702	0	61900
Myeloid_vs_CD8_Non-responder	CD8(NME1+ T)	Macro_NLRP3	GNAI2	C5AR1	8,67312E-03	38707,6	2,97711E-03	49707	1,27067E+00	13269	1,88544E+00	25264	8,90191E-01	43398	0	61900
Myeloid_vs_CD8_Non-responder	CD8(Terminal Tex)	Macro_ISG15	HMGB1	TLR2	8,68622E-03	42195,8	3,45580E-03	40055	9,13113E-01	29028	1,26472E+00	49722	9,09557E-01	30274	0	61900
Myeloid_vs_CD8_Non-responder	CD8(Temra)	Mono_CD16	CD52	SIGLEC10	8,68797E-03	39886,8	4,06692E-03	31477	9,55150E-01	26570	1,26470E+00	49724	9,10316E-01	29763	0	61900

Myeloid_vs_CD8_Post_NR

Myeloid_vs_CD8_Non-responder	CD8(NME1+ T)	Macro_LYVE1	ITGB3BP	ITGB5	8,71008E-03	67011,4	2,20717E-02	1776	9,80247E-01	25232	6,88226E-01	82669	6,41552E-01	163480	0	61900
Myeloid_vs_CD8_Non-responder	CD8(NME1+ T)	Mono_CD16	HLA-B	LILRB1	8,71070E-03	37690,4	3,45998E-03	39978	9,58868E-01	26349	1,26417E+00	49750	9,41889E-01	10475	0	61900
Myeloid_vs_CD8_Non-responder	Macro_FOLR2+APOE+	CD8(GZMK+ Tex)	HLA-C	CD8B	8,71333E-03	42664	3,01868E-03	48773	6,66804E-01	47934	1,26409E+00	49753	9,49332E-01	4960	0	61900
Myeloid_vs_CD8_Non-responder	CD8(Temra)	pDC_LILRA4	HMGB1	TLR9	8,73273E-03	32453	2,81800E-02	1089	2,02180E+00	2430	1,12587E+00	56948	8,95286E-01	39898	0	61900
Myeloid_vs_CD8_Non-responder	CD8(IL7R+ZNF683+ Tm)	Macro_ISG15	CCL5	CCRL2	8,74273E-03	36764,8	8,72249E-03	9983	1,02837E+00	22881	9,97377E-01	64086	9,18048E-01	24974	0	61900
Myeloid_vs_CD8_Non-responder	CD8(ID2+CXCR4+ T)	pDC_LILRA4	TGFB1	TGFBR1_TGFBR2	8,74578E-03	44898,4	3,82660E-03	34433	7,09591E-01	43998	1,61225E+00	34371	8,80585E-01	49790	0	61900
Myeloid_vs_CD8_Non-responder	Macro_FOLR2+APOE+	CD8(ID2+CXCR4+ T)	HLA-C	CD8A	8,74666E-03	40300,8	2,97332E-03	49791	7,53381E-01	40289	1,28105E+00	48902	9,59474E-01	622	0	61900
Myeloid_vs_CD8_Non-responder	CD8(Tn)	Macro_FOLR2+APOE+	HLA-C	LILRB2	8,75368E-03	35410,6	2,97281E-03	49799	1,07566E+00	20724	1,52724E+00	37770	9,46601E-01	6860	0	61900
Myeloid_vs_CD8_Non-responder	CD8(Temra)	pDC_LILRA4	IRAK4	TLR7	8,75517E-03	46318,6	8,93681E-03	9602	1,45093E+00	8544	1,82429E+00	27088	7,56354E-01	124459	0	61900
Myeloid_vs_CD8_Non-responder	cDC_CLEC9A	CD8(ISG+ T)	HLA-DPA1	LAG3	8,76581E-03	23579,4	4,73499E-03	24991	1,20357E+00	15553	2,70534E+00	8822	9,46895E-01	6631	0	61900
Myeloid_vs_CD8_Non-responder	Macro_IER3	CD8(Temra)	IL1B	ADRB2	8,77014E-03	26087,8	1,27536E-02	5277	1,69891E+00	4869	2,59718E+00	10218	8,83042E-01	48175	0	61900
Myeloid_vs_CD8_Non-responder	Macro_NLRP3	CD8(IL7R+ZNF683+ Tm)	LGALS1	CD69	8,79419E-03	41119,6	2,98930E-03	49432	7,84897E-01	37874	1,26212E+00	49845	9,46992E-01	6547	0	61900
Myeloid_vs_CD8_Non-responder	CD8(Tc17)	Macro_FOLR2+APOE+	B2M	LILRB1	8,79772E-03	40028,4	2,96995E-03	49849	8,91384E-01	30418	1,28703E+00	48603	9,43401E-01	9372	0	61900
Myeloid_vs_CD8_Non-responder	cDC_CLEC9A	CD8(ID2+CXCR4+ T)	HLA-DRB5	LAG3	8,79845E-03	26307,8	4,73131E-03	25015	1,09763E+00	19769	2,39328E+00	13446	9,40549E-01	11409	0	61900
Myeloid_vs_CD8_Non-responder	CD8(Terminal Tex)	Mono_CD16	HLA-B	LILRA1	8,81008E-03	28221,8	9,04546E-03	9441	1,63258E+00	5612	1,26181E+00	49863	9,36337E-01	14293	0	61900
Myeloid_vs_CD8_Non-responder	Macro_IER3	CD8(GZMK+ Tem)	HLA-DPB1	LAG3	8,81208E-03	25348,8	5,77635E-03	18580	1,18596E+00	16189	1,89395E+00	25025	9,49174E-01	5050	0	61900
Myeloid_vs_CD8_Non-responder	Macro_NLRP3	CD8(EOMES+ NK-like)	VEGFA	ITGB1	8,81450E-03	30250,8	7,82852E-03	11782	1,83678E+00	3642	1,92709E+00	24062	8,80457E-01	49868	0	61900
Myeloid_vs_CD8_Non-responder	cDC_CLEC9A	CD8(ISG+ T)	HLA-DRA	LAG3	8,81754E-03	24031,6	4,72900E-03	25029	1,15476E+00	17351	2,65285E+00	9504	9,47237E-01	6374	0	61900
Myeloid_vs_CD8_Non-responder	Macro_ISG15	CD8(Temra)	CD14	ITGB1	8,82846E-03	25328,6	4,72726E-03	25037	1,44904E+00	8574	2,65407E+00	9485	9,23388E-01	21647	0	61900
Myeloid_vs_CD8_Non-responder	CD8(IL7R+ZNF683+ Tm)	Macro_ISG15	TGFB1	ENG	8,83130E-03	45814,4	3,62981E-03	37297	7,91228E-01	37372	1,41490E+00	42616	8,80435E-01	49887	0	61900
Myeloid_vs_CD8_Non-responder	CD8(ZNF683+KLRB1+ T)	cDC(CD1C)	CD52	SIGLEC10	8,83573E-03	36328,8	4,74412E-03	24910	1,11720E+00	18919	1,26110E+00	49892	9,16407E-01	26023	0	61900
Myeloid_vs_CD8_Non-responder	CD8(Tc17)	cDC_CLEC9A	HMGB1	THBD	8,83661E-03	42246,6	3,74672E-03	35506	9,34058E-01	27795	1,26103E+00	49893	9,00829E-01	36139	0	61900
Myeloid_vs_CD8_Non-responder	Macro_ISG15	CD8(GZMK+ Tem)	CD14	ITGB1	8,84212E-03	25450,2	4,72624E-03	25047	1,44892E+00	8576	2,60679E+00	10078	9,23380E-01	21650	0	61900
Myeloid_vs_CD8_Non-responder	CD8(LAYN+ T)	Macro_OLFML3	HLA-A	LILRB2	8,85079E-03	34038	2,96708E-03	49909	1,19480E+00	15892	1,57908E+00	35658	9,46641E-01	6831	0	61900
Myeloid_vs_CD8_Non-responder	Macro_IER3	CD8(GZMK+ Tem)	HLA-DPA1	LAG3	8,85443E-03	25478	5,61976E-03	19364	1,16352E+00	17031	1,89262E+00	25056	9,51041E-01	4039	0	61900
Myeloid_vs_CD8_Non-responder	CD8(NME1+ T)	Macro_IER3	MIF	CD44_CD74	8,85563E-03	56508	1,75623E-03	93717	5,73369E-01	57867	9,53399E-01	66665	9,54695E-01	2391	0	61900
Myeloid_vs_CD8_Non-responder	Macro_FOLR2+APOE+	CD8(ZNF683+KLRB1+ T)	B2M	KLRD1	8,87387E-03	42801,4	2,96599E-03	49935	7,17954E-01	43270	1,32582E+00	46704	9,39457E-01	12198	0	61900
Myeloid_vs_CD8_Non-responder	CD8(GZMK+ Early Tem)	Macro_FOLR2+APOE+	B2M	LILRB1	8,88988E-03	40344,4	2,96558E-03	49953	8,85241E-01	30781	1,26550E+00	49685	9,43362E-01	9403	0	61900
Myeloid_vs_CD8_Non-responder	CD8(ISG+ T)	pDC_LILRA4	HLA-C	NOTCH4	8,89418E-03	33090,4	1,27973E-02	5229	1,26162E+00	13532	1,07657E+00	59706	9,17855E-01	25085	0	61900
Myeloid_vs_CD8_Non-responder	Macro_NLRP3	CD8(GZMK+ Tem)	HLA-DPA1	LAG3	8,90145E-03	42743,6	3,41408E-03	40790	6,67474E-01	47874	1,25976E+00	49966	9,38044E-01	13188	0	61900
Myeloid_vs_CD8_Non-responder	CD8(ISG+ T)	Mast	GPI	NTRK1	8,91578E-03	59384,4	1,88629E-02	2386	1,37463E+00	10276	2,50775E-01	110891	7,81246E-01	111469	0	61900
Myeloid_vs_CD8_Non-responder	Macro_FOLR2+APOE+	CD8(Terminal Tex)	B2M	KLRC2	8,91928E-03	48665,8	3,41975E-03	40683	6,84841E-01	46269	1,37424E+00	44491	8,80269E-01	49986	0	61900
Myeloid_vs_CD8_Non-responder	CD8(Temra)	Macro_LYVE1	TNFSF9	HLA-DPA1	8,92285E-03	42397,2	2,96400E-03	49990	9,16649E-01	28834	1,56404E+00	36270	9,02549E-01	34992	0	61900
Myeloid_vs_CD8_Non-responder	Macro_LYVE1	CD8(Temra)	CD14	ITGB1	8,93406E-03	28185,2	4,71876E-03	25114	1,44644E+00	8625	1,94290E+00	23602	9,23324E-01	21685	0	61900
Myeloid_vs_CD8_Non-responder	Mono_CD14	CD8(NME1+ T)	VCAN	SELL	8,94610E-03	28470,4	8,56543E-03	10288	2,44600E+00	966	2,65865E+00	9435	8,65584E-01	59763	0	61900
Myeloid_vs_CD8_Non-responder	Macro_LYVE1	CD8(GZMK+ Tem)	CD14	ITGB1	8,94647E-03	28461,8	4,71774E-03	25123	1,44632E+00	8629	1,89561E+00	24970	9,23316E-01	21687	0	61900
Myeloid_vs_CD8_Non-responder	CD8(Tc17)	Macro_FOLR2-APOE+	ADAM10	TREM2	8,94876E-03	32347,6	7,23059E-03	13275	1,51631E+00	7328	1,75647E+00	29216	8,80225E-01	50019	0	61900
Myeloid_vs_CD8_Non-responder	CD8(NME1+ T)	Macro_LYVE1	MFGE8	ITGB5	8,95087E-03	72662,8	1,91532E-02	2299	8,30240E-01	34476	5,81989E-01	89556	5,44931E-01	175083	0	61900
Myeloid_vs_CD8_Non-responder	Mono_CD16	CD8(ISG+ T)	HLA-DRA	LAG3	8,95234E-03	40653,6	3,28509E-03	43253	7,31119E-01	42166	1,42173E+00	42315	9,37355E-01	13634	0	61900
Myeloid_vs_CD8_Non-responder	Mono_CD16	CD8(ISG+ T)	HLA-DQA2	LAG3	8,95413E-03	41860,8	3,78630E-03	34967	7,05896E-01	44323	1,80404E+00	27720	8,94514E-01	40394	0	61900
Myeloid_vs_CD8_Non-responder	Macro_OLFML3	CD8(LAYN+ T)	CD14	ITGA4	8,96855E-03	27625,4	4,71587E-03	25139	1,27053E+00	13274	2,22122E+00	16799	9,24415E-01	21015	0	61900
Myeloid_vs_CD8_Non-responder	CD8(GZMK+ Tem)	Macro_FOLR2+APOE+	B2M	LILRB1	8,98011E-03	40306	2,96154E-03	50054	8,79564E-01	31154	1,27900E+00	48989	9,43325E-01	9433	0	61900
Myeloid_vs_CD8_Non-responder	Mono_CD16	CD8(ISG+ T)	HLA-DPA1	LAG3	8,98280E-03	43694	3,09853E-03	46976	6,65055E-01	48103	1,33060E+00	46472	9,35166E-01	15019	0	61900
Myeloid_vs_CD8_Non-responder	CD8(GZMK+ Early Tem)	Macro_OLFML3	HLA-B	LILRB2	8,98280E-03	32537,4	2,96136E-03	50057	1,26060E+00	13565	1,67631E+00	31971	9,48997E-01	5194	0	61900
Myeloid_vs_CD8_Non-responder	CD8(EOMES+ NK-like)	Macro_OLFML3	CD52	SIGLEC10	8,98729E-03	38322,8	4,38407E-03	28092	1,01061E+00	23679	1,25798E+00	50062	9,13334E-01	27881	0	61900
Myeloid_vs_CD8_Non-responder	CD8(Temra)	Macro_FOLR2+APOE+	TNF	TNFRSF1A	8,98998E-03	40178,4	4,85191E-03	24064	9,13548E-01	29005	1,57434E+00	35858	8,80155E-01	50065	0	61900
Myeloid_vs_CD8_Non-responder	Mono_CD16	CD8(ISG+ T)	HLA-DRB5	LAG3	9,00076E-03	40542,2	3,34404E-03	42066	7,21786E-01	42927	1,52145E+00	38017	9,30072E-01	17801	0	61900
Myeloid_vs_CD8_Non-responder	CD8(NME1+ T)	Mono_INHBA	HMGB1	TLR2	9,00076E-03	43305	3,14652E-03	45941	9,70939E-01	25700	1,25767E+00	50077	9,05626E-01	32907	0	61900
Myeloid_vs_CD8_Non-responder	Mono_CD16	CD8(ISG+ T)	VCAN	CD44	9,01425E-03	45581	3,20419E-03	44792	8,09339E-01	35973	1,28705E+00	48601	9,00111E-01	36639	0	61900
Myeloid_vs_CD8_Non-responder	CD8(EOMES+ NK-like)	Macro_FOLR2+APOE+	B2M	LILRB1	9,03136E-03	40407	2,95921E-03	50111	8,76278E-01	31365	1,27483E+00	49208	9,43304E-01	9451	0	61900
Myeloid_vs_CD8_Non-responder	CD8(GZMK+ Tem)	pDC_LILRA4	CXCL13	CXCR3	9,03587E-03	40207,4	1,21193E-02	5840	7,13020E-01	43711	1,48741E+00	39470	8,80087E-01	50116	0	61900
Myeloid_vs_CD8_Non-responder	CD8(Tc17)	Macro_FOLR2+APOE+	HLA-B	LILRB1	9,03857E-03	41774,4	2,95887E-03	50119	8,47377E-01	33316	1,25981E+00	49963	9,37456E-01	13574	0	61900
Myeloid_vs_CD8_Non-responder	Macro_NLRP3	CD8(ISG+ T)	S100A9	ITGB2	9,04037E-03	29130,8	2,95883E-03	50121	1,74442E+00	4425	2,51572E+00	11390	9,30050E-01	17818	0	61900
Myeloid_vs_CD8_Non-responder	Mono_CD16	CD8(ISG+ T)	S100A8	CD69	9,04128E-03	33630	4,40220E-03	27904	9,17478E-01	28779	1,71018E+00	30759	9,28307E-01	18808	0	61900
Myeloid_vs_CD8_Non-responder	Macro_ISG15	CD8(Terminal Tex)	HLA-DPA1	LAG3	9,04198E-03	27421,8	4,70975E-03	25192	1,00454E+00	24001	2,10818E+00	19294	9,46760E-01	6722	0	61900
Myeloid_vs_CD8_Non-responder	Macro_FOLR2+APOE+	CD8(GZMK+ Tex)	HLA-A	CD3D	9,05933E-03	40229	2,95786E-03	50142	7,58505E-01	39877	1,28183E+00	48867	9,60867E-01	359	0	61900
Myeloid_vs_CD8_Non-responder	Macro_LYVE1	CD8(GZMK+ Tem)	HLA-DPB1	LAG3	9,06145E-03	24128	6,22903E-03	16616	1,28505E+00	12839	1,88740E+00	25206	9,50963E-01	4079	0	61900
Myeloid_vs_CD8_Non-responder	CD8(Temra)	Mast	CALR	ITGA2B	9,07317E-03	54327,4	2,55334E-02	1328	1,51176E+00	7392	1,24551E-01	119163	8,31191E-01	81854	0	61900
Myeloid_vs_CD8_Non-responder	CD8(Temra)	Mast	COPA	NTRK1	9,07572E-03	60534,2	2,00074E-02	2094	1,42977E+00	8979	3,96343E-01	101401	7,48171E-01	128297	0	61900
Myeloid_vs_CD8_Non-responder	CD8(Temra)	Mast	AIMP1	NTRK1	9,07827E-03	66408,6	1,45736E-02	4042	1,38669E+00	9975	3,97253E-01	101347	6,77743E-01	154779	0	61900
Myeloid_vs_CD8_Non-responder	cDC(CD1C)	CD8(ID2+CXCR4+ T)	HLA-DPA1	LAG3	9,08095E-03	25571	4,70571E-03	25220	1,18133E+00	16356	2,18197E+00	17639	9,46739E-01	6740	0	61900
Myeloid_vs_CD8_Non-responder	CD8(NME1+ T)	pDC_LILRA4	HSP90B1	TLR7	9,08375E-03	33113,6	8,25018E-03	10890	1,47669E+00	8033	1,60722E+00	34576	8,79999E-01	50169	0	61900
Myeloid_vs_CD8_Non-responder	Mono_CD16	CD8(GZMK+ Early Tem)	VCAN	CD44	9,10368E-03	42716,2	3,45155E-03	40126	8,69964E-01	31795	1,35544E+00	45375	9,03404E-01	34385	0	61900
Myeloid_vs_CD8_Non-responder	CD8(ID2+CXCR4+ T)	Macro_FOLR2+APOE+	HSP90B1	LRP1	9,10822E-03	43539,8	2,95572E-03	50196	9,80405E-01	25223	1,47424E+00	40003	8,94536E-01	40377	0	61900
Myeloid_vs_CD8_Non-responder	CD8(LAYN+ T)	Macro_FOLR2+APOE+	B2M	LILRB1	9,12183E-03	40535,6	2,95527E-03	50211	8,70745E-01	31754	1,27236E+00	49330	9,43269E-01	9483	0	61900
Myeloid_vs_CD8_Non-responder	CD8(NME1+ T)	Macro_LYVE1	MIF	CD44_CD74	9,12799E-03	62211,8	1,75072E-03	93995	5,23234E-01	63958	5,93835E-01	88778	9,54627E-01	2428	0	61900
Myeloid_vs_CD8_Non-responder	Mono_CD16	CD8(GZMK+ Early Tem)	AGTRAP	RACK1	9,12819E-03	37658,4	4,65520E-03	25631	8,99629E-01	29910	1,26843E+00	49529	9,23895E-01	21322	0	61900
Myeloid_vs_CD8_Non-responder	Mono_CD16	CD8(GZMK+ Early Tem)	S100A8	CD69	9,13274E-03	31865,6	4,69575E-03	25292	9,62776E-01	26124	1,78268E+00	28424	9,30426E-01	17588	0	61900
Myeloid_vs_CD8_Non-responder	Macro_FOLR2+APOE+	CD8(IL7R+ZNF683+ Tm)	SECTM1	CD7	9,13365E-03	35924	9,02052E-03	9484	1,18749E+00	16136	1,25473E+00	50224	8,92455E-01	41876	0	61900
Myeloid_vs_CD8_Non-responder	CD8(Tc17)	pDC_LILRA4	HSP90B1	TLR9	9,13541E-03	35769,6	2,20507E-02	1780	2,02187E+00	2429	1,22455E+00	51767	8,63681E-01	60972	0	61900
Myeloid_vs_CD8_Non-responder	Mono_CD16	CD8(Temra)	LGALS9	PTPRC	9,13638E-03	39604	2,96477E-03	49969	9,38553E-01	27509	1,44445E+00	41313	9,30971E-01	17329	0	61900
Myeloid_vs_CD8_Non-responder	CD8(Terminal Tex)	Macro_OLFML3	HLA-F	LILRB1	9,13820E-03	45680,6	3,20846E-03	44719	9,85634E-01	24960	1,32828E+00	46595	8,79905E-01	50229	0	61900
Myeloid_vs_CD8_Non-responder	CD8(Tn)	pDC_LILRA4	HSP90B1	TLR9	9,15025E-03	35805,6	2,20471E-02	1781	2,02179E+00	2431	1,22122E+00	51932	8,63671E-01	60984	0	61900
Myeloid_vs_CD8_Non-responder	Mono_CD16	CD8(Temra)	CD55	ADGRE5	9,15914E-03	39774,6	4,42407E-03	27684	7,80697E-01	38186	1,32903E+00	46555	9,18666E-01	24548	0	61900
Myeloid_vs_CD8_Non-responder	CD8(ID2+CXCR4+ T)	Mono_CD16	HLA-F	LILRB1	9,15914E-03	43915,8	3,20675E-03	44754	1,05455E+00	21710	1,45284E+00	40963	8,79877E-01	50252	0	61900
Myeloid_vs_CD8_Non-responder	cDC_CLEC9A	CD8(ZNF683+KLRB1+ T)	B2M	CD3D	9,16133E-03	53289,8	2,58363E-03	60586	4,12810E-01	79257	9,87688E-01	64641	9,63951E-01	65	0	61900
Myeloid_vs_CD8_Non-responder	Macro_FOLR2+APOE+	CD8(Terminal Tex)	HLA-A	CD8B	9,16278E-03	40711,2	3,30459E-03	42881	7,01121E-01	44747	1,25402E+00	50256	9,51546E-01	3772	0	61900

Myeloid_vs_CD8_Post_NR

Myeloid_vs_CD8_Non-responder	CD8(ZNF683+KLRB1+ T)	Macro_IER3	CD99	PILRA	9,16461E-03	38948,6	3,60650E-03	37662	1,05864E+00	21512	1,25402E+00	50258	9,20550E-01	23411	0	61900
Myeloid_vs_CD8_Non-responder	Macro_FOLR2+APOE+	CD8(GZMK+ Tex)	HLA-E	KLRC1_KLRD1	9,17190E-03	40827,8	4,59171E-03	26129	6,43468E-01	50266	1,41445E+00	42640	9,20877E-01	23204	0	61900
Myeloid_vs_CD8_Non-responder	CD8(ISG+ T)	Macro_ISG15	IFNG	IFNGR1_IFNGR2	9,17646E-03	48490,8	3,76005E-03	35321	6,89598E-01	45820	1,25377E+00	50271	8,81579E-01	49142	0	61900
Myeloid_vs_CD8_Non-responder	Macro_OLFML3	CD8(Tc17)	S100A8	CD69	9,18595E-03	30117	4,69507E-03	25295	9,88251E-01	24809	2,03987E+00	20990	9,30421E-01	17591	0	61900
Myeloid_vs_CD8_Non-responder	CD8(LAYN+ T)	Macro_ISG15	HMGB1	THBD	9,19016E-03	49833,4	2,95221E-03	50286	7,17086E-01	43353	1,26150E+00	49881	8,89663E-01	43747	0	61900
Myeloid_vs_CD8_Non-responder	CD8(ZNF683+KLRB1+ T)	Macro_IER3	SPON2	ITGB2	9,20022E-03	41922,8	5,56019E-03	19701	8,21429E-01	35097	1,25338E+00	50297	8,91373E-01	42619	0	61900
Myeloid_vs_CD8_Non-responder	CD8(Temra)	Macro_FOLR2+APOE+	CCL5	CCR1	9,22678E-03	38228,8	8,44700E-03	10502	1,04625E+00	22091	8,74222E-01	71327	9,17480E-01	25324	0	61900
Myeloid_vs_CD8_Non-responder	Mono_CD16	CD8(Temra)	S100A8	CD69	9,23686E-03	31621,8	4,88975E-03	23791	9,92713E-01	24586	1,70481E+00	30929	9,31725E-01	16903	0	61900
Myeloid_vs_CD8_Non-responder	cDC(CD1C)	CD8(LAYN+ T)	HLA-DPB1	LAG3	9,23948E-03	26356,2	4,69124E-03	25333	1,19997E+00	15698	2,08396E+00	19877	9,43914E-01	8973	0	61900
Myeloid_vs_CD8_Non-responder	Mono_CD16	CD8(NME1+ T)	HLA-DRB5	LAG3	9,24145E-03	45943,6	2,95003E-03	50342	6,57116E-01	48892	1,29179E+00	48391	9,25884E-01	20193	0	61900
Myeloid_vs_CD8_Non-responder	Macro_ISG15	CD8(ZNF683+KLRB1+ T)	CXCL16	CXCR6	9,24230E-03	24743,4	8,50687E-03	10388	1,30746E+00	12191	2,36743E+00	13903	9,17463E-01	25335	0	61900
Myeloid_vs_CD8_Non-responder	Mono_CD16	CD8(Temra)	NAMPT	ITGA5_ITGB1	9,24237E-03	41846,6	3,21199E-03	44649	8,71502E-01	31706	1,74348E+00	29666	8,93254E-01	41312	0	61900
Myeloid_vs_CD8_Non-responder	Macro_OLFML3	CD8(Temra)	CXCL9	CXCR3	9,24525E-03	30259	9,88568E-03	8131	2,07944E+00	2136	2,58397E+00	10405	8,51693E-01	68723	0	61900
Myeloid_vs_CD8_Non-responder	cDC(CD1C)	CD8(ISG+ T)	C3	IFITM1	9,25502E-03	27902	6,38296E-03	16000	1,14981E+00	17525	1,88295E+00	25344	9,28424E-01	18741	0	61900
Myeloid_vs_CD8_Non-responder	CD8(Tn)	Mono_CD16	HLA-A	LILRB1	9,26258E-03	38330	3,47664E-03	39712	9,40996E-01	27372	1,25191E+00	50365	9,39308E-01	12301	0	61900
Myeloid_vs_CD8_Non-responder	CD8(Temra)	cDC_CLEC9A	ADAM10	CADM1	9,27112E-03	40498,8	1,02473E-02	7695	1,49371E+00	7735	1,64523E+00	33123	8,14791E-01	92041	0	61900
Myeloid_vs_CD8_Non-responder	pDC_LILRA4	CD8(GZMK+ Tem)	HLA-DQA1	LAG3	9,27199E-03	27116	5,39331E-03	20619	9,78061E-01	25356	2,39241E+00	13468	9,36411E-01	14237	0	61900
Myeloid_vs_CD8_Non-responder	CD8(EOMES+ NK-like)	Macro_OLFML3	HLA-A	LILRB2	9,27730E-03	34309,4	2,94824E-03	50381	1,17703E+00	16516	1,57581E+00	35789	9,46480E-01	6961	0	61900
Myeloid_vs_CD8_Non-responder	Mono_CD16	CD8(Tn)	VCAN	CD44	9,29481E-03	38909,2	3,75643E-03	35378	9,44687E-01	27148	1,51844E+00	38143	9,07035E-01	31977	0	61900
Myeloid_vs_CD8_Non-responder	Mono_INHBA	CD8(ID2+CXCR4+ T)	SPP1	CD44	9,29750E-03	27330,6	7,90086E-03	11646	1,16450E+00	16994	2,05033E+00	20739	9,17406E-01	25374	0	61900
Myeloid_vs_CD8_Non-responder	CD8(ITM2C+ T)	Mono_CD14	HLA-F	LILRB2	9,30034E-03	41550	2,94710E-03	50406	1,05480E+00	21692	1,59460E+00	35046	8,97048E-01	38706	0	61900
Myeloid_vs_CD8_Non-responder	CD8(GZMK+ Tem)	Mono_CD16	IFNG	IFNGR1_IFNGR2	9,30865E-03	41731,4	5,32366E-03	21005	7,87476E-01	37676	1,25106E+00	50415	8,98560E-01	37661	0	61900
Myeloid_vs_CD8_Non-responder	CD8(ID2+CXCR4+ T)	Macro_ISG15	CD52	SIGLEC10	9,31327E-03	41597,2	3,82786E-03	34419	9,01275E-01	29795	1,25099E+00	50420	9,07812E-01	31452	0	61900
Myeloid_vs_CD8_Non-responder	Mono_CD14	CD8(LAYN+ T)	THBS1	ITGA4	9,31737E-03	22495,8	1,01368E-02	7816	1,55799E+00	6663	2,56323E+00	10712	9,17391E-01	25388	0	61900
Myeloid_vs_CD8_Non-responder	Mono_CD16	CD8(Tn)	S100A8	CD69	9,31881E-03	31454,6	4,80801E-03	24413	9,80099E-01	25238	1,78004E+00	28517	9,31187E-01	17205	0	61900
Myeloid_vs_CD8_Non-responder	CD8(ID2+CXCR4+ T)	Macro_OLFML3	CD99	PILRA	9,32435E-03	42980,6	3,13325E-03	46245	9,03879E-01	29606	1,25073E+00	50432	9,15251E-01	26720	0	61900
Myeloid_vs_CD8_Non-responder	CD8(Terminal Tex)	Macro_FOLR2+APOE-	TNFSF9	HLA-DPA1	9,32620E-03	40944,2	3,52703E-03	38948	1,01653E+00	23400	1,25072E+00	50434	9,09934E-01	30039	0	61900
Myeloid_vs_CD8_Non-responder	CD8(LAYN+ T)	Macro_FOLR2+APOE+	ANXA1	FPR2_FPR3	9,33175E-03	34509,6	7,08359E-03	13717	1,16450E+00	16993	1,25051E+00	50440	9,10761E-01	29498	0	61900
Myeloid_vs_CD8_Non-responder	CD8(Tn)	Macro_LYVE1	ANXA1	FPR1	9,34286E-03	43499,2	2,94538E-03	50452	8,58106E-01	32584	1,31784E+00	47114	9,17303E-01	25446	0	61900
Myeloid_vs_CD8_Non-responder	CD8(ISG+ T)	cDC_CLEC9A	CRTAM	CADM1	9,34378E-03	43333,4	1,26899E-02	5333,5	1,36792E+00	10443,5	1,19997E+00	53067,5	8,24674E-01	85922,5	0	61900
Myeloid_vs_CD8_Non-responder	CD8(GZMK+ Tem)	Macro_FOLR2+APOE+	HLA-DRB1	CD4	9,35027E-03	34656	2,94514E-03	50460	1,17276E+00	16686	1,64647E+00	33090	9,40957E-01	11144	0	61900
Myeloid_vs_CD8_Non-responder	Mast	CD8(GZMK+ Early Tem)	ADCYAP1	ADRB2	9,35177E-03	56663,6	3,59657E-02	665	2,01645E+00	2458	9,15725E-01	68881	6,95101E-01	149414	0	61900
Myeloid_vs_CD8_Non-responder	Mast	CD8(IL7R+ZNF683+ Tm)	ADCYAP1	ADRB2	9,35927E-03	56491	3,59212E-02	666	2,01624E+00	2459	9,30989E-01	67970	6,94970E-01	149460	0	61900
Myeloid_vs_CD8_Non-responder	CD8(NME1+ T)	Mono_INHBA	MIF	CD44_CD74	9,35927E-03	53585,8	1,80198E-03	91559	6,87917E-01	45984	9,59017E-01	66316	9,55248E-01	2170	0	61900
Myeloid_vs_CD8_Non-responder	Macro_FOLR2+APOE+	CD8(Tn)	FN1	CD44	9,36139E-03	42917	5,11388E-03	22262	7,24162E-01	42728	1,54089E+00	37223	8,79533E-01	50472	0	61900
Myeloid_vs_CD8_Non-responder	Macro_ISG15	CD8(GZMK+ Tem)	S100A8	CD69	9,36290E-03	29347,2	5,01488E-03	22923	1,09178E+00	20017	1,88065E+00	25420	9,32524E-01	16476	0	61900
Myeloid_vs_CD8_Non-responder	Mono_CD16	CD8(IL7R+ZNF683+ Tm)	SECTM1	CD7	9,37902E-03	32711,2	1,04334E-02	7485	1,34176E+00	11147	1,34578E+00	45809	8,99241E-01	37215	0	61900
Myeloid_vs_CD8_Non-responder	CD8(ZNF683+KLRB1+ T)	Macro_OLFML3	HLA-B	LILRB2	9,37995E-03	33021,4	2,94356E-03	50492	1,24119E+00	14210	1,64247E+00	33238	9,48851E-01	5267	0	61900
Myeloid_vs_CD8_Non-responder	Mono_CD16	CD8(NME1+ T)	HLA-DQA2	LAG3	9,38553E-03	46997	3,34019E-03	42141	6,41227E-01	50498	1,57437E+00	35855	8,88452E-01	44591	0	61900
Myeloid_vs_CD8_Non-responder	Mono_CD16	CD8(IL7R+ZNF683+ Tm)	VCAN	CD44	9,38925E-03	42274,6	3,45923E-03	39994	8,71845E-01	31684	1,39663E+00	43480	9,03501E-01	34315	0	61900
Myeloid_vs_CD8_Non-responder	Mono_CD14	CD8(GZMK+ Tex)	VCAN	ITGA4	9,39069E-03	21586,8	9,83894E-03	8190	2,44181E+00	977	2,57904E+00	10461	9,15798E-01	26406	0	61900
Myeloid_vs_CD8_Non-responder	CD8(GZMK+ Tex)	Macro_OLFML3	CD52	SIGLEC10	9,39203E-03	37553,8	4,55924E-03	26434	1,04882E+00	21971	1,24916E+00	50505	9,14872E-01	26959	0	61900
Myeloid_vs_CD8_Non-responder	Macro_FOLR2+APOE+	CD8(GZMK+ Tem)	CXCL10	CXCR3	9,40897E-03	28013,2	1,27987E-02	5228	1,36695E+00	10468	2,80494E+00	7598	8,72874E-01	54872	0	61900
Myeloid_vs_CD8_Non-responder	Mono_CD16	CD8(IL7R+ZNF683+ Tm)	GNAI2	CXCR3	9,41158E-03	47263,8	3,80320E-03	34734	6,51072E-01	49498	1,42439E+00	42191	8,83305E-01	47996	0	61900
Myeloid_vs_CD8_Non-responder	CD8(ID2+CXCR4+ T)	Macro_OLFML3	HLA-B	LILRB2	9,41344E-03	33142,6	2,94206E-03	50528	1,23956E+00	14258	1,62777E+00	33748	9,48839E-01	5279	0	61900
Myeloid_vs_CD8_Non-responder	Mono_CD16	CD8(IL7R+ZNF683+ Tm)	S100A8	CD69	9,41717E-03	29191	5,28656E-03	21219	1,05395E+00	21730	1,87658E+00	25534	9,34165E-01	15572	0	61900
Myeloid_vs_CD8_Non-responder	Mono_CD16	CD8(GZMK+ Tem)	LGALS9	PTPRC	9,42089E-03	39248	2,96591E-03	49940	9,39028E-01	27490	1,48448E+00	39588	9,30984E-01	17322	0	61900
Myeloid_vs_CD8_Non-responder	Macro_ISG15	CD8(GZMK+ Tex)	HLA-DPA1	LAG3	9,42578E-03	27444,6	4,67552E-03	25464	9,99380E-01	24261	2,13285E+00	18721	9,46576E-01	6877	0	61900
Myeloid_vs_CD8_Non-responder	Mono_CD16	CD8(GZMK+ Tem)	HLA-DRA	LAG3	9,43488E-03	29806,8	4,80689E-03	24425	9,85377E-01	24976	1,68535E+00	31641	9,47644E-01	6092	0	61900
Myeloid_vs_CD8_Non-responder	Mono_CD16	CD8(GZMK+ Tem)	HLA-DQA2	LAG3	9,43582E-03	31508,6	5,54028E-03	19807	9,60155E-01	26283	2,06766E+00	20304	9,11172E-01	29249	0	61900
Myeloid_vs_CD8_Non-responder	Mono_CD16	CD8(GZMK+ Tem)	HLA-DRB1	LAG3	9,43768E-03	37768,8	3,94899E-03	32906	7,71946E-01	38834	1,34277E+00	45947	9,43568E-01	9257	0	61900
Myeloid_vs_CD8_Non-responder	Mono_CD16	CD8(GZMK+ Tem)	HLA-DPB1	LAG3	9,44048E-03	35155,2	4,18065E-03	30224	8,36643E-01	34014	1,51289E+00	38390	9,40784E-01	11248	0	61900
Myeloid_vs_CD8_Non-responder	CD8(LAYN+ T)	pDC_LILRA4	SIRPG	CD47	9,44142E-03	33100,2	8,60983E-03	10207,5	9,91147E-01	24658,5	2,15784E+00	18176,5	8,79396E-01	50558,5	0	61900
Myeloid_vs_CD8_Non-responder	CD8(ITM2C+ T)	Macro_NLRP3	HLA-B	LILRB2	9,44329E-03	32437,8	2,94084E-03	50560	1,32044E+00	11799	1,65738E+00	32646	9,48829E-01	5284	0	61900
Myeloid_vs_CD8_Non-responder	Mono_CD16	CD8(GZMK+ Tem)	CD55	ADGRE5	9,44982E-03	34789,2	5,19216E-03	21790	8,95654E-01	30141	1,49602E+00	39124	9,24450E-01	20991	0	61900
Myeloid_vs_CD8_Non-responder	CD8(Temra)	Macro_ISG15	CCL5	CCRL2	9,45447E-03	38349,4	8,53869E-03	10322	1,00759E+00	23840	8,92746E-01	70201	9,17244E-01	25484	0	61900
Myeloid_vs_CD8_Non-responder	CD8(Tn)	cDC(CD1C)	LTB	TNFRSF1A	9,45543E-03	41780,2	5,37934E-03	20691	8,76010E-01	31387	1,24783E+00	50573	8,88763E-01	44350	0	61900
Myeloid_vs_CD8_Non-responder	Mono_CD16	CD8(GZMK+ Tem)	HLA-DPA1	LAG3	9,46572E-03	31967,4	4,53389E-03	26676	9,19314E-01	28679	1,59421E+00	35062	9,45793E-01	7520	0	61900
Myeloid_vs_CD8_Non-responder	CD8(ZNF683+KLRB1+ T)	Macro_IER3	HMGB1	CD163	9,47227E-03	42032	2,99992E-03	49196	9,30185E-01	28023	1,24744E+00	50591	9,25416E-01	20450	0	61900
Myeloid_vs_CD8_Non-responder	cDC_CLEC9A	CD8(ISG+ T)	HLA-DRB5	LAG3	9,47315E-03	26261,6	4,67118E-03	25497	1,09057E+00	20075	2,46931E+00	12145	9,40190E-01	11691	0	61900
Myeloid_vs_CD8_Non-responder	CD8(ZNF683+KLRB1+ T)	Macro_NLRP3	LTB	TNFRSF1A	9,47789E-03	45438,8	4,47481E-03	27224	7,54015E-01	40235	1,31559E+00	47238	8,79331E-01	50597	0	61900
Myeloid_vs_CD8_Non-responder	Mono_CD16	CD8(GZMK+ Tem)	HLA-DRB5	LAG3	9,48538E-03	30049,4	4,89313E-03	23758	9,76044E-01	25453	1,78507E+00	28351	9,41482E-01	10785	0	61900
Myeloid_vs_CD8_Non-responder	CD8(NME1+ T)	Mono_INHBA	HMGB1	THBD	9,49757E-03	43768,6	3,34921E-03	41990	9,89622E-01	24741	1,24691E+00	50618	8,95705E-01	39594	0	61900
Myeloid_vs_CD8_Non-responder	Mono_CD16	CD8(GZMK+ Tem)	VCAN	CD44	9,49851E-03	42151,4	3,51009E-03	39224	8,84311E-01	30836	1,36545E+00	44897	9,04136E-01	33900	0	61900
Myeloid_vs_CD8_Non-responder	CD8(ZNF683+KLRB1+ T)	Macro_ISG15	LTB	CD40	9,50133E-03	33822,8	7,85033E-03	11744	1,24431E+00	14103	1,71071E+00	30745	8,79300E-01	50622	0	61900
Myeloid_vs_CD8_Non-responder	Mono_CD16	CD8(GZMK+ Tem)	LGALS3	LAG3	9,50133E-03	35261,4	5,18510E-03	21827	8,25044E-01	34860	1,53626E+00	37409	9,25666E-01	20311	0	61900
Myeloid_vs_CD8_Non-responder	CD8(Tn)	pDC_LILRA4	LTB	TNFRSF1A	9,50790E-03	39584,2	4,47124E-03	27260	7,60894E-01	39669	2,14449E+00	18463	8,79289E-01	50629	0	61900
Myeloid_vs_CD8_Non-responder	CD8(ID2+CXCR4+ T)	pDC_LILRA4	CXCL13	CXCR3	9,50884E-03	39808,2	1,19366E-02	6003	7,08580E-01	44083	1,56051E+00	36425	8,79283E-01	50630	0	61900
Myeloid_vs_CD8_Non-responder	Mono_CD16	CD8(GZMK+ Tem)	GNAI2	CXCR3	9,52857E-03	43839,6	4,35520E-03	28369	7,17912E-01	43274	1,42387E+00	42217	8,90111E-01	43438	0	61900
Myeloid_vs_CD8_Non-responder	Macro_IER3	CD8(GZMK+ Tex)	HLA-DQB1	LAG3	9,53223E-03	26624	6,01862E-03	17493	1,15849E+00	17218	1,87641E+00	25538	9,41211E-01	10971	0	61900
Myeloid_vs_CD8_Non-responder	Mono_CD16	CD8(GZMK+ Tem)	S100A8	CD69	9,53422E-03	37838	4,03922E-03	31802	8,61464E-01	32370	1,41412E+00	42653	9,25390E-01	20465	0	61900
Myeloid_vs_CD8_Non-responder	CD8(NME1+ T)	Mono_CD16	GNAI2	FPR1	9,53704E-03	40294,4	2,93599E-03	50660	1,15075E+00	17486	1,85334E+00	26194	8,87456E-01	45232	0	61900
Myeloid_vs_CD8_Non-responder	Mono_CD16	CD8(GZMK+ Tex)	HLA-DRA	LAG3	9,55211E-03	32849,2	4,21969E-03	29797	8,87269E-01	30666	1,64268E+00	33227	9,44316E-01	8656	0	61900
Myeloid_vs_CD8_Non-responder	Mono_CD16	CD8(GZMK+ Tex)	HLA-DQA2	LAG3	9,55305E-03	34479,4	4,86348E-03	23977	8,62046E-01	32316	2,02499E+00	21371	9,05756E-01	32833	0	61900
Myeloid_vs_CD8_Non-responder	Mono_CD16	CD8(GZMK+ Tex)	HLA-DRB1	LAG3	9,55494E-03	41777,6	3,46659E-03	39884	6,73837E-01	47289	1,30010E+00	47999	9,39997E-01	11816	0	61900
Myeloid_vs_CD8_Non-responder	Mono_CD16	CD8(GZMK+ Tex)	HLA-DPB1	LAG3	9,55871E-03	38811,6	3,66995E-03	36619	7,38535E-01	41535	1,47022E+00	40189	9,37049E-01	13815	0	61900
Myeloid_vs_CD8_Non-responder	Macro_FOLR2+APOE+	CD8(GZMK+ Tex)	HLA-C	CD3D	9,56626E-03	39272,4	2,93452E-03	50691	7,79222E-01	38306	1,36221E+00	45068	9,60651E-01	397	0	61900

Myeloid_vs_CD8_Post_NR

Myeloid_vs_CD8_Non-responder	Mono_CD16	CD8(GZMK+ Tex)	CD55	ADGRE5	9,56815E-03	41529,8	4,16613E-03	30399	7,42092E-01	41238	1,29818E+00	48083	9,16394E-01	26029	0	61900
Myeloid_vs_CD8_Non-responder	pDC_LILRA4	CD8(ITM2C+ T)	CDH1	KLRG1	9,58383E-03	38869,2	1,10800E-01	68	1,77880E+00	4157	1,88731E+00	25209	7,96455E-01	103012	0	61900
Myeloid_vs_CD8_Non-responder	cDC_LAMP3	CD8(GZMK+ Tex)	B2M	CD3D	9,58383E-03	57546,4	2,57995E-03	60687	4,85917E-01	68865	4,78124E-01	96212	9,63927E-01	68	0	61900
Myeloid_vs_CD8_Non-responder	Macro_NLRP3	CD8(Tc17)	S100A8	ITGB2	9,58893E-03	30736	2,93327E-03	50715	1,73262E+00	4554	2,33606E+00	14487	9,22793E-01	22024	0	61900
Myeloid_vs_CD8_Non-responder	Mono_CD16	CD8(GZMK+ Tex)	HLA-DPA1	LAG3	9,58987E-03	35300,4	3,98004E-03	32518	8,21205E-01	35119	1,55155E+00	36807	9,42355E-01	10158	0	61900
Myeloid_vs_CD8_Non-responder	CD8(Terminal Tex)	Macro_FOLR2+APOE+	HLA-A	LILRB1	9,59365E-03	40557,8	3,09337E-03	47103	9,22716E-01	28492	1,24518E+00	50720	9,35891E-01	14574	0	61900
Myeloid_vs_CD8_Non-responder	Mono_CD16	CD8(GZMK+ Tex)	LGALS9	PTPRC	9,59933E-03	39619,2	2,93290E-03	50726	9,25347E-01	28311	1,48266E+00	39660	9,30623E-01	17499	0	61900
Myeloid_vs_CD8_Non-responder	Macro_OLFML3	CD8(GZMK+ Tem)	CXCL11	CXCR3	9,60096E-03	32728,4	3,62576E-02	650	2,01056E+00	2491	1,87558E+00	25556	8,45098E-01	73045	0	61900
Myeloid_vs_CD8_Non-responder	CD8(ITM2C+ T)	Macro_NLRP3	HLA-A	LILRB2	9,60311E-03	33972,4	2,93272E-03	50730	1,22670E+00	14704	1,58453E+00	35453	9,46346E-01	7075	0	61900
Myeloid_vs_CD8_Non-responder	Mono_CD16	CD8(GZMK+ Tex)	HLA-DRB5	LAG3	9,60974E-03	33038,2	4,29540E-03	28968	8,77936E-01	31265	1,74240E+00	29699	9,37788E-01	13359	0	61900
Myeloid_vs_CD8_Non-responder	Mono_CD16	CD8(GZMK+ Tex)	SECTM1	CD7	9,61069E-03	28220	1,22959E-02	5680	1,49077E+00	7791	1,63971E+00	33359	9,06442E-01	32370	0	61900
Myeloid_vs_CD8_Non-responder	Mono_CD16	CD8(GZMK+ Tex)	LGALS3	LAG3	9,62585E-03	38645	4,55169E-03	26504	7,26936E-01	42496	1,49359E+00	39223	9,21057E-01	23102	0	61900
Myeloid_vs_CD8_Non-responder	Mono_CD16	CD8(GZMK+ Tex)	CXCL16	CXCR6	9,63249E-03	32092,2	6,12375E-03	17023	9,81600E-01	25163	1,98374E+00	22473	9,04134E-01	33902	0	61900
Myeloid_vs_CD8_Non-responder	CD8(ID2+CXCR4+ T)	Macro_ISG15	HMGB1	HAVCR2	9,63249E-03	45418	2,98249E-03	49575	7,26194E-01	42557	1,24422E+00	50761	9,22316E-01	22297	0	61900
Myeloid_vs_CD8_Non-responder	Macro_LYVE1	CD8(Tc17)	CXCL12	DPP4	9,63899E-03	43377	5,94906E-02	255	2,00925E+00	2496	1,43550E+00	41696	7,82892E-01	110538	0	61900
Myeloid_vs_CD8_Non-responder	cDC(CD1C)	CD8(LAYN+ T)	HLA-DRA	LAG3	9,64101E-03	26097	4,65669E-03	25613	1,15941E+00	17183	2,11449E+00	19132	9,46851E-01	6657	0	61900
Myeloid_vs_CD8_Non-responder	pDC_LILRA4	CD8(Terminal Tex)	HLA-DRA	LAG3	9,64830E-03	26698,2	4,65632E-03	25618	9,84434E-01	25018	2,34554E+00	14295	9,46849E-01	6660	0	61900
Myeloid_vs_CD8_Non-responder	Mono_CD16	CD8(GZMK+ Tex)	S100A8	CD69	9,65243E-03	35743	4,22950E-03	29695	8,90828E-01	30447	1,54365E+00	37098	9,26964E-01	19575	0	61900
Myeloid_vs_CD8_Non-responder	CD8(GZMK+ Tex)	Macro_OLFML3	CD99	PILRA	9,65243E-03	43457,4	3,09162E-03	47145	8,91489E-01	30412	1,24390E+00	50782	9,14731E-01	27048	0	61900
Myeloid_vs_CD8_Non-responder	Macro_OLFML3	CD8(ITM2C+ T)	CXCL11	CCR5	9,65422E-03	40828,4	3,71571E-02	618	2,00896E+00	2498	2,07100E+00	20213	7,67333E-01	118913	0	61900
Myeloid_vs_CD8_Non-responder	Macro_NLRP3	CD8(GZMK+ Tex)	TIMP1	CD63	9,66099E-03	27419	2,93017E-03	50791	1,68054E+00	5043	2,93762E+00	6266	9,38196E-01	13095	0	61900
Myeloid_vs_CD8_Non-responder	CD8(Temra)	Macro_ISG15	SPN	SIGLEC1	9,66203E-03	38929,6	1,04588E-02	7460	1,38522E+00	10014	1,59657E+00	34980	8,33620E-01	80294	0	61900
Myeloid_vs_CD8_Non-responder	Macro_FOLR2+APOE+	CD8(Tn)	LGALS1	CD69	9,66289E-03	37728,2	2,93013E-03	50793	7,89208E-01	37530	1,69002E+00	31467	9,46488E-01	6951	0	61900
Myeloid_vs_CD8_Non-responder	Macro_FOLR2+APOE+	CD8(GZMK+ Tem)	CD14	ITGB2	9,66670E-03	29740,8	2,92994E-03	50797	1,42762E+00	9026	2,53434E+00	11116	9,33655E-01	15865	0	61900
Myeloid_vs_CD8_Non-responder	CD8(Tn)	Mono_CD16	B2M	LILRB1	9,67526E-03	37825,6	3,42468E-03	40606	9,08652E-01	29335	1,24362E+00	50806	9,47087E-01	6481	0	61900
Myeloid_vs_CD8_Non-responder	CD8(ID2+CXCR4+ T)	Macro_ISG15	HMGB1	CD163	9,67526E-03	39363	2,92961E-03	50806	9,00072E-01	29882	1,64081E+00	33312	9,24593E-01	20915	0	61900
Myeloid_vs_CD8_Non-responder	Macro_NLRP3	CD8(ID2+CXCR4+ T)	CD14	ITGA4	9,68288E-03	39215	2,92883E-03	50814	9,86705E-01	24897	1,86788E+00	25781	9,05999E-01	32683	0	61900
Myeloid_vs_CD8_Non-responder	Macro_FOLR2+APOE+	CD8(GZMK+ Tex)	B2M	CD3G	9,68384E-03	38339,8	2,92877E-03	50815	8,51966E-01	32974	1,38649E+00	43913	9,55428E-01	2097	0	61900
Myeloid_vs_CD8_Non-responder	CD8(NME1+ T)	Macro_FOLR2+APOE+	CD28	CD86	9,68478E-03	39108,4	8,19577E-03	10992,5	1,29555E+00	12541,5	1,87232E+00	25643,5	8,26990E-01	84464,5	0	61900
Myeloid_vs_CD8_Non-responder	Macro_FOLR2+APOE+	CD8(ZNF683+KLRB1+ T)	SECTM1	CD7	9,68479E-03	36604	8,70221E-03	10026	1,15804E+00	17235	1,24339E+00	50816	8,90719E-01	43043	0	61900
Myeloid_vs_CD8_Non-responder	Macro_FOLR2+APOE+	CD8(IL7R+ZNF683+ Tm)	HLA-C	CD8A	9,69051E-03	40667	2,92835E-03	50822	7,40754E-01	41361	1,28841E+00	48539	9,59176E-01	713	0	61900
Myeloid_vs_CD8_Non-responder	Macro_OLFML3	CD8(ID2+CXCR4+ T)	HLA-DRB5	LAG3	9,69062E-03	28013,6	4,65194E-03	25647	1,07586E+00	20709	2,07833E+00	20041	9,40074E-01	11771	0	61900
Myeloid_vs_CD8_Non-responder	Macro_LYVE1	CD8(GZMK+ Tem)	F13A1	ITGA4	9,69355E-03	22259	1,14902E-02	6358	2,20839E+00	1626	2,27238E+00	15762	9,16973E-01	25649	0	61900
Myeloid_vs_CD8_Non-responder	CD8(Terminal Tex)	Mono_INHBA	IFNG	IFNGR1_IFNGR2	9,69528E-03	41970	5,28756E-03	21212	7,82727E-01	38052	1,24322E+00	50827	8,98250E-01	37859	0	61900
Myeloid_vs_CD8_Non-responder	CD8(Tc17)	Macro_NLRP3	TNF	TNFRSF1B	9,69528E-03	48042	3,35264E-03	41912	6,37828E-01	50827	1,29061E+00	48442	8,99343E-01	37129	0	61900
Myeloid_vs_CD8_Non-responder	Mono_CD16	CD8(GZMK+ Tex)	CD86	CTLA4	9,69814E-03	34663,2	7,83718E-03	11774	1,08083E+00	20481	1,78547E+00	28331	8,78961E-01	50830	0	61900
Myeloid_vs_CD8_Non-responder	CD8(ITM2C+ T)	Mono_CD14	IFNG	IFNGR1_IFNGR2	9,70386E-03	46780,8	4,25112E-03	29464	6,37749E-01	50836	1,32547E+00	46723	8,87838E-01	44981	0	61900
Myeloid_vs_CD8_Non-responder	Macro_OLFML3	CD8(GZMK+ Tex)	CXCL16	CXCR6	9,70964E-03	26356,6	8,39302E-03	10624	1,25937E+00	13603	2,08007E+00	19996	9,16952E-01	25660	0	61900
Myeloid_vs_CD8_Non-responder	Mono_CD16	CD8(ZNF683+KLRB1+ T)	SECTM1	CD7	9,71341E-03	33292,4	1,00652E-02	7895	1,31230E+00	12048	1,33445E+00	46301	8,97602E-01	38318	0	61900
Myeloid_vs_CD8_Non-responder	CD8(Temra)	Macro_ISG15	TGFB1	SDC2	9,71796E-03	34756,8	1,13325E-02	6493	1,41320E+00	9349	1,61433E+00	34300	8,62505E-01	61742	0	61900
Myeloid_vs_CD8_Non-responder	CD8(GZMK+ Tex)	Macro_OLFML3	HLA-B	LILRB2	9,72392E-03	33294,4	2,92695E-03	50857	1,22309E+00	14846	1,63479E+00	33517	9,48714E-01	5352	0	61900
Myeloid_vs_CD8_Non-responder	Mono_CD16	CD8(ZNF683+KLRB1+ T)	CXCL16	CXCR6	9,73062E-03	34208,6	5,59863E-03	19494	9,18088E-01	28743	1,91820E+00	24315	9,00177E-01	36591	0	61900
Myeloid_vs_CD8_Non-responder	CD8(Terminal Tex)	cDC_CLEC9A	CRTAM	CADM1	9,73157E-03	28895,6	3,02181E-02	955,5	1,65955E+00	5274,5	1,87875E+00	25482,5	8,78911E-01	50865,5	0	61900
Myeloid_vs_CD8_Non-responder	Mono_CD16	CD8(ZNF683+KLRB1+ T)	LGALS1	CD69	9,73157E-03	30766	3,82502E-03	34447	1,01478E+00	23484	1,70760E+00	30844	9,52849E-01	3155	0	61900
Myeloid_vs_CD8_Non-responder	CD8(IL7R+ZNF683+ Tm)	Macro_OLFML3	HLA-A	LILRB2	9,75072E-03	34830,2	2,92591E-03	50885	1,15597E+00	17309	1,54792E+00	36941	9,46287E-01	7116	0	61900
Myeloid_vs_CD8_Non-responder	cDC(CD1C)	CD8(ISG+ T)	HLA-DPA1	LAG3	9,76392E-03	25457,6	4,64591E-03	25697	1,17426E+00	16626	2,25800E+00	16048	9,46415E-01	7017	0	61900
Myeloid_vs_CD8_Non-responder	Macro_OLFML3	CD8(ID2+CXCR4+ T)	CXCL16	CXCR6	9,76539E-03	26644	8,37999E-03	10647	1,25822E+00	13645	2,02659E+00	21330	9,16893E-01	25698	0	61900
Myeloid_vs_CD8_Non-responder	Mono_CD16	CD8(EOMES+ NK-like)	HLA-C	KIR3DL1	9,77566E-03	41164,8	1,65043E-02	3116	6,71719E-01	47469	1,25742E+00	50088	8,90407E-01	43251	0	61900
Myeloid_vs_CD8_Non-responder	Mono_CD16	CD8(EOMES+ NK-like)	HLA-E	KLRC2_KLRD1	9,77854E-03	37331	6,54590E-03	15417	7,09608E-01	43996	1,32912E+00	46548	9,28332E-01	18794	0	61900
Myeloid_vs_CD8_Non-responder	Mono_CD16	CD8(EOMES+ NK-like)	HLA-E	KLRC3_KLRD1	9,77950E-03	35617	6,61117E-03	15177	7,69191E-01	39034	1,35854E+00	45219	9,32005E-01	16755	0	61900
Myeloid_vs_CD8_Non-responder	Macro_FOLR2+APOE+	CD8(Temra)	LGALS9	CD44	9,79872E-03	38431	2,92348E-03	50935	1,03961E+00	22373	1,59863E+00	34910	9,22771E-01	22037	0	61900
Myeloid_vs_CD8_Non-responder	Mono_CD16	CD8(EOMES+ NK-like)	SECTM1	CD7	9,80642E-03	25614	1,36765E-02	4580	1,60124E+00	6032	1,85587E+00	26128	9,10858E-01	29430	0	61900
Myeloid_vs_CD8_Non-responder	Macro_OLFML3	CD8(ID2+CXCR4+ T)	HLA-DRA	LAG3	9,82433E-03	25890	4,64025E-03	25738	1,11848E+00	18853	2,24943E+00	16239	9,46762E-01	6720	0	61900
Myeloid_vs_CD8_Non-responder	Macro_FOLR2+APOE+	CD8(IL7R+ZNF683+ Tm)	HLA-E	CD8B	9,82568E-03	41961,8	3,45624E-03	40043	6,62193E-01	48395	1,24054E+00	50963	9,44488E-01	8508	0	61900
Myeloid_vs_CD8_Non-responder	pDC_LILRA4	CD8(Tc17)	APP	LRP10	9,83583E-03	27471,8	8,39067E-03	10629	2,23725E+00	1528	2,96199E+00	6054	8,69337E-01	57248	0	61900
Myeloid_vs_CD8_Non-responder	Mono_CD16	CD8(EOMES+ NK-like)	S100A8	CD69	9,83919E-03	30321,2	5,07592E-03	22534	1,02144E+00	23178	1,80428E+00	27715	9,32904E-01	16279	0	61900
Myeloid_vs_CD8_Non-responder	Macro_ISG15	CD8(Terminal Tex)	HLA-DPB1	LAG3	9,84058E-03	28584,2	4,63823E-03	25749	9,76749E-01	25415	2,05473E+00	20625	9,43612E-01	9232	0	61900
Myeloid_vs_CD8_Non-responder	Mono_CD16	CD8(ID2+CXCR4+ T)	LGALS9	PTPRC	9,84112E-03	38591	3,01618E-03	48842	9,59862E-01	26300	1,50147E+00	38889	9,31522E-01	17024	0	61900
Myeloid_vs_CD8_Non-responder	Mono_CD16	CD8(ID2+CXCR4+ T)	HLA-DRA	LAG3	9,85174E-03	41014,6	3,32738E-03	42398	7,38183E-01	41567	1,34571E+00	45812	9,37730E-01	13396	0	61900
Myeloid_vs_CD8_Non-responder	Mono_CD16	CD8(ID2+CXCR4+ T)	HLA-DQA2	LAG3	9,85270E-03	42020	3,83504E-03	34307	7,12961E-01	43716	1,72802E+00	30172	8,95116E-01	40005	0	61900
Myeloid_vs_CD8_Non-responder	Mono_CD16	CD8(ID2+CXCR4+ T)	CD55	ADGRE5	9,86720E-03	42952,6	4,02047E-03	32035	7,20292E-01	43077	1,24210E+00	50888	9,15020E-01	26863	0	61900
Myeloid_vs_CD8_Non-responder	CD8(GZMK+ Tem)	Macro_OLFML3	HLA-B	LILRB2	9,87204E-03	33640,6	2,92074E-03	51011	1,21632E+00	15096	1,60123E+00	34803	9,48662E-01	5393	0	61900
Myeloid_vs_CD8_Non-responder	Macro_NLRP3	CD8(Temra)	ICAM1	ITGAM_ITGB2	9,87301E-03	39197,4	2,92072E-03	51012	1,13034E+00	18345	2,01252E+00	21684	8,90709E-01	43046	0	61900
Myeloid_vs_CD8_Non-responder	Mono_CD16	CD8(GZMK+ Tex)	LGALS9	HAVCR2	9,87494E-03	39959	3,41245E-03	40812	1,02019E+00	23229	1,97007E+00	22840	8,78692E-01	51014	0	61900
Myeloid_vs_CD8_Non-responder	Macro_FOLR2+APOE+	CD8(GZMK+ Early Tem)	CXCL12	CXCR4	9,87785E-03	35636,2	9,18354E-03	9206	1,20878E+00	15356	1,45877E+00	40702	8,78688E-01	51017	0	61900
Myeloid_vs_CD8_Non-responder	Mono_INHBA	CD8(GZMK+ Early Tem)	CCL2	CCR4	9,88162E-03	41468,8	4,58560E-02	408	1,45640E+00	8433	2,56782E+00	10646	7,53204E-01	125957	0	61900
Myeloid_vs_CD8_Non-responder	Mono_CD16	CD8(ID2+CXCR4+ T)	HLA-DPA1	LAG3	9,88753E-03	44095	3,13841E-03	46138	6,72120E-01	47429	1,25457E+00	50230	9,35552E-01	14778	0	61900
Myeloid_vs_CD8_Non-responder	CD8(GZMK+ Tem)	Macro_NLRP3	HLA-C	LILRB2	9,88753E-03	33436,8	2,92012E-03	51027	1,26735E+00	13359	1,63042E+00	33659	9,46148E-01	7239	0	61900
Myeloid_vs_CD8_Non-responder	CD8(Tn)	Mono_CD16	HLA-F	LILRB2	9,89529E-03	35580,2	2,91992E-03	51035	1,64738E+00	5416	2,05711E+00	20551	8,96620E-01	38999	0	61900
Myeloid_vs_CD8_Non-responder	Mono_CD16	CD8(ID2+CXCR4+ T)	HLA-DRB5	LAG3	9,90304E-03	40867,8	3,38708E-03	41250	7,28850E-01	42357	1,44543E+00	41269	9,30487E-01	17563	0	61900
Myeloid_vs_CD8_Non-responder	Mono_CD16	CD8(ID2+CXCR4+ T)	SECTM1	CD7	9,90401E-03	27295,4	1,31418E-02	4958	1,55846E+00	6652	1,66214E+00	32463	9,09226E-01	30504	0	61900
Myeloid_vs_CD8_Non-responder	Mono_CD16	CD8(IL7R+ZNF683+ Tm)	CD55	ADGRE5	9,90887E-03	44200	3,82970E-03	34395	6,91741E-01	45624	1,23894E+00	51049	9,13111E-01	28032	0	61900
Myeloid_vs_CD8_Non-responder	Mono_CD16	CD8(ID2+CXCR4+ T)	CXCL16	CXCR6	9,92149E-03	32422,4	6,11424E-03	17063	9,80450E-01	25221	1,93026E+00	23964	9,04066E-01	33964	0	61900
Myeloid_vs_CD8_Non-responder	CD8(EOMES+ NK-like)	cDC_CLEC9A	CD52	SIGLEC10	9,92926E-03	43192,2	3,64809E-03	37002	8,49167E-01	33177	1,23858E+00	51070	9,05779E-01	32812	0	61900
Myeloid_vs_CD8_Non-responder	CD8(ZNF683+KLRB1+ T)	pDC_LILRA4	HSP90B1	TLR9	9,93043E-03	35829,4	2,11360E-02	1897	2,00287E+00	2534	1,25497E+00	50209	8,61167E-01	62607	0	61900
Myeloid_vs_CD8_Non-responder	Macro_FOLR2+APOE+	CD8(Terminal Tex)	HLA-B	KLRD1	9,93315E-03	40533,2	4,04687E-03	31718	6,68420E-01	47775	1,23854E+00	51074	9,42296E-01	10199	0	61900
Myeloid_vs_CD8_Non-responder	Macro_NLRP3	CD8(NME1+ T)	CD14	ITGB1	9,93510E-03	39794	2,91843E-03	51076	9,90407E-01	24699	1,80751E+00	27622	9,04490E-01	33673	0	61900

Myeloid_vs_CD8_Post_NR

Myeloid_vs_CD8_Non-responder	Mono_CD16	CD8(ID2+CXCR4+ T)	S100A8	CD69	9,94191E-03	32153,6	4,65865E-03	25597	9,57052E-01	26448	1,76069E+00	29092	9,30169E-01	17731	0	61900
Myeloid_vs_CD8_Non-responder	CD8(ISG+ T)	Macro_ISG15	ANXA1	FPR1	9,94678E-03	42685	2,91802E-03	51088	8,29989E-01	34499	1,46837E+00	40273	9,16948E-01	25665	0	61900
Myeloid_vs_CD8_Non-responder	CD8(Temra)	Macro_OLFML3	CD99	PILRA	9,95749E-03	43126,4	3,13186E-03	46276	9,03467E-01	29627	1,23816E+00	51099	9,15234E-01	26730	0	61900
Myeloid_vs_CD8_Non-responder	CD8(ISG+ T)	Macro_FOLR2+APOE+	LTB	TNFRSF1A	9,96431E-03	43111	4,40973E-03	27831	8,30492E-01	34453	1,46854E+00	40265	8,78552E-01	51106	0	61900
Myeloid_vs_CD8_Non-responder	CD8(Terminal Tex)	Macro_ISG15	CCL3	CCR1	9,96529E-03	33069,2	7,25978E-03	13200	1,39860E+00	9681	1,74999E+00	29458	8,78546E-01	51107	0	61900
Myeloid_vs_CD8_Non-responder	CD8(GZMK+ Tem)	Macro_NLRP3	CIRBP	TREM1	9,97406E-03	44275	3,95278E-03	32866	9,11558E-01	29137	1,23772E+00	51116	8,85787E-01	46356	0	61900
Myeloid_vs_CD8_Non-responder	Macro_FOLR2+APOE+	CD8(GZMK+ Tex)	HLA-A	CD3G	9,98480E-03	42694,4	3,03973E-03	48289	6,74797E-01	47204	1,23733E+00	51127	9,49343E-01	4952	0	61900
Myeloid_vs_CD8_Non-responder	Macro_FOLR2+APOE+	CD8(ITM2C+ T)	LGALS9	CD44	9,98578E-03	38225	2,91647E-03	51128	1,03795E+00	22450	1,63360E+00	33555	9,22686E-01	22092	0	61900
Myeloid_vs_CD8_Non-responder	CD8(GZMK+ Tem)	Macro_OLFML3	CD99	PILRA	9,99164E-03	43203,6	3,12494E-03	46424	9,01406E-01	29782	1,23718E+00	51134	9,15148E-01	26778	0	61900
Myeloid_vs_CD8_Non-responder	CD8(NME1+ T)	Macro_OLFML3	HLA-F	LILRB1	9,99359E-03	46642,2	3,12470E-03	46433	9,73239E-01	25575	1,29651E+00	48167	8,78500E-01	51136	0	61900
Myeloid_vs_CD8_Non-responder	Mono_CD16	CD8(Tc17)	VCAN	CD44	9,99555E-03	46571,4	3,12270E-03	46466	7,89365E-01	37507	1,26752E+00	49583	8,98946E-01	37401	0	61900
Myeloid_vs_CD8_Non-responder	Macro_FOLR2+APOE+	CD8(ZNF683+KLRB1+ T)	TIMP2	CD44	9,99750E-03	48877,6	3,02473E-03	48612	8,20455E-01	35184	1,23703E+00	51140	8,84002E-01	47552	0	61900

Myeloid_vs_CD8_Post_R

Response	source	target	ligand.complex	receptor.complex	aggregate_rank	mean_rank	natmi.edge_specificity	natmi.rank	connectome.weight_sc	connectome.rank	logfc.logfc_comb	logfc.rank	sca.LRscore	sca.rank	cellphonedb.pvalue	cellphonedb.rank
Myeloid_vs_CD8_Responder	Mono_CD14	CD8(ZNF683+KLRB1+ T)	S100A8	CD69	1,15024E-07	10013,1	2,40215E-02	1309	4,53192E+00	233	4,81835E+00	59	9,64608E-01	83	0	48381,5
Myeloid_vs_CD8_Responder	CD8(EOMES+ NK-like)	cDC_CLEC9A	XCL1	XCR1	3,89164E-07	16323,7	1,84383E-01	14	8,54659E+00	9	2,62591E+00	3764	9,04938E-01	29450	0	48381,5
Myeloid_vs_CD8_Responder	CD8(EOMES+ NK-like)	cDC_CLEC9A	XCL2	XCR1	6,43279E-07	17016,5	1,73421E-01	18	8,50680E+00	11	2,59042E+00	4020	8,99142E-01	32652	0	48381,5
Myeloid_vs_CD8_Responder	Macro_OLFML3	CD8(ISG+ T)	C3	IFITM1	7,43124E-07	11096,3	2,05582E-02	2000	2,95774E+00	1647	2,93921E+00	2089	9,57421E-01	1364	0	48381,5
Myeloid_vs_CD8_Responder	Mono_CD14	CD8(Tc17)	S100A8	CD69	7,44544E-07	10265,1	2,01621E-02	2090	4,37640E+00	286	4,34343E+00	134	9,61494E-01	434	0	48381,5
Myeloid_vs_CD8_Responder	CD8(ISG+ T)	pDC_LILRA4	BST2	LILRA4	7,56846E-07	13112,3	4,53592E-02	355	6,43032E+00	66	3,80925E+00	393	9,28235E-01	16366	0	48381,5
Myeloid_vs_CD8_Responder	CD8(ITM2C+ T)	Macro_OLFML3	TNFSF9	HLA-DPA1	1,08893E-06	10763,7	1,92791E-02	2299	2,75095E+00	2189	3,60622E+00	574	9,61959E-01	375	0	48381,5
Myeloid_vs_CD8_Responder	Mono_CD14	CD8(EOMES+ NK-like)	S100A8	CD69	1,14475E-06	10348,5	1,91534E-02	2328	4,33575E+00	301	4,34907E+00	132	9,60532E-01	600	0	48381,5
Myeloid_vs_CD8_Responder	Mono_CD14	CD8(GZMK+ Early Tem)	S100A8	CD69	1,23143E-06	10367,9	1,90033E-02	2371	4,32970E+00	302	4,30625E+00	150	9,60383E-01	635	0	48381,5
Myeloid_vs_CD8_Responder	CD8(ITM2C+ T)	cDC_CLEC9A	TNFSF9	HLA-DPA1	1,37841E-06	10766,3	1,87536E-02	2439	2,70399E+00	2357	4,13287E+00	214	9,61450E-01	440	0	48381,5
Myeloid_vs_CD8_Responder	Mono_CD14	CD8(IL7R+ZNF683+ Tm)	S100A8	CD69	1,43795E-06	10411,5	1,86406E-02	2465	4,31508E+00	307	4,21674E+00	183	9,60015E-01	721	0	48381,5
Myeloid_vs_CD8_Responder	CD8(IL7R+ZNF683+ Tm)	cDC_CLEC9A	XCL1	XCR1	1,44721E-06	17833,9	1,48823E-01	27	8,42423E+00	15	2,36452E+00	5974	8,95314E-01	34772	0	48381,5
Myeloid_vs_CD8_Responder	CD8(ITM2C+ T)	cDC(CD1C)	TNFSF9	HLA-DPA1	1,60527E-06	10813,3	1,83930E-02	2534	2,67178E+00	2457	4,18858E+00	194	9,61088E-01	500	0	48381,5
Myeloid_vs_CD8_Responder	CD8(Terminal Tex)	cDC(CD1C)	B2M	CD1C	1,62782E-06	12465,7	2,31727E-02	1447	3,98049E+00	449	2,17833E+00	7999	9,50031E-01	4052	0	48381,5
Myeloid_vs_CD8_Responder	Mono_CD14	CD8(ISG+ T)	S100A8	CD69	1,89875E-06	10467,9	1,79998E-02	2643	4,28926E+00	314	4,40120E+00	124	9,59338E-01	877	0	48381,5
Myeloid_vs_CD8_Responder	Mono_CD14	CD8(ID2+CXCR4+ T)	S100A8	CD69	2,07956E-06	10512,3	1,77739E-02	2704	4,28016E+00	315	4,10071E+00	221	9,59091E-01	940	0	48381,5
Myeloid_vs_CD8_Responder	Mono_CD14	CD8(Terminal Tex)	S100A8	CD69	2,34254E-06	10527,5	1,75045E-02	2786	4,26930E+00	319	4,34831E+00	133	9,58790E-01	1018	0	48381,5
Myeloid_vs_CD8_Responder	CD8(GZMK+ Tex)	cDC(CD1C)	B2M	CD1C	2,40440E-06	12669,9	2,27115E-02	1543	3,87719E+00	505	2,13010E+00	8648	9,49552E-01	4272	0	48381,5
Myeloid_vs_CD8_Responder	CD8(Tn)	pDC_LILRA4	BST2	LILRA4	2,44817E-06	18231,9	1,74198E-02	2817	5,89215E+00	116	3,11041E+00	1508	8,89081E-01	38337	0	48381,5
Myeloid_vs_CD8_Responder	Mono_CD14	CD8(Tn)	S100A8	CD69	2,75231E-06	10578,5	1,71481E-02	2901	4,25494E+00	323	4,23450E+00	177	9,58382E-01	1110	0	48381,5
Myeloid_vs_CD8_Responder	cDC_LAMP3	CD8(Tn)	CCL19	CCR7	3,01904E-06	18866,9	1,32289E-01	32	6,84454E+00	39	2,60175E+00	3935	8,82794E-01	41947	0	48381,5
Myeloid_vs_CD8_Responder	Mono_CD14	CD8(Temra)	S100A8	CD69	3,29059E-06	10636,1	1,66868E-02	3034	4,23635E+00	329	4,20331E+00	187	9,57835E-01	1249	0	48381,5
Myeloid_vs_CD8_Responder	CD8(Terminal Tex)	pDC_LILRA4	BST2	LILRA4	3,54337E-06	14224,9	3,51930E-02	590	6,23450E+00	91	3,53284E+00	658	9,19310E-01	21404	0	48381,5
Myeloid_vs_CD8_Responder	CD8(ITM2C+ T)	Macro_IFI27	TNFSF9	HLA-DPA1	4,06351E-06	11399,3	1,63586E-02	3133	2,49001E+00	3199	3,19474E+00	1280	9,58836E-01	1003	0	48381,5
Myeloid_vs_CD8_Responder	CD8(ITM2C+ T)	Macro_FOLR2+APOE+	TNFSF9	HLA-DPA1	4,09396E-06	11619,9	1,63351E-02	3142	2,48791E+00	3205	2,86968E+00	2359	9,58808E-01	1012	0	48381,5
Myeloid_vs_CD8_Responder	CD8(ITM2C+ T)	Macro_LYVE1	TNFSF9	HLA-DPA1	4,44024E-06	11611,9	1,61982E-02	3194	2,47567E+00	3271	2,92134E+00	2165	9,58641E-01	1048	0	48381,5
Myeloid_vs_CD8_Responder	CD8(ZNF683+KLRB1+ T)	cDC_CLEC9A	XCL1	XCR1	4,76515E-06	20211,9	1,10625E-01	49	8,29279E+00	16	2,08196E+00	9344	8,80577E-01	43269	0	48381,5
Myeloid_vs_CD8_Responder	CD8(ID2+CXCR4+ T)	cDC_CLEC9A	XCL1	XCR1	4,96157E-06	20105,1	1,10099E-01	50	8,29098E+00	17	2,12859E+00	8668	8,80326E-01	43409	0	48381,5
Myeloid_vs_CD8_Responder	CD8(ITM2C+ T)	Macro_FOLR2+APOE-	TNFSF9	HLA-DPA1	5,23461E-06	11610,3	1,58852E-02	3291	2,44771E+00	3409	3,01069E+00	1828	9,58253E-01	1142	0	48381,5
Myeloid_vs_CD8_Responder	CD8(ZNF683+KLRB1+ T)	cDC_CLEC9A	XCL2	XCR1	6,22332E-06	21154,5	1,04970E-01	56	8,27759E+00	18	2,02605E+00	10170	8,73989E-01	47147	0	48381,5
Myeloid_vs_CD8_Responder	Mono_CD14	CD8(Tn)	VCAN	SELL	6,35952E-06	14954,1	2,98217E-02	800	3,75637E+00	587	3,52103E+00	674	9,14355E-01	24328	0	48381,5
Myeloid_vs_CD8_Responder	CD8(ID2+CXCR4+ T)	cDC_CLEC9A	XCL2	XCR1	7,62776E-06	21343,5	1,01393E-01	62	8,26562E+00	19	2,03589E+00	10013	8,72068E-01	48242	0	48381,5
Myeloid_vs_CD8_Responder	CD8(ID2+CXCR4+ T)	cDC(CD1C)	B2M	CD1C	7,64471E-06	13204,1	2,22256E-02	1636	3,76836E+00	575	1,98144E+00	10899	9,49031E-01	4529	0	48381,5
Myeloid_vs_CD8_Responder	Mono_CD14	CD8(GZMK+ Tex)	S100A8	CD69	7,74934E-06	10950,7	1,47501E-02	3762	4,15831E+00	357	4,06444E+00	241	9,55272E-01	2012	0	48381,5
Myeloid_vs_CD8_Responder	CD8(ITM2C+ T)	Macro_FOLR2-APOE+	TNFSF9	HLA-DPA1	8,17604E-06	11897,1	1,51174E-02	3624	2,37911E+00	3813	2,89915E+00	2257	9,57251E-01	1410	0	48381,5
Myeloid_vs_CD8_Responder	Mono_CD14	CD8(GZMK+ Tem)	S100A8	CD69	8,50521E-06	11000,9	1,45215E-02	3851	4,14909E+00	361	3,98575E+00	283	9,54937E-01	2128	0	48381,5
Myeloid_vs_CD8_Responder	CD8(NME1+ T)	pDC_LILRA4	BST2	LILRA4	1,12136E-05	14867,7	3,11277E-02	735	6,15620E+00	96	3,33789E+00	967	9,14639E-01	24159	0	48381,5
Myeloid_vs_CD8_Responder	cDC_LAMP3	CD8(Terminal Tex)	CCL19	CXCR3	1,26968E-05	18775,1	8,96584E-02	80	6,62312E+00	47	2,14131E+00	8515	8,91655E-01	36852	0	48381,5
Myeloid_vs_CD8_Responder	CD8(Tn)	pDC_LILRA4	HMGB1	TLR9	1,35431E-05	27268,7	2,67508E-02	1030	3,55160E+00	756	1,04829E+00	39162	8,74196E-01	47014	0	48381,5
Myeloid_vs_CD8_Responder	CD8(ITM2C+ T)	cDC_LAMP3	TNFSF9	HLA-DPA1	1,36603E-05	12199,1	1,42706E-02	3962	2,30345E+00	4338	2,82324E+00	2553	9,56056E-01	1761	0	48381,5
Myeloid_vs_CD8_Responder	Mono_CD14	CD8(ITM2C+ T)	S100A8	CD69	1,38745E-05	11215,3	1,34735E-02	4355	4,10686E+00	381	3,96880E+00	297	9,53297E-01	2662	0	48381,5
Myeloid_vs_CD8_Responder	CD8(ITM2C+ T)	Macro_IER3	TNFSF9	HLA-DPA1	1,45978E-05	12368,3	1,41811E-02	3997	2,29544E+00	4411	2,70252E+00	3251	9,55923E-01	1801	0	48381,5
Myeloid_vs_CD8_Responder	CD8(GZMK+ Early Tem)	cDC_CLEC9A	XCL2	XCR1	1,50146E-05	21975,1	8,82552E-02	87	8,22162E+00	20	2,12926E+00	8659	8,64125E-01	52728	0	48381,5
Myeloid_vs_CD8_Responder	CD8(Temra)	cDC_CLEC9A	XCL2	XCR1	1,53616E-05	22682,3	8,78859E-02	88	8,22039E+00	21	1,91673E+00	12056	8,63878E-01	52865	0	48381,5
Myeloid_vs_CD8_Responder	pDC_LILRA4	CD8(Temra)	SCT	ADRB2	1,75265E-05	26617,7	8,93566E-02	81	6,17125E+00	94	2,78738E+00	2761	8,11592E-01	81771	0	48381,5
Myeloid_vs_CD8_Responder	pDC_LILRA4	CD8(ITM2C+ T)	SCT	ADRB2	1,79011E-05	26815,9	8,72796E-02	89	6,16058E+00	95	2,77539E+00	2822	8,09787E-01	82692	0	48381,5
Myeloid_vs_CD8_Responder	CD8(GZMK+ Tex)	Macro_LYVE1	HMGB1	CD163	1,98149E-05	16219,1	6,71237E-03	13186	2,88200E+00	1833	1,94929E+00	11471	9,45777E-01	6224	0	48381,5
Myeloid_vs_CD8_Responder	Mono_CD14	CD8(LAYN+ T)	S100A8	CD69	2,10441E-05	11443,1	1,25560E-02	4836	4,06989E+00	393	3,97357E+00	294	9,51702E-01	3311	0	48381,5
Myeloid_vs_CD8_Responder	CD8(Tn)	cDC_CLEC9A	FLT3LG	FLT3	2,14887E-05	33285,9	2,49052E-02	1202	4,44503E+00	262	1,60391E+00	18793	7,80004E-01	97791	0	48381,5
Myeloid_vs_CD8_Responder	pDC_LILRA4	CD8(Tc17)	SCT	ADRB2	2,22835E-05	27456,9	8,12421E-02	106	6,12956E+00	99	2,73508E+00	3067	8,04205E-01	85631	0	48381,5
Myeloid_vs_CD8_Responder	cDC_LAMP3	CD8(NME1+ T)	CCL19	CXCR3	2,27057E-05	19832,5	8,11861E-02	107	6,56508E+00	51	1,97749E+00	10966	8,86766E-01	39657	0	48381,5
Myeloid_vs_CD8_Responder	CD8(ISG+ T)	cDC(CD1C)	B2M	CD1C	2,37111E-05	12665,5	2,27839E-02	1525	3,89341E+00	493	2,12727E+00	8695	9,49628E-01	4233	0	48381,5
Myeloid_vs_CD8_Responder	CD8(ITM2C+ T)	Macro_ISG15	TNFSF9	HLA-DPA1	2,46438E-05	12387,9	1,32995E-02	4433	2,21668E+00	5032	3,00788E+00	1841	9,54551E-01	2252	0	48381,5
Myeloid_vs_CD8_Responder	CD8(Tn)	Mono_CD16	ANXA1	FPR1	2,51773E-05	18165,5	7,67993E-03	10587	2,14369E+00	5663	1,82198E+00	13833	9,35016E-01	12363	0	48381,5
Myeloid_vs_CD8_Responder	CD8(Terminal Tex)	Macro_LYVE1	HMGB1	CD163	2,79196E-05	16925,7	6,56417E-03	13690	2,85702E+00	1905	1,80709E+00	14122	9,45202E-01	6530	0	48381,5
Myeloid_vs_CD8_Responder	Macro_NLRP3	CD8(ZNF683+KLRB1+ T)	S100A8	CD69	2,81434E-05	12583,1	1,19662E-02	5203	2,38711E+00	3759	3,02179E+00	1777	9,50584E-01	3795	0	48381,5
Myeloid_vs_CD8_Responder	Mono_CD14	CD8(Temra)	S100A8	ITGB2	2,92112E-05	11216,1	1,19229E-02	5252	4,43503E+00	266	4,59295E+00	86	9,55031E-01	2095	0	48381,5
Myeloid_vs_CD8_Responder	CD8(Tn)	Macro_NLRP3	ANXA1	FPR1	3,02473E-05	19093,1	6,91109E-03	12575	1,95470E+00	7702	1,89526E+00	12457	9,31737E-01	14350	0	48381,5
Myeloid_vs_CD8_Responder	CD8(ID2+CXCR4+ T)	pDC_LILRA4	BST2	LILRA4	3,34686E-05	21469,1	1,16829E-02	5435	5,78165E+00	126	2,80194E+00	2671	8,67801E-01	50732	0	48381,5
Myeloid_vs_CD8_Responder	CD8(GZMK+ Early Tem)	cDC_CLEC9A	XCL1	XCR1	3,55983E-05	23419,9	7,35030E-02	134	8,16505E+00	22	1,92106E+00	11965	8,57355E-01	56597	0	48381,5
Myeloid_vs_CD8_Responder	CD8(IL7R+ZNF683+ Tm)	cDC(CD1C)	B2M	CD1C	3,58126E-05	14224,9	2,10462E-02	1889	3,50422E+00	796	1,77221E+00	14843	9,47696E-01	5215	0	48381,5
Myeloid_vs_CD8_Responder	CD8(Tn)	cDC(CD1C)	B2M	CD1C	3,59820E-05	14504,5	2,05390E-02	2005	3,39062E+00	919	1,73404E+00	15682	9,47088E-01	5535	0	48381,5
Myeloid_vs_CD8_Responder	Mono_CD14	CD8(Terminal Tex)	S100A8	ITGB2	3,91809E-05	11367,5	1,14046E-02	5655	4,40372E+00	277	4,49998E+00	108	9,54067E-01	2416	0	48381,5
Myeloid_vs_CD8_Responder	CD8(ITM2C+ T)	Macro_NLRP3	TNFSF9	HLA-DPA1	4,19180E-05	13034,1	1,23617E-02	4958	2,13289E+00	5752	2,69739E+00	3285	9,52938E-01	2794	0	48381,5
Myeloid_vs_CD8_Responder	Mono_CD14	CD8(GZMK+ Tem)	S100A8	ITGB2	4,19180E-05	11402,5	1,13015E-02	5752	4,39749E+00	282	4,45559E+00	116	9,53868E-01	2481	0	48381,5
Myeloid_vs_CD8_Responder	CD8(NME1+ T)	cDC_CLEC9A	XCL2	XCR1	4,28334E-05	24961,3	6,94808E-02	147	8,15876E+00	23	1,75667E+00	15161	8,49462E-01	61094	0	48381,5
Myeloid_vs_CD8_Responder	CD8(ID2+CXCR4+ T)	Mono_CD14	RPS19	C5AR1	4,59840E-05	17146,7	6,03641E-03	15604	2,13496E+00	5737	2,00448E+00	10521	9,47173E-01	5490	0	48381,5
Myeloid_vs_CD8_Responder	CD8(GZMK+ Early Tem)	cDC(CD1C)	B2M	CD1C	4,90851E-05	13825,9	2,14301E-02	1796	3,59022E+00	721	1,85300E+00	13242	9,48142E-01	4989	0	48381,5
Myeloid_vs_CD8_Responder	CD8(ID2+CXCR4+ T)	Macro_LYVE1	HMGB1	CD163	5,04189E-05	17644,9	6,27152E-03	14709	2,80769E+00	2034	1,72464E+00	15894	9,44009E-01	7206	0	48381,5
Myeloid_vs_CD8_Responder	CD8(IL7R+ZNF683+ Tm)	Mono_CD14	ANXA1	FPR1	5,09445E-05	20190,5	6,35304E-03	14404	1,80910E+00	9661	1,88893E+00	12579	9,29011E-01	15927	0	48381,5
Myeloid_vs_CD8_Responder	CD8(IL7R+ZNF683+ Tm)	cDC_CLEC9A	XCL2	XCR1	5,26545E-05	25276,9	6,67924E-02	163	8,14976E+00	24	1,74996E+00	15339	8,46922E-01	62477	0	48381,5
Myeloid_vs_CD8_Responder	Mono_CD14	CD8(NME1+ T)	S100A8	ITGB2	5,34431E-05	11534,7	1,08869E-02	6115	4,37244E+00	288	4,37049E+00	129	9,53039E-01	2760	0	48381,5
Myeloid_vs_CD8_Responder	cDC_LAMP3	CD8(ISG+ T)	CCL19	CXCR3	5,46085E-05	21222,9	6,54587E-02	166	6,45736E+00	60	1,95973E+00	11288	8,75497E-01	46219	0	48381,5
Myeloid_vs_CD8_Responder	CD8(Tc17)	Mono_CD14	RPS19	C5AR1	5,48833E-05	17800,5	5,89575E-03	16166	2,10458E+00	6025	1,88675E+00	12625	9,46580E-01	5805	0	48381,5
Myeloid_vs_CD8_Responder	CD8(Tn)	Mono_CD14	ANXA1	FPR1	5,52919E-05	19284,3	6,26888E-03	14721	1,79684E+00	9871	2,24258E+00	7258	9,28570E-01	16190	0	48381,5
Myeloid_vs_CD8_Responder	CD8(Tn)	pDC_LILRA4	TNF	PTPRS	5,54476E-05	31761,1	2,21687E-02	1651	3,61794E+00	694	1,94346E+00	11555	7,82605E-01	96524	0	48381,5

Myeloid_vs_CD8_Responder	CD8(Tn)	Macro_ISG15	ANXA1	FPR1	5,66367E-05	20398,7	6,24095E-03	14824	1,78997E+00	9979	1,89100E+00	12541	9,28421E-01	16268	0	48381,5
Myeloid_vs_CD8_Responder	cDC_LAMP3	CD8(ITM2C+ T)	CCL19	CXCR3	5,86230E-05	21760,9	6,48722E-02	172	6,45334E+00	61	1,82972E+00	13676	8,75006E-01	46514	0	48381,5
Myeloid_vs_CD8_Responder	CD8(Tc17)	cDC(CD1C)	B2M	CD1C	6,17188E-05	14662,5	2,06035E-02	1989	3,40508E+00	900	1,69693E+00	16550	9,47167E-01	5492	0	48381,5
Myeloid_vs_CD8_Responder	Macro_ISG15	CD8(Tc17)	CXCL10	DPP4	6,17875E-05	20150,3	9,50140E-02	69	2,95375E+00	1662	3,04404E+00	1712	8,70853E-01	48927	0	48381,5
Myeloid_vs_CD8_Responder	CD8(IL7R+ZNF683+ Tm)	Mono_CD14	RPS19	C5AR1	6,40082E-05	17905,9	5,77842E-03	16671	2,07924E+00	6286	1,91377E+00	12116	9,46070E-01	6075	0	48381,5
Myeloid_vs_CD8_Responder	CD8(ITM2C+ T)	Mono_CD16	TNFSF9	HLA-DPA1	6,45471E-05	13631,3	1,24296E-02	4909	2,13895E+00	5699	2,32302E+00	6413	9,53061E-01	2754	0	48381,5
Myeloid_vs_CD8_Responder	CD8(ITM2C+ T)	Mono_INHBA	TNFSF9	HLA-DPA1	6,70170E-05	13380,9	1,15543E-02	5540	2,06075E+00	6474	2,73485E+00	3069	9,51400E-01	3440	0	48381,5
Myeloid_vs_CD8_Responder	CD8(ISG+ T)	Macro_LYVE1	HMGB1	CD163	6,78806E-05	18322,5	5,92016E-03	16060	2,74847E+00	2198	1,68241E+00	16868	9,42466E-01	8105	0	48381,5
Myeloid_vs_CD8_Responder	CD8(Terminal Tex)	pDC_LILRA4	TNF	PTPRS	6,90934E-05	27441,3	3,24076E-02	689	3,69545E+00	637	2,31130E+00	6524	8,13174E-01	80975	0	48381,5
Myeloid_vs_CD8_Responder	CD8(ITM2C+ T)	cDC_CLEC9A	XCL2	XCR1	7,00228E-05	25912,5	6,08778E-02	188	8,12995E+00	25	1,75913E+00	15113	8,40814E-01	65855	0	48381,5
Myeloid_vs_CD8_Responder	CD8(ITM2C+ T)	pDC_LILRA4	TNF	PTPRS	7,11605E-05	18195,7	1,02151E-01	61	4,22344E+00	336	3,01704E+00	1795	8,85421E-01	40405	0	48381,5
Myeloid_vs_CD8_Responder	CD8(LAYN+ T)	cDC(CD1C)	B2M	CD1C	7,28490E-05	13829,5	2,13914E-02	1804	3,58155E+00	726	1,85406E+00	13227	9,48098E-01	5009	0	48381,5
Myeloid_vs_CD8_Responder	Macro_FOLR2+APOE+	CD8(Tc17)	APOE	LSR	7,47670E-05	15395,9	2,38545E-02	1332	2,88372E+00	1825	3,26996E+00	1107	9,14337E-01	24334	0	48381,5
Myeloid_vs_CD8_Responder	Macro_FOLR2+APOE+	CD8(Tn)	APOE	LSR	7,52569E-05	15365,7	2,37790E-02	1347	2,88254E+00	1829	3,39175E+00	866	9,14213E-01	24405	0	48381,5
Myeloid_vs_CD8_Responder	CD8(LAYN+ T)	Mono_CD14	RPS19	C5AR1	7,56592E-05	18757,3	5,65366E-03	17238	2,05230E+00	6571	1,75358E+00	15229	9,45510E-01	6367	0	48381,5
Myeloid_vs_CD8_Responder	CD8(GZMK+ Tex)	Mono_CD16	HLA-A	LILRB2	7,56812E-05	17186,3	5,90149E-03	16136	2,76286E+00	2154	1,66660E+00	17239	9,55246E-01	2021	0	48381,5
Myeloid_vs_CD8_Responder	CD8(NME1+ T)	cDC_CLEC9A	XCL1	XCR1	7,61013E-05	26311,9	6,01196E-02	196	8,11900E+00	27	1,58760E+00	19225	8,44618E-01	63730	0	48381,5
Myeloid_vs_CD8_Responder	Macro_FOLR2+APOE+	CD8(IL7R+ZNF683+ Tm)	APOE	LSR	7,83666E-05	15535,3	2,32133E-02	1439	2,87369E+00	1854	3,28404E+00	1074	9,13264E-01	24928	0	48381,5
Myeloid_vs_CD8_Responder	CD8(GZMK+ Tex)	pDC_LILRA4	BST2	LILRA4	8,00167E-05	16953,5	2,11330E-02	1867	5,96368E+00	110	3,19565E+00	1278	8,98257E-01	33131	0	48381,5
Myeloid_vs_CD8_Responder	CD8(Terminal Tex)	cDC_CLEC9A	XCL2	XCR1	8,32412E-05	26054,7	5,86044E-02	205	8,12234E+00	26	1,79522E+00	14370	8,38251E-01	67291	0	48381,5
Myeloid_vs_CD8_Responder	cDC_LAMP3	CD8(GZMK+ Tem)	CCL19	CXCR3	8,73457E-05	22420,1	5,84421E-02	210	6,40929E+00	69	1,83708E+00	13543	8,69185E-01	49897	0	48381,5
Myeloid_vs_CD8_Responder	Macro_NLRP3	CD8(Tc17)	S100A8	CD69	8,74287E-05	14105,1	1,00437E-02	6923	2,23158E+00	4923	2,54687E+00	4358	9,46305E-01	5940	0	48381,5
Myeloid_vs_CD8_Responder	CD8(GZMK+ Early Tem)	Macro_OLFML3	TNFSF9	HLA-DPA1	9,17556E-05	20111,9	5,51029E-03	17916	1,50191E+00	15785	2,62459E+00	3777	9,31125E-01	14700	0	48381,5
Myeloid_vs_CD8_Responder	CD8(Terminal Tex)	Mono_CD16	HLA-C	LILRB2	9,20890E-05	17414,9	5,84750E-03	16374	2,78595E+00	2092	1,63881E+00	17929	9,54393E-01	2298	0	48381,5
Myeloid_vs_CD8_Responder	CD8(GZMK+ Tex)	Mono_CD16	HLA-C	LILRB2	9,28103E-05	17593,9	5,72853E-03	16915	2,72946E+00	2264	1,63805E+00	17957	9,53944E-01	2452	0	48381,5
Myeloid_vs_CD8_Responder	Mono_CD14	CD8(NME1+ T)	S100A8	CD69	9,59299E-05	12516,7	9,87599E-03	7087	3,96189E+00	456	3,70782E+00	484	9,45875E-01	6175	0	48381,5
Myeloid_vs_CD8_Responder	CD8(Tn)	pDC_LILRA4	HSP90B1	TLR9	1,00899E-04	31432,5	2,04987E-02	2018	3,60668E+00	707	1,11373E+00	36160	8,33694E-01	69896	0	48381,5
Myeloid_vs_CD8_Responder	Mono_CD14	CD8(ID2+CXCR4+ T)	S100A8	ITGB2	1,02586E-04	11971,5	9,75884E-03	7208	4,30429E+00	313	4,33823E+00	136	9,50529E-01	3819	0	48381,5
Myeloid_vs_CD8_Responder	CD8(GZMK+ Tex)	cDC_CLEC9A	XCL2	XCR1	1,02938E-04	26443,3	5,60307E-02	228	8,11372E+00	28	1,78584E+00	14533	8,35183E-01	69046	0	48381,5
Myeloid_vs_CD8_Responder	CD8(Terminal Tex)	Mono_CD16	HLA-A	LILRB2	1,03590E-04	17386,7	5,91774E-03	16072	2,77138E+00	2127	1,62233E+00	18356	9,55304E-01	1997	0	48381,5
Myeloid_vs_CD8_Responder	CD8(Temra)	cDC(CD1C)	B2M	CD1C	1,08975E-04	13057,1	2,22844E-02	1623	3,78155E+00	562	2,02320E+00	10217	9,49095E-01	4502	0	48381,5
Myeloid_vs_CD8_Responder	CD8(Terminal Tex)	Mono_CD16	B2M	LILRB2	1,11137E-04	17533,9	5,38188E-03	18616	2,83543E+00	1960	1,63825E+00	17950	9,59788E-01	762	0	48381,5
Myeloid_vs_CD8_Responder	cDC_LAMP3	CD8(ISG+ T)	CCL19	CCR7	1,11212E-04	26526,1	5,44025E-02	237	6,42258E+00	68	1,96810E+00	11148	8,28476E-01	72796	0	48381,5
Myeloid_vs_CD8_Responder	CD8(Tn)	Mono_CD14	RPS19	C5AR1	1,13667E-04	18984,5	5,36228E-03	18700	1,98937E+00	7291	1,84281E+00	13427	9,44131E-01	7123	0	48381,5
Myeloid_vs_CD8_Responder	CD8(GZMK+ Early Tem)	Mono_CD14	RPS19	C5AR1	1,14124E-04	19081,3	5,36048E-03	18715	1,98898E+00	7299	1,81944E+00	13880	9,44122E-01	7131	0	48381,5
Myeloid_vs_CD8_Responder	CD8(GZMK+ Early Tem)	cDC_CLEC9A	TNFSF9	HLA-DPA1	1,14185E-04	20153,7	5,36009E-03	18717	1,45495E+00	17054	3,15124E+00	1394	9,30233E-01	15222	0	48381,5
Myeloid_vs_CD8_Responder	Macro_NLRP3	CD8(EOMES+ NK-like)	S100A8	CD69	1,16676E-04	14410,9	9,54119E-03	7446	2,19093E+00	5248	2,55251E+00	4316	9,44986E-01	6663	0	48381,5
Myeloid_vs_CD8_Responder	CD8(GZMK+ Tem)	pDC_LILRA4	BST2	LILRA4	1,17529E-04	17351,9	2,00039E-02	2124	5,94193E+00	112	3,08023E+00	1599	8,95720E-01	34543	0	48381,5
Myeloid_vs_CD8_Responder	cDC_LAMP3	CD8(GZMK+ Tex)	CCL19	CXCR3	1,17868E-04	22717,5	5,41934E-02	244	6,38019E+00	73	1,89085E+00	12543	8,64834E-01	52346	0	48381,5
Myeloid_vs_CD8_Responder	CD8(ID2+CXCR4+ T)	Macro_NLRP3	RPS19	C5AR1	1,18708E-04	17412,3	6,93824E-03	12511	2,42950E+00	3497	1,60127E+00	18863	9,50550E-01	3809	0	48381,5
Myeloid_vs_CD8_Responder	cDC_LAMP3	CD8(IL7R+ZNF683+ Tm)	CCL19	CXCR3	1,22741E-04	22874,1	5,36408E-02	249	6,37641E+00	74	1,86674E+00	12999	8,64234E-01	52667	0	48381,5
Myeloid_vs_CD8_Responder	Macro_NLRP3	CD8(GZMK+ Early Tem)	S100A8	CD69	1,22748E-04	14527,5	9,46639E-03	7542	2,18488E+00	5294	2,50968E+00	4648	9,44781E-01	6772	0	48381,5
Myeloid_vs_CD8_Responder	CD8(GZMK+ Tex)	Mono_CD16	B2M	LILRB2	1,29327E-04	17967,5	5,27475E-03	19189	2,73213E+00	2255	1,59002E+00	19152	9,59398E-01	860	0	48381,5
Myeloid_vs_CD8_Responder	CD8(ISG+ T)	Mono_CD16	B2M	LILRB2	1,30817E-04	17950,9	5,29157E-03	19101	2,74835E+00	2199	1,58719E+00	19233	9,59460E-01	840	0	48381,5
Myeloid_vs_CD8_Responder	CD8(GZMK+ Early Tem)	cDC(CD1C)	TNFSF9	HLA-DPA1	1,33387E-04	20509,3	5,25704E-03	19308	1,42273E+00	17995	3,20694E+00	1246	9,29601E-01	15616	0	48381,5
Myeloid_vs_CD8_Responder	CD8(Terminal Tex)	cDC_CLEC9A	XCL1	XCR1	1,33806E-04	26476,7	5,30332E-02	260	8,09461E+00	29	1,75329E+00	15237	8,36209E-01	68476	0	48381,5
Myeloid_vs_CD8_Responder	CD8(Temra)	Macro_LYVE1	HMGB1	CD163	1,35368E-04	19047,7	5,62025E-03	17388	2,69792E+00	2374	1,62724E+00	18220	9,41040E-01	8875	0	48381,5
Myeloid_vs_CD8_Responder	CD8(ISG+ T)	Mono_CD16	HLA-B	LILRB2	1,37195E-04	17811,7	5,62021E-03	17390	2,74525E+00	2207	1,58014E+00	19417	9,56426E-01	1663	0	48381,5
Myeloid_vs_CD8_Responder	Macro_NLRP3	CD8(IL7R+ZNF683+ Tm)	S100A8	CD69	1,38043E-04	14809,7	9,28570E-03	7769	2,17027E+00	5419	2,42017E+00	5439	9,44276E-01	7040	0	48381,5
Myeloid_vs_CD8_Responder	CD8(Temra)	Mono_CD16	HLA-C	LILRB2	1,38650E-04	17562,5	5,78556E-03	16640	2,75654E+00	2171	1,62646E+00	18242	9,54161E-01	2378	0	48381,5
Myeloid_vs_CD8_Responder	Mono_CD14	CD8(LAYN+ T)	VCAN	CD44	1,45285E-04	13077,3	1,37445E-02	4208	3,45022E+00	846	2,58344E+00	4081	9,42874E-01	7870	0	48381,5
Myeloid_vs_CD8_Responder	CD8(ID2+CXCR4+ T)	Mono_CD14	ANXA1	FPR1	1,51614E-04	23928,9	5,24742E-03	19361	1,64809E+00	12439	1,56534E+00	19809	9,22442E-01	19654	0	48381,5
Myeloid_vs_CD8_Responder	CD8(LAYN+ T)	Macro_LYVE1	HMGB1	CD163	1,52074E-04	19489,7	5,54644E-03	17746	2,68548E+00	2416	1,56496E+00	19821	9,40672E-01	9084	0	48381,5
Myeloid_vs_CD8_Responder	CD8(NME1+ T)	Macro_LYVE1	HMGB1	CD163	1,52125E-04	13015,3	9,15197E-03	7962	3,29320E+00	1044	2,46932E+00	4986	9,53199E-01	2703	0	48381,5
Myeloid_vs_CD8_Responder	cDC_LAMP3	CD8(IL7R+ZNF683+ Tm)	CCL19	CCR7	1,54040E-04	27523,5	5,10563E-02	279	6,40445E+00	71	1,83257E+00	13630	8,23919E-01	75256	0	48381,5
Myeloid_vs_CD8_Responder	cDC_LAMP3	CD8(Tc17)	CCL22	DPP4	1,55144E-04	26531,5	1,83694E-01	15	4,40143E+00	280	2,45305E+00	5125	8,17169E-01	78856	0	48381,5
Myeloid_vs_CD8_Responder	Macro_FOLR2+APOE-	CD8(Terminal Tex)	CCL13	CXCR3	1,59601E-04	27264,5	6,83901E-02	154	4,38076E+00	284	1,40509E+00	24740	8,46372E-01	62763	0	48381,5
Myeloid_vs_CD8_Responder	CD8(IL7R+ZNF683+ Tm)	cDC(CD1C)	ANXA1	FPR1	1,61104E-04	23492,5	5,15110E-03	19914	1,51756E+00	15401	1,82753E+00	13715	9,21776E-01	20051	0	48381,5
Myeloid_vs_CD8_Responder	CD8(ID2+CXCR4+ T)	Mono_CD16	HLA-B	LILRB2	1,68342E-04	17926,7	5,65137E-03	17253	2,76501E+00	2148	1,55118E+00	20228	9,56541E-01	1623	0	48381,5
Myeloid_vs_CD8_Responder	Macro_ISG15	CD8(Terminal Tex)	CXCL10	CXCR3	1,69741E-04	15629,9	3,45039E-02	619	2,68960E+00	2403	2,98133E+00	1940	9,13450E-01	24806	0	48381,5
Myeloid_vs_CD8_Responder	CD8(Terminal Tex)	Macro_OLFML3	IFNG	IFNGR1_IFNGR2	1,70097E-04	20858,7	1,24286E-02	4910	1,58734E+00	13687	1,67469E+00	17045	9,21384E-01	20270	0	48381,5
Myeloid_vs_CD8_Responder	CD8(Tn)	cDC(CD1C)	ANXA1	FPR1	1,72927E-04	22536,9	5,08287E-03	20337	1,50530E+00	15696	2,18117E+00	7958	9,21294E-01	20312	0	48381,5
Myeloid_vs_CD8_Responder	Macro_NLRP3	CD8(ISG+ T)	S100A8	CD69	1,73254E-04	14757,3	8,96652E-03	8228	2,14445E+00	5659	2,60464E+00	3915	9,43348E-01	7603	0	48381,5
Myeloid_vs_CD8_Responder	CD8(GZMK+ Tex)	cDC_CLEC9A	XCL1	XCR1	1,76872E-04	27875,9	4,94199E-02	299	8,08218E+00	30	1,57836E+00	19458	8,31319E-01	71211	0	48381,5
Myeloid_vs_CD8_Responder	CD8(IL7R+ZNF683+ Tm)	Macro_NLRP3	ANXA1	FPR1	1,78394E-04	20562,9	7,00386E-03	12313	1,96696E+00	7556	1,54161E+00	20464	9,32160E-01	14100	0	48381,5
Myeloid_vs_CD8_Responder	Mono_INHBA	CD8(Tc17)	CXCL2	DPP4	1,80035E-04	19444,5	5,43911E-02	238	3,14432E+00	1280	2,84604E+00	2451	8,77797E-01	44872	0	48381,5
Myeloid_vs_CD8_Responder	Macro_FOLR2+APOE-	CD8(NME1+ T)	CCL13	CXCR3	1,81626E-04	29238,7	6,19276E-02	185	4,32272E+00	303	1,24128E+00	30885	8,39807E-01	66439	0	48381,5
Myeloid_vs_CD8_Responder	CD8(NME1+ T)	pDC_LILRA4	HMGB1	TLR9	1,82825E-04	17364,9	5,31600E-02	258	4,31793E+00	304	2,05479E+00	9739	9,07371E-01	28142	0	48381,5
Myeloid_vs_CD8_Responder	CD8(IL7R+ZNF683+ Tm)	Macro_ISG15	ANXA1	FPR1	1,83508E-04	21851,3	6,32473E-03	14507	1,80223E+00	9778	1,53735E+00	20580	9,28863E-01	16010	0	48381,5
Myeloid_vs_CD8_Responder	Macro_NLRP3	CD8(ID2+CXCR4+ T)	S100A8	CD69	1,86178E-04	15378,1	8,85397E-03	8379	2,13534E+00	5729	2,30415E+00	6611	9,43010E-01	7790	0	48381,5
Myeloid_vs_CD8_Responder	CD8(Tn)	Mast	HSPA8	ADRB2	1,87919E-04	21419,9	1,01524E-02	6810	1,33229E+00	20678	1,64882E+00	17674	9,33075E-01	13556	0	48381,5
Myeloid_vs_CD8_Responder	CD8(ITM2C+ T)	pDC_LILRA4	BST2	LILRA4	1,88020E-04	17867,1	1,85418E-02	2487	5,91376E+00	115	3,01949E+00	1788	8,92122E-01	36564	0	48381,5
Myeloid_vs_CD8_Responder	CD8(GZMK+ Tex)	Macro_FOLR2+APOE-	HMGB1	CD163	1,88101E-04	19883,3	5,02638E-03	20682	2,17590E+00	5374	1,80022E+00	14261	9,37864E-01	10718	0	48381,5
Myeloid_vs_CD8_Responder	Macro_FOLR2+APOE+	CD8(EOMES+ NK-like)	APOE	LSR	1,89374E-04	16838,9	1,85259E-02	2493	2,80039E+00	2055	3,20244E+00	1258	9,03905E-01	30007	0	48381,5
Myeloid_vs_CD8_Responder	CD8(Terminal Tex)	Mono_CD16	HLA-B	LILRB2	1,91398E-04	18146,5	5,57600E-03	17586	2,71722E+00	2304	1,53172E+00	20754	9,56261E-01	1707	0	48381,5
Myeloid_vs_CD8_Responder	CD8(GZMK+ Tem)	cDC(CD1C)	B2M	CD1C	1,95865E-04	12966,3	2,24642E-02	1591	3,82180E+00	533	2,04397E+00	9919	9,49289E-01	4407	0	48381,5
Myeloid_vs_CD8_Responder	CD8(GZMK+ Tem)	Mono_CD14	RPS19	C5AR1	2,03691E-04	19918,1	5,01863E-03	20727	1,91515E+00	8203	1,80708E+00	14123	9,42358E-01	8156	0	48381,5

Myeloid_vs_CD8_Responder	CD8(ISG+ T)	Mono_CD16	HLA-A	LILRB2	2,04710E-04	18228,1	5,71318E-03	16988	2,66405E+00	2483	1,52204E+00	21035	9,54547E-01	2253	0	48381,5
Myeloid_vs_CD8_Responder	Macro_OLFML3	CD8(Temra)	C3	IFITM1	2,05235E-04	13565,7	1,45875E-02	3828	2,56261E+00	2895	2,13585E+00	8588	9,49852E-01	4136	0	48381,5
Myeloid_vs_CD8_Responder	Macro_NLRP3	CD8(Terminal Tex)	S100A8	CD69	2,05802E-04	15028,3	8,71978E-03	8594	2,12449E+00	5819	2,55174E+00	4321	9,42598E-01	8026	0	48381,5
Myeloid_vs_CD8_Responder	CD8(ISG+ T)	Mono_CD16	HLA-C	LILRB2	2,08485E-04	18434,3	5,58405E-03	17553	2,66086E+00	2496	1,51986E+00	21112	9,53379E-01	2629	0	48381,5
Myeloid_vs_CD8_Responder	Macro_FOLR2+APOE-	CD8(NME1+ T)	CCL13	CCR5	2,08901E-04	39160,1	5,85014E-02	208	4,24926E+00	325	1,18038E+00	33295	7,45049E-01	113591	0	48381,5
Myeloid_vs_CD8_Responder	CD8(Tn)	Macro_OLFML3	CD40LG	CD40	2,08901E-04	33588,9	5,21739E-02	271	1,83096E+00	9361	1,29768E+00	28696	8,12642E-01	81235	0	48381,5
Myeloid_vs_CD8_Responder	cDC_LAMP3	CD8(LAYN+ T)	CCL19	CXCR3	2,08901E-04	24575,5	4,80932E-02	325	6,33841E+00	78	1,64787E+00	17695	8,57701E-01	56398	0	48381,5
Myeloid_vs_CD8_Responder	CD8(ISG+ T)	Mono_CD16	HLA-F	LILRB2	2,09276E-04	20337,5	7,28934E-03	11572	2,69216E+00	2393	1,62776E+00	18213	9,19796E-01	21128	0	48381,5
Myeloid_vs_CD8_Responder	CD8(ITM2C+ T)	pDC_LILRA4	TNF	TNFRSF21	2,09483E-04	17930,7	4,06354E-02	439	4,07887E+00	390	2,81978E+00	2579	8,89837E-01	37864	0	48381,5
Myeloid_vs_CD8_Responder	CD8(ITM2C+ T)	pDC_LILRA4	TNFSF9	HLA-DPA1	2,14458E-04	14231,9	9,50710E-03	7499	1,87783E+00	8684	3,39305E+00	860	9,46688E-01	5735	0	48381,5
Myeloid_vs_CD8_Responder	Macro_ISG15	CD8(NME1+ T)	CXCL10	CXCR3	2,15830E-04	16259,3	3,12434E-02	730	2,63157E+00	2605	2,81751E+00	2594	9,09445E-01	26986	0	48381,5
Myeloid_vs_CD8_Responder	CD8(Temra)	Mono_CD14	RPS19	C5AR1	2,18241E-04	19831,3	4,93208E-03	21306	1,89646E+00	8449	1,88735E+00	12613	9,41884E-01	8407	0	48381,5
Myeloid_vs_CD8_Responder	Macro_FOLR2+APOE-	CD8(Terminal Tex)	CCL13	CCR5	2,19289E-04	39392,9	5,42902E-02	241	4,22488E+00	333	1,22793E+00	31410	7,37889E-01	116599	0	48381,5
Myeloid_vs_CD8_Responder	Mono_CD14	CD8(ISG+ T)	S100A8	ITGB2	2,20606E-04	12703,3	8,43892E-03	9040	4,22455E+00	334	4,22824E+00	180	9,46999E-01	5581	0	48381,5
Myeloid_vs_CD8_Responder	CD8(IL7R+ZNF683+ Tm)	Macro_NLRP3	RPS19	C5AR1	2,21332E-04	18258,9	6,64171E-03	13411	2,37378E+00	3840	1,51056E+00	21366	9,49513E-01	4296	0	48381,5
Myeloid_vs_CD8_Responder	Mono_CD14	CD8(IL7R+ZNF683+ Tm)	VCAN	CD44	2,22577E-04	13284,1	1,29385E-02	4625	3,39425E+00	917	2,63162E+00	3731	9,41225E-01	8766	0	48381,5
Myeloid_vs_CD8_Responder	Macro_IFI27	CD8(ISG+ T)	C3	IFITM1	2,22979E-04	15134,7	1,13093E-02	5744	1,87133E+00	8770	2,44201E+00	5230	9,43431E-01	7548	0	48381,5
Myeloid_vs_CD8_Responder	CD8(Terminal Tex)	Macro_FOLR2+APOE-	HMGB1	CD163	2,24300E-04	20783,5	4,91540E-03	21423	2,15092E+00	5595	1,65802E+00	17437	9,37211E-01	11081	0	48381,5
Myeloid_vs_CD8_Responder	Mono_CD14	CD8(GZMK+ Tex)	S100A8	ITGB2	2,27246E-04	12749,5	8,36790E-03	9178	4,22026E+00	339	4,28018E+00	161	9,46786E-01	5688	0	48381,5
Myeloid_vs_CD8_Responder	Mono_CD14	CD8(GZMK+ Early Tem)	S100A8	ITGB2	2,28585E-04	12752,3	8,36765E-03	9179	4,22024E+00	340	4,24530E+00	172	9,46785E-01	5689	0	48381,5
Myeloid_vs_CD8_Responder	CD8(GZMK+ Early Tem)	Mono_CD16	HLA-B	LILRB2	2,29531E-04	18475,1	5,47435E-03	18126	2,65277E+00	2530	1,50553E+00	21522	9,55875E-01	1816	0	48381,5
Myeloid_vs_CD8_Responder	Macro_FOLR2+APOE-	CD8(ISG+ T)	CCL13	CXCR3	2,29929E-04	31083,1	4,99309E-02	293	4,21500E+00	341	1,22351E+00	31592	8,24788E-01	74808	0	48381,5
Myeloid_vs_CD8_Responder	Macro_FOLR2+APOE-	CD8(ITM2C+ T)	CCL13	CXCR3	2,32628E-04	32238,3	4,94835E-02	298	4,21098E+00	343	1,09350E+00	37037	8,24137E-01	75132	0	48381,5
Myeloid_vs_CD8_Responder	Macro_NLRP3	CD8(Tn)	S100A8	CD69	2,32890E-04	15360,5	8,54222E-03	8867	2,11012E+00	5970	2,43794E+00	5260	9,42039E-01	8324	0	48381,5
Myeloid_vs_CD8_Responder	CD8(Terminal Tex)	pDC_LILRA4	TNF	TNFRSF21	2,34347E-04	28023,1	1,28917E-02	4660	3,55089E+00	757	2,11404E+00	8881	8,19809E-01	77436	0	48381,5
Myeloid_vs_CD8_Responder	Macro_FOLR2+APOE-	CD8(ISG+ T)	CCL13	CCR5	2,36706E-04	40414,1	4,96433E-02	295	4,19797E+00	346	1,18849E+00	32989	7,29144E-01	120059	0	48381,5
Myeloid_vs_CD8_Responder	CD8(Tc17)	cDC_CLEC9A	FLT3LG	FLT3	2,36706E-04	27302,5	4,64469E-02	346	4,64730E+00	208	1,76441E+00	14995	8,28823E-01	72582	0	48381,5
Myeloid_vs_CD8_Responder	Mono_CD14	CD8(Tc17)	VCAN	CD44	2,37176E-04	13380,3	1,28239E-02	4693	3,38629E+00	926	2,59387E+00	3993	9,40978E-01	8908	0	48381,5
Myeloid_vs_CD8_Responder	CD8(ID2+CXCR4+ T)	pDC_LILRA4	HSP90B1	TLR9	2,37986E-04	30936,3	2,25585E-02	1574	3,64676E+00	675	1,07534E+00	37866	8,40226E-01	66185	0	48381,5
Myeloid_vs_CD8_Responder	Mono_CD14	CD8(ITM2C+ T)	S100A8	ITGB2	2,38074E-04	13099,3	7,90595E-03	10104	4,19235E+00	347	4,14209E+00	210	9,45337E-01	6454	0	48381,5
Myeloid_vs_CD8_Responder	CD8(ID2+CXCR4+ T)	pDC_LILRA4	HMGB1	TLR9	2,38776E-04	23060,3	3,64287E-02	547	3,83243E+00	525	1,31011E+00	28205	8,90219E-01	37643	0	48381,5
Myeloid_vs_CD8_Responder	Macro_FOLR2+APOE-	CD8(GZMK+ Tem)	CCL13	CCR5	2,39445E-04	40859,5	4,79868E-02	327	4,18838E+00	348	1,16618E+00	33881	7,25780E-01	121360	0	48381,5
Myeloid_vs_CD8_Responder	CD8(GZMK+ Tem)	Macro_LYVE1	HMGB1	CD163	2,41504E-04	18609,7	5,82892E-03	16453	2,73309E+00	2250	1,65026E+00	17641	9,42043E-01	8323	0	48381,5
Myeloid_vs_CD8_Responder	CD8(Tc17)	Mono_CD14	ANXA1	FPR1	2,41893E-04	24552,3	5,15430E-03	19891	1,63453E+00	12703	1,49793E+00	21749	9,21799E-01	20037	0	48381,5
Myeloid_vs_CD8_Responder	CD8(Tn)	Mast	CD40LG	CD9	2,43983E-04	22956,9	6,46552E-02	175	2,06358E+00	6446	2,10770E+00	8972	8,67660E-01	50810	0	48381,5
Myeloid_vs_CD8_Responder	CD8(GZMK+ Tem)	Mono_CD16	HLA-B	LILRB2	2,46601E-04	17363,7	5,73345E-03	16894	2,81704E+00	2010	1,63643E+00	17999	9,56840E-01	1534	0	48381,5
Myeloid_vs_CD8_Responder	CD8(GZMK+ Tem)	Mono_CD16	HLA-A	LILRB2	2,47110E-04	17361,5	5,95006E-03	15946	2,78834E+00	2086	1,61927E+00	18434	9,55420E-01	1960	0	48381,5
Myeloid_vs_CD8_Responder	CD8(GZMK+ Tem)	Mono_CD16	HLA-C	LILRB2	2,47392E-04	18339,9	5,59766E-03	17484	2,66732E+00	2478	1,53204E+00	20746	9,53434E-01	2610	0	48381,5
Myeloid_vs_CD8_Responder	CD8(GZMK+ Tem)	Mono_CD16	HLA-F	LILRB2	2,47902E-04	20681,5	7,17322E-03	11862	2,68183E+00	2422	1,58539E+00	19285	9,19202E-01	21457	0	48381,5
Myeloid_vs_CD8_Responder	CD8(GZMK+ Tem)	Mono_CD16	B2M	LILRB2	2,48243E-04	18566,7	5,21731E-03	19543	2,67674E+00	2438	1,50389E+00	21560	9,59185E-01	911	0	48381,5
Myeloid_vs_CD8_Responder	CD8(Terminal Tex)	Mono_CD16	HLA-F	LILRB2	2,51783E-04	20752,7	7,03240E-03	12238	2,66930E+00	2470	1,60540E+00	18750	9,18463E-01	21924	0	48381,5
Myeloid_vs_CD8_Responder	Mono_CD14	CD8(GZMK+ Early Tem)	VCAN	CD44	2,52144E-04	13329,9	1,27099E-02	4761	3,37837E+00	944	2,66378E+00	3516	9,40729E-01	9047	0	48381,5
Myeloid_vs_CD8_Responder	Mono_CD14	CD8(Temra)	S100A9	ITGB2	2,58369E-04	12227,1	8,40923E-03	9103	3,94229E+00	463	4,07974E+00	227	9,52520E-01	2961	0	48381,5
Myeloid_vs_CD8_Responder	CD8(Temra)	Macro_NLRP3	RPS19	C5AR1	2,65570E-04	19856,7	5,66893E-03	17171	2,19100E+00	5247	1,48414E+00	22159	9,45579E-01	6325	0	48381,5
Myeloid_vs_CD8_Responder	CD8(Tc17)	Macro_NLRP3	RPS19	C5AR1	2,66890E-04	18258,1	6,77657E-03	12976	2,39912E+00	3681	1,48354E+00	22181	9,49993E-01	4071	0	48381,5
Myeloid_vs_CD8_Responder	CD8(Temra)	Mono_CD16	B2M	LILRB2	2,67493E-04	18777,9	5,17557E-03	19771	2,63649E+00	2594	1,48312E+00	22191	9,59027E-01	952	0	48381,5
Myeloid_vs_CD8_Responder	Macro_FOLR2+APOE-	CD8(GZMK+ Tem)	CCL13	CXCR3	2,69144E-04	33014,7	4,45788E-02	369	4,16693E+00	354	1,10087E+00	36731	8,16444E-01	79238	0	48381,5
Myeloid_vs_CD8_Responder	Macro_NLRP3	CD8(Tn)	VCAN	SELL	2,69796E-04	16862,9	2,30482E-02	1469	3,01620E+00	1523	2,77822E+00	2808	9,03713E-01	30133	0	48381,5
Myeloid_vs_CD8_Responder	Mono_CD14	CD8(EOMES+ NK-like)	S100A8	ITGB2	2,74997E-04	14266,5	6,66891E-03	13317	4,11761E+00	373	4,07910E+00	228	9,40770E-01	9033	0	48381,5
Myeloid_vs_CD8_Responder	CD8(Temra)	Mono_CD16	CD99	PILRA	2,76161E-04	20691,9	6,86977E-03	12695	2,25759E+00	4704	1,47902E+00	22333	9,30044E-01	15346	0	48381,5
Myeloid_vs_CD8_Responder	Macro_NLRP3	CD8(Temra)	S100A8	CD69	2,78678E-04	15619,9	8,31244E-03	9279	2,09154E+00	6169	2,40674E+00	5544	9,41290E-01	8726	0	48381,5
Myeloid_vs_CD8_Responder	cDC_LAMP3	CD8(Tn)	CCL22	CCR4	2,79428E-04	30321,7	1,77160E-01	17	4,11478E+00	376	2,37509E+00	5862	7,81702E-01	96972	0	48381,5
Myeloid_vs_CD8_Responder	Mono_CD14	CD8(ZNF683+KLRB1+ T)	S100A8	ITGB2	2,83894E-04	14470,3	6,49854E-03	13920	4,10732E+00	379	4,06700E+00	239	9,40045E-01	9432	0	48381,5
Myeloid_vs_CD8_Responder	Macro_OLFML3	CD8(ITM2C+ T)	C3	IFITM1	2,87927E-04	13807,9	1,42776E-02	3956	2,54211E+00	2968	2,08112E+00	9356	9,49339E-01	4378	0	48381,5
Myeloid_vs_CD8_Responder	CD8(IL7R+ZNF683+ Tm)	Mono_CD16	ANXA1	FPR1	2,97111E-04	19821,1	7,78302E-03	10368	2,15595E+00	5554	1,46833E+00	22662	9,35420E-01	12140	0	48381,5
Myeloid_vs_CD8_Responder	CD8(Tn)	Macro_OLFML3	CD40LG	CD9	3,00570E-04	30288,7	4,31598E-02	390	1,49925E+00	15852	1,48915E+00	22010	8,42685E-01	64810	0	48381,5
Myeloid_vs_CD8_Responder	CD8(GZMK+ Tex)	Mono_CD16	HLA-F	LILRB2	3,02925E-04	20744,3	6,77235E-03	12992	2,64617E+00	2554	1,67470E+00	17044	9,17041E-01	22750	0	48381,5
Myeloid_vs_CD8_Responder	CD8(Temra)	cDC_CLEC9A	XCL1	XCR1	3,03653E-04	29183,7	4,30809E-02	392	8,06037E+00	32	1,53789E+00	20559	8,21475E-01	76554	0	48381,5
Myeloid_vs_CD8_Responder	CD8(ID2+CXCR4+ T)	Mono_CD16	HLA-A	LILRB2	3,07709E-04	18732,1	5,67109E-03	17164	2,64196E+00	2573	1,44968E+00	23241	9,54387E-01	2301	0	48381,5
Myeloid_vs_CD8_Responder	CD8(ID2+CXCR4+ T)	Macro_FOLR2+APOE-	HMGB1	CD163	3,10319E-04	21743,7	4,69626E-03	22860	2,10159E+00	6058	1,57557E+00	19525	9,35855E-01	11894	0	48381,5
Myeloid_vs_CD8_Responder	CD8(IL7R+ZNF683+ Tm)	Macro_LYVE1	HMGB1	CD163	3,11475E-04	20819,7	5,15512E-03	19887	2,61952E+00	2651	1,46181E+00	22877	9,38597E-01	10302	0	48381,5
Myeloid_vs_CD8_Responder	CD8(ID2+CXCR4+ T)	Mono_CD16	B2M	LILRB2	3,13036E-04	19071,3	5,16189E-03	19852	2,62330E+00	2630	1,44136E+00	23526	9,58975E-01	967	0	48381,5
Myeloid_vs_CD8_Responder	Macro_OLFML3	CD8(Terminal Tex)	B2M	CD3D	3,15102E-04	43360,5	2,91632E-03	45411	6,80644E-01	54146	5,60928E-01	68862	9,66809E-01	2	0	48381,5
Myeloid_vs_CD8_Responder	CD8(Tn)	Macro_OLFML3	ANXA1	FPR1	3,19660E-04	24977,3	5,26078E-03	19285	1,54904E+00	14615	1,45829E+00	22996	9,22532E-01	19609	0	48381,5
Myeloid_vs_CD8_Responder	CD8(GZMK+ Early Tem)	Macro_LYVE1	HMGB1	CD163	3,23572E-04	21194,9	4,98842E-03	20921	2,59143E+00	2769	1,45641E+00	23052	9,37643E-01	10851	0	48381,5
Myeloid_vs_CD8_Responder	pDC_LILRA4	CD8(ITM2C+ T)	APP	RPSA	3,25086E-04	14522,9	1,17347E-02	5396	2,17640E+00	5368	2,61894E+00	3821	9,39650E-01	9648	0	48381,5
Myeloid_vs_CD8_Responder	CD8(ID2+CXCR4+ T)	cDC_CLEC9A	CD52	SIGLEC10	3,26459E-04	24517,5	5,31716E-03	18959	1,51643E+00	15433	1,68870E+00	16721	9,16466E-01	23093	0	48381,5
Myeloid_vs_CD8_Responder	CD8(GZMK+ Early Tem)	Mono_CD16	HLA-C	LILRB2	3,33302E-04	19111,9	5,43162E-03	18350	2,58848E+00	2783	1,45168E+00	23189	9,52760E-01	2856	0	48381,5
Myeloid_vs_CD8_Responder	Macro_OLFML3	CD8(GZMK+ Tem)	HLA-DRA	LAG3	3,33414E-04	13870,7	8,40953E-03	9102	1,80623E+00	9710	3,07368E+00	1615	9,60838E-01	545	0	48381,5
Myeloid_vs_CD8_Responder	CD8(ISG+ T)	cDC_CLEC9A	XCL2	XCR1	3,33722E-04	30075,1	4,16806E-02	411	8,06567E+00	31	1,52641E+00	20901	8,13798E-01	80651	0	48381,5
Myeloid_vs_CD8_Responder	Mono_CD14	CD8(LAYN+ T)	S100A8	ITGB2	3,33722E-04	15937,3	5,46358E-03	18182	4,04479E+00	411	3,92666E+00	322	9,34966E-01	12390	0	48381,5
Myeloid_vs_CD8_Responder	Macro_ISG15	CD8(ISG+ T)	CXCL10	CXCR3	3,42254E-04	17473,9	2,51909E-02	1175	2,52384E+00	3042	2,79975E+00	2686	9,00179E-01	32085	0	48381,5
Myeloid_vs_CD8_Responder	CD8(Temra)	Mono_CD16	HLA-B	LILRB2	3,44370E-04	19011,9	5,37844E-03	18631	2,59197E+00	2765	1,44647E+00	23341	9,55500E-01	1941	0	48381,5
Myeloid_vs_CD8_Responder	Mono_CD14	CD8(Terminal Tex)	S100A9	ITGB2	3,45505E-04	12468,7	8,04370E-03	9798	3,91098E+00	482	3,98676E+00	282	9,51505E-01	3400	0	48381,5
Myeloid_vs_CD8_Responder	Macro_FOLR2+APOE-	CD8(GZMK+ Tex)	CCL13	CXCR3	3,46805E-04	33153,5	4,13379E-02	419	4,13783E+00	365	1,15464E+00	34385	8,10721E-01	82217	0	48381,5
Myeloid_vs_CD8_Responder	Macro_OLFML3	CD8(Terminal Tex)	C3	IFITM1	3,48437E-04	14260,3	1,32215E-02	4477	2,47222E+00	3285	2,05025E+00	9819	9,47458E-01	5339	0	48381,5
Myeloid_vs_CD8_Responder	pDC_LILRA4	CD8(Tc17)	APP	RPSA	3,48718E-04	14565,5	1,16223E-02	5487	2,16533E+00	5462	2,64113E+00	3676	9,39376E-01	9821	0	48381,5

Myeloid_vs_CD8_Responder	CD8(GZMK+ Tex)	pDC_LILRA4	HMGB1	THBD	3,52185E-04	18662,5	7,51604E-03	10967	2,11185E+00	5951	2,51820E+00	4567	9,15897E-01	23446	0	48381,5
Myeloid_vs_CD8_Responder	CD8(Tn)	pDC_LILRA4	COPA	P2RY6	3,52377E-04	31211,1	8,02604E-03	9847	2,30319E+00	4340	2,17501E+00	8048	8,04632E-01	85439	0	48381,5
Myeloid_vs_CD8_Responder	Mono_CD14	CD8(LAYN+ T)	VCAN	ITGB1	3,57859E-04	14337,3	1,80240E-02	2631	3,48237E+00	817	2,73161E+00	3088	9,27531E-01	16769	0	48381,5
Myeloid_vs_CD8_Responder	CD8(ID2+CXCR4+ T)	Mono_INHBA	RPS19	C5AR1	3,59837E-04	21641,7	5,19104E-03	19684	1,85886E+00	8948	1,44084E+00	23547	9,43269E-01	7648	0	48381,5
Myeloid_vs_CD8_Responder	CD8(Tn)	Macro_LYVE1	HMGB1	CD163	3,60219E-04	22005,7	4,60539E-03	23552	2,52687E+00	3028	1,46282E+00	22841	9,35266E-01	12226	0	48381,5
Myeloid_vs_CD8_Responder	CD8(Tn)	Macro_NLRP3	RPS19	C5AR1	3,62750E-04	19359,1	6,16340E-03	15114	2,28391E+00	4497	1,43960E+00	23585	9,47691E-01	5218	0	48381,5
Myeloid_vs_CD8_Responder	Macro_FOLR2+APOE-	CD8(IL7R+ZNF683+ Tm)	CCL13	CXCR3	3,63511E-04	33455,7	4,09164E-02	429	4,13405E+00	369	1,13053E+00	35477	8,09933E-01	82622	0	48381,5
Myeloid_vs_CD8_Responder	CD8(NME1+ T)	Macro_OLFML3	HMGB1	TLR2	3,64599E-04	21886,5	6,77935E-03	12966	2,00891E+00	7061	1,45130E+00	23197	9,25583E-01	17827	0	48381,5
Myeloid_vs_CD8_Responder	Mono_CD14	CD8(GZMK+ Tem)	S100A9	ITGB2	3,67589E-04	12524,9	7,97099E-03	9953	3,90475E+00	485	3,94237E+00	314	9,51295E-01	3491	0	48381,5
Myeloid_vs_CD8_Responder	cDC_LAMP3	CD8(Tc17)	CCL19	CCR7	3,68599E-04	29808,5	4,08899E-02	432	6,34937E+00	77	1,71546E+00	16115	8,07229E-01	84037	0	48381,5
Myeloid_vs_CD8_Responder	CD8(GZMK+ Early Tem)	Macro_IFI27	TNFSF9	HLA-DPA1	3,70897E-04	24090,5	4,67557E-03	23027	1,24096E+00	23690	2,21311E+00	7589	9,25667E-01	17765	0	48381,5
Myeloid_vs_CD8_Responder	cDC_LAMP3	CD8(Tn)	CCL19	CXCR3	3,72010E-04	25098,5	4,08352E-02	434	6,28869E+00	82	1,79176E+00	14425	8,47423E-01	62170	0	48381,5
Myeloid_vs_CD8_Responder	CD8(Tc17)	Mono_CD16	HLA-B	LILRB2	3,74275E-04	18984,5	5,43539E-03	18327	2,62807E+00	2616	1,43507E+00	23733	9,55724E-01	1865	0	48381,5
Myeloid_vs_CD8_Responder	pDC_LILRA4	CD8(GZMK+ Early Tem)	SCT	ADRB2	3,75438E-04	33460,7	4,06668E-02	436	5,92110E+00	113	2,55316E+00	4309	7,43983E-01	114064	0	48381,5
Myeloid_vs_CD8_Responder	CD8(GZMK+ Early Tem)	Macro_FOLR2+APOE+	TNFSF9	HLA-DPA1	3,75934E-04	25120,9	4,66885E-03	23070	1,23886E+00	23754	1,88805E+00	12594	9,25618E-01	17805	0	48381,5
Myeloid_vs_CD8_Responder	Mono_CD14	CD8(GZMK+ Tem)	VCAN	ITGA4	3,76767E-04	15011,7	1,71916E-02	2888	3,39860E+00	911	2,72198E+00	3142	9,22291E-01	19736	0	48381,5
Myeloid_vs_CD8_Responder	cDC_LAMP3	CD8(GZMK+ Early Tem)	CCL19	CXCR3	3,77157E-04	25615,3	4,06644E-02	437	6,28752E+00	83	1,68384E+00	16840	8,47152E-01	62335	0	48381,5
Myeloid_vs_CD8_Responder	CD8(NME1+ T)	cDC(CD1C)	B2M	CD1C	3,77440E-04	13049,5	2,21973E-02	1644	3,76204E+00	579	2,03022E+00	10098	9,49000E-01	4545	0	48381,5
Myeloid_vs_CD8_Responder	Mono_CD14	CD8(Tc17)	S100A8	ITGB2	3,80607E-04	17656,1	4,61477E-03	23474	3,99351E+00	439	3,80475E+00	396	9,29641E-01	15590	0	48381,5
Myeloid_vs_CD8_Responder	CD8(Tn)	Macro_LYVE1	HSPA1A	TLR4	3,81105E-04	19283,7	7,82692E-03	10273	1,94843E+00	7783	2,34365E+00	6162	9,15244E-01	23819	0	48381,5
Myeloid_vs_CD8_Responder	CD8(Terminal Tex)	Macro_FOLR2+APOE+	IFNG	IFNGR1_IFNGR2	3,87873E-04	23838,5	1,05122E-02	6459	1,46440E+00	16774	1,43681E+00	23675	9,15101E-01	23903	0	48381,5
Myeloid_vs_CD8_Responder	CD8(NME1+ T)	Mono_INHBA	HMGB1	CD163	3,88198E-04	19484,3	4,84895E-03	21855	1,97145E+00	7496	2,14999E+00	8386	9,36809E-01	11303	0	48381,5
Myeloid_vs_CD8_Responder	Mono_CD14	CD8(IL7R+ZNF683+ Tm)	S100A8	ITGB2	3,89302E-04	17984,9	4,47422E-03	24540	3,98501E+00	444	3,78964E+00	406	9,28622E-01	16153	0	48381,5
Myeloid_vs_CD8_Responder	CD8(Terminal Tex)	pDC_LILRA4	HMGB1	THBD	3,91048E-04	19160,7	7,35009E-03	11415	2,08687E+00	6213	2,37600E+00	5852	9,15033E-01	23942	0	48381,5
Myeloid_vs_CD8_Responder	CD8(Tn)	pDC_LILRA4	CD40LG	CD9	3,93019E-04	39278,1	1,82467E-02	2572	8,45196E-01	42979	2,71498E+00	3187	7,76933E-01	99271	0	48381,5
Myeloid_vs_CD8_Responder	CD8(LAYN+ T)	Mono_CD16	HLA-B	LILRB2	3,97956E-04	19068,3	5,42179E-03	18392	2,61945E+00	2652	1,42718E+00	24026	9,55671E-01	1890	0	48381,5
Myeloid_vs_CD8_Responder	CD8(GZMK+ Tex)	Mono_CD14	RPS19	C5AR1	3,99615E-04	21160,5	4,54005E-03	24046	1,81180E+00	9629	1,81031E+00	14057	9,39575E-01	9689	0	48381,5
Myeloid_vs_CD8_Responder	CD8(Tc17)	pDC_LILRA4	TNF	TNFRSF21	4,00094E-04	22687,1	2,29257E-02	1491	3,74184E+00	599	2,26604E+00	7010	8,58500E-01	55954	0	48381,5
Myeloid_vs_CD8_Responder	CD8(Tc17)	pDC_LILRA4	TNF	PTPRS	4,00560E-04	22656,3	5,76315E-02	217	3,88641E+00	496	2,46330E+00	5041	8,53035E-01	59146	0	48381,5
Myeloid_vs_CD8_Responder	CD8(Tc17)	pDC_LILRA4	BST2	LILRA4	4,03208E-04	22389,9	1,05660E-02	6416	5,76014E+00	128	2,74200E+00	3018	8,61930E-01	54006	0	48381,5
Myeloid_vs_CD8_Responder	CD8(Temra)	Mono_CD16	HLA-A	LILRB2	4,04625E-04	19136,9	5,52811E-03	17834	2,56694E+00	2874	1,42437E+00	24106	9,53828E-01	2489	0	48381,5
Myeloid_vs_CD8_Responder	CD8(Tc17)	pDC_LILRA4	COPA	P2RY6	4,04772E-04	31527,7	7,99798E-03	9890	2,30220E+00	4354	2,07575E+00	9456	8,04357E-01	85557	0	48381,5
Myeloid_vs_CD8_Responder	CD8(Temra)	Mono_CD16	HLA-F	LILRB2	4,08585E-04	22055,3	6,36506E-03	14348	2,60994E+00	2693	1,53318E+00	20701	9,14651E-01	24153	0	48381,5
Myeloid_vs_CD8_Responder	CD8(ZNF683+KLRB1+ T)	cDC_CLEC9A	FLT3LG	FLT3	4,10568E-04	28868,5	3,97476E-02	456	4,58439E+00	223	1,69340E+00	16619	8,17489E-01	78663	0	48381,5
Myeloid_vs_CD8_Responder	CD8(GZMK+ Early Tem)	Macro_LYVE1	TNFSF9	HLA-DPA1	4,12491E-04	25107,9	4,62971E-03	23361	1,22663E+00	24199	1,93971E+00	11622	9,25327E-01	17976	0	48381,5
Myeloid_vs_CD8_Responder	CD8(ISG+ T)	cDC_CLEC9A	FLT3LG	FLT3	4,14167E-04	28592,7	3,96902E-02	458	4,58386E+00	225	1,75624E+00	15174	8,17381E-01	78725	0	48381,5
Myeloid_vs_CD8_Responder	Mono_CD14	CD8(Tn)	S100A8	ITGB2	4,14167E-04	19138,9	4,04552E-03	28424	3,95911E+00	458	3,80724E+00	394	9,25211E-01	18037	0	48381,5
Myeloid_vs_CD8_Responder	CD8(GZMK+ Early Tem)	Macro_NLRP3	RPS19	C5AR1	4,27185E-04	19519,7	6,16133E-03	15123	2,28352E+00	4500	1,41623E+00	24369	9,47683E-01	5225	0	48381,5
Myeloid_vs_CD8_Responder	CD8(NME1+ T)	Macro_ISG15	HMGB1	TLR2	4,27273E-04	25009,5	5,11627E-03	20133	1,63535E+00	12689	1,55707E+00	20050	9,15290E-01	23794	0	48381,5
Myeloid_vs_CD8_Responder	Macro_FOLR2-APOE+	CD8(ISG+ T)	C3	IFITM1	4,28282E-04	16015,7	1,04122E-02	6542	1,76595E+00	10346	2,36020E+00	6016	9,41185E-01	8793	0	48381,5
Myeloid_vs_CD8_Responder	Macro_OLFML3	CD8(Terminal Tex)	HLA-DRA	LAG3	4,33532E-04	14240,3	7,99811E-03	9888	1,76381E+00	10378	3,01408E+00	1809	9,59883E-01	745	0	48381,5
Myeloid_vs_CD8_Responder	Macro_FOLR2+APOE-	CD8(GZMK+ Tex)	CCL13	CCR5	4,37940E-04	42164,5	3,91772E-02	471	4,13737E+00	366	1,19434E+00	32735	7,05141E-01	128869	0	48381,5
Myeloid_vs_CD8_Responder	CD8(NME1+ T)	Macro_NLRP3	HMGB1	CD163	4,40675E-04	17757,1	5,51516E-03	17889	2,17609E+00	5371	2,18046E+00	7969	9,40514E-01	9175	0	48381,5
Myeloid_vs_CD8_Responder	CD8(NME1+ T)	Macro_NLRP3	HMGB1	TLR2	4,41214E-04	24150,9	5,48369E-03	18066	1,71788E+00	11148	1,52619E+00	20906	9,17940E-01	22253	0	48381,5
Myeloid_vs_CD8_Responder	CD8(NME1+ T)	Macro_ISG15	HMGB1	CD163	4,43648E-04	20933,5	4,47291E-03	24554	1,85594E+00	8988	2,03971E+00	9966	9,34377E-01	12778	0	48381,5
Myeloid_vs_CD8_Responder	pDC_LILRA4	CD8(LAYN+ T)	APP	RPSA	4,44006E-04	14922,3	1,12085E-02	5839	2,12462E+00	5818	2,57800E+00	4132	9,38335E-01	10441	0	48381,5
Myeloid_vs_CD8_Responder	CD8(GZMK+ Tex)	Macro_NLRP3	RPS19	C5AR1	4,55425E-04	21235,5	5,21833E-03	19534	2,10633E+00	6009	1,40710E+00	24683	9,43409E-01	7570	0	48381,5
Myeloid_vs_CD8_Responder	CD8(NME1+ T)	pDC_LILRA4	HMGB1	THBD	4,57644E-04	15371,1	1,02477E-02	6707	2,52304E+00	3046	3,03822E+00	1731	9,27093E-01	16990	0	48381,5
Myeloid_vs_CD8_Responder	cDC(CD1C)	CD8(GZMK+ Tem)	HLA-DRA	LAG3	4,58091E-04	13942,3	8,15701E-03	9580	1,75494E+00	10524	3,62603E+00	558	9,60260E-01	668	0	48381,5
Myeloid_vs_CD8_Responder	CD8(GZMK+ Tex)	pDC_LILRA4	HMGB1	TLR9	4,62373E-04	21156,1	3,89894E-02	479	3,90673E+00	484	1,53477E+00	20647	8,93494E-01	35789	0	48381,5
Myeloid_vs_CD8_Responder	CD8(GZMK+ Tem)	Macro_NLRP3	RPS19	C5AR1	4,64257E-04	20212,3	5,76841E-03	16720	2,20969E+00	5083	1,40387E+00	24778	9,46025E-01	6099	0	48381,5
Myeloid_vs_CD8_Responder	Mono_CD14	CD8(NME1+ T)	S100A9	ITGB2	4,69526E-04	12740,9	7,67853E-03	10590	3,87970E+00	503	3,85727E+00	365	9,50422E-01	3865	0	48381,5
Myeloid_vs_CD8_Responder	CD8(ISG+ T)	Macro_FOLR2+APOE-	HMGB1	CD163	4,71326E-04	22708,9	4,43316E-03	24853	2,04237E+00	6678	1,53334E+00	20693	9,34103E-01	12939	0	48381,5
Myeloid_vs_CD8_Responder	CD8(ID2+CXCR4+ T)	Mono_CD16	RPS19	C5AR1	4,75227E-04	19715,7	6,20681E-03	14934	2,19061E+00	5250	1,40023E+00	24894	9,47865E-01	5119	0	48381,5
Myeloid_vs_CD8_Responder	CD8(ID2+CXCR4+ T)	pDC_LILRA4	HMGB1	THBD	4,80692E-04	19808,9	7,02240E-03	12259	2,03754E+00	6733	2,29355E+00	6720	9,13243E-01	24951	0	48381,5
Myeloid_vs_CD8_Responder	Macro_ISG15	CD8(GZMK+ Tex)	CXCL10	CXCR3	4,82492E-04	18762,9	2,08557E-02	1927	2,44667E+00	3415	2,73087E+00	3092	8,91367E-01	36999	0	48381,5
Myeloid_vs_CD8_Responder	CD8(GZMK+ Tex)	Mono_CD16	HLA-B	LILRB2	4,84170E-04	19422,1	5,33441E-03	18863	2,56405E+00	2891	1,39736E+00	24987	9,55325E-01	1988	0	48381,5
Myeloid_vs_CD8_Responder	Macro_ISG15	CD8(GZMK+ Tem)	CXCL10	CXCR3	4,88384E-04	18326,7	2,24907E-02	1585	2,47578E+00	3270	2,67710E+00	3429	8,94968E-01	34968	0	48381,5
Myeloid_vs_CD8_Responder	Macro_OLFML3	CD8(LAYN+ T)	C3	IFITM1	4,88874E-04	14685,3	1,25632E-02	4832	2,42865E+00	3501	1,99376E+00	10699	9,46172E-01	6013	0	48381,5
Myeloid_vs_CD8_Responder	CD8(Tn)	Mono_CD14	HSPA1A	TLR4	4,89718E-04	19121,5	7,40591E-03	11253	1,88158E+00	8644	2,89272E+00	2285	9,13075E-01	25044	0	48381,5
Myeloid_vs_CD8_Responder	Macro_ISG15	CD8(IL7R+ZNF683+ Tm)	CXCL10	CXCR3	4,92622E-04	18866,3	2,06430E-02	1979	2,44289E+00	3439	2,70676E+00	3226	8,90870E-01	37306	0	48381,5
Myeloid_vs_CD8_Responder	CD8(Terminal Tex)	pDC_LILRA4	HMGB1	TLR9	4,93349E-04	22183,1	3,81286E-02	495	3,88175E+00	500	1,39256E+00	25143	8,92427E-01	36396	0	48381,5
Myeloid_vs_CD8_Responder	CD8(NME1+ T)	Mono_CD16	HLA-B	LILRB2	4,96996E-04	19113,1	5,37766E-03	18632	2,59147E+00	2767	1,43227E+00	23841	9,55497E-01	1944	0	48381,5
Myeloid_vs_CD8_Responder	pDC_LILRA4	CD8(ID2+CXCR4+ T)	APP	RPSA	4,97214E-04	15080,9	1,10168E-02	6012	2,10576E+00	6012	2,56122E+00	4254	9,37834E-01	10745	0	48381,5
Myeloid_vs_CD8_Responder	CD8(NME1+ T)	Mono_CD16	HLA-A	LILRB2	4,97986E-04	18407,5	5,68377E-03	17127	2,64862E+00	2543	1,49989E+00	21698	9,54435E-01	2288	0	48381,5
Myeloid_vs_CD8_Responder	CD8(NME1+ T)	Mono_CD16	HLA-C	LILRB2	4,98779E-04	19145,9	5,43957E-03	18310	2,59225E+00	2763	1,44405E+00	23429	9,52793E-01	2846	0	48381,5
Myeloid_vs_CD8_Responder	CD8(NME1+ T)	Mono_CD16	CD99	PILRA	4,99275E-04	20402,1	7,03411E-03	12231	2,28029E+00	4528	1,48969E+00	21998	9,30809E-01	14872	0	48381,5
Myeloid_vs_CD8_Responder	Mono_CD14	CD8(ITM2C+ T)	VCAN	SELL	4,99453E-04	19350,1	1,55122E-02	3455	3,41120E+00	894	2,67992E+00	3405	8,85055E-01	40615	0	48381,5
Myeloid_vs_CD8_Responder	CD8(NME1+ T)	Mono_CD16	HLA-F	LILRB2	5,00070E-04	20608,7	7,38649E-03	11314	2,70080E+00	2364	1,55552E+00	20110	9,20283E-01	20874	0	48381,5
Myeloid_vs_CD8_Responder	CD8(NME1+ T)	Mono_CD16	B2M	LILRB2	5,00667E-04	18776,7	5,15534E-03	19885	2,61698E+00	2660	1,49014E+00	21984	9,58950E-01	973	0	48381,5
Myeloid_vs_CD8_Responder	Macro_ISG15	CD8(ITM2C+ T)	CXCL10	CXCR3	5,08948E-04	17680,7	2,49652E-02	1195	2,51982E+00	3061	2,66974E+00	3477	8,99774E-01	32289	0	48381,5
Myeloid_vs_CD8_Responder	CD8(Terminal Tex)	cDC_CLEC9A	CD52	SIGLEC10	5,11609E-04	26638,3	4,82584E-03	21999	1,41493E+00	18216	1,58390E+00	19331	9,12679E-01	25264	0	48381,5
Myeloid_vs_CD8_Responder	CD8(GZMK+ Early Tem)	Macro_FOLR2+APOE-	TNFSF9	HLA-DPA1	5,12217E-04	25237,9	4,54026E-03	24044	1,19866E+00	25270	2,02906E+00	10118	9,24651E-01	18376	0	48381,5
Myeloid_vs_CD8_Responder	CD8(GZMK+ Early Tem)	pDC_LILRA4	TNF	PTPRS	5,13215E-04	22767,1	5,49209E-02	236	3,86589E+00	510	2,60516E+00	3913	8,49990E-01	60795	0	48381,5
Myeloid_vs_CD8_Responder	Mast	CD8(LAYN+ T)	TIMP3	CD44	5,15223E-04	16396,7	4,03335E-02	444	3,86517E+00	511	1,75656E+00	15165	9,26150E-01	17482	0	48381,5
Myeloid_vs_CD8_Responder	CD8(Temra)	pDC_LILRA4	TNF	PTPRS	5,27353E-04	23073,7	5,35352E-02	255	3,85540E+00	517	2,52218E+00	4542	8,48353E-01	61673	0	48381,5
Myeloid_vs_CD8_Responder	CD8(NME1+ T)	Macro_FOLR2+APOE-	HMGB1	CD163	5,31765E-04	15259,7	6,85321E-03	12743	2,58709E+00	2793	2,32024E+00	6442	9,46307E-01	5939	0	48381,5

Myeloid_vs_CD8_Responder	CD8(GZMK+ Tem)	Macro_FOLR2+APOE-	HMGB1	CD163	5,32078E-04	23121,9	4,36483E-03	25463	2,02699E+00	6860	1,50118E+00	21656	9,33623E-01	13249	0	48381,5
Myeloid_vs_CD8_Responder	CD8(ISG+ T)	Mono_CD14	RPS19	C5AR1	5,39749E-04	21920,7	4,35726E-03	25536	1,77232E+00	10233	1,76263E+00	15042	9,38398E-01	10411	0	48381,5
Myeloid_vs_CD8_Responder	CD8(ZNF683+KLRB1+ T)	cDC(CD1C)	B2M	CD1C	5,46747E-04	13246,7	2,21145E-02	1658	3,74348E+00	598	1,97513E+00	11007	9,48910E-01	4589	0	48381,5
Myeloid_vs_CD8_Responder	CD8(NME1+ T)	Macro_FOLR2+APOE+	HMGB1	CD163	5,48578E-04	18215,5	5,95488E-03	15922	2,31115E+00	4279	1,78886E+00	14487	9,42624E-01	8008	0	48381,5
Myeloid_vs_CD8_Responder	Macro_ISG15	CD8(ZNF683+KLRB1+ T)	S100A8	CD69	5,61577E-04	16206,1	8,22405E-03	9460	1,72133E+00	11082	2,71088E+00	3207	9,40994E-01	8900	0	48381,5
Myeloid_vs_CD8_Responder	CD8(LAYN+ T)	Mono_CD16	HLA-A	LILRB2	5,66802E-04	19488,1	5,51821E-03	17875	2,56175E+00	2899	1,37413E+00	25787	9,53788E-01	2498	0	48381,5
Myeloid_vs_CD8_Responder	pDC_LILRA4	CD8(IL7R+ZNF683+ Tm)	APP	RPSA	5,68599E-04	15246,5	1,07686E-02	6220	2,08133E+00	6259	2,56100E+00	4255	9,37167E-01	11117	0	48381,5
Myeloid_vs_CD8_Responder	CD8(IL7R+ZNF683+ Tm)	Mono_CD16	HLA-B	LILRB2	5,68783E-04	19665,9	5,29136E-03	19103	2,53676E+00	2989	1,37374E+00	25805	9,55152E-01	2051	0	48381,5
Myeloid_vs_CD8_Responder	Mono_CD14	CD8(ID2+CXCR4+ T)	S100A9	ITGB2	5,68800E-04	13423,3	6,88293E-03	12653	3,81155E+00	537	3,82502E+00	382	9,47780E-01	5163	0	48381,5
Myeloid_vs_CD8_Responder	Macro_FOLR2+APOE-	CD8(LAYN+ T)	CCL13	CXCR3	5,70913E-04	46848,6	3,66848E-02	538	4,09605E+00	386	9,11653E-01	45942	8,01388E-01	87065	0,004	100312
Myeloid_vs_CD8_Responder	Macro_FOLR2+APOE+	CD8(ITM2C+ T)	APOE	LDLR	5,71227E-04	17999,5	1,51445E-02	3615	2,88422E+00	1821	3,22126E+00	1206	8,94956E-01	34974	0	48381,5
Myeloid_vs_CD8_Responder	cDC_LAMP3	CD8(EOMES+ NK-like)	CCL19	CXCR3	5,73030E-04	26816,5	3,66659E-02	539	6,26013E+00	87	1,59754E+00	18961	8,40329E-01	66114	0	48381,5
Myeloid_vs_CD8_Responder	Mast	CD8(IL7R+ZNF683+ Tm)	TIMP3	CD44	5,77276E-04	16463,1	3,79685E-02	502	3,80920E+00	541	1,80473E+00	14171	9,24057E-01	18720	0	48381,5
Myeloid_vs_CD8_Responder	pDC_LILRA4	CD8(EOMES+ NK-like)	APP	RPSA	5,77926E-04	15276,7	1,07360E-02	6251	2,07813E+00	6297	2,55565E+00	4291	9,37078E-01	11163	0	48381,5
Myeloid_vs_CD8_Responder	Macro_OLFML3	CD8(GZMK+ Tem)	HLA-DQB1	LAG3	5,79764E-04	14760,3	9,67785E-03	7298	1,71659E+00	11172	2,52202E+00	4543	9,54077E-01	2407	0	48381,5
Myeloid_vs_CD8_Responder	Macro_ISG15	CD8(NME1+ T)	CXCL10	SDC4	5,80649E-04	31274,9	1,53963E-02	3499	2,44766E+00	3410	2,64902E+00	3635	7,80701E-01	97449	0	48381,5
Myeloid_vs_CD8_Responder	Macro_OLFML3	CD8(GZMK+ Tem)	HLA-DRB5	LAG3	5,83454E-04	14424,7	9,25954E-03	7804	1,71559E+00	11190	2,74633E+00	2991	9,56068E-01	1757	0	48381,5
Myeloid_vs_CD8_Responder	cDC(CD1C)	CD8(Terminal Tex)	HLA-DRA	LAG3	5,95669E-04	14309,1	7,75794E-03	10417	1,71252E+00	11249	3,56643E+00	610	9,59292E-01	888	0	48381,5
Myeloid_vs_CD8_Responder	CD8(GZMK+ Early Tem)	Mono_CD16	HLA-A	LILRB2	5,95959E-04	19633,7	5,46647E-03	18172	2,53460E+00	2999	1,36673E+00	26047	9,53580E-01	2569	0	48381,5
Myeloid_vs_CD8_Responder	Mast	CD8(Tc17)	TIMP3	CD44	5,96575E-04	16662,1	3,76321E-02	513	3,80124E+00	550	1,76699E+00	14949	9,23744E-01	18917	0	48381,5
Myeloid_vs_CD8_Responder	Mast	CD8(GZMK+ Early Tem)	TIMP3	CD44	6,05253E-04	16420,9	3,72974E-02	523	3,79332E+00	554	1,83690E+00	13546	9,23429E-01	19100	0	48381,5
Myeloid_vs_CD8_Responder	Macro_FOLR2+APOE+	CD8(ISG+ T)	C3	IFITM1	6,09557E-04	16859,5	1,06739E-02	6311	1,79670E+00	9876	1,95779E+00	11315	9,41868E-01	8414	0	48381,5
Myeloid_vs_CD8_Responder	Mono_CD14	CD8(LAYN+ T)	VCAN	ITGA4	6,10506E-04	15908,5	1,49024E-02	3697	3,32108E+00	1002	2,63783E+00	3696	9,17013E-01	22766	0	48381,5
Myeloid_vs_CD8_Responder	CD8(ISG+ T)	pDC_LILRA4	HMGB1	THBD	6,13786E-04	20522,3	6,62898E-03	13446	1,97832E+00	7422	2,25132E+00	7161	9,10932E-01	26201	0	48381,5
Myeloid_vs_CD8_Responder	cDC_LAMP3	CD8(Temra)	CCL19	CXCR3	6,13994E-04	26681,5	3,61553E-02	558	6,25664E+00	88	1,64786E+00	17696	8,39386E-01	66684	0	48381,5
Myeloid_vs_CD8_Responder	Macro_OLFML3	CD8(GZMK+ Tex)	C3	IFITM1	6,17875E-04	15233,1	1,16370E-02	5473	2,36736E+00	3875	1,95582E+00	11354	9,44189E-01	7082	0	48381,5
Myeloid_vs_CD8_Responder	CD8(ISG+ T)	Macro_NLRP3	RPS19	C5AR1	6,23925E-04	22013,5	5,00823E-03	20797	2,06686E+00	6416	1,35941E+00	26287	9,42302E-01	8186	0	48381,5
Myeloid_vs_CD8_Responder	CD8(Tn)	cDC_CLEC9A	CD40LG	CD40	6,24980E-04	29796,9	5,35859E-02	252	1,86915E+00	8797	1,95402E+00	11387	8,14667E-01	80167	0	48381,5
Myeloid_vs_CD8_Responder	cDC_LAMP3	CD8(Tn)	CCL17	CCR4	6,27222E-04	33908,9	1,95146E-01	13	3,78082E+00	564	1,83399E+00	13598	7,60409E-01	106988	0	48381,5
Myeloid_vs_CD8_Responder	Macro_FOLR2+APOE+	CD8(LAYN+ T)	APOE	LSR	6,27798E-04	18314,1	1,48304E-02	3732	2,74260E+00	2218	3,07506E+00	1611	8,93798E-01	35628	0	48381,5
Myeloid_vs_CD8_Responder	CD8(Tn)	cDC(CD1C)	CD28	CD86	6,28446E-04	27713,7	1,15720E-02	5527,5	1,70389E+00	11403,5	1,97448E+00	11019,5	8,47337E-01	62236,5	0	48381,5
Myeloid_vs_CD8_Responder	Macro_FOLR2+APOE+	CD8(Terminal Tex)	B2M	CD3D	6,30187E-04	47656,5	2,85229E-03	46862	5,65506E-01	63507	4,27883E-01	79528	9,66450E-01	4	0	48381,5
Myeloid_vs_CD8_Responder	CD8(ZNF683+KLRB1+ T)	pDC_LILRA4	TNF	PTPRS	6,31662E-04	24944,7	4,32599E-02	389	3,77761E+00	566	2,38748E+00	5754	8,34130E-01	69633	0	48381,5
Myeloid_vs_CD8_Responder	Macro_NLRP3	CD8(GZMK+ Tex)	S100A8	CD69	6,33235E-04	16914,1	7,34766E-03	11425	2,01349E+00	7009	2,26788E+00	6990	9,37787E-01	10765	0	48381,5
Myeloid_vs_CD8_Responder	CD8(ID2+CXCR4+ T)	cDC(CD1C)	ANXA1	FPR1	6,40839E-04	28094,3	4,25466E-03	26428	1,35655E+00	19916	1,50394E+00	21558	9,14599E-01	24188	0	48381,5
Myeloid_vs_CD8_Responder	CD8(Terminal Tex)	Mono_CD16	CD99	PILRA	6,42174E-04	22607,1	6,13074E-03	15237	2,15553E+00	5556	1,35613E+00	26439	9,26250E-01	17422	0	48381,5
Myeloid_vs_CD8_Responder	CD8(LAYN+ T)	pDC_LILRA4	HSP90B1	TLR9	6,45076E-04	34648	3,57217E-02	572	3,90286E+00	487	1,41541E+00	24395	8,68725E-01	50176	0,001	97610
Myeloid_vs_CD8_Responder	cDC(CD1C)	CD8(GZMK+ Tem)	HLA-DPB1	LAG3	6,49099E-04	14322,7	8,02337E-03	9854	1,69858E+00	11497	3,48150E+00	728	9,58213E-01	1153	0	48381,5
Myeloid_vs_CD8_Responder	CD8(GZMK+ Tem)	pDC_LILRA4	HMGB1	THBD	6,55274E-04	20777,7	6,52681E-03	13840	1,96294E+00	7597	2,21916E+00	7524	9,10299E-01	26546	0	48381,5
Myeloid_vs_CD8_Responder	CD8(Tc17)	Mono_CD16	HLA-C	LILRB2	6,56509E-04	19895,7	5,37110E-03	18665	2,55974E+00	2910	1,35306E+00	26556	9,52508E-01	2966	0	48381,5
Myeloid_vs_CD8_Responder	CD8(EOMES+ NK-like)	cDC(CD1C)	B2M	CD1C	6,57127E-04	14005,3	2,11982E-02	1854	3,53826E+00	767	1,81803E+00	13912	9,47874E-01	5112	0	48381,5
Myeloid_vs_CD8_Responder	CD8(IL7R+ZNF683+ Tm)	Mono_CD16	HLA-C	LILRB2	6,64084E-04	20054,9	5,29153E-03	19102	2,52196E+00	3052	1,35131E+00	26617	9,52169E-01	3122	0	48381,5
Myeloid_vs_CD8_Responder	CD8(EOMES+ NK-like)	Mono_INHBA	RPS19	C5AR1	6,65332E-04	22700,3	4,95735E-03	21123	1,80018E+00	9815	1,37237E+00	25853	9,42023E-01	8329	0	48381,5
Myeloid_vs_CD8_Responder	CD8(NME1+ T)	pDC_LILRA4	HSP90B1	TLR9	6,67744E-04	24826,3	3,55080E-02	582	3,89871E+00	489	1,41905E+00	24279	8,68383E-01	50400	0	48381,5
Myeloid_vs_CD8_Responder	CD8(LAYN+ T)	Macro_NLRP3	RPS19	C5AR1	6,67835E-04	19504,9	6,49831E-03	13921	2,34683E+00	4026	1,35037E+00	26647	9,48988E-01	4549	0	48381,5
Myeloid_vs_CD8_Responder	CD8(IL7R+ZNF683+ Tm)	Mono_INHBA	RPS19	C5AR1	6,69591E-04	22831,3	4,96919E-03	21052	1,80315E+00	9763	1,35014E+00	26661	9,42089E-01	8299	0	48381,5
Myeloid_vs_CD8_Responder	pDC_LILRA4	CD8(Tn)	APP	RPSA	6,72532E-04	15342,3	1,04651E-02	6495	2,05148E+00	6580	2,64526E+00	3654	9,36320E-01	11601	0	48381,5
Myeloid_vs_CD8_Responder	CD8(Tn)	Macro_FOLR2-APOE+	CD40LG	CD40	6,74620E-04	38247,9	3,73306E-02	522	1,42950E+00	17805	1,27453E+00	29556	7,85816E-01	94975	0	48381,5
Myeloid_vs_CD8_Responder	Macro_FOLR2+APOE+	CD8(Tn)	APOE	LDLR	6,76354E-04	18196,1	1,45901E-02	3827	2,87225E+00	1857	3,34769E+00	948	8,93190E-01	35967	0	48381,5
Myeloid_vs_CD8_Responder	CD8(EOMES+ NK-like)	Mono_CD14	RPS19	C5AR1	6,77667E-04	17847,5	5,76465E-03	16741	2,07627E+00	6317	1,93600E+00	11692	9,46009E-01	6106	0	48381,5
Myeloid_vs_CD8_Responder	CD8(ID2+CXCR4+ T)	Mono_CD16	HLA-C	LILRA3	6,81604E-04	33469,7	1,45613E-02	3837	2,42874E+00	3500	3,15955E-01	89286	9,17765E-01	22344	0	48381,5
Myeloid_vs_CD8_Responder	Macro_OLFML3	CD8(GZMK+ Tem)	HLA-DPB1	LAG3	6,87028E-04	14898,9	7,97748E-03	9929	1,68870E+00	11664	2,68876E+00	3340	9,58098E-01	1180	0	48381,5
Myeloid_vs_CD8_Responder	pDC_LILRA4	CD8(GZMK+ Early Tem)	APP	RPSA	6,87957E-04	15470,7	1,04267E-02	6528	2,04770E+00	6625	2,57528E+00	4151	9,36210E-01	11668	0	48381,5
Myeloid_vs_CD8_Responder	Mono_INHBA	CD8(Temra)	IL1B	ADRB2	6,92717E-04	20055,1	1,45032E-02	3858	2,60091E+00	2728	3,02970E+00	1755	8,80074E-01	43553	0	48381,5
Myeloid_vs_CD8_Responder	CD8(Temra)	Macro_FOLR2+APOE-	HMGB1	CD163	6,94439E-04	23752,9	4,20857E-03	26856	1,99182E+00	7266	1,47817E+00	22356	9,32484E-01	13905	0	48381,5
Myeloid_vs_CD8_Responder	CD8(Tn)	Mono_CD16	HSPA1A	TLR4	6,99106E-04	20878,5	6,79954E-03	12908	1,78531E+00	10037	2,34285E+00	6174	9,09624E-01	26892	0	48381,5
Myeloid_vs_CD8_Responder	Macro_NLRP3	CD8(GZMK+ Tem)	S100A8	CD69	6,99402E-04	17221,1	7,23380E-03	11717	2,00428E+00	7114	2,18919E+00	7873	9,37330E-01	11020	0	48381,5
Myeloid_vs_CD8_Responder	pDC_LILRA4	CD8(NME1+ T)	APP	CD74	7,07664E-04	14372,9	7,21614E-03	11752	2,24989E+00	4779	2,91708E+00	2189	9,48551E-01	4763	0	48381,5
Myeloid_vs_CD8_Responder	CD8(ISG+ T)	cDC_CLEC9A	CD52	SIGLEC10	7,15771E-04	28519,3	4,44970E-03	24727	1,33723E+00	20517	1,49136E+00	21952	9,09390E-01	27019	0	48381,5
Myeloid_vs_CD8_Responder	Mono_CD14	CD8(Tn)	VCAN	CD44	7,20552E-04	14400,5	1,07905E-02	6198	3,24508E+00	1110	2,52589E+00	4507	9,35997E-01	11806	0	48381,5
Myeloid_vs_CD8_Responder	Mono_CD14	CD8(ISG+ T)	VCAN	SELL	7,20746E-04	19695,9	1,43787E-02	3910	3,38385E+00	932	2,87884E+00	2335	8,81139E-01	42921	0	48381,5
Myeloid_vs_CD8_Responder	CD8(EOMES+ NK-like)	Macro_NLRP3	RPS19	C5AR1	7,21618E-04	18148,9	6,62589E-03	13459	2,37081E+00	3858	1,53279E+00	20717	9,49456E-01	4329	0	48381,5
Myeloid_vs_CD8_Responder	Mono_CD14	CD8(ISG+ T)	S100A9	ITGB2	7,28499E-04	14515,9	5,95199E-03	15940	3,73180E+00	608	3,71503E+00	478	9,44065E-01	7172	0	48381,5
Myeloid_vs_CD8_Responder	CD8(Tc17)	cDC(CD1C)	ANXA1	FPR1	7,30729E-04	28820,5	4,17915E-03	27131	1,34299E+00	20353	1,43653E+00	23681	9,13897E-01	24556	0	48381,5
Myeloid_vs_CD8_Responder	CD8(EOMES+ NK-like)	cDC_CLEC9A	CD52	SIGLEC10	7,33291E-04	28826,3	4,44087E-03	24799	1,33541E+00	20579	1,44751E+00	23307	9,09309E-01	27065	0	48381,5
Myeloid_vs_CD8_Responder	Mono_CD14	CD8(GZMK+ Tex)	S100A9	ITGB2	7,35678E-04	14574,1	5,90190E-03	16133	3,72751E+00	611	3,76697E+00	433	9,43841E-01	7312	0	48381,5
Myeloid_vs_CD8_Responder	Mono_CD14	CD8(GZMK+ Early Tem)	S100A9	ITGB2	7,38078E-04	14579,1	5,90172E-03	16135	3,72750E+00	612	3,73209E+00	454	9,43840E-01	7313	0	48381,5
Myeloid_vs_CD8_Responder	CD8(EOMES+ NK-like)	pDC_LILRA4	HMGB1	THBD	7,42657E-04	20779,7	6,54178E-03	13783	1,96519E+00	7575	2,20619E+00	7672	9,10393E-01	26487	0	48381,5
Myeloid_vs_CD8_Responder	CD8(ISG+ T)	Mono_CD16	CD99	PILRA	7,44159E-04	22891,5	6,05558E-03	15536	2,14515E+00	5648	1,33625E+00	27230	9,25827E-01	17662	0	48381,5
Myeloid_vs_CD8_Responder	Macro_OLFML3	CD8(Terminal Tex)	HLA-DQB1	LAG3	7,51301E-04	15207,3	9,20437E-03	7884	1,67417E+00	11932	2,46242E+00	5049	9,52966E-01	2790	0	48381,5
Myeloid_vs_CD8_Responder	CD8(Terminal Tex)	cDC(CD1C)	IFNG	IFNGR1_IFNGR2	7,51842E-04	23679,9	9,01236E-03	8168	1,18375E+00	25838	2,12538E+00	8726	9,08927E-01	27286	0	48381,5
Myeloid_vs_CD8_Responder	Macro_OLFML3	CD8(Terminal Tex)	HLA-DRB5	LAG3	7,54030E-04	14847,5	8,80653E-03	8456	1,67317E+00	11943	2,68673E+00	3353	9,55002E-01	2104	0	48381,5
Myeloid_vs_CD8_Responder	Macro_ISG15	CD8(Tn)	CXCL10	CXCR3	7,55128E-04	20964,3	1,57149E-02	3376	2,35518E+00	3972	2,63178E+00	3729	8,76887E-01	45363	0	48381,5
Myeloid_vs_CD8_Responder	CD8(Temra)	pDC_LILRA4	HMGB1	THBD	7,61119E-04	21237,1	6,29315E-03	14640	1,92777E+00	8031	2,19615E+00	7780	9,08800E-01	27353	0	48381,5
Myeloid_vs_CD8_Responder	Macro_OLFML3	CD8(GZMK+ Tem)	HLA-DPA1	LAG3	7,61513E-04	14858,5	7,92597E-03	10049	1,67117E+00	11973	2,74996E+00	2973	9,59167E-01	916	0	48381,5
Myeloid_vs_CD8_Responder	CD8(LAYN+ T)	Macro_FOLR2+APOE-	HMGB1	CD163	7,66560E-04	24345,7	4,15330E-03	27392	1,97938E+00	7407	1,41588E+00	24383	9,32067E-01	14165	0	48381,5
Myeloid_vs_CD8_Responder	CD8(ISG+ T)	pDC_LILRA4	HMGB1	TLR9	7,67190E-04	23737,5	3,43878E-02	624	3,77321E+00	572	1,26789E+00	29805	8,87370E-01	39305	0	48381,5

Myeloid_vs_CD8_Responder	CD8(EOMES+ NK-like)	Macro_LYVE1	HMGB1	CD163	7,72455E-04	18657,1	5,84229E-03	16398	2,73535E+00	2241	1,63729E+00	17974	9,42105E-01	8291	0	48381,5
Myeloid_vs_CD8_Responder	Mono_INHBA	CD8(ITM2C+ T)	IL1B	ADRB2	7,75578E-04	20244,1	1,41661E-02	4008	2,59024E+00	2772	3,01772E+00	1791	8,78827E-01	44268	0	48381,5
Myeloid_vs_CD8_Responder	CD8(IL7R+ZNF683+ Tm)	pDC_LILRA4	TNF	PTPRS	7,79185E-04	28622,3	3,00502E-02	785	3,67761E+00	650	2,08277E+00	9335	8,07369E-01	83960	0	48381,5
Myeloid_vs_CD8_Responder	CD8(IL7R+ZNF683+ Tm)	pDC_LILRA4	BST2	LILRA4	7,84034E-04	23917,7	9,03565E-03	8133	5,73066E+00	130	2,67681E+00	3432	8,52353E-01	59512	0	48381,5
Myeloid_vs_CD8_Responder	Mono_CD14	CD8(ITM2C+ T)	S100A9	ITGB2	7,84430E-04	15064,5	5,57608E-03	17585	3,69960E+00	631	3,62888E+00	548	9,42317E-01	8177	0	48381,5
Myeloid_vs_CD8_Responder	CD8(EOMES+ NK-like)	Mono_CD16	HLA-C	LILRB2	7,86065E-04	19675,9	5,34165E-03	18823	2,54576E+00	2956	1,39078E+00	25199	9,52383E-01	3020	0	48381,5
Myeloid_vs_CD8_Responder	CD8(IL7R+ZNF683+ Tm)	pDC_LILRA4	COPA	P2RY6	7,86083E-04	32698,9	7,31812E-03	11491	2,27808E+00	4544	2,04070E+00	9950	7,97272E-01	89128	0	48381,5
Myeloid_vs_CD8_Responder	CD8(EOMES+ NK-like)	Mono_CD16	CD99	PILRA	7,86494E-04	22291,1	6,31916E-03	14533	2,18155E+00	5324	1,35882E+00	26316	9,27277E-01	16901	0	48381,5
Myeloid_vs_CD8_Responder	CD8(Tn)	Macro_FOLR2-APOE+	CD40LG	CD9	7,86908E-04	28140,7	4,73095E-02	340	1,60819E+00	13246	1,66681E+00	17235	8,48675E-01	61501	0	48381,5
Myeloid_vs_CD8_Responder	CD8(EOMES+ NK-like)	Mono_CD16	RPS19	C5AR1	7,89354E-04	20660,3	5,92739E-03	16035	2,13192E+00	5759	1,33176E+00	27401	9,46715E-01	5725	0	48381,5
Myeloid_vs_CD8_Responder	CD8(NME1+ T)	cDC_CLEC9A	CD52	SIGLEC10	7,98564E-04	29216,7	4,33606E-03	25724	1,31375E+00	21234	1,45367E+00	23127	9,08319E-01	27617	0	48381,5
Myeloid_vs_CD8_Responder	Macro_OLFML3	CD8(EOMES+ NK-like)	C3	IFITM1	8,02092E-04	15103,1	1,22520E-02	5025	2,40806E+00	3620	1,91318E+00	12132	9,45530E-01	6357	0	48381,5
Myeloid_vs_CD8_Responder	CD8(LAYN+ T)	pDC_LILRA4	HMGB1	THBD	8,02911E-04	21549,9	6,21051E-03	14919	1,91533E+00	8197	2,13386E+00	8605	9,08251E-01	27647	0	48381,5
Myeloid_vs_CD8_Responder	CD8(EOMES+ NK-like)	pDC_LILRA4	HMGB1	TLR9	8,04365E-04	24183,1	3,39354E-02	639	3,76008E+00	582	1,22276E+00	31624	8,86706E-01	39689	0	48381,5
Myeloid_vs_CD8_Responder	CD8(Temra)	Mono_INHBA	RPS19	C5AR1	8,10637E-04	25301,5	4,24137E-03	26547	1,62037E+00	13009	1,32372E+00	27700	9,37614E-01	10870	0	48381,5
Myeloid_vs_CD8_Responder	CD8(Tc17)	Mono_INHBA	RPS19	C5AR1	8,13421E-04	22785,5	5,07009E-03	20427	1,82849E+00	9396	1,32311E+00	27719	9,42634E-01	8004	0	48381,5
Myeloid_vs_CD8_Responder	CD8(GZMK+ Tem)	pDC_LILRA4	HMGB1	TLR9	8,14426E-04	24089,9	3,38578E-02	643	3,75783E+00	584	1,23573E+00	31096	8,86591E-01	39745	0	48381,5
Myeloid_vs_CD8_Responder	pDC_LILRA4	CD8(Terminal Tex)	APP	CD74	8,15704E-04	14621,7	7,05111E-03	12184	2,22162E+00	5002	2,83668E+00	2478	9,47984E-01	5063	0	48381,5
Myeloid_vs_CD8_Responder	CD8(EOMES+ NK-like)	Macro_FOLR2+APOE-	HMGB1	CD163	8,16065E-04	23164,9	4,37484E-03	25364	2,02924E+00	6831	1,48821E+00	22037	9,33694E-01	13211	0	48381,5
Myeloid_vs_CD8_Responder	CD8(Tc17)	Macro_LYVE1	HMGB1	CD163	8,21819E-04	23227,9	4,51086E-03	24277	2,51093E+00	3102	1,32175E+00	27776	9,34635E-01	12603	0	48381,5
Myeloid_vs_CD8_Responder	CD8(ITM2C+ T)	cDC_CLEC9A	FLT3LG	FLT3	8,37289E-04	30646,9	3,34172E-02	652	4,52495E+00	235	1,62318E+00	18327	8,04190E-01	85639	0	48381,5
Myeloid_vs_CD8_Responder	cDC(CD1C)	CD8(Terminal Tex)	HLA-DPB1	LAG3	8,39130E-04	14719,1	7,63083E-03	10708	1,65616E+00	12272	3,42190E+00	808	9,57197E-01	1426	0	48381,5
Myeloid_vs_CD8_Responder	cDC_LAMP3	CD8(GZMK+ Early Tem)	CCL19	CCR7	8,39849E-04	31755,5	3,33791E-02	653	6,30868E+00	81	1,66265E+00	17312	7,90944E-01	92350	0	48381,5
Myeloid_vs_CD8_Responder	CD8(Tc17)	cDC_CLEC9A	LTB	CD40	8,43712E-04	23477,9	1,29096E-02	4642	1,84714E+00	9117	1,90482E+00	12289	8,81076E-01	42960	0	48381,5
Myeloid_vs_CD8_Responder	CD8(ZNF683+KLRB1+ T)	pDC_LILRA4	HMGB1	TLR9	8,44980E-04	24270,3	3,32737E-02	655	3,74088E+00	600	1,22630E+00	31481	8,85713E-01	40234	0	48381,5
Myeloid_vs_CD8_Responder	CD8(EOMES+ NK-like)	Mono_CD16	HLA-A	LILRB2	8,50618E-04	20245,5	5,34133E-03	18825	2,46894E+00	3301	1,31634E+00	27968	9,53065E-01	2752	0	48381,5
Myeloid_vs_CD8_Responder	CD8(Tc17)	pDC_LILRA4	HSP90B1	TLR9	8,52706E-04	25629,5	3,32101E-02	658	3,85400E+00	518	1,36569E+00	26085	8,64512E-01	52505	0	48381,5
Myeloid_vs_CD8_Responder	CD8(Tn)	Macro_ISG15	CD40LG	CD40	8,57875E-04	37799,9	3,69367E-02	530	1,41884E+00	18098	1,35194E+00	26595	7,84922E-01	95395	0	48381,5
Myeloid_vs_CD8_Responder	CD8(LAYN+ T)	Mono_CD16	B2M	LILRB2	8,61316E-04	20419,9	4,96817E-03	21060	2,43649E+00	3468	1,31398E+00	28038	9,58216E-01	1152	0	48381,5
Myeloid_vs_CD8_Responder	CD8(Terminal Tex)	pDC_LILRA4	HLA-C	NOTCH4	8,65525E-04	21591,7	1,61532E-02	3213	1,68175E+00	11794	1,89942E+00	12369	8,99945E-01	32201	0	48381,5
Myeloid_vs_CD8_Responder	Macro_OLFML3	CD8(IL7R+ZNF683+ Tm)	FN1	CD44	8,66076E-04	16721,7	1,19902E-02	5189	2,16329E+00	5482	1,89931E+00	12371	9,35326E-01	12185	0	48381,5
Myeloid_vs_CD8_Responder	CD8(GZMK+ Early Tem)	Mono_CD16	B2M	LILRB2	8,68560E-04	20406,7	4,97716E-03	20999	2,44516E+00	3425	1,31292E+00	28085	9,58252E-01	1143	0	48381,5
Myeloid_vs_CD8_Responder	CD8(GZMK+ Early Tem)	Macro_FOLR2-APOE+	TNFSF9	HLA-DPA1	8,78969E-04	26766,9	4,32081E-03	25857	1,13006E+00	28152	1,91751E+00	12034	9,22907E-01	19410	0	48381,5
Myeloid_vs_CD8_Responder	CD8(Tn)	Macro_NLRP3	HSPA1A	TLR4	8,87904E-04	21482,3	6,42314E-03	14156	1,72554E+00	11005	2,39612E+00	5660	9,07256E-01	28209	0	48381,5
Myeloid_vs_CD8_Responder	Mono_CD14	CD8(GZMK+ Tex)	VCAN	ITGB1	8,88933E-04	15659,5	1,37693E-02	4197	3,32461E+00	998	2,83821E+00	2469	9,17944E-01	22252	0	48381,5
Myeloid_vs_CD8_Responder	CD8(ITM2C+ T)	cDC_CLEC9A	XCL1	XCR1	8,89220E-04	31740,3	3,29605E-02	672	8,02554E+00	33	1,47838E+00	22351	8,00989E-01	87264	0	48381,5
Myeloid_vs_CD8_Responder	CD8(IL7R+ZNF683+ Tm)	Mono_CD16	RPS19	C5AR1	8,91214E-04	20804,7	5,94154E-03	15981	2,13490E+00	5738	1,30953E+00	28230	9,46775E-01	5693	0	48381,5
Myeloid_vs_CD8_Responder	Macro_FOLR2-APOE+	CD8(ITM2C+ T)	SPP1	S1PR1	8,93328E-04	20542,7	4,23989E-02	400	2,32261E+00	4204	2,74587E+00	2992	8,74643E-01	46736	0	48381,5
Myeloid_vs_CD8_Responder	Macro_OLFML3	CD8(Terminal Tex)	HLA-DPB1	LAG3	8,94504E-04	15374,7	7,58719E-03	10805	1,64628E+00	12473	2,62916E+00	3745	9,57079E-01	1469	0	48381,5
Myeloid_vs_CD8_Responder	cDC(CD1C)	CD8(GZMK+ Tem)	HLA-DQB1	LAG3	9,00441E-04	14560,3	9,24685E-03	7823	1,64521E+00	12494	3,16914E+00	1356	9,53069E-01	2747	0	48381,5
Myeloid_vs_CD8_Responder	CD8(GZMK+ Tem)	cDC_CLEC9A	XCL1	XCR1	9,02444E-04	32309,1	3,27096E-02	677	8,02468E+00	34	1,40063E+00	24877	8,00379E-01	87576	0	48381,5
Myeloid_vs_CD8_Responder	CD8(LAYN+ T)	pDC_LILRA4	BST2	LILRA4	9,02794E-04	20149,7	1,37228E-02	4219	5,82094E+00	121	2,81923E+00	2584	8,76762E-01	45443	0	48381,5
Myeloid_vs_CD8_Responder	Macro_OLFML3	CD8(GZMK+ Early Tem)	FN1	CD44	9,04132E-04	16728,5	1,17783E-02	5356	2,14741E+00	5628	1,93148E+00	11770	9,34785E-01	12507	0	48381,5
Myeloid_vs_CD8_Responder	CD8(NME1+ T)	Mono_CD14	HMGB1	TLR2	9,07591E-04	27625,9	4,17183E-03	27196	1,42321E+00	17976	1,71034E+00	16243	9,07036E-01	28333	0	48381,5
Myeloid_vs_CD8_Responder	Macro_FOLR2+APOE+	CD8(ITM2C+ T)	APOE	LSR	9,09142E-04	18859,7	1,37070E-02	4229	2,72503E+00	2276	3,06492E+00	1643	8,90001E-01	37769	0	48381,5
Myeloid_vs_CD8_Responder	CD8(Temra)	cDC_CLEC9A	CD52	SIGLEC10	9,09354E-04	29968,7	4,20263E-03	26913	1,28619E+00	22142	1,42593E+00	24063	9,07009E-01	28344	0	48381,5
Myeloid_vs_CD8_Responder	CD8(Temra)	pDC_LILRA4	HMGB1	TLR9	9,10426E-04	24495,7	3,26457E-02	680	3,72266E+00	616	1,21272E+00	32015	8,84745E-01	40786	0	48381,5
Myeloid_vs_CD8_Responder	cDC_CLEC9A	CD8(GZMK+ Tem)	HLA-DQB1	LAG3	9,22751E-04	14664,9	9,22049E-03	7860	1,64085E+00	12572	3,03613E+00	1738	9,53005E-01	2773	0	48381,5
Myeloid_vs_CD8_Responder	CD8(GZMK+ Tex)	Macro_NLRP3	HMGB1	CD163	9,23072E-04	23835,9	4,04501E-03	28429	1,76489E+00	10357	1,66044E+00	17373	9,31226E-01	14639	0	48381,5
Myeloid_vs_CD8_Responder	CD8(Temra)	pDC_LILRA4	BST2	LILRA4	9,23851E-04	20128,3	1,36527E-02	4252	5,81959E+00	122	2,89718E+00	2266	8,76485E-01	45620	0	48381,5
Myeloid_vs_CD8_Responder	CD8(ZNF683+KLRB1+ T)	Mono_INHBA	RPS19	C5AR1	9,26486E-04	22991,9	4,90671E-03	21479	1,78746E+00	10006	1,35159E+00	26608	9,41742E-01	8485	0	48381,5
Myeloid_vs_CD8_Responder	CD8(ZNF683+KLRB1+ T)	Mono_CD14	RPS19	C5AR1	9,37611E-04	18037,1	5,70577E-03	17020	2,06355E+00	6448	1,91523E+00	12087	9,45746E-01	6249	0	48381,5
Myeloid_vs_CD8_Responder	Mono_CD14	CD8(EOMES+ NK-like)	S100A9	ITGB2	9,39988E-04	16682,3	4,70360E-03	22805	3,62487E+00	691	3,56589E+00	612	9,37515E-01	10922	0	48381,5
Myeloid_vs_CD8_Responder	CD8(IL7R+ZNF683+ Tm)	pDC_LILRA4	TNF	TNFRSF21	9,45174E-04	29496,3	1,19539E-02	5218	3,53304E+00	774	1,88551E+00	12649	8,14163E-01	80459	0	48381,5
Myeloid_vs_CD8_Responder	CD8(ID2+CXCR4+ T)	pDC_LILRA4	COPA	P2RY6	9,48999E-04	33481,7	6,87976E-03	12662	2,26253E+00	4659	2,03620E+00	10009	7,92235E-01	91697	0	48381,5
Myeloid_vs_CD8_Responder	CD8(GZMK+ Tex)	cDC_CLEC9A	CD52	SIGLEC10	9,51502E-04	29876,3	4,15556E-03	27359	1,27647E+00	22464	1,47051E+00	22575	9,06533E-01	28602	0	48381,5
Myeloid_vs_CD8_Responder	CD8(Terminal Tex)	Macro_FOLR2-APOE+	IFNG	IFNGR1_IFNGR2	9,52333E-04	26231,5	8,51035E-03	8936	1,25783E+00	23071	1,48411E+00	22162	9,06527E-01	28607	0	48381,5
Myeloid_vs_CD8_Responder	CD8(Temra)	Macro_OLFML3	SPON2	ITGB2	9,57170E-04	27404,3	1,18932E-02	5275	1,16834E+00	26458	1,29938E+00	28636	9,07163E-01	28271	0	48381,5
Myeloid_vs_CD8_Responder	CD8(LAYN+ T)	pDC_LILRA4	HMGB1	TLR9	9,59045E-04	36456,6	3,22170E-02	698	3,71021E+00	625	1,15043E+00	34587	8,84069E-01	41137	0,032	105236
Myeloid_vs_CD8_Responder	Macro_NLRP3	CD8(Temra)	IL1B	ADRB2	9,60977E-04	21002,7	1,35457E-02	4309	2,44628E+00	3419	2,70477E+00	3238	8,76423E-01	45666	0	48381,5
Myeloid_vs_CD8_Responder	Mono_CD14	CD8(ZNF683+KLRB1+ T)	S100A9	ITGB2	9,61782E-04	16957,1	4,58343E-03	23730	3,61457E+00	699	3,55378E+00	628	9,36752E-01	11347	0	48381,5
Myeloid_vs_CD8_Responder	CD8(LAYN+ T)	cDC_CLEC9A	XCL1	XCR1	9,64524E-04	43283,8	3,21825E-02	700	8,02287E+00	35	1,26732E+00	29830	7,99078E-01	88244	0,001	97610
Myeloid_vs_CD8_Responder	CD8(Terminal Tex)	Macro_NLRP3	IFNG	IFNGR1_IFNGR2	9,64547E-04	25997,7	8,48503E-03	8972	1,18816E+00	25667	1,62476E+00	18288	9,06401E-01	28680	0	48381,5
Myeloid_vs_CD8_Responder	CD8(GZMK+ Early Tem)	Mono_CD16	HLA-F	LILRB2	9,66566E-04	25175,1	5,19464E-03	19666	2,50584E+00	3127	1,36787E+00	26009	9,06378E-01	28692	0	48381,5
Myeloid_vs_CD8_Responder	CD8(ZNF683+KLRB1+ T)	Macro_NLRP3	RPS19	C5AR1	9,73831E-04	18365,1	6,55820E-03	13713	2,35809E+00	3955	1,51202E+00	21331	9,49209E-01	4445	0	48381,5
Myeloid_vs_CD8_Responder	CD8(NME1+ T)	Mono_CD16	HMGB1	TLR2	9,78755E-04	30036,7	4,19401E-03	26996	1,42819E+00	17836	1,29579E+00	28764	9,07260E-01	28206	0	48381,5
Myeloid_vs_CD8_Responder	CD8(ZNF683+KLRB1+ T)	cDC_CLEC9A	CD52	SIGLEC10	9,83016E-04	25811,5	5,06830E-03	20438	1,46502E+00	16756	1,58480E+00	19301	9,14612E-01	24181	0	48381,5
Myeloid_vs_CD8_Responder	CD8(NME1+ T)	Macro_NLRP3	RPS19	C5AR1	9,85750E-04	24069,3	4,39621E-03	25166	1,95186E+00	7743	1,29464E+00	28805	9,38655E-01	10251	0	48381,5
Myeloid_vs_CD8_Responder	CD8(ZNF683+KLRB1+ T)	pDC_LILRA4	HMGB1	THBD	9,91410E-04	20987,9	6,41422E-03	14185	1,94599E+00	7818	2,20973E+00	7643	9,09586E-01	26912	0	48381,5
Myeloid_vs_CD8_Responder	CD8(NME1+ T)	Macro_NLRP3	HMGB1	THBD	9,94680E-04	24620,3	5,89949E-03	16146	1,67634E+00	11895	1,64316E+00	17822	9,06087E-01	28857	0	48381,5
Myeloid_vs_CD8_Responder	Macro_FOLR2+APOE-	CD8(ITM2C+ T)	CCL13	CCR5	9,94938E-04	44694,3	3,18571E-02	711	4,09498E+00	387	1,06430E+00	38368	6,83193E-01	135624	0	48381,5
Myeloid_vs_CD8_Responder	Macro_OLFML3	CD8(Terminal Tex)	HLA-DPA1	LAG3	9,97329E-04	15322,3	7,53821E-03	10917	1,62875E+00	12823	2,69037E+00	3329	9,58173E-01	1161	0	48381,5
Myeloid_vs_CD8_Responder	CD8(Tn)	Macro_ISG15	HSPA1A	TLR4	9,99169E-04	21687,5	6,23720E-03	14831	1,69602E+00	11541	2,49143E+00	4801	9,06013E-01	28883	0	48381,5
Myeloid_vs_CD8_Responder	Mast	CD8(Tn)	TIMP3	CD44	1,00611E-03	17755,7	3,16651E-02	715	3,66003E+00	661	1,69900E+00	16497	9,17437E-01	22524	0	48381,5
Myeloid_vs_CD8_Responder	CD8(EOMES+ NK-like)	pDC_LILRA4	BST2	LILRA4	1,00858E-03	20293,9	1,34112E-02	4380	5,81494E+00	123	2,86282E+00	2378	8,75516E-01	46207	0	48381,5
Myeloid_vs_CD8_Responder	CD8(ZNF683+KLRB1+ T)	Macro_LYVE1	HMGB1	CD163	1,01396E-03	18810,3	5,72837E-03	16917	2,71614E+00	2307	1,64083E+00	17869	9,41566E-01	8577	0	48381,5
Myeloid_vs_CD8_Responder	pDC_LILRA4	CD8(GZMK+ Tem)	APP	RPSA	1,01766E-03	16090,7	9,74705E-03	7225	1,98083E+00	7390	2,51819E+00	4568	9,34168E-01	12889	0	48381,5

Myeloid_vs_CD8_Post_R

Myeloid_vs_CD8_Responder	CD8(GZMK+ Tex)	pDC_LILRA4	HLA-C	NOTCH4	1,01921E-03	21937,1	1,58246E-02	3315	1,62526E+00	12894	1,89865E+00	12385	8,99015E-01	32710	0	48381,5
Myeloid_vs_CD8_Responder	Macro_ISG15	CD8(Tc17)	DEFB1	CCR6	1,02017E-03	25819,1	2,25857E-01	9	3,59141E+00	720	2,02130E+00	10241	8,33981E-01	69744	0	48381,5
Myeloid_vs_CD8_Responder	CD8(ID2+CXCR4+ T)	cDC(CD1C)	CD52	SIGLEC10	1,02045E-03	30207,7	4,08366E-03	28035	1,22272E+00	24340	1,51379E+00	21277	9,05791E-01	29005	0	48381,5
Myeloid_vs_CD8_Responder	CD8(Terminal Tex)	Macro_LYVE1	IFNG	IFNGR1_IFNGR2	1,02168E-03	28012,3	8,46257E-03	9002	1,11055E+00	29012	1,39932E+00	24922	9,06288E-01	28744	0	48381,5
Myeloid_vs_CD8_Responder	CD8(ZNF683+KLRB1+ T)	Mono_CD16	HLA-C	LILRB2	1,02327E-03	19699,9	5,32695E-03	18901	2,53878E+00	2981	1,39105E+00	25188	9,52321E-01	3048	0	48381,5
Myeloid_vs_CD8_Responder	Mono_CD14	CD8(GZMK+ Tex)	VCAN	ITGA4	1,02363E-03	16505,7	1,33610E-02	4402	3,26889E+00	1073	2,67410E+00	3455	9,12763E-01	25217	0	48381,5
Myeloid_vs_CD8_Responder	CD8(ZNF683+KLRB1+ T)	Mono_CD16	CD99	PILRA	1,02380E-03	23737,3	5,75038E-03	16813	2,10300E+00	6041	1,29738E+00	28712	9,24032E-01	18739	0	48381,5
Myeloid_vs_CD8_Responder	CD8(ZNF683+KLRB1+ T)	Mono_CD16	B2M	LILRB2	1,02450E-03	19172,9	5,13610E-03	20020	2,59843E+00	2740	1,43505E+00	23734	9,58876E-01	989	0	48381,5
Myeloid_vs_CD8_Responder	cDC_CLEC9A	CD8(GZMK+ Tem)	HLA-DPA1	LAG3	1,02638E-03	14792,5	7,70992E-03	10525	1,62422E+00	12917	3,27661E+00	1089	9,58622E-01	1050	0	48381,5
Myeloid_vs_CD8_Responder	CD8(ZNF683+KLRB1+ T)	Mono_CD16	RPS19	C5AR1	1,02680E-03	20914,3	5,86684E-03	16272	2,11921E+00	5876	1,31098E+00	28176	9,46456E-01	5866	0	48381,5
Myeloid_vs_CD8_Responder	CD8(Tn)	Mono_INHBA	ANXA1	FPR1	1,03034E-03	29714,9	3,98585E-03	29061	1,23564E+00	23875	1,50214E+00	21620	9,12016E-01	25637	0	48381,5
Myeloid_vs_CD8_Responder	CD8(ITM2C+ T)	pDC_LILRA4	HMGB1	TLR9	1,03432E-03	35597,2	3,13393E-02	725	3,68475E+00	643	1,09494E+00	36977	8,82647E-01	42031	0,001	97610
Myeloid_vs_CD8_Responder	Macro_FOLR2+APOE+	CD8(GZMK+ Tem)	HLA-DRA	LAG3	1,03924E-03	15792,5	7,50329E-03	10999	1,62218E+00	12958	2,40395E+00	5575	9,58635E-01	1049	0	48381,5
Myeloid_vs_CD8_Responder	CD8(ZNF683+KLRB1+ T)	Macro_FOLR2+APOE-	HMGB1	CD163	1,04891E-03	23409,7	4,28953E-03	26127	2,01004E+00	7049	1,49175E+00	21938	9,33082E-01	13553	0	48381,5
Myeloid_vs_CD8_Responder	Macro_ISG15	CD8(Tc17)	TNFSF10	CCR6	1,04929E-03	15500,9	4,66158E-02	344	2,29089E+00	4439	2,67943E+00	3410	9,20185E-01	20930	0	48381,5
Myeloid_vs_CD8_Responder	Macro_FOLR2+APOE-	CD8(Tn)	CCL13	CXCR3	1,06004E-03	36435,7	3,11485E-02	734	4,04633E+00	406	1,05555E+00	38810	7,88047E-01	93847	0	48381,5
Myeloid_vs_CD8_Responder	CD8(Temra)	Mono_CD16	RPS19	C5AR1	1,06192E-03	22754,9	5,07131E-03	20418	1,95212E+00	7740	1,28311E+00	29237	9,42641E-01	7998	0	48381,5
Myeloid_vs_CD8_Responder	Macro_OLFML3	CD8(GZMK+ Tem)	HLA-DQA1	LAG3	1,06436E-03	15424,5	9,41302E-03	7609	1,61888E+00	13037	2,39426E+00	5680	9,54068E-01	2415	0	48381,5
Myeloid_vs_CD8_Responder	CD8(Tc17)	Mono_CD16	RPS19	C5AR1	1,06537E-03	20819,5	6,06219E-03	15514	2,16024E+00	5515	1,28250E+00	29256	9,47280E-01	5431	0	48381,5
Myeloid_vs_CD8_Responder	CD8(GZMK+ Early Tem)	Mono_CD16	CD99	PILRA	1,06939E-03	23952,1	5,70091E-03	17046	2,09617E+00	6127	1,28193E+00	29278	9,23728E-01	18928	0	48381,5
Myeloid_vs_CD8_Responder	Macro_NLRP3	CD8(ITM2C+ T)	IL1B	ADRB2	1,07111E-03	21214,1	1,32309E-02	4470	2,43561E+00	3472	2,69279E+00	3312	8,75143E-01	46435	0	48381,5
Myeloid_vs_CD8_Responder	CD8(ZNF683+KLRB1+ T)	Mono_CD16	HLA-F	LILRB2	1,07910E-03	25703,9	5,04879E-03	20556	2,49286E+00	3190	1,34018E+00	27061	9,05163E-01	29331	0	48381,5
Myeloid_vs_CD8_Responder	CD8(IL7R+ZNF683+ Tm)	pDC_LILRA4	HMGB1	THBD	1,08002E-03	22718,7	5,77234E-03	16700	1,84937E+00	9087	2,03072E+00	10089	9,05156E-01	29336	0	48381,5
Myeloid_vs_CD8_Responder	Macro_FOLR2+APOE-	CD8(GZMK+ Early Tem)	CCL13	CXCR3	1,08026E-03	37525,3	3,10182E-02	741	4,04516E+00	409	9,47621E-01	44055	7,87697E-01	94040	0	48381,5
Myeloid_vs_CD8_Responder	Macro_OLFML3	CD8(Tc17)	FN1	CD44	1,08082E-03	16930,5	1,18840E-02	5282	2,15533E+00	5558	1,86157E+00	13088	9,35057E-01	12343	0	48381,5
Myeloid_vs_CD8_Responder	CD8(Terminal Tex)	Macro_ISG15	CCL5	CCR1	1,08131E-03	26979,1	8,66756E-03	8687	1,14988E+00	27280	1,28002E+00	29343	9,19665E-01	21204	0	48381,5
Myeloid_vs_CD8_Responder	CD8(Tc17)	cDC(CD1C)	CD28	CD86	1,08309E-03	27976,9	1,17396E-02	5389,5	1,70948E+00	11301,5	1,86120E+00	13095,5	8,48265E-01	61716,5	0	48381,5
Myeloid_vs_CD8_Responder	CD8(Tn)	Mono_INHBA	RPS19	C5AR1	1,08722E-03	24387,5	4,61132E-03	23502	1,71328E+00	11228	1,27917E+00	29375	9,40016E-01	9451	0	48381,5
Myeloid_vs_CD8_Responder	CD8(Terminal Tex)	Macro_NLRP3	HMGB1	CD163	1,09130E-03	24952,3	3,95570E-03	29397	1,73991E+00	10772	1,51824E+00	21158	9,30508E-01	15053	0	48381,5
Myeloid_vs_CD8_Responder	Mono_INHBA	CD8(Tc17)	IL1B	ADRB2	1,09178E-03	20860,5	1,31862E-02	4499	2,55922E+00	2913	2,97740E+00	1959	8,74958E-01	46550	0	48381,5
Myeloid_vs_CD8_Responder	CD8(EOMES+ NK-like)	Mono_CD16	B2M	LILRB2	1,09520E-03	20816,5	4,92328E-03	21366	2,39320E+00	3716	1,27795E+00	29418	9,58034E-01	1201	0	48381,5
Myeloid_vs_CD8_Responder	CD8(NME1+ T)	Macro_NLRP3	CD99	PILRA	1,09725E-03	27083,3	4,82532E-03	22007	1,61158E+00	13189	1,27763E+00	29429	9,17642E-01	22410	0	48381,5
Myeloid_vs_CD8_Responder	Macro_OLFML3	CD8(GZMK+ Tem)	HLA-DQA2	LAG3	1,10404E-03	16785,7	1,24038E-02	4931	1,61312E+00	13159	2,26903E+00	6974	9,38266E-01	10483	0	48381,5
Myeloid_vs_CD8_Responder	Macro_ISG15	CD8(GZMK+ Early Tem)	CXCL10	CXCR3	1,10837E-03	21156,3	1,56492E-02	3396	2,35401E+00	3983	2,52386E+00	4522	8,76660E-01	45499	0	48381,5
Myeloid_vs_CD8_Responder	CD8(Tn)	Macro_FOLR2+APOE+	ANXA1	FPR1	1,11036E-03	27522,5	4,88566E-03	21627	1,45683E+00	17000	1,27602E+00	29499	9,19847E-01	21105	0	48381,5
Myeloid_vs_CD8_Responder	Mono_CD14	CD8(EOMES+ NK-like)	VCAN	CD44	1,11230E-03	15122,5	1,00153E-02	6944	3,19125E+00	1200	2,37091E+00	5903	9,33728E-01	13184	0	48381,5
Myeloid_vs_CD8_Responder	Macro_NLRP3	CD8(ITM2C+ T)	S100A8	CD69	1,11496E-03	17918,9	6,71174E-03	13192	1,96205E+00	7611	2,17223E+00	8088	9,35093E-01	12322	0	48381,5
Myeloid_vs_CD8_Responder	Mono_CD14	CD8(Temra)	IL1B	ADRB2	1,11636E-03	20956,3	1,31307E-02	4533	2,37926E+00	3810	3,16294E+00	1367	8,74728E-01	46690	0	48381,5
Myeloid_vs_CD8_Responder	Macro_NLRP3	CD8(IL7R+ZNF683+ Tm)	VCAN	CD44	1,12428E-03	16733,1	9,99979E-03	6960	2,65408E+00	2523	1,88881E+00	12581	9,33680E-01	13220	0	48381,5
Myeloid_vs_CD8_Responder	CD8(ISG+ T)	pDC_LILRA4	TNF	PTPRS	1,13312E-03	27949,9	3,06715E-02	759	3,68231E+00	646	2,28569E+00	6793	8,08956E-01	83170	0	48381,5
Myeloid_vs_CD8_Responder	CD8(Terminal Tex)	cDC(CD1C)	CSF1	CSF2RA	1,13568E-03	29007,3	1,11887E-02	5857	2,01003E+00	7050	1,85222E+00	13254	8,32580E-01	70494	0	48381,5
Myeloid_vs_CD8_Responder	cDC_CLEC9A	CD8(GZMK+ Tem)	HLA-DRA	LAG3	1,13602E-03	15018,1	7,43244E-03	11167	1,60779E+00	13255	3,23007E+00	1194	9,58447E-01	1093	0	48381,5
Myeloid_vs_CD8_Responder	Macro_OLFML3	CD8(LAYN+ T)	FN1	CD44	1,14176E-03	16510,9	1,27370E-02	4743	2,21926E+00	5017	1,85114E+00	13272	9,37130E-01	11141	0	48381,5
Myeloid_vs_CD8_Responder	CD8(ZNF683+KLRB1+ T)	Mono_CD16	HLA-A	LILRB2	1,14253E-03	20769,7	5,24899E-03	19357	2,42049E+00	3551	1,27135E+00	29668	9,52673E-01	2891	0	48381,5
Myeloid_vs_CD8_Responder	Mono_CD14	CD8(Temra)	VCAN	ITGA4	1,14573E-03	16781,7	1,30679E-02	4573	3,25896E+00	1092	2,57729E+00	4136	9,11876E-01	25726	0	48381,5
Myeloid_vs_CD8_Responder	CD8(ID2+CXCR4+ T)	Mono_CD16	HLA-C	LILRB2	1,14573E-03	23842,5	4,76937E-03	22376	2,27403E+00	4573	1,03705E+00	39707	9,49747E-01	4175	0	48381,5
Myeloid_vs_CD8_Responder	CD8(NME1+ T)	Mono_CD14	HMGB1	THBD	1,14658E-03	24055,5	5,68649E-03	17122	1,63486E+00	12697	1,89845E+00	12388	9,04511E-01	29689	0	48381,5
Myeloid_vs_CD8_Responder	pDC_LILRA4	CD8(GZMK+ Tem)	APP	CD74	1,15295E-03	15245,1	6,67326E-03	13305	2,15690E+00	5545	2,71223E+00	3200	9,46609E-01	5794	0	48381,5
Myeloid_vs_CD8_Responder	CD8(ITM2C+ T)	Macro_OLFML3	IFNG	IFNGR1_IFNGR2	1,16055E-03	26171,9	9,31973E-03	7726	1,38011E+00	19240	1,29043E+00	28969	9,10306E-01	26543	0	48381,5
Myeloid_vs_CD8_Responder	CD8(GZMK+ Tex)	Macro_FOLR2+APOE+	HMGB1	CD163	1,16192E-03	25044,9	4,36752E-03	25437	1,89996E+00	8404	1,26883E+00	29768	9,33642E-01	13234	0	48381,5
Myeloid_vs_CD8_Responder	CD8(ITM2C+ T)	Macro_OLFML3	HSPA1A	TLR4	1,16779E-03	21158,1	6,81947E-03	12849	1,89747E+00	8432	2,08538E+00	9296	9,09745E-01	26832	0	48381,5
Myeloid_vs_CD8_Responder	CD8(ITM2C+ T)	cDC(CD1C)	ANXA1	FPR1	1,17073E-03	29241,9	4,04824E-03	28398	1,31948E+00	21062	1,45544E+00	23087	9,12637E-01	25281	0	48381,5
Myeloid_vs_CD8_Responder	pDC_LILRA4	CD8(Temra)	APP	RPSA	1,17213E-03	16210,9	9,51488E-03	7493	1,95798E+00	7661	2,57447E+00	4158	9,33422E-01	13361	0	48381,5
Myeloid_vs_CD8_Responder	cDC(CD1C)	CD8(Terminal Tex)	HLA-DQB1	LAG3	1,17351E-03	14990,9	8,79446E-03	8479	1,60279E+00	13365	3,10954E+00	1514	9,51934E-01	3215	0	48381,5
Myeloid_vs_CD8_Responder	Macro_NLRP3	CD8(Tc17)	VCAN	CD44	1,17627E-03	16927,1	9,91120E-03	7052	2,64612E+00	2555	1,85107E+00	13274	9,33404E-01	13373	0	48381,5
Myeloid_vs_CD8_Responder	CD8(Temra)	Macro_NLRP3	CD99	PILRA	1,17722E-03	27509,7	4,71258E-03	22740	1,58889E+00	13654	1,26697E+00	29846	9,16745E-01	22927	0	48381,5
Myeloid_vs_CD8_Responder	CD8(ITM2C+ T)	cDC(CD1C)	B2M	CD1C	1,18038E-03	13896,3	2,12275E-02	1847	3,54483E+00	764	1,84447E+00	13393	9,47908E-01	5096	0	48381,5
Myeloid_vs_CD8_Responder	Macro_IFI27	CD8(GZMK+ Tem)	HLA-DRA	LAG3	1,18423E-03	15608,5	7,40187E-03	11264	1,60158E+00	13396	2,60971E+00	3885	9,58365E-01	1116	0	48381,5
Myeloid_vs_CD8_Responder	CD8(ITM2C+ T)	Mono_INHBA	RPS19	C5AR1	1,19049E-03	21689,5	5,25258E-03	19333	1,87432E+00	8732	1,41061E+00	24541	9,43583E-01	7460	0	48381,5
Myeloid_vs_CD8_Responder	CD8(ITM2C+ T)	Mono_INHBA	HSPA1A	TLR4	1,19448E-03	21066,9	6,54796E-03	13761	1,86479E+00	8856	2,30677E+00	6577	9,08063E-01	27759	0	48381,5
Myeloid_vs_CD8_Responder	CD8(ITM2C+ T)	Mono_CD14	ANXA1	FPR1	1,19788E-03	24867,3	4,99283E-03	20893	1,61102E+00	13195	1,51684E+00	21195	9,20644E-01	20672	0	48381,5
Myeloid_vs_CD8_Responder	CD8(ITM2C+ T)	Mono_CD14	TNFSF9	HLA-DPA1	1,20228E-03	19983,9	5,92594E-03	16041	1,55786E+00	14409	2,20118E+00	7726	9,33420E-01	13362	0	48381,5
Myeloid_vs_CD8_Responder	CD8(GZMK+ Tem)	cDC_CLEC9A	CD52	SIGLEC10	1,20268E-03	32239,9	3,91426E-03	29834	1,22662E+00	24200	1,29440E+00	28810	9,03968E-01	29974	0	48381,5
Myeloid_vs_CD8_Responder	cDC_CLEC9A	CD8(Terminal Tex)	HLA-DQB1	LAG3	1,20415E-03	15110,7	8,76939E-03	8513	1,59843E+00	13453	2,97653E+00	1961	9,51869E-01	3245	0	48381,5
Myeloid_vs_CD8_Responder	CD8(ITM2C+ T)	Mono_CD14	RPS19	C5AR1	1,20489E-03	17134,9	6,10796E-03	15333	2,15041E+00	5605	1,97425E+00	11023	9,47467E-01	5332	0	48381,5
Myeloid_vs_CD8_Responder	cDC_CLEC9A	CD8(GZMK+ Tem)	HLA-DPB1	LAG3	1,20661E-03	15077,1	7,55695E-03	10877	1,59816E+00	13460	3,23686E+00	1172	9,56997E-01	1495	0	48381,5
Myeloid_vs_CD8_Responder	CD8(ITM2C+ T)	Mono_CD14	HSPA1A	TLR4	1,20791E-03	16380,9	9,76857E-03	7200	2,25247E+00	4757	2,83677E+00	2477	9,23453E-01	19089	0	48381,5
Myeloid_vs_CD8_Responder	Macro_NLRP3	CD8(LAYN+ T)	VCAN	CD44	1,21013E-03	16522,9	1,06227E-02	6358	2,71005E+00	2335	1,84064E+00	13470	9,35526E-01	12070	0	48381,5
Myeloid_vs_CD8_Responder	CD8(ITM2C+ T)	Macro_FOLR2-APOE+	HSPA1A	TLR4	1,22349E-03	20679,3	6,99741E-03	12338	1,91889E+00	8151	2,15928E+00	8249	9,10796E-01	26277	0	48381,5
Myeloid_vs_CD8_Responder	CD8(Tn)	Mono_CD16	HLA-B	LILRB2	1,22369E-03	22665,7	4,85664E-03	21809	2,26115E+00	4676	1,12244E+00	35795	9,53280E-01	2667	0	48381,5
Myeloid_vs_CD8_Responder	CD8(GZMK+ Early Tem)	Mono_CD14	ANXA1	FPR1	1,22390E-03	29638,3	3,89053E-03	30079	1,45049E+00	17183	1,35686E+00	26408	9,11040E-01	26140	0	48381,5
Myeloid_vs_CD8_Responder	Macro_NLRP3	CD8(GZMK+ Early Tem)	VCAN	CD44	1,22894E-03	16721,3	9,82305E-03	7144	2,63820E+00	2588	1,92098E+00	11970	9,33125E-01	13523	0	48381,5
Myeloid_vs_CD8_Responder	CD8(GZMK+ Early Tem)	pDC_LILRA4	HMGB1	THBD	1,23062E-03	23142,5	5,58567E-03	17545	1,82127E+00	9487	2,02531E+00	10187	9,03736E-01	30112	0	48381,5
Myeloid_vs_CD8_Responder	CD8(ZNF683+KLRB1+ T)	cDC(CD1C)	CD52	SIGLEC10	1,23246E-03	31896,3	3,89254E-03	30060	1,17131E+00	26354	1,40989E+00	24565	9,03726E-01	30121	0	48381,5
Myeloid_vs_CD8_Responder	pDC_LILRA4	CD8(ZNF683+KLRB1+ T)	APP	RPSA	1,23251E-03	16506,3	9,42006E-03	7598	1,94865E+00	7778	2,44093E+00	5241	9,33111E-01	13533	0	48381,5
Myeloid_vs_CD8_Responder	CD8(EOMES+ NK-like)	Mono_CD16	HLA-B	LILRB2	1,23308E-03	21043,1	5,05043E-03	20545	2,38401E+00	3780	1,25938E+00	30124	9,54143E-01	2385	0	48381,5
Myeloid_vs_CD8_Responder	CD8(IL7R+ZNF683+ Tm)	pDC_LILRA4	HMGB1	TLR9	1,23328E-03	26503,5	2,99440E-02	792	3,64426E+00	677	1,04728E+00	39225	8,80267E-01	43442	0	48381,5

Myeloid_vs_CD8_Responder	Mono_CD14	CD8(ITM2C+ T)	IL1B	ADRB2	1,23610E-03	21163,5	1,28255E-02	4692	2,36859E+00	3870	3,15095E+00	1396	8,73434E-01	47478	0	48381,5
Myeloid_vs_CD8_Responder	CD8(ITM2C+ T)	Macro_ISG15	HSPA1A	TLR4	1,23779E-03	18441,5	8,22701E-03	9451	2,06690E+00	6414	2,43548E+00	5282	9,17158E-01	22679	0	48381,5
Myeloid_vs_CD8_Responder	CD8(EOMES+ NK-like)	Mono_CD16	HLA-F	LILRB2	1,23903E-03	26424,5	4,87877E-03	21669	2,47774E+00	3260	1,29869E+00	28659	9,03682E-01	30153	0	48381,5
Myeloid_vs_CD8_Responder	CD8(ITM2C+ T)	Macro_NLRP3	RPS19	C5AR1	1,24892E-03	17482,7	7,02049E-03	12262	2,44495E+00	3428	1,57104E+00	19654	9,50826E-01	3688	0	48381,5
Myeloid_vs_CD8_Responder	CD8(ITM2C+ T)	Macro_NLRP3	HSPA1A	TLR4	1,25306E-03	18349,3	8,47227E-03	8987	2,09643E+00	6124	2,34017E+00	6203	9,18267E-01	22051	0	48381,5
Myeloid_vs_CD8_Responder	cDC(CD1C)	CD8(GZMK+ Tem)	HLA-DPA1	LAG3	1,25338E-03	14993,3	7,56169E-03	10871	1,59200E+00	13591	3,33232E+00	977	9,58236E-01	1146	0	48381,5
Myeloid_vs_CD8_Responder	CD8(GZMK+ Tem)	Mono_CD16	CD99	PILRA	1,25493E-03	24133,1	5,70522E-03	17027	2,09676E+00	6118	1,25716E+00	30230	9,23755E-01	18909	0	48381,5
Myeloid_vs_CD8_Responder	CD8(ITM2C+ T)	cDC_CLEC9A	CD52	SIGLEC10	1,26117E-03	29723,9	4,32990E-03	25776	1,31248E+00	21270	1,38131E+00	25548	9,08260E-01	27644	0	48381,5
Myeloid_vs_CD8_Responder	CD8(GZMK+ Early Tem)	Mono_INHBA	RPS19	C5AR1	1,26638E-03	24574,1	4,60978E-03	23513	1,71289E+00	11238	1,25581E+00	30285	9,40006E-01	9453	0	48381,5
Myeloid_vs_CD8_Responder	CD8(ITM2C+ T)	pDC_LILRA4	HMGB1	THBD	1,27183E-03	22044,1	6,04132E-03	15580	1,88986E+00	8540	2,07838E+00	9411	9,07093E-01	28308	0	48381,5
Myeloid_vs_CD8_Responder	CD8(GZMK+ Tex)	pDC_LILRA4	HSP90B1	TLR9	1,27704E-03	26206,3	2,98004E-02	806	3,78766E+00	555	1,39478E+00	25071	8,58041E-01	56218	0	48381,5
Myeloid_vs_CD8_Responder	CD8(Terminal Tex)	Macro_LYVE1	HSPA1A	TLR4	1,28067E-03	27257,5	5,85539E-03	16330	1,65559E+00	12287	1,29124E+00	28936	9,03289E-01	30353	0	48381,5
Myeloid_vs_CD8_Responder	Macro_IFI27	CD8(GZMK+ Tem)	HLA-DQA2	LAG3	1,28555E-03	17075,7	1,21607E-02	5083	1,58770E+00	13679	2,22999E+00	7410	9,37690E-01	10825	0	48381,5
Myeloid_vs_CD8_Responder	CD8(IL7R+ZNF683+ Tm)	Mono_CD16	HLA-A	LILRB2	1,28870E-03	20879,9	5,26450E-03	19258	2,42862E+00	3502	1,25293E+00	30391	9,52740E-01	2867	0	48381,5
Myeloid_vs_CD8_Responder	CD8(IL7R+ZNF683+ Tm)	Macro_FOLR2+APOE-	HMGB1	CD163	1,29486E-03	26133,9	3,86028E-03	30420	1,91342E+00	8225	1,31274E+00	28100	9,29714E-01	15543	0	48381,5
Myeloid_vs_CD8_Responder	Macro_IER3	CD8(Temra)	IL1B	ADRB2	1,29619E-03	21149,3	1,37599E-02	4198	2,48087E+00	3244	2,49629E+00	4768	8,77270E-01	45155	0	48381,5
Myeloid_vs_CD8_Responder	CD8(ITM2C+ T)	Macro_LYVE1	HMGB1	CD163	1,29827E-03	20065,3	5,39534E-03	18545	2,66001E+00	2500	1,50947E+00	21392	9,39896E-01	9508	0	48381,5
Myeloid_vs_CD8_Responder	CD8(ITM2C+ T)	Macro_LYVE1	HSPA1A	TLR4	1,29891E-03	16789,7	1,03239E-02	6636	2,31931E+00	4222	2,28770E+00	6770	9,25384E-01	17939	0	48381,5
Myeloid_vs_CD8_Responder	CD8(ITM2C+ T)	Mono_CD16	HLA-B	LILRB2	1,31047E-03	19939,7	5,28806E-03	19116	2,53467E+00	2997	1,33798E+00	27149	9,55139E-01	2055	0	48381,5
Myeloid_vs_CD8_Responder	CD8(ITM2C+ T)	Mono_CD16	HLA-C	LILRB2	1,31348E-03	18769,7	5,56969E-03	17620	2,65404E+00	2524	1,46809E+00	22670	9,53322E-01	2653	0	48381,5
Myeloid_vs_CD8_Responder	CD8(ITM2C+ T)	Mono_CD16	CD99	PILRA	1,31456E-03	23682,9	5,93328E-03	16015	2,12826E+00	5790	1,25910E+00	30139	9,25124E-01	18089	0	48381,5
Myeloid_vs_CD8_Responder	CD8(ITM2C+ T)	Mono_CD16	HLA-F	LILRB2	1,31542E-03	23176,1	6,03980E-03	15590	2,58101E+00	2808	1,43372E+00	23785	9,12581E-01	25316	0	48381,5
Myeloid_vs_CD8_Responder	CD8(ITM2C+ T)	Mono_CD16	B2M	LILRB2	1,31607E-03	20604,1	4,93009E-03	21326	2,39977E+00	3675	1,30439E+00	28448	9,58062E-01	1190	0	48381,5
Myeloid_vs_CD8_Responder	CD8(ITM2C+ T)	Mono_CD16	RPS19	C5AR1	1,31758E-03	19817,1	6,28039E-03	14682	2,20607E+00	5112	1,37000E+00	25934	9,48156E-01	4976	0	48381,5
Myeloid_vs_CD8_Responder	CD8(ITM2C+ T)	Mono_CD16	HSPA1A	TLR4	1,32039E-03	17949,7	8,96875E-03	8225	2,15619E+00	5551	2,28690E+00	6780	9,20379E-01	20811	0	48381,5
Myeloid_vs_CD8_Responder	CD8(Terminal Tex)	Macro_NLRP3	RPS19	C5AR1	1,32039E-03	25851,5	3,84964E-03	30539	1,84916E+00	9090	1,29758E+00	28703	9,34719E-01	12544	0	48381,5
Myeloid_vs_CD8_Responder	CD8(Temra)	Mono_CD14	ANXA1	FPR1	1,32277E-03	30050,1	3,99339E-03	28969	1,46547E+00	16749	1,24924E+00	30550	9,12091E-01	25601	0	48381,5
Myeloid_vs_CD8_Responder	CD8(ITM2C+ T)	Mast	HSPA8	ADRB2	1,32428E-03	20401,5	1,06902E-02	6295	1,42327E+00	17973	1,68612E+00	16780	9,34668E-01	12578	0	48381,5
Myeloid_vs_CD8_Responder	CD8(Tn)	Mono_CD16	B2M	LILRB2	1,32531E-03	21935,1	4,77018E-03	22369	2,24556E+00	4804	1,19396E+00	32751	9,57394E-01	1370	0	48381,5
Myeloid_vs_CD8_Responder	CD8(LAYN+ T)	Mast	HSPA8	ADRB2	1,32754E-03	26326,1	8,92198E-03	8284	1,12412E+00	28422	1,24873E+00	30572	9,28926E-01	15971	0	48381,5
Myeloid_vs_CD8_Responder	CD8(NME1+ T)	Macro_FOLR2-APOE+	HMGB1	TLR2	1,33101E-03	30503,1	4,17027E-03	27216	1,42286E+00	17992	1,24845E+00	30588	9,07020E-01	28338	0	48381,5
Myeloid_vs_CD8_Responder	CD8(Terminal Tex)	pDC_LILRA4	HSP90B1	TLR9	1,33120E-03	26775,3	2,95202E-02	823	3,78221E+00	561	1,32725E+00	27573	8,57465E-01	56538	0	48381,5
Myeloid_vs_CD8_Responder	CD8(Tn)	pDC_LILRA4	TNF	TNFRSF21	1,33120E-03	33177,3	8,81869E-03	8434	3,47337E+00	823	1,74620E+00	15419	7,90045E-01	92829	0	48381,5
Myeloid_vs_CD8_Responder	CD8(NME1+ T)	Macro_LYVE1	HMGB1	TLR4	1,33254E-03	26821,9	6,26563E-03	14726	1,75099E+00	10586	1,24830E+00	30595	9,04260E-01	29821	0	48381,5
Myeloid_vs_CD8_Responder	CD8(Tn)	pDC_LILRA4	CD40LG	TRAF3	1,33442E-03	45019,7	3,43101E-02	627	7,06727E-01	52286	2,05954E+00	9671	7,43843E-01	114133	0	48381,5
Myeloid_vs_CD8_Responder	cDC_CLEC9A	CD8(Terminal Tex)	HLA-DPA1	LAG3	1,33646E-03	15235,3	7,33273E-03	11453	1,58180E+00	13815	3,21701E+00	1218	9,57616E-01	1309	0	48381,5
Myeloid_vs_CD8_Responder	CD8(ITM2C+ T)	Macro_IER3	HSPA1A	TLR4	1,33734E-03	20591,1	6,78953E-03	12935	1,89387E+00	8481	2,33775E+00	6230	9,09564E-01	26928	0	48381,5
Myeloid_vs_CD8_Responder	Mono_CD14	CD8(LAYN+ T)	S100A9	ITGB2	1,33764E-03	18996,7	3,85347E-03	30494	3,55205E+00	755	3,41345E+00	825	9,31415E-01	14528	0	48381,5
Myeloid_vs_CD8_Responder	cDC(CD1C)	CD8(GZMK+ Tem)	HLA-DQA1	LAG3	1,34178E-03	14825,9	9,18155E-03	7919	1,58135E+00	13829	3,14372E+00	1413	9,53519E-01	2587	0	48381,5
Myeloid_vs_CD8_Responder	CD8(ITM2C+ T)	Macro_FOLR2+APOE-	HMGB1	CD163	1,34456E-03	25098,7	4,04016E-03	28486	1,95391E+00	7712	1,36040E+00	26252	9,31187E-01	14662	0	48381,5
Myeloid_vs_CD8_Responder	CD8(GZMK+ Early Tem)	Macro_ISG15	CCL4	CCR1	1,34500E-03	28679,1	7,69257E-03	10560	1,07513E+00	30652	1,41046E+00	24547	9,05325E-01	29255	0	48381,5
Myeloid_vs_CD8_Responder	CD8(ITM2C+ T)	Macro_FOLR2+APOE+	TNF	VSIR	1,34719E-03	25112,3	1,24189E-02	4917	1,32522E+00	20878	1,43270E+00	23829	9,08439E-01	27556	0	48381,5
Myeloid_vs_CD8_Responder	Mast	CD8(EOMES+ NK-like)	TIMP3	CD44	1,34733E-03	18904,3	2,93902E-02	828	3,60619E+00	708	1,54402E+00	20399	9,14569E-01	24205	0	48381,5
Myeloid_vs_CD8_Responder	CD8(GZMK+ Tex)	Mono_INHBA	RPS19	C5AR1	1,34873E-03	27241,3	3,90424E-03	29939	1,53570E+00	14930	1,24667E+00	30669	9,35147E-01	12287	0	48381,5
Myeloid_vs_CD8_Responder	Macro_ISG15	CD8(LAYN+ T)	CXCL10	CXCR3	1,35073E-03	19948,3	1,85081E-02	2498	2,40489E+00	3645	2,48789E+00	4835	8,85449E-01	40382	0	48381,5
Myeloid_vs_CD8_Responder	Macro_ISG15	CD8(Temra)	CXCL10	CXCR3	1,35156E-03	22176,7	1,39139E-02	4120	2,32312E+00	4201	2,48788E+00	4836	8,70164E-01	49345	0	48381,5
Myeloid_vs_CD8_Responder	CD8(GZMK+ Tex)	Mono_CD16	CD99	PILRA	1,35314E-03	24963,1	5,33907E-03	18834	2,04620E+00	6642	1,24627E+00	30689	9,21386E-01	20269	0	48381,5
Myeloid_vs_CD8_Responder	Macro_FOLR2+APOE+	CD8(Terminal Tex)	HLA-DRA	LAG3	1,35669E-03	16334,3	7,13620E-03	11960	1,57976E+00	13868	2,34435E+00	6157	9,57629E-01	1305	0	48381,5
Myeloid_vs_CD8_Responder	CD8(ITM2C+ T)	Macro_FOLR2+APOE+	HSPA1A	TLR4	1,36043E-03	22143,1	6,36633E-03	14343	1,84293E+00	9189	2,01321E+00	10390	9,06882E-01	28412	0	48381,5
Myeloid_vs_CD8_Responder	pDC_LILRA4	CD8(ITM2C+ T)	APP	PTGER2	1,36730E-03	24562,3	2,24056E-02	1605	2,23871E+00	4855	2,79552E+00	2707	8,41906E-01	65263	0	48381,5
Myeloid_vs_CD8_Responder	Macro_OLFML3	CD8(ISG+ T)	HLA-DRA	LAG3	1,36902E-03	15958,1	6,50423E-03	13900	1,60978E+00	13218	2,85401E+00	2418	9,55708E-01	1873	0	48381,5
Myeloid_vs_CD8_Responder	Macro_OLFML3	CD8(Tn)	FN1	CD44	1,37176E-03	18468,7	9,99962E-03	6961	2,01412E+00	7000	1,79359E+00	14397	9,29614E-01	15604	0	48381,5
Myeloid_vs_CD8_Responder	CD8(GZMK+ Tex)	pDC_LILRA4	COPA	P2RY6	1,37212E-03	34150,5	6,50204E-03	13908	2,24913E+00	4782	2,06866E+00	9559	7,87549E-01	94122	0	48381,5
Myeloid_vs_CD8_Responder	Macro_OLFML3	CD8(Tn)	APOE	LSR	1,37287E-03	17551,5	1,82291E-02	2579	2,23234E+00	4920	3,12122E+00	1473	9,03201E-01	30404	0	48381,5
Myeloid_vs_CD8_Responder	CD8(ID2+CXCR4+ T)	Macro_LYVE1	RPS19	C5AR1	1,37689E-03	21223,9	6,02589E-03	15658	2,13152E+00	5764	1,24351E+00	30796	9,47129E-01	5520	0	48381,5
Myeloid_vs_CD8_Responder	CD8(GZMK+ Tem)	Mono_INHBA	RPS19	C5AR1	1,37756E-03	25652,9	4,31580E-03	25898	1,63906E+00	12617	1,24345E+00	30799	9,38121E-01	10569	0	48381,5
Myeloid_vs_CD8_Responder	Macro_IER3	CD8(ITM2C+ T)	IL1B	ADRB2	1,37981E-03	21369,9	1,34401E-02	4368	2,47020E+00	3295	2,48431E+00	4870	8,75998E-01	45935	0	48381,5
Myeloid_vs_CD8_Responder	CD8(NME1+ T)	Mono_CD14	RPS19	C5AR1	1,38092E-03	24131,7	3,82479E-03	30814	1,65733E+00	12251	1,69785E+00	16526	9,34521E-01	12686	0	48381,5
Myeloid_vs_CD8_Responder	Macro_OLFML3	CD8(Tn)	C3	IFITM1	1,38137E-03	16452,9	1,03967E-02	6558	2,28528E+00	4481	1,81190E+00	14022	9,41144E-01	8822	0	48381,5
Myeloid_vs_CD8_Responder	Macro_OLFML3	CD8(ID2+CXCR4+ T)	HLA-DRA	LAG3	1,38833E-03	22190,3	3,98923E-03	29024	1,35048E+00	20119	2,33237E+00	6303	9,44129E-01	7124	0	48381,5
Myeloid_vs_CD8_Responder	Macro_OLFML3	CD8(ID2+CXCR4+ T)	HLA-DQA2	LAG3	1,38855E-03	27519,5	5,88398E-03	16208	1,15737E+00	26945	1,52772E+00	20867	9,12800E-01	25196	0	48381,5
Myeloid_vs_CD8_Responder	Macro_OLFML3	CD8(ID2+CXCR4+ T)	HLA-DQA1	LAG3	1,38878E-03	25970,7	4,46525E-03	24615	1,16313E+00	26703	1,65294E+00	17572	9,34667E-01	12582	0	48381,5
Myeloid_vs_CD8_Responder	Macro_OLFML3	CD8(ID2+CXCR4+ T)	HLA-DQB1	LAG3	1,38923E-03	24448,9	4,59088E-03	23661	1,26084E+00	22972	1,78071E+00	14658	9,34680E-01	12572	0	48381,5
Myeloid_vs_CD8_Responder	CD8(Tn)	Mono_CD16	HLA-F	LILRB2	1,39035E-03	26192,9	4,73268E-03	22606	2,46474E+00	3320	1,37385E+00	25801	9,02351E-01	30856	0	48381,5
Myeloid_vs_CD8_Responder	Macro_OLFML3	CD8(Terminal Tex)	HLA-DQA1	LAG3	1,39236E-03	15929,7	8,95250E-03	8246	1,57646E+00	13960	2,33466E+00	6270	9,52956E-01	2791	0	48381,5
Myeloid_vs_CD8_Responder	Mono_CD14	CD8(Terminal Tex)	VCAN	ITGA4	1,39240E-03	17097,1	1,24671E-02	4885	3,23862E+00	1120	2,54092E+00	4395	9,09967E-01	26704	0	48381,5
Myeloid_vs_CD8_Responder	CD8(Terminal Tex)	cDC_LAMP3	IFNG	IFNGR1_IFNGR2	1,39509E-03	28083,3	7,72002E-03	10504	1,16943E+00	26416	1,42017E+00	24238	9,02315E-01	30877	0	48381,5
Myeloid_vs_CD8_Responder	Macro_OLFML3	CD8(ID2+CXCR4+ T)	HLA-DRB5	LAG3	1,40030E-03	23610,9	4,39245E-03	25203	1,25984E+00	23009	2,00501E+00	10511	9,37456E-01	10950	0	48381,5
Myeloid_vs_CD8_Responder	Macro_OLFML3	CD8(ID2+CXCR4+ T)	FN1	CD44	1,40279E-03	21698,5	8,11533E-03	9656	1,87291E+00	8752	1,48747E+00	22063	9,22469E-01	19640	0	48381,5
Myeloid_vs_CD8_Responder	pDC_LILRA4	CD8(Temra)	APP	PTGER2	1,40337E-03	24555,9	2,22494E-02	1629	2,23561E+00	4898	2,88183E+00	2321	8,41440E-01	65550	0	48381,5
Myeloid_vs_CD8_Responder	CD8(NME1+ T)	Macro_IER3	HMGB1	THBD	1,40597E-03	27027,1	5,39748E-03	18532	1,57858E+00	13904	1,44502E+00	23393	9,02235E-01	30925	0	48381,5
Myeloid_vs_CD8_Responder	Macro_OLFML3	CD8(ID2+CXCR4+ T)	GRN	TNFRSF1B	1,40620E-03	23597,5	3,83480E-03	30711	1,75206E+00	10570	1,75023E+00	15325	9,34012E-01	13000	0	48381,5
Myeloid_vs_CD8_Responder	Macro_OLFML3	CD8(ID2+CXCR4+ T)	FN1	ITGA4_ITGB1	1,40688E-03	21305,5	1,11978E-02	5848	2,01284E+00	7013	1,79570E+00	14356	9,02231E-01	30929	0	48381,5
Myeloid_vs_CD8_Responder	Macro_OLFML3	CD8(ID2+CXCR4+ T)	CXCL16	CXCR6	1,40824E-03	24648,9	1,03488E-02	6614	1,43921E+00	17535	1,35520E+00	26477	9,14523E-01	24237	0	48381,5
Myeloid_vs_CD8_Responder	Macro_OLFML3	CD8(ID2+CXCR4+ T)	C1QB	C1QBP	1,41189E-03	21468,9	4,12624E-03	27636	2,15271E+00	5578	2,80404E+00	2665	9,16483E-01	23084	0	48381,5
Myeloid_vs_CD8_Responder	Macro_OLFML3	CD8(ID2+CXCR4+ T)	C3	IFITM1	1,41212E-03	15764,7	1,17313E-02	5401	2,37360E+00	3841	1,80195E+00	14224	9,44401E-01	6976	0	48381,5
Myeloid_vs_CD8_Responder	Macro_OLFML3	CD8(Tc17)	APOE	LSR	1,41695E-03	17608,9	1,82870E-02	2563	2,23352E+00	4914	2,99943E+00	1865	9,03339E-01	30321	0	48381,5

Myeloid_vs_CD8_Post_R

Myeloid_vs_CD8_Responder	Macro_OLFML3	CD8(Terminal Tex)	HLA-DQA2	LAG3	1,41875E-03	17356,3	1,17969E-02	5345	1,57070E+00	14097	2,20943E+00	7646	9,36797E-01	11312	0	48381,5
Myeloid_vs_CD8_Responder	Macro_OLFML3	CD8(GZMK+ Early Tem)	C3	IFITM1	1,41917E-03	15865,7	1,14662E-02	5616	2,35606E+00	3962	1,81154E+00	14028	9,43798E-01	7341	0	48381,5
Myeloid_vs_CD8_Responder	Macro_OLFML3	CD8(Terminal Tex)	HLA-DRB1	LAG3	1,41920E-03	16512,5	7,01666E-03	12275	1,50999E+00	15587	2,45953E+00	5077	9,57855E-01	1242	0	48381,5
Myeloid_vs_CD8_Responder	Macro_OLFML3	CD8(Terminal Tex)	CD14	ITGA4	1,42287E-03	27894,3	3,81728E-03	30912	1,65091E+00	12390	1,57489E+00	19545	9,07202E-01	28243	0	48381,5
Myeloid_vs_CD8_Responder	CD8(Tn)	Mono_CD16	RPS19	C5AR1	1,42356E-03	22132,1	5,51365E-03	17896	2,04502E+00	6650	1,23856E+00	31002	9,44860E-01	6731	0	48381,5
Myeloid_vs_CD8_Responder	CD8(Terminal Tex)	Mono_CD14	CSF1	CSF3R	1,42473E-03	30667,1	1,26056E-02	4811	1,82403E+00	9450	1,81092E+00	14042	8,21265E-01	76651	0	48381,5
Myeloid_vs_CD8_Responder	CD8(IL7R+ZNF683+ Tm)	Mono_CD16	CD99	PILRA	1,42793E-03	24207,1	5,74550E-03	16836	2,10233E+00	6046	1,23779E+00	31021	9,24002E-01	18751	0	48381,5
Myeloid_vs_CD8_Responder	CD8(GZMK+ Early Tem)	pDC_LILRA4	HMGB1	TLR9	1,42947E-03	26765,1	2,89757E-02	853	3,61616E+00	697	1,04188E+00	39462	8,78524E-01	44432	0	48381,5
Myeloid_vs_CD8_Responder	CD8(Terminal Tex)	Macro_IER3	IFNG	IFNGR1_IFNGR2	1,43185E-03	28735,3	7,67301E-03	10603	1,13519E+00	27919	1,37566E+00	25735	9,02045E-01	31038	0	48381,5
Myeloid_vs_CD8_Responder	Macro_OLFML3	CD8(Terminal Tex)	FN1	CD44	1,43277E-03	23857,3	6,65308E-03	13366	1,76334E+00	10392	1,45033E+00	23221	9,15059E-01	23926	0	48381,5
Myeloid_vs_CD8_Responder	Macro_OLFML3	CD8(Terminal Tex)	HLA-DQB2	LAG3	1,43438E-03	20993,1	1,61919E-02	3198	1,84399E+00	9173	1,59951E+00	18907	9,12589E-01	25306	0	48381,5
Myeloid_vs_CD8_Responder	Macro_OLFML3	CD8(Terminal Tex)	C1QB	C1QBP	1,44481E-03	21985,3	3,97048E-03	29241	2,14021E+00	5693	2,80607E+00	2656	9,14999E-01	23955	0	48381,5
Myeloid_vs_CD8_Responder	Macro_OLFML3	CD8(GZMK+ Tem)	C3	IFITM1	1,45119E-03	16434,3	1,04548E-02	6501	2,28913E+00	4459	1,80794E+00	14108	9,41298E-01	8722	0	48381,5
Myeloid_vs_CD8_Responder	Macro_OLFML3	CD8(GZMK+ Tex)	HLA-DRA	LAG3	1,45226E-03	17418,1	5,56004E-03	17665	1,51244E+00	15516	2,84531E+00	2455	9,52267E-01	3073	0	48381,5
Myeloid_vs_CD8_Responder	Macro_OLFML3	CD8(GZMK+ Tex)	HLA-DQA2	LAG3	1,45249E-03	21394,9	8,20088E-03	9499	1,31933E+00	21065	2,04067E+00	9952	9,25139E-01	18077	0	48381,5
Myeloid_vs_CD8_Responder	Macro_OLFML3	CD8(GZMK+ Tex)	HLA-DQA1	LAG3	1,45273E-03	19888,9	6,22351E-03	14871	1,32509E+00	20881	2,16589E+00	8162	9,44101E-01	7149	0	48381,5
Myeloid_vs_CD8_Responder	Macro_OLFML3	CD8(GZMK+ Tex)	HLA-DRB1	LAG3	1,45296E-03	20796,3	4,87777E-03	21679	1,25862E+00	23052	2,29077E+00	6747	9,49873E-01	4122	0	48381,5
Myeloid_vs_CD8_Responder	Macro_OLFML3	CD8(GZMK+ Tex)	HLA-DQB1	LAG3	1,45319E-03	18893,3	6,39861E-03	14237	1,42280E+00	17993	2,29366E+00	6717	9,44113E-01	7138	0	48381,5
Myeloid_vs_CD8_Responder	Macro_OLFML3	CD8(GZMK+ Tex)	HLA-DPB1	LAG3	1,45459E-03	19198,5	5,27439E-03	19191	1,39491E+00	18792	2,46040E+00	5064	9,48959E-01	4564	0	48381,5
Myeloid_vs_CD8_Responder	Macro_OLFML3	CD8(GZMK+ Tex)	CD14	ITGB1	1,45646E-03	24525,9	4,21600E-03	26790	1,73690E+00	10821	1,87219E+00	12906	9,15401E-01	23731	0	48381,5
Myeloid_vs_CD8_Responder	Macro_OLFML3	CD8(GZMK+ Tex)	CD14	ITGA4	1,45670E-03	26219,7	4,09097E-03	27971	1,68118E+00	11804	1,70807E+00	16293	9,10076E-01	26649	0	48381,5
Myeloid_vs_CD8_Responder	CD8(NME1+ T)	Mono_CD14	HMGB1	TLR4	1,45693E-03	24323,5	5,92861E-03	16029	1,68414E+00	11747	1,79737E+00	14314	9,01840E-01	31146	0	48381,5
Myeloid_vs_CD8_Responder	Macro_OLFML3	CD8(Terminal Tex)	LYZ	ITGAL	1,45857E-03	27469,3	3,79779E-03	31153	1,56758E+00	14169	1,89356E+00	12495	9,01837E-01	31148	0	48381,5
Myeloid_vs_CD8_Responder	Macro_OLFML3	CD8(GZMK+ Tex)	HLA-DPA1	LAG3	1,46044E-03	19121,5	5,24034E-03	19409	1,37738E+00	19328	2,52160E+00	4546	9,50249E-01	3943	0	48381,5
Myeloid_vs_CD8_Responder	Macro_OLFML3	CD8(GZMK+ Tex)	HLA-DRB5	LAG3	1,46443E-03	18416,7	6,12203E-03	15274	1,42180E+00	18021	2,51796E+00	4569	9,46511E-01	5838	0	48381,5
Myeloid_vs_CD8_Responder	Macro_OLFML3	CD8(GZMK+ Tex)	FN1	ITGA4_ITGB1	1,46631E-03	19608,3	1,25807E-02	4818	2,06578E+00	6425	2,02385E+00	10203	9,07248E-01	28214	0	48381,5
Myeloid_vs_CD8_Responder	Macro_OLFML3	CD8(GZMK+ Tex)	FN1	CD44	1,46678E-03	20624,5	8,33269E-03	9247	1,88920E+00	8557	1,64319E+00	17821	9,23409E-01	19116	0	48381,5
Myeloid_vs_CD8_Responder	Macro_OLFML3	CD8(GZMK+ Tex)	CXCL16	CXCR6	1,47432E-03	26514,3	8,23537E-03	9426	1,30560E+00	21498	1,42956E+00	23934	9,05161E-01	29332	0	48381,5
Myeloid_vs_CD8_Responder	Macro_NLRP3	CD8(Tc17)	IL1B	ADRB2	1,47912E-03	21874,7	1,23156E-02	4986	2,40459E+00	3646	2,65247E+00	3602	8,71174E-01	48758	0	48381,5
Myeloid_vs_CD8_Responder	Macro_OLFML3	CD8(IL7R+ZNF683+ Tm)	APOE	LSR	1,48087E-03	17772,3	1,77955E-02	2697	2,22350E+00	4988	3,01351E+00	1814	9,02143E-01	30981	0	48381,5
Myeloid_vs_CD8_Responder	CD8(IL7R+ZNF683+ Tm)	Mono_CD16	B2M	LILRB2	1,48118E-03	21283,1	4,88798E-03	21609	2,35916E+00	3942	1,23213E+00	31249	9,57889E-01	1234	0	48381,5
Myeloid_vs_CD8_Responder	CD8(Terminal Tex)	Macro_ISG15	IFNG	IFNGR1_IFNGR2	1,48260E-03	26944,1	7,60580E-03	10753	1,17594E+00	26132	1,62828E+00	18199	9,01656E-01	31255	0	48381,5
Myeloid_vs_CD8_Responder	CD8(ZNF683+KLRB1+ T)	pDC_LILRA4	BST2	LILRA4	1,48526E-03	20942,3	1,23092E-02	4993	5,79371E+00	125	2,90595E+00	2237	8,70768E-01	48975	0	48381,5
Myeloid_vs_CD8_Responder	Mono_CD14	CD8(ID2+CXCR4+ T)	VCAN	ITGB1	1,48701E-03	16680,3	1,23039E-02	4995	3,27027E+00	1071	2,56301E+00	4247	9,13605E-01	24707	0	48381,5
Myeloid_vs_CD8_Responder	CD8(GZMK+ Tem)	pDC_LILRA4	HSP90B1	TLR9	1,49010E-03	27236,7	2,87136E-02	871	3,76651E+00	577	1,29293E+00	28860	8,55764E-01	57494	0	48381,5
Myeloid_vs_CD8_Responder	Macro_OLFML3	CD8(ID2+CXCR4+ T)	HLA-DPB1	LAG3	1,49235E-03	24887,7	3,78428E-03	31296	1,23295E+00	23958	1,94745E+00	11497	9,40292E-01	9306	0	48381,5
Myeloid_vs_CD8_Responder	Macro_ISG15	CD8(Tc17)	S100A8	CD69	1,49278E-03	18874,9	6,90275E-03	12604	1,56580E+00	14210	2,23596E+00	7336	9,35940E-01	11843	0	48381,5
Myeloid_vs_CD8_Responder	cDC_CLEC9A	CD8(Terminal Tex)	HLA-DRA	LAG3	1,49691E-03	15489,5	7,06882E-03	12139	1,56537E+00	14220	3,17047E+00	1347	9,57436E-01	1360	0	48381,5
Myeloid_vs_CD8_Responder	Macro_OLFML3	CD8(Tc17)	APOE	SORL1	1,49976E-03	20121,1	5,83412E-03	16428	2,20494E+00	5122	3,02294E+00	1774	9,05976E-01	28900	0	48381,5
Myeloid_vs_CD8_Responder	Macro_OLFML3	CD8(Tc17)	C3	IFITM1	1,50263E-03	16992,1	1,07073E-02	6281	2,30584E+00	4317	1,65124E+00	17614	9,41954E-01	8367	0	48381,5
Myeloid_vs_CD8_Responder	CD8(Tn)	Macro_IFI27	CD40LG	CD9	1,50374E-03	30137,5	4,09670E-02	426	1,44168E+00	17469	1,65057E+00	17639	8,39198E-01	66772	0	48381,5
Myeloid_vs_CD8_Responder	CD8(Terminal Tex)	Mono_CD16	IFNG	IFNGR1_IFNGR2	1,50551E-03	29176,9	7,57598E-03	10833	1,06406E+00	31110	1,42113E+00	24209	9,01482E-01	31351	0	48381,5
Myeloid_vs_CD8_Responder	CD8(ID2+CXCR4+ T)	Macro_NLRP3	HMGB1	CD163	1,50671E-03	26199,9	3,77934E-03	31356	1,69058E+00	11628	1,43578E+00	23709	9,29018E-01	15925	0	48381,5
Myeloid_vs_CD8_Responder	cDC_LAMP3	CD8(ITM2C+ T)	CCL19	CCR7	1,50716E-03	33645,1	2,86368E-02	876	6,28298E+00	84	1,55459E+00	20137	7,77993E-01	98747	0	48381,5
Myeloid_vs_CD8_Responder	Macro_OLFML3	CD8(Tc17)	TNFSF10	CCR6	1,50768E-03	20474,7	3,17729E-02	714	1,88318E+00	8625	1,75542E+00	15193	9,04926E-01	29460	0	48381,5
Myeloid_vs_CD8_Responder	Macro_FOLR2+APOE+	CD8(NME1+ T)	C1QB	C1QBP	1,51183E-03	16479,1	6,41103E-03	14196	2,51090E+00	3103	2,84232E+00	2459	9,31873E-01	14256	0	48381,5
Myeloid_vs_CD8_Responder	CD8(ISG+ T)	pDC_LILRA4	TNF	TNFRSF21	1,51391E-03	28616,5	1,22011E-02	5054	3,53774E+00	768	2,08843E+00	9251	8,15706E-01	79628	0	48381,5
Myeloid_vs_CD8_Responder	pDC_LILRA4	CD8(IL7R+ZNF683+ Tm)	APP	PTGER2	1,51709E-03	24946,7	2,13096E-02	1829	2,21693E+00	5029	2,87633E+00	2344	8,38540E-01	67150	0	48381,5
Myeloid_vs_CD8_Responder	Mono_CD14	CD8(Temra)	VCAN	ITGB1	1,51887E-03	16669,1	1,22334E-02	5031	3,26765E+00	1077	2,59230E+00	4009	9,13378E-01	24847	0	48381,5
Myeloid_vs_CD8_Responder	CD8(NME1+ T)	Macro_LYVE1	HSP90B1	LRP1	1,52264E-03	25424,3	5,49321E-03	18017	2,30315E+00	4341	1,22761E+00	31422	9,13228E-01	24960	0	48381,5
Myeloid_vs_CD8_Responder	Macro_NLRP3	CD8(Tc17)	CXCL2	DPP4	1,53048E-03	21535,3	4,44825E-02	370	2,66808E+00	2476	2,46301E+00	5044	8,66594E-01	51405	0	48381,5
Myeloid_vs_CD8_Responder	CD8(EOMES+ NK-like)	pDC_LILRA4	HSP90B1	TLR9	1,53466E-03	27587,9	2,84215E-02	884	3,76083E+00	580	1,25792E+00	30199	8,55132E-01	57895	0	48381,5
Myeloid_vs_CD8_Responder	Macro_OLFML3	CD8(IL7R+ZNF683+ Tm)	C3	IFITM1	1,53479E-03	15754,9	1,18970E-02	5272	2,38457E+00	3776	1,78442E+00	14567	9,44768E-01	6778	0	48381,5
Myeloid_vs_CD8_Responder	Macro_OLFML3	CD8(Terminal Tex)	FN1	ITGA4_ITGB1	1,53625E-03	21458,5	1,09506E-02	6058	2,00357E+00	7122	1,80049E+00	14253	9,01242E-01	31478	0	48381,5
Myeloid_vs_CD8_Responder	Macro_OLFML3	CD8(ID2+CXCR4+ T)	CD14	ITGB1	1,53967E-03	27363,9	3,76731E-03	31492	1,68256E+00	11784	1,59699E+00	18970	9,10941E-01	26192	0	48381,5
Myeloid_vs_CD8_Responder	CD8(EOMES+ NK-like)	Mono_CD14	ANXA1	FPR1	1,54016E-03	30714,9	3,76716E-03	31494	1,43253E+00	17715	1,28576E+00	29143	9,09725E-01	26841	0	48381,5
Myeloid_vs_CD8_Responder	Macro_OLFML3	CD8(ISG+ T)	HLA-DQA2	LAG3	1,54236E-03	19777,1	9,59352E-03	7394	1,41668E+00	18159	2,04936E+00	9830	9,30393E-01	15121	0	48381,5
Myeloid_vs_CD8_Responder	Macro_OLFML3	CD8(ISG+ T)	HLA-DQA1	LAG3	1,54261E-03	18208,9	7,28036E-03	11593	1,42244E+00	18008	2,17459E+00	8054	9,48099E-01	5008	0	48381,5
Myeloid_vs_CD8_Responder	Macro_OLFML3	CD8(ISG+ T)	HLA-DRB1	LAG3	1,54285E-03	18919,1	5,70609E-03	17017	1,35597E+00	19944	2,29946E+00	6653	9,53478E-01	2600	0	48381,5
Myeloid_vs_CD8_Responder	Macro_OLFML3	CD8(ISG+ T)	HLA-DQB1	LAG3	1,54310E-03	17275,7	7,48519E-03	11032	1,52015E+00	15330	2,30235E+00	6631	9,48109E-01	5004	0	48381,5
Myeloid_vs_CD8_Responder	Macro_OLFML3	CD8(ISG+ T)	HLA-DPB1	LAG3	1,54432E-03	17483,5	6,17007E-03	15088	1,49226E+00	16040	2,46909E+00	4989	9,52626E-01	2919	0	48381,5
Myeloid_vs_CD8_Responder	Macro_OLFML3	CD8(ISG+ T)	HLA-DPA1	LAG3	1,54972E-03	17417,3	6,13023E-03	15239	1,47473E+00	16511	2,53029E+00	4467	9,53828E-01	2488	0	48381,5
Myeloid_vs_CD8_Responder	Macro_OLFML3	CD8(ISG+ T)	HLA-DRB5	LAG3	1,55292E-03	16805,7	7,16165E-03	11892	1,51915E+00	15355	2,52665E+00	4500	9,50345E-01	3900	0	48381,5
Myeloid_vs_CD8_Responder	Macro_OLFML3	CD8(ISG+ T)	FN1	CD44	1,55489E-03	20459,1	8,48856E-03	8964	1,90088E+00	8389	1,64202E+00	17846	9,24062E-01	18715	0	48381,5
Myeloid_vs_CD8_Responder	CD8(Temra)	pDC_LILRA4	HSP90B1	TLR9	1,55544E-03	27366,1	2,83925E-02	890	3,76026E+00	581	1,28814E+00	29048	8,55068E-01	57930	0	48381,5
Myeloid_vs_CD8_Responder	Macro_OLFML3	CD8(ISG+ T)	HLA-DQB2	LAG3	1,55662E-03	23617,5	1,31676E-02	4508	1,68996E+00	11639	1,43943E+00	23593	9,03983E-01	29966	0	48381,5
Myeloid_vs_CD8_Responder	CD8(GZMK+ Early Tem)	cDC_LAMP3	TNFSF9	HLA-DPA1	1,55958E-03	28410,5	4,07879E-03	28078	1,05440E+00	31573	1,84160E+00	13453	9,20831E-01	20567	0	48381,5
Myeloid_vs_CD8_Responder	Macro_IFI27	CD8(Terminal Tex)	HLA-DRA	LAG3	1,56023E-03	16135,7	7,03975E-03	12215	1,55916E+00	14371	2,55011E+00	4332	9,57352E-01	1379	0	48381,5
Myeloid_vs_CD8_Responder	CD8(LAYN+ T)	Macro_LYVE1	HSP90B1	LRP1	1,56056E-03	25379,3	5,52625E-03	17841	2,30731E+00	4307	1,22396E+00	31577	9,13465E-01	24790	0	48381,5
Myeloid_vs_CD8_Responder	Macro_OLFML3	CD8(ID2+CXCR4+ T)	HLA-DPA1	LAG3	1,56106E-03	24700,5	3,75985E-03	31579	1,21542E+00	24620	2,00865E+00	10466	9,41788E-01	8456	0	48381,5
Myeloid_vs_CD8_Responder	Mono_CD14	CD8(GZMK+ Tem)	VCAN	CD44	1,56449E-03	15520,9	9,38181E-03	7647	3,14725E+00	1274	2,36940E+00	5921	9,31677E-01	14381	0	48381,5
Myeloid_vs_CD8_Responder	Mono_CD14	CD8(NME1+ T)	VCAN	ITGB1	1,56931E-03	16839,3	1,21562E-02	5087	3,26479E+00	1085	2,51163E+00	4627	9,13127E-01	25016	0	48381,5
Myeloid_vs_CD8_Responder	Macro_ISG15	CD8(Terminal Tex)	B2M	CD3D	1,57535E-03	45321,9	2,79168E-03	48347	4,56531E-01	73377	7,34711E-01	56494	9,66101E-01	10	0	48381,5
Myeloid_vs_CD8_Responder	CD8(IL7R+ZNF683+ Tm)	pDC_LILRA4	HSP90B1	TLR9	1,57637E-03	27741,5	2,82816E-02	896	3,75811E+00	583	1,24415E+00	30769	8,54826E-01	58078	0	48381,5
Myeloid_vs_CD8_Responder	CD8(Terminal Tex)	Mono_INHBA	CCL5	CCRL2	1,57644E-03	27774,1	8,87217E-03	8353	1,08973E+00	29934	1,22214E+00	31641	9,20840E-01	20561	0	48381,5
Myeloid_vs_CD8_Responder	Macro_FOLR2+APOE-	CD8(GZMK+ Tem)	HLA-DRA	LAG3	1,57819E-03	16253,7	7,18622E-03	11828	1,55779E+00	14413	2,42548E+00	5378	9,57771E-01	1268	0	48381,5
Myeloid_vs_CD8_Responder	CD8(ID2+CXCR4+ T)	cDC_CLEC9A	CALM1	MYLK	1,58666E-03	31721,9	2,69668E-02	1006	2,20668E+00	5106	1,08780E+00	37304	8,39124E-01	66812	0	48381,5

Myeloid_vs_CD8_Post_R

Myeloid_vs_CD8_Responder	CD8(Terminal Tex)	Mono_CD14	HSPA1A	TLR4	1,59321E-03	25000,5	5,54044E-03	17779	1,58875E+00	13657	1,84031E+00	13477	9,00846E-01	31708	0	48381,5
Myeloid_vs_CD8_Responder	cDC_CLEC9A	CD8(Terminal Tex)	HLA-DPB1	LAG3	1,60020E-03	15556,7	7,18724E-03	11825	1,55574E+00	14464	3,17726E+00	1320	9,55953E-01	1793	0	48381,5
Myeloid_vs_CD8_Responder	CD8(GZMK+ Tex)	Macro_ISG15	CCL5	CCR1	1,60025E-03	28421,3	8,23904E-03	9422	1,08402E+00	30229	1,21971E+00	31736	9,17772E-01	22338	0	48381,5
Myeloid_vs_CD8_Responder	Macro_OLFML3	CD8(LAYN+ T)	HLA-DQA2	LAG3	1,60530E-03	31986,7	4,50020E-03	24355	1,06064E+00	31287	1,40929E+00	24590	9,01522E-01	31320	0	48381,5
Myeloid_vs_CD8_Responder	Macro_OLFML3	CD8(LAYN+ T)	CD14	ITGB1	1,60783E-03	21540,9	5,51871E-03	17874	1,89466E+00	8469	1,76559E+00	14971	9,25261E-01	18009	0	48381,5
Myeloid_vs_CD8_Responder	Macro_OLFML3	CD8(LAYN+ T)	CD14	ITGA4	1,60808E-03	24908,5	4,56292E-03	23878	1,73337E+00	10881	1,67180E+00	17119	9,14445E-01	24283	0	48381,5
Myeloid_vs_CD8_Responder	Macro_OLFML3	CD8(LAYN+ T)	FN1	ITGAV_ITGB1	1,61087E-03	19607,5	1,66998E-02	3029	2,25339E+00	4746	2,02656E+00	10160	9,00824E-01	31721	0	48381,5
Myeloid_vs_CD8_Responder	CD8(ZNF683+KLRB1+ T)	Mono_CD16	HLA-B	LILRB2	1,61087E-03	21600,7	4,95641E-03	21130	2,32440E+00	4196	1,21848E+00	31778	9,53730E-01	2518	0	48381,5
Myeloid_vs_CD8_Responder	Macro_OLFML3	CD8(LAYN+ T)	FN1	ITGA4_ITGB1	1,61112E-03	18511,9	1,53313E-02	3533	2,17076E+00	5415	1,95242E+00	11408	9,15240E-01	23822	0	48381,5
Myeloid_vs_CD8_Responder	Macro_LYVE1	CD8(GZMK+ Tem)	HLA-DRA	LAG3	1,61413E-03	16507,5	7,17033E-03	11869	1,55456E+00	14496	2,31269E+00	6514	9,57726E-01	1277	0	48381,5
Myeloid_vs_CD8_Responder	Macro_OLFML3	CD8(LAYN+ T)	GRN	TNFRSF1B	1,61417E-03	21251,1	4,36219E-03	25488	1,84201E+00	9202	1,89451E+00	12471	9,37873E-01	10713	0	48381,5
Myeloid_vs_CD8_Responder	Macro_OLFML3	CD8(LAYN+ T)	LGALS3BP	ITGB1	1,61518E-03	23769,9	1,10478E-02	5982	1,94875E+00	7776	1,25980E+00	30108	9,10183E-01	26602	0	48381,5
Myeloid_vs_CD8_Responder	CD8(ID2+CXCR4+ T)	Macro_NLRP3	ANXA1	FPR1	1,61620E-03	24835,9	5,78498E-03	16643	1,80595E+00	9717	1,21802E+00	31799	9,25859E-01	17639	0	48381,5
Myeloid_vs_CD8_Responder	Macro_OLFML3	CD8(LAYN+ T)	C1QB	C1QBP	1,61696E-03	18094,5	5,48879E-03	18037	2,26204E+00	4666	2,90718E+00	2231	9,26774E-01	17157	0	48381,5
Myeloid_vs_CD8_Responder	Macro_OLFML3	CD8(LAYN+ T)	LYZ	ITGAL	1,61772E-03	27939,5	3,77532E-03	31403	1,56530E+00	14223	1,79280E+00	14407	9,01574E-01	31283	0	48381,5
Myeloid_vs_CD8_Responder	CD8(ZNF683+KLRB1+ T)	pDC_LILRA4	HSP90B1	TLR9	1,62573E-03	27551,1	2,81462E-02	910	3,75547E+00	589	1,27345E+00	29592	8,54528E-01	58283	0	48381,5
Myeloid_vs_CD8_Responder	Macro_OLFML3	CD8(Temra)	CD14	ITGB1	1,62818E-03	27306,3	3,74573E-03	31758	1,67995E+00	11828	1,62628E+00	18246	9,10708E-01	26318	0	48381,5
Myeloid_vs_CD8_Responder	Macro_OLFML3	CD8(Temra)	CD14	ITGA4	1,62843E-03	27000,5	4,00121E-03	28880	1,67125E+00	11970	1,61126E+00	18612	9,09164E-01	27159	0	48381,5
Myeloid_vs_CD8_Responder	Macro_OLFML3	CD8(GZMK+ Tem)	HLA-DRB1	LAG3	1,63034E-03	15962,9	7,37760E-03	11345	1,55241E+00	14533	2,51913E+00	4559	9,58856E-01	996	0	48381,5
Myeloid_vs_CD8_Responder	CD8(Terminal Tex)	Mono_INHBA	IFNG	IFNGR1_IFNGR2	1,63253E-03	27933,9	7,42103E-03	11198	1,10711E+00	29163	1,59329E+00	19064	9,00560E-01	31863	0	48381,5
Myeloid_vs_CD8_Responder	Mono_INHBA	CD8(Temra)	INHBA	TGFBR3	1,63640E-03	24077,9	6,74519E-02	159	3,39844E+00	913	2,40110E+00	5610	8,41798E-01	65326	0	48381,5
Myeloid_vs_CD8_Responder	CD8(GZMK+ Early Tem)	Macro_FOLR2+APOE-	HMGB1	CD163	1,63740E-03	26666,7	3,73544E-03	31882	1,88532E+00	8602	1,30733E+00	28324	9,28632E-01	16144	0	48381,5
Myeloid_vs_CD8_Responder	Macro_OLFML3	CD8(Temra)	FN1	ITGA4_ITGB1	1,63920E-03	20744,9	1,17034E-02	5419	2,03234E+00	6798	1,85249E+00	13250	9,04162E-01	29876	0	48381,5
Myeloid_vs_CD8_Responder	CD8(LAYN+ T)	Macro_NLRP3	ANXA1	FPR1	1,63946E-03	25283,5	5,57550E-03	17589	1,77828E+00	10148	1,21603E+00	31890	9,24583E-01	18409	0	48381,5
Myeloid_vs_CD8_Responder	Macro_OLFML3	CD8(Temra)	FN1	CD44	1,63971E-03	20754,7	8,49715E-03	8957	1,90153E+00	8382	1,58246E+00	19367	9,24097E-01	18686	0	48381,5
Myeloid_vs_CD8_Responder	CD8(GZMK+ Early Tem)	Mono_CD16	RPS19	C5AR1	1,64564E-03	22319,5	5,51180E-03	17909	2,04464E+00	6654	1,21520E+00	31914	9,44852E-01	6739	0	48381,5
Myeloid_vs_CD8_Responder	Macro_OLFML3	CD8(Temra)	APOE	SORL1	1,64873E-03	20867,9	5,44406E-03	18283	2,18030E+00	5333	3,01127E+00	1823	9,02987E-01	30519	0	48381,5
Myeloid_vs_CD8_Responder	cDC(CD1C)	CD8(ISG+ T)	HLA-DRA	LAG3	1,65023E-03	16053,5	6,30892E-03	14578	1,55850E+00	14387	3,40636E+00	834	9,55058E-01	2087	0	48381,5
Myeloid_vs_CD8_Responder	Macro_FOLR2+APOE+	CD8(LAYN+ T)	CD14	ITGB1	1,65156E-03	18945,1	6,62045E-03	13483	2,23999E+00	4847	1,84257E+00	13433	9,31316E-01	14581	0	48381,5
Myeloid_vs_CD8_Responder	Macro_OLFML3	CD8(Temra)	LYZ	ITGAL	1,65313E-03	26879,7	3,92920E-03	29670	1,58094E+00	13836	1,91048E+00	12183	9,03333E-01	30328	0	48381,5
Myeloid_vs_CD8_Responder	cDC_CLEC9A	CD8(GZMK+ Tem)	HLA-DQA2	LAG3	1,65378E-03	16423,5	1,18027E-02	5338	1,55027E+00	14586	2,82977E+00	2511	9,36811E-01	11301	0	48381,5
Myeloid_vs_CD8_Responder	CD8(GZMK+ Early Tem)	Macro_IER3	TNFSF9	HLA-DPA1	1,65857E-03	29070,9	4,05319E-03	28341	1,04640E+00	31964	1,72089E+00	15975	9,20601E-01	20693	0	48381,5
Myeloid_vs_CD8_Responder	CD8(ID2+CXCR4+ T)	Macro_ISG15	ANXA1	FPR1	1,65961E-03	26433,1	5,22404E-03	19500	1,64122E+00	12569	1,21377E+00	31968	9,22282E-01	19747	0	48381,5
Myeloid_vs_CD8_Responder	CD8(Tn)	cDC(CD1C)	CD40LG	CD40	1,66144E-03	40510,5	2,80489E-02	920	1,17846E+00	26048	1,54455E+00	20386	7,60779E-01	106817	0	48381,5
Myeloid_vs_CD8_Responder	cDC(CD1C)	CD8(Terminal Tex)	HLA-DPA1	LAG3	1,66314E-03	15464,7	7,19174E-03	11815	1,54958E+00	14607	3,27272E+00	1102	9,57220E-01	1418	0	48381,5
Myeloid_vs_CD8_Responder	CD8(ITM2C+ T)	Macro_LYVE1	RPS19	C5AR1	1,66506E-03	21346,3	6,09732E-03	15372	2,14698E+00	5633	1,21329E+00	31989	9,47424E-01	5356	0	48381,5
Myeloid_vs_CD8_Responder	Macro_OLFML3	CD8(GZMK+ Tem)	CD14	ITGA4	1,66689E-03	22711,1	5,26385E-03	19261	1,81089E+00	9639	1,75596E+00	15180	9,19872E-01	21094	0	48381,5
Myeloid_vs_CD8_Responder	Macro_OLFML3	CD8(GZMK+ Tem)	FN1	ITGA4_ITGB1	1,67628E-03	20877,5	1,17832E-02	5354	2,03628E+00	6747	1,80436E+00	14181	9,04456E-01	29724	0	48381,5
Myeloid_vs_CD8_Responder	Macro_OLFML3	CD8(Temra)	C1QB	C1QBP	1,67655E-03	22918,5	3,72361E-03	32033	2,12040E+00	5868	2,75641E+00	2933	9,12469E-01	25377	0	48381,5
Myeloid_vs_CD8_Responder	Macro_OLFML3	CD8(GZMK+ Tem)	FN1	CD44	1,67681E-03	20283,7	8,69414E-03	8642	1,91629E+00	8179	1,63710E+00	17978	9,24897E-01	18238	0	48381,5
Myeloid_vs_CD8_Responder	Macro_OLFML3	CD8(GZMK+ Tem)	HLA-DQB2	LAG3	1,67864E-03	20304,9	1,70248E-02	2935	1,88641E+00	8594	1,65910E+00	17408	9,14569E-01	24206	0	48381,5
Myeloid_vs_CD8_Responder	Macro_FOLR2+APOE-	CD8(EOMES+ NK-like)	CCL13	CXCR3	1,67944E-03	39347,3	2,79682E-02	925	4,01777E+00	424	8,61324E-01	48696	7,78913E-01	98310	0	48381,5
Myeloid_vs_CD8_Responder	CD8(ID2+CXCR4+ T)	Mono_CD16	CD99	PILRA	1,68074E-03	24695,3	5,59844E-03	17481	2,08202E+00	6256	1,21180E+00	32049	9,23087E-01	19309	0	48381,5
Myeloid_vs_CD8_Responder	Macro_IER3	CD8(Tc17)	IL1B	ADRB2	1,68486E-03	22032,5	1,25104E-02	4861	2,43918E+00	3455	2,44399E+00	5211	8,72052E-01	48254	0	48381,5
Myeloid_vs_CD8_Responder	CD8(Tn)	pDC_LILRA4	HMGB1	THBD	1,68546E-03	24179,5	5,15679E-03	19876	1,75671E+00	10502	2,03172E+00	10071	9,00204E-01	32067	0	48381,5
Myeloid_vs_CD8_Responder	Macro_FOLR2+APOE+	CD8(GZMK+ Tem)	HLA-DQA1	LAG3	1,68647E-03	16549,7	8,97065E-03	8220	1,54714E+00	14659	2,12606E+00	8711	9,53002E-01	2777	0	48381,5
Myeloid_vs_CD8_Responder	CD8(ISG+ T)	pDC_LILRA4	HLA-C	NOTCH4	1,68918E-03	22864,5	1,54255E-02	3490	1,55666E+00	14439	1,78047E+00	14665	8,97850E-01	33347	0	48381,5
Myeloid_vs_CD8_Responder	Macro_OLFML3	CD8(GZMK+ Tem)	C1QB	C1QBP	1,68941E-03	22947,1	3,72236E-03	32045	2,12030E+00	5869	2,73672E+00	3057	9,12455E-01	25383	0	48381,5
Myeloid_vs_CD8_Responder	Macro_OLFML3	CD8(GZMK+ Tem)	LYZ	ITGAL	1,69073E-03	26446,5	4,03469E-03	28545	1,59166E+00	13597	1,91916E+00	12005	9,04483E-01	29704	0	48381,5
Myeloid_vs_CD8_Responder	Macro_ISG15	CD8(ITM2C+ T)	SPP1	S1PR1	1,69633E-03	20983,9	3,92408E-02	469	2,19345E+00	5223	3,07318E+00	1617	8,70337E-01	49229	0	48381,5
Myeloid_vs_CD8_Responder	Macro_OLFML3	CD8(NME1+ T)	HLA-DRA	LAG3	1,69785E-03	16993,7	5,93556E-03	16002	1,55115E+00	14566	2,66639E+00	3500	9,53730E-01	2519	0	48381,5
Myeloid_vs_CD8_Responder	Macro_OLFML3	CD8(NME1+ T)	HLA-DQA2	LAG3	1,69838E-03	21346,5	8,75475E-03	8536	1,35804E+00	19879	1,86174E+00	13085	9,27371E-01	16851	0	48381,5
Myeloid_vs_CD8_Responder	Macro_OLFML3	CD8(NME1+ T)	HLA-DQA1	LAG3	1,69891E-03	19701,9	6,64383E-03	13400	1,36381E+00	19708	1,98696E+00	10810	9,45801E-01	6210	0	48381,5
Myeloid_vs_CD8_Responder	Macro_OLFML3	CD8(NME1+ T)	HLA-DRB1	LAG3	1,69944E-03	20423,1	5,20720E-03	19595	1,29734E+00	21794	2,11184E+00	8907	9,51406E-01	3438	0	48381,5
Myeloid_vs_CD8_Responder	Macro_OLFML3	CD8(NME1+ T)	HLA-DQB1	LAG3	1,69997E-03	18624,5	6,83075E-03	12805	1,46152E+00	16857	2,11473E+00	8873	9,45812E-01	6206	0	48381,5
Myeloid_vs_CD8_Responder	Macro_OLFML3	CD8(NME1+ T)	HLA-DPB1	LAG3	1,70182E-03	18810,3	5,63061E-03	17334	1,43363E+00	17682	2,28147E+00	6830	9,50519E-01	3824	0	48381,5
Myeloid_vs_CD8_Responder	Macro_OLFML3	CD8(NME1+ T)	CD14	ITGB1	1,70473E-03	27824,5	3,72208E-03	32049	1,67708E+00	11883	1,54560E+00	20356	9,10450E-01	26453	0	48381,5
Myeloid_vs_CD8_Responder	Macro_OLFML3	CD8(NME1+ T)	HLA-DPA1	LAG3	1,70978E-03	18703,5	5,59426E-03	17500	1,41609E+00	18179	2,34267E+00	6176	9,51771E-01	3281	0	48381,5
Myeloid_vs_CD8_Responder	Mono_CD14	CD8(Tc17)	IL1B	ADRB2	1,71074E-03	21800,1	1,19383E-02	5238	2,33758E+00	4103	3,11063E+00	1506	8,69418E-01	49772	0	48381,5
Myeloid_vs_CD8_Responder	Macro_FOLR2-APOE+	CD8(GZMK+ Tem)	HLA-DRA	LAG3	1,71141E-03	16324,3	7,12550E-03	11990	1,54546E+00	14714	2,44352E+00	5219	9,57599E-01	1317	0	48381,5
Myeloid_vs_CD8_Responder	CD8(Tc17)	Mono_CD16	HLA-F	LILRB2	1,71164E-03	28002,5	4,49202E-03	24412	2,44334E+00	3436	1,22289E+00	31617	9,00027E-01	32166	0	48381,5
Myeloid_vs_CD8_Responder	Macro_IFI27	CD8(Terminal Tex)	HLA-DQA2	LAG3	1,71278E-03	17683,9	1,15658E-02	5531	1,54528E+00	14717	2,17039E+00	8119	9,36208E-01	11671	0	48381,5
Myeloid_vs_CD8_Responder	Macro_OLFML3	CD8(NME1+ T)	HLA-DRB5	LAG3	1,71564E-03	18057,7	6,53550E-03	13807	1,46051E+00	16895	2,33903E+00	6215	9,48141E-01	4990	0	48381,5
Myeloid_vs_CD8_Responder	Macro_OLFML3	CD8(NME1+ T)	FN1	ITGA4_ITGB1	1,71777E-03	21562,5	1,11438E-02	5889	2,01080E+00	7042	1,74490E+00	15444	9,02018E-01	31056	0	48381,5
Myeloid_vs_CD8_Responder	Macro_OLFML3	CD8(NME1+ T)	FN1	CD44	1,71830E-03	24999,3	6,44504E-03	14082	1,74775E+00	10641	1,33482E+00	27292	9,13816E-01	24600	0	48381,5
Myeloid_vs_CD8_Responder	Macro_OLFML3	CD8(NME1+ T)	HLA-DQB2	LAG3	1,72204E-03	25794,3	1,20163E-02	5168	1,63133E+00	12768	1,25181E+00	30449	8,99938E-01	32205	0	48381,5
Myeloid_vs_CD8_Responder	Macro_OLFML3	CD8(GZMK+ Tem)	FN1	ITGA4_ITGB7	1,72338E-03	20247,7	1,63552E-02	3134	2,12131E+00	5854	1,93794E+00	11659	8,99920E-01	32210	0	48381,5
Myeloid_vs_CD8_Responder	CD8(Terminal Tex)	cDC(CD1C)	CD52	SIGLEC10	1,73411E-03	33012,3	3,70632E-03	32250	1,12122E+00	28541	1,40899E+00	24604	9,01572E-01	31285	0	48381,5
Myeloid_vs_CD8_Responder	Macro_OLFML3	CD8(NME1+ T)	C1QB	C1QBP	1,73438E-03	17475,9	5,83154E-03	16439	2,28954E+00	4453	2,94970E+00	2059	9,28803E-01	16047	0	48381,5
Myeloid_vs_CD8_Responder	Macro_OLFML3	CD8(NME1+ T)	C3	IFITM1	1,73491E-03	17340,1	9,66985E-03	7310	2,23718E+00	4874	1,71418E+00	16143	9,39104E-01	9992	0	48381,5
Myeloid_vs_CD8_Responder	Macro_ISG15	CD8(EOMES+ NK-like)	CXCL10	CXCR3	1,73493E-03	22145,9	1,41104E-02	4035	2,32662E+00	4179	2,43756E+00	5263	8,70955E-01	48871	0	48381,5
Myeloid_vs_CD8_Responder	Macro_NLRP3	CD8(LAYN+ T)	S100A8	CD69	1,73893E-03	18576,5	6,25470E-03	14774	1,92507E+00	8057	2,17700E+00	8020	9,32920E-01	13650	0	48381,5
Myeloid_vs_CD8_Responder	CD8(NME1+ T)	Macro_ISG15	CD99	PILRA	1,73976E-03	31706,1	3,89708E-03	30015	1,33056E+00	20731	1,20665E+00	32271	9,09201E-01	27132	0	48381,5
Myeloid_vs_CD8_Responder	Macro_OLFML3	CD8(EOMES+ NK-like)	HLA-DQA2	LAG3	1,74381E-03	31073,1	4,73085E-03	22616	1,07676E+00	30567	1,43689E+00	23672	9,03719E-01	30129	0	48381,5
Myeloid_vs_CD8_Responder	CD8(Tn)	Macro_LYVE1	CD40LG	CD9	1,74505E-03	31784,5	3,96613E-02	459	1,40740E+00	18421	1,43800E+00	23639	8,37001E-01	68022	0	48381,5
Myeloid_vs_CD8_Responder	CD8(GZMK+ Tex)	Mono_CD16	RPS19	C5AR1	1,74624E-03	24366,9	4,66821E-03	23077	1,86745E+00	8817	1,20606E+00	32295	9,40360E-01	9264	0	48381,5
Myeloid_vs_CD8_Responder	Macro_OLFML3	CD8(ISG+ T)	C1QB	C1QBP	1,74813E-03	23004,3	3,70184E-03	32302	2,11865E+00	5881	2,75454E+00	2941	9,12234E-01	25516	0	48381,5

Myeloid_vs_CD8_Responder	Mono_CD14	CD8(Temra)	VCAN	CD44	1,74956E-03	15782,3	9,16924E-03	7936	3,13249E+00	1304	2,31477E+00	6493	9,30944E-01	14797	0	48381,5
Myeloid_vs_CD8_Responder	Mono_CD14	CD8(ISG+ T)	VCAN	CD44	1,75652E-03	15662,3	9,15997E-03	7948	3,13185E+00	1306	2,37433E+00	5864	9,30911E-01	14812	0	48381,5
Myeloid_vs_CD8_Responder	Mono_CD14	CD8(NME1+ T)	VCAN	ITGA4	1,75739E-03	17566,9	1,18799E-02	5286	3,21873E+00	1147	2,44278E+00	5226	9,07971E-01	27794	0	48381,5
Myeloid_vs_CD8_Responder	Macro_OLFML3	CD8(EOMES+ NK-like)	FN1	CD44	1,76361E-03	19737,9	9,28121E-03	7771	1,96028E+00	7628	1,63860E+00	17938	9,27135E-01	16971	0	48381,5
Myeloid_vs_CD8_Responder	CD8(Tn)	pDC_LILRA4	MAML2	NOTCH4	1,76490E-03	42293,3	2,26059E-02	1563	1,53946E+00	14830	1,91994E+00	11989	6,86380E-01	134703	0	48381,5
Myeloid_vs_CD8_Responder	Mono_INHBA	CD8(LAYN+ T)	INHBA	TGFBR3	1,77089E-03	24468,7	6,43566E-02	176	3,37556E+00	950	2,38800E+00	5745	8,38645E-01	67091	0	48381,5
Myeloid_vs_CD8_Responder	cDC(CD1C)	CD8(Terminal Tex)	HLA-DQA1	LAG3	1,77144E-03	15279,9	8,73236E-03	8577	1,53893E+00	14844	3,08412E+00	1582	9,52395E-01	3015	0	48381,5
Myeloid_vs_CD8_Responder	CD8(GZMK+ Tex)	Macro_LYVE1	HSP90B1	LRP1	1,77344E-03	27680,1	4,61022E-03	23509	2,19210E+00	5236	1,20333E+00	32395	9,06029E-01	28879	0	48381,5
Myeloid_vs_CD8_Responder	pDC_LILRA4	CD8(GZMK+ Tex)	APP	CD74	1,77472E-03	15970,1	6,23175E-03	14851	2,08127E+00	6261	2,65043E+00	3619	9,44853E-01	6738	0	48381,5
Myeloid_vs_CD8_Responder	CD8(GZMK+ Tem)	Mono_CD16	RPS19	C5AR1	1,77700E-03	23179,3	5,16031E-03	19861	1,97081E+00	7510	1,20284E+00	32408	9,43109E-01	7736	0	48381,5
Myeloid_vs_CD8_Responder	Mono_CD14	CD8(ID2+CXCR4+ T)	VCAN	ITGA4	1,78102E-03	17501,7	1,18389E-02	5310	3,21734E+00	1150	2,49299E+00	4791	9,07826E-01	27876	0	48381,5
Myeloid_vs_CD8_Responder	Macro_OLFML3	CD8(LAYN+ T)	C3	CD46	1,78414E-03	20000,9	1,03876E-02	6564	2,45156E+00	3387	2,08919E+00	9238	8,99521E-01	32434	0	48381,5
Myeloid_vs_CD8_Responder	CD8(NME1+ T)	Macro_LYVE1	CALR	LRP1	1,78524E-03	24463,9	5,71993E-03	16953	2,27663E+00	4553	1,20192E+00	32438	9,21875E-01	19994	0	48381,5
Myeloid_vs_CD8_Responder	Macro_FOLR2+APOE-	CD8(Temra)	CCL13	CXCR3	1,78575E-03	38919,9	2,75787E-02	954	4,01428E+00	426	9,11647E-01	45943	7,77703E-01	98895	0	48381,5
Myeloid_vs_CD8_Responder	Macro_OLFML3	CD8(EOMES+ NK-like)	HLA-DQB1	LAG3	1,78910E-03	28037,1	3,69117E-03	32452	1,18024E+00	25975	1,68987E+00	16704	9,27697E-01	16673	0	48381,5
Myeloid_vs_CD8_Responder	CD8(Terminal Tex)	Mono_CD16	HLA-G	LILRB2	1,79258E-03	25682,1	1,08131E-02	6177	2,77119E+00	2129	1,77021E+00	14889	8,56930E-01	56834	0	48381,5
Myeloid_vs_CD8_Responder	CD8(Temra)	pDC_LILRA4	HLA-C	NOTCH4	1,79352E-03	21819,5	1,59821E-02	3266	1,65234E+00	12365	1,88707E+00	12617	8,99464E-01	32468	0	48381,5
Myeloid_vs_CD8_Responder	Mono_CD14	CD8(GZMK+ Early Tem)	VCAN	ITGA4	1,79490E-03	17463,3	1,18214E-02	5324	3,21675E+00	1152	2,52158E+00	4547	9,07764E-01	27912	0	48381,5
Myeloid_vs_CD8_Responder	Macro_OLFML3	CD8(ZNF683+KLRB1+ T)	FN1	CD44	1,79628E-03	21167,5	8,16238E-03	9568	1,87644E+00	8704	1,57120E+00	19648	9,22675E-01	19536	0	48381,5
Myeloid_vs_CD8_Responder	CD8(Tn)	cDC_LAMP3	CD40LG	CD40	1,79693E-03	32636,9	5,23380E-02	267	1,83539E+00	9298	1,42398E+00	24115	8,12881E-01	81123	0	48381,5
Myeloid_vs_CD8_Responder	Macro_OLFML3	CD8(ZNF683+KLRB1+ T)	CXCL16	CXCR6	1,80015E-03	27137,5	8,97371E-03	8216	1,35228E+00	20058	1,22123E+00	31670	9,08783E-01	27362	0	48381,5
Myeloid_vs_CD8_Responder	Macro_OLFML3	CD8(ZNF683+KLRB1+ T)	C3	IFITM1	1,80376E-03	16872,9	1,02097E-02	6755	2,27291E+00	4583	1,74035E+00	15540	9,40639E-01	9105	0	48381,5
Myeloid_vs_CD8_Responder	Mono_CD14	CD8(ISG+ T)	VCAN	ITGA4	1,80386E-03	17481,1	1,18088E-02	5333	3,21632E+00	1154	2,51479E+00	4597	9,07719E-01	27940	0	48381,5
Myeloid_vs_CD8_Responder	CD8(GZMK+ Tex)	Macro_LYVE1	CALR	LRP1	1,80681E-03	25780,9	5,09362E-03	20276	2,18500E+00	5293	1,19999E+00	32516	9,17595E-01	22438	0	48381,5
Myeloid_vs_CD8_Responder	Macro_OLFML3	CD8(ITM2C+ T)	HLA-DQA2	LAG3	1,80931E-03	32296,7	4,46573E-03	24613	1,05823E+00	31398	1,38040E+00	25579	9,01180E-01	31512	0	48381,5
Myeloid_vs_CD8_Responder	CD8(ID2+CXCR4+ T)	Macro_OLFML3	CD52	SIGLEC10	1,81154E-03	24474,7	6,61116E-03	13526	1,82455E+00	9443	1,19954E+00	32533	9,24434E-01	18490	0	48381,5
Myeloid_vs_CD8_Responder	Mast	CD8(GZMK+ Tem)	TIMP3	CD44	1,81564E-03	19236,1	2,75312E-02	962	3,56220E+00	744	1,54252E+00	20439	9,11982E-01	25654	0	48381,5
Myeloid_vs_CD8_Responder	CD8(ISG+ T)	Mono_INHBA	RPS19	C5AR1	1,81795E-03	28341,3	3,74705E-03	31739	1,49623E+00	15947	1,19899E+00	32556	9,33890E-01	13083	0	48381,5
Myeloid_vs_CD8_Responder	Macro_OLFML3	CD8(ITM2C+ T)	FN1	CD44	1,81907E-03	22073,7	7,99510E-03	9895	1,86390E+00	8870	1,44927E+00	23254	9,21933E-01	19968	0	48381,5
Myeloid_vs_CD8_Responder	Macro_OLFML3	CD8(EOMES+ NK-like)	FN1	ITGA4_ITGB1	1,81991E-03	22003,9	1,04875E-02	6477	1,98580E+00	7334	1,75236E+00	15264	8,99303E-01	32563	0	48381,5
Myeloid_vs_CD8_Responder	CD8(Tc17)	pDC_LILRA4	HMGB1	THBD	1,82326E-03	24959,7	5,05094E-03	20542	1,74078E+00	10753	1,89065E+00	12547	8,99269E-01	32575	0	48381,5
Myeloid_vs_CD8_Responder	Macro_OLFML3	CD8(ITM2C+ T)	C1QB	C1QBP	1,82943E-03	21396,1	4,16674E-03	27248	2,15596E+00	5553	2,75680E+00	2930	9,16856E-01	22868	0	48381,5
Myeloid_vs_CD8_Responder	cDC_LAMP3	CD8(ZNF683+KLRB1+ T)	CCL19	CXCR3	1,83822E-03	29535,5	2,74765E-02	968	6,19719E+00	93	1,52598E+00	20911	8,20011E-01	77324	0	48381,5
Myeloid_vs_CD8_Responder	CD8(Terminal Tex)	Macro_NLRP3	CCL5	CCRL2	1,84548E-03	26616,1	9,75097E-03	7217	1,17421E+00	26207	1,19651E+00	32654	9,24214E-01	18621	0	48381,5
Myeloid_vs_CD8_Responder	CD8(Temra)	Macro_ISG15	CD99	PILRA	1,85256E-03	32236,7	3,80603E-03	31039	1,30786E+00	21418	1,19598E+00	32679	9,08220E-01	27666	0	48381,5
Myeloid_vs_CD8_Responder	CD8(Terminal Tex)	cDC_CLEC9A	FLT3LG	FLT3	1,85519E-03	33579,3	2,47757E-02	1221	4,44381E+00	263	1,55704E+00	20051	7,79557E-01	97980	0	48381,5
Myeloid_vs_CD8_Responder	CD8(LAYN+ T)	Mono_CD16	HLA-C	LILRB2	1,85596E-03	21859,7	5,00739E-03	20803	2,38705E+00	3760	1,19567E+00	32691	9,50896E-01	3663	0	48381,5
Myeloid_vs_CD8_Responder	CD8(IL7R+ZNF683+ Tm)	cDC_CLEC9A	FLT3LG	FLT3	1,85714E-03	32598,7	2,73461E-02	973	4,46795E+00	251	1,57736E+00	19478	7,87921E-01	93910	0	48381,5
Myeloid_vs_CD8_Responder	Mono_CD14	CD8(EOMES+ NK-like)	VCAN	ITGA4	1,86843E-03	17576,5	1,17347E-02	5397	3,21382E+00	1158	2,48554E+00	4853	9,07456E-01	28093	0	48381,5
Myeloid_vs_CD8_Responder	CD8(Tn)	Macro_FOLR2-APOE+	HSPA1A	TLR4	1,88280E-03	24478,9	5,30500E-03	19028	1,54801E+00	14637	2,21523E+00	7563	8,98890E-01	32785	0	48381,5
Myeloid_vs_CD8_Responder	CD8(Tn)	Mono_CD16	HLA-A	LILRB2	1,88891E-03	23849,9	4,77269E-03	22353	2,17057E+00	5417	1,04625E+00	39261	9,50482E-01	3837	0	48381,5
Myeloid_vs_CD8_Responder	CD8(Terminal Tex)	cDC_CLEC9A	CALM3	MYLK	1,89406E-03	38213,9	2,75579E-02	958	2,22932E+00	4943	1,27525E+00	29522	7,59732E-01	107265	0	48381,5
Myeloid_vs_CD8_Responder	CD8(Tn)	Mono_CD16	HLA-C	LILRB2	1,91058E-03	24630,3	4,54563E-03	24009	2,16779E+00	5438	1,01800E+00	40585	9,48588E-01	4738	0	48381,5
Myeloid_vs_CD8_Responder	CD8(Tn)	Mono_INHBA	CD40LG	CD9	1,91063E-03	36776,9	2,71699E-02	987	1,07946E+00	30449	1,51594E+00	21223	8,09528E-01	82844	0	48381,5
Myeloid_vs_CD8_Responder	CD8(LAYN+ T)	Mono_INHBA	RPS19	C5AR1	1,92217E-03	24376,1	4,86190E-03	21775	1,77620E+00	10177	1,18995E+00	32921	9,41490E-01	8626	0	48381,5
Myeloid_vs_CD8_Responder	Mono_CD14	CD8(GZMK+ Tex)	VCAN	CD44	1,92883E-03	15786,1	8,99177E-03	8194	3,12017E+00	1326	2,37549E+00	5859	9,30313E-01	15170	0	48381,5
Myeloid_vs_CD8_Responder	Macro_OLFML3	CD8(GZMK+ Tex)	LGALS3BP	ITGB1	1,93476E-03	25281,7	8,43993E-03	9038	1,79099E+00	9966	1,36639E+00	26059	8,98552E-01	32964	0	48381,5
Myeloid_vs_CD8_Responder	Macro_ISG15	CD8(EOMES+ NK-like)	S100A8	CD69	1,94832E-03	19468,9	6,55742E-03	13719	1,52516E+00	15209	2,24160E+00	7268	9,34384E-01	12767	0	48381,5
Myeloid_vs_CD8_Responder	CD8(ISG+ T)	cDC_CLEC9A	LTB	CD40	1,95435E-03	29245,5	8,03500E-03	9819	1,56845E+00	14149	1,75425E+00	15221	8,53906E-01	58657	0	48381,5
Myeloid_vs_CD8_Responder	CD8(ID2+CXCR4+ T)	Macro_FOLR2+APOE+	ENTPD1	TMIGD3	1,95965E-03	49474,9	1,44179E-02	3889	2,16301E+00	5485	4,24195E-01	79817	7,53864E-01	109802	0	48381,5
Myeloid_vs_CD8_Responder	CD8(Tn)	Mono_CD16	TNFSF8	TNFRSF8	1,96487E-03	59234,3	2,96013E-02	816	1,18969E+00	25596	5,06312E-01	73081	6,27885E-01	148297	0	48381,5
Myeloid_vs_CD8_Responder	CD8(Temra)	Macro_FOLR2+APOE+	SPON2	ITGB2	1,97113E-03	28976,7	1,13796E-02	5676	1,12304E+00	28468	1,18618E+00	33087	9,05288E-01	29271	0	48381,5
Myeloid_vs_CD8_Responder	Macro_OLFML3	CD8(ITM2C+ T)	APOE	LDLR	1,97231E-03	20670,7	1,16099E-02	5497	2,23402E+00	4909	2,95073E+00	2054	8,81791E-01	42512	0	48381,5
Myeloid_vs_CD8_Responder	CD8(Terminal Tex)	Macro_IFI27	IFNG	IFNGR1_IFNGR2	1,98337E-03	29890,1	7,05297E-03	12179	1,02571E+00	32957	1,46418E+00	22805	8,98259E-01	33128	0	48381,5
Myeloid_vs_CD8_Responder	CD8(NME1+ T)	Mono_INHBA	HLA-F	LILRB2	1,98397E-03	29829,7	4,32001E-03	25866	1,61116E+00	13194	1,30054E+00	28577	8,98257E-01	33130	0	48381,5
Myeloid_vs_CD8_Responder	Macro_OLFML3	CD8(NME1+ T)	CD14	ITGA4	1,98487E-03	29204,9	3,63747E-03	33133	1,63102E+00	12775	1,47676E+00	22397	9,05151E-01	29338	0	48381,5
Myeloid_vs_CD8_Responder	CD8(NME1+ T)	Mono_INHBA	HMGB1	THBD	1,98666E-03	28157,5	4,93876E-03	21257	1,48926E+00	16127	1,49364E+00	21883	8,98247E-01	33139	0	48381,5
Myeloid_vs_CD8_Responder	Mono_INHBA	CD8(ZNF683+KLRB1+ T)	S100A8	CD69	1,99438E-03	18940,1	7,10078E-03	12063	1,52148E+00	15300	2,21008E+00	7639	9,36782E-01	11317	0	48381,5
Myeloid_vs_CD8_Responder	CD8(ITM2C+ T)	Mono_CD16	HLA-A	LILRB2	2,00019E-03	21848,9	5,08185E-03	20341	2,33279E+00	4129	1,18371E+00	33184	9,51938E-01	3209	0	48381,5
Myeloid_vs_CD8_Responder	CD8(NME1+ T)	Mast	ARPC5	ADRB2	2,00109E-03	25573,5	9,61739E-03	7368	1,39138E+00	18902	1,55791E+00	20029	8,98157E-01	33187	0	48381,5
Myeloid_vs_CD8_Responder	CD8(Terminal Tex)	cDC(CD1C)	B2M	CD1A	2,00848E-03	24503,3	2,91218E-02	845	2,15837E+00	5531	1,21070E+00	32101	8,93733E-01	35658	0	48381,5
Myeloid_vs_CD8_Responder	CD8(Tc17)	Macro_FOLR2+APOE+	ADAM10	GPNMB	2,01128E-03	25111,7	8,31643E-03	9276	3,00105E+00	1560	1,75010E+00	15333	8,67311E-01	51008	0	48381,5
Myeloid_vs_CD8_Responder	CD8(GZMK+ Early Tem)	pDC_LILRA4	TNF	TNFRSF21	2,01383E-03	22776,9	2,18474E-02	1713	3,72132E+00	617	2,40790E+00	5536	8,55549E-01	57637	0	48381,5
Myeloid_vs_CD8_Responder	Mast	CD8(Temra)	TIMP3	CD44	2,01590E-03	19666,5	2,69074E-02	1014	3,54744E+00	760	1,48788E+00	22045	9,11057E-01	26132	0	48381,5
Myeloid_vs_CD8_Responder	CD8(ID2+CXCR4+ T)	Macro_FOLR2+APOE+	CD52	SIGLEC10	2,01955E-03	22756,5	7,73489E-03	10465	2,09213E+00	6162	1,18144E+00	33248	9,29738E-01	15526	0	48381,5
Myeloid_vs_CD8_Responder	Macro_ISG15	CD8(GZMK+ Early Tem)	S100A8	CD69	2,02312E-03	19655,7	6,50601E-03	13895	1,51911E+00	15356	2,19877E+00	7743	9,34142E-01	12903	0	48381,5
Myeloid_vs_CD8_Responder	CD8(NME1+ T)	Mono_CD16	HMGB1	TLR4	2,02472E-03	28849,5	5,44319E-03	18292	1,58787E+00	13675	1,24750E+00	30634	8,97993E-01	33265	0	48381,5
Myeloid_vs_CD8_Responder	Macro_OLFML3	CD8(ID2+CXCR4+ T)	CD14	ITGA4	2,02868E-03	28954,3	3,62492E-03	33278	1,62963E+00	12801	1,52697E+00	20892	9,05003E-01	29419	0	48381,5
Myeloid_vs_CD8_Responder	CD8(GZMK+ Tem)	pDC_LILRA4	HLA-C	NOTCH4	2,03112E-03	22766,1	1,54631E-02	3477	1,56312E+00	14275	1,79264E+00	14411	8,97962E-01	33286	0	48381,5
Myeloid_vs_CD8_Responder	Mast	CD8(ISG+ T)	TIMP3	CD44	2,03173E-03	19326,5	2,68802E-02	1018	3,54679E+00	762	1,54744E+00	20317	9,11016E-01	26154	0	48381,5
Myeloid_vs_CD8_Responder	CD8(IL7R+ZNF683+ Tm)	Mono_CD16	HLA-F	LILRB2	2,03265E-03	28685,1	4,29132E-03	26117	2,42549E+00	3522	1,21033E+00	32114	8,97952E-01	33291	0	48381,5
Myeloid_vs_CD8_Responder	CD8(Tc17)	Macro_LYVE1	ADAM10	GPNMB	2,03294E-03	26308,7	7,49206E-03	11018	2,70799E+00	2344	1,74857E+00	15375	8,61188E-01	54425	0	48381,5
Myeloid_vs_CD8_Responder	CD8(ISG+ T)	cDC(CD1C)	CD28	CD86	2,03450E-03	33357,5	7,61900E-03	10733,5	1,57203E+00	14065,5	1,74850E+00	15378,5	8,18303E-01	78228,5	0	48381,5
Myeloid_vs_CD8_Responder	Macro_OLFML3	CD8(GZMK+ Tem)	B2M	CD3D	2,04788E-03	46984,3	2,76474E-03	49044	6,24869E-01	58523	4,34670E-01	78960	9,65941E-01	13	0	48381,5
Myeloid_vs_CD8_Responder	Macro_OLFML3	CD8(GZMK+ Early Tem)	CD14	ITGA4	2,05503E-03	28824,1	3,61958E-03	33364	1,62904E+00	12817	1,55555E+00	20109	9,04939E-01	29449	0	48381,5
Myeloid_vs_CD8_Responder	CD8(Tn)	cDC_CLEC9A	LTB	CD40	2,07153E-03	27982,3	8,94492E-03	8258	1,62047E+00	13002	1,74473E+00	15449	8,60471E-01	54821	0	48381,5
Myeloid_vs_CD8_Responder	CD8(Temra)	cDC_CLEC9A	FLT3LG	FLT3	2,07159E-03	32824,9	2,67607E-02	1028	4,46245E+00	258	1,57208E+00	19608	7,86108E-01	94849	0	48381,5

Myeloid_vs_CD8_Responder	CD8(ITM2C+ T)	pDC_LILRA4	HLA-C	NOTCH4	2,07171E-03	23140,7	1,53858E-02	3506	1,54984E+00	14600	1,72869E+00	15798	8,97732E-01	33418	0	48381,5
Myeloid_vs_CD8_Responder	Macro_OLFML3	CD8(ISG+ T)	CD14	ITGA4	2,07202E-03	28879,3	3,61570E-03	33419	1,62861E+00	12829	1,54876E+00	20287	9,04893E-01	29480	0	48381,5
Myeloid_vs_CD8_Responder	CD8(Tn)	Macro_OLFML3	HSPA1A	TLR4	2,07730E-03	25060,3	5,17009E-03	19801	1,52659E+00	15169	2,14133E+00	8514	8,97714E-01	33436	0	48381,5
Myeloid_vs_CD8_Responder	Macro_FOLR2+APOE-	CD8(Terminal Tex)	HLA-DRA	LAG3	2,07836E-03	16830,7	6,83464E-03	12793	1,51537E+00	15462	2,36588E+00	5958	9,56745E-01	1559	0	48381,5
Myeloid_vs_CD8_Responder	Macro_OLFML3	CD8(EOMES+ NK-like)	APOE	LSR	2,09087E-03	19378,5	1,42021E-02	3986	2,15020E+00	5607	2,93191E+00	2116	8,91726E-01	36802	0	48381,5
Myeloid_vs_CD8_Responder	CD8(ISG+ T)	Mono_INHBA	HLA-F	LILRB2	2,09132E-03	29483,5	4,26319E-03	26348	1,60252E+00	13369	1,37279E+00	25838	8,97651E-01	33481	0	48381,5
Myeloid_vs_CD8_Responder	CD8(EOMES+ NK-like)	Macro_LYVE1	RPS19	C5AR1	2,09726E-03	22229,7	5,75461E-03	16783	2,07283E+00	6350	1,17504E+00	33500	9,45964E-01	6134	0	48381,5
Myeloid_vs_CD8_Responder	CD8(Tc17)	Macro_LYVE1	HSP90B1	LRP1	2,10572E-03	26602,5	5,13772E-03	20007	2,25844E+00	4697	1,17425E+00	33527	9,10540E-01	26400	0	48381,5
Myeloid_vs_CD8_Responder	Macro_LYVE1	CD8(Terminal Tex)	HLA-DRA	LAG3	2,11011E-03	17093,1	6,81954E-03	12847	1,51214E+00	15522	2,25309E+00	7143	9,56699E-01	1572	0	48381,5
Myeloid_vs_CD8_Responder	CD8(Tn)	Macro_IER3	HSPA1A	TLR4	2,11611E-03	24565,1	5,14739E-03	19943	1,52298E+00	15259	2,39370E+00	5682	8,97511E-01	33560	0	48381,5
Myeloid_vs_CD8_Responder	Mono_CD14	CD8(Tn)	S100A9	ITGB2	2,13208E-03	23493,3	2,85331E-03	46825	3,46637E+00	830	3,29402E+00	1043	9,21172E-01	20387	0	48381,5
Myeloid_vs_CD8_Responder	CD8(NME1+ T)	cDC_CLEC9A	HMGB1	THBD	2,14368E-03	27804,1	4,84281E-03	21897	1,47057E+00	16616	1,61669E+00	18479	8,97347E-01	33647	0	48381,5
Myeloid_vs_CD8_Responder	Mono_CD14	CD8(ZNF683+KLRB1+ T)	VCAN	CD44	2,14600E-03	16077,9	8,80799E-03	8451	3,10740E+00	1350	2,30350E+00	6618	9,29641E-01	15589	0	48381,5
Myeloid_vs_CD8_Responder	Macro_FOLR2+APOE+	CD8(LAYN+ T)	C1QB	C1QBP	2,15734E-03	17065,7	6,03421E-03	15610	2,48339E+00	3229	2,79980E+00	2684	9,29925E-01	15424	0	48381,5
Myeloid_vs_CD8_Responder	CD8(Temra)	cDC(CD1C)	SPON2	ITGB2	2,16157E-03	26615,9	1,01084E-02	6853	1,01093E+00	33703	1,91897E+00	12007	9,00086E-01	32135	0	48381,5
Myeloid_vs_CD8_Responder	CD8(Terminal Tex)	Macro_LYVE1	CALR	LRP1	2,16157E-03	25471,9	5,33393E-03	18867	2,22016E+00	5009	1,17053E+00	33703	9,19321E-01	21399	0	48381,5
Myeloid_vs_CD8_Responder	CD8(Terminal Tex)	Macro_LYVE1	SPN	SIGLEC1	2,16307E-03	39402,5	1,27526E-02	4734	2,42410E+00	3532	7,65117E-01	54544	8,03827E-01	85821	0	48381,5
Myeloid_vs_CD8_Responder	cDC_CLEC9A	CD8(Terminal Tex)	HLA-DQA2	LAG3	2,16438E-03	16972,5	1,12253E-02	5818	1,50785E+00	15623	2,77017E+00	2845	9,35310E-01	12195	0	48381,5
Myeloid_vs_CD8_Responder	Macro_OLFML3	CD8(EOMES+ NK-like)	CD14	ITGA4	2,16960E-03	29147,5	3,59303E-03	33728	1,62611E+00	12874	1,51951E+00	21126	9,04622E-01	29628	0	48381,5
Myeloid_vs_CD8_Responder	CD8(ITM2C+ T)	Macro_NLRP3	ANXA1	FPR1	2,17411E-03	25807,3	5,50431E-03	17957	1,76888E+00	10294	1,16952E+00	33742	9,24134E-01	18662	0	48381,5
Myeloid_vs_CD8_Responder	Mono_CD14	CD8(Tc17)	S100A9	ITGB2	2,18109E-03	21357,1	3,25481E-03	38885	3,50076E+00	799	3,29154E+00	1055	9,25821E-01	17665	0	48381,5
Myeloid_vs_CD8_Responder	Macro_OLFML3	CD8(EOMES+ NK-like)	HLA-DQA1	LAG3	2,18185E-03	29805,1	3,59016E-03	33766	1,08253E+00	30296	1,56211E+00	19900	9,27683E-01	16682	0	48381,5
Myeloid_vs_CD8_Responder	CD8(Tn)	Macro_IER3	CD40LG	CD40	2,18931E-03	44472,5	2,74800E-02	966	1,16307E+00	26707	1,05825E+00	38666	7,58909E-01	107642	0	48381,5
Myeloid_vs_CD8_Responder	Mast	CD8(GZMK+ Tex)	TIMP3	CD44	2,18931E-03	19414,3	2,63866E-02	1057	3,53511E+00	771	1,54860E+00	20291	9,10262E-01	26571	0	48381,5
Myeloid_vs_CD8_Responder	Mono_CD14	CD8(ID2+CXCR4+ T)	VCAN	CD44	2,20808E-03	16298,7	8,75722E-03	8533	3,10388E+00	1359	2,21978E+00	7517	9,29451E-01	15703	0	48381,5
Myeloid_vs_CD8_Responder	Macro_FOLR2+APOE+	CD8(Terminal Tex)	HLA-DQA1	LAG3	2,21359E-03	17165,3	8,53177E-03	8892	1,50473E+00	15713	2,06646E+00	9593	9,51866E-01	3247	0	48381,5
Myeloid_vs_CD8_Responder	CD8(Terminal Tex)	Mono_CD16	HSPA1A	TLR4	2,21697E-03	29515,5	5,08680E-03	20319	1,49247E+00	16036	1,29044E+00	28967	8,96966E-01	33874	0	48381,5
Myeloid_vs_CD8_Responder	Macro_ISG15	CD8(IL7R+ZNF683+ Tm)	S100A8	CD69	2,21746E-03	20127,3	6,38183E-03	14288	1,50449E+00	15720	2,10927E+00	8951	9,33547E-01	13296	0	48381,5
Myeloid_vs_CD8_Responder	CD8(ID2+CXCR4+ T)	Mono_CD16	ANXA1	FPR1	2,21856E-03	24065,1	6,42855E-03	14144	1,99494E+00	7223	1,14474E+00	34855	9,29399E-01	15722	0	48381,5
Myeloid_vs_CD8_Responder	Macro_OLFML3	CD8(GZMK+ Tex)	HLA-DQB2	LAG3	2,21861E-03	25104,7	1,12561E-02	5791	1,59261E+00	13578	1,43074E+00	23894	8,96957E-01	33879	0	48381,5
Myeloid_vs_CD8_Responder	CD8(GZMK+ Early Tem)	cDC(CD1C)	CD28	CD86	2,22741E-03	28036,5	1,13945E-02	5661,5	1,69797E+00	11501,5	1,92649E+00	11855,5	8,46335E-01	62782,5	0	48381,5
Myeloid_vs_CD8_Responder	CD8(GZMK+ Tem)	Mono_INHBA	HLA-F	LILRB2	2,22812E-03	30061,9	4,19528E-03	26984	1,59219E+00	13585	1,33041E+00	27451	8,96910E-01	33908	0	48381,5
Myeloid_vs_CD8_Responder	Macro_FOLR2-APOE+	CD8(Terminal Tex)	HLA-DRA	LAG3	2,23351E-03	16900,9	6,77689E-03	12974	1,50304E+00	15749	2,38392E+00	5783	9,56569E-01	1617	0	48381,5
Myeloid_vs_CD8_Responder	CD8(ITM2C+ T)	Macro_ISG15	ANXA1	FPR1	2,23404E-03	27488,5	4,97058E-03	21045	1,60415E+00	13333	1,16527E+00	33926	9,20480E-01	20757	0	48381,5
Myeloid_vs_CD8_Responder	CD8(ISG+ T)	Macro_LYVE1	LGALS9	LRP1	2,24463E-03	25758,9	6,28217E-03	14676	2,66549E+00	2480	1,73004E+00	15769	8,73420E-01	47488	0	48381,5
Myeloid_vs_CD8_Responder	CD8(ID2+CXCR4+ T)	Macro_ISG15	CD52	SIGLEC10	2,24889E-03	32360,1	4,16538E-03	27262	1,24218E+00	23647	1,16438E+00	33971	9,06633E-01	28539	0	48381,5
Myeloid_vs_CD8_Responder	cDC(CD1C)	CD8(ISG+ T)	HLA-DPB1	LAG3	2,24909E-03	16617,5	6,20556E-03	14941	1,50214E+00	15777	3,26183E+00	1130	9,52756E-01	2858	0	48381,5
Myeloid_vs_CD8_Responder	CD8(Tc17)	pDC_LILRA4	HMGB1	TLR9	2,26399E-03	28817,5	2,62017E-02	1075	3,53567E+00	770	9,07221E-01	46172	8,73051E-01	47689	0	48381,5
Myeloid_vs_CD8_Responder	CD8(NME1+ T)	Mono_INHBA	CALR	LRP1	2,26416E-03	32206,3	3,68264E-03	32557	1,47992E+00	16362	1,16308E+00	34017	9,04472E-01	29714	0	48381,5
Myeloid_vs_CD8_Responder	CD8(Terminal Tex)	Mast	IL16	CD9	2,27149E-03	25568,3	8,46762E-03	8998	1,75390E+00	10545	1,72804E+00	15817	8,79144E-01	44100	0	48381,5
Myeloid_vs_CD8_Responder	CD8(NME1+ T)	pDC_LILRA4	HLA-C	NOTCH4	2,27416E-03	23719,3	1,50264E-02	3655	1,48806E+00	16150	1,70466E+00	16363	8,96642E-01	34047	0	48381,5
Myeloid_vs_CD8_Responder	CD8(GZMK+ Tex)	Mono_INHBA	CCL5	CCRL2	2,28219E-03	29238,5	8,43353E-03	9055	1,02387E+00	33062	1,16183E+00	34071	9,18972E-01	21623	0	48381,5
Myeloid_vs_CD8_Responder	CD8(GZMK+ Early Tem)	pDC_LILRA4	HLA-C	NOTCH4	2,28655E-03	23713,5	1,50044E-02	3660	1,48428E+00	16249	1,71229E+00	16193	8,96574E-01	34084	0	48381,5
Myeloid_vs_CD8_Responder	CD8(ISG+ T)	Macro_NLRP3	HMGB1	CD163	2,28688E-03	27467,5	3,56761E-03	34085	1,63136E+00	12767	1,39356E+00	25115	9,27094E-01	16989	0	48381,5
Myeloid_vs_CD8_Responder	cDC(CD1C)	CD8(GZMK+ Tem)	HLA-DRB5	LAG3	2,28952E-03	15658,9	7,99275E-03	9897	1,49933E+00	15849	3,16982E+00	1353	9,52873E-01	2814	0	48381,5
Myeloid_vs_CD8_Responder	pDC_LILRA4	CD8(ISG+ T)	APP	RPSA	2,30142E-03	17555,3	8,31864E-03	9273	1,84028E+00	9221	2,46429E+00	5031	9,29124E-01	15870	0	48381,5
Myeloid_vs_CD8_Responder	Mono_INHBA	CD8(Tn)	VCAN	SELL	2,30295E-03	20783,9	1,49182E-02	3692	2,12779E+00	5794	2,55817E+00	4272	8,83054E-01	41780	0	48381,5
Myeloid_vs_CD8_Responder	Macro_OLFML3	CD8(GZMK+ Early Tem)	HLA-DQA2	LAG3	2,32470E-03	34393,3	4,01758E-03	28706	1,02690E+00	32886	1,32137E+00	27796	8,96370E-01	34197	0	48381,5
Myeloid_vs_CD8_Responder	CD8(NME1+ T)	Macro_NLRP3	HLA-F	LILRB2	2,32572E-03	31330,9	4,14618E-03	27460	1,54939E+00	14611	1,21295E+00	32002	8,96365E-01	34200	0	48381,5
Myeloid_vs_CD8_Responder	Mono_CD14	CD8(IL7R+ZNF683+ Tm)	S100A9	ITGB2	2,32718E-03	21844,3	3,15568E-03	40630	3,49227E+00	806	3,27643E+00	1090	9,24752E-01	18314	0	48381,5
Myeloid_vs_CD8_Responder	Mono_CD14	CD8(Terminal Tex)	VCAN	ITGB1	2,32760E-03	17312,1	1,12303E-02	5815	3,23046E+00	1126	2,52468E+00	4516	9,09933E-01	26722	0	48381,5
Myeloid_vs_CD8_Responder	CD8(ISG+ T)	Mono_CD16	RPS19	C5AR1	2,33117E-03	25284,7	4,48026E-03	24492	1,82797E+00	9404	1,15838E+00	34216	9,39198E-01	9930	0	48381,5
Myeloid_vs_CD8_Responder	CD8(GZMK+ Tex)	Mono_INHBA	HMGB1	CD163	2,34106E-03	26431,3	3,55639E-03	34245	1,56025E+00	14346	1,62997E+00	18151	9,26987E-01	17033	0	48381,5
Myeloid_vs_CD8_Responder	Mono_CD14	CD8(ITM2C+ T)	VCAN	CD44	2,35060E-03	16482,5	8,62748E-03	8748	3,09487E+00	1373	2,18158E+00	7954	9,28960E-01	15956	0	48381,5
Myeloid_vs_CD8_Responder	CD8(ISG+ T)	pDC_LILRA4	MAML2	NOTCH4	2,35579E-03	44201,5	1,89009E-02	2393	1,49557E+00	15965	1,80036E+00	14258	6,66801E-01	140010	0	48381,5
Myeloid_vs_CD8_Responder	CD8(Tc17)	Mono_CD16	B2M	LILRB2	2,35580E-03	22195,7	4,78517E-03	22275	2,26002E+00	4683	1,15685E+00	34288	9,57458E-01	1351	0	48381,5
Myeloid_vs_CD8_Responder	Macro_NLRP3	CD8(Temra)	S100A8	ITGB2	2,36910E-03	16493,1	5,93932E-03	15988	2,29022E+00	4447	2,79638E+00	2700	9,37459E-01	10949	0	48381,5
Myeloid_vs_CD8_Responder	Macro_OLFML3	CD8(Tn)	APOE	LDLR	2,38341E-03	20906,3	1,11849E-02	5862	2,22205E+00	4998	3,07716E+00	1604	8,79833E-01	43686	0	48381,5
Myeloid_vs_CD8_Responder	CD8(Tc17)	pDC_LILRA4	HLA-C	NOTCH4	2,38376E-03	24413,3	1,48372E-02	3731	1,45554E+00	17036	1,61367E+00	18549	8,96053E-01	34369	0	48381,5
Myeloid_vs_CD8_Responder	Macro_IER3	CD8(GZMK+ Tem)	HLA-DRA	LAG3	2,39471E-03	17305,3	6,86560E-03	12707	1,49267E+00	16032	2,18944E+00	7869	9,56838E-01	1537	0	48381,5
Myeloid_vs_CD8_Responder	CD8(Terminal Tex)	Mono_INHBA	HLA-F	LILRB2	2,39487E-03	30211,5	4,11292E-03	27759	1,57967E+00	13871	1,35043E+00	26645	8,95990E-01	34401	0	48381,5
Myeloid_vs_CD8_Responder	CD8(ZNF683+KLRB1+ T)	Macro_LYVE1	RPS19	C5AR1	2,39592E-03	22521,3	5,69583E-03	17074	2,06012E+00	6481	1,15426E+00	34404	9,45701E-01	6266	0	48381,5
Myeloid_vs_CD8_Responder	Macro_OLFML3	CD8(ZNF683+KLRB1+ T)	C1QB	C1QBP	2,39731E-03	23639,1	3,54440E-03	34408	2,10602E+00	6010	2,75215E+00	2958	9,10479E-01	26438	0	48381,5
Myeloid_vs_CD8_Responder	CD8(NME1+ T)	Macro_LYVE1	HMGB1	TLR2	2,39975E-03	33972,9	3,60975E-03	33508	1,29696E+00	21800	1,15410E+00	34415	9,00753E-01	31760	0	48381,5
Myeloid_vs_CD8_Responder	CD8(Tc17)	Mono_CD16	ANXA1	FPR1	2,40173E-03	24826,3	6,31446E-03	14553	1,98138E+00	7383	1,07734E+00	37770	9,28810E-01	16044	0	48381,5
Myeloid_vs_CD8_Responder	Mast	CD8(ZNF683+KLRB1+ T)	TIMP3	CD44	2,41707E-03	19936,9	2,58473E-02	1111	3,52235E+00	786	1,47662E+00	22400	9,09415E-01	27006	0	48381,5
Myeloid_vs_CD8_Responder	CD8(Tn)	Mono_INHBA	HSPA1A	TLR4	2,42109E-03	25186,9	4,96425E-03	21087	1,49391E+00	16002	2,36272E+00	5988	8,95833E-01	34476	0	48381,5
Myeloid_vs_CD8_Responder	CD8(IL7R+ZNF683+ Tm)	Macro_LYVE1	RPS19	C5AR1	2,42355E-03	22401,5	5,76836E-03	16721	2,07581E+00	6322	1,15281E+00	34483	9,46025E-01	6100	0	48381,5
Myeloid_vs_CD8_Responder	CD8(Terminal Tex)	Mono_CD16	HLA-C	LILRA3	2,42554E-03	23285,7	1,78529E-02	2677	2,94066E+00	1682	9,17719E-01	45607	9,25136E-01	18081	0	48381,5
Myeloid_vs_CD8_Responder	pDC_LILRA4	CD8(ISG+ T)	APP	CD74	2,42642E-03	16755,3	5,91501E-03	16086	2,02702E+00	6858	2,47653E+00	4931	9,43478E-01	7520	0	48381,5
Myeloid_vs_CD8_Responder	CD8(EOMES+ NK-like)	pDC_LILRA4	HLA-C	NOTCH4	2,42813E-03	24343,3	1,47559E-02	3758	1,44156E+00	17472	1,65139E+00	17609	8,95797E-01	34496	0	48381,5
Myeloid_vs_CD8_Responder	CD8(IL7R+ZNF683+ Tm)	cDC_CLEC9A	CD52	SIGLEC10	2,43129E-03	34344,5	3,72678E-03	31992	1,18789E+00	25677	1,15221E+00	34505	9,01816E-01	31167	0	48381,5
Myeloid_vs_CD8_Responder	CD8(ISG+ T)	Macro_NLRP3	HLA-F	LILRB2	2,44012E-03	30970,3	4,09164E-03	27966	1,54075E+00	14803	1,28520E+00	29171	8,95748E-01	34530	0	48381,5
Myeloid_vs_CD8_Responder	CD8(ZNF683+KLRB1+ T)	pDC_LILRA4	HLA-C	NOTCH4	2,45215E-03	24396,5	1,47153E-02	3778	1,43458E+00	17655	1,65166E+00	17604	8,95668E-01	34564	0	48381,5
Myeloid_vs_CD8_Responder	CD8(Tc17)	Macro_NLRP3	ANXA1	FPR1	2,45606E-03	25610,9	5,68232E-03	17131	1,79239E+00	9946	1,15061E+00	34575	9,25242E-01	18021	0	48381,5
Myeloid_vs_CD8_Responder	Macro_OLFML3	CD8(EOMES+ NK-like)	HLA-DRB5	LAG3	2,45783E-03	27197,7	3,53163E-03	34580	1,17923E+00	26016	1,91418E+00	12105	9,30748E-01	14906	0	48381,5
Myeloid_vs_CD8_Responder	CD8(LAYN+ T)	Mono_CD16	RPS19	C5AR1	2,47780E-03	22302,1	5,81326E-03	16511	2,10795E+00	5993	1,14934E+00	34636	9,46223E-01	5989	0	48381,5

Myeloid_vs_CD8_Responder	cDC_CLEC9A	CD8(GZMK+ Tem)	HLA-DRB5	LAG3	2,48175E-03	15892,9	7,91952E-03	10070	1,48683E+00	16179	2,98190E+00	1937	9,52665E-01	2897	0	48381,5
Myeloid_vs_CD8_Responder	cDC(CD1C)	CD8(GZMK+ Tem)	HLA-DRB1	LAG3	2,48535E-03	15845,1	7,08040E-03	12108	1,48672E+00	16185	3,16999E+00	1352	9,58037E-01	1199	0	48381,5
Myeloid_vs_CD8_Responder	Macro_OLFML3	CD8(Terminal Tex)	FN1	ITGA4_ITGB7	2,48819E-03	20829,5	1,48560E-02	3719	2,10189E+00	6053	1,95715E+00	11329	8,95507E-01	34665	0	48381,5
Myeloid_vs_CD8_Responder	CD8(GZMK+ Tem)	Macro_LYVE1	CALR	LRP1	2,48819E-03	25924,7	5,21775E-03	19540	2,20316E+00	5133	1,14875E+00	34665	9,18501E-01	21904	0	48381,5
Myeloid_vs_CD8_Responder	CD8(IL7R+ZNF683+ Tm)	Mono_INHBA	ANXA1	FPR1	2,49250E-03	32065,3	4,03936E-03	28498	1,24790E+00	23443	1,14849E+00	34677	9,12549E-01	25327	0	48381,5
Myeloid_vs_CD8_Responder	CD8(Tc17)	Macro_LYVE1	RPS19	C5AR1	2,49498E-03	22427,3	5,88548E-03	16201	2,10115E+00	6062	1,12579E+00	35665	9,46536E-01	5827	0	48381,5
Myeloid_vs_CD8_Responder	CD8(Tn)	Macro_FOLR2+APOE+	CD40LG	CD40	2,49985E-03	38275,3	4,09291E-02	428	1,52682E+00	15164	1,10954E+00	36326	7,93459E-01	91077	0	48381,5
Myeloid_vs_CD8_Responder	CD8(NME1+ T)	Mono_INHBA	HMGB1	TLR2	2,50222E-03	32458,9	3,52202E-03	34704	1,27725E+00	22443	1,41507E+00	24404	8,99647E-01	32362	0	48381,5
Myeloid_vs_CD8_Responder	Mast	CD8(ID2+CXCR4+ T)	TIMP3	CD44	2,50424E-03	20516,7	2,56983E-02	1131	3,51882E+00	787	1,39289E+00	25134	9,09177E-01	27150	0	48381,5
Myeloid_vs_CD8_Responder	CD8(EOMES+ NK-like)	Macro_NLRP3	HMGB1	CD163	2,51052E-03	28021,5	3,52068E-03	34727	1,61824E+00	13050	1,34843E+00	26735	9,26645E-01	17214	0	48381,5
Myeloid_vs_CD8_Responder	CD8(IL7R+ZNF683+ Tm)	pDC_LILRA4	HLA-C	NOTCH4	2,51776E-03	24734,7	1,46174E-02	3821	1,41777E+00	18125	1,61192E+00	18599	8,95356E-01	34747	0	48381,5
Myeloid_vs_CD8_Responder	CD8(NME1+ T)	Macro_NLRP3	HMGB1	TLR4	2,51921E-03	29362,9	5,14187E-03	19983	1,52810E+00	15133	1,30077E+00	28566	8,95355E-01	34751	0	48381,5
Myeloid_vs_CD8_Responder	CD8(GZMK+ Tem)	Macro_ISG15	HLA-A	LILRB1	2,52247E-03	29304,3	3,51812E-03	34760	1,40730E+00	18423	1,22668E+00	31460	9,33154E-01	13497	0	48381,5
Myeloid_vs_CD8_Responder	Macro_IER3	CD8(Terminal Tex)	ICAM1	IL2RG	2,52340E-03	19146,5	8,63073E-03	8742	1,71854E+00	11132	1,96315E+00	11229	9,28470E-01	16248	0	48381,5
Myeloid_vs_CD8_Responder	CD8(EOMES+ NK-like)	Macro_NLRP3	CD99	PILRA	2,52465E-03	29831,1	4,33487E-03	25732	1,51285E+00	15508	1,14677E+00	34766	9,13500E-01	24768	0	48381,5
Myeloid_vs_CD8_Responder	CD8(NME1+ T)	cDC(CD1C)	HMGB1	TLR2	2,52465E-03	31134,5	3,51758E-03	34766	1,27625E+00	22472	1,64960E+00	17657	8,99590E-01	32396	0	48381,5
Myeloid_vs_CD8_Responder	CD8(Tc17)	Macro_ISG15	ANXA1	FPR1	2,53156E-03	27235,9	5,13133E-03	20043	1,62766E+00	12850	1,14636E+00	34785	9,21637E-01	20120	0	48381,5
Myeloid_vs_CD8_Responder	CD8(Temra)	Macro_ISG15	CCL4	CCR1	2,53702E-03	31463,9	7,33448E-03	11451	1,03818E+00	32323	1,14614E+00	34800	9,03262E-01	30364	0	48381,5
Myeloid_vs_CD8_Responder	CD8(Tc17)	Mono_CD16	HLA-A	LILRB2	2,54286E-03	22140,9	5,09802E-03	20247	2,34127E+00	4080	1,14578E+00	34816	9,52011E-01	3180	0	48381,5
Myeloid_vs_CD8_Responder	pDC_LILRA4	CD8(NME1+ T)	APP	RPSA	2,54901E-03	17541,5	8,13657E-03	9617	1,82237E+00	9471	2,59944E+00	3948	9,28392E-01	16290	0	48381,5
Myeloid_vs_CD8_Responder	CD8(GZMK+ Tem)	Macro_NLRP3	HMGB1	CD163	2,55053E-03	27960,3	3,51262E-03	34837	1,61598E+00	13102	1,36140E+00	26222	9,26567E-01	17259	0	48381,5
Myeloid_vs_CD8_Responder	Macro_FOLR2+APOE-	CD8(GZMK+ Tem)	HLA-DRB5	LAG3	2,55452E-03	17017,9	7,89301E-03	10132	1,48230E+00	16299	2,23508E+00	7344	9,52590E-01	2933	0	48381,5
Myeloid_vs_CD8_Responder	Macro_OLFML3	CD8(LAYN+ T)	HLA-DQB1	LAG3	2,55676E-03	28955,1	3,51121E-03	34854	1,16411E+00	26659	1,66228E+00	17324	9,26003E-01	17557	0	48381,5
Myeloid_vs_CD8_Responder	Macro_OLFML3	CD8(ISG+ T)	FN1	ITGA4_ITGB1	2,56154E-03	22800,9	9,58417E-03	7404	1,95145E+00	7745	1,73748E+00	15607	8,95150E-01	34867	0	48381,5
Myeloid_vs_CD8_Responder	CD8(Terminal Tex)	Macro_NLRP3	CD99	PILRA	2,56963E-03	30363,1	4,20562E-03	26888	1,48683E+00	16180	1,14407E+00	34889	9,12297E-01	25477	0	48381,5
Myeloid_vs_CD8_Responder	Mono_CD16	CD8(ZNF683+KLRB1+ T)	S100A8	CD69	2,57357E-03	19963,9	6,87388E-03	12680	1,48111E+00	16330	2,00510E+00	10509	9,35814E-01	11919	0	48381,5
Myeloid_vs_CD8_Responder	Macro_OLFML3	CD8(EOMES+ NK-like)	GRN	TNFRSF1B	2,57811E-03	25366,7	3,50732E-03	34912	1,69620E+00	11536	1,66124E+00	17353	9,31207E-01	14651	0	48381,5
Myeloid_vs_CD8_Responder	CD8(GZMK+ Early Tem)	pDC_LILRA4	BST2	LILRA4	2,58530E-03	23463,1	9,43875E-03	7581	5,73842E+00	129	2,69348E+00	3303	8,55078E-01	57921	0	48381,5
Myeloid_vs_CD8_Responder	CD8(LAYN+ T)	Mono_CD16	ANXA1	FPR1	2,58957E-03	24449,7	6,19576E-03	14985	1,96727E+00	7548	1,14275E+00	34943	9,28180E-01	16391	0	48381,5
Myeloid_vs_CD8_Responder	CD8(GZMK+ Early Tem)	pDC_LILRA4	COPA	P2RY6	2,59149E-03	34651,1	6,34004E-03	14461	2,24338E+00	4824	2,01086E+00	10424	7,85431E-01	95165	0	48381,5
Myeloid_vs_CD8_Responder	CD8(GZMK+ Tem)	Macro_NLRP3	HLA-F	LILRB2	2,59477E-03	31570,3	4,02646E-03	28615	1,53042E+00	15072	1,24282E+00	30826	8,94996E-01	34957	0	48381,5
Myeloid_vs_CD8_Responder	pDC_LILRA4	CD8(Tc17)	APP	PTGER2	2,60142E-03	28076,3	1,55849E-02	3417	2,10318E+00	6039	2,71787E+00	3172	8,16224E-01	79372	0	48381,5
Myeloid_vs_CD8_Responder	CD8(Tn)	Macro_ISG15	ANXA1	FPR2_FPR3	2,60369E-03	26599,9	7,30317E-03	11535	1,59353E+00	13559	1,41059E+00	24543	8,94948E-01	34981	0	48381,5
Myeloid_vs_CD8_Responder	Macro_ISG15	CD8(ISG+ T)	S100A8	CD69	2,60825E-03	20104,5	6,16247E-03	15119	1,47867E+00	16386	2,29373E+00	6714	9,32454E-01	13922	0	48381,5
Myeloid_vs_CD8_Responder	Macro_OLFML3	CD8(ID2+CXCR4+ T)	HLA-DRB1	LAG3	2,61823E-03	27291,7	3,49971E-03	35020	1,09666E+00	29637	1,77782E+00	14723	9,41352E-01	8697	0	48381,5
Myeloid_vs_CD8_Responder	CD8(Terminal Tex)	Macro_ISG15	HLA-A	LILRB1	2,62235E-03	29459,7	3,49901E-03	35031	1,39034E+00	18948	1,22975E+00	31334	9,32984E-01	13604	0	48381,5
Myeloid_vs_CD8_Responder	Mast	CD8(ITM2C+ T)	TIMP3	CD44	2,63334E-03	20864,7	2,53175E-02	1160	3,50981E+00	794	1,35469E+00	26496	9,08559E-01	27492	0	48381,5
Myeloid_vs_CD8_Responder	CD8(Terminal Tex)	Mono_CD14	IFNG	IFNGR1_IFNGR2	2,63773E-03	28585,9	6,54558E-03	13768	1,05508E+00	31536	1,80467E+00	14172	8,94797E-01	35072	0	48381,5
Myeloid_vs_CD8_Responder	Mono_CD14	CD8(EOMES+ NK-like)	VCAN	ITGB1	2,63834E-03	17559,3	1,09405E-02	6068	3,21971E+00	1144	2,48380E+00	4876	9,08856E-01	27327	0	48381,5
Myeloid_vs_CD8_Responder	CD8(ID2+CXCR4+ T)	Mono_CD16	HLA-F	LILRB2	2,63923E-03	29839,5	4,00842E-03	28801	2,40032E+00	3669	1,18095E+00	33270	8,94785E-01	35076	0	48381,5
Myeloid_vs_CD8_Responder	Macro_OLFML3	CD8(GZMK+ Tem)	GRN	TNFRSF1B	2,64074E-03	25328,3	3,49599E-03	35080	1,69427E+00	11572	1,68155E+00	16896	9,31103E-01	14712	0	48381,5
Myeloid_vs_CD8_Responder	cDC_LAMP3	CD8(LAYN+ T)	CCL19	CCR7	2,64236E-03	34822,9	2,53123E-02	1162	6,26497E+00	86	1,53736E+00	20579	7,67153E-01	103906	0	48381,5
Myeloid_vs_CD8_Responder	CD8(Terminal Tex)	Mono_CD14	RPS19	C5AR1	2,65146E-03	26355,7	3,34926E-03	37294	1,55463E+00	14495	1,70079E+00	16455	9,30340E-01	15153	0	48381,5
Myeloid_vs_CD8_Responder	Macro_OLFML3	CD8(IL7R+ZNF683+ Tm)	C1QB	C1QBP	2,65469E-03	23903,7	3,49271E-03	35117	2,10187E+00	6055	2,70874E+00	3214	9,09878E-01	26751	0	48381,5
Myeloid_vs_CD8_Responder	CD8(GZMK+ Tex)	Macro_ISG15	HLA-A	LILRB1	2,67365E-03	29195,9	3,48940E-03	35167	1,38182E+00	19189	1,27402E+00	29577	9,32898E-01	13665	0	48381,5
Myeloid_vs_CD8_Responder	Macro_OLFML3	CD8(GZMK+ Tex)	B2M	CD3D	2,67786E-03	44294,3	2,74438E-03	49540	6,17377E-01	59147	6,20869E-01	64386	9,65820E-01	17	0	48381,5
Myeloid_vs_CD8_Responder	Mono_INHBA	CD8(Tc17)	CCL20	CCR6	2,67946E-03	20216,7	9,52189E-02	68	2,09797E+00	6100	2,69981E+00	3267	8,80583E-01	43267	0	48381,5
Myeloid_vs_CD8_Responder	CD8(Tn)	Macro_FOLR2+APOE+	HSPA1A	TLR4	2,69424E-03	26345,1	4,82655E-03	21996	1,47204E+00	16577	2,06915E+00	9550	8,94513E-01	35221	0	48381,5
Myeloid_vs_CD8_Responder	CD8(GZMK+ Tex)	Macro_NLRP3	CCL5	CCRL2	2,69883E-03	28033,7	9,26889E-03	7789	1,10835E+00	29098	1,13620E+00	35233	9,22420E-01	19667	0	48381,5
Myeloid_vs_CD8_Responder	CD8(Terminal Tex)	Mast	HSPA8	ADRB2	2,70036E-03	27664,9	8,74126E-03	8560	1,09355E+00	29788	1,13608E+00	35237	9,28248E-01	16358	0	48381,5
Myeloid_vs_CD8_Responder	Macro_OLFML3	CD8(ITM2C+ T)	HLA-DQB1	LAG3	2,70113E-03	29232,1	3,48431E-03	35239	1,16170E+00	26757	1,63338E+00	18067	9,25739E-01	17716	0	48381,5
Myeloid_vs_CD8_Responder	CD8(Terminal Tex)	Macro_LYVE1	HSP90B1	LRP1	2,70573E-03	28371,9	4,56687E-03	23851	2,18665E+00	5280	1,13580E+00	35251	9,05626E-01	29096	0	48381,5
Myeloid_vs_CD8_Responder	CD8(LAYN+ T)	Mono_INHBA	HSP90B1	LRP1	2,71649E-03	33318,3	3,55795E-03	34230	1,51059E+00	15572	1,18513E+00	33129	8,94404E-01	35279	0	48381,5
Myeloid_vs_CD8_Responder	CD8(GZMK+ Tem)	Mono_INHBA	HLA-A	LILRB2	2,72922E-03	25880,5	3,47991E-03	35312	1,69870E+00	11494	1,36429E+00	26133	9,42496E-01	8082	0	48381,5
Myeloid_vs_CD8_Responder	CD8(NME1+ T)	Mono_INHBA	CD99	PILRA	2,72961E-03	34065,9	3,58824E-03	33786	1,23706E+00	23811	1,13425E+00	35313	9,05734E-01	29038	0	48381,5
Myeloid_vs_CD8_Responder	Macro_NLRP3	CD8(Tn)	VCAN	CD44	2,73107E-03	18385,1	8,33967E-03	9228	2,50491E+00	3130	1,78309E+00	14606	9,27833E-01	16580	0	48381,5
Myeloid_vs_CD8_Responder	CD8(NME1+ T)	Macro_LYVE1	CD99	PILRA	2,73464E-03	26305,7	5,45633E-03	18219	1,80262E+00	9772	1,13391E+00	35326	9,22169E-01	19830	0	48381,5
Myeloid_vs_CD8_Responder	CD8(ID2+CXCR4+ T)	cDC_CLEC9A	CALM2	MYLK	2,73669E-03	35459,9	2,26834E-02	1549	2,09409E+00	6144	9,98004E-01	41586	8,15688E-01	79639	0	48381,5
Myeloid_vs_CD8_Responder	CD8(Terminal Tex)	Macro_NLRP3	HSPA1A	TLR4	2,74084E-03	30102,1	4,80521E-03	22145	1,43270E+00	17707	1,34371E+00	26935	8,94304E-01	35342	0	48381,5
Myeloid_vs_CD8_Responder	CD8(Terminal Tex)	Mono_INHBA	HMGB1	CD163	2,74278E-03	27632,3	3,47787E-03	35347	1,53527E+00	14945	1,48777E+00	22050	9,26228E-01	17438	0	48381,5
Myeloid_vs_CD8_Responder	Macro_FOLR2+APOE+	CD8(GZMK+ Tem)	HLA-DPB1	LAG3	2,75497E-03	17679,7	6,96427E-03	12439	1,47056E+00	16617	2,10908E+00	8954	9,55285E-01	2007	0	48381,5
Myeloid_vs_CD8_Responder	Macro_OLFML3	CD8(LAYN+ T)	APOE	LSR	2,75507E-03	21238,1	1,13691E-02	5685	2,09241E+00	6158	2,80453E+00	2663	8,80508E-01	43303	0	48381,5
Myeloid_vs_CD8_Responder	CD8(ITM2C+ T)	pDC_LILRA4	ADM	CALCRL	2,75507E-03	40475,7	1,08394E-02	6158	2,31751E+00	4236	2,35566E+00	6058	6,76380E-01	137545	0	48381,5
Myeloid_vs_CD8_Responder	CD8(GZMK+ Tex)	Mono_INHBA	HLA-F	LILRB2	2,76067E-03	30361,9	3,96083E-03	29341	1,55654E+00	14443	1,41973E+00	24251	8,94221E-01	35393	0	48381,5
Myeloid_vs_CD8_Responder	Macro_FOLR2-APOE+	CD8(Temra)	SPP1	S1PR1	2,76693E-03	23710,5	2,98132E-02	803	2,09185E+00	6167	2,51252E+00	4619	8,54029E-01	58582	0	48381,5
Myeloid_vs_CD8_Responder	CD8(ITM2C+ T)	cDC(CD1C)	IFNG	IFNGR1_IFNGR2	2,77200E-03	29322,1	6,75801E-03	13043	9,76520E-01	35422	1,74112E+00	15519	8,96290E-01	34245	0	48381,5
Myeloid_vs_CD8_Responder	Macro_ISG15	CD8(ID2+CXCR4+ T)	S100A8	CD69	2,77576E-03	21064,9	6,08511E-03	15416	1,46956E+00	16649	1,99324E+00	10708	9,32055E-01	14170	0	48381,5
Myeloid_vs_CD8_Responder	CD8(NME1+ T)	Mono_INHBA	HSP90B1	LRP1	2,78062E-03	33397,9	3,53667E-03	34522	1,50644E+00	15664	1,18877E+00	32978	8,94120E-01	35444	0	48381,5
Myeloid_vs_CD8_Responder	CD8(Terminal Tex)	Macro_NLRP3	HLA-F	LILRB2	2,79398E-03	31746,3	3,94742E-03	29476	1,51790E+00	15391	1,26284E+00	30005	8,94061E-01	35478	0	48381,5
Myeloid_vs_CD8_Responder	CD8(Tn)	cDC_LAMP3	CD40LG	TRAF3	2,80270E-03	58357,1	2,49543E-02	1197	6,09704E-01	59787	7,41743E-01	56041	7,12354E-01	126379	0	48381,5
Myeloid_vs_CD8_Responder	CD8(Tn)	cDC_CLEC9A	ANXA1	DYSF	2,80783E-03	22659,9	1,41092E-02	4036	1,93445E+00	7959	1,69010E+00	16698	8,92754E-01	36225	0	48381,5
Myeloid_vs_CD8_Responder	CD8(GZMK+ Early Tem)	Macro_LYVE1	CALR	LRP1	2,80897E-03	26439,9	5,06853E-03	20435	2,18133E+00	5326	1,12960E+00	35516	9,17408E-01	22541	0	48381,5
Myeloid_vs_CD8_Responder	CD8(NME1+ T)	Macro_ISG15	HMGB1	TLR4	2,81927E-03	29144,5	4,99302E-03	20891	1,49858E+00	15875	1,39607E+00	25033	8,93971E-01	35542	0	48381,5
Myeloid_vs_CD8_Responder	CD8(NME1+ T)	Macro_ISG15	HLA-F	LILRB2	2,82125E-03	30862,7	3,93956E-03	29558	1,47598E+00	16477	1,41671E+00	24350	8,93966E-01	35547	0	48381,5
Myeloid_vs_CD8_Responder	CD8(ID2+CXCR4+ T)	Mast	HSPA8	ADRB2	2,82324E-03	26681,3	9,21647E-03	7869	1,17394E+00	26221	1,12868E+00	35552	9,29991E-01	15383	0	48381,5
Myeloid_vs_CD8_Responder	Macro_LYVE1	CD8(GZMK+ Tem)	HLA-DRB5	LAG3	2,82892E-03	17464,5	7,79771E-03	10342	1,46603E+00	16730	2,11806E+00	8818	9,52315E-01	3051	0	48381,5
Myeloid_vs_CD8_Responder	Macro_ISG15	CD8(LAYN+ T)	CD14	ITGB1	2,83421E-03	19045,7	5,90835E-03	16111	2,01679E+00	6972	2,26462E+00	7026	9,27586E-01	16738	0	48381,5

Myeloid_vs_CD8_Responder	Mono_CD16	CD8(Terminal Tex)	B2M	CD3D	2,83534E-03	54567,9	2,74364E-03	49560	3,70138E-01	82004	2,76563E-01	92876	9,65815E-01	18	0	48381,5
Myeloid_vs_CD8_Responder	CD8(ITM2C+ T)	Macro_ISG15	TNF	VSIR	2,83676E-03	28394,5	8,95134E-03	8249	1,05033E+00	31762	1,63662E+00	17994	8,93882E-01	35586	0	48381,5
Myeloid_vs_CD8_Responder	CD8(Terminal Tex)	Mono_INHBA	HLA-A	LILRB2	2,84235E-03	25992,7	3,46101E-03	35600	1,68175E+00	11795	1,36736E+00	26026	9,42348E-01	8161	0	48381,5
Myeloid_vs_CD8_Responder	CD8(ZNF683+KLRB1+ T)	Mono_CD14	ANXA1	FPR1	2,84674E-03	32550,5	3,46014E-03	35611	1,38782E+00	19022	1,24038E+00	30929	9,06173E-01	28809	0	48381,5
Myeloid_vs_CD8_Responder	CD8(LAYN+ T)	cDC_CLEC9A	TGFB1	LPP	2,85154E-03	27539,3	8,28092E-03	9350	1,12564E+00	28342	1,71985E+00	16000	8,93810E-01	35623	0	48381,5
Myeloid_vs_CD8_Responder	CD8(ISG+ T)	Mast	IL16	CD9	2,85278E-03	26478,1	7,91886E-03	10073	1,72671E+00	10979	1,68694E+00	16766	8,75539E-01	46191	0	48381,5
Myeloid_vs_CD8_Responder	cDC(CD1C)	CD8(NME1+ T)	HLA-DRA	LAG3	2,85411E-03	16991,7	5,75732E-03	16768	1,49987E+00	15840	3,21874E+00	1213	9,53053E-01	2756	0	48381,5
Myeloid_vs_CD8_Responder	CD8(Terminal Tex)	Macro_FOLR2+APOE+	HMGB1	CD163	2,85435E-03	26529,3	4,27109E-03	26283	1,87498E+00	8722	1,12663E+00	35630	9,32947E-01	13630	0	48381,5
Myeloid_vs_CD8_Responder	CD8(Temra)	Macro_LYVE1	RPS19	C5AR1	2,86036E-03	24463,7	4,92349E-03	21365	1,89303E+00	8493	1,12639E+00	35645	9,41836E-01	8434	0	48381,5
Myeloid_vs_CD8_Responder	CD8(Terminal Tex)	Macro_ISG15	HLA-C	LILRB1	2,86237E-03	29522,7	3,45747E-03	35650	1,40490E+00	18491	1,24623E+00	30694	9,31651E-01	14397	0	48381,5
Myeloid_vs_CD8_Responder	CD8(ISG+ T)	Macro_NLRP3	CD99	PILRA	2,89139E-03	30740,5	4,15406E-03	27377	1,47645E+00	16462	1,12419E+00	35722	9,11802E-01	25760	0	48381,5
Myeloid_vs_CD8_Responder	CD8(ZNF683+KLRB1+ T)	Macro_NLRP3	HMGB1	CD163	2,89341E-03	28344,9	3,45203E-03	35727	1,59904E+00	13441	1,35197E+00	26594	9,25973E-01	17581	0	48381,5
Myeloid_vs_CD8_Responder	CD8(GZMK+ Tex)	Mono_INHBA	HLA-A	LILRB2	2,89665E-03	25751,9	3,45151E-03	35735	1,67322E+00	11942	1,41163E+00	24500	9,42274E-01	8201	0	48381,5
Myeloid_vs_CD8_Responder	CD8(Temra)	Mono_INHBA	CD99	PILRA	2,89909E-03	34659,5	3,50440E-03	34944	1,21436E+00	24653	1,12358E+00	35741	9,04720E-01	29578	0	48381,5
Myeloid_vs_CD8_Responder	Macro_IFI27	CD8(GZMK+ Tem)	HLA-DRB5	LAG3	2,90499E-03	16887,5	7,77347E-03	10391	1,46190E+00	16844	2,38907E+00	5735	9,52244E-01	3086	0	48381,5
Myeloid_vs_CD8_Responder	CD8(Temra)	Macro_LYVE1	CD99	PILRA	2,90558E-03	26690,5	5,32885E-03	18894	1,77992E+00	10122	1,12324E+00	35757	9,21316E-01	20298	0	48381,5
Myeloid_vs_CD8_Responder	CD8(Tn)	Macro_FOLR2+APOE-	HMGB1	CD163	2,91168E-03	27861,3	3,44862E-03	35772	1,82076E+00	9496	1,31374E+00	28054	9,25939E-01	17603	0	48381,5
Myeloid_vs_CD8_Responder	Macro_LYVE1	CD8(LAYN+ T)	CD14	ITGB1	2,92458E-03	20739,7	5,86435E-03	16282	2,00300E+00	7127	1,76299E+00	15035	9,27335E-01	16873	0	48381,5
Myeloid_vs_CD8_Responder	CD8(ITM2C+ T)	Macro_NLRP3	IFNG	IFNGR1_IFNGR2	2,93004E-03	32938,1	6,36258E-03	14363	9,80934E-01	35204	1,24050E+00	30925	8,93455E-01	35817	0	48381,5
Myeloid_vs_CD8_Responder	Mono_CD14	CD8(Tn)	VCAN	ITGB1	2,94053E-03	17584,3	1,06894E-02	6296	3,21040E+00	1166	2,56535E+00	4228	9,07890E-01	27850	0	48381,5
Myeloid_vs_CD8_Responder	CD8(IL7R+ZNF683+ Tm)	cDC_CLEC9A	ANXA1	DYSF	2,94192E-03	24640,7	1,42986E-02	3947	1,94670E+00	7806	1,33645E+00	27223	8,93391E-01	35846	0	48381,5
Myeloid_vs_CD8_Responder	pDC_LILRA4	CD8(GZMK+ Tex)	APP	RPSA	2,95176E-03	18370,7	7,86380E-03	10206	1,79553E+00	9894	2,31846E+00	6459	9,27250E-01	16913	0	48381,5
Myeloid_vs_CD8_Responder	CD8(ISG+ T)	Macro_ISG15	HLA-F	LILRB2	2,95837E-03	30617,9	3,88775E-03	30113	1,46733E+00	16694	1,48896E+00	22015	8,93337E-01	35886	0	48381,5
Myeloid_vs_CD8_Responder	Macro_ISG15	CD8(Terminal Tex)	S100A8	CD69	2,97021E-03	20561,9	5,99288E-03	15776	1,45871E+00	16940	2,24084E+00	7277	9,31569E-01	14435	0	48381,5
Myeloid_vs_CD8_Responder	CD8(GZMK+ Early Tem)	Macro_ISG15	TNFSF9	HLA-DPA1	2,97034E-03	29532,1	3,80124E-03	31107	9,67638E-01	35915	2,02625E+00	10168	9,18224E-01	22089	0	48381,5
Myeloid_vs_CD8_Responder	Macro_OLFML3	CD8(Terminal Tex)	CD14	ITGB1	2,97200E-03	29023,3	3,43858E-03	35919	1,64275E+00	12538	1,55865E+00	20009	9,07168E-01	28269	0	48381,5
Myeloid_vs_CD8_Responder	CD8(Terminal Tex)	Macro_FOLR2+APOE+	ENTPD1	TMIGD3	2,97774E-03	41218,5	2,28010E-02	1519	2,31041E+00	4288	6,67283E-01	61049	7,93884E-01	90855	0	48381,5
Myeloid_vs_CD8_Responder	CD8(NME1+ T)	Macro_FOLR2+APOE+	HMGB1	TLR2	2,97780E-03	31564,9	4,17169E-03	27199	1,42318E+00	17977	1,11879E+00	35933	9,07035E-01	28334	0	48381,5
Myeloid_vs_CD8_Responder	CD8(Terminal Tex)	Mono_INHBA	CALR	LRP1	3,00483E-03	33834,3	3,43413E-03	35998	1,42345E+00	17969	1,13169E+00	35426	9,01411E-01	31397	0	48381,5
Myeloid_vs_CD8_Responder	cDC(CD1C)	CD8(ID2+CXCR4+ T)	HLA-DRA	LAG3	3,00733E-03	22069,9	3,86944E-03	30314	1,29919E+00	21725	2,88472E+00	2309	9,43319E-01	7620	0	48381,5
Myeloid_vs_CD8_Responder	cDC(CD1C)	CD8(ID2+CXCR4+ T)	HLA-DQA1	LAG3	3,00817E-03	24190,1	4,35545E-03	25554	1,12560E+00	28344	2,40240E+00	5597	9,33903E-01	13074	0	48381,5
Myeloid_vs_CD8_Responder	cDC(CD1C)	CD8(Terminal Tex)	HLA-DRB5	LAG3	3,00875E-03	16190,9	7,60172E-03	10768	1,45691E+00	16996	3,11022E+00	1510	9,51733E-01	3299	0	48381,5
Myeloid_vs_CD8_Responder	cDC(CD1C)	CD8(ID2+CXCR4+ T)	HLA-DQB1	LAG3	3,00900E-03	23607,1	4,38643E-03	25255	1,18946E+00	25611	2,42783E+00	5350	9,33275E-01	13438	0	48381,5
Myeloid_vs_CD8_Responder	cDC(CD1C)	CD8(ID2+CXCR4+ T)	HLA-DPB1	LAG3	3,00942E-03	23057,7	3,80605E-03	31038	1,24283E+00	23624	2,74019E+00	3034	9,40453E-01	9211	0	48381,5
Myeloid_vs_CD8_Responder	cDC(CD1C)	CD8(ID2+CXCR4+ T)	HLA-DPA1	LAG3	3,01862E-03	24652,1	3,58704E-03	33803	1,13625E+00	27873	2,59100E+00	4015	9,40485E-01	9188	0	48381,5
Myeloid_vs_CD8_Responder	cDC(CD1C)	CD8(ID2+CXCR4+ T)	CD86	CTLA4	3,01988E-03	21148,1	1,27112E-02	4760	1,81897E+00	9524	2,06121E+00	9648	8,97722E-01	33427	0	48381,5
Myeloid_vs_CD8_Responder	cDC(CD1C)	CD8(ID2+CXCR4+ T)	HLA-DRB5	LAG3	3,02365E-03	26122,9	3,79152E-03	31218	1,04358E+00	32072	2,42850E+00	5344	9,33002E-01	13599	0	48381,5
Myeloid_vs_CD8_Responder	CD8(Terminal Tex)	Mono_CD14	CCL5	CCR1	3,03458E-03	30688,3	6,88177E-03	12657	9,64347E-01	36069	1,26250E+00	30022	9,10719E-01	26312	0	48381,5
Myeloid_vs_CD8_Responder	Macro_OLFML3	CD8(Temra)	LGALS3BP	ITGB1	3,03584E-03	28438,5	7,49849E-03	11006	1,73403E+00	10867	1,12048E+00	35866	8,93033E-01	36072	0	48381,5
Myeloid_vs_CD8_Responder	cDC(CD1C)	CD8(ID2+CXCR4+ T)	CXCL16	CXCR6	3,03668E-03	22271,9	9,61822E-03	7367	1,34876E+00	20160	2,06595E+00	9598	9,11617E-01	25853	0	48381,5
Myeloid_vs_CD8_Responder	CD8(NME1+ T)	Macro_LYVE1	CD99	CD81	3,03963E-03	29607,3	4,91062E-03	21451	1,22035E+00	24432	1,11558E+00	36081	9,25782E-01	17691	0	48381,5
Myeloid_vs_CD8_Responder	Macro_FOLR2-APOE+	CD8(LAYN+ T)	SPP1	CD44	3,04069E-03	18093,5	8,62296E-03	8756	1,92226E+00	8099	2,16403E+00	8189	9,26978E-01	17042	0	48381,5
Myeloid_vs_CD8_Responder	CD8(Tc17)	Mono_CD16	CD99	PILRA	3,04764E-03	26236,3	5,25192E-03	19337	2,03416E+00	6770	1,11512E+00	36100	9,20787E-01	20593	0	48381,5
Myeloid_vs_CD8_Responder	cDC(CD1C)	CD8(Terminal Tex)	HLA-DQA2	LAG3	3,05398E-03	18657,1	9,87654E-03	7085	1,35955E+00	19834	2,67845E+00	3414	9,31328E-01	14571	0	48381,5
Myeloid_vs_CD8_Responder	cDC(CD1C)	CD8(Terminal Tex)	HLA-DRB1	LAG3	3,05482E-03	16374,3	6,73400E-03	13115	1,44430E+00	17381	3,11039E+00	1509	9,57017E-01	1485	0	48381,5
Myeloid_vs_CD8_Responder	CD8(ISG+ T)	cDC(CD1C)	B2M	CD1A	3,05863E-03	25183,3	2,86331E-02	877	2,07128E+00	6364	1,15963E+00	34168	8,92927E-01	36126	0	48381,5
Myeloid_vs_CD8_Responder	CD8(NME1+ T)	cDC(CD1C)	HMGB1	THBD	3,06032E-03	28431,7	4,40661E-03	25081	1,38564E+00	19091	1,84035E+00	13475	8,92917E-01	36130	0	48381,5
Myeloid_vs_CD8_Responder	cDC(CD1C)	CD8(Terminal Tex)	ICAM1	IL2RG	3,06287E-03	31645,9	4,07409E-03	28119	9,63043E-01	36136	1,86918E+00	12959	8,99175E-01	32634	0	48381,5
Myeloid_vs_CD8_Responder	CD8(Terminal Tex)	Macro_ISG15	HSPA1A	TLR4	3,06414E-03	29953,9	4,66611E-03	23093	1,40318E+00	18551	1,43902E+00	23605	8,92908E-01	36139	0	48381,5
Myeloid_vs_CD8_Responder	cDC(CD1C)	CD8(Terminal Tex)	HLA-DQB2	LAG3	3,07858E-03	17610,9	1,70480E-02	2930	1,92217E+00	8101	2,52992E+00	4469	9,14622E-01	24173	0	48381,5
Myeloid_vs_CD8_Responder	CD8(Temra)	pDC_LILRA4	TNF	TNFRSF21	3,07988E-03	23170,5	2,12962E-02	1831	3,71083E+00	624	2,32492E+00	6396	8,53963E-01	58620	0	48381,5
Myeloid_vs_CD8_Responder	CD8(Temra)	Macro_ISG15	HLA-C	LILRB1	3,08667E-03	29944,9	3,42085E-03	36192	1,37550E+00	19391	1,23388E+00	31174	9,31311E-01	14586	0	48381,5
Myeloid_vs_CD8_Responder	CD8(LAYN+ T)	cDC(CD1C)	CSF1	CSF2RA	3,08765E-03	32423,7	8,50100E-03	8953	1,91850E+00	8155	1,74915E+00	15356	8,12550E-01	81273	0	48381,5
Myeloid_vs_CD8_Responder	Macro_ISG15	CD8(ZNF683+KLRB1+ T)	CXCL10	CXCR3	3,09263E-03	24817,5	1,05740E-02	6405	2,26367E+00	4653	2,36600E+00	5957	8,53856E-01	58691	0	48381,5
Myeloid_vs_CD8_Responder	CD8(Tn)	Macro_OLFML3	CD40LG	ITGAM_ITGB2	3,09324E-03	37386,7	2,44940E-02	1258	9,87005E-01	34918	1,02640E+00	40155	8,47353E-01	62221	0	48381,5
Myeloid_vs_CD8_Responder	CD8(Terminal Tex)	Mono_INHBA	HLA-C	LILRB2	3,09350E-03	26072,7	3,41993E-03	36208	1,69631E+00	11533	1,38384E+00	25456	9,41190E-01	8785	0	48381,5
Myeloid_vs_CD8_Responder	CD8(GZMK+ Tex)	cDC(CD1C)	B2M	CD1A	3,09393E-03	25210,7	2,85421E-02	880	2,05507E+00	6537	1,16247E+00	34046	8,92775E-01	36209	0	48381,5
Myeloid_vs_CD8_Responder	Mono_CD14	CD8(IL7R+ZNF683+ Tm)	VCAN	ITGA4	3,09547E-03	18435,9	1,05726E-02	6407	3,17446E+00	1232	2,39856E+00	5635	9,02983E-01	30524	0	48381,5
Myeloid_vs_CD8_Responder	cDC(CD1C)	CD8(Terminal Tex)	LYZ	ITGAL	3,09735E-03	22898,9	4,30189E-03	26007	1,77199E+00	10239	3,06873E+00	1635	9,07218E-01	28232	0	48381,5
Myeloid_vs_CD8_Responder	CD8(NME1+ T)	Mono_CD14	HLA-F	LILRB2	3,10377E-03	31167,5	3,84007E-03	30663	1,44062E+00	17501	1,45623E+00	23060	8,92748E-01	36232	0	48381,5
Myeloid_vs_CD8_Responder	cDC(CD1C)	CD8(GZMK+ Tex)	HLA-DRA	LAG3	3,10591E-03	17601,7	5,39308E-03	18553	1,46115E+00	16867	3,39767E+00	845	9,51569E-01	3362	0	48381,5
Myeloid_vs_CD8_Responder	cDC(CD1C)	CD8(GZMK+ Tex)	HLA-DQA2	LAG3	3,10634E-03	23319,5	6,86588E-03	12705	1,10818E+00	29107	2,50969E+00	4647	9,18749E-01	21757	0	48381,5
Myeloid_vs_CD8_Responder	Macro_NLRP3	CD8(Terminal Tex)	S100A8	ITGB2	3,10672E-03	17035,5	5,68115E-03	17136	2,25891E+00	4693	2,70341E+00	3247	9,36143E-01	11720	0	48381,5
Myeloid_vs_CD8_Responder	cDC(CD1C)	CD8(GZMK+ Tex)	HLA-DQA1	LAG3	3,10677E-03	19139,5	6,07047E-03	15482	1,28755E+00	22095	2,91535E+00	2196	9,43441E-01	7543	0	48381,5
Myeloid_vs_CD8_Responder	cDC(CD1C)	CD8(GZMK+ Tex)	HLA-DRB1	LAG3	3,10719E-03	20702,1	4,68128E-03	22973	1,19293E+00	25477	2,94163E+00	2078	9,48885E-01	4601	0	48381,5
Myeloid_vs_CD8_Responder	cDC(CD1C)	CD8(GZMK+ Tex)	HLA-DQB1	LAG3	3,10762E-03	18741,7	6,11365E-03	15311	1,35142E+00	20081	2,94078E+00	2080	9,42899E-01	7855	0	48381,5
Myeloid_vs_CD8_Responder	cDC(CD1C)	CD8(GZMK+ Tex)	HLA-DPB1	LAG3	3,10848E-03	18310,9	5,30473E-03	19031	1,40479E+00	18497	3,25314E+00	1145	9,49098E-01	4500	0	48381,5
Myeloid_vs_CD8_Responder	CD8(ISG+ T)	cDC(CD1C)	CD52	SIGLEC10	3,10934E-03	35595,5	3,41744E-03	36245	1,04352E+00	32075	1,31645E+00	27964	8,97913E-01	33312	0	48381,5
Myeloid_vs_CD8_Responder	cDC(CD1C)	CD8(GZMK+ Tex)	HLA-DPA1	LAG3	3,11922E-03	19402,9	4,99948E-03	20852	1,29821E+00	21763	3,10395E+00	1530	9,49125E-01	4488	0	48381,5
Myeloid_vs_CD8_Responder	CD8(NME1+ T)	Macro_IER3	HMGB1	CD163	3,12094E-03	27157,5	3,41573E-03	36272	1,53121E+00	15056	1,62573E+00	18266	9,25610E-01	17812	0	48381,5
Myeloid_vs_CD8_Responder	cDC(CD1C)	CD8(GZMK+ Tex)	HLA-DRB5	LAG3	3,12524E-03	20515,7	5,28448E-03	19138	1,20554E+00	24999	2,94145E+00	2079	9,42662E-01	7981	0	48381,5
Myeloid_vs_CD8_Responder	Macro_OLFML3	CD8(LAYN+ T)	HLA-DQA1	LAG3	3,12524E-03	30780,7	3,41513E-03	36282	1,06640E+00	31010	1,53452E+00	20660	9,25988E-01	17570	0	48381,5
Myeloid_vs_CD8_Responder	cDC(CD1C)	CD8(GZMK+ Tex)	HLA-DQB2	LAG3	3,13084E-03	20845,1	1,18512E-02	5305	1,67080E+00	11979	2,36116E+00	6008	8,99314E-01	32552	0	48381,5
Myeloid_vs_CD8_Responder	CD8(LAYN+ T)	Mono_CD14	CSF1	CSF3R	3,13442E-03	34132,1	9,57753E-03	7411	1,73250E+00	10896	1,70785E+00	16299	8,00206E-01	87673	0	48381,5
Myeloid_vs_CD8_Responder	cDC(CD1C)	CD8(GZMK+ Tex)	CXCL16	CXCR6	3,14121E-03	24652,3	7,65398E-03	10646	1,21515E+00	24628	2,14031E+00	8529	9,01972E-01	31077	0	48381,5
Myeloid_vs_CD8_Responder	CD8(GZMK+ Tem)	Macro_ISG15	HLA-F	LILRB2	3,14467E-03	31168,9	3,82581E-03	30802	1,45701E+00	16995	1,44658E+00	23339	8,92570E-01	36327	0	48381,5
Myeloid_vs_CD8_Responder	Macro_IER3	CD8(Terminal Tex)	HLA-DRA	LAG3	3,14656E-03	17980,3	6,52971E-03	13827	1,45025E+00	17192	2,12984E+00	8651	9,55790E-01	1850	0	48381,5

Myeloid_vs_CD8_Post_R

Myeloid_vs_CD8_Responder	CD8(EOMES+ NK-like)	cDC(CD1C)	CD52	SIGLEC10	3,15811E-03	35979,5	3,41066E-03	36358	1,04169E+00	32167	1,27260E+00	29625	8,97821E-01	33366	0	48381,5
Myeloid_vs_CD8_Responder	Macro_OLFML3	CD8(ITM2C+ T)	APOE	LSR	3,17417E-03	21926,3	1,05078E-02	6462	2,07484E+00	6334	2,79439E+00	2716	8,76301E-01	45738	0	48381,5
Myeloid_vs_CD8_Responder	cDC(CD1C)	CD8(ISG+ T)	HLA-DQB1	LAG3	3,18172E-03	17039,5	7,15184E-03	11920	1,44877E+00	17241	2,94947E+00	2060	9,46977E-01	5595	0	48381,5
Myeloid_vs_CD8_Responder	Macro_IER3	CD8(Tc17)	CXCL2	DPP4	3,20166E-03	21422,5	4,66991E-02	343	2,77462E+00	2120	2,31680E+00	6481	8,69380E-01	49787	0	48381,5
Myeloid_vs_CD8_Responder	CD8(Temra)	Mono_CD14	SPON2	ITGB2	3,20969E-03	28473,3	9,49360E-03	7515	9,56707E-01	36476	1,70901E+00	16280	8,97229E-01	33714	0	48381,5
Myeloid_vs_CD8_Responder	pDC_LILRA4	CD8(Temra)	CDH1	KLRG1	3,21619E-03	28698,9	1,16940E-01	40	2,05873E+00	6491	2,60459E+00	3916	8,06005E-01	84666	0	48381,5
Myeloid_vs_CD8_Responder	CD8(GZMK+ Tex)	Macro_NLRP3	HLA-F	LILRB2	3,21718E-03	31867,9	3,80144E-03	31102	1,49476E+00	15981	1,33214E+00	27382	8,92263E-01	36493	0	48381,5
Myeloid_vs_CD8_Responder	CD8(LAYN+ T)	cDC_CLEC9A	NECTIN3	CADM1	3,21936E-03	37647,9	8,84730E-02	84	1,63218E+00	12749	1,66624E+00	17245	7,53930E-01	109780	0	48381,5
Myeloid_vs_CD8_Responder	CD8(Terminal Tex)	Macro_FOLR2+APOE+	CD52	SIGLEC10	3,22081E-03	24604,3	7,02016E-03	12263	1,99063E+00	7278	1,07664E+00	37804	9,26504E-01	17295	0	48381,5
Myeloid_vs_CD8_Responder	CD8(LAYN+ T)	pDC_LILRA4	COPA	P2RY6	3,23027E-03	29031,3	9,52631E-03	7471	2,35642E+00	3957	2,28389E+00	6806	8,17751E-01	78541	0	48381,5
Myeloid_vs_CD8_Responder	CD8(NME1+ T)	Macro_NLRP3	GNAI2	C5AR1	3,23043E-03	25683,7	4,63650E-03	23309	2,38254E+00	3787	1,70256E+00	16418	8,92190E-01	36523	0	48381,5
Myeloid_vs_CD8_Responder	CD8(Temra)	Macro_LYVE1	CD99	CD81	3,23396E-03	30125,1	4,79588E-03	22219	1,19766E+00	25308	1,10492E+00	36531	9,24966E-01	18186	0	48381,5
Myeloid_vs_CD8_Responder	CD8(Temra)	pDC_LILRA4	SELPLG	SELL	3,23618E-03	25503,7	8,81155E-03	8445	1,13654E+00	27865	2,33326E+00	6291	8,92166E-01	36536	0	48381,5
Myeloid_vs_CD8_Responder	CD8(IL7R+ZNF683+ Tm)	Macro_OLFML3	ANXA1	FPR1	3,23928E-03	27494,9	5,33140E-03	18881	1,56129E+00	14318	1,10464E+00	36543	9,23008E-01	19351	0	48381,5
Myeloid_vs_CD8_Responder	cDC(CD1C)	CD8(ISG+ T)	HLA-DQA2	LAG3	3,24416E-03	21255,1	8,03181E-03	9828	1,20553E+00	25000	2,51838E+00	4565	9,24415E-01	18501	0	48381,5
Myeloid_vs_CD8_Responder	cDC(CD1C)	CD8(ISG+ T)	HLA-DQA1	LAG3	3,24460E-03	17406,1	7,10134E-03	12061	1,38490E+00	19114	2,92405E+00	2152	9,47483E-01	5322	0	48381,5
Myeloid_vs_CD8_Responder	cDC(CD1C)	CD8(ISG+ T)	HLA-DRB1	LAG3	3,24505E-03	18701,5	5,47623E-03	18115	1,29028E+00	22006	2,95032E+00	2056	9,52558E-01	2949	0	48381,5
Myeloid_vs_CD8_Responder	CD8(LAYN+ T)	Macro_LYVE1	TNFSF12	CD163	3,24999E-03	29905,7	6,17928E-03	15047	2,89028E+00	1807	1,95103E+00	11434	8,28348E-01	72859	0	48381,5
Myeloid_vs_CD8_Responder	CD8(ISG+ T)	Mono_CD14	HLA-F	LILRB2	3,25126E-03	30952,1	3,78956E-03	31236	1,43198E+00	17731	1,52847E+00	20842	8,92112E-01	36570	0	48381,5
Myeloid_vs_CD8_Responder	Macro_NLRP3	CD8(GZMK+ Tem)	S100A8	ITGB2	3,25219E-03	17182,5	5,62980E-03	17338	2,25268E+00	4754	2,65902E+00	3556	9,35871E-01	11883	0	48381,5
Myeloid_vs_CD8_Responder	cDC(CD1C)	CD8(ISG+ T)	HLA-DPA1	LAG3	3,25304E-03	17575,1	5,84848E-03	16372	1,39556E+00	18776	3,11264E+00	1496	9,52781E-01	2850	0	48381,5
Myeloid_vs_CD8_Responder	cDC(CD1C)	CD8(ISG+ T)	HLA-DRB5	LAG3	3,25803E-03	18555,7	6,18188E-03	15035	1,30289E+00	21605	2,95015E+00	2057	9,46757E-01	5700	0	48381,5
Myeloid_vs_CD8_Responder	cDC(CD1C)	CD8(ISG+ T)	HLA-DQB2	LAG3	3,26328E-03	19510,5	1,38638E-02	4156	1,76815E+00	10306	2,36985E+00	5914	9,06196E-01	28795	0	48381,5
Myeloid_vs_CD8_Responder	CD8(GZMK+ Early Tem)	cDC_CLEC9A	CRTAM	CADM1	3,26686E-03	24499,7	2,83668E-02	892,5	1,44491E+00	17358,5	1,90642E+00	12249,5	8,79964E-01	43616,5	0	48381,5
Myeloid_vs_CD8_Responder	CD8(GZMK+ Tex)	Macro_NLRP3	HMGB1	THBD	3,26953E-03	33876,7	4,32689E-03	25804	1,26514E+00	22824	1,12313E+00	35763	8,92041E-01	36611	0	48381,5
Myeloid_vs_CD8_Responder	CD8(LAYN+ T)	Macro_LYVE1	RPS19	C5AR1	3,26980E-03	24100,3	5,64381E-03	17284	2,04886E+00	6611	9,92620E-01	41838	9,45465E-01	6387	0	48381,5
Myeloid_vs_CD8_Responder	CD8(LAYN+ T)	Macro_LYVE1	ADAM10	GPNMB	3,27495E-03	23459,7	9,50488E-03	7502	2,79359E+00	2071	1,88285E+00	12706	8,74810E-01	46638	0	48381,5
Myeloid_vs_CD8_Responder	cDC_CLEC9A	CD8(Terminal Tex)	HLA-DRB5	LAG3	3,27863E-03	16448,3	7,53207E-03	10934	1,44441E+00	17374	2,92230E+00	2161	9,51522E-01	3391	0	48381,5
Myeloid_vs_CD8_Responder	cDC_CLEC9A	CD8(ISG+ T)	HLA-DQB1	LAG3	3,28011E-03	17192,7	7,13145E-03	11974	1,44440E+00	17376	2,81646E+00	2606	9,46905E-01	5626	0	48381,5
Myeloid_vs_CD8_Responder	cDC(CD1C)	CD8(ISG+ T)	C3	IFITM1	3,28071E-03	24850,9	5,07087E-03	20422	1,13855E+00	27771	2,42693E+00	5361	9,17814E-01	22319	0	48381,5
Myeloid_vs_CD8_Responder	Macro_ISG15	CD8(Tn)	S100A8	CD69	3,28232E-03	21106,1	5,87085E-03	16259	1,44434E+00	17379	2,12703E+00	8698	9,30911E-01	14813	0	48381,5
Myeloid_vs_CD8_Responder	cDC(CD1C)	CD8(ISG+ T)	LYZ	ITGAL	3,28250E-03	25187,7	3,68005E-03	32593	1,71619E+00	11179	3,00375E+00	1851	9,00434E-01	31934	0	48381,5
Myeloid_vs_CD8_Responder	CD8(Terminal Tex)	Mono_CD14	HLA-G	LILRB2	3,28453E-03	36007,1	5,62148E-03	17382	1,51102E+00	15559	1,67092E+00	17139	8,11982E-01	81574	0	48381,5
Myeloid_vs_CD8_Responder	CD8(Temra)	Macro_FOLR2-APOE+	SPON2	ITGB2	3,28474E-03	31946,7	9,45033E-03	7568	9,52890E-01	36645	1,18039E+00	33293	8,97018E-01	33846	0	48381,5
Myeloid_vs_CD8_Responder	Macro_FOLR2+APOE+	CD8(GZMK+ Tem)	CD14	ITGA4	3,29117E-03	19899,7	6,31471E-03	14552	2,15623E+00	5550	1,83294E+00	13624	9,26329E-01	17391	0	48381,5
Myeloid_vs_CD8_Responder	Macro_OLFML3	CD8(Terminal Tex)	CXCL9	CXCR3	3,29147E-03	21792,1	1,82941E-02	2561	1,79023E+00	9976	1,95444E+00	11382	8,91945E-01	36660	0	48381,5
Myeloid_vs_CD8_Responder	CD8(GZMK+ Early Tem)	pDC_LILRA4	HSP90B1	TLR9	3,29645E-03	29704,1	2,40961E-02	1299	3,67667E+00	651	1,15344E+00	34452	8,44603E-01	63737	0	48381,5
Myeloid_vs_CD8_Responder	CD8(GZMK+ Tem)	Macro_ISG15	HLA-B	LILRB1	3,29686E-03	29138,3	3,39004E-03	36672	1,43600E+00	17614	1,24385E+00	30782	9,35234E-01	12242	0	48381,5
Myeloid_vs_CD8_Responder	Macro_OLFML3	CD8(ITM2C+ T)	HLA-DQA1	LAG3	3,30226E-03	31084,9	3,38897E-03	36684	1,06399E+00	31113	1,50562E+00	21520	9,25724E-01	17726	0	48381,5
Myeloid_vs_CD8_Responder	Macro_LYVE1	CD8(Terminal Tex)	B2M	CD3D	3,30777E-03	55498,9	2,72327E-03	50068	3,33501E-01	85778	2,72045E-01	93246	9,65692E-01	21	0	48381,5
Myeloid_vs_CD8_Responder	CD8(GZMK+ Tem)	Macro_LYVE1	HSP90B1	LRP1	3,30856E-03	29003,1	4,44208E-03	24789	2,17096E+00	5408	1,10148E+00	36698	9,04435E-01	29739	0	48381,5
Myeloid_vs_CD8_Responder	cDC(CD1C)	CD8(NME1+ T)	HLA-DPB1	LAG3	3,31042E-03	17675,1	5,66300E-03	17195	1,44350E+00	17417	3,07421E+00	1612	9,50653E-01	3770	0	48381,5
Myeloid_vs_CD8_Responder	CD8(LAYN+ T)	cDC_CLEC9A	COPA	P2RY6	3,31191E-03	30642,9	9,90773E-03	7058	2,44942E+00	3399	1,65871E+00	17419	8,20658E-01	76957	0	48381,5
Myeloid_vs_CD8_Responder	CD8(GZMK+ Tex)	Macro_ISG15	HLA-C	LILRB1	3,31262E-03	30150,5	3,38713E-03	36707	1,34841E+00	20171	1,24546E+00	30726	9,30993E-01	14767	0	48381,5
Myeloid_vs_CD8_Responder	CD8(Temra)	Macro_NLRP3	HMGB1	CD163	3,31488E-03	28808,5	3,38687E-03	36712	1,58081E+00	13841	1,33839E+00	27129	9,25317E-01	17979	0	48381,5
Myeloid_vs_CD8_Responder	CD8(Terminal Tex)	Macro_ISG15	CCL4	CCR1	3,33388E-03	31776,3	7,37214E-03	11359	1,04207E+00	32149	1,10041E+00	36754	9,03485E-01	30238	0	48381,5
Myeloid_vs_CD8_Responder	CD8(Temra)	Mono_INHBA	HLA-C	LILRB2	3,33570E-03	26407,3	3,38370E-03	36758	1,66690E+00	12056	1,37149E+00	25884	9,40894E-01	8957	0	48381,5
Myeloid_vs_CD8_Responder	CD8(ID2+CXCR4+ T)	Macro_LYVE1	CALR	LRP1	3,33888E-03	26430,7	5,17924E-03	19745	2,19753E+00	5172	1,10020E+00	36765	9,18223E-01	22090	0	48381,5
Myeloid_vs_CD8_Responder	Macro_OLFML3	CD8(NME1+ T)	FN1	ITGA4_ITGB7	3,33978E-03	21888,5	1,37383E-02	4211	2,06618E+00	6424	1,83118E+00	13659	8,91790E-01	36767	0	48381,5
Myeloid_vs_CD8_Responder	cDC(CD1C)	CD8(LAYN+ T)	CD86	CTLA4	3,34296E-03	21459,1	1,28964E-02	4656	1,82784E+00	9406	1,92928E+00	11804	8,98385E-01	33048	0	48381,5
Myeloid_vs_CD8_Responder	CD8(ITM2C+ T)	Macro_FOLR2-APOE+	IFNG	IFNGR1_IFNGR2	3,34342E-03	33384,7	6,38157E-03	14291	1,05059E+00	31746	1,09985E+00	36775	8,93596E-01	35730	0	48381,5
Myeloid_vs_CD8_Responder	cDC(CD1C)	CD8(LAYN+ T)	GRN	TNFRSF1B	3,35070E-03	22966,5	3,67991E-03	32597	1,56590E+00	14204	2,37158E+00	5895	9,32730E-01	13755	0	48381,5
Myeloid_vs_CD8_Responder	cDC(CD1C)	CD8(LAYN+ T)	CXCL16	CXCR6	3,35207E-03	27448,9	6,92892E-03	12530	1,16583E+00	26578	1,71294E+00	16176	8,97483E-01	33579	0	48381,5
Myeloid_vs_CD8_Responder	CD8(ID2+CXCR4+ T)	pDC_LILRA4	GZMB	IGF2R	3,35617E-03	25986,3	1,09022E-02	6104	9,50105E-01	36803	2,16298E+00	8200	9,03129E-01	30443	0	48381,5
Myeloid_vs_CD8_Responder	cDC(CD1C)	CD8(LAYN+ T)	LYZ	ITGAL	3,35799E-03	23049,5	4,27643E-03	26240	1,76970E+00	10279	2,96797E+00	1985	9,06967E-01	28362	0	48381,5
Myeloid_vs_CD8_Responder	CD8(LAYN+ T)	Macro_FOLR2+APOE+	ADAM10	GPNMB	3,36567E-03	22446,7	1,05507E-02	6426	3,08665E+00	1386	1,88437E+00	12675	8,80415E-01	43365	0	48381,5
Myeloid_vs_CD8_Responder	Mono_INHBA	CD8(GZMK+ Early Tem)	INHBA	TGFBR3	3,36729E-03	31039,1	3,10795E-02	736	3,12954E+00	1313	2,14097E+00	8522	7,83169E-01	96243	0	48381,5
Myeloid_vs_CD8_Responder	CD8(ISG+ T)	Macro_ISG15	HLA-A	LILRB1	3,37765E-03	31448,7	3,37806E-03	36850	1,28301E+00	22235	1,12946E+00	35524	9,31876E-01	14253	0	48381,5
Myeloid_vs_CD8_Responder	CD8(Tc17)	Macro_FOLR2+APOE-	HMGB1	CD163	3,37994E-03	29320,7	3,37783E-03	36855	1,80483E+00	9738	1,17267E+00	33601	9,25225E-01	18028	0	48381,5
Myeloid_vs_CD8_Responder	Macro_FOLR2+APOE-	CD8(Terminal Tex)	HLA-DRB5	LAG3	3,38373E-03	17669,9	7,50686E-03	10991	1,43989E+00	17515	2,17548E+00	8039	9,51444E-01	3423	0	48381,5
Myeloid_vs_CD8_Responder	Mono_INHBA	CD8(ID2+CXCR4+ T)	INHBA	TGFBR3	3,38766E-03	31164,5	3,08460E-02	754	3,12781E+00	1317	2,11867E+00	8812	7,82528E-01	96558	0	48381,5
Myeloid_vs_CD8_Responder	CD8(Terminal Tex)	Macro_ISG15	HLA-F	LILRB2	3,38867E-03	31408,7	3,75071E-03	31693	1,44448E+00	17370	1,46659E+00	22725	8,91616E-01	36874	0	48381,5
Myeloid_vs_CD8_Responder	CD8(Temra)	Macro_LYVE1	HSP90B1	LRP1	3,39787E-03	29185,9	4,39241E-03	25204	2,16471E+00	5469	1,09670E+00	36894	9,03948E-01	29981	0	48381,5
Myeloid_vs_CD8_Responder	CD8(Temra)	pDC_LILRA4	GZMB	IGF2R	3,39833E-03	25951,3	1,08754E-02	6126	9,48228E-01	36895	2,19068E+00	7853	9,03022E-01	30501	0	48381,5
Myeloid_vs_CD8_Responder	Macro_OLFML3	CD8(GZMK+ Tex)	GRN	TNFRSF1B	3,39971E-03	25398,1	3,37449E-03	36898	1,67354E+00	11940	1,79469E+00	14375	9,29960E-01	15396	0	48381,5
Myeloid_vs_CD8_Responder	CD8(Terminal Tex)	Mono_CD16	RPS19	C5AR1	3,40247E-03	29794,5	3,44381E-03	35847	1,61028E+00	13207	1,09655E+00	36904	9,31237E-01	14633	0	48381,5
Myeloid_vs_CD8_Responder	cDC(CD1C)	CD8(Temra)	LYZ	ITGAL	3,40709E-03	22429,5	4,45073E-03	24720	1,78534E+00	10035	3,08565E+00	1574	9,08639E-01	27437	0	48381,5
Myeloid_vs_CD8_Responder	CD8(ITM2C+ T)	Mono_CD16	ANXA1	FPR1	3,40986E-03	24979,1	6,11665E-03	15298	1,95787E+00	7665	1,09624E+00	36920	9,27750E-01	16631	0	48381,5
Myeloid_vs_CD8_Responder	Macro_IFI27	CD8(GZMK+ Tem)	HLA-DPB1	LAG3	3,41323E-03	17531,3	6,81490E-03	12863	1,43841E+00	17554	2,29505E+00	6692	9,54820E-01	2166	0	48381,5
Myeloid_vs_CD8_Responder	cDC(CD1C)	CD8(GZMK+ Tem)	HLA-DQA2	LAG3	3,41956E-03	18045,7	1,03846E-02	6571	1,40197E+00	18575	2,73805E+00	3048	9,32915E-01	13653	0	48381,5
Myeloid_vs_CD8_Responder	CD8(ZNF683+KLRB1+ T)	Macro_OLFML3	CD52	SIGLEC10	3,42188E-03	25930,7	6,30174E-03	14607	1,77314E+00	10223	1,09564E+00	36946	9,22743E-01	19496	0	48381,5
Myeloid_vs_CD8_Responder	CD8(Terminal Tex)	cDC(CD1C)	B2M	CD1B	3,42415E-03	32859,3	1,87218E-02	2448	2,04735E+00	6631	6,63019E-01	61355	8,76690E-01	45481	0	48381,5
Myeloid_vs_CD8_Responder	cDC(CD1C)	CD8(Terminal Tex)	CXCL16	CXCR6	3,42419E-03	28981,7	6,10028E-03	15363	1,10946E+00	29043	1,75635E+00	15170	8,91473E-01	36951	0	48381,5
Myeloid_vs_CD8_Responder	CD8(Terminal Tex)	Macro_OLFML3	CD52	SIGLEC10	3,44137E-03	26531,9	6,00028E-03	15749	1,72305E+00	11056	1,09473E+00	36988	9,20977E-01	20485	0	48381,5
Myeloid_vs_CD8_Responder	cDC(CD1C)	CD8(GZMK+ Tem)	HLA-DQB2	LAG3	3,44277E-03	17138,3	1,79249E-02	2660	1,96459E+00	7580	2,58952E+00	4030	9,16560E-01	23040	0	48381,5
Myeloid_vs_CD8_Responder	CD8(Terminal Tex)	pDC_LILRA4	HLA-DPA1	CD4	3,44904E-03	27523,5	2,68377E-03	51161	1,43645E+00	17601	2,43143E+00	5316	9,30329E-01	15158	0	48381,5
Myeloid_vs_CD8_Responder	cDC(CD1C)	CD8(ID2+CXCR4+ T)	HLA-DQA2	LAG3	3,44929E-03	29315,3	4,92614E-03	21350	9,46224E-01	37005	1,99674E+00	10654	9,05464E-01	29186	0	48381,5

Myeloid_vs_CD8_Responder	cDC(CD1C)	CD8(GZMK+ Tem)	CD86	CTLA4	3,45069E-03	22603,5	1,11058E-02	5928	1,74209E+00	10733	1,97747E+00	10967	8,91355E-01	37008	0	48381,5
Myeloid_vs_CD8_Responder	CD8(GZMK+ Tem)	Mono_CD14	HLA-F	LILRB2	3,45442E-03	31495,7	3,72919E-03	31954	1,42165E+00	18025	1,48610E+00	22102	8,91337E-01	37016	0	48381,5
Myeloid_vs_CD8_Responder	CD8(NME1+ T)	Mono_CD16	RPS19	C5AR1	3,46236E-03	27688,1	3,93276E-03	29627	1,71298E+00	11233	1,09361E+00	37033	9,35368E-01	12166	0	48381,5
Myeloid_vs_CD8_Responder	cDC(CD1C)	CD8(GZMK+ Tem)	LYZ	ITGAL	3,46283E-03	22097,5	4,57024E-03	23825	1,79607E+00	9888	3,09434E+00	1555	9,09733E-01	26838	0	48381,5
Myeloid_vs_CD8_Responder	CD8(LAYN+ T)	pDC_LILRA4	NECTIN3	NECTIN1	3,46524E-03	60931,8	1,56402E-01	22	1,00582E+00	33964	1,70170E+00	16436	5,46019E-01	156627	0,001	97610
Myeloid_vs_CD8_Responder	cDC(CD1C)	CD8(NME1+ T)	HLA-DQA2	LAG3	3,47406E-03	22797,5	7,32958E-03	11466	1,14690E+00	27421	2,33076E+00	6323	9,21155E-01	20396	0	48381,5
Myeloid_vs_CD8_Responder	cDC(CD1C)	CD8(NME1+ T)	HLA-DQA1	LAG3	3,47500E-03	18565,5	6,48046E-03	13985	1,32627E+00	20845	2,73642E+00	3058	9,45159E-01	6558	0	48381,5
Myeloid_vs_CD8_Responder	cDC(CD1C)	CD8(NME1+ T)	HLA-DRB1	LAG3	3,47594E-03	19998,9	4,99744E-03	20868	1,23164E+00	24001	2,76270E+00	2890	9,50447E-01	3854	0	48381,5
Myeloid_vs_CD8_Responder	cDC(CD1C)	CD8(NME1+ T)	HLA-DQB1	LAG3	3,47687E-03	18183,7	6,52655E-03	13841	1,39014E+00	18951	2,76185E+00	2894	9,44633E-01	6851	0	48381,5
Myeloid_vs_CD8_Responder	CD8(Temra)	Mono_INHBA	HLA-F	LILRB2	3,47922E-03	32445,3	3,72262E-03	32041	1,52031E+00	15325	1,27821E+00	29410	8,91252E-01	37069	0	48381,5
Myeloid_vs_CD8_Responder	cDC(CD1C)	CD8(NME1+ T)	HLA-DPA1	LAG3	3,48909E-03	18732,7	5,33714E-03	18845	1,33692E+00	20532	2,92502E+00	2148	9,50680E-01	3757	0	48381,5
Myeloid_vs_CD8_Responder	Macro_FOLR2+APOE-	CD8(LAYN+ T)	CD14	ITGB1	3,49360E-03	20691,9	5,61094E-03	17431	1,92357E+00	8079	1,92365E+00	11909	9,25832E-01	17659	0	48381,5
Myeloid_vs_CD8_Responder	cDC(CD1C)	CD8(NME1+ T)	HLA-DRB5	LAG3	3,49850E-03	19819,9	5,64139E-03	17290	1,24425E+00	23563	2,76252E+00	2891	9,44403E-01	6974	0	48381,5
Myeloid_vs_CD8_Responder	CD8(NME1+ T)	Macro_ISG15	HLA-A	LILRB1	3,49945E-03	31806,1	3,36067E-03	37112	1,26758E+00	22731	1,10731E+00	36441	9,31712E-01	14365	0	48381,5
Myeloid_vs_CD8_Responder	cDC(CD1C)	CD8(NME1+ T)	HLA-DQB2	LAG3	3,50416E-03	20669,5	1,26517E-02	4791	1,70951E+00	11299	2,18223E+00	7950	9,02234E-01	30926	0	48381,5
Myeloid_vs_CD8_Responder	Macro_OLFML3	CD8(LAYN+ T)	HLA-DRB5	LAG3	3,50889E-03	28143,9	3,35944E-03	37132	1,16311E+00	26704	1,88659E+00	12628	9,29120E-01	15874	0	48381,5
Myeloid_vs_CD8_Responder	CD8(GZMK+ Tem)	Mono_INHBA	CALR	LRP1	3,50983E-03	34443,1	3,35932E-03	37134	1,40645E+00	18449	1,10991E+00	36312	9,00428E-01	31939	0	48381,5
Myeloid_vs_CD8_Responder	cDC(CD1C)	CD8(ID2+CXCR4+ T)	HLA-DRB1	LAG3	3,51267E-03	26578,9	3,35873E-03	37140	1,03097E+00	32679	2,42868E+00	5342	9,40207E-01	9352	0	48381,5
Myeloid_vs_CD8_Responder	Macro_OLFML3	CD8(ID2+CXCR4+ T)	LGALS3BP	ITGB1	3,51929E-03	28634,9	7,54170E-03	10913	1,73665E+00	10825	1,09120E+00	37154	8,93307E-01	35901	0	48381,5
Myeloid_vs_CD8_Responder	CD8(Terminal Tex)	pDC_LILRA4	COPA	P2RY6	3,52769E-03	36573,1	5,55404E-03	17703	2,21549E+00	5039	1,97135E+00	11075	7,74067E-01	100667	0	48381,5
Myeloid_vs_CD8_Responder	cDC(CD1C)	CD8(NME1+ T)	LYZ	ITGAL	3,52877E-03	25243,9	3,67929E-03	32608	1,71612E+00	11182	2,93314E+00	2108	9,00425E-01	31940	0	48381,5
Myeloid_vs_CD8_Responder	CD8(GZMK+ Tem)	Mono_CD14	CIRBP	TREM1	3,53685E-03	26531,1	7,38985E-03	11303	1,79893E+00	9835	1,36974E+00	25945	8,91053E-01	37191	0	48381,5
Myeloid_vs_CD8_Responder	cDC(CD1C)	CD8(EOMES+ NK-like)	HLA-DQA1	LAG3	3,53923E-03	27806,5	3,50188E-03	34990	1,04499E+00	32017	2,31157E+00	6522	9,26843E-01	17122	0	48381,5
Myeloid_vs_CD8_Responder	CD8(IL7R+ZNF683+ Tm)	pDC_LILRA4	MAML2	NOTCH4	3,54015E-03	47030,1	1,35688E-02	4288	1,43240E+00	17719	1,69142E+00	16668	6,29024E-01	148094	0	48381,5
Myeloid_vs_CD8_Responder	cDC(CD1C)	CD8(EOMES+ NK-like)	HLA-DQB1	LAG3	3,54018E-03	27162,9	3,52679E-03	34636	1,10886E+00	29078	2,33700E+00	6239	9,26154E-01	17480	0	48381,5
Myeloid_vs_CD8_Responder	Mono_INHBA	CD8(IL7R+ZNF683+ Tm)	INHBA	TGFBR3	3,54241E-03	32091,9	2,81535E-02	908	3,10791E+00	1347	2,07586E+00	9453	7,74656E-01	100370	0	48381,5
Myeloid_vs_CD8_Responder	cDC(CD1C)	CD8(LAYN+ T)	HLA-DQB1	LAG3	3,54447E-03	28090,1	3,35484E-03	37207	1,09273E+00	29820	2,30940E+00	6550	9,24427E-01	18492	0	48381,5
Myeloid_vs_CD8_Responder	CD8(GZMK+ Tem)	Mono_INHBA	HLA-B	LILRB2	3,55400E-03	25827,3	3,35323E-03	37227	1,72741E+00	10966	1,38146E+00	25540	9,44303E-01	7022	0	48381,5
Myeloid_vs_CD8_Responder	CD8(GZMK+ Tex)	Mono_INHBA	HLA-C	LILRB2	3,57410E-03	26570,5	3,35035E-03	37269	1,63982E+00	12595	1,38307E+00	25481	9,40618E-01	9126	0	48381,5
Myeloid_vs_CD8_Responder	Macro_OLFML3	CD8(EOMES+ NK-like)	CD14	ITGB1	3,57985E-03	29689,7	3,34983E-03	37281	1,63200E+00	12752	1,51777E+00	21170	9,06061E-01	28864	0	48381,5
Myeloid_vs_CD8_Responder	Macro_OLFML3	CD8(Temra)	CD14	ITGB2	3,58129E-03	24330,9	3,34973E-03	37284	1,84476E+00	9159	1,92145E+00	11956	9,30801E-01	14874	0	48381,5
Myeloid_vs_CD8_Responder	cDC_LAMP3	CD8(GZMK+ Tem)	HLA-DRA	LAG3	3,60608E-03	17718,7	6,55462E-03	13729	1,42952E+00	17803	2,27871E+00	6862	9,55871E-01	1818	0	48381,5
Myeloid_vs_CD8_Responder	cDC(CD1C)	CD8(ZNF683+KLRB1+ T)	CXCL16	CXCR6	3,61407E-03	24270,7	8,34018E-03	9227	1,26183E+00	22937	1,93198E+00	11754	9,05702E-01	29054	0	48381,5
Myeloid_vs_CD8_Responder	cDC(CD1C)	CD8(ZNF683+KLRB1+ T)	LGALS1	CD69	3,61504E-03	25278,9	3,77994E-03	31344	1,00363E+00	34079	2,27211E+00	6938	9,46848E-01	5652	0	48381,5
Myeloid_vs_CD8_Responder	Macro_FOLR2+APOE+	CD8(Terminal Tex)	HLA-DPB1	LAG3	3,63302E-03	18376,9	6,62355E-03	13471	1,42814E+00	17837	2,04948E+00	9829	9,54201E-01	2366	0	48381,5
Myeloid_vs_CD8_Responder	CD8(LAYN+ T)	Macro_NLRP3	HMGB1	CD163	3,63833E-03	29538,7	3,34239E-03	37402	1,56837E+00	14151	1,27610E+00	29497	9,24859E-01	18262	0	48381,5
Myeloid_vs_CD8_Responder	Macro_OLFML3	CD8(Tc17)	C1QB	C1QBP	3,64027E-03	24609,3	3,34224E-03	37406	2,08980E+00	6179	2,69213E+00	3318	9,08056E-01	27762	0	48381,5
Myeloid_vs_CD8_Responder	CD8(ZNF683+KLRB1+ T)	Macro_NLRP3	CD99	PILRA	3,64368E-03	31962,5	3,94470E-03	29507	1,43430E+00	17660	1,08532E+00	37413	9,09701E-01	26851	0	48381,5
Myeloid_vs_CD8_Responder	cDC_CLEC9A	CD8(ISG+ T)	HLA-DPA1	LAG3	3,64575E-03	17296,3	5,96313E-03	15885	1,42777E+00	17853	3,05694E+00	1667	9,53216E-01	2695	0	48381,5
Myeloid_vs_CD8_Responder	CD8(ID2+CXCR4+ T)	Macro_ISG15	HLA-B	LILRB1	3,64709E-03	30328,9	3,34151E-03	37420	1,38397E+00	19135	1,15859E+00	34204	9,34796E-01	12504	0	48381,5
Myeloid_vs_CD8_Responder	CD8(ISG+ T)	Mono_INHBA	HLA-A	LILRB2	3,64855E-03	27665,5	3,34137E-03	37423	1,57442E+00	14004	1,26707E+00	29842	9,41385E-01	8677	0	48381,5
Myeloid_vs_CD8_Responder	CD8(GZMK+ Tem)	Macro_NLRP3	HLA-A	LILRB2	3,66467E-03	27330,9	3,33988E-03	37456	1,63693E+00	12660	1,27670E+00	29472	9,41373E-01	8685	0	48381,5
Myeloid_vs_CD8_Responder	Macro_OLFML3	CD8(GZMK+ Tex)	FN1	ITGA4_ITGB7	3,67201E-03	21667,5	1,33938E-02	4388	2,04314E+00	6670	1,95137E+00	11427	8,90559E-01	37471	0	48381,5
Myeloid_vs_CD8_Responder	Macro_ISG15	CD8(Temra)	S100A8	CD69	3,68575E-03	21552,9	5,71293E-03	16991	1,42576E+00	17903	2,09584E+00	9132	9,30029E-01	15357	0	48381,5
Myeloid_vs_CD8_Responder	Macro_FOLR2+APOE+	CD8(ISG+ T)	HLA-DRA	LAG3	3,68655E-03	18691,5	5,80331E-03	16553	1,42574E+00	17904	2,18428E+00	7931	9,53231E-01	2688	0	48381,5
Myeloid_vs_CD8_Responder	CD8(Terminal Tex)	Mono_CD16	SEMA4A	LILRB2	3,69781E-03	31443,9	6,30677E-03	14592	2,67474E+00	2448	1,63908E+00	17918	8,26502E-01	73880	0	48381,5
Myeloid_vs_CD8_Responder	Macro_IFI27	CD8(Temra)	C3	IFITM1	3,71312E-03	21187,1	8,02474E-03	9851	1,47621E+00	16472	1,63865E+00	17937	9,33548E-01	13294	0	48381,5
Myeloid_vs_CD8_Responder	CD8(Terminal Tex)	Mono_CD14	HLA-F	LILRB2	3,71484E-03	31747,3	3,65598E-03	32919	1,40913E+00	18375	1,50611E+00	21503	8,90373E-01	37558	0	48381,5
Myeloid_vs_CD8_Responder	CD8(ZNF683+KLRB1+ T)	Macro_LYVE1	HSP90B1	LRP1	3,71830E-03	29446,3	4,35431E-03	25563	2,15992E+00	5517	1,08201E+00	37565	9,03569E-01	30205	0	48381,5
Myeloid_vs_CD8_Responder	Macro_OLFML3	CD8(ITM2C+ T)	HLA-DRB5	LAG3	3,71880E-03	28382,3	3,33371E-03	37566	1,16070E+00	26801	1,85769E+00	13157	9,28866E-01	16006	0	48381,5
Myeloid_vs_CD8_Responder	Macro_IFI27	CD8(GZMK+ Tem)	HLA-DQB1	LAG3	3,72282E-03	17674,5	7,91346E-03	10083	1,42438E+00	17949	2,21030E+00	7634	9,49461E-01	4325	0	48381,5
Myeloid_vs_CD8_Responder	CD8(Tn)	Macro_LYVE1	RPS19	C5AR1	3,72325E-03	23840,9	5,35294E-03	18758	1,98594E+00	7333	1,08184E+00	37575	9,44085E-01	7157	0	48381,5
Myeloid_vs_CD8_Responder	Macro_LYVE1	CD8(Terminal Tex)	HLA-DRB5	LAG3	3,73741E-03	18160,5	7,41622E-03	11218	1,42362E+00	17967	2,05846E+00	9693	9,51163E-01	3543	0	48381,5
Myeloid_vs_CD8_Responder	cDC(CD1C)	CD8(LAYN+ T)	HLA-DQA1	LAG3	3,73814E-03	28731,1	3,33115E-03	37605	1,02887E+00	32781	2,28398E+00	6805	9,25130E-01	18083	0	48381,5
Myeloid_vs_CD8_Responder	CD8(GZMK+ Tex)	Mono_CD14	HMGB1	THBD	3,74411E-03	32632,7	4,17067E-03	27211	1,22366E+00	24300	1,37842E+00	25654	8,90258E-01	37617	0	48381,5
Myeloid_vs_CD8_Responder	CD8(NME1+ T)	cDC(CD1C)	CD52	SIGLEC10	3,74411E-03	36527,1	3,33016E-03	37617	1,02004E+00	33235	1,27876E+00	29392	8,96721E-01	34010	0	48381,5
Myeloid_vs_CD8_Responder	cDC(CD1C)	CD8(ITM2C+ T)	HLA-DQB1	LAG3	3,75257E-03	28284,1	3,32914E-03	37634	1,09032E+00	29915	2,28051E+00	6841	9,24158E-01	18649	0	48381,5
Myeloid_vs_CD8_Responder	Macro_FOLR2+APOE+	CD8(GZMK+ Tem)	B2M	CD3D	3,78017E-03	51593,5	2,70404E-03	50588	5,09731E-01	68391	3,01624E-01	90583	9,65574E-01	24	0	48381,5
Myeloid_vs_CD8_Responder	CD8(ITM2C+ T)	cDC(CD1C)	CD52	SIGLEC10	3,78359E-03	37138,1	3,32543E-03	37696	1,01877E+00	33296	1,20640E+00	32279	8,96655E-01	34038	0	48381,5
Myeloid_vs_CD8_Responder	Mono_INHBA	CD8(ITM2C+ T)	INHBA	TGFBR3	3,78628E-03	33400,3	2,48831E-02	1203	3,08373E+00	1393	2,00489E+00	10512	7,63696E-01	105512	0	48381,5
Myeloid_vs_CD8_Responder	CD8(ISG+ T)	pDC_LILRA4	COPA	P2RY6	3,78632E-03	36696,3	5,49090E-03	18027	2,21325E+00	5053	1,98221E+00	10884	7,73066E-01	101136	0	48381,5
Myeloid_vs_CD8_Responder	Macro_FOLR2-APOE+	CD8(Tn)	SPP1	S1PR1	3,78770E-03	24686,1	2,62679E-02	1068	2,02685E+00	6863	2,57962E+00	4121	8,45960E-01	62997	0	48381,5
Myeloid_vs_CD8_Responder	CD8(GZMK+ Tex)	Mono_CD16	HLA-G	LILRB2	3,78960E-03	32020,5	6,36238E-03	14366	2,61538E+00	2669	1,63515E+00	18031	8,21251E-01	76655	0	48381,5
Myeloid_vs_CD8_Responder	CD8(NME1+ T)	Mono_INHBA	HLA-A	LILRB2	3,79313E-03	27993,7	3,32417E-03	37715	1,55898E+00	14379	1,24492E+00	30744	9,41243E-01	8749	0	48381,5
Myeloid_vs_CD8_Responder	CD8(ISG+ T)	Macro_ISG15	HLA-B	LILRB1	3,80018E-03	30291,5	3,32309E-03	37729	1,36421E+00	19702	1,18755E+00	33038	9,34627E-01	12607	0	48381,5
Myeloid_vs_CD8_Responder	Macro_OLFML3	CD8(Tn)	FN1	ITGA4_ITGB1	3,80169E-03	23890,9	8,61864E-03	8760	1,91384E+00	8216	1,70462E+00	16365	8,90062E-01	37732	0	48381,5
Myeloid_vs_CD8_Responder	CD8(ID2+CXCR4+ T)	Mono_INHBA	HMGB1	CD163	3,80370E-03	29084,3	3,32281E-03	37736	1,48595E+00	16200	1,40532E+00	24730	9,24655E-01	18374	0	48381,5
Myeloid_vs_CD8_Responder	CD8(Terminal Tex)	Macro_NLRP3	HLA-A	LILRB2	3,81430E-03	27454,5	3,32174E-03	37757	1,61997E+00	13015	1,27976E+00	29351	9,41223E-01	8768	0	48381,5
Myeloid_vs_CD8_Responder	CD8(ZNF683+KLRB1+ T)	Macro_FOLR2+APOE+	CD52	SIGLEC10	3,81582E-03	24118,7	7,37288E-03	11357	2,04072E+00	6691	1,07754E+00	37760	9,28156E-01	16404	0	48381,5
Myeloid_vs_CD8_Responder	Macro_IFI27	CD8(Terminal Tex)	HLA-DRB5	LAG3	3,83157E-03	17533,3	7,39316E-03	11292	1,41948E+00	18082	2,32947E+00	6335	9,51090E-01	3576	0	48381,5
Myeloid_vs_CD8_Responder	Mono_INHBA	CD8(Tc17)	INHBA	TGFBR3	3,83493E-03	33484,1	2,44312E-02	1263	3,08039E+00	1402	2,03012E+00	10100	7,62038E-01	106274	0	48381,5
Myeloid_vs_CD8_Responder	CD8(GZMK+ Tem)	cDC(CD1C)	B2M	CD1A	3,84775E-03	26154,3	2,82313E-02	900	1,99968E+00	7167	1,07633E+00	37823	8,92249E-01	36500	0	48381,5
Myeloid_vs_CD8_Responder	Macro_ISG15	CD8(ZNF683+KLRB1+ T)	DEFB1	CCR6	3,85121E-03	31442,3	1,40521E-01	28	3,07917E+00	1405	1,60058E+00	18879	7,98483E-01	88518	0	48381,5
Myeloid_vs_CD8_Responder	CD8(ID2+CXCR4+ T)	Mono_INHBA	HLA-A	LILRB2	3,85437E-03	28454,1	3,31675E-03	37836	1,55233E+00	14536	1,19471E+00	32722	9,41181E-01	8795	0	48381,5
Myeloid_vs_CD8_Responder	CD8(EOMES+ NK-like)	Macro_ISG15	CD99	PILRA	3,85794E-03	34964,1	3,50098E-03	35003	1,23182E+00	23996	1,07578E+00	37843	9,04678E-01	29597	0	48381,5
Myeloid_vs_CD8_Responder	CD8(Terminal Tex)	Macro_ISG15	HLA-G	LILRB2	3,86057E-03	35695,9	5,76713E-03	16727	1,54637E+00	14681	1,63140E+00	18117	8,13927E-01	80573	0	48381,5
Myeloid_vs_CD8_Responder	Mono_CD14	CD8(Tc17)	VCAN	ITGA4	3,86428E-03	18835,1	1,00589E-02	6910	3,15707E+00	1262	2,37277E+00	5879	9,00779E-01	31743	0	48381,5

Myeloid_vs_CD8_Responder	Macro_OLFML3	CD8(IL7R+ZNF683+ Tm)	FN1	ITGA4_ITGB1	3,86508E-03	24376,9	8,58209E-03	8808	1,91306E+00	8229	1,61135E+00	18609	8,89854E-01	37857	0	48381,5
Myeloid_vs_CD8_Responder	Macro_OLFML3	CD8(GZMK+ Tem)	LGALS9	HAVCR2	3,86712E-03	32069,9	4,98711E-03	20929	1,32755E+00	20809	1,20393E+00	32369	8,89842E-01	37861	0	48381,5
Myeloid_vs_CD8_Responder	CD8(NME1+ T)	cDC_CLEC9A	FLT3LG	FLT3	3,86753E-03	34502,5	2,34138E-02	1408	4,43103E+00	268	1,48661E+00	22087	7,74661E-01	100368	0	48381,5
Myeloid_vs_CD8_Responder	Macro_FOLR2+APOE+	CD8(GZMK+ Tem)	HLA-DQB1	LAG3	3,88139E-03	18974,5	7,87106E-03	10187	1,41736E+00	18142	1,82455E+00	13778	9,49332E-01	4384	0	48381,5
Myeloid_vs_CD8_Responder	CD8(GZMK+ Tex)	Macro_ISG15	HLA-F	LILRB2	3,88965E-03	31682,7	3,61201E-03	33472	1,42135E+00	18035	1,53590E+00	20620	8,89781E-01	37905	0	48381,5
Myeloid_vs_CD8_Responder	CD8(GZMK+ Tex)	Macro_NLRP3	HLA-A	LILRB2	3,89016E-03	27197,7	3,31262E-03	37906	1,61145E+00	13190	1,32403E+00	27692	9,41146E-01	8819	0	48381,5
Myeloid_vs_CD8_Responder	Macro_FOLR2-APOE+	CD8(LAYN+ T)	CD14	ITGB1	3,89810E-03	21267,9	5,47896E-03	18095	1,88220E+00	8639	1,86379E+00	13062	9,25011E-01	18162	0	48381,5
Myeloid_vs_CD8_Responder	Macro_OLFML3	CD8(Temra)	FN1	ITGA4_ITGB7	3,90867E-03	22070,3	1,31676E-02	4507	2,03717E+00	6737	1,87865E+00	12784	8,89726E-01	37942	0	48381,5
Myeloid_vs_CD8_Responder	CD8(Terminal Tex)	Macro_ISG15	CD99	PILRA	3,91537E-03	35638,3	3,39659E-03	36561	1,20580E+00	24987	1,07308E+00	37955	9,03365E-01	30307	0	48381,5
Myeloid_vs_CD8_Responder	CD8(GZMK+ Tem)	Macro_ISG15	HLA-C	LILRB1	3,92001E-03	31756,3	3,30975E-03	37964	1,28628E+00	22138	1,13945E+00	35084	9,30247E-01	15214	0	48381,5
Myeloid_vs_CD8_Responder	Macro_NLRP3	CD8(EOMES+ NK-like)	VCAN	CD44	3,93252E-03	19684,5	7,74051E-03	10453	2,45107E+00	3390	1,62810E+00	18203	9,25297E-01	17995	0	48381,5
Myeloid_vs_CD8_Responder	CD8(Tc17)	Mono_INHBA	HSP90B1	LRP1	3,93656E-03	35154,3	3,30779E-03	37996	1,46173E+00	16848	1,13541E+00	35267	8,90911E-01	37279	0	48381,5
Myeloid_vs_CD8_Responder	CD8(Temra)	Macro_LYVE1	CALR	LRP1	3,94641E-03	27558,7	4,81834E-03	22057	2,14473E+00	5655	1,07174E+00	38015	9,15470E-01	23685	0	48381,5
Myeloid_vs_CD8_Responder	CD8(Tn)	Macro_LYVE1	CD59	STAB1	3,94940E-03	30605,9	6,46352E-03	14029	2,57010E+00	2861	1,62719E+00	18223	8,34304E-01	69535	0	48381,5
Myeloid_vs_CD8_Responder	Macro_FOLR2-APOE+	CD8(IL7R+ZNF683+ Tm)	SPP1	CD44	3,95447E-03	18539,1	8,11734E-03	9653	1,86629E+00	8828	2,21221E+00	7604	9,24906E-01	18229	0	48381,5
Myeloid_vs_CD8_Responder	cDC(CD1C)	CD8(ITM2C+ T)	HLA-DQA1	LAG3	3,95784E-03	28942,3	3,30563E-03	38037	1,02646E+00	32916	2,25508E+00	7119	9,24864E-01	18258	0	48381,5
Myeloid_vs_CD8_Responder	CD8(ID2+CXCR4+ T)	Mono_INHBA	HLA-B	LILRB2	3,96149E-03	26870,5	3,30522E-03	38044	1,67537E+00	11914	1,29620E+00	28750	9,43922E-01	7263	0	48381,5
Myeloid_vs_CD8_Responder	Macro_OLFML3	CD8(IL7R+ZNF683+ Tm)	HLA-DQA2	LAG3	3,98183E-03	37803,3	3,47915E-03	35325	9,89267E-01	34809	1,20260E+00	32418	8,89494E-01	38083	0	48381,5
Myeloid_vs_CD8_Responder	CD8(GZMK+ Early Tem)	Macro_NLRP3	CD99	PILRA	3,98864E-03	32248,9	3,91076E-03	29866	1,42747E+00	17860	1,06987E+00	38096	9,09345E-01	27041	0	48381,5
Myeloid_vs_CD8_Responder	CD8(ISG+ T)	Macro_ISG15	HLA-C	LILRB1	3,99073E-03	31940,1	3,30171E-03	38100	1,27982E+00	22349	1,12728E+00	35609	9,30168E-01	15261	0	48381,5
Myeloid_vs_CD8_Responder	cDC_CLEC9A	CD8(ISG+ T)	HLA-DRA	LAG3	4,02085E-03	17621,5	5,74851E-03	16819	1,41135E+00	18307	3,01040E+00	1830	9,53019E-01	2770	0	48381,5
Myeloid_vs_CD8_Responder	CD8(Terminal Tex)	Macro_ISG15	HLA-B	LILRB1	4,03333E-03	30993,7	3,29694E-03	38181	1,33618E+00	20554	1,13913E+00	35090	9,34386E-01	12762	0	48381,5
Myeloid_vs_CD8_Responder	CD8(Terminal Tex)	cDC_CLEC9A	IFNG	IFNGR1_IFNGR2	4,03545E-03	31347,5	5,84318E-03	16397	9,42532E-01	37232	1,69717E+00	16542	8,89333E-01	38185	0	48381,5
Myeloid_vs_CD8_Responder	Macro_IFI27	CD8(GZMK+ Tem)	HLA-DPA1	LAG3	4,05176E-03	17584,3	6,72532E-03	13142	1,41023E+00	18343	2,33848E+00	6222	9,55826E-01	1833	0	48381,5
Myeloid_vs_CD8_Responder	CD8(ITM2C+ T)	Macro_ISG15	HLA-C	LILRB1	4,06459E-03	32469,1	3,29322E-03	38240	1,27300E+00	22557	1,07550E+00	37857	9,30084E-01	15310	0	48381,5
Myeloid_vs_CD8_Responder	CD8(Temra)	Macro_NLRP3	HLA-F	LILRB2	4,07470E-03	34095,7	3,57282E-03	34004	1,45854E+00	16946	1,19062E+00	32888	8,89245E-01	38259	0	48381,5
Myeloid_vs_CD8_Responder	cDC_CLEC9A	CD8(GZMK+ Tem)	HLA-DQA1	LAG3	4,07765E-03	16551,7	8,11979E-03	9646	1,40916E+00	18373	2,81425E+00	2617	9,50719E-01	3741	0	48381,5
Myeloid_vs_CD8_Responder	CD8(EOMES+ NK-like)	Macro_LYVE1	HSP90B1	LRP1	4,08109E-03	29447,7	4,39690E-03	25160	2,16528E+00	5464	1,06647E+00	38271	9,03992E-01	29962	0	48381,5
Myeloid_vs_CD8_Responder	Macro_NLRP3	CD8(NME1+ T)	S100A8	ITGB2	4,08544E-03	17682,1	5,42324E-03	18382	2,22763E+00	4953	2,57392E+00	4162	9,34741E-01	12532	0	48381,5
Myeloid_vs_CD8_Responder	Macro_FOLR2+APOE-	CD8(GZMK+ Tex)	F13A1	ITGB1	4,09324E-03	17877,3	1,35087E-02	4335	3,48437E+00	816	2,26287E+00	7047	9,06175E-01	28807	0	48381,5
Myeloid_vs_CD8_Responder	CD8(NME1+ T)	Mono_INHBA	RPS19	C5AR1	4,09391E-03	31343,3	3,28915E-03	38295	1,38123E+00	19208	1,13422E+00	35314	9,29751E-01	15518	0	48381,5
Myeloid_vs_CD8_Responder	Macro_IFI27	CD8(Terminal Tex)	B2M	CD3D	4,09508E-03	55577,3	2,68982E-03	51005	2,73349E-01	92429	3,52252E-01	86045	9,65487E-01	26	0	48381,5
Myeloid_vs_CD8_Responder	CD8(Terminal Tex)	Mono_CD14	HMGB1	THBD	4,09604E-03	34221,9	4,07858E-03	28082	1,19868E+00	25269	1,23622E+00	31078	8,89162E-01	38299	0	48381,5
Myeloid_vs_CD8_Responder	Macro_FOLR2-APOE+	CD8(Tc17)	SPP1	CD44	4,09932E-03	18716,9	8,04543E-03	9794	1,85833E+00	8955	2,17446E+00	8056	9,24597E-01	18398	0	48381,5
Myeloid_vs_CD8_Responder	Macro_FOLR2+APOE+	CD8(GZMK+ Tem)	HLA-DPA1	LAG3	4,10366E-03	18437,3	6,71565E-03	13173	1,40813E+00	18403	2,01342E+00	10381	9,55796E-01	1848	0	48381,5
Myeloid_vs_CD8_Responder	CD8(ISG+ T)	Mono_INHBA	HLA-B	LILRB2	4,11533E-03	26800,1	3,28700E-03	38335	1,65561E+00	12286	1,32516E+00	27648	9,43776E-01	7350	0	48381,5
Myeloid_vs_CD8_Responder	CD8(Tn)	Macro_FOLR2+APOE+	CD40LG	ITGAM_ITGB2	4,15010E-03	39452,3	2,31140E-02	1459	9,14173E-01	38804	9,42818E-01	44301	8,43565E-01	64316	0	48381,5
Myeloid_vs_CD8_Responder	CD8(Terminal Tex)	Macro_NLRP3	HLA-C	LILRB2	4,15466E-03	27534,3	3,28231E-03	38408	1,63454E+00	12702	1,29625E+00	28746	9,40042E-01	9434	0	48381,5
Myeloid_vs_CD8_Responder	CD8(Tn)	Mast	CD55	ADGRE2	4,15655E-03	26239,9	9,87787E-03	7084	2,08625E+00	6218	1,44394E+00	23431	8,75724E-01	46085	0	48381,5
Myeloid_vs_CD8_Responder	CD8(NME1+ T)	cDC(CD1C)	B2M	CD1A	4,17309E-03	26498,1	2,78960E-02	932	1,93992E+00	7894	1,06259E+00	38442	8,91674E-01	36841	0	48381,5
Myeloid_vs_CD8_Responder	Macro_IFI27	CD8(ISG+ T)	HLA-DRA	LAG3	4,17363E-03	18463,1	5,72487E-03	16930	1,40514E+00	18483	2,39004E+00	5726	9,52926E-01	2795	0	48381,5
Myeloid_vs_CD8_Responder	CD8(GZMK+ Tex)	Macro_ISG15	HMGB1	CD163	4,17471E-03	28786,7	3,28059E-03	38445	1,44475E+00	17362	1,51968E+00	21120	9,24208E-01	18625	0	48381,5
Myeloid_vs_CD8_Responder	CD8(GZMK+ Tex)	Macro_ISG15	CCL4	CCR1	4,17797E-03	33193,1	6,89474E-03	12623	9,92804E-01	34628	1,06246E+00	38451	9,00527E-01	31882	0	48381,5
Myeloid_vs_CD8_Responder	CD8(GZMK+ Tex)	Mono_INHBA	CALR	LRP1	4,18558E-03	34499,5	3,27940E-03	38465	1,38829E+00	19010	1,16115E+00	34107	8,99343E-01	32534	0	48381,5
Myeloid_vs_CD8_Responder	CD8(IL7R+ZNF683+ Tm)	cDC(CD1C)	CD28	CD86	4,18596E-03	36623,3	6,24160E-03	14821,5	1,52608E+00	15179,5	1,61594E+00	18497,5	8,03006E-01	86236,5	0	48381,5
Myeloid_vs_CD8_Responder	CD8(GZMK+ Tex)	Mono_CD16	HLA-C	LILRA3	4,18684E-03	23428,1	1,74897E-02	2793	2,88417E+00	1822	9,16951E-01	45646	9,24421E-01	18498	0	48381,5
Myeloid_vs_CD8_Responder	CD8(LAYN+ T)	Macro_OLFML3	ADAM10	TREM2	4,19655E-03	26325,3	9,13423E-03	7985	2,16486E+00	5467	1,61561E+00	18509	8,66811E-01	51284	0	48381,5
Myeloid_vs_CD8_Responder	CD8(Terminal Tex)	pDC_LILRA4	HLA-DPB1	CD4	4,19744E-03	29097,3	2,53293E-03	55486	1,40430E+00	18510	2,38702E+00	5758	9,26398E-01	17351	0	48381,5
Myeloid_vs_CD8_Responder	Macro_OLFML3	CD8(ID2+CXCR4+ T)	CD86	CTLA4	4,19811E-03	28570,5	1,05496E-02	6427	1,54312E+00	14762	1,14630E+00	34794	8,88843E-01	38488	0	48381,5
Myeloid_vs_CD8_Responder	CD8(ID2+CXCR4+ T)	Mono_INHBA	CALR	LRP1	4,20958E-03	35037,7	3,33453E-03	37556	1,40081E+00	18612	1,06137E+00	38509	9,00095E-01	32130	0	48381,5
Myeloid_vs_CD8_Responder	Macro_OLFML3	CD8(ID2+CXCR4+ T)	FN1	ITGA4_ITGB7	4,21286E-03	22282,1	1,29250E-02	4635	2,03739E+00	6734	1,85814E+00	13145	8,88810E-01	38515	0	48381,5
Myeloid_vs_CD8_Responder	Macro_OLFML3	CD8(Terminal Tex)	C3	CD46	4,23313E-03	22177,5	8,27147E-03	9368	2,37108E+00	3854	1,99152E+00	10732	8,88748E-01	38552	0	48381,5
Myeloid_vs_CD8_Responder	CD8(ZNF683+KLRB1+ T)	Macro_ISG15	CD52	SIGLEC10	4,23698E-03	34278,7	3,97043E-03	29243	1,19077E+00	25561	1,06048E+00	38559	9,04584E-01	29649	0	48381,5
Myeloid_vs_CD8_Responder	CD8(GZMK+ Tem)	Mono_INHBA	HLA-C	LILRB2	4,23753E-03	27958,1	3,27381E-03	38560	1,57768E+00	13929	1,27706E+00	29458	9,39969E-01	9462	0	48381,5
Myeloid_vs_CD8_Responder	Macro_ISG15	CD8(LAYN+ T)	SPP1	CD44	4,24358E-03	18319,5	7,98066E-03	9923	1,79311E+00	9929	2,49135E+00	4802	9,24314E-01	18562	0	48381,5
Myeloid_vs_CD8_Responder	Macro_OLFML3	CD8(ZNF683+KLRB1+ T)	FN1	ITGA4_ITGB1	4,24413E-03	24207,9	8,38486E-03	9142	1,90525E+00	8332	1,69395E+00	16612	8,88709E-01	38572	0	48381,5
Myeloid_vs_CD8_Responder	Macro_OLFML3	CD8(Tn)	CD14	ITGB1	4,24413E-03	29638,9	3,27297E-03	38572	1,62269E+00	12952	1,59932E+00	18911	9,05068E-01	29378	0	48381,5
Myeloid_vs_CD8_Responder	Macro_FOLR2-APOE+	CD8(GZMK+ Tem)	HLA-DRB5	LAG3	4,24447E-03	17678,5	7,42559E-03	11187	1,40251E+00	18563	2,29289E+00	6730	9,51192E-01	3531	0	48381,5
Myeloid_vs_CD8_Responder	Macro_FOLR2+APOE+	CD8(GZMK+ Tex)	B2M	CD3D	4,25253E-03	48664,3	2,68412E-03	51149	5,02239E-01	69123	4,87824E-01	74641	9,65451E-01	27	0	48381,5
Myeloid_vs_CD8_Responder	Macro_OLFML3	CD8(Tc17)	FN1	DPP4	4,25358E-03	21529,3	7,91119E-02	116	2,56136E+00	2902	2,25320E+00	7140	8,70542E-01	49107	0	48381,5
Myeloid_vs_CD8_Responder	CD8(ITM2C+ T)	Mono_INHBA	HLA-F	LILRB2	4,25459E-03	34192,3	3,53240E-03	34568	1,49138E+00	16065	1,17875E+00	33356	8,88684E-01	38591	0	48381,5
Myeloid_vs_CD8_Responder	pDC_LILRA4	CD8(ISG+ T)	APP	LRP10	4,25533E-03	24239,9	1,12564E-02	5790	2,00153E+00	7141	2,67569E+00	3443	8,57618E-01	56444	0	48381,5
Myeloid_vs_CD8_Responder	CD8(Terminal Tex)	Macro_ISG15	CD52	SIGLEC10	4,25845E-03	35359,5	3,78049E-03	31337	1,14068E+00	27678	1,05958E+00	38598	9,02448E-01	30803	0	48381,5
Myeloid_vs_CD8_Responder	cDC_CLEC9A	CD8(ISG+ T)	HLA-DPB1	LAG3	4,25964E-03	17718,3	5,84481E-03	16389	1,40172E+00	18580	3,01719E+00	1794	9,51389E-01	3447	0	48381,5
Myeloid_vs_CD8_Responder	Macro_FOLR2+APOE+	CD8(GZMK+ Early Tem)	SPP1	CD44	4,27037E-03	18646,7	7,97387E-03	9946	1,85041E+00	9074	2,24437E+00	7240	9,24285E-01	18592	0	48381,5
Myeloid_vs_CD8_Responder	CD8(ISG+ T)	Mono_CD14	CIRBP	TREM1	4,27281E-03	27181,3	7,03352E-03	12234	1,76004E+00	10439	1,36125E+00	26228	8,88631E-01	38624	0	48381,5
Myeloid_vs_CD8_Responder	Macro_OLFML3	CD8(IL7R+ZNF683+ Tm)	APOE	SORL1	4,28278E-03	24885,3	3,99876E-03	28913	2,08898E+00	6189	2,88651E+00	2301	8,88608E-01	38642	0	48381,5
Myeloid_vs_CD8_Responder	CD8(GZMK+ Early Tem)	Macro_LYVE1	RPS19	C5AR1	4,29054E-03	24060,7	5,35114E-03	18767	1,98555E+00	7339	1,05848E+00	38656	9,44076E-01	7160	0	48381,5
Myeloid_vs_CD8_Responder	Macro_OLFML3	CD8(Tn)	APOE	SORL1	4,29832E-03	24839,5	3,99465E-03	28956	2,08872E+00	6193	2,96482E+00	1997	8,88557E-01	38670	0	48381,5
Myeloid_vs_CD8_Responder	cDC(CD1C)	CD8(GZMK+ Tex)	CD86	CTLA4	4,30833E-03	22622,1	1,04835E-02	6483	1,71230E+00	11254	2,15592E+00	8304	8,88532E-01	38688	0	48381,5
Myeloid_vs_CD8_Responder	CD8(GZMK+ Tex)	Mono_CD14	HLA-F	LILRB2	4,31111E-03	32081,9	3,52079E-03	34725	1,38599E+00	19082	1,57541E+00	19528	8,88521E-01	38693	0	48381,5
Myeloid_vs_CD8_Responder	CD8(ISG+ T)	Mono_INHBA	HLA-C	LILRB2	4,31334E-03	28117,5	3,26585E-03	38697	1,57122E+00	14082	1,26489E+00	29920	9,39901E-01	9507	0	48381,5
Myeloid_vs_CD8_Responder	CD8(ID2+CXCR4+ T)	Macro_ISG15	HLA-A	LILRB1	4,32897E-03	32342,9	3,35317E-03	37229	1,26092E+00	22970	1,05709E+00	38725	9,31641E-01	14409	0	48381,5
Myeloid_vs_CD8_Responder	CD8(IL7R+ZNF683+ Tm)	Macro_ISG15	ANXA1	FPR2_FPR3	4,33232E-03	29261,5	7,40121E-03	11266	1,60578E+00	13302	1,05694E+00	38731	8,95574E-01	34627	0	48381,5
Myeloid_vs_CD8_Responder	CD8(GZMK+ Early Tem)	Mono_INHBA	CALR	LRP1	4,34072E-03	35217,5	3,26325E-03	38746	1,38462E+00	19122	1,09077E+00	37175	8,99120E-01	32663	0	48381,5
Myeloid_vs_CD8_Responder	CD8(Terminal Tex)	Mono_INHBA	HLA-B	LILRB2	4,35699E-03	27388,5	3,26114E-03	38775	1,62758E+00	12851	1,27674E+00	29468	9,43566E-01	7467	0	48381,5
Myeloid_vs_CD8_Responder	CD8(Temra)	Mono_CD14	SPON2	ITGAM	4,36056E-03	31484,9	2,68293E-02	1023	1,70579E+00	11369	1,67634E+00	17021	8,15704E-01	79630	0	48381,5

Myeloid_vs_CD8_Post_R

Myeloid_vs_CD8_Responder	CD8(Temra)	cDC(CD1C)	B2M	CD1A	4,37613E-03	26495,5	2,80054E-02	923	1,95943E+00	7641	1,05557E+00	38809	8,91863E-01	36723	0	48381,5
Myeloid_vs_CD8_Responder	Macro_IER3	CD8(LAYN+ T)	CD14	ITGB1	4,38789E-03	22386,7	5,35892E-03	18722	1,84457E+00	9161	1,67401E+00	17060	9,24239E-01	18609	0	48381,5
Myeloid_vs_CD8_Responder	CD8(ITM2C+ T)	Mono_INHBA	HLA-C	LILRB2	4,39420E-03	28601,5	3,25745E-03	38841	1,56440E+00	14239	1,21311E+00	31997	9,39828E-01	9549	0	48381,5
Myeloid_vs_CD8_Responder	Macro_IFI27	CD8(Terminal Tex)	HLA-DPB1	LAG3	4,42728E-03	18197,3	6,48149E-03	13978	1,39599E+00	18765	2,23545E+00	7340	9,53725E-01	2522	0	48381,5
Myeloid_vs_CD8_Responder	Macro_LYVE1	CD8(GZMK+ Tem)	HLA-DPA1	LAG3	4,42820E-03	18400,5	6,65935E-03	13343	1,39589E+00	18766	2,06508E+00	9605	9,55617E-01	1907	0	48381,5
Myeloid_vs_CD8_Responder	CD8(ITM2C+ T)	Macro_ISG15	IFNG	IFNGR1_IFNGR2	4,42881E-03	34190,5	5,70328E-03	17035	9,68713E-01	35855	1,24402E+00	30779	8,88135E-01	38902	0	48381,5
Myeloid_vs_CD8_Responder	Macro_OLFML3	CD8(Tc17)	HLA-DQA2	LAG3	4,43565E-03	38428,7	3,38329E-03	36771	9,82566E-01	35123	1,18940E+00	32954	8,88114E-01	38914	0	48381,5
Myeloid_vs_CD8_Responder	CD8(ISG+ T)	Macro_ISG15	CD99	PILRA	4,43565E-03	36098,9	3,35495E-03	37206	1,19542E+00	25388	1,05320E+00	38914	9,02825E-01	30605	0	48381,5
Myeloid_vs_CD8_Responder	Macro_OLFML3	CD8(Terminal Tex)	LGALS3BP	ITGB1	4,44648E-03	29990,7	6,88362E-03	12651	1,69684E+00	11524	1,05286E+00	38933	8,88878E-01	38464	0	48381,5
Myeloid_vs_CD8_Responder	CD8(IL7R+ZNF683+ Tm)	Macro_LYVE1	HSP90B1	LRP1	4,44877E-03	29651,5	4,37525E-03	25359	2,16255E+00	5488	1,05270E+00	38937	9,03778E-01	30092	0	48381,5
Myeloid_vs_CD8_Responder	CD8(ITM2C+ T)	Macro_NLRP3	HMGB1	CD163	4,45048E-03	30517,5	3,25134E-03	38940	1,54290E+00	14766	1,22062E+00	31695	9,23894E-01	18805	0	48381,5
Myeloid_vs_CD8_Responder	CD8(ITM2C+ T)	Macro_FOLR2+APOE+	IFNG	IFNGR1_IFNGR2	4,45449E-03	30191,1	7,88266E-03	10154	1,25717E+00	23092	1,05255E+00	38947	9,03230E-01	30381	0	48381,5
Myeloid_vs_CD8_Responder	Macro_OLFML3	CD8(ZNF683+KLRB1+ T)	HLA-DQA2	LAG3	4,46307E-03	38027,7	3,37634E-03	36874	9,82080E-01	35147	1,24407E+00	30774	8,88011E-01	38962	0	48381,5
Myeloid_vs_CD8_Responder	Macro_OLFML3	CD8(ISG+ T)	LYZ	ITGAL	4,47339E-03	30341,9	3,24882E-03	38980	1,51178E+00	15532	1,82858E+00	13700	8,94707E-01	35116	0	48381,5
Myeloid_vs_CD8_Responder	Macro_OLFML3	CD8(NME1+ T)	LYZ	ITGAL	4,48028E-03	30632,9	3,24815E-03	38992	1,51172E+00	15536	1,75797E+00	15134	8,94697E-01	35121	0	48381,5
Myeloid_vs_CD8_Responder	CD8(Temra)	Macro_NLRP3	HLA-C	LILRB2	4,48430E-03	27901,5	3,24754E-03	38999	1,60513E+00	13318	1,28390E+00	29214	9,39742E-01	9595	0	48381,5
Myeloid_vs_CD8_Responder	CD8(GZMK+ Tex)	Macro_LYVE1	RPS19	C5AR1	4,55023E-03	26200,7	4,53214E-03	24103	1,80836E+00	9676	1,04935E+00	39113	9,39525E-01	9730	0	48381,5
Myeloid_vs_CD8_Responder	Macro_IFI27	CD8(ISG+ T)	HLA-DQA2	LAG3	4,56015E-03	20172,7	9,40552E-03	7615	1,39126E+00	18908	2,01032E+00	10438	9,29749E-01	15521	0	48381,5
Myeloid_vs_CD8_Responder	Macro_LYVE1	CD8(GZMK+ Tem)	HLA-DRB1	LAG3	4,57990E-03	18337,7	6,64592E-03	13391	1,39068E+00	18929	2,07712E+00	9429	9,56745E-01	1558	0	48381,5
Myeloid_vs_CD8_Responder	CD8(Temra)	cDC(CD1C)	ANXA1	FPR1	4,57997E-03	35426,7	3,23788E-03	39164	1,17393E+00	26223	1,18783E+00	33022	9,03312E-01	30343	0	48381,5
Myeloid_vs_CD8_Responder	Macro_OLFML3	CD8(IL7R+ZNF683+ Tm)	CD14	ITGA4	4,58465E-03	31451,3	3,23720E-03	39172	1,58675E+00	13703	1,43253E+00	23833	9,00027E-01	32167	0	48381,5
Myeloid_vs_CD8_Responder	CD8(GZMK+ Early Tem)	Macro_ISG15	HLA-B	LILRB1	4,59168E-03	31897,9	3,23684E-03	39184	1,27173E+00	22599	1,11294E+00	36203	9,33819E-01	13122	0	48381,5
Myeloid_vs_CD8_Responder	CD8(ISG+ T)	Macro_FOLR2+APOE+	CD52	SIGLEC10	4,60350E-03	26367,1	6,47299E-03	14006	1,91293E+00	8231	9,84101E-01	42263	9,23693E-01	18954	0	48381,5
Myeloid_vs_CD8_Responder	cDC(CD1C)	CD8(ID2+CXCR4+ T)	GRN	TNFRSF1B	4,60634E-03	25539,1	3,23501E-03	39209	1,47595E+00	16479	2,22730E+00	7440	9,28573E-01	16186	0	48381,5
Myeloid_vs_CD8_Responder	CD8(GZMK+ Tex)	Mono_CD16	HLA-A	LILRB1	4,61281E-03	31403,7	3,44388E-03	35846	1,36561E+00	19669	1,04735E+00	39220	9,32486E-01	13902	0	48381,5
Myeloid_vs_CD8_Responder	CD8(Tn)	Mono_CD14	CD40LG	ITGAM_ITGB2	4,61302E-03	31333,5	2,27305E-02	1539	1,23193E+00	23993	1,64386E+00	17800	8,42458E-01	64954	0	48381,5
Myeloid_vs_CD8_Responder	CD8(ITM2C+ T)	Macro_NLRP3	CD99	PILRA	4,62046E-03	31773,3	4,07017E-03	28162	1,45956E+00	16912	1,04705E+00	39233	9,10978E-01	26178	0	48381,5
Myeloid_vs_CD8_Responder	CD8(Temra)	Mono_INHBA	HLA-A	LILRB2	4,62458E-03	29398,7	3,23313E-03	39240	1,47731E+00	16429	1,16940E+00	33748	9,40470E-01	9195	0	48381,5
Myeloid_vs_CD8_Responder	CD8(Terminal Tex)	pDC_LILRA4	HLA-G	CD4	4,63289E-03	31617,9	6,05702E-03	15530	1,38922E+00	18985	2,50638E+00	4684	8,32559E-01	70509	0	48381,5
Myeloid_vs_CD8_Responder	CD8(GZMK+ Tem)	Macro_LYVE1	RPS19	C5AR1	4,63933E-03	24970,3	5,00989E-03	20787	1,91172E+00	8240	1,04612E+00	39265	9,42311E-01	8178	0	48381,5
Myeloid_vs_CD8_Responder	Mono_INHBA	CD8(ISG+ T)	INHBA	TGFBR3	4,64275E-03	34033,3	2,27095E-02	1544	3,06766E+00	1423	2,06315E+00	9625	7,55349E-01	109193	0	48381,5
Myeloid_vs_CD8_Responder	pDC_LILRA4	CD8(GZMK+ Early Tem)	APP	CD74	4,64526E-03	18678,7	5,31051E-03	18998	1,92347E+00	8080	2,12173E+00	8768	9,40534E-01	9166	0	48381,5
Myeloid_vs_CD8_Responder	Macro_FOLR2+APOE+	CD8(GZMK+ Tem)	HLA-DRB1	LAG3	4,64812E-03	18552,7	6,63598E-03	13431	1,38849E+00	19001	2,01349E+00	10380	9,56714E-01	1570	0	48381,5
Myeloid_vs_CD8_Responder	Macro_OLFML3	CD8(Terminal Tex)	CXCL16	CXCR6	4,65531E-03	32310,7	6,56365E-03	13693	1,19992E+00	25218	1,04560E+00	39292	8,94965E-01	34969	0	48381,5
Myeloid_vs_CD8_Responder	pDC_LILRA4	CD8(GZMK+ Tem)	APP	LRP10	4,66012E-03	24893,3	1,06321E-02	6351	1,98287E+00	7366	2,61836E+00	3828	8,54099E-01	58540	0	48381,5
Myeloid_vs_CD8_Responder	CD8(GZMK+ Tem)	Macro_NLRP3	CD99	PILRA	4,66776E-03	32480,3	3,91372E-03	29841	1,42806E+00	17839	1,04511E+00	39313	9,09376E-01	27027	0	48381,5
Myeloid_vs_CD8_Responder	CD8(Temra)	Macro_IER3	SPON2	ITGB2	4,67251E-03	33786,3	8,90822E-03	8300	9,05080E-01	39321	1,08224E+00	37556	8,94257E-01	35373	0	48381,5
Myeloid_vs_CD8_Responder	Macro_OLFML3	CD8(ZNF683+KLRB1+ T)	C3	CD46	4,68083E-03	22378,5	8,03657E-03	9815	2,36215E+00	3914	2,00957E+00	10447	8,87316E-01	39335	0	48381,5
Myeloid_vs_CD8_Responder	CD8(Temra)	cDC(CD1C)	CD52	SIGLEC10	4,68560E-03	37530,3	3,22768E-03	39343	9,92477E-01	34648	1,25102E+00	30487	8,95264E-01	34792	0	48381,5
Myeloid_vs_CD8_Responder	CD8(LAYN+ T)	Mono_INHBA	HLA-A	LILRB2	4,69155E-03	29890,7	3,22734E-03	39353	1,47211E+00	16576	1,11916E+00	35915	9,40420E-01	9228	0	48381,5
Myeloid_vs_CD8_Responder	CD8(ID2+CXCR4+ T)	Macro_FOLR2+APOE+	HMGB1	CD163	4,69334E-03	27942,3	4,08067E-03	28068	1,82565E+00	9429	1,04418E+00	39356	9,31506E-01	14477	0	48381,5
Myeloid_vs_CD8_Responder	cDC_LAMP3	CD8(Terminal Tex)	HLA-DRA	LAG3	4,69593E-03	18395,3	6,23394E-03	14843	1,38710E+00	19051	2,21911E+00	7525	9,54801E-01	2176	0	48381,5
Myeloid_vs_CD8_Responder	CD8(ISG+ T)	Mono_CD16	HLA-C	LILRA3	4,69785E-03	24952,1	1,70486E-02	2929	2,81557E+00	2016	7,98768E-01	52381	9,23524E-01	19053	0	48381,5
Myeloid_vs_CD8_Responder	CD8(GZMK+ Tex)	Mono_CD14	HMGB1	TLR4	4,70767E-03	33076,3	4,34825E-03	25616	1,27295E+00	22558	1,27734E+00	29446	8,87237E-01	39380	0	48381,5
Myeloid_vs_CD8_Responder	CD8(GZMK+ Tex)	Macro_OLFML3	ENTPD1	ADORA3	4,71031E-03	27873,9	1,14171E-02	5649	2,05371E+00	6553	1,40062E+00	24878	8,62103E-01	53908	0	48381,5
Myeloid_vs_CD8_Responder	Macro_ISG15	CD8(GZMK+ Tem)	HLA-DRA	LAG3	4,71226E-03	17668,1	6,34250E-03	14453	1,38644E+00	19068	2,54117E+00	4394	9,55172E-01	2044	0	48381,5
Myeloid_vs_CD8_Responder	Macro_FOLR2+APOE-	CD8(GZMK+ Tem)	HLA-DPB1	LAG3	4,71708E-03	18395,1	6,57237E-03	13655	1,38619E+00	19073	2,14650E+00	8437	9,54032E-01	2429	0	48381,5
Myeloid_vs_CD8_Responder	cDC_CLEC9A	CD8(NME1+ T)	HLA-DQB1	LAG3	4,73154E-03	18399,7	6,50794E-03	13890	1,38577E+00	19088	2,62884E+00	3749	9,44558E-01	6890	0	48381,5
Myeloid_vs_CD8_Responder	Macro_OLFML3	CD8(NME1+ T)	CXCL9	CXCR3	4,76171E-03	23256,1	1,65654E-02	3083	1,73220E+00	10901	1,79063E+00	14445	8,87068E-01	39470	0	48381,5
Myeloid_vs_CD8_Responder	CD8(Tn)	Mono_INHBA	CD40LG	ITGA5_ITGB1	4,77467E-03	43892,3	2,25995E-02	1566	8,03117E-01	45629	1,07684E+00	37797	8,03319E-01	86088	0	48381,5
Myeloid_vs_CD8_Responder	CD8(GZMK+ Tem)	Macro_NLRP3	HLA-B	LILRB2	4,78407E-03	27295,7	3,21829E-03	39507	1,66564E+00	12080	1,29387E+00	28829	9,43213E-01	7681	0	48381,5
Myeloid_vs_CD8_Responder	CD8(Terminal Tex)	Mono_CD16	HLA-C	LILRA1	4,78908E-03	27724,3	1,02082E-02	6757	1,97714E+00	7435	7,75444E-01	53877	9,18089E-01	22171	0	48381,5
Myeloid_vs_CD8_Responder	CD8(Terminal Tex)	Macro_OLFML3	ENTPD1	ADORA3	4,79097E-03	32464,5	9,58111E-03	7409	1,97689E+00	7436	1,05076E+00	39034	8,51348E-01	60062	0	48381,5
Myeloid_vs_CD8_Responder	Macro_OLFML3	CD8(GZMK+ Early Tem)	FN1	ITGA4_ITGB1	4,79255E-03	25025,1	8,10136E-03	9685	1,89504E+00	8465	1,59301E+00	19073	8,86997E-01	39521	0	48381,5
Myeloid_vs_CD8_Responder	Macro_OLFML3	CD8(ID2+CXCR4+ T)	C3	CD46	4,79437E-03	22581,7	7,98481E-03	9913	2,36018E+00	3936	1,96759E+00	11154	8,86992E-01	39524	0	48381,5
Myeloid_vs_CD8_Responder	CD8(Terminal Tex)	pDC_LILRA4	HLA-DRB1	CD4	4,79750E-03	28619,9	2,55567E-03	54825	1,38303E+00	19156	2,39065E+00	5712	9,30554E-01	15025	0	48381,5
Myeloid_vs_CD8_Responder	CD8(Temra)	pDC_LILRA4	COPA	P2RY6	4,80140E-03	32944,7	7,15161E-03	11921	2,27217E+00	4585	2,05373E+00	9754	7,95406E-01	90082	0	48381,5
Myeloid_vs_CD8_Responder	CD8(Tn)	cDC_LAMP3	CD28	CD80	4,81096E-03	49652,1	2,25695E-02	1572,5	1,00435E+00	34042,5	8,49977E-01	49316,5	7,41926E-01	114947,5	0	48381,5
Myeloid_vs_CD8_Responder	CD8(NME1+ T)	Macro_ISG15	HLA-C	LILRB1	4,81381E-03	33499,3	3,21628E-03	39556	1,21121E+00	24786	1,05146E+00	39005	9,29312E-01	15768	0	48381,5
Myeloid_vs_CD8_Responder	Macro_OLFML3	CD8(NME1+ T)	LGALS3BP	ITGB1	4,81503E-03	29241,3	7,45115E-03	11107	1,73117E+00	10919	1,03981E+00	39558	8,92730E-01	36241	0	48381,5
Myeloid_vs_CD8_Responder	CD8(GZMK+ Tex)	Macro_NLRP3	HLA-C	LILRB2	4,82234E-03	28084,1	3,21553E-03	39570	1,57805E+00	13922	1,29548E+00	28777	9,39461E-01	9770	0	48381,5
Myeloid_vs_CD8_Responder	CD8(ID2+CXCR4+ T)	Mono_CD14	HMGB1	THBD	4,82600E-03	35942,5	3,89675E-03	30017	1,14936E+00	27305	1,15377E+00	34433	8,86895E-01	39576	0	48381,5
Myeloid_vs_CD8_Responder	Macro_IFI27	CD8(Terminal Tex)	HLA-DQB1	LAG3	4,82780E-03	18362,7	7,52631E-03	10943	1,38196E+00	19187	2,15070E+00	8377	9,48244E-01	4925	0	48381,5
Myeloid_vs_CD8_Responder	CD8(Tc17)	Macro_ISG15	HLA-B	LILRB1	4,83820E-03	32825,9	3,21381E-03	39596	1,24703E+00	23462	1,04249E+00	39427	9,33598E-01	13263	0	48381,5
Myeloid_vs_CD8_Responder	CD8(Tc17)	Mast	CD55	ADGRE2	4,84615E-03	24532,5	1,22171E-02	5043	2,21628E+00	5036	1,40919E+00	24593	8,86836E-01	39609	0	48381,5
Myeloid_vs_CD8_Responder	CD8(ITM2C+ T)	Mono_INHBA	IFNG	IFNGR1_IFNGR2	4,85901E-03	35478,1	5,56473E-03	17643	8,99879E-01	39630	1,20903E+00	32168	8,86907E-01	39568	0	48381,5
Myeloid_vs_CD8_Responder	CD8(GZMK+ Early Tem)	Macro_ISG15	HLA-C	LILRB1	4,86453E-03	33473,5	3,21157E-03	39639	1,20744E+00	24927	1,05910E+00	38626	9,29264E-01	15794	0	48381,5
Myeloid_vs_CD8_Responder	CD8(Terminal Tex)	Mono_CD14	CIRBP	TREM1	4,86637E-03	28133,5	6,77831E-03	12969	1,73219E+00	10904	1,29562E+00	28771	8,86789E-01	39642	0	48381,5
Myeloid_vs_CD8_Responder	CD8(NME1+ T)	cDC_CLEC9A	HMGB1	HAVCR2	4,88789E-03	29254,5	3,72575E-03	32006	8,99058E-01	39677	1,90226E+00	12327	9,32519E-01	13881	0	48381,5
Myeloid_vs_CD8_Responder	CD8(NME1+ T)	Mono_CD16	GNAI2	C5AR1	4,89036E-03	28563,3	4,14772E-03	27446	2,14365E+00	5664	1,50153E+00	21644	8,86714E-01	39681	0	48381,5
Myeloid_vs_CD8_Responder	CD8(Temra)	Macro_ISG15	HLA-F	LILRB2	4,89714E-03	33771,3	3,39478E-03	36596	1,38512E+00	19106	1,39438E+00	25081	8,86703E-01	39692	0	48381,5
Myeloid_vs_CD8_Responder	CD8(Terminal Tex)	Macro_ISG15	HMGB1	CD163	4,89837E-03	30189,3	3,20815E-03	39694	1,41977E+00	18074	1,37748E+00	25690	9,23422E-01	19107	0	48381,5
Myeloid_vs_CD8_Responder	CD8(GZMK+ Tex)	Macro_ISG15	HMGB1	TLR2	4,90702E-03	34966,1	3,75245E-03	31672	1,22416E+00	24281	1,03704E+00	39708	9,02472E-01	30788	0	48381,5
Myeloid_vs_CD8_Responder	CD8(GZMK+ Tex)	Mono_CD14	CCL5	CCR1	4,90763E-03	32347,5	6,54154E-03	13784	8,98486E-01	39709	1,20219E+00	32424	9,08636E-01	27439	0	48381,5
Myeloid_vs_CD8_Responder	Macro_OLFML3	CD8(EOMES+ NK-like)	HLA-DRA	LAG3	4,90825E-03	25719,5	3,20743E-03	39710	1,26987E+00	22653	2,24153E+00	7270	9,38089E-01	10583	0	48381,5
Myeloid_vs_CD8_Responder	CD8(Tn)	Mono_CD16	CD99	PILRA	4,90949E-03	30209,9	4,08002E-03	28071	1,87232E+00	8761	1,03692E+00	39712	9,11076E-01	26124	0	48381,5
Myeloid_vs_CD8_Responder	CD8(ISG+ T)	Macro_NLRP3	HLA-A	LILRB2	4,91382E-03	29255,1	3,20692E-03	39719	1,51264E+00	15512	1,17948E+00	33329	9,40242E-01	9334	0	48381,5

Myeloid_vs_CD8_Responder	CD8(ITM2C+ T)	Macro_NLRP3	HLA-F	LILRB2	4,91505E-03	35945,9	3,39025E-03	36669	1,42961E+00	17799	1,09116E+00	37159	8,86636E-01	39721	0	48381,5
Myeloid_vs_CD8_Responder	Macro_IER3	CD8(ISG+ T)	ICAM1	IL2RG	4,91655E-03	21503,7	7,39005E-03	11301	1,59032E+00	13623	1,76776E+00	14936	9,23142E-01	19277	0	48381,5
Myeloid_vs_CD8_Responder	CD8(ITM2C+ T)	cDC_LAMP3	IFNG	IFNGR1_IFNGR2	4,93613E-03	35884,1	5,78894E-03	16627	9,62204E-01	36187	1,03591E+00	39755	8,88873E-01	38470	0	48381,5
Myeloid_vs_CD8_Responder	Macro_OLFML3	CD8(Terminal Tex)	CD14	ITGB2	4,94420E-03	25438,5	3,20413E-03	39768	1,81345E+00	9595	1,82847E+00	13702	9,29356E-01	15746	0	48381,5
Myeloid_vs_CD8_Responder	Macro_OLFML3	CD8(Terminal Tex)	CXCL10	CXCR3	4,95540E-03	22489,5	1,89058E-02	2392	1,59497E+00	13521	2,15142E+00	8367	8,86523E-01	39786	0	48381,5
Myeloid_vs_CD8_Responder	CD8(LAYN+ T)	Macro_ISG15	HLA-B	LILRB1	4,96226E-03	33000,9	3,20576E-03	39739	1,23841E+00	23774	1,03460E+00	39797	9,33521E-01	13313	0	48381,5
Myeloid_vs_CD8_Responder	CD8(NME1+ T)	Macro_FOLR2-APOE+	CD99	CD81	4,96288E-03	34949,5	3,93623E-03	29594	9,92370E-01	34656	1,03456E+00	39798	9,17817E-01	22318	0	48381,5
Myeloid_vs_CD8_Responder	Macro_IFI27	CD8(ITM2C+ T)	C3	IFITM1	4,96837E-03	21729,1	7,85429E-03	10226	1,45571E+00	17031	1,58392E+00	19329	9,32879E-01	13678	0	48381,5
Myeloid_vs_CD8_Responder	cDC(CD1C)	CD8(GZMK+ Tem)	LGALS3	LAG3	4,96912E-03	26940,3	5,58556E-03	17546	8,96685E-01	39808	1,97586E+00	10995	9,25332E-01	17971	0	48381,5
Myeloid_vs_CD8_Responder	CD8(Temra)	Macro_ISG15	SPON2	ITGB2	4,96974E-03	31931,9	8,81280E-03	8443	8,96664E-01	39809	1,33239E+00	27376	8,93747E-01	35650	0	48381,5
Myeloid_vs_CD8_Responder	CD8(GZMK+ Early Tem)	Mono_INHBA	HLA-B	LILRB2	4,97099E-03	28144,9	3,20169E-03	39811	1,56314E+00	14274	1,25055E+00	30505	9,43074E-01	7753	0	48381,5
Myeloid_vs_CD8_Responder	CD8(GZMK+ Tex)	Macro_NLRP3	CD99	PILRA	4,97349E-03	33778,5	3,66254E-03	32817	1,37749E+00	19325	1,03421E+00	39815	9,06606E-01	28554	0	48381,5
Myeloid_vs_CD8_Responder	Macro_ISG15	CD8(ISG+ T)	C3	IFITM1	4,97538E-03	20685,1	7,10270E-03	12055	1,37721E+00	19336	2,17301E+00	8076	9,29661E-01	15577	0	48381,5
Myeloid_vs_CD8_Responder	CD8(LAYN+ T)	cDC_CLEC9A	ADAM10	CADM1	4,97838E-03	32499,9	1,20275E-02	5162	1,37713E+00	19339	1,66886E+00	17190	8,29082E-01	72427	0	48381,5
Myeloid_vs_CD8_Responder	CD8(NME1+ T)	Macro_NLRP3	HSP90B1	LRP1	4,99037E-03	35581,7	3,41454E-03	36290	1,45670E+00	17004	1,03369E+00	39842	8,92445E-01	36391	0	48381,5
Myeloid_vs_CD8_Responder	Macro_NLRP3	CD8(GZMK+ Tem)	VCAN	CD44	4,99343E-03	20254,9	7,25090E-03	11672	2,40708E+00	3627	1,62660E+00	18240	9,23007E-01	19354	0	48381,5
Myeloid_vs_CD8_Responder	Macro_OLFML3	CD8(Tn)	LGALS3BP	ITGB1	4,99413E-03	30179,5	6,55208E-03	13746	1,67678E+00	11887	1,09353E+00	37035	8,86416E-01	39848	0	48381,5
Myeloid_vs_CD8_Responder	CD8(Temra)	Macro_LYVE1	ADAM10	GPNMB	4,99644E-03	27653,3	6,69191E-03	13250	2,67396E+00	2450	1,73045E+00	15761	8,54298E-01	58424	0	48381,5
Myeloid_vs_CD8_Responder	CD8(GZMK+ Early Tem)	Mono_INHBA	HLA-A	LILRB2	5,01170E-03	30246,7	3,19709E-03	39876	1,44497E+00	17357	1,11176E+00	36248	9,40156E-01	9371	0	48381,5
Myeloid_vs_CD8_Responder	Macro_OLFML3	CD8(ISG+ T)	FN1	ITGA4_ITGB7	5,01296E-03	22770,3	1,23025E-02	4997	2,01535E+00	6986	1,83352E+00	13609	8,86348E-01	39878	0	48381,5
Myeloid_vs_CD8_Responder	CD8(Tc17)	Mono_CD14	CIRBP	TREM1	5,01925E-03	28772,1	6,71628E-03	13170	1,72542E+00	11009	1,22787E+00	31412	8,86327E-01	39888	0	48381,5
Myeloid_vs_CD8_Responder	CD8(Temra)	Macro_NLRP3	SPON2	ITGB2	5,02869E-03	33164,9	8,79533E-03	8476	8,95123E-01	39903	1,17864E+00	33367	8,93653E-01	35697	0	48381,5
Myeloid_vs_CD8_Responder	CD8(LAYN+ T)	Mono_CD16	CD99	PILRA	5,02932E-03	30243,1	4,08151E-03	28057	1,87253E+00	8758	1,03211E+00	39904	9,11091E-01	26115	0	48381,5
Myeloid_vs_CD8_Responder	CD8(GZMK+ Tex)	Mono_CD14	CIRBP	TREM1	5,03815E-03	27738,3	6,70990E-03	13197	1,72472E+00	11023	1,36294E+00	26172	8,86279E-01	39918	0	48381,5
Myeloid_vs_CD8_Responder	CD8(Temra)	Macro_ISG15	HLA-A	LILRB1	5,04068E-03	33511,5	3,26863E-03	38643	1,18590E+00	25746	1,03179E+00	39922	9,30823E-01	14865	0	48381,5
Myeloid_vs_CD8_Responder	Macro_FOLR2+APOE+	CD8(Terminal Tex)	HLA-DQB1	LAG3	5,04888E-03	19761,3	7,48598E-03	11030	1,37494E+00	19409	1,76495E+00	14983	9,48112E-01	5003	0	48381,5
Myeloid_vs_CD8_Responder	Macro_OLFML3	CD8(Tc17)	C3	CD46	5,05395E-03	22830,9	7,86499E-03	10203	2,35562E+00	3965	1,93784E+00	11662	8,86232E-01	39943	0	48381,5
Myeloid_vs_CD8_Responder	Macro_OLFML3	CD8(Temra)	GRN	TNFRSF1B	5,05965E-03	26967,7	3,19298E-03	39952	1,64259E+00	12543	1,65379E+00	17550	9,28138E-01	16412	0	48381,5
Myeloid_vs_CD8_Responder	CD8(GZMK+ Tex)	cDC(CD1C)	CD52	SIGLEC10	5,07296E-03	37468,7	3,19153E-03	39973	9,82753E-01	35115	1,29560E+00	28773	8,94735E-01	35101	0	48381,5
Myeloid_vs_CD8_Responder	CD8(GZMK+ Tem)	Mono_INHBA	CIRBP	TREM1	5,08312E-03	30660,7	6,69206E-03	13248	1,63641E+00	12670	1,05125E+00	39015	8,86145E-01	39989	0	48381,5
Myeloid_vs_CD8_Responder	Macro_IER3	CD8(NME1+ T)	ICAM1	IL2RG	5,08338E-03	21894,7	7,32757E-03	11469	1,58386E+00	13778	1,70329E+00	16402	9,22841E-01	19443	0	48381,5
Myeloid_vs_CD8_Responder	cDC_LAMP3	CD8(GZMK+ Tem)	HLA-DPB1	LAG3	5,08338E-03	18449,1	6,51462E-03	13878	1,37376E+00	19443	2,17440E+00	8057	9,53838E-01	2486	0	48381,5
Myeloid_vs_CD8_Responder	CD8(NME1+ T)	Macro_NLRP3	HLA-A	LILRB2	5,08375E-03	29588,5	3,19041E-03	39990	1,49721E+00	15908	1,15733E+00	34266	9,40097E-01	9397	0	48381,5
Myeloid_vs_CD8_Responder	CD8(LAYN+ T)	Macro_NLRP3	HSP90B1	LRP1	5,08757E-03	35495,7	3,43508E-03	35980	1,46086E+00	16881	1,03005E+00	39996	8,92733E-01	36240	0	48381,5
Myeloid_vs_CD8_Responder	CD8(Terminal Tex)	Mono_CD14	HMGB1	TLR4	5,09330E-03	34710,3	4,25224E-03	26448	1,24797E+00	23440	1,13514E+00	35277	8,86116E-01	40005	0	48381,5
Myeloid_vs_CD8_Responder	CD8(Temra)	Mono_CD16	HLA-C	LILRA3	5,09968E-03	23489,7	1,76638E-02	2737	2,91125E+00	1749	9,05368E-01	46278	9,24766E-01	18303	0	48381,5
Myeloid_vs_CD8_Responder	Macro_ISG15	CD8(Temra)	SPP1	S1PR1	5,10681E-03	24133,1	2,75925E-02	952	1,96270E+00	7600	2,83984E+00	2468	8,49138E-01	61264	0	48381,5
Myeloid_vs_CD8_Responder	CD8(Terminal Tex)	Mono_CD16	HLA-A	LILRA1	5,10877E-03	27749,7	1,03309E-02	6628	1,96257E+00	7601	7,58958E-01	54933	9,19664E-01	21205	0	48381,5
Myeloid_vs_CD8_Responder	CD8(NME1+ T)	Macro_FOLR2-APOE+	HMGB1	TLR4	5,11306E-03	34182,9	4,24678E-03	26497	1,35057E+00	20115	1,11987E+00	35885	8,86051E-01	40036	0	48381,5
Myeloid_vs_CD8_Responder	Macro_OLFML3	CD8(Terminal Tex)	LGALS9	HAVCR2	5,11561E-03	33546,9	4,62059E-03	23430	1,28259E+00	22245	1,17212E+00	33638	8,86045E-01	40040	0	48381,5
Myeloid_vs_CD8_Responder	Macro_FOLR2+APOE-	CD8(NME1+ T)	C1QB	C1QBP	5,12010E-03	19638,5	5,22661E-03	19479	2,05846E+00	6494	2,38948E+00	5733	9,25096E-01	18105	0	48381,5
Myeloid_vs_CD8_Responder	CD8(Tc17)	Mono_CD14	THBS1	CD36	5,13444E-03	37326,9	5,44045E-03	18306	1,82777E+00	9407	1,57688E+00	19493	7,93505E-01	91047	0	48381,5
Myeloid_vs_CD8_Responder	CD8(Temra)	Macro_IFI27	SPON2	ITGB2	5,14378E-03	33042,7	8,76392E-03	8519	8,92353E-01	40084	1,20208E+00	32431	8,93483E-01	35798	0	48381,5
Myeloid_vs_CD8_Responder	CD8(EOMES+ NK-like)	Macro_LYVE1	CALR	LRP1	5,15534E-03	27998,3	4,80936E-03	22109	2,14341E+00	5667	1,02756E+00	40102	9,15398E-01	23732	0	48381,5
Myeloid_vs_CD8_Responder	CD8(NME1+ T)	Macro_IFI27	CD99	PILRA	5,16886E-03	34250,1	3,71940E-03	32080	1,27677E+00	22456	1,02716E+00	40123	9,07256E-01	28210	0	48381,5
Myeloid_vs_CD8_Responder	CD8(ID2+CXCR4+ T)	Macro_NLRP3	HLA-A	LILRB2	5,16886E-03	30096,5	3,18329E-03	40123	1,49056E+00	16088	1,10711E+00	36451	9,40034E-01	9439	0	48381,5
Myeloid_vs_CD8_Responder	CD8(ZNF683+KLRB1+ T)	Mono_CD14	CIRBP	TREM1	5,17788E-03	28407,7	6,66129E-03	13339	1,71942E+00	11120	1,28784E+00	29061	8,85912E-01	40137	0	48381,5
Myeloid_vs_CD8_Responder	CD8(Terminal Tex)	Macro_ISG15	B2M	LILRB1	5,18756E-03	29204,5	3,18217E-03	40152	1,45439E+00	17074	1,24567E+00	30715	9,39565E-01	9700	0	48381,5
Myeloid_vs_CD8_Responder	Mono_CD14	CD8(Terminal Tex)	VCAN	CD44	5,19724E-03	17859,5	7,17930E-03	11841	2,99430E+00	1575	2,18263E+00	7946	9,22653E-01	19554	0	48381,5
Myeloid_vs_CD8_Responder	cDC_CLEC9A	CD8(GZMK+ Tem)	HLA-DRB1	LAG3	5,19724E-03	17189,1	6,54938E-03	13758	1,36935E+00	19554	2,81732E+00	2595	9,56441E-01	1657	0	48381,5
Myeloid_vs_CD8_Responder	CD8(NME1+ T)	Mono_INHBA	HLA-C	LILRB2	5,20050E-03	29446,7	3,18135E-03	40172	1,50262E+00	15760	1,18907E+00	32964	9,39156E-01	9956	0	48381,5
Myeloid_vs_CD8_Responder	cDC_CLEC9A	CD8(NME1+ T)	HLA-DPA1	LAG3	5,20241E-03	18431,7	5,44177E-03	18300	1,36914E+00	19559	2,86932E+00	2360	9,51133E-01	3558	0	48381,5
Myeloid_vs_CD8_Responder	CD8(IL7R+ZNF683+ Tm)	Macro_NLRP3	CD99	PILRA	5,20697E-03	32531,7	3,94135E-03	29540	1,43362E+00	17683	1,02574E+00	40182	9,09666E-01	26872	0	48381,5
Myeloid_vs_CD8_Responder	CD8(IL7R+ZNF683+ Tm)	Mast	CD55	ADGRE2	5,21151E-03	24337,7	1,19735E-02	5198	2,20274E+00	5135	1,46487E+00	22785	8,85821E-01	40189	0	48381,5
Myeloid_vs_CD8_Responder	CD8(Temra)	Macro_ISG15	HLA-B	LILRB1	5,21540E-03	33140,3	3,18013E-03	40195	1,21093E+00	24798	1,05388E+00	38887	9,33271E-01	13440	0	48381,5
Myeloid_vs_CD8_Responder	CD8(NME1+ T)	Macro_ISG15	HLA-B	LILRB1	5,22124E-03	33283,7	3,17967E-03	40204	1,21043E+00	24824	1,03969E+00	39566	9,33267E-01	13443	0	48381,5
Myeloid_vs_CD8_Responder	CD8(Terminal Tex)	pDC_LILRA4	CCL5	CXCR3	5,22729E-03	35341,7	5,21067E-03	19583	4,39363E-01	74999	1,66949E+00	17172	9,27846E-01	16573	0	48381,5
Myeloid_vs_CD8_Responder	CD8(Tc17)	Mono_INHBA	HLA-B	LILRB2	5,23164E-03	28926,5	3,17891E-03	40220	1,53844E+00	14859	1,18010E+00	33305	9,42882E-01	7867	0	48381,5
Myeloid_vs_CD8_Responder	Macro_FOLR2+APOE-	CD8(GZMK+ Tem)	HLA-DPA1	LAG3	5,23560E-03	18431,3	6,53068E-03	13823	1,36793E+00	19591	2,15443E+00	8329	9,55202E-01	2032	0	48381,5
Myeloid_vs_CD8_Responder	CD8(GZMK+ Early Tem)	Mono_CD16	TNFSF9	HLA-DPA1	5,23945E-03	34700,3	3,55260E-03	34283	8,89912E-01	40232	1,34139E+00	27020	9,15648E-01	23585	0	48381,5
Myeloid_vs_CD8_Responder	Macro_IFI27	CD8(Terminal Tex)	HLA-DPA1	LAG3	5,24080E-03	18251,9	6,39629E-03	14243	1,36781E+00	19596	2,27888E+00	6855	9,54755E-01	2184	0	48381,5
Myeloid_vs_CD8_Responder	CD8(GZMK+ Early Tem)	Mono_CD16	HLA-C	LILRA3	5,24913E-03	26008,3	1,65832E-02	3078	2,74319E+00	2214	7,30583E-01	56764	9,22541E-01	19604	0	48381,5
Myeloid_vs_CD8_Responder	CD8(GZMK+ Early Tem)	Mono_CD14	CCL4	CCR1	5,25053E-03	32813,3	6,10766E-03	15335	8,89602E-01	40249	1,39295E+00	25131	8,94964E-01	34970	0	48381,5
Myeloid_vs_CD8_Responder	CD8(GZMK+ Early Tem)	Mono_INHBA	HLA-C	LILRB2	5,25640E-03	29426,7	3,17670E-03	40258	1,49884E+00	15863	1,19671E+00	32646	9,39114E-01	9985	0	48381,5
Myeloid_vs_CD8_Responder	CD8(Temra)	Mast	IL16	CD9	5,26164E-03	29778,9	6,28420E-03	14669	1,64573E+00	12484	1,57189E+00	19616	8,62385E-01	53744	0	48381,5
Myeloid_vs_CD8_Responder	Macro_FOLR2-APOE+	CD8(LAYN+ T)	SPP1	ITGAV_ITGB1	5,26365E-03	21671,9	1,13057E-02	5748	1,95639E+00	7679	2,33945E+00	6210	8,85526E-01	40341	0	48381,5
Myeloid_vs_CD8_Responder	CD8(ID2+CXCR4+ T)	Mono_CD16	HLA-B	LILRA1	5,26565E-03	28566,1	9,86585E-03	7096	1,95620E+00	7680	6,87804E-01	59640	9,21806E-01	20033	0	48381,5
Myeloid_vs_CD8_Responder	CD8(Temra)	Macro_FOLR2-APOE+	CD99	CD81	5,27012E-03	35578,9	3,84426E-03	30602	9,69673E-01	35806	1,02390E+00	40279	9,16921E-01	22826	0	48381,5
Myeloid_vs_CD8_Responder	Macro_OLFML3	CD8(GZMK+ Tem)	CD14	ITGB2	5,27012E-03	25767,9	3,17516E-03	40279	1,80722E+00	9696	1,78408E+00	14581	9,29057E-01	15902	0	48381,5
Myeloid_vs_CD8_Responder	Macro_FOLR2+APOE+	CD8(NME1+ T)	HLA-DRA	LAG3	5,27104E-03	20257,5	5,29592E-03	19077	1,36710E+00	19625	1,99666E+00	10655	9,51148E-01	3549	0	48381,5
Myeloid_vs_CD8_Responder	cDC_CLEC9A	CD8(Terminal Tex)	HLA-DQA1	LAG3	5,27836E-03	17146,7	7,72254E-03	10497	1,36674E+00	19632	2,75465E+00	2940	9,49531E-01	4283	0	48381,5
Myeloid_vs_CD8_Responder	CD8(GZMK+ Tex)	pDC_LILRA4	HLA-DRB1	CD4	5,29301E-03	29093,3	2,50345E-03	56443	1,36637E+00	19646	2,40642E+00	5549	9,29884E-01	15447	0	48381,5
Myeloid_vs_CD8_Responder	CD8(GZMK+ Tem)	Macro_ISG15	HLA-A	LILRB2	5,29503E-03	26942,3	3,17344E-03	40317	1,56352E+00	14262	1,48046E+00	22274	9,39946E-01	9477	0	48381,5
Myeloid_vs_CD8_Responder	CD8(Tc17)	cDC_CLEC9A	TGFB1	LPP	5,29766E-03	31718,7	6,99959E-03	12330	1,01595E+00	33439	1,42362E+00	24122	8,85564E-01	40321	0	48381,5
Myeloid_vs_CD8_Responder	Mono_INHBA	CD8(Tc17)	S100A8	CD69	5,30350E-03	22826,9	5,95995E-03	15899	1,36596E+00	19656	1,73516E+00	15660	9,31394E-01	14538	0	48381,5
Myeloid_vs_CD8_Responder	CD8(ID2+CXCR4+ T)	Macro_NLRP3	HLA-B	LILRB2	5,31015E-03	28388,5	3,17222E-03	40340	1,61360E+00	13146	1,20861E+00	32183	9,42825E-01	7892	0	48381,5
Myeloid_vs_CD8_Responder	Macro_FOLR2+APOE+	CD8(Terminal Tex)	HLA-DPA1	LAG3	5,31085E-03	19180,9	6,38709E-03	14275	1,36571E+00	19663	1,95382E+00	11392	9,54724E-01	2193	0	48381,5

Myeloid_vs_CD8_Post_R

Myeloid_vs_CD8_Responder	CD8(LAYN+ T)	Mono_INHBA	HLA-B	LILRB2	5,32399E-03	29072,1	3,17095E-03	40361	1,52982E+00	15086	1,17221E+00	33632	9,42815E-01	7900	0	48381,5
Myeloid_vs_CD8_Responder	CD8(ID2+CXCR4+ T)	Mono_CD16	CD52	SIGLEC10	5,34314E-03	33012,1	4,30282E-03	26002	1,27490E+00	22508	1,02180E+00	40390	9,07998E-01	27779	0	48381,5
Myeloid_vs_CD8_Responder	CD8(LAYN+ T)	Mono_CD16	HLA-F	LILRB2	5,34645E-03	34040,7	3,31057E-03	37943	2,33825E+00	4098	1,04354E+00	39386	8,85435E-01	40395	0	48381,5
Myeloid_vs_CD8_Responder	CD8(Temra)	Mono_CD14	HLA-F	LILRB2	5,35572E-03	34136,9	3,30905E-03	37976	1,34977E+00	20137	1,43389E+00	23781	8,85412E-01	40409	0	48381,5
Myeloid_vs_CD8_Responder	Macro_ISG15	CD8(GZMK+ Tem)	CD14	ITGA4	5,35829E-03	20098,3	5,63549E-03	17310	1,93302E+00	7972	2,25499E+00	7120	9,22350E-01	19708	0	48381,5
Myeloid_vs_CD8_Responder	Macro_OLFML3	CD8(GZMK+ Tex)	C1QB	C1QBP	5,36501E-03	25479,9	3,16765E-03	40423	2,07579E+00	6323	2,69969E+00	3268	9,05792E-01	29004	0	48381,5
Myeloid_vs_CD8_Responder	CD8(NME1+ T)	Mono_CD14	GNAI2	C5AR1	5,39627E-03	26522,1	4,03384E-03	28555	2,08800E+00	6202	2,10578E+00	9002	8,85309E-01	40470	0	48381,5
Myeloid_vs_CD8_Responder	CD8(ITM2C+ T)	Mast	IL16	CD9	5,40761E-03	24076,7	9,52968E-03	7467	1,80652E+00	9706	1,79612E+00	14342	8,85282E-01	40487	0	48381,5
Myeloid_vs_CD8_Responder	CD8(GZMK+ Tex)	Mono_CD14	HLA-A	LILRB2	5,41455E-03	27249,9	3,06805E-03	42286	1,50268E+00	15757	1,56731E+00	19761	9,38986E-01	10064	0	48381,5
Myeloid_vs_CD8_Responder	CD8(Terminal Tex)	Mono_CD16	HLA-C	LILRB1	5,41496E-03	31768,9	3,41237E-03	36334	1,38870E+00	18996	1,01956E+00	40498	9,31231E-01	14635	0	48381,5
Myeloid_vs_CD8_Responder	Macro_OLFML3	CD8(EOMES+ NK-like)	C1QB	C1QBP	5,41563E-03	25541,9	3,16345E-03	40499	2,07545E+00	6328	2,67159E+00	3464	9,05735E-01	29037	0	48381,5
Myeloid_vs_CD8_Responder	CD8(Temra)	Macro_FOLR2+APOE+	ADAM10	GPNMB	5,42415E-03	26322,9	7,42824E-03	11179	2,96702E+00	1627	1,73198E+00	15722	8,60676E-01	54705	0	48381,5
Myeloid_vs_CD8_Responder	CD8(NME1+ T)	Macro_LYVE1	GNAI2	C5AR1	5,42835E-03	30125,5	4,02682E-03	28610	2,08457E+00	6234	1,34481E+00	26884	8,85220E-01	40518	0	48381,5
Myeloid_vs_CD8_Responder	CD8(LAYN+ T)	Macro_FOLR2-APOE+	ADAM10	GPNMB	5,42842E-03	31285,7	5,95374E-03	15928	1,79853E+00	9840	1,56666E+00	19774	8,46872E-01	62505	0	48381,5
Myeloid_vs_CD8_Responder	Macro_FOLR2+APOE-	CD8(ISG+ T)	HLA-DRA	LAG3	5,43590E-03	19319,9	5,55808E-03	17680	1,36134E+00	19781	2,20581E+00	7680	9,52259E-01	3077	0	48381,5
Myeloid_vs_CD8_Responder	CD8(GZMK+ Tex)	Mono_CD16	HLA-C	LILRB1	5,44176E-03	32400,9	3,34294E-03	37392	1,33221E+00	20680	1,01880E+00	40538	9,30570E-01	15013	0	48381,5
Myeloid_vs_CD8_Responder	Macro_NLRP3	CD8(Temra)	VCAN	CD44	5,47018E-03	20724,7	7,08661E-03	12093	2,39232E+00	3724	1,57196E+00	19612	9,22188E-01	19813	0	48381,5
Myeloid_vs_CD8_Responder	Macro_FOLR2-APOE+	CD8(Terminal Tex)	HLA-DRB5	LAG3	5,47233E-03	18356,9	7,06230E-03	12157	1,36009E+00	19815	2,23329E+00	7369	9,50014E-01	4062	0	48381,5
Myeloid_vs_CD8_Responder	Mono_INHBA	CD8(Tn)	SPP1	CD44	5,47540E-03	26343,5	4,85432E-03	21822	1,22654E+00	24203	2,18802E+00	7885	9,04985E-01	29426	0	48381,5
Myeloid_vs_CD8_Responder	Mono_INHBA	CD8(Tn)	VCAN	CD44	5,47945E-03	25124,1	5,39793E-03	18528	1,61650E+00	13093	1,56303E+00	19875	9,11844E-01	25743	0	48381,5
Myeloid_vs_CD8_Responder	CD8(GZMK+ Early Tem)	Macro_NLRP3	TNFSF9	HLA-DPA1	5,48147E-03	32668,5	3,53320E-03	34557	8,83847E-01	40597	1,71576E+00	16106	9,15436E-01	23701	0	48381,5
Myeloid_vs_CD8_Responder	Macro_ISG15	CD8(IL7R+ZNF683+ Tm)	SPP1	CD44	5,48200E-03	18880,1	7,51270E-03	10977	1,73714E+00	10815	2,53952E+00	4403	9,22174E-01	19824	0	48381,5
Myeloid_vs_CD8_Responder	Mono_INHBA	CD8(Tn)	HBEGF	CD44	5,48485E-03	28145,5	5,31945E-03	18944	1,57338E+00	14028	1,23892E+00	30987	9,06917E-01	28387	0	48381,5
Myeloid_vs_CD8_Responder	Mono_INHBA	CD8(Tn)	LGALS1	CD69	5,48620E-03	29399,3	3,41644E-03	36257	9,94527E-01	34548	1,53185E+00	20752	9,44245E-01	7058	0	48381,5
Myeloid_vs_CD8_Responder	Macro_OLFML3	CD8(ZNF683+KLRB1+ T)	GRN	TNFRSF1B	5,49566E-03	27420,5	3,15657E-03	40618	1,63638E+00	12671	1,60336E+00	18805	9,27754E-01	16627	0	48381,5
Myeloid_vs_CD8_Responder	CD8(GZMK+ Early Tem)	Macro_LYVE1	CCL4	CCR1	5,49769E-03	35704,5	6,17494E-03	15067	8,97478E-01	39768	1,01726E+00	40621	8,95478E-01	34685	0	48381,5
Myeloid_vs_CD8_Responder	CD8(Terminal Tex)	Macro_ISG15	HLA-A	LILRB2	5,49837E-03	27085,7	3,15621E-03	40622	1,54656E+00	14677	1,48352E+00	22182	9,39793E-01	9566	0	48381,5
Myeloid_vs_CD8_Responder	Macro_NLRP3	CD8(ISG+ T)	VCAN	CD44	5,50030E-03	20435,3	7,07945E-03	12112	2,39167E+00	3728	1,63152E+00	18114	9,22152E-01	19841	0	48381,5
Myeloid_vs_CD8_Responder	Mono_INHBA	CD8(Tn)	S100A8	CD69	5,50108E-03	25632,1	5,06899E-03	20432	1,24450E+00	23554	1,62623E+00	18249	9,26036E-01	17544	0	48381,5
Myeloid_vs_CD8_Responder	CD8(GZMK+ Tem)	Mono_CD16	HLA-B	LILRB1	5,50311E-03	31379,9	3,34581E-03	37347	1,41980E+00	18073	1,01718E+00	40629	9,34835E-01	12469	0	48381,5
Myeloid_vs_CD8_Responder	cDC(CD1C)	CD8(EOMES+ NK-like)	B2M	KIR3DL1	5,50379E-03	26630,9	2,04966E-02	2019	8,83400E-01	40630	1,44303E+00	23461	9,24132E-01	18663	0	48381,5
Myeloid_vs_CD8_Responder	Mono_INHBA	CD8(Tn)	VEGFA	CD44	5,50446E-03	23437,3	7,72037E-03	10502	2,15118E+00	5592	1,42734E+00	24020	9,06382E-01	28691	0	48381,5
Myeloid_vs_CD8_Responder	CD8(Tc17)	Mono_CD16	HLA-C	LILRA3	5,51433E-03	27455,9	1,63984E-02	3119	2,71446E+00	2313	6,31962E-01	63612	9,22139E-01	19854	0	48381,5
Myeloid_vs_CD8_Responder	CD8(ISG+ T)	Macro_NLRP3	HLA-B	LILRB2	5,51667E-03	28315,7	3,15473E-03	40649	1,59384E+00	13546	1,23757E+00	31030	9,42676E-01	7972	0	48381,5
Myeloid_vs_CD8_Responder	Macro_LYVE1	CD8(GZMK+ Tem)	HLA-DPB1	LAG3	5,51865E-03	19344,5	6,44576E-03	14078	1,35893E+00	19858	1,92718E+00	11844	9,53603E-01	2561	0	48381,5
Myeloid_vs_CD8_Responder	CD8(GZMK+ Early Tem)	cDC(CD1C)	ANXA1	FPR1	5,51938E-03	35129,3	3,15448E-03	40653	1,15896E+00	26868	1,29545E+00	28779	9,02167E-01	30965	0	48381,5
Myeloid_vs_CD8_Responder	cDC(CD1C)	CD8(GZMK+ Tem)	CXCL16	CXCR6	5,52685E-03	31405,7	5,34939E-03	18780	1,05839E+00	31388	1,64341E+00	17815	8,84954E-01	40664	0	48381,5
Myeloid_vs_CD8_Responder	CD8(Temra)	Macro_IFI27	CD99	PILRA	5,52753E-03	34842,5	3,63250E-03	33195	1,25407E+00	23209	1,01650E+00	40665	9,06256E-01	28762	0	48381,5
Myeloid_vs_CD8_Responder	CD8(ID2+CXCR4+ T)	cDC(CD1C)	CSF1	CSF2RA	5,53271E-03	39927,7	5,15838E-03	19871	1,80467E+00	9741	1,56763E+00	19750	7,71515E-01	101895	0	48381,5
Myeloid_vs_CD8_Responder	Macro_LYVE1	CD8(ISG+ T)	HLA-DRA	LAG3	5,53813E-03	19657,7	5,54579E-03	17749	1,35812E+00	19876	2,09302E+00	9180	9,52208E-01	3102	0	48381,5
Myeloid_vs_CD8_Responder	CD8(ID2+CXCR4+ T)	cDC(CD1C)	B2M	CD1A	5,53885E-03	26948,3	2,79314E-02	927	1,94624E+00	7814	1,01380E+00	40822	8,91735E-01	36797	0	48381,5
Myeloid_vs_CD8_Responder	CD8(Terminal Tex)	Macro_FOLR2-APOE+	HSPA1A	TLR4	5,54455E-03	35105,9	3,96872E-03	29256	1,25517E+00	23173	1,16282E+00	34029	8,84918E-01	40690	0	48381,5
Myeloid_vs_CD8_Responder	Mono_INHBA	CD8(ID2+CXCR4+ T)	SPP1	CD44	5,54455E-03	31082,3	3,93959E-03	29557	1,08533E+00	30153	1,88190E+00	12724	8,95621E-01	34596	0	48381,5
Myeloid_vs_CD8_Responder	Mono_INHBA	CD8(ID2+CXCR4+ T)	SPP1	PTGER4	5,54523E-03	24937,3	8,20805E-03	9489	1,33075E+00	20722	2,30481E+00	6601	8,87035E-01	39493	0	48381,5
Myeloid_vs_CD8_Responder	Macro_OLFML3	CD8(EOMES+ NK-like)	FN1	ITGA4_ITGB7	5,54727E-03	23131,9	1,19591E-02	5212	2,00361E+00	7121	1,80056E+00	14251	8,84914E-01	40694	0	48381,5
Myeloid_vs_CD8_Responder	Mono_INHBA	CD8(ID2+CXCR4+ T)	VCAN	CD44	5,54727E-03	30179,7	4,38077E-03	25308	1,47529E+00	16497	1,25692E+00	30240	9,03084E-01	30472	0	48381,5
Myeloid_vs_CD8_Responder	Macro_LYVE1	CD8(GZMK+ Tem)	CD14	ITGA4	5,55114E-03	21829,7	5,59353E-03	17501	1,91923E+00	8144	1,75336E+00	15234	9,22082E-01	19888	0	48381,5
Myeloid_vs_CD8_Responder	Mono_INHBA	CD8(Temra)	IL1B	SIGIRR	5,55337E-03	18403,3	9,24889E-03	7821	2,43448E+00	3478	2,88142E+00	2323	9,03888E-01	30013	0	48381,5
Myeloid_vs_CD8_Responder	Mono_INHBA	CD8(ID2+CXCR4+ T)	CXCL16	CXCR6	5,55409E-03	29671,1	7,40048E-03	11269	1,07418E+00	30689	1,36525E+00	26103	9,00472E-01	31913	0	48381,5
Myeloid_vs_CD8_Responder	Mono_INHBA	CD8(ID2+CXCR4+ T)	LGALS1	CD69	5,55545E-03	29518,9	3,54112E-03	34461	1,01975E+00	33245	1,39806E+00	24963	9,45181E-01	6544	0	48381,5
Myeloid_vs_CD8_Responder	Mono_INHBA	CD8(ID2+CXCR4+ T)	TIMP1	CD63	5,55614E-03	21178,3	3,16798E-03	40416	2,23505E+00	4902	3,00498E+00	1847	9,38526E-01	10345	0	48381,5
Myeloid_vs_CD8_Responder	CD8(NME1+ T)	Mono_CD14	CALR	LRP1	5,55955E-03	34927,3	3,15222E-03	40712	1,27249E+00	22570	1,27750E+00	29435	8,97539E-01	33538	0	48381,5
Myeloid_vs_CD8_Responder	cDC(CD1C)	CD8(Tc17)	CXCL16	CXCR6	5,56228E-03	31840,1	5,33958E-03	18832	1,05772E+00	31421	1,56386E+00	19850	8,84861E-01	40716	0	48381,5
Myeloid_vs_CD8_Responder	Mono_INHBA	CD8(ID2+CXCR4+ T)	S100A8	CD69	5,57117E-03	25838,3	5,25398E-03	19325	1,26972E+00	22656	1,49244E+00	21919	9,27254E-01	16910	0	48381,5
Myeloid_vs_CD8_Responder	Mono_INHBA	CD8(ID2+CXCR4+ T)	VEGFA	CD44	5,57254E-03	27955,9	6,26557E-03	14728	2,00998E+00	7052	1,12122E+00	35844	8,97140E-01	33774	0	48381,5
Myeloid_vs_CD8_Responder	CD8(LAYN+ T)	pDC_LILRA4	GZMB	IGF2R	5,57459E-03	27994,7	9,92438E-03	7038	8,81596E-01	40734	1,97141E+00	11074	8,98940E-01	32746	0	48381,5
Myeloid_vs_CD8_Responder	CD8(ITM2C+ T)	Macro_LYVE1	IFNG	IFNGR1_IFNGR2	5,57938E-03	35777,1	6,34574E-03	14436	9,03318E-01	39435	1,01506E+00	40741	8,93328E-01	35892	0	48381,5
Myeloid_vs_CD8_Responder	Mono_INHBA	CD8(Terminal Tex)	HLA-DRA	LAG3	5,58143E-03	19646,5	5,74447E-03	16843	1,28258E+00	22246	2,17913E+00	7988	9,53003E-01	2774	0	48381,5
Myeloid_vs_CD8_Responder	Mono_INHBA	CD8(Terminal Tex)	HLA-DRB1	LAG3	5,58349E-03	23077,3	5,12813E-03	20068	1,07108E+00	30815	1,89157E+00	12530	9,51051E-01	3592	0	48381,5
Myeloid_vs_CD8_Responder	Mono_INHBA	CD8(Terminal Tex)	HLA-DPB1	LAG3	5,58486E-03	26528,3	4,44967E-03	24729	9,36044E-01	37581	1,75877E+00	15121	9,44680E-01	6829	0	48381,5
Myeloid_vs_CD8_Responder	Mono_INHBA	CD8(Terminal Tex)	ICAM1	ITGAL_ITGB2	5,58829E-03	33010,7	3,64223E-03	33075	1,19131E+00	25538	1,57572E+00	19522	8,88771E-01	38537	0	48381,5
Myeloid_vs_CD8_Responder	Mono_INHBA	CD8(Terminal Tex)	ICAM1	IL2RG	5,58966E-03	22376,3	6,90492E-03	12594	1,43240E+00	17720	1,89075E+00	12546	9,20699E-01	20640	0	48381,5
Myeloid_vs_CD8_Responder	Mono_INHBA	CD8(Terminal Tex)	HLA-DPA1	LAG3	5,59721E-03	25944,1	4,51779E-03	24209	9,38549E-01	37455	1,81899E+00	13893	9,46623E-01	5782	0	48381,5
Myeloid_vs_CD8_Responder	CD8(GZMK+ Tex)	cDC(CD1C)	B2M	CD1B	5,60333E-03	33946,5	1,83492E-02	2546	1,94405E+00	7845	6,14792E-01	64815	8,75599E-01	46145	0	48381,5
Myeloid_vs_CD8_Responder	Mono_INHBA	CD8(Terminal Tex)	HLA-DRB5	LAG3	5,60545E-03	24510,9	5,14134E-03	19986	1,01528E+00	33477	1,90243E+00	12324	9,41915E-01	8386	0	48381,5
Myeloid_vs_CD8_Responder	Macro_OLFML3	CD8(LAYN+ T)	CD86	CTLA4	5,60682E-03	29605,9	1,07034E-02	6283	1,55199E+00	14543	1,01437E+00	40781	8,89556E-01	38041	0	48381,5
Myeloid_vs_CD8_Responder	CD8(ZNF683+KLRB1+ T)	Macro_ISG15	CD99	PILRA	5,60820E-03	37648,9	3,18586E-03	40067	1,15327E+00	27136	1,01433E+00	40783	9,00533E-01	31877	0	48381,5
Myeloid_vs_CD8_Responder	CD8(NME1+ T)	Macro_OLFML3	HMGB1	TLR4	5,61232E-03	35345,9	4,13878E-03	27520	1,32915E+00	20764	1,04598E+00	39275	8,84744E-01	40789	0	48381,5
Myeloid_vs_CD8_Responder	Mono_INHBA	CD8(Terminal Tex)	IL1B	SIGIRR	5,61301E-03	21832,9	6,50476E-03	13899	2,30369E+00	4332	2,69490E+00	3297	8,87475E-01	39255	0	48381,5
Myeloid_vs_CD8_Responder	Mono_INHBA	CD8(Terminal Tex)	S100A8	ITGB2	5,61370E-03	27703,5	3,37122E-03	36949	1,39328E+00	18850	1,89171E+00	12527	9,18653E-01	21810	0	48381,5
Myeloid_vs_CD8_Responder	Macro_FOLR2-APOE+	CD8(IL7R+ZNF683+ Tm)	SPP1	PTGER4	5,61378E-03	18424,5	1,39419E-02	4099	1,94382E+00	7850	2,40067E+00	5615	9,10983E-01	26177	0	48381,5
Myeloid_vs_CD8_Responder	Mono_INHBA	CD8(Terminal Tex)	S100A9	ITGB2	5,61508E-03	24066,1	3,47966E-03	35316	1,75737E+00	10487	2,05770E+00	9706	9,28083E-01	16440	0	48381,5
Myeloid_vs_CD8_Responder	pDC_LILRA4	CD8(ZNF683+KLRB1+ T)	APP	LRP10	5,61587E-03	26229,9	9,32384E-03	7718	1,94377E+00	7851	2,58336E+00	4082	8,45726E-01	63117	0	48381,5
Myeloid_vs_CD8_Responder	CD8(Terminal Tex)	Mono_INHBA	B2M	LILRB2	5,61783E-03	26068,9	3,14761E-03	40797	1,74580E+00	10672	1,38328E+00	25471	9,48061E-01	5023	0	48381,5
Myeloid_vs_CD8_Responder	CD8(IL7R+ZNF683+ Tm)	Mono_CD14	ANXA1	DYSF	5,61852E-03	27982,5	1,19898E-02	5190	1,68361E+00	11756	1,16842E+00	33787	8,84709E-01	40798	0	48381,5
Myeloid_vs_CD8_Responder	Mono_INHBA	CD8(Terminal Tex)	SPP1	CD44	5,61852E-03	35326,9	3,22974E-03	39308	9,75751E-01	35464	1,84476E+00	13383	8,85963E-01	40098	0	48381,5
Myeloid_vs_CD8_Responder	CD8(GZMK+ Tex)	Macro_ISG15	HLA-A	LILRB2	5,61852E-03	26904,7	3,14754E-03	40798	1,53803E+00	14871	1,52779E+00	20865	9,39715E-01	9608	0	48381,5

Myeloid_vs_CD8_Responder	Mono_INHBA	CD8(Terminal Tex)	VCAN	CD44	5,62265E-03	33804,1	3,59142E-03	33754	1,36571E+00	19662	1,21977E+00	31732	8,94034E-01	35491	0	48381,5
Myeloid_vs_CD8_Responder	pDC_LILRA4	CD8(EOMES+ NK-like)	APP	LRP10	5,62633E-03	26305,9	9,31824E-03	7729	1,94361E+00	7856	2,53581E+00	4428	8,45687E-01	63135	0	48381,5
Myeloid_vs_CD8_Responder	Mono_INHBA	CD8(Terminal Tex)	LGALS3	LAG3	5,62817E-03	19996,5	8,34430E-03	9219	1,28440E+00	22193	2,06611E+00	9595	9,38069E-01	10594	0	48381,5
Myeloid_vs_CD8_Responder	CD8(ZNF683+KLRB1+ T)	pDC_LILRA4	TNF	TNFRSF21	5,63052E-03	25205,7	1,72087E-02	2884	3,63304E+00	684	2,19022E+00	7858	8,40166E-01	66221	0	48381,5
Myeloid_vs_CD8_Responder	Mono_INHBA	CD8(Terminal Tex)	LGALS1	CD69	5,63437E-03	28384,5	3,48745E-03	35201	1,00889E+00	33817	1,64566E+00	17754	9,44784E-01	6769	0	48381,5
Myeloid_vs_CD8_Responder	Mono_INHBA	CD8(Terminal Tex)	TIMP1	CD63	5,63644E-03	20430,5	3,31498E-03	37869	2,26144E+00	4670	3,05275E+00	1677	9,39822E-01	9555	0	48381,5
Myeloid_vs_CD8_Responder	CD8(Temra)	Mono_INHBA	HLA-B	LILRB2	5,63990E-03	29172,9	3,14560E-03	40829	1,50233E+00	15771	1,19150E+00	32856	9,42598E-01	8027	0	48381,5
Myeloid_vs_CD8_Responder	CD8(EOMES+ NK-like)	cDC_CLEC9A	TGFB1	LPP	5,64335E-03	31706,9	6,87105E-03	12690	1,00494E+00	34015	1,46945E+00	22614	8,84621E-01	40834	0	48381,5
Myeloid_vs_CD8_Responder	Mono_INHBA	CD8(Terminal Tex)	LYZ	ITGAL	5,64473E-03	24228,7	4,20887E-03	26855	1,73427E+00	10861	2,33211E+00	6305	9,06294E-01	28741	0	48381,5
Myeloid_vs_CD8_Responder	CD8(NME1+ T)	Mono_INHBA	HLA-B	LILRB2	5,64473E-03	29290,9	3,14514E-03	40836	1,50184E+00	15788	1,17730E+00	33419	9,42594E-01	8030	0	48381,5
Myeloid_vs_CD8_Responder	Mono_INHBA	CD8(Terminal Tex)	S100A8	CD69	5,64957E-03	24783,7	5,17435E-03	19774	1,25886E+00	23042	1,74004E+00	15548	9,26738E-01	17173	0	48381,5
Myeloid_vs_CD8_Responder	Mono_INHBA	CD8(Terminal Tex)	VEGFA	CD44	5,65303E-03	30689,9	5,13661E-03	20015	1,90040E+00	8399	1,08408E+00	37472	8,87605E-01	39182	0	48381,5
Myeloid_vs_CD8_Responder	Mono_INHBA	CD8(GZMK+ Tex)	HLA-DRA	LAG3	5,66342E-03	25511,7	3,99338E-03	28970	1,03120E+00	32665	2,01036E+00	10435	9,44156E-01	7107	0	48381,5
Myeloid_vs_CD8_Responder	Mono_INHBA	CD8(GZMK+ Tem)	PTGS2	CAV1	5,66619E-03	30464,3	4,81584E-02	323	2,31774E+00	4235	2,18912E+00	7875	7,92583E-01	91507	0	48381,5
Myeloid_vs_CD8_Responder	CD8(ITM2C+ T)	Mono_CD14	CIRBP	TREM1	5,66827E-03	29934,7	6,48502E-03	13963	1,70018E+00	11476	1,14189E+00	34983	8,84550E-01	40870	0	48381,5
Myeloid_vs_CD8_Responder	Macro_ISG15	CD8(GZMK+ Tem)	B2M	CD3D	5,66940E-03	48937,3	2,64658E-03	52183	4,00756E-01	78786	6,08452E-01	65300	9,65216E-01	36	0	48381,5
Myeloid_vs_CD8_Responder	Mono_INHBA	CD8(GZMK+ Tex)	ICAM1	IL2RG	5,67105E-03	27866,3	4,95099E-03	21176	1,17999E+00	25983	1,72783E+00	15821	9,07674E-01	27970	0	48381,5
Myeloid_vs_CD8_Responder	CD8(ISG+ T)	Macro_IER3	HLA-F	LILRB2	5,67174E-03	38494,1	3,25284E-03	38922	1,23872E+00	23766	1,01897E+00	40526	8,84540E-01	40875	0	48381,5
Myeloid_vs_CD8_Responder	Mono_INHBA	CD8(Terminal Tex)	HBEGF	CD82	5,67521E-03	23686,9	1,22984E-02	4998	1,89312E+00	8491	1,73395E+00	15684	8,84524E-01	40880	0	48381,5
Myeloid_vs_CD8_Responder	Macro_ISG15	CD8(Tc17)	SPP1	CD44	5,67589E-03	19036,3	7,44615E-03	11122	1,72918E+00	10948	2,50177E+00	4728	9,21854E-01	20002	0	48381,5
Myeloid_vs_CD8_Responder	CD8(GZMK+ Tem)	Macro_NLRP3	CIRBP	TREM1	5,67660E-03	28498,3	7,94980E-03	10004	1,92934E+00	8019	1,01243E+00	40882	8,94548E-01	35205	0	48381,5
Myeloid_vs_CD8_Responder	CD8(NME1+ T)	Macro_FOLR2-APOE+	HMGB1	CD163	5,67660E-03	29079,5	3,14244E-03	40882	1,44726E+00	17284	1,58423E+00	19320	9,22687E-01	19530	0	48381,5
Myeloid_vs_CD8_Responder	CD8(NME1+ T)	Macro_IER3	HMGB1	TLR4	5,67868E-03	33298,1	4,12061E-03	27689	1,32554E+00	20866	1,29835E+00	28669	8,84519E-01	40885	0	48381,5
Myeloid_vs_CD8_Responder	CD8(GZMK+ Tem)	Macro_NLRP3	HLA-C	LILRB2	5,67868E-03	29564,5	3,14207E-03	40885	1,51591E+00	15445	1,18947E+00	32951	9,38800E-01	10160	0	48381,5
Myeloid_vs_CD8_Responder	pDC_LILRA4	CD8(Tc17)	APP	LRP10	5,68514E-03	26412,5	9,23562E-03	7840	1,94114E+00	7884	2,52687E+00	4498	8,45105E-01	63459	0	48381,5
Myeloid_vs_CD8_Responder	CD8(Tc17)	Mono_INHBA	HLA-C	LILRB2	5,68771E-03	30581,7	3,14130E-03	40898	1,47011E+00	16633	1,09809E+00	36831	9,38793E-01	10165	0	48381,5
Myeloid_vs_CD8_Responder	cDC_CLEC9A	CD8(ISG+ T)	HLA-DQA2	LAG3	5,68804E-03	19266,3	9,12866E-03	7993	1,35383E+00	20013	2,61010E+00	3879	9,28767E-01	16065	0	48381,5
Myeloid_vs_CD8_Responder	CD8(ISG+ T)	cDC(CD1C)	CSF1	CSF2RA	5,69025E-03	39336,1	5,38482E-03	18606	1,81238E+00	9621	1,55856E+00	20015	7,75279E-01	100057	0	48381,5
Myeloid_vs_CD8_Responder	Macro_OLFML3	CD8(EOMES+ NK-like)	LGALS3BP	ITGB1	5,69050E-03	30678,7	6,70596E-03	13212	1,68609E+00	11707	1,01198E+00	40902	8,87580E-01	39191	0	48381,5
Myeloid_vs_CD8_Responder	pDC_LILRA4	CD8(LAYN+ T)	COL24A1	ITGA1_ITGB1	5,69592E-03	43110,1	6,54464E-02	167	2,92936E+00	1712	8,27951E-01	50600	7,42581E-01	114690	0	48381,5
Myeloid_vs_CD8_Responder	Macro_LYVE1	CD8(Terminal Tex)	HLA-DPA1	LAG3	5,69799E-03	19128,7	6,33355E-03	14478	1,35347E+00	20022	2,00548E+00	10505	9,54541E-01	2257	0	48381,5
Myeloid_vs_CD8_Responder	Mono_INHBA	CD8(GZMK+ Tex)	SPP1	CD44	5,70024E-03	30028,9	4,04511E-03	28426	1,10162E+00	29408	2,03761E+00	9985	8,96850E-01	33944	0	48381,5
Myeloid_vs_CD8_Responder	CD8(Terminal Tex)	Mono_CD16	B2M	LILRB1	5,70024E-03	31461,9	3,14065E-03	40916	1,43819E+00	17557	1,01900E+00	40523	9,39192E-01	9932	0	48381,5
Myeloid_vs_CD8_Responder	Mono_INHBA	CD8(GZMK+ Tex)	VCAN	ITGB1	5,70233E-03	24897,3	6,88807E-03	12639	1,69602E+00	11540	1,87535E+00	12852	8,87794E-01	39074	0	48381,5
Myeloid_vs_CD8_Responder	Mono_INHBA	CD8(GZMK+ Tex)	VCAN	CD44	5,70373E-03	28622,1	4,49810E-03	24364	1,49158E+00	16055	1,41263E+00	24466	9,04234E-01	29844	0	48381,5
Myeloid_vs_CD8_Responder	Mono_INHBA	CD8(GZMK+ Tex)	LGALS3	LAG3	5,70930E-03	25432,3	5,80071E-03	16571	1,03302E+00	32579	1,89735E+00	12409	9,26627E-01	17221	0	48381,5
Myeloid_vs_CD8_Responder	Mono_INHBA	CD8(GZMK+ Tex)	HBEGF	CD44	5,71279E-03	32102,1	4,43270E-03	24860	1,44847E+00	17245	1,08852E+00	37267	8,98929E-01	32757	0	48381,5
Myeloid_vs_CD8_Responder	Mono_INHBA	CD8(GZMK+ Tex)	CXCL16	CXCR6	5,71349E-03	32684,3	5,88914E-03	16190	9,40570E-01	37344	1,43961E+00	23584	8,89758E-01	37922	0	48381,5
Myeloid_vs_CD8_Responder	CD8(LAYN+ T)	Macro_OLFML3	TNFSF9	HLA-DPA1	5,71558E-03	29360,3	3,13942E-03	40938	1,28683E+00	22121	2,10034E+00	9063	9,10748E-01	26298	0	48381,5
Myeloid_vs_CD8_Responder	CD8(Tn)	Macro_OLFML3	CD40LG	CD53	5,72227E-03	38028,3	2,18376E-02	1716	8,20166E-01	44530	8,84026E-01	47445	8,72367E-01	48069	0	48381,5
Myeloid_vs_CD8_Responder	CD8(NME1+ T)	Macro_FOLR2+APOE+	CD99	CD81	5,72676E-03	30406,7	4,95513E-03	21141	1,23077E+00	24044	1,01110E+00	40954	9,26092E-01	17513	0	48381,5
Myeloid_vs_CD8_Responder	CD8(Tn)	Macro_FOLR2+APOE+	CD40LG	CD53	5,72887E-03	39269,9	2,18315E-02	1717	8,19922E-01	44539	7,79484E-01	53630	8,72352E-01	48082	0	48381,5
Myeloid_vs_CD8_Responder	cDC_CLEC9A	CD8(NME1+ T)	HLA-DRA	LAG3	5,72903E-03	18801,1	5,24592E-03	19370	1,35272E+00	20050	2,82278E+00	2556	9,50927E-01	3648	0	48381,5
Myeloid_vs_CD8_Responder	Mono_INHBA	CD8(GZMK+ Tex)	S100A8	CD69	5,73026E-03	28992,3	4,36013E-03	25507	1,14786E+00	27375	1,45617E+00	23063	9,20709E-01	20635	0	48381,5
Myeloid_vs_CD8_Responder	Mono_INHBA	CD8(GZMK+ Tex)	VEGFA	CD44	5,73166E-03	26381,9	6,43339E-03	14128	2,02626E+00	6872	1,27694E+00	29462	8,98354E-01	33066	0	48381,5
Myeloid_vs_CD8_Responder	Macro_FOLR2-APOE+	CD8(Temra)	C3	IFITM1	5,73903E-03	22813,5	7,38815E-03	11309	1,37083E+00	19516	1,55684E+00	20059	9,30938E-01	14802	0	48381,5
Myeloid_vs_CD8_Responder	CD8(ISG+ T)	Mono_INHBA	HMGB1	CD163	5,75268E-03	30598,3	3,13665E-03	40991	1,42673E+00	17883	1,36309E+00	26168	9,22622E-01	19568	0	48381,5
Myeloid_vs_CD8_Responder	Mono_INHBA	CD8(Tc17)	SPP1	CD44	5,77306E-03	23473,7	5,76908E-03	16715	1,36775E+00	19601	2,25600E+00	7104	9,12153E-01	25567	0	48381,5
Myeloid_vs_CD8_Responder	Mono_INHBA	CD8(Tc17)	SPP1	PTGER4	5,77376E-03	25434,9	7,98053E-03	9924	1,31517E+00	21190	2,23113E+00	7397	8,85619E-01	40282	0	48381,5
Myeloid_vs_CD8_Responder	Mono_INHBA	CD8(Tc17)	VCAN	CD44	5,77587E-03	22608,9	6,41512E-03	14181	1,75771E+00	10484	1,63101E+00	18127	9,18541E-01	21871	0	48381,5
Myeloid_vs_CD8_Responder	Macro_OLFML3	CD8(GZMK+ Early Tem)	HLA-DQB1	LAG3	5,77939E-03	31406,9	3,13466E-03	41029	1,13038E+00	28142	1,57436E+00	19557	9,22021E-01	19925	0	48381,5
Myeloid_vs_CD8_Responder	CD8(GZMK+ Early Tem)	Macro_NLRP3	ANXA1	FPR1	5,78150E-03	30557,9	4,28909E-03	26131	1,60835E+00	13242	1,00954E+00	41032	9,14913E-01	24003	0	48381,5
Myeloid_vs_CD8_Responder	Mono_INHBA	CD8(Tc17)	HBEGF	CD44	5,78221E-03	25398,5	6,32185E-03	14525	1,71459E+00	11201	1,30690E+00	28355	9,13952E-01	24530	0	48381,5
Myeloid_vs_CD8_Responder	CD8(ISG+ T)	Macro_NLRP3	HLA-C	LILRB2	5,78221E-03	29727,3	3,13443E-03	41033	1,50945E+00	15595	1,17730E+00	33418	9,38730E-01	10209	0	48381,5
Myeloid_vs_CD8_Responder	Mono_INHBA	CD8(Tc17)	LGALS1	CD69	5,78573E-03	25725,3	4,01693E-03	28717	1,11599E+00	28783	1,64078E+00	17871	9,48357E-01	4874	0	48381,5
Myeloid_vs_CD8_Responder	Macro_FOLR2+APOE+	CD8(ISG+ T)	HLA-DQA1	LAG3	5,79372E-03	19776,3	6,93822E-03	12512	1,35070E+00	20108	1,90639E+00	12251	9,46902E-01	5629	0	48381,5
Myeloid_vs_CD8_Responder	CD8(Temra)	Macro_FOLR2+APOE+	CD52	SIGLEC10	5,79484E-03	27651,7	6,11357E-03	15312	1,86188E+00	8906	9,18668E-01	45550	9,21655E-01	20109	0	48381,5
Myeloid_vs_CD8_Responder	CD8(Temra)	cDC_CLEC9A	TGFB1	LPP	5,79631E-03	31419,9	6,81492E-03	12862	1,00014E+00	34259	1,53835E+00	20544	8,84202E-01	41053	0	48381,5
Myeloid_vs_CD8_Responder	CD8(ISG+ T)	Macro_FOLR2-APOE+	HLA-F	LILRB2	5,79843E-03	38356,5	3,28501E-03	38365	1,25031E+00	23357	1,00914E+00	41056	8,85042E-01	40623	0	48381,5
Myeloid_vs_CD8_Responder	Mono_INHBA	CD8(Tc17)	VEGFA	CD44	5,80762E-03	21473,7	9,17520E-03	7927	2,29239E+00	4430	1,49532E+00	21831	9,13455E-01	24799	0	48381,5
Myeloid_vs_CD8_Responder	Macro_FOLR2-APOE+	CD8(GZMK+ Tem)	HLA-DRB1	LAG3	5,81279E-03	18563,7	6,46263E-03	14032	1,35017E+00	20125	2,13907E+00	8549	9,56163E-01	1731	0	48381,5
Myeloid_vs_CD8_Responder	CD8(ISG+ T)	Mono_CD16	HLA-B	LILRA1	5,82177E-03	28255,3	9,81145E-03	7160	1,93644E+00	7939	7,16766E-01	57663	9,21607E-01	20133	0	48381,5
Myeloid_vs_CD8_Responder	cDC(CD1C)	CD8(Terminal Tex)	B2M	CD3D	5,82681E-03	48314,1	2,64497E-03	52224	1,92704E-01	102246	1,05799E+00	38682	9,65205E-01	37	0	48381,5
Myeloid_vs_CD8_Responder	CD8(IL7R+ZNF683+ Tm)	Mono_CD16	HLA-C	LILRA3	5,83077E-03	27582,1	1,61555E-02	3212	2,67668E+00	2439	6,30215E-01	63737	9,21602E-01	20141	0	48381,5
Myeloid_vs_CD8_Responder	Macro_FOLR2+APOE+	CD8(LAYN+ T)	GRN	TNFRSF1B	5,83527E-03	18492,1	5,11439E-03	20145	2,14641E+00	5638	2,02795E+00	10136	9,42350E-01	8160	0	48381,5
Myeloid_vs_CD8_Responder	CD8(NME1+ T)	Macro_NLRP3	CALR	LRP1	5,83738E-03	34415,1	3,55547E-03	34256	1,43019E+00	17787	1,00801E+00	41111	9,02943E-01	30540	0	48381,5
Myeloid_vs_CD8_Responder	CD8(Terminal Tex)	Macro_NLRP3	HLA-B	LILRB2	5,83880E-03	28954,5	3,12991E-03	41113	1,56581E+00	14209	1,18915E+00	32961	9,42463E-01	8108	0	48381,5
Myeloid_vs_CD8_Responder	Mono_INHBA	CD8(IL7R+ZNF683+ Tm)	IL1B	SIGIRR	5,84519E-03	22310,3	6,24730E-03	14798	2,29142E+00	4435	2,65985E+00	3547	8,85442E-01	40390	0	48381,5
Myeloid_vs_CD8_Responder	Macro_FOLR2-APOE+	CD8(ISG+ T)	HLA-DRA	LAG3	5,84541E-03	19414,5	5,51111E-03	17911	1,34901E+00	20154	2,22384E+00	7470	9,52065E-01	3156	0	48381,5
Myeloid_vs_CD8_Responder	Mono_INHBA	CD8(IL7R+ZNF683+ Tm)	SPP1	CD44	5,85088E-03	23262,1	5,82064E-03	16480	1,37571E+00	19385	2,29374E+00	6713	9,12509E-01	25351	0	48381,5
Myeloid_vs_CD8_Responder	Mono_INHBA	CD8(IL7R+ZNF683+ Tm)	SPP1	PTGER4	5,85159E-03	22288,3	9,99719E-03	6965	1,45323E+00	17105	2,48221E+00	4888	8,96544E-01	34102	0	48381,5
Myeloid_vs_CD8_Responder	CD8(Terminal Tex)	pDC_LILRA4	HLA-DRA	CD4	5,85331E-03	33567,7	2,06167E-03	71352	1,34872E+00	20161	2,16286E+00	8201	9,22285E-01	19743	0	48381,5
Myeloid_vs_CD8_Responder	Mono_INHBA	CD8(IL7R+ZNF683+ Tm)	VCAN	CD44	5,85372E-03	22321,9	6,47246E-03	14008	1,76567E+00	10349	1,66876E+00	17196	9,18873E-01	21675	0	48381,5
Myeloid_vs_CD8_Responder	CD8(ISG+ T)	Macro_ISG15	B2M	LILRB1	5,85515E-03	30374,5	3,12877E-03	41136	1,36731E+00	19620	1,19460E+00	32725	9,39083E-01	10010	0	48381,5
Myeloid_vs_CD8_Responder	CD8(ZNF683+KLRB1+ T)	cDC(CD1C)	B2M	CD1A	5,85728E-03	27103,3	2,77919E-02	940	1,92136E+00	8113	1,00750E+00	41139	8,91493E-01	36943	0	48381,5
Myeloid_vs_CD8_Responder	Mono_INHBA	CD8(IL7R+ZNF683+ Tm)	HBEGF	CD44	5,85799E-03	24997,9	6,37836E-03	14297	1,72255E+00	11065	1,34465E+00	26891	9,14302E-01	24355	0	48381,5
Myeloid_vs_CD8_Responder	Mono_INHBA	CD8(IL7R+ZNF683+ Tm)	LGALS1	CD69	5,86084E-03	27849,9	3,71379E-03	32144	1,05467E+00	31559	1,51409E+00	21270	9,46402E-01	5895	0	48381,5

Myeloid_vs_CD8_Post_R

Myeloid_vs_CD8_Responder	pDC_LILRA4	CD8(ID2+CXCR4+ T)	COL24A1	ITGA1_ITGB1	5,86163E-03	43935,7	5,80473E-02	214	2,91665E+00	1737	8,38925E-01	49971	7,30948E-01	119375	0	48381,5
Myeloid_vs_CD8_Responder	CD8(Temra)	Mono_CD16	HLA-C	LILRB1	5,86512E-03	32216,9	3,37622E-03	36876	1,35929E+00	19847	1,00721E+00	41150	9,30890E-01	14830	0	48381,5
Myeloid_vs_CD8_Responder	Macro_IFI27	CD8(Terminal Tex)	C3	IFITM1	5,86573E-03	22868,5	7,27330E-03	11609	1,38581E+00	19087	1,55306E+00	20172	9,30433E-01	15093	0	48381,5
Myeloid_vs_CD8_Responder	CD8(ITM2C+ T)	Macro_ISG15	HLA-F	LILRB2	5,86726E-03	35542,9	3,22131E-03	39453	1,35619E+00	19932	1,29492E+00	28795	8,84042E-01	41153	0	48381,5
Myeloid_vs_CD8_Responder	Macro_LYVE1	CD8(Terminal Tex)	HLA-DRB1	LAG3	5,86799E-03	19054,5	6,32078E-03	14529	1,34826E+00	20174	2,01752E+00	10309	9,55695E-01	1879	0	48381,5
Myeloid_vs_CD8_Responder	CD8(LAYN+ T)	Macro_OLFML3	ANXA2	TLR2	5,87153E-03	31741,5	4,27303E-03	26265	1,75808E+00	10477	1,20215E+00	32425	8,84033E-01	41159	0	48381,5
Myeloid_vs_CD8_Responder	CD8(Tn)	Mono_CD14	ANXA1	DYSF	5,87581E-03	25572,9	1,18310E-02	5317	1,67136E+00	11968	1,52207E+00	21033	8,84027E-01	41165	0	48381,5
Myeloid_vs_CD8_Responder	Mono_INHBA	CD8(IL7R+ZNF683+ Tm)	S100A8	CD69	5,87653E-03	24501,9	5,51018E-03	17917	1,30464E+00	21526	1,60847E+00	18666	9,28844E-01	16019	0	48381,5
Myeloid_vs_CD8_Responder	Mono_CD14	CD8(ZNF683+KLRB1+ T)	VCAN	ITGA4	5,87689E-03	19538,3	9,14338E-03	7974	3,12606E+00	1318	2,37576E+00	5857	8,96432E-01	34161	0	48381,5
Myeloid_vs_CD8_Responder	Macro_ISG15	CD8(GZMK+ Early Tem)	SPP1	CD44	5,87817E-03	19030,9	7,37992E-03	11334	1,72126E+00	11084	2,57169E+00	4172	9,21531E-01	20183	0	48381,5
Myeloid_vs_CD8_Responder	Mono_INHBA	CD8(IL7R+ZNF683+ Tm)	VEGFA	CD44	5,88010E-03	21175,1	9,25721E-03	7808	2,30035E+00	4375	1,53307E+00	20707	9,13806E-01	24604	0	48381,5
Myeloid_vs_CD8_Responder	Mono_CD14	CD8(NME1+ T)	VCAN	CD44	5,88950E-03	18444,1	6,95481E-03	12463	2,97871E+00	1601	2,06712E+00	9582	9,21512E-01	20193	0	48381,5
Myeloid_vs_CD8_Responder	Mono_INHBA	CD8(ISG+ T)	HLA-DRA	LAG3	5,89010E-03	22984,1	4,67152E-03	23052	1,12855E+00	28222	2,01905E+00	10285	9,48150E-01	4980	0	48381,5
Myeloid_vs_CD8_Responder	Mono_INHBA	CD8(ISG+ T)	HLA-DRB1	LAG3	5,89225E-03	27213,5	4,17031E-03	27215	9,17059E-01	38631	1,73150E+00	15733	9,46008E-01	6107	0	48381,5
Myeloid_vs_CD8_Responder	CD8(GZMK+ Tex)	Macro_NLRP3	HMGB1	TLR2	5,89582E-03	33772,9	4,02193E-03	28655	1,30669E+00	21459	1,00616E+00	41193	9,05482E-01	29176	0	48381,5
Myeloid_vs_CD8_Responder	Mono_INHBA	CD8(ISG+ T)	ICAM1	IL2RG	5,89726E-03	25330,5	5,91232E-03	16097	1,30418E+00	21547	1,69536E+00	16583	9,14845E-01	24044	0	48381,5
Myeloid_vs_CD8_Responder	CD8(ISG+ T)	Mono_CD14	HMGB1	THBD	5,89726E-03	37675,5	3,67844E-03	32620	1,09014E+00	29925	1,11154E+00	36256	8,83971E-01	41195	0	48381,5
Myeloid_vs_CD8_Responder	CD8(ITM2C+ T)	Macro_NLRP3	HLA-C	LILRB2	5,89797E-03	30252,5	3,12637E-03	41196	1,50263E+00	15759	1,12552E+00	35676	9,38656E-01	10250	0	48381,5
Myeloid_vs_CD8_Responder	CD8(GZMK+ Tex)	Macro_NLRP3	CALR	LRP1	5,90084E-03	36784,1	3,16616E-03	40454	1,33855E+00	20475	1,00607E+00	41200	8,97741E-01	33410	0	48381,5
Myeloid_vs_CD8_Responder	CD8(ID2+CXCR4+ T)	cDC_CLEC9A	ANXA1	DYSF	5,90370E-03	29162,5	1,18102E-02	5332	1,78570E+00	10027	1,01286E+00	40868	8,83937E-01	41204	0	48381,5
Myeloid_vs_CD8_Responder	CD8(GZMK+ Tex)	Macro_OLFML3	IFNG	IFNGR1_IFNGR2	5,90370E-03	32755,5	6,55071E-03	13755	1,19554E+00	25380	1,00604E+00	41204	8,94833E-01	35057	0	48381,5
Myeloid_vs_CD8_Responder	CD8(IL7R+ZNF683+ Tm)	cDC_CLEC9A	TGFB1	LPP	5,91087E-03	32090,9	6,77801E-03	12971	9,96978E-01	34422	1,44290E+00	23466	8,83924E-01	41214	0	48381,5
Myeloid_vs_CD8_Responder	Mono_INHBA	CD8(ISG+ T)	IL1B	SIGIRR	5,91589E-03	19579,1	8,05784E-03	9768	2,37771E+00	3819	2,82999E+00	2508	8,97731E-01	33419	0	48381,5
Myeloid_vs_CD8_Responder	Macro_NLRP3	CD8(GZMK+ Tex)	VCAN	CD44	5,91903E-03	20592,5	6,94945E-03	12476	2,37999E+00	3802	1,63268E+00	18084	9,21484E-01	20219	0	48381,5
Myeloid_vs_CD8_Responder	cDC_CLEC9A	CD8(GZMK+ Tex)	HLA-DQB1	LAG3	5,92017E-03	18903,5	6,09622E-03	15374	1,34705E+00	20220	2,80777E+00	2648	9,42822E-01	7894	0	48381,5
Myeloid_vs_CD8_Responder	CD8(GZMK+ Tex)	Macro_NLRP3	CIRBP	TREM1	5,92020E-03	29636,5	7,21833E-03	11748	1,85513E+00	9006	1,00563E+00	41227	8,89908E-01	37820	0	48381,5
Myeloid_vs_CD8_Responder	CD8(NME1+ T)	cDC(CD1C)	CD99	PILRA	5,92020E-03	35288,5	3,12458E-03	41227	1,09669E+00	29636	1,40186E+00	24841	8,99660E-01	32357	0	48381,5
Myeloid_vs_CD8_Responder	Mono_INHBA	CD8(ISG+ T)	SPP1	CD44	5,92235E-03	29683,3	4,12078E-03	27686	1,11330E+00	28901	2,03645E+00	10002	8,97705E-01	33446	0	48381,5
Myeloid_vs_CD8_Responder	CD8(EOMES+ NK-like)	Mono_INHBA	HLA-C	LILRB2	5,92594E-03	30430,9	3,12408E-03	41235	1,45612E+00	17018	1,13580E+00	35250	9,38635E-01	10270	0	48381,5
Myeloid_vs_CD8_Responder	Mono_INHBA	CD8(ISG+ T)	VCAN	CD44	5,92738E-03	28354,3	4,58224E-03	23741	1,50326E+00	15745	1,41147E+00	24508	9,05034E-01	29396	0	48381,5
Myeloid_vs_CD8_Responder	CD8(GZMK+ Early Tem)	Macro_ISG15	ANXA1	FPR1	5,92810E-03	32708,3	3,87319E-03	30268	1,44363E+00	17410	1,00528E+00	41238	9,10859E-01	26244	0	48381,5
Myeloid_vs_CD8_Responder	Macro_LYVE1	CD8(NME1+ T)	C1QB	C1QBP	5,92814E-03	20239,5	5,10185E-03	20227	2,01080E+00	7041	2,27160E+00	6942	9,24255E-01	18606	0	48381,5
Myeloid_vs_CD8_Responder	CD8(EOMES+ NK-like)	Mono_INHBA	HLA-A	LILRB2	5,93169E-03	31429,1	3,12389E-03	41243	1,37930E+00	19267	1,06137E+00	38508	9,39501E-01	9746	0	48381,5
Myeloid_vs_CD8_Responder	Mono_INHBA	CD8(ISG+ T)	LGALS3	LAG3	5,93241E-03	23210,5	6,78576E-03	12946	1,13037E+00	28143	1,90604E+00	12261	9,31784E-01	14321	0	48381,5
Myeloid_vs_CD8_Responder	Mono_INHBA	CD8(Terminal Tex)	INHBA	ACTR2	5,93522E-03	22267,9	1,25570E-02	4834	3,13705E+00	1292	2,17802E+00	8001	8,71016E-01	48831	0	48381,5
Myeloid_vs_CD8_Responder	CD8(ISG+ T)	pDC_LILRA4	HSP90B1	TLR9	5,93528E-03	31197,9	2,16435E-02	1748	3,62896E+00	689	1,08584E+00	37393	8,37427E-01	67778	0	48381,5
Myeloid_vs_CD8_Responder	Mono_INHBA	CD8(ISG+ T)	HBEGF	CD44	5,93745E-03	31827,9	4,51562E-03	24233	1,46015E+00	16904	1,08736E+00	37328	8,99768E-01	32293	0	48381,5
Myeloid_vs_CD8_Responder	Mono_INHBA	CD8(ISG+ T)	LGALS1	CD69	5,93961E-03	27572,1	3,58614E-03	33818	1,02885E+00	32783	1,69855E+00	16510	9,45508E-01	6368	0	48381,5
Myeloid_vs_CD8_Responder	Macro_IFI27	CD8(NME1+ T)	HLA-DRA	LAG3	5,94752E-03	19904,3	5,22434E-03	19496	1,34651E+00	20244	2,20242E+00	7715	9,50831E-01	3685	0	48381,5
Myeloid_vs_CD8_Responder	Mono_INHBA	CD8(ISG+ T)	LYZ	ITGAL	5,94969E-03	26669,5	3,60048E-03	33630	1,67847E+00	11857	2,26713E+00	7004	8,99450E-01	32475	0	48381,5
Myeloid_vs_CD8_Responder	CD8(GZMK+ Tex)	Macro_ISG15	HLA-B	LILRB1	5,94969E-03	33953,5	3,15410E-03	40665	1,18301E+00	25861	1,00477E+00	41268	9,33015E-01	13592	0	48381,5
Myeloid_vs_CD8_Responder	Mono_INHBA	CD8(ISG+ T)	S100A8	CD69	5,95474E-03	24159,3	5,32077E-03	18938	1,27882E+00	22388	1,79293E+00	14405	9,27679E-01	16684	0	48381,5
Myeloid_vs_CD8_Responder	Mono_INHBA	CD8(ISG+ T)	VEGFA	CD44	5,95979E-03	26193,3	6,55373E-03	13737	2,03794E+00	6724	1,27578E+00	29504	8,99197E-01	32620	0	48381,5
Myeloid_vs_CD8_Responder	CD8(Tn)	cDC_CLEC9A	COPA	P2RY6	5,96581E-03	33072,3	8,34739E-03	9215	2,39620E+00	3701	1,54983E+00	20260	8,07699E-01	83804	0	48381,5
Myeloid_vs_CD8_Responder	Macro_FOLR2+APOE+	CD8(Terminal Tex)	HLA-DRB1	LAG3	5,96581E-03	19298,3	6,31132E-03	14568	1,34607E+00	20260	1,95389E+00	11390	9,55664E-01	1892	0	48381,5
Myeloid_vs_CD8_Responder	CD8(ID2+CXCR4+ T)	Mono_CD14	HMGB1	TLR4	5,96846E-03	36424,5	4,06267E-03	28237	1,19864E+00	25272	1,05269E+00	38938	8,83794E-01	41294	0	48381,5
Myeloid_vs_CD8_Responder	CD8(Terminal Tex)	pDC_LILRA4	IL16	CD4	5,97039E-03	29639,5	5,19106E-03	19683	1,34592E+00	20264	2,45944E+00	5081	8,60527E-01	54788	0	48381,5
Myeloid_vs_CD8_Responder	CD8(ISG+ T)	Macro_NLRP3	CIRBP	TREM1	5,97569E-03	29119,5	7,56648E-03	10859	1,89045E+00	8534	1,00394E+00	41304	8,92194E-01	36519	0	48381,5
Myeloid_vs_CD8_Responder	CD8(GZMK+ Tex)	Mono_INHBA	HLA-B	LILRB2	5,98293E-03	29864,5	3,11985E-03	41314	1,47442E+00	16518	1,14238E+00	34965	9,42375E-01	8144	0	48381,5
Myeloid_vs_CD8_Responder	CD8(GZMK+ Tem)	Macro_LYVE1	PMCH	MERTK	5,98421E-03	37415,1	1,18059E-01	38	2,77613E+00	2118	1,25581E+00	30284	7,62082E-01	106254	0	48381,5
Myeloid_vs_CD8_Responder	CD8(ZNF683+KLRB1+ T)	Macro_LYVE1	CALR	LRP1	5,99090E-03	28959,1	4,53968E-03	24047	2,10396E+00	6031	1,00352E+00	41325	9,13136E-01	25011	0	48381,5
Myeloid_vs_CD8_Responder	CD8(Tn)	Macro_FOLR2+APOE+	CD40LG	CD9	5,99587E-03	44248,1	2,16195E-02	1757	9,33743E-01	37720	1,00588E+00	41213	7,91285E-01	92169	0	48381,5
Myeloid_vs_CD8_Responder	Mono_INHBA	CD8(GZMK+ Early Tem)	IL1B	SIGIRR	5,99597E-03	20799,5	7,12596E-03	11988	2,33330E+00	4126	2,76991E+00	2846	8,91949E-01	36656	0	48381,5
Myeloid_vs_CD8_Responder	CD8(EOMES+ NK-like)	Mono_INHBA	CD99	PILRA	5,99742E-03	37700,9	3,22353E-03	39422	1,13832E+00	27786	1,00338E+00	41334	9,01058E-01	31581	0	48381,5
Myeloid_vs_CD8_Responder	CD8(GZMK+ Tex)	Macro_ISG15	B2M	LILRB1	5,99742E-03	30497,7	3,11882E-03	41334	1,35109E+00	20095	1,19744E+00	32619	9,38992E-01	10059	0	48381,5
Myeloid_vs_CD8_Responder	CD8(Terminal Tex)	Macro_ISG15	HLA-C	LILRB2	5,99960E-03	27208,7	3,11874E-03	41337	1,56112E+00	14321	1,50001E+00	21692	9,38586E-01	10312	0	48381,5
Myeloid_vs_CD8_Responder	CD8(NME1+ T)	Macro_IER3	HMGB1	TLR2	6,00032E-03	37855,7	3,11870E-03	41338	1,18666E+00	25719	1,06496E+00	38338	8,94022E-01	35502	0	48381,5
Myeloid_vs_CD8_Responder	Mono_INHBA	CD8(GZMK+ Early Tem)	SPP1	CD44	6,00105E-03	23462,9	5,71776E-03	16963	1,35982E+00	19824	2,32591E+00	6382	9,11794E-01	25764	0	48381,5
Myeloid_vs_CD8_Responder	Mono_INHBA	CD8(GZMK+ Early Tem)	VCAN	CD44	6,00395E-03	22384,1	6,35806E-03	14376	1,74979E+00	10605	1,70092E+00	16452	9,18206E-01	22106	0	48381,5
Myeloid_vs_CD8_Responder	CD8(Terminal Tex)	Mono_CD16	HLA-B	LILRA1	6,00596E-03	29035,7	9,73427E-03	7240	1,90841E+00	8284	6,68346E-01	60978	9,21321E-01	20295	0	48381,5
Myeloid_vs_CD8_Responder	CD8(Terminal Tex)	Mono_CD16	HLA-A	LILRB1	6,00686E-03	31739,9	3,45336E-03	35697	1,37413E+00	19427	1,00308E+00	41347	9,32572E-01	13847	0	48381,5
Myeloid_vs_CD8_Responder	CD8(EOMES+ NK-like)	Macro_LYVE1	CD99	PILRA	6,00904E-03	28943,3	4,90174E-03	21504	1,70388E+00	11405	1,00304E+00	41350	9,18234E-01	22076	0	48381,5
Myeloid_vs_CD8_Responder	Mono_INHBA	CD8(GZMK+ Early Tem)	HBEGF	CD44	6,01122E-03	24974,9	6,26563E-03	14727	1,70667E+00	11345	1,37681E+00	25709	9,13601E-01	24712	0	48381,5
Myeloid_vs_CD8_Responder	Mono_INHBA	CD8(ID2+CXCR4+ T)	LGALS3	LAG3	6,01195E-03	33343,5	4,16190E-03	27296	8,71066E-01	41354	1,38440E+00	25438	9,14510E-01	24248	0	48381,5
Myeloid_vs_CD8_Responder	Mono_INHBA	CD8(GZMK+ Early Tem)	LGALS1	CD69	6,01340E-03	26996,5	3,78606E-03	31277	1,06929E+00	30885	1,60359E+00	18801	9,46889E-01	5638	0	48381,5
Myeloid_vs_CD8_Responder	Macro_FOLR2+APOE+	CD8(LAYN+ T)	CD14	ITGA4	6,01517E-03	21694,3	5,47384E-03	18129	2,07871E+00	6291	1,74878E+00	15367	9,21301E-01	20303	0	48381,5
Myeloid_vs_CD8_Responder	Macro_OLFML3	CD8(Temra)	FN1	ITGA5_ITGB1	6,01849E-03	22606,7	1,15948E-02	5511	2,10481E+00	6022	1,93193E+00	11756	8,83698E-01	41363	0	48381,5
Myeloid_vs_CD8_Responder	Macro_OLFML3	CD8(GZMK+ Tem)	C3	CD81	6,02213E-03	22763,3	6,62812E-03	13449	2,49301E+00	3189	2,22813E+00	7429	8,83694E-01	41368	0	48381,5
Myeloid_vs_CD8_Responder	Mono_INHBA	CD8(GZMK+ Early Tem)	S100A8	CD69	6,02868E-03	23811,9	5,61739E-03	17403	1,31926E+00	21066	1,69797E+00	16523	9,29478E-01	15686	0	48381,5
Myeloid_vs_CD8_Responder	Mono_INHBA	CD8(GZMK+ Early Tem)	VEGFA	CD44	6,03087E-03	21150,9	9,09359E-03	8038	2,28447E+00	4489	1,56523E+00	19816	9,13101E-01	25030	0	48381,5
Myeloid_vs_CD8_Responder	Macro_ISG15	CD8(Terminal Tex)	HLA-DRA	LAG3	6,03823E-03	18325,5	6,03220E-03	15624	1,34402E+00	20323	2,48157E+00	4895	9,54085E-01	2404	0	48381,5
Myeloid_vs_CD8_Responder	CD8(ISG+ T)	Macro_OLFML3	CD52	SIGLEC10	6,04181E-03	28462,9	5,53260E-03	17818	1,64535E+00	12491	1,00220E+00	41395	9,17973E-01	22229	0	48381,5
Myeloid_vs_CD8_Responder	Macro_OLFML3	CD8(LAYN+ T)	CXCL16	CXCR6	6,04254E-03	31147,1	7,45524E-03	11101	1,25628E+00	23127	1,00220E+00	41396	9,00802E-01	31730	0	48381,5
Myeloid_vs_CD8_Responder	CD8(ISG+ T)	Mono_INHBA	CIRBP	TREM1	6,04327E-03	31400,9	6,36938E-03	14334	1,59753E+00	13475	1,04276E+00	39417	8,83628E-01	41397	0	48381,5
Myeloid_vs_CD8_Responder	Mono_INHBA	CD8(LAYN+ T)	ICAM1	IL2RG	6,04838E-03	32333,7	4,27073E-03	26286	1,09212E+00	29841	1,37678E+00	25711	9,01291E-01	31449	0	48381,5
Myeloid_vs_CD8_Responder	CD8(ZNF683+KLRB1+ T)	Mono_INHBA	HLA-C	LILRB2	6,05057E-03	30517,7	3,11548E-03	41407	1,44914E+00	17231	1,13608E+00	35238	9,38555E-01	10331	0	48381,5
Myeloid_vs_CD8_Responder	Macro_FOLR2+APOE-	CD8(Terminal Tex)	HLA-DPB1	LAG3	6,05094E-03	19114,3	6,25083E-03	14784	1,34377E+00	20334	2,08691E+00	9274	9,52919E-01	2798	0	48381,5

Myeloid_vs_CD8_Responder	CD8(ISG+ T)	Macro_FOLR2+APOE+	HMGB1	CD163	6,05130E-03	29244,9	3,85205E-03	30508	1,76643E+00	10340	1,00195E+00	41408	9,29644E-01	15587	0	48381,5
Myeloid_vs_CD8_Responder	Mono_INHBA	CD8(LAYN+ T)	CD14	ITGB1	6,05203E-03	28511,3	3,84376E-03	30614	1,36965E+00	19545	1,62704E+00	18228	9,11753E-01	25788	0	48381,5
Myeloid_vs_CD8_Responder	Mono_INHBA	CD8(LAYN+ T)	CD14	ITGA4	6,05276E-03	33365,5	3,17805E-03	40237	1,20836E+00	24888	1,53325E+00	20698	8,99194E-01	32623	0	48381,5
Myeloid_vs_CD8_Responder	cDC(CD1C)	CD8(GZMK+ Tem)	LGALS9	HAVCR2	6,05349E-03	30815,9	4,40513E-03	25093	1,19509E+00	25400	1,82376E+00	13794	8,83612E-01	41411	0	48381,5
Myeloid_vs_CD8_Responder	CD8(Terminal Tex)	Macro_OLFML3	HSPA1A	TLR4	6,05642E-03	36261,5	3,86780E-03	30328	1,23375E+00	23933	1,08892E+00	37250	8,83600E-01	41415	0	48381,5
Myeloid_vs_CD8_Responder	CD8(GZMK+ Tex)	Macro_FOLR2+APOE+	CD52	SIGLEC10	6,05788E-03	27326,1	6,04510E-03	15569	1,85216E+00	9049	9,63246E-01	43291	9,21248E-01	20340	0	48381,5
Myeloid_vs_CD8_Responder	CD8(ISG+ T)	Macro_LYVE1	RPS19	C5AR1	6,06154E-03	27226,5	4,34966E-03	25602	1,76889E+00	10293	1,00166E+00	41422	9,38347E-01	10434	0	48381,5
Myeloid_vs_CD8_Responder	Mono_INHBA	CD8(LAYN+ T)	LGALS1	ITGB1	6,06813E-03	26110,9	3,91352E-03	29842	1,22452E+00	24266	1,73444E+00	15673	9,34961E-01	12392	0	48381,5
Myeloid_vs_CD8_Responder	cDC_CLEC9A	CD8(NME1+ T)	HLA-DPB1	LAG3	6,06830E-03	18910,1	5,33379E-03	18869	1,34309E+00	20349	2,82957E+00	2513	9,49229E-01	4438	0	48381,5
Myeloid_vs_CD8_Responder	Mono_INHBA	CD8(LAYN+ T)	SPP1	ITGA4_ITGB1	6,07032E-03	24951,9	7,44261E-03	11135	1,38318E+00	19152	2,34685E+00	6132	8,86198E-01	39959	0	48381,5
Myeloid_vs_CD8_Responder	Mono_INHBA	CD8(LAYN+ T)	SPP1	CD44	6,07106E-03	22478,5	6,18320E-03	15027	1,43167E+00	17744	2,24557E+00	7223	9,14891E-01	24017	0	48381,5
Myeloid_vs_CD8_Responder	Mono_INHBA	CD8(LAYN+ T)	VCAN	ITGB1	6,07252E-03	22475,1	9,01642E-03	8159	1,85378E+00	9028	1,76875E+00	14919	9,00522E-01	31888	0	48381,5
Myeloid_vs_CD8_Responder	Mono_INHBA	CD8(LAYN+ T)	VCAN	ITGA4	6,07325E-03	25577,5	7,45485E-03	11102	1,69249E+00	11597	1,67497E+00	17040	8,86564E-01	39767	0	48381,5
Myeloid_vs_CD8_Responder	Mono_INHBA	CD8(LAYN+ T)	VCAN	CD44	6,07399E-03	21873,5	6,87562E-03	12676	1,82164E+00	9480	1,62058E+00	18404	9,21097E-01	20426	0	48381,5
Myeloid_vs_CD8_Responder	Mono_INHBA	CD8(LAYN+ T)	VEGFA	ITGB1	6,07545E-03	22024,5	1,28957E-02	4658	2,38847E+00	3751	1,63306E+00	18076	8,94440E-01	35256	0	48381,5
Myeloid_vs_CD8_Responder	Mono_INHBA	CD8(LAYN+ T)	HBEGF	CD44	6,08059E-03	24646,1	6,77566E-03	12979	1,77852E+00	10145	1,29647E+00	28739	9,16640E-01	22986	0	48381,5
Myeloid_vs_CD8_Responder	Mono_INHBA	CD8(LAYN+ T)	CXCL16	CXCR6	6,08132E-03	37813,7	5,33127E-03	18883	8,91251E-01	40147	1,01225E+00	40888	8,84781E-01	40769	0	48381,5
Myeloid_vs_CD8_Responder	Mono_INHBA	CD8(LAYN+ T)	TIMP1	CD63	6,08279E-03	21276,7	3,16183E-03	40526	2,23394E+00	4910	2,91595E+00	2193	9,38470E-01	10373	0	48381,5
Myeloid_vs_CD8_Responder	Mono_INHBA	CD8(LAYN+ T)	LYZ	ITGAL	6,09013E-03	24525,7	4,18396E-03	27079	1,73199E+00	10907	2,23134E+00	7392	9,06041E-01	28869	0	48381,5
Myeloid_vs_CD8_Responder	CD8(Terminal Tex)	Mono_INHBA	CD99	PILRA	6,09160E-03	38458,9	3,12741E-03	41171	1,11230E+00	28947	1,00069E+00	41463	8,99701E-01	32332	0	48381,5
Myeloid_vs_CD8_Responder	Mono_INHBA	CD8(LAYN+ T)	S100A8	CD69	6,09380E-03	32432,7	3,71157E-03	32174	1,05945E+00	31341	1,36530E+00	26100	9,14627E-01	24167	0	48381,5
Myeloid_vs_CD8_Responder	Macro_OLFML3	CD8(LAYN+ T)	FN1	ITGA4_ITGB7	6,09527E-03	23691,3	1,16423E-02	5469	1,96935E+00	7530	1,73747E+00	15608	8,83540E-01	41468	0	48381,5
Myeloid_vs_CD8_Responder	Mono_INHBA	CD8(LAYN+ T)	VEGFA	CD44	6,09600E-03	20981,5	9,83383E-03	7132	2,35632E+00	3961	1,48489E+00	22141	9,16156E-01	23292	0	48381,5
Myeloid_vs_CD8_Responder	CD8(Temra)	Macro_FOLR2+APOE+	CD99	CD81	6,09747E-03	30935,5	4,83935E-03	21920	1,20807E+00	24901	1,00043E+00	41471	9,25278E-01	18004	0	48381,5
Myeloid_vs_CD8_Responder	CD8(Terminal Tex)	Macro_LYVE1	CD99	PILRA	6,10115E-03	29381,5	4,75559E-03	22460	1,67786E+00	11870	1,00034E+00	41476	9,17091E-01	22720	0	48381,5
Myeloid_vs_CD8_Responder	CD8(Temra)	Mono_CD14	CIRBP	TREM1	6,10777E-03	29349,3	6,35519E-03	14393	1,68601E+00	11710	1,24404E+00	30777	8,83513E-01	41485	0	48381,5
Myeloid_vs_CD8_Responder	CD8(GZMK+ Tem)	Mono_CD16	HLA-A	LILRB1	6,11146E-03	31594,1	3,47222E-03	35440	1,39109E+00	18914	1,00001E+00	41490	9,32743E-01	13745	0	48381,5
Myeloid_vs_CD8_Responder	Mono_INHBA	CD8(Temra)	ICAM1	ITGAL_ITGB2	6,11367E-03	31937,7	3,79762E-03	31154	1,21364E+00	24685	1,63066E+00	18132	8,90819E-01	37336	0	48381,5
Myeloid_vs_CD8_Responder	Mono_INHBA	CD8(Temra)	ICAM1	IL2RG	6,11514E-03	30039,1	4,69505E-03	22869	1,14693E+00	27417	1,47923E+00	22327	9,05426E-01	29201	0	48381,5
Myeloid_vs_CD8_Responder	cDC(CD1C)	CD8(EOMES+ NK-like)	HLA-DRA	LAG3	6,11514E-03	25636,9	3,11112E-03	41495	1,21859E+00	24499	2,79389E+00	2720	9,37198E-01	11089	0	48381,5
Myeloid_vs_CD8_Responder	CD8(EOMES+ NK-like)	Mono_CD14	CIRBP	TREM1	6,11588E-03	29691,9	6,35242E-03	14407	1,68571E+00	11717	1,20147E+00	32458	8,83491E-01	41496	0	48381,5
Myeloid_vs_CD8_Responder	CD8(ID2+CXCR4+ T)	Macro_NLRP3	CD99	PILRA	6,11809E-03	33252,5	3,84047E-03	30661	1,41331E+00	18251	9,99747E-01	41499	9,08595E-01	27470	0	48381,5
Myeloid_vs_CD8_Responder	Mono_INHBA	CD8(Temra)	S100A8	ITGB2	6,13580E-03	26539,5	3,52442E-03	34669	1,42459E+00	17937	1,98468E+00	10844	9,20298E-01	20866	0	48381,5
Myeloid_vs_CD8_Responder	Mono_INHBA	CD8(Temra)	S100A9	ITGB2	6,13654E-03	23105,1	3,63779E-03	33128	1,78868E+00	9989	2,15067E+00	8378	9,29552E-01	15649	0	48381,5
Myeloid_vs_CD8_Responder	Mono_INHBA	CD8(Temra)	SPP1	CD44	6,14097E-03	29854,3	4,12495E-03	27646	1,11394E+00	28863	1,97689E+00	10977	8,97751E-01	33404	0	48381,5
Myeloid_vs_CD8_Responder	Mono_INHBA	CD8(Temra)	VCAN	CD44	6,14467E-03	28756,9	4,58688E-03	23702	1,50390E+00	15729	1,35191E+00	26598	9,05077E-01	29374	0	48381,5
Myeloid_vs_CD8_Responder	CD8(NME1+ T)	Mono_CD14	CD99	PILRA	6,14763E-03	36106,9	3,10884E-03	41539	1,09192E+00	29853	1,30835E+00	28273	8,99432E-01	32488	0	48381,5
Myeloid_vs_CD8_Responder	CD8(GZMK+ Early Tem)	Macro_ISG15	CD99	PILRA	6,14837E-03	38008,7	3,15845E-03	40580	1,14644E+00	27441	9,98882E-01	41540	9,00145E-01	32101	0	48381,5
Myeloid_vs_CD8_Responder	Mono_INHBA	CD8(Temra)	HBEGF	CD44	6,15281E-03	32363,9	4,52019E-03	24192	1,46079E+00	16885	1,02780E+00	40091	8,99813E-01	32270	0	48381,5
Myeloid_vs_CD8_Responder	Mono_INHBA	CD8(Temra)	LGALS1	CD69	6,15429E-03	30142,9	3,32454E-03	37709	9,75939E-01	35451	1,50066E+00	21672	9,43523E-01	7501	0	48381,5
Myeloid_vs_CD8_Responder	CD8(GZMK+ Tem)	Macro_ISG15	CCL5	CCR1	6,15577E-03	31418,1	7,81238E-03	10308	1,01844E+00	33313	9,98573E-01	41550	9,15743E-01	23538	0	48381,5
Myeloid_vs_CD8_Responder	CD8(ZNF683+KLRB1+ T)	Macro_ISG15	HLA-C	LILRB1	6,16096E-03	34755,3	3,14969E-03	40755	1,15774E+00	26929	9,98470E-01	41557	9,28622E-01	16154	0	48381,5
Myeloid_vs_CD8_Responder	Mono_INHBA	CD8(Temra)	LYZ	ITGAL	6,16763E-03	23725,3	4,35450E-03	25562	1,74763E+00	10644	2,34902E+00	6108	9,07728E-01	27931	0	48381,5
Myeloid_vs_CD8_Responder	CD8(GZMK+ Tex)	cDC_CLEC9A	CALM1	MYLK	6,16784E-03	34882,5	2,33531E-02	1422	2,01888E+00	6951	9,23537E-01	45289	8,29175E-01	72369	0	48381,5
Myeloid_vs_CD8_Responder	CD8(Terminal Tex)	Macro_IER3	HSPA1A	TLR4	6,16838E-03	34312,5	3,85081E-03	30521	1,23015E+00	24067	1,34129E+00	27026	8,83373E-01	41567	0	48381,5
Myeloid_vs_CD8_Responder	CD8(GZMK+ Tex)	cDC_CLEC9A	CALM2	MYLK	6,17006E-03	35265,5	2,17260E-02	1733	2,05359E+00	6559	1,06535E+00	38318	8,12424E-01	81336	0	48381,5
Myeloid_vs_CD8_Responder	CD8(GZMK+ Tex)	cDC_CLEC9A	CALM3	MYLK	6,17229E-03	42191,5	2,05614E-02	1998	2,08191E+00	6257	1,13359E+00	35348	7,31996E-01	118973	0	48381,5
Myeloid_vs_CD8_Responder	CD8(IL7R+ZNF683+ Tm)	Macro_NLRP3	HMGB1	CD163	6,17283E-03	31812,1	3,10658E-03	41573	1,50241E+00	15767	1,17296E+00	33589	9,22277E-01	19750	0	48381,5
Myeloid_vs_CD8_Responder	Mono_INHBA	CD8(Temra)	S100A8	CD69	6,17431E-03	26206,5	4,93264E-03	21301	1,22591E+00	24226	1,59504E+00	19020	9,25097E-01	18104	0	48381,5
Myeloid_vs_CD8_Responder	CD8(EOMES+ NK-like)	Macro_ISG15	HLA-C	LILRB1	6,17431E-03	34651,1	3,15838E-03	40582	1,16472E+00	26618	9,98194E-01	41575	9,28713E-01	16099	0	48381,5
Myeloid_vs_CD8_Responder	Mono_INHBA	CD8(Temra)	NAMPT	ITGA5_ITGB1	6,17877E-03	35712,7	3,42958E-03	36061	9,99755E-01	34280	1,47088E+00	22563	8,90912E-01	37278	0	48381,5
Myeloid_vs_CD8_Responder	Mono_INHBA	CD8(Temra)	VEGFA	CD44	6,18026E-03	26655,5	6,56036E-03	13704	2,03859E+00	6717	1,21622E+00	31883	8,99242E-01	32592	0	48381,5
Myeloid_vs_CD8_Responder	Mono_INHBA	CD8(GZMK+ Tem)	HLA-DRA	LAG3	6,18992E-03	18911,1	6,03996E-03	15589	1,32500E+00	20886	2,23873E+00	7304	9,54113E-01	2395	0	48381,5
Myeloid_vs_CD8_Responder	Mono_CD14	CD8(Temra)	VCAN	SELL	6,19009E-03	24069,3	9,04518E-03	8117	3,25520E+00	1098	2,51909E+00	4560	8,54644E-01	58190	0	48381,5
Myeloid_vs_CD8_Responder	Mono_INHBA	CD8(GZMK+ Tem)	HLA-DQA2	LAG3	6,19067E-03	31897,9	5,31542E-03	18972	8,71887E-01	41310	1,44474E+00	23404	9,08670E-01	27422	0	48381,5
Myeloid_vs_CD8_Responder	Mono_INHBA	CD8(GZMK+ Tem)	HLA-DRB1	LAG3	6,19216E-03	22072,5	5,39193E-03	18559	1,11350E+00	28889	1,95117E+00	11430	9,52206E-01	3103	0	48381,5
Myeloid_vs_CD8_Responder	Mono_INHBA	CD8(GZMK+ Tem)	HLA-DQB1	LAG3	6,19290E-03	29915,5	4,55159E-03	23968	8,67592E-01	41578	1,46102E+00	22905	9,34417E-01	12745	0	48381,5
Myeloid_vs_CD8_Responder	Mono_INHBA	CD8(GZMK+ Tem)	HLA-DPB1	LAG3	6,19365E-03	25349,1	4,67856E-03	23003	9,78464E-01	35333	1,81837E+00	13902	9,45976E-01	6126	0	48381,5
Myeloid_vs_CD8_Responder	Mono_INHBA	CD8(GZMK+ Tem)	ICAM1	ITGAL_ITGB2	6,19811E-03	32856,3	3,67599E-03	32647	1,20024E+00	25206	1,56632E+00	19778	8,89226E-01	38269	0	48381,5
Myeloid_vs_CD8_Responder	Mono_INHBA	CD8(GZMK+ Tem)	ICAM1	IL2RG	6,19960E-03	29267,5	4,91854E-03	21401	1,17580E+00	26142	1,48023E+00	22281	9,07398E-01	28132	0	48381,5
Myeloid_vs_CD8_Responder	Mono_INHBA	CD8(GZMK+ Tem)	CD14	ITGA4	6,20482E-03	30185,7	3,66625E-03	32764	1,28588E+00	22148	1,61741E+00	18463	9,05489E-01	29172	0	48381,5
Myeloid_vs_CD8_Responder	Mono_INHBA	CD8(GZMK+ Tem)	HLA-DPA1	LAG3	6,20780E-03	24795,3	4,75018E-03	22496	9,80968E-01	35202	1,87859E+00	12786	9,47876E-01	5111	0	48381,5
Myeloid_vs_CD8_Responder	CD8(EOMES+ NK-like)	Mono_CD14	HMGB1	THBD	6,20780E-03	38411,7	3,63005E-03	33228	1,07701E+00	30555	1,06642E+00	38274	8,83290E-01	41620	0	48381,5
Myeloid_vs_CD8_Responder	CD8(NME1+ T)	Macro_OLFML3	CD99	PILRA	6,21079E-03	30072,3	4,67430E-03	23037	1,56586E+00	14205	9,97339E-01	41624	9,16433E-01	23114	0	48381,5
Myeloid_vs_CD8_Responder	CD8(Temra)	Macro_NLRP3	HLA-A	LILRB2	6,21302E-03	31129,1	3,10303E-03	41627	1,41554E+00	18193	1,08181E+00	37579	9,39310E-01	9865	0	48381,5
Myeloid_vs_CD8_Responder	Mono_INHBA	CD8(GZMK+ Tem)	HLA-DRB5	LAG3	6,21676E-03	23438,1	5,40581E-03	18487	1,05770E+00	31424	1,96202E+00	11254	9,43272E-01	7644	0	48381,5
Myeloid_vs_CD8_Responder	CD8(Terminal Tex)	pDC_LILRA4	SEMA4A	NRP1_PLXNA4	6,22132E-03	44699,1	9,03741E-03	8131	2,13546E+00	5727	1,30148E+00	28538	6,93241E-01	132718	0	48381,5
Myeloid_vs_CD8_Responder	CD8(Temra)	Mono_INHBA	CALR	LRP1	6,22198E-03	36810,1	3,10217E-03	41639	1,34801E+00	20187	1,03290E+00	39872	8,96800E-01	33971	0	48381,5
Myeloid_vs_CD8_Responder	cDC(CD1C)	CD8(ID2+CXCR4+ T)	HLA-DQB2	LAG3	6,22273E-03	25579,3	8,50306E-03	8950	1,50884E+00	15605	1,84821E+00	13320	8,83255E-01	41640	0	48381,5
Myeloid_vs_CD8_Responder	Mono_INHBA	CD8(GZMK+ Tem)	IL1B	SIGIRR	6,22348E-03	20958,9	7,06474E-03	12151	2,33038E+00	4142	2,71313E+00	3197	8,91533E-01	36923	0	48381,5
Myeloid_vs_CD8_Responder	Mono_INHBA	CD8(GZMK+ Tem)	S100A8	ITGB2	6,22423E-03	28046,7	3,34075E-03	37441	1,38705E+00	19052	1,84731E+00	13338	9,18313E-01	22021	0	48381,5
Myeloid_vs_CD8_Responder	Mono_INHBA	CD8(GZMK+ Tem)	S100A9	ITGB2	6,22572E-03	24346,7	3,44821E-03	35778	1,75115E+00	10582	2,01331E+00	10386	9,27779E-01	16606	0	48381,5
Myeloid_vs_CD8_Responder	Mono_INHBA	CD8(GZMK+ Tem)	SPP1	CD44	6,22946E-03	29249,7	4,22058E-03	26746	1,12871E+00	28215	2,03153E+00	10075	8,98798E-01	32831	0	48381,5
Myeloid_vs_CD8_Responder	Mono_INHBA	CD8(GZMK+ Tem)	VCAN	ITGA4	6,23320E-03	23661,3	8,60003E-03	8784	1,77002E+00	10272	1,75912E+00	15114	8,93553E-01	35755	0	48381,5
Myeloid_vs_CD8_Responder	Mono_INHBA	CD8(GZMK+ Tem)	VCAN	CD44	6,23395E-03	28038,3	4,69322E-03	22883	1,51867E+00	15369	1,40654E+00	24692	9,06057E-01	28866	0	48381,5
Myeloid_vs_CD8_Responder	Macro_FOLR2+APOE+	CD8(GZMK+ Tex)	CD14	ITGB1	6,23685E-03	21283,1	5,05767E-03	20493	2,08223E+00	6252	1,94916E+00	11475	9,22188E-01	19814	0	48381,5
Myeloid_vs_CD8_Responder	Mono_INHBA	CD8(GZMK+ Tem)	LGALS3	LAG3	6,23919E-03	19235,3	8,77354E-03	8507	1,32682E+00	20829	2,12571E+00	8719	9,39510E-01	9740	0	48381,5

Myeloid_vs_CD8_Responder	pDC_LILRA4	CD8(Terminal Tex)	APP	LRP10	6,24145E-03	26612,3	9,02627E-03	8140	1,93488E+00	7954	2,55453E+00	4299	8,43598E-01	64287	0	48381,5
Myeloid_vs_CD8_Responder	CD8(LAYN+ T)	Mono_CD14	ANXA1	FPR1	6,24158E-03	34280,6	5,05741E-03	20497	1,62042E+00	13006	1,56335E+00	19869	9,21112E-01	20421	0,001	97610
Myeloid_vs_CD8_Responder	Mono_INHBA	CD8(GZMK+ Tem)	HBEGF	CD44	6,24293E-03	31505,1	4,62498E-03	23395	1,47555E+00	16489	1,08243E+00	37548	9,00841E-01	31712	0	48381,5
Myeloid_vs_CD8_Responder	cDC(CD1C)	CD8(EOMES+ NK-like)	HLA-DQA2	LAG3	6,25568E-03	33245,3	3,96073E-03	29344	8,65617E-01	41684	1,90591E+00	12265	8,95707E-01	34552	0	48381,5
Myeloid_vs_CD8_Responder	Mono_INHBA	CD8(GZMK+ Tem)	LYZ	ITGAL	6,26018E-03	23359,5	4,47142E-03	24569	1,75835E+00	10472	2,35771E+00	6037	9,08832E-01	27338	0	48381,5
Myeloid_vs_CD8_Responder	Mono_INHBA	CD8(GZMK+ Tem)	S100A8	CD69	6,26694E-03	29777,9	4,29257E-03	26098	1,13865E+00	27766	1,37748E+00	25689	9,20137E-01	20955	0	48381,5
Myeloid_vs_CD8_Responder	CD8(Terminal Tex)	Mono_CD14	HLA-C	LILRB2	6,26765E-03	27537,7	3,03998E-03	42861	1,52577E+00	15191	1,53952E+00	20519	9,37844E-01	10736	0	48381,5
Myeloid_vs_CD8_Responder	Mono_INHBA	CD8(GZMK+ Tem)	VEGFA	CD44	6,26995E-03	25968,7	6,71245E-03	13184	2,05335E+00	6560	1,27085E+00	29692	9,00276E-01	32026	0	48381,5
Myeloid_vs_CD8_Responder	CD8(GZMK+ Tem)	Mono_CD14	HMGB1	THBD	6,27220E-03	38353,9	3,62174E-03	33335	1,07476E+00	30666	1,07938E+00	37681	8,83172E-01	41706	0	48381,5
Myeloid_vs_CD8_Responder	Mono_CD14	CD8(ISG+ T)	VCAN	ITGB1	6,27285E-03	19013,1	9,02010E-03	8154	3,14850E+00	1270	2,42479E+00	5385	9,00540E-01	31875	0	48381,5
Myeloid_vs_CD8_Responder	CD8(Terminal Tex)	Mono_CD14	B2M	LILRB2	6,27714E-03	27538,5	2,79792E-03	48196	1,57526E+00	13985	1,53896E+00	20527	9,45084E-01	6603	0	48381,5
Myeloid_vs_CD8_Responder	Mono_INHBA	CD8(Tc17)	IL1B	SIGIRR	6,28048E-03	22839,1	5,97384E-03	15840	2,27838E+00	4541	2,63443E+00	3716	8,83153E-01	41717	0	48381,5
Myeloid_vs_CD8_Responder	Mono_INHBA	CD8(NME1+ T)	HLA-DRA	LAG3	6,28500E-03	25085,3	4,26309E-03	26350	1,06992E+00	30855	1,83143E+00	13654	9,45854E-01	6186	0	48381,5
Myeloid_vs_CD8_Responder	CD8(GZMK+ Tex)	Mono_CD14	HLA-C	LILRB2	6,28546E-03	28142,1	2,97813E-03	44071	1,46928E+00	16653	1,53876E+00	20534	9,37242E-01	11071	0	48381,5
Myeloid_vs_CD8_Responder	CD8(LAYN+ T)	Macro_NLRP3	HLA-A	LILRB2	6,28650E-03	31655,1	3,09748E-03	41725	1,41034E+00	18342	1,03157E+00	39931	9,39259E-01	9896	0	48381,5
Myeloid_vs_CD8_Responder	CD8(GZMK+ Tex)	pDC_LILRA4	CXCL13	CXCR3	6,28858E-03	29498,5	1,24109E-02	4927	5,64993E-01	63553	2,68006E+00	3403	9,09020E-01	27228	0	48381,5
Myeloid_vs_CD8_Responder	Mono_INHBA	CD8(NME1+ T)	ICAM1	ITGAL_ITGB2	6,29781E-03	35312,3	3,38414E-03	36749	1,14774E+00	27380	1,44318E+00	23458	8,85086E-01	40593	0	48381,5
Myeloid_vs_CD8_Responder	Mono_INHBA	CD8(NME1+ T)	ICAM1	IL2RG	6,29932E-03	25766,3	5,86234E-03	16295	1,29772E+00	21780	1,63089E+00	18130	9,14514E-01	24245	0	48381,5
Myeloid_vs_CD8_Responder	Macro_ISG15	CD8(GZMK+ Tex)	S100A8	CD69	6,30332E-03	23616,5	5,04986E-03	20549	1,34771E+00	20198	1,95697E+00	11335	9,25907E-01	17619	0	48381,5
Myeloid_vs_CD8_Responder	CD8(GZMK+ Tex)	pDC_LILRA4	GZMB	IGF2R	6,31788E-03	28041,9	9,18472E-03	7916	8,29771E-01	43902	2,43684E+00	5270	8,95367E-01	34740	0	48381,5
Myeloid_vs_CD8_Responder	CD8(EOMES+ NK-like)	Mono_INHBA	HMGB1	CD163	6,32123E-03	31232,7	3,09539E-03	41771	1,41360E+00	18245	1,31796E+00	27918	9,22148E-01	19848	0	48381,5
Myeloid_vs_CD8_Responder	Mono_INHBA	CD8(NME1+ T)	IL1B	SIGIRR	6,32653E-03	21129,5	6,99869E-03	12332	2,32723E+00	4173	2,65371E+00	3591	8,91078E-01	37170	0	48381,5
Myeloid_vs_CD8_Responder	Mono_INHBA	CD8(NME1+ T)	S100A8	ITGB2	6,32729E-03	29108,1	3,21818E-03	39510	1,36200E+00	19764	1,76222E+00	15047	9,16900E-01	22838	0	48381,5
Myeloid_vs_CD8_Responder	Mono_INHBA	CD8(NME1+ T)	S100A9	ITGB2	6,32880E-03	25247,7	3,32170E-03	37758	1,72609E+00	10993	1,92821E+00	11820	9,26517E-01	17286	0	48381,5
Myeloid_vs_CD8_Responder	CD8(ISG+ T)	Mono_INHBA	B2M	LILRB2	6,33183E-03	27001,9	3,09479E-03	41785	1,65871E+00	12221	1,33222E+00	27379	9,47643E-01	5243	0	48381,5
Myeloid_vs_CD8_Responder	Mono_INHBA	CD8(NME1+ T)	SPP1	CD44	6,33259E-03	36514,7	3,12875E-03	41137	9,60161E-01	36291	1,72925E+00	15782	8,84349E-01	40982	0	48381,5
Myeloid_vs_CD8_Responder	CD8(IL7R+ZNF683+ Tm)	Mono_INHBA	HLA-C	LILRB2	6,33335E-03	31047,1	3,09477E-03	41787	1,43233E+00	17723	1,09634E+00	36915	9,38363E-01	10429	0	48381,5
Myeloid_vs_CD8_Responder	CD8(IL7R+ZNF683+ Tm)	Mono_INHBA	HLA-B	LILRB2	6,33486E-03	30331,3	3,09467E-03	41789	1,44712E+00	17289	1,11877E+00	35934	9,42155E-01	8263	0	48381,5
Myeloid_vs_CD8_Responder	Mono_INHBA	CD8(NME1+ T)	VCAN	CD44	6,33790E-03	35350,7	3,47912E-03	35326	1,35012E+00	20129	1,10426E+00	36557	8,92520E-01	36360	0	48381,5
Myeloid_vs_CD8_Responder	Mono_INHBA	CD8(NME1+ T)	LGALS3	LAG3	6,34397E-03	25247,9	6,19248E-03	14993	1,07174E+00	30788	1,71842E+00	16038	9,28818E-01	16039	0	48381,5
Myeloid_vs_CD8_Responder	Macro_IFI27	CD8(GZMK+ Tem)	HLA-DRB1	LAG3	6,34632E-03	18586,3	6,39455E-03	14250	1,33512E+00	20585	2,18503E+00	7916	9,55940E-01	1799	0	48381,5
Myeloid_vs_CD8_Responder	CD8(GZMK+ Tem)	Mono_CD14	HLA-A	LILRB2	6,35612E-03	27270,1	3,09330E-03	41817	1,52816E+00	15129	1,51997E+00	21108	9,39220E-01	9915	0	48381,5
Myeloid_vs_CD8_Responder	CD8(ITM2C+ T)	Mono_INHBA	HLA-B	LILRB2	6,36372E-03	30670,5	3,09274E-03	41827	1,44503E+00	17354	1,08301E+00	37518	9,42138E-01	8272	0	48381,5
Myeloid_vs_CD8_Responder	Mono_INHBA	CD8(NME1+ T)	LYZ	ITGAL	6,36676E-03	26826,3	3,59973E-03	33640	1,67840E+00	11860	2,19652E+00	7769	8,99441E-01	32481	0	48381,5
Myeloid_vs_CD8_Responder	Macro_OLFML3	CD8(GZMK+ Tex)	LGALS9	HAVCR2	6,37438E-03	32647,3	4,34950E-03	25606	1,24933E+00	23392	1,42739E+00	24016	8,82957E-01	41841	0	48381,5
Myeloid_vs_CD8_Responder	CD8(ISG+ T)	pDC_LILRA4	IRAK4	TLR7	6,37511E-03	41147,5	1,03615E-02	6597	1,66279E+00	12146	1,53625E+00	20609	7,34408E-01	118004	0	48381,5
Myeloid_vs_CD8_Responder	Macro_NLRP3	CD8(ZNF683+KLRB1+ T)	VCAN	CD44	6,38954E-03	21140,5	6,80741E-03	12885	2,36723E+00	3879	1,56070E+00	19936	9,20734E-01	20621	0	48381,5
Myeloid_vs_CD8_Responder	cDC(CD1C)	CD8(LAYN+ T)	LGALS1	ITGB1	6,39268E-03	31219,1	3,09096E-03	41865	9,56645E-01	36478	1,89085E+00	12545	9,27408E-01	16826	0	48381,5
Myeloid_vs_CD8_Responder	Mono_INHBA	CD8(EOMES+ NK-like)	ICAM1	IL2RG	6,39803E-03	38349,3	3,40544E-03	36424	9,80345E-01	35240	1,15564E+00	34339	8,90753E-01	37362	0	48381,5
Myeloid_vs_CD8_Responder	CD8(GZMK+ Early Tem)	Mono_CD14	CIRBP	TREM1	6,41256E-03	29682,5	6,27816E-03	14690	1,67761E+00	11874	1,22398E+00	31576	8,82884E-01	41891	0	48381,5
Myeloid_vs_CD8_Responder	CD8(ITM2C+ T)	Macro_IER3	IFNG	IFNGR1_IFNGR2	6,42021E-03	36751,7	5,75368E-03	16788	9,27964E-01	38027	9,91399E-01	41901	8,88571E-01	38661	0	48381,5
Myeloid_vs_CD8_Responder	Mono_INHBA	CD8(EOMES+ NK-like)	IL1B	SIGIRR	6,42174E-03	21335,9	6,81228E-03	12870	2,31835E+00	4229	2,69775E+00	3282	8,89761E-01	37917	0	48381,5
Myeloid_vs_CD8_Responder	Mono_INHBA	CD8(EOMES+ NK-like)	SPP1	CD44	6,42788E-03	28050,3	4,50557E-03	24314	1,17270E+00	26292	2,03303E+00	10052	9,01731E-01	31212	0	48381,5
Myeloid_vs_CD8_Responder	Mono_INHBA	CD8(EOMES+ NK-like)	VCAN	CD44	6,43095E-03	27089,7	5,01012E-03	20785	1,56266E+00	14287	1,40805E+00	24643	9,08801E-01	27352	0	48381,5
Myeloid_vs_CD8_Responder	CD8(GZMK+ Tem)	Mono_INHBA	HMGB1	CD163	6,43095E-03	31187,7	3,08831E-03	41915	1,41135E+00	18308	1,33093E+00	27434	9,22065E-01	19900	0	48381,5
Myeloid_vs_CD8_Responder	CD8(ITM2C+ T)	Macro_IER3	TNF	VSIR	6,43785E-03	34416,7	7,16287E-03	11890	9,08559E-01	39129	1,24447E+00	30759	8,82837E-01	41924	0	48381,5
Myeloid_vs_CD8_Responder	Mono_INHBA	CD8(EOMES+ NK-like)	HBEGF	CD44	6,43862E-03	30520,3	4,93728E-03	21270	1,51955E+00	15347	1,08394E+00	37477	9,03722E-01	30126	0	48381,5
Myeloid_vs_CD8_Responder	Mono_INHBA	CD8(EOMES+ NK-like)	LGALS1	CD69	6,44016E-03	26644,1	3,81597E-03	30927	1,07534E+00	30637	1,64642E+00	17737	9,47086E-01	5538	0	48381,5
Myeloid_vs_CD8_Responder	CD8(GZMK+ Early Tem)	Mono_CD16	HLA-B	LILRA1	6,44507E-03	29703,1	9,55681E-03	7432	1,84397E+00	9174	6,42155E-01	62861	9,20652E-01	20667	0	48381,5
Myeloid_vs_CD8_Responder	CD8(ITM2C+ T)	Macro_IFI27	HSPA1A	TLR4	6,44553E-03	29173,5	3,80970E-03	30991	1,53517E+00	14946	2,06390E+00	9615	8,82819E-01	41934	0	48381,5
Myeloid_vs_CD8_Responder	Macro_IFI27	CD8(NME1+ T)	HLA-DQA2	LAG3	6,45355E-03	21773,1	8,58318E-03	8803	1,33263E+00	20674	1,82270E+00	13822	9,26702E-01	17185	0	48381,5
Myeloid_vs_CD8_Responder	Macro_OLFML3	CD8(NME1+ T)	B2M	CD3D	6,45641E-03	49881,1	2,63948E-03	52386	5,78781E-01	62353	3,50079E-01	86244	9,65170E-01	41	0	48381,5
Myeloid_vs_CD8_Responder	Mono_INHBA	CD8(EOMES+ NK-like)	S100A8	CD69	6,45861E-03	23503,3	5,66178E-03	17201	1,32531E+00	20873	1,74080E+00	15531	9,29736E-01	15530	0	48381,5
Myeloid_vs_CD8_Responder	Mono_INHBA	CD8(EOMES+ NK-like)	VEGFA	CD44	6,46015E-03	25284,1	7,16570E-03	11881	2,09734E+00	6110	1,27236E+00	29629	9,03171E-01	30419	0	48381,5
Myeloid_vs_CD8_Responder	Mono_INHBA	CD8(GZMK+ Tem)	THBS1	ITGA4	6,46015E-03	33144,7	5,85333E-03	16342	9,15692E-01	38706	1,54638E+00	20341	8,82783E-01	41953	0	48381,5
Myeloid_vs_CD8_Responder	Mono_CD14	CD8(ZNF683+KLRB1+ T)	VCAN	ITGB1	6,46339E-03	18973,1	8,96214E-03	8238	3,14635E+00	1276	2,47674E+00	4930	9,00251E-01	32040	0	48381,5
Myeloid_vs_CD8_Responder	Mono_INHBA	CD8(LAYN+ T)	SPP1	PTGER4	6,46785E-03	26227,1	7,54741E-03	10898	1,28552E+00	22163	2,20051E+00	7730	8,82762E-01	41963	0	48381,5
Myeloid_vs_CD8_Responder	Mono_INHBA	CD8(ZNF683+KLRB1+ T)	ICAM1	IL2RG	6,47556E-03	34369,1	3,85596E-03	30459	1,03854E+00	32305	1,35018E+00	26659	8,96653E-01	34041	0	48381,5
Myeloid_vs_CD8_Responder	CD8(Temra)	Macro_ISG15	HLA-C	LILRB2	6,47633E-03	27584,5	3,08571E-03	41974	1,53171E+00	15039	1,48766E+00	22057	9,38278E-01	10471	0	48381,5
Myeloid_vs_CD8_Responder	Mono_INHBA	CD8(ISG+ T)	HLA-DRB5	LAG3	6,48328E-03	28955,3	4,18105E-03	27108	8,61259E-01	41983	1,74235E+00	15495	9,35994E-01	11809	0	48381,5
Myeloid_vs_CD8_Responder	CD8(GZMK+ Tex)	Mono_INHBA	B2M	LILRB2	6,48637E-03	27096,5	3,08495E-03	41987	1,64249E+00	12545	1,33505E+00	27285	9,47564E-01	5284	0	48381,5
Myeloid_vs_CD8_Responder	CD8(GZMK+ Tem)	Macro_ISG15	B2M	LILRB1	6,48791E-03	31739,3	3,08486E-03	41989	1,29570E+00	21829	1,11130E+00	36263	9,38678E-01	10234	0	48381,5
Myeloid_vs_CD8_Responder	CD8(Tn)	Macro_FOLR2+APOE+	ADAM10	GPNMB	6,48878E-03	31540,7	5,02325E-03	20703	2,87488E+00	1853	1,63978E+00	17897	8,35526E-01	68869	0	48381,5
Myeloid_vs_CD8_Responder	CD8(ITM2C+ T)	Mono_CD14	HLA-F	LILRB2	6,48946E-03	35923,5	3,13995E-03	40926	1,32083E+00	21012	1,33443E+00	27307	8,82724E-01	41991	0	48381,5
Myeloid_vs_CD8_Responder	cDC_LAMP3	CD8(Terminal Tex)	HLA-DPB1	LAG3	6,49365E-03	19163,7	6,19590E-03	14984	1,33134E+00	20707	2,11480E+00	8871	9,52721E-01	2875	0	48381,5
Myeloid_vs_CD8_Responder	Mono_INHBA	CD8(ZNF683+KLRB1+ T)	SPP1	CD44	6,49487E-03	30664,9	3,96243E-03	29325	1,08886E+00	29987	1,96563E+00	11188	8,95891E-01	34443	0	48381,5
Myeloid_vs_CD8_Responder	Mono_INHBA	CD8(ZNF683+KLRB1+ T)	SPP1	PTGER4	6,49564E-03	25686,9	7,68523E-03	10575	1,29496E+00	21856	2,33601E+00	6256	8,83695E-01	41366	0	48381,5
Myeloid_vs_CD8_Responder	Mono_INHBA	CD8(ZNF683+KLRB1+ T)	VCAN	CD44	6,49796E-03	29445,5	4,40616E-03	25087	1,47882E+00	16382	1,34064E+00	27051	9,03336E-01	30326	0	48381,5
Myeloid_vs_CD8_Responder	CD8(Terminal Tex)	Macro_IER3	HLA-F	LILRB2	6,50183E-03	39521,1	3,13818E-03	40957	1,21587E+00	24604	9,96608E-01	41656	8,82695E-01	42007	0	48381,5
Myeloid_vs_CD8_Responder	Mono_INHBA	CD8(ZNF683+KLRB1+ T)	HBEGF	CD44	6,50338E-03	33121,5	4,34211E-03	25668	1,43570E+00	17621	1,01653E+00	40662	8,97987E-01	33275	0	48381,5
Myeloid_vs_CD8_Responder	Mono_INHBA	CD8(ZNF683+KLRB1+ T)	CXCL16	CXCR6	6,50415E-03	32864,1	6,41713E-03	14172	9,87247E-01	34906	1,23128E+00	31283	8,93899E-01	35578	0	48381,5
Myeloid_vs_CD8_Responder	Mono_INHBA	CD8(ZNF683+KLRB1+ T)	LGALS1	CD69	6,50493E-03	21019,1	4,78584E-03	22273	1,27151E+00	22603	2,11570E+00	8855	9,52482E-01	2983	0	48381,5
Myeloid_vs_CD8_Responder	CD8(Terminal Tex)	Macro_NLRP3	GNAI2	C5AR1	6,51267E-03	28411,1	3,83163E-03	30740	2,29659E+00	4398	1,69841E+00	16515	8,82672E-01	42021	0	48381,5
Myeloid_vs_CD8_Responder	Macro_NLRP3	CD8(Temra)	S100A9	ITGB2	6,51438E-03	17024,7	5,01961E-03	20724	2,41237E+00	3596	2,81488E+00	2614	9,39392E-01	9808	0	48381,5
Myeloid_vs_CD8_Responder	CD8(NME1+ T)	Mono_INHBA	HMGB1	TLR4	6,51500E-03	34248,5	3,97400E-03	29203	1,29646E+00	21807	1,26737E+00	29827	8,82656E-01	42024	0	48381,5
Myeloid_vs_CD8_Responder	Mono_INHBA	CD8(ZNF683+KLRB1+ T)	VEGFA	CD44	6,51888E-03	27190,3	6,30190E-03	14606	2,01350E+00	7008	1,20495E+00	32338	8,97407E-01	33618	0	48381,5
Myeloid_vs_CD8_Responder	cDC_CLEC9A	CD8(GZMK+ Tex)	HLA-DPA1	LAG3	6,52904E-03	19062,7	5,09749E-03	20249	1,33042E+00	20736	3,04825E+00	1695	9,49592E-01	4252	0	48381,5

Myeloid_vs_CD8_Responder	CD8(NME1+ T)	Macro_ISG15	HLA-F	LILRB1	6,52974E-03	34187,1	4,36744E-03	25438	1,31976E+00	21050	1,16293E+00	34023	8,82623E-01	42043	0	48381,5
Myeloid_vs_CD8_Responder	CD8(EOMES+ NK-like)	Mono_INHBA	CALR	LRP1	6,53673E-03	37286,9	3,09639E-03	41750	1,34670E+00	20233	9,88723E-01	42052	8,96714E-01	34018	0	48381,5
Myeloid_vs_CD8_Responder	Mono_INHBA	CD8(ITM2C+ T)	ICAM1	IL2RG	6,53751E-03	32284,7	4,41874E-03	24984	1,11124E+00	28987	1,30434E+00	28449	9,02797E-01	30622	0	48381,5
Myeloid_vs_CD8_Responder	Macro_OLFML3	CD8(NME1+ T)	C3	CD46	6,55307E-03	23855,5	7,32210E-03	11485	2,33497E+00	4117	1,85419E+00	13221	8,82577E-01	42073	0	48381,5
Myeloid_vs_CD8_Responder	Mono_INHBA	CD8(ITM2C+ T)	IL1B	SIGIRR	6,55307E-03	21665,9	6,64862E-03	13382	2,31055E+00	4285	2,65121E+00	3613	8,88562E-01	38668	0	48381,5
Myeloid_vs_CD8_Responder	Macro_FOLR2+APOE-	CD8(GZMK+ Tem)	CD14	ITGA4	6,55598E-03	21848,7	5,35182E-03	18764	1,83980E+00	9230	1,91402E+00	12110	9,20480E-01	20758	0	48381,5
Myeloid_vs_CD8_Responder	Mono_INHBA	CD8(ITM2C+ T)	SPP1	CD44	6,56008E-03	31513,3	3,88123E-03	30190	1,07632E+00	30588	1,84370E+00	13407	8,94922E-01	35000	0	48381,5
Myeloid_vs_CD8_Responder	CD8(GZMK+ Tem)	cDC(CD1C)	CD28	CD86	6,56457E-03	27008,5	1,26575E-02	4787,5	1,74010E+00	10767,5	1,92100E+00	11968,5	8,53046E-01	59137,5	0	48381,5
Myeloid_vs_CD8_Responder	Mono_INHBA	CD8(ITM2C+ T)	VCAN	CD44	6,56476E-03	30717,7	4,31586E-03	25896	1,46628E+00	16725	1,21871E+00	31773	9,02428E-01	30813	0	48381,5
Myeloid_vs_CD8_Responder	Macro_OLFML3	CD8(Tc17)	CD14	ITGA4	6,56710E-03	32537,5	3,07990E-03	42091	1,56936E+00	14129	1,40674E+00	24688	8,97764E-01	33398	0	48381,5
Myeloid_vs_CD8_Responder	CD8(IL7R+ZNF683+ Tm)	Mono_INHBA	HLA-A	LILRB2	6,57491E-03	32506,1	3,07896E-03	42101	1,33899E+00	20455	9,97955E-01	41588	9,39088E-01	10005	0	48381,5
Myeloid_vs_CD8_Responder	Mono_INHBA	CD8(ITM2C+ T)	S100A8	CD69	6,58897E-03	31194,3	3,98278E-03	29108	1,09642E+00	29648	1,36053E+00	26249	9,17341E-01	22585	0	48381,5
Myeloid_vs_CD8_Responder	Mono_INHBA	CD8(ITM2C+ T)	NAMPT	ITGA5_ITGB1	6,59367E-03	36697,3	3,11035E-03	41517	1,10580E+00	29219	1,41700E+00	24341	8,86073E-01	40028	0	48381,5
Myeloid_vs_CD8_Responder	Mono_INHBA	CD8(ITM2C+ T)	VEGFA	CD44	6,59523E-03	28456,5	6,17275E-03	15079	2,00097E+00	7150	1,08302E+00	37517	8,96450E-01	34155	0	48381,5
Myeloid_vs_CD8_Responder	Macro_OLFML3	CD8(ZNF683+KLRB1+ T)	TNFSF10	CCR6	6,59758E-03	27901,3	1,97681E-02	2184	1,37094E+00	19512	1,33470E+00	27299	8,82459E-01	42130	0	48381,5
Myeloid_vs_CD8_Responder	Macro_FOLR2+APOE-	CD8(LAYN+ T)	F13A1	ITGB1	6,59943E-03	16570,5	1,76827E-02	2728	3,64213E+00	678	2,15628E+00	8297	9,17013E-01	22768	0	48381,5
Myeloid_vs_CD8_Responder	CD8(Terminal Tex)	Mono_CD14	HLA-A	LILRB2	6,60228E-03	27418,3	3,07650E-03	42136	1,51120E+00	15553	1,52304E+00	21001	9,39065E-01	10020	0	48381,5
Myeloid_vs_CD8_Responder	CD8(Terminal Tex)	Macro_FOLR2-APOE+	HLA-F	LILRB2	6,60620E-03	39353,3	3,16922E-03	40396	1,22745E+00	24168	9,86776E-01	42141	8,83203E-01	41680	0	48381,5
Myeloid_vs_CD8_Responder	CD8(Tc17)	Mono_CD16	HLA-B	LILRA1	6,60640E-03	30849,5	9,48880E-03	7519	1,81927E+00	9517	5,71700E-01	68031	9,20391E-01	20799	0	48381,5
Myeloid_vs_CD8_Responder	CD8(Temra)	Macro_OLFML3	CD99	PILRA	6,61090E-03	30559,1	4,56509E-03	23862	1,54316E+00	14758	9,86673E-01	42147	9,15523E-01	23647	0	48381,5
Myeloid_vs_CD8_Responder	Mono_INHBA	CD8(NME1+ T)	HLA-DRB1	LAG3	6,61169E-03	29880,9	3,80569E-03	31043	8,58427E-01	42148	1,54388E+00	20403	9,43624E-01	7429	0	48381,5
Myeloid_vs_CD8_Responder	CD8(GZMK+ Tex)	Macro_LYVE1	SPN	SIGLEC1	6,61802E-03	40547,7	1,14626E-02	5620	2,39077E+00	3734	7,60287E-01	54847	7,95283E-01	90156	0	48381,5
Myeloid_vs_CD8_Responder	cDC_CLEC9A	CD8(Terminal Tex)	HLA-DRB1	LAG3	6,63976E-03	17791,7	6,22896E-03	14859	1,32693E+00	20826	2,75772E+00	2920	9,55385E-01	1972	0	48381,5
Myeloid_vs_CD8_Responder	cDC(CD1C)	CD8(LAYN+ T)	LGALS9	CD44	6,64391E-03	33593,9	3,07318E-03	42189	1,10378E+00	29320	1,41618E+00	24372	9,15429E-01	23707	0	48381,5
Myeloid_vs_CD8_Responder	CD8(GZMK+ Early Tem)	Macro_NLRP3	HLA-B	LILRB2	6,65100E-03	29752,9	3,07285E-03	42198	1,50137E+00	15800	1,16296E+00	34021	9,41962E-01	8364	0	48381,5
Myeloid_vs_CD8_Responder	CD8(ZNF683+KLRB1+ T)	Mono_CD14	HMGB1	THBD	6,67388E-03	38866,1	3,55927E-03	34213	1,05781E+00	31418	1,06995E+00	38091	8,82271E-01	42227	0	48381,5
Myeloid_vs_CD8_Responder	Macro_OLFML3	CD8(LAYN+ T)	SPP1	CD44	6,68654E-03	39224,3	3,32510E-03	37703	8,56963E-01	42243	1,30211E+00	28518	8,87425E-01	39276	0	48381,5
Myeloid_vs_CD8_Responder	Mono_CD16	CD8(Tc17)	S100A8	CD69	6,68693E-03	24380,5	5,76950E-03	16713	1,32559E+00	20864	1,53018E+00	20796	9,30349E-01	15148	0	48381,5
Myeloid_vs_CD8_Responder	CD8(LAYN+ T)	Mono_CD16	HLA-B	LILRA1	6,68817E-03	31014,1	9,46506E-03	7545	1,81064E+00	9644	5,63808E-01	68635	9,20299E-01	20865	0	48381,5
Myeloid_vs_CD8_Responder	CD8(ZNF683+KLRB1+ T)	Mono_INHBA	HLA-A	LILRB2	6,68891E-03	32414,1	3,06989E-03	42246	1,33085E+00	20719	1,01637E+00	40672	9,39003E-01	10052	0	48381,5
Myeloid_vs_CD8_Responder	CD8(EOMES+ NK-like)	Macro_LYVE1	CD99	CD81	6,68970E-03	32825,5	4,41150E-03	25044	1,12162E+00	28526	9,84716E-01	42247	9,22015E-01	19929	0	48381,5
Myeloid_vs_CD8_Responder	Macro_FOLR2+APOE-	CD8(Terminal Tex)	HLA-DPA1	LAG3	6,69066E-03	19141,5	6,21118E-03	14914	1,32551E+00	20867	2,09483E+00	9152	9,54116E-01	2393	0	48381,5
Myeloid_vs_CD8_Responder	CD8(ITM2C+ T)	Mono_CD16	IFNG	IFNGR1_IFNGR2	6,69525E-03	37297,5	5,68093E-03	17137	8,56834E-01	42254	1,03687E+00	39715	8,87939E-01	39000	0	48381,5
Myeloid_vs_CD8_Responder	CD8(Terminal Tex)	Mono_CD14	CCL4	CCR1	6,71031E-03	36128,7	5,85325E-03	16344	8,56537E-01	42273	1,08289E+00	37526	8,92948E-01	36119	0	48381,5
Myeloid_vs_CD8_Responder	CD8(GZMK+ Tem)	Macro_LYVE1	HLA-A	APLP2	6,71111E-03	31413,7	3,06848E-03	42274	1,22886E+00	24116	1,06086E+00	38533	9,50662E-01	3764	0	48381,5
Myeloid_vs_CD8_Responder	CD8(Temra)	Mono_CD14	HLA-C	LILRB2	6,71185E-03	27925,9	3,00778E-03	43509	1,49636E+00	15943	1,52717E+00	20884	9,37533E-01	10912	0	48381,5
Myeloid_vs_CD8_Responder	CD8(GZMK+ Early Tem)	Macro_NLRP3	HLA-A	LILRB2	6,71190E-03	32026,9	3,06843E-03	42275	1,38320E+00	19151	1,02416E+00	40265	9,38990E-01	10062	0	48381,5
Myeloid_vs_CD8_Responder	CD8(ISG+ T)	pDC_LILRA4	SELPLG	SELL	6,71746E-03	28398,9	7,21617E-03	11751	1,04224E+00	32142	2,22737E+00	7438	8,82175E-01	42282	0	48381,5
Myeloid_vs_CD8_Responder	CD8(NME1+ T)	Macro_OLFML3	HMGB1	HAVCR2	6,71905E-03	36313,3	3,53474E-03	34538	8,68736E-01	41509	9,83703E-01	42284	9,30844E-01	14854	0	48381,5
Myeloid_vs_CD8_Responder	CD8(GZMK+ Tex)	cDC_CLEC9A	HMGB1	THBD	6,72382E-03	38641,9	3,55188E-03	34295	1,05938E+00	31346	1,09667E+00	36897	8,82163E-01	42290	0	48381,5
Myeloid_vs_CD8_Responder	Macro_IFI27	CD8(ITM2C+ T)	APOE	LDLR	6,73262E-03	23516,3	9,81420E-03	7154	1,90370E+00	8354	2,37752E+00	5838	8,72749E-01	47854	0	48381,5
Myeloid_vs_CD8_Responder	Macro_FOLR2-APOE+	CD8(GZMK+ Tem)	HLA-DPB1	LAG3	6,73308E-03	19319,3	6,28579E-03	14663	1,32449E+00	20901	2,04519E+00	9892	9,53044E-01	2759	0	48381,5
Myeloid_vs_CD8_Responder	CD8(ID2+CXCR4+ T)	Mono_CD14	CSF1	CSF3R	6,73433E-03	41430,5	5,81162E-03	16525	1,61866E+00	13041	1,52633E+00	20902	7,57276E-01	108303	0	48381,5
Myeloid_vs_CD8_Responder	CD8(ITM2C+ T)	Mono_CD14	HSPA8	LDLR	6,73973E-03	34152,5	5,44617E-03	18271	8,55921E-01	42310	1,35093E+00	26630	8,94615E-01	35170	0	48381,5
Myeloid_vs_CD8_Responder	Macro_IFI27	CD8(Tc17)	APOE	LSR	6,74438E-03	20023,9	1,54586E-02	3479	1,90321E+00	8359	2,42622E+00	5372	8,95751E-01	34528	0	48381,5
Myeloid_vs_CD8_Responder	Macro_LYVE1	CD8(GZMK+ Tem)	LGALS3	LAG3	6,74685E-03	20069,3	8,75464E-03	8537	1,32427E+00	20912	1,88125E+00	12736	9,39448E-01	9780	0	48381,5
Myeloid_vs_CD8_Responder	CD8(ID2+CXCR4+ T)	Macro_ISG15	HMGB1	CD163	6,76606E-03	31816,7	3,06512E-03	42343	1,37044E+00	19524	1,29503E+00	28794	9,21794E-01	20041	0	48381,5
Myeloid_vs_CD8_Responder	Mono_INHBA	CD8(ID2+CXCR4+ T)	VCAN	ITGB1	6,77245E-03	27465,9	6,15500E-03	15147	1,64168E+00	12559	1,60015E+00	18891	8,82066E-01	42351	0	48381,5
Myeloid_vs_CD8_Responder	CD8(Terminal Tex)	Macro_LYVE1	CD99	CD81	6,77805E-03	33432,1	4,27996E-03	26205	1,09560E+00	29694	9,82018E-01	42358	9,20920E-01	20522	0	48381,5
Myeloid_vs_CD8_Responder	Macro_IFI27	CD8(Tn)	APOE	LSR	6,78212E-03	19843,3	1,54096E-02	3496	1,90203E+00	8375	2,54801E+00	4352	8,95603E-01	34612	0	48381,5
Myeloid_vs_CD8_Responder	CD8(LAYN+ T)	Macro_ISG15	HLA-A	LILRB1	6,80209E-03	34075,7	3,26278E-03	38753	1,18071E+00	25957	9,81548E-01	42388	9,30765E-01	14899	0	48381,5
Myeloid_vs_CD8_Responder	Macro_OLFML3	CD8(GZMK+ Tem)	CD86	CTLA4	6,80931E-03	30763,1	9,21722E-03	7865	1,46625E+00	16727	1,06257E+00	38445	8,81996E-01	42397	0	48381,5
Myeloid_vs_CD8_Responder	CD8(GZMK+ Tex)	Macro_OLFML3	CD52	SIGLEC10	6,80931E-03	29621,9	5,16687E-03	19816	1,58459E+00	13758	9,81341E-01	42397	9,15362E-01	23757	0	48381,5
Myeloid_vs_CD8_Responder	CD8(GZMK+ Tem)	Mono_CD14	HLA-B	LILRB2	6,80969E-03	27253,3	2,98069E-03	44032	1,55687E+00	14436	1,53714E+00	20586	9,41124E-01	8831	0	48381,5
Myeloid_vs_CD8_Responder	CD8(IL7R+ZNF683+ Tm)	Macro_ISG15	HLA-B	LILRB1	6,81895E-03	34538,5	3,12865E-03	41143	1,15572E+00	27023	9,81156E-01	42409	9,32761E-01	13736	0	48381,5
Myeloid_vs_CD8_Responder	cDC(CD1C)	CD8(Terminal Tex)	LGALS3	LAG3	6,82860E-03	28175,1	5,31229E-03	18983	8,54266E-01	42421	1,91626E+00	12067	9,23580E-01	19023	0	48381,5
Myeloid_vs_CD8_Responder	CD8(Terminal Tex)	Macro_NLRP3	HMGB1	THBD	6,83182E-03	35680,9	4,23136E-03	26636	1,24016E+00	23715	9,80931E-01	42425	8,90961E-01	37247	0	48381,5
Myeloid_vs_CD8_Responder	CD8(Temra)	Macro_ISG15	B2M	LILRB1	6,83505E-03	32308,1	3,06018E-03	42429	1,25545E+00	23158	1,09054E+00	37187	9,38446E-01	10385	0	48381,5
Myeloid_vs_CD8_Responder	cDC(CD1C)	CD8(EOMES+ NK-like)	HLA-DPB1	LAG3	6,83585E-03	26831,5	3,06015E-03	42430	1,16222E+00	26738	2,64936E+00	3632	9,34044E-01	12976	0	48381,5
Myeloid_vs_CD8_Responder	CD8(ISG+ T)	Mono_INHBA	CD99	PILRA	6,84069E-03	38952,3	3,08907E-03	41902	1,10192E+00	29391	9,80808E-01	42436	8,99143E-01	32651	0	48381,5
Myeloid_vs_CD8_Responder	CD8(GZMK+ Tex)	Mono_CD14	HMGB1	TLR2	6,84552E-03	38671,5	3,05977E-03	42442	1,01202E+00	33628	1,19031E+00	32894	8,93115E-01	36012	0	48381,5
Myeloid_vs_CD8_Responder	CD8(ISG+ T)	Macro_ISG15	HLA-F	LILRB1	6,84633E-03	33840,9	4,31000E-03	25951	1,31112E+00	21310	1,23518E+00	31119	8,81936E-01	42443	0	48381,5
Myeloid_vs_CD8_Responder	CD8(ISG+ T)	Macro_LYVE1	CD99	PILRA	6,85521E-03	29744,5	4,69728E-03	22848	1,66748E+00	12041	9,80467E-01	42454	9,16620E-01	22998	0	48381,5
Myeloid_vs_CD8_Responder	CD8(NME1+ T)	Mono_CD16	GNAI2	FPR1	6,85682E-03	30807,1	4,02090E-03	28667	1,98996E+00	7285	1,33590E+00	27246	8,81920E-01	42456	0	48381,5
Myeloid_vs_CD8_Responder	Macro_OLFML3	CD8(NME1+ T)	CD14	ITGB2	6,85763E-03	26803,9	3,05867E-03	42457	1,78217E+00	10092	1,69899E+00	16499	9,27815E-01	16590	0	48381,5
Myeloid_vs_CD8_Responder	CD8(Tc17)	Macro_NLRP3	HSP90B1	LRP1	6,86086E-03	37471,9	3,19357E-03	39942	1,41200E+00	18287	9,80332E-01	42461	8,89192E-01	38288	0	48381,5
Myeloid_vs_CD8_Responder	Mast	CD8(Terminal Tex)	TIMP3	CD44	6,86125E-03	21886,3	2,10679E-02	1881	3,40924E+00	896	1,35575E+00	26452	9,00634E-01	31821	0	48381,5
Myeloid_vs_CD8_Responder	CD8(GZMK+ Tem)	Macro_ISG15	HLA-B	LILRB2	6,87056E-03	26926,9	3,05792E-03	42473	1,59222E+00	13583	1,49762E+00	21761	9,41828E-01	8436	0	48381,5
Myeloid_vs_CD8_Responder	CD8(GZMK+ Tex)	pDC_LILRA4	HLA-DPA1	CD4	6,87169E-03	31343,3	2,31535E-03	62457	1,32091E+00	21011	2,27288E+00	6932	9,25389E-01	17935	0	48381,5
Myeloid_vs_CD8_Responder	CD8(Temra)	Mono_CD16	HLA-B	LILRA1	6,88693E-03	30859,3	9,38938E-03	7640	1,78316E+00	10071	5,83097E-01	67181	9,20004E-01	21023	0	48381,5
Myeloid_vs_CD8_Responder	CD8(Temra)	Mono_CD14	CCL4	CCR1	6,90622E-03	35835,1	5,82335E-03	16470	8,52651E-01	42517	1,12862E+00	35553	8,92703E-01	36254	0	48381,5
Myeloid_vs_CD8_Responder	Macro_ISG15	CD8(GZMK+ Tem)	S100A8	CD69	6,90730E-03	24127,7	4,97161E-03	21039	1,33850E+00	20476	1,87828E+00	12793	9,25369E-01	17949	0	48381,5
Myeloid_vs_CD8_Responder	Macro_ISG15	CD8(Tn)	SPP1	S1PR1	6,90810E-03	25231,1	2,43113E-02	1273	1,89770E+00	8428	2,90693E+00	2234	8,40848E-01	65839	0	48381,5
Myeloid_vs_CD8_Responder	CD8(NME1+ T)	Mono_CD14	CIRBP	TREM1	6,91272E-03	30856,1	6,14540E-03	15188	1,66312E+00	12139	1,11646E+00	36047	8,81774E-01	42525	0	48381,5
Myeloid_vs_CD8_Responder	CD8(GZMK+ Tex)	Macro_ISG15	HLA-C	LILRB2	6,91435E-03	27795,3	3,05529E-03	42527	1,50463E+00	15715	1,49924E+00	21715	9,37990E-01	10638	0	48381,5
Myeloid_vs_CD8_Responder	Mono_INHBA	CD8(Temra)	VCAN	ITGB1	6,91760E-03	27395,3	6,11973E-03	15287	1,63907E+00	12616	1,62944E+00	18161	8,81767E-01	42531	0	48381,5
Myeloid_vs_CD8_Responder	Macro_FOLR2+APOE-	CD8(GZMK+ Tem)	F13A1	ITGA4	6,91767E-03	17331,9	1,68661E-02	2979	3,55837E+00	749	2,14664E+00	8432	9,11086E-01	26118	0	48381,5

Myeloid_vs_CD8_Responder	Macro_OLFML3	CD8(EOMES+ NK-like)	CXCL9	KIR2DL3	6,92857E-03	29823,7	1,15945E-01	44	2,15945E+00	5521	2,04429E+00	9911	8,04948E-01	85261	0	48381,5
Myeloid_vs_CD8_Responder	Macro_ISG15	CD8(GZMK+ Tex)	B2M	CD3D	6,92857E-03	46681,3	2,62709E-03	52747	3,93264E-01	79566	7,94651E-01	52668	9,65091E-01	44	0	48381,5
Myeloid_vs_CD8_Responder	CD8(EOMES+ NK-like)	cDC(CD1C)	ANXA1	FPR1	6,92899E-03	36380,7	3,05445E-03	42545	1,14099E+00	27659	1,22435E+00	31549	9,00736E-01	31769	0	48381,5
Myeloid_vs_CD8_Responder	Mono_INHBA	CD8(ZNF683+KLRB1+ T)	IL1B	SIGIRR	6,94122E-03	23137,5	5,81086E-03	16526	2,27062E+00	4599	2,65029E+00	3621	8,81718E-01	42560	0	48381,5
Myeloid_vs_CD8_Responder	CD8(LAYN+ T)	cDC_CLEC9A	TNFSF9	HLA-DPA1	6,94529E-03	29065,1	3,05385E-03	42565	1,23988E+00	23723	2,62699E+00	3760	9,09619E-01	26896	0	48381,5
Myeloid_vs_CD8_Responder	CD8(Tn)	Mono_CD14	CIRBP	TREM1	6,94611E-03	29548,1	6,13725E-03	15210	1,66223E+00	12163	1,27791E+00	29420	8,81705E-01	42566	0	48381,5
Myeloid_vs_CD8_Responder	CD8(NME1+ T)	Macro_NLRP3	HLA-C	LILRB2	6,95182E-03	31161,5	3,05333E-03	42573	1,44085E+00	17497	1,10148E+00	36700	9,37972E-01	10656	0	48381,5
Myeloid_vs_CD8_Responder	CD8(Tn)	Macro_FOLR2-APOE+	CD40LG	ITGAM_ITGB2	6,95527E-03	37854,7	2,10125E-02	1894	1,02566E+00	32959	1,06980E+00	38103	8,37172E-01	67936	0	48381,5
Myeloid_vs_CD8_Responder	CD8(ID2+CXCR4+ T)	Macro_ISG15	B2M	LILRB1	6,97308E-03	32837,1	3,05209E-03	42599	1,24226E+00	23641	1,04878E+00	39139	9,38370E-01	10425	0	48381,5
Myeloid_vs_CD8_Responder	CD8(Tn)	cDC(CD1C)	CD40LG	ITGAM_ITGB2	6,97706E-03	36168,5	2,10023E-02	1897	8,96811E-01	39801	1,46377E+00	22810	8,37139E-01	67953	0	48381,5
Myeloid_vs_CD8_Responder	CD8(Terminal Tex)	Macro_LYVE1	HLA-A	APLP2	6,97881E-03	31589,9	3,05181E-03	42606	1,21190E+00	24766	1,06393E+00	38379	9,50534E-01	3817	0	48381,5
Myeloid_vs_CD8_Responder	CD8(Temra)	cDC(CD1C)	CD99	PILRA	6,98290E-03	35962,7	3,05157E-03	42611	1,07399E+00	30697	1,39119E+00	25181	8,98587E-01	32943	0	48381,5
Myeloid_vs_CD8_Responder	Macro_FOLR2+APOE-	CD8(GZMK+ Tem)	HLA-DRB1	LAG3	6,98664E-03	19083,7	6,31753E-03	14543	1,31810E+00	21101	2,07286E+00	9510	9,55684E-01	1883	0	48381,5
Myeloid_vs_CD8_Responder	CD8(GZMK+ Tem)	Mono_INHBA	B2M	LILRB2	6,98700E-03	28136,7	3,05136E-03	42616	1,58710E+00	13694	1,24892E+00	30565	9,47291E-01	5427	0	48381,5
Myeloid_vs_CD8_Responder	CD8(Terminal Tex)	Mono_INHBA	CIRBP	TREM1	6,98864E-03	32578,5	6,13827E-03	15209	1,56967E+00	14121	9,77127E-01	42618	8,81714E-01	42563	0	48381,5
Myeloid_vs_CD8_Responder	Macro_FOLR2+APOE+	CD8(GZMK+ Tex)	HLA-DRA	LAG3	6,99049E-03	20509,9	4,96087E-03	21104	1,32839E+00	20783	2,17559E+00	8037	9,49607E-01	4244	0	48381,5
Myeloid_vs_CD8_Responder	Macro_OLFML3	CD8(LAYN+ T)	MDK	ITGA4_ITGB1	6,99192E-03	28903,1	1,40620E-02	4061	1,77503E+00	10192	1,04631E+00	39259	8,81613E-01	42622	0	48381,5
Myeloid_vs_CD8_Responder	Macro_OLFML3	CD8(LAYN+ T)	HLA-DRA	LAG3	6,99274E-03	26643,9	3,05105E-03	42623	1,25375E+00	23217	2,21394E+00	7578	9,36622E-01	11420	0	48381,5
Myeloid_vs_CD8_Responder	Macro_FOLR2+APOE-	CD8(GZMK+ Tem)	HLA-DQA2	LAG3	6,99435E-03	20367,5	9,58138E-03	7408	1,31798E+00	21107	2,05151E+00	9794	9,30352E-01	15147	0	48381,5
Myeloid_vs_CD8_Responder	CD8(Tc17)	Macro_NLRP3	HLA-B	LILRB2	6,99438E-03	30604,1	3,05099E-03	42625	1,47667E+00	16453	1,09250E+00	37091	9,41766E-01	8470	0	48381,5
Myeloid_vs_CD8_Responder	CD8(Terminal Tex)	Macro_NLRP3	CALR	LRP1	7,01821E-03	36118,9	3,31554E-03	37856	1,37371E+00	19444	9,76611E-01	42654	8,99838E-01	32259	0	48381,5
Myeloid_vs_CD8_Responder	Macro_NLRP3	CD8(GZMK+ Tem)	HLA-DRA	LAG3	7,01881E-03	19240,1	6,00163E-03	15746	1,31721E+00	21126	2,14206E+00	8504	9,53974E-01	2443	0	48381,5
Myeloid_vs_CD8_Responder	CD8(GZMK+ Tem)	Macro_IER3	HLA-F	LILRB2	7,02068E-03	39266,5	3,20102E-03	39818	1,22839E+00	24129	9,76594E-01	42657	8,83717E-01	41347	0	48381,5
Myeloid_vs_CD8_Responder	Macro_OLFML3	CD8(GZMK+ Early Tem)	HLA-DQA1	LAG3	7,02479E-03	33382,3	3,04888E-03	42662	1,03267E+00	32594	1,44660E+00	23338	9,22006E-01	19936	0	48381,5
Myeloid_vs_CD8_Responder	CD8(GZMK+ Early Tem)	Macro_NLRP3	HLA-C	LILRB2	7,02562E-03	31131,3	3,04887E-03	42663	1,43707E+00	17585	1,10911E+00	36349	9,37929E-01	10678	0	48381,5
Myeloid_vs_CD8_Responder	cDC(CD1C)	CD8(EOMES+ NK-like)	HLA-DRB5	LAG3	7,03550E-03	30214,1	3,04847E-03	42675	9,62974E-01	36140	2,33767E+00	6231	9,25854E-01	17643	0	48381,5
Myeloid_vs_CD8_Responder	CD8(GZMK+ Tex)	Mono_CD16	HLA-A	LILRA1	7,03559E-03	27228,5	1,03025E-02	6657	1,95405E+00	7709	8,03230E-01	52132	9,19563E-01	21263	0	48381,5
Myeloid_vs_CD8_Responder	CD8(ITM2C+ T)	Macro_ISG15	CD99	PILRA	7,03962E-03	37306,7	3,28719E-03	38332	1,17853E+00	26043	9,76060E-01	42680	9,01926E-01	31097	0	48381,5
Myeloid_vs_CD8_Responder	CD8(NME1+ T)	Macro_ISG15	B2M	LILRB1	7,04375E-03	32446,3	3,04822E-03	42685	1,23594E+00	23859	1,09756E+00	36863	9,38333E-01	10443	0	48381,5
Myeloid_vs_CD8_Responder	Macro_LYVE1	CD8(Terminal Tex)	HLA-DPB1	LAG3	7,04593E-03	20146,7	6,13041E-03	15238	1,31651E+00	21147	1,86758E+00	12982	9,52481E-01	2985	0	48381,5
Myeloid_vs_CD8_Responder	CD8(Terminal Tex)	Mono_INHBA	HSPA1A	TLR4	7,04788E-03	35318,9	3,71381E-03	32143	1,20107E+00	25179	1,31031E+00	28201	8,81494E-01	42690	0	48381,5
Myeloid_vs_CD8_Responder	CD8(GZMK+ Tex)	Mono_CD16	HLA-C	LILRA1	7,05011E-03	27997,1	1,00005E-02	6959	1,92065E+00	8122	7,74676E-01	53922	9,17313E-01	22601	0	48381,5
Myeloid_vs_CD8_Responder	CD8(ISG+ T)	Macro_LYVE1	CALR	LRP1	7,05283E-03	29795,5	4,34787E-03	25621	2,07589E+00	6321	9,75735E-01	42696	9,11409E-01	25958	0	48381,5
Myeloid_vs_CD8_Responder	cDC(CD1C)	CD8(LAYN+ T)	HLA-DQA2	LAG3	7,05283E-03	34247,3	3,76763E-03	31489	8,49494E-01	42696	1,87831E+00	12792	8,93349E-01	35878	0	48381,5
Myeloid_vs_CD8_Responder	Macro_IFI27	CD8(IL7R+ZNF683+ Tm)	APOE	LSR	7,05738E-03	20200,7	1,50431E-02	3647	1,89318E+00	8490	2,44030E+00	5247	8,94472E-01	35238	0	48381,5
Myeloid_vs_CD8_Responder	CD8(ISG+ T)	Macro_ISG15	HLA-A	LILRB2	7,06357E-03	28853,3	3,04711E-03	42709	1,43923E+00	17534	1,38324E+00	25473	9,38790E-01	10169	0	48381,5
Myeloid_vs_CD8_Responder	CD8(GZMK+ Tex)	pDC_LILRA4	CCL5	CXCR3	7,06405E-03	37465,5	4,95306E-03	21161	3,73503E-01	81648	1,60918E+00	18646	9,26130E-01	17491	0	48381,5
Myeloid_vs_CD8_Responder	Macro_FOLR2+APOE-	CD8(ZNF683+KLRB1+ T)	CCL13	CXCR3	7,06453E-03	42766,3	2,09587E-02	1909	3,95483E+00	460	7,89766E-01	52946	7,53076E-01	110135	0	48381,5
Myeloid_vs_CD8_Responder	Mono_INHBA	CD8(Tc17)	ICAM1	IL2RG	7,06523E-03	41676,3	3,04706E-03	42711	9,34050E-01	37705	1,05011E+00	39067	8,85223E-01	40517	0	48381,5
Myeloid_vs_CD8_Responder	CD8(IL7R+ZNF683+ Tm)	Macro_FOLR2+APOE+	ANXA1	FPR1	7,07701E-03	30481,9	4,95125E-03	21171	1,46908E+00	16659	9,22375E-01	45363	9,20338E-01	20835	0	48381,5
Myeloid_vs_CD8_Responder	Mono_INHBA	CD8(NME1+ T)	VCAN	ITGB1	7,08344E-03	27902,1	6,08109E-03	15435	1,63620E+00	12675	1,54877E+00	20286	8,81436E-01	42733	0	48381,5
Myeloid_vs_CD8_Responder	CD8(Tc17)	cDC_CLEC9A	CD52	SIGLEC10	7,08344E-03	38935,3	3,24920E-03	38973	1,08923E+00	29963	9,74870E-01	42733	8,95575E-01	34626	0	48381,5
Myeloid_vs_CD8_Responder	Macro_FOLR2+APOE+	CD8(EOMES+ NK-like)	CXCL9	KIR2DL3	7,08594E-03	31520,3	1,13973E-01	45	2,13343E+00	5746	1,65608E+00	17495	8,03597E-01	85934	0	48381,5
Myeloid_vs_CD8_Responder	Macro_OLFML3	CD8(GZMK+ Tex)	HLA-B	CD8A	7,08594E-03	47318,1	3,42604E-03	36111	4,08637E-01	78051	4,95630E-01	74002	9,65079E-01	45	0	48381,5
Myeloid_vs_CD8_Responder	Macro_OLFML3	CD8(NME1+ T)	CXCL10	CXCR3	7,08676E-03	23943,9	1,71193E-02	2906	1,53694E+00	14900	1,98761E+00	10795	8,81433E-01	42737	0	48381,5
Myeloid_vs_CD8_Responder	CD8(ISG+ T)	Mono_CD14	CSF1	CSF3R	7,08869E-03	40911,3	6,06673E-03	15494	1,62638E+00	12870	1,51726E+00	21180	7,61202E-01	106631	0	48381,5
Myeloid_vs_CD8_Responder	Macro_IER3	CD8(Temra)	IL1B	SIGIRR	7,09138E-03	19708,1	8,77488E-03	8504	2,31444E+00	4255	2,34801E+00	6118	9,01578E-01	31282	0	48381,5
Myeloid_vs_CD8_Responder	CD8(LAYN+ T)	Mono_CD14	HSP90B1	LRP1	7,09173E-03	36189,9	3,04548E-03	42743	1,30316E+00	21588	1,29955E+00	28623	8,86831E-01	39614	0	48381,5
Myeloid_vs_CD8_Responder	Macro_FOLR2+APOE+	CD8(Tc17)	APOE	SORL1	7,09381E-03	17007,1	7,61033E-03	10745	2,85514E+00	1913	3,29347E+00	1044	9,16702E-01	22952	0	48381,5
Myeloid_vs_CD8_Responder	CD8(GZMK+ Tex)	Mono_CD16	HLA-B	LILRA1	7,09389E-03	31754,3	9,31252E-03	7742	1,75525E+00	10518	5,33984E-01	70946	9,19701E-01	21184	0	48381,5
Myeloid_vs_CD8_Responder	CD8(GZMK+ Early Tem)	Macro_ISG15	HLA-A	LILRB1	7,10834E-03	34521,9	3,23219E-03	39265	1,15356E+00	27118	9,74146E-01	42763	9,30461E-01	15082	0	48381,5
Myeloid_vs_CD8_Responder	CD8(GZMK+ Tem)	Macro_ISG15	CD99	PILRA	7,11084E-03	38237,1	3,16084E-03	40542	1,14704E+00	27412	9,74117E-01	42766	9,00179E-01	32084	0	48381,5
Myeloid_vs_CD8_Responder	Macro_IFI27	CD8(Tn)	APOE	LDLR	7,11573E-03	23640,1	9,45493E-03	7562	1,89174E+00	8514	2,50395E+00	4711	8,70664E-01	49032	0	48381,5
Myeloid_vs_CD8_Responder	cDC_LAMP3	CD8(GZMK+ Tem)	HLA-DQB1	LAG3	7,11991E-03	19697,9	7,25175E-03	11668	1,31479E+00	21204	1,92801E+00	11829	9,47324E-01	5407	0	48381,5
Myeloid_vs_CD8_Responder	cDC(CD1C)	CD8(Tc17)	LGALS1	CD69	7,12248E-03	30804,5	3,17264E-03	40334	8,48111E-01	42780	1,79719E+00	14319	9,42264E-01	8208	0	48381,5
Myeloid_vs_CD8_Responder	cDC(CD1C)	CD8(LAYN+ T)	HBEGF	CD44	7,12498E-03	41166,5	3,09208E-03	41838	9,06846E-01	39220	1,17255E+00	33610	8,81352E-01	42783	0	48381,5
Myeloid_vs_CD8_Responder	CD8(GZMK+ Tex)	Macro_LYVE1	HLA-A	APLP2	7,13081E-03	31298,3	3,04344E-03	42790	1,20338E+00	25085	1,10820E+00	36391	9,50469E-01	3844	0	48381,5
Myeloid_vs_CD8_Responder	CD8(GZMK+ Tex)	Mono_INHBA	HMGB1	THBD	7,13164E-03	39340,1	3,62226E-03	33326	1,07806E+00	30500	9,73611E-01	42791	8,83179E-01	41702	0	48381,5
Myeloid_vs_CD8_Responder	CD8(LAYN+ T)	Macro_NLRP3	HLA-B	LILRB2	7,13248E-03	30763,3	3,04335E-03	42792	1,46804E+00	16679	1,08461E+00	37447	9,41697E-01	8517	0	48381,5
Myeloid_vs_CD8_Responder	Macro_OLFML3	CD8(Temra)	C3	CD46	7,14165E-03	23832,7	7,14803E-03	11926	2,32835E+00	4161	1,92472E+00	11892	8,81324E-01	42803	0	48381,5
Myeloid_vs_CD8_Responder	CD8(GZMK+ Tem)	Macro_ISG15	SEMA4A	LILRB2	7,14209E-03	36948,3	5,01733E-03	20738	1,58273E+00	13796	1,59770E+00	18956	8,09486E-01	82870	0	48381,5
Myeloid_vs_CD8_Responder	Macro_OLFML3	CD8(EOMES+ NK-like)	HLA-DPB1	LAG3	7,14415E-03	28926,3	3,04264E-03	42806	1,15234E+00	27174	1,85662E+00	13174	9,33867E-01	13096	0	48381,5
Myeloid_vs_CD8_Responder	CD8(GZMK+ Tem)	cDC(CD1C)	CSF1	CSF2RA	7,14993E-03	40721,9	4,94373E-03	21227	1,79736E+00	9856	1,53981E+00	20510	7,67747E-01	103635	0	48381,5
Myeloid_vs_CD8_Responder	Macro_FOLR2-APOE+	CD8(GZMK+ Tem)	CD14	ITGA4	7,16431E-03	22433,7	5,22594E-03	19485	1,79843E+00	9842	1,85416E+00	13222	9,19605E-01	21238	0	48381,5
Myeloid_vs_CD8_Responder	CD8(Temra)	Mono_CD14	HMGB1	THBD	7,16922E-03	39475,3	3,49209E-03	35126	1,03958E+00	32259	1,05637E+00	38774	8,81278E-01	42836	0	48381,5
Myeloid_vs_CD8_Responder	CD8(Terminal Tex)	Mono_INHBA	HLA-G	LILRB2	7,16955E-03	34560,1	6,32407E-03	14510	1,68156E+00	11796	1,51524E+00	21242	8,20807E-01	76871	0	48381,5
Myeloid_vs_CD8_Responder	Macro_FOLR2+APOE-	CD8(GZMK+ Tem)	HLA-DQB1	LAG3	7,17086E-03	19560,1	7,24286E-03	11696	1,31332E+00	21243	1,97216E+00	11055	9,47294E-01	5425	0	48381,5
Myeloid_vs_CD8_Responder	cDC(CD1C)	CD8(ITM2C+ T)	HLA-DQA2	LAG3	7,17341E-03	34495,3	3,73876E-03	31845	8,47084E-01	42841	1,84942E+00	13302	8,92982E-01	36107	0	48381,5
Myeloid_vs_CD8_Responder	CD8(ISG+ T)	Mono_CD16	HLA-F	LILRB1	7,18095E-03	36123,1	4,25377E-03	26436	1,29491E+00	21861	1,00851E+00	41087	8,81250E-01	42850	0	48381,5
Myeloid_vs_CD8_Responder	CD8(GZMK+ Tex)	Macro_FOLR2-APOE+	HLA-F	LILRB2	7,18262E-03	39533,7	3,05202E-03	42601	1,20432E+00	25043	1,05608E+00	38791	8,81246E-01	42852	0	48381,5
Myeloid_vs_CD8_Responder	CD8(Tn)	Mono_CD14	HSPA4	TLR4	7,18659E-03	36758,7	6,75827E-03	13041	1,37162E+00	19489	1,51476E+00	21255	8,11850E-01	81627	0	48381,5
Myeloid_vs_CD8_Responder	CD8(Terminal Tex)	pDC_LILRA4	IRAK4	TLR7	7,19315E-03	41897,7	9,78397E-03	7184	1,64694E+00	12460	1,51440E+00	21260	7,28776E-01	120203	0	48381,5
Myeloid_vs_CD8_Responder	Macro_OLFML3	CD8(ID2+CXCR4+ T)	MDK	NCL	7,20276E-03	27414,7	1,15206E-02	5578	1,72194E+00	11075	9,71641E-01	42876	9,05514E-01	29163	0	48381,5
Myeloid_vs_CD8_Responder	CD8(NME1+ T)	Macro_FOLR2+APOE+	HMGB1	TLR4	7,20696E-03	37387,3	3,86377E-03	30375	1,27460E+00	22515	9,73803E-01	42784	8,81192E-01	42881	0	48381,5
Myeloid_vs_CD8_Responder	CD8(GZMK+ Tex)	Mono_INHBA	CIRBP	TREM1	7,21368E-03	32074,1	6,07631E-03	15456	1,56221E+00	14300	1,04445E+00	39344	8,81184E-01	42889	0	48381,5
Myeloid_vs_CD8_Responder	CD8(GZMK+ Tex)	Mono_CD16	B2M	LILRB1	7,24063E-03	32858,5	3,07813E-03	42109	1,33488E+00	20594	9,70773E-01	42921	9,38615E-01	10287	0	48381,5
Myeloid_vs_CD8_Responder	CD8(ZNF683+KLRB1+ T)	Macro_ISG15	B2M	LILRB1	7,24401E-03	33158,5	3,03684E-03	42925	1,21738E+00	24542	1,04247E+00	39429	9,38225E-01	10515	0	48381,5

Myeloid_vs_CD8_Post_R

Myeloid_vs_CD8_Responder	CD8(Temra)	Mono_CD14	CD99	PILRA	7,25583E-03	36792,9	3,03621E-03	42939	1,06923E+00	30888	1,29768E+00	28694	8,98357E-01	33062	0	48381,5
Myeloid_vs_CD8_Responder	CD8(GZMK+ Tem)	Macro_ISG15	HLA-F	LILRB1	7,26259E-03	34467,9	4,24134E-03	26548	1,30079E+00	21667	1,19280E+00	32796	8,81097E-01	42947	0	48381,5
Myeloid_vs_CD8_Responder	CD8(GZMK+ Tem)	Macro_FOLR2+APOE+	HMGB1	CD163	7,27359E-03	29799,1	3,79268E-03	31206	1,75105E+00	10584	9,69796E-01	42960	9,29135E-01	15864	0	48381,5
Myeloid_vs_CD8_Responder	CD8(ZNF683+KLRB1+ T)	cDC_CLEC9A	TGFB1	LPP	7,27444E-03	32894,9	6,41493E-03	14182	9,65896E-01	36008	1,45969E+00	22942	8,81069E-01	42961	0	48381,5
Myeloid_vs_CD8_Responder	CD8(ZNF683+KLRB1+ T)	Mono_INHBA	HMGB1	CD163	7,27698E-03	31637,9	3,03504E-03	42964	1,39440E+00	18812	1,32150E+00	27787	9,21438E-01	20245	0	48381,5
Myeloid_vs_CD8_Responder	CD8(NME1+ T)	Macro_ISG15	HMGB1	HAVCR2	7,28037E-03	34262,1	3,38728E-03	36705	8,45326E-01	42968	1,32760E+00	27561	9,29460E-01	15695	0	48381,5
Myeloid_vs_CD8_Responder	Macro_NLRP3	CD8(ISG+ T)	VCAN	SELL	7,28520E-03	23327,7	1,11128E-02	5922	2,64368E+00	2565	2,13603E+00	8583	8,66971E-01	51187	0	48381,5
Myeloid_vs_CD8_Responder	Mono_INHBA	CD8(GZMK+ Tex)	VEGFA	ITGB1	7,29054E-03	23794,3	9,85163E-03	7110	2,23070E+00	4934	1,73966E+00	15566	8,81037E-01	42980	0	48381,5
Myeloid_vs_CD8_Responder	CD8(ITM2C+ T)	Macro_ISG15	RPS19	C5AR1	7,29393E-03	33331,3	3,03427E-03	42984	1,15831E+00	26903	1,22846E+00	31392	9,27071E-01	16996	0	48381,5
Myeloid_vs_CD8_Responder	CD8(GZMK+ Early Tem)	Mono_INHBA	HLA-F	LILRB2	7,30582E-03	37730,3	3,03810E-03	42890	1,41620E+00	18176	1,11290E+00	36206	8,81006E-01	42998	0	48381,5
Myeloid_vs_CD8_Responder	Mono_INHBA	CD8(NME1+ T)	VEGFA	CD44	7,31857E-03	32214,3	4,97600E-03	21007	1,88481E+00	8609	9,68570E-01	43013	8,86011E-01	40061	0	48381,5
Myeloid_vs_CD8_Responder	CD8(IL7R+ZNF683+ Tm)	Mono_CD16	HLA-B	LILRA1	7,31999E-03	32260,3	9,23736E-03	7836	1,72795E+00	10962	5,10368E-01	72766	9,19401E-01	21356	0	48381,5
Myeloid_vs_CD8_Responder	Mono_INHBA	CD8(GZMK+ Early Tem)	ICAM1	IL2RG	7,32453E-03	41438,9	3,03243E-03	43020	9,32161E-01	37793	1,08683E+00	37347	8,84979E-01	40653	0	48381,5
Myeloid_vs_CD8_Responder	Mono_INHBA	CD8(GZMK+ Tex)	VCAN	ITGA4	7,33475E-03	26699,3	6,68379E-03	13280	1,64030E+00	12584	1,71124E+00	16219	8,80957E-01	43032	0	48381,5
Myeloid_vs_CD8_Responder	CD8(ZNF683+KLRB1+ T)	pDC_LILRA4	COPA	P2RY6	7,34226E-03	30294,7	8,71385E-03	8606	2,32760E+00	4169	2,15955E+00	8245	8,11014E-01	82072	0	48381,5
Myeloid_vs_CD8_Responder	CD8(ISG+ T)	Mono_CD16	B2M	LILRB1	7,34242E-03	32731,7	3,08795E-03	41925	1,35110E+00	20093	9,67937E-01	43041	9,38707E-01	10218	0	48381,5
Myeloid_vs_CD8_Responder	CD8(NME1+ T)	Macro_ISG15	HLA-A	LILRB2	7,34413E-03	29175,7	3,03142E-03	43043	1,42380E+00	17961	1,36108E+00	26232	9,38641E-01	10261	0	48381,5
Myeloid_vs_CD8_Responder	Macro_NLRP3	CD8(NME1+ T)	S100A8	CD69	7,36530E-03	21929,7	4,91967E-03	21390	1,81707E+00	9543	1,91126E+00	12169	9,25006E-01	18165	0	48381,5
Myeloid_vs_CD8_Responder	Macro_FOLR2+APOE-	CD8(LAYN+ T)	C1QB	C1QBP	7,36797E-03	20418,9	4,91941E-03	21392	2,03096E+00	6819	2,34697E+00	6129	9,22970E-01	19373	0	48381,5
Myeloid_vs_CD8_Responder	CD8(ISG+ T)	Macro_ISG15	CD52	SIGLEC10	7,38517E-03	38137,5	3,48583E-03	35225	1,06298E+00	31169	9,67038E-01	43091	8,98817E-01	32821	0	48381,5
Myeloid_vs_CD8_Responder	CD8(NME1+ T)	pDC_LILRA4	TNF	PTPRS	7,38982E-03	32359,9	2,07664E-02	1953	3,60732E+00	706	1,94601E+00	11526	7,76995E-01	99233	0	48381,5
Myeloid_vs_CD8_Responder	CD8(GZMK+ Tem)	Macro_FOLR2-APOE+	HLA-F	LILRB2	7,39374E-03	39100,9	3,23268E-03	39251	1,23998E+00	23720	9,66762E-01	43101	8,84222E-01	41051	0	48381,5
Myeloid_vs_CD8_Responder	Macro_OLFML3	CD8(ITM2C+ T)	HLA-DRA	LAG3	7,39975E-03	26854,9	3,02768E-03	43108	1,25134E+00	23309	2,18504E+00	7915	9,36393E-01	11561	0	48381,5
Myeloid_vs_CD8_Responder	CD8(ZNF683+KLRB1+ T)	Mono_INHBA	CIRBP	TREM1	7,40490E-03	32904,9	6,03230E-03	15622	1,55690E+00	14432	9,69350E-01	42975	8,80803E-01	43114	0	48381,5
Myeloid_vs_CD8_Responder	CD8(NME1+ T)	Mono_CD14	HSP90B1	LRP1	7,40576E-03	36300,3	3,02727E-03	43115	1,29901E+00	21733	1,30319E+00	28491	8,86530E-01	39781	0	48381,5
Myeloid_vs_CD8_Responder	CD8(ISG+ T)	Mono_CD14	HMGB1	TLR4	7,40662E-03	38183,7	3,83506E-03	30709	1,13942E+00	27728	1,01046E+00	40984	8,80801E-01	43116	0	48381,5
Myeloid_vs_CD8_Responder	Macro_FOLR2-APOE+	CD8(Terminal Tex)	HLA-DRB1	LAG3	7,41082E-03	19287,3	6,14646E-03	15177	1,30775E+00	21424	2,07947E+00	9385	9,55099E-01	2069	0	48381,5
Myeloid_vs_CD8_Responder	CD8(Temra)	Mono_INHBA	B2M	LILRB2	7,41177E-03	28620,7	3,02695E-03	43122	1,54685E+00	14663	1,22815E+00	31403	9,47091E-01	5534	0	48381,5
Myeloid_vs_CD8_Responder	pDC_LILRA4	CD8(ITM2C+ T)	APP	CD74	7,41618E-03	20075,5	4,91516E-03	21428	1,85575E+00	8993	1,96887E+00	11133	9,38333E-01	10442	0	48381,5
Myeloid_vs_CD8_Responder	cDC_CLEC9A	CD8(GZMK+ Tex)	HLA-DRA	LAG3	7,41887E-03	19449,9	4,91403E-03	21430	1,31400E+00	21227	3,00170E+00	1856	9,49380E-01	4355	0	48381,5
Myeloid_vs_CD8_Responder	CD8(ITM2C+ T)	pDC_LILRA4	HSP90B1	TLR9	7,42723E-03	43934,6	2,07397E-02	1958	3,61137E+00	701	9,80660E-01	42443	8,34503E-01	69424	0,031	105147
Myeloid_vs_CD8_Responder	CD8(Tc17)	Macro_ISG15	LTB	TNFRSF1A	7,43845E-03	30889,3	8,45996E-03	9004	1,11756E+00	28705	1,39072E+00	25203	8,80765E-01	43153	0	48381,5
Myeloid_vs_CD8_Responder	CD8(GZMK+ Tem)	cDC_CLEC9A	LTB	CD40	7,43903E-03	29676,7	8,34227E-03	9224	1,58602E+00	13719	1,56511E+00	19817	8,56231E-01	57242	0	48381,5
Myeloid_vs_CD8_Responder	pDC_LILRA4	CD8(NME1+ T)	APP	LRP10	7,43968E-03	27123,7	8,69334E-03	8645	1,92493E+00	8059	2,48912E+00	4821	8,41103E-01	65712	0	48381,5
Myeloid_vs_CD8_Responder	CD8(GZMK+ Tem)	cDC_CLEC9A	CRTAM	CADM1	7,44038E-03	25275,7	2,72811E-02	977,5	1,42367E+00	17964,5	1,80093E+00	14245,5	8,77887E-01	44809,5	0	48381,5
Myeloid_vs_CD8_Responder	pDC_LILRA4	CD8(GZMK+ Early Tem)	APP	LRP10	7,45223E-03	27032,3	8,68940E-03	8650	1,92481E+00	8062	2,54886E+00	4342	8,41073E-01	65726	0	48381,5
Myeloid_vs_CD8_Responder	CD8(ID2+CXCR4+ T)	Macro_ISG15	HLA-A	LILRB2	7,45484E-03	29638,7	3,02466E-03	43172	1,41714E+00	18146	1,31087E+00	28179	9,38577E-01	10315	0	48381,5
Myeloid_vs_CD8_Responder	CD8(GZMK+ Tem)	pDC_LILRA4	TNF	TNFRSF21	7,47003E-03	38645,1	5,94382E-03	15974	3,41866E+00	888	1,60240E+00	18829	7,55458E-01	109153	0	48381,5
Myeloid_vs_CD8_Responder	CD8(GZMK+ Tem)	pDC_LILRA4	TNF	PTPRS	7,47138E-03	35957,3	1,49418E-02	3684	3,56323E+00	743	1,79966E+00	14273	7,47185E-01	112705	0	48381,5
Myeloid_vs_CD8_Responder	CD8(GZMK+ Tem)	pDC_LILRA4	HLA-DRB1	CD4	7,47948E-03	29658,1	2,46164E-03	57843	1,35304E+00	20039	2,33441E+00	6272	9,29333E-01	15755	0	48381,5
Myeloid_vs_CD8_Responder	Macro_OLFML3	CD8(EOMES+ NK-like)	HLA-DPA1	LAG3	7,48338E-03	28726,9	3,02300E-03	43205	1,13481E+00	27938	1,91782E+00	12028	9,35513E-01	12082	0	48381,5
Myeloid_vs_CD8_Responder	CD8(GZMK+ Tem)	pDC_LILRA4	HLA-DPB1	CD4	7,48623E-03	31124,7	2,35322E-03	61316	1,34846E+00	20170	2,27435E+00	6914	9,23849E-01	18842	0	48381,5
Myeloid_vs_CD8_Responder	Mono_CD14	CD8(GZMK+ Tem)	VCAN	ITGB1	7,49248E-03	19452,1	8,67962E-03	8666	3,13588E+00	1296	2,35134E+00	6091	8,98803E-01	32826	0	48381,5
Myeloid_vs_CD8_Responder	CD8(NME1+ T)	Macro_OLFML3	CD52	SIGLEC10	7,49377E-03	29181,3	5,39130E-03	18566	1,62187E+00	12964	9,64505E-01	43217	9,16994E-01	22778	0	48381,5
Myeloid_vs_CD8_Responder	CD8(GZMK+ Tem)	pDC_LILRA4	HLA-DPA1	CD4	7,49570E-03	31940,7	2,27869E-03	63681	1,30941E+00	21372	2,17864E+00	7994	9,24836E-01	18275	0	48381,5
Myeloid_vs_CD8_Responder	CD8(GZMK+ Tex)	Macro_IER3	HLA-F	LILRB2	7,49724E-03	39713,1	3,02213E-03	43221	1,19274E+00	25484	1,06591E+00	38298	8,80730E-01	43181	0	48381,5
Myeloid_vs_CD8_Responder	CD8(ITM2C+ T)	cDC_CLEC9A	ANXA1	DYSF	7,50245E-03	30164,3	1,12372E-02	5809	1,74862E+00	10624	9,64359E-01	43227	8,81362E-01	42780	0	48381,5
Myeloid_vs_CD8_Responder	Mono_INHBA	CD8(NME1+ T)	INHBA	ACTR2	7,50509E-03	21618,7	1,38513E-02	4161	3,18276E+00	1218	2,12831E+00	8671	8,76427E-01	45662	0	48381,5
Myeloid_vs_CD8_Responder	Macro_OLFML3	CD8(ZNF683+KLRB1+ T)	FN1	ITGA4_ITGB7	7,50852E-03	23859,9	1,10110E-02	6015	1,98845E+00	7307	1,79550E+00	14362	8,80641E-01	43234	0	48381,5
Myeloid_vs_CD8_Responder	cDC(CD1C)	CD8(GZMK+ Early Tem)	LGALS9	PTPRC	7,51026E-03	33803,1	3,02128E-03	43236	9,93616E-01	34586	1,45085E+00	23209	9,22542E-01	19603	0	48381,5
Myeloid_vs_CD8_Responder	CD8(Terminal Tex)	Macro_NLRP3	B2M	LILRB2	7,51374E-03	27540,7	3,02095E-03	43240	1,68403E+00	11751	1,29569E+00	28768	9,47041E-01	5563	0	48381,5
Myeloid_vs_CD8_Responder	CD8(GZMK+ Tem)	pDC_LILRA4	COPA	P2RY6	7,53094E-03	32526,1	7,38294E-03	11324	2,28038E+00	4526	2,06380E+00	9617	7,97984E-01	88782	0	48381,5
Myeloid_vs_CD8_Responder	pDC_LILRA4	CD8(EOMES+ NK-like)	COL24A1	ITGA1_ITGB1	7,53249E-03	46963,9	4,82654E-02	321	2,83125E+00	1972	7,14567E-01	57793	7,12420E-01	126352	0	48381,5
Myeloid_vs_CD8_Responder	pDC_LILRA4	CD8(GZMK+ Tex)	APP	LRP10	7,53288E-03	27012,5	8,67121E-03	8682	1,92427E+00	8069	2,57781E+00	4134	8,40933E-01	65796	0	48381,5
Myeloid_vs_CD8_Responder	Macro_OLFML3	CD8(GZMK+ Early Tem)	C1QB	C1QBP	7,53809E-03	26329,1	3,01966E-03	43268	2,06392E+00	6442	2,67641E+00	3437	9,03731E-01	30117	0	48381,5
Myeloid_vs_CD8_Responder	cDC(CD1C)	CD8(GZMK+ Early Tem)	LYZ	ITGAL	7,54419E-03	28623,1	3,01939E-03	43275	1,65691E+00	12257	2,93290E+00	2110	8,91207E-01	37092	0	48381,5
Myeloid_vs_CD8_Responder	Macro_FOLR2+APOE+	CD8(Temra)	APOE	SORL1	7,54758E-03	17599,3	7,10151E-03	12060	2,83049E+00	1974	3,28180E+00	1080	9,14022E-01	24501	0	48381,5
Myeloid_vs_CD8_Responder	CD8(GZMK+ Tem)	pDC_LILRA4	PSEN1	NOTCH4	7,54996E-03	47036,1	1,18355E-02	5315	1,45235E+00	17123	1,74412E+00	15458	6,24535E-01	148903	0	48381,5
Myeloid_vs_CD8_Responder	CD8(Temra)	Macro_NLRP3	HLA-B	LILRB2	7,55117E-03	30875,3	3,01902E-03	43283	1,44056E+00	17502	1,10390E+00	36578	9,41477E-01	8632	0	48381,5
Myeloid_vs_CD8_Responder	CD8(ID2+CXCR4+ T)	Mono_INHBA	B2M	LILRB2	7,55204E-03	29061,7	3,01895E-03	43284	1,53367E+00	14993	1,18639E+00	33080	9,47024E-01	5570	0	48381,5
Myeloid_vs_CD8_Responder	CD8(NME1+ T)	Macro_NLRP3	HLA-B	LILRB2	7,55553E-03	31008,3	3,01858E-03	43288	1,44007E+00	17511	1,08970E+00	37225	9,41473E-01	8636	0	48381,5
Myeloid_vs_CD8_Responder	Mono_CD14	CD8(LAYN+ T)	RETN	GPR25	7,55805E-03	32683,5	1,10866E-01	48	2,50384E+00	3136	1,59655E+00	18984	7,89974E-01	92868	0	48381,5
Myeloid_vs_CD8_Responder	cDC_CLEC9A	CD8(GZMK+ Tex)	HLA-DPB1	LAG3	7,56766E-03	19576,7	4,99635E-03	20874	1,30437E+00	21540	3,00850E+00	1837	9,47631E-01	5251	0	48381,5
Myeloid_vs_CD8_Responder	CD8(LAYN+ T)	Macro_FOLR2+APOE+	CD52	SIGLEC10	7,57039E-03	30844,1	5,69948E-03	17054	1,80308E+00	9766	7,19486E-01	57477	9,19085E-01	21542	0	48381,5
Myeloid_vs_CD8_Responder	CD8(Terminal Tex)	cDC_CLEC9A	CALR	SCARF1	7,57341E-03	35118,9	1,15418E-02	5559	1,87678E+00	8698	8,17086E-01	51266	8,48313E-01	61690	0	48381,5
Myeloid_vs_CD8_Responder	Macro_IFI27	CD8(GZMK+ Tex)	HLA-DRA	LAG3	7,58812E-03	20315,3	4,89382E-03	21555	1,30779E+00	21422	2,38135E+00	5807	9,49281E-01	4411	0	48381,5
Myeloid_vs_CD8_Responder	Macro_IFI27	CD8(GZMK+ Tem)	HLA-DQA1	LAG3	7,59495E-03	19224,3	7,47073E-03	11066	1,30390E+00	21560	2,00979E+00	10443	9,48730E-01	4671	0	48381,5
Myeloid_vs_CD8_Responder	CD8(ITM2C+ T)	Mono_CD14	IFNG	IFNGR1_IFNGR2	7,59927E-03	36040,9	4,90827E-03	21466	8,47853E-01	42792	1,42041E+00	24227	8,80457E-01	43338	0	48381,5
Myeloid_vs_CD8_Responder	CD8(Temra)	Mono_CD16	ANXA1	FPR1	7,60178E-03	30242,9	4,89225E-03	21565	1,81232E+00	9622	8,28638E-01	50563	9,19897E-01	21083	0	48381,5
Myeloid_vs_CD8_Responder	CD8(IL7R+ZNF683+ Tm)	Mono_CD14	CIRBP	TREM1	7,60541E-03	31028,7	5,99143E-03	15781	1,64631E+00	12472	1,13748E+00	35164	8,80445E-01	43345	0	48381,5
Myeloid_vs_CD8_Responder	CD8(ISG+ T)	Macro_LYVE1	CD99	CD81	7,61067E-03	33866,3	4,22749E-03	26677	1,08522E+00	30159	9,62141E-01	43351	9,20469E-01	20763	0	48381,5
Myeloid_vs_CD8_Responder	CD8(GZMK+ Early Tem)	Macro_LYVE1	HSP90B1	LRP1	7,61946E-03	32811,3	3,72774E-03	31975	2,08112E+00	6264	9,61990E-01	43361	8,96585E-01	34075	0	48381,5
Myeloid_vs_CD8_Responder	CD8(NME1+ T)	Mono_INHBA	B2M	LILRB2	7,62121E-03	28720,5	3,01512E-03	43363	1,52735E+00	15152	1,23517E+00	31120	9,46992E-01	5586	0	48381,5
Myeloid_vs_CD8_Responder	CD8(GZMK+ Tem)	Mono_CD16	HLA-F	LILRB1	7,62473E-03	36825,7	4,18601E-03	27059	1,28458E+00	22188	9,66136E-01	43133	8,80407E-01	43367	0	48381,5
Myeloid_vs_CD8_Responder	CD8(Tc17)	Macro_NLRP3	HLA-C	LILRB2	7,62561E-03	32400,5	3,01490E-03	43368	1,40833E+00	18394	1,01049E+00	40982	9,37602E-01	10877	0	48381,5
Myeloid_vs_CD8_Responder	CD8(GZMK+ Tem)	Mono_CD14	SEMA4A	LILRB2	7,62916E-03	37298,5	4,89062E-03	21585	1,54738E+00	14651	1,63722E+00	17976	8,07506E-01	83899	0	48381,5
Myeloid_vs_CD8_Responder	CD8(GZMK+ Tex)	pDC_LILRA4	HLA-DRA	CD4	7,63053E-03	35699,9	1,90628E-03	77343	1,30318E+00	21586	2,04815E+00	9849	9,19430E-01	21340	0	48381,5

Myeloid_vs_CD8_Post_R

Myeloid_vs_CD8_Responder	CD8(ID2+CXCR4+ T)	Macro_ISG15	HLA-B	LILRB2	7,63704E-03	27942,7	3,01414E-03	43381	1,54018E+00	14816	1,41237E+00	24476	9,41432E-01	8659	0	48381,5
Myeloid_vs_CD8_Responder	Macro_NLRP3	CD8(Temra)	IL1B	SIGIRR	7,65745E-03	19523,5	8,63828E-03	8731	2,27985E+00	4532	2,55649E+00	4285	9,00880E-01	31688	0	48381,5
Myeloid_vs_CD8_Responder	CD8(ISG+ T)	Mono_CD16	HLA-B	LILRB1	7,65996E-03	32657,9	3,27973E-03	38460	1,34800E+00	20188	9,60886E-01	43407	9,34225E-01	12853	0	48381,5
Myeloid_vs_CD8_Responder	pDC_LILRA4	CD8(GZMK+ Tex)	COL24A1	ITGA1_ITGB1	7,66127E-03	44936,1	5,07757E-02	282	2,82573E+00	1989	8,45854E-01	49576	7,17586E-01	124452	0	48381,5
Myeloid_vs_CD8_Responder	Macro_FOLR2+APOE-	CD8(NME1+ T)	HLA-DRA	LAG3	7,67036E-03	20942,1	5,07213E-03	20417	1,30271E+00	21615	2,01819E+00	10303	9,50135E-01	3994	0	48381,5
Myeloid_vs_CD8_Responder	CD8(Tc17)	Macro_ISG15	HLA-C	LILRB1	7,67408E-03	34819,1	3,17579E-03	40271	1,17870E+00	26033	9,60475E-01	43423	9,28895E-01	15987	0	48381,5
Myeloid_vs_CD8_Responder	Macro_FOLR2-APOE+	CD8(ITM2C+ T)	C3	IFITM1	7,67999E-03	23412,1	7,23123E-03	11719	1,35033E+00	20123	1,50211E+00	21622	9,30245E-01	15215	0	48381,5
Myeloid_vs_CD8_Responder	Macro_FOLR2-APOE+	CD8(LAYN+ T)	SPP1	ITGA4_ITGB1	7,68048E-03	20364,1	1,03793E-02	6578	1,87377E+00	8740	2,26531E+00	7021	9,01923E-01	31100	0	48381,5
Myeloid_vs_CD8_Responder	CD8(ZNF683+KLRB1+ T)	Macro_FOLR2+APOE+	HMGB1	CD163	7,68116E-03	30171,1	3,72726E-03	31984	1,73410E+00	10864	9,60365E-01	43431	9,28559E-01	16195	0	48381,5
Myeloid_vs_CD8_Responder	CD8(GZMK+ Early Tem)	cDC_CLEC9A	CD52	SIGLEC10	7,72992E-03	40766,5	3,00859E-03	43486	1,03952E+00	32263	9,68367E-01	43019	8,91923E-01	36683	0	48381,5
Myeloid_vs_CD8_Responder	Macro_OLFML3	CD8(ISG+ T)	C3	CD46	7,73347E-03	24076,3	6,99282E-03	12356	2,32245E+00	4206	1,92220E+00	11948	8,80171E-01	43490	0	48381,5
Myeloid_vs_CD8_Responder	CD8(IL7R+ZNF683+ Tm)	Macro_ISG15	HLA-C	LILRB1	7,73970E-03	35393,3	3,12875E-03	41138	1,14092E+00	27665	9,58727E-01	43497	9,28400E-01	16285	0	48381,5
Myeloid_vs_CD8_Responder	CD8(EOMES+ NK-like)	Macro_OLFML3	CD52	SIGLEC10	7,75840E-03	28911,5	5,52162E-03	17860	1,64353E+00	12521	9,58343E-01	43518	9,17899E-01	22277	0	48381,5
Myeloid_vs_CD8_Responder	Macro_OLFML3	CD8(IL7R+ZNF683+ Tm)	C3	CD46	7,76821E-03	24185,7	6,98448E-03	12384	2,32213E+00	4209	1,89665E+00	12425	8,80108E-01	43529	0	48381,5
Myeloid_vs_CD8_Responder	CD8(EOMES+ NK-like)	Mono_CD14	HMGB1	TLR4	7,77089E-03	38938,9	3,78461E-03	31294	1,12629E+00	28320	9,65339E-01	43167	8,80104E-01	43532	0	48381,5
Myeloid_vs_CD8_Responder	CD8(ISG+ T)	Macro_OLFML3	TNFSF9	HLA-DPA1	7,77446E-03	29765,7	3,00633E-03	43536	1,27476E+00	22513	2,25334E+00	7137	9,08972E-01	27261	0	48381,5
Myeloid_vs_CD8_Responder	CD8(GZMK+ Tem)	cDC(CD1C)	CD52	SIGLEC10	7,77803E-03	40457,7	3,00621E-03	43540	9,32906E-01	37757	1,11949E+00	35905	8,91885E-01	36705	0	48381,5
Myeloid_vs_CD8_Responder	CD8(GZMK+ Early Tem)	Macro_NLRP3	HMGB1	CD163	7,78160E-03	32538,7	3,00612E-03	43544	1,47432E+00	16520	1,16755E+00	33820	9,21091E-01	20428	0	48381,5
Myeloid_vs_CD8_Responder	CD8(Terminal Tex)	Macro_ISG15	HLA-F	LILRB1	7,79860E-03	34673,7	4,15808E-03	27344	1,28826E+00	22069	1,21282E+00	32011	8,80054E-01	43563	0	48381,5
Myeloid_vs_CD8_Responder	CD8(Terminal Tex)	Macro_ISG15	B2M	LILRB2	7,80051E-03	27193,9	2,87041E-03	46428	1,61061E+00	13201	1,49944E+00	21709	9,45744E-01	6250	0	48381,5
Myeloid_vs_CD8_Responder	Macro_LYVE1	CD8(NME1+ T)	HLA-DRA	LAG3	7,80748E-03	21374,5	5,06092E-03	20478	1,29949E+00	21714	1,90539E+00	12274	9,50083E-01	4025	0	48381,5
Myeloid_vs_CD8_Responder	CD8(ZNF683+KLRB1+ T)	Mono_INHBA	B2M	LILRB2	7,81382E-03	29301,1	3,00386E-03	43580	1,50879E+00	15606	1,18008E+00	33306	9,46898E-01	5632	0	48381,5
Myeloid_vs_CD8_Responder	CD8(Terminal Tex)	Macro_FOLR2+APOE+	HSPA1A	TLR4	7,81651E-03	38425,1	3,61079E-03	33495	1,17920E+00	26018	1,01675E+00	40648	8,80017E-01	43583	0	48381,5
Myeloid_vs_CD8_Responder	Macro_FOLR2-APOE+	CD8(GZMK+ Tem)	HLA-DPA1	LAG3	7,82003E-03	19464,5	6,21503E-03	14899	1,29933E+00	21723	2,04288E+00	9929	9,54130E-01	2390	0	48381,5
Myeloid_vs_CD8_Responder	CD8(GZMK+ Tem)	Mono_CD14	CSF1	CSF3R	7,82841E-03	42187,9	5,56978E-03	17617	1,61135E+00	13192	1,49851E+00	21729	7,53348E-01	110020	0	48381,5
Myeloid_vs_CD8_Responder	Macro_IER3	CD8(GZMK+ Tem)	CD14	ITGA4	7,82841E-03	23596,9	5,11144E-03	20170	1,76080E+00	10429	1,66437E+00	17275	9,18782E-01	21729	0	48381,5
Myeloid_vs_CD8_Responder	cDC(CD1C)	CD8(ISG+ T)	ICAM1	IL2RG	7,83087E-03	36184,3	3,48843E-03	35188	8,34822E-01	43599	1,67380E+00	17067	8,91919E-01	36686	0	48381,5
Myeloid_vs_CD8_Responder	CD8(GZMK+ Tem)	Mono_CD14	HMGB1	TLR4	7,83536E-03	38874,3	3,77595E-03	31395	1,12404E+00	28426	9,78307E-01	42565	8,79983E-01	43604	0	48381,5
Myeloid_vs_CD8_Responder	Macro_ISG15	CD8(NME1+ T)	CCL8	CCR5	7,83719E-03	34047,3	4,61488E-02	350	2,81647E+00	2012	1,93646E+00	11682	7,58487E-01	107811	0	48381,5
Myeloid_vs_CD8_Responder	CD8(EOMES+ NK-like)	Macro_FOLR2+APOE+	HMGB1	CD163	7,83986E-03	29893,1	3,80138E-03	31104	1,75330E+00	10549	9,56828E-01	43609	9,29210E-01	15822	0	48381,5
Myeloid_vs_CD8_Responder	CD8(Temra)	cDC_CLEC9A	SPON2	ITGB2	7,85515E-03	31742,9	8,10392E-03	9681	8,34145E-01	43626	1,59314E+00	19066	8,89699E-01	37960	0	48381,5
Myeloid_vs_CD8_Responder	Macro_OLFML3	CD8(GZMK+ Early Tem)	HLA-DRB5	LAG3	7,87949E-03	30501,9	2,99916E-03	43653	1,12937E+00	28182	1,79867E+00	14296	9,25292E-01	17997	0	48381,5
Myeloid_vs_CD8_Responder	CD8(ID2+CXCR4+ T)	Macro_ISG15	RPS19	C5AR1	7,88310E-03	33395,9	2,99872E-03	43657	1,14286E+00	27582	1,25869E+00	30157	9,26671E-01	17202	0	48381,5
Myeloid_vs_CD8_Responder	CD8(EOMES+ NK-like)	Macro_NLRP3	HLA-C	LILRB2	7,88852E-03	32195,7	2,99837E-03	43663	1,39435E+00	18813	1,04821E+00	39166	9,37441E-01	10955	0	48381,5
Myeloid_vs_CD8_Responder	CD8(EOMES+ NK-like)	Macro_NLRP3	HLA-A	LILRB2	7,89303E-03	33279,5	2,99819E-03	43668	1,31753E+00	21116	9,73774E-01	42785	9,38323E-01	10447	0	48381,5
Myeloid_vs_CD8_Responder	Macro_NLRP3	CD8(ID2+CXCR4+ T)	S100A8	ITGB2	7,89707E-03	18928,5	4,86132E-03	21778	2,15947E+00	5520	2,54167E+00	4390	9,31324E-01	14573	0	48381,5
Myeloid_vs_CD8_Responder	Mono_INHBA	CD8(LAYN+ T)	HBEGF	CD82	7,89936E-03	25414,1	1,12425E-02	5805	1,83812E+00	9252	1,56006E+00	19957	8,79860E-01	43675	0	48381,5
Myeloid_vs_CD8_Responder	Mono_INHBA	CD8(LAYN+ T)	CD86	CTLA4	7,90117E-03	30957,5	8,84792E-03	8391	1,31860E+00	21091	1,18152E+00	33247	8,79851E-01	43677	0	48381,5
Myeloid_vs_CD8_Responder	CD8(Tc17)	cDC_LAMP3	LTB	CD40	7,90750E-03	26447,5	1,26090E-02	4810	1,81339E+00	9598	1,37478E+00	25764	8,79835E-01	43684	0	48381,5
Myeloid_vs_CD8_Responder	CD8(ISG+ T)	Macro_ISG15	HLA-B	LILRB2	7,90750E-03	27928,7	2,99752E-03	43684	1,52043E+00	15322	1,44133E+00	23528	9,41279E-01	8728	0	48381,5
Myeloid_vs_CD8_Responder	CD8(GZMK+ Tem)	Mono_CD14	CCL5	CCR1	7,91928E-03	35625,5	6,20278E-03	14954	8,32912E-01	43697	9,81056E-01	42419	9,06405E-01	28676	0	48381,5
Myeloid_vs_CD8_Responder	Mono_INHBA	CD8(ID2+CXCR4+ T)	ICAM1	IL2RG	7,92018E-03	41932,7	2,99685E-03	43698	9,27564E-01	38047	1,06022E+00	38574	8,84377E-01	40963	0	48381,5
Myeloid_vs_CD8_Responder	Macro_IFI27	CD8(LAYN+ T)	C3	IFITM1	7,92099E-03	23837,3	6,91115E-03	12574	1,34225E+00	20369	1,49657E+00	21795	9,28762E-01	16067	0	48381,5
Myeloid_vs_CD8_Responder	cDC(CD1C)	CD8(Temra)	LGALS9	PTPRC	7,92653E-03	34257,5	2,99666E-03	43705	9,83348E-01	35096	1,41687E+00	24343	9,22249E-01	19762	0	48381,5
Myeloid_vs_CD8_Responder	Mono_INHBA	CD8(Temra)	VCAN	ITGA4	7,93197E-03	27440,9	6,53714E-03	13799	1,63037E+00	12779	1,61443E+00	18534	8,79789E-01	43711	0	48381,5
Myeloid_vs_CD8_Responder	CD8(GZMK+ Tem)	Macro_NLRP3	CALR	LRP1	7,93833E-03	36777,9	3,24332E-03	39069	1,35671E+00	19910	9,54834E-01	43718	8,98841E-01	32811	0	48381,5
Myeloid_vs_CD8_Responder	CD8(IL7R+ZNF683+ Tm)	Macro_ISG15	CD99	PILRA	7,94014E-03	38258,1	3,18316E-03	40127	1,15260E+00	27160	9,54746E-01	43720	9,00495E-01	31902	0	48381,5
Myeloid_vs_CD8_Responder	Mono_INHBA	CD8(EOMES+ NK-like)	TIMP1	CD63	7,94105E-03	22126,5	2,99613E-03	43721	2,20419E+00	5126	2,92287E+00	2156	9,36898E-01	11248	0	48381,5
Myeloid_vs_CD8_Responder	Macro_IER3	CD8(ISG+ T)	HLA-DRA	LAG3	7,94497E-03	20765,9	5,31010E-03	19000	1,29623E+00	21812	1,96977E+00	11111	9,51210E-01	3525	0	48381,5
Myeloid_vs_CD8_Responder	CD8(GZMK+ Tex)	Macro_FOLR2+APOE+	ENTPD1	TMIGD3	7,94979E-03	35495,5	2,71702E-02	986	2,38724E+00	3758	1,01714E+00	40633	8,07858E-01	83719	0	48381,5
Myeloid_vs_CD8_Responder	CD8(Terminal Tex)	cDC_CLEC9A	HMGB1	THBD	7,95195E-03	40609,1	3,47346E-03	35418	1,03440E+00	32511	9,54468E-01	43733	8,80998E-01	43002	0	48381,5
Myeloid_vs_CD8_Responder	CD8(ISG+ T)	pDC_LILRA4	IL16	CD4	7,95627E-03	30774,5	4,85464E-03	21820	1,31874E+00	21087	2,41834E+00	5458	8,56457E-01	57126	0	48381,5
Myeloid_vs_CD8_Responder	CD8(LAYN+ T)	cDC(CD1C)	TNFSF9	HLA-DPA1	7,95923E-03	29555,3	2,99513E-03	43741	1,20766E+00	24921	2,68269E+00	3389	9,08818E-01	27344	0	48381,5
Myeloid_vs_CD8_Responder	cDC(CD1C)	CD8(GZMK+ Early Tem)	HLA-DQB1	LAG3	7,96014E-03	30360,3	2,99506E-03	43742	1,05900E+00	31364	2,22148E+00	7496	9,20367E-01	20818	0	48381,5
Myeloid_vs_CD8_Responder	CD8(GZMK+ Tem)	Mono_CD16	HLA-B	LILRA1	7,97325E-03	27229,5	1,00091E-02	6951	2,00824E+00	7070	7,73061E-01	54019	9,22325E-01	19726	0	48381,5
Myeloid_vs_CD8_Responder	CD8(GZMK+ Tex)	Macro_NLRP3	HLA-B	LILRB2	7,97471E-03	31600,5	2,99431E-03	43758	1,41265E+00	18272	1,05479E+00	38844	9,41250E-01	8747	0	48381,5
Myeloid_vs_CD8_Responder	CD8(GZMK+ Early Tem)	Mast	HSPA8	ADRB2	7,97927E-03	30799,3	8,14687E-03	9598	9,92992E-01	34621	9,53809E-01	43763	9,25867E-01	17633	0	48381,5
Myeloid_vs_CD8_Responder	CD8(GZMK+ Tem)	Mono_CD16	HLA-A	LILRA1	7,98458E-03	27713,5	1,03873E-02	6565	1,97953E+00	7404	7,55895E-01	55120	9,19865E-01	21097	0	48381,5
Myeloid_vs_CD8_Responder	Macro_FOLR2-APOE+	CD8(GZMK+ Tem)	HLA-DQA2	LAG3	7,98458E-03	21484,7	9,36478E-03	7675	1,29533E+00	21840	1,81766E+00	13918	9,29607E-01	15609	0	48381,5
Myeloid_vs_CD8_Responder	CD8(Tc17)	Macro_OLFML3	LTB	CD40	7,98565E-03	27446,5	1,25695E-02	4829	1,80895E+00	9665	1,24848E+00	30587	8,79669E-01	43770	0	48381,5
Myeloid_vs_CD8_Responder	cDC_CLEC9A	CD8(NME1+ T)	HLA-DQA2	LAG3	7,98883E-03	20528,9	8,33053E-03	9257	1,29519E+00	21843	2,42247E+00	5409	9,25680E-01	17754	0	48381,5
Myeloid_vs_CD8_Responder	CD8(GZMK+ Tem)	Mono_CD16	HLA-C	LILRA3	7,99592E-03	24789,7	1,70901E-02	2915	2,82203E+00	1998	8,10939E-01	51656	9,23610E-01	18998	0	48381,5
Myeloid_vs_CD8_Responder	Macro_OLFML3	CD8(GZMK+ Early Tem)	MDK	NCL	8,00665E-03	28265,9	1,07460E-02	6239	1,66299E+00	12142	9,53203E-01	43793	9,02494E-01	30774	0	48381,5
Myeloid_vs_CD8_Responder	cDC(CD1C)	CD8(Terminal Tex)	LGALS9	HAVCR2	8,00939E-03	32385,5	4,08139E-03	28059	1,15013E+00	27271	1,79194E+00	14420	8,79629E-01	43796	0	48381,5
Myeloid_vs_CD8_Responder	CD8(Terminal Tex)	Mono_CD14	HMGB1	TLR2	8,01305E-03	40576,3	2,99221E-03	43800	9,87037E-01	34916	1,04811E+00	39176	8,92045E-01	36608	0	48381,5
Myeloid_vs_CD8_Responder	CD8(GZMK+ Tem)	Mono_CD16	SEMA4A	LILRB2	8,02433E-03	26529,5	9,40726E-03	7614	2,80756E+00	2036	1,73651E+00	15631	8,53331E-01	58985	0	48381,5
Myeloid_vs_CD8_Responder	Macro_ISG15	CD8(Tc17)	CXCL11	DPP4	8,03013E-03	35281,5	1,09325E-01	51	2,07467E+00	6337	1,63248E+00	18090	7,67924E-01	103548	0	48381,5
Myeloid_vs_CD8_Responder	CD8(ID2+CXCR4+ T)	cDC(CD1C)	IFNG	IFNGR1_IFNGR2	8,04604E-03	36038,9	5,17224E-03	19783	8,30749E-01	43836	1,35465E+00	26499	8,83187E-01	41695	0	48381,5
Myeloid_vs_CD8_Responder	CD8(Temra)	pDC_LILRA4	IRAK4	TLR7	8,04996E-03	43198,1	8,80236E-03	8470	1,61999E+00	13014	1,49359E+00	21886	7,18202E-01	124239	0	48381,5
Myeloid_vs_CD8_Responder	CD8(ZNF683+KLRB1+ T)	Macro_NLRP3	HLA-C	LILRB2	8,05522E-03	32284,9	2,99011E-03	43846	1,38737E+00	19040	1,04849E+00	39156	9,37360E-01	11001	0	48381,5
Myeloid_vs_CD8_Responder	pDC_LILRA4	CD8(LAYN+ T)	APP	CD74	8,05567E-03	30274,7	4,84405E-03	21890	1,84357E+00	9177	2,04493E+00	9895	9,37910E-01	10693	0,003	99718,5
Myeloid_vs_CD8_Responder	Mono_INHBA	CD8(GZMK+ Tex)	LGALS1	ITGB1	8,06441E-03	30821,1	2,98972E-03	43856	1,06676E+00	30991	1,84103E+00	13468	9,26279E-01	17409	0	48381,5
Myeloid_vs_CD8_Responder	Macro_IFI27	CD8(GZMK+ Tex)	HLA-DQA2	LAG3	8,06565E-03	21824,9	8,04017E-03	9808	1,29391E+00	21897	2,00162E+00	10563	9,24451E-01	18475	0	48381,5
Myeloid_vs_CD8_Responder	Mast	CD8(NME1+ T)	TIMP3	CD44	8,06957E-03	22978,1	2,04091E-02	2042	3,39365E+00	918	1,24024E+00	30936	8,99203E-01	32613	0	48381,5
Myeloid_vs_CD8_Responder	CD8(Temra)	Mono_CD16	SPON2	ITGB2	8,07361E-03	36736,7	8,08843E-03	9715	8,32779E-01	43705	9,51634E-01	43866	8,89605E-01	38016	0	48381,5
Myeloid_vs_CD8_Responder	Mono_INHBA	CD8(GZMK+ Tex)	IL1B	SIGIRR	8,08650E-03	23629,3	5,57320E-03	17600	2,25929E+00	4688	2,65295E+00	3597	8,79523E-01	43880	0	48381,5
Myeloid_vs_CD8_Responder	Mast	CD8(Tc17)	TNFSF10	CCR6	8,08671E-03	18994,9	3,27694E-02	674	1,91055E+00	8256	2,11302E+00	8896	9,06246E-01	28767	0	48381,5

Myeloid_vs_CD8_Responder	CD8(ID2+CXCR4+ T)	Macro_FOLR2-APOE+	RPS19	C5AR1	8,10771E-03	33215,5	3,40366E-03	36446	1,27511E+00	22502	9,50865E-01	43903	9,30860E-01	14845	0	48381,5
Myeloid_vs_CD8_Responder	pDC_LILRA4	CD8(Tn)	APP	LRP10	8,11056E-03	27160,3	8,52242E-03	8905	1,91982E+00	8133	2,60380E+00	3920	8,39772E-01	66462	0	48381,5
Myeloid_vs_CD8_Responder	Macro_IFI27	CD8(Terminal Tex)	HLA-DRB1	LAG3	8,11717E-03	19323,1	6,08170E-03	15432	1,29270E+00	21933	2,12543E+00	8724	9,54872E-01	2145	0	48381,5
Myeloid_vs_CD8_Responder	Mono_INHBA	CD8(Terminal Tex)	HLA-DQA2	LAG3	8,12897E-03	33361,1	5,05537E-03	20510	8,29467E-01	43926	1,38514E+00	25409	9,06567E-01	28579	0	48381,5
Myeloid_vs_CD8_Responder	Macro_FOLR2+APOE+	CD8(NME1+ T)	HLA-DQA1	LAG3	8,13583E-03	21545,9	6,33160E-03	14484	1,29207E+00	21946	1,71877E+00	16024	9,44554E-01	6894	0	48381,5
Myeloid_vs_CD8_Responder	CD8(ID2+CXCR4+ T)	cDC(CD1C)	MIF	CD74_CXCR4	8,13993E-03	44671,3	1,82365E-03	80617	4,92030E-01	70065	1,47994E+00	22297	9,55305E-01	1996	0	48381,5
Myeloid_vs_CD8_Responder	CD8(GZMK+ Tem)	Macro_ISG15	HLA-C	LILRB2	8,14657E-03	29184,9	2,98549E-03	43945	1,44249E+00	17450	1,39323E+00	25124	9,37315E-01	11024	0	48381,5
Myeloid_vs_CD8_Responder	CD8(Tc17)	cDC_CLEC9A	ADAM10	CADM1	8,16605E-03	36066,5	9,48049E-03	7530	1,29153E+00	21967	1,53458E+00	20656	8,11557E-01	81798	0	48381,5
Myeloid_vs_CD8_Responder	CD8(GZMK+ Tex)	Mono_CD14	B2M	LILRB2	8,16749E-03	28683,1	2,74222E-03	49597	1,47195E+00	16580	1,49073E+00	21968	9,44560E-01	6889	0	48381,5
Myeloid_vs_CD8_Responder	CD8(Terminal Tex)	Mono_CD16	HLA-F	LILRB1	8,17627E-03	36994,9	4,10383E-03	27859	1,27206E+00	22585	9,86150E-01	42172	8,79360E-01	43977	0	48381,5
Myeloid_vs_CD8_Responder	Macro_NLRP3	CD8(LAYN+ T)	VCAN	ITGB1	8,18190E-03	17492,9	1,39301E-02	4109	2,74220E+00	2220	1,98881E+00	10776	9,18380E-01	21978	0	48381,5
Myeloid_vs_CD8_Responder	cDC(CD1C)	CD8(NME1+ T)	ICAM1	IL2RG	8,18837E-03	36714,1	3,45894E-03	35625	8,28366E-01	43990	1,60933E+00	18643	8,91509E-01	36931	0	48381,5
Myeloid_vs_CD8_Responder	CD8(LAYN+ T)	Macro_IFI27	ADAM10	GPNMB	8,19634E-03	35435,3	4,91390E-03	21431	1,50716E+00	15650	1,48994E+00	21988	8,34008E-01	69726	0	48381,5
Myeloid_vs_CD8_Responder	CD8(ISG+ T)	Mono_CD16	HLA-A	LILRA1	8,19779E-03	29613,1	9,97375E-03	6988	1,85524E+00	9003	6,58673E-01	61704	9,18355E-01	21989	0	48381,5
Myeloid_vs_CD8_Responder	Macro_FOLR2-APOE+	CD8(Tn)	SPP1	CD44	8,20212E-03	20705,3	6,76973E-03	13003	1,71712E+00	11162	2,10648E+00	8988	9,18354E-01	21992	0	48381,5
Myeloid_vs_CD8_Responder	Macro_OLFML3	CD8(Tc17)	CXCL10	DPP4	8,21176E-03	27079,7	5,20612E-02	272	1,85913E+00	8943	2,21413E+00	7577	8,33094E-01	70225	0	48381,5
Myeloid_vs_CD8_Responder	cDC_CLEC9A	CD8(ISG+ T)	HLA-DRB5	LAG3	8,21658E-03	18872,9	6,12524E-03	15258	1,29039E+00	22002	2,76223E+00	2893	9,46524E-01	5830	0	48381,5
Myeloid_vs_CD8_Responder	Macro_FOLR2-APOE+	CD8(NME1+ T)	HLA-DRA	LAG3	8,21803E-03	21030,1	5,02927E-03	20663	1,29038E+00	22003	2,03622E+00	10007	9,49934E-01	4096	0	48381,5
Myeloid_vs_CD8_Responder	Macro_OLFML3	CD8(EOMES+ NK-like)	C3	CD46	8,22940E-03	24346,1	6,87337E-03	12683	2,31790E+00	4233	1,89769E+00	12399	8,79260E-01	44034	0	48381,5
Myeloid_vs_CD8_Responder	CD8(ISG+ T)	Macro_ISG15	HLA-C	LILRB2	8,26028E-03	29337,1	2,97824E-03	44067	1,43603E+00	17613	1,38106E+00	25555	9,37243E-01	11069	0	48381,5
Myeloid_vs_CD8_Responder	CD8(Temra)	Mono_INHBA	HMGB1	CD163	8,26778E-03	32146,5	2,97775E-03	44075	1,37617E+00	19369	1,30792E+00	28292	9,20745E-01	20615	0	48381,5
Myeloid_vs_CD8_Responder	Mono_INHBA	CD8(ID2+CXCR4+ T)	S100A9	ITGB2	8,27060E-03	27330,9	2,97752E-03	44078	1,65794E+00	12233	1,89595E+00	12441	9,22705E-01	19521	0	48381,5
Myeloid_vs_CD8_Responder	CD8(ISG+ T)	Mono_CD14	B2M	LILRB2	8,27753E-03	28559,7	2,75097E-03	49381	1,48817E+00	16147	1,48790E+00	22044	9,44643E-01	6845	0	48381,5
Myeloid_vs_CD8_Responder	CD8(GZMK+ Early Tem)	Macro_FOLR2+APOE+	TNF	VSIR	8,28374E-03	36423,9	6,67699E-03	13295	9,67665E-01	35914	1,02082E+00	40437	8,79155E-01	44092	0	48381,5
Myeloid_vs_CD8_Responder	CD8(Temra)	Macro_FOLR2+APOE+	HMGB1	CD163	8,28938E-03	30620,9	3,65691E-03	32901	1,71588E+00	11184	9,46783E-01	44098	9,27925E-01	16540	0	48381,5
Myeloid_vs_CD8_Responder	CD8(GZMK+ Tex)	pDC_LILRA4	SELPLG	SELL	8,29032E-03	28955,7	6,81212E-03	12871	1,01836E+00	33317	2,34867E+00	6110	8,79147E-01	44099	0	48381,5
Myeloid_vs_CD8_Responder	CD8(NME1+ T)	Macro_IER3	HLA-F	LILRB2	8,29126E-03	38929,1	3,29620E-03	38192	1,24736E+00	23451	9,46724E-01	44100	8,85214E-01	40521	0	48381,5
Myeloid_vs_CD8_Responder	Mono_INHBA	CD8(ZNF683+KLRB1+ T)	INHBA	ACTR2	8,29223E-03	23080,3	1,16748E-02	5445	3,10589E+00	1356	2,10753E+00	8973	8,66868E-01	51246	0	48381,5
Myeloid_vs_CD8_Responder	CD8(GZMK+ Tex)	cDC_CLEC9A	CRTAM	CADM1	8,29646E-03	28758,3	2,03686E-02	2049,5	1,28846E+00	22057,5	1,67718E+00	16994,5	8,61341E-01	54308,5	0	48381,5
Myeloid_vs_CD8_Responder	CD8(NME1+ T)	Macro_FOLR2+APOE+	CD52	SIGLEC10	8,30537E-03	27023,1	6,30767E-03	14587	1,88945E+00	8551	9,46410E-01	44115	9,22776E-01	19481	0	48381,5
Myeloid_vs_CD8_Responder	Mono_INHBA	CD8(ID2+CXCR4+ T)	CD86	CTLA4	8,31761E-03	30106,7	8,72082E-03	8592	1,30973E+00	21362	1,31345E+00	28070	8,79084E-01	44128	0	48381,5
Myeloid_vs_CD8_Responder	CD8(GZMK+ Tex)	Macro_ISG15	CD52	SIGLEC10	8,31761E-03	40023,3	3,25540E-03	38875	1,00221E+00	34167	9,46183E-01	44128	8,95665E-01	34565	0	48381,5
Myeloid_vs_CD8_Responder	CD8(Tn)	Macro_FOLR2-APOE+	CD40LG	ITGA5_ITGB1	8,32109E-03	49144,5	2,02519E-02	2074	7,08589E-01	52156	7,95890E-01	52572	7,94511E-01	90539	0	48381,5
Myeloid_vs_CD8_Responder	CD8(NME1+ T)	Macro_FOLR2-APOE+	HSP90B1	LRP1	8,32327E-03	37216,1	3,30699E-03	38015	1,41291E+00	18262	9,46085E-01	44134	8,90899E-01	37288	0	48381,5
Myeloid_vs_CD8_Responder	Macro_OLFML3	CD8(EOMES+ NK-like)	B2M	KIR3DL1	8,32893E-03	26075,3	2,25994E-02	1567	1,37134E+00	19501	9,45966E-01	44140	9,27486E-01	16787	0	48381,5
Myeloid_vs_CD8_Responder	CD8(ZNF683+KLRB1+ T)	Mono_CD14	HMGB1	TLR4	8,33082E-03	39374,5	3,71081E-03	32186	1,10709E+00	29164	9,68876E-01	42999	8,79061E-01	44142	0	48381,5
Myeloid_vs_CD8_Responder	CD8(Terminal Tex)	Macro_ISG15	HLA-B	LILRB2	8,33176E-03	28511,7	2,97394E-03	44143	1,49240E+00	16039	1,39291E+00	25132	9,41061E-01	8863	0	48381,5
Myeloid_vs_CD8_Responder	cDC(CD1C)	CD8(GZMK+ Early Tem)	HLA-DQA1	LAG3	8,33270E-03	31050,3	2,97390E-03	44144	9,95130E-01	34518	2,19606E+00	7783	9,21106E-01	20425	0	48381,5
Myeloid_vs_CD8_Responder	Macro_ISG15	CD8(Terminal Tex)	CCL8	CCR5	8,33693E-03	34490,5	4,28268E-02	394	2,79208E+00	2076	1,98401E+00	10854	7,51579E-01	110747	0	48381,5
Myeloid_vs_CD8_Responder	Mono_INHBA	CD8(IL7R+ZNF683+ Tm)	ICAM1	IL2RG	8,33742E-03	42190,1	2,97355E-03	44149	9,24554E-01	38198	1,05087E+00	39028	8,83977E-01	41194	0	48381,5
Myeloid_vs_CD8_Responder	Mono_INHBA	CD8(LAYN+ T)	INHBA	ACTR2	8,33806E-03	21703,7	1,38159E-02	4176	3,18151E+00	1219	2,10645E+00	8990	8,76288E-01	45752	0	48381,5
Myeloid_vs_CD8_Responder	pDC_LILRA4	CD8(Temra)	APP	LRP10	8,33806E-03	27338,7	8,47041E-03	8990	1,91827E+00	8158	2,53031E+00	4466	8,39359E-01	66698	0	48381,5
Myeloid_vs_CD8_Responder	CD8(Tc17)	cDC_CLEC9A	ANXA1	DYSF	8,35916E-03	30020,9	1,16006E-02	5504	1,77214E+00	10238	9,45450E-01	44172	8,83015E-01	41809	0	48381,5
Myeloid_vs_CD8_Responder	CD8(ITM2C+ T)	Macro_ISG15	HLA-B	LILRB1	8,36200E-03	34920,9	3,12669E-03	41187	1,15362E+00	27115	9,45401E-01	44175	9,32742E-01	13746	0	48381,5
Myeloid_vs_CD8_Responder	Mono_INHBA	CD8(Terminal Tex)	HLA-DQB1	LAG3	8,37716E-03	31379,5	4,32891E-03	25786	8,25172E-01	44191	1,40142E+00	24849	9,32863E-01	13690	0	48381,5
Myeloid_vs_CD8_Responder	Macro_OLFML3	CD8(GZMK+ Tex)	CD86	CTLA4	8,38569E-03	29941,7	8,70076E-03	8629	1,43645E+00	17602	1,24101E+00	30896	8,78962E-01	44200	0	48381,5
Myeloid_vs_CD8_Responder	CD8(ITM2C+ T)	Macro_ISG15	HLA-C	LILRB2	8,39518E-03	29804,1	2,97058E-03	44210	1,42922E+00	17809	1,32928E+00	27504	9,37168E-01	11116	0	48381,5
Myeloid_vs_CD8_Responder	CD8(NME1+ T)	Macro_IFI27	HMGB1	TLR2	8,39898E-03	38232,9	2,97037E-03	44214	1,15334E+00	27130	1,14995E+00	34610	8,91692E-01	36829	0	48381,5
Myeloid_vs_CD8_Responder	CD8(ISG+ T)	Macro_NLRP3	B2M	LILRB2	8,40088E-03	28524,5	2,97026E-03	44216	1,59694E+00	13484	1,24462E+00	30752	9,46615E-01	5789	0	48381,5
Myeloid_vs_CD8_Responder	CD8(IL7R+ZNF683+ Tm)	Macro_NLRP3	HLA-C	LILRB2	8,40183E-03	32863,7	2,97024E-03	44217	1,37056E+00	19522	1,00874E+00	41075	9,37164E-01	11123	0	48381,5
Myeloid_vs_CD8_Responder	CD8(ISG+ T)	Mono_CD14	HLA-A	LILRB2	8,40278E-03	29175,7	2,97015E-03	44218	1,40387E+00	18520	1,42275E+00	24155	9,38050E-01	10604	0	48381,5
Myeloid_vs_CD8_Responder	CD8(IL7R+ZNF683+ Tm)	Macro_NLRP3	HLA-B	LILRB2	8,40468E-03	32105,7	2,97014E-03	44220	1,38535E+00	19099	1,03117E+00	39949	9,41025E-01	8879	0	48381,5
Myeloid_vs_CD8_Responder	CD8(ID2+CXCR4+ T)	cDC_CLEC9A	CALR	SCARF1	8,42204E-03	36342,5	1,12071E-02	5842	1,85415E+00	9021	7,46765E-01	55727	8,46410E-01	62741	0	48381,5
Myeloid_vs_CD8_Responder	Macro_FOLR2+APOE-	CD8(ISG+ T)	HLA-DRB5	LAG3	8,43282E-03	20420,1	6,10474E-03	15345	1,28586E+00	22150	2,01540E+00	10344	9,46439E-01	5880	0	48381,5
Myeloid_vs_CD8_Responder	CD8(ITM2C+ T)	Macro_NLRP3	HLA-B	LILRB2	8,44849E-03	32479,1	2,96829E-03	44266	1,38326E+00	19148	9,95418E-01	41707	9,41008E-01	8893	0	48381,5
Myeloid_vs_CD8_Responder	CD8(ID2+CXCR4+ T)	Macro_LYVE1	SPN	SIGLEC1	8,44925E-03	46541,9	8,44720E-03	9031	2,31287E+00	4269	5,70371E-01	68129	7,69314E-01	102899	0	48381,5
Myeloid_vs_CD8_Responder	CD8(GZMK+ Tex)	Mono_INHBA	HSP90B1	LRP1	8,45230E-03	37144,9	2,96818E-03	44270	1,39539E+00	18780	1,16450E+00	33962	8,85534E-01	40331	0	48381,5
Myeloid_vs_CD8_Responder	Macro_LYVE1	CD8(LAYN+ T)	C1QB	C1QBP	8,46384E-03	21041,3	4,80199E-03	22171	1,98330E+00	7363	2,22909E+00	7418	9,22107E-01	19873	0	48381,5
Myeloid_vs_CD8_Responder	Macro_NLRP3	CD8(Terminal Tex)	S100A9	ITGB2	8,46680E-03	17613,1	4,80142E-03	22173	2,38106E+00	3795	2,72191E+00	3144	9,38115E-01	10572	0	48381,5
Myeloid_vs_CD8_Responder	Mono_CD16	CD8(EOMES+ NK-like)	S100A8	CD69	8,46828E-03	25077,1	5,48087E-03	18084	1,28494E+00	22174	1,53582E+00	20622	9,28667E-01	16124	0	48381,5
Myeloid_vs_CD8_Responder	CD8(ID2+CXCR4+ T)	Mono_CD16	HLA-A	LILRA1	8,47420E-03	30780,7	9,90026E-03	7065	1,83316E+00	9325	5,86307E-01	66954	9,18078E-01	22178	0	48381,5
Myeloid_vs_CD8_Responder	CD8(GZMK+ Tem)	Macro_FOLR2+APOE+	CD52	SIGLEC10	8,47716E-03	29985,1	5,69409E-03	17085	1,80231E+00	9775	7,87142E-01	53118	9,19050E-01	21566	0	48381,5
Myeloid_vs_CD8_Responder	CD8(GZMK+ Tem)	Macro_FOLR2+APOE+	ADAM10	GPNMB	8,48456E-03	32216,7	4,88545E-03	21631	2,86960E+00	1864	1,58642E+00	19258	8,33606E-01	69949	0	48381,5
Myeloid_vs_CD8_Responder	CD8(Terminal Tex)	Mono_CD14	GNAI2	C5AR1	8,50383E-03	29698,9	3,33360E-03	37568	2,00206E+00	7137	2,10162E+00	9051	8,75267E-01	46357	0	48381,5
Myeloid_vs_CD8_Responder	CD8(NME1+ T)	Macro_FOLR2-APOE+	CD99	PILRA	8,50782E-03	34964,9	3,74099E-03	31814	1,28330E+00	22227	9,42443E-01	44328	9,07499E-01	28074	0	48381,5
Myeloid_vs_CD8_Responder	CD8(LAYN+ T)	Macro_FOLR2-APOE+	HSP90B1	LRP1	8,50974E-03	37126,9	3,32688E-03	37670	1,41706E+00	18150	9,42441E-01	44330	8,91190E-01	37103	0	48381,5
Myeloid_vs_CD8_Responder	Macro_IER3	CD8(GZMK+ Tem)	HLA-DRB1	LAG3	8,51423E-03	20033,9	6,16279E-03	15118	1,28389E+00	22205	1,89717E+00	12416	9,55156E-01	2049	0	48381,5
Myeloid_vs_CD8_Responder	CD8(GZMK+ Tex)	Mast	IL16	CD9	8,51423E-03	33968,3	4,79833E-03	22205	1,57211E+00	14063	1,48990E+00	21990	8,45582E-01	63202	0	48381,5
Myeloid_vs_CD8_Responder	Mono_INHBA	CD8(Tn)	IL1B	SIGIRR	8,53857E-03	23788,5	5,48350E-03	18068	2,25501E+00	4734	2,68072E+00	3399	8,78661E-01	44360	0	48381,5
Myeloid_vs_CD8_Responder	CD8(NME1+ T)	Macro_IFI27	CD99	CD81	8,55109E-03	39544,7	3,21038E-03	39657	8,22539E-01	44373	1,06119E+00	38520	9,09795E-01	26792	0	48381,5
Myeloid_vs_CD8_Responder	Macro_OLFML3	CD8(IL7R+ZNF683+ Tm)	GRN	TNFRSF1B	8,55109E-03	29081,1	2,96326E-03	44373	1,60340E+00	13348	1,50656E+00	21490	9,25608E-01	17813	0	48381,5
Myeloid_vs_CD8_Responder	CD8(GZMK+ Tex)	pDC_LILRA4	HSP90B1	TLR7	8,55739E-03	31619,9	6,80190E-03	12901	1,59804E+00	13462	1,48160E+00	22234	8,49407E-01	61121	0	48381,5
Myeloid_vs_CD8_Responder	CD8(ZNF683+KLRB1+ T)	Macro_LYVE1	CD99	PILRA	8,55976E-03	30894,5	4,46054E-03	24648	1,62533E+00	12891	9,41596E-01	44382	9,14623E-01	24170	0	48381,5
Myeloid_vs_CD8_Responder	CD8(GZMK+ Tem)	Mono_INHBA	SEMA4A	LILRB2	8,56186E-03	35788,1	5,50186E-03	17967	1,71792E+00	11144	1,48153E+00	22237	8,16494E-01	79211	0	48381,5
Myeloid_vs_CD8_Responder	CD8(Tc17)	Macro_OLFML3	ADAM10	TREM2	8,57380E-03	29631,7	7,19990E-03	11794	2,07926E+00	6284	1,48133E+00	22245	8,52466E-01	59454	0	48381,5
Myeloid_vs_CD8_Responder	Macro_OLFML3	CD8(ITM2C+ T)	FN1	ITGA5_ITGB1	8,57520E-03	23421,3	1,05155E-02	6456	2,21085E+00	5073	1,87805E+00	12798	8,78582E-01	44398	0	48381,5
Myeloid_vs_CD8_Responder	cDC(CD1C)	CD8(EOMES+ NK-like)	CXCL16	CXCR6	8,58487E-03	33958,9	4,73231E-03	22609	1,01641E+00	33418	1,52362E+00	20978	8,78566E-01	44408	0	48381,5

Myeloid_vs_CD8_Responder	CD8(EOMES+ NK-like)	pDC_LILRA4	GZMB	IGF2R	8,58680E-03	28974,5	9,07265E-03	8069	8,21919E-01	44410	2,11021E+00	8938	8,94791E-01	35074	0	48381,5
Myeloid_vs_CD8_Responder	CD8(ISG+ T)	Mono_CD14	HLA-B	LILRB2	8,59472E-03	28261,7	2,92182E-03	45300	1,48507E+00	16224	1,48085E+00	22259	9,40568E-01	9144	0	48381,5
Myeloid_vs_CD8_Responder	Macro_FOLR2-APOE+	CD8(Terminal Tex)	HLA-DPB1	LAG3	8,59771E-03	20104,7	5,97827E-03	15825	1,28207E+00	22261	1,98559E+00	10830	9,51909E-01	3226	0	48381,5
Myeloid_vs_CD8_Responder	CD8(NME1+ T)	Macro_LYVE1	HLA-DRB5	CD4	8,60518E-03	30782,5	2,96084E-03	44429	1,53937E+00	14833	1,34142E+00	27019	9,23207E-01	19250	0	48381,5
Myeloid_vs_CD8_Responder	CD8(GZMK+ Tex)	Macro_NLRP3	B2M	LILRB2	8,60615E-03	28624,5	2,96081E-03	44430	1,58072E+00	13845	1,24746E+00	30637	9,46534E-01	5829	0	48381,5
Myeloid_vs_CD8_Responder	CD8(GZMK+ Tem)	Mono_INHBA	CCL5	CCRL2	8,60712E-03	32377,3	7,99680E-03	9892	9,58298E-01	36391	9,40699E-01	44431	9,16970E-01	22791	0	48381,5
Myeloid_vs_CD8_Responder	Macro_FOLR2+APOE-	CD8(GZMK+ Tex)	F13A1	ITGA4	8,60817E-03	18975,9	1,31080E-02	4546	3,42865E+00	872	2,09876E+00	9089	9,00332E-01	31991	0	48381,5
Myeloid_vs_CD8_Responder	Macro_LYVE1	CD8(Terminal Tex)	LGALS3	LAG3	8,60969E-03	20875,1	8,32633E-03	9260	1,28185E+00	22269	1,82166E+00	13840	9,38006E-01	10625	0	48381,5
Myeloid_vs_CD8_Responder	pDC_LILRA4	CD8(NME1+ T)	COL24A1	ITGA1_ITGB1	8,61659E-03	48898,5	4,44531E-02	371	2,77863E+00	2111	6,21554E-01	64341	7,03919E-01	129288	0	48381,5
Myeloid_vs_CD8_Responder	Macro_IER3	CD8(ISG+ T)	C3	IFITM1	8,61868E-03	23669,9	6,28983E-03	14650	1,28173E+00	22275	1,75440E+00	15215	9,25582E-01	17828	0	48381,5
Myeloid_vs_CD8_Responder	CD8(EOMES+ NK-like)	Macro_FOLR2+APOE+	CD52	SIGLEC10	8,62651E-03	26822,7	6,46014E-03	14041	1,91110E+00	8248	9,40247E-01	44451	9,23623E-01	18992	0	48381,5
Myeloid_vs_CD8_Responder	cDC(CD1C)	CD8(LAYN+ T)	HLA-DRA	LAG3	8,64108E-03	26563,3	2,95944E-03	44466	1,20246E+00	25129	2,76629E+00	2869	9,35711E-01	11971	0	48381,5
Myeloid_vs_CD8_Responder	CD8(Terminal Tex)	Macro_LYVE1	RPS19	C5AR1	8,64205E-03	31995,5	3,34343E-03	37382	1,55119E+00	14563	9,39829E-01	44467	9,30284E-01	15184	0	48381,5
Myeloid_vs_CD8_Responder	CD8(EOMES+ NK-like)	Macro_ISG15	CCL5	CCR1	8,65858E-03	32093,5	7,77604E-03	10384	1,01286E+00	33589	9,39391E-01	44484	9,15563E-01	23629	0	48381,5
Myeloid_vs_CD8_Responder	cDC(CD1C)	CD8(EOMES+ NK-like)	GRN	TNFRSF1B	8,65858E-03	27466,7	2,95875E-03	44484	1,42009E+00	18068	2,13831E+00	8556	9,25555E-01	17844	0	48381,5
Myeloid_vs_CD8_Responder	CD8(GZMK+ Tex)	Macro_ISG15	CD99	PILRA	8,67806E-03	39917,1	2,95798E-03	44504	1,09647E+00	29645	9,63223E-01	43295	8,97159E-01	33760	0	48381,5
Myeloid_vs_CD8_Responder	CD8(EOMES+ NK-like)	Macro_NLRP3	ANXA1	FPR1	8,70539E-03	31722,9	4,15308E-03	27395	1,59039E+00	13621	9,38438E-01	44532	9,13651E-01	24685	0	48381,5
Myeloid_vs_CD8_Responder	CD8(Terminal Tex)	Macro_NLRP3	CIRBP	TREM1	8,71322E-03	30184,5	7,29192E-03	11563	1,86260E+00	8892	9,38306E-01	44540	8,90404E-01	37546	0	48381,5
Myeloid_vs_CD8_Responder	Mono_INHBA	CD8(GZMK+ Tex)	HLA-DRB1	LAG3	8,72300E-03	30281,7	3,56492E-03	34116	8,19710E-01	44550	1,72281E+00	15941	9,41860E-01	8420	0	48381,5
Myeloid_vs_CD8_Responder	CD8(Tn)	Macro_NLRP3	CD40LG	ITGAM_ITGB2	8,72971E-03	37869,5	2,00023E-02	2125	1,02644E+00	32917	1,11584E+00	36070	8,33786E-01	69854	0	48381,5
Myeloid_vs_CD8_Responder	CD8(NME1+ T)	Mono_CD14	HLA-A	LILRB2	8,73672E-03	29505,5	2,95486E-03	44564	1,38844E+00	19004	1,40060E+00	24880	9,37900E-01	10698	0	48381,5
Myeloid_vs_CD8_Responder	Macro_NLRP3	CD8(GZMK+ Tex)	VCAN	ITGB1	8,75786E-03	18923,1	1,06419E-02	6338	2,58443E+00	2799	2,09541E+00	9143	9,07703E-01	27954	0	48381,5
Myeloid_vs_CD8_Responder	Mono_INHBA	CD8(GZMK+ Early Tem)	LYZ	ITGAL	8,75830E-03	30293,1	2,95411E-03	44586	1,61919E+00	13032	2,19627E+00	7775	8,90142E-01	37691	0	48381,5
Myeloid_vs_CD8_Responder	CD8(EOMES+ NK-like)	Mono_INHBA	HLA-B	LILRB2	8,76125E-03	33021,7	2,95376E-03	44589	1,29437E+00	21884	1,00441E+00	41282	9,40872E-01	8972	0	48381,5
Myeloid_vs_CD8_Responder	Macro_NLRP3	CD8(ID2+CXCR4+ T)	VCAN	CD44	8,77862E-03	21680,7	6,76818E-03	13007	2,36371E+00	3902	1,47697E+00	22381	9,20523E-01	20732	0	48381,5
Myeloid_vs_CD8_Responder	CD8(NME1+ T)	Macro_FOLR2-APOE+	HLA-F	LILRB2	8,78092E-03	38780,5	3,32880E-03	37639	1,25895E+00	23040	9,36892E-01	44609	8,85713E-01	40233	0	48381,5
Myeloid_vs_CD8_Responder	CD8(ZNF683+KLRB1+ T)	Mono_INHBA	HLA-F	LILRB2	8,78092E-03	38569,1	2,95280E-03	44609	1,40323E+00	18547	1,08520E+00	37417	8,79505E-01	43891	0	48381,5
Myeloid_vs_CD8_Responder	CD8(Temra)	Mono_CD16	HLA-C	LILRA1	8,78166E-03	28015,7	1,01001E-02	6861	1,94773E+00	7791	7,63093E-01	54662	9,17688E-01	22383	0	48381,5
Myeloid_vs_CD8_Responder	CD8(NME1+ T)	Macro_LYVE1	RPS19	C5AR1	8,78190E-03	29787,7	3,81813E-03	30902	1,65389E+00	12329	9,36891E-01	44610	9,34468E-01	12716	0	48381,5
Myeloid_vs_CD8_Responder	Mono_CD16	CD8(GZMK+ Early Tem)	S100A8	CD69	8,78318E-03	25453,7	5,43790E-03	18319	1,27889E+00	22384	1,49299E+00	21901	9,28406E-01	16283	0	48381,5
Myeloid_vs_CD8_Responder	CD8(EOMES+ NK-like)	pDC_LILRA4	TNF	PTPRS	8,78653E-03	32736,5	1,99616E-02	2132	3,60123E+00	714	1,94366E+00	11554	7,73552E-01	100901	0	48381,5
Myeloid_vs_CD8_Responder	CD8(Temra)	Macro_OLFML3	CD52	SIGLEC10	8,78781E-03	29907,9	5,22539E-03	19489	1,59431E+00	13537	9,36764E-01	44616	9,15797E-01	23516	0	48381,5
Myeloid_vs_CD8_Responder	CD8(ITM2C+ T)	Macro_IFI27	IFNG	IFNGR1_IFNGR2	8,78978E-03	38151,9	5,28874E-03	19112	8,18476E-01	44618	1,07993E+00	37657	8,84331E-01	40991	0	48381,5
Myeloid_vs_CD8_Responder	CD8(GZMK+ Early Tem)	Mono_CD16	ANXA1	FPR1	8,79686E-03	29385,9	4,76624E-03	22393	1,79735E+00	9857	9,36262E-01	44651	9,18930E-01	21647	0	48381,5
Myeloid_vs_CD8_Responder	Macro_OLFML3	CD8(GZMK+ Early Tem)	GRN	TNFRSF1B	8,79766E-03	28820,5	2,95228E-03	44626	1,60153E+00	13399	1,56544E+00	19808	9,25480E-01	17888	0	48381,5
Myeloid_vs_CD8_Responder	CD8(Tn)	Macro_IER3	ANXA1	FPR1	8,80161E-03	39489,5	2,95194E-03	44630	9,81494E-01	35165	1,10249E+00	36654	8,99199E-01	32617	0	48381,5
Myeloid_vs_CD8_Responder	cDC(CD1C)	CD8(GZMK+ Tex)	LYZ	ITGAL	8,80259E-03	29029,3	2,95189E-03	44631	1,65085E+00	12392	2,95729E+00	2028	8,90106E-01	37714	0	48381,5
Myeloid_vs_CD8_Responder	Mono_CD14	CD8(Temra)	IL1B	SIGIRR	8,81092E-03	19374,1	8,37365E-03	9162	2,21283E+00	5056	3,01465E+00	1808	8,99482E-01	32463	0	48381,5
Myeloid_vs_CD8_Responder	CD8(NME1+ T)	Mono_CD16	HLA-F	LILRB1	8,82134E-03	36597,3	4,31046E-03	25947	1,30355E+00	21572	9,36266E-01	44650	8,81941E-01	42436	0	48381,5
Myeloid_vs_CD8_Responder	Mono_INHBA	CD8(Terminal Tex)	CXCL16	CXCR6	8,83815E-03	39664,5	4,69369E-03	22878	8,34885E-01	43592	1,05565E+00	38804	8,78128E-01	44667	0	48381,5
Myeloid_vs_CD8_Responder	CD8(GZMK+ Early Tem)	Macro_NLRP3	CALR	LRP1	8,85003E-03	37589,1	3,15056E-03	40737	1,33488E+00	20593	9,35688E-01	44679	8,97515E-01	33555	0	48381,5
Myeloid_vs_CD8_Responder	cDC(CD1C)	CD8(GZMK+ Tem)	GRN	TNFRSF1B	8,85300E-03	27469,3	2,94919E-03	44682	1,41816E+00	18112	2,15862E+00	8260	9,25444E-01	17911	0	48381,5
Myeloid_vs_CD8_Responder	pDC_LILRA4	CD8(Terminal Tex)	COL24A1	ITGA1_ITGB1	8,85986E-03	48696,3	4,24211E-02	398	2,76800E+00	2141	6,59439E-01	61646	6,99020E-01	130915	0	48381,5
Myeloid_vs_CD8_Responder	CD8(GZMK+ Tex)	Macro_ISG15	HLA-F	LILRB1	8,87879E-03	34804,7	4,00431E-03	28842	1,26513E+00	22825	1,28212E+00	29267	8,78051E-01	44708	0	48381,5
Myeloid_vs_CD8_Responder	CD8(Temra)	Macro_ISG15	HLA-A	LILRB2	8,87978E-03	30671,9	2,94840E-03	44709	1,34212E+00	20373	1,28557E+00	29153	9,37837E-01	10743	0	48381,5
Myeloid_vs_CD8_Responder	Macro_NLRP3	CD8(GZMK+ Tem)	S100A9	ITGB2	8,88083E-03	17763,7	4,75802E-03	22448	2,37483E+00	3833	2,67752E+00	3425	9,37851E-01	10731	0	48381,5
Myeloid_vs_CD8_Responder	CD8(Temra)	Mono_CD14	HMGB1	TLR4	8,88177E-03	39972,9	3,64077E-03	33091	1,08887E+00	29985	9,55294E-01	43696	8,78044E-01	44711	0	48381,5
Myeloid_vs_CD8_Responder	CD8(ID2+CXCR4+ T)	Mono_CD14	HLA-A	LILRB2	8,88673E-03	29935,9	2,94827E-03	44716	1,38178E+00	19192	1,35039E+00	26646	9,37835E-01	10744	0	48381,5
Myeloid_vs_CD8_Responder	Macro_ISG15	CD8(ISG+ T)	CCL8	CCR5	8,90891E-03	35396,7	3,91610E-02	472	2,76518E+00	2147	1,94456E+00	11541	7,43132E-01	114442	0	48381,5
Myeloid_vs_CD8_Responder	CD8(NME1+ T)	Macro_LYVE1	HLA-DRB1	CD4	8,91858E-03	30507,9	2,94693E-03	44748	1,52701E+00	15158	1,21596E+00	31891	9,35018E-01	12361	0	48381,5
Myeloid_vs_CD8_Responder	CD8(Tn)	Mono_INHBA	CD40LG	CD40	8,92529E-03	48155,3	1,98943E-02	2149	9,57905E-01	36414	1,17880E+00	33354	7,28138E-01	120478	0	48381,5
Myeloid_vs_CD8_Responder	CD8(EOMES+ NK-like)	Macro_ISG15	ANXA1	FPR1	8,92755E-03	33936,9	3,75037E-03	31699	1,42566E+00	17907	9,34181E-01	44757	9,09542E-01	26940	0	48381,5
Myeloid_vs_CD8_Responder	CD8(ISG+ T)	Macro_LYVE1	HLA-A	APLP2	8,93054E-03	33976,7	2,94632E-03	44760	1,10457E+00	29285	9,63640E-01	43264	9,49700E-01	4193	0	48381,5
Myeloid_vs_CD8_Responder	CD8(Terminal Tex)	cDC_LAMP3	CSF1	CSF2RA	8,93460E-03	33702,5	1,01733E-02	6790	1,84165E+00	9206	1,26404E+00	29952	8,25844E-01	74183	0	48381,5
Myeloid_vs_CD8_Responder	CD8(GZMK+ Tex)	cDC_CLEC9A	CALR	SCARF1	8,93742E-03	35296,5	1,10218E-02	6008	1,84162E+00	9207	8,46547E-01	49538	8,45323E-01	63348	0	48381,5
Myeloid_vs_CD8_Responder	pDC_LILRA4	CD8(Terminal Tex)	APP	RPSA	8,93764E-03	21390,5	5,98759E-03	15796	1,61092E+00	13197	2,25671E+00	7093	9,17504E-01	22485	0	48381,5
Myeloid_vs_CD8_Responder	Mono_CD16	CD8(EOMES+ NK-like)	CFP	NCR1	8,94168E-03	36381,3	5,84655E-02	209	2,76404E+00	2151	1,29515E+00	28791	7,70440E-01	102374	0	48381,5
Myeloid_vs_CD8_Responder	Macro_FOLR2+APOE-	CD8(Terminal Tex)	HLA-DRB1	LAG3	8,94842E-03	19843,1	6,00845E-03	15721	1,27568E+00	22492	2,01326E+00	10389	9,54610E-01	2232	0	48381,5
Myeloid_vs_CD8_Responder	Macro_FOLR2+APOE-	CD8(Terminal Tex)	HLA-DQA2	LAG3	8,94996E-03	21142,9	9,11262E-03	8014	1,27556E+00	22493	1,99191E+00	10724	9,28709E-01	16102	0	48381,5
Myeloid_vs_CD8_Responder	cDC(CD1C)	CD8(GZMK+ Early Tem)	HLA-DQA2	LAG3	8,97352E-03	36750,7	3,36357E-03	37057	8,15758E-01	44803	1,79039E+00	14451	8,87823E-01	39061	0	48381,5
Myeloid_vs_CD8_Responder	Macro_OLFML3	CD8(Tc17)	FN1	ITGA4_ITGB1	8,97553E-03	27562,3	6,79706E-03	12914	1,84504E+00	9157	1,47127E+00	22554	8,77896E-01	44805	0	48381,5
Myeloid_vs_CD8_Responder	CD8(GZMK+ Tem)	pDC_LILRA4	HLA-DRA	CD4	8,97618E-03	36859,3	1,80959E-03	81213	1,27484E+00	22510	2,05833E+00	9696	9,17481E-01	22496	0	48381,5
Myeloid_vs_CD8_Responder	Macro_NLRP3	CD8(Terminal Tex)	HLA-DRA	LAG3	8,97773E-03	20012,7	5,70801E-03	17012	1,27479E+00	22511	2,08246E+00	9340	9,52860E-01	2819	0	48381,5
Myeloid_vs_CD8_Responder	Mono_INHBA	CD8(ID2+CXCR4+ T)	HBEGF	CD44	8,99257E-03	34048,1	4,31708E-03	25883	1,43218E+00	17726	9,32806E-01	44822	8,97722E-01	33428	0	48381,5
Myeloid_vs_CD8_Responder	Macro_OLFML3	CD8(GZMK+ Tem)	CXCL16	CXCR6	8,99758E-03	35187,3	5,75573E-03	16771	1,14884E+00	27330	9,32666E-01	44827	8,88629E-01	38627	0	48381,5
Myeloid_vs_CD8_Responder	Macro_FOLR2+APOE+	CD8(ISG+ T)	HLA-DPB1	LAG3	9,00091E-03	21279,7	5,38641E-03	18601	1,27412E+00	22526	1,88941E+00	12570	9,49466E-01	4320	0	48381,5
Myeloid_vs_CD8_Responder	CD8(LAYN+ T)	Macro_ISG15	HLA-A	LILRB2	9,00160E-03	31123,9	2,94312E-03	44831	1,33693E+00	20531	1,23532E+00	31110	9,37784E-01	10766	0	48381,5
Myeloid_vs_CD8_Responder	Macro_FOLR2-APOE+	CD8(GZMK+ Tem)	HLA-DQB1	LAG3	9,02259E-03	20496,1	7,00264E-03	12316	1,27353E+00	22540	1,84597E+00	13365	9,46445E-01	5878	0	48381,5
Myeloid_vs_CD8_Responder	cDC_LAMP3	CD8(GZMK+ Tem)	HLA-DQA2	LAG3	9,03500E-03	21673,1	9,15324E-03	7959	1,27321E+00	22548	1,84137E+00	13462	9,28856E-01	16015	0	48381,5
Myeloid_vs_CD8_Responder	CD8(ID2+CXCR4+ T)	Mono_CD16	HLA-B	LILRB1	9,03780E-03	32754,1	3,29792E-03	38162	1,36776E+00	19600	9,31924E-01	44867	9,34395E-01	12760	0	48381,5
Myeloid_vs_CD8_Responder	CD8(Temra)	Macro_FOLR2+APOE+	TNF	VSIR	9,04586E-03	37630,9	6,50854E-03	13888	9,57176E-01	36451	9,37839E-01	44559	8,77791E-01	44875	0	48381,5
Myeloid_vs_CD8_Responder	CD8(Temra)	Macro_FOLR2-APOE+	CD99	PILRA	9,04586E-03	35563,5	3,65359E-03	32952	1,26061E+00	22983	9,31777E-01	44875	9,06502E-01	28626	0	48381,5
Myeloid_vs_CD8_Responder	Macro_FOLR2-APOE+	CD8(Terminal Tex)	C3	IFITM1	9,04897E-03	24633,5	6,69633E-03	13235	1,28043E+00	22330	1,47124E+00	22557	9,27710E-01	16664	0	48381,5
Myeloid_vs_CD8_Responder	CD8(Terminal Tex)	Mono_INHBA	HSP90B1	LRP1	9,06301E-03	37946,9	2,94027E-03	44892	1,38994E+00	18958	1,09696E+00	36887	8,85055E-01	40616	0	48381,5
Myeloid_vs_CD8_Responder	CD8(GZMK+ Tex)	Macro_OLFML3	HMGB1	TLR2	9,07108E-03	30442,1	4,97221E-03	21036	1,59772E+00	13471	9,31275E-01	44900	9,14176E-01	24422	0	48381,5
Myeloid_vs_CD8_Responder	cDC_LAMP3	CD8(Terminal Tex)	HLA-DQB1	LAG3	9,07695E-03	20524,7	6,89697E-03	12619	1,27237E+00	22575	1,86841E+00	12969	9,46059E-01	6079	0	48381,5
Myeloid_vs_CD8_Responder	CD8(Tc17)	Macro_FOLR2+APOE+	LTB	TNFRSF1A	9,07816E-03	35511,5	7,99155E-03	9899	1,07357E+00	30718	9,56049E-01	43652	8,77742E-01	44907	0	48381,5

Myeloid_vs_CD8_Responder	CD8(Terminal Tex)	Mono_CD14	CALR	LRP1	9,07917E-03	36788,7	2,93950E-03	44908	1,21602E+00	24599	1,24611E+00	30699	8,94281E-01	35356	0	48381,5
Myeloid_vs_CD8_Responder	Mono_INHBA	CD8(GZMK+ Tem)	INHBA	ACTR2	9,09076E-03	22967,7	1,19113E-02	5259	3,11424E+00	1336	2,08773E+00	9261	8,68021E-01	50601	0	48381,5
Myeloid_vs_CD8_Responder	Mono_INHBA	CD8(GZMK+ Tex)	LGALS1	CD69	9,10244E-03	33727,9	2,93868E-03	44931	8,97893E-01	39747	1,36179E+00	26206	9,40145E-01	9374	0	48381,5
Myeloid_vs_CD8_Responder	CD8(LAYN+ T)	Mono_INHBA	HMGB1	CD163	9,10447E-03	32925,7	2,93864E-03	44933	1,36373E+00	19711	1,24563E+00	30717	9,20262E-01	20886	0	48381,5
Myeloid_vs_CD8_Responder	CD8(ITM2C+ T)	Macro_ISG15	TNF	TNFRSF1A	9,11156E-03	28801,5	8,61814E-03	8762	1,21213E+00	24750	1,66944E+00	17174	8,77680E-01	44940	0	48381,5
Myeloid_vs_CD8_Responder	CD8(ZNF683+KLRB1+ T)	Macro_NLRP3	CIRBP	TREM1	9,11156E-03	30465,5	7,16604E-03	11880	1,84983E+00	9082	9,30529E-01	44940	8,89551E-01	38044	0	48381,5
Myeloid_vs_CD8_Responder	CD8(GZMK+ Tem)	cDC(CD1C)	IFNG	IFNGR1_IFNGR2	9,11258E-03	36793,5	4,98731E-03	20928	8,13750E-01	44941	1,34459E+00	26894	8,81295E-01	42823	0	48381,5
Myeloid_vs_CD8_Responder	CD8(Terminal Tex)	Macro_OLFML3	TNFSF9	HLA-DPA1	9,11967E-03	30148,5	2,93802E-03	44948	1,26856E+00	22700	2,27154E+00	6943	9,08017E-01	27770	0	48381,5
Myeloid_vs_CD8_Responder	CD8(ID2+CXCR4+ T)	Mono_CD14	HLA-B	LILRB2	9,12069E-03	28254,7	2,93802E-03	44949	1,50483E+00	15710	1,45188E+00	23182	9,40723E-01	9051	0	48381,5
Myeloid_vs_CD8_Responder	Mono_CD16	CD8(GZMK+ Tem)	B2M	CD3D	9,13150E-03	58890,9	2,60104E-03	53466	3,14363E-01	87889	1,50305E-01	104660	9,64923E-01	58	0	48381,5
Myeloid_vs_CD8_Responder	cDC(CD1C)	CD8(ITM2C+ T)	HLA-DRA	LAG3	9,13490E-03	26743,7	2,93677E-03	44963	1,20005E+00	25214	2,73740E+00	3055	9,35479E-01	12105	0	48381,5
Myeloid_vs_CD8_Responder	CD8(ITM2C+ T)	Macro_LYVE1	CALR	LRP1	9,13897E-03	30213,7	4,35955E-03	25511	2,07760E+00	6304	9,30042E-01	44967	9,11517E-01	25905	0	48381,5
Myeloid_vs_CD8_Responder	CD8(GZMK+ Tex)	pDC_LILRA4	HLA-DPB1	CD4	9,14094E-03	33966,9	2,10448E-03	69806	1,27118E+00	22616	2,18470E+00	7922	9,19826E-01	21109	0	48381,5
Myeloid_vs_CD8_Responder	Mono_INHBA	CD8(GZMK+ Tex)	CD14	ITGB1	9,14710E-03	33163,7	2,93643E-03	44975	1,21189E+00	24768	1,73364E+00	15691	9,00303E-01	32003	0	48381,5
Myeloid_vs_CD8_Responder	Macro_ISG15	CD8(GZMK+ Tem)	CCL8	CCR5	9,14784E-03	35767,9	3,78543E-02	506	2,75559E+00	2176	1,92226E+00	11946	7,39879E-01	115830	0	48381,5
Myeloid_vs_CD8_Responder	Macro_FOLR2+APOE-	CD8(Terminal Tex)	HLA-DQB1	LAG3	9,15347E-03	20376,3	6,88852E-03	12638	1,27090E+00	22624	1,91256E+00	12141	9,46027E-01	6097	0	48381,5
Myeloid_vs_CD8_Responder	Macro_FOLR2+APOE+	CD8(GZMK+ Tex)	CD14	ITGA4	9,15503E-03	22779,9	4,90768E-03	21470	2,02651E+00	6869	1,78505E+00	14554	9,17251E-01	22625	0	48381,5
Myeloid_vs_CD8_Responder	CD8(IL7R+ZNF683+ Tm)	Macro_LYVE1	CALR	LRP1	9,15829E-03	29751,1	4,51859E-03	24204	2,10087E+00	6067	9,29758E-01	44986	9,12952E-01	25117	0	48381,5
Myeloid_vs_CD8_Responder	Macro_OLFML3	CD8(ISG+ T)	LGALS3BP	ITGB1	9,16440E-03	33625,9	5,52887E-03	17828	1,61488E+00	13119	9,52967E-01	43809	8,77584E-01	44992	0	48381,5
Myeloid_vs_CD8_Responder	CD8(NME1+ T)	Macro_ISG15	CD52	SIGLEC10	9,17867E-03	39139,5	3,39680E-03	36554	1,03950E+00	32267	9,29346E-01	45006	8,97635E-01	33489	0	48381,5
Myeloid_vs_CD8_Responder	Mono_CD14	CD8(Tn)	VCAN	ITGA4	9,19397E-03	21085,1	7,75953E-03	10413	3,07920E+00	1404	2,30849E+00	6560	8,88562E-01	38667	0	48381,5
Myeloid_vs_CD8_Responder	CD8(IL7R+ZNF683+ Tm)	Macro_FOLR2+APOE+	CD52	SIGLEC10	9,19426E-03	32499,7	5,42136E-03	18394	1,76358E+00	10385	6,44953E-01	62688	9,17206E-01	22650	0	48381,5
Myeloid_vs_CD8_Responder	CD8(ID2+CXCR4+ T)	cDC(CD1C)	B2M	CD1B	9,19968E-03	36732,5	1,79566E-02	2654	1,83522E+00	9299	4,66127E-01	76438	8,74416E-01	46890	0	48381,5
Myeloid_vs_CD8_Responder	Mono_CD14	CD8(Tn)	HBEGF	CD44	9,20010E-03	28736,5	4,71638E-03	22717	1,39161E+00	18898	1,47453E+00	22459	9,01712E-01	31227	0	48381,5
Myeloid_vs_CD8_Responder	CD8(Tn)	Macro_FOLR2+APOE-	CD59	STAB1	9,20054E-03	36459,5	4,72574E-03	22654	1,90715E+00	8299	1,51908E+00	21143	8,11512E-01	81820	0	48381,5
Myeloid_vs_CD8_Responder	CD8(ZNF683+KLRB1+ T)	Macro_NLRP3	HLA-A	LILRB2	9,20419E-03	34321,5	2,94635E-03	44758	1,26908E+00	22682	9,28779E-01	45031	9,37816E-01	10755	0	48381,5
Myeloid_vs_CD8_Responder	Mono_INHBA	CD8(GZMK+ Early Tem)	LGALS1	PTPRC	9,20521E-03	28958,3	2,93392E-03	45032	1,08221E+00	30311	1,62094E+00	18392	9,53266E-01	2675	0	48381,5
Myeloid_vs_CD8_Responder	CD8(ID2+CXCR4+ T)	Macro_ISG15	CD99	PILRA	9,20725E-03	39130,9	3,10168E-03	41643	1,13229E+00	28052	9,28758E-01	45034	8,99327E-01	32544	0	48381,5
Myeloid_vs_CD8_Responder	CD8(ITM2C+ T)	Mono_INHBA	HLA-A	LILRB2	9,20930E-03	34360,1	2,97213E-03	44182	1,24315E+00	23608	9,28736E-01	45036	9,38070E-01	10593	0	48381,5
Myeloid_vs_CD8_Responder	Macro_LYVE1	CD8(ISG+ T)	HLA-DRB5	LAG3	9,21942E-03	21021,9	6,03103E-03	15634	1,26959E+00	22666	1,89838E+00	12389	9,46131E-01	6039	0	48381,5
Myeloid_vs_CD8_Responder	CD8(ZNF683+KLRB1+ T)	Mono_INHBA	CD99	PILRA	9,22055E-03	40619,1	2,93338E-03	45047	1,05977E+00	31325	9,41937E-01	44358	8,96773E-01	33984	0	48381,5
Myeloid_vs_CD8_Responder	Mono_CD14	CD8(ID2+CXCR4+ T)	CD14	ITGB1	9,24309E-03	29715,1	3,16839E-03	40407	1,40755E+00	18418	1,96471E+00	11209	9,03664E-01	30160	0	48381,5
Myeloid_vs_CD8_Responder	Mono_CD14	CD8(ID2+CXCR4+ T)	CD14	ITGA4	9,24411E-03	31436,1	3,04863E-03	42668	1,35463E+00	19989	1,89468E+00	12466	8,97295E-01	33676	0	48381,5
Myeloid_vs_CD8_Responder	CD8(GZMK+ Early Tem)	Mono_INHBA	TNFSF9	HLA-DPA1	9,24514E-03	34397,1	3,30242E-03	38091	8,11704E-01	45071	1,75321E+00	15240	9,12784E-01	25202	0	48381,5
Myeloid_vs_CD8_Responder	CD8(ID2+CXCR4+ T)	Macro_NLRP3	CD52	SIGLEC10	9,24822E-03	41959,3	3,00371E-03	43585	9,65566E-01	36019	9,28097E-01	45074	8,91844E-01	36737	0	48381,5
Myeloid_vs_CD8_Responder	CD8(NME1+ T)	Macro_LYVE1	HLA-A	APLP2	9,26259E-03	34416,9	2,93116E-03	45088	1,08914E+00	29969	9,41488E-01	44389	9,49577E-01	4257	0	48381,5
Myeloid_vs_CD8_Responder	CD8(NME1+ T)	Mono_INHBA	GNAI2	C5AR1	9,26361E-03	31806,7	3,46893E-03	35489	1,81191E+00	9625	1,54214E+00	20449	8,77423E-01	45089	0	48381,5
Myeloid_vs_CD8_Responder	Mono_CD14	CD8(ID2+CXCR4+ T)	HBEGF	CD44	9,27286E-03	34579,1	3,82764E-03	30785	1,25040E+00	23349	1,16842E+00	33786	8,92064E-01	36594	0	48381,5
Myeloid_vs_CD8_Responder	Macro_OLFML3	CD8(Tn)	C1QB	C1QBP	9,27595E-03	26811,7	2,93058E-03	45101	2,05677E+00	6515	2,70461E+00	3240	9,02420E-01	30821	0	48381,5
Myeloid_vs_CD8_Responder	CD8(Temra)	Mono_CD16	HLA-A	LILRA1	9,28256E-03	31550,5	9,65067E-03	7332	1,75814E+00	10476	5,61003E-01	68857	9,17112E-01	22706	0	48381,5
Myeloid_vs_CD8_Responder	Mono_CD14	CD8(ID2+CXCR4+ T)	AGTRAP	RACK1	9,28932E-03	33343,3	4,60956E-03	23515	9,05084E-01	39320	1,14895E+00	34655	9,20329E-01	20845	0	48381,5
Myeloid_vs_CD8_Responder	CD8(GZMK+ Tex)	Mono_CD16	HLA-F	LILRB1	9,29653E-03	37031,9	3,95207E-03	29436	1,24892E+00	23403	1,05545E+00	38818	8,77347E-01	45121	0	48381,5
Myeloid_vs_CD8_Responder	Mono_CD14	CD8(Terminal Tex)	ICAM1	IL2RG	9,31612E-03	27117,5	5,10378E-03	20214	1,13377E+00	27989	1,93374E+00	11722	9,08939E-01	27281	0	48381,5
Myeloid_vs_CD8_Responder	Macro_ISG15	CD8(LAYN+ T)	CD14	ITGA4	9,32218E-03	21971,9	4,88507E-03	21633	1,85550E+00	9000	2,17083E+00	8114	9,17076E-01	22731	0	48381,5
Myeloid_vs_CD8_Responder	CD8(Tc17)	Macro_ISG15	RPS19	C5AR1	9,32232E-03	35023,9	2,92885E-03	45146	1,11248E+00	28937	1,14096E+00	35021	9,25866E-01	17634	0	48381,5
Myeloid_vs_CD8_Responder	Mono_CD14	CD8(Terminal Tex)	CD14	ITGA4	9,32335E-03	30268,1	3,21041E-03	39656	1,37590E+00	19377	1,94261E+00	11567	8,99653E-01	32359	0	48381,5
Myeloid_vs_CD8_Responder	Mono_INHBA	CD8(Terminal Tex)	VCAN	ITGA4	9,32748E-03	28207,9	6,23663E-03	14833	1,61003E+00	13212	1,57806E+00	19462	8,77278E-01	45151	0	48381,5
Myeloid_vs_CD8_Responder	CD8(LAYN+ T)	Mono_INHBA	HLA-C	LILRB2	9,32955E-03	34221,9	2,92859E-03	45153	1,29741E+00	21792	9,40694E-01	44432	9,36747E-01	11351	0	48381,5
Myeloid_vs_CD8_Responder	CD8(GZMK+ Tem)	Macro_NLRP3	B2M	LILRB2	9,33058E-03	29760,9	2,92857E-03	45154	1,52533E+00	15200	1,16132E+00	34100	9,46257E-01	5969	0	48381,5
Myeloid_vs_CD8_Responder	CD8(GZMK+ Tex)	Macro_FOLR2+APOE+	ADAM10	GPNMB	9,33171E-03	32631,7	4,71266E-03	22737	2,86298E+00	1880	1,60174E+00	18848	8,31093E-01	71312	0	48381,5
Myeloid_vs_CD8_Responder	Mono_INHBA	CD8(ITM2C+ T)	SPP1	S1PR1	9,33265E-03	23771,3	3,04027E-02	769	1,83202E+00	9345	2,82740E+00	2527	8,55246E-01	57834	0	48381,5
Myeloid_vs_CD8_Responder	CD8(GZMK+ Early Tem)	Macro_LYVE1	CD99	PILRA	9,34505E-03	31181,7	4,42217E-03	24948	1,61850E+00	13047	9,26146E-01	45168	9,14285E-01	24364	0	48381,5
Myeloid_vs_CD8_Responder	CD8(LAYN+ T)	Mono_CD16	HLA-A	LILRA1	9,34602E-03	32356,5	9,63339E-03	7355	1,75294E+00	10558	5,10760E-01	72742	9,17044E-01	22746	0	48381,5
Myeloid_vs_CD8_Responder	Macro_OLFML3	CD8(ZNF683+KLRB1+ T)	LGALS3BP	ITGB1	9,34609E-03	33199,5	5,49335E-03	18015	1,61273E+00	13169	1,00492E+00	41263	8,77237E-01	45169	0	48381,5
Myeloid_vs_CD8_Responder	Mono_CD14	CD8(Terminal Tex)	IL1B	SIGIRR	9,35023E-03	23100,9	5,88920E-03	16189	2,08205E+00	6254	2,82813E+00	2521	8,82415E-01	42159	0	48381,5
Myeloid_vs_CD8_Responder	Mono_INHBA	CD8(Terminal Tex)	VCAN	ITGB1	9,35230E-03	28850,7	5,61792E-03	17398	1,60187E+00	13391	1,56181E+00	19908	8,77234E-01	45175	0	48381,5
Myeloid_vs_CD8_Responder	Mono_CD14	CD8(Terminal Tex)	HBEGF	CD44	9,36783E-03	38955,7	3,13796E-03	40965	1,14082E+00	27672	1,13128E+00	35443	8,82120E-01	42317	0	48381,5
Myeloid_vs_CD8_Responder	CD8(Temra)	Mono_INHBA	CIRBP	TREM1	9,37509E-03	34034,5	5,75510E-03	16780	1,52350E+00	15243	9,25551E-01	45197	8,78311E-01	44571	0	48381,5
Myeloid_vs_CD8_Responder	Mono_CD14	CD8(Terminal Tex)	HBEGF	CD82	9,37820E-03	24297,1	1,09041E-02	6101	1,71134E+00	11271	1,96956E+00	11121	8,78235E-01	44611	0	48381,5
Myeloid_vs_CD8_Responder	Mono_CD14	CD8(Terminal Tex)	LYZ	ITGAL	9,38132E-03	19880,1	5,16351E-03	19841	2,12136E+00	5853	3,25066E+00	1150	9,14621E-01	24175	0	48381,5
Myeloid_vs_CD8_Responder	CD8(ID2+CXCR4+ T)	cDC_CLEC9A	TGFB1	LPP	9,38858E-03	34952,5	5,96092E-03	15896	9,27028E-01	38077	1,33709E+00	27198	8,77169E-01	45210	0	48381,5
Myeloid_vs_CD8_Responder	CD8(GZMK+ Tex)	cDC(CD1C)	HMGB1	THBD	9,38962E-03	39246,1	3,23196E-03	39270	9,74441E-01	35540	1,32032E+00	27828	8,77168E-01	45211	0	48381,5
Myeloid_vs_CD8_Responder	CD8(GZMK+ Tex)	Macro_IER3	HMGB1	THBD	9,39377E-03	37720,1	3,95870E-03	29366	1,16739E+00	26499	9,25000E-01	45215	8,87684E-01	39139	0	48381,5
Myeloid_vs_CD8_Responder	CD8(GZMK+ Tex)	pDC_LILRA4	HLA-DQA1	CD4	9,39861E-03	28389,3	2,78993E-03	48381	1,39144E+00	18901	2,66492E+00	3504	9,16993E-01	22779	0	48381,5
Myeloid_vs_CD8_Responder	Mono_CD14	CD8(GZMK+ Tex)	ICAM1	IL2RG	9,40729E-03	34455,1	3,65953E-03	32863	8,81364E-01	40757	1,77082E+00	14867	8,94205E-01	35407	0	48381,5
Myeloid_vs_CD8_Responder	CD8(GZMK+ Tex)	Macro_LYVE1	HLA-DQA1	CD4	9,40937E-03	31528,5	2,92562E-03	45230	1,45422E+00	17079	1,39017E+00	25226	9,18783E-01	21726	0	48381,5
Myeloid_vs_CD8_Responder	Mono_CD14	CD8(GZMK+ Tex)	CD14	ITGB1	9,41353E-03	26890,5	3,54574E-03	34387	1,46189E+00	16845	2,23990E+00	7292	9,08452E-01	27547	0	48381,5
Myeloid_vs_CD8_Responder	Mono_CD14	CD8(GZMK+ Tex)	CD14	ITGA4	9,41457E-03	28566,1	3,44059E-03	35886	1,40617E+00	18457	2,07579E+00	9455	9,02735E-01	30651	0	48381,5
Myeloid_vs_CD8_Responder	CD8(ISG+ T)	cDC_CLEC9A	TNFSF9	HLA-DPA1	9,43436E-03	29693,3	2,92438E-03	45254	1,22780E+00	24151	2,77999E+00	2801	9,07822E-01	27879	0	48381,5
Myeloid_vs_CD8_Responder	Mono_CD14	CD8(GZMK+ Tem)	LYZ	ITGAL	9,43699E-03	19194,7	5,48560E-03	18053	2,14544E+00	5645	3,27626E+00	1091	9,16954E-01	22803	0	48381,5
Myeloid_vs_CD8_Responder	Mono_CD14	CD8(GZMK+ Tex)	HBEGF	CD44	9,45105E-03	32876,9	3,93016E-03	29658	1,26669E+00	22767	1,32413E+00	27688	8,93330E-01	35890	0	48381,5
Myeloid_vs_CD8_Responder	CD8(EOMES+ NK-like)	Macro_ISG15	HLA-A	LILRB1	9,45836E-03	35964,7	3,15819E-03	40586	1,08790E+00	30030	9,23757E-01	45277	9,29708E-01	15549	0	48381,5
Myeloid_vs_CD8_Responder	CD8(Temra)	Macro_LYVE1	SPON2	ITGB2	9,45940E-03	36280,1	8,43497E-03	9048	8,63342E-01	41837	9,23718E-01	45278	8,91648E-01	36856	0	48381,5
Myeloid_vs_CD8_Responder	Macro_IFI27	CD8(ISG+ T)	HLA-DRB5	LAG3	9,45943E-03	20224,5	6,01227E-03	15711	1,26545E+00	22817	2,16940E+00	8128	9,46051E-01	6085	0	48381,5
Myeloid_vs_CD8_Responder	Mono_CD14	CD8(GZMK+ Tex)	LYZ	ITGAL	9,46463E-03	24853,3	3,54312E-03	34426	2,00023E+00	7159	3,13922E+00	1428	8,98722E-01	32872	0	48381,5
Myeloid_vs_CD8_Responder	CD8(ZNF683+KLRB1+ T)	Mono_INHBA	CALR	LRP1	9,46567E-03	38764,9	2,92276E-03	45284	1,30724E+00	21439	9,64684E-01	43206	8,94011E-01	35514	0	48381,5
Myeloid_vs_CD8_Responder	CD8(Temra)	Macro_OLFML3	ADAM10	TREM2	9,47709E-03	31079,7	6,43095E-03	14138	2,04523E+00	6649	1,46321E+00	22828	8,45221E-01	63402	0	48381,5

Myeloid_vs_CD8_Responder	CD8(ZNF683+KLRB1+ T)	Macro_LYVE1	CD99	CD81	9,48973E-03	35278,5	4,01443E-03	28744	1,04307E+00	32098	9,23269E-01	45307	9,18556E-01	21862	0	48381,5
Myeloid_vs_CD8_Responder	CD8(EOMES+ NK-like)	Macro_ISG15	CD52	SIGLEC10	9,49916E-03	38632,7	3,47891E-03	35327	1,06115E+00	31268	9,23184E-01	45316	8,98727E-01	32871	0	48381,5
Myeloid_vs_CD8_Responder	Mono_CD14	CD8(Tc17)	IL1B	SIGIRR	9,50440E-03	24201,9	5,40852E-03	18470	2,05674E+00	6516	2,76766E+00	2858	8,77925E-01	44784	0	48381,5
Myeloid_vs_CD8_Responder	Macro_OLFML3	CD8(LAYN+ T)	MDK	NCL	9,51804E-03	28449,9	1,08853E-02	6117	1,67360E+00	11939	9,22931E-01	45334	9,03059E-01	30478	0	48381,5
Myeloid_vs_CD8_Responder	Mono_CD14	CD8(Tc17)	HBEGF	CD44	9,51804E-03	25697,5	5,60514E-03	17459	1,53282E+00	15012	1,54251E+00	20440	9,09102E-01	27195	0	48381,5
Myeloid_vs_CD8_Responder	cDC(CD1C)	CD8(EOMES+ NK-like)	LGALS1	CD69	9,51804E-03	32060,3	3,01392E-03	43387	8,07464E-01	45334	1,80283E+00	14210	9,40852E-01	8989	0	48381,5
Myeloid_vs_CD8_Responder	pDC_LILRA4	CD8(IL7R+ZNF683+ Tm)	COL24A1	ITGA1_ITGB1	9,52466E-03	51163,9	3,64914E-02	546	2,74196E+00	2221	5,59440E-01	68972	6,82947E-01	135699	0	48381,5
Myeloid_vs_CD8_Responder	CD8(GZMK+ Early Tem)	Macro_ISG15	HLA-B	LILRB2	9,52749E-03	29355,7	2,91973E-03	45343	1,42795E+00	17848	1,36672E+00	26050	9,40548E-01	9156	0	48381,5
Myeloid_vs_CD8_Responder	Macro_FOLR2+APOE-	CD8(GZMK+ Tex)	HLA-DRA	LAG3	9,53022E-03	21249,9	4,75124E-03	22492	1,26399E+00	22861	2,19712E+00	7762	9,48564E-01	4753	0	48381,5
Myeloid_vs_CD8_Responder	CD8(GZMK+ Tex)	Mono_CD14	CCL4	CCR1	9,53065E-03	37826,1	5,47420E-03	18127	8,07273E-01	45346	1,04494E+00	39322	8,89705E-01	37954	0	48381,5
Myeloid_vs_CD8_Responder	Mono_CD14	CD8(Tc17)	AGTRAP	RACK1	9,53380E-03	31768,5	4,82283E-03	22022	9,50961E-01	36754	1,21987E+00	31727	9,21971E-01	19958	0	48381,5
Myeloid_vs_CD8_Responder	Macro_ISG15	CD8(LAYN+ T)	SPP1	ITGAV_ITGB1	9,53442E-03	22089,3	1,04636E-02	6497	1,82724E+00	9414	2,66677E+00	3495	8,81543E-01	42659	0	48381,5
Myeloid_vs_CD8_Responder	Mono_INHBA	CD8(NME1+ T)	S100A8	CD69	9,54011E-03	39360,3	2,91936E-03	45355	9,51449E-01	36730	1,09955E+00	36791	9,04775E-01	29544	0	48381,5
Myeloid_vs_CD8_Responder	CD8(ISG+ T)	cDC_CLEC9A	LGALS9	MRC2	9,54151E-03	38887,1	9,26882E-03	7790	1,77043E+00	10264	1,46207E+00	22868	7,64533E-01	105132	0	48381,5
Myeloid_vs_CD8_Responder	Mono_CD14	CD8(Tc17)	CXCL2	DPP4	9,54915E-03	26504,5	2,69710E-02	1005	1,82642E+00	9419	2,52321E+00	4532	8,34934E-01	69185	0	48381,5
Myeloid_vs_CD8_Responder	CD8(Tn)	Macro_LYVE1	HSP90B1	LRP1	9,55484E-03	35926,1	3,17122E-03	40356	2,01113E+00	7038	9,22285E-01	45369	8,88845E-01	38486	0	48381,5
Myeloid_vs_CD8_Responder	Macro_FOLR2+APOE+	CD8(IL7R+ZNF683+ Tm)	APOE	SORL1	9,56699E-03	20618,3	5,21619E-03	19550	2,73918E+00	2226	3,15704E+00	1377	9,01098E-01	31557	0	48381,5
Myeloid_vs_CD8_Responder	Macro_ISG15	CD8(Tn)	CCL2	CCR4	9,57275E-03	32929,7	7,03770E-02	143	2,17642E+00	5367	2,07717E+00	9427	7,72680E-01	101330	0	48381,5
Myeloid_vs_CD8_Responder	Mono_CD14	CD8(IL7R+ZNF683+ Tm)	IL1B	SIGIRR	9,57381E-03	23625,9	5,65611E-03	17226	2,06977E+00	6379	2,79308E+00	2722	8,80303E-01	43421	0	48381,5
Myeloid_vs_CD8_Responder	Macro_NLRP3	CD8(GZMK+ Tem)	LYZ	ITGAL	9,57385E-03	19628,1	5,46680E-03	18169	2,13827E+00	5703	2,74366E+00	2999	9,16823E-01	22888	0	48381,5
Myeloid_vs_CD8_Responder	CD8(EOMES+ NK-like)	Mono_CD14	CCL5	CCR1	9,57698E-03	36336,3	6,17393E-03	15071	8,27327E-01	44047	9,21873E-01	45390	9,06207E-01	28792	0	48381,5
Myeloid_vs_CD8_Responder	Mono_CD14	CD8(IL7R+ZNF683+ Tm)	VCAN	ITGB1	9,57909E-03	20178,5	8,07835E-03	9729	3,11358E+00	1337	2,28875E+00	6766	8,95491E-01	34679	0	48381,5
Myeloid_vs_CD8_Responder	Macro_FOLR2+APOE+	CD8(Tn)	APOE	SORL1	9,58395E-03	20590,1	5,21082E-03	19579	2,73892E+00	2228	3,23535E+00	1176	9,01052E-01	31586	0	48381,5
Myeloid_vs_CD8_Responder	Mono_CD14	CD8(IL7R+ZNF683+ Tm)	HBEGF	CD44	9,58647E-03	25360,9	5,65524E-03	17230	1,54078E+00	14802	1,58026E+00	19413	9,09469E-01	26978	0	48381,5
Myeloid_vs_CD8_Responder	Macro_FOLR2+APOE+	CD8(GZMK+ Tem)	HLA-DRB5	LAG3	9,59653E-03	21148,5	6,60671E-03	13543	1,26271E+00	22902	1,71692E+00	16076	9,48407E-01	4840	0	48381,5
Myeloid_vs_CD8_Responder	Macro_LYVE1	CD8(LAYN+ T)	CD14	ITGA4	9,59653E-03	23905,3	4,84869E-03	21857	1,84171E+00	9204	1,66921E+00	17182	9,16791E-01	22902	0	48381,5
Myeloid_vs_CD8_Responder	Mono_CD14	CD8(IL7R+ZNF683+ Tm)	AGTRAP	RACK1	9,59915E-03	35415,9	4,22685E-03	26682	8,22758E-01	44347	1,14257E+00	34951	9,17093E-01	22718	0	48381,5
Myeloid_vs_CD8_Responder	CD8(LAYN+ T)	Macro_ISG15	B2M	LILRB1	9,60126E-03	36265,5	2,93755E-03	44955	1,05545E+00	31515	9,21395E-01	45413	9,37254E-01	11063	0	48381,5
Myeloid_vs_CD8_Responder	Macro_FOLR2+APOE-	CD8(Terminal Tex)	B2M	CD3D	9,60345E-03	63493,7	2,59675E-03	53589	1,06001E-01	113445	1,78893E-01	101992	9,64895E-01	61	0	48381,5
Myeloid_vs_CD8_Responder	CD8(NME1+ T)	Macro_ISG15	HSP90B1	TLR2	9,60444E-03	39741,3	3,41740E-03	36246	1,21613E+00	24594	9,21329E-01	45416	8,79191E-01	44069	0	48381,5
Myeloid_vs_CD8_Responder	CD8(GZMK+ Early Tem)	Macro_NLRP3	HLA-F	LILRB2	9,61078E-03	39652,7	2,91585E-03	45422	1,35443E+00	19996	1,02530E+00	40200	8,78837E-01	44264	0	48381,5
Myeloid_vs_CD8_Responder	CD8(Terminal Tex)	Mono_CD16	GNAI2	C5AR1	9,61607E-03	31634,9	3,42770E-03	36092	2,05771E+00	6503	1,49737E+00	21771	8,76779E-01	45427	0	48381,5
Myeloid_vs_CD8_Responder	CD8(GZMK+ Early Tem)	Macro_ISG15	HLA-A	LILRB2	9,61925E-03	31501,7	2,91553E-03	45430	1,30978E+00	21359	1,22792E+00	31411	9,37509E-01	10927	0	48381,5
Myeloid_vs_CD8_Responder	CD8(ISG+ T)	Macro_ISG15	CCL5	CCR1	9,62137E-03	34297,1	7,04157E-03	12210	8,99975E-01	39627	9,20951E-01	45432	9,11648E-01	25835	0	48381,5
Myeloid_vs_CD8_Responder	Mono_CD14	CD8(ISG+ T)	ICAM1	IL2RG	9,62666E-03	30847,7	4,37010E-03	25411	1,00555E+00	33978	1,73836E+00	15588	9,02310E-01	30880	0	48381,5
Myeloid_vs_CD8_Responder	CD8(ID2+CXCR4+ T)	Macro_ISG15	CCL5	CCR1	9,62772E-03	31920,9	7,91869E-03	10074	1,03478E+00	32494	9,20866E-01	45438	9,16263E-01	23217	0	48381,5
Myeloid_vs_CD8_Responder	Mono_CD14	CD8(ISG+ T)	CD14	ITGA4	9,63302E-03	31410,9	3,04088E-03	42844	1,35361E+00	20017	1,91648E+00	12063	8,97177E-01	33749	0	48381,5
Myeloid_vs_CD8_Responder	CD8(ITM2C+ T)	Macro_FOLR2-APOE+	RPS19	C5AR1	9,63938E-03	33260,1	3,44400E-03	35845	1,29056E+00	21994	9,20638E-01	45449	9,31239E-01	14631	0	48381,5
Myeloid_vs_CD8_Responder	CD8(Tn)	Macro_NLRP3	CIRBP	TREM1	9,64044E-03	31541,1	6,60229E-03	13562	1,79264E+00	9938	9,20597E-01	45450	8,85462E-01	40374	0	48381,5
Myeloid_vs_CD8_Responder	Mono_CD14	CD8(ISG+ T)	IL1B	SIGIRR	9,64787E-03	20706,1	7,29531E-03	11555	2,15607E+00	5552	2,96322E+00	2004	8,93076E-01	36038	0	48381,5
Myeloid_vs_CD8_Responder	CD8(LAYN+ T)	Macro_FOLR2+APOE-	ADAM10	GPNMB	9,65014E-03	35628,7	4,91277E-03	21440	1,50684E+00	15654	1,45988E+00	22935	8,33992E-01	69733	0	48381,5
Myeloid_vs_CD8_Responder	CD8(NME1+ T)	Macro_FOLR2-APOE+	CALR	LRP1	9,65955E-03	36016,3	3,44347E-03	35851	1,38639E+00	19069	9,20398E-01	45468	9,01531E-01	31312	0	48381,5
Myeloid_vs_CD8_Responder	CD8(GZMK+ Early Tem)	Macro_ISG15	B2M	LILRB1	9,66273E-03	36164,5	2,94287E-03	44838	1,06412E+00	31105	9,20340E-01	45471	9,37307E-01	11027	0	48381,5
Myeloid_vs_CD8_Responder	Mono_CD14	CD8(ISG+ T)	HBEGF	CD44	9,66698E-03	32554,7	4,00368E-03	28849	1,27837E+00	22414	1,32297E+00	27725	8,94210E-01	35404	0	48381,5
Myeloid_vs_CD8_Responder	Macro_IFI27	CD8(Terminal Tex)	HLA-DQA1	LAG3	9,67948E-03	20029,9	7,10523E-03	12049	1,26148E+00	22953	1,95019E+00	11447	9,47496E-01	5319	0	48381,5
Myeloid_vs_CD8_Responder	Mono_CD14	CD8(ISG+ T)	LYZ	ITGAL	9,68081E-03	21743,7	4,41712E-03	24997	2,06557E+00	6427	3,18568E+00	1301	9,08324E-01	27612	0	48381,5
Myeloid_vs_CD8_Responder	cDC(CD1C)	CD8(Terminal Tex)	CD86	CTLA4	9,69359E-03	26136,5	8,33505E-03	9242	1,60943E+00	13226	1,79657E+00	14333	8,76659E-01	45500	0	48381,5
Myeloid_vs_CD8_Responder	CD8(GZMK+ Early Tem)	Mono_CD16	HLA-A	LILRA1	9,69581E-03	32626,5	9,54306E-03	7444	1,72580E+00	11000	5,03358E-01	73344	9,16685E-01	22963	0	48381,5
Myeloid_vs_CD8_Responder	Mono_CD14	CD8(ID2+CXCR4+ T)	SECTM1	CD7	9,69998E-03	35231,7	8,61212E-03	8769	1,06676E+00	30989	9,79456E-01	42513	8,76647E-01	45506	0	48381,5
Myeloid_vs_CD8_Responder	CD8(Terminal Tex)	Macro_FOLR2+APOE+	ADAM10	GPNMB	9,70234E-03	32797,5	4,68186E-03	22967	2,86180E+00	1891	1,58958E+00	19166	8,30633E-01	71582	0	48381,5
Myeloid_vs_CD8_Responder	cDC(CD1C)	CD8(LAYN+ T)	HLA-DPB1	LAG3	9,70638E-03	27810,3	2,91095E-03	45512	1,14610E+00	27459	2,62176E+00	3800	9,32488E-01	13899	0	48381,5
Myeloid_vs_CD8_Responder	CD8(GZMK+ Early Tem)	Mono_INHBA	B2M	LILRB2	9,70851E-03	31720,1	2,91091E-03	45514	1,35552E+00	19960	1,05795E+00	38685	9,46103E-01	6060	0	48381,5
Myeloid_vs_CD8_Responder	Macro_LYVE1	CD8(GZMK+ Tex)	HLA-DRA	LAG3	9,71379E-03	21600,9	4,74074E-03	22547	1,26077E+00	22974	2,08432E+00	9314	9,48510E-01	4788	0	48381,5
Myeloid_vs_CD8_Responder	CD8(LAYN+ T)	Macro_LYVE1	CALR	LRP1	9,71384E-03	31154,1	4,09793E-03	27912	2,03932E+00	6708	9,19329E-01	45519	9,08990E-01	27250	0	48381,5
Myeloid_vs_CD8_Responder	Mono_CD14	CD8(GZMK+ Early Tem)	CD14	ITGA4	9,71384E-03	31360,9	3,04414E-03	42775	1,35403E+00	20008	1,92327E+00	11922	8,97227E-01	33718	0	48381,5
Myeloid_vs_CD8_Responder	cDC(CD1C)	CD8(IL7R+ZNF683+ Tm)	HBEGF	CD44	9,71491E-03	42571,7	2,91077E-03	45520	8,50878E-01	42622	1,22073E+00	31685	8,78156E-01	44650	0	48381,5
Myeloid_vs_CD8_Responder	Mono_CD14	CD8(GZMK+ Early Tem)	IL1B	SIGIRR	9,72345E-03	22023,7	6,45161E-03	14067	2,11165E+00	5953	2,90314E+00	2244	8,87065E-01	39473	0	48381,5
Myeloid_vs_CD8_Responder	CD8(Tn)	Macro_IFI27	CD40LG	CD40	9,72866E-03	49040,1	1,94906E-02	2245	9,46988E-01	36959	1,10832E+00	36387	7,26105E-01	121228	0	48381,5
Myeloid_vs_CD8_Responder	Mono_CD14	CD8(GZMK+ Early Tem)	VCAN	ITGB1	9,72879E-03	23550,5	5,96606E-03	15868	3,03526E+00	1486	2,12906E+00	8661	8,80435E-01	43356	0	48381,5
Myeloid_vs_CD8_Responder	Macro_IER3	CD8(GZMK+ Tem)	HLA-DPB1	LAG3	9,73179E-03	20548,9	5,98900E-03	15792	1,26059E+00	22985	1,89866E+00	12384	9,51950E-01	3202	0	48381,5
Myeloid_vs_CD8_Responder	CD8(GZMK+ Tem)	Mono_CD14	HLA-C	LILRB2	9,73306E-03	29525,9	2,91009E-03	45537	1,40714E+00	18436	1,43274E+00	23824	9,36559E-01	11451	0	48381,5
Myeloid_vs_CD8_Responder	Macro_IFI27	CD8(GZMK+ Tex)	C3	IFITM1	9,73343E-03	25074,3	6,40163E-03	14228	1,28096E+00	22309	1,45862E+00	22986	9,26186E-01	17467	0	48381,5
Myeloid_vs_CD8_Responder	Mono_INHBA	CD8(Temra)	LGALS1	PTPRC	9,73413E-03	29336,9	2,91001E-03	45538	1,07194E+00	30781	1,58696E+00	19241	9,53084E-01	2743	0	48381,5
Myeloid_vs_CD8_Responder	Mono_CD14	CD8(GZMK+ Early Tem)	HBEGF	CD44	9,73841E-03	25453,9	5,55528E-03	17697	1,52490E+00	15215	1,61243E+00	18587	9,08732E-01	27389	0	48381,5
Myeloid_vs_CD8_Responder	Macro_FOLR2-APOE+	CD8(ID2+CXCR4+ T)	SPP1	PTGER4	9,74196E-03	20332,3	1,14468E-02	5630	1,82134E+00	9484	2,22327E+00	7478	9,02657E-01	30688	0	48381,5
Myeloid_vs_CD8_Responder	Mono_INHBA	CD8(NME1+ T)	HBEGF	CD82	9,74482E-03	26180,7	1,05740E-02	6404	1,80330E+00	9759	1,52984E+00	20811	8,76582E-01	45548	0	48381,5
Myeloid_vs_CD8_Responder	Mono_CD14	CD8(GZMK+ Early Tem)	LYZ	ITGAL	9,74803E-03	24512,1	3,62414E-03	33300	2,00628E+00	7087	3,11482E+00	1487	8,99746E-01	32305	0	48381,5
Myeloid_vs_CD8_Responder	Mono_CD14	CD8(GZMK+ Early Tem)	AGTRAP	RACK1	9,75232E-03	35329,1	4,19996E-03	26939	8,16975E-01	44726	1,17002E+00	33725	9,16850E-01	22874	0	48381,5
Myeloid_vs_CD8_Responder	CD8(GZMK+ Tex)	Macro_FOLR2-APOE+	CALR	LRP1	9,76088E-03	38477,1	3,06643E-03	42315	1,29476E+00	21867	9,18464E-01	45563	8,96263E-01	34259	0	48381,5
Myeloid_vs_CD8_Responder	Mono_CD14	CD8(LAYN+ T)	THBS1	ITGA4	9,76731E-03	34216,5	5,30762E-03	19013	8,76917E-01	41012	1,64750E+00	17707	8,77624E-01	44969	0	48381,5
Myeloid_vs_CD8_Responder	Mono_CD14	CD8(LAYN+ T)	CD14	ITGB1	9,77267E-03	22971,9	4,64135E-03	23278	1,61965E+00	13021	2,13331E+00	8612	9,19050E-01	21567	0	48381,5
Myeloid_vs_CD8_Responder	Mono_CD14	CD8(LAYN+ T)	CD14	ITGA4	9,77374E-03	26819,3	3,83750E-03	30685	1,45836E+00	16950	2,03952E+00	9968	9,07424E-01	28112	0	48381,5
Myeloid_vs_CD8_Responder	Macro_IER3	CD8(GZMK+ Tex)	ICAM1	IL2RG	9,77445E-03	23477,7	6,18844E-03	15007	1,46613E+00	16729	1,80023E+00	14260	9,16606E-01	23011	0	48381,5
Myeloid_vs_CD8_Responder	Mono_CD14	CD8(LAYN+ T)	SECTM1	CD7	9,77803E-03	33383,5	9,16700E-03	7939	1,13088E+00	28117	1,05411E+00	38877	8,79984E-01	43603	0	48381,5
Myeloid_vs_CD8_Responder	CD8(GZMK+ Early Tem)	Mono_INHBA	CD99	PILRA	9,77803E-03	40988,5	2,90815E-03	45579	1,05294E+00	31636	9,26487E-01	45150	8,96373E-01	34196	0	48381,5
Myeloid_vs_CD8_Responder	Macro_OLFML3	CD8(EOMES+ NK-like)	B2M	KLRC2	9,77910E-03	28875,5	1,22754E-02	5013	1,00273E+00	34142	9,18159E-01	45580	9,36869E-01	11261	0	48381,5
Myeloid_vs_CD8_Responder	pDC_LILRA4	CD8(ZNF683+KLRB1+ T)	COL24A1	ITGA1_ITGB1	9,77999E-03	50017,9	3,65606E-02	542	2,73289E+00	2251	6,36444E-01	63278	6,83152E-01	135637	0	48381,5

Myeloid_vs_CD8_Responder	Mono_CD14	CD8(LAYN+ T)	HBEGF	CD44	9,79198E-03	24807,5	6,00749E-03	15724	1,59675E+00	13490	1,53209E+00	20742	9,11926E-01	25700	0	48381,5
Myeloid_vs_CD8_Responder	CD8(ZNF683+KLRB1+ T)	Mono_CD16	CD52	SIGLEC10	9,79735E-03	35014,5	4,10144E-03	27883	1,22349E+00	24310	9,17901E-01	45597	9,05976E-01	28901	0	48381,5
Myeloid_vs_CD8_Responder	Mono_CD14	CD8(LAYN+ T)	LYZ	ITGAL	9,80058E-03	20002,3	5,13295E-03	20038	2,11908E+00	5878	3,14990E+00	1399	9,14389E-01	24315	0	48381,5
Myeloid_vs_CD8_Responder	Macro_OLFML3	CD8(ID2+CXCR4+ T)	HLA-DQB2	LAG3	9,80273E-03	32952,7	8,07607E-03	9735	1,43066E+00	17772	9,17791E-01	45602	8,80572E-01	43273	0	48381,5
Myeloid_vs_CD8_Responder	CD8(GZMK+ Early Tem)	Mast	IL16	CD9	9,80901E-03	34440,3	4,67482E-03	23032	1,56599E+00	14200	1,47463E+00	22456	8,43872E-01	64132	0	48381,5
Myeloid_vs_CD8_Responder	CD8(LAYN+ T)	Macro_ISG15	HSP90B1	TLR2	9,80918E-03	39648,5	3,43795E-03	35928	1,22028E+00	24436	9,17685E-01	45608	8,79509E-01	43889	0	48381,5
Myeloid_vs_CD8_Responder	Macro_IFI27	CD8(EOMES+ NK-like)	APOE	LSR	9,81379E-03	22136,3	1,20055E-02	5181	1,81988E+00	9508	2,35870E+00	6026	8,83343E-01	41585	0	48381,5
Myeloid_vs_CD8_Responder	Mono_CD14	CD8(Temra)	ICAM1	IL2RG	9,82640E-03	36896,9	3,47035E-03	35466	8,48302E-01	42768	1,52222E+00	21025	8,91668E-01	36844	0	48381,5
Myeloid_vs_CD8_Responder	CD8(Terminal Tex)	pDC_LILRA4	GZMB	IGF2R	9,82879E-03	30417,1	8,19594E-03	9513	7,60494E-01	48507	2,18966E+00	7867	8,89910E-01	37817	0	48381,5
Myeloid_vs_CD8_Responder	CD8(Tn)	Macro_ISG15	CD40LG	ITGAM_ITGB2	9,83144E-03	38090,9	1,94607E-02	2257	9,55393E-01	36529	1,20346E+00	32391	8,31875E-01	70896	0	48381,5
Myeloid_vs_CD8_Responder	Mono_CD14	CD8(Temra)	CD14	ITGB1	9,83178E-03	29716,9	3,15023E-03	40744	1,40494E+00	18487	1,99399E+00	10693	9,03413E-01	30279	0	48381,5
Myeloid_vs_CD8_Responder	Mono_CD14	CD8(Temra)	CD14	ITGA4	9,83286E-03	29262,9	3,36510E-03	37032	1,39624E+00	18759	1,97898E+00	10941	9,01757E-01	31201	0	48381,5
Myeloid_vs_CD8_Responder	CD8(Terminal Tex)	Mono_INHBA	FADD	ABCA1	9,83705E-03	42103,5	7,68909E-03	10567	1,45794E+00	16965	1,45649E+00	23049	7,49839E-01	111555	0	48381,5
Myeloid_vs_CD8_Responder	CD8(LAYN+ T)	Mono_INHBA	B2M	LILRB2	9,84148E-03	31791,3	2,90565E-03	45638	1,34685E+00	20226	1,05901E+00	38631	9,46056E-01	6080	0	48381,5
Myeloid_vs_CD8_Responder	CD8(Terminal Tex)	Mono_CD16	CD52	SIGLEC10	9,84795E-03	36047,1	3,90523E-03	29927	1,17341E+00	26249	9,16999E-01	45644	9,03867E-01	30034	0	48381,5
Myeloid_vs_CD8_Responder	CD8(Temra)	Macro_NLRP3	B2M	LILRB2	9,85011E-03	30273,1	2,90514E-03	45646	1,48508E+00	16223	1,14056E+00	35032	9,46052E-01	6083	0	48381,5
Myeloid_vs_CD8_Responder	CD8(Tn)	Mono_INHBA	CIRBP	TREM1	9,85227E-03	34205,9	5,55774E-03	17683	1,49971E+00	15845	9,59418E-01	43472	8,76434E-01	45648	0	48381,5
Myeloid_vs_CD8_Responder	Mono_INHBA	CD8(NME1+ T)	HLA-DRB5	LAG3	9,85982E-03	31714,3	3,81549E-03	30932	8,02626E-01	45655	1,55473E+00	20129	9,33199E-01	13474	0	48381,5
Myeloid_vs_CD8_Responder	Mono_CD14	CD8(Temra)	HBEGF	CD44	9,86847E-03	32984,9	4,00773E-03	28810	1,27901E+00	22382	1,26341E+00	29979	8,94257E-01	35372	0	48381,5
Myeloid_vs_CD8_Responder	CD8(ISG+ T)	Mono_CD14	ANXA1	FPR1	9,86955E-03	36737,3	2,90444E-03	45664	1,30689E+00	21450	1,13691E+00	35192	8,98461E-01	32999	0	48381,5
Myeloid_vs_CD8_Responder	Mono_CD14	CD8(Temra)	LYZ	ITGAL	9,88144E-03	19494,9	5,34216E-03	18820	2,13472E+00	5740	3,26757E+00	1114	9,15940E-01	23419	0	48381,5
Myeloid_vs_CD8_Responder	cDC(CD1C)	CD8(GZMK+ Tex)	LGALS9	HAVCR2	9,88577E-03	32661,7	3,84193E-03	30644	1,11688E+00	28740	2,04721E+00	9864	8,76391E-01	45679	0	48381,5
Myeloid_vs_CD8_Responder	CD8(Temra)	cDC_CLEC9A	ADAM10	CADM1	9,88833E-03	37610,9	8,46798E-03	8997	1,25750E+00	23080	1,51646E+00	21209	8,02768E-01	86387	0	48381,5
Myeloid_vs_CD8_Responder	Mono_CD14	CD8(Temra)	NAMPT	ITGA5_ITGB1	9,89551E-03	26570,7	4,38336E-03	25284	1,32405E+00	20918	2,23273E+00	7374	9,02277E-01	30896	0	48381,5
Myeloid_vs_CD8_Responder	CD8(Tc17)	Macro_OLFML3	LTB	TNFRSF1A	9,89984E-03	35398,7	7,79052E-03	10353	1,05469E+00	31558	1,01005E+00	41009	8,76368E-01	45692	0	48381,5
Myeloid_vs_CD8_Responder	CD8(ISG+ T)	Mono_CD14	HLA-C	LILRB2	9,90418E-03	29684,5	2,90302E-03	45696	1,40068E+00	18620	1,42057E+00	24223	9,36487E-01	11502	0	48381,5
Myeloid_vs_CD8_Responder	cDC_CLEC9A	CD8(Terminal Tex)	B2M	CD3D	9,91807E-03	52469,9	2,59123E-03	53763	9,60658E-02	114774	9,22298E-01	45368	9,64859E-01	63	0	48381,5
Myeloid_vs_CD8_Responder	Mono_CD14	CD8(GZMK+ Tem)	ICAM1	IL2RG	9,91936E-03	35821,3	3,63555E-03	33154	8,77173E-01	40996	1,52322E+00	20996	8,93894E-01	35579	0	48381,5
Myeloid_vs_CD8_Responder	Mono_CD14	CD8(GZMK+ Tem)	THBS1	ITGA4	9,92044E-03	31307,5	6,12295E-03	15269	9,54438E-01	36571	1,73165E+00	15728	8,85093E-01	40588	0	48381,5
Myeloid_vs_CD8_Responder	Mono_CD14	CD8(GZMK+ Tem)	CD14	ITGA4	9,92696E-03	24378,9	4,42700E-03	24904	1,53588E+00	14925	2,12367E+00	8745	9,13254E-01	24939	0	48381,5
Myeloid_vs_CD8_Responder	Macro_FOLR2-APOE+	CD8(Terminal Tex)	HLA-DPA1	LAG3	9,92816E-03	20244,9	5,91097E-03	16101	1,25691E+00	23104	1,98329E+00	10871	9,53020E-01	2767	0	48381,5
Myeloid_vs_CD8_Responder	CD8(NME1+ T)	Macro_NLRP3	GNAI2	FPR1	9,93130E-03	32375,5	3,61837E-03	33378	1,80097E+00	9801	1,40918E+00	24596	8,76316E-01	45721	0	48381,5
Myeloid_vs_CD8_Responder	cDC(CD1C)	CD8(GZMK+ Early Tem)	LGALS1	CD69	9,93673E-03	32430,5	2,99029E-03	43839	8,01413E-01	45726	1,76001E+00	15095	9,40633E-01	9111	0	48381,5
Myeloid_vs_CD8_Responder	Mono_CD14	CD8(GZMK+ Tem)	IL1B	SIGIRR	9,94325E-03	22156,5	6,39619E-03	14245	2,10873E+00	5983	2,84636E+00	2449	8,86632E-01	39724	0	48381,5
Myeloid_vs_CD8_Responder	CD8(NME1+ T)	Macro_ISG15	HLA-C	LILRB2	9,94325E-03	30731,9	2,90118E-03	45732	1,36743E+00	19617	1,30524E+00	28417	9,36468E-01	11512	0	48381,5
Myeloid_vs_CD8_Responder	CD8(GZMK+ Tem)	Mono_CD16	HLA-C	LILRA1	9,94812E-03	29720,1	9,77207E-03	7195	1,85851E+00	8953	6,68664E-01	60955	9,16432E-01	23116	0	48381,5
Myeloid_vs_CD8_Responder	cDC_CLEC9A	CD8(GZMK+ Tex)	HLA-DQA2	LAG3	9,95312E-03	20978,1	7,80350E-03	10329	1,25648E+00	23119	2,60140E+00	3936	9,23401E-01	19125	0	48381,5
Myeloid_vs_CD8_Responder	Mono_CD14	CD8(GZMK+ Tem)	HBEGF	CD44	9,96066E-03	32169,7	4,10064E-03	27886	1,29378E+00	21902	1,31805E+00	27916	8,95336E-01	34763	0	48381,5
Myeloid_vs_CD8_Responder	Macro_OLFML3	CD8(IL7R+ZNF683+ Tm)	SPP1	CD44	9,96610E-03	40571,9	3,13012E-03	41106	8,00995E-01	45753	1,35028E+00	26652	8,84371E-01	40967	0	48381,5
Myeloid_vs_CD8_Responder	Macro_OLFML3	CD8(GZMK+ Early Tem)	FN1	ITGA4_ITGB7	9,97264E-03	25015,7	1,01454E-02	6816	1,93812E+00	7915	1,71170E+00	16207	8,76270E-01	45759	0	48381,5
Myeloid_vs_CD8_Responder	cDC(CD1C)	CD8(LAYN+ T)	HLA-DRB5	LAG3	9,97264E-03	31265,1	2,89984E-03	45759	9,46851E-01	36967	2,31008E+00	6545	9,24120E-01	18673	0	48381,5
Myeloid_vs_CD8_Responder	CD8(GZMK+ Tem)	Macro_NLRP3	CCL5	CCRL2	9,98136E-03	31098,7	8,78890E-03	8486	1,04278E+00	32110	9,15068E-01	45767	9,20495E-01	20749	0	48381,5
Myeloid_vs_CD8_Responder	Macro_FOLR2+APOE+	CD8(Temra)	CD14	ITGA4	9,98312E-03	23483,3	4,80000E-03	22193	2,01658E+00	6973	1,68824E+00	16732	9,16405E-01	23137	0	48381,5
Myeloid_vs_CD8_Responder	CD8(Tc17)	Macro_ISG15	HLA-B	LILRB2	9,99227E-03	30143,9	2,89895E-03	45777	1,40325E+00	18544	1,29626E+00	28745	9,40348E-01	9272	0	48381,5
Myeloid_vs_CD8_Responder	CD8(Terminal Tex)	Mono_CD14	HLA-B	LILRB2	9,99554E-03	28852,5	2,89883E-03	45780	1,45704E+00	16992	1,43243E+00	23836	9,40347E-01	9273	0	48381,5
Myeloid_vs_CD8_Responder	CD8(ZNF683+KLRB1+ T)	Mono_INHBA	HLA-B	LILRB2	9,99664E-03	34123,1	2,89877E-03	45781	1,23476E+00	23905	9,63512E-01	43273	9,40347E-01	9275	0	48381,5
